# Supplementary material for: Clonal dynamics of haematopoiesis across the human lifespan
Source: Nature. 2022 Jun 1;606(7913):343–50. doi: 10.1038/s41586-022-04786-y (PMC9177428; doi:10.1038/s41586-022-04786-y)
Supplement: Supplementary file 1 — This file contains the following sections. Supplementary Methods. Further details on variant calling, phylogenetic tree reconstruction, approximate Bayesian computation modelling and inference of positive selection not included in the Methods. Supplementary Background. A detailed exposition of the concepts involved in phylodynamic inference and how phylogenies can be used to reconstruct clonal histories. Supplementary Simulations. An exploration of the patterns of phylogenetic changes seen with simulations of different scenarios of population size dynamics, and how these compare with the observed trees. Supplementary Results. Further results of analyses performed in the study that were not fully documented in the main paper. Supplementary Note 1. An explanation for the intuition underpinning approximate Bayesian computation methods used in the analyses. Supplementary Note 2. A supplementary discussion comparing the data reported here for blood with what has been observed in solid organs. Data Availability. A detailed description of the file formats available for the data archive deposited at Mendeley Data. [file 41586_2022_4786_MOESM1_ESM.pdf]

nature portfolio

https://doi.org/10.1038/s41586-022-04786-y

Supplementary information

Clonal dynamics of haematopoiesis across the human lifespan

In the format provided by the authors and unedited

Supplementary information

Clonal dynamics of haematopoiesis across the human lifespan

Emily Mitchell[1,2,3], Michael Spencer Chapman[1,#], Nicholas Williams[1,#], Kevin J Dawson[1,#], Nicole Mende[2], Emily F Calderbank[2], Hyunchul Jung[1], Thomas Mitchell[1], Tim H H Coorens[1], David H Spencer[4], Heather Machado[1], Henry Lee-Six[1], Megan Davies[5], Daniel Hayler[2], Margarete Fabre[1,2,3], Krishnaa Mahbubani[6,7], Federico Abascal[1], Alex Cagan[1], George Vassiliou[1,2,3], Joanna Baxter[3], Inigo Martincorena[1], Michael R Stratton[1], David G Kent[8], Krishna Chatterjee[9], Kourosh Saeb Parsy[6,7], Anthony R Green[2,3], Jyoti Nangalia[1,2,3*], Elisa Laurenti[2,3*], Peter J Campbell[1,2*].

Table of Contents

Supplementary Methods **3**

Substitution and indel calling *3*

Structural variants and copy number *4*

Construction of phylogenetic trees *4*

Validation of the phylogeny *10*

Using Rsimpop to simulate HSC populations *14*

HSC population size modelling *15*

HSC population size estimate *17*

dN/dS analysis *17*

Amino acid variant annotation *20*

Driver mutation acquisition rate estimation *21*

Modelling positive selection in the HSC population *22*

Phylofit estimation of selection coefficients *23*

Supplementary Background **27**

Definition of phylodynamics *27*

Definition of $N\tau$ *27*

Phylodynamic principles *27*

Application of phylodynamics to stem cell populations *28*

Supplementary Simulations **28**

Effect of population size *29*

Effect of age *30*

Population decline *31*

Population growth *32*

Population bottlenecks *33*

Positive selection *34*

Supplementary Results **38**

Phylodyn trajectories for the older individuals *38*

Phylogeny annotated with BM HSC/MPP vs BM HPC cell type *39*

Posterior distributions for 'driver modelling' parameters *39*

Putative additional novel drivers *40*

Supplementary Note 1: Intuition for Approximate Bayesian Computation **41**

Supplementary Note 2: Comparison with clonal dynamics in solid organs **42**

Data Availability **44**

References **45**

Supplementary Methods

Substitution and indel calling

CaVEMan (used for calling SNVs) and *Pindel* (used for calling small indels) were run against an unmatched synthetic normal genome using in-house pipelines[1,2]. *CaVEMan* was run with the 'normal contamination of tumour' set to 0.05, otherwise standard settings were used. Default filters were also used, one of which excludes putative SNVs that are present in a large panel of normal samples, so excluding most of the germline single nucleotide polymorphisms (SNPs) from subsequent analysis. It leaves around 30,000-40,000 germline SNPs in most individuals, which represent inherited SNPs that are rare within the population. In addition to the default *CaVEMan* filters, thresholds were set to require putative variants to have a mean mapping score (ASMD) of at least 140 and fewer than half supporting reads being clipped (CLPM=0). *Pindel* was run with standard settings. A custom filter was then used to remove artefacts associated with the 'low input' library preparation method, including those due to cruciform DNA structures[3].

Specifically, the custom 'low input' filter incorporates two additional filtering strategies[4]. Firstly, a fragment-based filter, designed to remove overlapping reads that result from the relatively shorter insert sizes produced by this protocol, which can result in the double counting of variants. Secondly, a cruciform filter, which removes erroneous variants introduced due to the incorrect processing of cruciform DNA. For each variant, the standard deviation (s.d.) and median absolute deviation (MAD) of the variant position within the read was calculated separately for positive and negative strands reads. Where a variant was supported by a low number of reads for one strand, the filtering used statistics calculated from the reads derived from the other strand. It was required that either: (a) ≤90% of supporting reads report the variant within the first 15% of the read as determined from the alignment start, or (b) MAD > 0 and s.d. > 4. Where both strands were supported by sufficient reads, it was required for both strands separately to either: (a) ≤90% of supporting reads report the variant within the first 15% of the read as determined from the alignment start, (b) MAD > 2 and s.d. > 2, or (c) at least one strand has MAD > 1 and s.d. > 10.

Following this, *cgpVAF* (another bespoke algorithm) was used to generate a matrix of variant and normal reads at all sites that had a detected variant in any sample from a given individual. These algorithms are available from the Sanger Institute's Cancer IT GitHub repository (https://github.com/cancerit).

Additional filtering on the read count and depth matrices containing several hundred samples per individual was then performed as follows: a) An exact binomial filter was used to remove variants with aggregated count distributions consistent with germline single nucleotide

polymorphisms (SNPs)[5]. b) A beta-binomial filter was used to remove low-frequency artefacts, i.e. variants present at low frequencies across samples in a way not consistent with the sample-to-sample variation expected for acquired somatic mutations[6]. c) Sites with a mean depth below 8 and over 40 were removed. d) Thresholds for read count and VAF were used to filter out *in vitro* variants from the remaining mutations using a bespoke script. The thresholds were set to require a minimum variant read count of 2 or more and a variant allele fraction of 0.2 for autosomes and 0.4 for XY chromosomes (**Extended Fig. 2a**). e) For each site normal and variant read counts were aggregated from samples with ≥ 3 variant reads. A one-sided exact binomial test was used to filter mutations inconsistent with a true somatic mutation (p-value < 0.001). f) A final filtering step was the removal of mutations that best mapped to the 'ancestral' branch of the SNV-derived phylogenetic tree (only the case for 8 mutations in one individual). Custom R scripts, used for these filtering steps were adapted from Spencer Chapman *et al*[7] (see **Code Availability**).

Structural variants and copy number

Structural variants (SVs) were called using GRIDDS[8], with all variants confirmed by visual inspection and by checking if they fit the distribution expected based on the SNV-derived phylogenetic tree. Specifically, GRIDSS with a default setting (version 2.9.4) was used to call SVs. SVs larger than 1kb in size with QUAL >=250 were included. For SVs smaller than 30kb, SVs with QUAL >=300 were only included. Furthermore, SVs that had assemblies from both sides of the breakpoint were only considered if they were supported by at least four discordant and two split reads. We further filtered out SVs for which the standard deviation of the alignment positions at either ends of the discordant read pairs was smaller than five. To remove potential germline SVs and artefacts, we generated the panel of normal by adding in-house normal samples (n=350) to the GRIDSS panel of normal. SVs found in at least three different samples in the panel of normal were removed.

Autosomal copy number aberrations (CNAs) and X chromosome CNAs in females were called using ASCAT (Allele-Specific Copy number Analysis of Tumours)[9], which was run against a single sample selected from each individual. The matched sample was selected to have a coverage > 15X, no loss of Y and to be a singleton in the phylogenetic tree (no coalescences post birth). The ASCAT output was manually interpreted through visual inspection. ASCAT was unable to accurately call copy number changes on the haploid sex chromosomes in males. Therefore, we ran the in-house algorithm BRASS (BReakpoint AnalySiS)[10] to generate an intermediate file containing information on binned read counts across 500bp segments of the genome. A comparison of the mean coverage of the X and Y chromosomes was used to call X or Y CNAs in individual samples, which were then validated by visual inspection of read depth.

Construction of phylogenetic trees

The key steps to generate the phylogenies shown in **Figures 2-3 and Extended Figure 5** are as follows:

1. Generate a 'genotype matrix' of mutation calls for every colony within a donor
2. Reconstruct phylogenetic trees from the genotype matrix
3. Correct terminal branch lengths for sensitivity to detect mutations in each colony
4. Make phylogenetic trees
5. Scale trees to chronological age
6. Overlay phenotypic and genotypic information on the tree

More detailed information on these steps is provided below:

MPBoot, a maximum parsimony tree approximation method[11], was used to build phylogenetic trees of the relationships between the sampled cells. Variants were genotyped as 'present' (coded as 1) in a sample if 2 or more variant reads supported the variant. Variants were genotyped as 'absent' (coded as 0) in a sample if 0 variant reads were present at a given site and depth at that site was 6 or more. Sites that did not fall into either of the above categories were marked as 'unknown' (coded as 0.5). In all cases only a small minority of sites (< 5%) were categorised as 'unknown' or 'missing data' as shown in the table below.

Sample_ID	Genotype 0 'absent'	Genotype 0.5 'unknown'	Genotype 1 'present'	% sites 'missing data'
**KX001**	3068118	42512	22049	1.38
**KX002**	2719616	28599	20085	1.05
**SX001**	4003266	127878	32218	3.17
**AX001**	5086607	173657	39577	3.39
**KX007**	9081044	48041	97580	0.52
**KX008**	10248698	78247	157878	0.75
**KX004**	21179417	197625	249761	0.92
**KX003**	8993397	107236	187015	1.17

The genotype matrix of shared variants was converted to a 'DNA string' for each sample with 'W' representing a 'wildtype' position, 'V' a 'variant' position and '?' representing 'unknown'. The DNA strings were then used as the input for *MPBoot*, which outputs unscaled trees with uninformative branch lengths (**Extended Fig. 3a**). We explicitly added a 'dummy sample' (called "Ancestral") into the DNA strings that *MPBoot* used, which has non-mutant genotypes across all sites i.e. representing the genotypes of the reference genome. After tree construction the 'ancestral' branch was dropped prior to downstream analyses."A maximum likelihood approach and the original count data was then used to assign each mutation in an individual's dataset to a branch in their *MPBoot* generated phylogenetic tree (https://github.com/NickWilliamsSanger/treemut). Tree edge lengths were then made proportional to the number of mutations assigned to the branch (**Extended Fig. 3b**).

The sensitivity of mutation calling in each sample was used to correct phylogeny branch lengths for sequencing coverage. Sensitivity was calculated as the fraction of known germline variants identified by CaVEMan in a specific sample. Mutation burden was corrected by multiplying the number of variants by 1/sensitivity for private branches. The sensitivity was adjusted to allow for the higher sensitivity on shared branches due to multiple samples containing the variant. Specifically, sensitivity was assessed by measuring the ability of the mutation-calling algorithms to detect heterozygous germline single nucleotide polymorphisms (SNPs) in each sample. Heterozygous SNPs should have the same VAF distribution and sensitivity as true somatic mutations. For private branches, the SNV component of branch lengths was scaled according to:

$$n_{cSNV} = \frac{n_{SNV}}{p_i}$$

Where n_{cSNV} is the corrected number of SNVs in sample *i*, n_{SNV} is the uncorrected number of SNVs called in sample *i* and p_i is the proportion of germline SNPs called by the Caveman algorithm in sample *i*.

For shared branches, it was assumed that (1) the regions of low sensitivity were independent between samples, (2) if a somatic mutation was called in at least one sample within the clade, it would also be correctly called (or 'rescued') in other samples in the clade (even in lower sensitivity samples). Shared branches were therefore scaled according to:

$$\frac{n_{SNV}}{1 - \pi_i(1 - p_i)}$$

Where the product is taken for $1 - p_i$ for each sample *i* within the clade. However, both of these assumptions will not hold true in all cases. Firstly, regions with low coverage are not randomly distributed, with some genomic regions likely to have low coverage in multiple samples. Secondly, while many mutations will be 'rescued' in subsequent samples once they have been called in a first sample - because the *treemut* algorithm for mutation assignment uses original read count data, meaning that even a single variant read in a subsequent sample is likely to result in the mutation being correctly assigned - this will not be true in every case. Some samples with very low coverage have 0 variant reads at a given site will by chance. In this situation, a mutation may not be correctly placed. While these factors may lead to an under-correction of shared branches, this approach provides a reasonable approximation. Corrected SNV burdens for each sample can then be calculated as the sum of corrected ancestral branch lengths back to the root of the phylogeny. **Supplementary Figs. 1-2** show the phylogenies with branch lengths corrected for differences in sequencing depth.

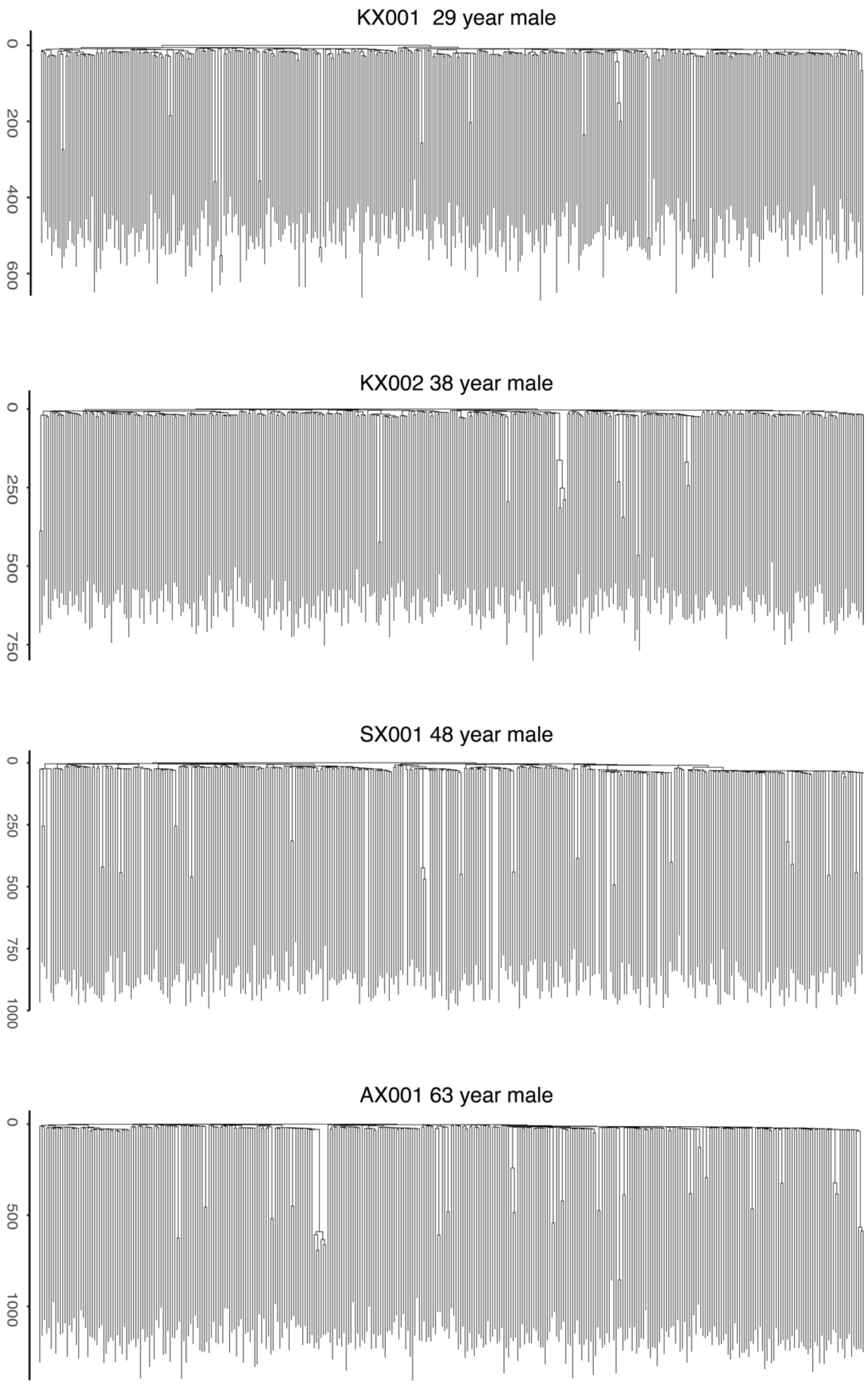

Supplementary Fig.1| Raw phylogenies for the four youngest adult donors. Phylogenies shown with raw mutation count branch lengths adjusted for sequencing depth of the sample using sensitivity.

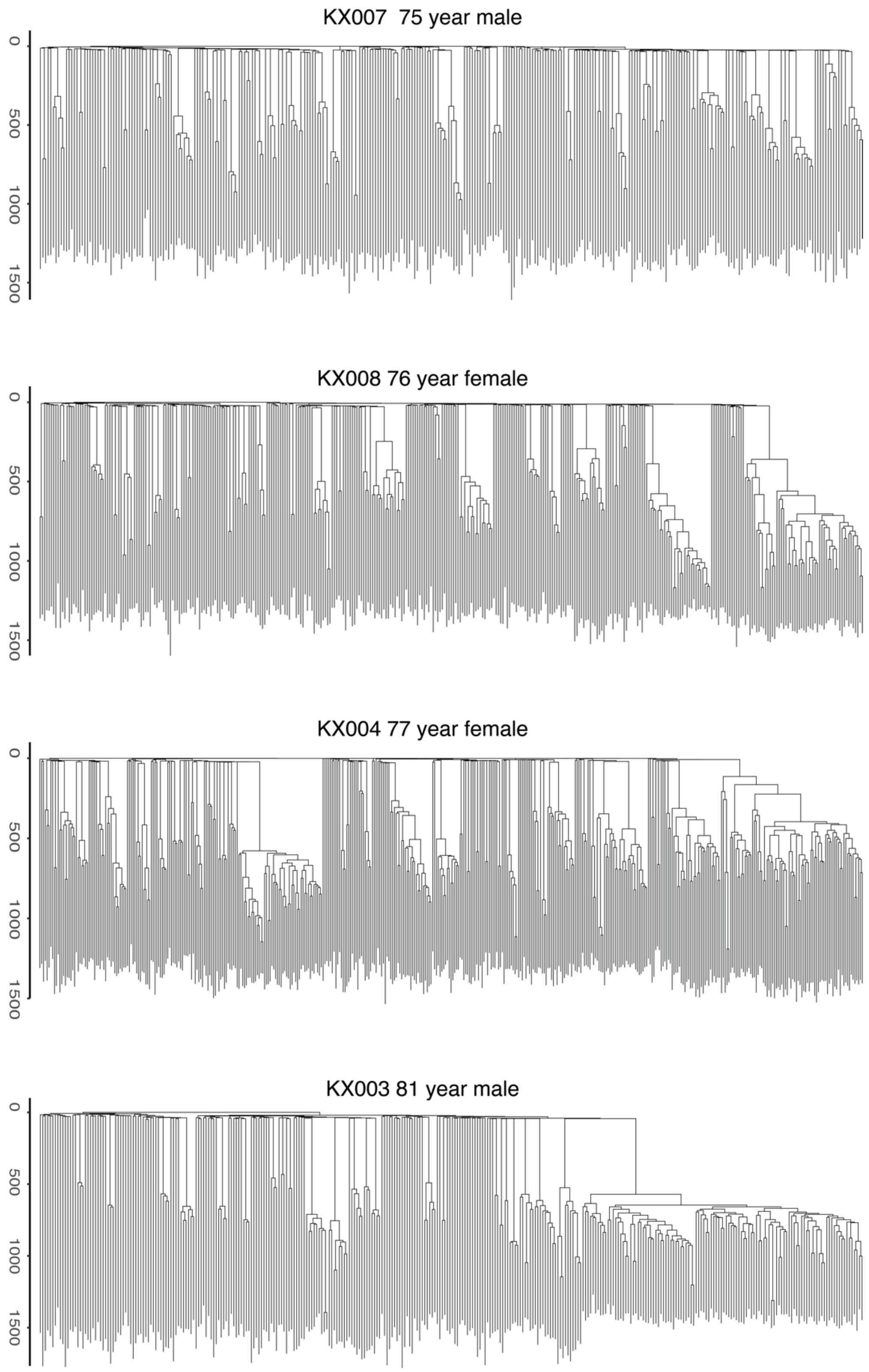

Supplementary Fig.2| Raw phylogenies for the four elderly adult donors. Phylogenies shown with raw mutation count branch lengths adjusted for sequencing depth of the sample using sensitivity.

The phylogenies were then made ultrametric (or linearised) using a bespoke algorithm to make all branch lengths equal, which we call the 'iteratively reweighted means approach' (**Extended Fig. 3c**, **Supplementary Code**). Starting from the root of the tree and moving progressively towards each tip, the fraction of time for the given shared branch is calculated as the fraction of remaining time times the number of mutations on the given shared branch divided by the mean number of mutations of all descendants from that shared branch. The function is called recursively, updating the fraction of remaining time, as the algorithm moves from root to tip. This algorithm therefore has the property that the most confident timings (nodes near the root) are defined first, anchoring the timings of subsequent, less confident nodes.

We compared the results obtained using our custom method for linearising the phylogenies and an alternative Bayesian approach (Rtreefit) utilised by Williams *et al*[12]. In brief, Rtreefit is a Bayesian model for converting mutation count based trees into time-based trees. The method jointly fits a global constant mutation acquisition rate and absolute time branch lengths under the assumption that the observed mutation count based branch lengths are Poisson distributed with $Mean = Duration \times Sensitivity \times Mutation\ Rate$ and subject to the constraint that the root to tip duration is the age at colony sampling. The mean branch timings are directly sampled from the posterior distribution and by construction the resulting trees are guaranteed to have a root to tip distance that matches the sampling age of the colony. The model is coded in R and Rstan and inferred using the Rstan implementation of Stan's No-U-Turn sampler variant of Hamiltonian Monte Carlo method*. For each patient tree the model was fitted across four chains each with 20,000 iterations including 10,000 burn-in iterations. The code is available as an R package "Rtreefit" at https://github.com/NickWilliamsSanger/rtreefit.

We found extremely high concordance between our custom 'iteratively reweighted means' approach and the Bayesian approach described above. In all phylogenies the R^2 for branch length comparisons between the two approaches was > 0.99 (**Supplementary Fig. 3**).

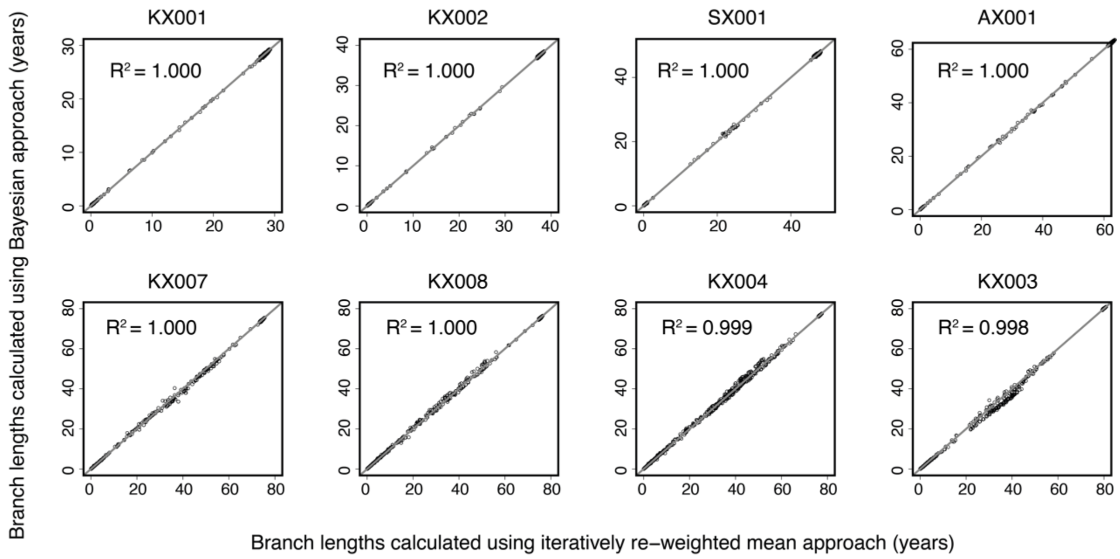

Supplementary Fig.3| Comparison of phylogeny linearisation methods. Plot comparing phylogeny branch lengths (in years) between the custom iteratively reweighted means approach for phylogeny linearization used in this manuscript and an alternative Bayesian approach (Williams *et al*[12]).

Given the tight linear accumulation of mutations in HSPCs with age, the mutation branch lengths correspond to molecular time, which can be converted to time in years (**Extended Fig. 3d**). Due to the known higher mutation rate during *in utero* development, which generates on average 55 somatic mutations in our cord blood HSC/MPPs, the first 55 mutations on the axis were assigned to the period between conception and birth (age 0), with the remaining mutation time evenly split between the years of age of the individual.

Additional information in the form of driver mutations, copy number changes and Y loss was then overlaid on the final ultrametric version of each phylogeny (as in **Extended Fig. 3d**) to generate the final phylogenies depicted in **Figures 2 and 3 and Extended Figure 5**. Driver mutations (which had already been assigned to a phylogenetic node using the tree_mut script above) were identified in the dataset by searching the VAGRENT annotations in the filtered_muts$COMB_mats.tree.build$mat matrix (in Rdata file annotated_mut_set_XXX). Copy number changes and Y loss events were identified as described in the relevant section above on a per sample basis, with this information read in to the tree_cut_analysis.Rmd script in .csv format and subsequently used in phylogeny annotation as described in tree_cut_analysis.Rmd.
(https://github.com/emily-mitchell/normal_haematopoiesis/4_phylogeny_analysis/scripts/tree_cut_analysis.Rmd)

Validation of the phylogeny

To assess the robustness, internal consistency and stability of the shared variants and inferred phylogenies we used several approaches:

(1) *Bootstrapping of the original read counts.*

One well-established approach to assessing the robustness of individual clades in a phylogeny is to repeatedly bootstrap the mutation matrix and re-build the phylogeny, observing in what proportion of bootstraps each clade is retained. *MPBoot* incorporates a bootstrap approximation method. However, somatic data has a well-established 'root' (the human reference genome) which makes this approach less applicable in our setting where we have high confidence in early splits that our supported by multiple samples, even if the numbers of mutations on the branch are low. With our data type the major cause of uncertainty is knowing exactly which cells carry a mutation, and what impact this would have on the inferred tree structure. Therefore, to better assess this type of uncertainty, we used an alternative bootstrapping approach as per Spencer Chapman *et al*[7]. Specifically, we used a partially-filtered mutation set and bootstrapped the read counts for each colony at each locus. We then subjected the raw read count data to the same filtering and phylogeny-building approach as was used on the original data, with 1000 replicates per individual. The only exception was the beta-binomial filter. This was applied to the simulated data before the read count boot-strapping step. As with conventional approaches, the bootstrap phylogenies were then compared to the observed phylogeny to assess the proportion of bootstraps in which each clade is retained or lost. This was compared to the conventional mutation bootstrapping approximation performed by *MPBoot* (**Supplementary Fig. 4a**). Quartet divergence and Robinson-Foulds similarities were calculated using the tqDist algorithm40 implemented in the R package Quartet v1.2.041. The bootstrapping analysis was performed for one of our elderly adult HSCPC phylogenies to ensure the finding of clonal expansions in the phylogeny was robust. We found the bootstrap phylogenies had high correlation to the observed phylogeny (**Supplementary Fig. 4b**) with a median Robinson-Fould similarity of 0.951 and quartet divergence of 0.999.

(2) *Assessment of internal consistency of genotype matrices using the disagreement score.*

This score is based on the observation that in a perfect phylogeny any pair of mutations should either be in discrete clades or nested one within the other. To test the consistency of our data with this assumption we calculated a 'disagreement score'. For every pair of loci the number of cells in disagreement with this assumption was calculated. The mean score across all pairs was then calculated in such a way that cells with unknown genotypes were assumed to be in agreement. These scores from all the observed phylogenies were then compared to scores generated from random shuffles of the corresponding genotype table, internal to each locus. In this way the disagreement score in the observed genotype table can be compared to one that has been randomly generated. In all the observed trees the 'disagreement score' was extremely low compared to that obtained after random shuffling (**Supplementary Fig. 4d**), showing our data has high internal consistency and the phylogenies are close to that expected in a perfect phylogeny (which would have a disagreement score of 0).

(3) *Comparison of the MPBoot phylogeny with other phylogeny inference methods.*
To assess the reliability and stability of the phylogeny generated by MPBoot we used the same genotype data as input into two alternative algorithms: IQ-TREE37 and SCITE38. IQ-TREE is a stochastic algorithm which infers phylogenies using maximum-likelihood. The Jukes-Cantor-type model for binary data was used as an appropriate model for single-cell whole-genome data. SCITE is an algorithm designed for somatic single-cell data. It uses Markov chain Monte Carlo sampling with an error model that takes potential false positives and false negatives into account for tree scoring. We used false positive and false negative rates of 0.001. Both these alternative algorithms produced phylogenies for KX003 (81 year male) with high agreement to the original MPBoot phylogeny with Robinson-Foulds distances of <0.05 for both comparisons (**Supplementary Fig. 4e,f**).

We further explored whether using SCITE as an alternative phylogeny bulidling approach would materially alter any conclusions of our paper. We were able to run SCITE over all but one of the phylogenies. The largest phylogeny (KX004) cannot complete within the timeframe required for our compute farm 'basement queue', which terminates jobs after 4 weeks. To be confident that the two methods give concordant trees, we have measured the similarity of trees estimated with MPBoot and SCITE. Reassuringly, in all cases there was high concordance in the phylogenies produced by the two approaches (Robinsons-Foulds distance < 0.07) as shown in the table below.

Individual	Robinsons-Foulds Similarity of SCITE tree	Quartet Similarity of SCITE tree	Comparison of 32 summary statistics
**KX001**	0.934	1.000	Unchanged
**KX002**	0.949	0.999	Unchanged
**SX001**	0.945	0.999	Unchanged
**AX001**	0.947	1.000	Unchanged
**KX007**	0.977	0.999	Subtle changes (see below)
**KX008**	0.960	0.998	Unchanged
**KX003**	0.954	1.000	Subtle changes (see below)

Most differences that did emerge affected the precise arrangement of some early embryonic branch points – these differences would not be anticipated to have an impact on any of the key downstream analyses in the manuscript.

We have also formally compared the summary statistics obtained from the phylogenies inferred using MPBoot vs SCITE. We found that when the range of summary statistics we utilise for the driver ABC modelling are assessed at 4 timepoints, in 5 out of 7 individuals the statistics for the MPBoot and SCITE phylogenies are identical. For KX007, 6 of 32, and for

KX003, 4 of 32 summary statistics calculated for each phylogeny are discordant but by a negligible amount (2 or less). These overall highly concordant findings further confirm that the choice of tree-building approach would not have altered the conclusions of the downstream modelling analyses.

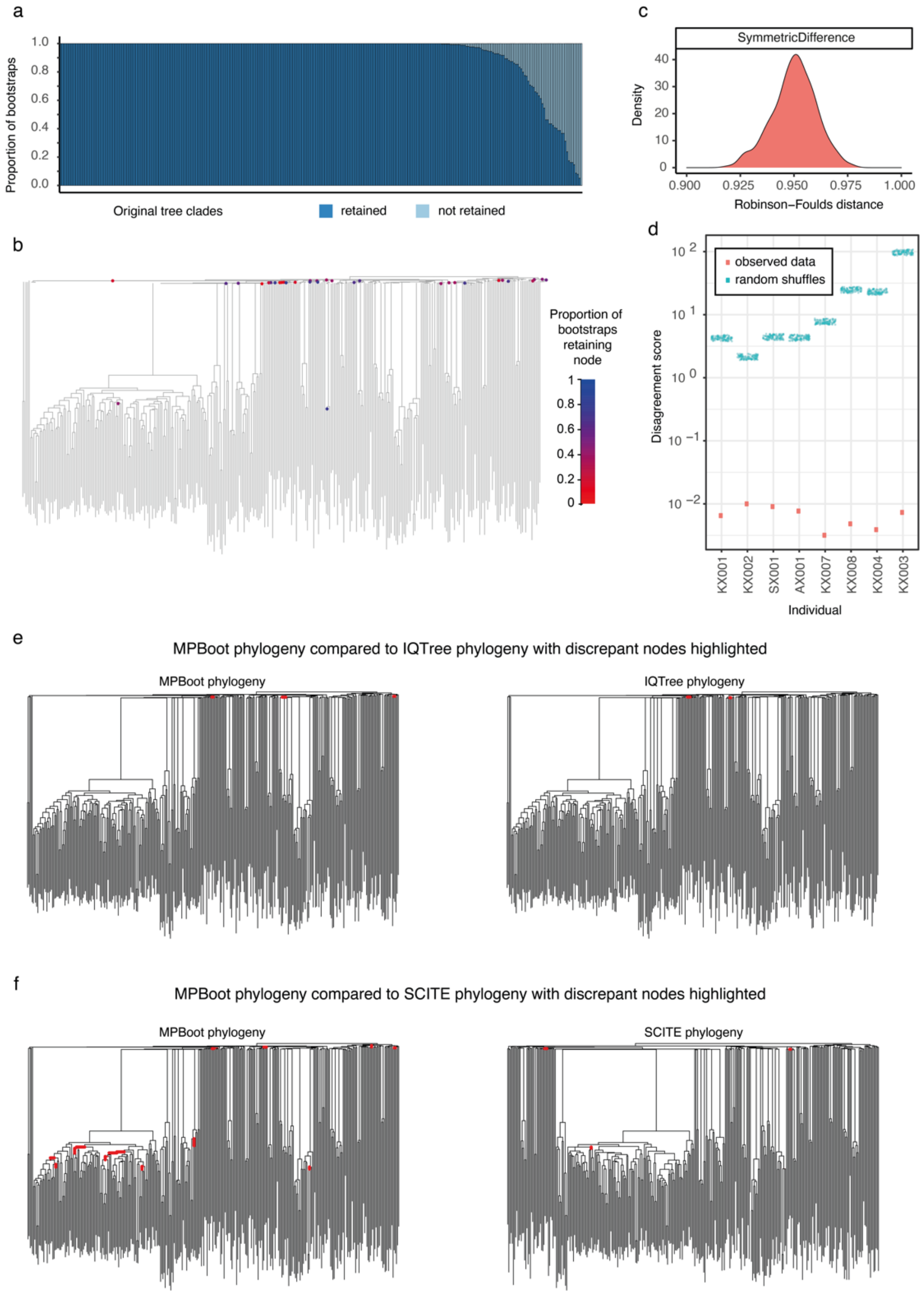

Supplementary Fig.4|Phylogeny benchmarking. a, Robustness of each clade in the KX003 phylogeny (81-year male) using bootstrapping of the raw sequencing read count data. The proportion of bootstraps in which a clade is retained is shown, ordered by decreasing robustness. **b,** KX003 phylogeny annotated to show all nodes that have <90% bootstrap support using bootstrapping of the raw sequencing read count data. The nodes are highlighted with the average bootstrap support value. **c,** Comparison of the sequencing read count bootstrap trees to the

original trees by Robinson-Foulds similarity. **d,** Internal consistency of the genotype matrix for each adult individual as demonstrated by the disagreement score. The random shuffles have been displayed 'jittered'. A perfect phylogeny has a score of zero. **e-f,** Comparison of KX003 phylogenies generated by *MPBoot* and by the alternative phylogeny inference methods IQTree and SCITE. The nodes highlighted in red are those that differ between the inference methods, showing that the phylogenies are only minimally impacted by the inference method utilised.

Using Rsimpop to simulate HSC populations

Simulations of complete HSC populations from conception to the age of sampling were performed for each individual using the R package *rsimpop*[12] (https://github.com/NickWilliamsSanger/rsimpop*).* *Rsimpop* utilises a birth-death model with specified somatic mutation accumulation rate and symmetric cell division rate, to simulate a complete HSC population. Each cell within the population has a rate of symmetric division and a rate of symmetric differentiation (or death). Asymmetric divisions do not impact on the HSC phylogeny and are not accounted for in the model.

Let α be the background rate of symmetric self-renewal cell divisions, measured in divisions per day. We model selective advantage of driver containing clone i as s_i. The increased rate of symmetric division $\alpha_i = \alpha(1 + s_i)$. We assume during the early population growth phase that the total population grows unrestrained by death. Once the specified population size, N, is reached (within the first few years of life) then the death rate, β, for each cell matches the average division rate in the full population:

$$\sum^{cells} \beta = \sum^{\text{wild type cells}} \alpha + \sum_i \sum^{\text{cells in clone } i} (1 + s_i)\alpha$$

Thus giving

$$\beta = \frac{(N - \sum_i \mathrm{N}_i)\alpha + \sum_i \mathrm{N}_i(1 + s_i)\alpha}{N}$$

In the case of a single driver mutation containing clone with selection coefficient s then the deterministic phase behaviour is governed by a logistic growth function:

$$N_m = N \frac{1}{1 + exp(-\alpha s(t - t_m))}$$

For some constant t_m (see Williams *et al)*[12].

In the early stages of the exponential growth process, it exhibits an annual rate of growth S:

$$S = exp(\alpha s) - 1.$$

For multiple competing driver mutation containing clones, each with modest population sizes, it is expected that the above single clone approximation will apply for the individual competing clones. Once one or more of the competing clones represents a significant fraction of the overall population then the dynamics will be more complex. For cells containing more than one driver mutation the fitness effect on S is additive.

The above model is implemented using the Gillespie algorithm. The waiting time until the next event is exponentially distributed, with a rate given by the total division rate + total death rate. This event is then 'division' with probability=total division rate/(total division rate + total death rate). If the event is 'division' then the choice of which cell divides is given by a probability proportional to the cell's division rate, whereas if the event is 'death' then all cells are equally likely to be chosen.

Implementation was in C++ with an R based wrapper as an R package *rsimpop*. The simulator maintains a genealogy of the extant cells, together with a record of the number of symmetric divisions on each branch, the absolute timing of any acquired drivers and the absolute timings of branch start and end. The package also provides mechanisms for sub-setting simulated genealogies whilst preserving the above per branch information.

HSC population size modelling

We first investigated simple neutral models of HSC populations (from which selection is absent). The cell phylogenies, constructed from singe cell genomes, include estimated branch lengths, from which we can calculate node heights, and hence the time intervals between successive coalescent events. In the case of a neutral model, the genomic data provides information about the trajectory of the product $N\tau$ (population size x time between symmetric self-renewal cell divisions).

However, the genomic data cannot provide information separately about N (population size), or τ (time between symmetric cell divisions). Furthermore, in the case of a neutral model, all the information provided by the genomic data, about the trajectory of the product $N\tau$, is contained in sequence of inter-coalescent intervals calculated from the phylogeny. This sequence of inter-coalescent intervals is precisely the information which the *phylodyn* package uses to infer the trajectory of the product $N\tau$.

Here, our aim was to perform additional Bayesian inferences about the parameters of neutral models from the phylogenies. Specifically, we want to compute marginal posterior densities (providing point estimates accompanied by credible intervals) for the 'LT-HSC $N\tau$' parameter for the first 2-3 decades of life, and two additional parameters representing the midlife fold-change in $N\tau$ (elderly donors only), and late-life fold-change in $N\tau$ (all donors). We chose flat prior densities on wide intervals (**Extended Fig. 8a**) to represent prior

uncertainty about the values of these parameters, so that the resulting the marginal posterior densities could be compared with the inferences from the *phylodyn* package.

An additional motivation for performing these Bayesian inferences on neutral models, was to enable us to perform posterior predictive checks (PPC), in order to decide if the observed phylogenies are compatible with neutral models. Note that a separate donor-specific posterior distribution was generated (sampled) for each donor (donor-specific ABC), and a separate donor-specific posterior predictive p-value was computed for each donor (donor-specific PPC). Each donor-specific ABC for the neutral model was performed using the ABC rejection method (R package *abc*)[13,14].

We used the population trajectory from *phylodyn* to identify the time period prior to the increase related to a ST-HSC/MPP contribution, and the timing of the midlife and late-life fold-change in $N\tau$ (**Fig. 4a and Supplementary Fig. 12**). We used our data to inform our choices for the time between symmetric cell divisions, which was set at 1 year (after the initial population growth phase in the first few years of life). We set the rate of mutation accumulation at 15 mutations per year with an additional 1 mutation for every cell division (both of these were drawn from a Poisson distribution centred on the input value).

In the younger individuals (aged < 65) estimates of $N\tau$ in the first few decades of life could be made due to the absence of the effect of positive selection (**Extended Fig. 12**). However, in the older individuals (aged > 75), estimates of $N\tau$ could not be reliably calculated in the phylogenies due to the confounding effect of positive selection. Here we focussed on using the PPC method to decide whether the neutral model changes in population size (in the form of a bottleneck in the population in mid-life) is compatible with each of the observed trees.

The Bayesian inferences about the parameters of these neutral models were performed using Approximate Bayesian Computation (ABC) methods (in which large numbers of simulations of the data are performed using *rsimpop*, in place of computation of the likelihood function).

In order to apply these methods, the sequence of inter-coalescent intervals was replaced by a set of summary statistics (the 'number of lineages' in the tree through time at three points). For each donor, the marginal posterior densities for the parameters of interest are plotted alongside the corresponding prior densities, to illustrate how the data has reduced our uncertainty about the values of these parameters. For each donor, we can also use the sample from the (approximate) posterior distribution (generated by donor-specific ABC) for the parameters of the neutral model, to perform donor-specific PPC.

We first generate a large sample of simulated data sets from the posterior predictive distribution, and from this we can estimate a donor-specific posterior predictive p-value. The purpose of this donor-specific PPC is to decide if the observed phylogeny obtained from each

donor is compatible with the proposed neutral model (while taking account of our uncertainty about the parameter values in the model). Here we are concerned with all features of the observed phylogenies (not only those features which are informative about the parameters of the neutral model). For observed phylogenies and simulated phylogenies, we can compute a chi-squared discrepancy variable which incorporates many summary statistics (including clade size statistics). The posterior predictive p-value is computed from the upper tail-area probability under the distribution of the difference between the simulated chi-squared discrepancy and the observed chi-squared discrepancy[15]. If the p-value is close to zero, then the observed data is extreme (an outlier) compared to the data predicted under the proposed model (taking account of our uncertainty about the parameter values in the model). Thus, when the p-value is close to zero, this is evidence that the observed phylogeny is not compatible with the neutral model.

HSC population size estimate

Using a Monte Carlo simulation approach, we sampled from the distributions of each variable 500,000 times, calculating the value of N for each set of randomly sampled variables. This was done using the following distributions: telomeric shortening rate per division: uniform(minimum = 30, maximum = 100); symmetric division rate: uniform(minimum = 0.8, maximum = 1.0); $N\tau$: uniform(minimum = 50,000, maximum = 250,000); average telomere loss per year: uniform(minimum = 30, maximum = 40).
(https://github.com/emily-mitchell/normal_haematopoiesis/6_population_modelling/scripts/estimating_N.Rmd)

dN/dS analysis

We used the R package *dndscv*[16] (https://github.com/im3sanger/dndscv) to look for evidence of positive selection in our dataset (https://github.com/emily-mitchell/normal_haematopoiesis/5_dNdS/scripts/all_DNDScv_final.Rmd). The *dndscv* package compares the observed ratio of missense, truncating and nonsense to synonymous mutations, with that expected under a neutral model. It incorporates information on the background mutation rate of each gene and uses trinucleotide-context substitution matrices. The approach provides a global estimate of selection in the coding variant dataset (**Table S7**), from which the number of excess protein coding, or 'driver mutations' can be estimated. In addition, it identifies specific genes that are under significant positive selection.

While a small bias in the estimated dN/dS ratio could lead to an apparently significant excess when the dataset contains large numbers of mutations, as our does. In defence of the significant excess of non-synonymous mutations we found, we proffer four lines of argument:

1. Correction for confounders in the dN/dS algorithm

The dN/dS algorithm[16] is one of the best-in-class algorithms for quantifying somatic selection, as demonstrated by a recent pan-cancer comparison of different methods[17]. One of the reasons for this is the rigorous approach it takes to correcting for the known variables influencing mutation rate across the genome, including replication timing, chromatin state and DNAse accessibility[18]. In addition, the model balances the predicted mutation rates from these global covariates with the observed synonymous mutation rate within a gene – this latter correction captures many of the unknown variables affecting mutation rates acting at a local level.

Furthermore, the algorithm corrects for the observed mutational spectrum[16] – this is important because, for example, transitions are more likely to generate a synonymous mutation than transversions. The model parameterises all 192 rates representing the 6 different types of base substitution, the 16 combinations of bases 3' and 5' to the mutated base, and transcribed versus non-transcribed gene. This means that trinucleotide mutational signatures do not bias the overall dN/dS estimate.

2. Running dN/dS algorithm with greater stringency

In addition to running the dN/dS algorithm in its standard implementation, we have checked whether the overall estimates are materially altered if we run it using two adaptations to impose greater stringency.

The first adaptation was to run the algorithm excluding sites that are masked by our variant caller in both the numerator and the denominator (a total of 175 million sites genome-wide). Essentially, most somatic mutation callers, including ours[1], have a 'normal panel' or equivalent where sites that are frequently non-reference because of sequencing artefact or germline polymorphism are masked. Since germline polymorphisms have a dN/dS ratio << 1, this can lead to under-calling of synonymous somatic mutations relative to non-synonymous mutations. Running our algorithm with sites in this normal panel excluded from both numerator and denominator had minimal impact on the estimated overall value of dN/dS (1.0548, $CI_{95\%}$=1.02488-1.0856; versus 1.0586, $CI_{95\%}$=.02861-1.0895 for the standard implementation). This argues that there is no bias arising from masking of true somatic mutations at germline polymorphisms.

The second adaptation was to run the dN/dS algorithm using correction for pentanucleotide sequence context. While a trinucleotide context captures virtually all of the effects of mutational signatures[19], there remains the theoretical possibility that any signature extending beyond that may affect synonymous mutations differently to non-synonymous mutations. To test this, we repeated the analysis using rates for the 6 mutation classes and 256 different combinations of 2 bases each side of the mutated base. This also had minimal impact on the estimated value of dN/dS for missense variants (1.0472, $CI_{95\%}$=1.0155-1.0799; versus 1.0589,

$CI_{95\%}$=1.02852-1.0902 for the standard implementation) or the dN/dS for truncating variants (1.0788, $CI_{95\%}$=1.0106-1.1516; versus 1.0569, $CI_{95\%}$=0.99558-1.1220 for the standard implementation). Importantly, a pentanucleotide context covers the whole of the codon, no matter which base in the codon is mutated (whereas a trinucleotide context only covers the whole codon if the middle base is mutated) – this means that even if there were residual effects of mutational signatures beyond the pentanucleotide, they would not affect the mutated codon.

3. Measuring dN/dS on simulated mutations

As a further check, we have now generated simulated mutations in the sequencing data and run the dN/dS algorithm. We took 19 BAM files from cord blood HSC/MPPs in our dataset for which zero coding mutations were identified by our variant caller. For each BAM file, we randomly chose 2000 sites in the exome to have simulated mutations, with the mutations following the same mutational spectrum as observed in the whole dataset. At each position with a mutation, we then extracted the reads reporting that base, and changed the base-call recorded at that base with 0.5 probability (to get average VAF of 50%), according to the following rules: change to mutant base if read reported reference base; change to reference base if read reported mutant base; change to the other non-reference, non-mutant base if read reported non-reference, non-mutant base.

The modified BAM files then underwent exactly the same process of variant calling as our real data. We verified that the majority of the simulated mutations were correctly called (the proportion dependent on sequencing coverage), and that the mutation spectrum was the same as that observed in the real data. In total, we called 29008, which was very close to our real dataset of 25,888 coding mutations. We ran the dN/dS algorithm over the simulated dataset and found no bias in the results, with a dN/dS ratio for all randomly simulated variants of 1.00. For the simulated missense mutations, the estimated dN/dS was 1.001 ($CI_{95\%}$=0.974-1.028); and for simulated truncating mutations, it was 1.001 (0.956-1.067).

These simulations would have captured any biases in the estimation of dN/dS that arose from, for example, differential sequencing coverage across the genome, variant calling, variant filtering, variant annotation or the dN/dS algorithm. Instead, the estimates of dN/dS are almost exactly 1.00, as expected, with confidence intervals that do not overlap with those for our real data.

4. Frequency of clonal expansions concords with estimates of numbers of driver mutations

Our argument that there is pervasive positive selection is not purely based on the genetic analysis. We also observe a number of clonal expansions in our dataset. We tested Approximate Bayesian Computation (ABC) models to see whether these expansions could be

explained by neutral drift (for example, programmed changes in HSC population size). However, these neutral models were unable to capture the asymmetry of branching across the clades in the elderly subjects (many singleton branches interspersed with 10-20 considerably expanded clones – see **Extended Figure 8**).

Instead, to capture this asymmetry, we had to use models which included positive selection. The estimates of driver mutation rates that were required to generate trees that matched those we observed were about 2.0 x 10^{-3}/HSC/year. These models considered only driver mutations with fitness coefficient s>5%. Note that the ABC modelling uses no genetic data to arrive at this estimate – it is purely based on how many drivers are required to generate the observed branching patterns in the real phylogenies of the older individuals.

Reassuringly, this estimate of the rate of drivers from the ABC modelling is broadly comparable with the estimate obtained from the dN/dS analysis. Overall, non-synonymous mutations accumulated in HSC/MPPs at a rate of 0.12/HSC/year ($CI_{95\%}$=0.11-0.13), with dN/dS estimates suggesting that 1/34 to 1/12 non-synonymous mutations were drivers (approximately 5%). This computes to a driver rate of 3.6-10 x10^{-3}/HSC/year estimated from direct genetic analysis, an estimate which would include drivers with s<5% present in sequenced colonies.

Amino acid variant annotation

We performed amino acid variant annotation using SIFT4G (https://sift.bii.a-star.edu.sg/sift4g/AnnotateVariants.html)[20] and Polyphen2 (http://genetics.bwh.harvard.edu/pph2/bgi.shtml)[21]. Of a total 16536 missense mutations in our dataset, 5088 could be annotated by SIFT4G (38 in myeloid driver genes) and 4551 could be annotated by Polyphen2 (35 in myeloid driver genes). Approximately 42% and 45% of the annotated mutations were deemed to be 'deleterious' respectively (see Table below). If the same proportion of missense mutations is present in the dataset as a whole we would predict approximately 7000 'deleterious mutations', equating to around 1000 per adult individual.

	SIFT4G	Polyphen2
**Total missense mutations in dataset**	16536	16536
**Number annotated**	5088	4551
**Number in known driver (excluded)**	38	35
**Deleterious**	1998	2049
**Possibly deleterious**	319	762
**Tolerated**	2495	1709
**Fraction deleterious**	0.42	0.45
**Predicted deleterious in whole dataset**	6945	7441

Table S7 lists all coding variants used to run dN/dS with annotation including the SIFT and Polyphen2 scores.

Driver mutation acquisition rate estimation

The dN/dS parameter is widely used in evolutionary genetics to infer patterns of selection[22,23], recently adapted for cancer and somatic mutations[16]. It is essentially a measure of how far the observed number of non-synonymous mutations diverges from the number that would be expected from the synonymous mutation rate, after correction for mutation spectrum[16]. It is underpinned by the assumption that synonymous mutations evolve neutrally, and selection only acts on non-synonymous mutations. For example, a dN/dS ratio of 1 means that we observed exactly the same number of non-synonymous mutations as we would have expected for the number of synonymous variants. A dN/dS ratio of 2 means we observed twice as many non-synonymous mutations as expected, implying that half of the observed non-synonymous mutations occurred as expected for the background mutational processes, while the other half have accumulated through positive selection. From this, with a total number of observed non-synonymous mutations, we can estimate the number of driver mutations in excess of the background expectation (noting that this is an underestimate of the true number in the presence of any negative selection).

This, then, is the intuition for the formal mathematical exposition. Given an observed number of non-synonymous mutations, n_{NS}, and an estimated dN/dS ratio, ω_{NS}, the formula for the expected number of drivers, n_{D}, is as follows:

$$n_{\mathrm{D}} = \frac{(\omega_{\mathrm{NS}} - 1)}{\omega_{\mathrm{NS}}} n_{\mathrm{NS}}$$

To give a worked example using missense substitutions in our dataset, we estimated the overall dN/dS ratio to be 1.06 with the 95% confidence interval to be 1.03 – 1.09 (**Extended Fig. 9b**). We observed a total of 16,536 non-synonymous mutations. The number of excess missense mutations, then, is calculated as (1.06 – 1)/1.06 * 16536, which works out at 936, with the lower bound on the confidence interval as (1.03 – 1)/1.03 * 16536, which equals 482. This then equates to the estimation in the manuscript that 1 mutation in every 18 (16536/936=17.7) occurring missense mutations is under positive selection.

Linear mixed effects models were used to test for a linear relationship between age and the number of non-synonymous mutations. Colonies with a sequencing depth <14 were excluded.

```
age.non_syn.depth <- lmer(number_non_syn ~ age + (age | donor_id), data = subset(summ_cut, mean_depth > 14), REML = F)
```

This linear regression analysis found that non-synonymous mutations are acquired at a rate of 0.12/HSC/year ($CI_{95\%}$=0.11-0.13) and the dN/dS estimates inform that 1 in 12 to 1 in 34

non-synonymous mutations in the dataset are drivers. We used these estimates in a Monte Carlo simulation approach, sampling from the distributions of each variable 500,000 times, calculating the value of N for each set of randomly sampled variables. This was done using the following distributions: non-synonymous mutation acquisition per year: uniform(minimum = 0.11, maximum = 0.13); fraction of drivers: uniform(minimum = 0.029 (1/34), maximum = 0.083 (1/12)).
(https://github.com/emily-mitchell/normal_haematopoiesis/5_dNdS/scripts/estimating_driver_acquisition_rate.Rmd)

Modelling positive selection in the HSC population

Considering evidence from the dN/dS analysis, we aimed to investigate more elaborate models of HSC population dynamics, by incorporating positive selection acting on driver mutations. Here, as before, we use ABC methods to make inferences about the parameters of the model (incorporating positive selection), and posterior predictive checks (PPC), in order to decide if the observed phylogenies are compatible with this relatively simple non-neutral model (incorporating positive selection). In this non-neutral model, a static HSC population of 100,000 cells undergoing 1 symmetric self-renewal division per year, we explored a range of parameter values for the number of drivers introduced into the population per year, as well as the shape and rate of the gamma distribution used to define the distribution of fitness effects these drivers were drawn from (**Extended Fig. 8a**).

We used a threshold of 5% for the minimum fitness effect of these drivers (equivalent to a selection coefficient of 0.05) as Watson *et al* predicted drivers with a fitness effect of 4% or less could not expand to a VAF > 1% over the human lifespan[24]. We chose flat prior densities on wide intervals (**Extended Fig. 8a**) to represent prior uncertainty about the values of these parameters.

First, a separate donor-specific posterior distribution was generated (sampled) for each donor (donor-specific ABC). The simulations were performed using *rsimpop*, and the donor-specific ABC (ridge regression on the re-scaled, and logit-transformed, parameter values) was performed using the R package *abc*.

Second, we used a sequence of four ABC regression steps to generate a sample from the (approximate) multiple-donor posterior distribution on the combined data from the four oldest donors. The simulations were again performed using *rsimpop*, and the ABC regression steps were again performed using the R package *abc*[13]. In the case of non-neutral models, it is no longer the case that all the information provided by the genomic data, about the parameters of the model, is contained in sequence of inter-coalescent intervals (calculated from the phylogeny). Therefore, additional summary statistics (including clade size statistics) were used in the ABC steps.

A separate donor-specific posterior predictive p-value (donor-specific PPC) was computed for each donor (not only for the four oldest donors), based on the (approximate) multiple-donor posterior distribution on the combined data from the 4 oldest donors. In this case, the sample from each donor-specific posterior predictive distribution was generated by repeated sampling of parameter values from the multiple-donor posterior distribution, and then re-simulating the model (using *rsimpop*) conditional on the donor-specific sample size (number of single cell genomes) and donor age. As before, the posterior predictive p-value is computed from the upper tail-area probability under the distribution of the difference between the simulated chi-squared discrepancy and the observed chi-squared discrepancy[15].

The purpose of this donor-specific PPC is to decide if the observed phylogeny obtained from each donor is compatible with the simple non-neutral model (while taking account of our uncertainty about the parameter values in the model). If the p-value is close to zero, then the observed data is extreme (an outlier) compared to the data predicted under the simple non-neutral model. This is interpreted as evidence that the observed phylogeny is not compatible with the simple non-neutral model, and that more elaborate models need to be considered.

Phylofit estimation of selection coefficients

We used the algorithm *phylofit* to estimate the selection coefficients of known and unknown drivers in our phylogenies. *Phylofit* uses an efficient MCMC approach to model selection within a clade using the probability density of coalescence times and the population size trajectory. As such it can be thought of as a parametric adaptation of the *phylodyn* model.

The starting point for *phylofit* is Equation 1 in Lan *et al* 'An Efficient Bayesian Inference Framework for Coalescent-Based Nonparametric Phylodynamics'[25]:

$$P(t_1,..,t_n|N(t)) = \prod_{k=2}^{n} \binom{k}{2} \frac{1}{N(t_{k-1})} e^{-\int_{t_k}^{t_{k-1}} \binom{k}{2} \frac{1}{N(t_{k-1})} dt}$$

Where $\{t_k|k \in 1..n\}$ are the timings of the time ordered coalescences belonging to the driver mutation containing clade, $t_1$is the first coalescence of the expansion and t_n is the sampling time. These times are expressed as the interval between the event and the sampling time (assumed to be isochronous).

Substituting our formula for the cell count of the driver mutation containing clade $N(t)$ (in our case aberrant cell count refers to expanded clades both with and without known drivers) and performing the integral, eliminating terms that do not depend on overall population size, N , the trajectory midpoint, $t^{(m)}$, and the selective coefficient, $\hat{s} = \alpha s$, we arrive at the following log-likelihood:

$$L\big(t_1,..,t_n|\hat{s},t^{(m)},N\big) =$$

$$(n-1)\log(\mathrm{N}) + \sum_{k=2}^{n}\left(\log\left(1+\exp\left(\hat{s}\big(t_{k-1}-T+t^{(m)}\big)\right)\right)\right) -$$

$$\frac{1}{\hat{s}N}\sum_{k=2}^{n}\left(\binom{k}{2}\exp\left(\hat{s}\big(t_{k-1}-T+t^{(m)}\big)\right)\left(\exp\big(\hat{s}(t_{k-1}-t_k)\big)-1\right)\right) +$$

$$\frac{1}{N}\sum_{k=2}^{n}\left(\binom{k}{2}\hat{s}(t_{k-1}-t_k)\right)$$

Where recall the annualised selective coefficient is $S = \exp(\alpha s) - 1 = \exp(\hat{s}) - 1$

We incorporate this central likelihood equation into a Bayesian model with uniform priors on log(N), $\hat{s}$ and $t^{(m)}$.

$$\hat{s} \sim U(0.001,2)$$
$$t^{(m)} \sim U(a,b)$$
$$\log10(\mathrm{N}) \sim U(4,6)$$
$$\boldsymbol{t} \sim Phylo(\hat{s},t^{(m)},N)$$

Here $Phylo$ is the probability distribution described by the log-likelihood function specified above.

Additionally, assuming unbiased sampling, we can optionally incorporate the number of sampled driver mutation containing colonies n_{mut} out of n_{tot} total colonies as an additional layer in the model:

$$n_{mut} \sim \mathrm{Binomial}\left(n_{tot}, \frac{1}{1+\exp\big(-\hat{s}(T-t^{(m)})\big)}\right)$$

The parameters, a and b, setting the realistic range for the midpoint depend on whether the last component of the model is active and are detailed in the code.

The above models were coded in R and Rstan and inferred using the Rstan implementation of Stan's No-U-Turn sampler variant of Hamiltonian Monte Carlo method*. Models were fitted across three chains each with 20,000 iterations including 10,000 burn-in iterations.

The input data for this approach is an ultrametric tree. We obtain the ultrametric tree for this analysis using the methods already outlined. The code used to run *phylofit* can be found at (https://github.com/emily-mitchell/normal_haematopoiesis/7_phylofit/scripts/phylofit.R)

The *phylofit* algorithm was validated by assessing the correctness of the selection coefficient inference when the algorithm was run on single driver mutation clones with a known selective coefficient. The procedure was as follows:

Simulate population with initial division rate of 0.1 per day ($\alpha = 0.1$) until population has grown to the target equilibrium population size.

- Set symmetric division rate to 1 per year ($\alpha = 0.5/365$) and simulate neutral evolution until time T=5 years.
- Save the state of the simulation (*)
- Introduce the driver with the specified selection coefficient.
- If the driver lineage dies out before the sampling age is reached, or has less than 2% clonal fraction at the sampling age, then return to the saved state (*) and continue.

An unbiased sub-sample of cells is taken from the extant population of cells. The *phylofit* algorithm was then applied to the mutant clade in the sub-sampled simulated ultrametric phylogenetic tree.

The algorithm was found to recover the selection coefficients over a range of values of selection coefficient (**Supplementary Fig. 5**).

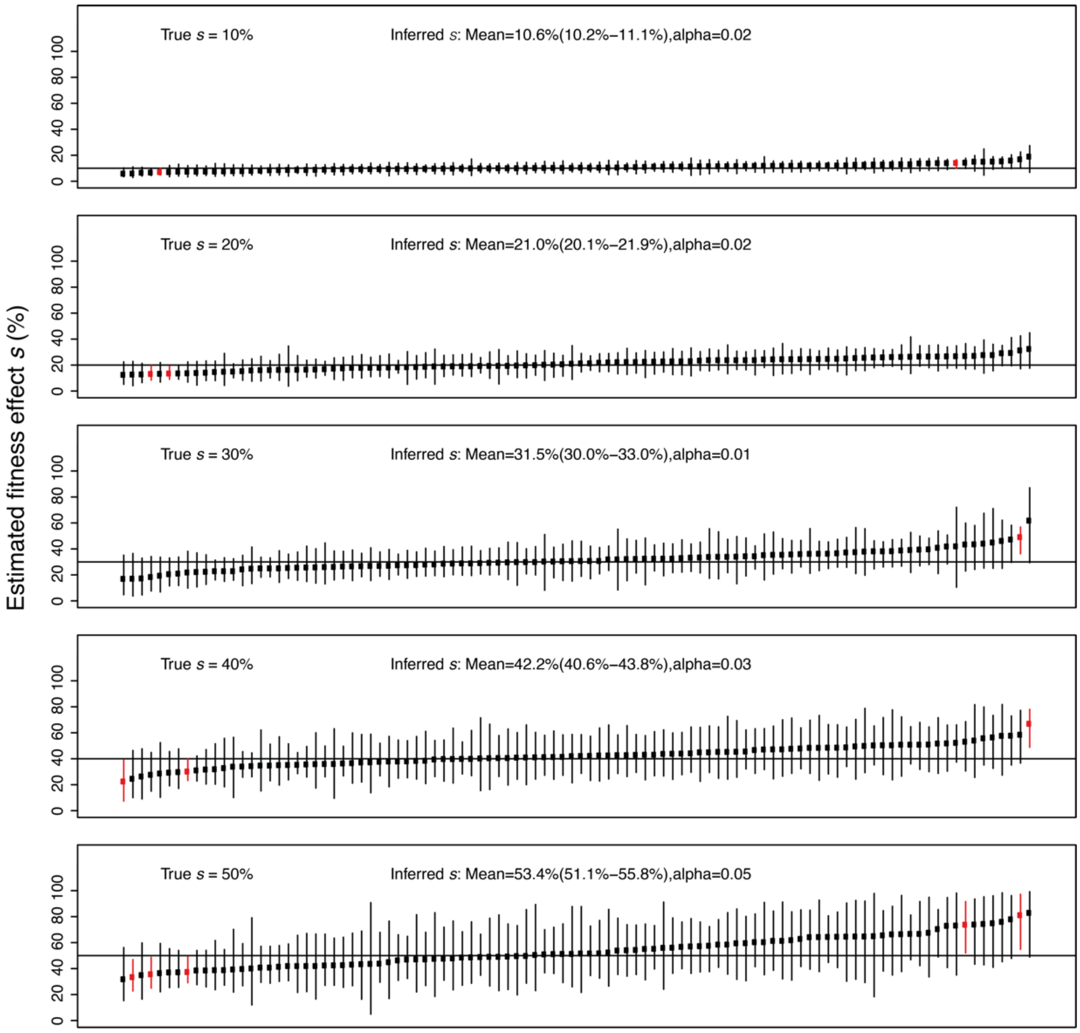

Supplementary Fig.5|Phylofit Benchmarking. The inference of annualised fitness effect, *s*. The *phylofit* results (prior *s* range is 0-100% and log10(N) is 4 to 6) are shown for one hundred simulations for each of five values of *s* (=10%, 20%, 30%, 40% and 50%) and N=100,000 cells. The vertical lines show the 95% credibility intervals of the inferred selection coefficients with red lines highlighting instances where the true selection coefficient lies outside the 95% credibility interval ("alpha" is the proportion of such cases). The sample mean estimate of *s* and the corresponding 95% confidence interval are also shown. The benchmarking shows that on average the selection coefficient is accurately recovered with little bias.

Supplementary Background

Definition of phylodynamics

Phylodynamics is defined as the study of how population level evolutionary processes act to shape phylogenies. To date the phylodynamic approach has been applied most commonly to rapidly evolving viral populations, where it has been used to characterise transmission dynamics[26].

Definition of $N\tau$

One fundamental tenet of phylodynamics is that the frequency of coalescent events in the trees is defined by $N\tau$, where N is the population size and τ is the generation time. This means that the same phylogeny could be obtained from a population of 100,000 with a generation time of 1 year ($N\tau$ = 100,000) and a population of 25,000 with a generation of 4 years (again $N\tau$ = 100,000).

Phylodynamic principles

It has been shown that in a neutrally evolving population the pattern of coalescent events in a phylogeny created from a random sample of individuals can be used to infer historic population size changes[25]. Specifically, in populations of a constant size (N) and generation time (τ) there will be more coalescent events (which define individuals that are related) observed in a small compared to a large population. The reason for this difference in phylogenies from small and large populations can be understood by imagining the predicted phylogeny obtained from sampling 10 random individuals from a population of 50 individuals, compared to sampling the same number from a population of 500. We would expect to have a higher chance of sampling siblings and cousins from the smaller population than the larger, which manifests as more coalescent events in the phylogeny from the smaller population (**Supplementary Fig. 6**). This concept can be taken a step further, such that in a population with a fluctuating population size, more coalescent events will be observed in time 'windows' where the population size is small compared to when it is larger.

The action of genetic drift in a population means that a proportion of lineages are lost stochastically per unit time, and therefore the older the lineages in a population the more coalescent events will be observed per unit time. This is accounted for in phylodynamic models such as *phylodyn*.

The action of positive selection in a population will alter the pattern of coalescent events in a phylogeny if it results in detectable clonal expansion. This means that inferences of population size and historic population dynamics are only valid in populations that do not

have evidence high levels of positive selection. The approach also relies on the random sampling of cells within the population.

Application of phylodynamics to stem cell populations

When applied to stem cell populations, N is the number of stem cells in the population and τ is the generation time. Stem cells can divide in three distinct ways. The first is a symmetric self-renewal division that creates 2 daughter stem cells, so increasing the stem cell population and being the equivalent of stem cell birth. The second is a symmetric differentiation division that results in 2 differentiated daughter cells, which is the equivalent of stem cell death. The final type is an asymmetric division, which produces one stem cell and one differentiated cell and therefore does not alter the size of the stem cell population. It can be seen that only the symmetric self-renewal division results in a daughter progeny that increases the size of the stem cell population. From the phylodynamic perspective stem cell generation time is therefore defined as the time between symmetric self-renewal divisions.

Due to the requirement to sample random cells within a population, it is impossible to robustly apply phylodynamic methodology to somatic stem cells in solid organs. However, the haematopoietic system is the one example of a somatic stem cell population that can be randomly sampled, either through peripheral or cord blood sampling, or by taking a large bone marrow sample from multiple bones. The sampling of large volumes of bone marrow (50-80ml) from deceased organ donors provides the additional advantage that these individuals are highly likely to have had high levels of circulating cytokines at the time of sampling which is known to mobilise HSCs within the bone marrow[27,28]. This makes the haematopoietic stem cell population sampled in these ways the ideal candidate for the application of phylodynamic methods. Nevertheless, interpretation of the phylogenies created by sampling HSCs can be non-intuitive. The next section therefore expands on the simulated phylogenies provided in **Extended Figures 6 and 7** to aid interpretation of our results.

Supplementary Simulations

In all the simulated phylogenies illustrated below, the R package *rsimpop* was used to simulate a full neutrally evolving HSC population of size N. At a given age 380 cells were sampled at random from the full population to allow creation of comparable phylogenies to those we have obtained from real HSC/MPPs. In all simulations the generation time (τ) was set at 1 year meaning $N\tau = N$. A *phylodyn* plot is also shown for each phylogeny to show how accurately the population trajectory could be recreated from the pattern of coalescent events. In *phylodyn* plots the downward dips in the trajectory (black line) represent coalescent events in the phylogeny.

Effect of population size

Increasing *N* reduces the number of coalescent events in the phylogeny of cells with a fixed generation time sampled from an individual of a given age (**Supplementary Fig. 6**). At age 30 there is loss of resolution in the *phylodyn* output between $N\tau$ = 500,000 and $N\tau$ = 750,000.

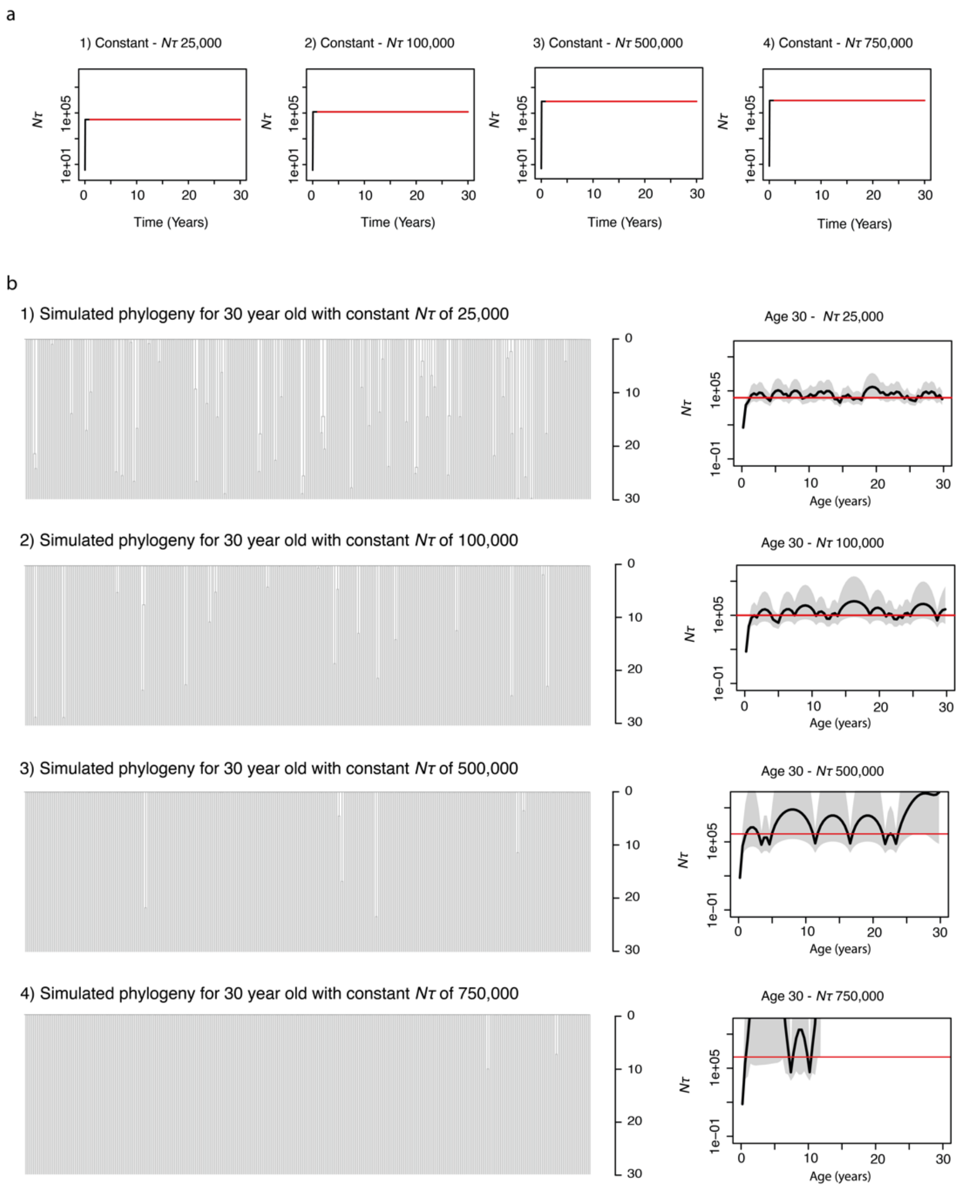

Supplementary Fig.6|Effect of population size. a, Trajectories of $N\tau$ used as input to *rsimpop* for the simulations to create phylogenies in b. Note the Y axis depicting $N\tau$ is on a log scale. **b,** Phylogenies created by randomly sampling 380 cells from the final full simulated population of between 25,000 cells (Phylogeny 1) and 750,000 cells (Phylogeny 4). Phylogenies 1 to 4 are all derived from simulations of the HSC population up to the age of 30 years. Each simulation has an $N\tau$ of 100,000. In all cases $N\tau$ is the same as the population size (*N*), as the generation time (τ) is 1 year. The *phylodyn* trajectories

to the right of each simulated phylogeny use the pattern of coalescent events to recover the input trajectories for $N\tau$.

Effect of age

Increasing age allows a more accurate estimate of $N\tau$ due to the higher number of coalescent events per unit time (**Supplementary Fig. 7**). This increase in the number of coalescent events per unit time for a given population size occurs as a result of genetic drift.

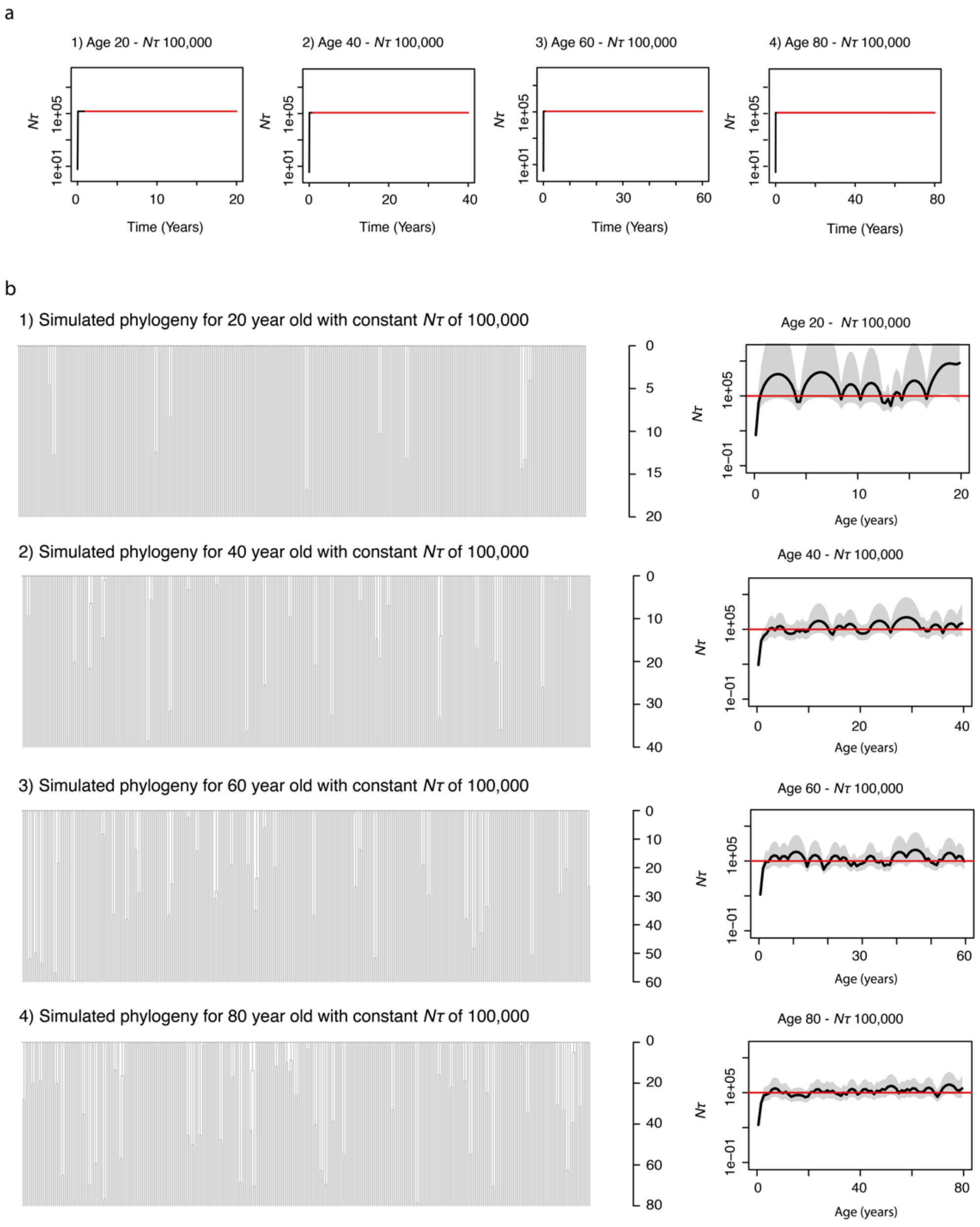

Supplementary Fig.7|Effect of age. a, Trajectories of $N\tau$ used as input to *rsimpop* for the simulations to create phylogenies in b. Note the Y axis depicting $N\tau$ is on a log scale. **b,** Phylogenies created by randomly sampling 380 cells from the final full simulated population of 100,000 cells at between age 20 (Phylogeny 1), age 40 (Phylogeny 2), age 60 (Phylogeny3) and age 80 (Phylogeny

4). Each simulation has a constant $N\tau$ of 100,000 In all cases $N\tau$ is the same as the population size (N), as the generation time (τ) is 1 year. The *phylodyn* trajectories to the right of each simulated phylogeny use the pattern of coalescent events to recover the input trajectories for $N\tau$.

Population decline

A decline in population size is reflected by an increase in the number of coalescent events captured per unit time as compared to when the population was larger (**Supplementary Fig. 8**). Again, the older the individual the more accurately *phylodyn* is able to recover the true simulated population size trajectory. Decreases in $N\tau$ to less than 25,000 can be reasonably accurately captured by *phylodyn*. In younger individuals this is best observed as an increase in the frequency of bumps in the trajectory.

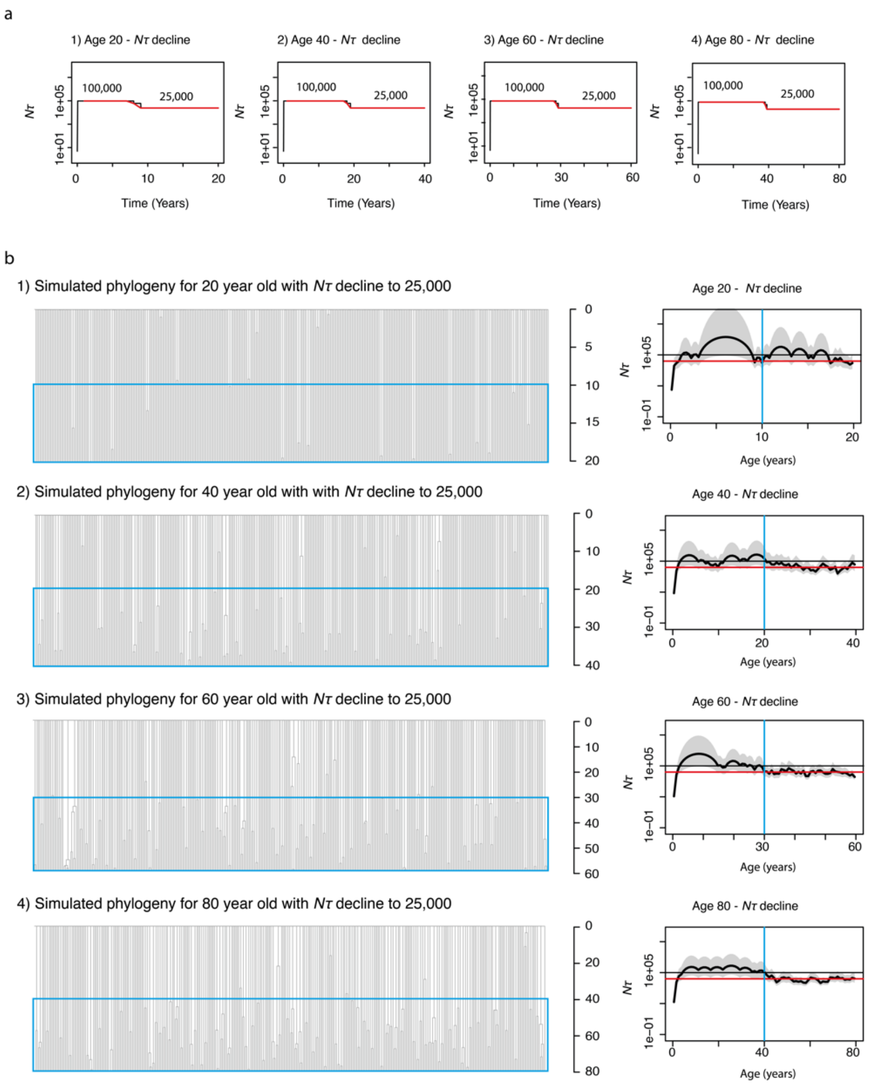

Supplementary Fig.8|Effect of population decline. a, Trajectories of $N\tau$ used as input to *rsimpop* for the simulations to create phylogenies in b. Note the Y axis depicting $N\tau$ is on a log scale. **b,**

Phylogenies created by randomly sampling 380 cells from the final full simulated population of 25,000. Each simulation has an initial $N\tau$ of 100,000 with a decline to 25,000. In all cases $N\tau$ is the same as the population size (N), as the generation time (τ) is 1 year. The blue boxes indicate the period of time in which the population size is decreased. The *phylodyn* trajectories to the right of each simulated phylogeny use the pattern of coalescent events to recover the input trajectories for $N\tau$. The blue line marks the time of change in $N\tau$.

Population growth

An increase in population size is reflected by a decrease in the number of coalescent events captured per unit time as compared to when the population was smaller (**Supplementary Fig. 9**). Again, the older the individual, the more accurately *phylodyn* is able to recover the true simulated population size trajectory. Increases in population size to over 500,000 result in a loss of resolution (and overestimation of $N\tau$ in individuals < 40). In younger individuals the change in population size is best observed as a reduction in the frequency of coalescent events (bumps in the trajectory), but the magnitude of the change cannot be accurately determined.

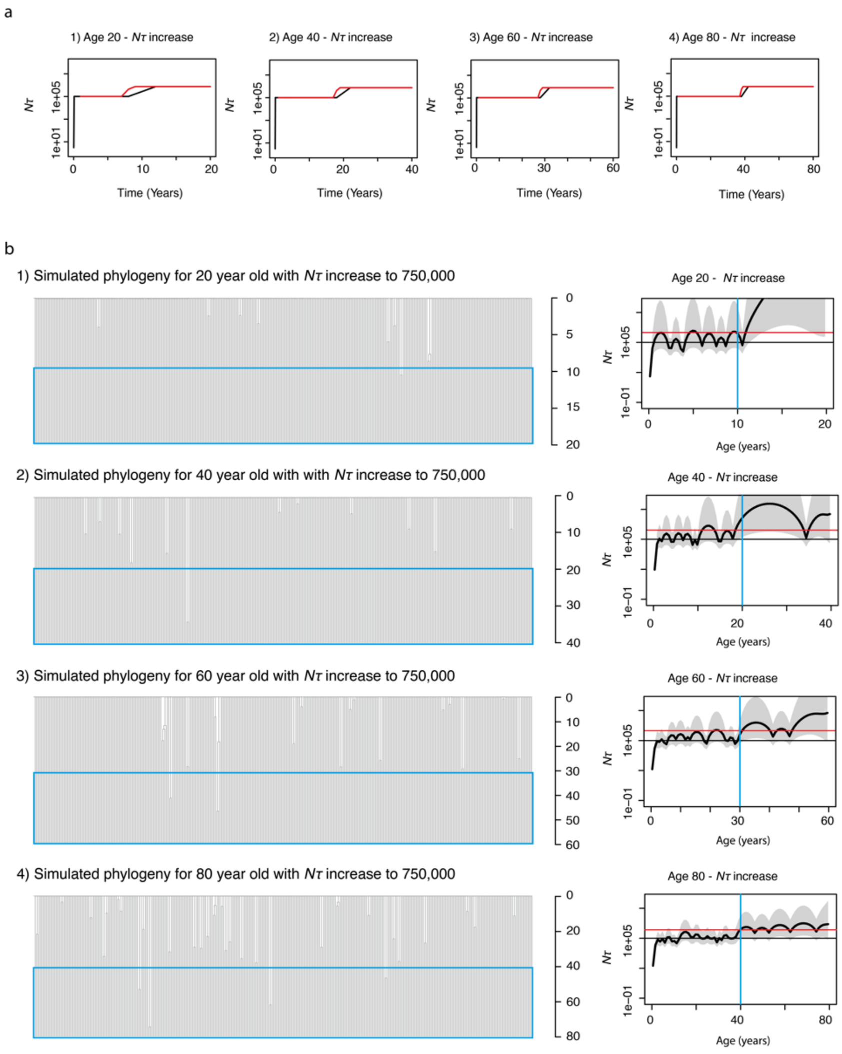

Supplementary Fig.9|Effect of population increase. a, Trajectories of $N\tau$ used as input to *rsimpop* for the simulations to create phylogenies in b. Note the Y axis depicting $N\tau$ is on a log scale. **b,** Phylogenies created by randomly sampling 380 cells from the final full simulated population of 750,000. Each simulation has an initial $N\tau$ of 100,000 with an increase to 750,000 in midlife. In all cases $N\tau$ is the same as the population size (*N*), as the generation time (τ) is 1 year. The blue boxes indicate the period of time in which the population size is increased. The *phylodyn* trajectories to the right of each simulated phylogeny use the pattern of coalescent events to recover the input trajectories for $N\tau$. The blue line marks the time of change in $N\tau$.

Population bottlenecks

'Bottlenecks' in the population represent periods of time with a reduced population size compared to baseline. These can be recovered accurately by *phylodyn* at all ages, given a reduction to in $N\tau$ from 100,000 to 10,000 during the bottleneck period (**Supplementary Fig. 10**).

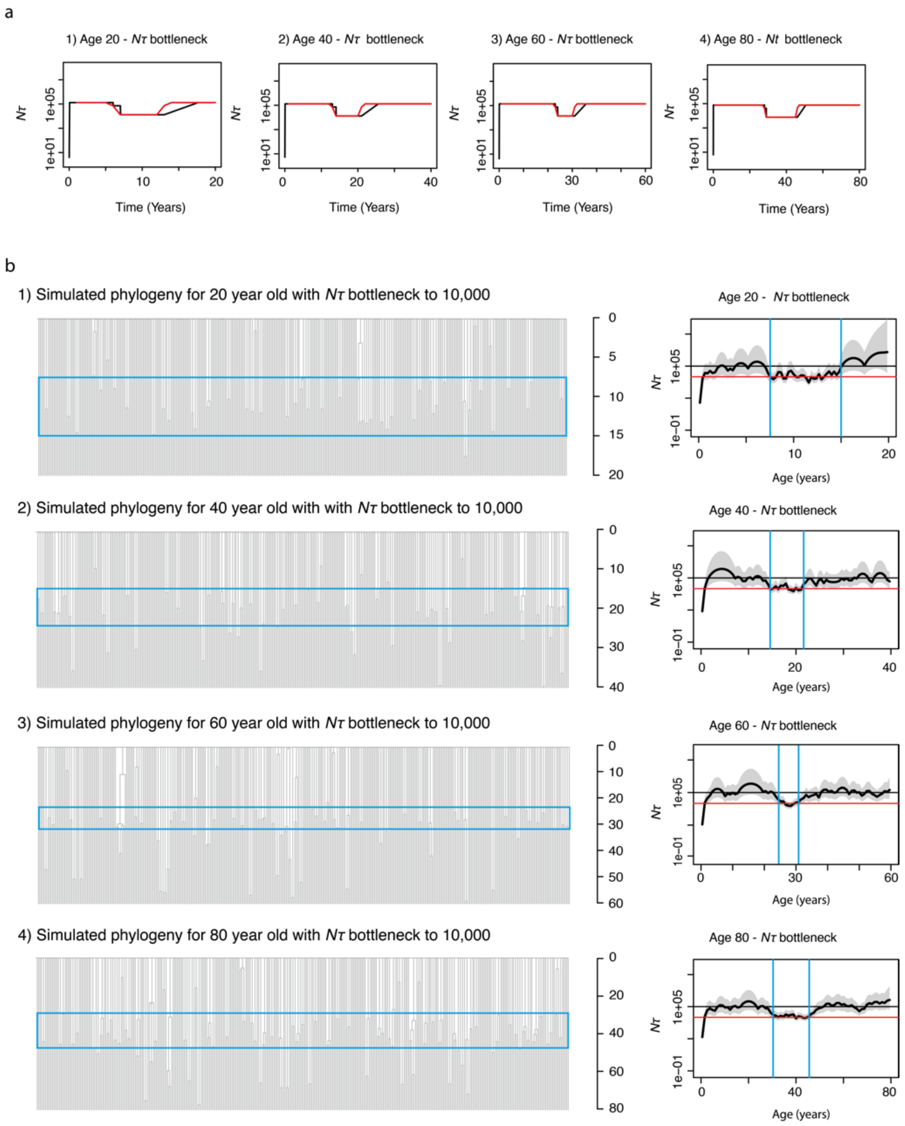

Supplementary Fig.10|Effect of population 'bottleneck'. a, Trajectories of $N\tau$ used as input to *rsimpop* for the simulations to create phylogenies in b. Note the Y axis depicting $N\tau$ is on a log scale. **b,** Phylogenies created by randomly sampling 380 cells from the final full simulated population of 100,000. Each simulation has an initial $N\tau$ of 100,000 with a decline to 10,000 during a period of midlife. In all cases $N\tau$ is the same as the population size (N), as the generation time (τ) is 1 year. The blue boxes indicate the period of time in which the population size is decreased. The *phylodyn* trajectories to the right of each simulated phylogeny use the pattern of coalescent events to recover the input trajectories for $N\tau$. The blue lines mark the times of change in $N\tau$.

Positive selection

Positive selection can also be simulated in the phylogenies as illustrated in **Supplementary Fig. 11 and Extended Fig. 11**. These figures show phylogenies drawn from HSC populations where N is 100,00 and τ is 1 year, with the population as a whole acquiring 200 driver mutations per year, although not all of these will be fixed in the population. The fitness effect of the driver mutations is drawn from a fitness effect gamma distribution (with shape = 0.47

and rate = 34) that incorporates a fitness effect threshold of 5% (**Fig. 5f**). These parameters allow accurate recapitulation of the observed phylogenies across the human lifespan. The simulations illustrate how, although driver mutations are present in the phylogenies of individuals aged below 40, they do not typically impact the pattern of observed coalescences until later in life. This observation provides support for the accuracy of our estimates of $N\tau$ in the two youngest individuals in our cohort. In addition, the simulations demonstrate how large clones typically only become detectable after the age of 60, despite the founding driver mutations having been acquired decades earlier (typically in the first 3-4 decades of life). They also illustrate the range of older phylogenies (similar to the range of topologies in our real phylogenies) that can be generated from the stochastic process of driver acquisition. The simulations show how by age 115 years the haematopoietic system could commonly be sustained by just two clones with no known driver mutations, as has been previously reported in a single real individual[29].

The simple model we use predicts that by age 80, typically > 90% HSCs contain at least 1 driver mutation. In addition, there is a high prevalence of cells containing multiple drivers, such that in later life clonal competition between driver containing clones with different fitness effects can cause complex clonal dynamics. This is illustrated by that fact that some of the highlighted clades remain stable in size over the last few decades of life, while others may even decline in size. In all illustrated cases one or more 'fittest' clones continues to expand into extreme old age.

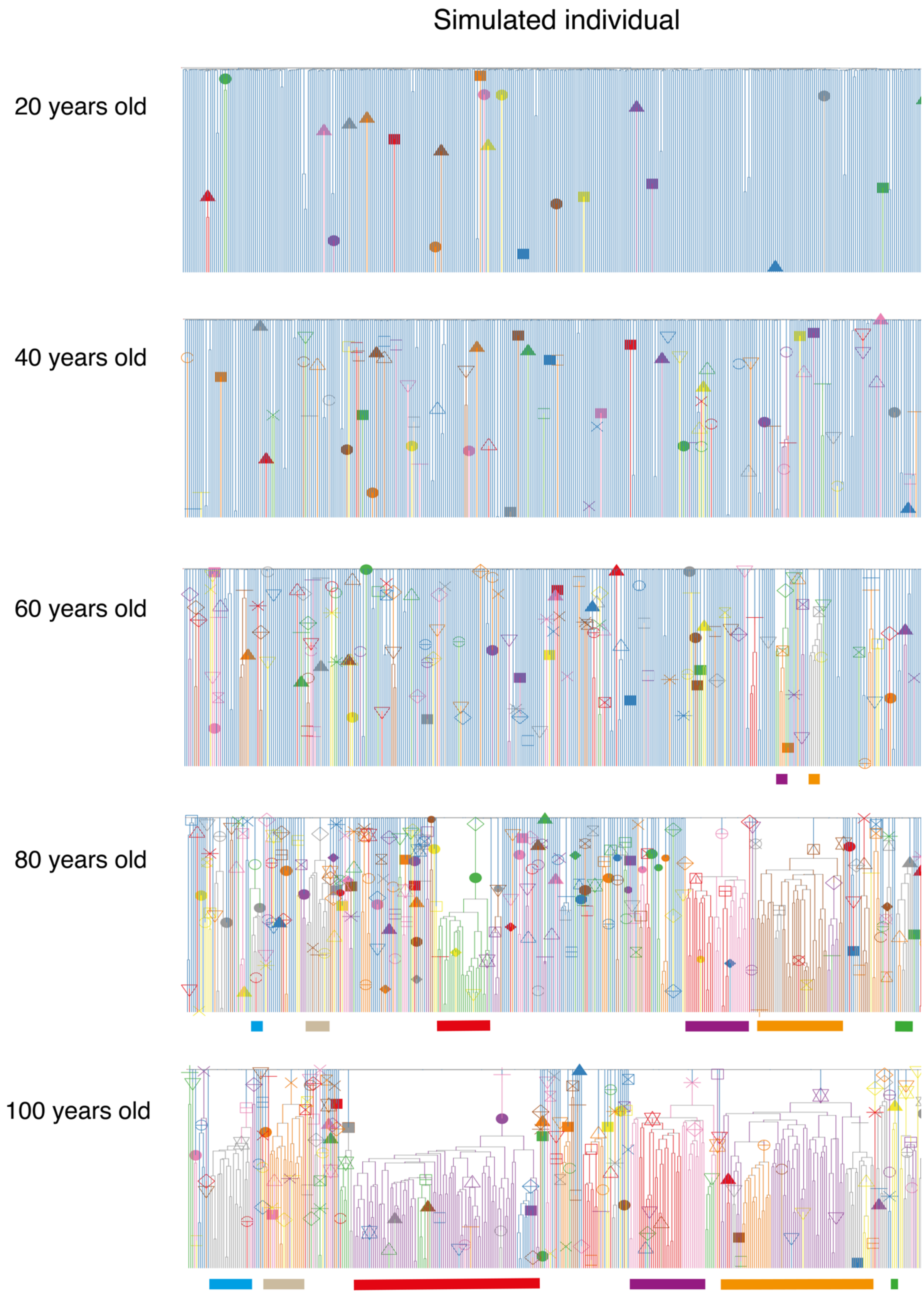

Supplementary Fig.11| Phylogenies of 380 cells sampled from a population of 100,000 cells that has been maintained at a constant $N\tau$ over life, with incorporation of positively selected 'driver mutations'. The driver mutations have a fitness effect > 5% (drawn from a gamma distribution with

shape = 0.47 and rate = 34) and enter the population at a rate of 200 per year. These are the optimal estimates of these parameters based on our ABC modelling. The inclusion of these driver mutations is able to recapitulate a similar clade size distribution to that observed in the real HSPC phylogenies of the observed individuals across the whole age range. However, including driver mutations does not fully recapitulate the observed lack of coalescent events in the last 10-15 years of life, showing that an increase in $N\tau$ over this time is also required to fully recreate the patterns of coalescences in the real phylogenies. Driver mutations are marked with a symbol and their descendent clades are coloured. In all cases $N\tau$ is the same as the population size (N) as the generation time (τ) in all simulations is fixed at 1 year. The symbols / colours are not consistent for driver mutations between plots. The largest clades are therefore coloured in a consistent way beneath the plots to show how their size changes over time. The simulated phylogenies illustrate the complex clonal dynamics that can occur in later life as a result of clonal competition. While the majority of clades continue to expand, others stay relatively stable and some reduce in size. The phylogenies also show that by the age of 80 typically > 90% of HSCs in the population carry at least one driver mutation.

Supplementary Results

Phylodyn trajectories for the older individuals

Phylodyn trajectories for the older individuals (age > 75) (**Supplementary Fig. 12**) cannot be reliably interpreted due to the presence of multiple positively selected clades in each case. However, the trajectories were used to inform the timing of populations size changes in the ABC modelling approach for HSC population size (as below).

Time period	75 year old	76 year old	77 year old	81 year old
**Change 1**	10-19	15-24	10-19	15-19
**Mid-life bottleneck**	20-45	25-50	20-45	20-50
**Change 2**	46-60	51-60	46-60	51-60

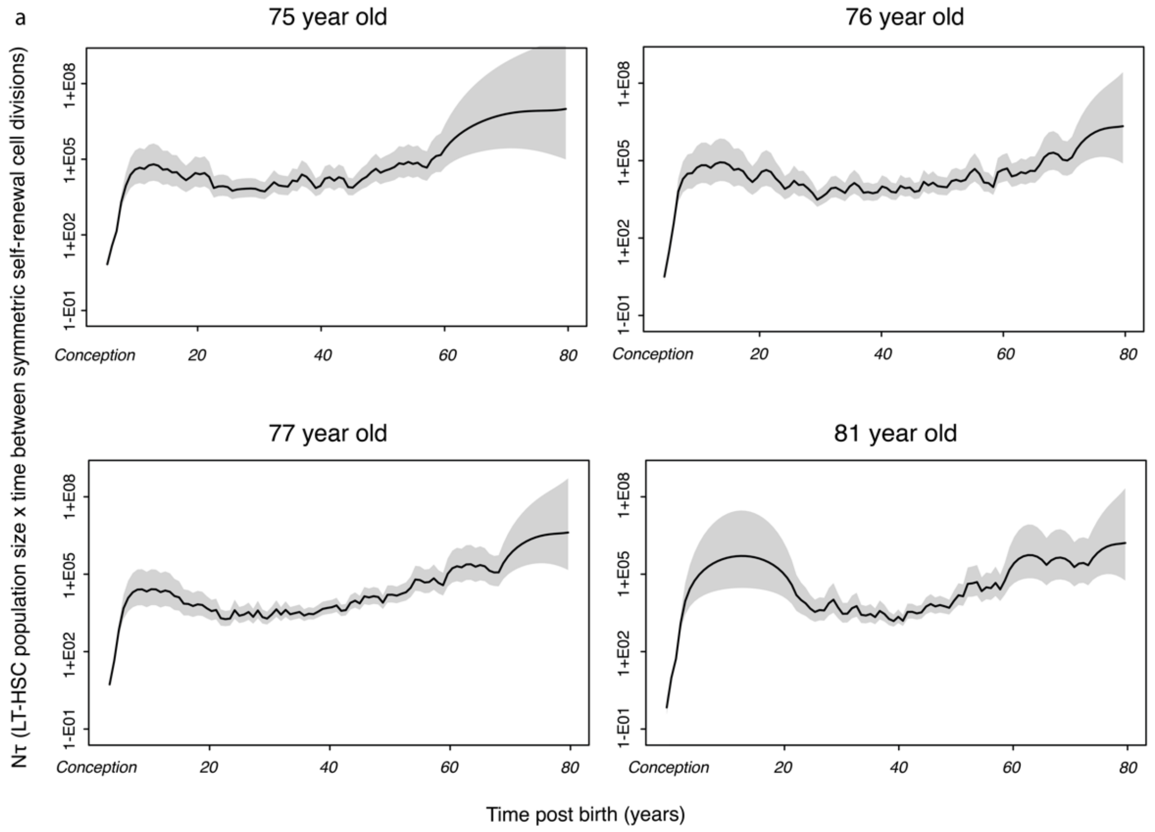

Supplementary Fig.12| *Phylodyn* plots illustrating the trajectory of $N\tau$ for human LT-HSCs in the four adult donors aged >75 if the pattern of coalescent events in their respective phylogenies was not confounded by the presence of positive selection. The black line represents the trajectory of LT-HSC $N\tau$, with the shaded grey area on either side representing the 95% credibility interval.

Phylogeny annotated with BM HSC/MPP vs BM HPC cell type

For the KX004 phylogeny (77-year-old female), we sequenced 352 BM HSC/MPPs and 99 HPCs, both bone marrow derived. **Supplementary Fig. 13** shows the phylogeny with the terminal progenitor cells lineages highlighted blue. For 2 clades the clonal fraction in the HSC/MPPs and HPCs is quite different, providing some evidence that drivers may differ in the compartment in which they exert their fitness effects. Some for example could cause increased proliferation in the HSC/MPP compartment, while others may confer an advantage at the progenitor level.

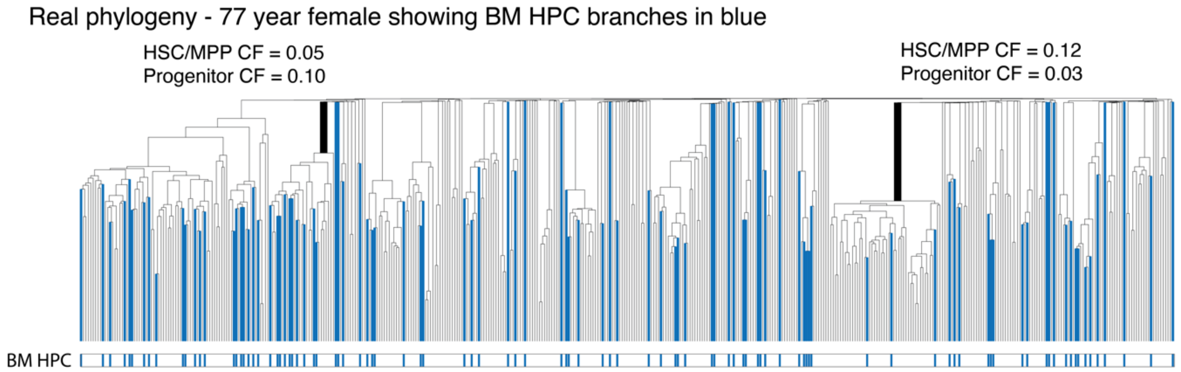

Supplementary Fig.13| Real phylogeny for KX004 (77 year female) annotated by cell type (BM HSC/MPP vs BM HPC). Two clades with differing clonal fractions of these cell types are highlighted.

Posterior distributions for 'driver modelling' parameters

Three parameters were included in the ABC driver modelling: 1) rate of the gamma distribution of fitness effects, 2) shape of the gamma distribution of fitness effects 3) Drivers (with s>5%) entering the HSC population of size 100,000 per year. The posterior distributions for all three parameters are shown in **Extended Fig. 13a**. 2D plots showing the relationship between the three parameters are shown in **Supplementary Fig. 14** below.

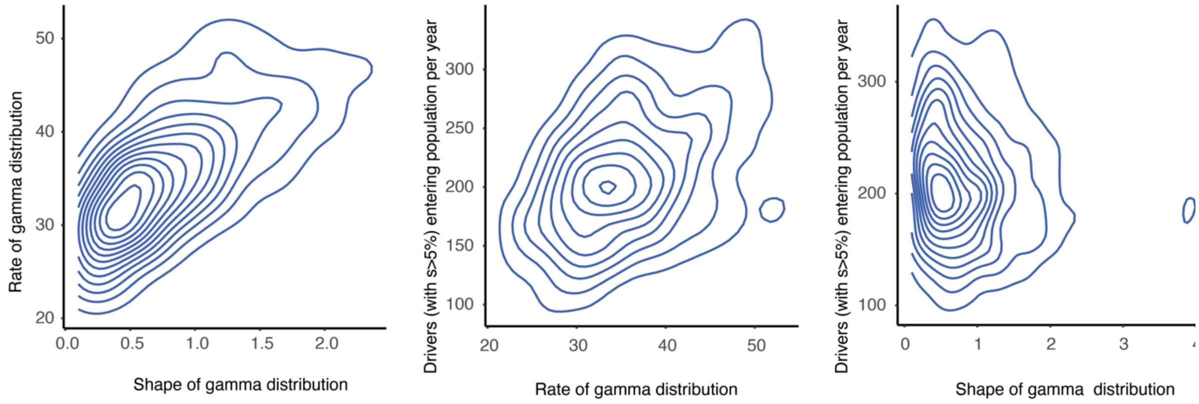

Supplementary Fig.14| 2D plots showing the relationship between the posterior distributions of the three parameters estimated in the driver modelling approach: 1) rate of the gamma distribution of fitness effects, 2) shape of the gamma distribution of fitness effects 3) Drivers (with s>5%) entering the HSC population of size 100,000 per year.

Putative additional novel drivers

Additional possible novel driver genes were identified on the branches of phylogenies leading to expanded clades (**Supplementary Fig. 15** and **Extended Fig. 11b**). Cancer gene variants, as included in the Cosmic Cancer Census v.92 gene set (**Table S4**), which comprises a set of 723 genes causally implicated in cancer development. The top 1500 dN/dS gene hits (**Table S6**) were also interrogated and included only where a manual check of gene function was not incompatible with a possible mechanism to explain clonal expansion.

Clade	Fitness effect (%)	Possible drivers
KX001_Clade1	31 (11-54)	CDC26 p.R23*
KX002_Clade1	35 (13-51)	SPEN p.P2019H<br>ACSM2B p.N193K
SX001_Clade1	NA	MAP3K1 p.T522fs*35
AX001_Clade1	34 (17-43)	KMT2D p.A4236E
KX007_Clade1	29 (19-35)	ST6GALNAC1 p.R590C
KX007_Clade3	22 (12-29)	FGFR1 p.M307I
KX007_Clade6	11 (7-18)	CSNK1A1 p.A216G
KX008_Clade3	23 (15-28)	PPP6C p.A189V<br>LSP1 p.P61T

Clade	Fitness effect (%)	Possible drivers
KX008_Clade4	20 (14-23)	NOTCH3 p.D317N
KX008_Clade5	19 (14-22)	PPIP5K2 p.E940fs*12
KX008_Clade8	17 (9-21)	CIITA p.G516R
KX008_Clade9	11 (7-20)	RFWD3 p.P21fs*15
KX004_Clade1	24 (19-29)	KCNMA1 p.R909Q
KX004_Clade3	25 (17-30)	ZNF331 p.V343I
KX004_Clade5	12 (7-18)	ZNF318 p.Q779*
KX004_Clade6	16 (10-21)	KPNB1 p.S50fs*15
KX003_Clade6	21 (12-27)	LPHN2 p.E1290K

Supplementary Fig.15| Table showing putative identified drivers for all four expanded clades in the young adult individuals (clade size 3 – 5). There are strong candidate drivers for these four individuals, whose expansions all have relatively high fitness effects, as would be expected for them to have been able to expand to a detectable level by a young age. The fitness effect was not calculated for SX001_Clade1 due to the clade size being 3 (too small for a meaningful *phylofit* analysis). For the elderly adult individuals a clade size cut off of ≥ 5 was used as clade sizes of up to 4 can occasionally be seen under a neutral model by this age.

Supplementary Note 1: Intuition for Approximate Bayesian Computation

The Approximate Bayesian Computation framework we have used for modelling different haematopoiesis scenarios is relatively intuitive. In the initial phase, we generate hundreds of thousands of different simulations of haematopoietic stem cell compartments. Each simulation follows exactly the same assumptions – constant population size of HSCs during adulthood; linear entry of driver mutations into the HSC compartment across life; fitness coefficients of drivers drawn from a gamma distribution; fitness coefficient is constant with time. We do not know *a priori* the values for several of these key parameters (distribution of fitness coefficients, rate of driver mutation entry), so each simulation takes a draw for the parameters from relatively uninformative prior distributions.

We then take the huge number of simulations and the phylogenetic trees that they produce, and compare informative summary statistics from simulated trees against the same summary statistics generated from our real phylogeny data. Clearly, many of the simulations will generate trees that are very different to those observed – for example, low driver mutation rates generate many trees with no clonal expansions; fitness coefficients that are too high generate single, massive clonal expansions rather than the oligoclonal patterns we observe.

From the small fraction of simulated trees that best match the observed data, then, we can extract posterior distributions of the parameters we are most interested in. The formal mathematics for this is well-established[14,30] – reassuringly, the posterior distributions we extract using these methods are a well-defined subspace of the prior distribution, suggesting that the observed phylogenetic trees contain considerable information about, and constraints upon, the underlying distribution of the key parameters.

With this intuition for how the modelling works, then, we can see that the inflection point in clonal diversity from the age of 70 years observed in the simulations is not an outcome by design – we do not build such an inflection point into the models as an explicit feature. Rather, it is a data-driven outcome of the simulations. That is, only a relatively narrow window in estimates for the rate of driver mutation acquisition and distribution of fitness coefficients are compatible with the observed phylogenies (**Extended Fig. 9b**; **Supplementary Fig. 12**) – pleasingly, this narrow window of parameter estimates generates simulations that match the inflection point of sharply reduced clonal diversity after the age of 70 years that we observe in the real data (**Fig. 5g**).

Supplementary Note 2: Comparison with clonal dynamics in solid organs

The haematopoietic system is distinctive among organ systems for being well-mixed. When we compare variant allele fraction of mutations in a single bone marrow draw with peripheral blood, we find strong correlation[31], confirming that recirculation of stem cells is sufficiently frequent that spatial biases are negligible over the timescales we are interested in here.

In contrast, studies in solid tissues have revealed that stem cell clones exhibit considerable spatial organisation[32]. For example, human colonic epithelium is organised by crypt, with 5-15 independent stem cells at the base of each crypt dividing neutrally to generate clonal sweeps every 1-2 years[33,34] – with ~10 million crypts per colon[35], which undergo crypt fission events only every 2-3 decades[34], this implies many tens of millions of independent colonic stem cells per adult human. Likewise, detailed lineage tracing of human prostate has revealed that each of the 24-30 independent glandular subunits are laid down *in utero* by 5-10 embryonic cells – these then proliferate to seed stem cells throughout the ductal tree which, following a wave of further proliferation and duct formation during puberty, enter a relatively quiescent phase of local stem/progenitor cell tissue maintenance in adulthood[36]. In squamous tissues, such as oesophagus[37,38], bronchial epithelium[39,40] and skin[41], driver mutations accumulate steadily with ageing, causing exponential clonal expansions[42], with competition occurring predominantly at clone boundaries[43].

The effects of ageing on stem cell clonal dynamics in solid tissues have not been extensively studied to date. However, the effects of disease and of toxicity have had some initial evaluation. In intestine, it is clear that inflammatory bowel disease leads to considerably larger clonal expansions than seen in normal individuals, partially driven by selection for driver mutations that protect against the inflammatory process and partially driven by the regenerative pressure of a relapsing-remitting disease course[44–46]. Likewise, while normal liver is a tightly knit patchwork of clones as small as 100-1000 hepatocytes, chronic liver disease is characterised by considerably larger clones, millimetres to centimetres in size, often accounting for entire cirrhotic nodules[47–50] – again, these clonal expansions are driven by a combination of selection for protective driver mutations and the regenerative milieu arising from sustained hepatocyte toxicity. Interestingly, cellular toxicity, such as that arising from tobacco smoke in bronchus[39] or ultraviolet light in skin[51], also increases the rate of driver mutations and clonal expansion in solid tissues.

Taken together, these data show that tissue maintenance in adults in solid organs is typically a hugely polyclonal process, strongly shaped by the spatial organisation of the tissue. Clonal competition therefore remains local, and opportunities for massive clonal expansion remain limited under normal physiological conditions. However, with disease or toxicity, when selective pressures are more pronounced and coupled with increased regenerative pressure, clonal expansions can be sizable, encompassing (square or cubic) millimetres to centimetres

of tissue. Furthermore, convergent evolution, where the same genes are recurrently mutated and positively selected in independent clones, can lead to an analogous situation to that we have observed here in blood, where 20-80% of all epithelial cells within, say, skin[41,51], oesophagus[37,38], endometrium[3] or bronchus[39] carry mutations in specific driver genes.

Data Availability

The main data needed to reanalyse / reproduce the results presented is available on Mendeley Data (https://data.mendeley.com/datasets/np54zjkvxr/1). The following files and folders are found at the Mendeley Data archive:

dNdS_input folder
Contains all raw input files for the dN/dS analysis.

Filtering_output_XXXX folders (one for each individual)
Contains four files:

a) annotated_mut_set_XXXX_01_standard_rho01
This is an R data object and is uploaded into an R workspace using load()
The genotype matrix used for MPBoot tree building is available in the matrix: filtered_muts$Genotype_shared_bin
The dna strings used as input for MPboot are available in the vector: filtered_muts$dna_strings
The annotated variant calls with tree node information are available in the matrix: filtered_muts$COMB_mats.tree.build$mat
The genotype matrix of mutations calls per sample is available in: filtered_muts$COMB_mats.tree.build$Genotype_bin
Information on whether the variant is an SNV or indel is available in: filtered_muts$COMB_mats.tree.build$mat$Mut_type
A summary of total numbers of shared and private SNVs and indels is available in:
filtered_muts$summary

b) XXXX_sensitivity
This file contains information on the sensitivity of SNV and Indel calls per sample.

c) tree_XXXX_01_standard_rho01.tree
The raw tree with branch lengths equal to number of mutations assigned (without adjustment for sequencing coverage).

metadata_matrix folder
Contains file "Summary_cut.csv" which records metadata on each sample in the dataset including cell_type sorted, sequencing depth, sequencing_platform, SNV burdens, indel burdens and telomere length.

References

1. Jones, D. *et al.* cgpCaVEManWrapper: Simple Execution of CaVEMan in Order to Detect Somatic Single Nucleotide Variants in NGS Data. in *Current Protocols in Bioinformatics* **2016**, 15.10.1-15.10.18 (John Wiley & Sons, Inc., 2016).
2. Raine, K. M. *et al.* cgpPindel: Identifying Somatically Acquired Insertion and Deletion Events from Paired End Sequencing. *Curr. Protoc. Bioinformatics* **52**, 15.7.1-15.7.12 (2015).
3. Moore, L. *et al.* The mutational landscape of normal human endometrial epithelium. *Nature* **580**, (2020).
4. Ellis, P. *et al.* Reliable detection of somatic mutations in solid tissues by laser-capture microdissection and low-input DNA sequencing. *Nat. Protoc.* 1–31 (2020). doi:10.1038/s41596-020-00437-6
5. Coorens, T. H. H. *et al.* Extensive phylogenies of human development inferred from somatic mutations. *Nature* **597**, 387–392 (2021).
6. Tim H Coorens, A. H. *et al.* TITLE Extensive phylogenies of human development reveal variable embryonic patterns. doi:10.1101/2020.11.25.397828
7. Spencer Chapman, M. *et al.* Lineage tracing of human development through somatic mutations. *Nature* **595**, 85–90 (2021).
8. Cameron, D. L. *et al.* GRIDSS: sensitive and specific genomic rearrangement detection using positional de Bruijn graph assembly. (2017). doi:10.1101/gr.222109.117
9. Van Loo, P. *et al.* Allele-specific copy number analysis of tumors. *Proc Natl Acad Sci U S A* **107**, 16910–16915 (2010).
10. Campbell, P. J. *et al.* Identification of somatically acquired rearrangements in cancer using genome-wide massively parallel paired-end sequencing. *Nat. Genet.* **40**, 722–9 (2008).
11. Thi Hoang, D. *et al.* MPBoot: fast phylogenetic maximum parsimony tree inference and bootstrap approximation. doi:10.1186/s12862-018-1131-3
12. Williams, N. *et al.* Phylogenetic reconstruction of myeloproliferative neoplasm reveals very early origins and lifelong evolution. doi:10.1101/2020.11.09.374710
13. Csilléry, K., François, O. & Blum, M. G. B. Abc: An R package for approximate Bayesian computation (ABC). *Methods Ecol. Evol.* **3**, 475–479 (2012).
14. Beaumont, M. A., Zhang, W. & Balding, D. J. Approximate Bayesian Computation in Population Genetics. *Genetics* **162**, 2025–2035 (2002).
15. Gelman, A. *et al. Bayesian data analysis*. (Chapman and Hall, CRC Press, 2004).
16. Martincorena, I. *et al.* Universal Patterns of Selection in Cancer and Somatic Tissues. *Cell* **171**, 1029-1041.e21 (2017).
17. Rheinbay, E. *et al.* Analyses of non-coding somatic drivers in 2,658 cancer whole genomes. *Nature* **578**, 102–111 (2020).

18. Lawrence, M. S. *et al.* Mutational heterogeneity in cancer and the search for new cancer-associated genes. *Nature* **499**, 214–8 (2013).
19. Alexandrov, L. B. *et al.* The repertoire of mutational signatures in human cancer. *Nature* **578**, 94–101 (2020).
20. Vaser, R., Adusumalli, S., Ngak Leng, S., Sikic, M. & Ng, P. C. SIFT missense predictions for genomes. *Nat. Protoc.* (2015). doi:10.1038/nprot.2015.123
21. Adzhubei, I., Jordan, D. M. & Sunyaev, S. R. Predicting Functional Effect of Human Missense Mutations Using PolyPhen-2. doi:10.1002/0471142905.hg0720s76
22. Nei, M. & Gojobori, T. Simple methods for estimating the numbers of synonymous and nonsynonymous nucleotide substitutions. *Mol. Biol. Evol.* **3**, 418–426 (1986).
23. Greenman, C., Wooster, R., Futreal, P. A., Stratton, M. R. & Easton, D. F. Statistical analysis of pathogenicity of somatic mutations in cancer. *Genetics* **173**, 2187–2198 (2006).
24. Watson, C. J. *et al.* The evolutionary dynamics and fitness landscape of clonal hematopoiesis. *Science (80-.).* **367**, 1449–1454 (2020).
25. Lan, S., Palacios, J. A., Karcher, M., Minin, V. N. & Shahbaba, B. An efficient Bayesian inference framework for coalescent-based nonparametric phylodynamics. *Bioinformatics* **31**, 3282–3289 (2015).
26. Rife, B. D. *et al.* Phylodynamic applications in 21st century global infectious disease research. *Glob. Heal. Res. Policy* **2**, 13 (2017).
27. Mimasaka, S. Postmortem cytokine levels and the cause of death. *Tohoku J. Exp. Med.* **197**, 145–150 (2002).
28. Schwarz, P. *et al.* Brain Death-Induced Inflammatory Activity is Similar to Sepsis-Induced Cytokine Release. *Cell Transplant.* **27**, 1417–1424 (2018).
29. Holstege, H. *et al.* Somatic mutations found in the healthy blood compartment of a 115-yr-old woman demonstrate oligoclonal hematopoiesis. *Genome Res.* **24**, 733–742 (2014).
30. Bertorelle, G., Benazzo, A. & Mona, S. ABC as a flexible framework to estimate demography over space and time: Some cons, many pros. *Mol. Ecol.* **19**, 2609–2625 (2010).
31. Lee-Six, H. *et al.* Population dynamics of normal human blood inferred from somatic mutations. *Nature* **561**, 473–478 (2018).
32. Li, R. *et al.* A body map of somatic mutagenesis in morphologically normal human tissues. *Nature* **597**, 398–403 (2021).
33. Lopez-Garcia, C., Klein, A. M., Simons, B. D. & Winton, D. J. Intestinal stem cell replacement follows a pattern of neutral drift. *Science (80-.).* **330**, 822–825 (2010).
34. Lee-Six, H. *et al.* The landscape of somatic mutation in normal colorectal epithelial cells. *Nature* **574**, 532–537 (2019).
35. Nguyen, H. *et al.* Deficient Pms2, ERCC1, Ku86, CcOI in field defects during progression to colon cancer. *J. Vis. Exp.* 2–6 (2010). doi:10.3791/1931
36. Grossmann, S. *et al.* Development, maturation, and maintenance of human prostate

inferred from somatic mutations. *Cell Stem Cell* **28**, 1262-1274.e5 (2021).
37. Yokoyama, A. *et al.* Age-related remodelling of oesophageal epithelia by mutated cancer drivers. *Nature* **565**, 312–317 (2019).
38. Martincorena, I. *et al.* Somatic mutant clones colonize the human esophagus with age. *Science (80-.).* **917**, 911–917 (2018).
39. Yoshida, K. *et al.* Tobacco smoking and somatic mutations in human bronchial epithelium. *Nature* **578**, 266–272 (2020).
40. Teixeira, V. H. *et al.* Stochastic homeostasis in human airway epithelium is achieved by neutral competition of basal cell progenitors. *Elife* **2**, e00966 (2013).
41. Martincorena, I. *et al.* High burden and pervasive positive selection of somatic mutations in normal human skin. *Science (80-.).* **348**, 880–886 (2015).
42. Williams, M. J. *et al.* Measuring the distribution of fitness effects in somatic evolution by combining clonal dynamics with dN/dS ratios. *Elife* **9**, 1–19 (2020).
43. Colom, B. *et al.* Spatial competition shapes the dynamic mutational landscape of normal esophageal epithelium. *Nat. Genet.* **52**, 604–614 (2020).
44. Kakiuchi, N. *et al.* Frequent mutations that converge on the NFKBIZ pathway in ulcerative colitis. *Nature* **577**, 260–265 (2020).
45. Nanki, K. *et al.* Somatic inflammatory gene mutations in human ulcerative colitis epithelium. *Nature* **577**, 254–259 (2020).
46. Olafsson, S. *et al.* Somatic Evolution in Non-neoplastic IBD-Affected Colon. *Cell* **182**, 672-684.e11 (2020).
47. Kim, S. K. *et al.* Comprehensive analysis of genetic aberrations linked to tumorigenesis in regenerative nodules of liver cirrhosis. *J. Gastroenterol.* (2019). doi:10.1007/s00535-019-01555-z
48. Zhu, M. *et al.* Somatic Mutations Increase Hepatic Clonal Fitness and Regeneration in Chronic Liver Disease. *Cell* **177**, 608–621 (2019).
49. Brunner, S. F. *et al.* Somatic mutations and clonal dynamics in healthy and cirrhotic human liver. *Nature* **574**, 538–542 (2019).
50. Ng, S. W. K. *et al.* Convergent somatic mutations in metabolism genes in chronic liver disease. *Nature* **598**, 473–478 (2021).
51. Fowler, J. C. *et al.* Selection of oncogenic mutant clones in normal human skin varies with body site. *Cancer Discov.* **11**, 340–361 (2021).
