# Supplementary material for: Clonal dynamics of haematopoiesis across the human lifespan
Source: Nature. 2022 Jun 1;606(7913):343–50. doi: 10.1038/s41586-022-04786-y (PMC9177428; doi:10.1038/s41586-022-04786-y)
Supplement: Supplementary file 2 — Reporting Summary [file 41586_2022_4786_MOESM2_ESM.pdf]

nature portfolio

Corresponding author(s): Peter Campbell
Elisa Laurenti
Jyoti Nangalia

Last updated by author(s): Feb 20, 2022

Reporting Summary

Nature Portfolio wishes to improve the reproducibility of the work that we publish. This form provides structure for consistency and transparency in reporting. For further information on Nature Portfolio policies, see our Editorial Policies and the Editorial Policy Checklist.

Statistics

For all statistical analyses, confirm that the following items are present in the figure legend, table legend, main text, or Methods section.

n/a	Confirmed	
☐	☒	The exact sample size (*n*) for each experimental group/condition, given as a discrete number and unit of measurement
☐	☒	A statement on whether measurements were taken from distinct samples or whether the same sample was measured repeatedly
☐	☒	The statistical test(s) used AND whether they are one- or two-sided<br>*Only common tests should be described solely by name; describe more complex techniques in the Methods section.*
☐	☒	A description of all covariates tested
☐	☒	A description of any assumptions or corrections, such as tests of normality and adjustment for multiple comparisons
☐	☒	A full description of the statistical parameters including central tendency (e.g. means) or other basic estimates (e.g. regression coefficient) AND variation (e.g. standard deviation) or associated estimates of uncertainty (e.g. confidence intervals)
☐	☒	For null hypothesis testing, the test statistic (e.g. *F*, *t*, *r*) with confidence intervals, effect sizes, degrees of freedom and *P* value noted<br>*Give P values as exact values whenever suitable.*
☐	☒	For Bayesian analysis, information on the choice of priors and Markov chain Monte Carlo settings
☐	☒	For hierarchical and complex designs, identification of the appropriate level for tests and full reporting of outcomes
☒	☐	Estimates of effect sizes (e.g. Cohen's *d*, Pearson's *r*), indicating how they were calculated

Our web collection on statistics for biologists contains articles on many of the points above.

Software and code

Policy information about availability of computer code

Data collection	None
Data analysis	List of programs and softwares:<br>• R: version 3.6.1<br>• BWA-MEM: version 0.7.17 (https://sourceforge.net/projects/bio-bwa/)<br>• cgpCaVEMan: version 1.11.2/1.13.14/1.14.1 (https://github.com/cancerit/CaVEMan)<br>• cgpPindel: version 2.2.5/3.2.0/3.3.0 (https://github.com/cancerit/cgpPindel)<br>• Brass: version 6.1.2/6.2.0/6.3.0/6.3.4 (https://github.com/cancerit/BRASS)<br>• ASCAT NGS: version 4.2.1/4.3.3 (https://github.com/cancerit/ascatNgs)<br>• VAGrENT: version 3.5.2/3.6.0/3.6.1 (https://github.com/cancerit/VAGrENT)<br>• GRIDSS: version 2.9.4 (https://github.com/PapenfussLab/gridss)<br>• MPBoot: version 1.1.0 (https://github.com/diepthihoang/mpboot)<br>• cgpVAF: version 2.4.0 (https://github.com/cancerit/vafCorrect)<br>• dNdScv: version 0.0.1 (https://github.com/im3sanger/dndscv)<br>• Telomerecat: version 3.4.0 (https://github.com/jhrf/telomerecat)<br>• Rsimpop: version 2.0.4 (https://github.com/NickWilliamsSanger/rsimpop)<br>• Phylodyn: version 0.9.02 (https://github.com/mdkarcher/phylodyn)<br>• FlowJo: version 10<br>Custom code made available (also stated in manuscript): https://github.com/emily-mitchell/normal_haematopoiesis<br>No commercial software used.

For manuscripts utilizing custom algorithms or software that are central to the research but not yet described in published literature, software must be made available to editors and reviewers. We strongly encourage code deposition in a community repository (e.g. GitHub). See the Nature Portfolio guidelines for submitting code & software for further information.

March 2021

Data

Policy information about availability of data

All manuscripts must include a data availability statement. This statement should provide the following information, where applicable:

- Accession codes, unique identifiers, or web links for publicly available datasets
- A description of any restrictions on data availability
- For clinical datasets or third party data, please ensure that the statement adheres to our policy

Sequence data that support the findings of this study have been deposited in the European Genome-Phenome Archive (https://www.ebi.ac.uk/ega/home; accession number EGAD00001007851).
The main data needed to reanalyse / reproduce the results presented is available on Mendeley Data (https://data.mendeley.com/datasets/np54zjkvxr/1).
All scripts and some smaller data matrices are available on github (https://github.com/emily-mitchell/normal _haematopoiesis).
hg37 human reference genome has been used in this study.

Field-specific reporting

Please select the one below that is the best fit for your research. If you are not sure, read the appropriate sections before making your selection.

☒ Life sciences ☐ Behavioural & social sciences ☐ Ecological, evolutionary & environmental sciences

For a reference copy of the document with all sections, see nature.com/documents/nr-reporting-summary-flat.pdf

Life sciences study design

All studies must disclose on these points even when the disclosure is negative.

Sample size	We optimised the number of individuals (10) and number of haematopoietic stem cells sequenced per individual (average of 358 cells per individual) to describe the mutation burden, telomere lengths and clonal structure of normal haematopoietic stem cell populations across a range of ages. No power calculation was performed, and there was no target effect size.
Data exclusions	Per pre-established criteria, genomes with a sequencing depth of less than 7x (17 samples) or with a VAF distribution showing evidence of non-clonality or contamination (peak VAF < 40%) (7 samples) were excluded from the analysis.
Replication	While the specific donor samples used have been exhausted, the results from this study should be generally reproducible in separate healthy individuals of the same age, using the protocols and code included in this manuscript.
Randomization	This is not relevant to our study. All individuals were haematopoietically normal, and there was no test versus control groups.
Blinding	Blinding was not relevant to our study. There was no test performed that required blinding.

Reporting for specific materials, systems and methods

We require information from authors about some types of materials, experimental systems and methods used in many studies. Here, indicate whether each material, system or method listed is relevant to your study. If you are not sure if a list item applies to your research, read the appropriate section before selecting a response.

Materials & experimental systems

n/a	Involved in the study	
☐	☒	Antibodies
☒	☐	Eukaryotic cell lines
☒	☐	Palaeontology and archaeology
☒	☐	Animals and other organisms
☐	☒	Human research participants
☒	☐	Clinical data
☒	☐	Dual use research of concern

Methods

n/a	Involved in the study	
☒	☐	ChIP-seq
☐	☒	Flow cytometry
☒	☐	MRI-based neuroimaging

Antibodies

Antibodies used

Marker ; Fluorochrome ; Manufacter ; Catalogue Number ; Clone ; Dilution
CD3 ; FITC ; BD ; 555339 ; HIT3a ; 1 in 500
CD90 ; PE ; Biolegend ; 328110 ; 5E10 ; 1 in 50
CD49f ; PECy5 ; BD ; 551129 ; GoH3 ; 1 in 100
CD19 ; A700 ; Biolegend ; 302226 ; HIB19 ; 1 in 300

CD34 ; APCCy7 ; Biolegend ; 343514 ; 581 ; 1 in 100
Zombie ; Aqua ; Biolegend ; 423101 ; NA ; 1 in 2000
CD38 ; PECy7 ; Biolegend ; 303516 ; HIT2 ; 1 in 100
CD45RA ; BV421 ; Biolegend ; 304130 ; HI100 ; 1 in 100
CD33; APC; BD; 571817; WM53; 1 in 200

Validation

These were all previously validated commercially available antibodies.
CD3 FITC: Validated by supplier with the following notes - species reactivity: human; application - flow cytometry
CD90 PE: Validated by supplier with the following notes - species reactivity: human, rhesus, baboon, macaque, pig; application - flow cytometry
CD49f PECy5: Validated by the supplier with the following notes - species reactivity: human, mouse, pig; application: flow cytometry
CD19 A700: Validated by the supplier with the following notes - species reactivity: human, chimpanzee, rhesus; application: flow cytometry
CD34 APCCy7: Validated by the supplier with the following notes - species reactivity: human, cynomolgus; application: flow cytometry
Zombie aqua: Validated by the supplier with the following notes - species reactivity: NA; application: flow cytometry and immunofluorescence microscopy
CD38 PECy7: Validated by the supplier with the following notes - species reactivity: human, chimpanzee, horse, cow; application: flow cytometry
CD45RA BV421: Validated by the supplier with the following notes - species reactivity: human, chimpanzee; application: flow cytometry and immunohistochemistry
CD33 APC: Validated by the supplier with the following notes - species reactivity: human, chimpanzee; application: flow cytometry

Human research participants

Policy information about studies involving human research participants

Population characteristics

The dataset comprised 3759 whole genomes from the bone marrow, blood or cord blood of 10 individuals aged 0 years to 81 years. All individuals were haematologically normal. One individual (38 year male) had inflammatory bowel disease (Crohn's disease) and one individual (48 year male) had selenoprotein deficiency, a rare genetic disorder not known to impact haematopoietic stem cell population dynamics. In total 4 females and 6 males were included in the study.

Recruitment

Cord blood mononuclear cell samples were obtained from Stem Cell Technologies. These had been collected with informed consent including for whole genome sequencing (catalog #70007). Cambridge Blood and Stem Cell Biobank (CBSB) provided fresh peripheral blood samples taken with informed consent from two patients at Addenbrooke's Hospital (NHS Cambridgeshire 4 Research Ethics Committee reference 07/MRE05/44 for samples collected pre-November 2019 and Cambridge East Ethics Committee reference 18/EE/0199 for samples collected from November 2019 onwards. Cambridge Biorepository for Translational Medicine (CBTM) provided frozen bone marrow +/- peripheral blood MNCs taken with informed consent from six deceased organ donors. Samples were collected at the time of abdominal organ harvest (Cambridgeshire 4 Research Ethics Committee reference 15/EE/0152).

Ethics oversight

Cambridgeshire 4 Research Ethics Committee reference 07/MRE05/44; Cambridge East Ethics Committee reference 18/EE/0199; Cambridgeshire 4 Research Ethics Committee reference 15/EE/0152.

Note that full information on the approval of the study protocol must also be provided in the manuscript.

Flow Cytometry

Plots

Confirm that:

☒ The axis labels state the marker and fluorochrome used (e.g. CD4-FITC).

☒ The axis scales are clearly visible. Include numbers along axes only for bottom left plot of group (a 'group' is an analysis of identical markers).

☒ All plots are contour plots with outliers or pseudocolor plots.

☒ A numerical value for number of cells or percentage (with statistics) is provided.

Methodology

Sample preparation

Mononuclear cells (MNCs) were isolated using lymphoprepTM density gradient centrifugation (STEMCELL Technologies), after diluting whole blood 1:1 with PBS. The red blood cell and granulocyte fraction of the blood was then removed. The MNC fraction underwent red cell lysis using 1 incubation at 4C for 15 mins with RBC lysis buffer (BioLegend). CD34 positive cell selection of peripheral blood and cord blood MNC samples was undertaken using the EasySep human whole blood CD34 positive selection kit (STEMCELL Technologies). The kit was used as per the manufacturer's instructions, but with only a single round of magnetic selection. Bone marrow MNCs did not undergo CD34 positive selection prior to cell sorting.

Instrument

Stained MNC or CD34+ enriched cell fractions were single cell index sorted on either a BD Aria III or BD Aria fusion cell sorter.

Software

No analysis of flow cytometry data is presented in this manuscript. FlowJo v10 was used to generate the gating strategy image.

Cell population abundance

Typical cell population abundancies are shown in the sorting strategy ED Fig. 1a. In BM samples and PB CD34+ selected

Cell population abundance

samples, myeloid cells were 20-50% of live cells and CD34+ cells were 1-5% of myeloid cells. In CB CD34+ selected samples, myeloid cells were 10-30% of live cells and CD34+ cells were 5-15% of myeloid cells. Approximately 10 x 96 well plates of HSC/MPP cells could be sorted from 5+E07 BM MNCs and 2-3 x 96 well plates of HSC/MPP cells could be sorted from 10+E07 PB MNCs.

Gating strategy

The gating strategy used for cell sorting is illustrated in ED Fig. 1a.
To summarise:
1. FSC-A vs SSC-A showing all events: gate on cell population (to exclude dead cells and debris)
2. FSC-A vs FSC-W showing cell population: gate on singlets (to exclude doublets)
3. ZOMBIE aqua vs SSC-A showing singlets: gate on live cells (to exclude dead cells)
4. CD19 A700 vs CD3 FITC showing live cells: gate on CD19-CD3- myeloid cells (to exclude T and B cells)
5. CD34 APCCy7 vs CD38 PECy7 showing myeloid cells: gate on CD34+
6. CD34 APCCy7 vs CD38 PECy7 showing CD34+ cells: gate on CD34+CD38- (approx 20% of population)
7. CD45RA BV421 vs CD90 PE showing CD34+CD38- cells: gate on CD45RA neg cells = sorted "HSC/MPP" population

☒ Tick this box to confirm that a figure exemplifying the gating strategy is provided in the Supplementary Information.
