# Supplementary material for: Clonal dynamics of haematopoiesis across the human lifespan
Source: Nature. 2022 Jun 1;606(7913):343–50. doi: 10.1038/s41586-022-04786-y (PMC9177428; doi:10.1038/s41586-022-04786-y)
Supplement: Supplementary file 4 — HTMLs of notebooks outlining key statistical analyses presented in the manuscript, including analysis of phylogenetic trees. [file 41586_2022_4786_MOESM4_ESM.zip › Supplementary_code/SNV_indel_analysis/AX001_sample_vaf_plots.pdf]

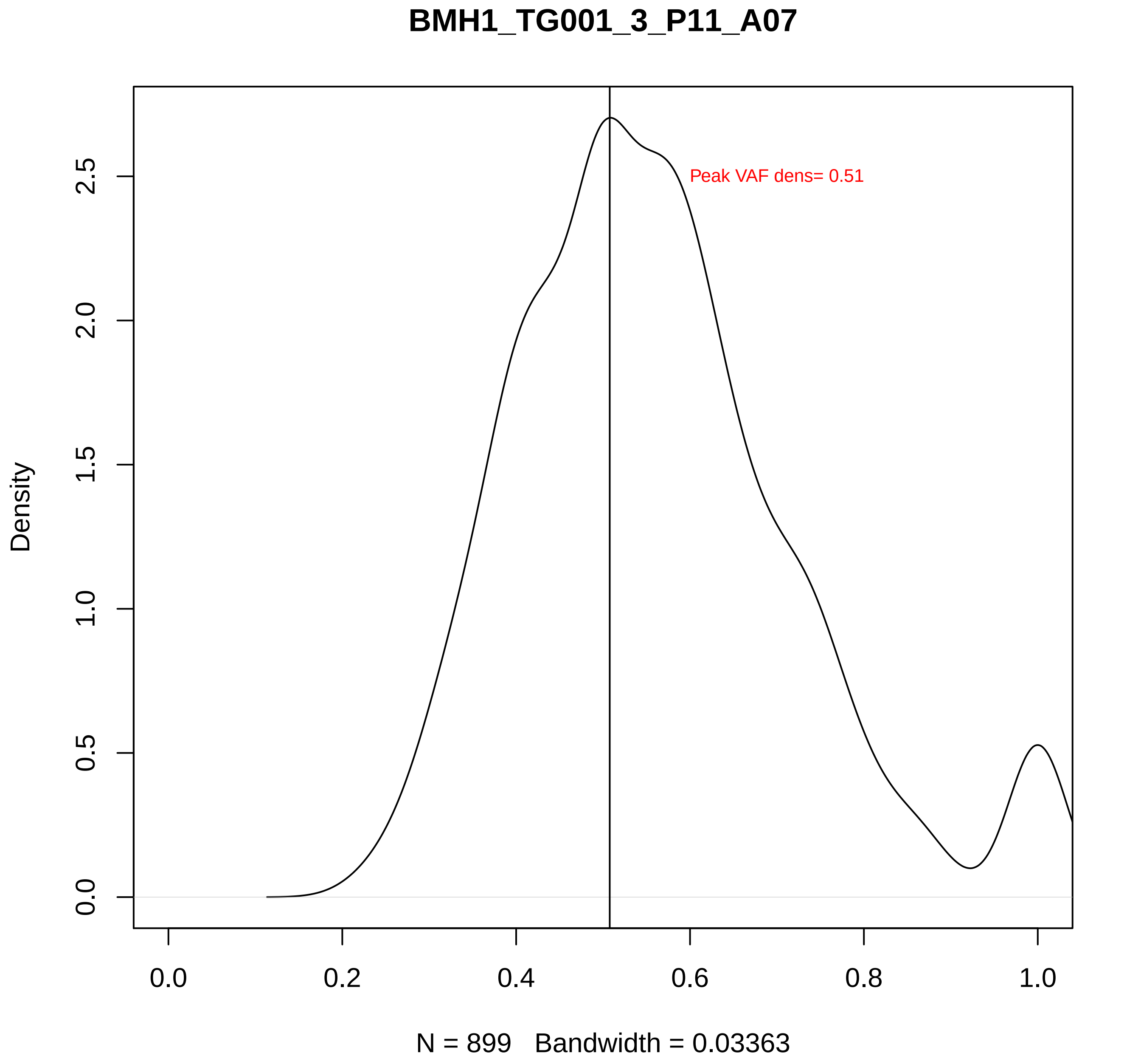
BMH1_TG001_3_P11_A07
Peak VAF dens= 0.51
Density
0.0
0.5
1.0
1.5
2.0
2.5
0.0
0.2
0.4
0.6
0.8
1.0
N = 899   Bandwidth = 0.03363

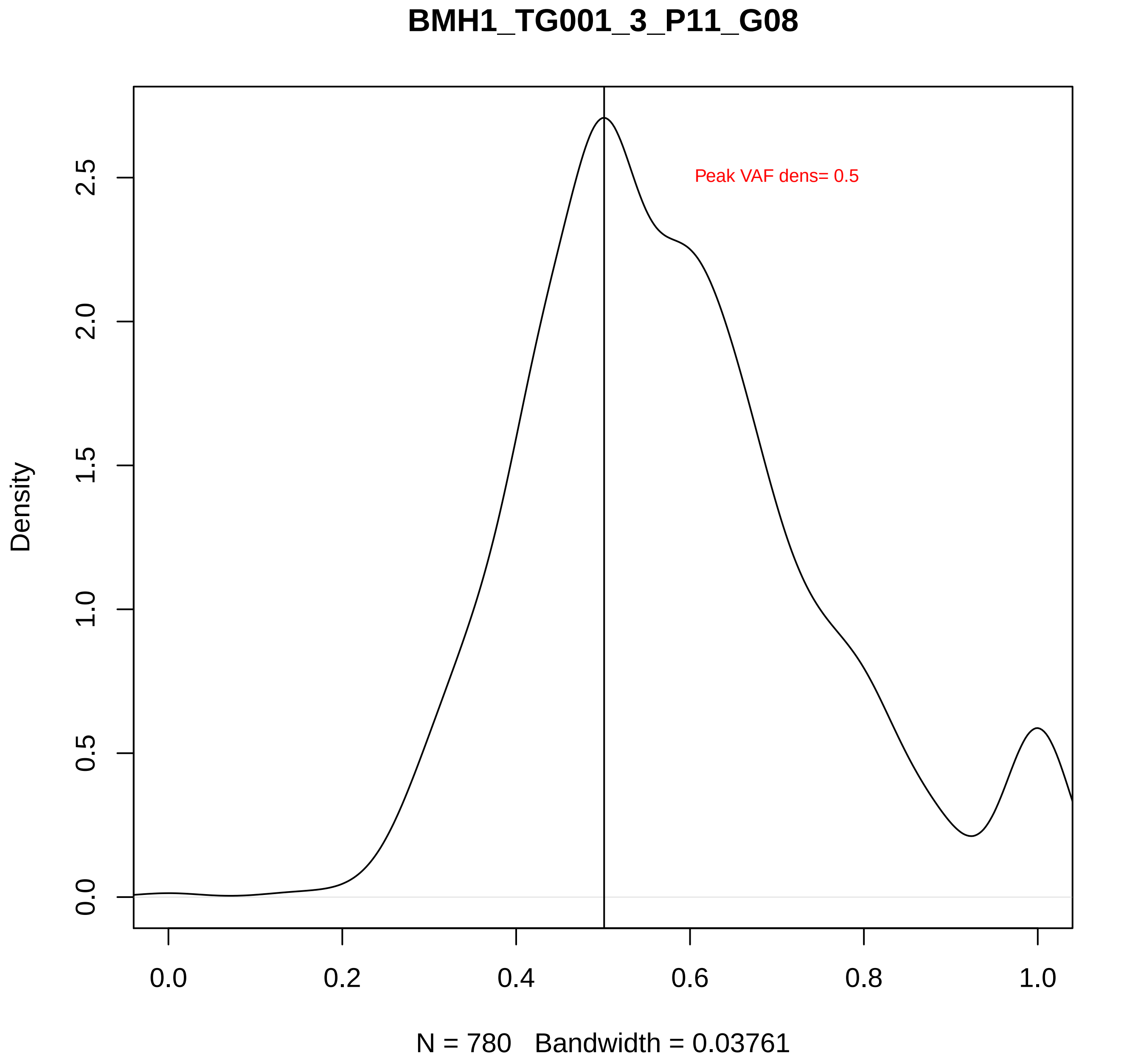

BMH1_TG001_3_P11_G08
Peak VAF dens= 0.5
Density
0.0
0.5
1.0
1.5
2.0
2.5
0.0
0.2
0.4
0.6
0.8
1.0
N = 780   Bandwidth = 0.03761

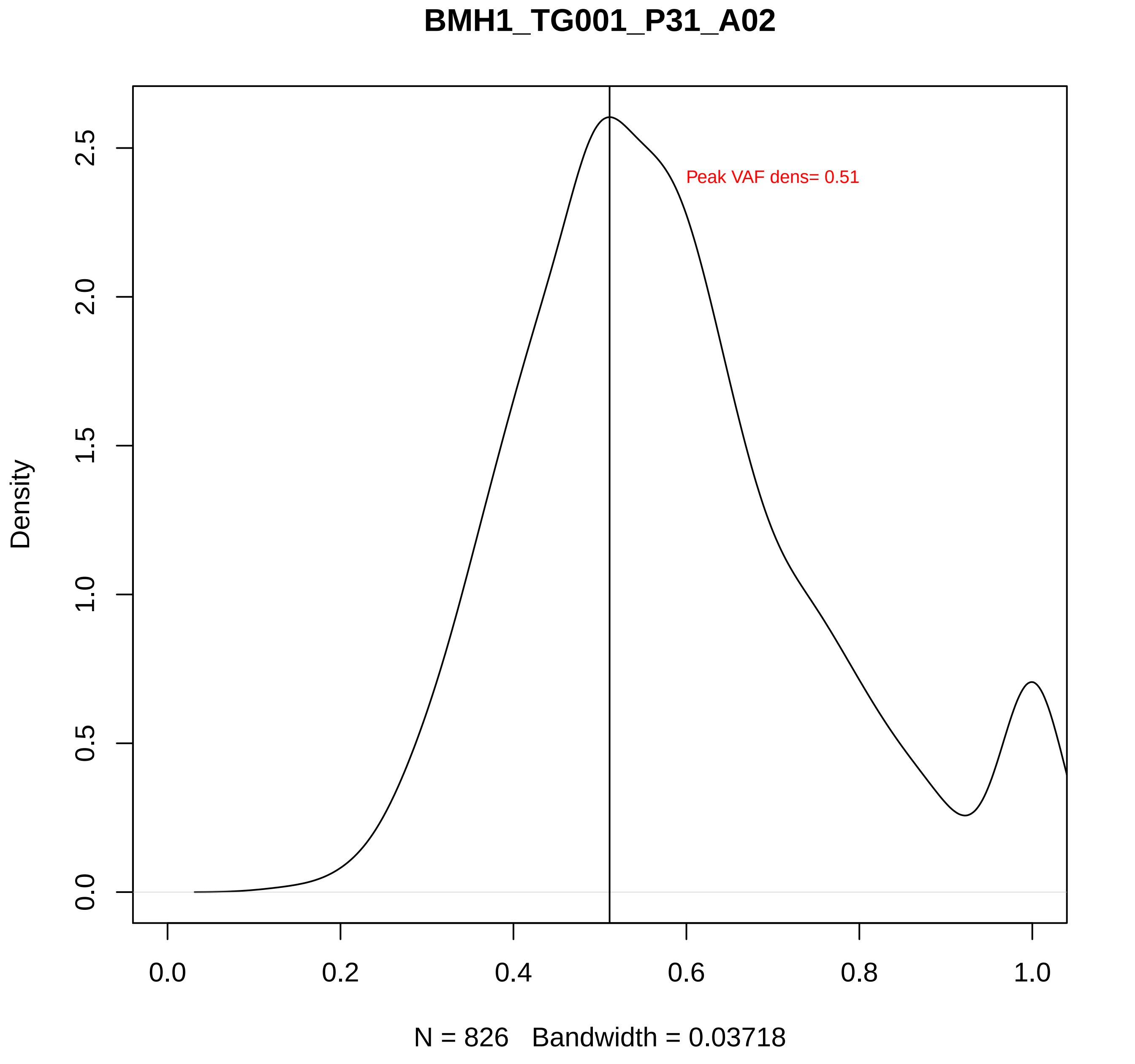
BMH1_TG001_P31_A02
Peak VAF dens= 0.51
Density
0.0
0.5
1.0
1.5
2.0
2.5
0.0
0.2
0.4
0.6
0.8
1.0
N = 826   Bandwidth = 0.03718

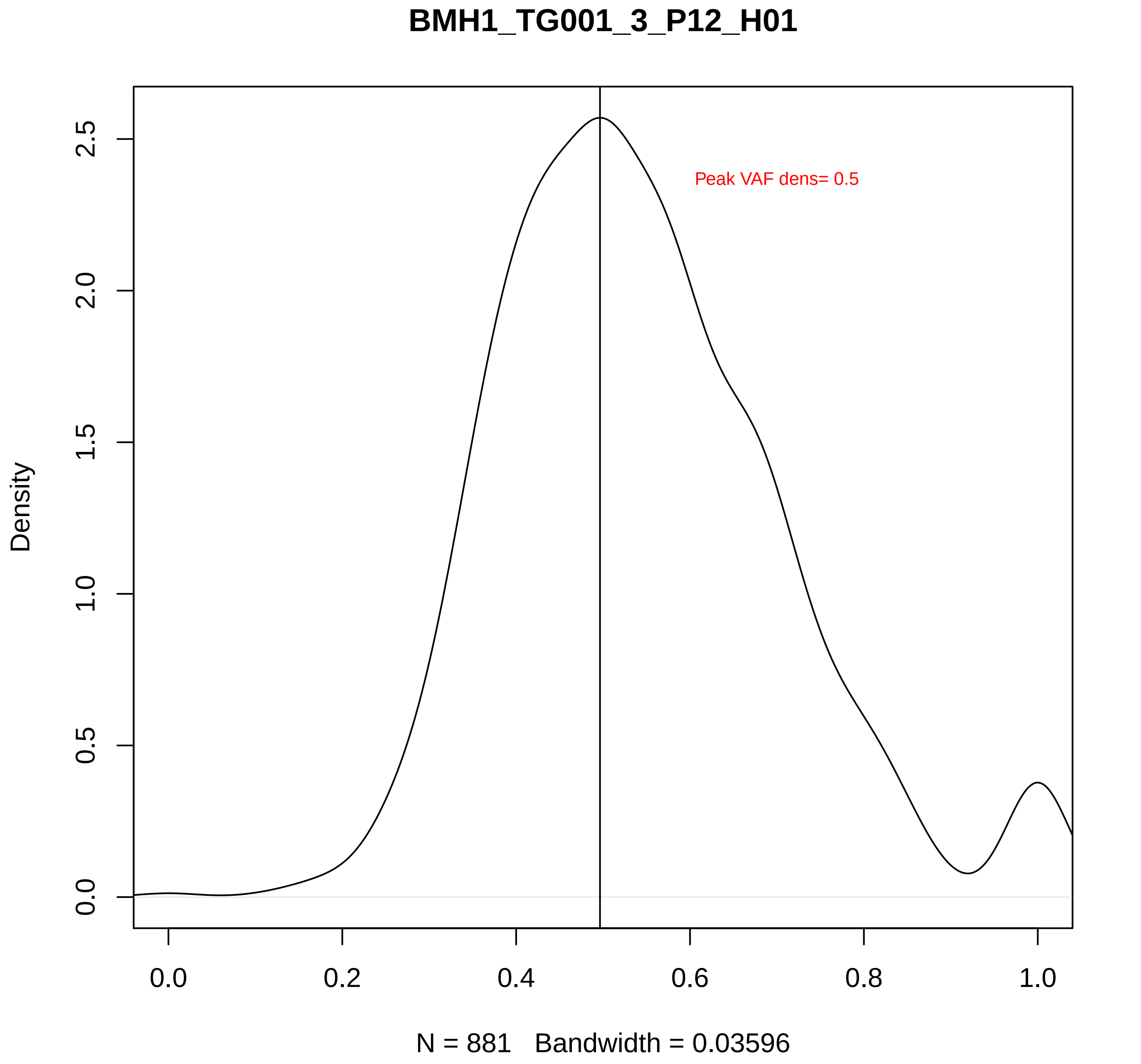
BMH1_TG001_3_P12_H01
Peak VAF dens= 0.5
Density
0.0
0.5
1.0
1.5
2.0
2.5
0.0
0.2
0.4
0.6
0.8
1.0
N = 881   Bandwidth = 0.03596

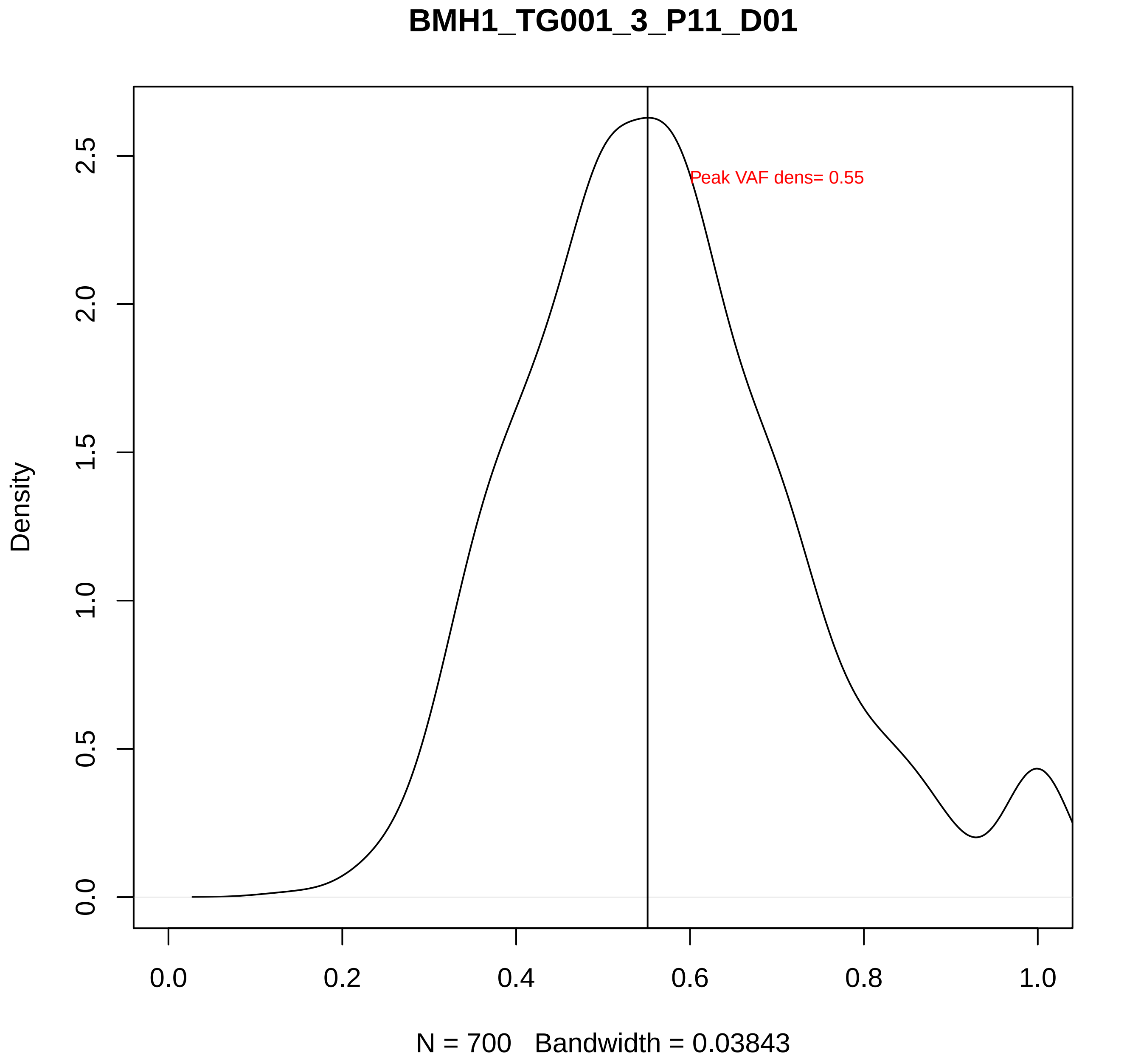

BMH1_TG001_3_P11_D01
Peak VAF dens= 0.55
Density
0.0
0.5
1.0
1.5
2.0
2.5
0.0
0.2
0.4
0.6
0.8
1.0
N = 700   Bandwidth = 0.03843

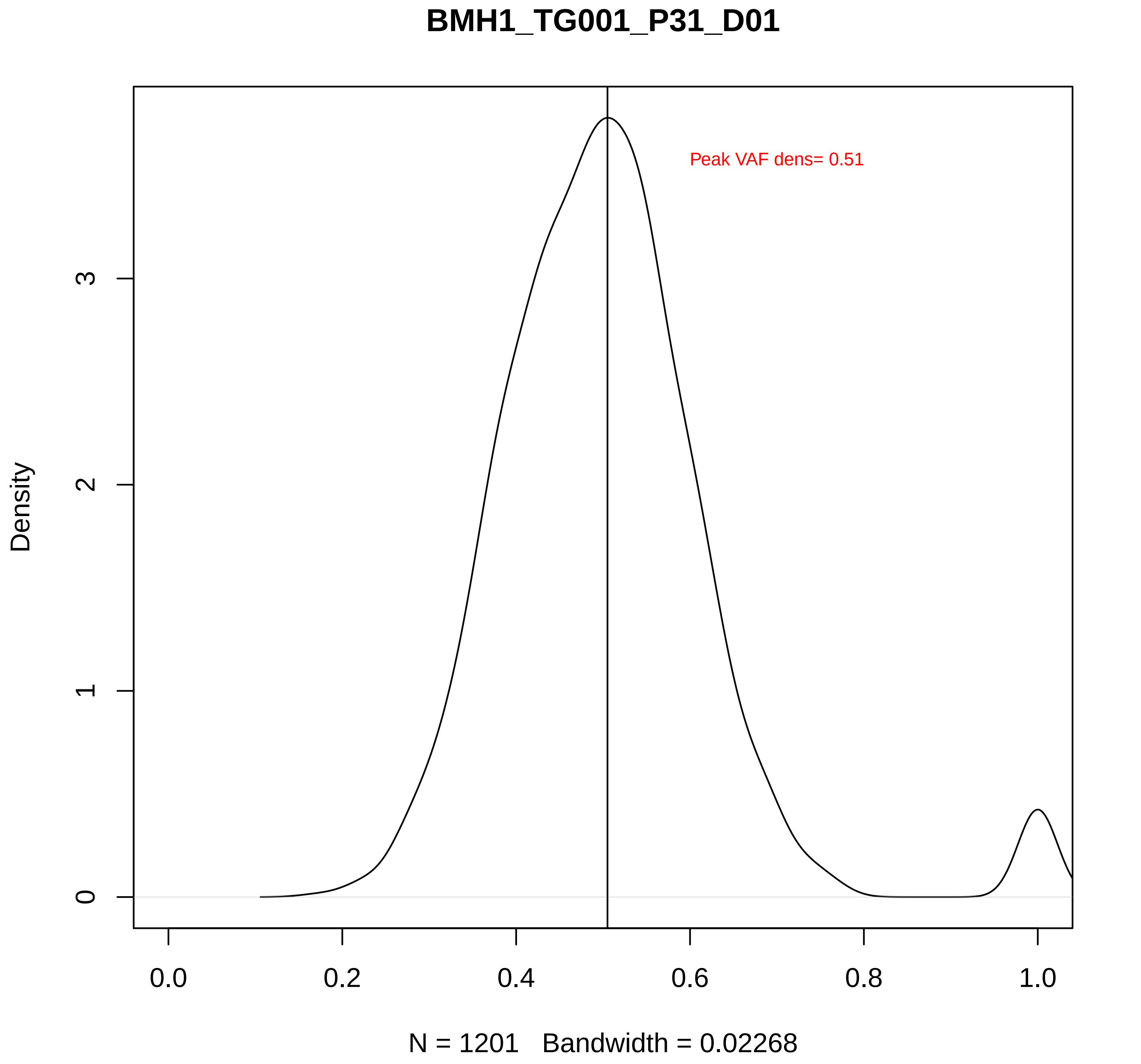

BMH1_TG001_P31_D01
Peak VAF dens= 0.51
Density
0
1
2
3
0.0
0.2
0.4
0.6
0.8
1.0
N = 1201   Bandwidth = 0.02268

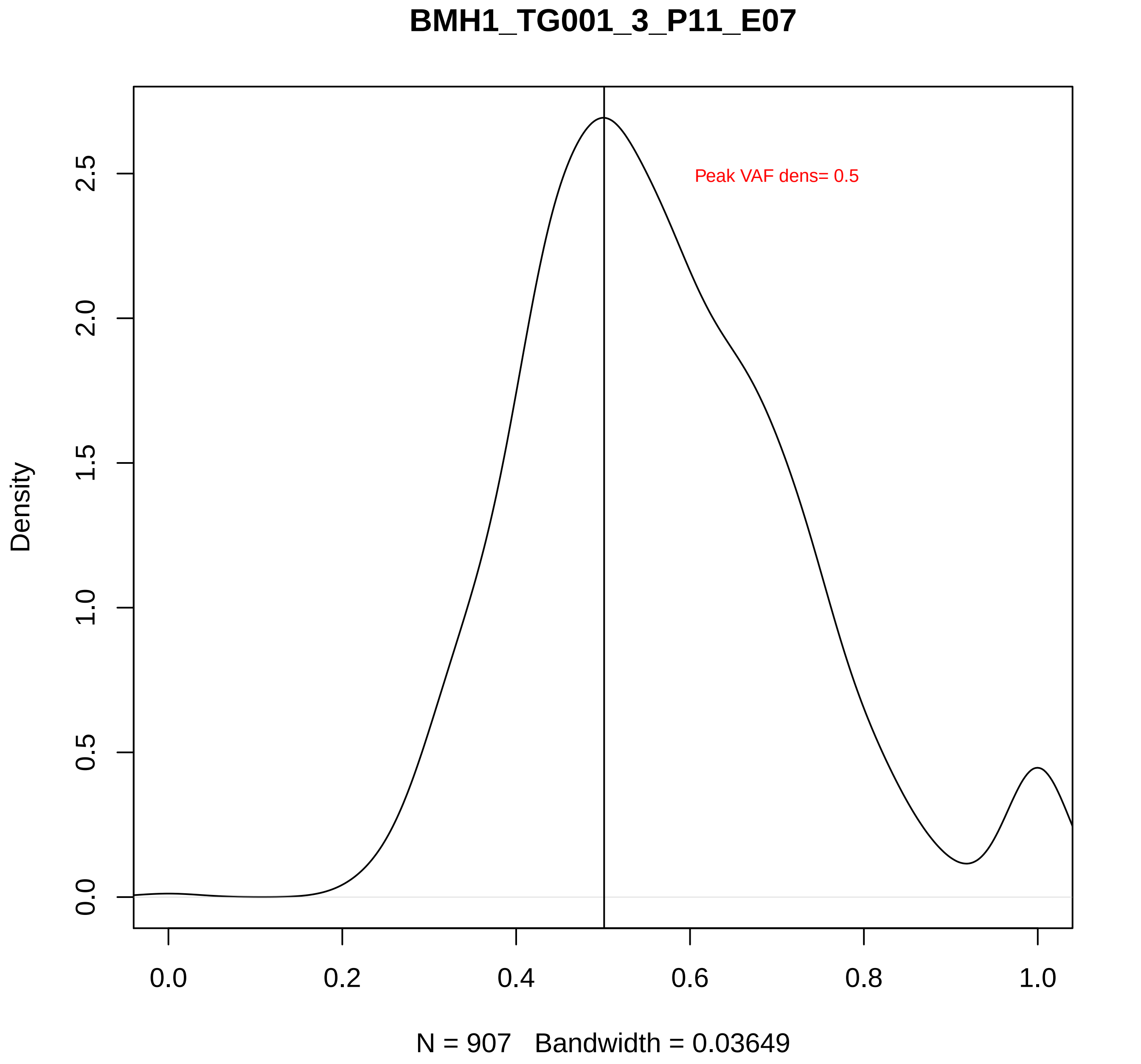
BMH1_TG001_3_P11_E07
Peak VAF dens= 0.5
Density
0.0
0.5
1.0
1.5
2.0
2.5
0.0
0.2
0.4
0.6
0.8
1.0
N = 907 Bandwidth = 0.03649

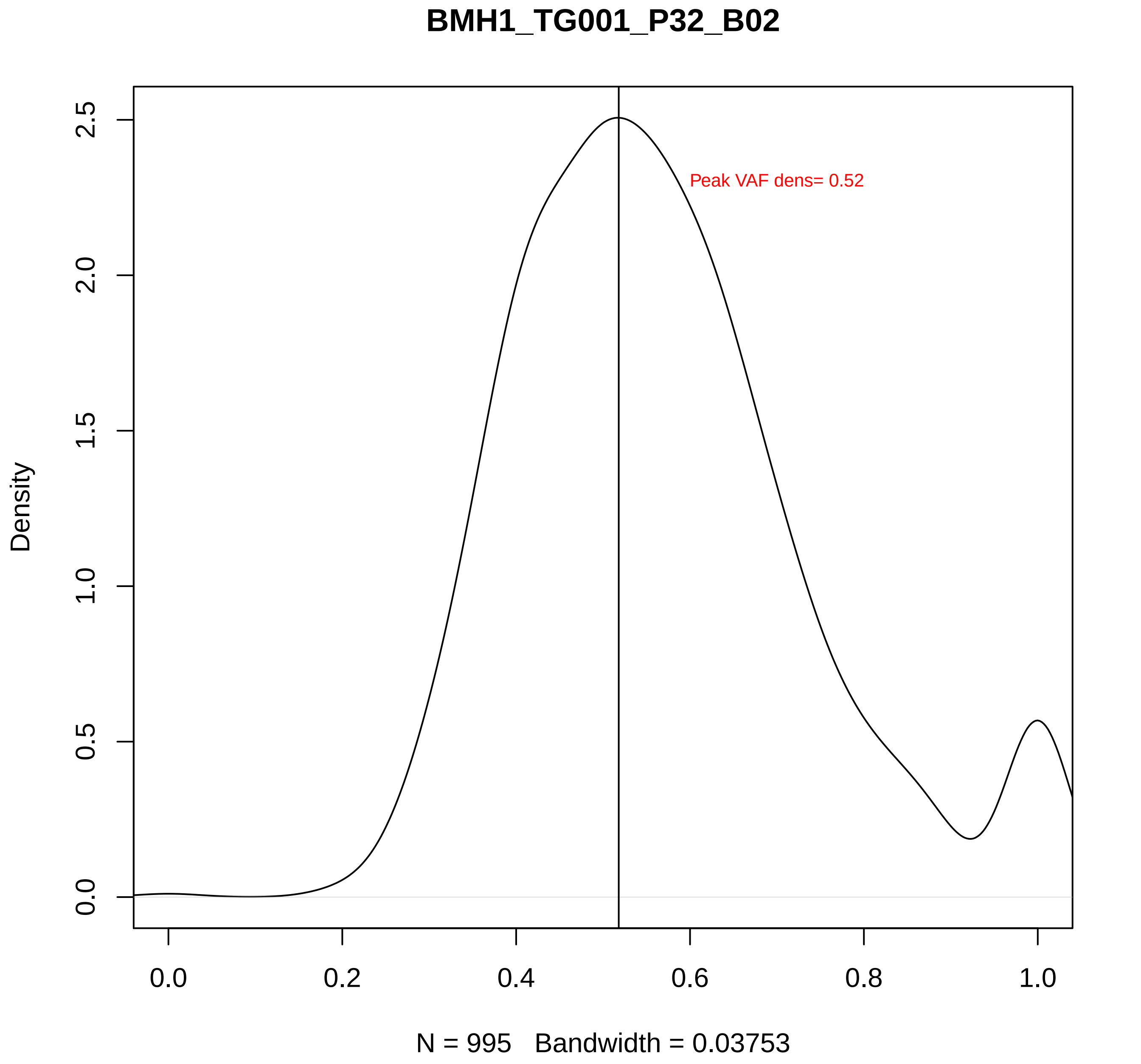

BMH1_TG001_P32_B02
Peak VAF dens= 0.52
Density
2.5
2.0
1.5
1.0
0.5
0.0
0.0
0.2
0.4
0.6
0.8
1.0
N = 995 Bandwidth = 0.03753

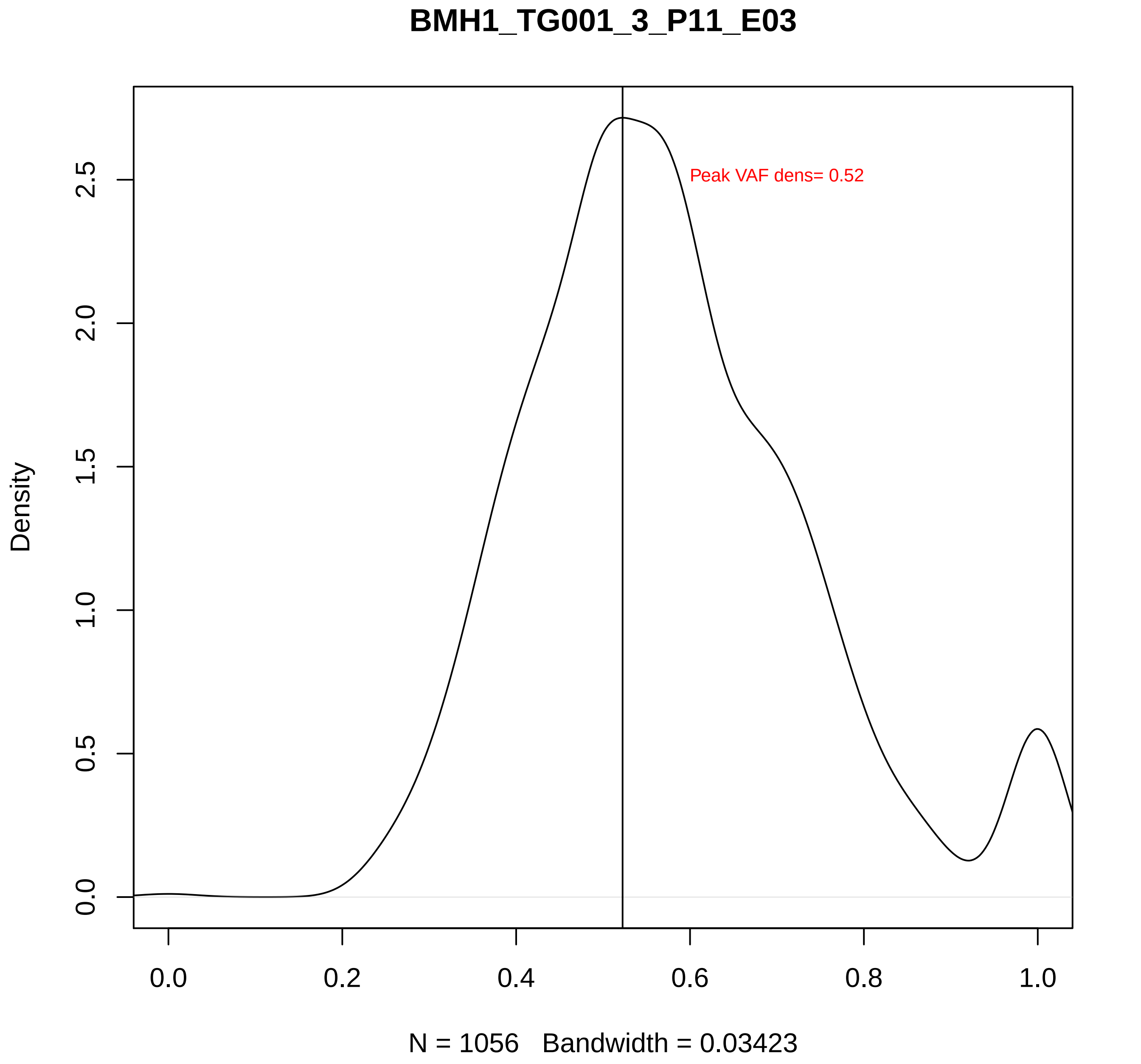

BMH1_TG001_3_P11_E03
Peak VAF dens= 0.52
Density
0.0
0.5
1.0
1.5
2.0
2.5
0.0
0.2
0.4
0.6
0.8
1.0
N = 1056   Bandwidth = 0.03423

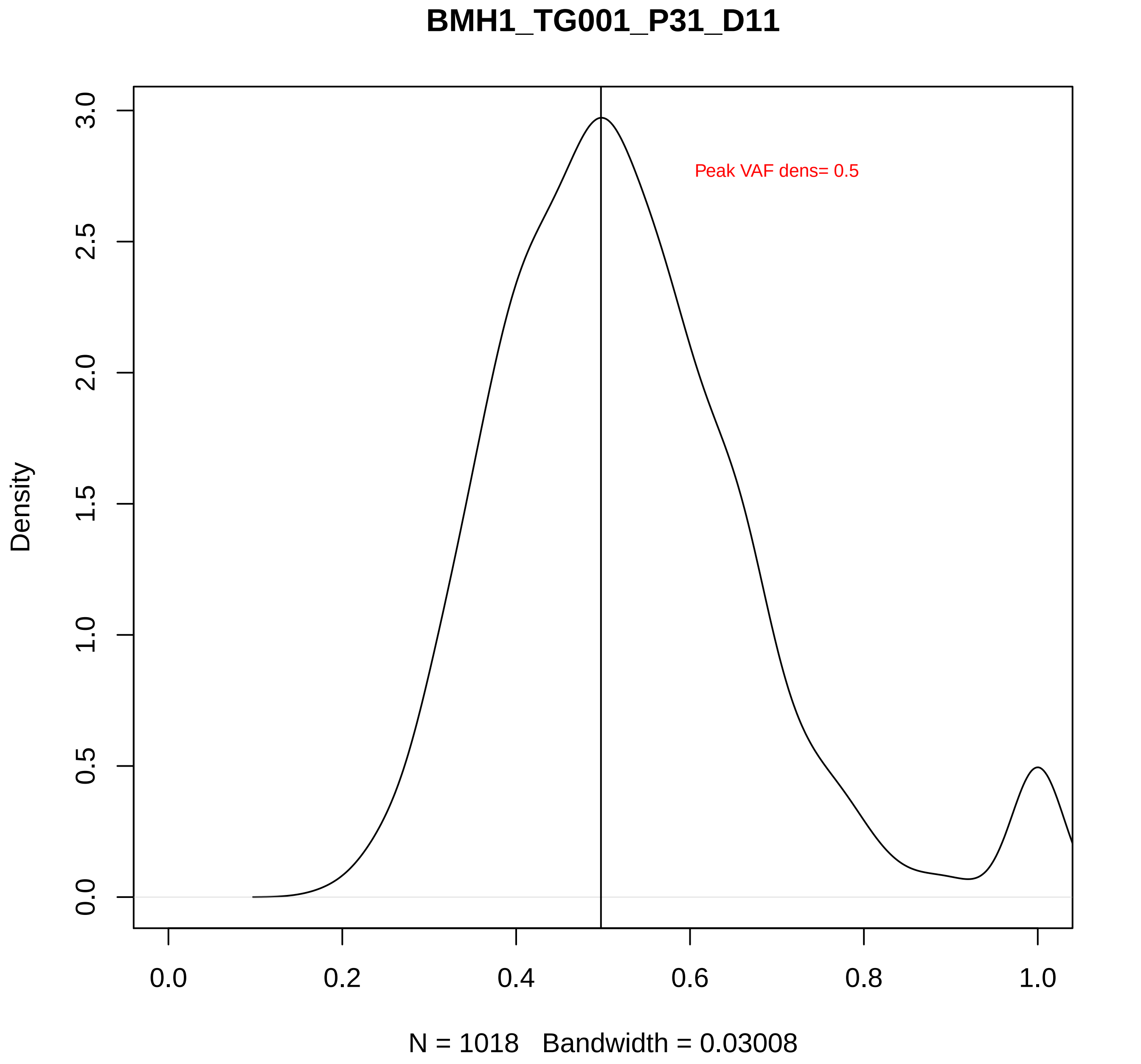
BMH1_TG001_P31_D11
Peak VAF dens= 0.5
Density
0.0
0.5
1.0
1.5
2.0
2.5
3.0
0.0
0.2
0.4
0.6
0.8
1.0
N = 1018   Bandwidth = 0.03008

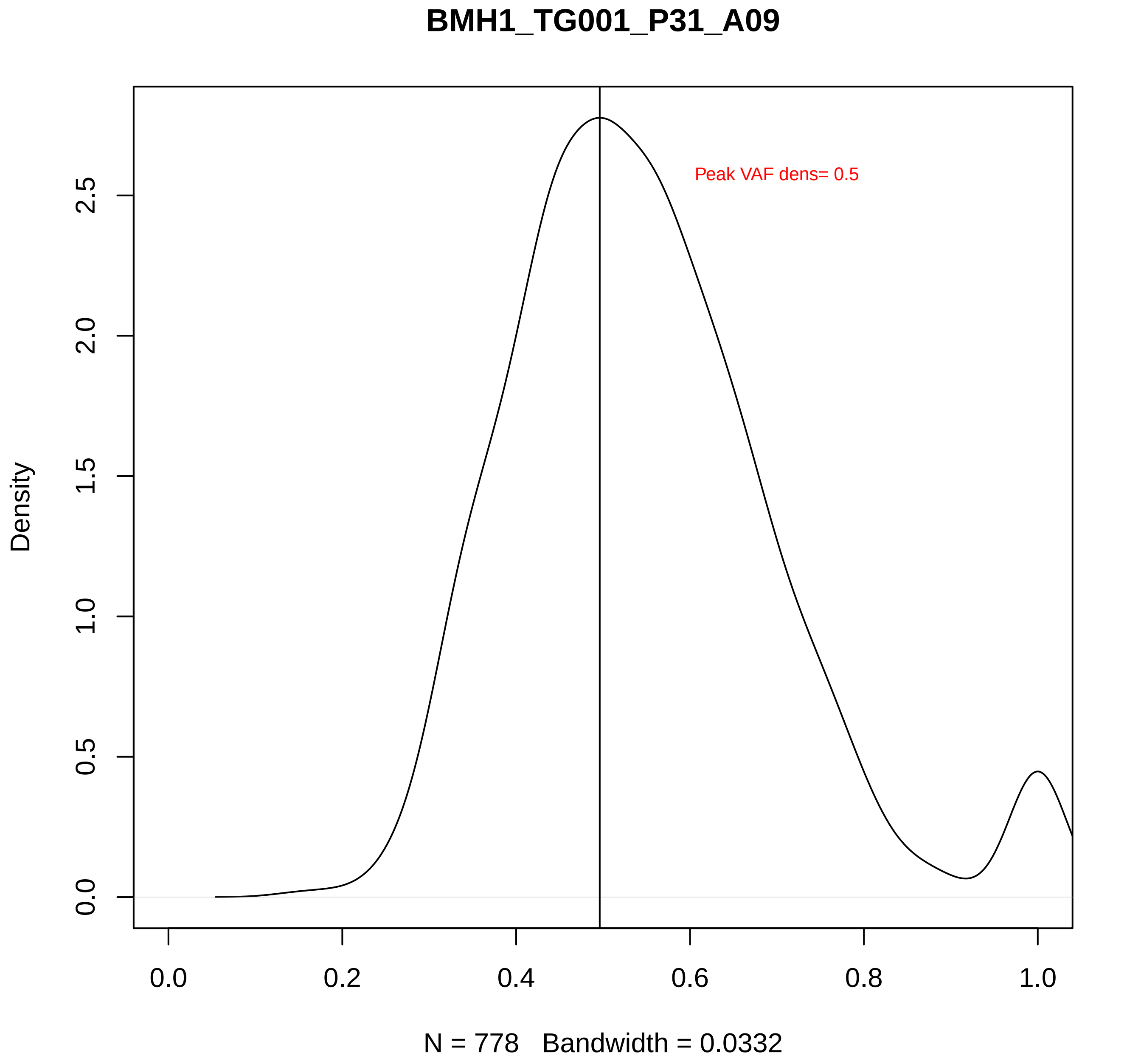
BMH1_TG001_P31_A09
Peak VAF dens= 0.5
Density
0.0
0.5
1.0
1.5
2.0
2.5
0.0
0.2
0.4
0.6
0.8
1.0
N = 778   Bandwidth = 0.0332

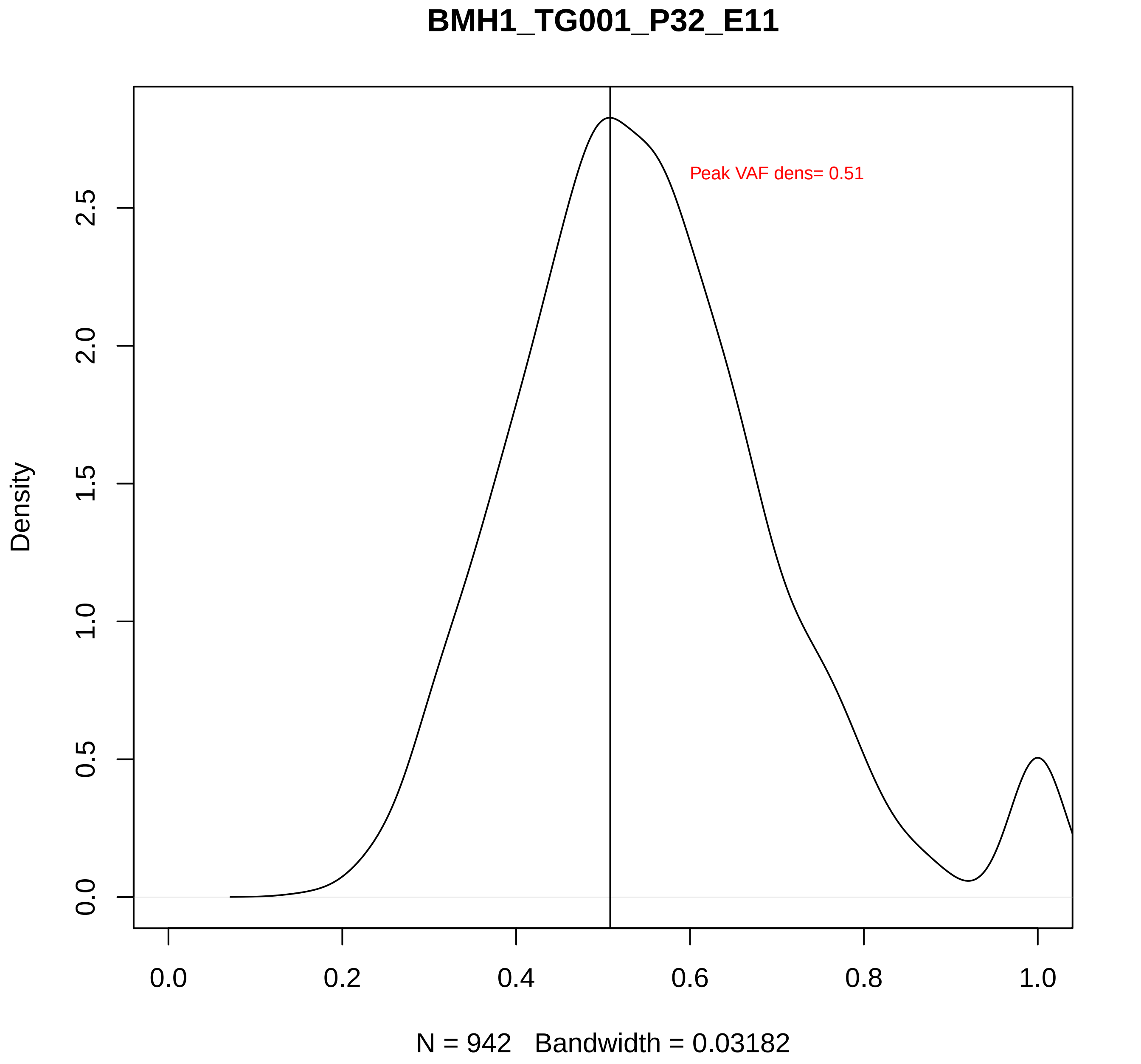

BMH1_TG001_P32_E11
Peak VAF dens= 0.51
Density
0.0
0.5
1.0
1.5
2.0
2.5
0.0
0.2
0.4
0.6
0.8
1.0
N = 942   Bandwidth = 0.03182

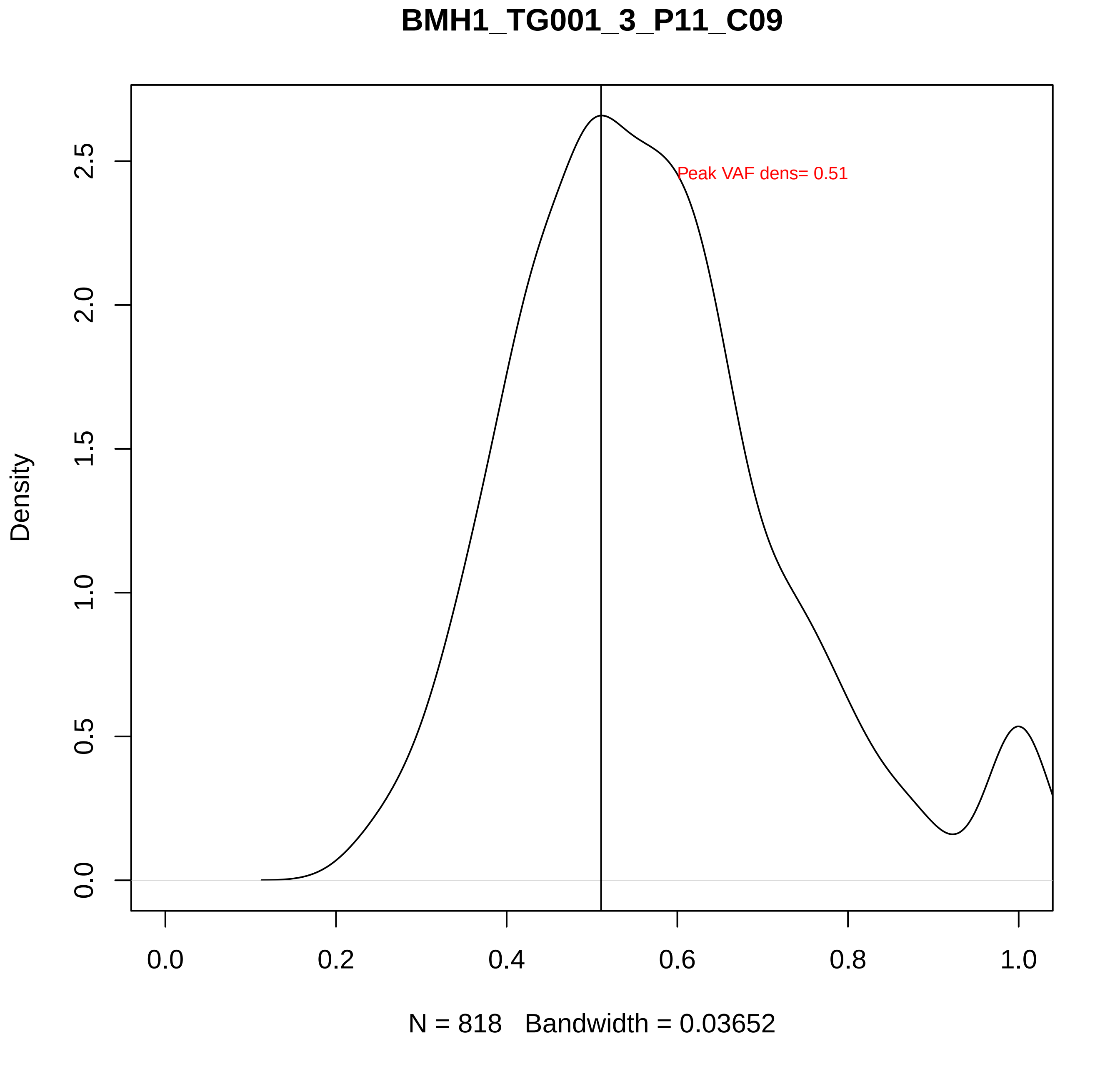
BMH1_TG001_3_P11_C09
Peak VAF dens= 0.51
Density
0.0
0.5
1.0
1.5
2.0
2.5
0.0
0.2
0.4
0.6
0.8
1.0
N = 818 Bandwidth = 0.03652

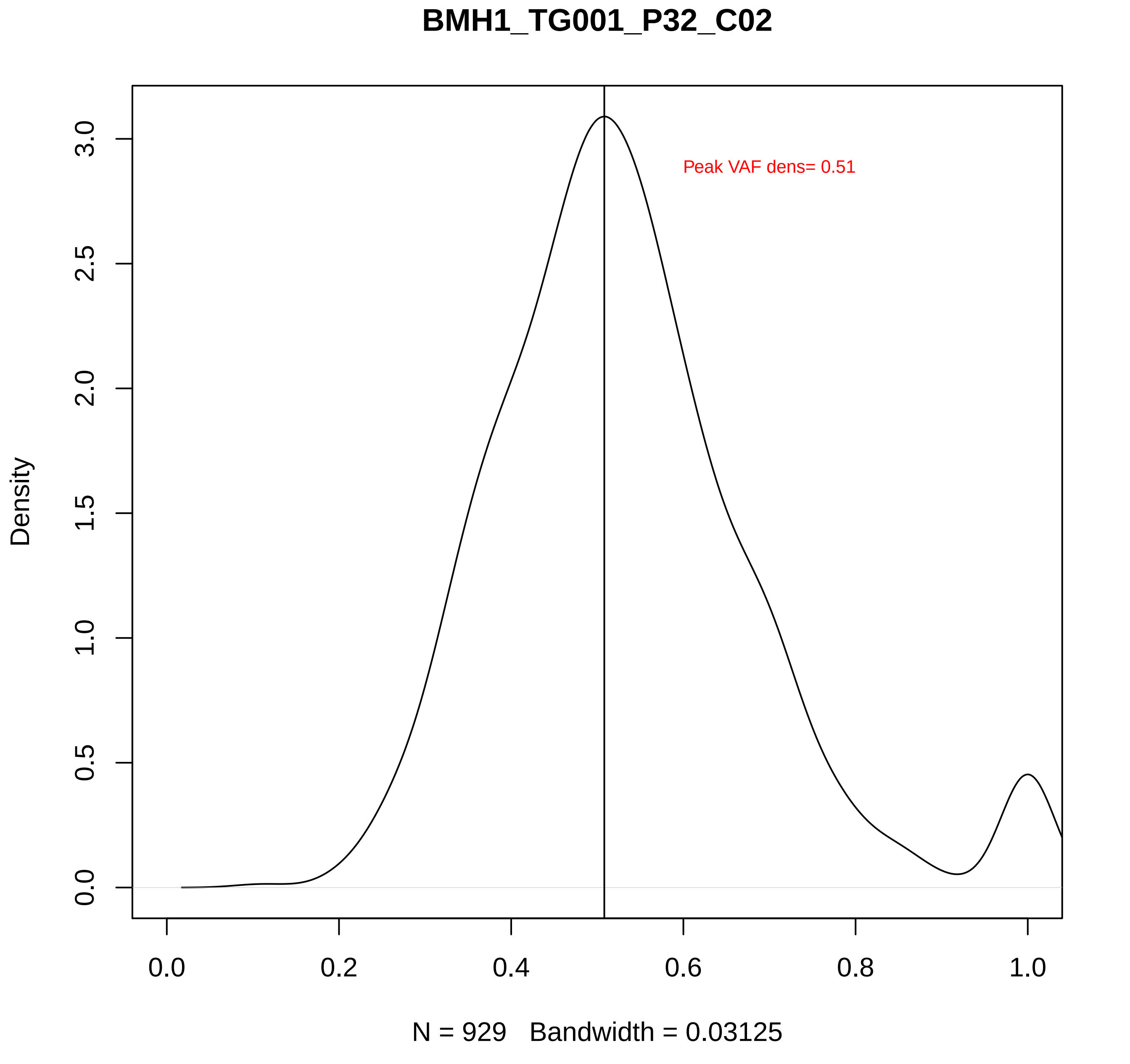
BMH1_TG001_P32_C02
Peak VAF dens= 0.51
Density
0.0
0.5
1.0
1.5
2.0
2.5
3.0
0.0
0.2
0.4
0.6
0.8
1.0
N = 929   Bandwidth = 0.03125

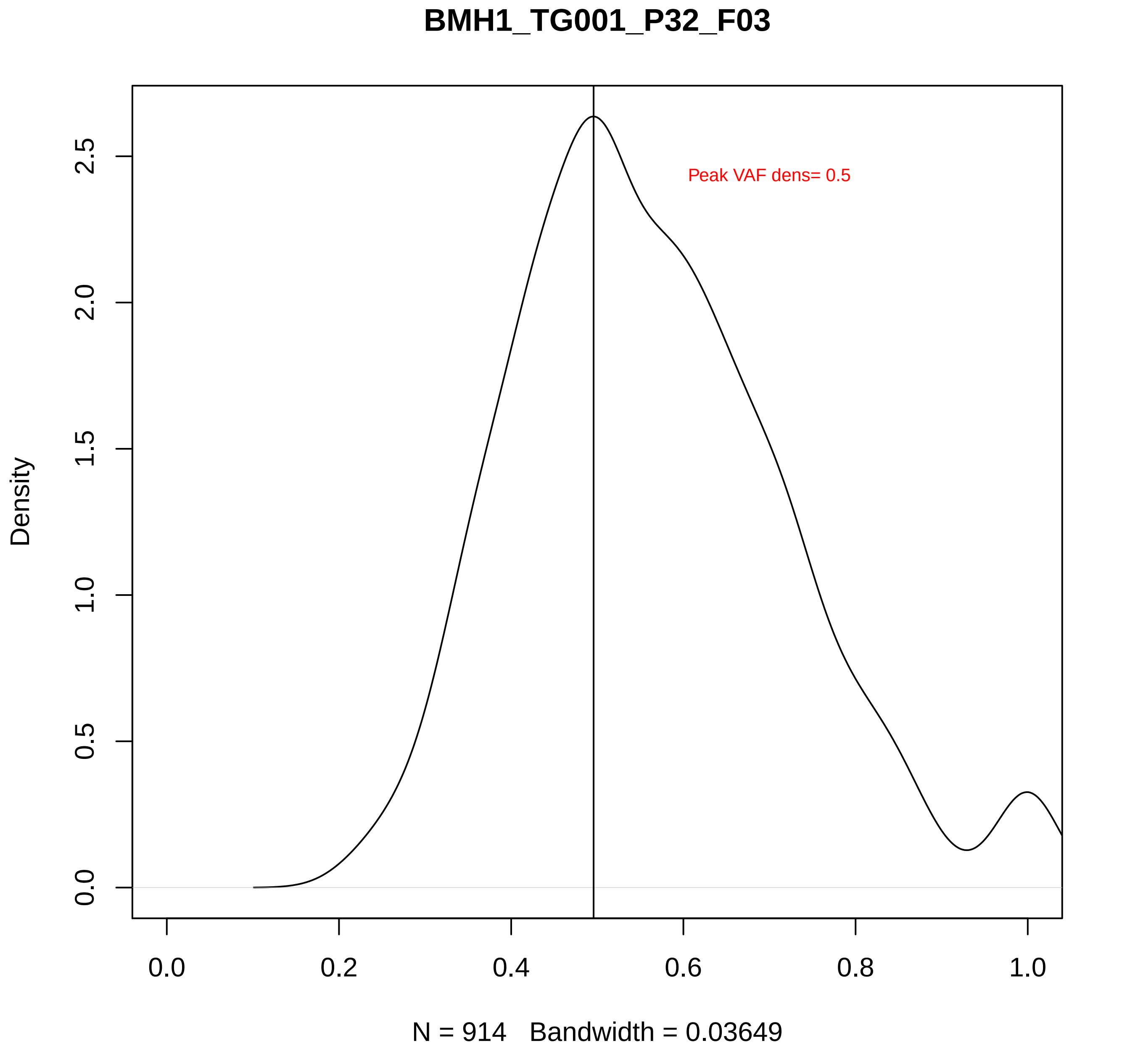
BMH1_TG001_P32_F03
Peak VAF dens= 0.5
Density
0.0
0.5
1.0
1.5
2.0
2.5
0.0
0.2
0.4
0.6
0.8
1.0
N = 914   Bandwidth = 0.03649

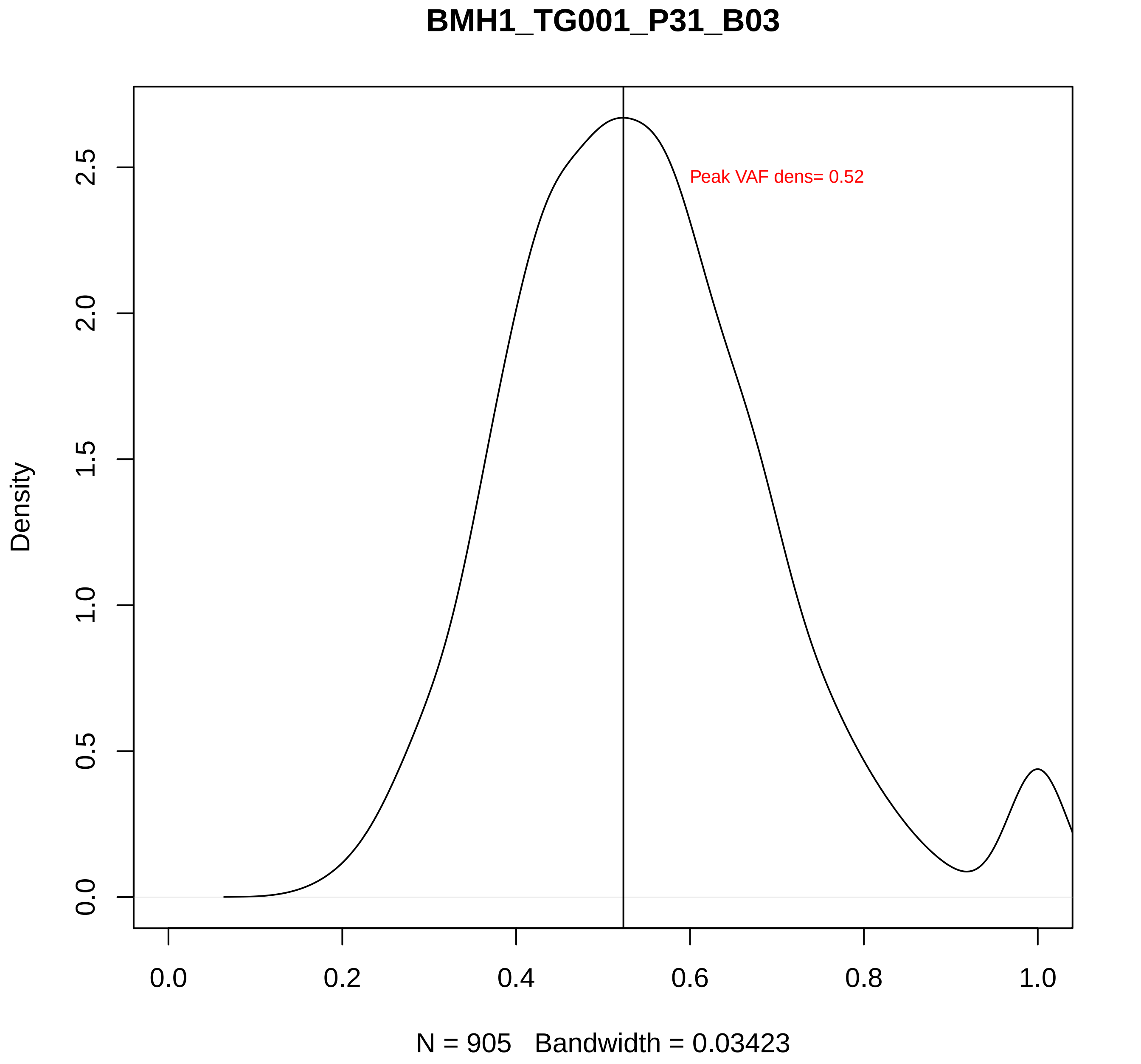
BMH1_TG001_P31_B03
Peak VAF dens= 0.52
Density
0.0
0.5
1.0
1.5
2.0
2.5
0.0
0.2
0.4
0.6
0.8
1.0
N = 905   Bandwidth = 0.03423

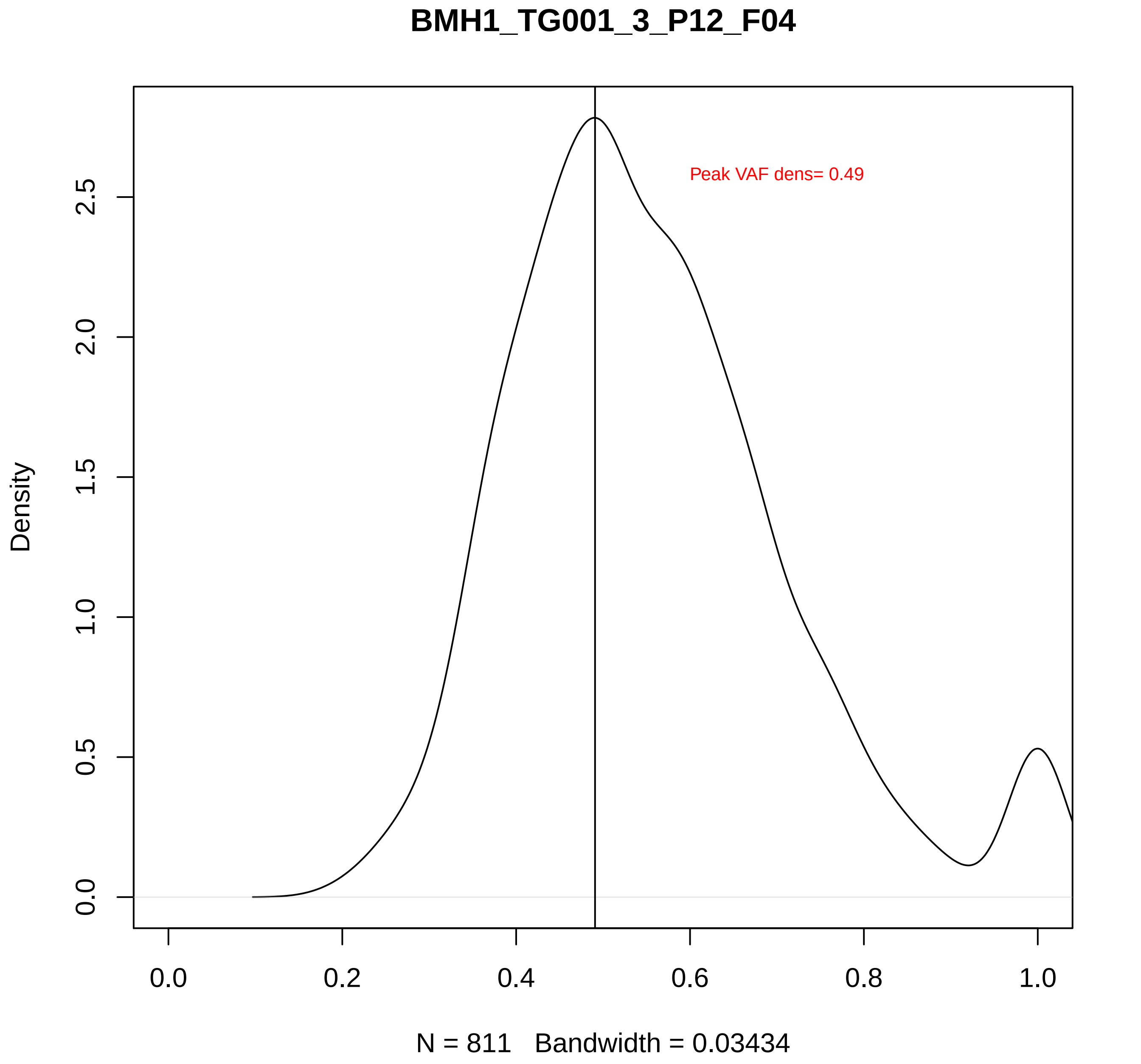
BMH1_TG001_3_P12_F04
Peak VAF dens= 0.49
Density
0.0
0.5
1.0
1.5
2.0
2.5
0.0
0.2
0.4
0.6
0.8
1.0
N = 811   Bandwidth = 0.03434

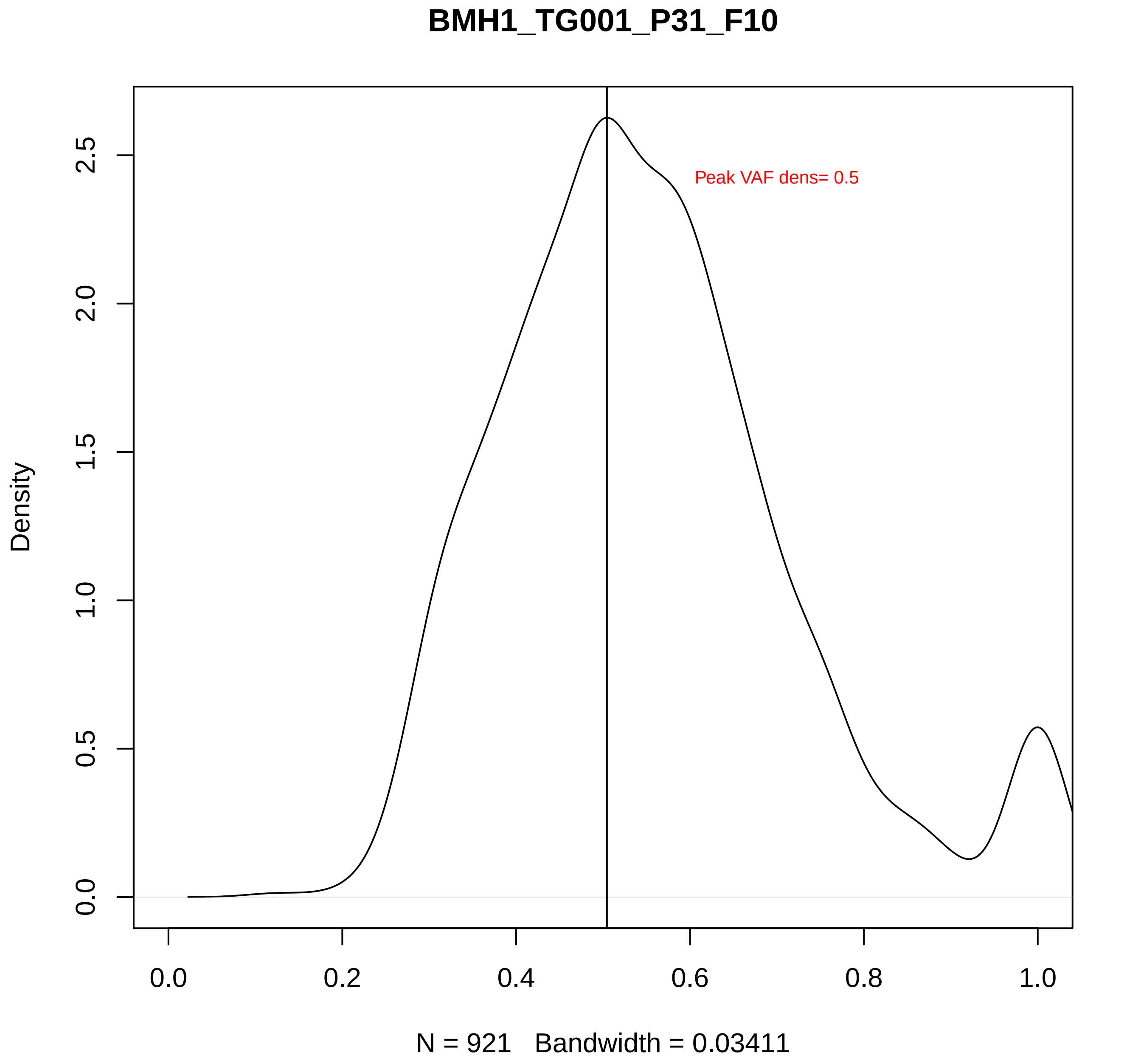
BMH1_TG001_P31_F10
Peak VAF dens= 0.5
Density
0.0
0.5
1.0
1.5
2.0
2.5
0.0
0.2
0.4
0.6
0.8
1.0
N = 921 Bandwidth = 0.03411

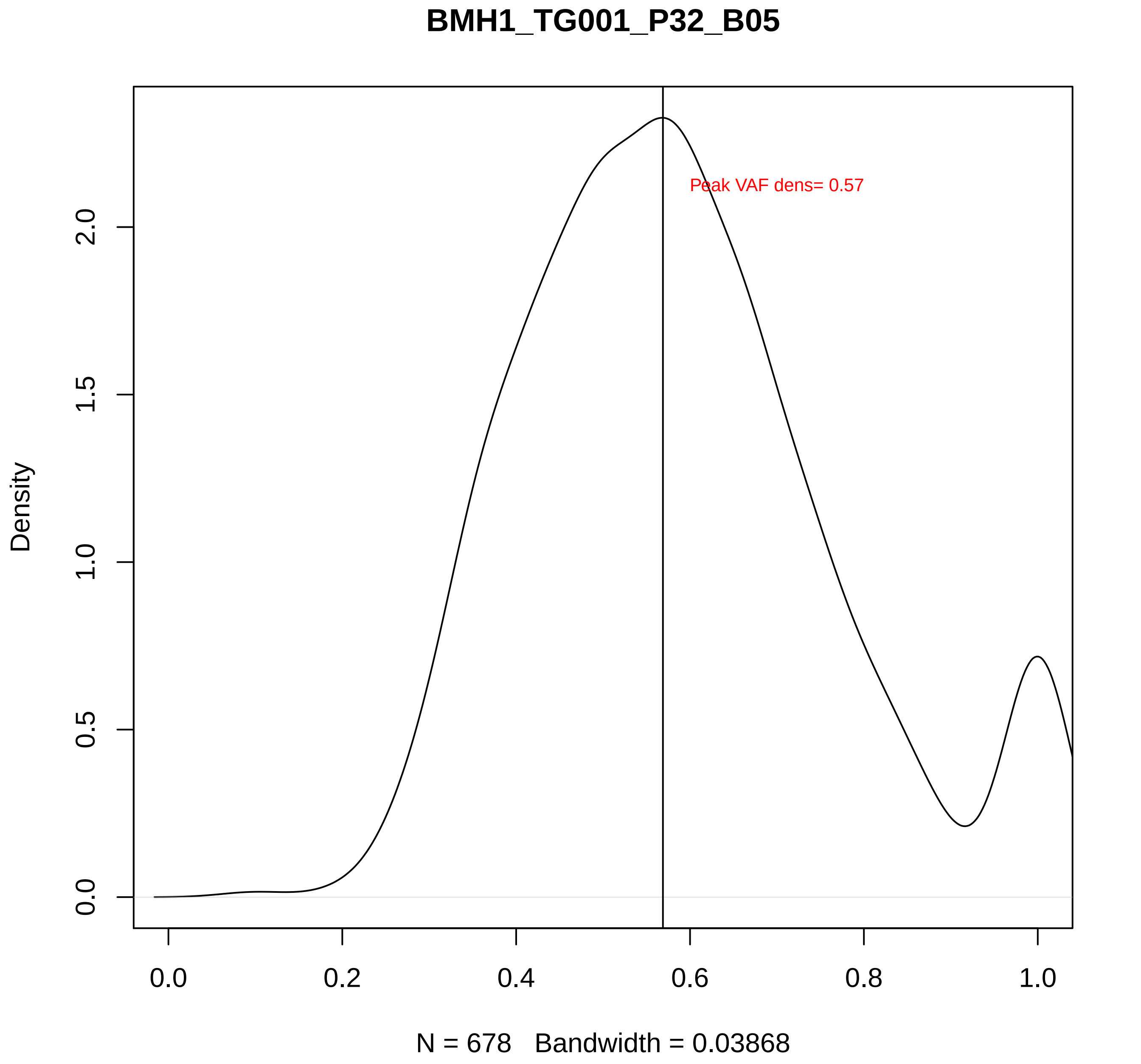

BMH1_TG001_P32_B05
Peak VAF dens= 0.57
Density
0.0
0.5
1.0
1.5
2.0
0.0
0.2
0.4
0.6
0.8
1.0
N = 678   Bandwidth = 0.03868

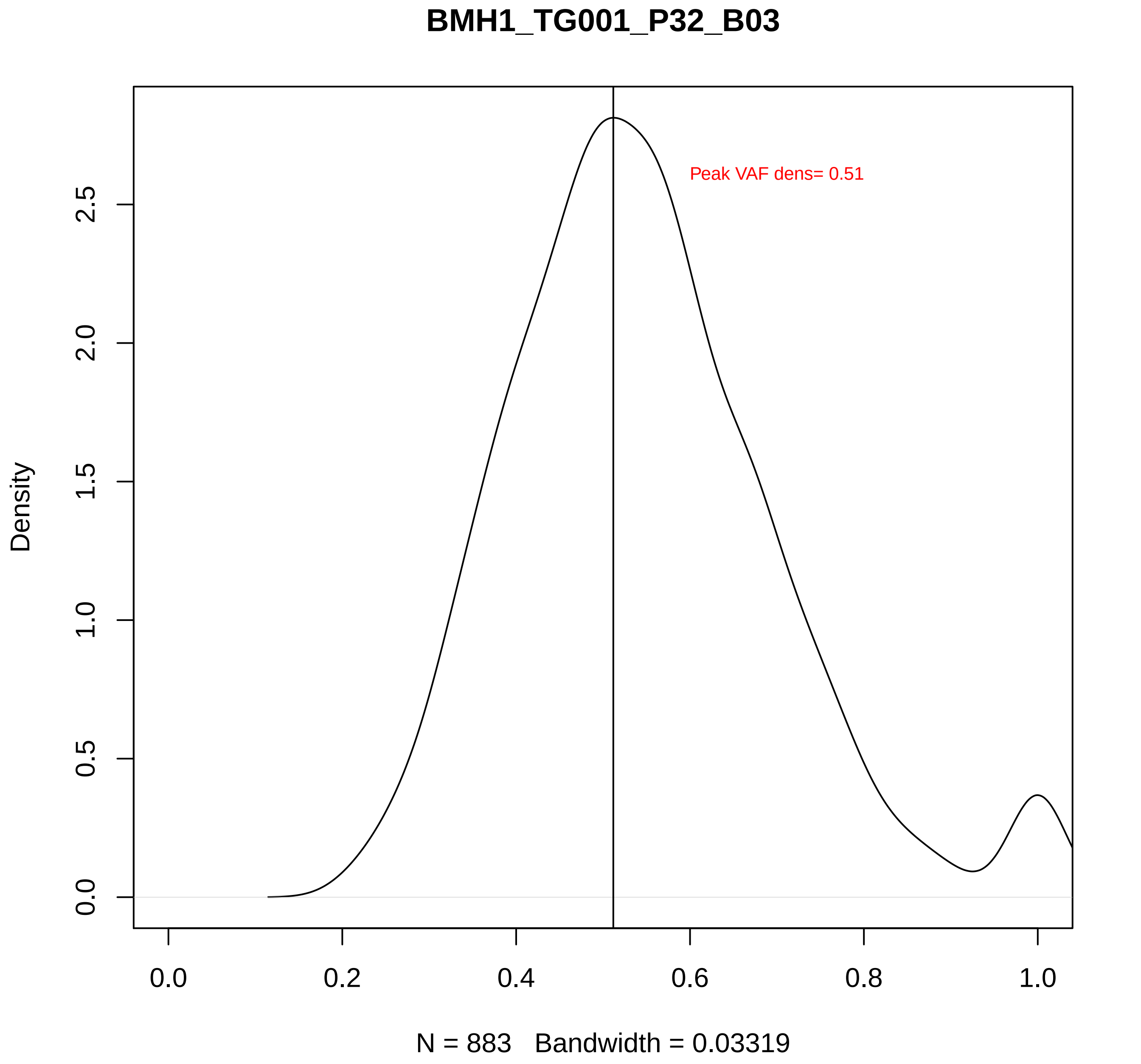

BMH1_TG001_P32_B03
Peak VAF dens= 0.51
Density
0.0
0.5
1.0
1.5
2.0
2.5
0.0
0.2
0.4
0.6
0.8
1.0
N = 883 Bandwidth = 0.03319

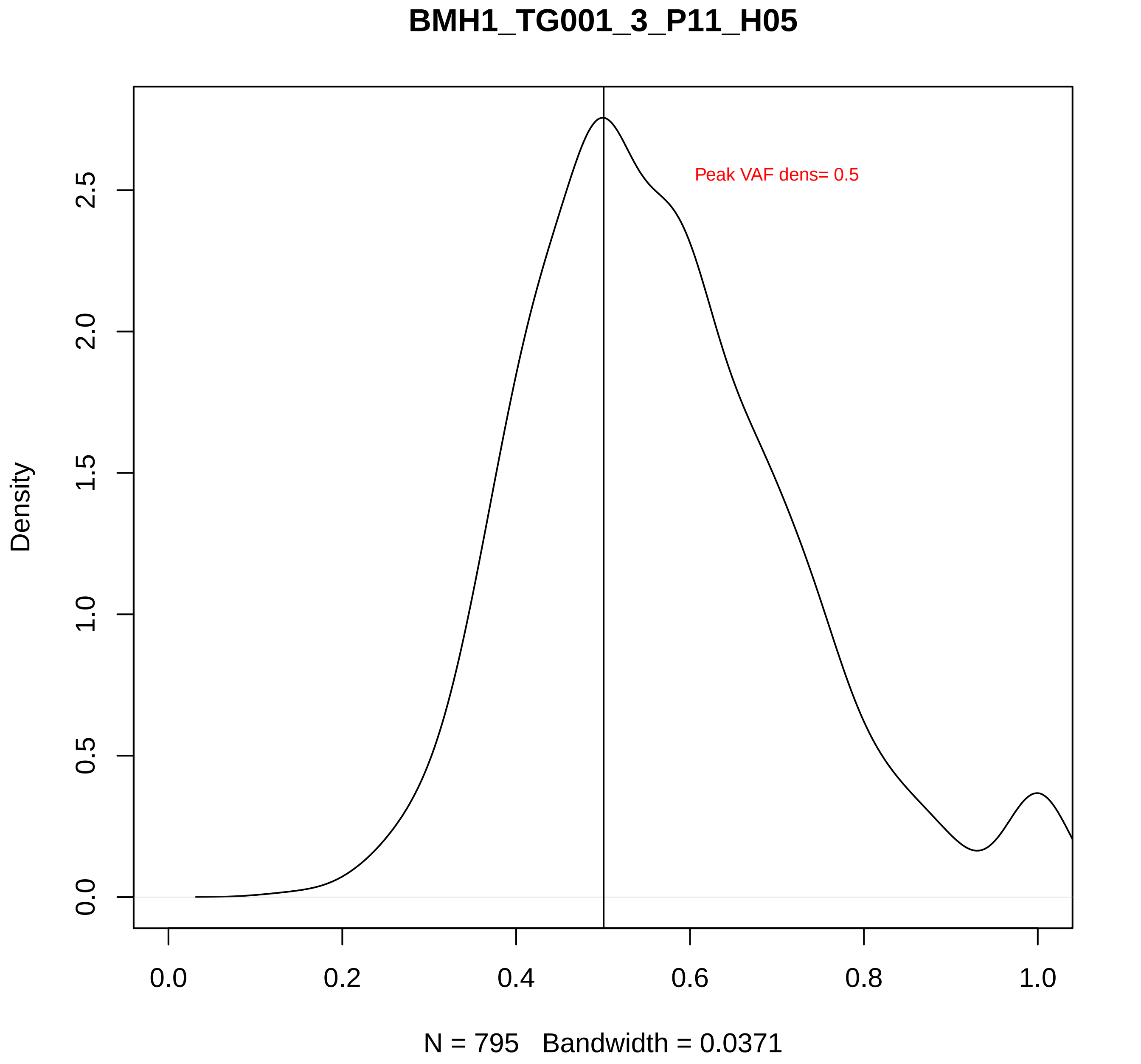

BMH1_TG001_3_P11_H05
Peak VAF dens= 0.5
Density
0.0
0.5
1.0
1.5
2.0
2.5
0.0
0.2
0.4
0.6
0.8
1.0
N = 795   Bandwidth = 0.0371

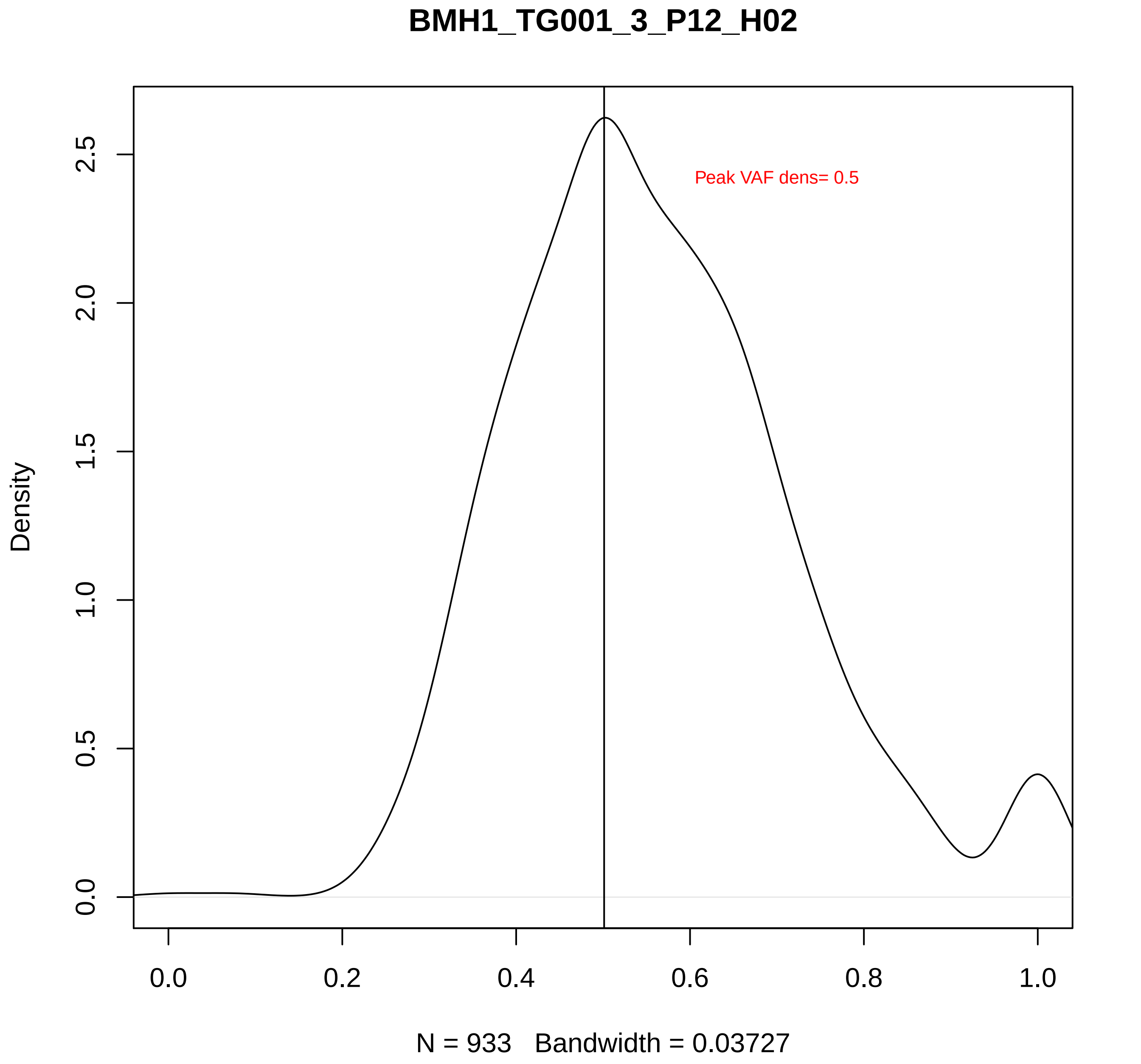
BMH1_TG001_3_P12_H02
Peak VAF dens= 0.5
Density
0.0
0.5
1.0
1.5
2.0
2.5
0.0
0.2
0.4
0.6
0.8
1.0
N = 933   Bandwidth = 0.03727

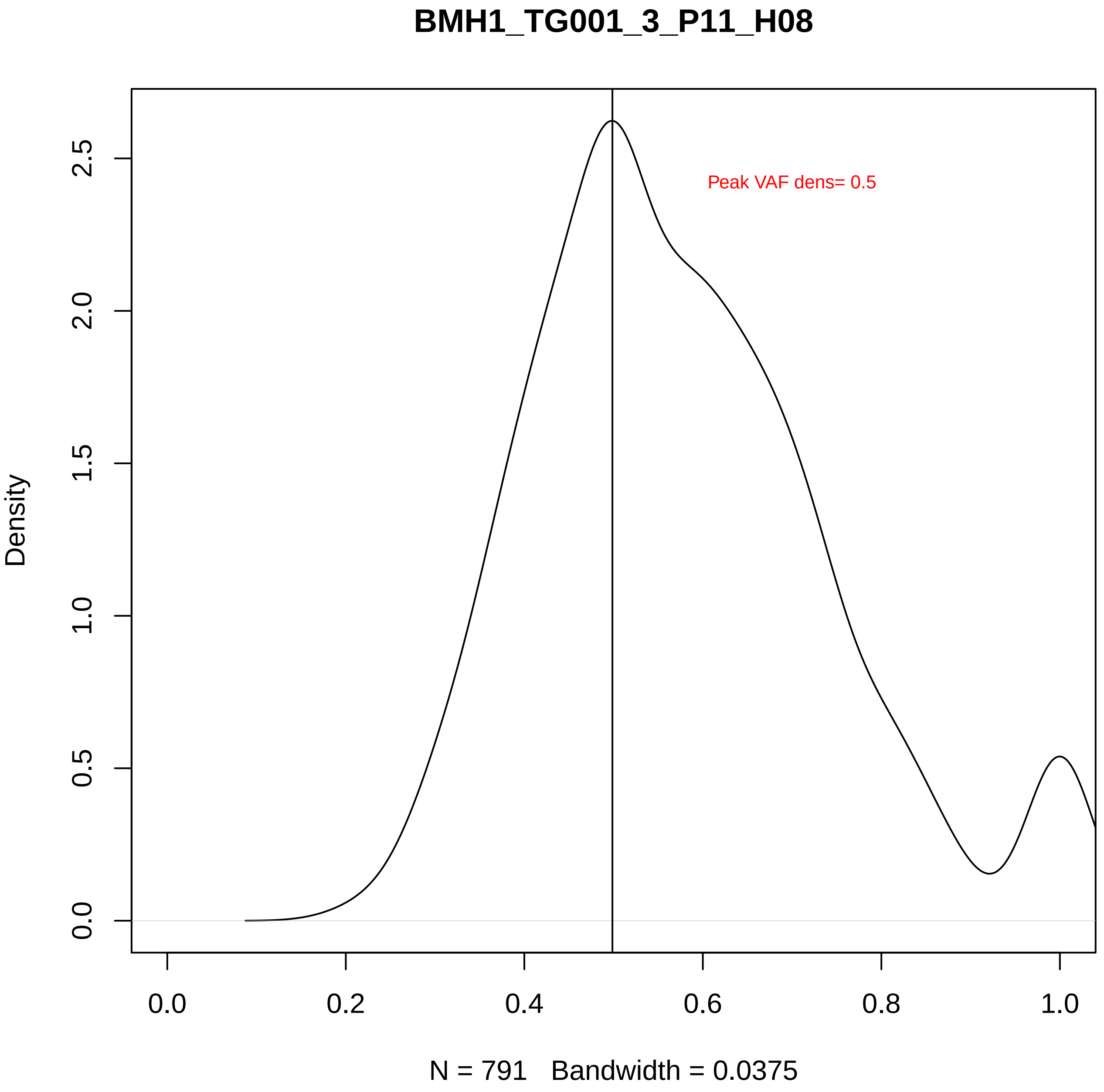
BMH1_TG001_3_P11_H08
Peak VAF dens= 0.5
Density
0.0
0.5
1.0
1.5
2.0
2.5
0.0
0.2
0.4
0.6
0.8
1.0
N = 791 Bandwidth = 0.0375

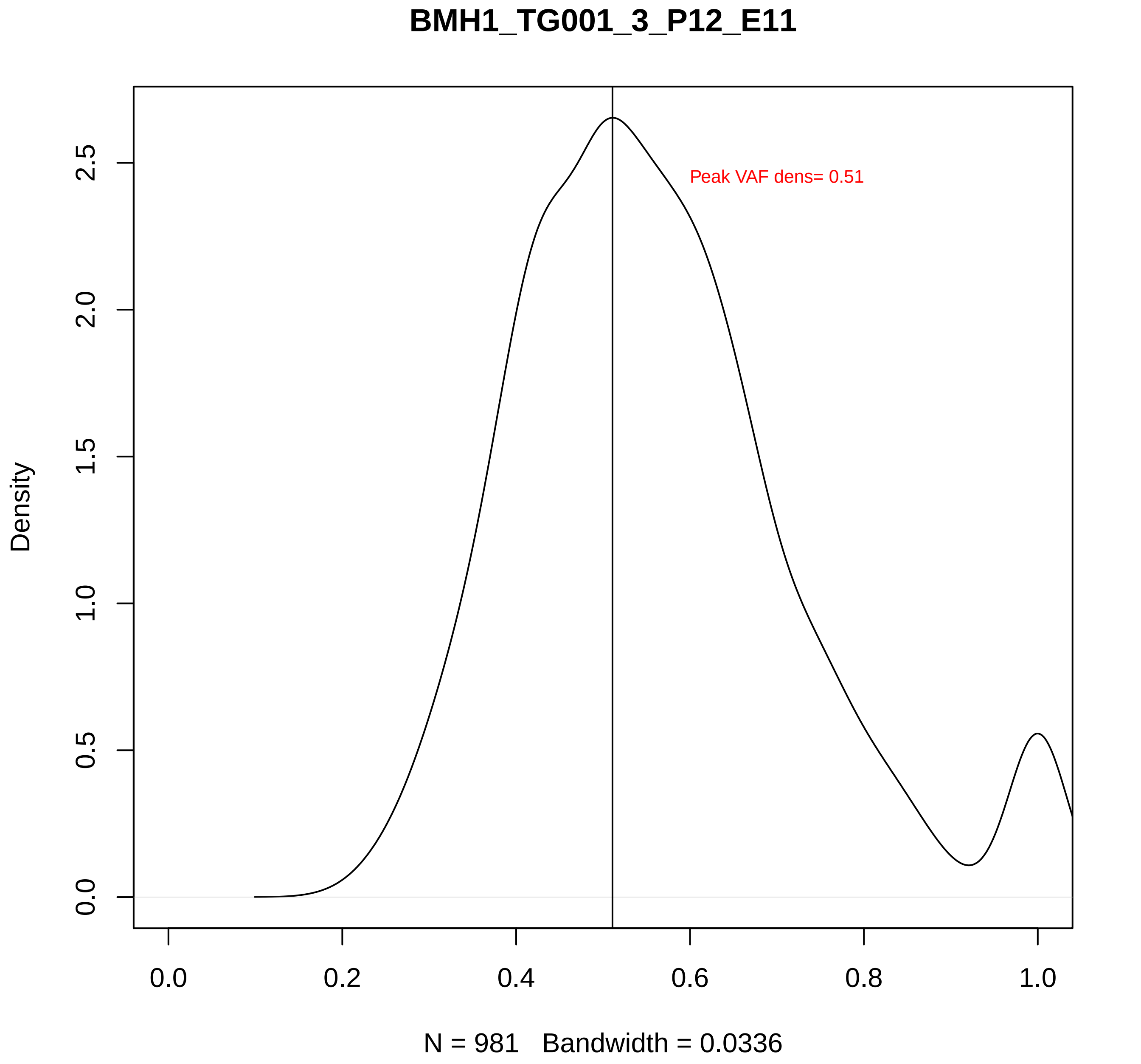
BMH1_TG001_3_P12_E11
Peak VAF dens= 0.51
Density
0.0
0.5
1.0
1.5
2.0
2.5
0.0
0.2
0.4
0.6
0.8
1.0
N = 981   Bandwidth = 0.0336

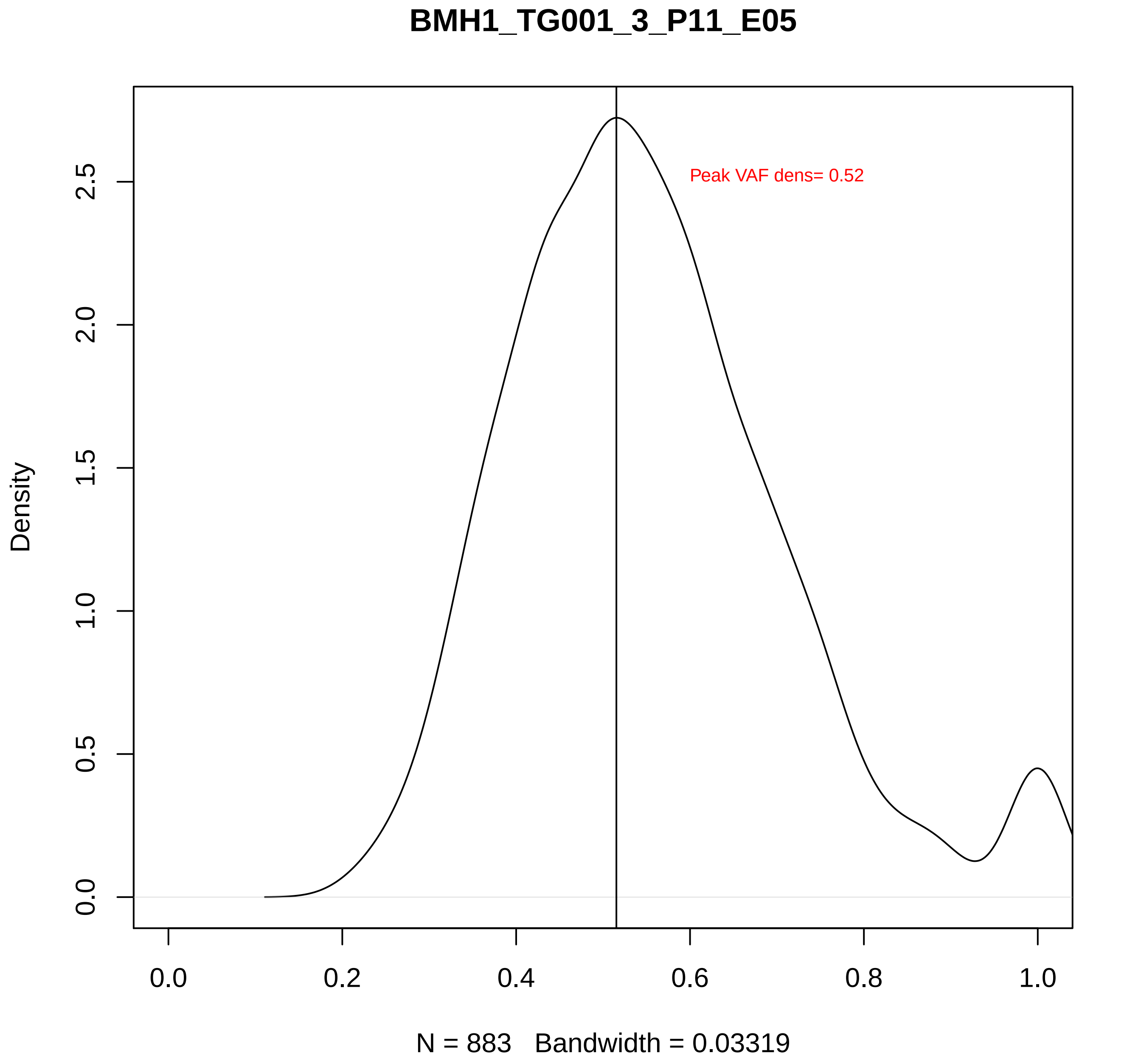
BMH1_TG001_3_P11_E05
Peak VAF dens= 0.52
Density
0.0
0.5
1.0
1.5
2.0
2.5
0.0
0.2
0.4
0.6
0.8
1.0
N = 883   Bandwidth = 0.03319

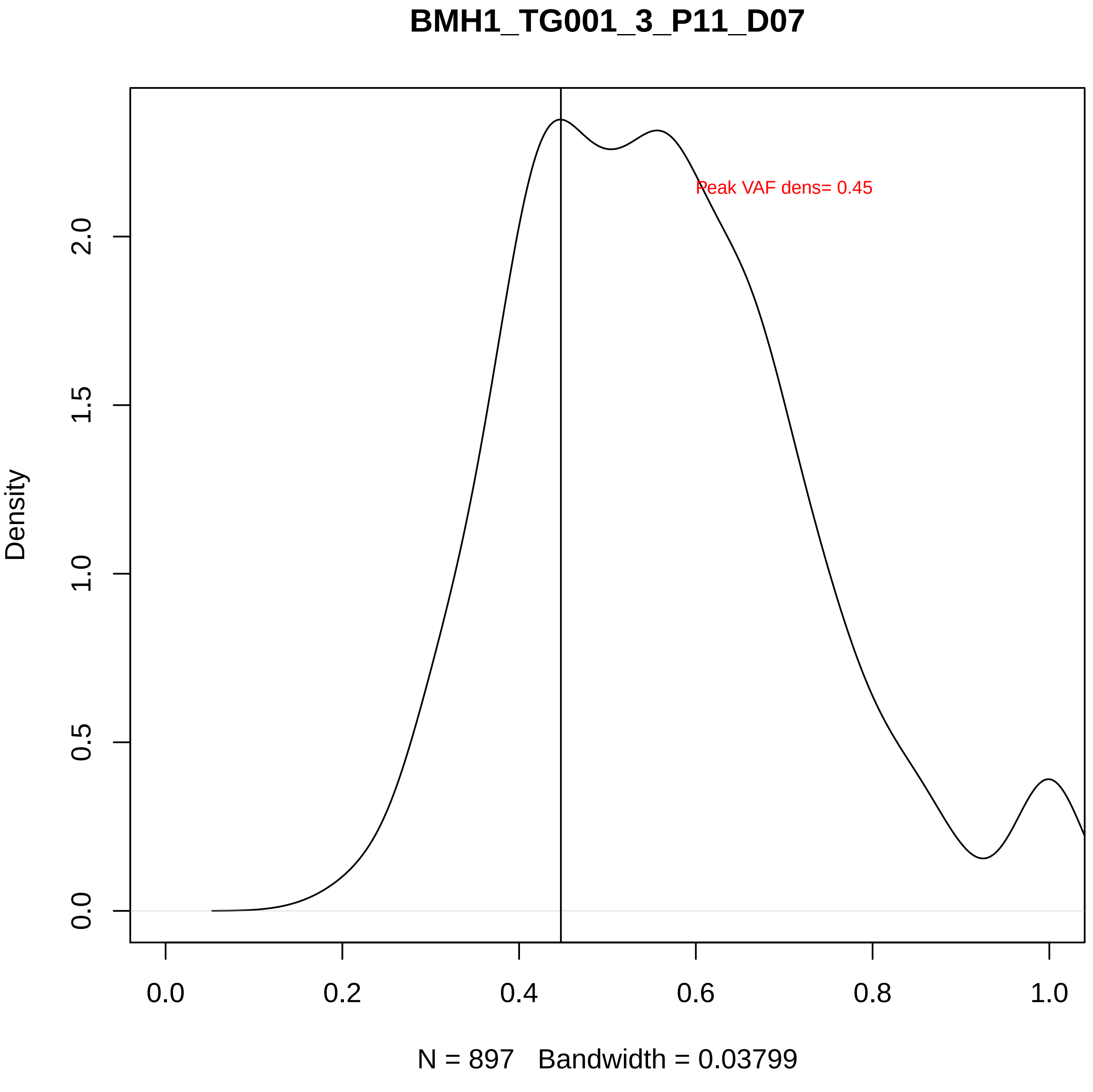
BMH1_TG001_3_P11_D07
Peak VAF dens= 0.45
Density
0.0
0.5
1.0
1.5
2.0
0.0
0.2
0.4
0.6
0.8
1.0
N = 897   Bandwidth = 0.03799

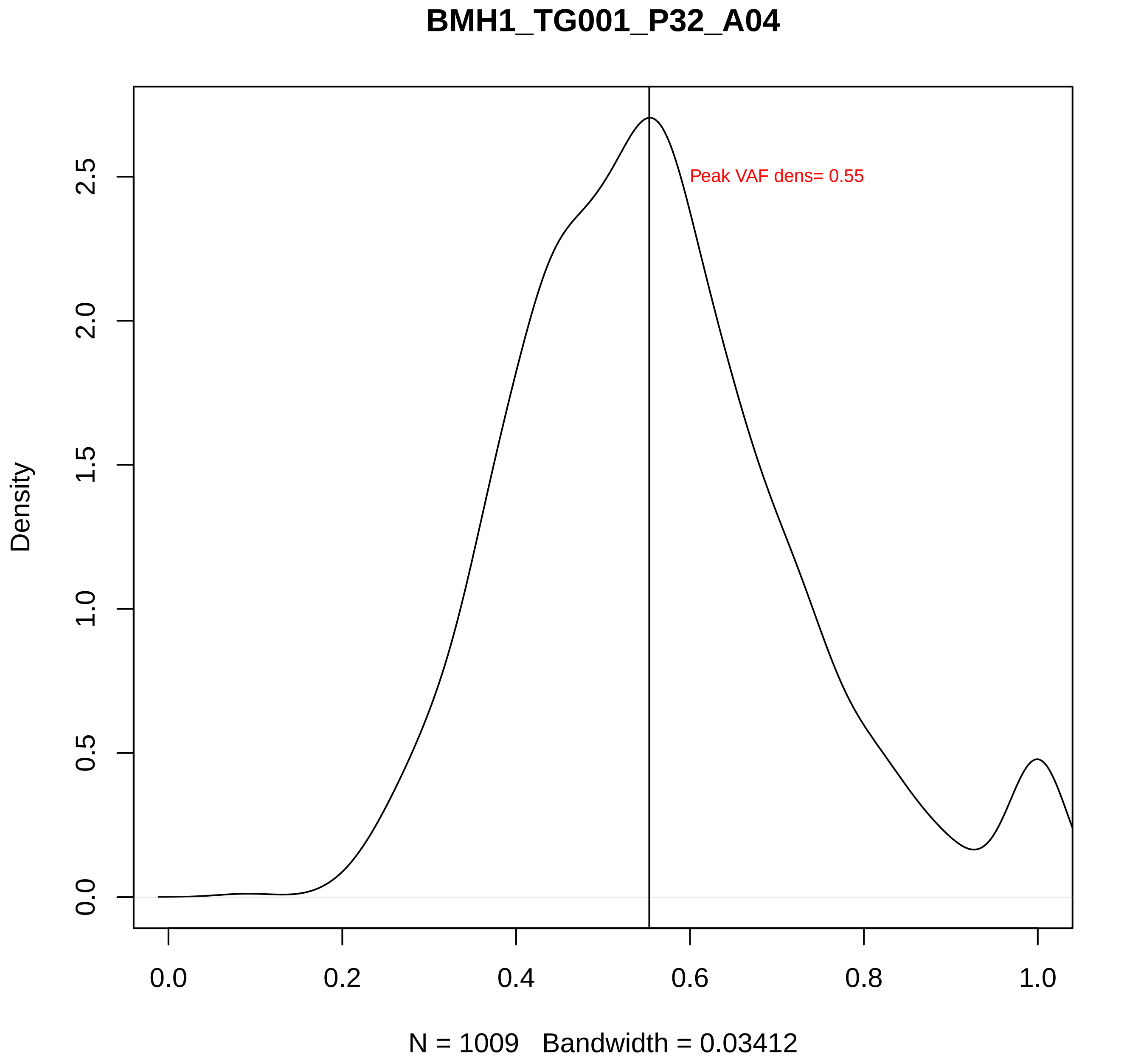

BMH1_TG001_P32_A04
Peak VAF dens= 0.55
Density
0.0
0.5
1.0
1.5
2.0
2.5
0.0
0.2
0.4
0.6
0.8
1.0
N = 1009   Bandwidth = 0.03412

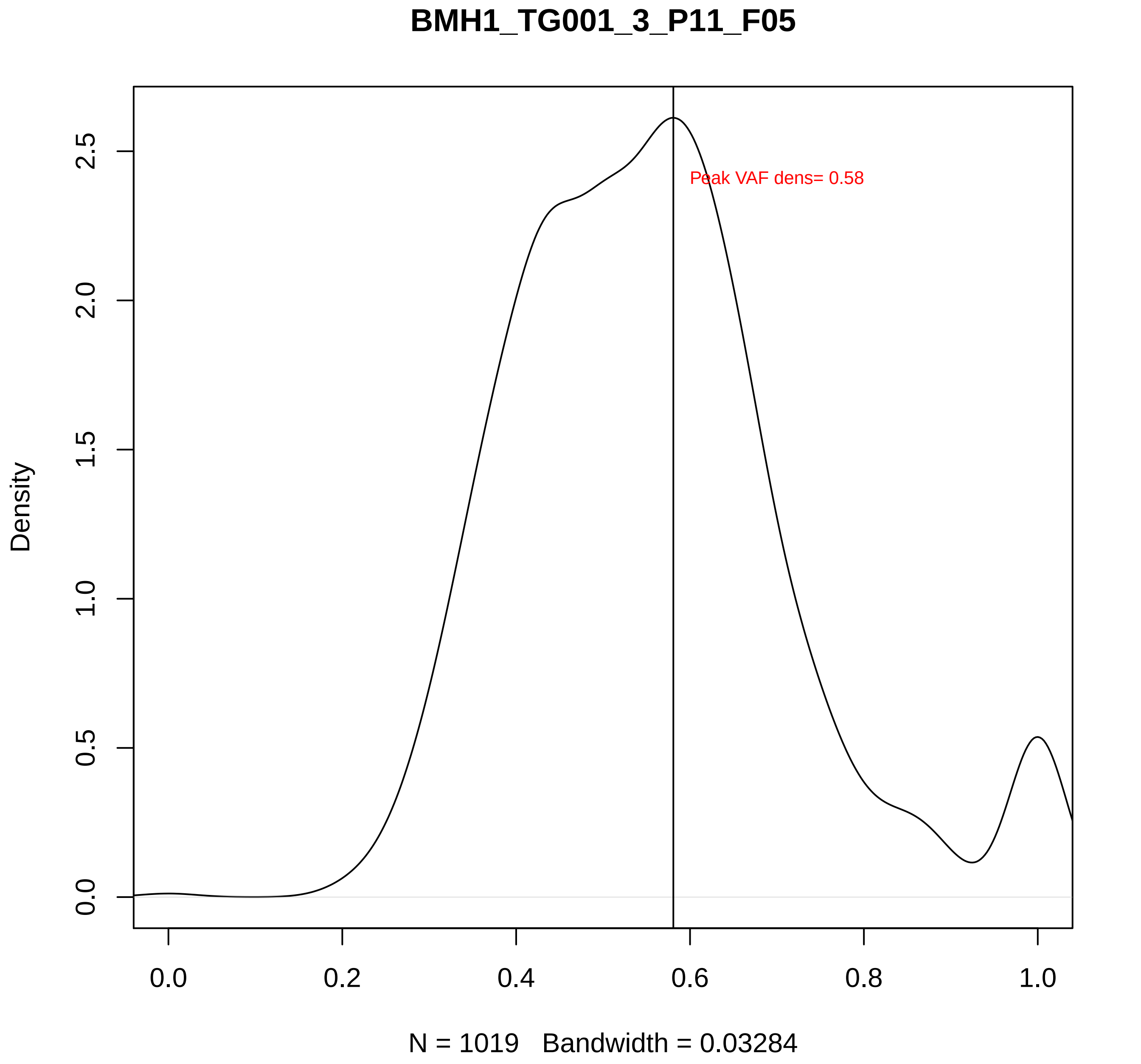
BMH1_TG001_3_P11_F05
Peak VAF dens= 0.58
Density
0.0
0.5
1.0
1.5
2.0
2.5
0.0
0.2
0.4
0.6
0.8
1.0
N = 1019   Bandwidth = 0.03284

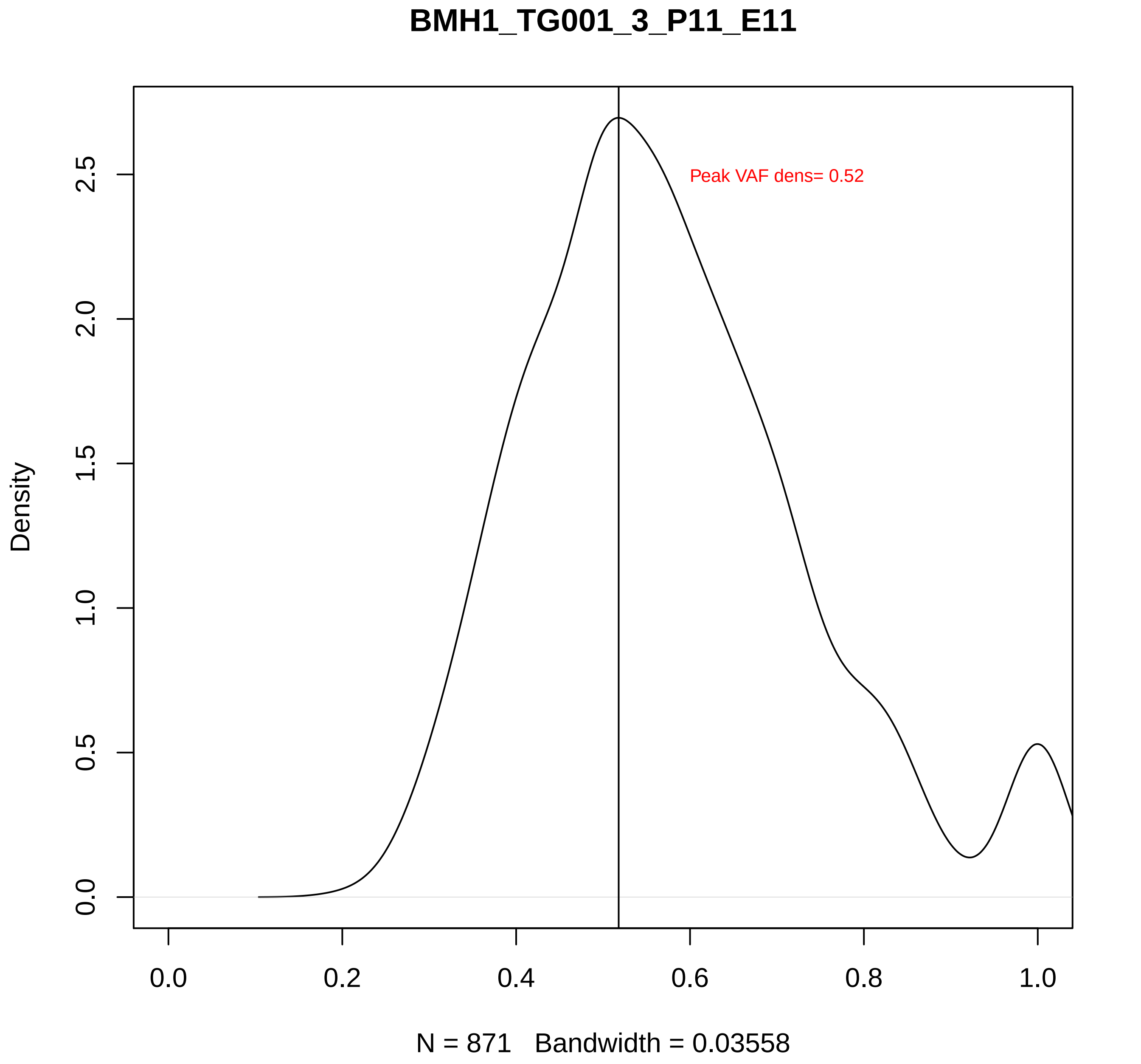

BMH1_TG001_3_P11_E11
Peak VAF dens= 0.52
Density
0.0
0.5
1.0
1.5
2.0
2.5
0.0
0.2
0.4
0.6
0.8
1.0
N = 871   Bandwidth = 0.03558

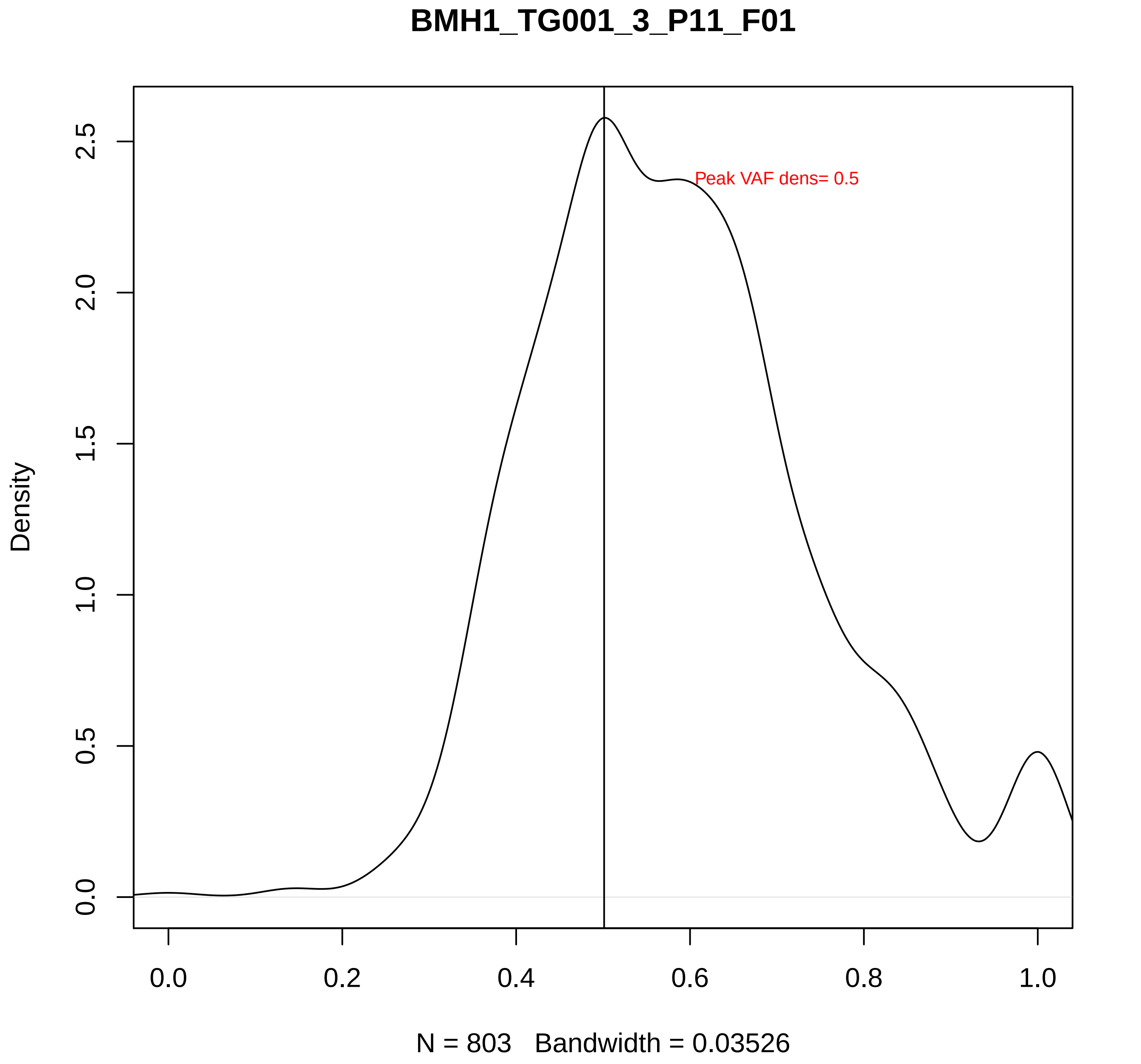
BMH1_TG001_3_P11_F01
Peak VAF dens= 0.5
Density
0.0
0.5
1.0
1.5
2.0
2.5
0.0
0.2
0.4
0.6
0.8
1.0
N = 803   Bandwidth = 0.03526

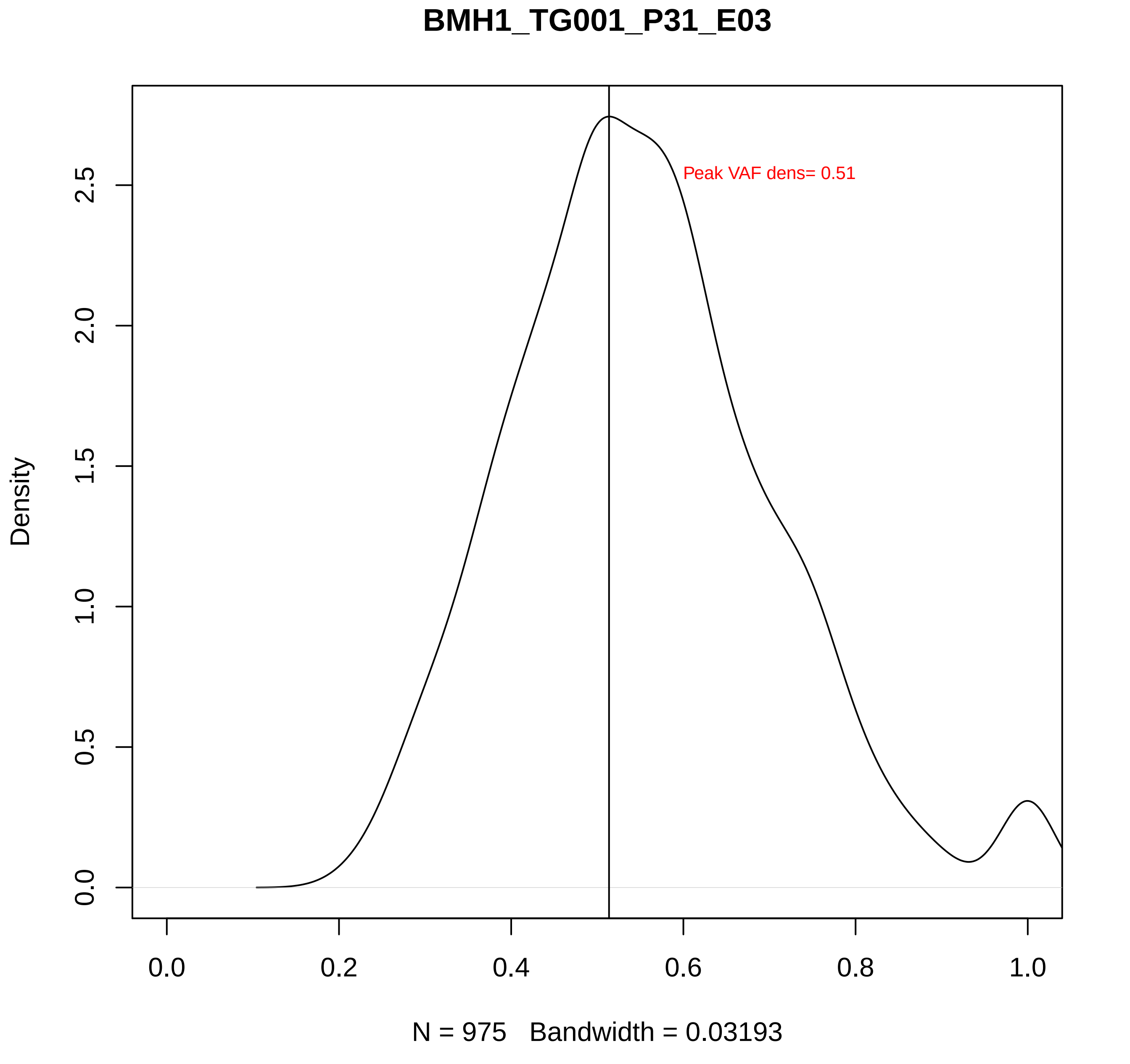

BMH1_TG001_P31_E03
Peak VAF dens= 0.51
Density
0.0
0.5
1.0
1.5
2.0
2.5
0.0
0.2
0.4
0.6
0.8
1.0
N = 975 Bandwidth = 0.03193

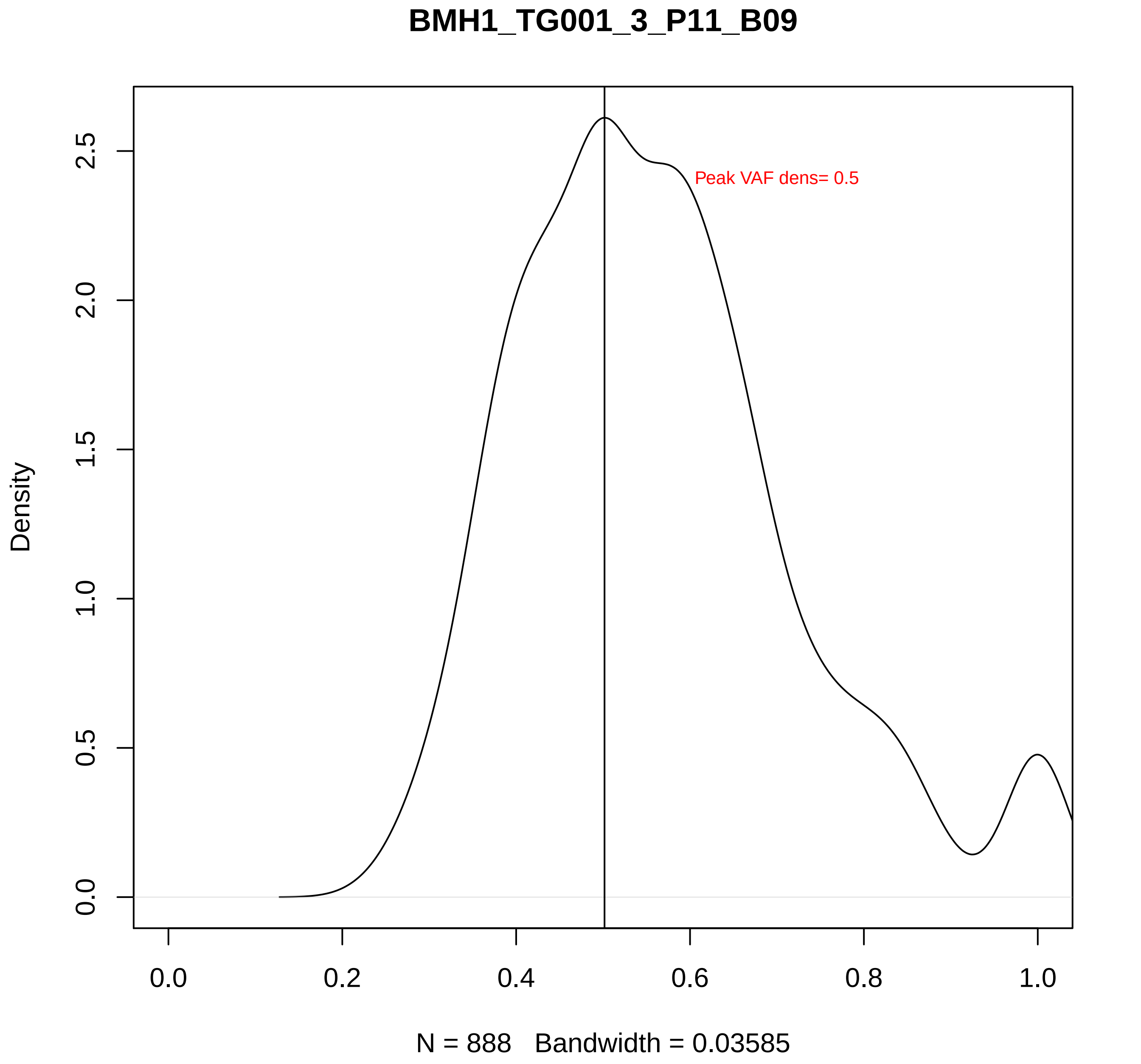
BMH1_TG001_3_P11_B09
Peak VAF dens= 0.5
Density
0.0
0.5
1.0
1.5
2.0
2.5
0.0
0.2
0.4
0.6
0.8
1.0
N = 888   Bandwidth = 0.03585

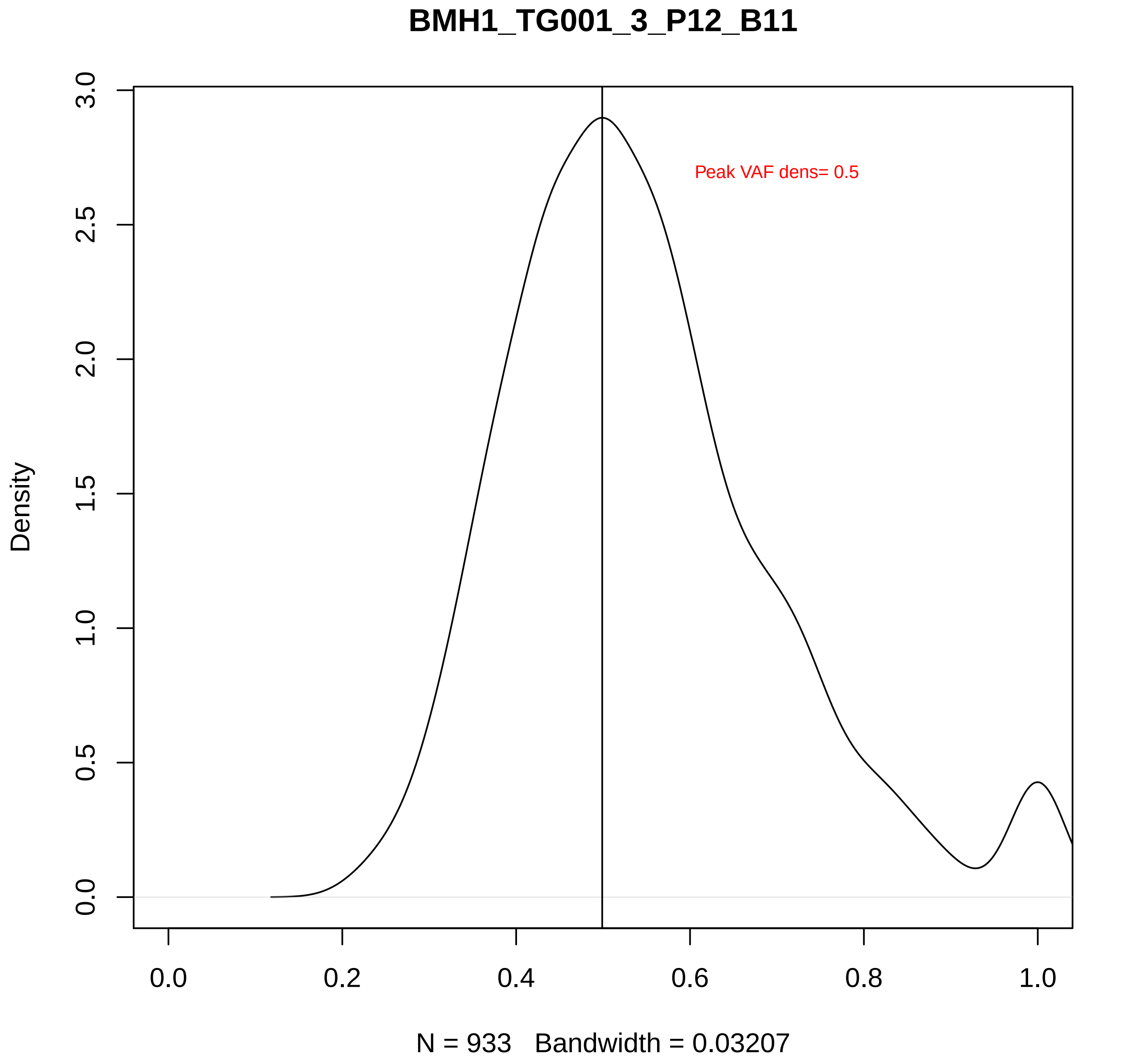
BMH1_TG001_3_P12_B11
Peak VAF dens= 0.5
Density
0.0
0.5
1.0
1.5
2.0
2.5
3.0
0.0
0.2
0.4
0.6
0.8
1.0
N = 933   Bandwidth = 0.03207

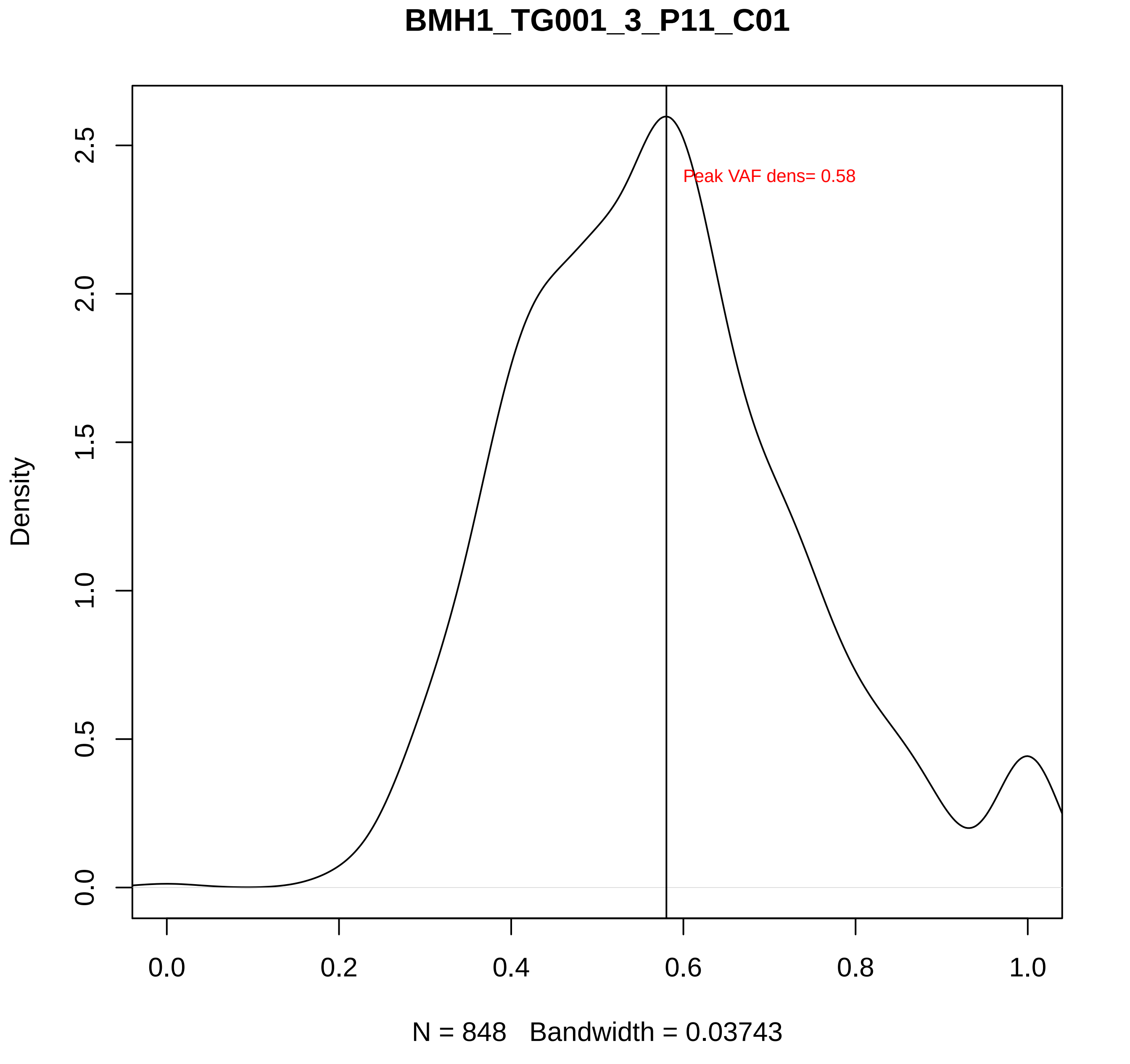

BMH1_TG001_3_P11_C01
Peak VAF dens= 0.58
Density
0.0
0.5
1.0
1.5
2.0
2.5
0.0
0.2
0.4
0.6
0.8
1.0
N = 848   Bandwidth = 0.03743

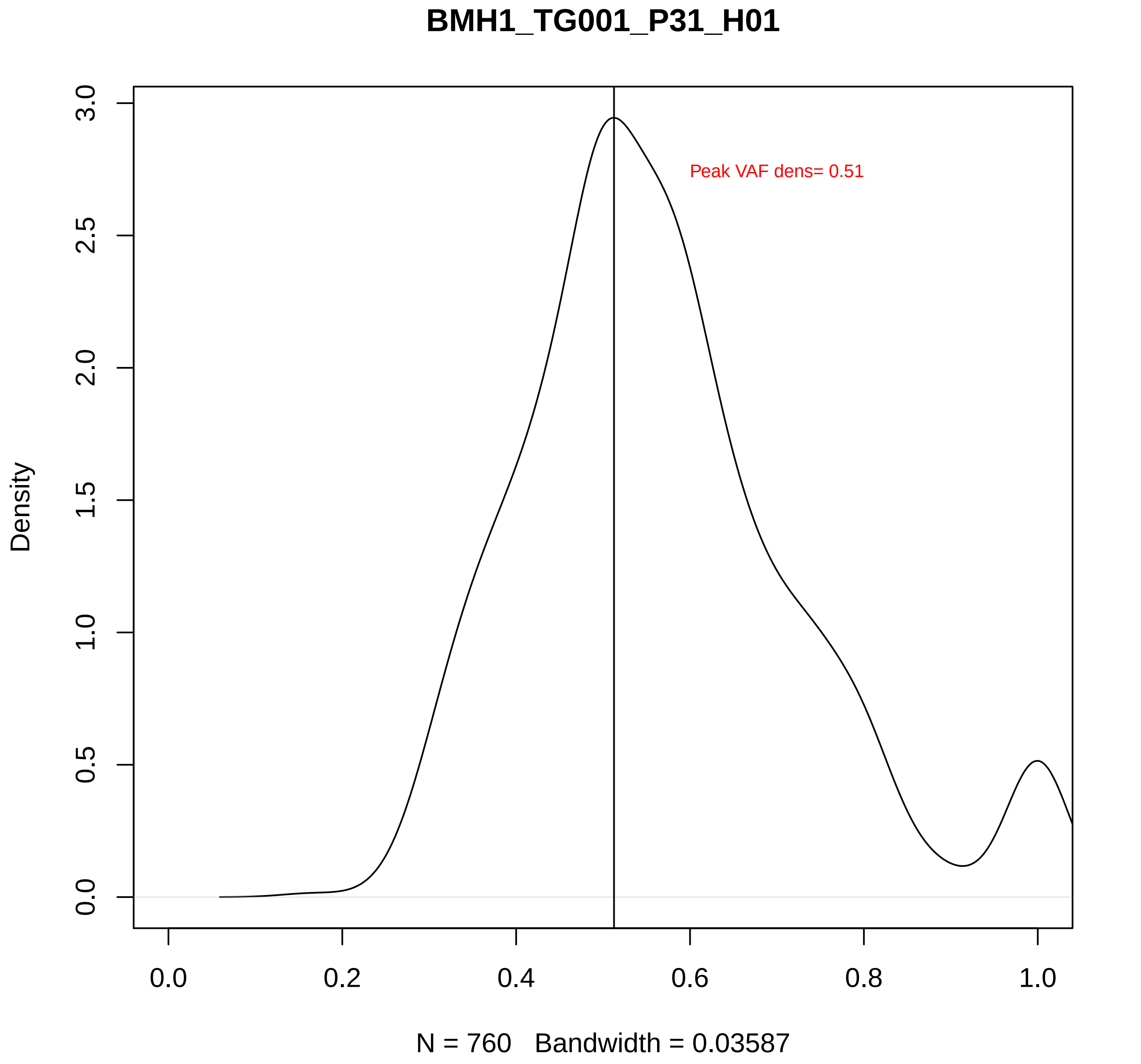
BMH1_TG001_P31_H01
Peak VAF dens= 0.51
Density
0.0
0.5
1.0
1.5
2.0
2.5
3.0
0.0
0.2
0.4
0.6
0.8
1.0
N = 760   Bandwidth = 0.03587

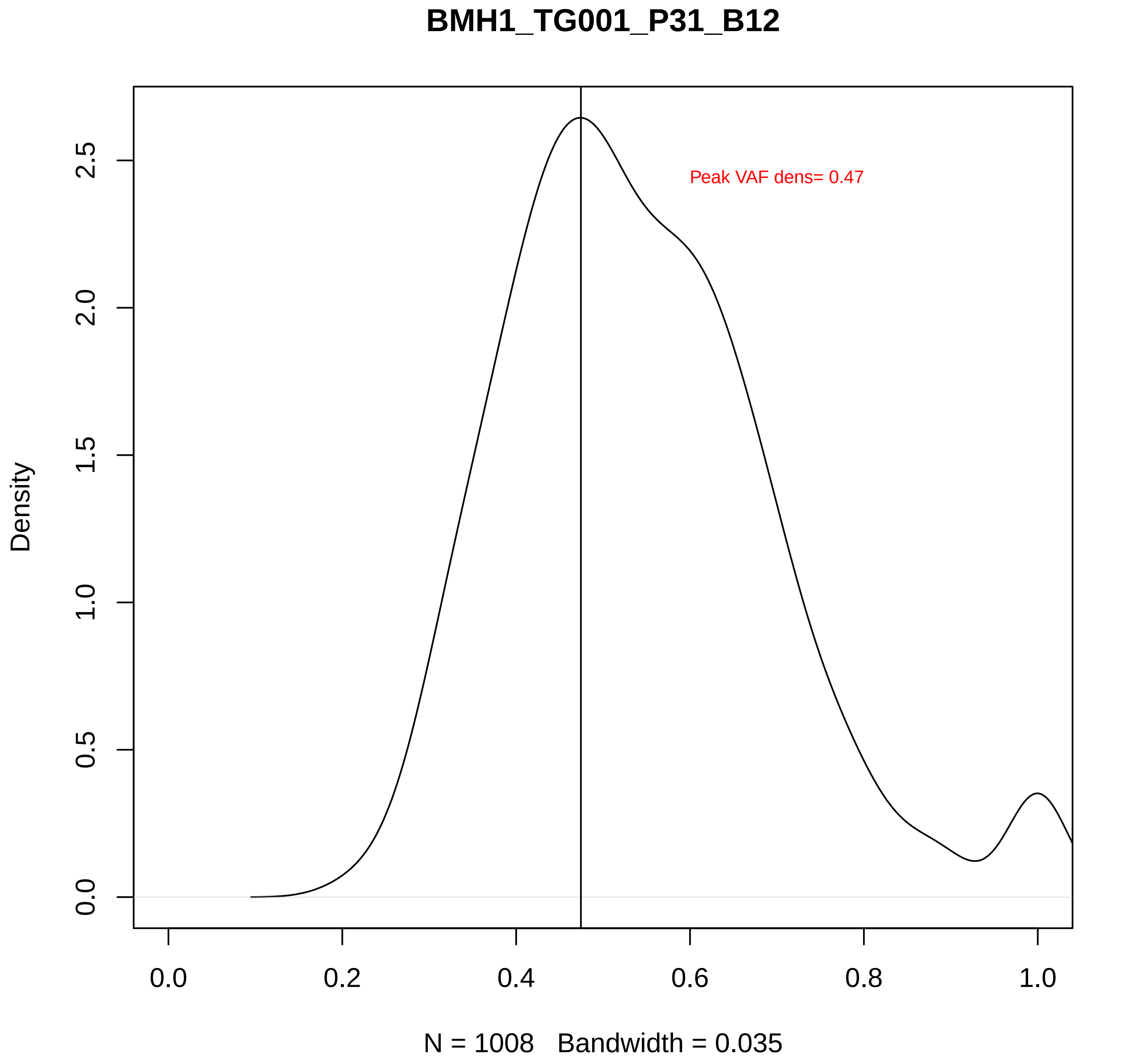
BMH1_TG001_P31_B12
Peak VAF dens= 0.47
Density
0.0
0.5
1.0
1.5
2.0
2.5
0.0
0.2
0.4
0.6
0.8
1.0
N = 1008   Bandwidth = 0.035

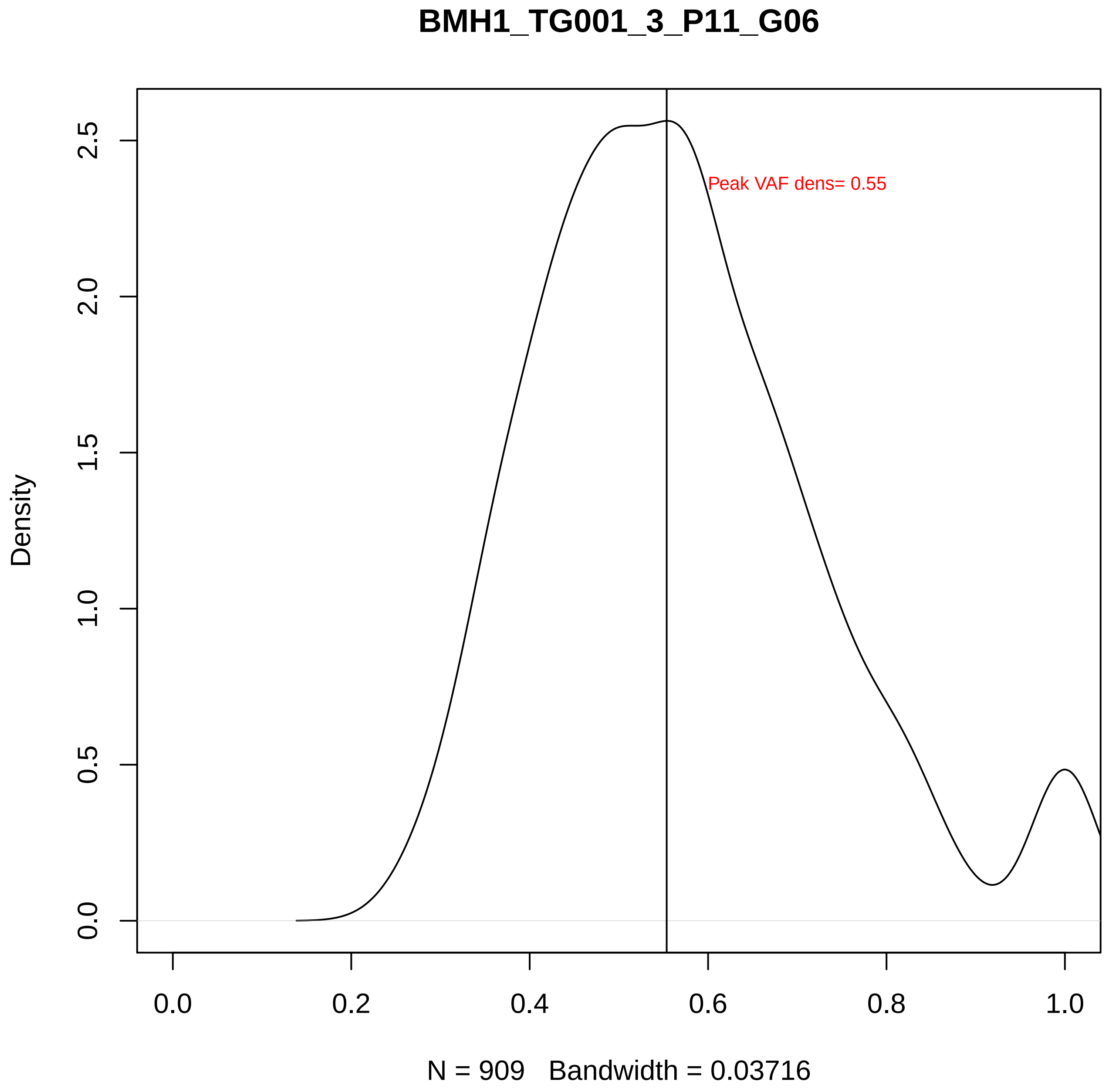
BMH1_TG001_3_P11_G06
Peak VAF dens= 0.55
Density
0.0
0.5
1.0
1.5
2.0
2.5
0.0
0.2
0.4
0.6
0.8
1.0
N = 909   Bandwidth = 0.03716

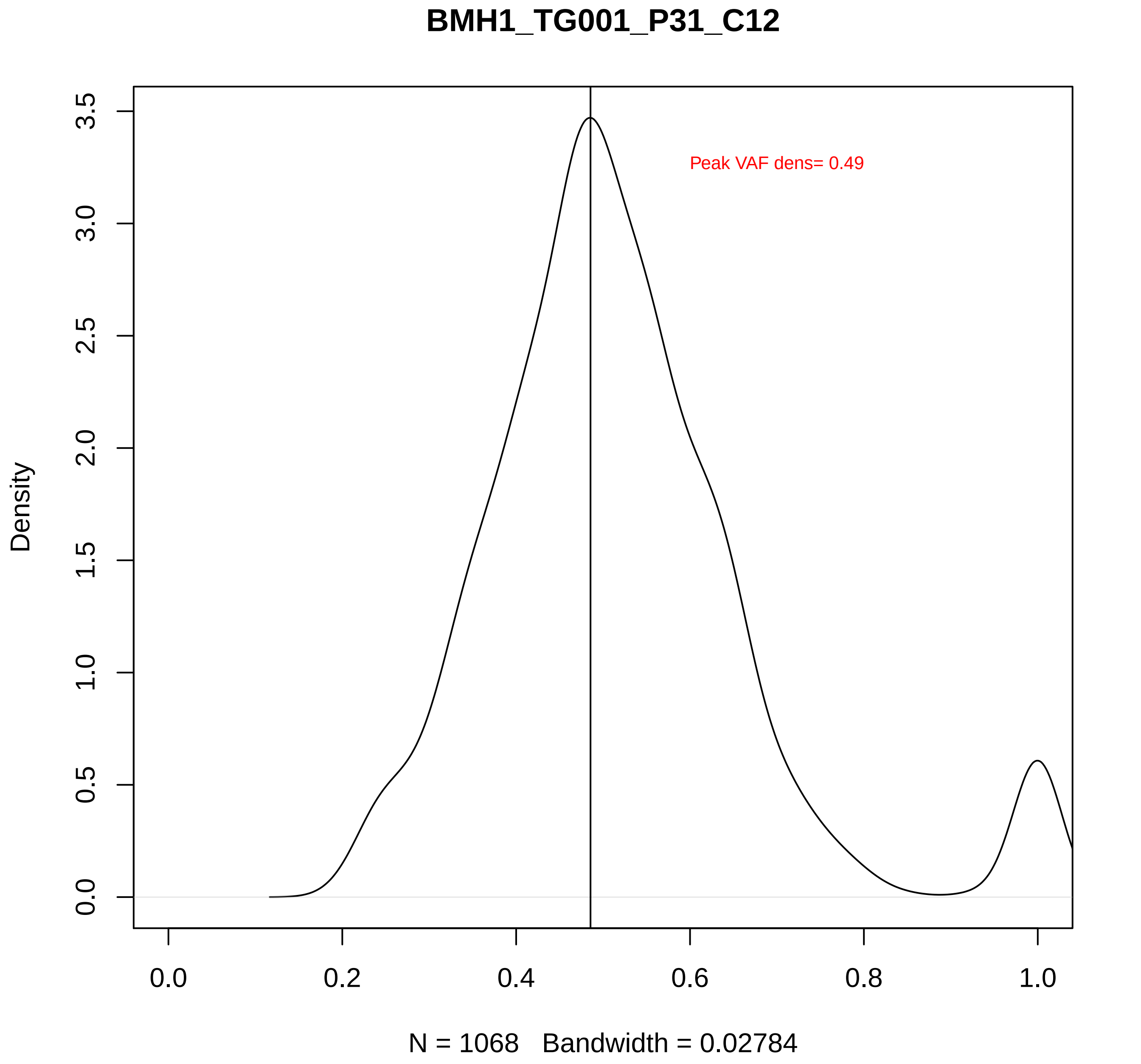
BMH1_TG001_P31_C12
Peak VAF dens= 0.49
Density
0.0
0.5
1.0
1.5
2.0
2.5
3.0
3.5
0.0
0.2
0.4
0.6
0.8
1.0
N = 1068   Bandwidth = 0.02784

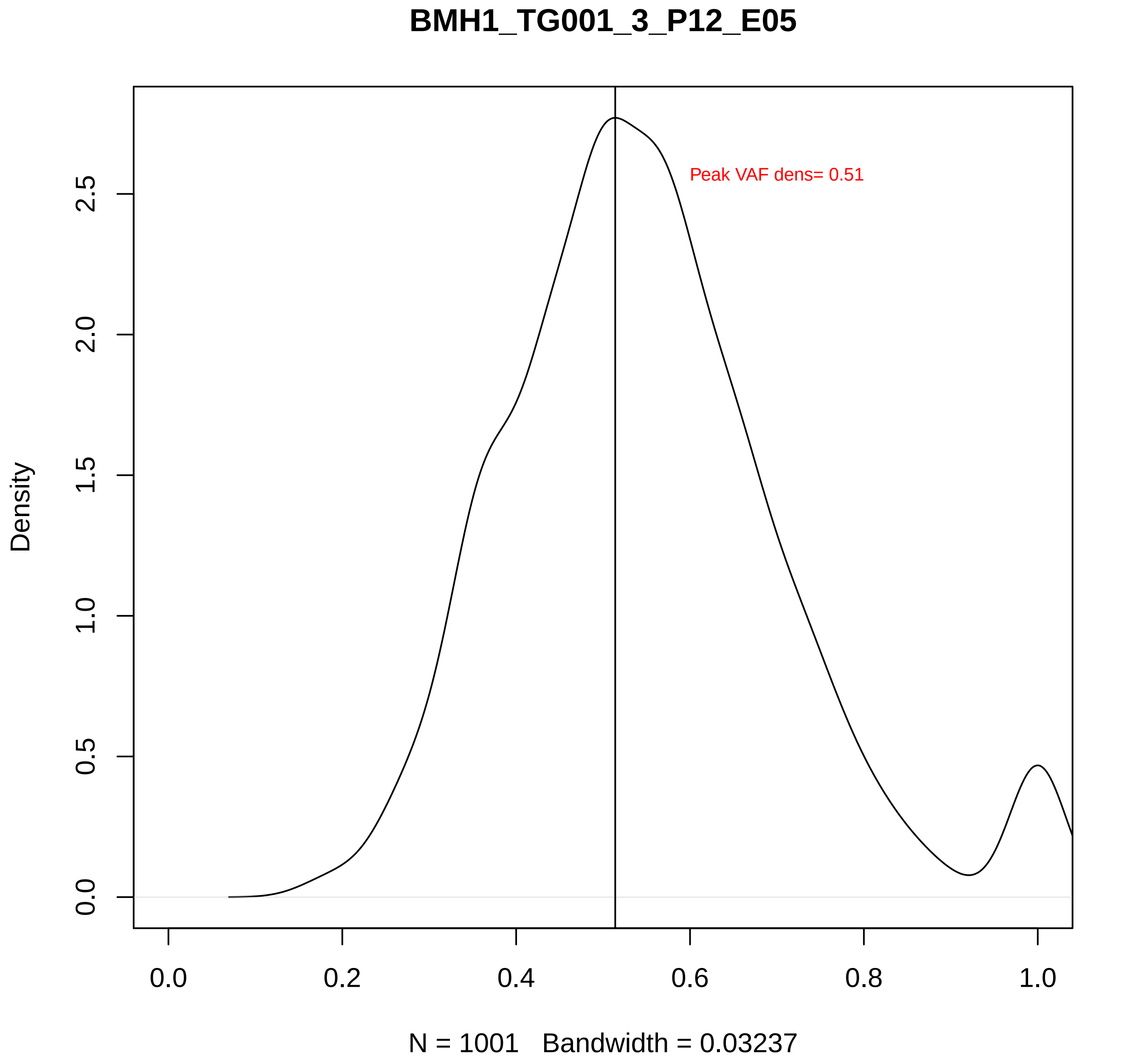

BMH1_TG001_3_P12_E05
Peak VAF dens= 0.51
Density
0.0
0.5
1.0
1.5
2.0
2.5
0.0
0.2
0.4
0.6
0.8
1.0
N = 1001   Bandwidth = 0.03237

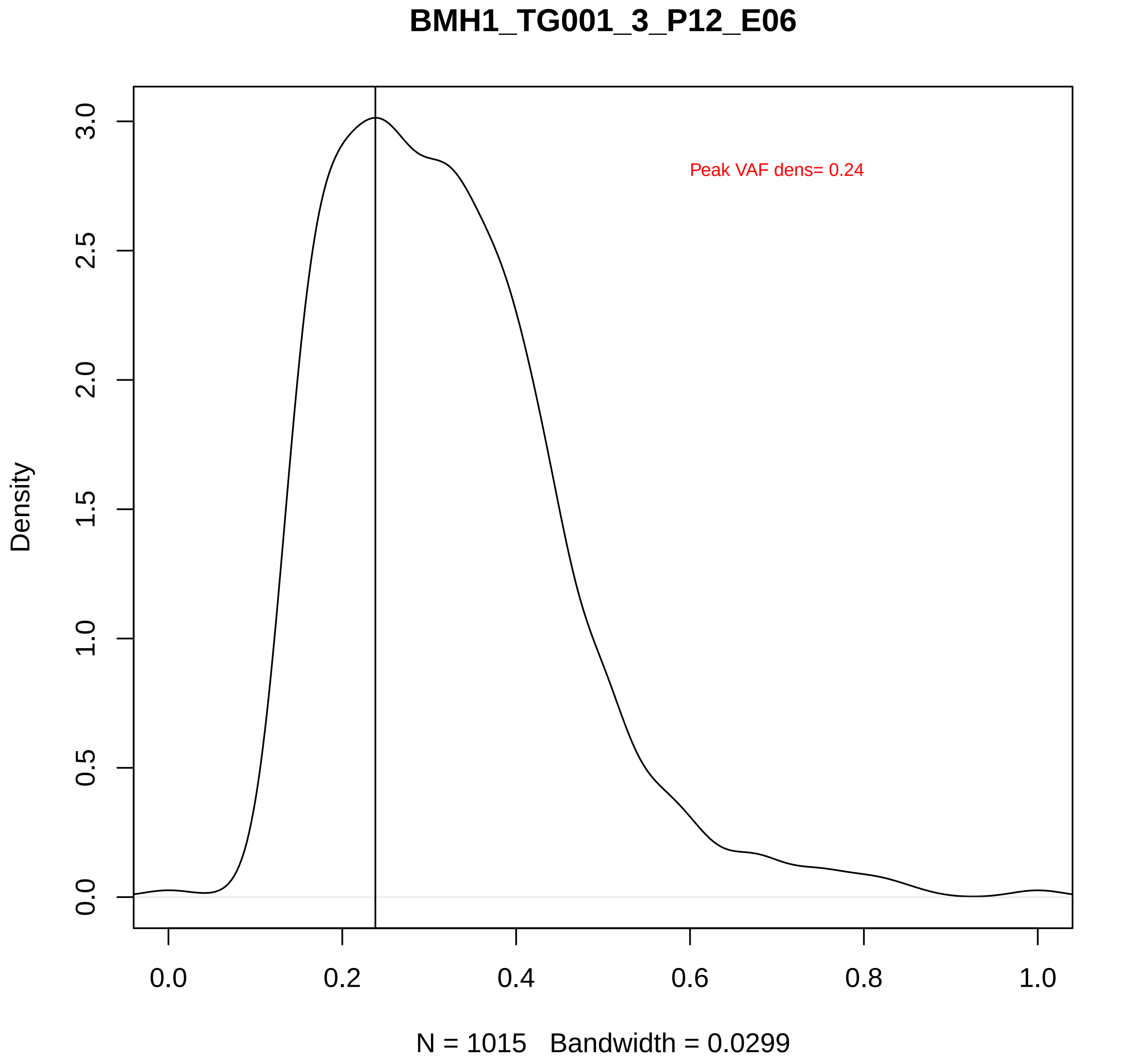
BMH1_TG001_3_P12_E06
Peak VAF dens= 0.24
Density
0.0
0.5
1.0
1.5
2.0
2.5
3.0
0.0
0.2
0.4
0.6
0.8
1.0
N = 1015   Bandwidth = 0.0299

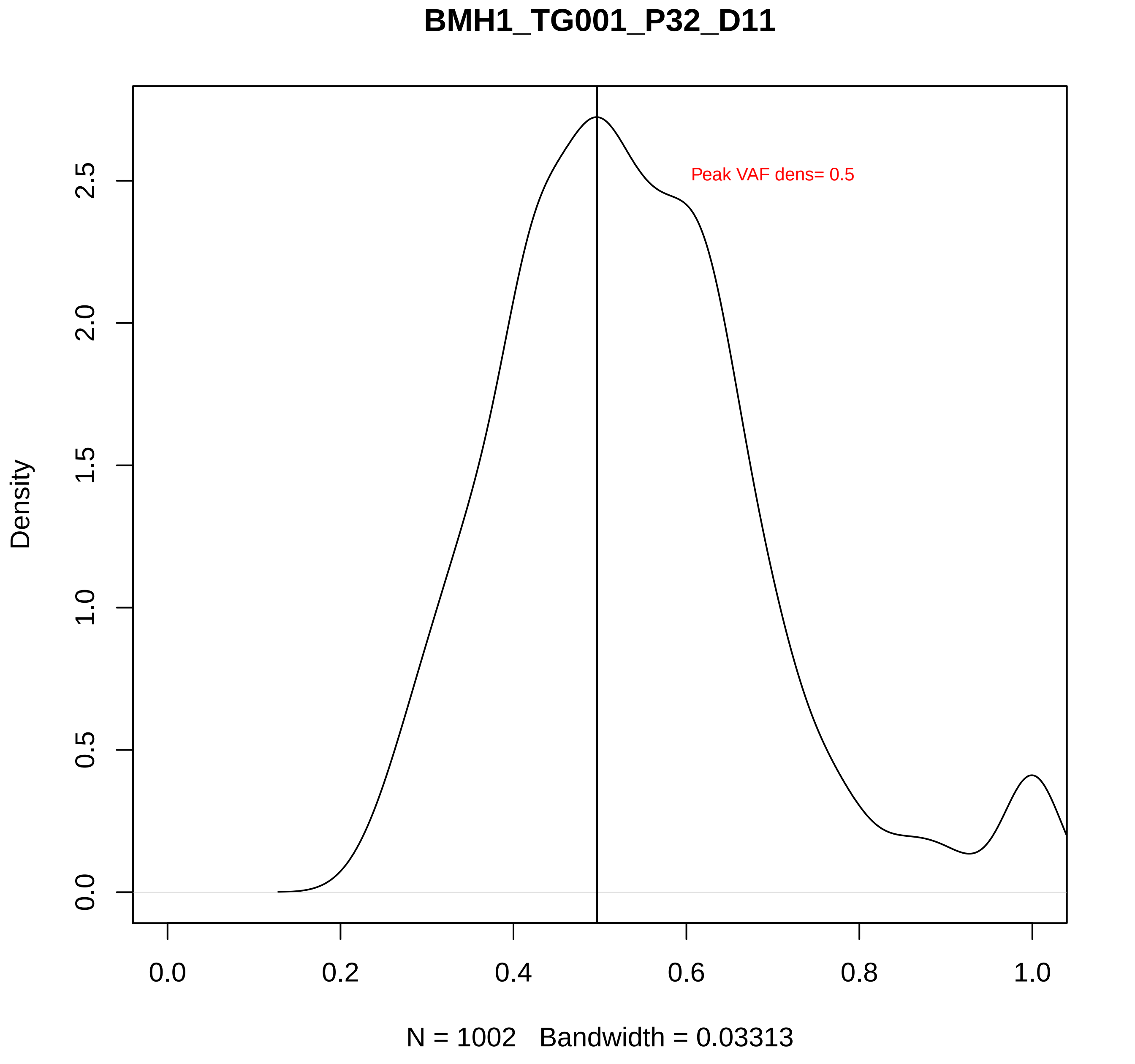
BMH1_TG001_P32_D11
Peak VAF dens= 0.5
Density
0.0
0.5
1.0
1.5
2.0
2.5
0.0
0.2
0.4
0.6
0.8
1.0
N = 1002   Bandwidth = 0.03313

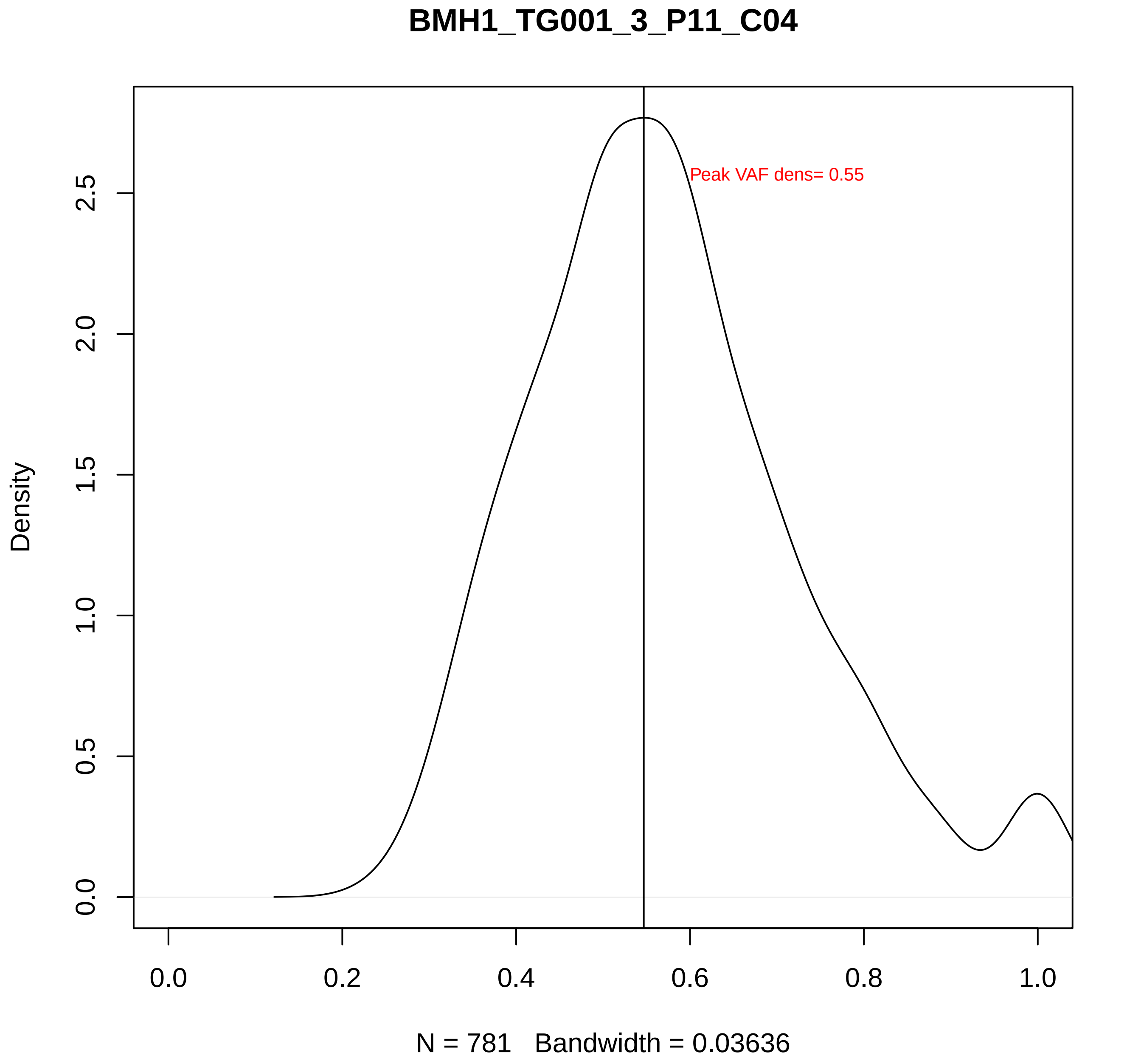

BMH1_TG001_3_P11_C04
Peak VAF dens= 0.55
Density
0.0
0.5
1.0
1.5
2.0
2.5
0.0
0.2
0.4
0.6
0.8
1.0
N = 781   Bandwidth = 0.03636

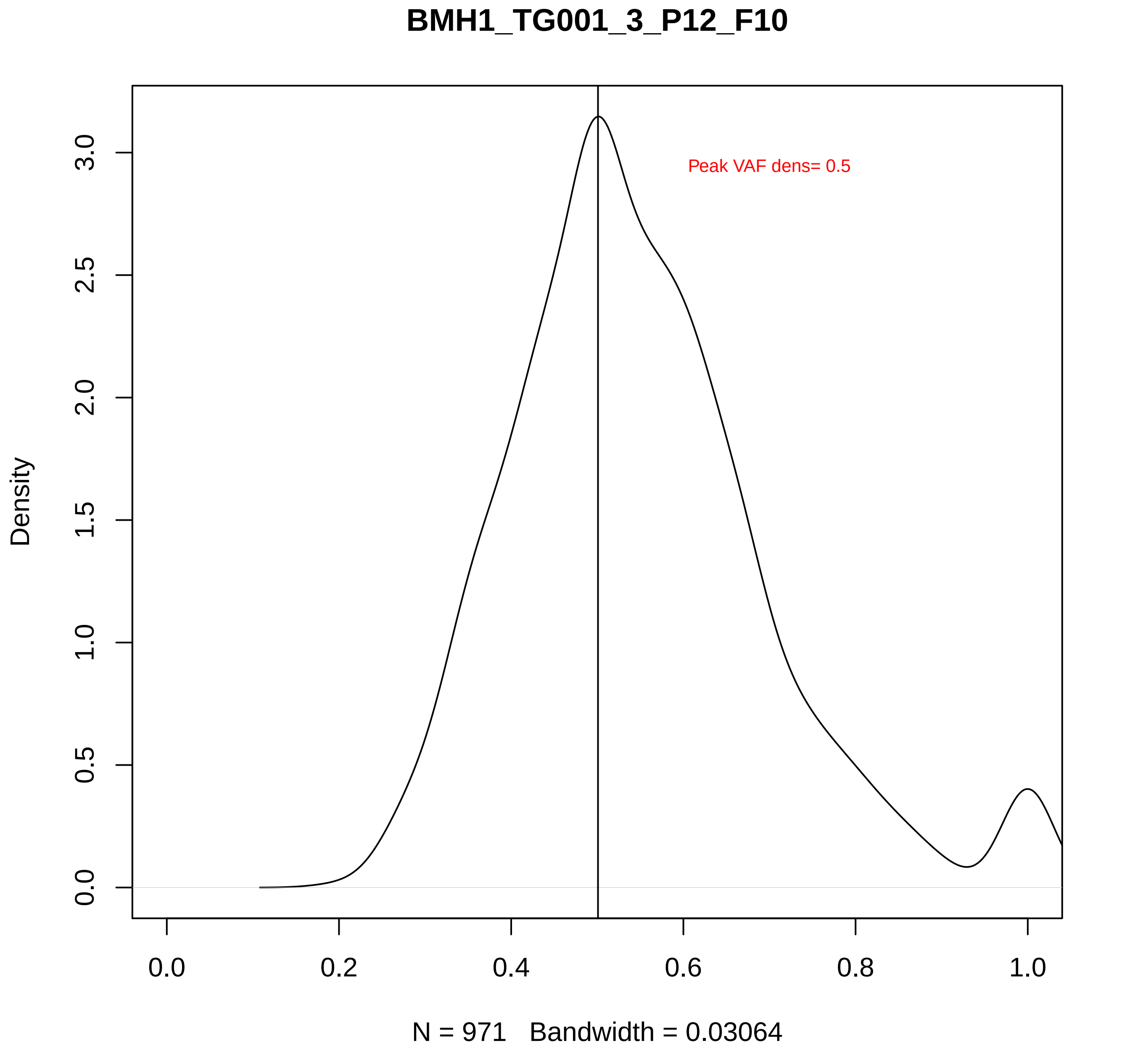
BMH1_TG001_3_P12_F10
Peak VAF dens= 0.5
Density
0.0
0.5
1.0
1.5
2.0
2.5
3.0
0.0
0.2
0.4
0.6
0.8
1.0
N = 971   Bandwidth = 0.03064

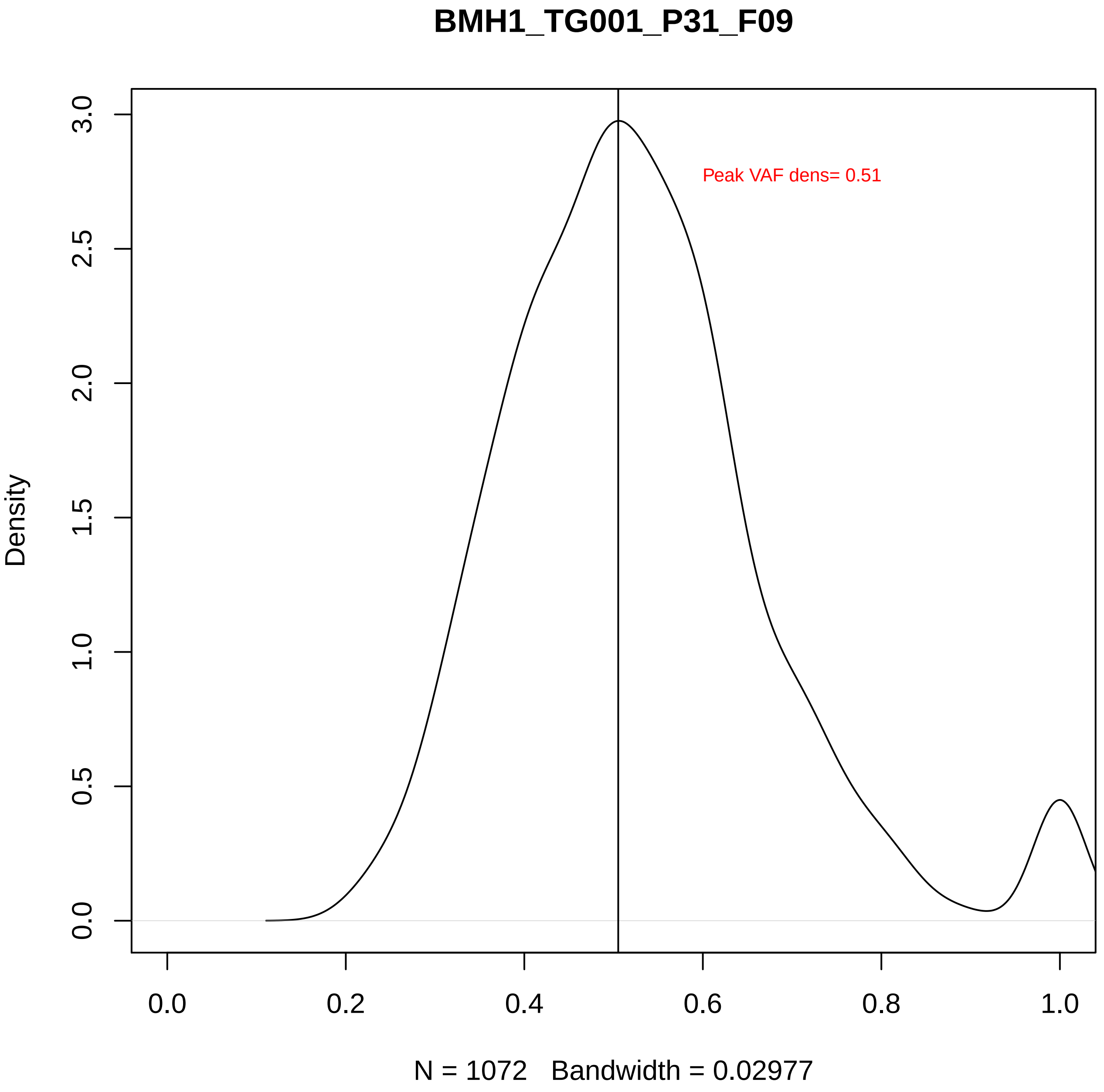
BMH1_TG001_P31_F09
Peak VAF dens= 0.51
Density
0.0
0.5
1.0
1.5
2.0
2.5
3.0
0.0
0.2
0.4
0.6
0.8
1.0
N = 1072   Bandwidth = 0.02977

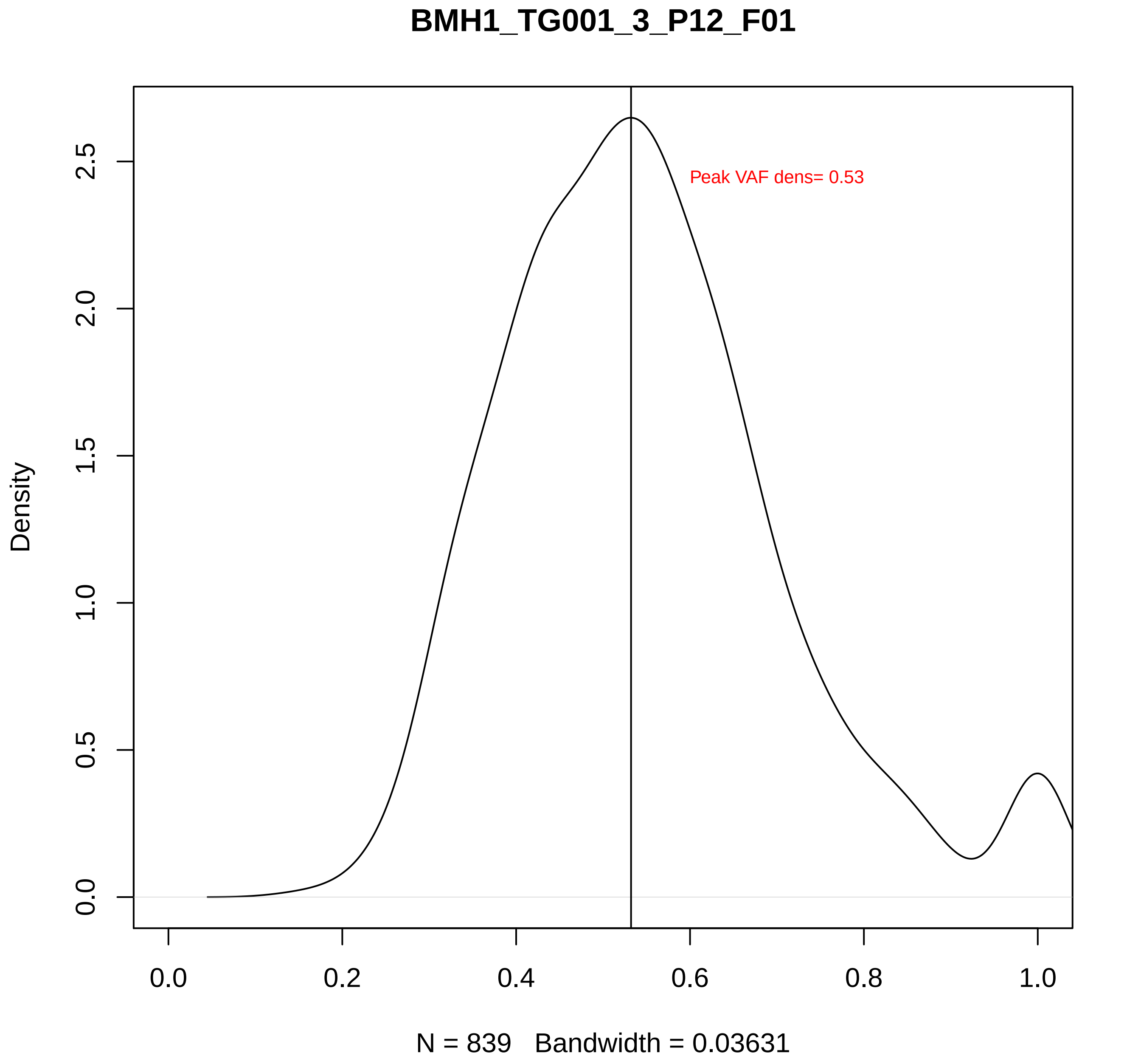

BMH1_TG001_3_P12_F01
Peak VAF dens= 0.53
Density
0.0
0.5
1.0
1.5
2.0
2.5
0.0
0.2
0.4
0.6
0.8
1.0
N = 839   Bandwidth = 0.03631

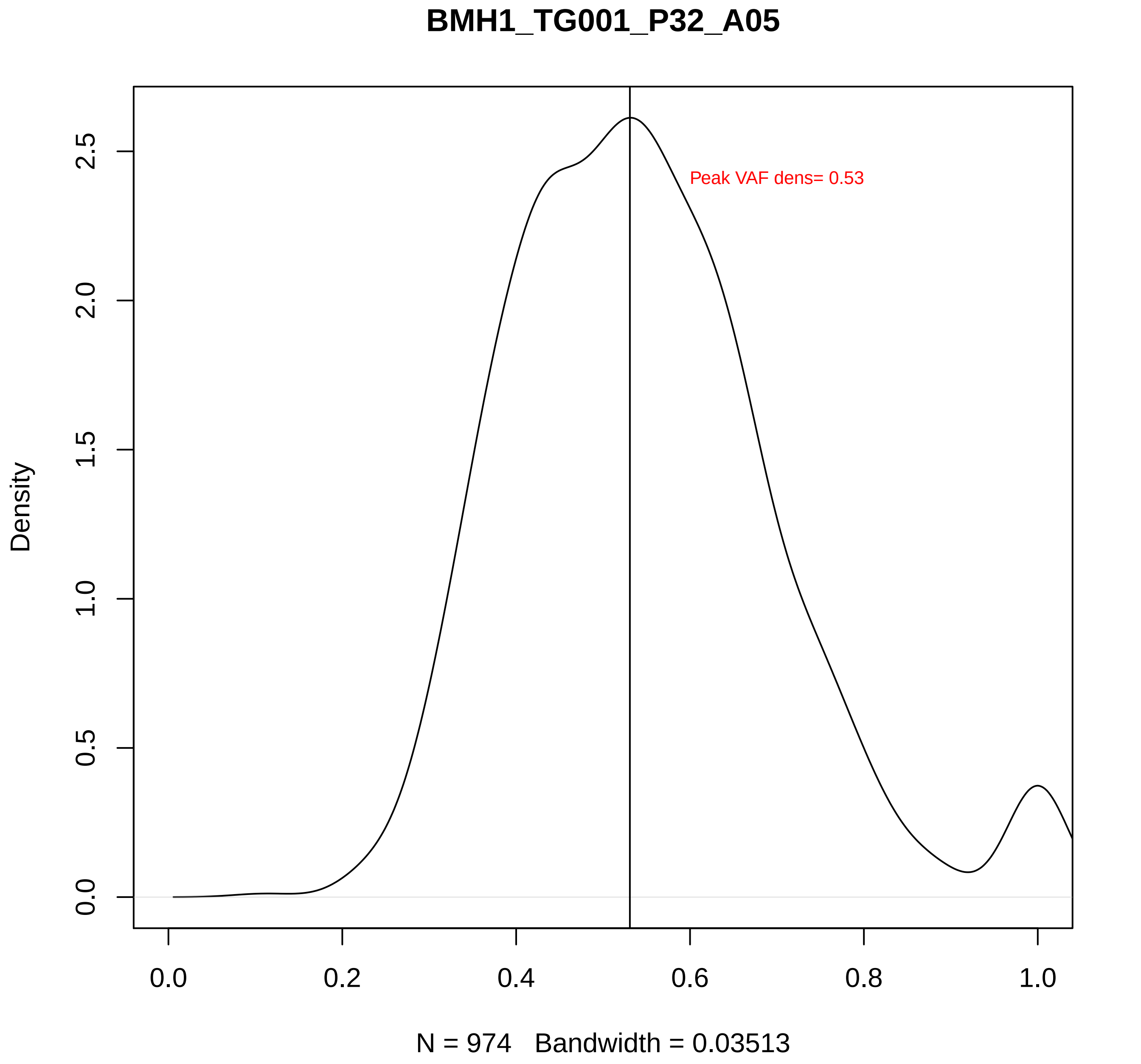
BMH1_TG001_P32_A05
Peak VAF dens= 0.53
Density
0.0
0.5
1.0
1.5
2.0
2.5
0.0
0.2
0.4
0.6
0.8
1.0
N = 974 Bandwidth = 0.03513

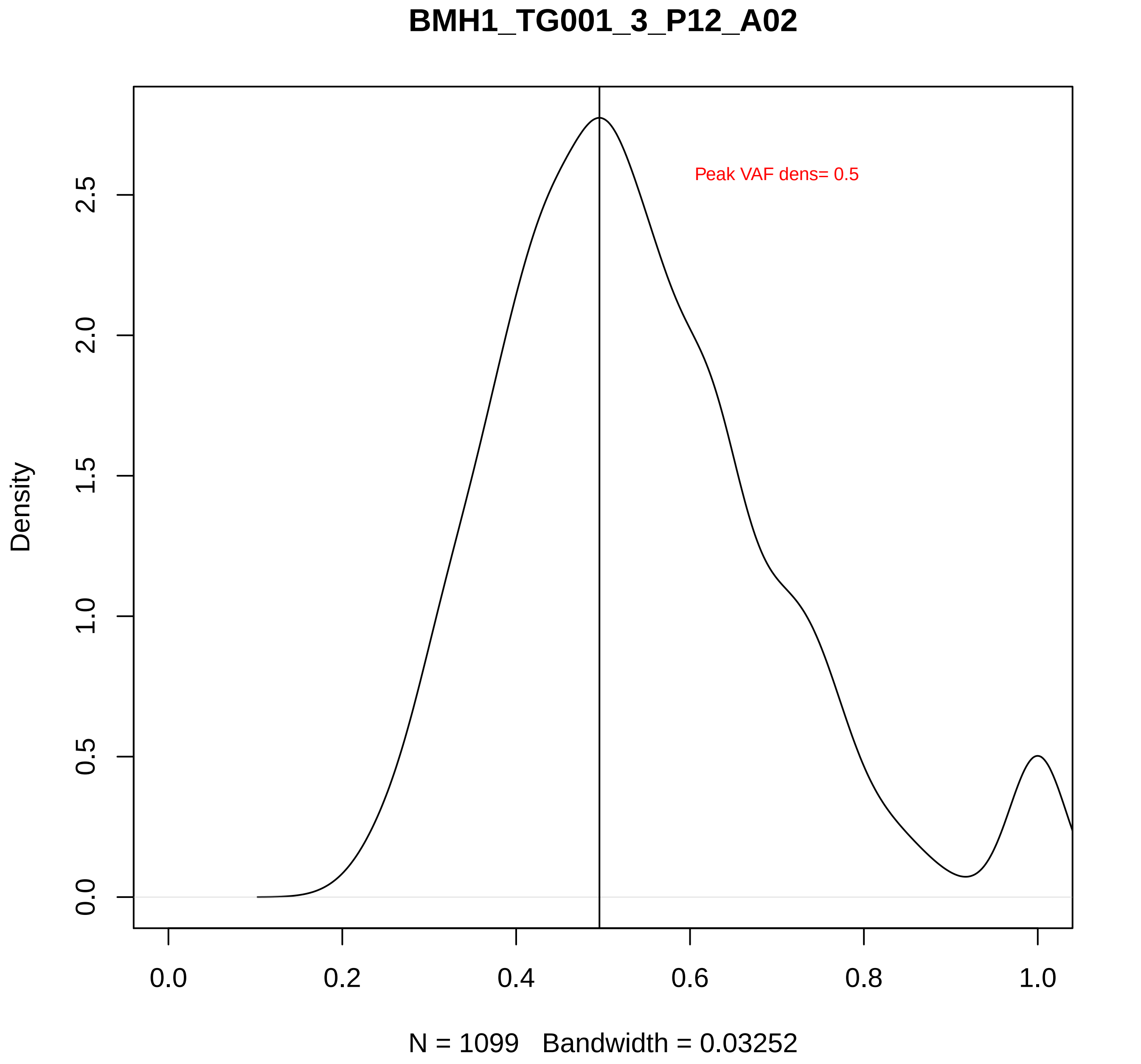
BMH1_TG001_3_P12_A02
Peak VAF dens= 0.5
Density
0.0
0.5
1.0
1.5
2.0
2.5
0.0
0.2
0.4
0.6
0.8
1.0
N = 1099   Bandwidth = 0.03252

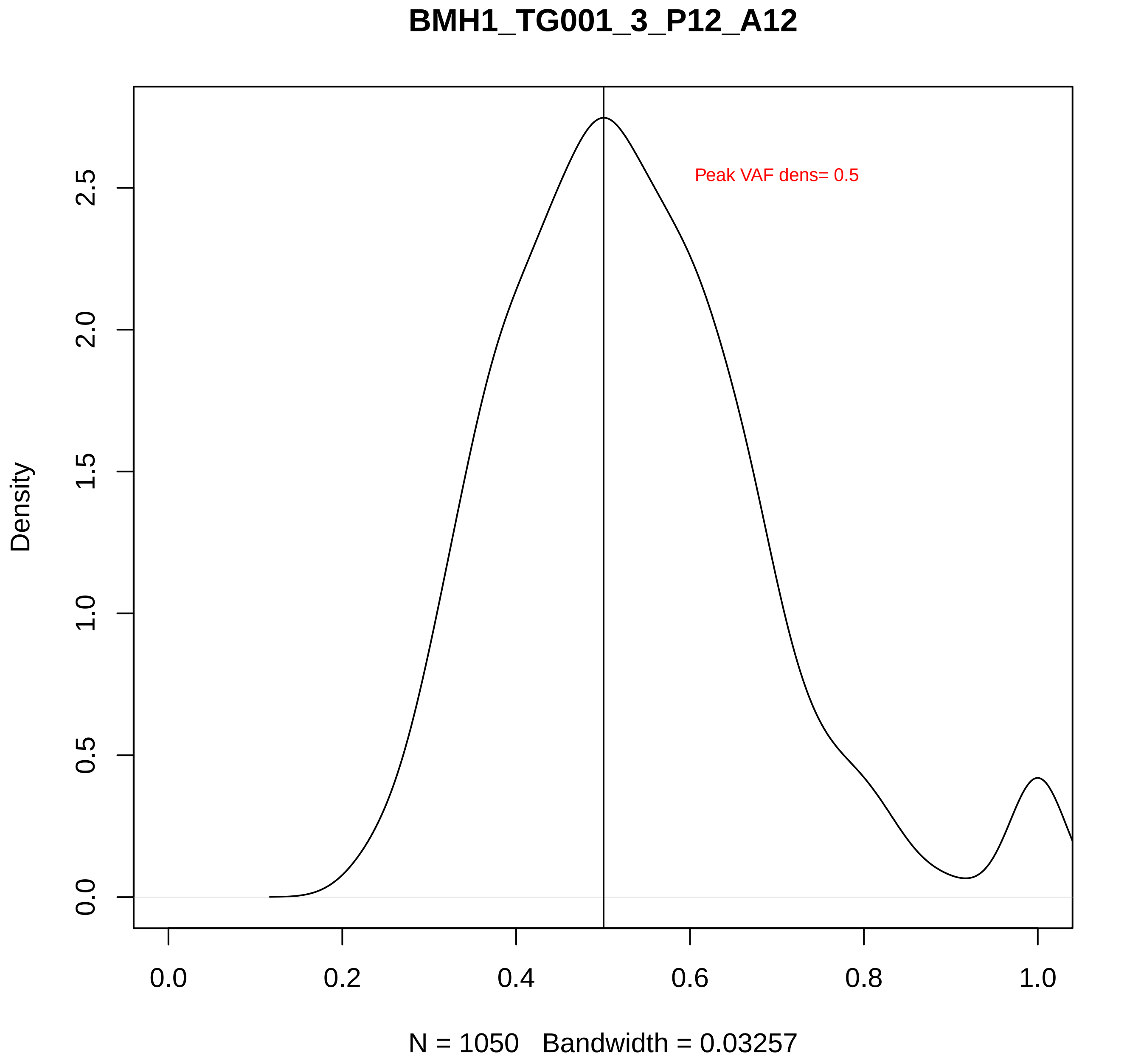
BMH1_TG001_3_P12_A12
Peak VAF dens= 0.5
Density
0.0
0.5
1.0
1.5
2.0
2.5
0.0
0.2
0.4
0.6
0.8
1.0
N = 1050   Bandwidth = 0.03257

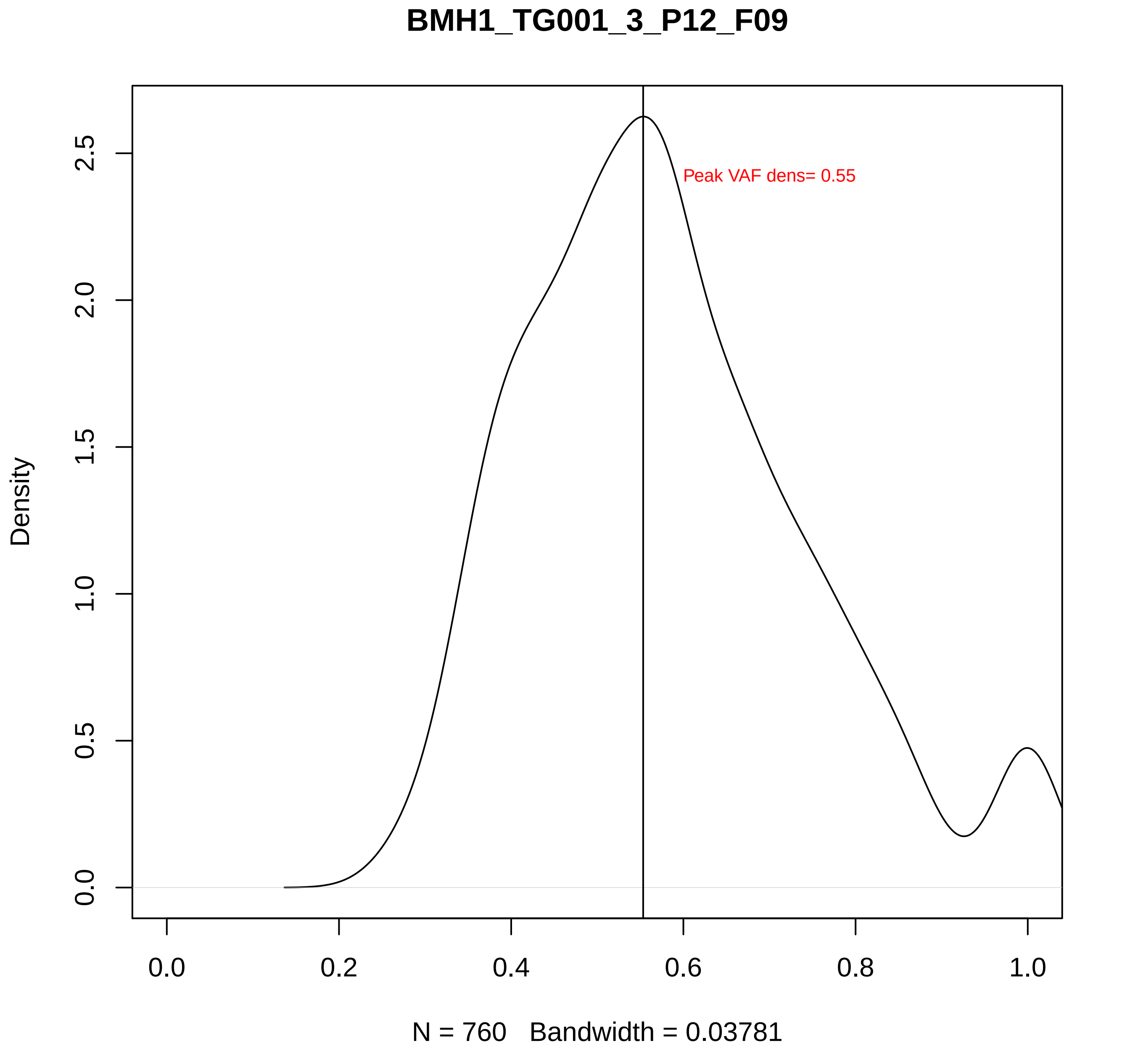

BMH1_TG001_3_P12_F09
Peak VAF dens= 0.55
Density
0.0
0.5
1.0
1.5
2.0
2.5
0.0
0.2
0.4
0.6
0.8
1.0
N = 760 Bandwidth = 0.03781

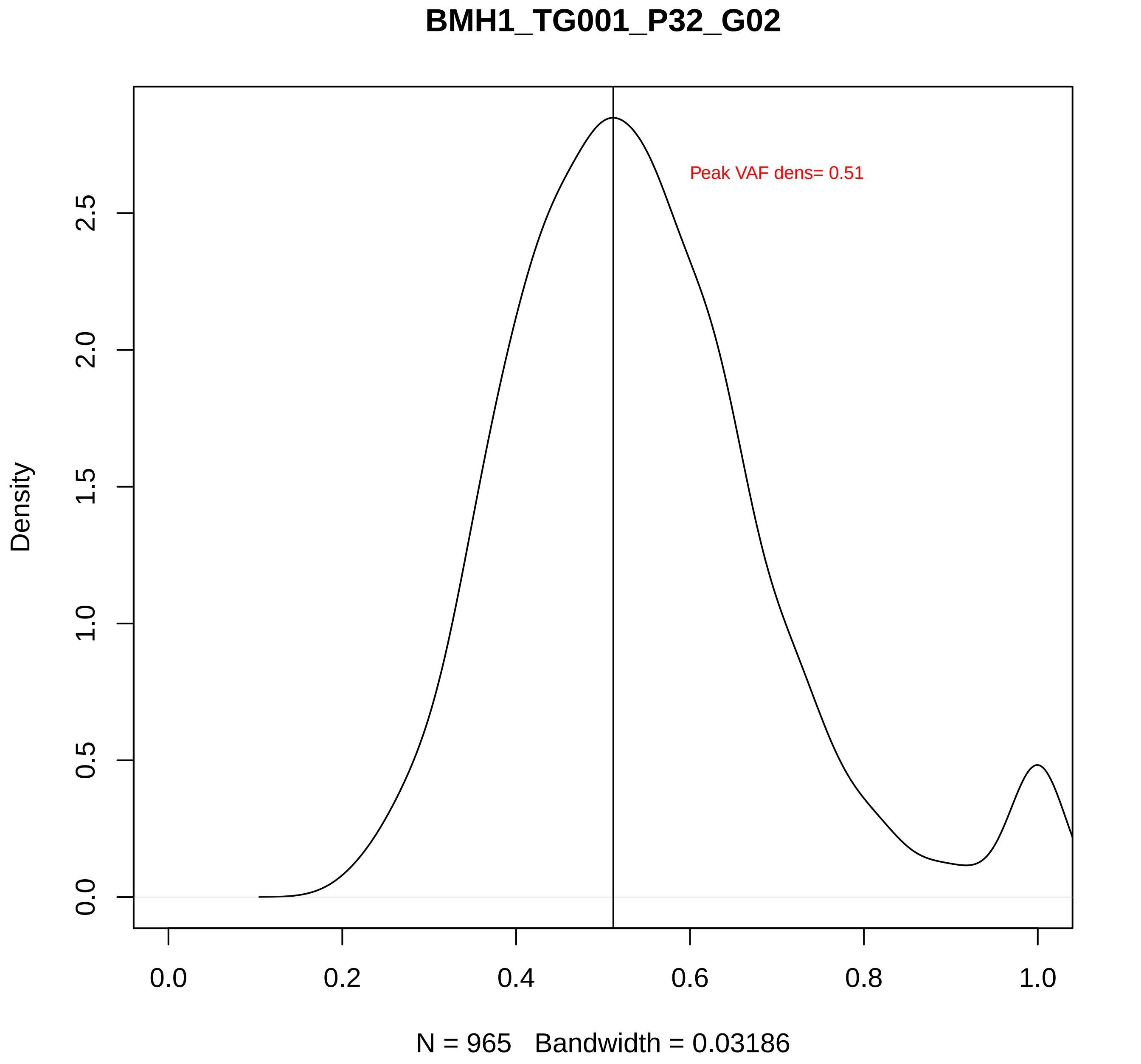
BMH1_TG001_P32_G02
Peak VAF dens= 0.51
Density
0.0
0.5
1.0
1.5
2.0
2.5
0.0
0.2
0.4
0.6
0.8
1.0
N = 965   Bandwidth = 0.03186

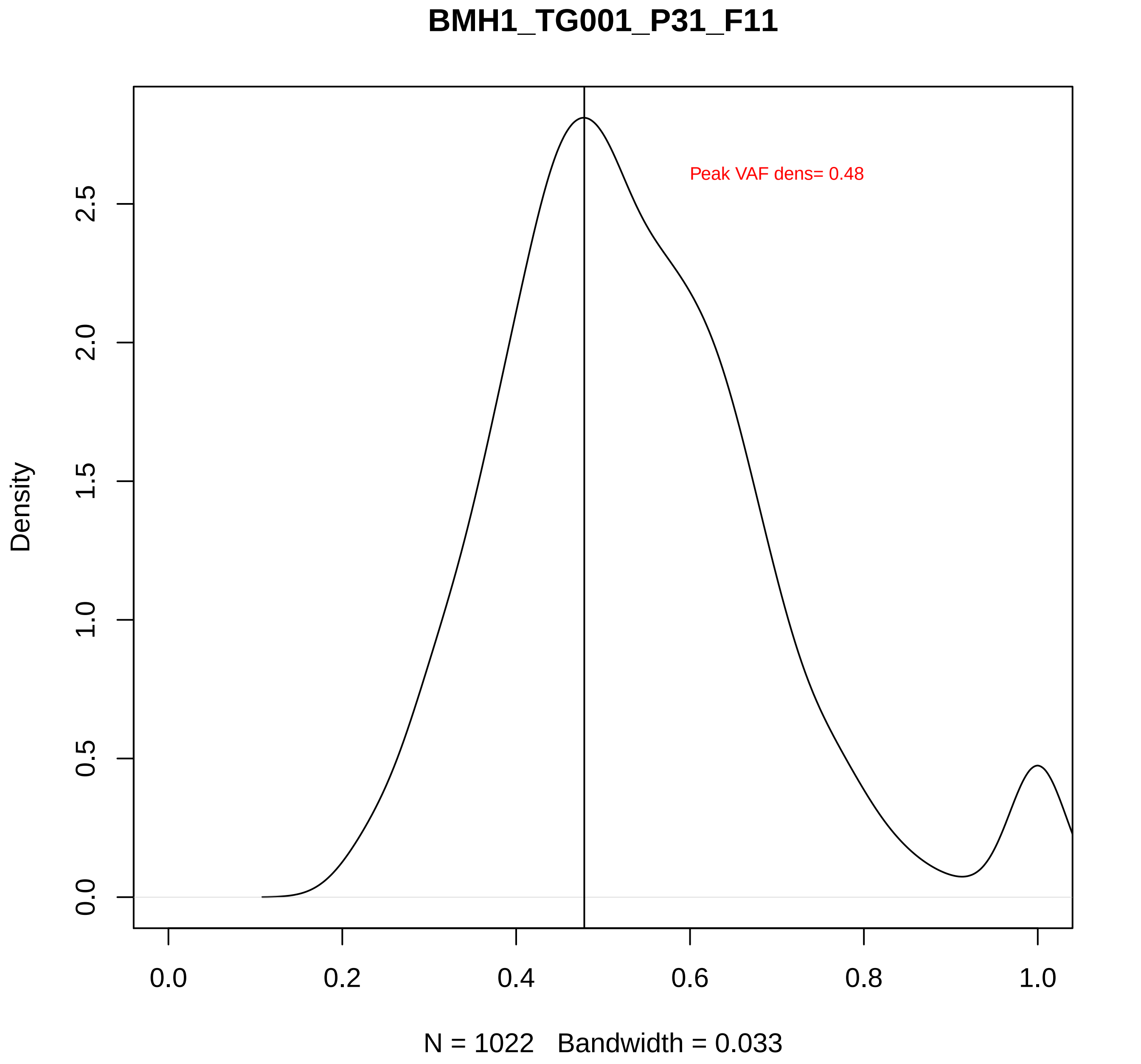
BMH1_TG001_P31_F11
Peak VAF dens= 0.48
Density
0.0
0.5
1.0
1.5
2.0
2.5
0.0
0.2
0.4
0.6
0.8
1.0
N = 1022   Bandwidth = 0.033

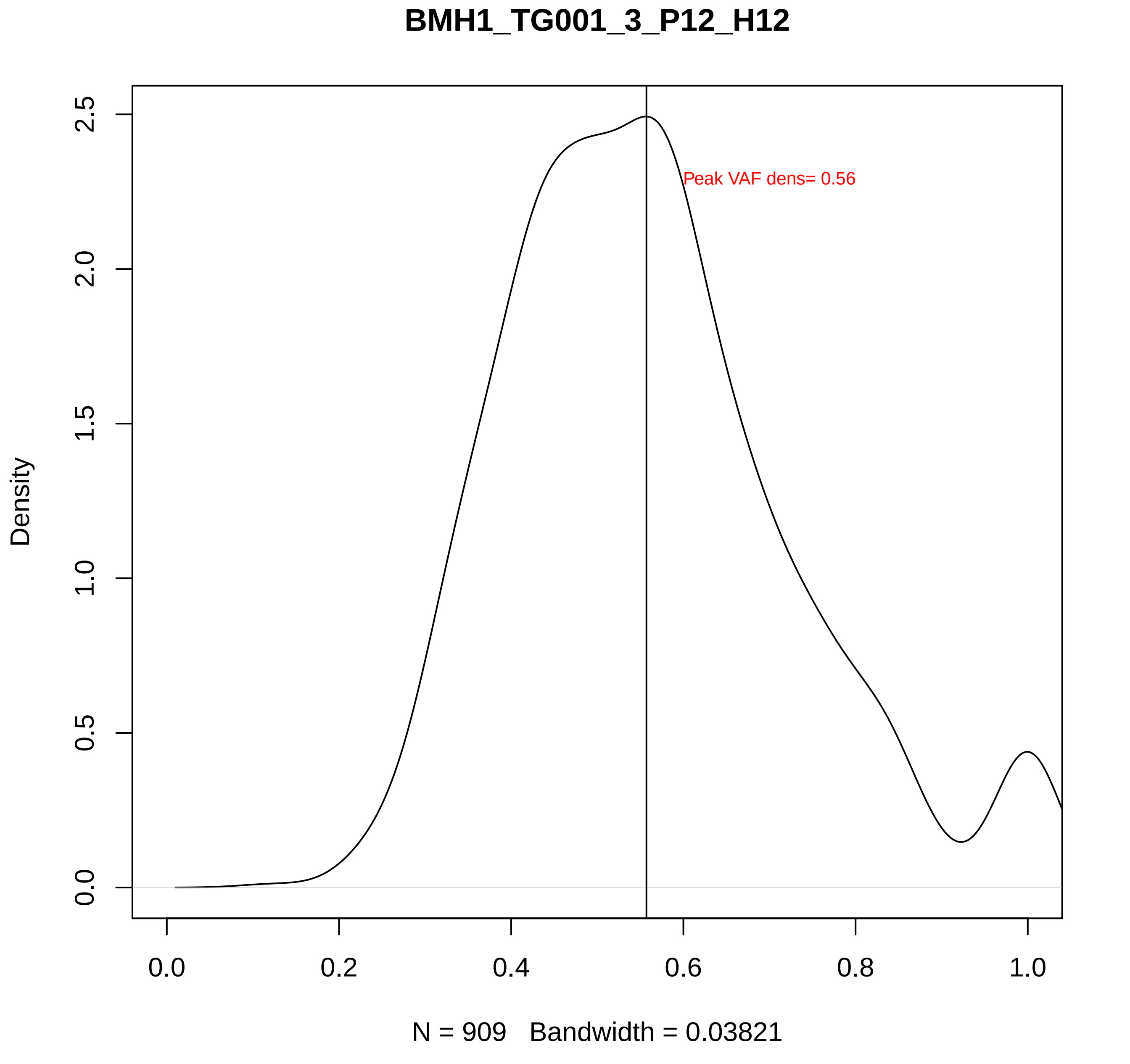

BMH1_TG001_3_P12_H12
Peak VAF dens= 0.56
Density
0.0
0.5
1.0
1.5
2.0
2.5
0.0
0.2
0.4
0.6
0.8
1.0
N = 909 Bandwidth = 0.03821

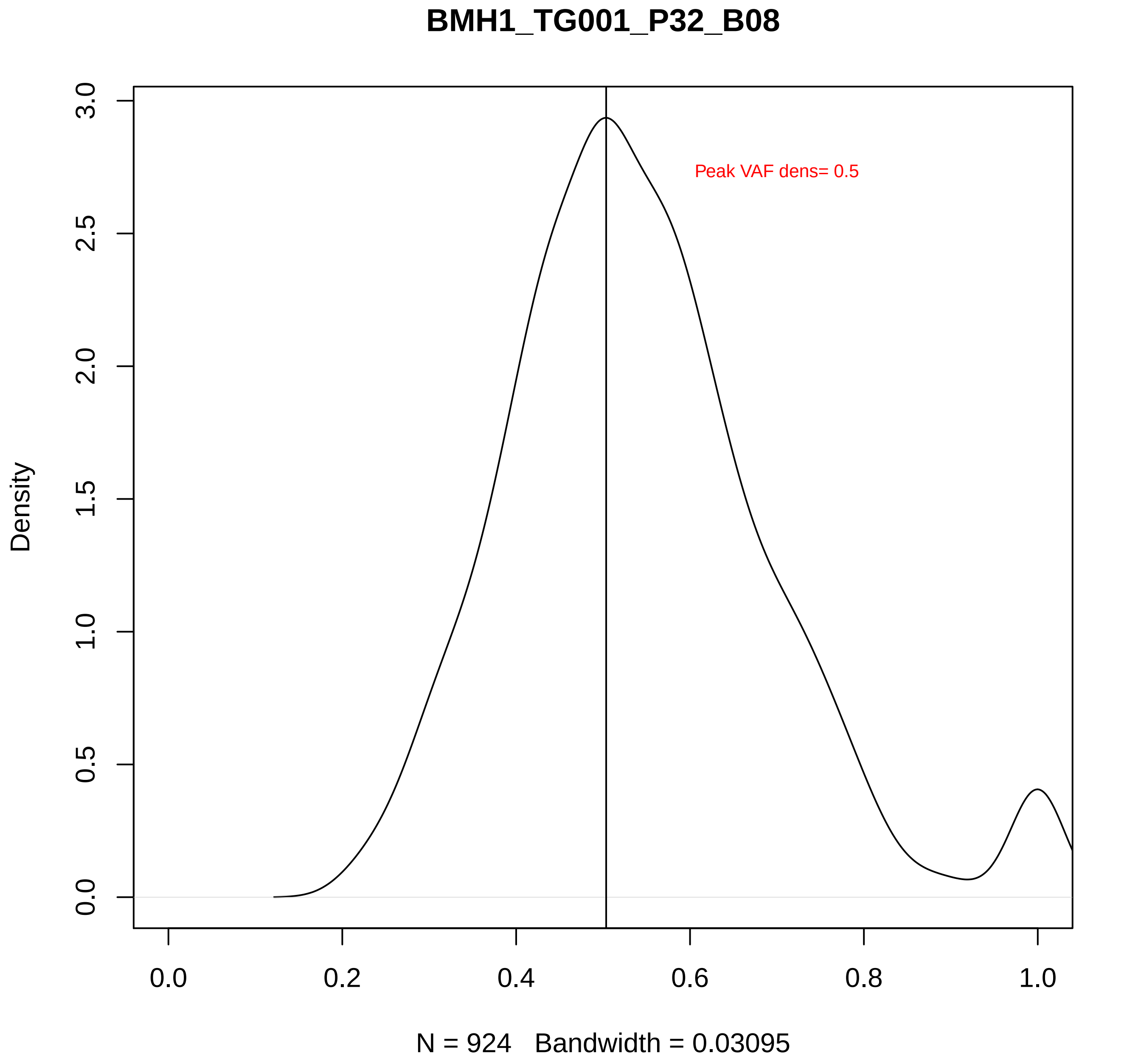
BMH1_TG001_P32_B08
Peak VAF dens= 0.5
Density
0.0
0.5
1.0
1.5
2.0
2.5
3.0
0.0
0.2
0.4
0.6
0.8
1.0
N = 924 Bandwidth = 0.03095

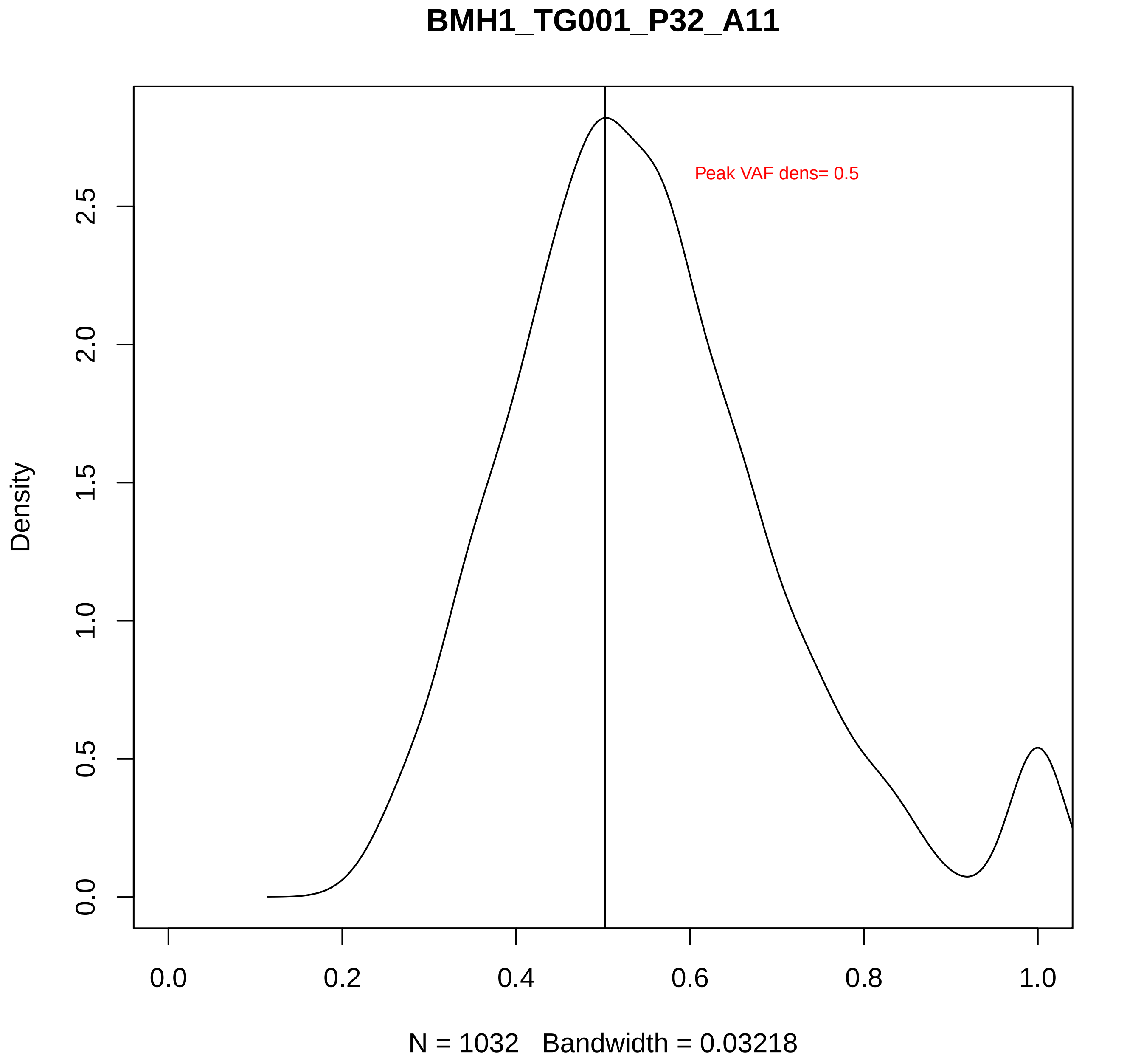

BMH1_TG001_P32_A11
Peak VAF dens= 0.5
Density
0.0
0.5
1.0
1.5
2.0
2.5
0.0
0.2
0.4
0.6
0.8
1.0
N = 1032   Bandwidth = 0.03218

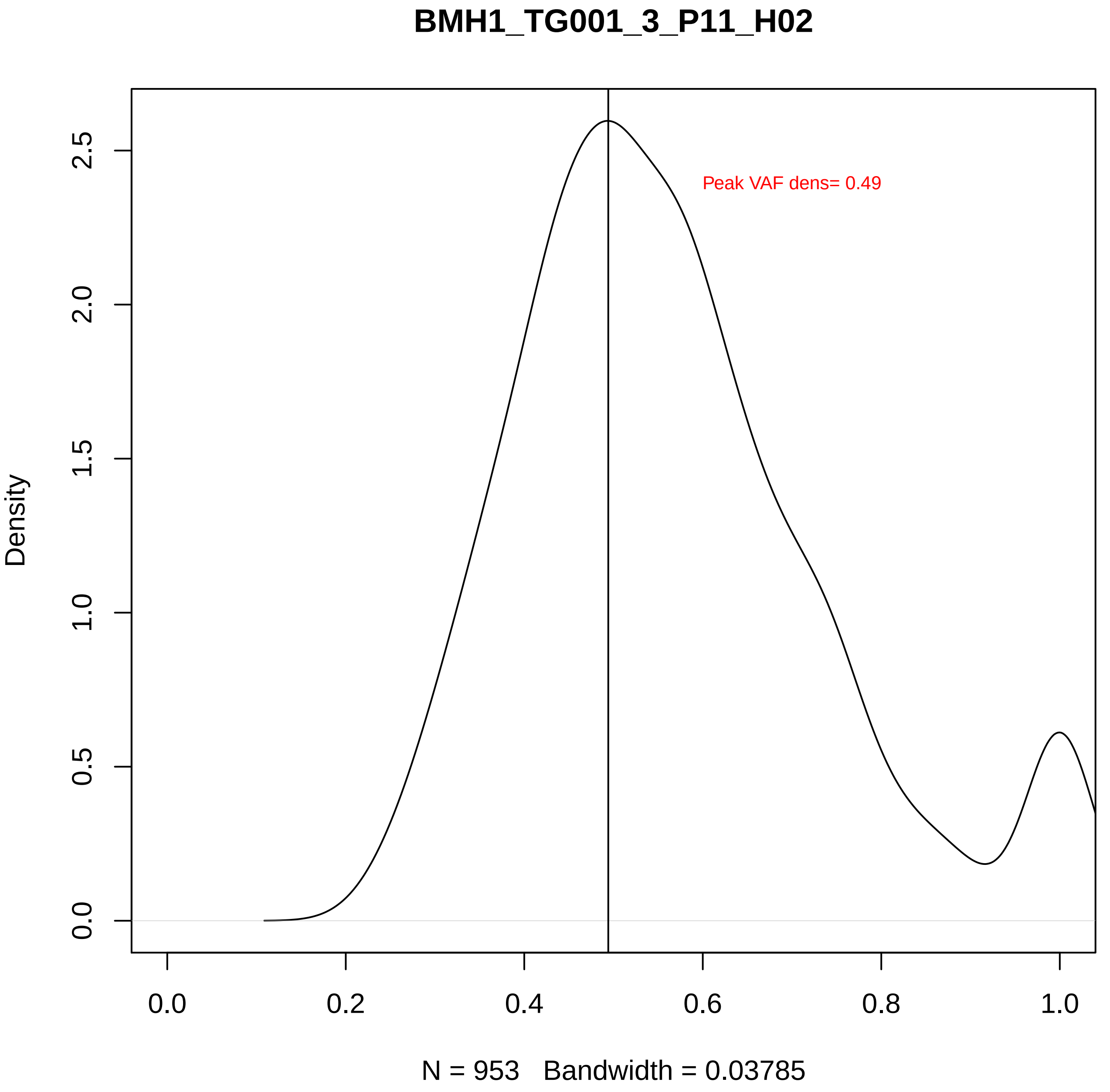
BMH1_TG001_3_P11_H02
Peak VAF dens= 0.49
Density
0.0
0.5
1.0
1.5
2.0
2.5
0.0
0.2
0.4
0.6
0.8
1.0
N = 953 Bandwidth = 0.03785

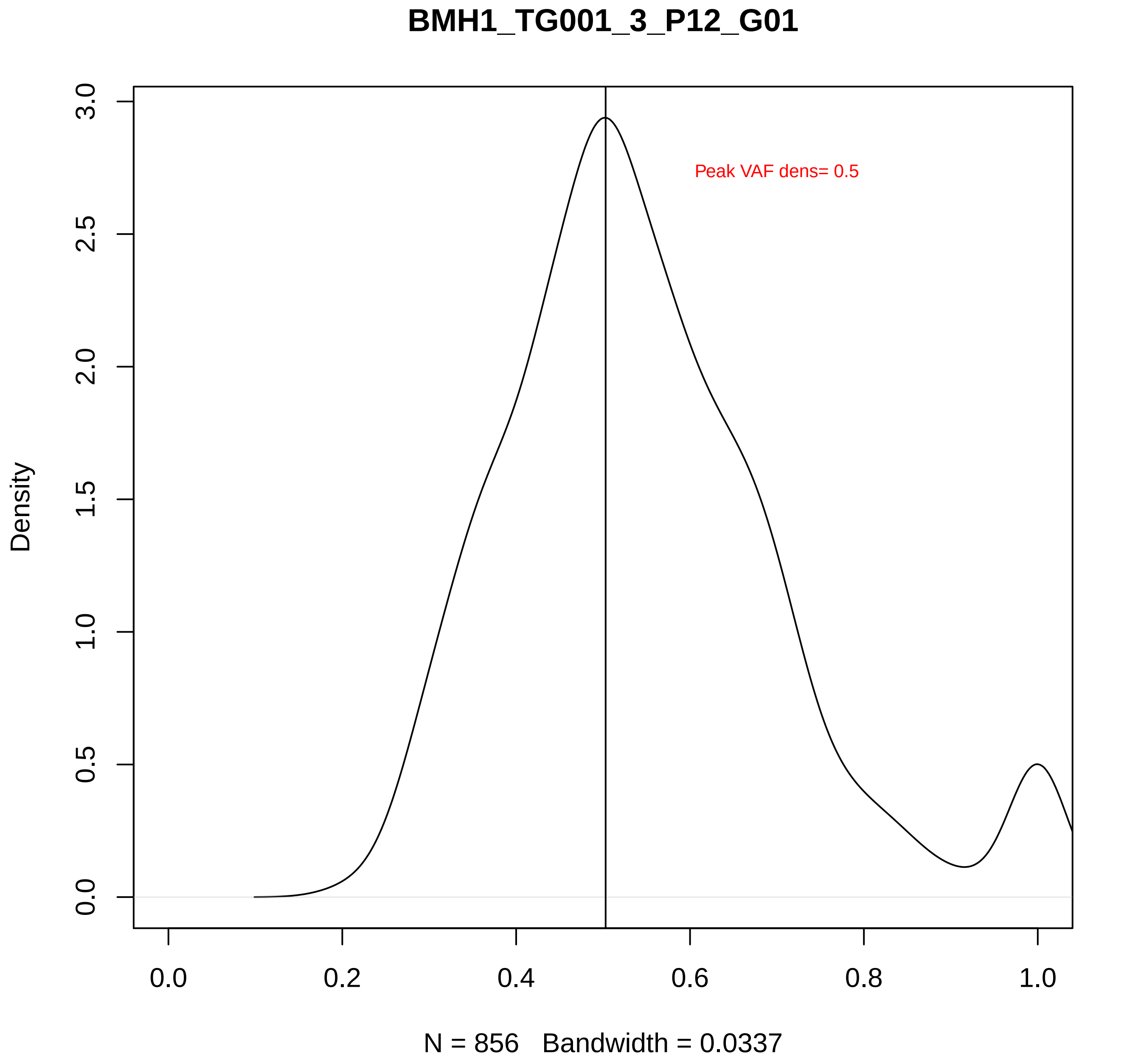

BMH1_TG001_3_P12_G01
Peak VAF dens= 0.5
Density
0.0
0.5
1.0
1.5
2.0
2.5
3.0
0.0
0.2
0.4
0.6
0.8
1.0
N = 856   Bandwidth = 0.0337

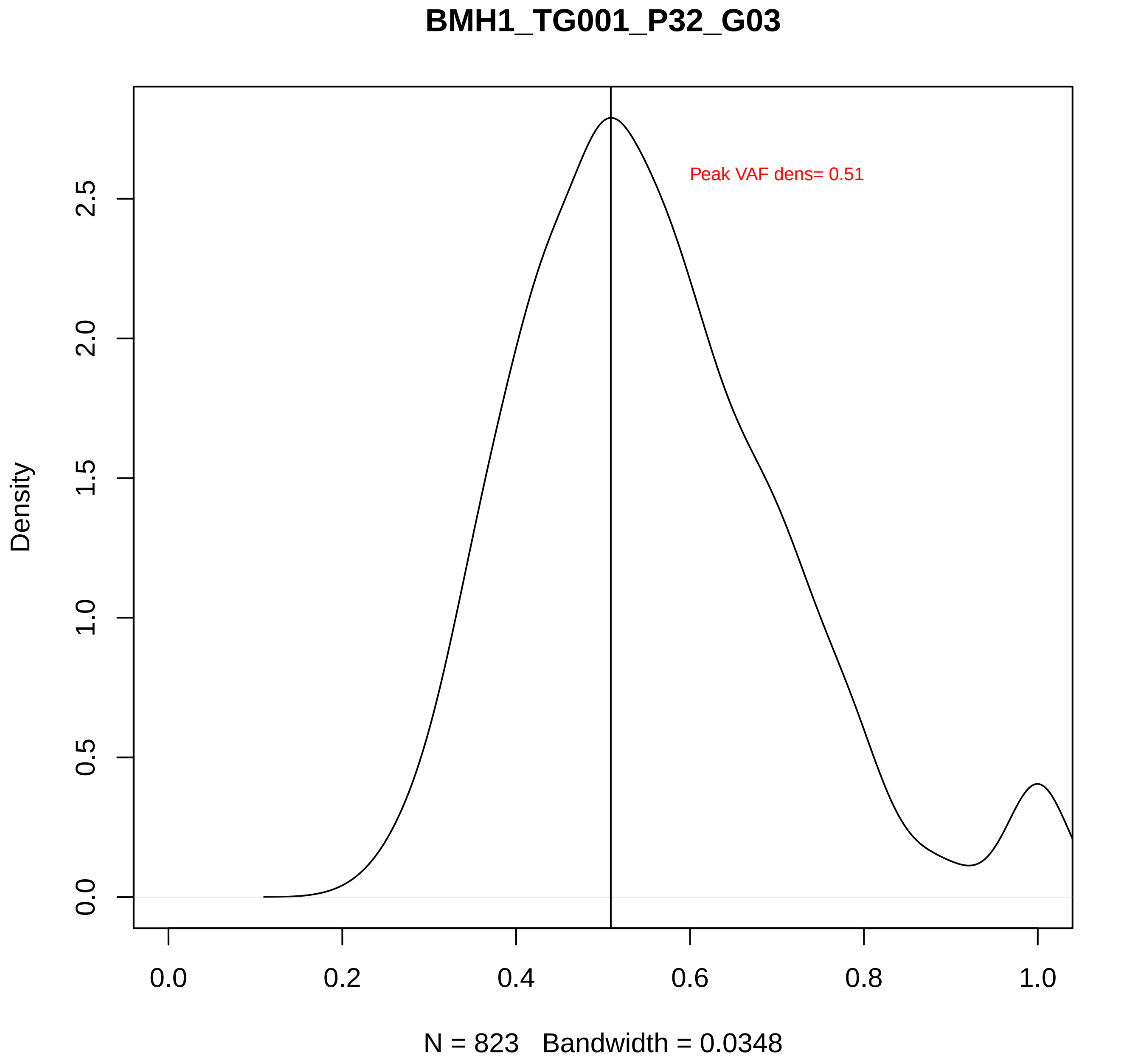

BMH1_TG001_P32_G03
Peak VAF dens= 0.51
Density
0.0
0.5
1.0
1.5
2.0
2.5
0.0
0.2
0.4
0.6
0.8
1.0
N = 823   Bandwidth = 0.0348

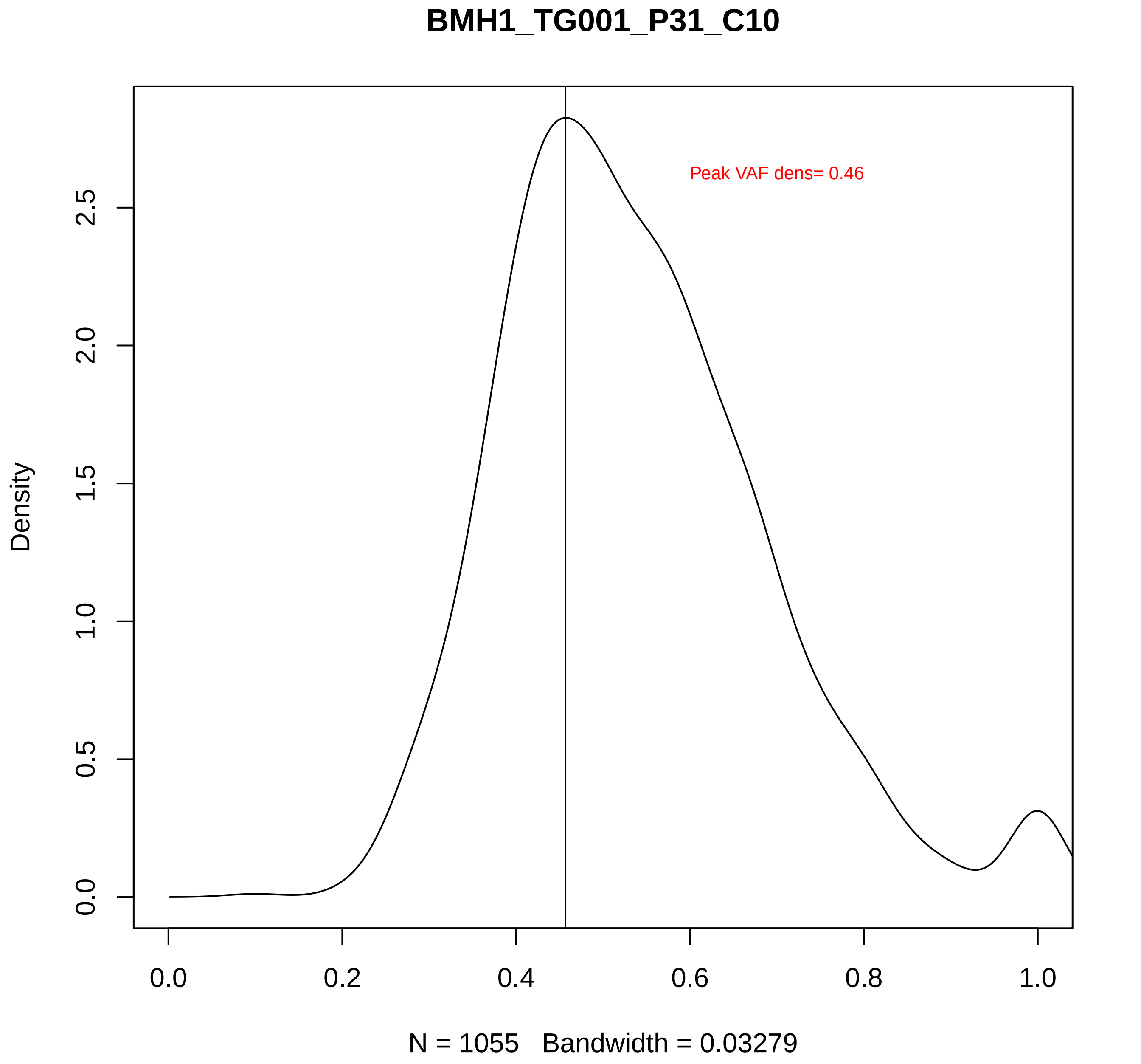

BMH1_TG001_P31_C10
Peak VAF dens= 0.46
Density
0.0
0.5
1.0
1.5
2.0
2.5
0.0
0.2
0.4
0.6
0.8
1.0
N = 1055 Bandwidth = 0.03279

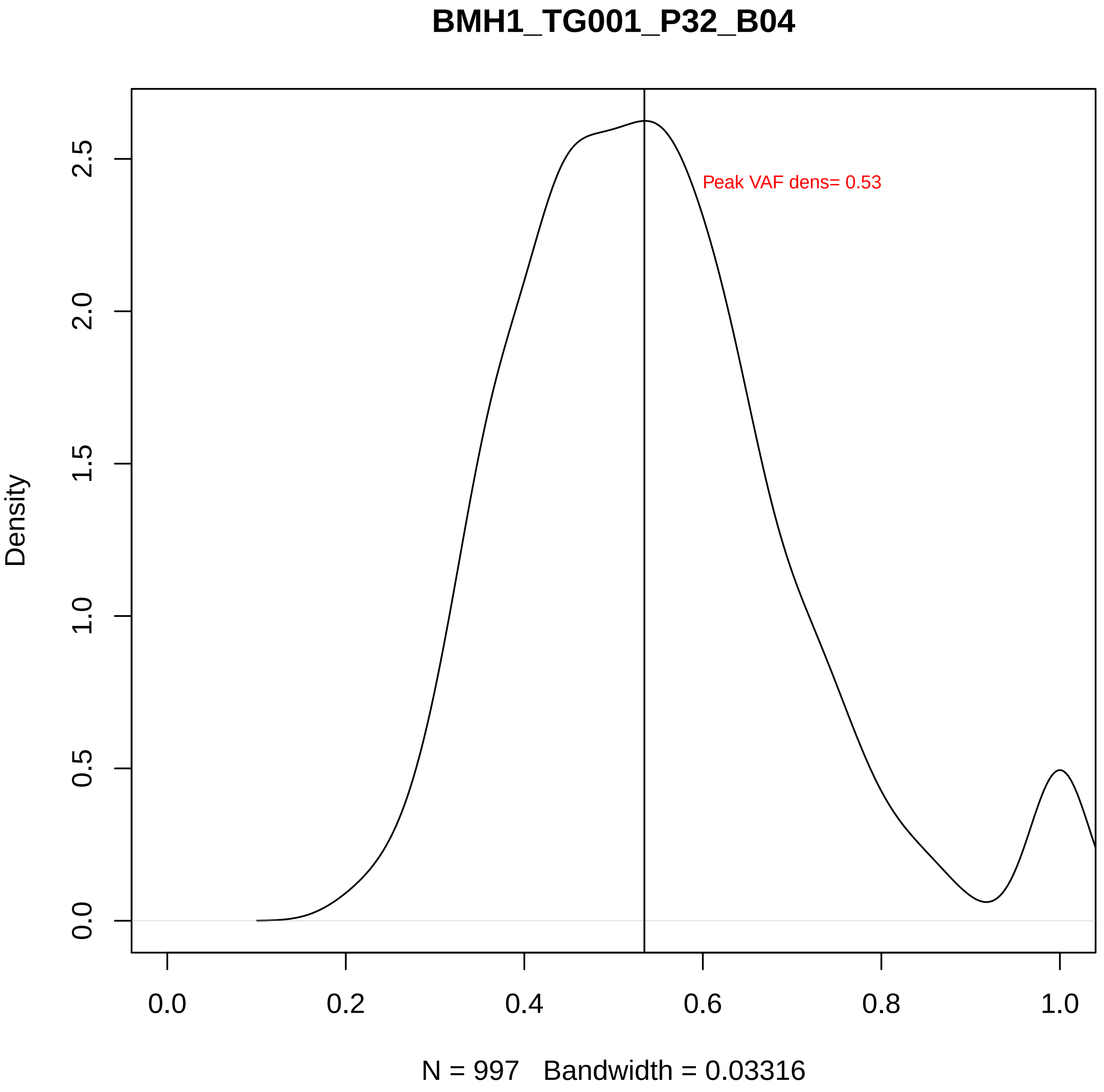

BMH1_TG001_P32_B04
Peak VAF dens= 0.53
Density
0.0
0.5
1.0
1.5
2.0
2.5
0.0
0.2
0.4
0.6
0.8
1.0
N = 997 Bandwidth = 0.03316

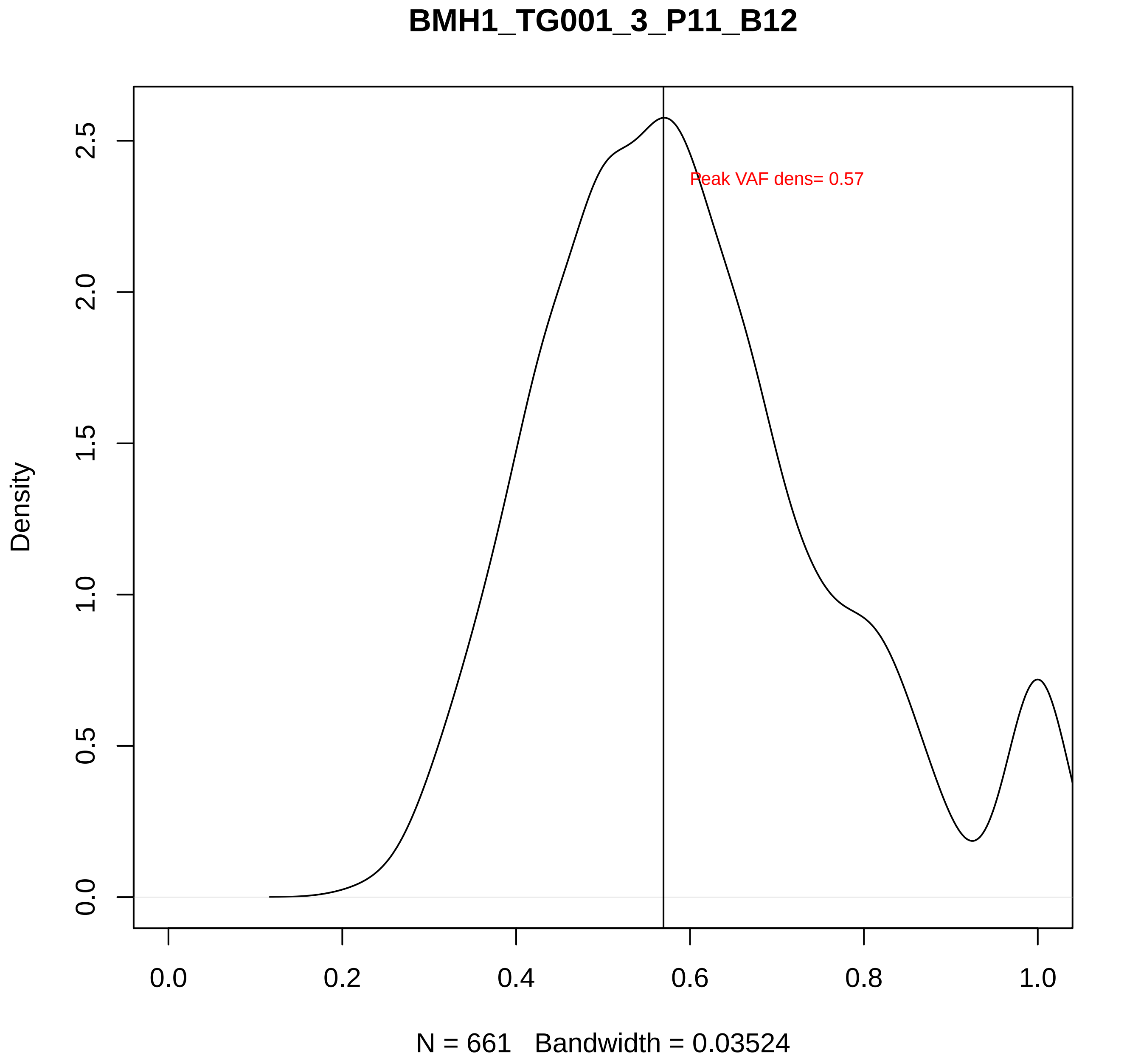
BMH1_TG001_3_P11_B12
Density
0.0
0.5
1.0
1.5
2.0
2.5
0.0
0.2
0.4
0.6
0.8
1.0
Peak VAF dens= 0.57
N = 661 Bandwidth = 0.03524

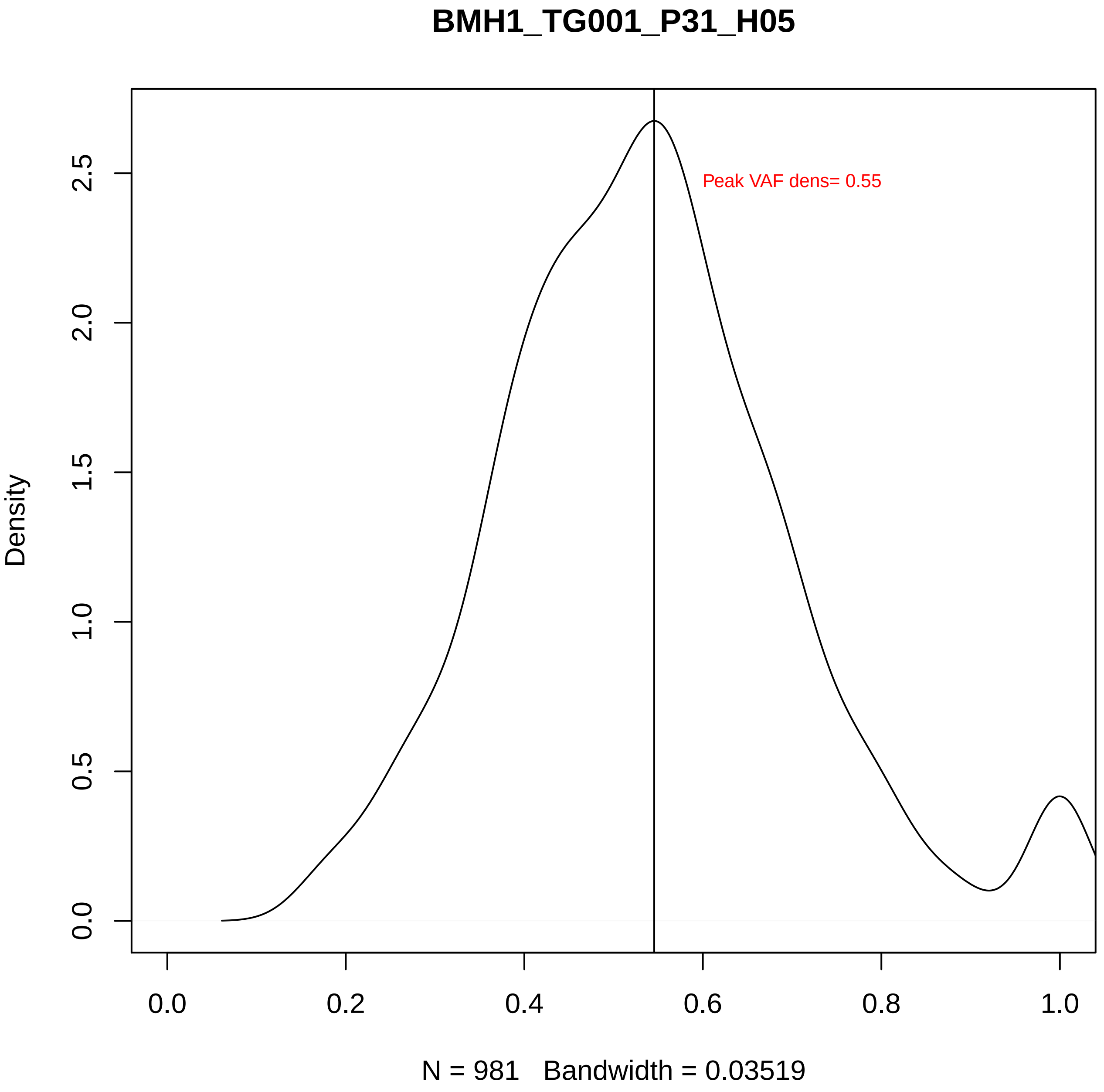
BMH1_TG001_P31_H05
Peak VAF dens= 0.55
Density
0.0
0.5
1.0
1.5
2.0
2.5
0.0
0.2
0.4
0.6
0.8
1.0
N = 981 Bandwidth = 0.03519

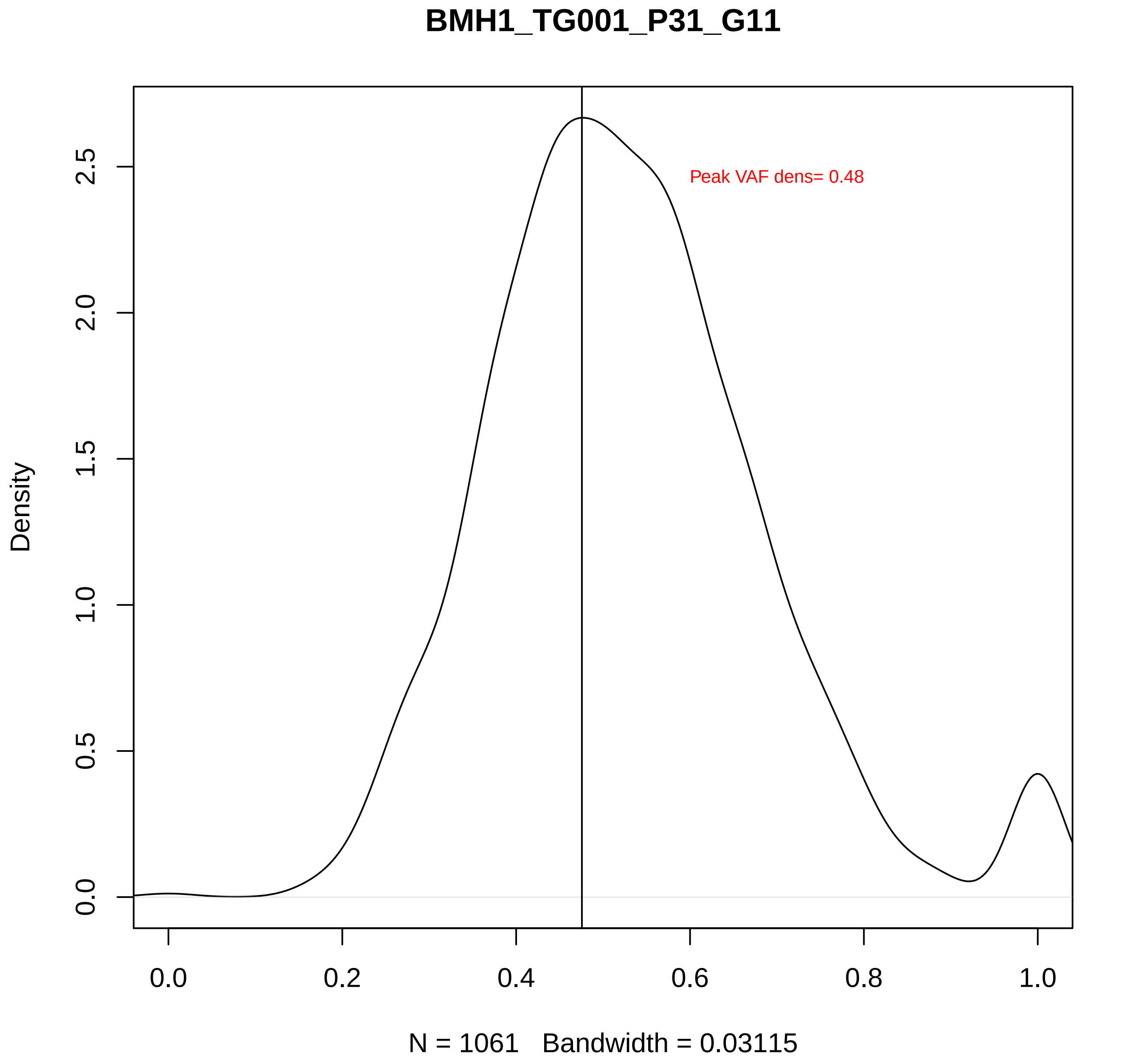
BMH1_TG001_P31_G11
Peak VAF dens= 0.48
Density
0.0
0.5
1.0
1.5
2.0
2.5
0.0
0.2
0.4
0.6
0.8
1.0
N = 1061   Bandwidth = 0.03115

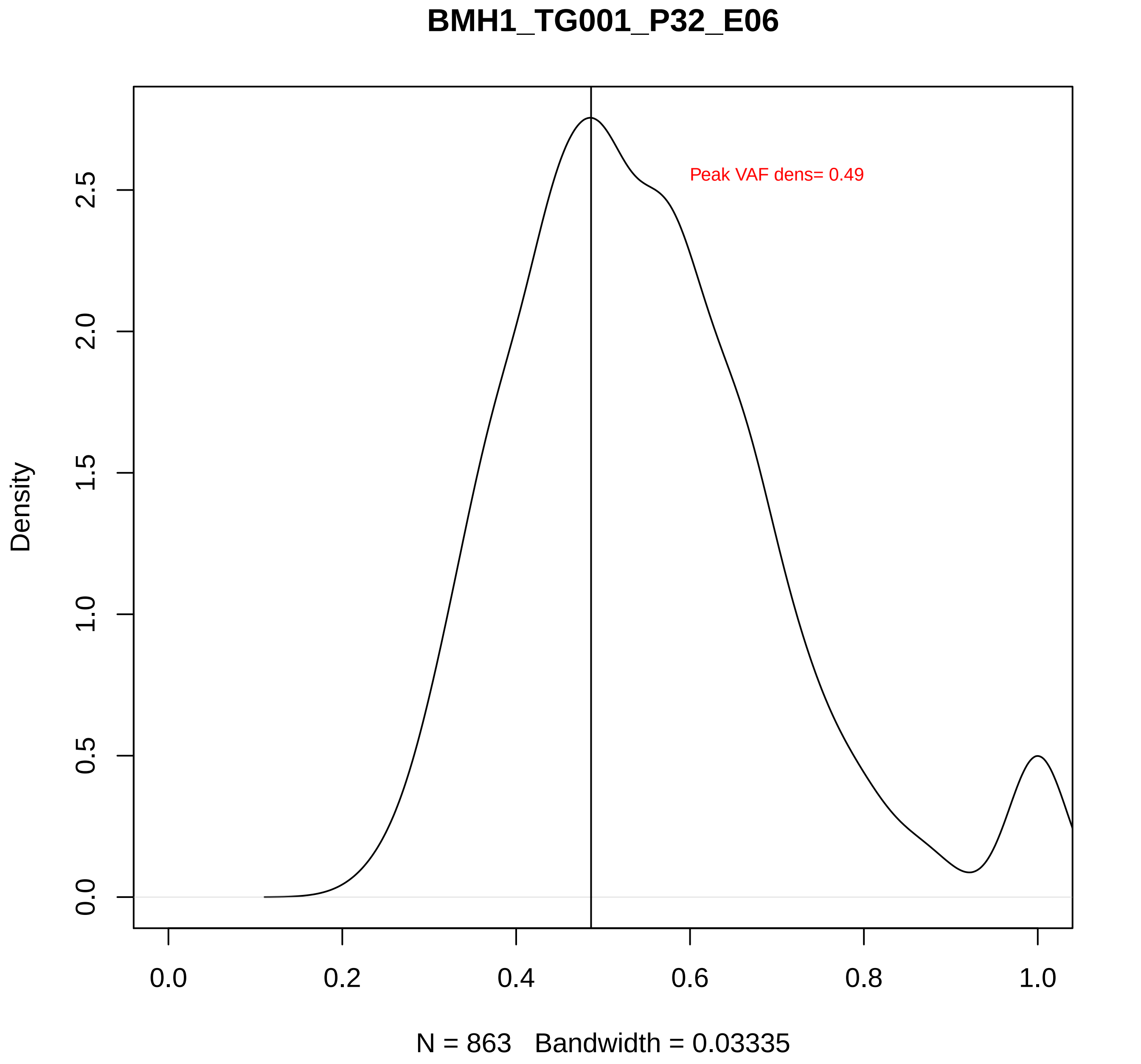

BMH1_TG001_P32_E06
Peak VAF dens= 0.49
Density
0.0
0.5
1.0
1.5
2.0
2.5
0.0
0.2
0.4
0.6
0.8
1.0
N = 863   Bandwidth = 0.03335

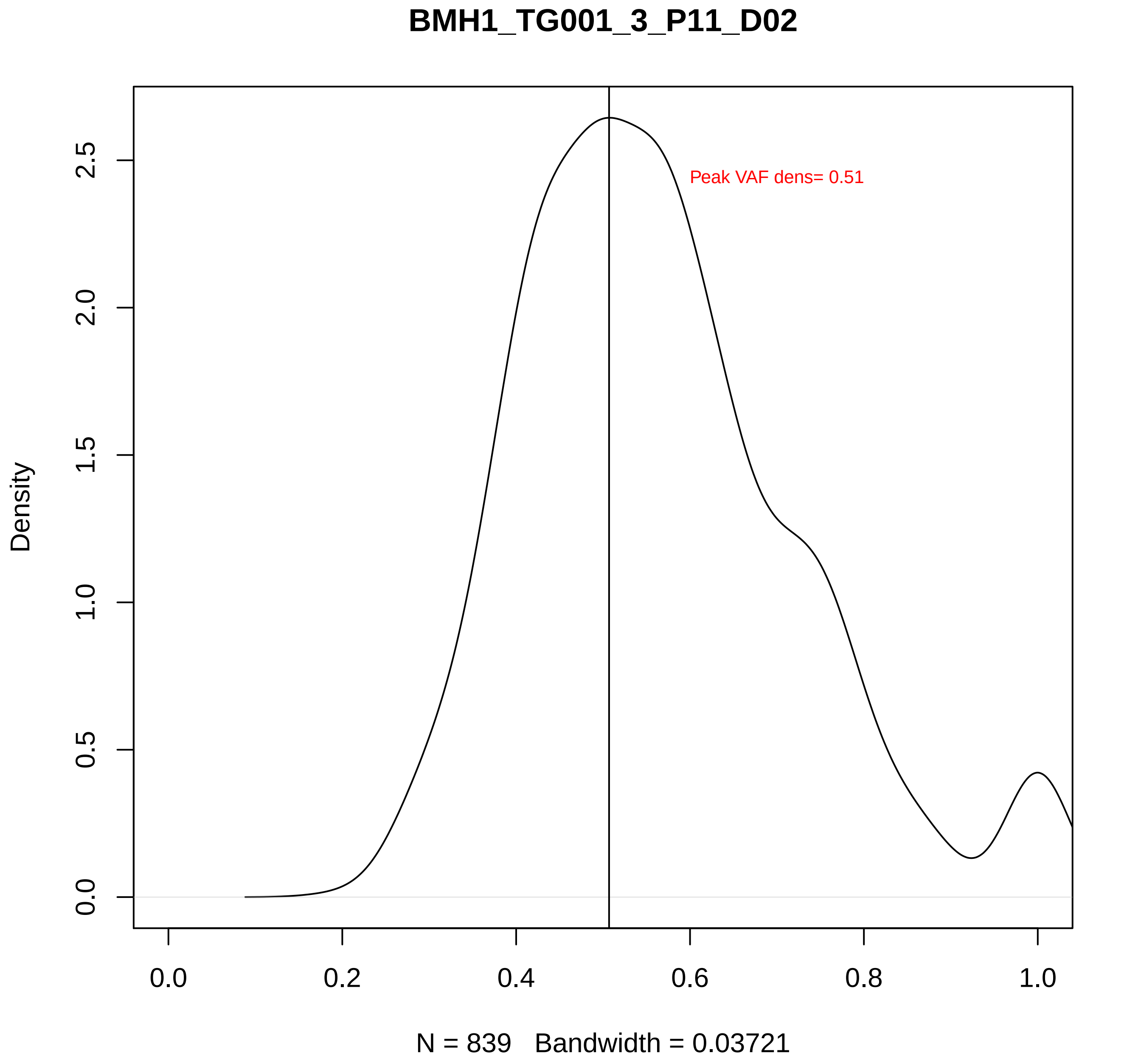
BMH1_TG001_3_P11_D02
Peak VAF dens= 0.51
Density
0.0
0.5
1.0
1.5
2.0
2.5
0.0
0.2
0.4
0.6
0.8
1.0
N = 839   Bandwidth = 0.03721

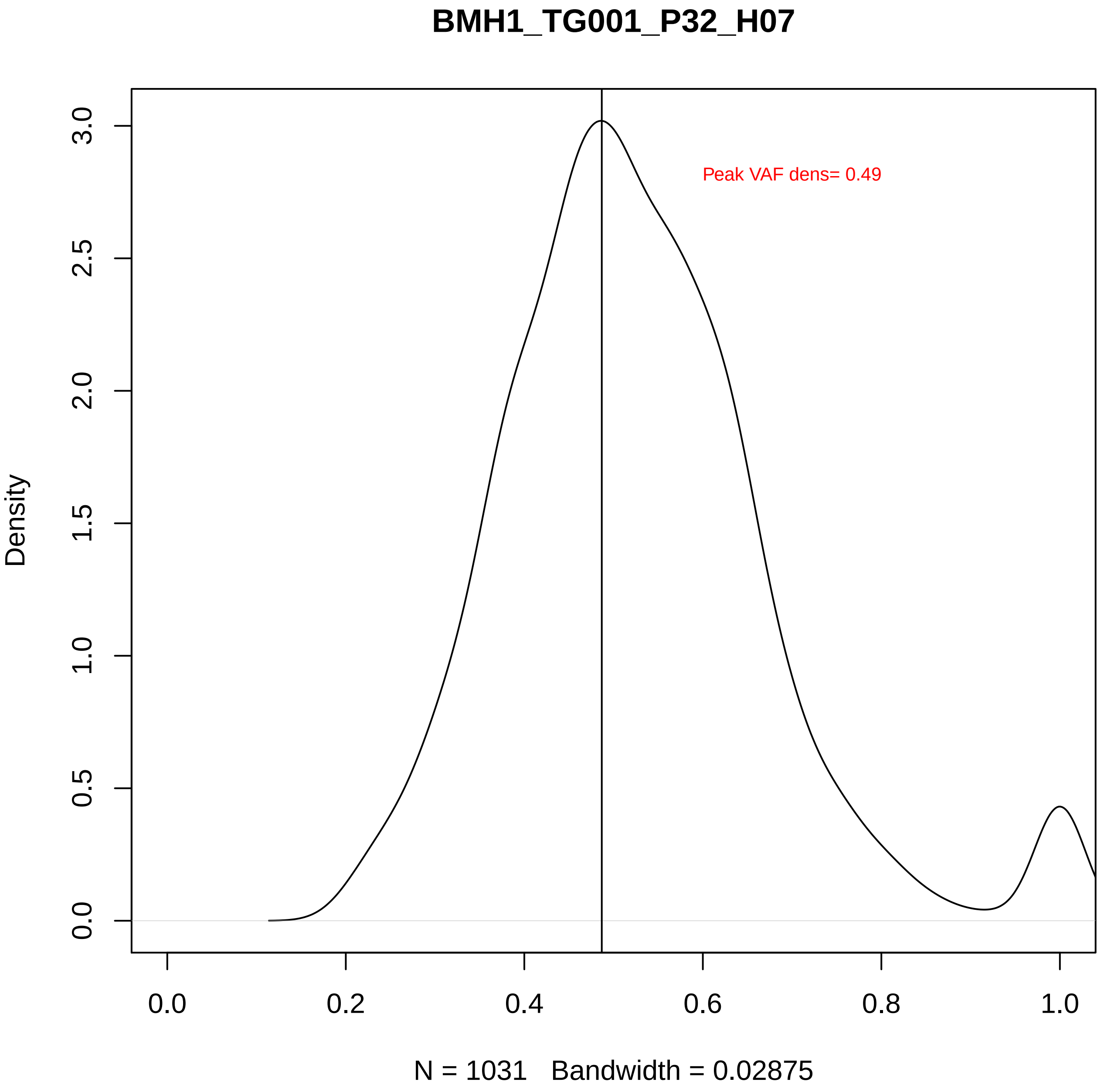

BMH1_TG001_P32_H07
Peak VAF dens= 0.49
Density
0.0
0.5
1.0
1.5
2.0
2.5
3.0
0.0
0.2
0.4
0.6
0.8
1.0
N = 1031 Bandwidth = 0.02875

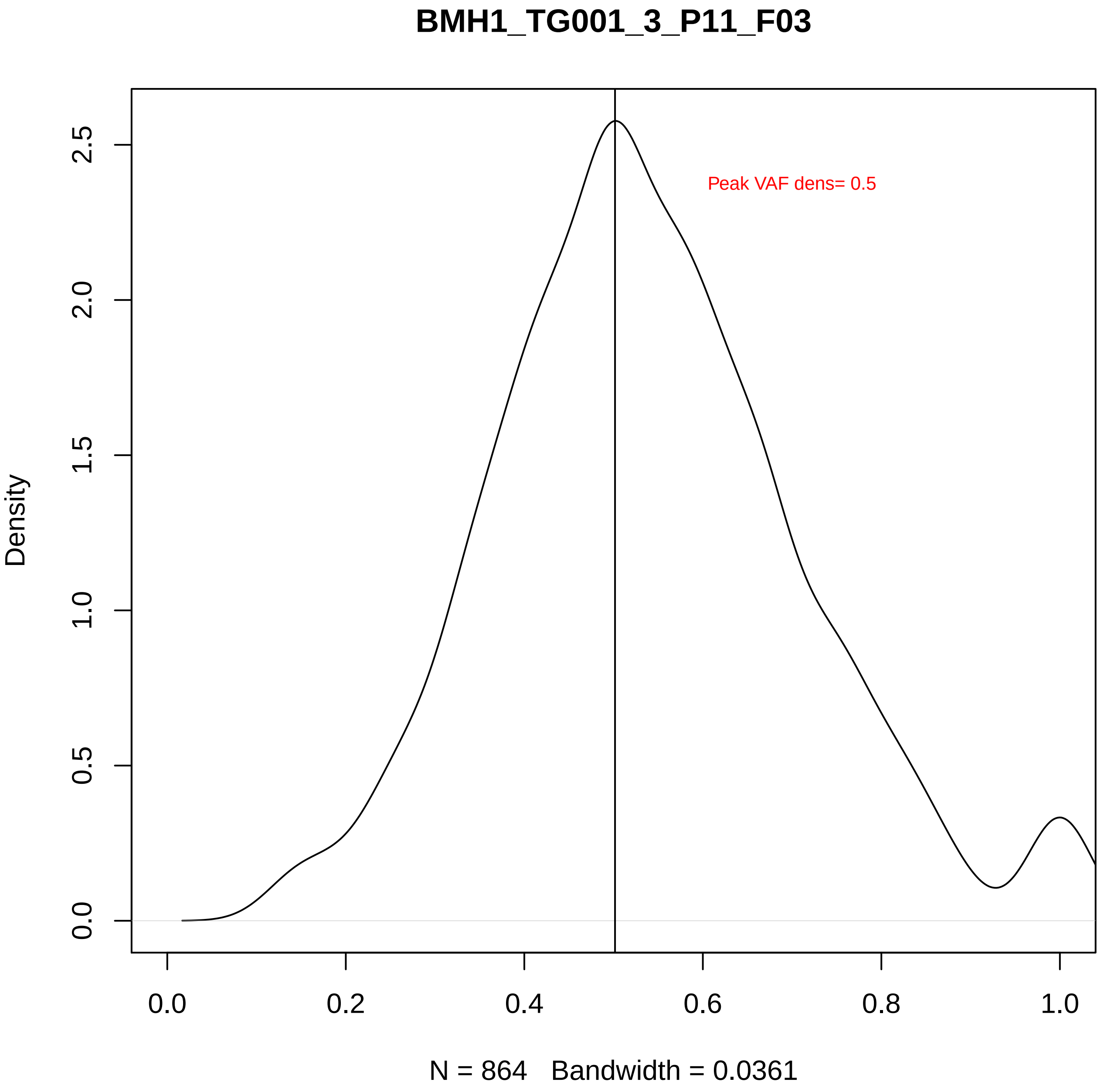
BMH1_TG001_3_P11_F03
Peak VAF dens= 0.5
Density
0.0
0.5
1.0
1.5
2.0
2.5
0.0
0.2
0.4
0.6
0.8
1.0
N = 864   Bandwidth = 0.0361

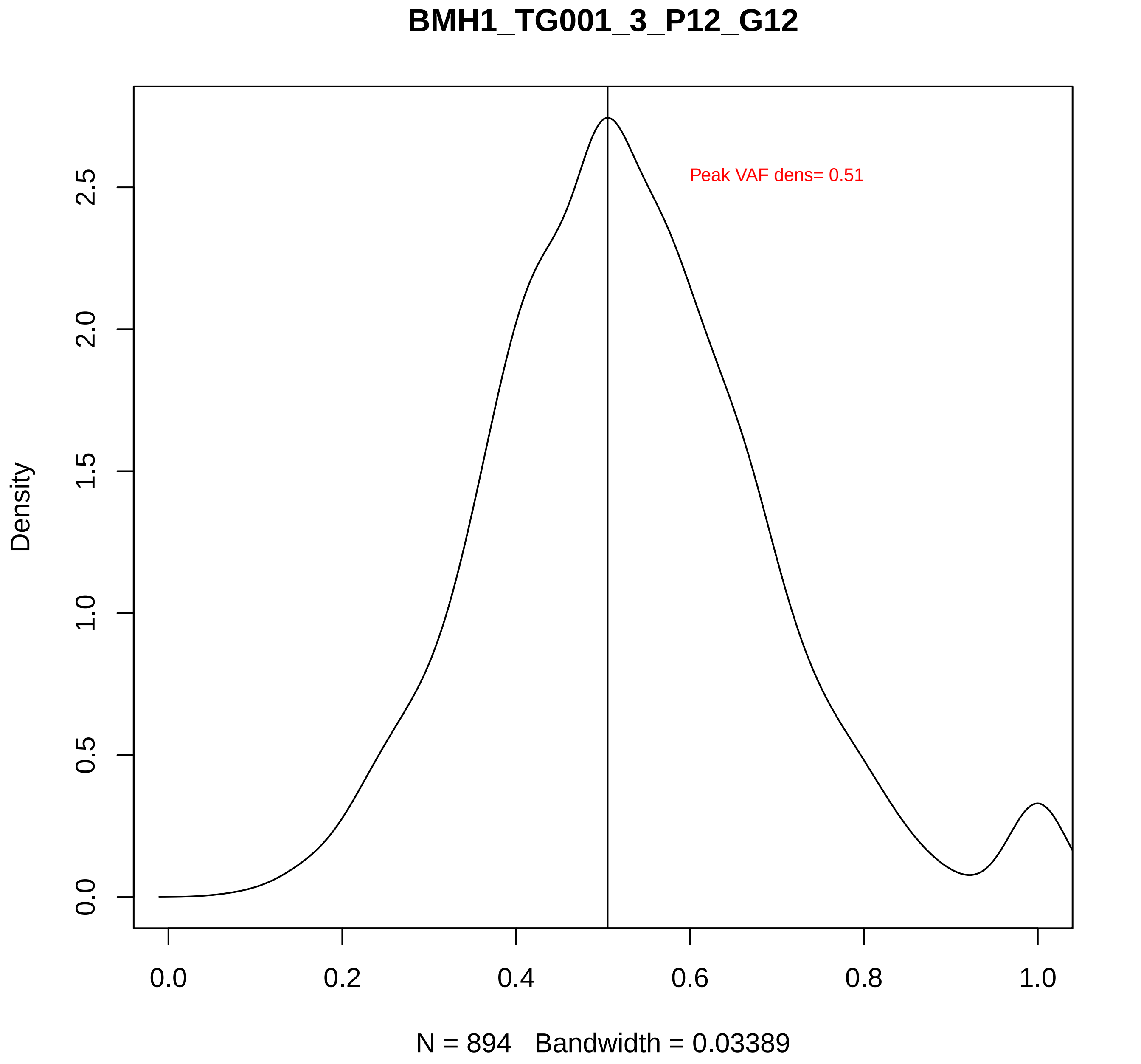

BMH1_TG001_3_P12_G12
Peak VAF dens= 0.51
Density
0.0
0.5
1.0
1.5
2.0
2.5
0.0
0.2
0.4
0.6
0.8
1.0
N = 894 Bandwidth = 0.03389

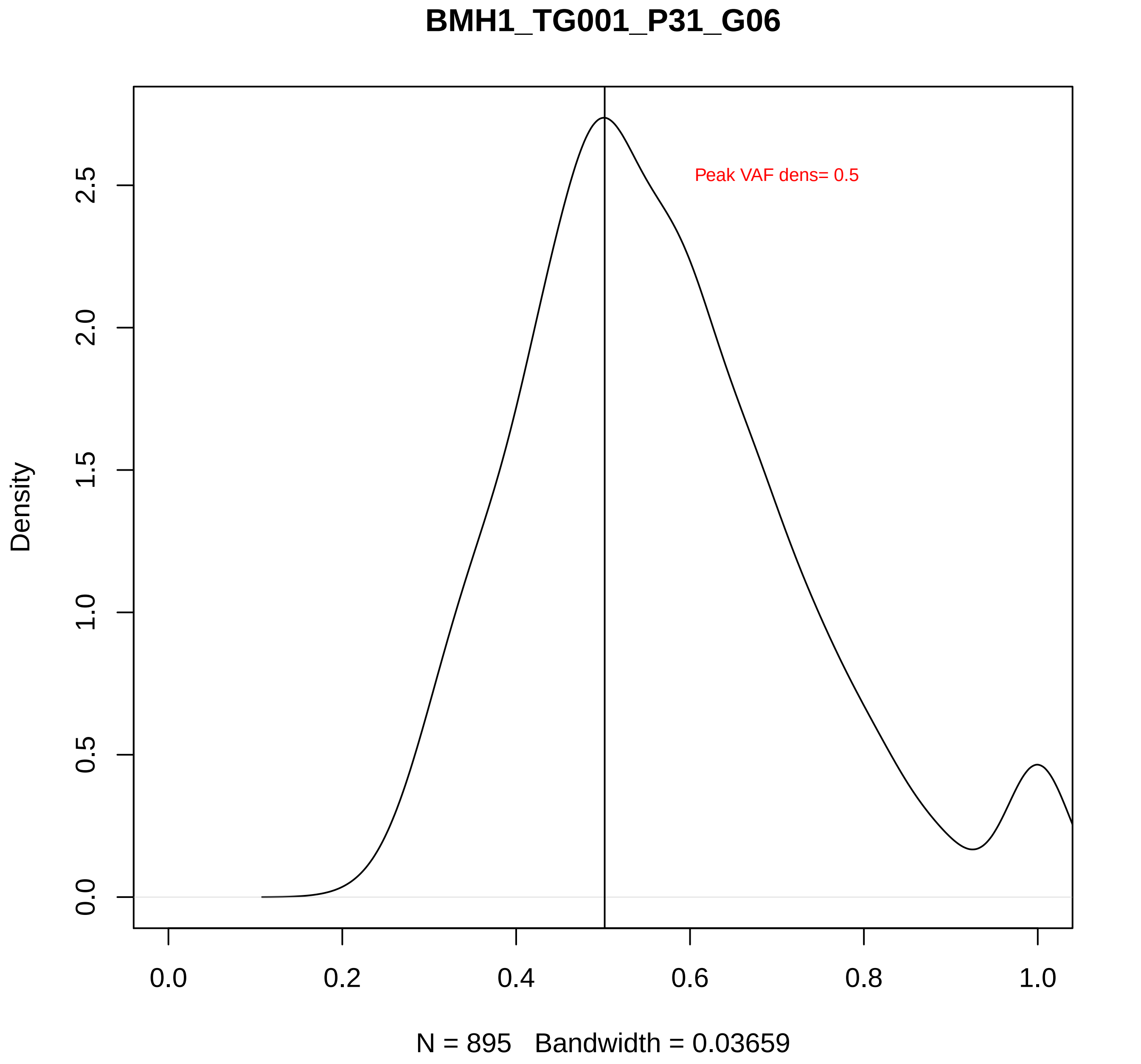
BMH1_TG001_P31_G06
Peak VAF dens= 0.5
Density
0.0
0.5
1.0
1.5
2.0
2.5
0.0
0.2
0.4
0.6
0.8
1.0
N = 895   Bandwidth = 0.03659

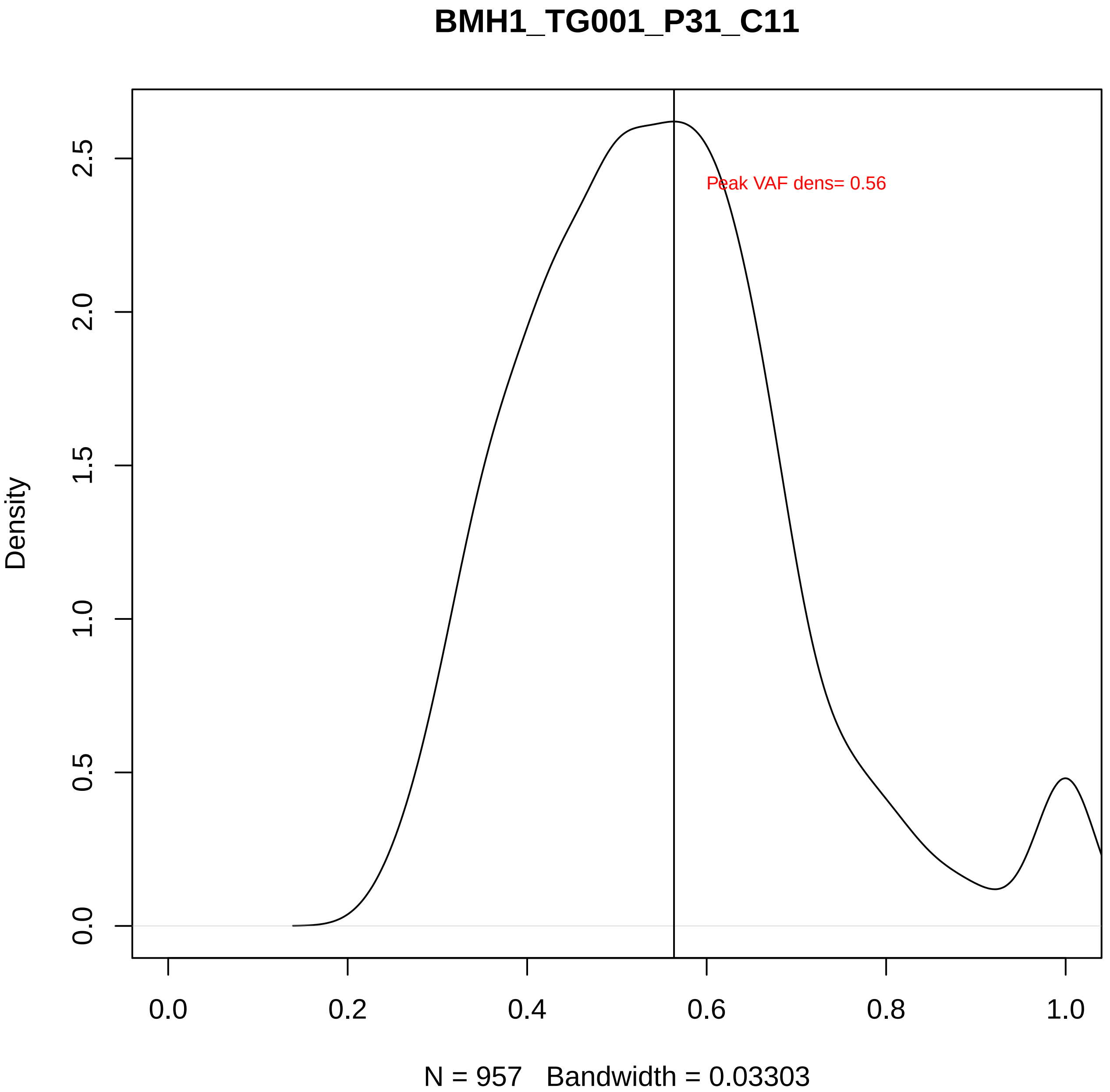
BMH1_TG001_P31_C11
Peak VAF dens= 0.56
Density
0.0
0.5
1.0
1.5
2.0
2.5
0.0
0.2
0.4
0.6
0.8
1.0
N = 957   Bandwidth = 0.03303

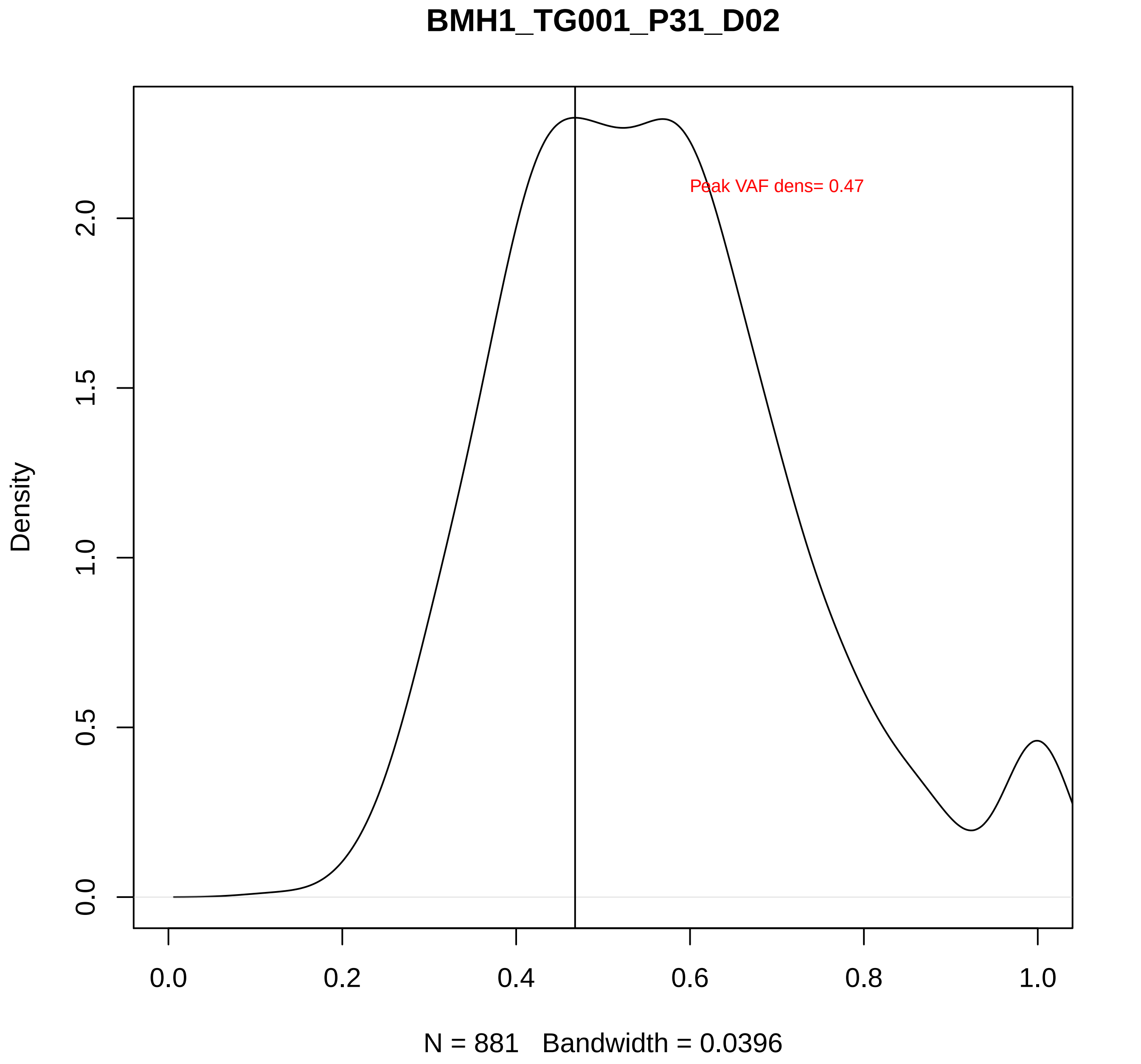
BMH1_TG001_P31_D02
Peak VAF dens= 0.47
Density
0.0
0.5
1.0
1.5
2.0
0.0
0.2
0.4
0.6
0.8
1.0
N = 881   Bandwidth = 0.0396

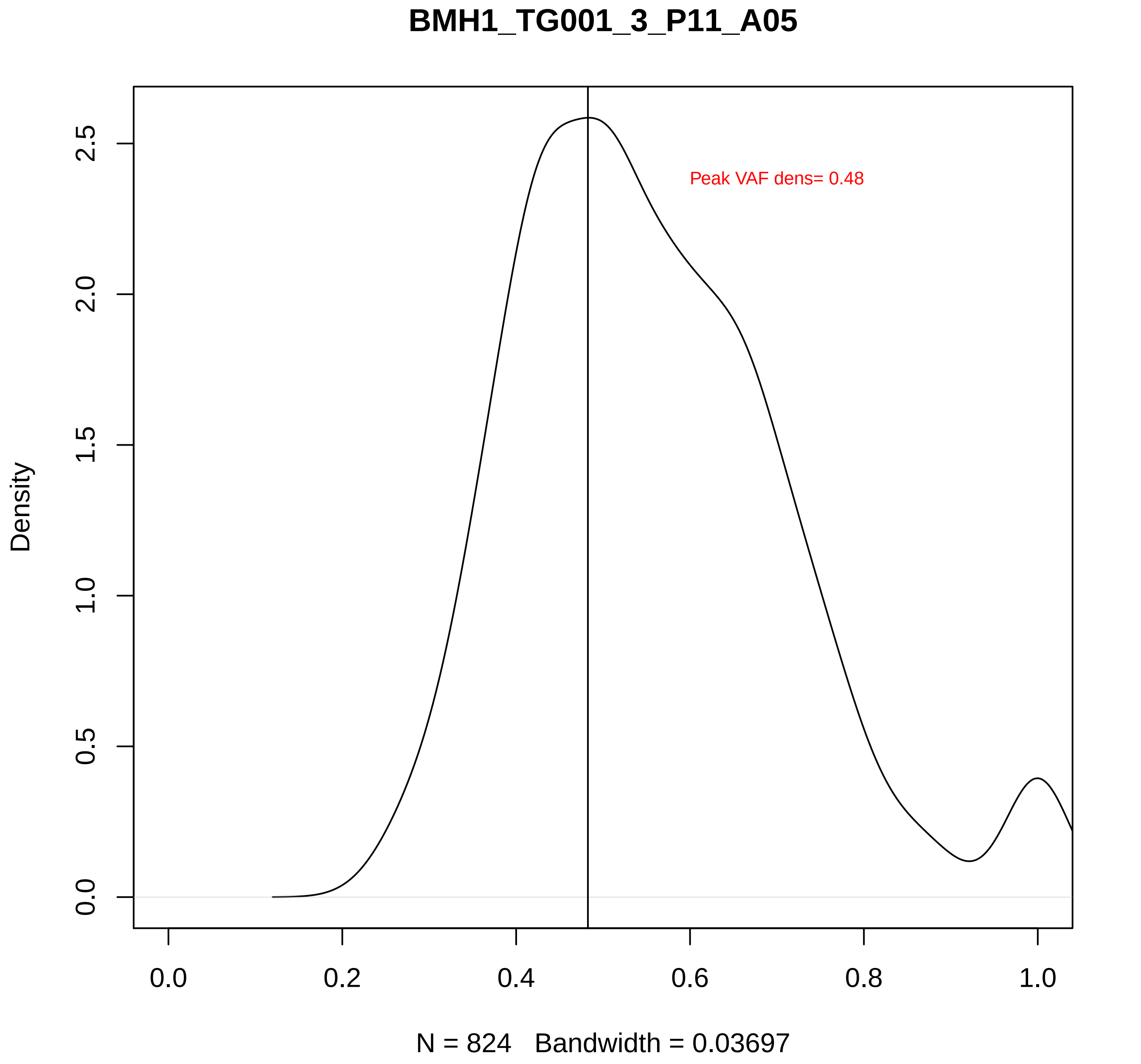
BMH1_TG001_3_P11_A05
Peak VAF dens= 0.48
Density
0.0
0.5
1.0
1.5
2.0
2.5
0.0
0.2
0.4
0.6
0.8
1.0
N = 824   Bandwidth = 0.03697

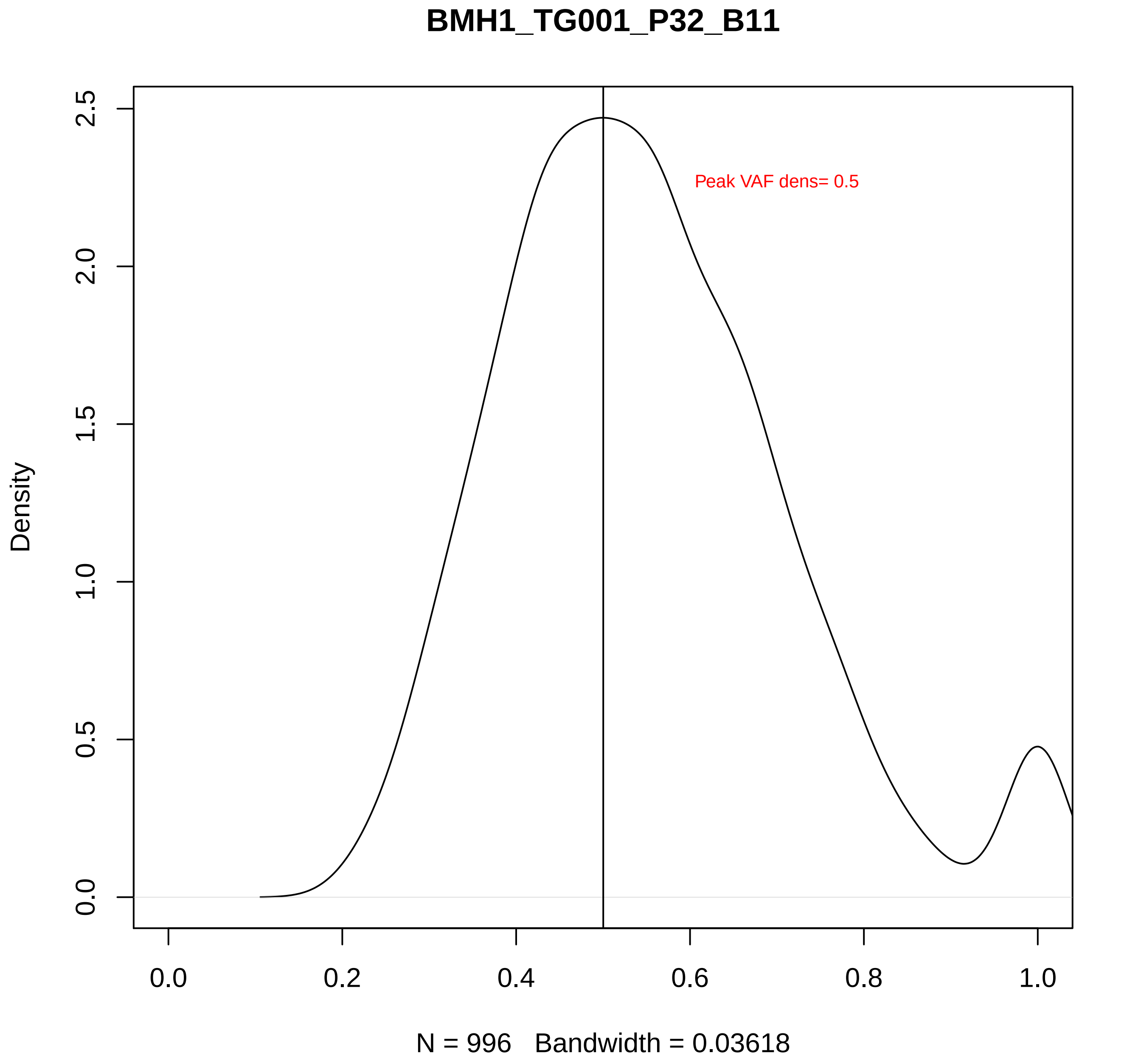
BMH1_TG001_P32_B11
Peak VAF dens= 0.5
Density
0.0
0.5
1.0
1.5
2.0
2.5
0.0
0.2
0.4
0.6
0.8
1.0
N = 996   Bandwidth = 0.03618

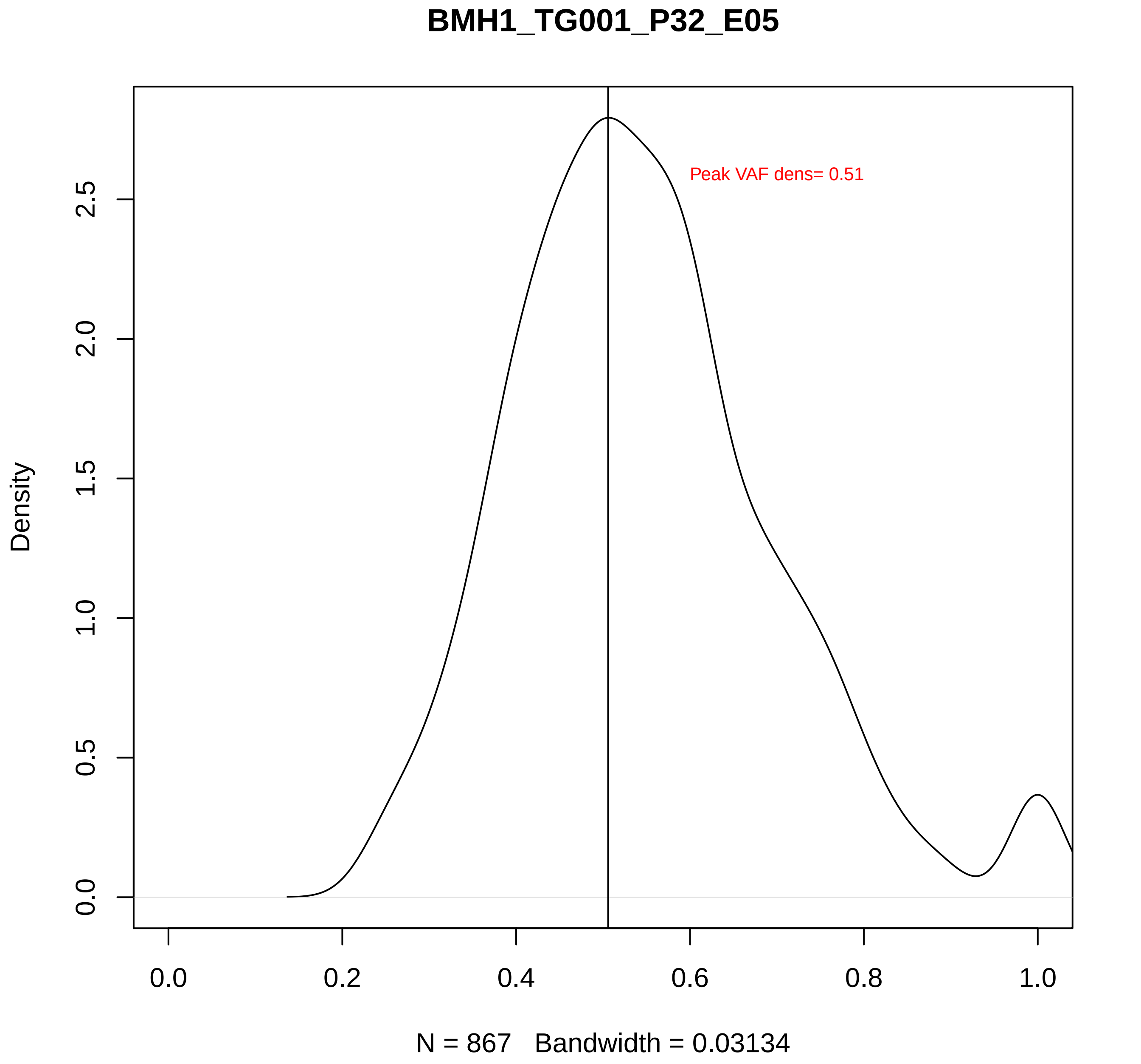

BMH1_TG001_P32_E05
Peak VAF dens= 0.51
Density
0.0
0.5
1.0
1.5
2.0
2.5
0.0
0.2
0.4
0.6
0.8
1.0
N = 867 Bandwidth = 0.03134

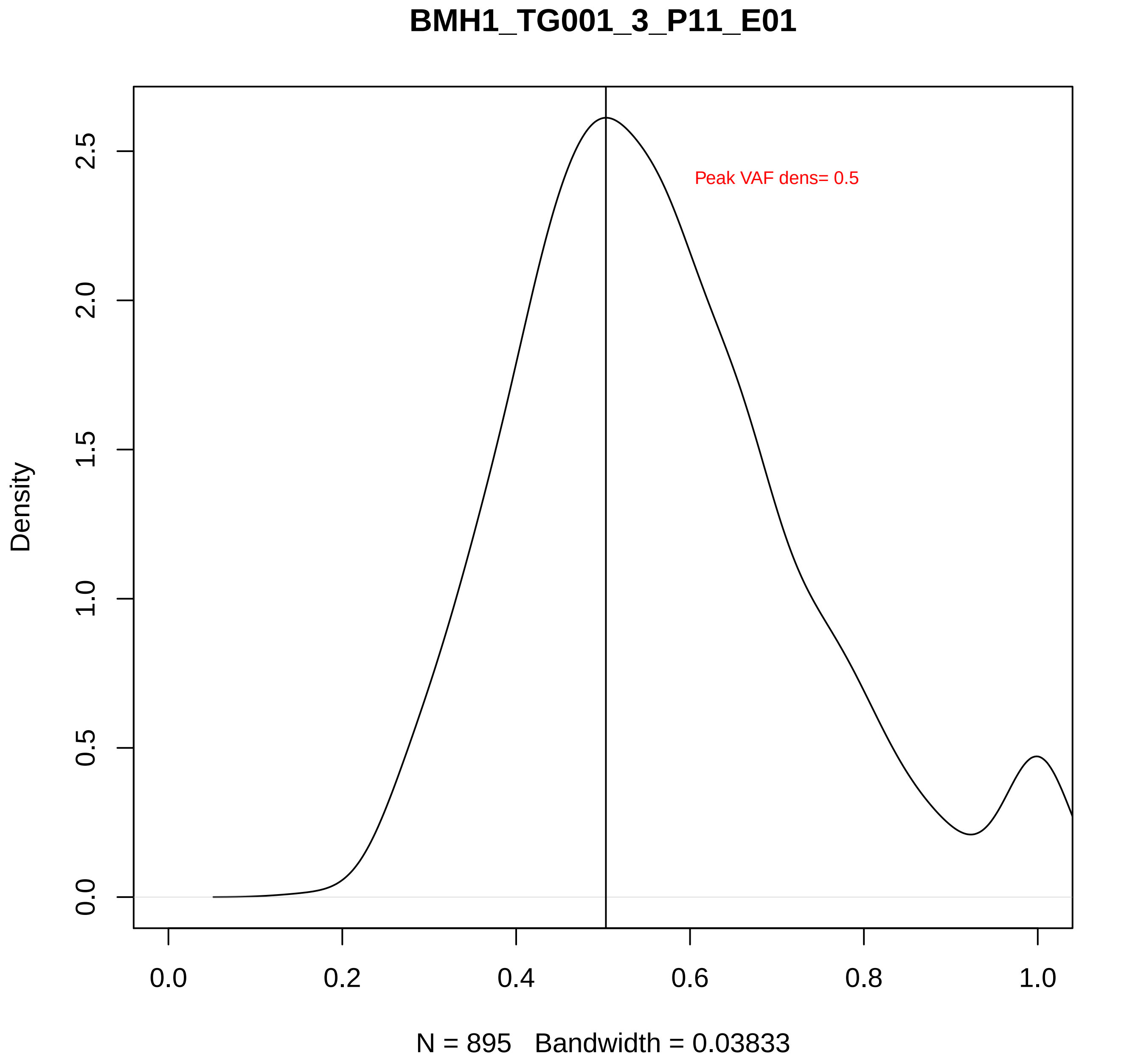
BMH1_TG001_3_P11_E01
Peak VAF dens= 0.5
Density
0.0
0.5
1.0
1.5
2.0
2.5
0.0
0.2
0.4
0.6
0.8
1.0
N = 895 Bandwidth = 0.03833

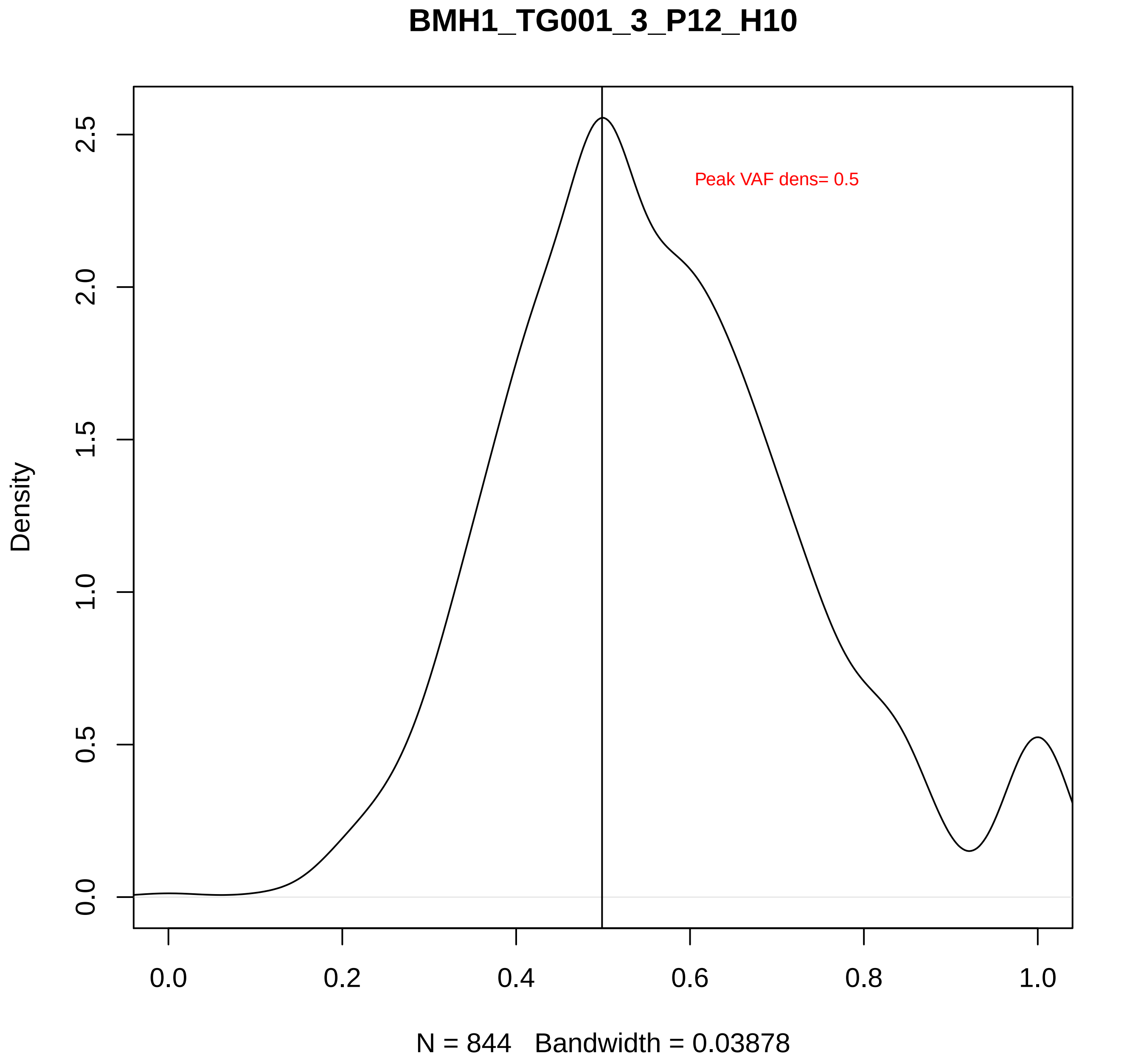

BMH1_TG001_3_P12_H10
Peak VAF dens= 0.5
Density
0.0
0.5
1.0
1.5
2.0
2.5
0.0
0.2
0.4
0.6
0.8
1.0
N = 844 Bandwidth = 0.03878

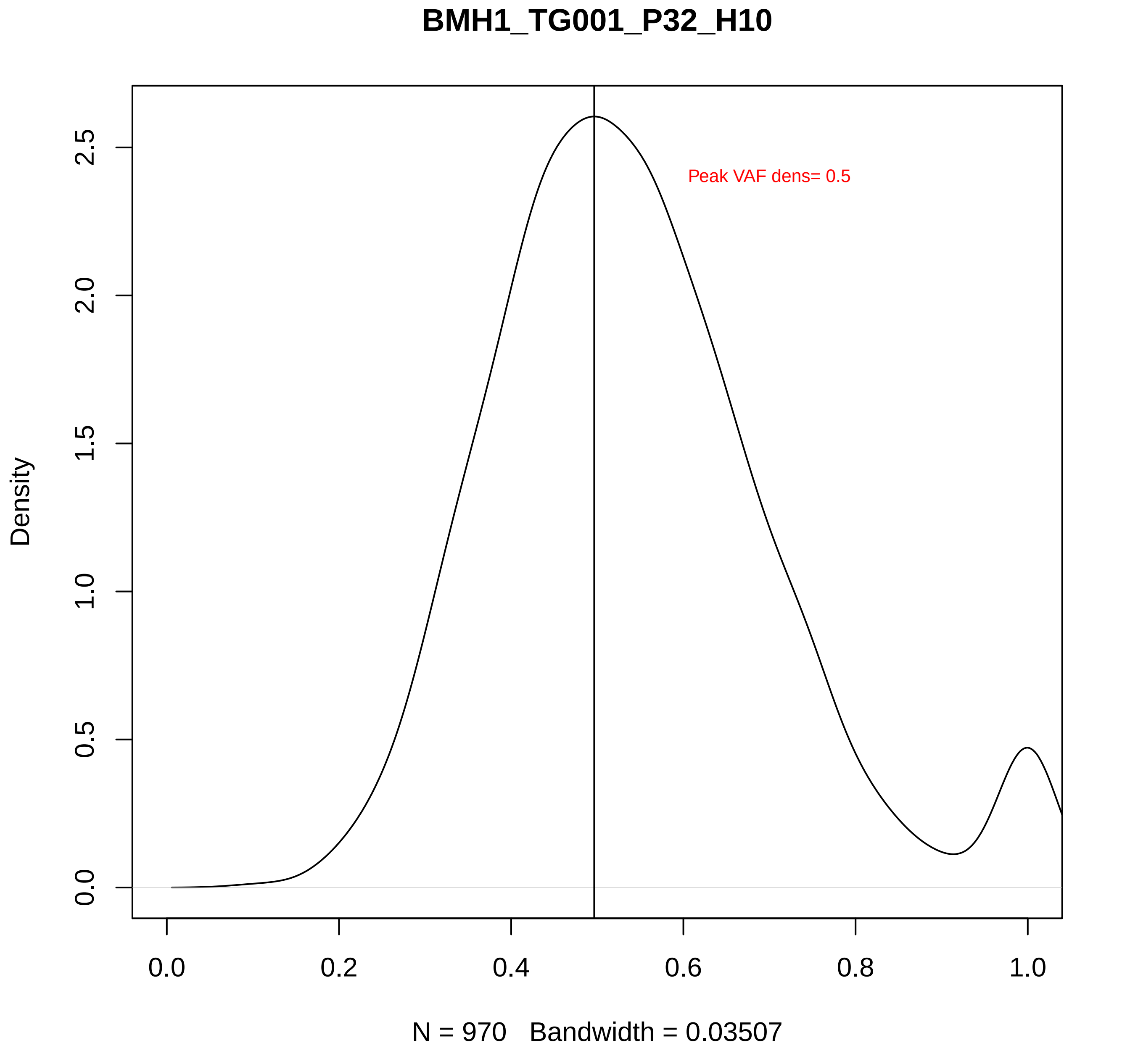
BMH1_TG001_P32_H10
Peak VAF dens= 0.5
Density
0.0
0.5
1.0
1.5
2.0
2.5
0.0
0.2
0.4
0.6
0.8
1.0
N = 970 Bandwidth = 0.03507

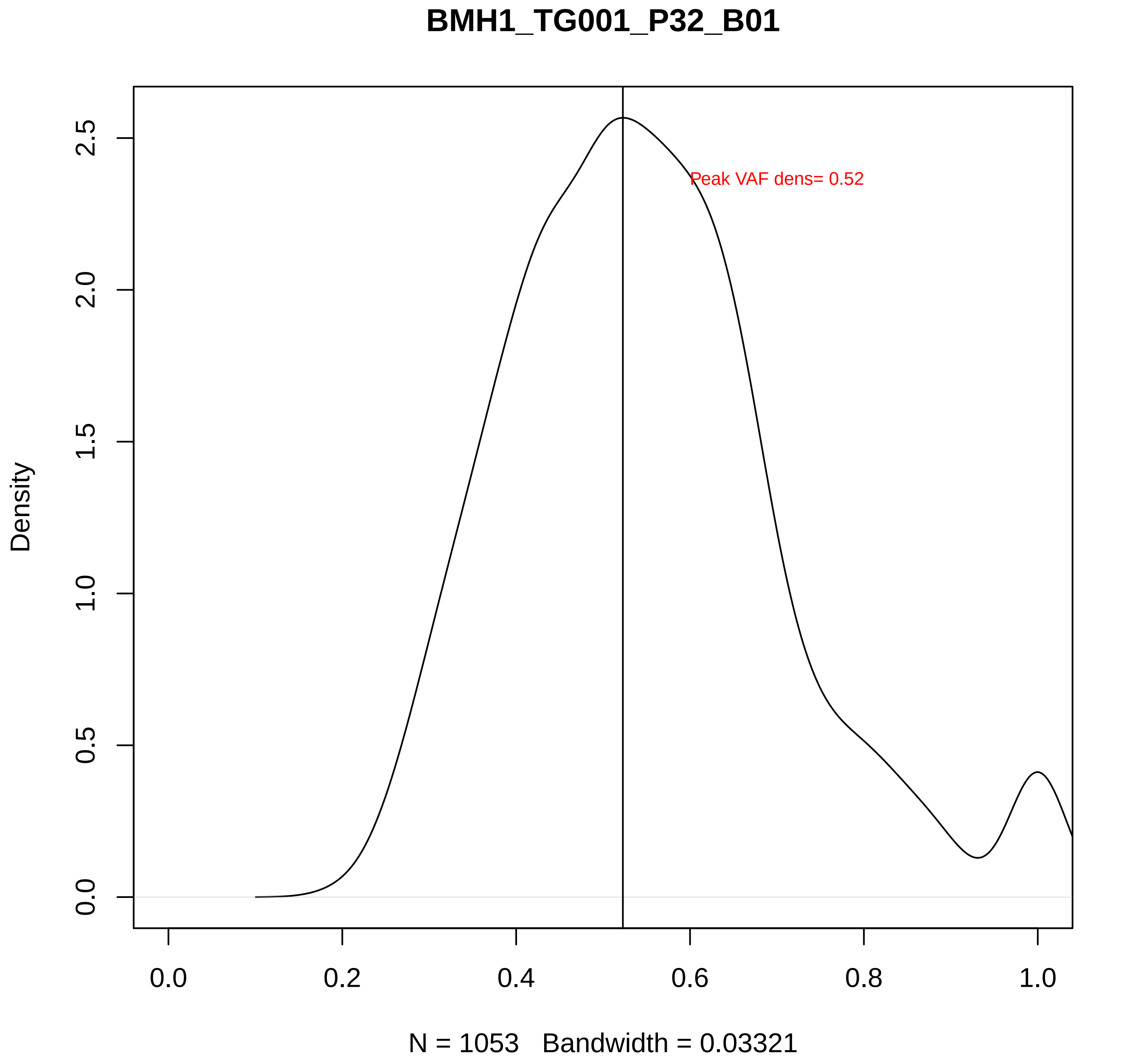

BMH1_TG001_P32_B01
Peak VAF dens= 0.52
Density
0.0
0.5
1.0
1.5
2.0
2.5
0.0
0.2
0.4
0.6
0.8
1.0
N = 1053   Bandwidth = 0.03321

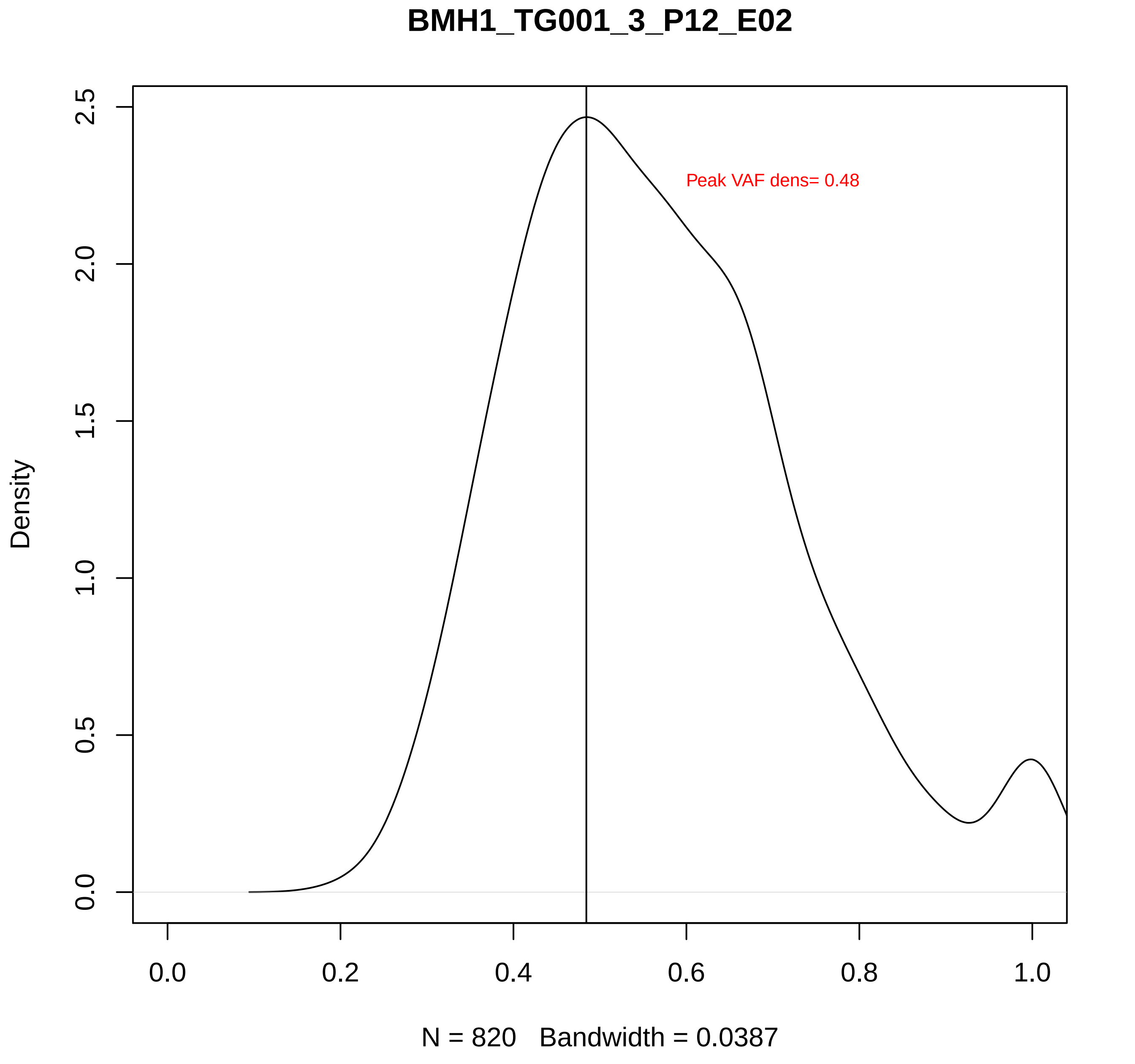
BMH1_TG001_3_P12_E02
Peak VAF dens= 0.48
Density
0.0
0.5
1.0
1.5
2.0
2.5
0.0
0.2
0.4
0.6
0.8
1.0
N = 820   Bandwidth = 0.0387

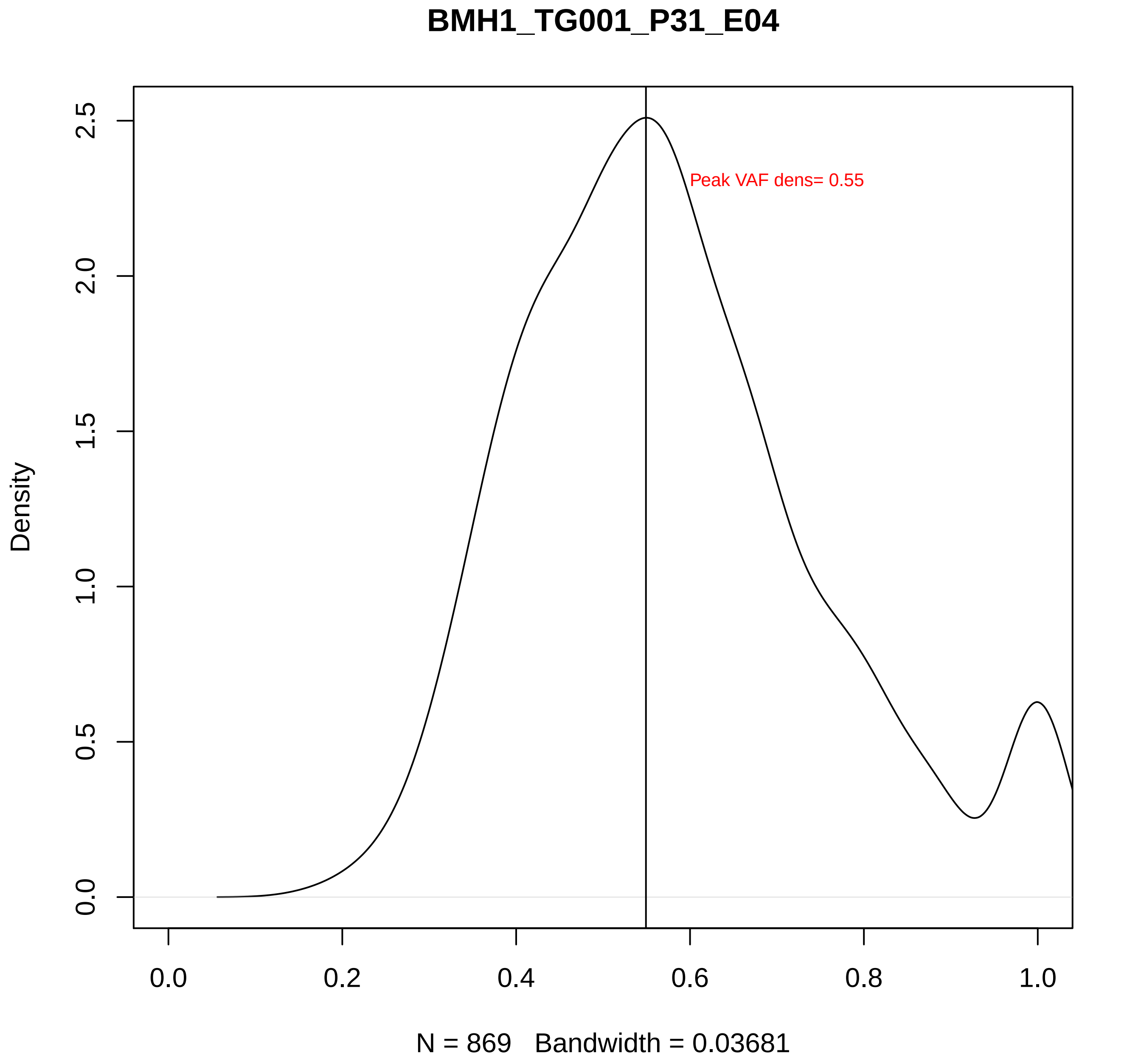
BMH1_TG001_P31_E04
Peak VAF dens= 0.55
Density
0.0
0.5
1.0
1.5
2.0
2.5
0.0
0.2
0.4
0.6
0.8
1.0
N = 869   Bandwidth = 0.03681

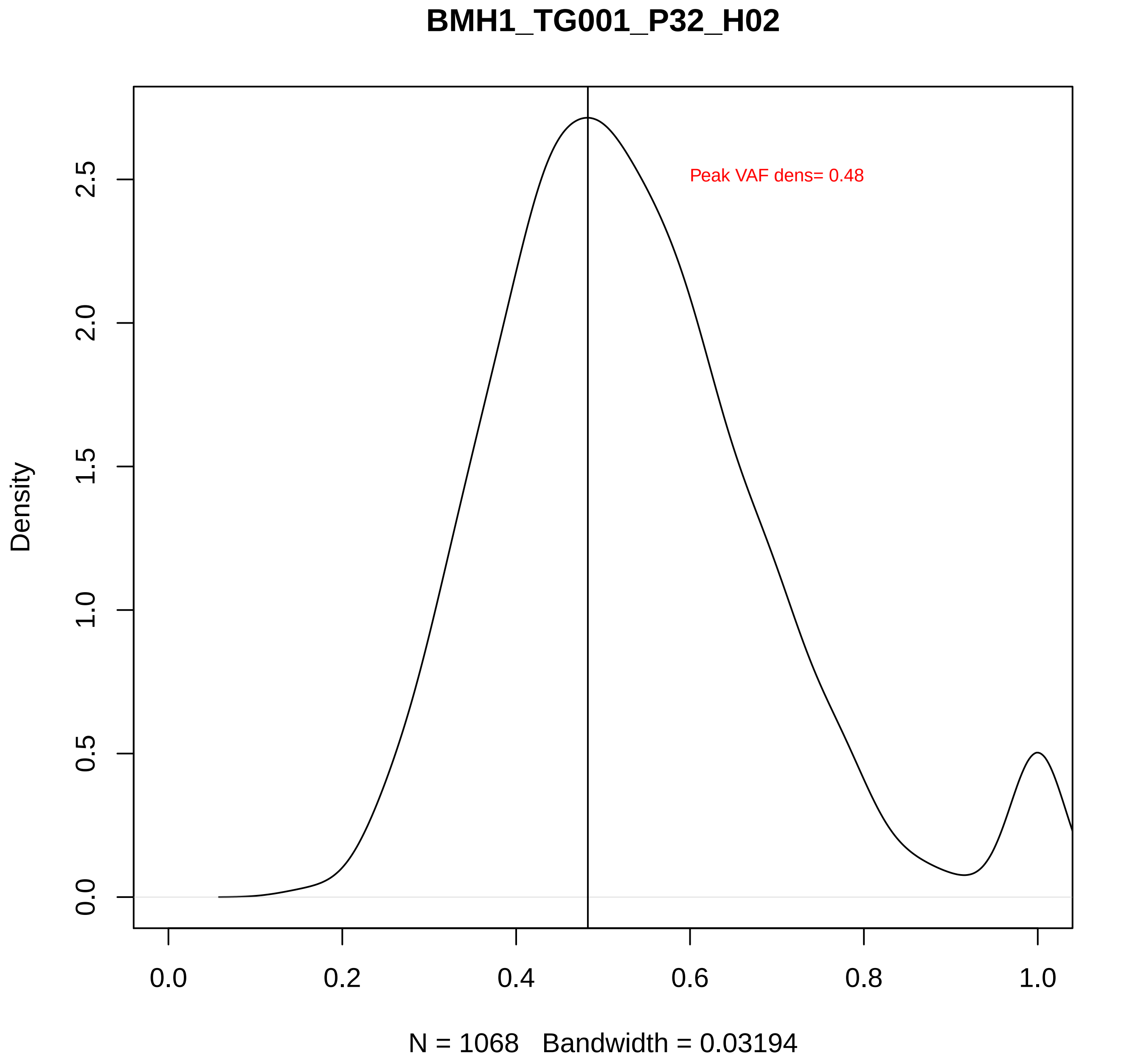

BMH1_TG001_P32_H02
Peak VAF dens= 0.48
Density
0.0
0.5
1.0
1.5
2.0
2.5
0.0
0.2
0.4
0.6
0.8
1.0
N = 1068   Bandwidth = 0.03194

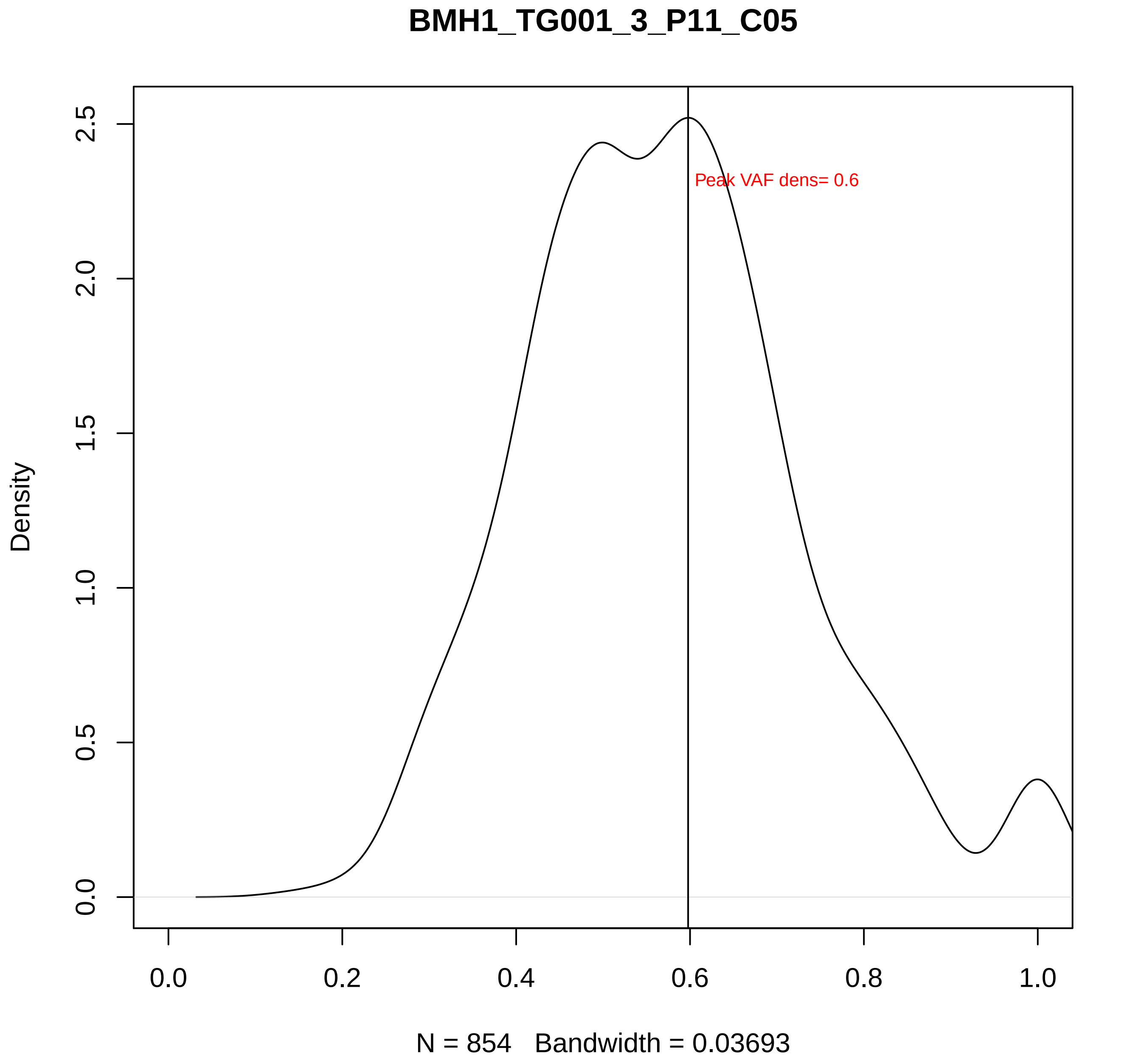
BMH1_TG001_3_P11_C05
Peak VAF dens= 0.6
Density
0.0
0.5
1.0
1.5
2.0
2.5
0.0
0.2
0.4
0.6
0.8
1.0
N = 854   Bandwidth = 0.03693

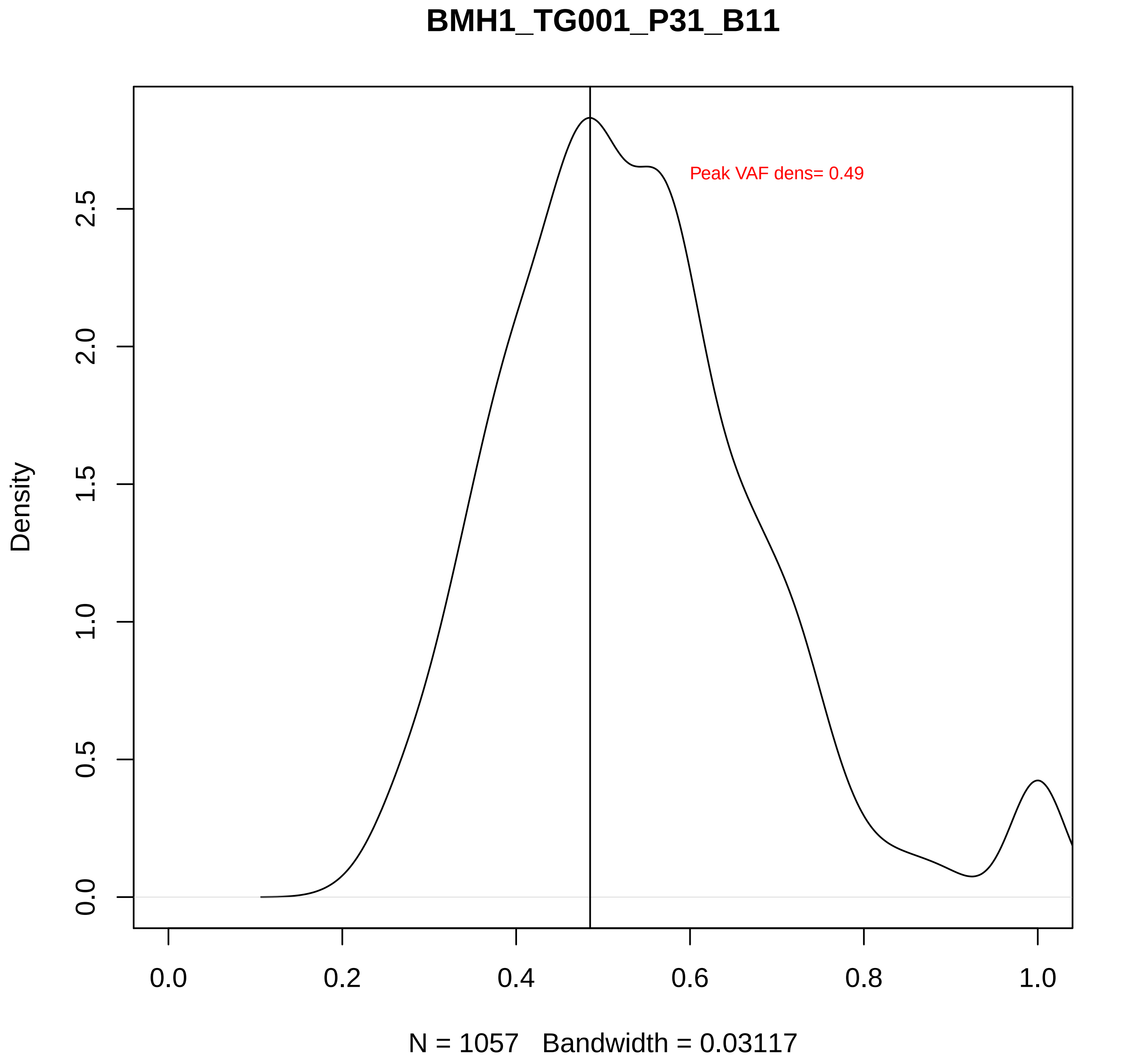
BMH1_TG001_P31_B11
Peak VAF dens= 0.49
Density
0.0
0.5
1.0
1.5
2.0
2.5
0.0
0.2
0.4
0.6
0.8
1.0
N = 1057   Bandwidth = 0.03117

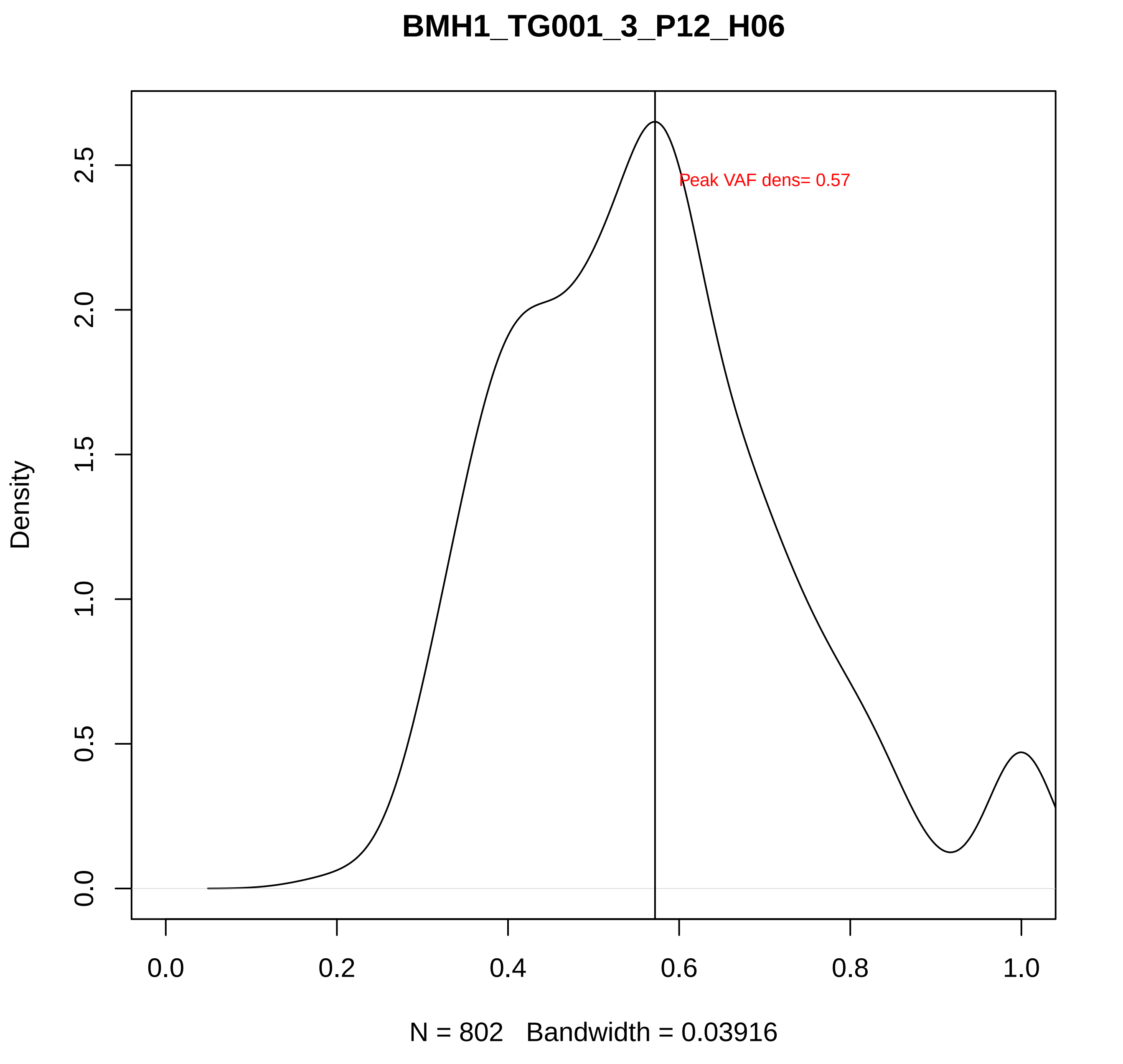
BMH1_TG001_3_P12_H06
Peak VAF dens= 0.57
Density
0.0
0.5
1.0
1.5
2.0
2.5
0.0
0.2
0.4
0.6
0.8
1.0
N = 802 Bandwidth = 0.03916

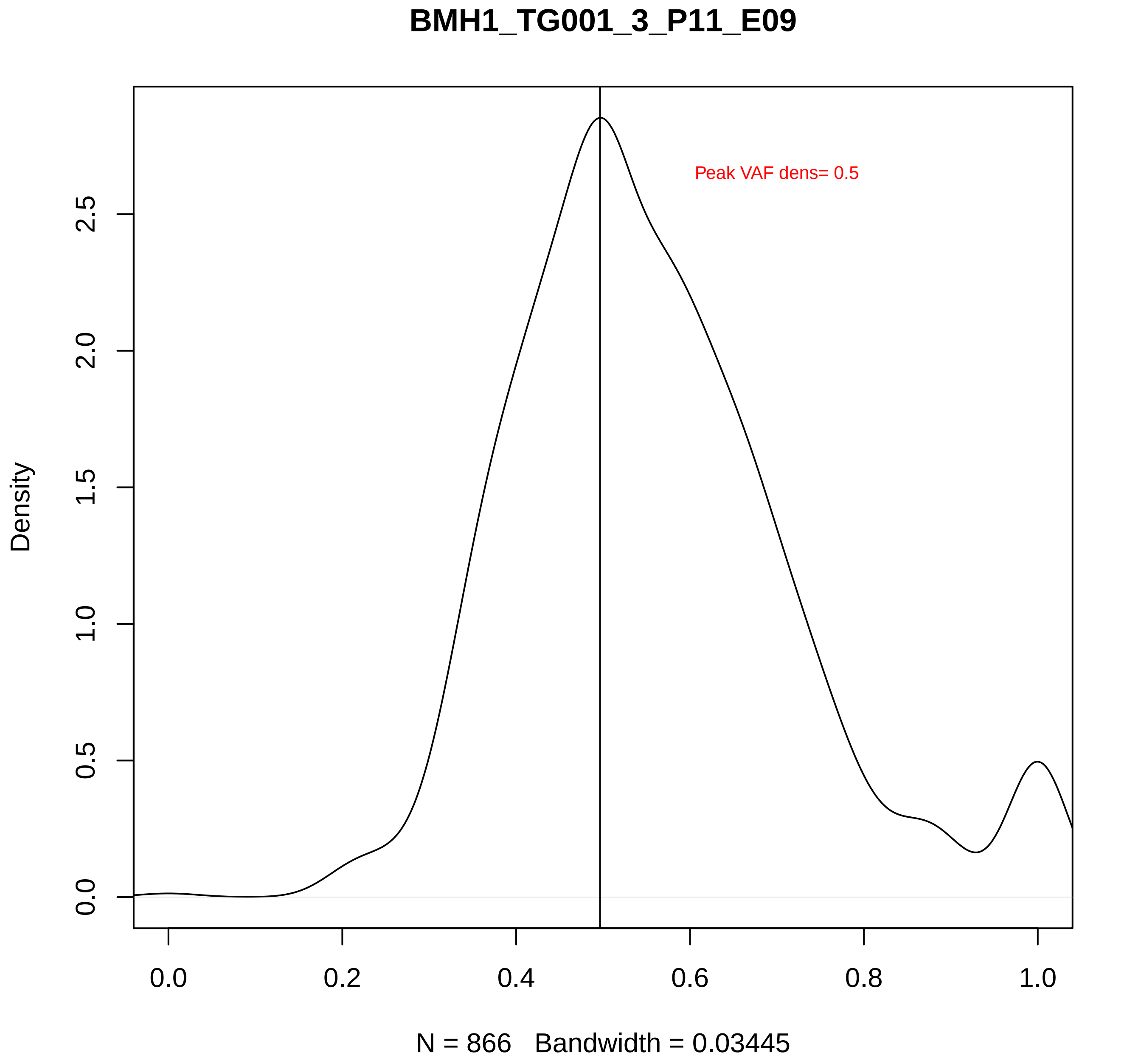
BMH1_TG001_3_P11_E09
Peak VAF dens= 0.5
Density
0.0
0.5
1.0
1.5
2.0
2.5
0.0
0.2
0.4
0.6
0.8
1.0
N = 866   Bandwidth = 0.03445

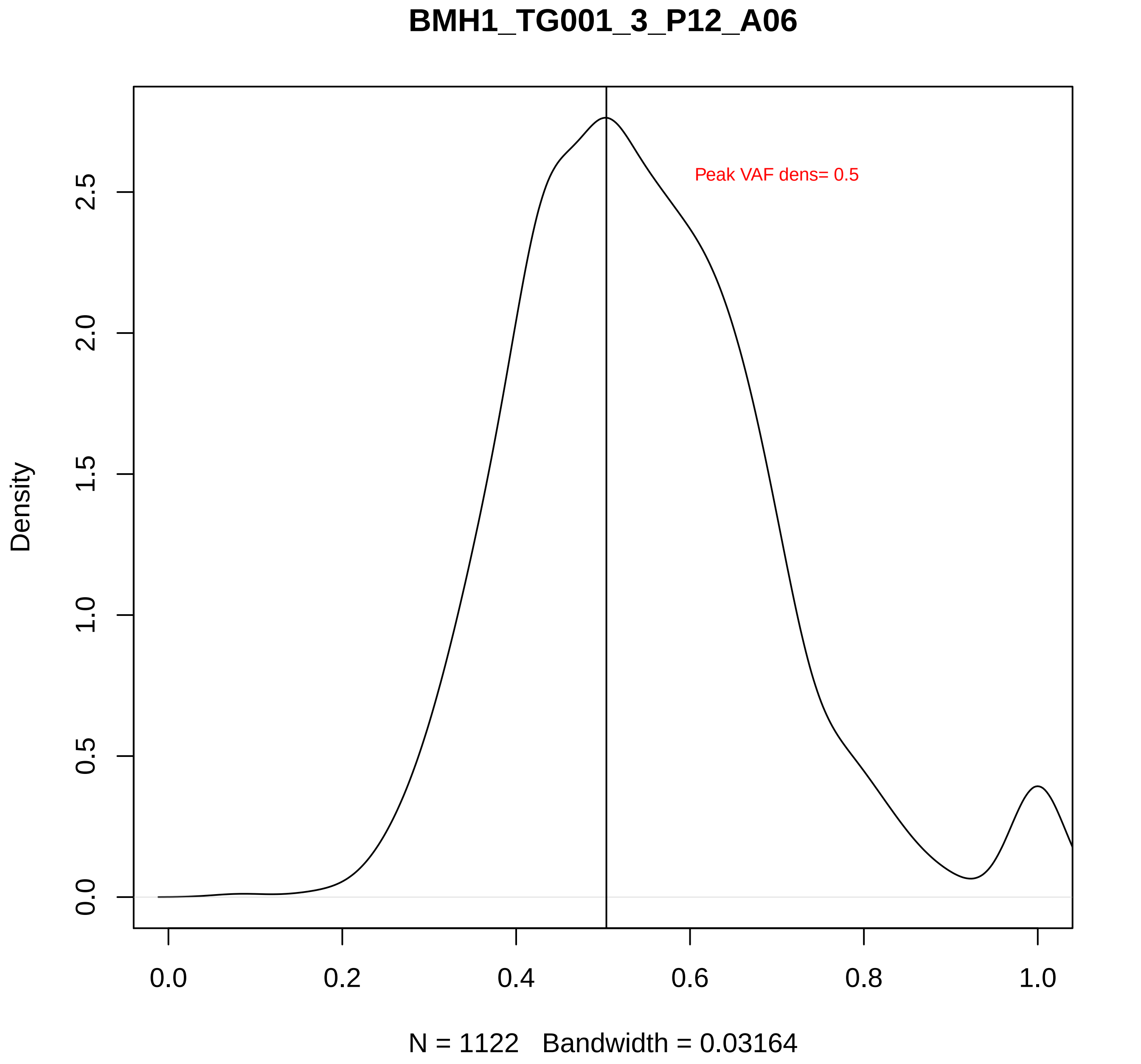

BMH1_TG001_3_P12_A06
Peak VAF dens= 0.5
Density
0.0
0.5
1.0
1.5
2.0
2.5
0.0
0.2
0.4
0.6
0.8
1.0
N = 1122 Bandwidth = 0.03164

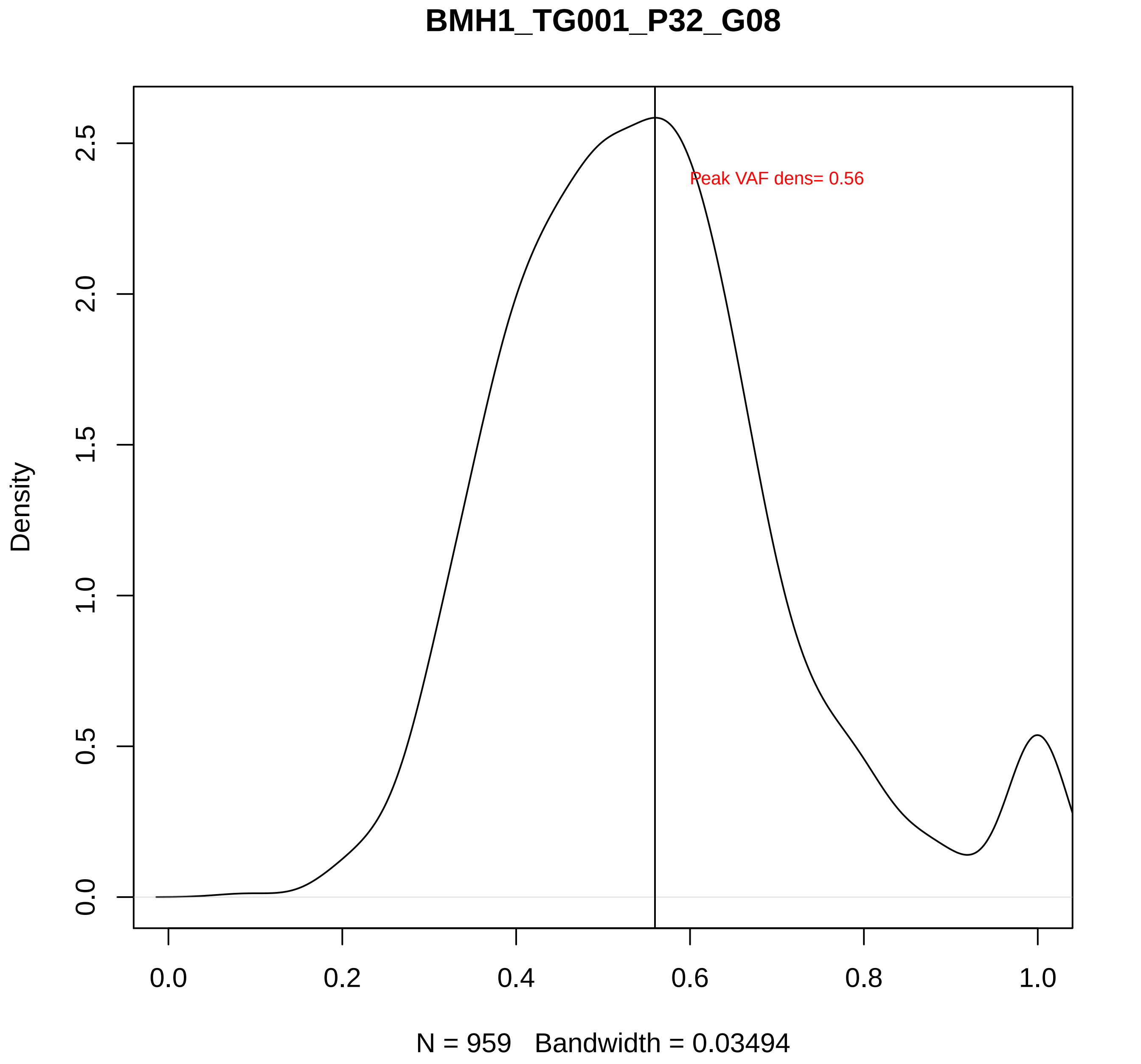
BMH1_TG001_P32_G08
Peak VAF dens= 0.56
Density
0.0
0.5
1.0
1.5
2.0
2.5
0.0
0.2
0.4
0.6
0.8
1.0
N = 959   Bandwidth = 0.03494

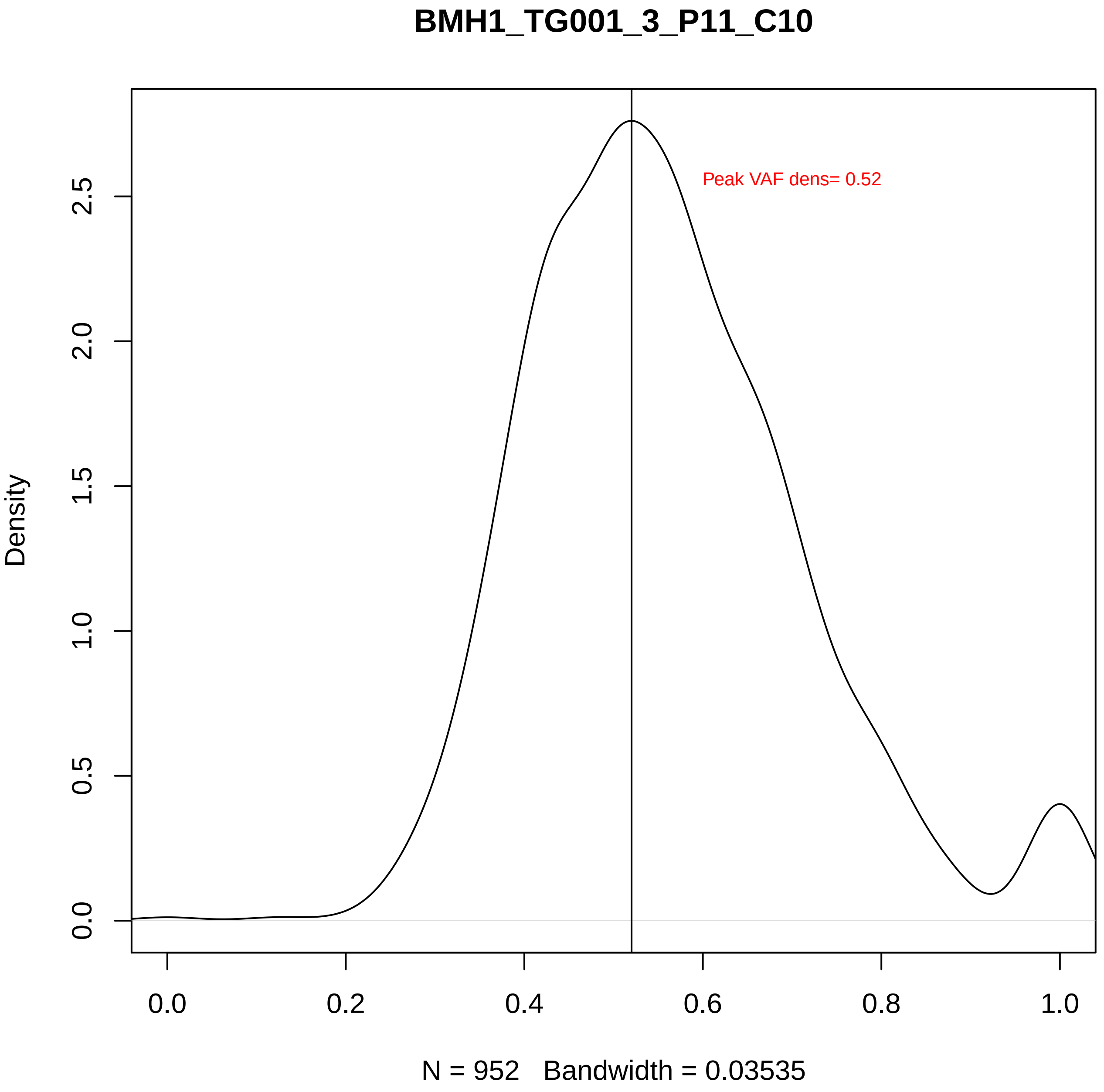
BMH1_TG001_3_P11_C10
Peak VAF dens= 0.52
Density
0.0
0.5
1.0
1.5
2.0
2.5
0.0
0.2
0.4
0.6
0.8
1.0
N = 952   Bandwidth = 0.03535

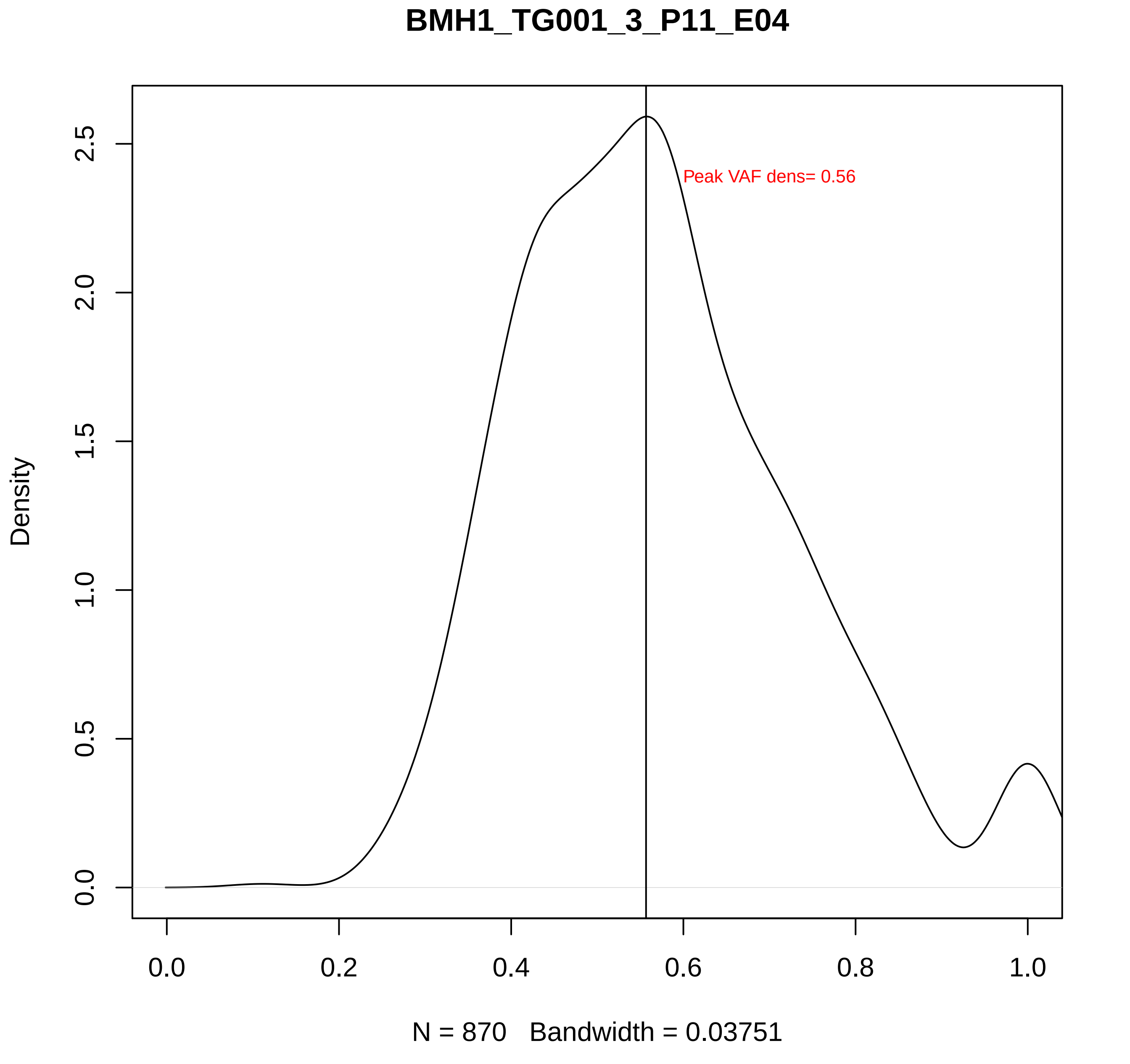

BMH1_TG001_3_P11_E04
Peak VAF dens= 0.56
Density
0.0
0.5
1.0
1.5
2.0
2.5
0.0
0.2
0.4
0.6
0.8
1.0
N = 870 Bandwidth = 0.03751

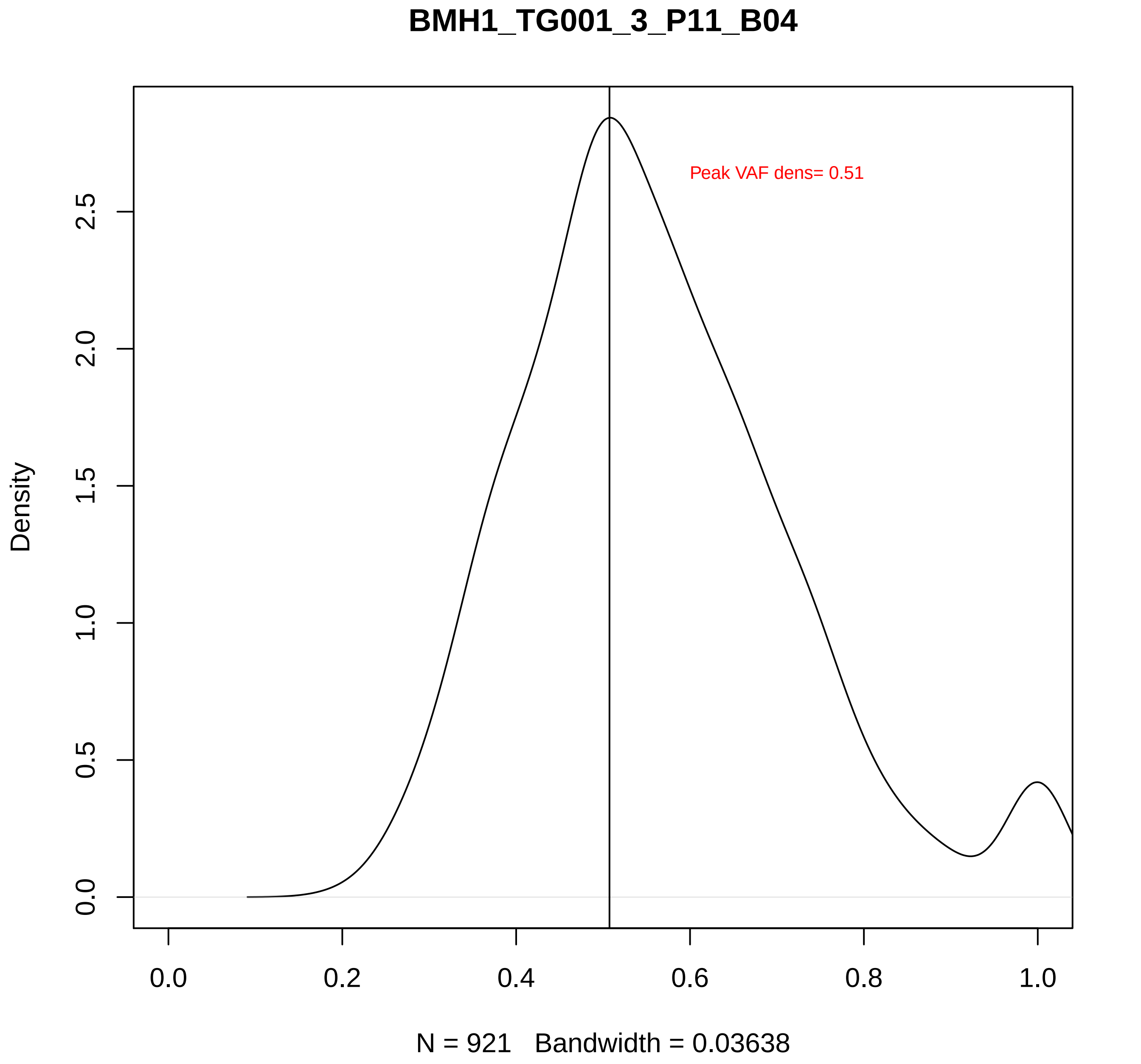

BMH1_TG001_3_P11_B04
Peak VAF dens= 0.51
Density
0.0
0.5
1.0
1.5
2.0
2.5
0.0
0.2
0.4
0.6
0.8
1.0
N = 921   Bandwidth = 0.03638

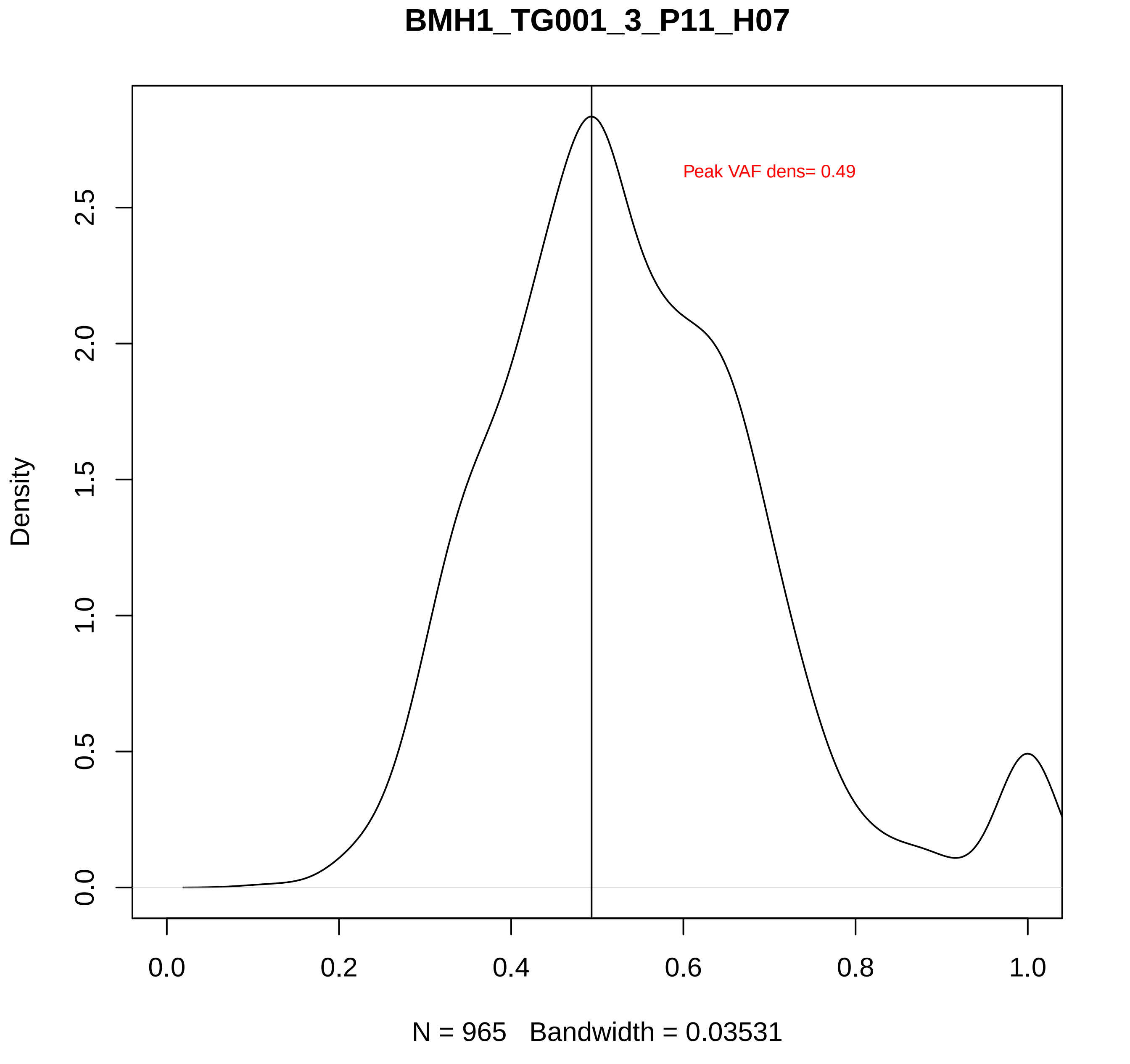
BMH1_TG001_3_P11_H07
Peak VAF dens= 0.49
Density
0.0
0.5
1.0
1.5
2.0
2.5
0.0
0.2
0.4
0.6
0.8
1.0
N = 965   Bandwidth = 0.03531

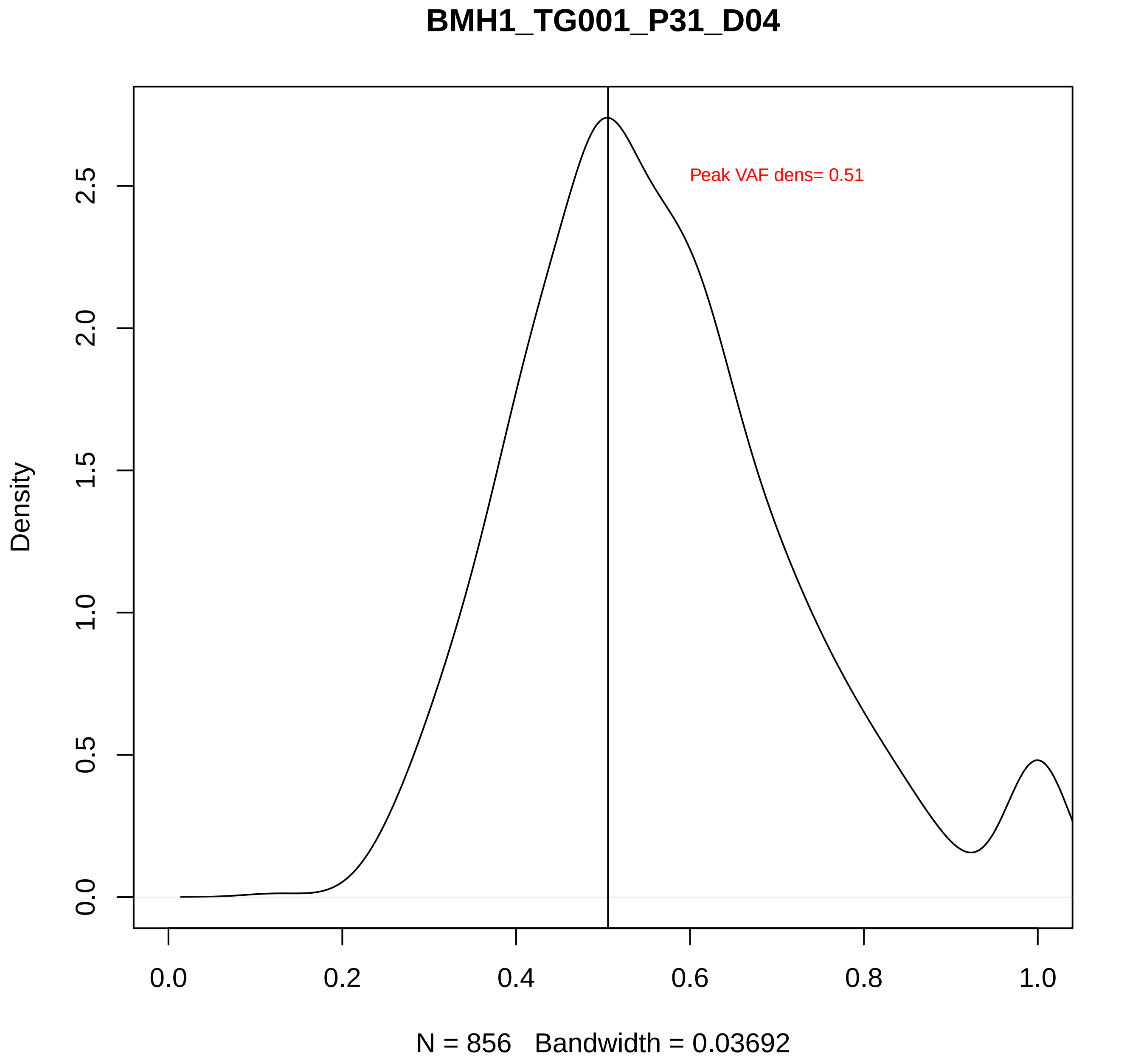
BMH1_TG001_P31_D04
Peak VAF dens= 0.51
Density
0.0
0.5
1.0
1.5
2.0
2.5
0.0
0.2
0.4
0.6
0.8
1.0
N = 856   Bandwidth = 0.03692

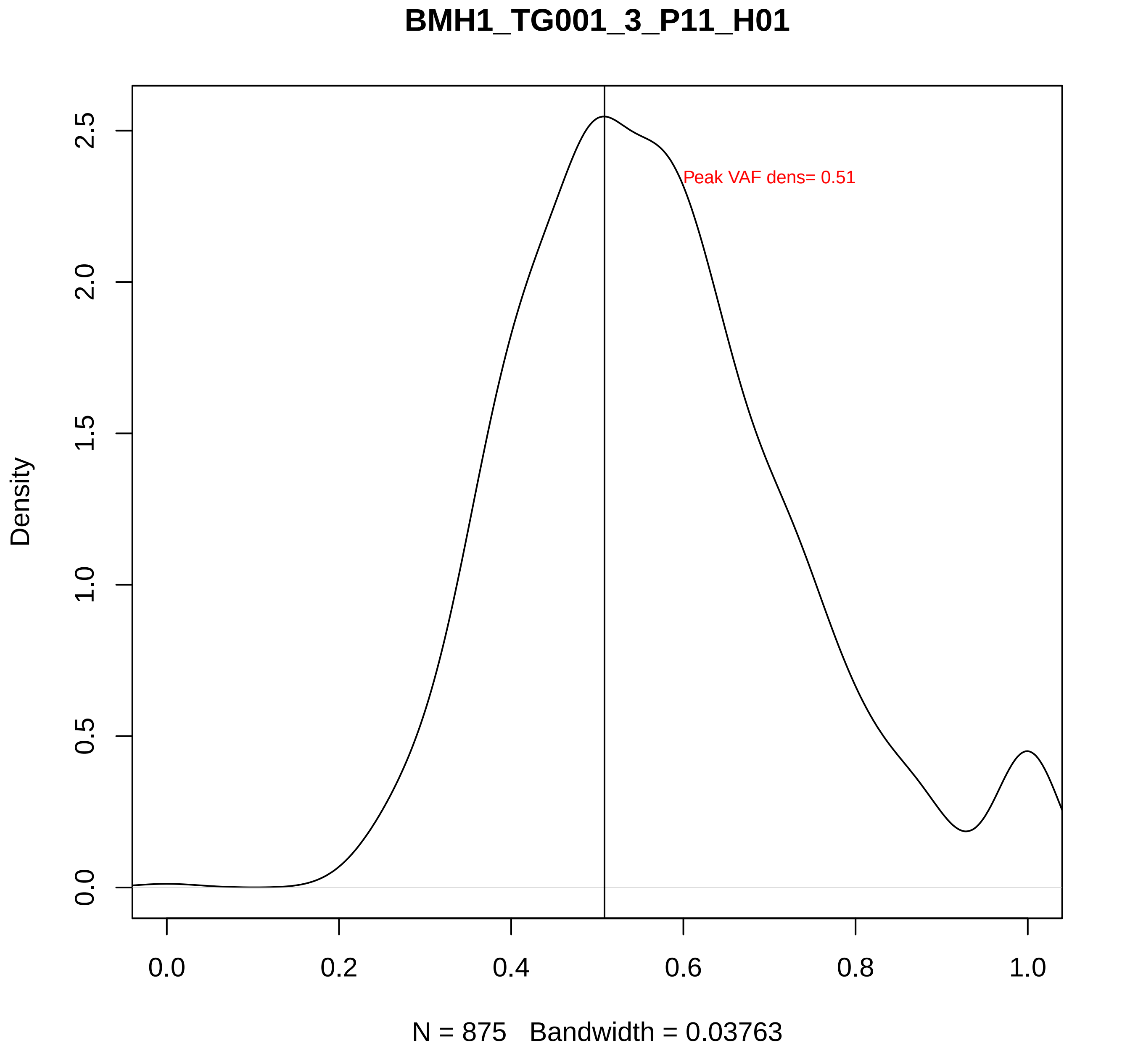

BMH1_TG001_3_P11_H01
Peak VAF dens= 0.51
Density
0.0
0.5
1.0
1.5
2.0
2.5
0.0
0.2
0.4
0.6
0.8
1.0
N = 875   Bandwidth = 0.03763

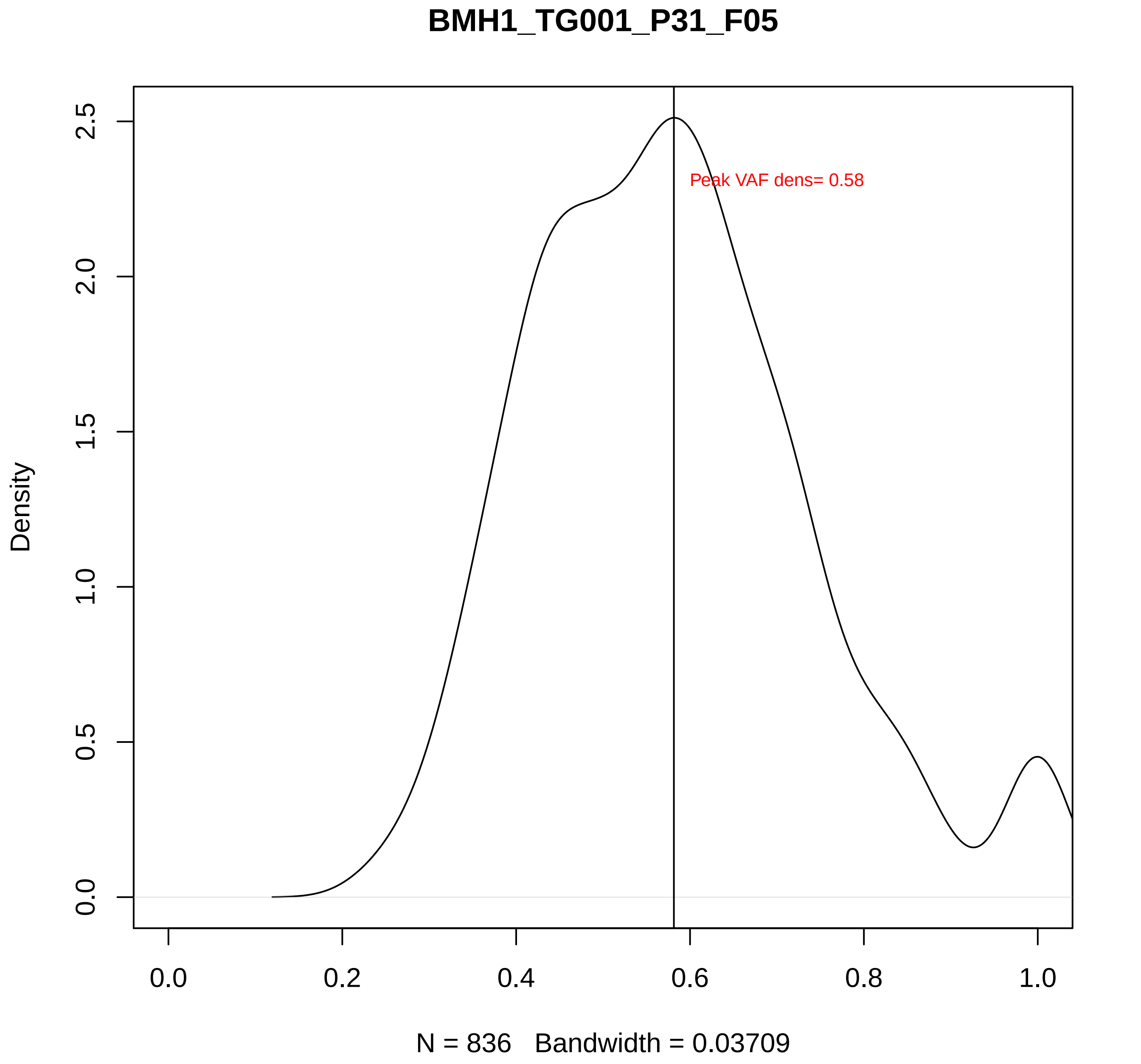
BMH1_TG001_P31_F05
Peak VAF dens= 0.58
Density
0.0
0.5
1.0
1.5
2.0
2.5
0.0
0.2
0.4
0.6
0.8
1.0
N = 836 Bandwidth = 0.03709

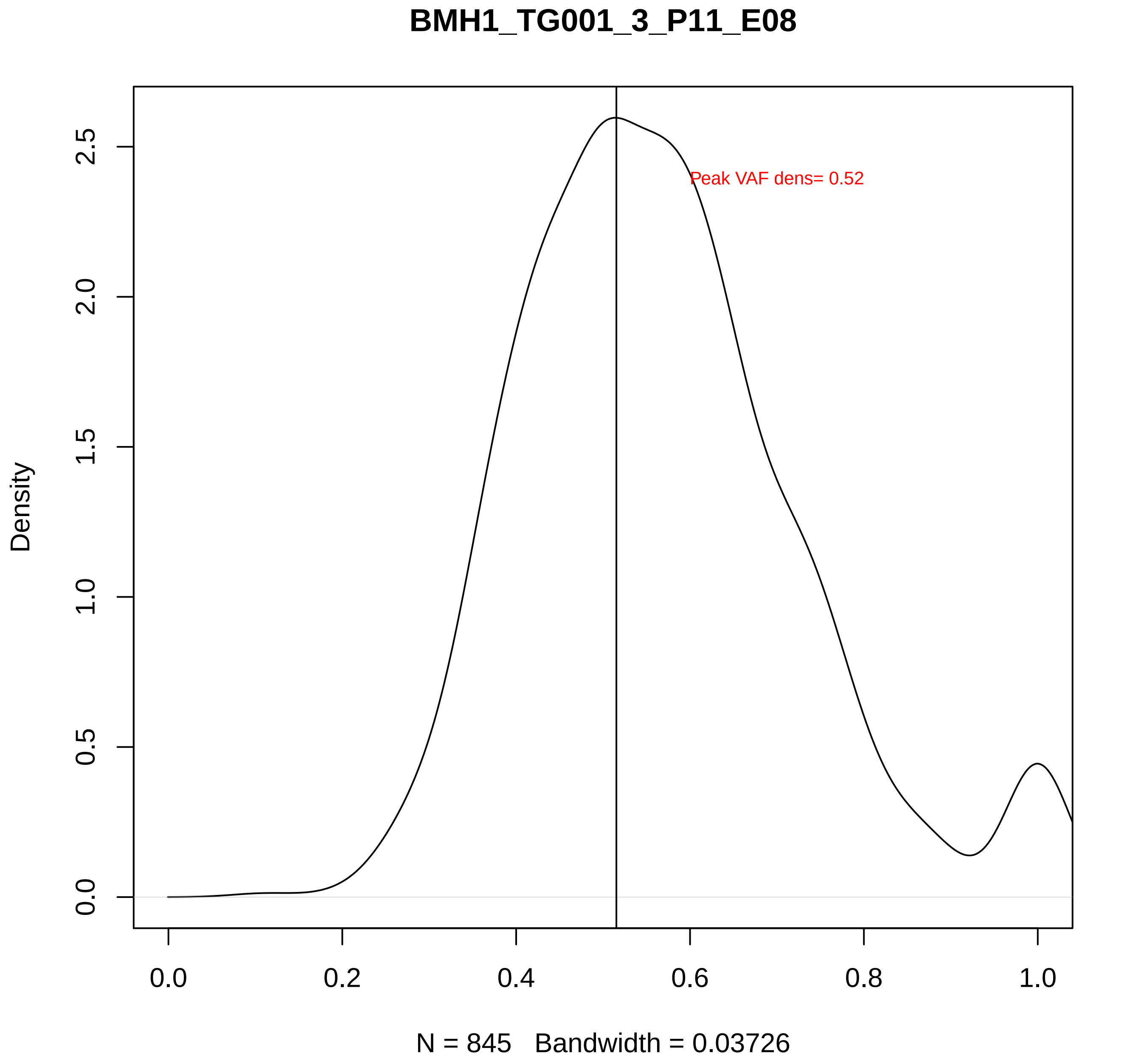
BMH1_TG001_3_P11_E08
Peak VAF dens= 0.52
Density
0.0
0.5
1.0
1.5
2.0
2.5
0.0
0.2
0.4
0.6
0.8
1.0
N = 845   Bandwidth = 0.03726

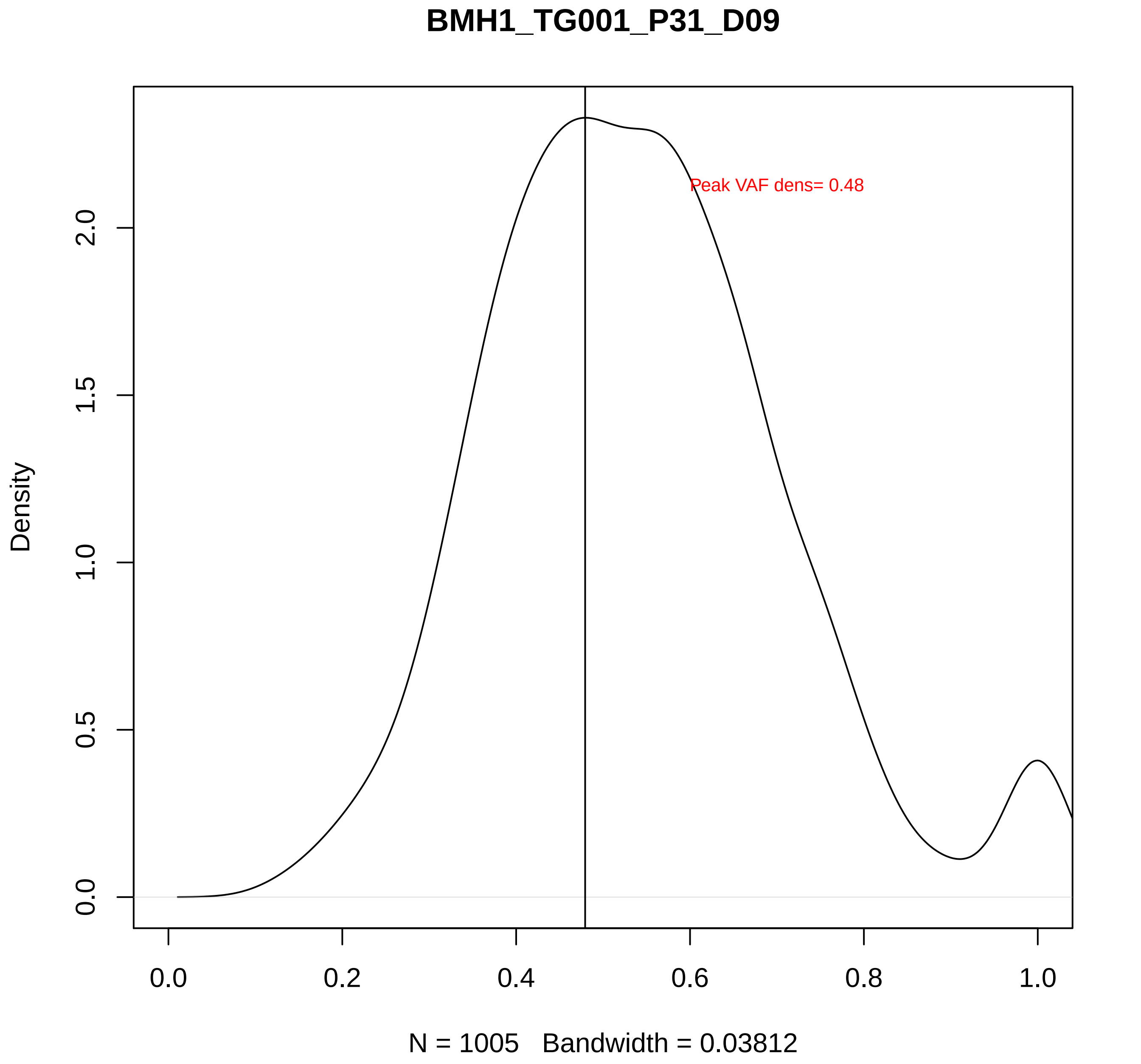

BMH1_TG001_P31_D09
Peak VAF dens= 0.48
Density
0.0
0.5
1.0
1.5
2.0
0.0
0.2
0.4
0.6
0.8
1.0
N = 1005   Bandwidth = 0.03812

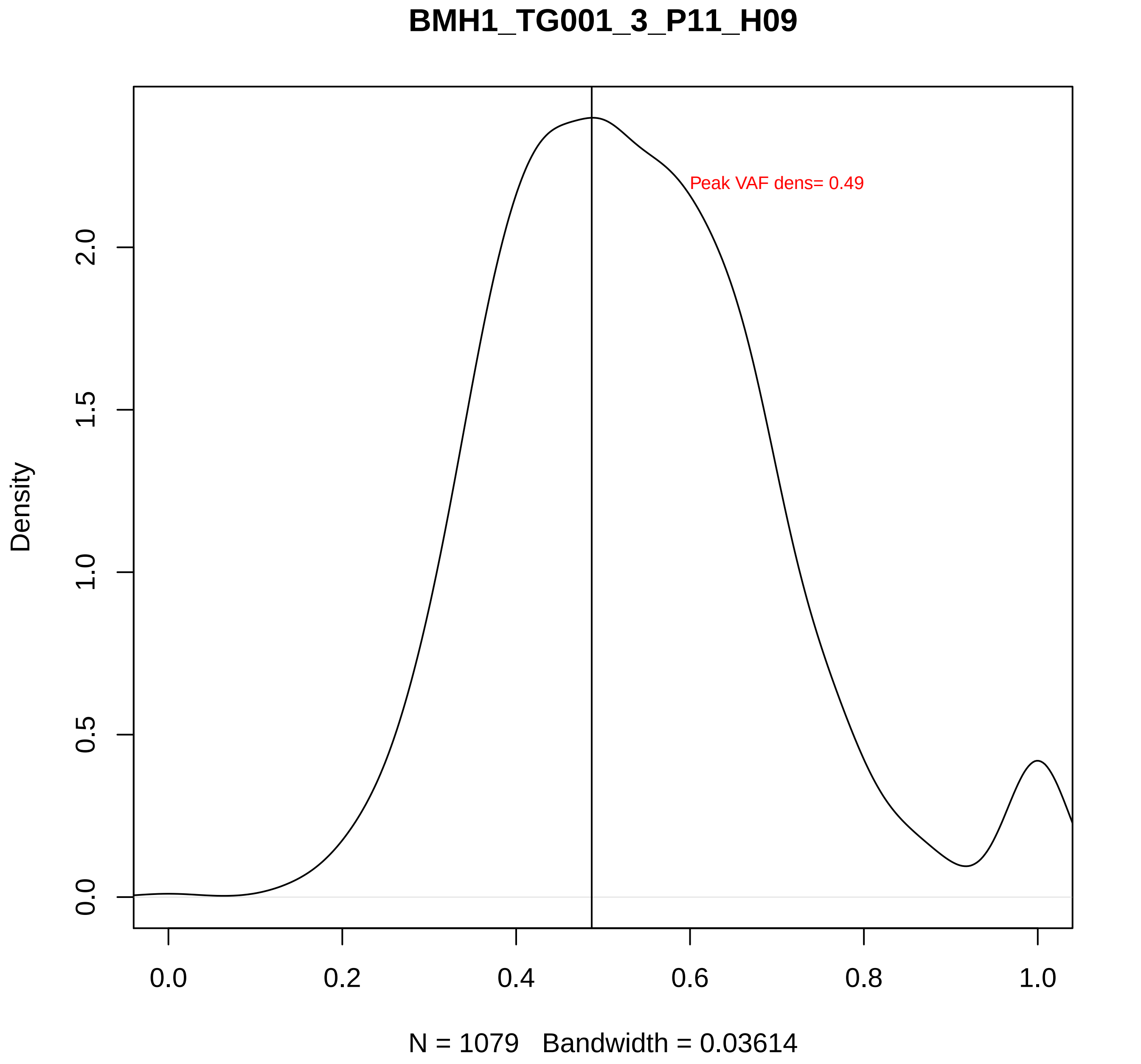
BMH1_TG001_3_P11_H09
Peak VAF dens= 0.49
Density
0.0
0.5
1.0
1.5
2.0
0.0
0.2
0.4
0.6
0.8
1.0
N = 1079   Bandwidth = 0.03614

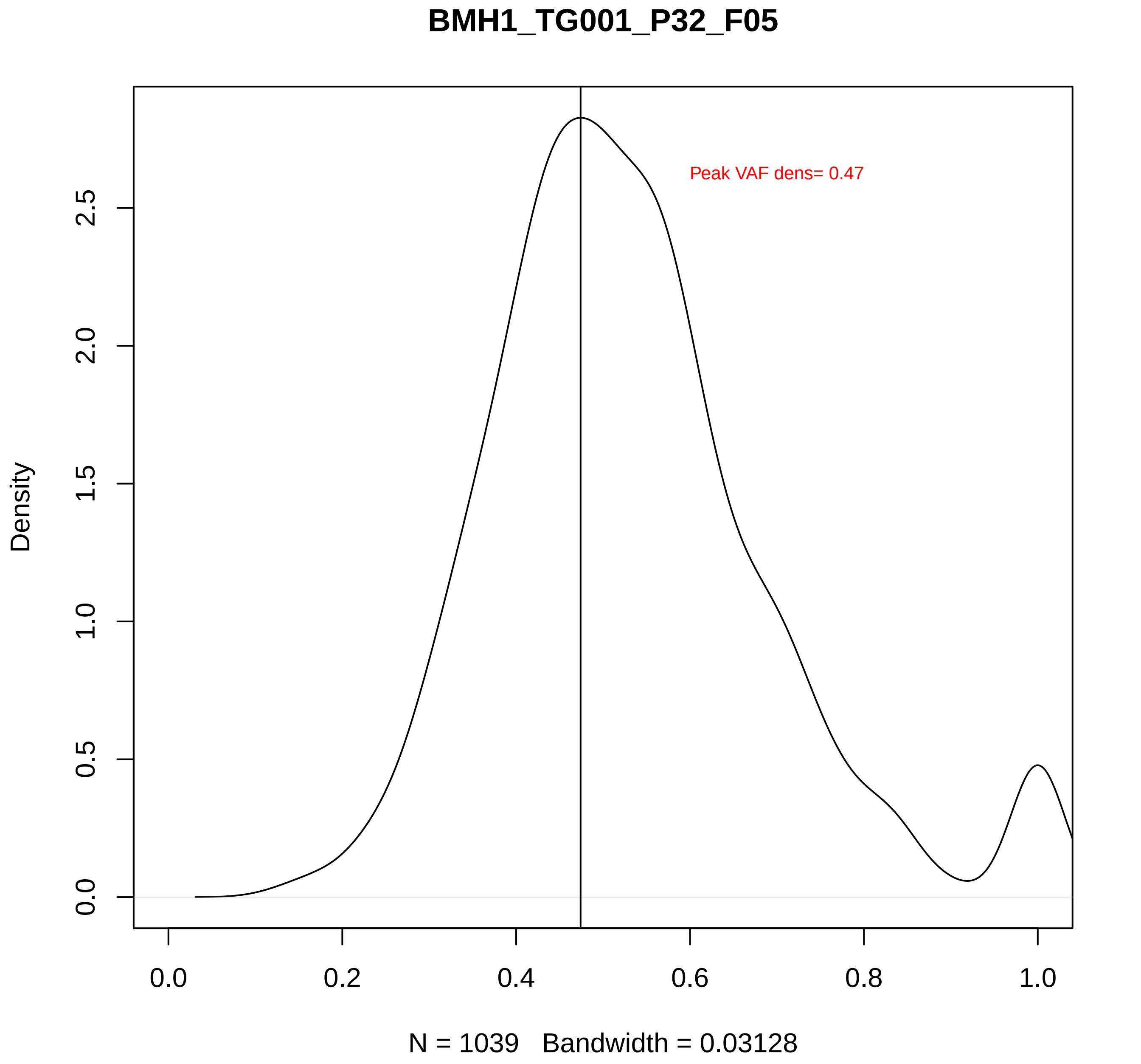
BMH1_TG001_P32_F05
Peak VAF dens= 0.47
Density
0.0
0.5
1.0
1.5
2.0
2.5
0.0
0.2
0.4
0.6
0.8
1.0
N = 1039   Bandwidth = 0.03128

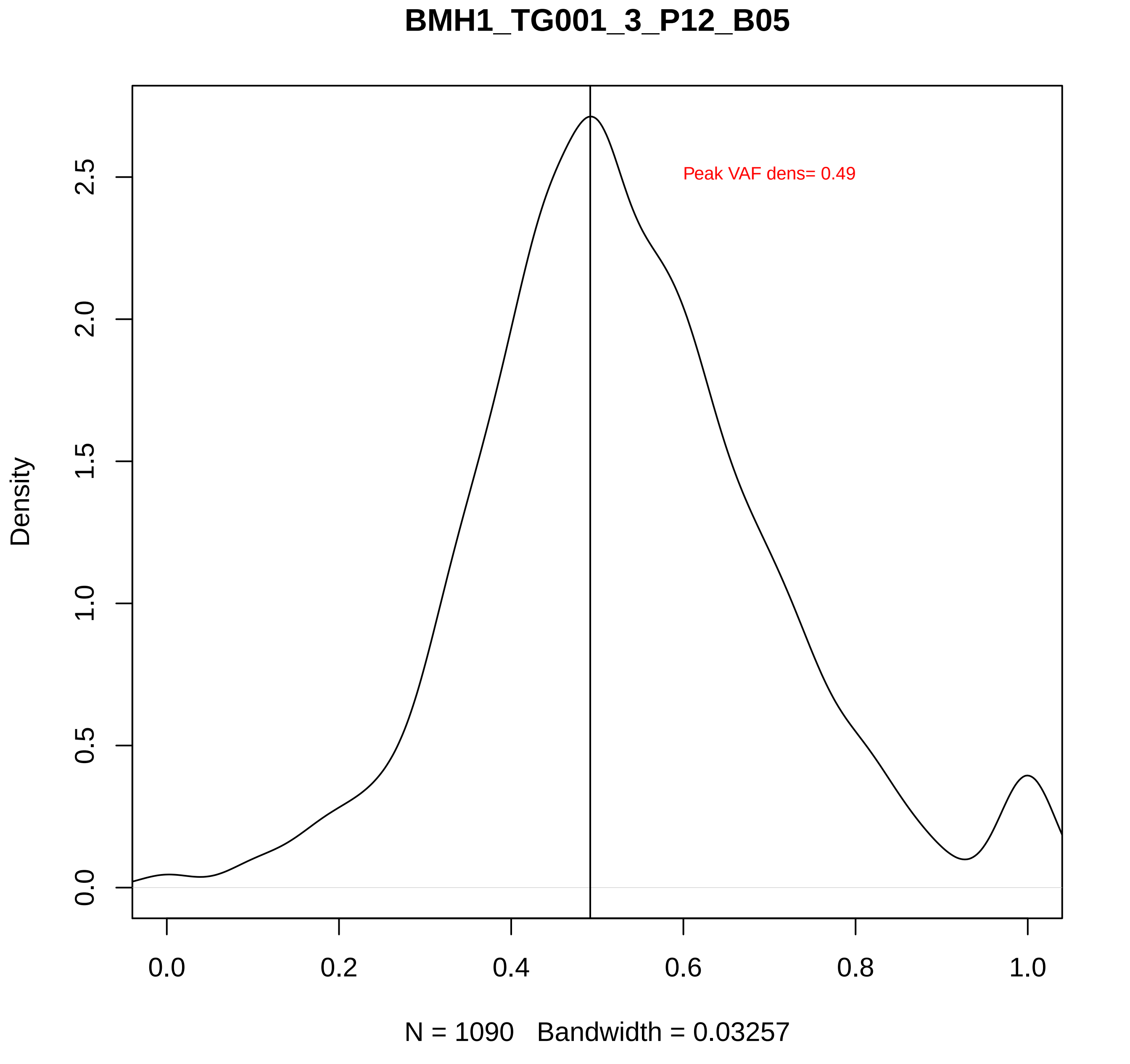
BMH1_TG001_3_P12_B05
Peak VAF dens= 0.49
Density
0.0
0.5
1.0
1.5
2.0
2.5
0.0
0.2
0.4
0.6
0.8
1.0
N = 1090   Bandwidth = 0.03257

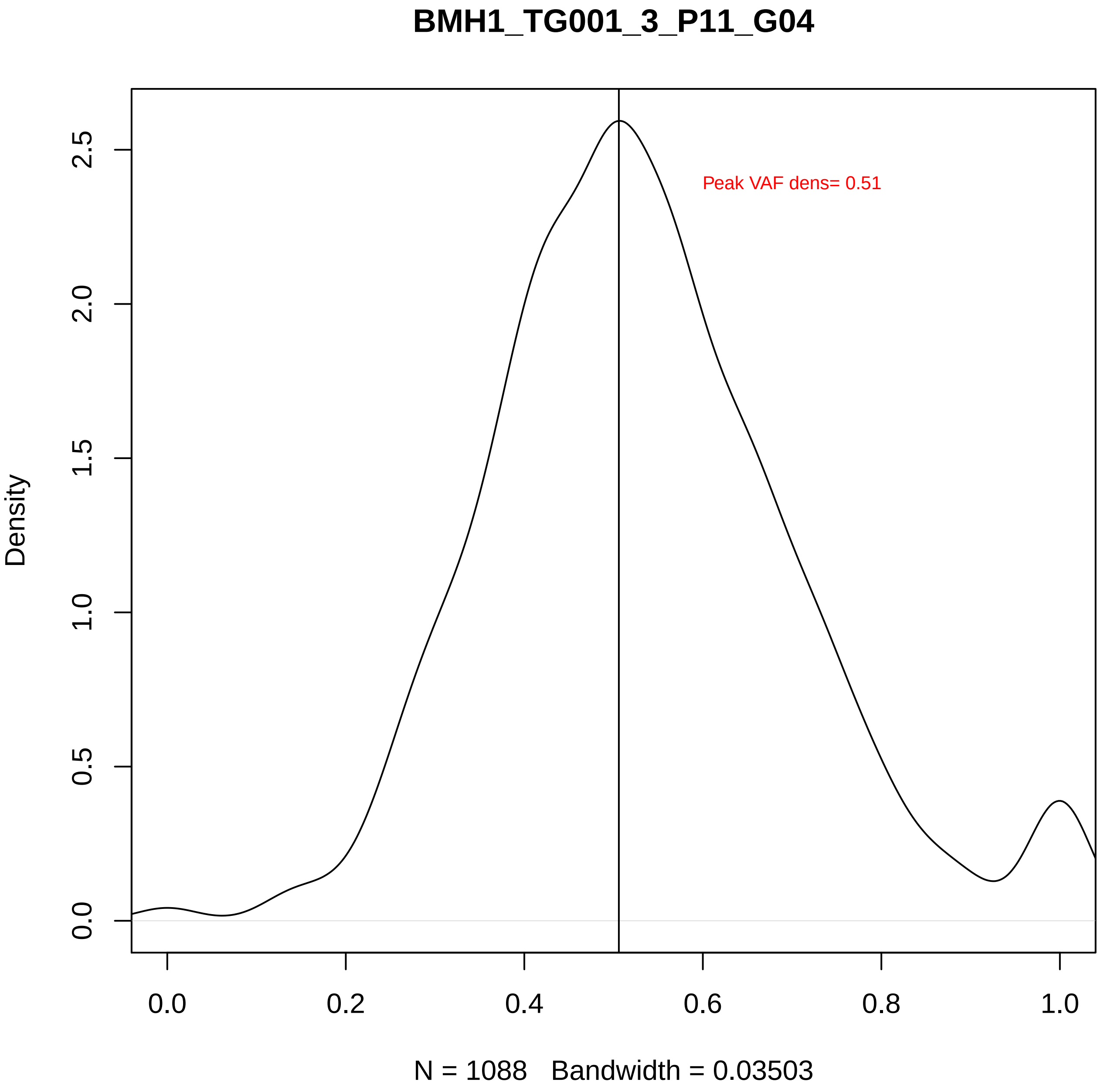

BMH1_TG001_3_P11_G04
Peak VAF dens= 0.51
Density
0.0
0.5
1.0
1.5
2.0
2.5
0.0
0.2
0.4
0.6
0.8
1.0
N = 1088   Bandwidth = 0.03503

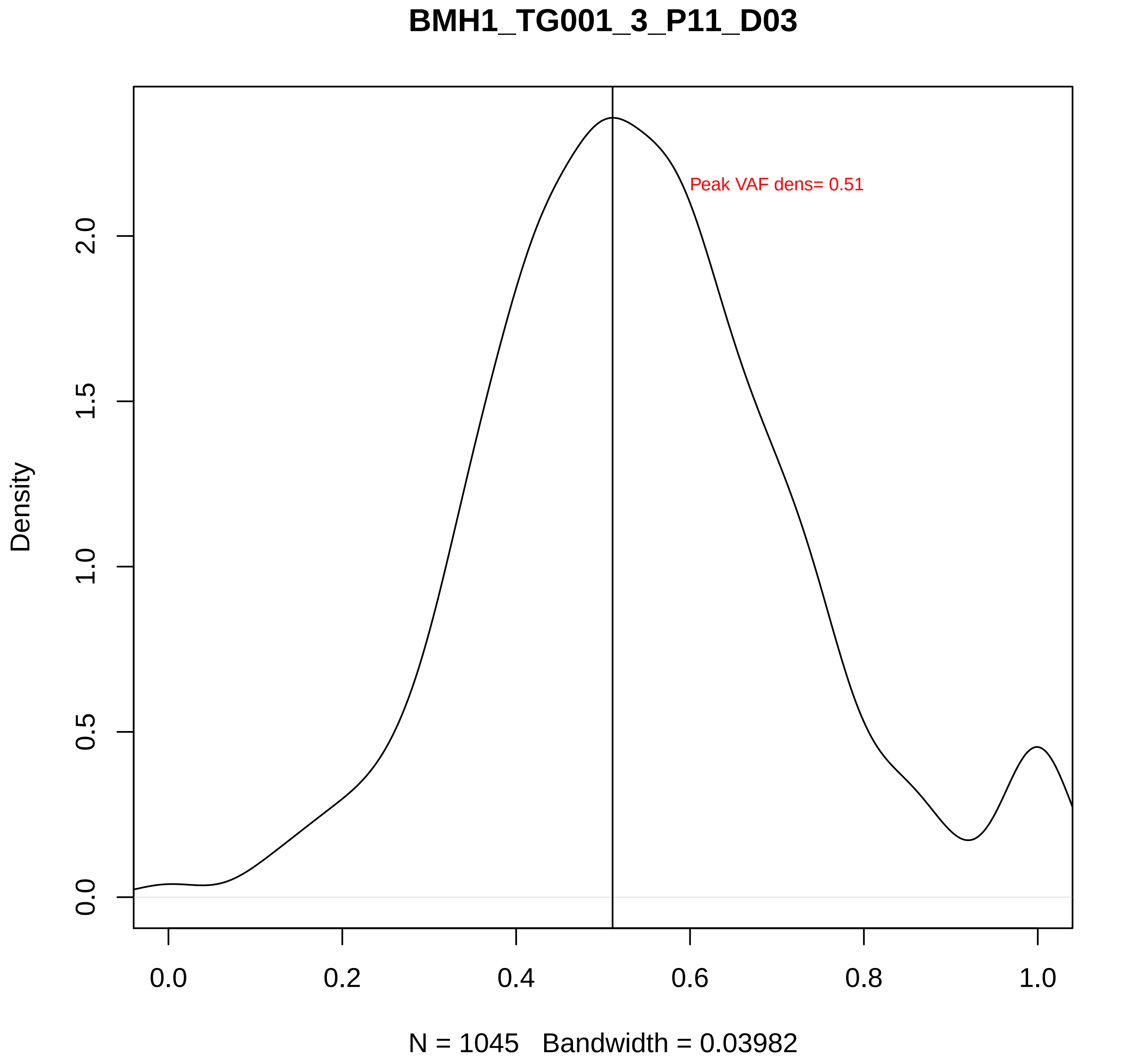
BMH1_TG001_3_P11_D03
Peak VAF dens= 0.51
Density
0.0
0.5
1.0
1.5
2.0
0.0
0.2
0.4
0.6
0.8
1.0
N = 1045   Bandwidth = 0.03982

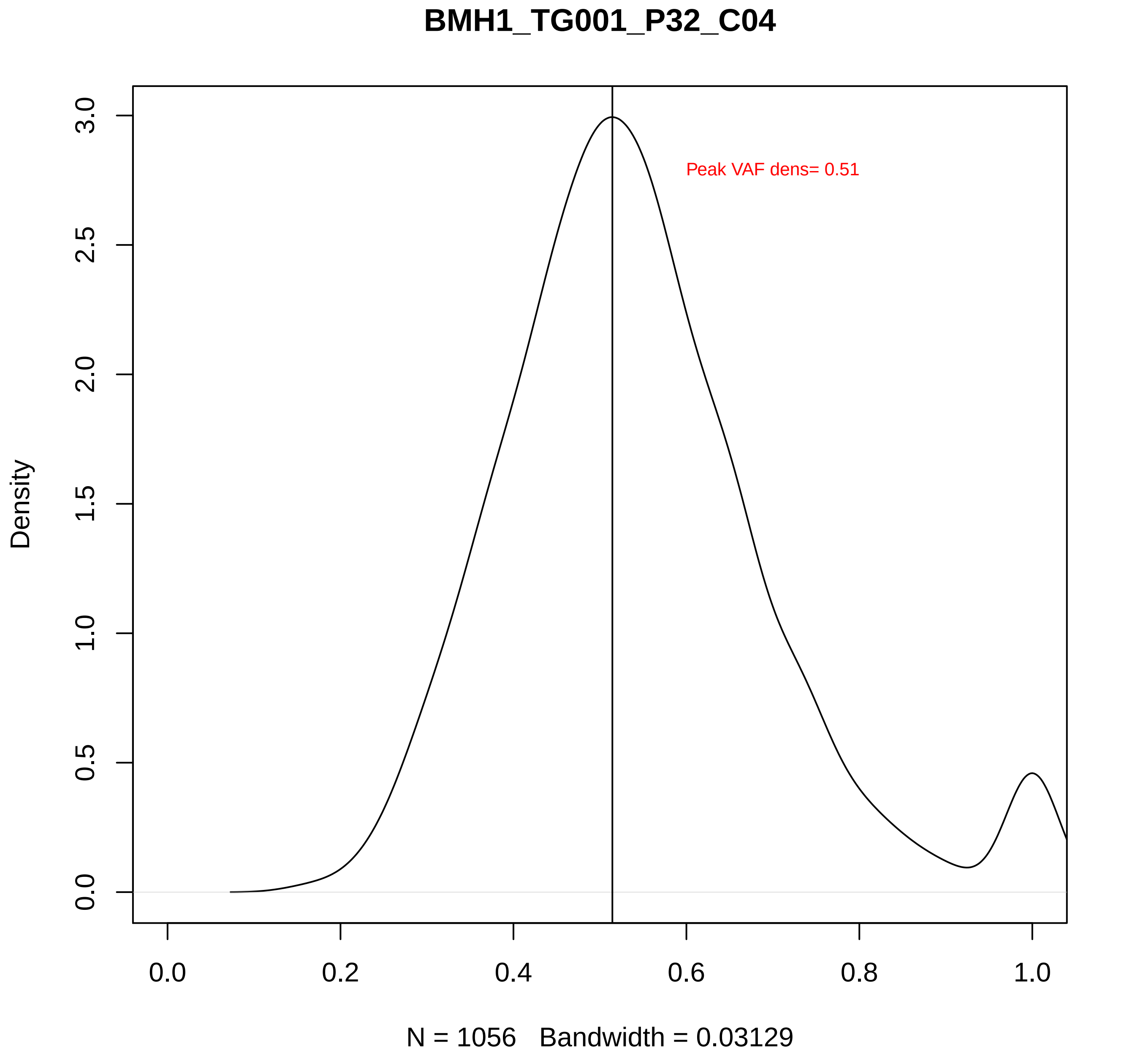
BMH1_TG001_P32_C04
Peak VAF dens= 0.51
Density
0.0
0.5
1.0
1.5
2.0
2.5
3.0
0.0
0.2
0.4
0.6
0.8
1.0
N = 1056   Bandwidth = 0.03129

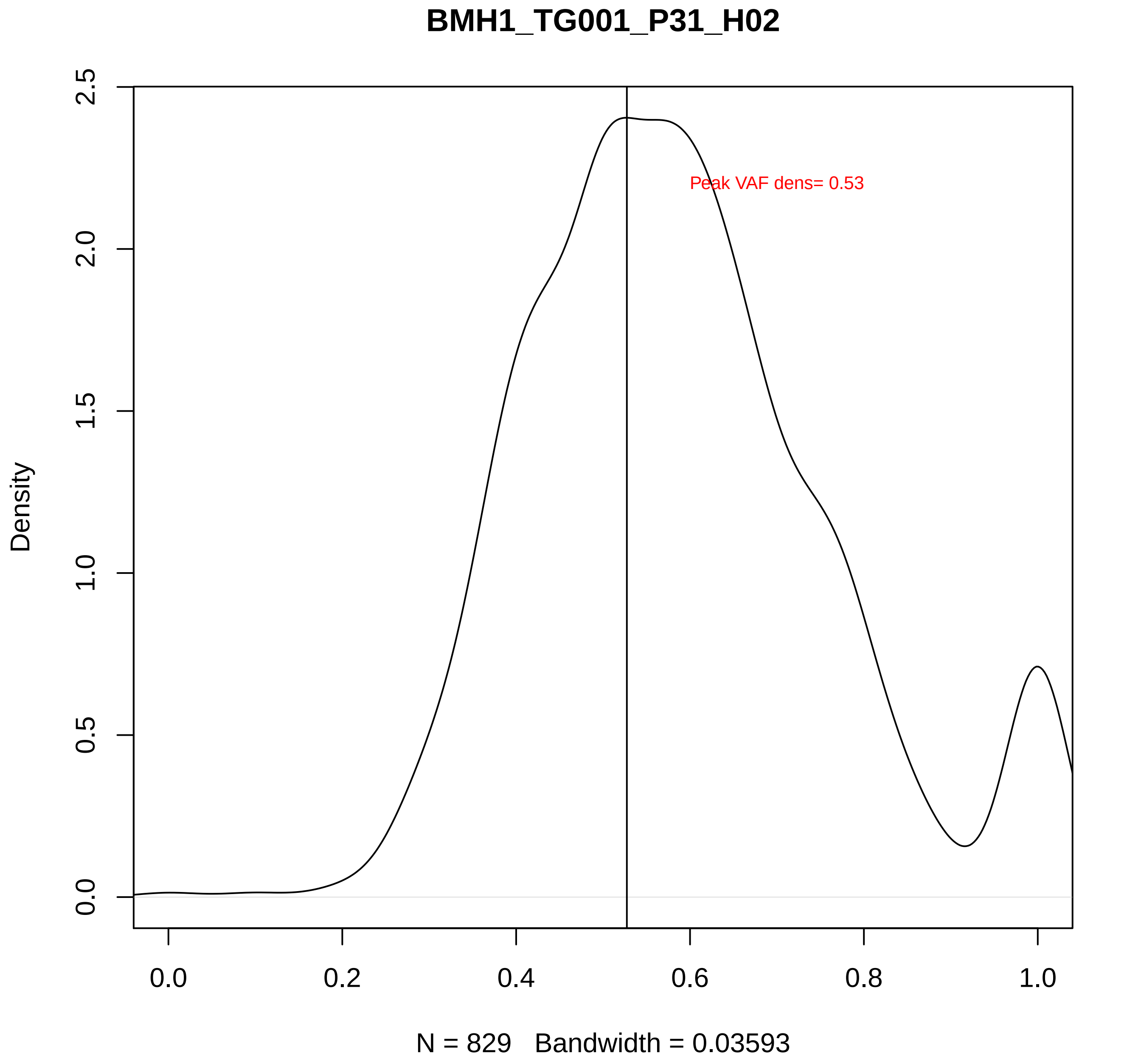
BMH1_TG001_P31_H02
Density
2.5
2.0
1.5
1.0
0.5
0.0
0.0
0.2
0.4
0.6
0.8
1.0
Peak VAF dens= 0.53
N = 829 Bandwidth = 0.03593

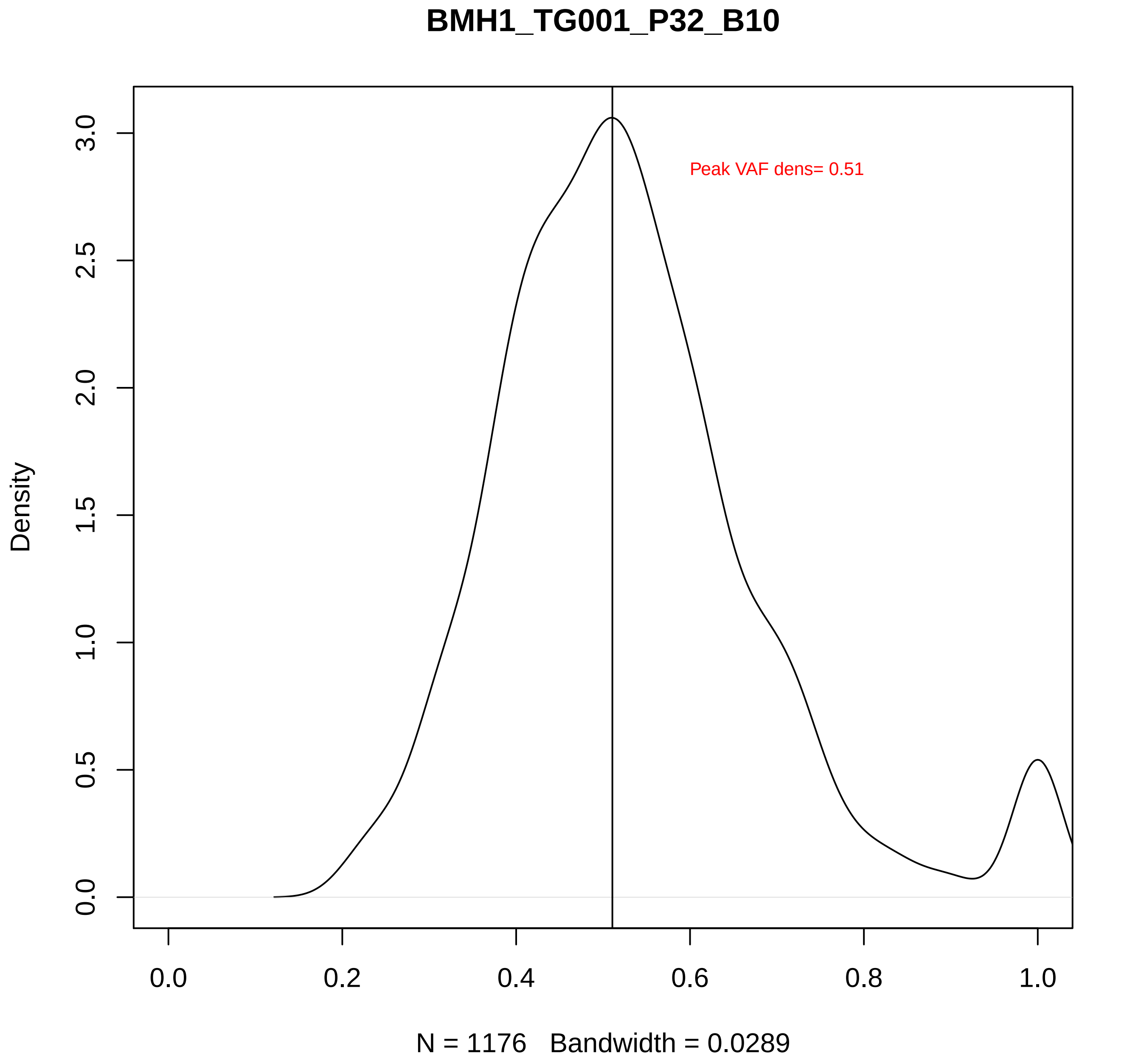

BMH1_TG001_P32_B10
Peak VAF dens= 0.51
Density
3.0
2.5
2.0
1.5
1.0
0.5
0.0
0.0
0.2
0.4
0.6
0.8
1.0
N = 1176   Bandwidth = 0.0289

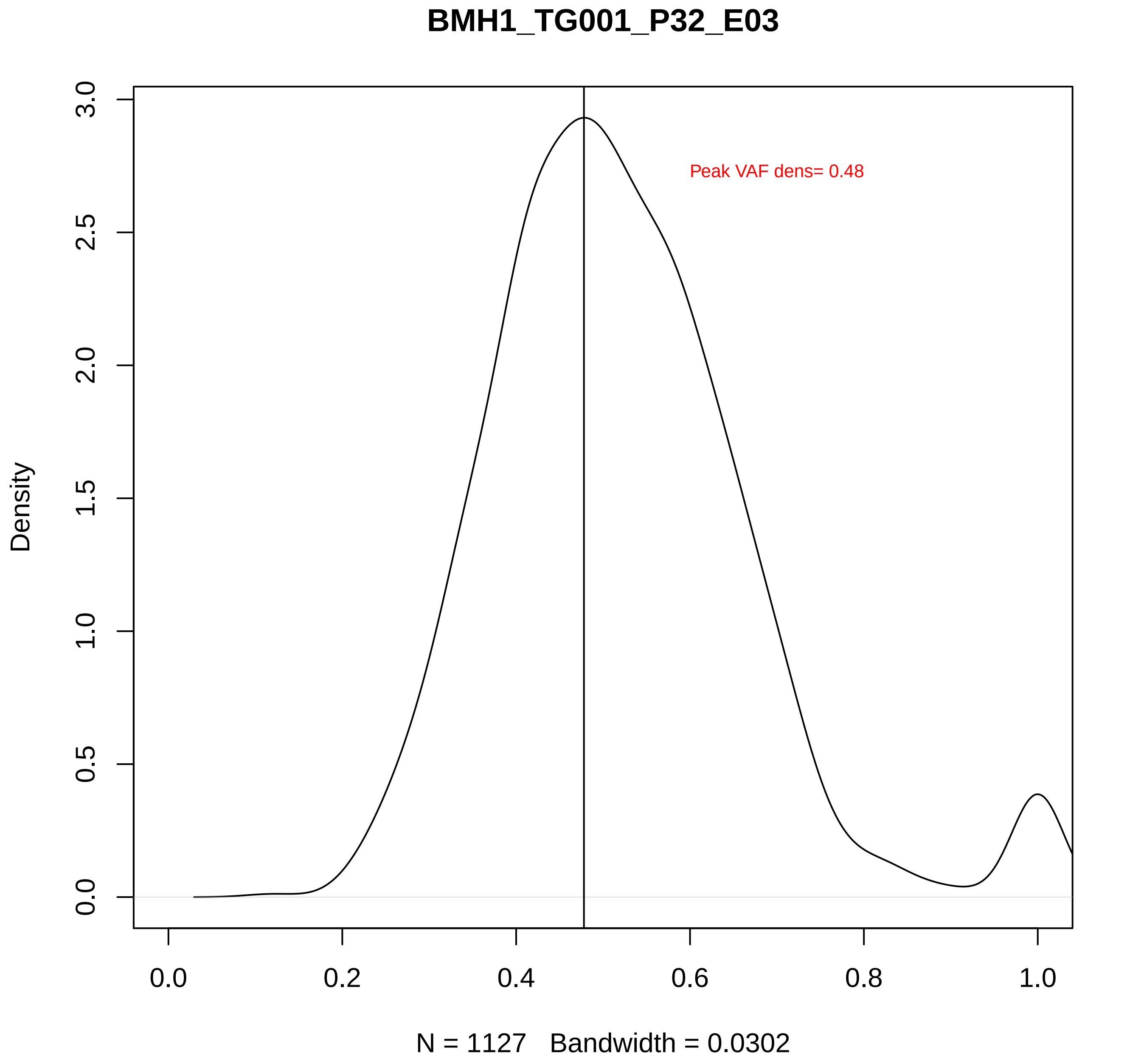
BMH1_TG001_P32_E03
Peak VAF dens= 0.48
Density
0.0
0.5
1.0
1.5
2.0
2.5
3.0
0.0
0.2
0.4
0.6
0.8
1.0
N = 1127   Bandwidth = 0.0302

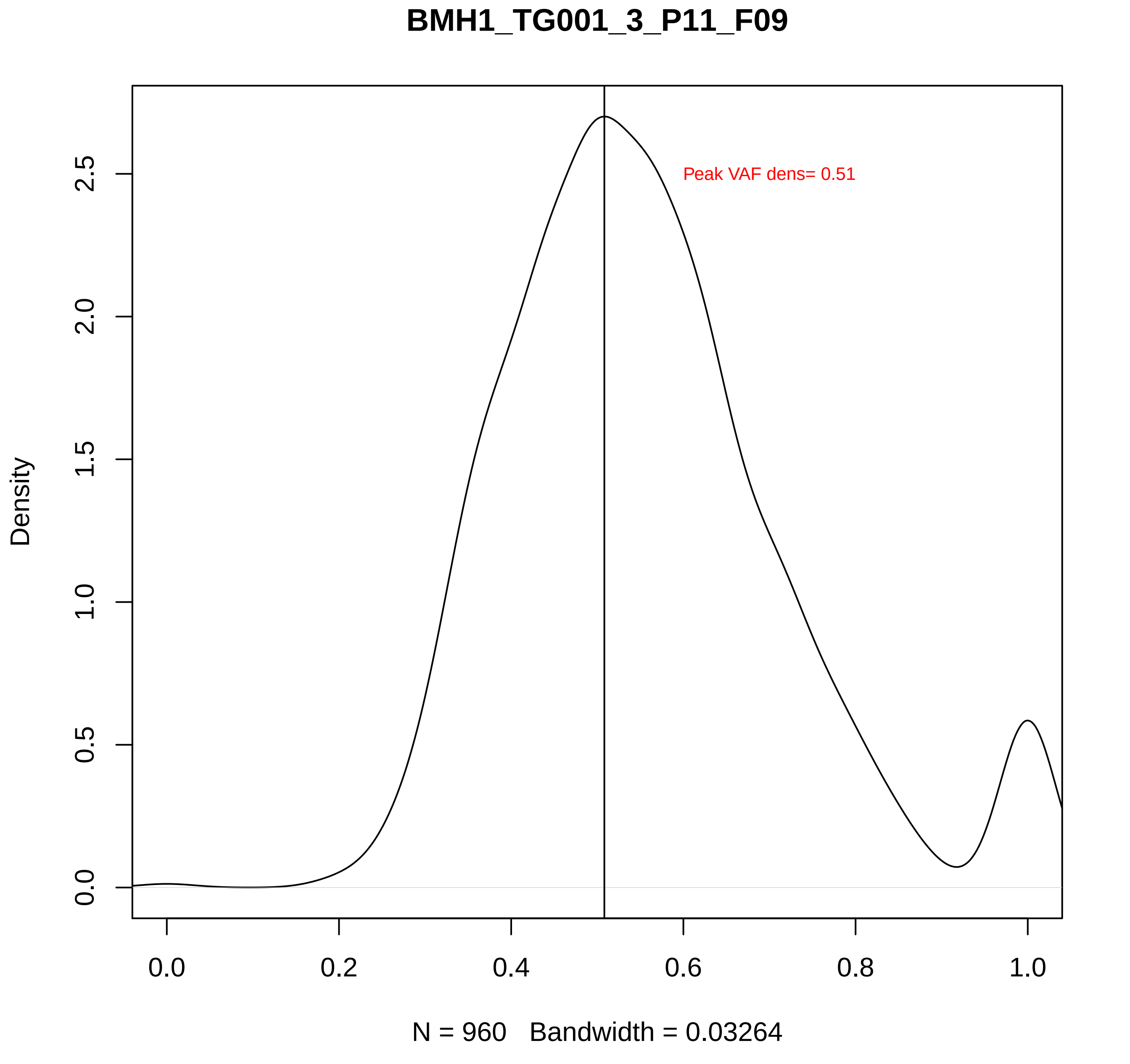
BMH1_TG001_3_P11_F09
Peak VAF dens= 0.51
Density
0.0
0.5
1.0
1.5
2.0
2.5
0.0
0.2
0.4
0.6
0.8
1.0
N = 960   Bandwidth = 0.03264

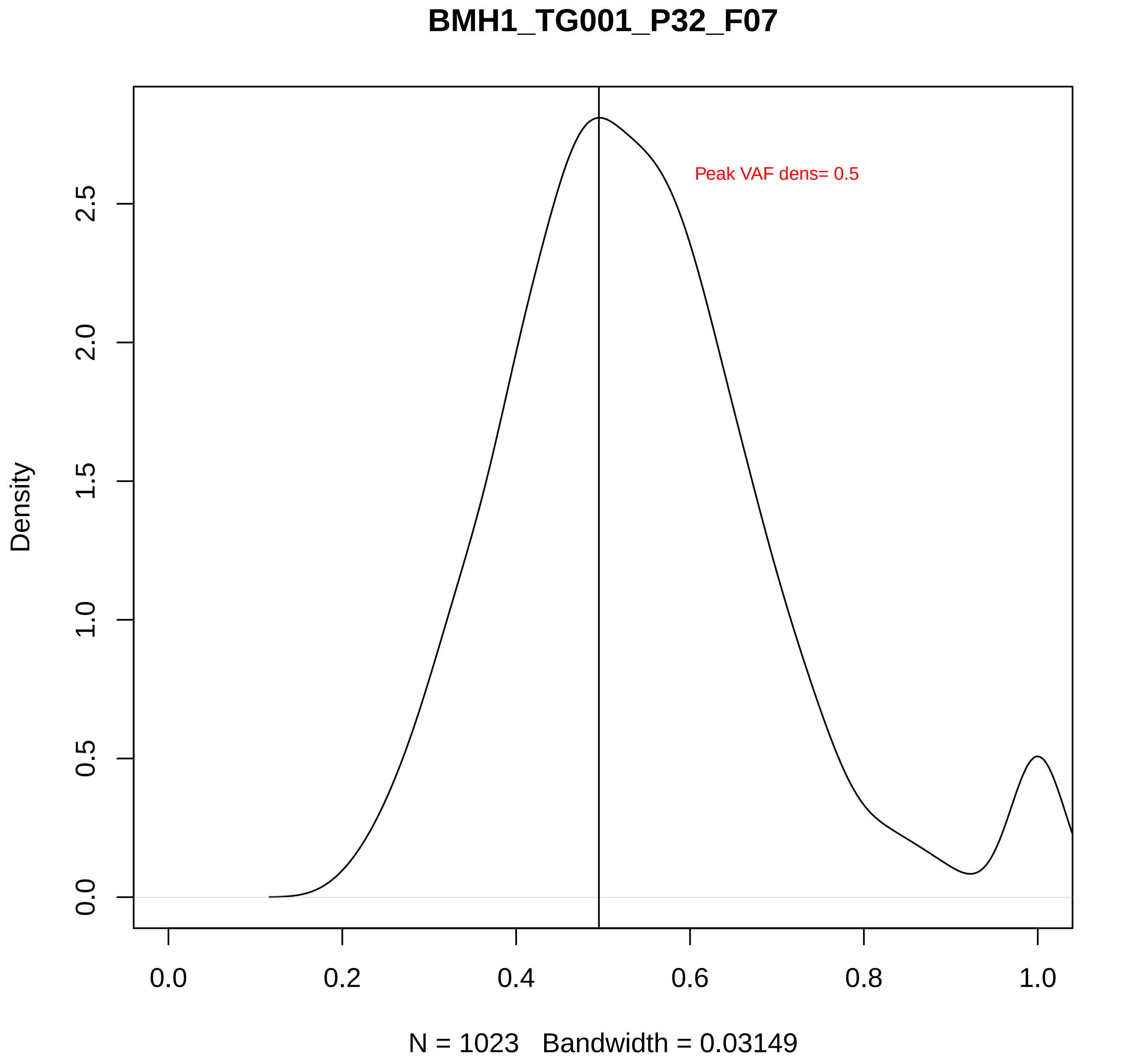
BMH1_TG001_P32_F07
Peak VAF dens= 0.5
Density
0.0
0.5
1.0
1.5
2.0
2.5
0.0
0.2
0.4
0.6
0.8
1.0
N = 1023   Bandwidth = 0.03149

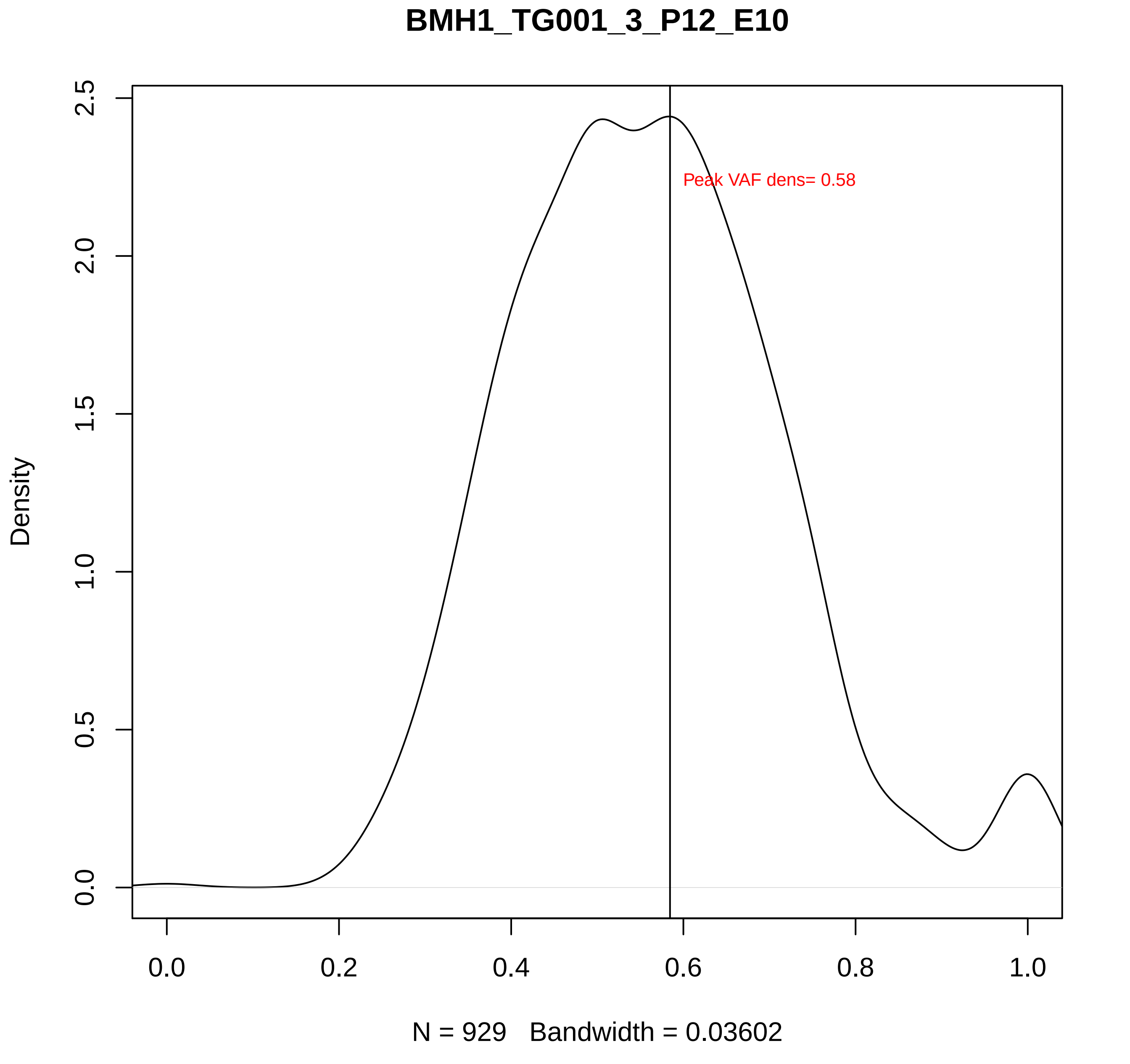

BMH1_TG001_3_P12_E10
Peak VAF dens= 0.58
Density
0.0
0.5
1.0
1.5
2.0
2.5
0.0
0.2
0.4
0.6
0.8
1.0
N = 929   Bandwidth = 0.03602

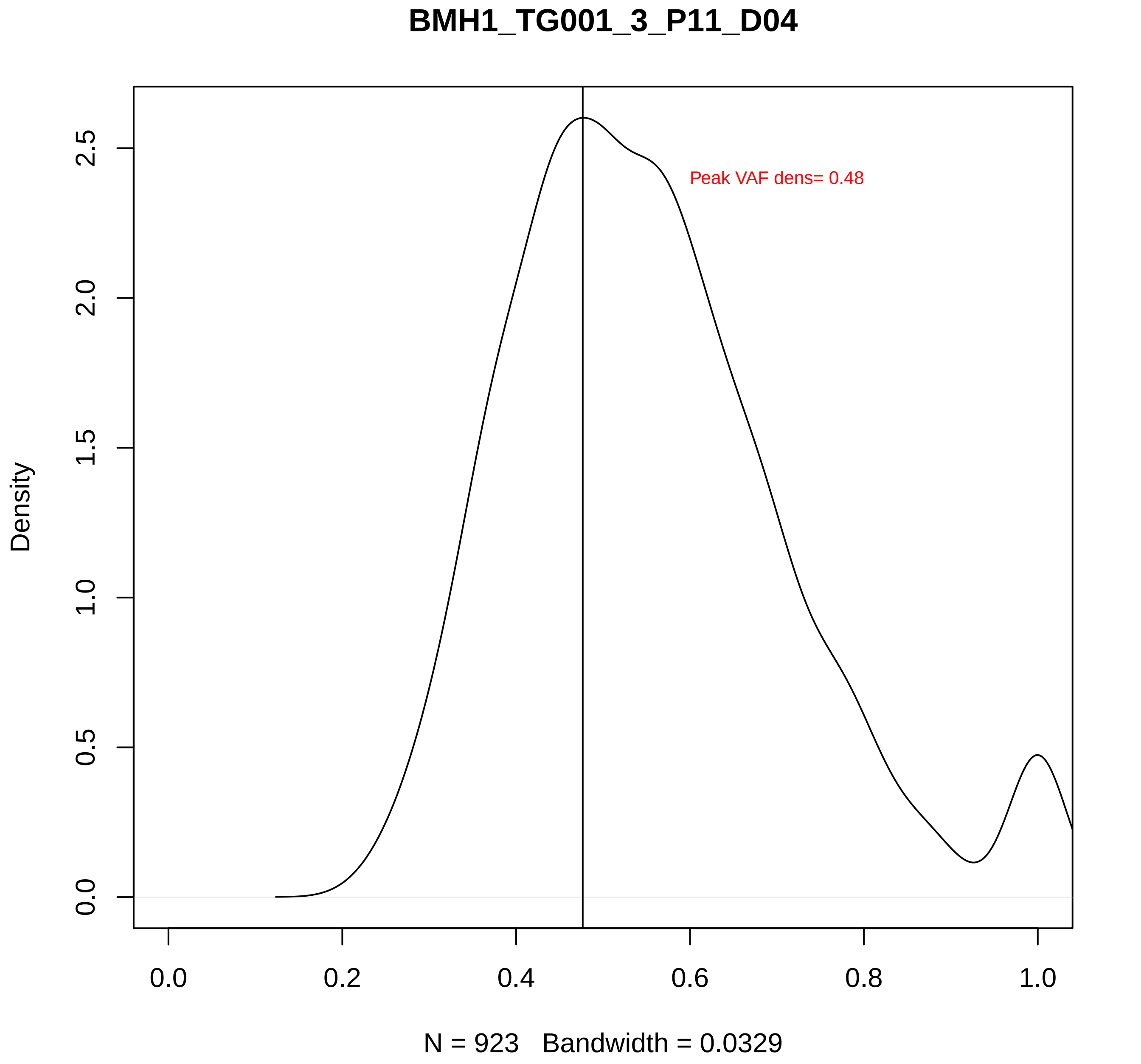

BMH1_TG001_3_P11_D04
Peak VAF dens= 0.48
Density
2.5
2.0
1.5
1.0
0.5
0.0
0.0
0.2
0.4
0.6
0.8
1.0
N = 923   Bandwidth = 0.0329

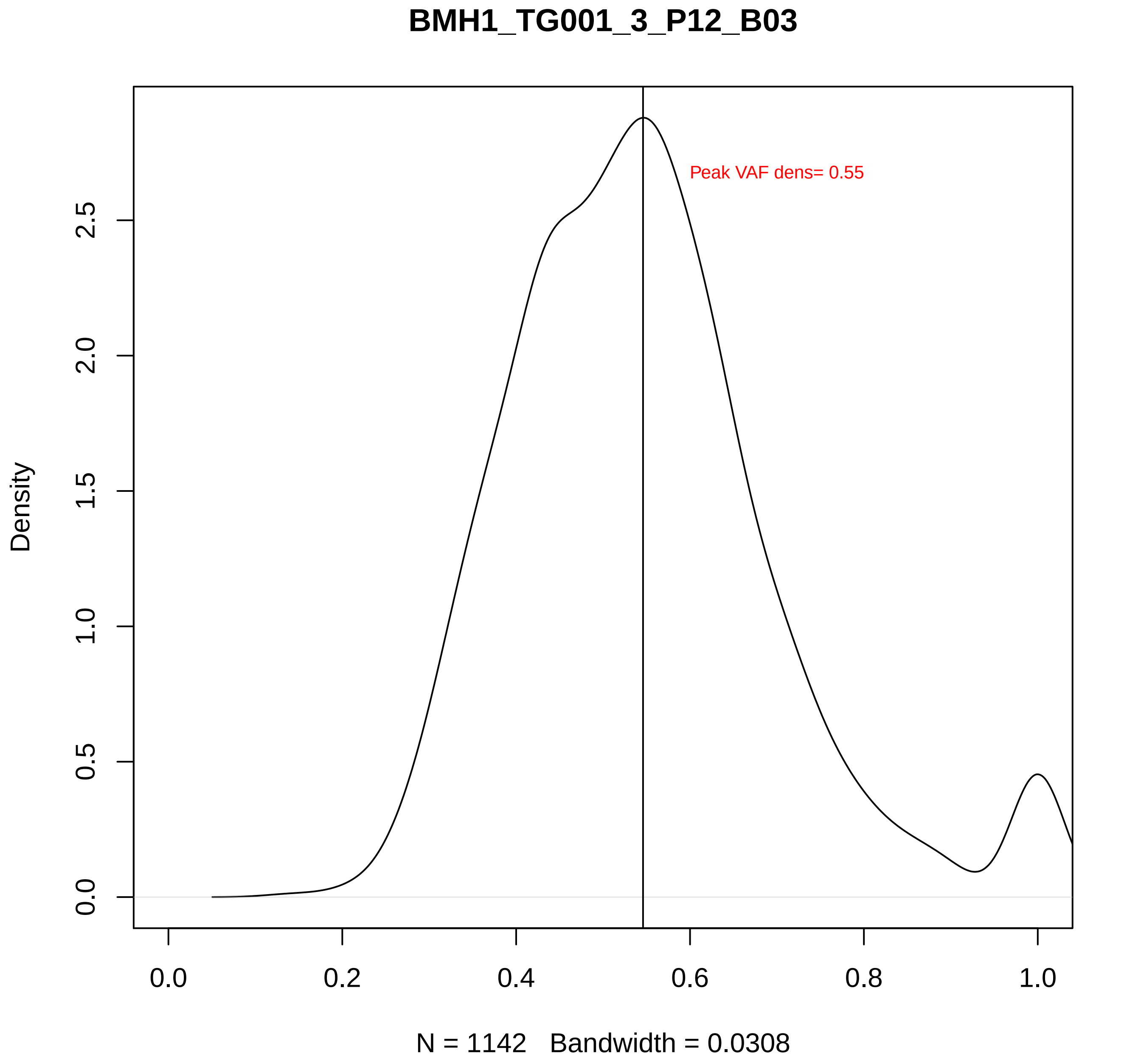
BMH1_TG001_3_P12_B03
Peak VAF dens= 0.55
Density
0.0
0.5
1.0
1.5
2.0
2.5
0.0
0.2
0.4
0.6
0.8
1.0
N = 1142 Bandwidth = 0.0308

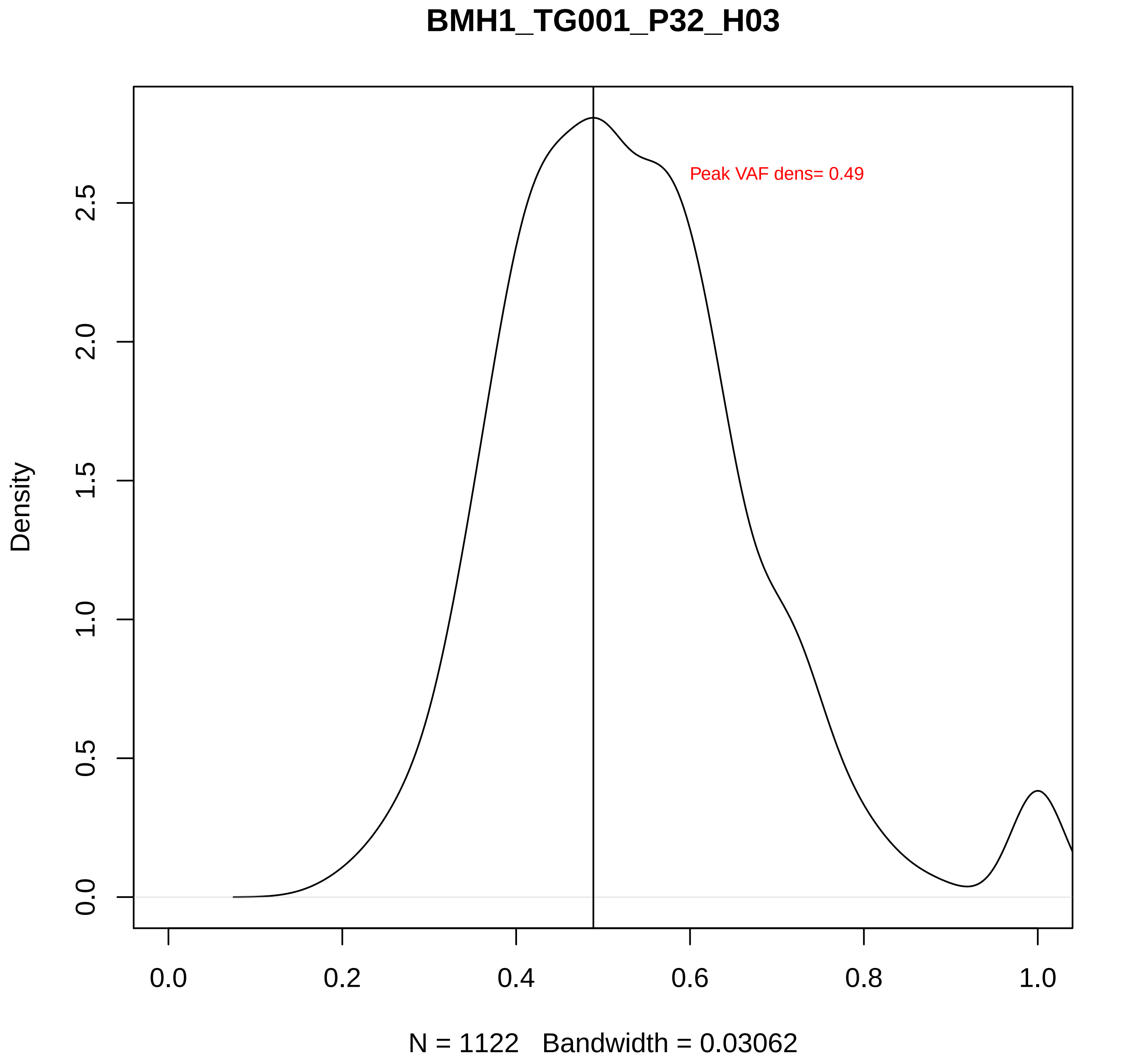

BMH1_TG001_P32_H03
Density
0.0
0.5
1.0
1.5
2.0
2.5
0.0
0.2
0.4
0.6
0.8
1.0
Peak VAF dens= 0.49
N = 1122   Bandwidth = 0.03062

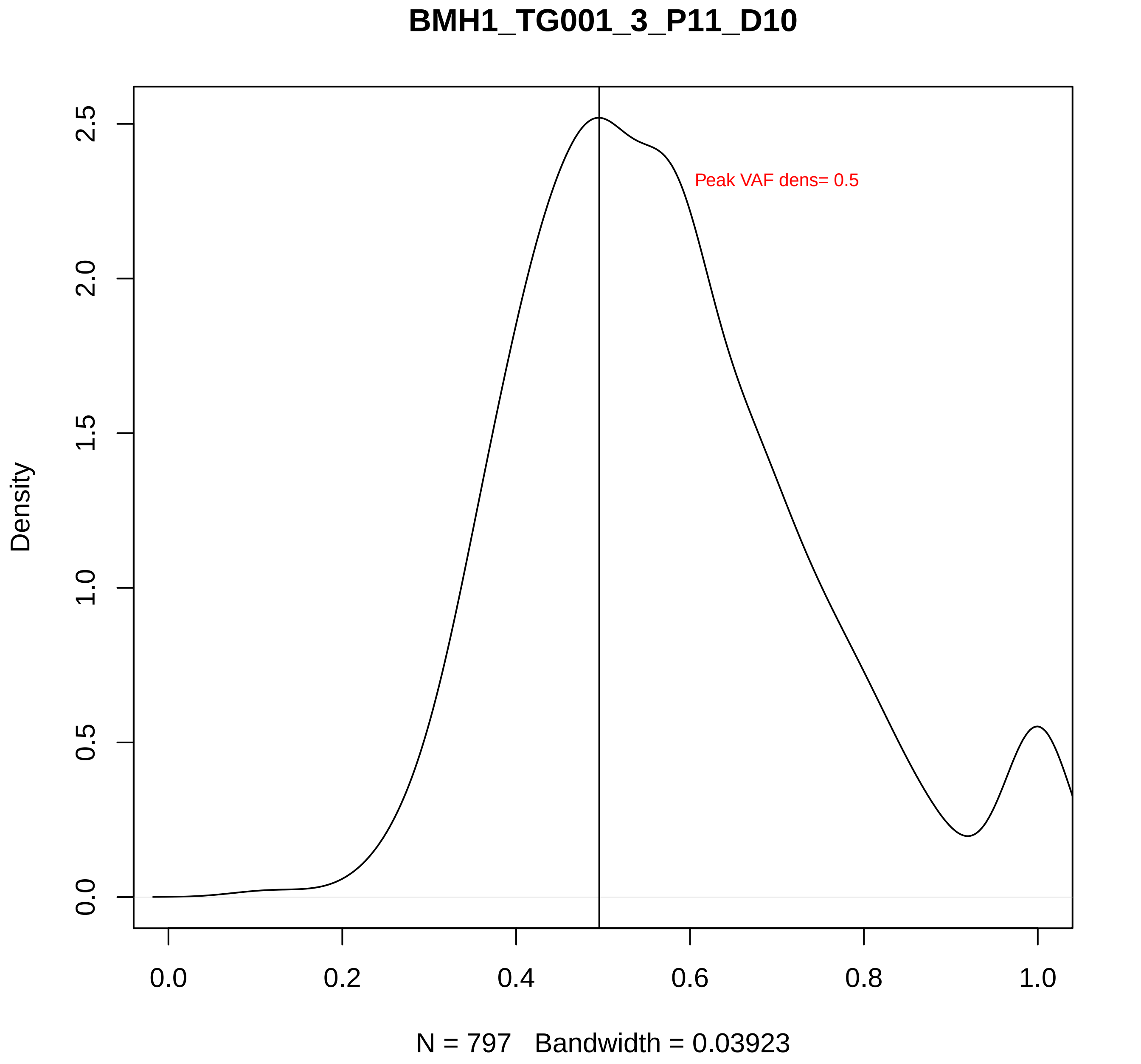

BMH1_TG001_3_P11_D10
Peak VAF dens= 0.5
Density
2.5
2.0
1.5
1.0
0.5
0.0
0.0
0.2
0.4
0.6
0.8
1.0
N = 797 Bandwidth = 0.03923

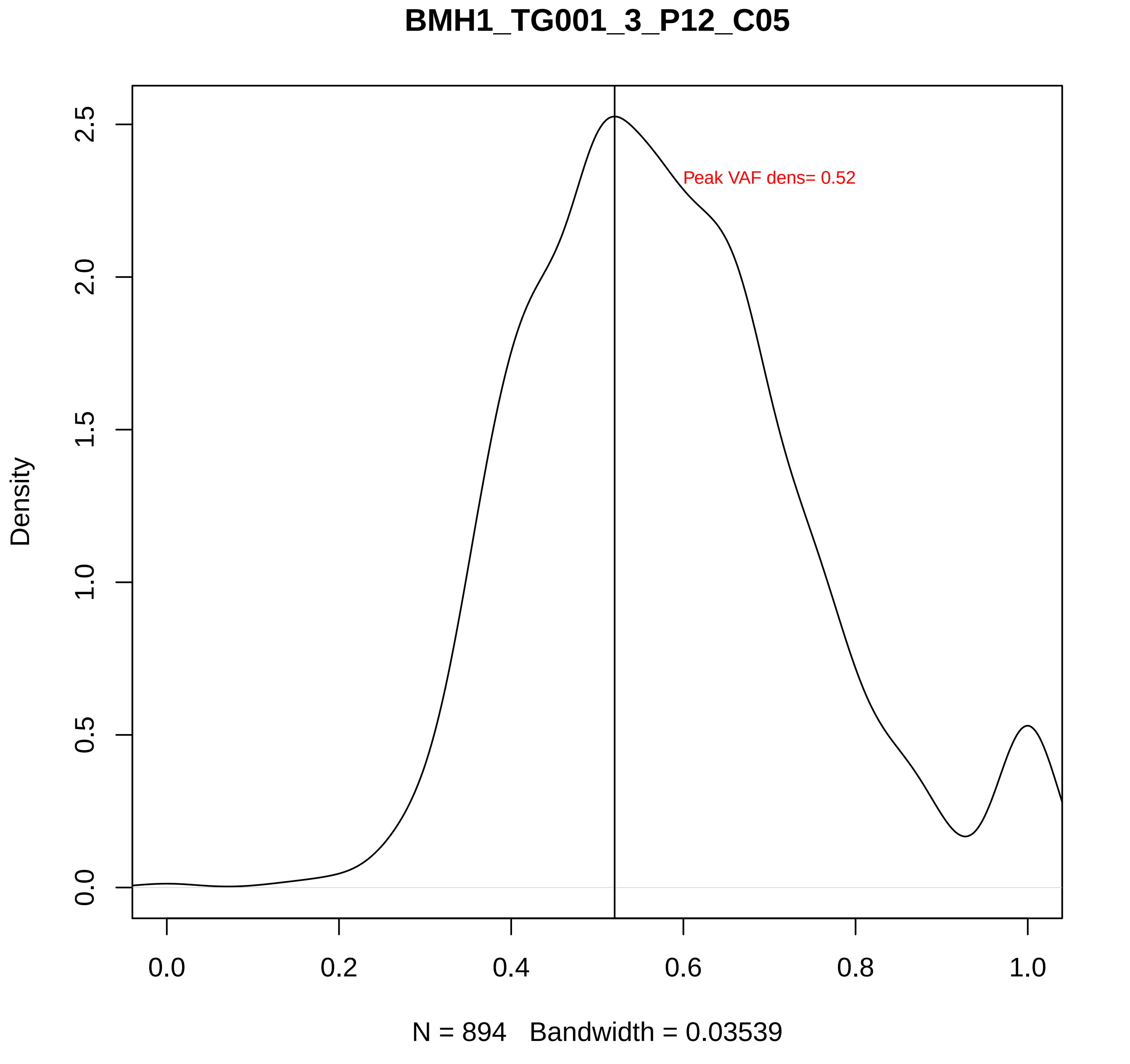

BMH1_TG001_3_P12_C05
Peak VAF dens= 0.52
Density
0.0
0.5
1.0
1.5
2.0
2.5
0.0
0.2
0.4
0.6
0.8
1.0
N = 894   Bandwidth = 0.03539

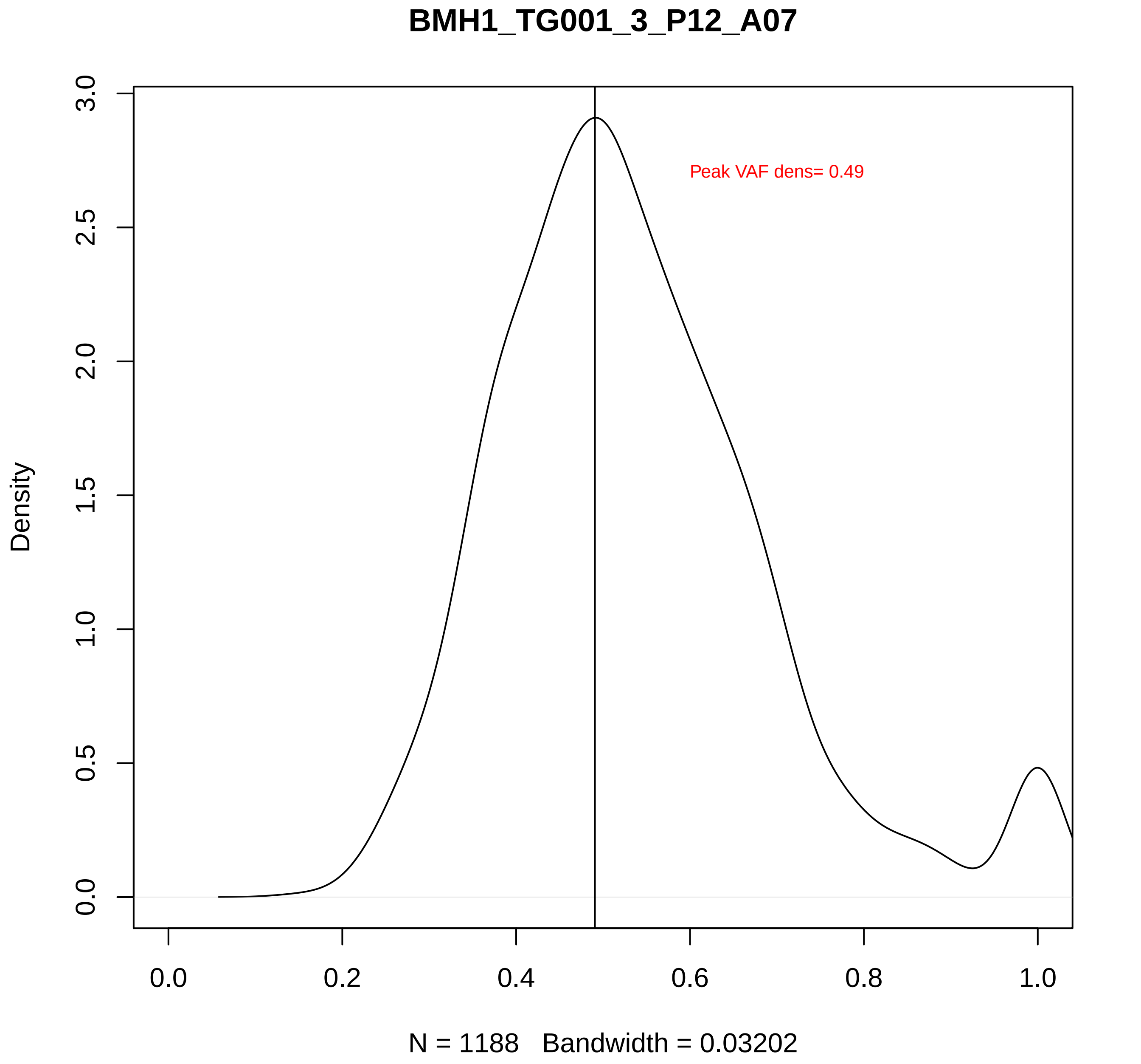

BMH1_TG001_3_P12_A07
Peak VAF dens= 0.49
Density
0.0
0.5
1.0
1.5
2.0
2.5
3.0
0.0
0.2
0.4
0.6
0.8
1.0
N = 1188 Bandwidth = 0.03202

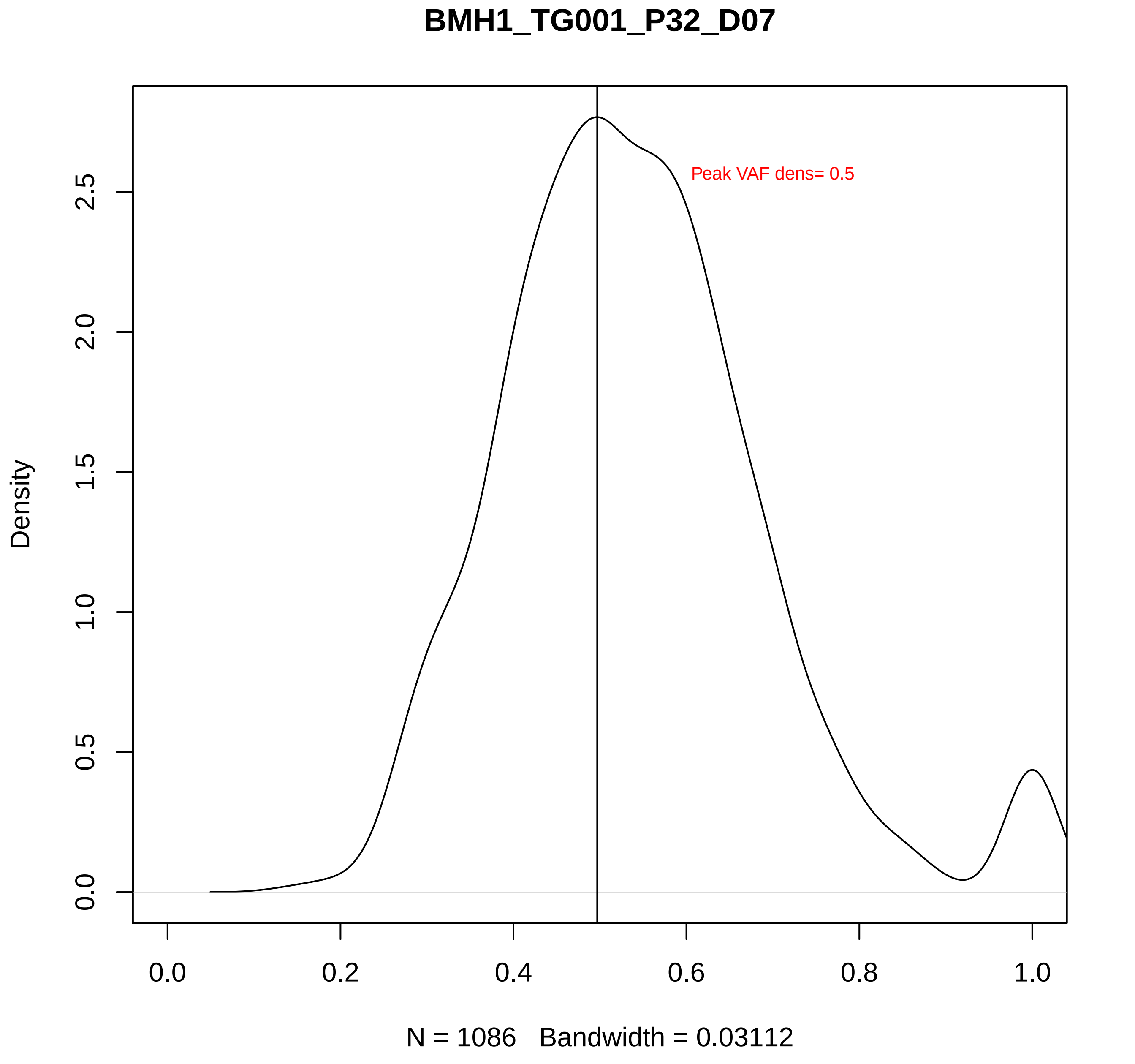
BMH1_TG001_P32_D07
Peak VAF dens= 0.5
Density
0.0
0.5
1.0
1.5
2.0
2.5
0.0
0.2
0.4
0.6
0.8
1.0
N = 1086 Bandwidth = 0.03112

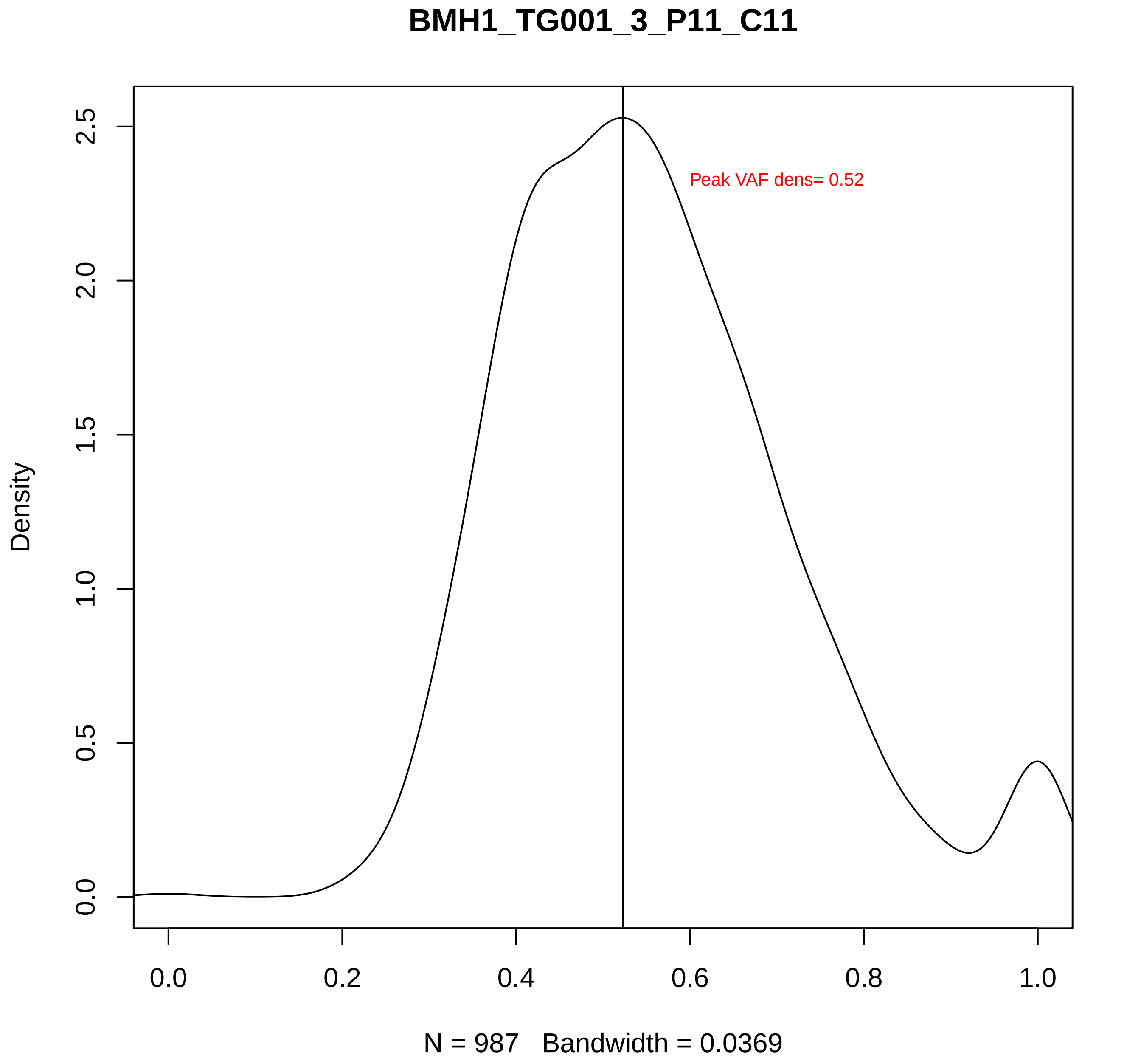

BMH1_TG001_3_P11_C11
Peak VAF dens= 0.52
Density
0.0
0.5
1.0
1.5
2.0
2.5
0.0
0.2
0.4
0.6
0.8
1.0
N = 987 Bandwidth = 0.0369

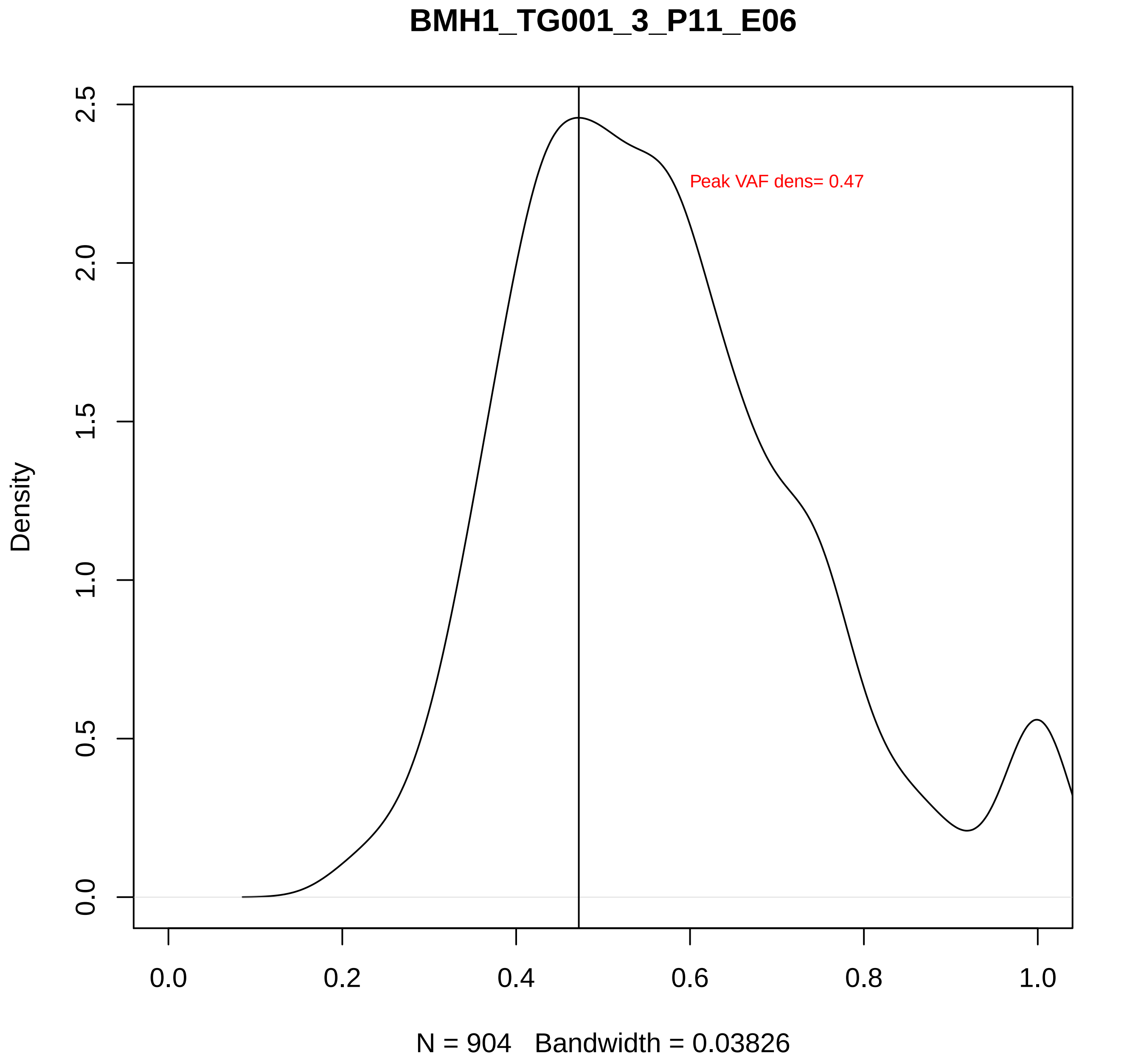

BMH1_TG001_3_P11_E06
Peak VAF dens= 0.47
Density
0.0
0.5
1.0
1.5
2.0
2.5
0.0
0.2
0.4
0.6
0.8
1.0
N = 904 Bandwidth = 0.03826

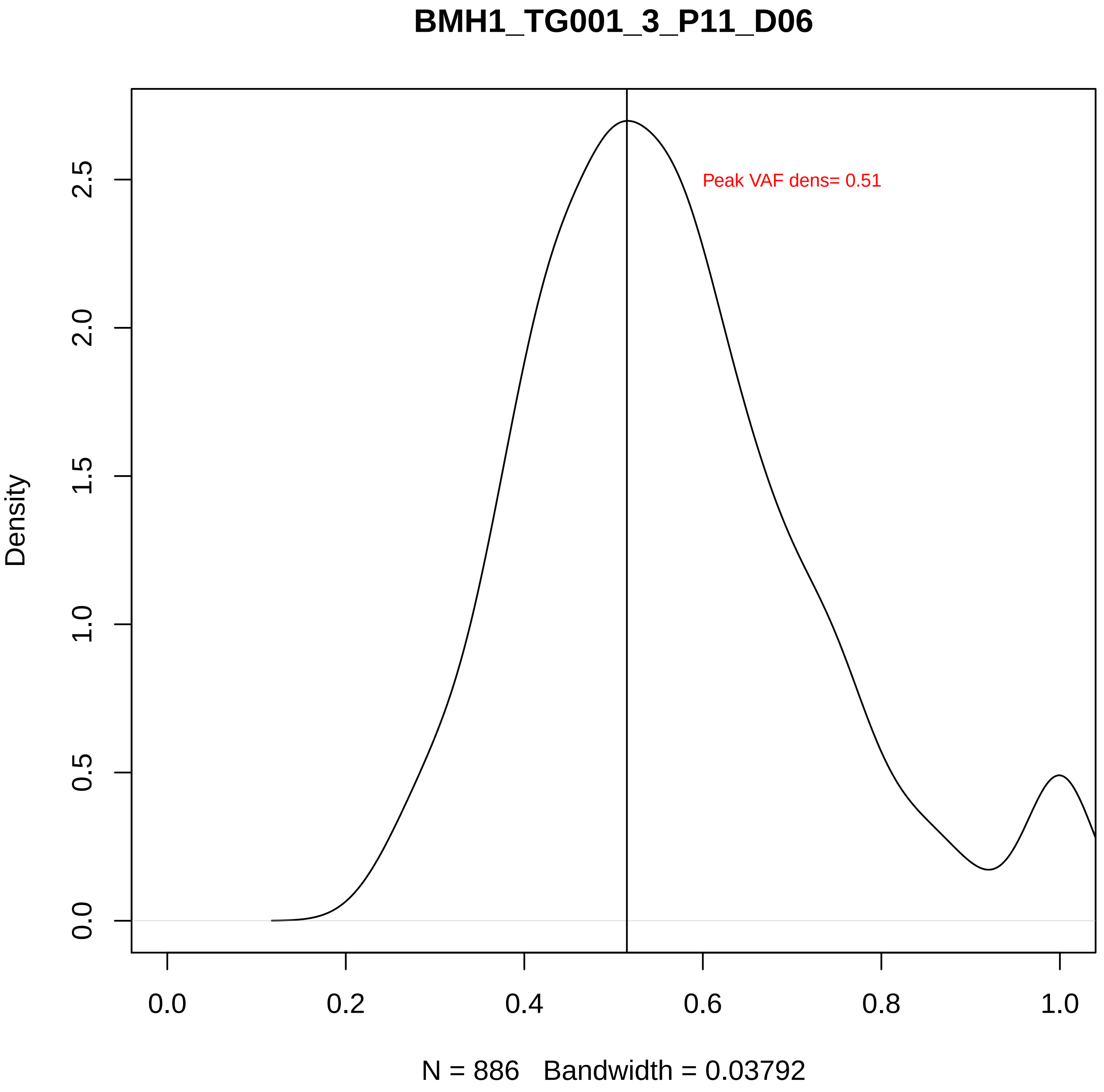

BMH1_TG001_3_P11_D06
Peak VAF dens= 0.51
Density
0.0
0.5
1.0
1.5
2.0
2.5
0.0
0.2
0.4
0.6
0.8
1.0
N = 886   Bandwidth = 0.03792

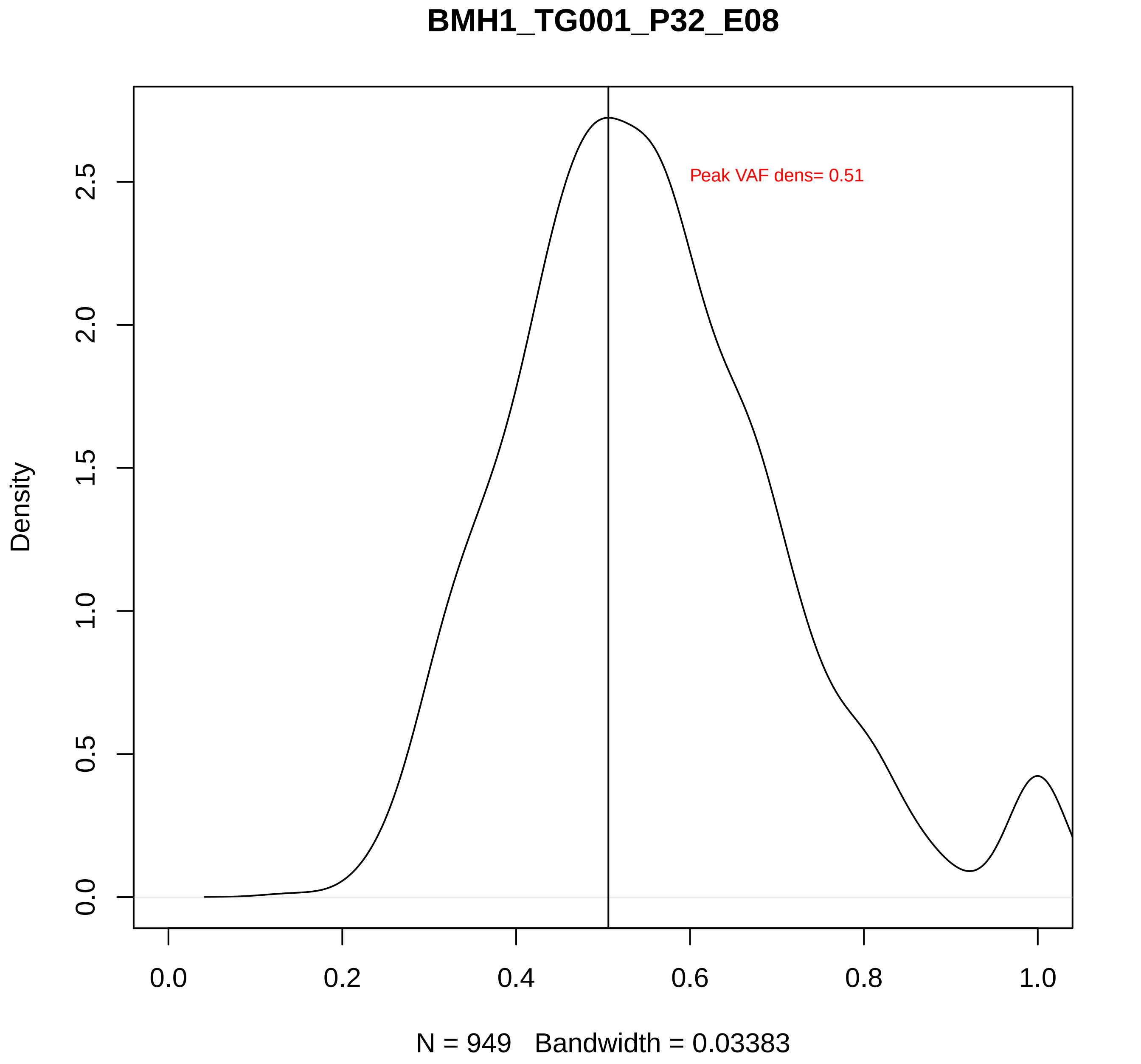
BMH1_TG001_P32_E08
Peak VAF dens= 0.51
Density
0.0
0.5
1.0
1.5
2.0
2.5
0.0
0.2
0.4
0.6
0.8
1.0
N = 949   Bandwidth = 0.03383

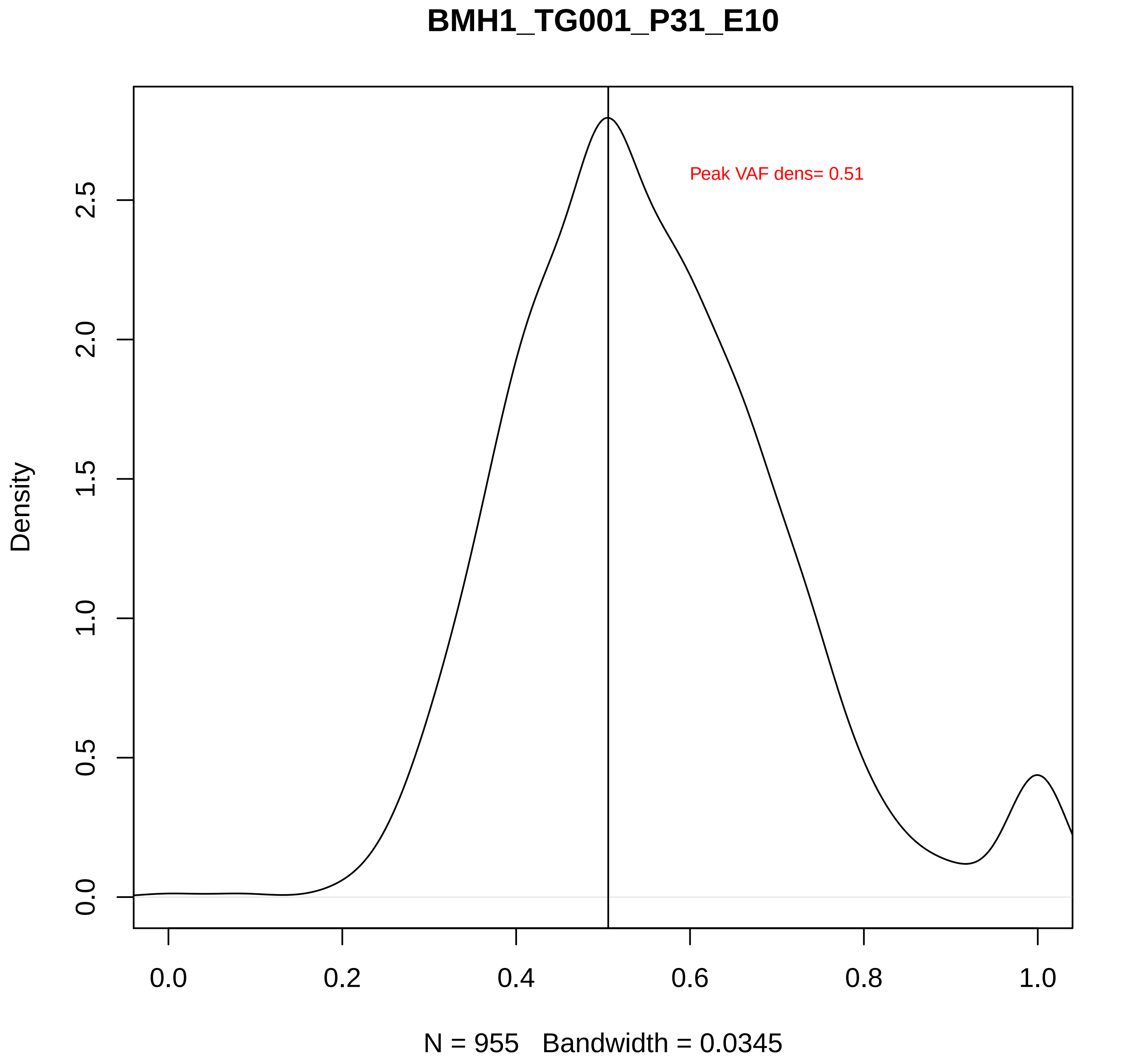
BMH1_TG001_P31_E10
Peak VAF dens= 0.51
Density
0.0
0.5
1.0
1.5
2.0
2.5
0.0
0.2
0.4
0.6
0.8
1.0
N = 955   Bandwidth = 0.0345

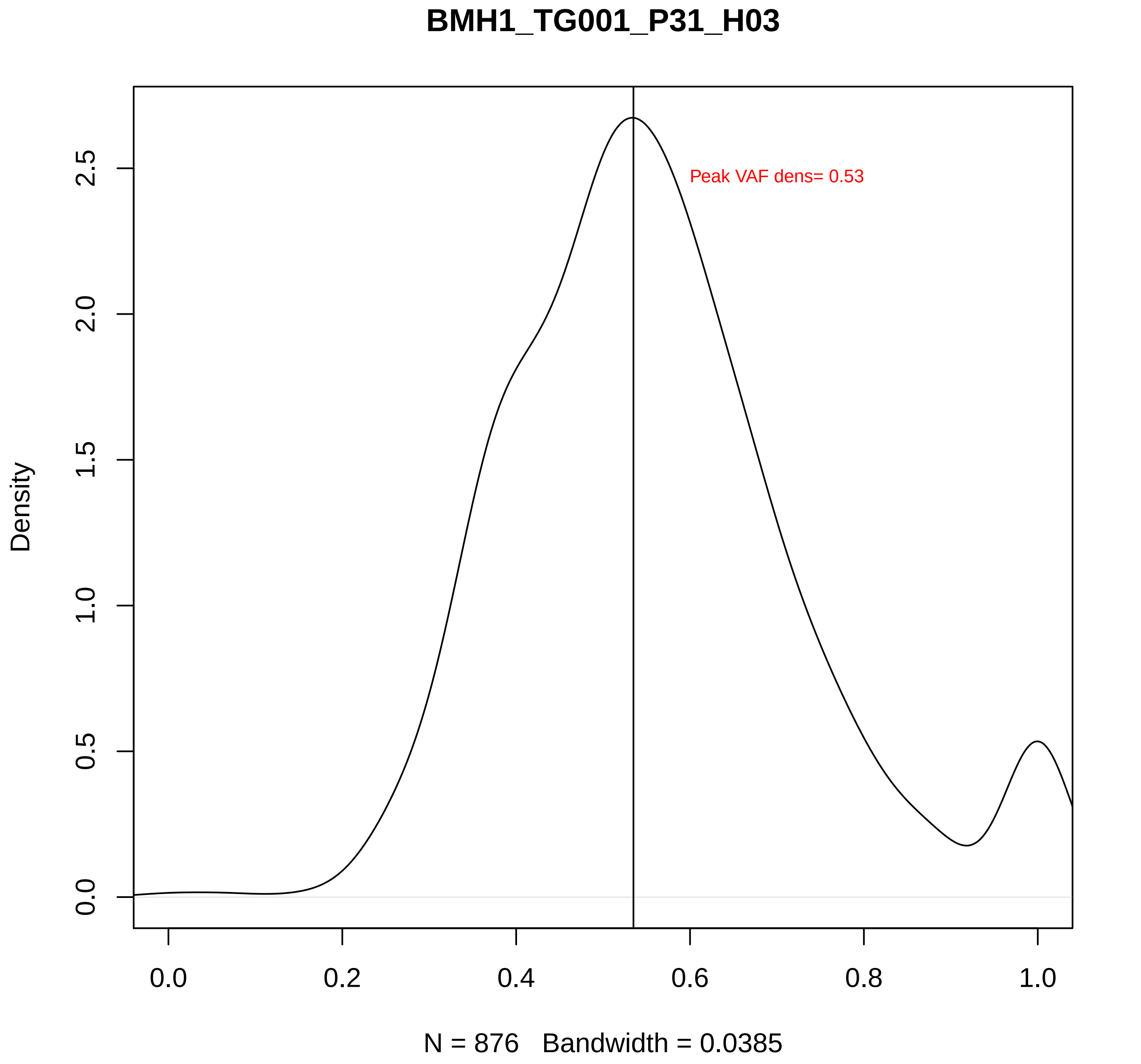
BMH1_TG001_P31_H03
Peak VAF dens= 0.53
Density
0.0
0.5
1.0
1.5
2.0
2.5
0.0
0.2
0.4
0.6
0.8
1.0
N = 876   Bandwidth = 0.0385

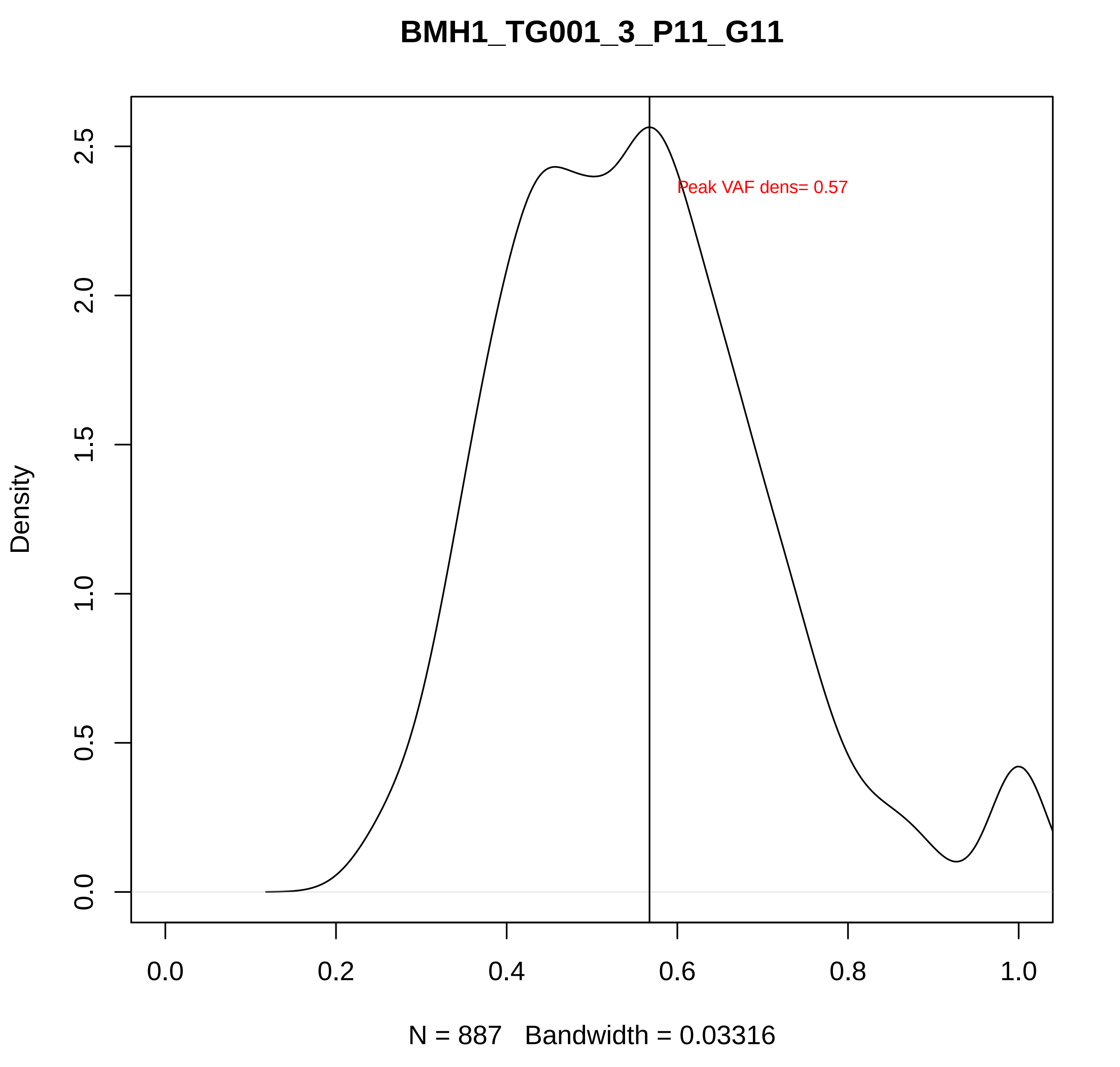

BMH1_TG001_3_P11_G11
Peak VAF dens= 0.57
Density
0.0
0.5
1.0
1.5
2.0
2.5
0.0
0.2
0.4
0.6
0.8
1.0
N = 887 Bandwidth = 0.03316

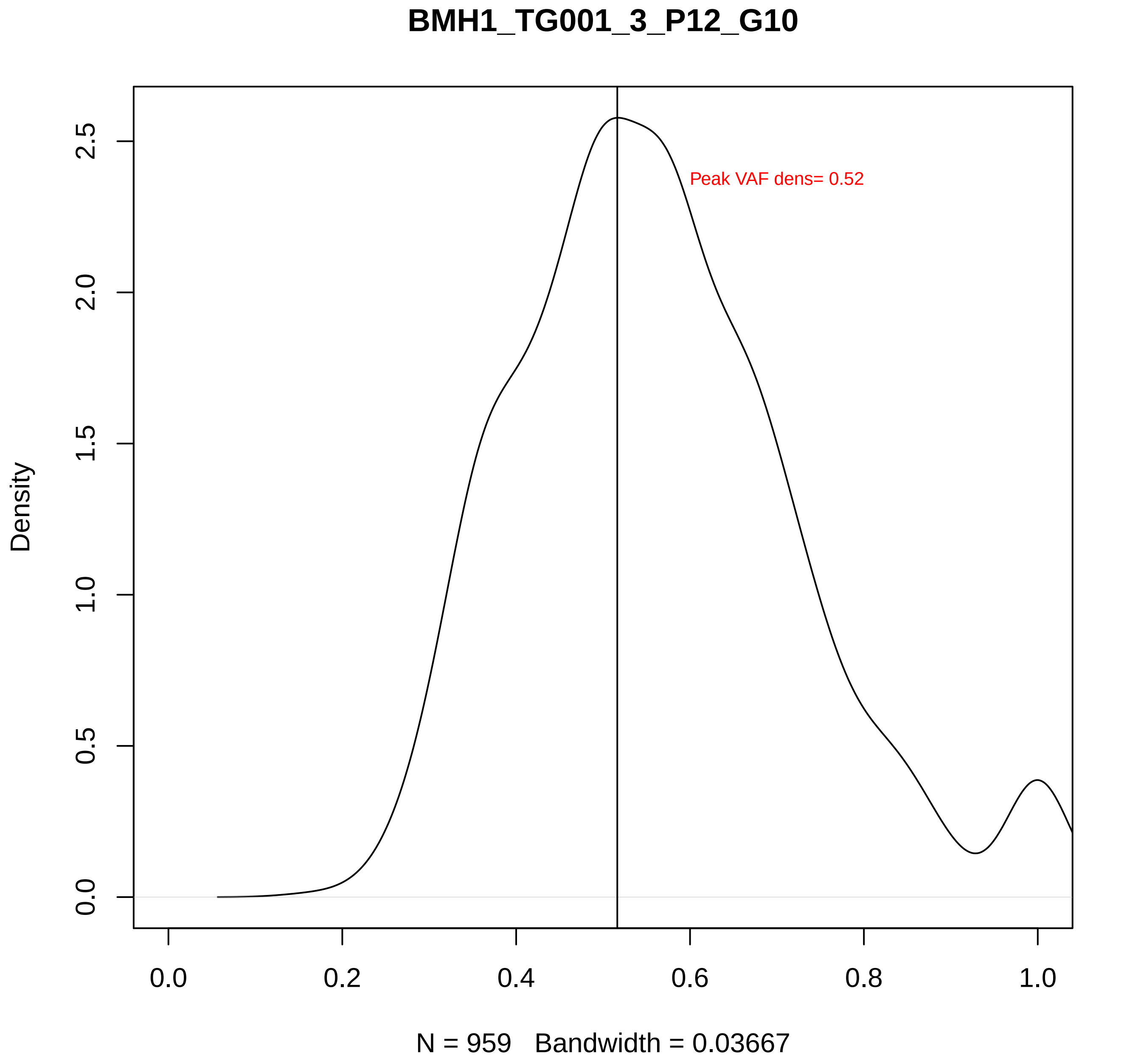
BMH1_TG001_3_P12_G10
Peak VAF dens= 0.52
Density
0.0
0.5
1.0
1.5
2.0
2.5
0.0
0.2
0.4
0.6
0.8
1.0
N = 959   Bandwidth = 0.03667

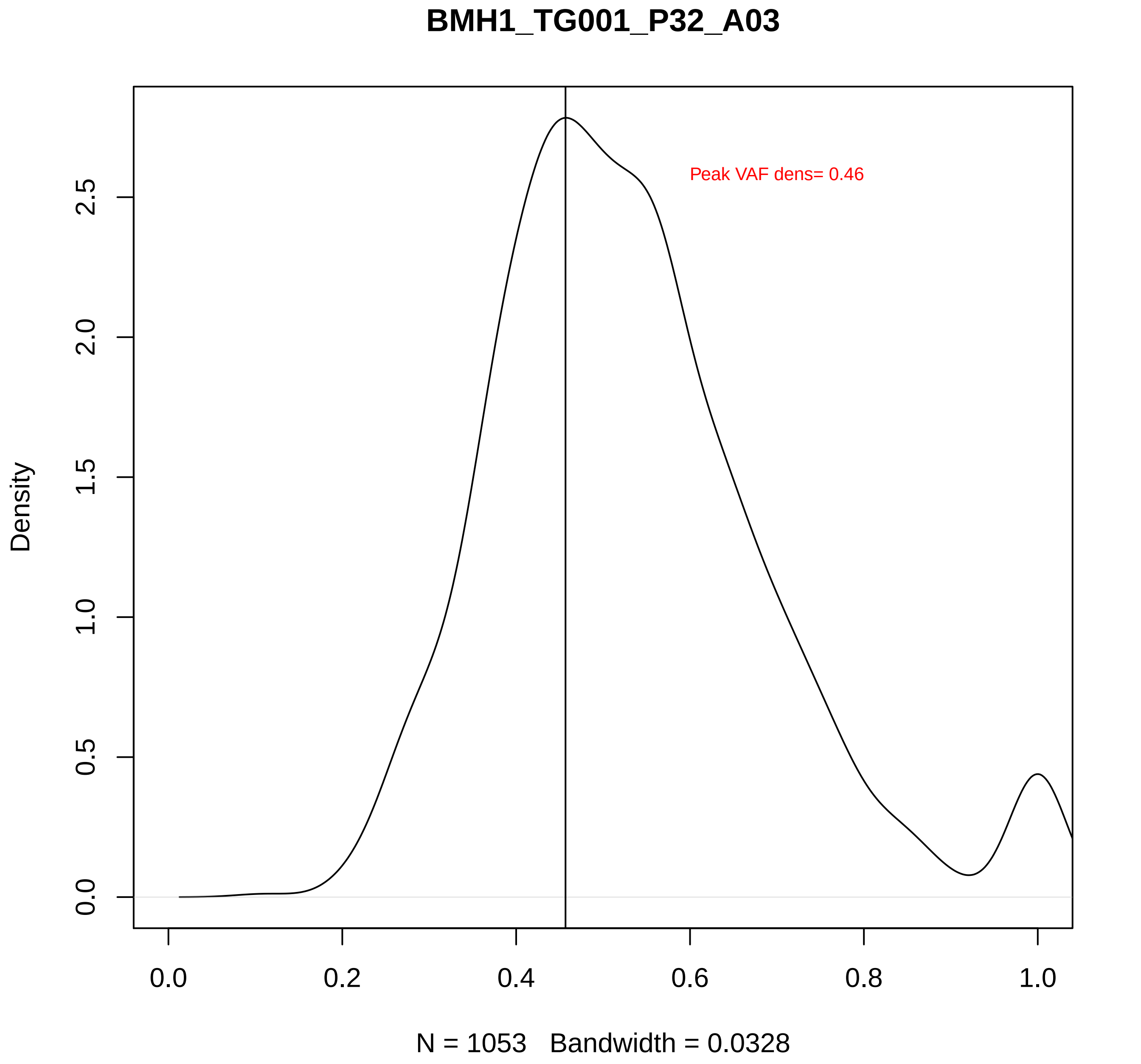
BMH1_TG001_P32_A03
Peak VAF dens= 0.46
Density
0.0
0.5
1.0
1.5
2.0
2.5
0.0
0.2
0.4
0.6
0.8
1.0
N = 1053   Bandwidth = 0.0328

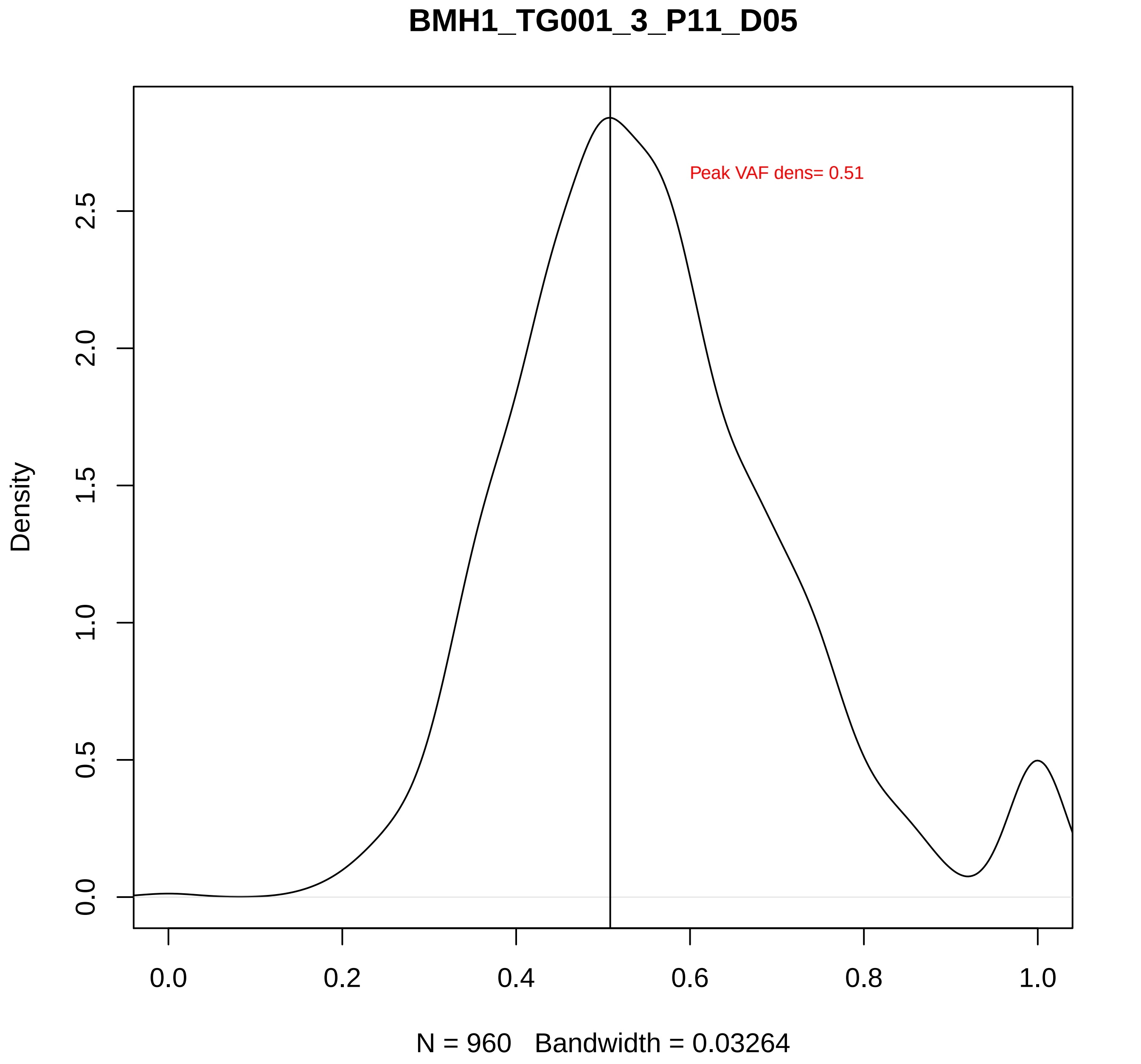
BMH1_TG001_3_P11_D05
Peak VAF dens= 0.51
Density
0.0
0.5
1.0
1.5
2.0
2.5
0.0
0.2
0.4
0.6
0.8
1.0
N = 960   Bandwidth = 0.03264

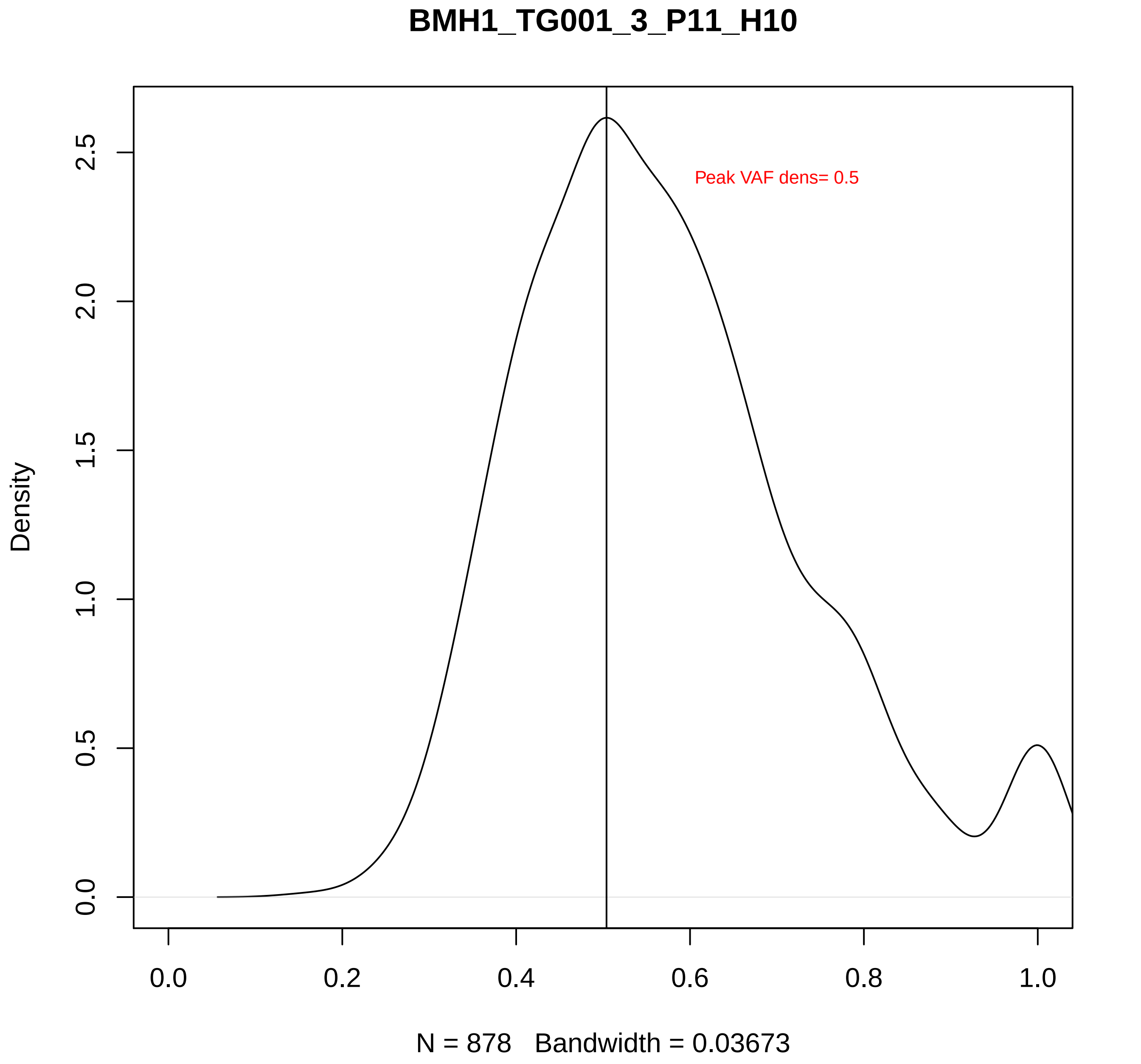
BMH1_TG001_3_P11_H10
Peak VAF dens= 0.5
Density
0.0
0.5
1.0
1.5
2.0
2.5
0.0
0.2
0.4
0.6
0.8
1.0
N = 878 Bandwidth = 0.03673

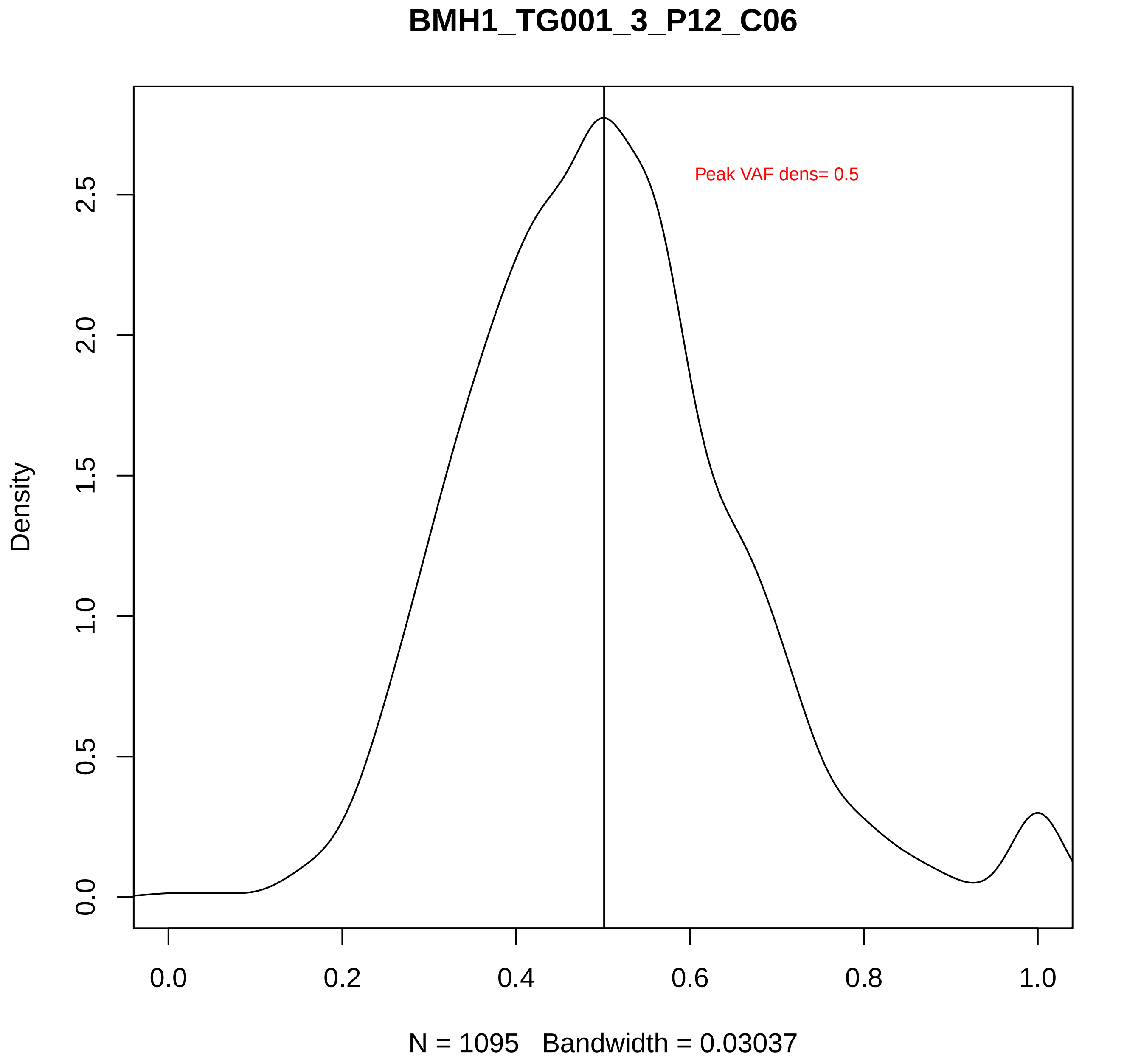

BMH1_TG001_3_P12_C06
Peak VAF dens= 0.5
Density
0.0
0.5
1.0
1.5
2.0
2.5
0.0
0.2
0.4
0.6
0.8
1.0
N = 1095   Bandwidth = 0.03037

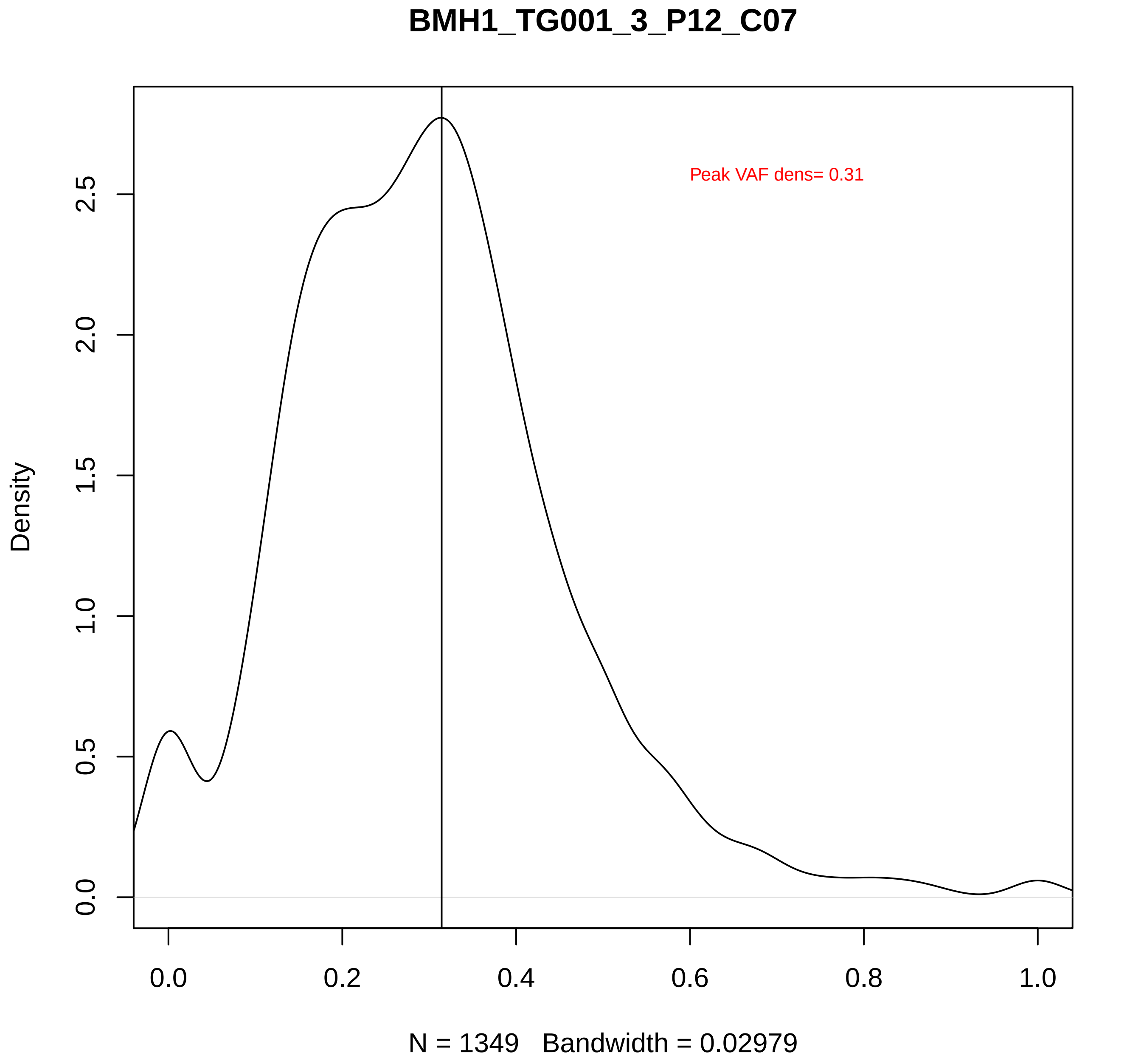
BMH1_TG001_3_P12_C07
Peak VAF dens= 0.31
Density
0.0
0.5
1.0
1.5
2.0
2.5
0.0
0.2
0.4
0.6
0.8
1.0
N = 1349 Bandwidth = 0.02979

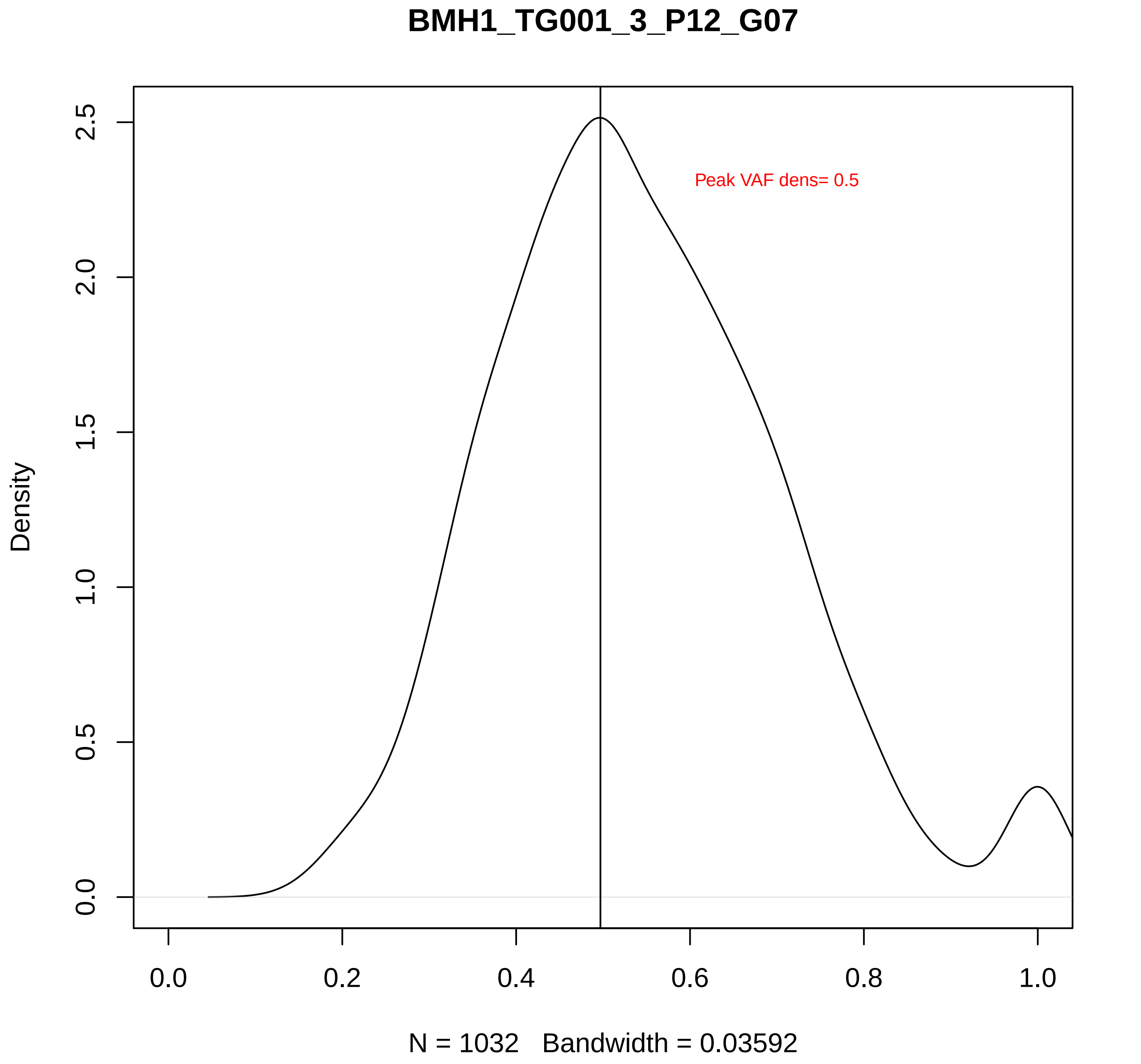
BMH1_TG001_3_P12_G07
Peak VAF dens= 0.5
Density
0.0
0.5
1.0
1.5
2.0
2.5
0.0
0.2
0.4
0.6
0.8
1.0
N = 1032   Bandwidth = 0.03592

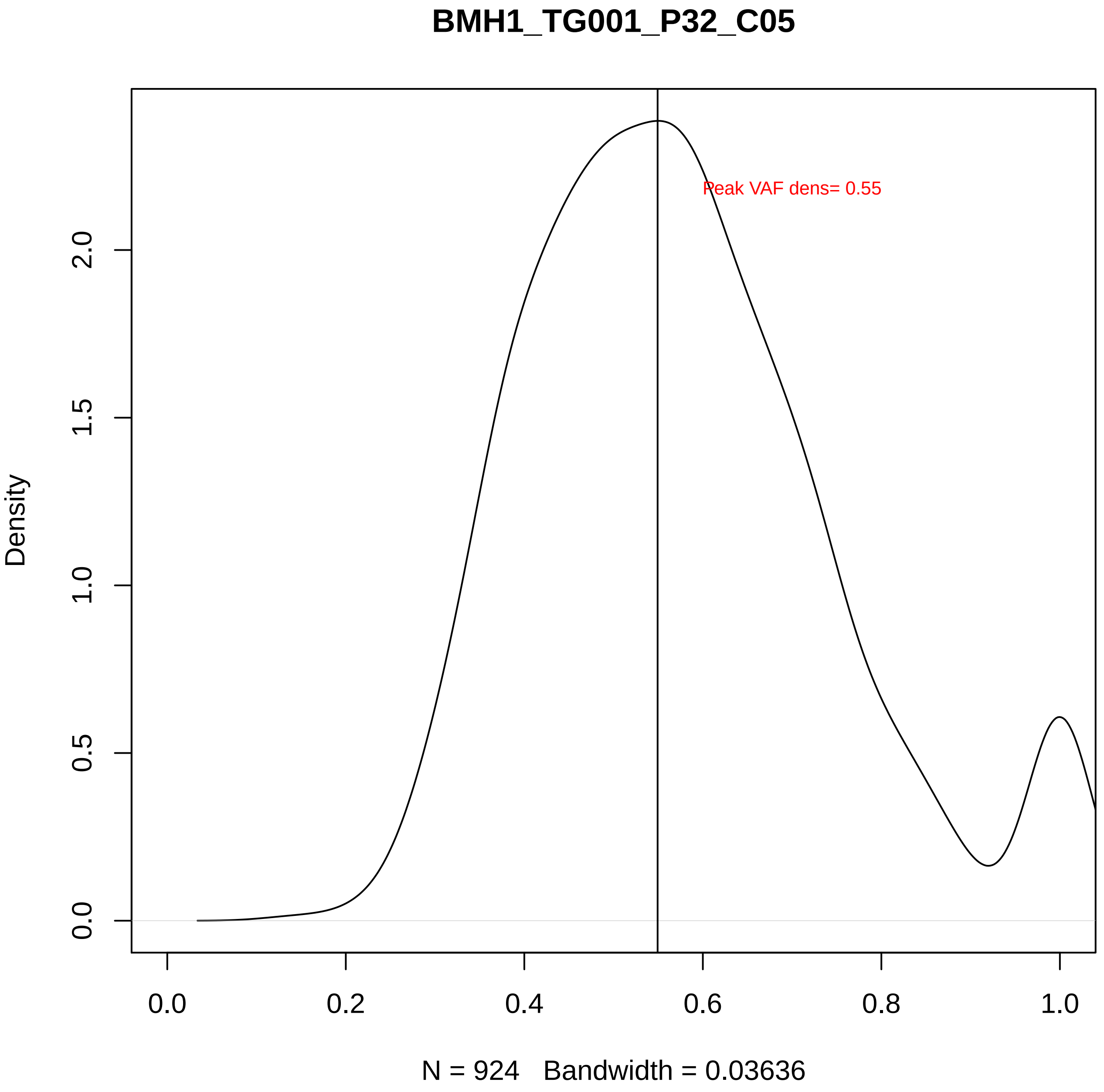

BMH1_TG001_P32_C05
Peak VAF dens= 0.55
Density
0.0
0.5
1.0
1.5
2.0
0.0
0.2
0.4
0.6
0.8
1.0
N = 924   Bandwidth = 0.03636

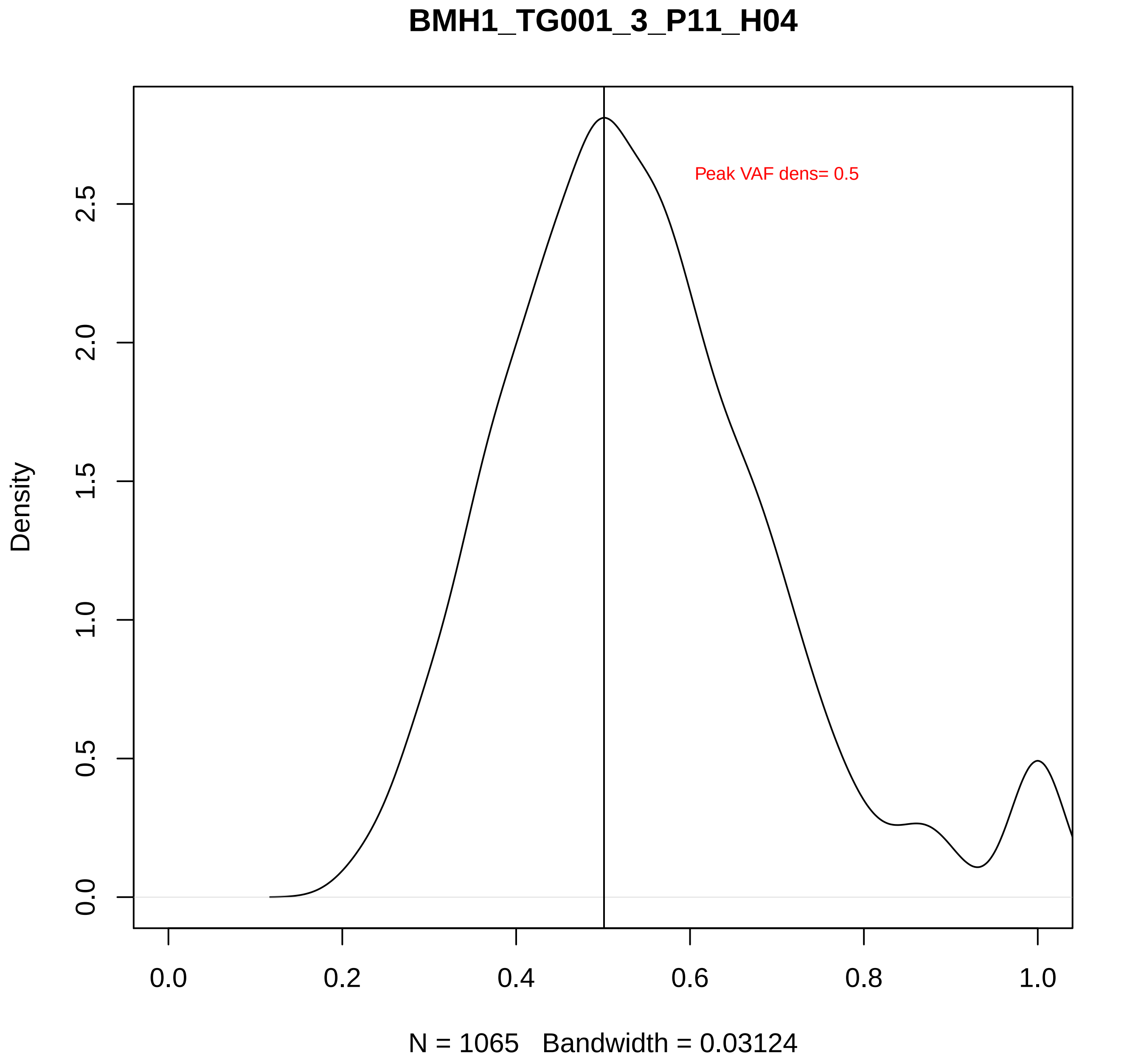
BMH1_TG001_3_P11_H04
Peak VAF dens= 0.5
Density
0.0
0.5
1.0
1.5
2.0
2.5
0.0
0.2
0.4
0.6
0.8
1.0
N = 1065   Bandwidth = 0.03124

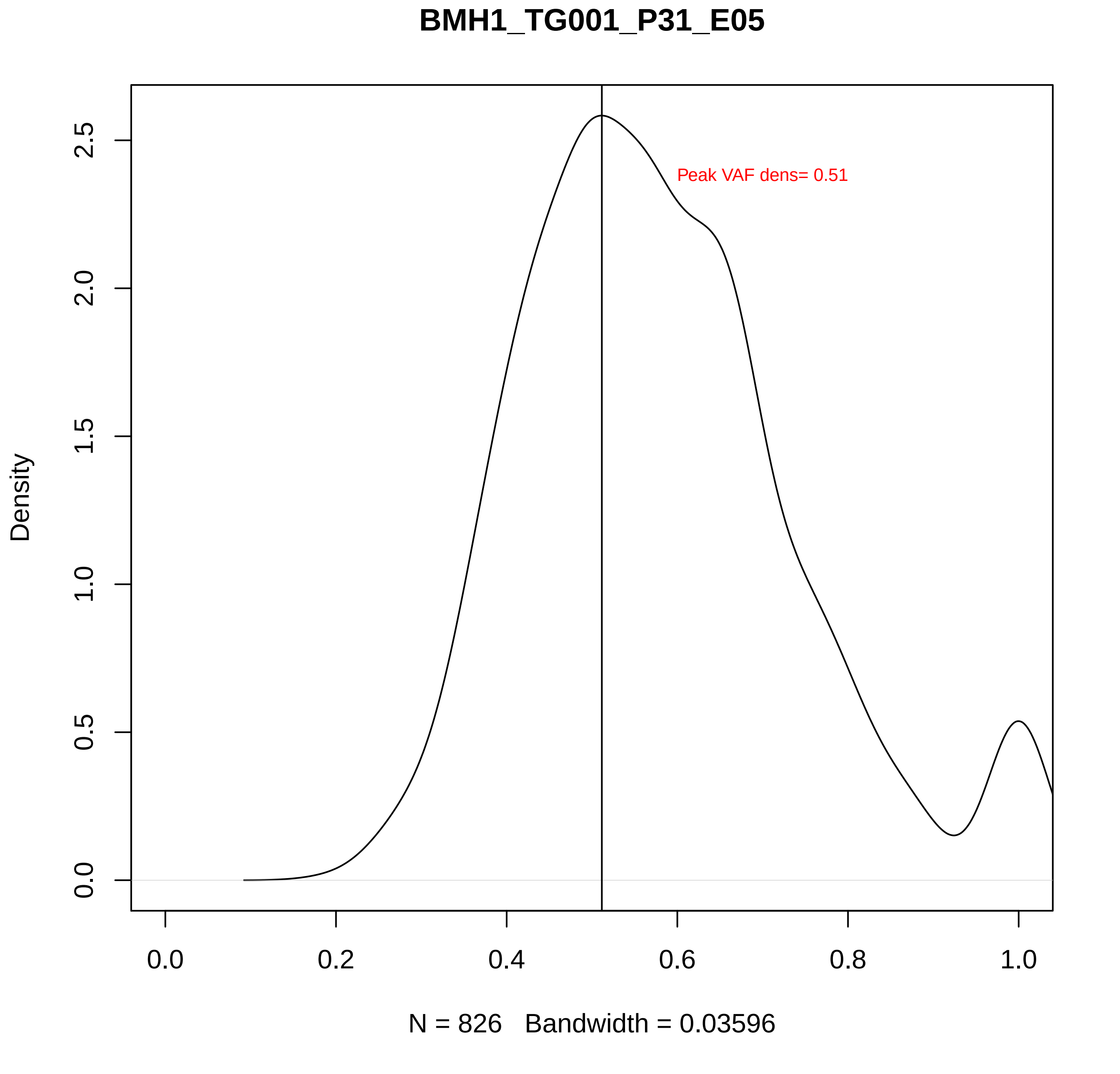

BMH1_TG001_P31_E05
Peak VAF dens= 0.51
Density
0.0
0.5
1.0
1.5
2.0
2.5
0.0
0.2
0.4
0.6
0.8
1.0
N = 826 Bandwidth = 0.03596

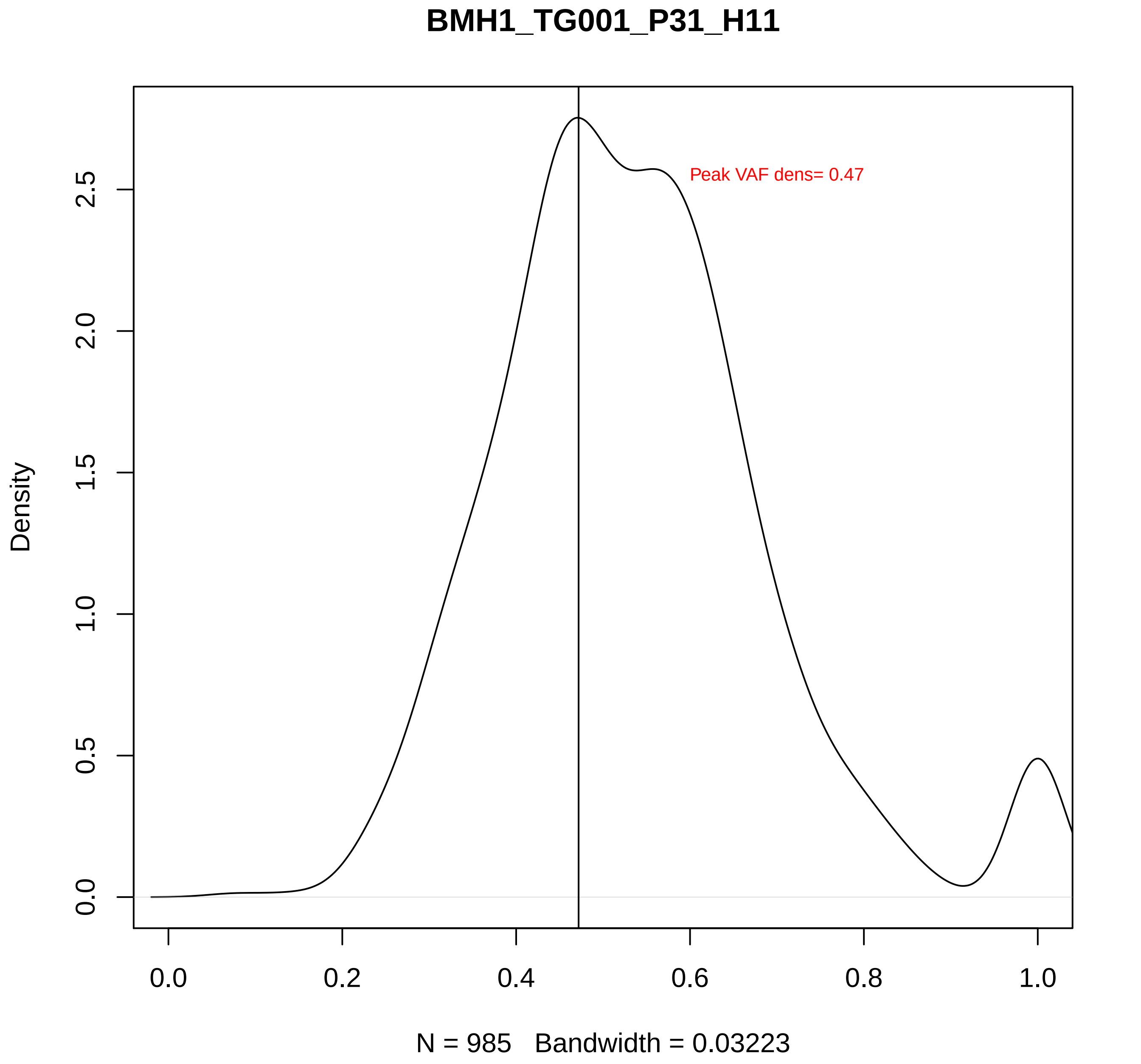

BMH1_TG001_P31_H11
Peak VAF dens= 0.47
Density
0.0
0.5
1.0
1.5
2.0
2.5
0.0
0.2
0.4
0.6
0.8
1.0
N = 985   Bandwidth = 0.03223

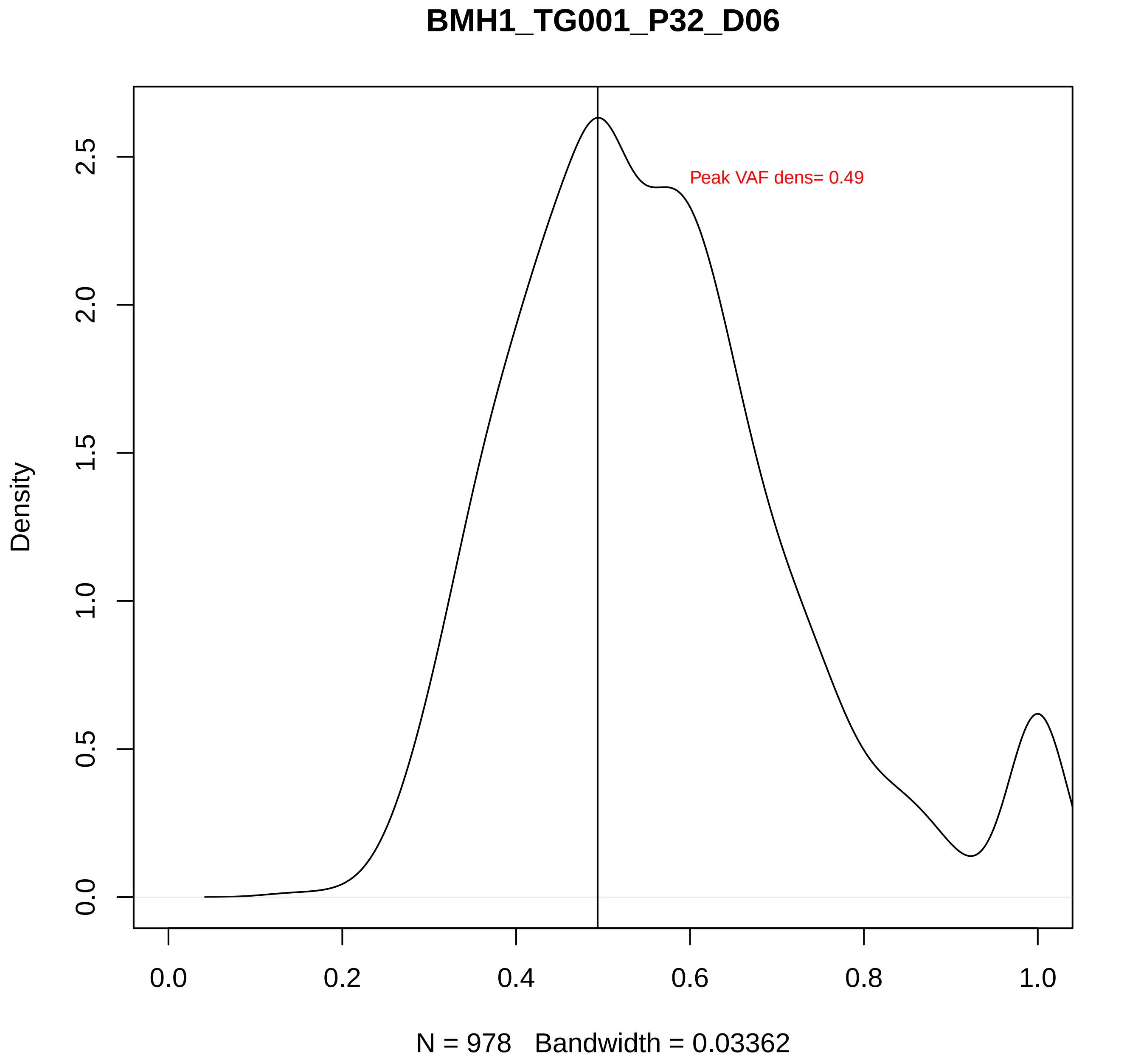
BMH1_TG001_P32_D06
Peak VAF dens= 0.49
Density
0.0
0.5
1.0
1.5
2.0
2.5
0.0
0.2
0.4
0.6
0.8
1.0
N = 978   Bandwidth = 0.03362

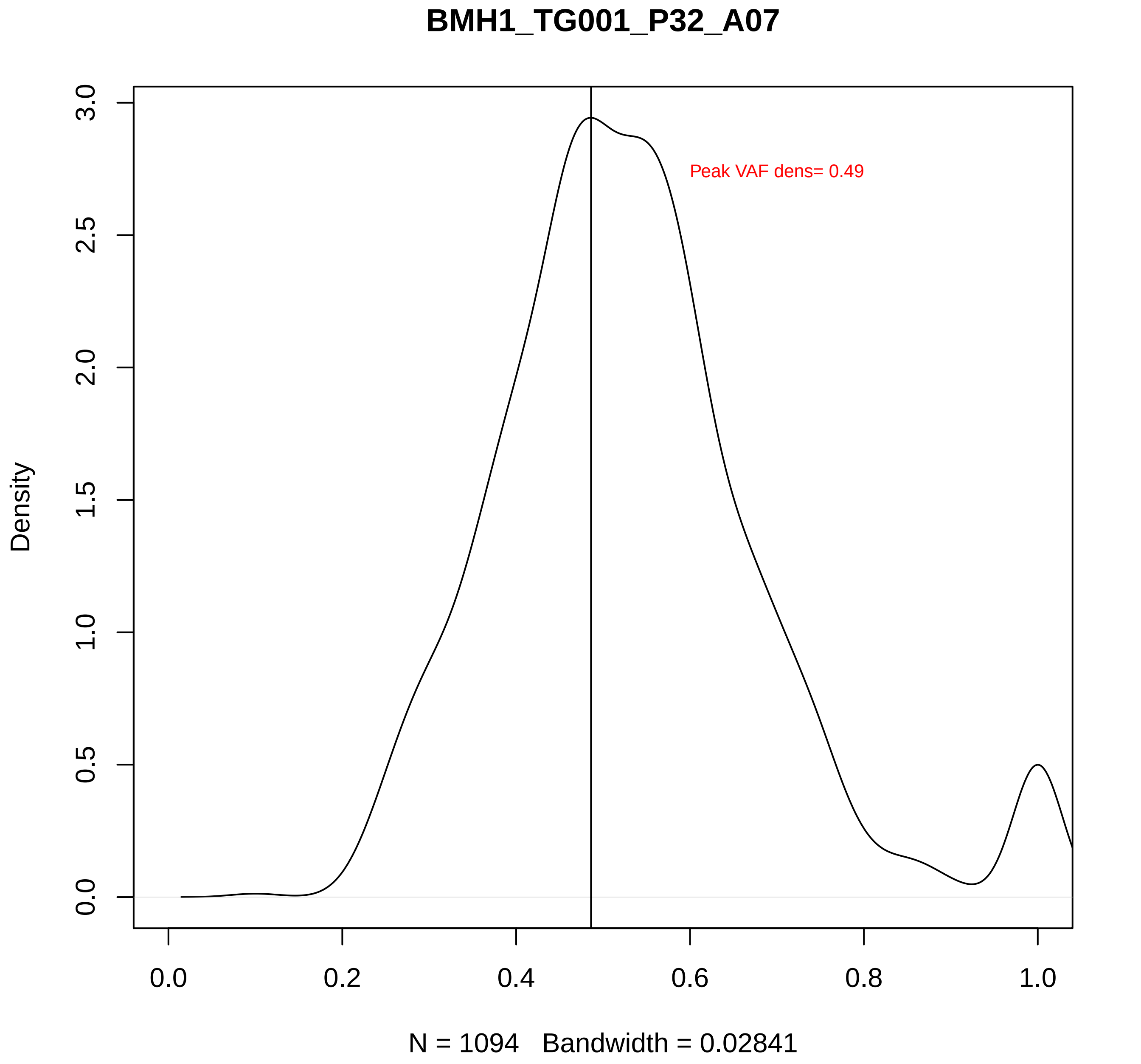

BMH1_TG001_P32_A07
Peak VAF dens= 0.49
Density
0.0
0.5
1.0
1.5
2.0
2.5
3.0
0.0
0.2
0.4
0.6
0.8
1.0
N = 1094 Bandwidth = 0.02841

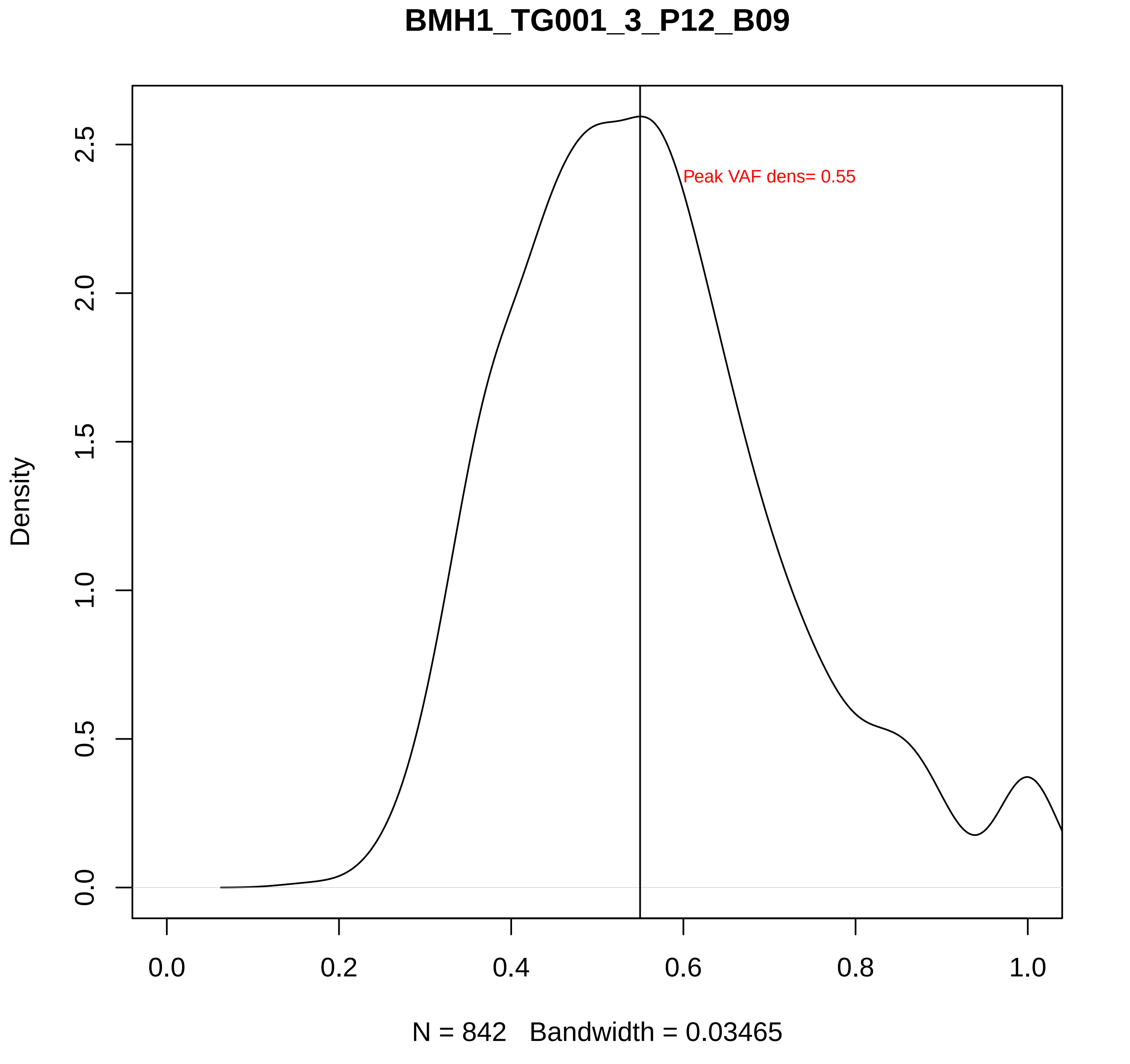
BMH1_TG001_3_P12_B09
Peak VAF dens= 0.55
Density
0.0
0.5
1.0
1.5
2.0
2.5
0.0
0.2
0.4
0.6
0.8
1.0
N = 842 Bandwidth = 0.03465

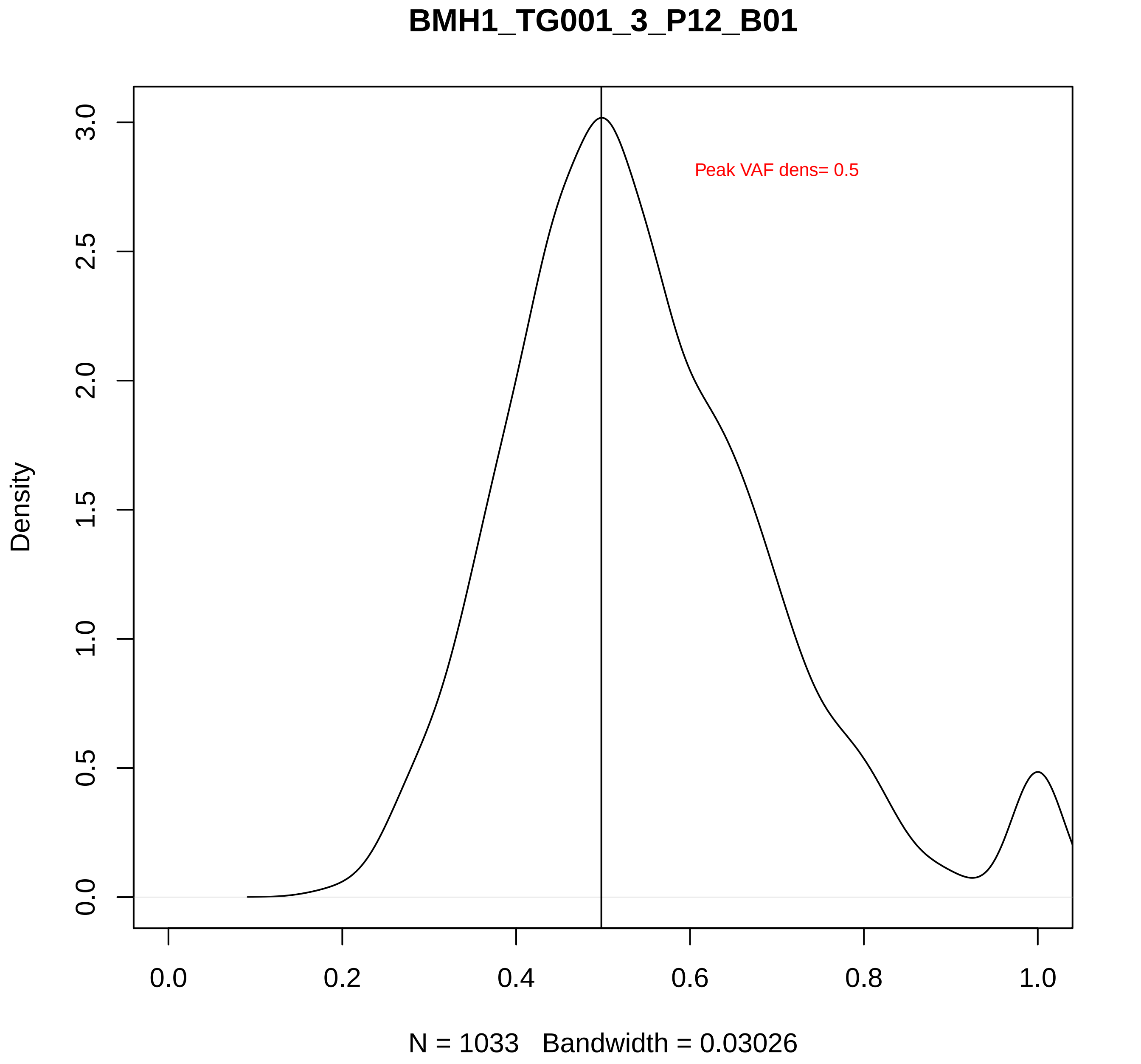
BMH1_TG001_3_P12_B01
Peak VAF dens= 0.5
Density
0.0
0.5
1.0
1.5
2.0
2.5
3.0
0.0
0.2
0.4
0.6
0.8
1.0
N = 1033 Bandwidth = 0.03026

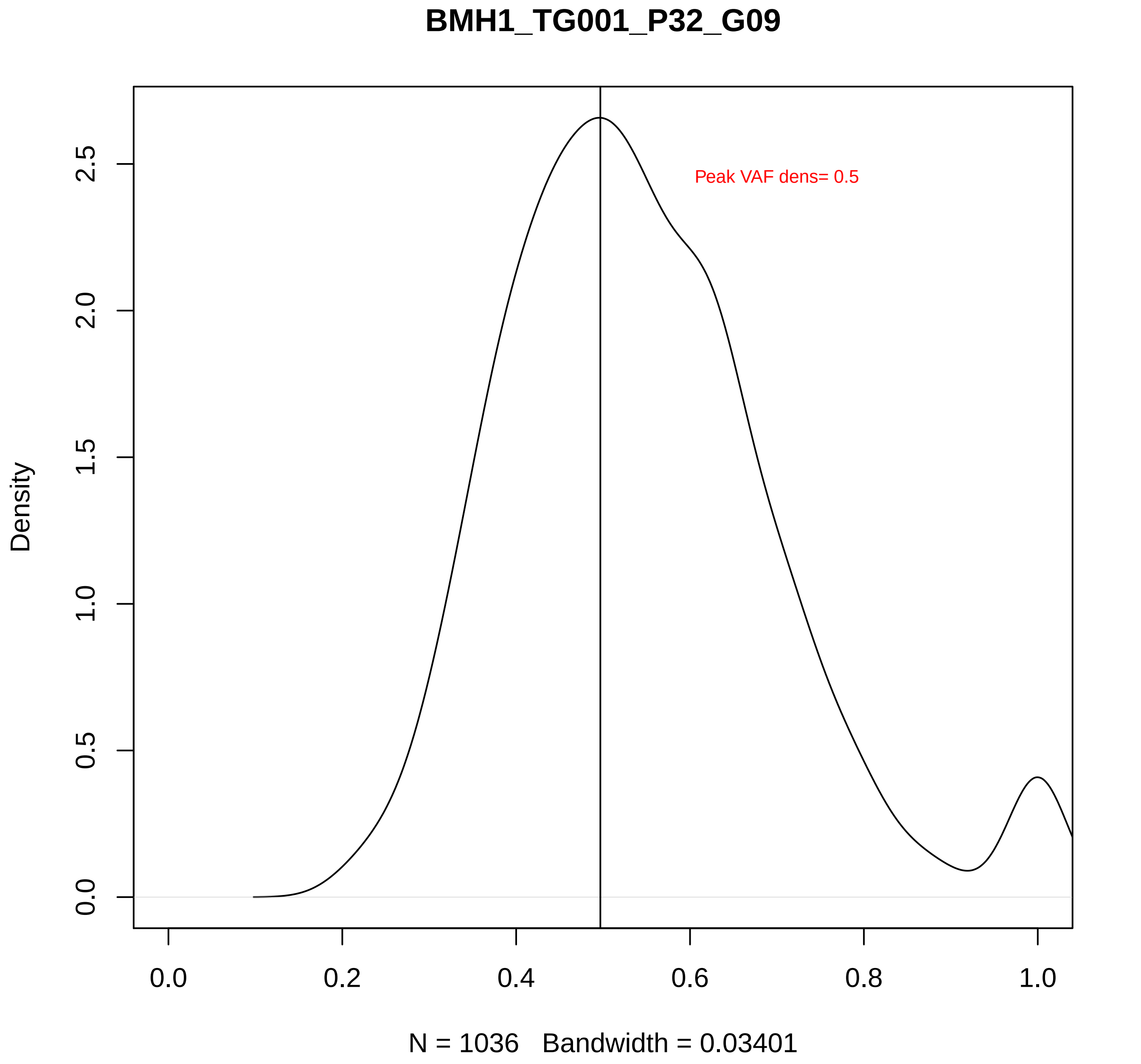
BMH1_TG001_P32_G09
Peak VAF dens= 0.5
Density
0.0
0.5
1.0
1.5
2.0
2.5
0.0
0.2
0.4
0.6
0.8
1.0
N = 1036   Bandwidth = 0.03401

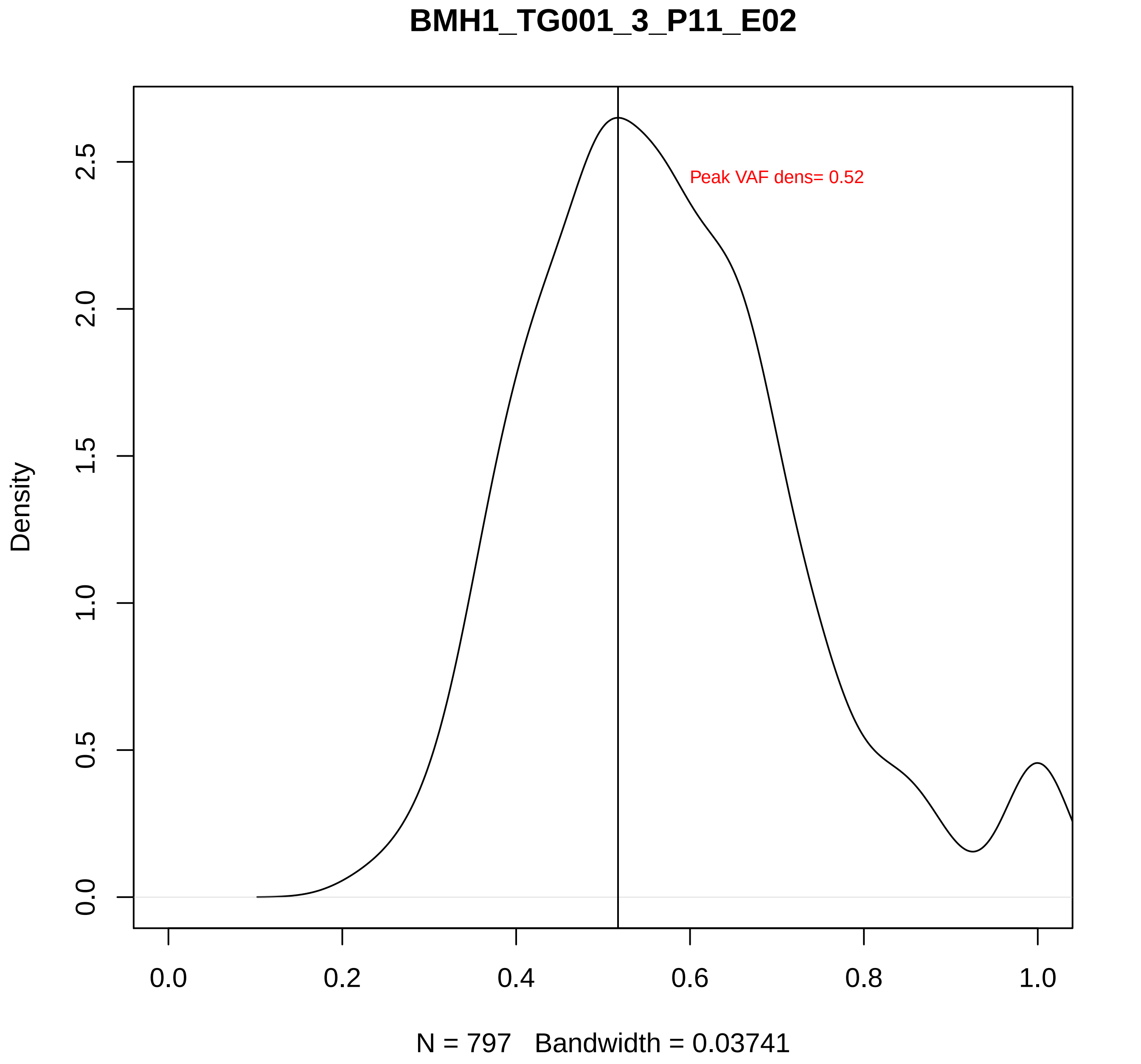

BMH1_TG001_3_P11_E02
Peak VAF dens= 0.52
Density
0.0
0.5
1.0
1.5
2.0
2.5
0.0
0.2
0.4
0.6
0.8
1.0
N = 797   Bandwidth = 0.03741

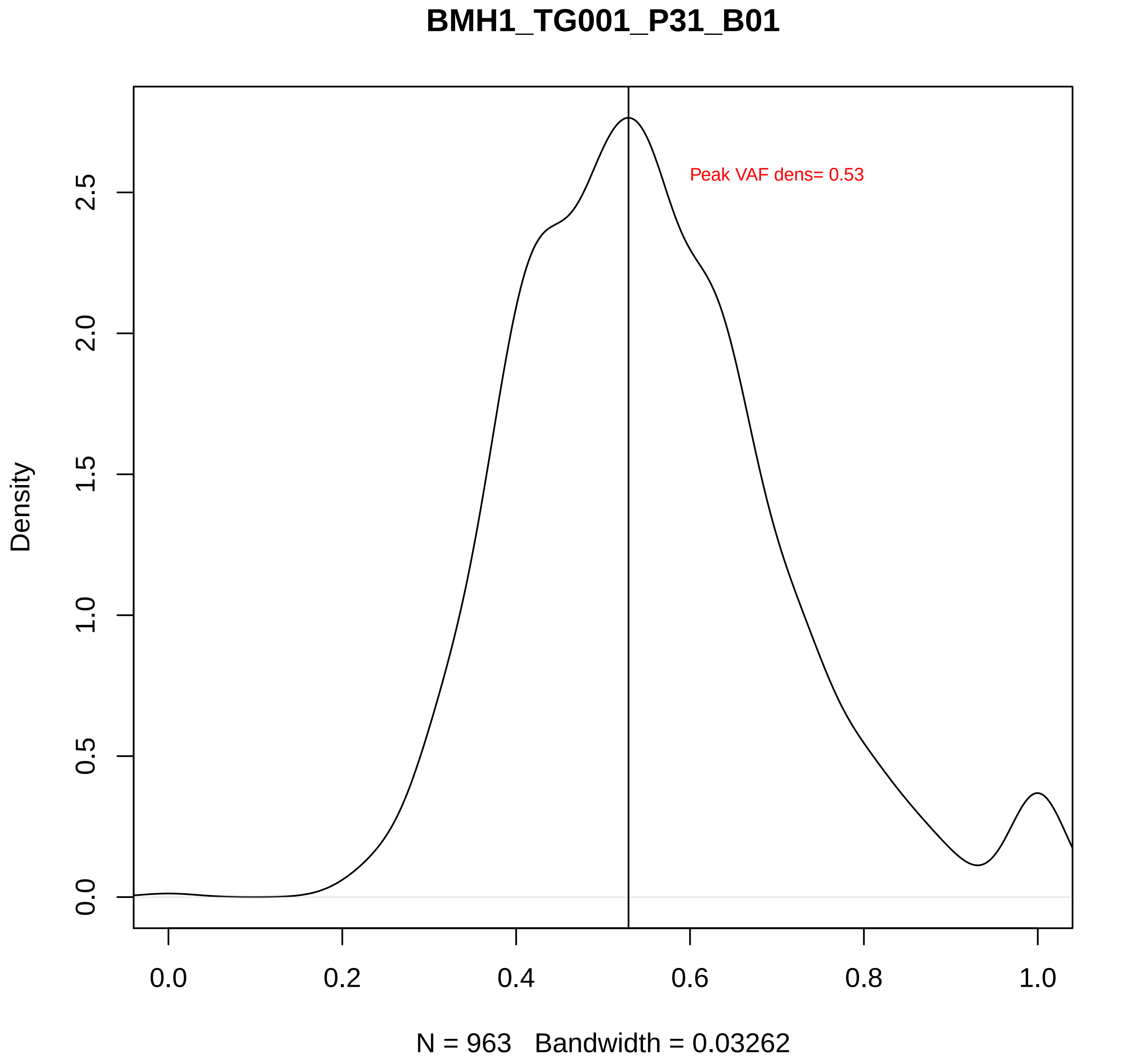

BMH1_TG001_P31_B01
Peak VAF dens= 0.53
Density
0.0
0.5
1.0
1.5
2.0
2.5
0.0
0.2
0.4
0.6
0.8
1.0
N = 963 Bandwidth = 0.03262

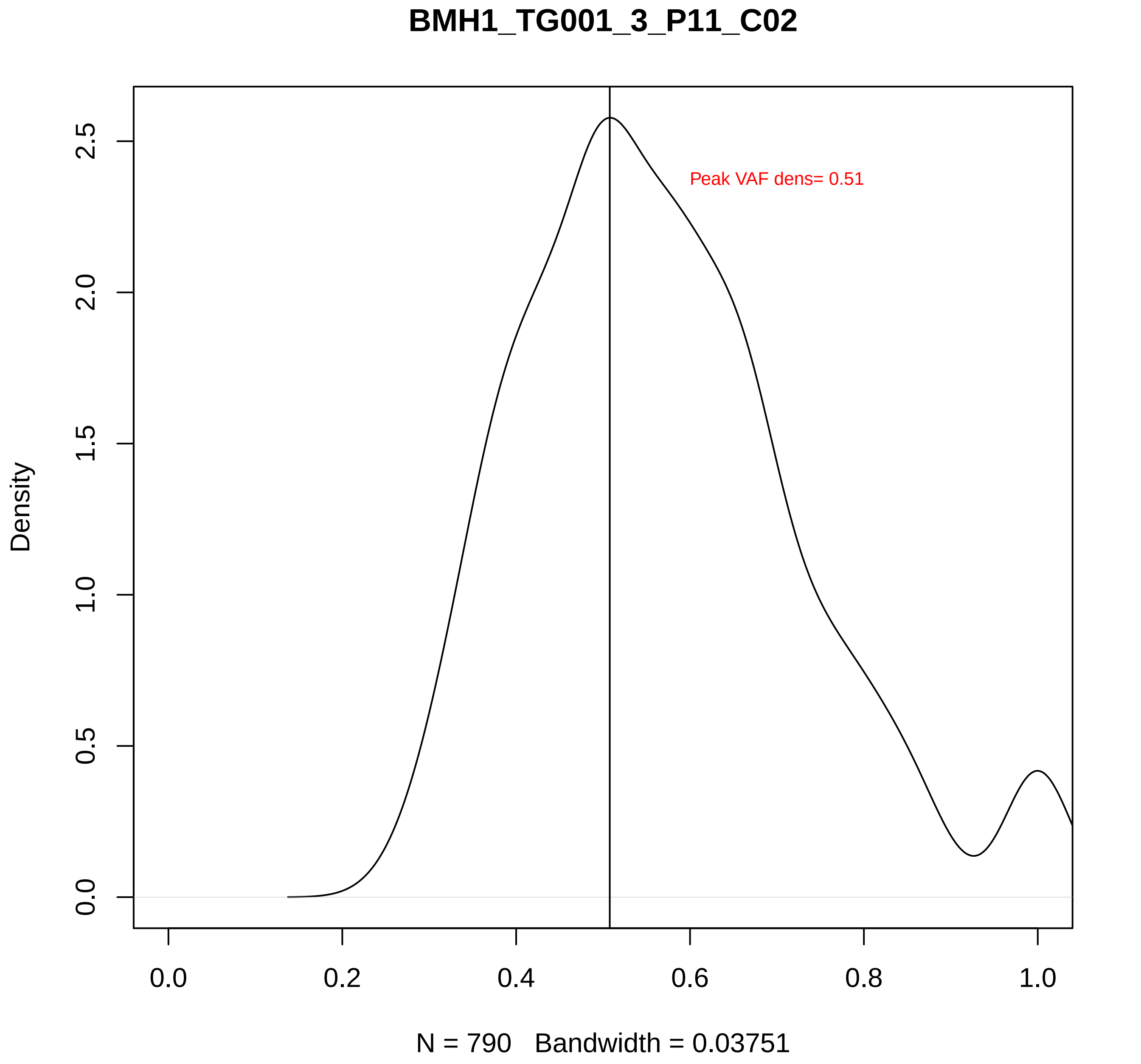
BMH1_TG001_3_P11_C02
Peak VAF dens= 0.51
Density
0.0
0.5
1.0
1.5
2.0
2.5
0.0
0.2
0.4
0.6
0.8
1.0
N = 790   Bandwidth = 0.03751

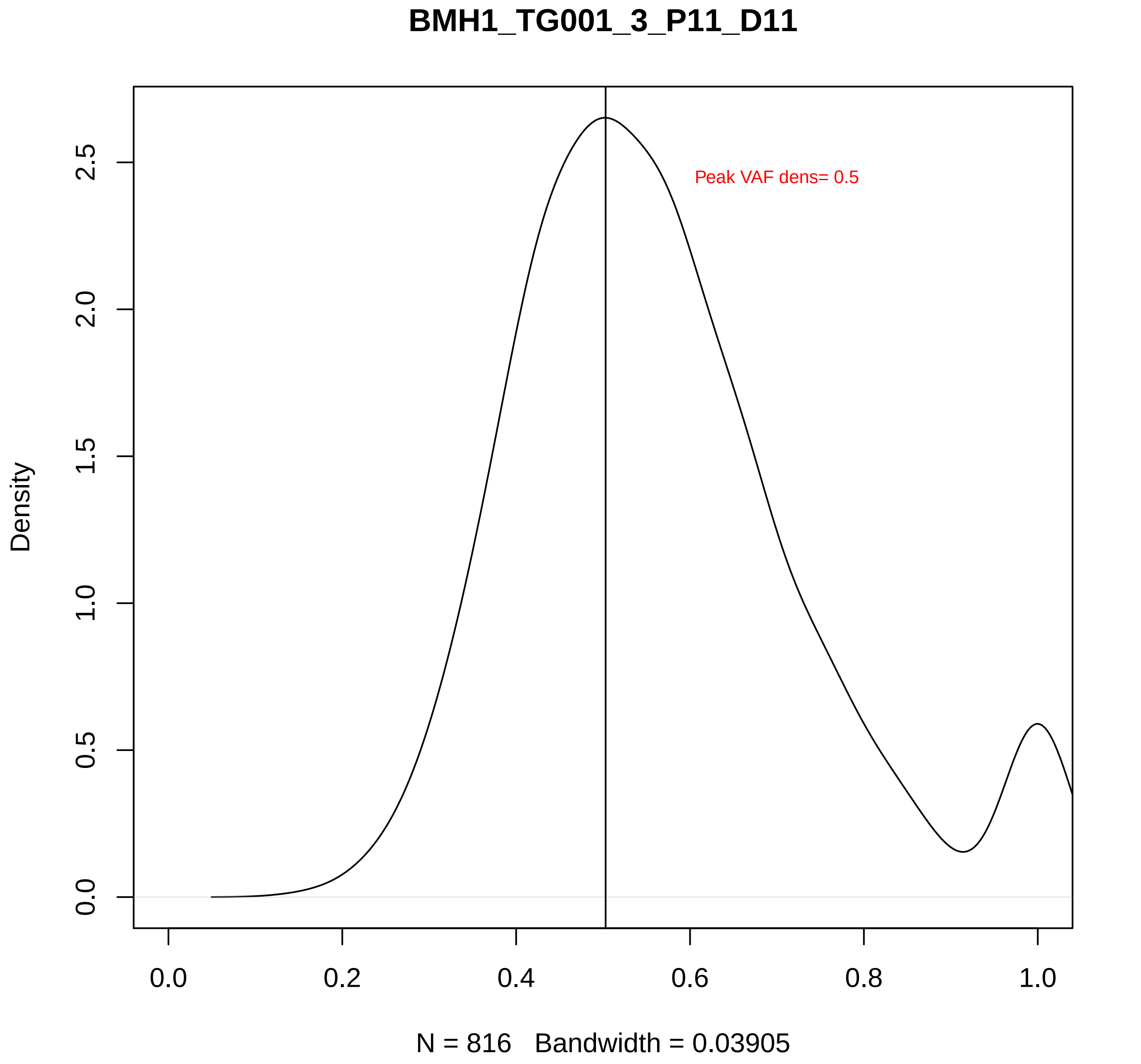
BMH1_TG001_3_P11_D11
Peak VAF dens= 0.5
Density
0.0
0.5
1.0
1.5
2.0
2.5
0.0
0.2
0.4
0.6
0.8
1.0
N = 816 Bandwidth = 0.03905

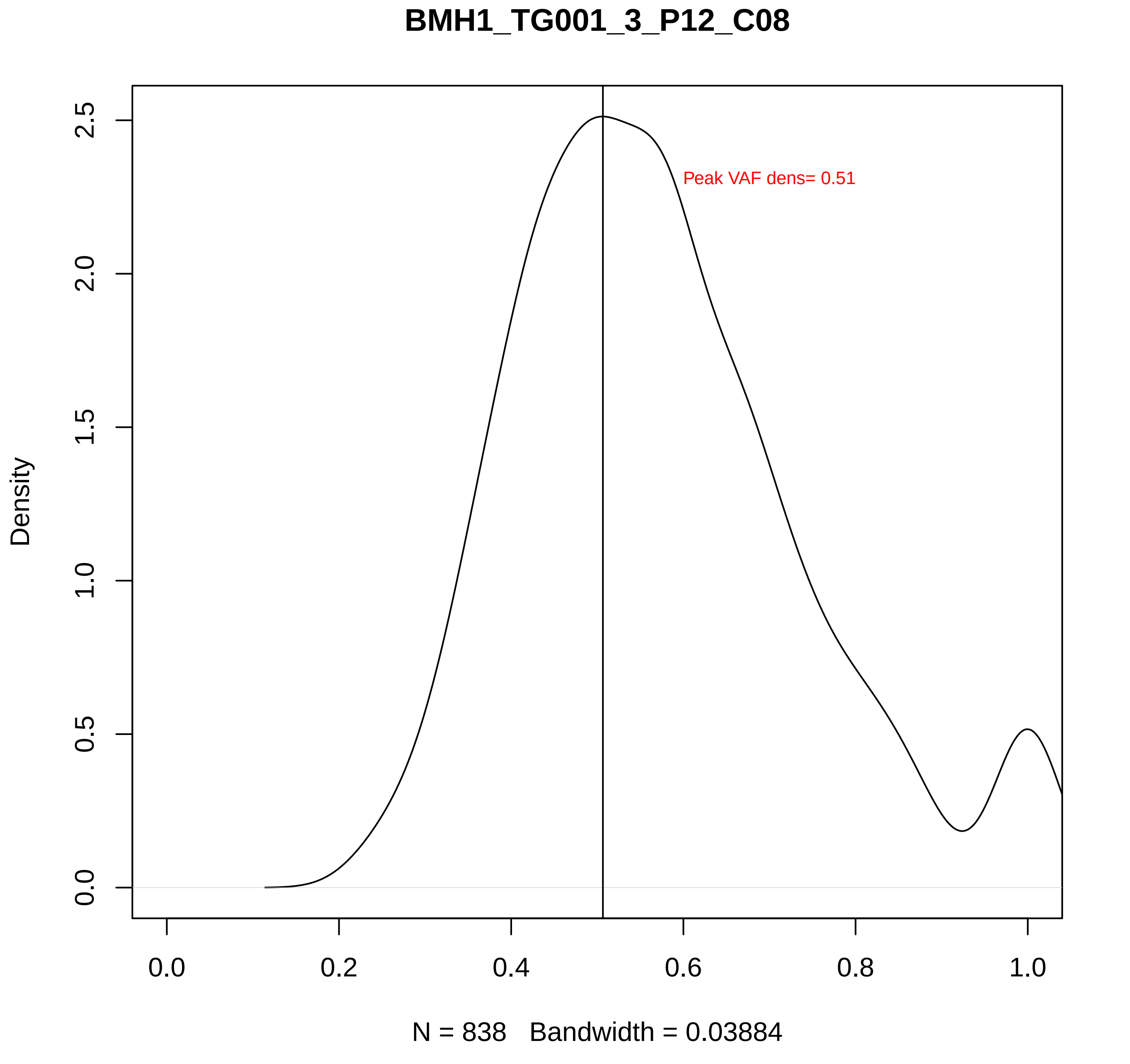

BMH1_TG001_3_P12_C08
Peak VAF dens= 0.51
Density
0.0
0.5
1.0
1.5
2.0
2.5
0.0
0.2
0.4
0.6
0.8
1.0
N = 838   Bandwidth = 0.03884

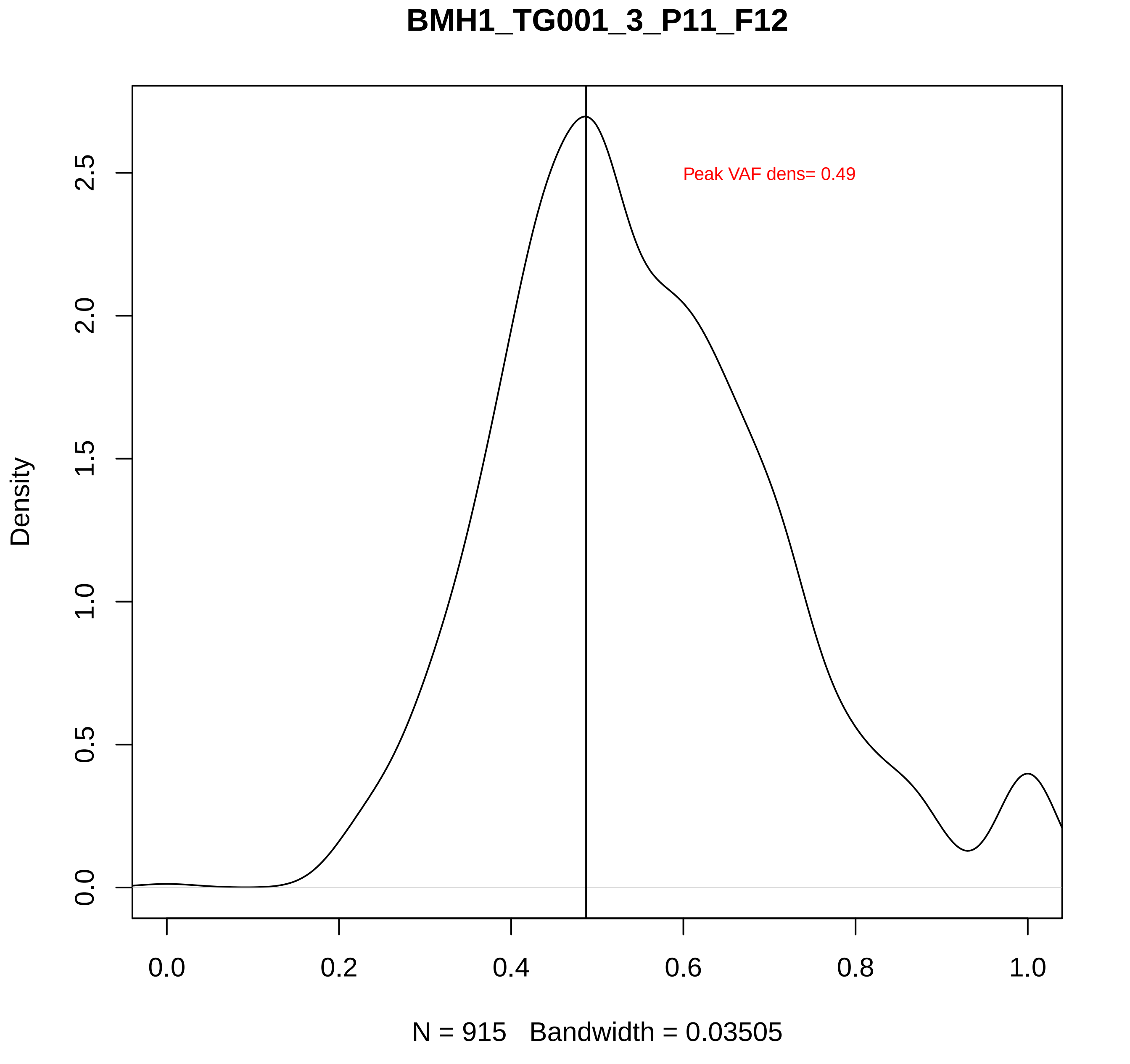

BMH1_TG001_3_P11_F12
Peak VAF dens= 0.49
Density
0.0
0.5
1.0
1.5
2.0
2.5
0.0
0.2
0.4
0.6
0.8
1.0
N = 915 Bandwidth = 0.03505

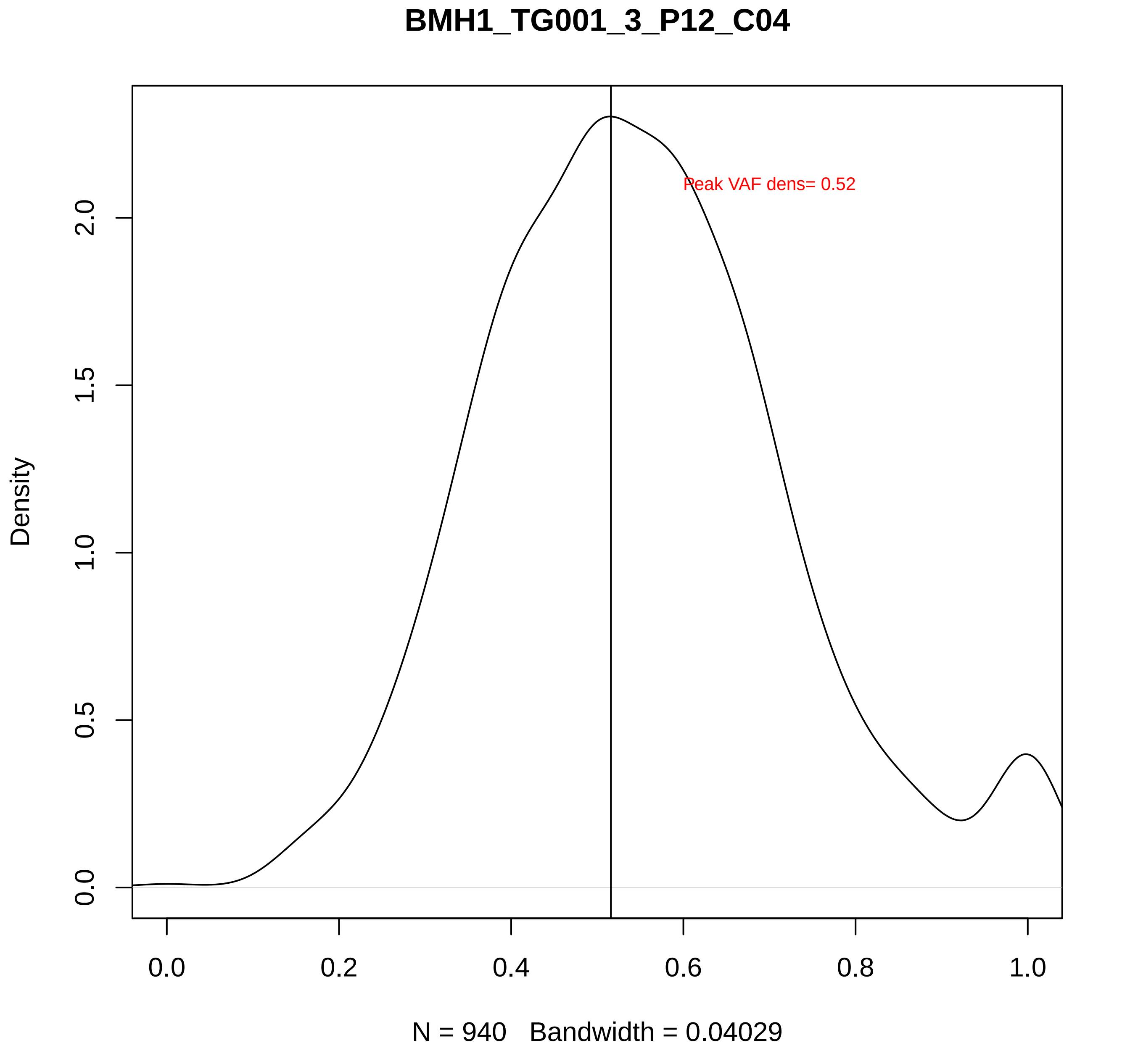
BMH1_TG001_3_P12_C04
Peak VAF dens= 0.52
Density
0.0
0.5
1.0
1.5
2.0
0.0
0.2
0.4
0.6
0.8
1.0
N = 940 Bandwidth = 0.04029

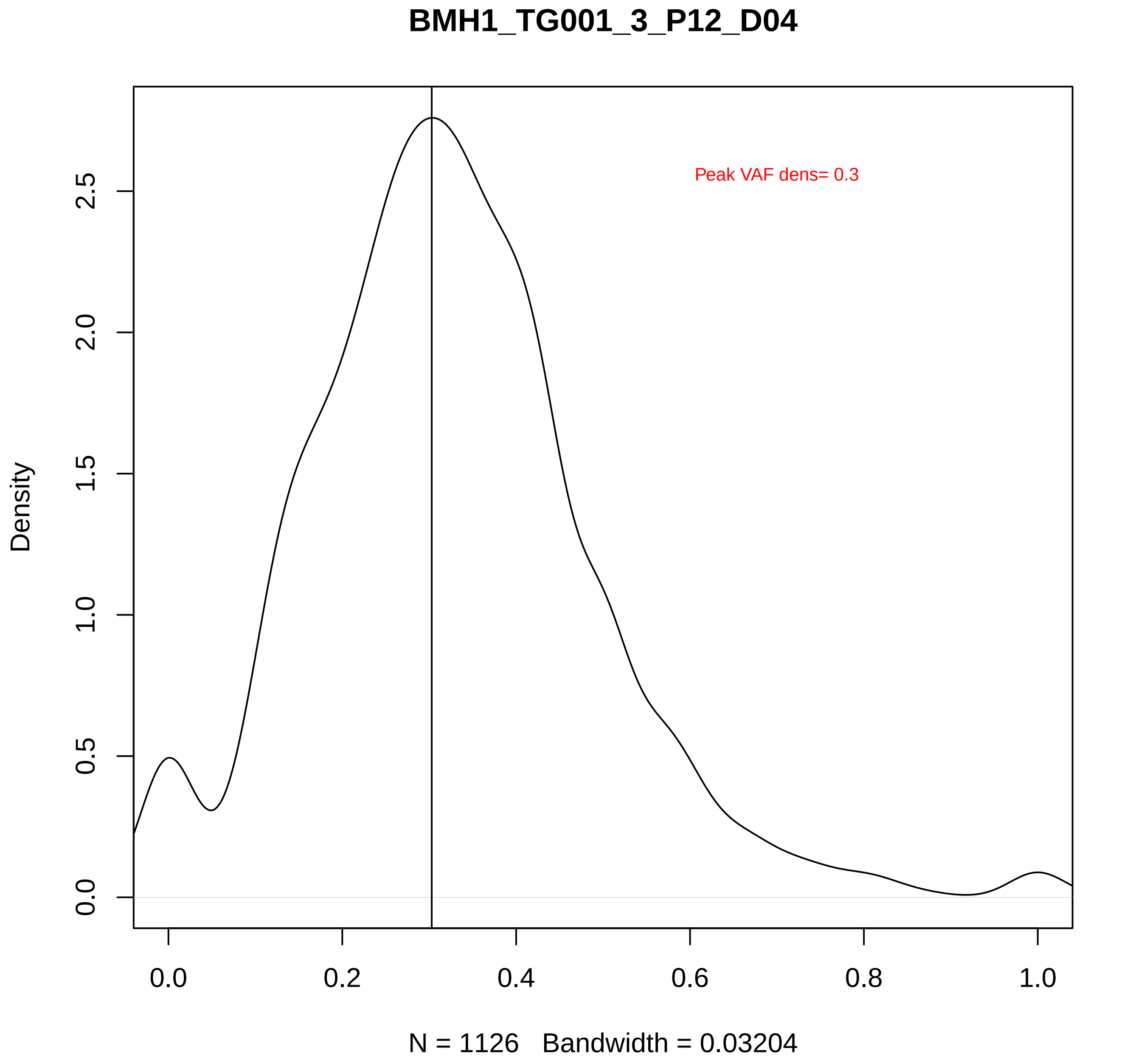

BMH1_TG001_3_P12_D04
Peak VAF dens= 0.3
Density
0.0
0.5
1.0
1.5
2.0
2.5
0.0
0.2
0.4
0.6
0.8
1.0
N = 1126   Bandwidth = 0.03204

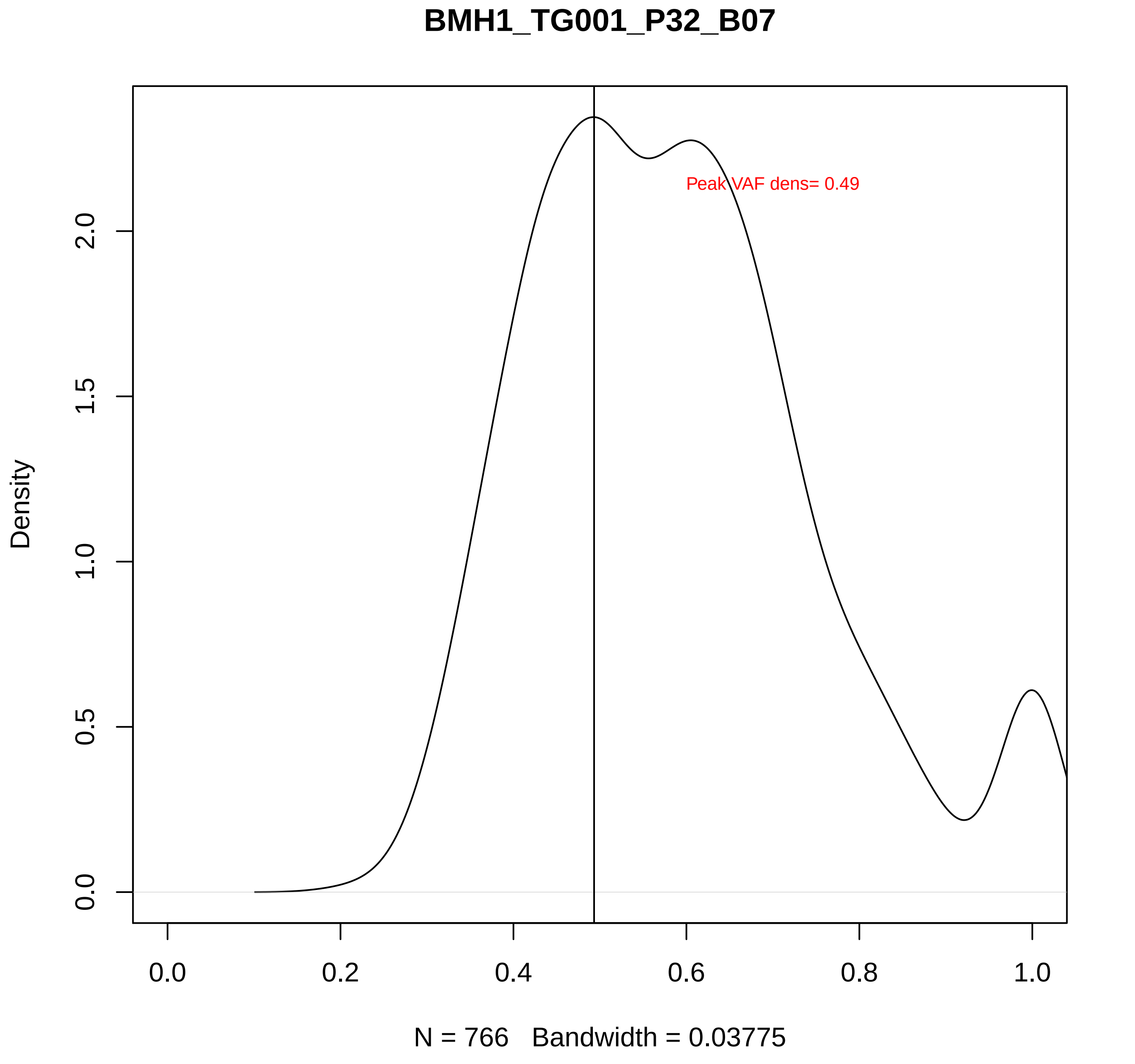
BMH1_TG001_P32_B07
Peak VAF dens= 0.49
Density
0.0
0.5
1.0
1.5
2.0
0.0
0.2
0.4
0.6
0.8
1.0
N = 766   Bandwidth = 0.03775

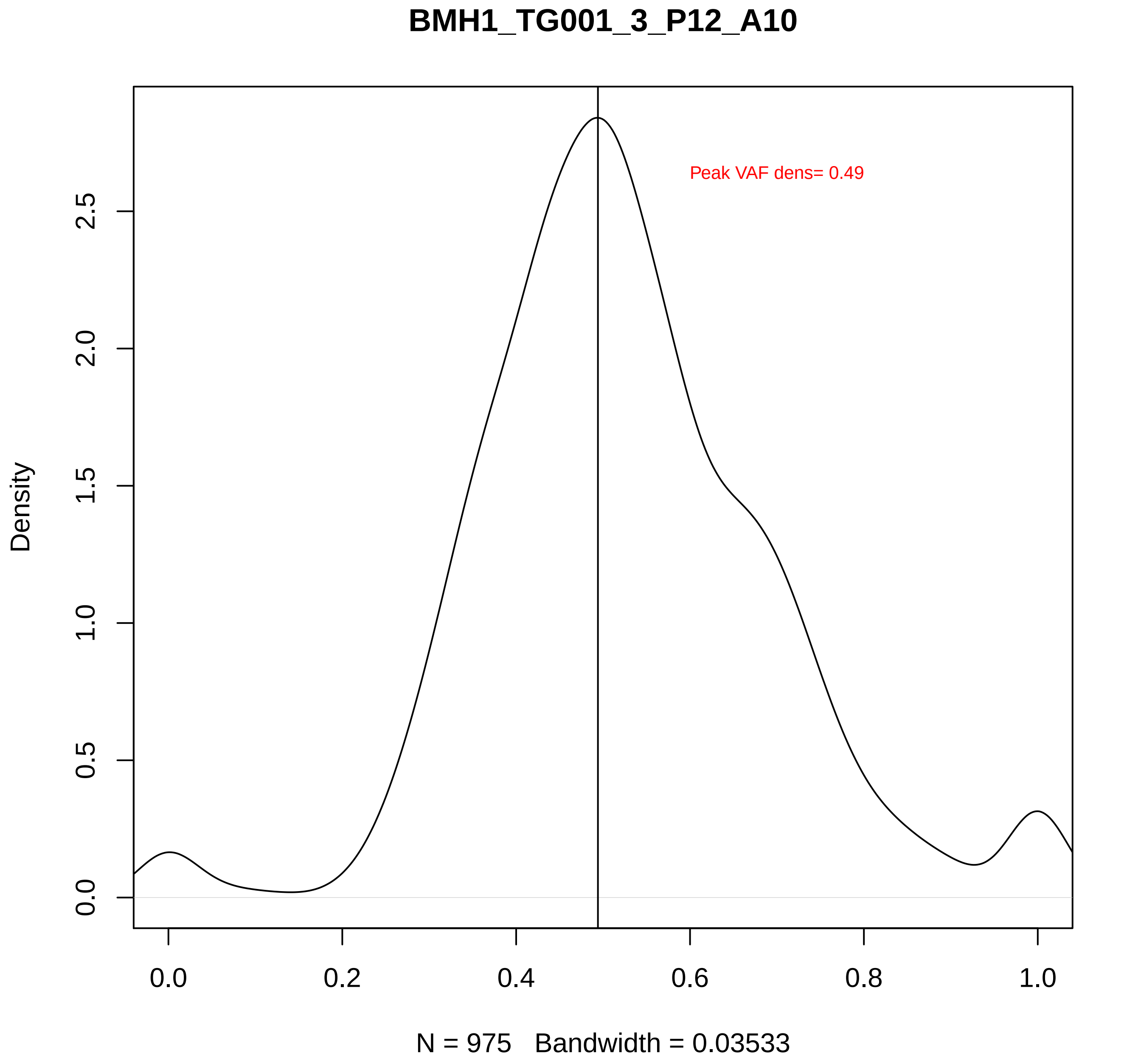

BMH1_TG001_3_P12_A10
Peak VAF dens= 0.49
Density
0.0
0.5
1.0
1.5
2.0
2.5
0.0
0.2
0.4
0.6
0.8
1.0
N = 975   Bandwidth = 0.03533

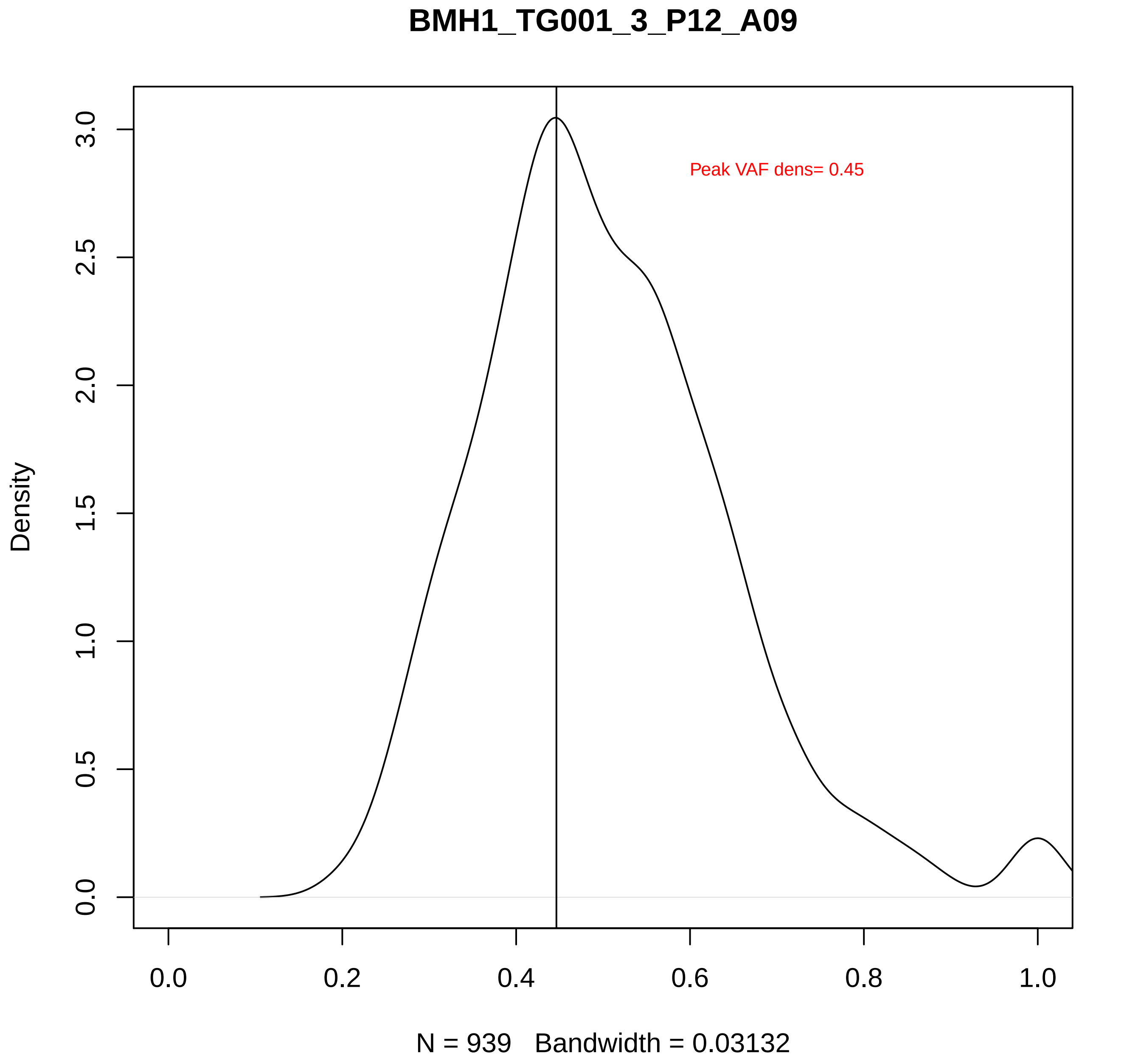

BMH1_TG001_3_P12_A09
Peak VAF dens= 0.45
Density
0.0
0.5
1.0
1.5
2.0
2.5
3.0
0.0
0.2
0.4
0.6
0.8
1.0
N = 939 Bandwidth = 0.03132

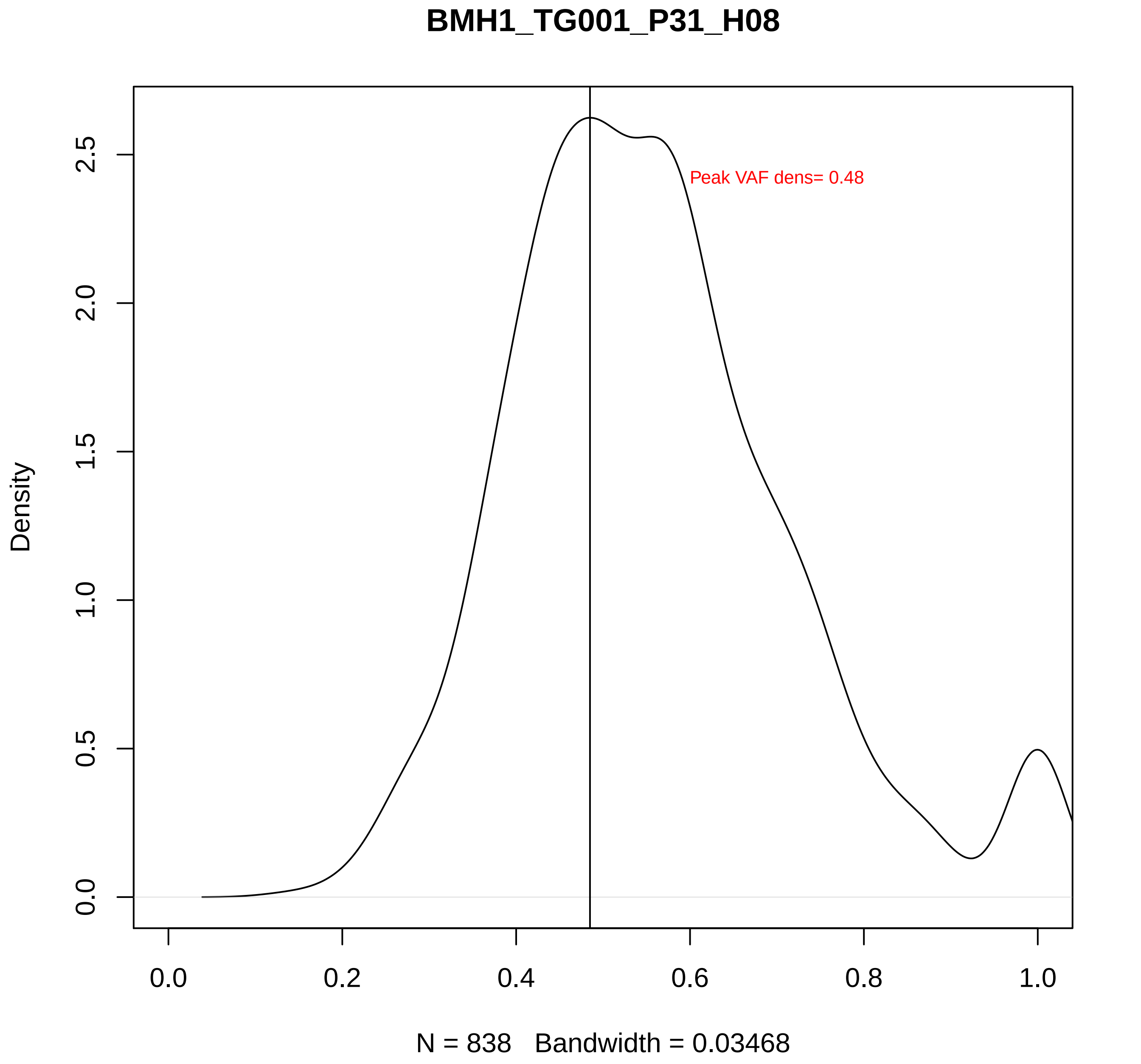

BMH1_TG001_P31_H08
Peak VAF dens= 0.48
Density
0.0
0.5
1.0
1.5
2.0
2.5
0.0
0.2
0.4
0.6
0.8
1.0
N = 838   Bandwidth = 0.03468

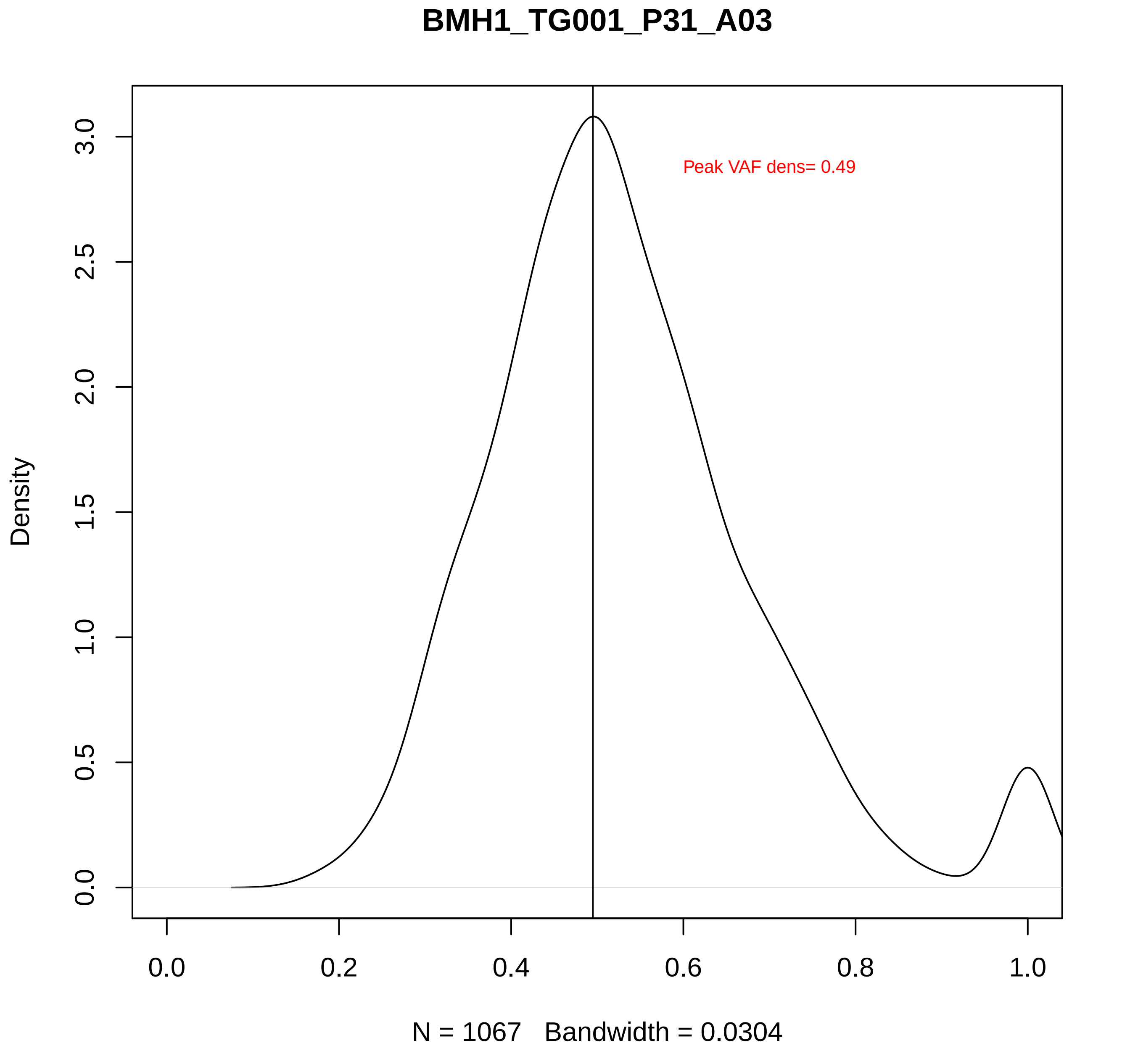
BMH1_TG001_P31_A03
Peak VAF dens= 0.49
Density
0.0
0.5
1.0
1.5
2.0
2.5
3.0
0.0
0.2
0.4
0.6
0.8
1.0
N = 1067   Bandwidth = 0.0304

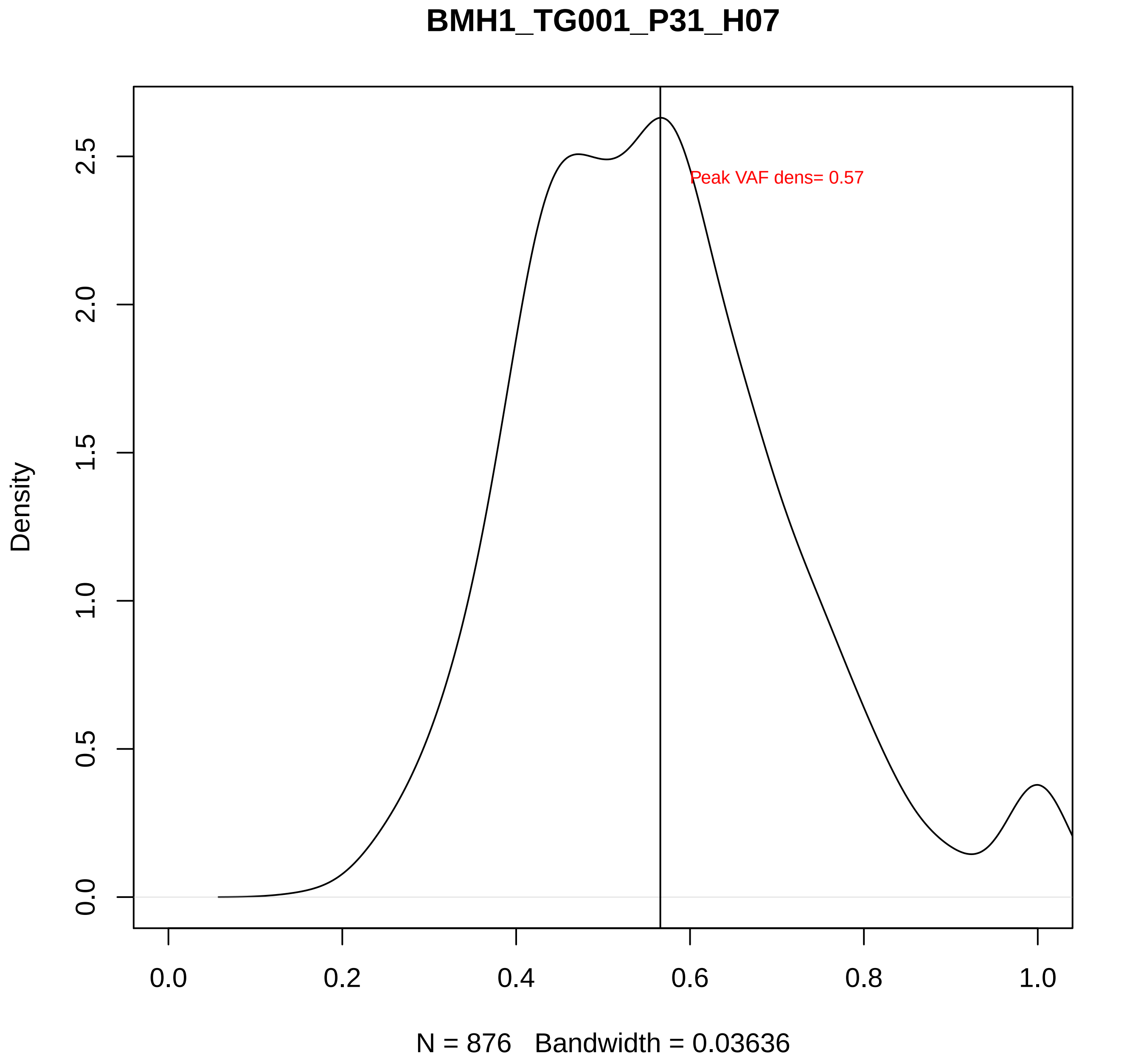
BMH1_TG001_P31_H07
Peak VAF dens= 0.57
Density
0.0
0.5
1.0
1.5
2.0
2.5
0.0
0.2
0.4
0.6
0.8
1.0
N = 876   Bandwidth = 0.03636

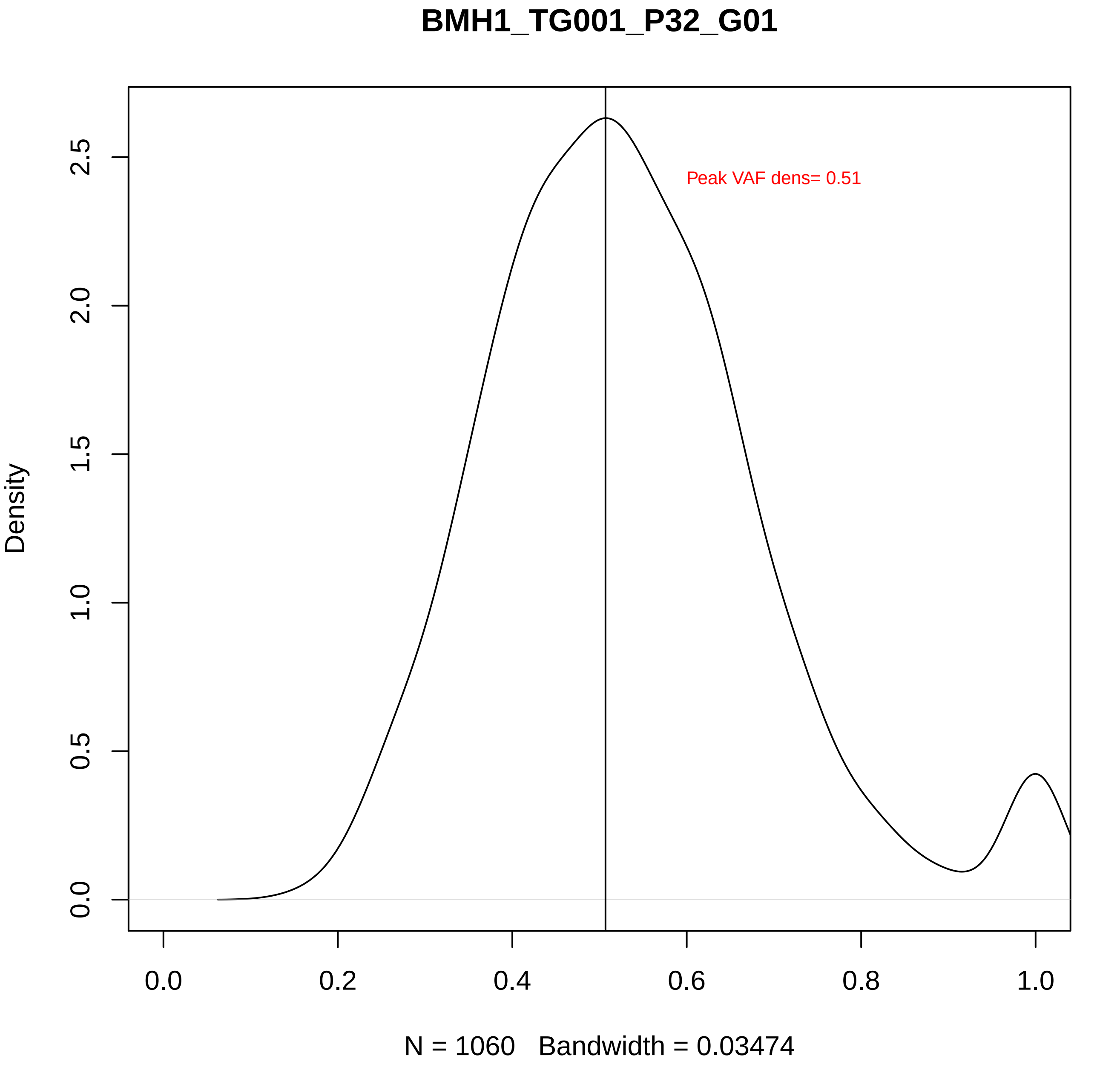

BMH1_TG001_P32_G01
Peak VAF dens= 0.51
Density
0.0
0.5
1.0
1.5
2.0
2.5
0.0
0.2
0.4
0.6
0.8
1.0
N = 1060   Bandwidth = 0.03474

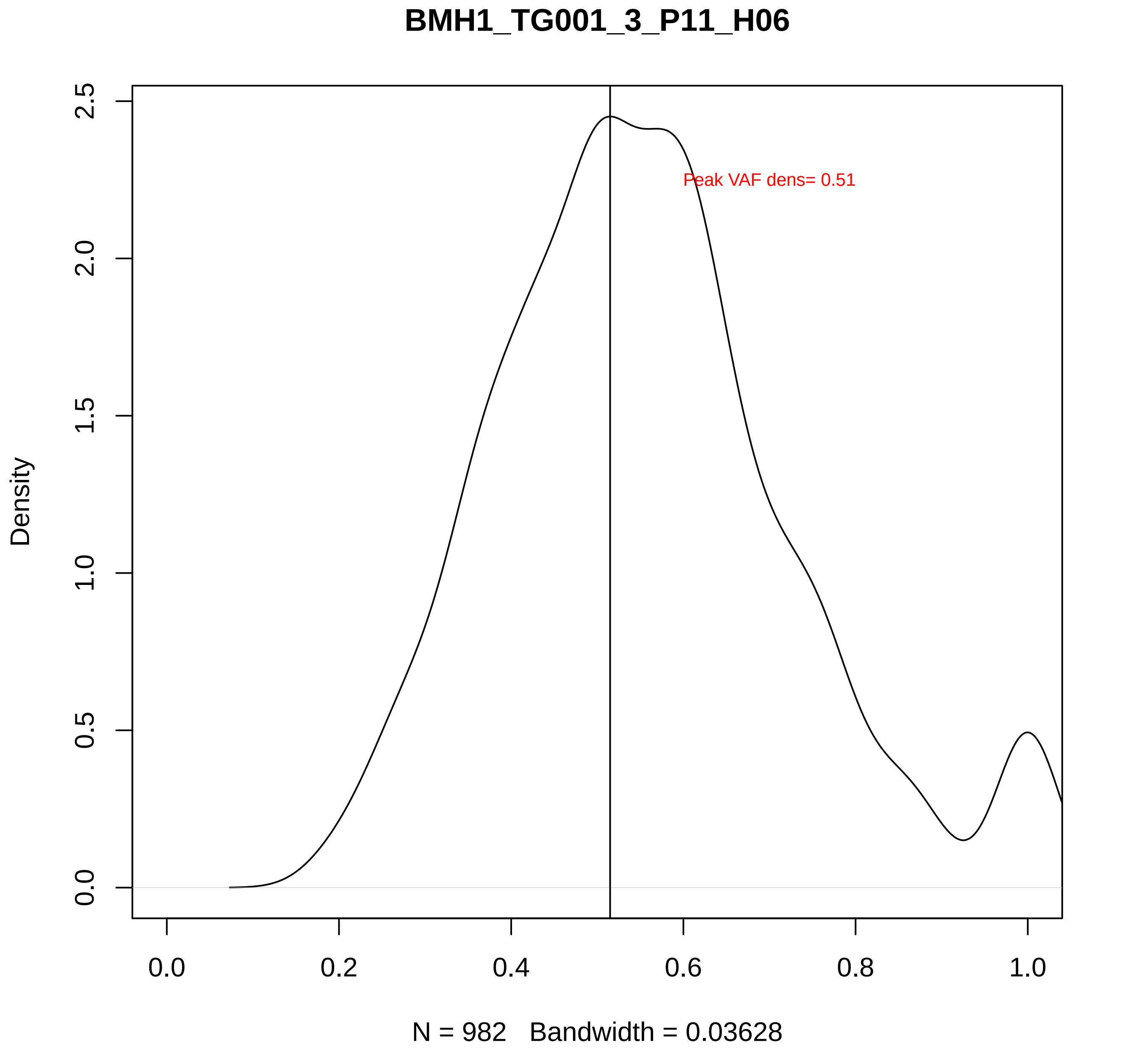

BMH1_TG001_3_P11_H06
Density
Peak VAF dens= 0.51
0.0
0.5
1.0
1.5
2.0
2.5
0.0
0.2
0.4
0.6
0.8
1.0
N = 982   Bandwidth = 0.03628

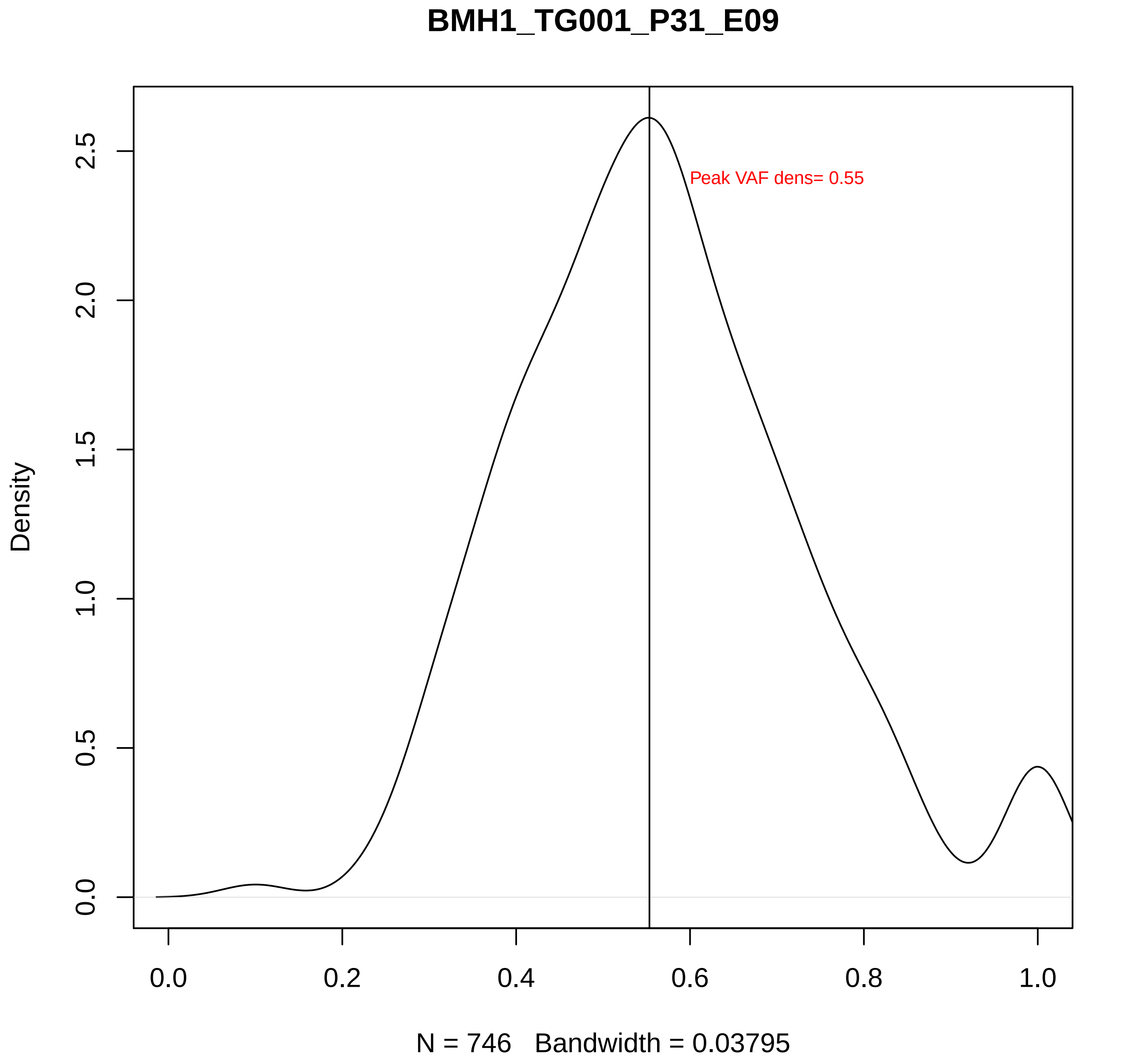

BMH1_TG001_P31_E09
Peak VAF dens= 0.55
Density
0.0
0.5
1.0
1.5
2.0
2.5
0.0
0.2
0.4
0.6
0.8
1.0
N = 746 Bandwidth = 0.03795

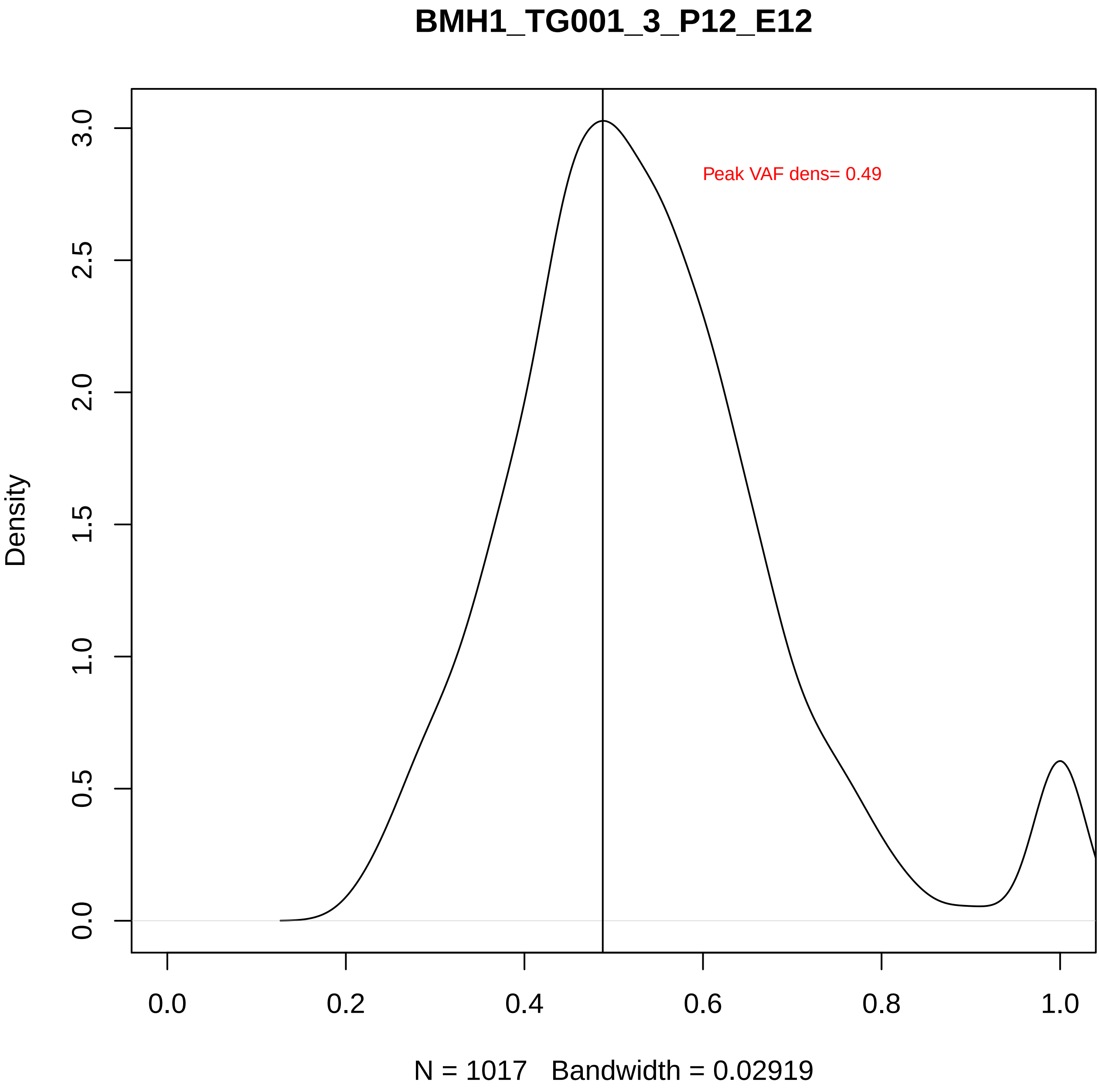

BMH1_TG001_3_P12_E12
Peak VAF dens= 0.49
Density
0.0
0.5
1.0
1.5
2.0
2.5
3.0
0.0
0.2
0.4
0.6
0.8
1.0
N = 1017   Bandwidth = 0.02919

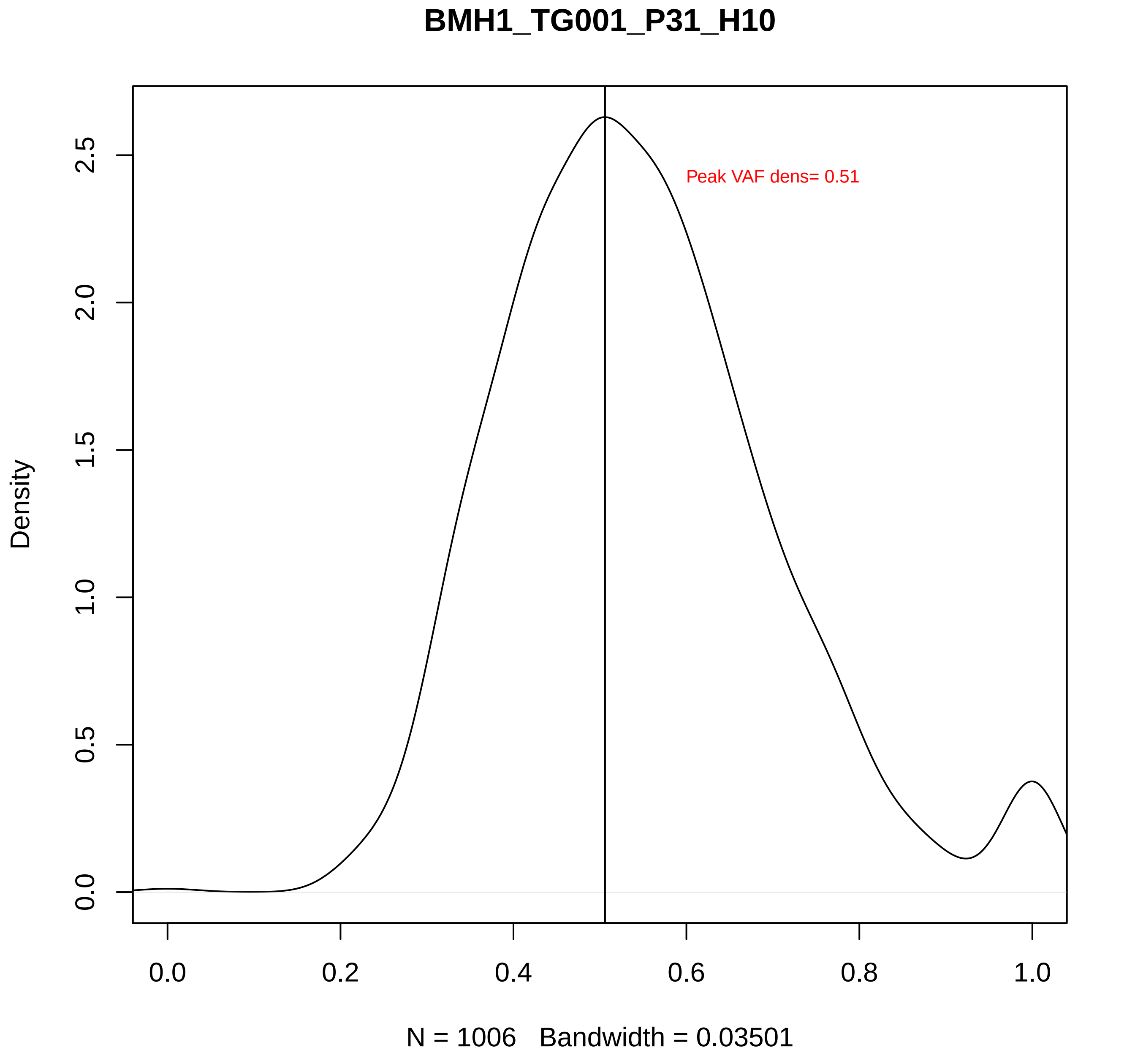
BMH1_TG001_P31_H10
Peak VAF dens= 0.51
Density
0.0
0.5
1.0
1.5
2.0
2.5
0.0
0.2
0.4
0.6
0.8
1.0
N = 1006   Bandwidth = 0.03501

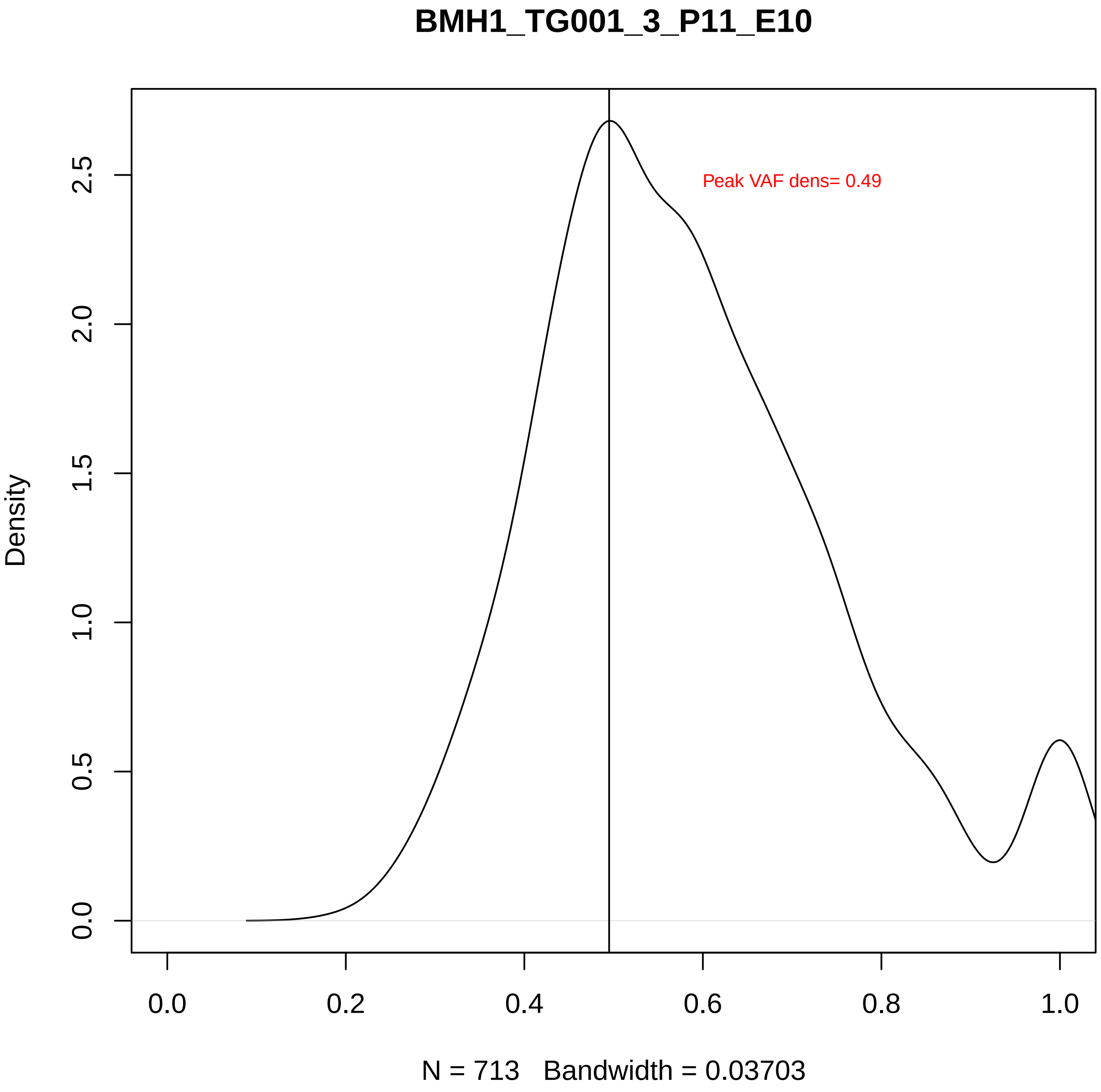
BMH1_TG001_3_P11_E10
Peak VAF dens= 0.49
Density
0.0
0.5
1.0
1.5
2.0
2.5
0.0
0.2
0.4
0.6
0.8
1.0
N = 713   Bandwidth = 0.03703

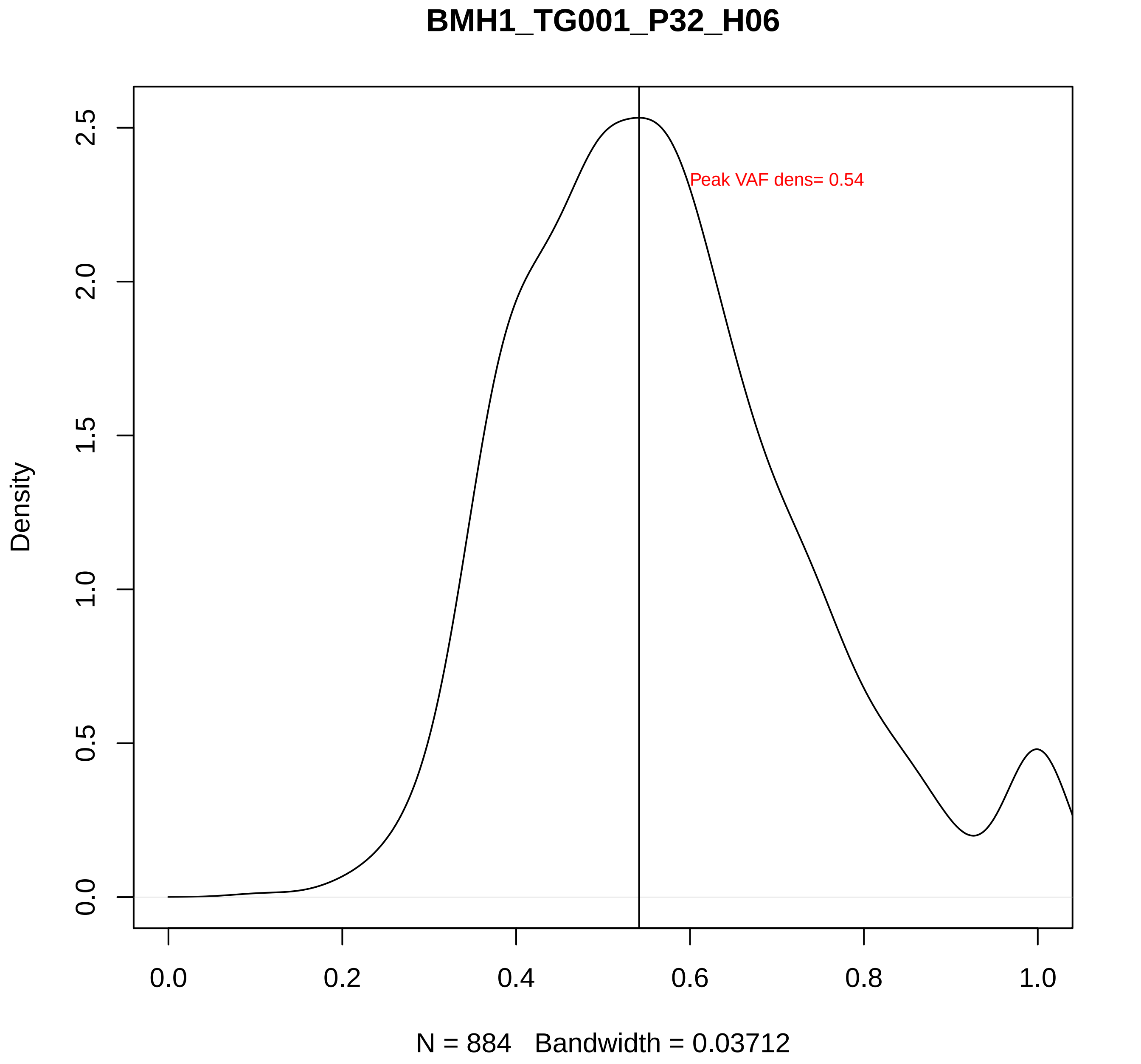
BMH1_TG001_P32_H06
Peak VAF dens= 0.54
Density
0.0
0.5
1.0
1.5
2.0
2.5
0.0
0.2
0.4
0.6
0.8
1.0
N = 884   Bandwidth = 0.03712

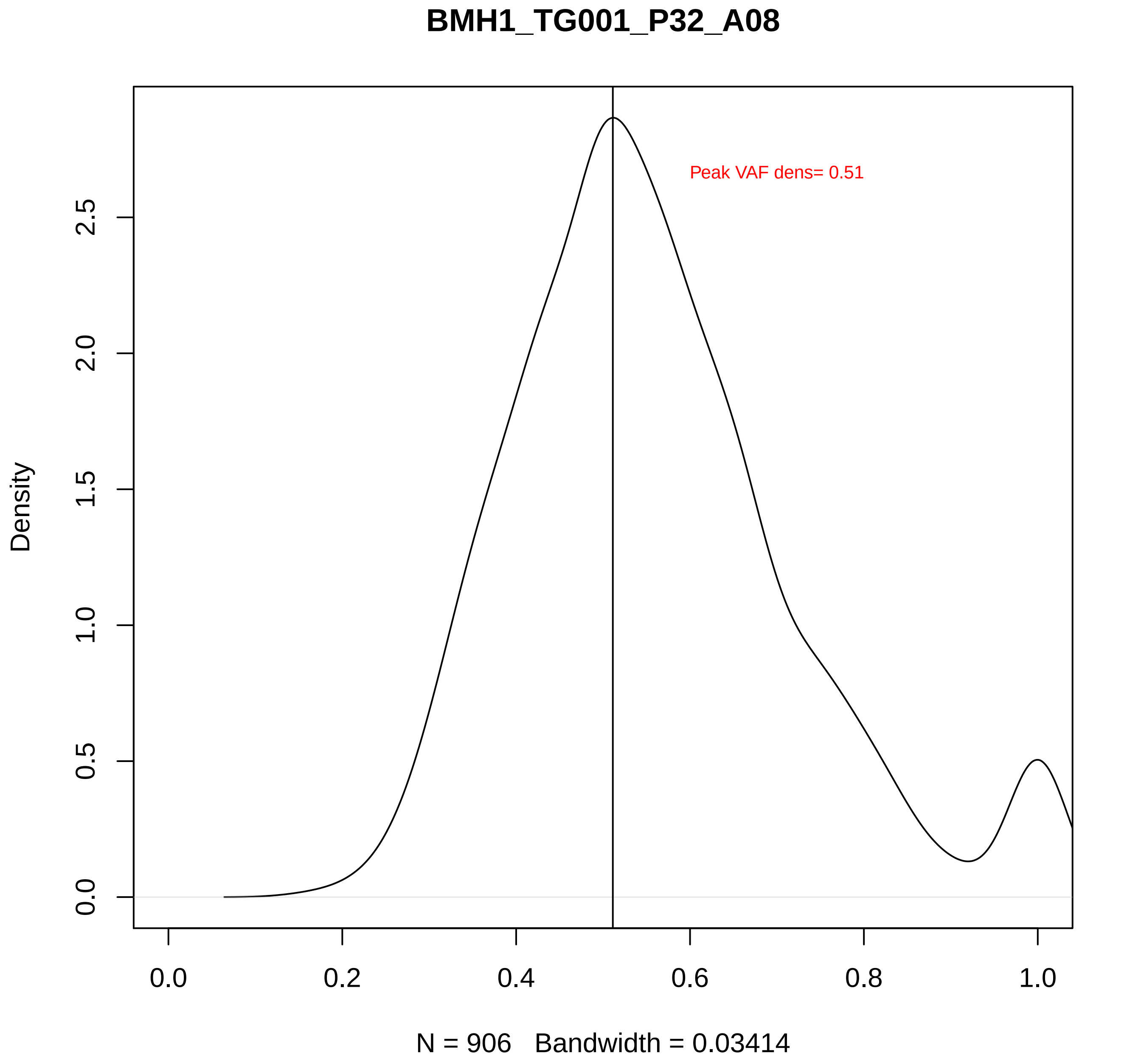
BMH1_TG001_P32_A08
Peak VAF dens= 0.51
Density
0.0
0.5
1.0
1.5
2.0
2.5
0.0
0.2
0.4
0.6
0.8
1.0
N = 906 Bandwidth = 0.03414

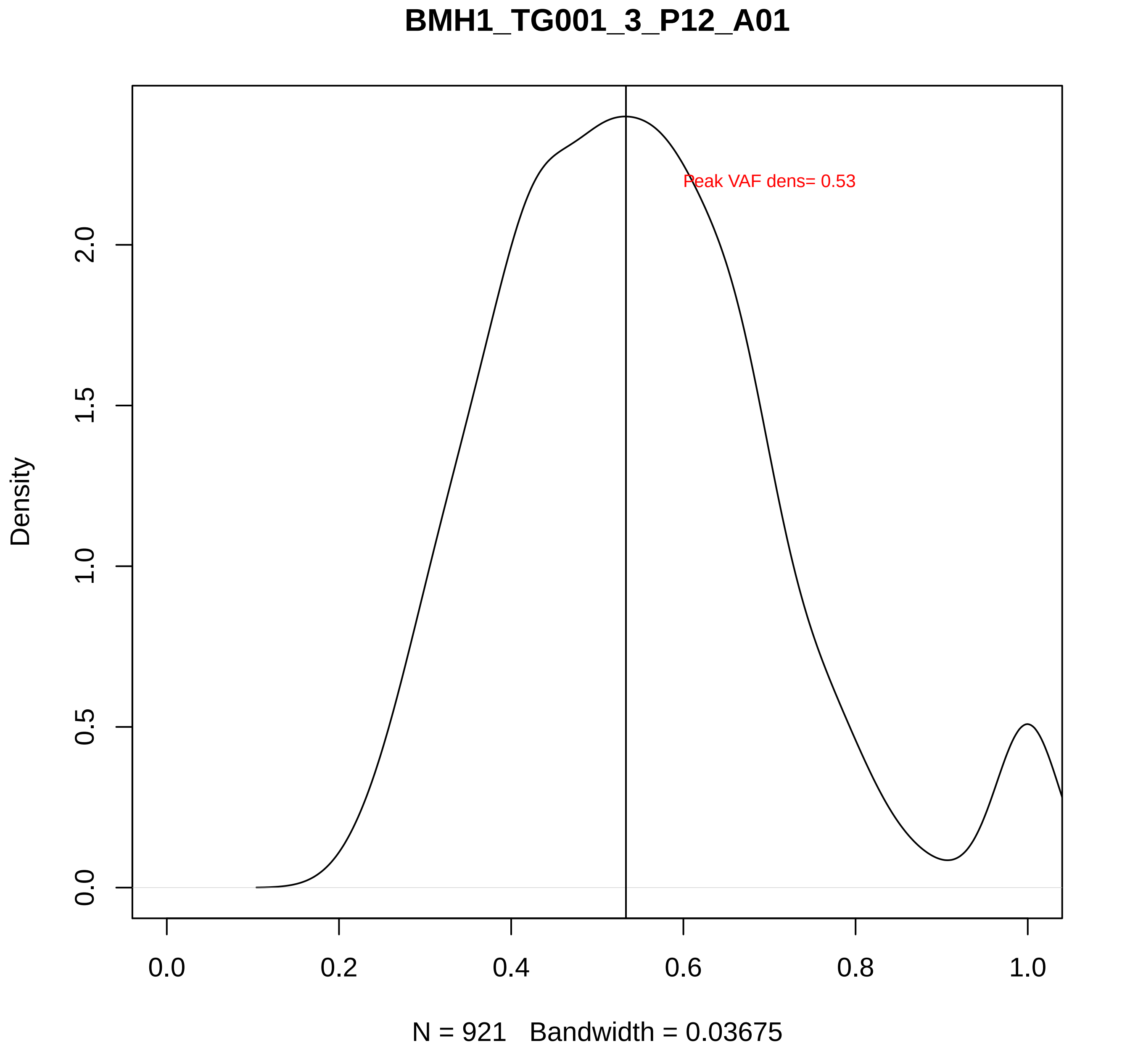
BMH1_TG001_3_P12_A01
Peak VAF dens= 0.53
Density
0.0
0.5
1.0
1.5
2.0
0.0
0.2
0.4
0.6
0.8
1.0
N = 921 Bandwidth = 0.03675

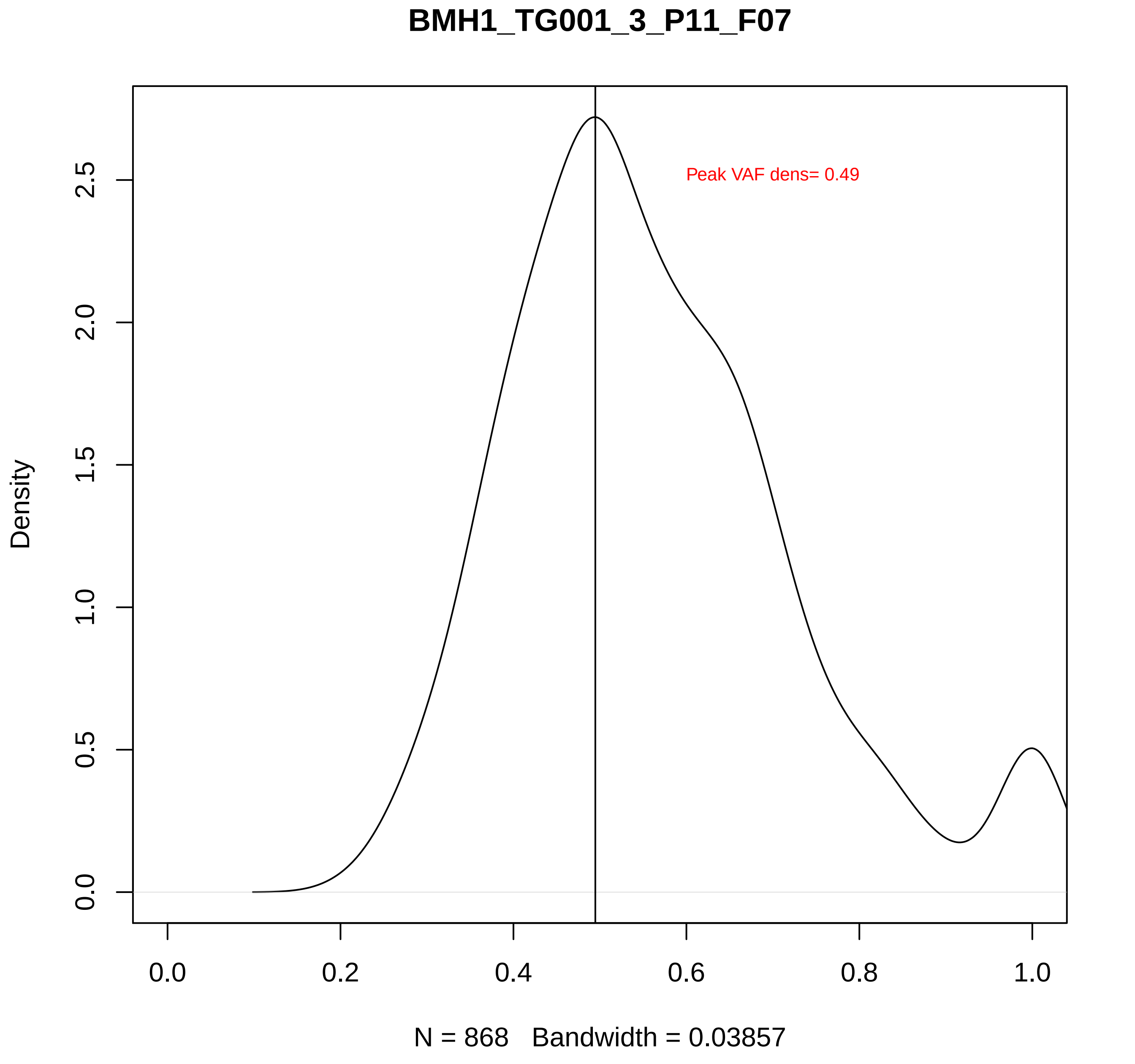

BMH1_TG001_3_P11_F07
Peak VAF dens= 0.49
Density
0.0
0.5
1.0
1.5
2.0
2.5
0.0
0.2
0.4
0.6
0.8
1.0
N = 868 Bandwidth = 0.03857

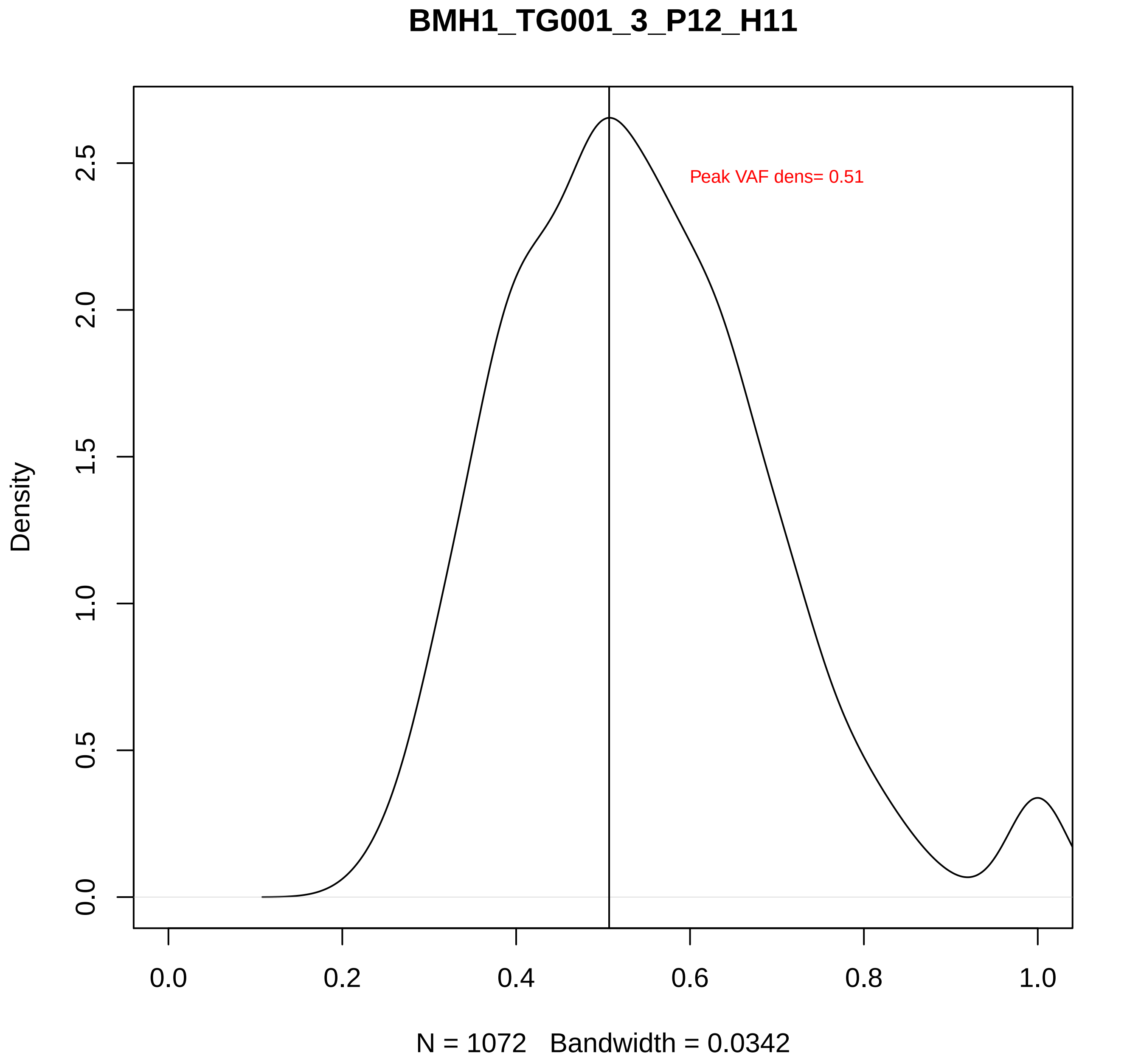

BMH1_TG001_3_P12_H11
Peak VAF dens= 0.51
Density
0.0
0.5
1.0
1.5
2.0
2.5
0.0
0.2
0.4
0.6
0.8
1.0
N = 1072 Bandwidth = 0.0342

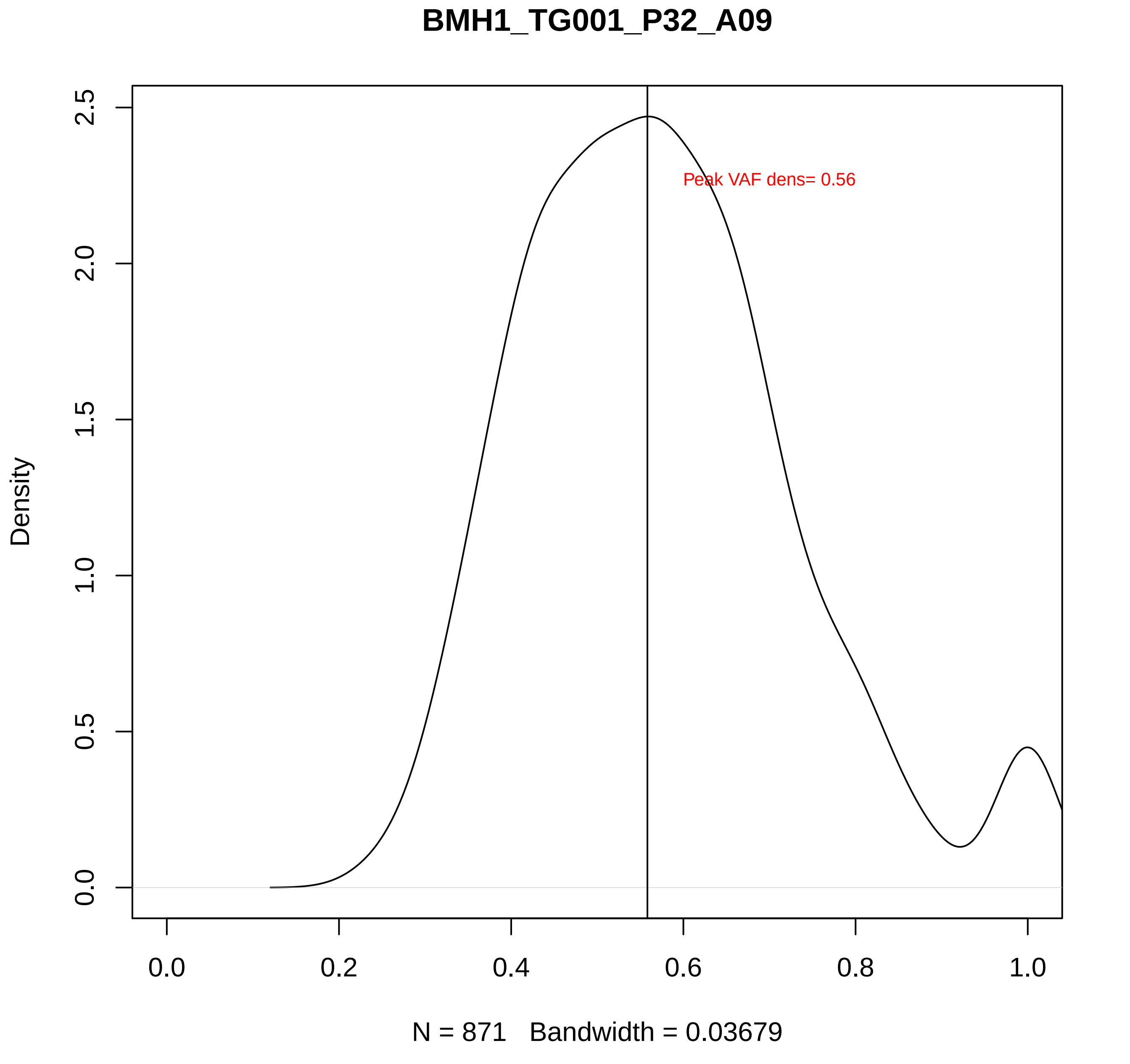
BMH1_TG001_P32_A09
Peak VAF dens= 0.56
Density
0.0
0.5
1.0
1.5
2.0
2.5
0.0
0.2
0.4
0.6
0.8
1.0
N = 871   Bandwidth = 0.03679

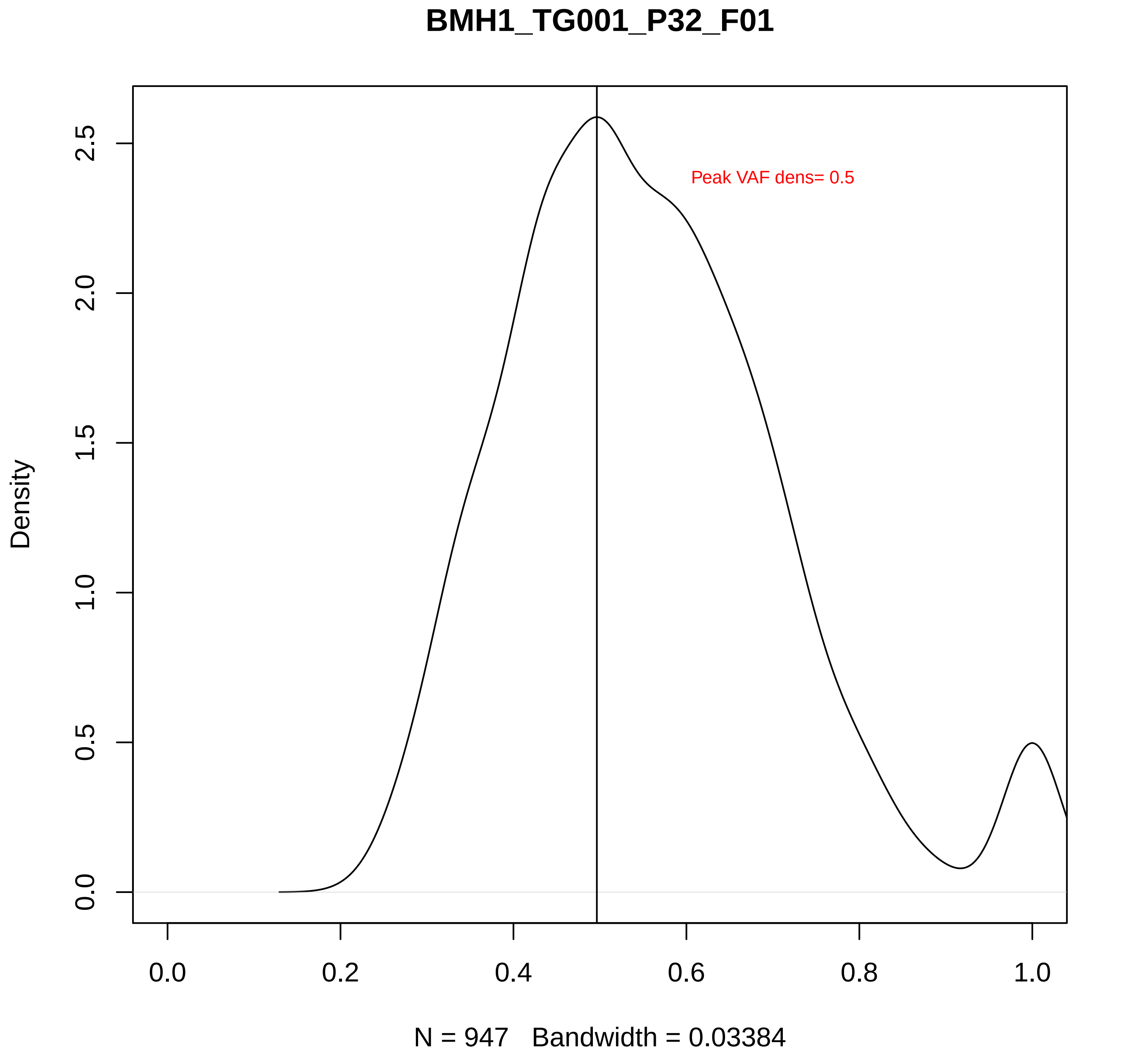
BMH1_TG001_P32_F01
Peak VAF dens= 0.5
Density
0.0
0.5
1.0
1.5
2.0
2.5
0.0
0.2
0.4
0.6
0.8
1.0
N = 947   Bandwidth = 0.03384

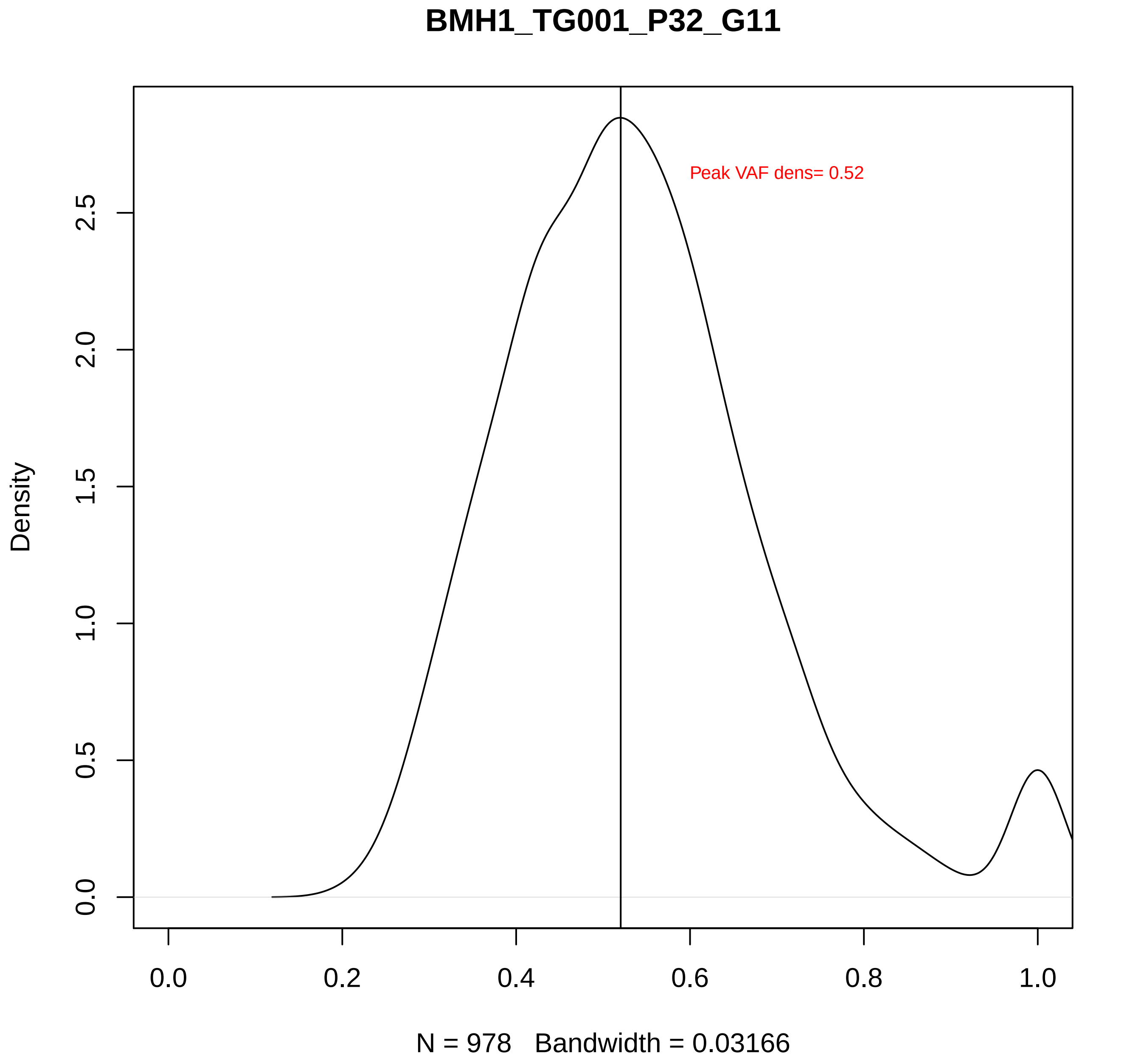

BMH1_TG001_P32_G11
Peak VAF dens= 0.52
Density
0.0
0.5
1.0
1.5
2.0
2.5
0.0
0.2
0.4
0.6
0.8
1.0
N = 978   Bandwidth = 0.03166

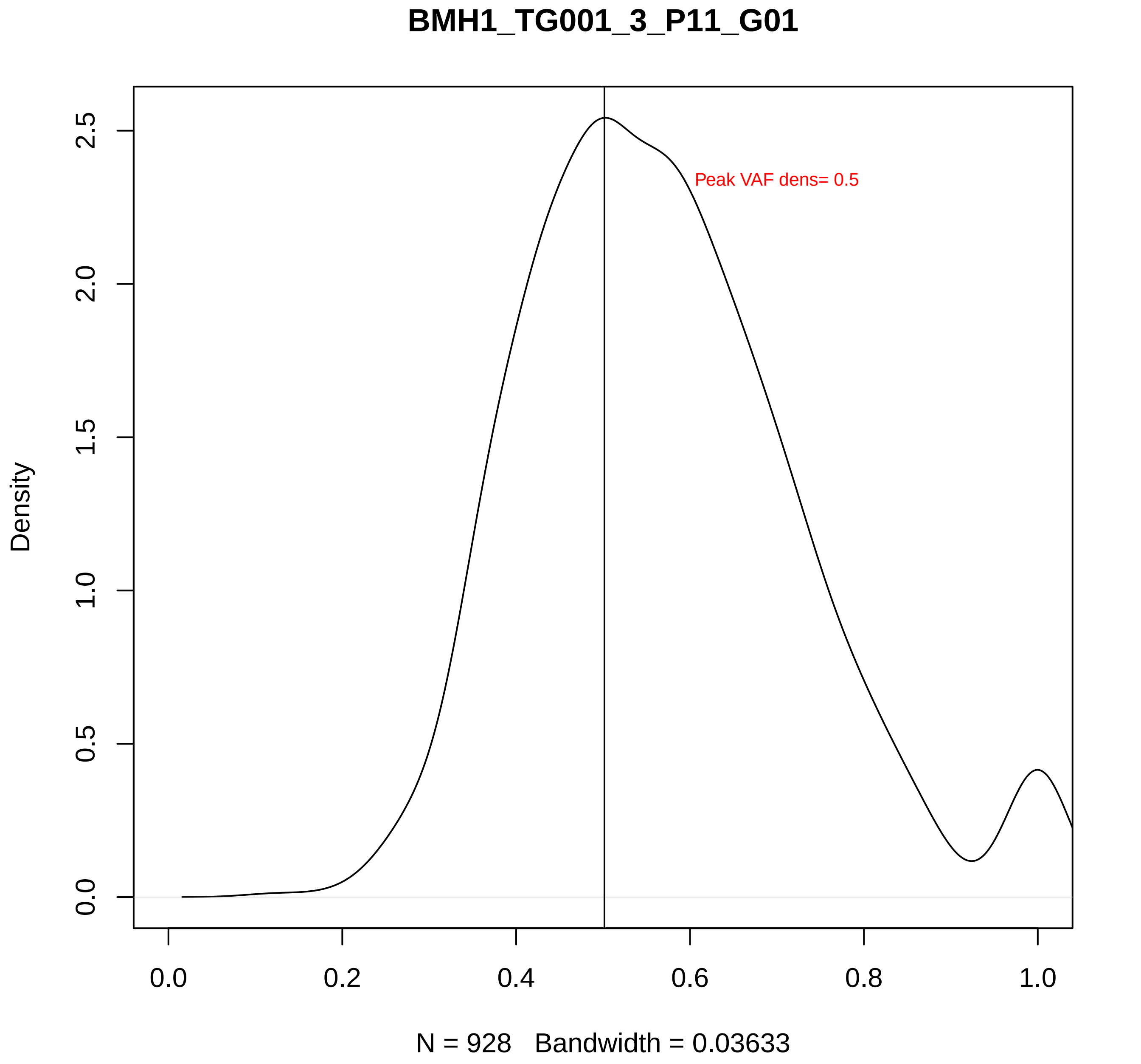
BMH1_TG001_3_P11_G01
Peak VAF dens= 0.5
Density
0.0
0.5
1.0
1.5
2.0
2.5
0.0
0.2
0.4
0.6
0.8
1.0
N = 928   Bandwidth = 0.03633

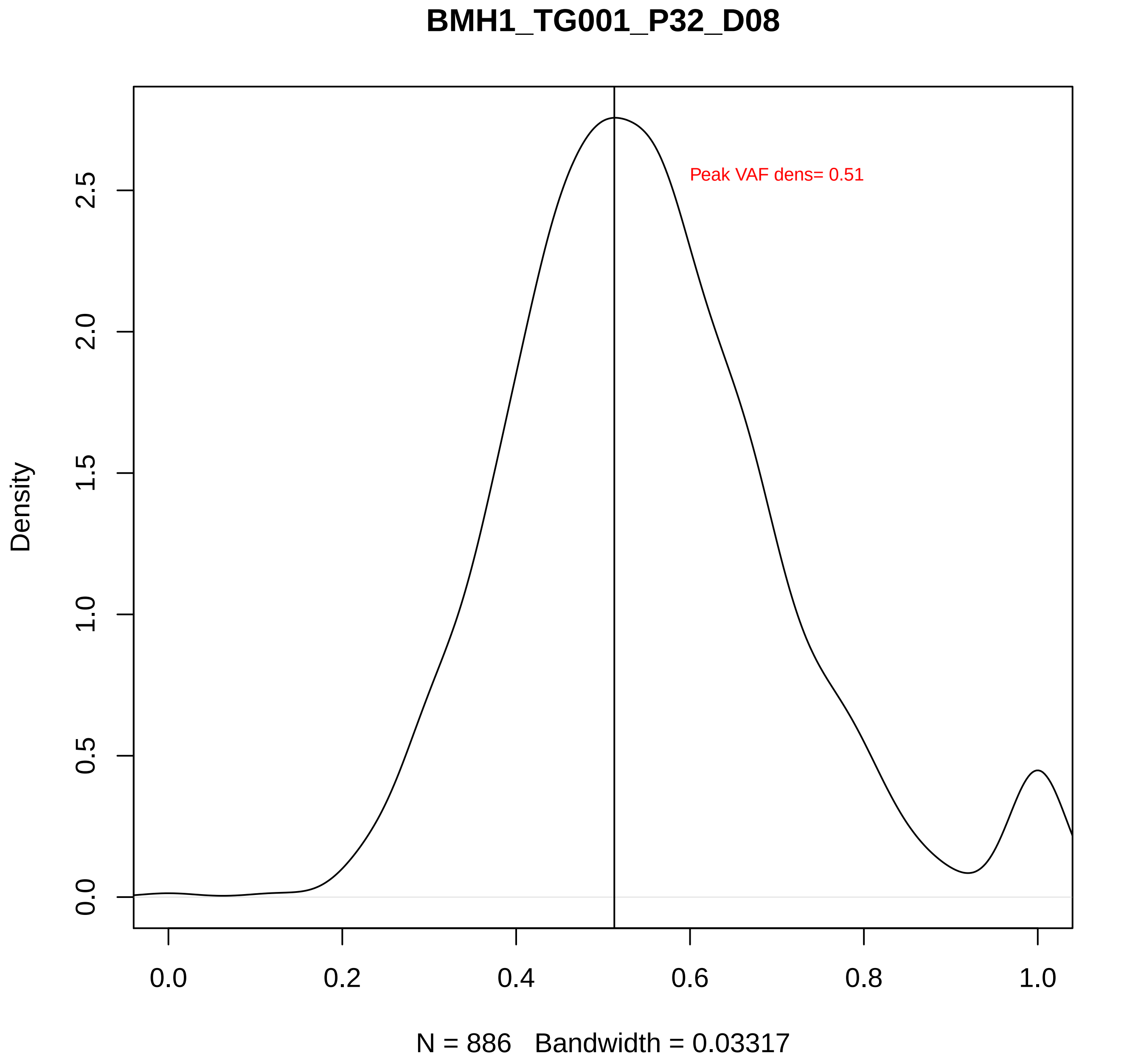

BMH1_TG001_P32_D08
Peak VAF dens= 0.51
Density
0.0
0.5
1.0
1.5
2.0
2.5
0.0
0.2
0.4
0.6
0.8
1.0
N = 886   Bandwidth = 0.03317

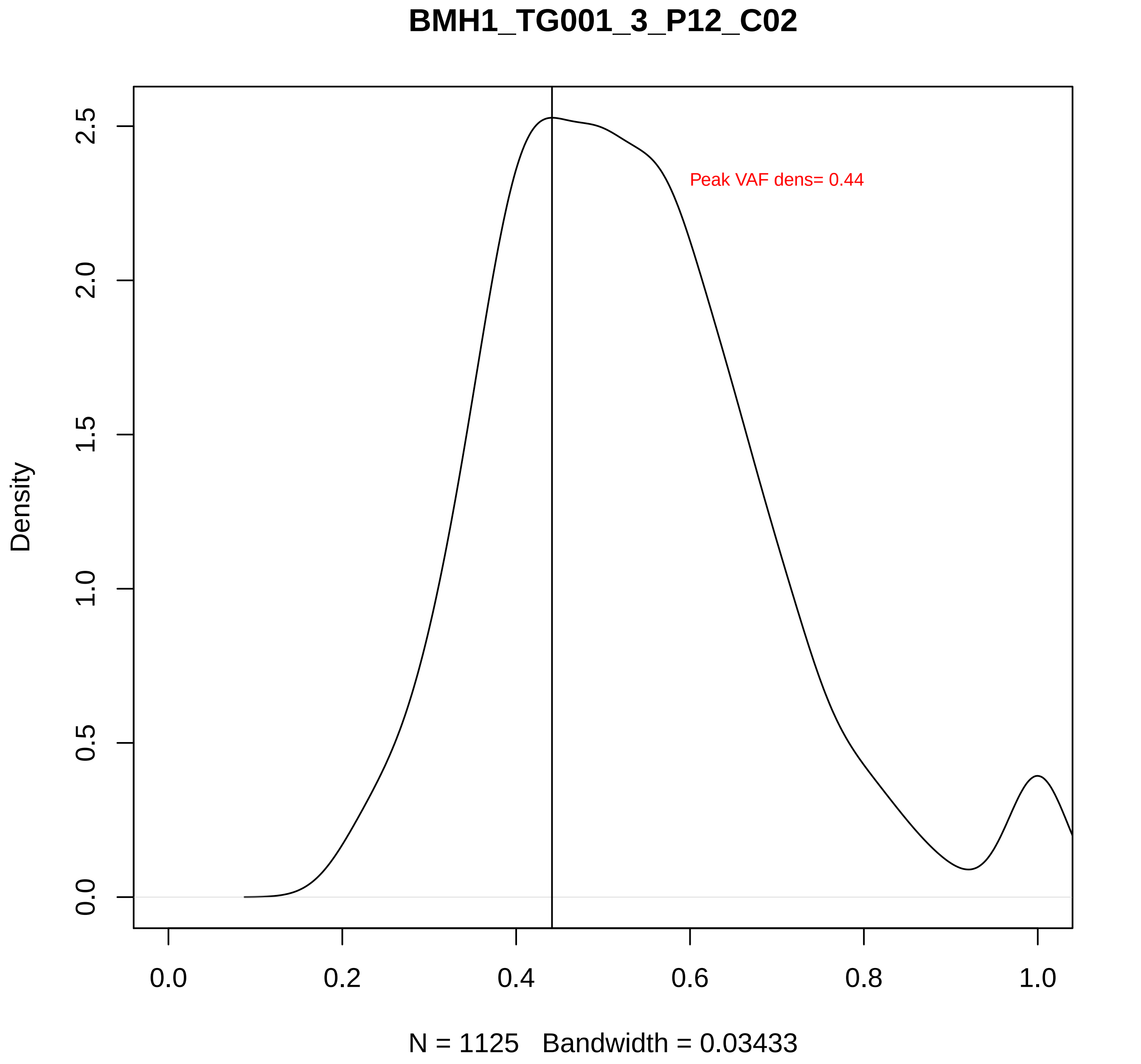
BMH1_TG001_3_P12_C02
Peak VAF dens= 0.44
Density
0.0
0.5
1.0
1.5
2.0
2.5
0.0
0.2
0.4
0.6
0.8
1.0
N = 1125 Bandwidth = 0.03433

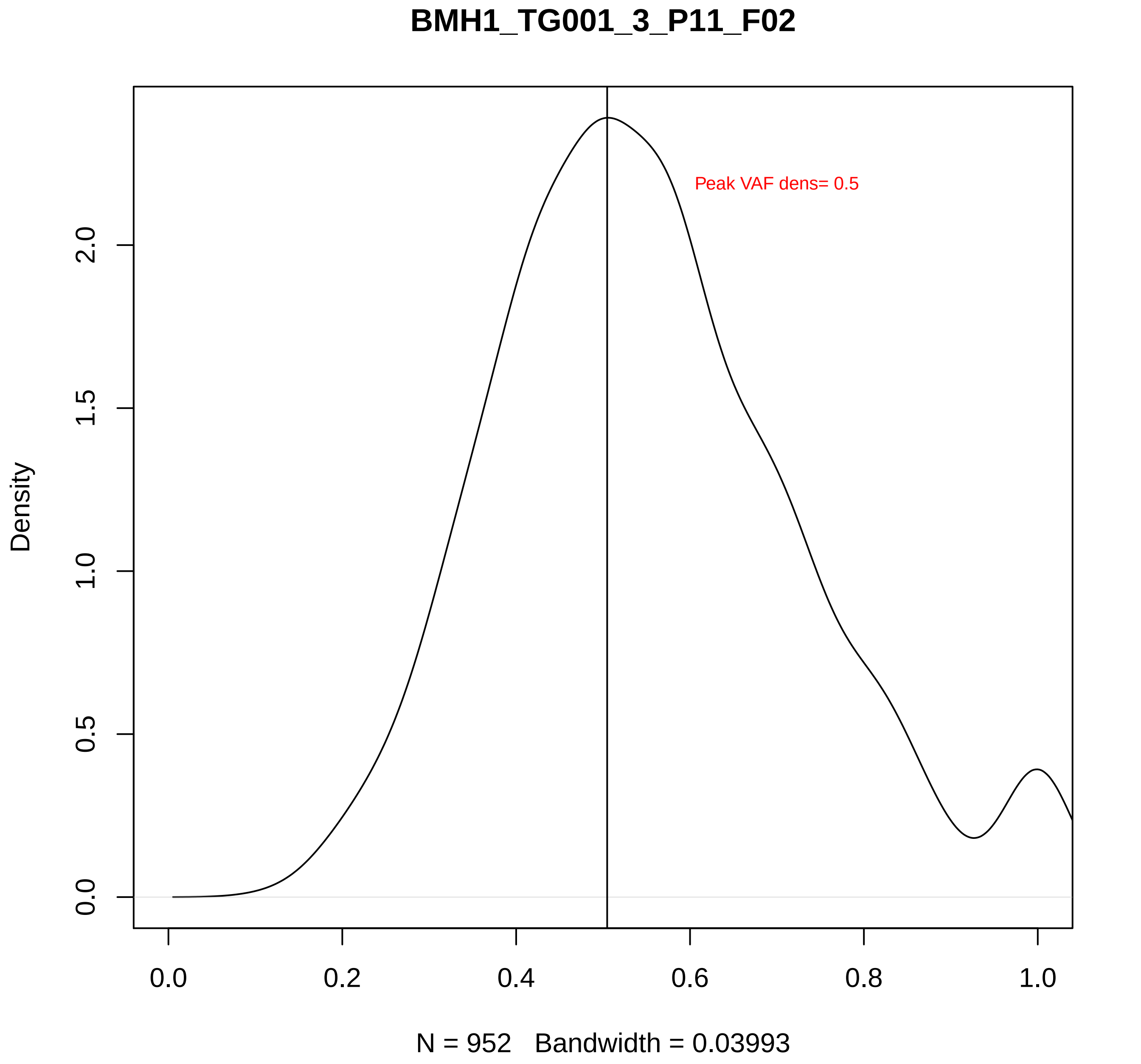
BMH1_TG001_3_P11_F02
Peak VAF dens= 0.5
Density
0.0
0.5
1.0
1.5
2.0
0.0
0.2
0.4
0.6
0.8
1.0
N = 952   Bandwidth = 0.03993

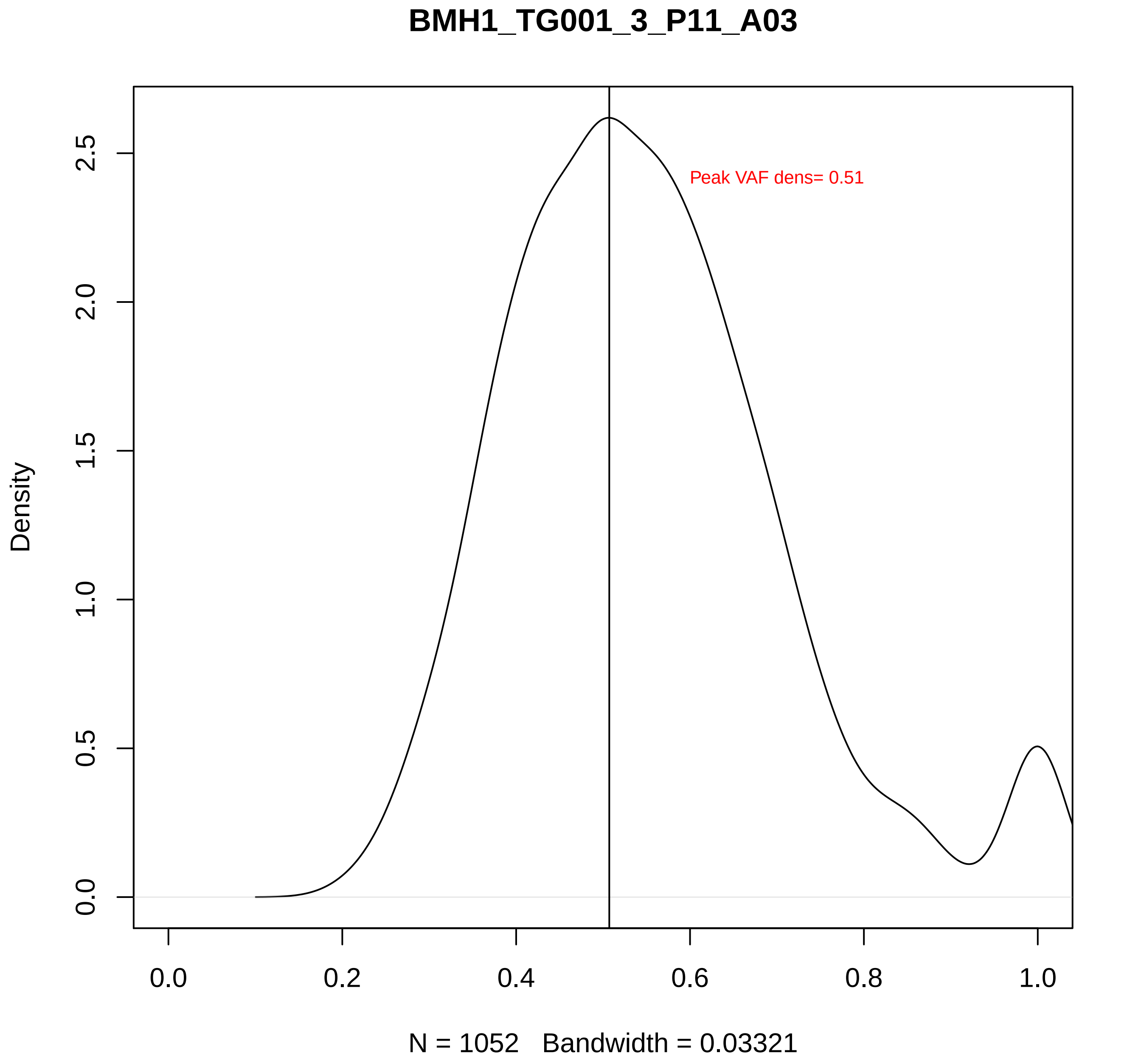

BMH1_TG001_3_P11_A03
Peak VAF dens= 0.51
Density
0.0
0.5
1.0
1.5
2.0
2.5
0.0
0.2
0.4
0.6
0.8
1.0
N = 1052   Bandwidth = 0.03321

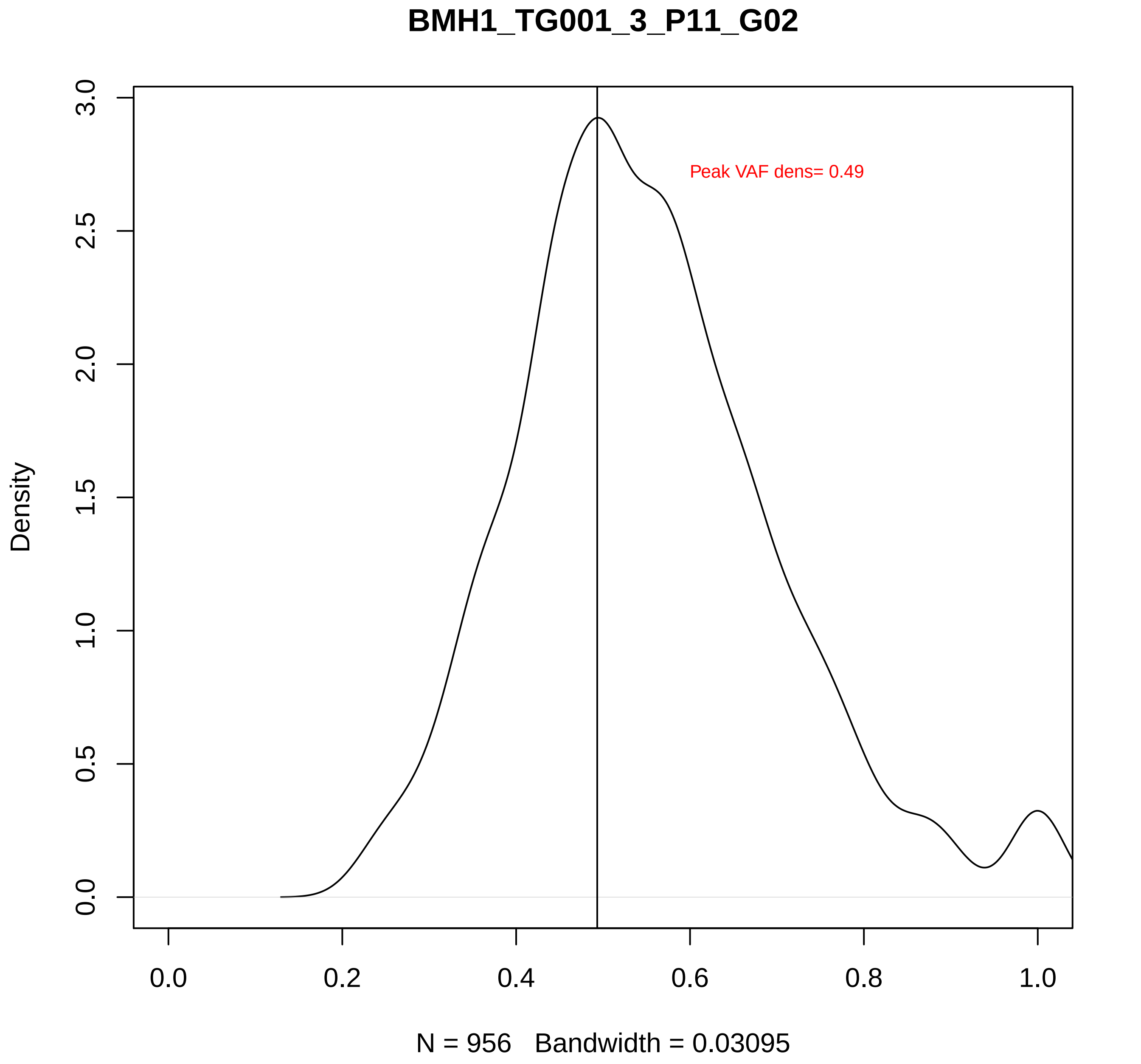

BMH1_TG001_3_P11_G02
Peak VAF dens= 0.49
Density
0.0
0.5
1.0
1.5
2.0
2.5
3.0
0.0
0.2
0.4
0.6
0.8
1.0
N = 956   Bandwidth = 0.03095

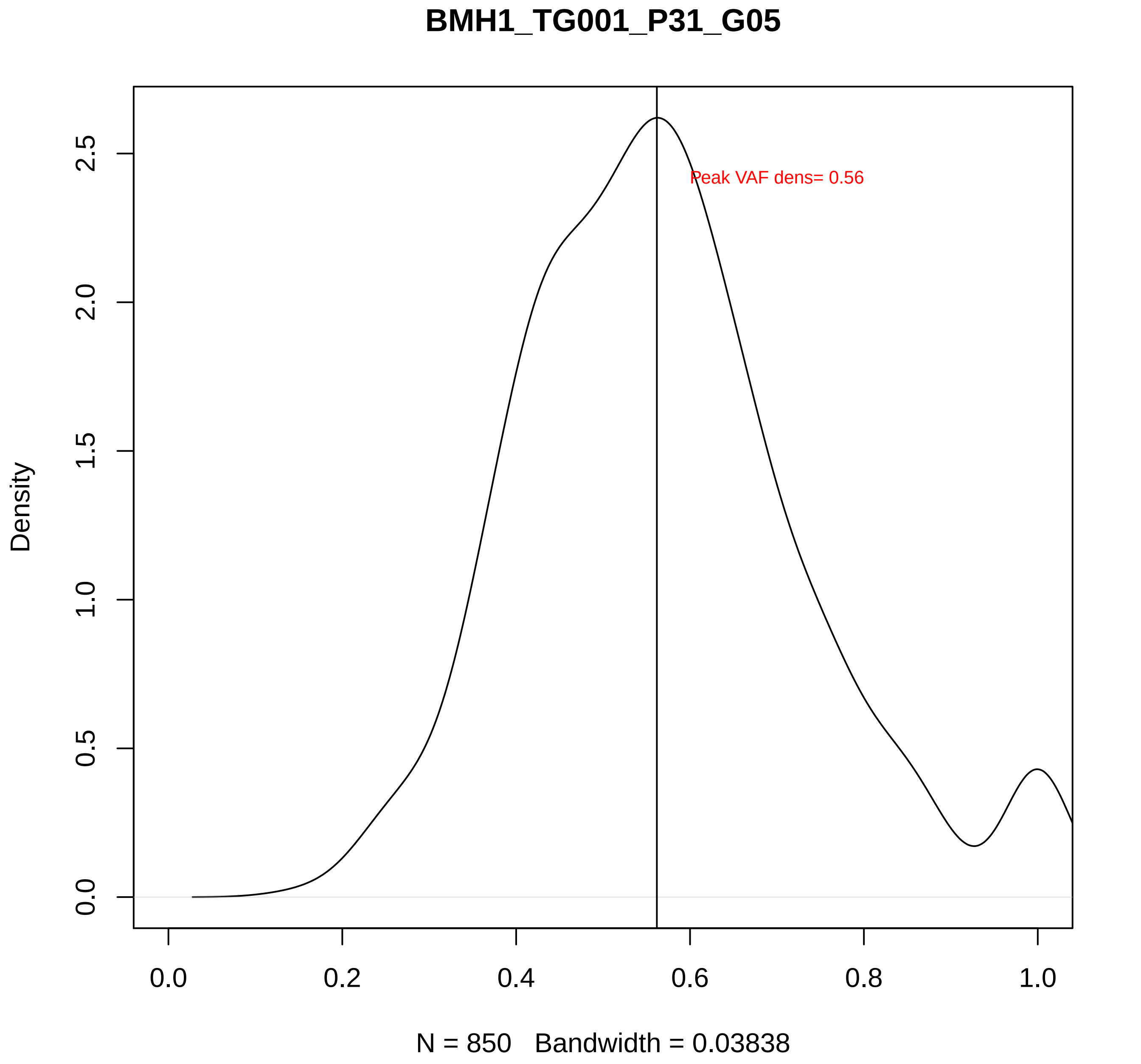

BMH1_TG001_P31_G05
Peak VAF dens= 0.56
Density
0.0
0.5
1.0
1.5
2.0
2.5
0.0
0.2
0.4
0.6
0.8
1.0
N = 850 Bandwidth = 0.03838

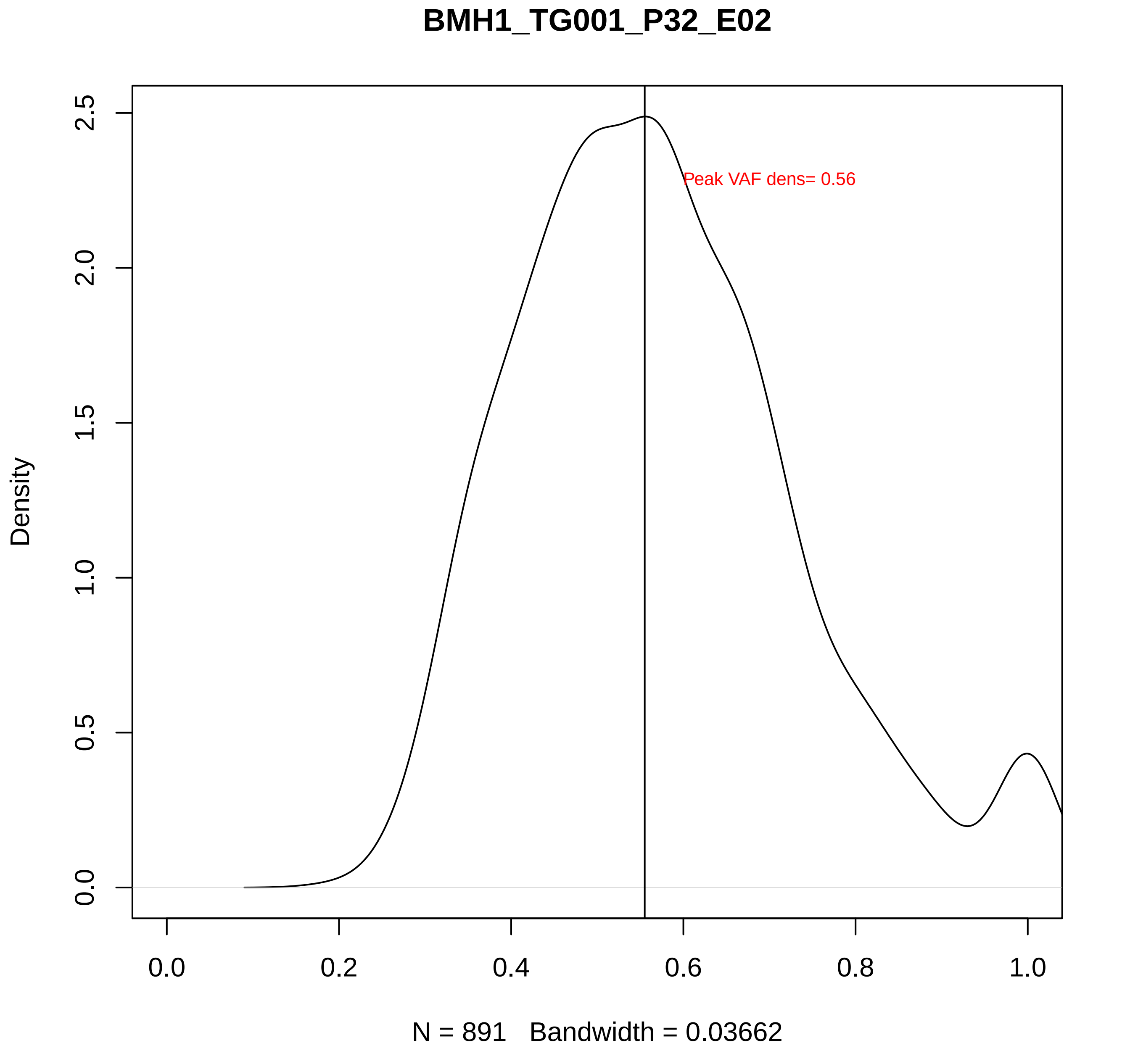
BMH1_TG001_P32_E02
Density
2.5
2.0
1.5
1.0
0.5
0.0
Peak VAF dens= 0.56
0.0
0.2
0.4
0.6
0.8
1.0
N = 891 Bandwidth = 0.03662

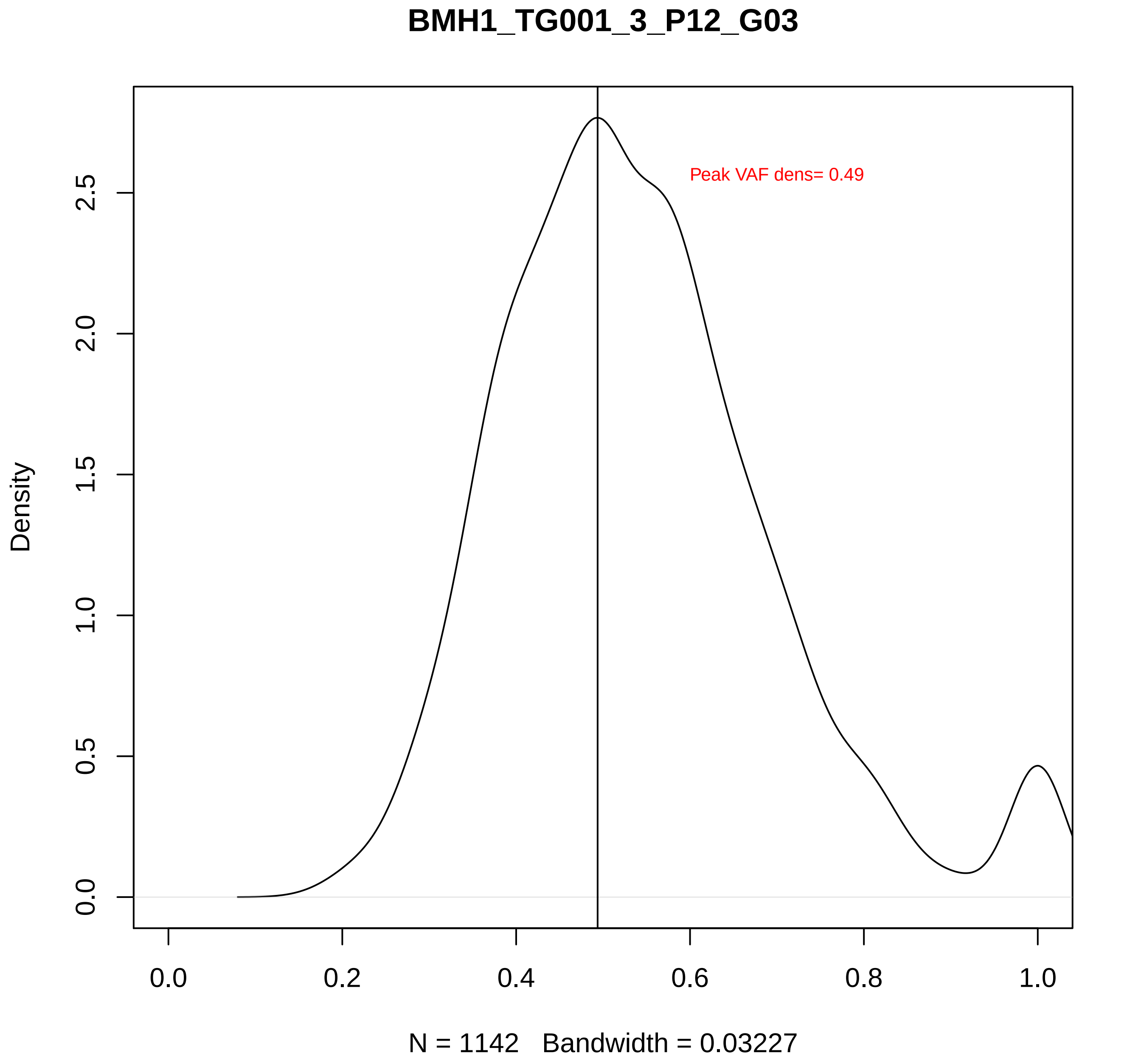
BMH1_TG001_3_P12_G03
Peak VAF dens= 0.49
Density
0.0
0.5
1.0
1.5
2.0
2.5
0.0
0.2
0.4
0.6
0.8
1.0
N = 1142   Bandwidth = 0.03227

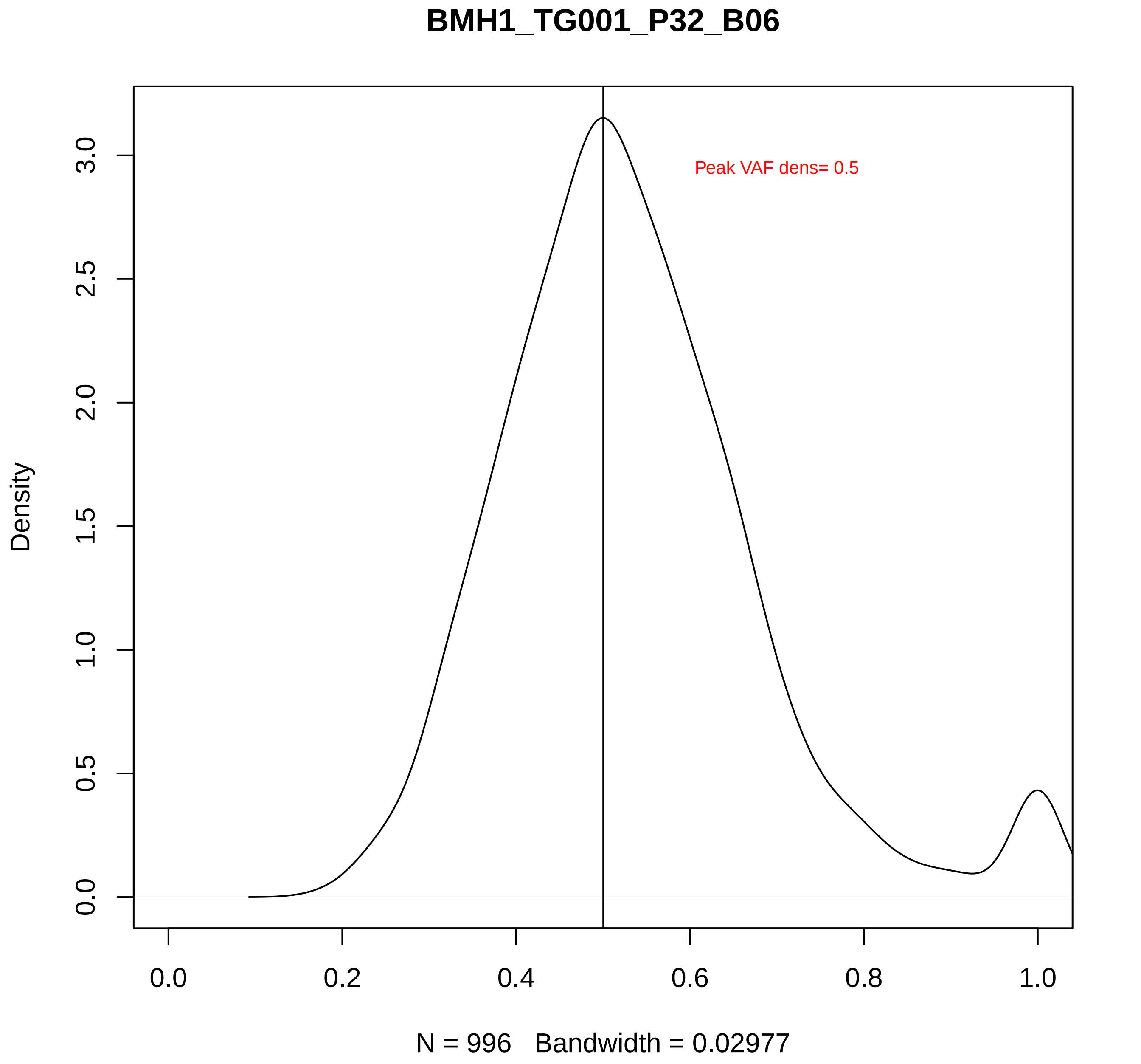
BMH1_TG001_P32_B06
Peak VAF dens= 0.5
Density
0.0
0.5
1.0
1.5
2.0
2.5
3.0
0.0
0.2
0.4
0.6
0.8
1.0
N = 996   Bandwidth = 0.02977

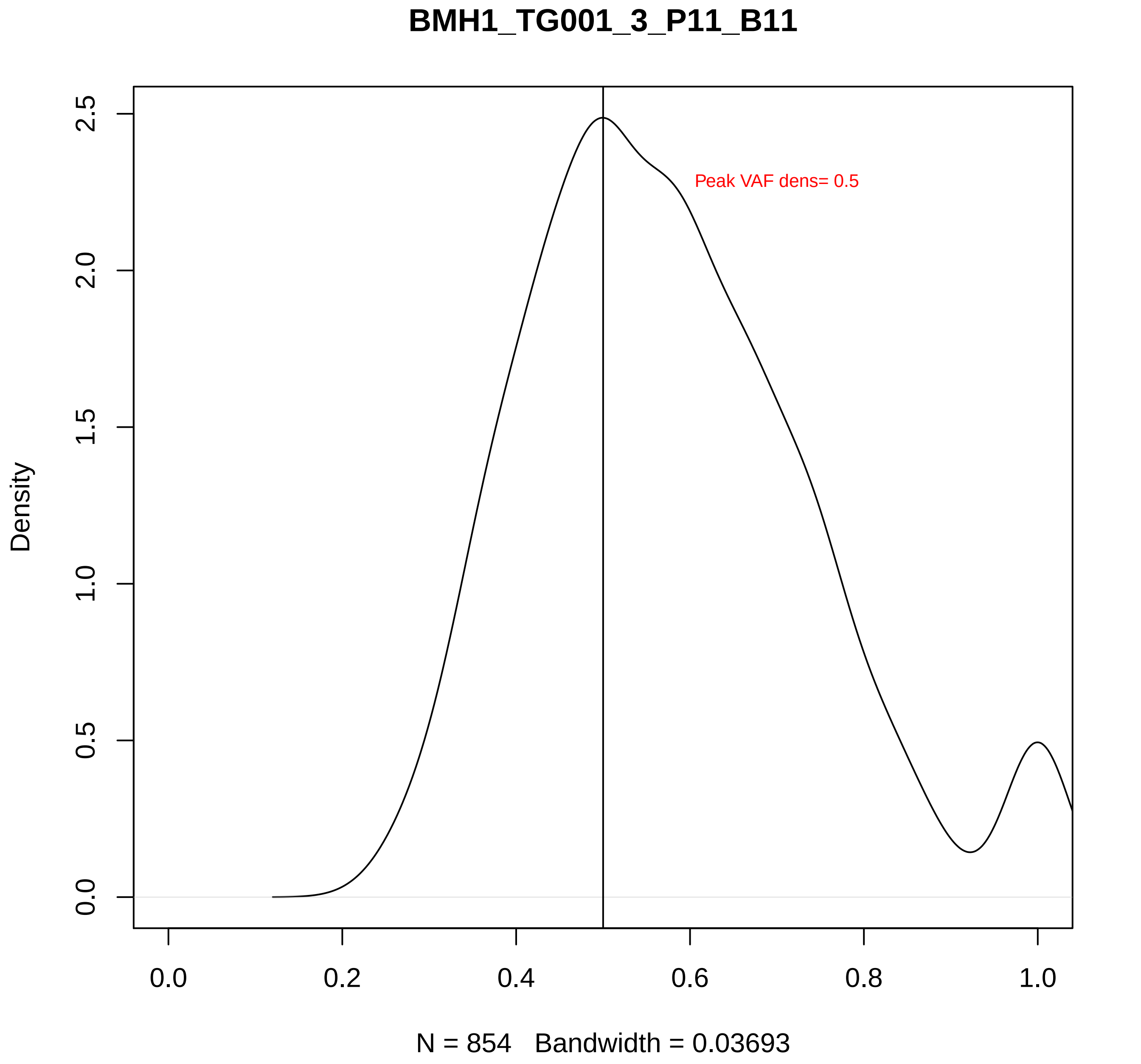

BMH1_TG001_3_P11_B11
Peak VAF dens= 0.5
Density
0.0
0.5
1.0
1.5
2.0
2.5
0.0
0.2
0.4
0.6
0.8
1.0
N = 854   Bandwidth = 0.03693

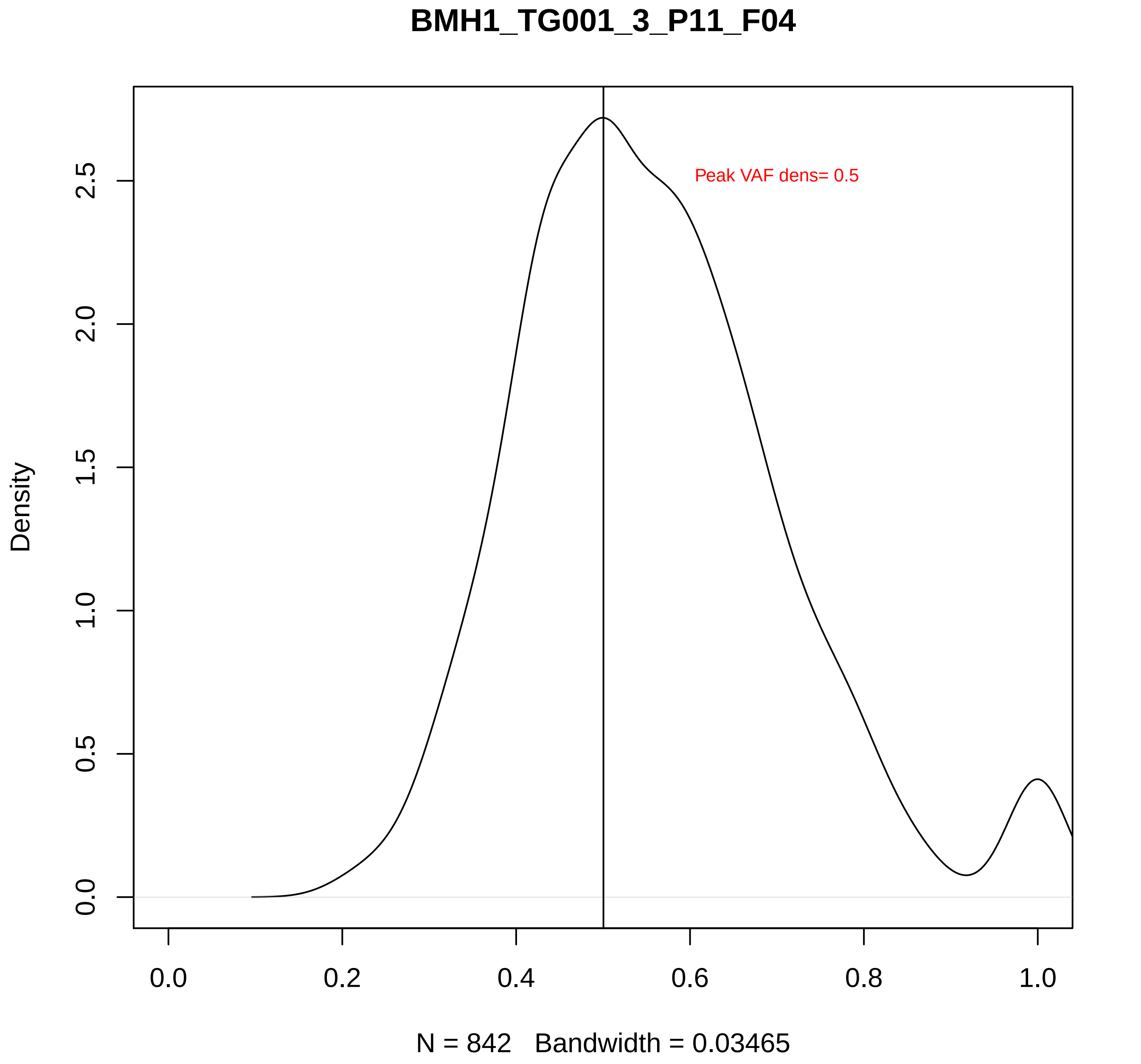
BMH1_TG001_3_P11_F04
Peak VAF dens= 0.5
Density
0.0
0.5
1.0
1.5
2.0
2.5
0.0
0.2
0.4
0.6
0.8
1.0
N = 842   Bandwidth = 0.03465

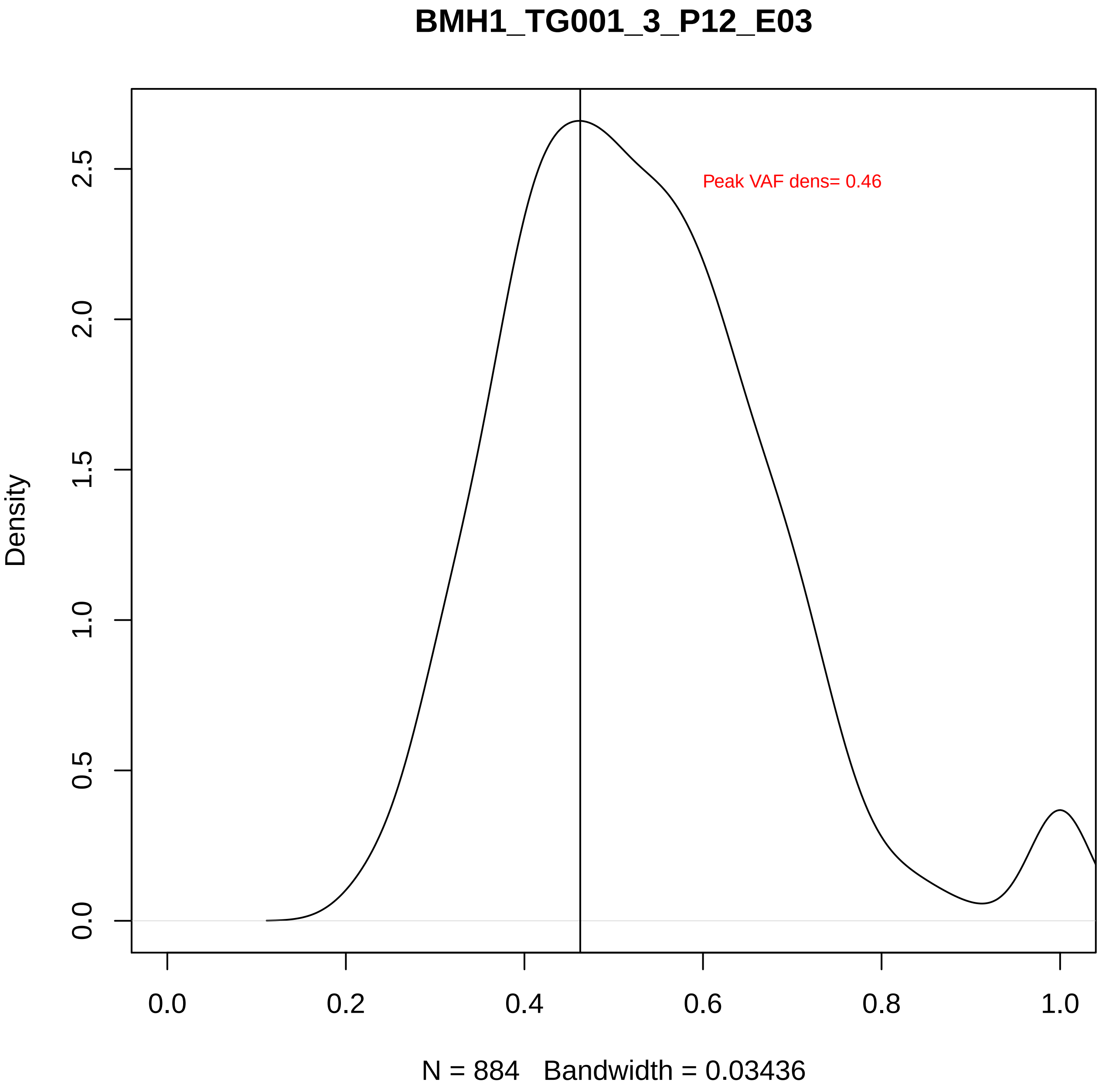

BMH1_TG001_3_P12_E03
Peak VAF dens= 0.46
Density
0.0
0.5
1.0
1.5
2.0
2.5
0.0
0.2
0.4
0.6
0.8
1.0
N = 884   Bandwidth = 0.03436

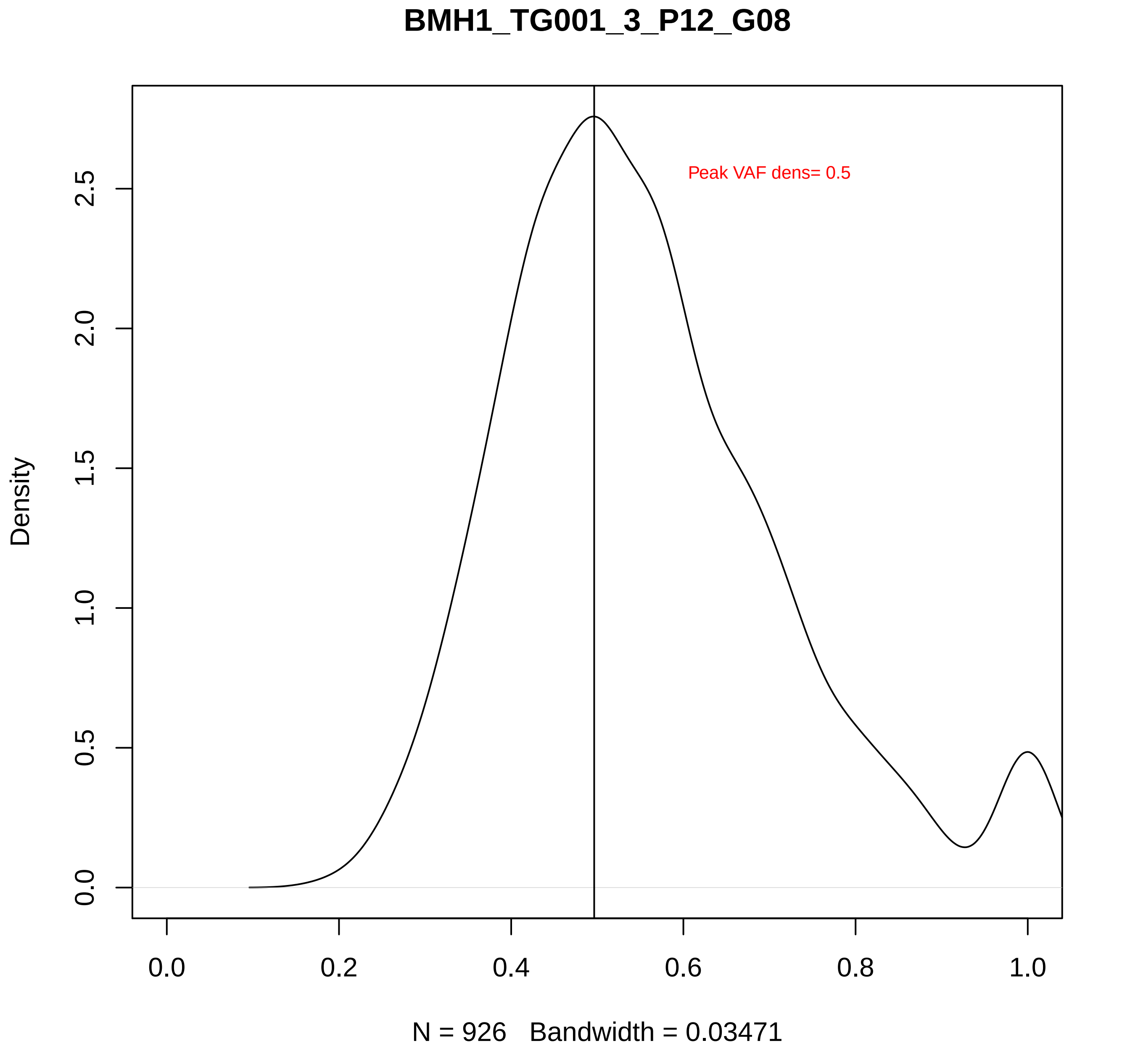
BMH1_TG001_3_P12_G08
Peak VAF dens= 0.5
Density
0.0
0.5
1.0
1.5
2.0
2.5
0.0
0.2
0.4
0.6
0.8
1.0
N = 926   Bandwidth = 0.03471

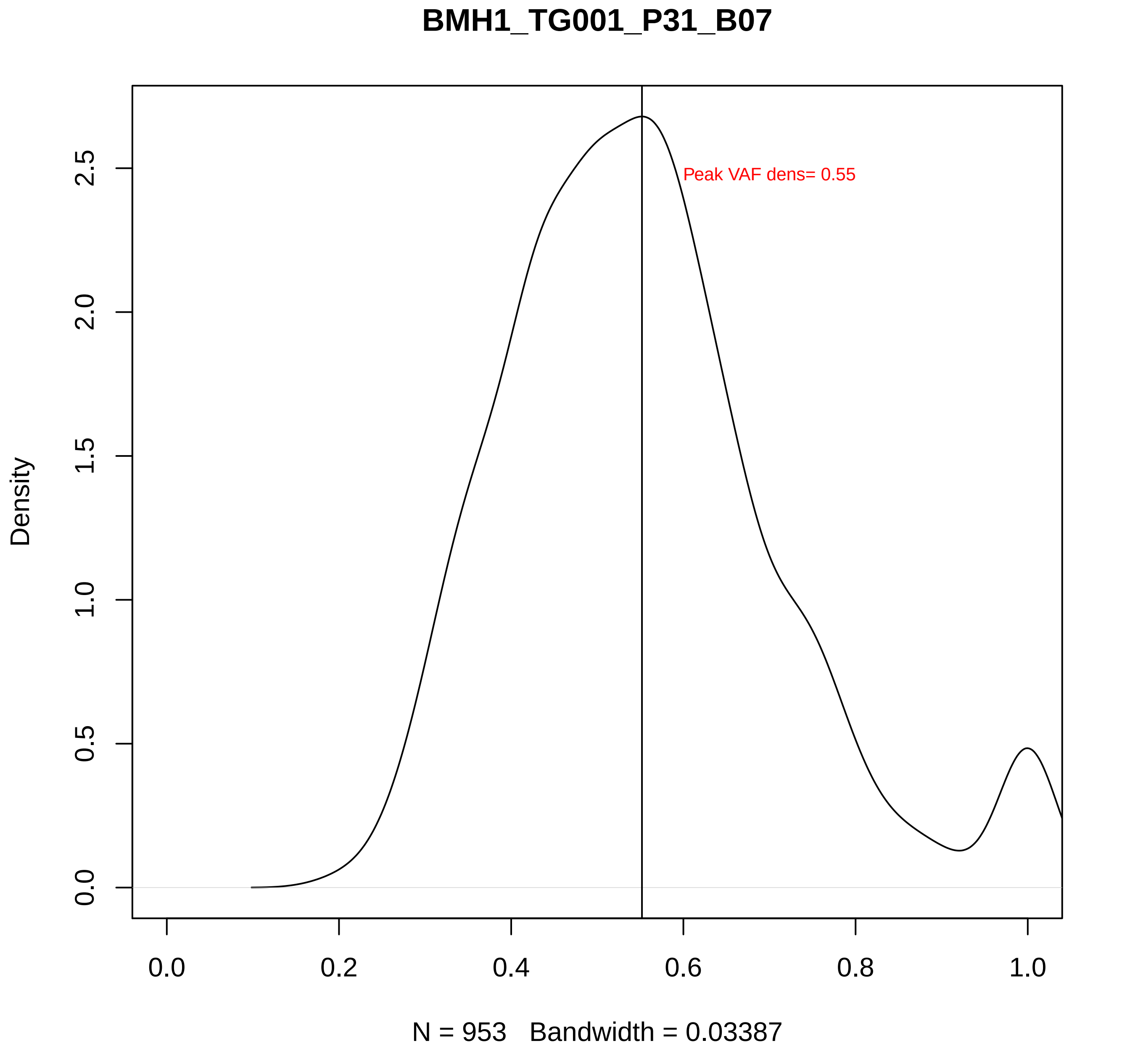
BMH1_TG001_P31_B07
Peak VAF dens= 0.55
Density
0.0
0.5
1.0
1.5
2.0
2.5
0.0
0.2
0.4
0.6
0.8
1.0
N = 953 Bandwidth = 0.03387

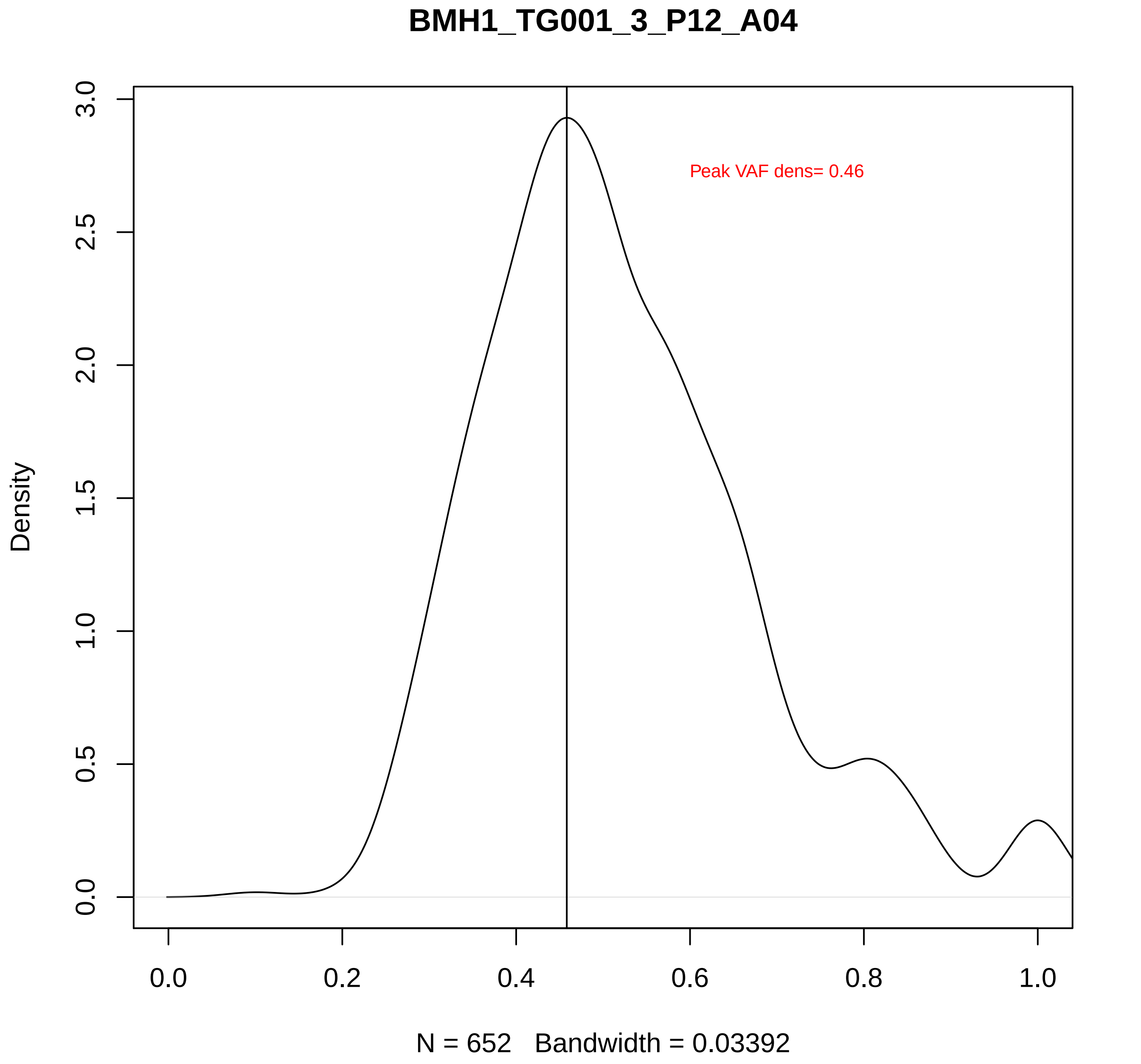

BMH1_TG001_3_P12_A04
Peak VAF dens= 0.46
Density
0.0
0.5
1.0
1.5
2.0
2.5
3.0
0.0
0.2
0.4
0.6
0.8
1.0
N = 652 Bandwidth = 0.03392

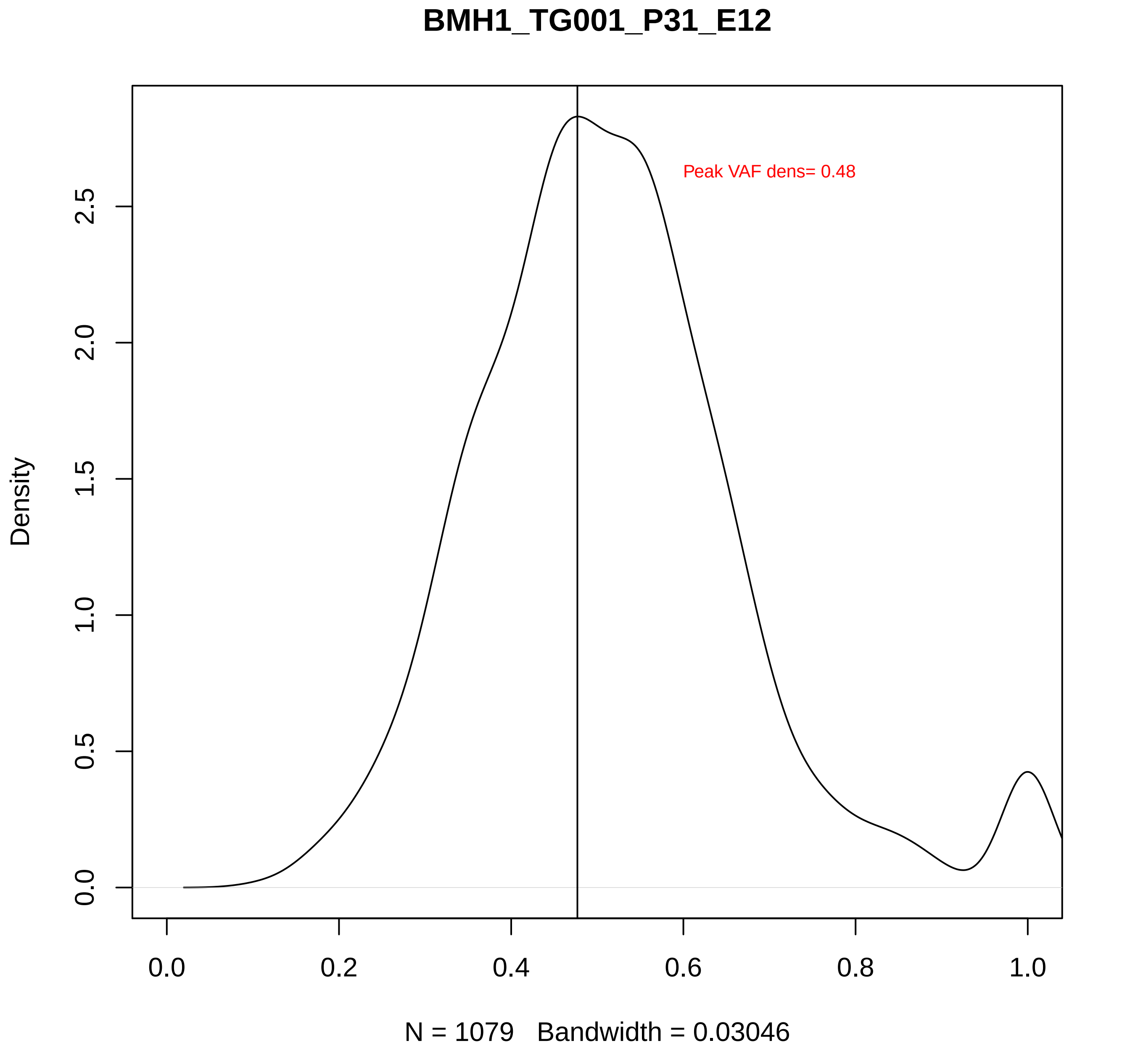
BMH1_TG001_P31_E12
Peak VAF dens= 0.48
Density
0.0
0.5
1.0
1.5
2.0
2.5
0.0
0.2
0.4
0.6
0.8
1.0
N = 1079   Bandwidth = 0.03046

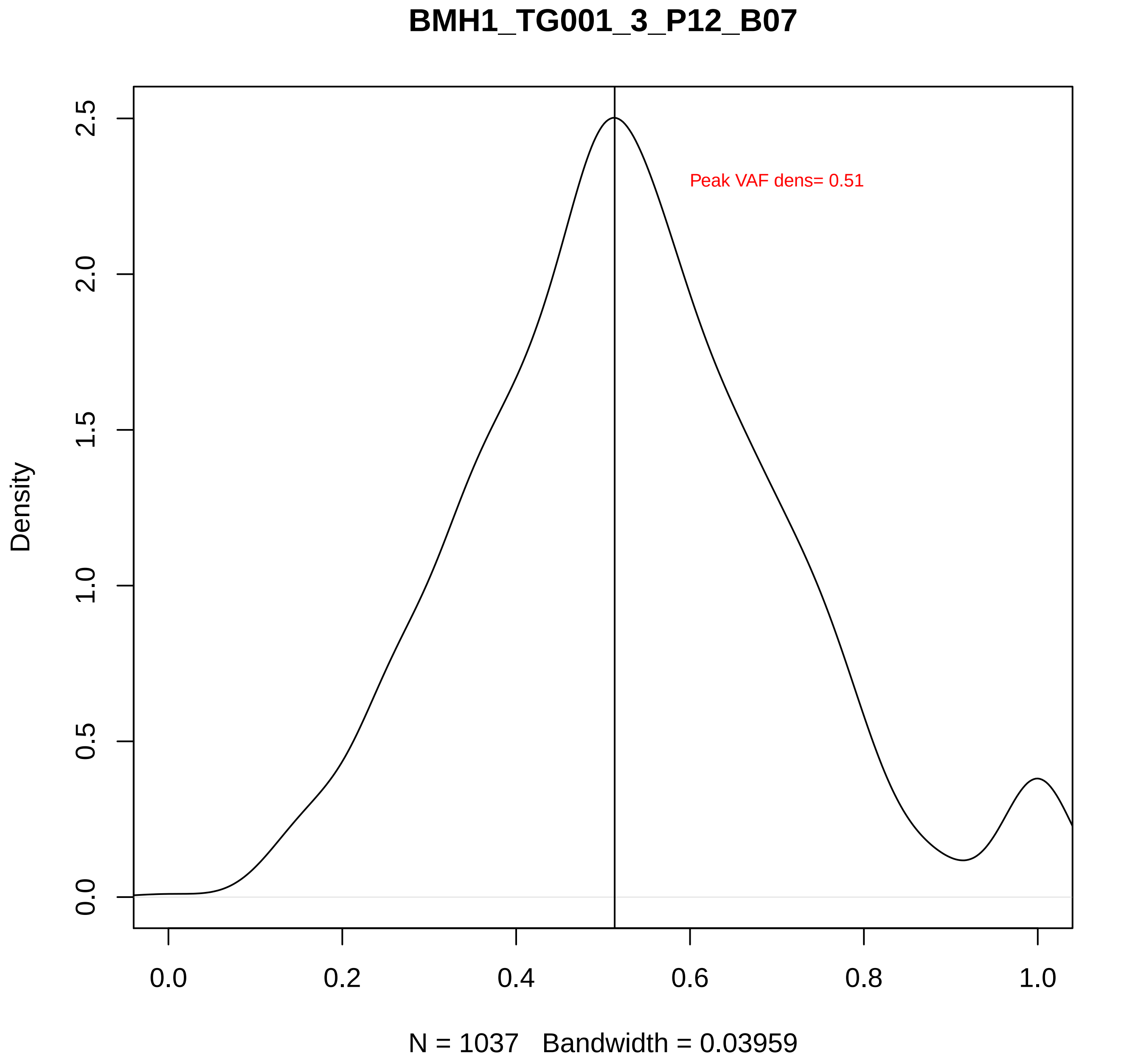

BMH1_TG001_3_P12_B07
Peak VAF dens= 0.51
Density
0.0
0.5
1.0
1.5
2.0
2.5
0.0
0.2
0.4
0.6
0.8
1.0
N = 1037   Bandwidth = 0.03959

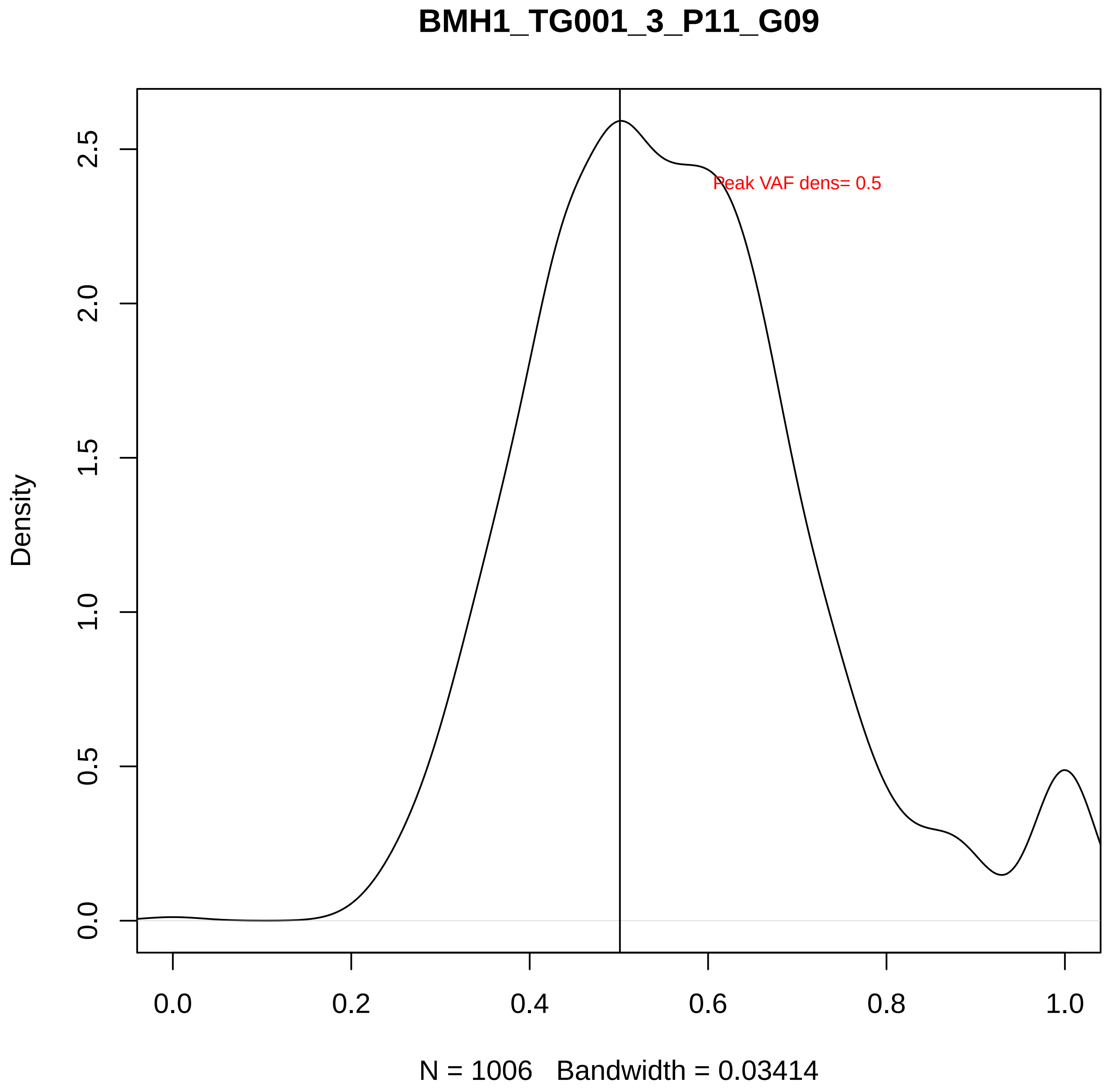
BMH1_TG001_3_P11_G09
Peak VAF dens= 0.5
Density
0.0
0.5
1.0
1.5
2.0
2.5
0.0
0.2
0.4
0.6
0.8
1.0
N = 1006   Bandwidth = 0.03414

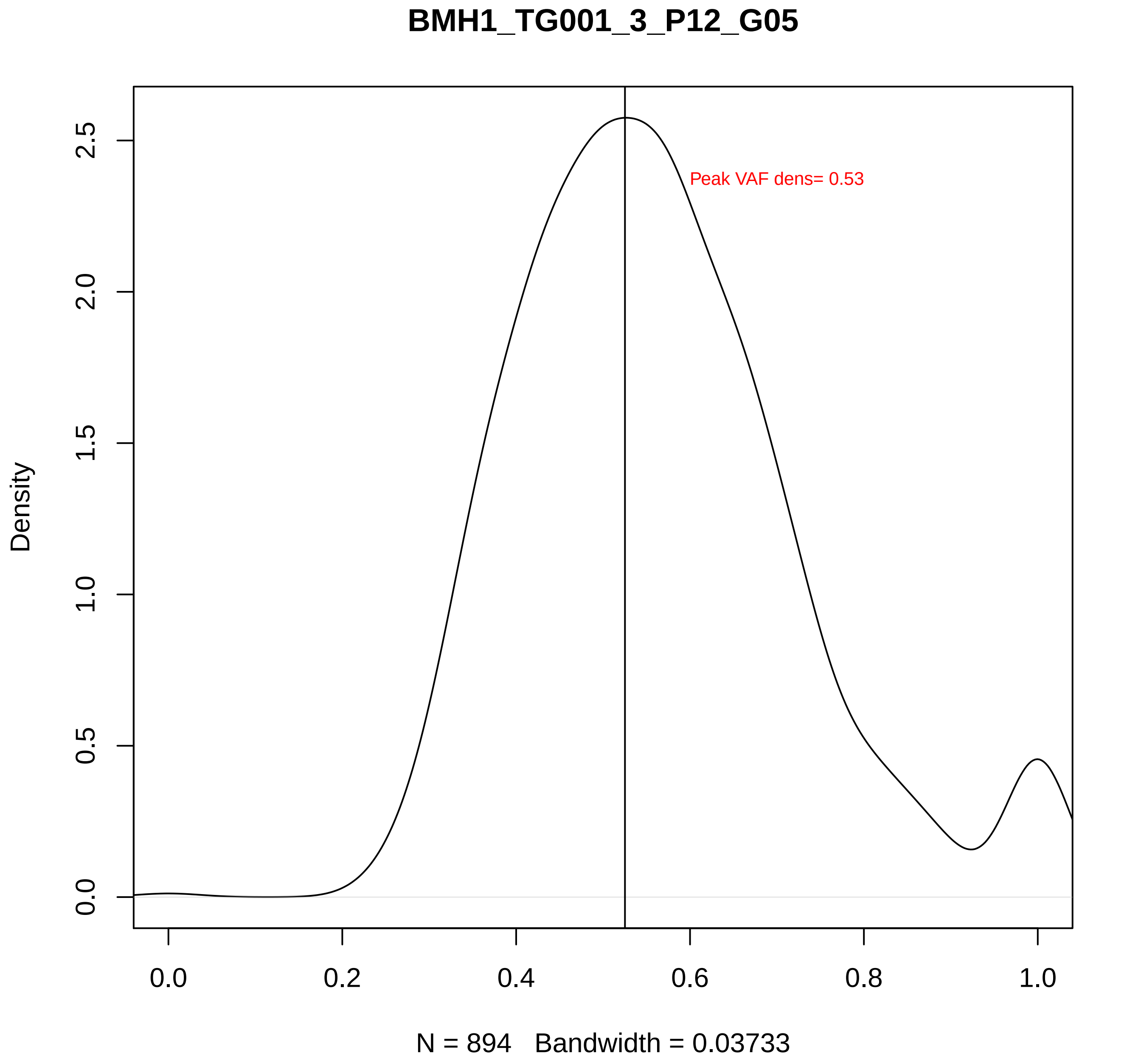
BMH1_TG001_3_P12_G05
Peak VAF dens= 0.53
Density
0.0
0.5
1.0
1.5
2.0
2.5
0.0
0.2
0.4
0.6
0.8
1.0
N = 894   Bandwidth = 0.03733

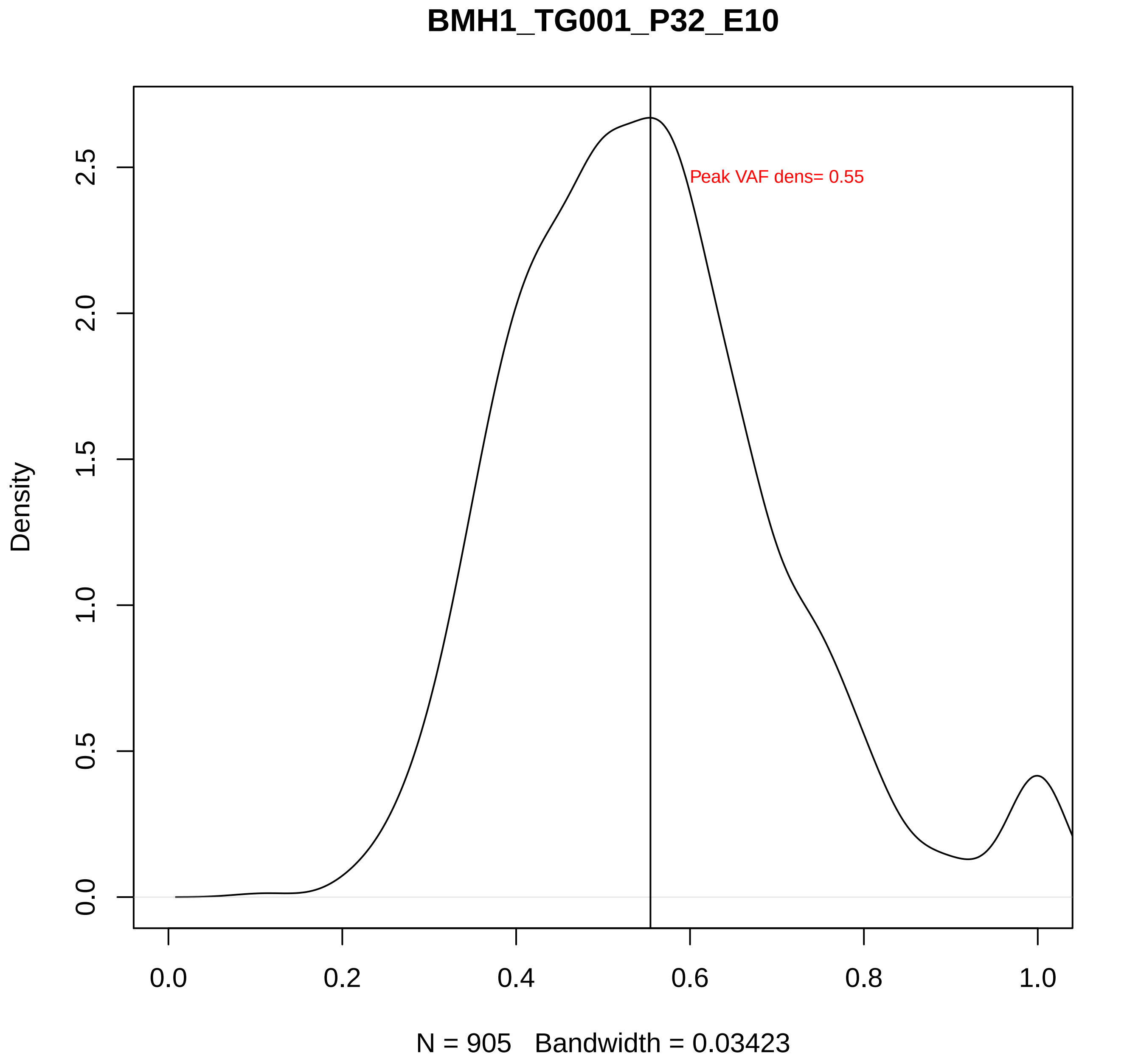

BMH1_TG001_P32_E10
Peak VAF dens= 0.55
Density
0.0
0.5
1.0
1.5
2.0
2.5
0.0
0.2
0.4
0.6
0.8
1.0
N = 905   Bandwidth = 0.03423

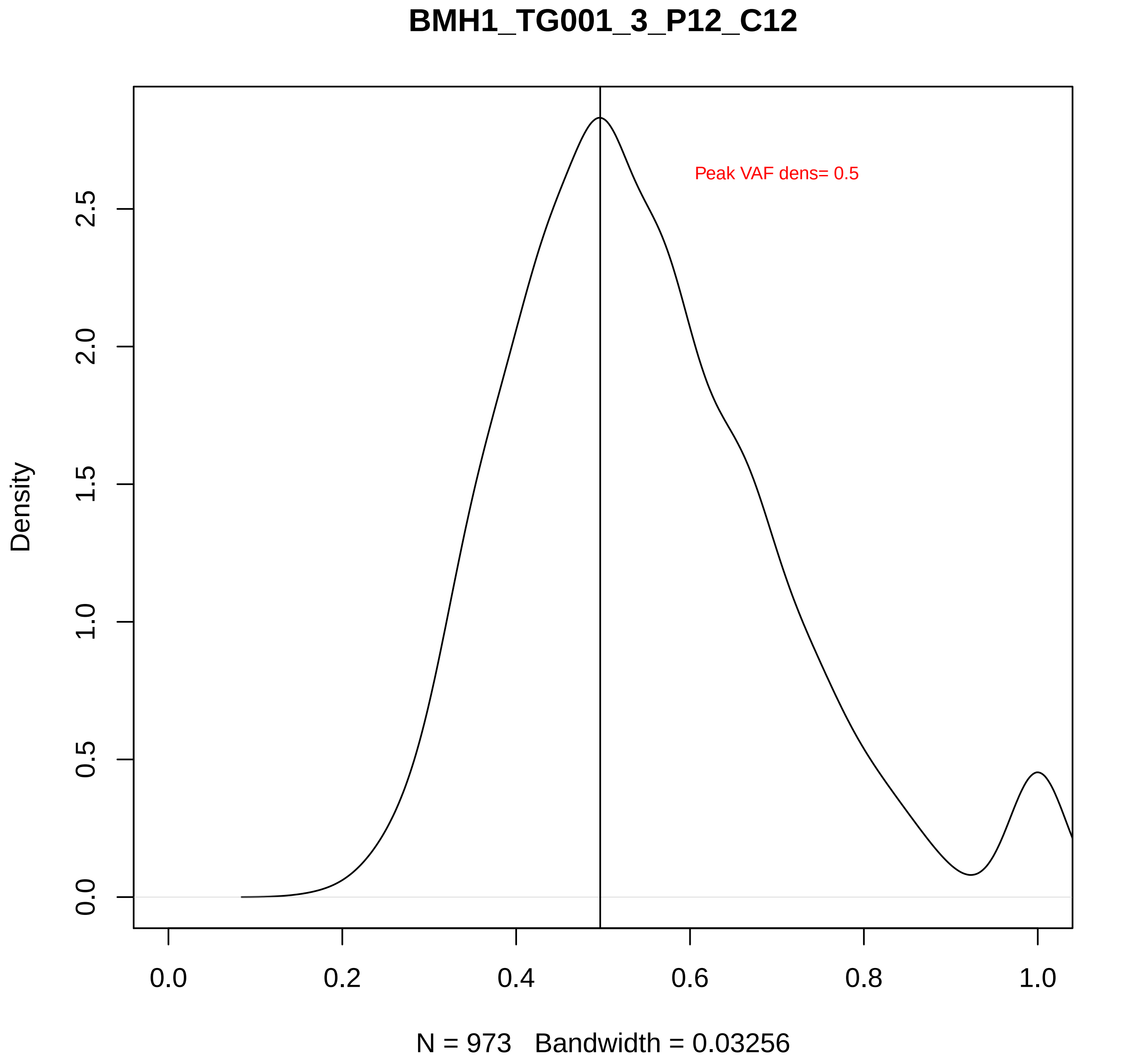
BMH1_TG001_3_P12_C12
Peak VAF dens= 0.5
Density
0.0
0.5
1.0
1.5
2.0
2.5
0.0
0.2
0.4
0.6
0.8
1.0
N = 973   Bandwidth = 0.03256

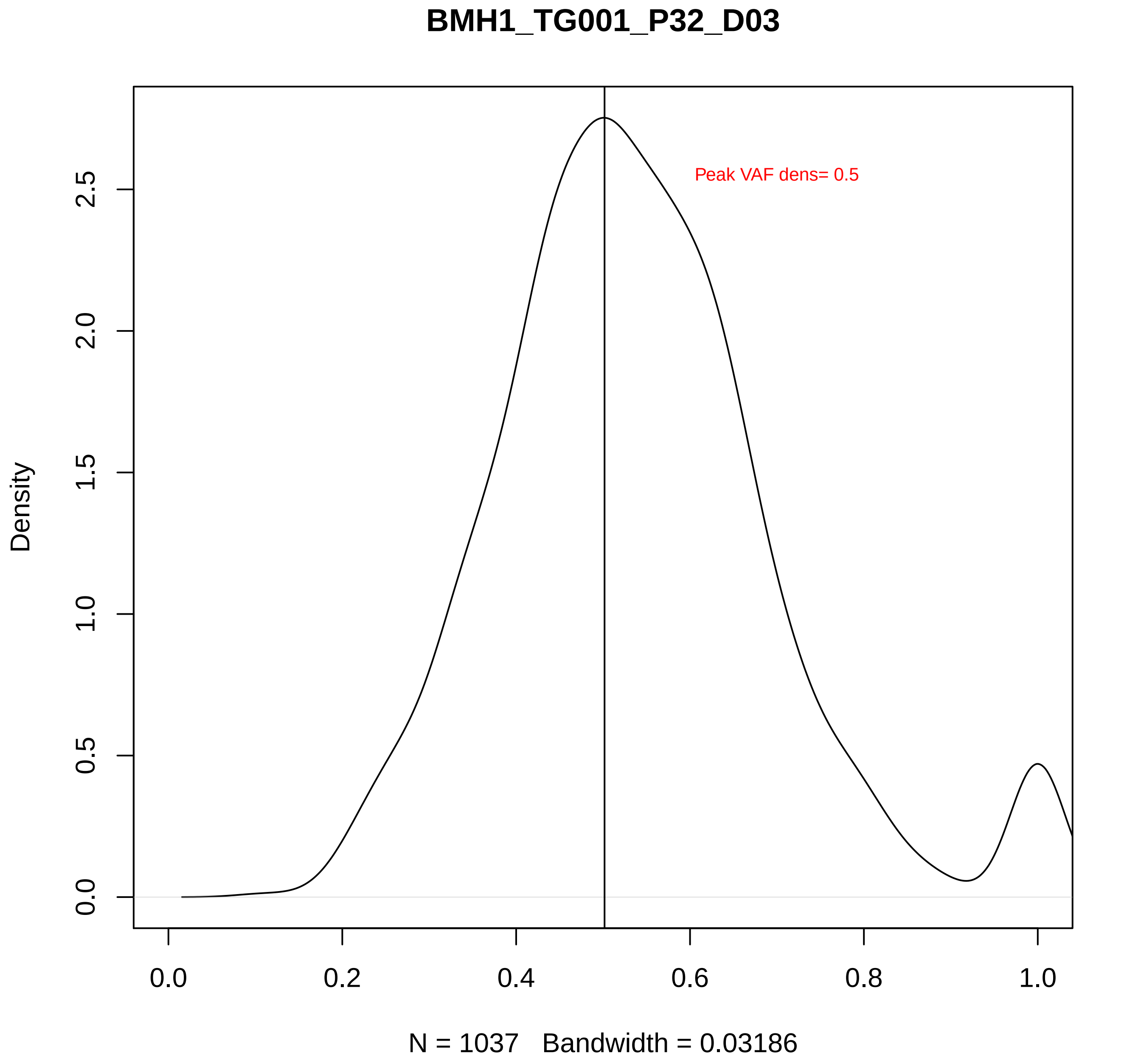

BMH1_TG001_P32_D03
Peak VAF dens= 0.5
Density
0.0
0.5
1.0
1.5
2.0
2.5
0.0
0.2
0.4
0.6
0.8
1.0
N = 1037 Bandwidth = 0.03186

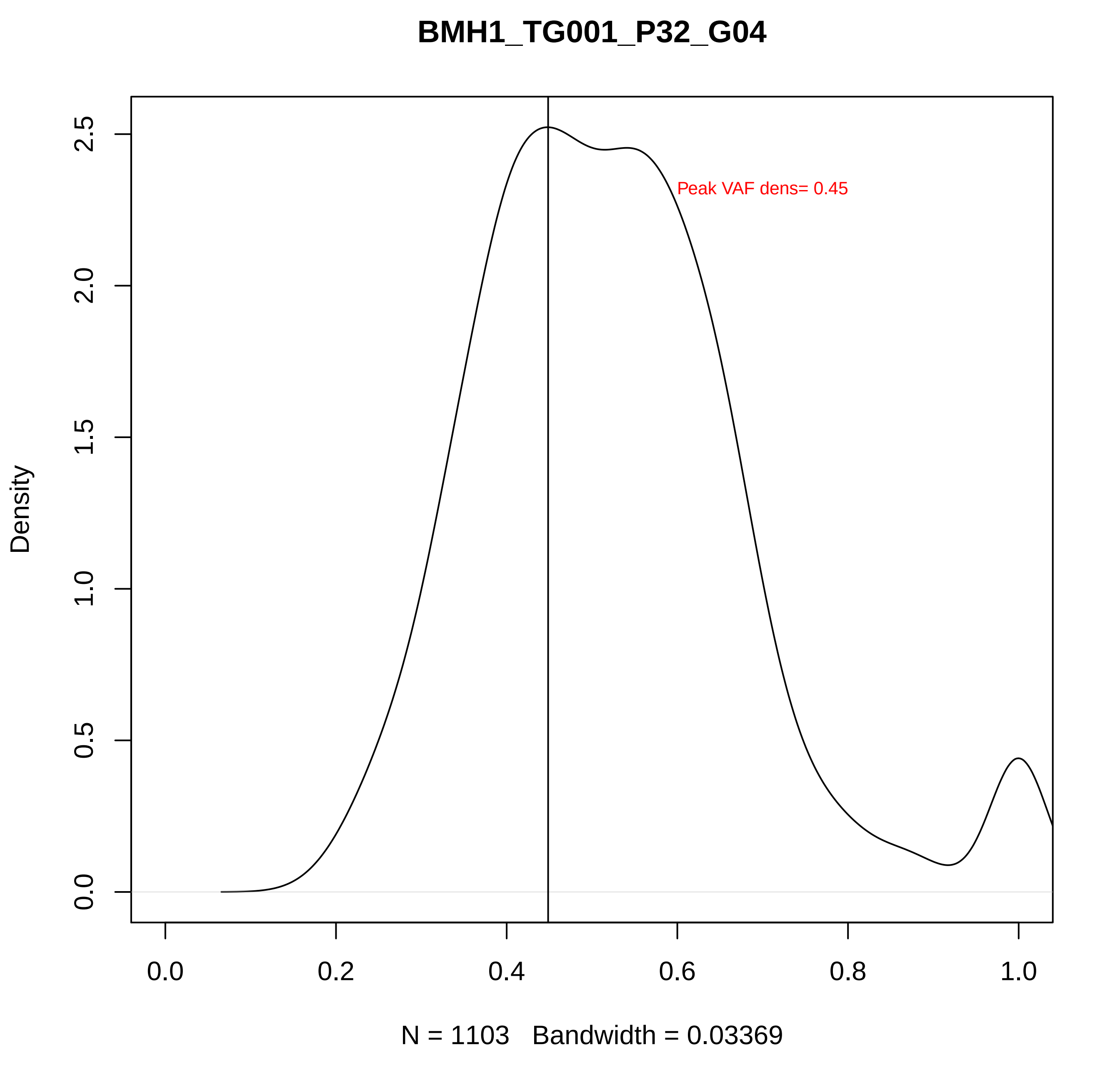

BMH1_TG001_P32_G04
Peak VAF dens= 0.45
Density
2.5
2.0
1.5
1.0
0.5
0.0
0.0
0.2
0.4
0.6
0.8
1.0
N = 1103   Bandwidth = 0.03369

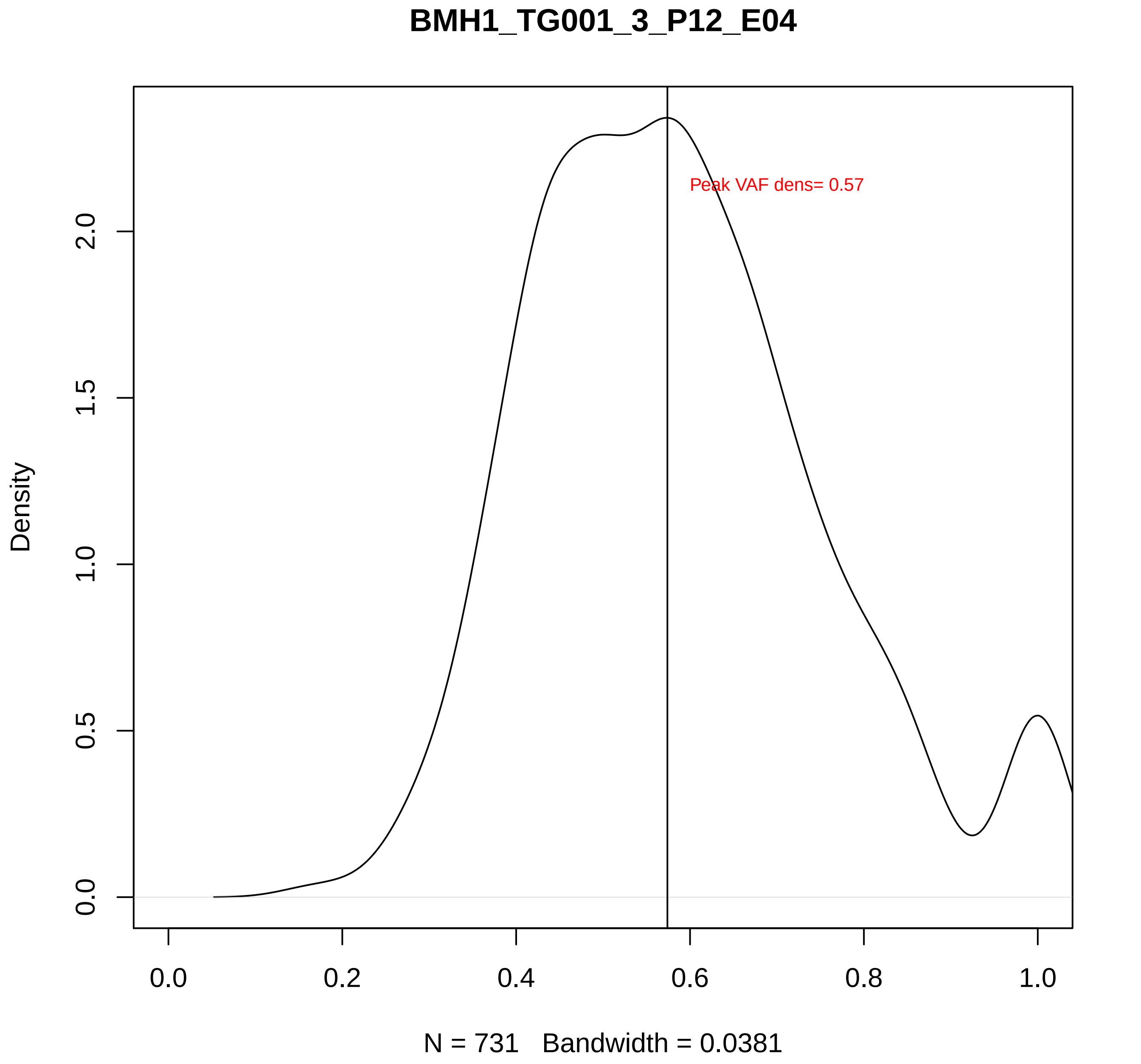
BMH1_TG001_3_P12_E04
Peak VAF dens= 0.57
Density
0.0
0.5
1.0
1.5
2.0
0.0
0.2
0.4
0.6
0.8
1.0
N = 731   Bandwidth = 0.0381

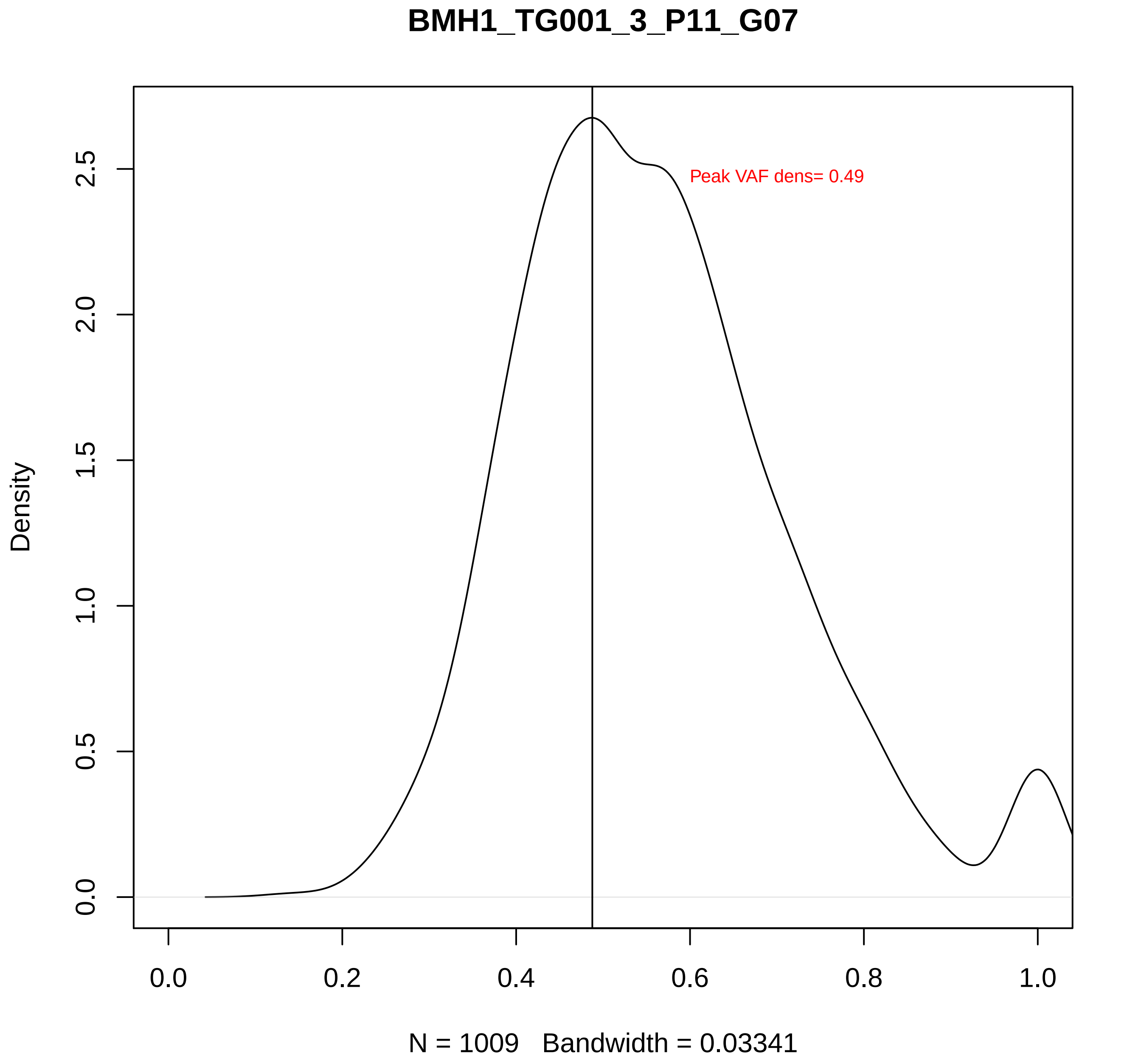
BMH1_TG001_3_P11_G07
Peak VAF dens= 0.49
Density
0.0
0.5
1.0
1.5
2.0
2.5
0.0
0.2
0.4
0.6
0.8
1.0
N = 1009   Bandwidth = 0.03341

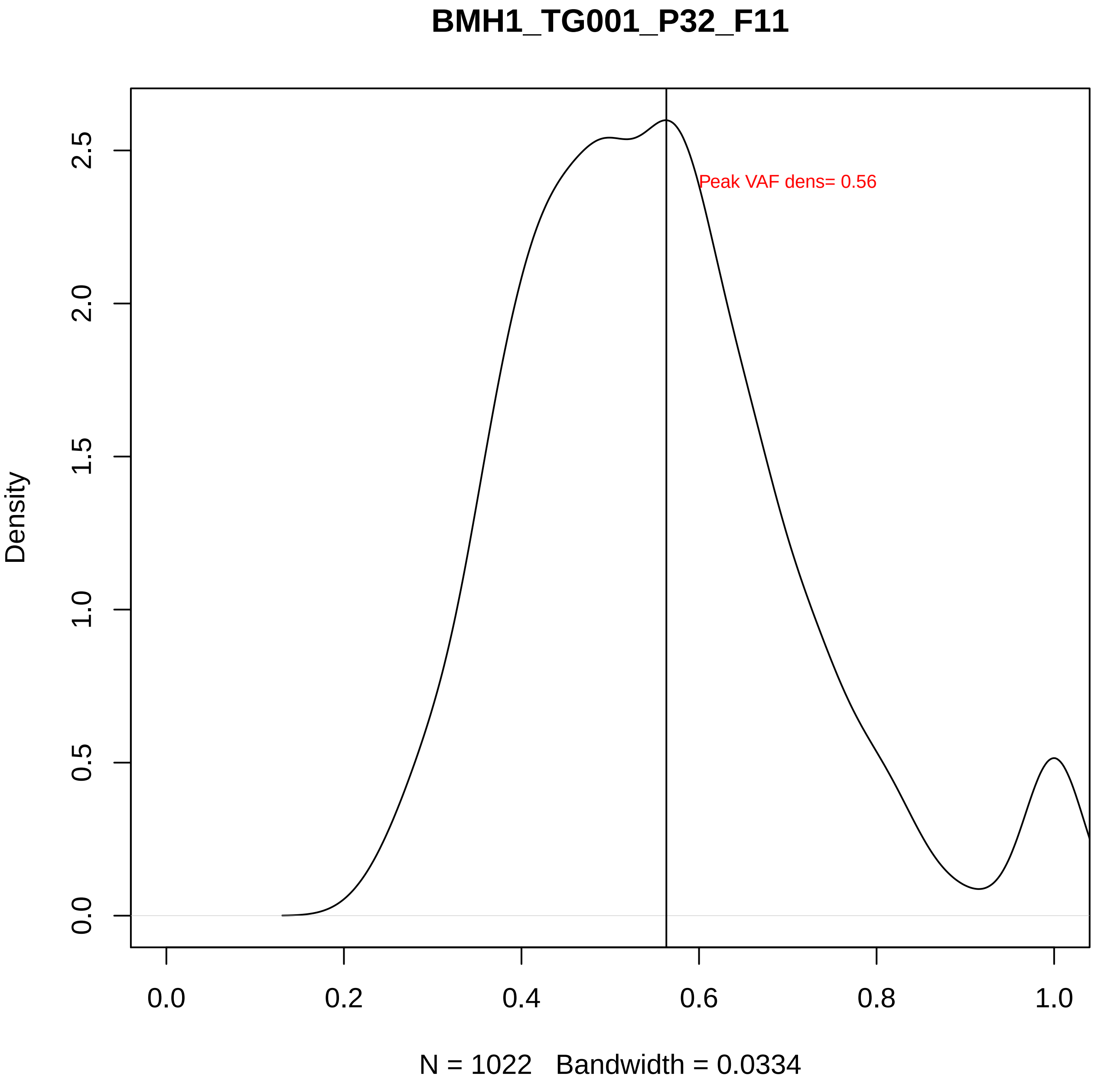
BMH1_TG001_P32_F11
Peak VAF dens= 0.56
Density
0.0
0.5
1.0
1.5
2.0
2.5
0.0
0.2
0.4
0.6
0.8
1.0
N = 1022   Bandwidth = 0.0334

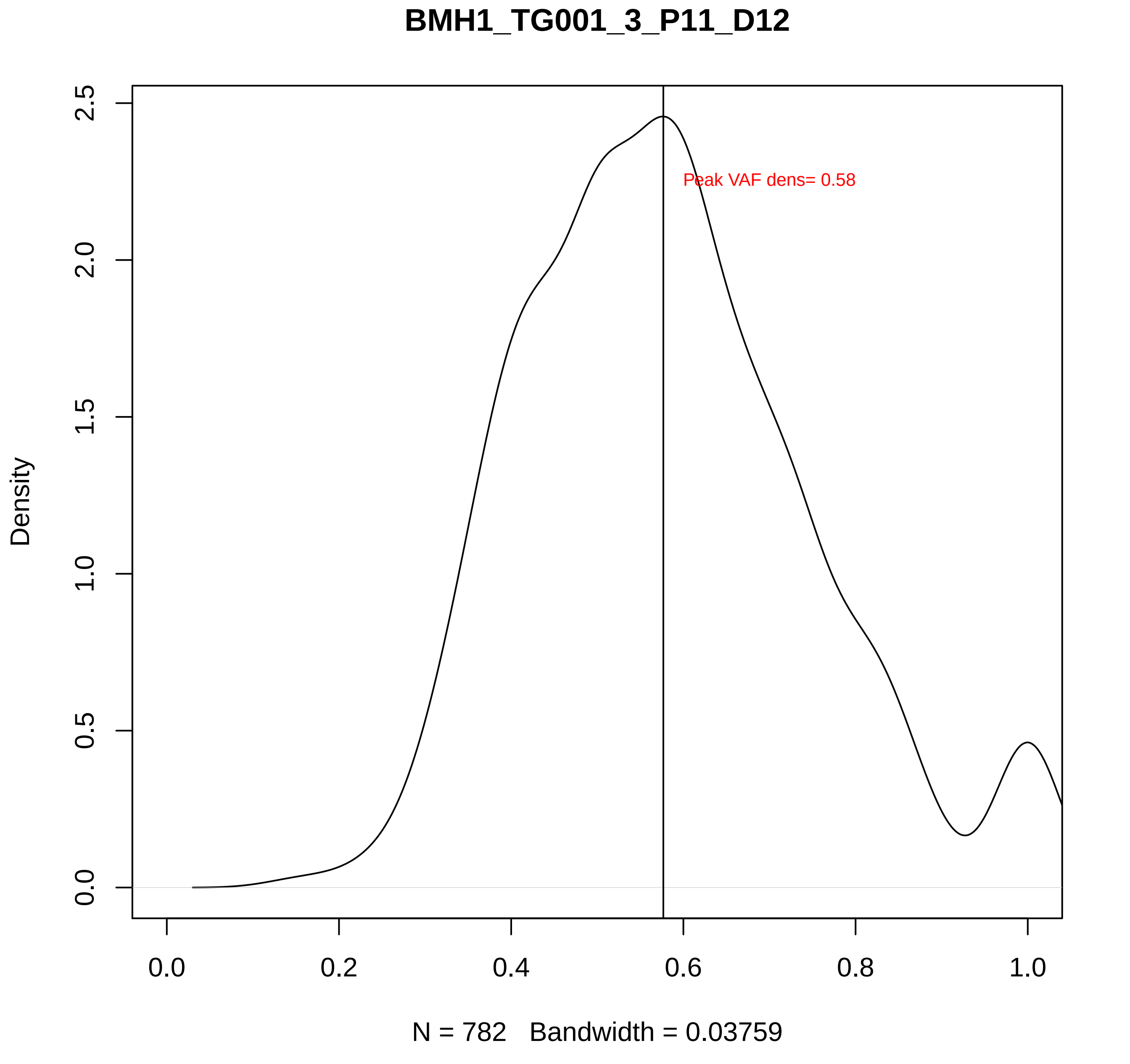

BMH1_TG001_3_P11_D12
Density
0.0
0.5
1.0
1.5
2.0
2.5
0.0
0.2
0.4
0.6
0.8
1.0
Peak VAF dens= 0.58
N = 782 Bandwidth = 0.03759

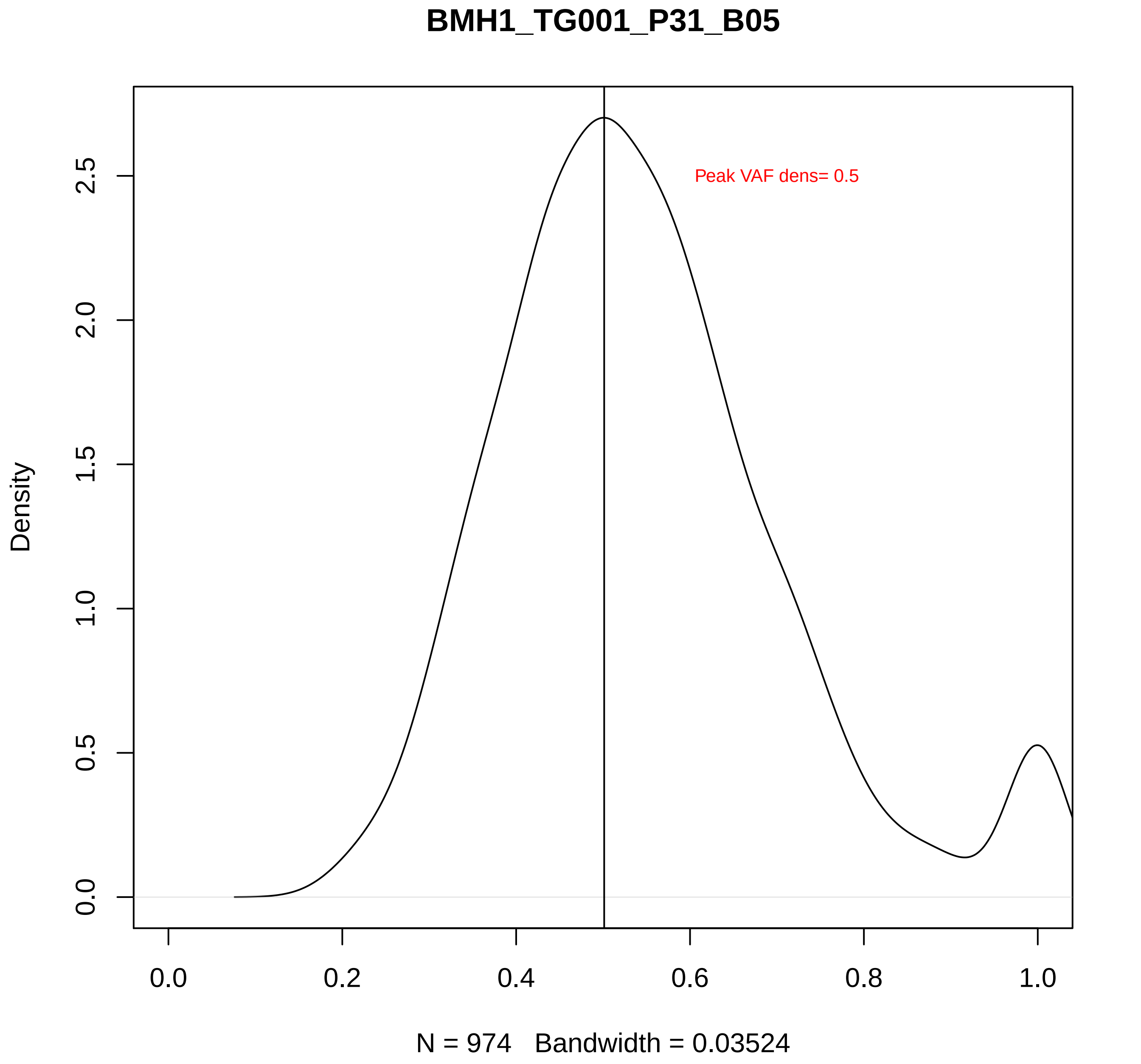
BMH1_TG001_P31_B05
Peak VAF dens= 0.5
Density
0.0
0.5
1.0
1.5
2.0
2.5
0.0
0.2
0.4
0.6
0.8
1.0
N = 974   Bandwidth = 0.03524

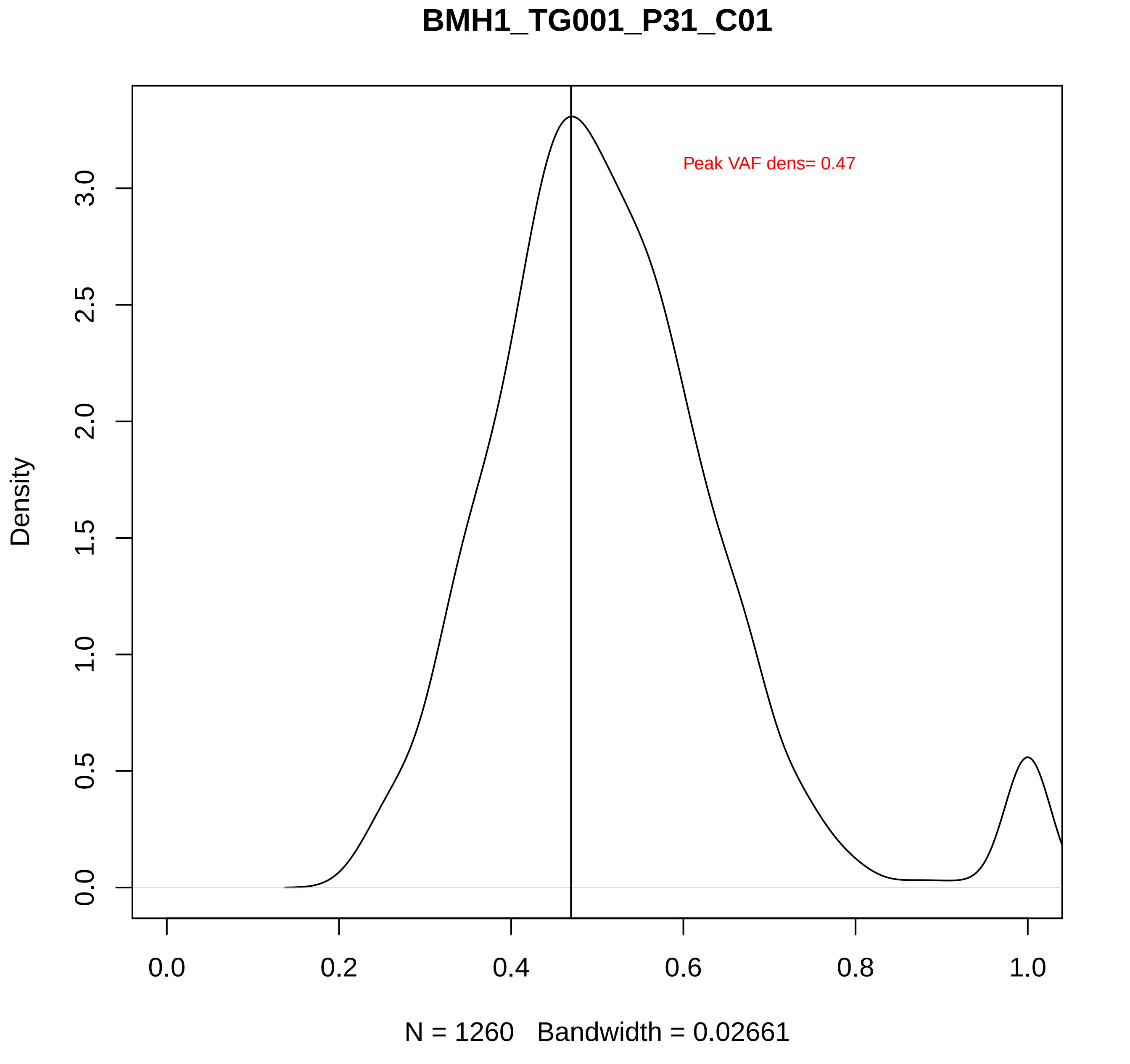
BMH1_TG001_P31_C01
Peak VAF dens= 0.47
Density
0.0
0.5
1.0
1.5
2.0
2.5
3.0
0.0
0.2
0.4
0.6
0.8
1.0
N = 1260 Bandwidth = 0.02661

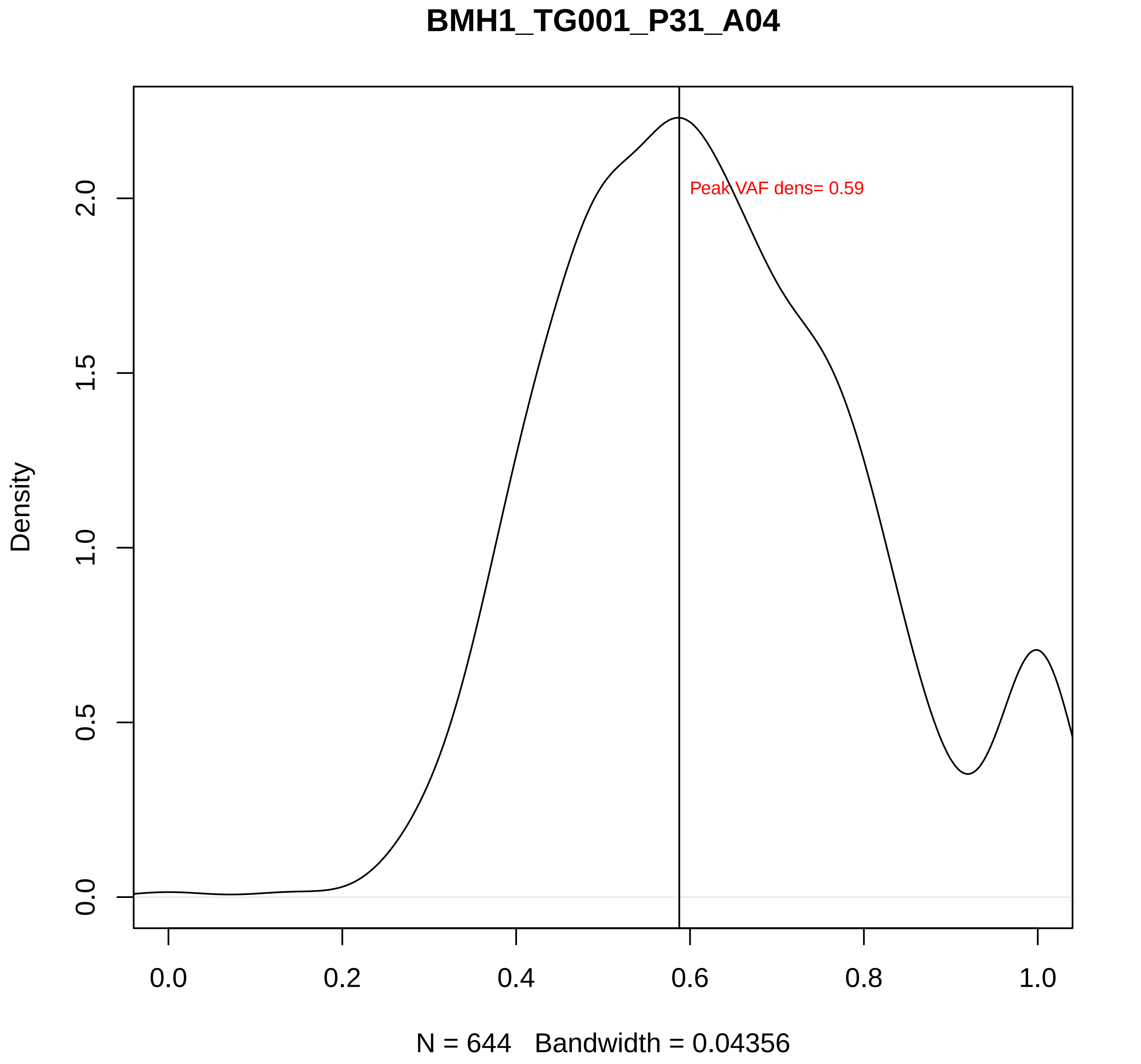

BMH1_TG001_P31_A04
Peak VAF dens= 0.59
Density
0.0
0.5
1.0
1.5
2.0
0.0
0.2
0.4
0.6
0.8
1.0
N = 644   Bandwidth = 0.04356

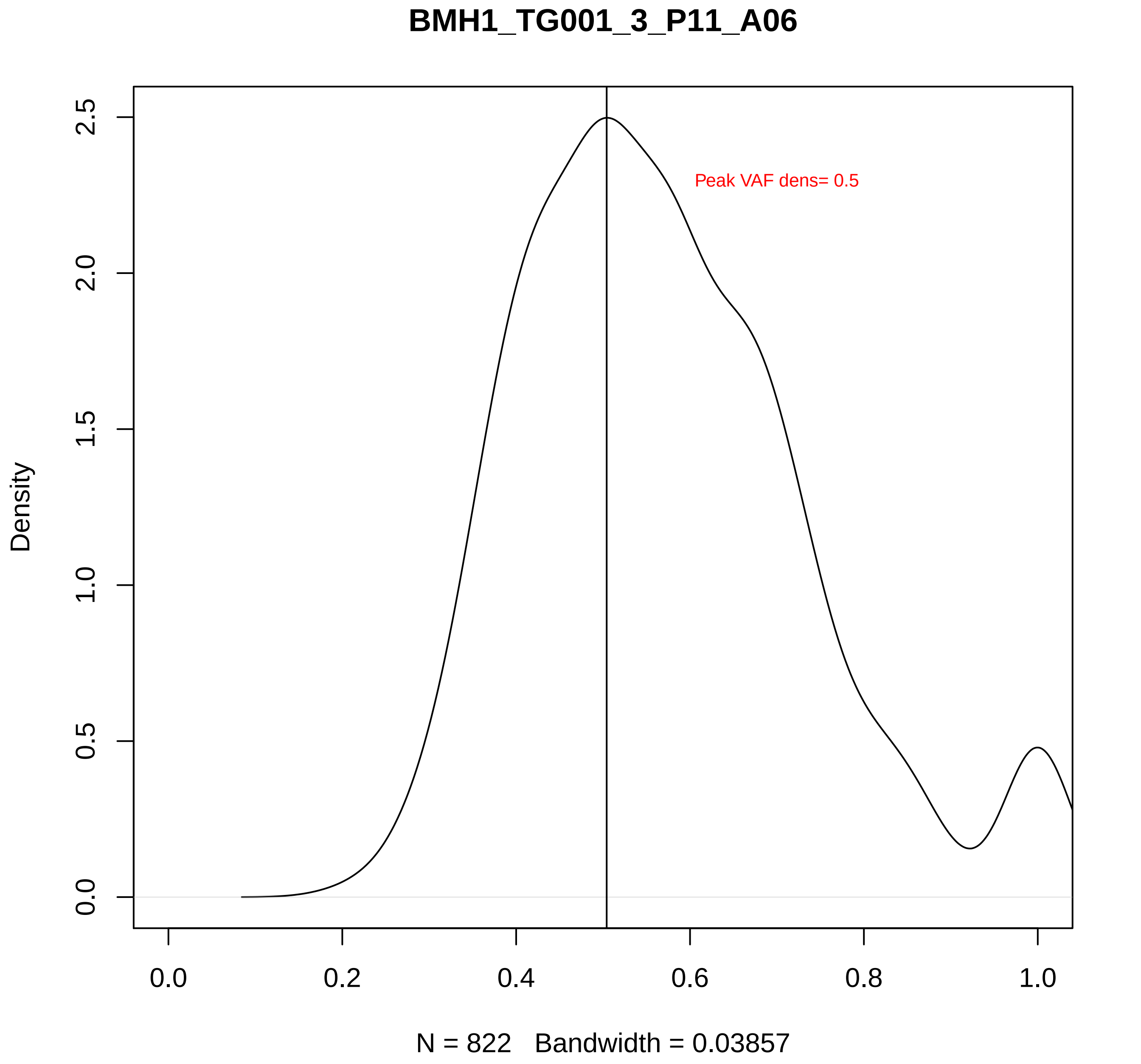

BMH1_TG001_3_P11_A06
Peak VAF dens= 0.5
Density
0.0
0.5
1.0
1.5
2.0
2.5
0.0
0.2
0.4
0.6
0.8
1.0
N = 822   Bandwidth = 0.03857

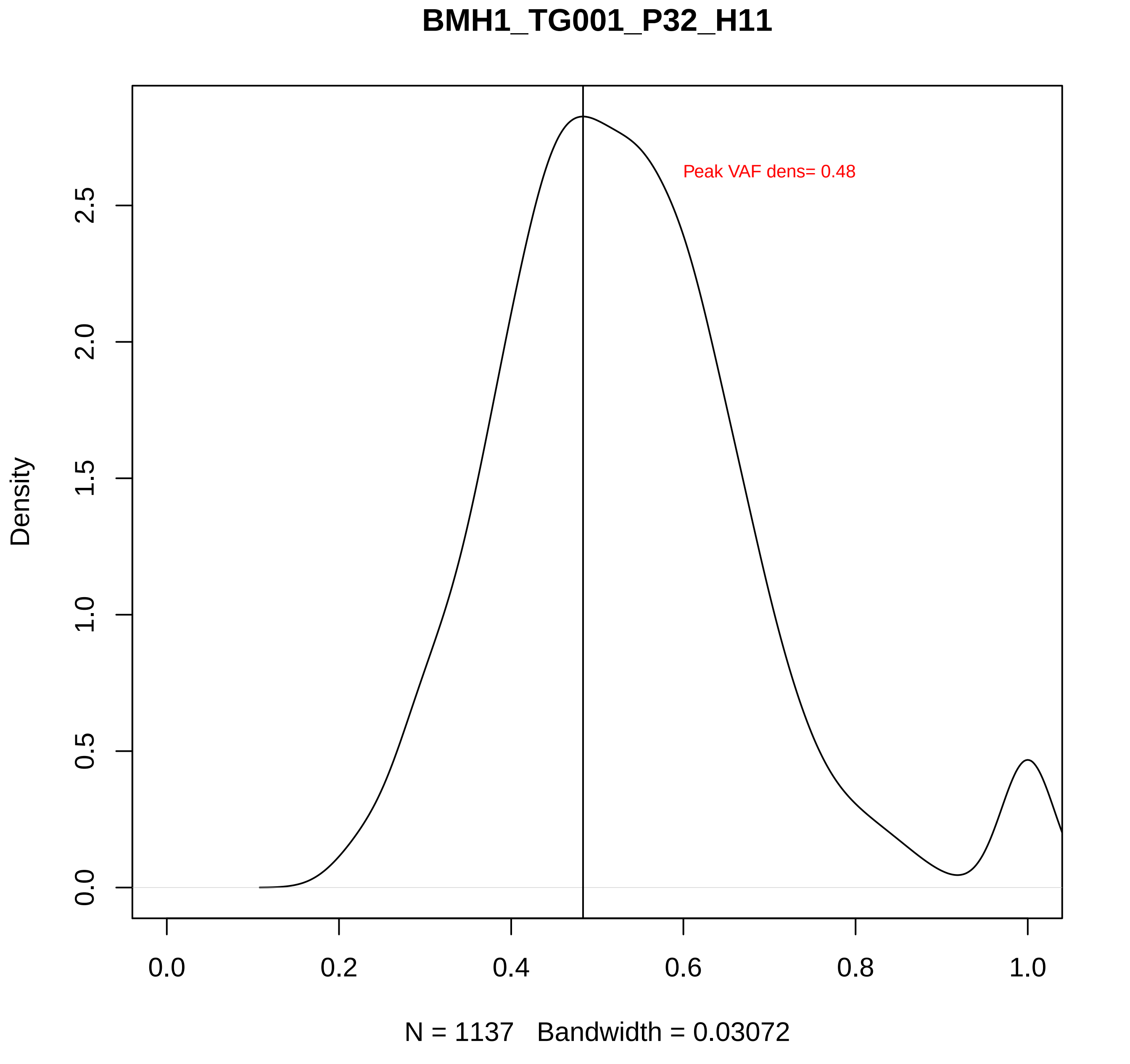
BMH1_TG001_P32_H11
Peak VAF dens= 0.48
Density
0.0
0.5
1.0
1.5
2.0
2.5
0.0
0.2
0.4
0.6
0.8
1.0
N = 1137 Bandwidth = 0.03072

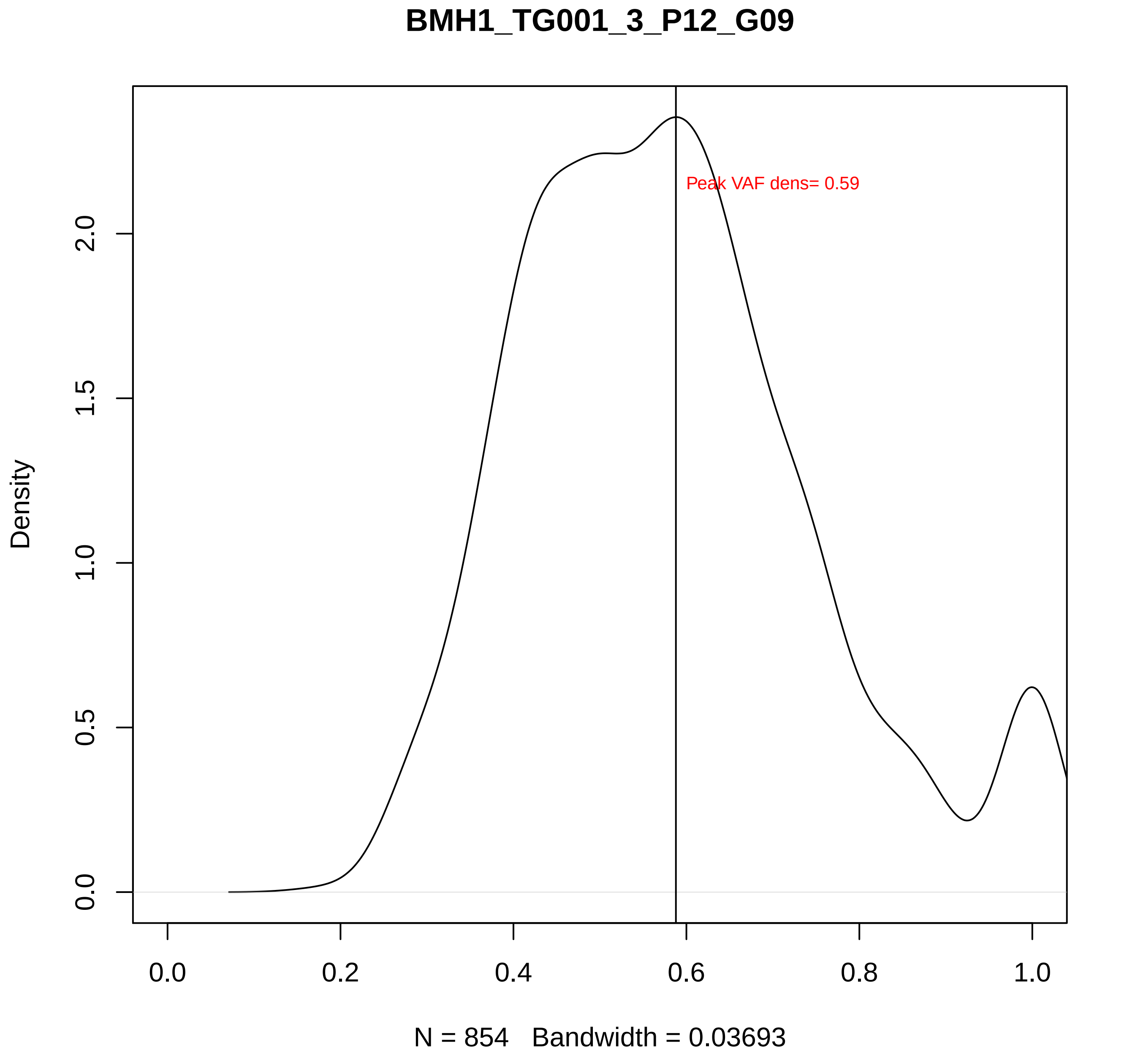
BMH1_TG001_3_P12_G09
Peak VAF dens= 0.59
Density
0.0
0.5
1.0
1.5
2.0
0.0
0.2
0.4
0.6
0.8
1.0
N = 854   Bandwidth = 0.03693

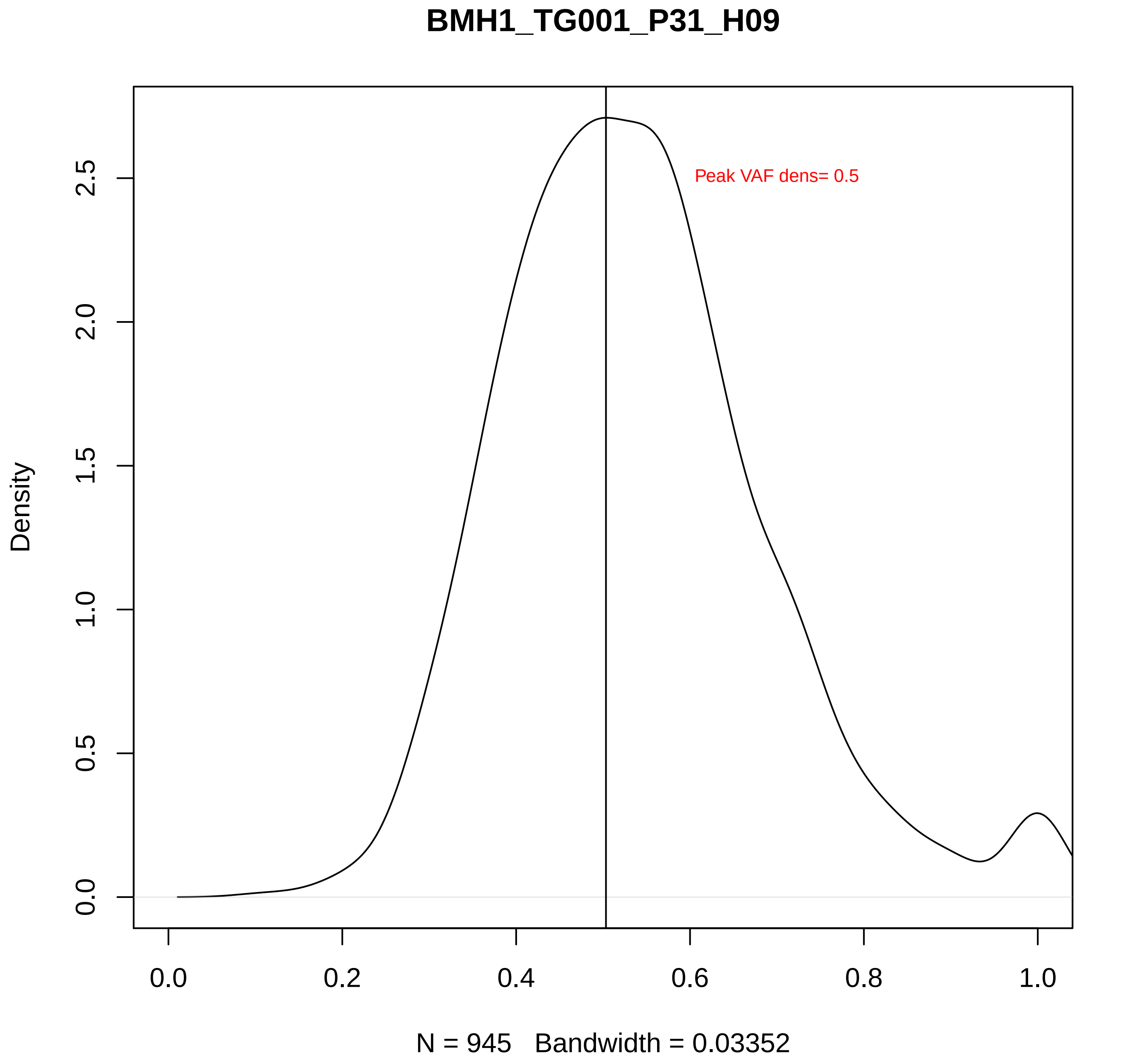
BMH1_TG001_P31_H09
Peak VAF dens= 0.5
Density
0.0
0.5
1.0
1.5
2.0
2.5
0.0
0.2
0.4
0.6
0.8
1.0
N = 945   Bandwidth = 0.03352

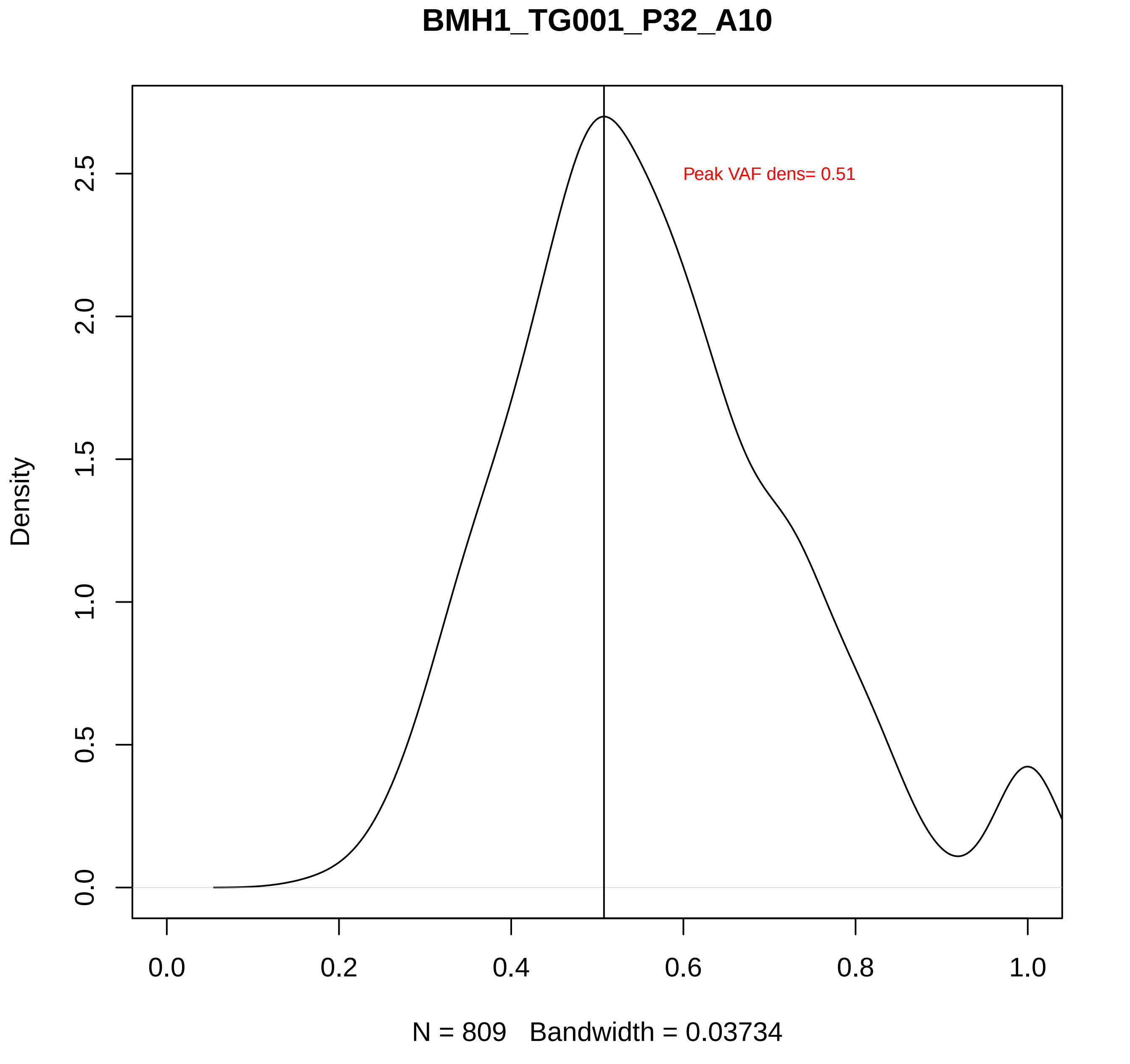
BMH1_TG001_P32_A10
Peak VAF dens= 0.51
Density
0.0
0.5
1.0
1.5
2.0
2.5
0.0
0.2
0.4
0.6
0.8
1.0
N = 809   Bandwidth = 0.03734

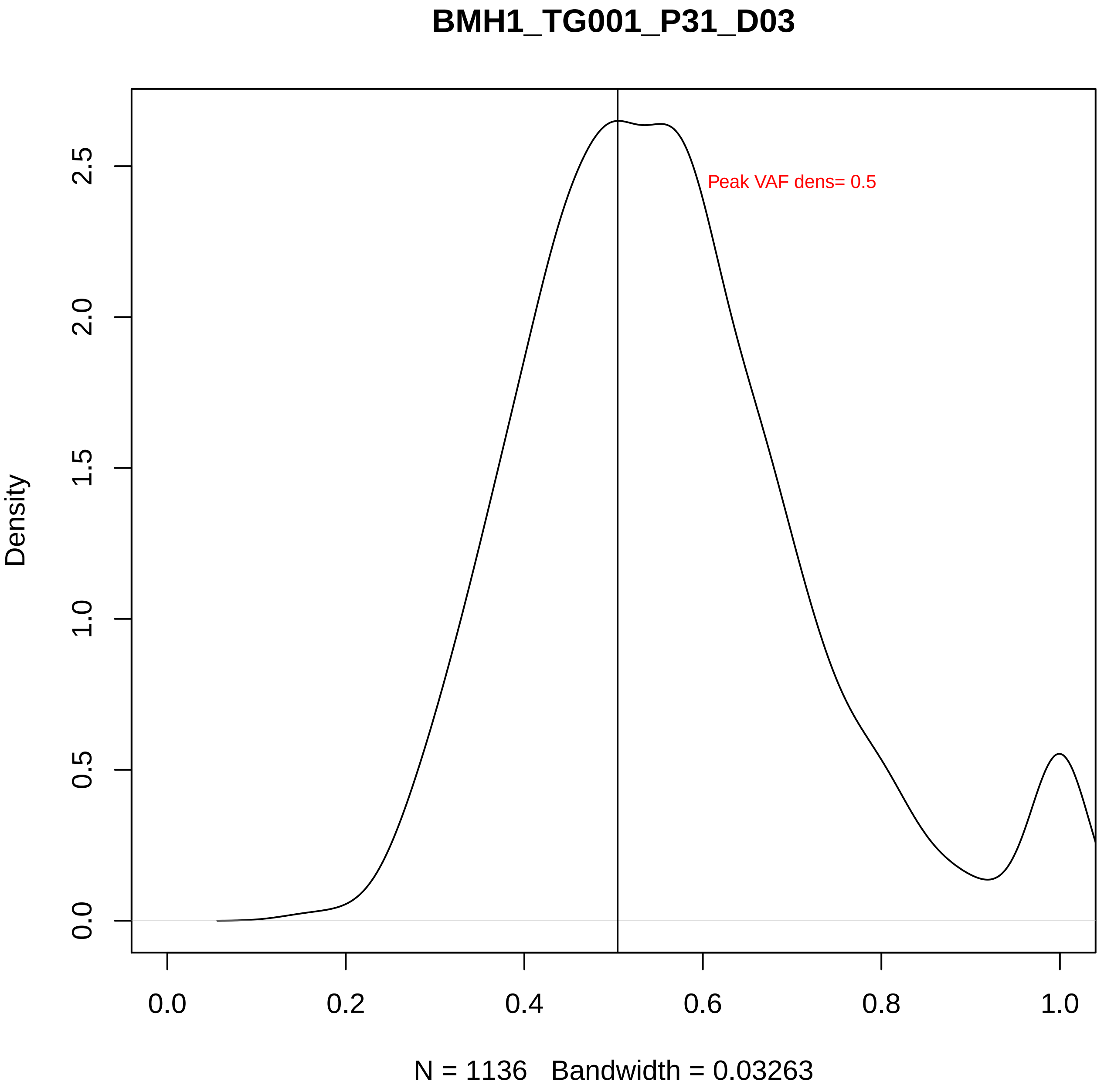

BMH1_TG001_P31_D03
Peak VAF dens= 0.5
Density
0.0
0.5
1.0
1.5
2.0
2.5
0.0
0.2
0.4
0.6
0.8
1.0
N = 1136   Bandwidth = 0.03263

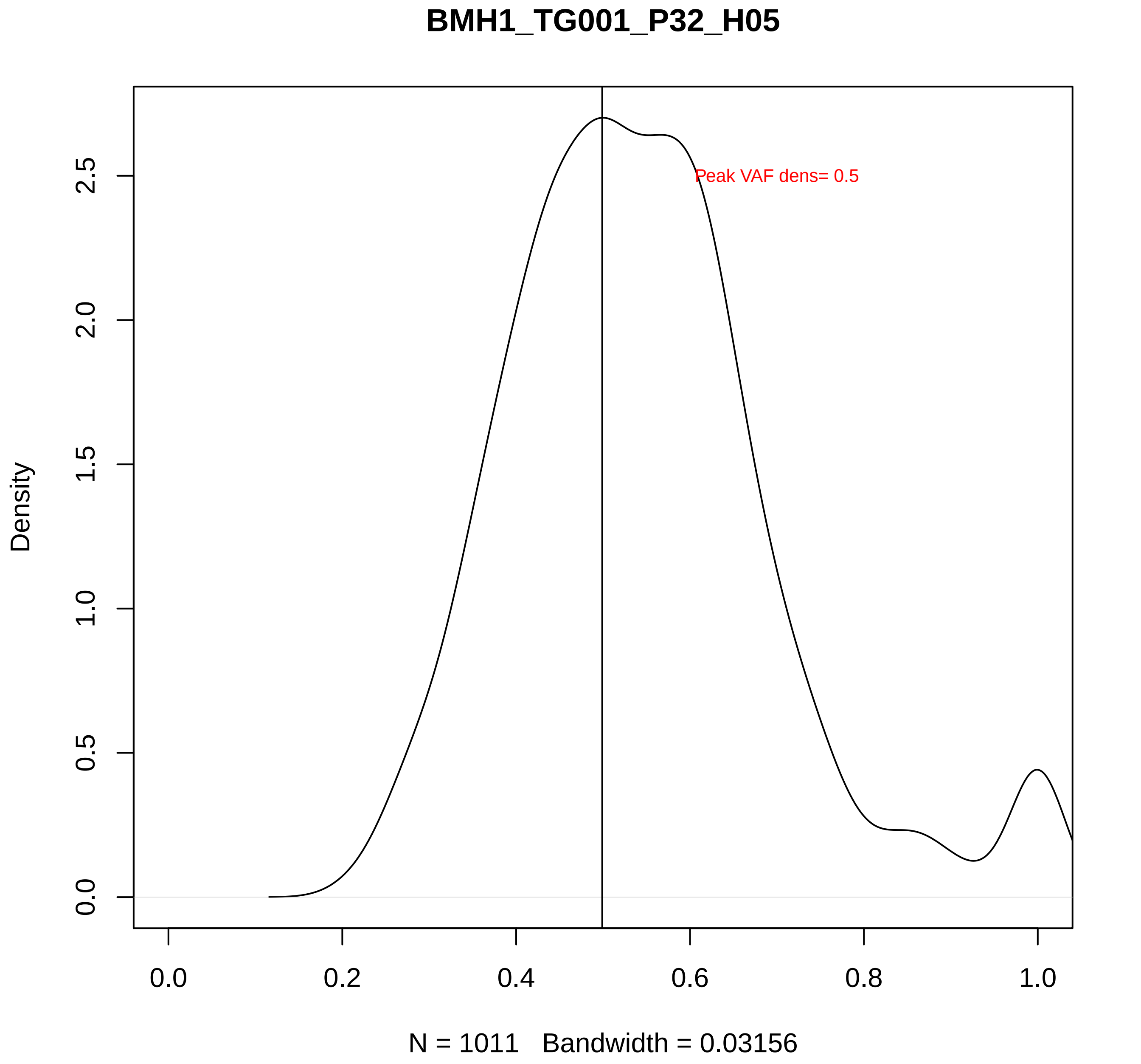

BMH1_TG001_P32_H05
Peak VAF dens= 0.5
Density
0.0
0.5
1.0
1.5
2.0
2.5
0.0
0.2
0.4
0.6
0.8
1.0
N = 1011   Bandwidth = 0.03156

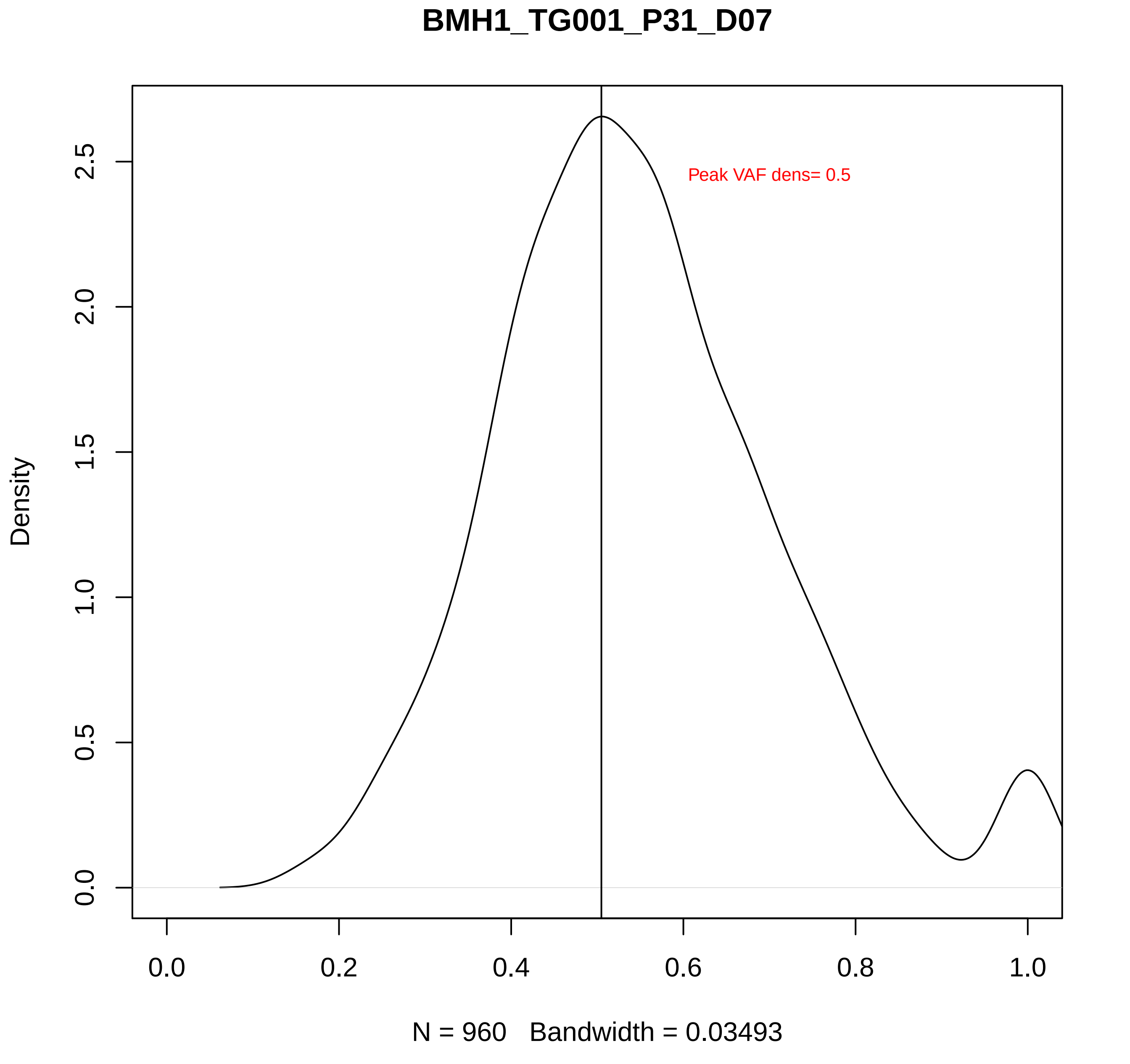
BMH1_TG001_P31_D07
Peak VAF dens= 0.5
Density
0.0
0.5
1.0
1.5
2.0
2.5
0.0
0.2
0.4
0.6
0.8
1.0
N = 960   Bandwidth = 0.03493

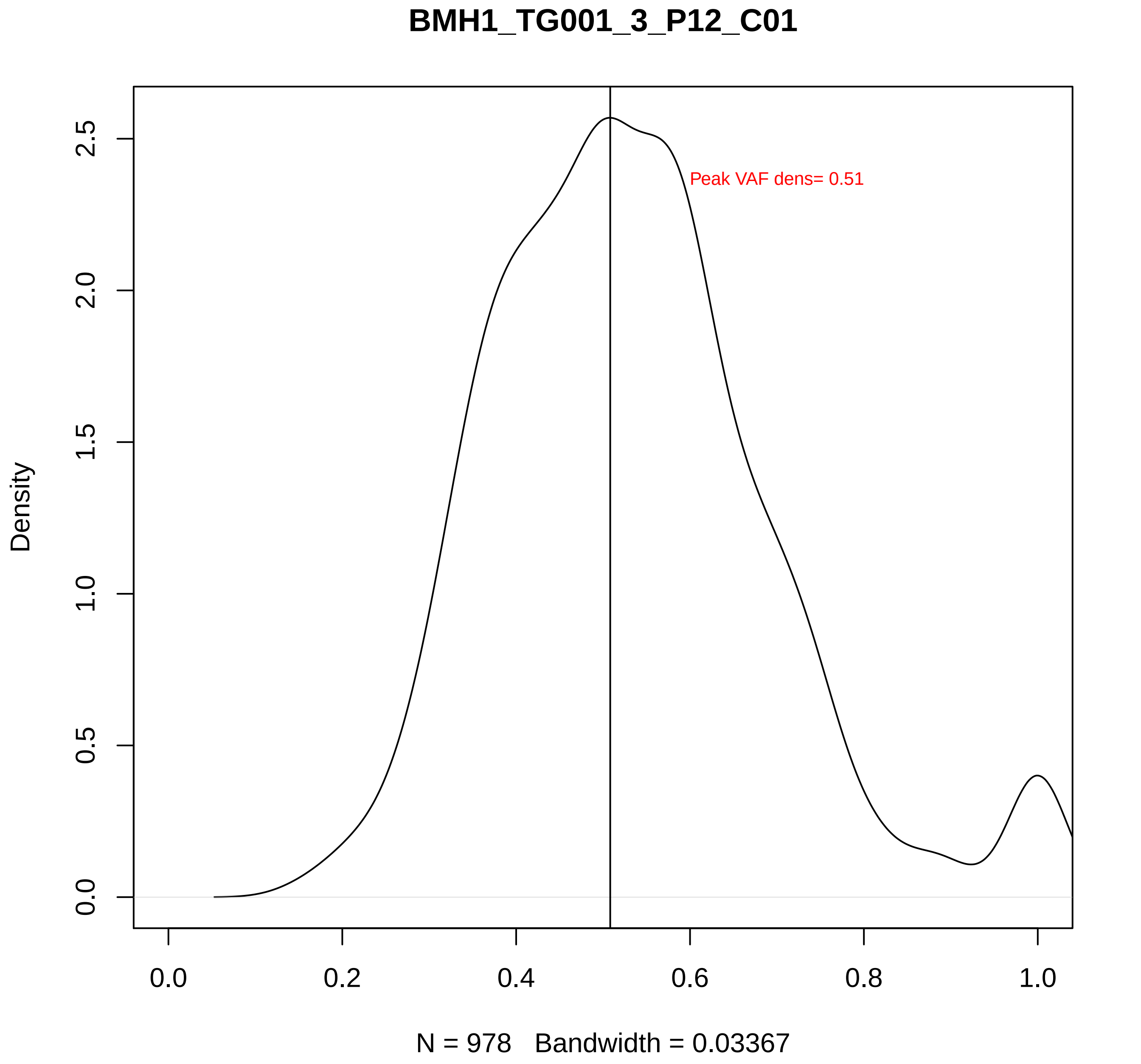
BMH1_TG001_3_P12_C01
Peak VAF dens= 0.51
Density
0.0
0.5
1.0
1.5
2.0
2.5
0.0
0.2
0.4
0.6
0.8
1.0
N = 978   Bandwidth = 0.03367

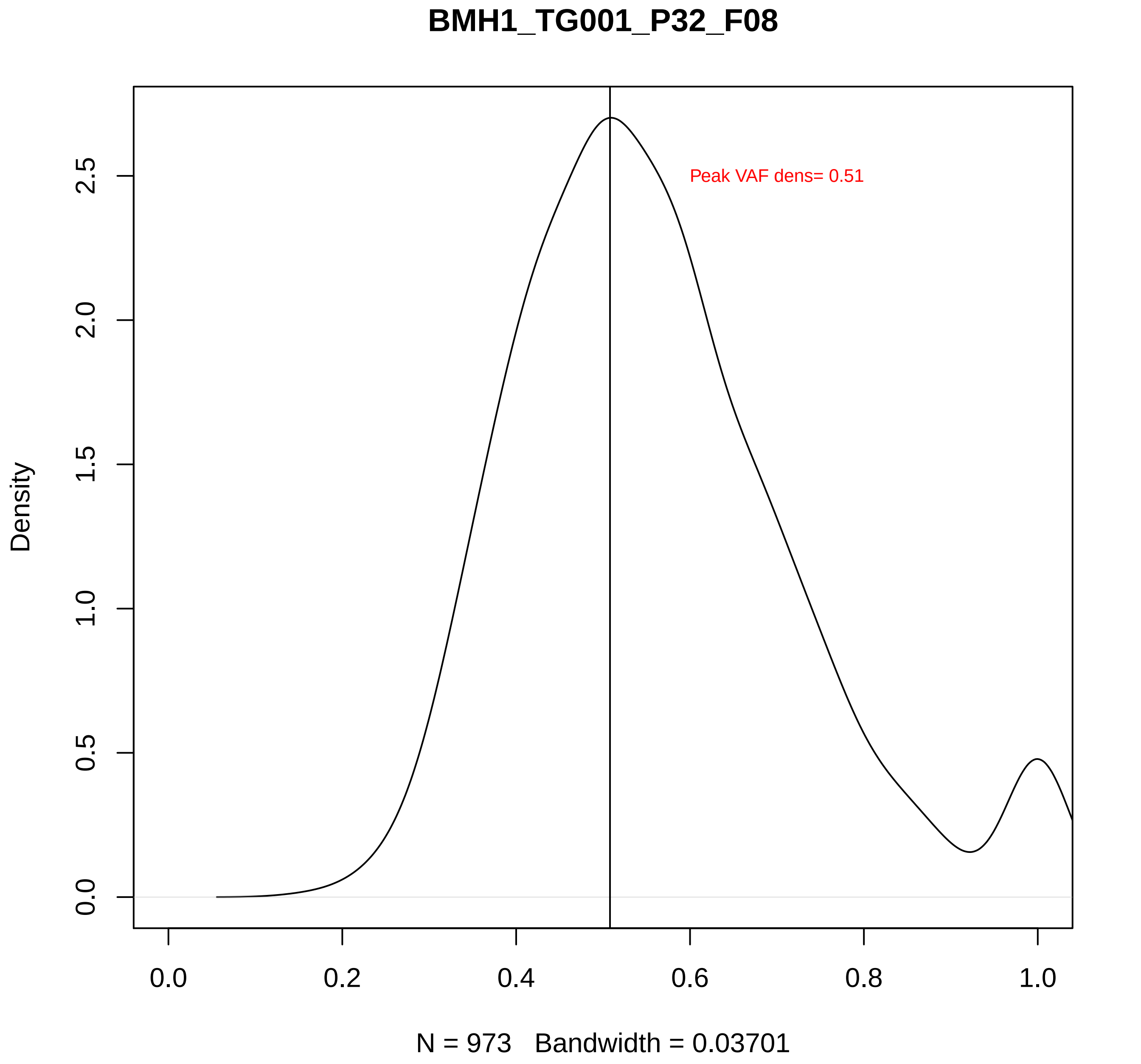

BMH1_TG001_P32_F08
Peak VAF dens= 0.51
Density
0.0
0.5
1.0
1.5
2.0
2.5
0.0
0.2
0.4
0.6
0.8
1.0
N = 973 Bandwidth = 0.03701

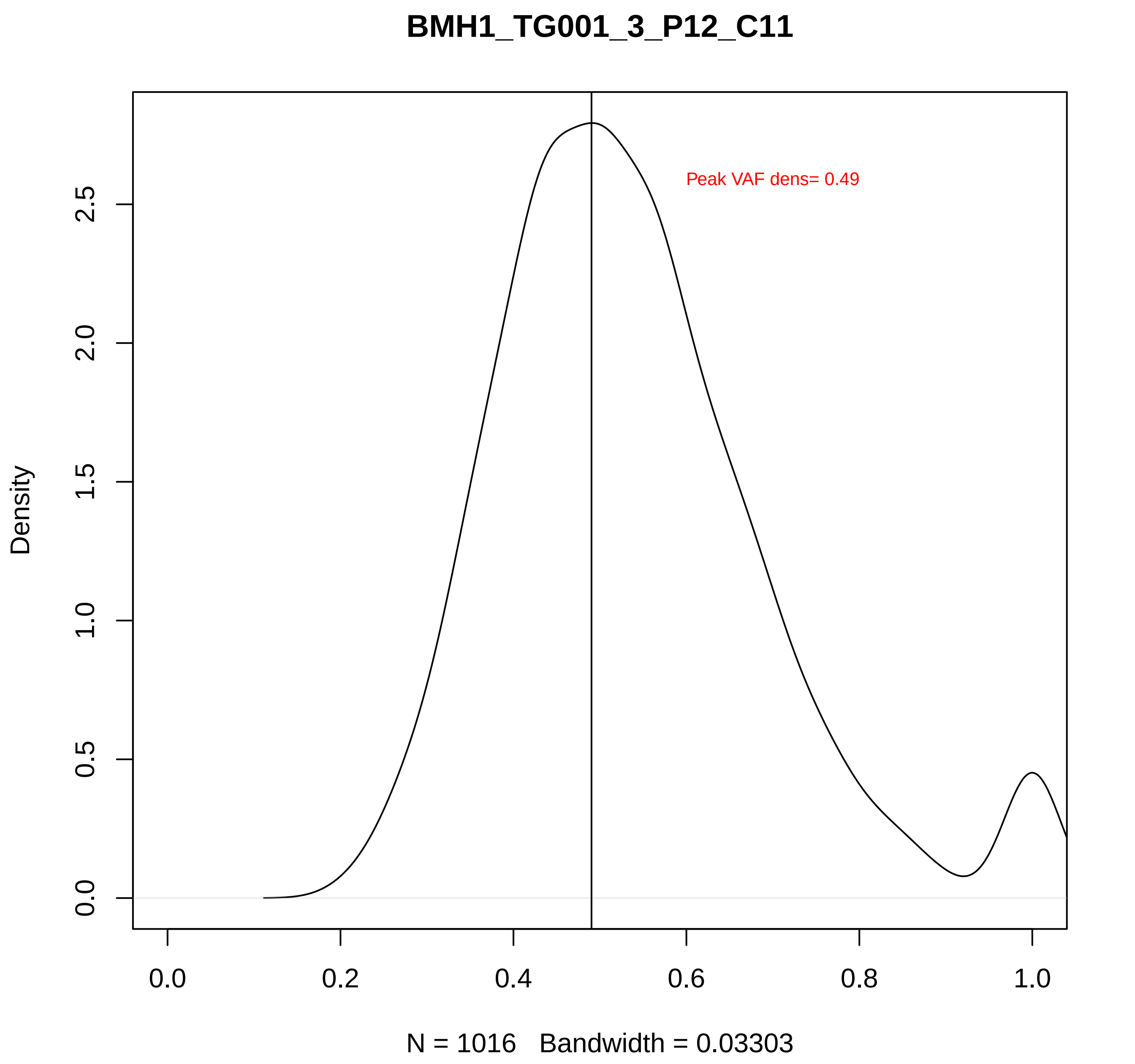

BMH1_TG001_3_P12_C11
Peak VAF dens= 0.49
Density
0.0
0.5
1.0
1.5
2.0
2.5
0.0
0.2
0.4
0.6
0.8
1.0
N = 1016   Bandwidth = 0.03303

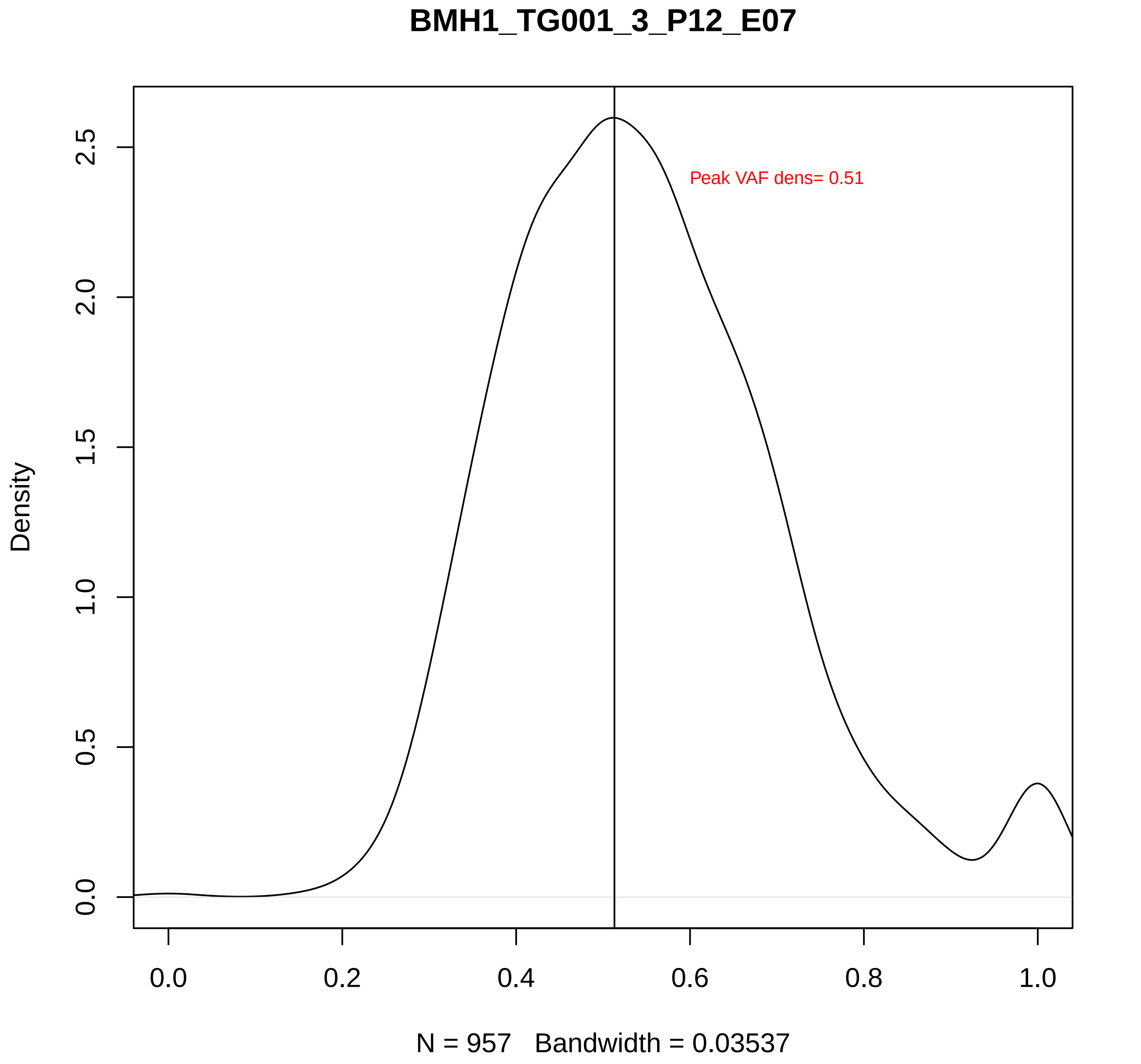
BMH1_TG001_3_P12_E07
Peak VAF dens= 0.51
Density
0.0
0.5
1.0
1.5
2.0
2.5
0.0
0.2
0.4
0.6
0.8
1.0
N = 957   Bandwidth = 0.03537

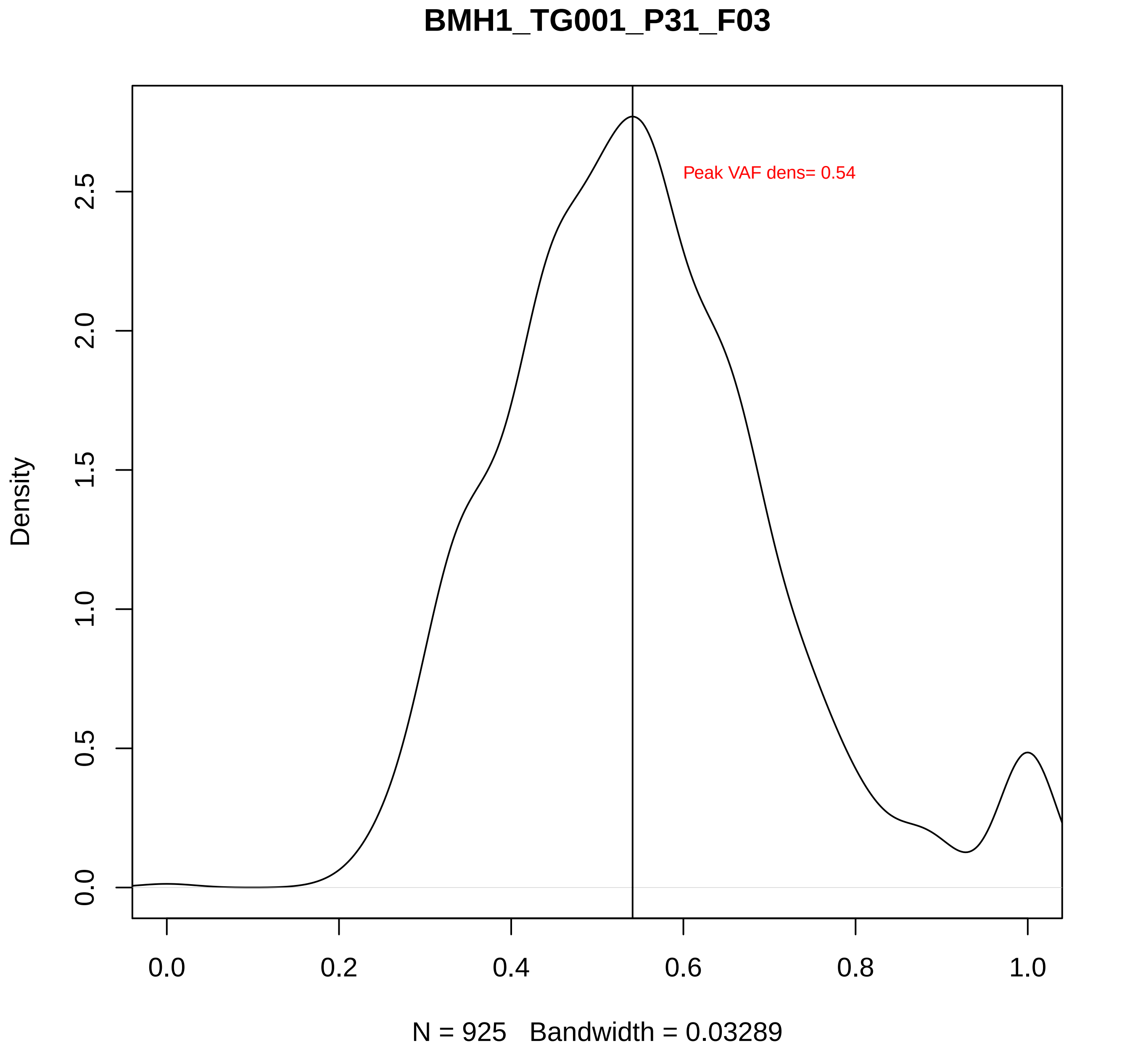

BMH1_TG001_P31_F03
Peak VAF dens= 0.54
Density
0.0
0.5
1.0
1.5
2.0
2.5
0.0
0.2
0.4
0.6
0.8
1.0
N = 925 Bandwidth = 0.03289

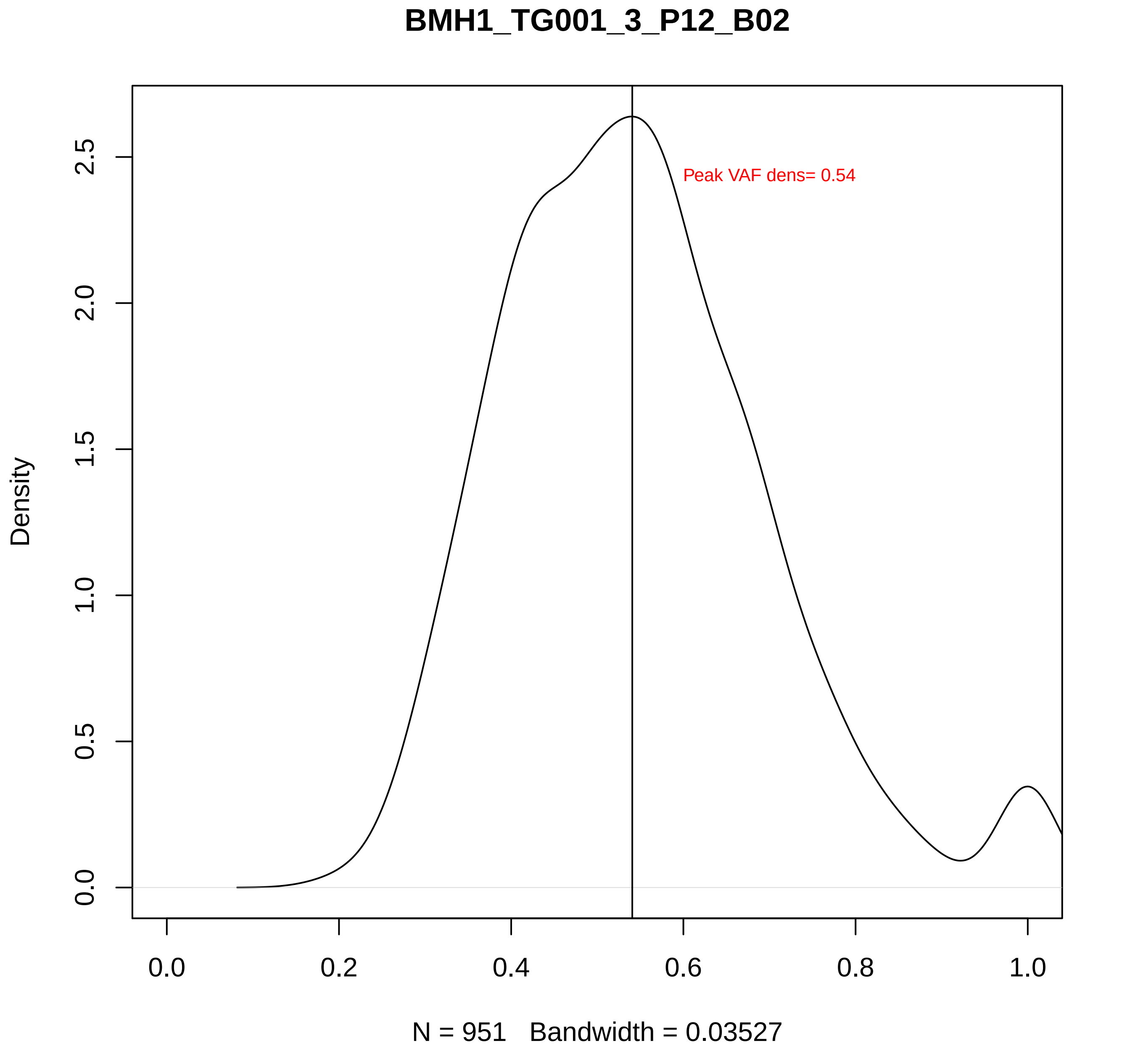
BMH1_TG001_3_P12_B02
Peak VAF dens= 0.54
Density
0.0
0.5
1.0
1.5
2.0
2.5
0.0
0.2
0.4
0.6
0.8
1.0
N = 951 Bandwidth = 0.03527

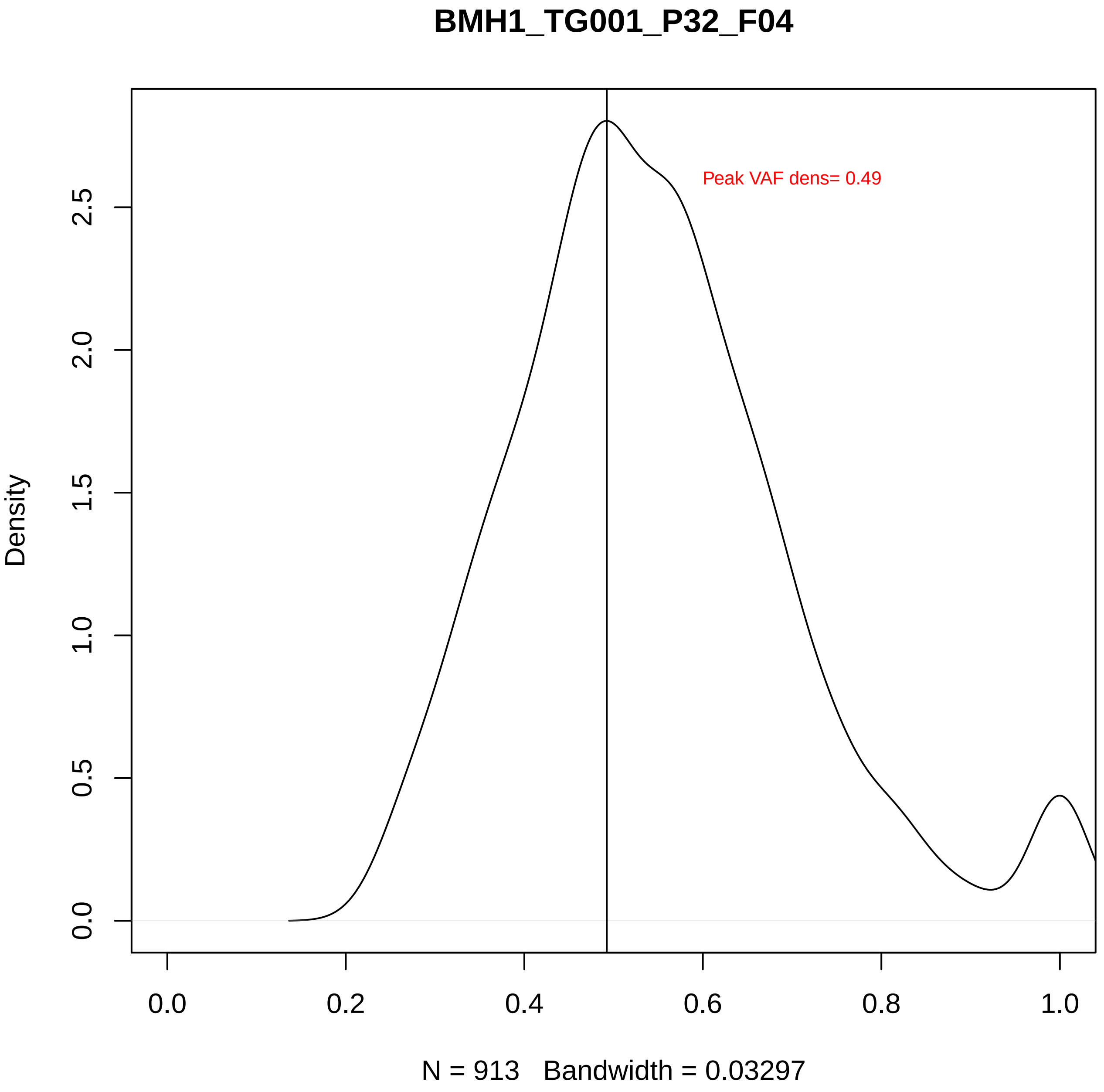

BMH1_TG001_P32_F04
Peak VAF dens= 0.49
Density
0.0
0.5
1.0
1.5
2.0
2.5
0.0
0.2
0.4
0.6
0.8
1.0
N = 913   Bandwidth = 0.03297

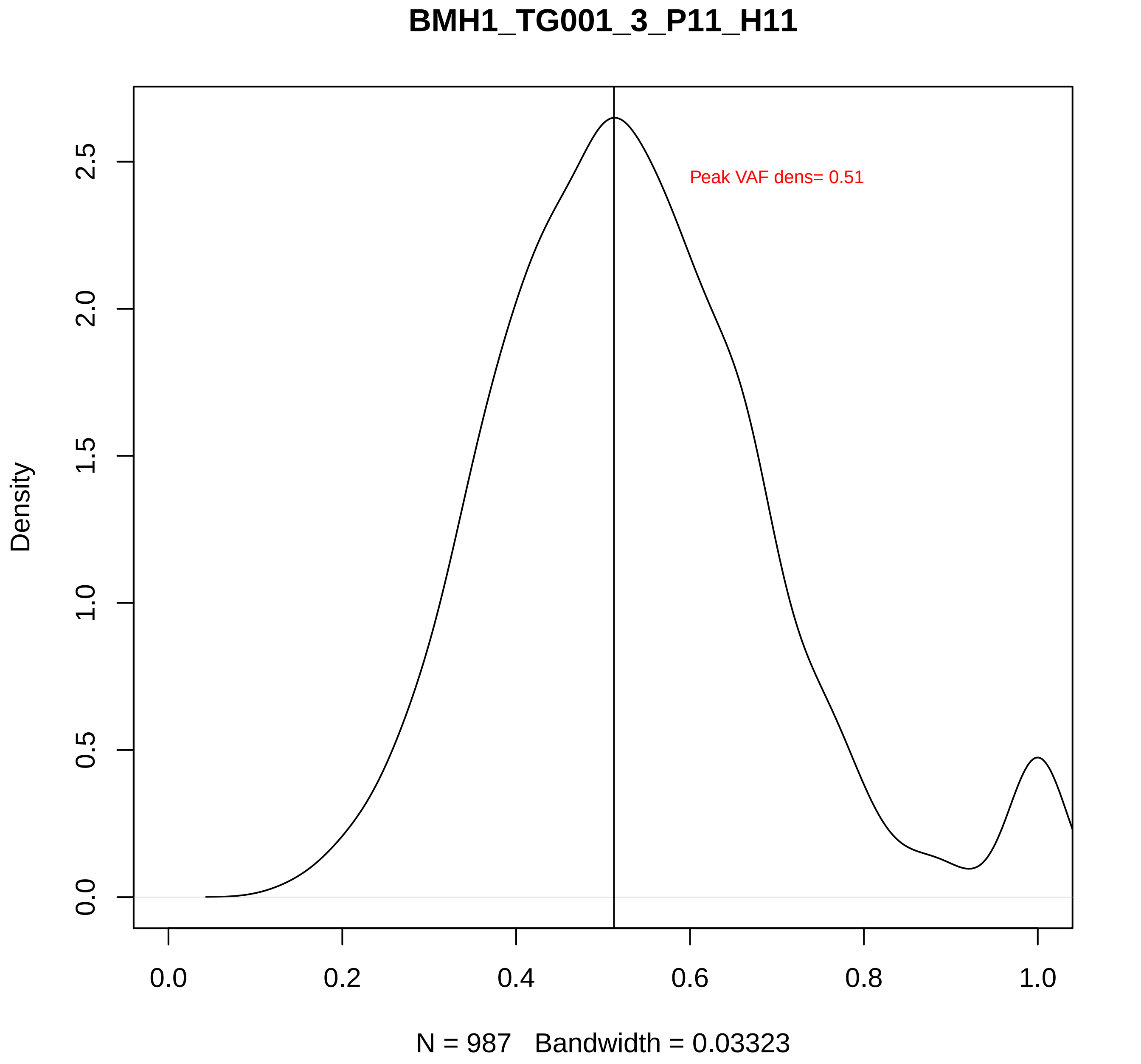
BMH1_TG001_3_P11_H11
Peak VAF dens= 0.51
Density
0.0
0.5
1.0
1.5
2.0
2.5
0.0
0.2
0.4
0.6
0.8
1.0
N = 987   Bandwidth = 0.03323

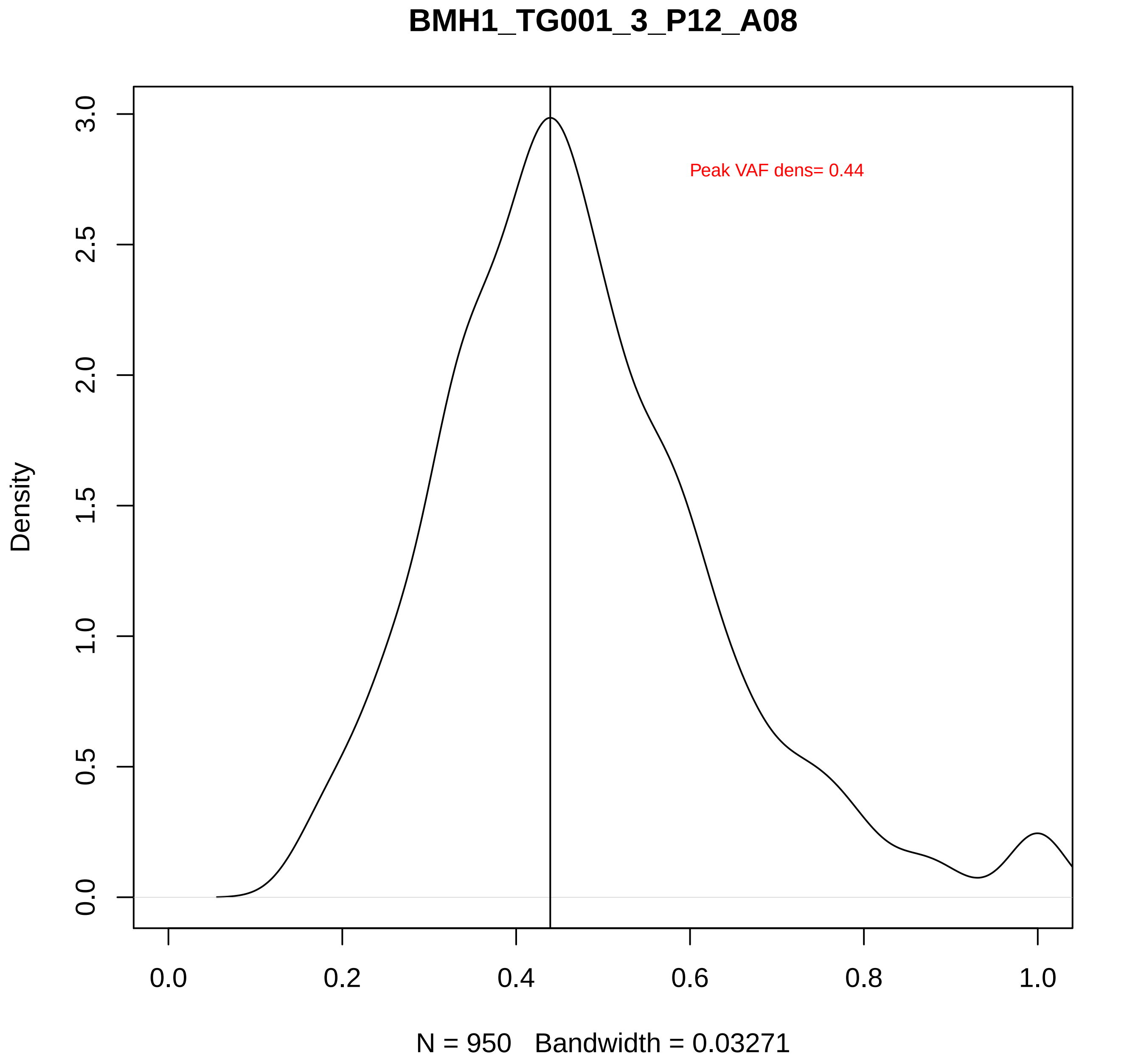
BMH1_TG001_3_P12_A08
Peak VAF dens= 0.44
Density
0.0
0.5
1.0
1.5
2.0
2.5
3.0
0.0
0.2
0.4
0.6
0.8
1.0
N = 950   Bandwidth = 0.03271

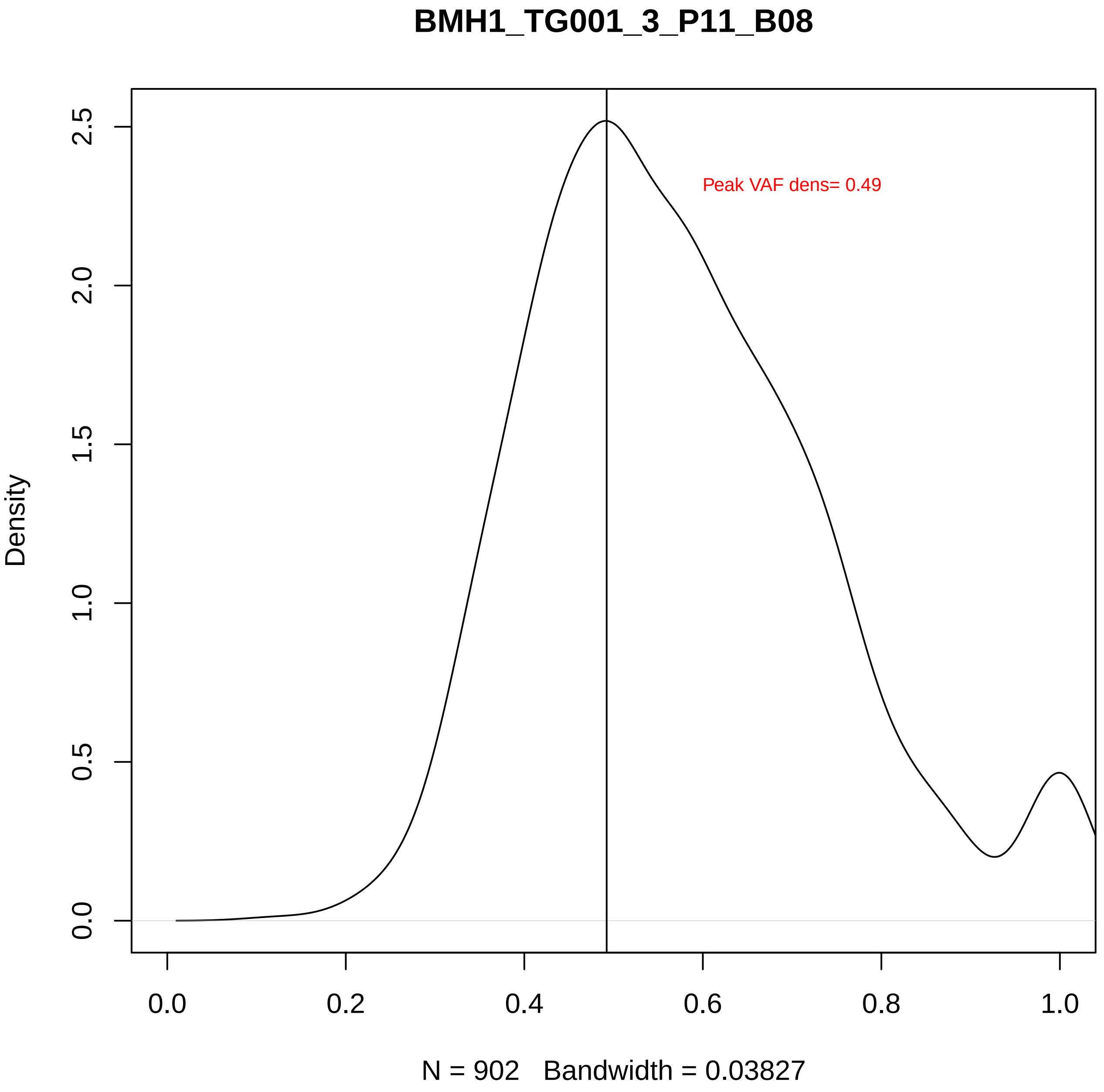

BMH1_TG001_3_P11_B08
Peak VAF dens= 0.49
Density
0.0
0.5
1.0
1.5
2.0
2.5
0.0
0.2
0.4
0.6
0.8
1.0
N = 902   Bandwidth = 0.03827

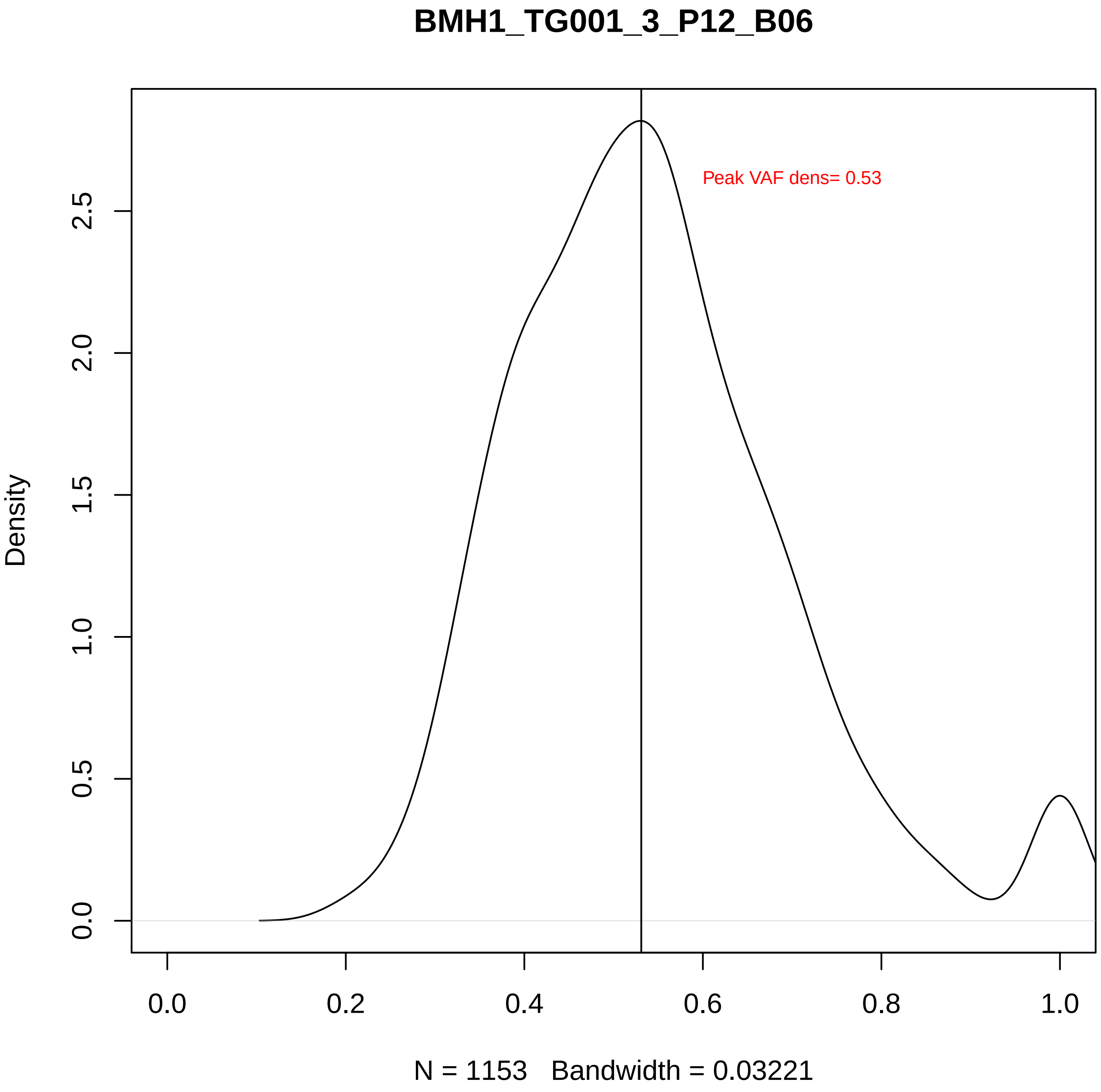

BMH1_TG001_3_P12_B06
Peak VAF dens= 0.53
Density
0.0
0.5
1.0
1.5
2.0
2.5
0.0
0.2
0.4
0.6
0.8
1.0
N = 1153   Bandwidth = 0.03221

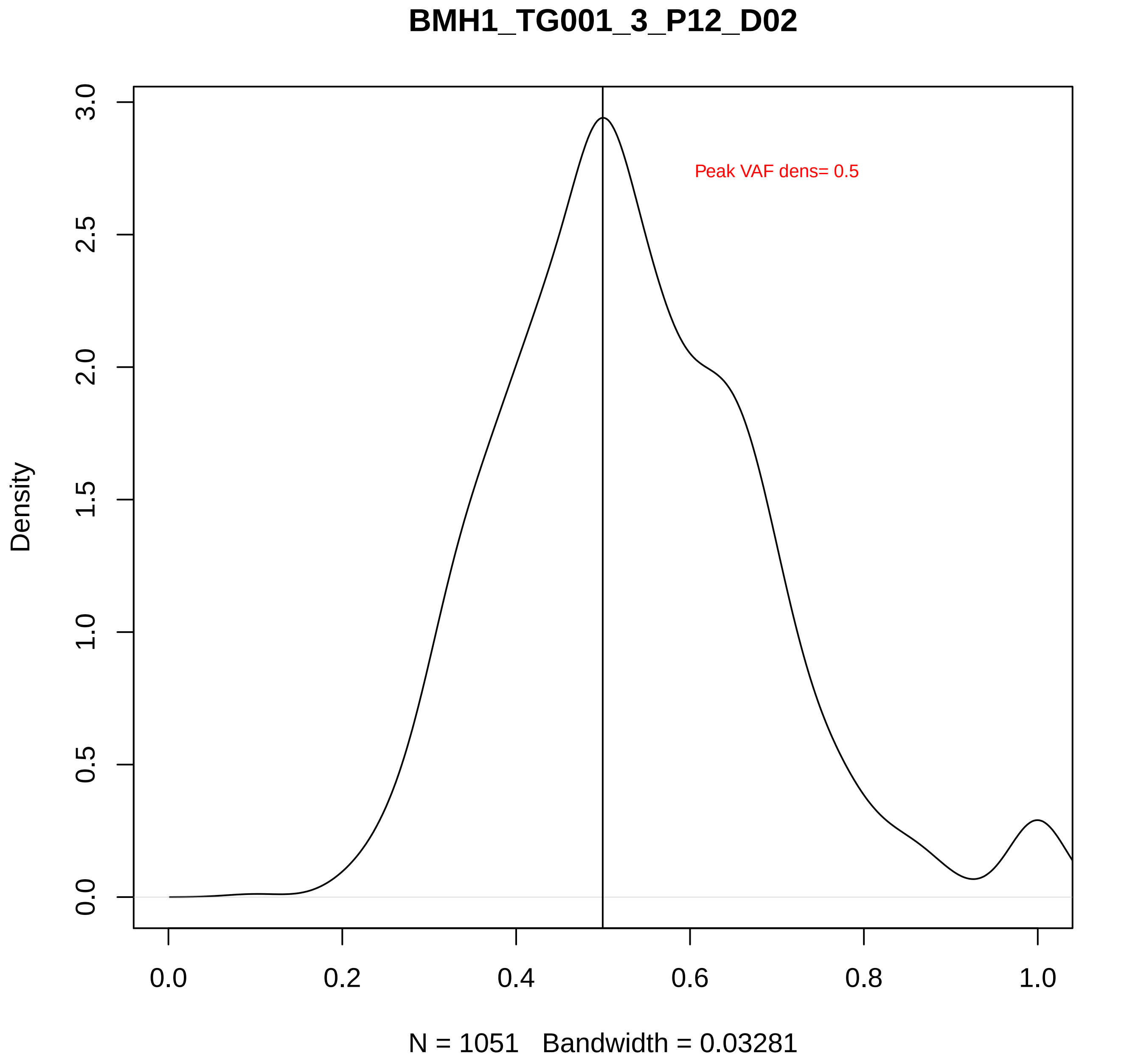

BMH1_TG001_3_P12_D02
Peak VAF dens= 0.5
Density
0.0
0.5
1.0
1.5
2.0
2.5
3.0
0.0
0.2
0.4
0.6
0.8
1.0
N = 1051   Bandwidth = 0.03281

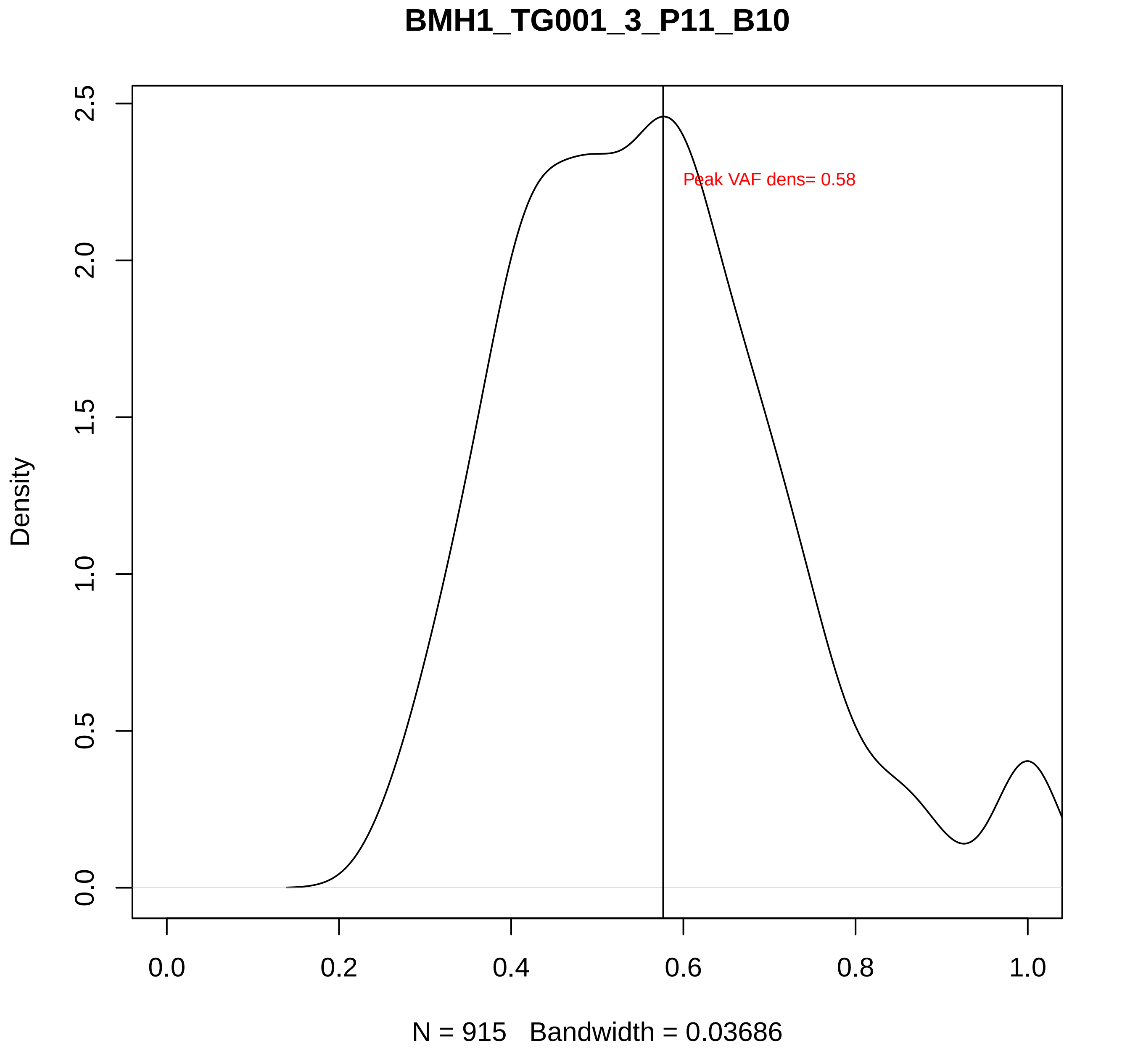
BMH1_TG001_3_P11_B10
Peak VAF dens= 0.58
Density
0.0
0.5
1.0
1.5
2.0
2.5
0.0
0.2
0.4
0.6
0.8
1.0
N = 915   Bandwidth = 0.03686

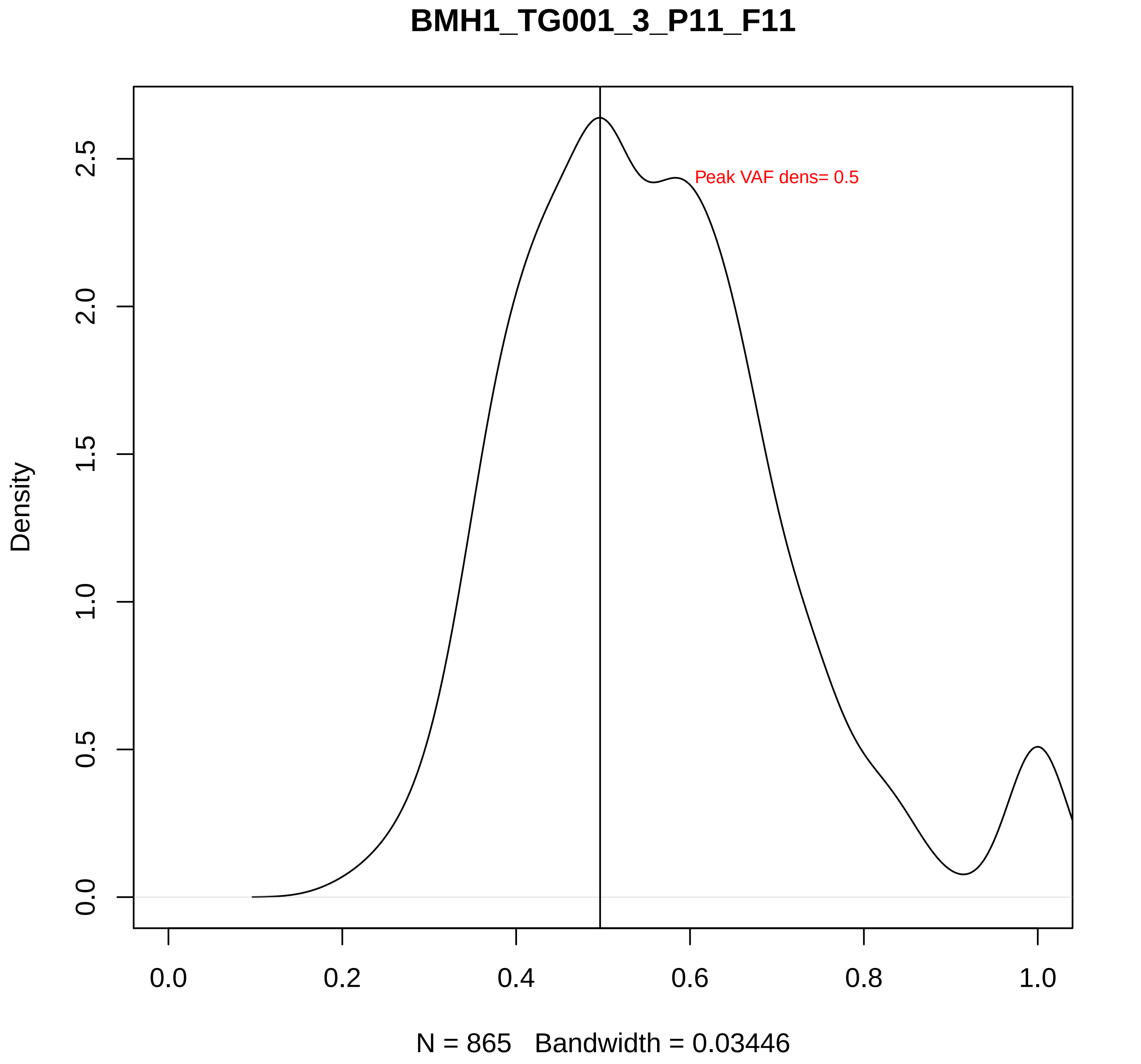

BMH1_TG001_3_P11_F11
Peak VAF dens= 0.5
Density
0.0
0.5
1.0
1.5
2.0
2.5
0.0
0.2
0.4
0.6
0.8
1.0
N = 865   Bandwidth = 0.03446

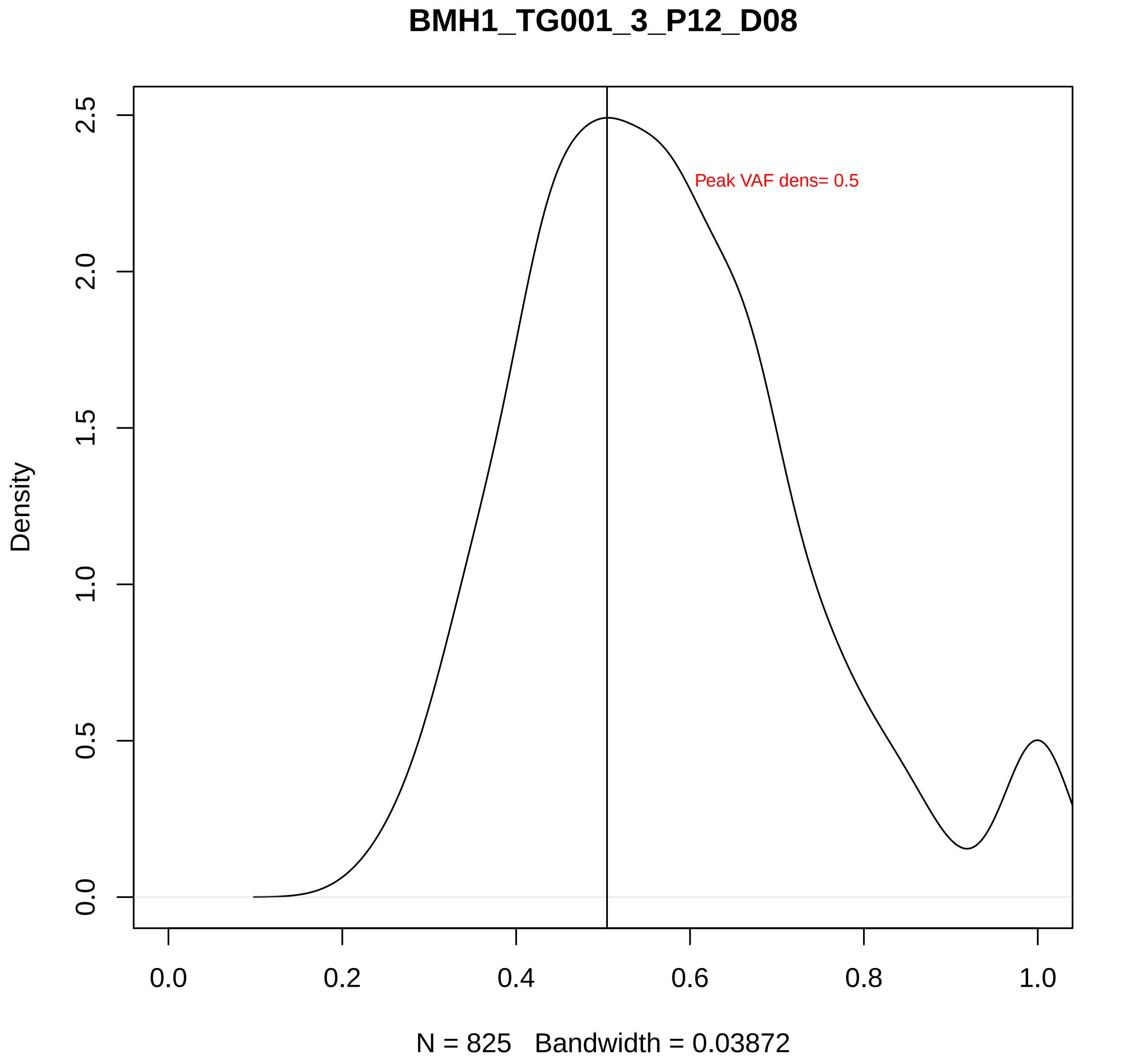
BMH1_TG001_3_P12_D08
Peak VAF dens= 0.5
Density
0.0
0.5
1.0
1.5
2.0
2.5
0.0
0.2
0.4
0.6
0.8
1.0
N = 825   Bandwidth = 0.03872

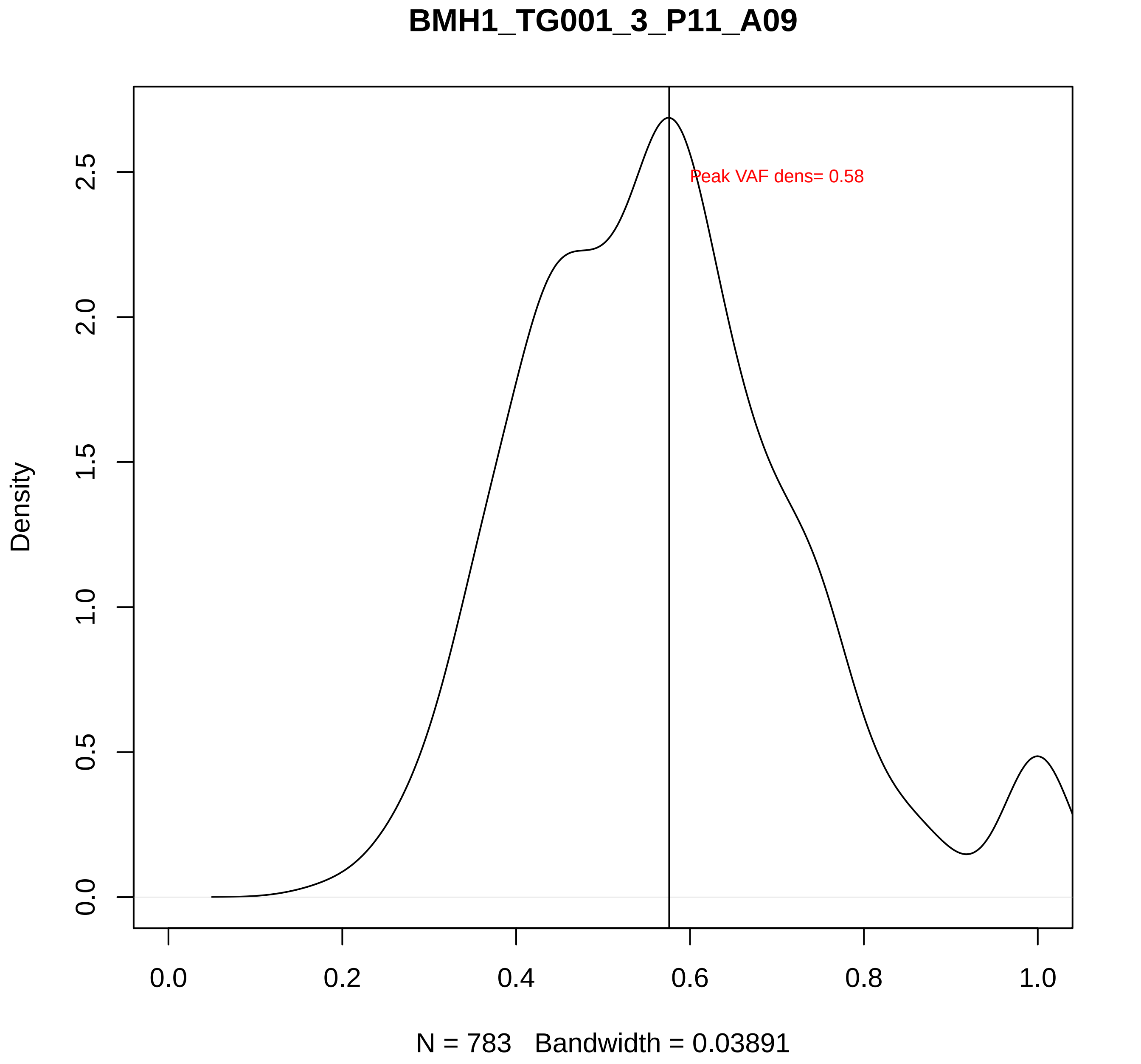
BMH1_TG001_3_P11_A09
Peak VAF dens= 0.58
Density
0.0
0.5
1.0
1.5
2.0
2.5
0.0
0.2
0.4
0.6
0.8
1.0
N = 783   Bandwidth = 0.03891

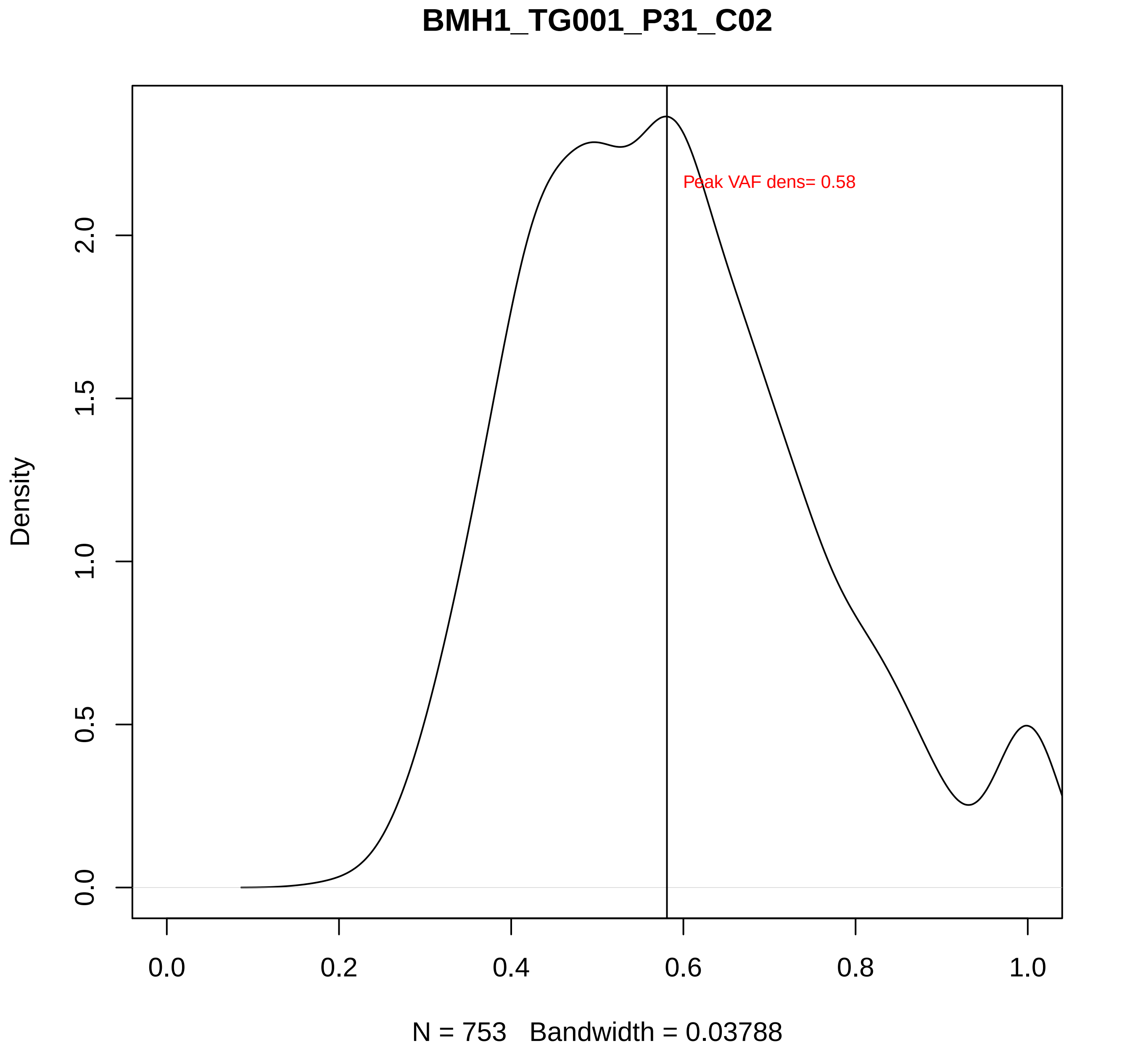
BMH1_TG001_P31_C02
Peak VAF dens= 0.58
Density
0.0
0.5
1.0
1.5
2.0
0.0
0.2
0.4
0.6
0.8
1.0
N = 753   Bandwidth = 0.03788

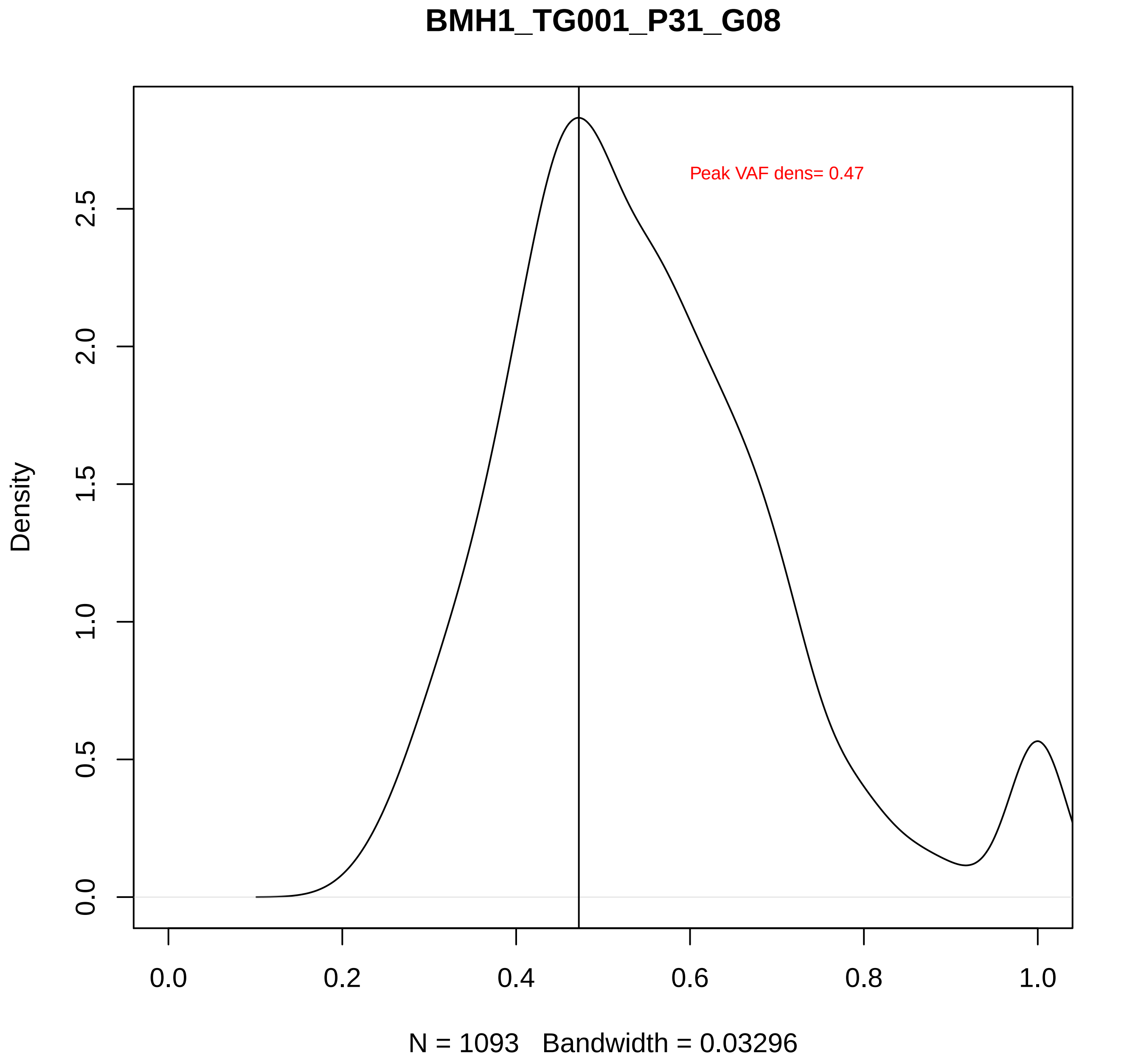

BMH1_TG001_P31_G08
Peak VAF dens= 0.47
Density
0.0
0.5
1.0
1.5
2.0
2.5
0.0
0.2
0.4
0.6
0.8
1.0
N = 1093   Bandwidth = 0.03296

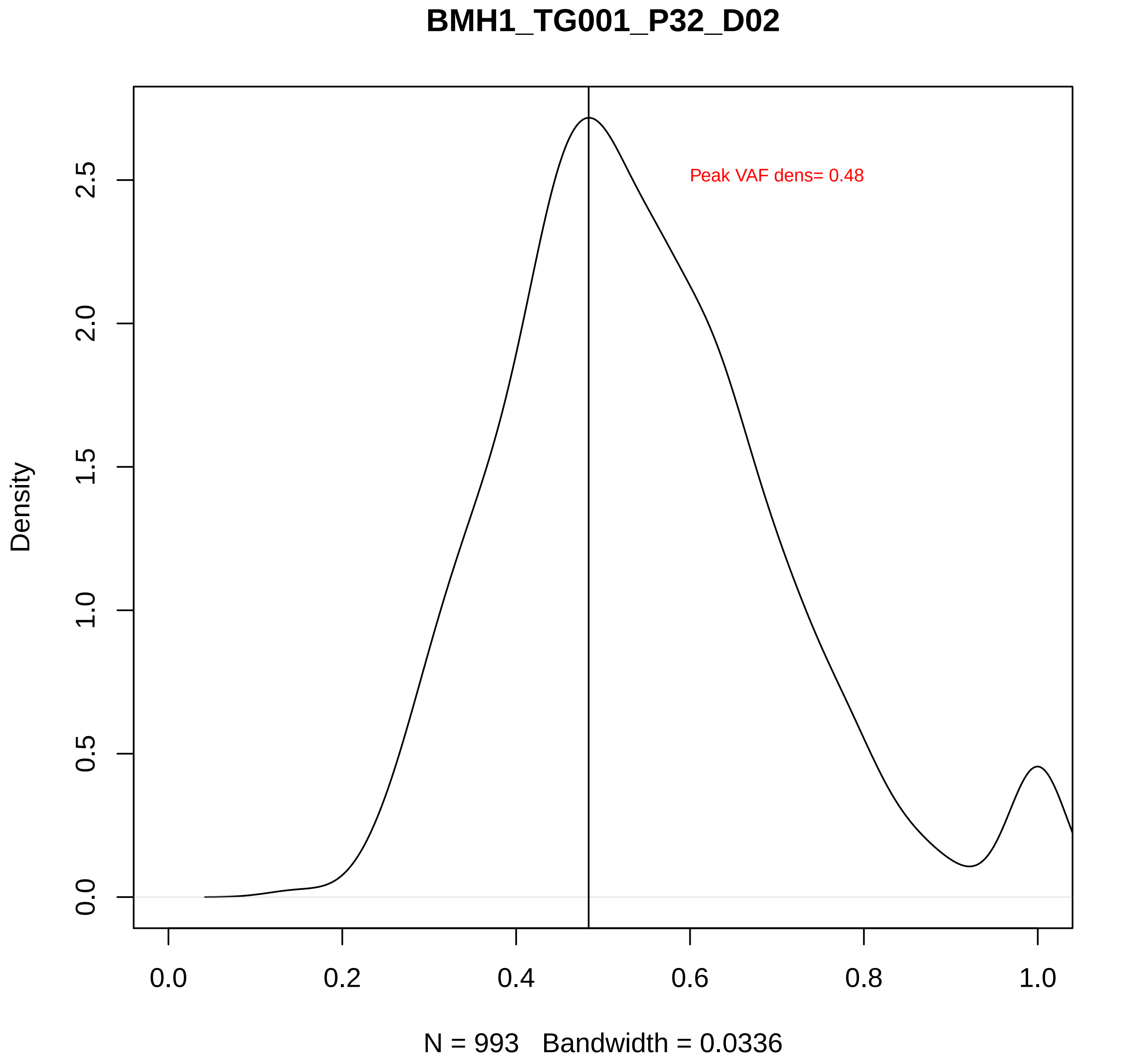
BMH1_TG001_P32_D02
Peak VAF dens= 0.48
Density
0.0
0.5
1.0
1.5
2.0
2.5
0.0
0.2
0.4
0.6
0.8
1.0
N = 993 Bandwidth = 0.0336

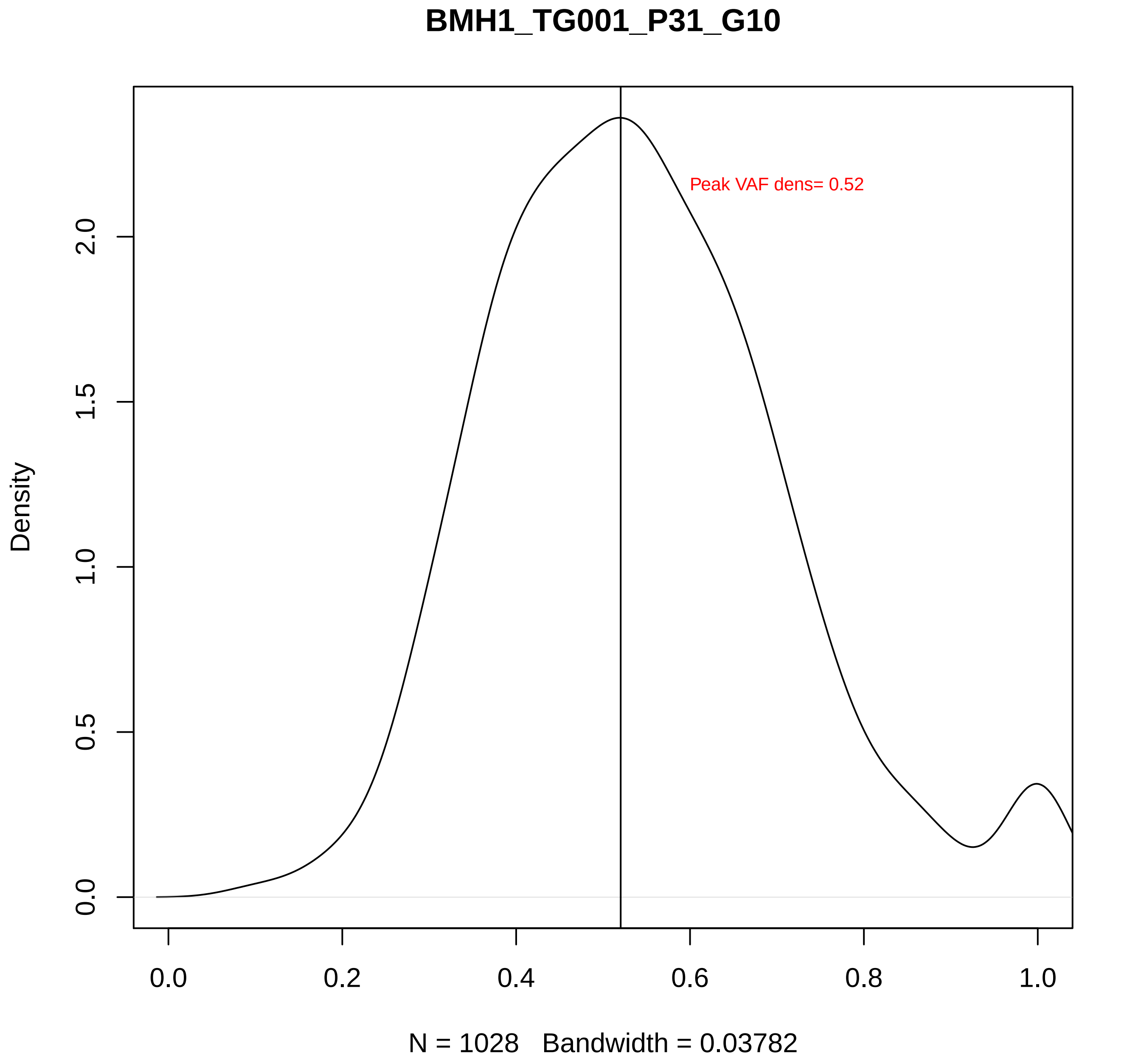

BMH1_TG001_P31_G10
Peak VAF dens= 0.52
Density
0.0
0.5
1.0
1.5
2.0
0.0
0.2
0.4
0.6
0.8
1.0
N = 1028   Bandwidth = 0.03782

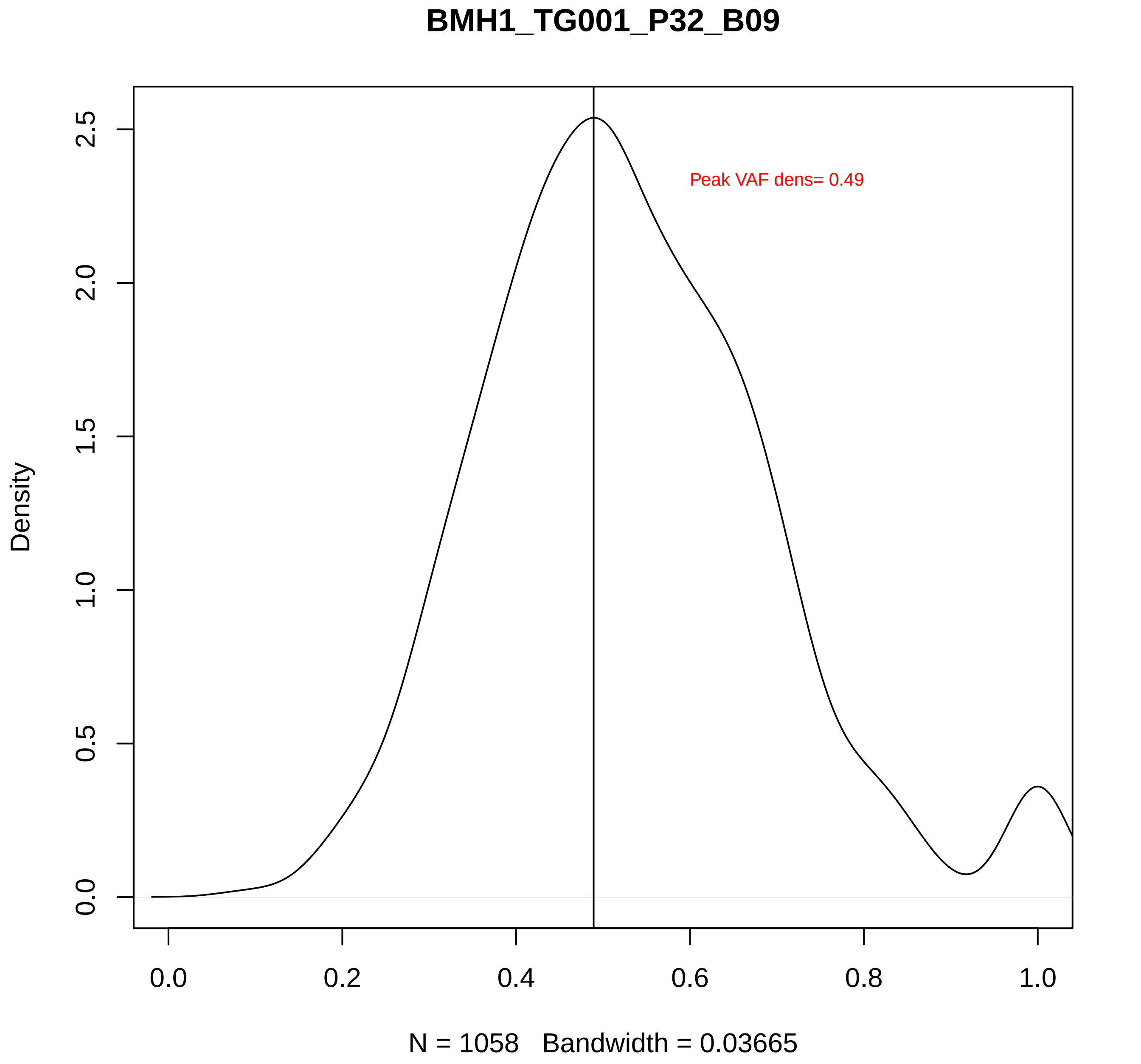

BMH1_TG001_P32_B09
Peak VAF dens= 0.49
Density
0.0
0.5
1.0
1.5
2.0
2.5
0.0
0.2
0.4
0.6
0.8
1.0
N = 1058   Bandwidth = 0.03665

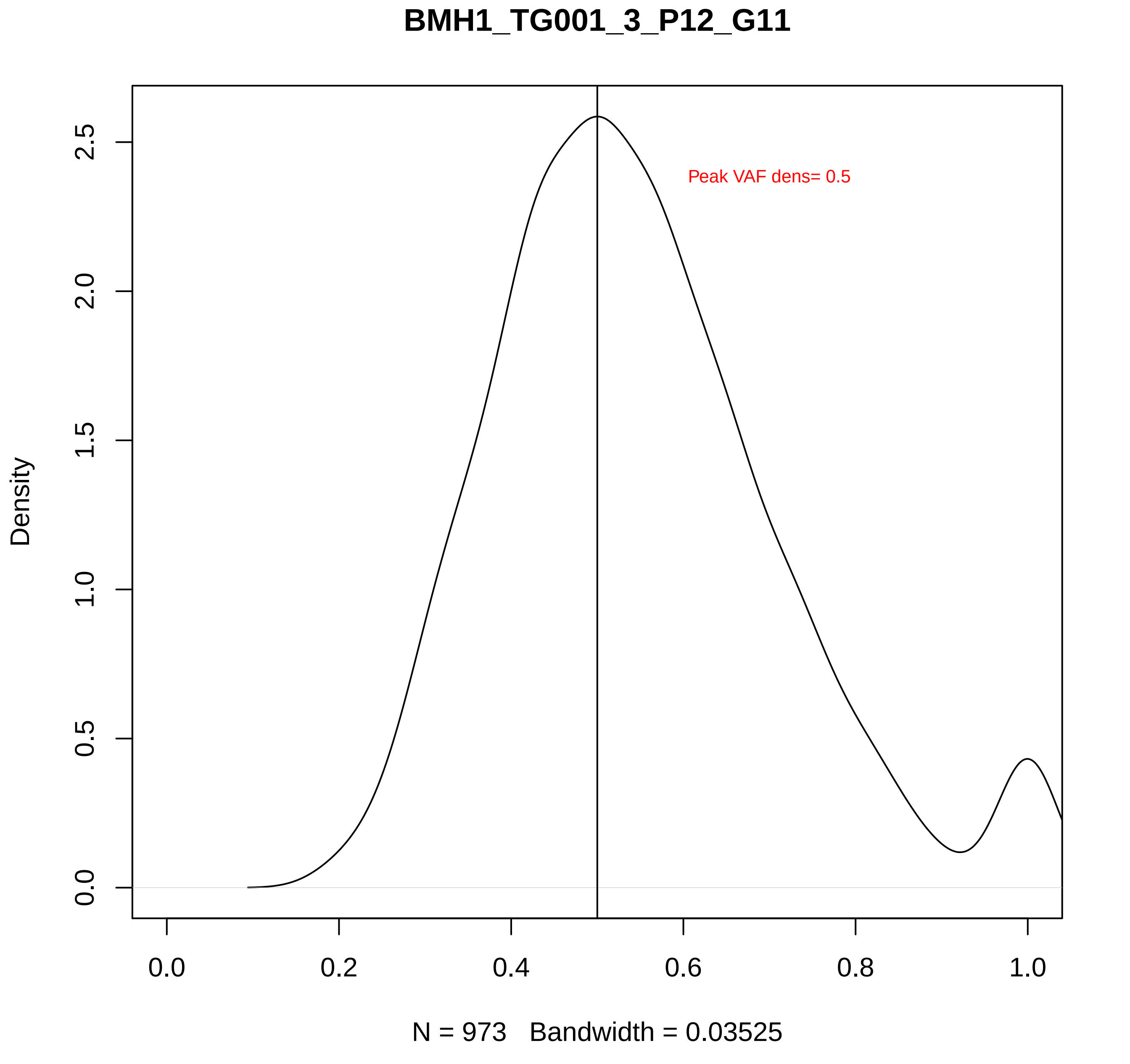
BMH1_TG001_3_P12_G11
Peak VAF dens= 0.5
Density
0.0
0.5
1.0
1.5
2.0
2.5
0.0
0.2
0.4
0.6
0.8
1.0
N = 973   Bandwidth = 0.03525

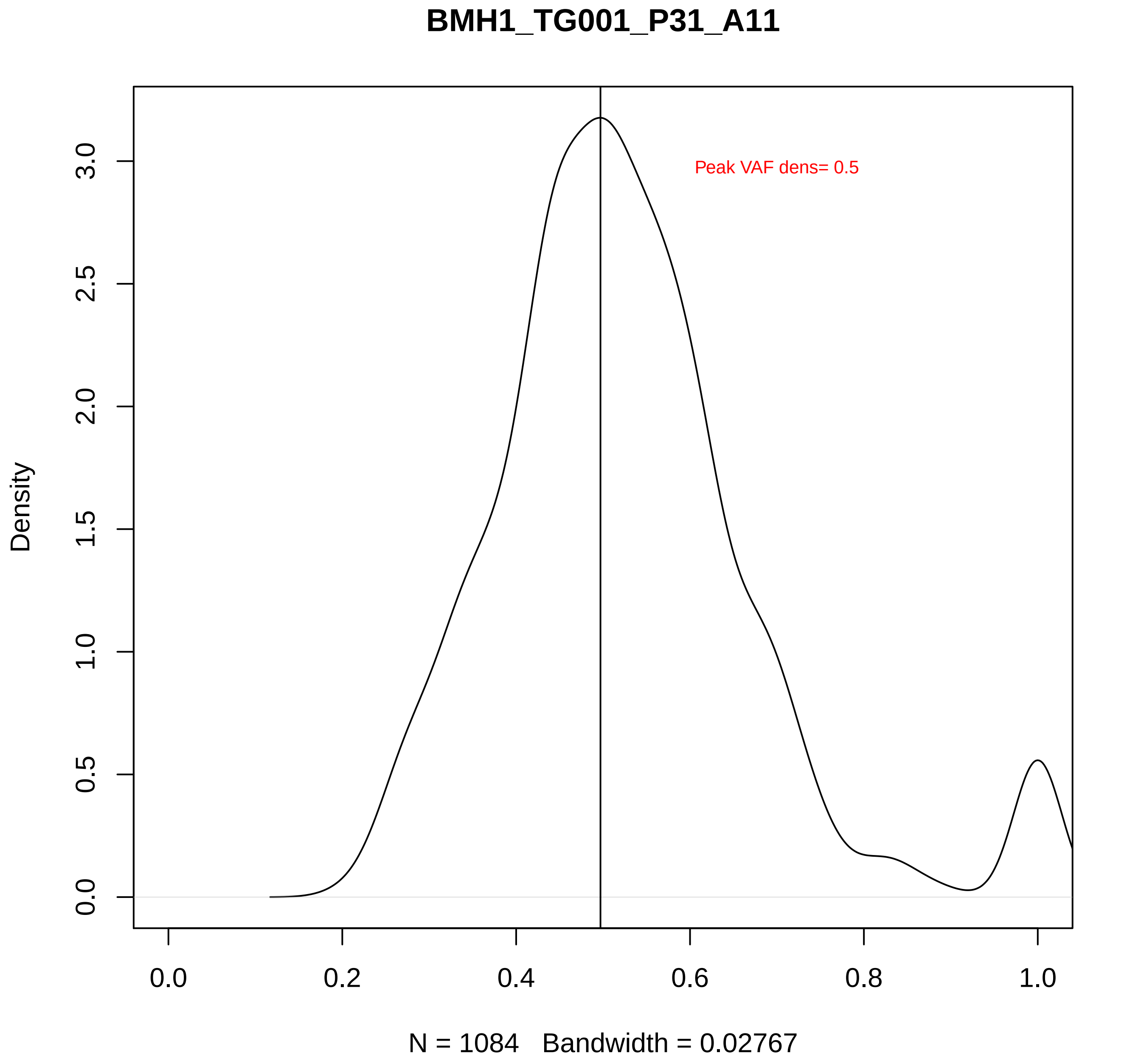
BMH1_TG001_P31_A11
Peak VAF dens= 0.5
Density
0.0
0.5
1.0
1.5
2.0
2.5
3.0
0.0
0.2
0.4
0.6
0.8
1.0
N = 1084 Bandwidth = 0.02767

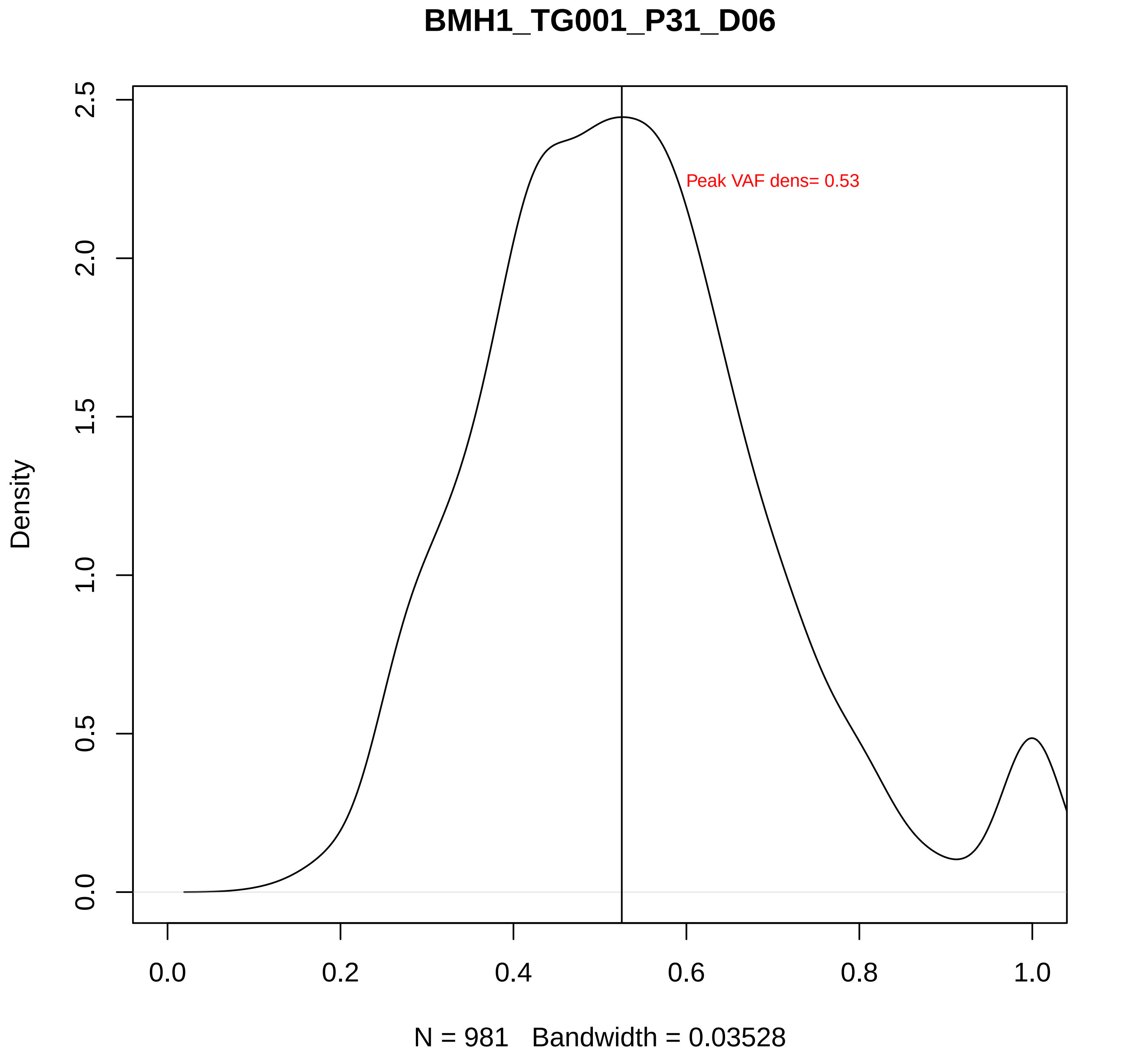
BMH1_TG001_P31_D06
Peak VAF dens= 0.53
Density
0.0
0.5
1.0
1.5
2.0
2.5
0.0
0.2
0.4
0.6
0.8
1.0
N = 981 Bandwidth = 0.03528

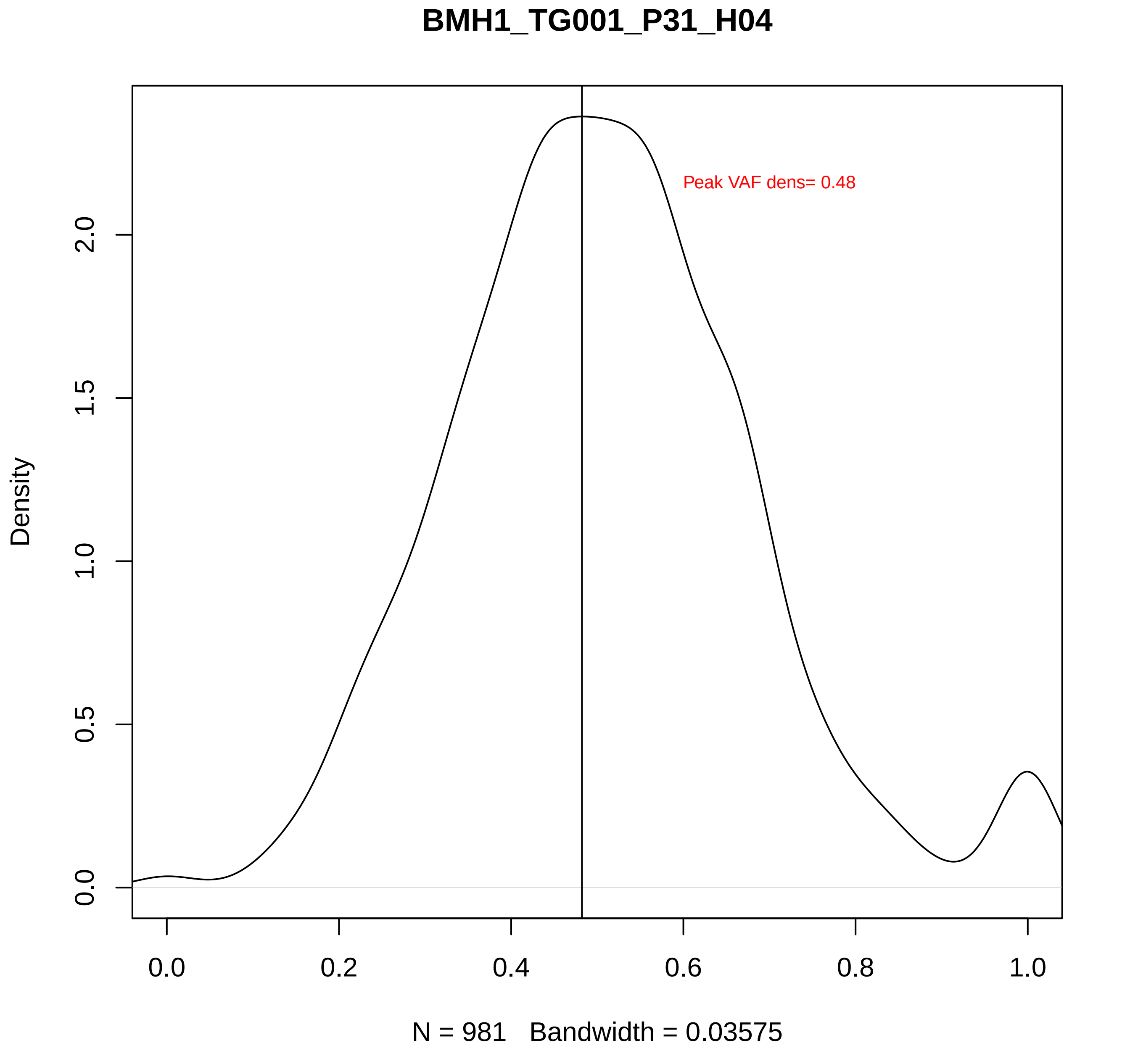
BMH1_TG001_P31_H04
Peak VAF dens= 0.48
Density
0.0
0.5
1.0
1.5
2.0
0.0
0.2
0.4
0.6
0.8
1.0
N = 981   Bandwidth = 0.03575

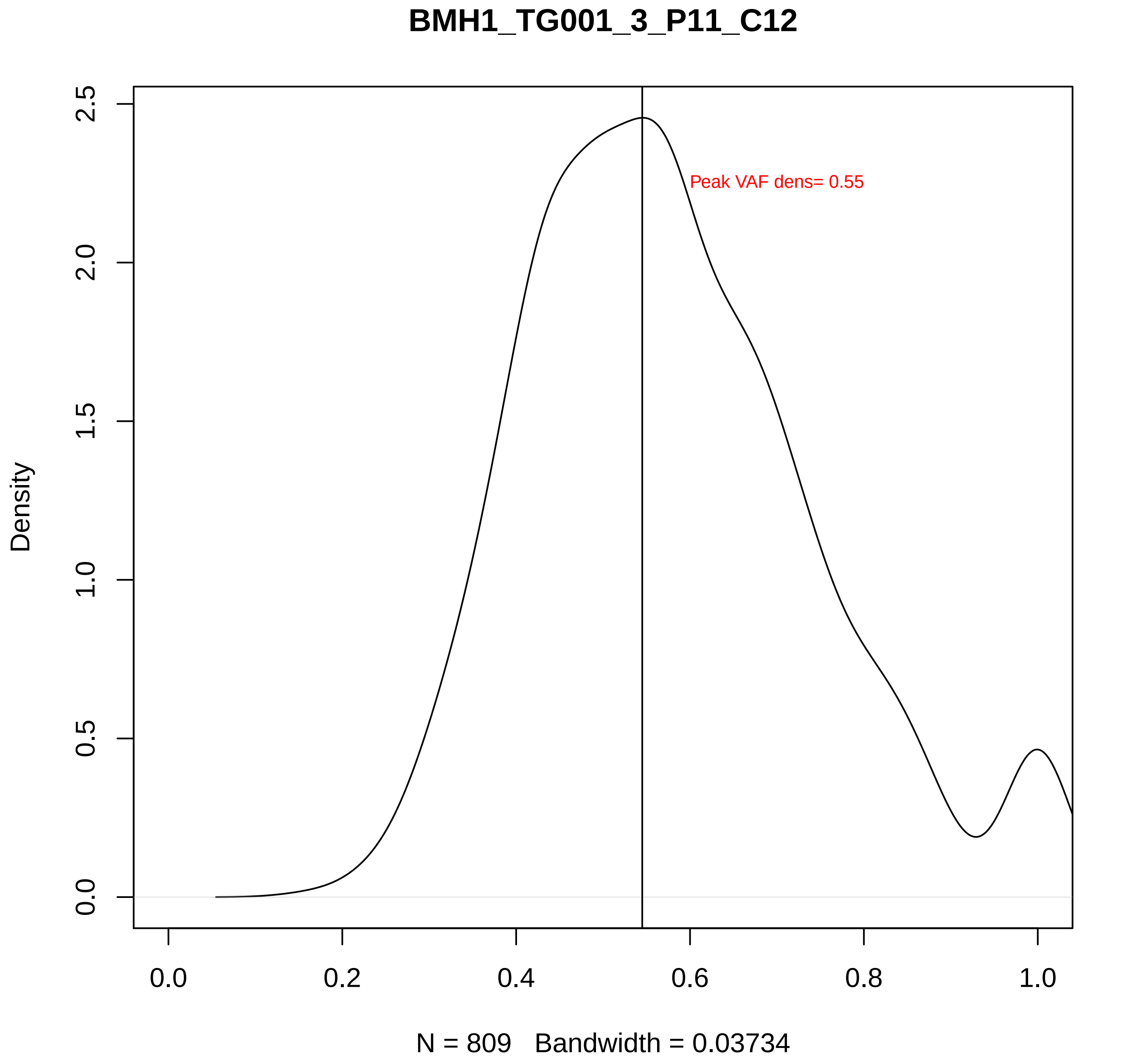
BMH1_TG001_3_P11_C12
Density
2.5
2.0
1.5
1.0
0.5
0.0
0.0
0.2
0.4
0.6
0.8
1.0
Peak VAF dens= 0.55
N = 809   Bandwidth = 0.03734

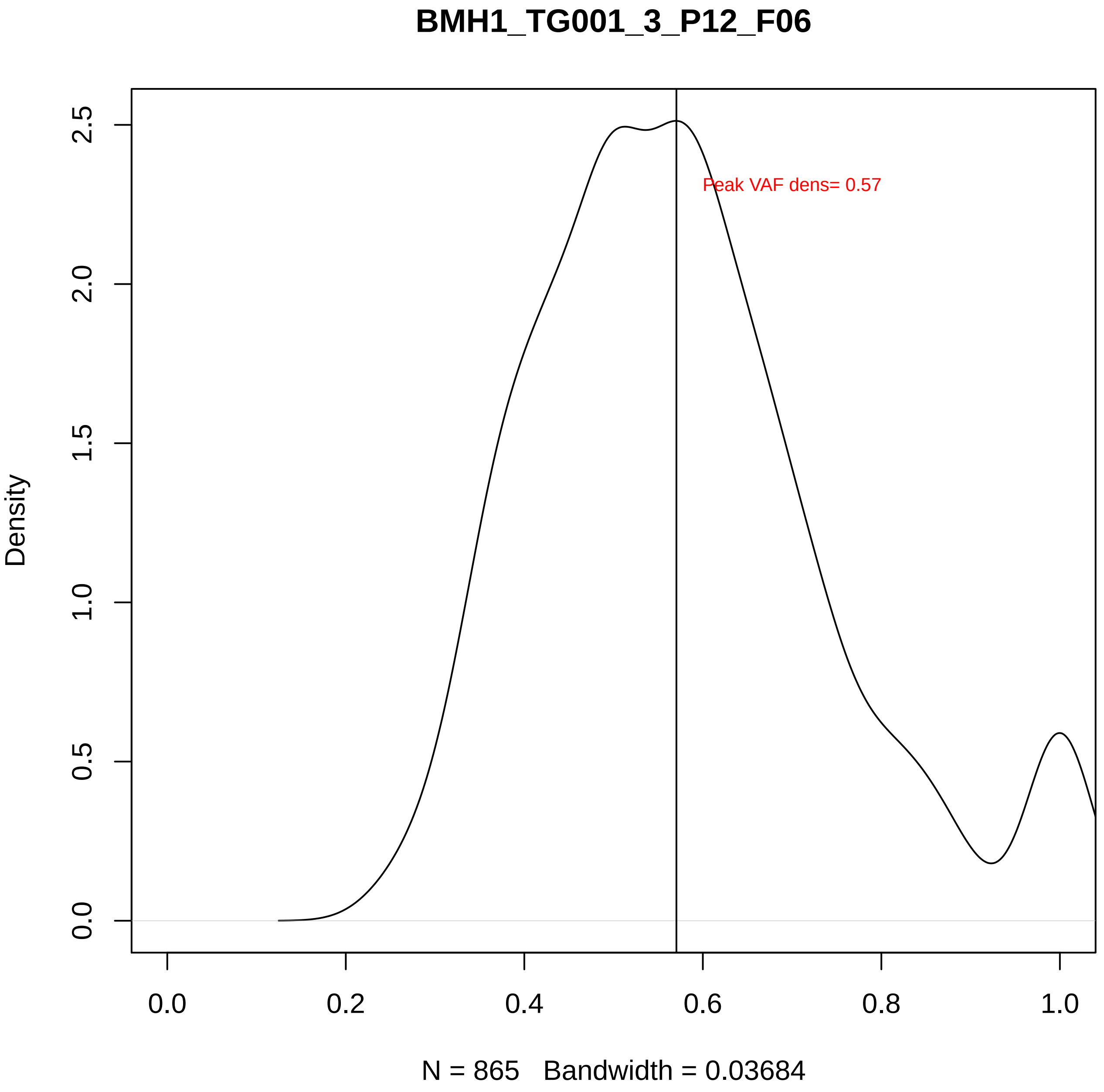

BMH1_TG001_3_P12_F06
Density
0.0
0.5
1.0
1.5
2.0
2.5
0.0
0.2
0.4
0.6
0.8
1.0
Peak VAF dens= 0.57
N = 865   Bandwidth = 0.03684

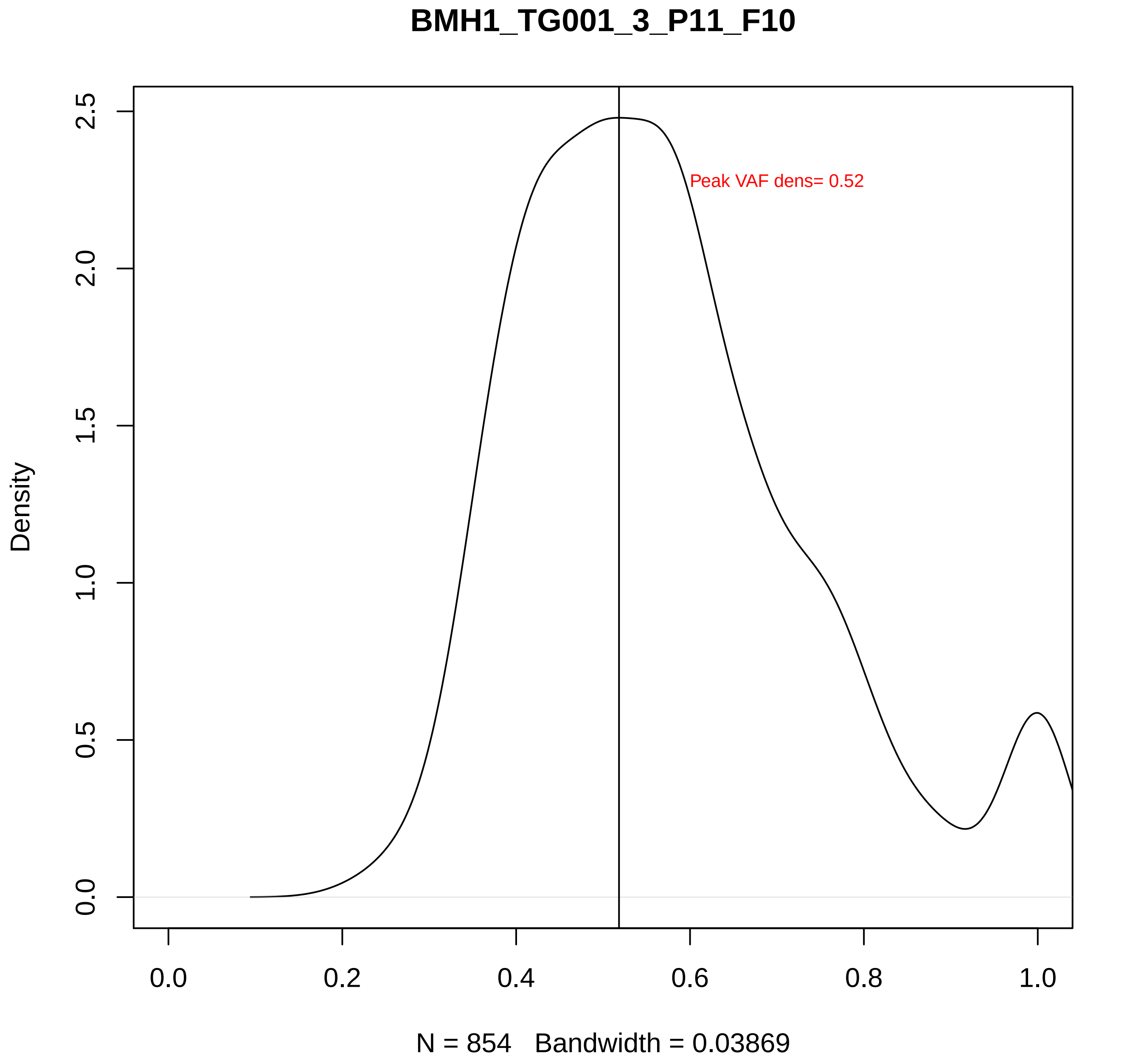

BMH1_TG001_3_P11_F10
Density
Peak VAF dens= 0.52
0.0
0.5
1.0
1.5
2.0
2.5
0.0
0.2
0.4
0.6
0.8
1.0
N = 854 Bandwidth = 0.03869

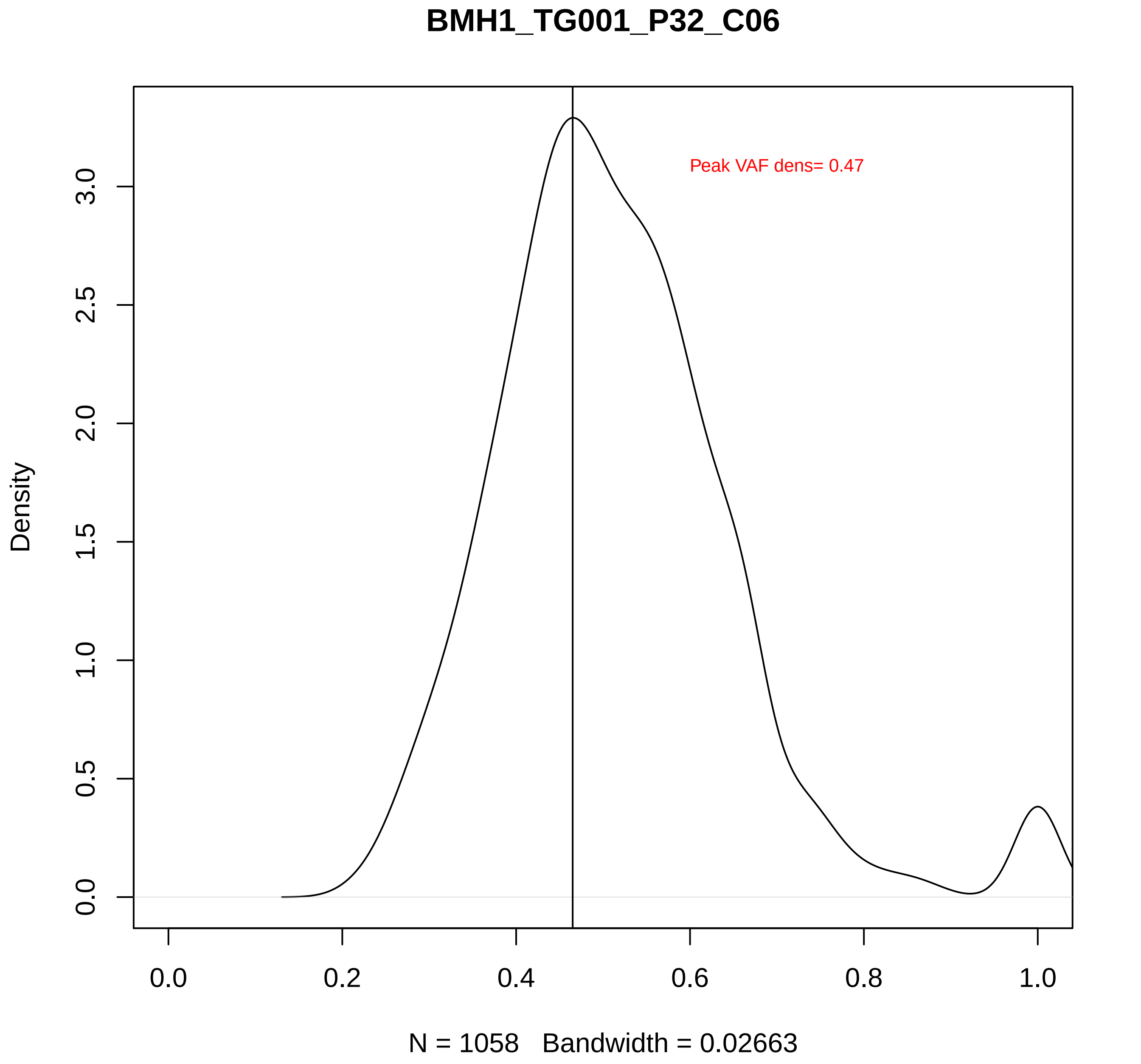
BMH1_TG001_P32_C06
Peak VAF dens= 0.47
Density
0.0
0.5
1.0
1.5
2.0
2.5
3.0
0.0
0.2
0.4
0.6
0.8
1.0
N = 1058   Bandwidth = 0.02663

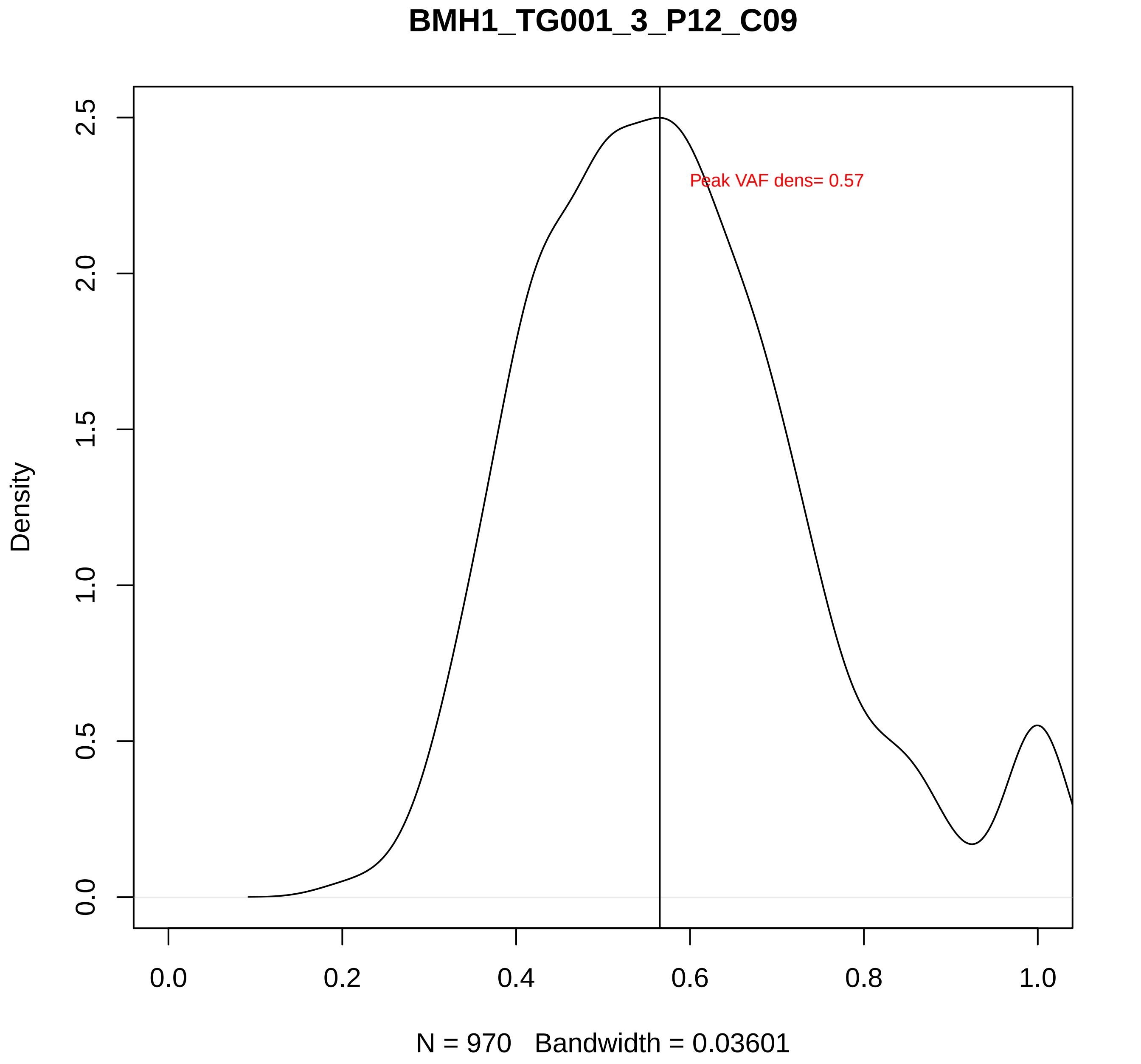
BMH1_TG001_3_P12_C09
Peak VAF dens= 0.57
Density
2.5
2.0
1.5
1.0
0.5
0.0
0.0
0.2
0.4
0.6
0.8
1.0
N = 970   Bandwidth = 0.03601

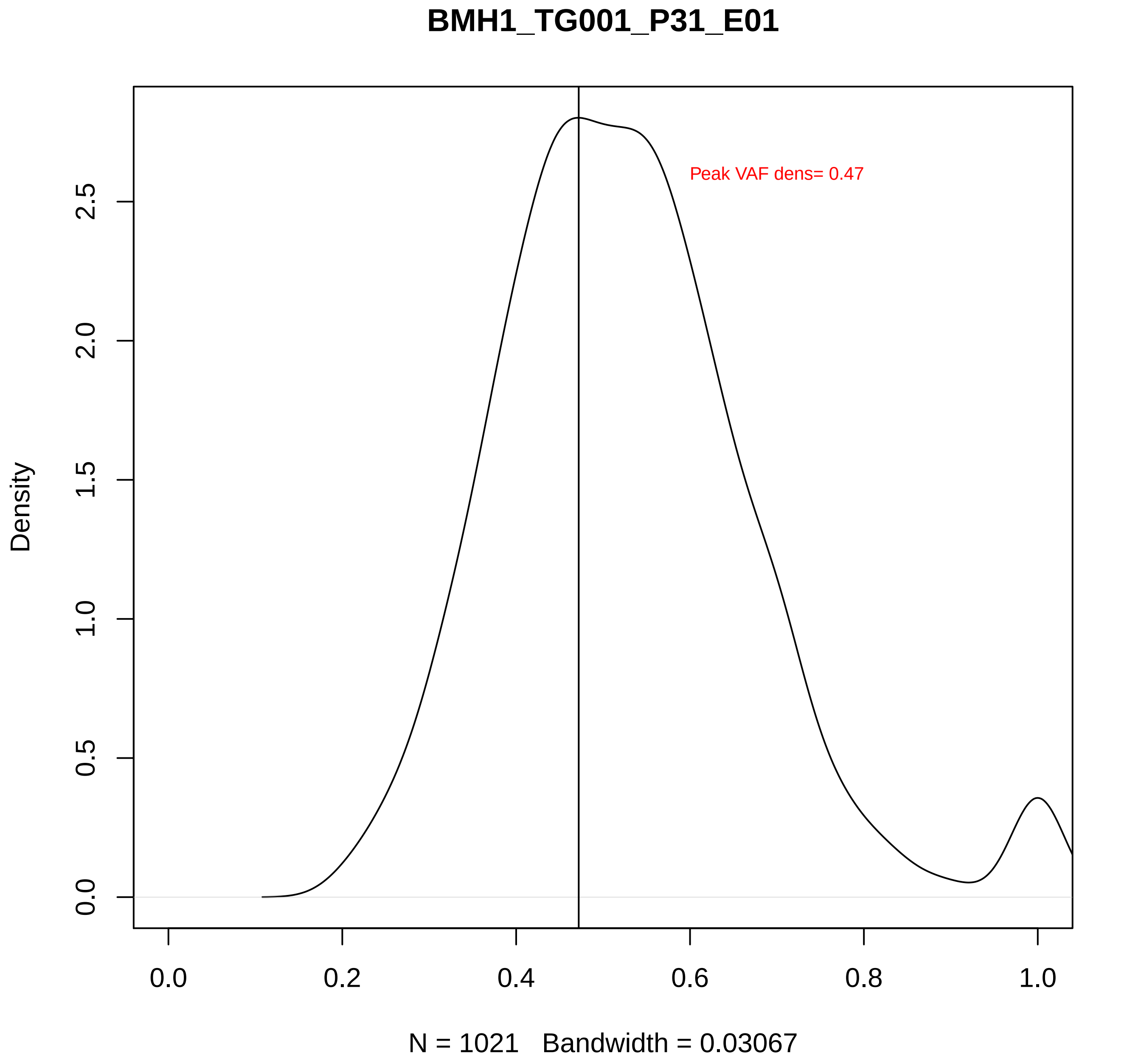
BMH1_TG001_P31_E01
Peak VAF dens= 0.47
Density
0.0
0.5
1.0
1.5
2.0
2.5
0.0
0.2
0.4
0.6
0.8
1.0
N = 1021   Bandwidth = 0.03067

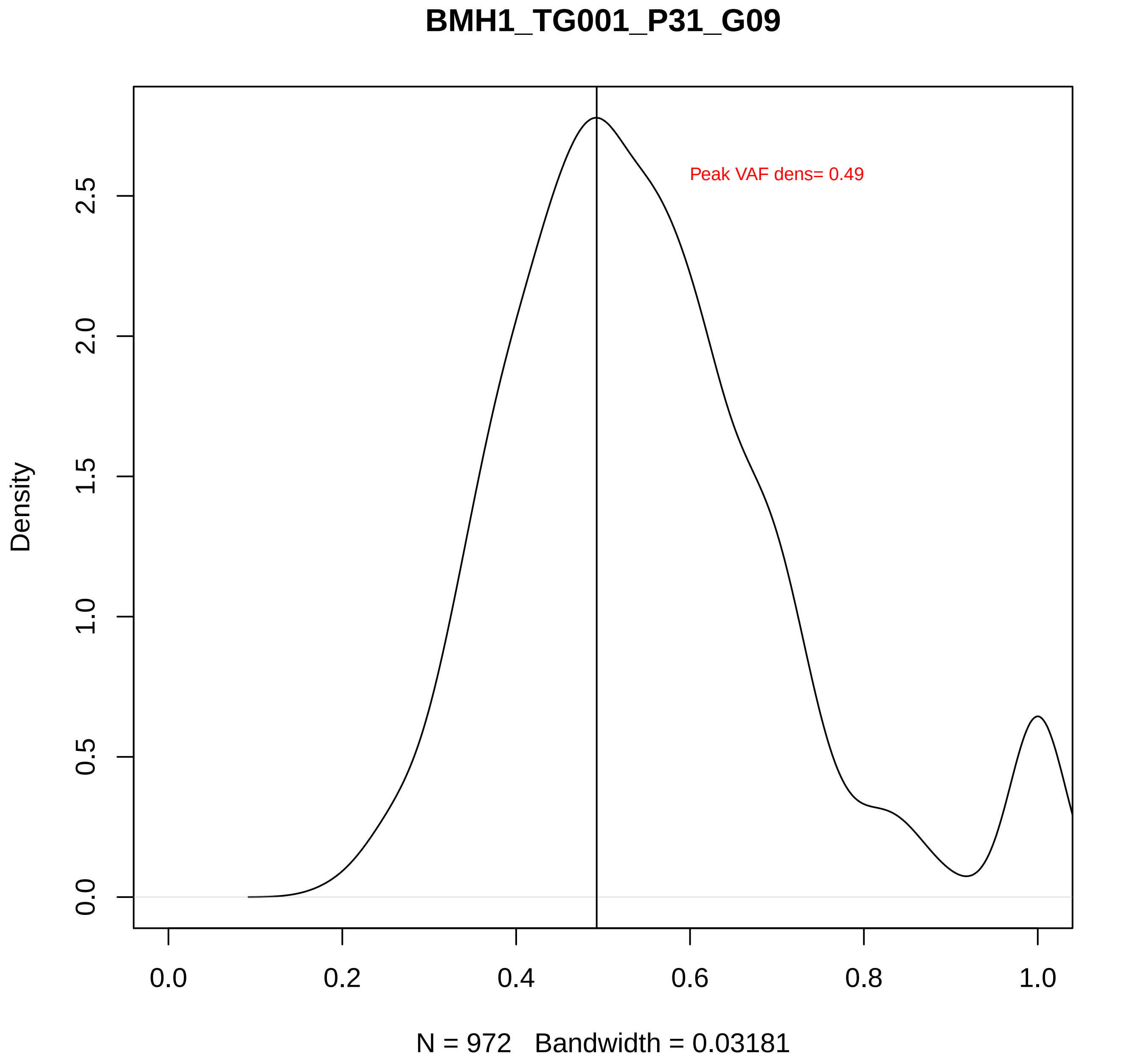
BMH1_TG001_P31_G09
Peak VAF dens= 0.49
Density
0.0
0.5
1.0
1.5
2.0
2.5
0.0
0.2
0.4
0.6
0.8
1.0
N = 972   Bandwidth = 0.03181

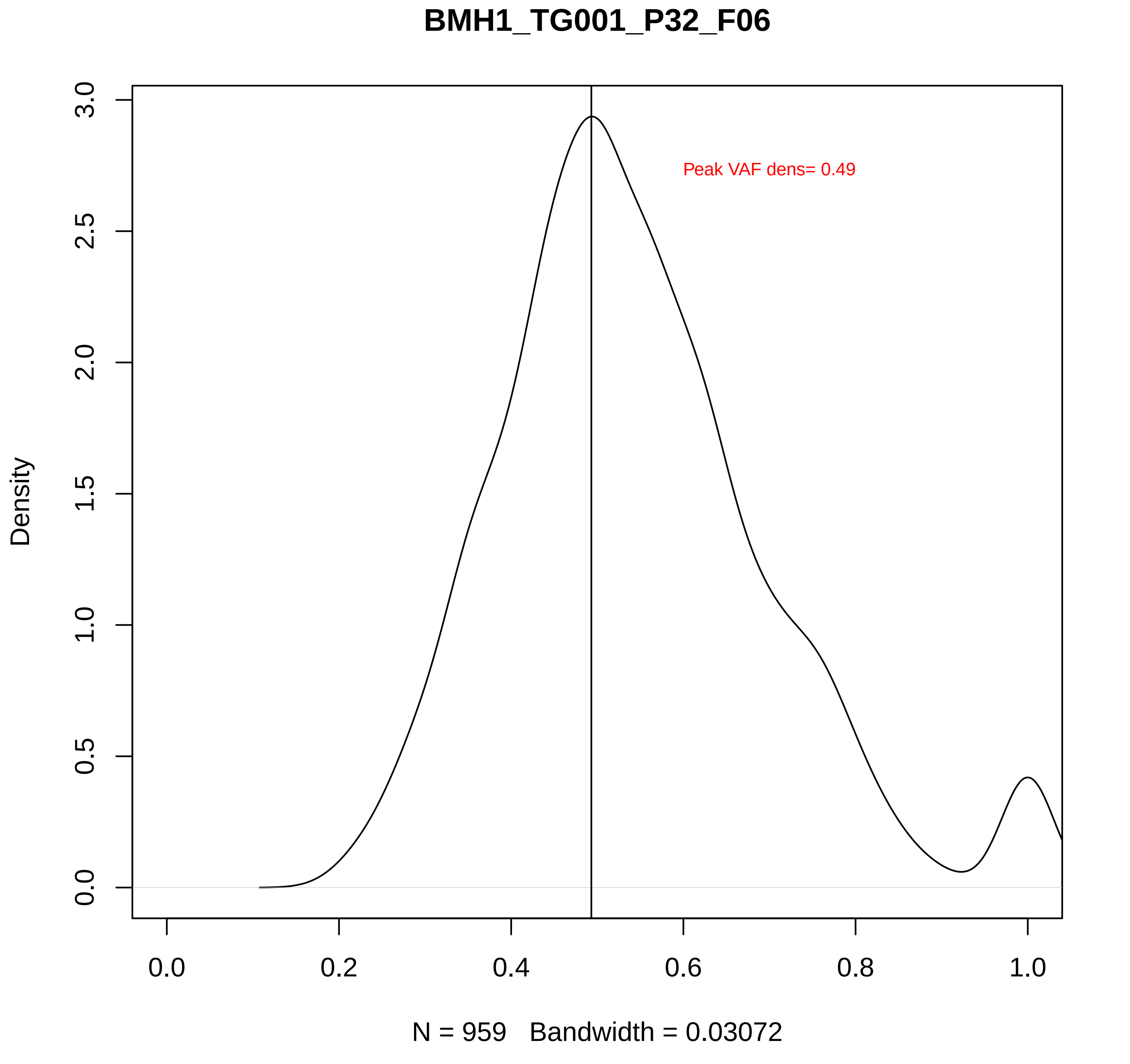
BMH1_TG001_P32_F06
Peak VAF dens= 0.49
Density
0.0
0.5
1.0
1.5
2.0
2.5
3.0
0.0
0.2
0.4
0.6
0.8
1.0
N = 959   Bandwidth = 0.03072

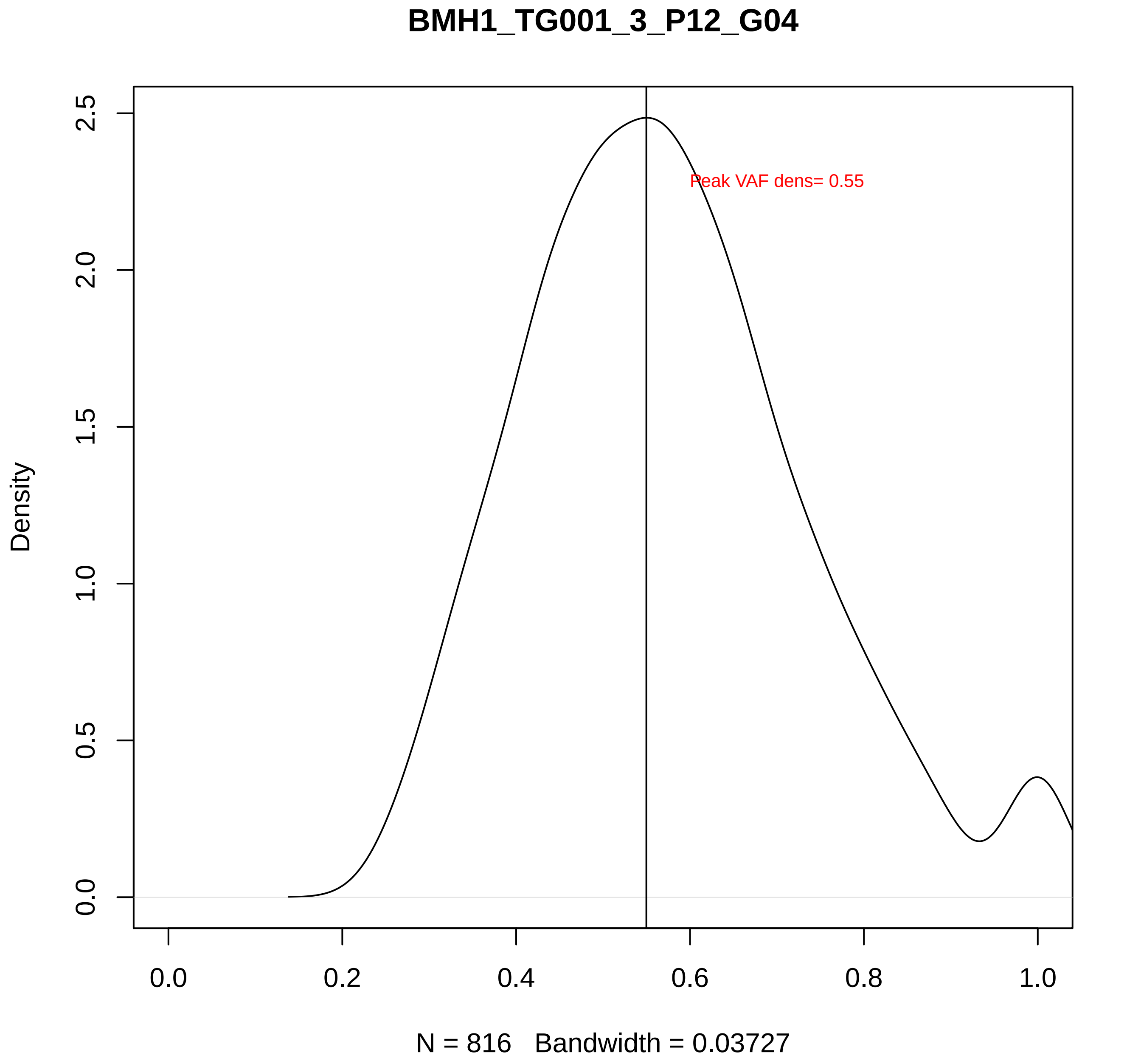
BMH1_TG001_3_P12_G04
Peak VAF dens= 0.55
Density
0.0
0.5
1.0
1.5
2.0
2.5
0.0
0.2
0.4
0.6
0.8
1.0
N = 816 Bandwidth = 0.03727

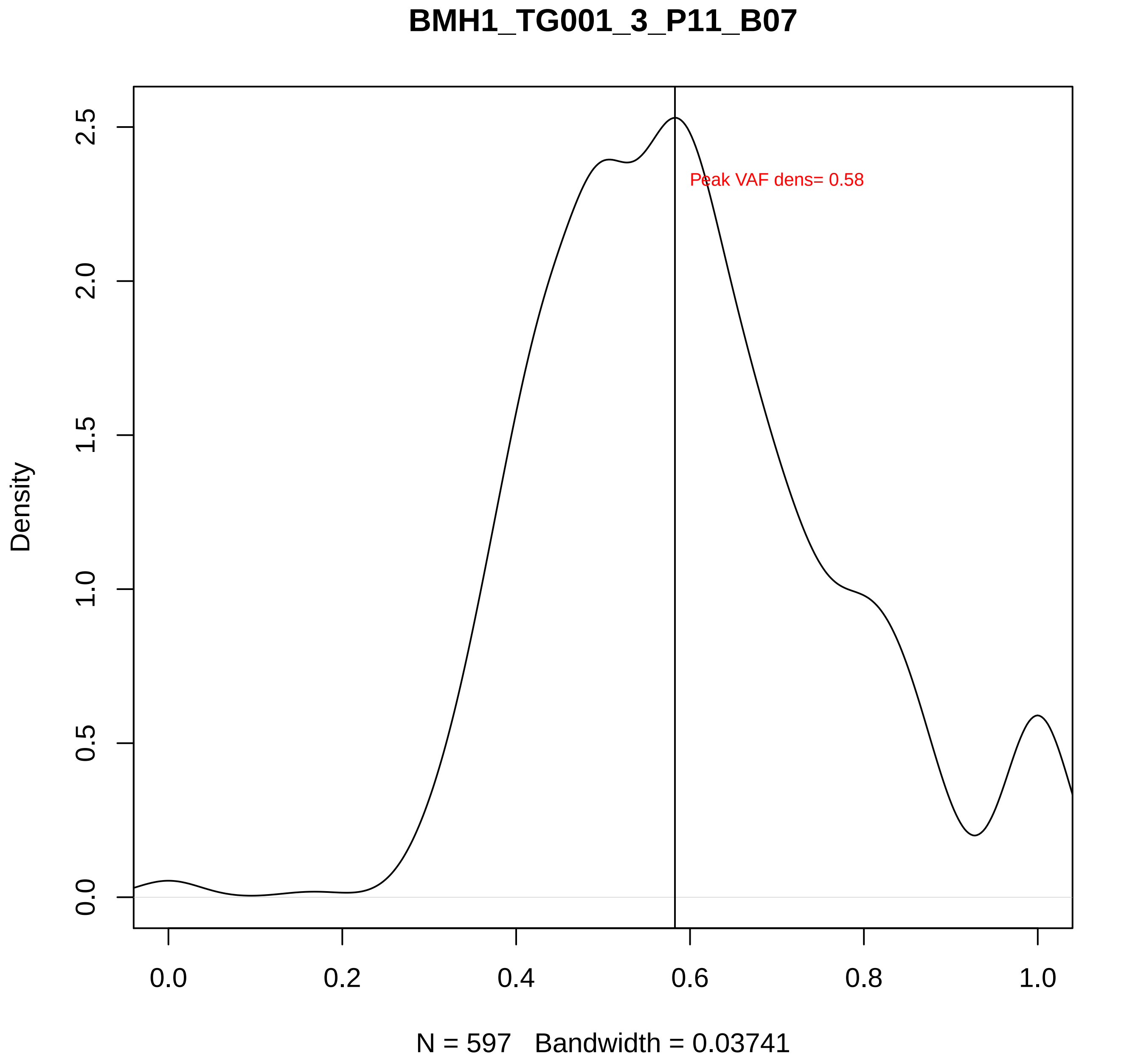
BMH1_TG001_3_P11_B07
Peak VAF dens= 0.58
Density
0.0
0.5
1.0
1.5
2.0
2.5
0.0
0.2
0.4
0.6
0.8
1.0
N = 597   Bandwidth = 0.03741

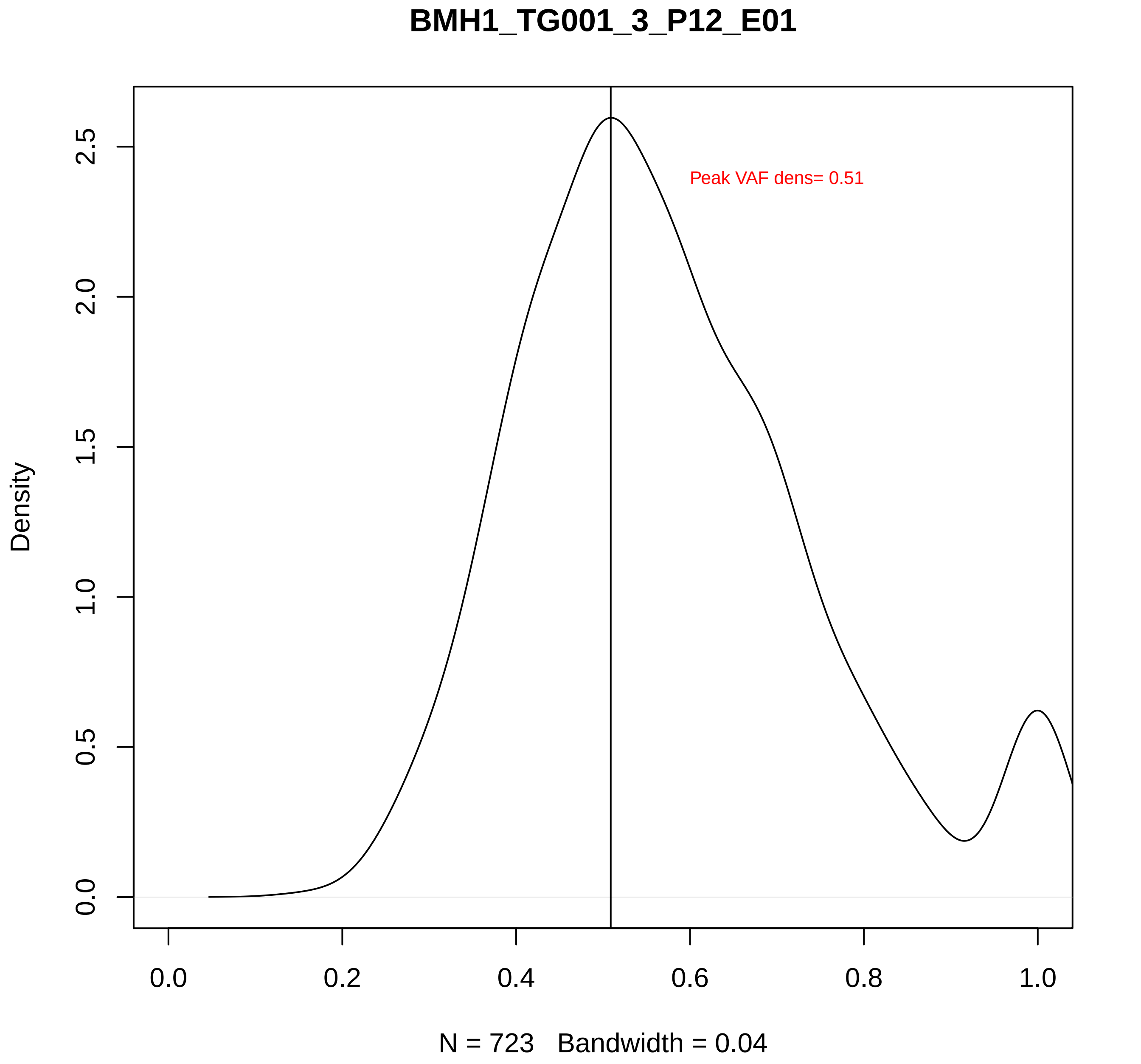
BMH1_TG001_3_P12_E01
Peak VAF dens= 0.51
Density
0.0
0.5
1.0
1.5
2.0
2.5
0.0
0.2
0.4
0.6
0.8
1.0
N = 723   Bandwidth = 0.04

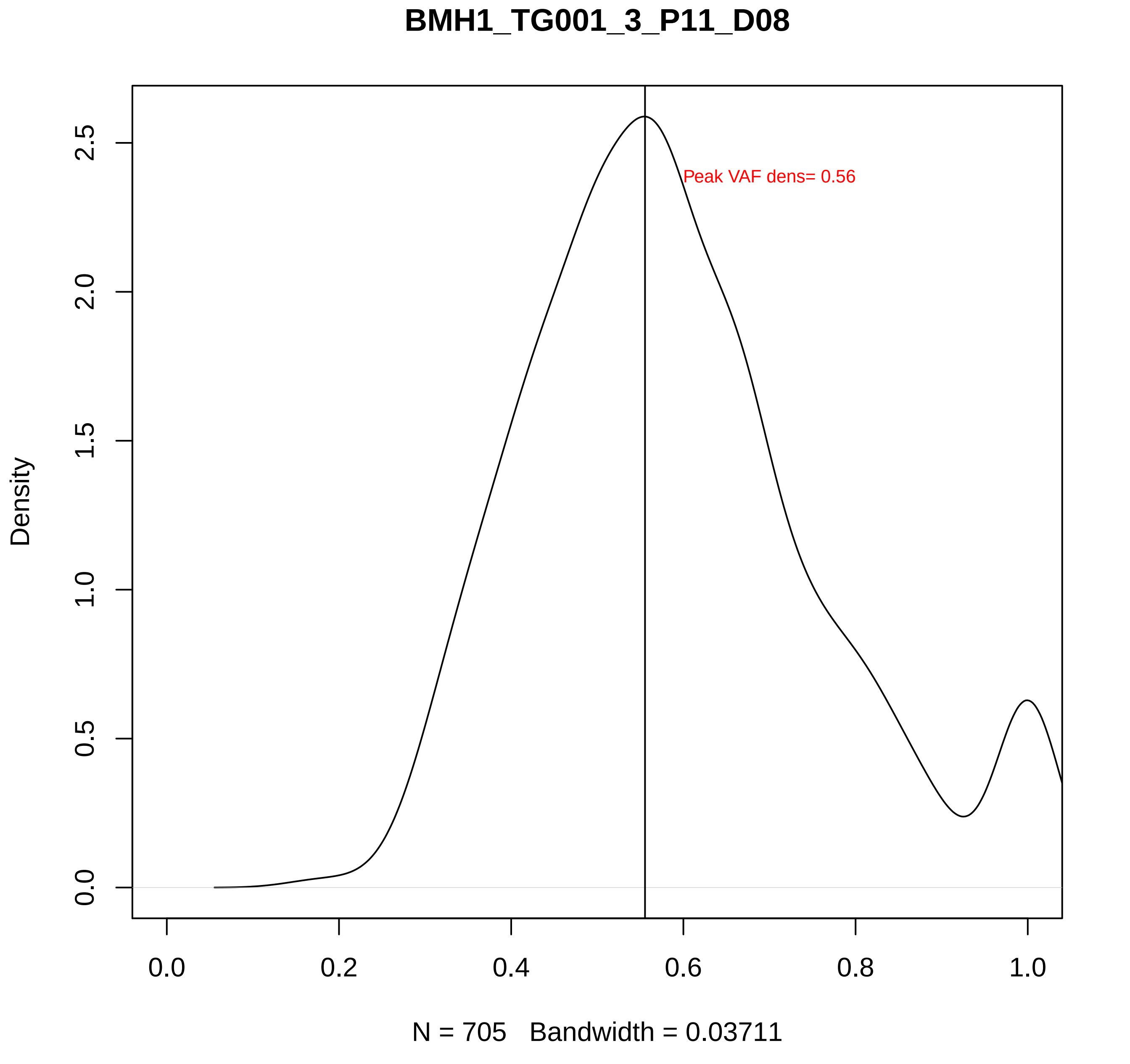
BMH1_TG001_3_P11_D08
Peak VAF dens= 0.56
Density
0.0
0.5
1.0
1.5
2.0
2.5
0.0
0.2
0.4
0.6
0.8
1.0
N = 705 Bandwidth = 0.03711

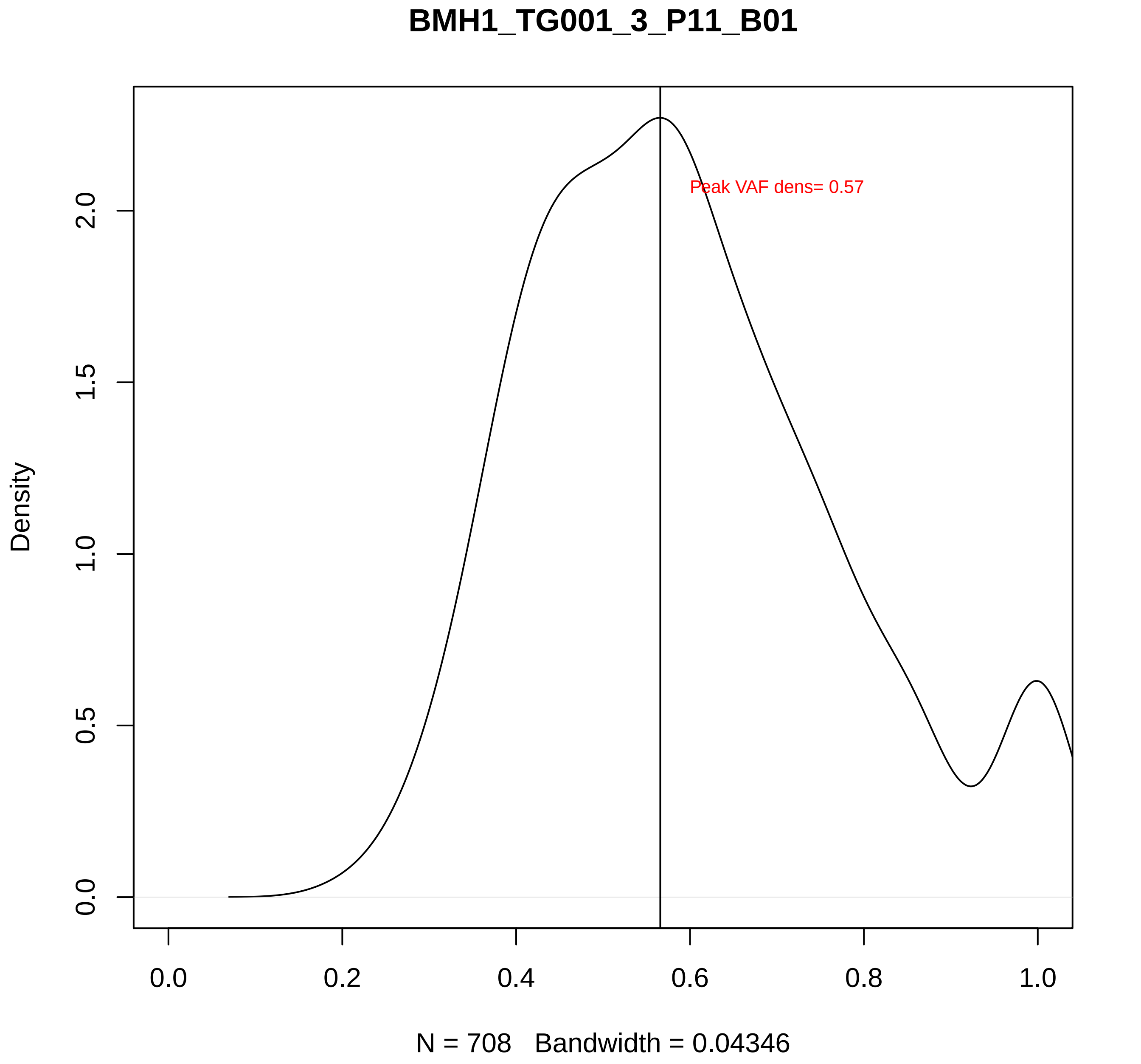

BMH1_TG001_3_P11_B01
Peak VAF dens= 0.57
Density
0.0
0.5
1.0
1.5
2.0
0.0
0.2
0.4
0.6
0.8
1.0
N = 708   Bandwidth = 0.04346

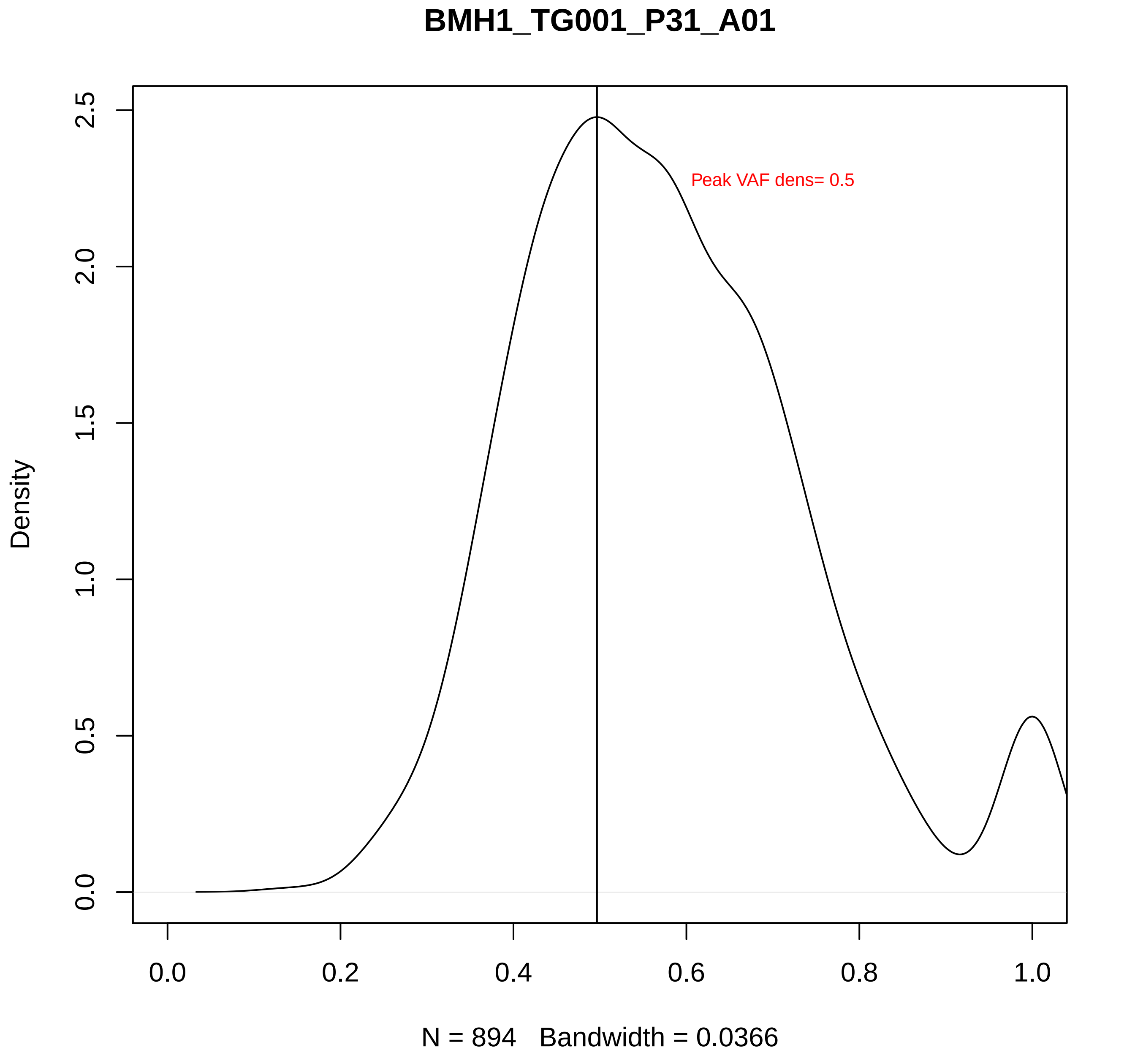

BMH1_TG001_P31_A01
Peak VAF dens= 0.5
Density
0.0
0.5
1.0
1.5
2.0
2.5
0.0
0.2
0.4
0.6
0.8
1.0
N = 894 Bandwidth = 0.0366

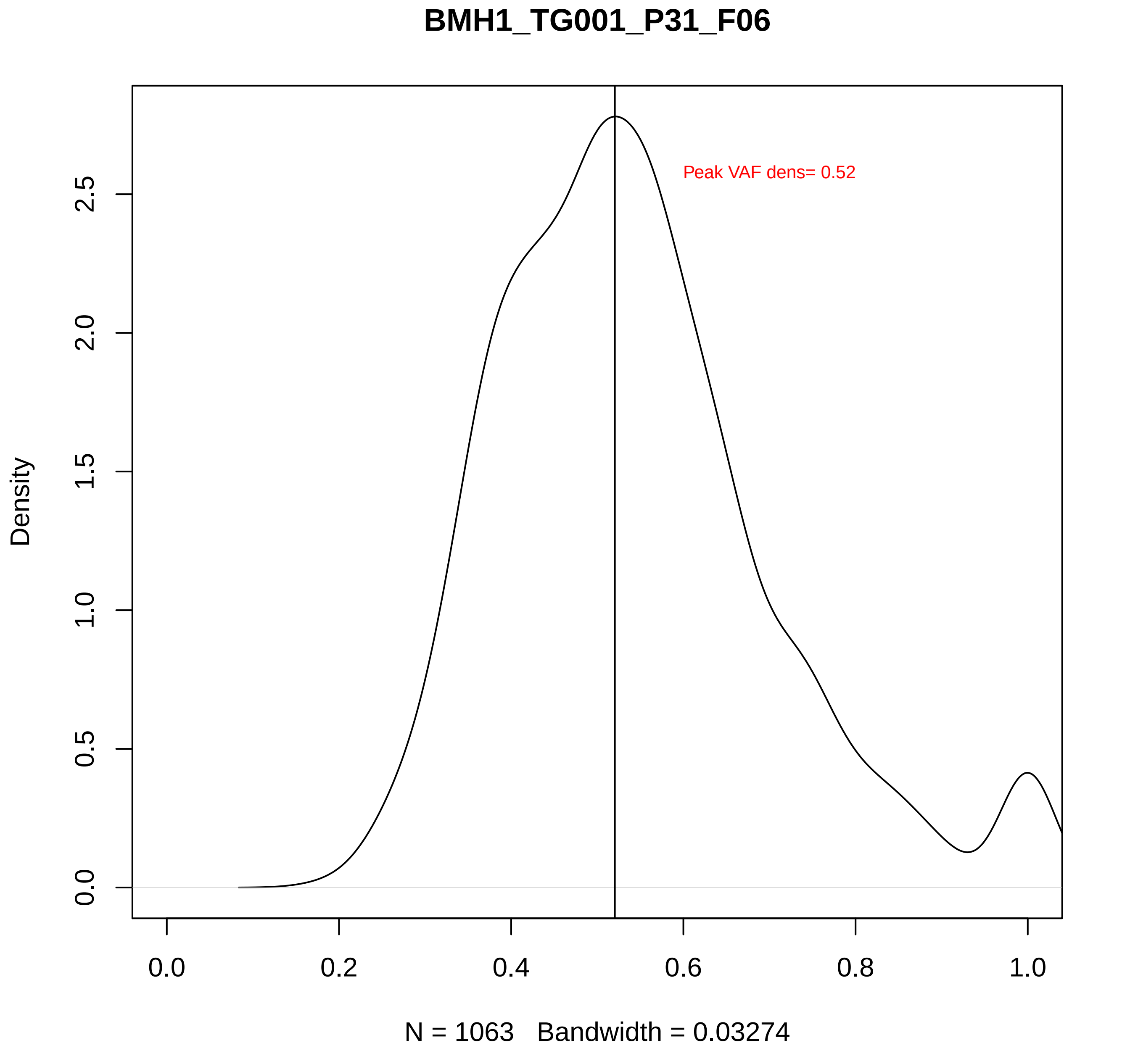
BMH1_TG001_P31_F06
Peak VAF dens= 0.52
Density
0.0
0.5
1.0
1.5
2.0
2.5
0.0
0.2
0.4
0.6
0.8
1.0
N = 1063   Bandwidth = 0.03274

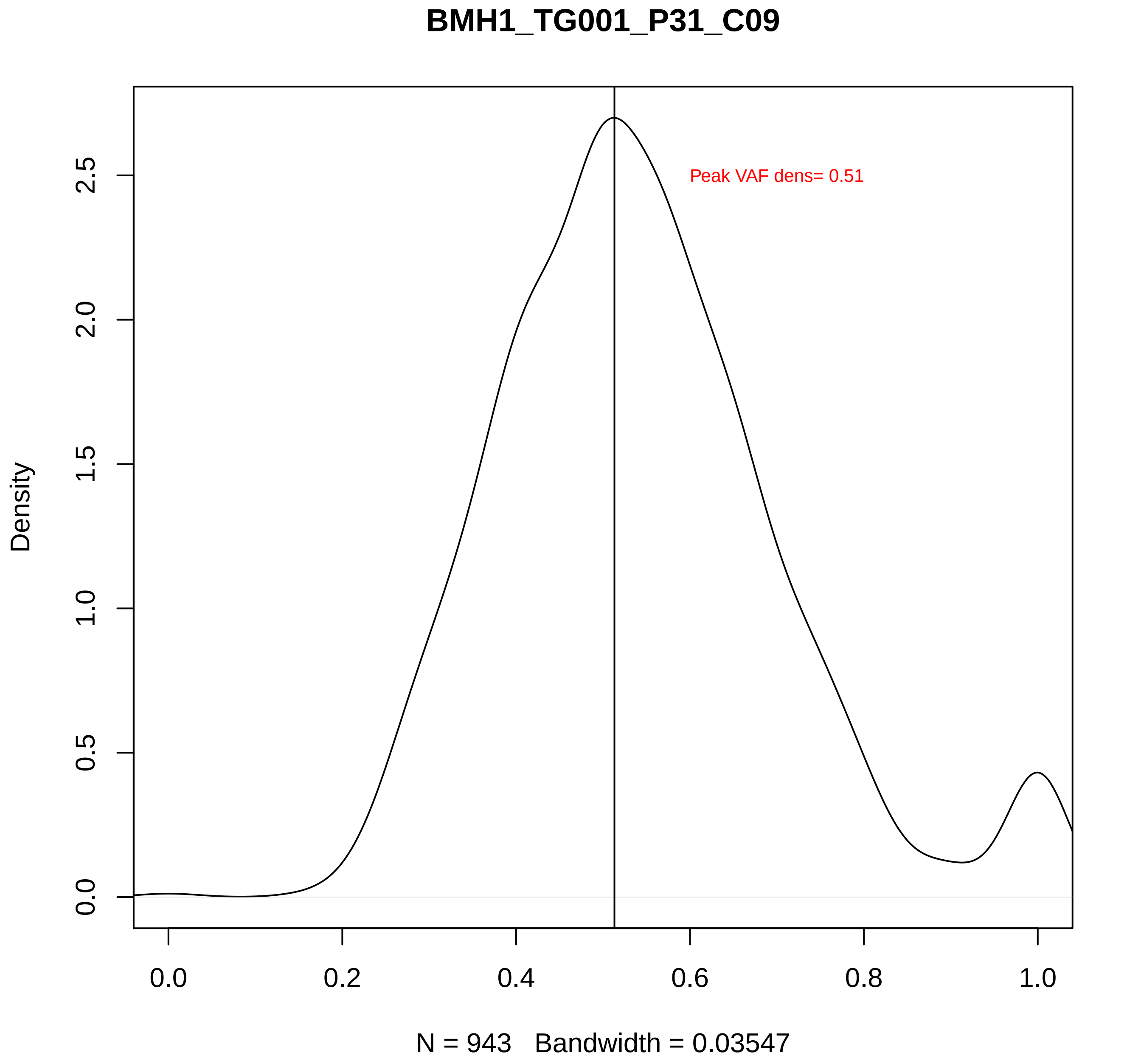

BMH1_TG001_P31_C09
Peak VAF dens= 0.51
Density
0.0
0.5
1.0
1.5
2.0
2.5
0.0
0.2
0.4
0.6
0.8
1.0
N = 943   Bandwidth = 0.03547

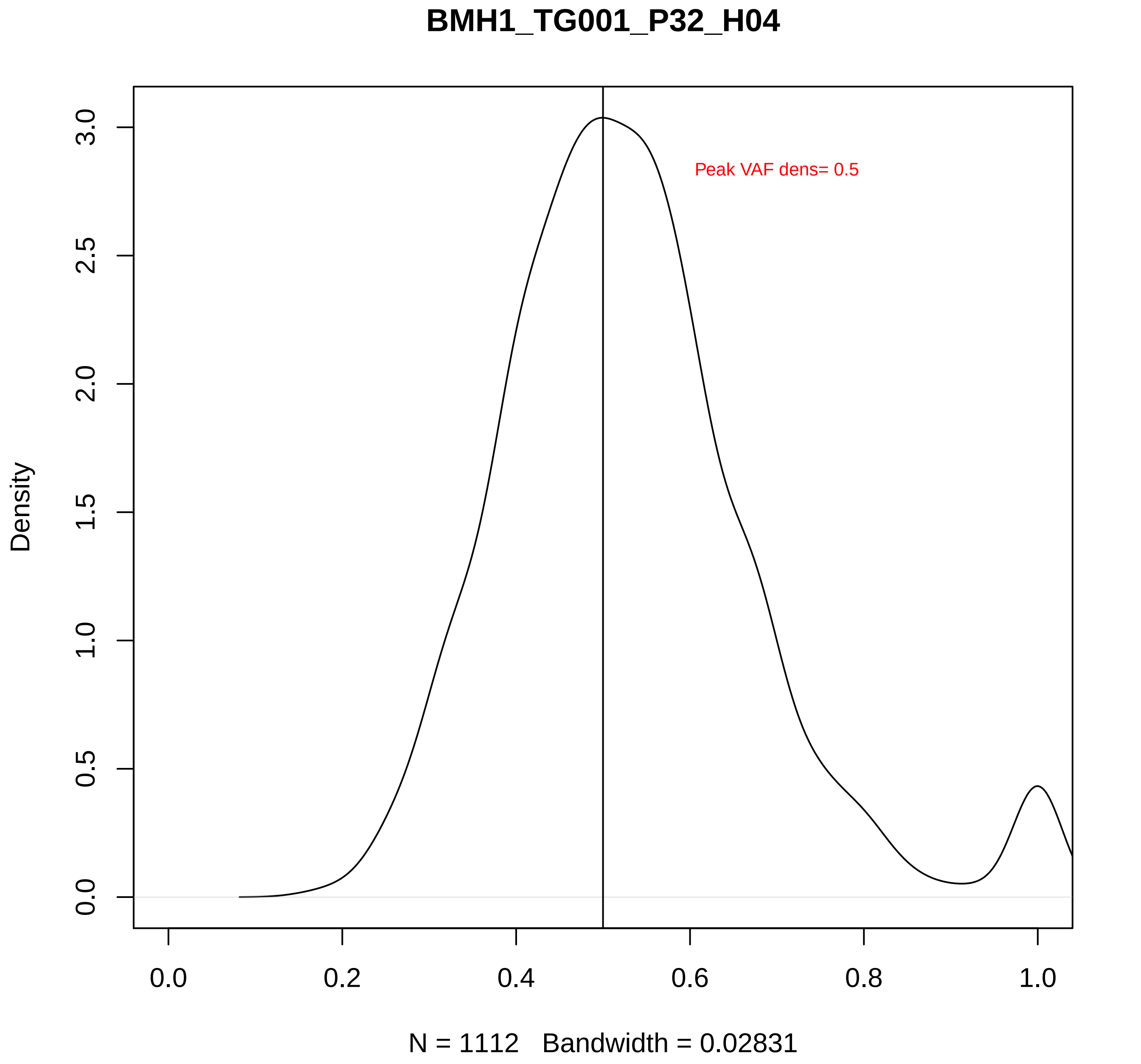

BMH1_TG001_P32_H04
Peak VAF dens= 0.5
Density
3.0
2.5
2.0
1.5
1.0
0.5
0.0
0.0
0.2
0.4
0.6
0.8
1.0
N = 1112   Bandwidth = 0.02831

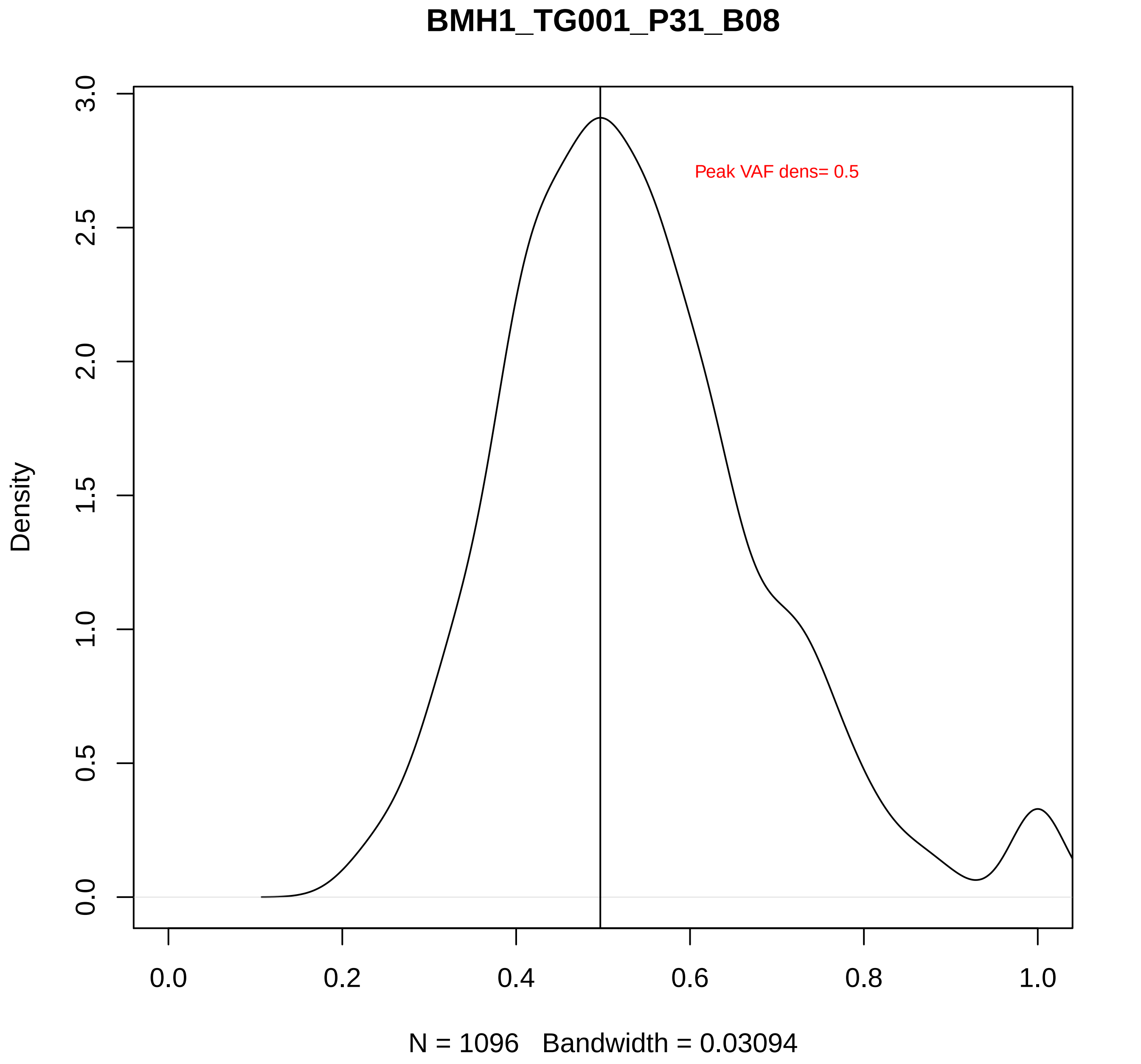

BMH1_TG001_P31_B08
Peak VAF dens= 0.5
Density
0.0
0.5
1.0
1.5
2.0
2.5
3.0
0.0
0.2
0.4
0.6
0.8
1.0
N = 1096 Bandwidth = 0.03094

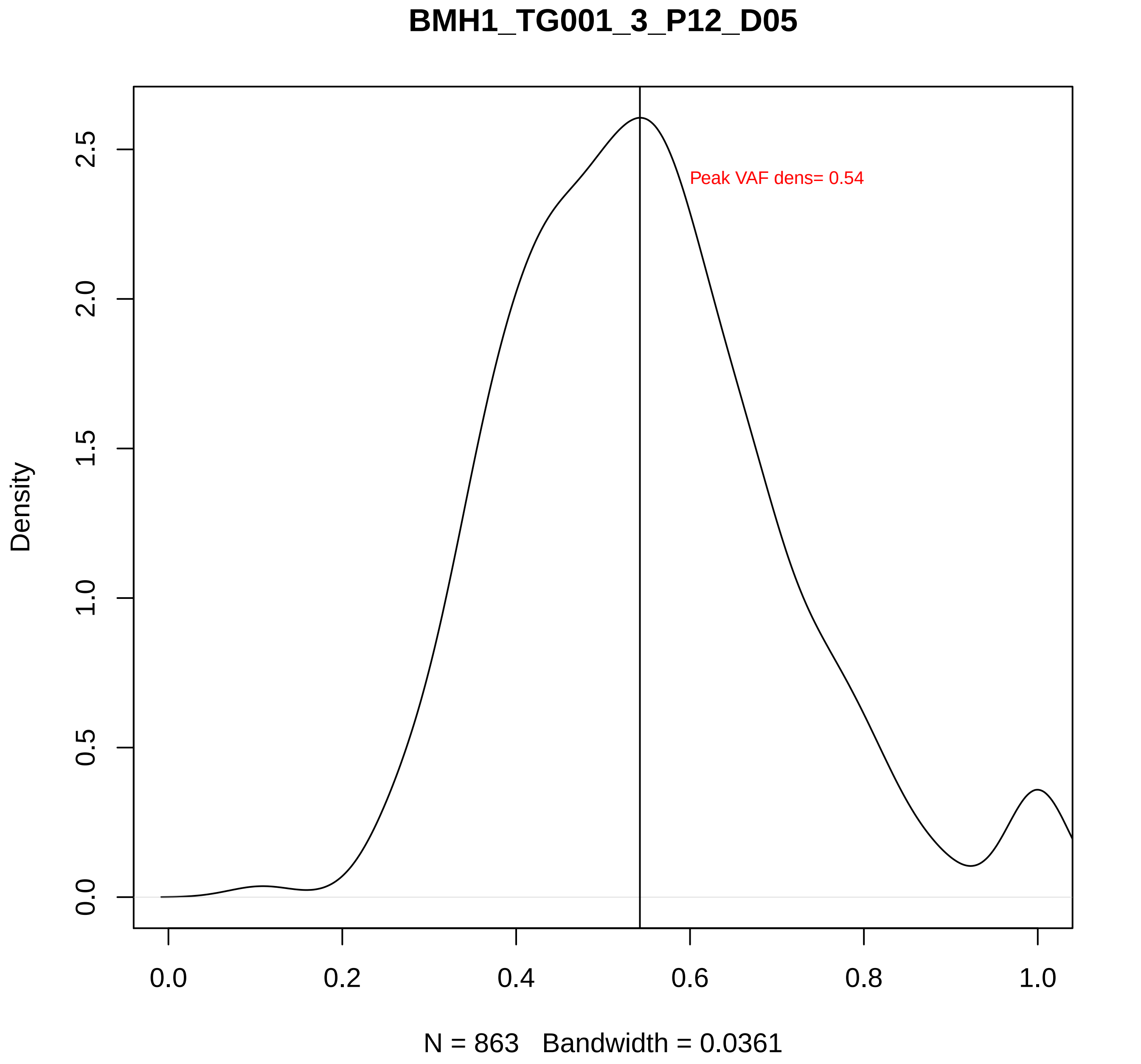

BMH1_TG001_3_P12_D05
Peak VAF dens= 0.54
Density
0.0
0.5
1.0
1.5
2.0
2.5
0.0
0.2
0.4
0.6
0.8
1.0
N = 863   Bandwidth = 0.0361

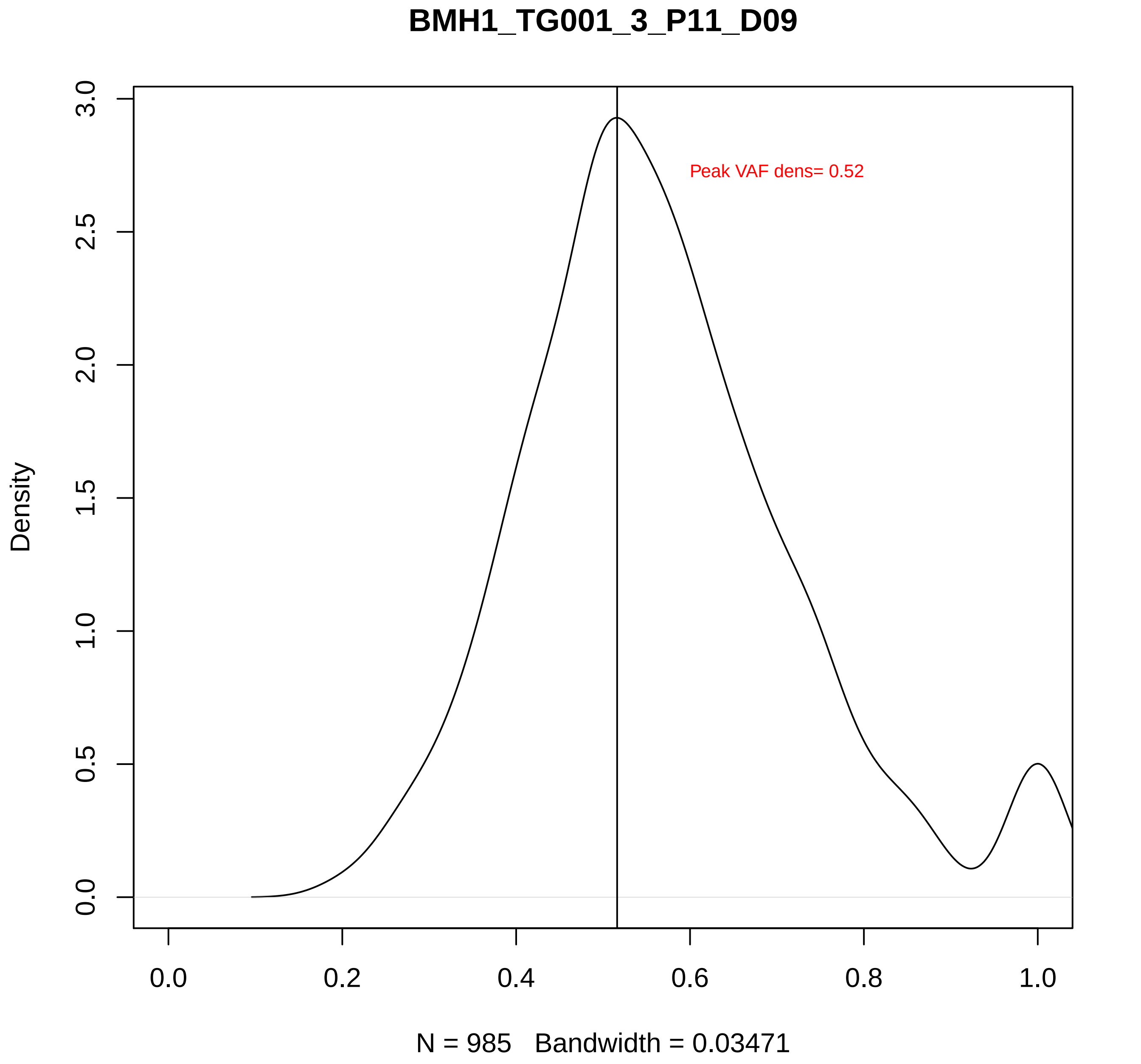
BMH1_TG001_3_P11_D09
Peak VAF dens= 0.52
Density
0.0
0.5
1.0
1.5
2.0
2.5
3.0
0.0
0.2
0.4
0.6
0.8
1.0
N = 985   Bandwidth = 0.03471

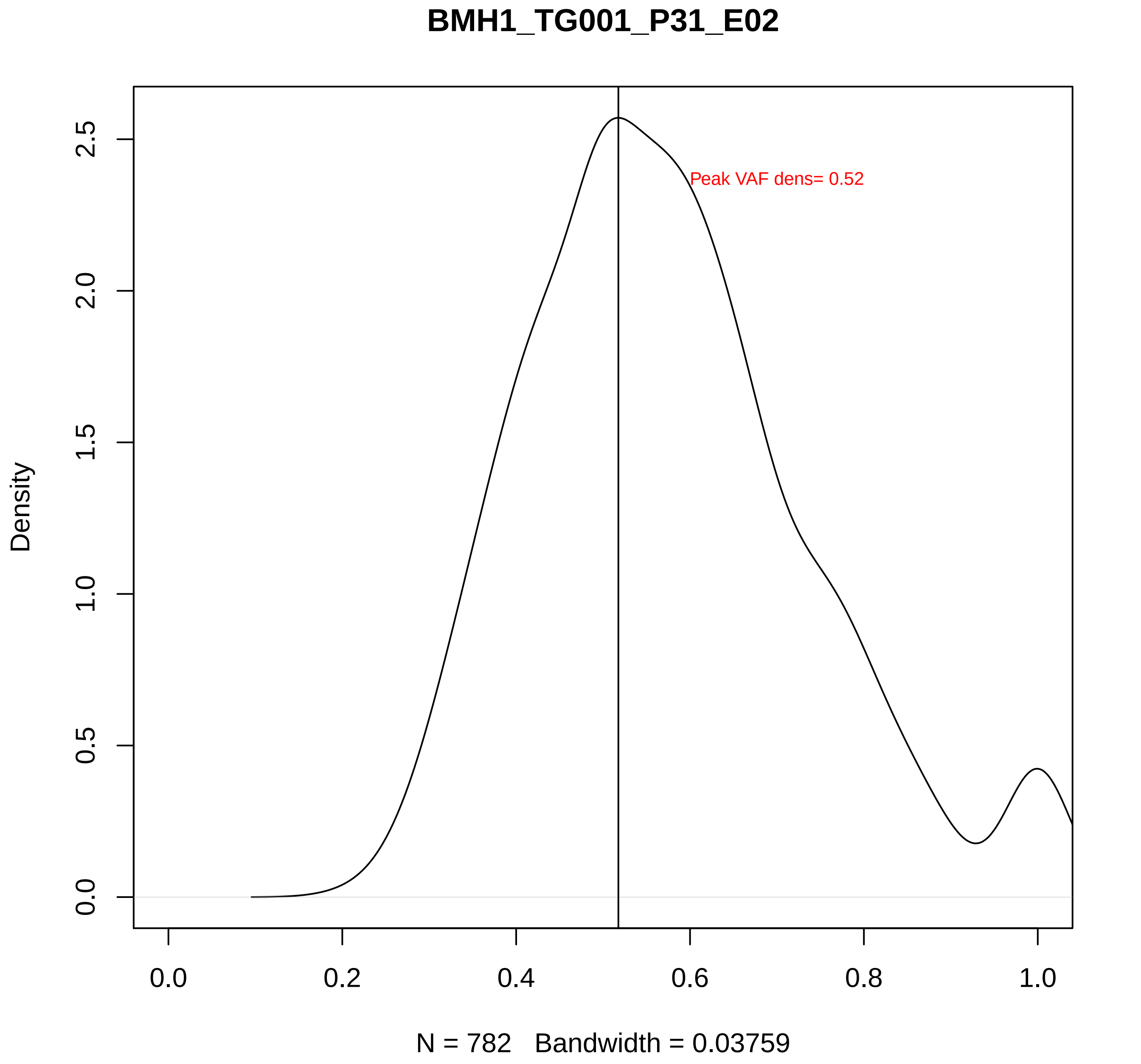

BMH1_TG001_P31_E02
Peak VAF dens= 0.52
Density
0.0
0.5
1.0
1.5
2.0
2.5
0.0
0.2
0.4
0.6
0.8
1.0
N = 782   Bandwidth = 0.03759

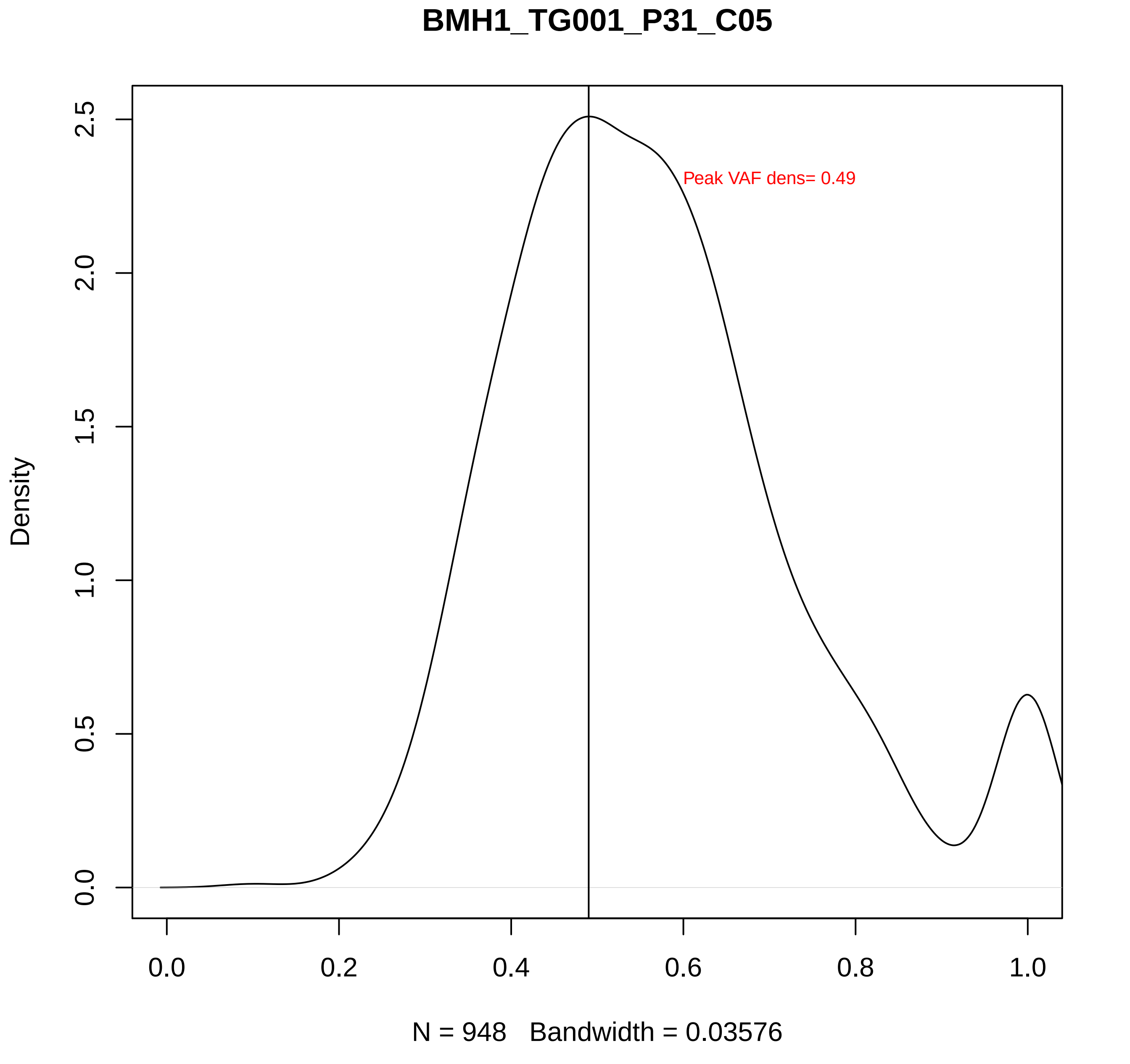
BMH1_TG001_P31_C05
Peak VAF dens= 0.49
Density
0.0
0.5
1.0
1.5
2.0
2.5
0.0
0.2
0.4
0.6
0.8
1.0
N = 948   Bandwidth = 0.03576

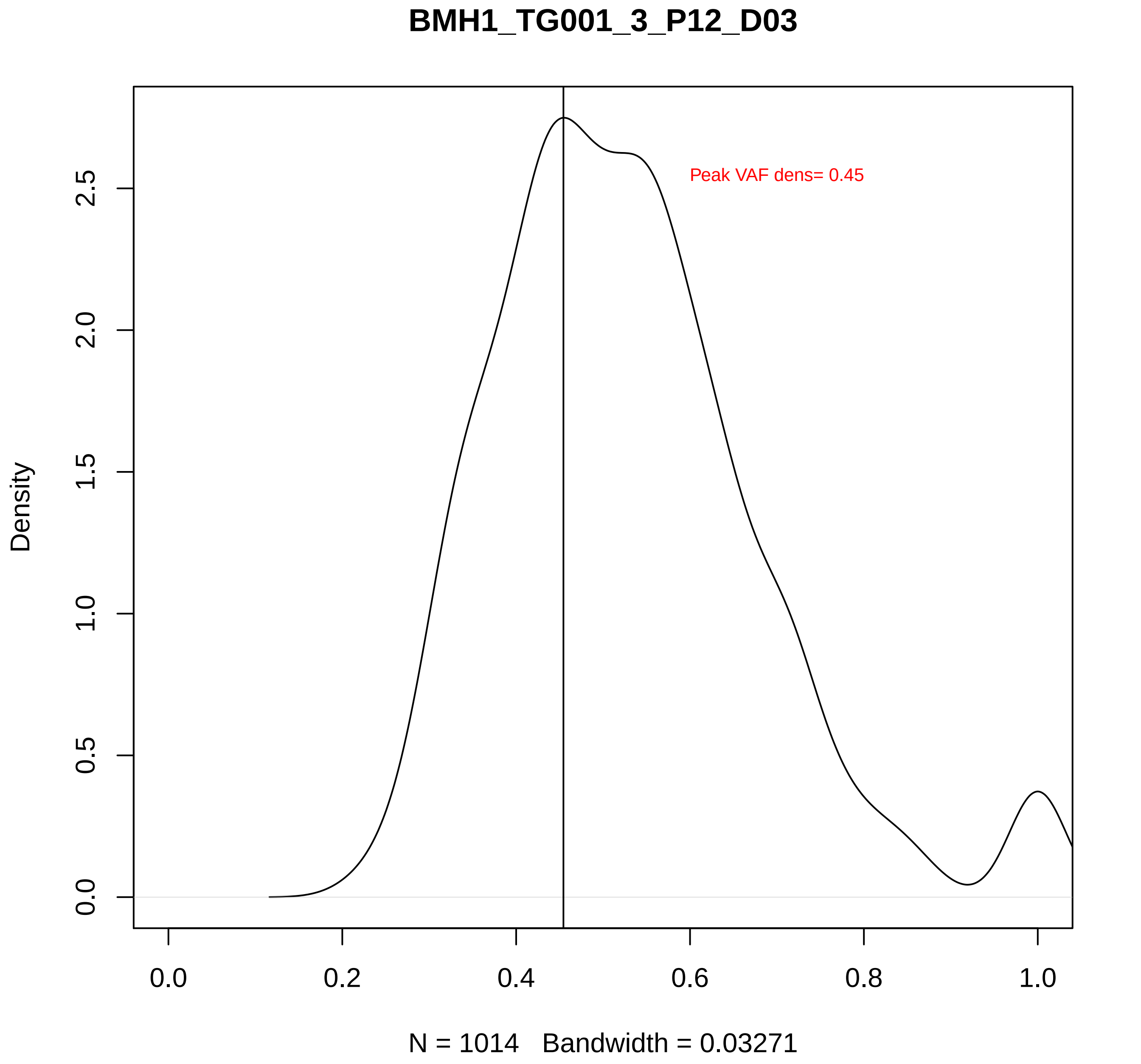
BMH1_TG001_3_P12_D03
Peak VAF dens= 0.45
Density
0.0
0.5
1.0
1.5
2.0
2.5
0.0
0.2
0.4
0.6
0.8
1.0
N = 1014   Bandwidth = 0.03271

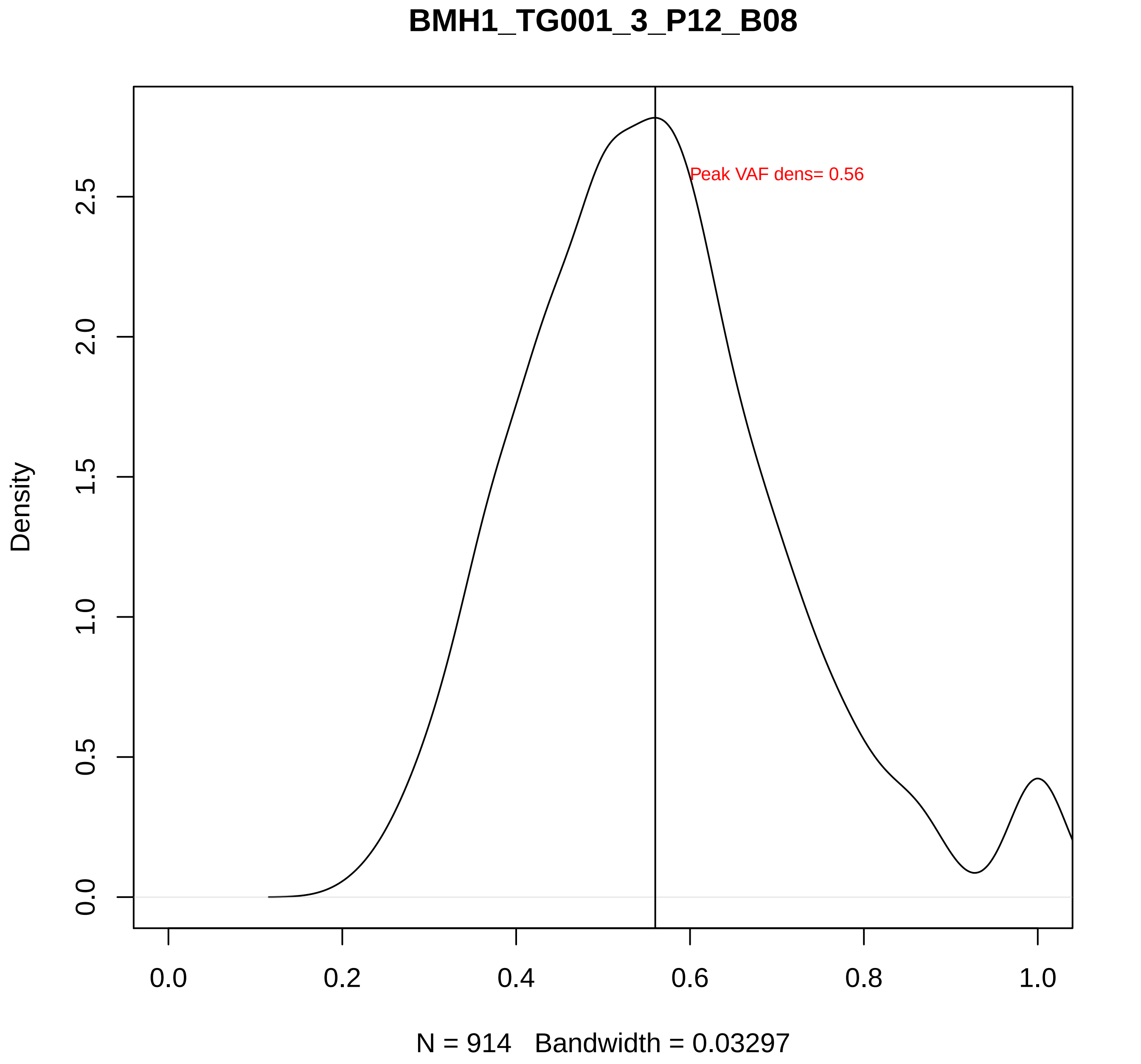
BMH1_TG001_3_P12_B08
Peak VAF dens= 0.56
Density
0.0
0.5
1.0
1.5
2.0
2.5
0.0
0.2
0.4
0.6
0.8
1.0
N = 914 Bandwidth = 0.03297

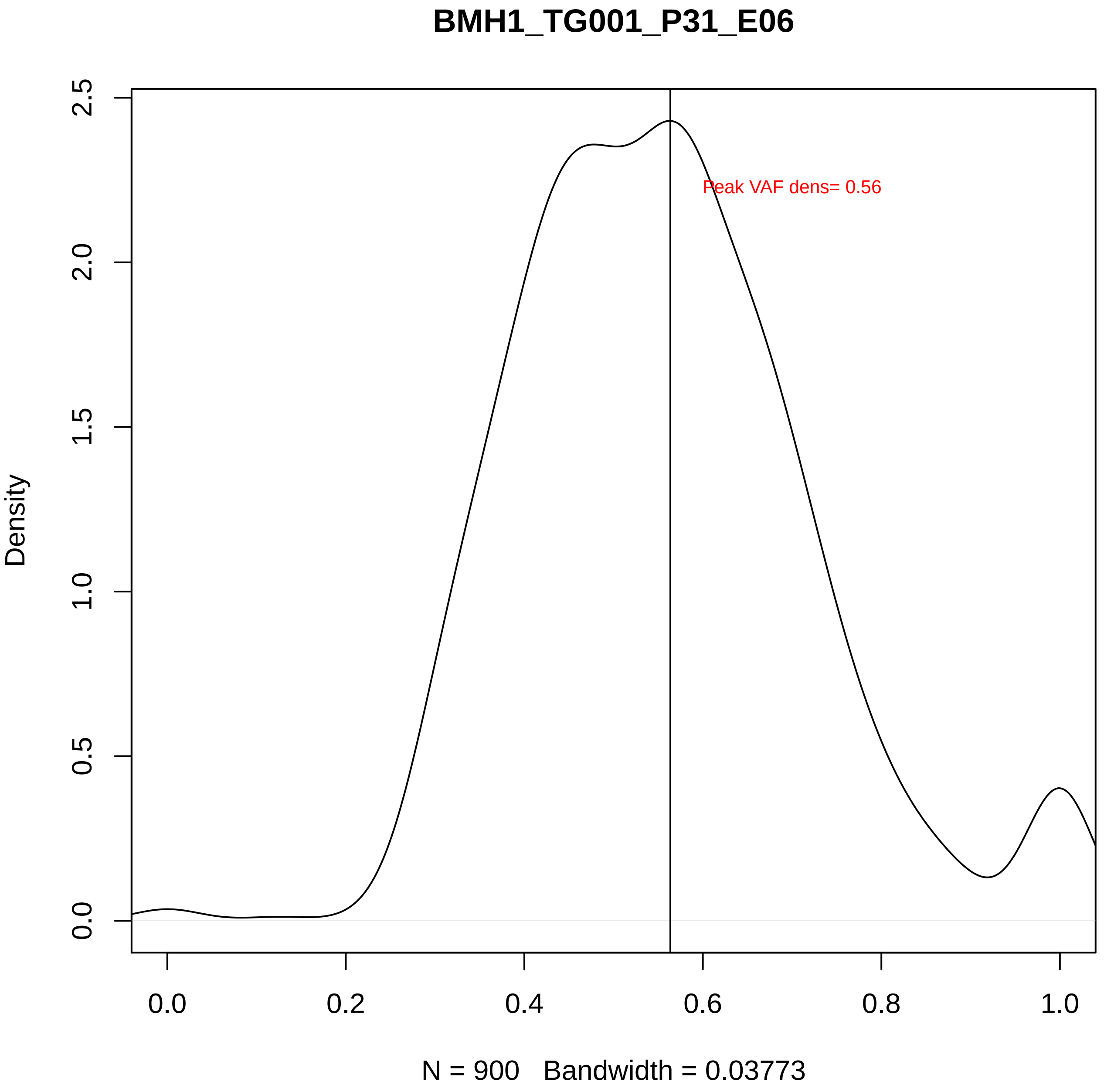
BMH1_TG001_P31_E06
Peak VAF dens= 0.56
Density
0.0
0.5
1.0
1.5
2.0
2.5
0.0
0.2
0.4
0.6
0.8
1.0
N = 900 Bandwidth = 0.03773

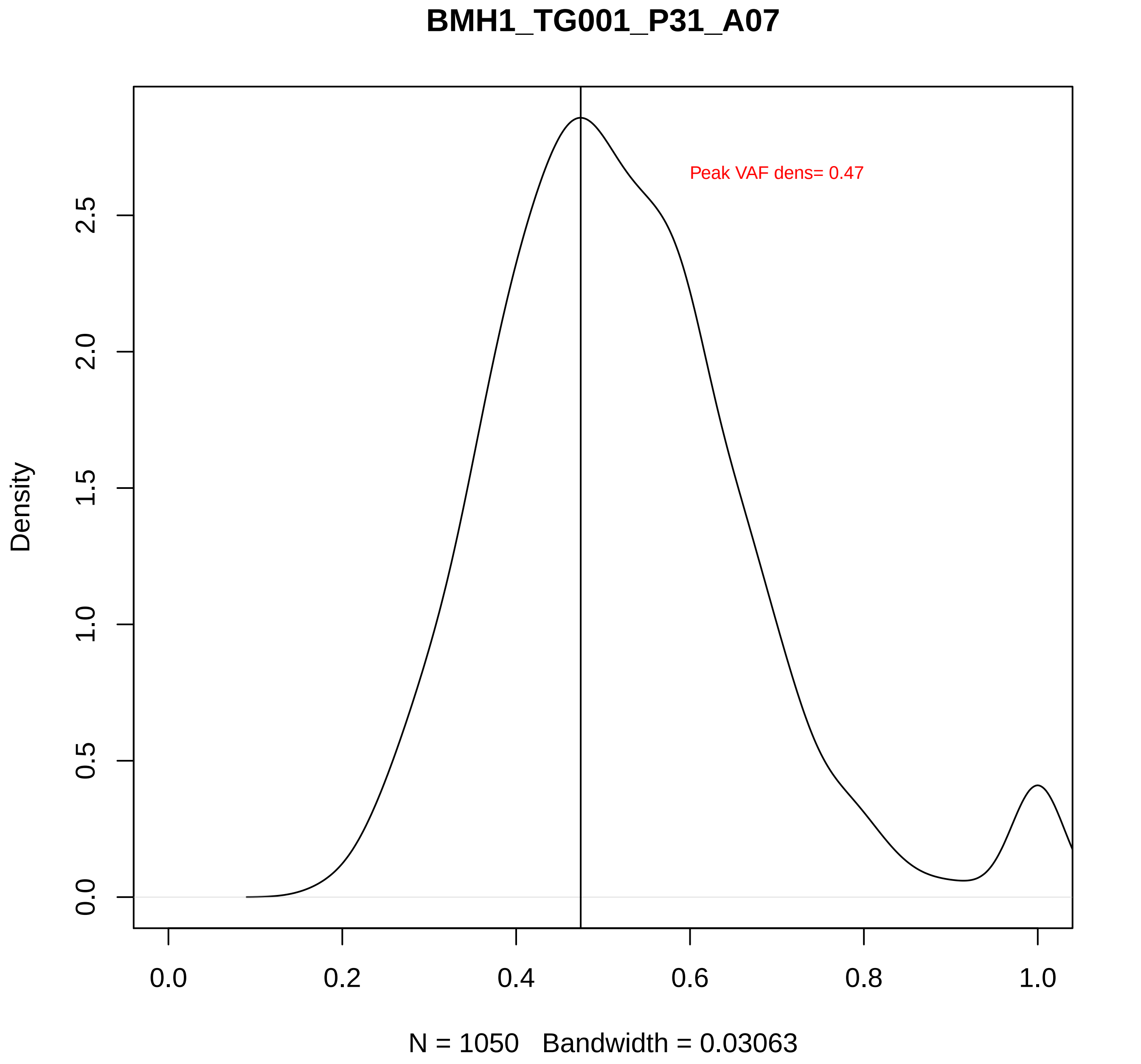
BMH1_TG001_P31_A07
Peak VAF dens= 0.47
Density
0.0
0.5
1.0
1.5
2.0
2.5
0.0
0.2
0.4
0.6
0.8
1.0
N = 1050   Bandwidth = 0.03063

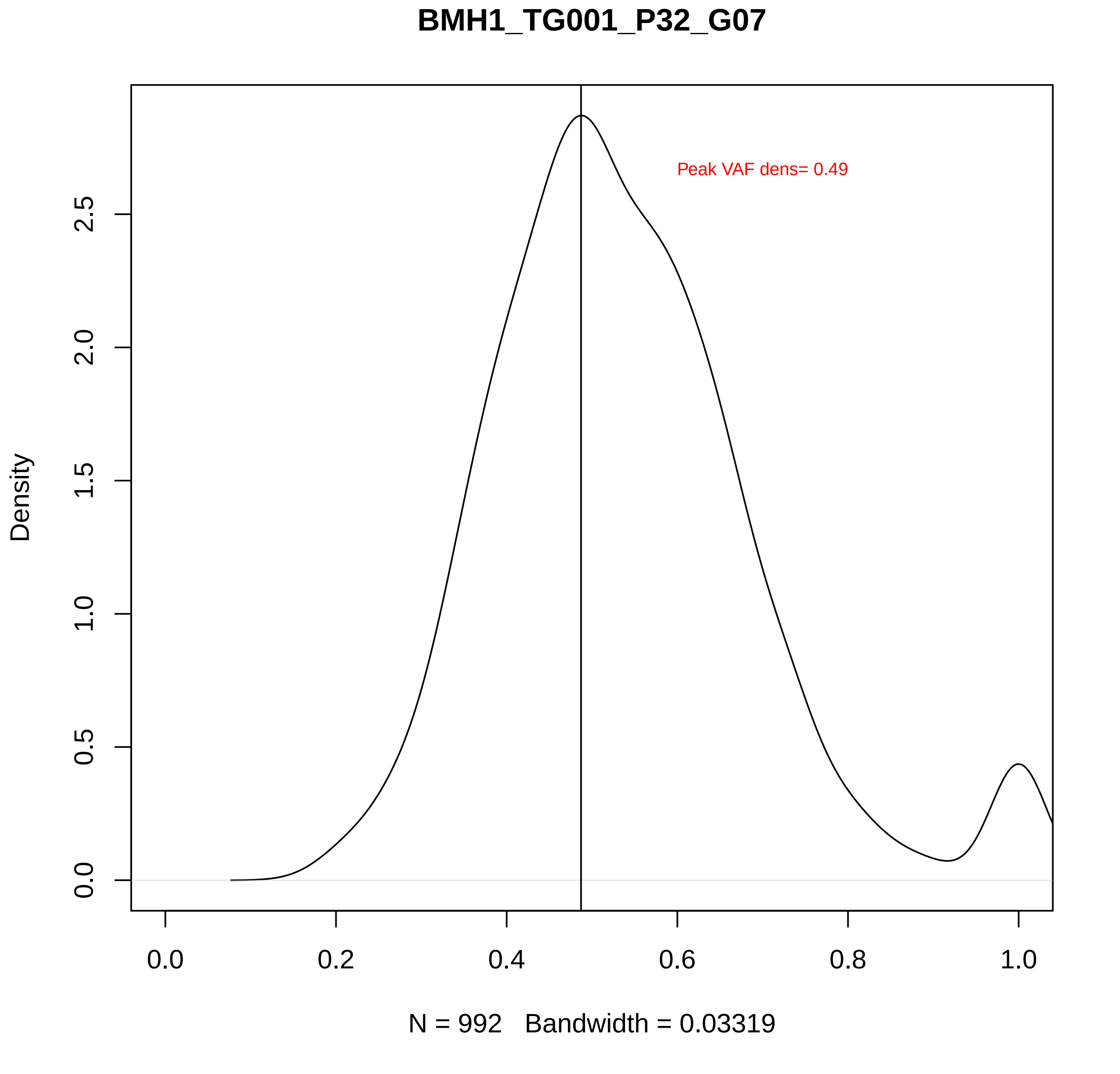
BMH1_TG001_P32_G07
Peak VAF dens= 0.49
Density
0.0
0.5
1.0
1.5
2.0
2.5
0.0
0.2
0.4
0.6
0.8
1.0
N = 992 Bandwidth = 0.03319

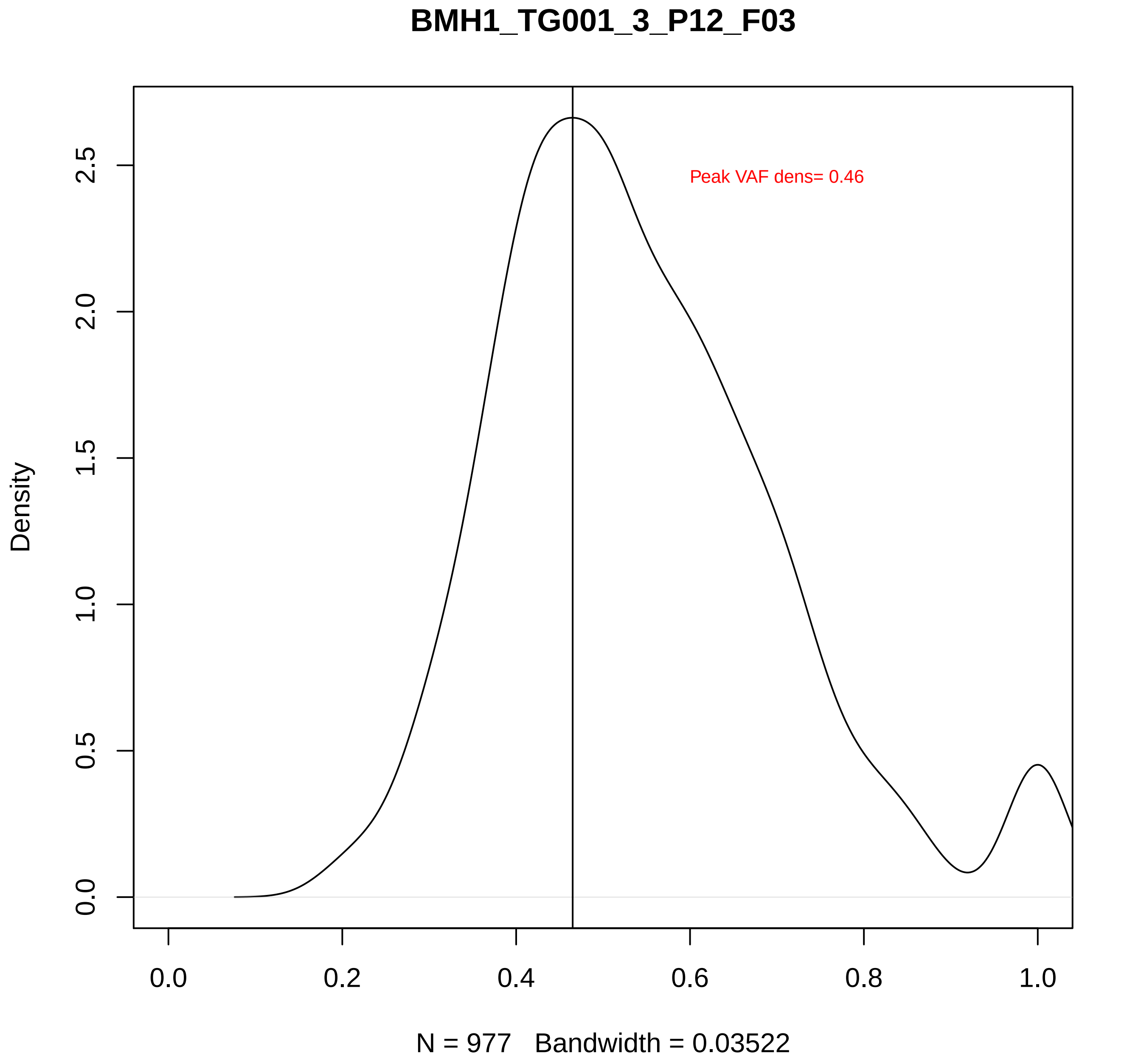
BMH1_TG001_3_P12_F03
Peak VAF dens= 0.46
Density
0.0
0.5
1.0
1.5
2.0
2.5
0.0
0.2
0.4
0.6
0.8
1.0
N = 977   Bandwidth = 0.03522

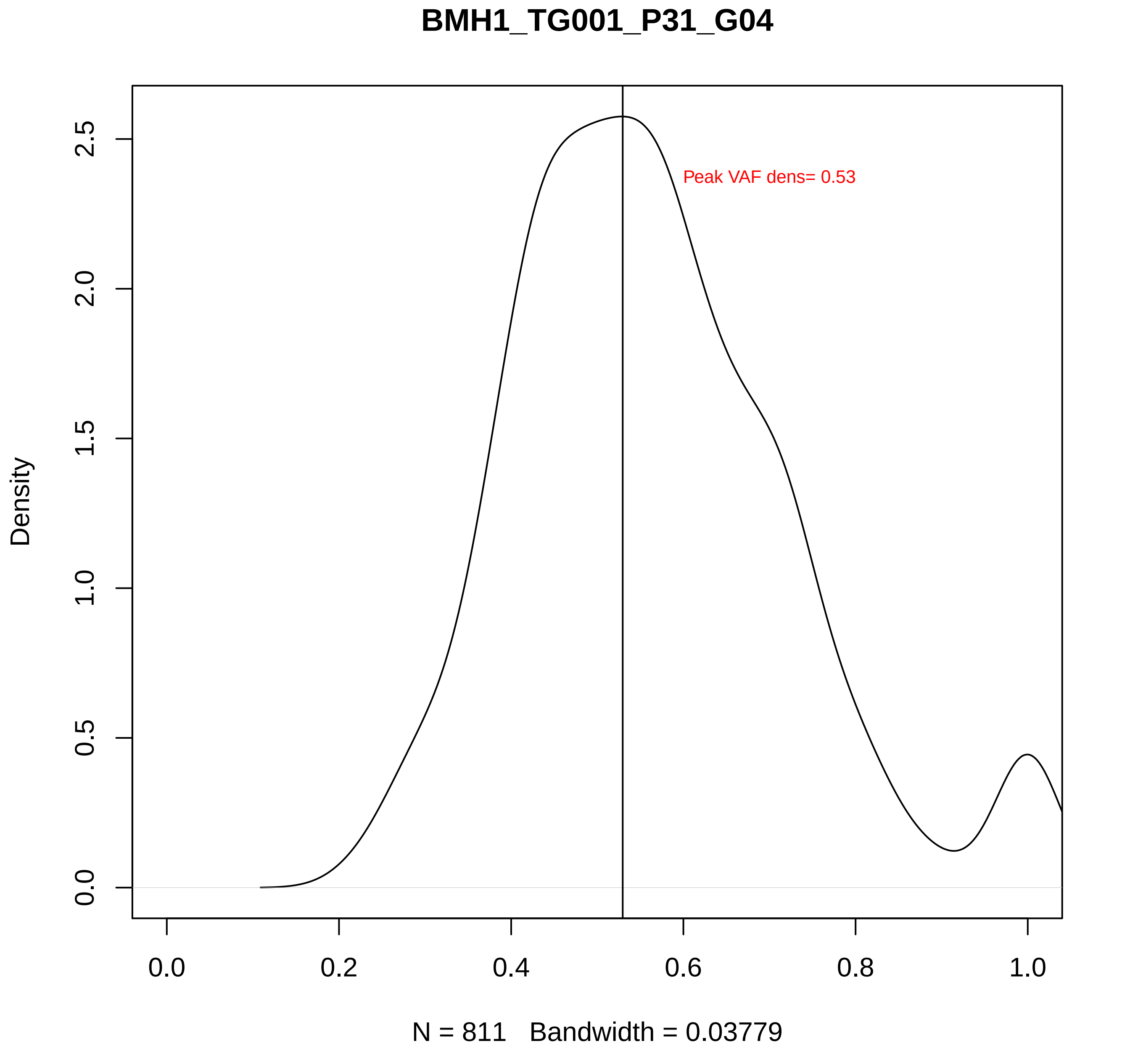

BMH1_TG001_P31_G04
Peak VAF dens= 0.53
Density
0.0
0.5
1.0
1.5
2.0
2.5
0.0
0.2
0.4
0.6
0.8
1.0
N = 811   Bandwidth = 0.03779

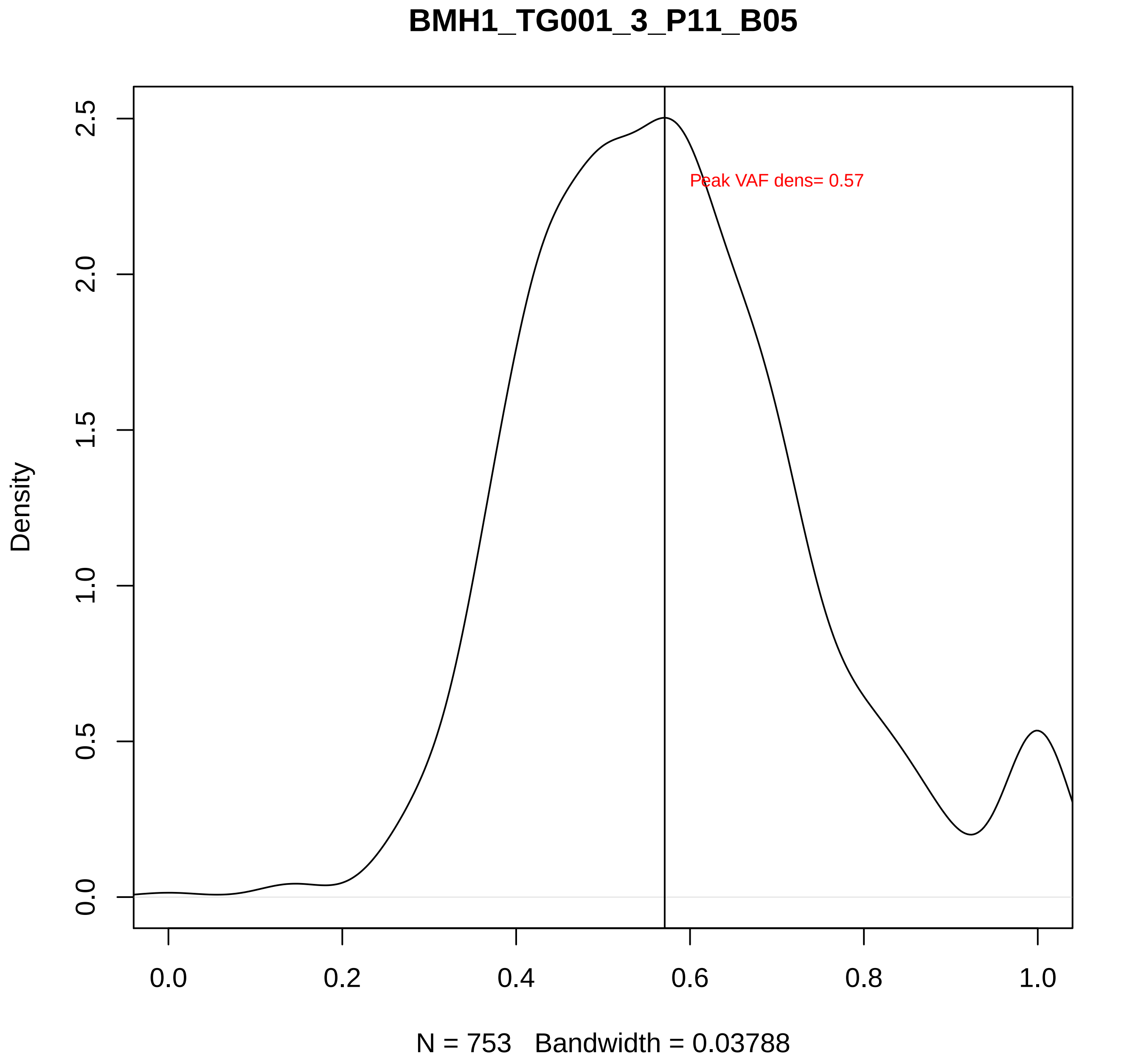
BMH1_TG001_3_P11_B05
Peak VAF dens= 0.57
Density
0.0
0.5
1.0
1.5
2.0
2.5
0.0
0.2
0.4
0.6
0.8
1.0
N = 753   Bandwidth = 0.03788

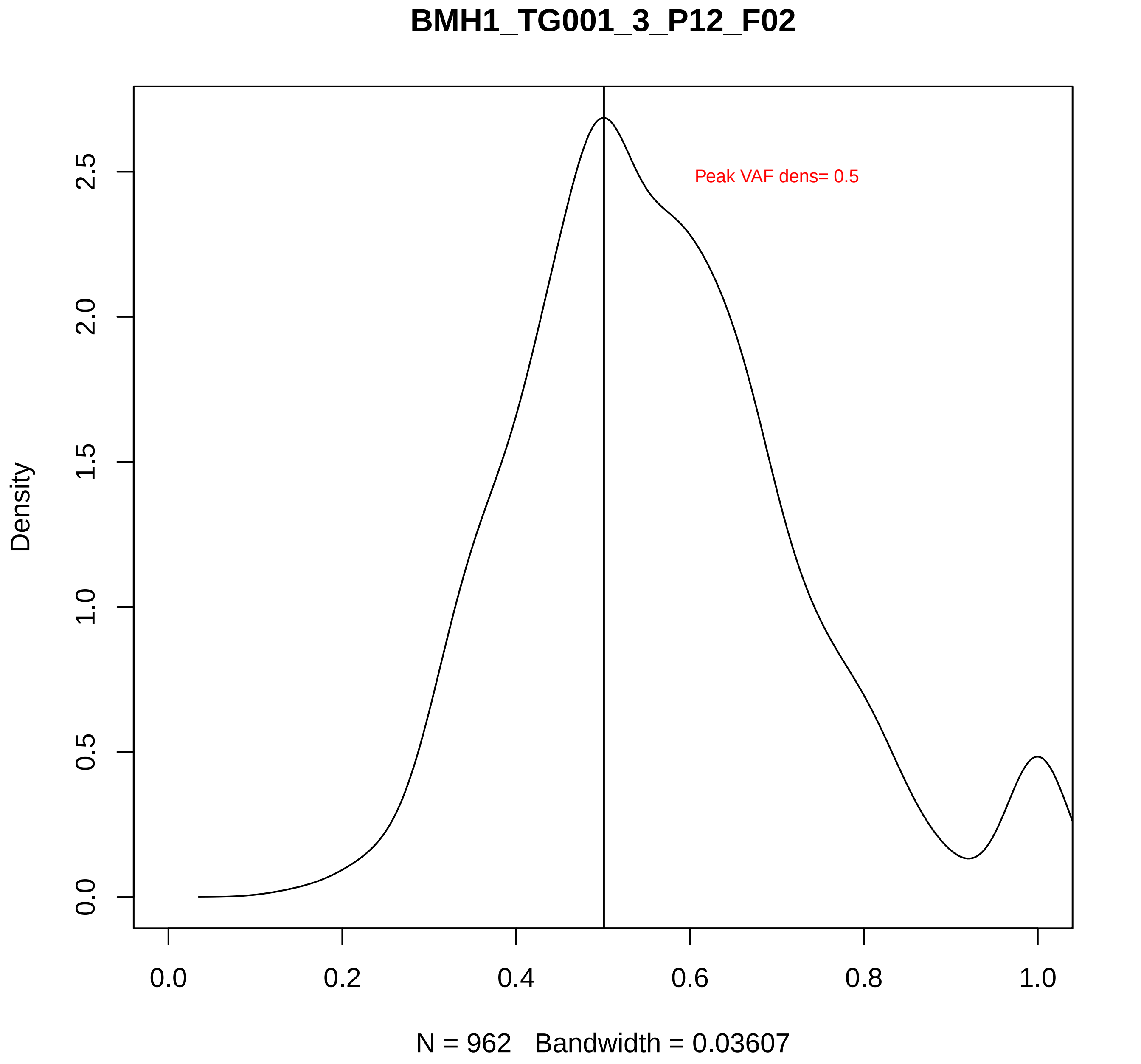
BMH1_TG001_3_P12_F02
Peak VAF dens= 0.5
Density
0.0
0.5
1.0
1.5
2.0
2.5
0.0
0.2
0.4
0.6
0.8
1.0
N = 962   Bandwidth = 0.03607

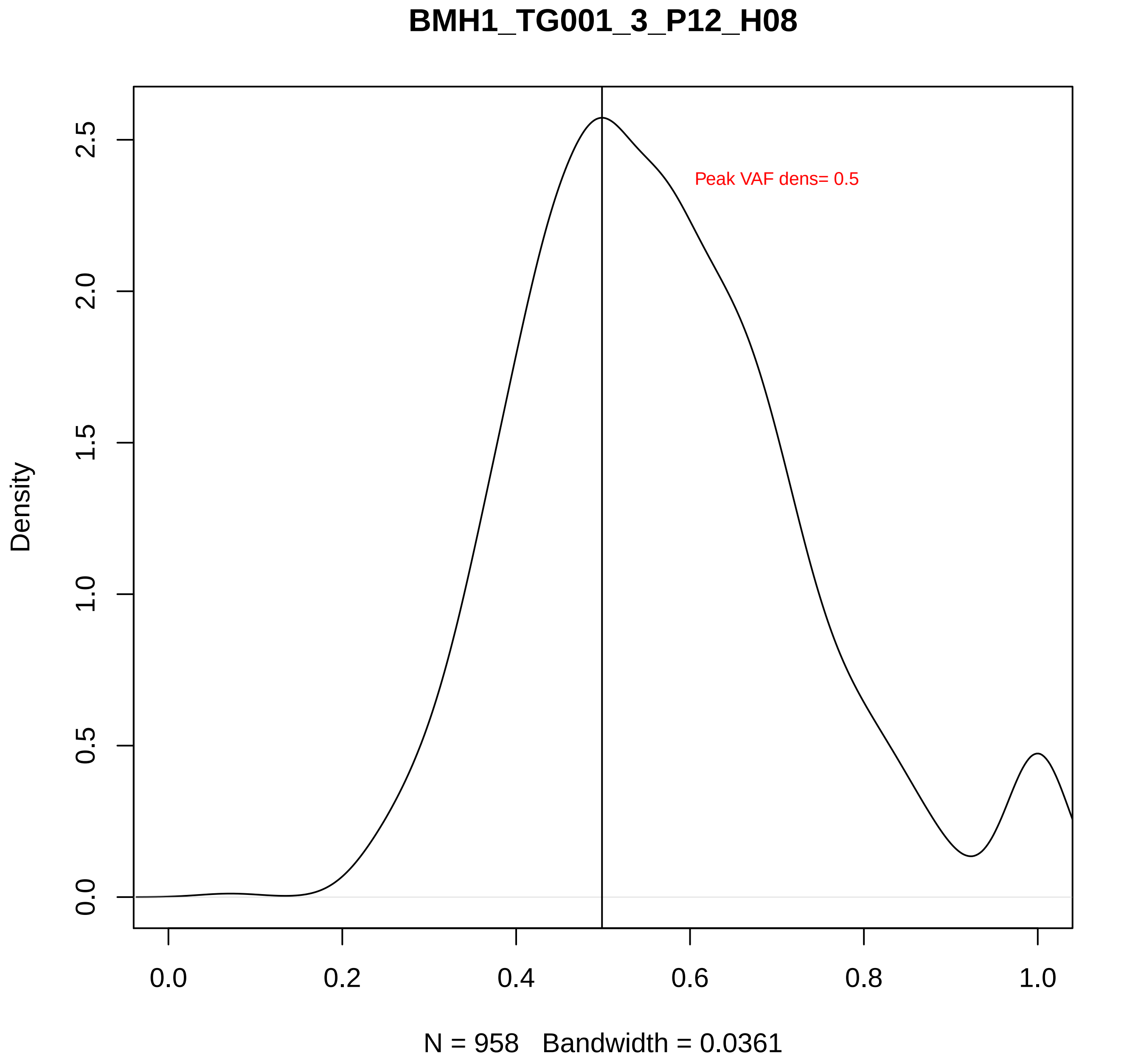

BMH1_TG001_3_P12_H08
Peak VAF dens= 0.5
Density
0.0
0.5
1.0
1.5
2.0
2.5
0.0
0.2
0.4
0.6
0.8
1.0
N = 958   Bandwidth = 0.0361

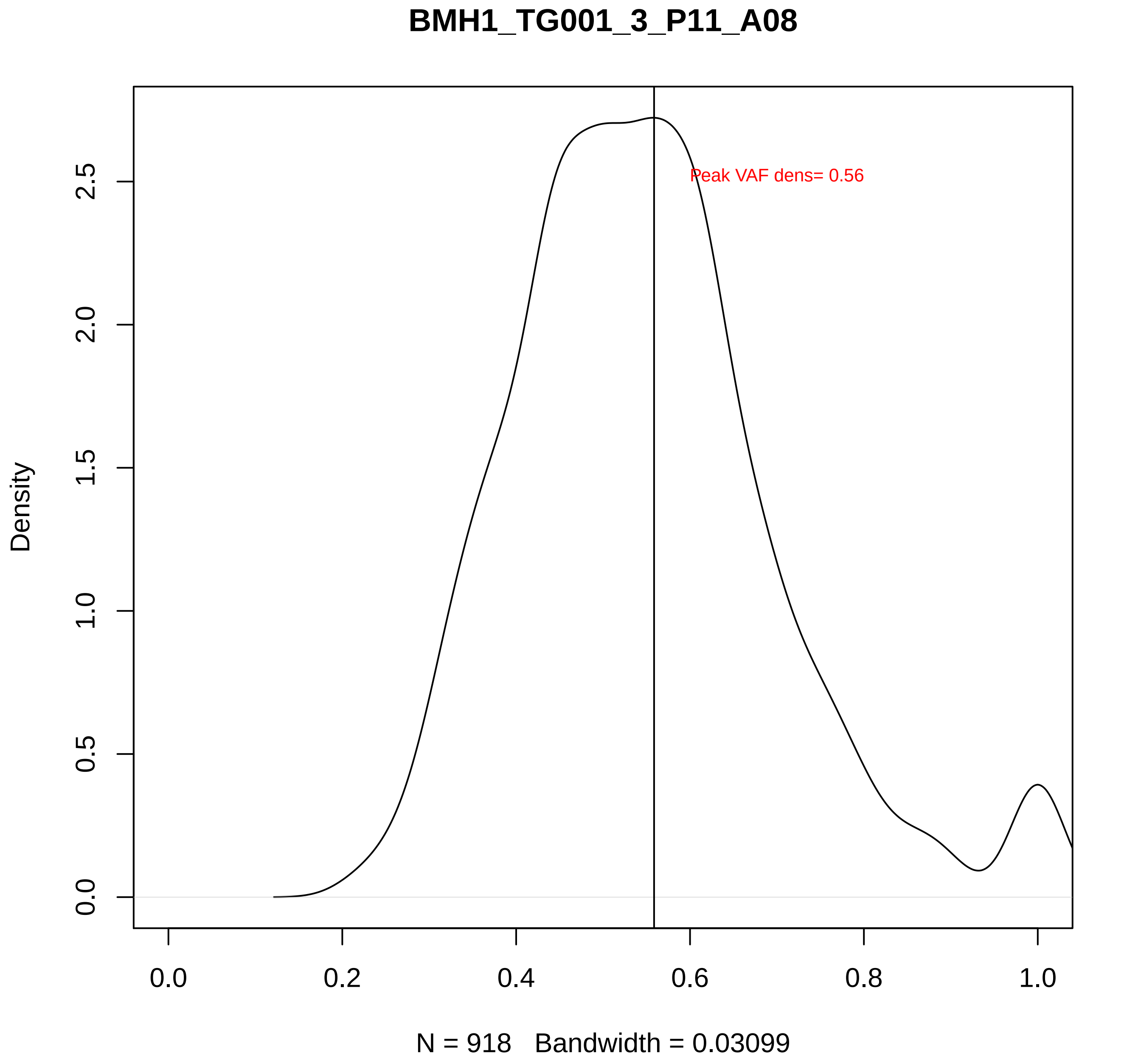
BMH1_TG001_3_P11_A08
Peak VAF dens= 0.56
Density
0.0
0.5
1.0
1.5
2.0
2.5
0.0
0.2
0.4
0.6
0.8
1.0
N = 918   Bandwidth = 0.03099

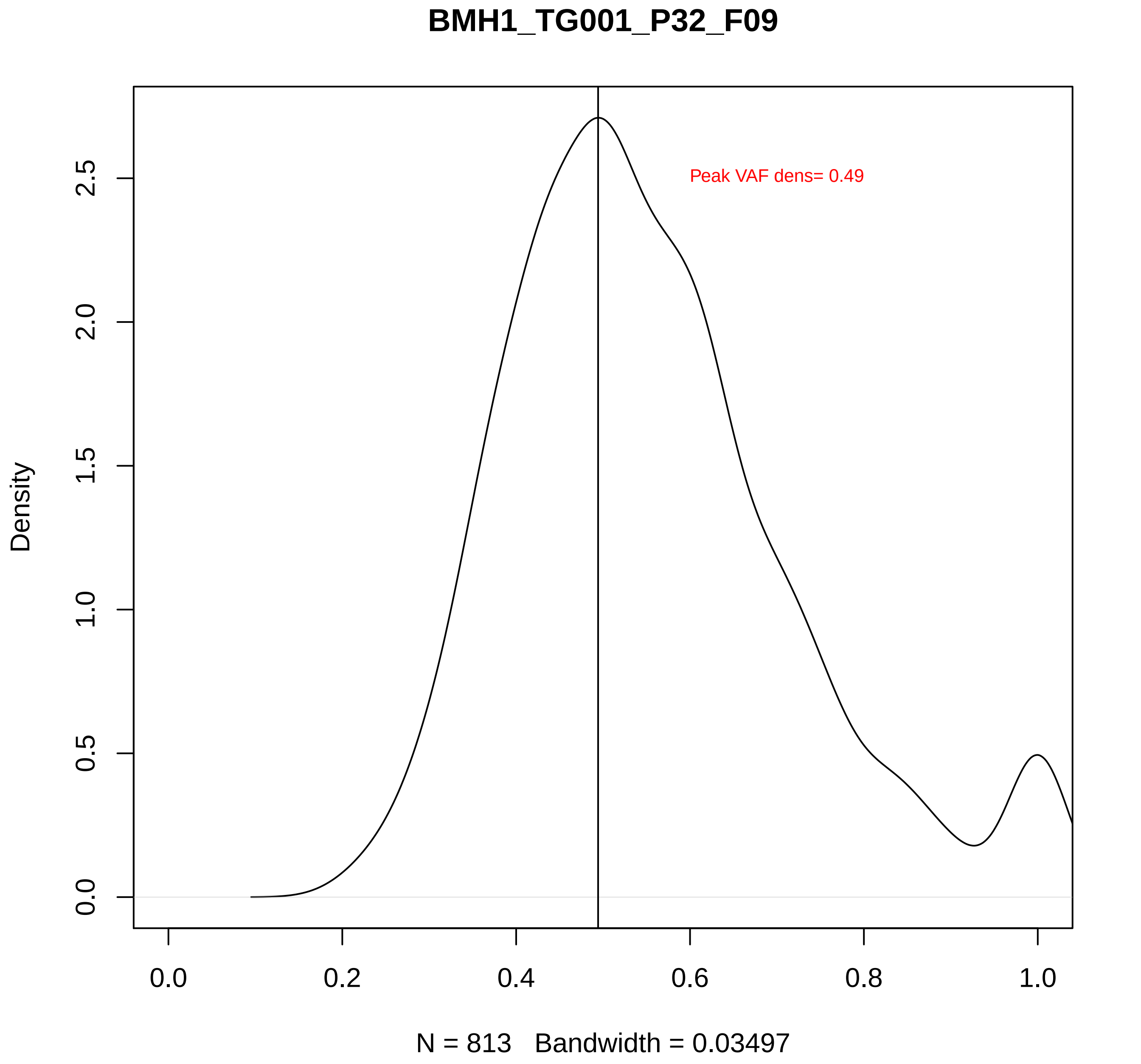
BMH1_TG001_P32_F09
Peak VAF dens= 0.49
Density
0.0
0.5
1.0
1.5
2.0
2.5
0.0
0.2
0.4
0.6
0.8
1.0
N = 813   Bandwidth = 0.03497

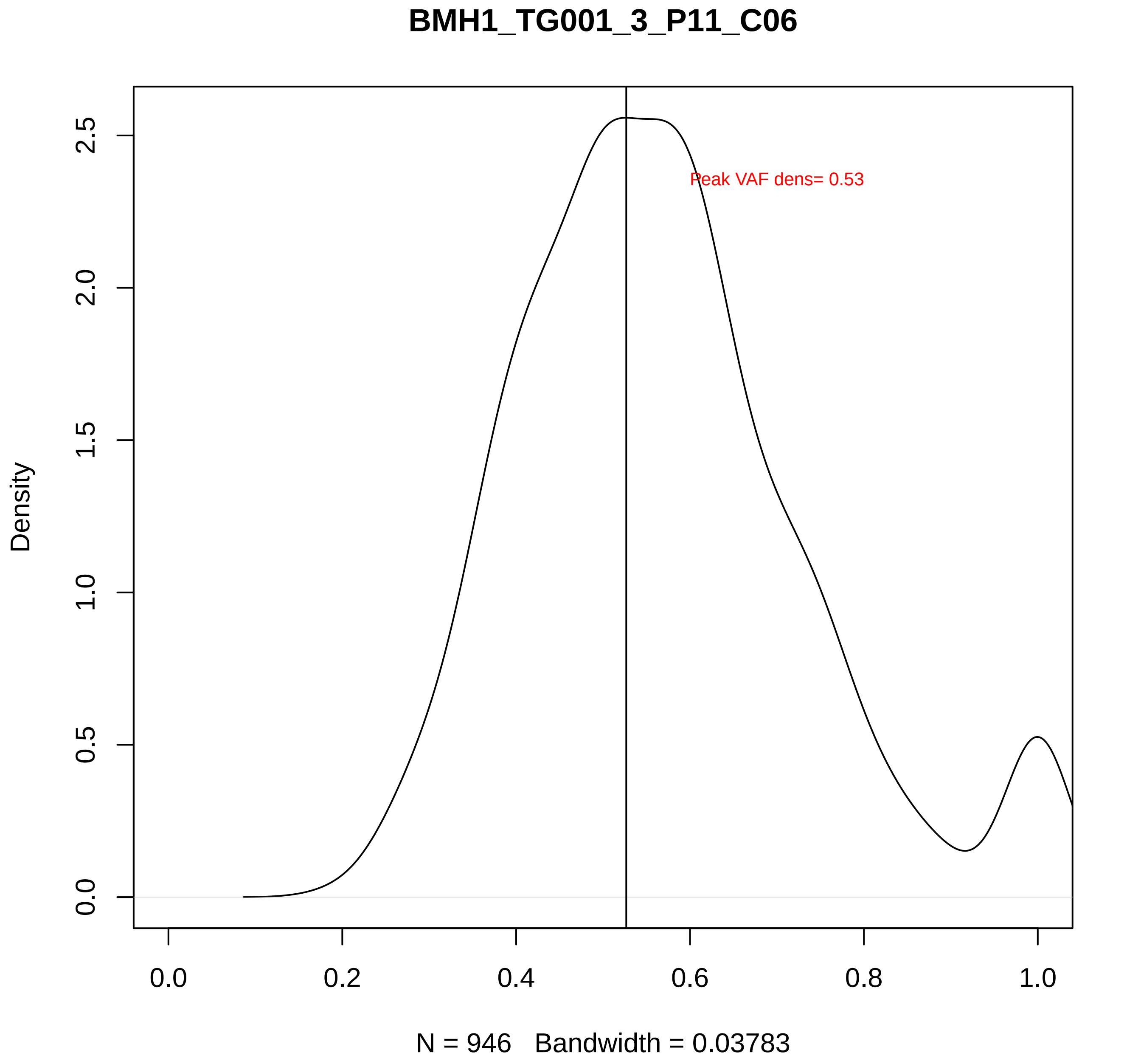

BMH1_TG001_3_P11_C06
Peak VAF dens= 0.53
Density
2.5
2.0
1.5
1.0
0.5
0.0
0.0
0.2
0.4
0.6
0.8
1.0
N = 946   Bandwidth = 0.03783

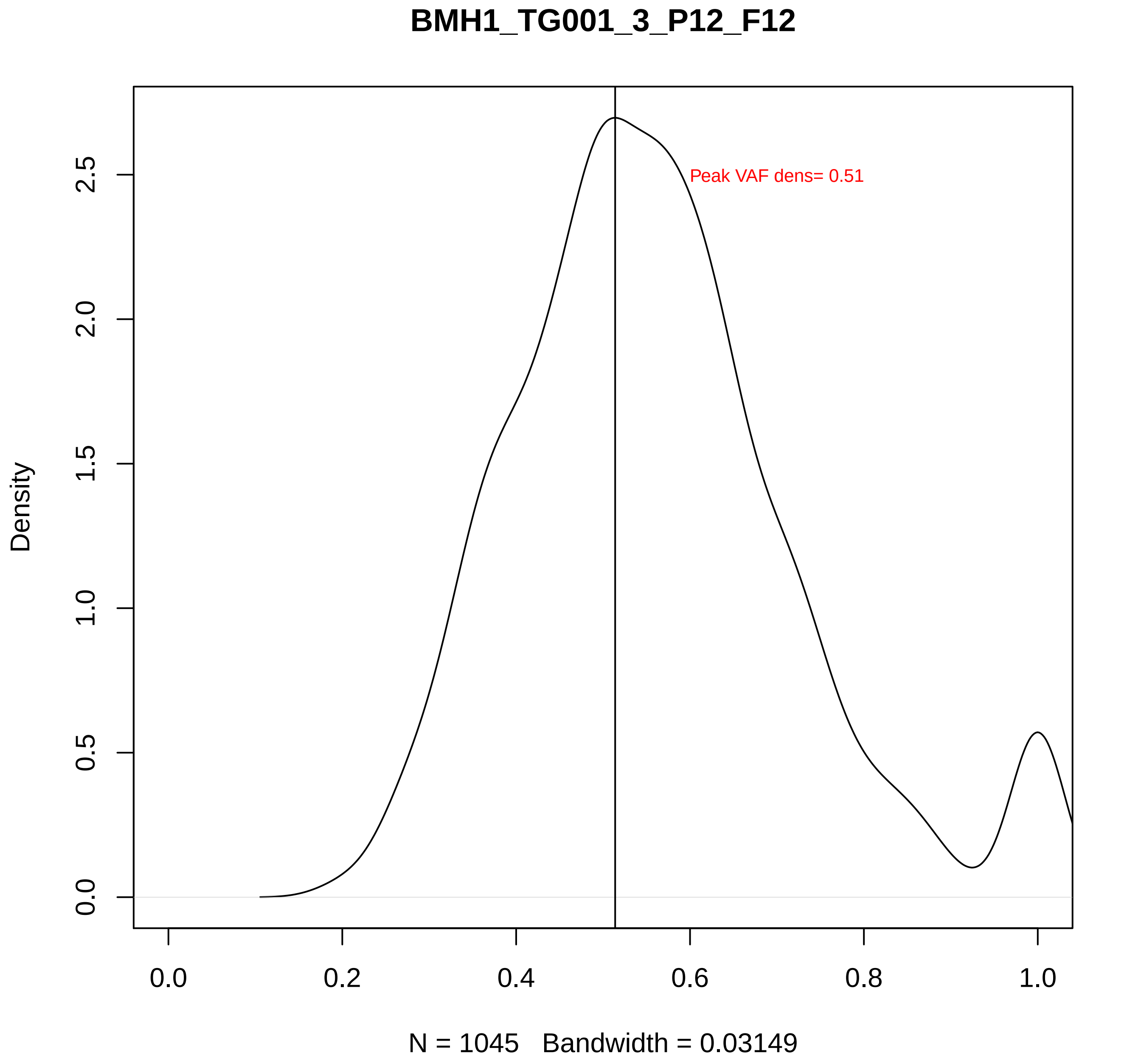
BMH1_TG001_3_P12_F12
Peak VAF dens= 0.51
Density
0.0
0.5
1.0
1.5
2.0
2.5
0.0
0.2
0.4
0.6
0.8
1.0
N = 1045   Bandwidth = 0.03149

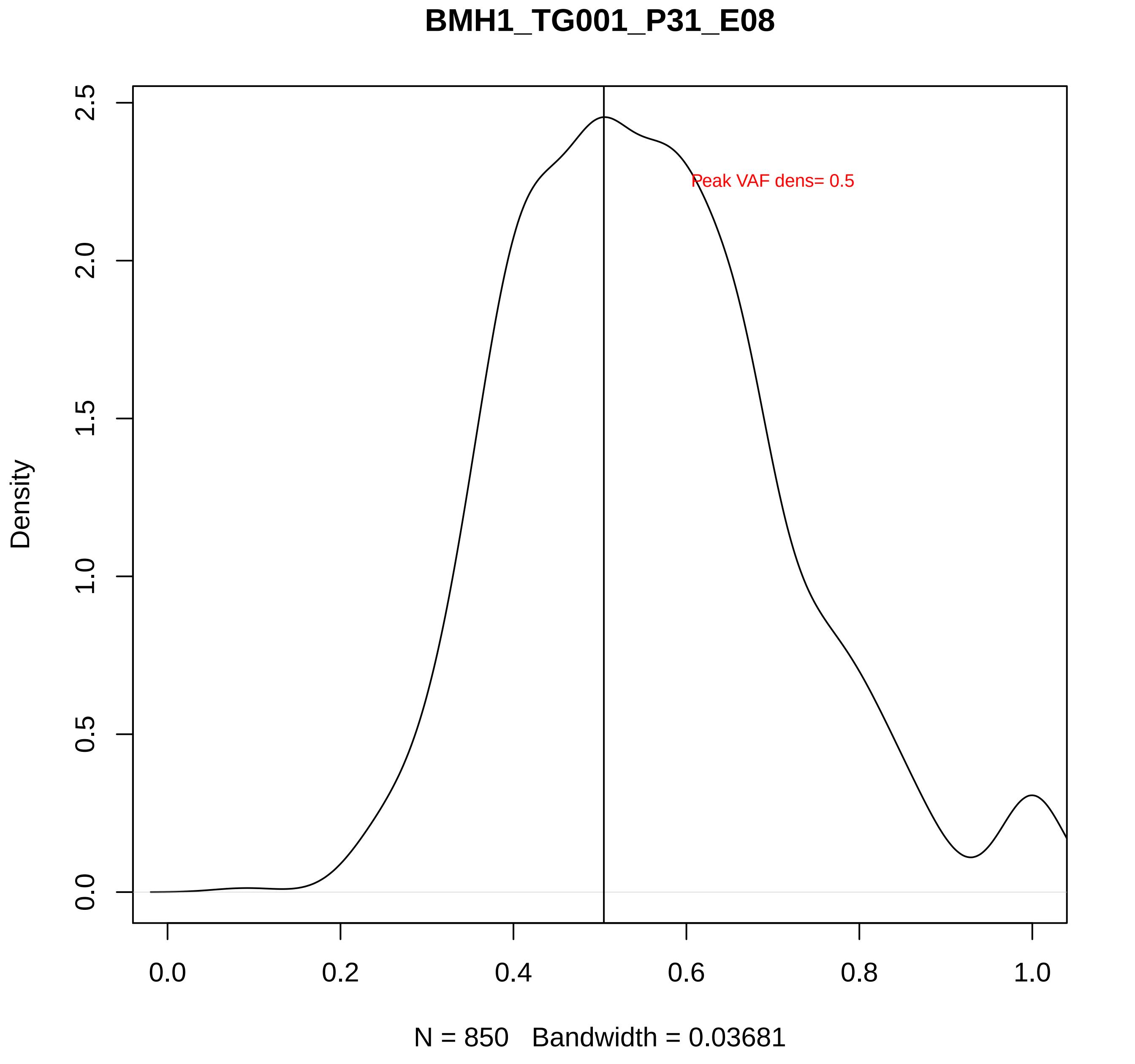

BMH1_TG001_P31_E08
Density
2.5
2.0
1.5
1.0
0.5
0.0
Peak VAF dens= 0.5
0.0
0.2
0.4
0.6
0.8
1.0
N = 850 Bandwidth = 0.03681

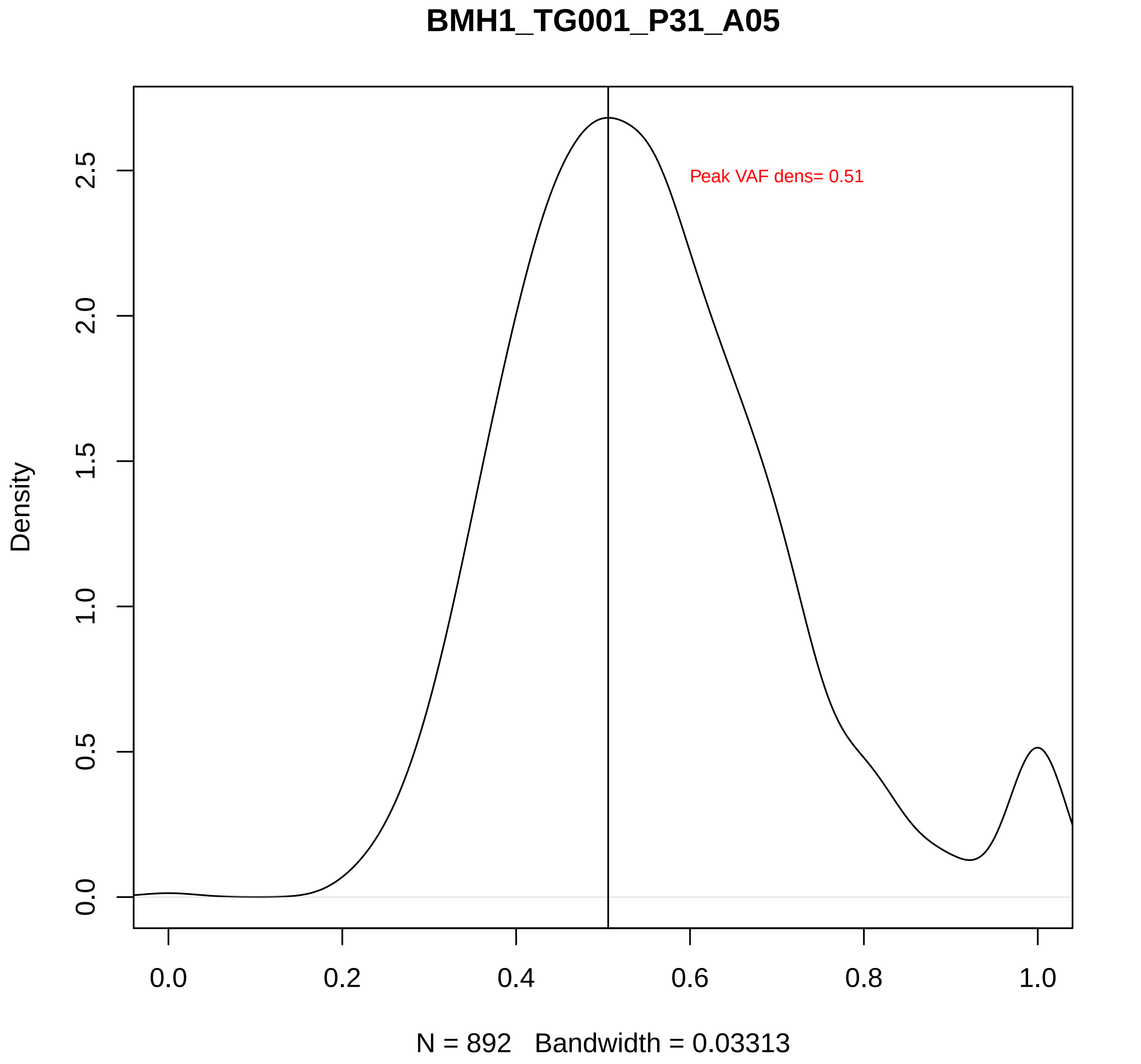
BMH1_TG001_P31_A05
Peak VAF dens= 0.51
Density
0.0
0.5
1.0
1.5
2.0
2.5
0.0
0.2
0.4
0.6
0.8
1.0
N = 892 Bandwidth = 0.03313

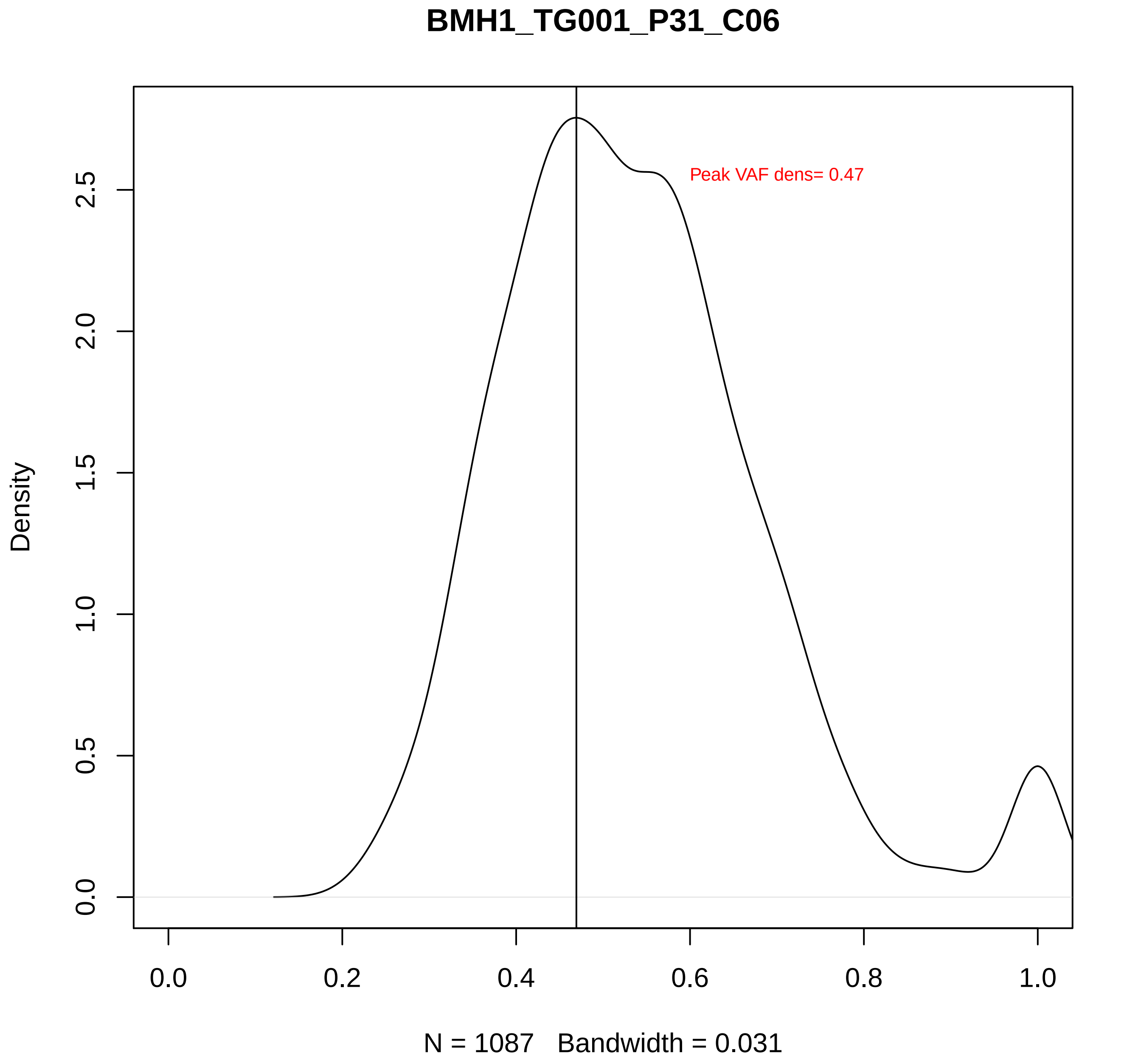
BMH1_TG001_P31_C06
Peak VAF dens= 0.47
Density
0.0
0.5
1.0
1.5
2.0
2.5
0.0
0.2
0.4
0.6
0.8
1.0
N = 1087 Bandwidth = 0.031

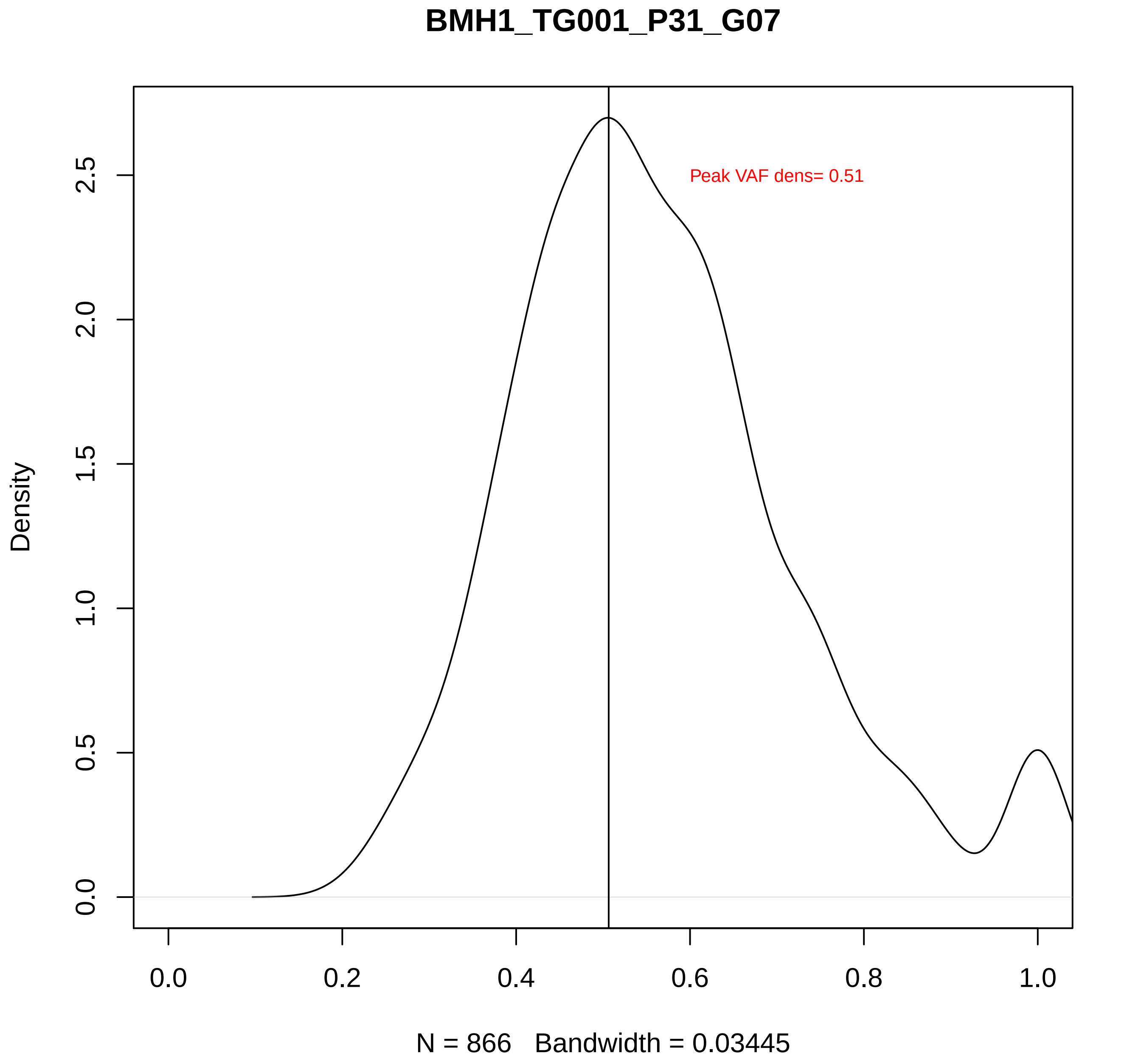

BMH1_TG001_P31_G07
Peak VAF dens= 0.51
Density
0.0
0.5
1.0
1.5
2.0
2.5
0.0
0.2
0.4
0.6
0.8
1.0
N = 866   Bandwidth = 0.03445

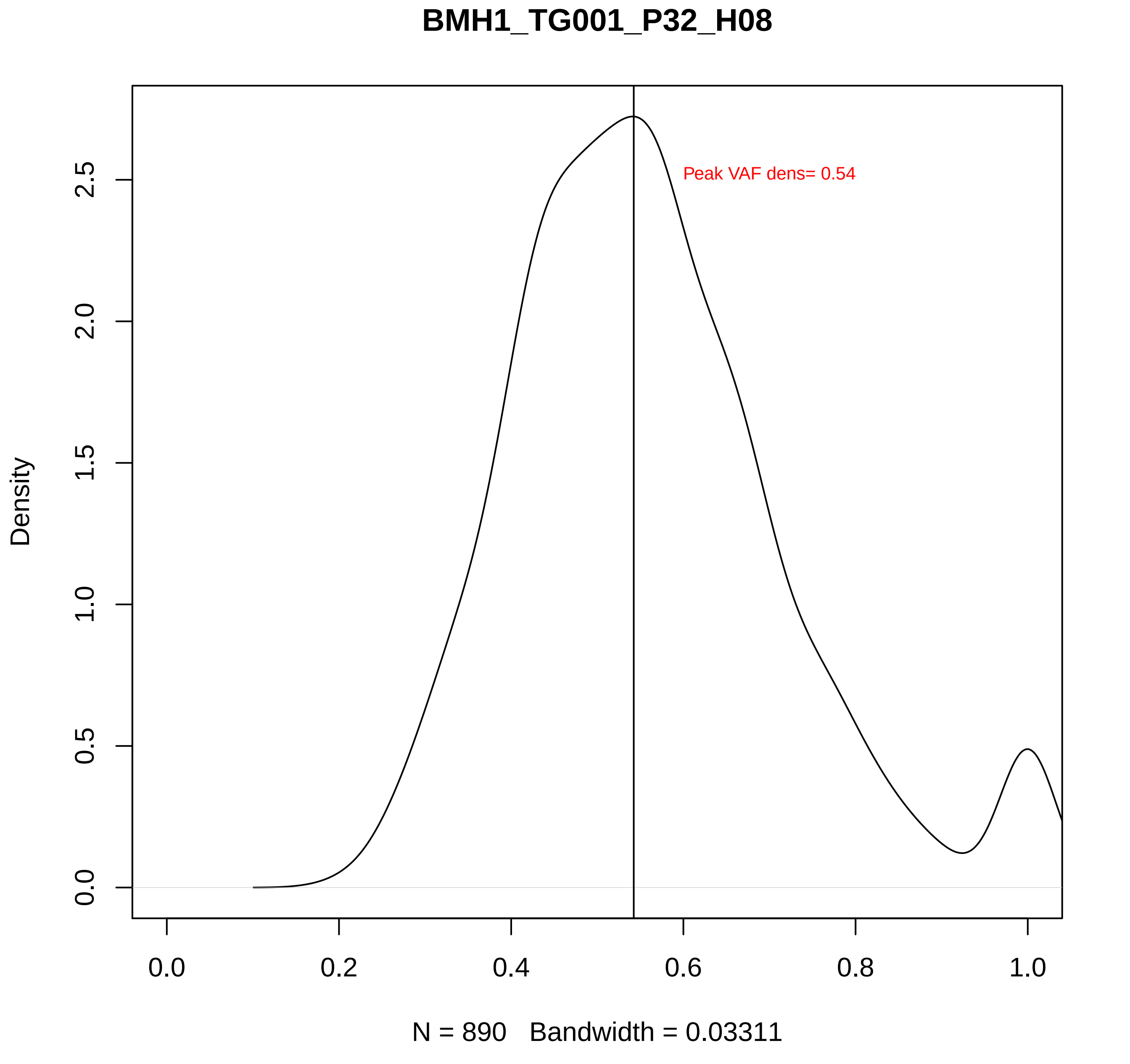

BMH1_TG001_P32_H08
Peak VAF dens= 0.54
Density
0.0
0.5
1.0
1.5
2.0
2.5
0.0
0.2
0.4
0.6
0.8
1.0
N = 890   Bandwidth = 0.03311

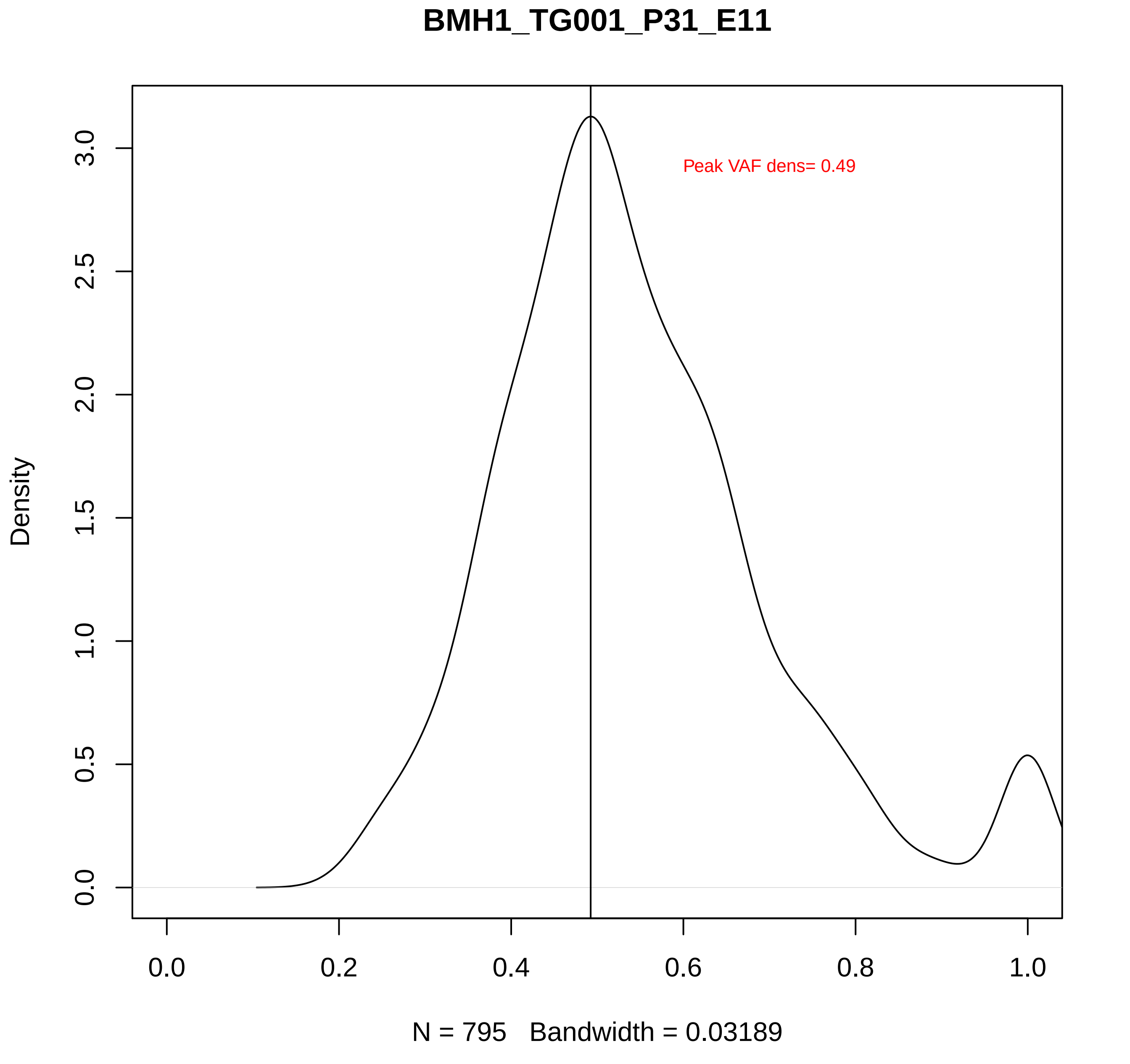

BMH1_TG001_P31_E11
Peak VAF dens= 0.49
Density
0.0
0.5
1.0
1.5
2.0
2.5
3.0
0.0
0.2
0.4
0.6
0.8
1.0
N = 795 Bandwidth = 0.03189

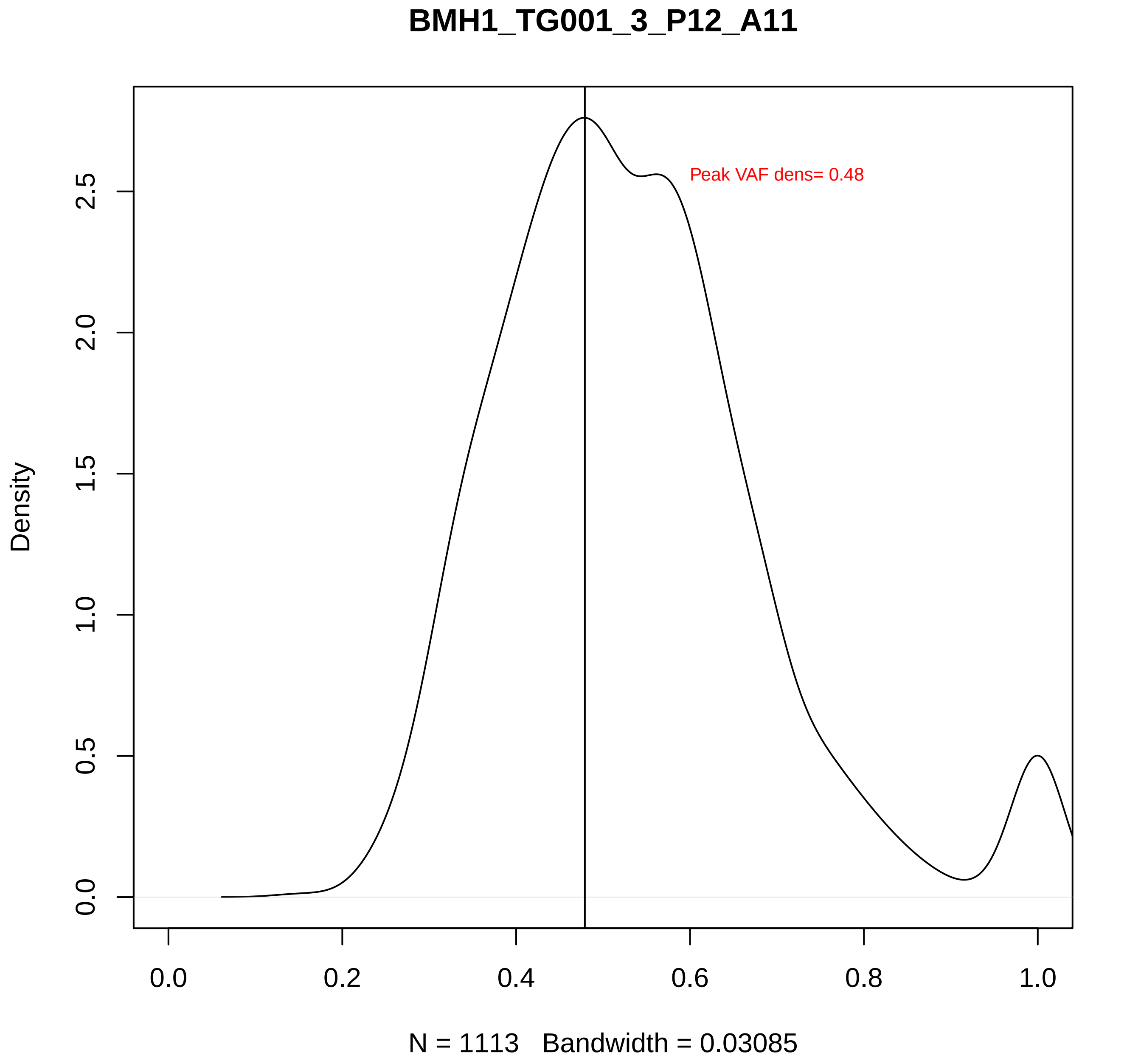

BMH1_TG001_3_P12_A11
Peak VAF dens= 0.48
Density
0.0
0.5
1.0
1.5
2.0
2.5
0.0
0.2
0.4
0.6
0.8
1.0
N = 1113   Bandwidth = 0.03085

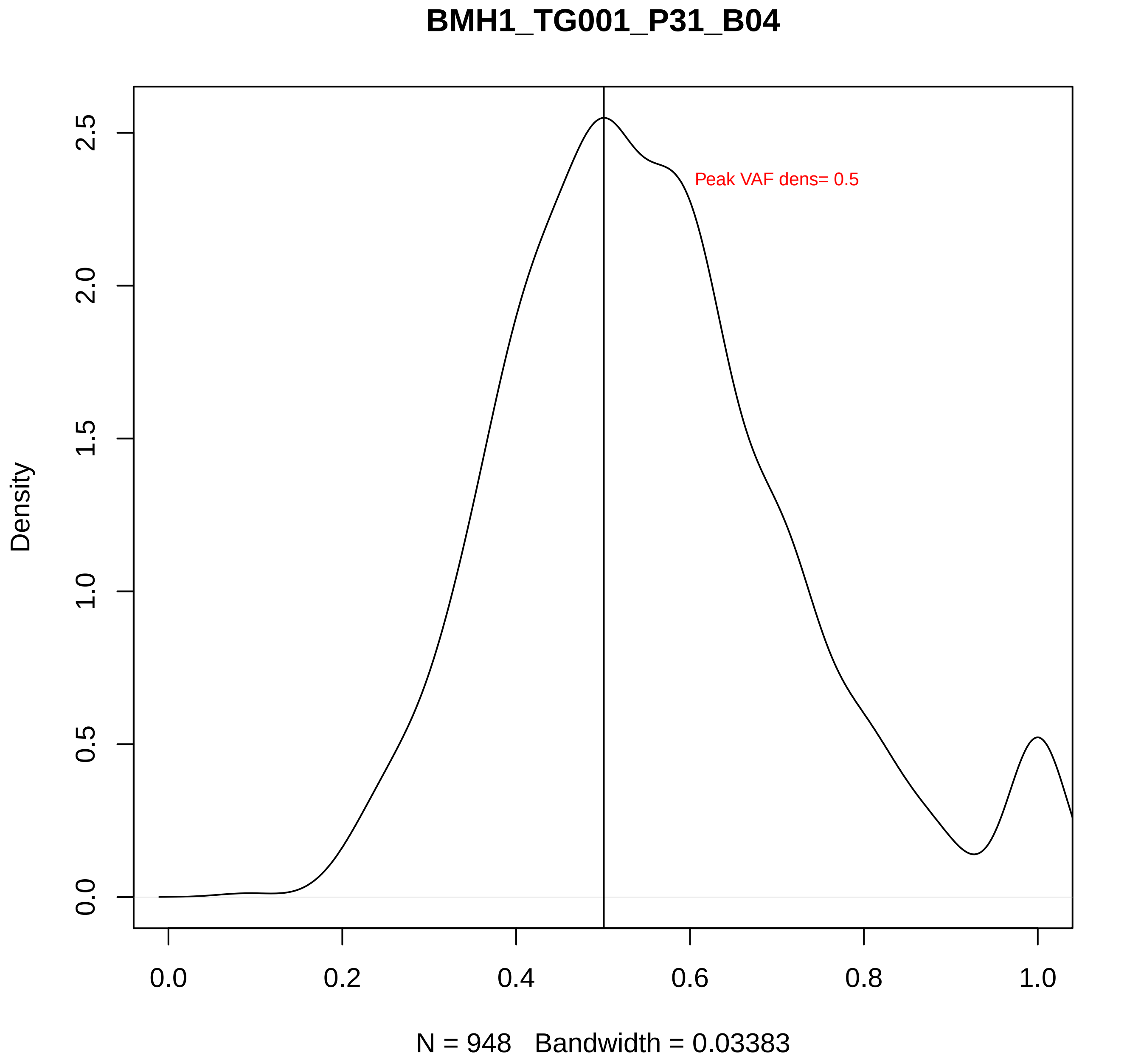
BMH1_TG001_P31_B04
Peak VAF dens= 0.5
Density
0.0
0.5
1.0
1.5
2.0
2.5
0.0
0.2
0.4
0.6
0.8
1.0
N = 948   Bandwidth = 0.03383

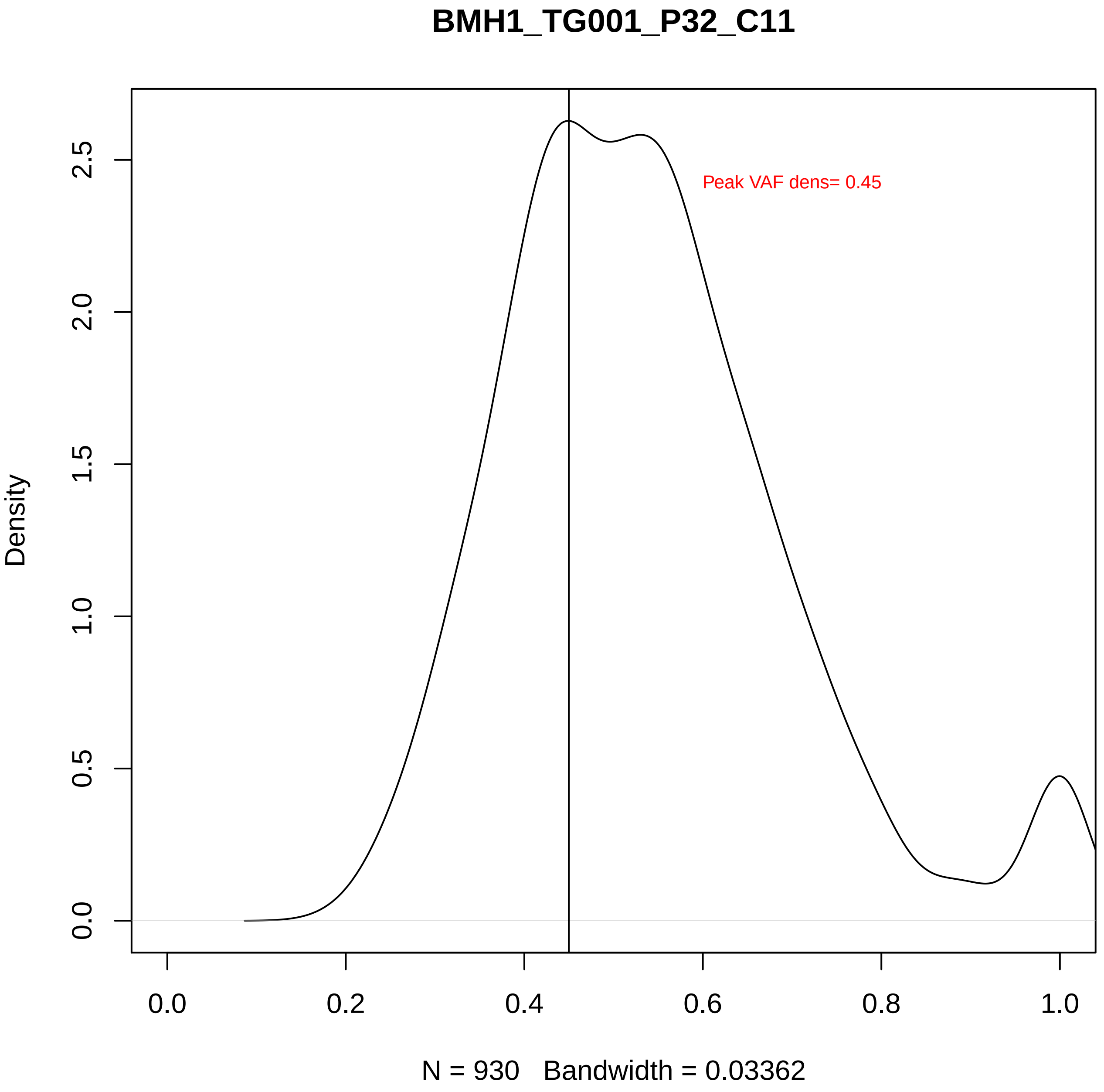

BMH1_TG001_P32_C11
Peak VAF dens= 0.45
Density
0.0
0.5
1.0
1.5
2.0
2.5
0.0
0.2
0.4
0.6
0.8
1.0
N = 930   Bandwidth = 0.03362

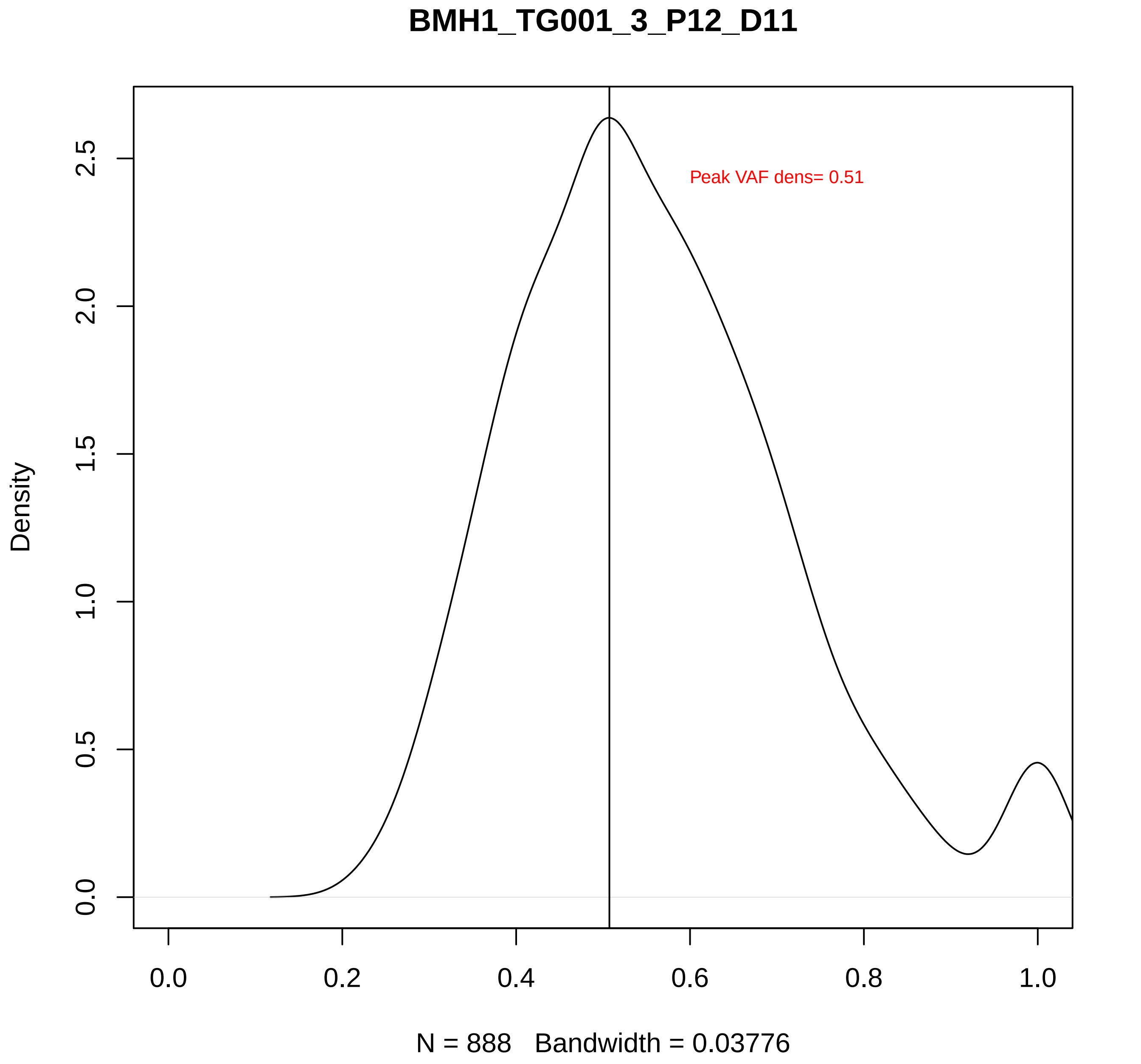

BMH1_TG001_3_P12_D11
Peak VAF dens= 0.51
Density
0.0
0.5
1.0
1.5
2.0
2.5
0.0
0.2
0.4
0.6
0.8
1.0
N = 888   Bandwidth = 0.03776

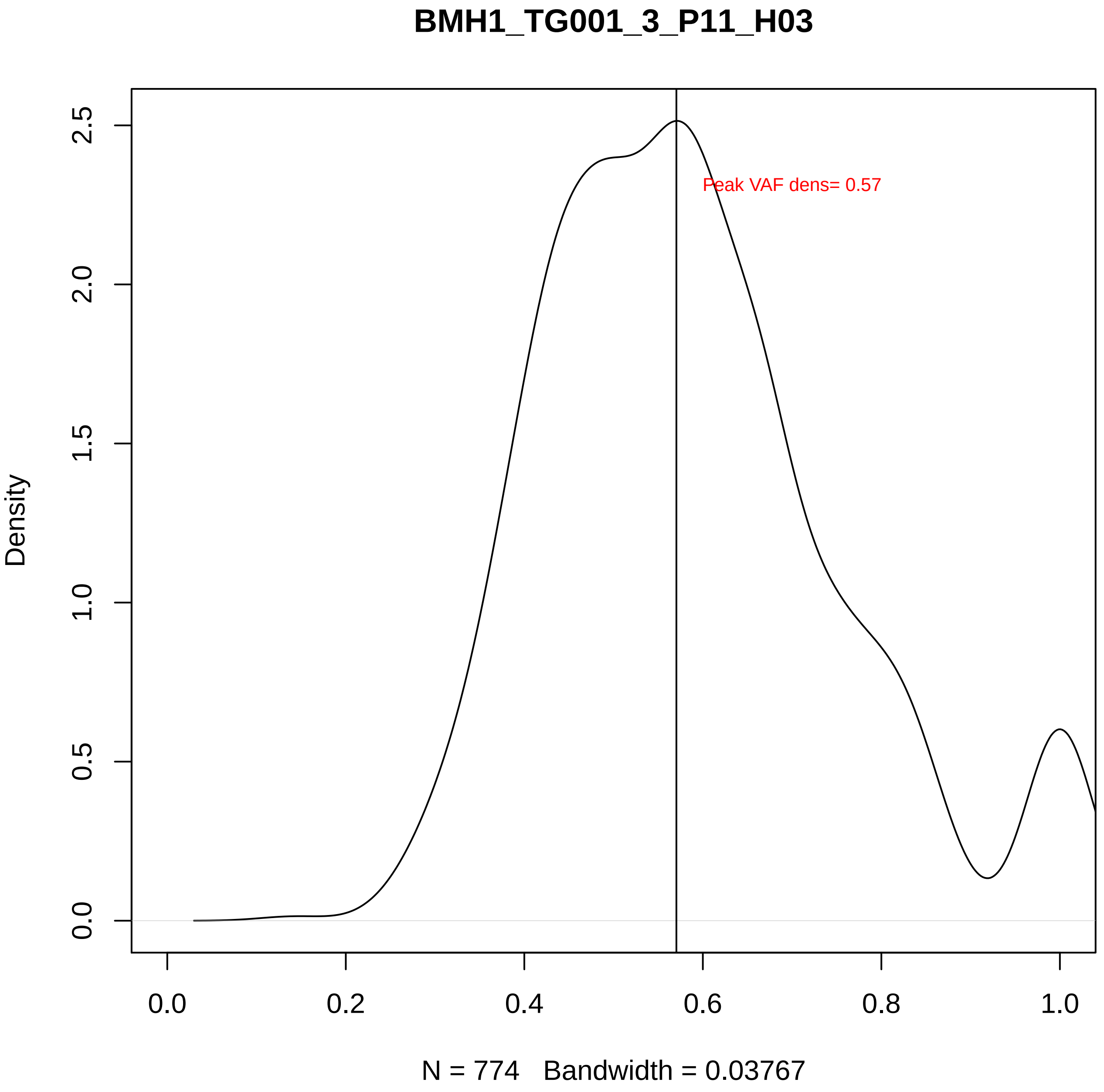

BMH1_TG001_3_P11_H03
Peak VAF dens= 0.57
Density
0.0
0.5
1.0
1.5
2.0
2.5
0.0
0.2
0.4
0.6
0.8
1.0
N = 774 Bandwidth = 0.03767

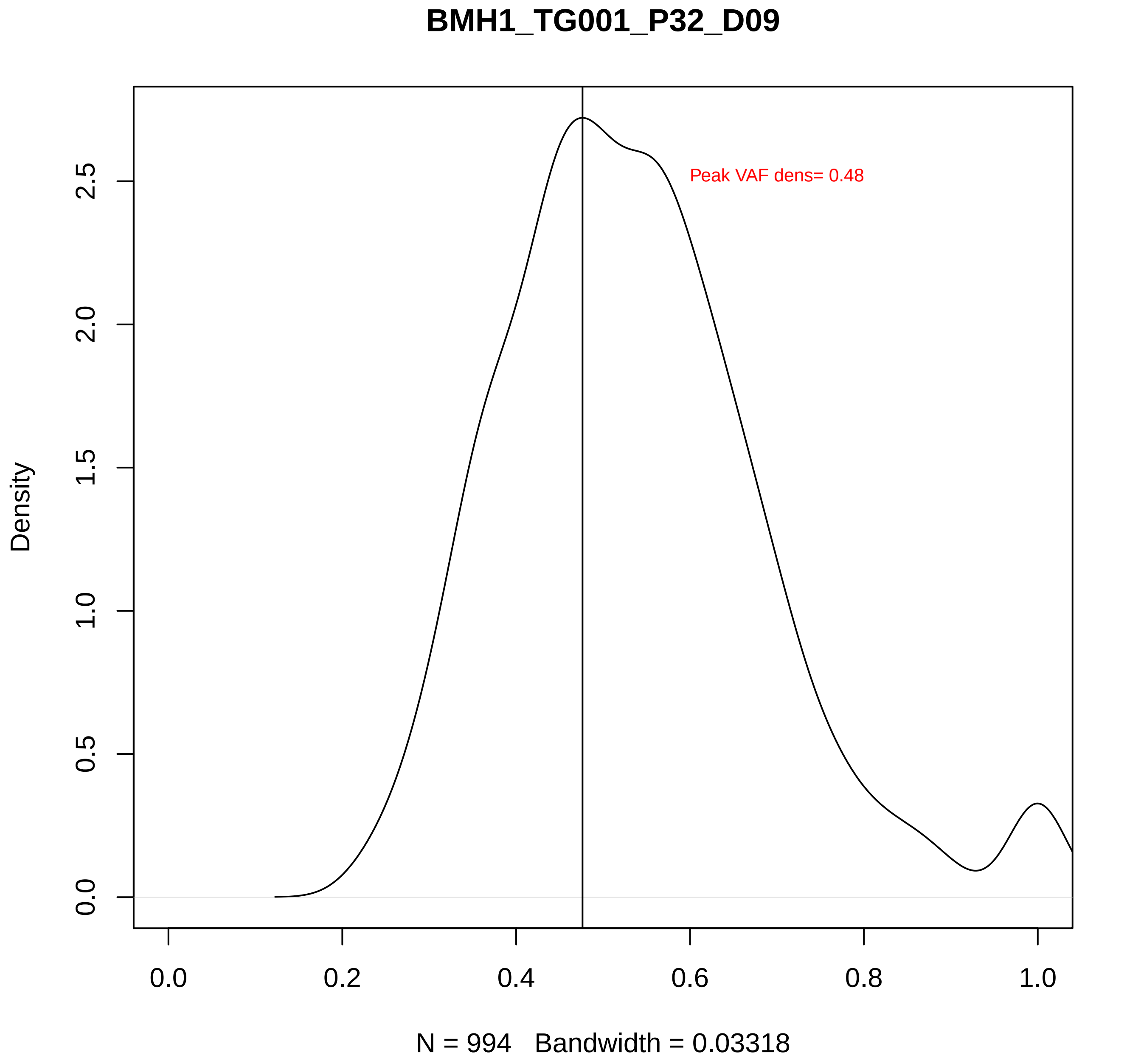
BMH1_TG001_P32_D09
Peak VAF dens= 0.48
Density
0.0
0.5
1.0
1.5
2.0
2.5
0.0
0.2
0.4
0.6
0.8
1.0
N = 994   Bandwidth = 0.03318

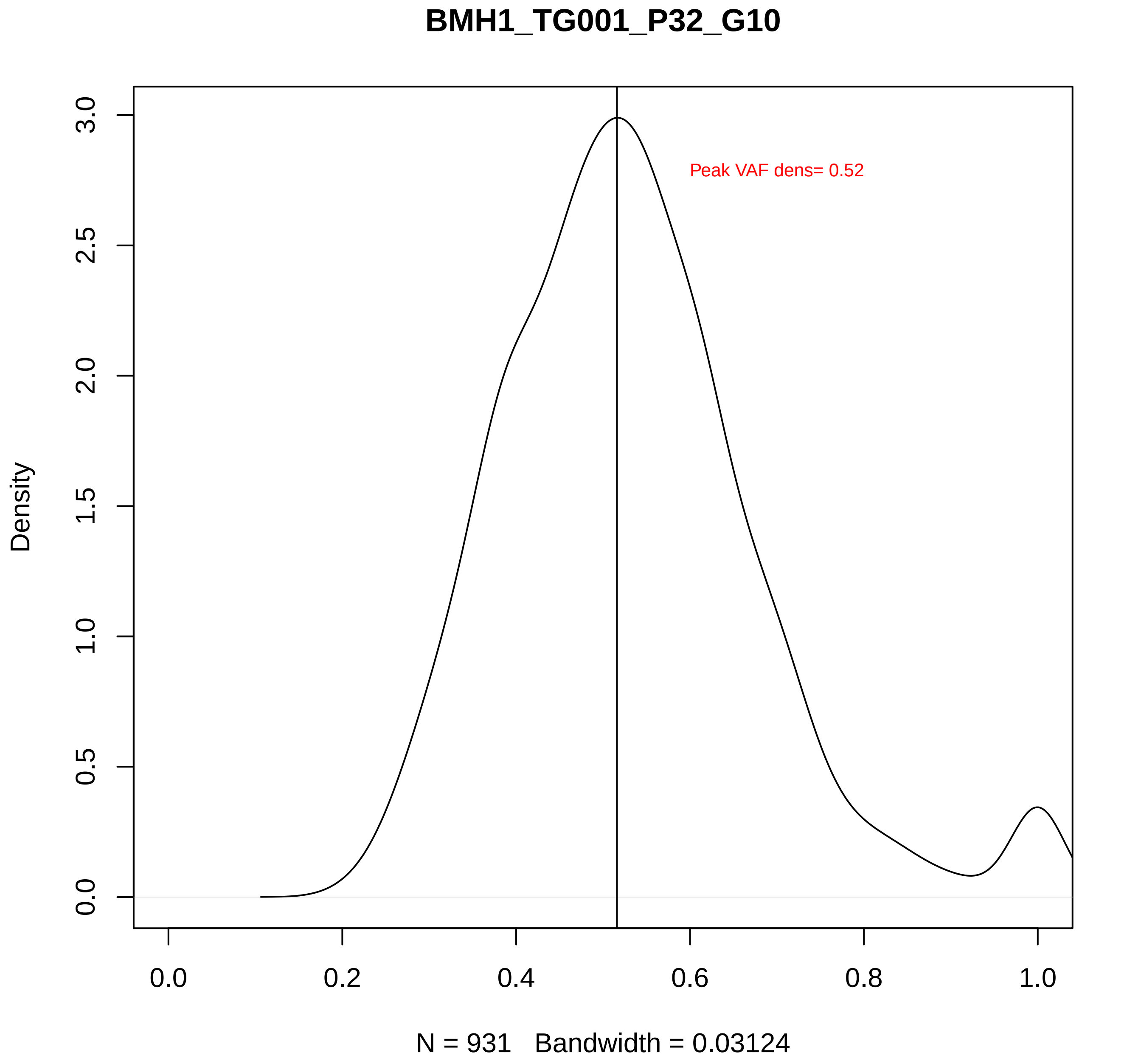

BMH1_TG001_P32_G10
Peak VAF dens= 0.52
Density
0.0
0.5
1.0
1.5
2.0
2.5
3.0
0.0
0.2
0.4
0.6
0.8
1.0
N = 931 Bandwidth = 0.03124

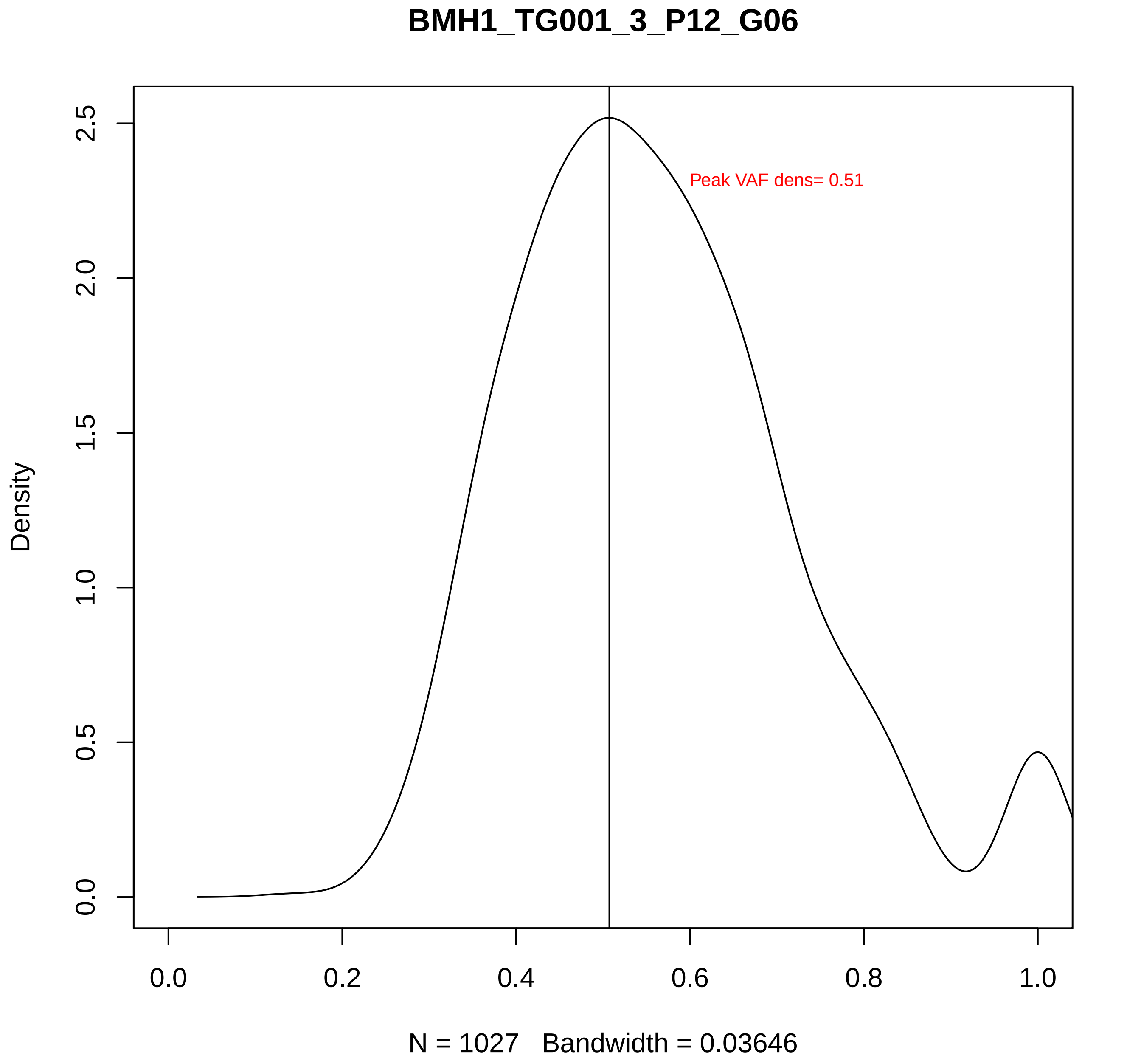

BMH1_TG001_3_P12_G06
Peak VAF dens= 0.51
Density
0.0
0.5
1.0
1.5
2.0
2.5
0.0
0.2
0.4
0.6
0.8
1.0
N = 1027   Bandwidth = 0.03646

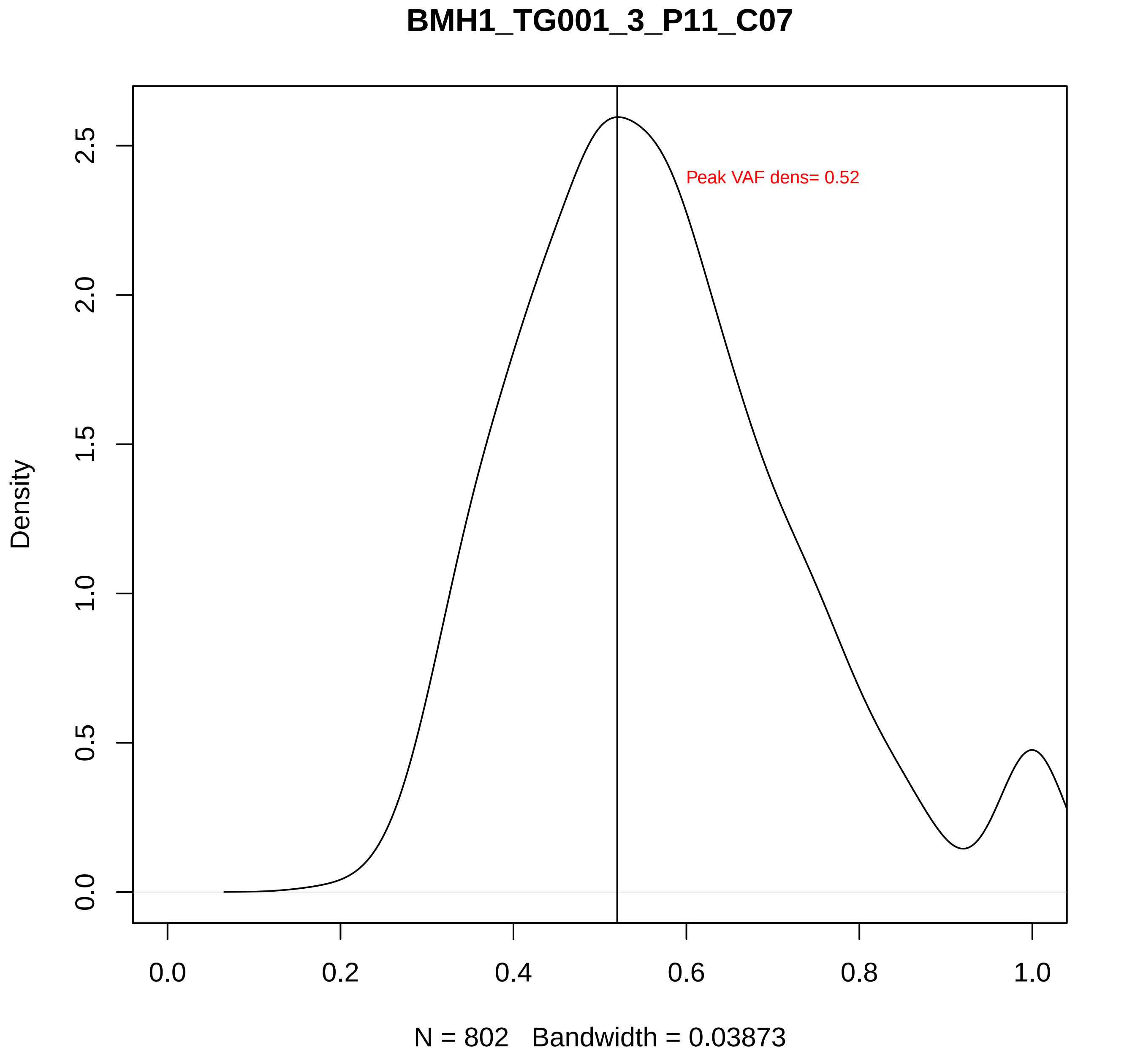
BMH1_TG001_3_P11_C07
Peak VAF dens= 0.52
Density
0.0
0.5
1.0
1.5
2.0
2.5
0.0
0.2
0.4
0.6
0.8
1.0
N = 802   Bandwidth = 0.03873

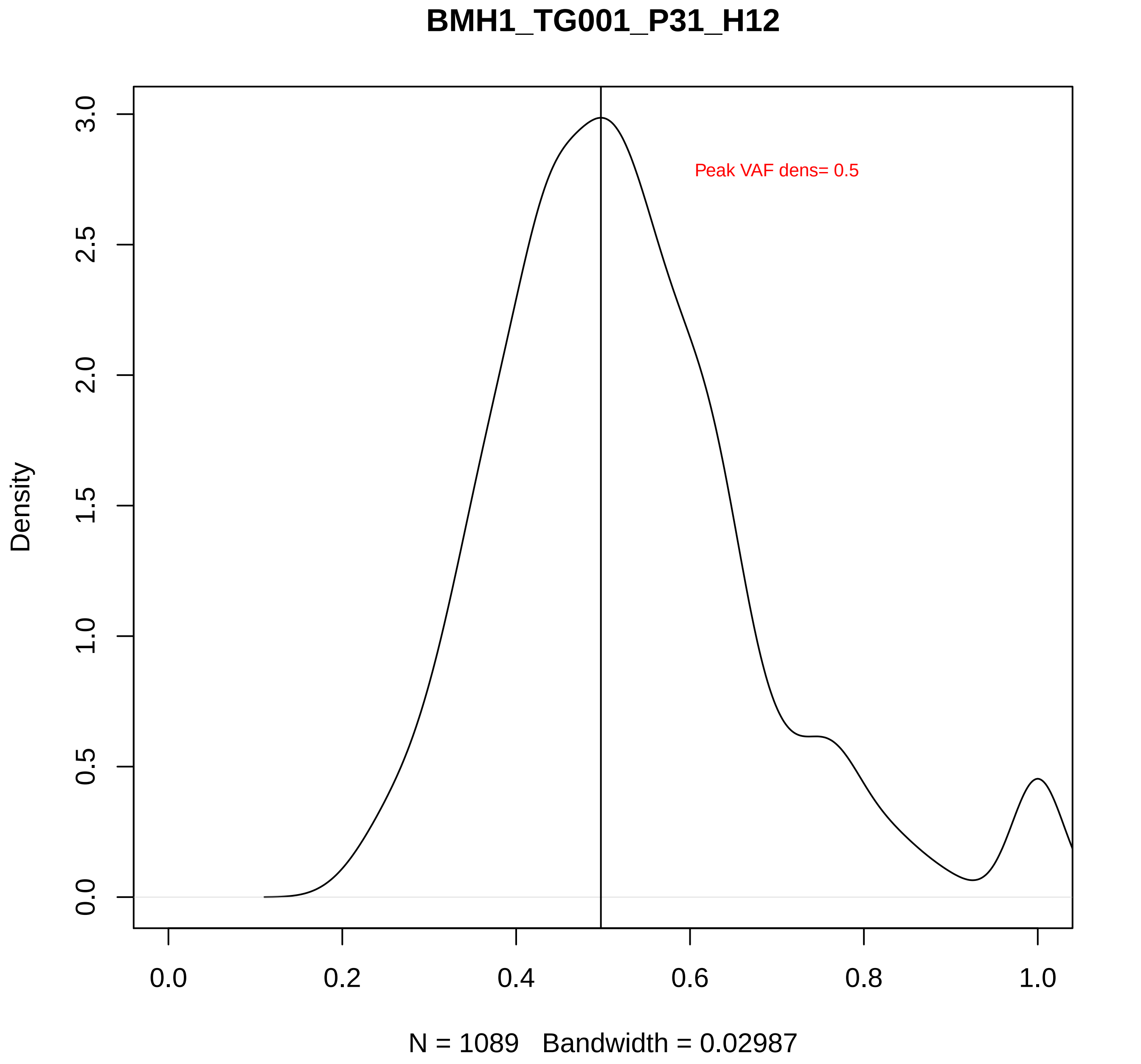
BMH1_TG001_P31_H12
Peak VAF dens= 0.5
Density
3.0
2.5
2.0
1.5
1.0
0.5
0.0
0.0
0.2
0.4
0.6
0.8
1.0
N = 1089   Bandwidth = 0.02987

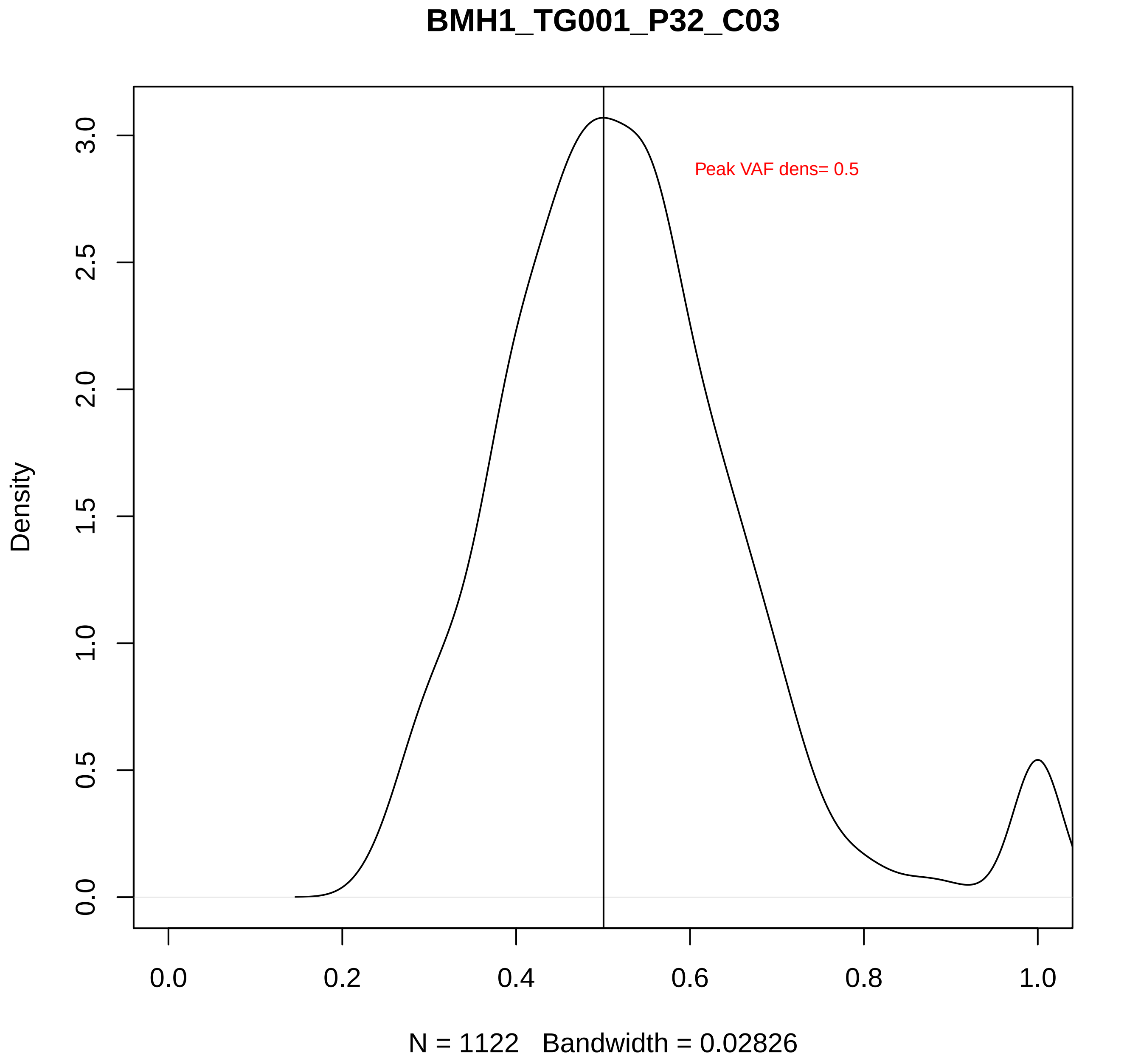
BMH1_TG001_P32_C03
Peak VAF dens= 0.5
Density
3.0
2.5
2.0
1.5
1.0
0.5
0.0
0.0
0.2
0.4
0.6
0.8
1.0
N = 1122   Bandwidth = 0.02826

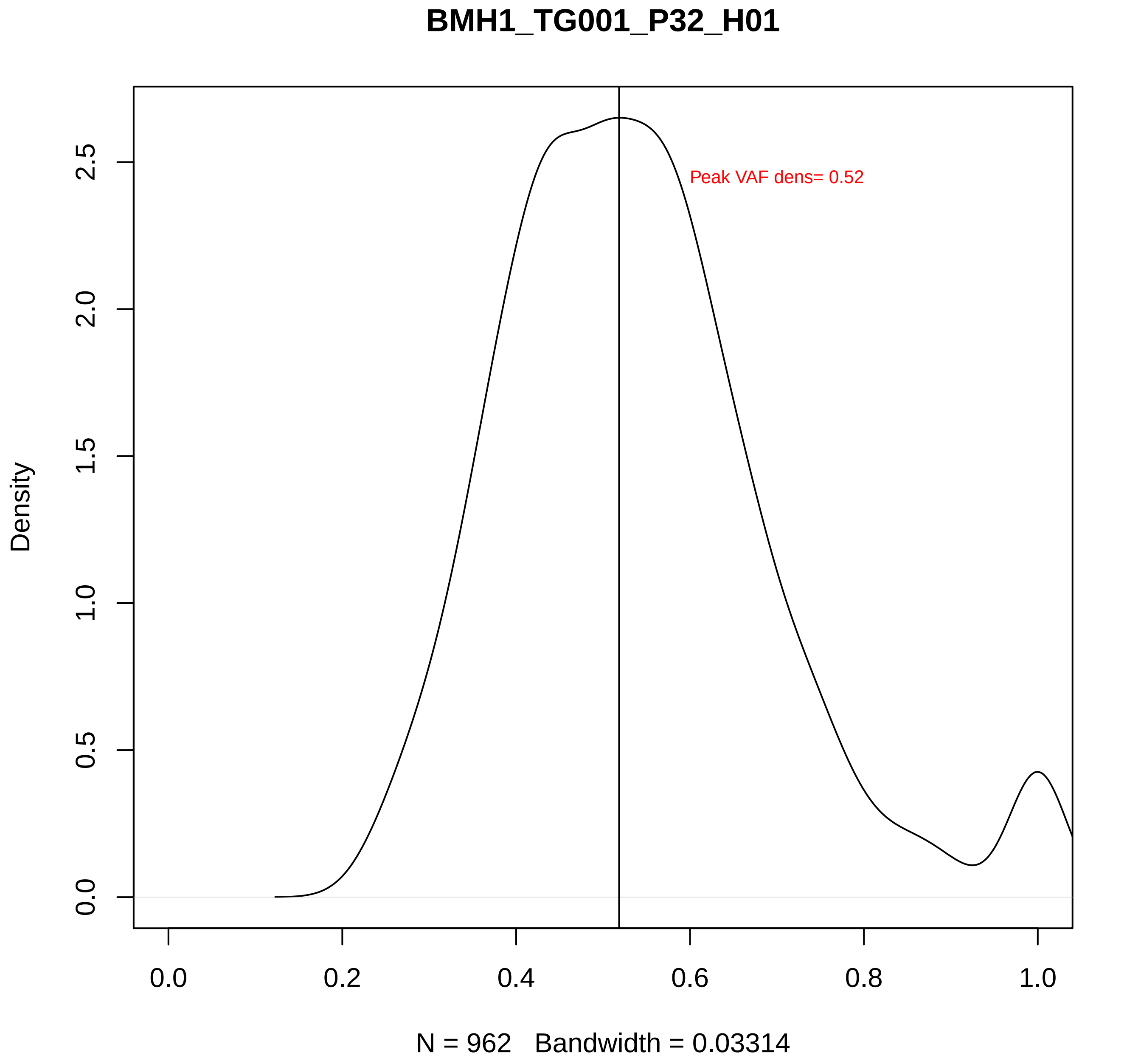

BMH1_TG001_P32_H01
Peak VAF dens= 0.52
Density
0.0
0.5
1.0
1.5
2.0
2.5
0.0
0.2
0.4
0.6
0.8
1.0
N = 962   Bandwidth = 0.03314

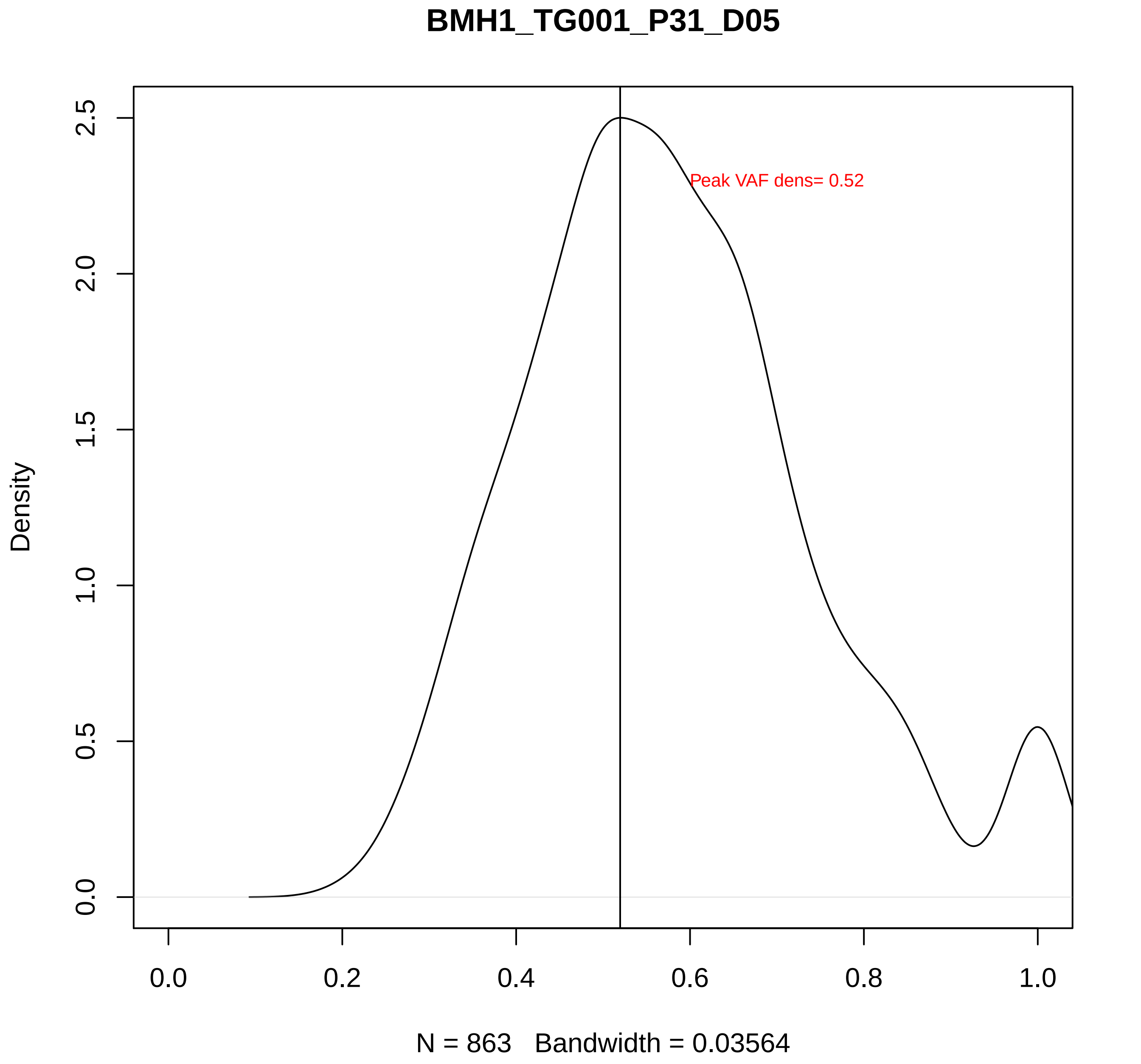
BMH1_TG001_P31_D05
Peak VAF dens= 0.52
Density
0.0
0.5
1.0
1.5
2.0
2.5
0.0
0.2
0.4
0.6
0.8
1.0
N = 863   Bandwidth = 0.03564

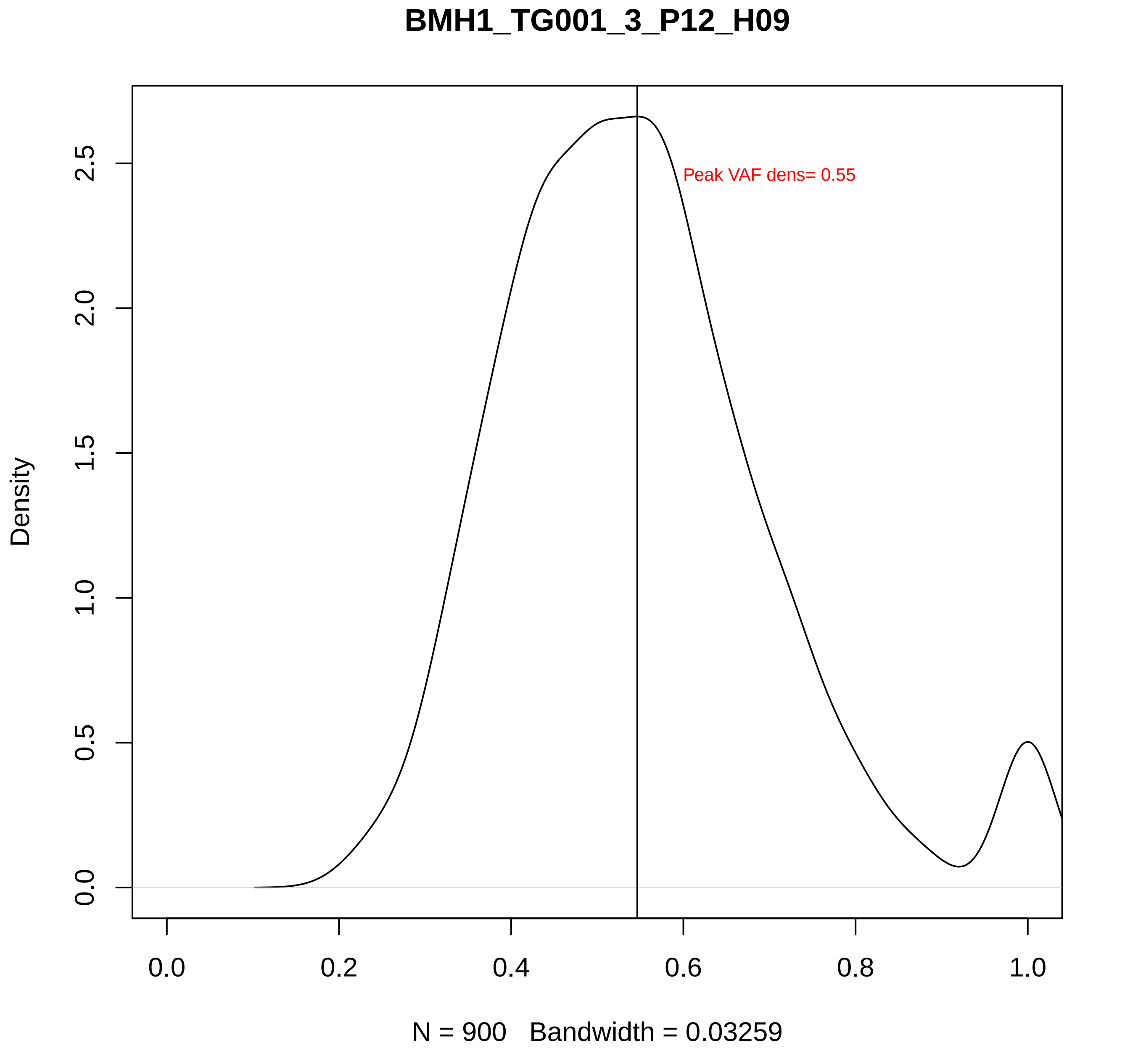
BMH1_TG001_3_P12_H09
Peak VAF dens= 0.55
Density
0.0
0.5
1.0
1.5
2.0
2.5
0.0
0.2
0.4
0.6
0.8
1.0
N = 900   Bandwidth = 0.03259

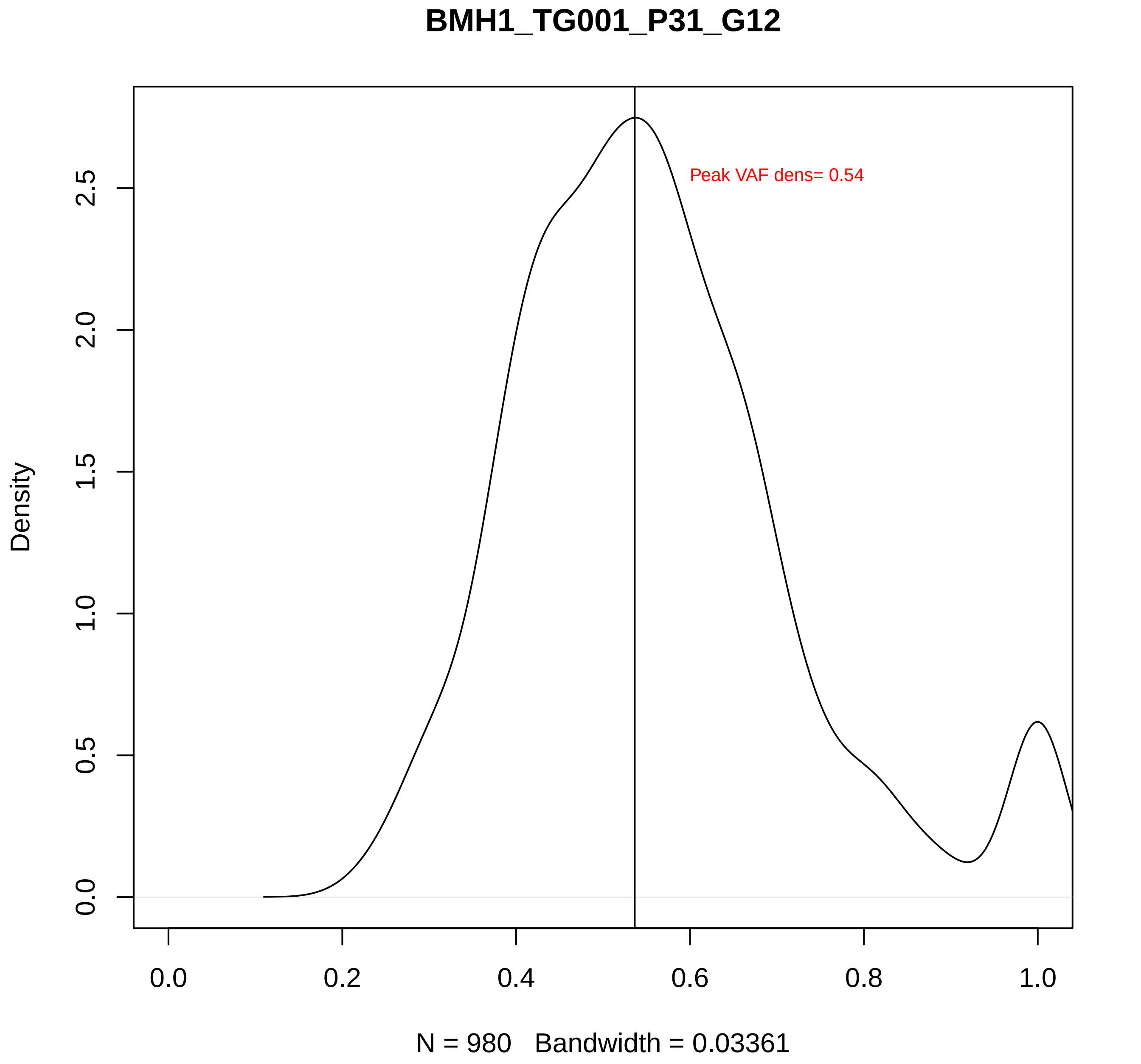
BMH1_TG001_P31_G12
Peak VAF dens= 0.54
Density
0.0
0.5
1.0
1.5
2.0
2.5
0.0
0.2
0.4
0.6
0.8
1.0
N = 980 Bandwidth = 0.03361

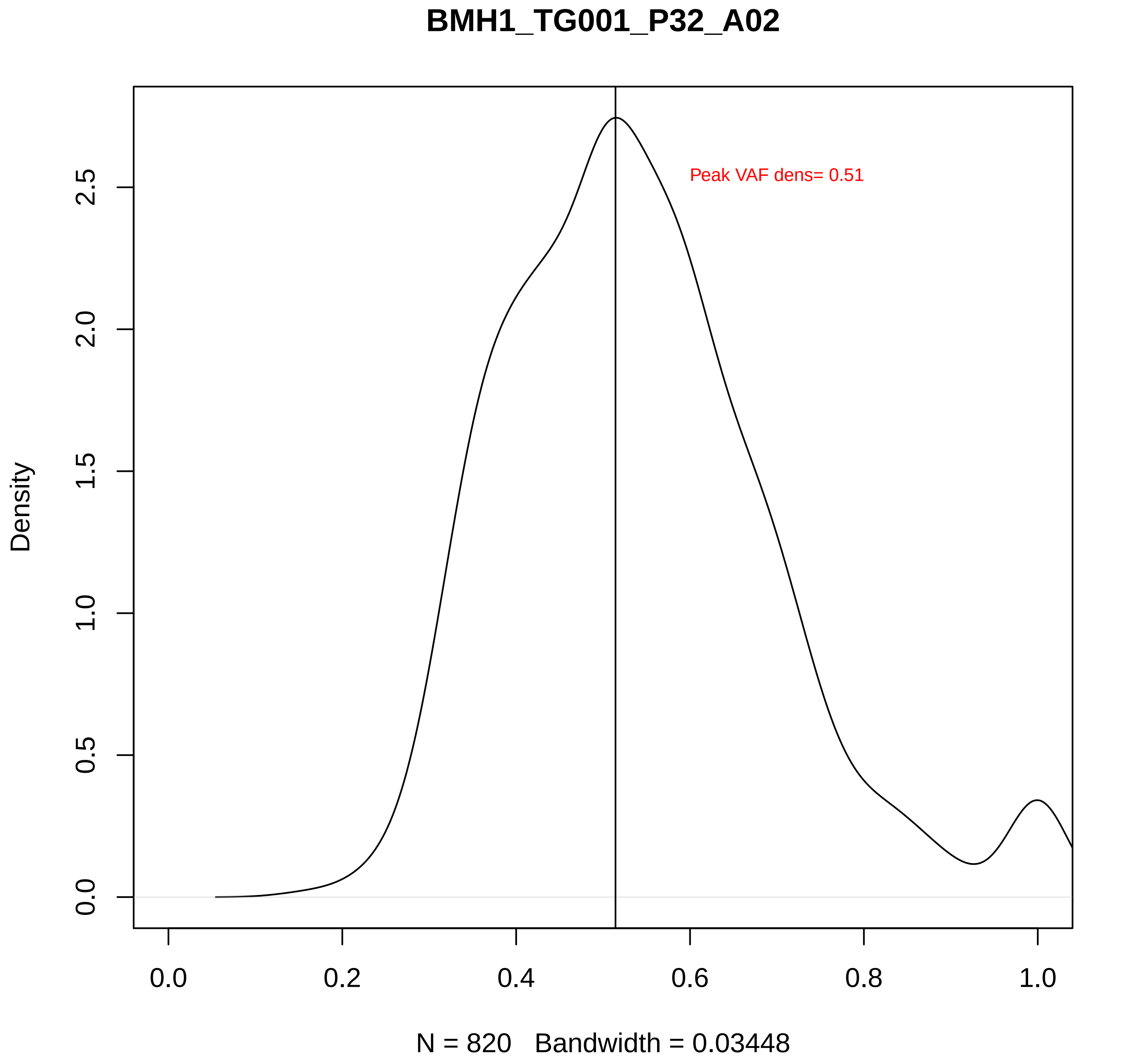
BMH1_TG001_P32_A02
Peak VAF dens= 0.51
Density
0.0
0.5
1.0
1.5
2.0
2.5
0.0
0.2
0.4
0.6
0.8
1.0
N = 820   Bandwidth = 0.03448

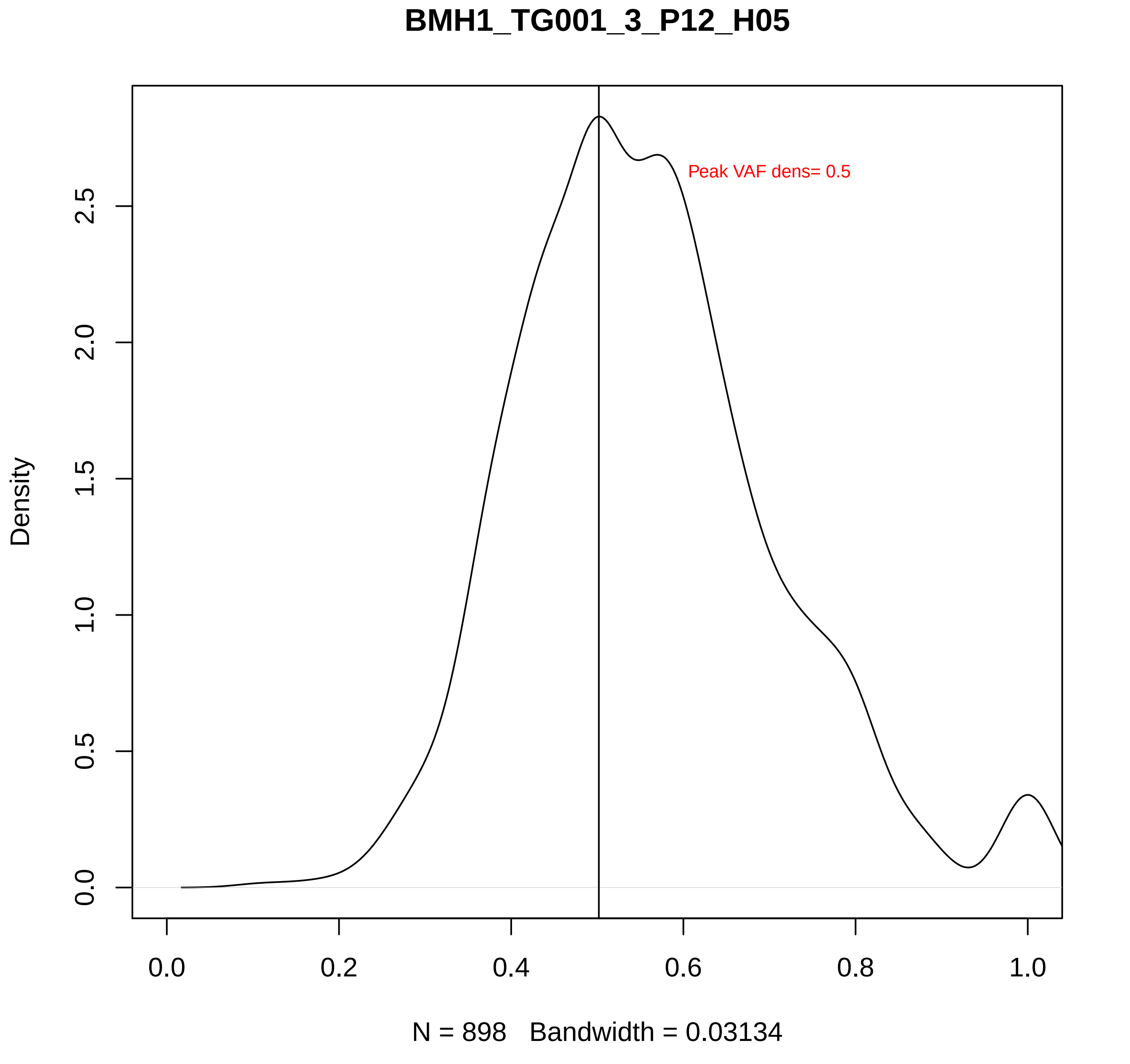
BMH1_TG001_3_P12_H05
Peak VAF dens= 0.5
Density
0.0
0.5
1.0
1.5
2.0
2.5
0.0
0.2
0.4
0.6
0.8
1.0
N = 898 Bandwidth = 0.03134

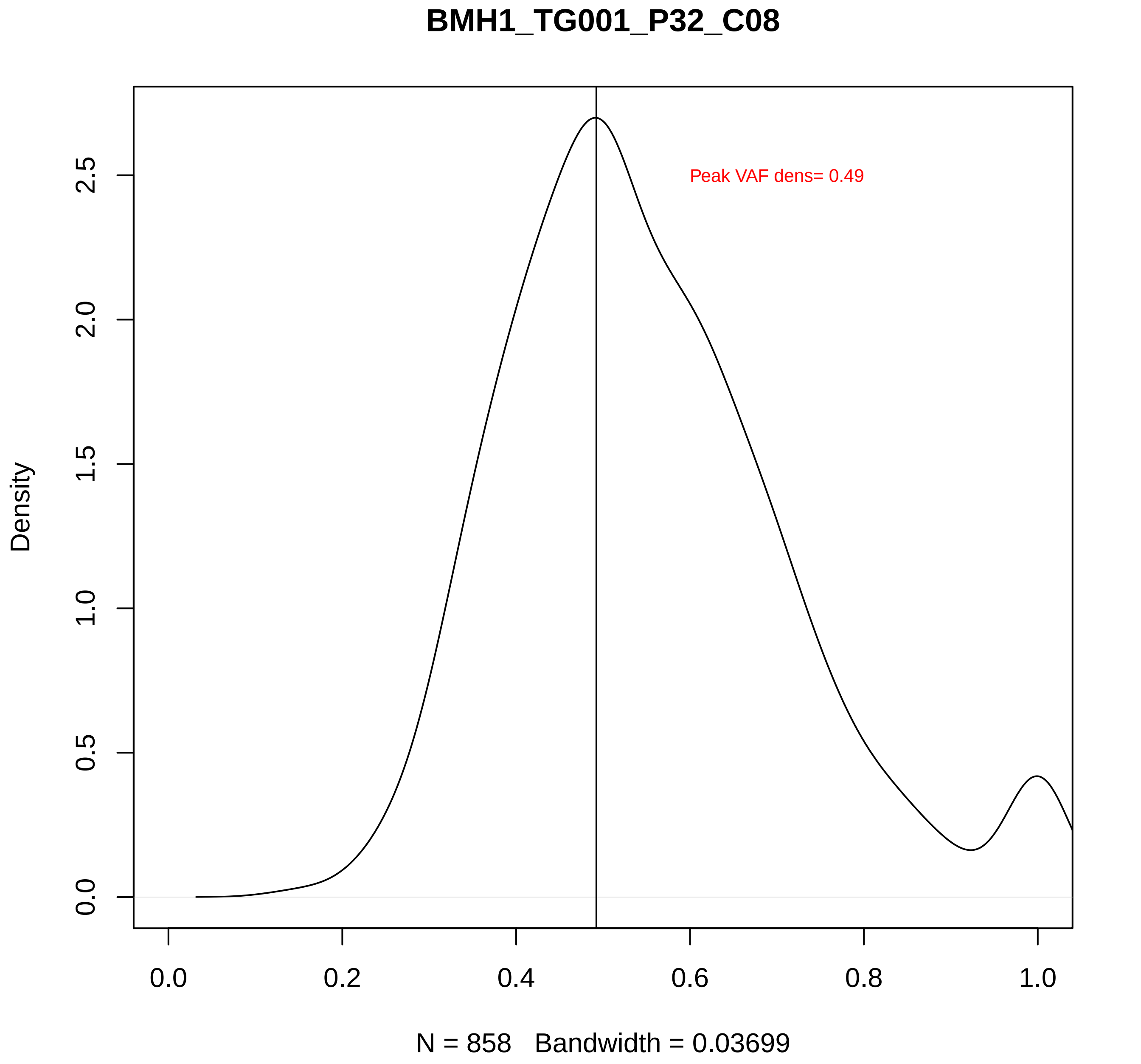

BMH1_TG001_P32_C08
Peak VAF dens= 0.49
Density
0.0
0.5
1.0
1.5
2.0
2.5
0.0
0.2
0.4
0.6
0.8
1.0
N = 858   Bandwidth = 0.03699

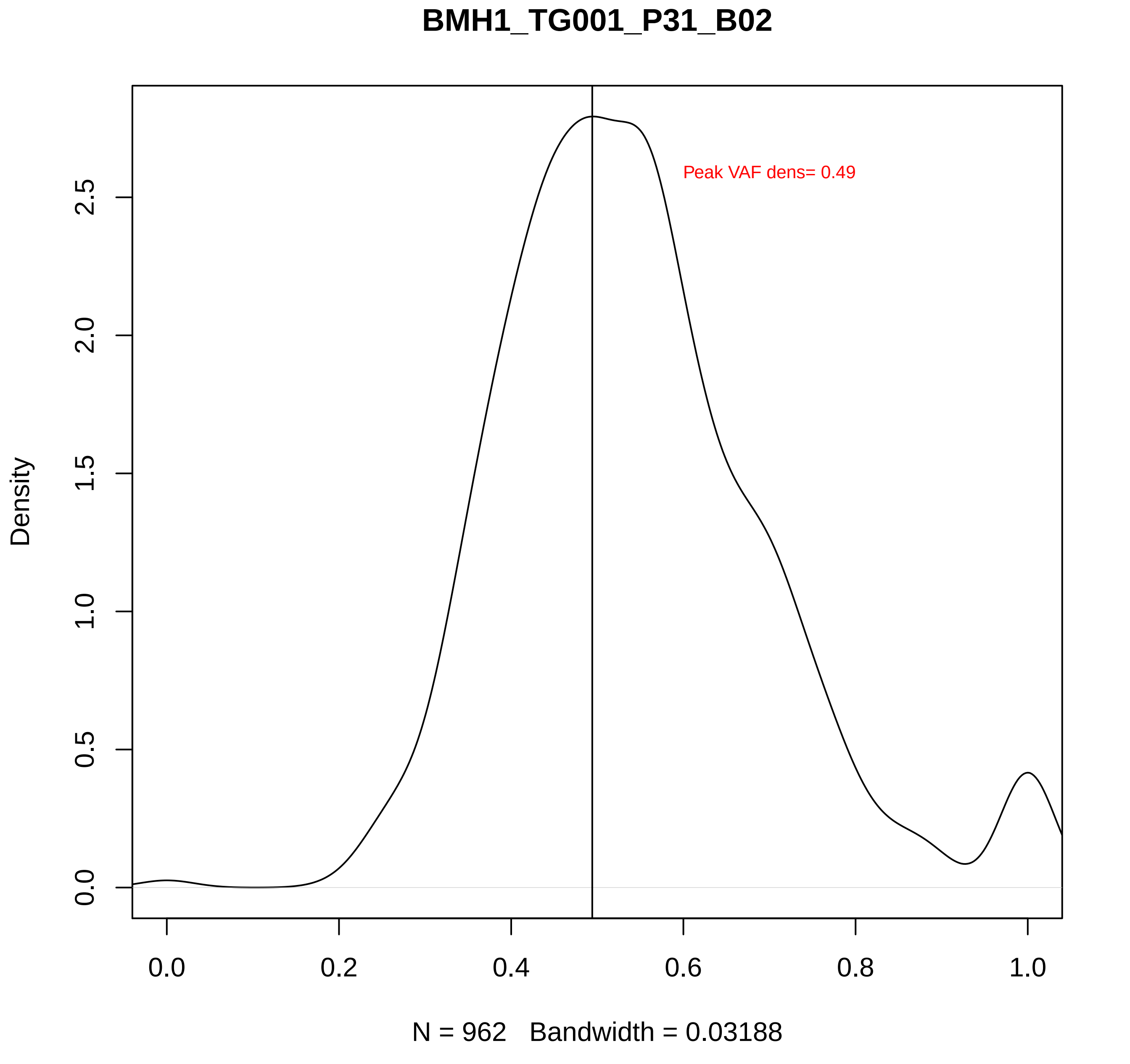

BMH1_TG001_P31_B02
Peak VAF dens= 0.49
Density
0.0
0.5
1.0
1.5
2.0
2.5
0.0
0.2
0.4
0.6
0.8
1.0
N = 962   Bandwidth = 0.03188

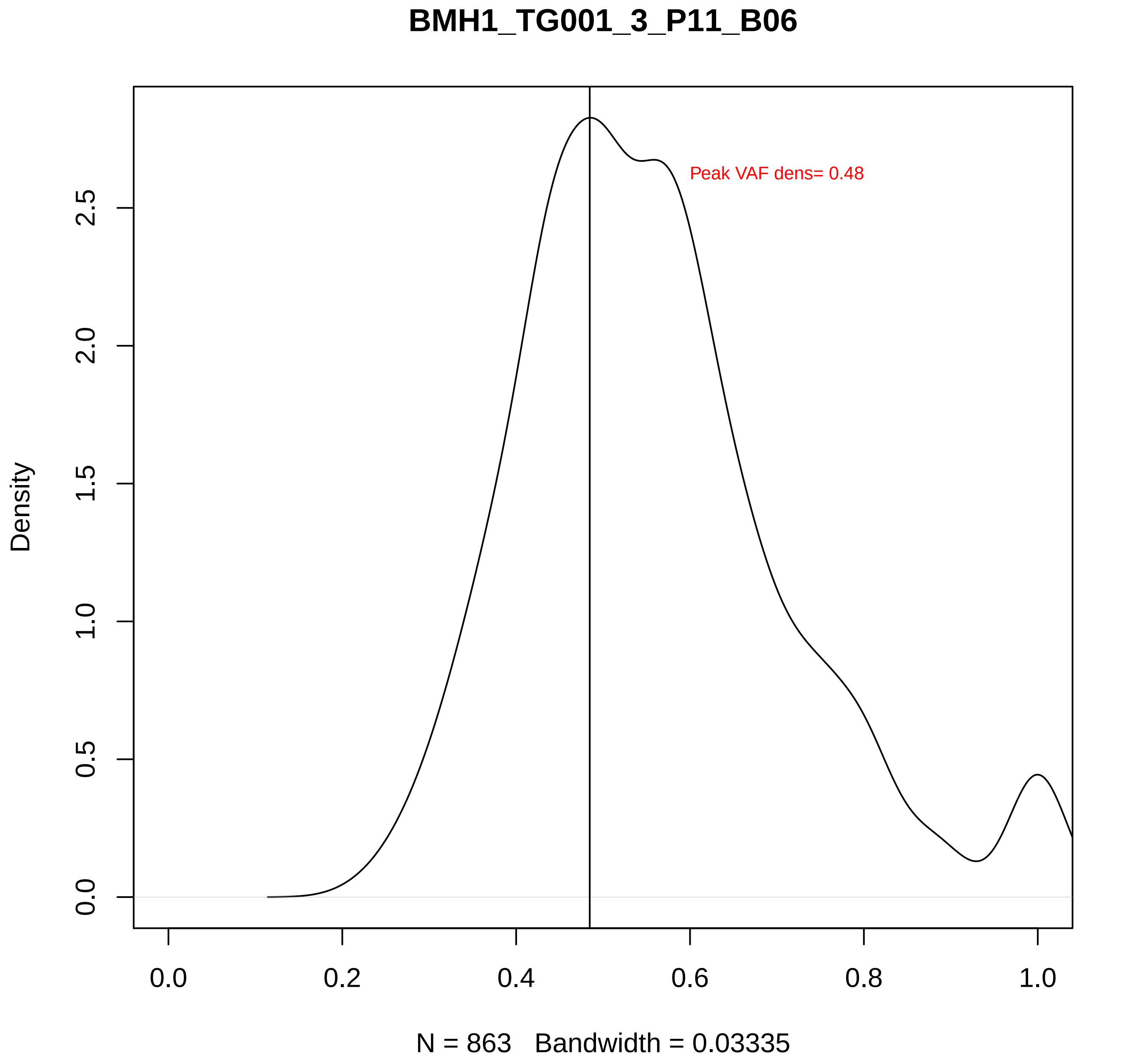

BMH1_TG001_3_P11_B06
Peak VAF dens= 0.48
Density
0.0
0.5
1.0
1.5
2.0
2.5
0.0
0.2
0.4
0.6
0.8
1.0
N = 863 Bandwidth = 0.03335

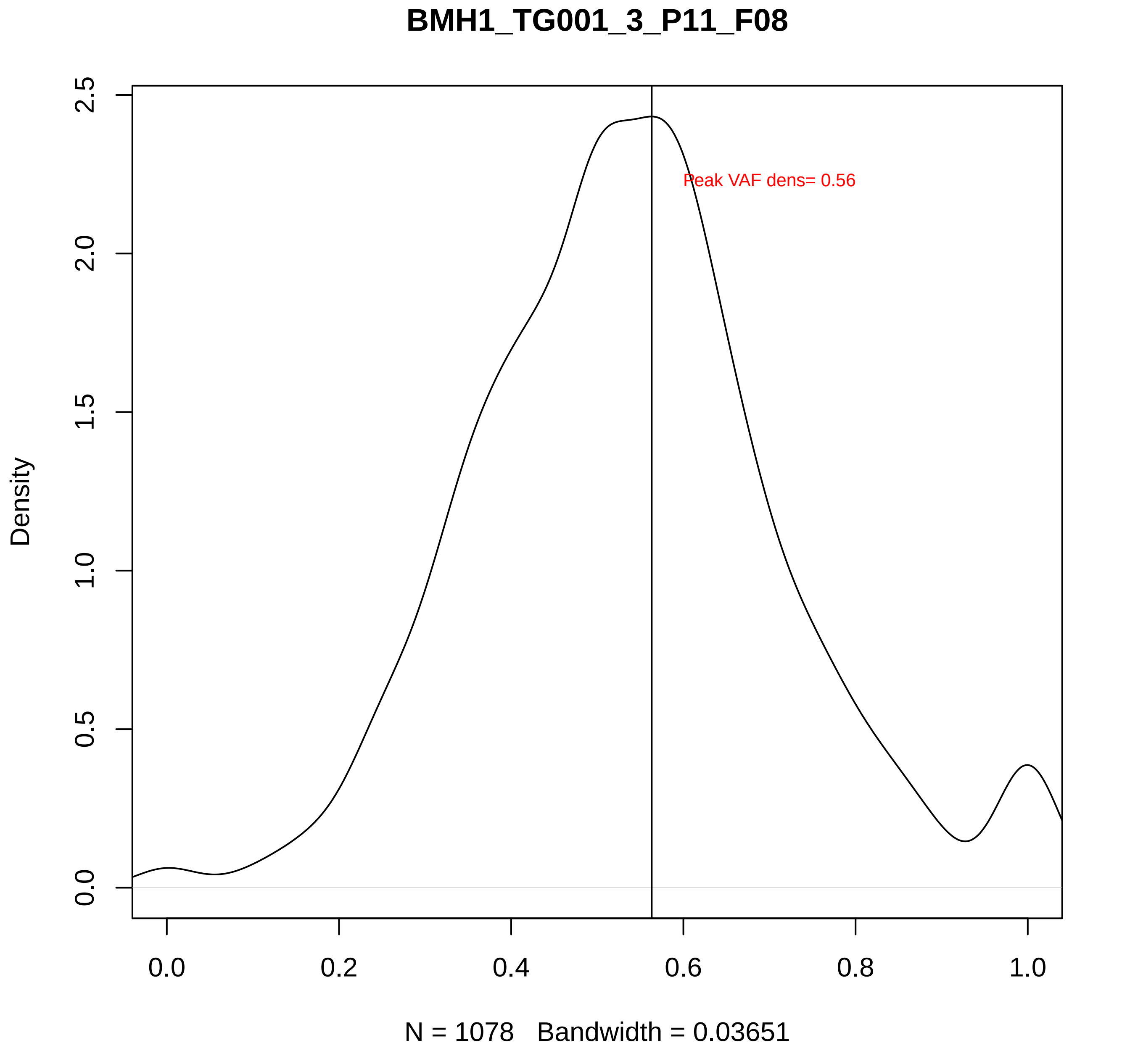
BMH1_TG001_3_P11_F08
Density
0.0
0.5
1.0
1.5
2.0
2.5
0.0
0.2
0.4
0.6
0.8
1.0
Peak VAF dens= 0.56
N = 1078 Bandwidth = 0.03651

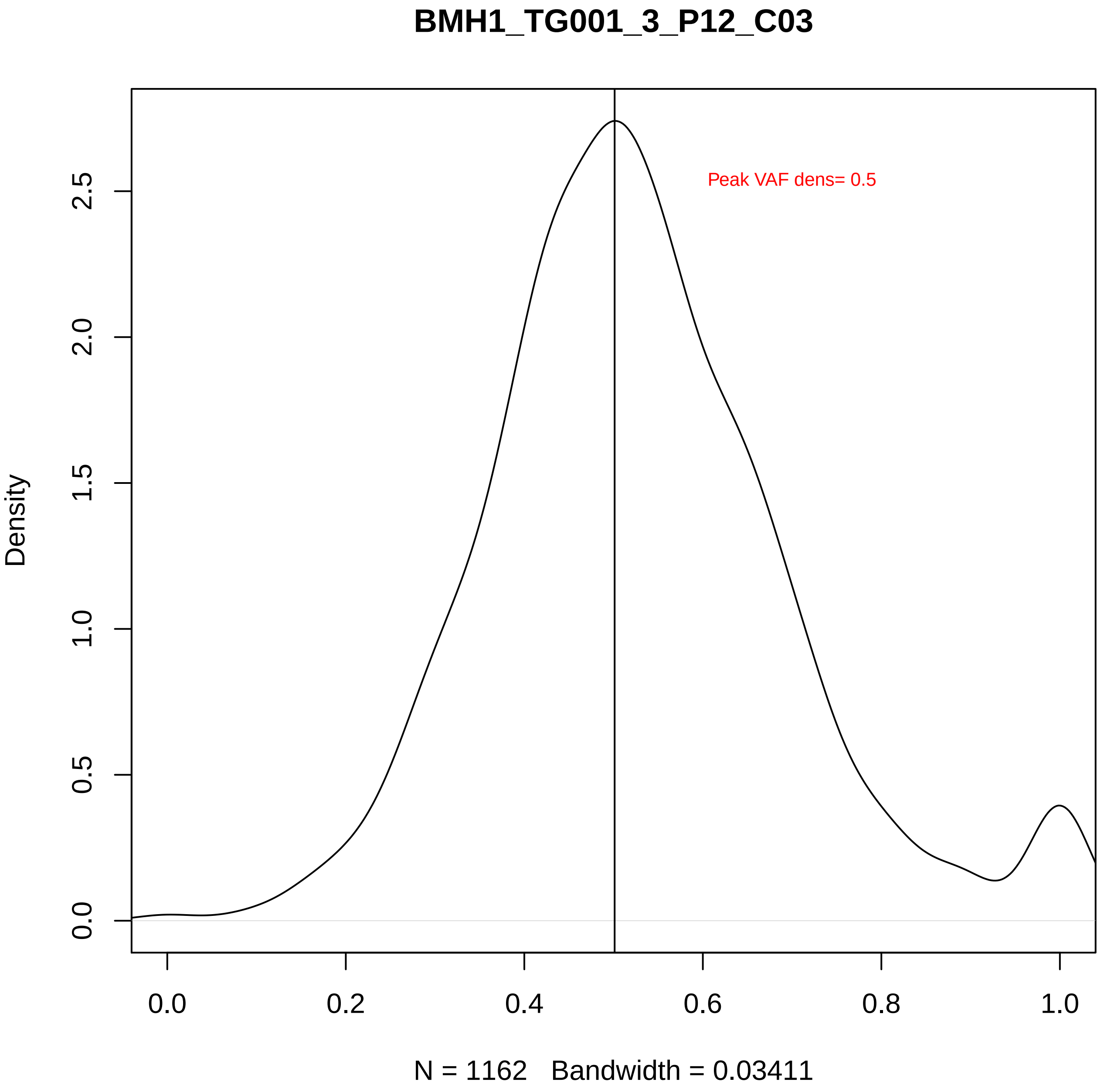
BMH1_TG001_3_P12_C03
Peak VAF dens= 0.5
Density
0.0
0.5
1.0
1.5
2.0
2.5
0.0
0.2
0.4
0.6
0.8
1.0
N = 1162   Bandwidth = 0.03411

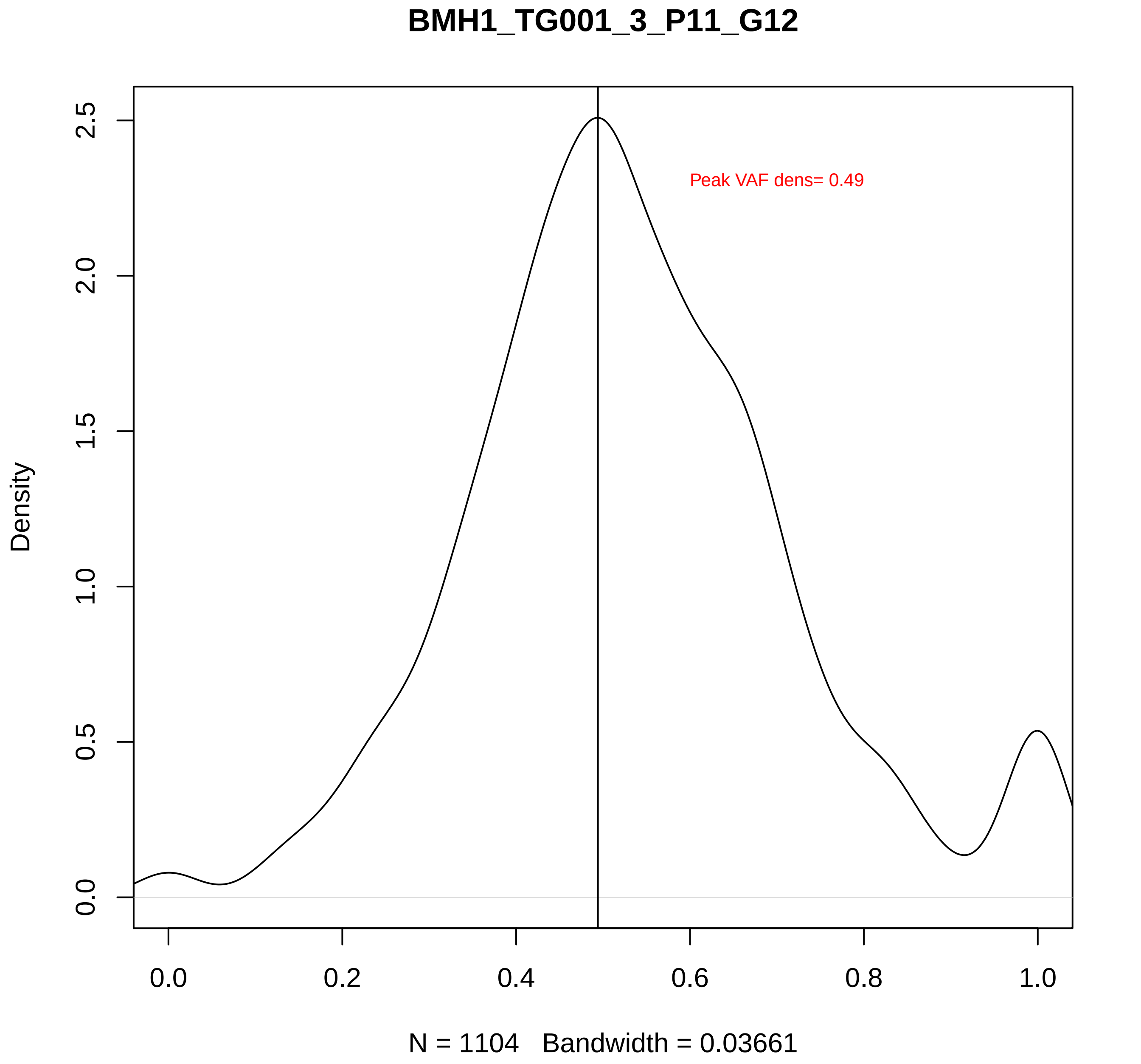
BMH1_TG001_3_P11_G12
Peak VAF dens= 0.49
Density
0.0
0.5
1.0
1.5
2.0
2.5
0.0
0.2
0.4
0.6
0.8
1.0
N = 1104   Bandwidth = 0.03661

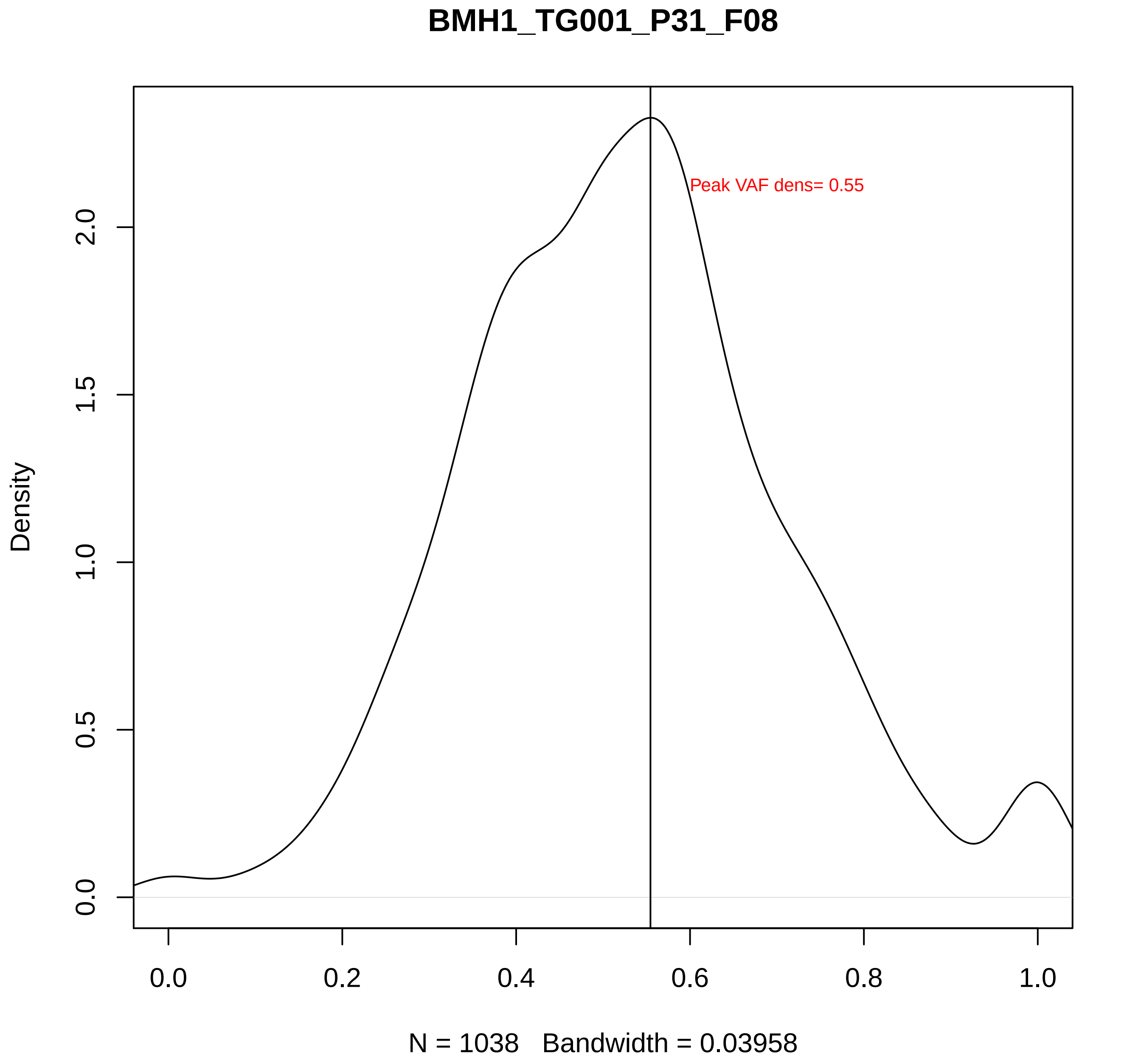
BMH1_TG001_P31_F08
Peak VAF dens= 0.55
Density
0.0
0.5
1.0
1.5
2.0
0.0
0.2
0.4
0.6
0.8
1.0
N = 1038   Bandwidth = 0.03958

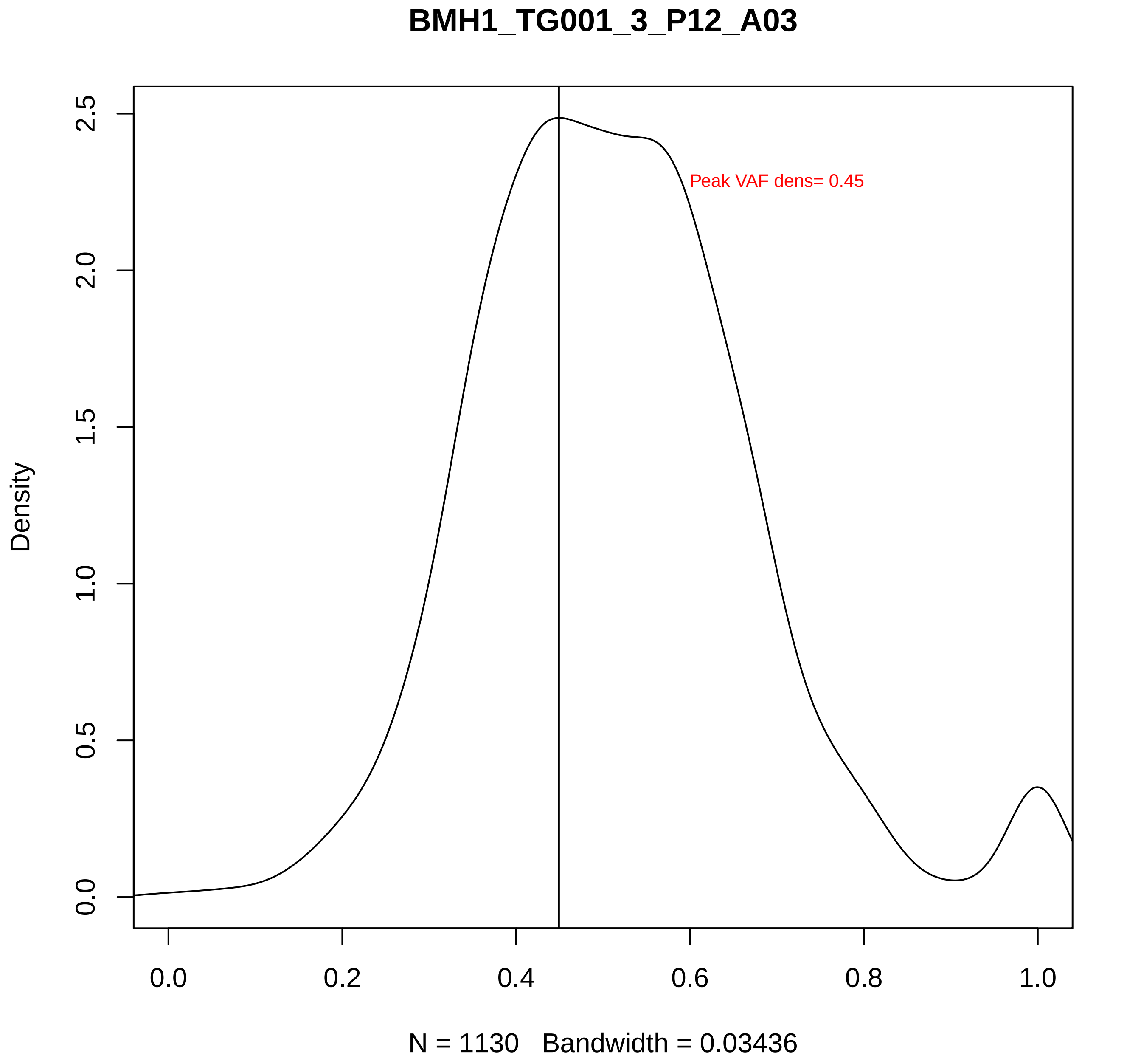
BMH1_TG001_3_P12_A03
Peak VAF dens= 0.45
Density
0.0
0.5
1.0
1.5
2.0
2.5
0.0
0.2
0.4
0.6
0.8
1.0
N = 1130   Bandwidth = 0.03436

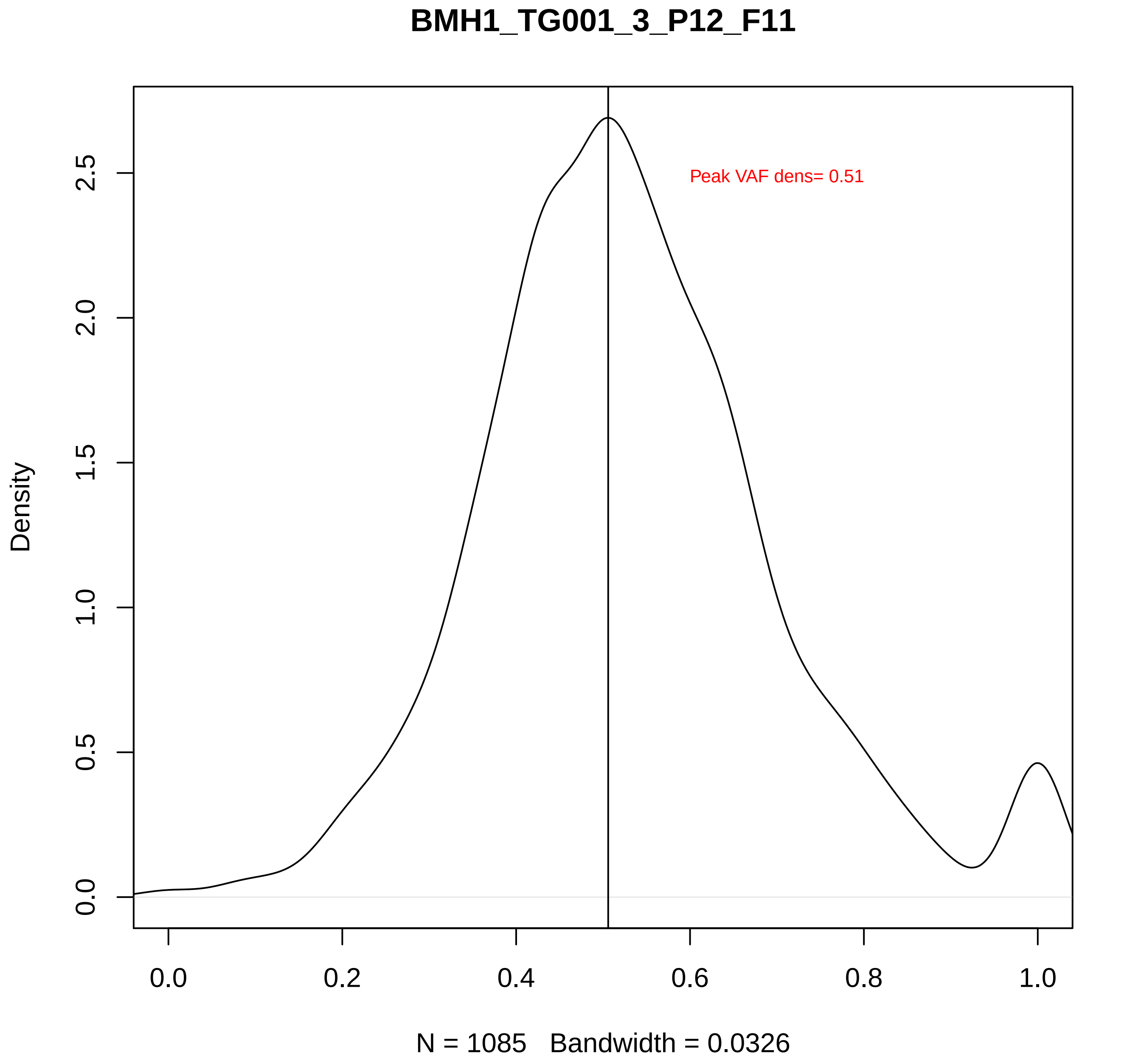

BMH1_TG001_3_P12_F11
Peak VAF dens= 0.51
Density
0.0
0.5
1.0
1.5
2.0
2.5
0.0
0.2
0.4
0.6
0.8
1.0
N = 1085   Bandwidth = 0.0326

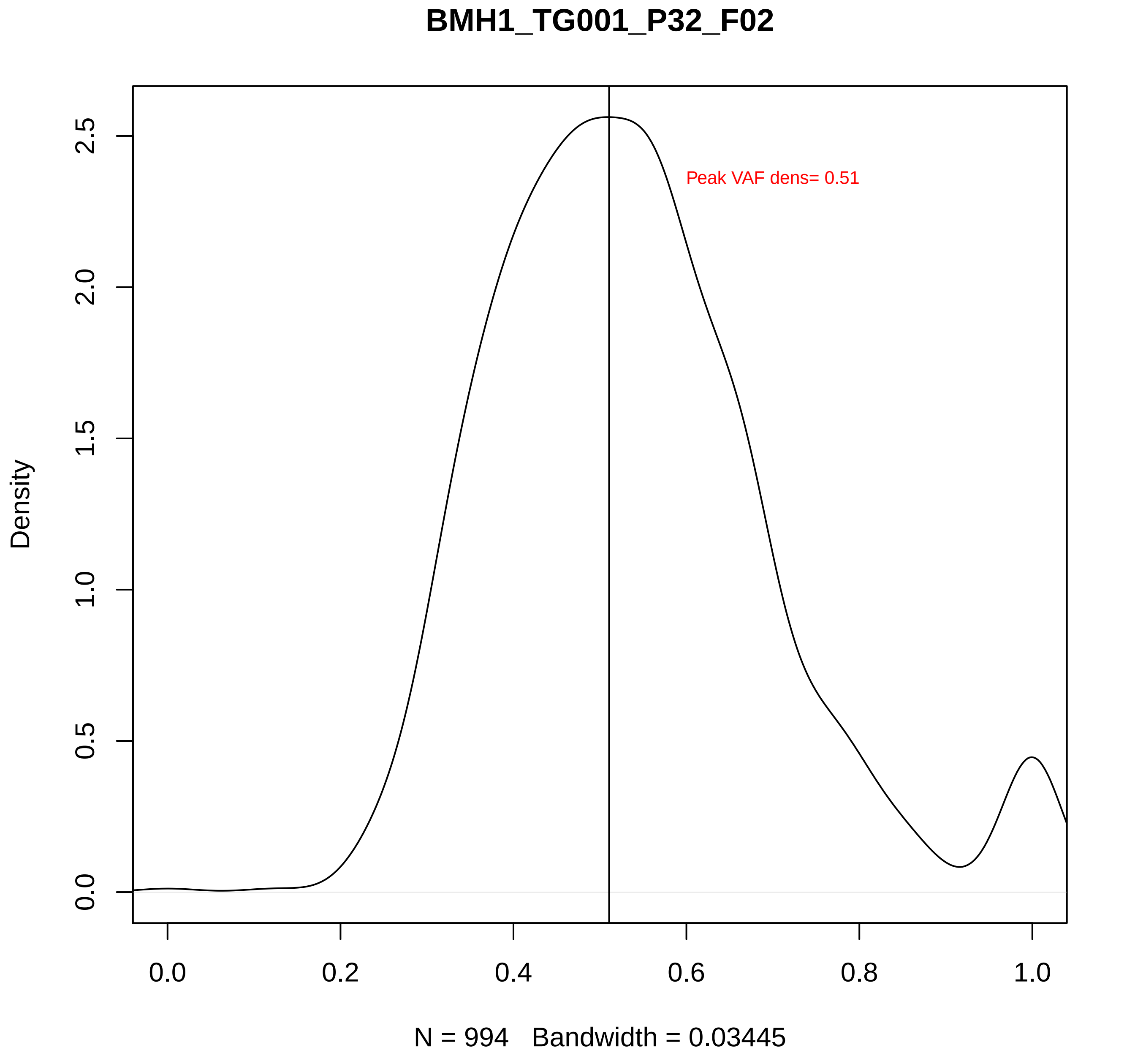
BMH1_TG001_P32_F02
Peak VAF dens= 0.51
Density
0.0
0.5
1.0
1.5
2.0
2.5
0.0
0.2
0.4
0.6
0.8
1.0
N = 994   Bandwidth = 0.03445

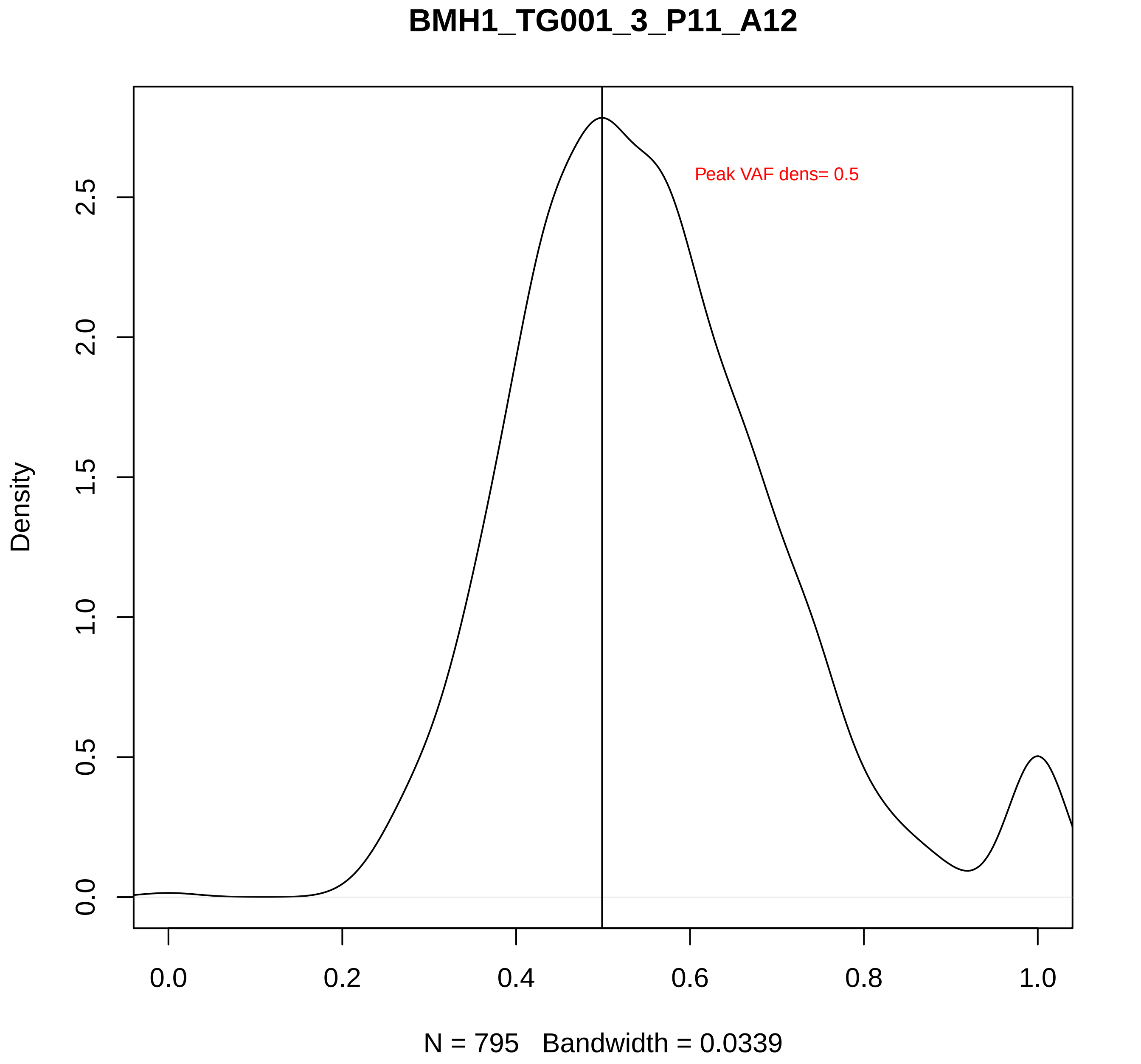

BMH1_TG001_3_P11_A12
Peak VAF dens= 0.5
Density
0.0
0.5
1.0
1.5
2.0
2.5
0.0
0.2
0.4
0.6
0.8
1.0
N = 795 Bandwidth = 0.0339

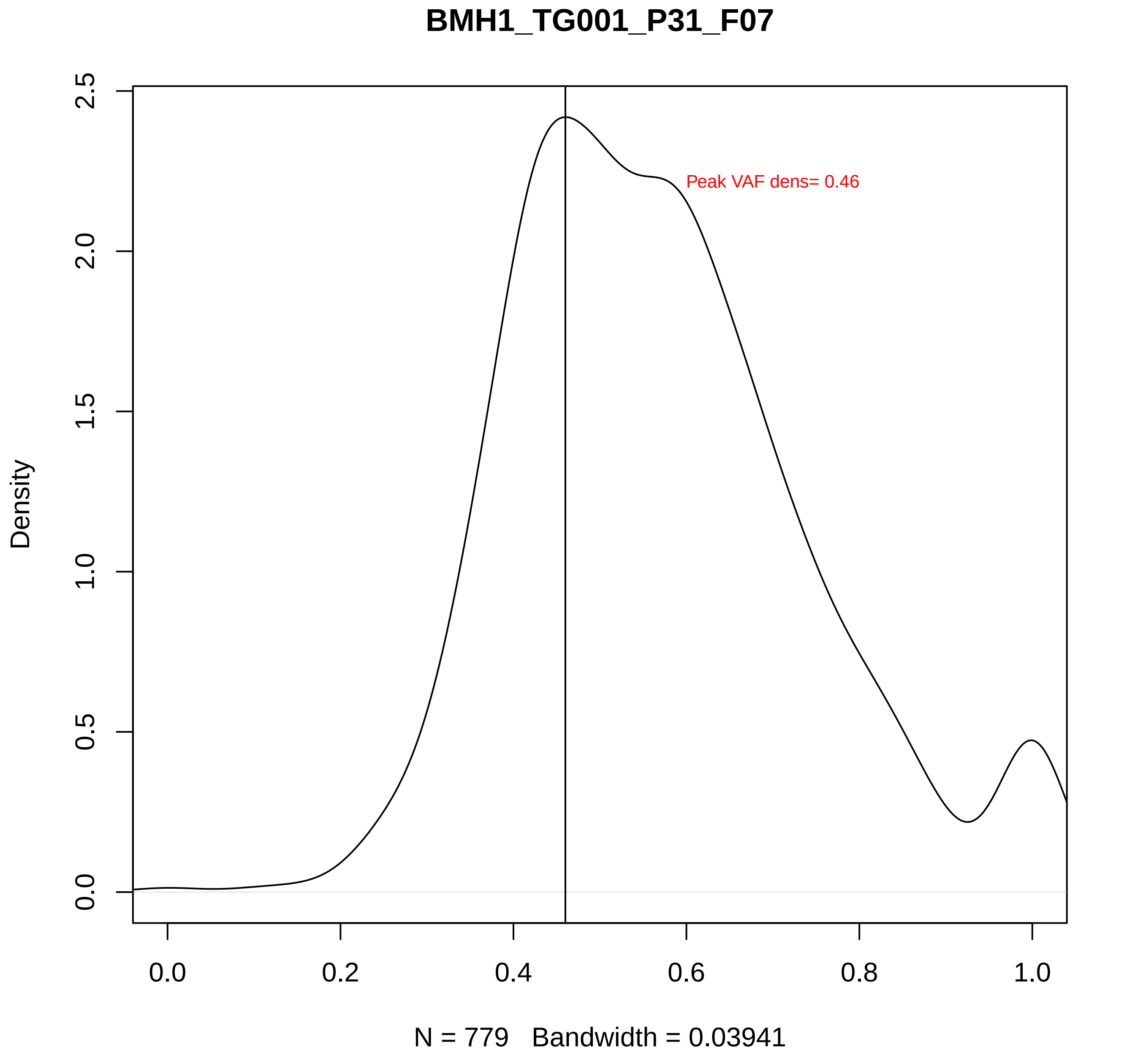

BMH1_TG001_P31_F07
Peak VAF dens= 0.46
Density
2.5
2.0
1.5
1.0
0.5
0.0
0.0
0.2
0.4
0.6
0.8
1.0
N = 779 Bandwidth = 0.03941

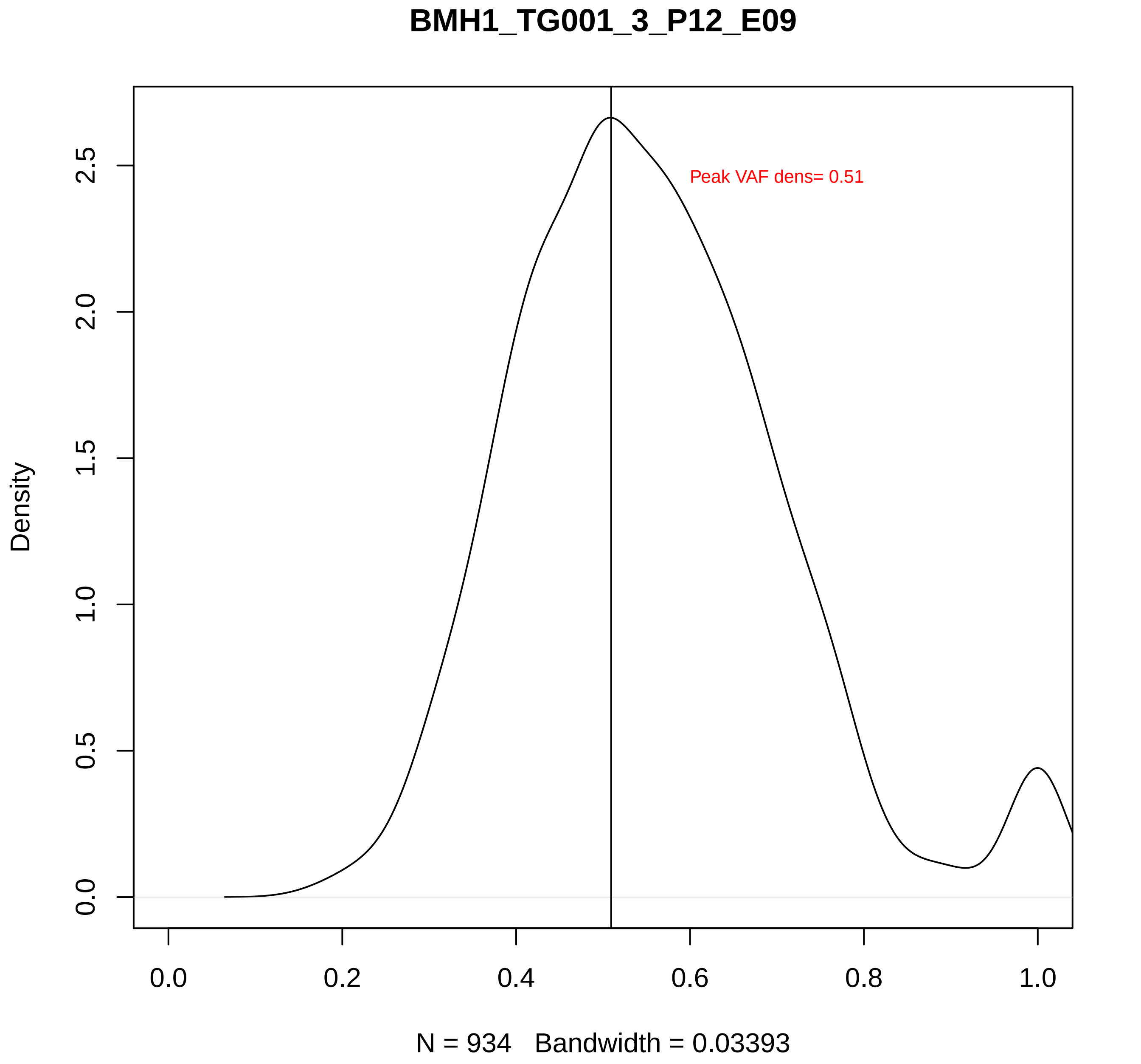
BMH1_TG001_3_P12_E09
Peak VAF dens= 0.51
Density
0.0
0.5
1.0
1.5
2.0
2.5
0.0
0.2
0.4
0.6
0.8
1.0
N = 934   Bandwidth = 0.03393

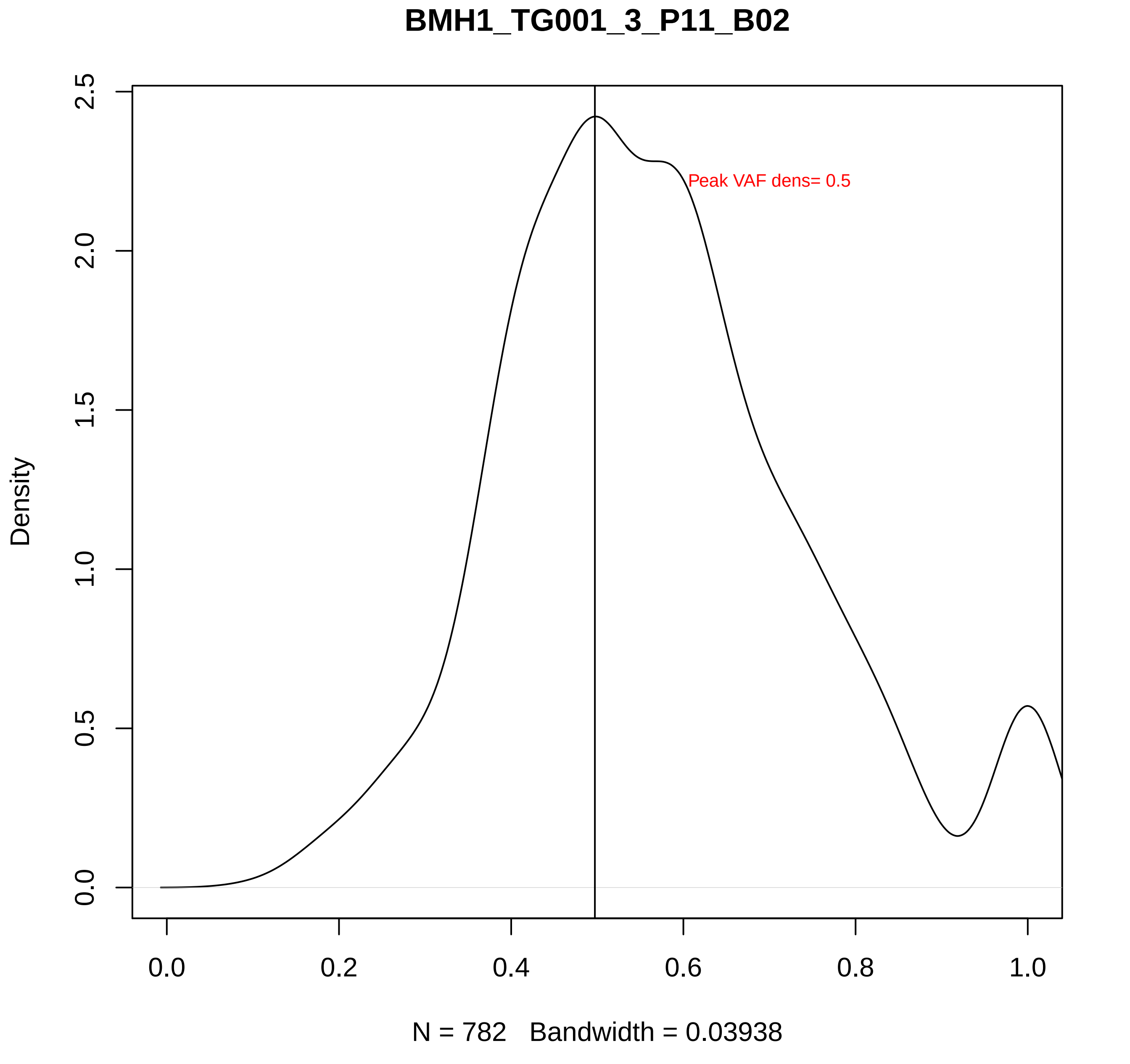

BMH1_TG001_3_P11_B02
Density
0.0
0.5
1.0
1.5
2.0
2.5
0.0
0.2
0.4
0.6
0.8
1.0
Peak VAF dens= 0.5
N = 782   Bandwidth = 0.03938

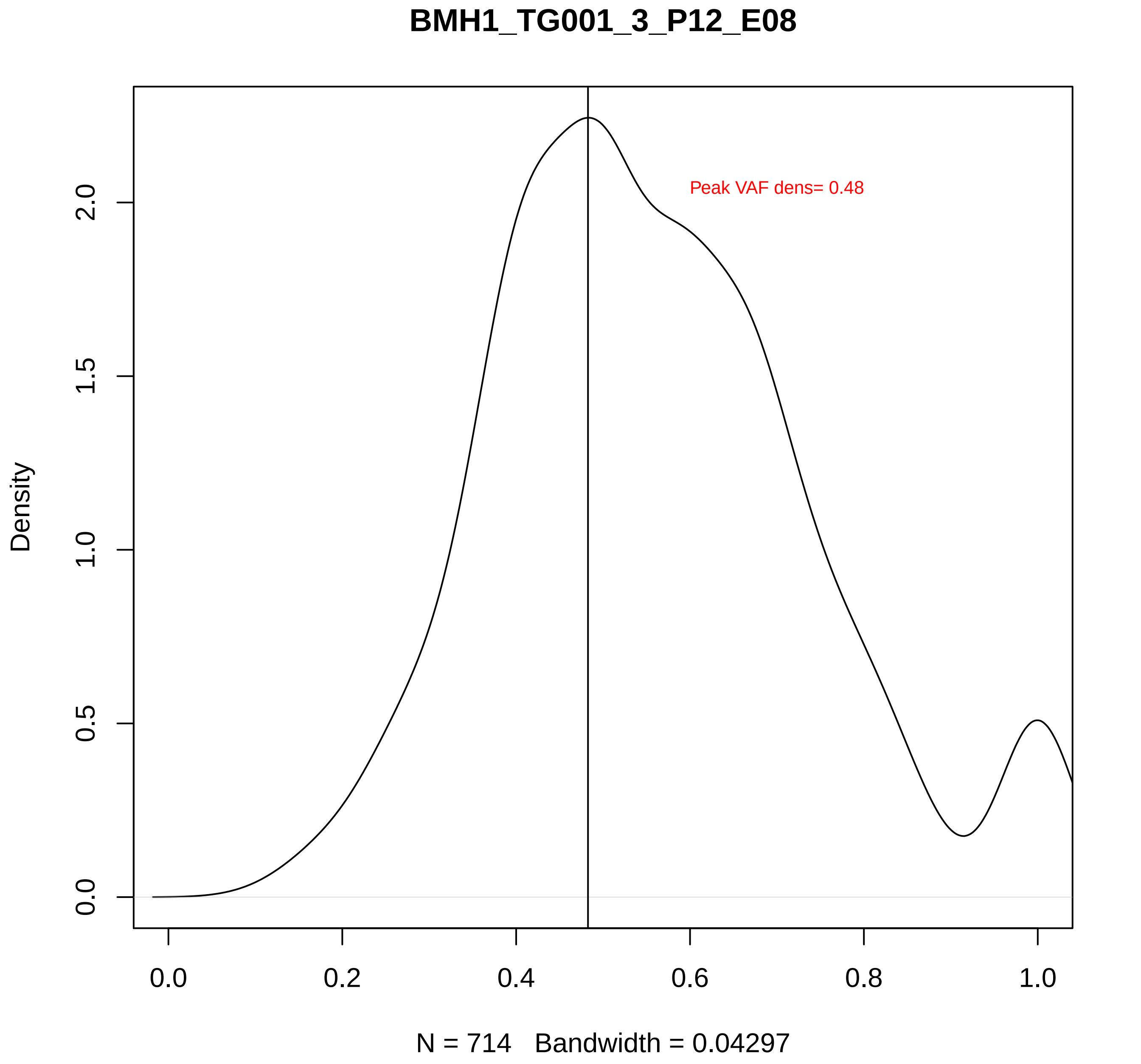
BMH1_TG001_3_P12_E08
Peak VAF dens= 0.48
Density
0.0
0.5
1.0
1.5
2.0
0.0
0.2
0.4
0.6
0.8
1.0
N = 714   Bandwidth = 0.04297

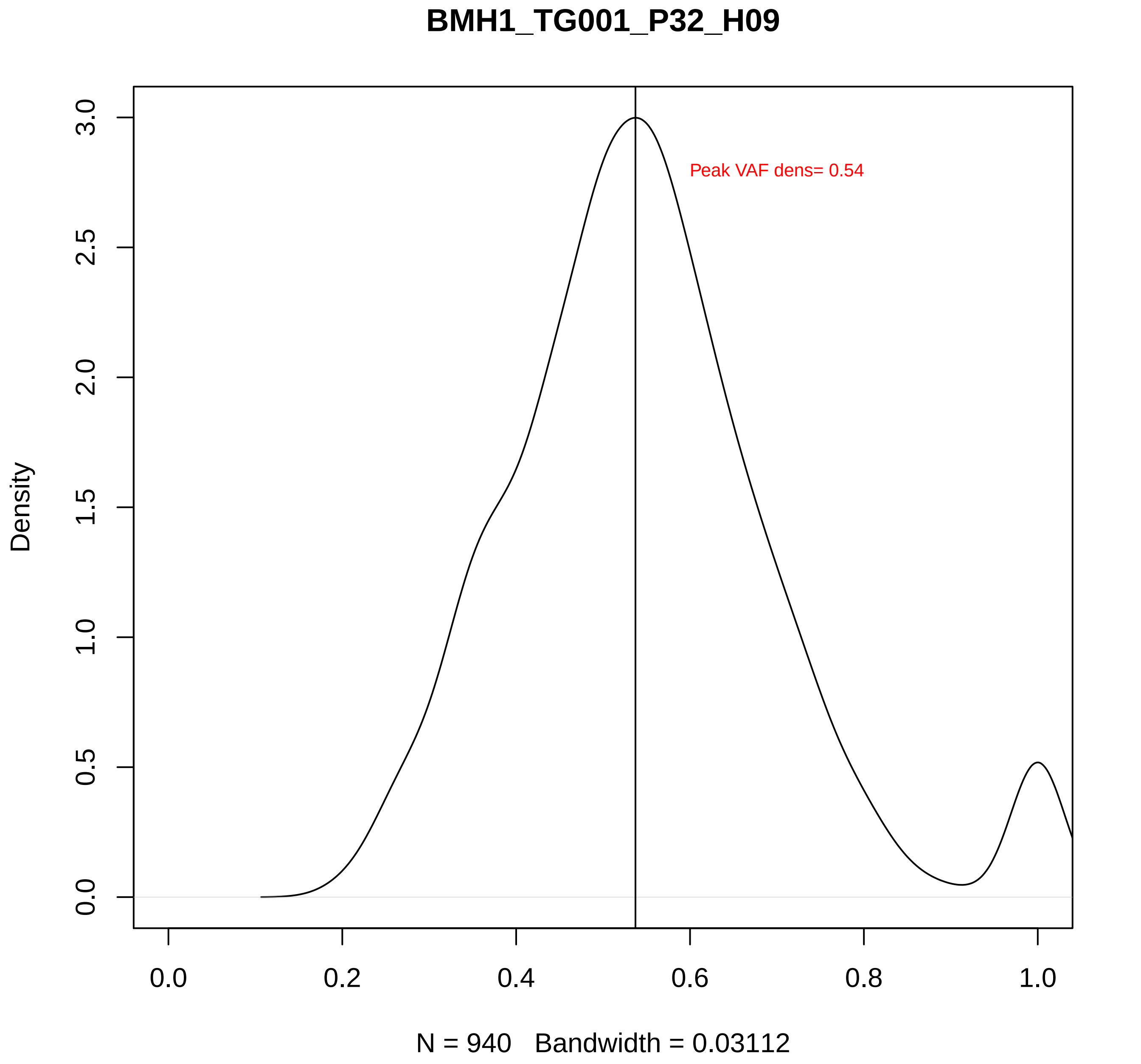

BMH1_TG001_P32_H09
Peak VAF dens= 0.54
Density
0.0
0.5
1.0
1.5
2.0
2.5
3.0
0.0
0.2
0.4
0.6
0.8
1.0
N = 940 Bandwidth = 0.03112

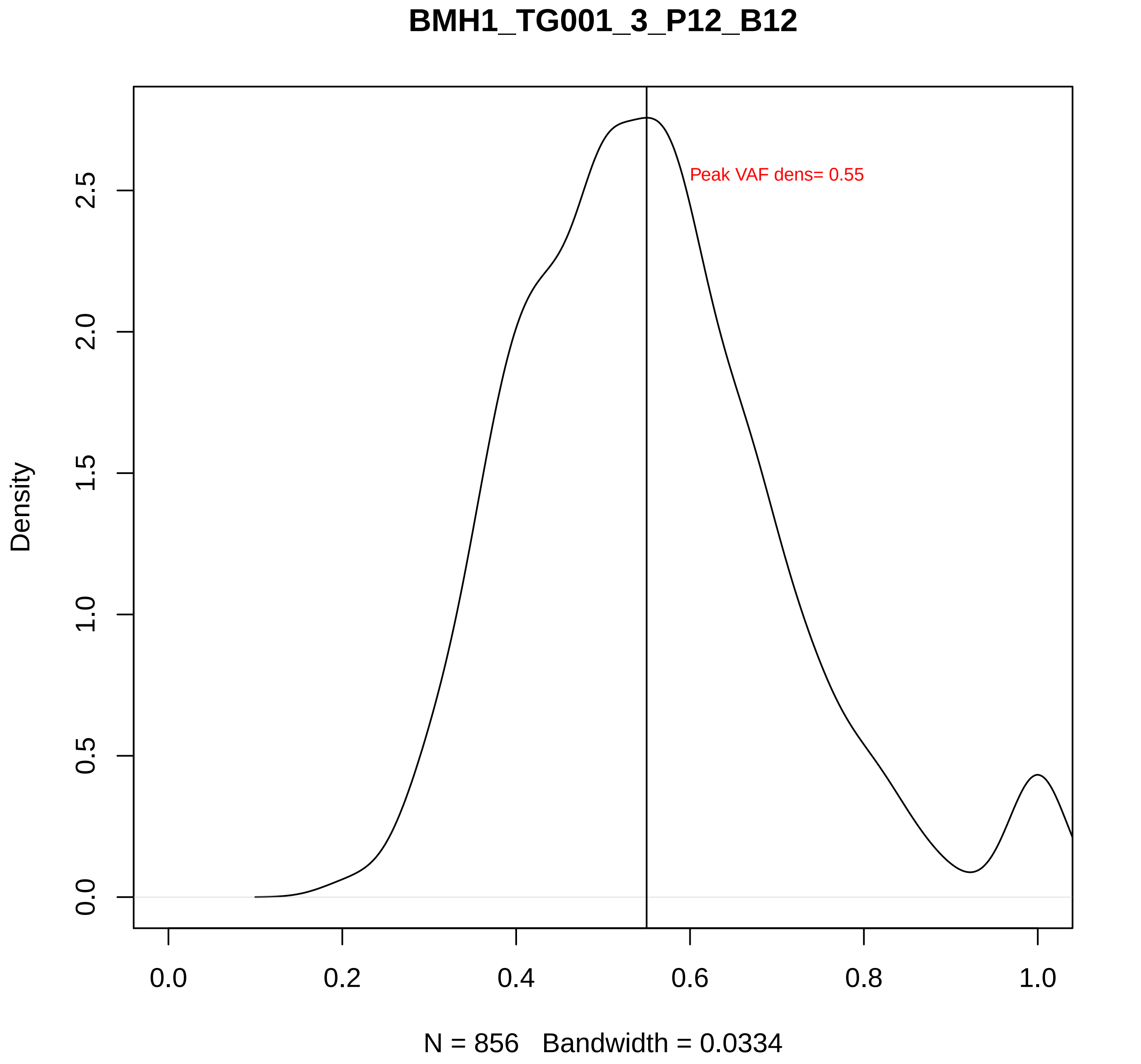
BMH1_TG001_3_P12_B12
Peak VAF dens= 0.55
Density
0.0
0.5
1.0
1.5
2.0
2.5
0.0
0.2
0.4
0.6
0.8
1.0
N = 856   Bandwidth = 0.0334

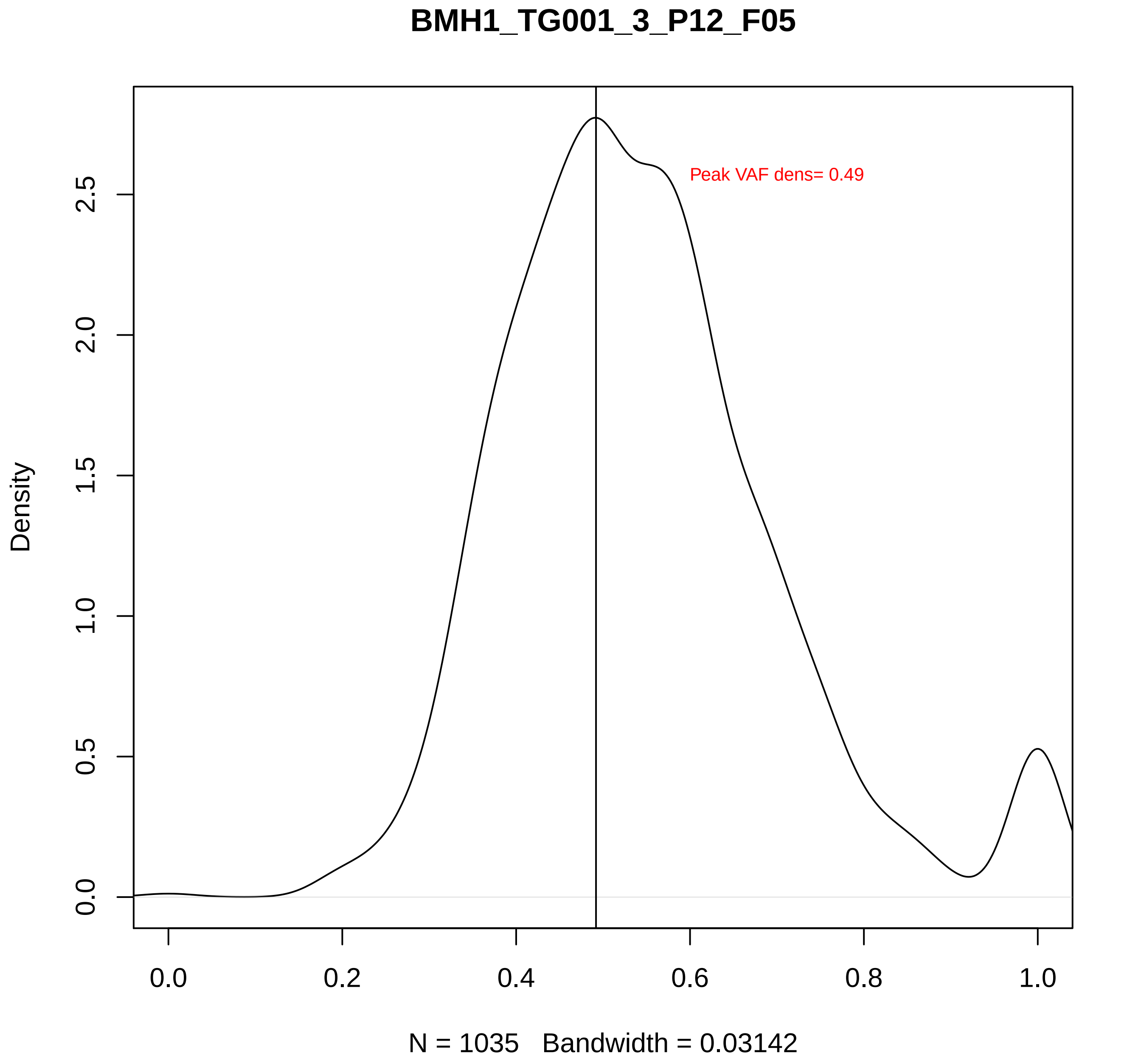
BMH1_TG001_3_P12_F05
Peak VAF dens= 0.49
Density
0.0
0.5
1.0
1.5
2.0
2.5
0.0
0.2
0.4
0.6
0.8
1.0
N = 1035   Bandwidth = 0.03142

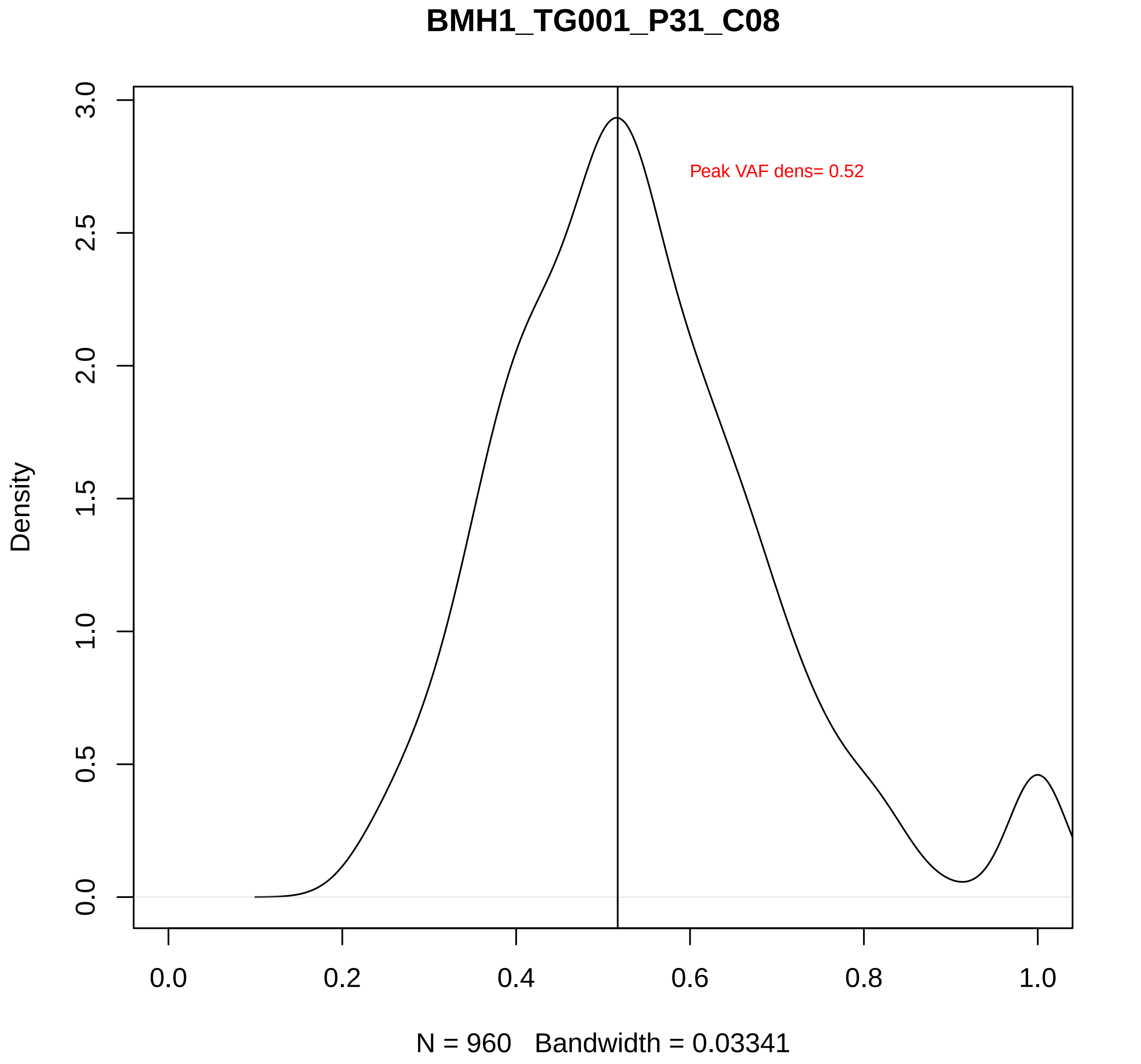

BMH1_TG001_P31_C08
Peak VAF dens= 0.52
Density
0.0
0.5
1.0
1.5
2.0
2.5
3.0
0.0
0.2
0.4
0.6
0.8
1.0
N = 960   Bandwidth = 0.03341

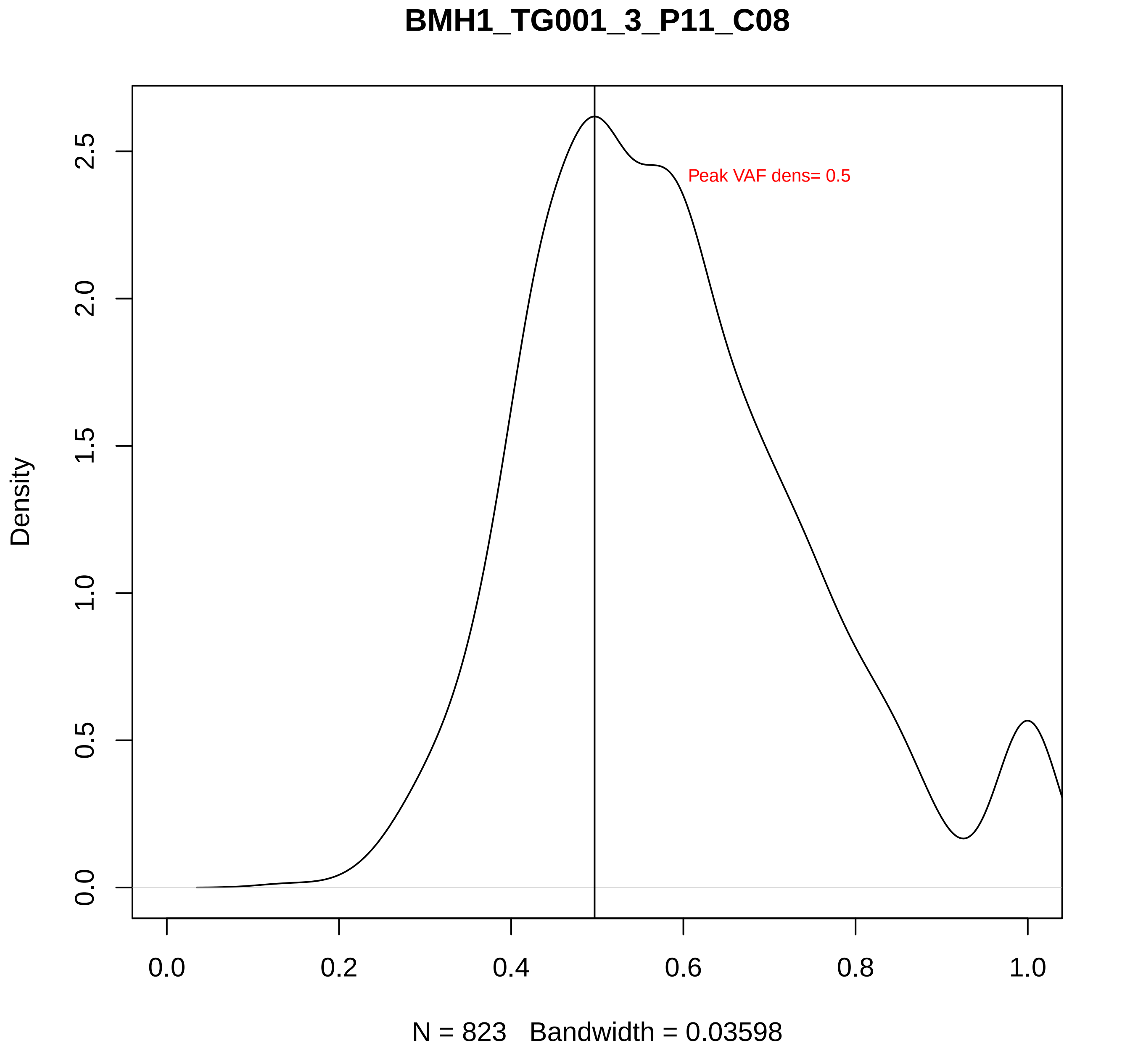

BMH1_TG001_3_P11_C08
Peak VAF dens= 0.5
Density
0.0
0.5
1.0
1.5
2.0
2.5
0.0
0.2
0.4
0.6
0.8
1.0
N = 823   Bandwidth = 0.03598

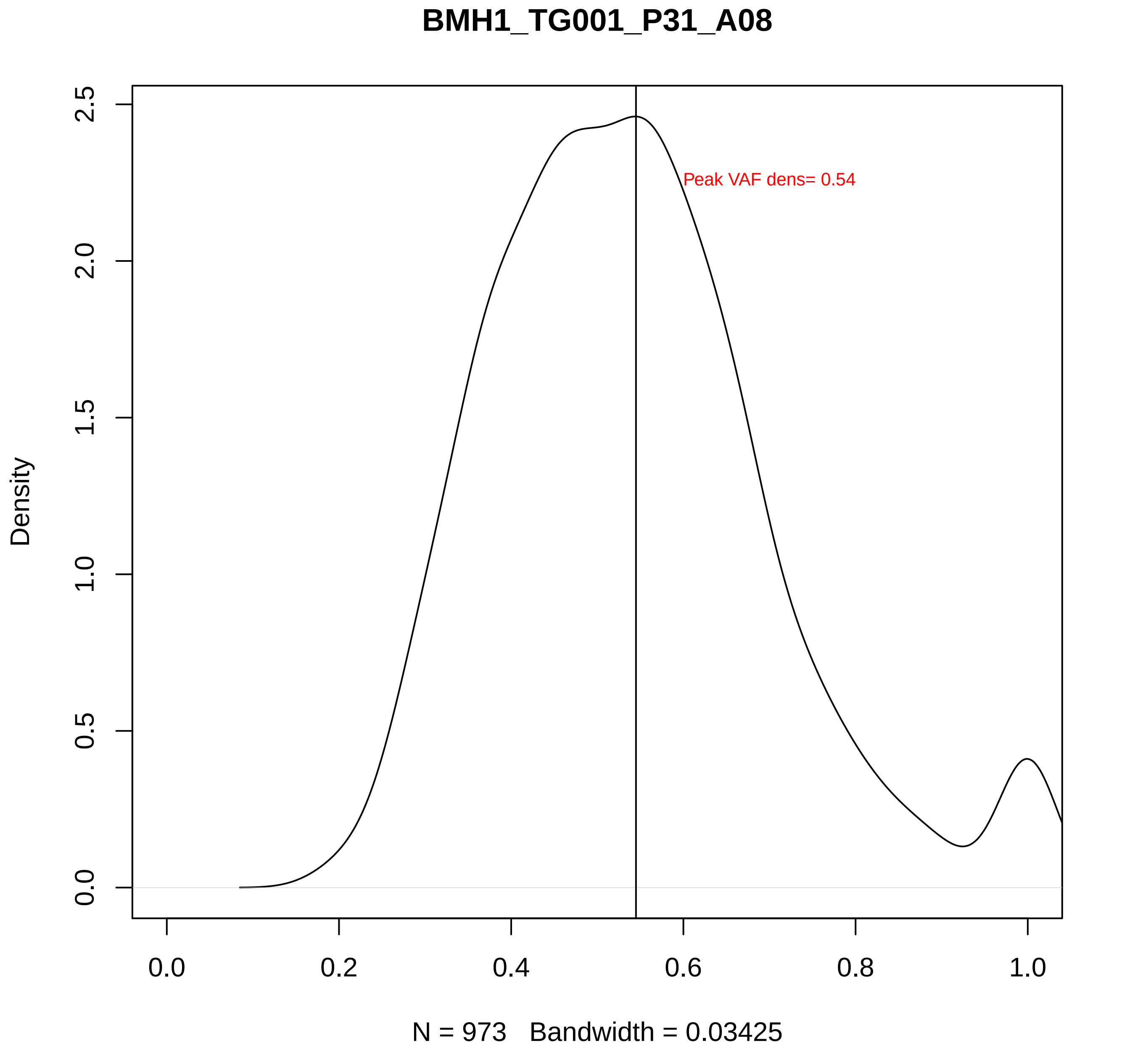
BMH1_TG001_P31_A08
Density
0.0
0.5
1.0
1.5
2.0
2.5
0.0
0.2
0.4
0.6
0.8
1.0
Peak VAF dens= 0.54
N = 973   Bandwidth = 0.03425

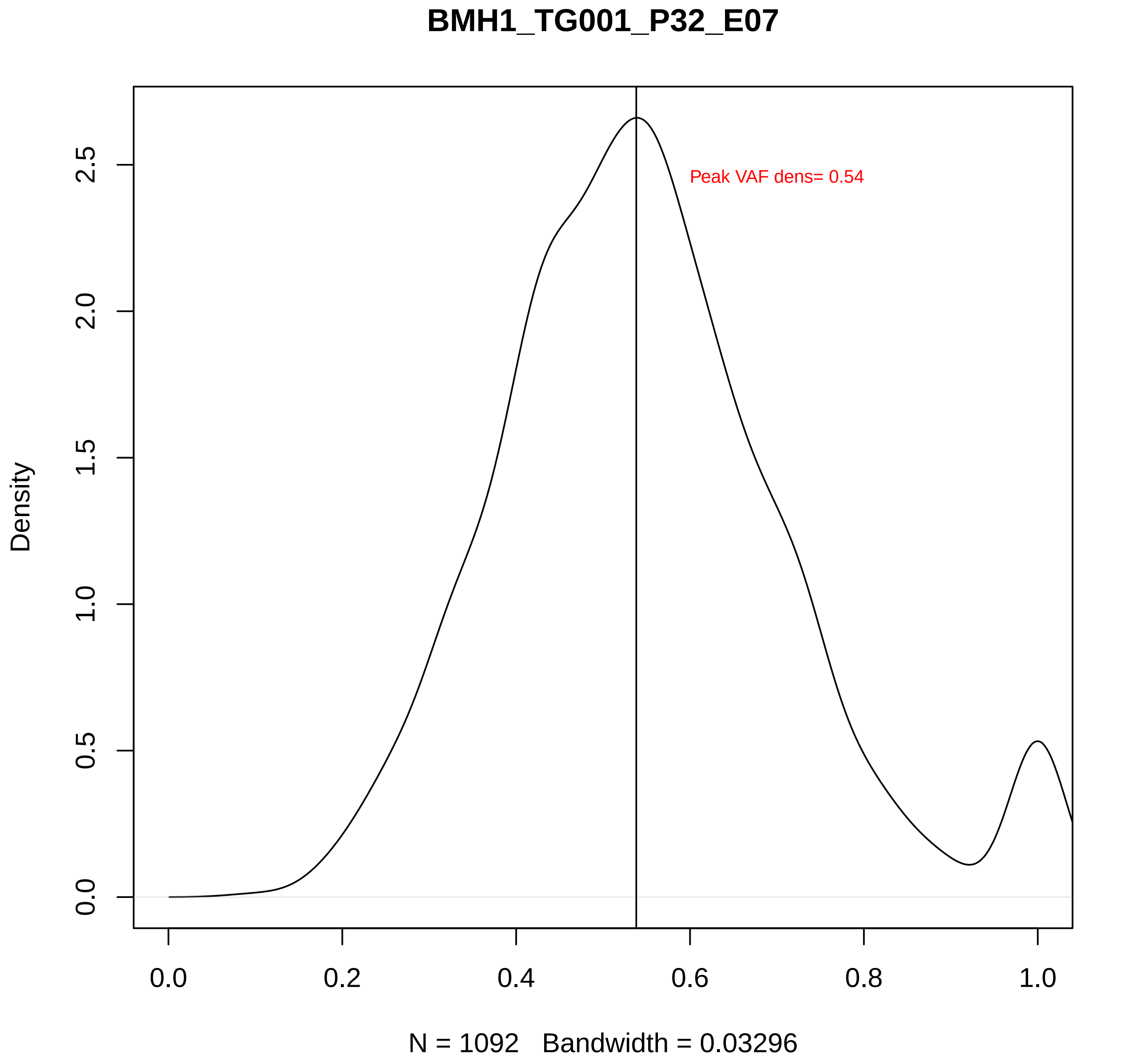
BMH1_TG001_P32_E07
Peak VAF dens= 0.54
Density
0.0
0.5
1.0
1.5
2.0
2.5
0.0
0.2
0.4
0.6
0.8
1.0
N = 1092   Bandwidth = 0.03296

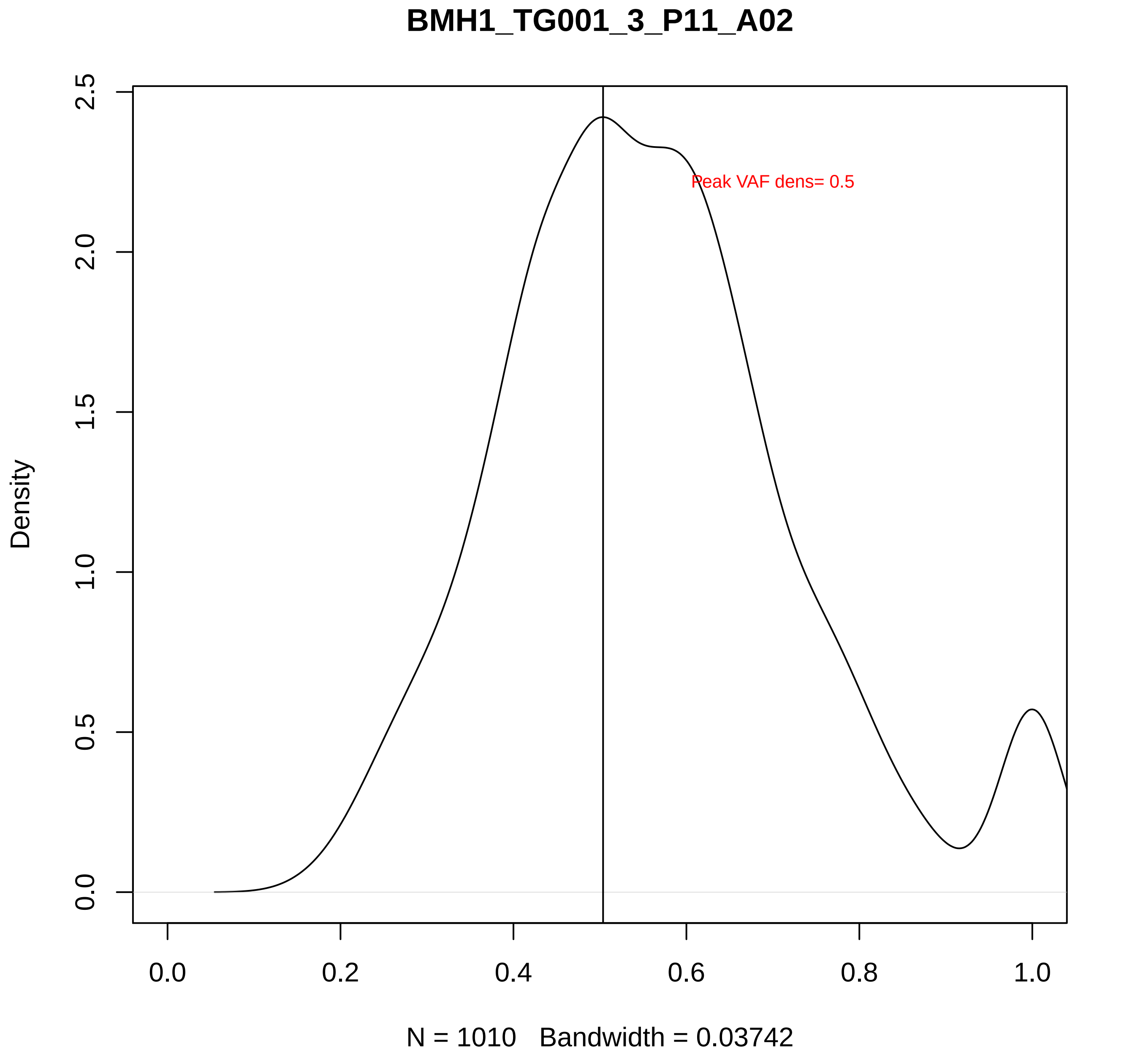
BMH1_TG001_3_P11_A02
Peak VAF dens= 0.5
Density
0.0
0.5
1.0
1.5
2.0
2.5
0.0
0.2
0.4
0.6
0.8
1.0
N = 1010   Bandwidth = 0.03742

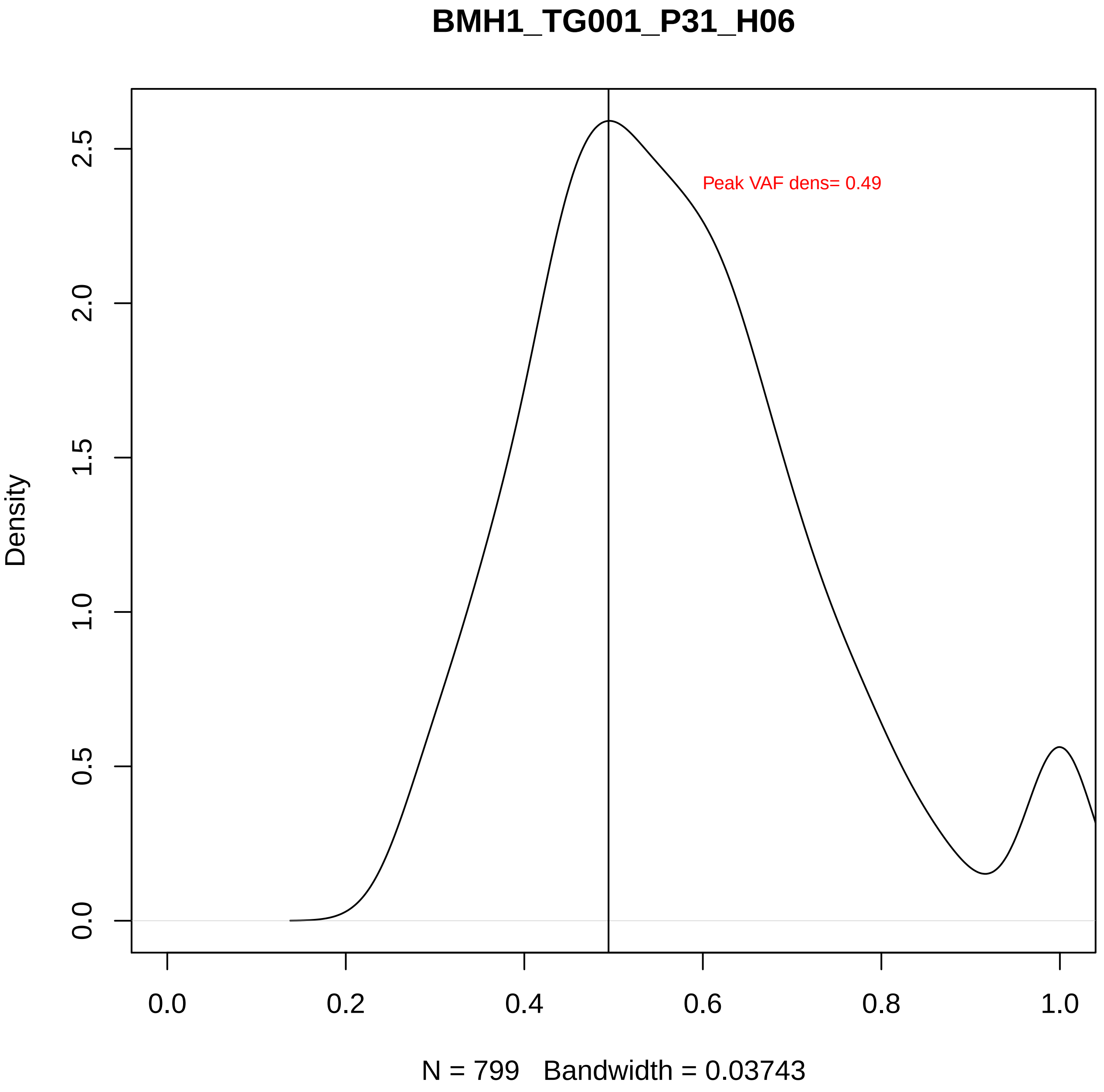
BMH1_TG001_P31_H06
Peak VAF dens= 0.49
Density
0.0
0.5
1.0
1.5
2.0
2.5
0.0
0.2
0.4
0.6
0.8
1.0
N = 799 Bandwidth = 0.03743

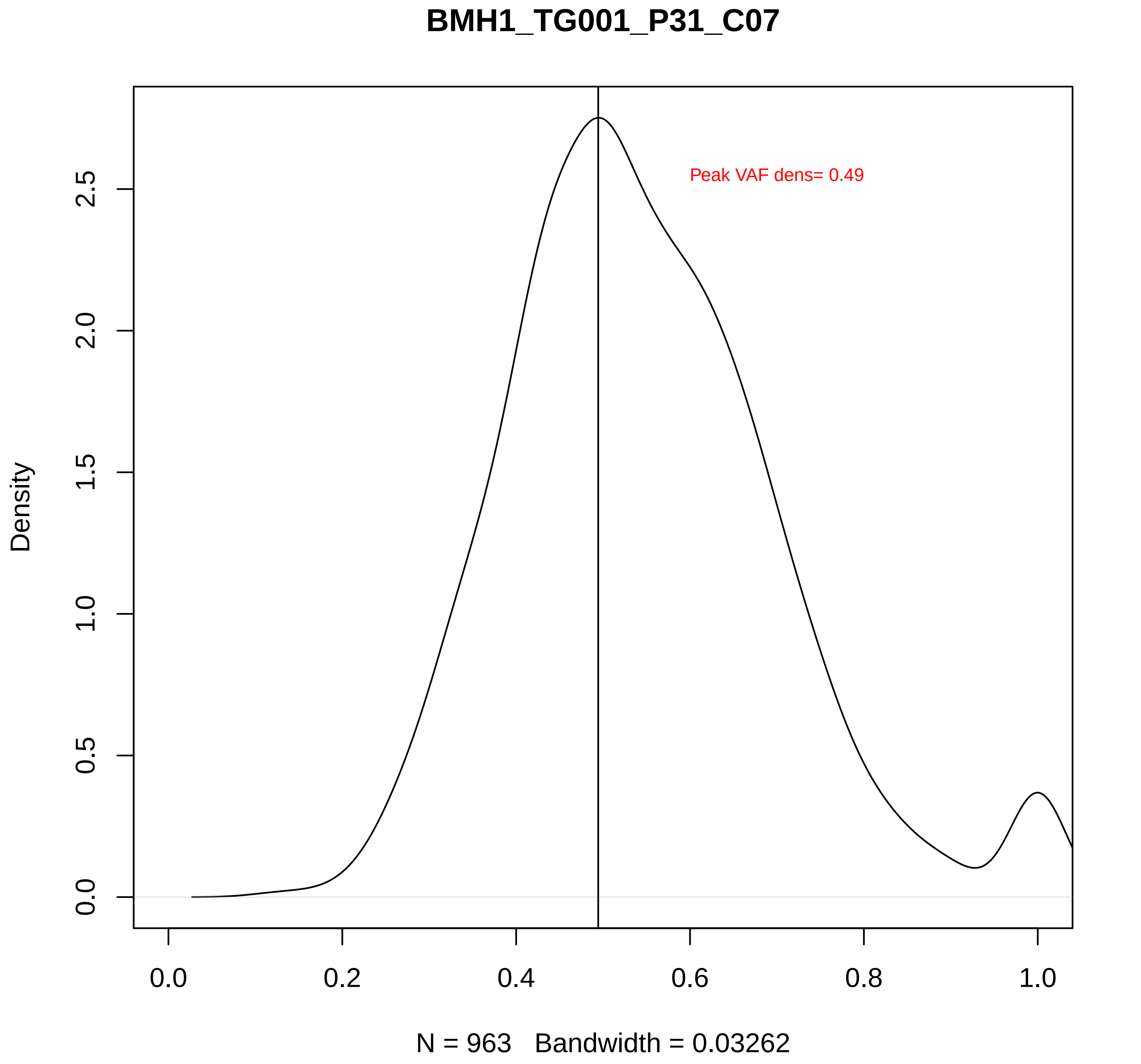
BMH1_TG001_P31_C07
Peak VAF dens= 0.49
Density
0.0
0.5
1.0
1.5
2.0
2.5
0.0
0.2
0.4
0.6
0.8
1.0
N = 963   Bandwidth = 0.03262

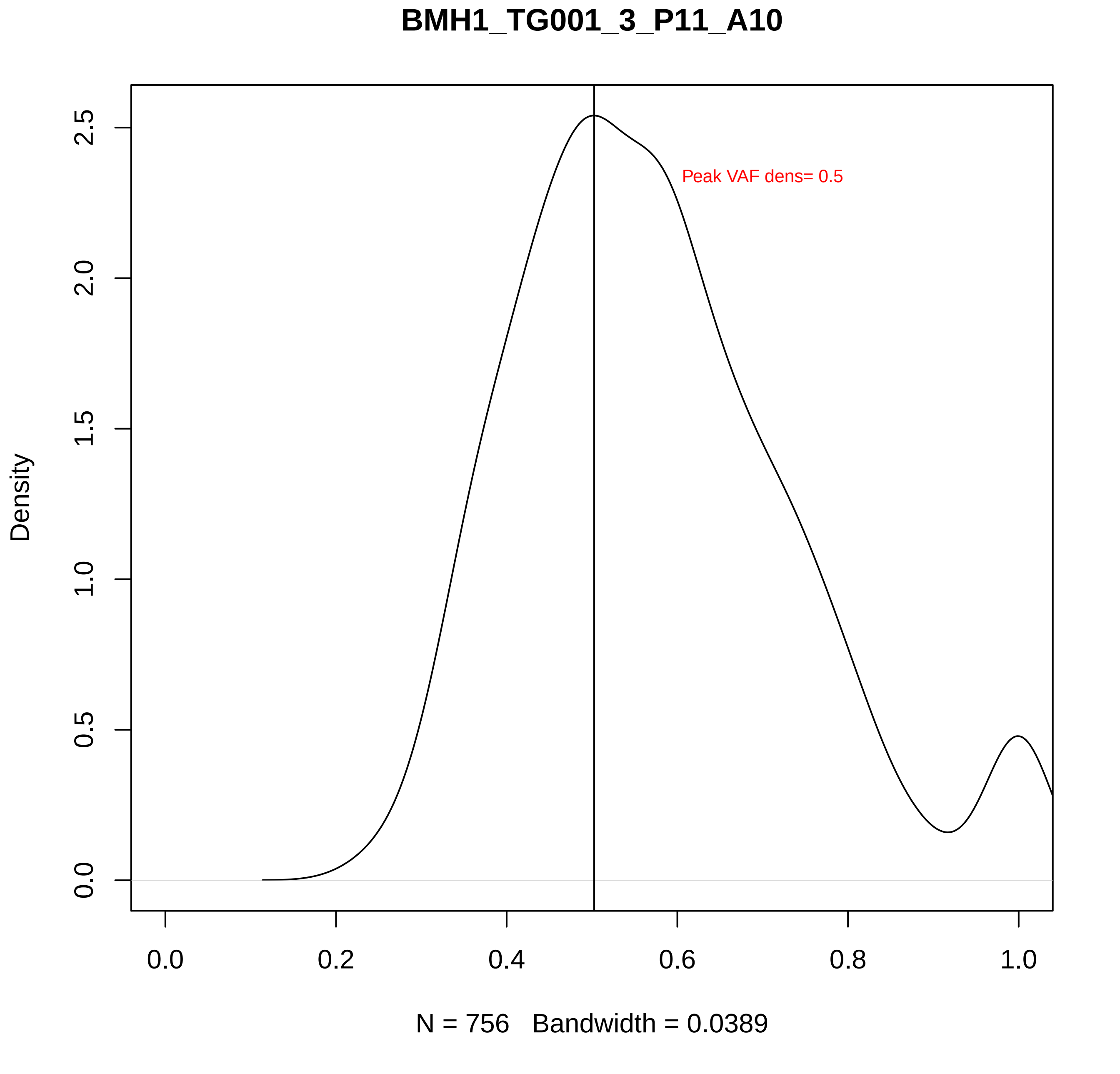

BMH1_TG001_3_P11_A10
Peak VAF dens= 0.5
Density
0.0
0.5
1.0
1.5
2.0
2.5
0.0
0.2
0.4
0.6
0.8
1.0
N = 756   Bandwidth = 0.0389

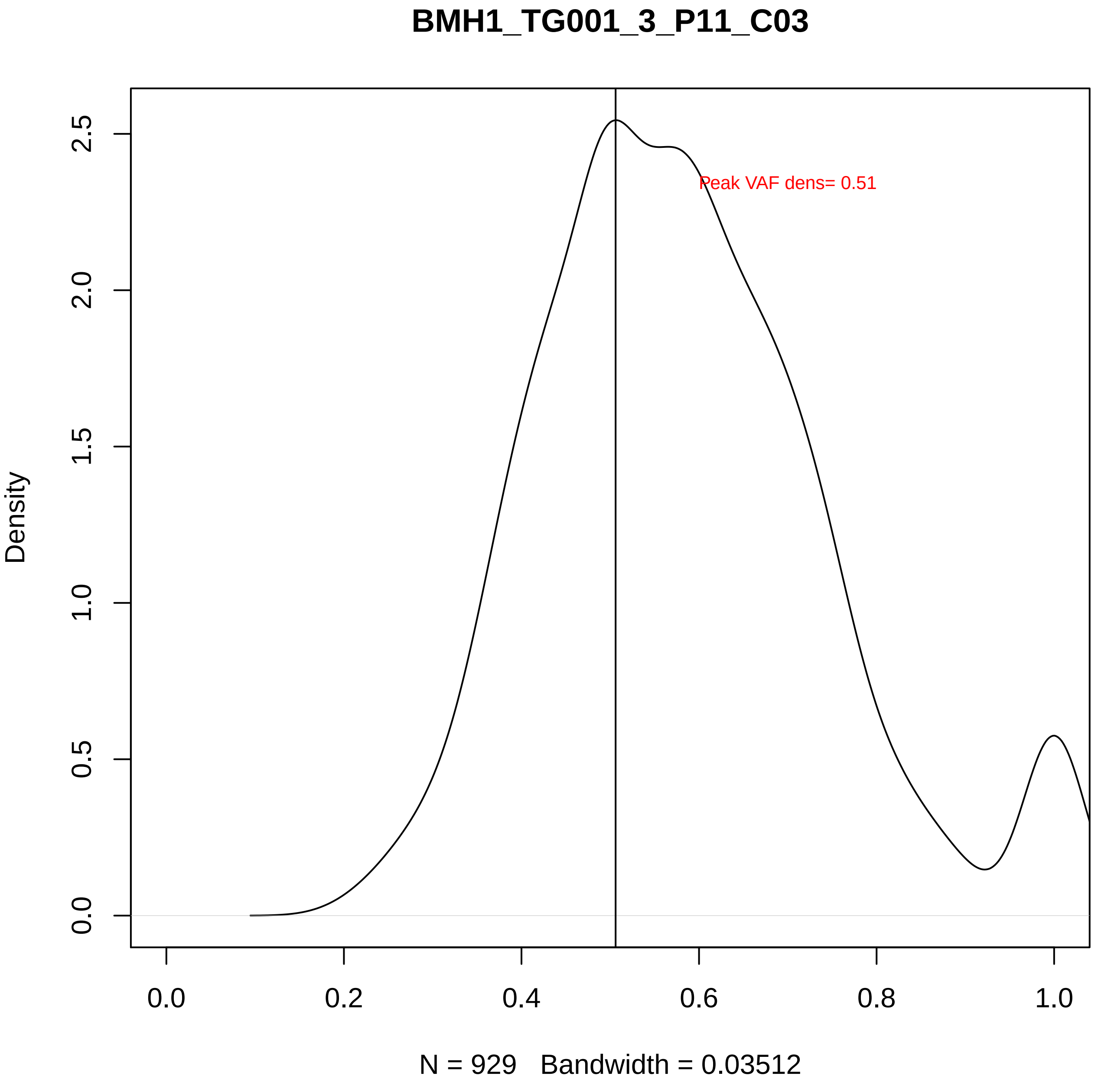
BMH1_TG001_3_P11_C03
Peak VAF dens= 0.51
Density
0.0
0.5
1.0
1.5
2.0
2.5
0.0
0.2
0.4
0.6
0.8
1.0
N = 929   Bandwidth = 0.03512

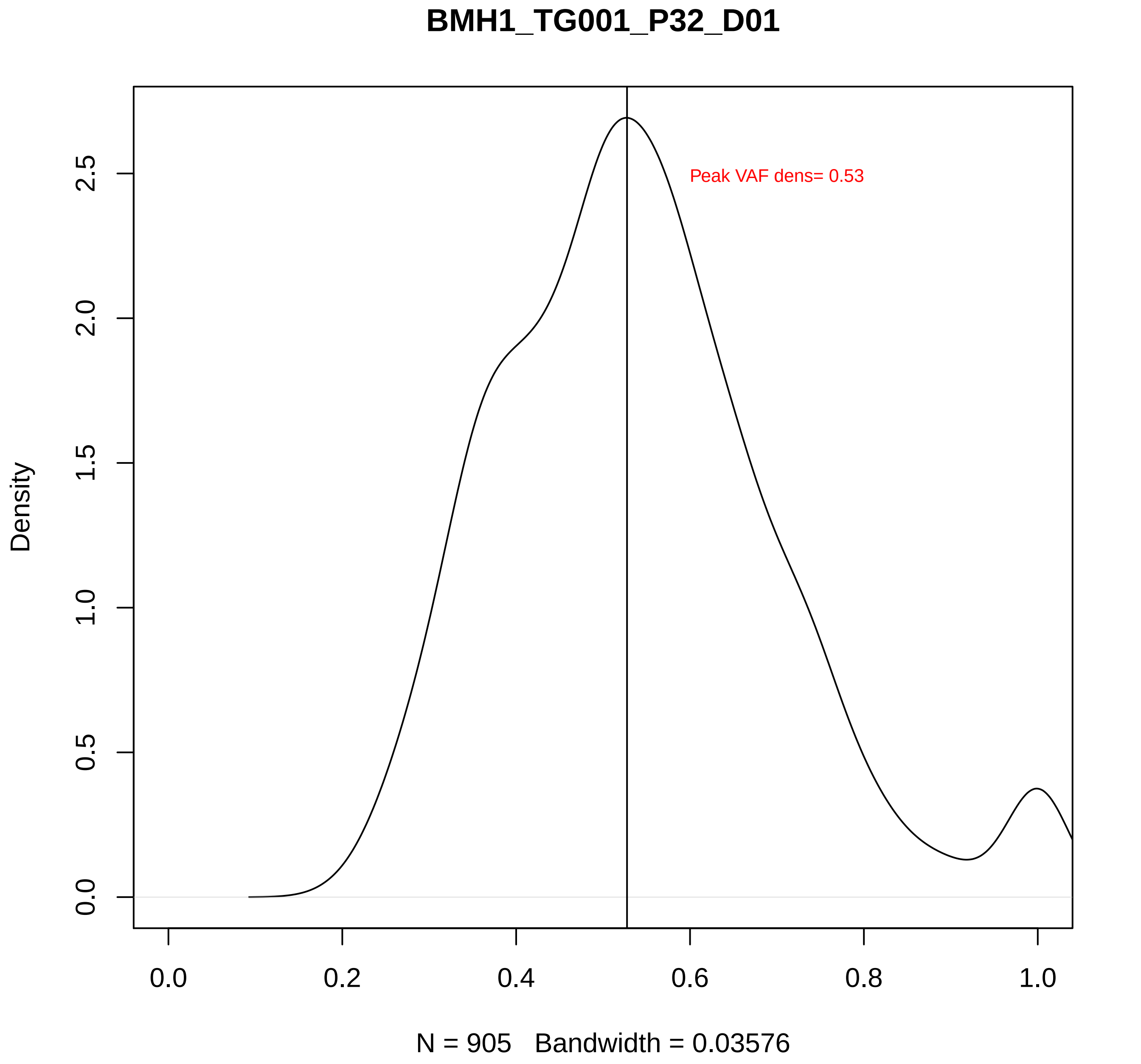
BMH1_TG001_P32_D01
Peak VAF dens= 0.53
Density
0.0
0.5
1.0
1.5
2.0
2.5
0.0
0.2
0.4
0.6
0.8
1.0
N = 905   Bandwidth = 0.03576

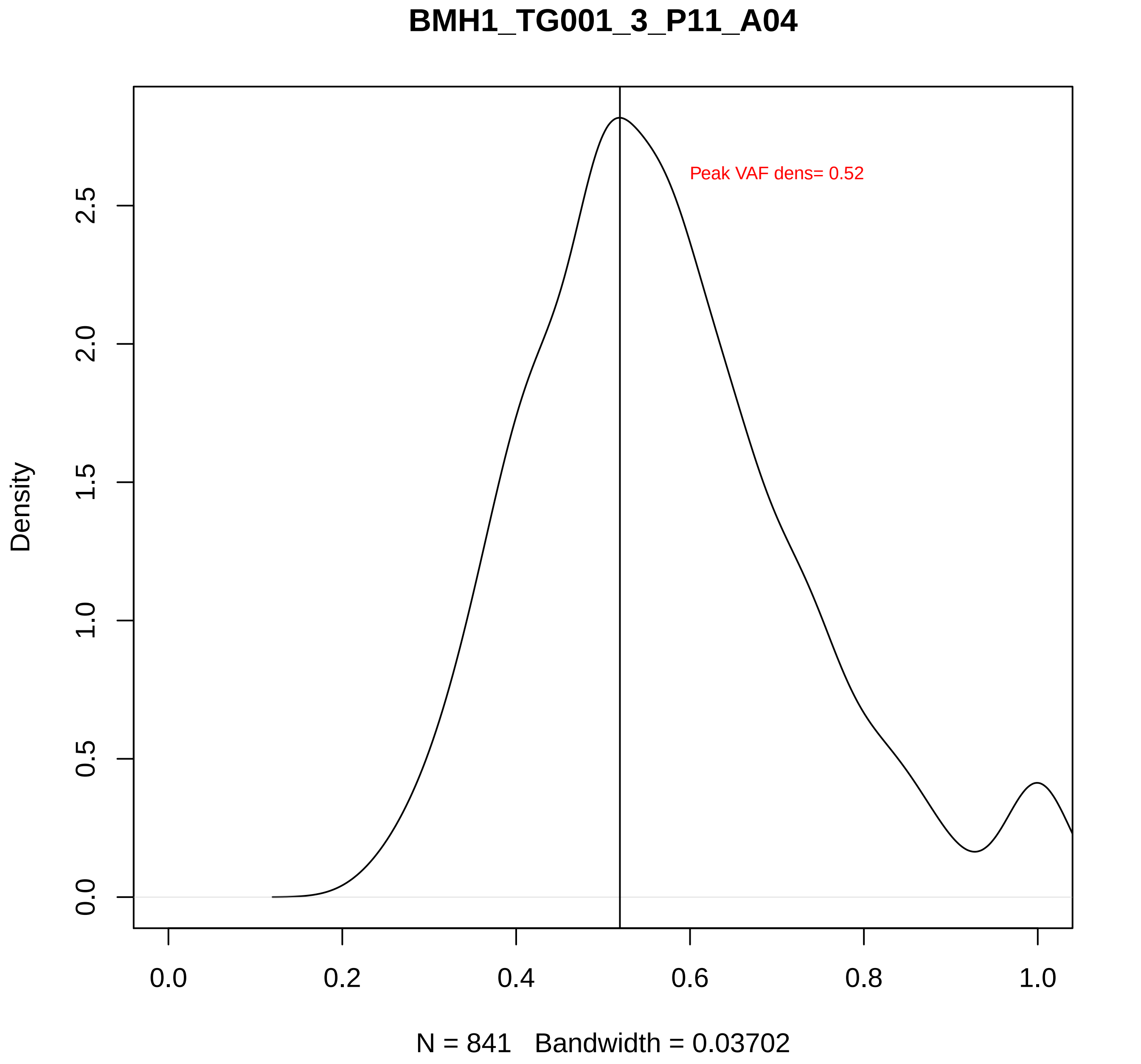

BMH1_TG001_3_P11_A04
Peak VAF dens= 0.52
Density
0.0
0.5
1.0
1.5
2.0
2.5
0.0
0.2
0.4
0.6
0.8
1.0
N = 841   Bandwidth = 0.03702

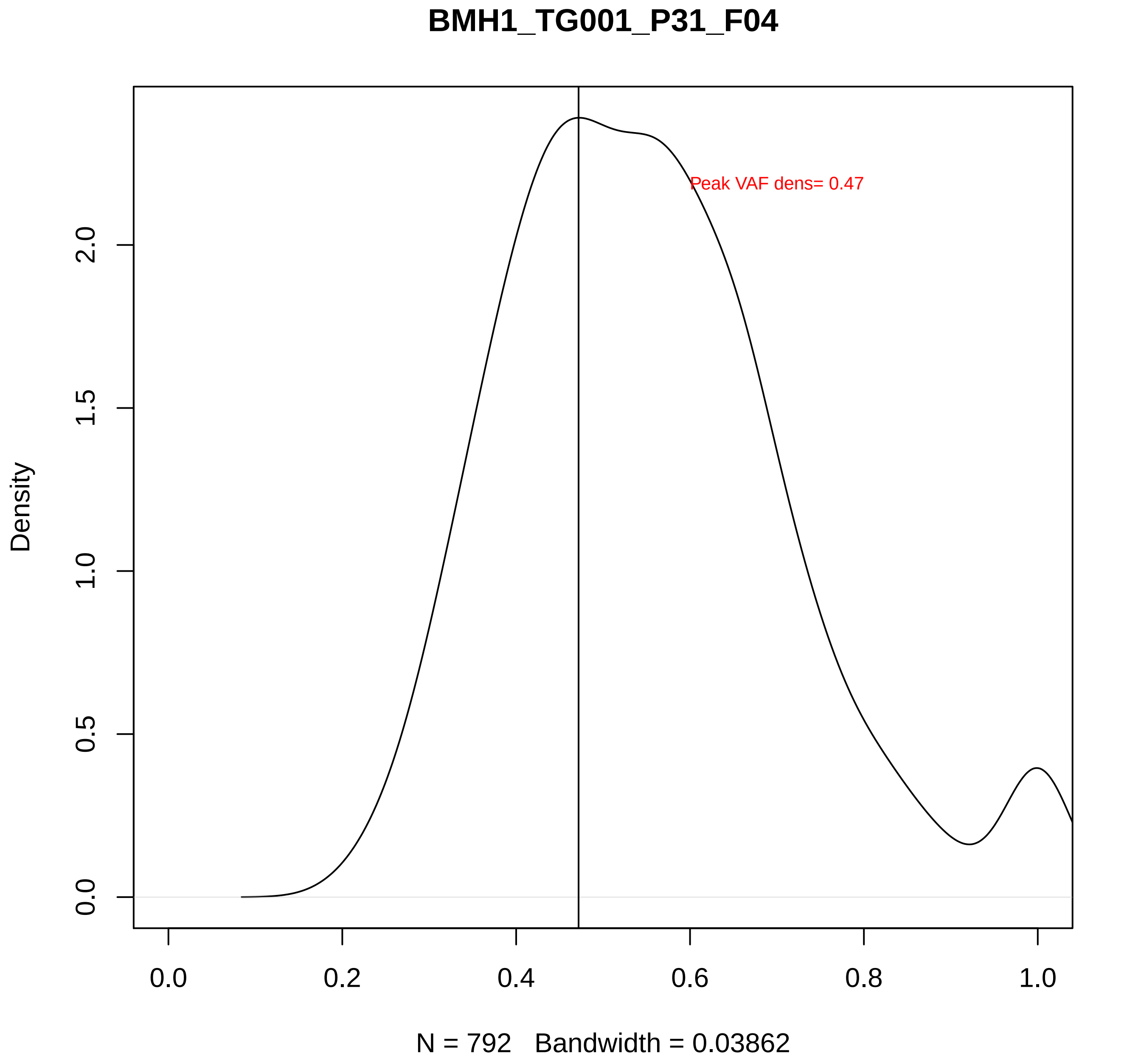

BMH1_TG001_P31_F04
Peak VAF dens= 0.47
Density
2.0
1.5
1.0
0.5
0.0
0.0
0.2
0.4
0.6
0.8
1.0
N = 792   Bandwidth = 0.03862

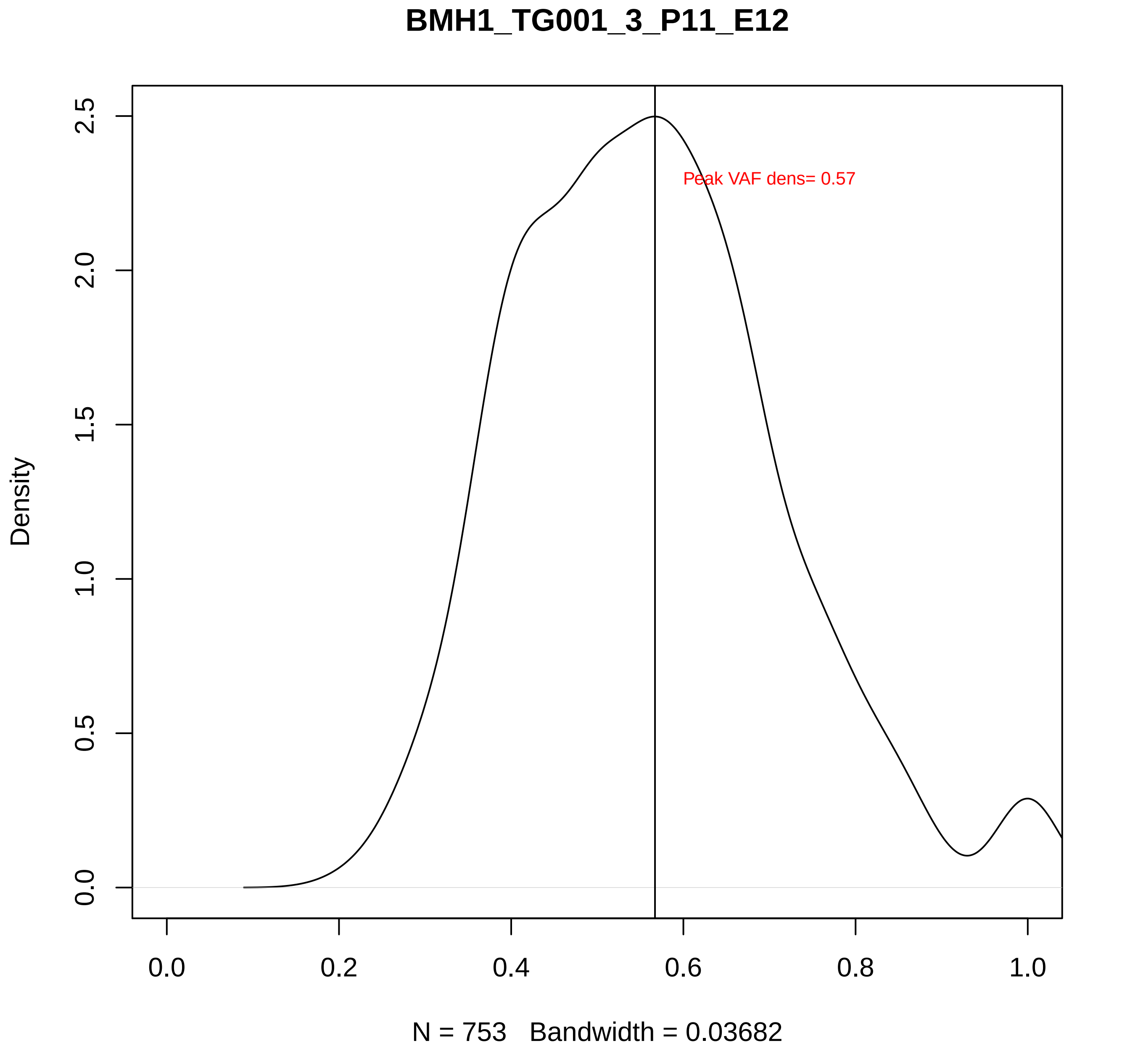
BMH1_TG001_3_P11_E12
Peak VAF dens= 0.57
Density
0.0
0.5
1.0
1.5
2.0
2.5
0.0
0.2
0.4
0.6
0.8
1.0
N = 753   Bandwidth = 0.03682

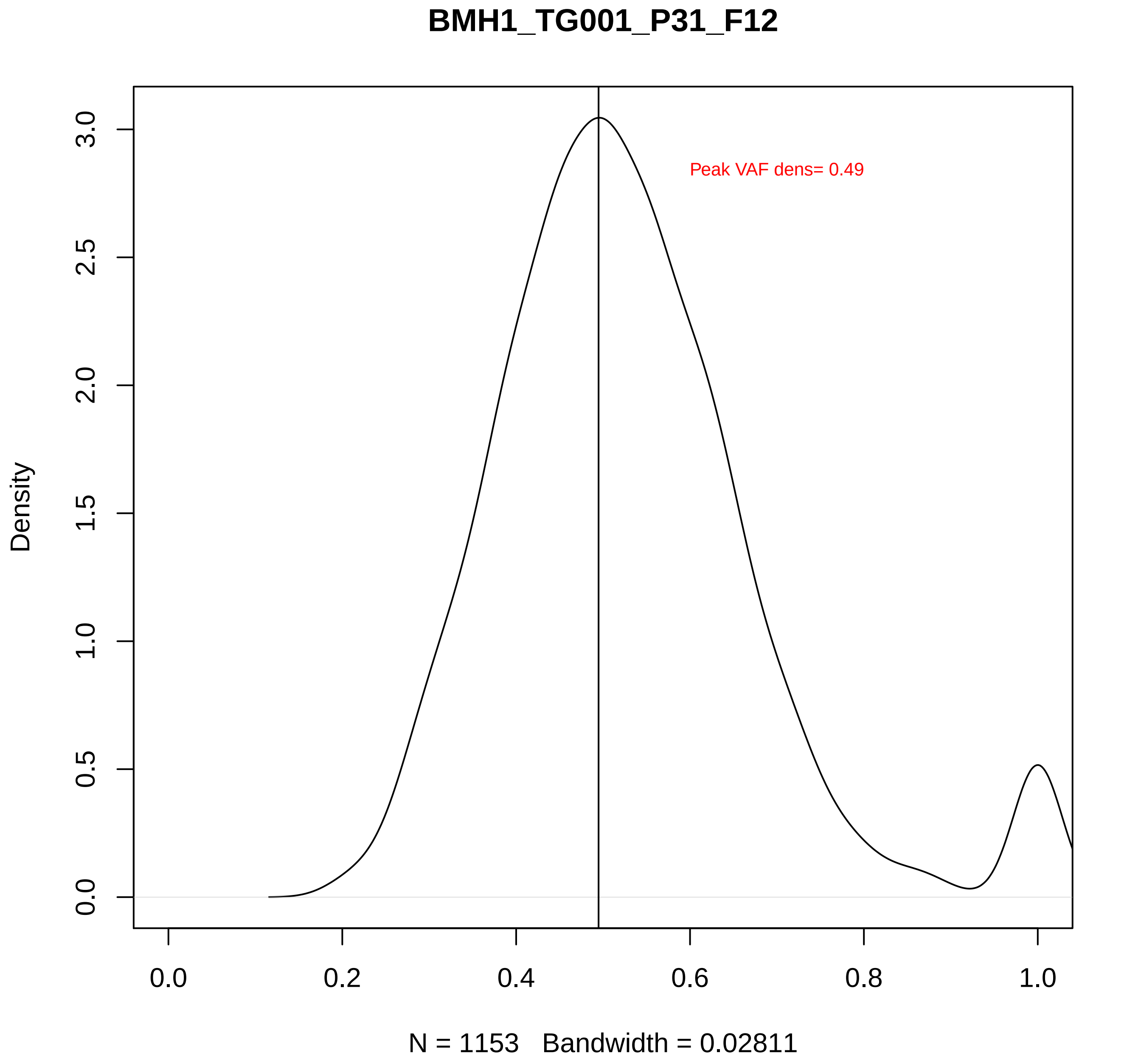

BMH1_TG001_P31_F12
Peak VAF dens= 0.49
Density
3.0
2.5
2.0
1.5
1.0
0.5
0.0
0.0
0.2
0.4
0.6
0.8
1.0
N = 1153 Bandwidth = 0.02811

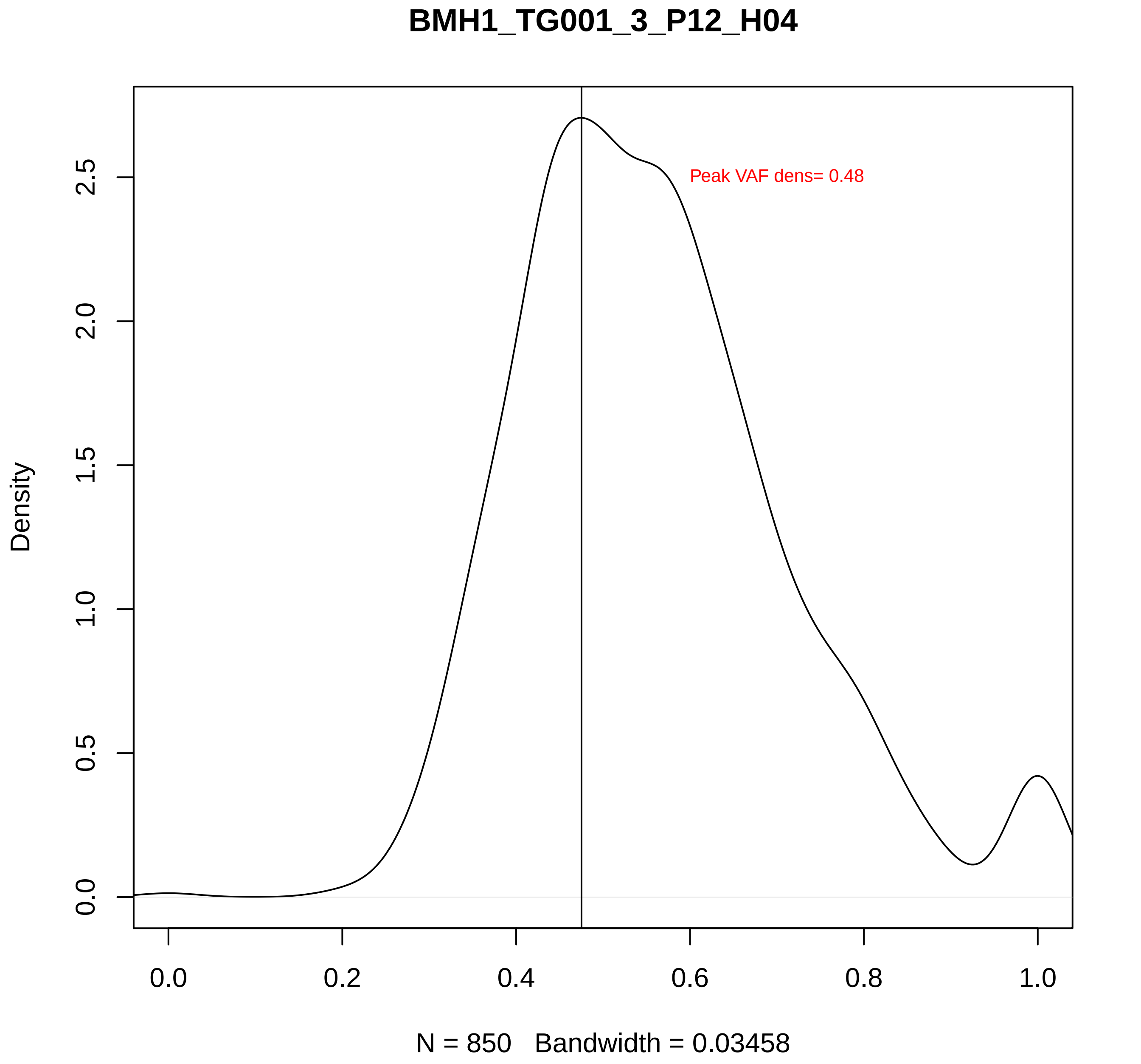
BMH1_TG001_3_P12_H04
Peak VAF dens= 0.48
Density
0.0
0.5
1.0
1.5
2.0
2.5
0.0
0.2
0.4
0.6
0.8
1.0
N = 850   Bandwidth = 0.03458

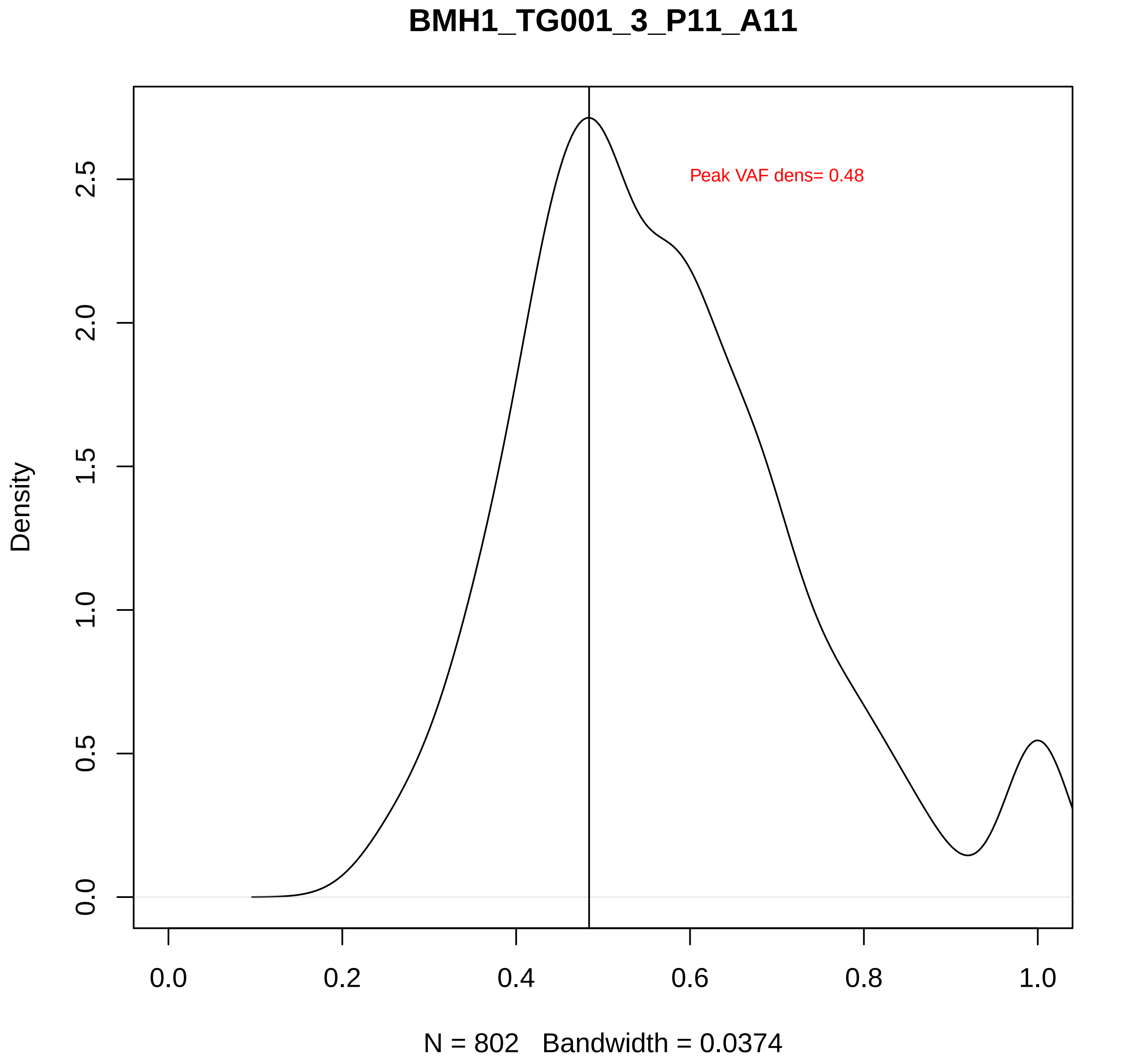
BMH1_TG001_3_P11_A11
Peak VAF dens= 0.48
Density
0.0
0.5
1.0
1.5
2.0
2.5
0.0
0.2
0.4
0.6
0.8
1.0
N = 802   Bandwidth = 0.0374

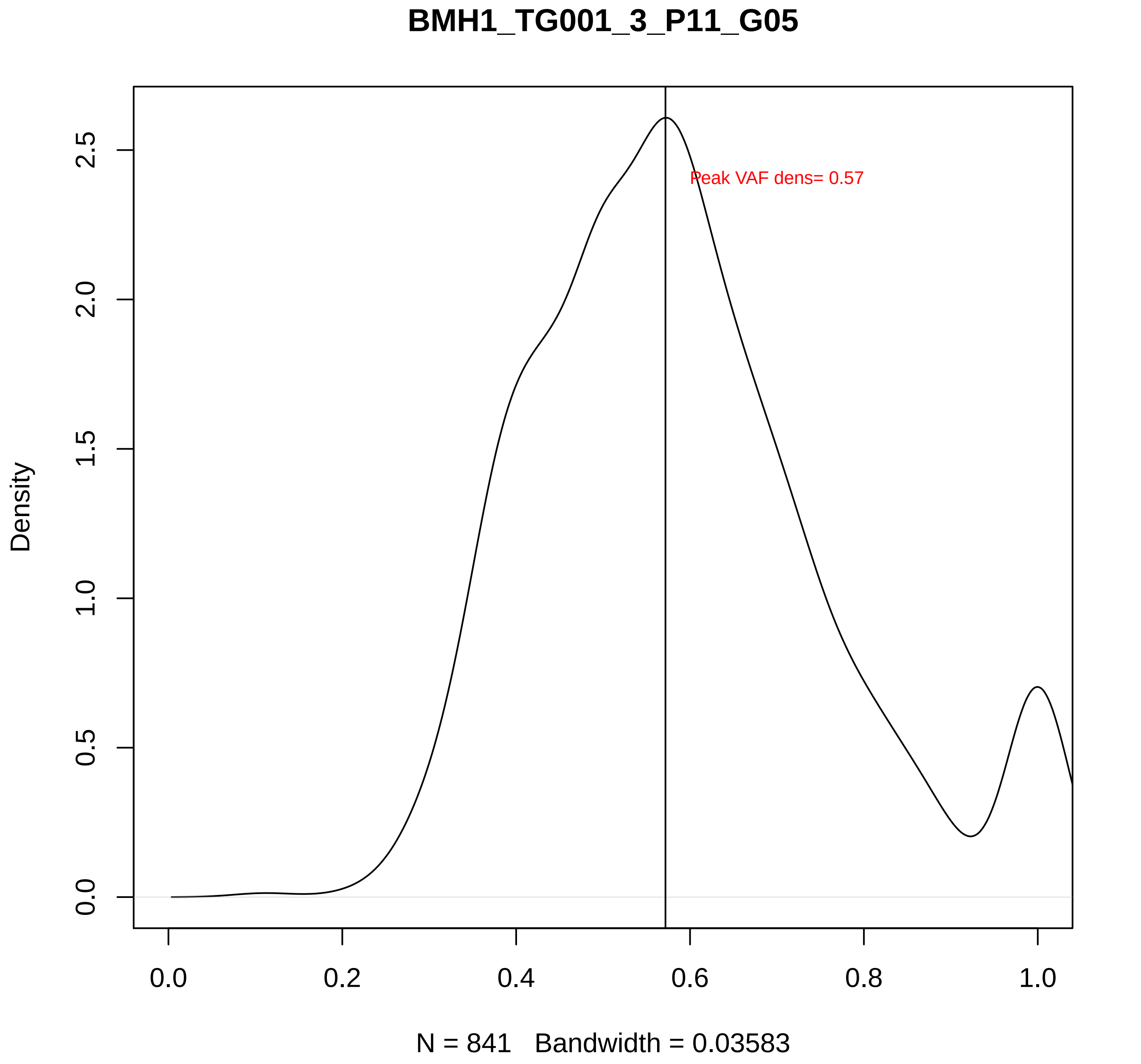
BMH1_TG001_3_P11_G05
Density
Peak VAF dens= 0.57
0.0
0.5
1.0
1.5
2.0
2.5
0.0
0.2
0.4
0.6
0.8
1.0
N = 841   Bandwidth = 0.03583

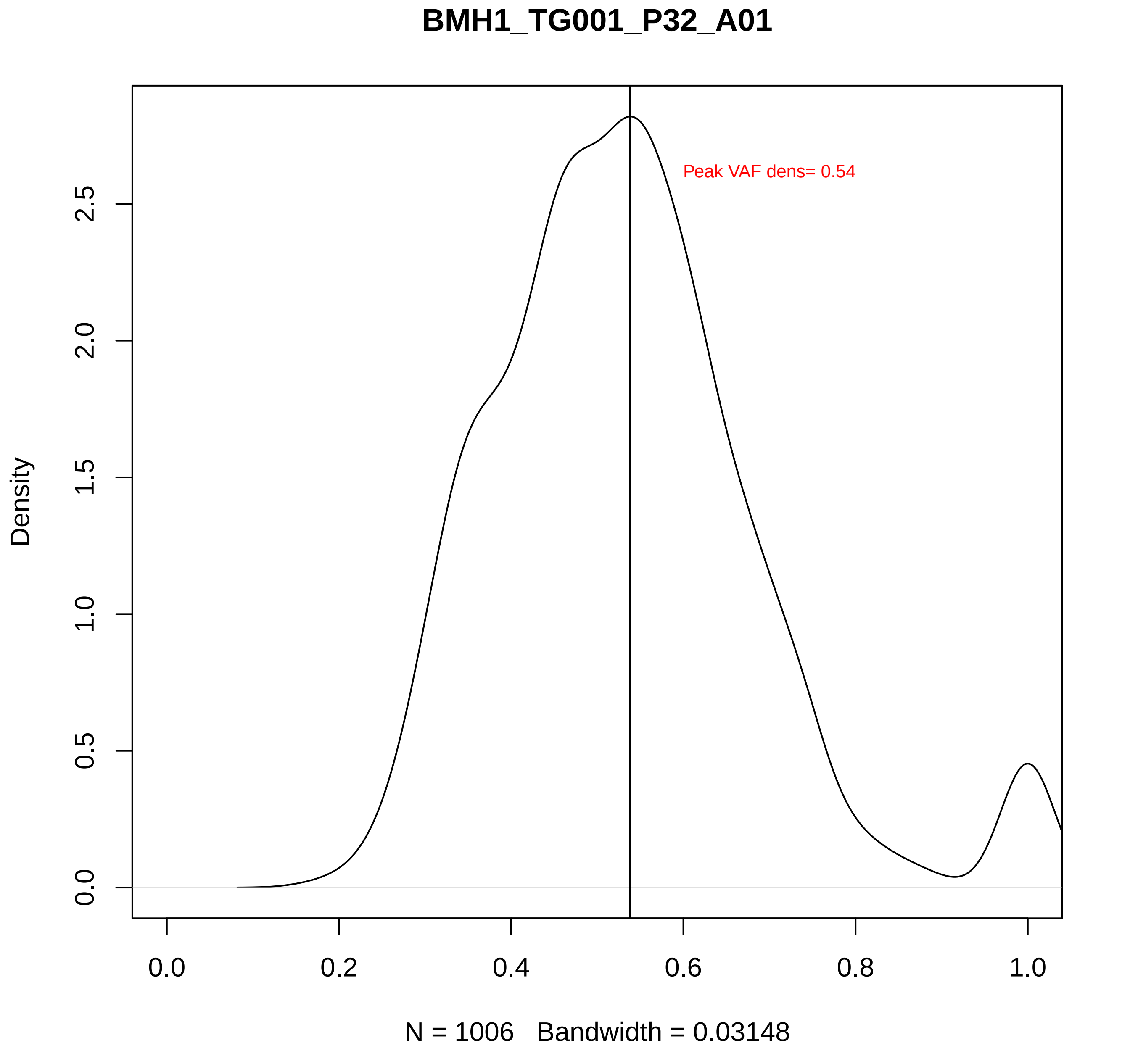
BMH1_TG001_P32_A01
Peak VAF dens= 0.54
Density
0.0
0.5
1.0
1.5
2.0
2.5
0.0
0.2
0.4
0.6
0.8
1.0
N = 1006   Bandwidth = 0.03148

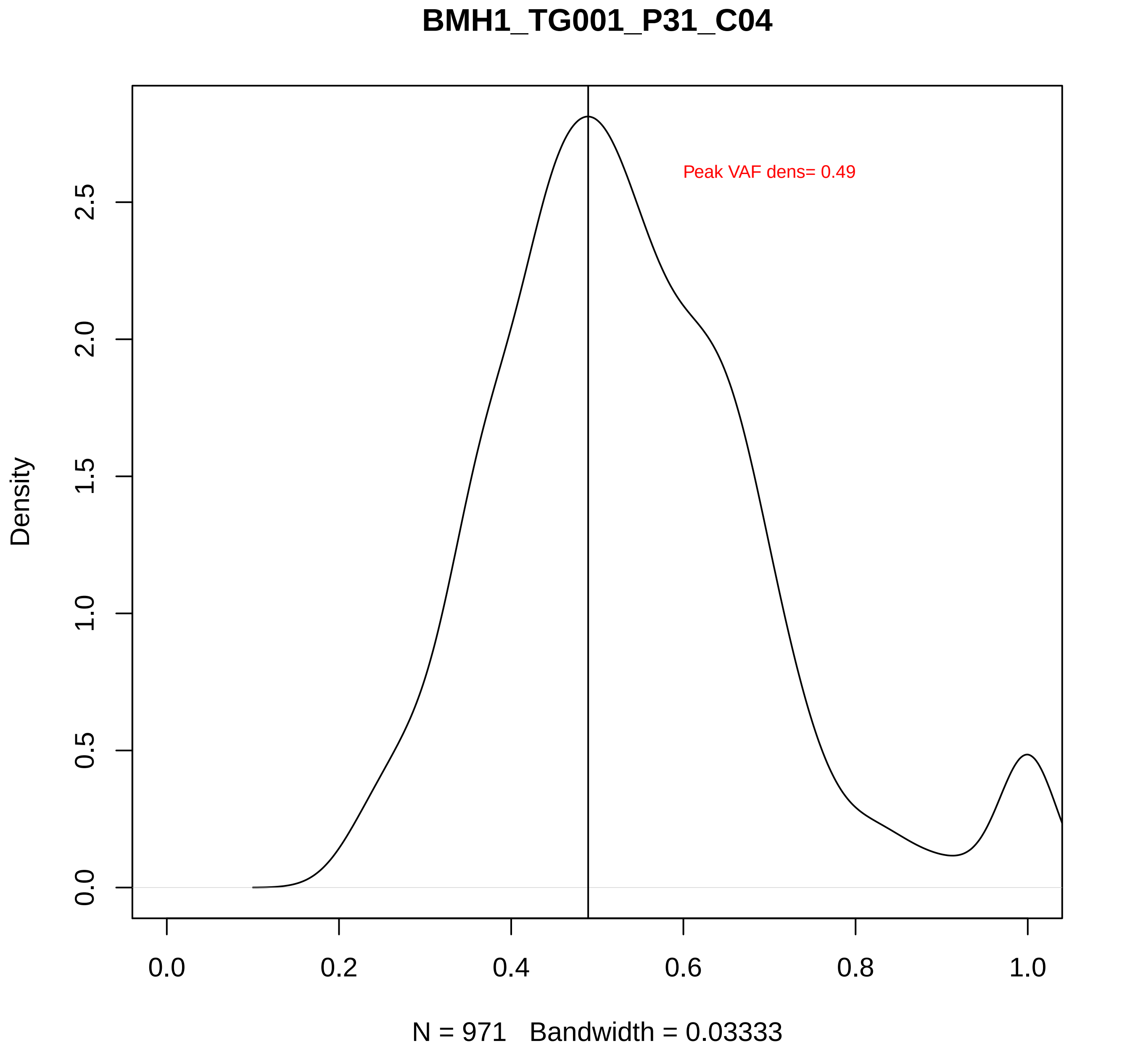

BMH1_TG001_P31_C04
Peak VAF dens= 0.49
Density
0.0
0.5
1.0
1.5
2.0
2.5
0.0
0.2
0.4
0.6
0.8
1.0
N = 971 Bandwidth = 0.03333

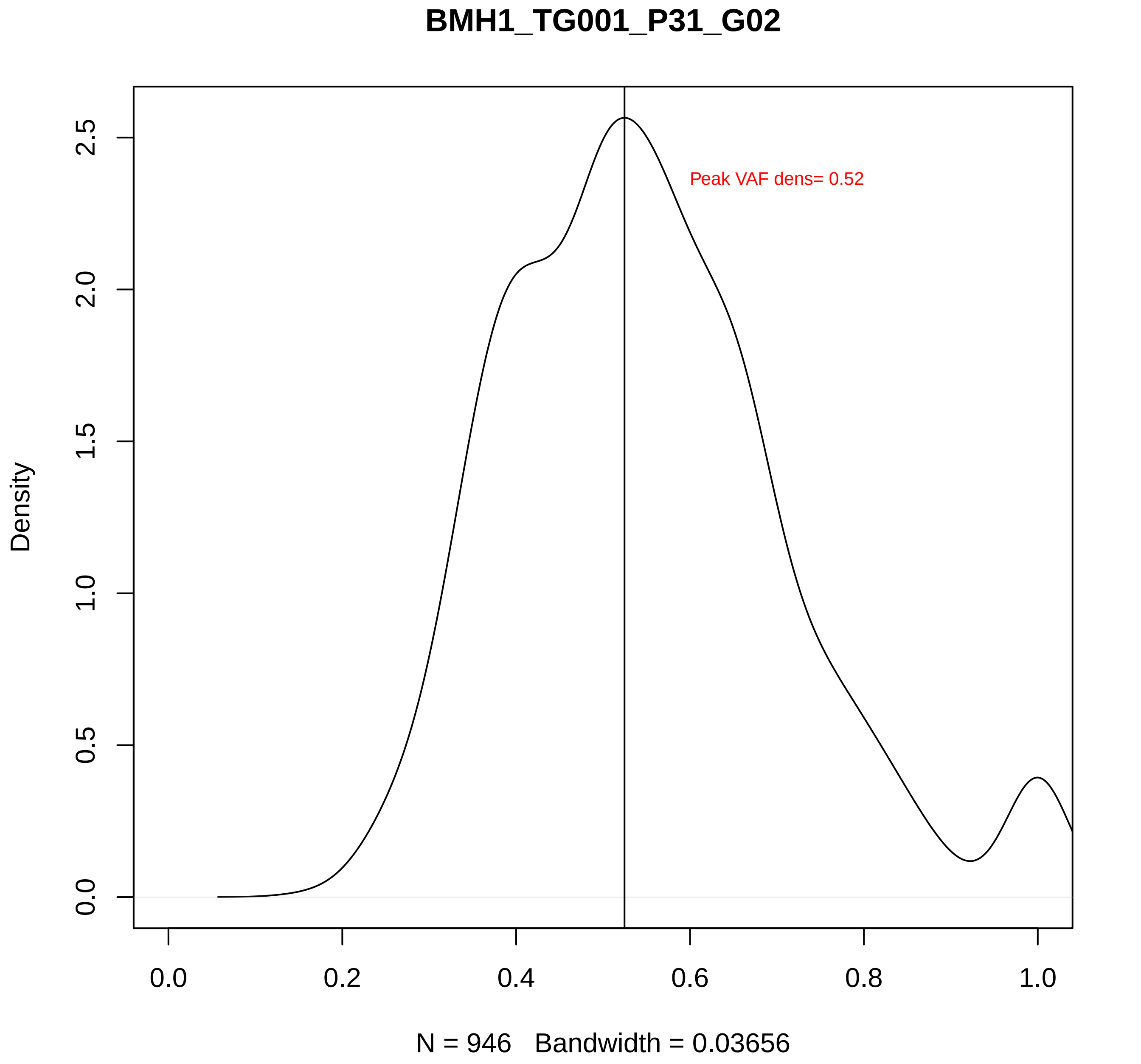
BMH1_TG001_P31_G02
Peak VAF dens= 0.52
Density
0.0
0.5
1.0
1.5
2.0
2.5
0.0
0.2
0.4
0.6
0.8
1.0
N = 946 Bandwidth = 0.03656

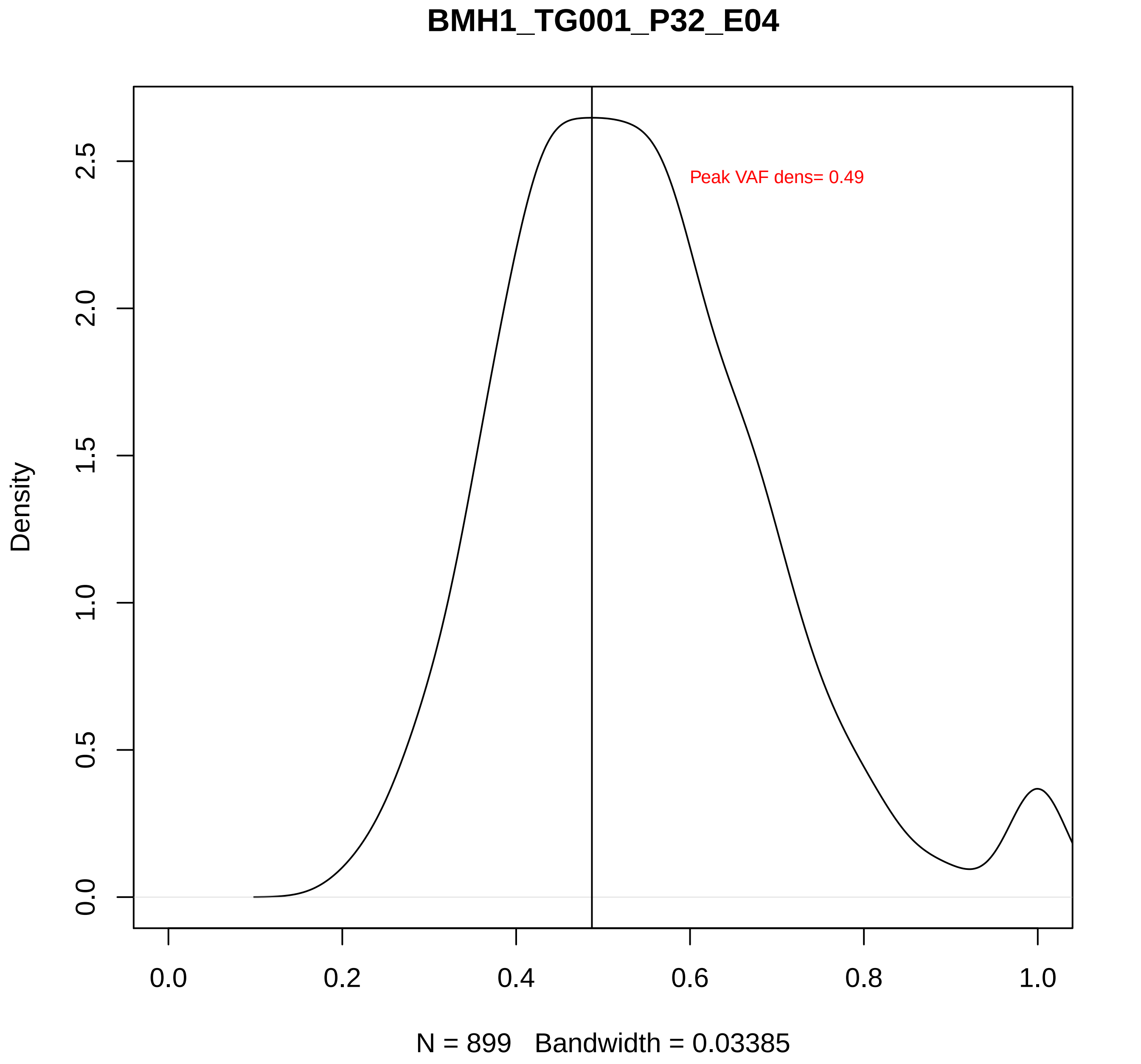
BMH1_TG001_P32_E04
Peak VAF dens= 0.49
Density
0.0
0.5
1.0
1.5
2.0
2.5
0.0
0.2
0.4
0.6
0.8
1.0
N = 899 Bandwidth = 0.03385

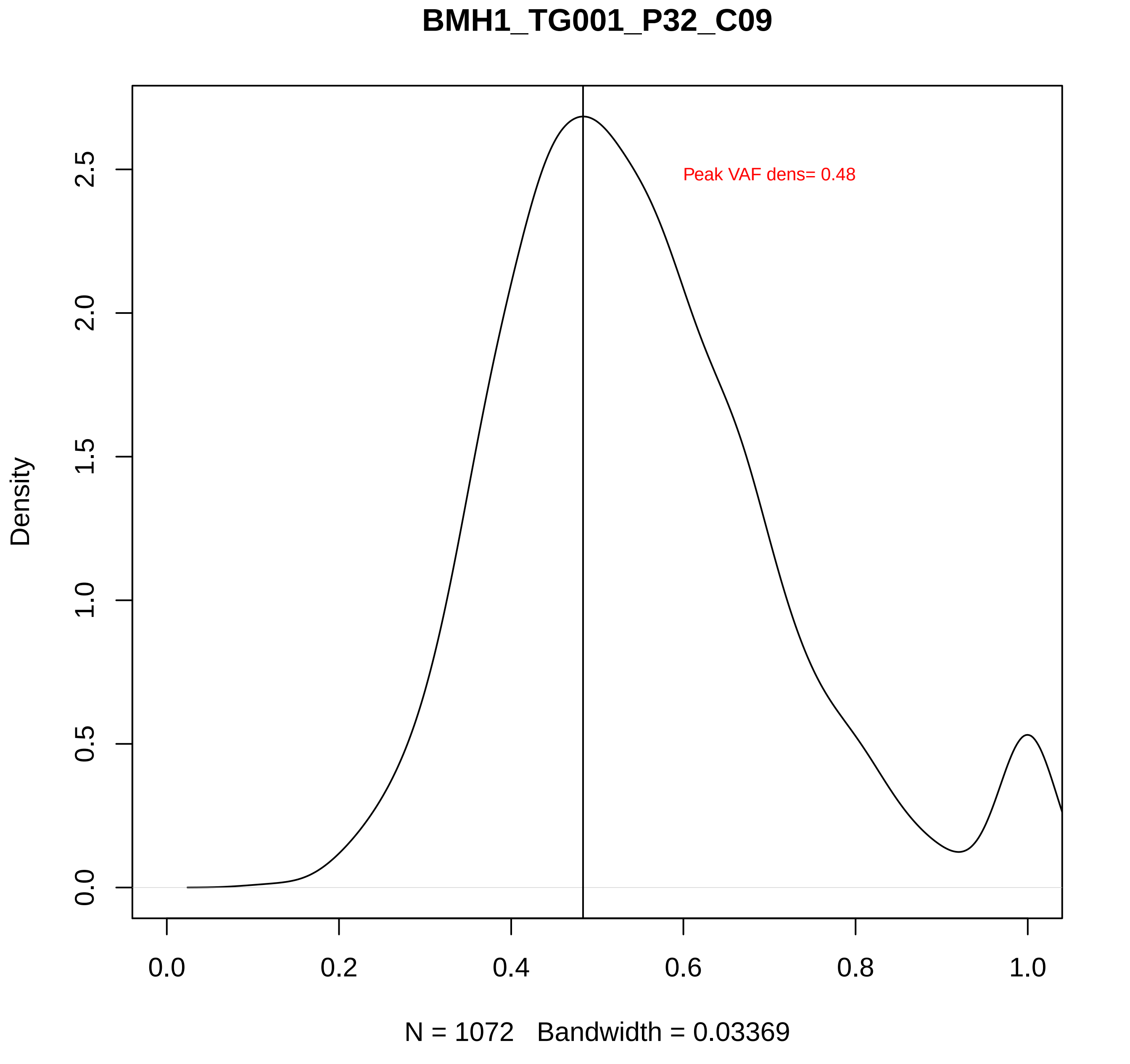

BMH1_TG001_P32_C09
Peak VAF dens= 0.48
Density
0.0
0.5
1.0
1.5
2.0
2.5
0.0
0.2
0.4
0.6
0.8
1.0
N = 1072   Bandwidth = 0.03369

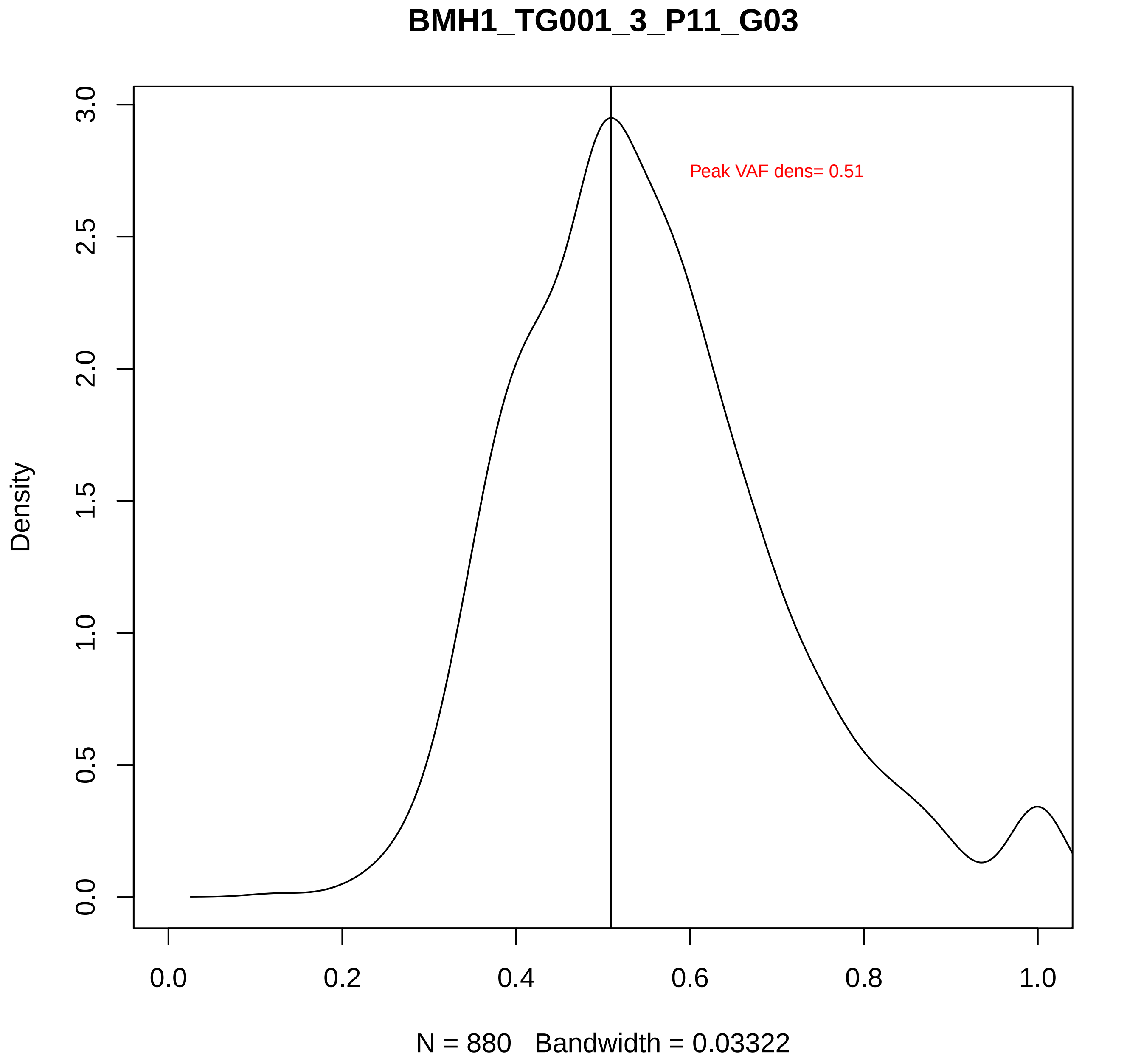

BMH1_TG001_3_P11_G03
Peak VAF dens= 0.51
Density
0.0
0.5
1.0
1.5
2.0
2.5
3.0
0.0
0.2
0.4
0.6
0.8
1.0
N = 880 Bandwidth = 0.03322

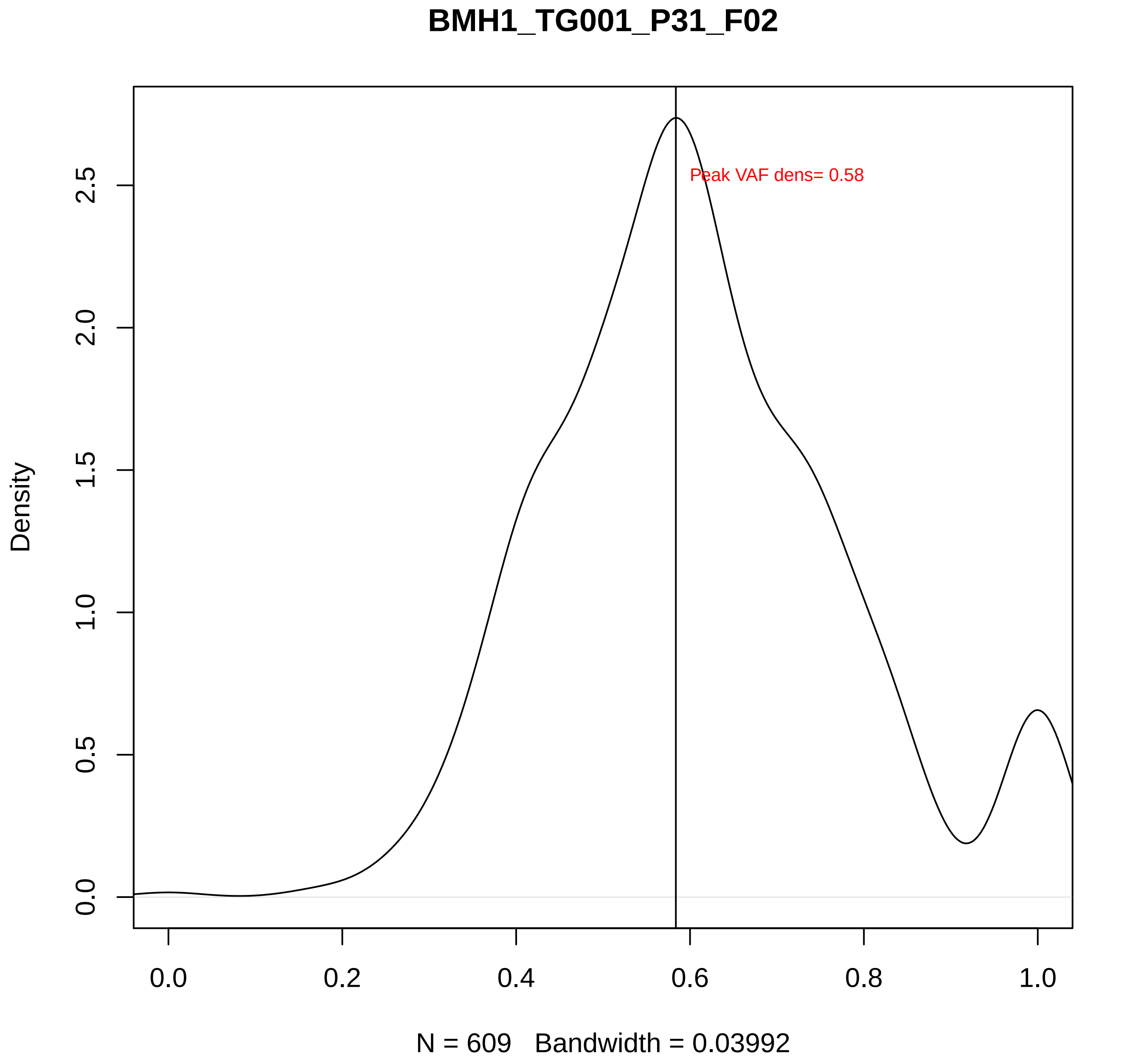
BMH1_TG001_P31_F02
Peak VAF dens= 0.58
Density
0.0
0.5
1.0
1.5
2.0
2.5
0.0
0.2
0.4
0.6
0.8
1.0
N = 609 Bandwidth = 0.03992

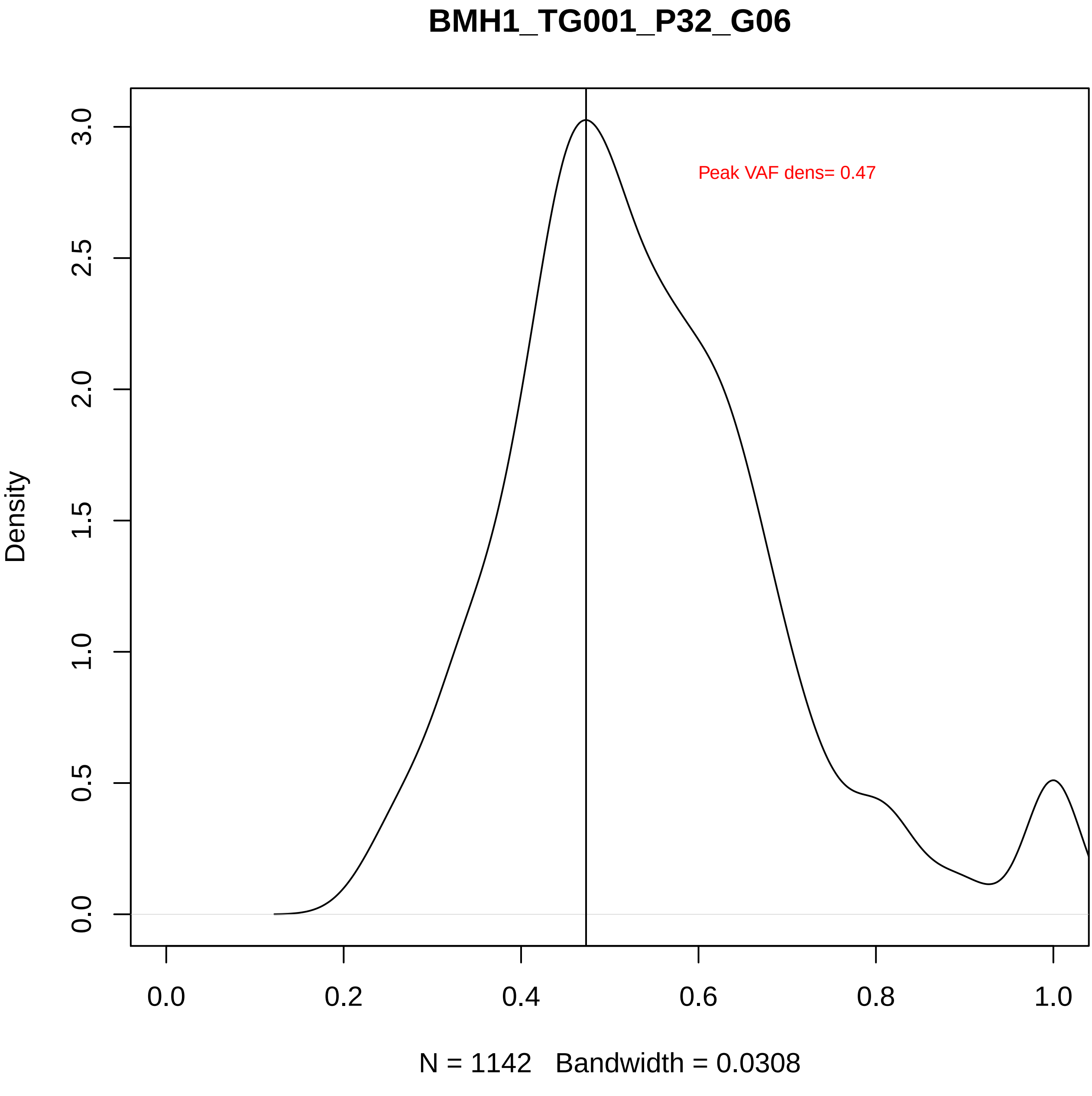
BMH1_TG001_P32_G06
Peak VAF dens= 0.47
Density
0.0
0.5
1.0
1.5
2.0
2.5
3.0
0.0
0.2
0.4
0.6
0.8
1.0
N = 1142 Bandwidth = 0.0308

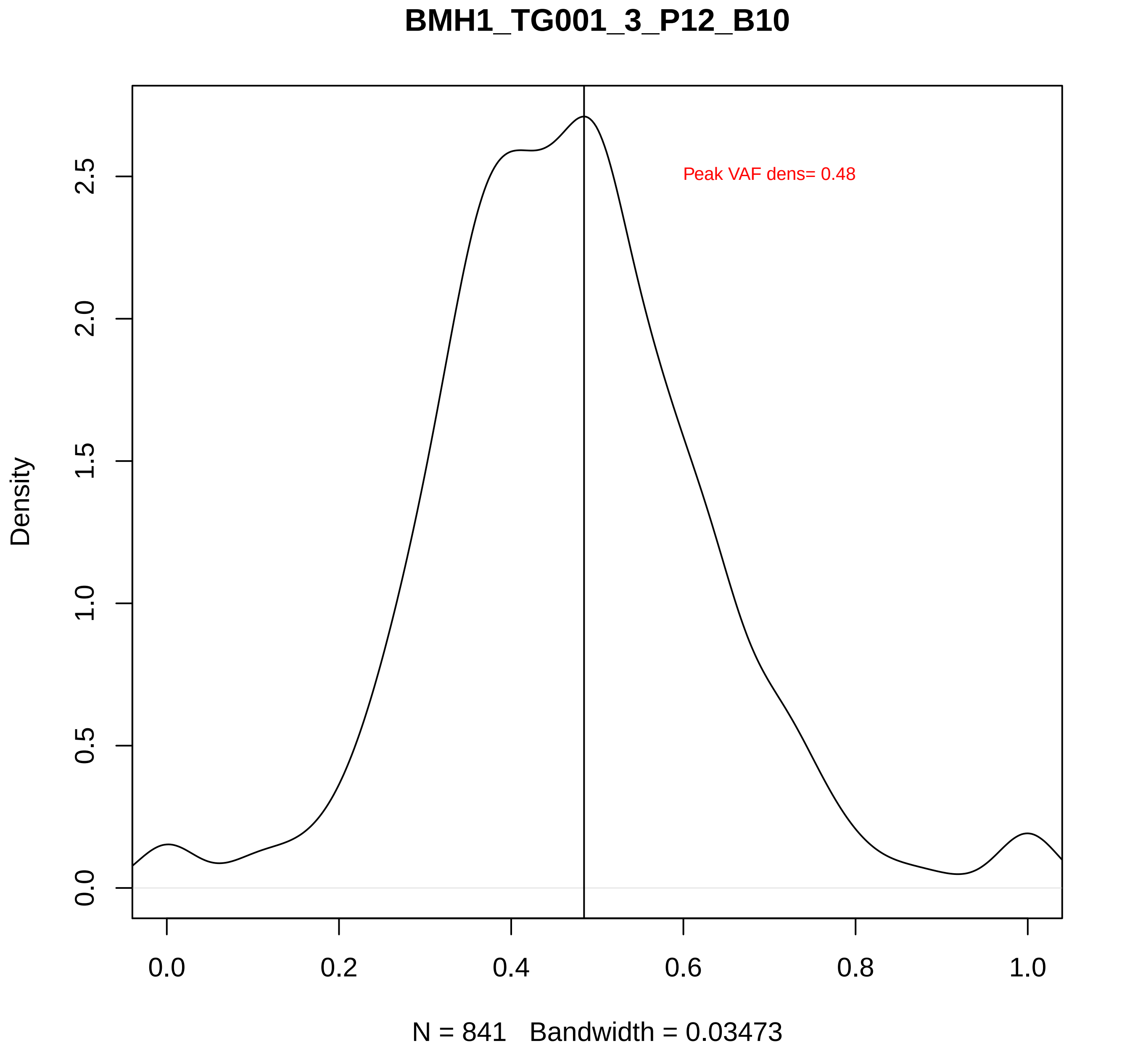

BMH1_TG001_3_P12_B10
Peak VAF dens= 0.48
Density
2.5
2.0
1.5
1.0
0.5
0.0
0.0
0.2
0.4
0.6
0.8
1.0
N = 841   Bandwidth = 0.03473

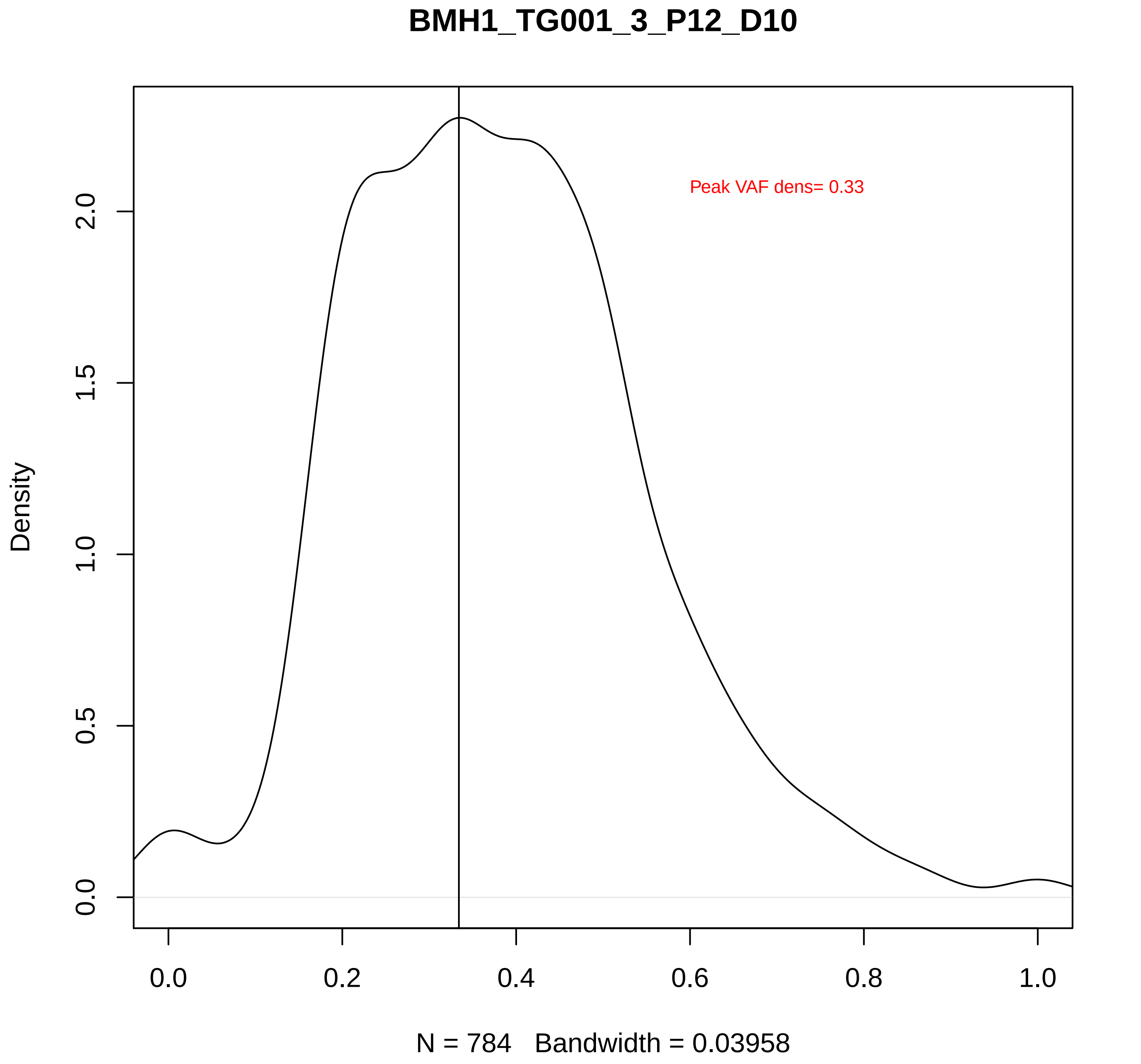
BMH1_TG001_3_P12_D10
Peak VAF dens= 0.33
Density
0.0
0.5
1.0
1.5
2.0
0.0
0.2
0.4
0.6
0.8
1.0
N = 784   Bandwidth = 0.03958

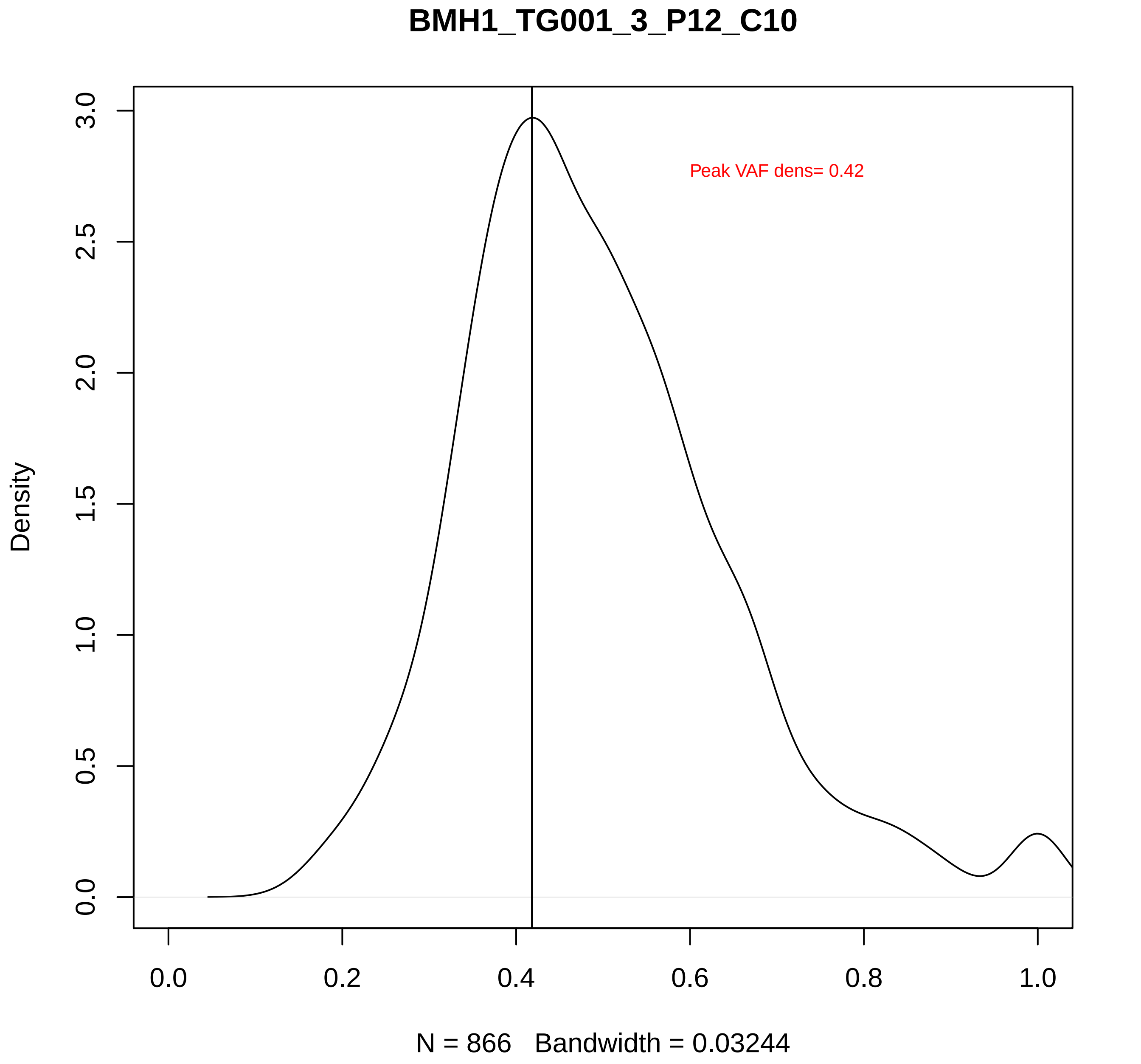

BMH1_TG001_3_P12_C10
Peak VAF dens= 0.42
Density
3.0
2.5
2.0
1.5
1.0
0.5
0.0
0.0
0.2
0.4
0.6
0.8
1.0
N = 866   Bandwidth = 0.03244

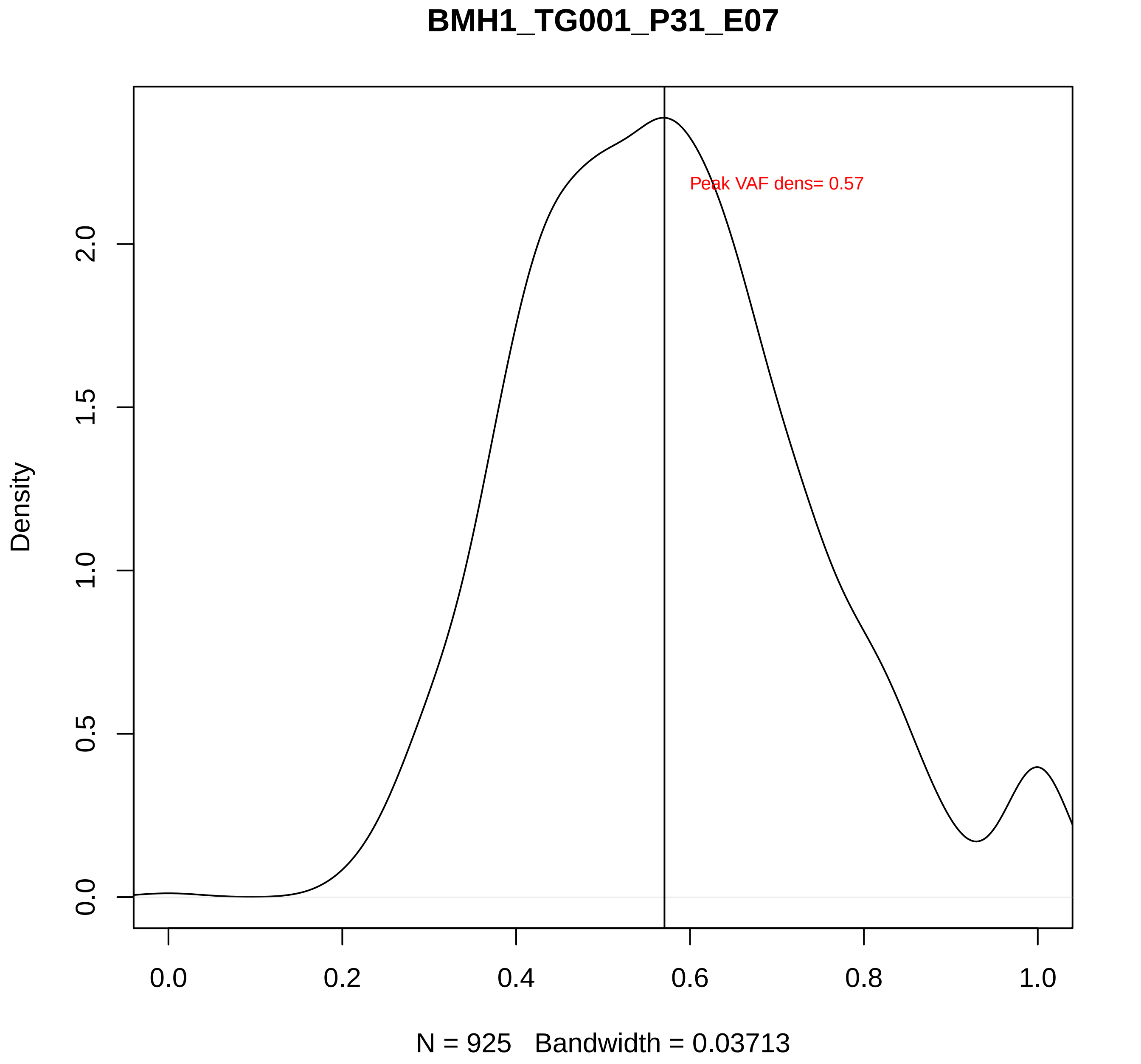

BMH1_TG001_P31_E07
Peak VAF dens= 0.57
Density
0.0
0.5
1.0
1.5
2.0
0.0
0.2
0.4
0.6
0.8
1.0
N = 925 Bandwidth = 0.03713

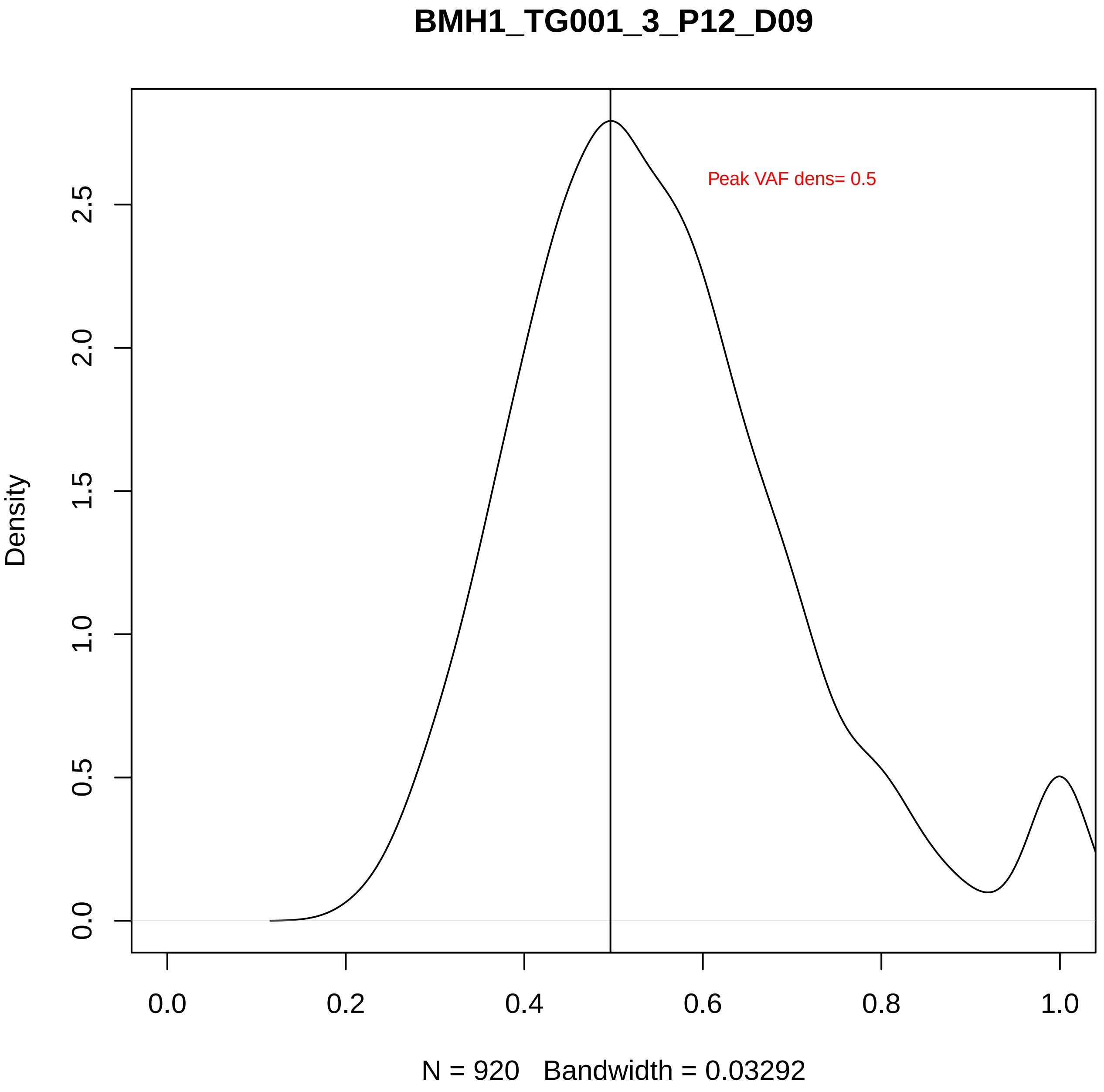

BMH1_TG001_3_P12_D09
Peak VAF dens= 0.5
Density
0.0
0.5
1.0
1.5
2.0
2.5
0.0
0.2
0.4
0.6
0.8
1.0
N = 920   Bandwidth = 0.03292

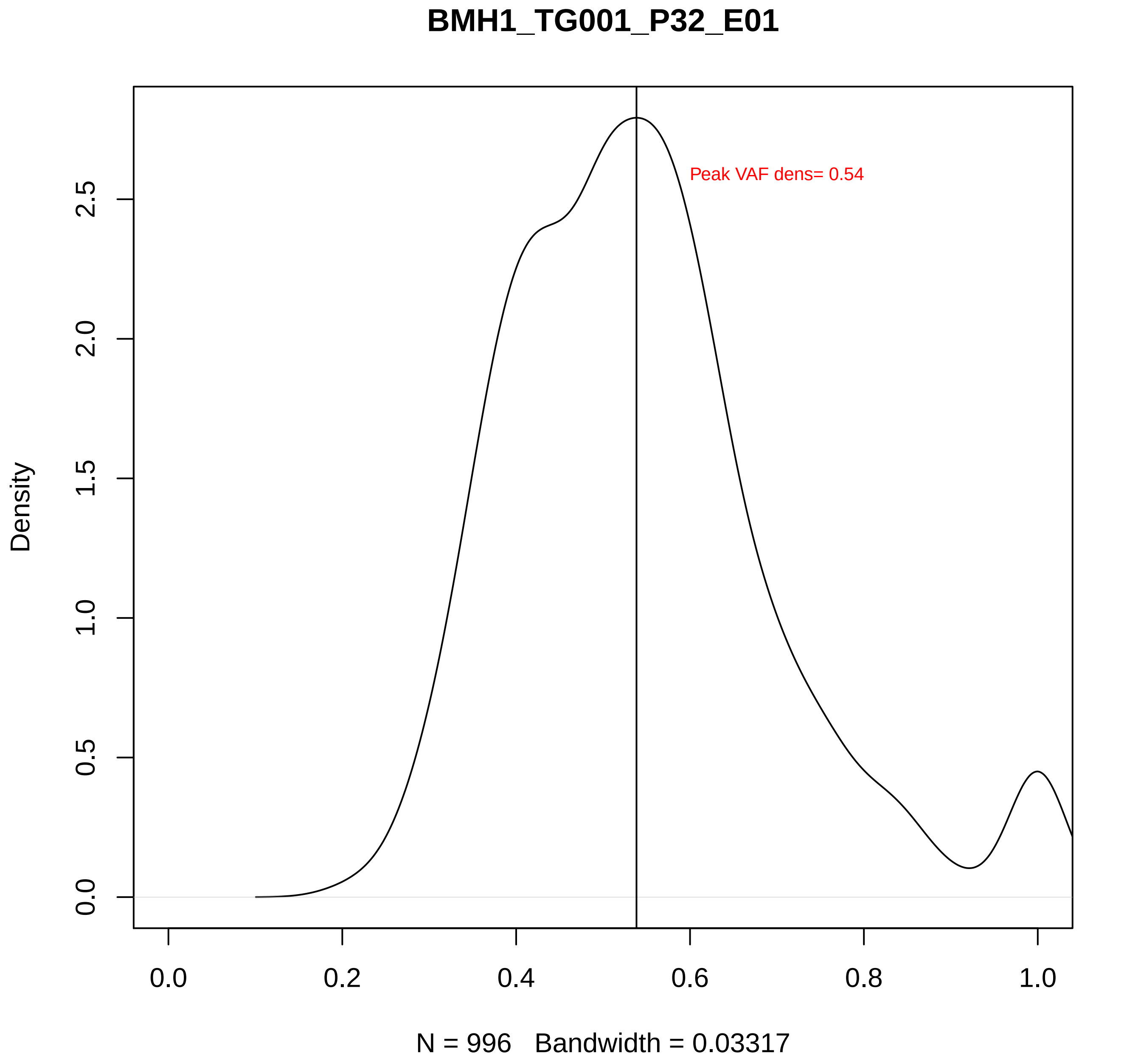
BMH1_TG001_P32_E01
Peak VAF dens= 0.54
Density
0.0
0.5
1.0
1.5
2.0
2.5
0.0
0.2
0.4
0.6
0.8
1.0
N = 996   Bandwidth = 0.03317

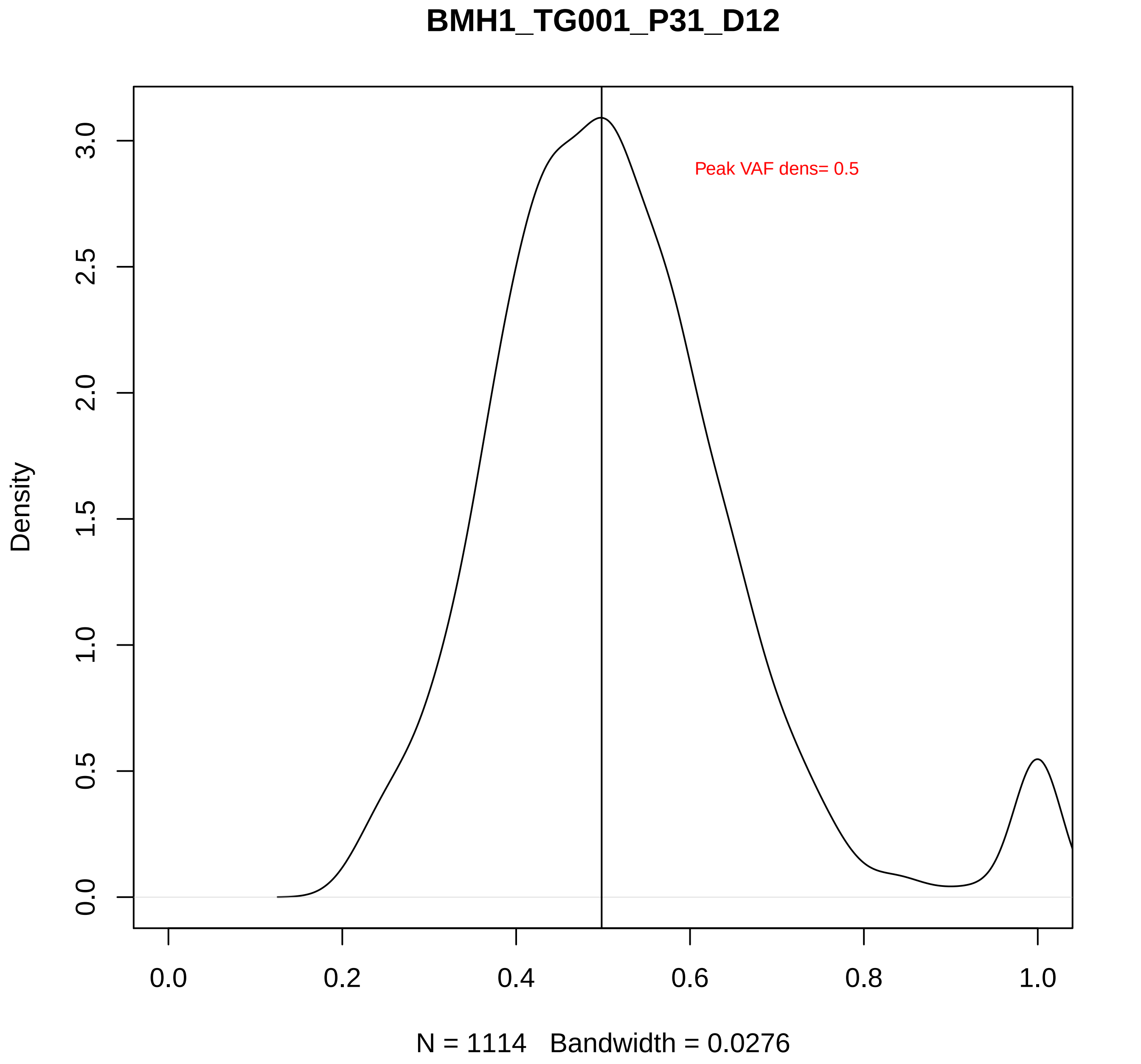
BMH1_TG001_P31_D12
Peak VAF dens= 0.5
Density
0.0
0.5
1.0
1.5
2.0
2.5
3.0
0.0
0.2
0.4
0.6
0.8
1.0
N = 1114   Bandwidth = 0.0276

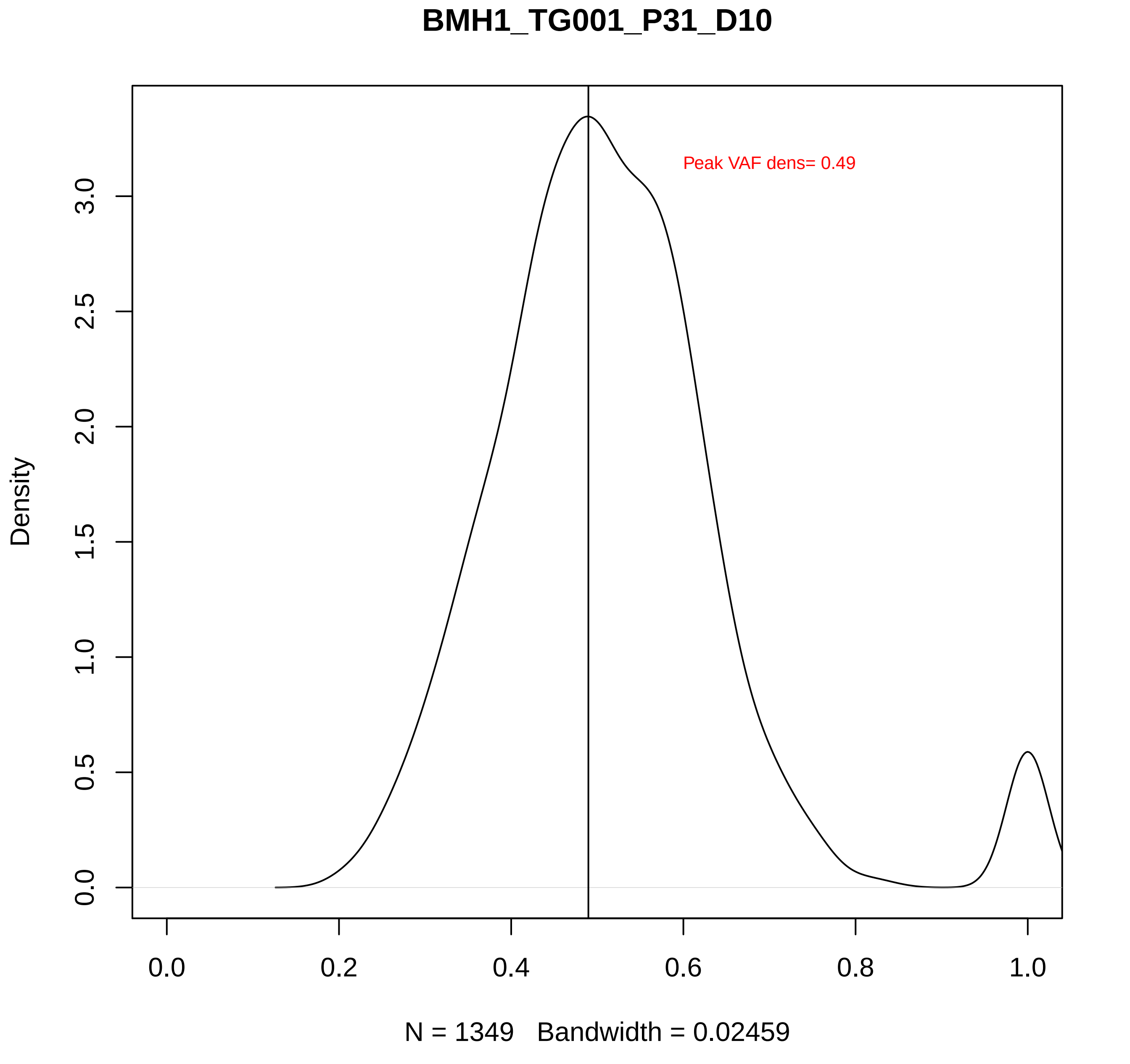
BMH1_TG001_P31_D10
Peak VAF dens= 0.49
Density
0.0
0.5
1.0
1.5
2.0
2.5
3.0
0.0
0.2
0.4
0.6
0.8
1.0
N = 1349   Bandwidth = 0.02459

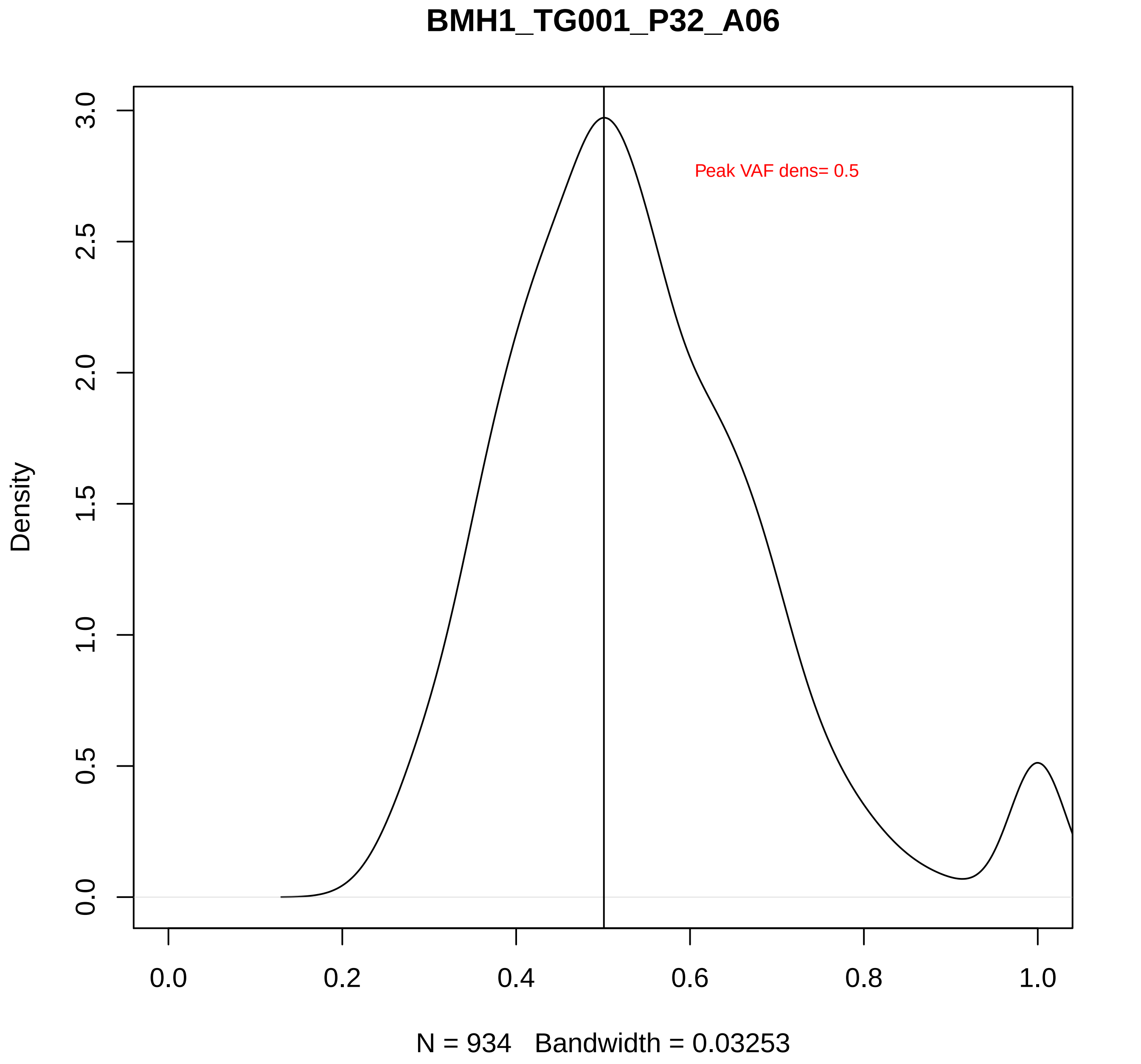

BMH1_TG001_P32_A06
Peak VAF dens= 0.5
Density
3.0
2.5
2.0
1.5
1.0
0.5
0.0
0.0
0.2
0.4
0.6
0.8
1.0
N = 934 Bandwidth = 0.03253

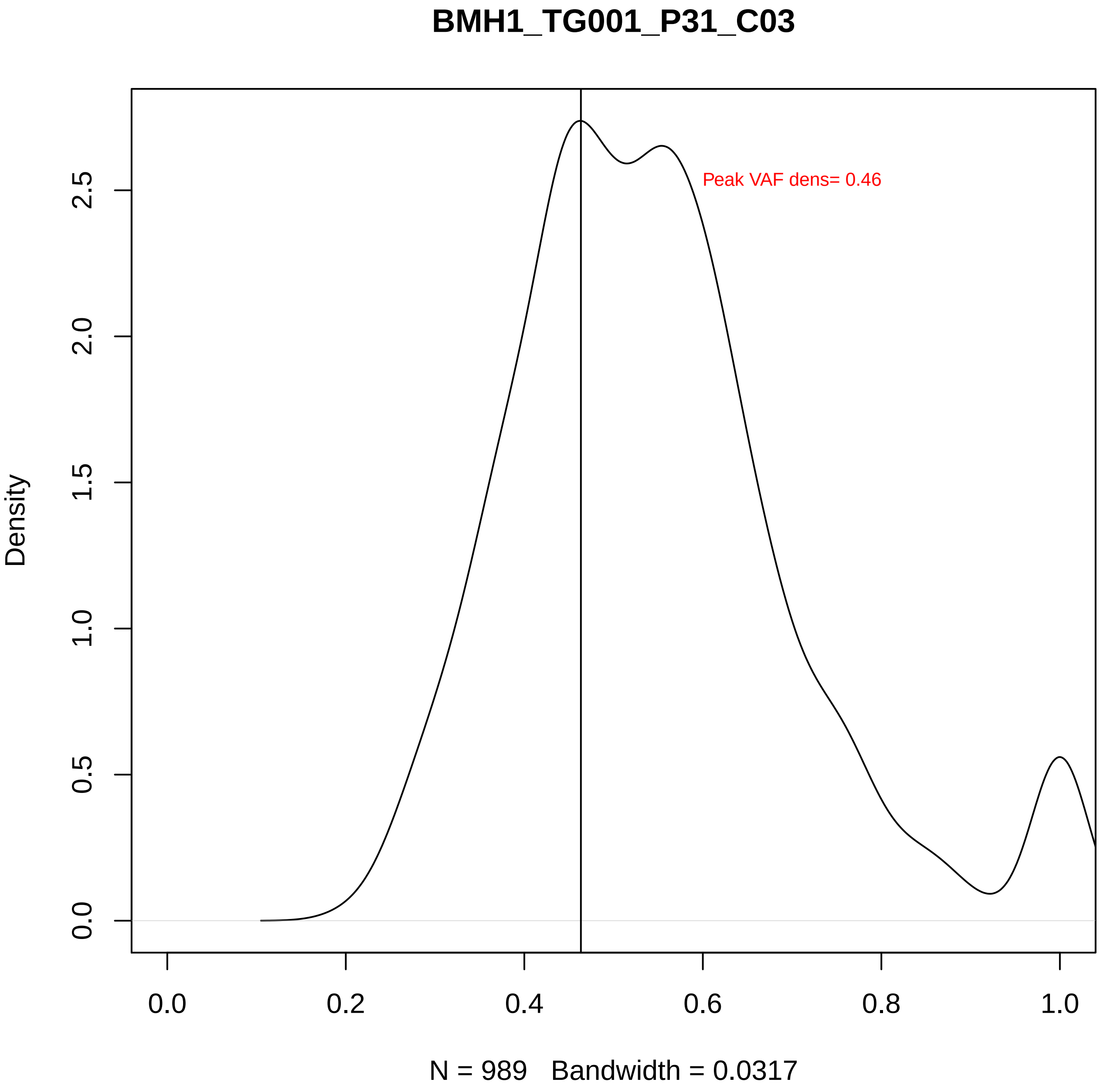

BMH1_TG001_P31_C03
Peak VAF dens= 0.46
Density
0.0
0.5
1.0
1.5
2.0
2.5
0.0
0.2
0.4
0.6
0.8
1.0
N = 989 Bandwidth = 0.0317

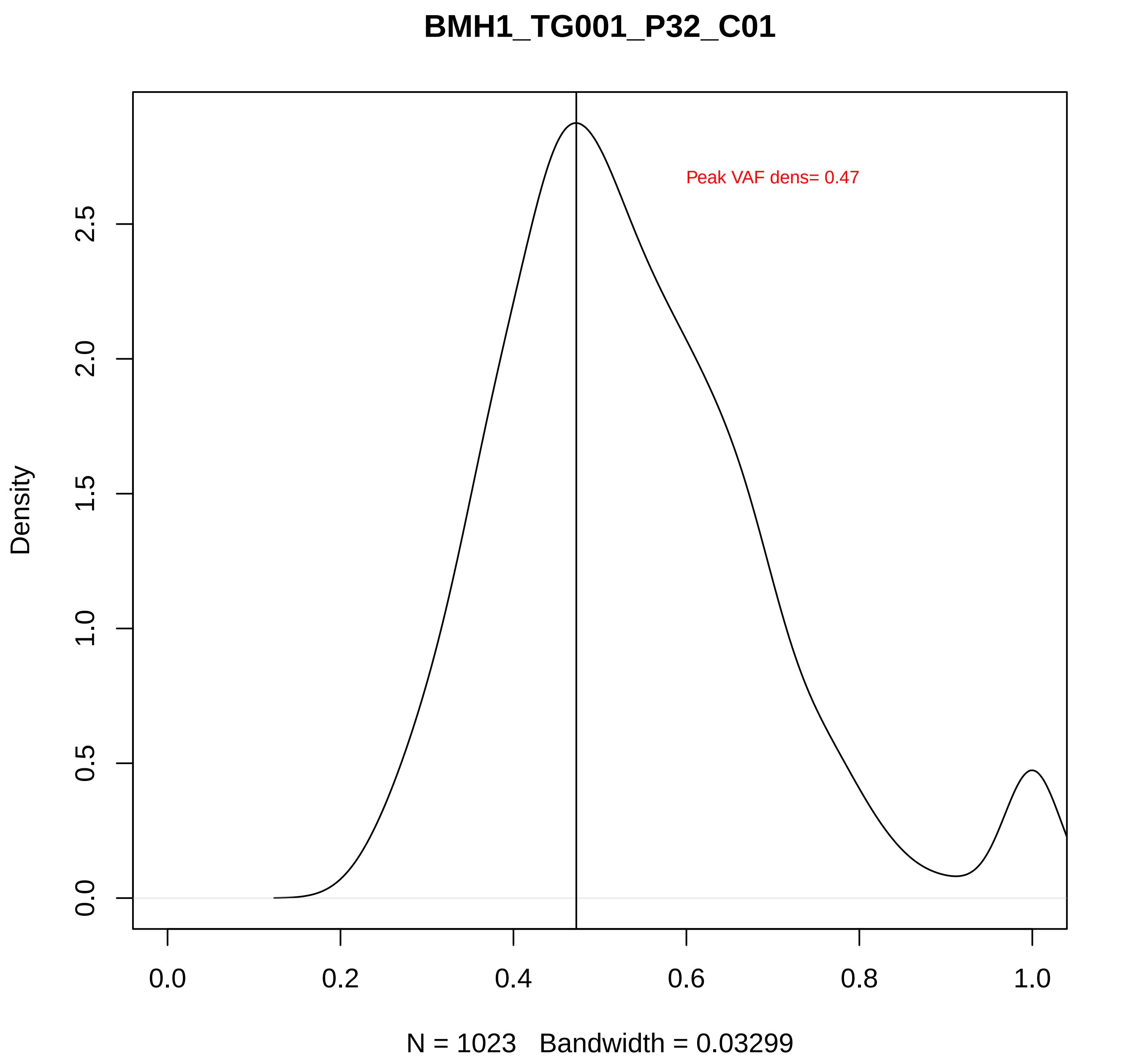
BMH1_TG001_P32_C01
Peak VAF dens= 0.47
Density
0.0
0.5
1.0
1.5
2.0
2.5
0.0
0.2
0.4
0.6
0.8
1.0
N = 1023 Bandwidth = 0.03299

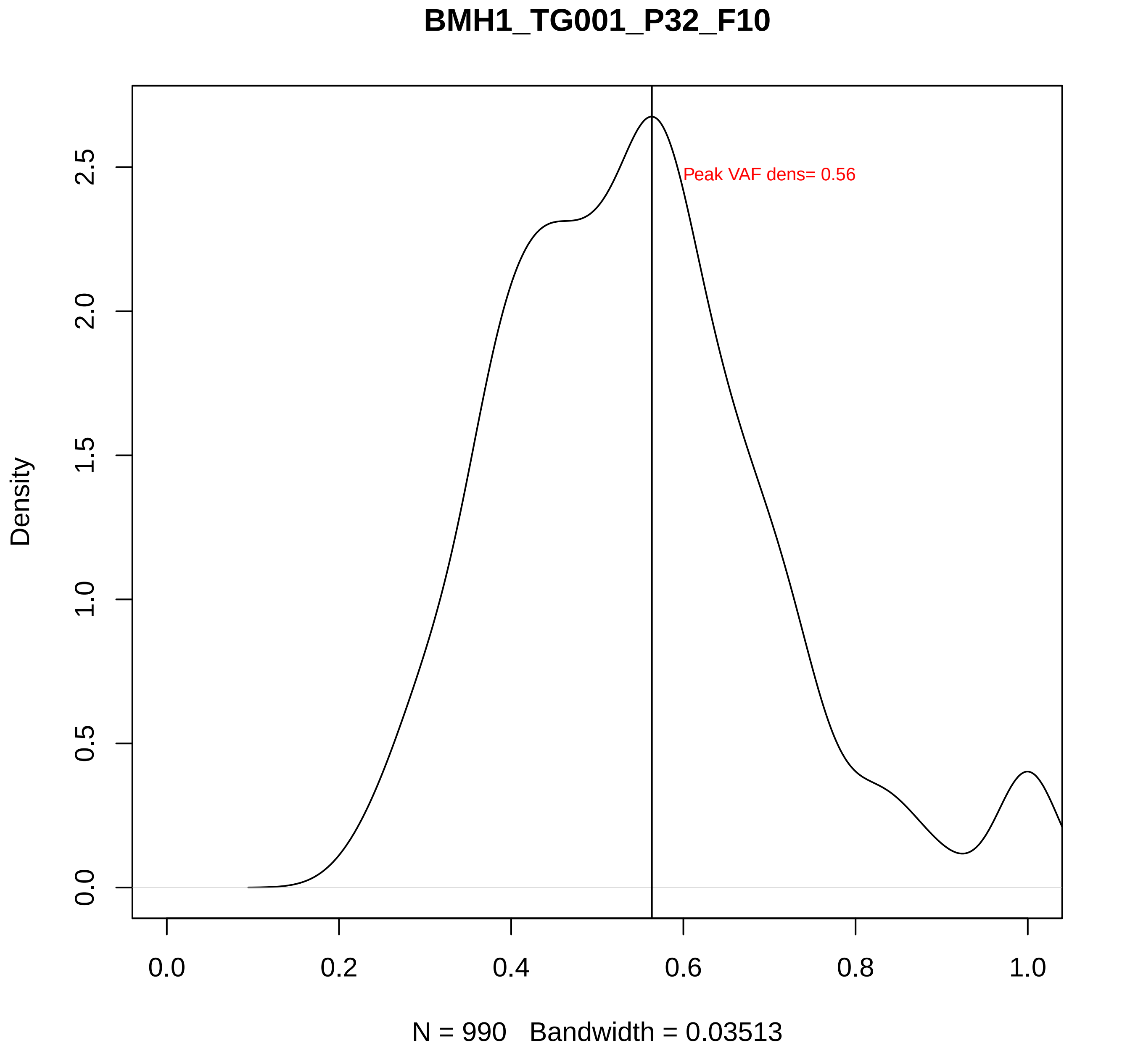

BMH1_TG001_P32_F10
Peak VAF dens= 0.56
Density
0.0
0.5
1.0
1.5
2.0
2.5
0.0
0.2
0.4
0.6
0.8
1.0
N = 990 Bandwidth = 0.03513

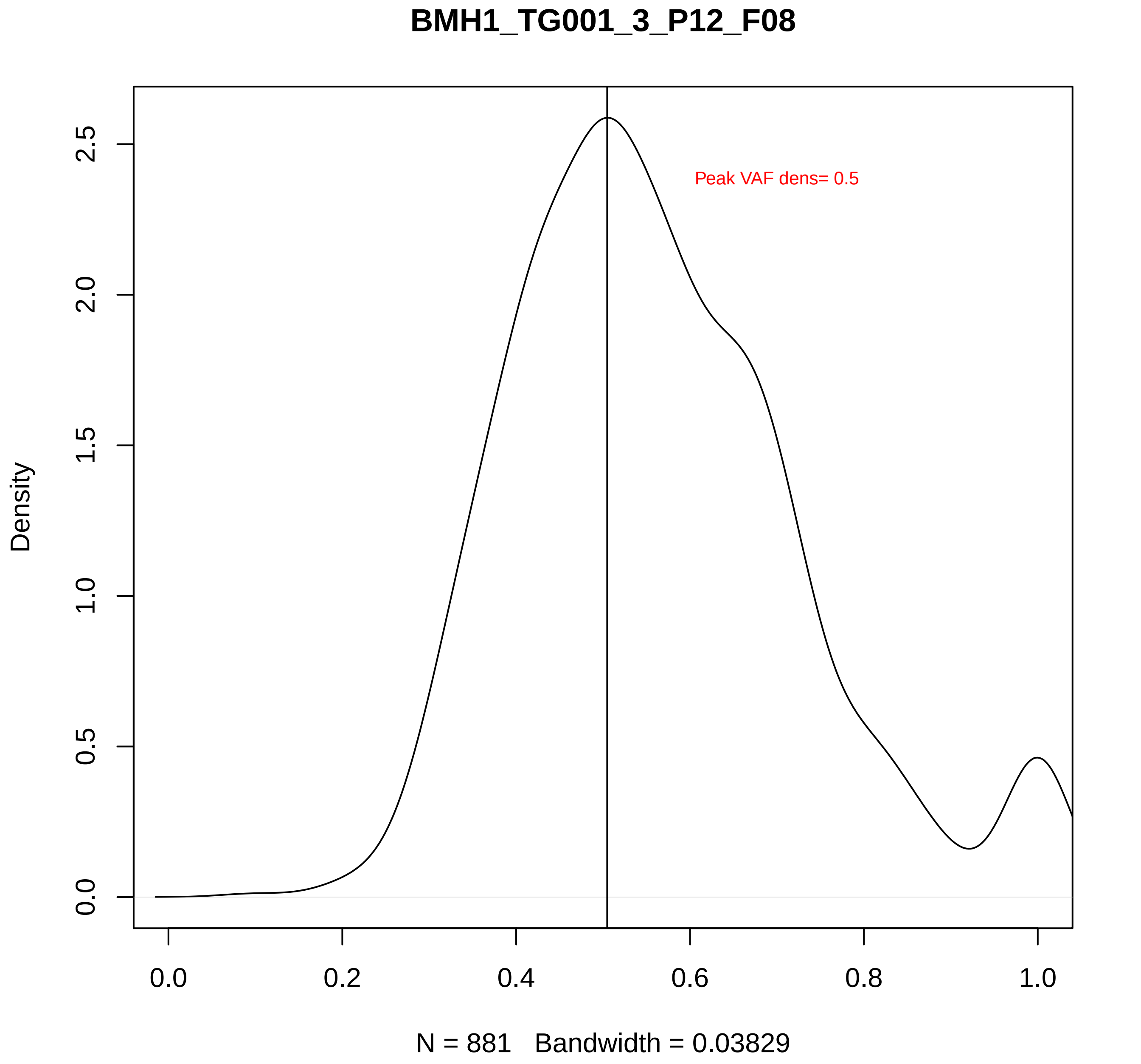

BMH1_TG001_3_P12_F08
Peak VAF dens= 0.5
Density
0.0
0.5
1.0
1.5
2.0
2.5
0.0
0.2
0.4
0.6
0.8
1.0
N = 881 Bandwidth = 0.03829

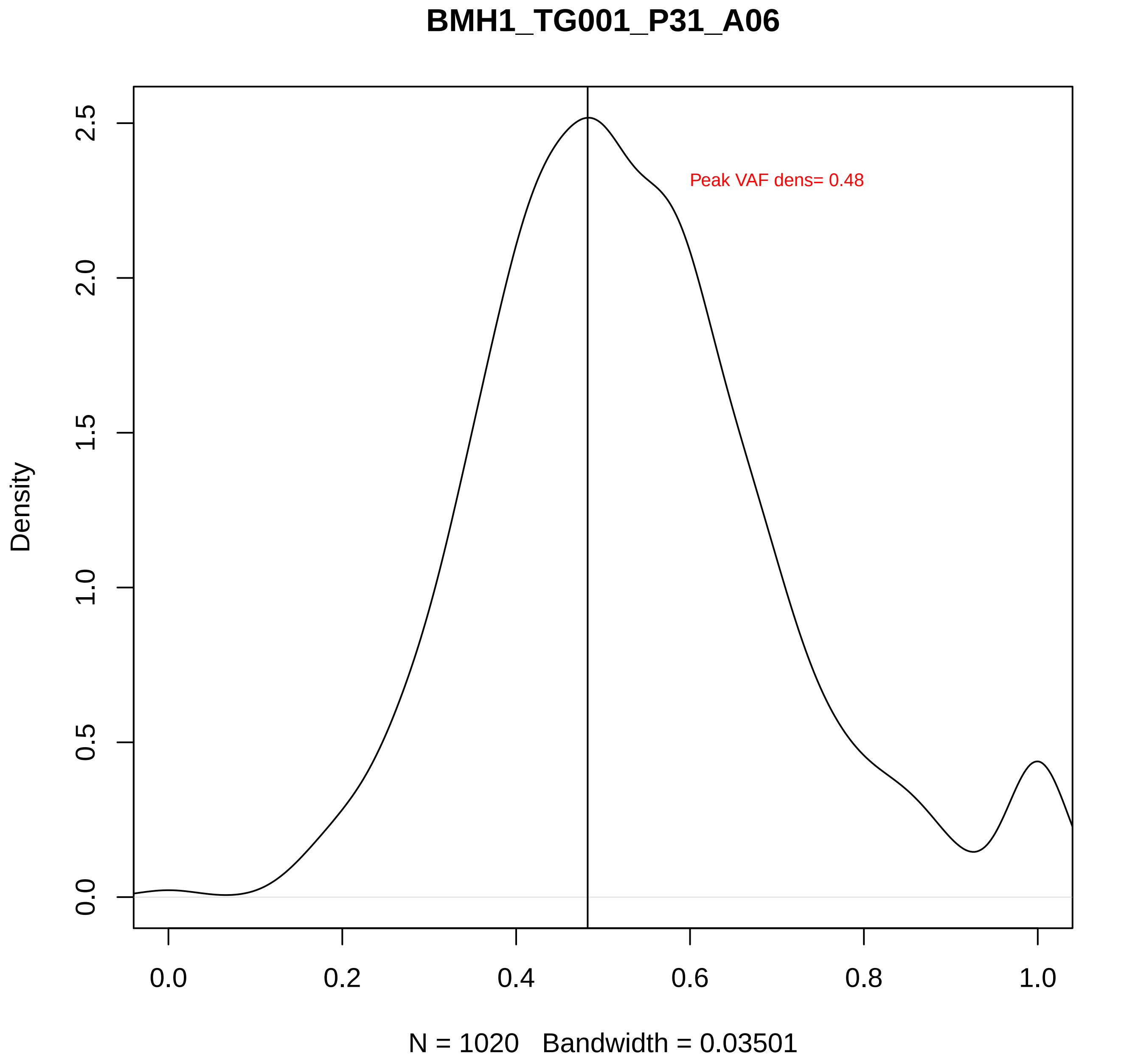
BMH1_TG001_P31_A06
Peak VAF dens= 0.48
Density
0.0
0.5
1.0
1.5
2.0
2.5
0.0
0.2
0.4
0.6
0.8
1.0
N = 1020   Bandwidth = 0.03501

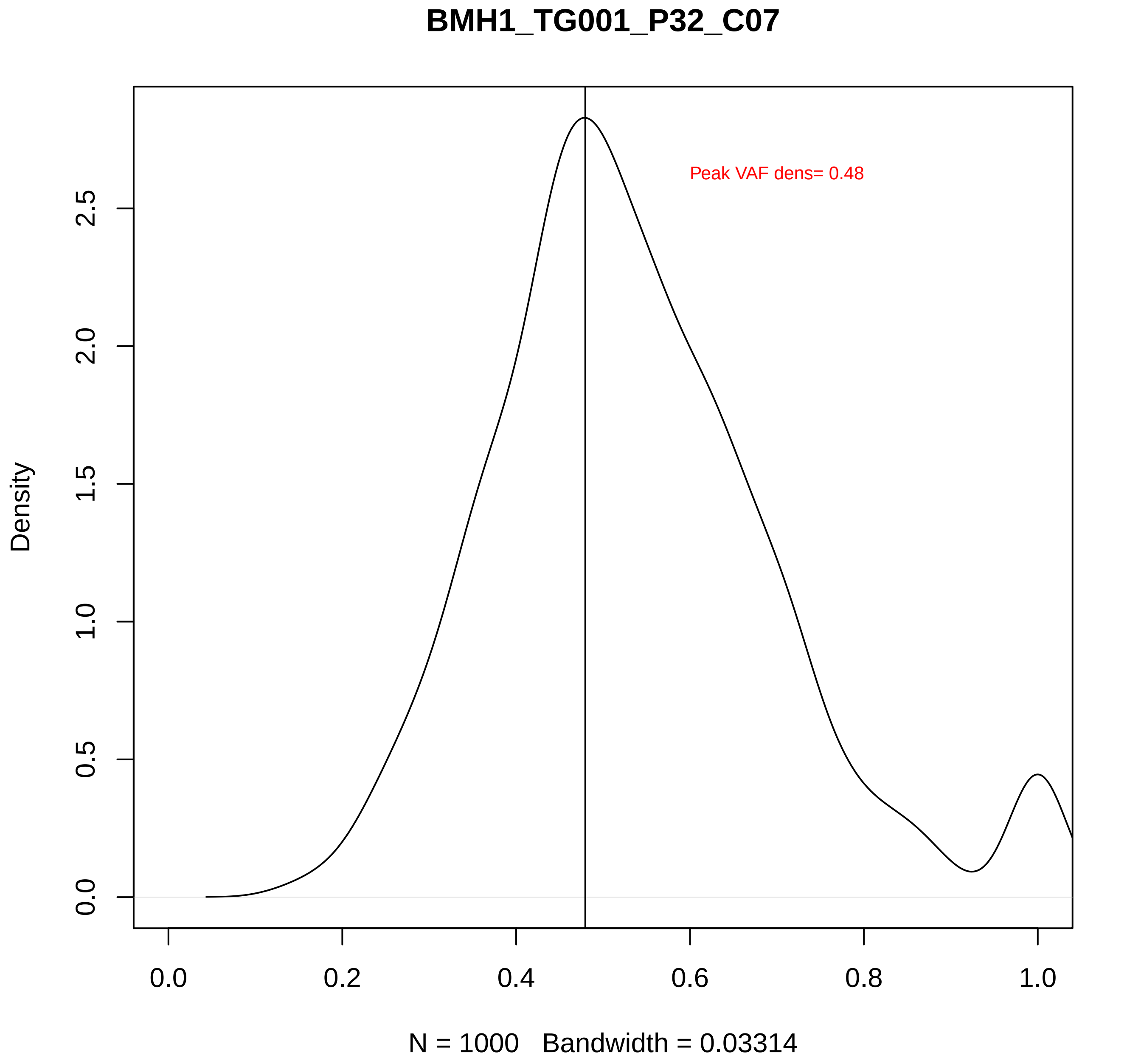

BMH1_TG001_P32_C07
Peak VAF dens= 0.48
Density
0.0
0.5
1.0
1.5
2.0
2.5
0.0
0.2
0.4
0.6
0.8
1.0
N = 1000   Bandwidth = 0.03314

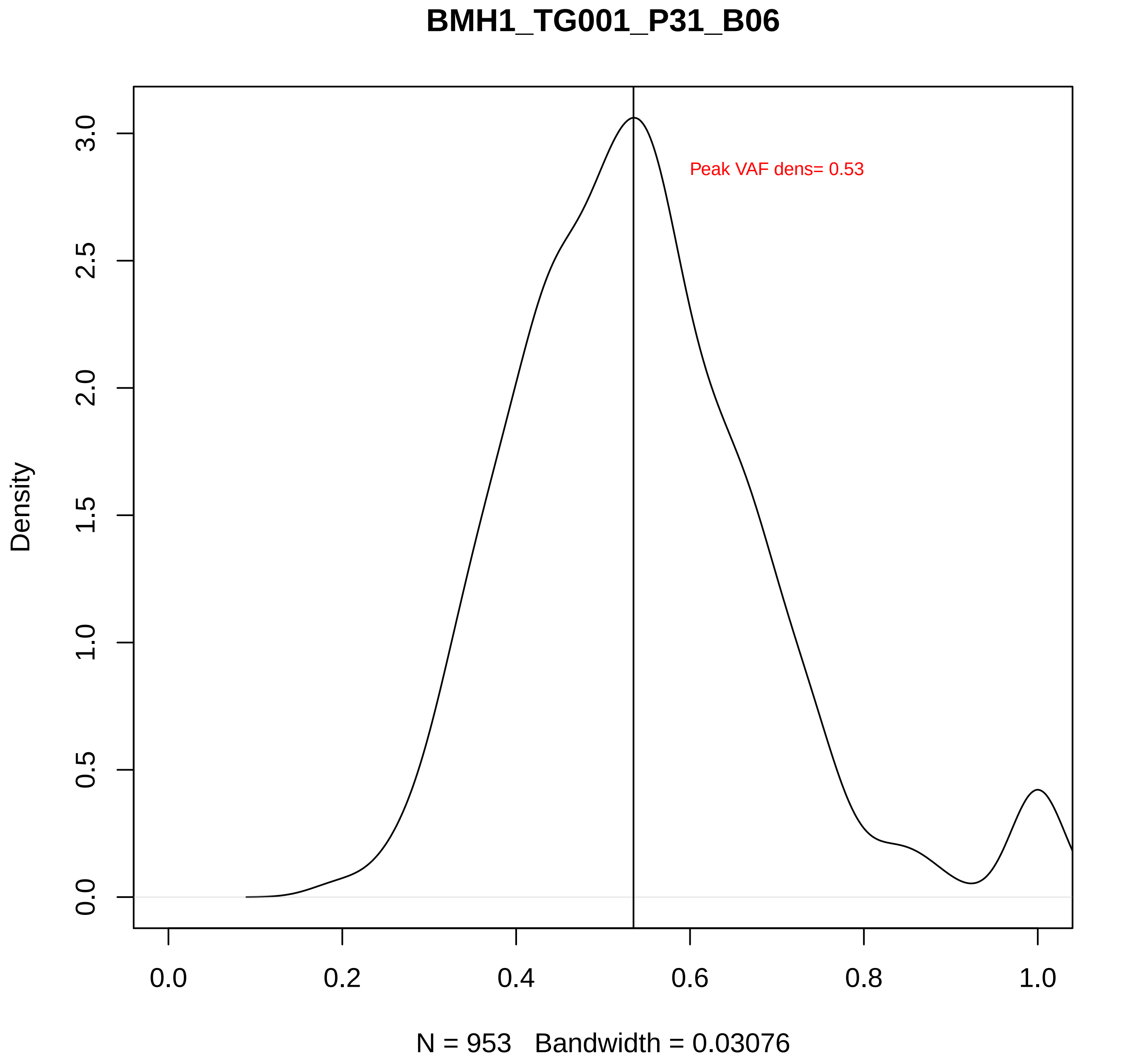
BMH1_TG001_P31_B06
Peak VAF dens= 0.53
Density
0.0
0.5
1.0
1.5
2.0
2.5
3.0
0.0
0.2
0.4
0.6
0.8
1.0
N = 953   Bandwidth = 0.03076

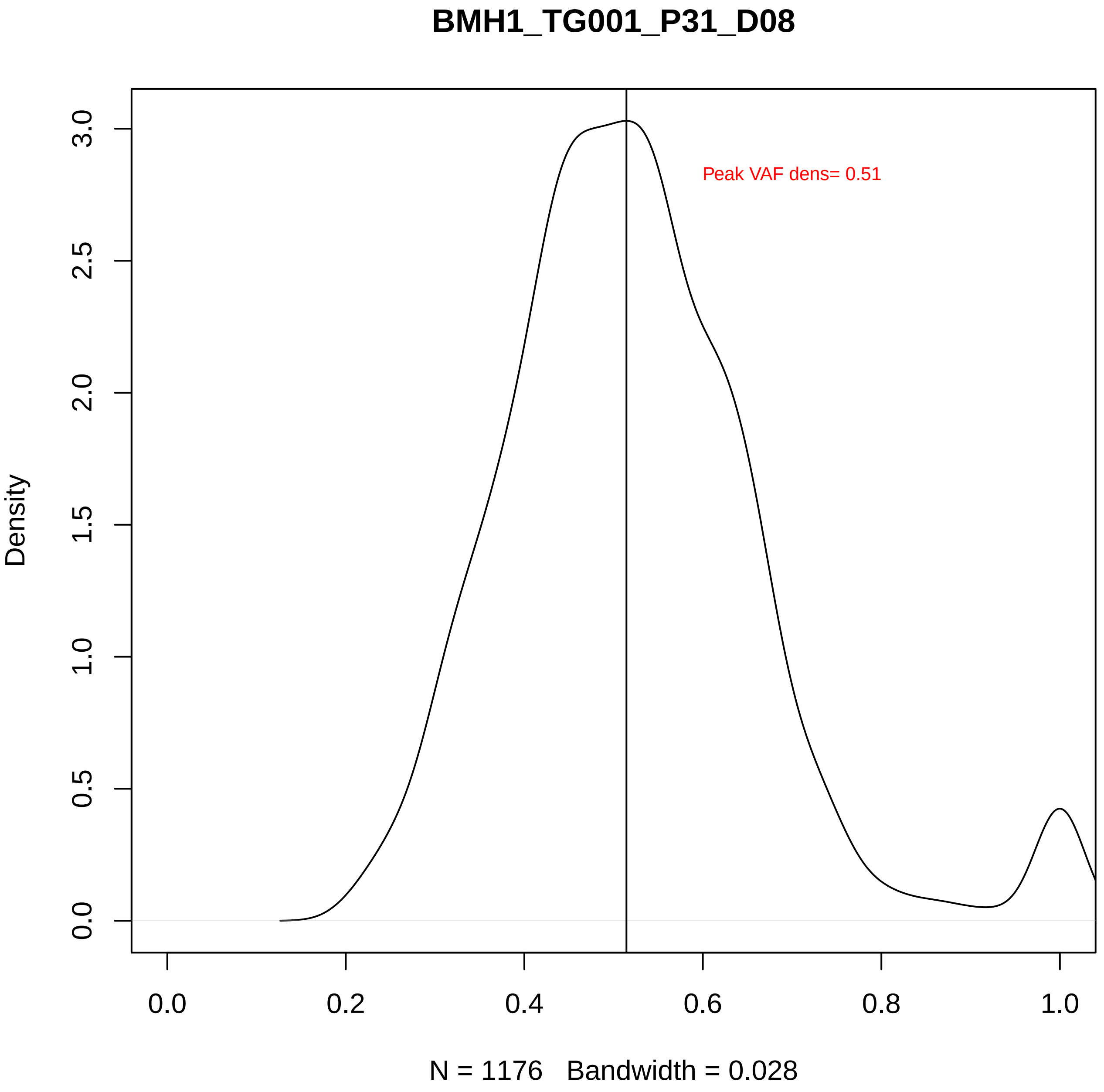

BMH1_TG001_P31_D08
Peak VAF dens= 0.51
Density
0.0
0.5
1.0
1.5
2.0
2.5
3.0
0.0
0.2
0.4
0.6
0.8
1.0
N = 1176   Bandwidth = 0.028

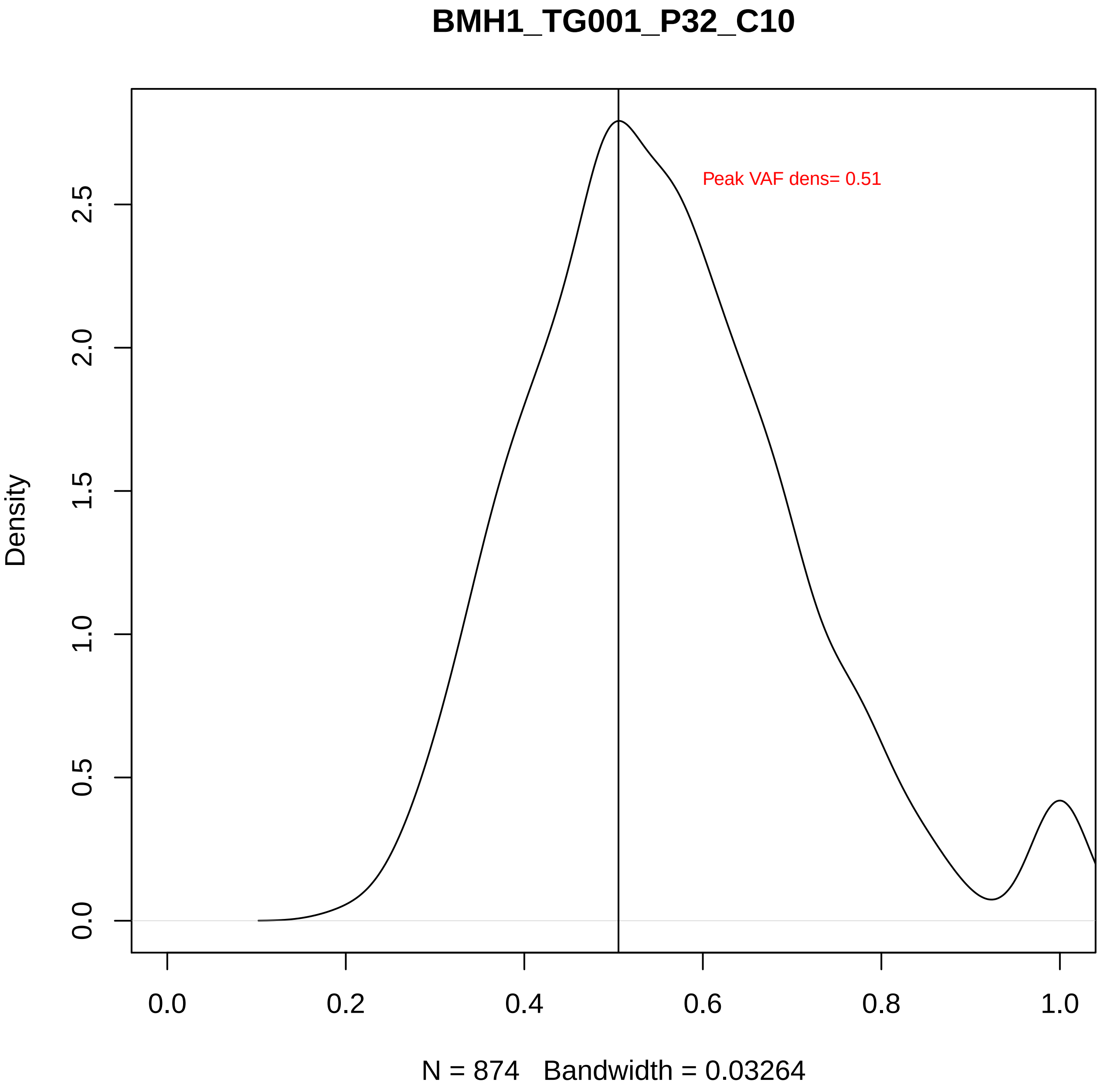
BMH1_TG001_P32_C10
Peak VAF dens= 0.51
Density
0.0
0.5
1.0
1.5
2.0
2.5
0.0
0.2
0.4
0.6
0.8
1.0
N = 874   Bandwidth = 0.03264

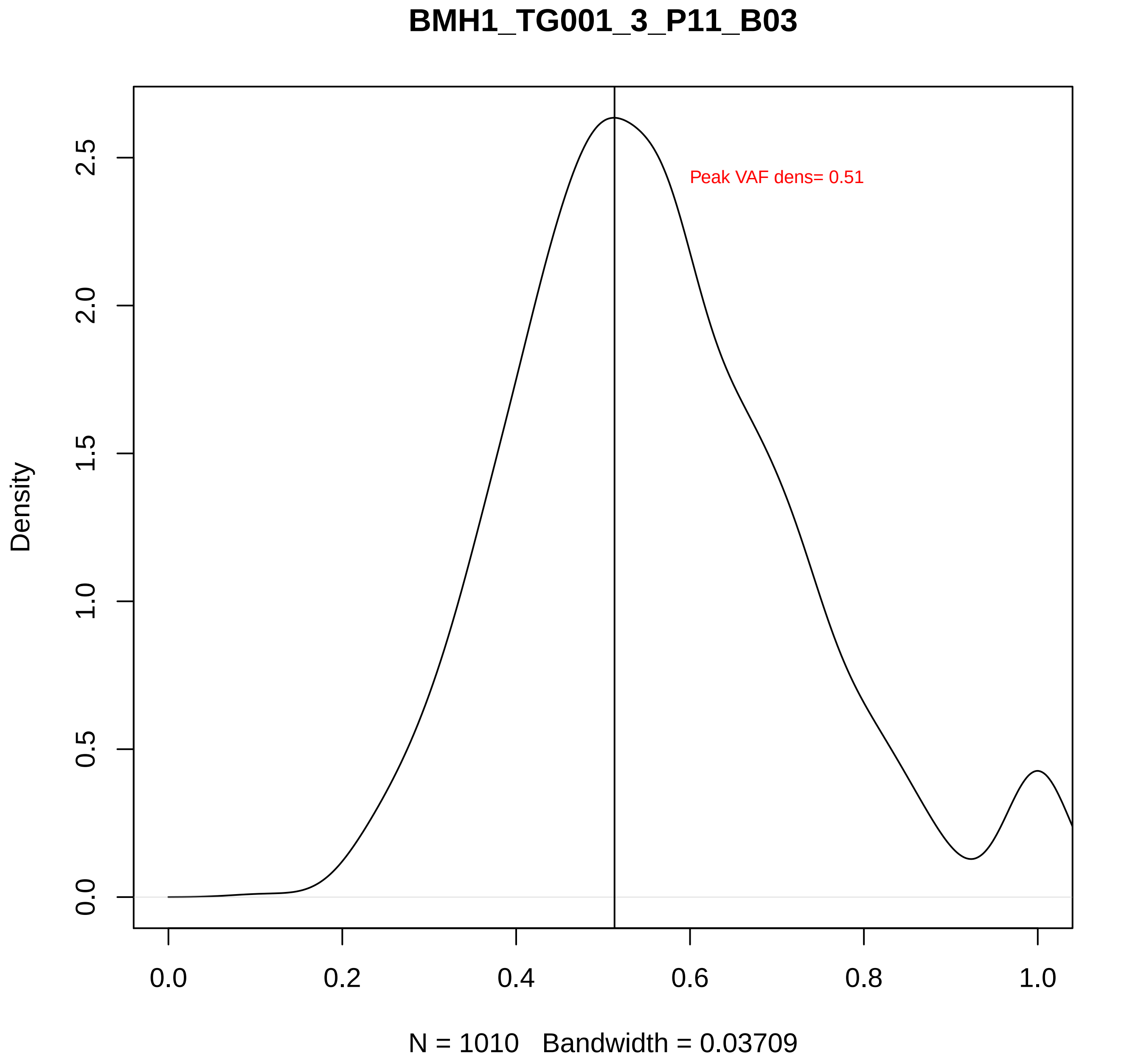

BMH1_TG001_3_P11_B03
Peak VAF dens= 0.51
Density
0.0
0.5
1.0
1.5
2.0
2.5
0.0
0.2
0.4
0.6
0.8
1.0
N = 1010   Bandwidth = 0.03709

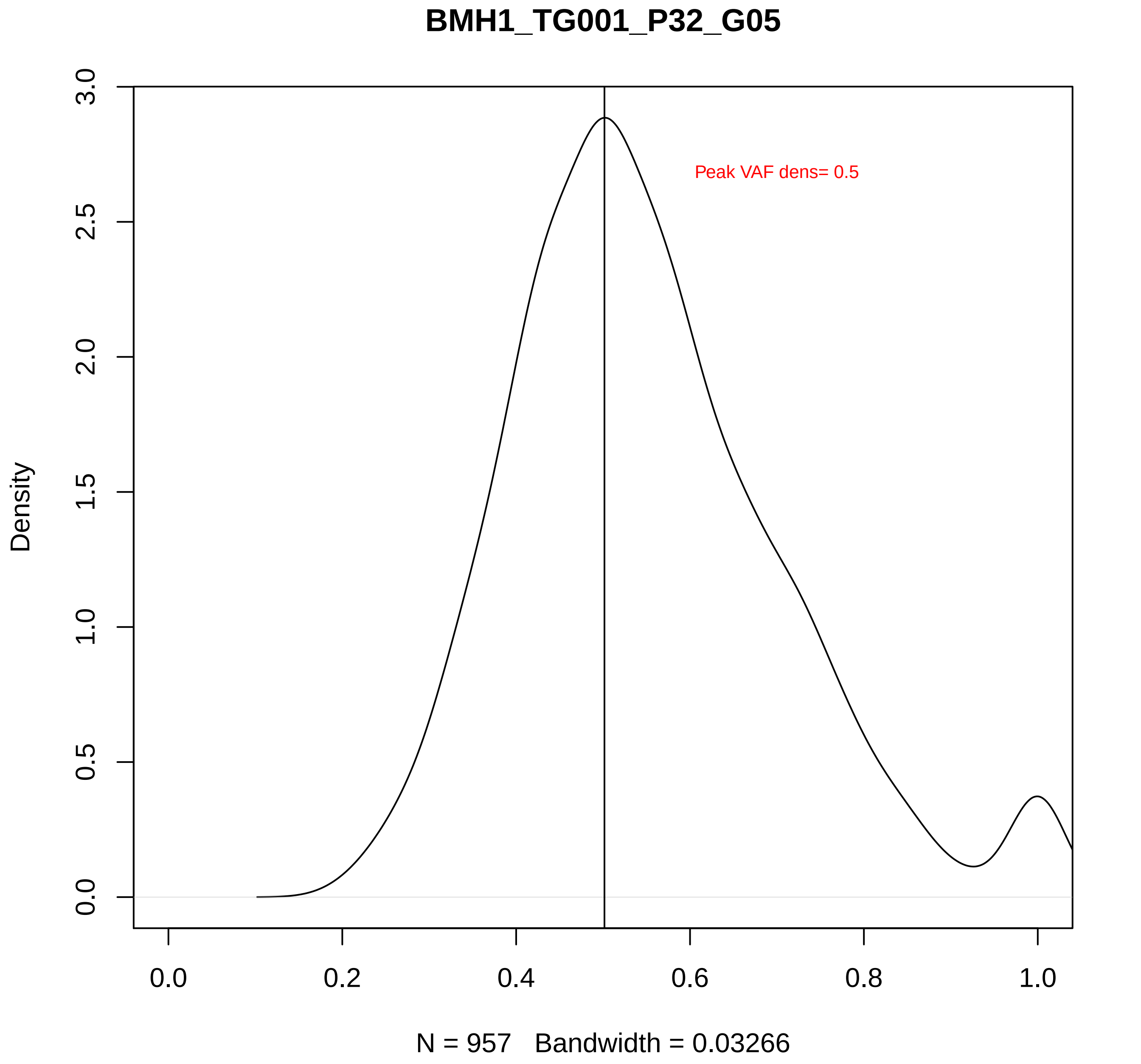
BMH1_TG001_P32_G05
Peak VAF dens= 0.5
Density
0.0
0.5
1.0
1.5
2.0
2.5
3.0
0.0
0.2
0.4
0.6
0.8
1.0
N = 957   Bandwidth = 0.03266

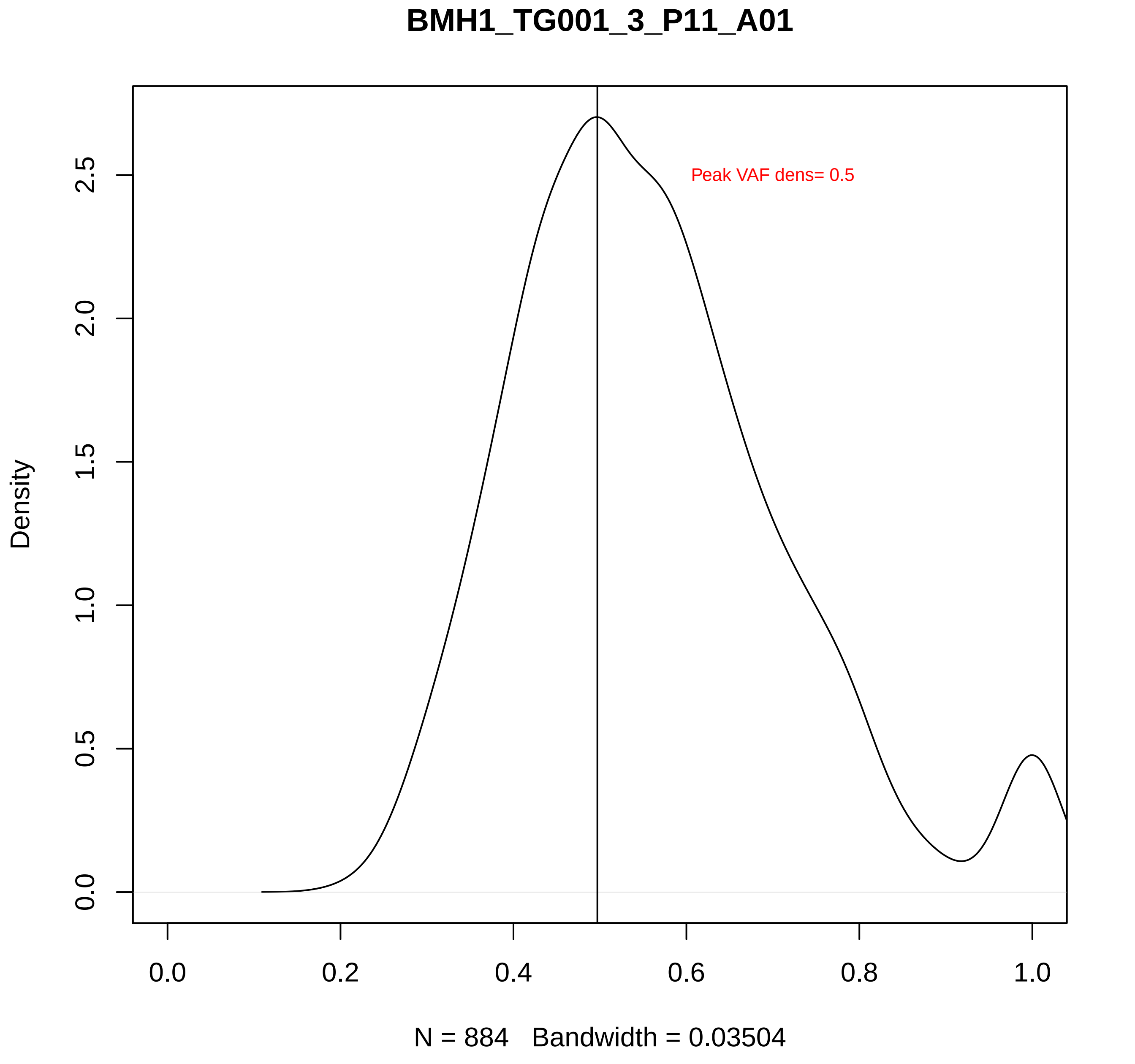

BMH1_TG001_3_P11_A01
Peak VAF dens= 0.5
Density
0.0
0.5
1.0
1.5
2.0
2.5
0.0
0.2
0.4
0.6
0.8
1.0
N = 884   Bandwidth = 0.03504

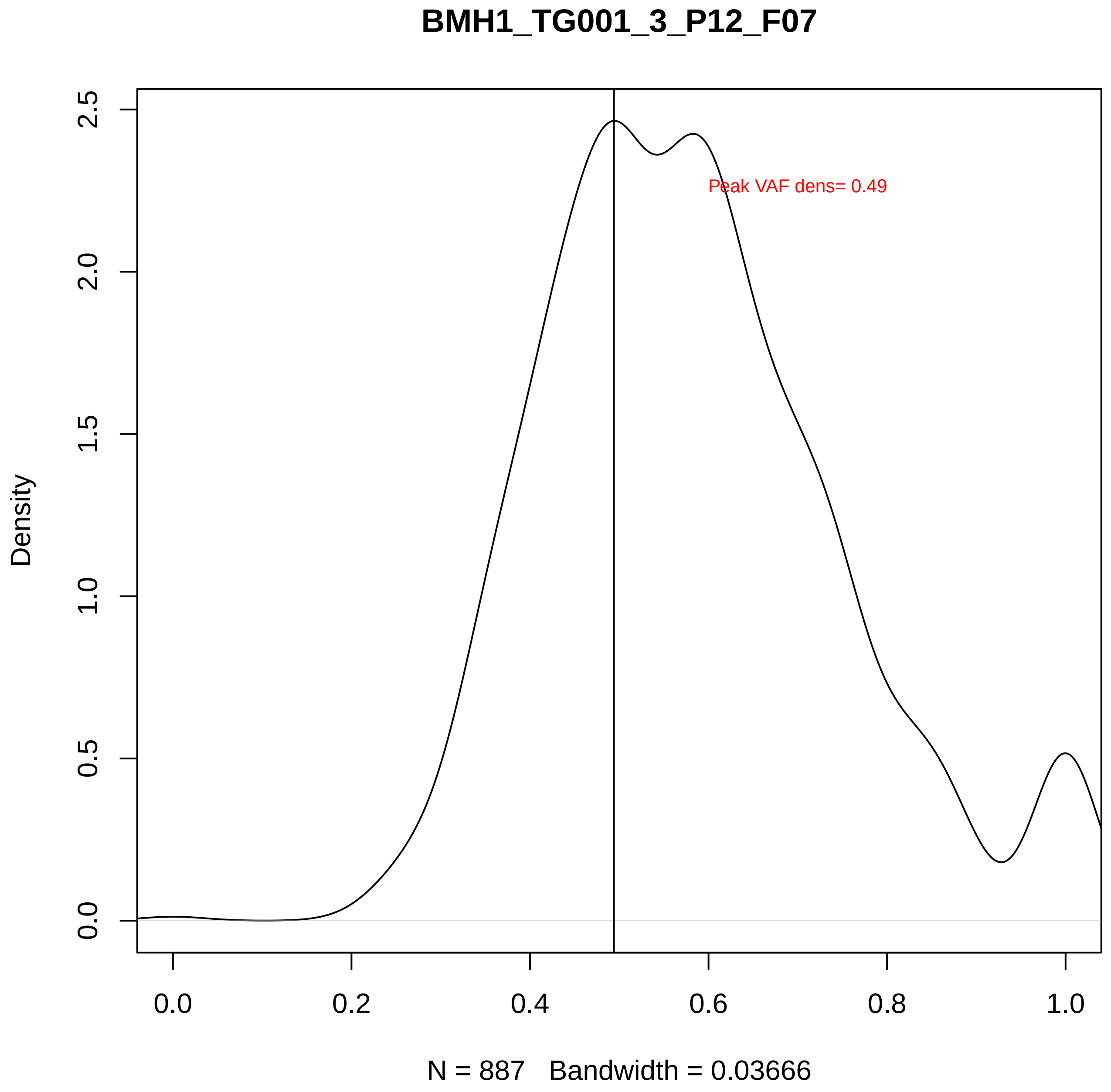

BMH1_TG001_3_P12_F07
Peak VAF dens= 0.49
Density
2.5
2.0
1.5
1.0
0.5
0.0
0.0
0.2
0.4
0.6
0.8
1.0
N = 887 Bandwidth = 0.03666

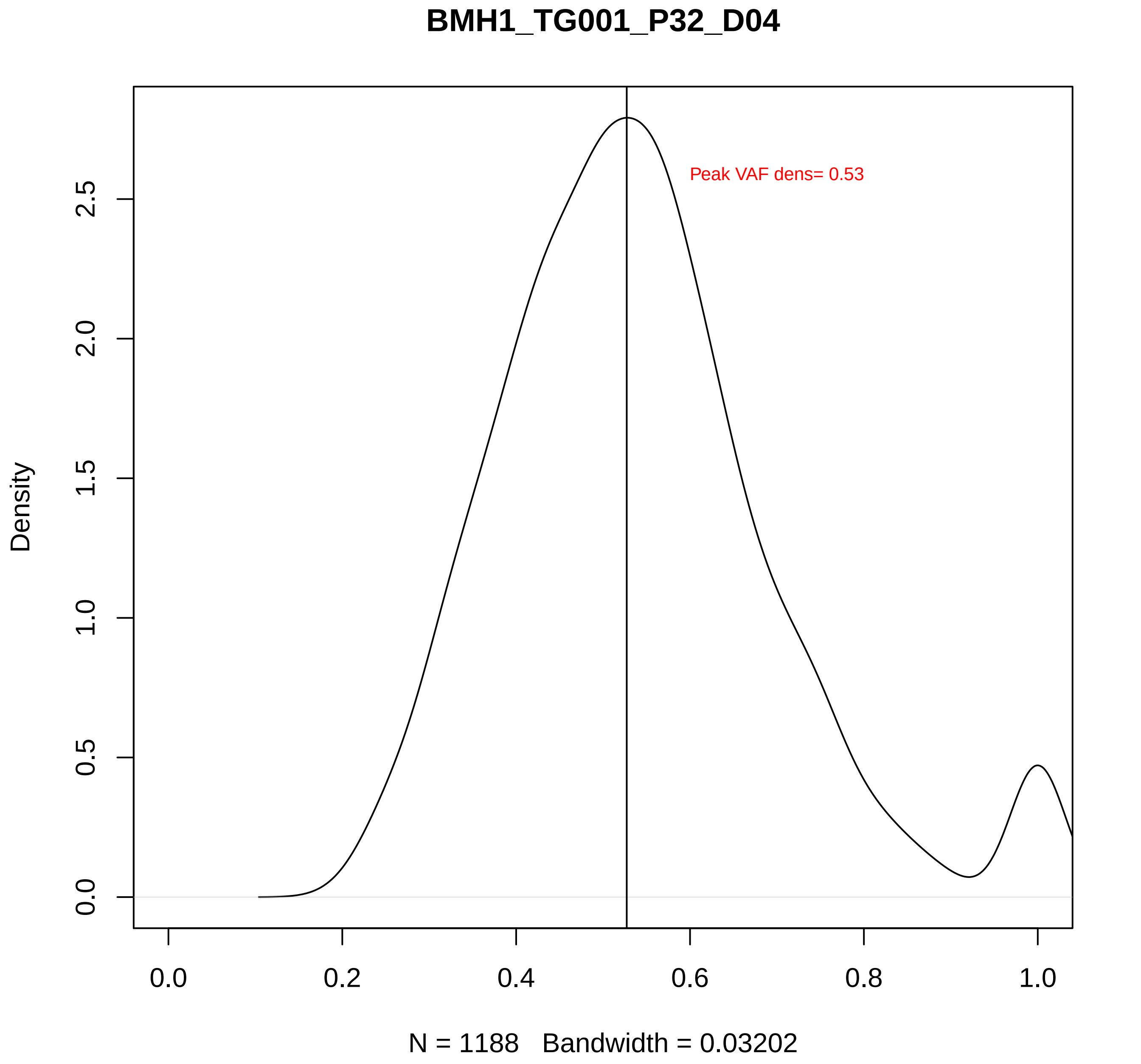

BMH1_TG001_P32_D04
Peak VAF dens= 0.53
Density
0.0
0.5
1.0
1.5
2.0
2.5
0.0
0.2
0.4
0.6
0.8
1.0
N = 1188   Bandwidth = 0.03202

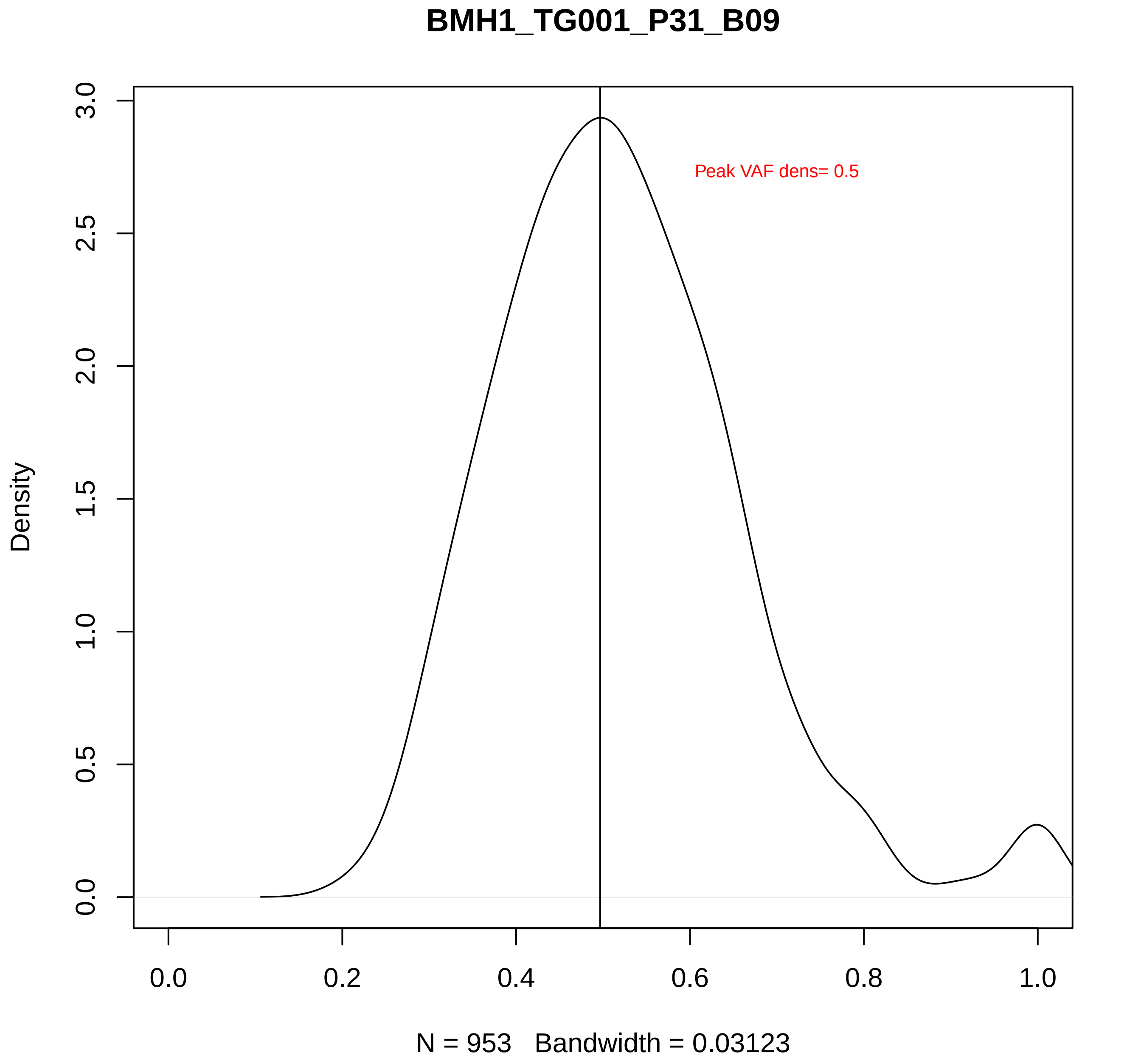

BMH1_TG001_P31_B09
Peak VAF dens= 0.5
Density
0.0
0.5
1.0
1.5
2.0
2.5
3.0
0.0
0.2
0.4
0.6
0.8
1.0
N = 953   Bandwidth = 0.03123

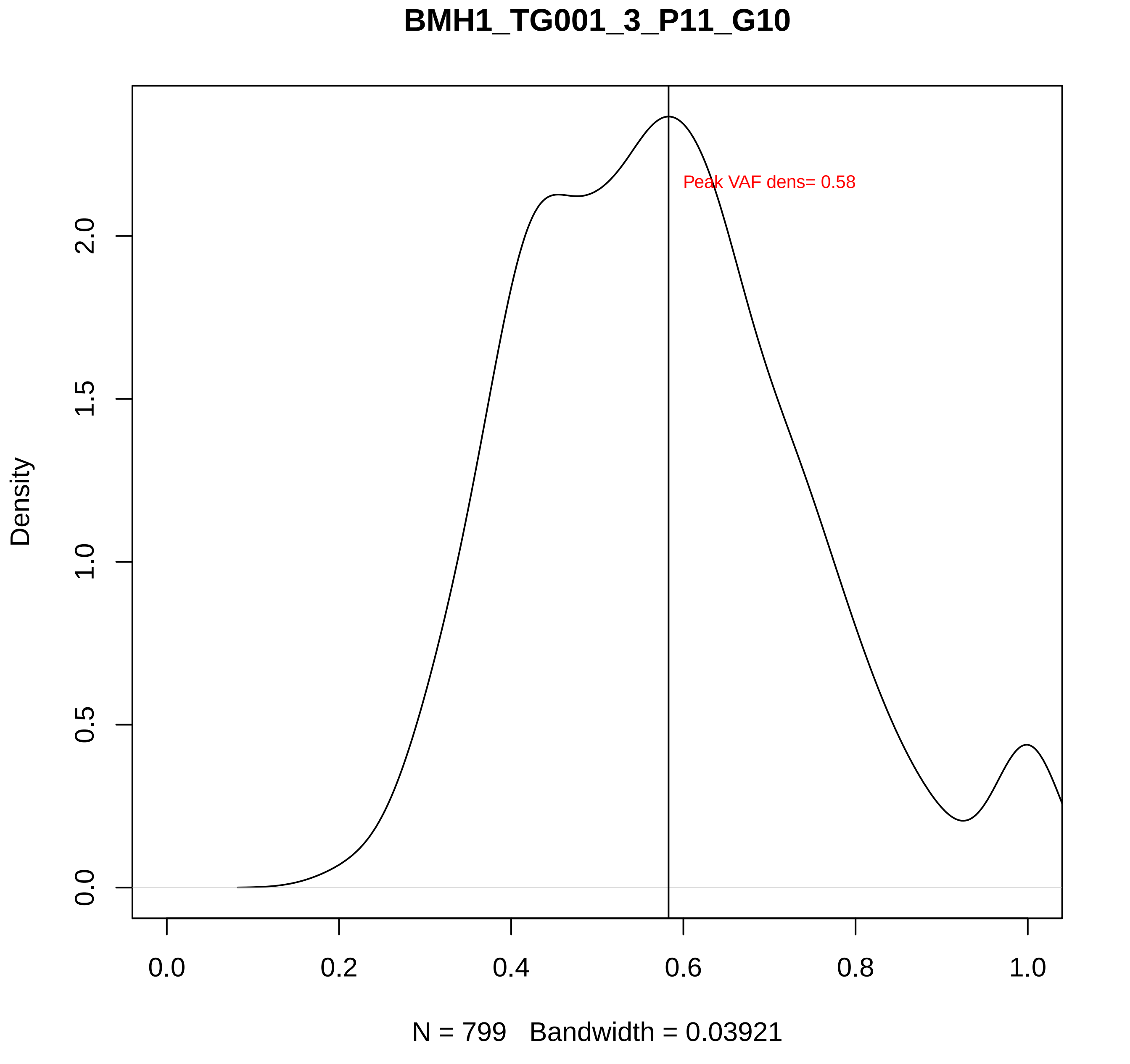
BMH1_TG001_3_P11_G10
Peak VAF dens= 0.58
Density
0.0
0.5
1.0
1.5
2.0
0.0
0.2
0.4
0.6
0.8
1.0
N = 799   Bandwidth = 0.03921

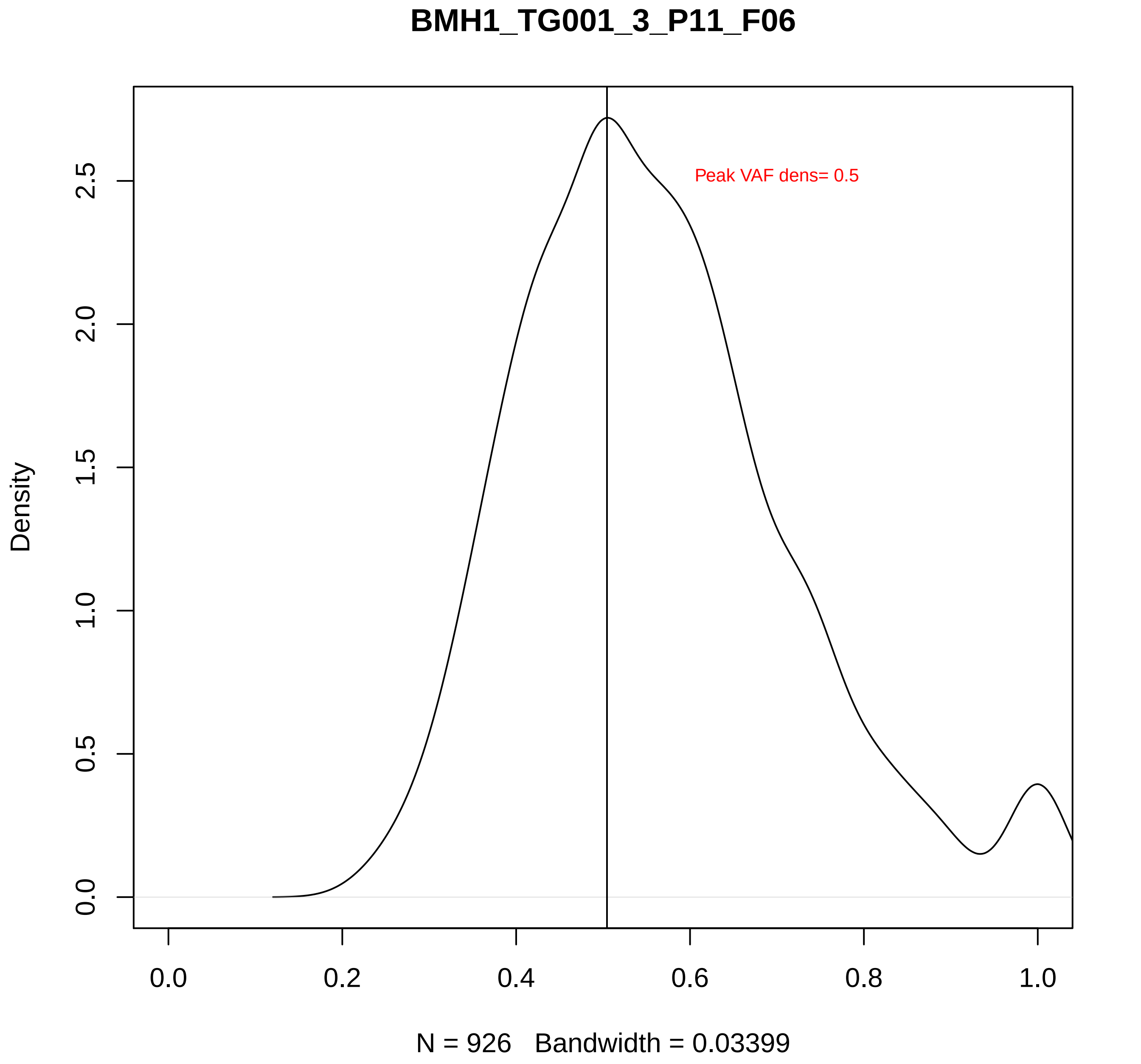
BMH1_TG001_3_P11_F06
Peak VAF dens= 0.5
Density
0.0
0.5
1.0
1.5
2.0
2.5
0.0
0.2
0.4
0.6
0.8
1.0
N = 926   Bandwidth = 0.03399

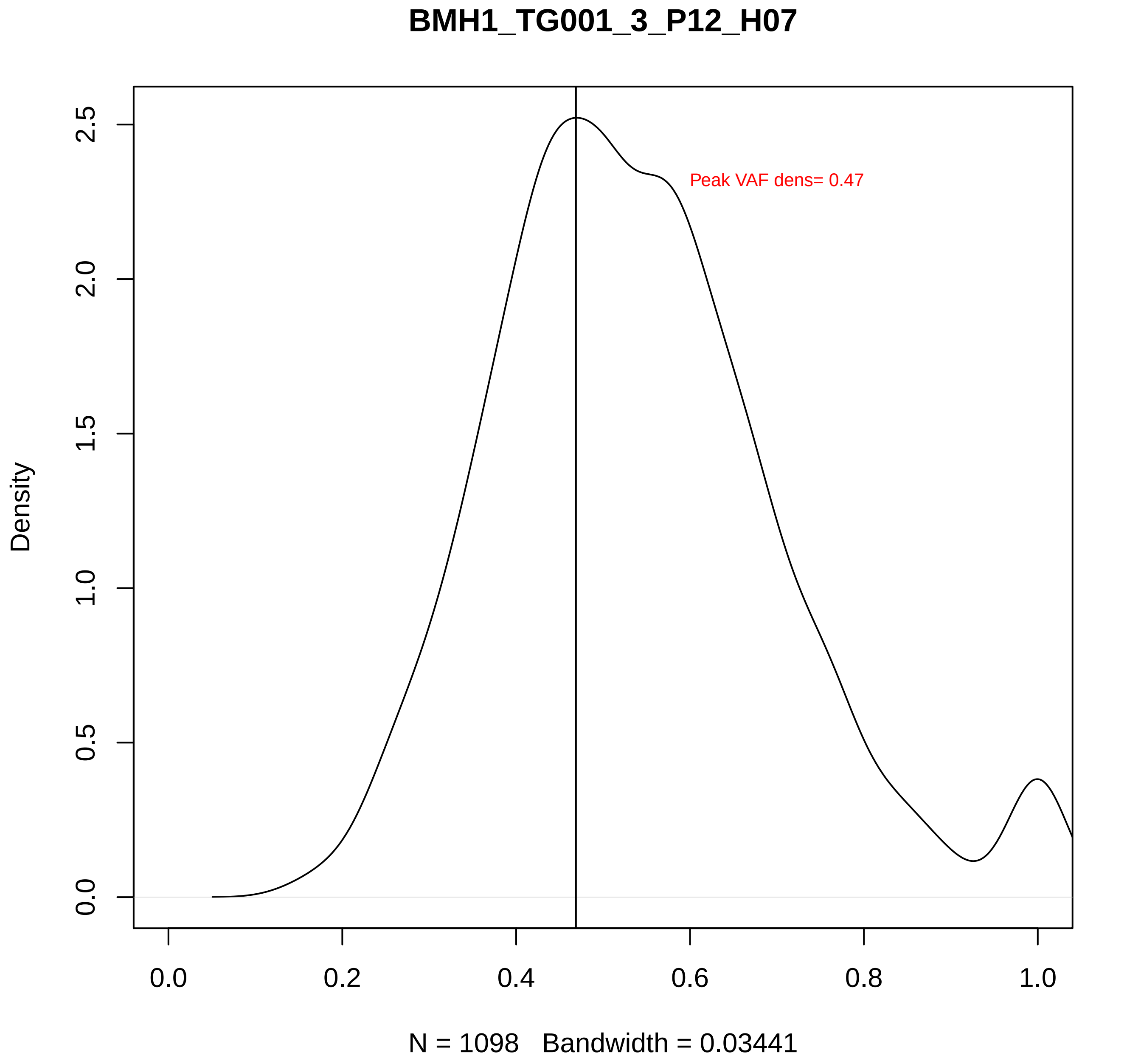
BMH1_TG001_3_P12_H07
Peak VAF dens= 0.47
Density
0.0
0.5
1.0
1.5
2.0
2.5
0.0
0.2
0.4
0.6
0.8
1.0
N = 1098   Bandwidth = 0.03441

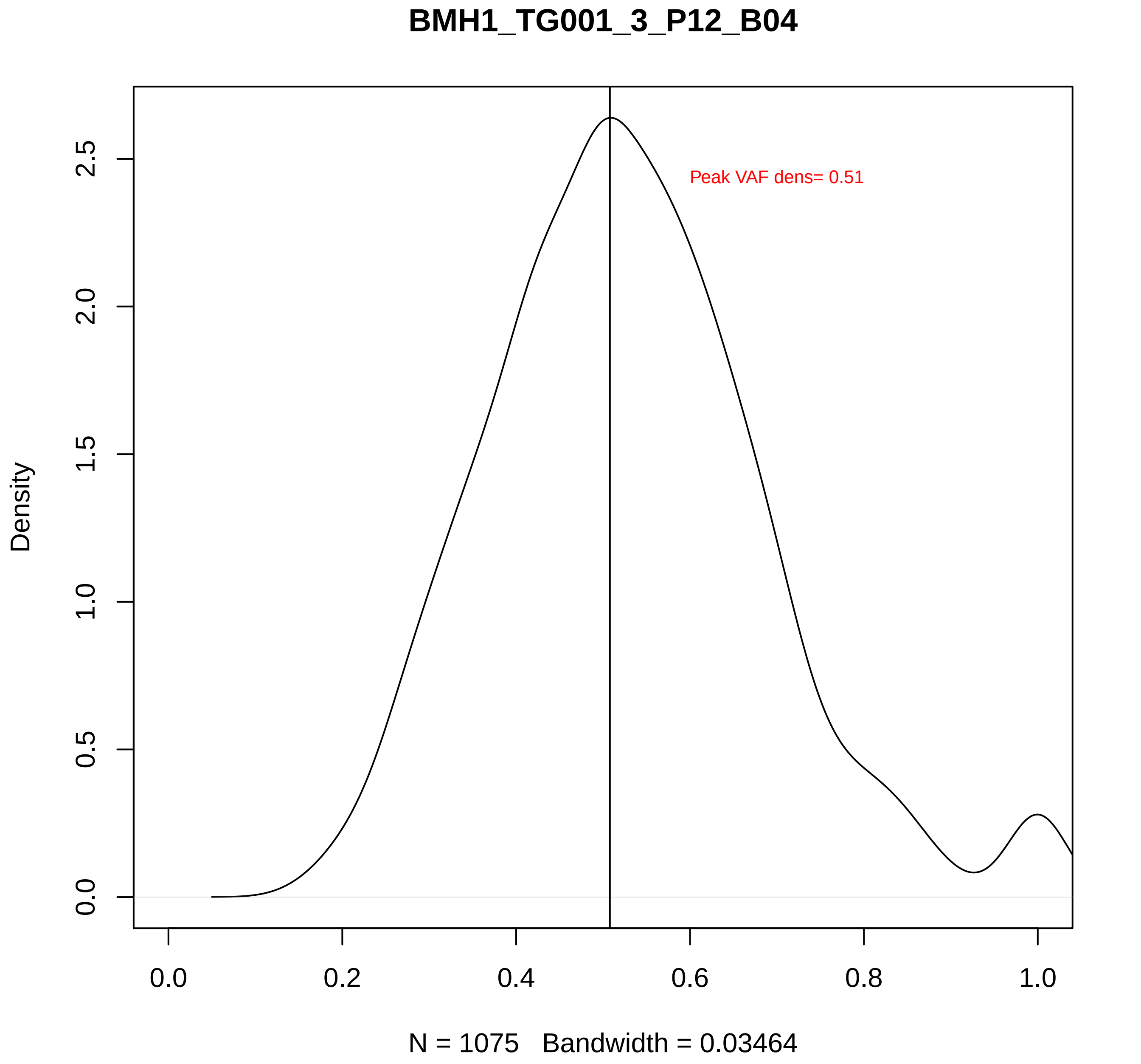

BMH1_TG001_3_P12_B04
Peak VAF dens= 0.51
Density
0.0
0.5
1.0
1.5
2.0
2.5
0.0
0.2
0.4
0.6
0.8
1.0
N = 1075   Bandwidth = 0.03464

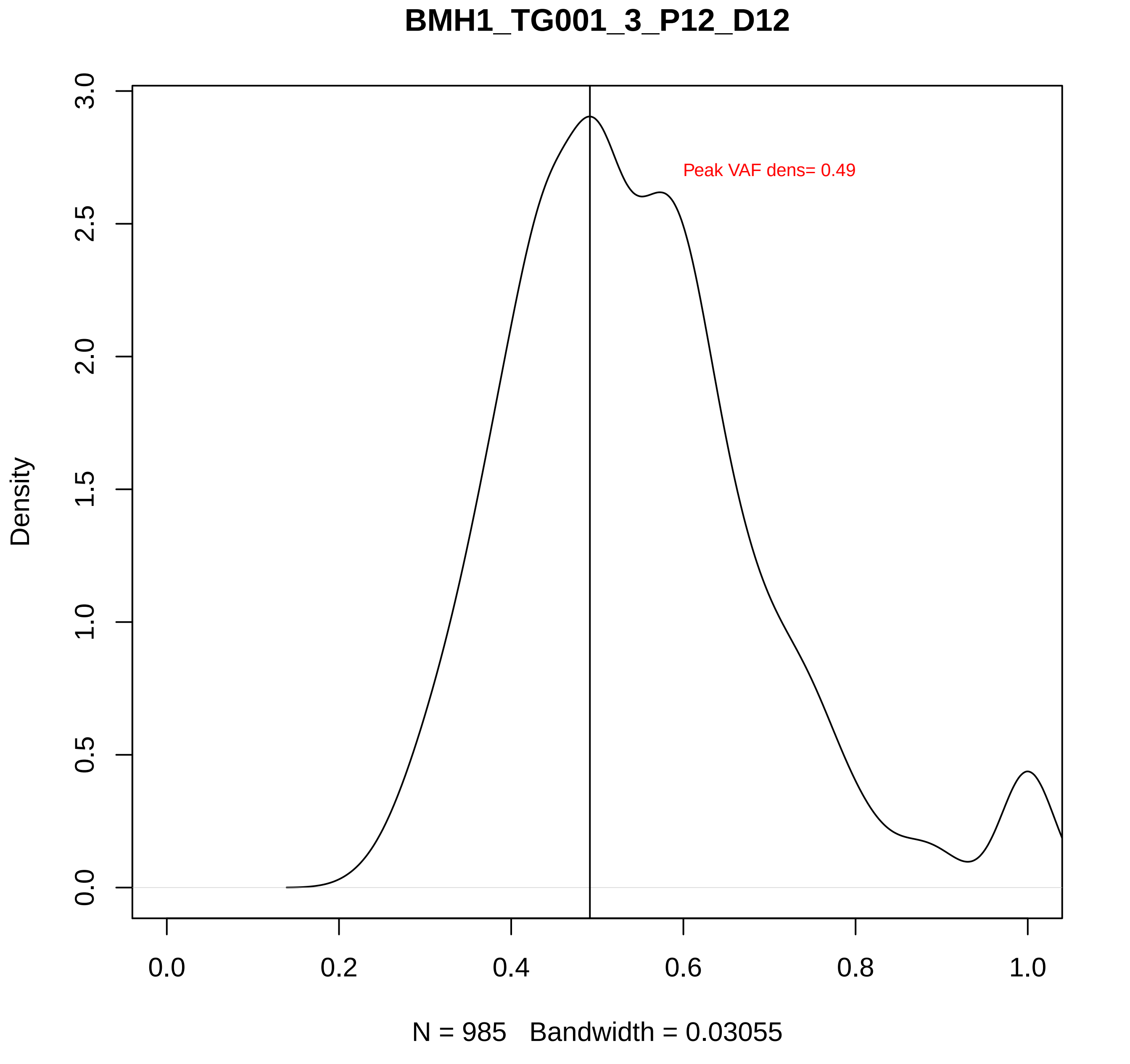

BMH1_TG001_3_P12_D12
Peak VAF dens= 0.49
Density
0.0
0.5
1.0
1.5
2.0
2.5
3.0
0.0
0.2
0.4
0.6
0.8
1.0
N = 985   Bandwidth = 0.03055

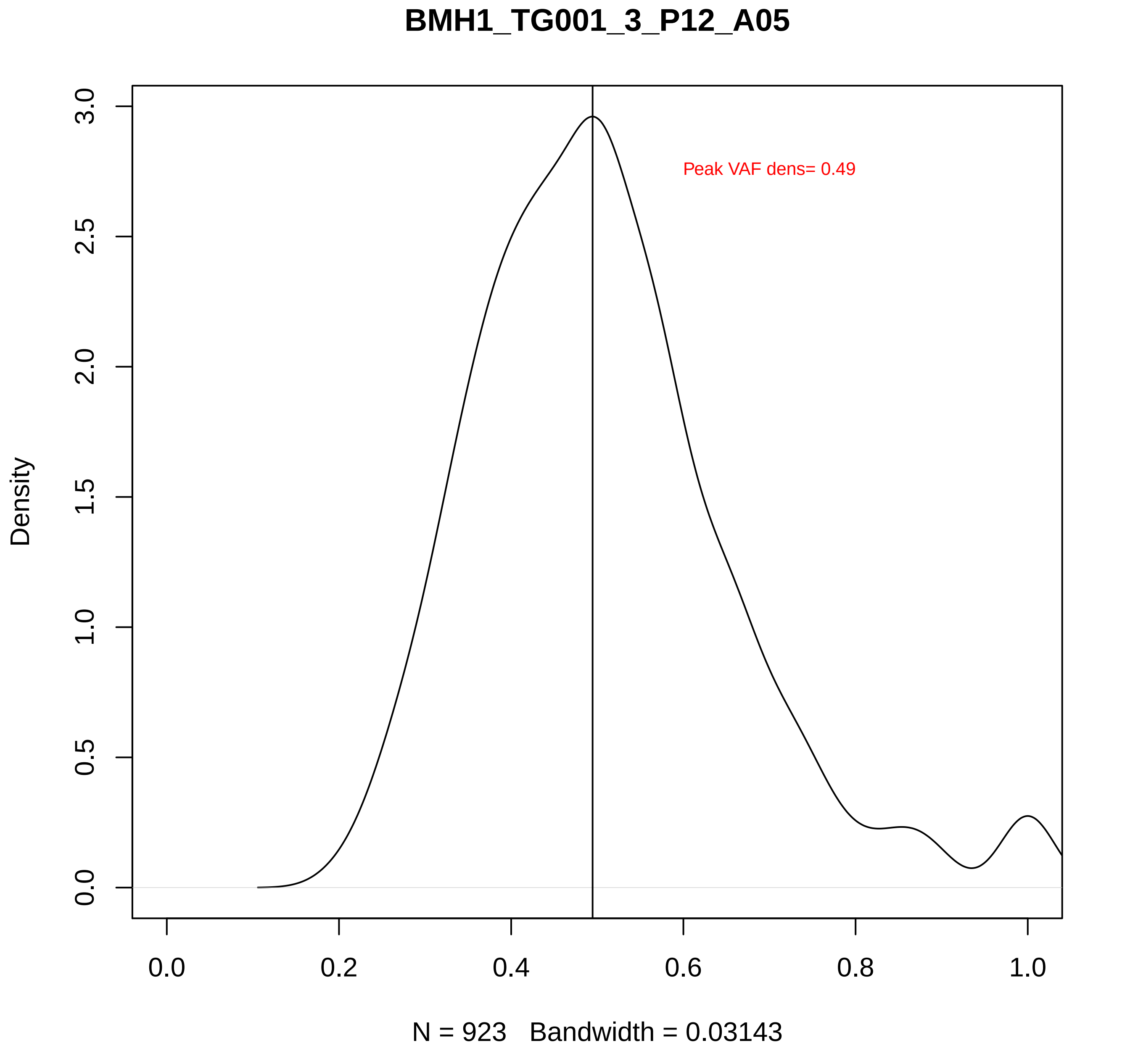

BMH1_TG001_3_P12_A05
Peak VAF dens= 0.49
Density
0.0
0.5
1.0
1.5
2.0
2.5
3.0
0.0
0.2
0.4
0.6
0.8
1.0
N = 923 Bandwidth = 0.03143

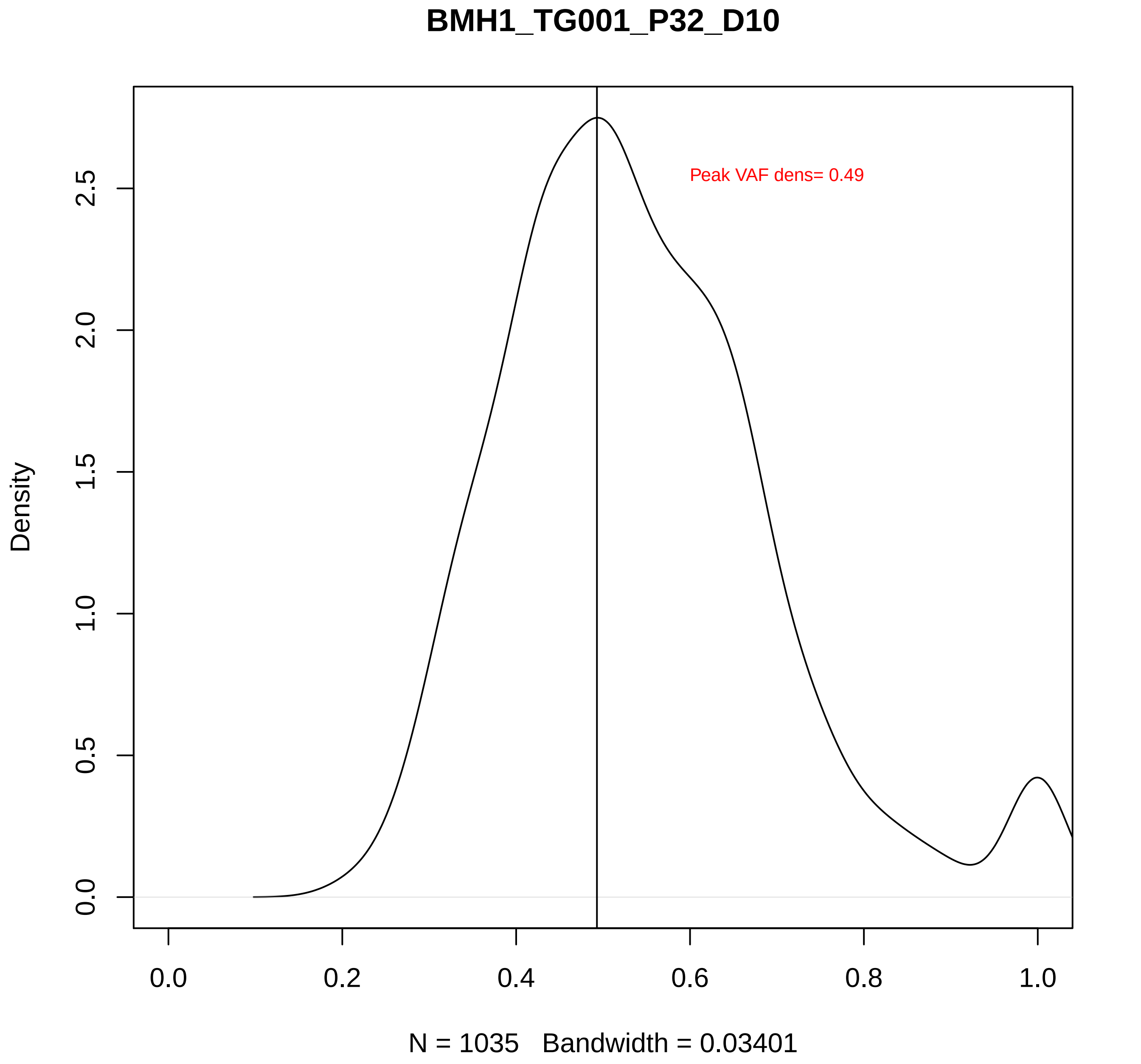
BMH1_TG001_P32_D10
Peak VAF dens= 0.49
Density
0.0
0.5
1.0
1.5
2.0
2.5
0.0
0.2
0.4
0.6
0.8
1.0
N = 1035   Bandwidth = 0.03401

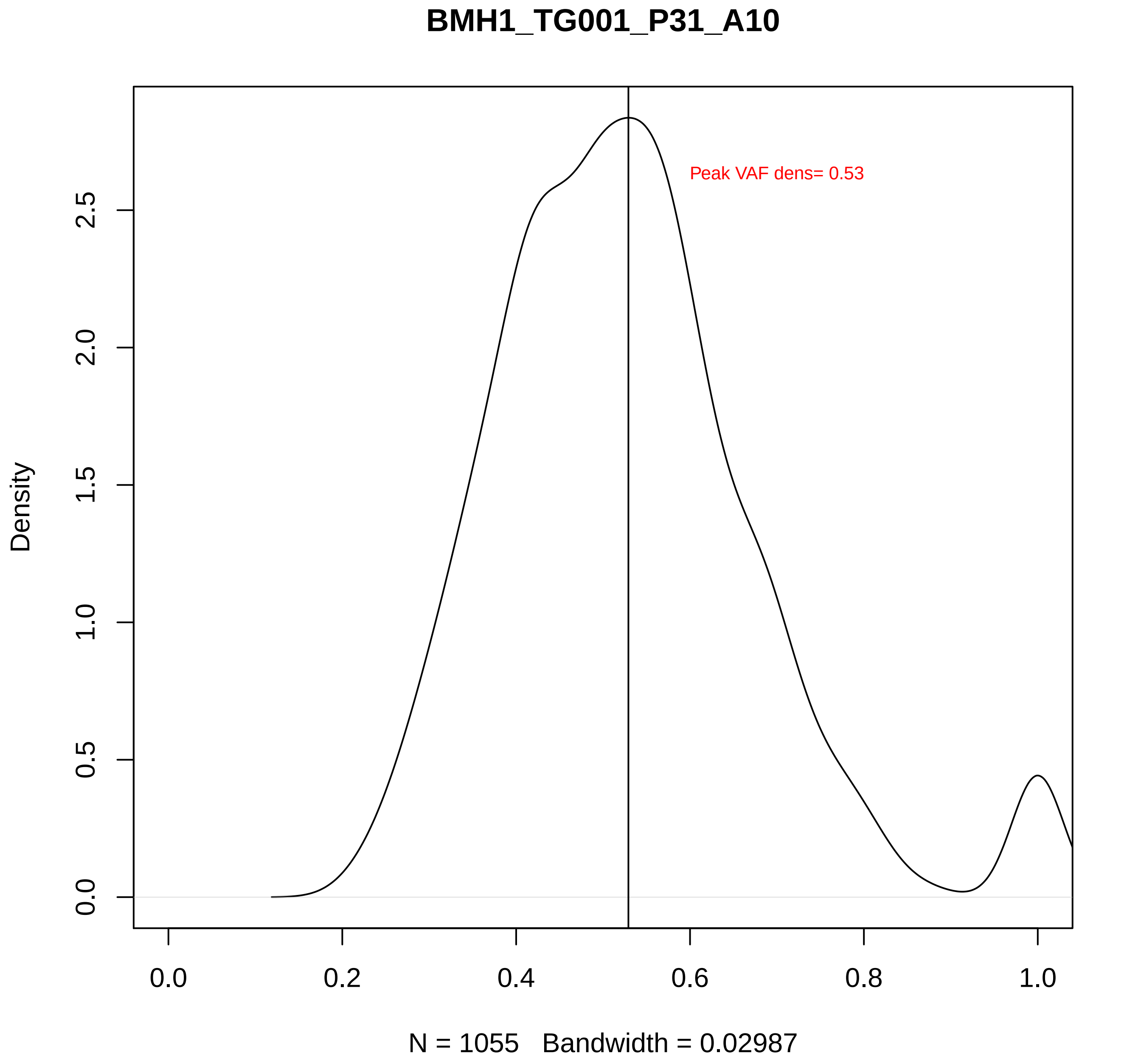
BMH1_TG001_P31_A10
Peak VAF dens= 0.53
Density
0.0
0.5
1.0
1.5
2.0
2.5
0.0
0.2
0.4
0.6
0.8
1.0
N = 1055   Bandwidth = 0.02987

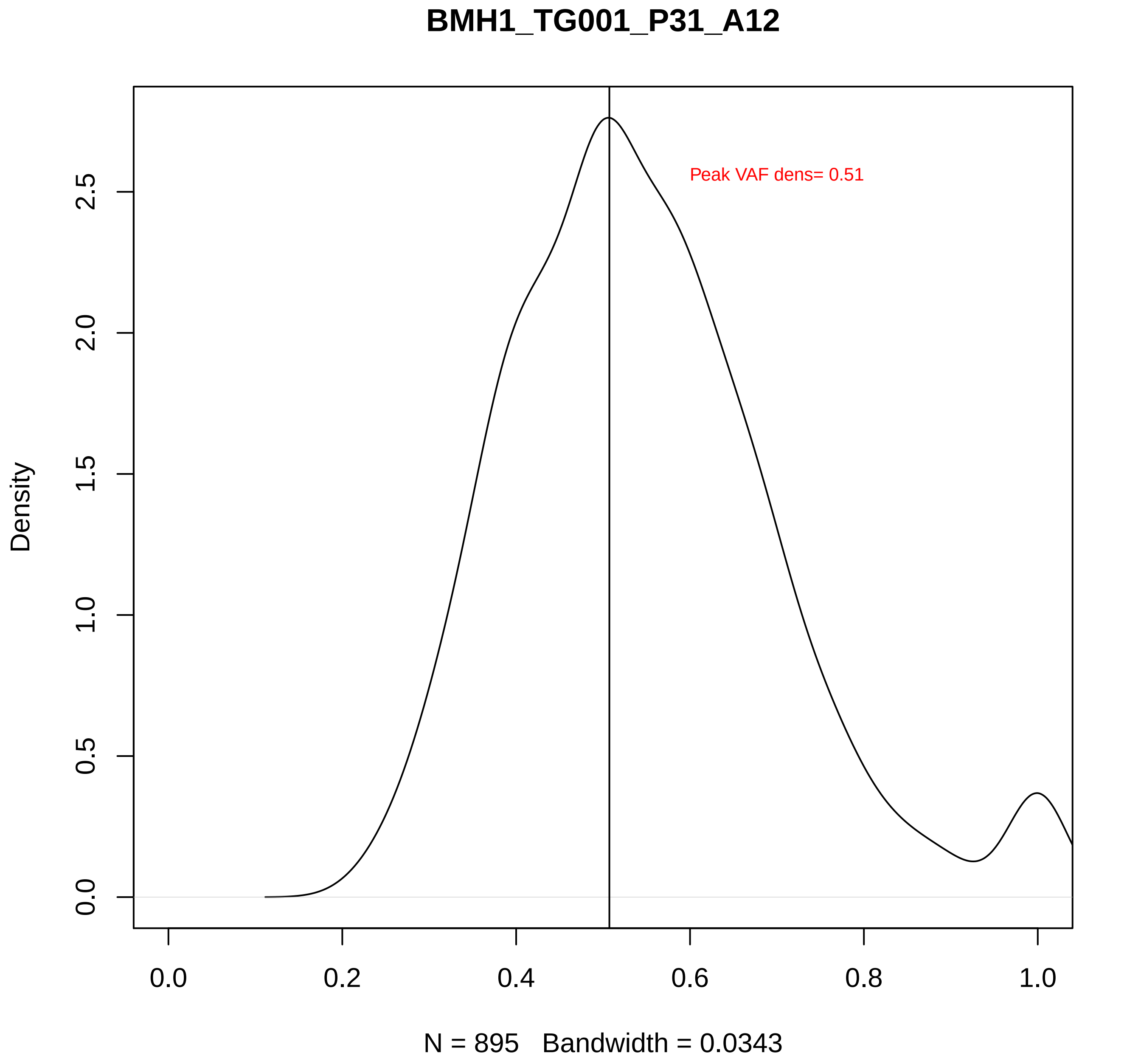
BMH1_TG001_P31_A12
Peak VAF dens= 0.51
Density
0.0
0.5
1.0
1.5
2.0
2.5
0.0
0.2
0.4
0.6
0.8
1.0
N = 895 Bandwidth = 0.0343

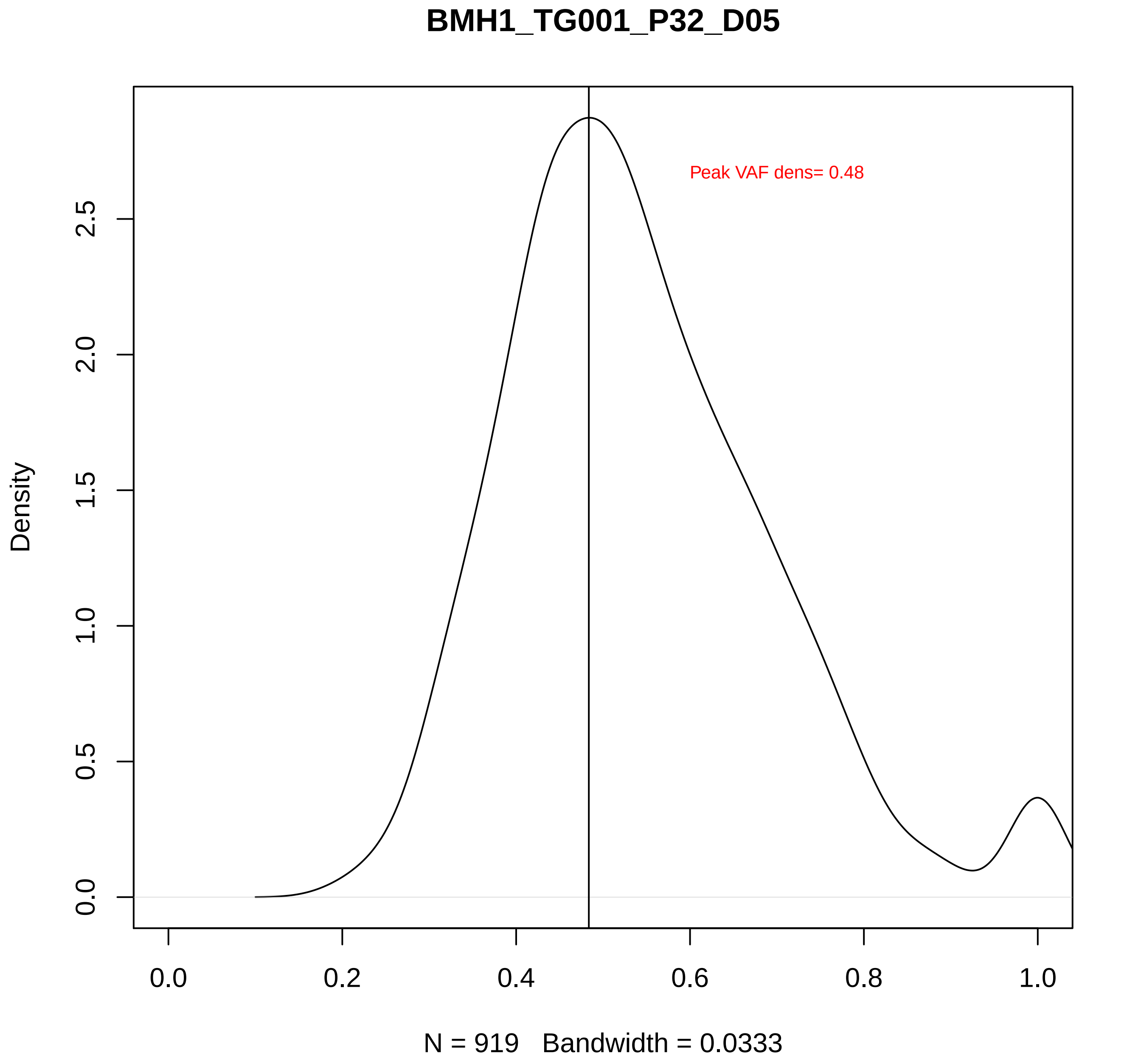

BMH1_TG001_P32_D05
Peak VAF dens= 0.48
Density
0.0
0.5
1.0
1.5
2.0
2.5
0.0
0.2
0.4
0.6
0.8
1.0
N = 919   Bandwidth = 0.0333

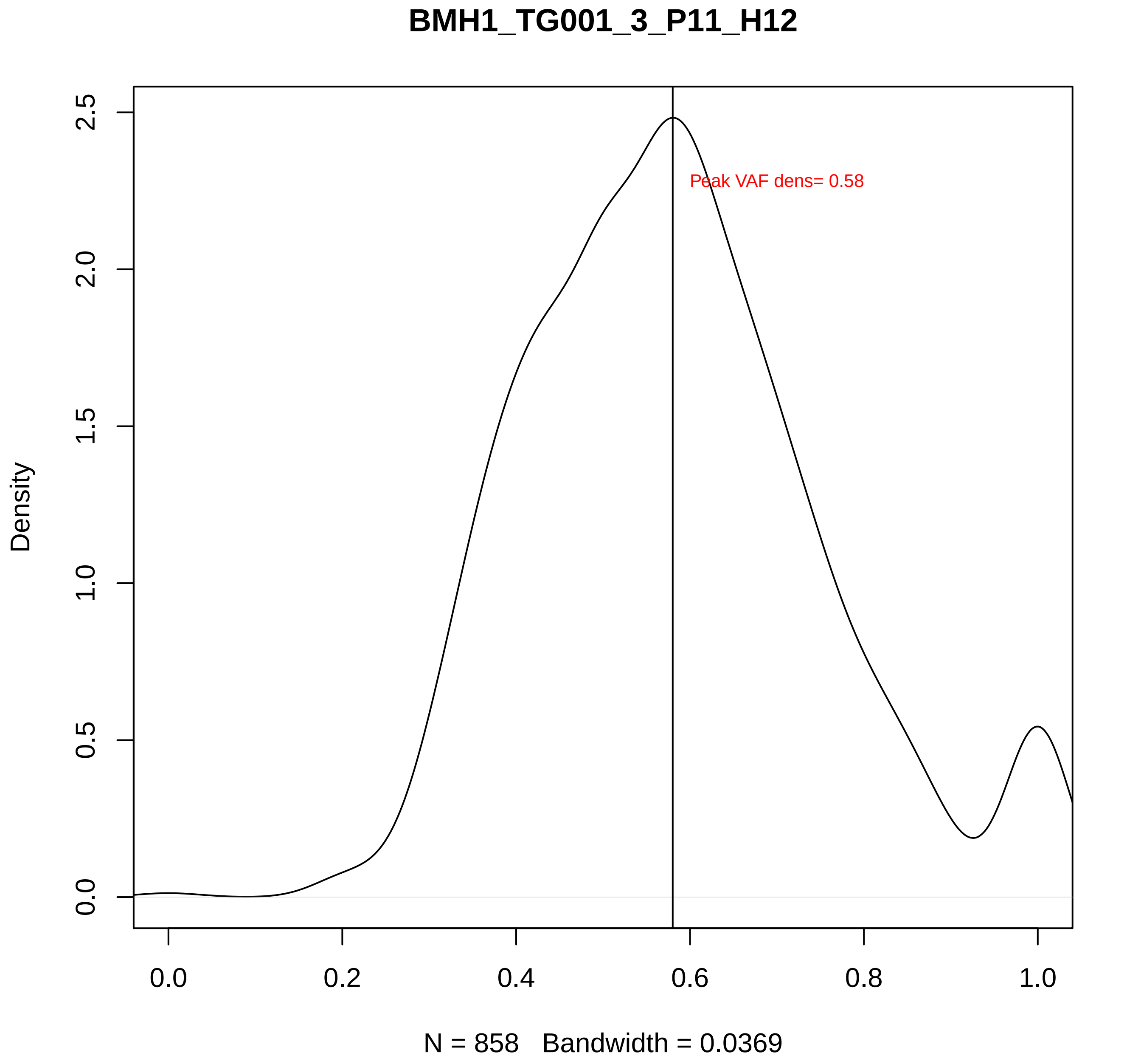
BMH1_TG001_3_P11_H12
Peak VAF dens= 0.58
Density
0.0
0.5
1.0
1.5
2.0
2.5
0.0
0.2
0.4
0.6
0.8
1.0
N = 858   Bandwidth = 0.0369

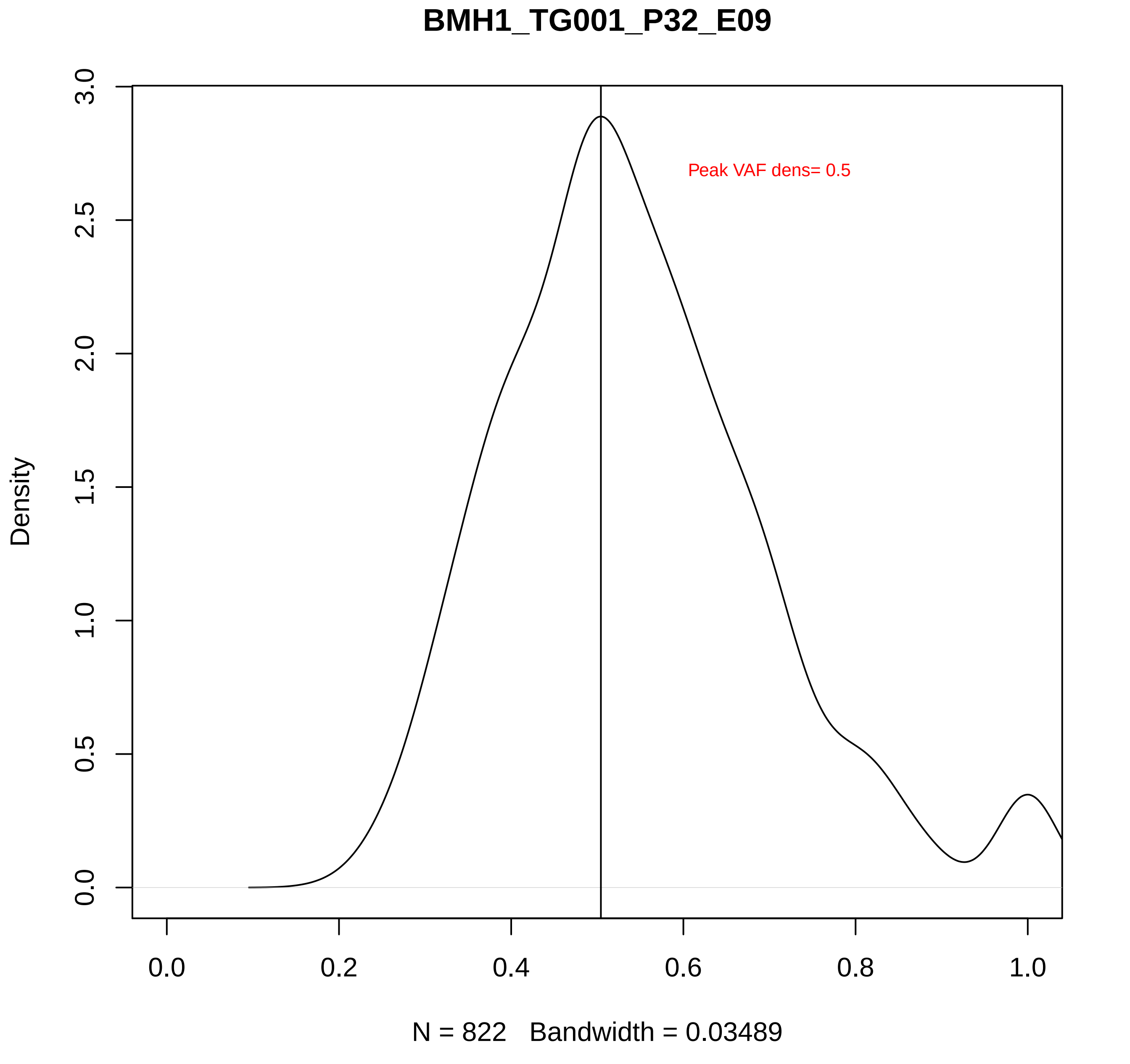
BMH1_TG001_P32_E09
Peak VAF dens= 0.5
Density
0.0
0.5
1.0
1.5
2.0
2.5
3.0
0.0
0.2
0.4
0.6
0.8
1.0
N = 822   Bandwidth = 0.03489

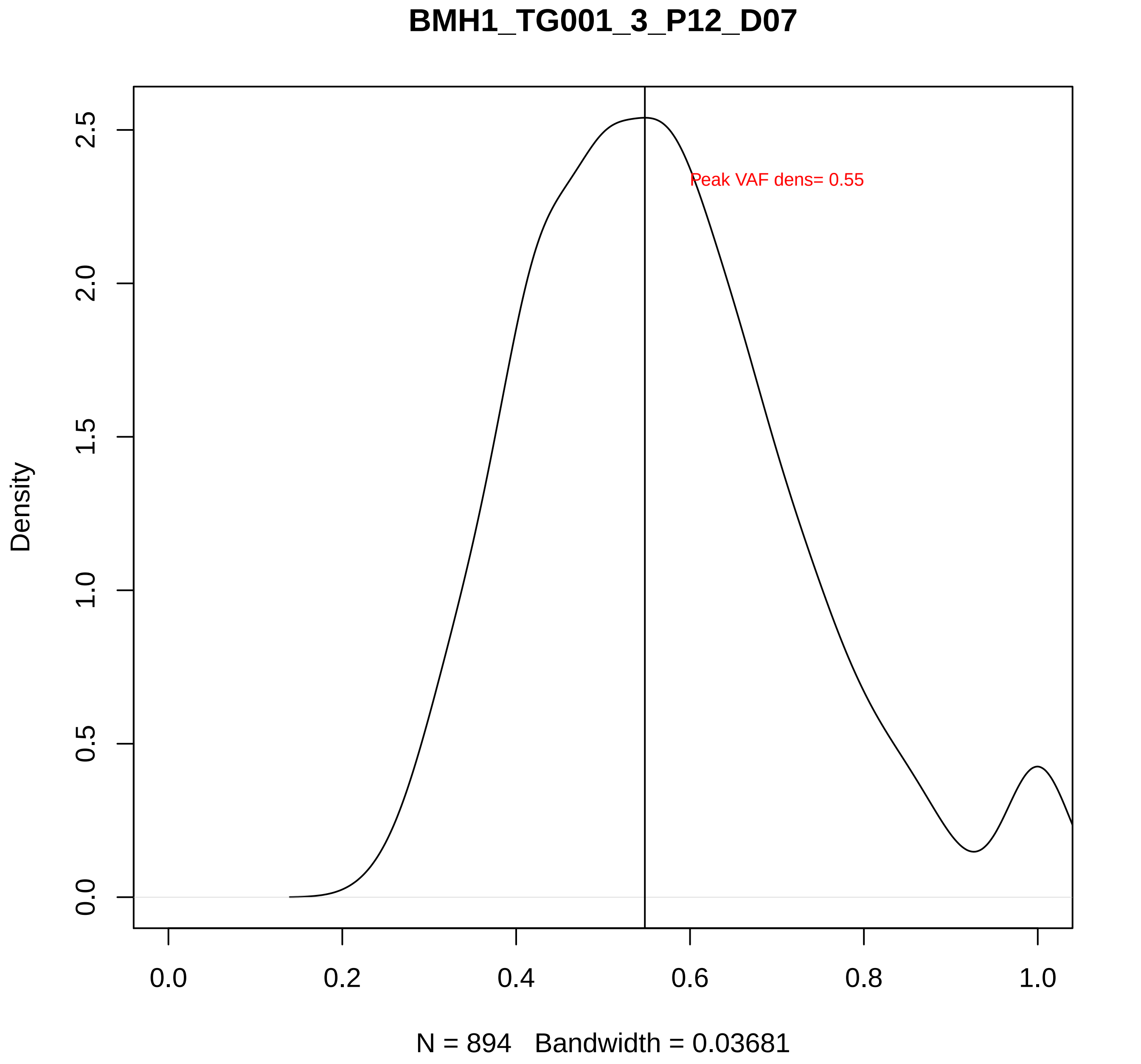
BMH1_TG001_3_P12_D07
Peak VAF dens= 0.55
Density
0.0
0.5
1.0
1.5
2.0
2.5
0.0
0.2
0.4
0.6
0.8
1.0
N = 894   Bandwidth = 0.03681
