# Supplementary material for: Clonal dynamics of haematopoiesis across the human lifespan
Source: Nature. 2022 Jun 1;606(7913):343–50. doi: 10.1038/s41586-022-04786-y (PMC9177428; doi:10.1038/s41586-022-04786-y)
Supplement: Supplementary file 4 — HTMLs of notebooks outlining key statistical analyses presented in the manuscript, including analysis of phylogenetic trees. [file 41586_2022_4786_MOESM4_ESM.zip › Supplementary_code/SNV_indel_analysis/CB002_sample_vaf_plots.pdf]

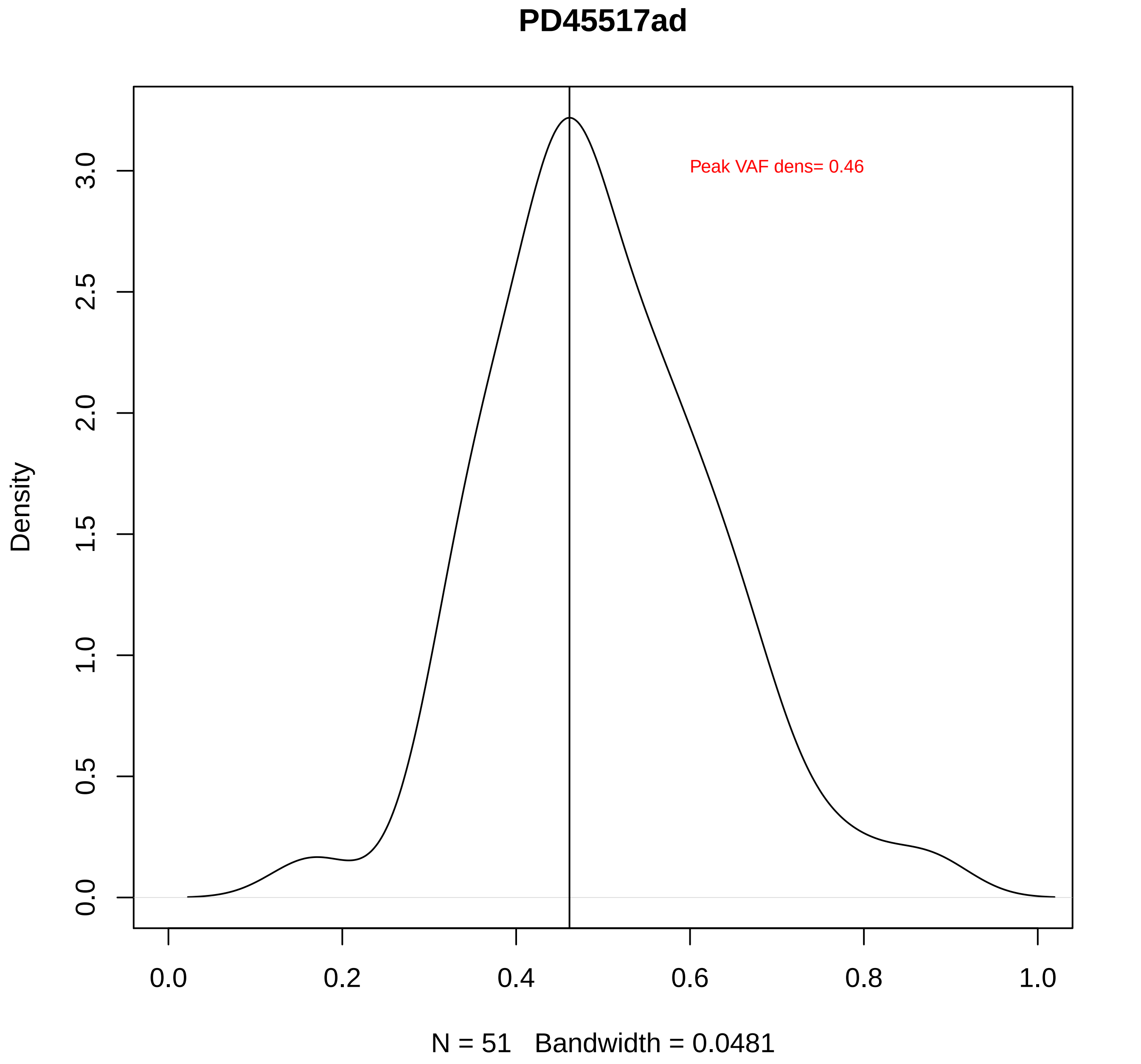
PD45517ad
Peak VAF dens= 0.46
Density
0.0
0.5
1.0
1.5
2.0
2.5
3.0
0.0
0.2
0.4
0.6
0.8
1.0
N = 51   Bandwidth = 0.0481

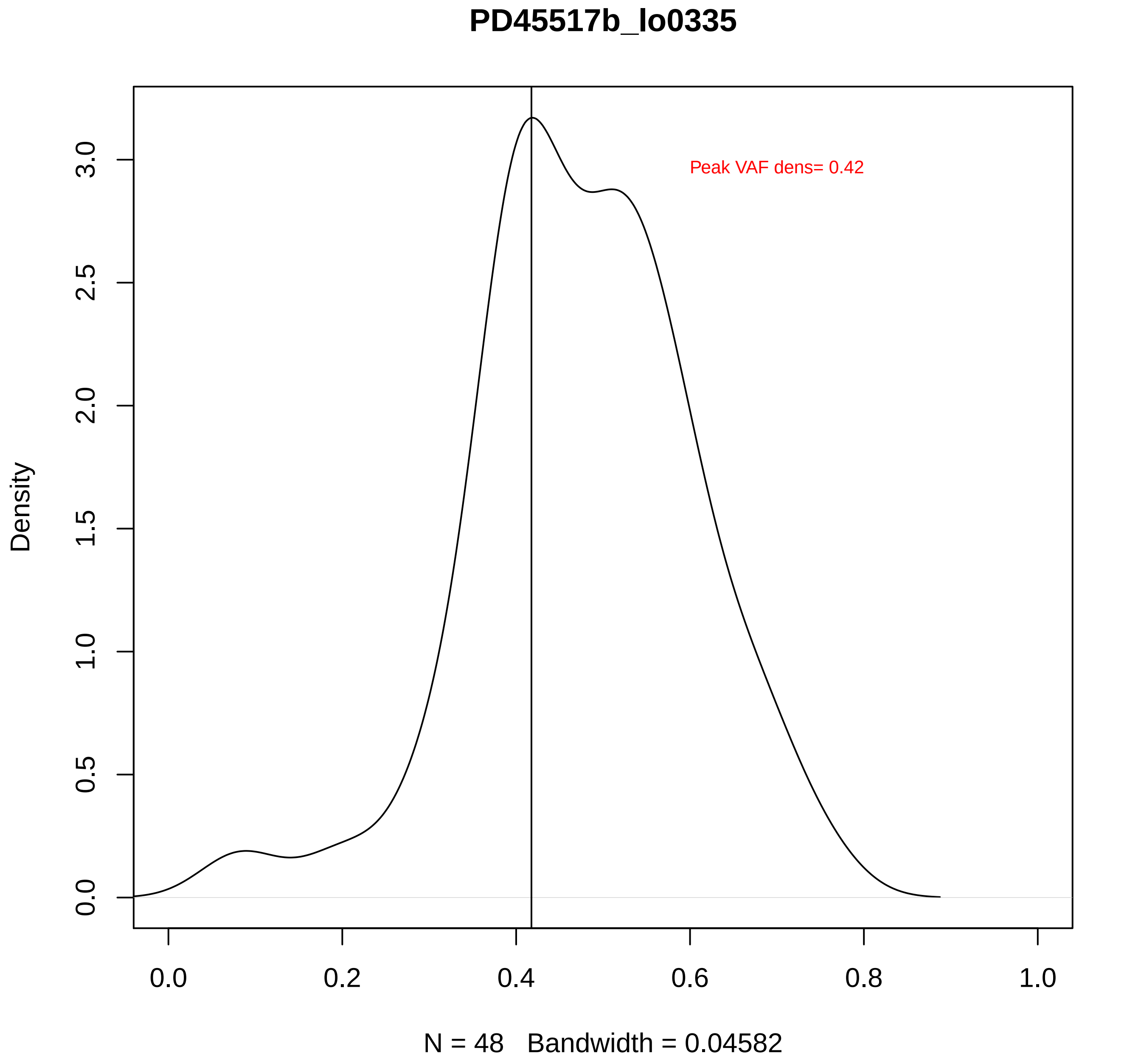
PD45517b_lo0335
Peak VAF dens= 0.42
Density
0.0
0.5
1.0
1.5
2.0
2.5
3.0
0.0
0.2
0.4
0.6
0.8
1.0
N = 48   Bandwidth = 0.04582

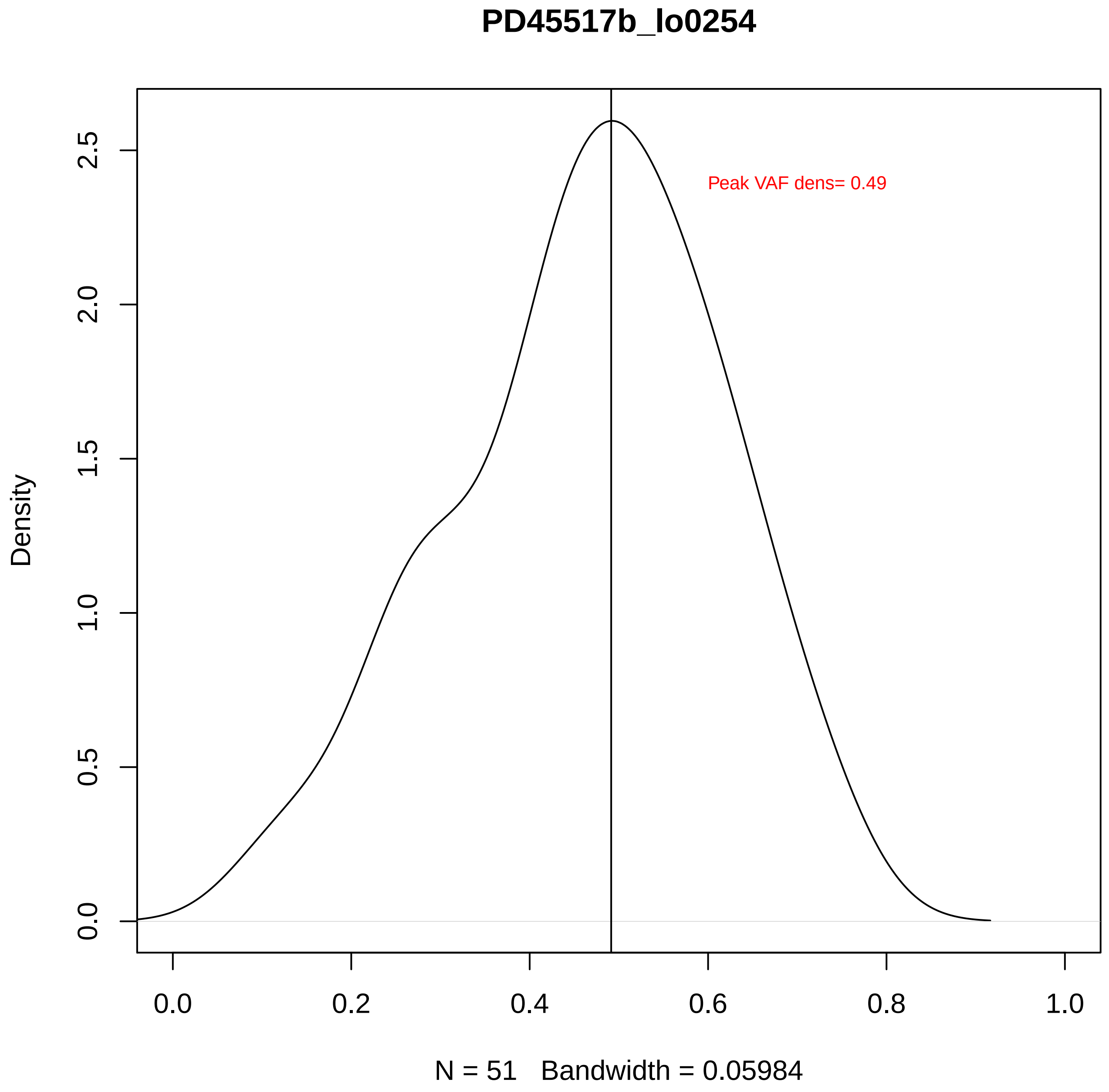
PD45517b_lo0254
Peak VAF dens= 0.49
Density
0.0
0.5
1.0
1.5
2.0
2.5
0.0
0.2
0.4
0.6
0.8
1.0
N = 51   Bandwidth = 0.05984

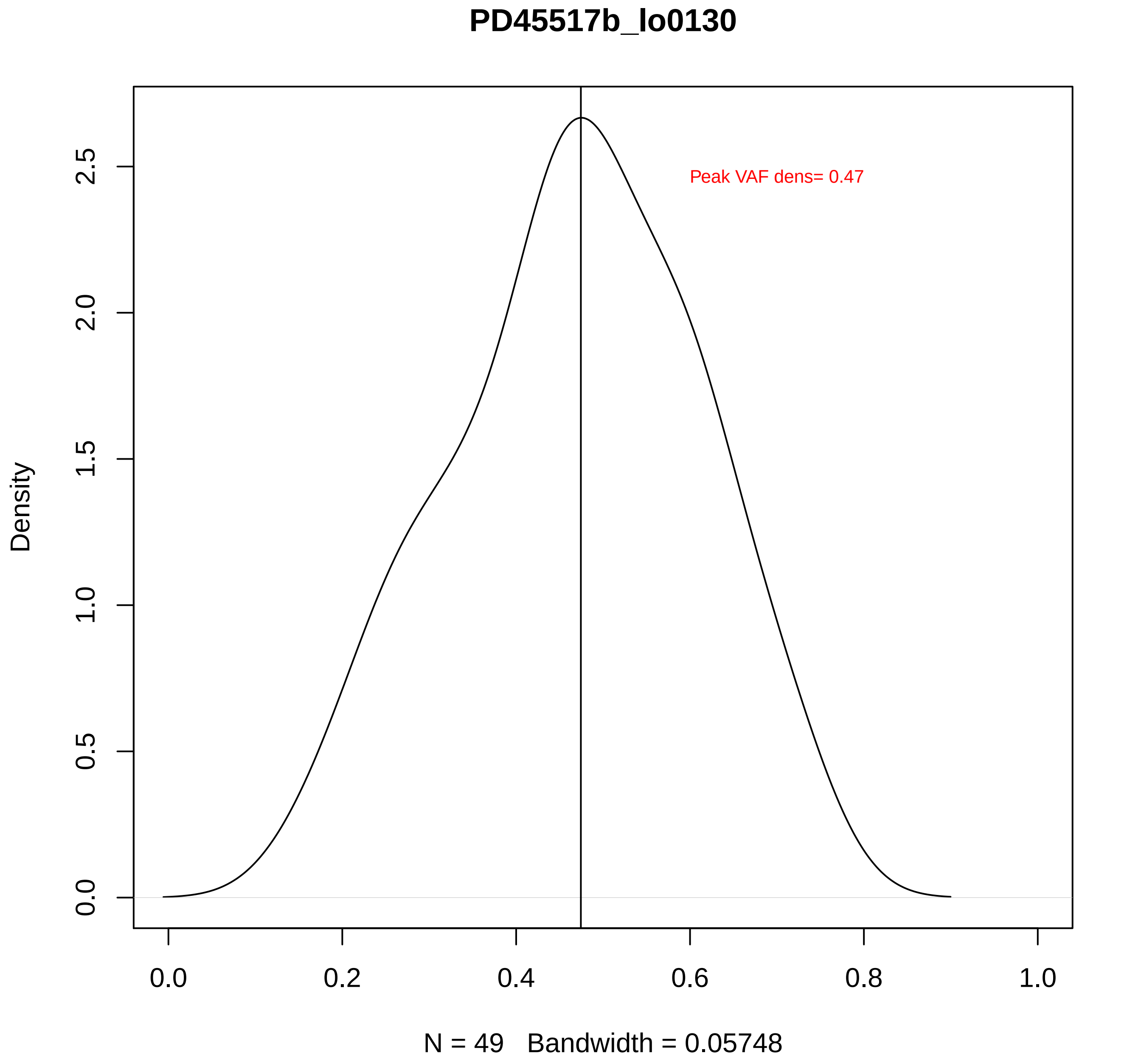
PD45517b_lo0130
Peak VAF dens= 0.47
Density
0.0
0.5
1.0
1.5
2.0
2.5
0.0
0.2
0.4
0.6
0.8
1.0
N = 49   Bandwidth = 0.05748

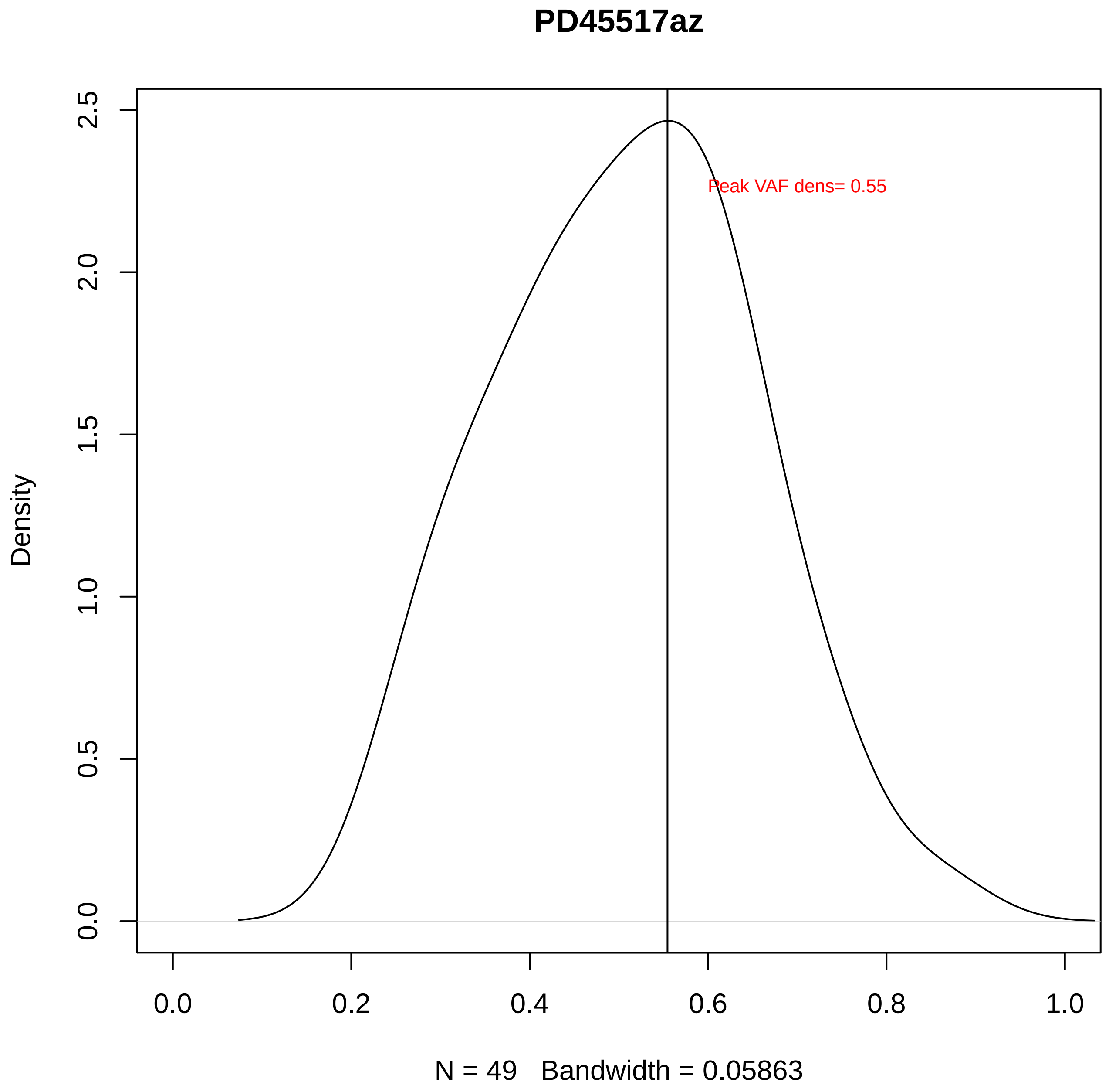
PD45517az
Peak VAF dens= 0.55
Density
0.0
0.5
1.0
1.5
2.0
2.5
0.0
0.2
0.4
0.6
0.8
1.0
N = 49 Bandwidth = 0.05863

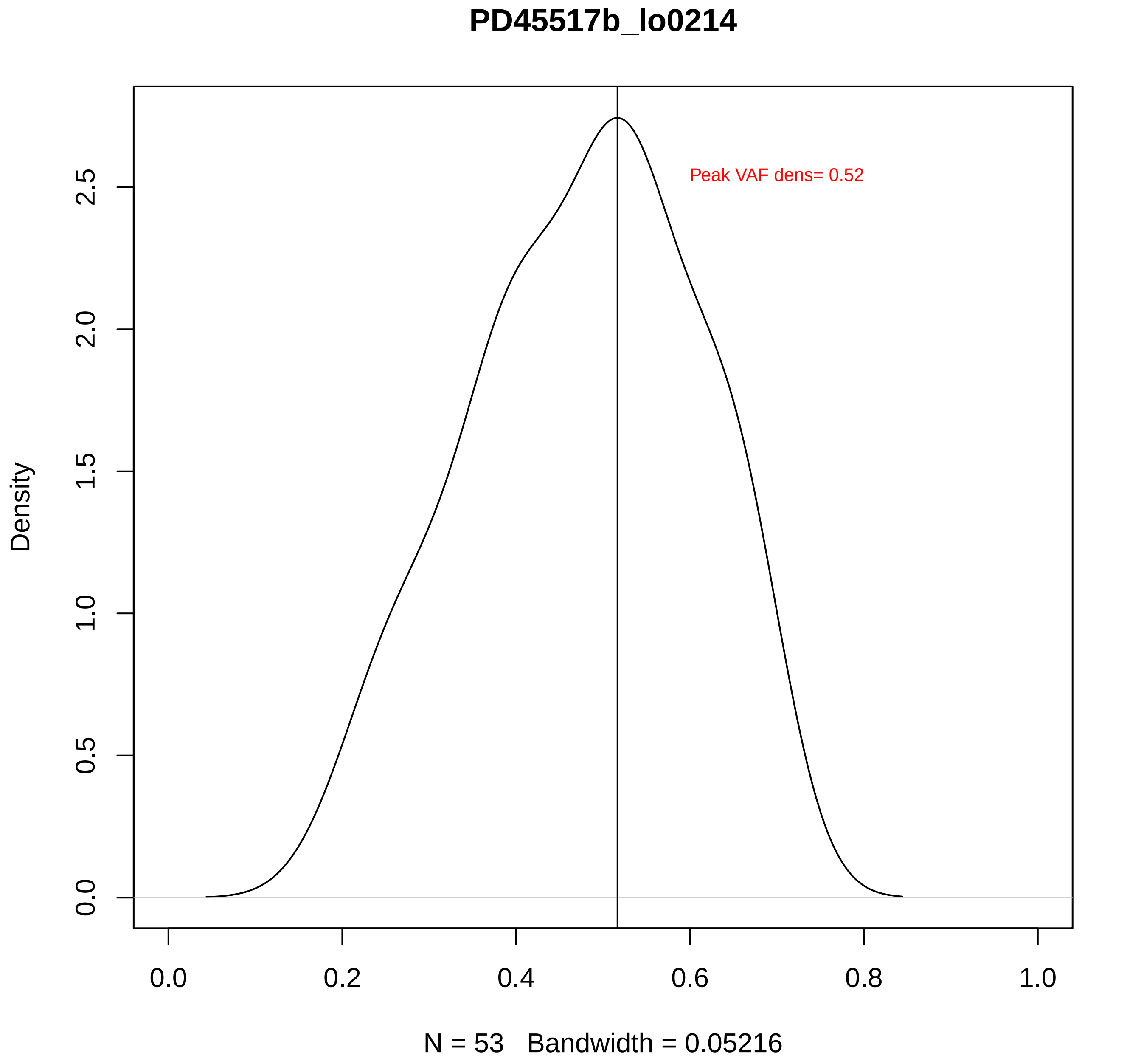

PD45517b_lo0214
Peak VAF dens= 0.52
Density
0.0
0.5
1.0
1.5
2.0
2.5
0.0
0.2
0.4
0.6
0.8
1.0
N = 53 Bandwidth = 0.05216

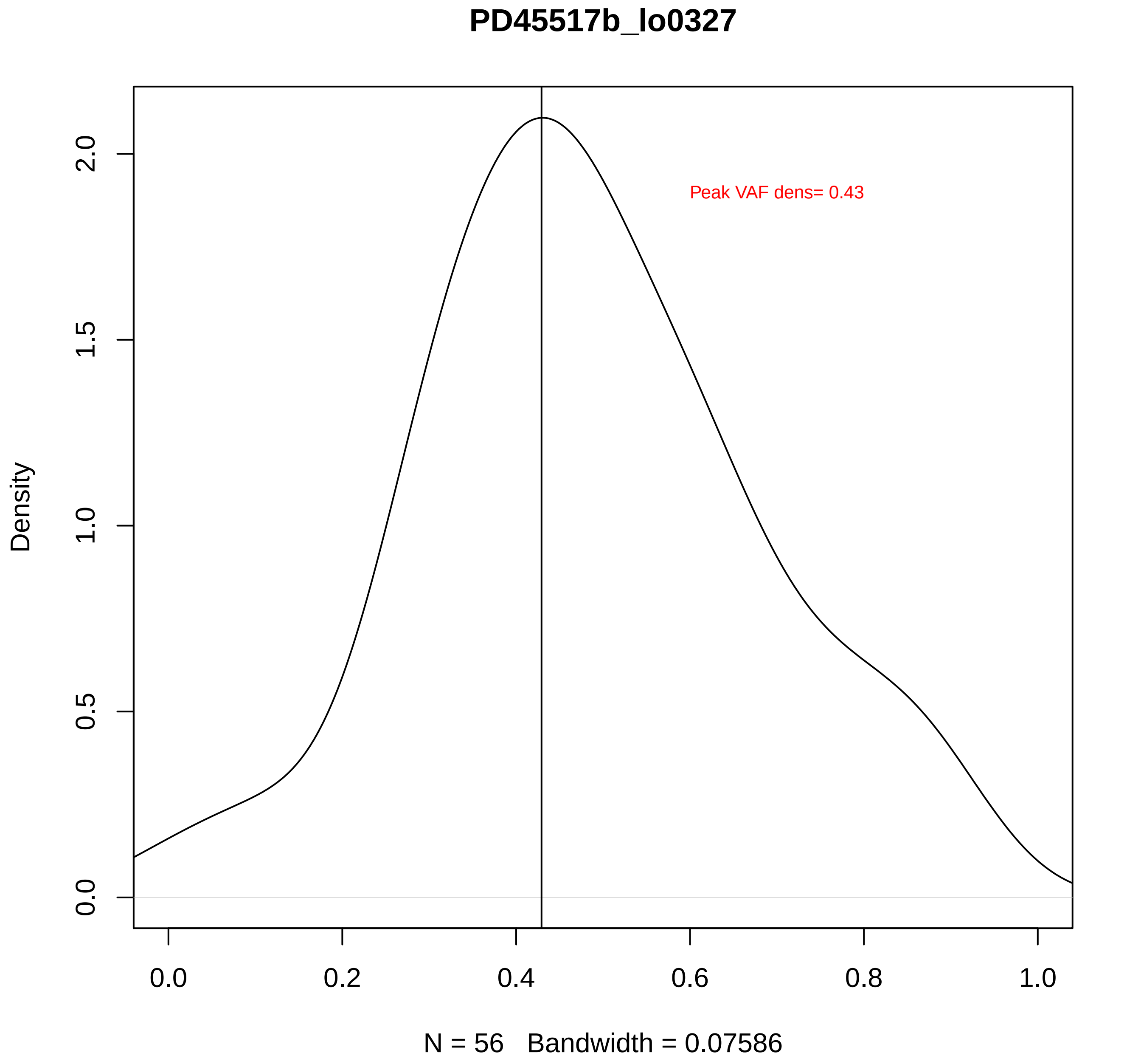
PD45517b_lo0327
Peak VAF dens= 0.43
Density
0.0
0.5
1.0
1.5
2.0
0.0
0.2
0.4
0.6
0.8
1.0
N = 56   Bandwidth = 0.07586

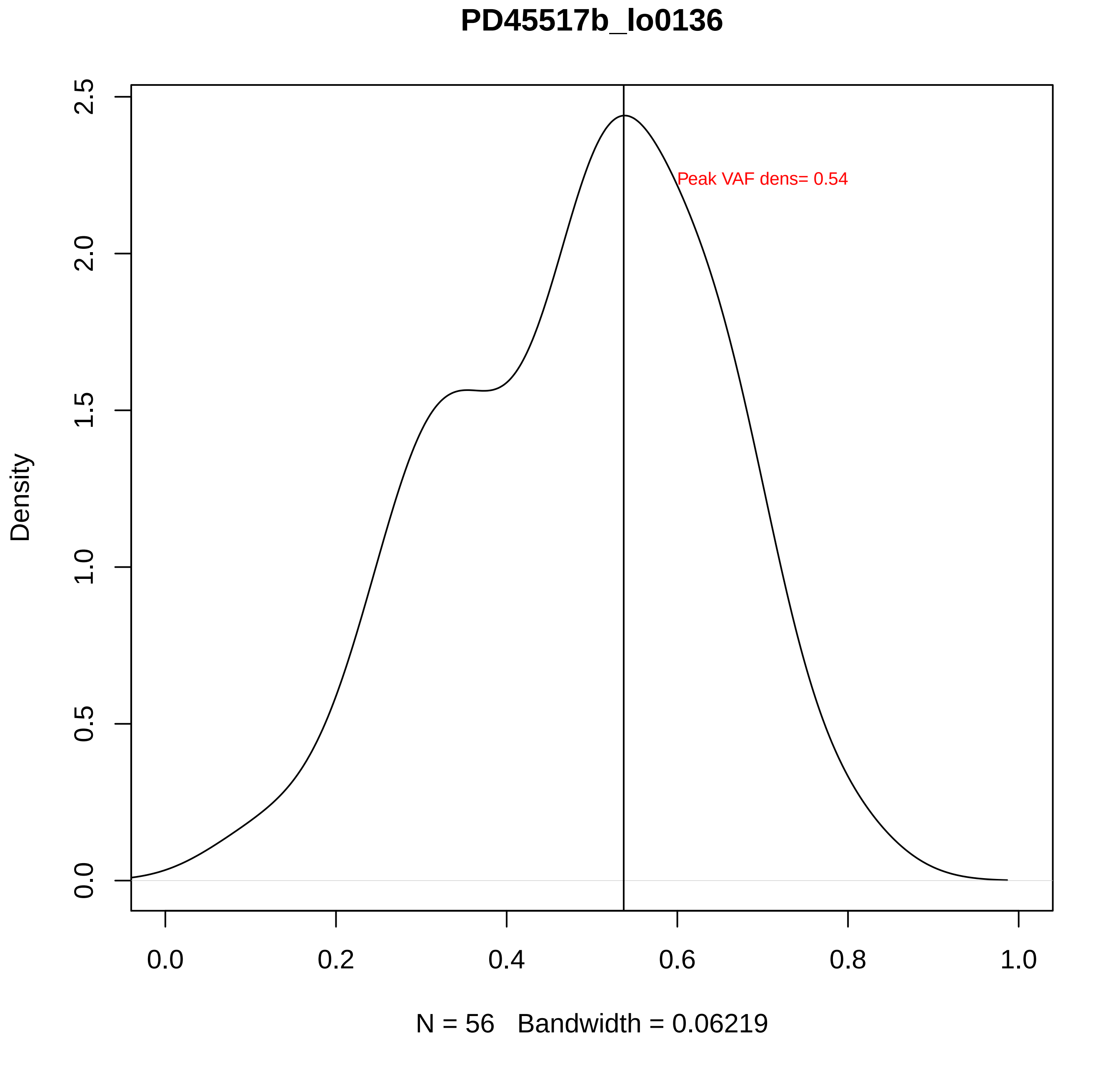
PD45517b_lo0136
Peak VAF dens= 0.54
Density
0.0
0.5
1.0
1.5
2.0
2.5
0.0
0.2
0.4
0.6
0.8
1.0
N = 56   Bandwidth = 0.06219

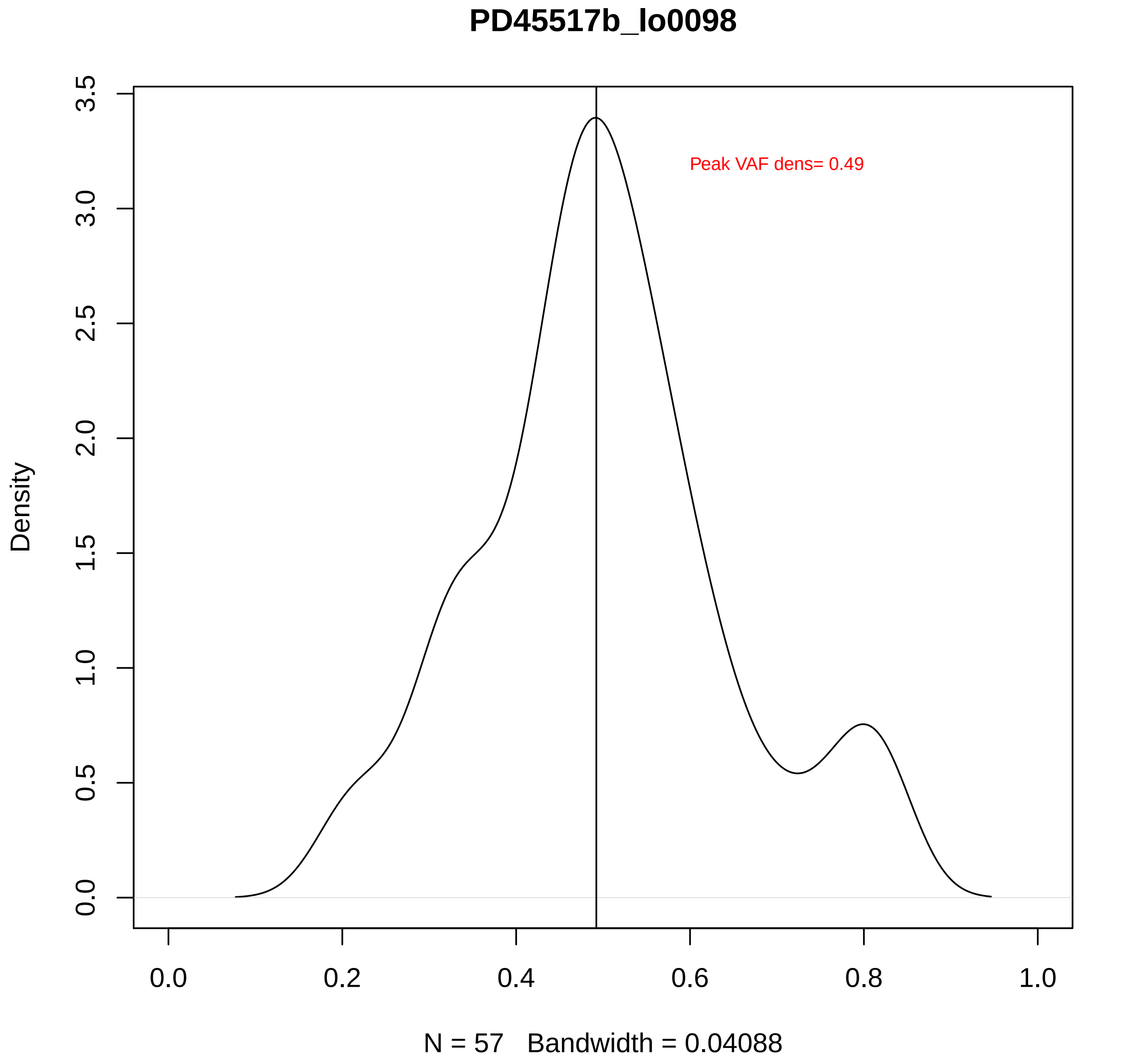
PD45517b_lo0098
Peak VAF dens= 0.49
Density
0.0
0.5
1.0
1.5
2.0
2.5
3.0
3.5
0.0
0.2
0.4
0.6
0.8
1.0
N = 57 Bandwidth = 0.04088

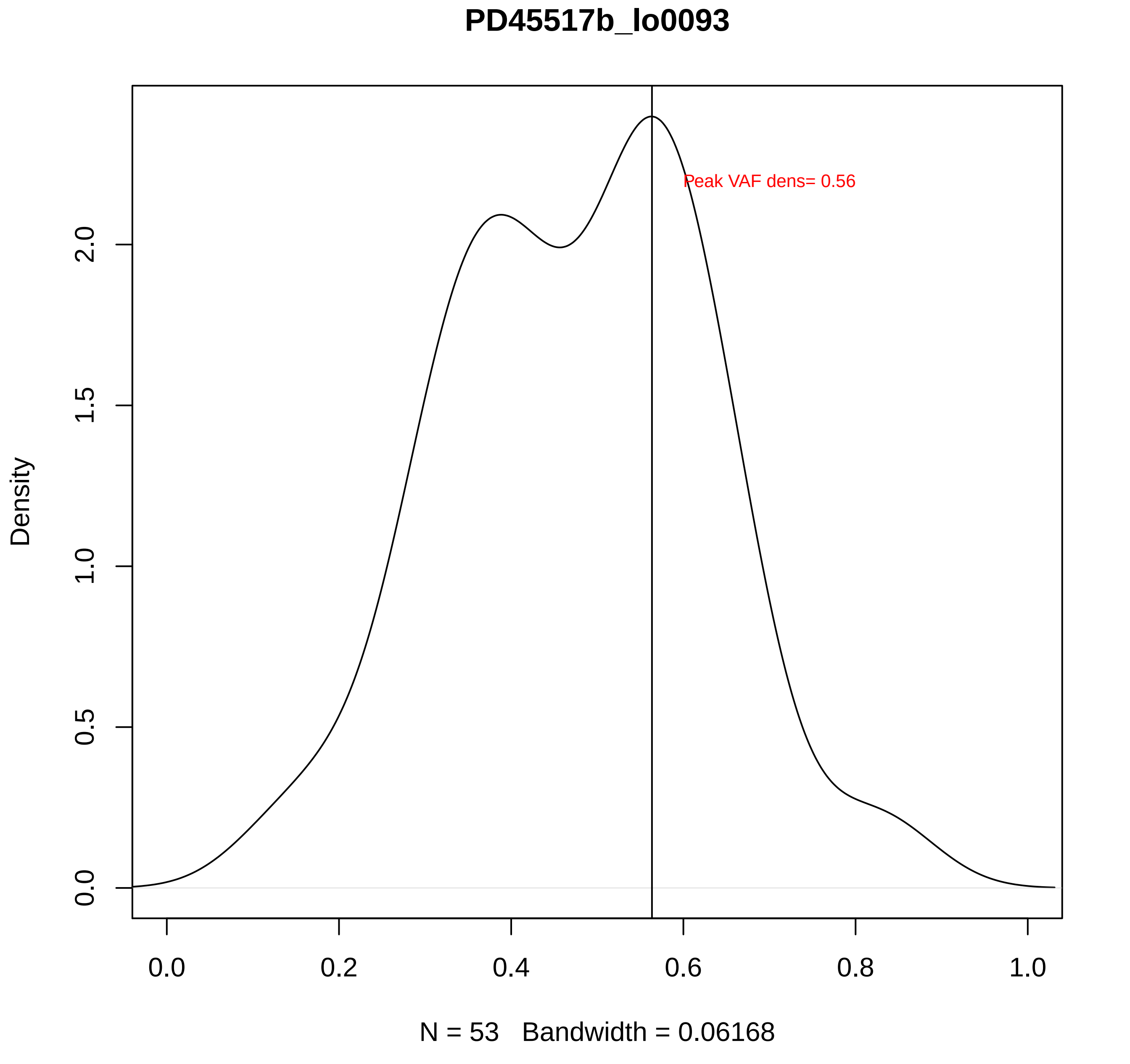
PD45517b_lo0093
Peak VAF dens= 0.56
Density
0.0
0.5
1.0
1.5
2.0
0.0
0.2
0.4
0.6
0.8
1.0
N = 53 Bandwidth = 0.06168

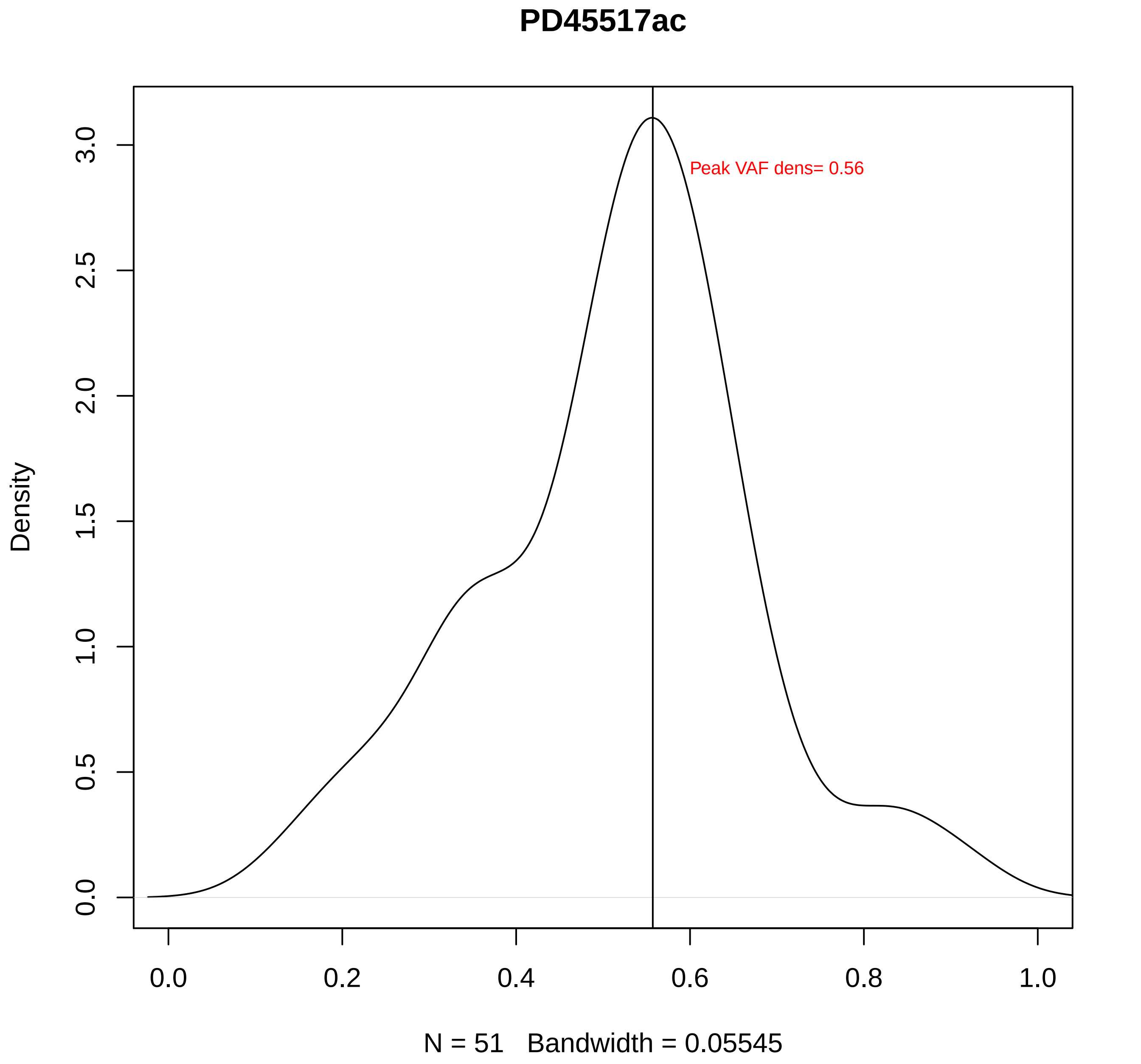
PD45517ac
Peak VAF dens= 0.56
Density
0.0
0.5
1.0
1.5
2.0
2.5
3.0
0.0
0.2
0.4
0.6
0.8
1.0
N = 51   Bandwidth = 0.05545

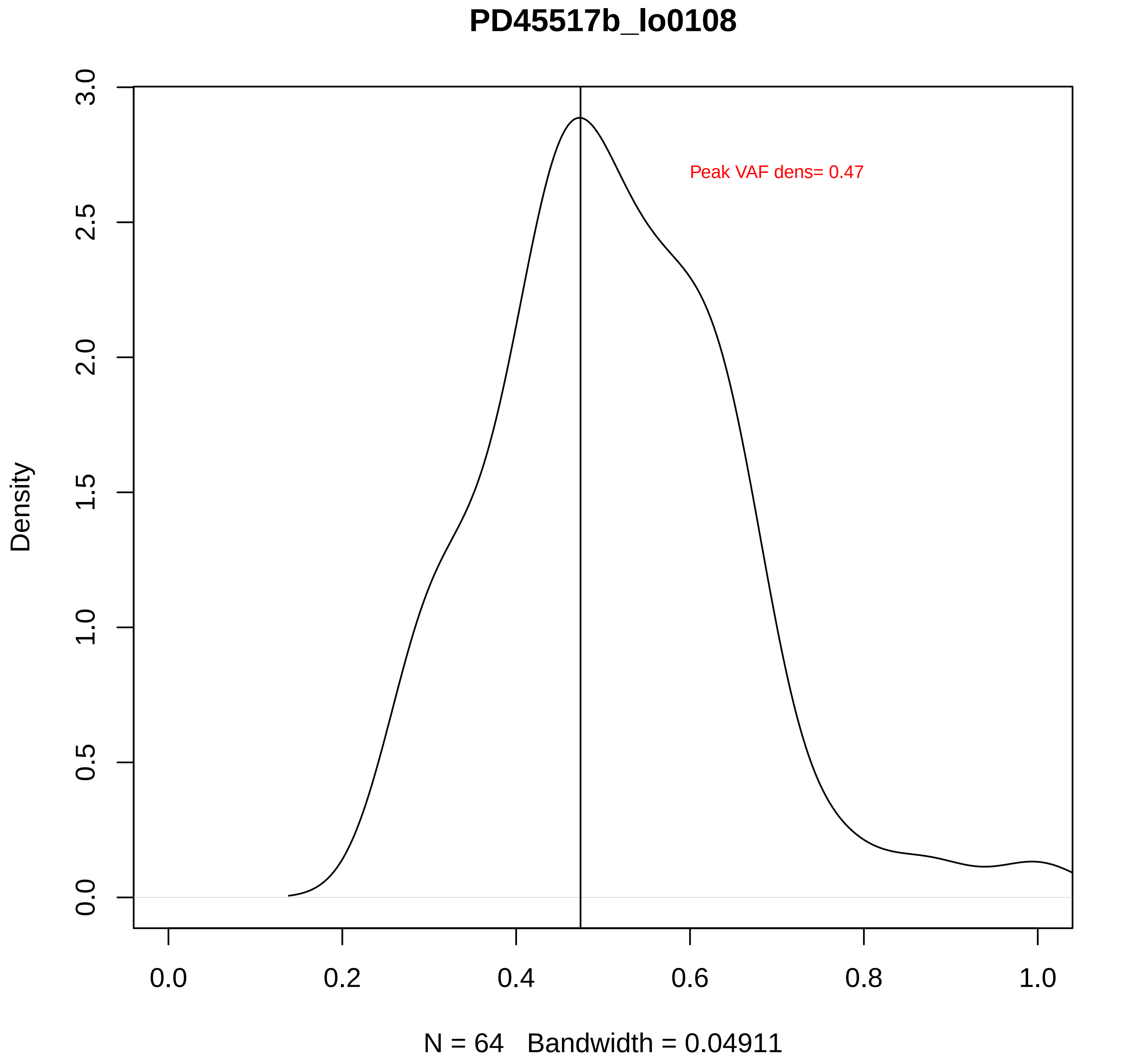

PD45517b_lo0108
Peak VAF dens= 0.47
Density
0.0
0.5
1.0
1.5
2.0
2.5
3.0
0.0
0.2
0.4
0.6
0.8
1.0
N = 64   Bandwidth = 0.04911

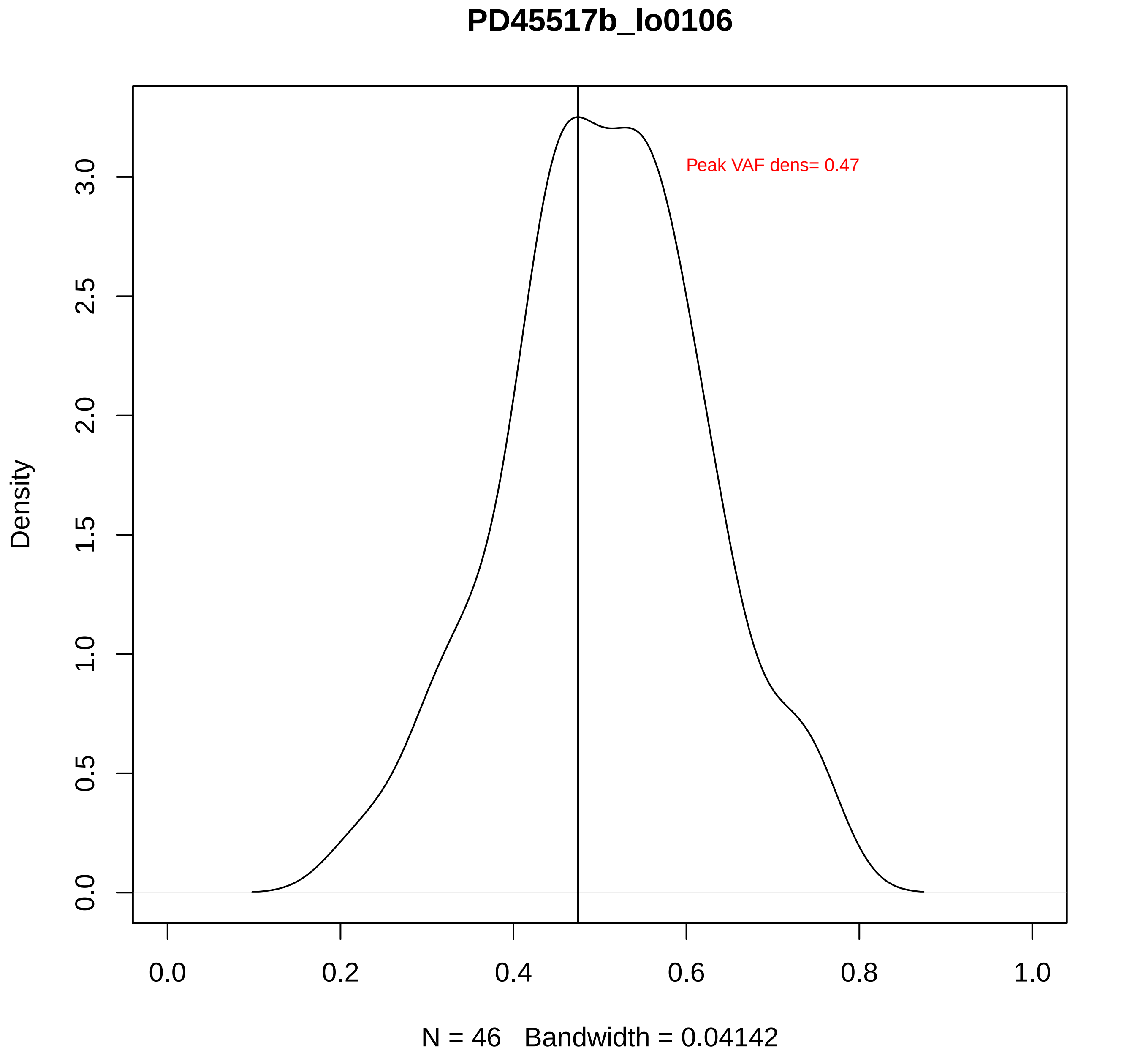
PD45517b_lo0106
Peak VAF dens= 0.47
Density
0.0
0.5
1.0
1.5
2.0
2.5
3.0
0.0
0.2
0.4
0.6
0.8
1.0
N = 46   Bandwidth = 0.04142

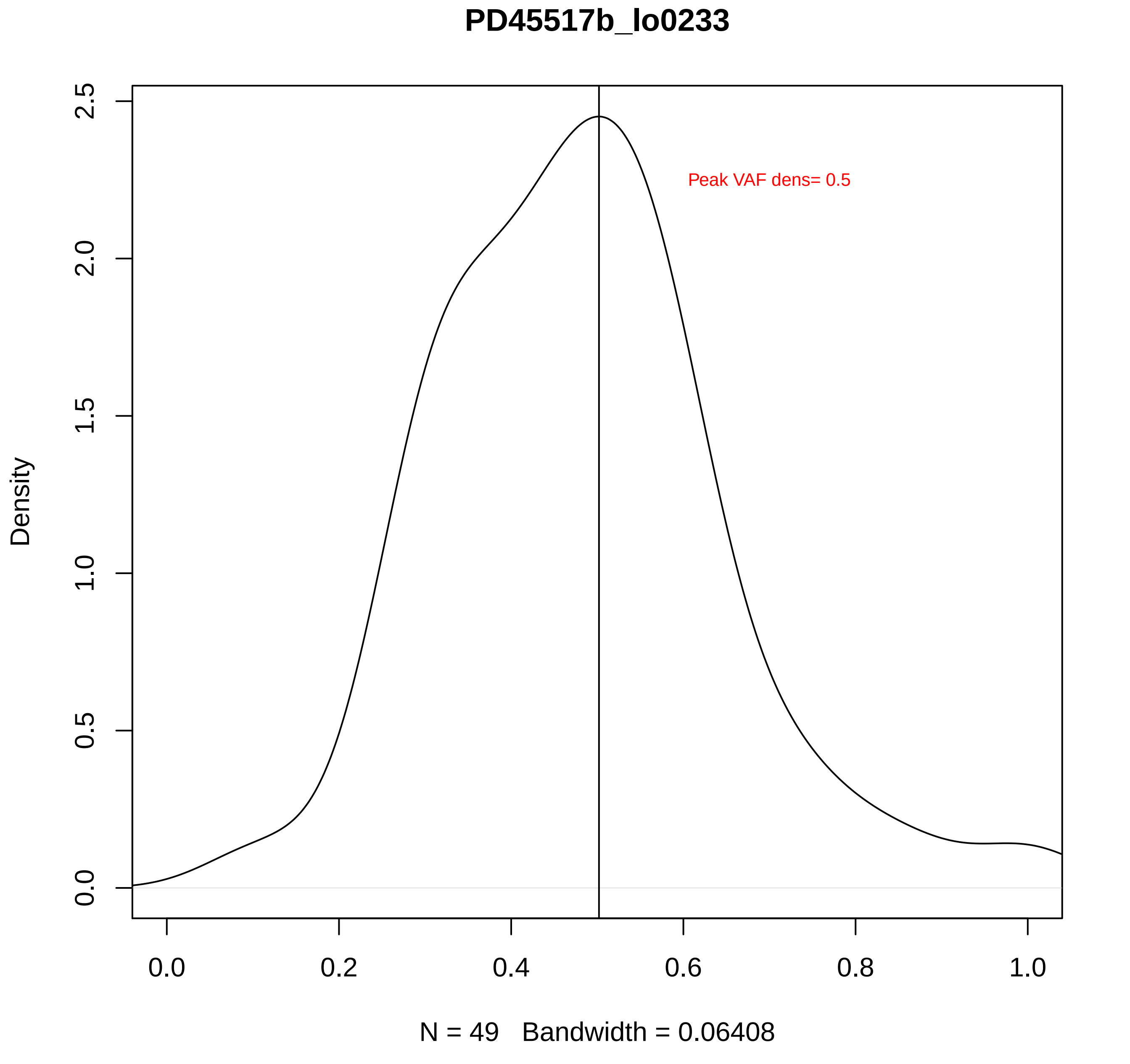

PD45517b_lo0233
Peak VAF dens= 0.5
Density
0.0
0.5
1.0
1.5
2.0
2.5
0.0
0.2
0.4
0.6
0.8
1.0
N = 49 Bandwidth = 0.06408

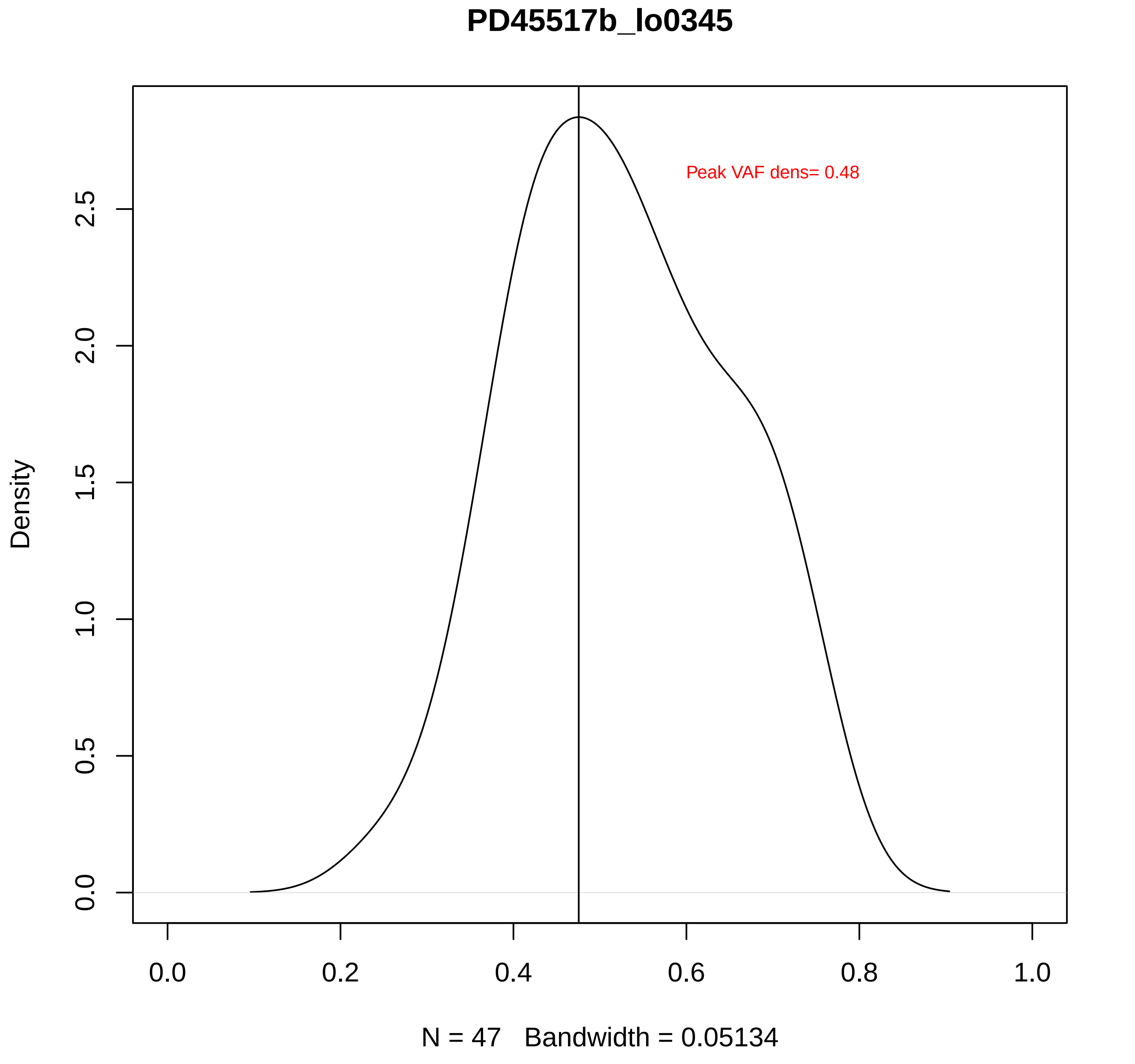
PD45517b_lo0345
Peak VAF dens= 0.48
Density
0.0
0.5
1.0
1.5
2.0
2.5
0.0
0.2
0.4
0.6
0.8
1.0
N = 47   Bandwidth = 0.05134

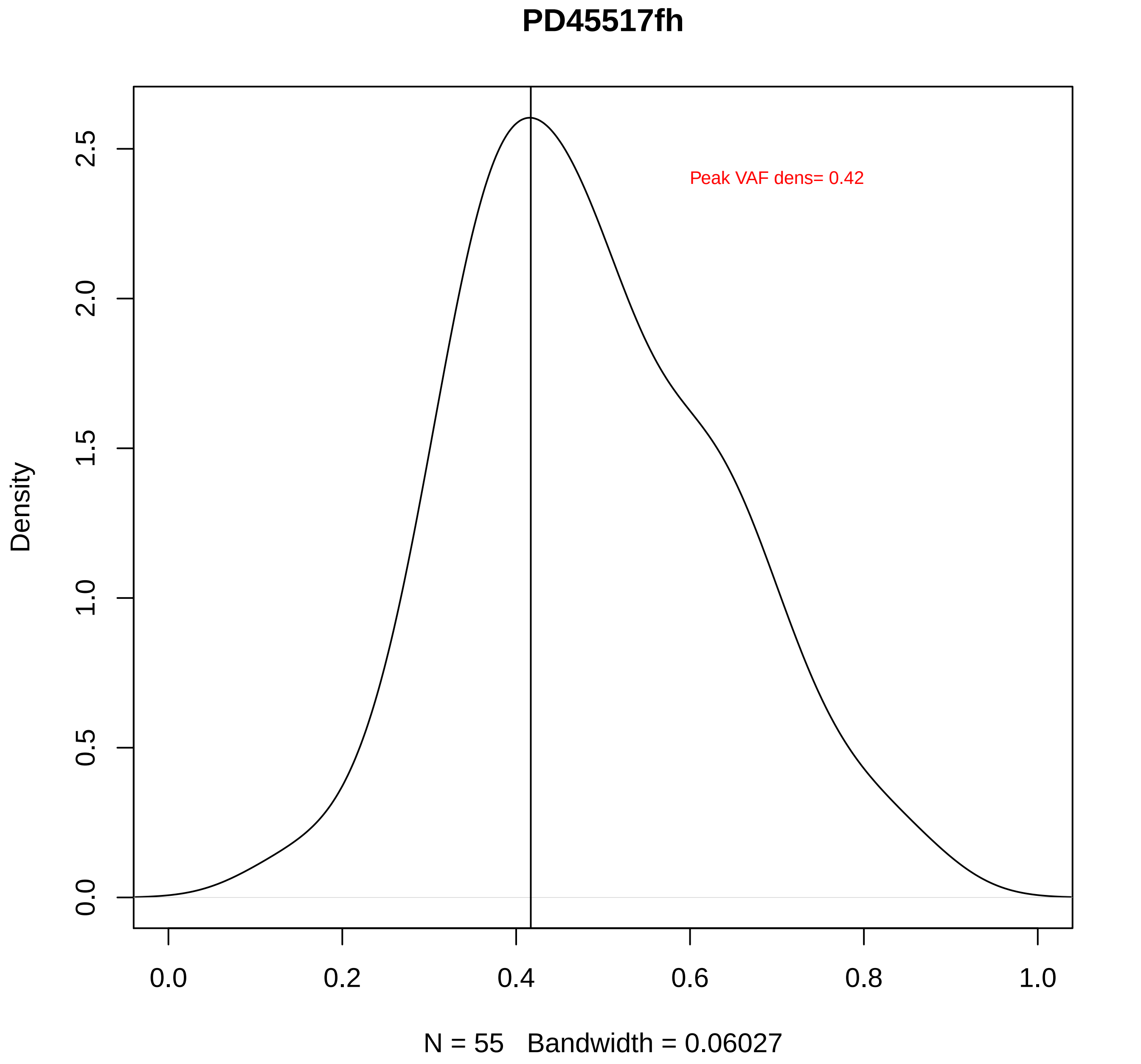
PD45517fh
Peak VAF dens= 0.42
Density
0.0
0.5
1.0
1.5
2.0
2.5
0.0
0.2
0.4
0.6
0.8
1.0
N = 55   Bandwidth = 0.06027

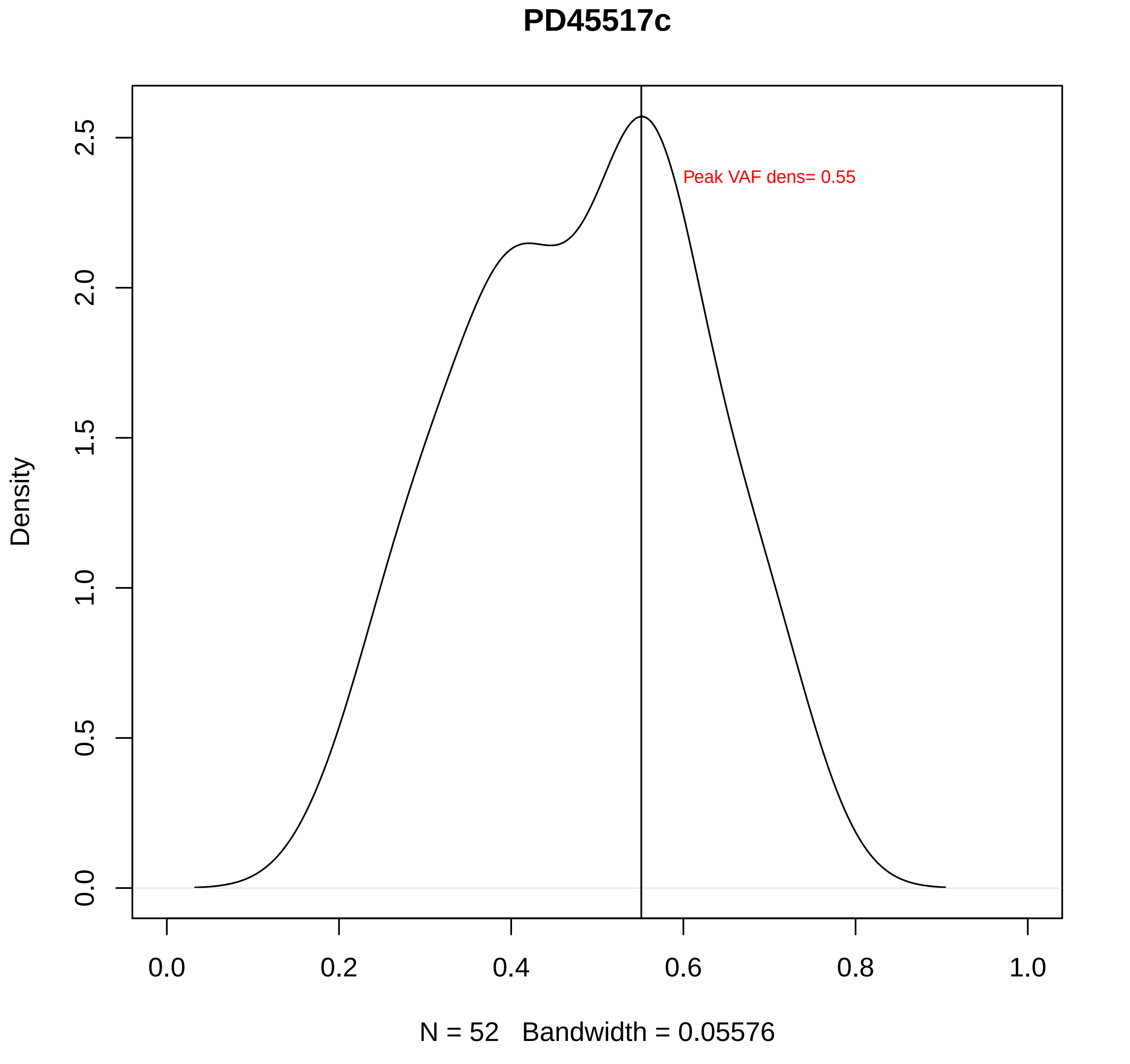
PD45517c
Peak VAF dens= 0.55
Density
0.0
0.5
1.0
1.5
2.0
2.5
0.0
0.2
0.4
0.6
0.8
1.0
N = 52   Bandwidth = 0.05576

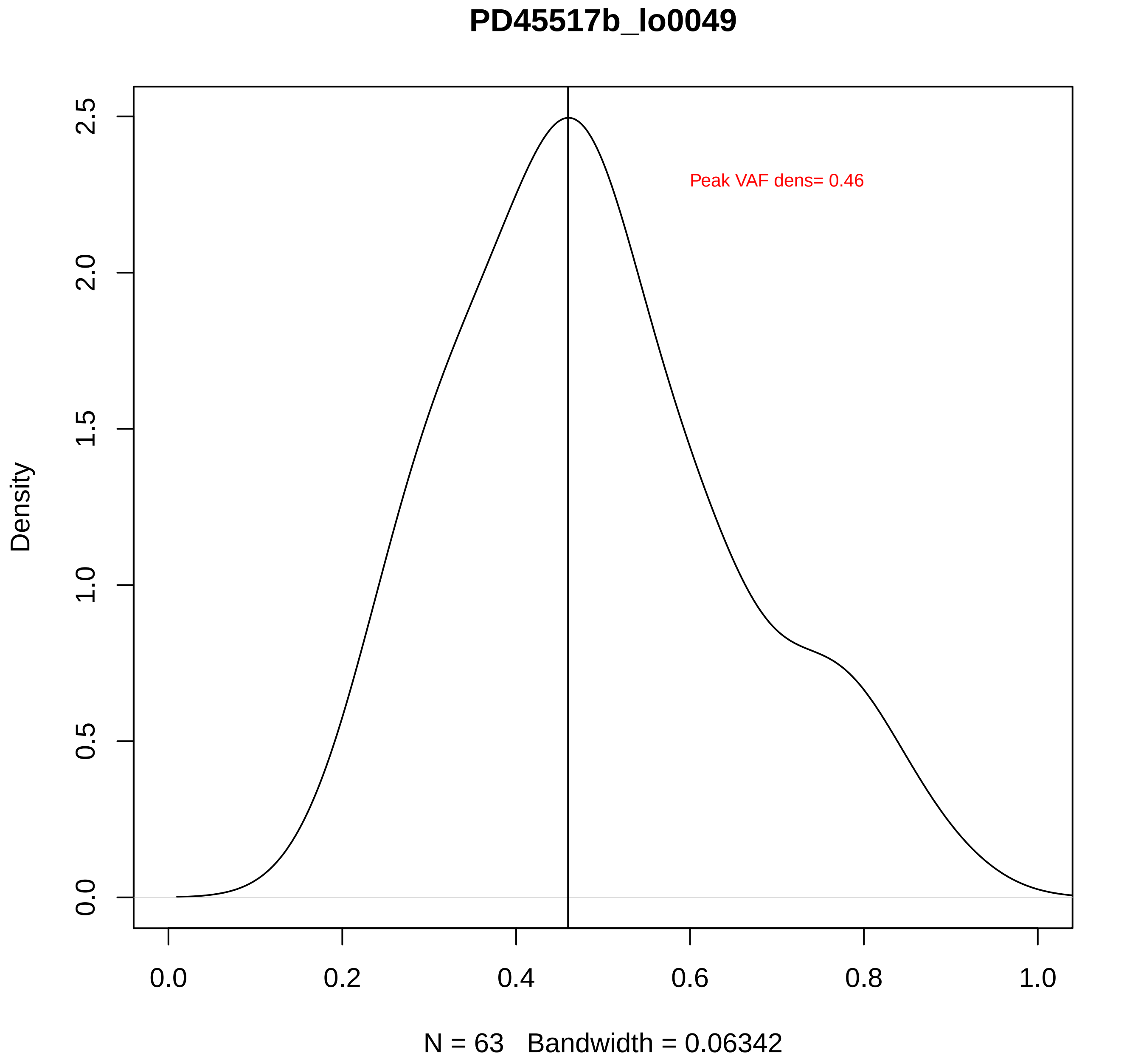
PD45517b_lo0049
Peak VAF dens= 0.46
Density
0.0
0.5
1.0
1.5
2.0
2.5
0.0
0.2
0.4
0.6
0.8
1.0
N = 63   Bandwidth = 0.06342

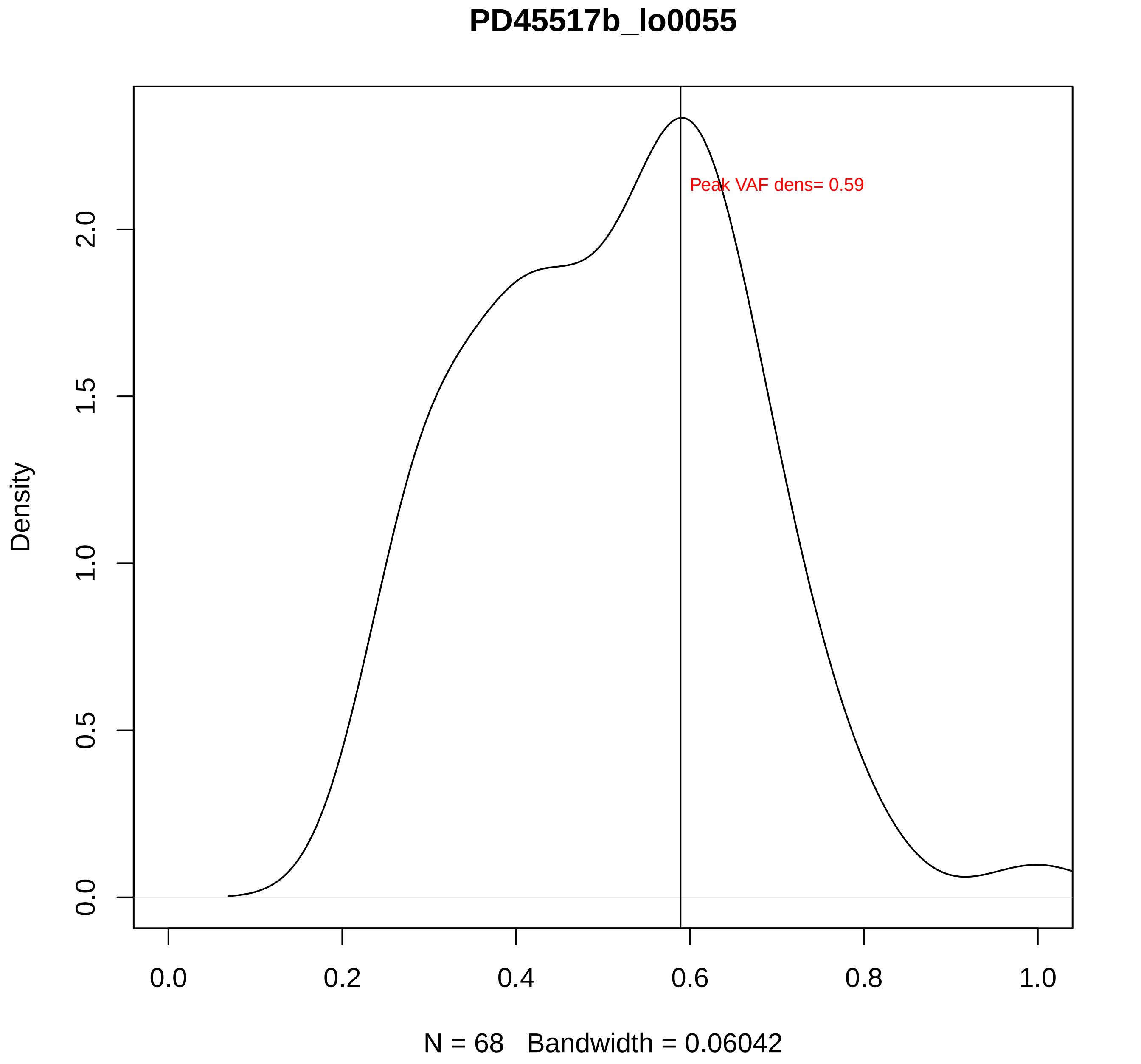
PD45517b_lo0055
Peak VAF dens= 0.59
Density
0.0
0.5
1.0
1.5
2.0
0.0
0.2
0.4
0.6
0.8
1.0
N = 68   Bandwidth = 0.06042

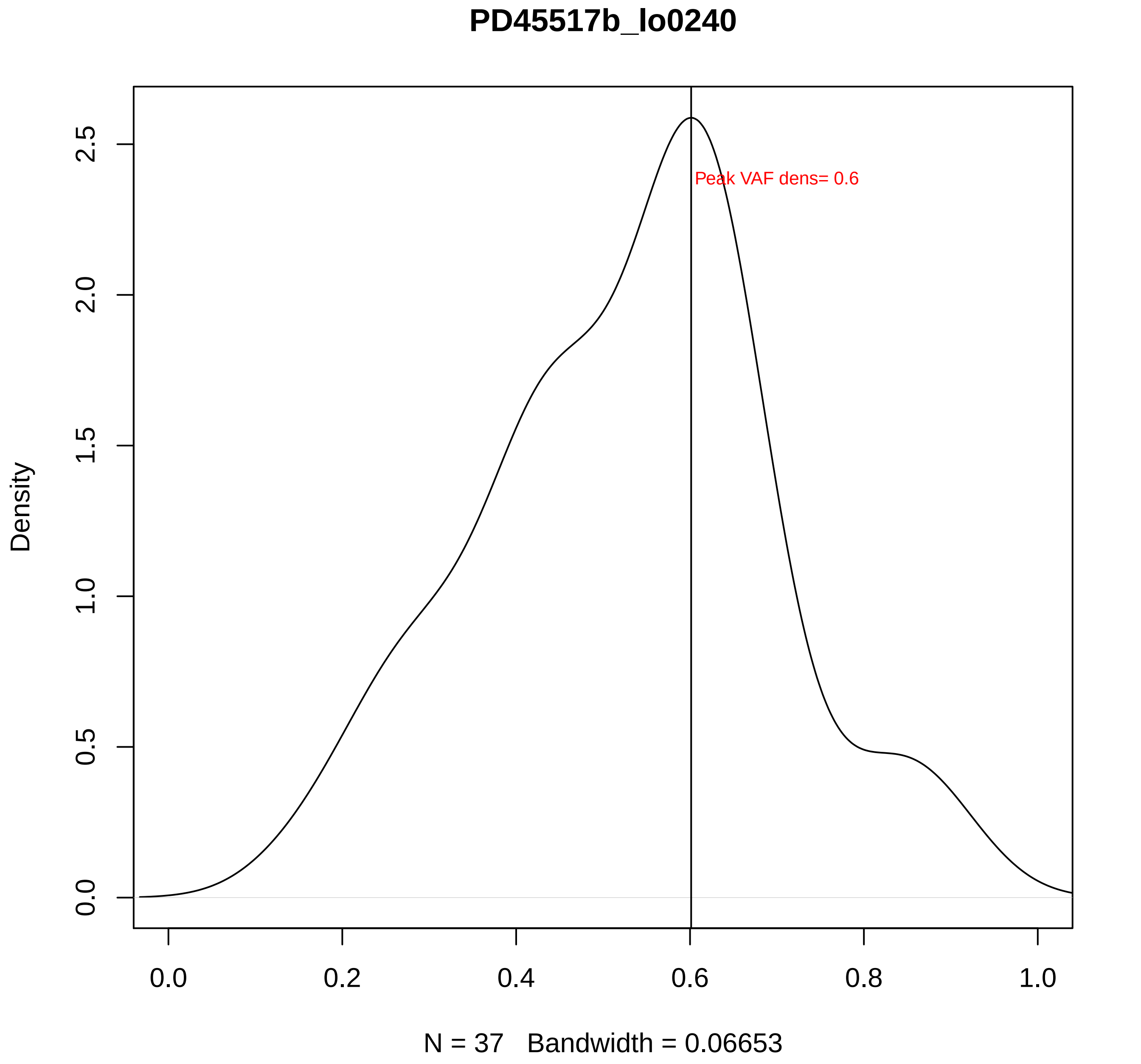

PD45517b_lo0240
Peak VAF dens= 0.6
Density
0.0
0.5
1.0
1.5
2.0
2.5
0.0
0.2
0.4
0.6
0.8
1.0
N = 37 Bandwidth = 0.06653

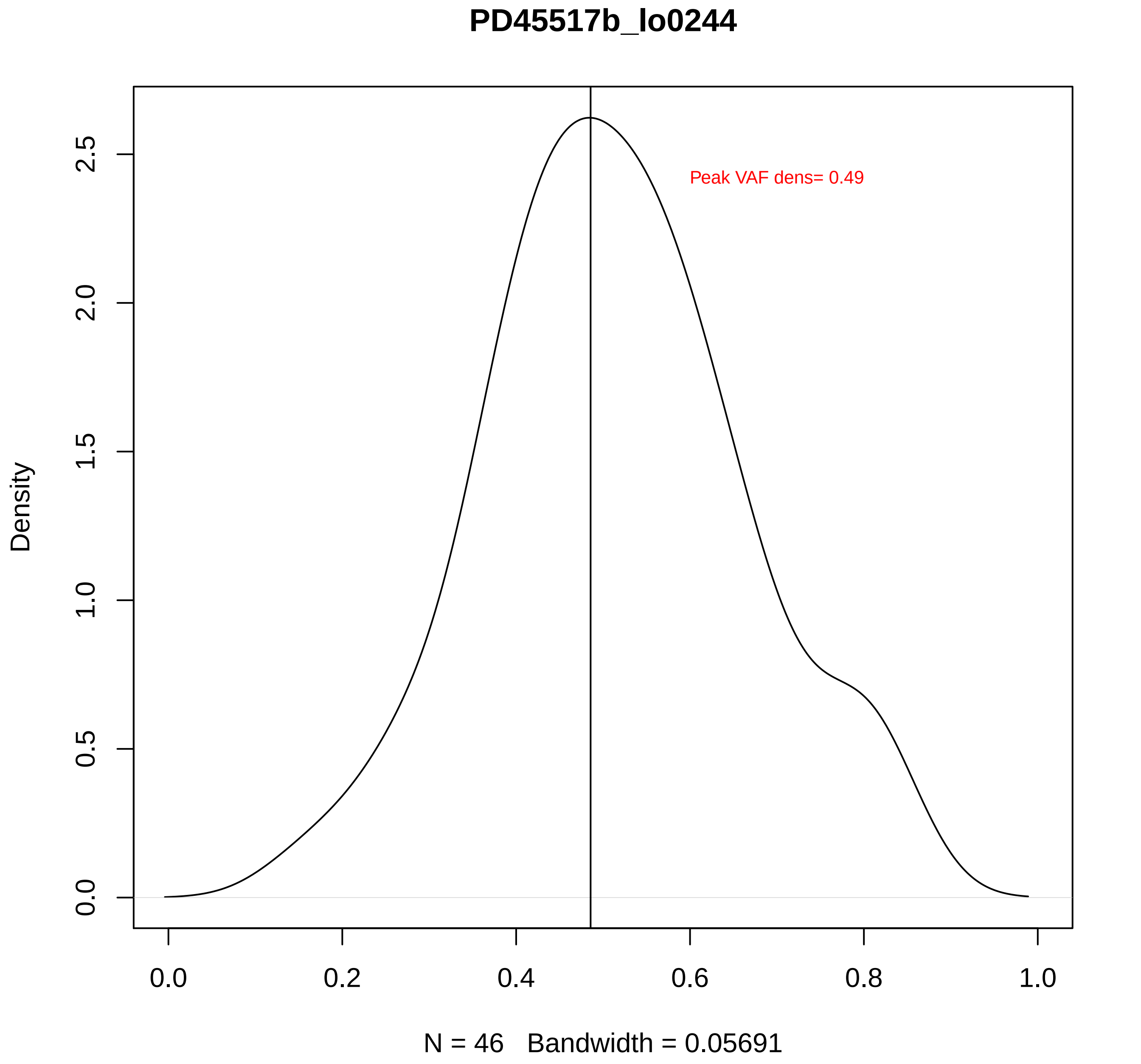
PD45517b_lo0244
Peak VAF dens= 0.49
Density
0.0
0.5
1.0
1.5
2.0
2.5
0.0
0.2
0.4
0.6
0.8
1.0
N = 46 Bandwidth = 0.05691

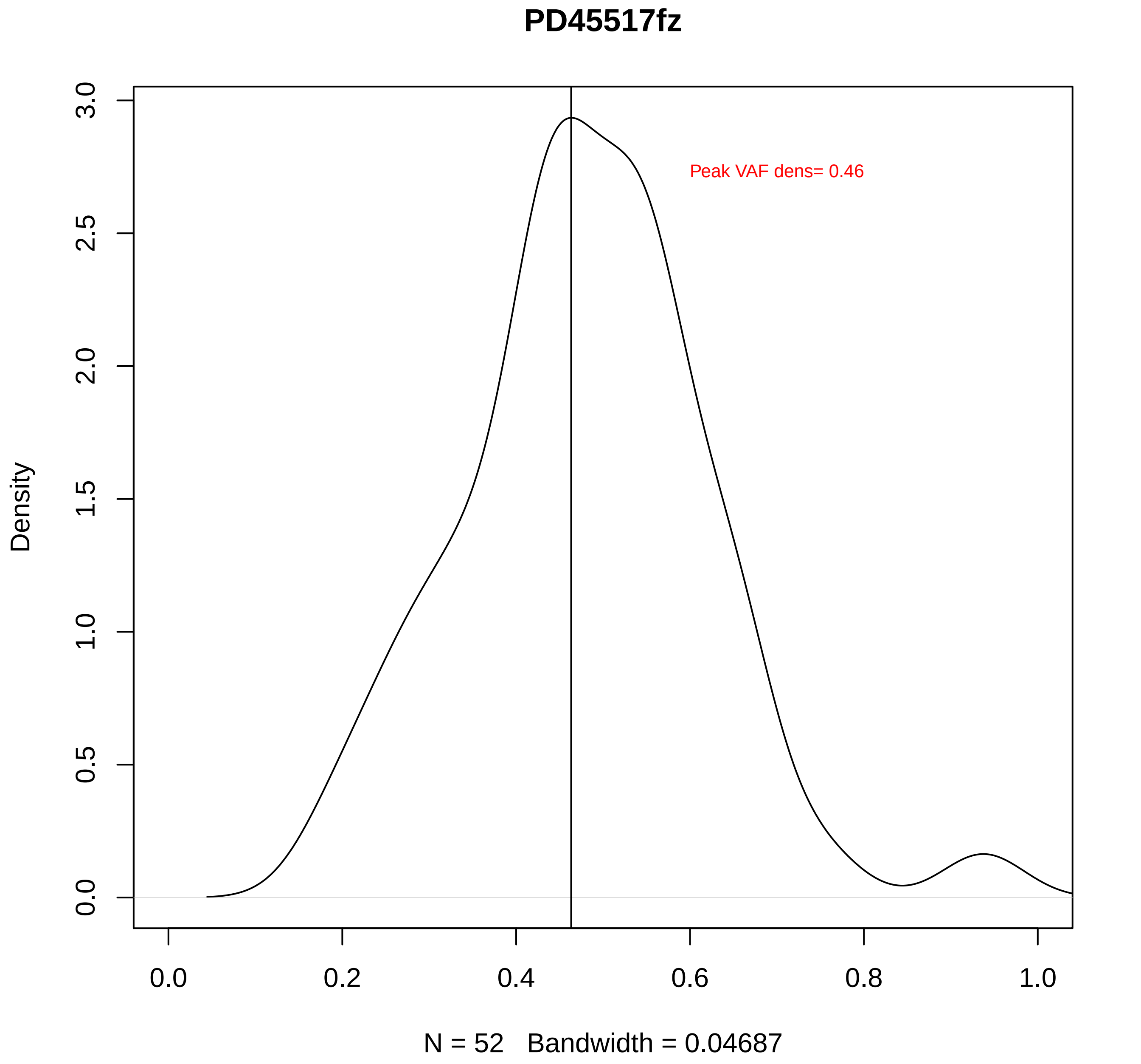
PD45517fz
Peak VAF dens= 0.46
Density
3.0
2.5
2.0
1.5
1.0
0.5
0.0
0.0
0.2
0.4
0.6
0.8
1.0
N = 52   Bandwidth = 0.04687

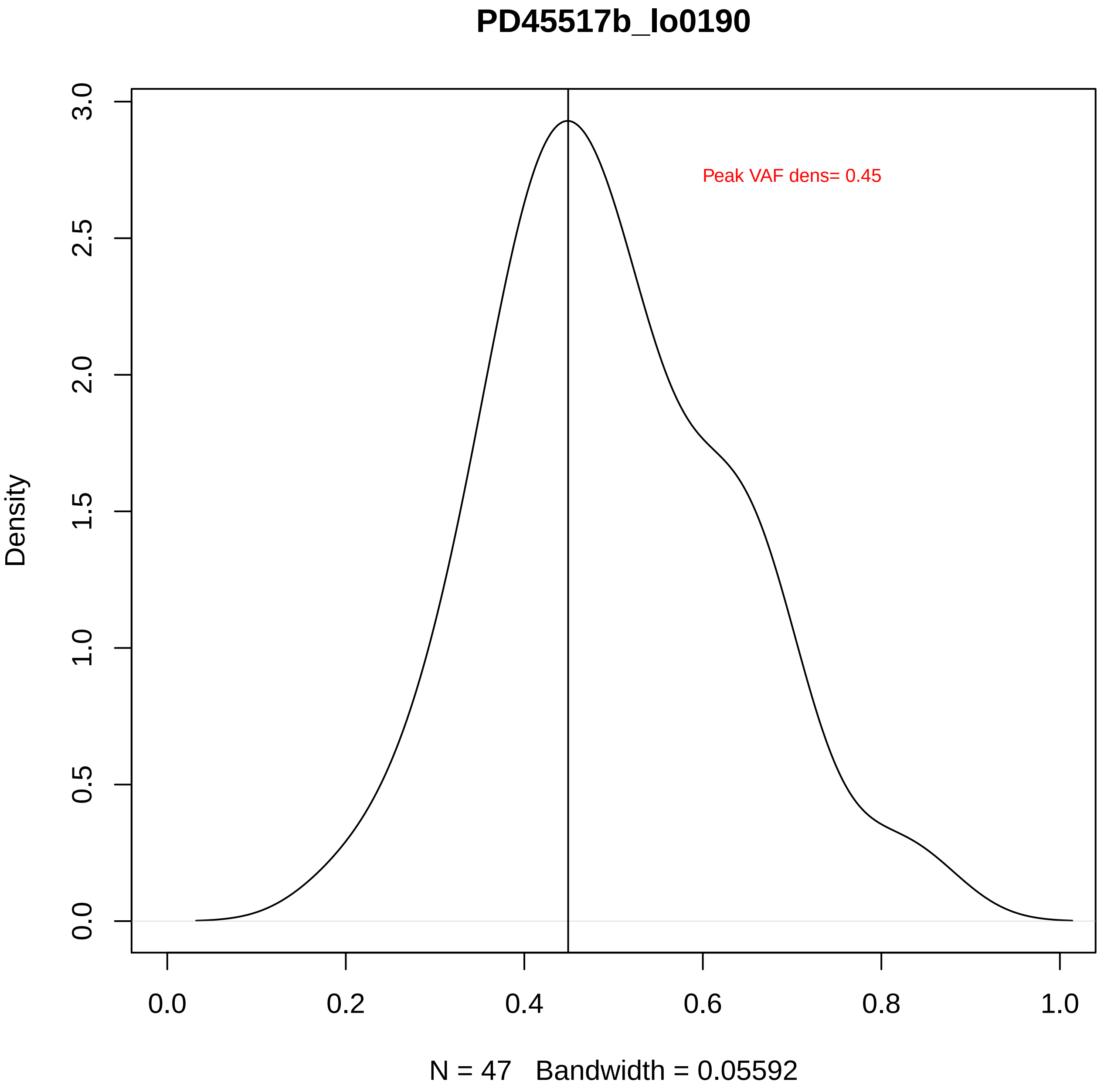
PD45517b_lo0190
Peak VAF dens= 0.45
Density
0.0
0.5
1.0
1.5
2.0
2.5
3.0
0.0
0.2
0.4
0.6
0.8
1.0
N = 47 Bandwidth = 0.05592

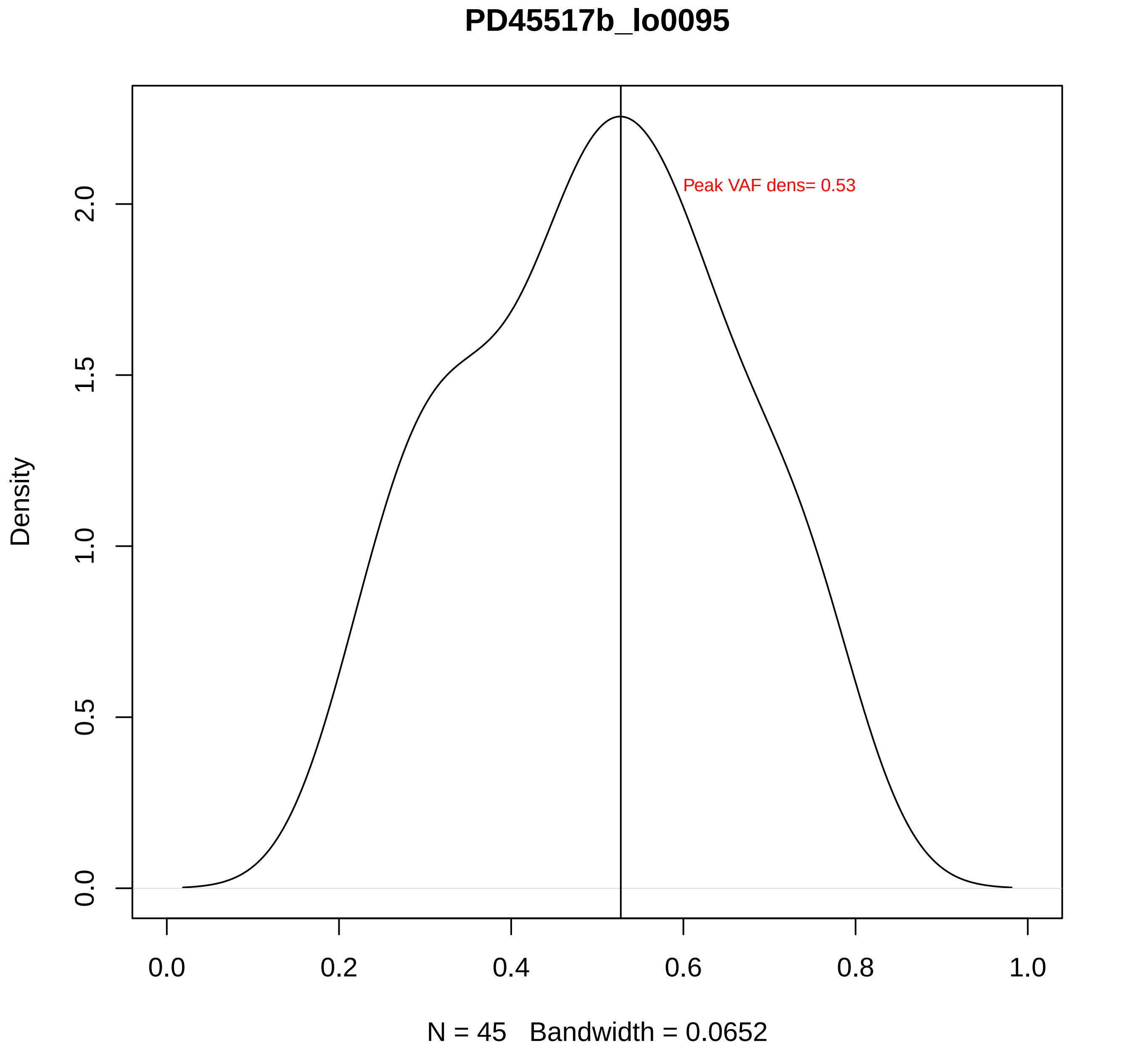

PD45517b_lo0095
Peak VAF dens= 0.53
Density
0.0
0.5
1.0
1.5
2.0
0.0
0.2
0.4
0.6
0.8
1.0
N = 45   Bandwidth = 0.0652

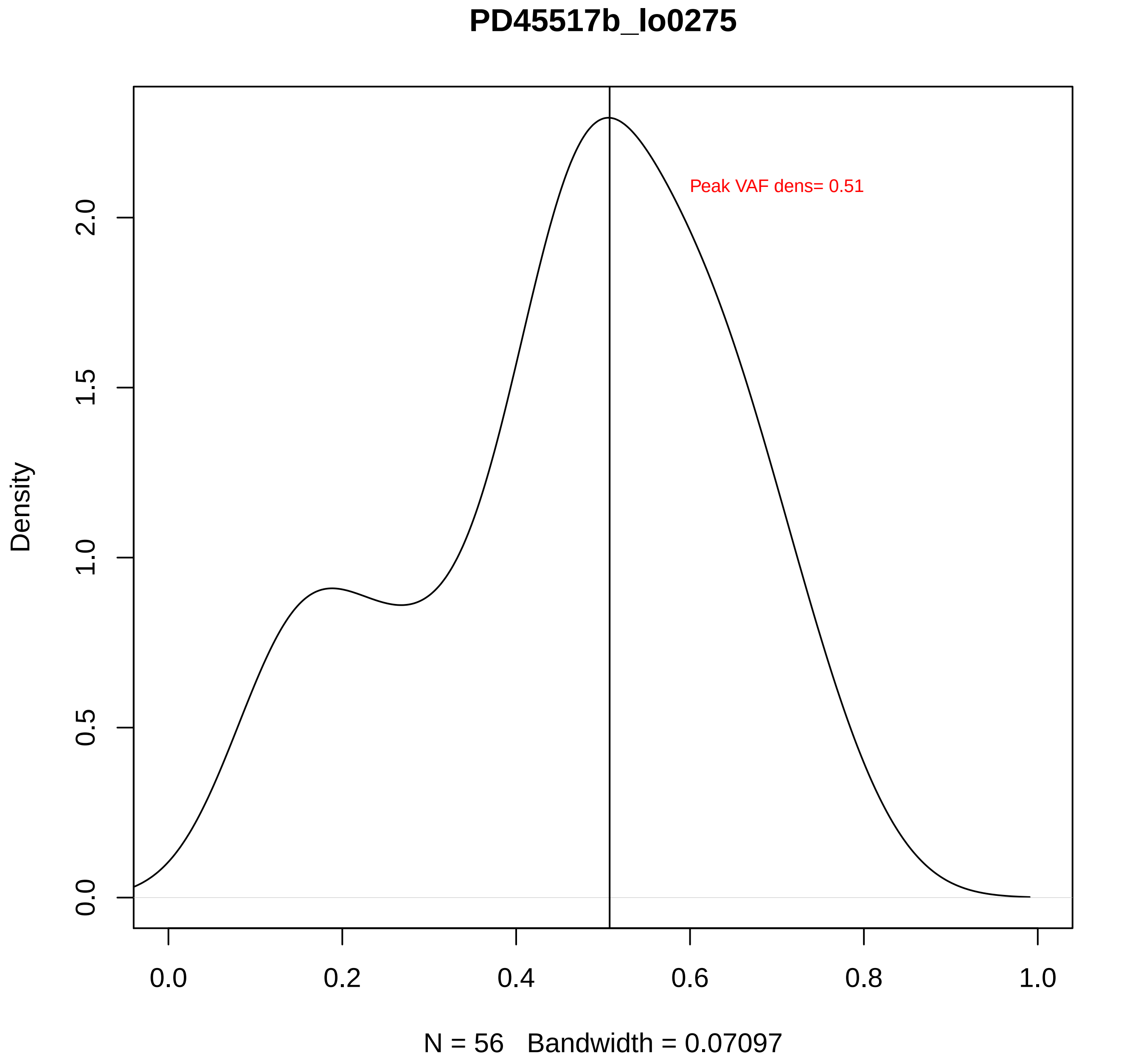

PD45517b_lo0275
Peak VAF dens= 0.51
Density
0.0
0.5
1.0
1.5
2.0
0.0
0.2
0.4
0.6
0.8
1.0
N = 56   Bandwidth = 0.07097

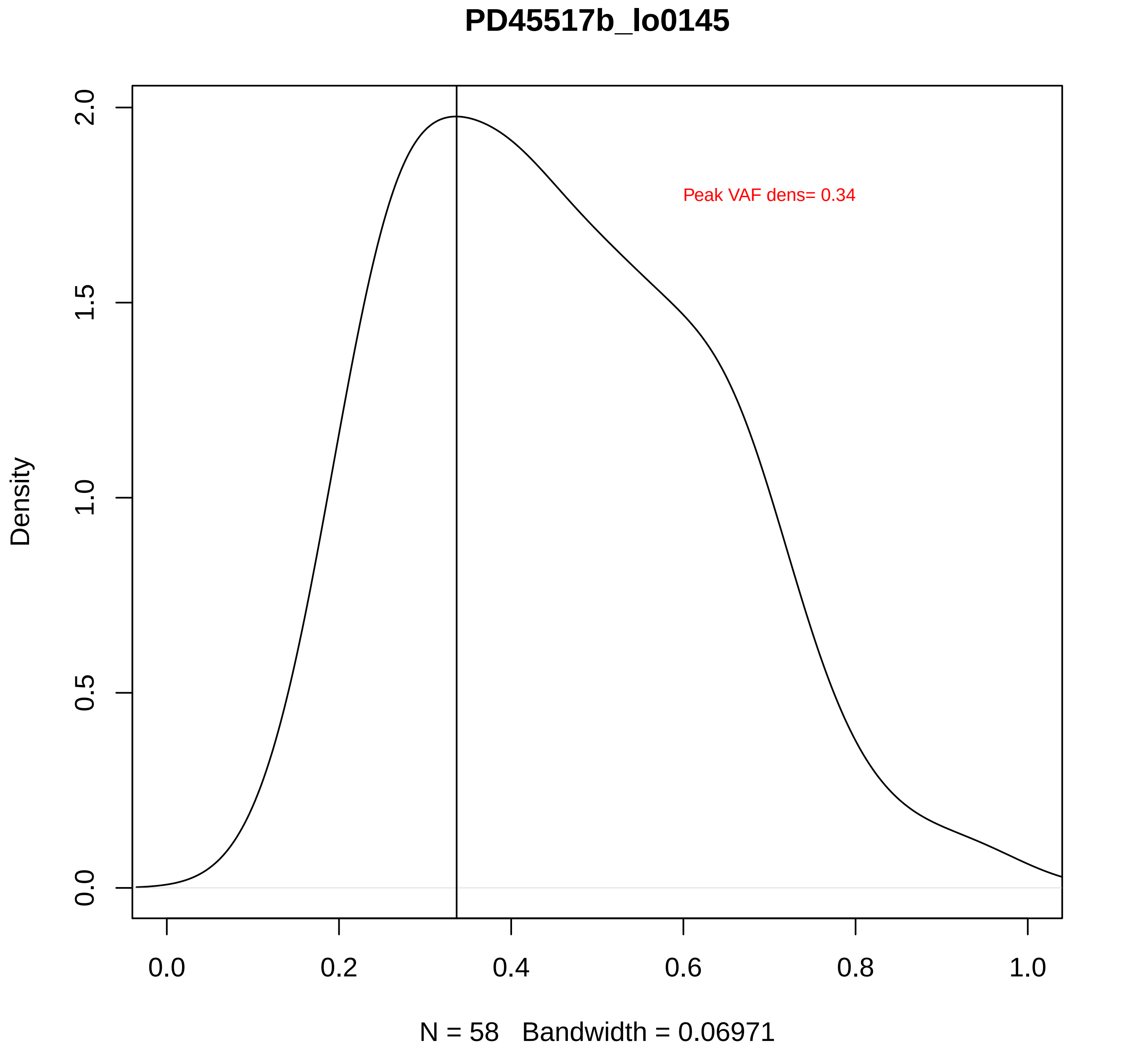
PD45517b_lo0145
Density
N = 58 Bandwidth = 0.06971
Peak VAF dens= 0.34
0.0
0.5
1.0
1.5
2.0
0.0
0.2
0.4
0.6
0.8
1.0

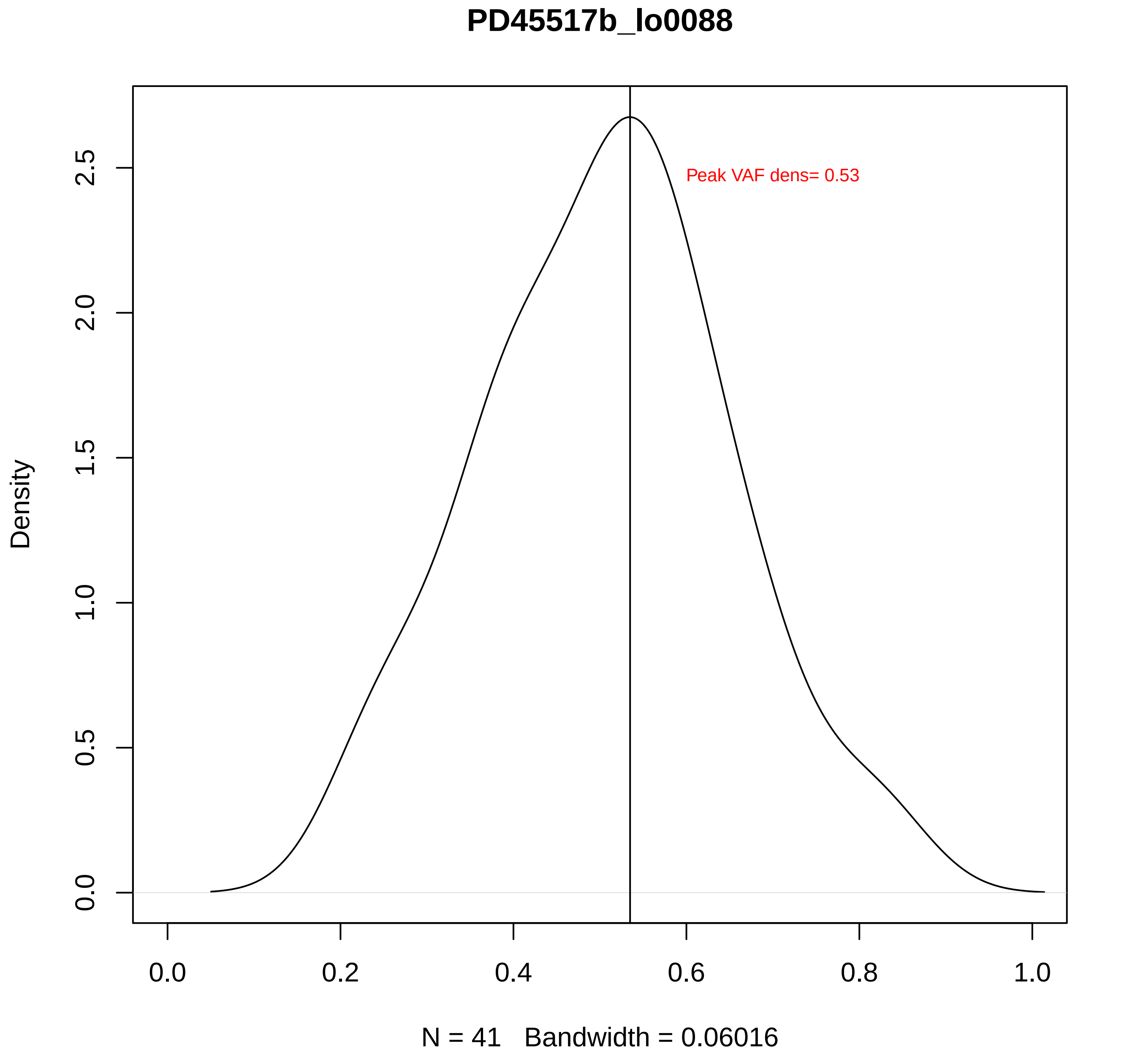
PD45517b_lo0088
Peak VAF dens= 0.53
Density
0.0
0.5
1.0
1.5
2.0
2.5
0.0
0.2
0.4
0.6
0.8
1.0
N = 41   Bandwidth = 0.06016

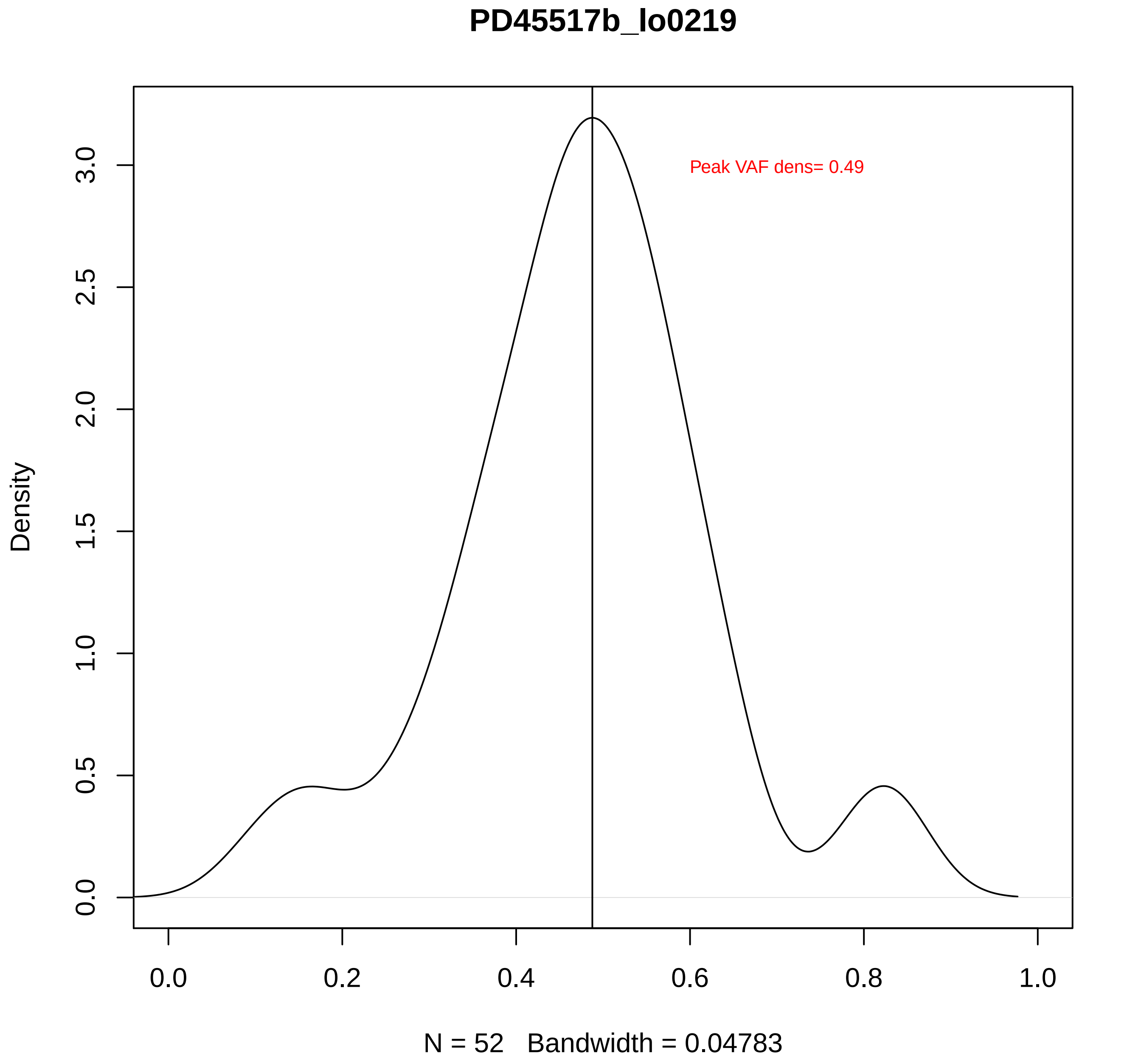

PD45517b_lo0219
Peak VAF dens= 0.49
Density
0.0
0.5
1.0
1.5
2.0
2.5
3.0
0.0
0.2
0.4
0.6
0.8
1.0
N = 52 Bandwidth = 0.04783

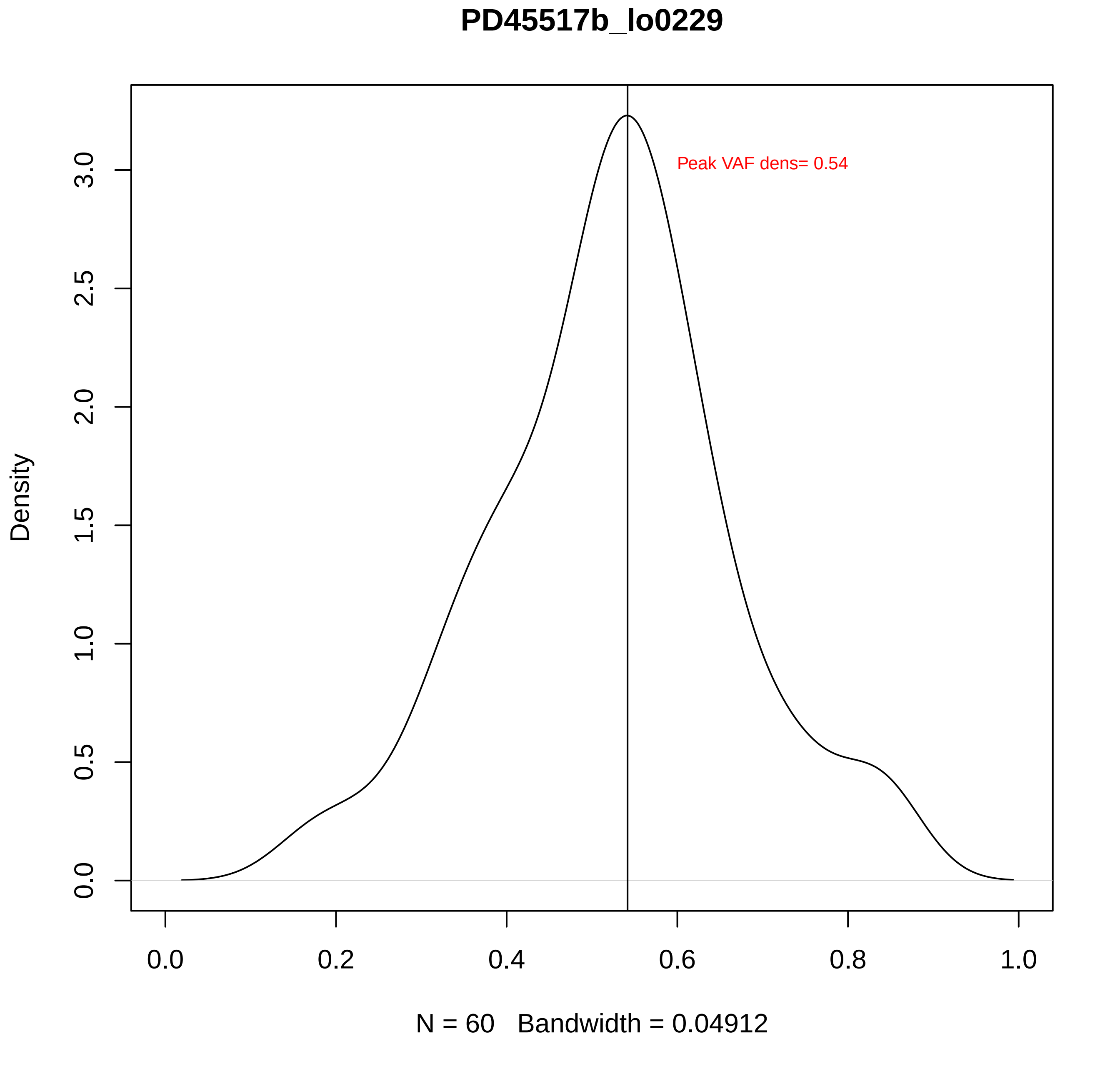

PD45517b_lo0229
Peak VAF dens= 0.54
Density
0.0
0.5
1.0
1.5
2.0
2.5
3.0
0.0
0.2
0.4
0.6
0.8
1.0
N = 60 Bandwidth = 0.04912

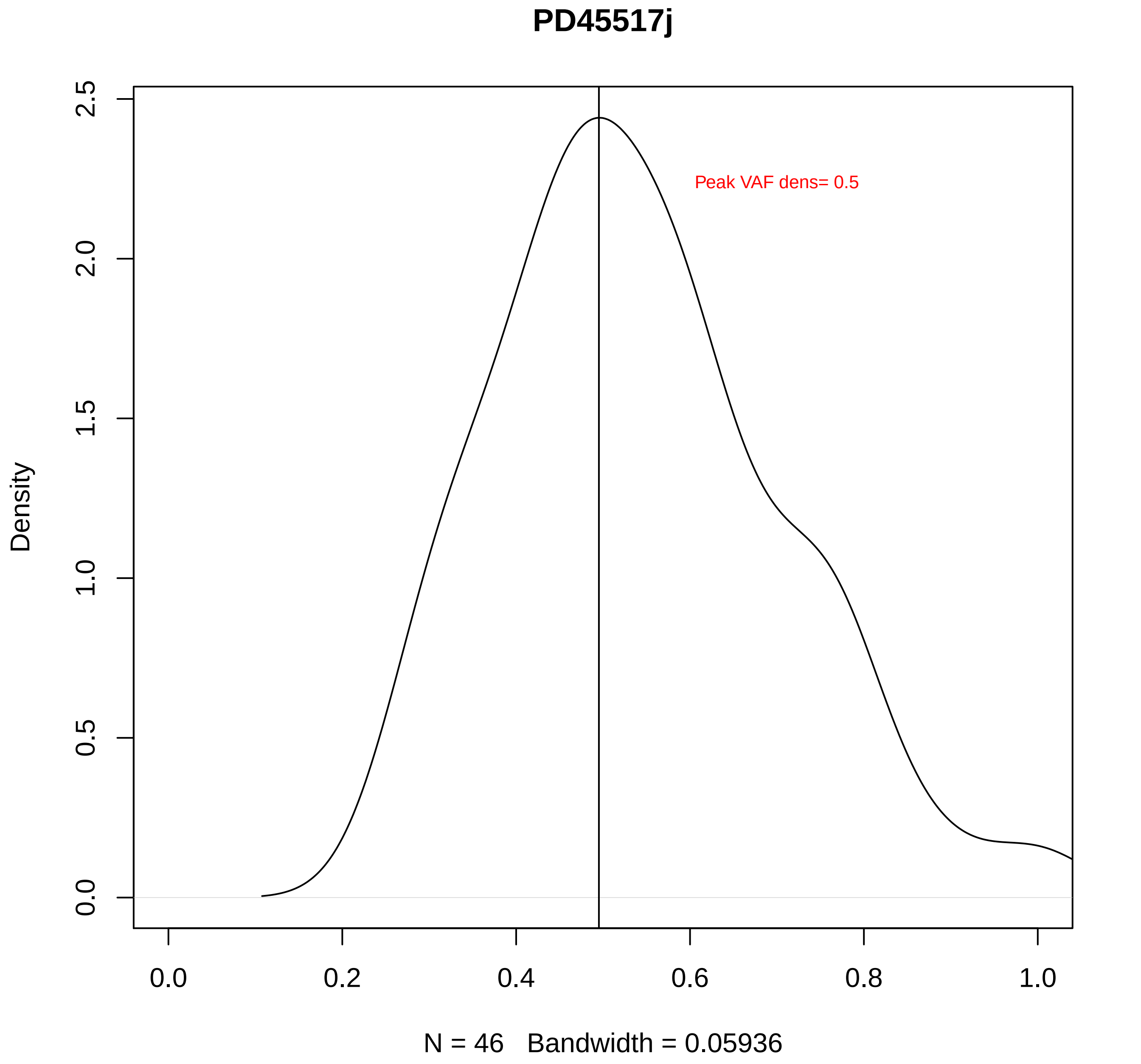
PD45517j
Peak VAF dens= 0.5
Density
0.0
0.5
1.0
1.5
2.0
2.5
0.0
0.2
0.4
0.6
0.8
1.0
N = 46   Bandwidth = 0.05936

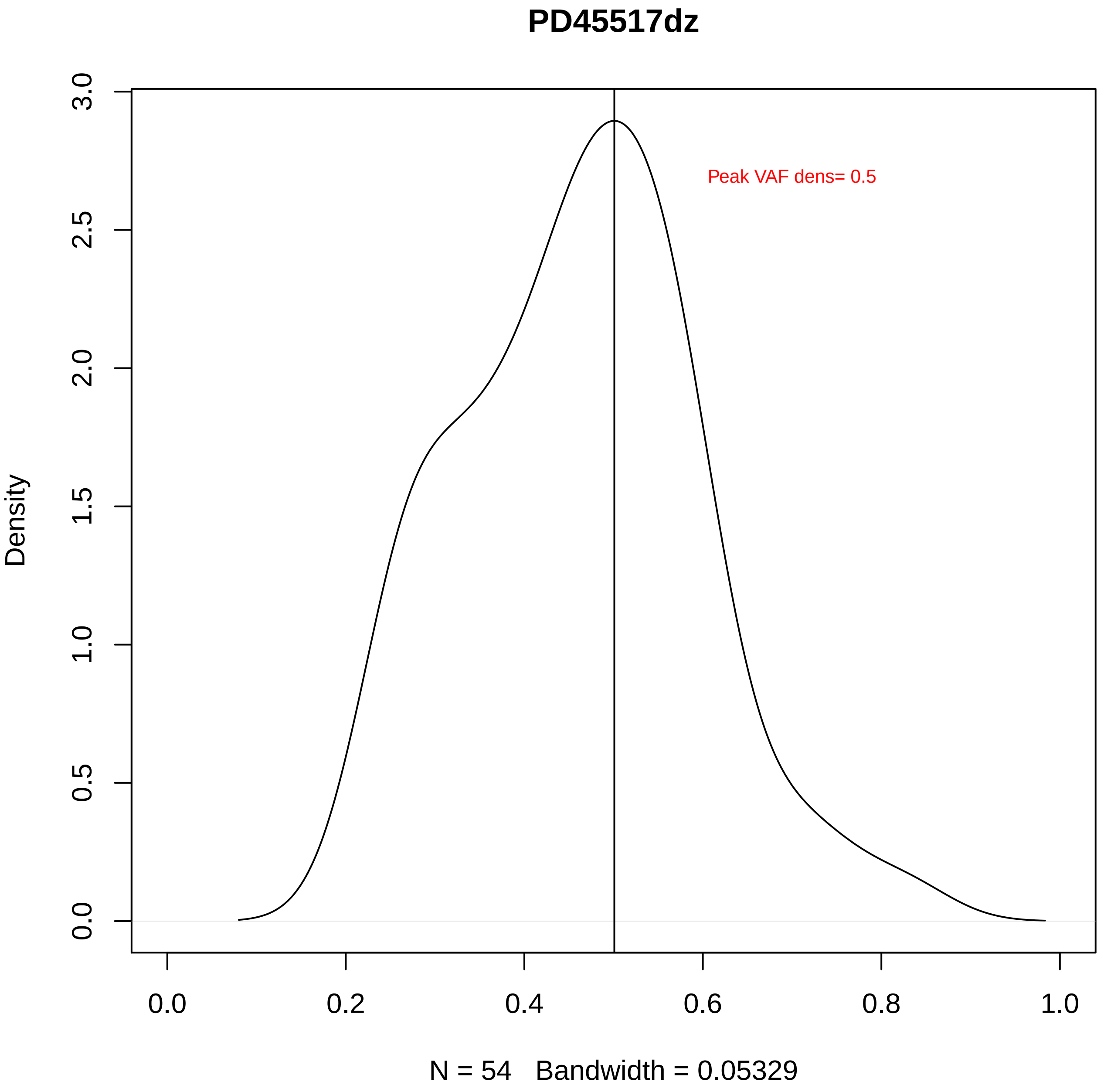
PD45517dz
Peak VAF dens= 0.5
Density
0.0
0.5
1.0
1.5
2.0
2.5
3.0
0.0
0.2
0.4
0.6
0.8
1.0
N = 54   Bandwidth = 0.05329

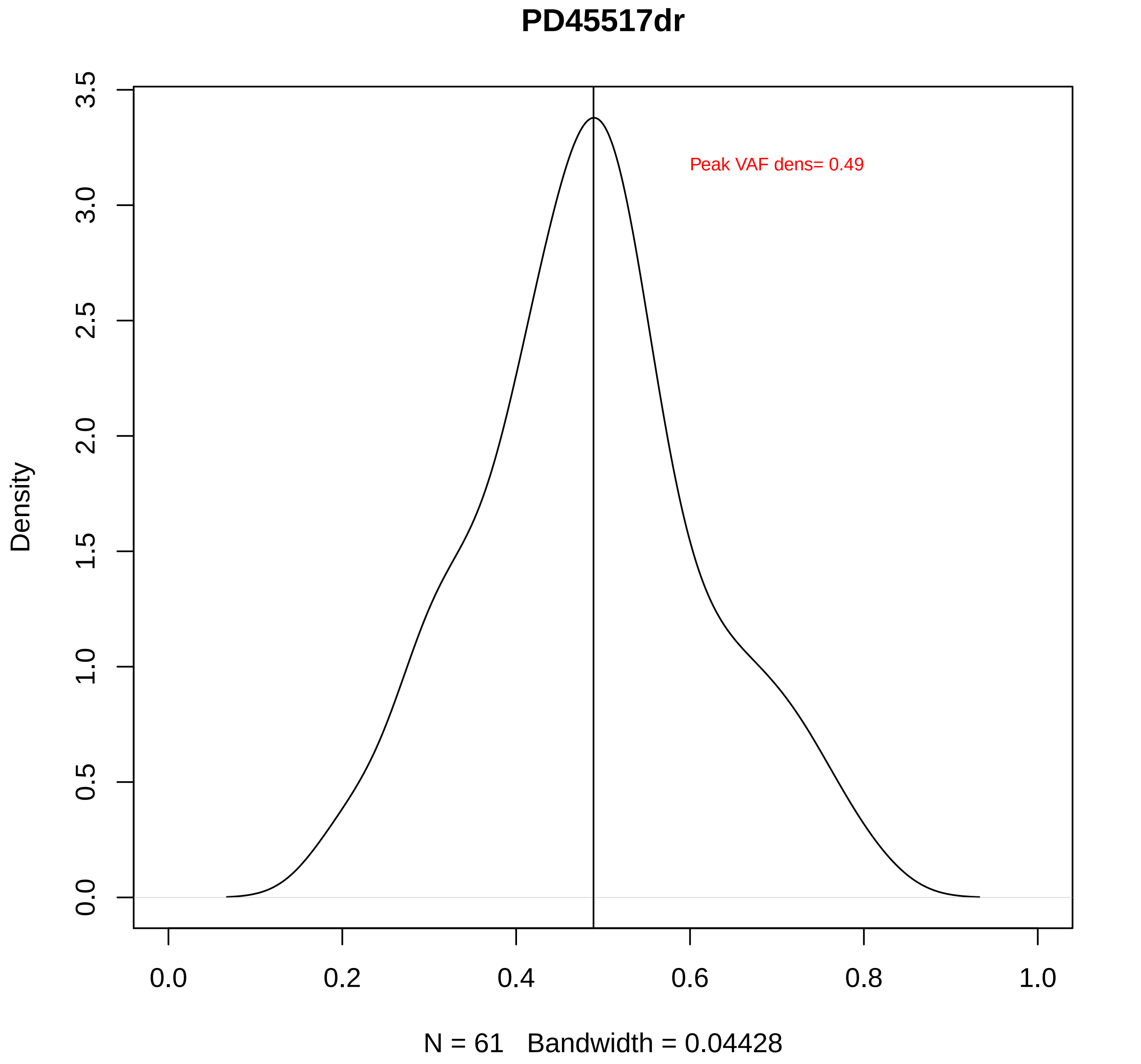
PD45517dr
Peak VAF dens= 0.49
Density
0.0
0.5
1.0
1.5
2.0
2.5
3.0
3.5
0.0
0.2
0.4
0.6
0.8
1.0
N = 61   Bandwidth = 0.04428

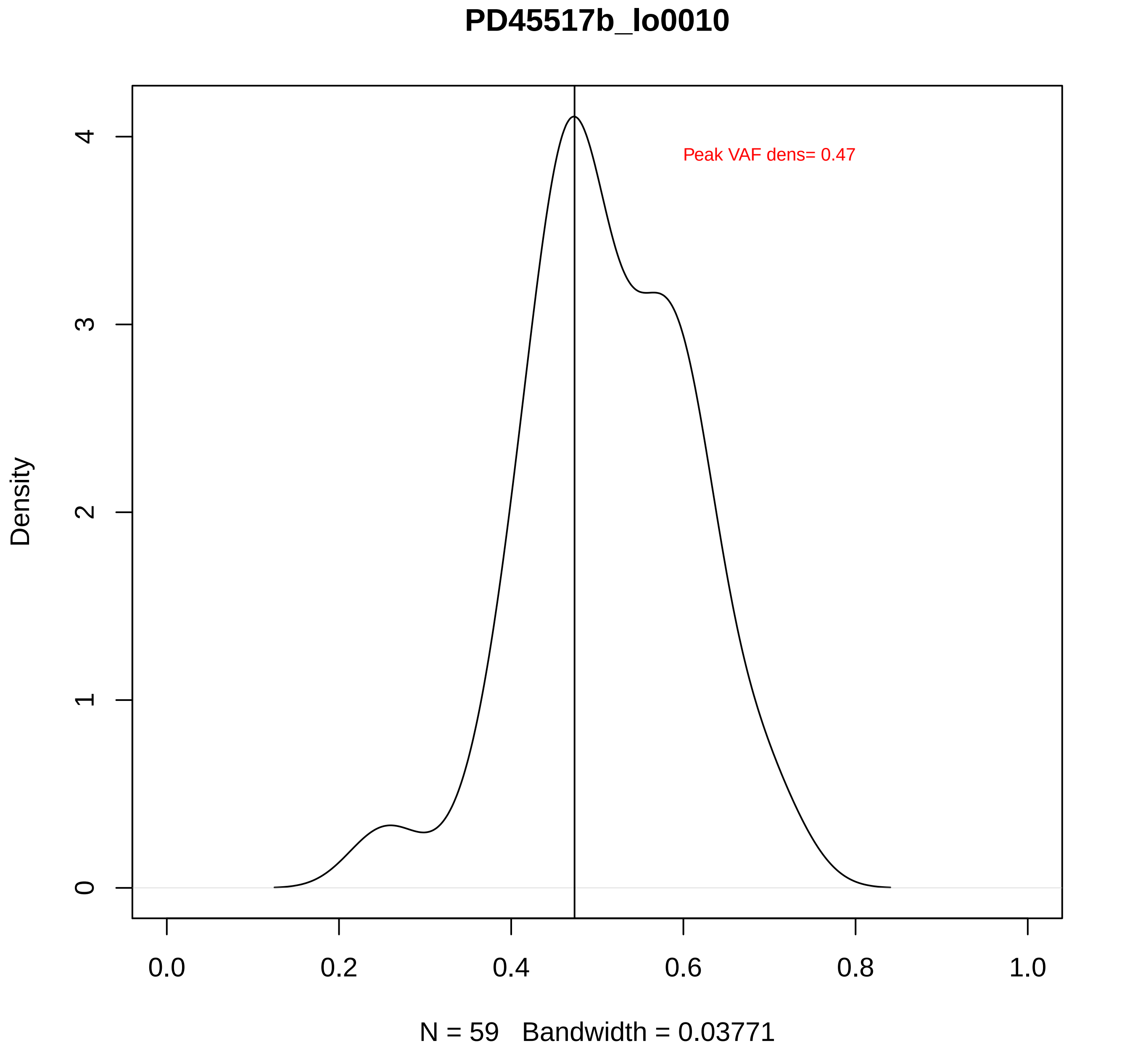
PD45517b_lo0010
Peak VAF dens= 0.47
Density
0
1
2
3
4
0.0
0.2
0.4
0.6
0.8
1.0
N = 59 Bandwidth = 0.03771

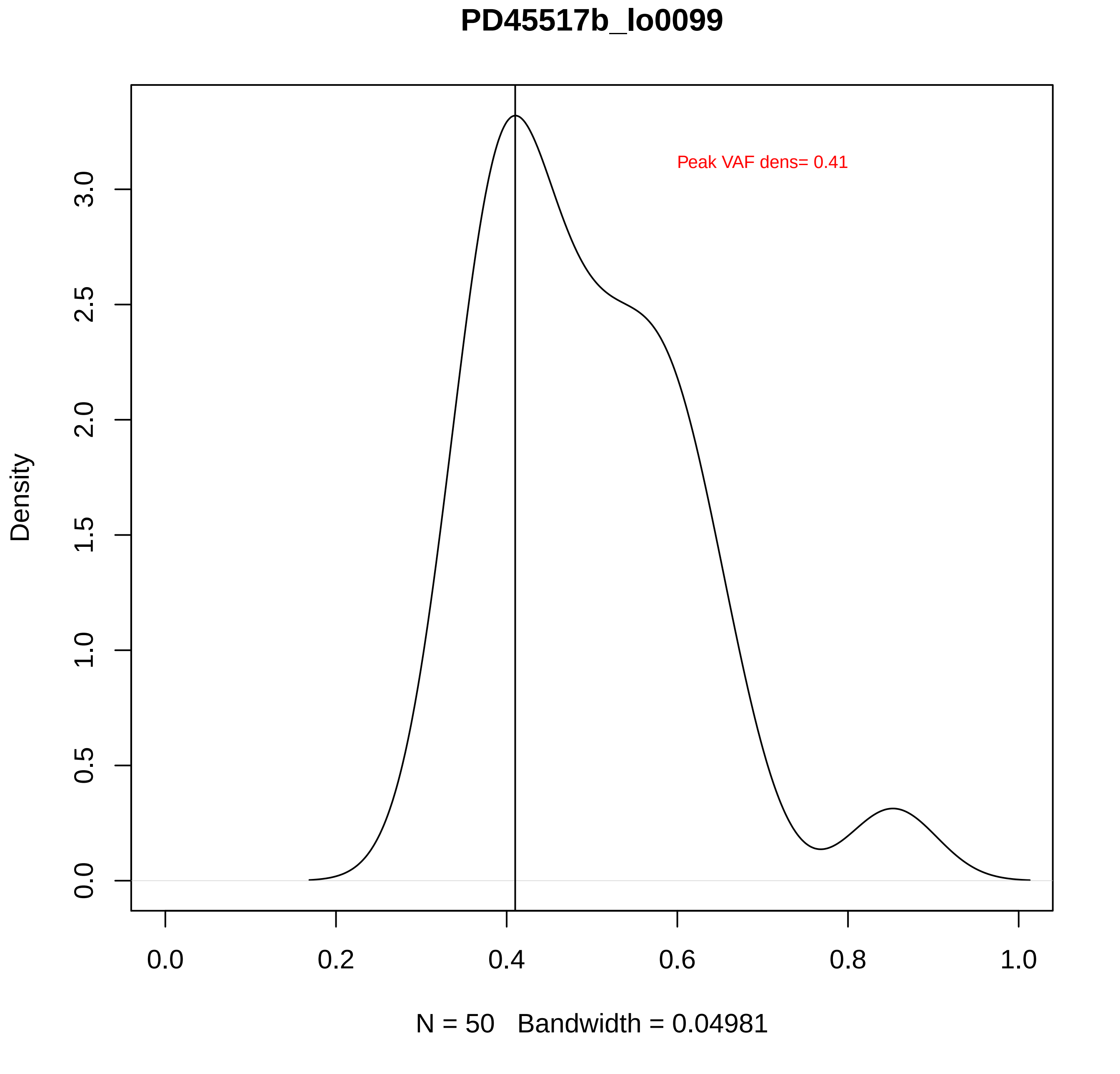

PD45517b_lo0099
Peak VAF dens= 0.41
Density
0.0
0.5
1.0
1.5
2.0
2.5
3.0
0.0
0.2
0.4
0.6
0.8
1.0
N = 50 Bandwidth = 0.04981

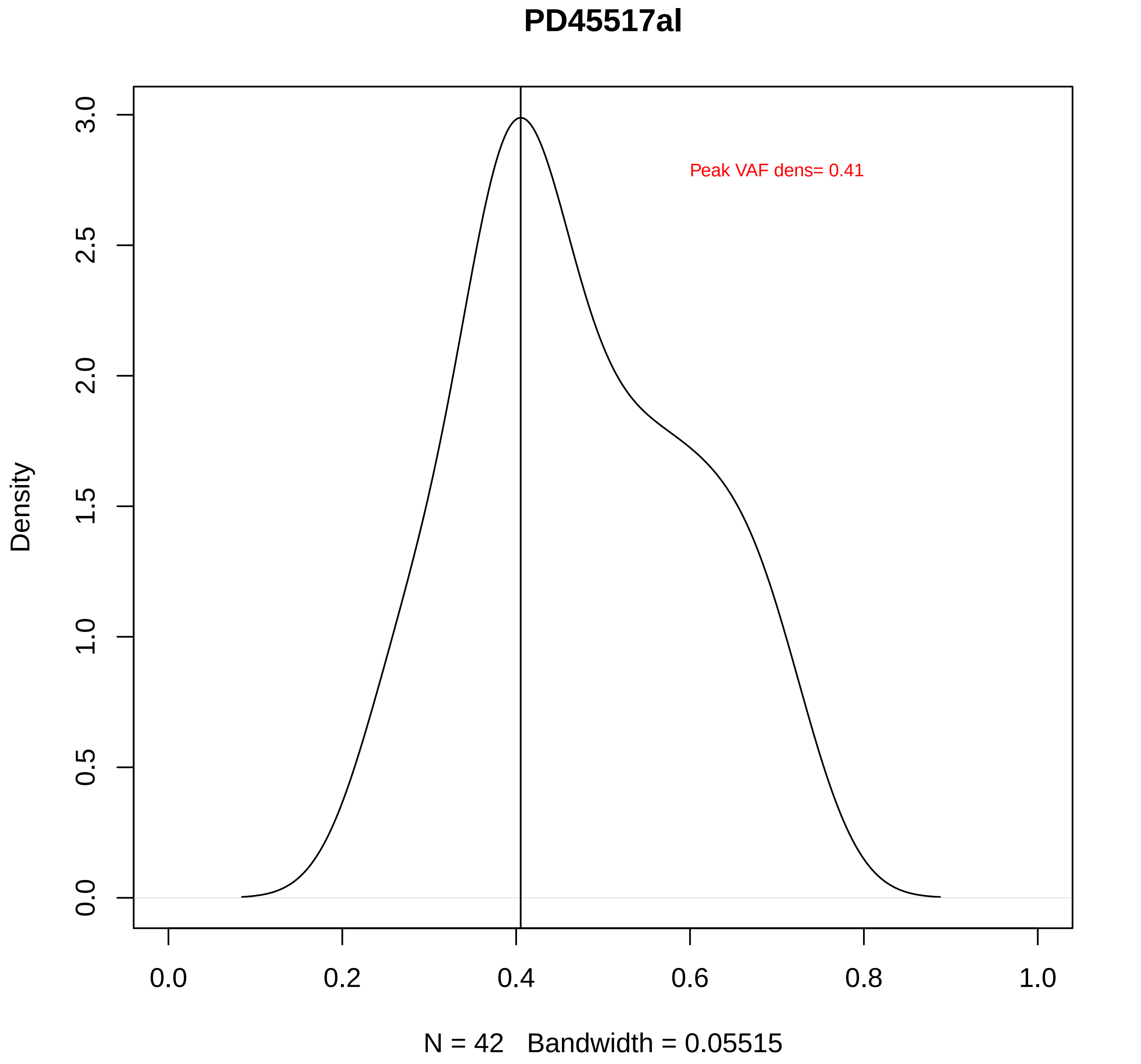

PD45517al
Peak VAF dens= 0.41
Density
0.0
0.5
1.0
1.5
2.0
2.5
3.0
0.0
0.2
0.4
0.6
0.8
1.0
N = 42   Bandwidth = 0.05515

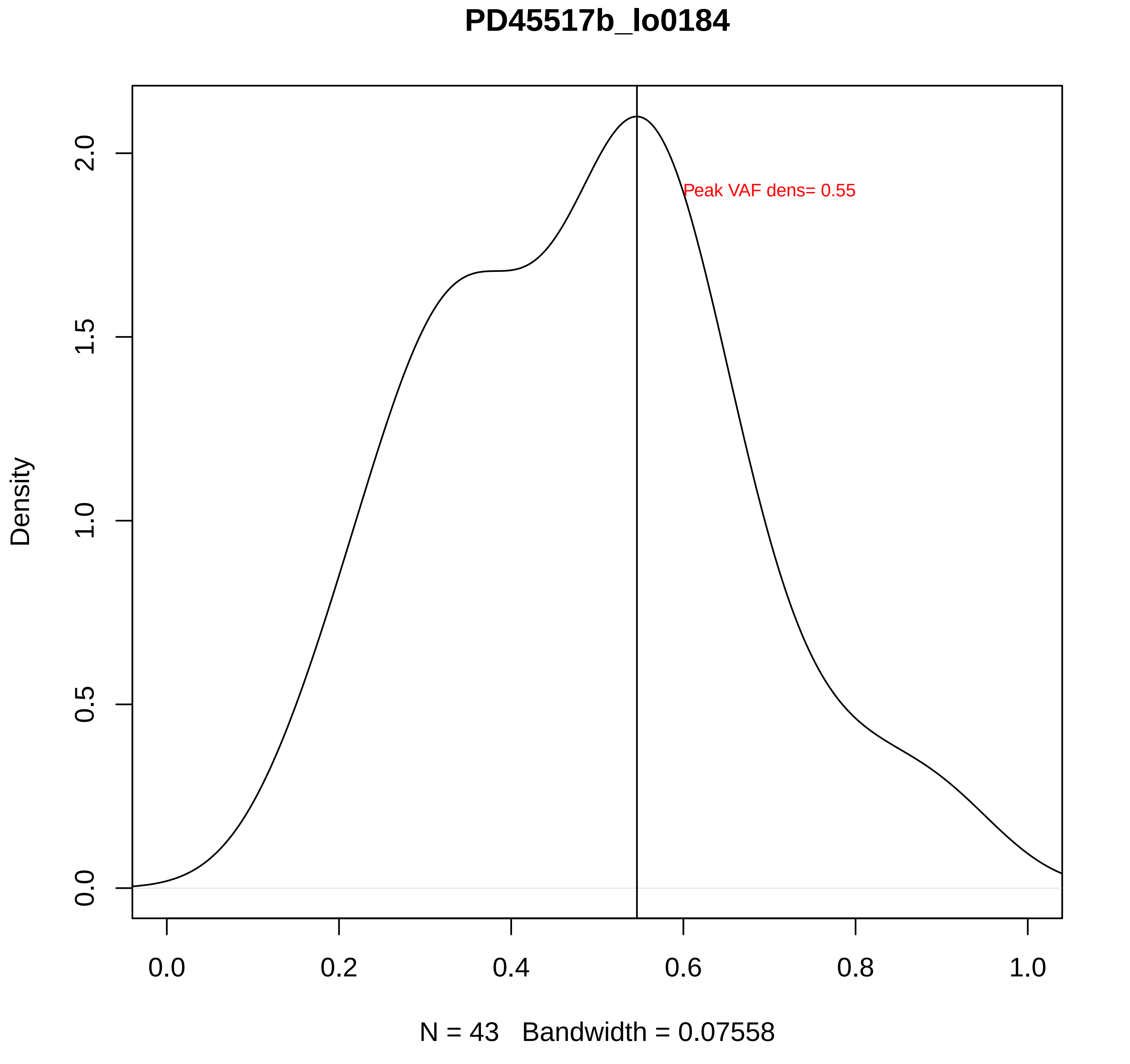
PD45517b_lo0184
Peak VAF dens= 0.55
Density
0.0
0.5
1.0
1.5
2.0
0.0
0.2
0.4
0.6
0.8
1.0
N = 43   Bandwidth = 0.07558

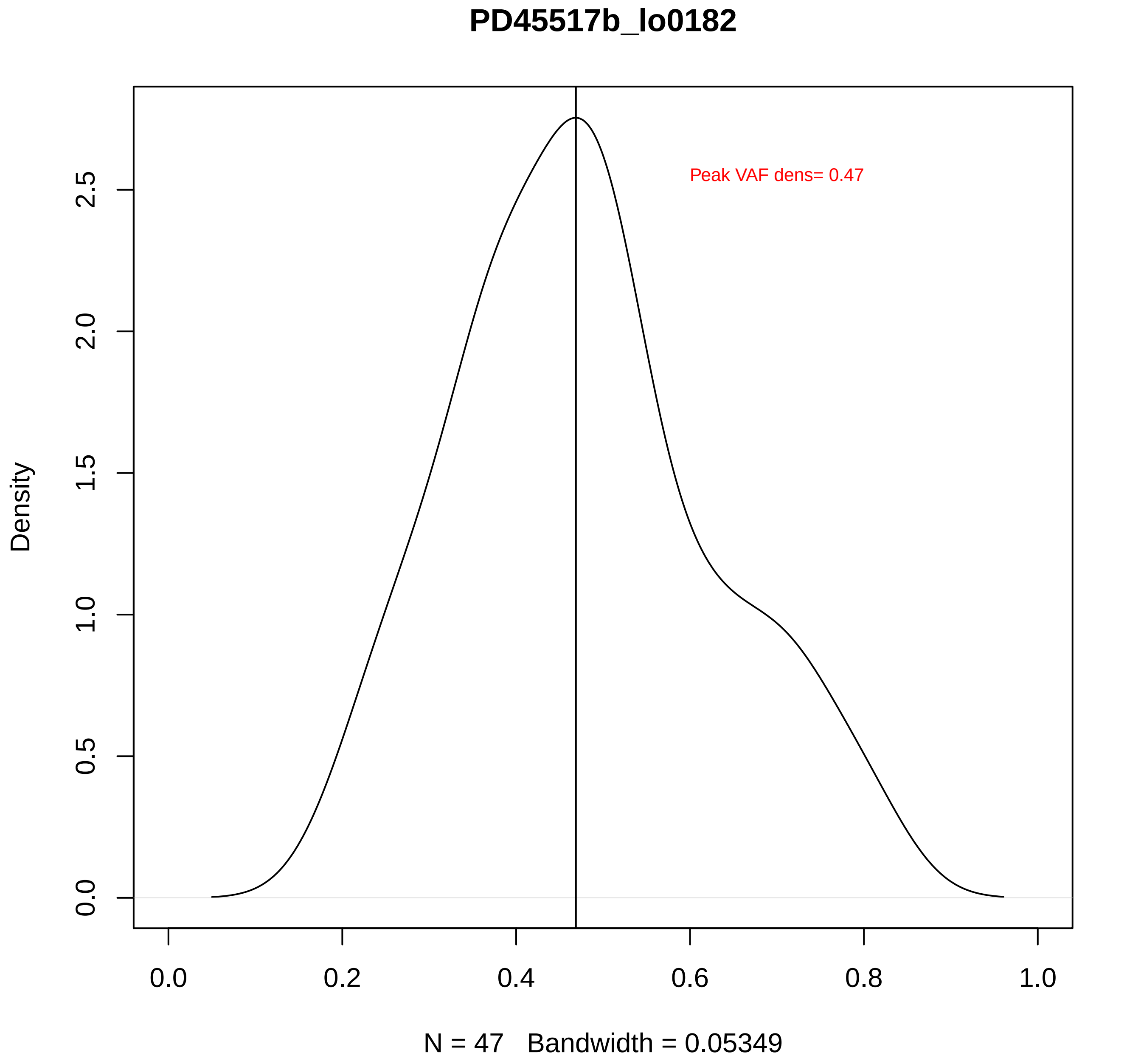

PD45517b_lo0182
Peak VAF dens= 0.47
Density
0.0
0.5
1.0
1.5
2.0
2.5
0.0
0.2
0.4
0.6
0.8
1.0
N = 47 Bandwidth = 0.05349

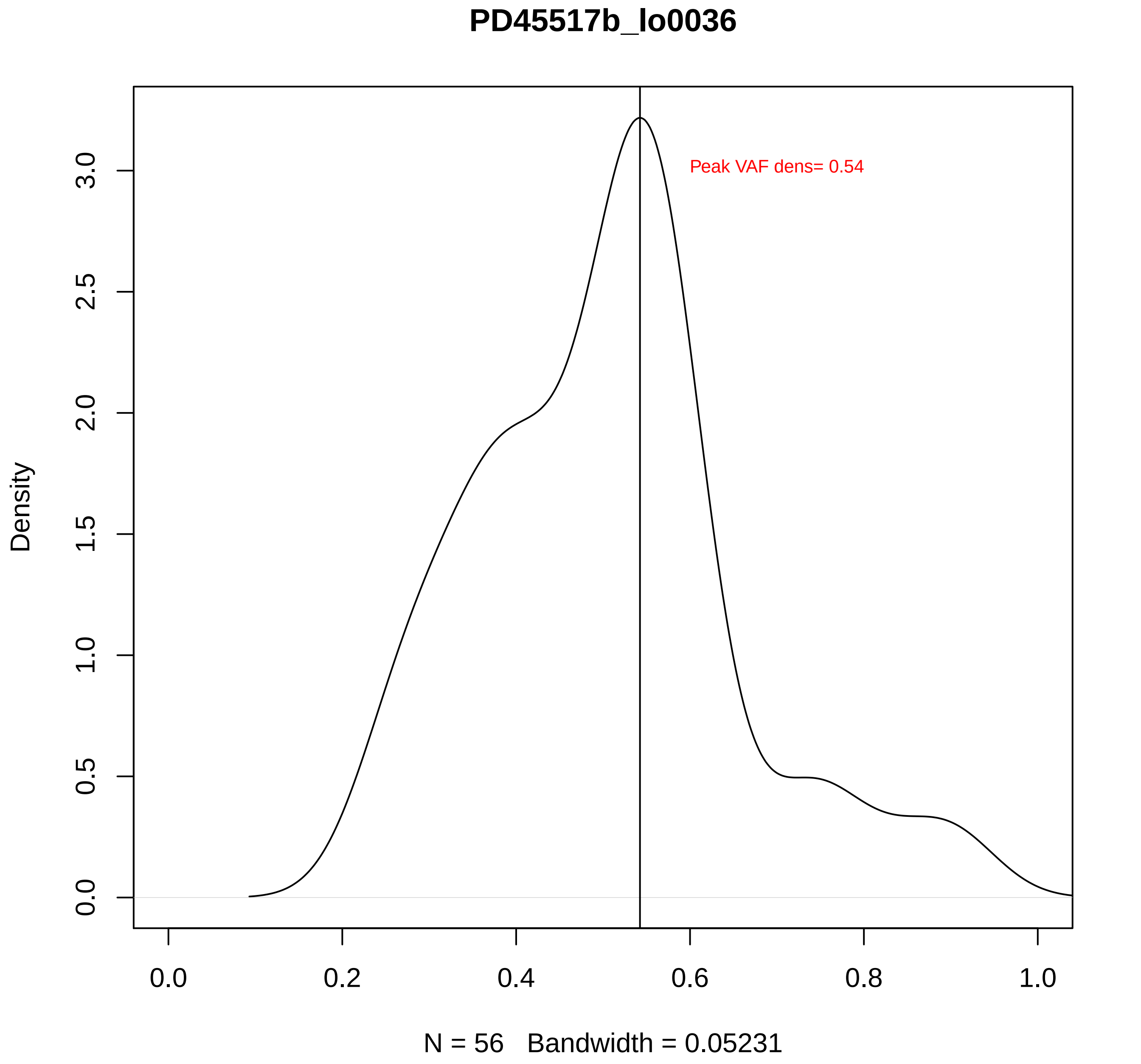
PD45517b_lo0036
Peak VAF dens= 0.54
Density
0.0
0.5
1.0
1.5
2.0
2.5
3.0
0.0
0.2
0.4
0.6
0.8
1.0
N = 56 Bandwidth = 0.05231

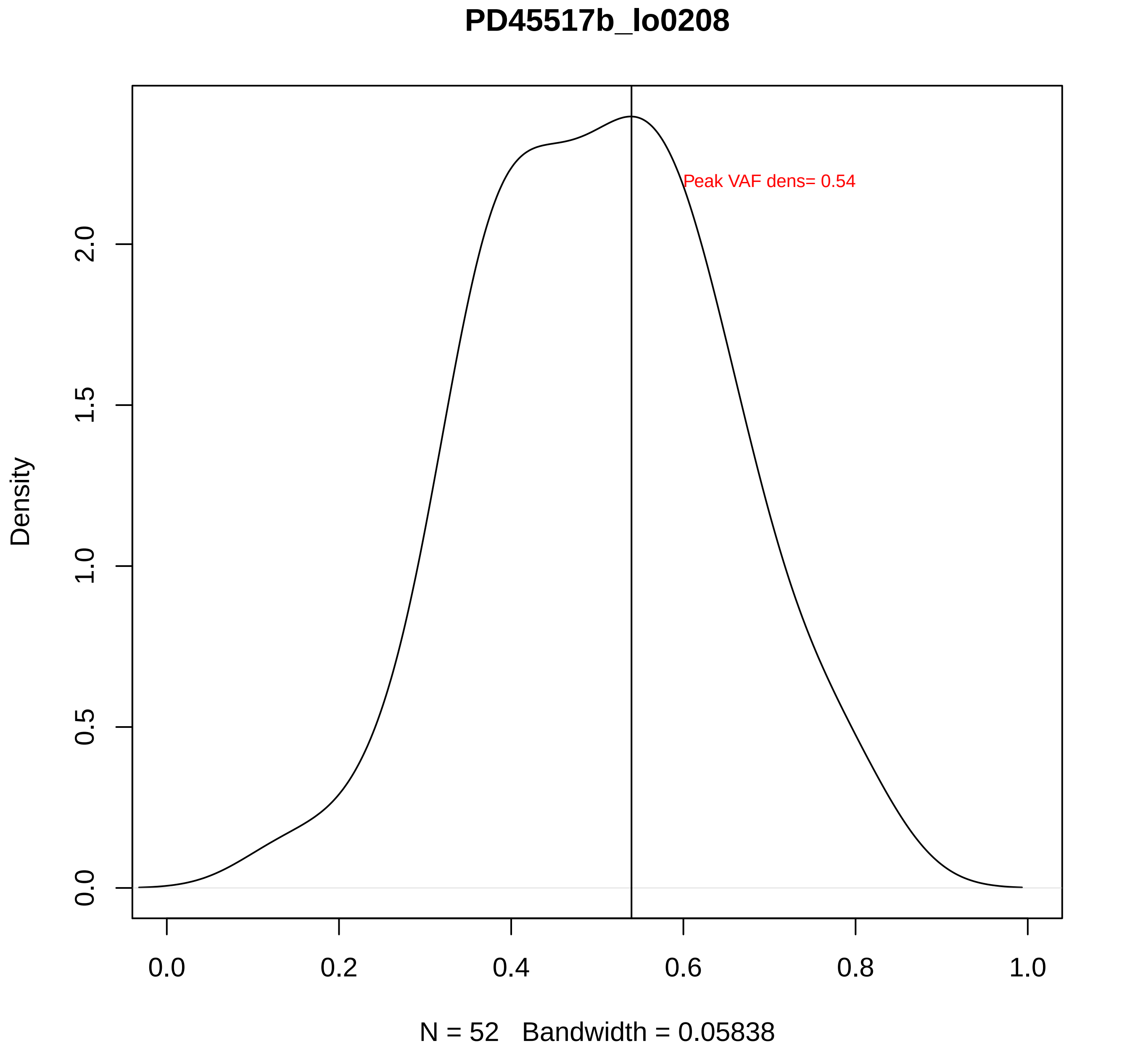

PD45517b_lo0208
Peak VAF dens= 0.54
Density
0.0
0.5
1.0
1.5
2.0
0.0
0.2
0.4
0.6
0.8
1.0
N = 52 Bandwidth = 0.05838

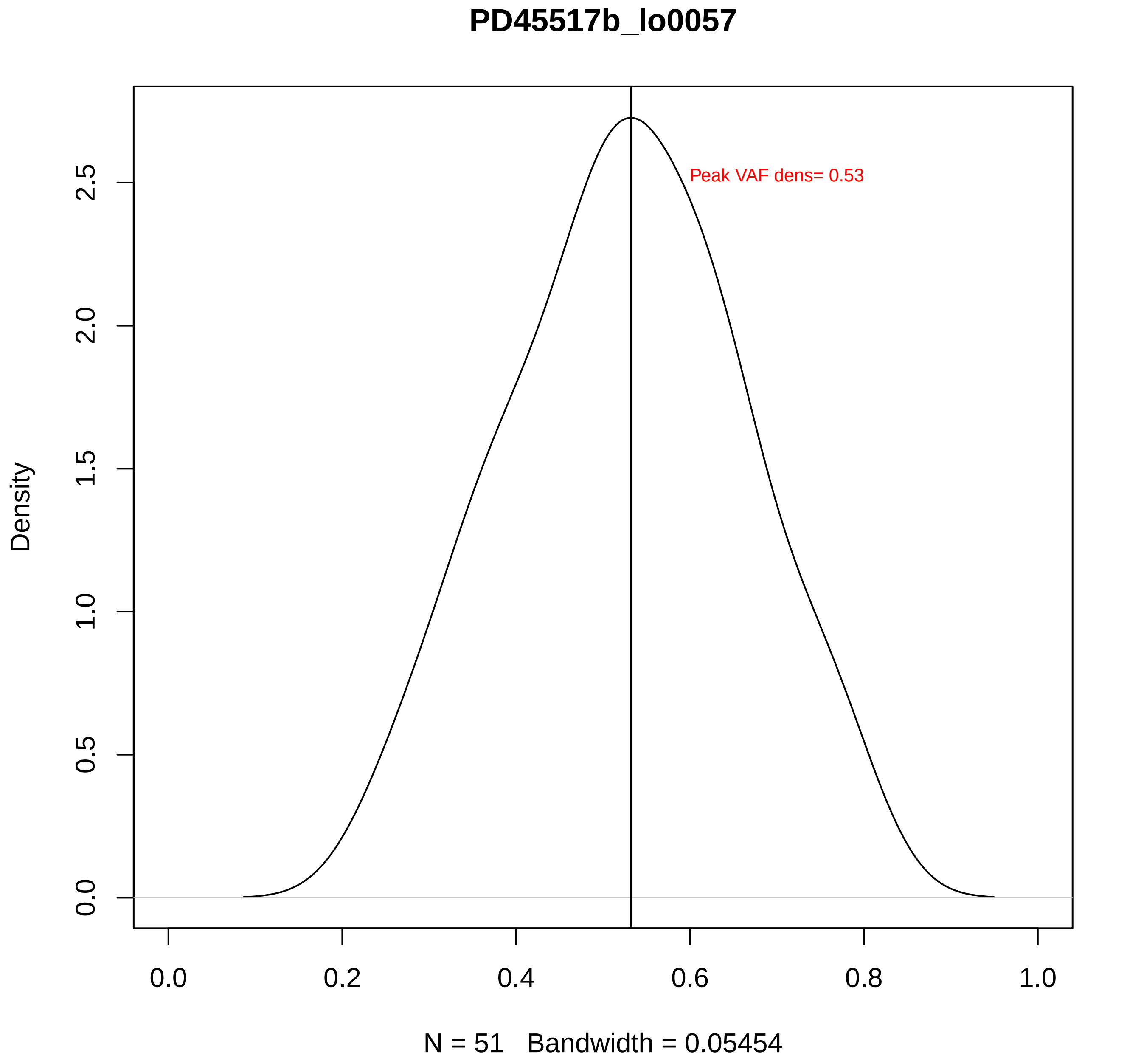
PD45517b_lo0057
Peak VAF dens= 0.53
Density
0.0
0.5
1.0
1.5
2.0
2.5
0.0
0.2
0.4
0.6
0.8
1.0
N = 51   Bandwidth = 0.05454

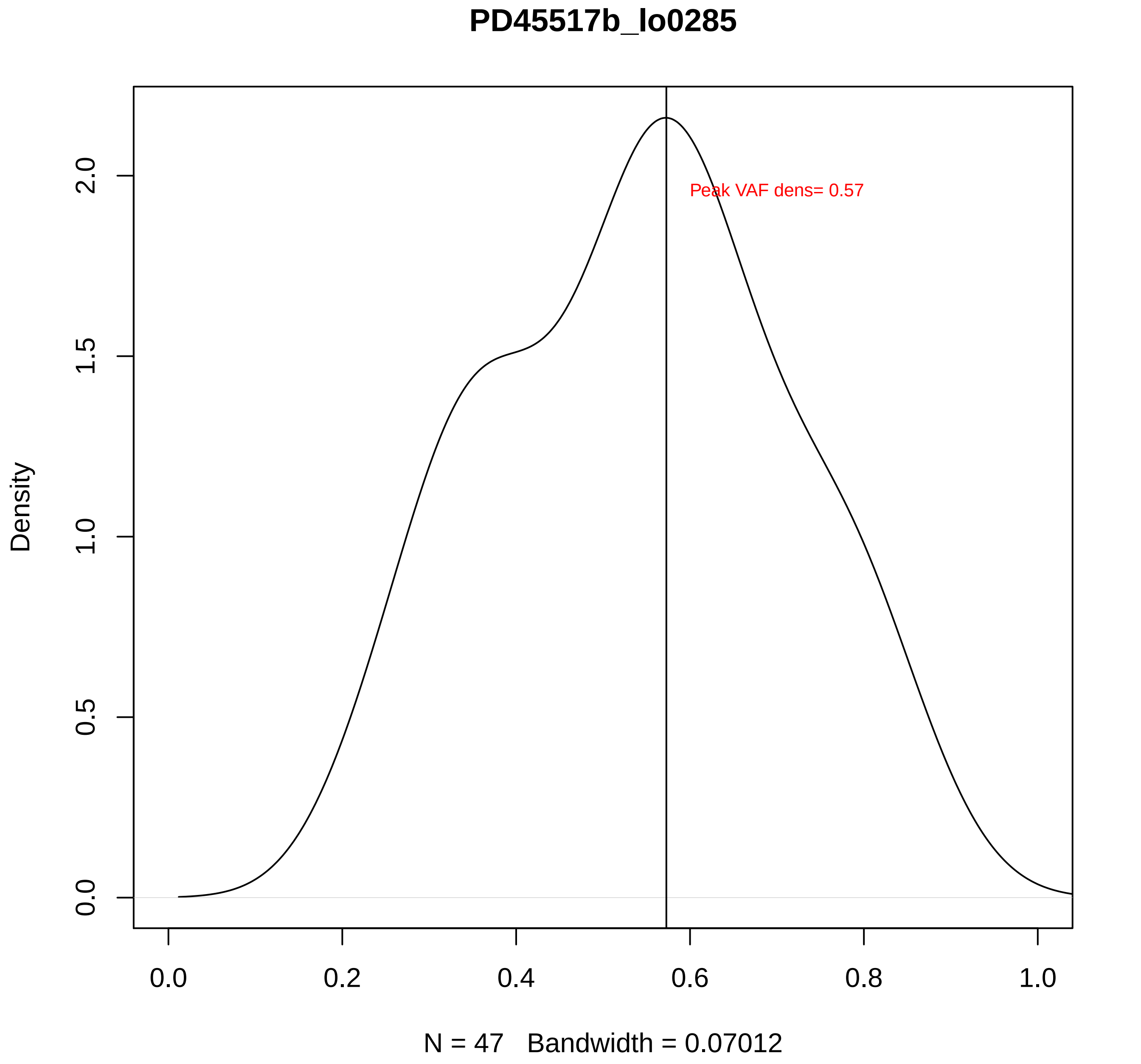

PD45517b_lo0285
Peak VAF dens= 0.57
Density
0.0
0.5
1.0
1.5
2.0
0.0
0.2
0.4
0.6
0.8
1.0
N = 47 Bandwidth = 0.07012

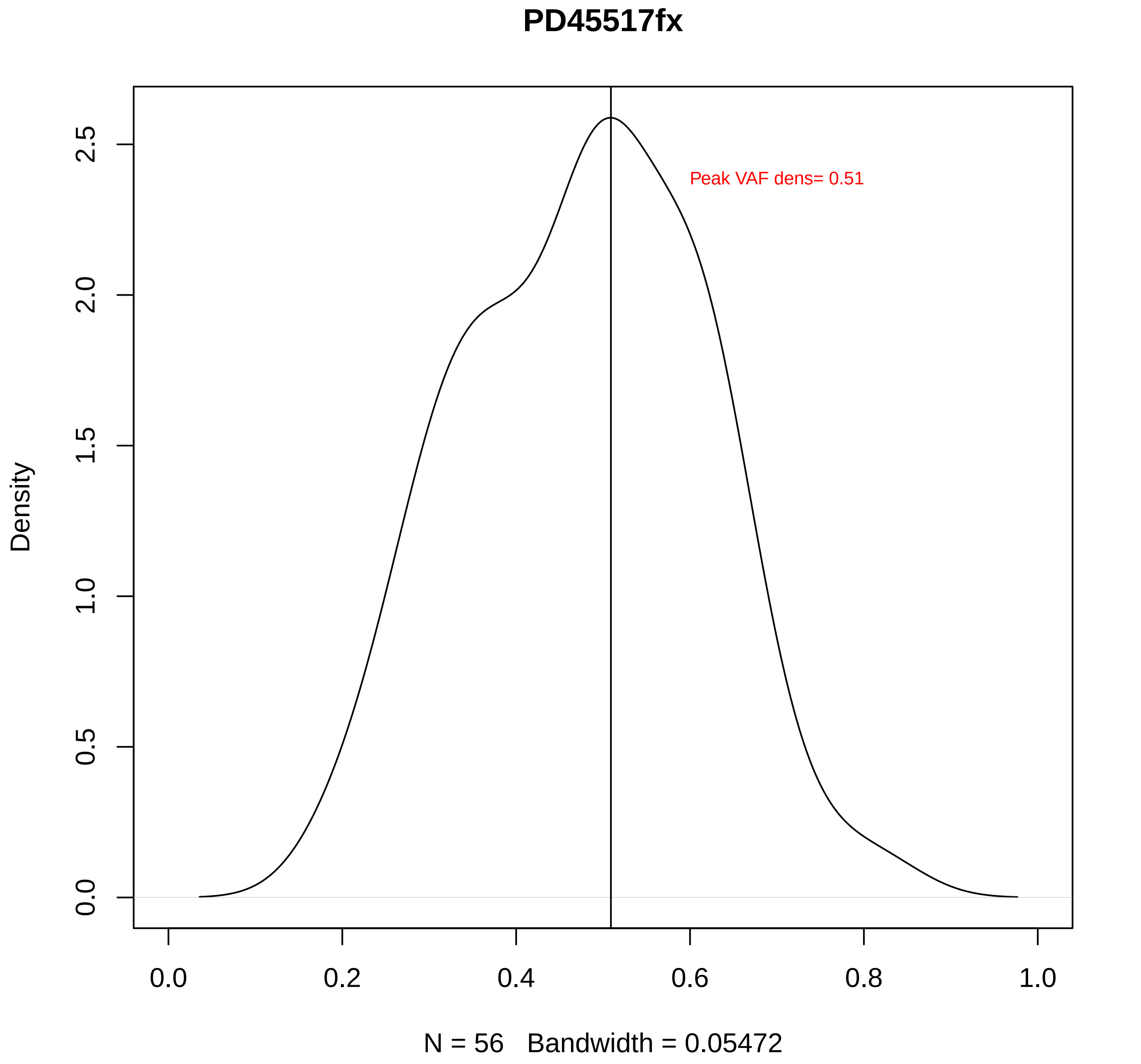
PD45517fx
Peak VAF dens= 0.51
Density
0.0
0.5
1.0
1.5
2.0
2.5
0.0
0.2
0.4
0.6
0.8
1.0
N = 56 Bandwidth = 0.05472

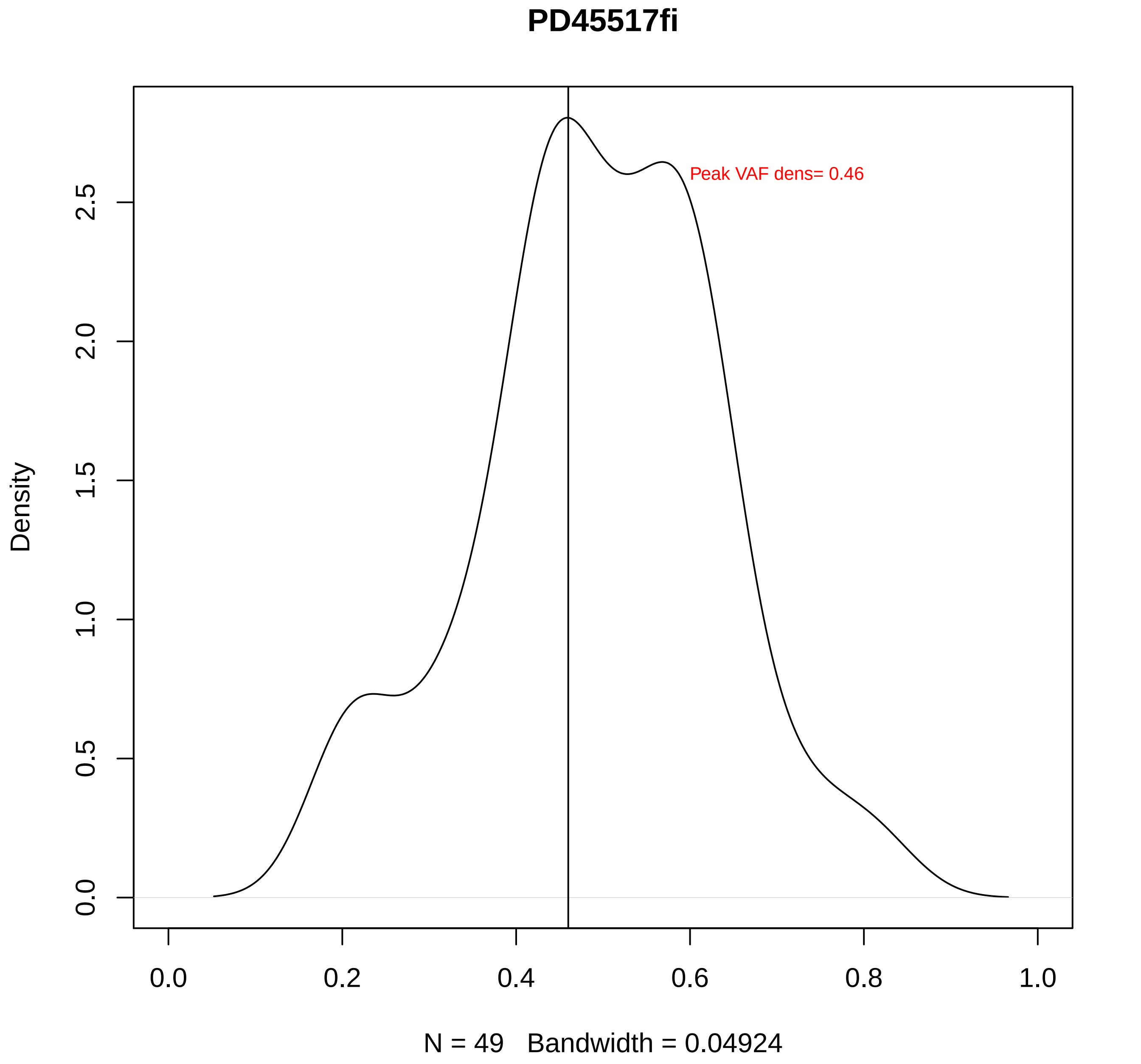
PD45517fi
Peak VAF dens= 0.46
Density
0.0
0.5
1.0
1.5
2.0
2.5
0.0
0.2
0.4
0.6
0.8
1.0
N = 49   Bandwidth = 0.04924

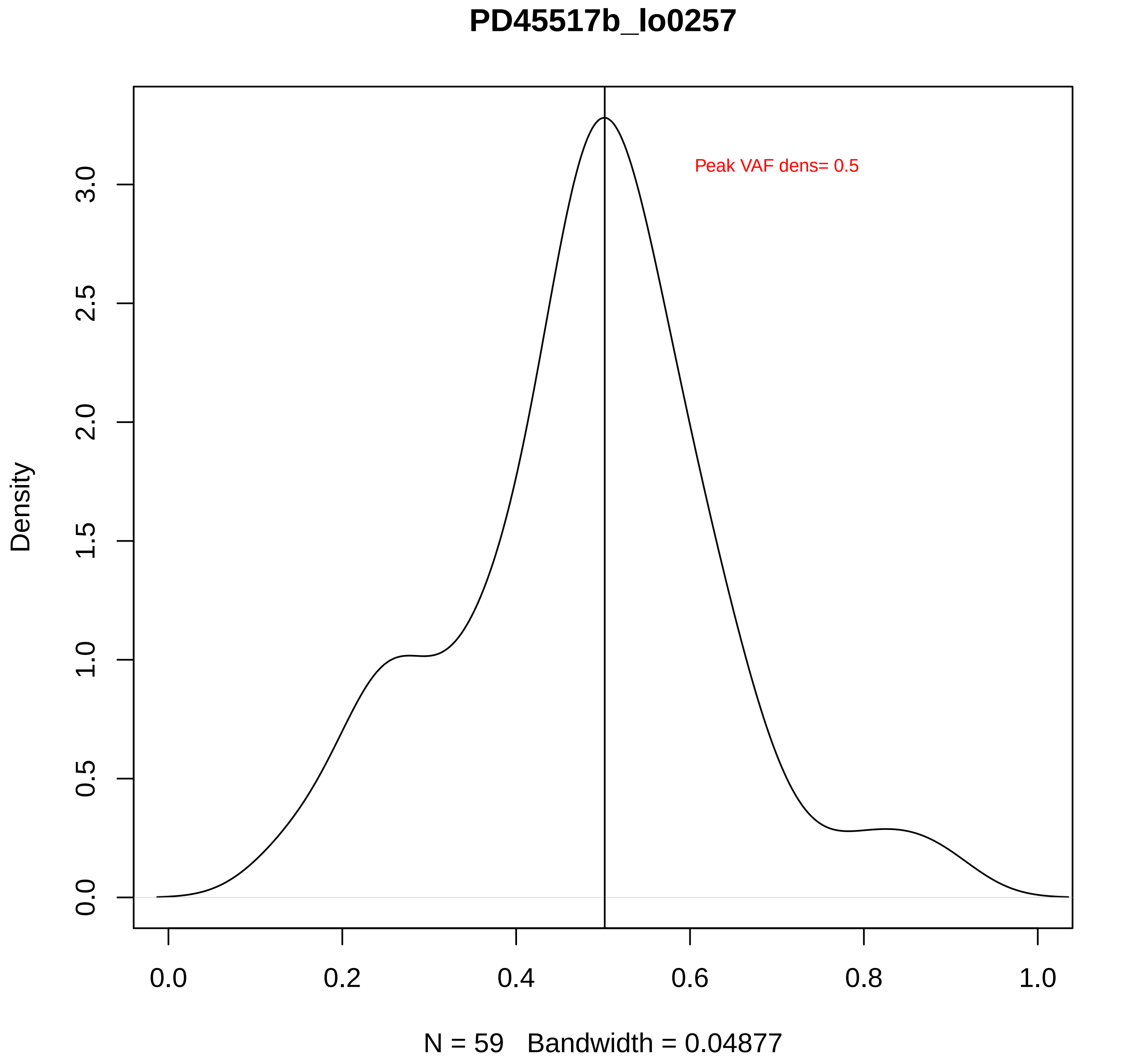
PD45517b_lo0257
Peak VAF dens= 0.5
Density
0.0
0.5
1.0
1.5
2.0
2.5
3.0
0.0
0.2
0.4
0.6
0.8
1.0
N = 59   Bandwidth = 0.04877

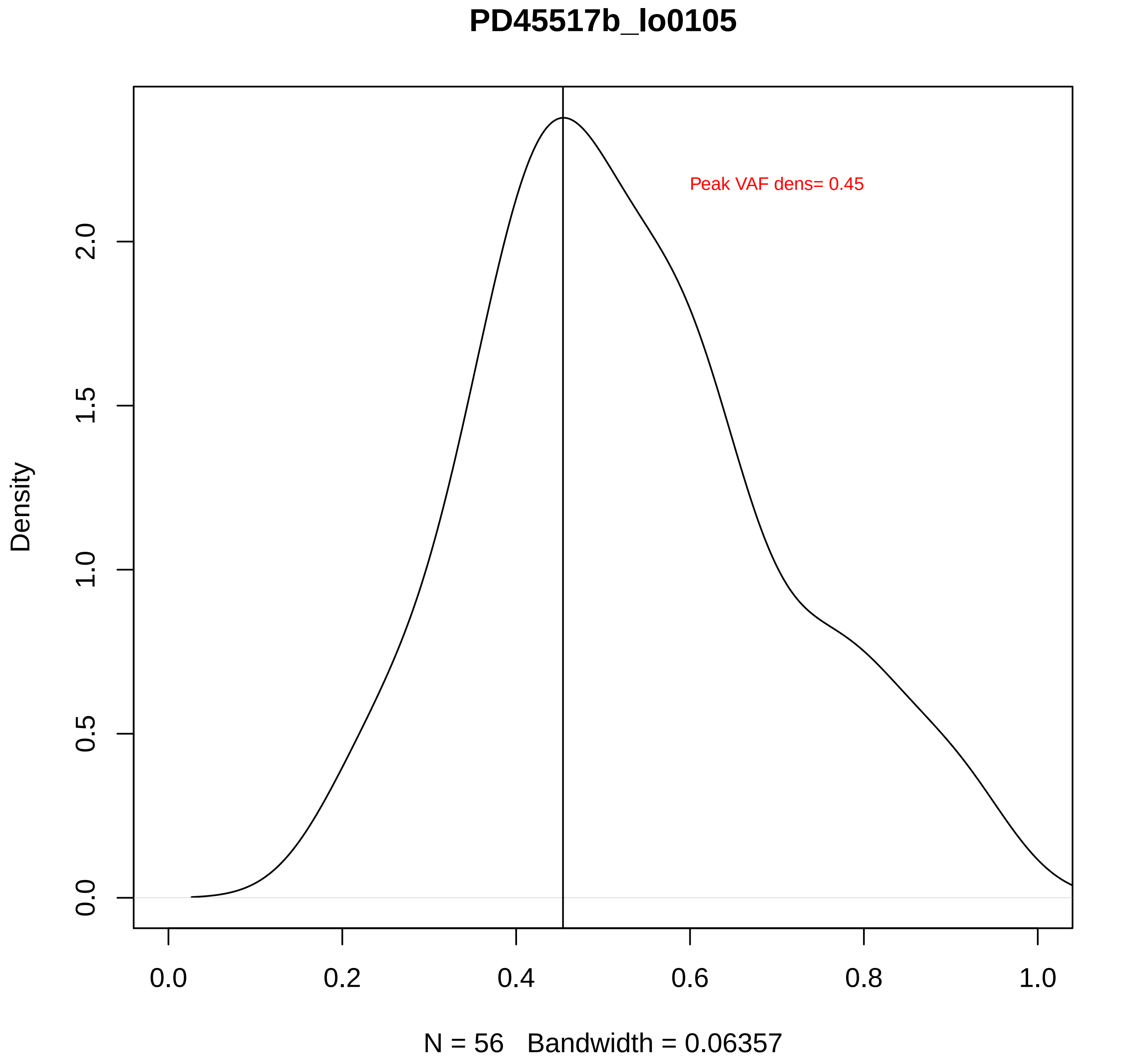
PD45517b_lo0105
Peak VAF dens= 0.45
Density
0.0
0.5
1.0
1.5
2.0
0.0
0.2
0.4
0.6
0.8
1.0
N = 56 Bandwidth = 0.06357

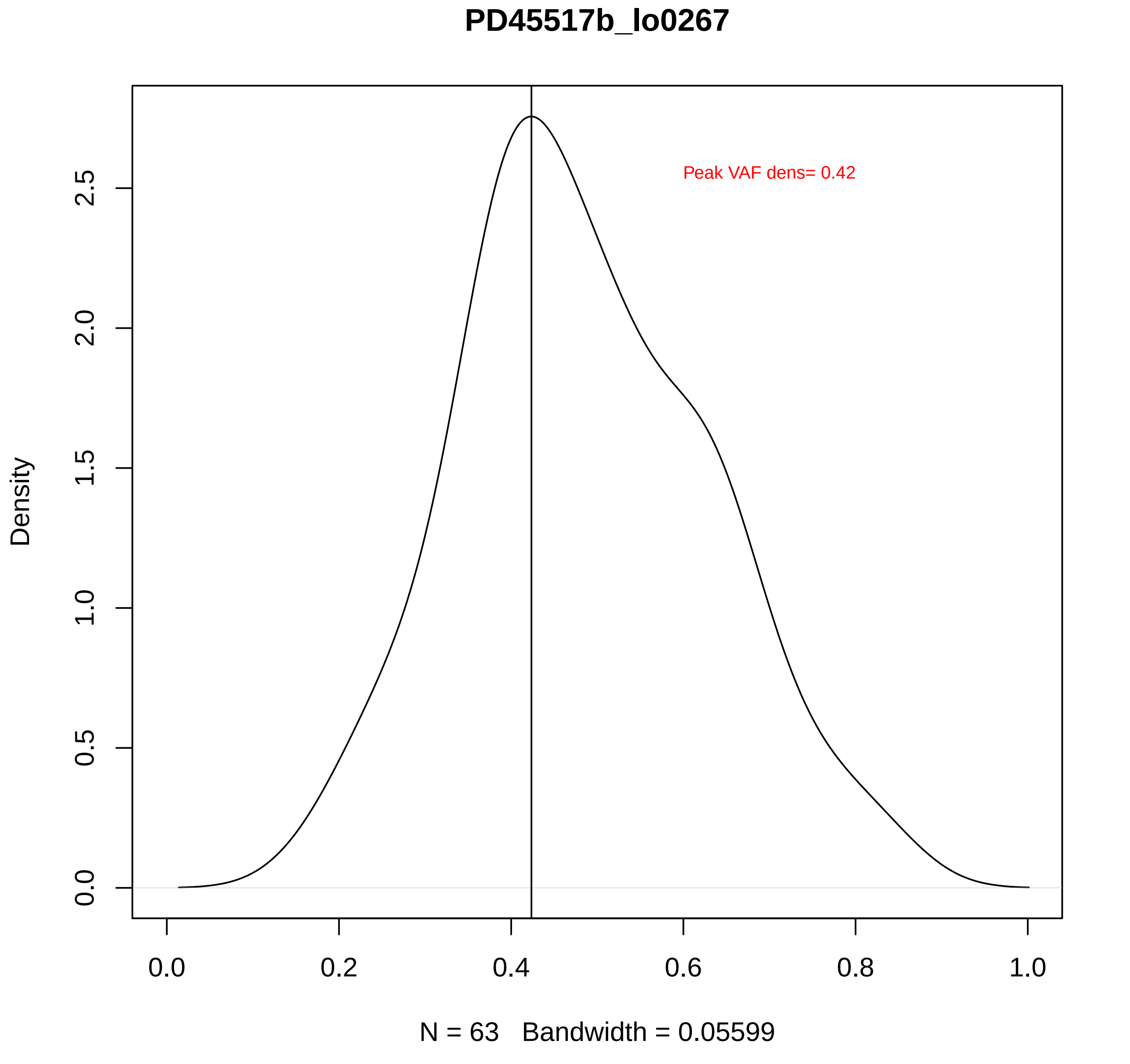
PD45517b_lo0267
Peak VAF dens= 0.42
Density
0.0
0.5
1.0
1.5
2.0
2.5
0.0
0.2
0.4
0.6
0.8
1.0
N = 63   Bandwidth = 0.05599

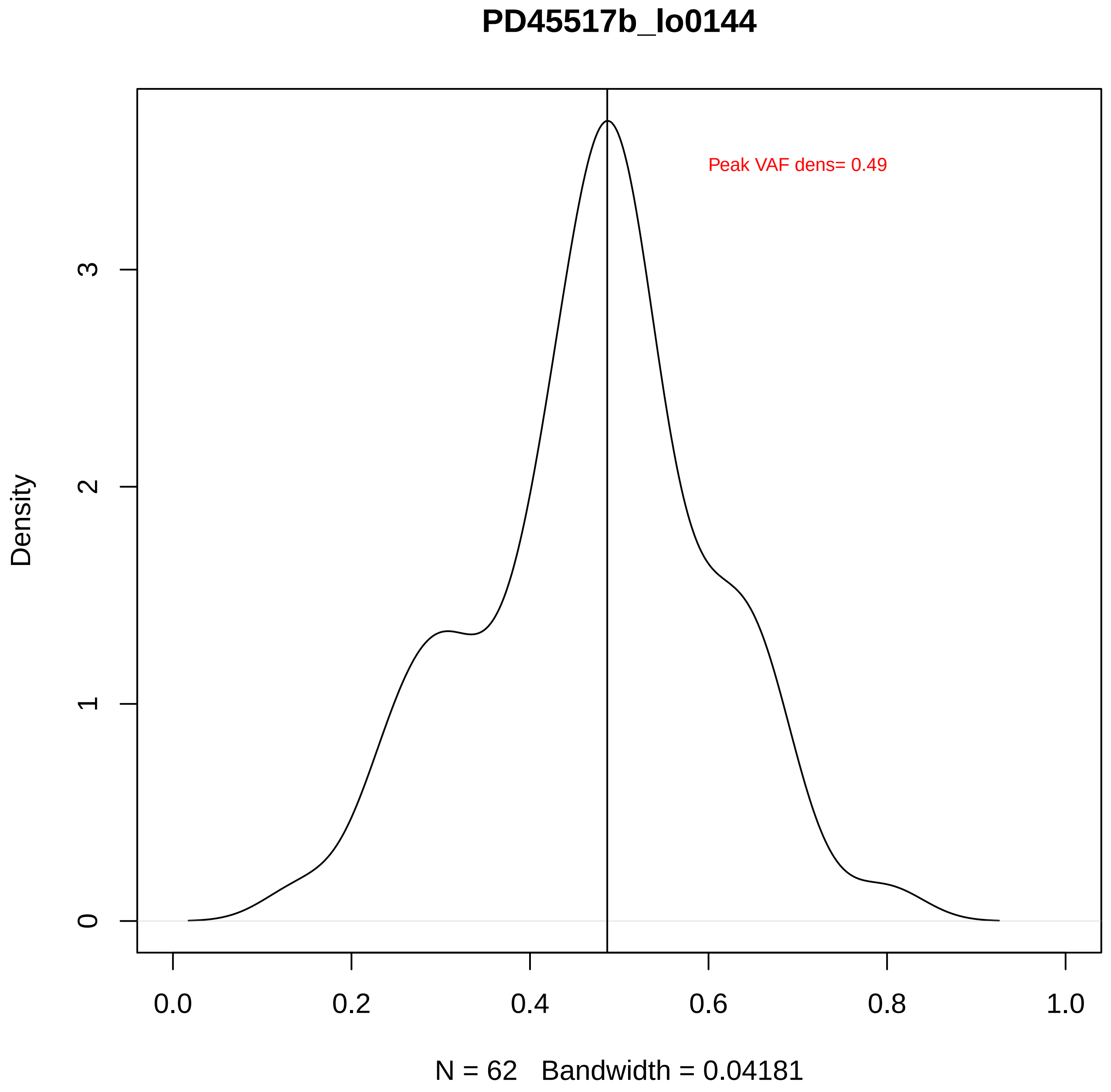

PD45517b_lo0144
Peak VAF dens= 0.49
Density
0
1
2
3
0.0
0.2
0.4
0.6
0.8
1.0
N = 62   Bandwidth = 0.04181

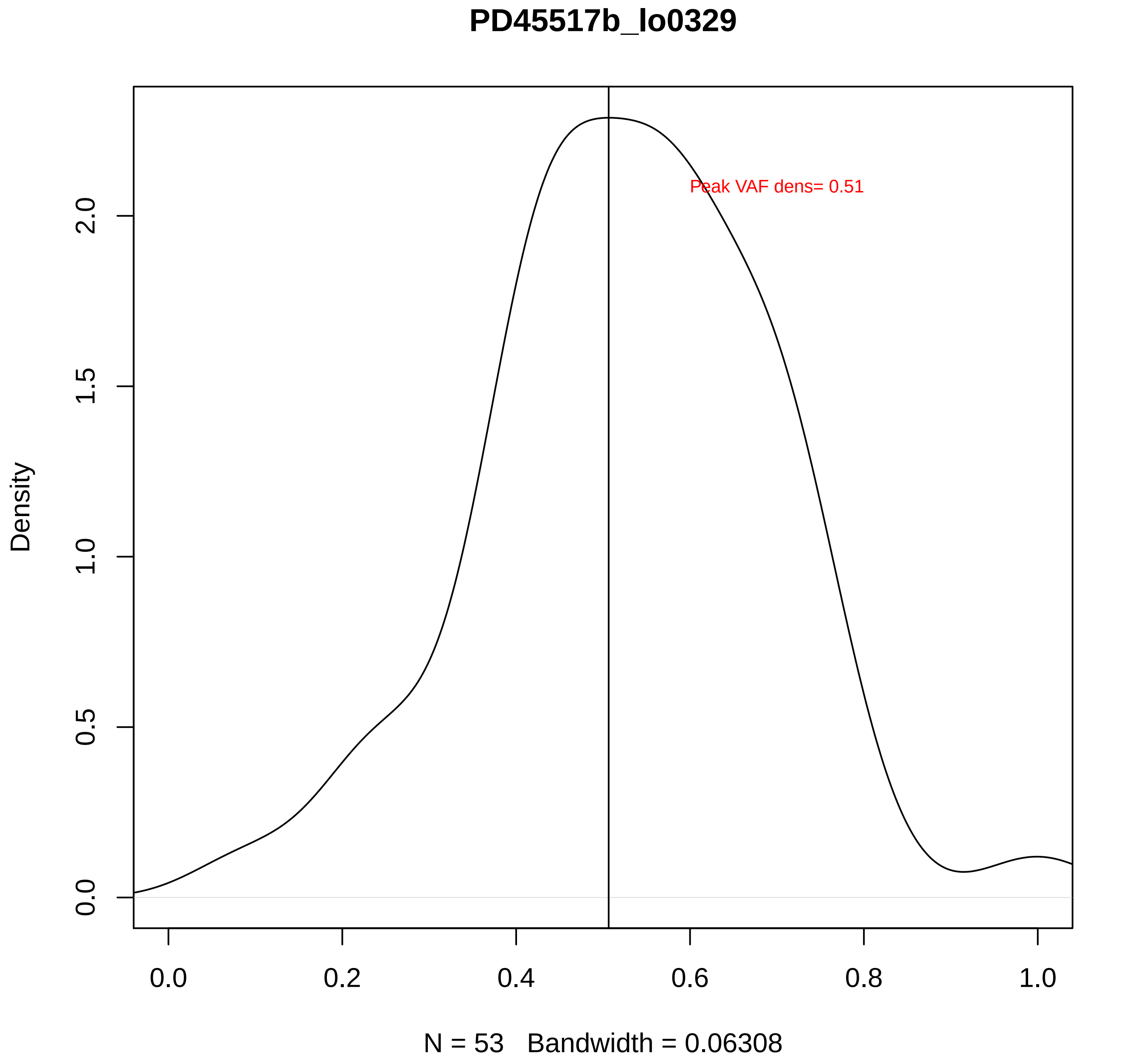
PD45517b_lo0329
Peak VAF dens= 0.51
Density
0.0
0.5
1.0
1.5
2.0
0.0
0.2
0.4
0.6
0.8
1.0
N = 53   Bandwidth = 0.06308

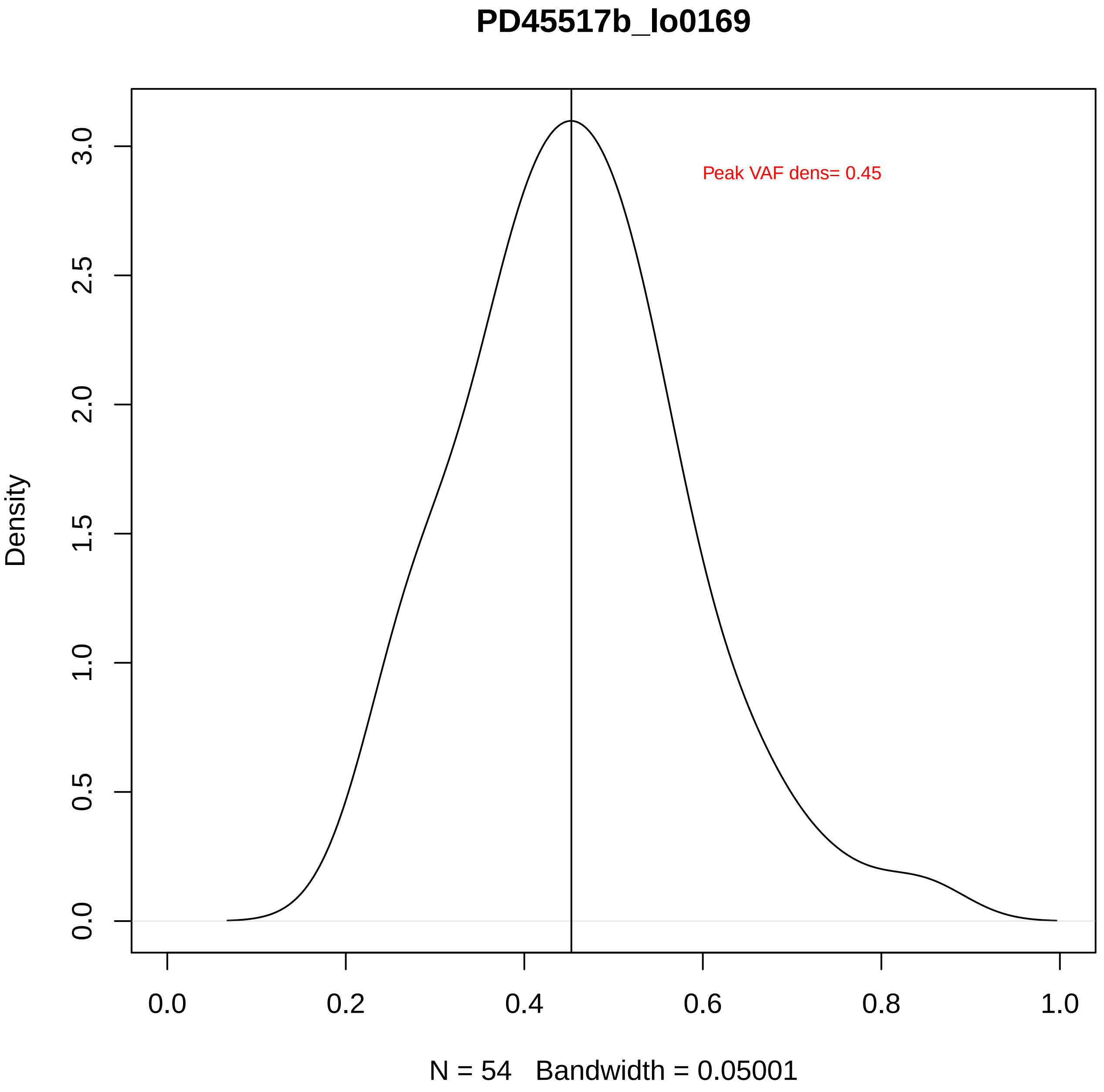
PD45517b_lo0169
Peak VAF dens= 0.45
Density
0.0
0.5
1.0
1.5
2.0
2.5
3.0
0.0
0.2
0.4
0.6
0.8
1.0
N = 54   Bandwidth = 0.05001

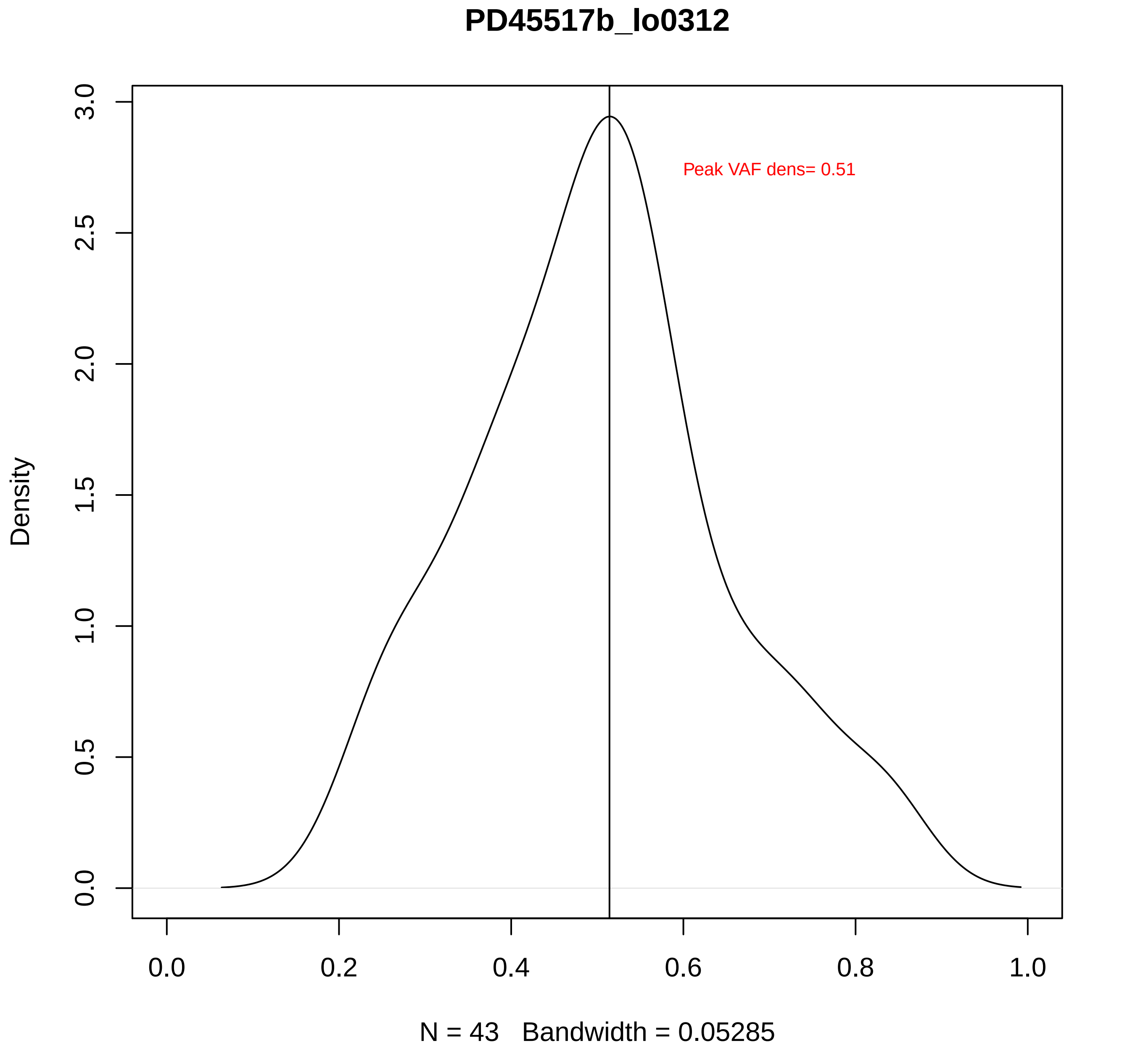
PD45517b_lo0312
Peak VAF dens= 0.51
Density
0.0
0.5
1.0
1.5
2.0
2.5
3.0
0.0
0.2
0.4
0.6
0.8
1.0
N = 43   Bandwidth = 0.05285

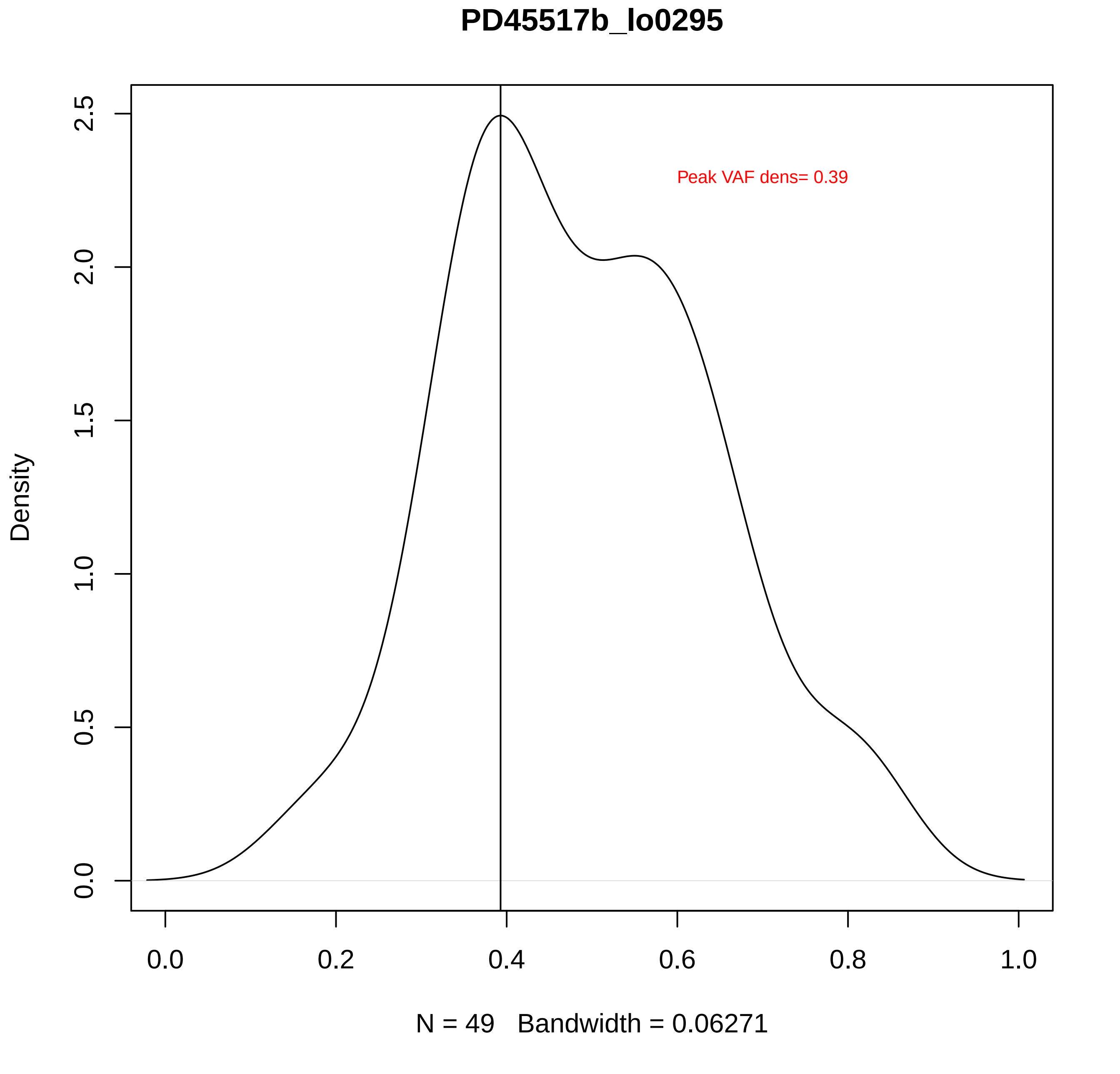
PD45517b_lo0295
Peak VAF dens= 0.39
Density
0.0
0.5
1.0
1.5
2.0
2.5
0.0
0.2
0.4
0.6
0.8
1.0
N = 49   Bandwidth = 0.06271

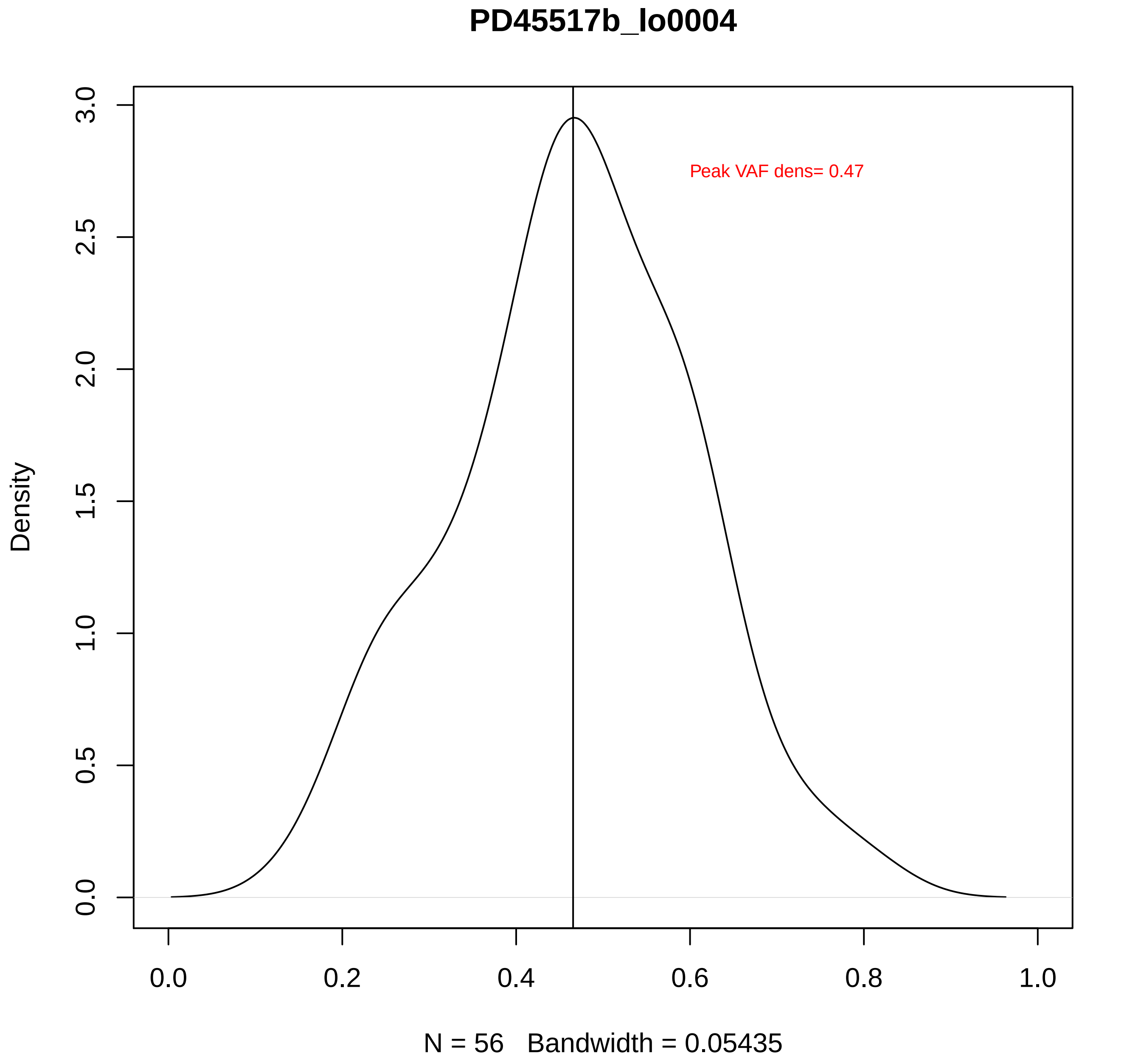
PD45517b_lo0004
Peak VAF dens= 0.47
Density
0.0
0.5
1.0
1.5
2.0
2.5
3.0
0.0
0.2
0.4
0.6
0.8
1.0
N = 56   Bandwidth = 0.05435

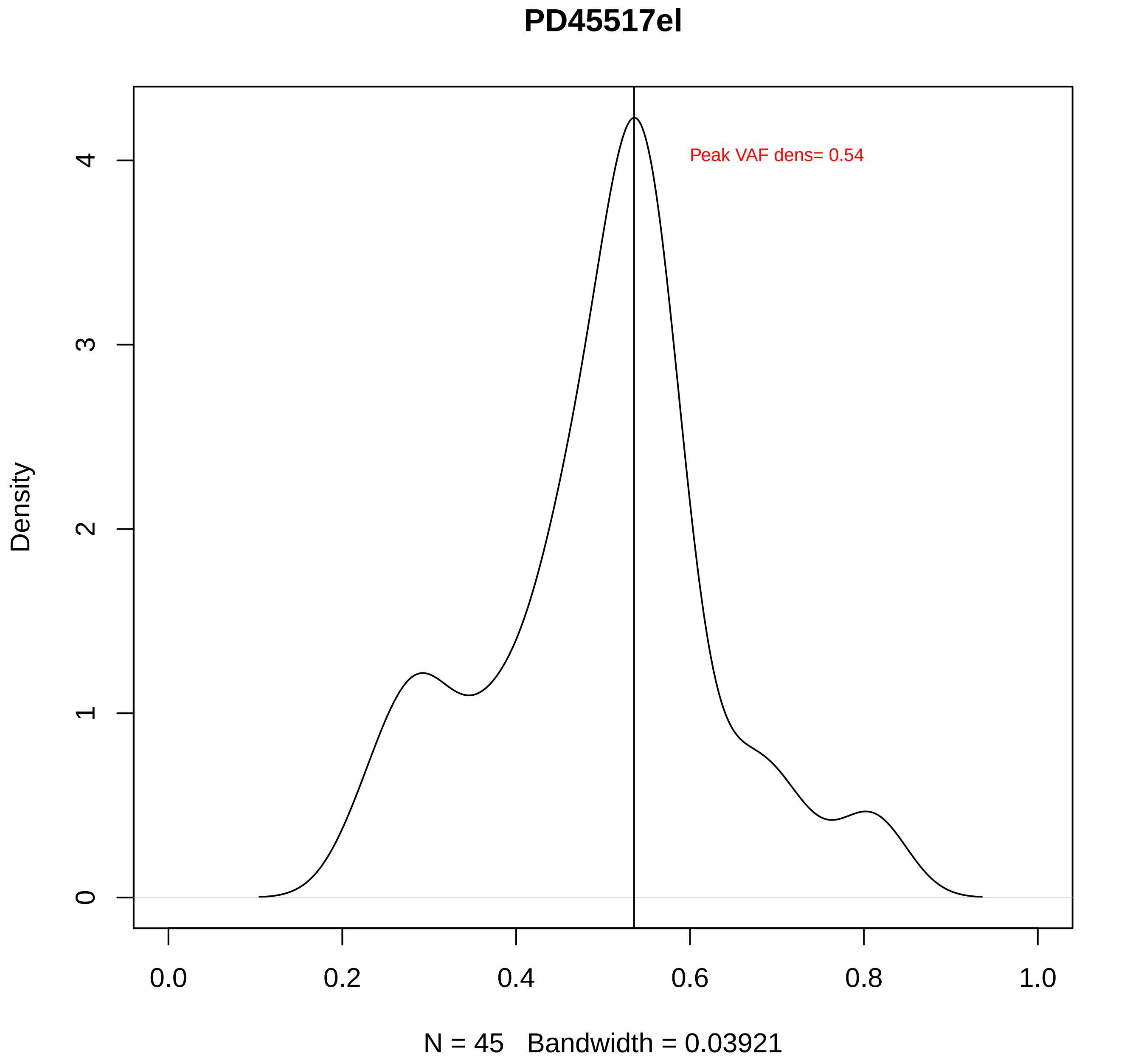
PD45517el
Peak VAF dens= 0.54
Density
0
1
2
3
4
0.0
0.2
0.4
0.6
0.8
1.0
N = 45 Bandwidth = 0.03921

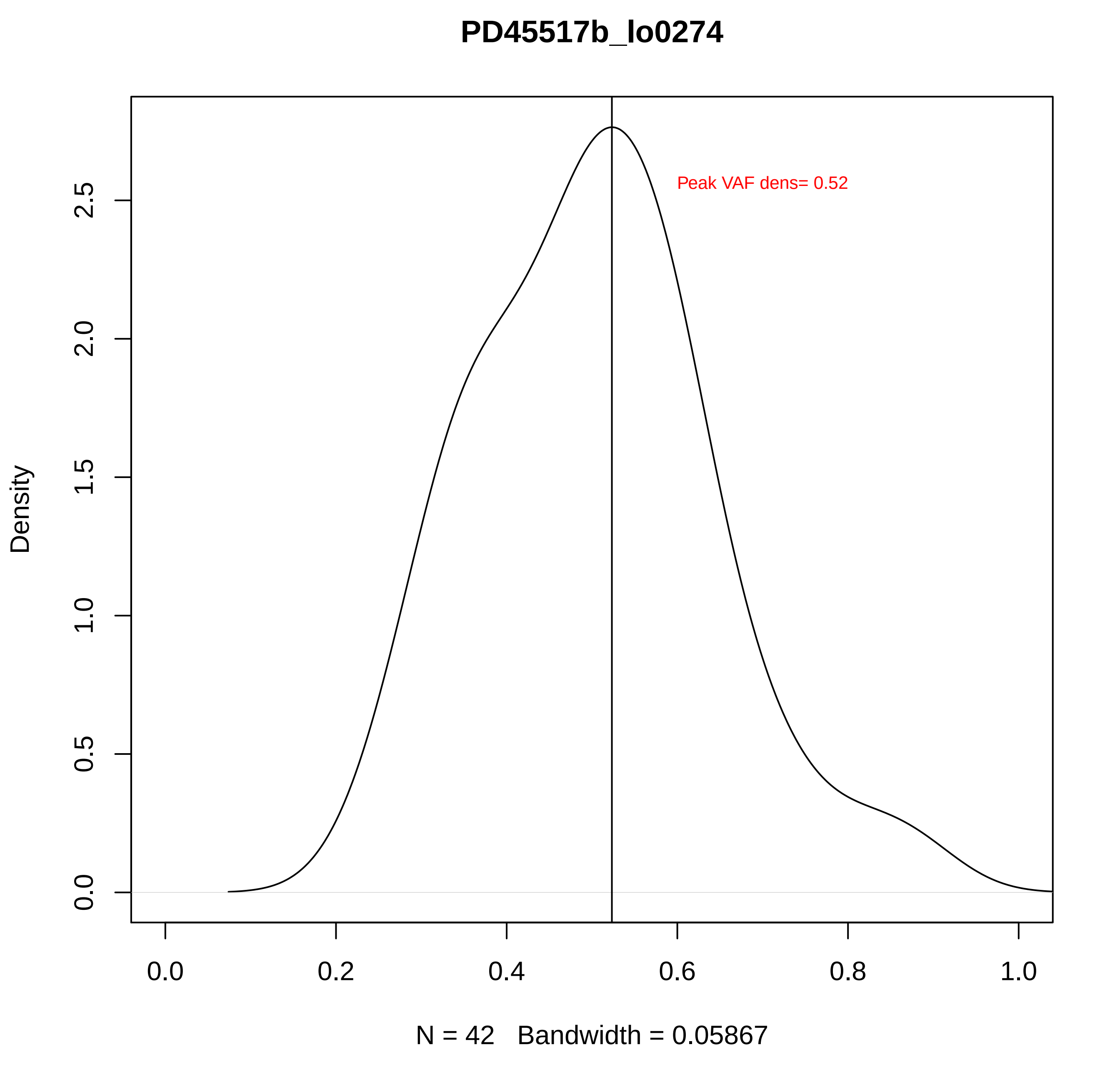
PD45517b_lo0274
Peak VAF dens= 0.52
Density
0.0
0.5
1.0
1.5
2.0
2.5
0.0
0.2
0.4
0.6
0.8
1.0
N = 42   Bandwidth = 0.05867

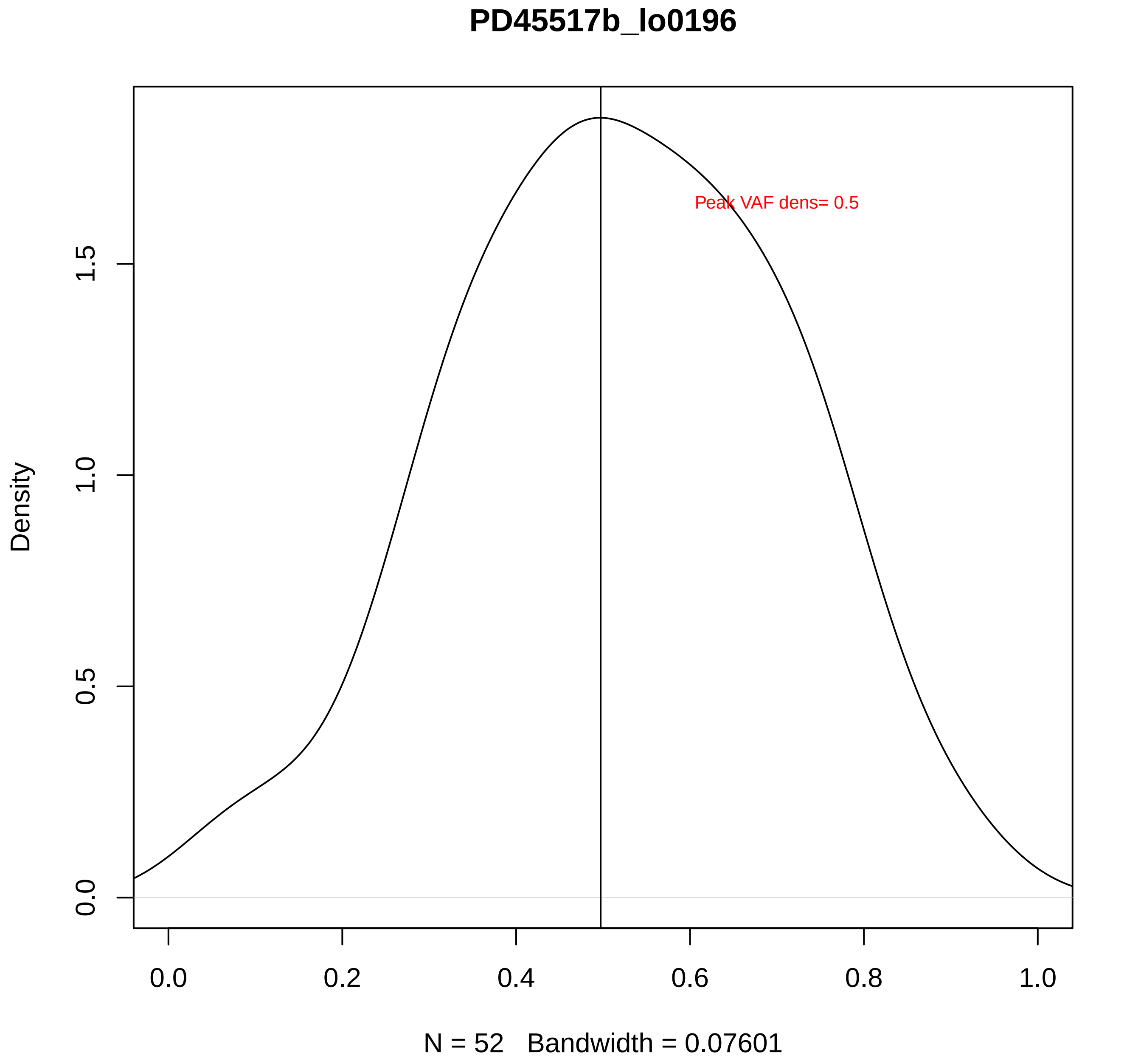
PD45517b_lo0196
Peak VAF dens= 0.5
Density
0.0
0.5
1.0
1.5
0.0
0.2
0.4
0.6
0.8
1.0
N = 52 Bandwidth = 0.07601

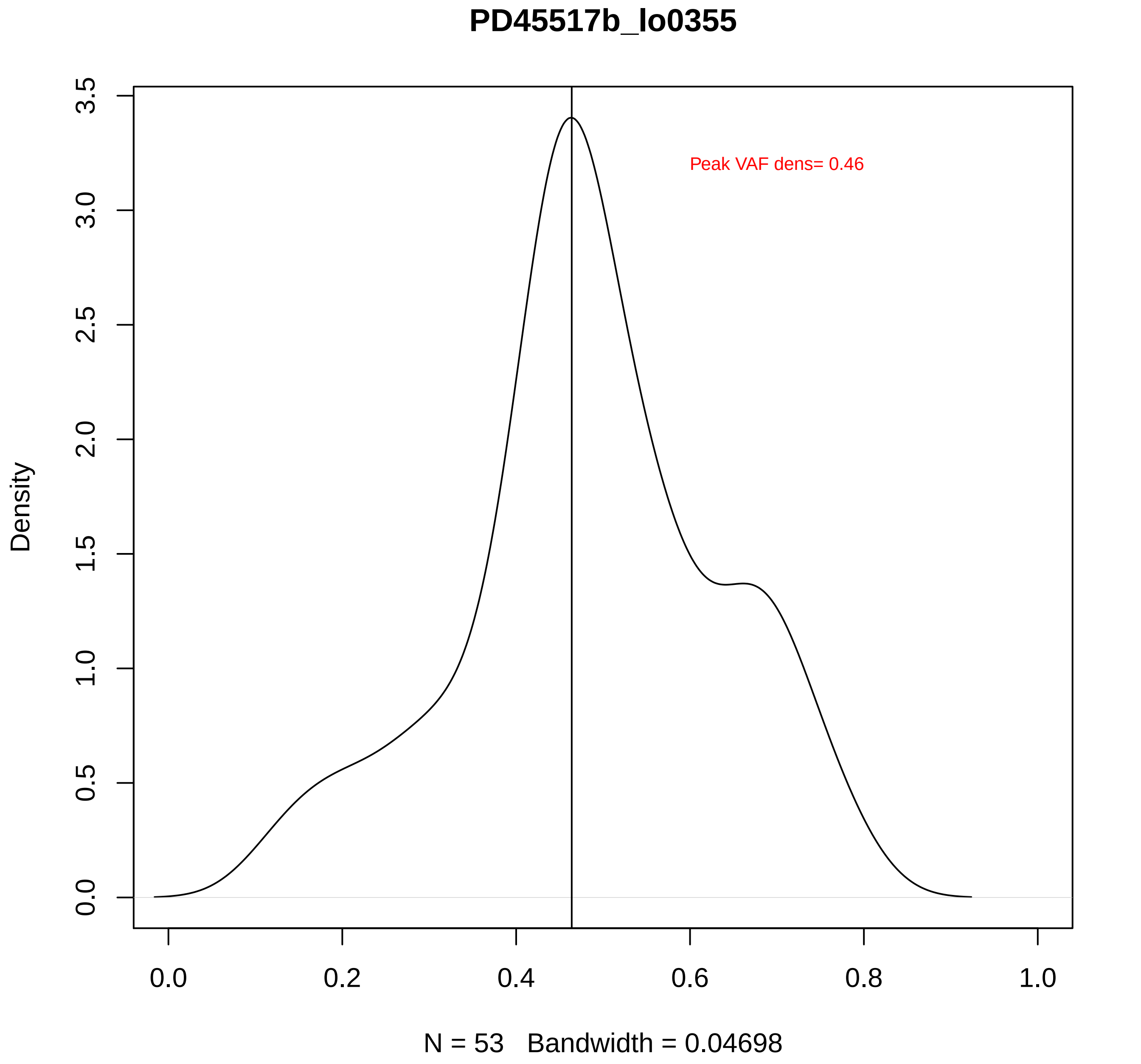
PD45517b_lo0355
Peak VAF dens= 0.46
Density
0.0
0.5
1.0
1.5
2.0
2.5
3.0
3.5
0.0
0.2
0.4
0.6
0.8
1.0
N = 53 Bandwidth = 0.04698

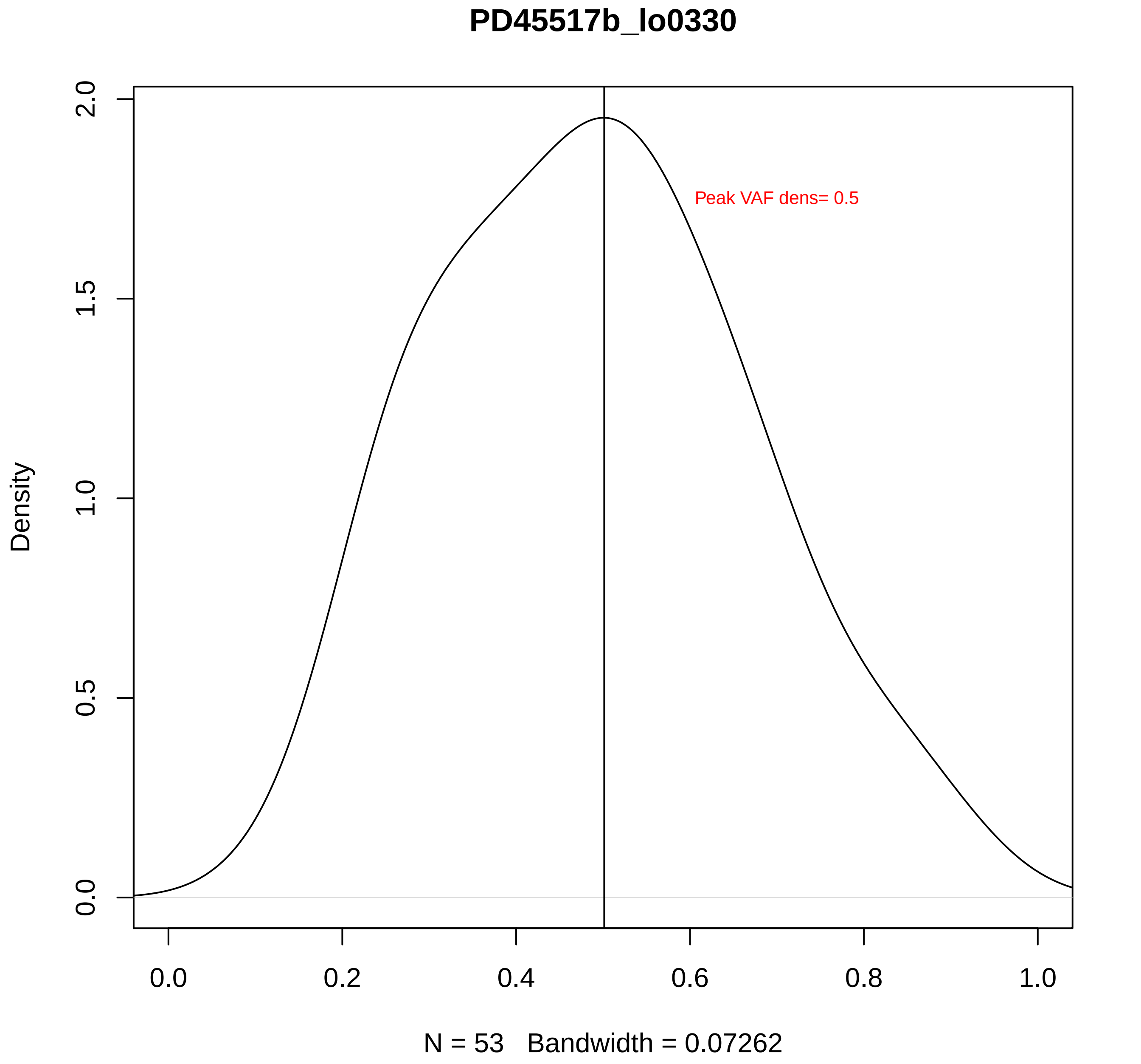

PD45517b_lo0330
Peak VAF dens= 0.5
Density
0.0
0.5
1.0
1.5
2.0
0.0
0.2
0.4
0.6
0.8
1.0
N = 53   Bandwidth = 0.07262

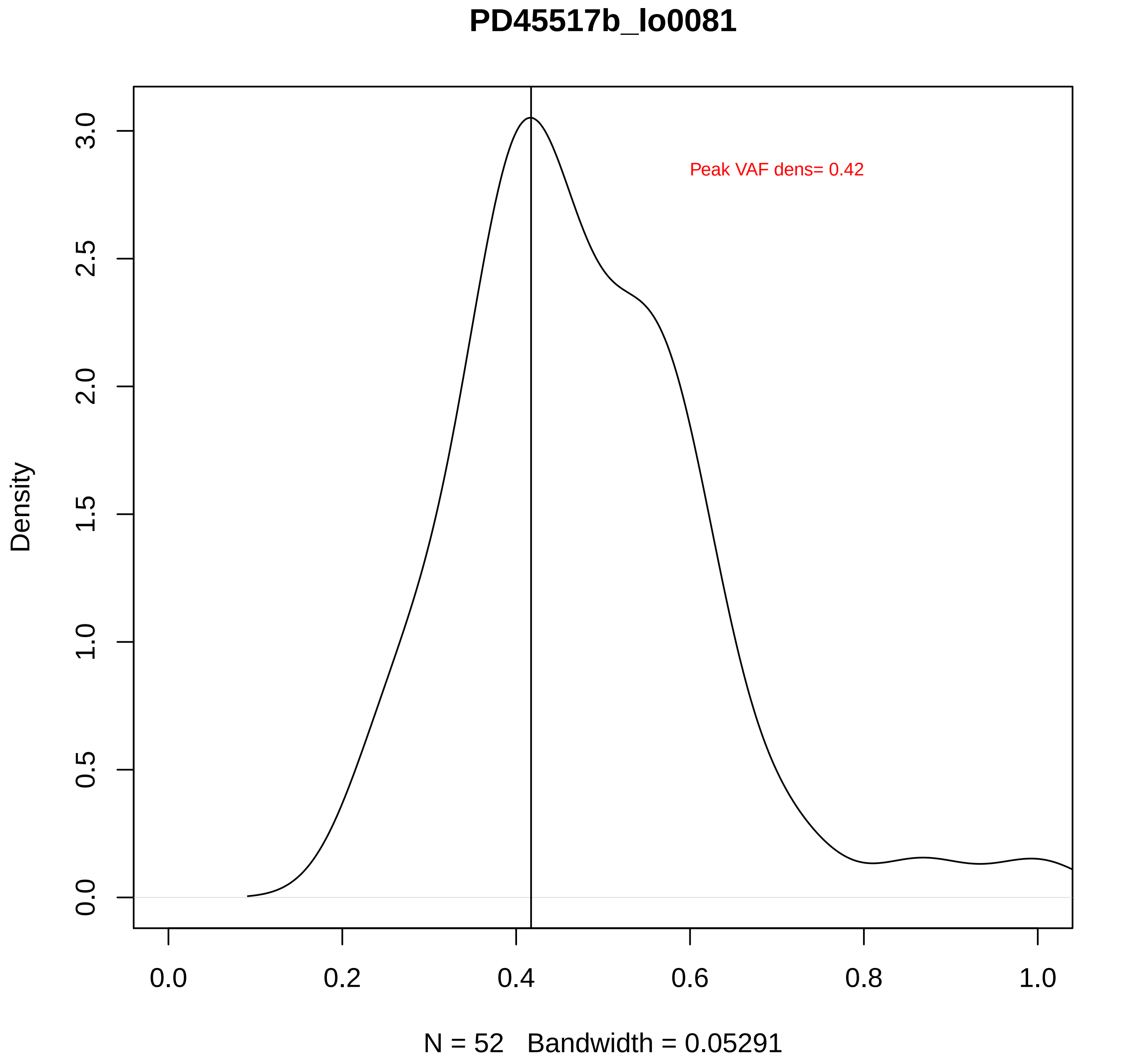
PD45517b_lo0081
Peak VAF dens= 0.42
Density
0.0
0.5
1.0
1.5
2.0
2.5
3.0
0.0
0.2
0.4
0.6
0.8
1.0
N = 52   Bandwidth = 0.05291

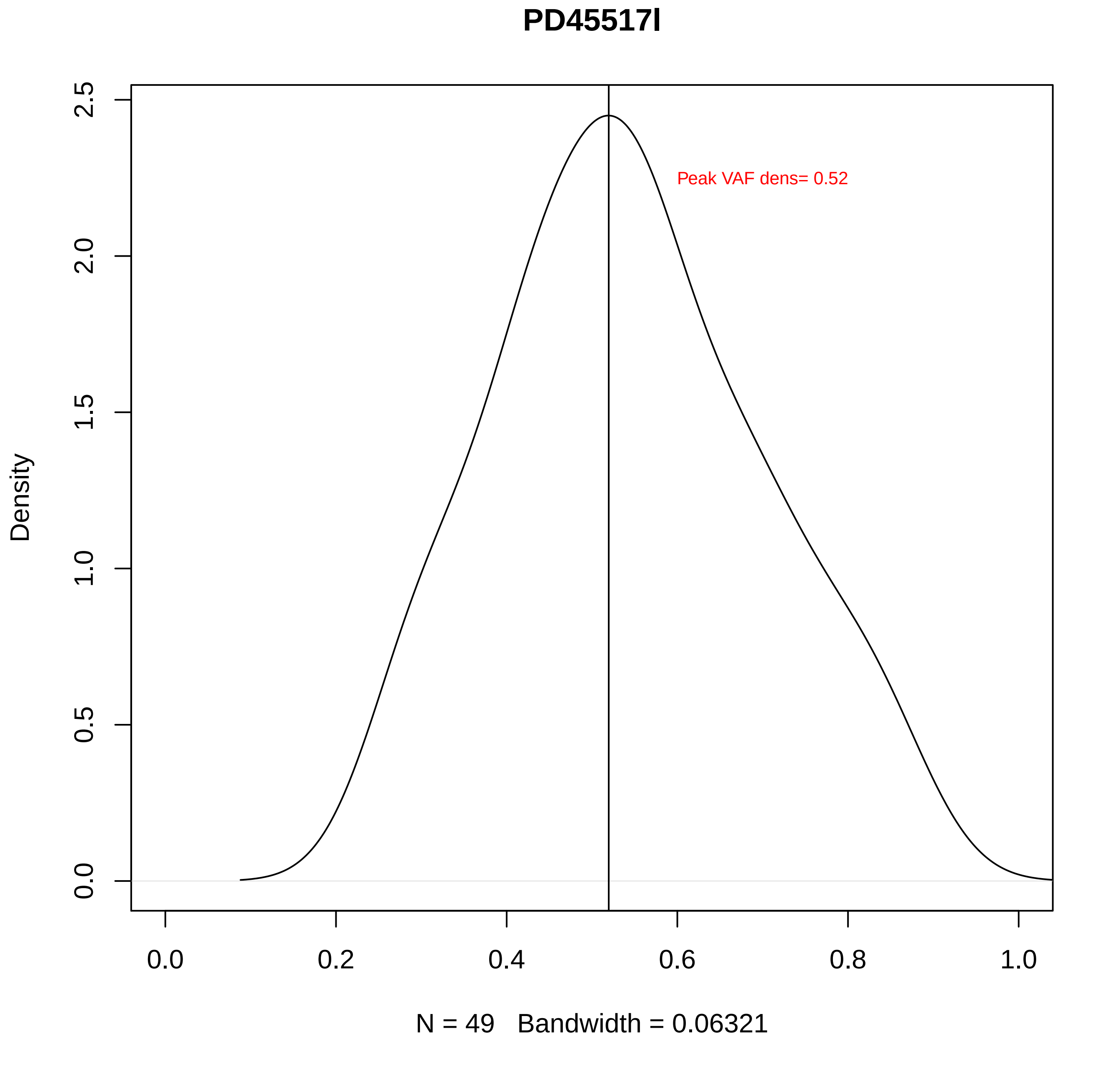

PD45517l
Peak VAF dens= 0.52
Density
0.0
0.5
1.0
1.5
2.0
2.5
0.0
0.2
0.4
0.6
0.8
1.0
N = 49 Bandwidth = 0.06321

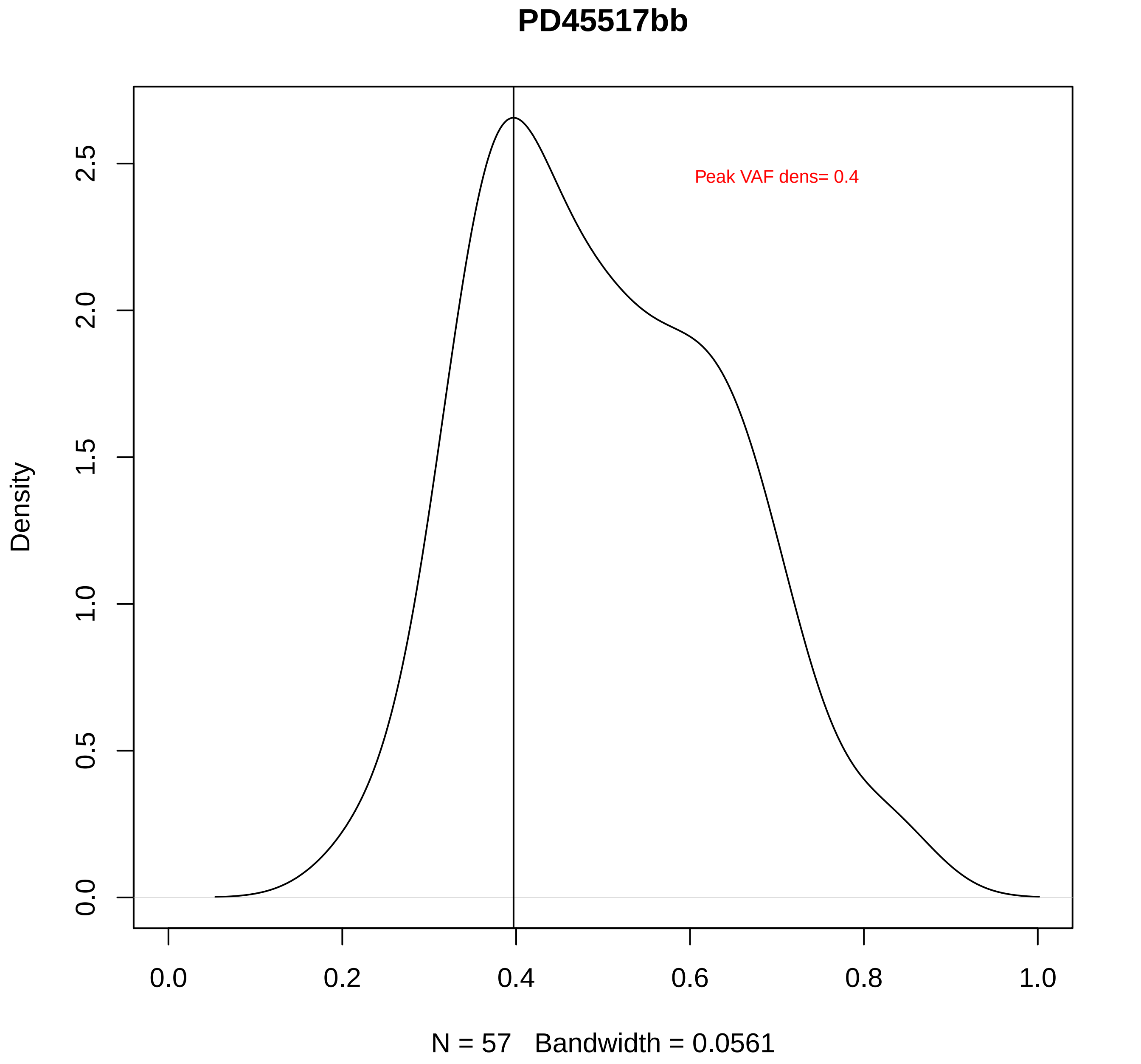
PD45517bb
Peak VAF dens= 0.4
Density
0.0
0.5
1.0
1.5
2.0
2.5
0.0
0.2
0.4
0.6
0.8
1.0
N = 57   Bandwidth = 0.0561

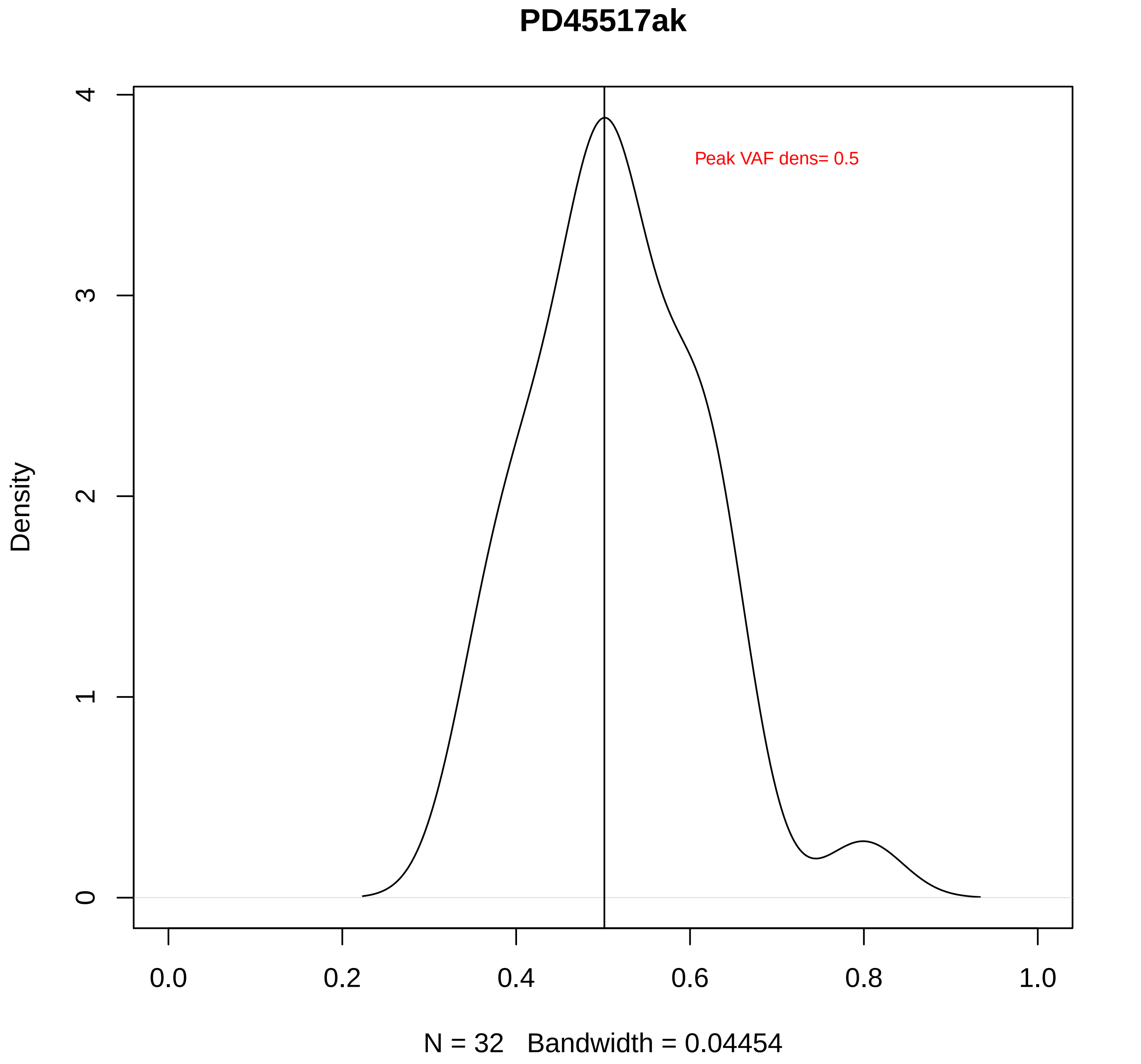

PD45517ak
Peak VAF dens= 0.5
Density
0
1
2
3
4
0.0
0.2
0.4
0.6
0.8
1.0
N = 32   Bandwidth = 0.04454

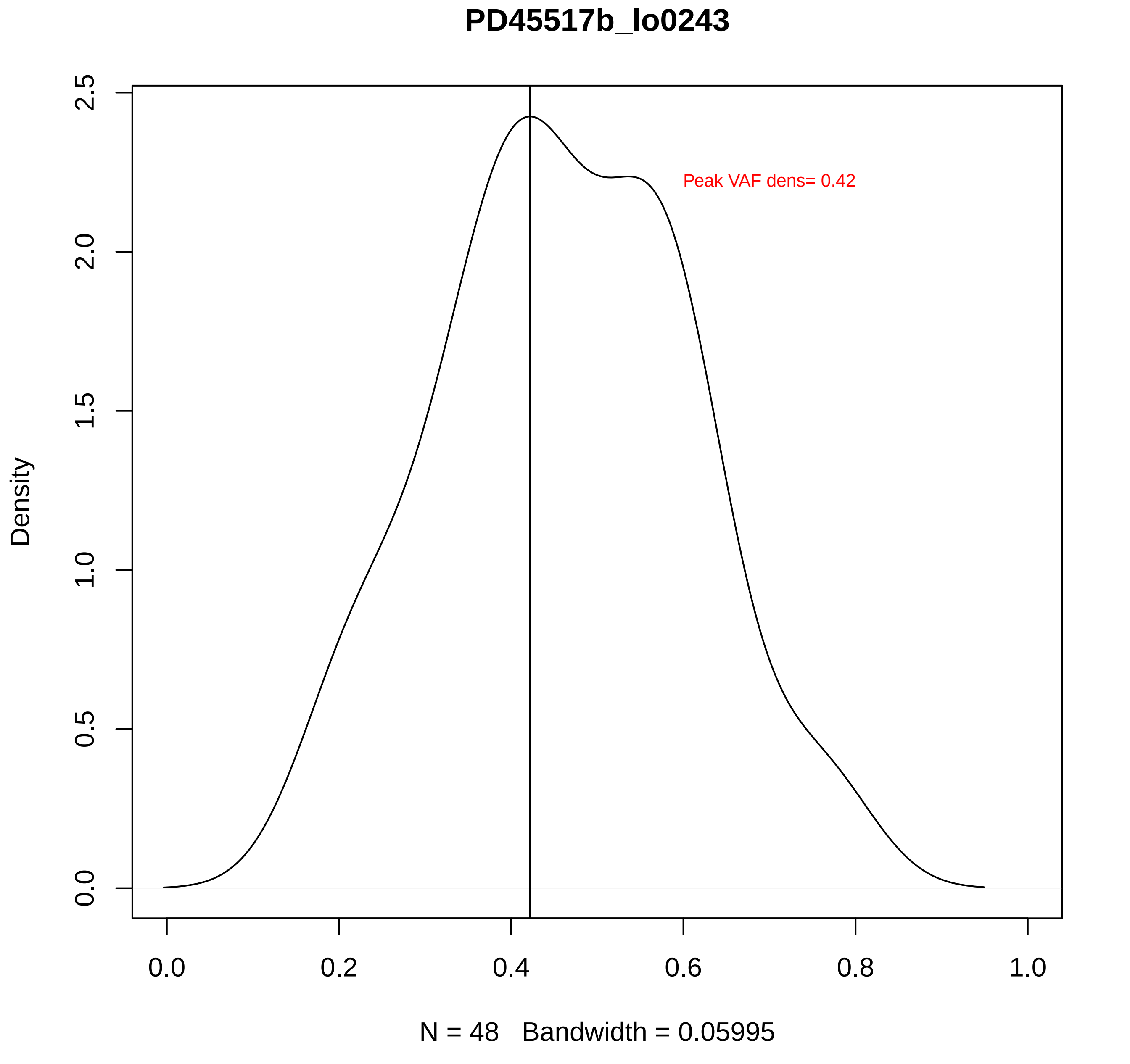
PD45517b_lo0243
Peak VAF dens= 0.42
Density
0.0
0.5
1.0
1.5
2.0
2.5
0.0
0.2
0.4
0.6
0.8
1.0
N = 48   Bandwidth = 0.05995

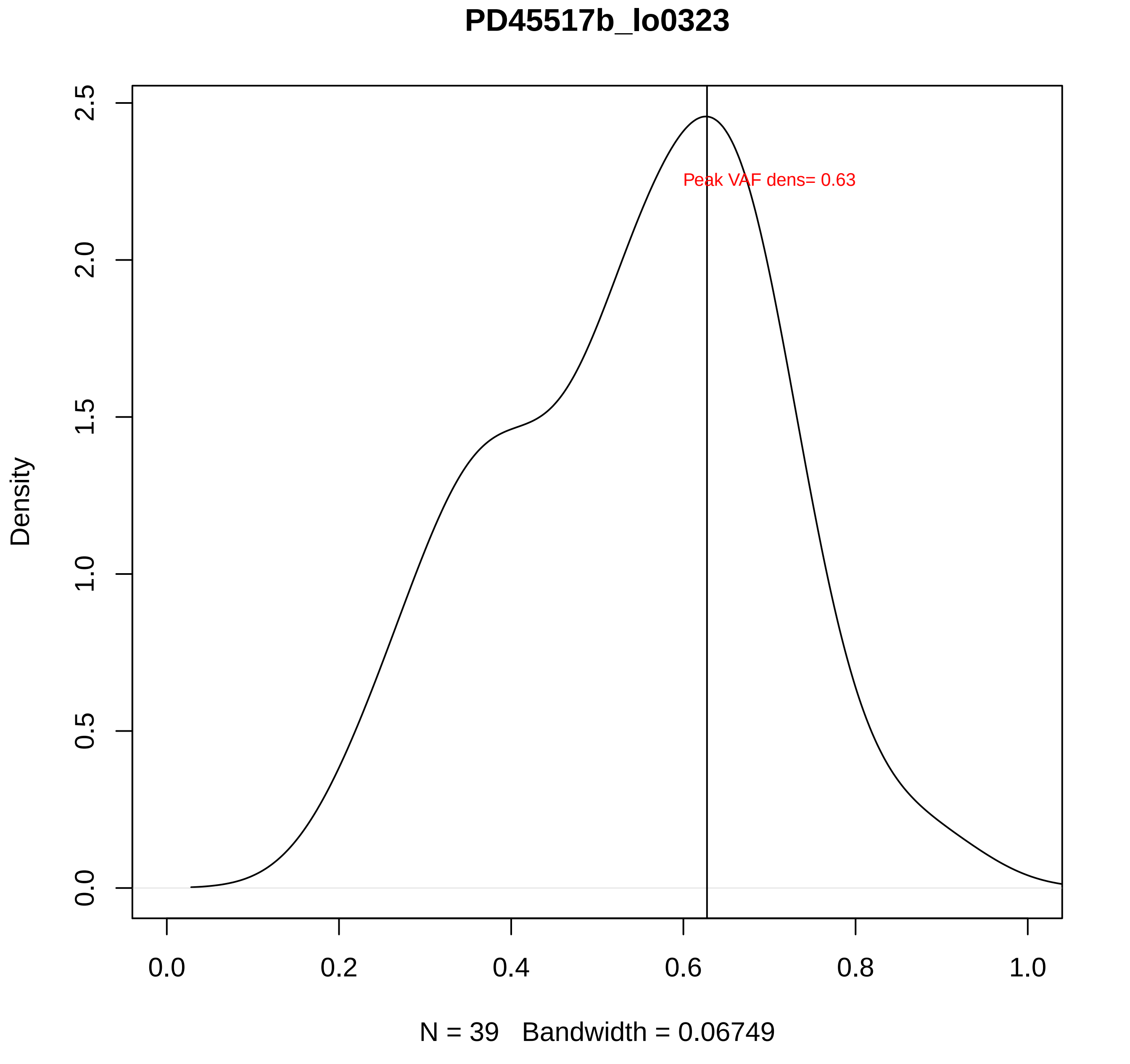
PD45517b_lo0323
Peak VAF dens= 0.63
Density
0.0
0.5
1.0
1.5
2.0
2.5
0.0
0.2
0.4
0.6
0.8
1.0
N = 39 Bandwidth = 0.06749

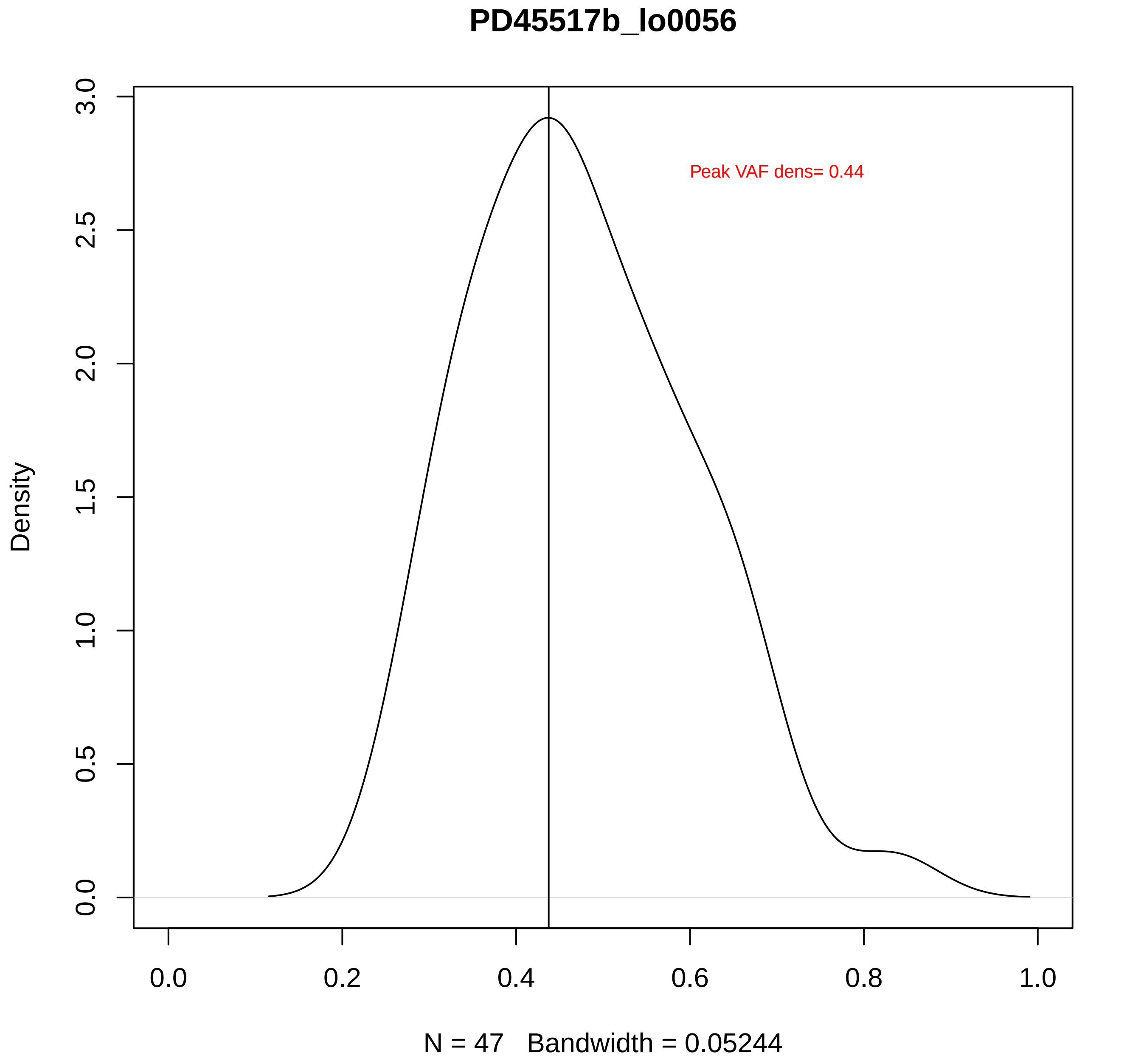

PD45517b_lo0056
Peak VAF dens= 0.44
Density
0.0
0.5
1.0
1.5
2.0
2.5
3.0
0.0
0.2
0.4
0.6
0.8
1.0
N = 47 Bandwidth = 0.05244

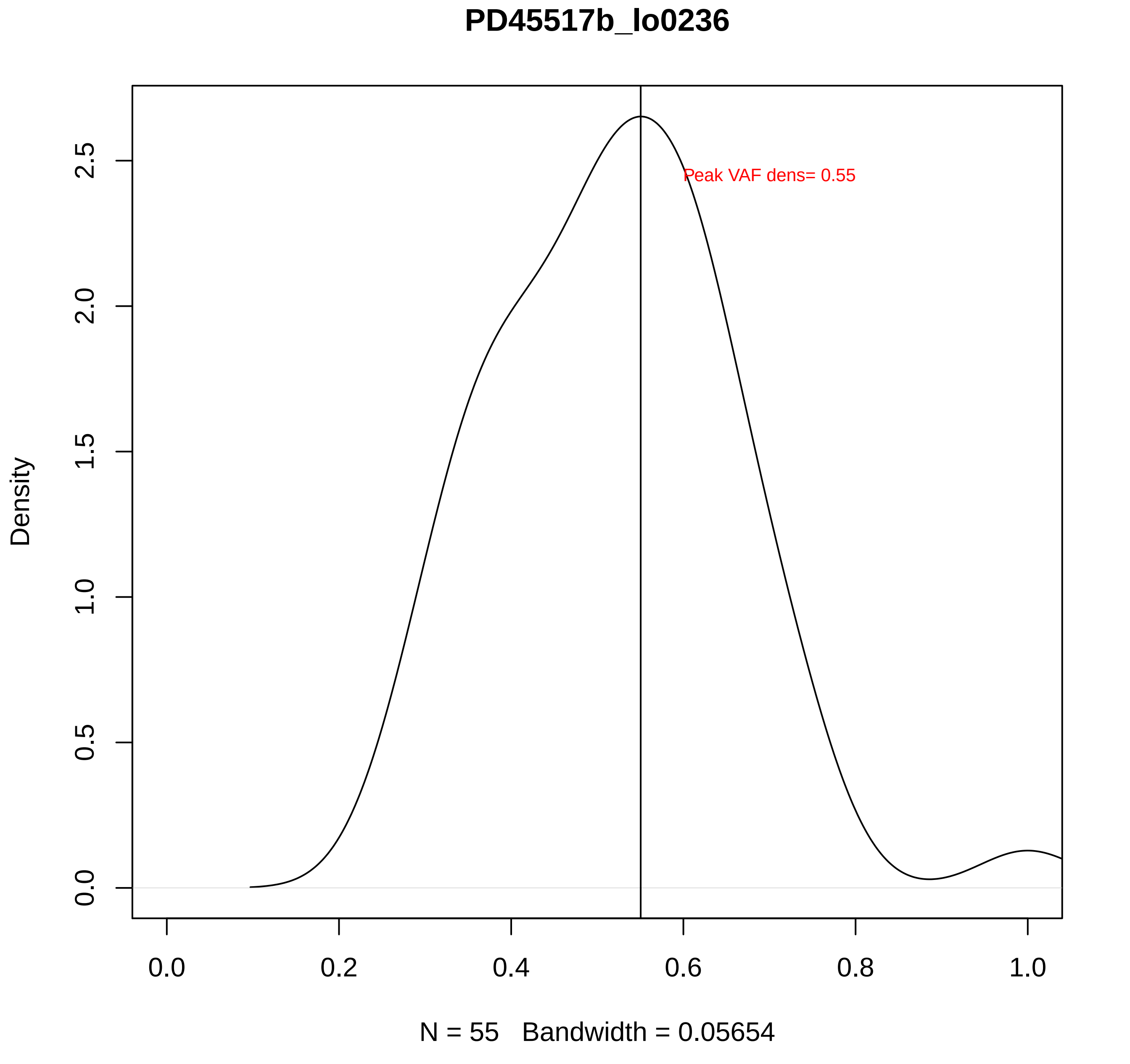

PD45517b_lo0236
Peak VAF dens= 0.55
Density
0.0
0.5
1.0
1.5
2.0
2.5
0.0
0.2
0.4
0.6
0.8
1.0
N = 55 Bandwidth = 0.05654

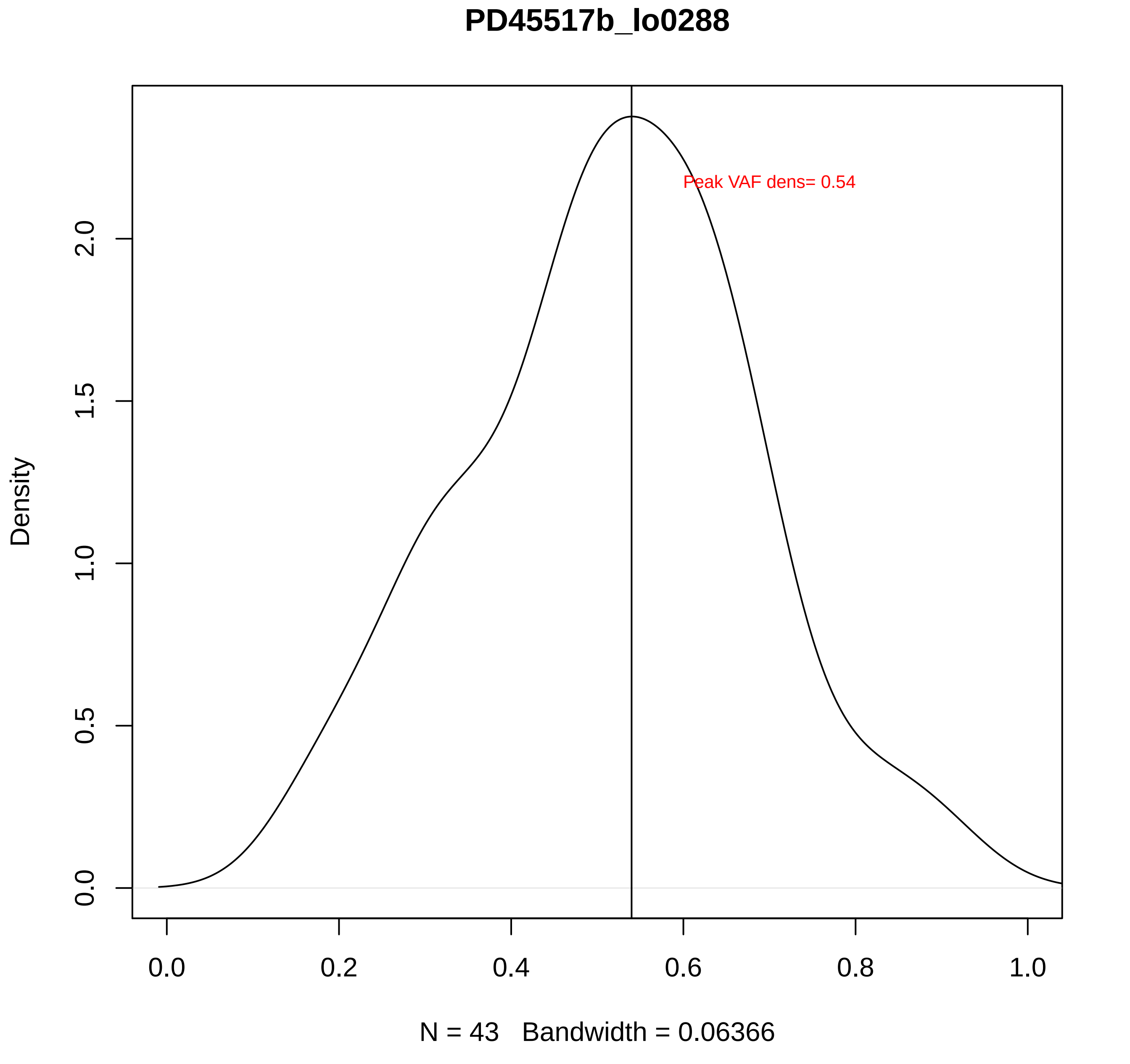
PD45517b_lo0288
Peak VAF dens= 0.54
Density
0.0
0.5
1.0
1.5
2.0
0.0
0.2
0.4
0.6
0.8
1.0
N = 43   Bandwidth = 0.06366

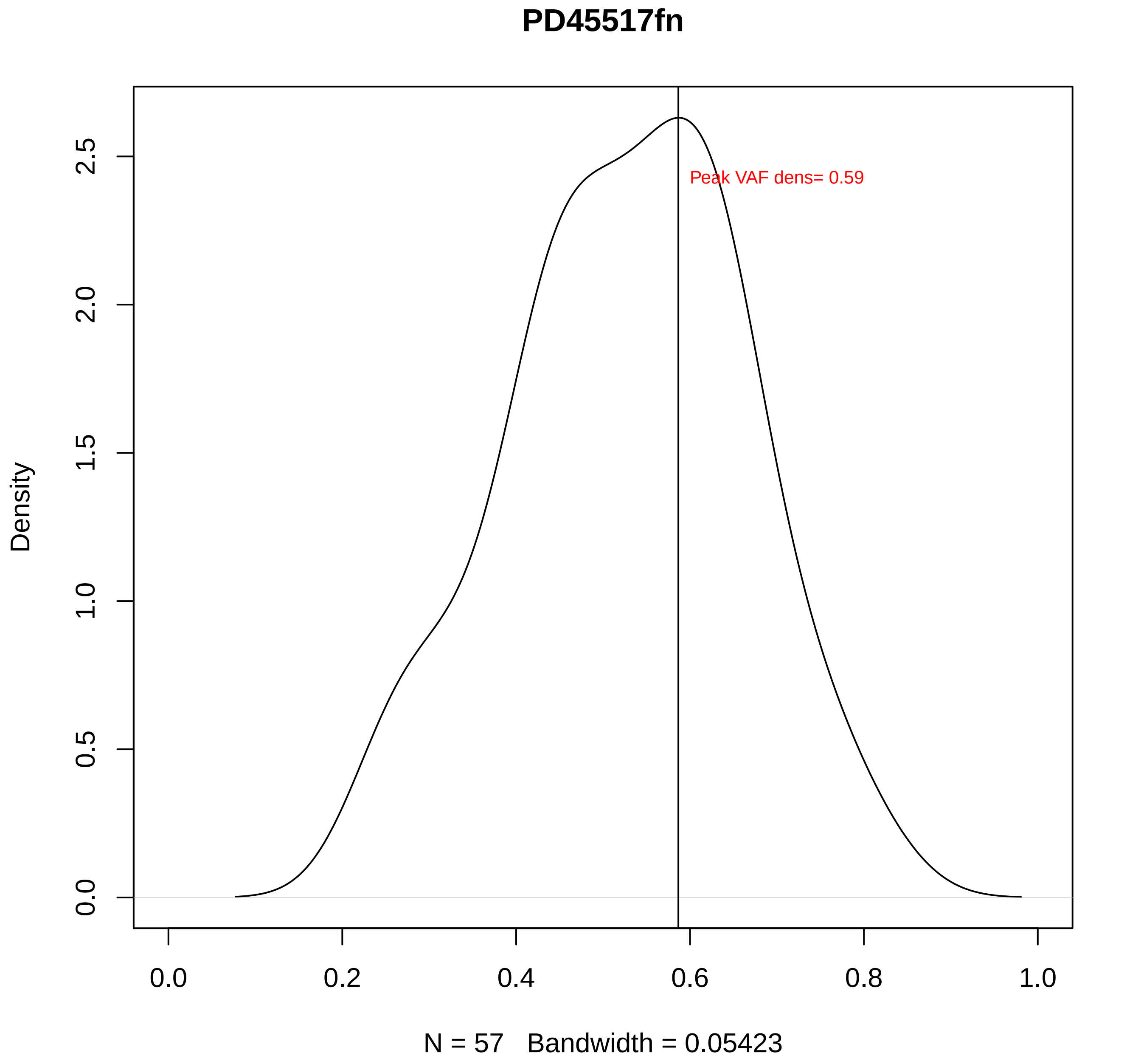
PD45517fn
Peak VAF dens= 0.59
Density
0.0
0.5
1.0
1.5
2.0
2.5
0.0
0.2
0.4
0.6
0.8
1.0
N = 57 Bandwidth = 0.05423

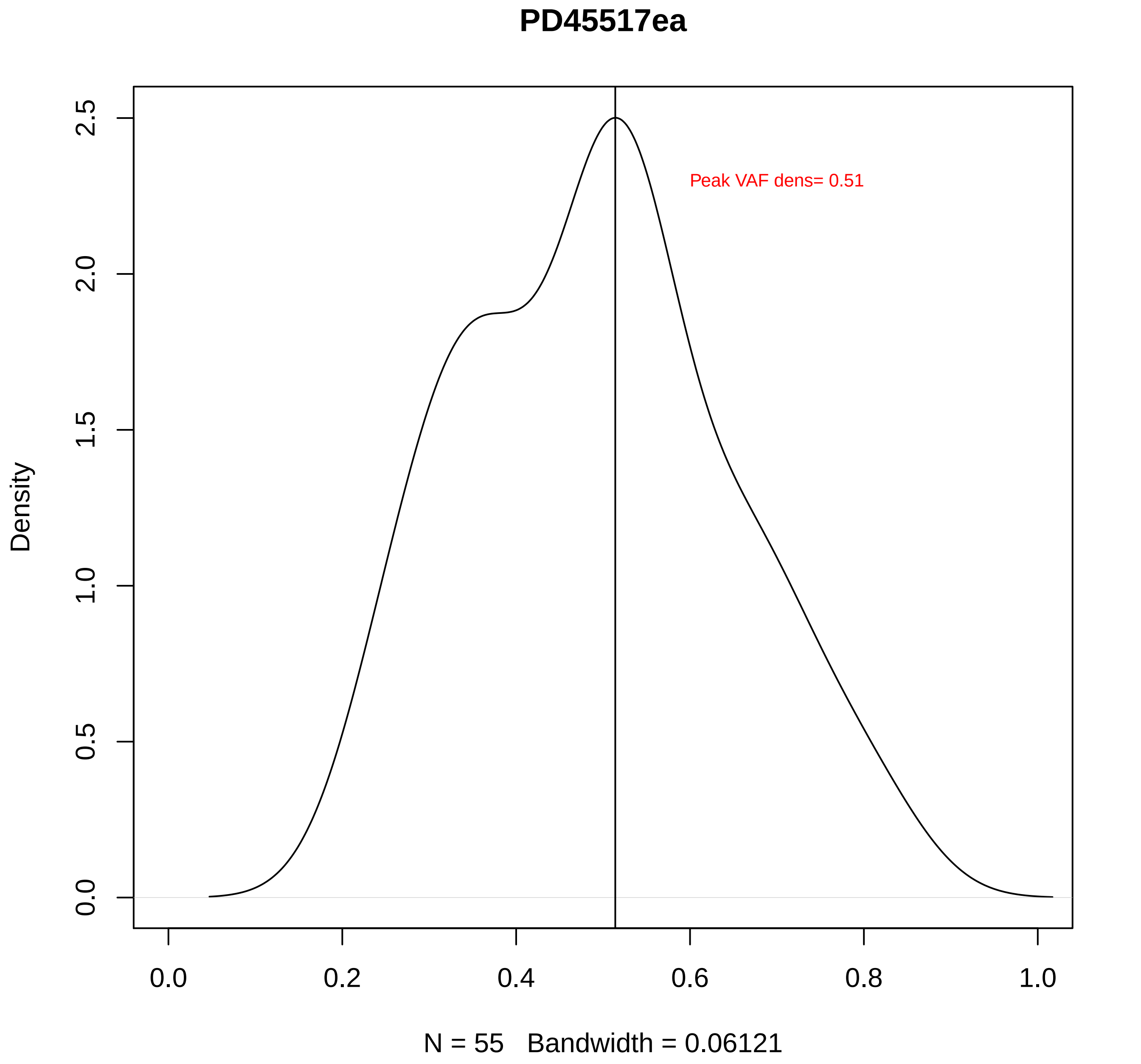
PD45517ea
Peak VAF dens= 0.51
Density
0.0
0.5
1.0
1.5
2.0
2.5
0.0
0.2
0.4
0.6
0.8
1.0
N = 55   Bandwidth = 0.06121

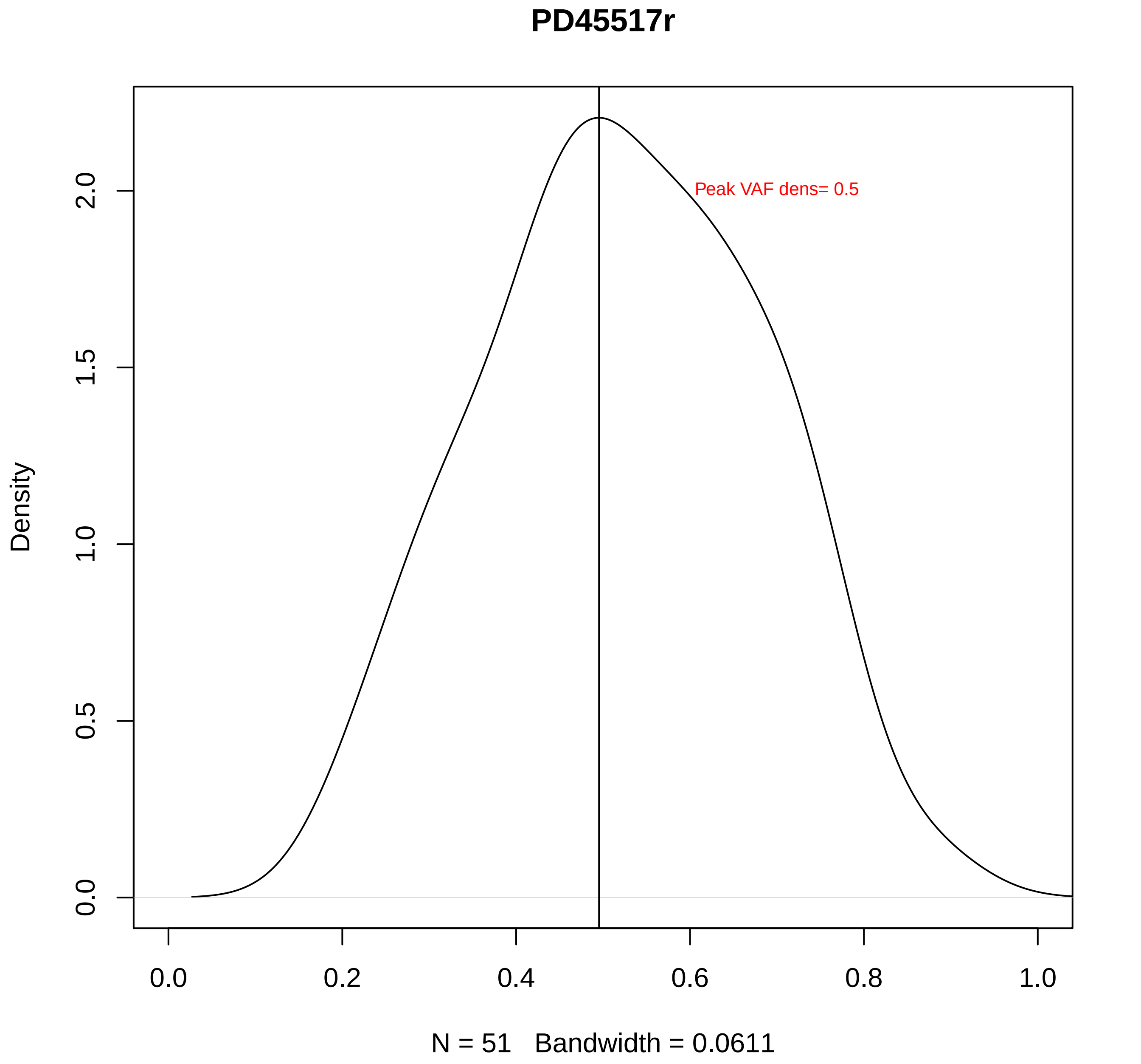
PD45517r
Peak VAF dens= 0.5
Density
0.0
0.5
1.0
1.5
2.0
0.0
0.2
0.4
0.6
0.8
1.0
N = 51   Bandwidth = 0.0611

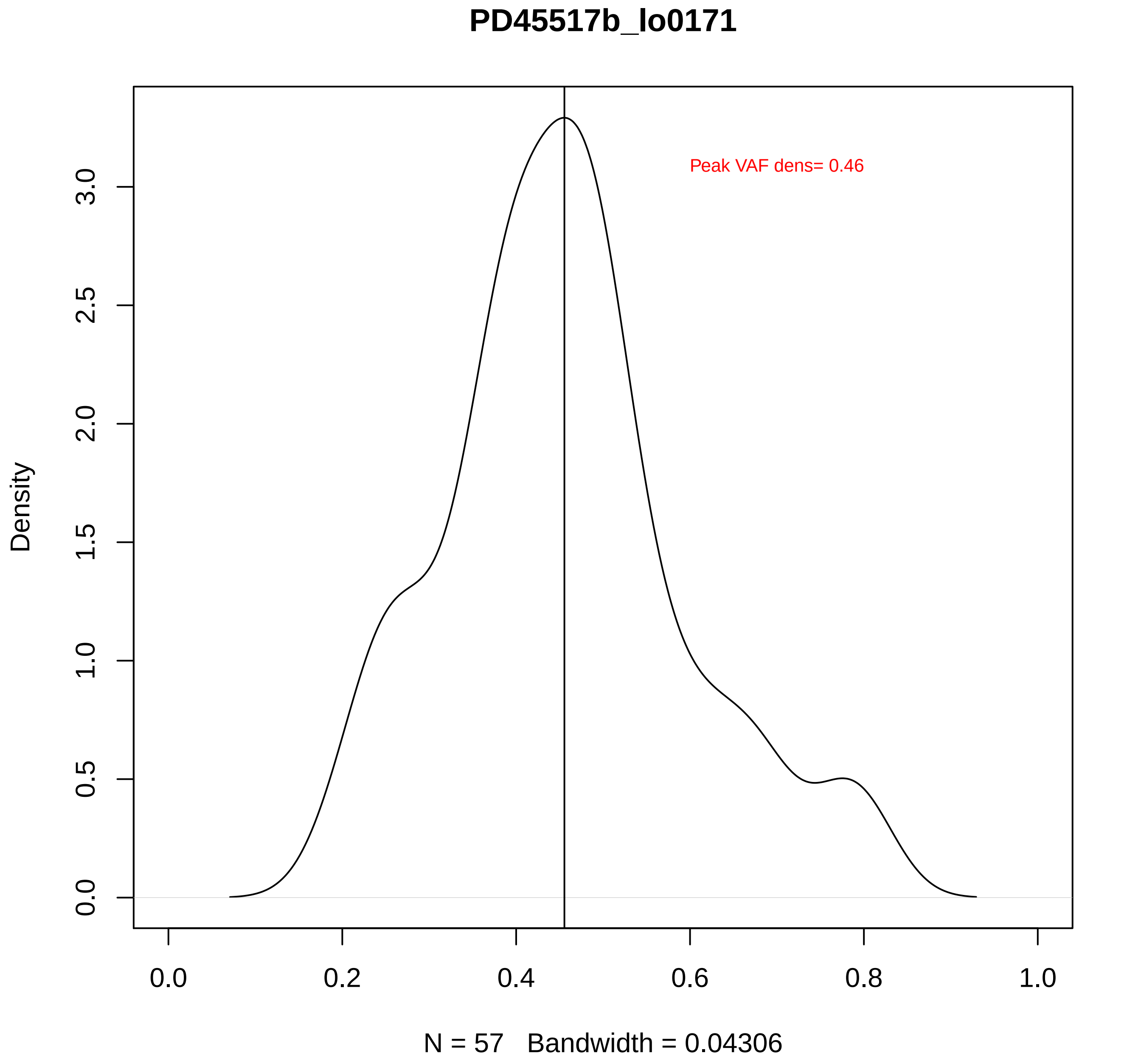
PD45517b_lo0171
Peak VAF dens= 0.46
Density
0.0
0.5
1.0
1.5
2.0
2.5
3.0
0.0
0.2
0.4
0.6
0.8
1.0
N = 57   Bandwidth = 0.04306

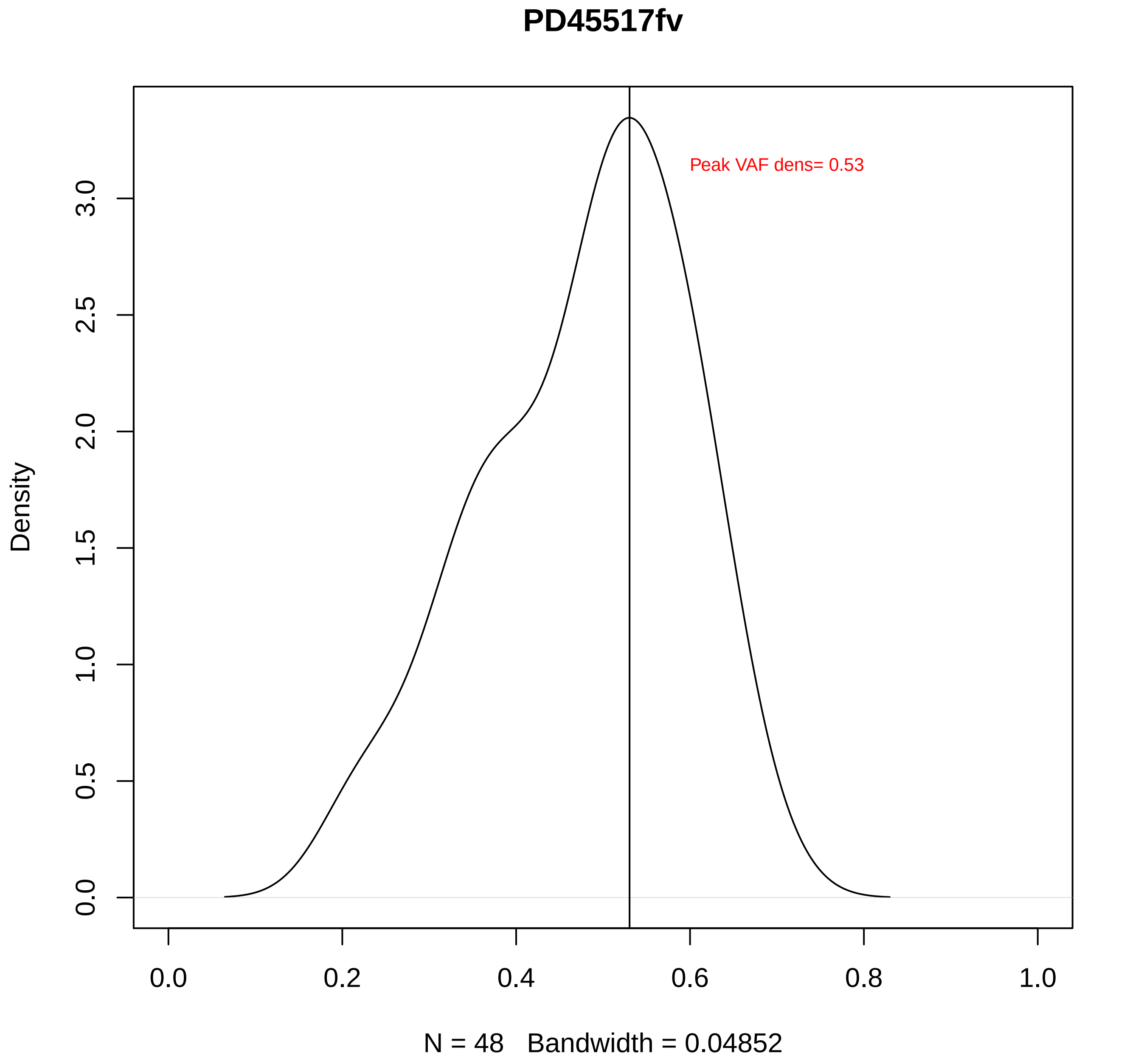
PD45517fv
Peak VAF dens= 0.53
Density
0.0
0.5
1.0
1.5
2.0
2.5
3.0
0.0
0.2
0.4
0.6
0.8
1.0
N = 48 Bandwidth = 0.04852

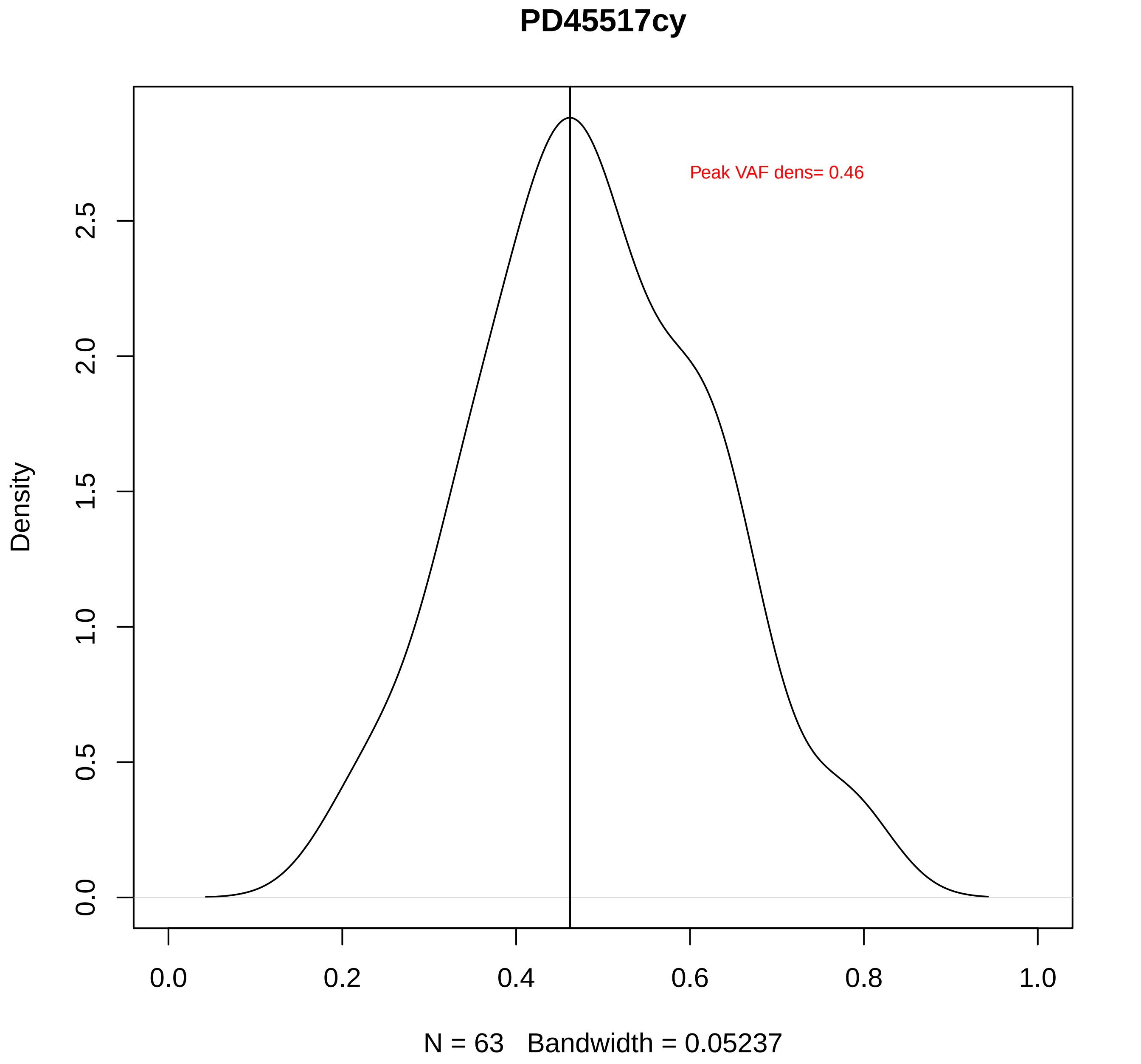
PD45517cy
Peak VAF dens= 0.46
Density
0.0
0.5
1.0
1.5
2.0
2.5
0.0
0.2
0.4
0.6
0.8
1.0
N = 63   Bandwidth = 0.05237

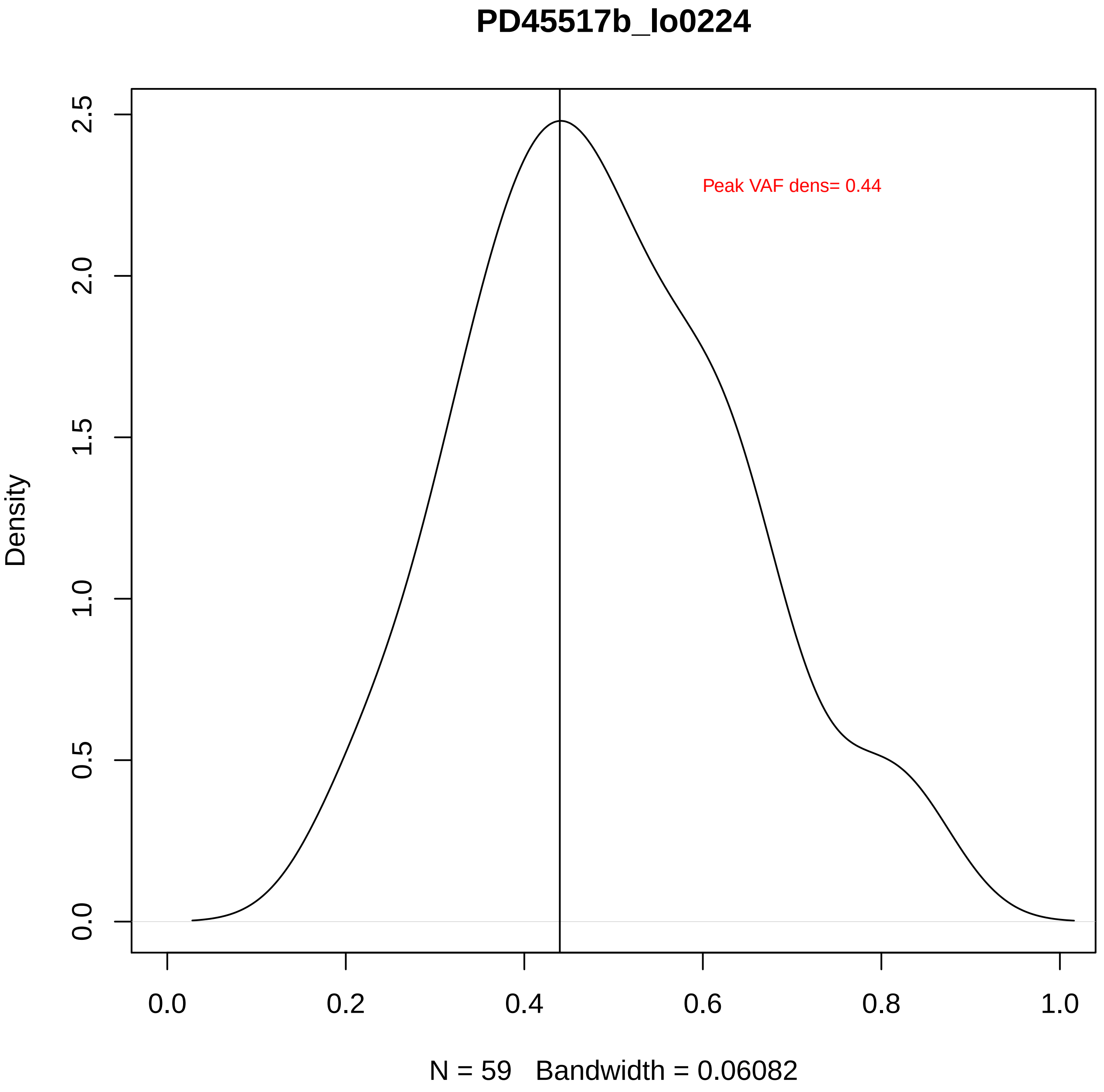

PD45517b_lo0224
Peak VAF dens= 0.44
Density
0.0
0.5
1.0
1.5
2.0
2.5
0.0
0.2
0.4
0.6
0.8
1.0
N = 59 Bandwidth = 0.06082

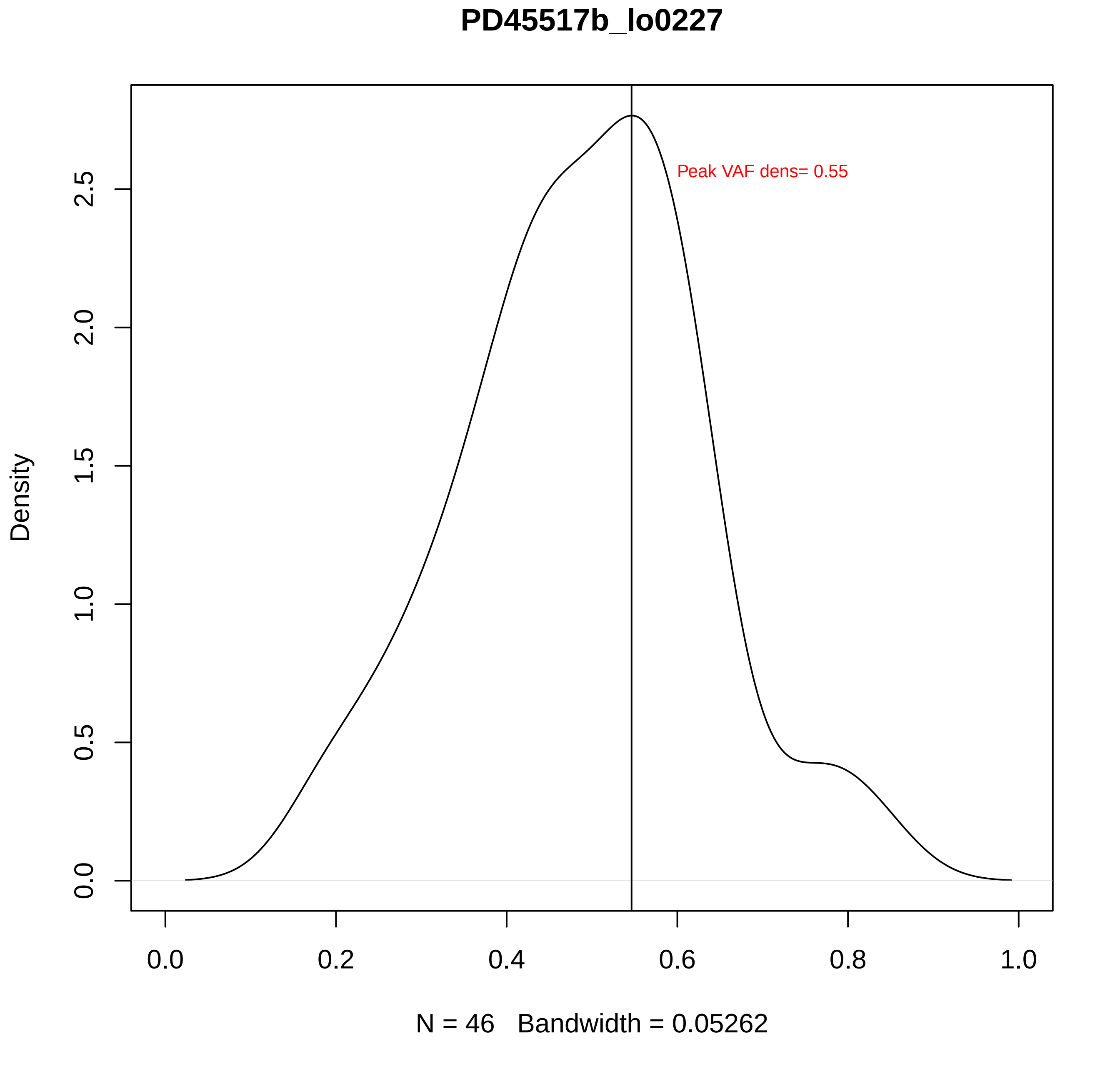
PD45517b_lo0227
Peak VAF dens= 0.55
Density
0.0
0.5
1.0
1.5
2.0
2.5
0.0
0.2
0.4
0.6
0.8
1.0
N = 46 Bandwidth = 0.05262

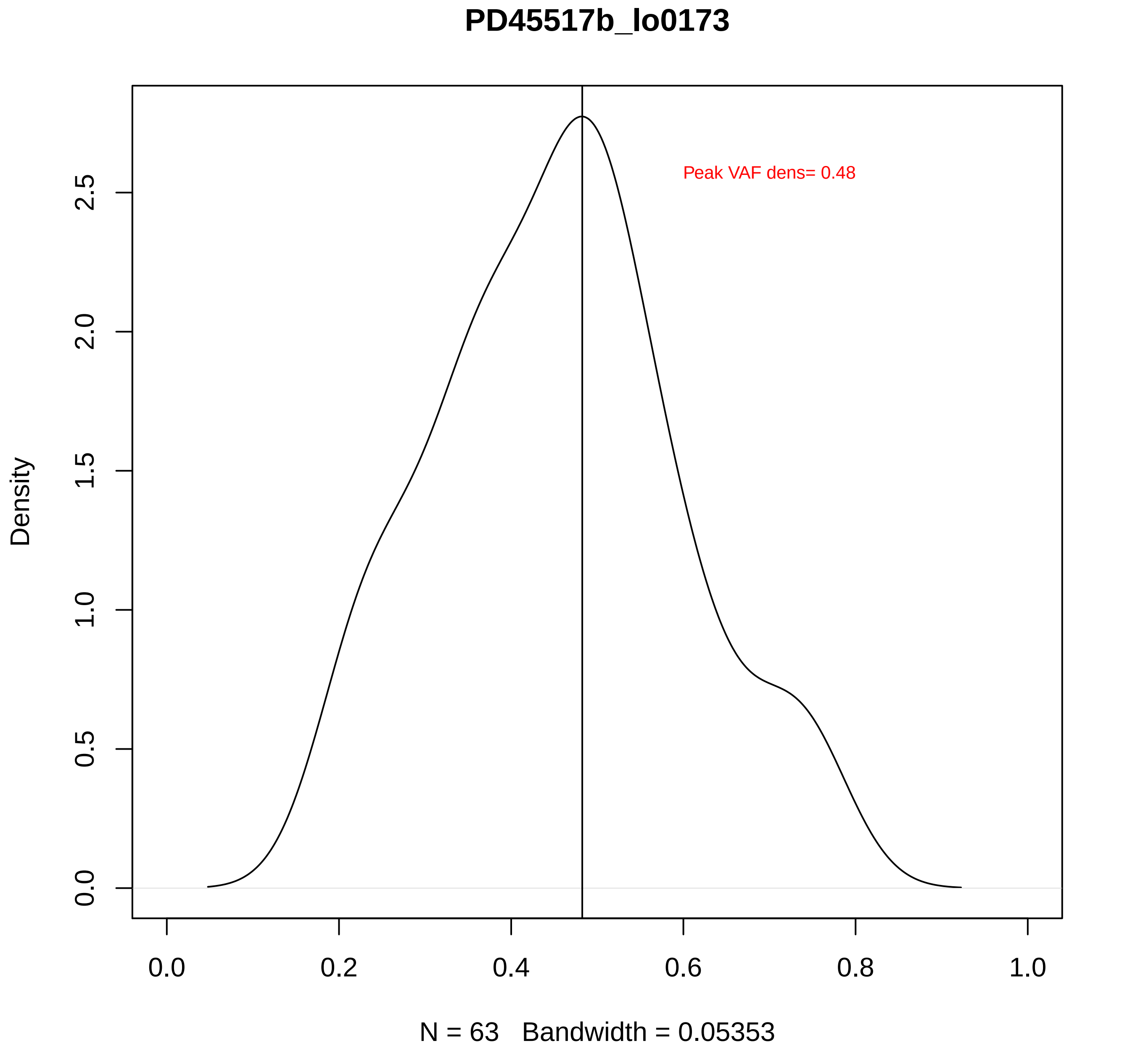
PD45517b_lo0173
Peak VAF dens= 0.48
Density
0.0
0.5
1.0
1.5
2.0
2.5
0.0
0.2
0.4
0.6
0.8
1.0
N = 63   Bandwidth = 0.05353

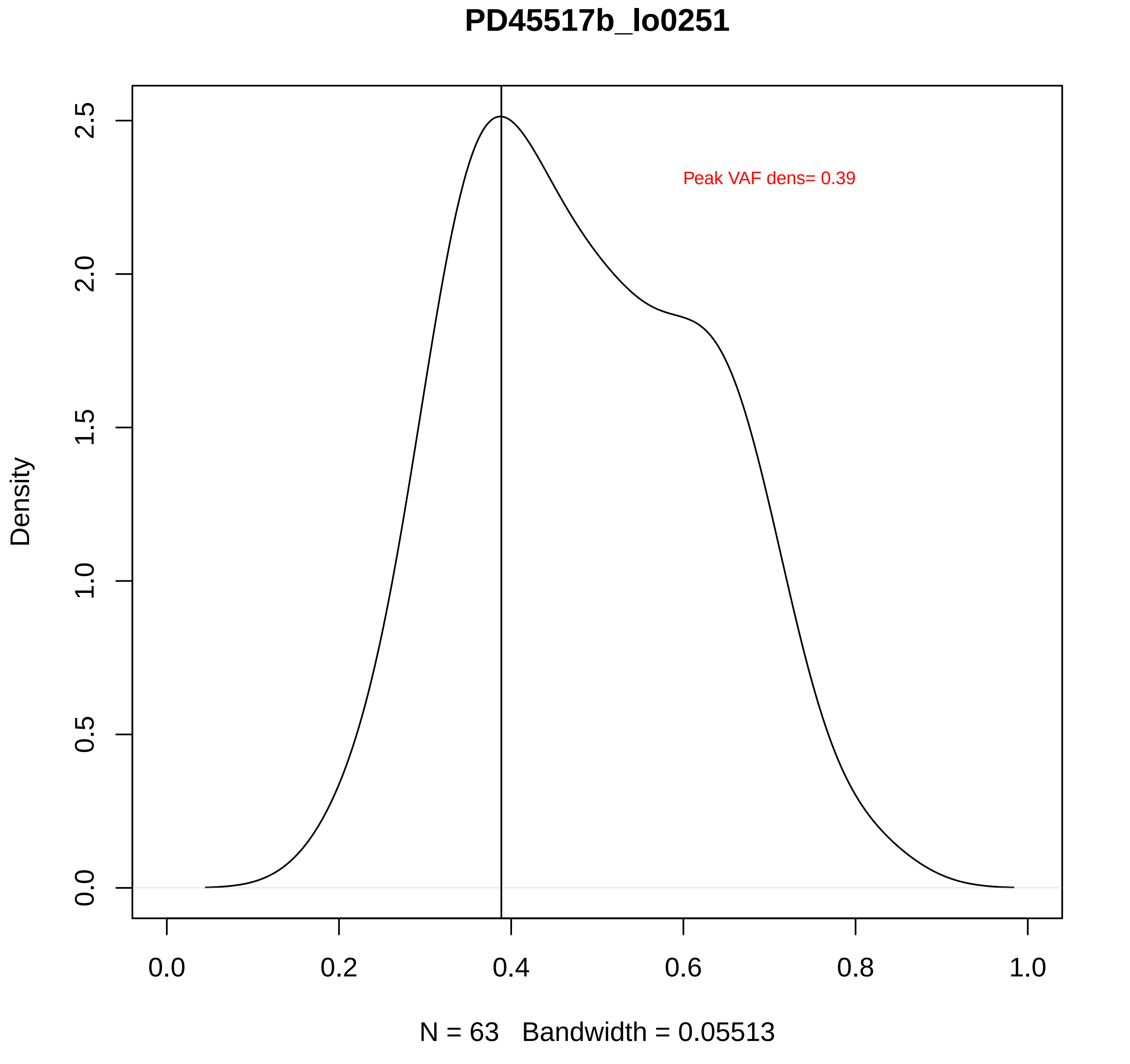
PD45517b_lo0251
Peak VAF dens= 0.39
Density
0.0
0.5
1.0
1.5
2.0
2.5
0.0
0.2
0.4
0.6
0.8
1.0
N = 63   Bandwidth = 0.05513

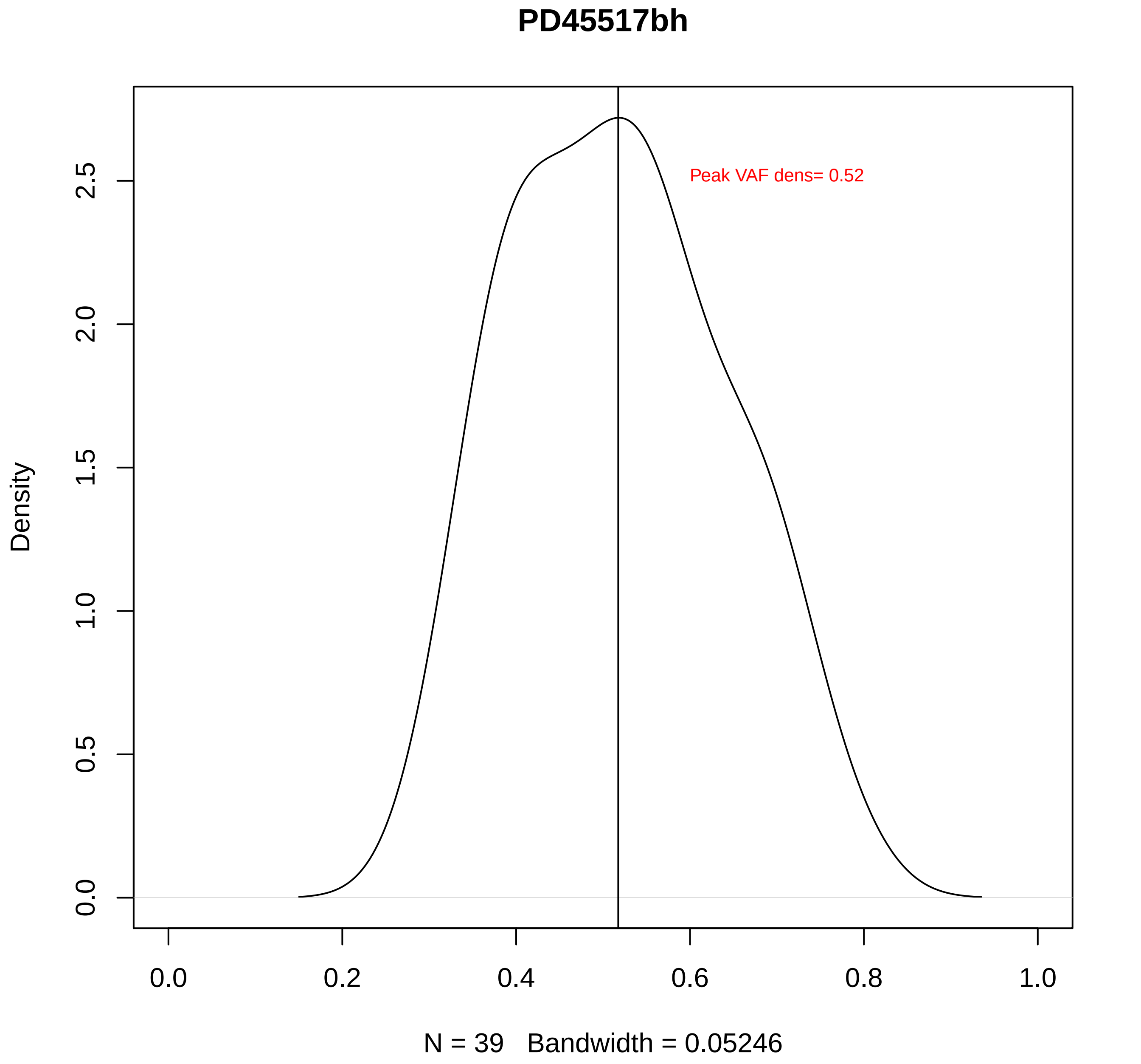

PD45517bh
Peak VAF dens= 0.52
Density
0.0
0.5
1.0
1.5
2.0
2.5
0.0
0.2
0.4
0.6
0.8
1.0
N = 39 Bandwidth = 0.05246

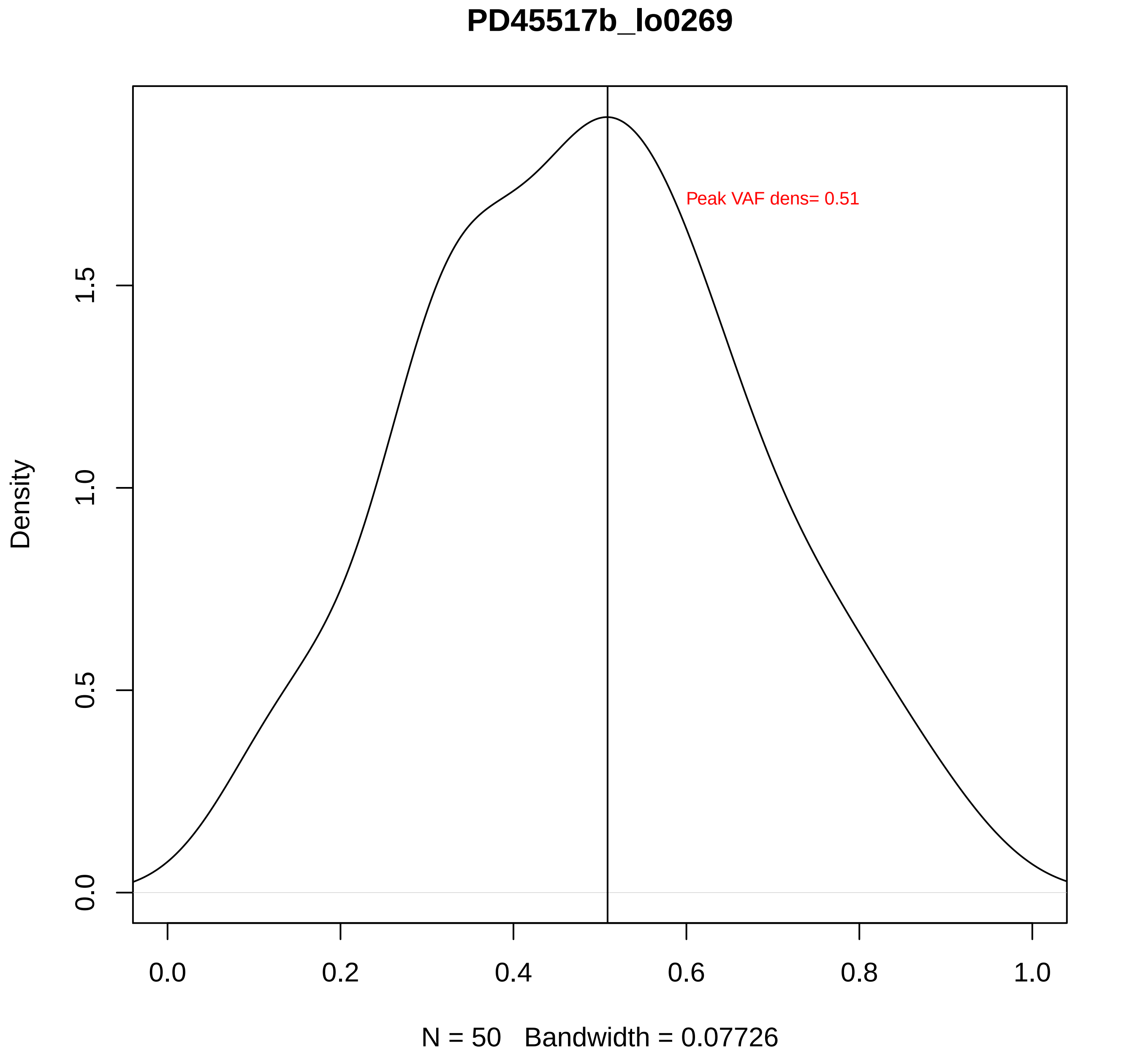
PD45517b_lo0269
Peak VAF dens= 0.51
Density
0.0
0.5
1.0
1.5
0.0
0.2
0.4
0.6
0.8
1.0
N = 50   Bandwidth = 0.07726

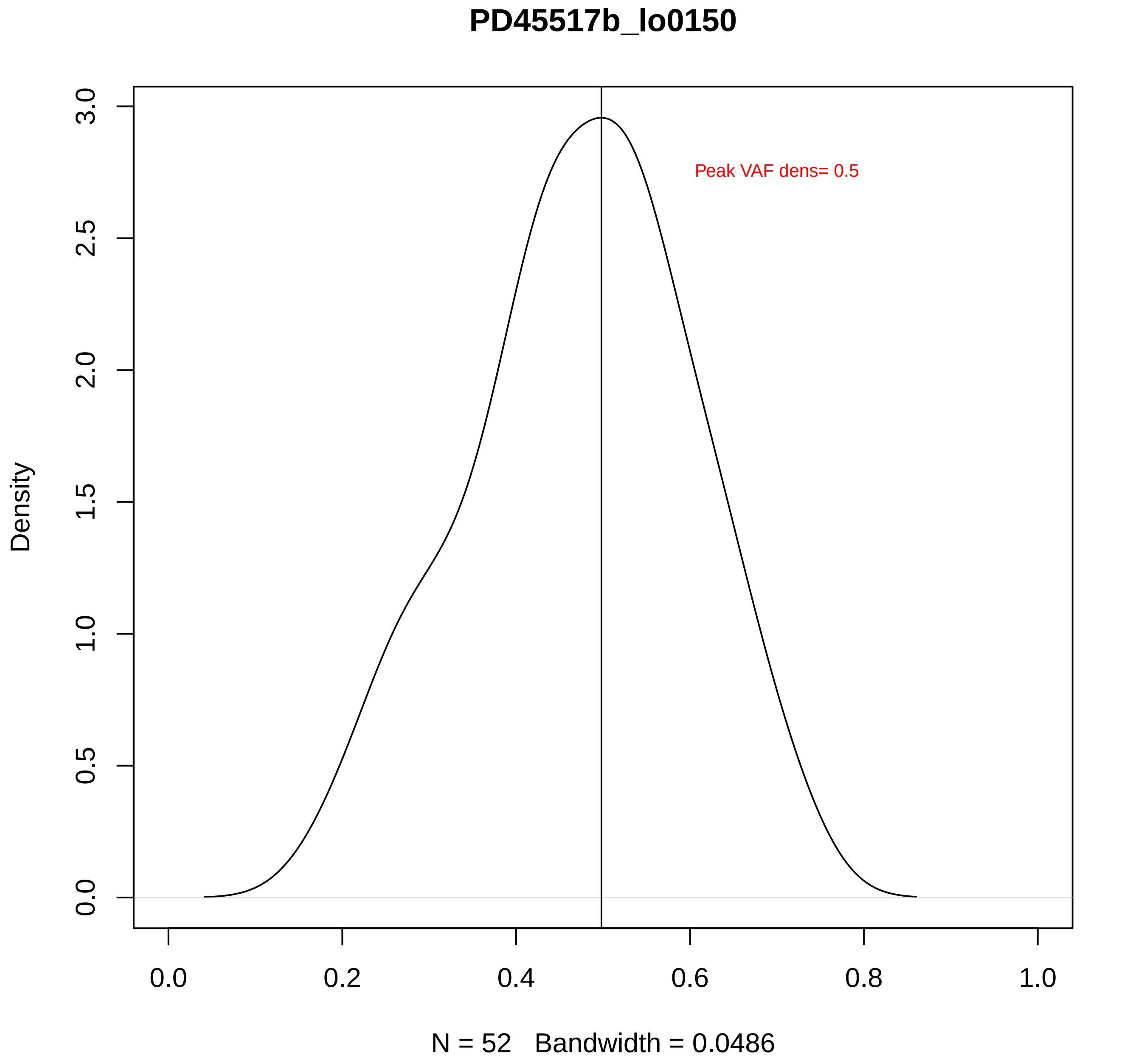
PD45517b_lo0150
Peak VAF dens= 0.5
Density
3.0
2.5
2.0
1.5
1.0
0.5
0.0
0.0
0.2
0.4
0.6
0.8
1.0
N = 52 Bandwidth = 0.0486

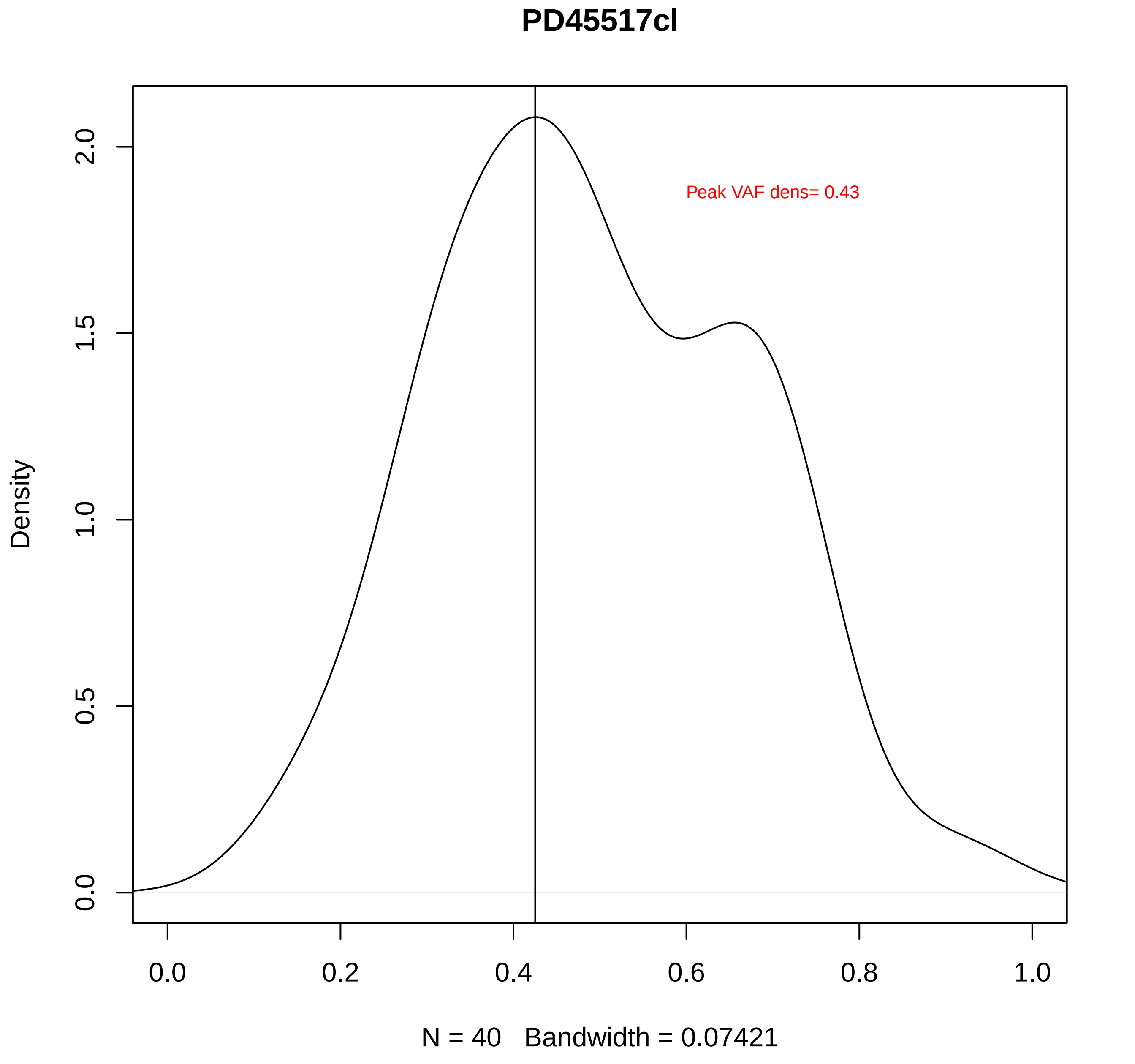
PD45517cl
Peak VAF dens= 0.43
Density
0.0
0.5
1.0
1.5
2.0
0.0
0.2
0.4
0.6
0.8
1.0
N = 40   Bandwidth = 0.07421

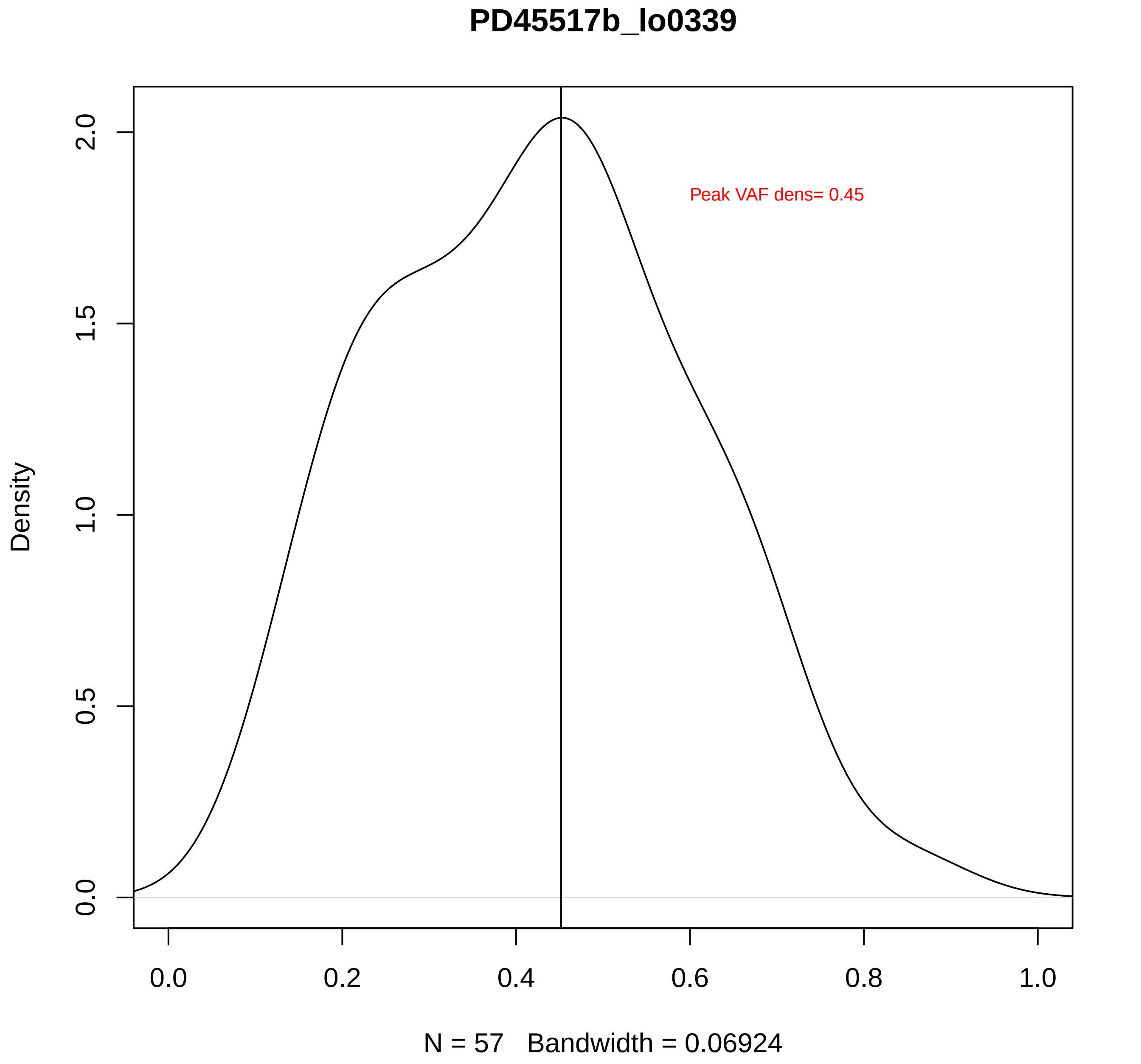
PD45517b_lo0339
Peak VAF dens= 0.45
Density
2.0
1.5
1.0
0.5
0.0
0.0
0.2
0.4
0.6
0.8
1.0
N = 57 Bandwidth = 0.06924

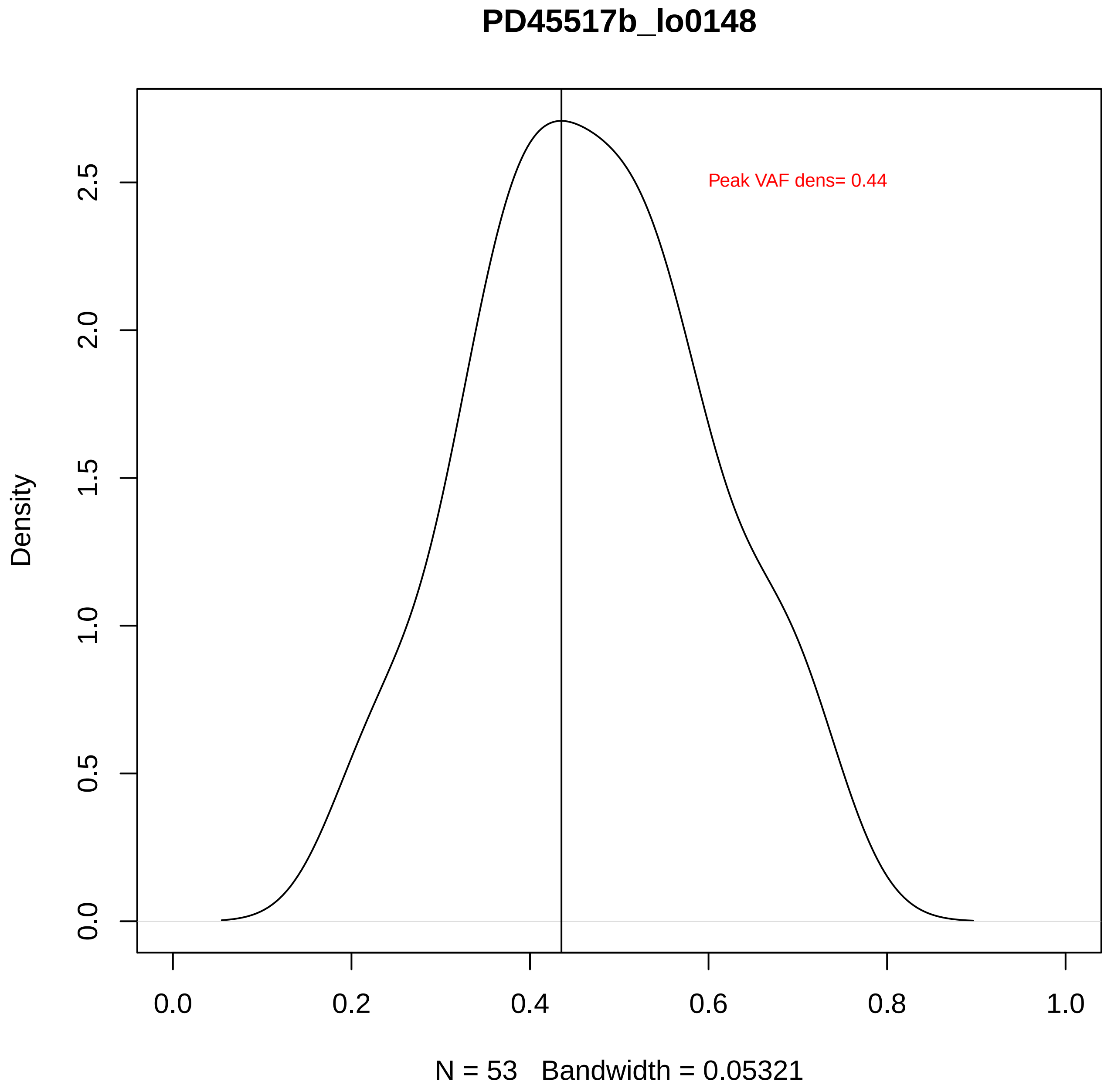
PD45517b_lo0148
Peak VAF dens= 0.44
Density
0.0
0.5
1.0
1.5
2.0
2.5
0.0
0.2
0.4
0.6
0.8
1.0
N = 53   Bandwidth = 0.05321

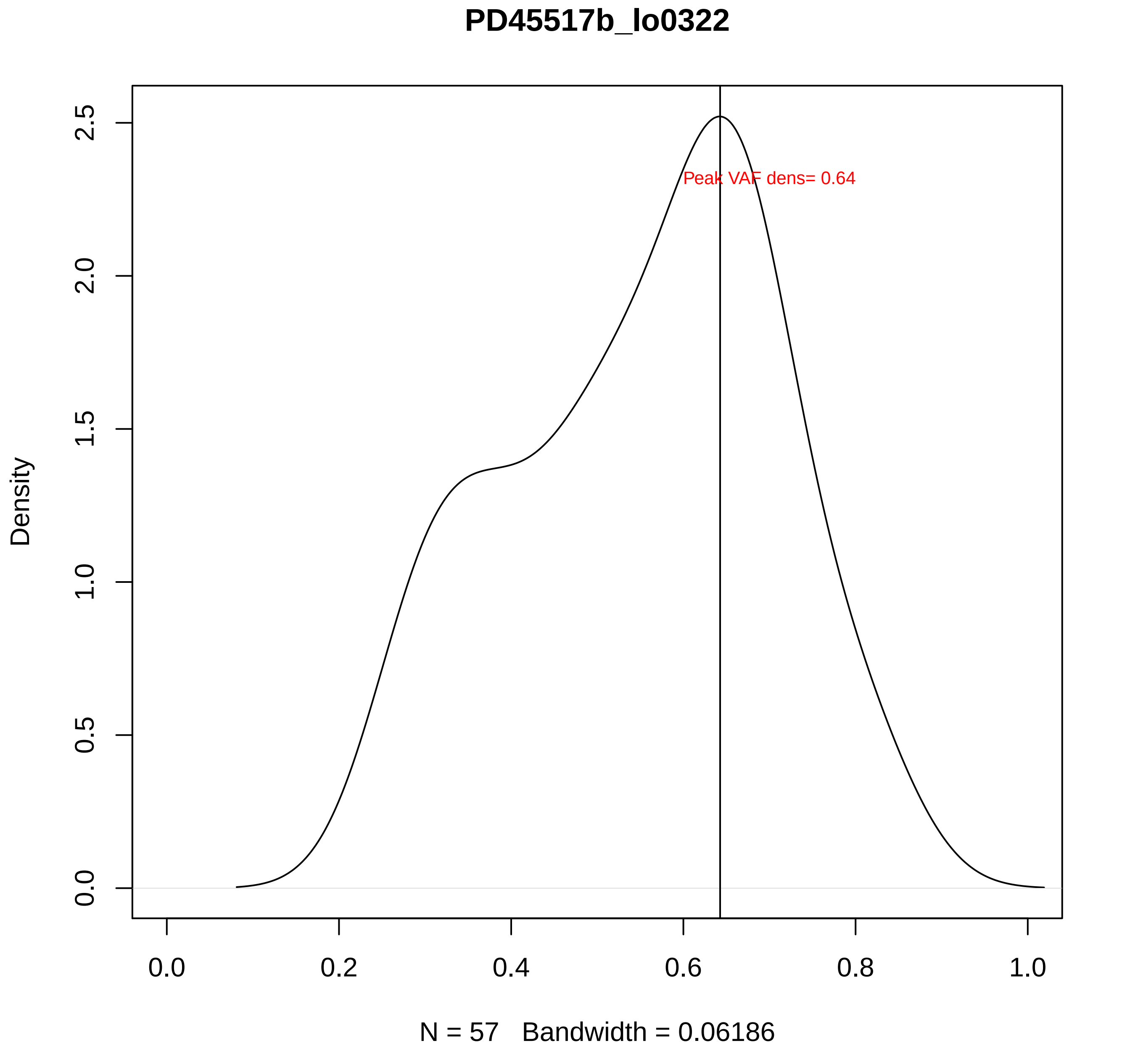
PD45517b_lo0322
Peak VAF dens= 0.64
Density
0.0
0.5
1.0
1.5
2.0
2.5
0.0
0.2
0.4
0.6
0.8
1.0
N = 57 Bandwidth = 0.06186

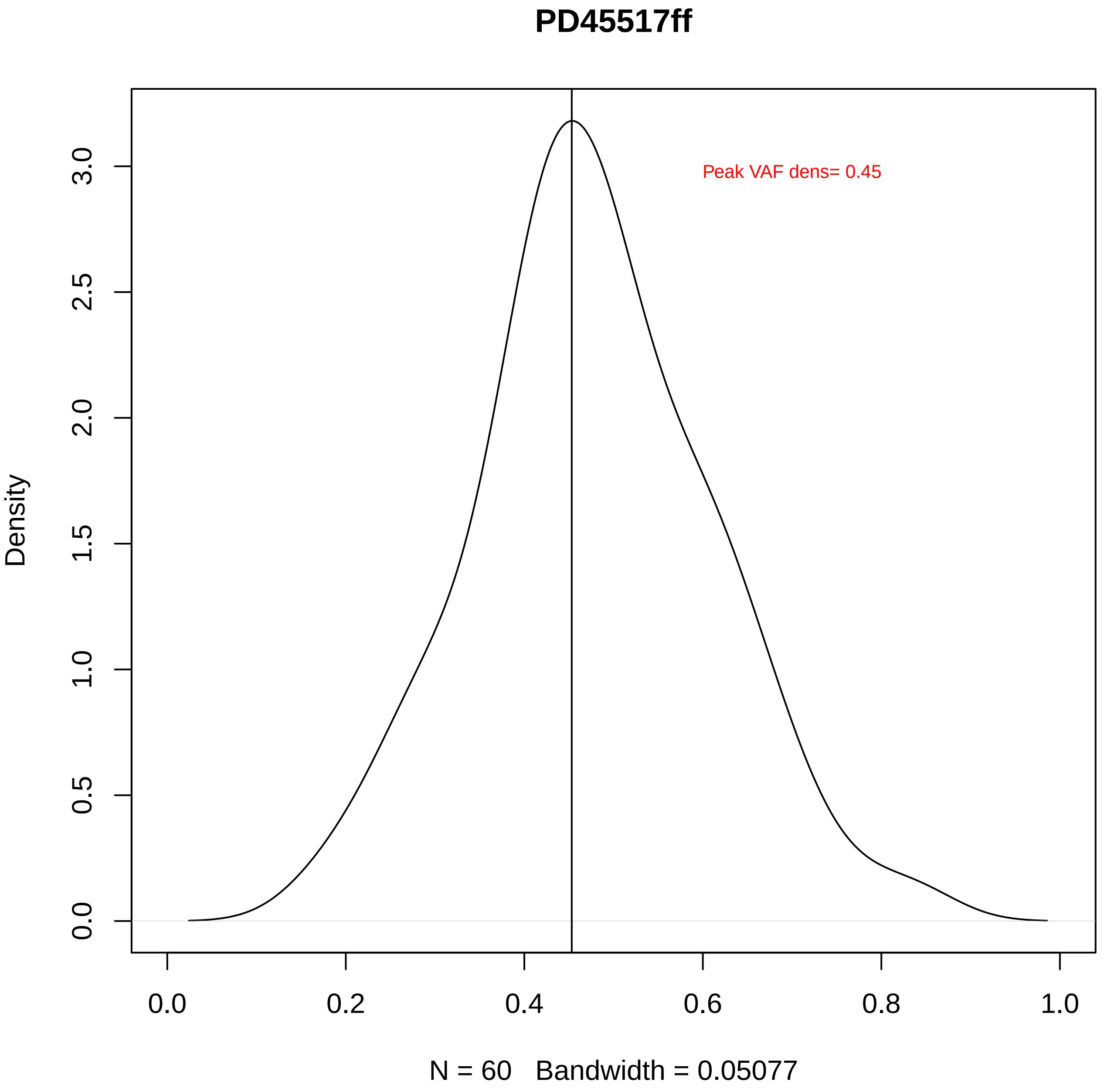
PD45517ff
Peak VAF dens= 0.45
Density
0.0
0.5
1.0
1.5
2.0
2.5
3.0
0.0
0.2
0.4
0.6
0.8
1.0
N = 60 Bandwidth = 0.05077

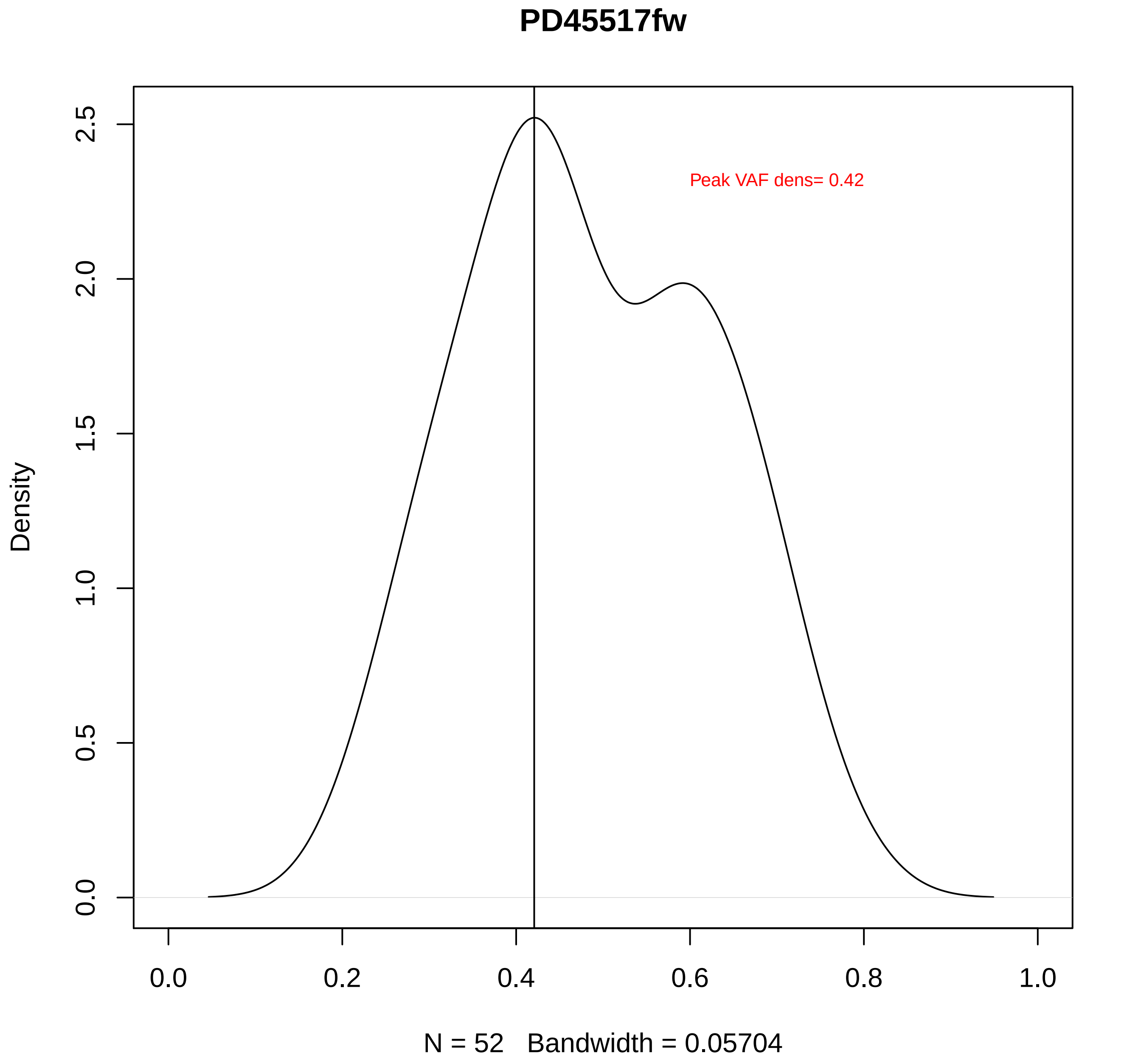

PD45517fw
Peak VAF dens= 0.42
Density
0.0
0.5
1.0
1.5
2.0
2.5
0.0
0.2
0.4
0.6
0.8
1.0
N = 52   Bandwidth = 0.05704

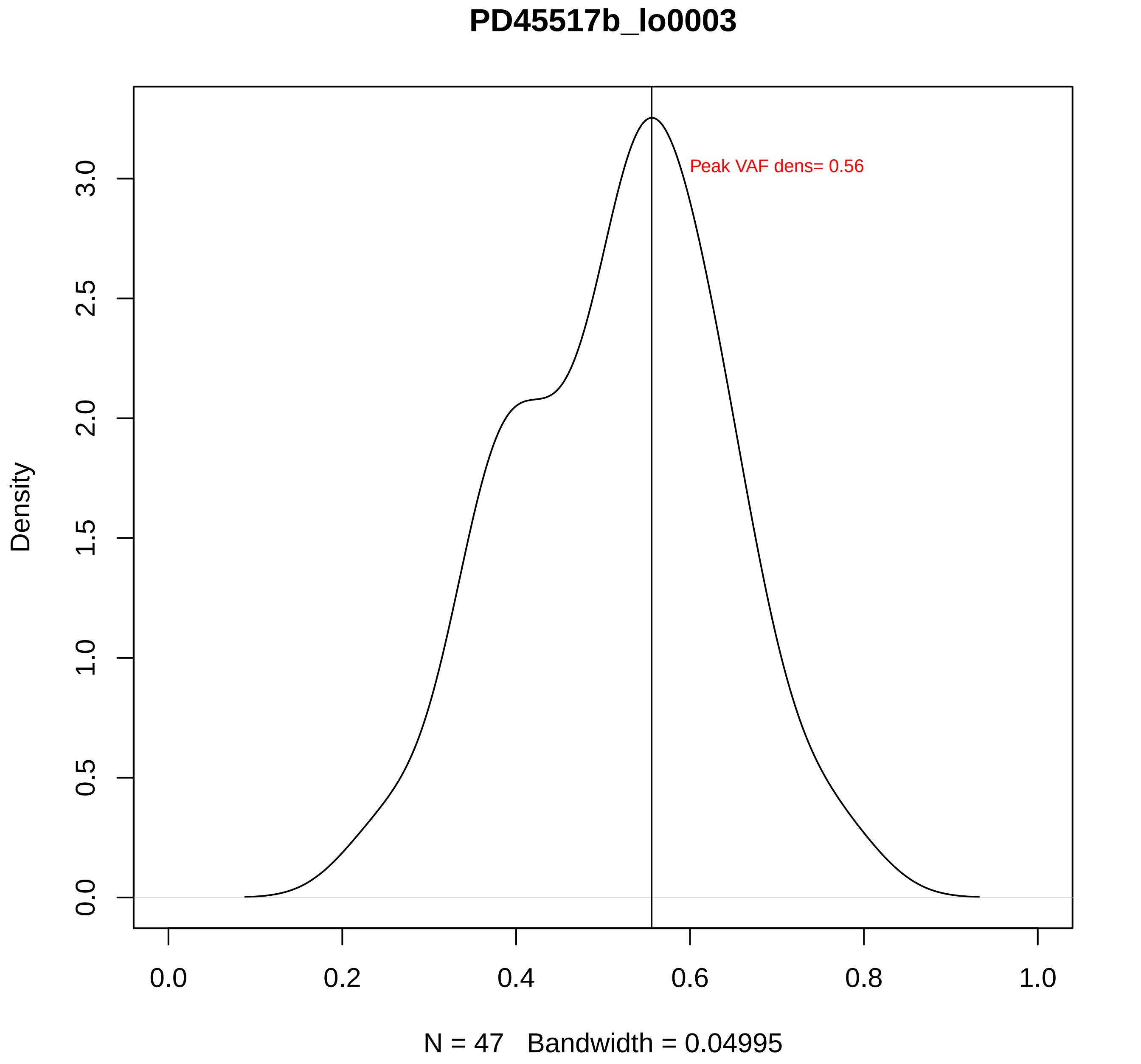
PD45517b_lo0003
Peak VAF dens= 0.56
Density
0.0
0.5
1.0
1.5
2.0
2.5
3.0
0.0
0.2
0.4
0.6
0.8
1.0
N = 47 Bandwidth = 0.04995

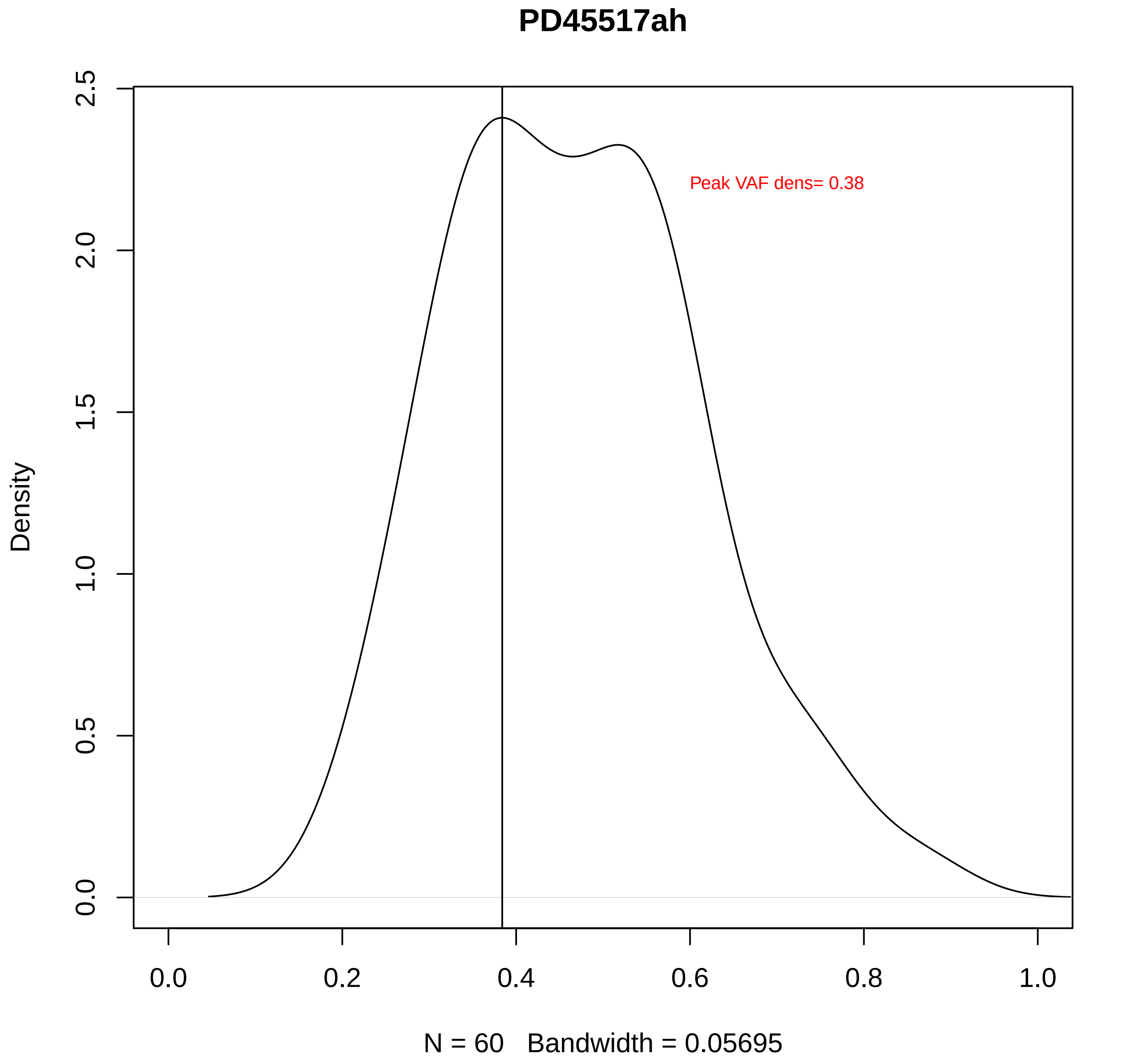

PD45517ah
Peak VAF dens= 0.38
Density
0.0
0.5
1.0
1.5
2.0
2.5
0.0
0.2
0.4
0.6
0.8
1.0
N = 60   Bandwidth = 0.05695

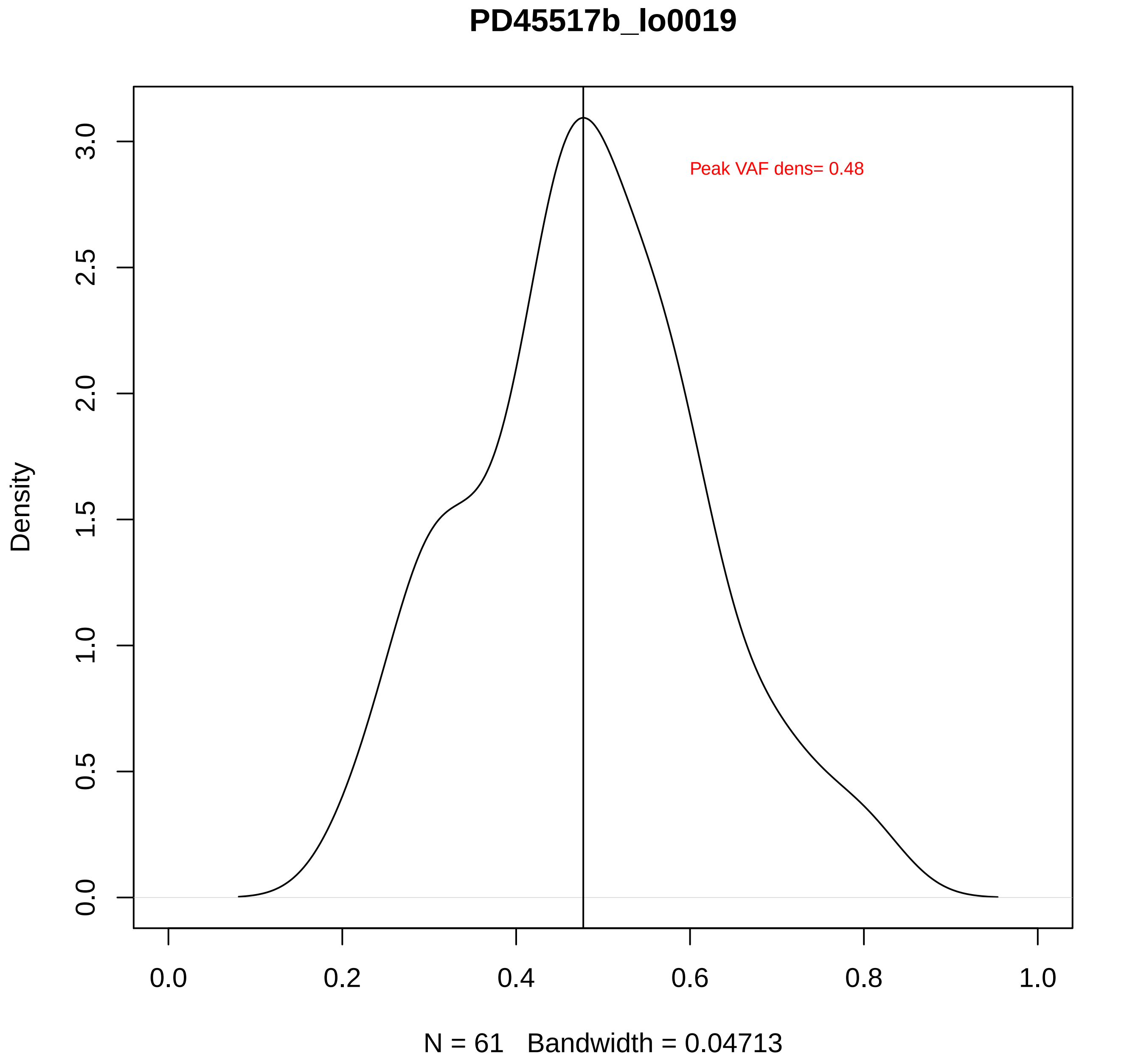
PD45517b_lo0019
Peak VAF dens= 0.48
Density
0.0
0.5
1.0
1.5
2.0
2.5
3.0
0.0
0.2
0.4
0.6
0.8
1.0
N = 61   Bandwidth = 0.04713

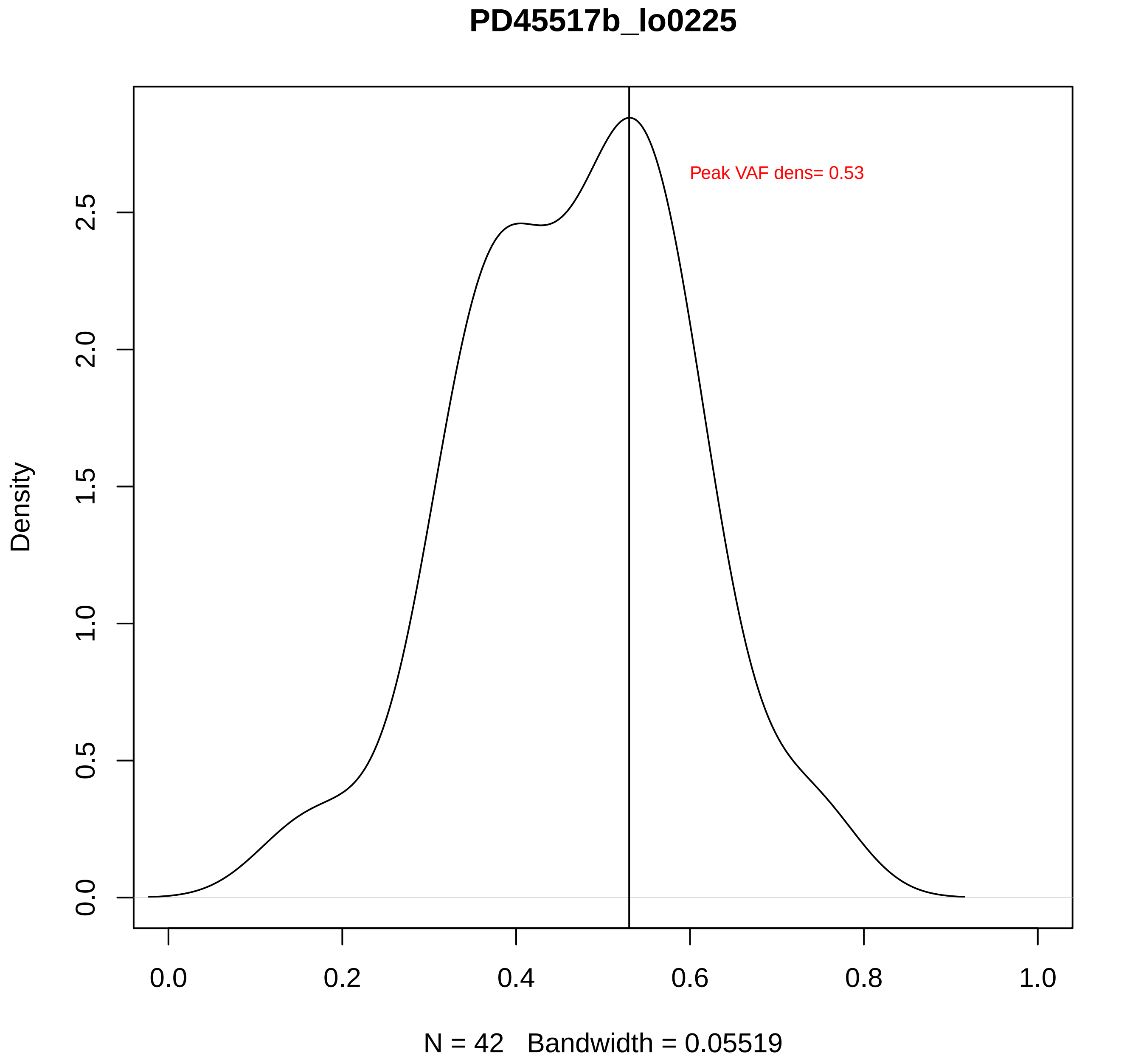
PD45517b_lo0225
Peak VAF dens= 0.53
Density
0.0
0.5
1.0
1.5
2.0
2.5
0.0
0.2
0.4
0.6
0.8
1.0
N = 42 Bandwidth = 0.05519

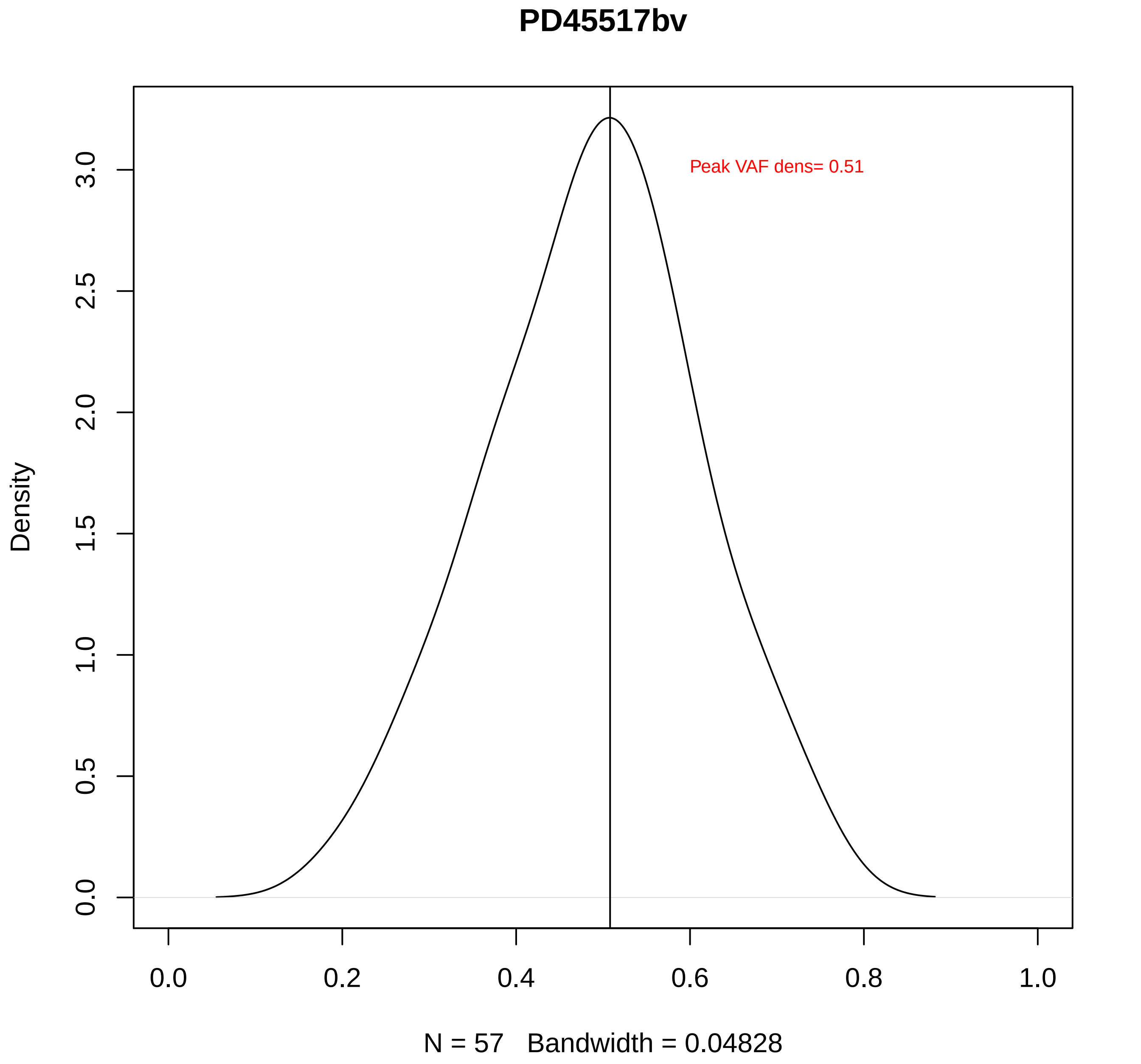
PD45517bv
Peak VAF dens= 0.51
Density
0.0
0.5
1.0
1.5
2.0
2.5
3.0
0.0
0.2
0.4
0.6
0.8
1.0
N = 57   Bandwidth = 0.04828

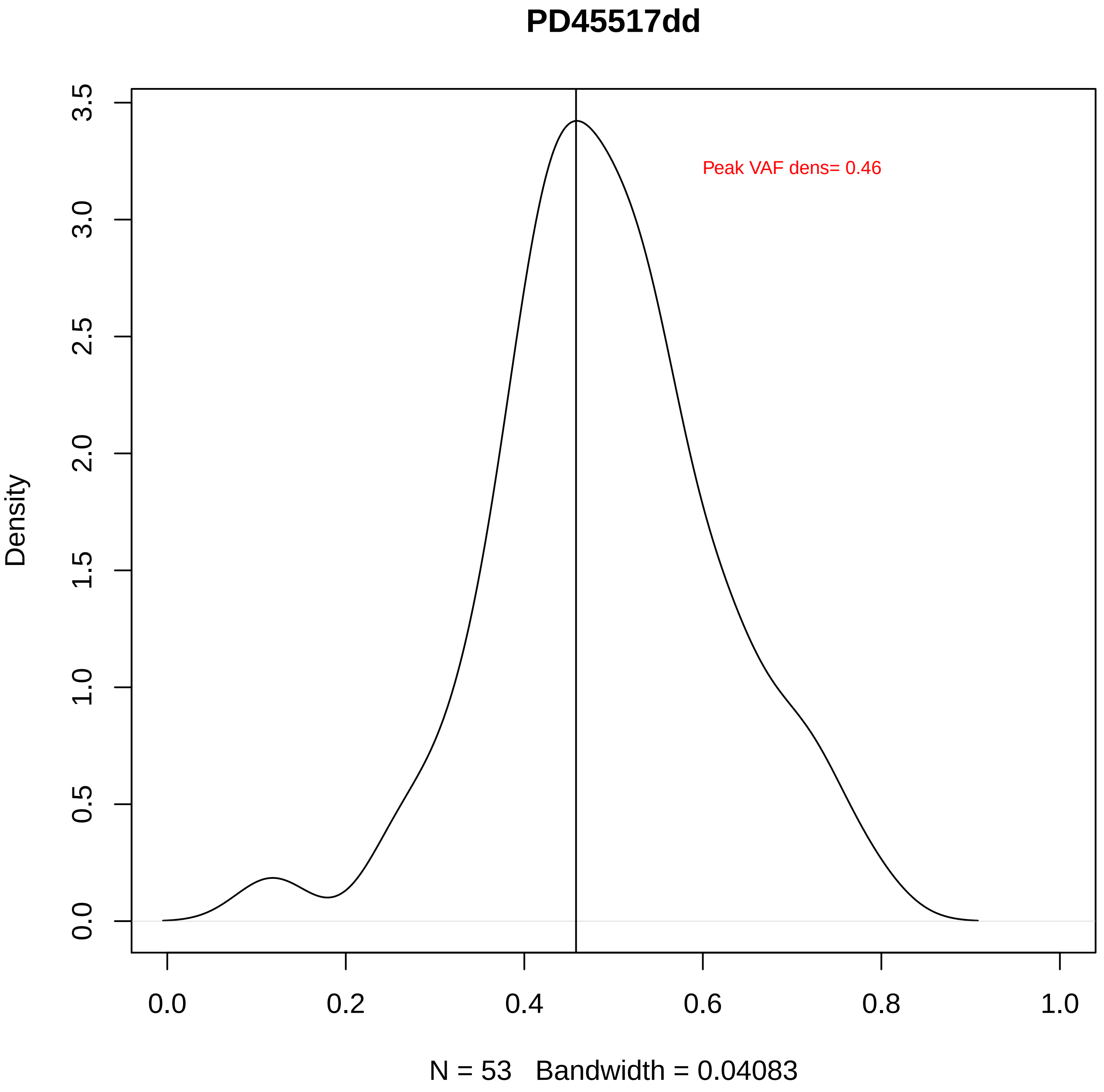
PD45517dd
Peak VAF dens= 0.46
Density
0.0
0.5
1.0
1.5
2.0
2.5
3.0
3.5
0.0
0.2
0.4
0.6
0.8
1.0
N = 53 Bandwidth = 0.04083

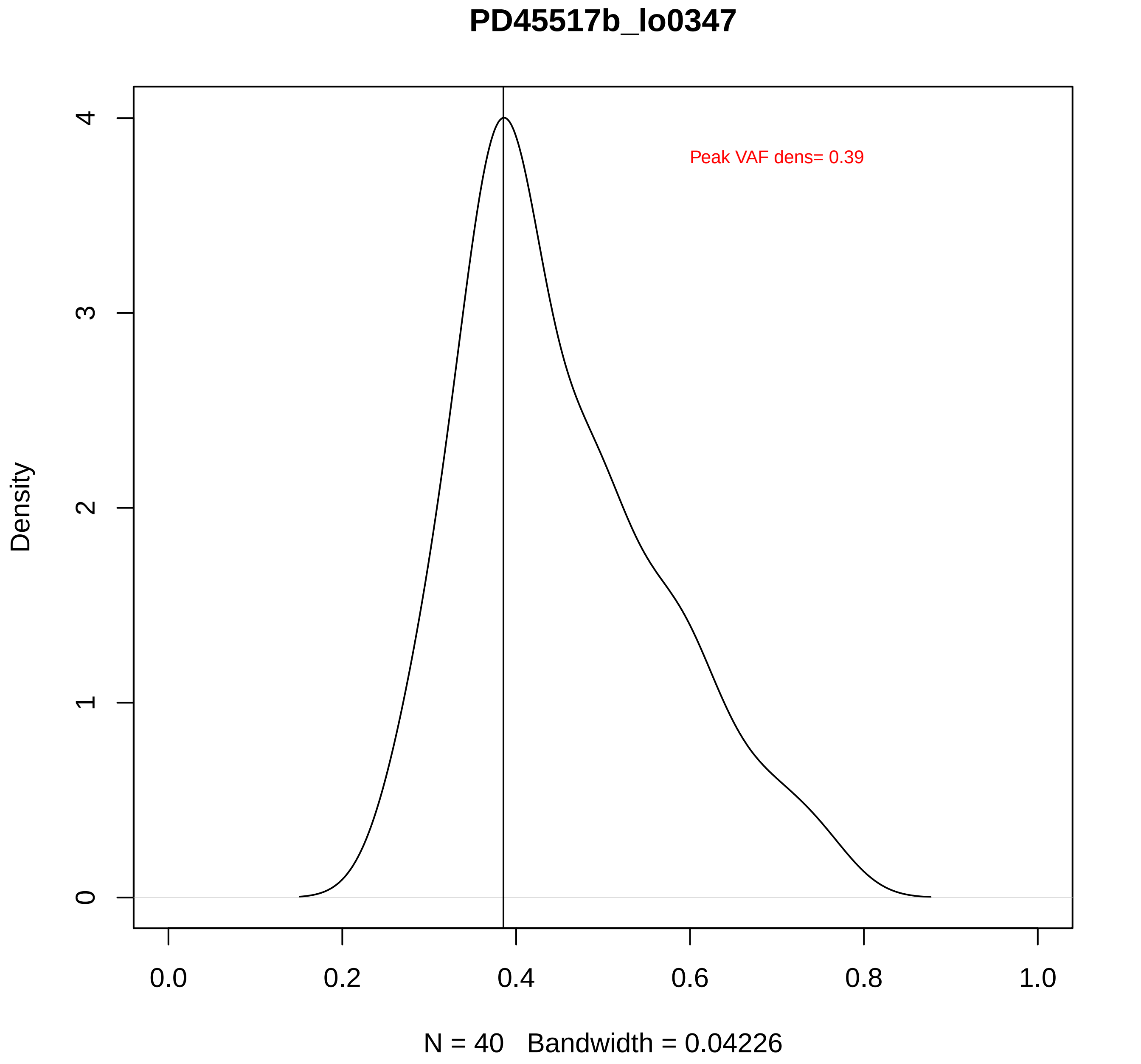
PD45517b_lo0347
Peak VAF dens= 0.39
Density
0
1
2
3
4
0.0
0.2
0.4
0.6
0.8
1.0
N = 40   Bandwidth = 0.04226

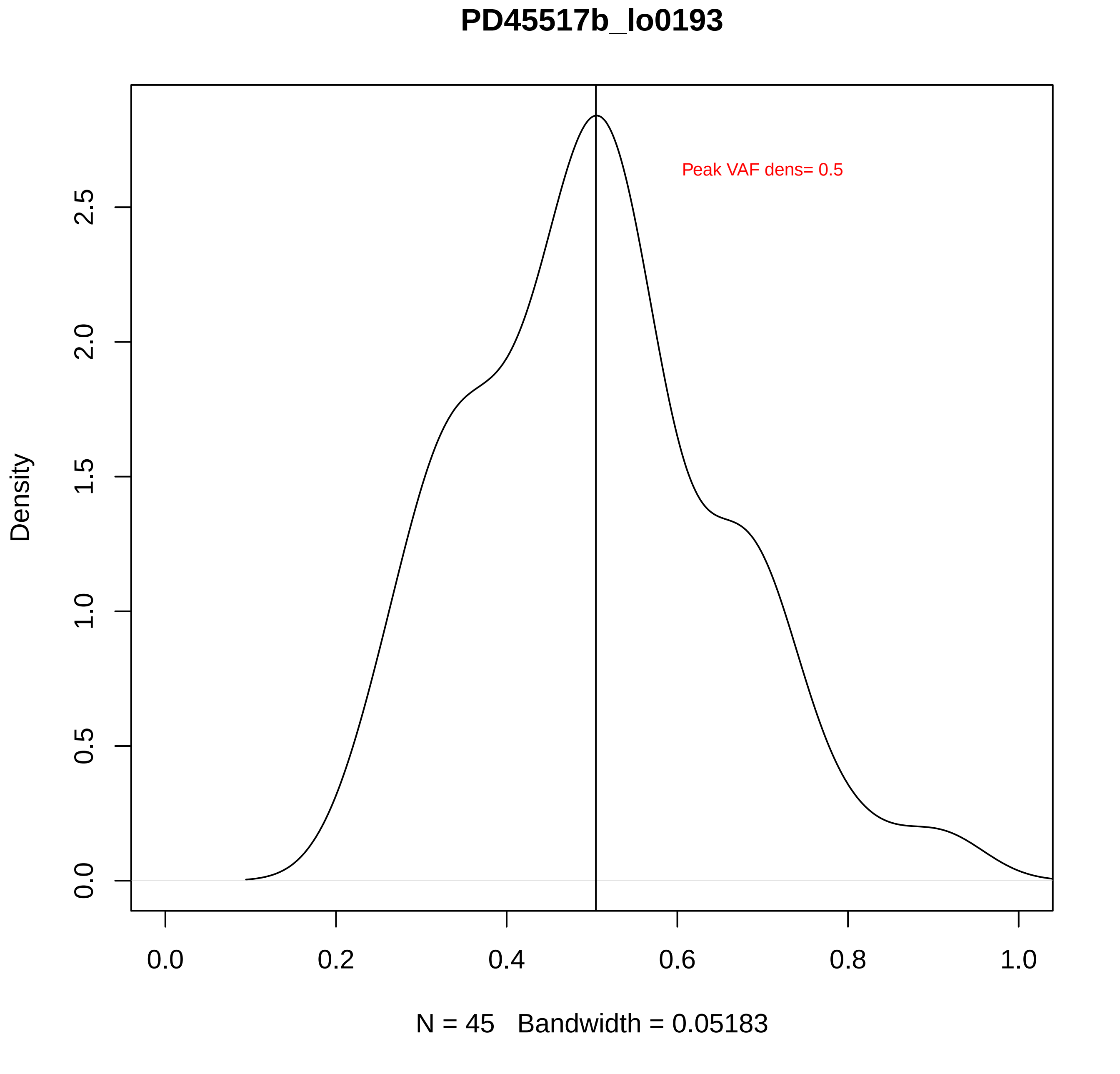

PD45517b_lo0193
Peak VAF dens= 0.5
Density
0.0
0.5
1.0
1.5
2.0
2.5
0.0
0.2
0.4
0.6
0.8
1.0
N = 45   Bandwidth = 0.05183

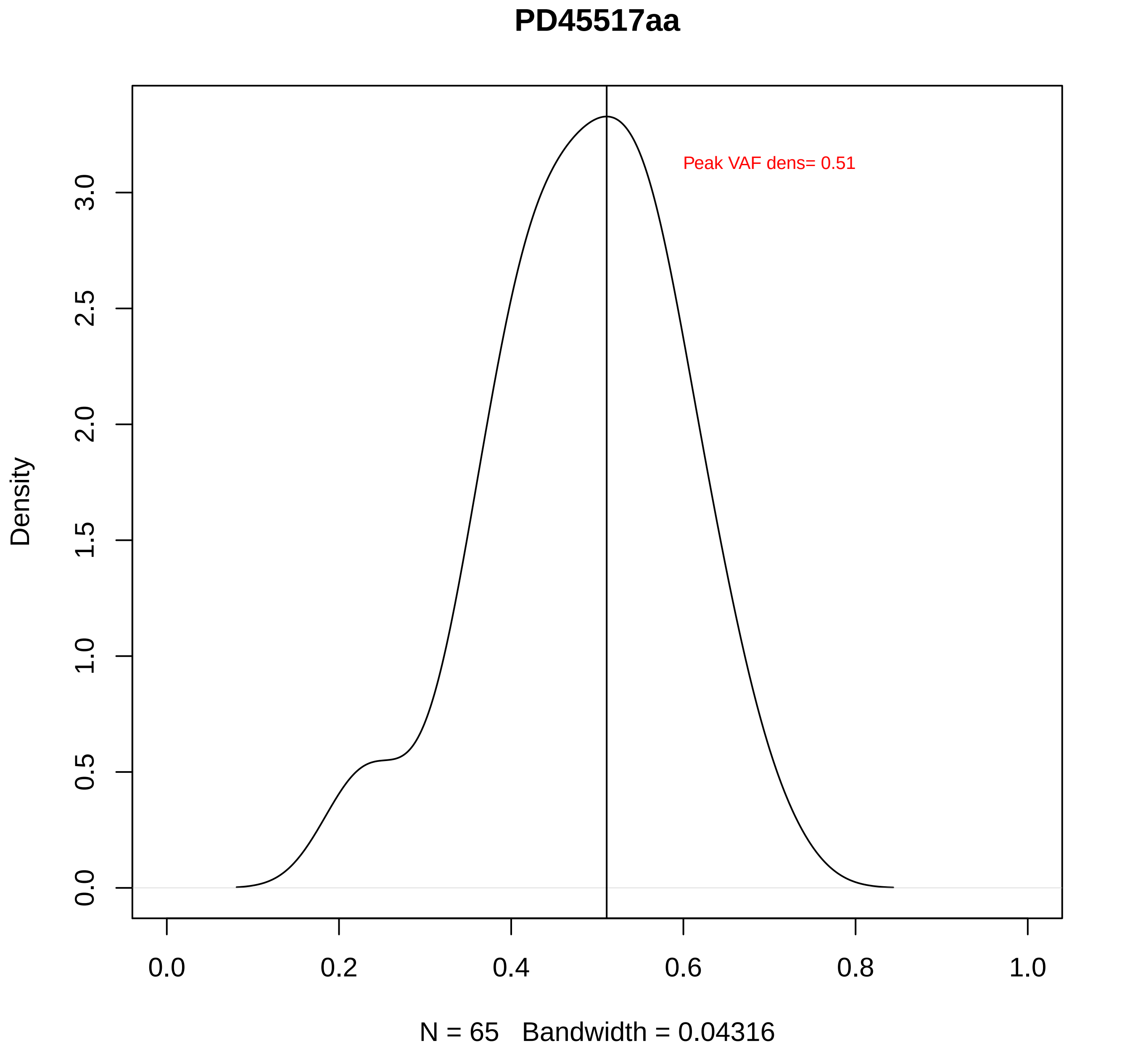
PD45517aa
Peak VAF dens= 0.51
Density
0.0
0.5
1.0
1.5
2.0
2.5
3.0
0.0
0.2
0.4
0.6
0.8
1.0
N = 65 Bandwidth = 0.04316

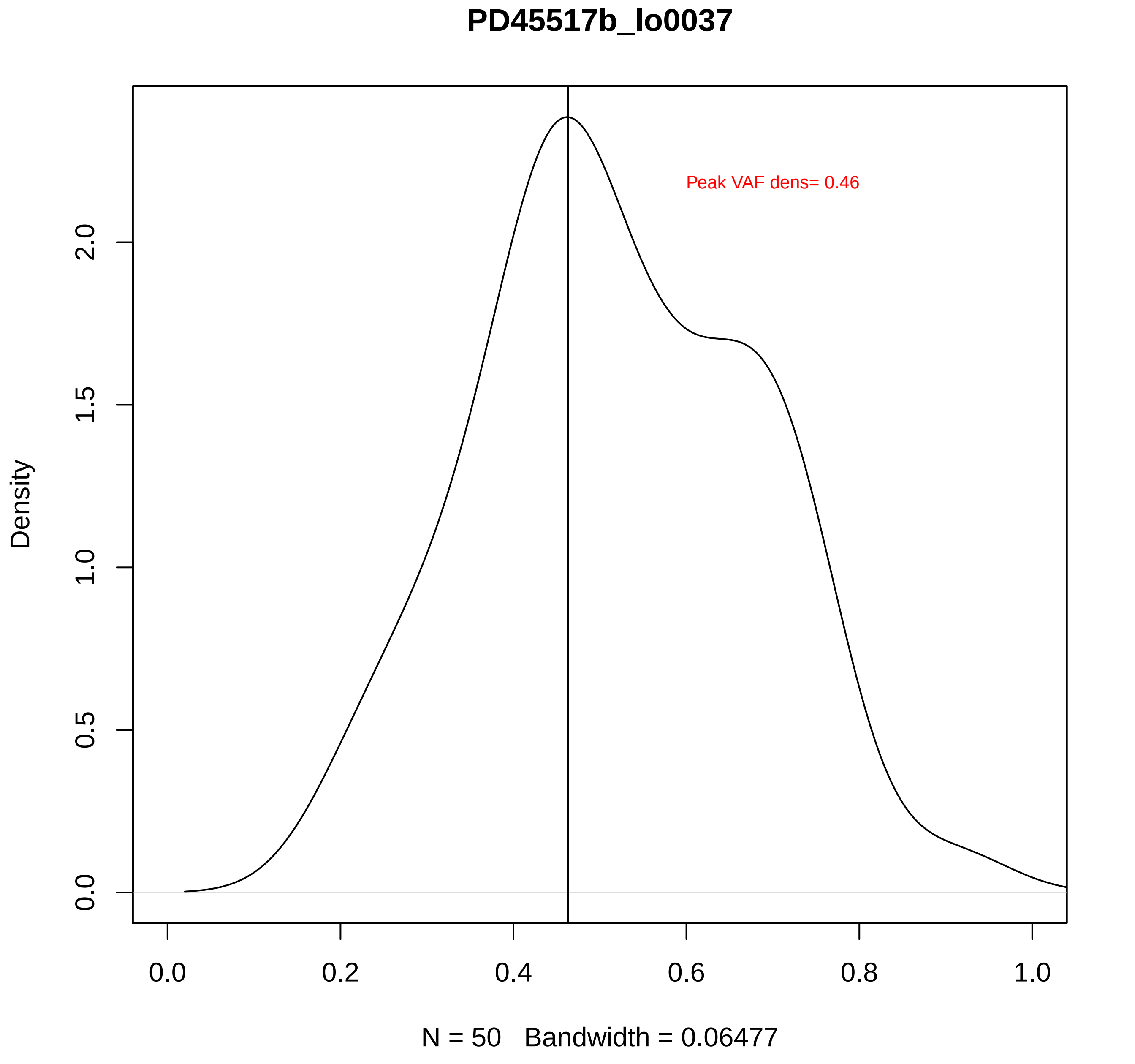
PD45517b_lo0037
Peak VAF dens= 0.46
Density
0.0
0.5
1.0
1.5
2.0
0.0
0.2
0.4
0.6
0.8
1.0
N = 50   Bandwidth = 0.06477

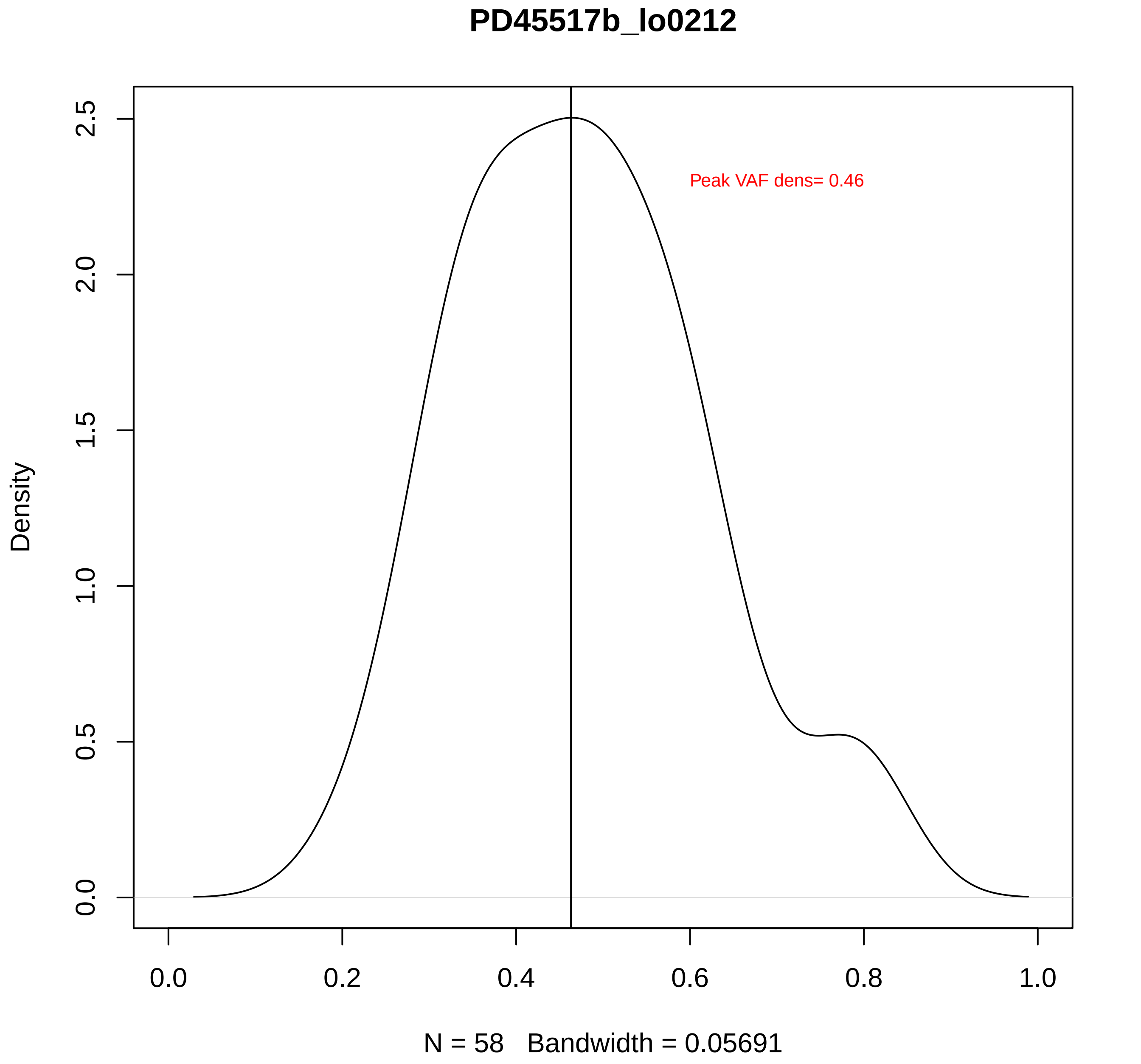
PD45517b_lo0212
Peak VAF dens= 0.46
Density
0.0
0.5
1.0
1.5
2.0
2.5
0.0
0.2
0.4
0.6
0.8
1.0
N = 58 Bandwidth = 0.05691

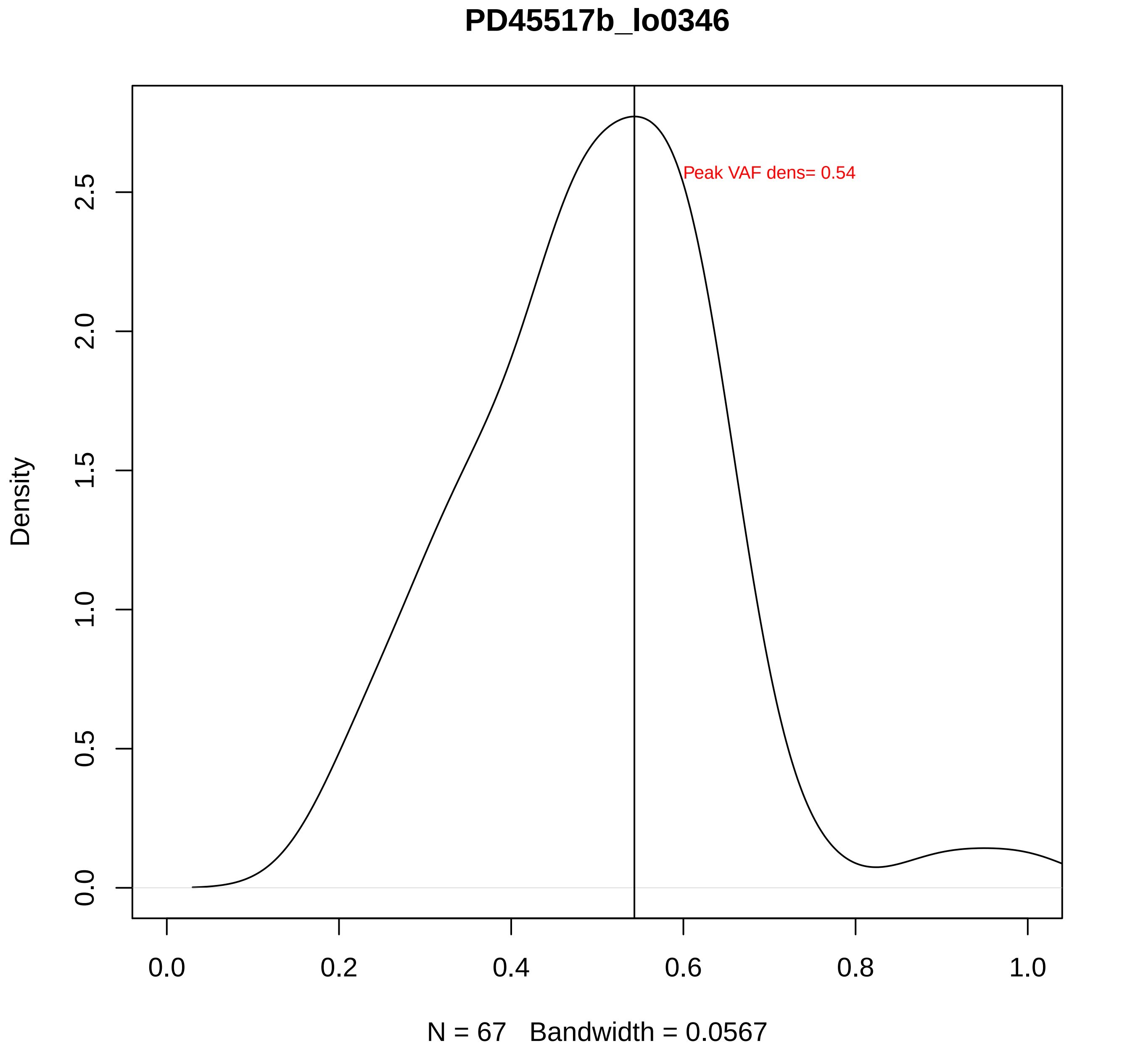
PD45517b_lo0346
Peak VAF dens= 0.54
Density
0.0
0.5
1.0
1.5
2.0
2.5
0.0
0.2
0.4
0.6
0.8
1.0
N = 67   Bandwidth = 0.0567

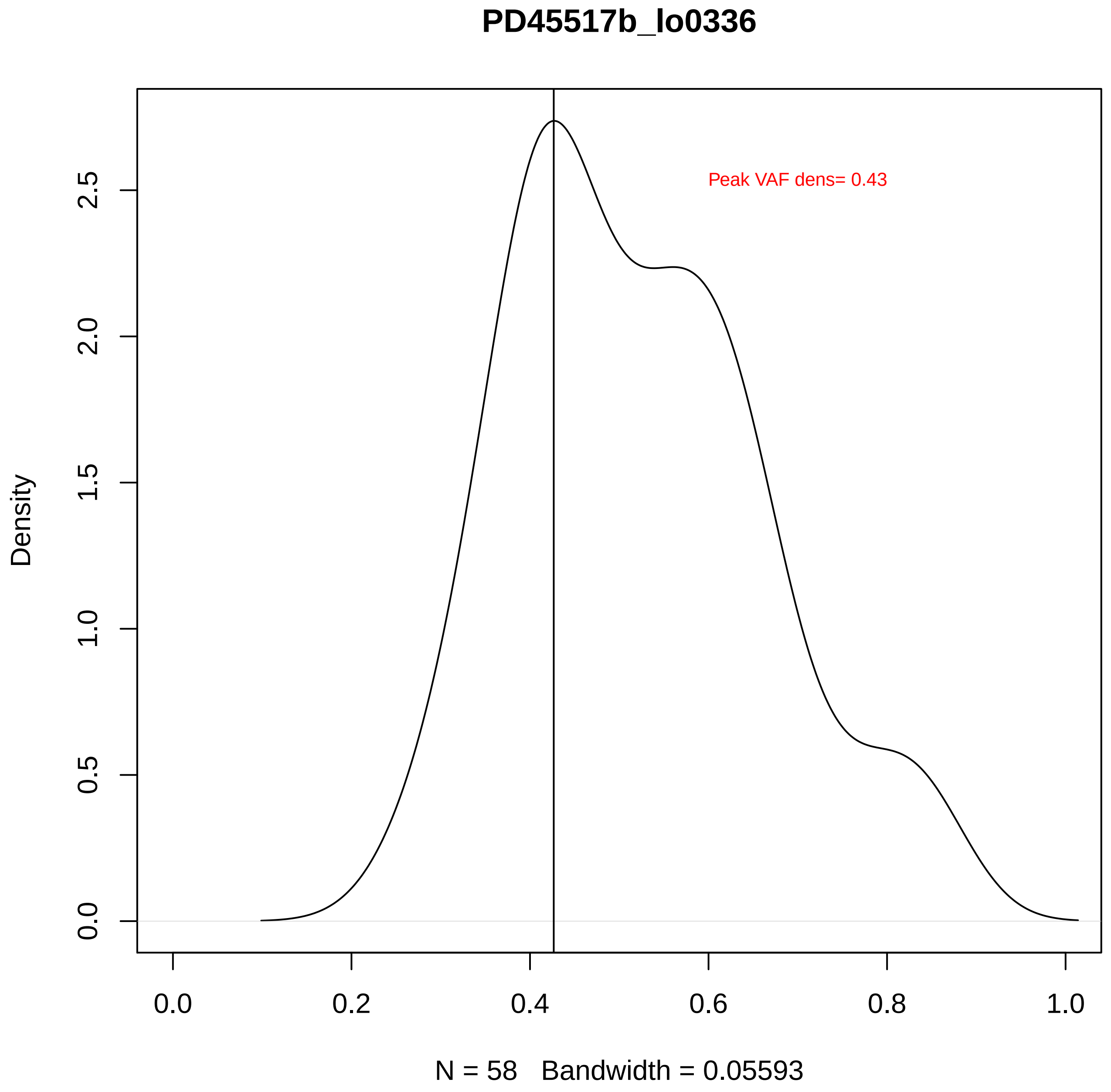
PD45517b_lo0336
Peak VAF dens= 0.43
Density
0.0
0.5
1.0
1.5
2.0
2.5
0.0
0.2
0.4
0.6
0.8
1.0
N = 58   Bandwidth = 0.05593

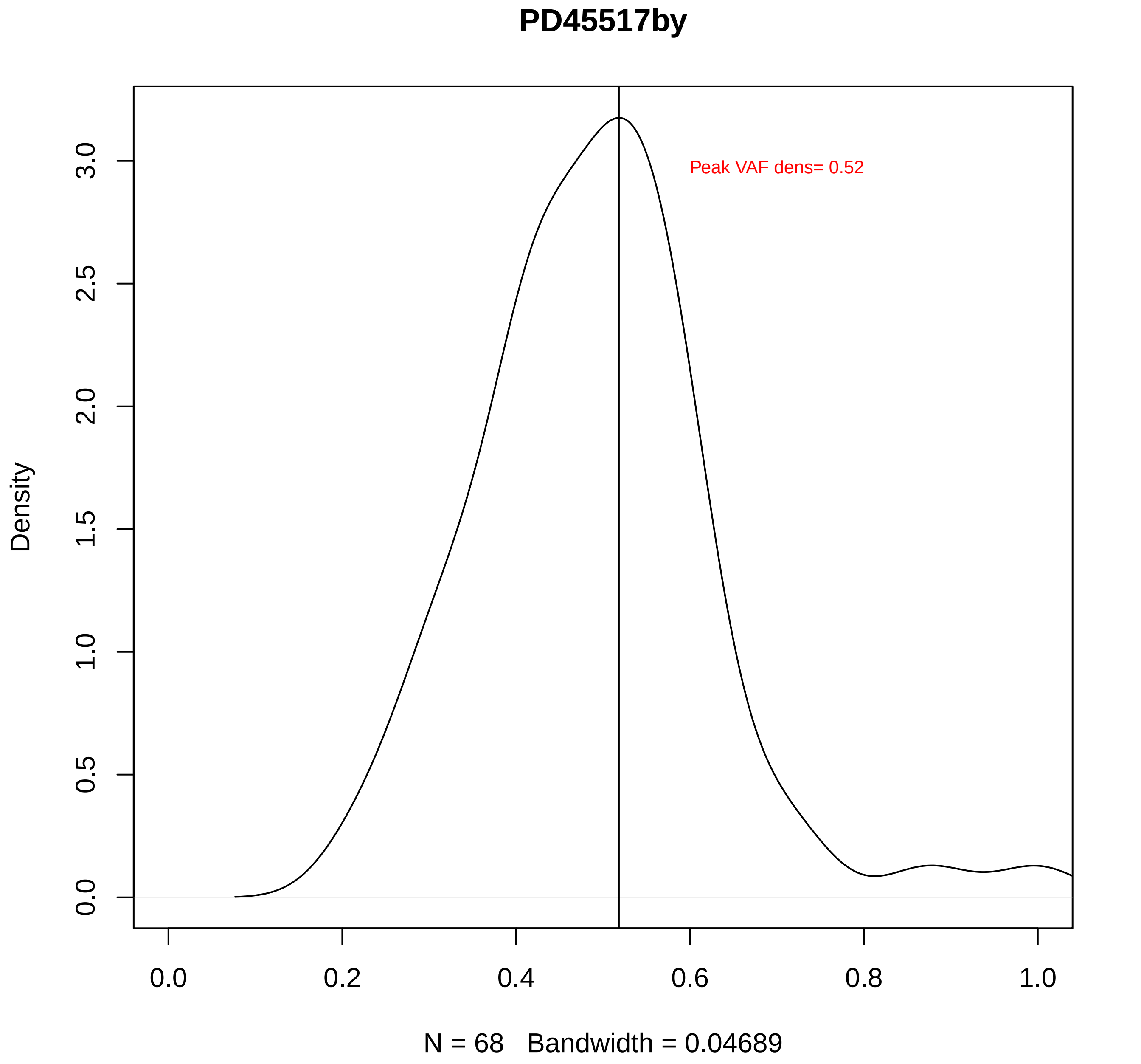
PD45517by
Peak VAF dens= 0.52
Density
0.0
0.5
1.0
1.5
2.0
2.5
3.0
0.0
0.2
0.4
0.6
0.8
1.0
N = 68   Bandwidth = 0.04689

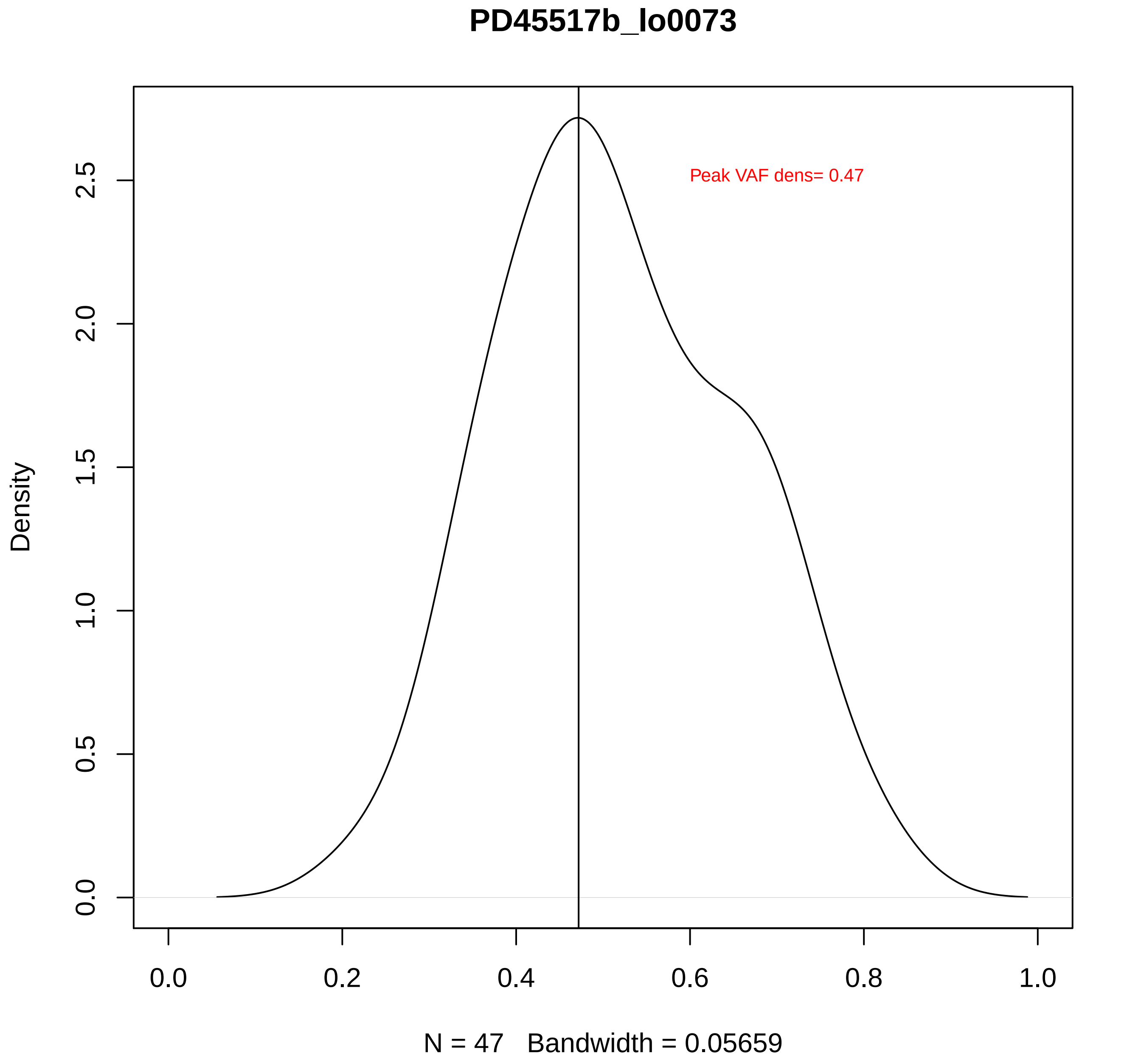
PD45517b_lo0073
Peak VAF dens= 0.47
Density
0.0
0.5
1.0
1.5
2.0
2.5
0.0
0.2
0.4
0.6
0.8
1.0
N = 47 Bandwidth = 0.05659

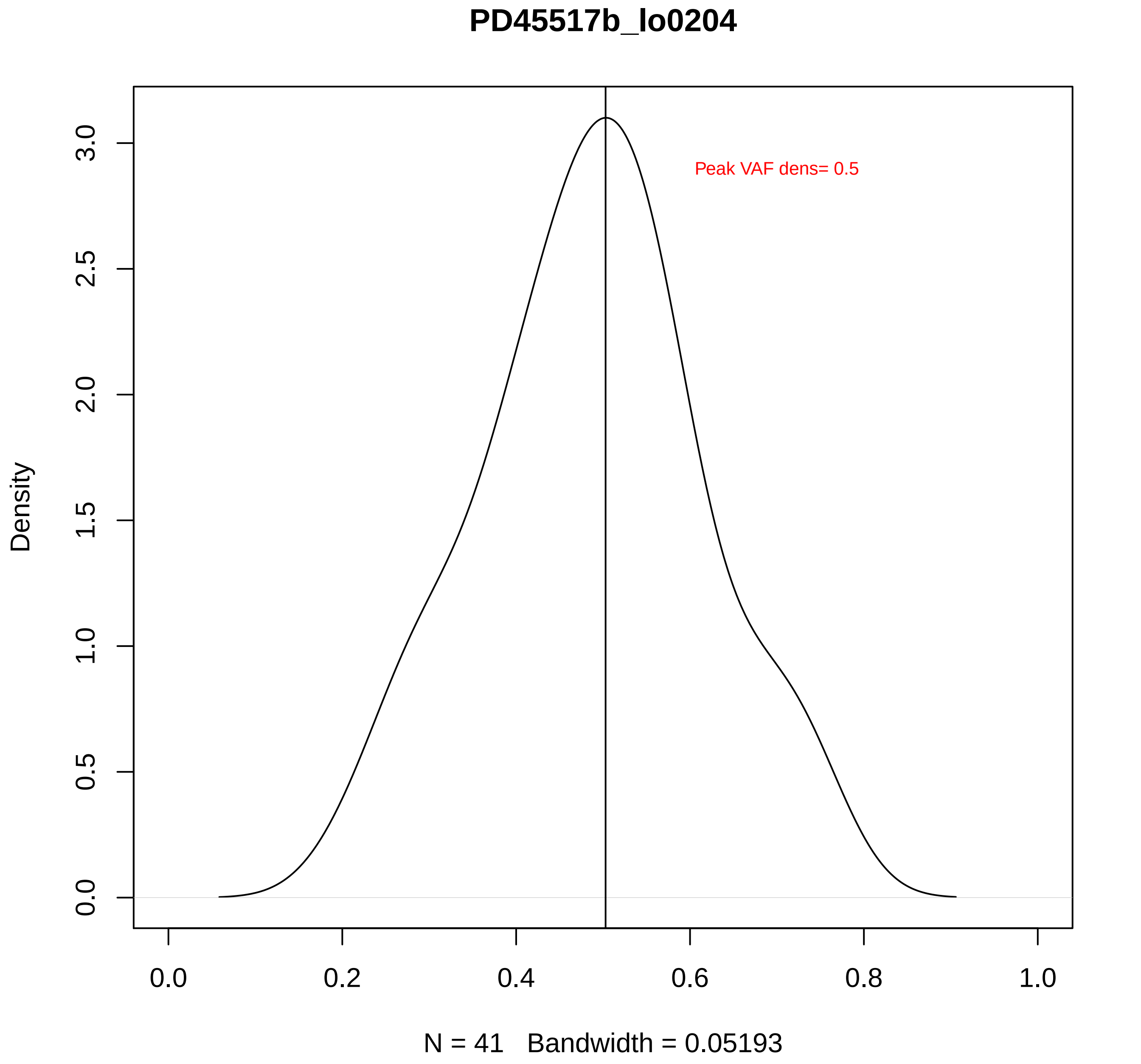
PD45517b_lo0204
Peak VAF dens= 0.5
Density
0.0
0.5
1.0
1.5
2.0
2.5
3.0
0.0
0.2
0.4
0.6
0.8
1.0
N = 41   Bandwidth = 0.05193

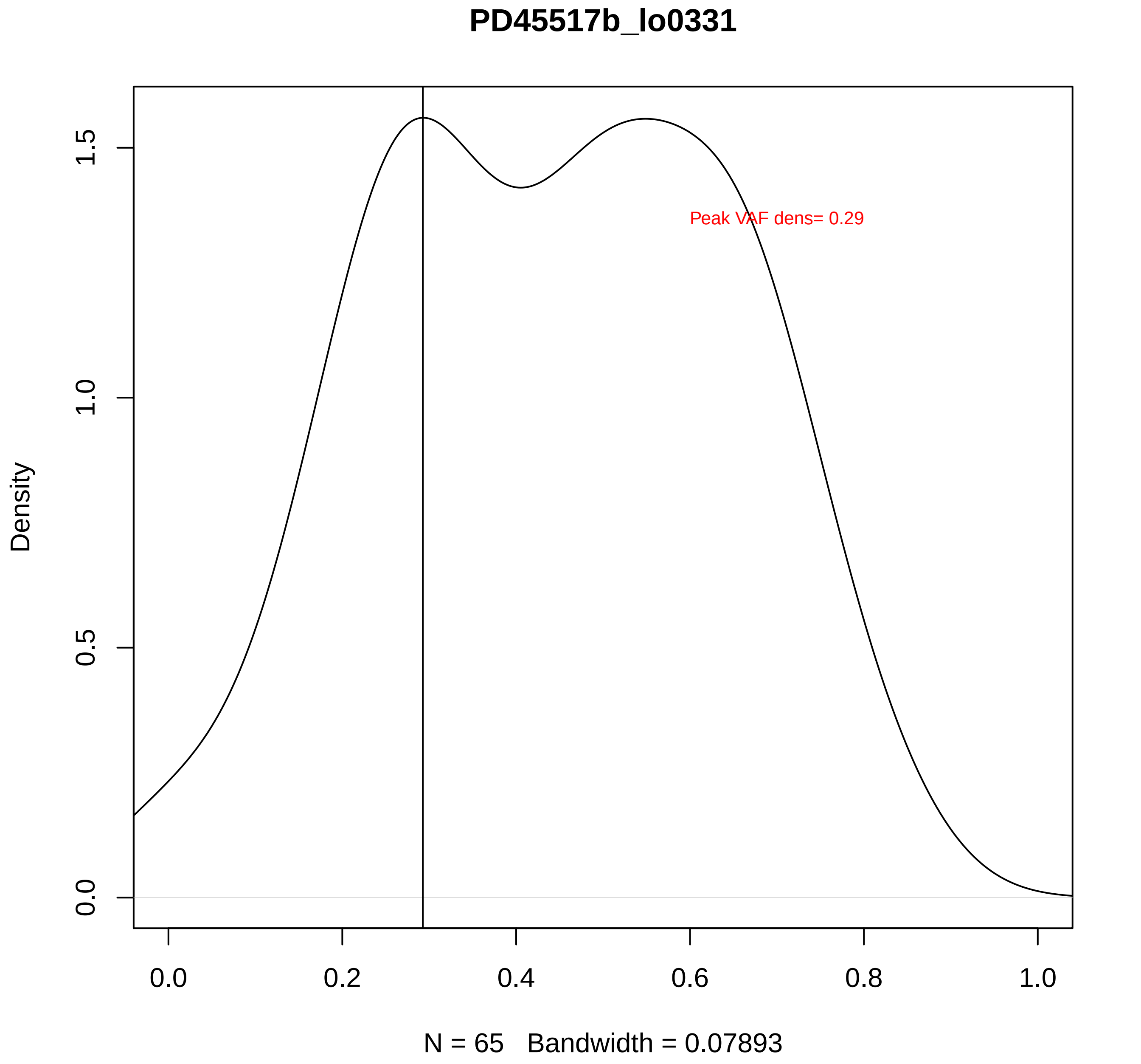
PD45517b_lo0331
Density
1.5
1.0
0.5
0.0
0.0
0.2
0.4
0.6
0.8
1.0
Peak VAF dens= 0.29
N = 65 Bandwidth = 0.07893

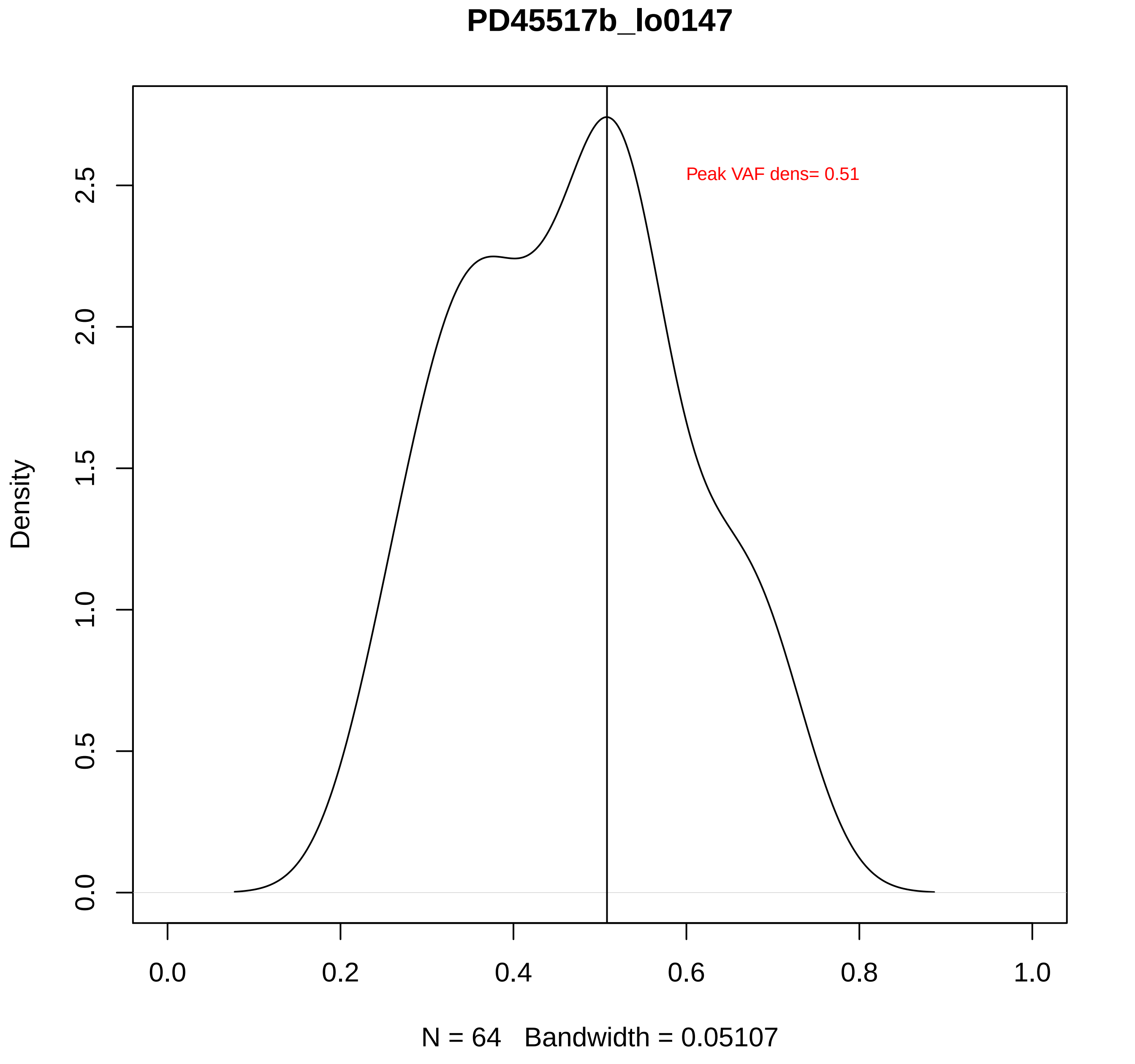
PD45517b_lo0147
Peak VAF dens= 0.51
Density
0.0
0.5
1.0
1.5
2.0
2.5
0.0
0.2
0.4
0.6
0.8
1.0
N = 64 Bandwidth = 0.05107

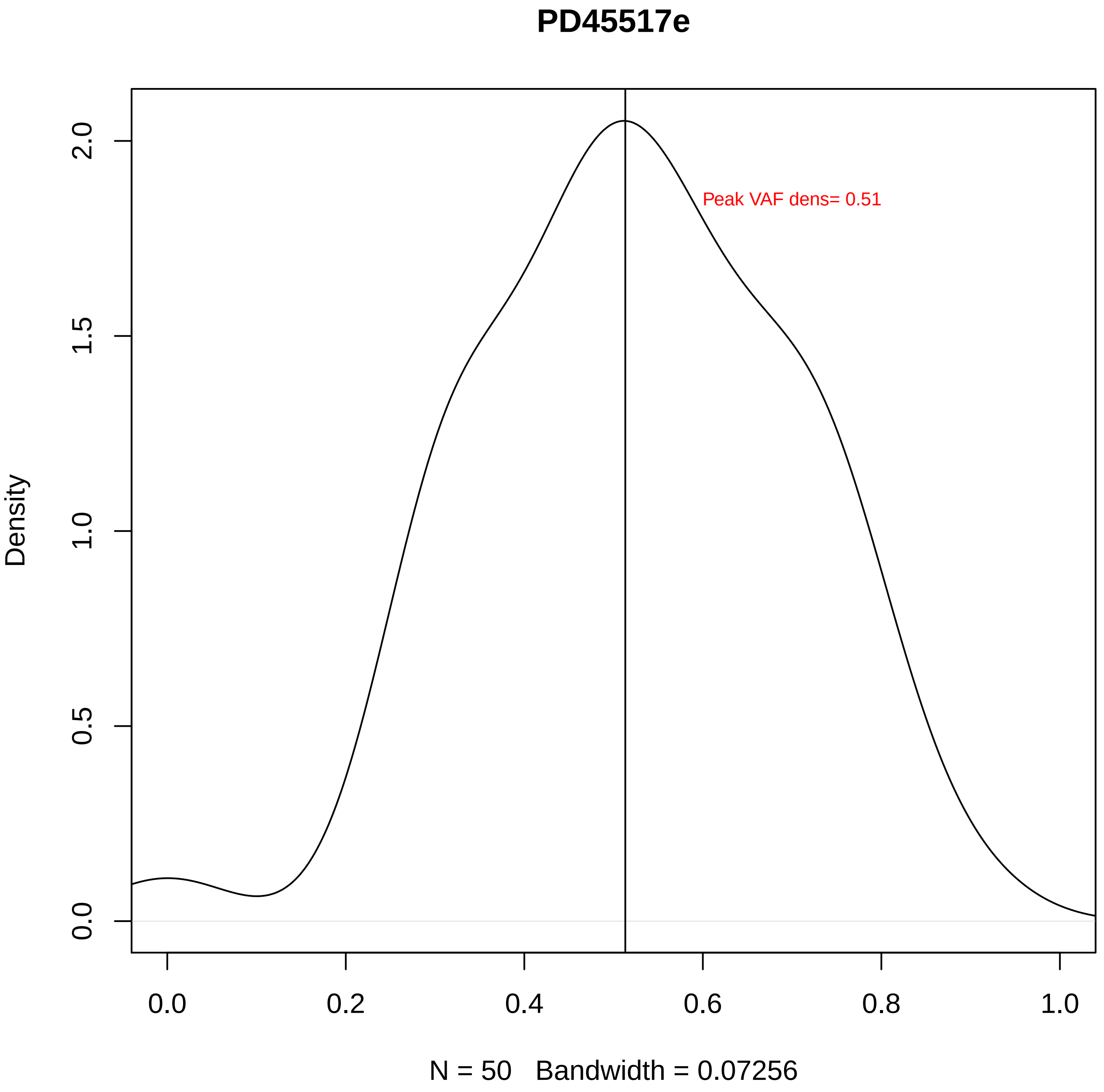
PD45517e
Peak VAF dens= 0.51
Density
0.0
0.5
1.0
1.5
2.0
0.0
0.2
0.4
0.6
0.8
1.0
N = 50   Bandwidth = 0.07256

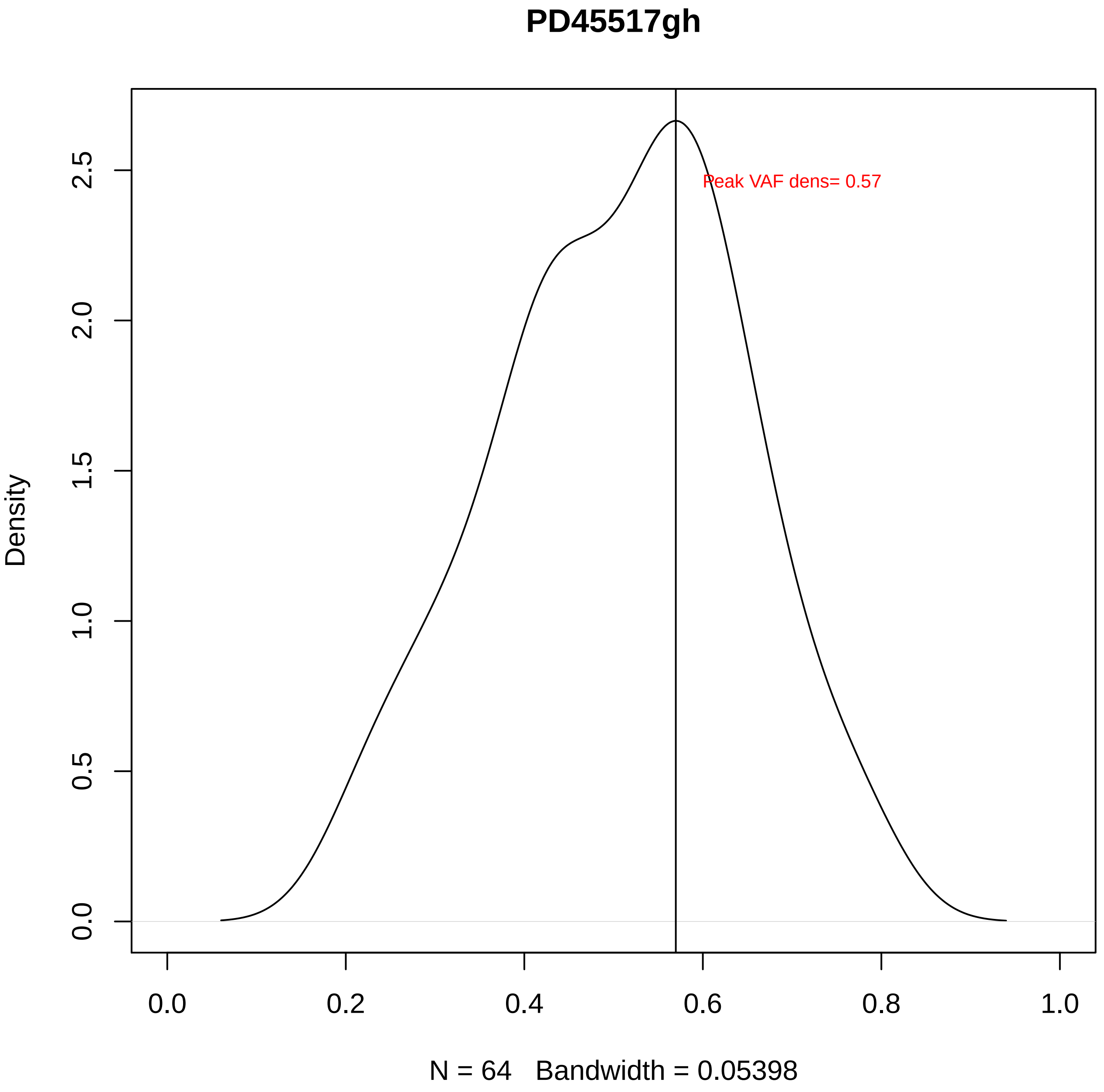

PD45517gh
Peak VAF dens= 0.57
Density
0.0
0.5
1.0
1.5
2.0
2.5
0.0
0.2
0.4
0.6
0.8
1.0
N = 64   Bandwidth = 0.05398

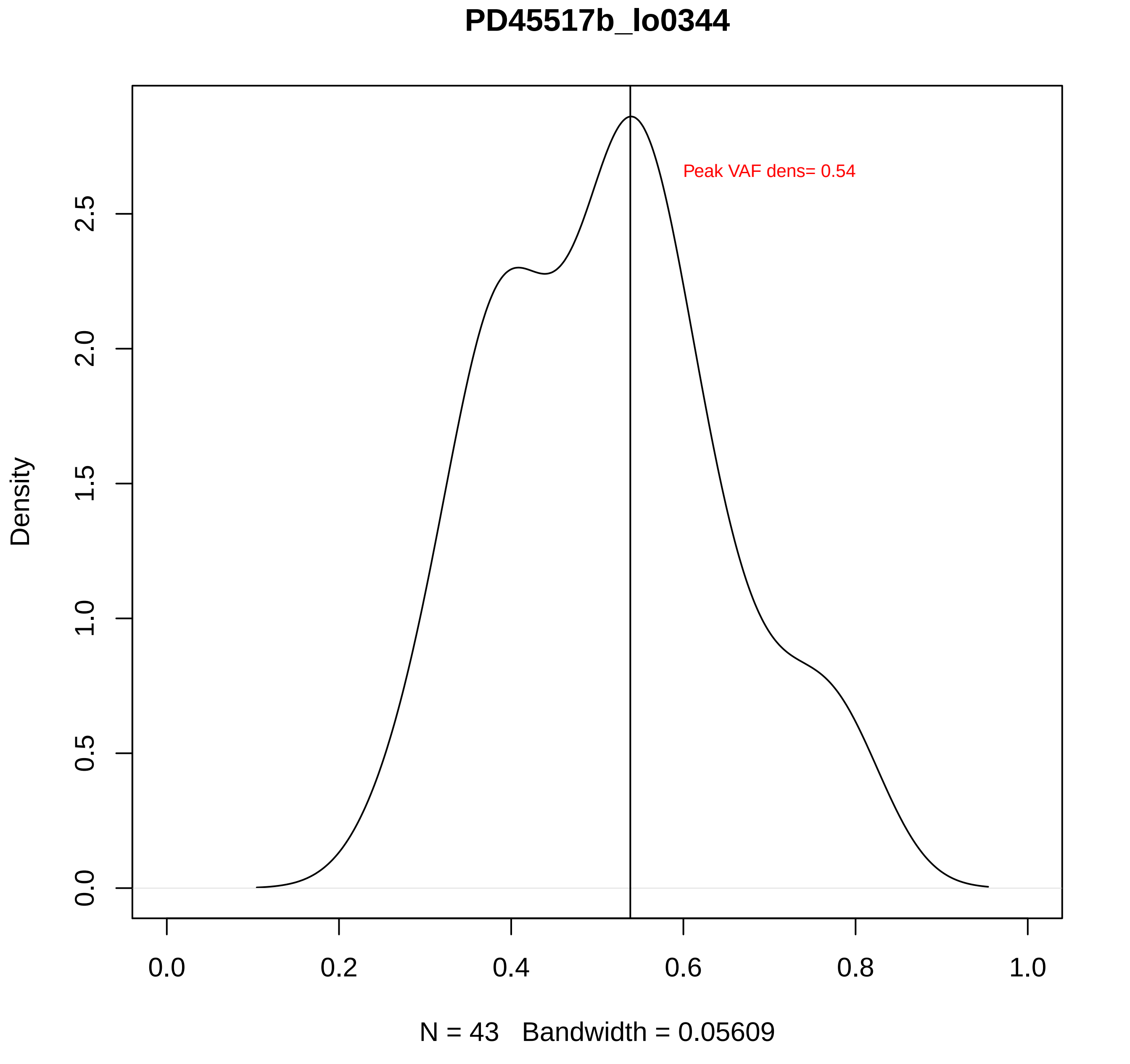
PD45517b_lo0344
Peak VAF dens= 0.54
Density
0.0
0.5
1.0
1.5
2.0
2.5
0.0
0.2
0.4
0.6
0.8
1.0
N = 43   Bandwidth = 0.05609

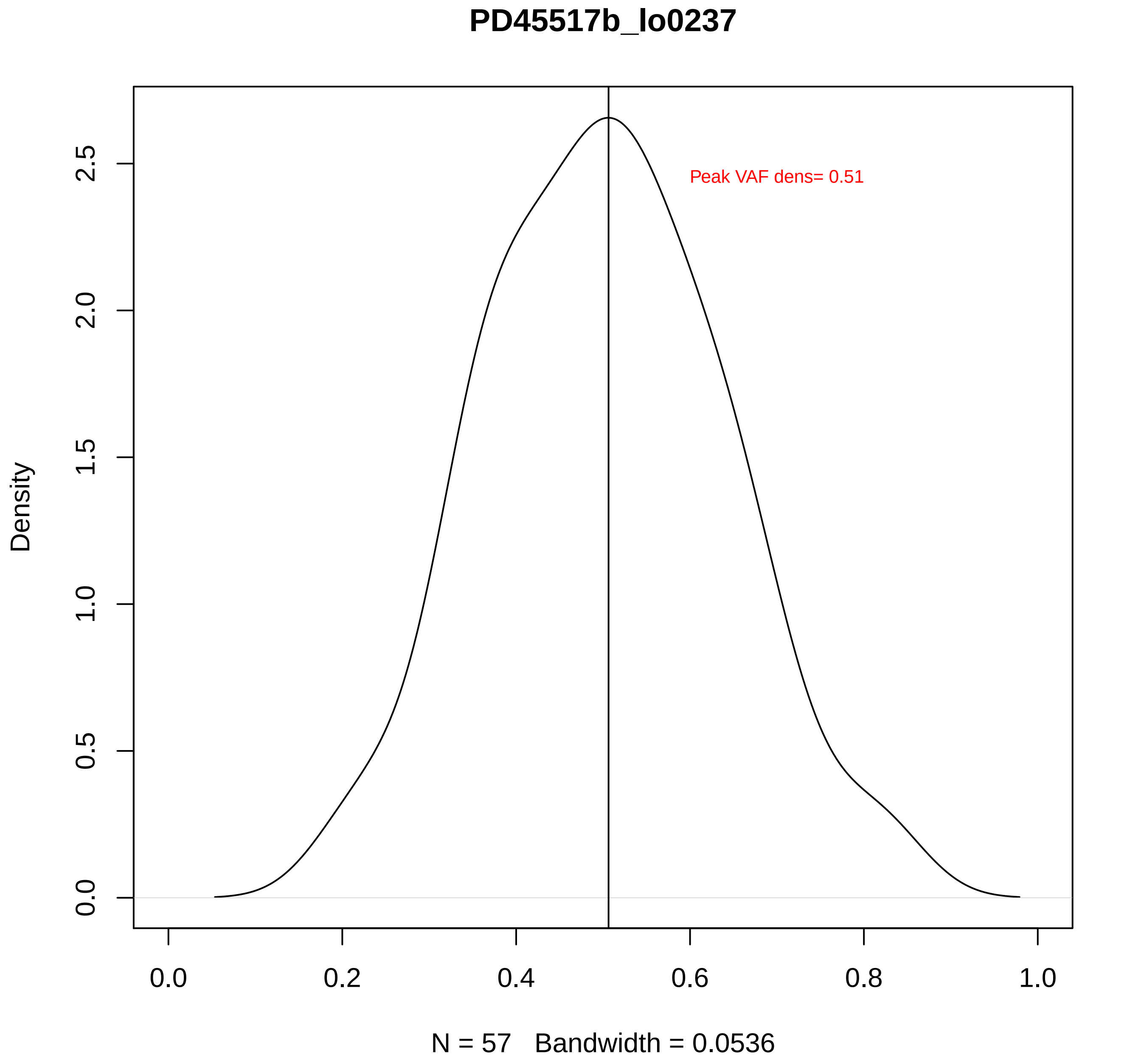
PD45517b_lo0237
Peak VAF dens= 0.51
Density
0.0
0.5
1.0
1.5
2.0
2.5
0.0
0.2
0.4
0.6
0.8
1.0
N = 57   Bandwidth = 0.0536

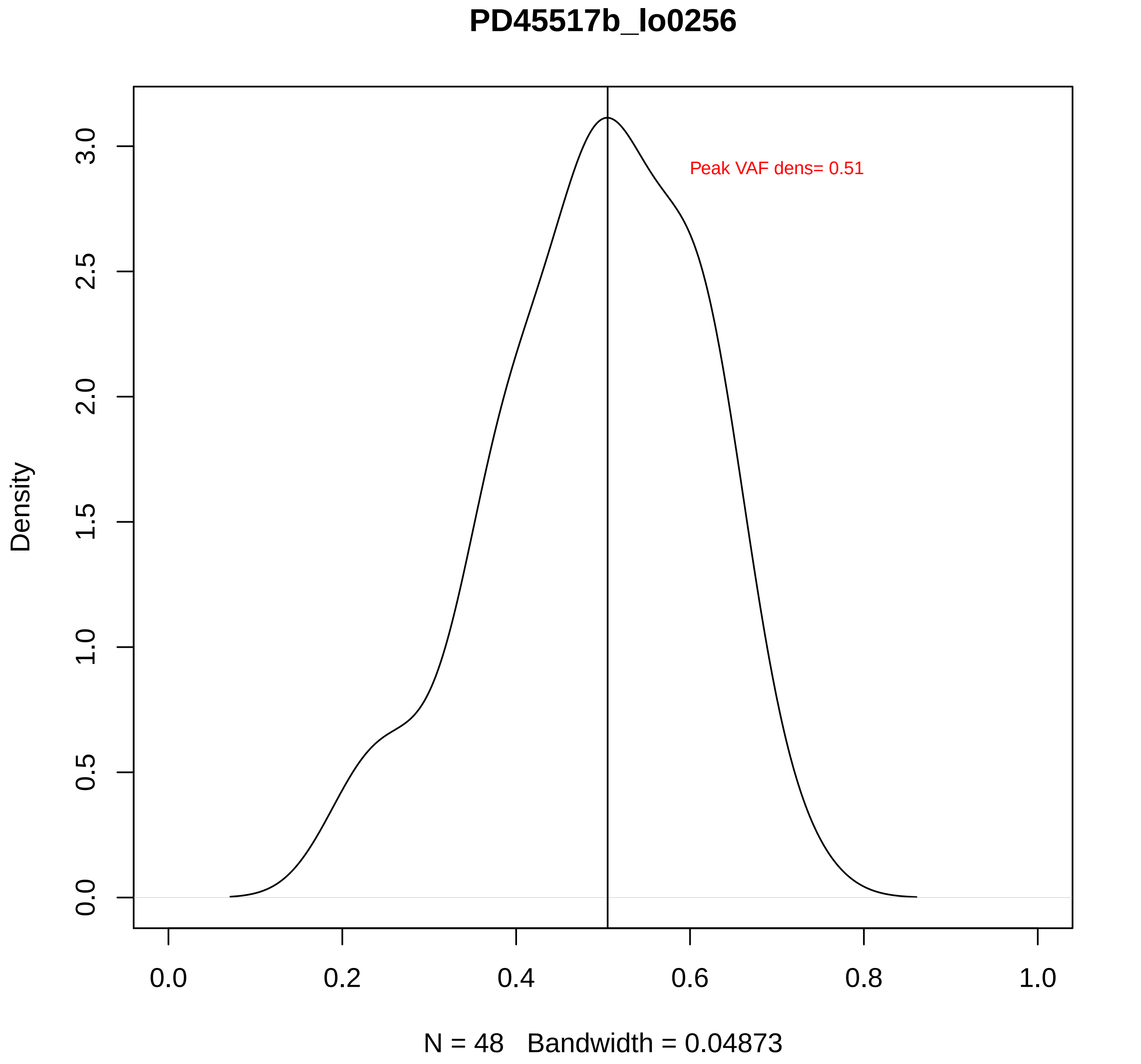
PD45517b_lo0256
Peak VAF dens= 0.51
Density
0.0
0.5
1.0
1.5
2.0
2.5
3.0
0.0
0.2
0.4
0.6
0.8
1.0
N = 48   Bandwidth = 0.04873

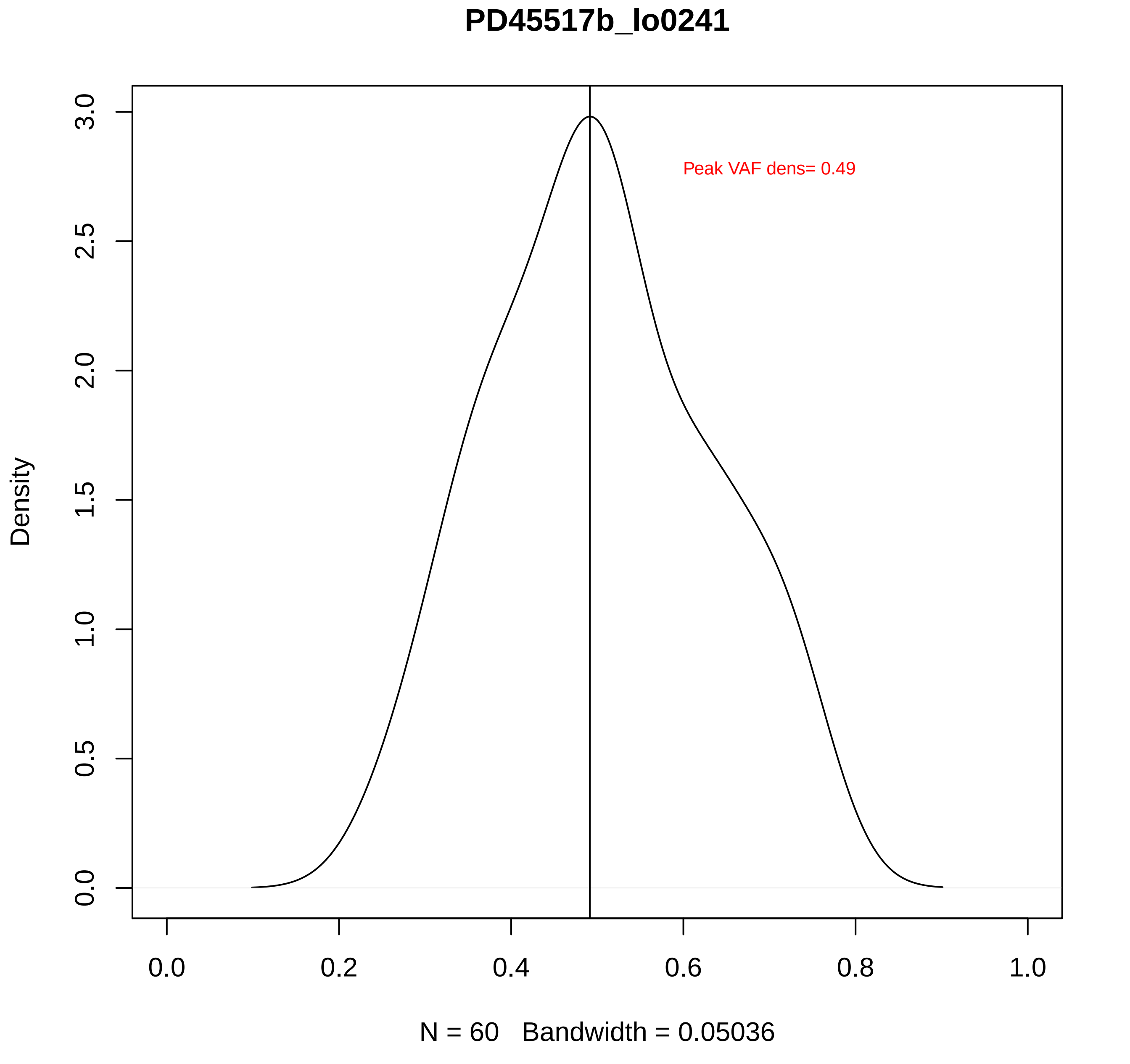
PD45517b_lo0241
Peak VAF dens= 0.49
Density
0.0
0.5
1.0
1.5
2.0
2.5
3.0
0.0
0.2
0.4
0.6
0.8
1.0
N = 60   Bandwidth = 0.05036

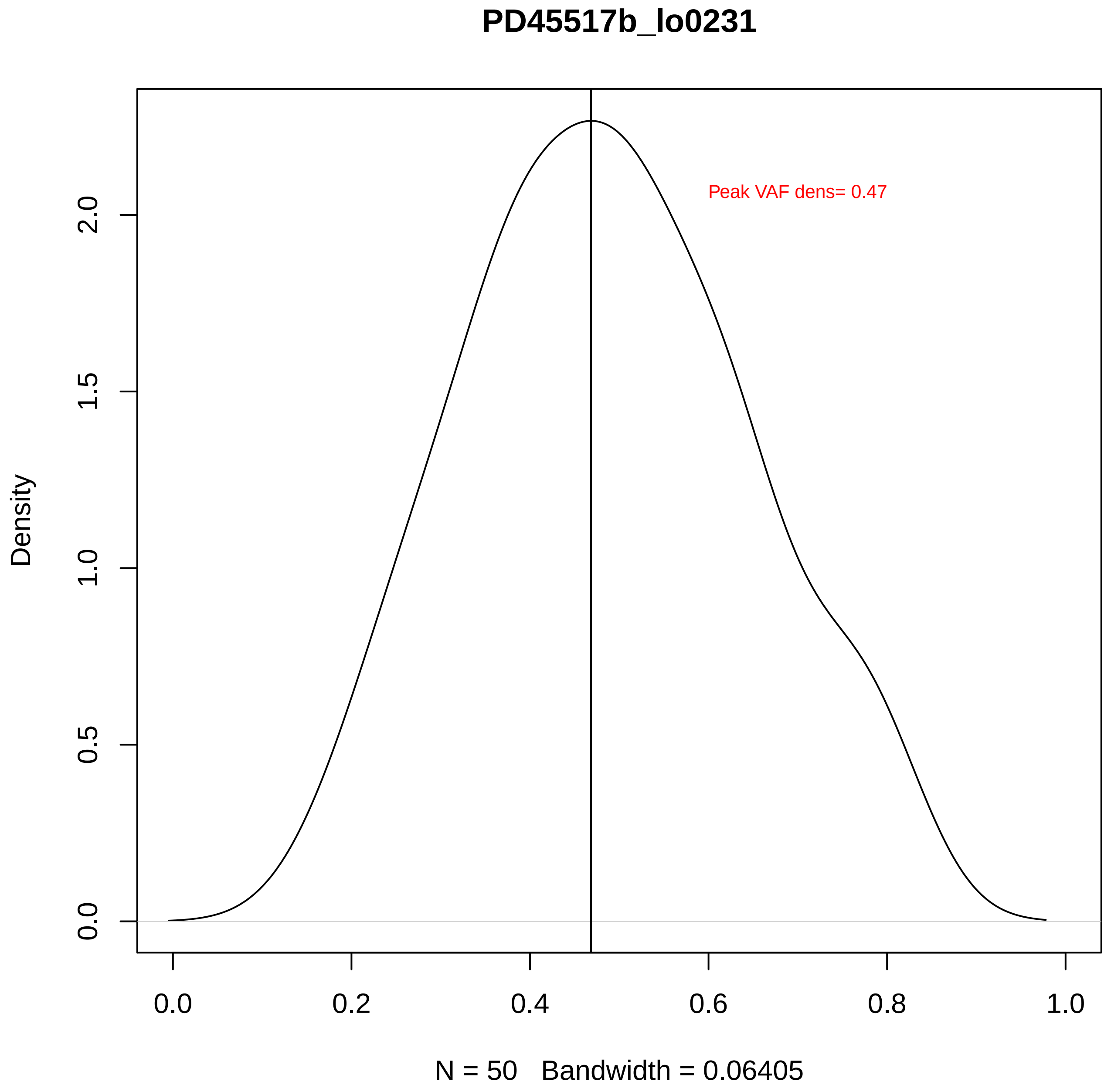

PD45517b_lo0231
Peak VAF dens= 0.47
Density
0.0
0.5
1.0
1.5
2.0
0.0
0.2
0.4
0.6
0.8
1.0
N = 50   Bandwidth = 0.06405

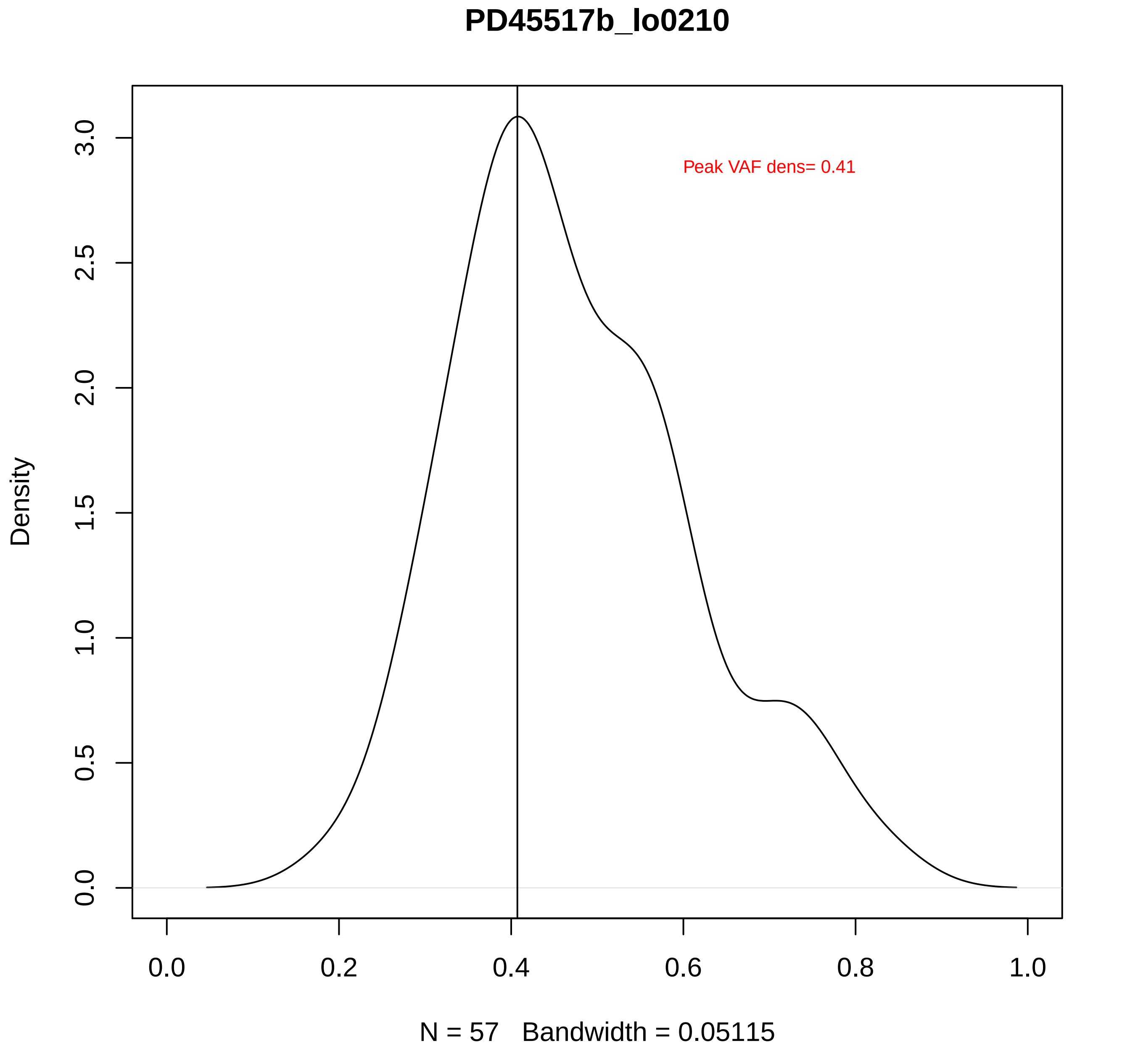
PD45517b_lo0210
Peak VAF dens= 0.41
Density
0.0
0.5
1.0
1.5
2.0
2.5
3.0
0.0
0.2
0.4
0.6
0.8
1.0
N = 57   Bandwidth = 0.05115

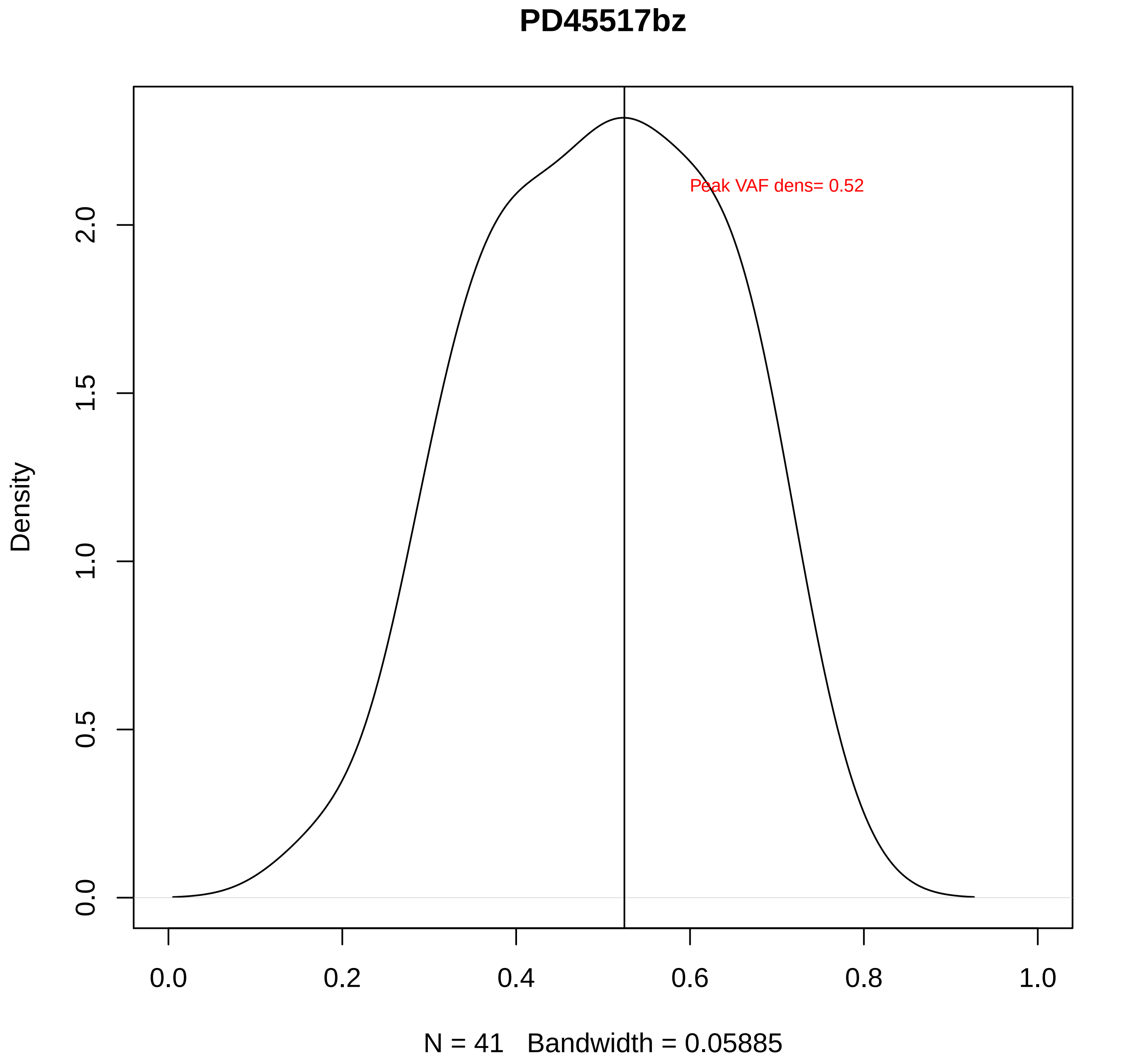
PD45517bz
Peak VAF dens= 0.52
Density
0.0
0.5
1.0
1.5
2.0
0.0
0.2
0.4
0.6
0.8
1.0
N = 41   Bandwidth = 0.05885

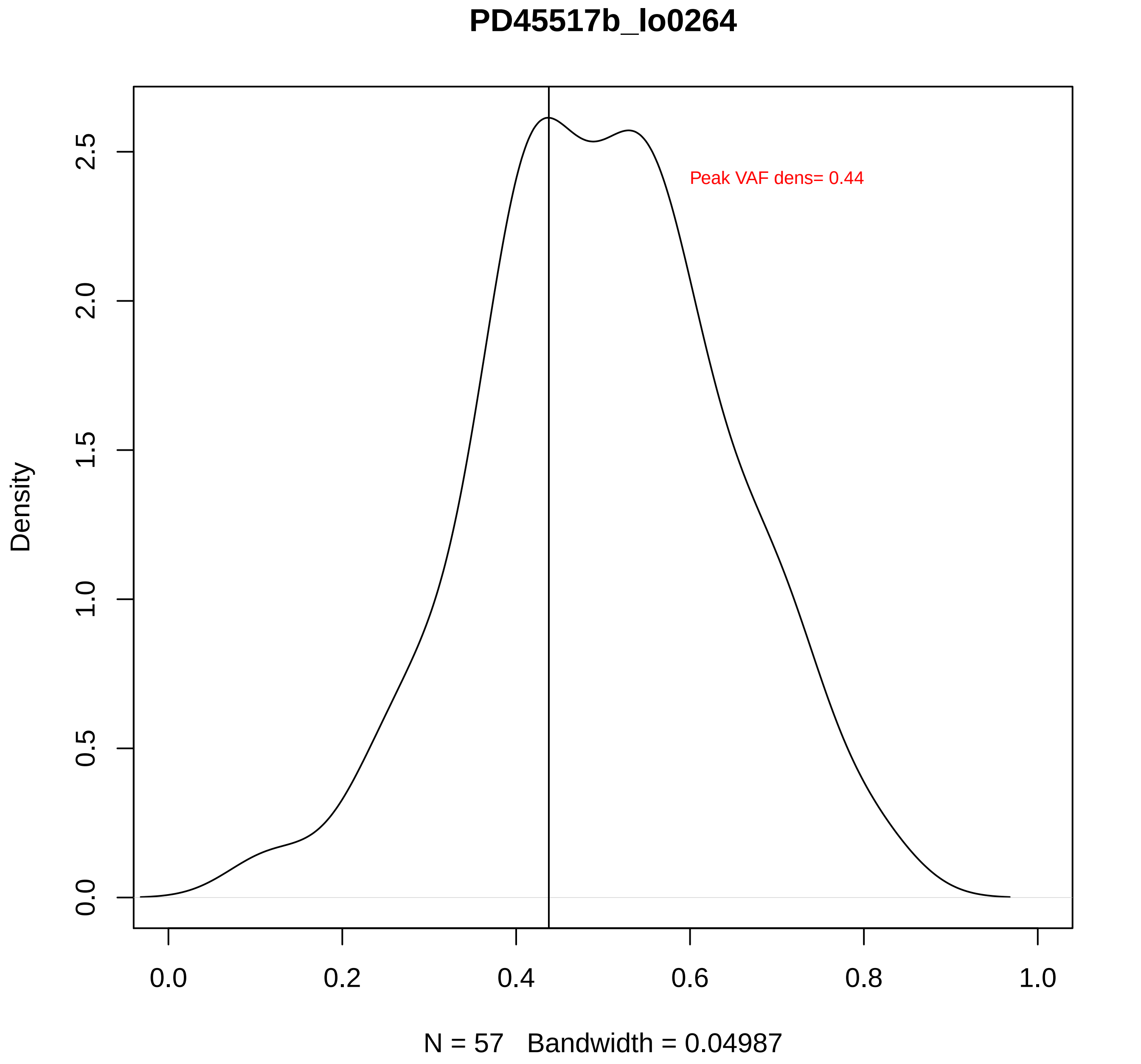
PD45517b_lo0264
Peak VAF dens= 0.44
Density
0.0
0.5
1.0
1.5
2.0
2.5
0.0
0.2
0.4
0.6
0.8
1.0
N = 57   Bandwidth = 0.04987

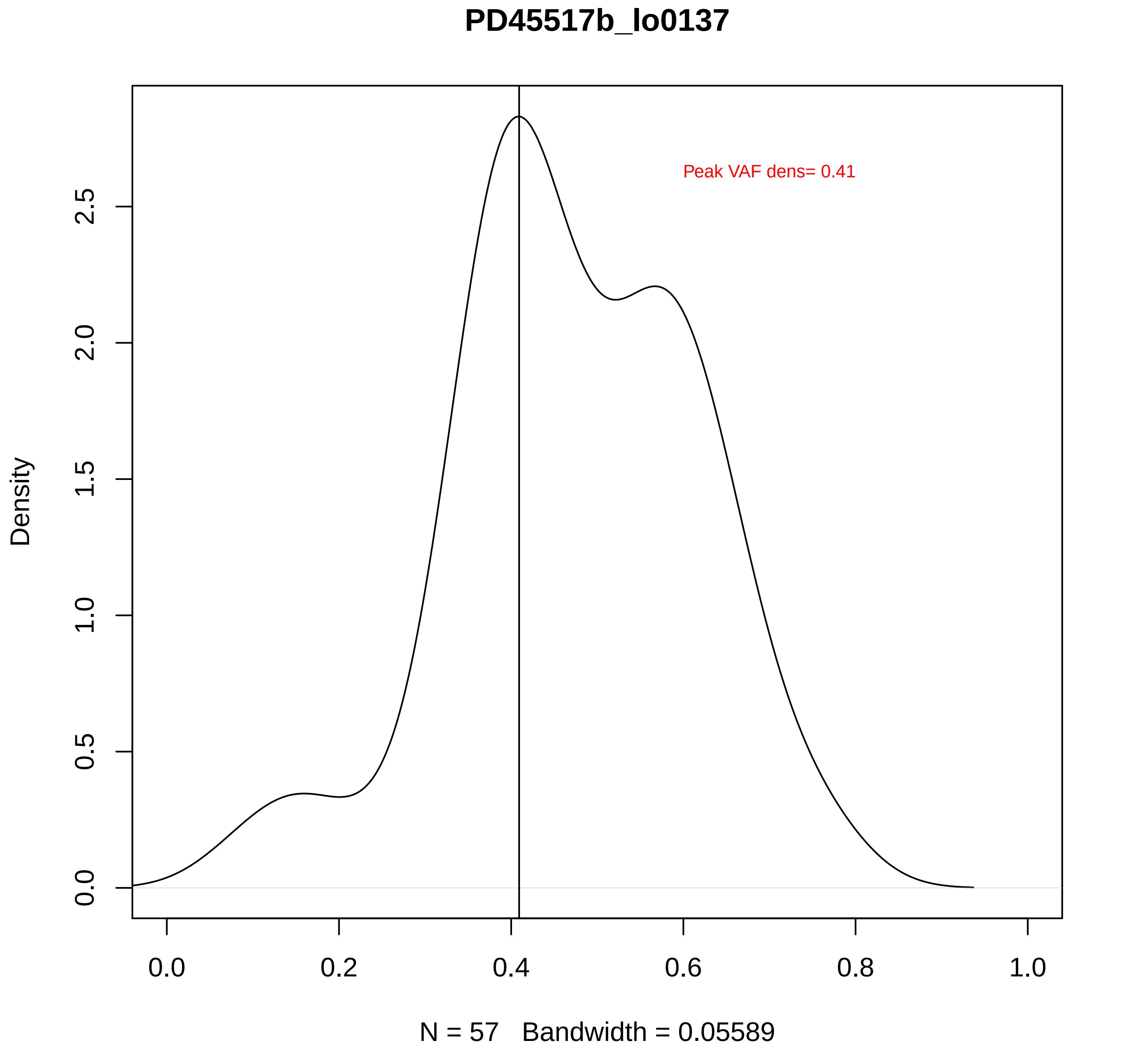

PD45517b_lo0137
Peak VAF dens= 0.41
Density
0.0
0.5
1.0
1.5
2.0
2.5
0.0
0.2
0.4
0.6
0.8
1.0
N = 57   Bandwidth = 0.05589

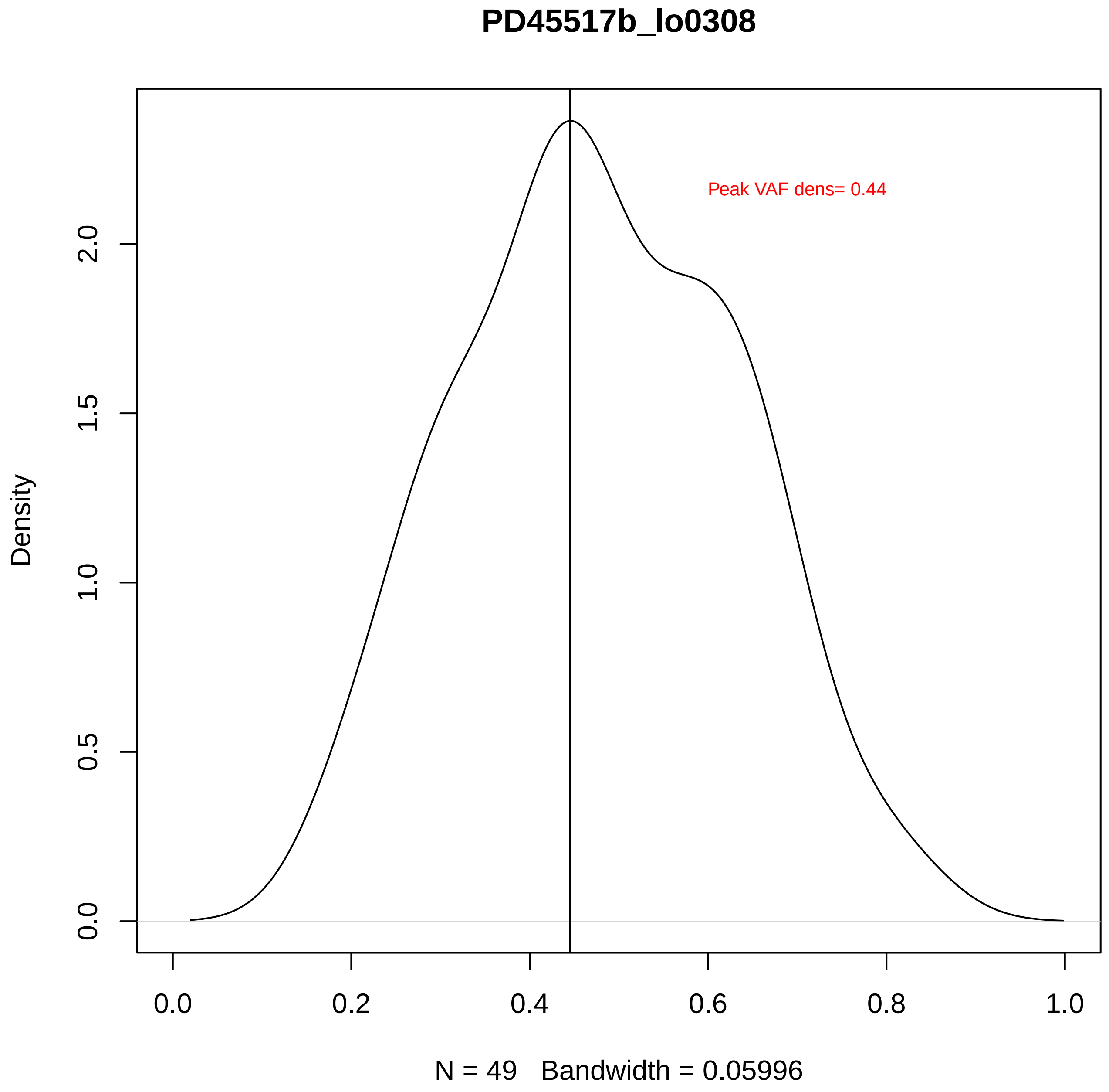
PD45517b_lo0308
Peak VAF dens= 0.44
Density
0.0
0.5
1.0
1.5
2.0
0.0
0.2
0.4
0.6
0.8
1.0
N = 49   Bandwidth = 0.05996

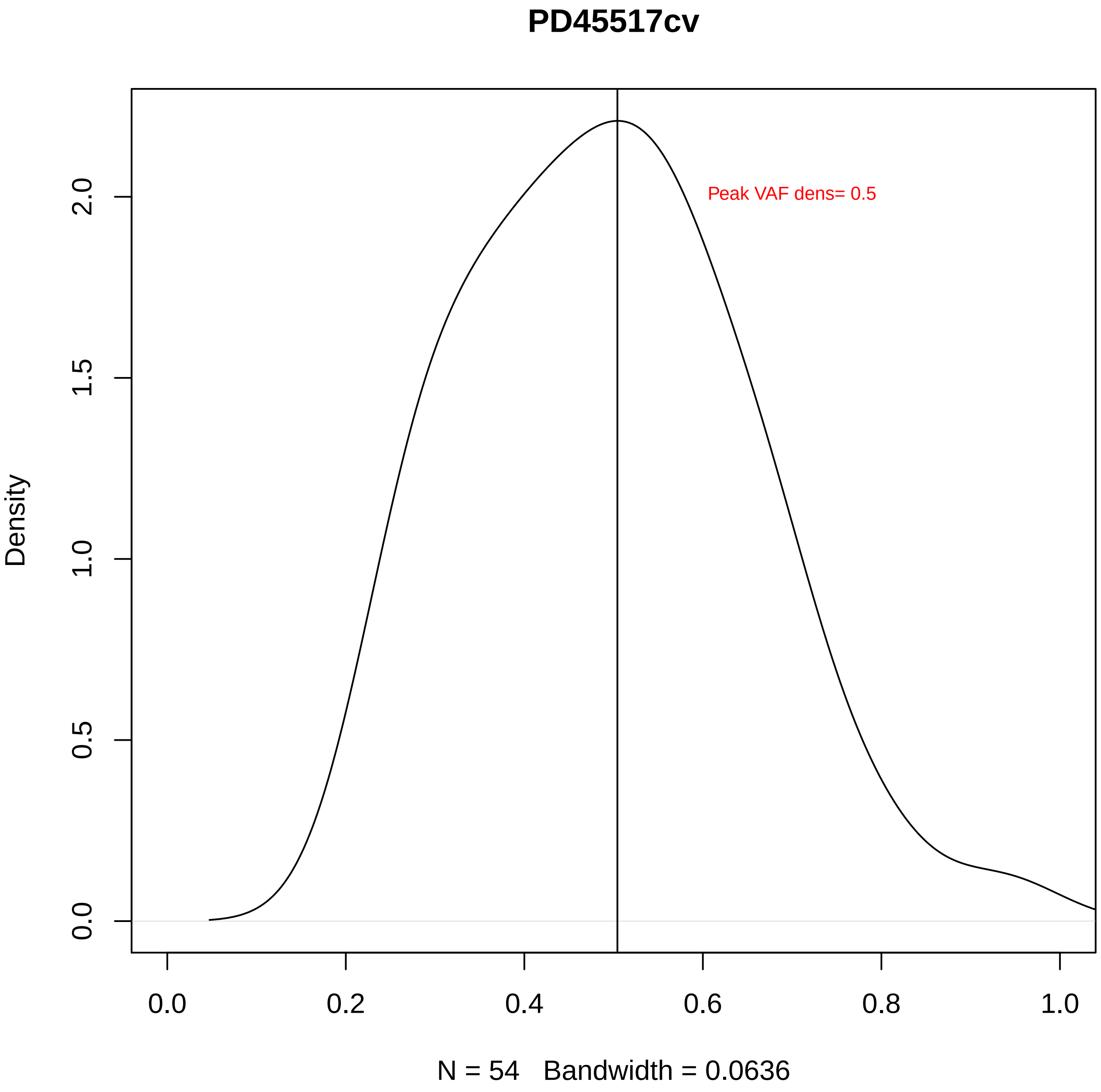

PD45517cv
Peak VAF dens= 0.5
Density
0.0
0.5
1.0
1.5
2.0
0.0
0.2
0.4
0.6
0.8
1.0
N = 54 Bandwidth = 0.0636

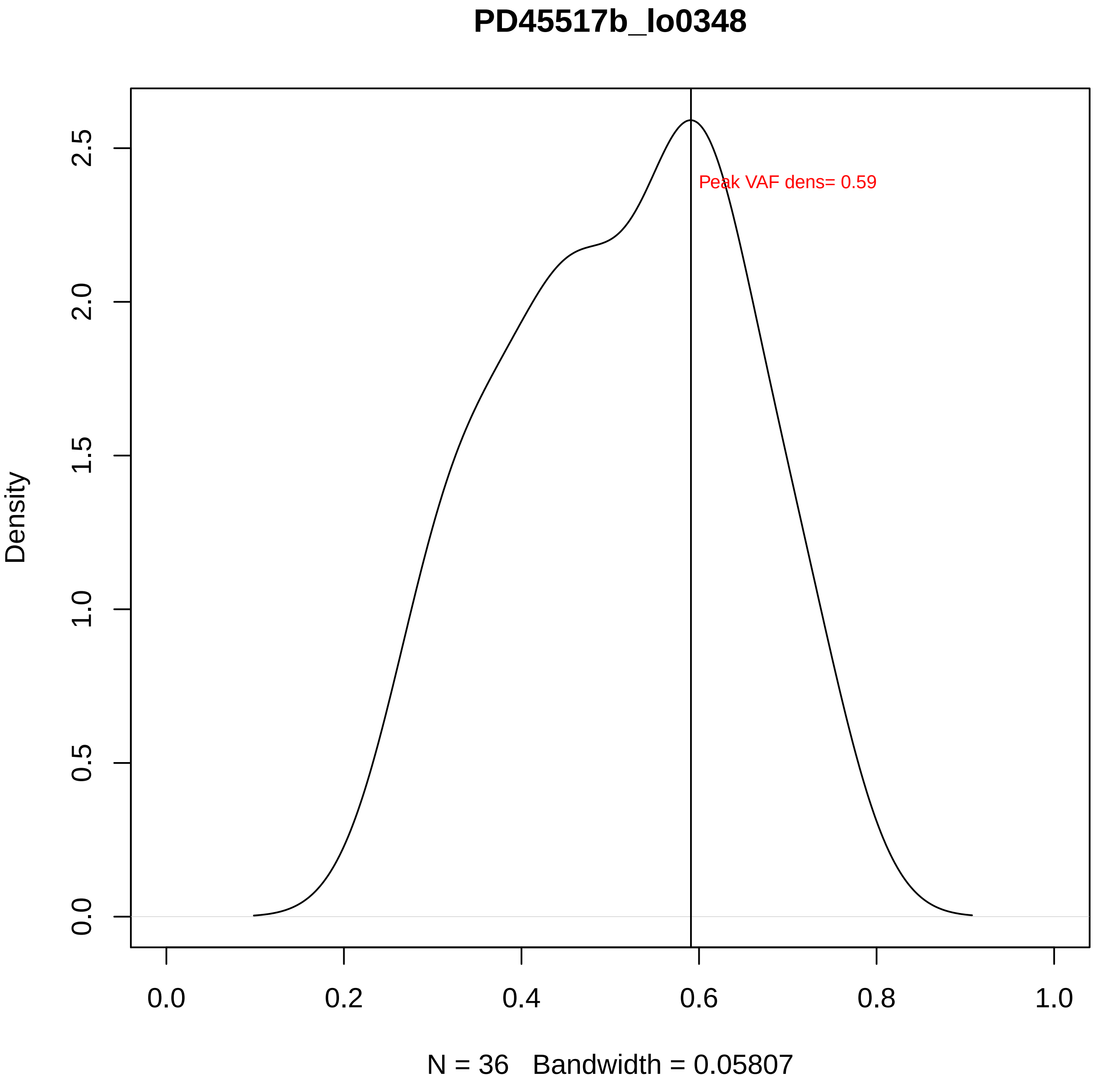
PD45517b_lo0348
Peak VAF dens= 0.59
Density
0.0
0.5
1.0
1.5
2.0
2.5
0.0
0.2
0.4
0.6
0.8
1.0
N = 36   Bandwidth = 0.05807

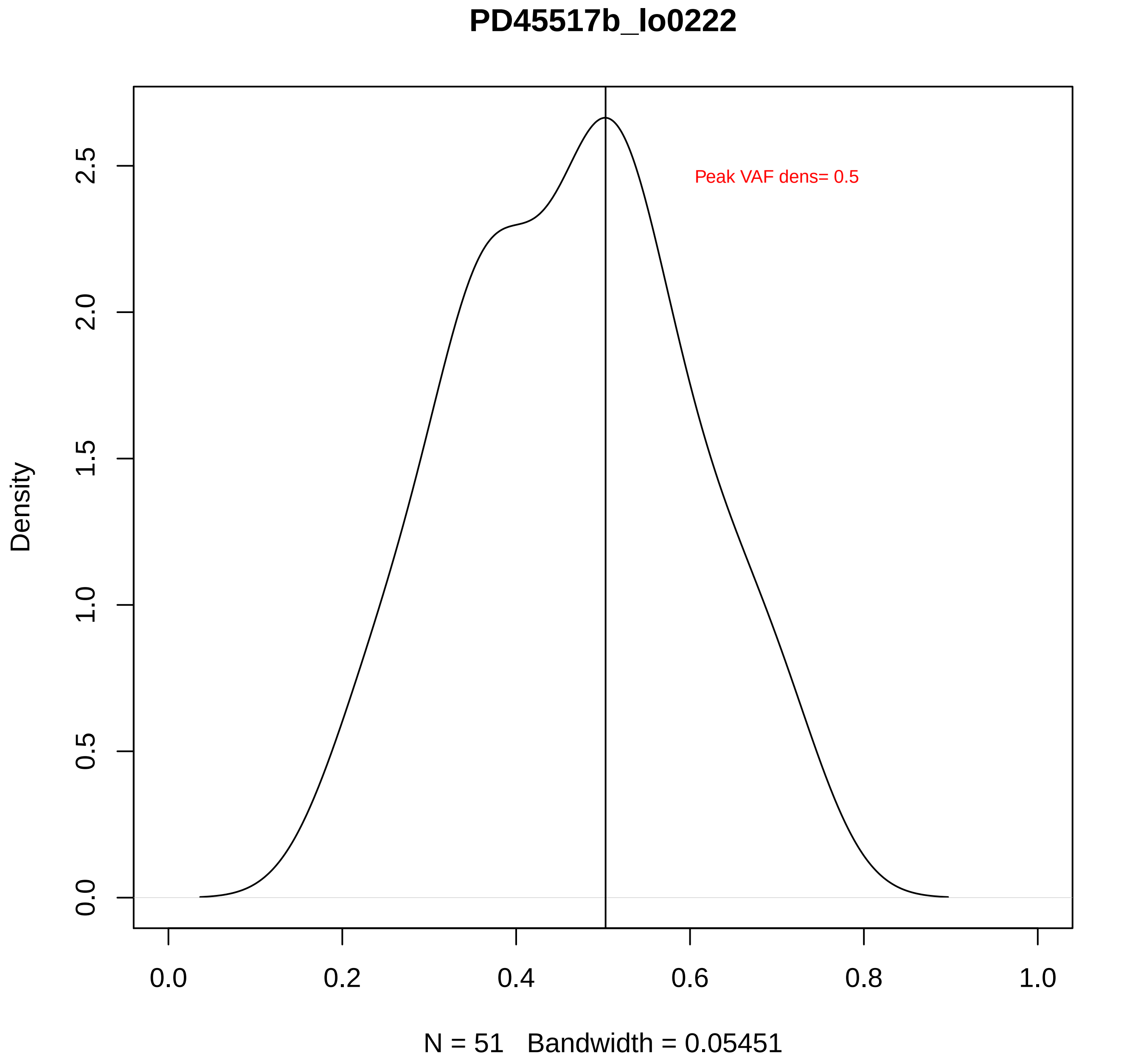

PD45517b_lo0222
Peak VAF dens= 0.5
Density
0.0
0.5
1.0
1.5
2.0
2.5
0.0
0.2
0.4
0.6
0.8
1.0
N = 51 Bandwidth = 0.05451

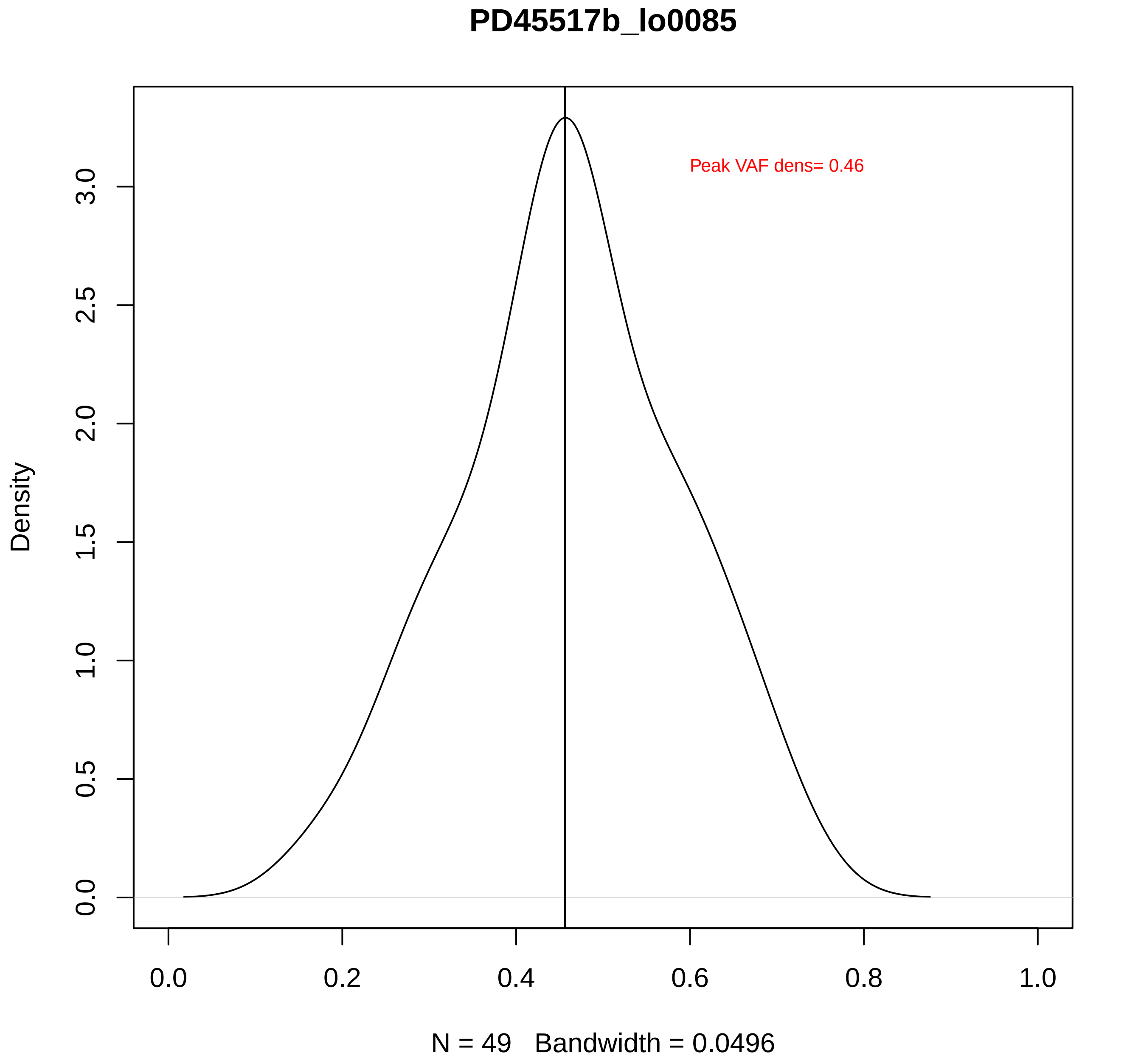

PD45517b_lo0085
Peak VAF dens= 0.46
Density
0.0
0.5
1.0
1.5
2.0
2.5
3.0
0.0
0.2
0.4
0.6
0.8
1.0
N = 49   Bandwidth = 0.0496

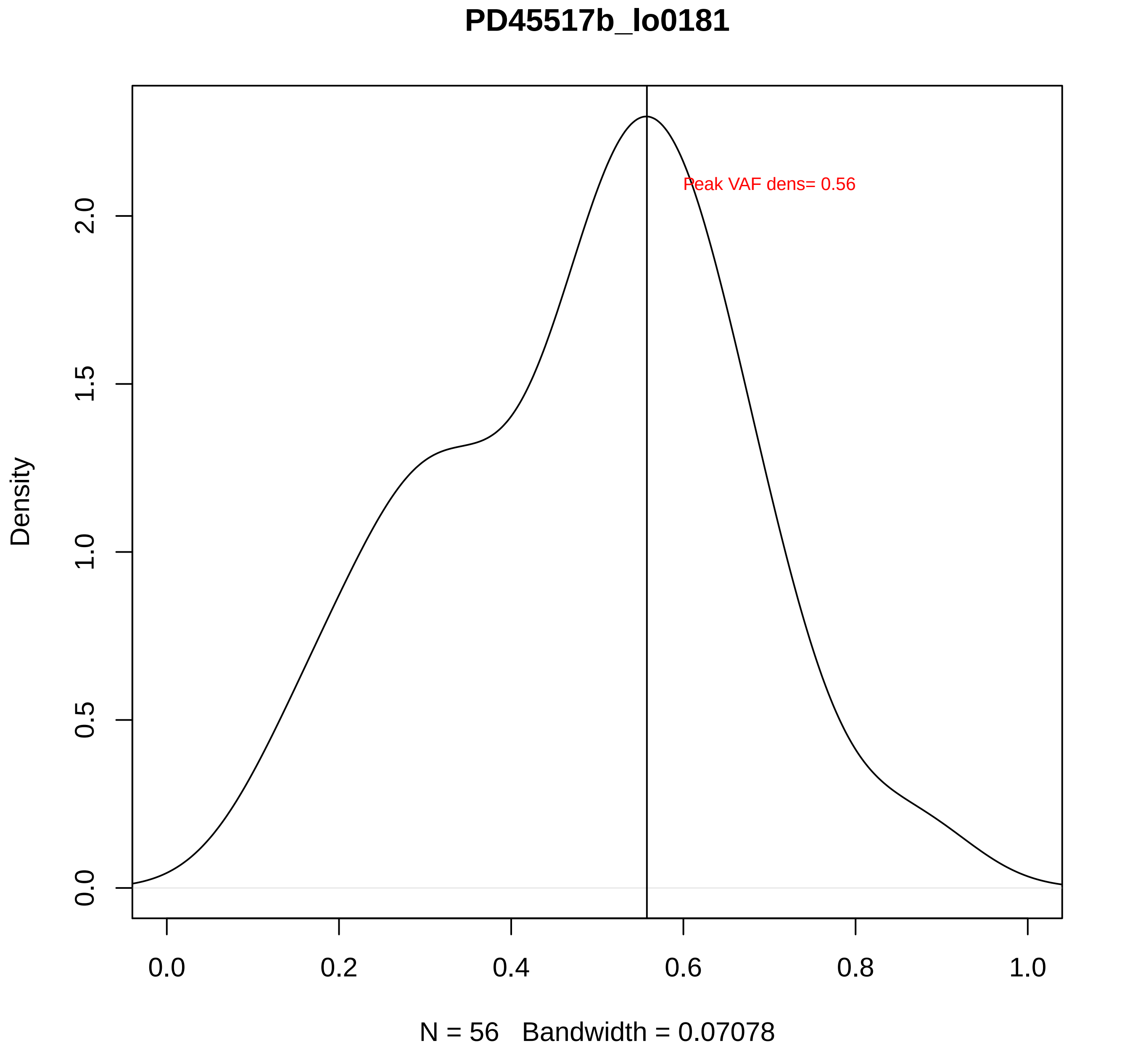

PD45517b_lo0181
Peak VAF dens= 0.56
Density
0.0
0.5
1.0
1.5
2.0
0.0
0.2
0.4
0.6
0.8
1.0
N = 56   Bandwidth = 0.07078

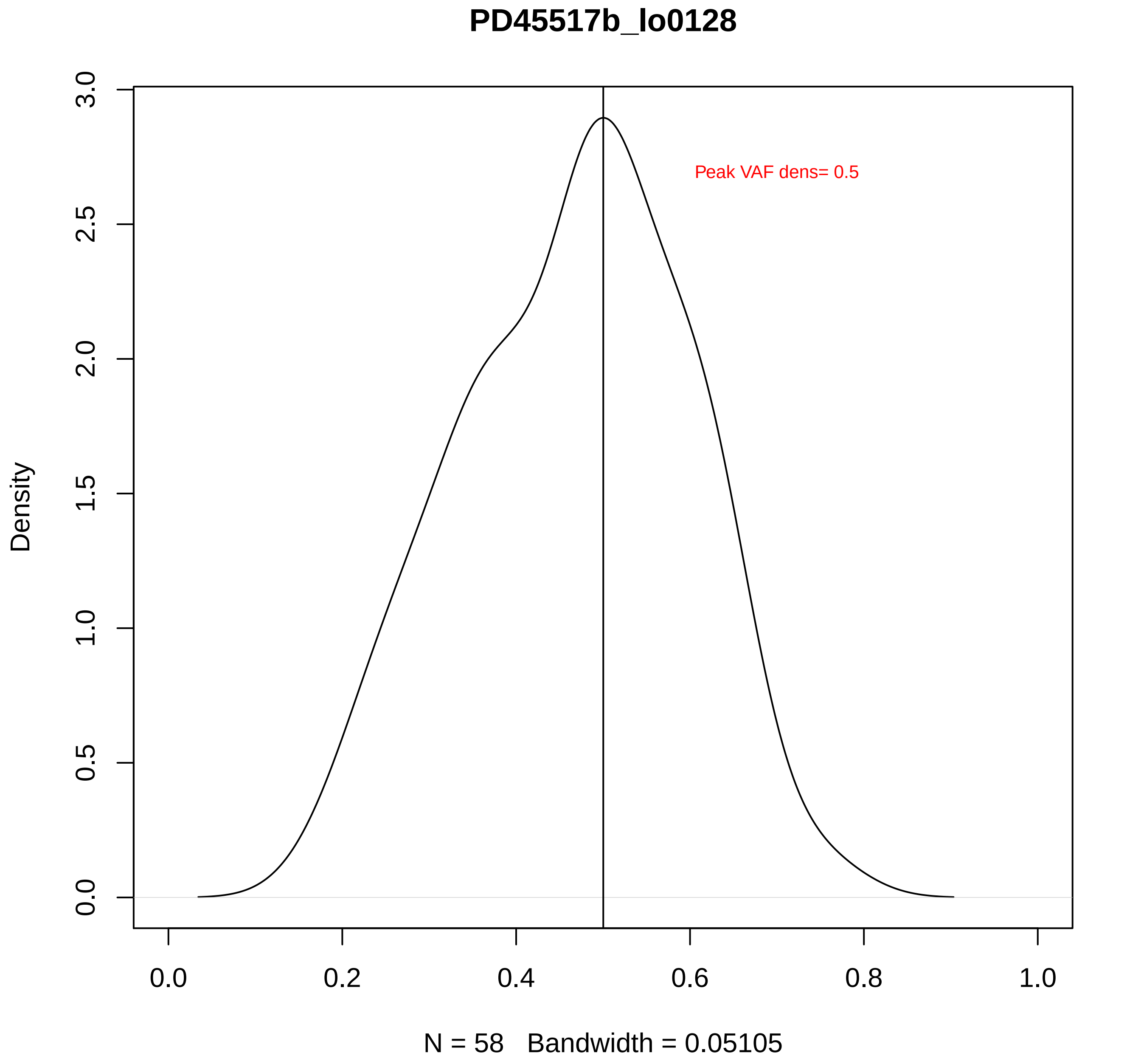

PD45517b_lo0128
Peak VAF dens= 0.5
Density
0.0
0.5
1.0
1.5
2.0
2.5
3.0
0.0
0.2
0.4
0.6
0.8
1.0
N = 58   Bandwidth = 0.05105

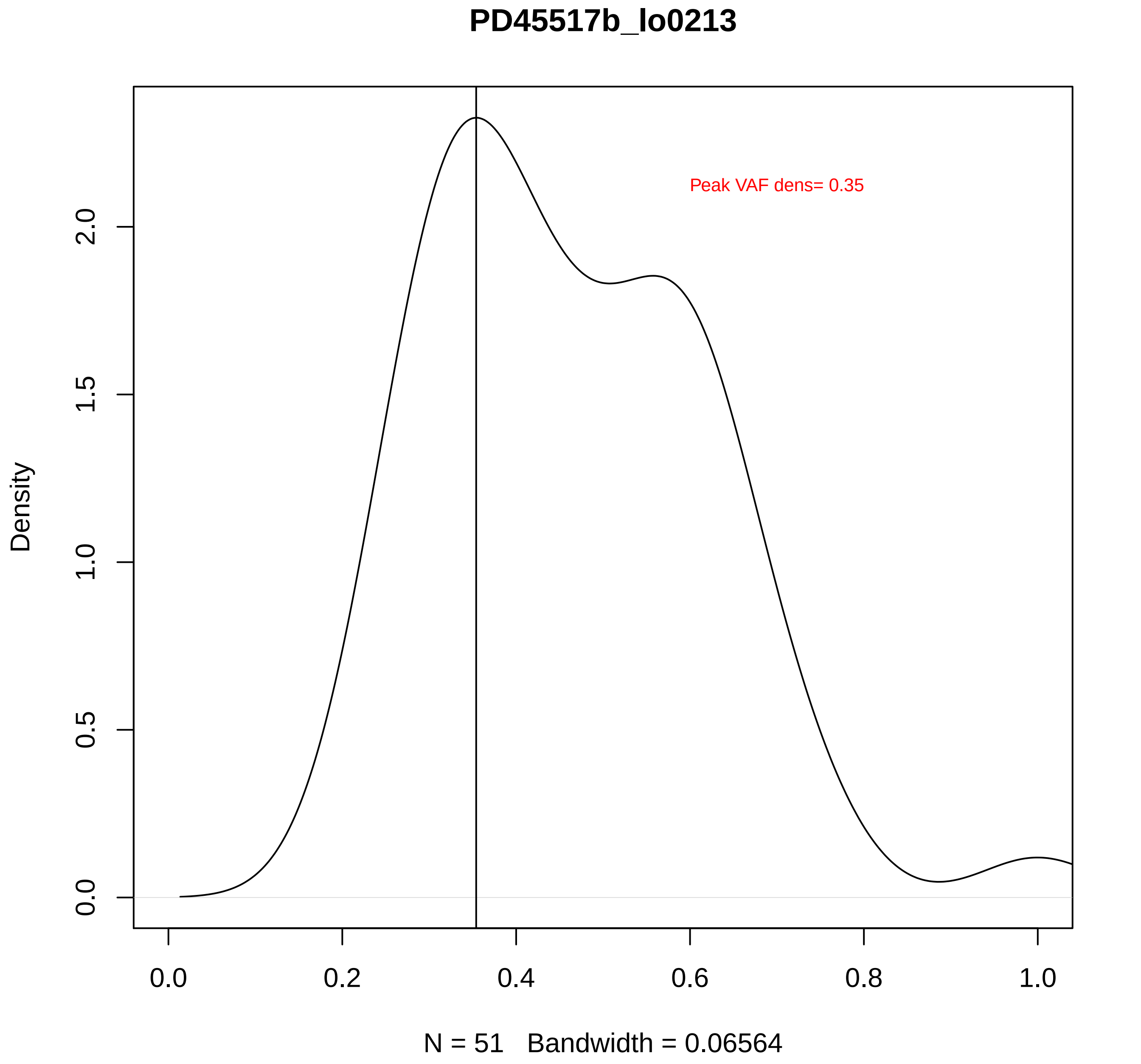

PD45517b_lo0213
Peak VAF dens= 0.35
Density
0.0
0.5
1.0
1.5
2.0
0.0
0.2
0.4
0.6
0.8
1.0
N = 51 Bandwidth = 0.06564

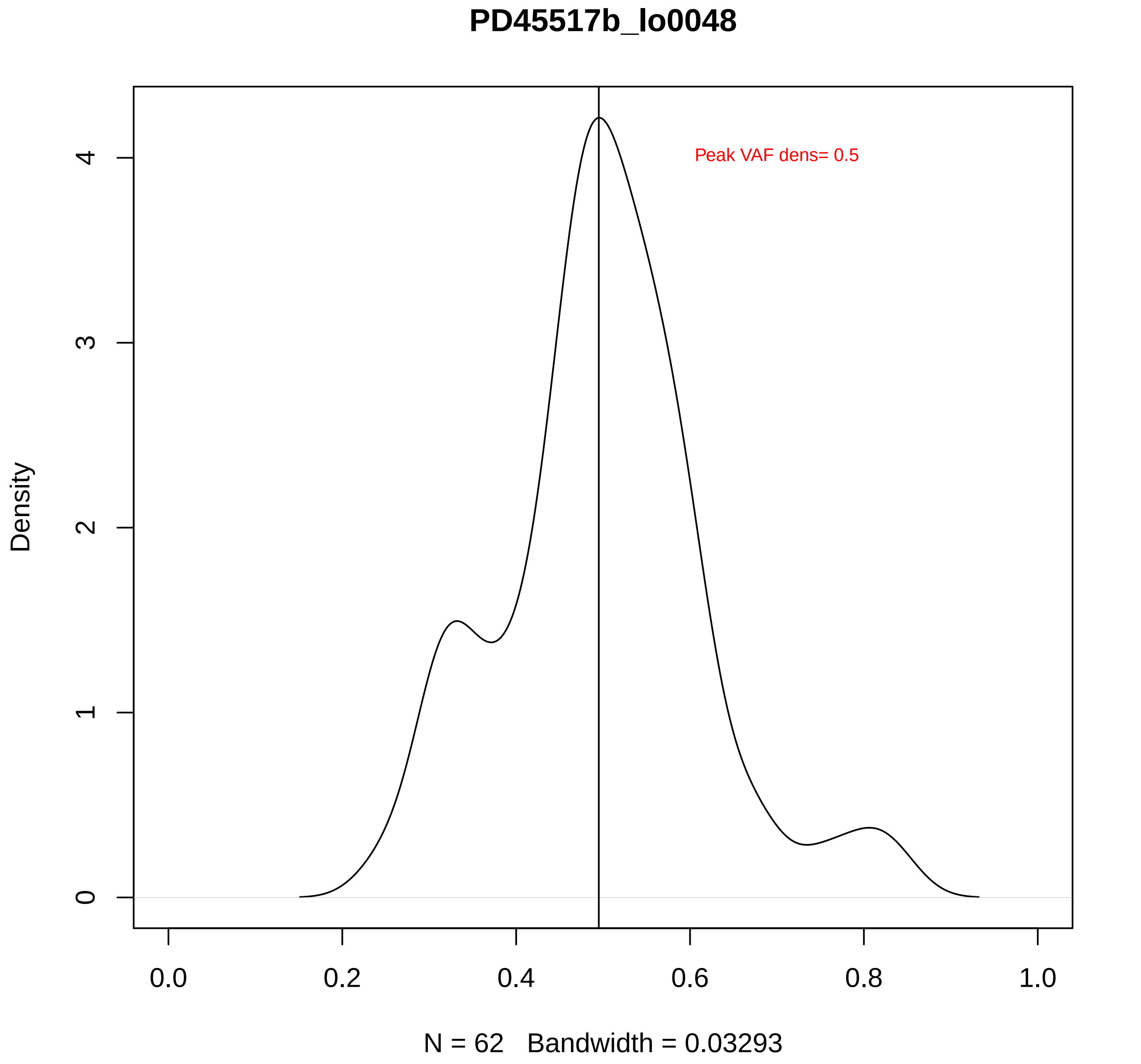

PD45517b_lo0048
Peak VAF dens= 0.5
Density
0
1
2
3
4
0.0
0.2
0.4
0.6
0.8
1.0
N = 62   Bandwidth = 0.03293

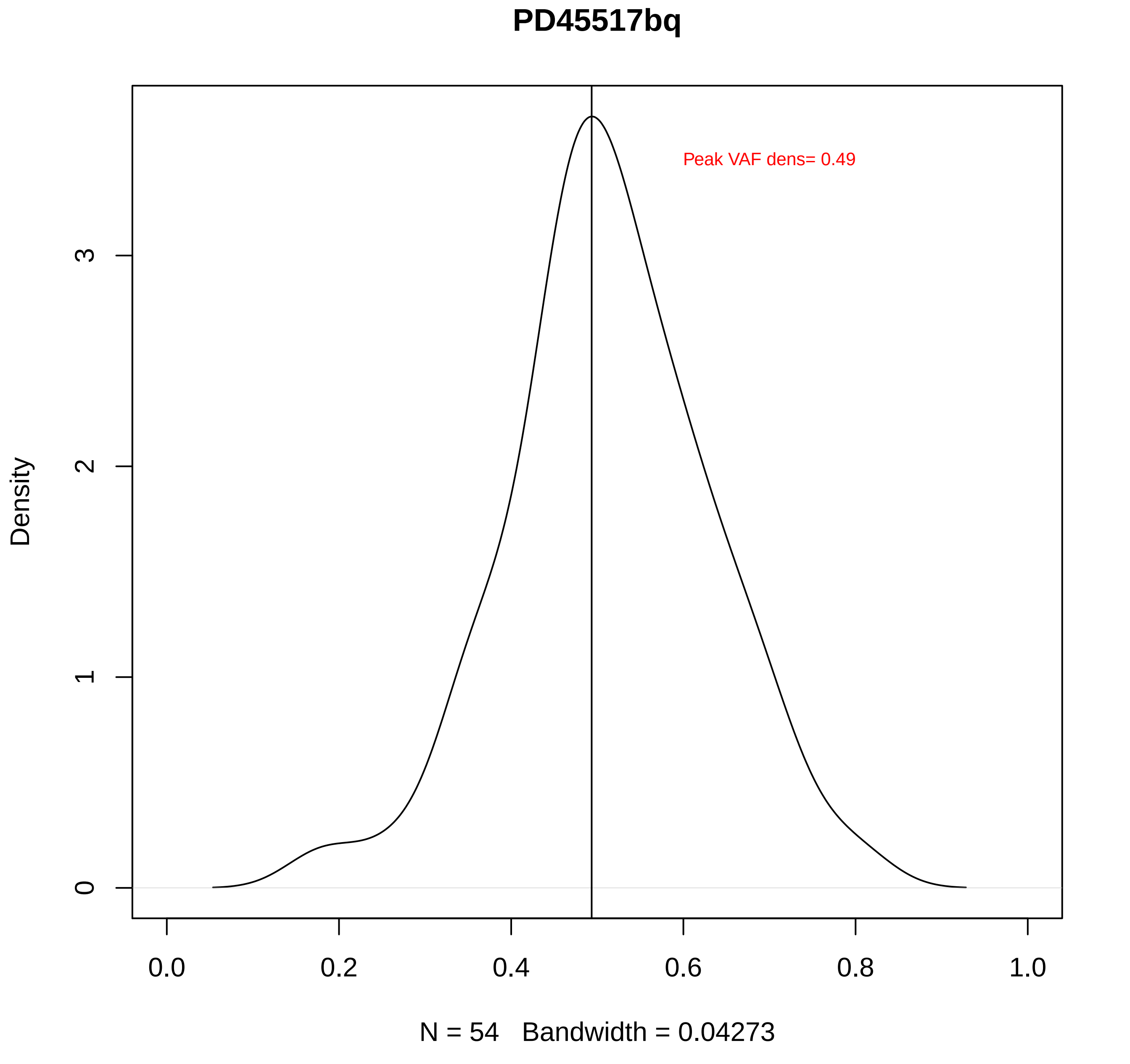
PD45517bq
Peak VAF dens= 0.49
Density
0
1
2
3
0.0
0.2
0.4
0.6
0.8
1.0
N = 54   Bandwidth = 0.04273

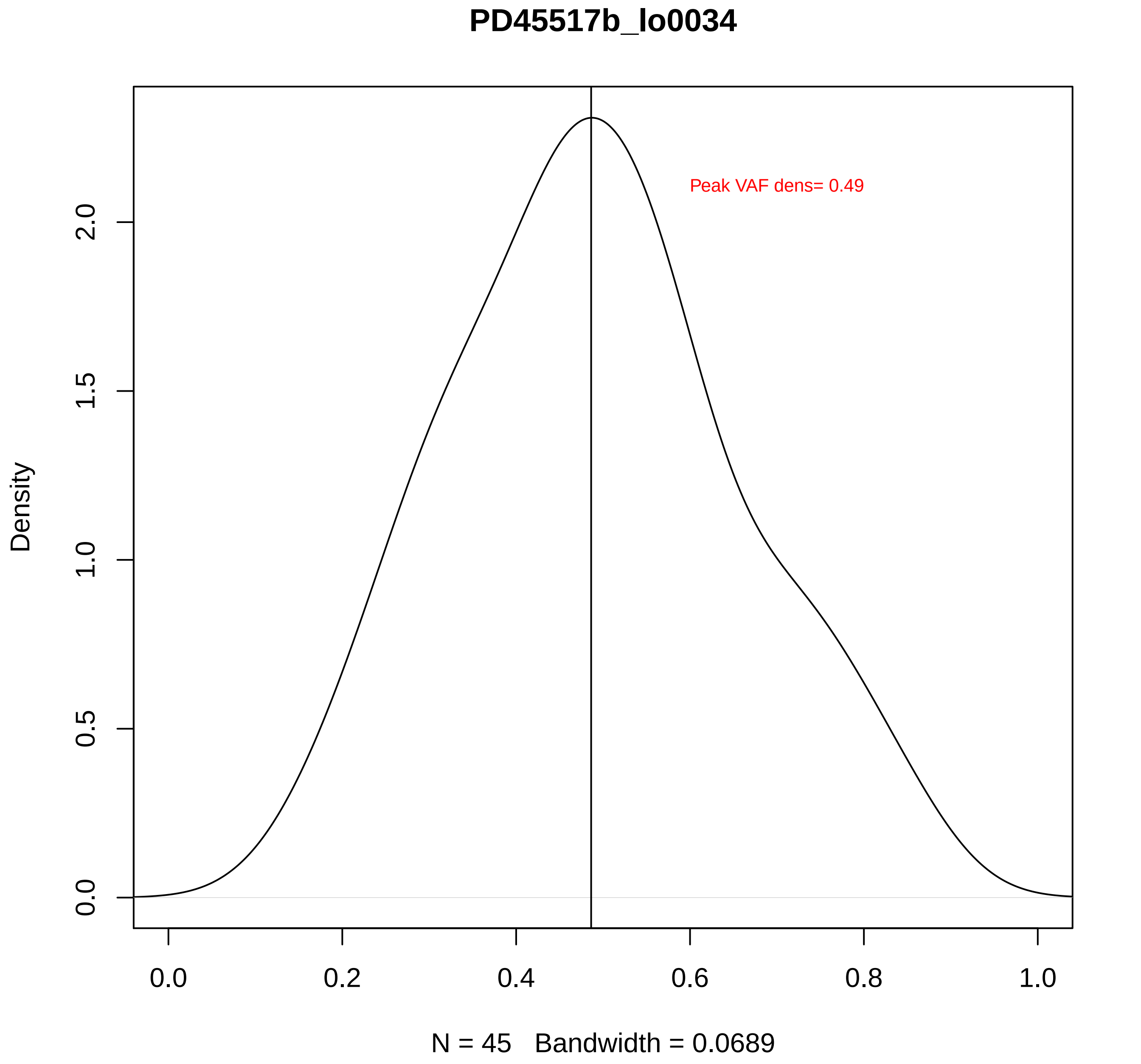
PD45517b_lo0034
Peak VAF dens= 0.49
Density
0.0
0.5
1.0
1.5
2.0
0.0
0.2
0.4
0.6
0.8
1.0
N = 45 Bandwidth = 0.0689

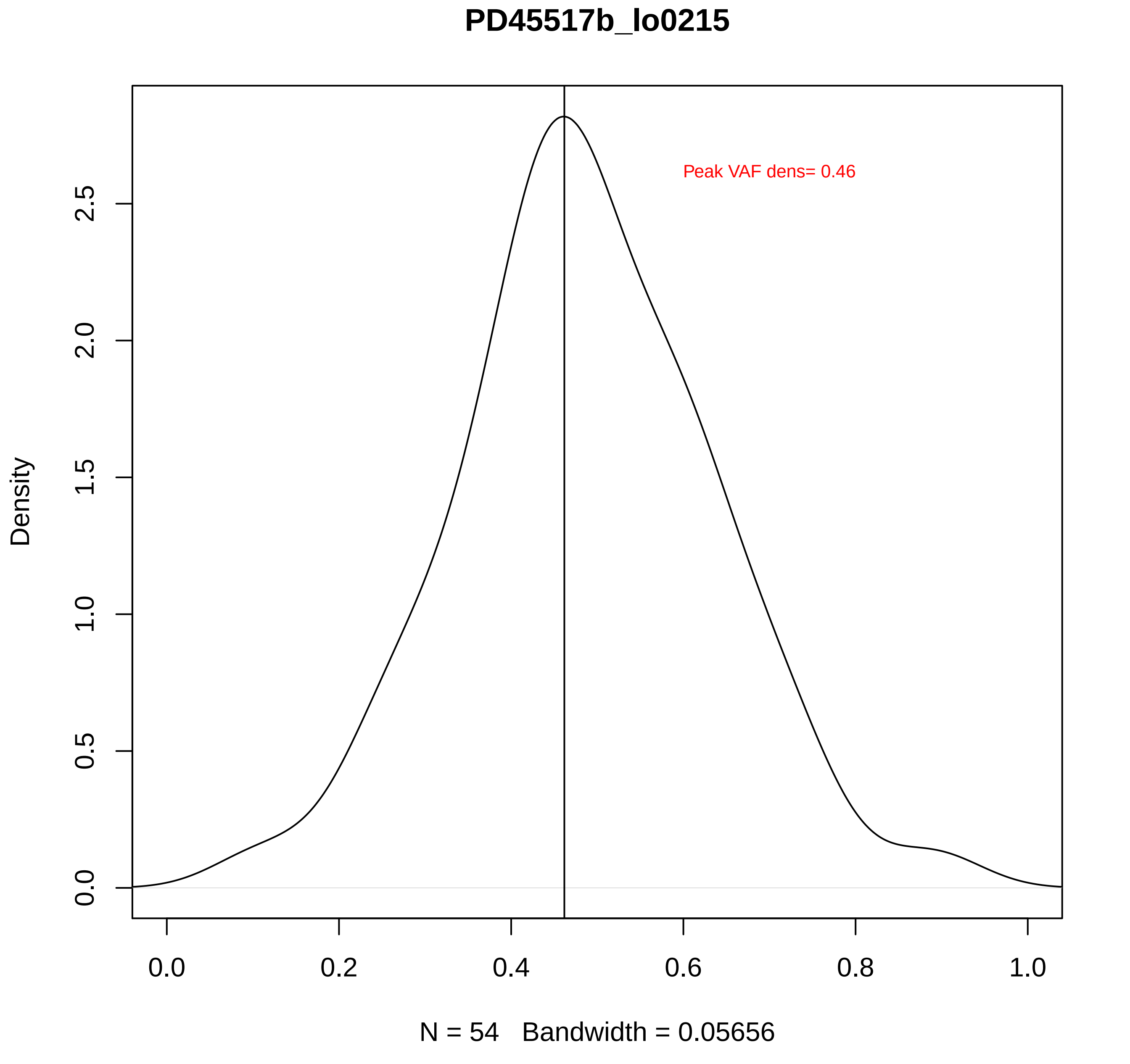
PD45517b_lo0215
Peak VAF dens= 0.46
Density
0.0
0.5
1.0
1.5
2.0
2.5
0.0
0.2
0.4
0.6
0.8
1.0
N = 54   Bandwidth = 0.05656

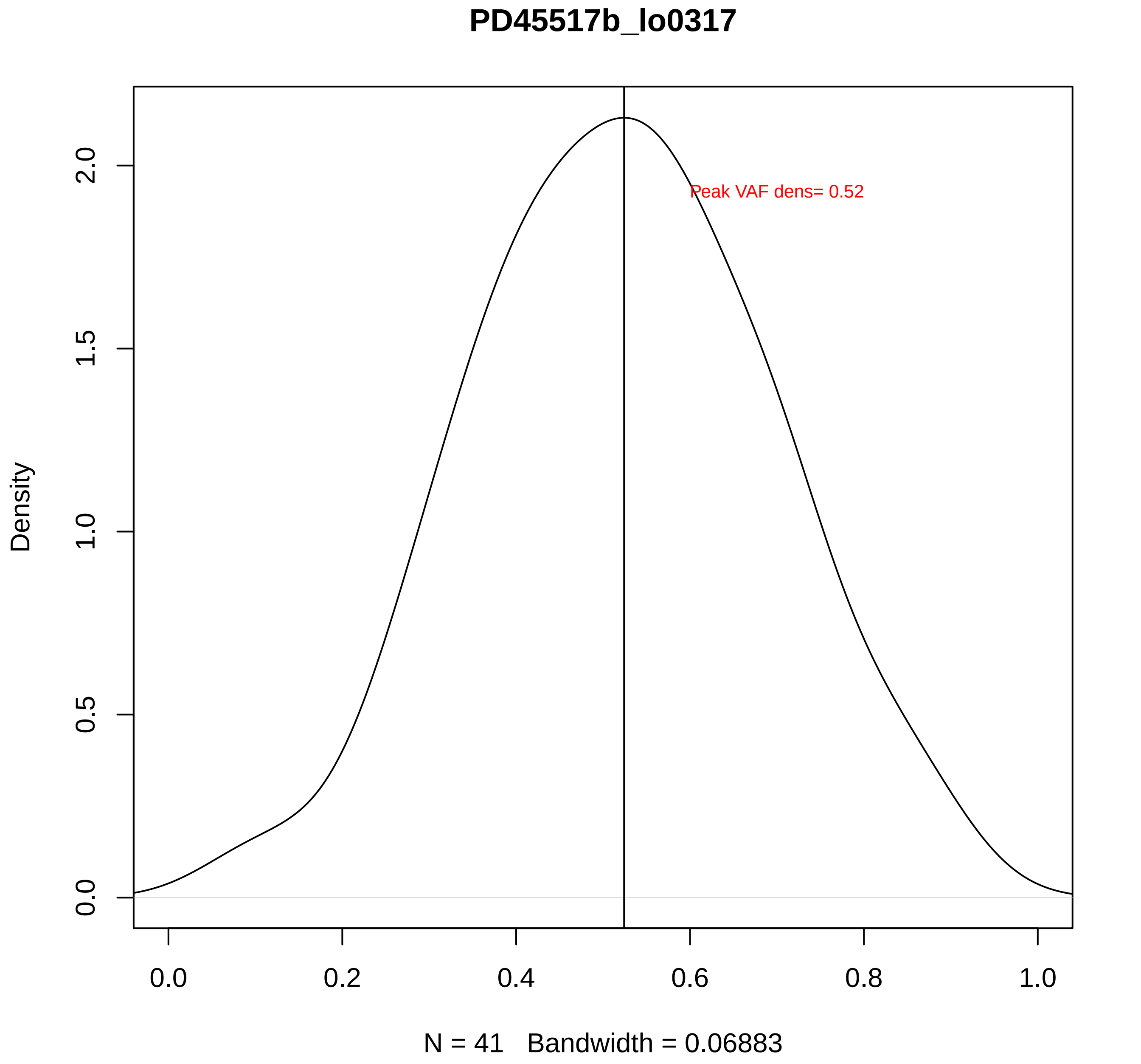
PD45517b_lo0317
Peak VAF dens= 0.52
Density
0.0
0.5
1.0
1.5
2.0
0.0
0.2
0.4
0.6
0.8
1.0
N = 41 Bandwidth = 0.06883

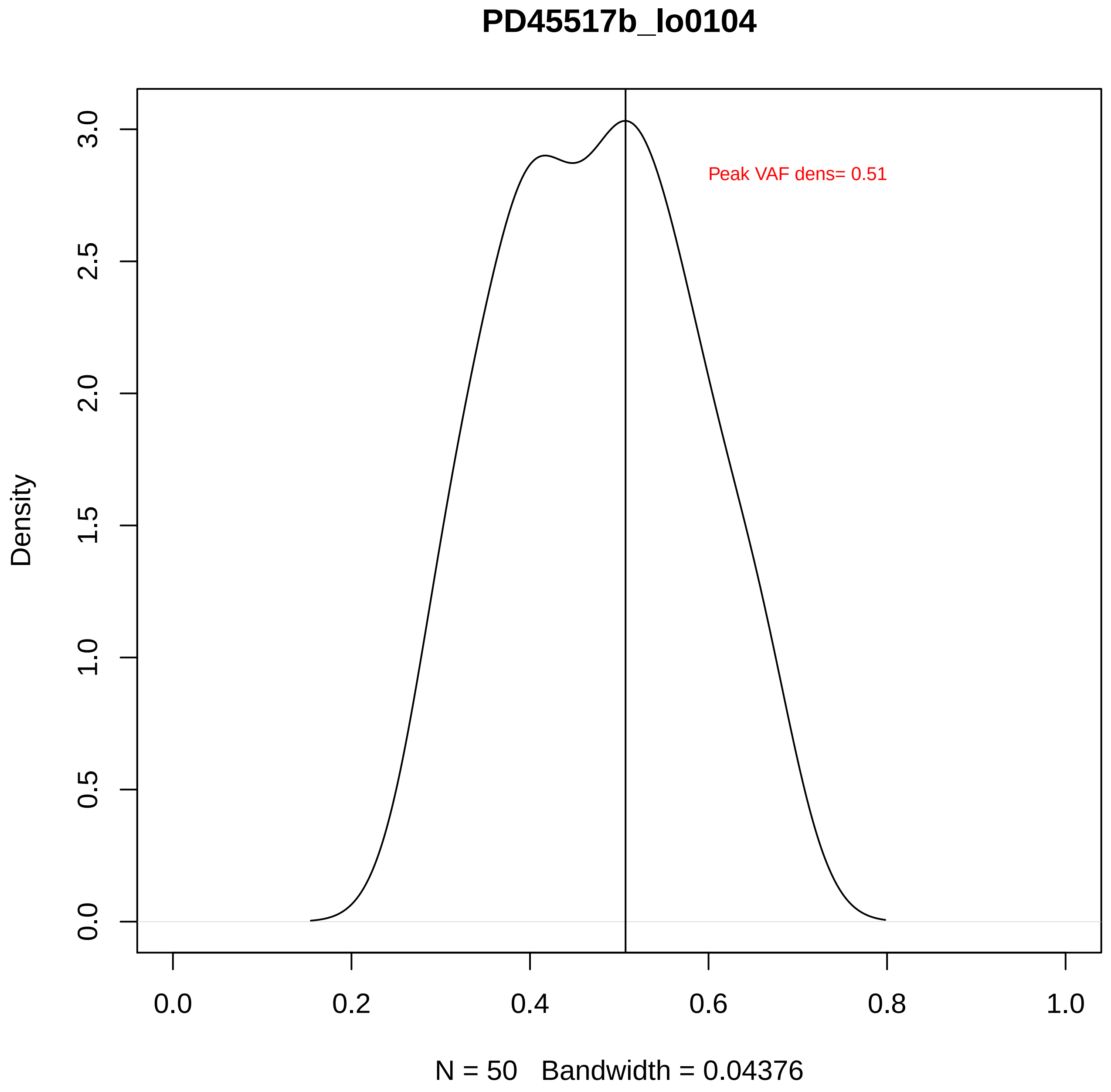
PD45517b_lo0104
Peak VAF dens= 0.51
Density
0.0
0.5
1.0
1.5
2.0
2.5
3.0
0.0
0.2
0.4
0.6
0.8
1.0
N = 50   Bandwidth = 0.04376

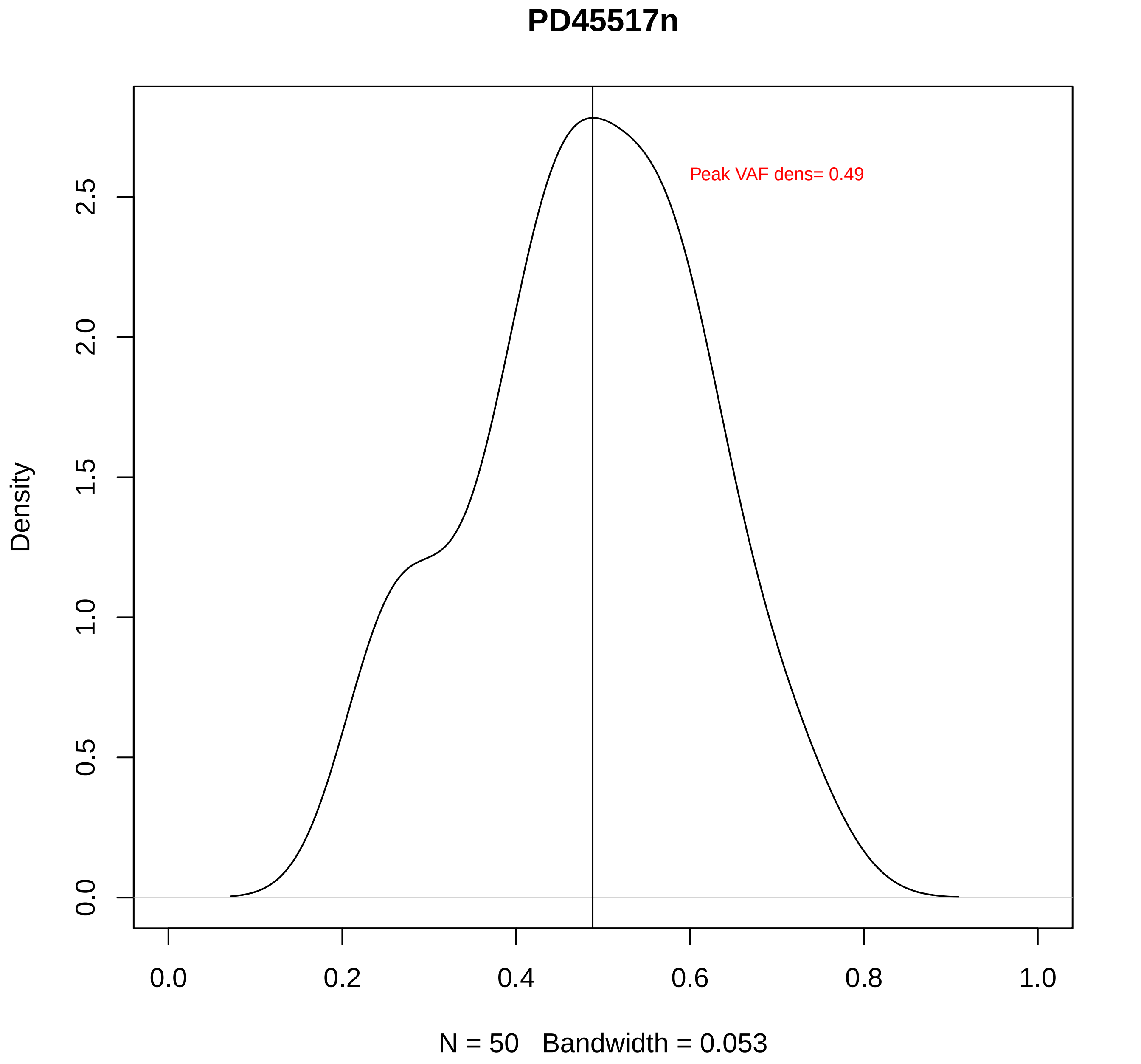
PD45517n
Peak VAF dens= 0.49
Density
0.0
0.5
1.0
1.5
2.0
2.5
0.0
0.2
0.4
0.6
0.8
1.0
N = 50   Bandwidth = 0.053

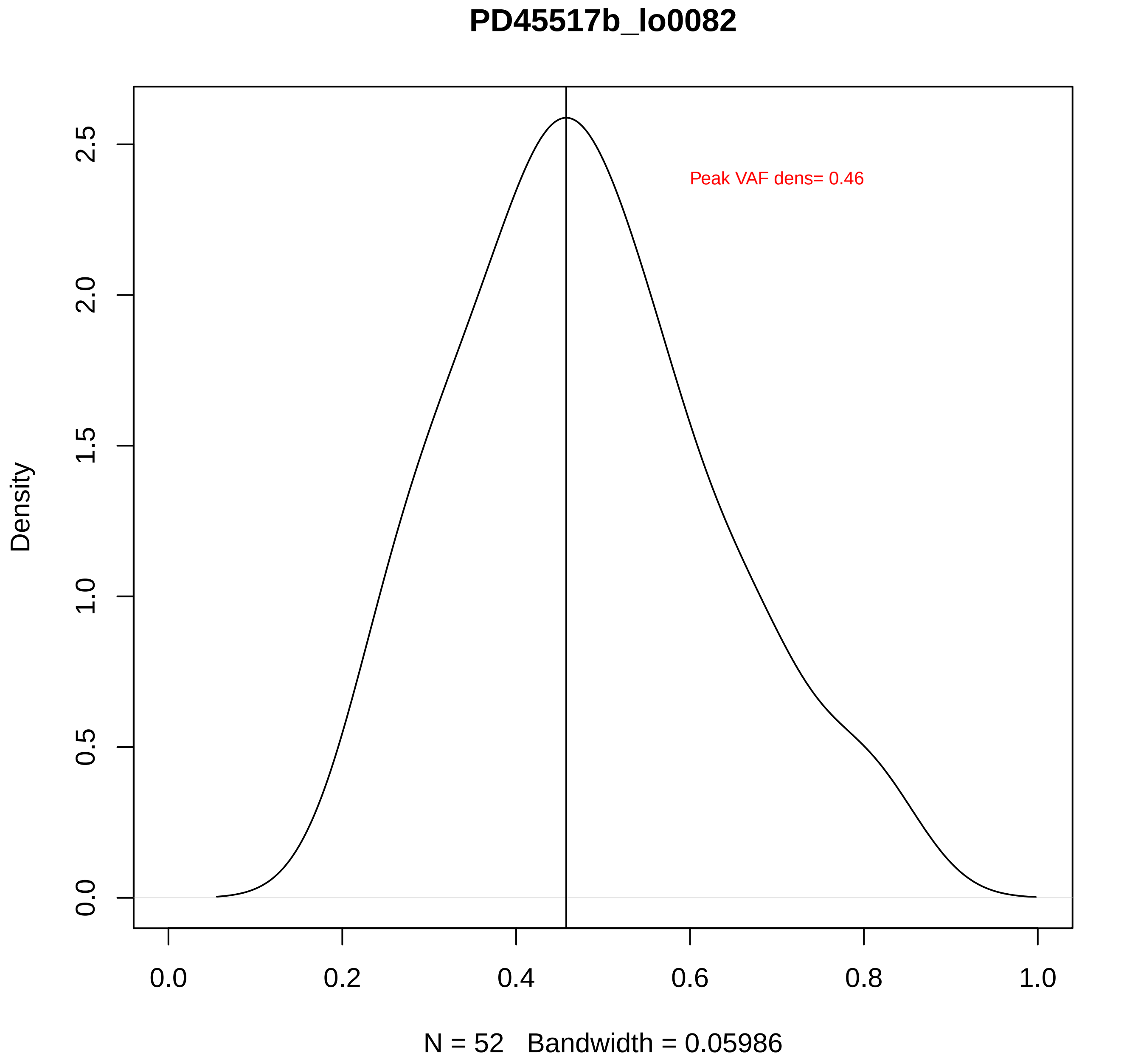
PD45517b_lo0082
Peak VAF dens= 0.46
Density
0.0
0.5
1.0
1.5
2.0
2.5
0.0
0.2
0.4
0.6
0.8
1.0
N = 52   Bandwidth = 0.05986

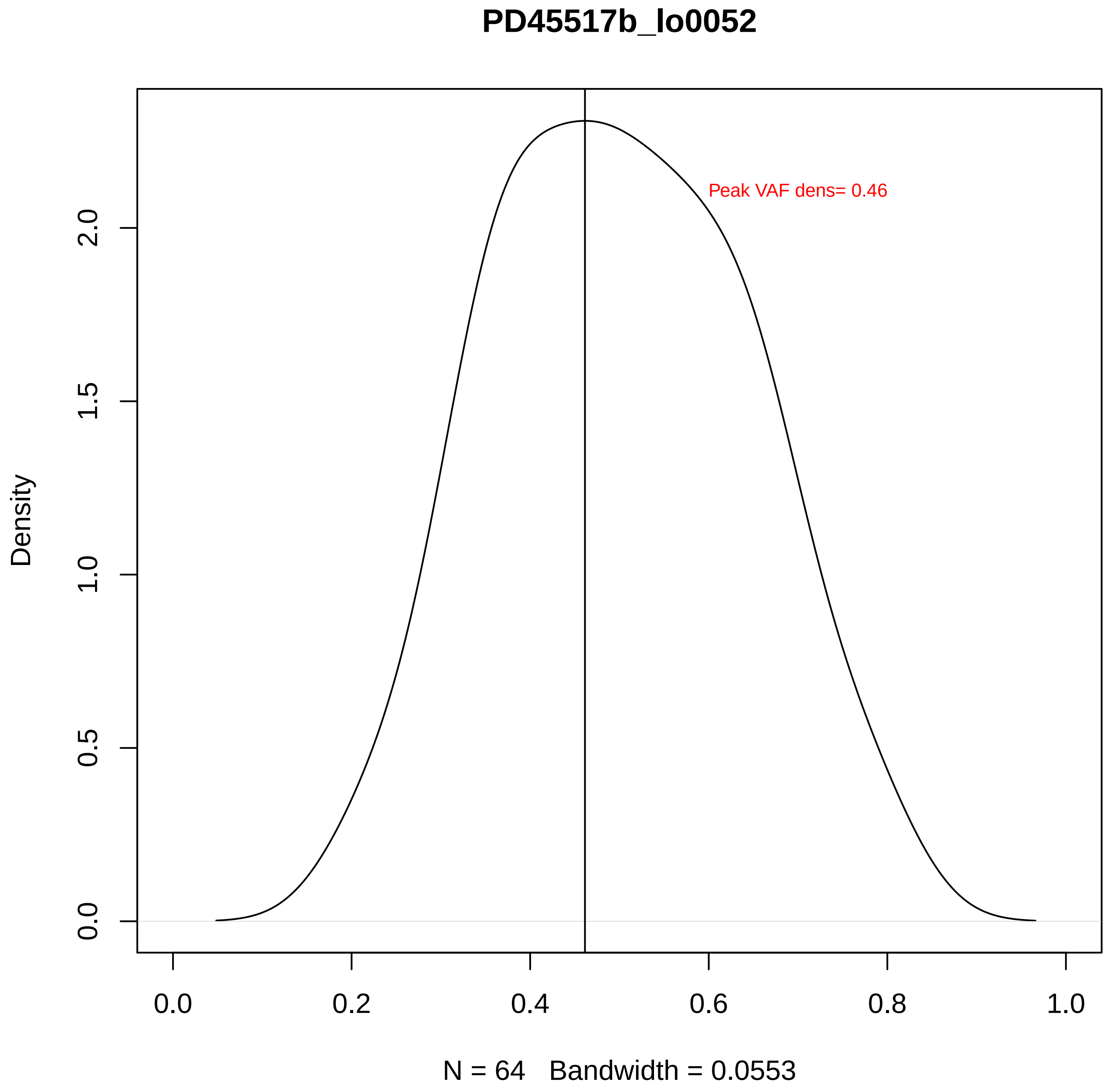
PD45517b_lo0052
Peak VAF dens= 0.46
Density
0.0
0.5
1.0
1.5
2.0
0.0
0.2
0.4
0.6
0.8
1.0
N = 64   Bandwidth = 0.0553

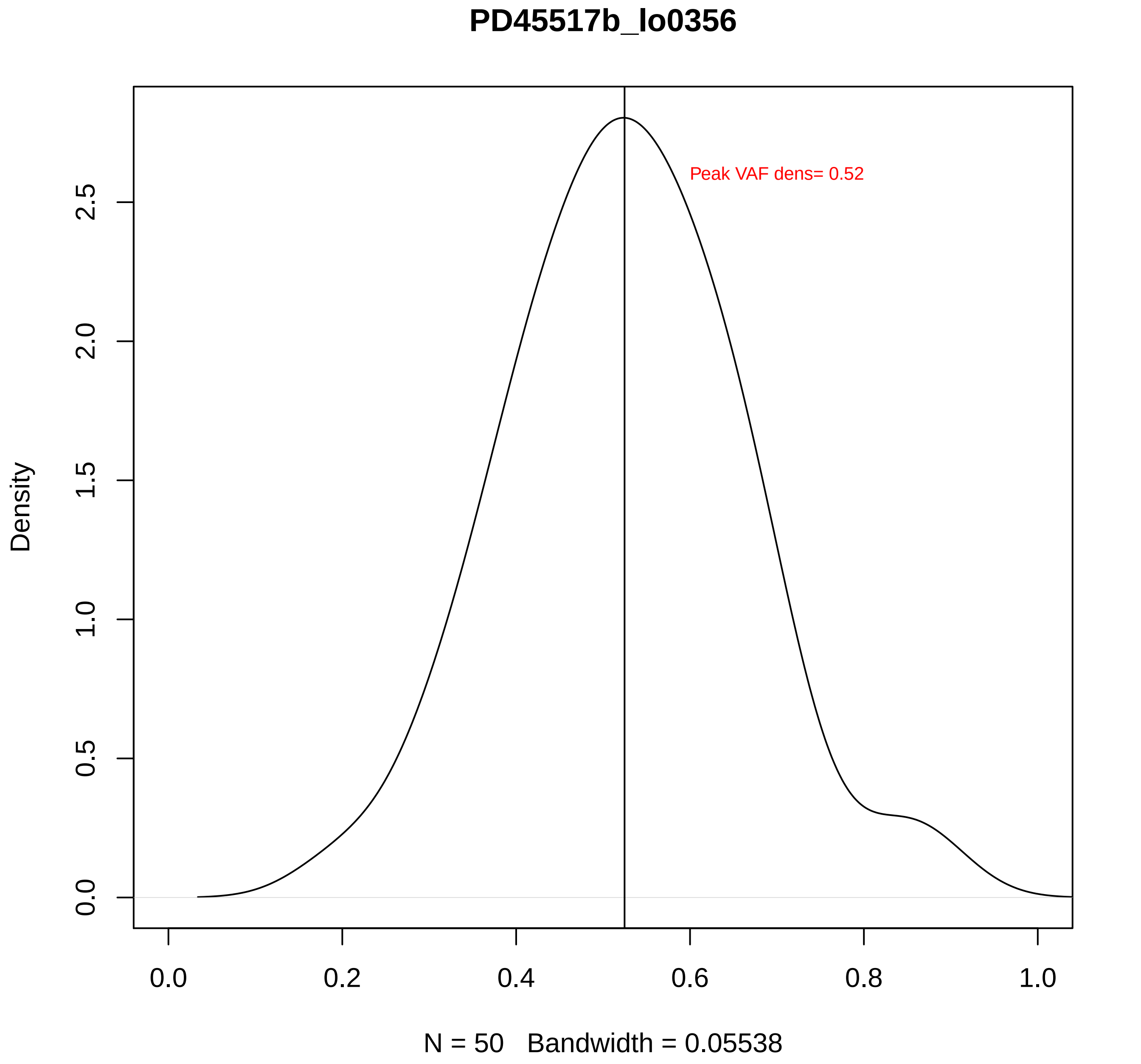
PD45517b_lo0356
Peak VAF dens= 0.52
Density
0.0
0.5
1.0
1.5
2.0
2.5
0.0
0.2
0.4
0.6
0.8
1.0
N = 50 Bandwidth = 0.05538

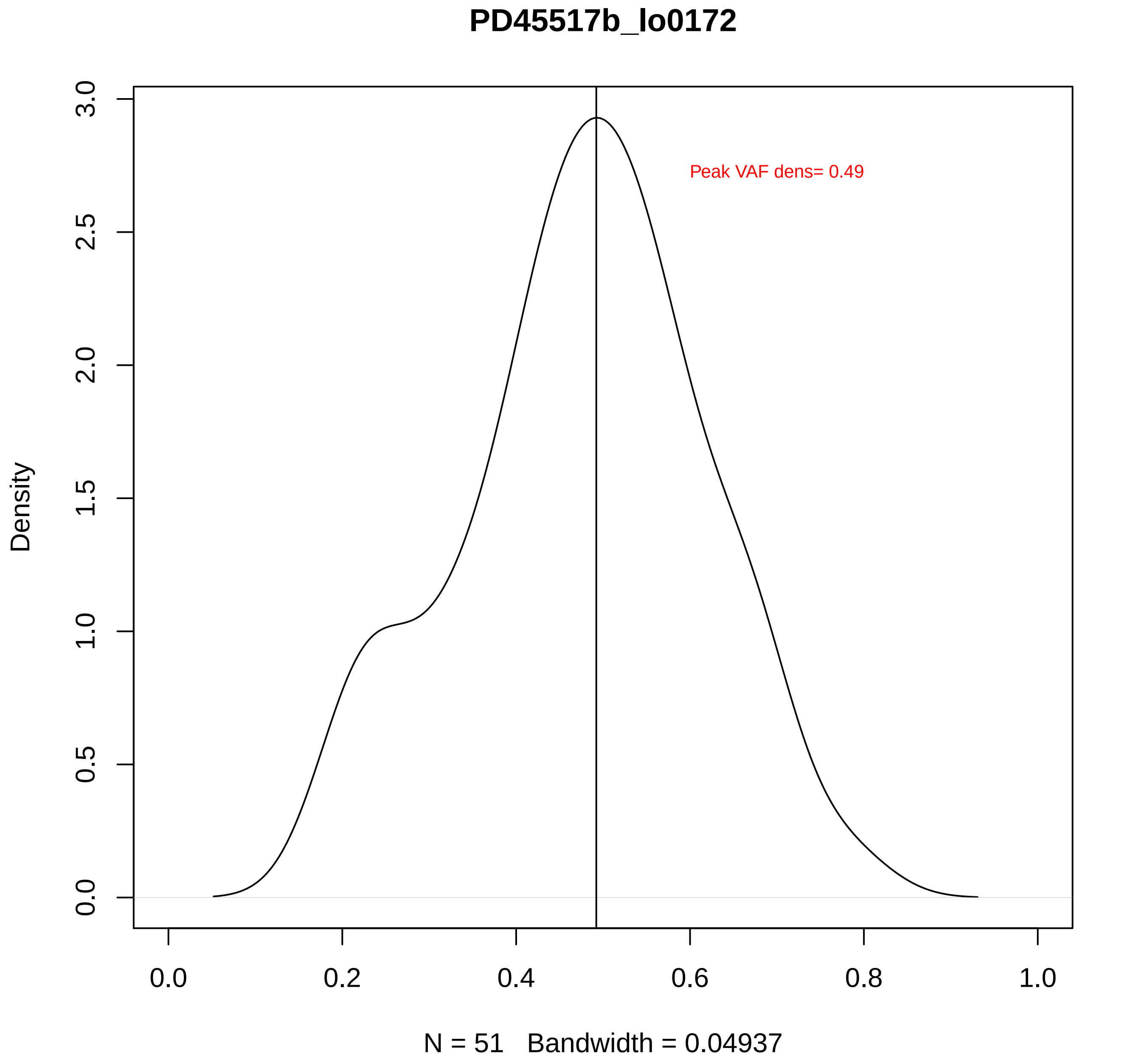
PD45517b_lo0172
Peak VAF dens= 0.49
Density
0.0
0.5
1.0
1.5
2.0
2.5
3.0
0.0
0.2
0.4
0.6
0.8
1.0
N = 51   Bandwidth = 0.04937

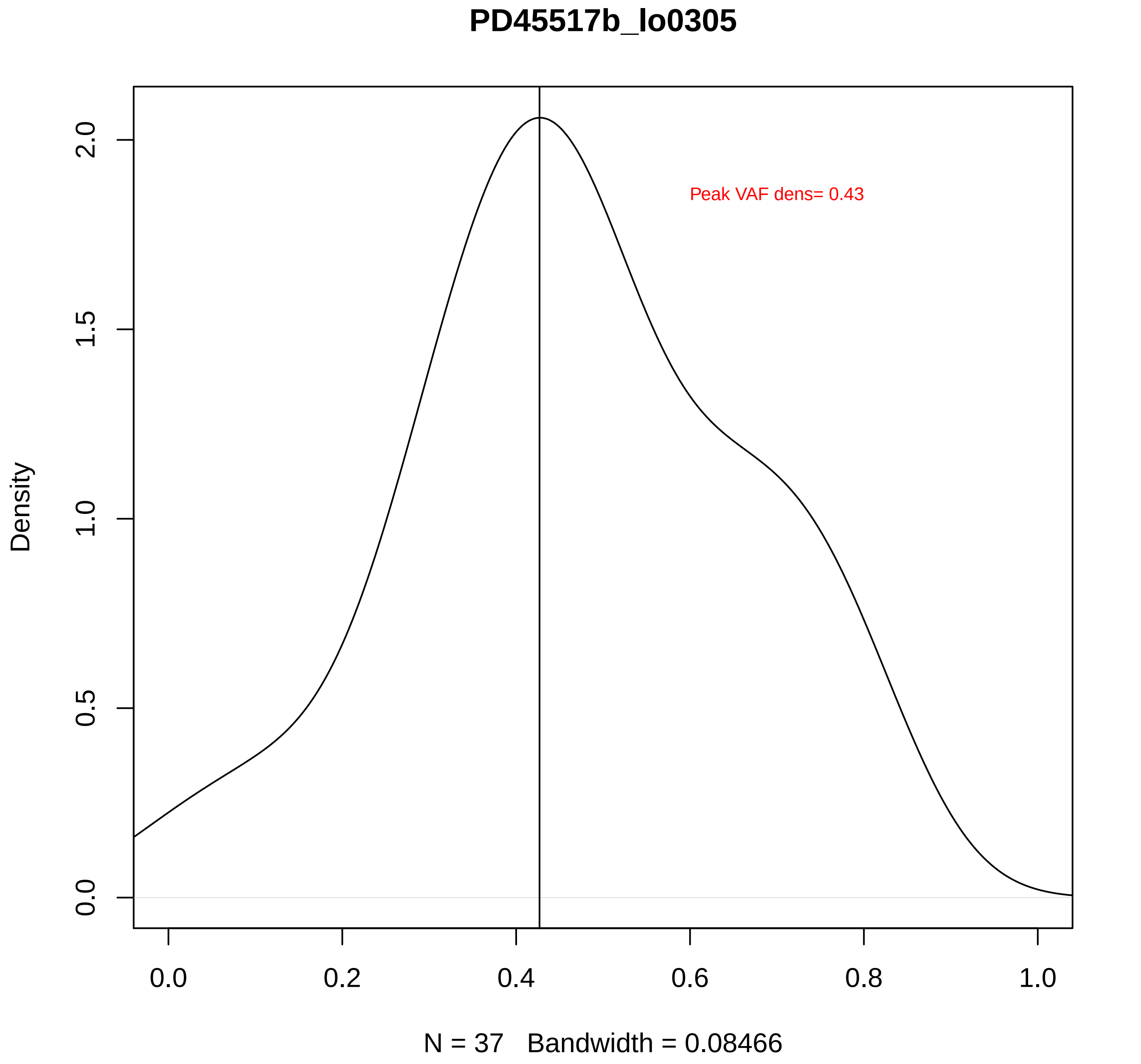
PD45517b_lo0305
Peak VAF dens= 0.43
Density
0.0
0.5
1.0
1.5
2.0
0.0
0.2
0.4
0.6
0.8
1.0
N = 37   Bandwidth = 0.08466

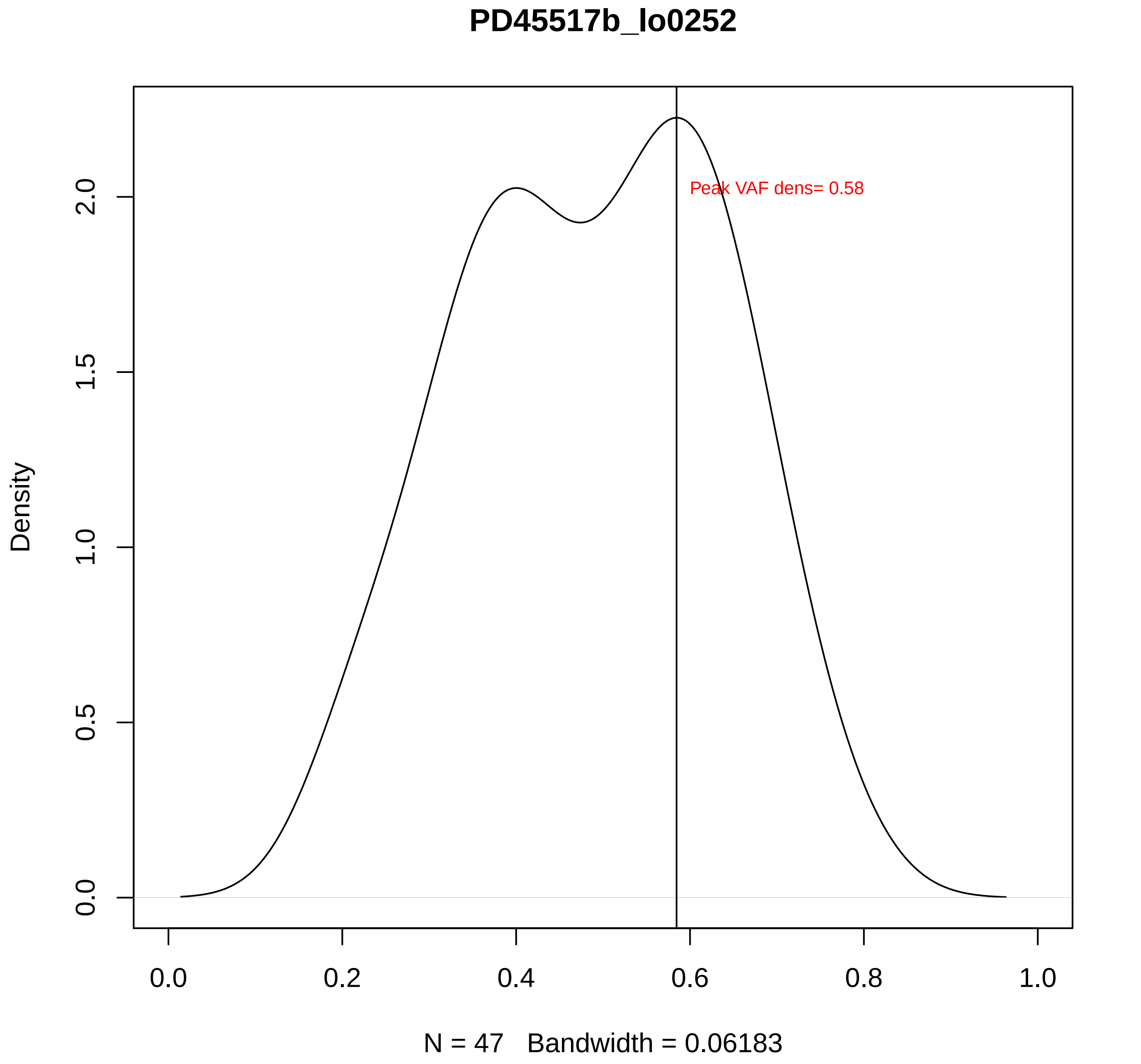

PD45517b_lo0252
Peak VAF dens= 0.58
Density
0.0
0.5
1.0
1.5
2.0
0.0
0.2
0.4
0.6
0.8
1.0
N = 47 Bandwidth = 0.06183

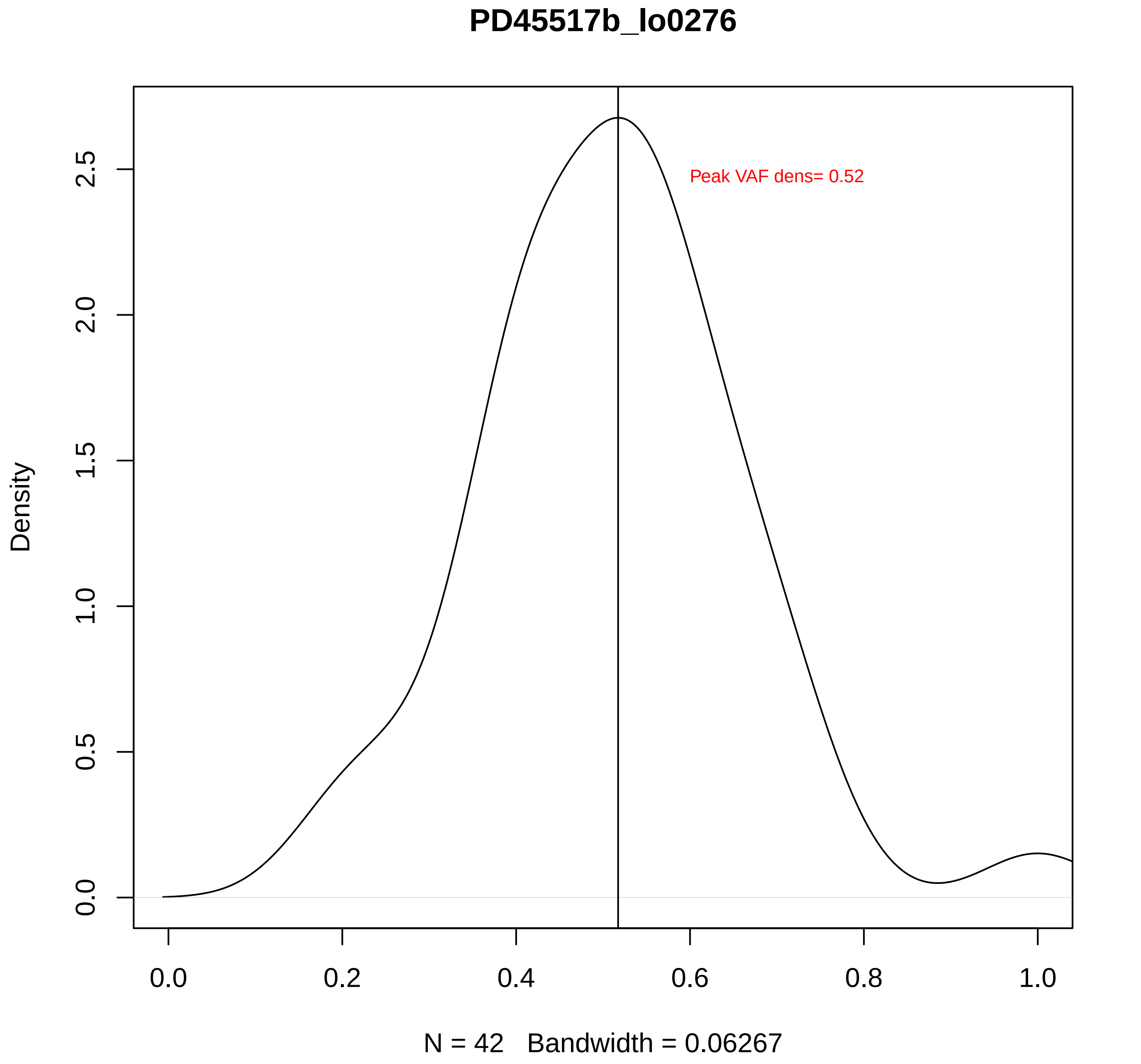

PD45517b_lo0276
Peak VAF dens= 0.52
Density
0.0
0.5
1.0
1.5
2.0
2.5
0.0
0.2
0.4
0.6
0.8
1.0
N = 42 Bandwidth = 0.06267

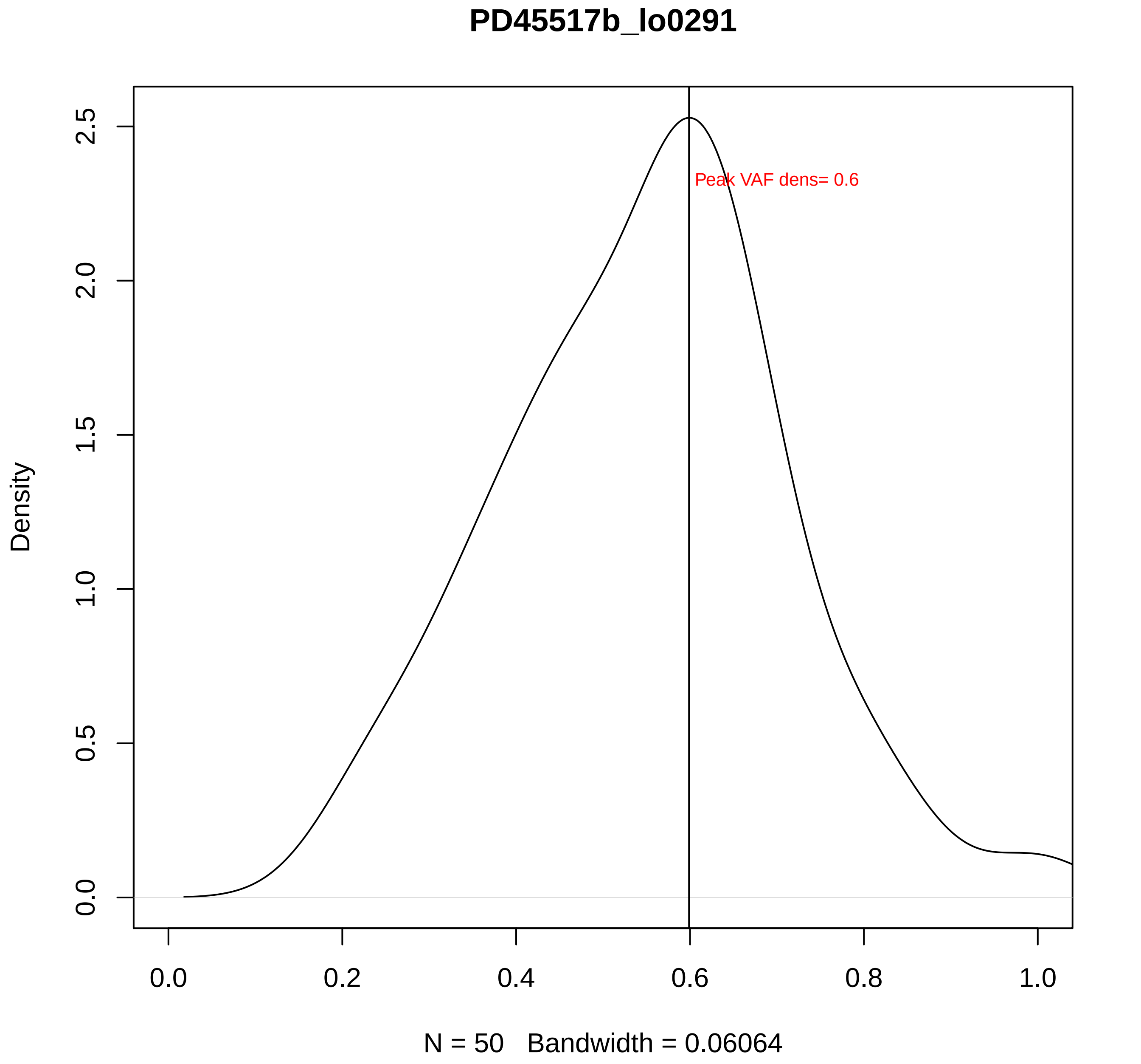
PD45517b_lo0291
Peak VAF dens= 0.6
Density
0.0
0.5
1.0
1.5
2.0
2.5
0.0
0.2
0.4
0.6
0.8
1.0
N = 50   Bandwidth = 0.06064

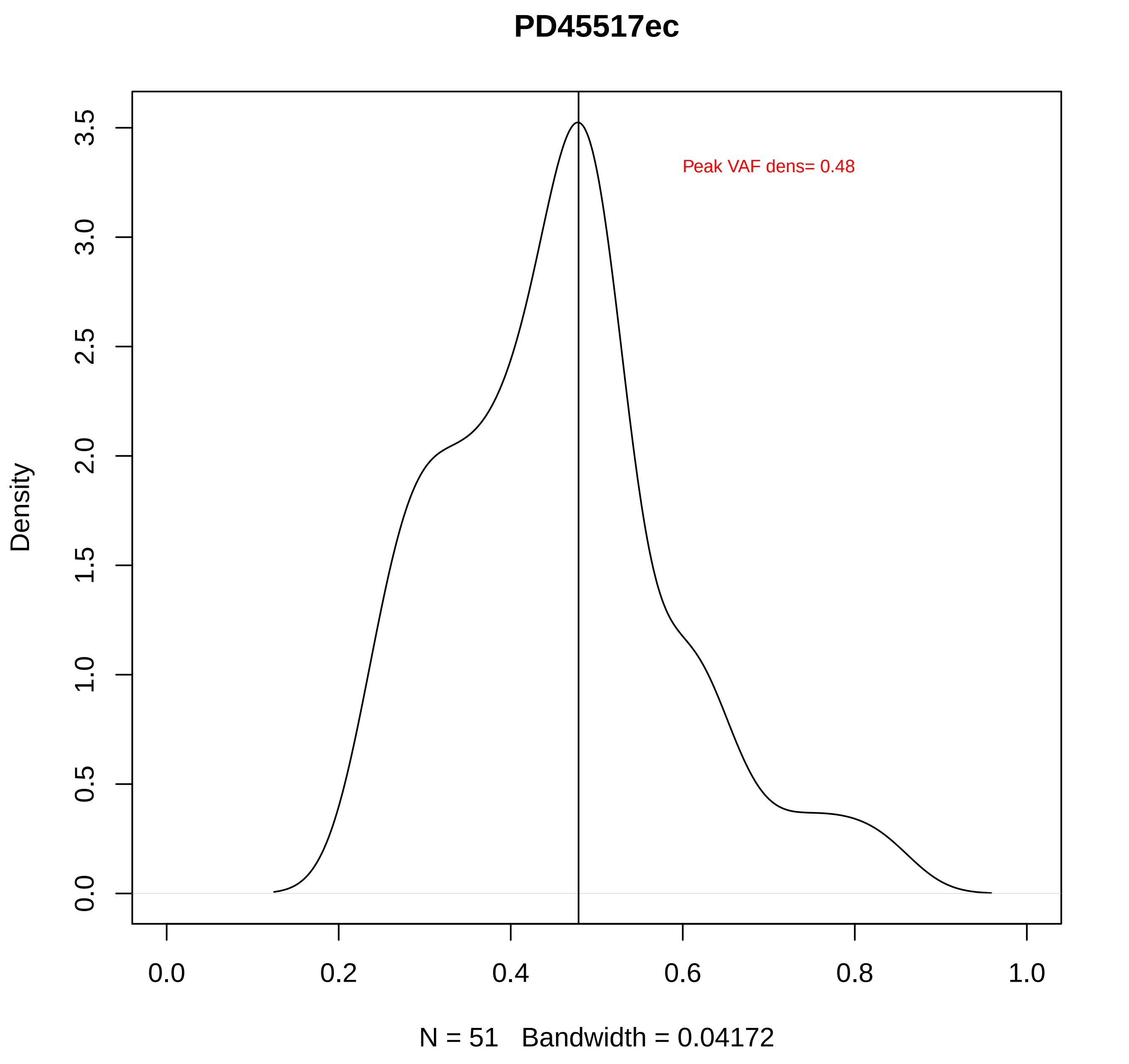
PD45517ec
Peak VAF dens= 0.48
Density
0.0
0.5
1.0
1.5
2.0
2.5
3.0
3.5
0.0
0.2
0.4
0.6
0.8
1.0
N = 51   Bandwidth = 0.04172

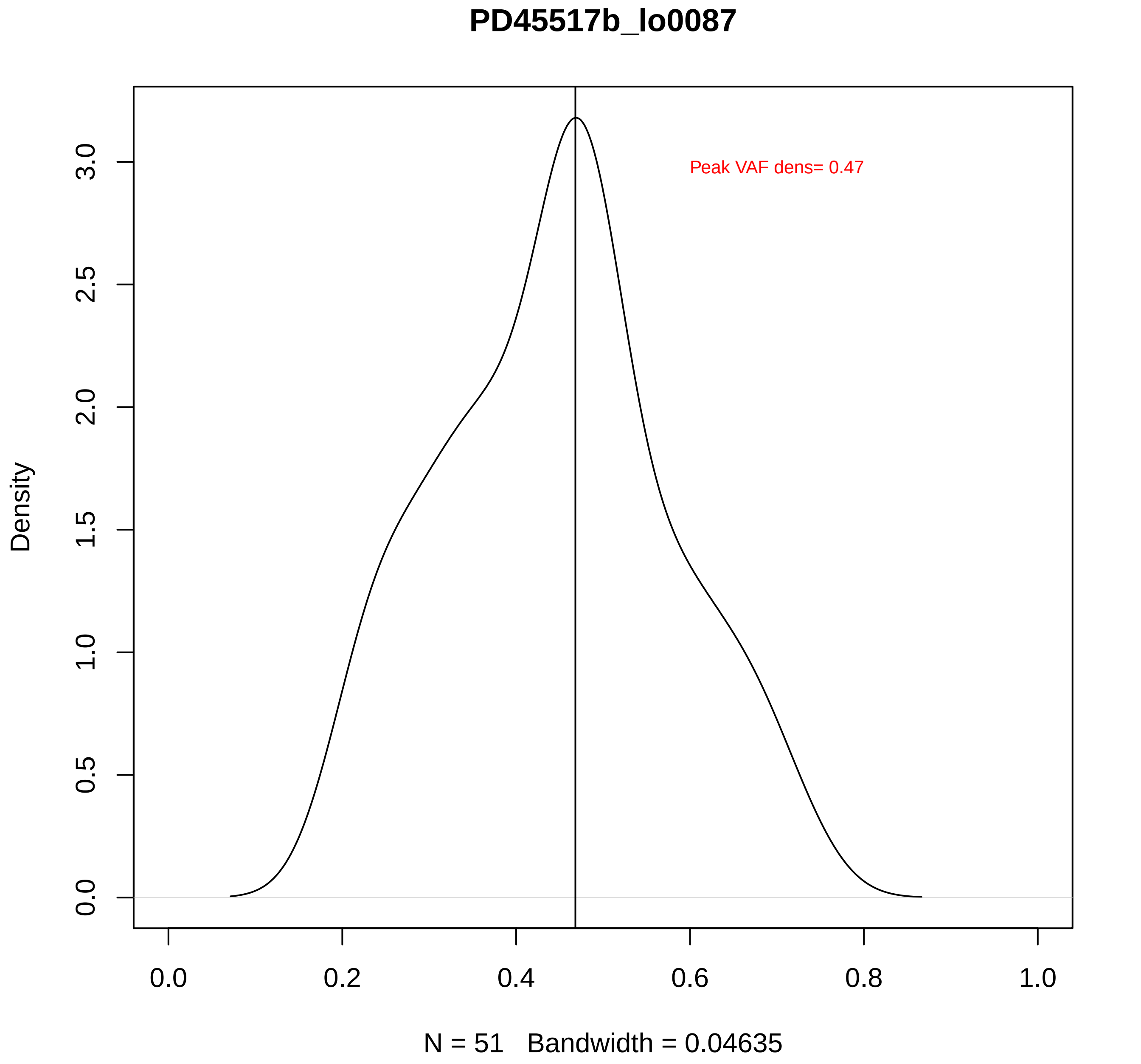
PD45517b_lo0087
Peak VAF dens= 0.47
Density
0.0
0.5
1.0
1.5
2.0
2.5
3.0
0.0
0.2
0.4
0.6
0.8
1.0
N = 51   Bandwidth = 0.04635

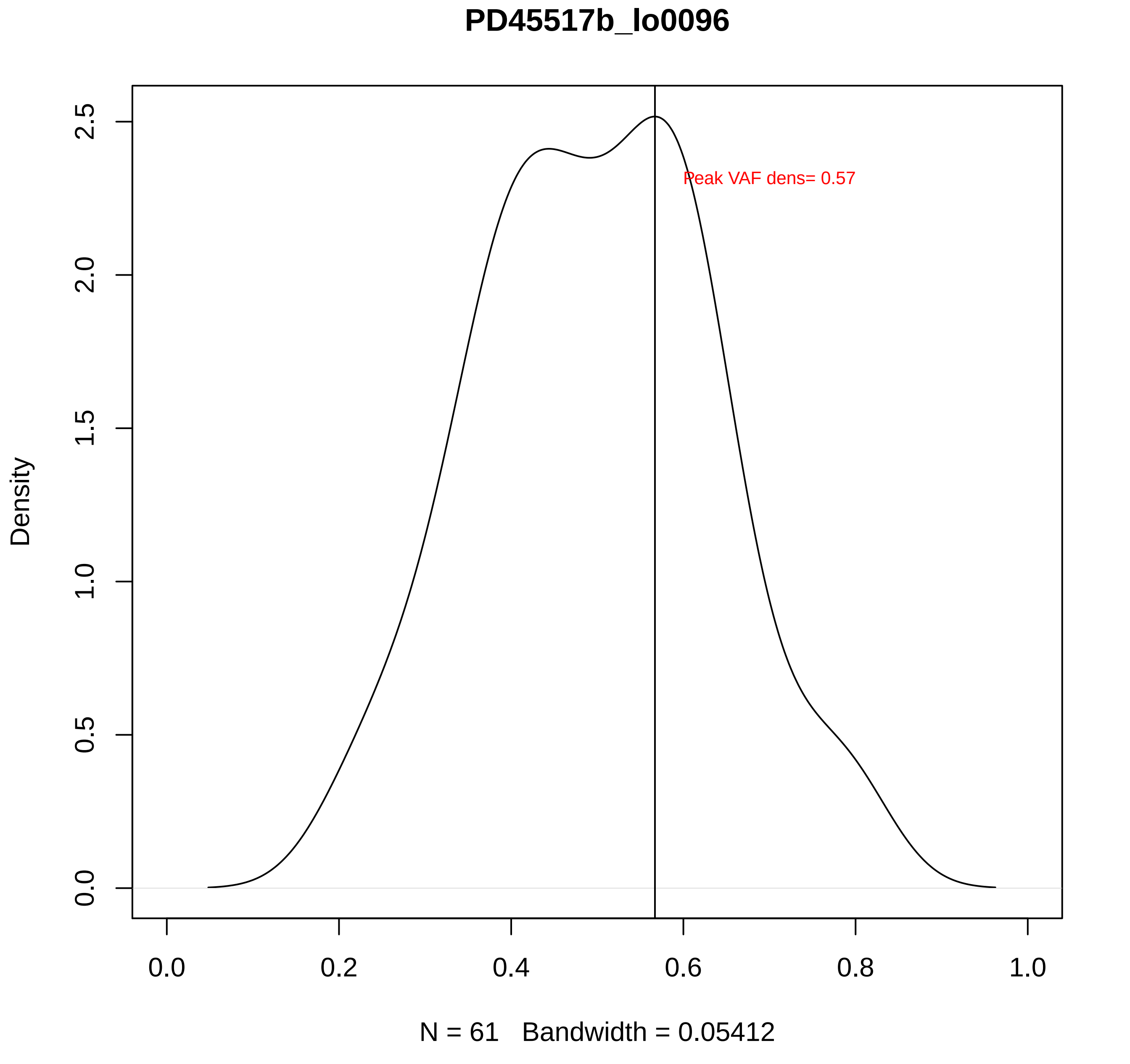

PD45517b_lo0096
Peak VAF dens= 0.57
Density
0.0
0.5
1.0
1.5
2.0
2.5
0.0
0.2
0.4
0.6
0.8
1.0
N = 61 Bandwidth = 0.05412

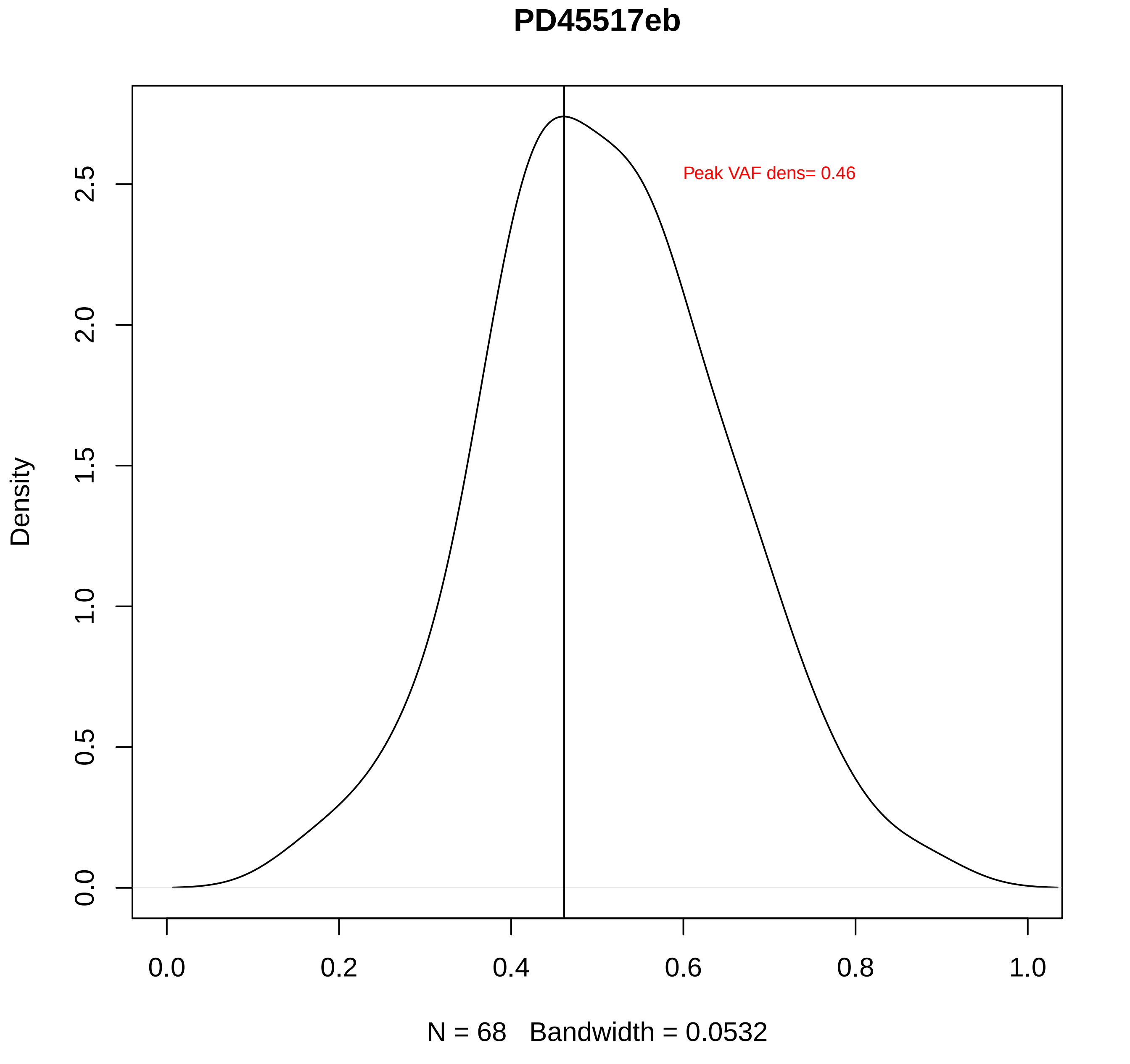
PD45517eb
Peak VAF dens= 0.46
Density
0.0
0.5
1.0
1.5
2.0
2.5
0.0
0.2
0.4
0.6
0.8
1.0
N = 68 Bandwidth = 0.0532

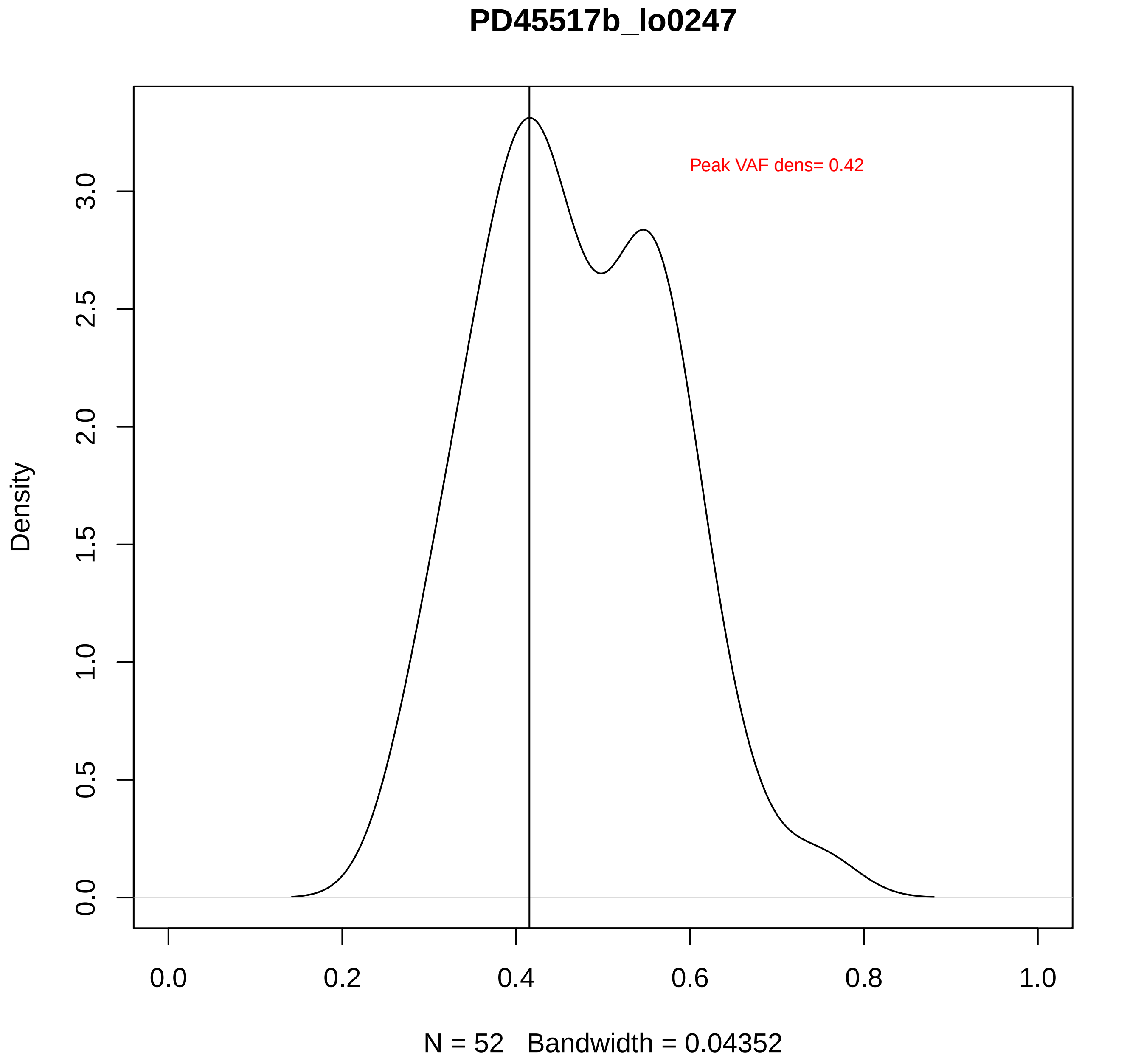

PD45517b_lo0247
Peak VAF dens= 0.42
Density
0.0
0.5
1.0
1.5
2.0
2.5
3.0
0.0
0.2
0.4
0.6
0.8
1.0
N = 52 Bandwidth = 0.04352

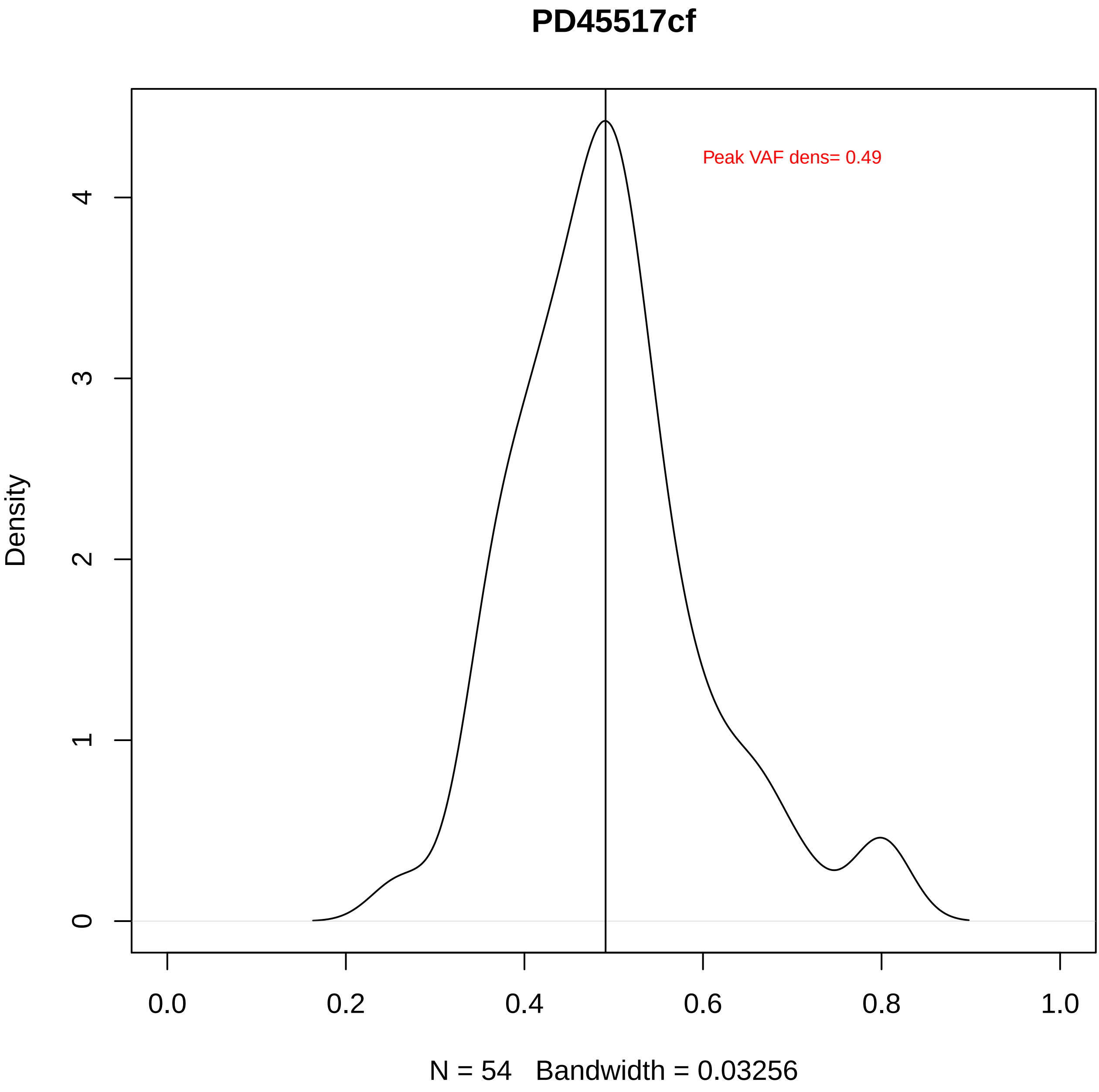
PD45517cf
Peak VAF dens= 0.49
Density
0
1
2
3
4
0.0
0.2
0.4
0.6
0.8
1.0
N = 54   Bandwidth = 0.03256

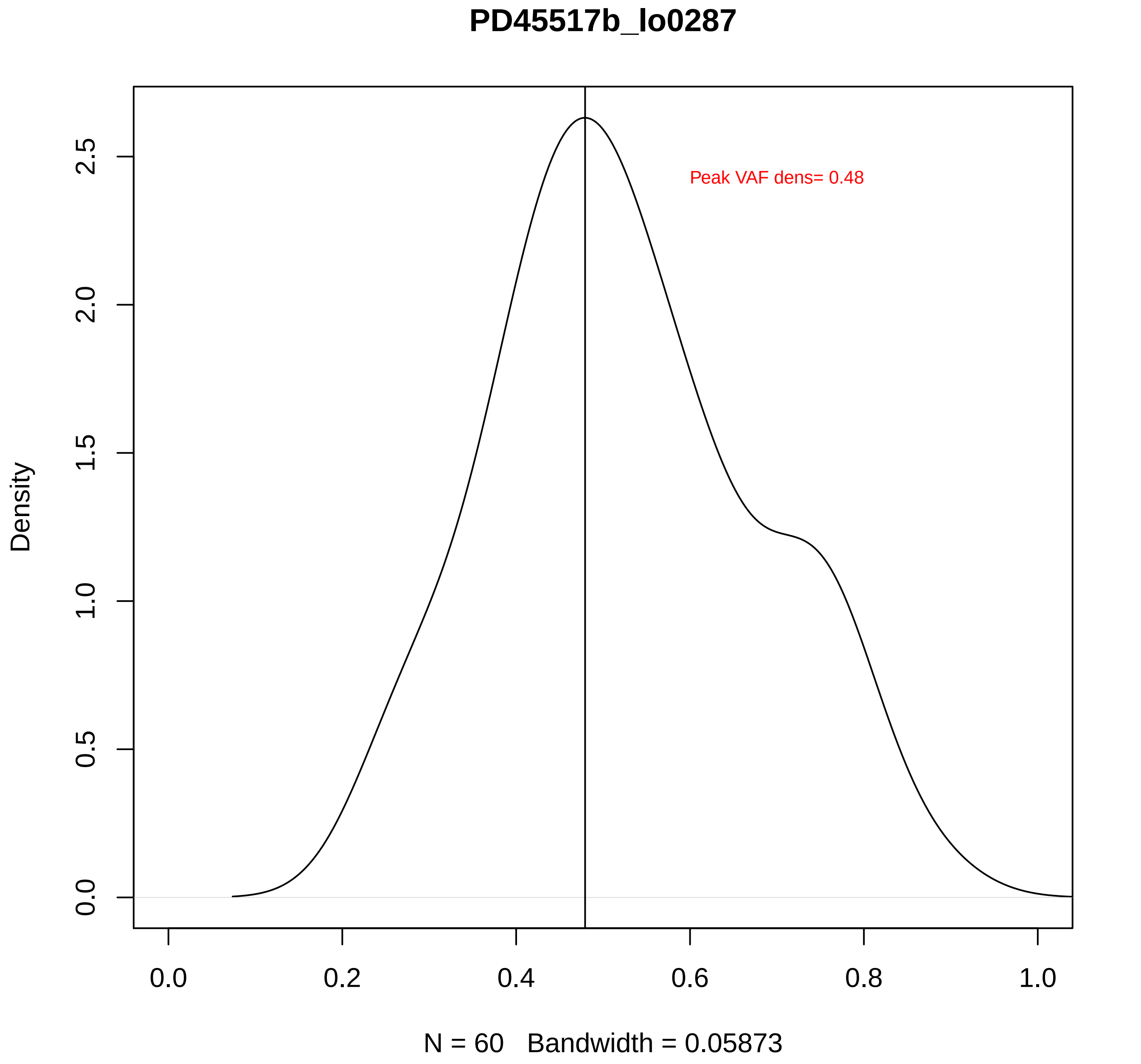
PD45517b_lo0287
Peak VAF dens= 0.48
Density
0.0
0.5
1.0
1.5
2.0
2.5
0.0
0.2
0.4
0.6
0.8
1.0
N = 60   Bandwidth = 0.05873

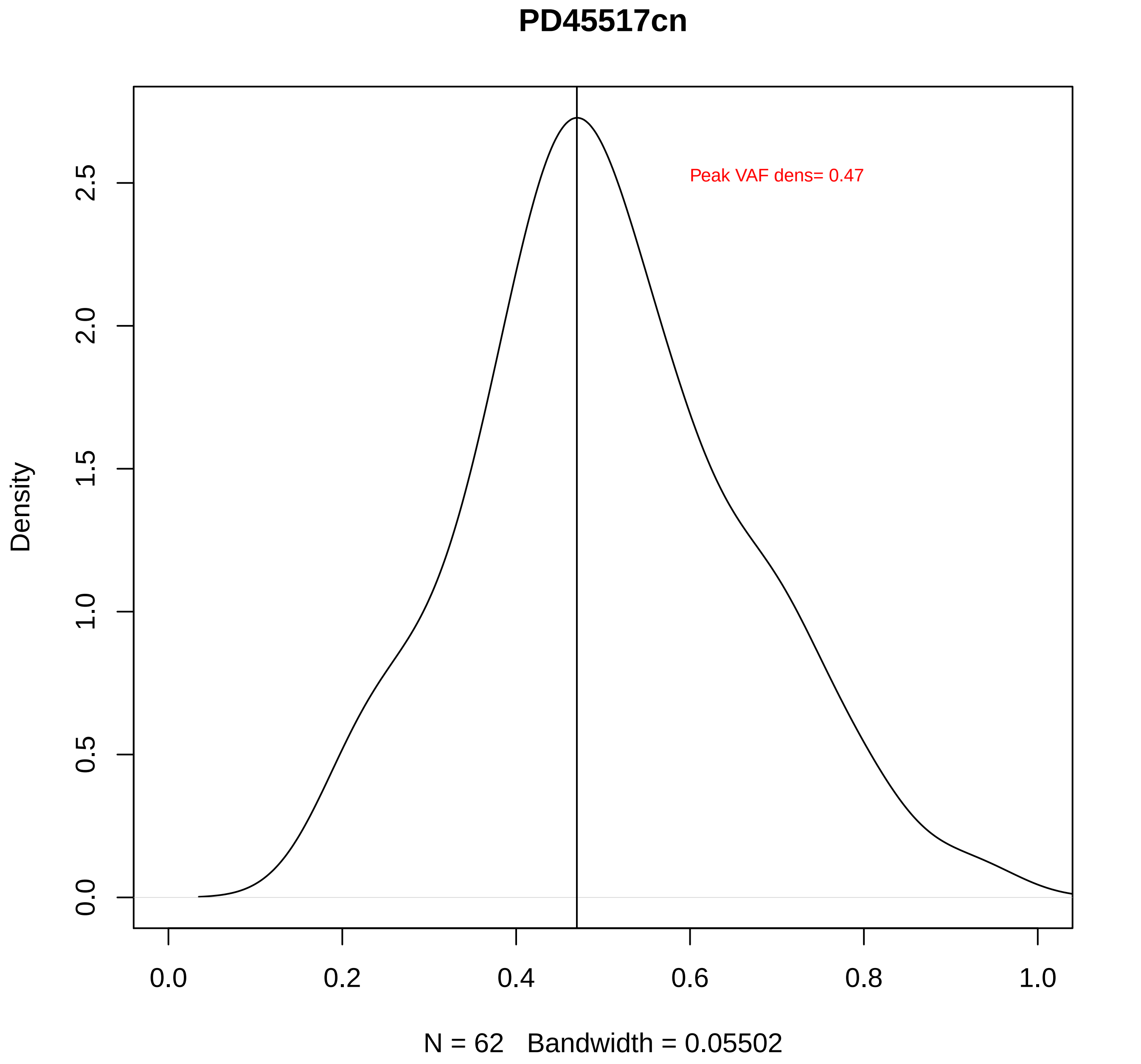
PD45517cn
Peak VAF dens= 0.47
Density
0.0
0.5
1.0
1.5
2.0
2.5
0.0
0.2
0.4
0.6
0.8
1.0
N = 62   Bandwidth = 0.05502

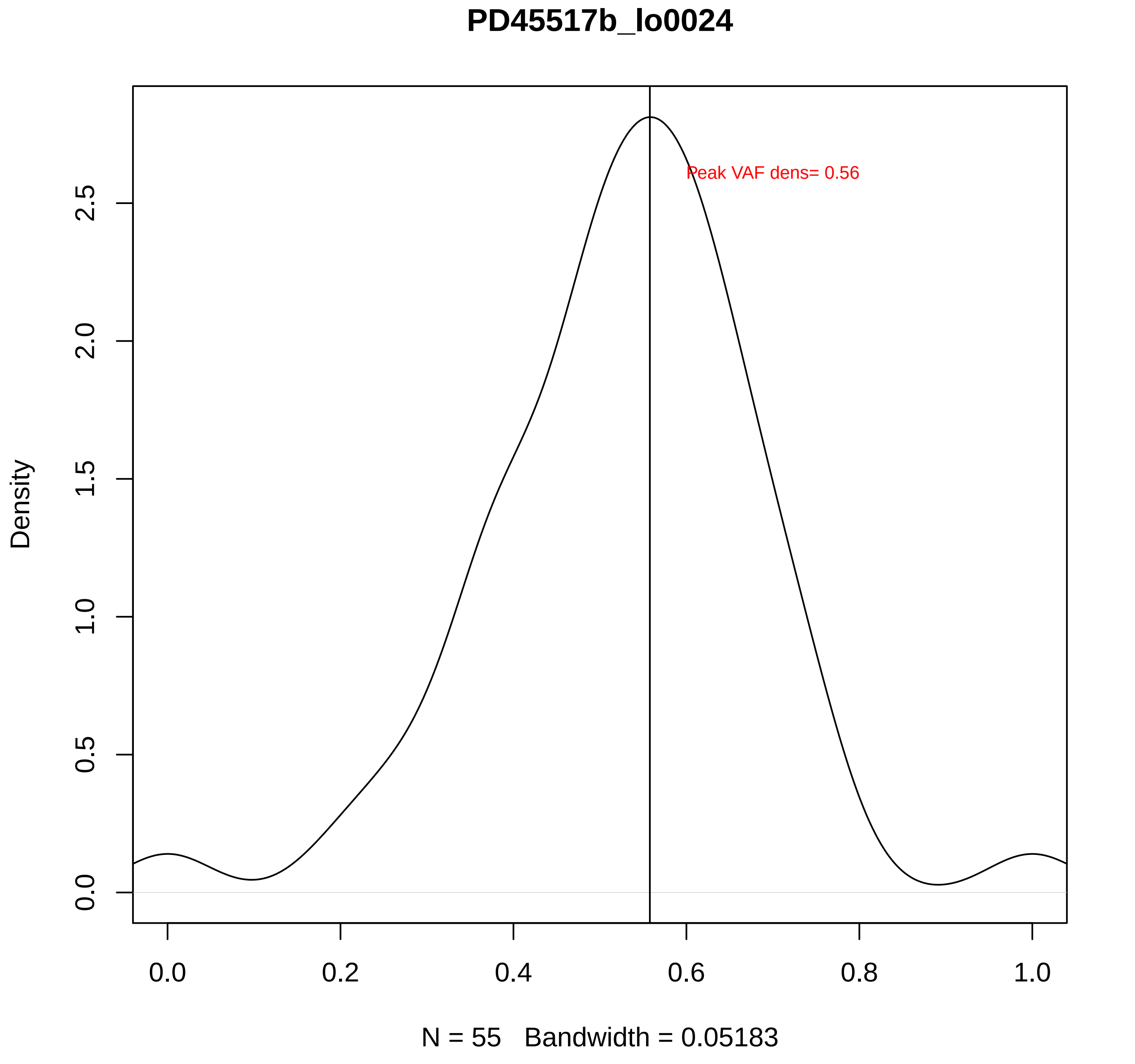
PD45517b_lo0024
Peak VAF dens= 0.56
Density
0.0
0.5
1.0
1.5
2.0
2.5
0.0
0.2
0.4
0.6
0.8
1.0
N = 55 Bandwidth = 0.05183

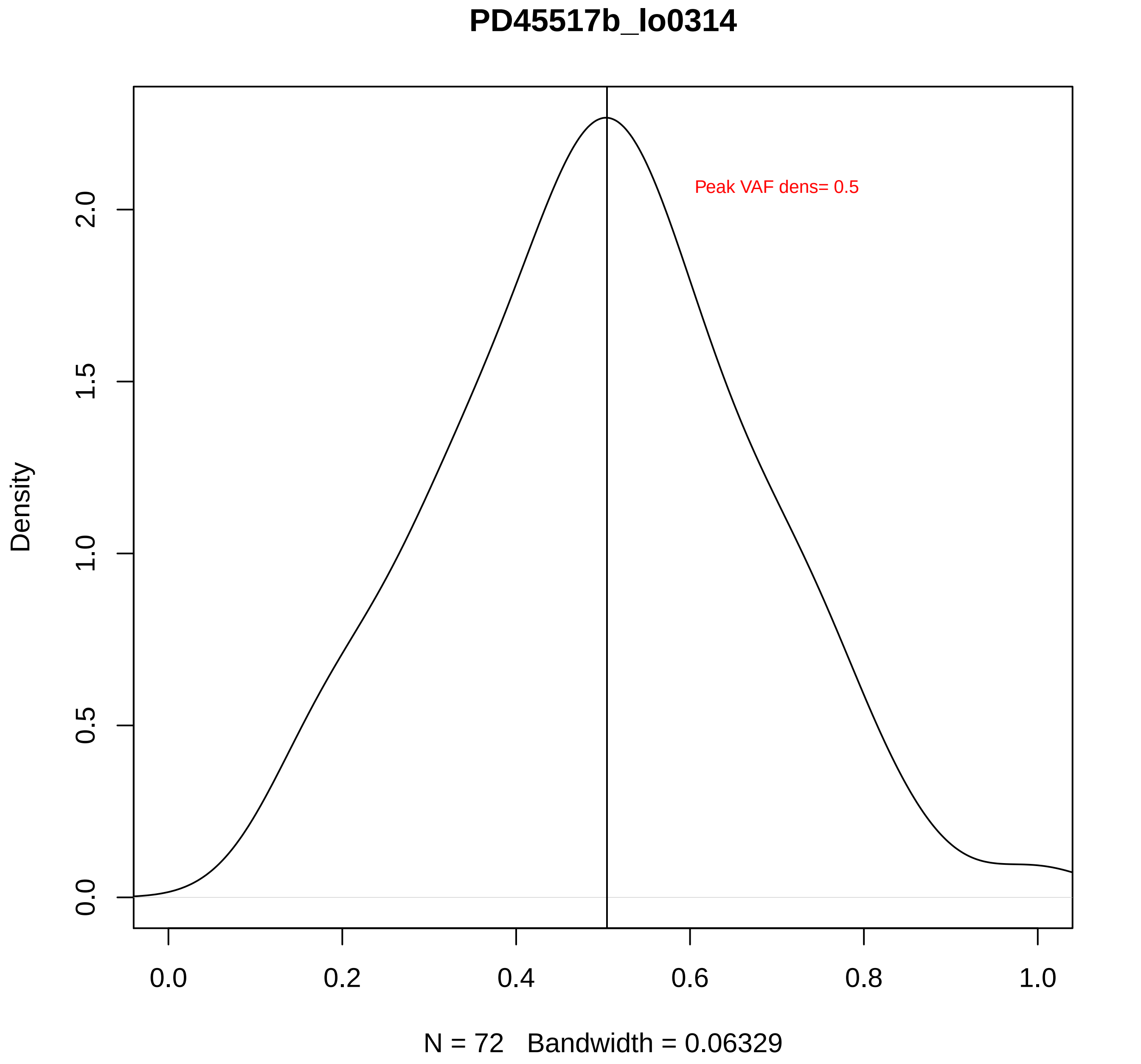
PD45517b_lo0314
Peak VAF dens= 0.5
Density
0.0
0.5
1.0
1.5
2.0
0.0
0.2
0.4
0.6
0.8
1.0
N = 72   Bandwidth = 0.06329

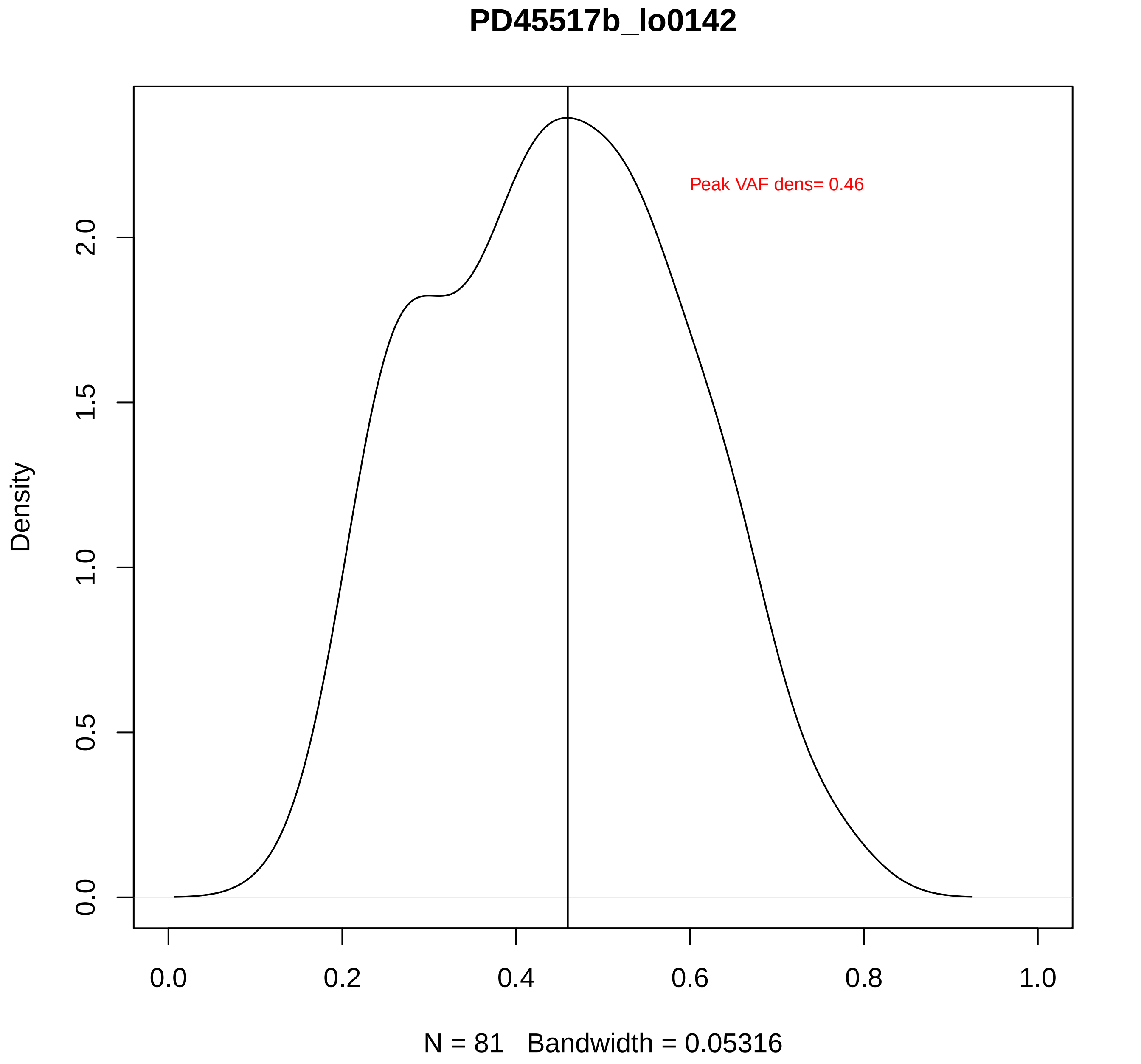
PD45517b_lo0142
Peak VAF dens= 0.46
Density
0.0
0.5
1.0
1.5
2.0
0.0
0.2
0.4
0.6
0.8
1.0
N = 81 Bandwidth = 0.05316

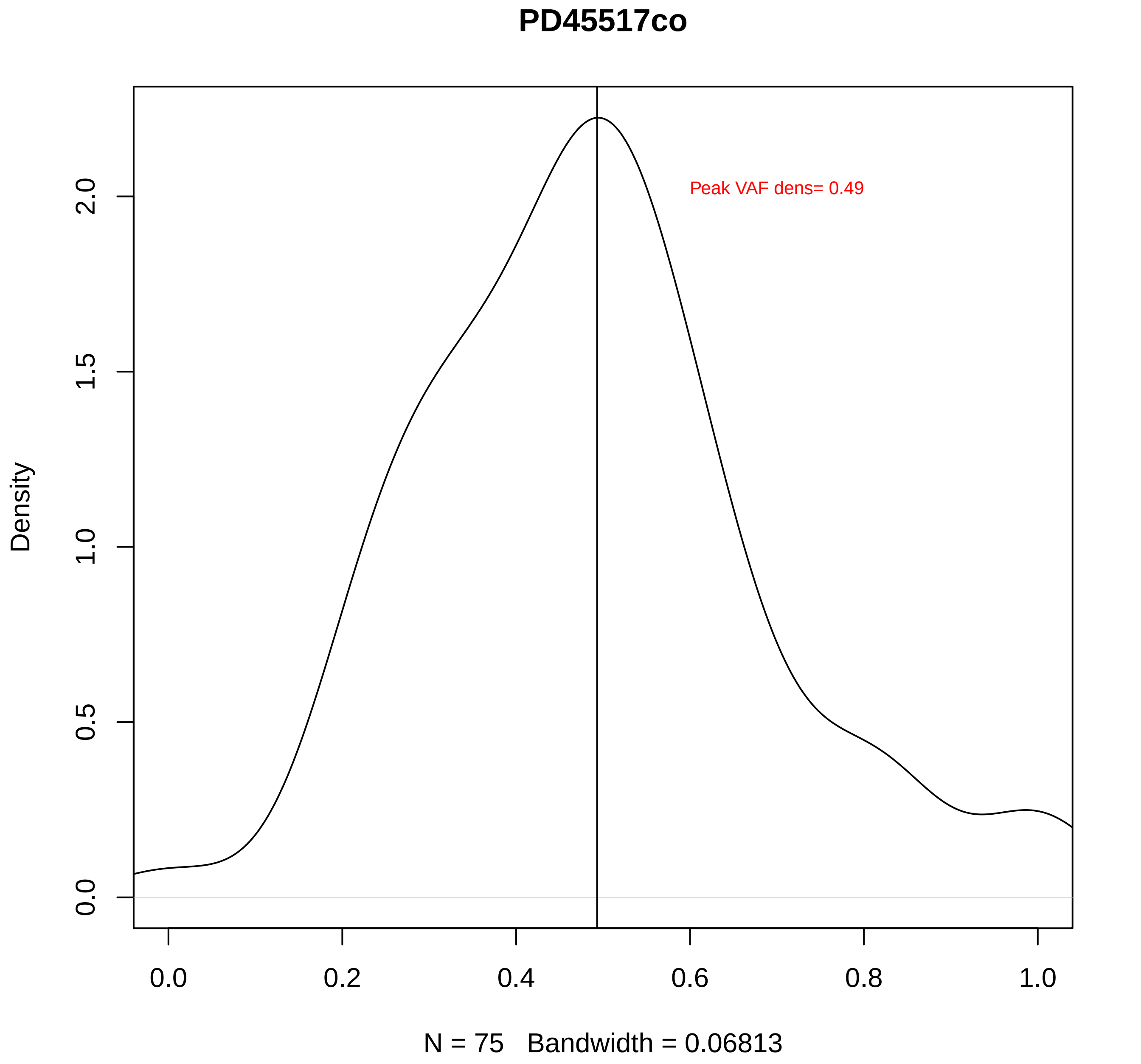
PD45517co
Peak VAF dens= 0.49
Density
0.0
0.5
1.0
1.5
2.0
0.0
0.2
0.4
0.6
0.8
1.0
N = 75   Bandwidth = 0.06813

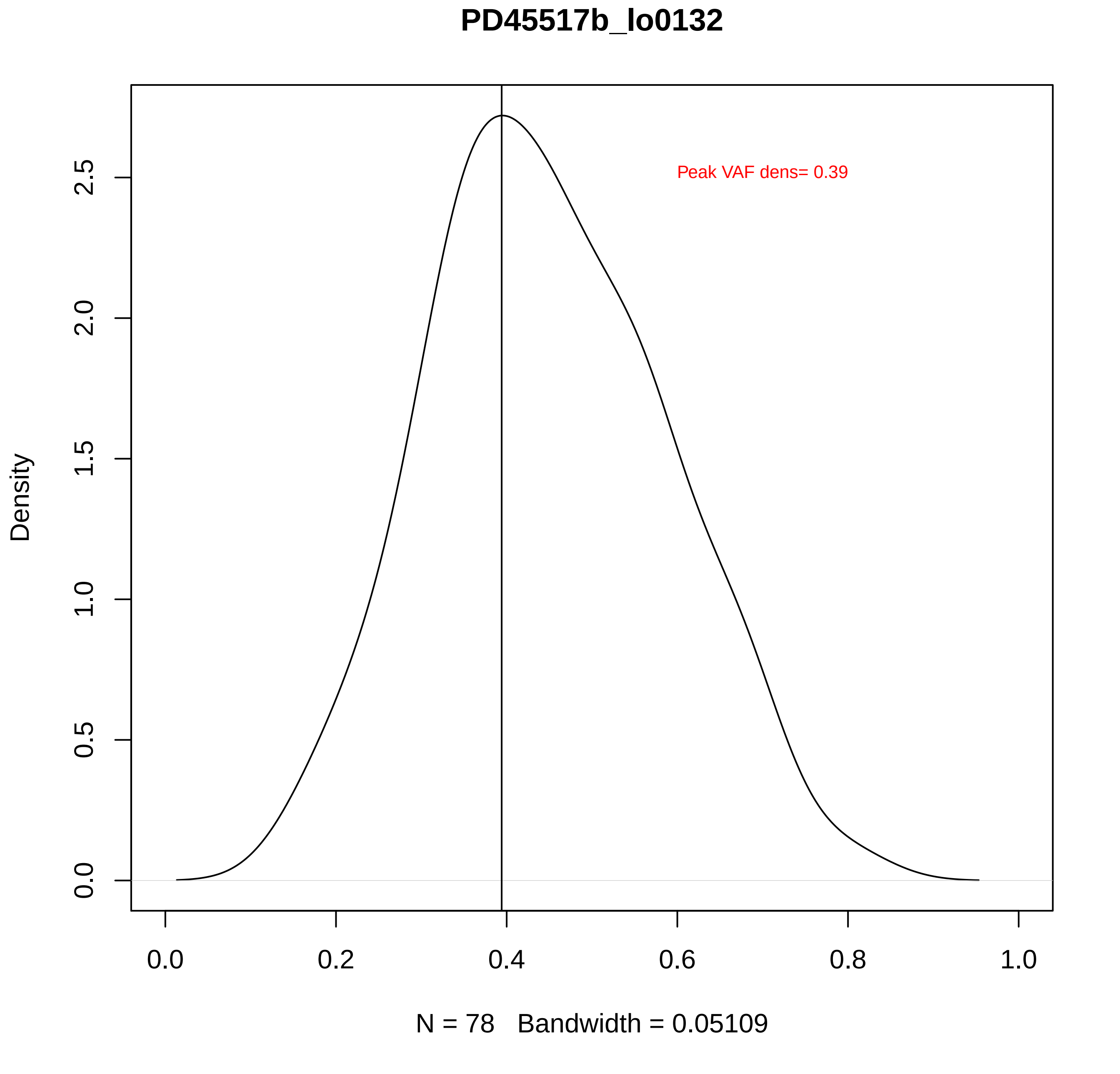
PD45517b_lo0132
Peak VAF dens= 0.39
Density
0.0
0.5
1.0
1.5
2.0
2.5
0.0
0.2
0.4
0.6
0.8
1.0
N = 78 Bandwidth = 0.05109

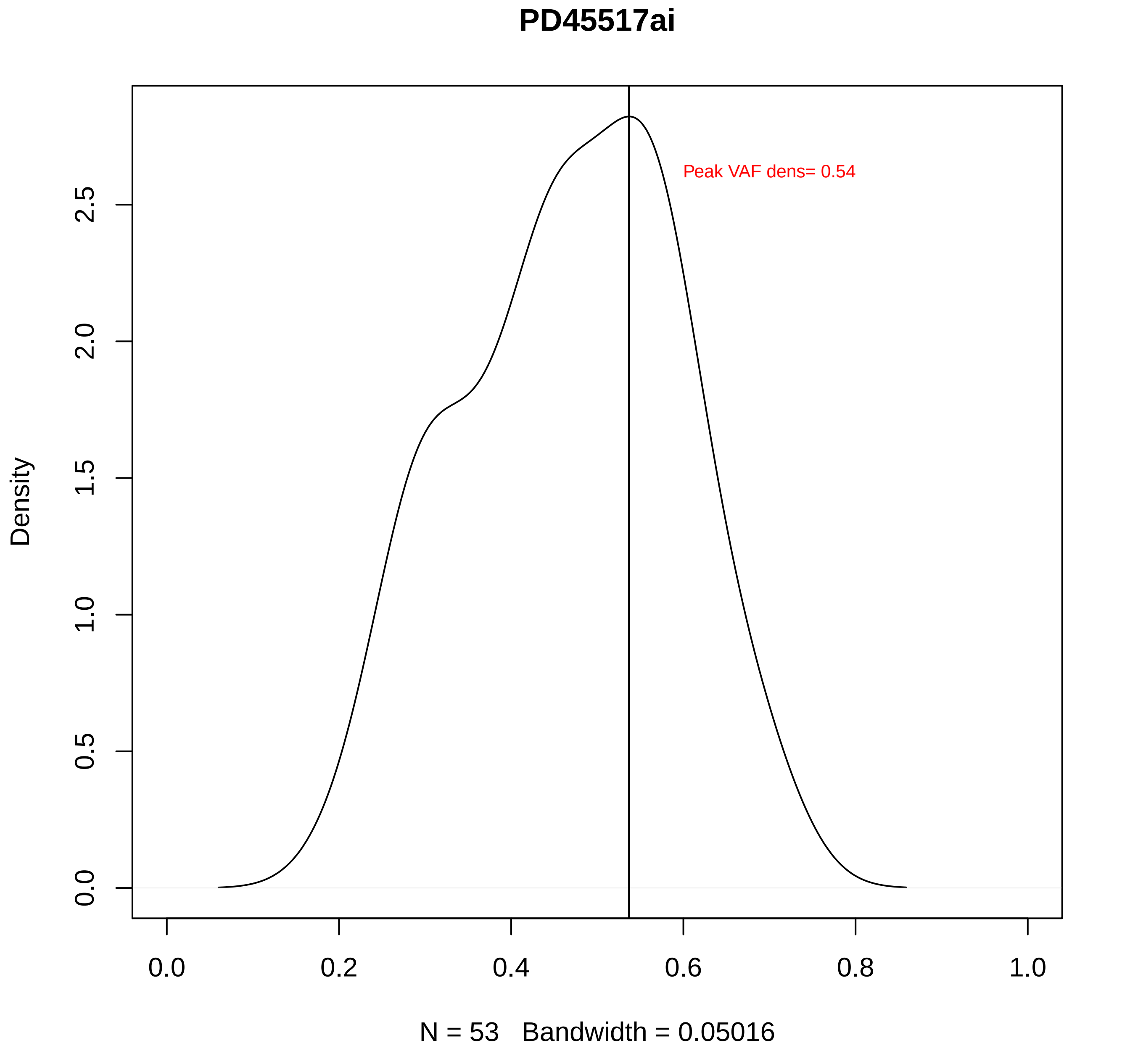
PD45517ai
Peak VAF dens= 0.54
Density
0.0
0.5
1.0
1.5
2.0
2.5
0.0
0.2
0.4
0.6
0.8
1.0
N = 53   Bandwidth = 0.05016

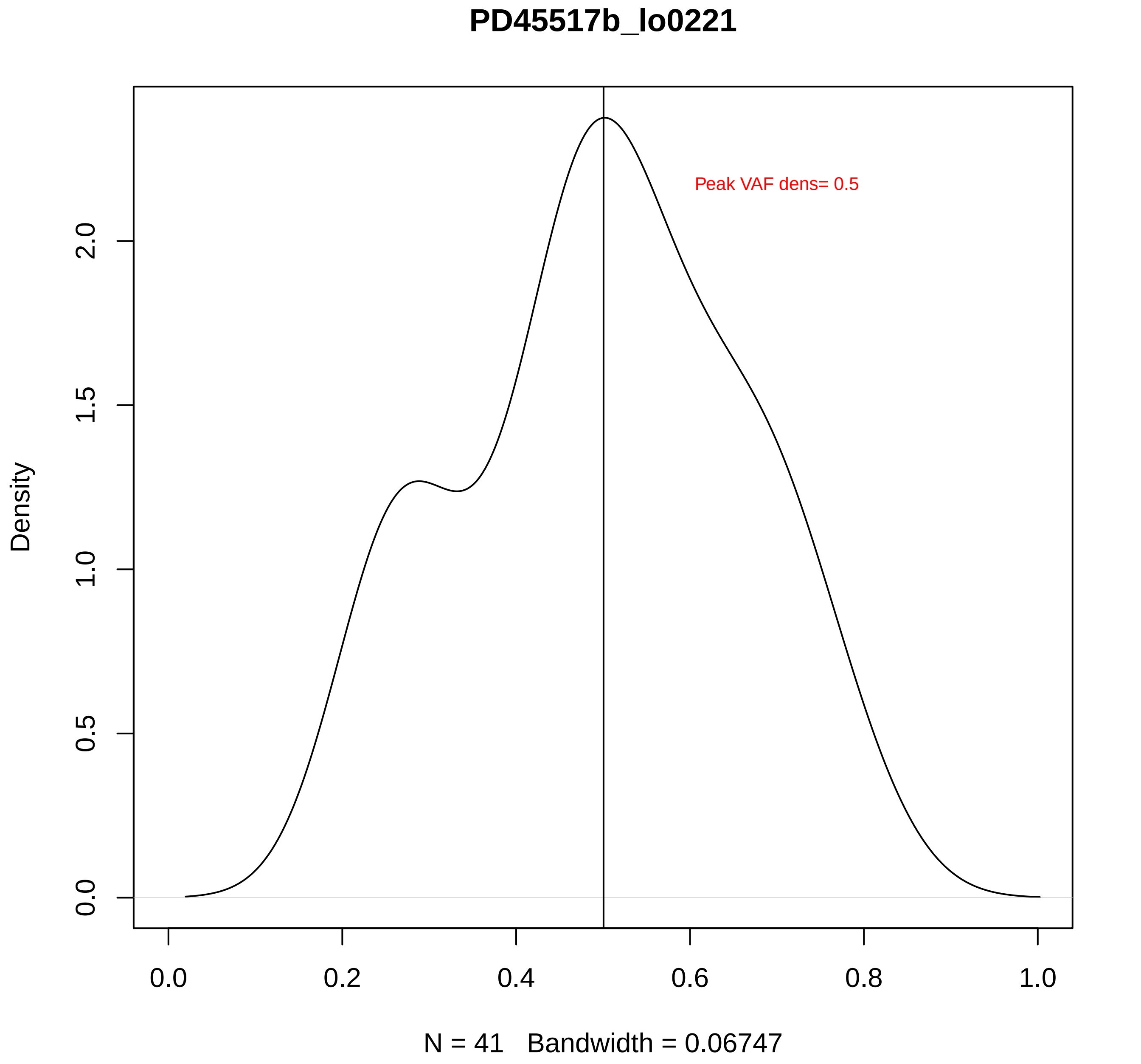
PD45517b_lo0221
Peak VAF dens= 0.5
Density
0.0
0.5
1.0
1.5
2.0
0.0
0.2
0.4
0.6
0.8
1.0
N = 41   Bandwidth = 0.06747

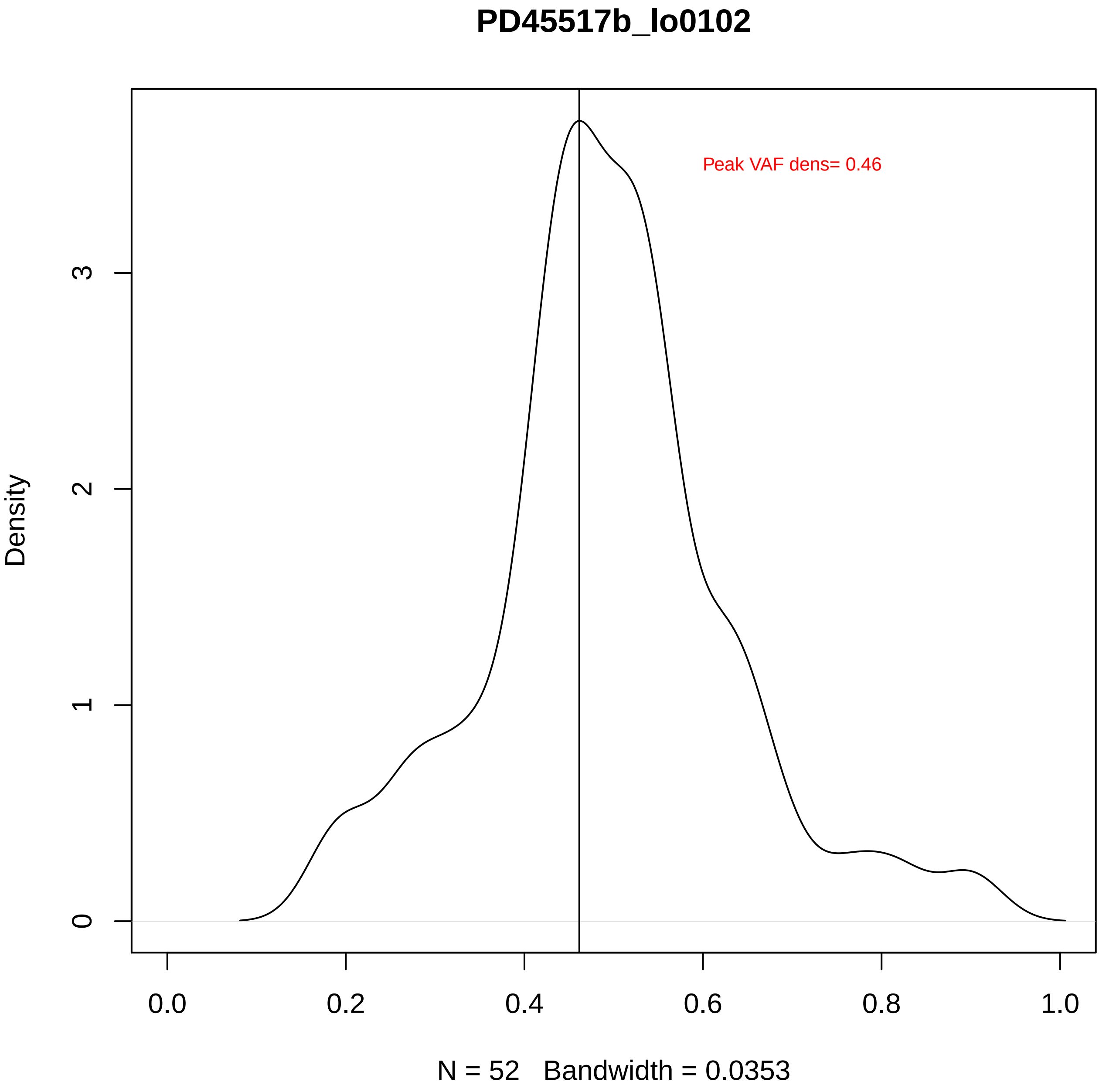
PD45517b_lo0102
Peak VAF dens= 0.46
Density
0
1
2
3
0.0
0.2
0.4
0.6
0.8
1.0
N = 52   Bandwidth = 0.0353

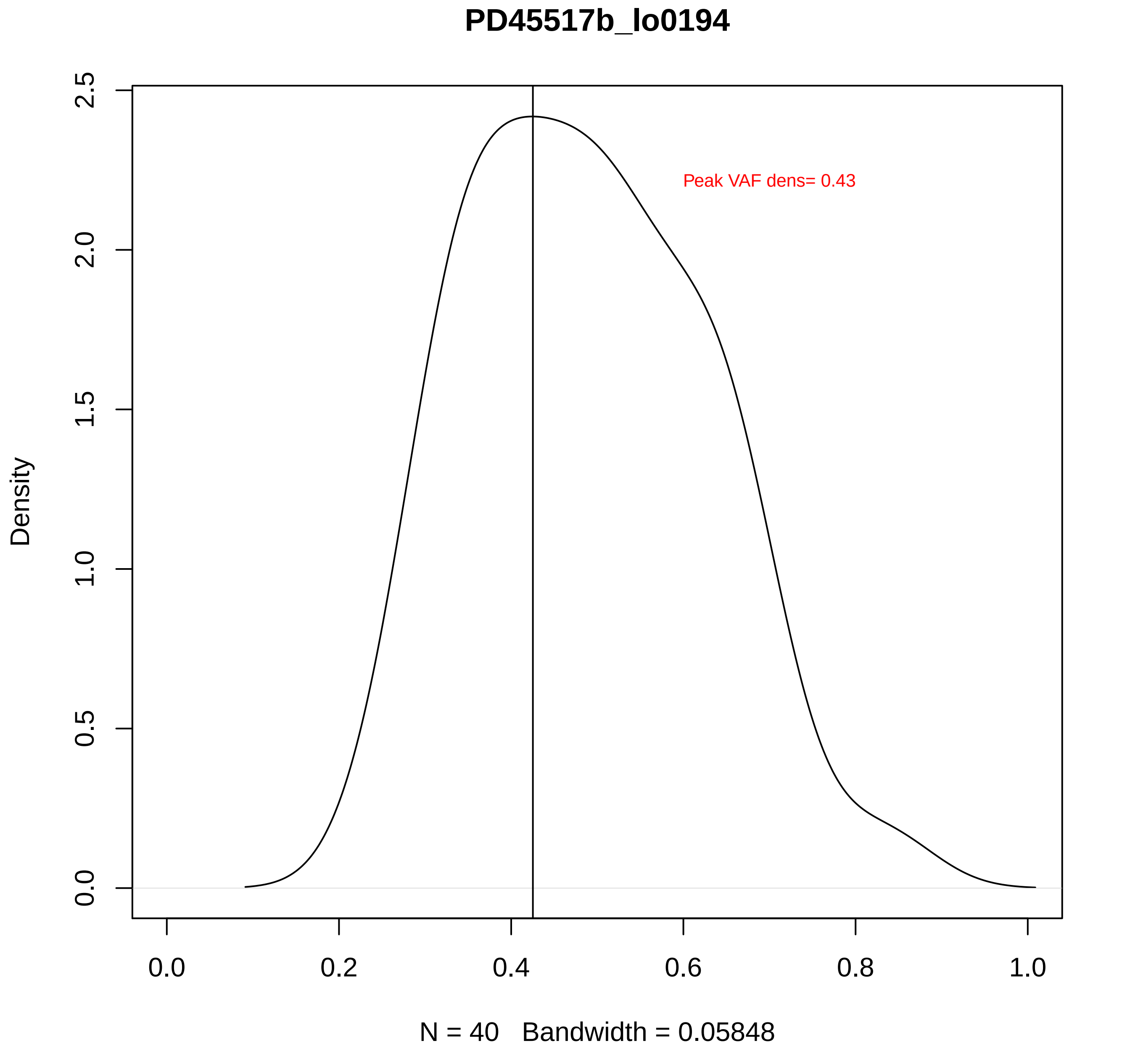
PD45517b_lo0194
Peak VAF dens= 0.43
Density
0.0
0.5
1.0
1.5
2.0
2.5
0.0
0.2
0.4
0.6
0.8
1.0
N = 40   Bandwidth = 0.05848

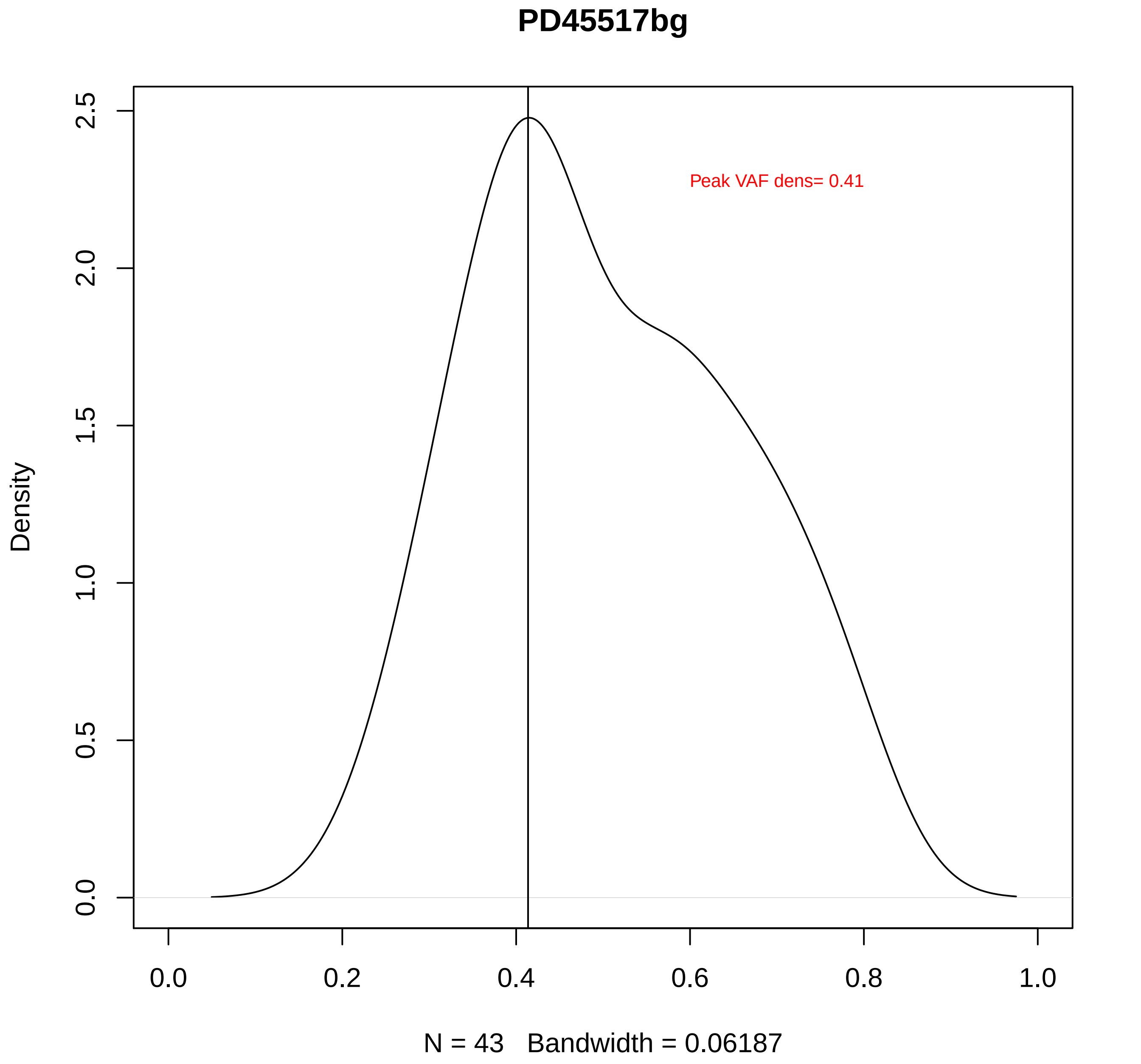
PD45517bg
Peak VAF dens= 0.41
Density
0.0
0.5
1.0
1.5
2.0
2.5
0.0
0.2
0.4
0.6
0.8
1.0
N = 43 Bandwidth = 0.06187

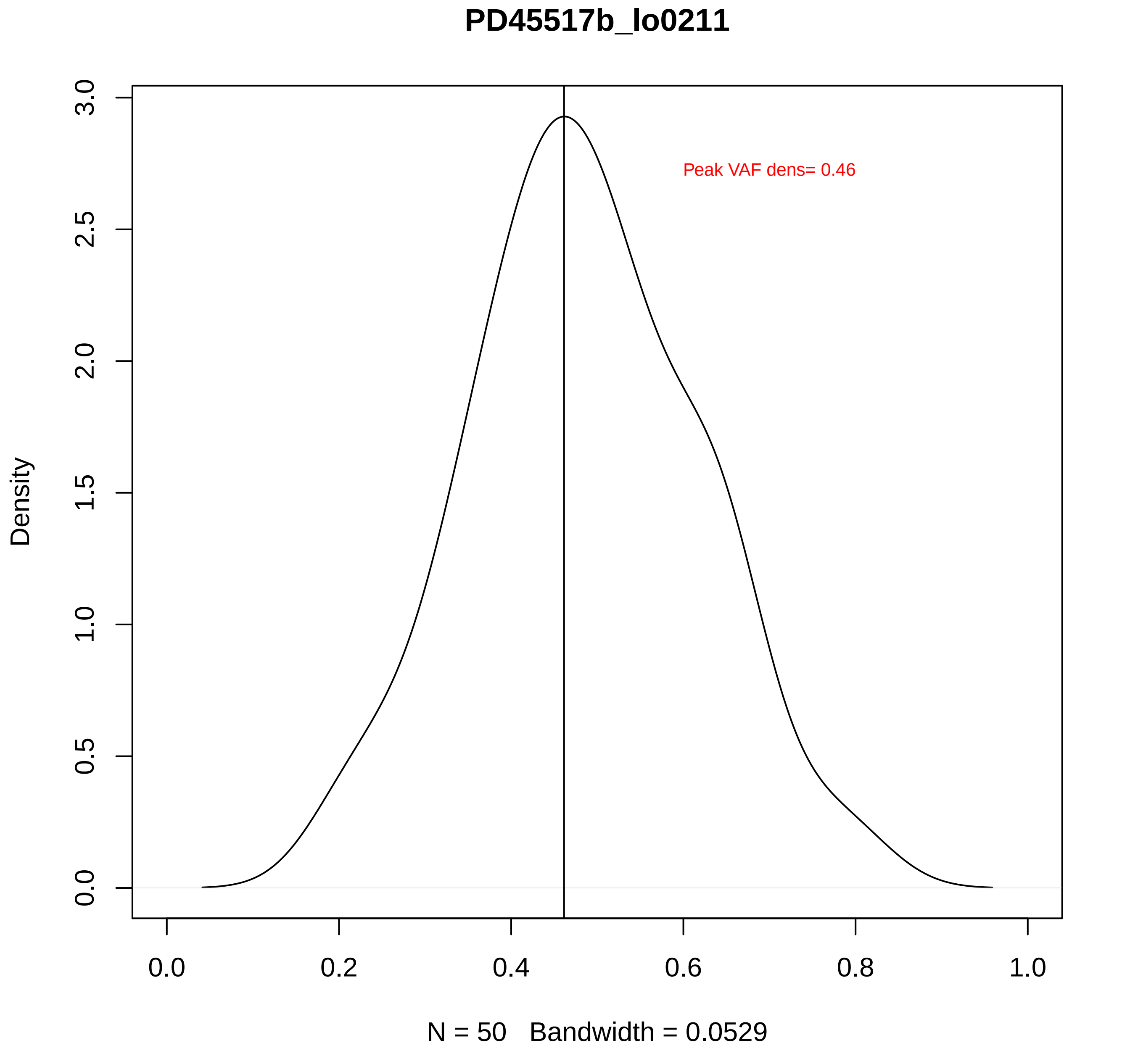
PD45517b_lo0211
Peak VAF dens= 0.46
Density
3.0
2.5
2.0
1.5
1.0
0.5
0.0
0.0
0.2
0.4
0.6
0.8
1.0
N = 50   Bandwidth = 0.0529

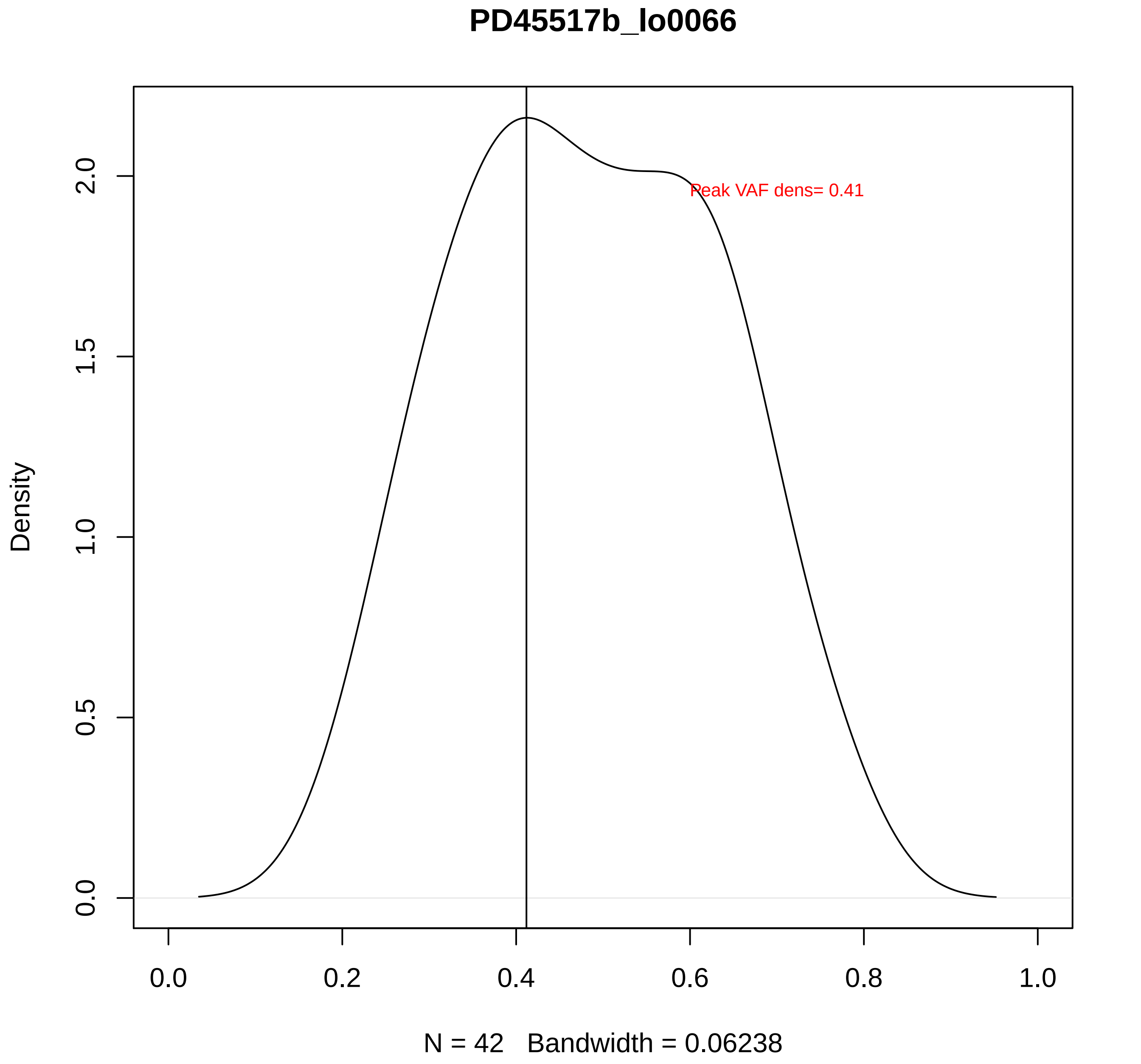
PD45517b_lo0066
Peak VAF dens= 0.41
Density
0.0
0.5
1.0
1.5
2.0
0.0
0.2
0.4
0.6
0.8
1.0
N = 42 Bandwidth = 0.06238

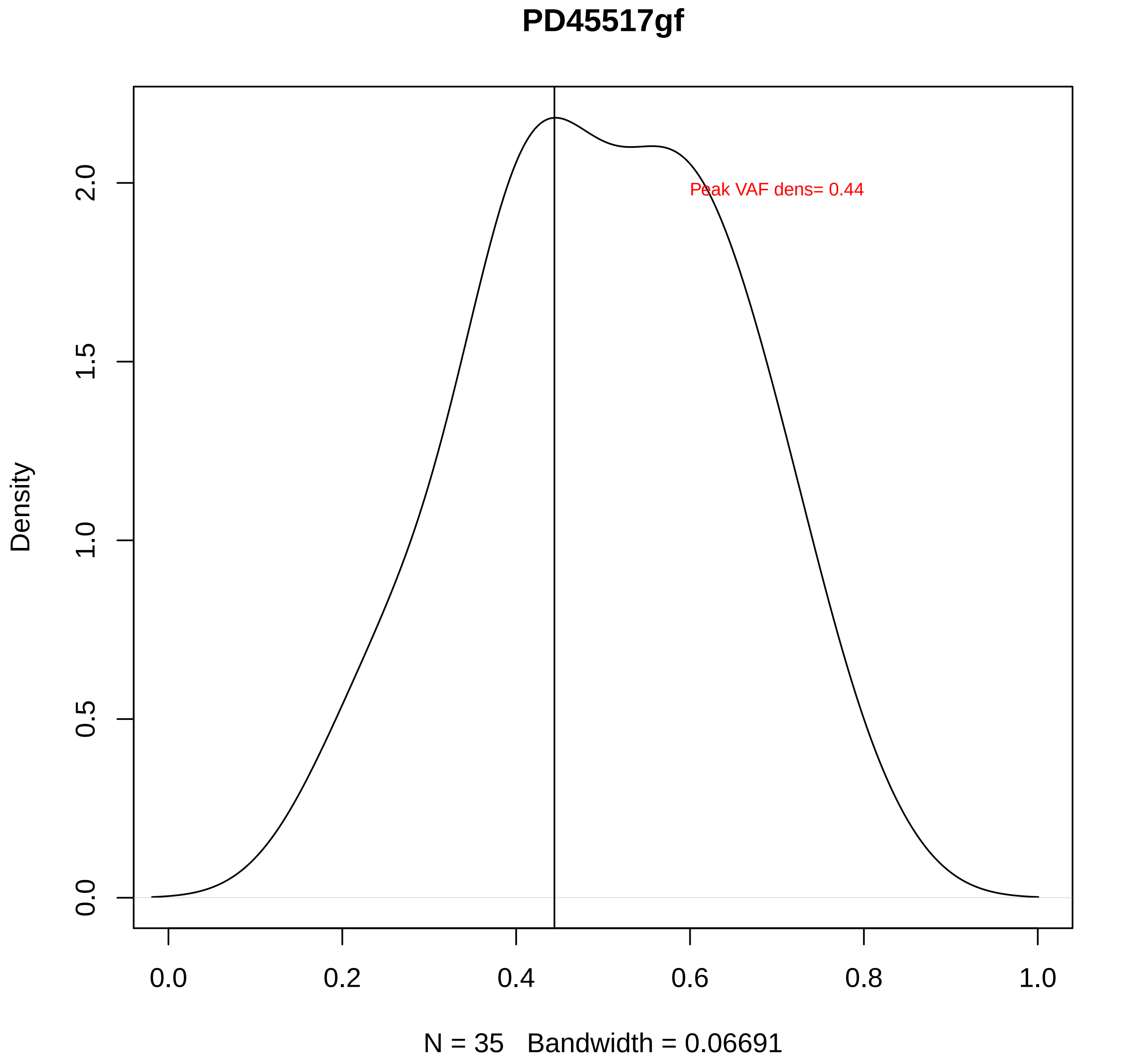

PD45517gf
Peak VAF dens= 0.44
Density
0.0
0.5
1.0
1.5
2.0
0.0
0.2
0.4
0.6
0.8
1.0
N = 35   Bandwidth = 0.06691

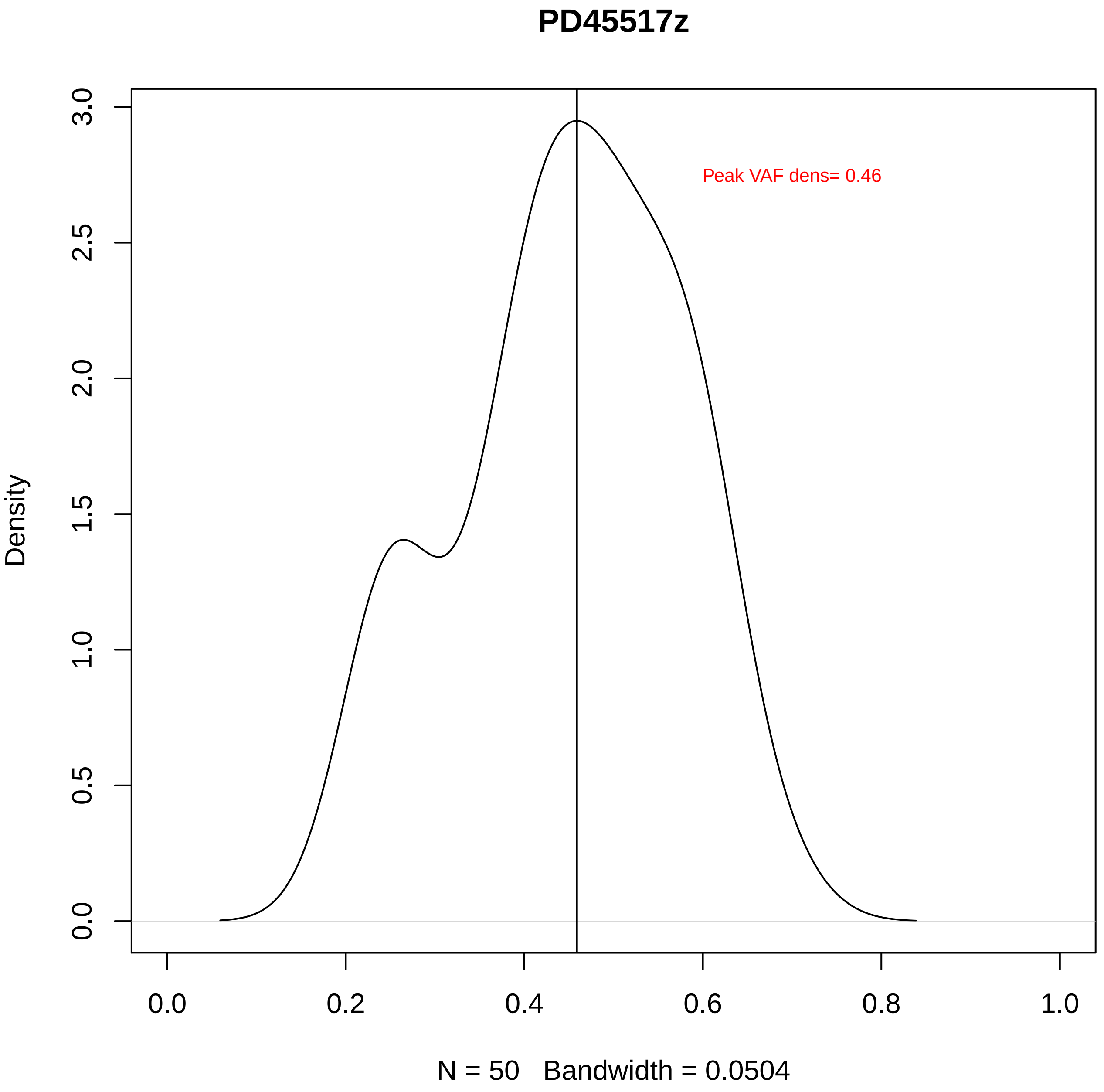
PD45517z
Peak VAF dens= 0.46
Density
0.0
0.5
1.0
1.5
2.0
2.5
3.0
0.0
0.2
0.4
0.6
0.8
1.0
N = 50   Bandwidth = 0.0504

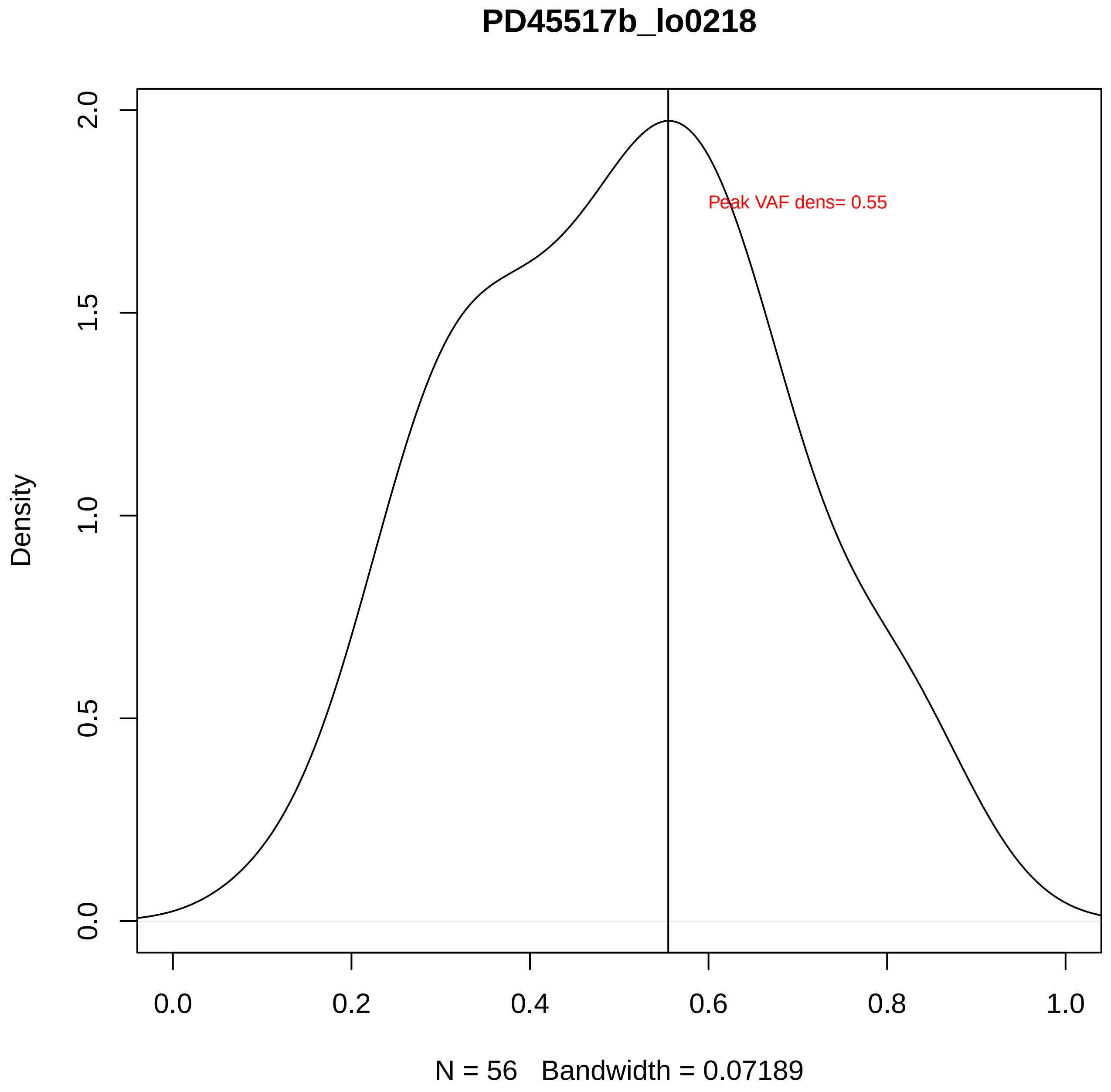

PD45517b_lo0218
Peak VAF dens= 0.55
Density
0.0
0.5
1.0
1.5
2.0
0.0
0.2
0.4
0.6
0.8
1.0
N = 56 Bandwidth = 0.07189

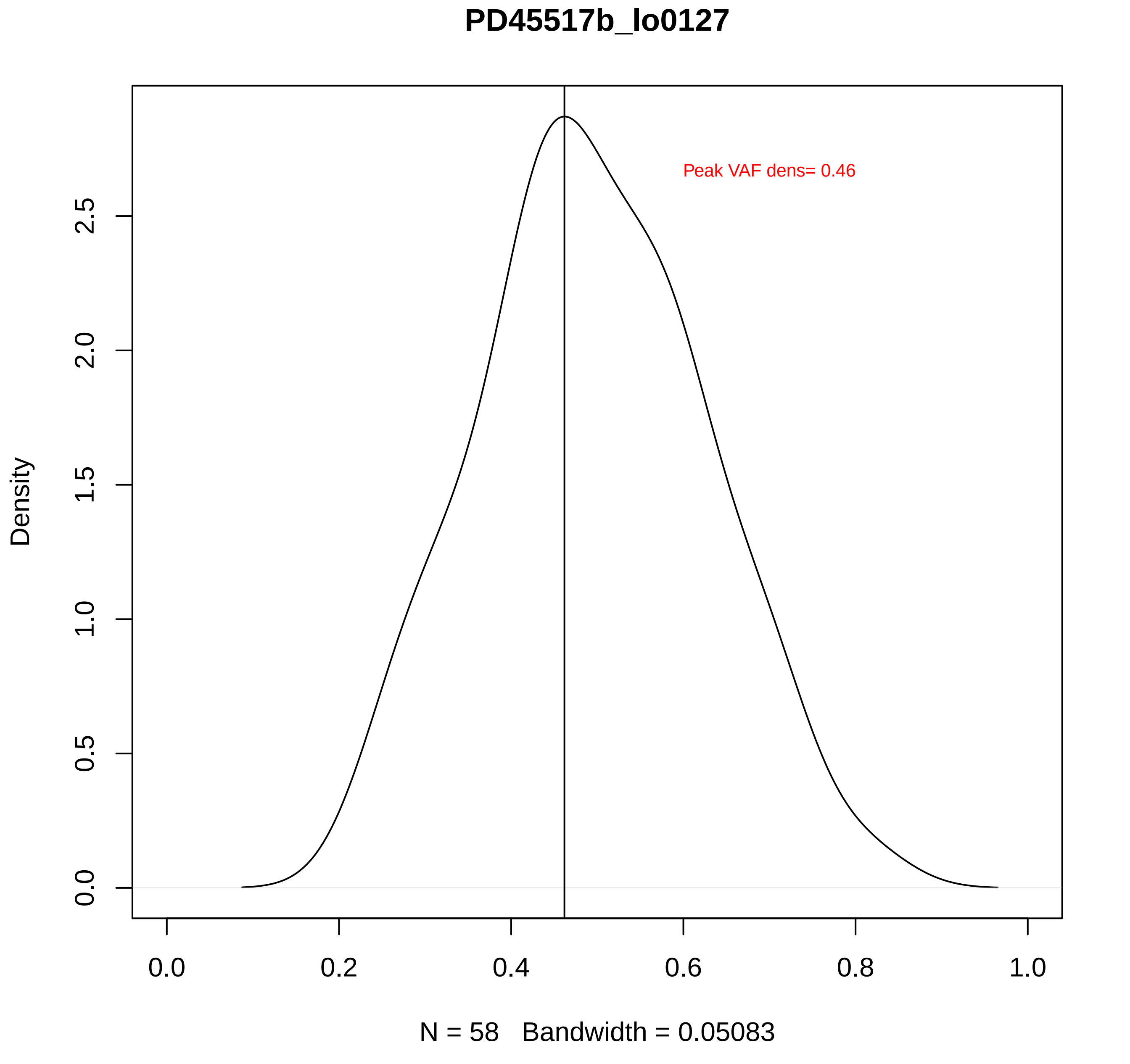

PD45517b_lo0127
Peak VAF dens= 0.46
Density
0.0
0.5
1.0
1.5
2.0
2.5
0.0
0.2
0.4
0.6
0.8
1.0
N = 58 Bandwidth = 0.05083

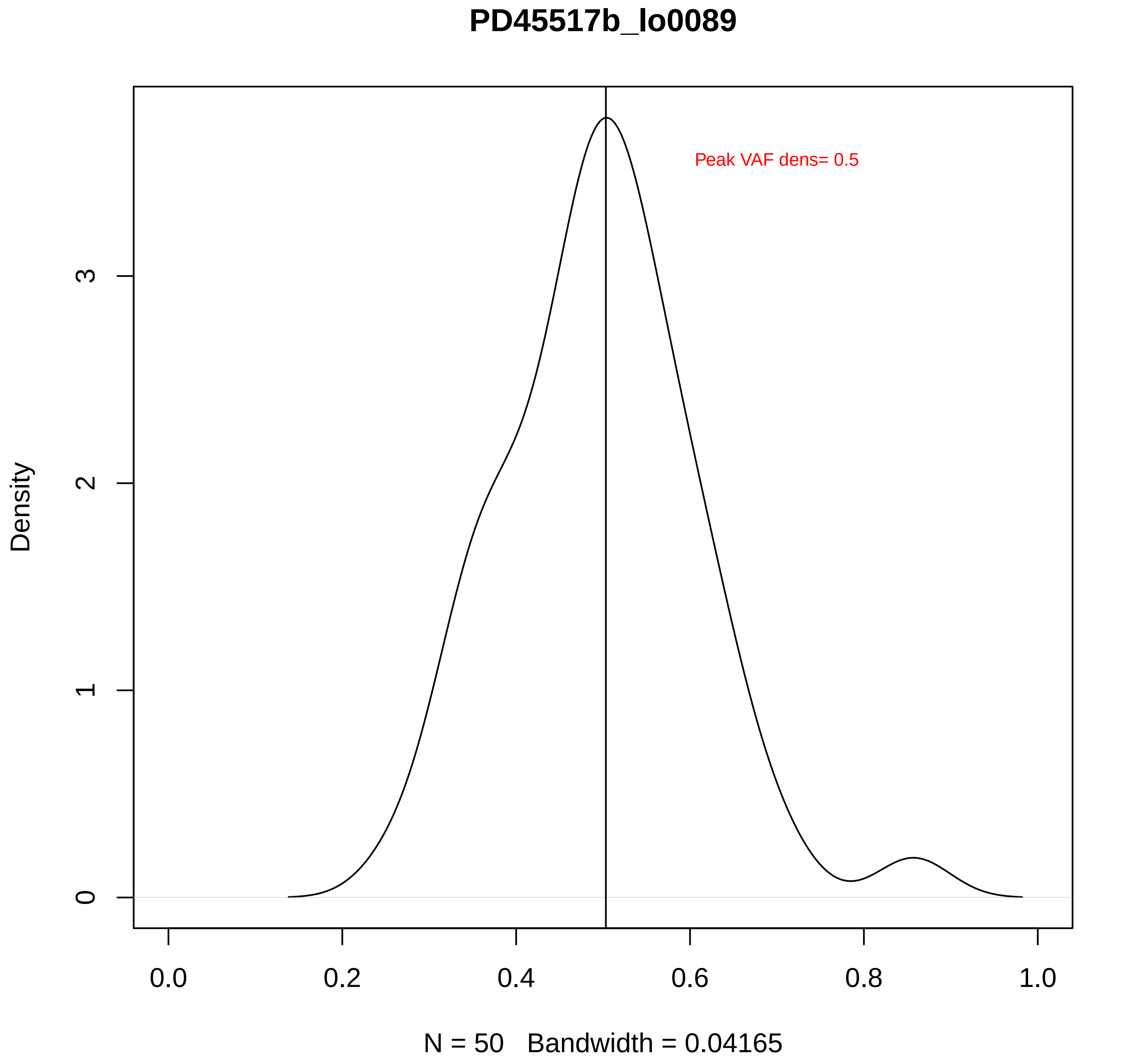
PD45517b_lo0089
Peak VAF dens= 0.5
Density
0
1
2
3
0.0
0.2
0.4
0.6
0.8
1.0
N = 50 Bandwidth = 0.04165

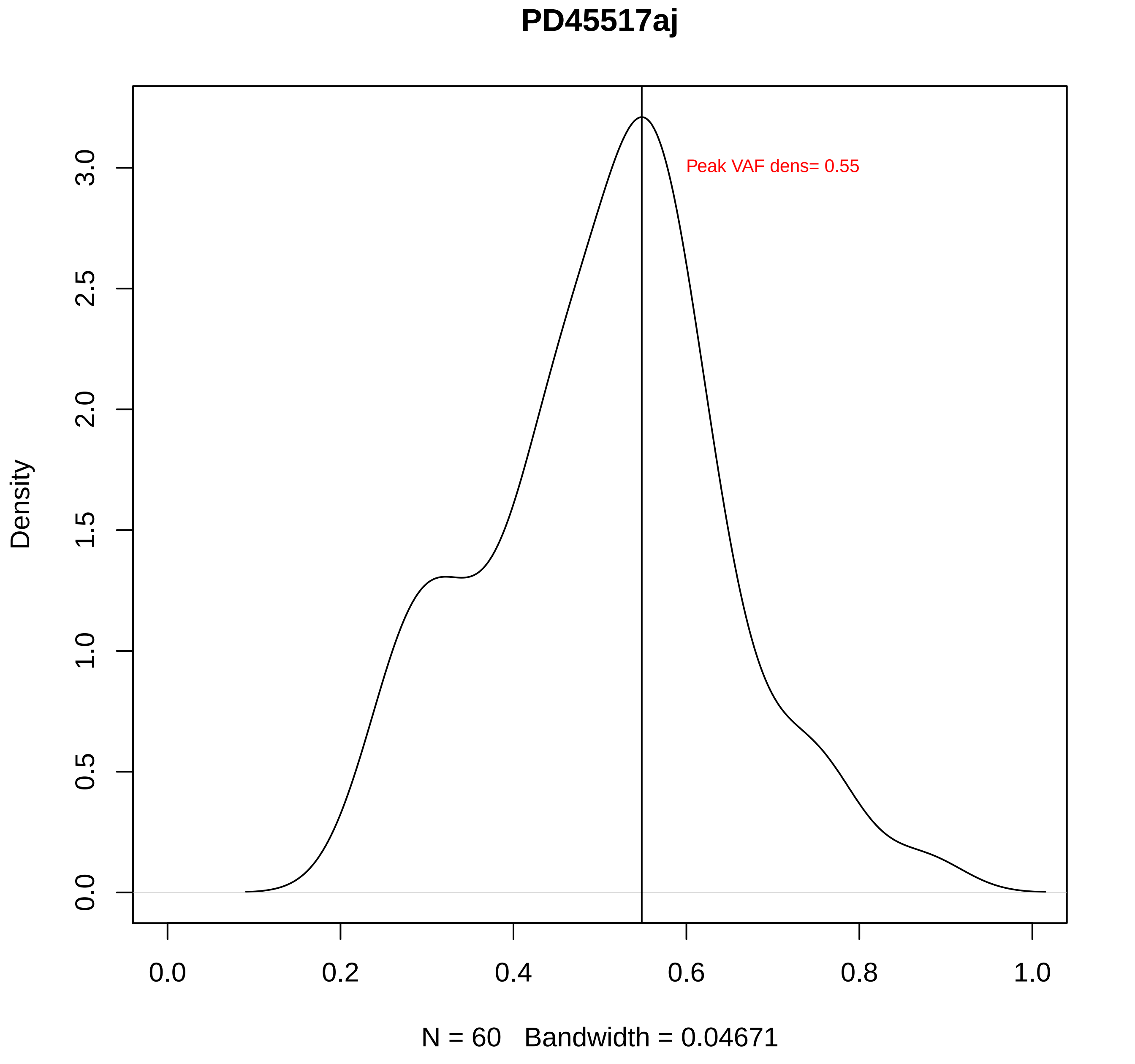
PD45517aj
Peak VAF dens= 0.55
Density
0.0
0.5
1.0
1.5
2.0
2.5
3.0
0.0
0.2
0.4
0.6
0.8
1.0
N = 60   Bandwidth = 0.04671

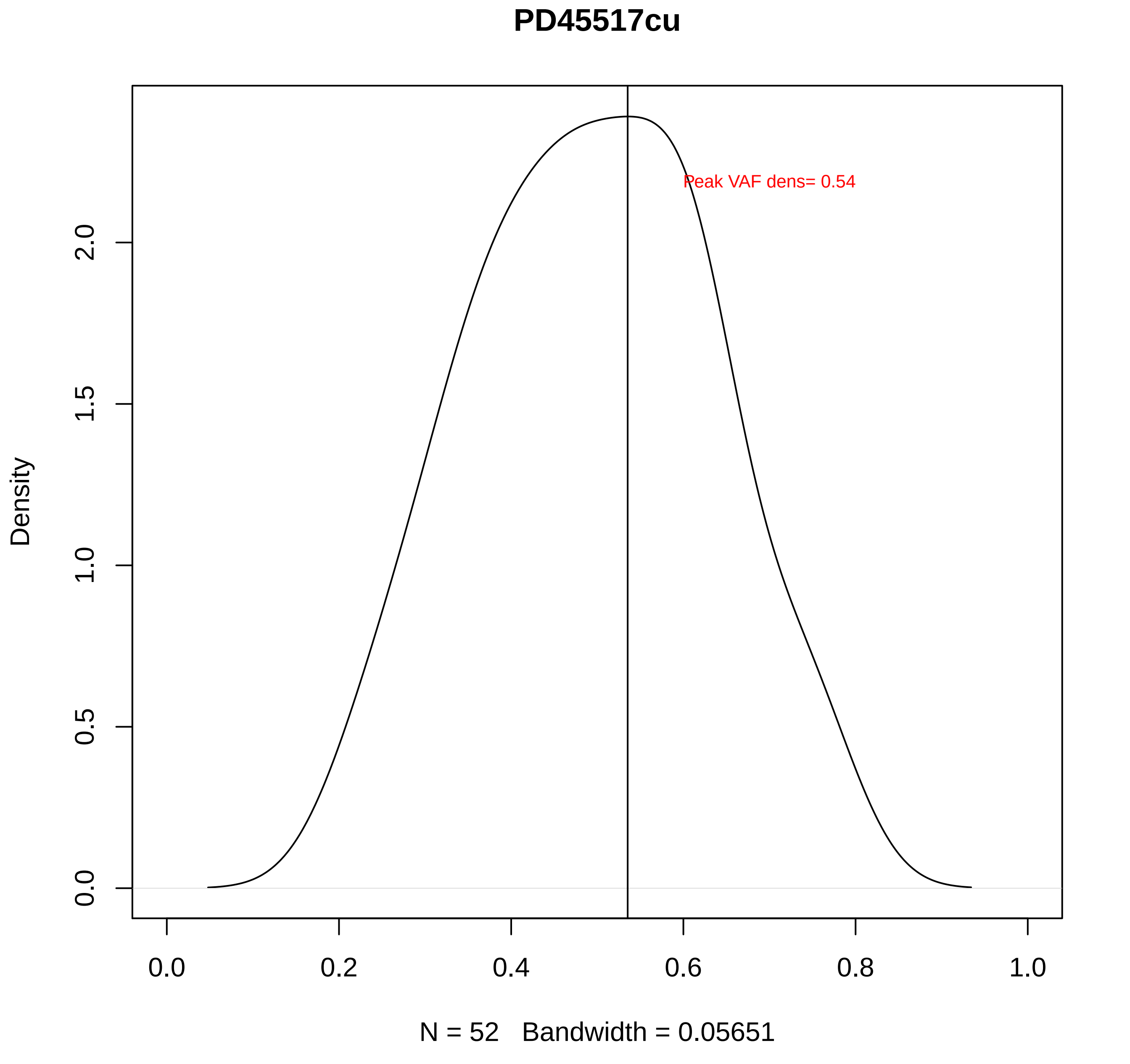
PD45517cu
Peak VAF dens= 0.54
Density
0.0
0.5
1.0
1.5
2.0
0.0
0.2
0.4
0.6
0.8
1.0
N = 52   Bandwidth = 0.05651

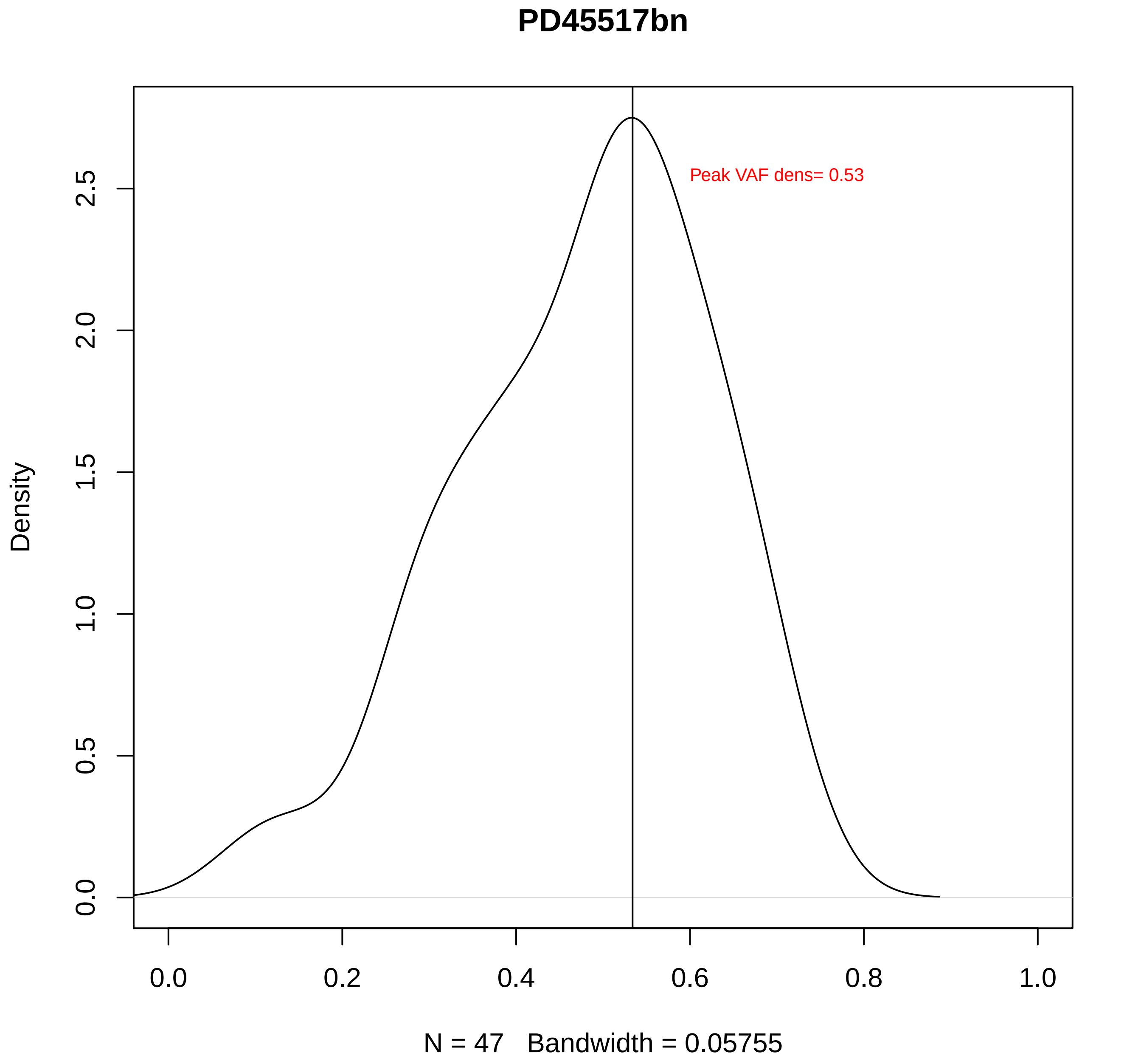

PD45517bn
Peak VAF dens= 0.53
Density
0.0
0.5
1.0
1.5
2.0
2.5
0.0
0.2
0.4
0.6
0.8
1.0
N = 47   Bandwidth = 0.05755

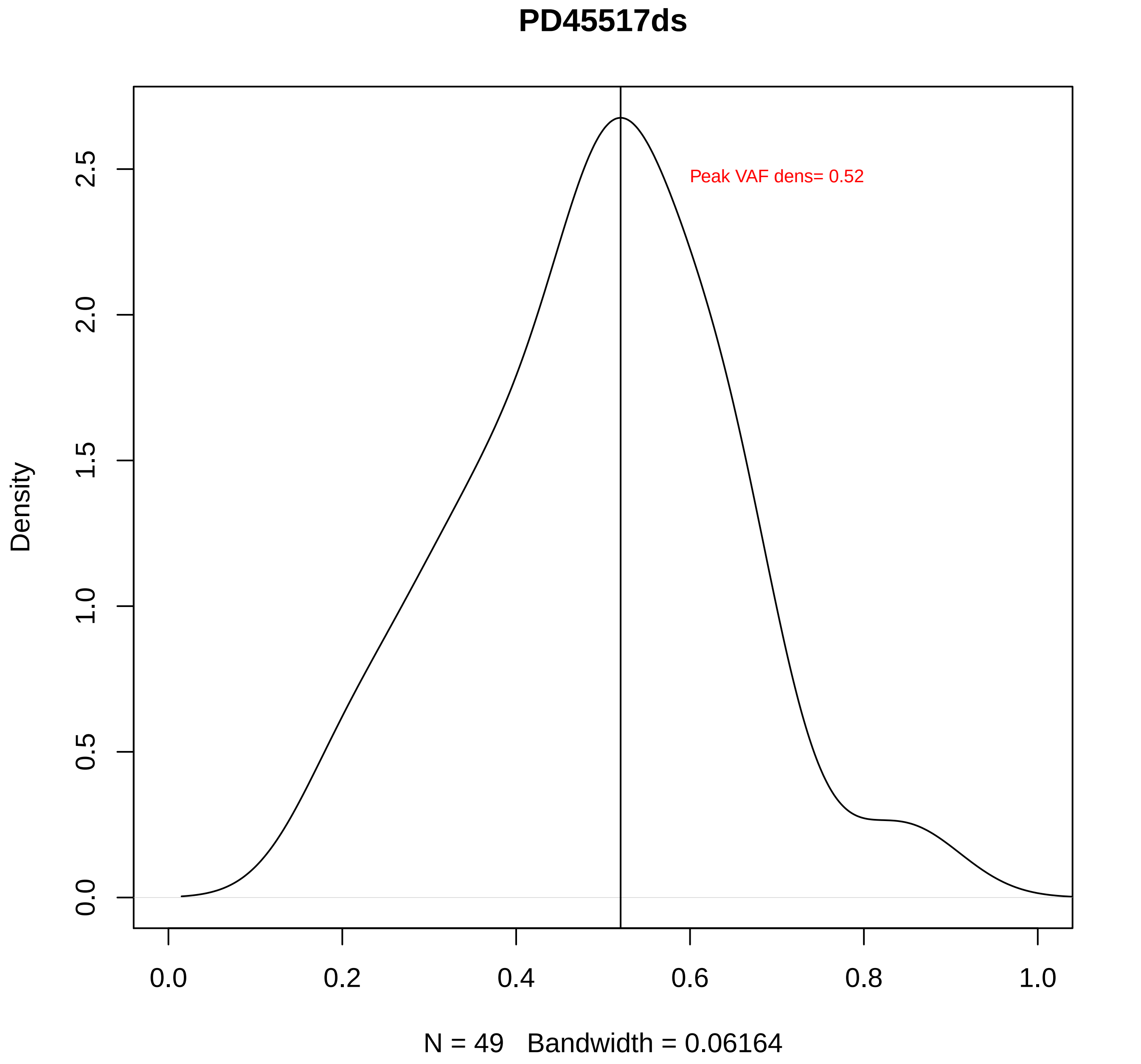
PD45517ds
Peak VAF dens= 0.52
Density
0.0
0.5
1.0
1.5
2.0
2.5
0.0
0.2
0.4
0.6
0.8
1.0
N = 49   Bandwidth = 0.06164

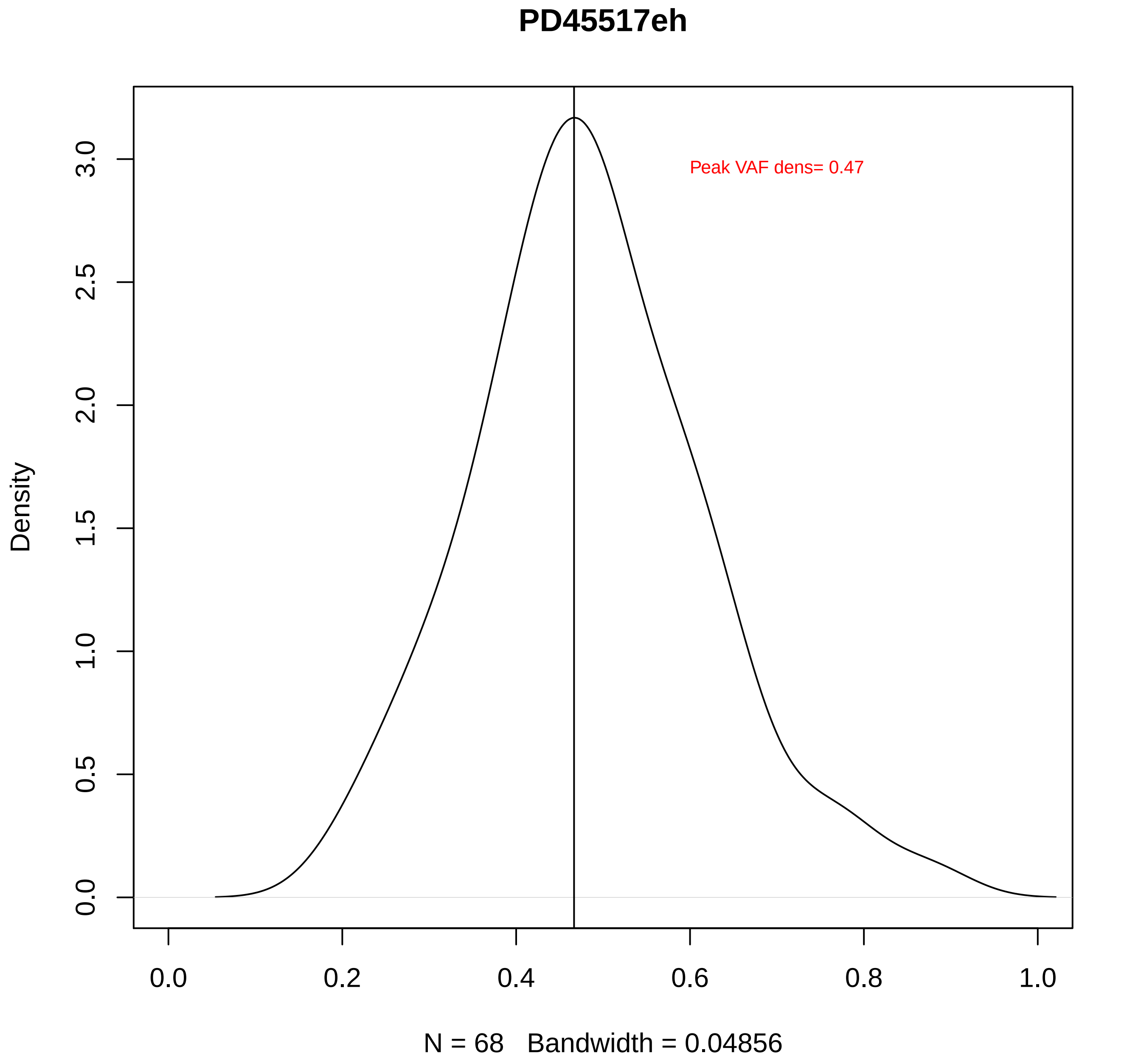
PD45517eh
Peak VAF dens= 0.47
Density
0.0
0.5
1.0
1.5
2.0
2.5
3.0
0.0
0.2
0.4
0.6
0.8
1.0
N = 68 Bandwidth = 0.04856

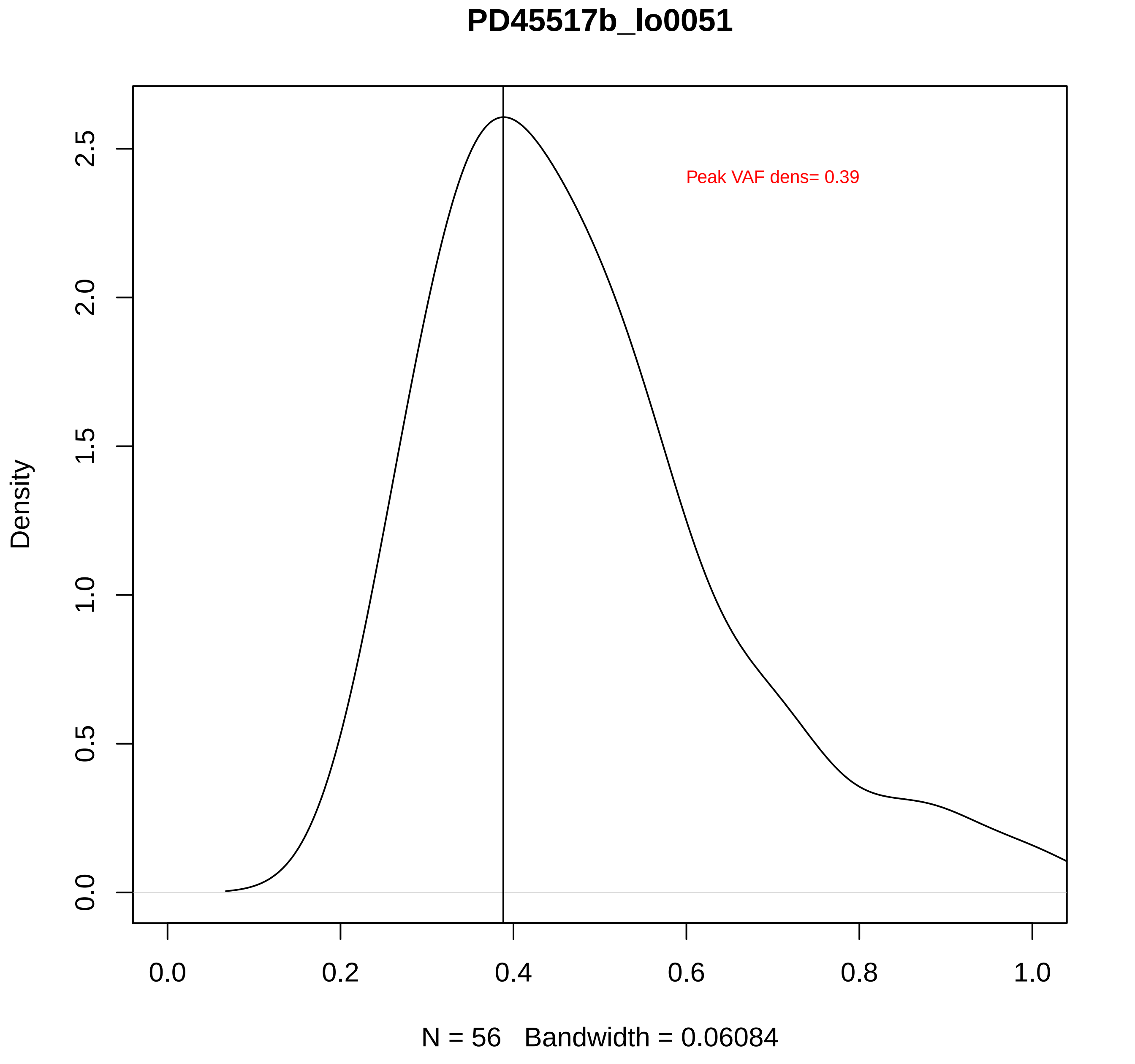
PD45517b_lo0051
Peak VAF dens= 0.39
Density
0.0
0.5
1.0
1.5
2.0
2.5
0.0
0.2
0.4
0.6
0.8
1.0
N = 56 Bandwidth = 0.06084

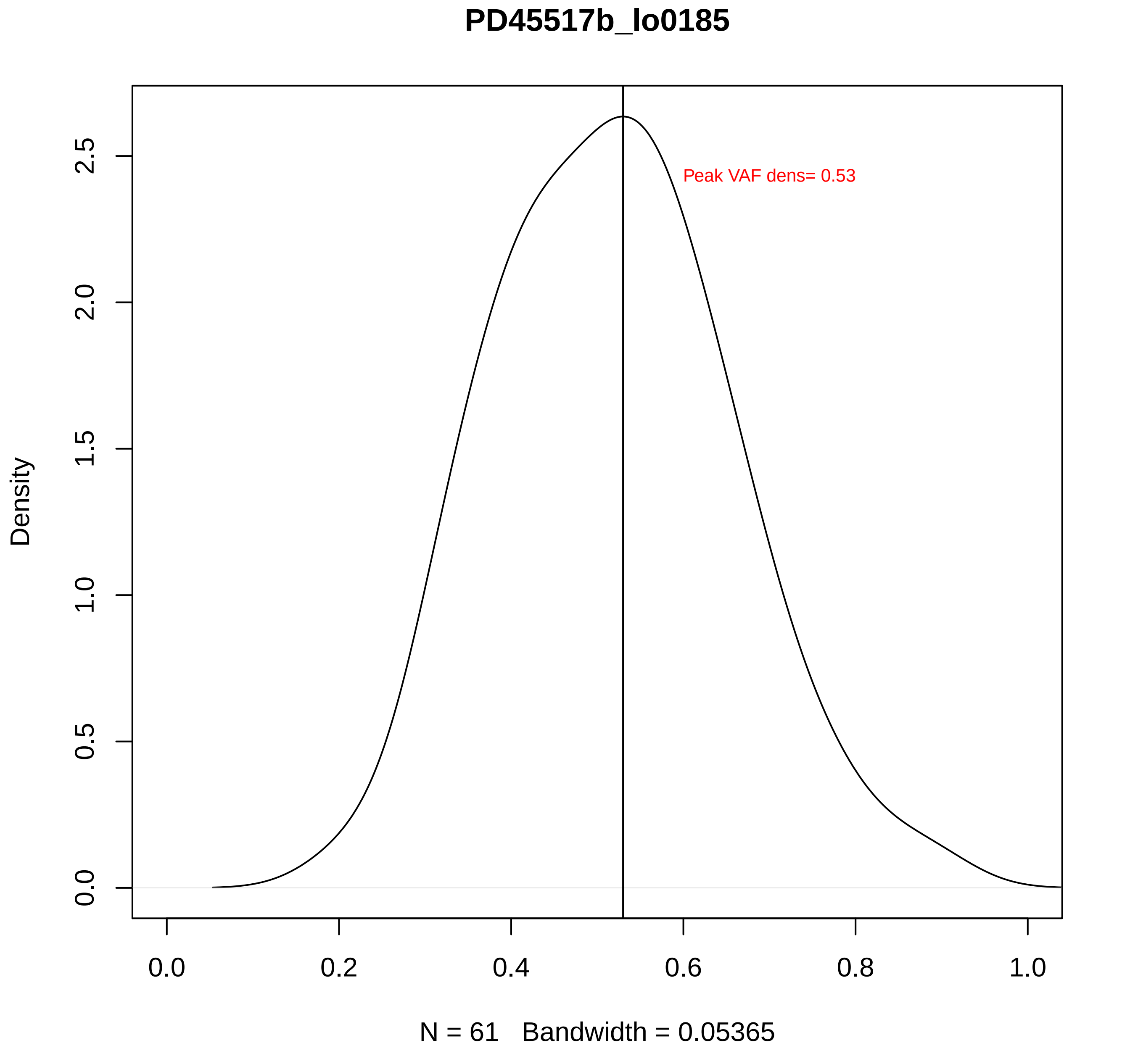

PD45517b_lo0185
Peak VAF dens= 0.53
Density
0.0
0.5
1.0
1.5
2.0
2.5
0.0
0.2
0.4
0.6
0.8
1.0
N = 61 Bandwidth = 0.05365

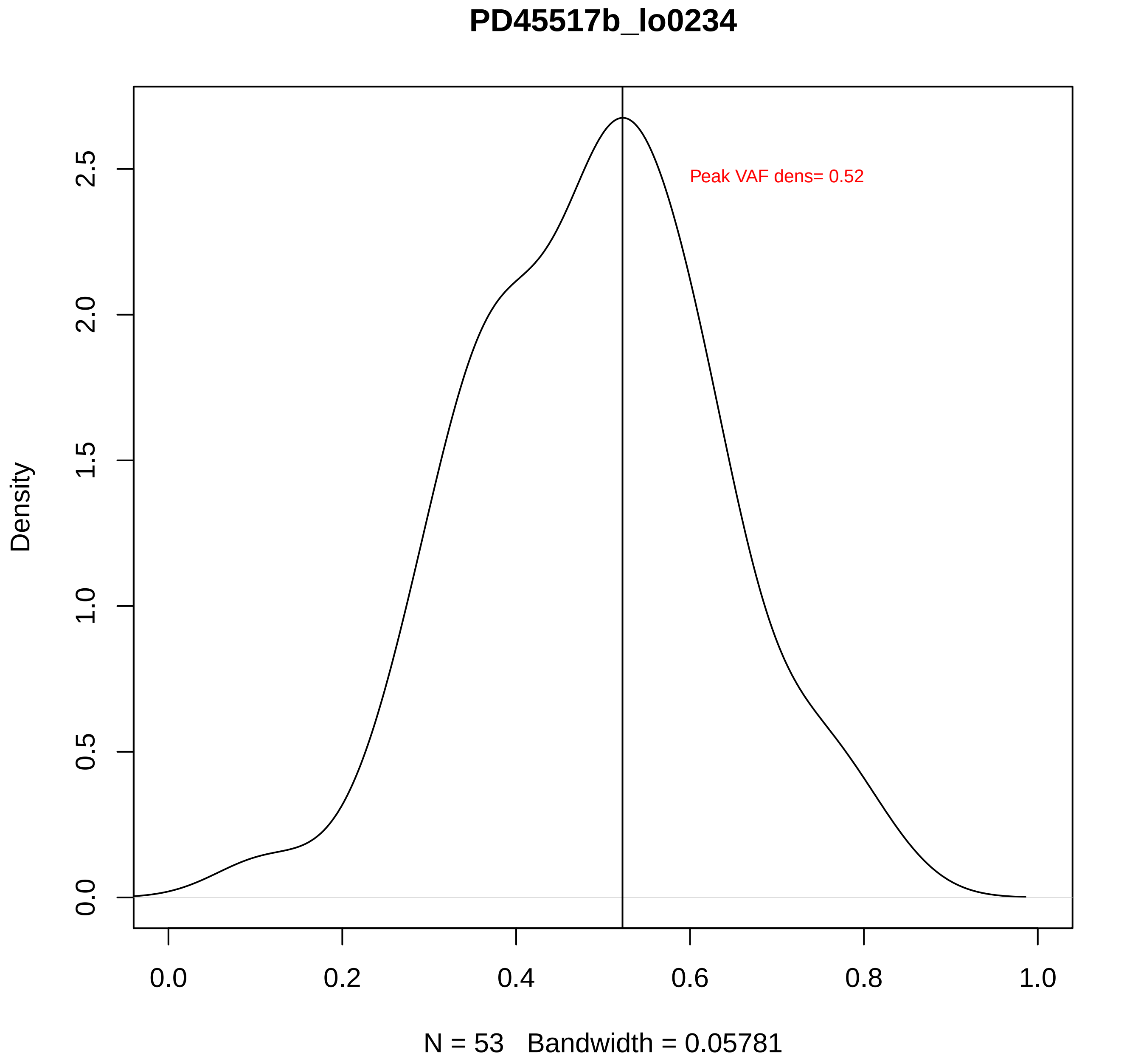

PD45517b_lo0234
Peak VAF dens= 0.52
Density
0.0
0.5
1.0
1.5
2.0
2.5
0.0
0.2
0.4
0.6
0.8
1.0
N = 53 Bandwidth = 0.05781

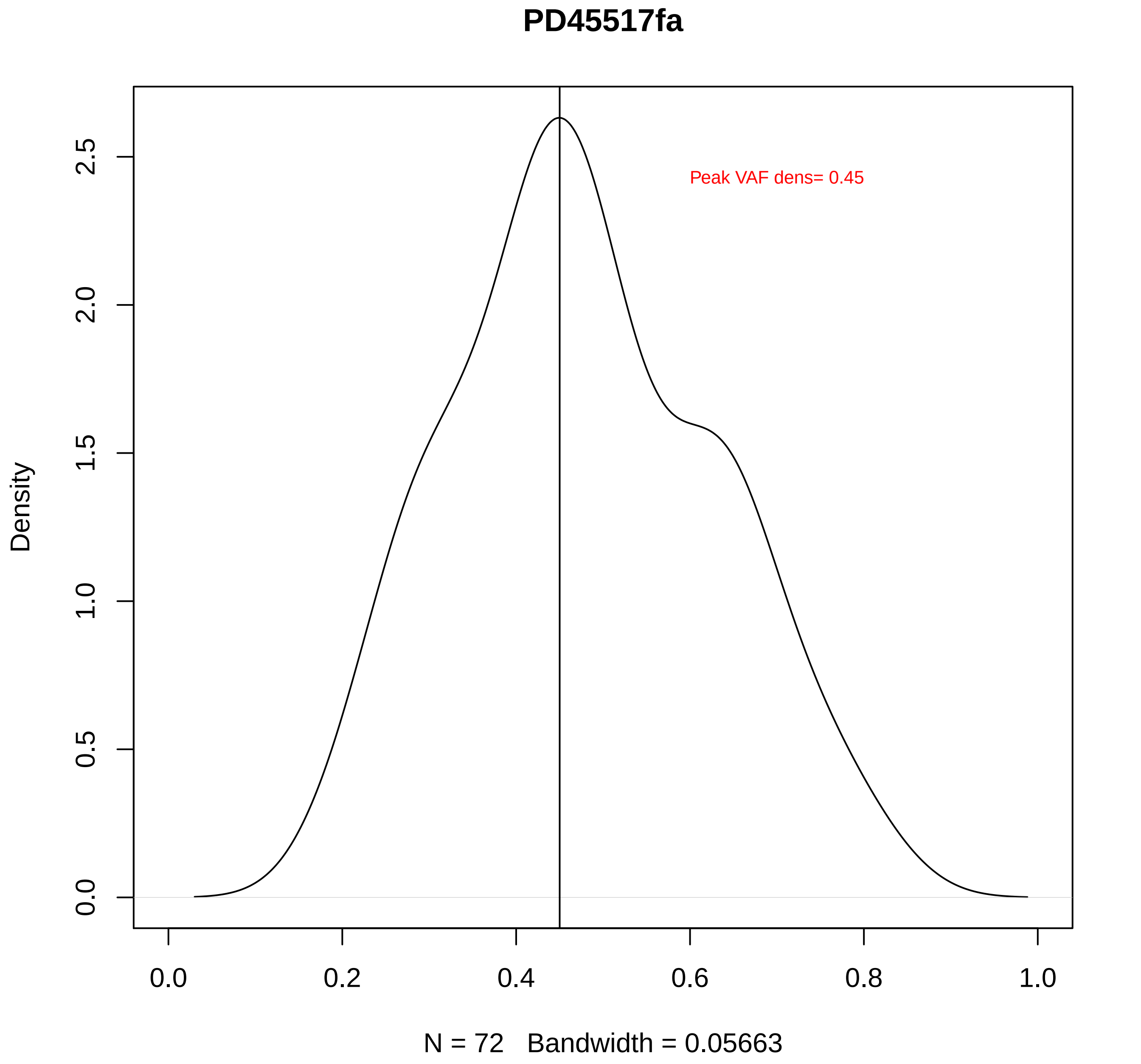
PD45517fa
Peak VAF dens= 0.45
Density
0.0
0.5
1.0
1.5
2.0
2.5
0.0
0.2
0.4
0.6
0.8
1.0
N = 72   Bandwidth = 0.05663

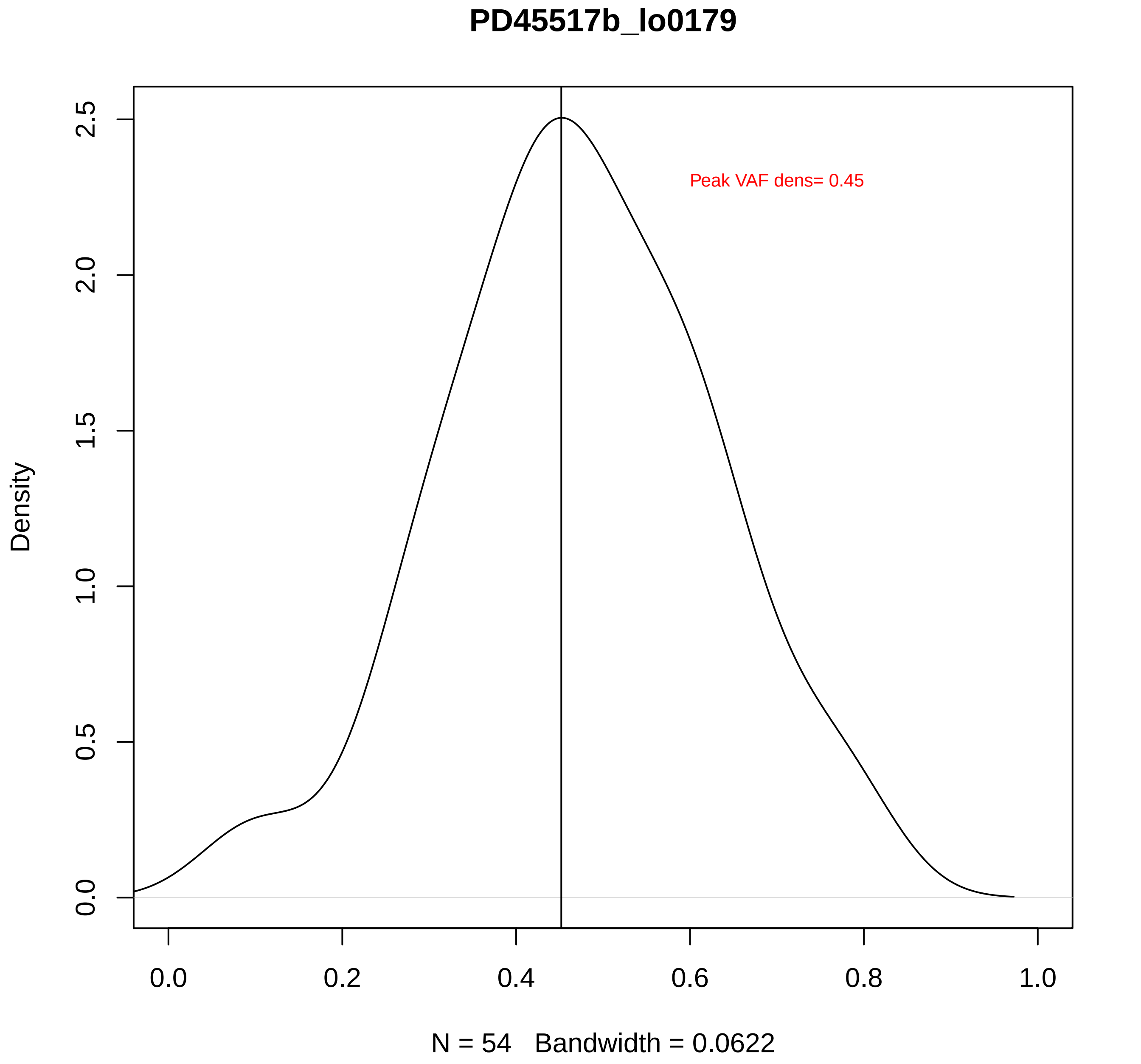
PD45517b_lo0179
Peak VAF dens= 0.45
Density
0.0
0.5
1.0
1.5
2.0
2.5
0.0
0.2
0.4
0.6
0.8
1.0
N = 54 Bandwidth = 0.0622

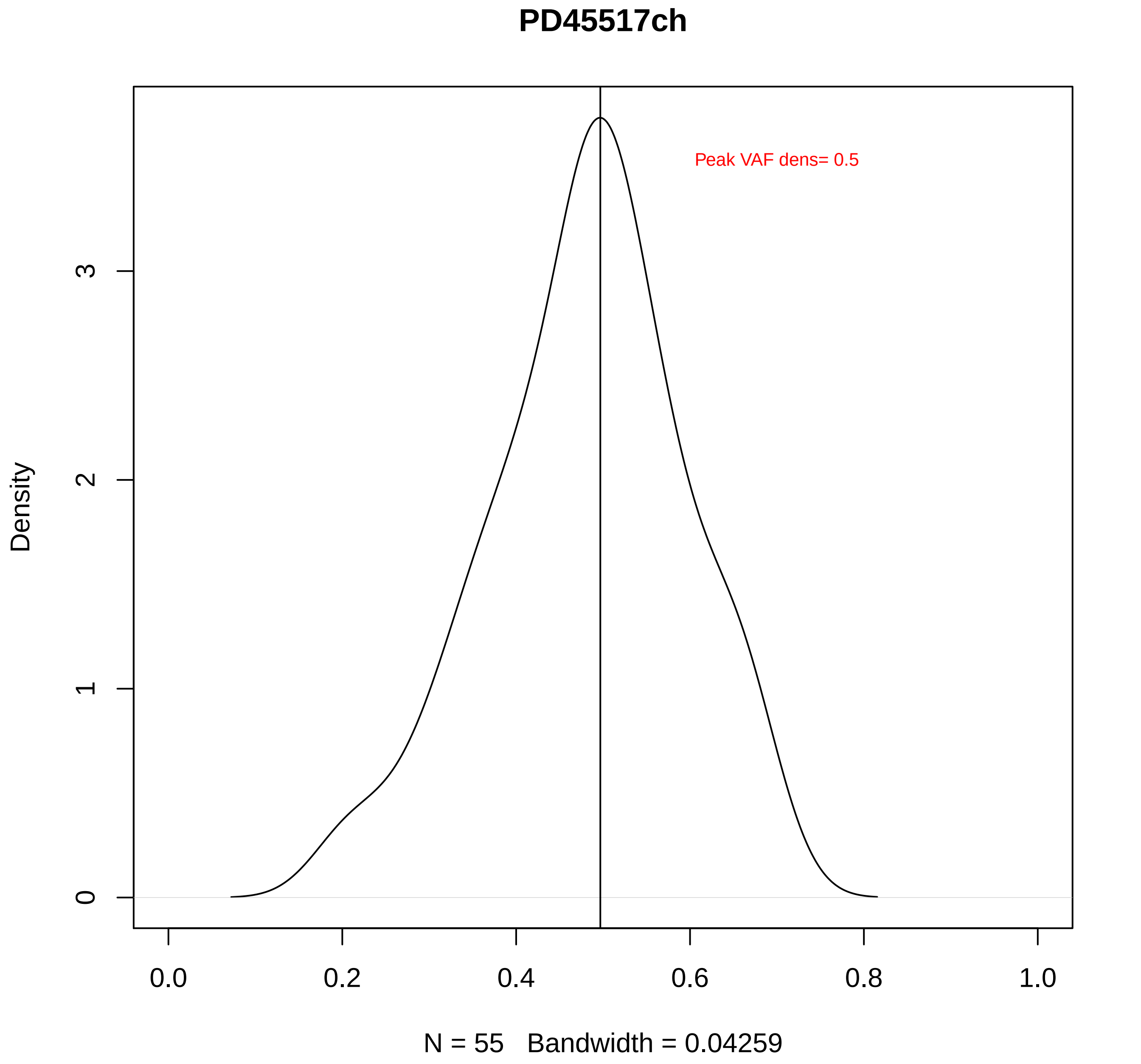
PD45517ch
Peak VAF dens= 0.5
Density
0
1
2
3
0.0
0.2
0.4
0.6
0.8
1.0
N = 55   Bandwidth = 0.04259

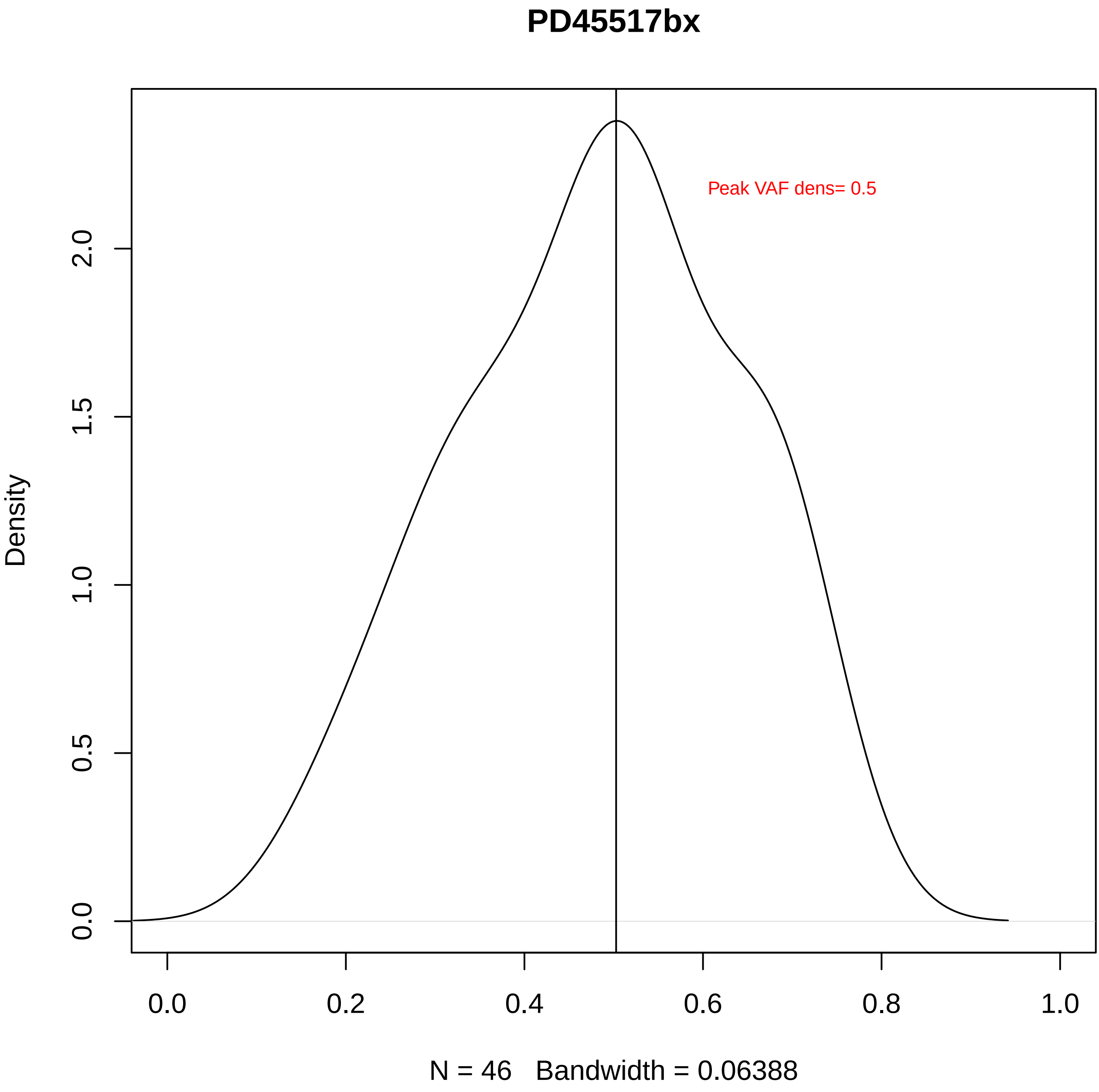
PD45517bx
Peak VAF dens= 0.5
Density
0.0
0.5
1.0
1.5
2.0
0.0
0.2
0.4
0.6
0.8
1.0
N = 46 Bandwidth = 0.06388

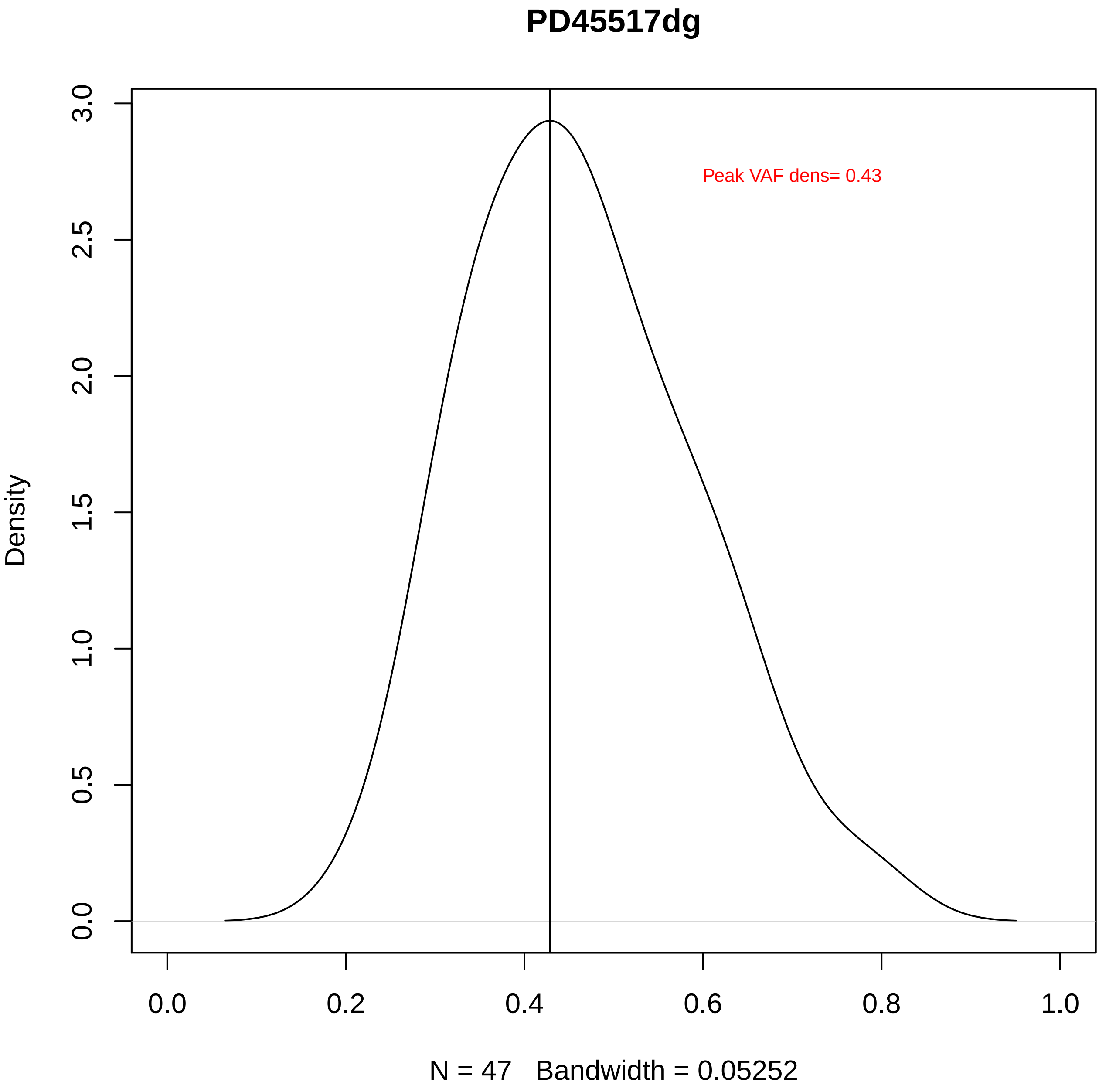

PD45517dg
Peak VAF dens= 0.43
Density
3.0
2.5
2.0
1.5
1.0
0.5
0.0
0.0
0.2
0.4
0.6
0.8
1.0
N = 47 Bandwidth = 0.05252

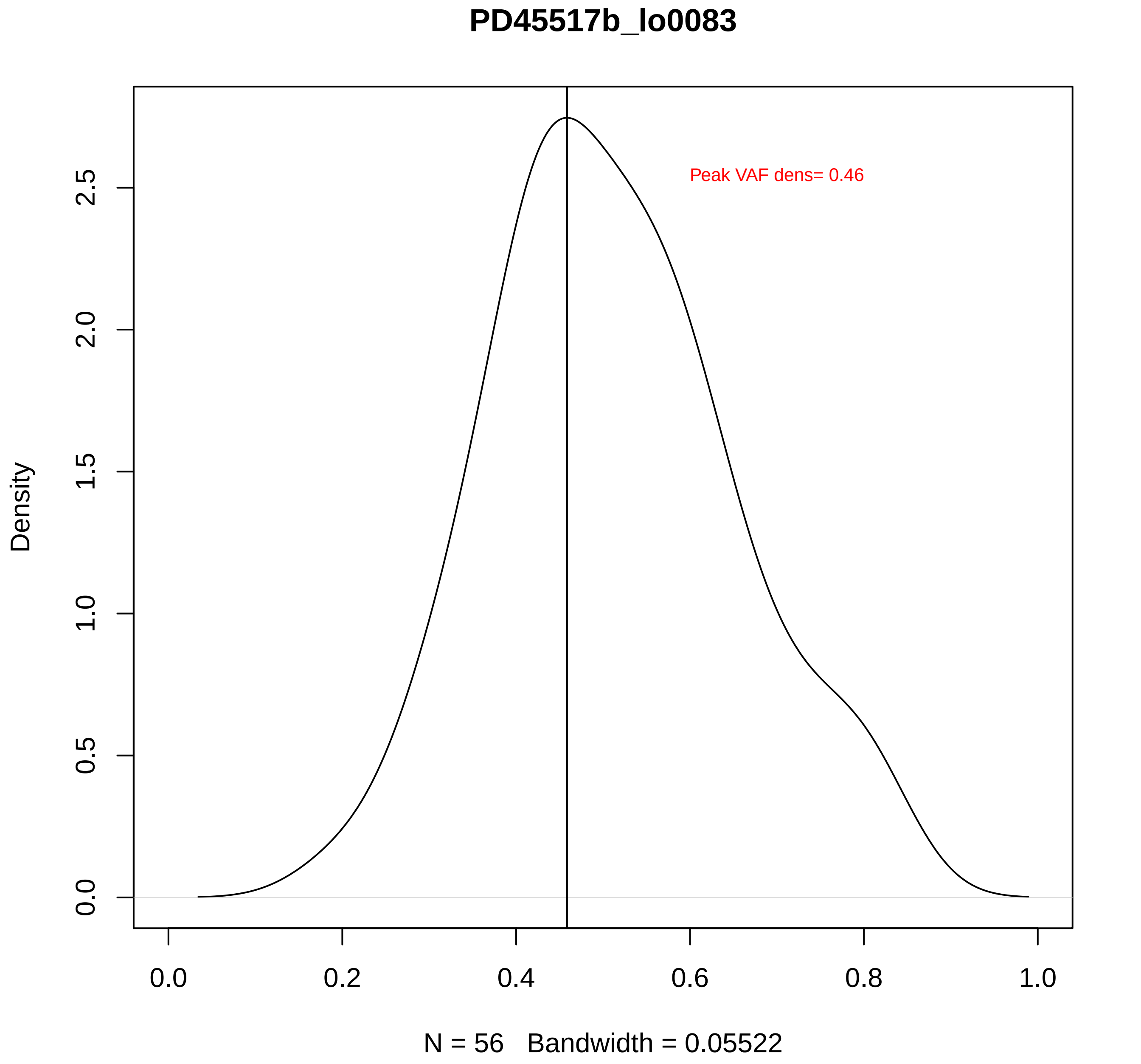

PD45517b_lo0083
Peak VAF dens= 0.46
Density
0.0
0.5
1.0
1.5
2.0
2.5
0.0
0.2
0.4
0.6
0.8
1.0
N = 56   Bandwidth = 0.05522

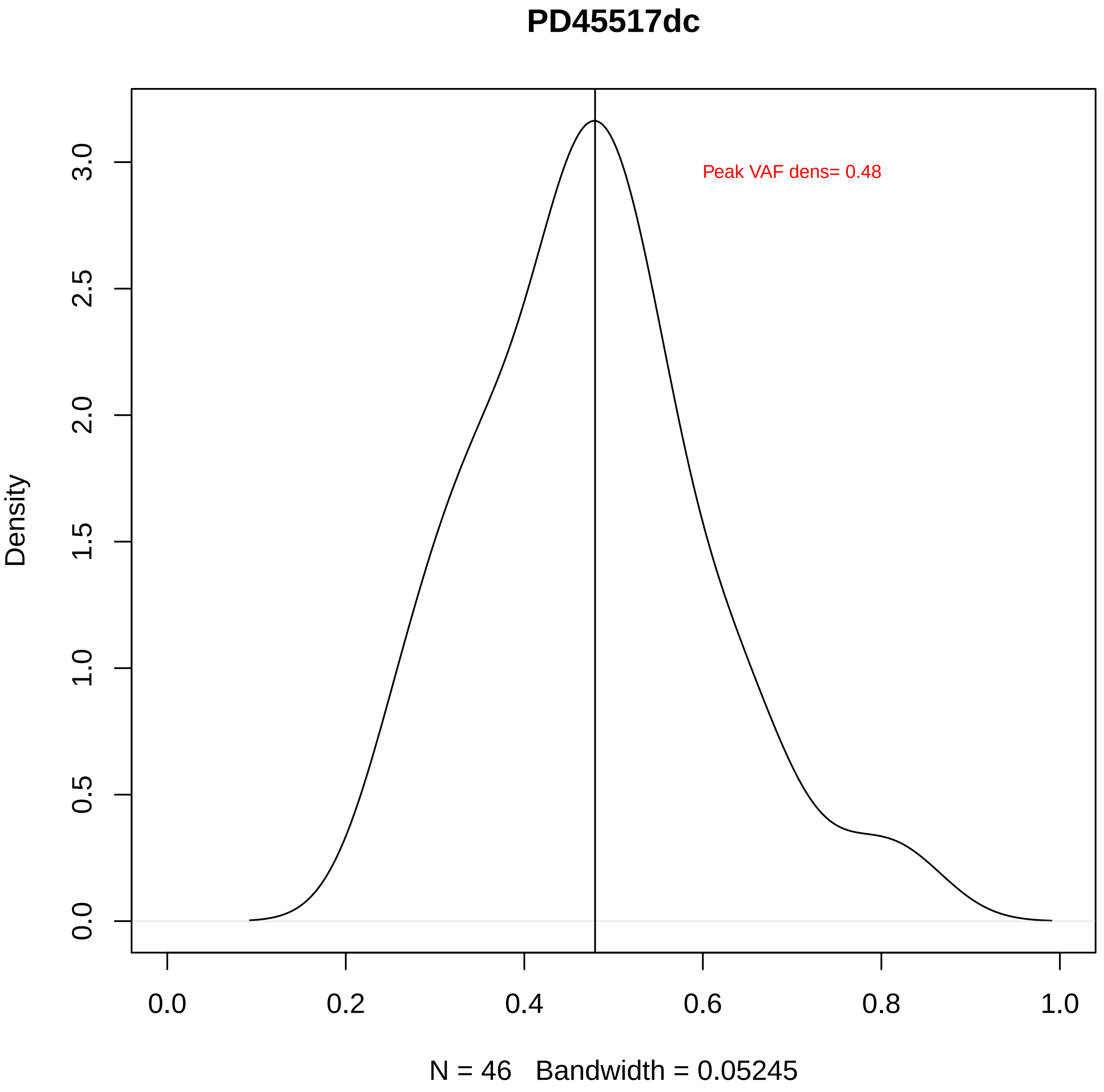
PD45517dc
Peak VAF dens= 0.48
Density
0.0
0.5
1.0
1.5
2.0
2.5
3.0
0.0
0.2
0.4
0.6
0.8
1.0
N = 46   Bandwidth = 0.05245

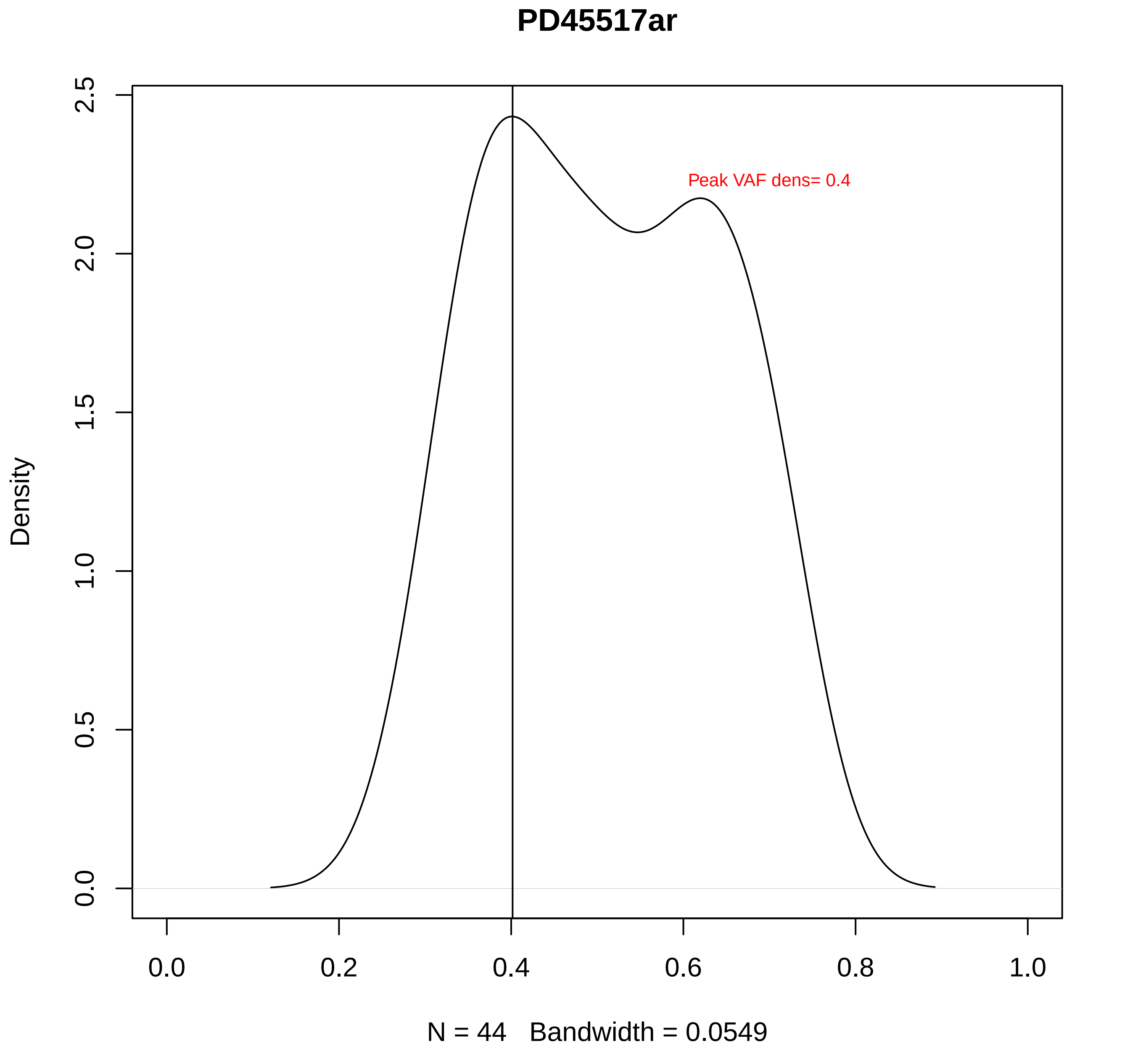
PD45517ar
Peak VAF dens= 0.4
Density
0.0
0.5
1.0
1.5
2.0
2.5
0.0
0.2
0.4
0.6
0.8
1.0
N = 44   Bandwidth = 0.0549

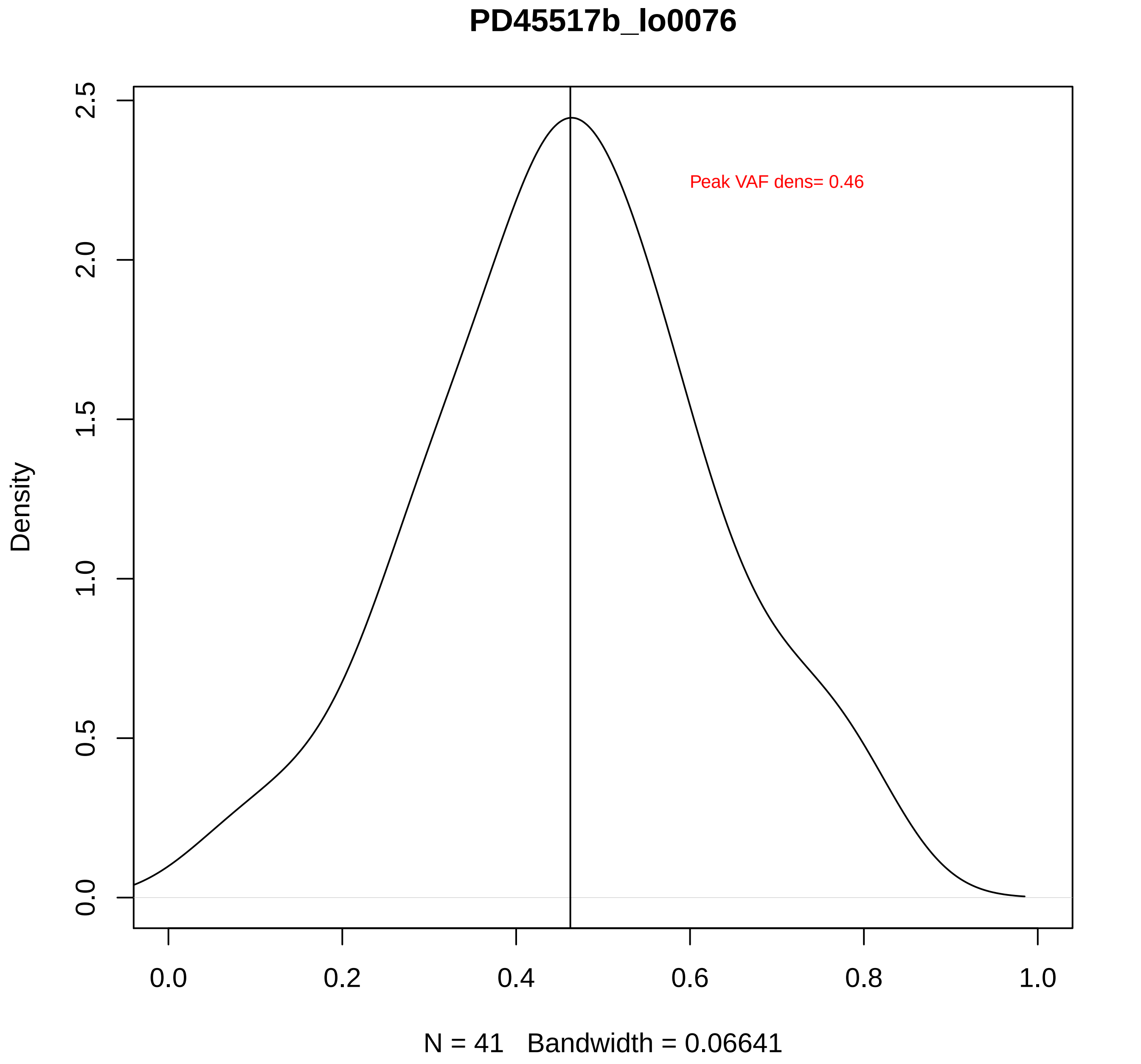
PD45517b_lo0076
Peak VAF dens= 0.46
Density
0.0
0.5
1.0
1.5
2.0
2.5
0.0
0.2
0.4
0.6
0.8
1.0
N = 41   Bandwidth = 0.06641

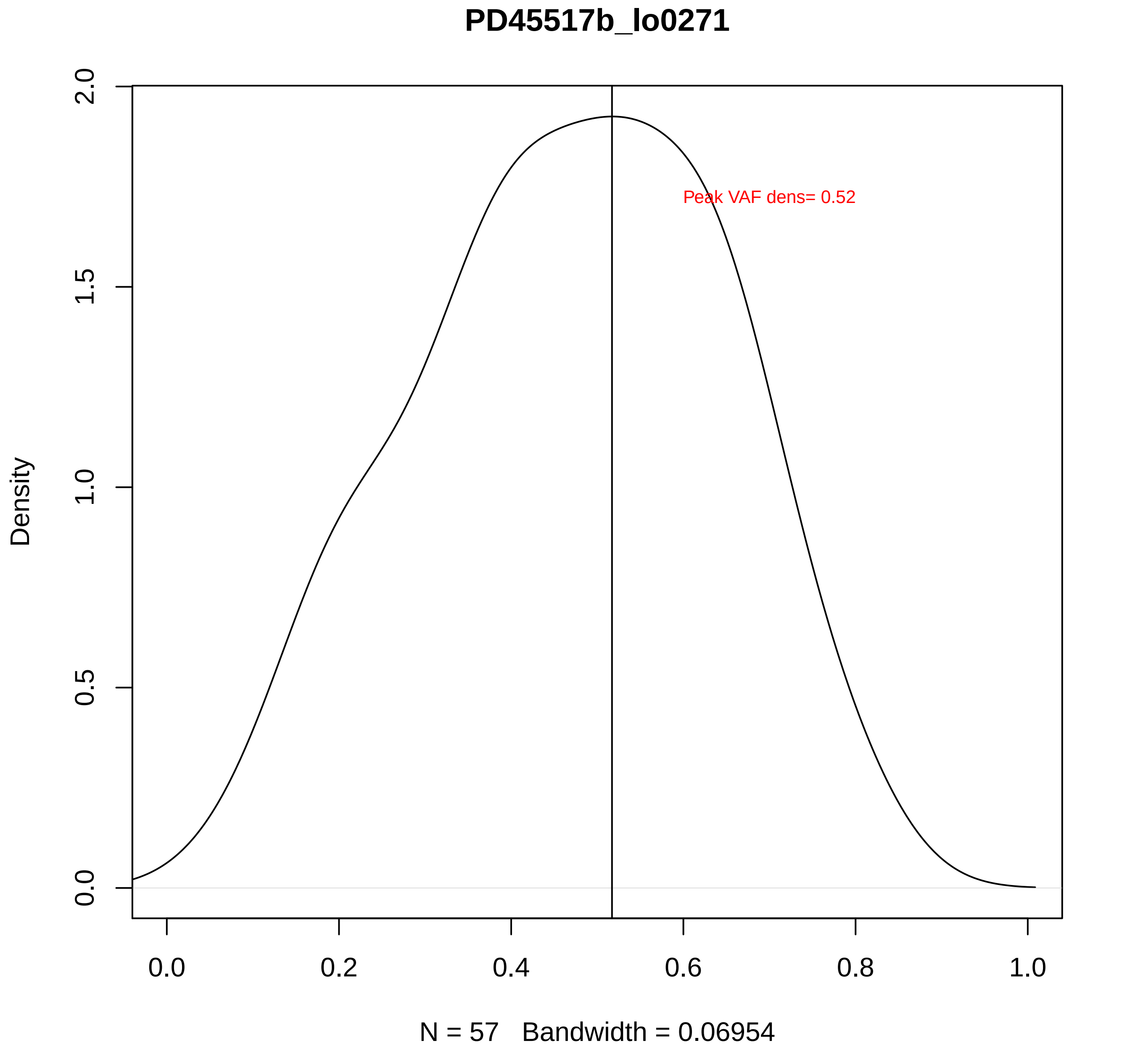
PD45517b_lo0271
Density
2.0
1.5
1.0
0.5
0.0
0.0
0.2
0.4
0.6
0.8
1.0
Peak VAF dens= 0.52
N = 57 Bandwidth = 0.06954

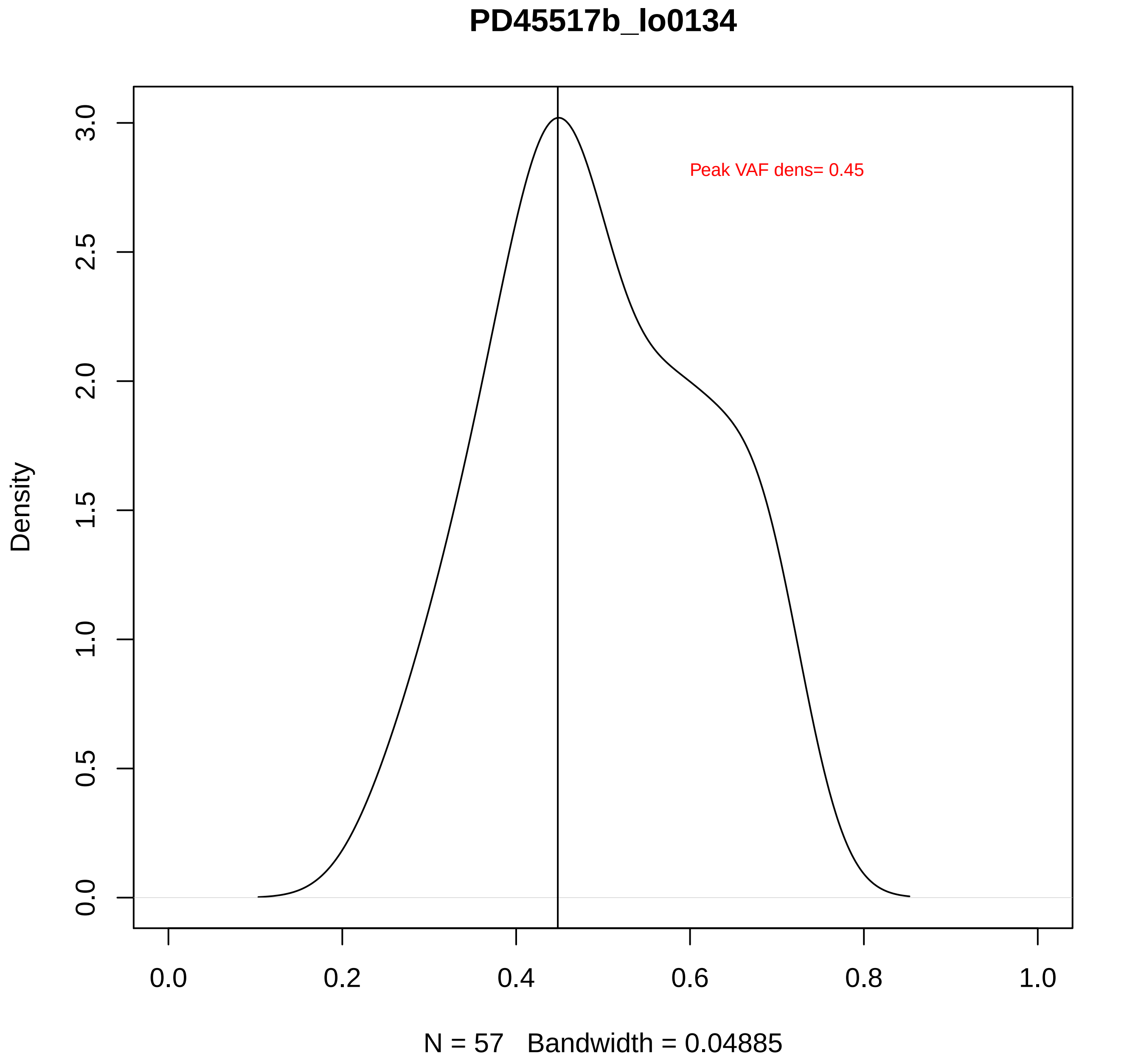
PD45517b_lo0134
Peak VAF dens= 0.45
Density
0.0
0.5
1.0
1.5
2.0
2.5
3.0
0.0
0.2
0.4
0.6
0.8
1.0
N = 57 Bandwidth = 0.04885

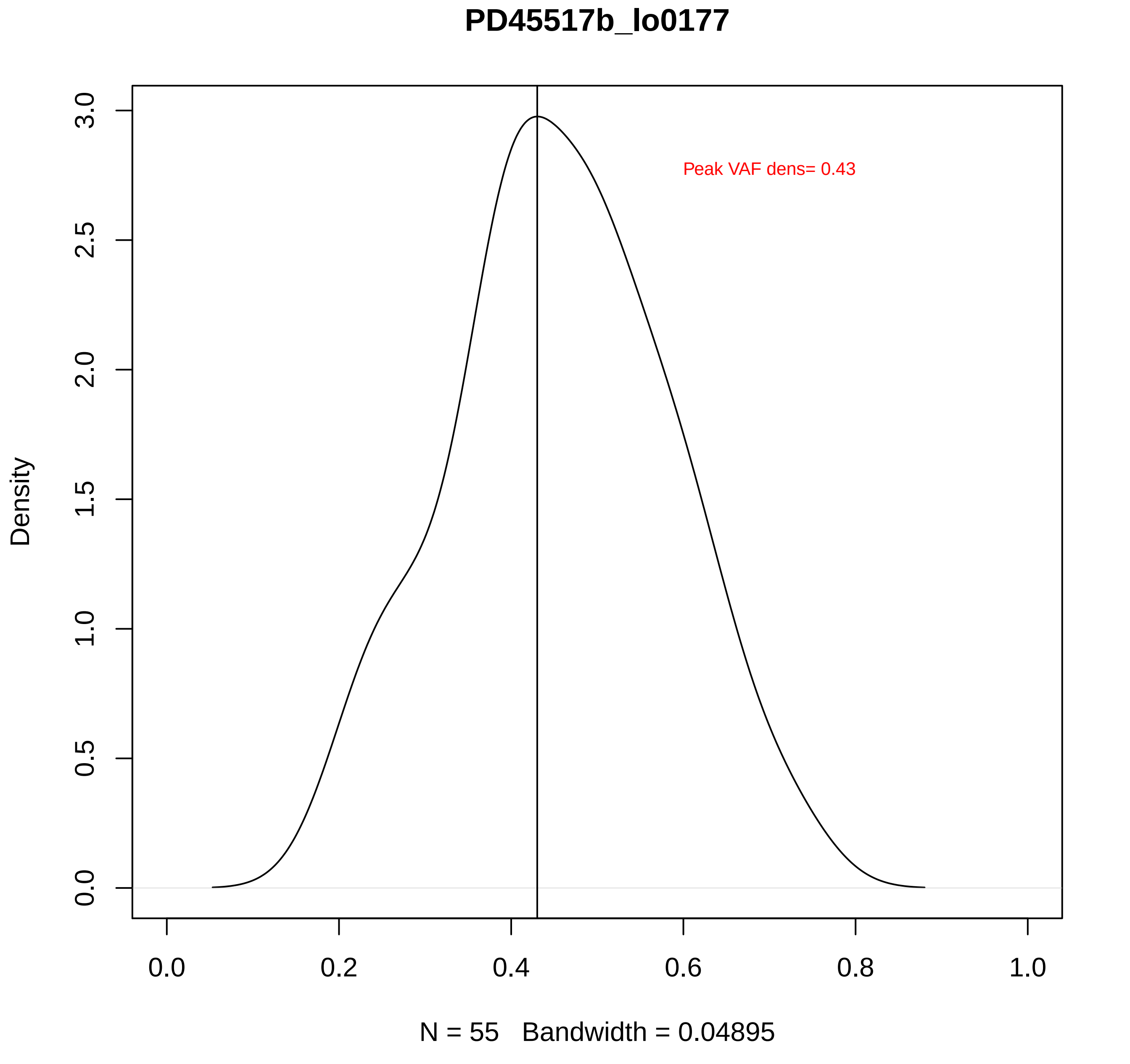
PD45517b_lo0177
Peak VAF dens= 0.43
Density
3.0
2.5
2.0
1.5
1.0
0.5
0.0
0.0
0.2
0.4
0.6
0.8
1.0
N = 55   Bandwidth = 0.04895

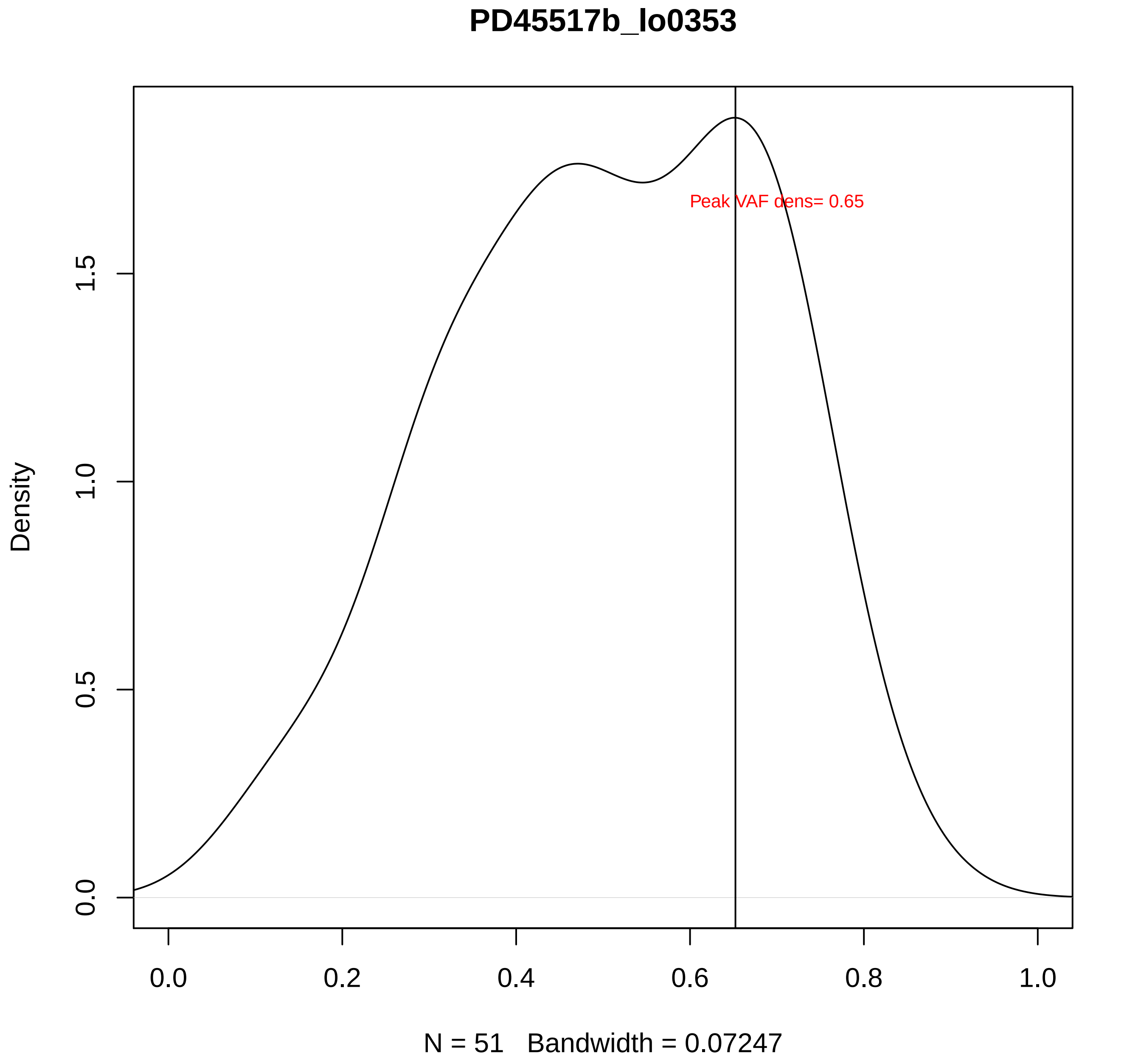
PD45517b_lo0353
Peak VAF dens= 0.65
Density
0.0
0.5
1.0
1.5
0.0
0.2
0.4
0.6
0.8
1.0
N = 51 Bandwidth = 0.07247

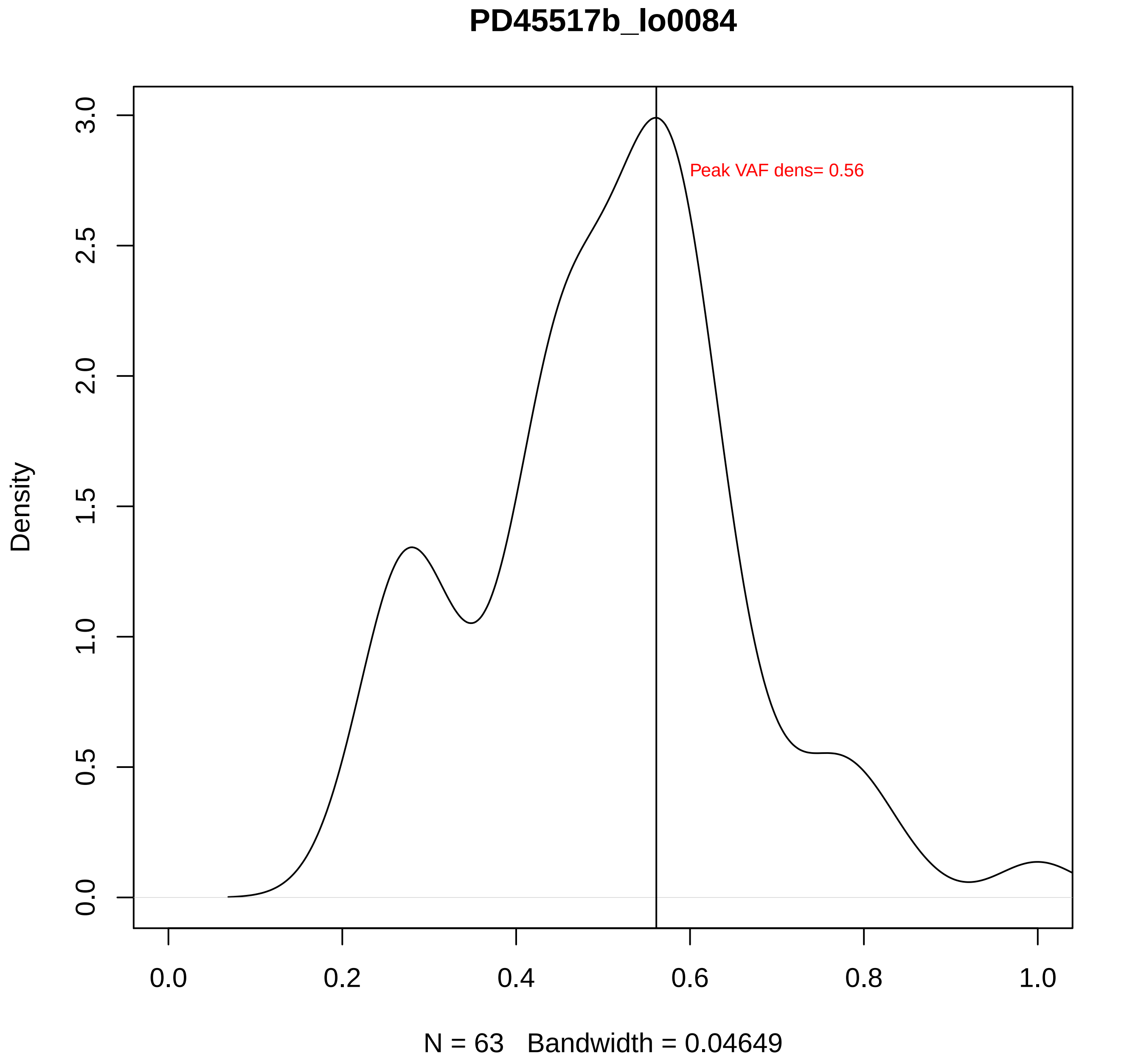

PD45517b_lo0084
Peak VAF dens= 0.56
Density
0.0
0.5
1.0
1.5
2.0
2.5
3.0
0.0
0.2
0.4
0.6
0.8
1.0
N = 63 Bandwidth = 0.04649

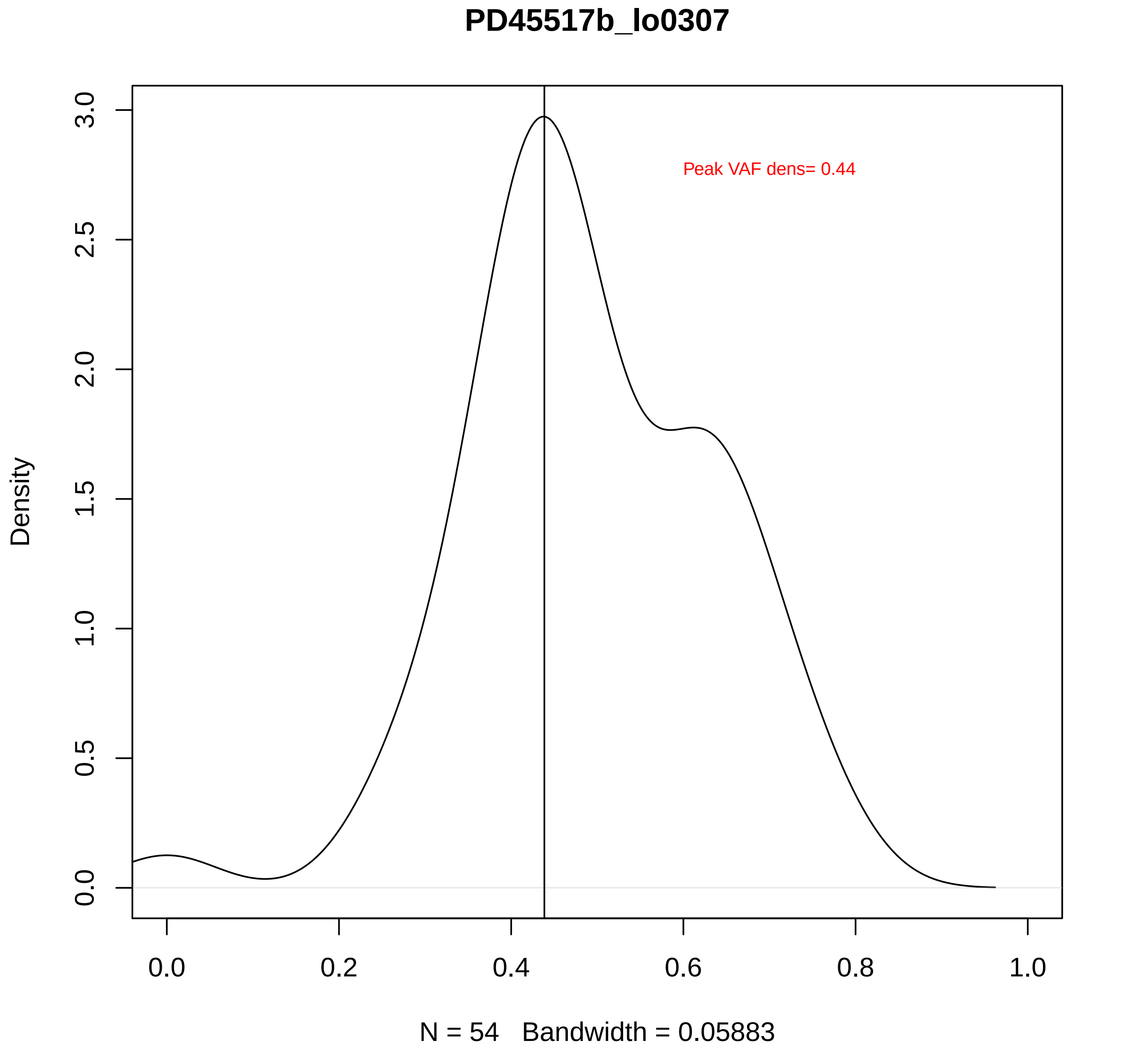

PD45517b_lo0307
Peak VAF dens= 0.44
Density
3.0
2.5
2.0
1.5
1.0
0.5
0.0
0.0
0.2
0.4
0.6
0.8
1.0
N = 54   Bandwidth = 0.05883

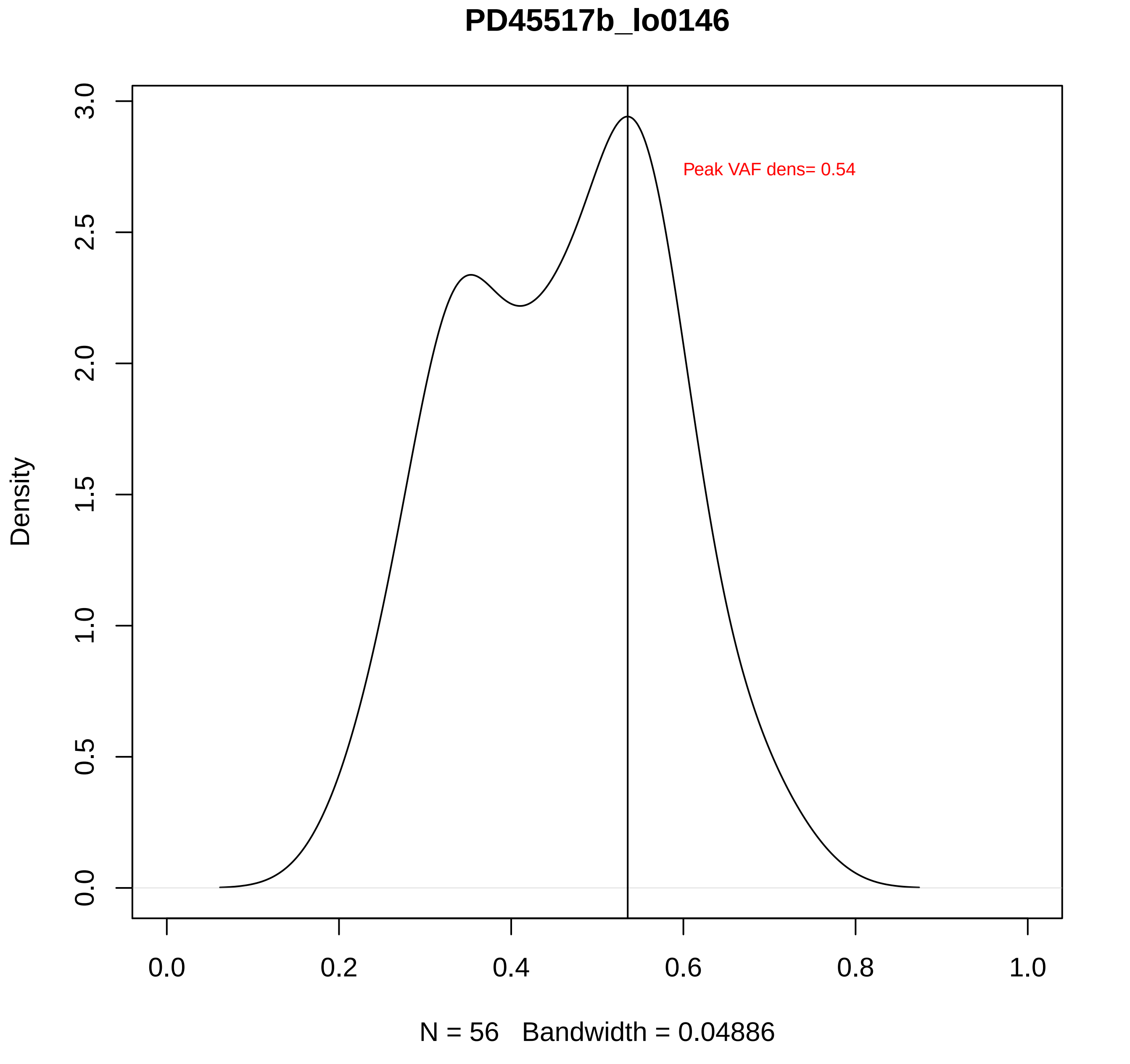
PD45517b_lo0146
Density
3.0
2.5
2.0
1.5
1.0
0.5
0.0
Peak VAF dens= 0.54
0.0
0.2
0.4
0.6
0.8
1.0
N = 56   Bandwidth = 0.04886

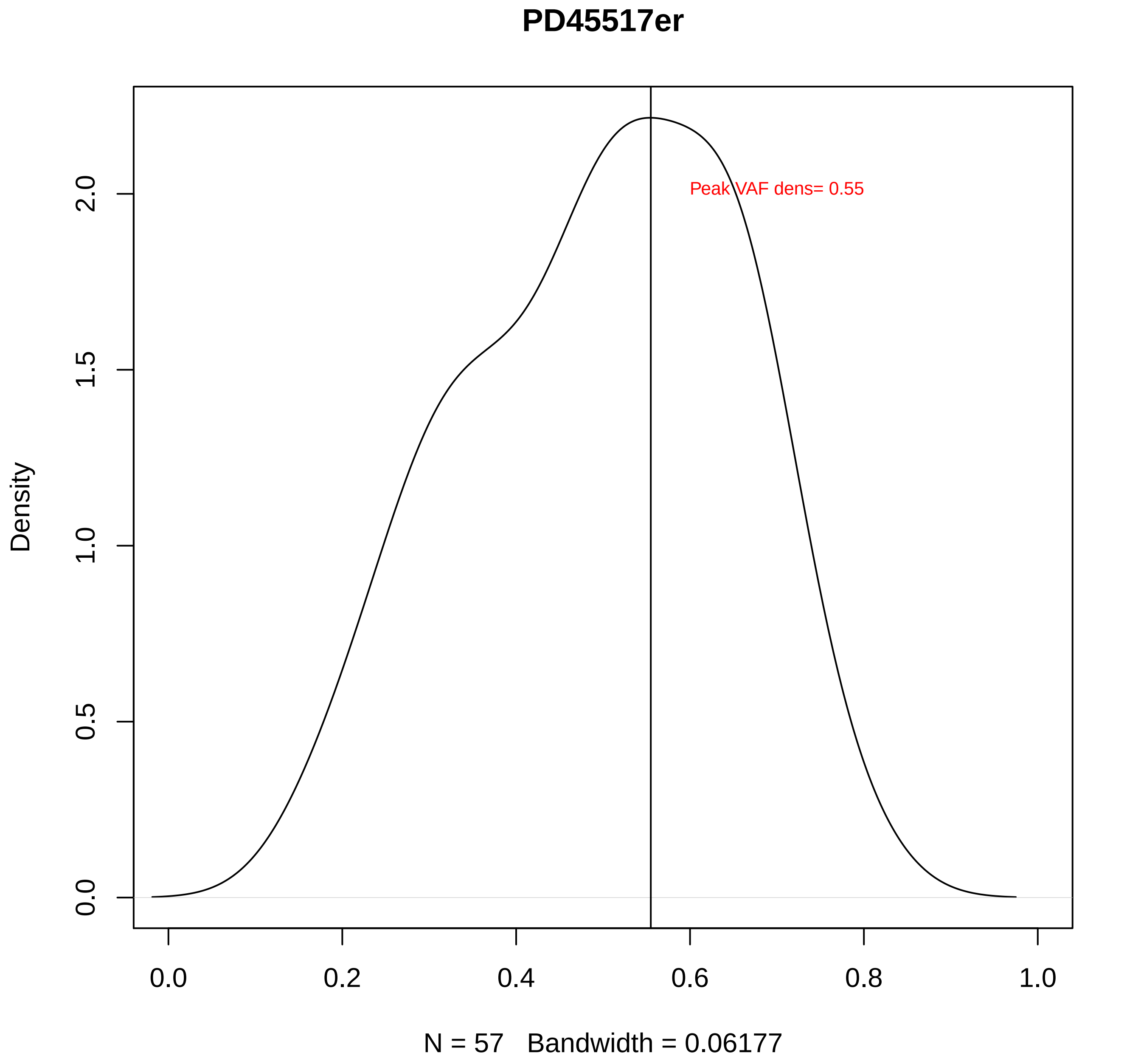
PD45517er
Peak VAF dens= 0.55
Density
0.0
0.5
1.0
1.5
2.0
0.0
0.2
0.4
0.6
0.8
1.0
N = 57   Bandwidth = 0.06177

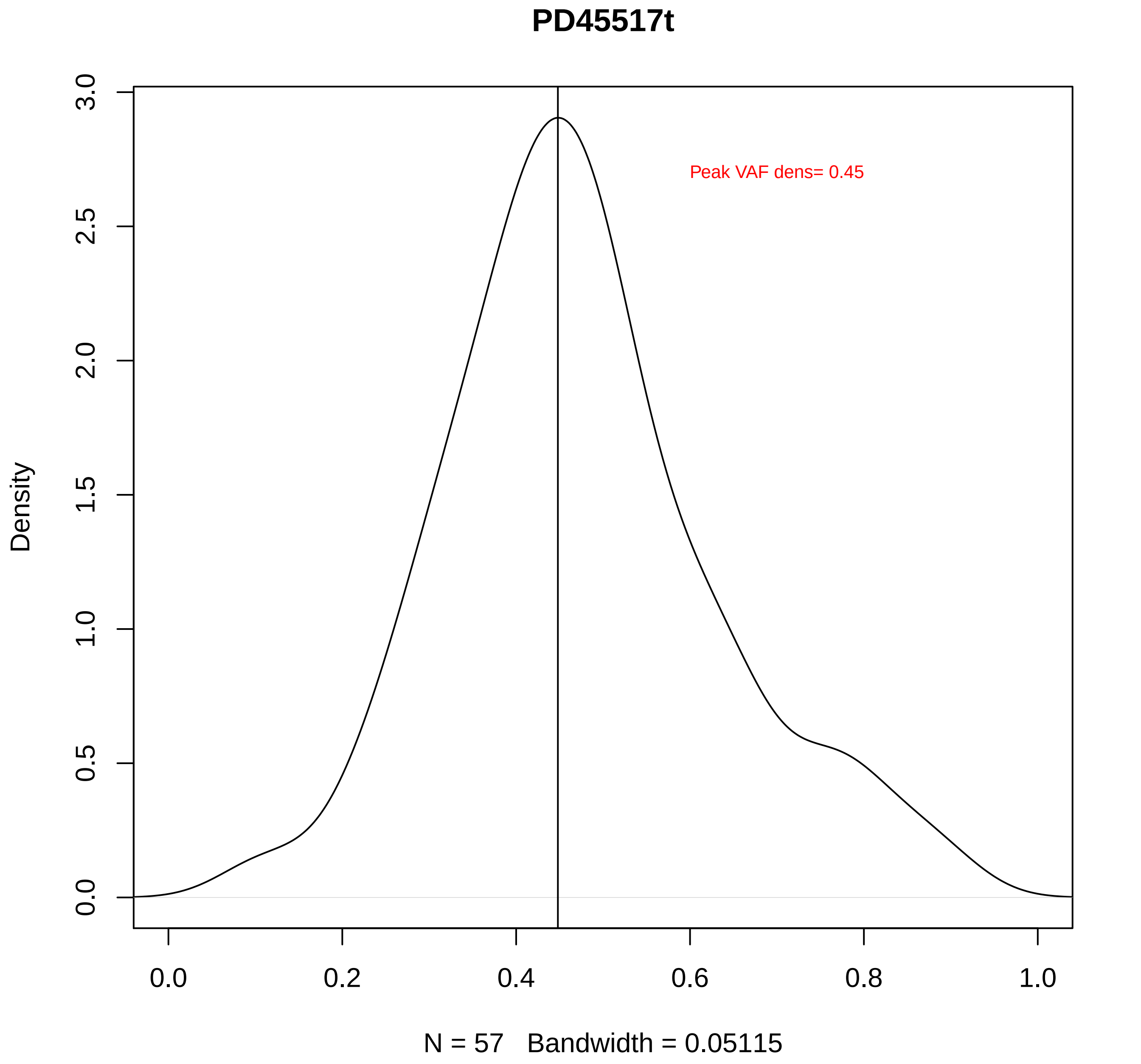
PD45517t
Peak VAF dens= 0.45
Density
0.0
0.5
1.0
1.5
2.0
2.5
3.0
0.0
0.2
0.4
0.6
0.8
1.0
N = 57   Bandwidth = 0.05115

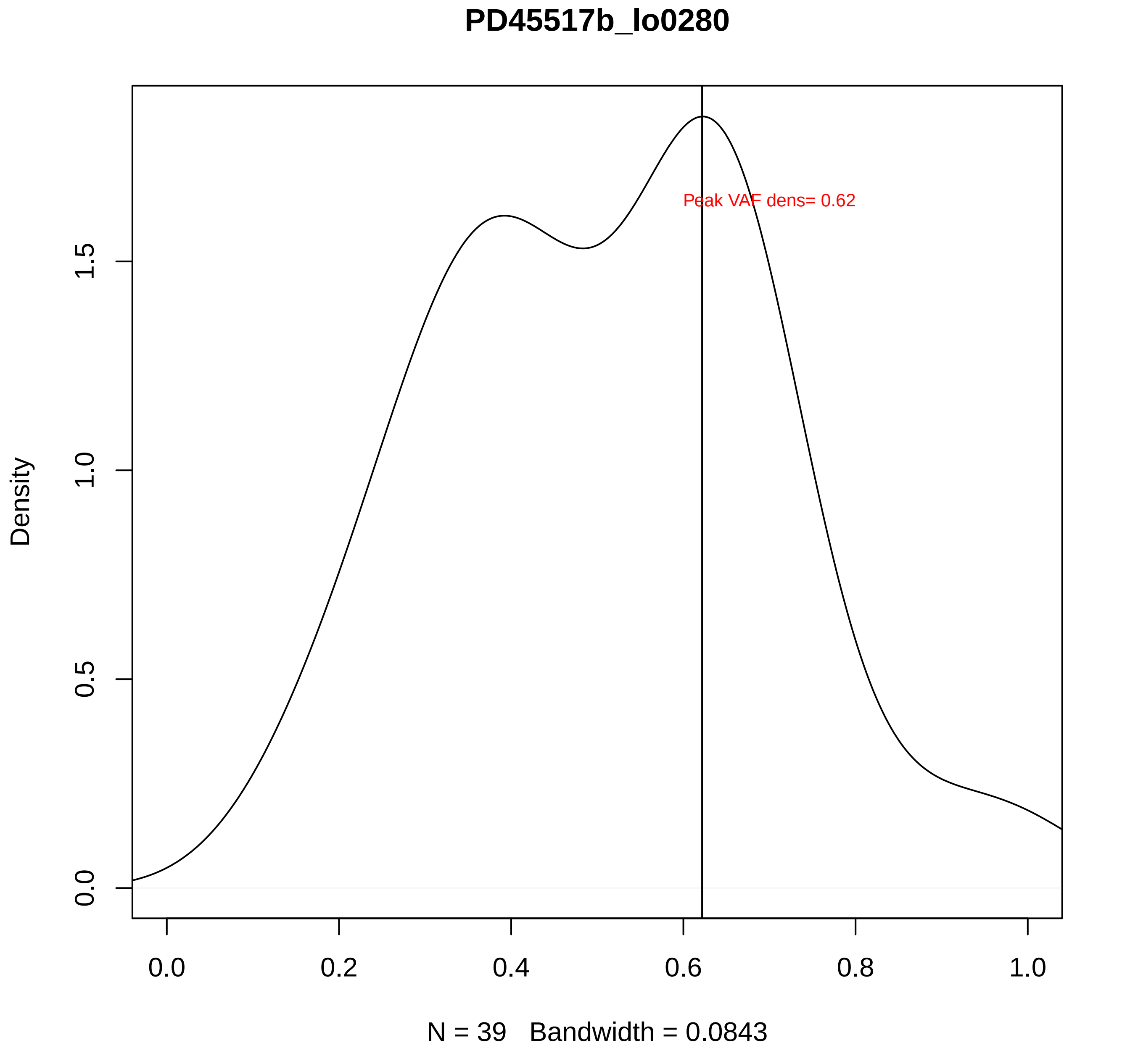

PD45517b_lo0280
Peak VAF dens= 0.62
Density
0.0
0.5
1.0
1.5
0.0
0.2
0.4
0.6
0.8
1.0
N = 39 Bandwidth = 0.0843

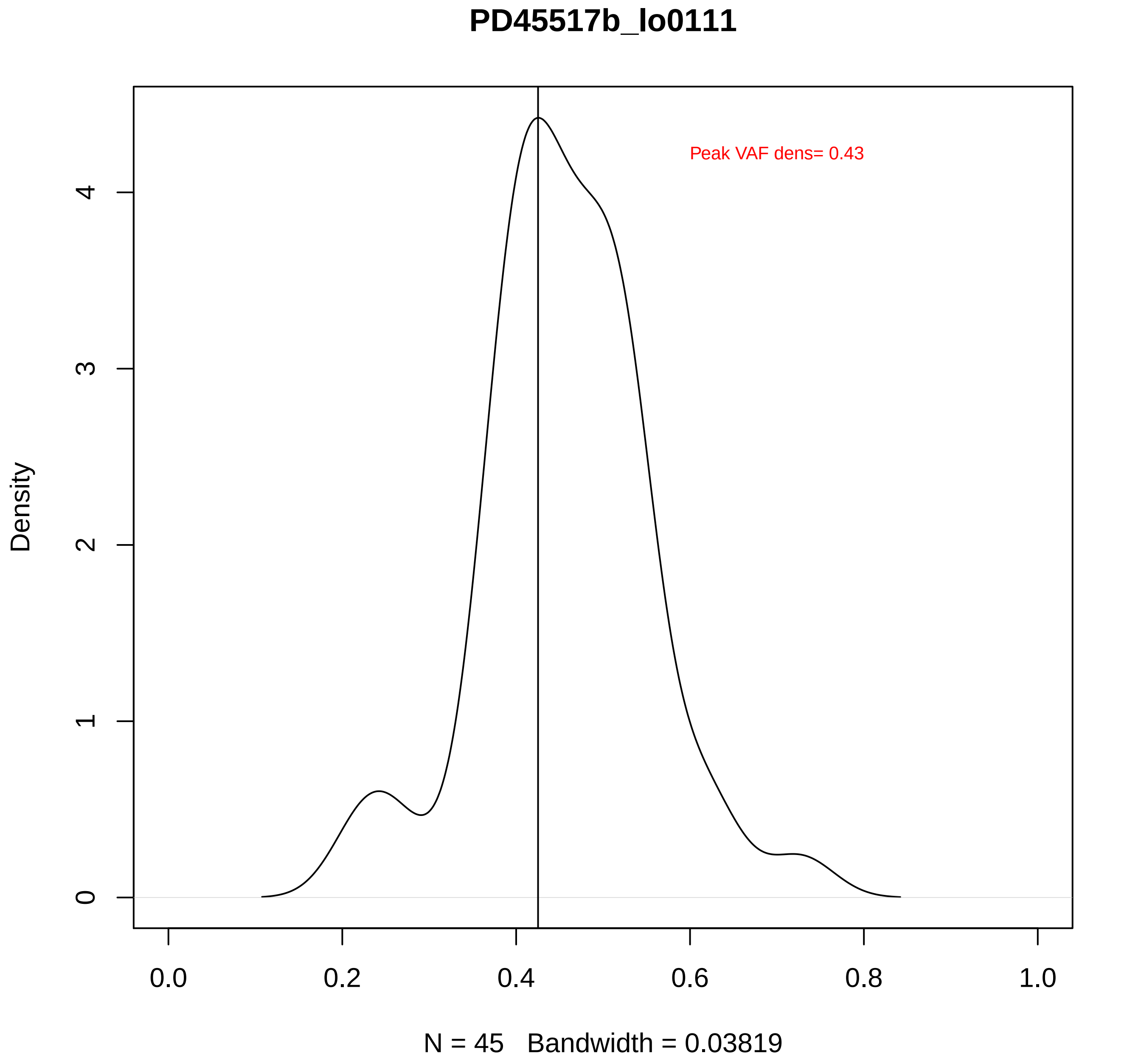
PD45517b_lo0111
Peak VAF dens= 0.43
Density
0
1
2
3
4
0.0
0.2
0.4
0.6
0.8
1.0
N = 45   Bandwidth = 0.03819

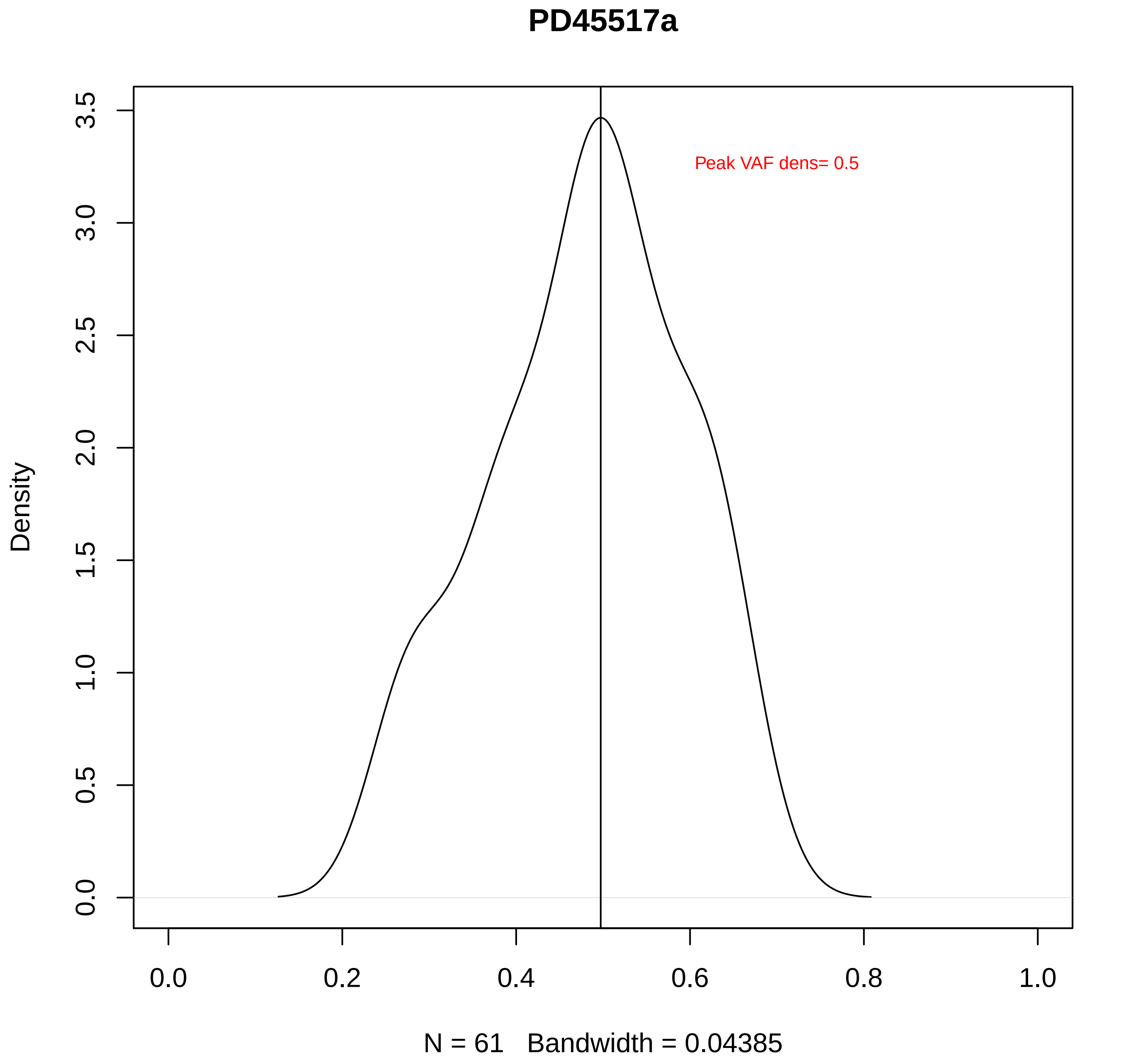
PD45517a
Peak VAF dens= 0.5
Density
0.0
0.5
1.0
1.5
2.0
2.5
3.0
3.5
0.0
0.2
0.4
0.6
0.8
1.0
N = 61   Bandwidth = 0.04385

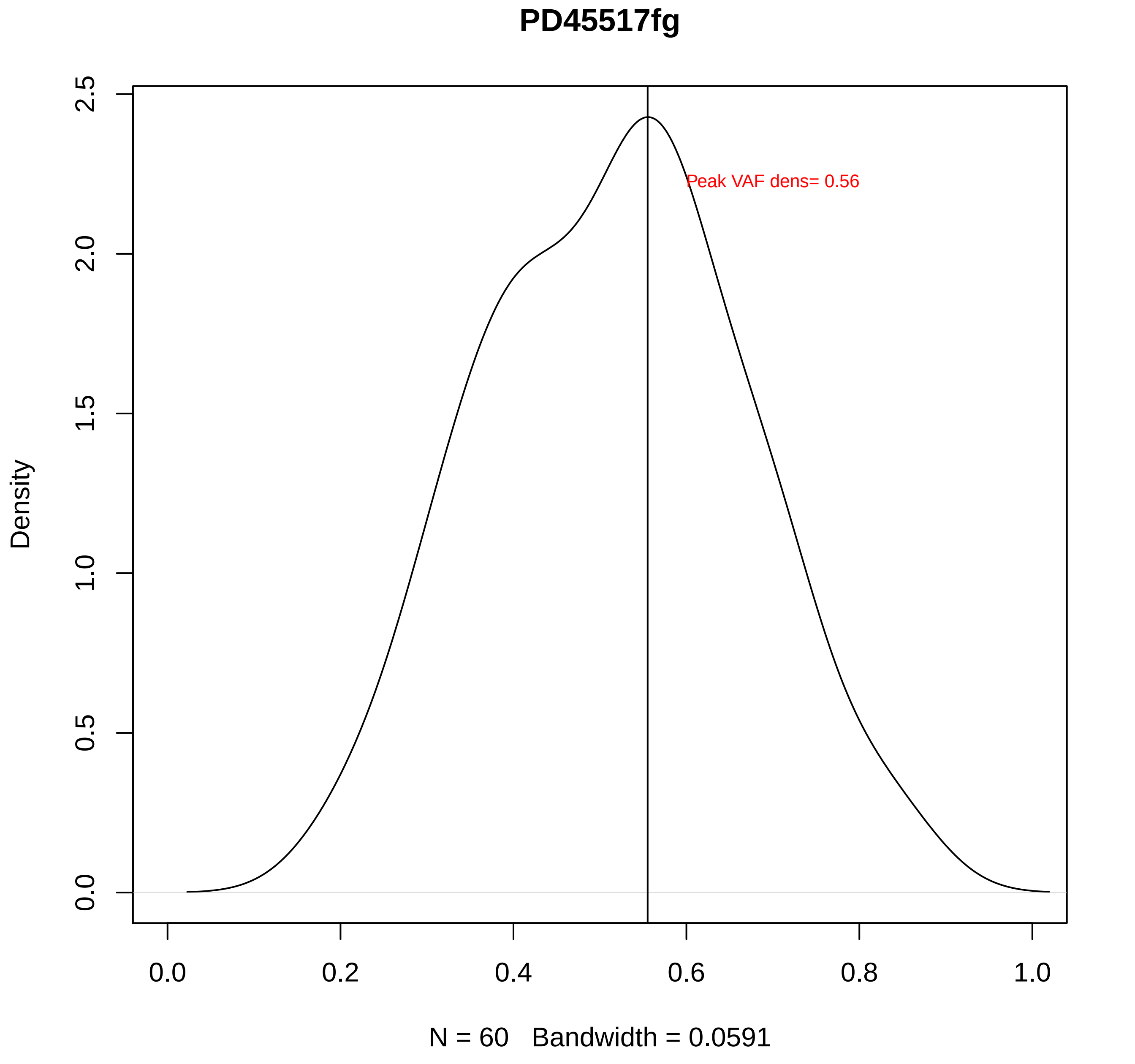
PD45517fg
Peak VAF dens= 0.56
Density
2.5
2.0
1.5
1.0
0.5
0.0
0.0
0.2
0.4
0.6
0.8
1.0
N = 60   Bandwidth = 0.0591

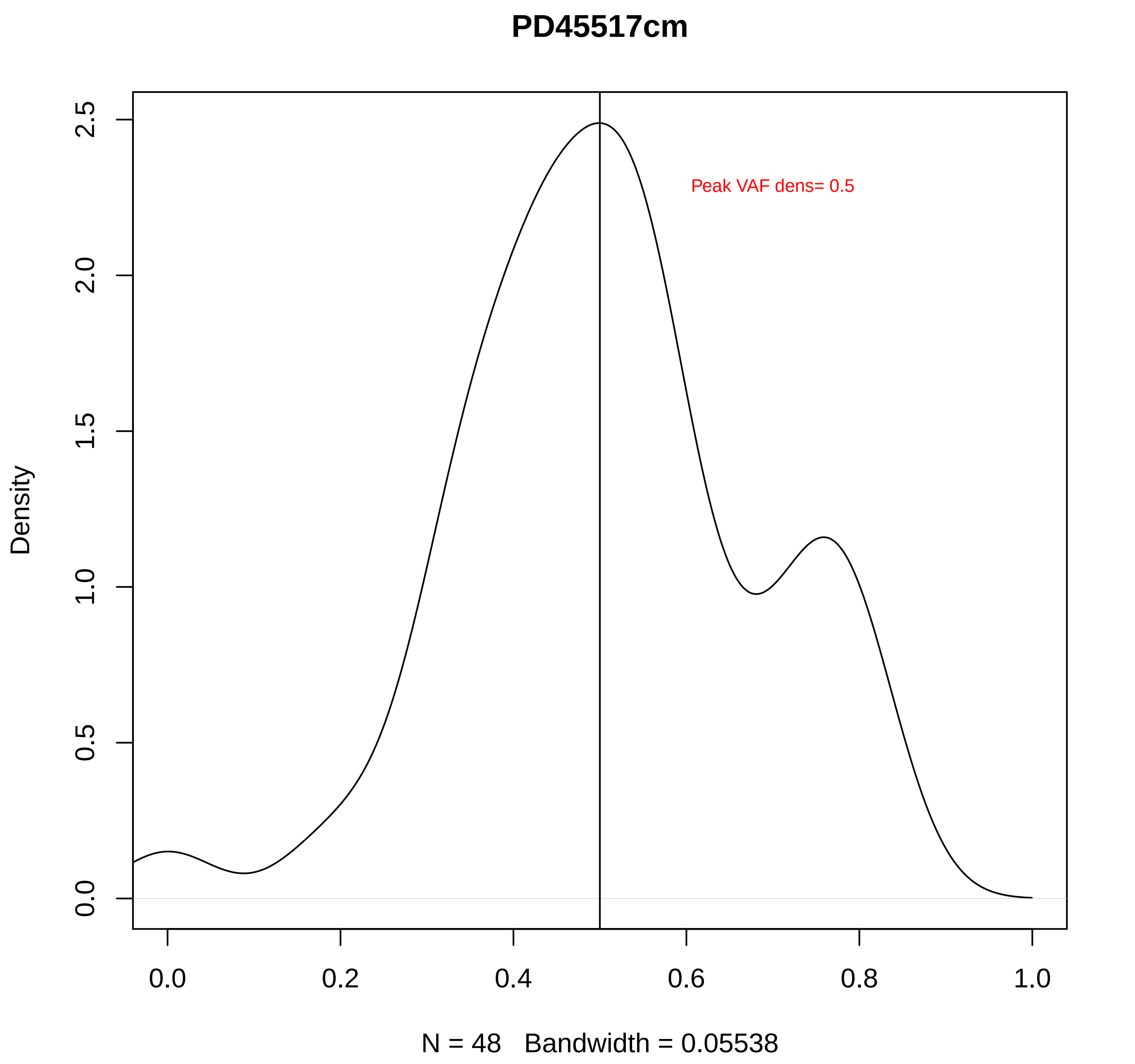

PD45517cm
Peak VAF dens= 0.5
Density
0.0
0.5
1.0
1.5
2.0
2.5
0.0
0.2
0.4
0.6
0.8
1.0
N = 48   Bandwidth = 0.05538

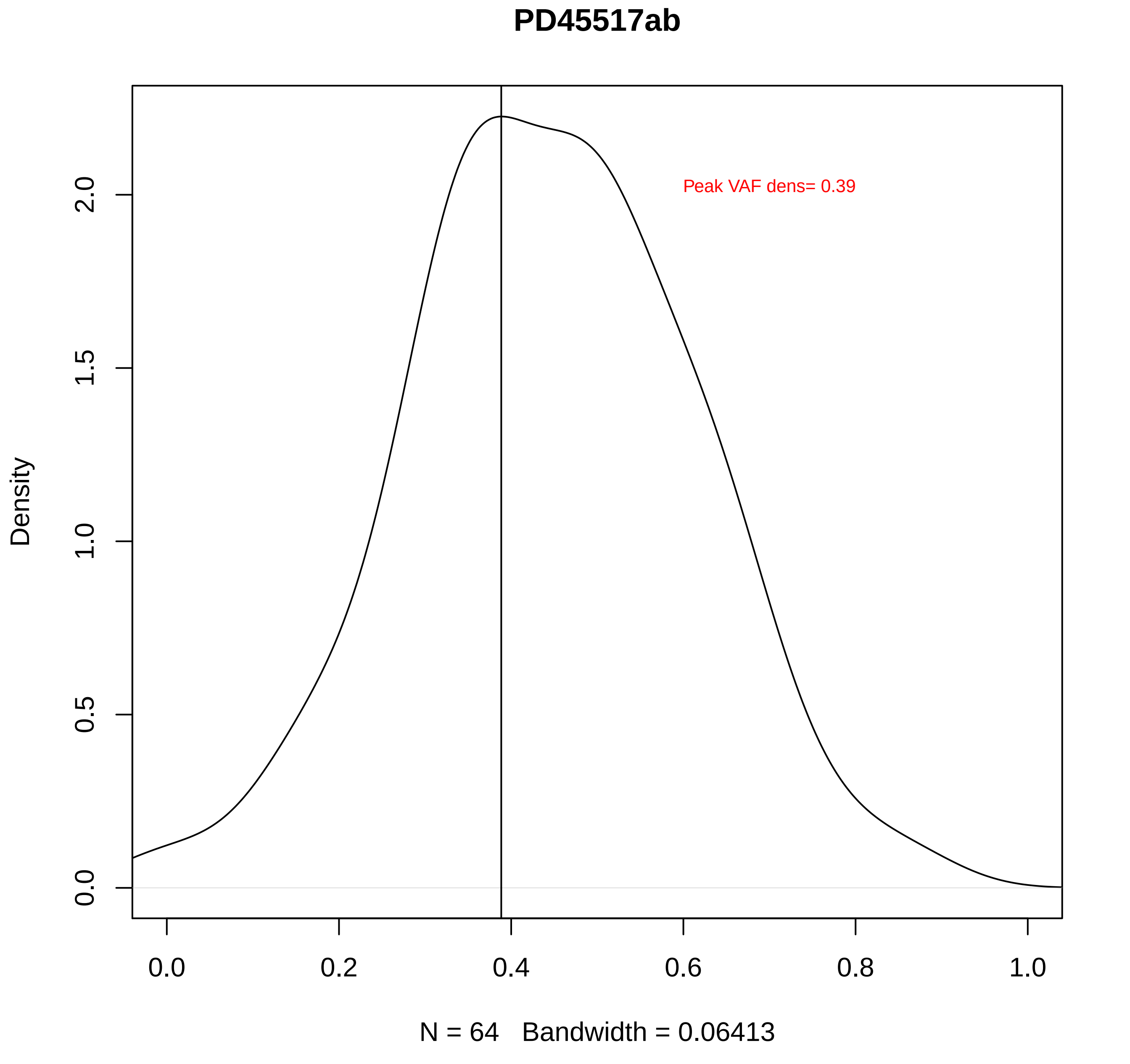
PD45517ab
Peak VAF dens= 0.39
Density
0.0
0.5
1.0
1.5
2.0
0.0
0.2
0.4
0.6
0.8
1.0
N = 64   Bandwidth = 0.06413

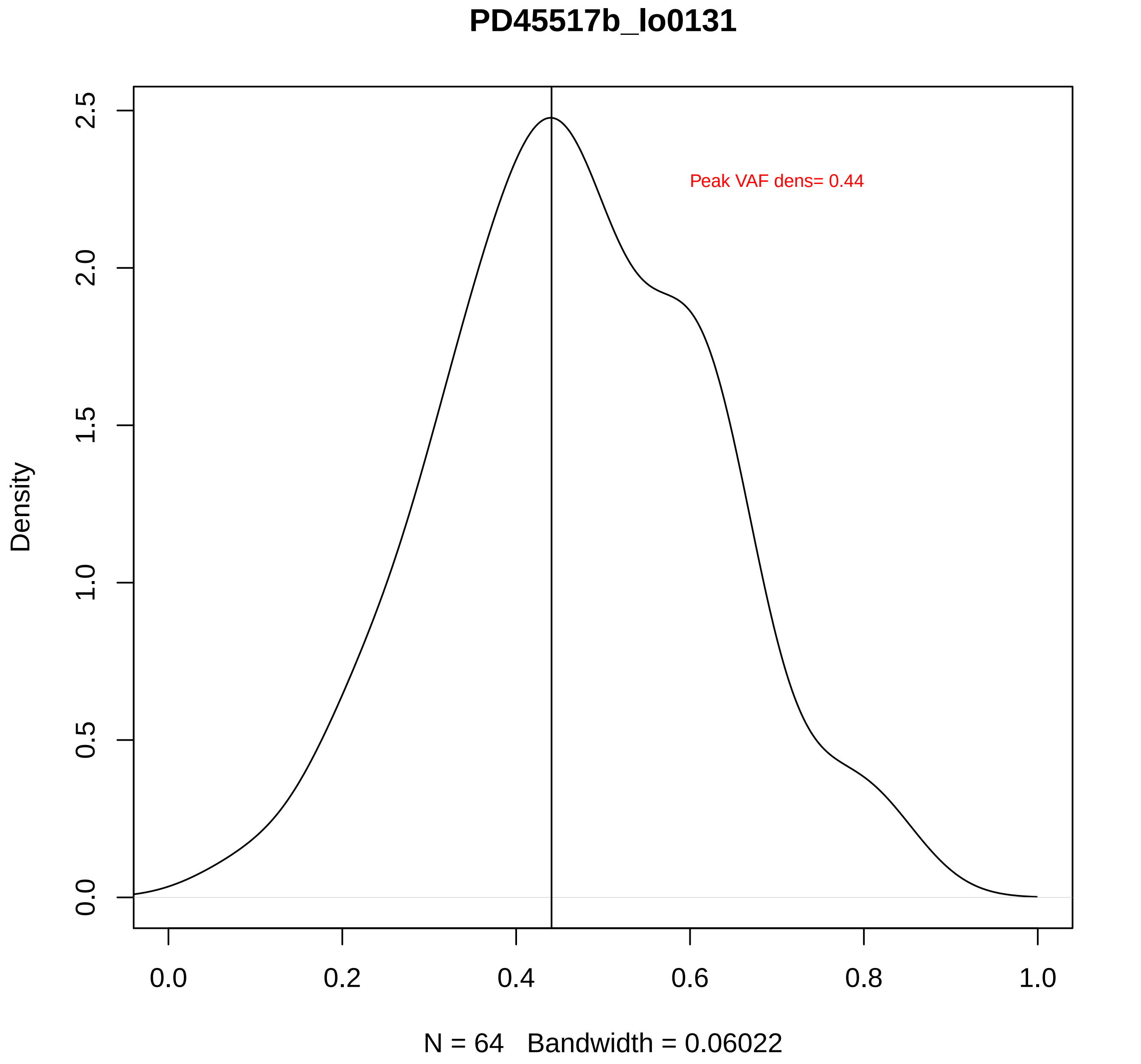

PD45517b_lo0131
Peak VAF dens= 0.44
Density
0.0
0.5
1.0
1.5
2.0
2.5
0.0
0.2
0.4
0.6
0.8
1.0
N = 64   Bandwidth = 0.06022

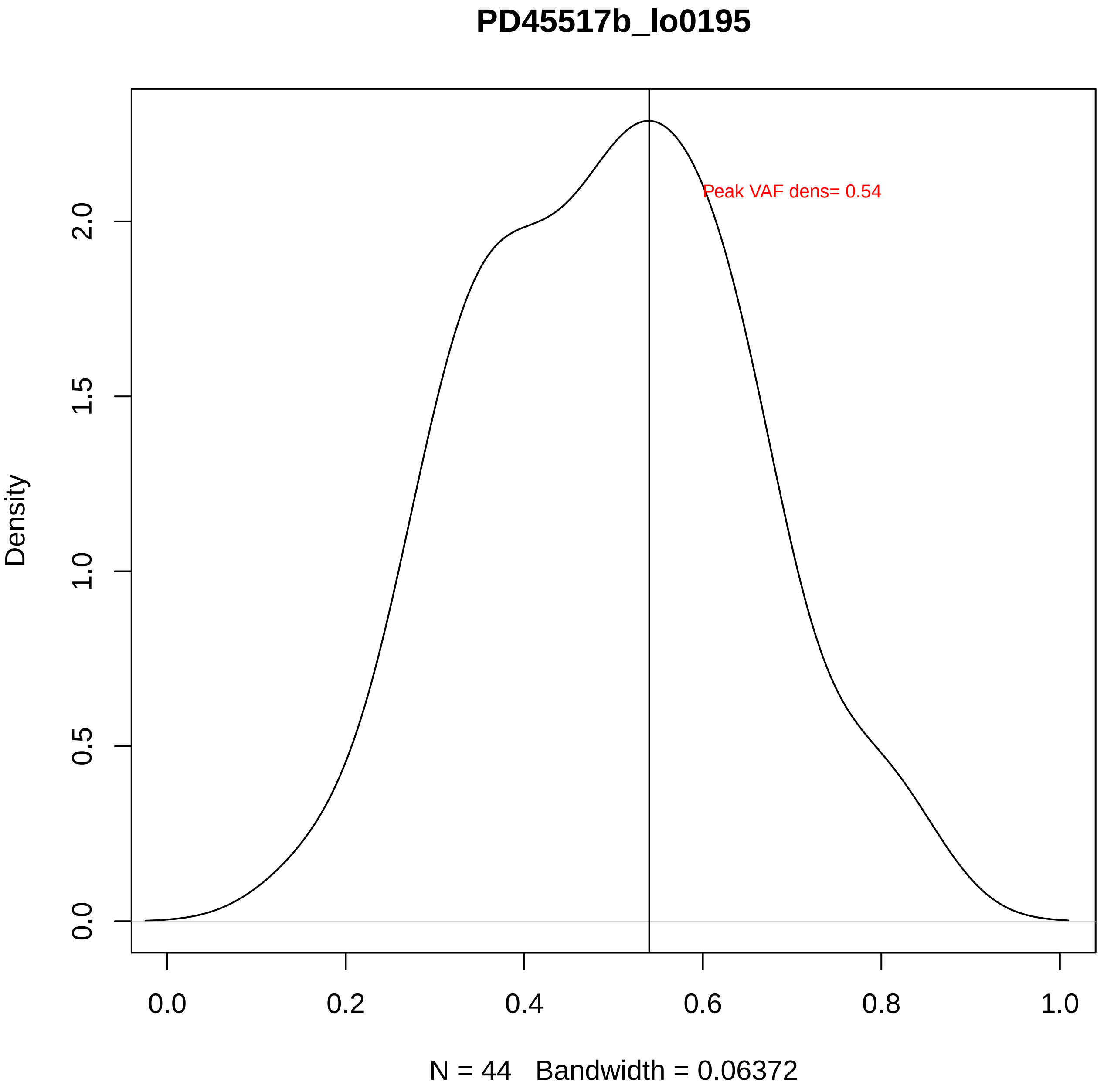
PD45517b_lo0195
Peak VAF dens= 0.54
Density
0.0
0.5
1.0
1.5
2.0
0.0
0.2
0.4
0.6
0.8
1.0
N = 44   Bandwidth = 0.06372

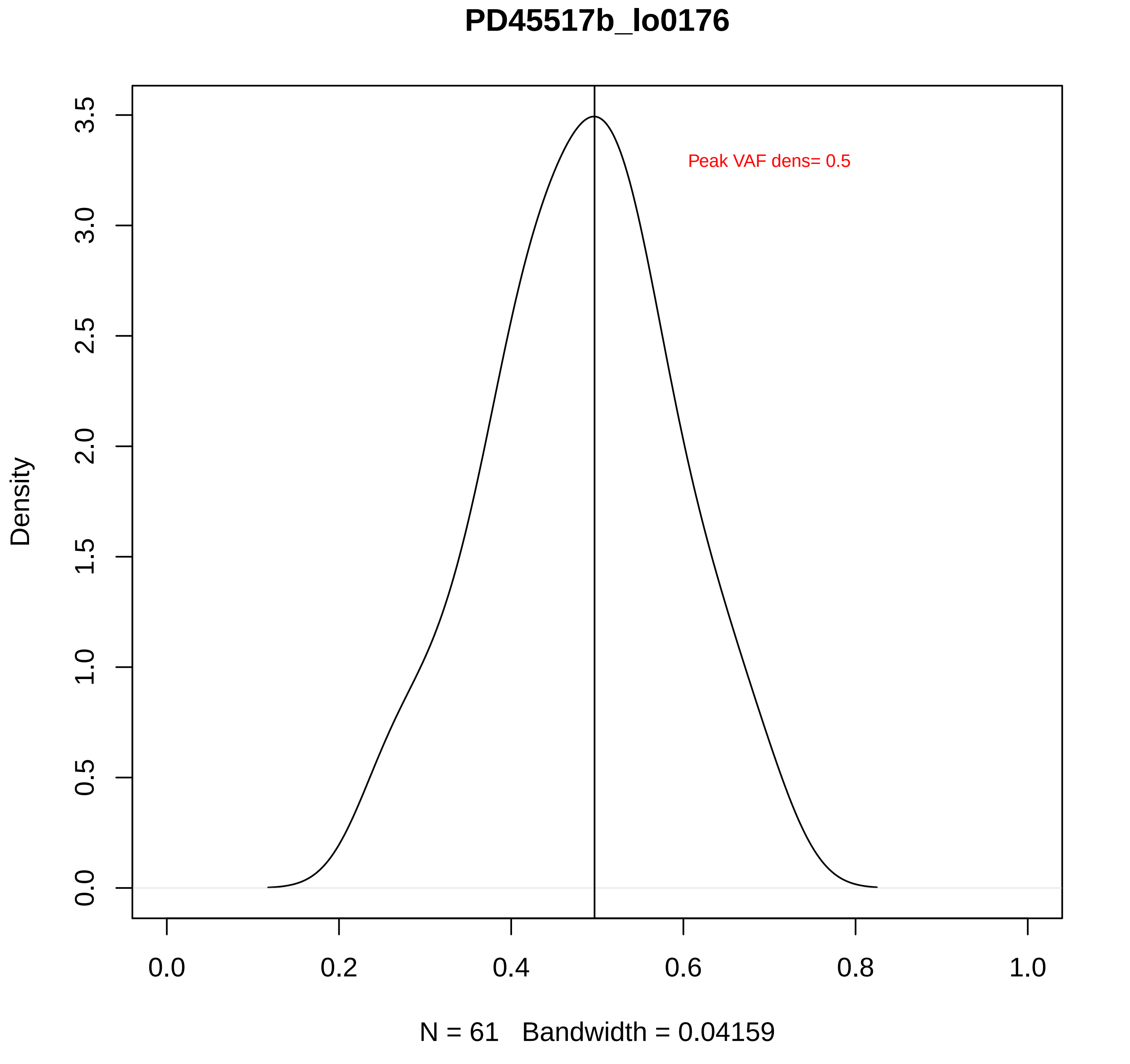
PD45517b_lo0176
Peak VAF dens= 0.5
Density
0.0
0.5
1.0
1.5
2.0
2.5
3.0
3.5
0.0
0.2
0.4
0.6
0.8
1.0
N = 61   Bandwidth = 0.04159

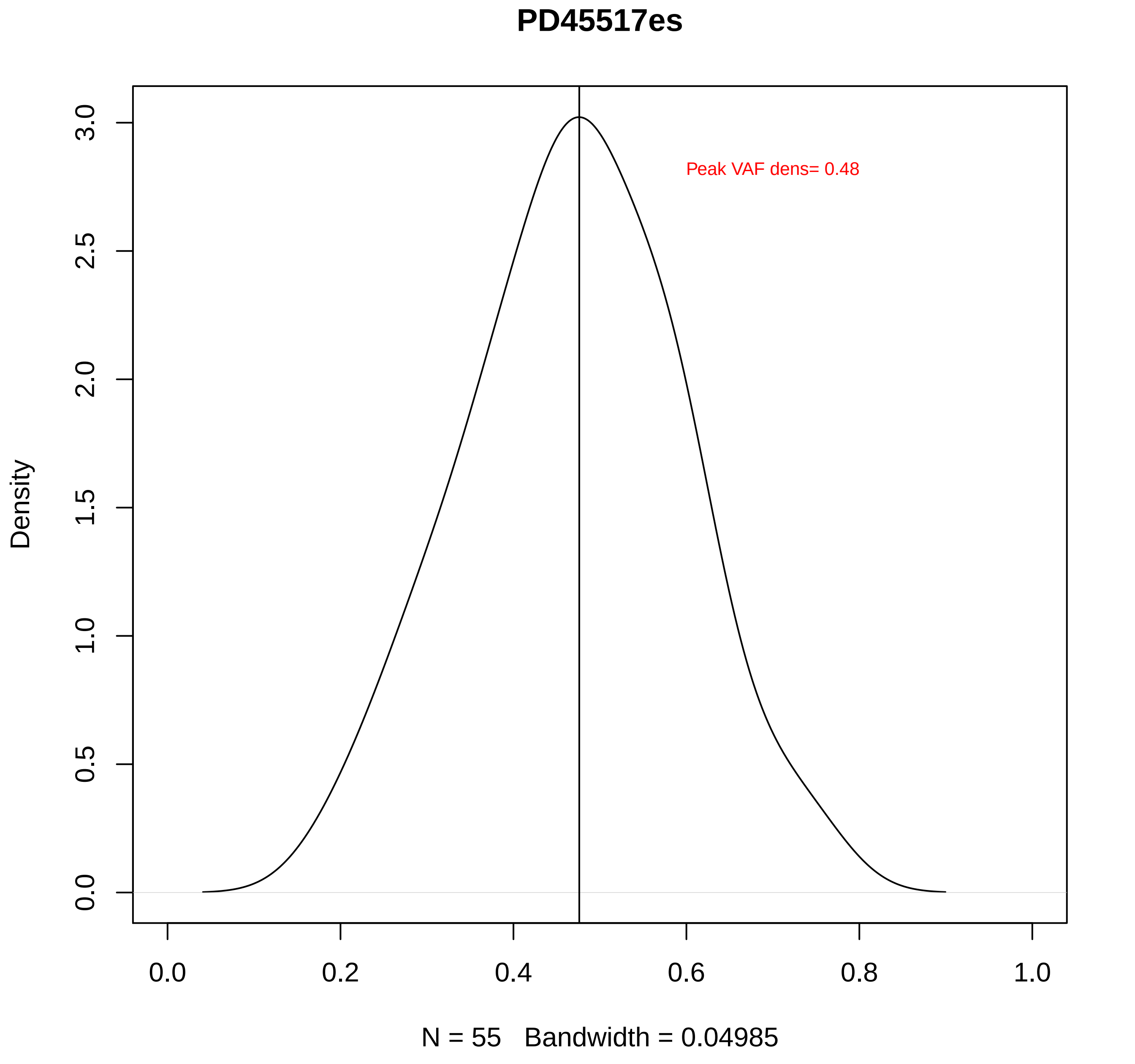

PD45517es
Peak VAF dens= 0.48
Density
0.0
0.5
1.0
1.5
2.0
2.5
3.0
0.0
0.2
0.4
0.6
0.8
1.0
N = 55   Bandwidth = 0.04985

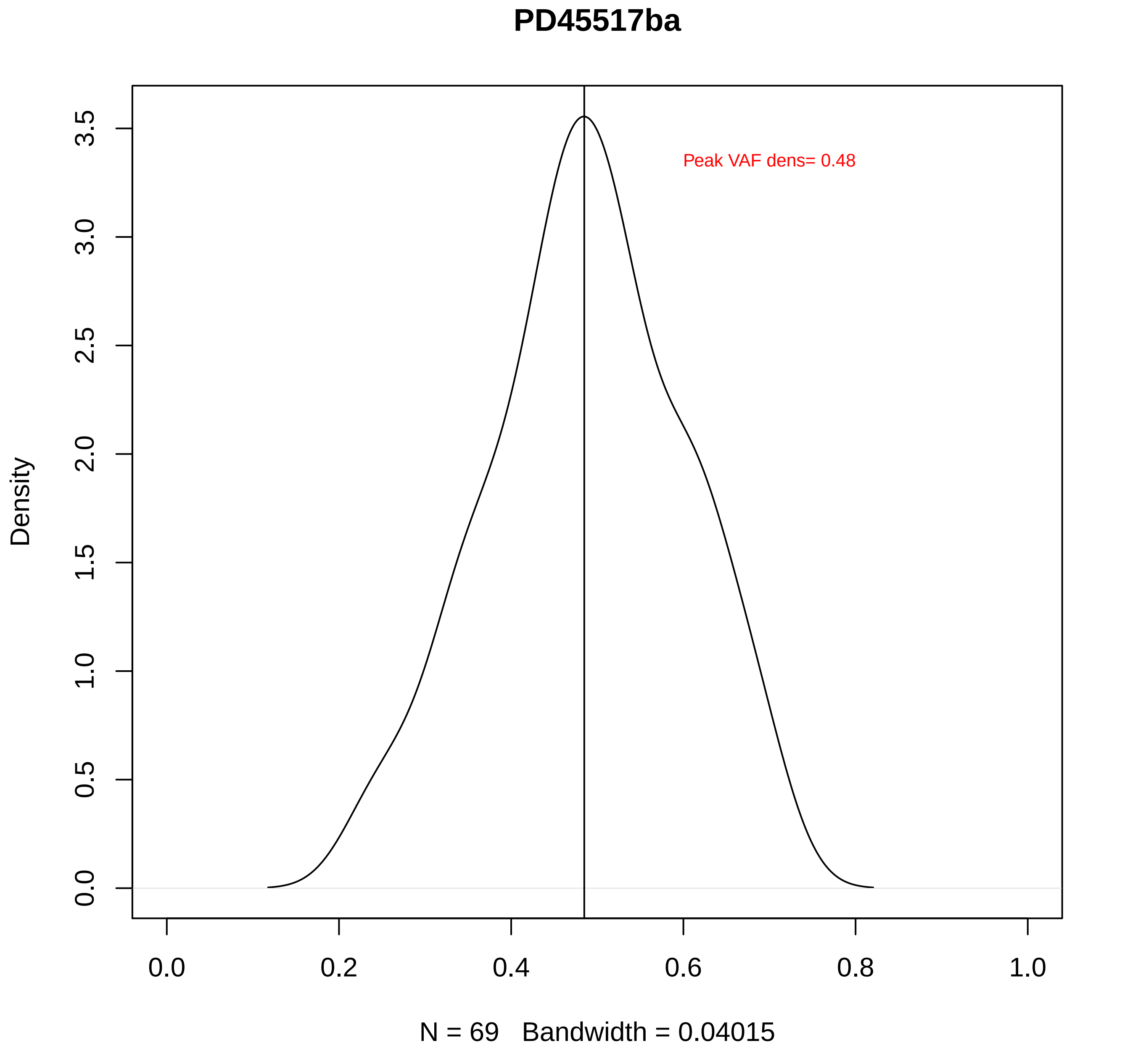
PD45517ba
Peak VAF dens= 0.48
Density
0.0
0.5
1.0
1.5
2.0
2.5
3.0
3.5
0.0
0.2
0.4
0.6
0.8
1.0
N = 69   Bandwidth = 0.04015

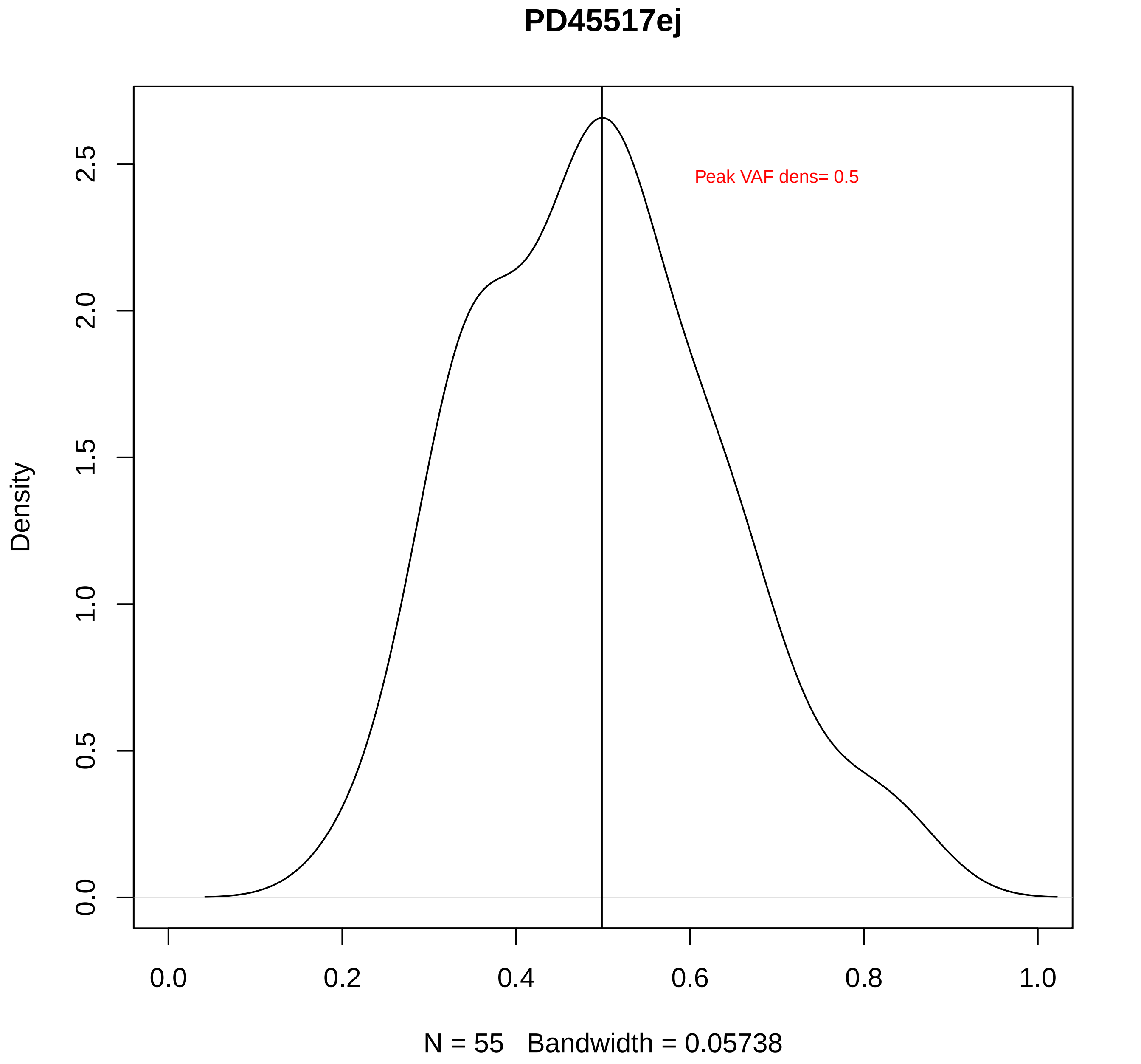
PD45517ej
Peak VAF dens= 0.5
Density
0.0
0.5
1.0
1.5
2.0
2.5
0.0
0.2
0.4
0.6
0.8
1.0
N = 55 Bandwidth = 0.05738

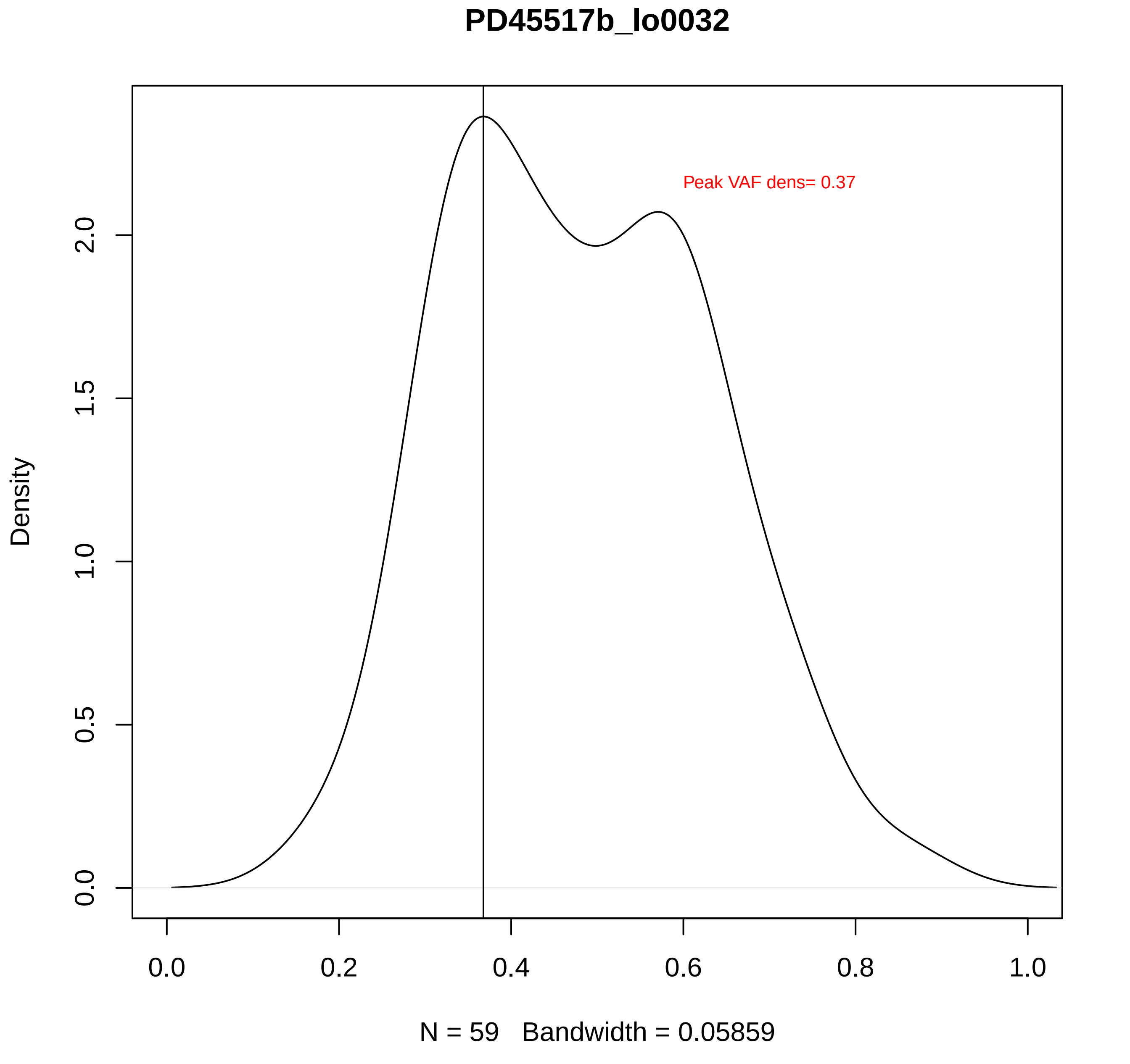
PD45517b_lo0032
Peak VAF dens= 0.37
Density
0.0
0.5
1.0
1.5
2.0
0.0
0.2
0.4
0.6
0.8
1.0
N = 59   Bandwidth = 0.05859

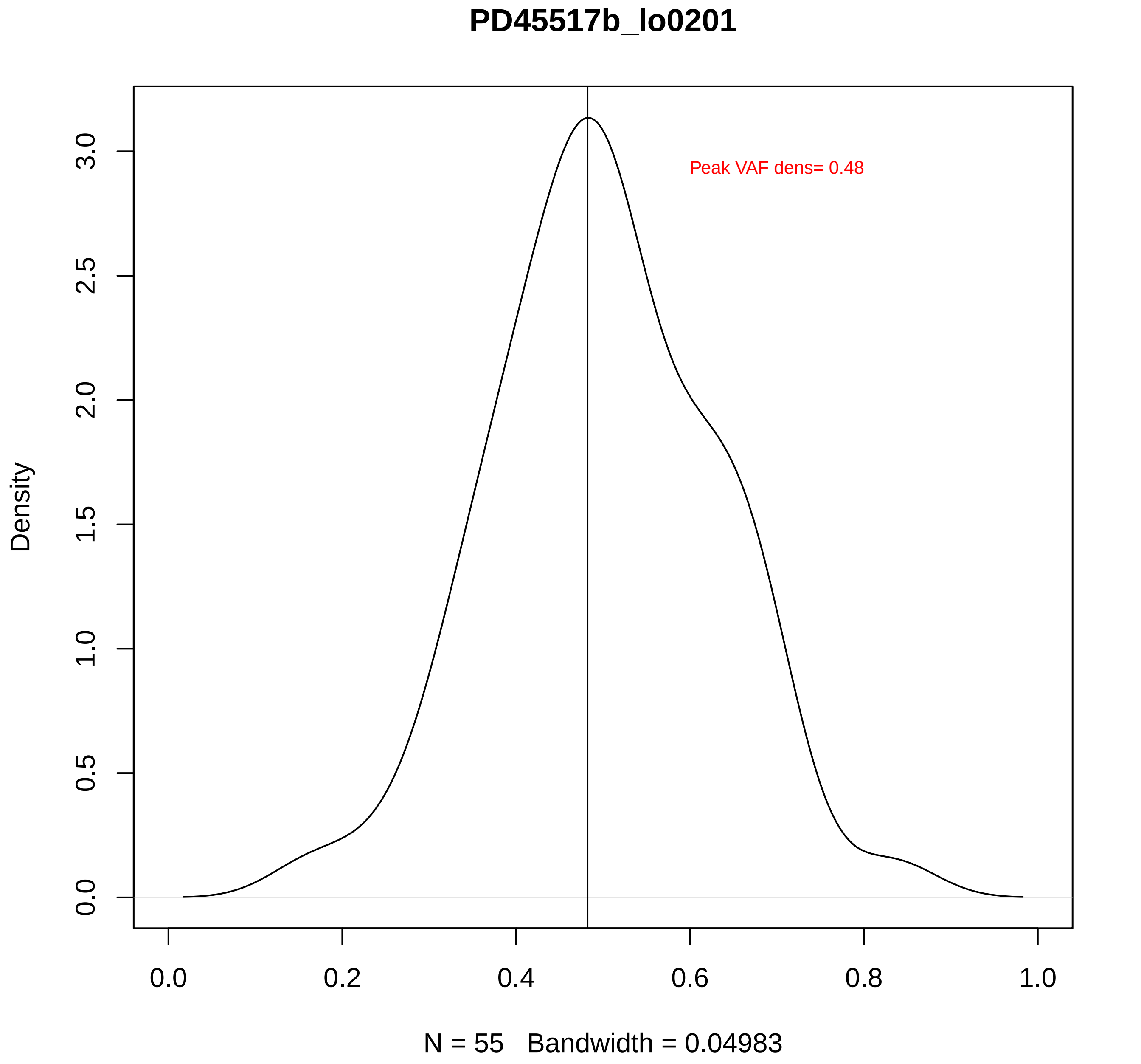
PD45517b_lo0201
Peak VAF dens= 0.48
Density
0.0
0.5
1.0
1.5
2.0
2.5
3.0
0.0
0.2
0.4
0.6
0.8
1.0
N = 55   Bandwidth = 0.04983

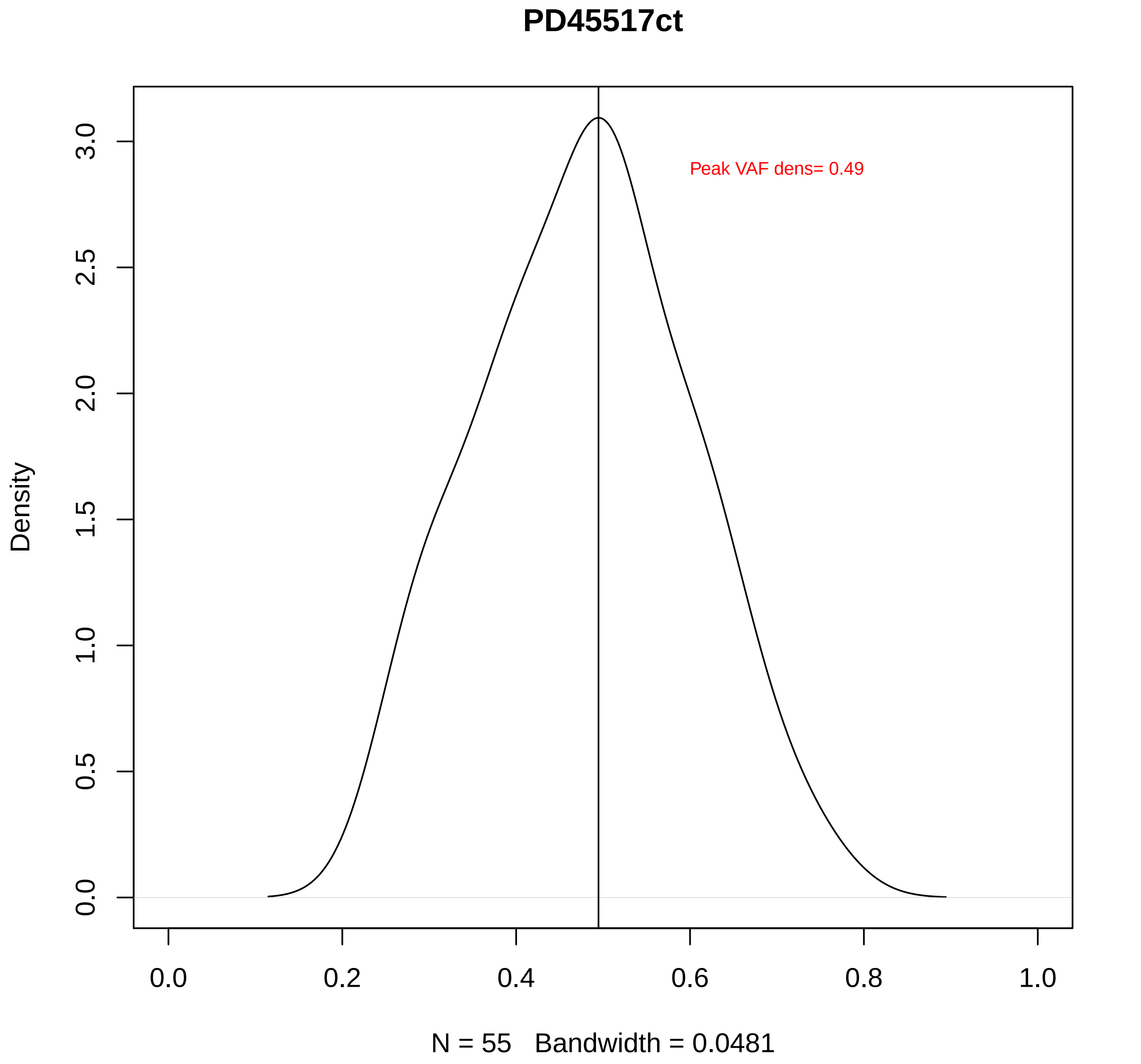
PD45517ct
Peak VAF dens= 0.49
Density
0.0
0.5
1.0
1.5
2.0
2.5
3.0
0.0
0.2
0.4
0.6
0.8
1.0
N = 55   Bandwidth = 0.0481

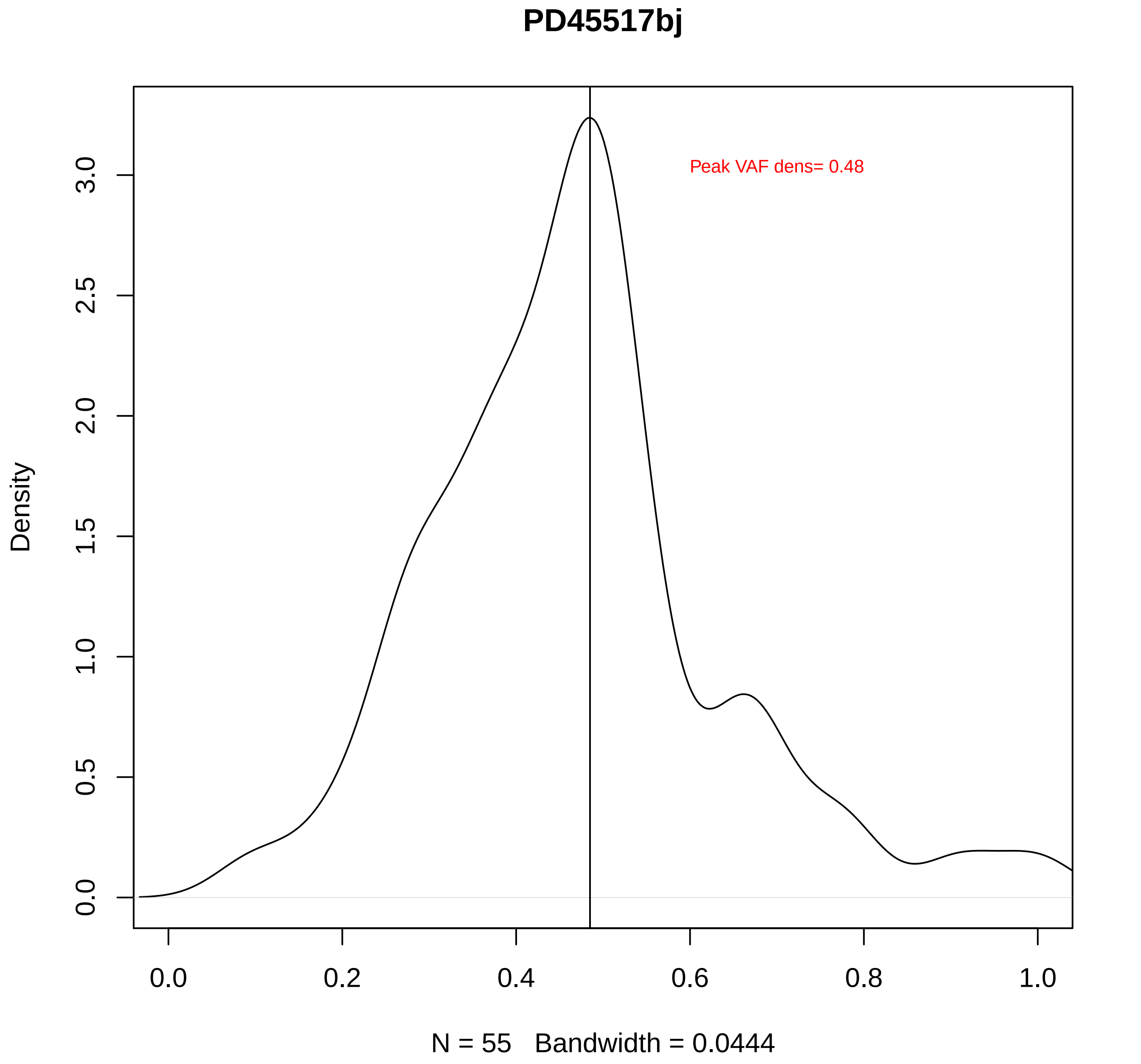
PD45517bj
Peak VAF dens= 0.48
Density
0.0
0.5
1.0
1.5
2.0
2.5
3.0
0.0
0.2
0.4
0.6
0.8
1.0
N = 55   Bandwidth = 0.0444

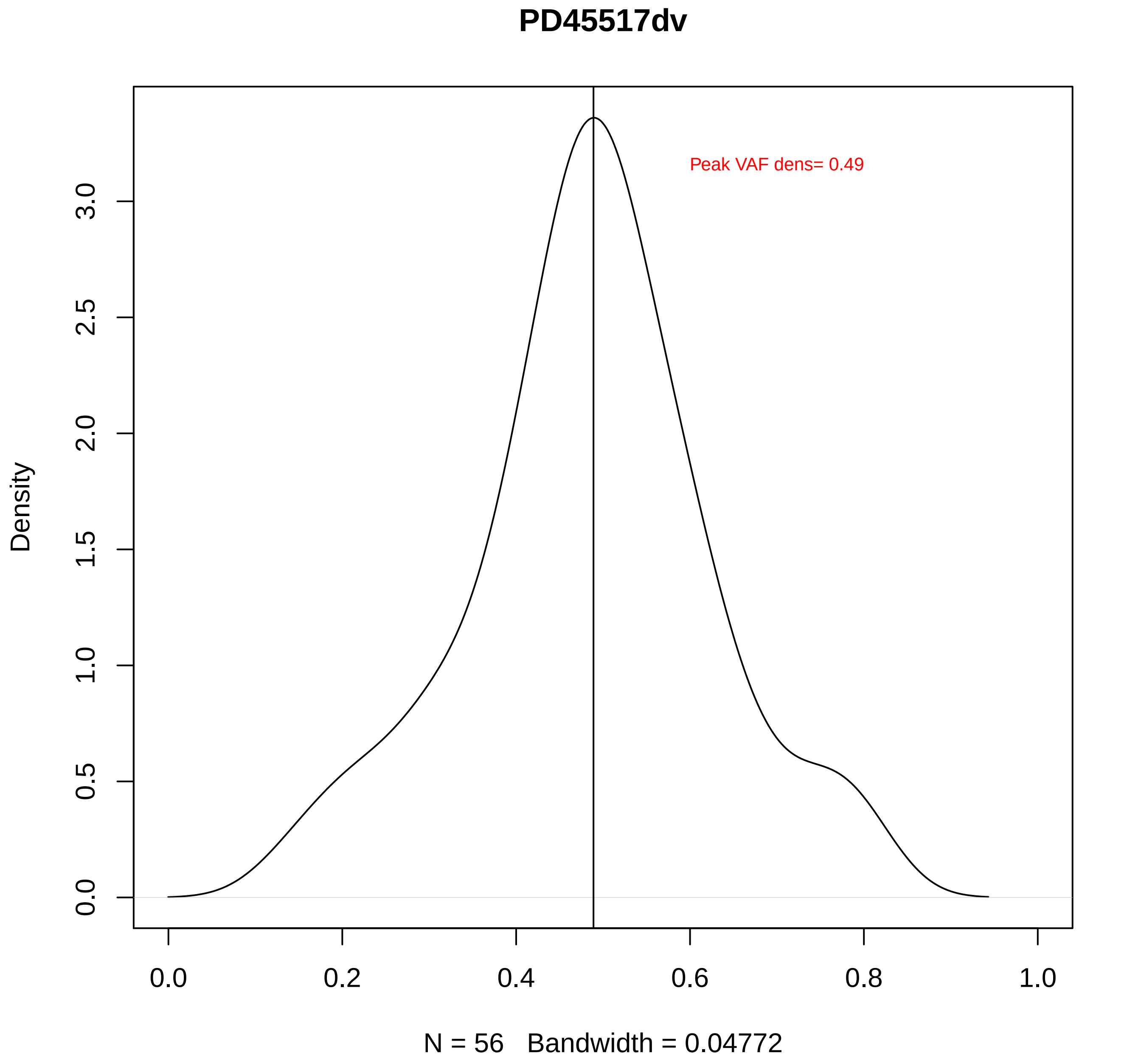

PD45517dv
Peak VAF dens= 0.49
Density
0.0
0.5
1.0
1.5
2.0
2.5
3.0
0.0
0.2
0.4
0.6
0.8
1.0
N = 56 Bandwidth = 0.04772

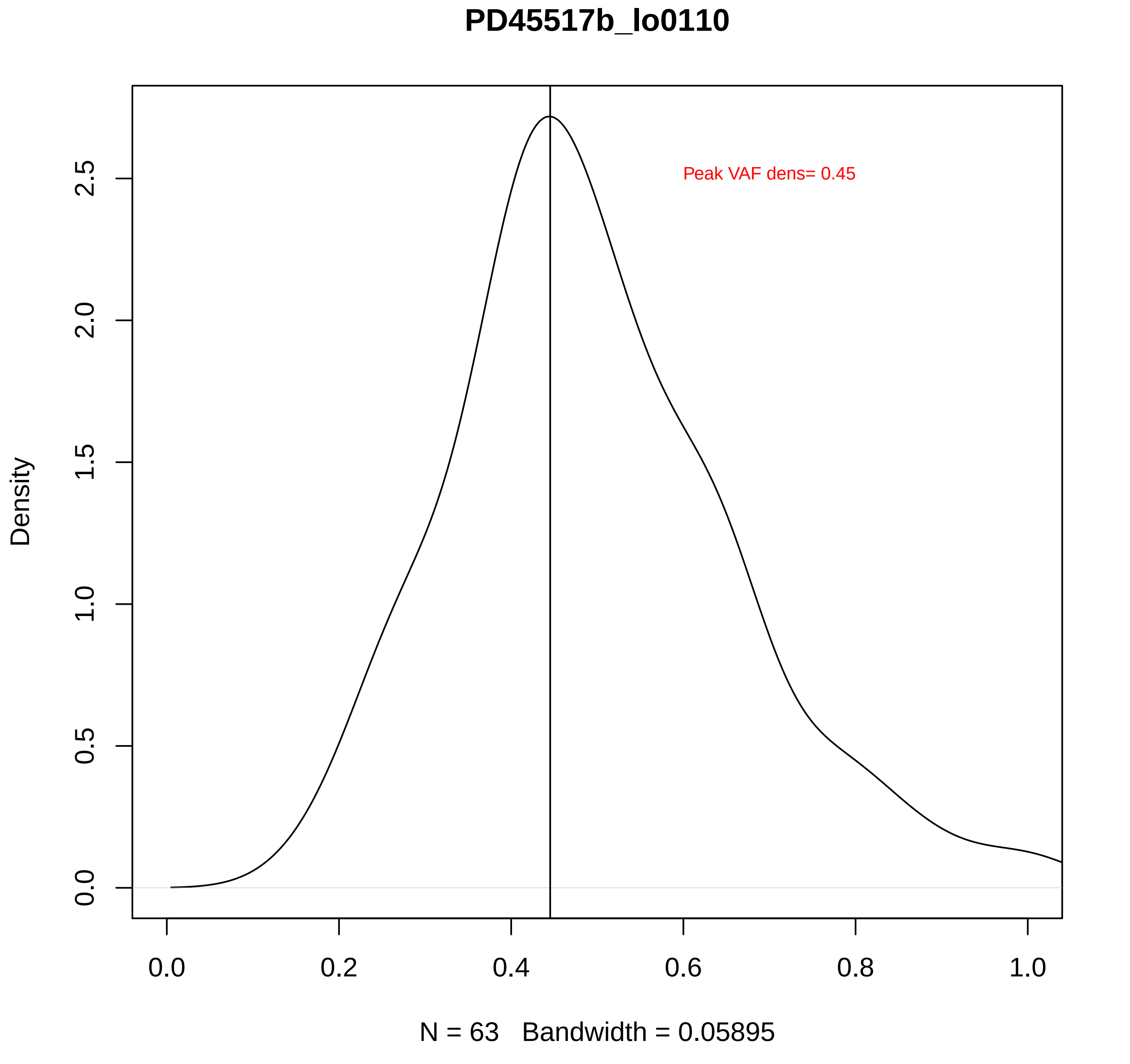
PD45517b_lo0110
Peak VAF dens= 0.45
Density
0.0
0.5
1.0
1.5
2.0
2.5
0.0
0.2
0.4
0.6
0.8
1.0
N = 63   Bandwidth = 0.05895

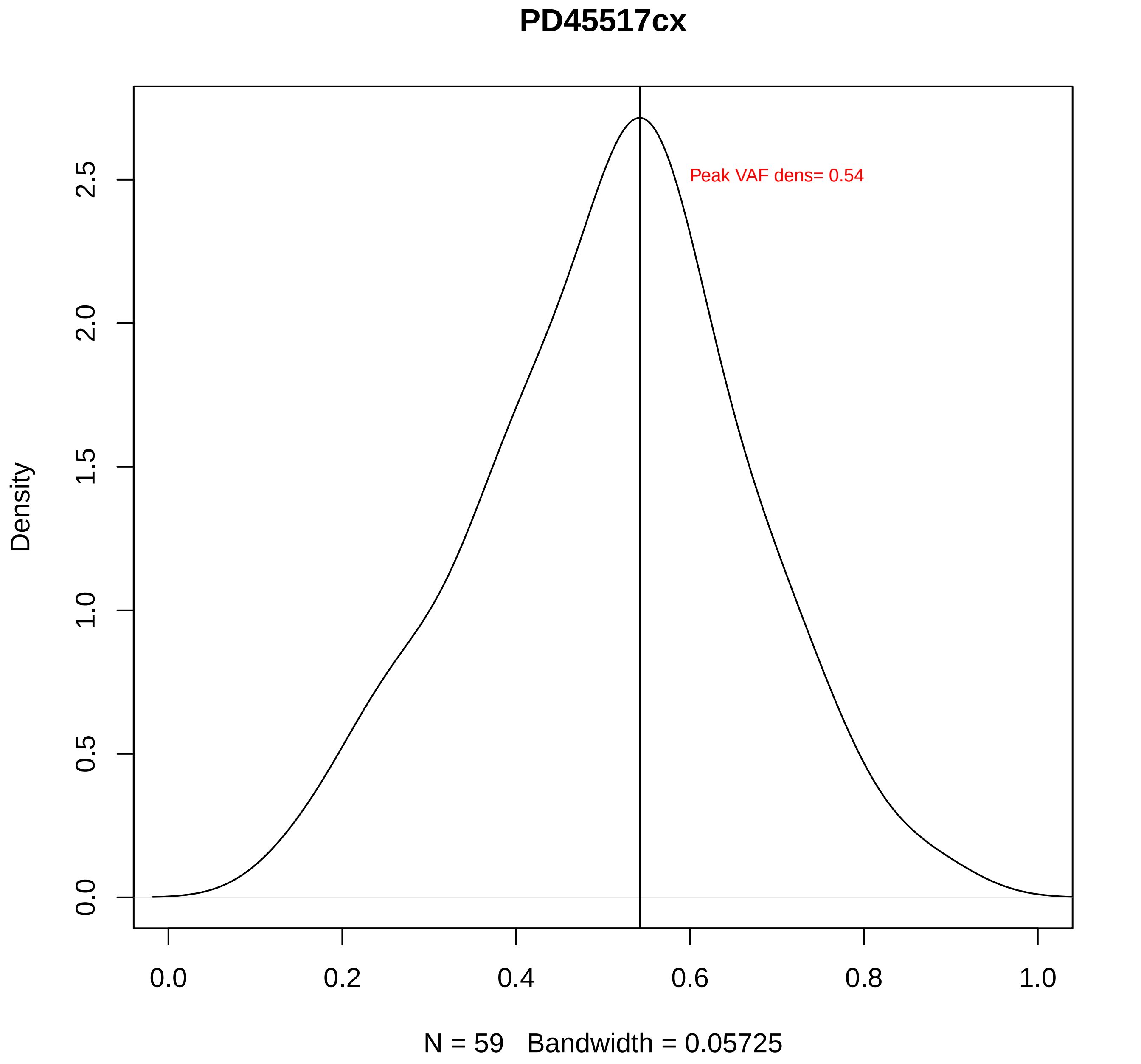
PD45517cx
Density
Peak VAF dens= 0.54
0.0
0.5
1.0
1.5
2.0
2.5
0.0
0.2
0.4
0.6
0.8
1.0
N = 59   Bandwidth = 0.05725

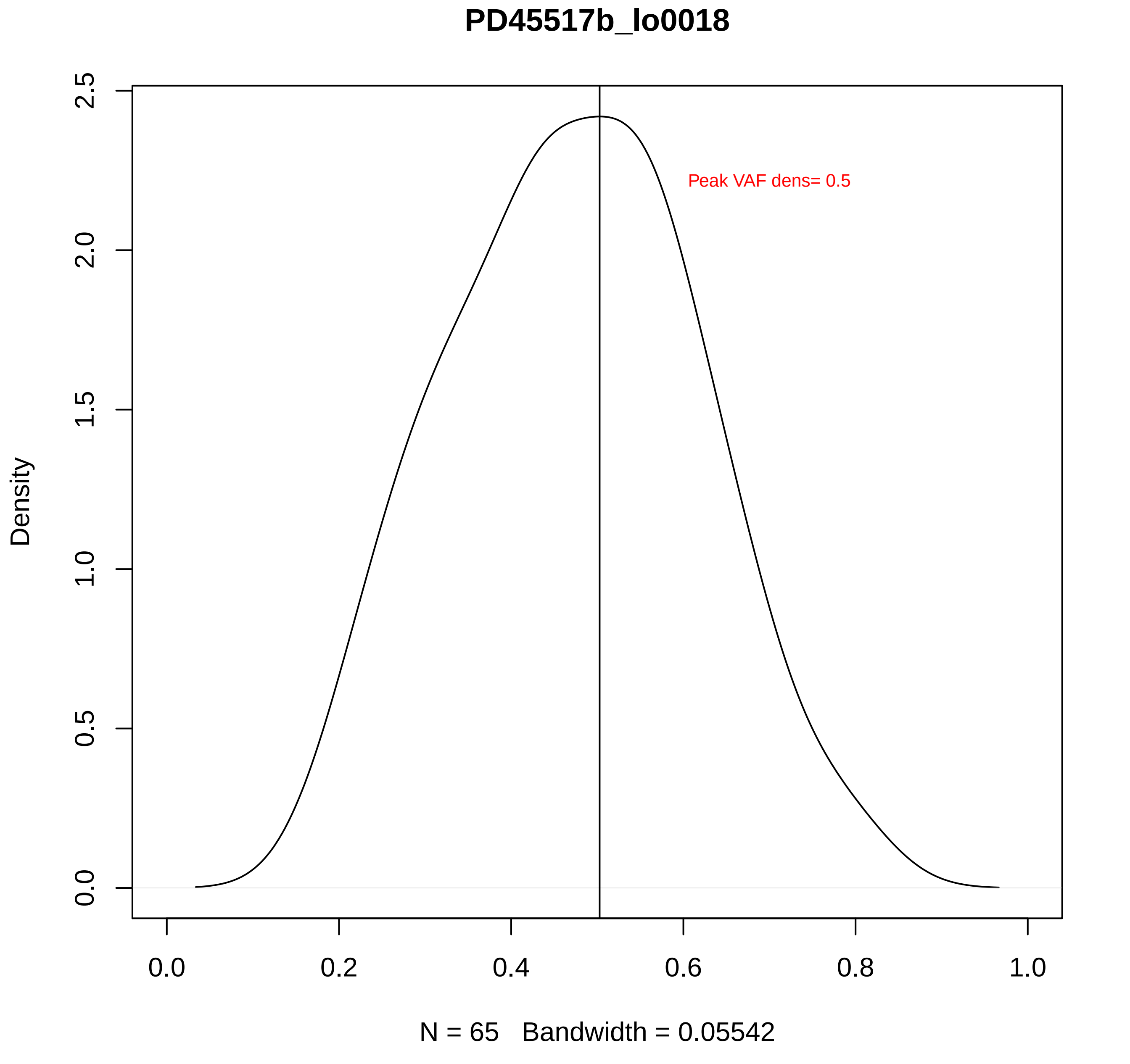

PD45517b_lo0018
Peak VAF dens= 0.5
Density
0.0
0.5
1.0
1.5
2.0
2.5
0.0
0.2
0.4
0.6
0.8
1.0
N = 65 Bandwidth = 0.05542

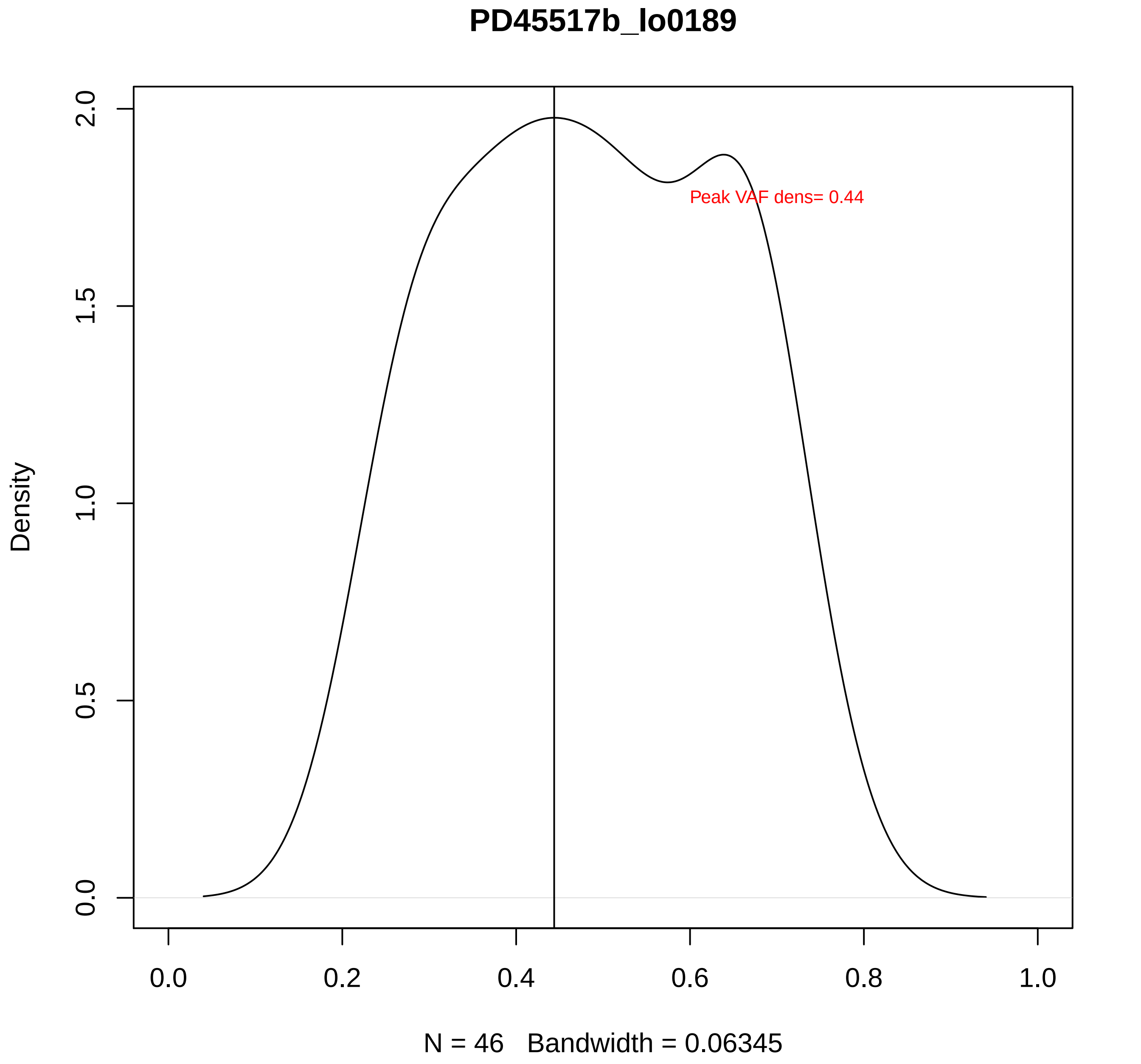
PD45517b_lo0189
Peak VAF dens= 0.44
Density
0.0
0.5
1.0
1.5
2.0
0.0
0.2
0.4
0.6
0.8
1.0
N = 46 Bandwidth = 0.06345

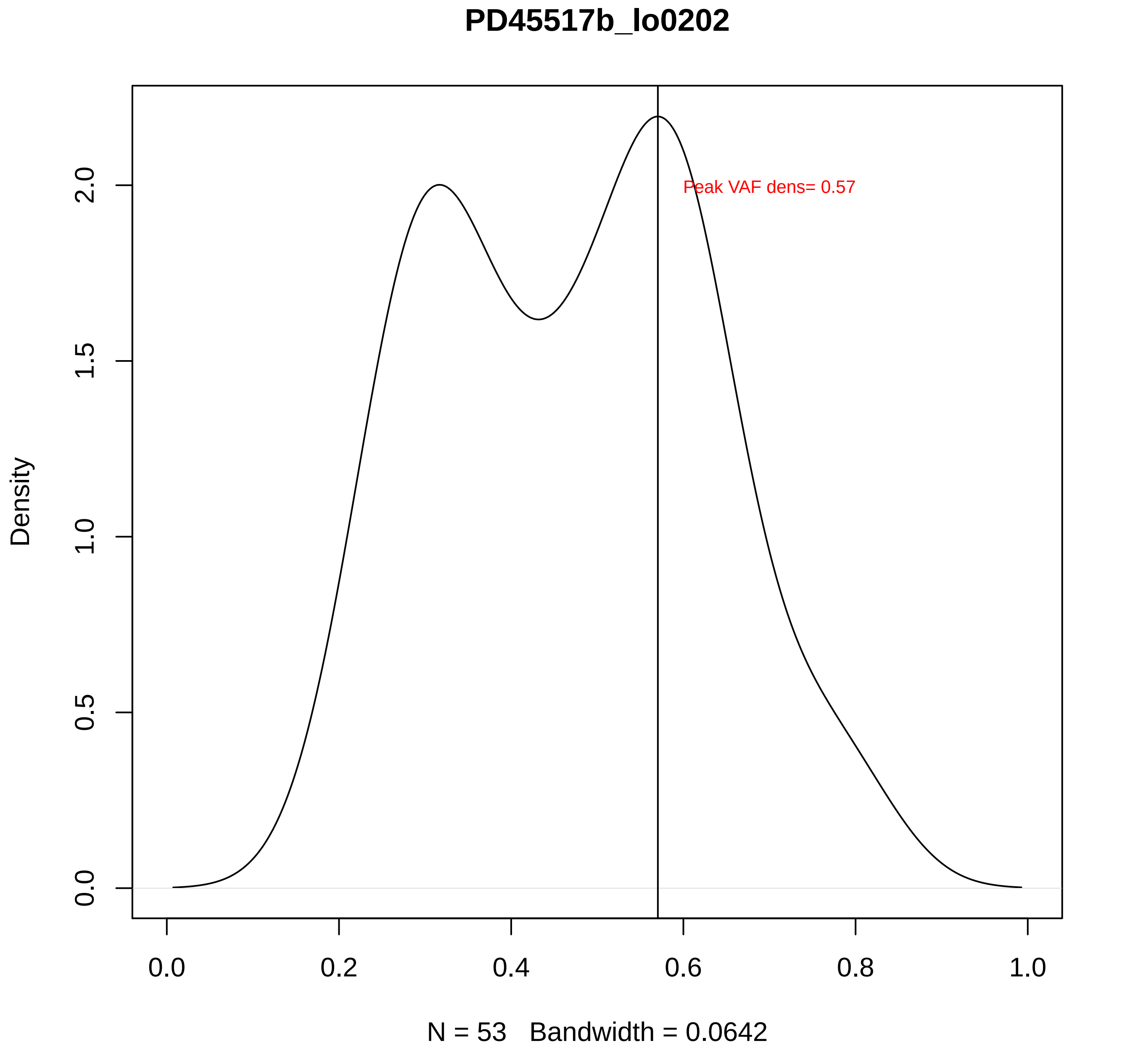
PD45517b_lo0202
Peak VAF dens= 0.57
Density
0.0
0.5
1.0
1.5
2.0
0.0
0.2
0.4
0.6
0.8
1.0
N = 53   Bandwidth = 0.0642

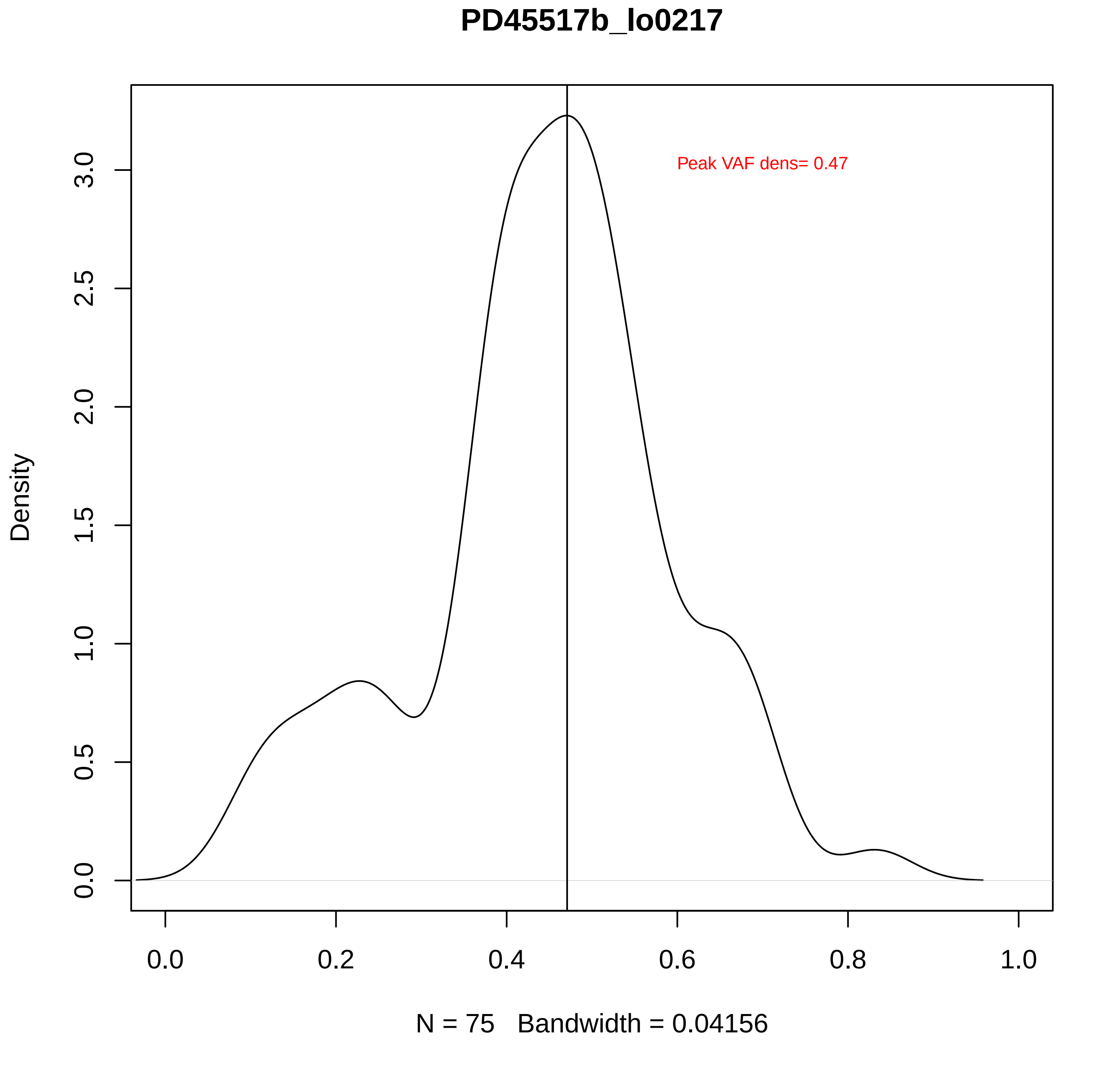
PD45517b_lo0217
Peak VAF dens= 0.47
Density
0.0
0.5
1.0
1.5
2.0
2.5
3.0
0.0
0.2
0.4
0.6
0.8
1.0
N = 75   Bandwidth = 0.04156

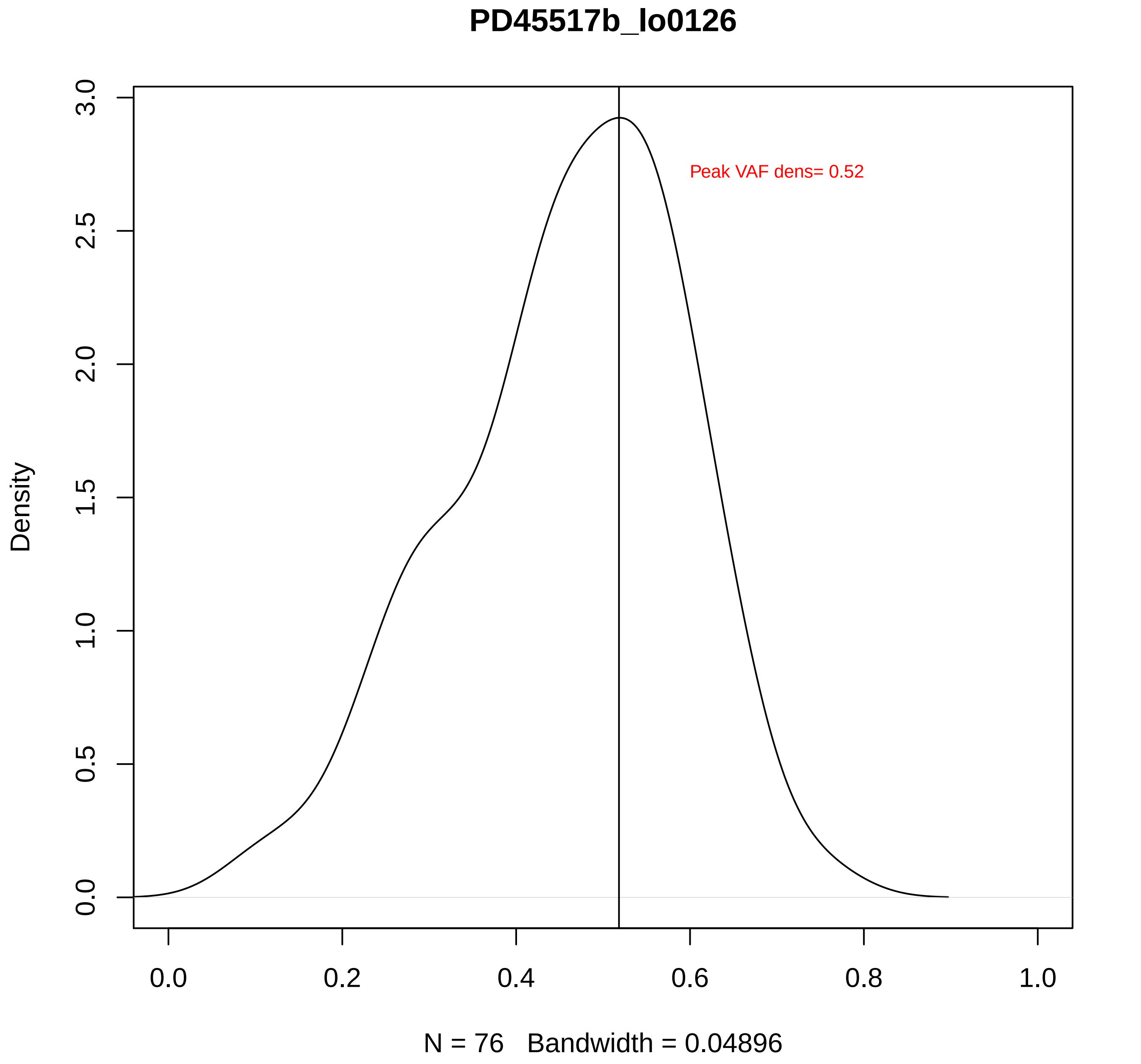
PD45517b_lo0126
Peak VAF dens= 0.52
Density
0.0
0.5
1.0
1.5
2.0
2.5
3.0
0.0
0.2
0.4
0.6
0.8
1.0
N = 76 Bandwidth = 0.04896

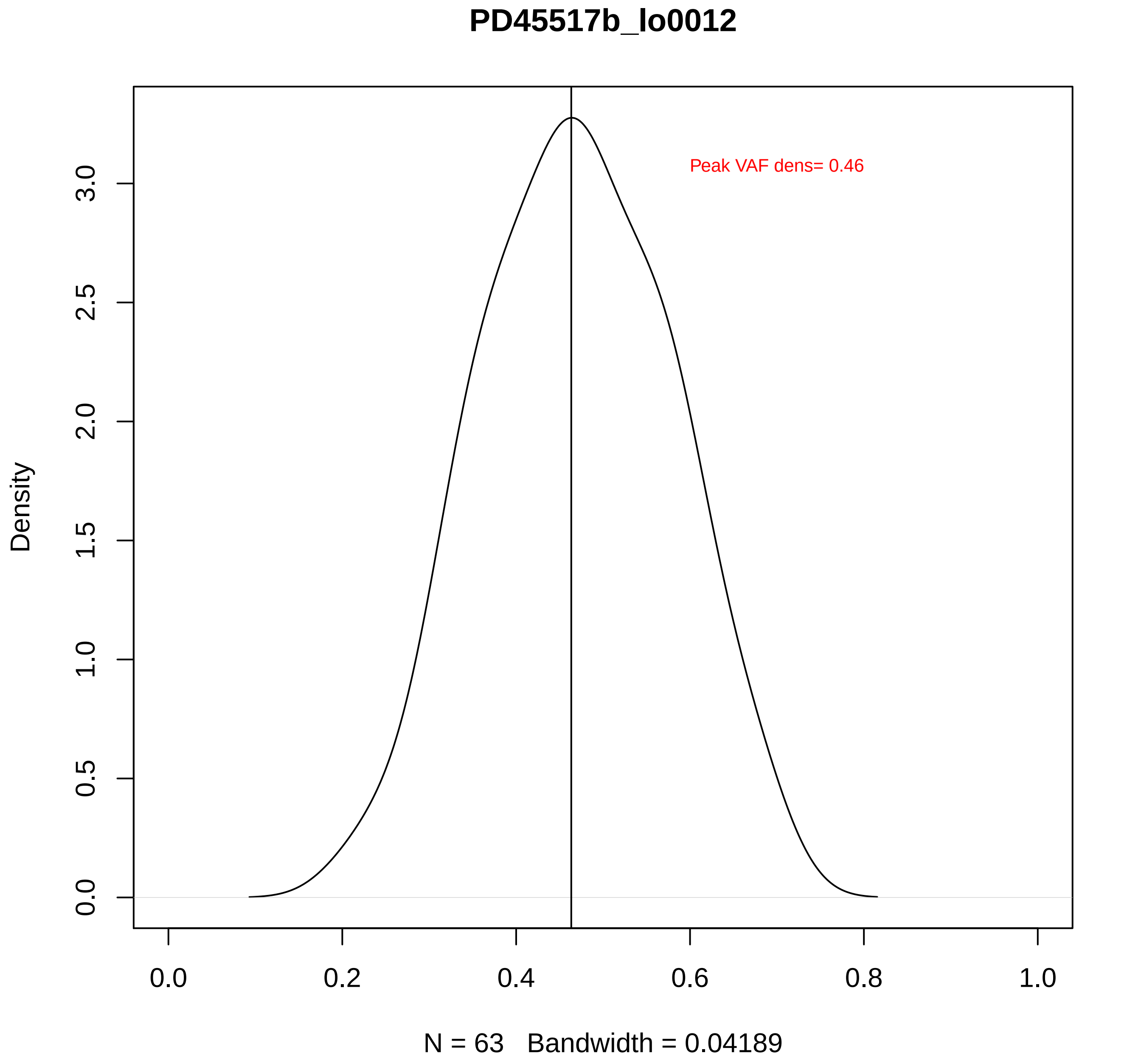
PD45517b_lo0012
Peak VAF dens= 0.46
Density
0.0
0.5
1.0
1.5
2.0
2.5
3.0
0.0
0.2
0.4
0.6
0.8
1.0
N = 63   Bandwidth = 0.04189

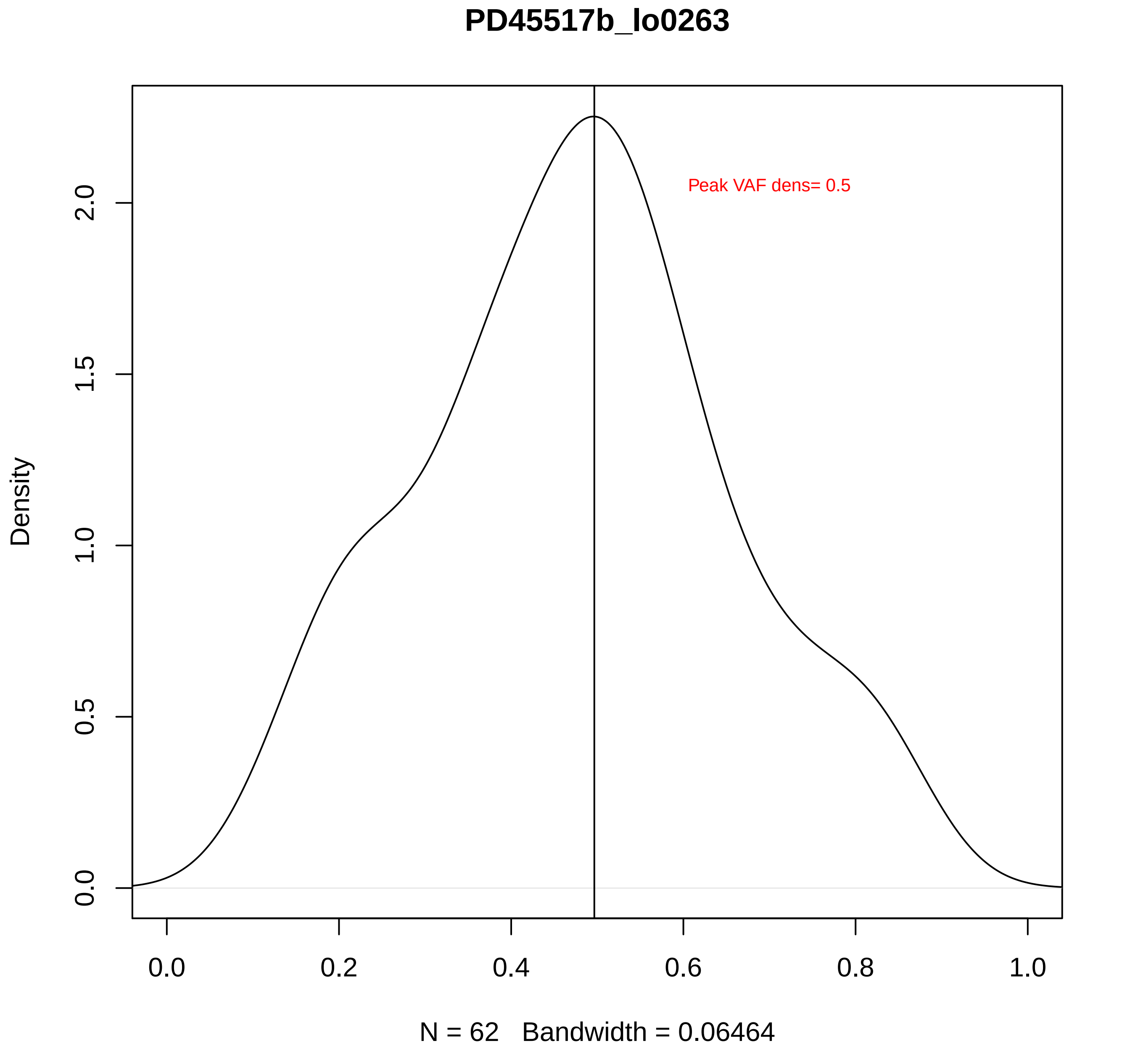

PD45517b_lo0263
Peak VAF dens= 0.5
Density
0.0
0.5
1.0
1.5
2.0
0.0
0.2
0.4
0.6
0.8
1.0
N = 62   Bandwidth = 0.06464

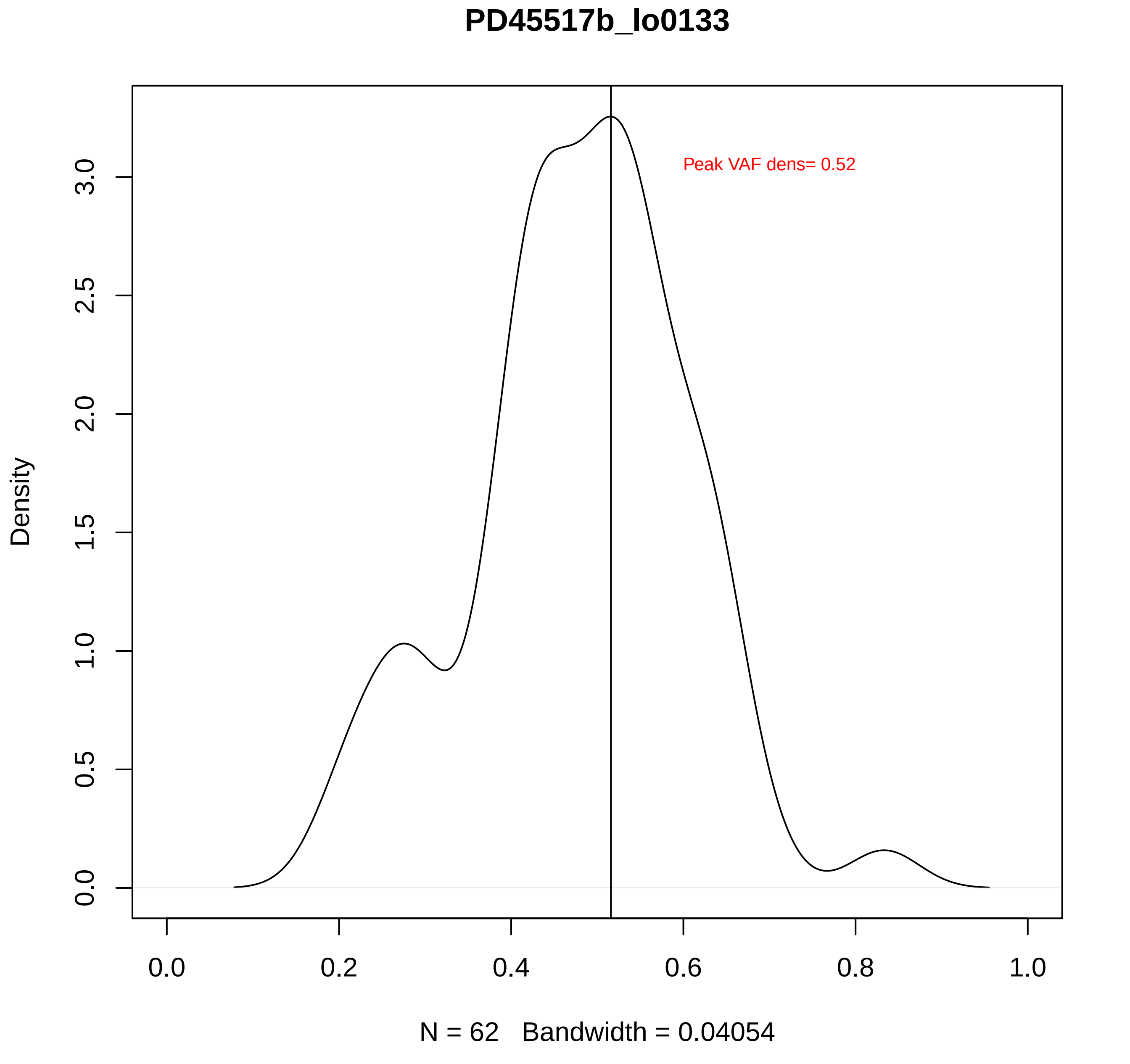

PD45517b_lo0133
Peak VAF dens= 0.52
Density
0.0
0.5
1.0
1.5
2.0
2.5
3.0
0.0
0.2
0.4
0.6
0.8
1.0
N = 62 Bandwidth = 0.04054

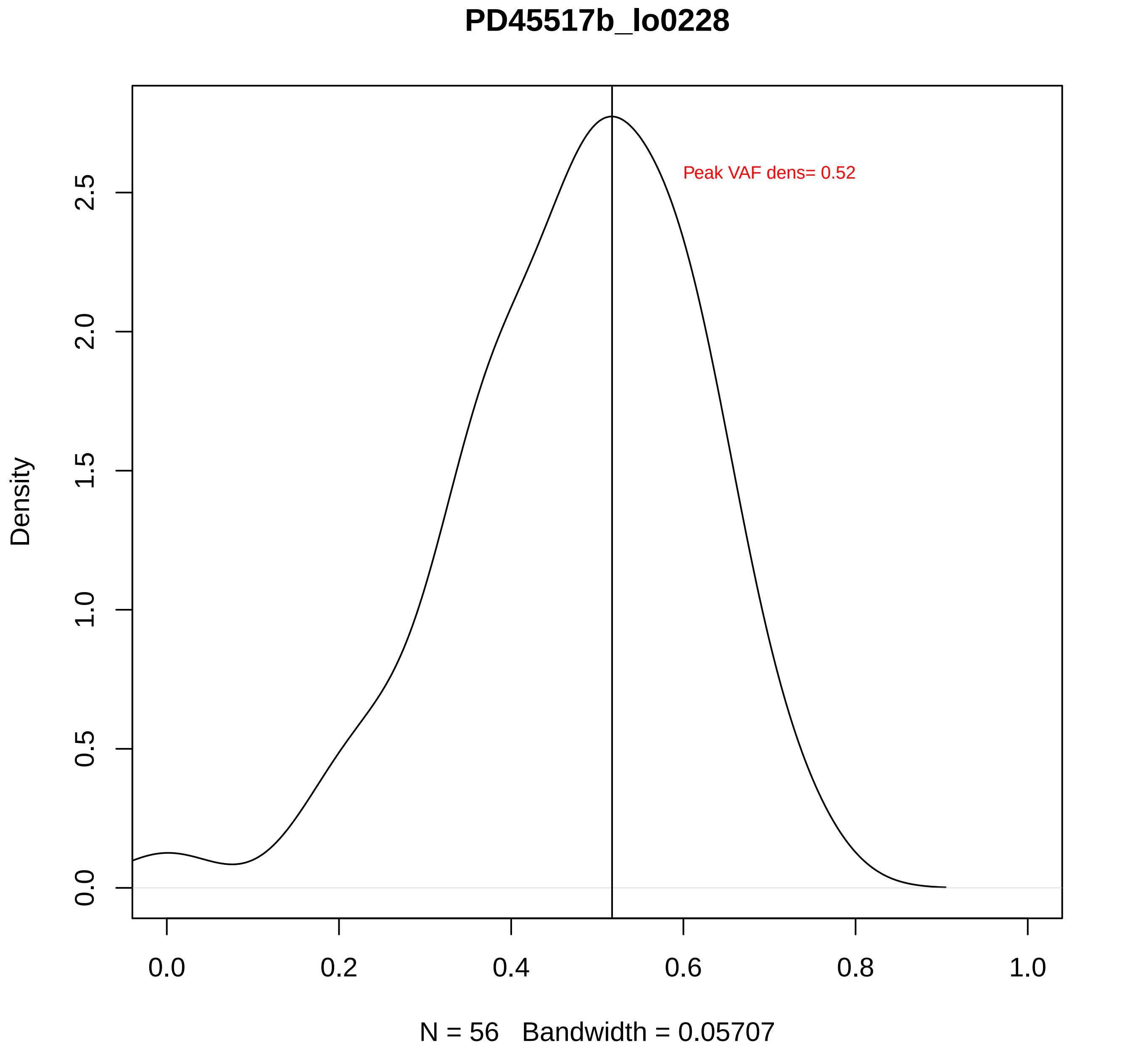
PD45517b_lo0228
Peak VAF dens= 0.52
Density
0.0
0.5
1.0
1.5
2.0
2.5
0.0
0.2
0.4
0.6
0.8
1.0
N = 56   Bandwidth = 0.05707

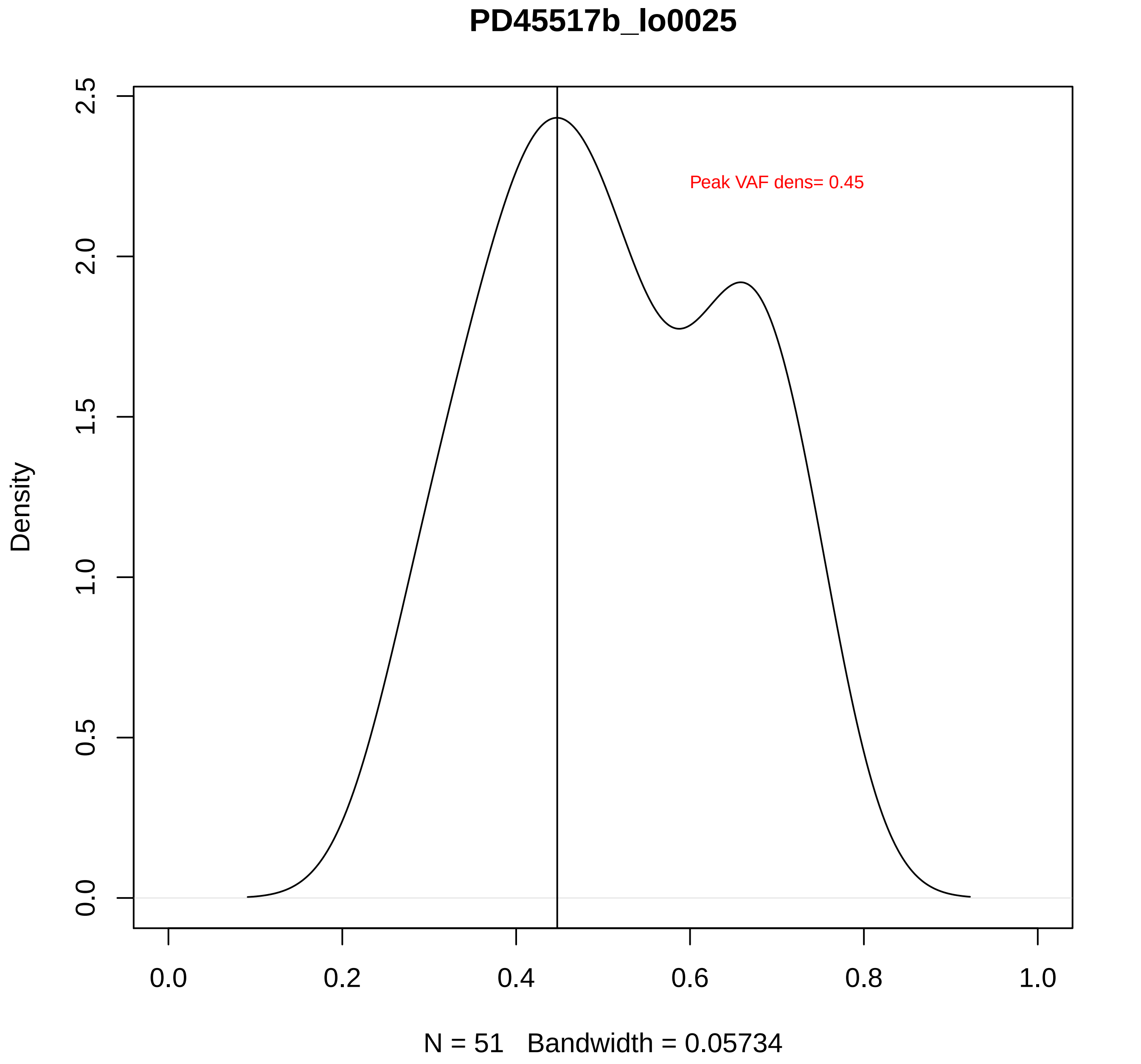

PD45517b_lo0025
Peak VAF dens= 0.45
Density
0.0
0.5
1.0
1.5
2.0
2.5
0.0
0.2
0.4
0.6
0.8
1.0
N = 51   Bandwidth = 0.05734

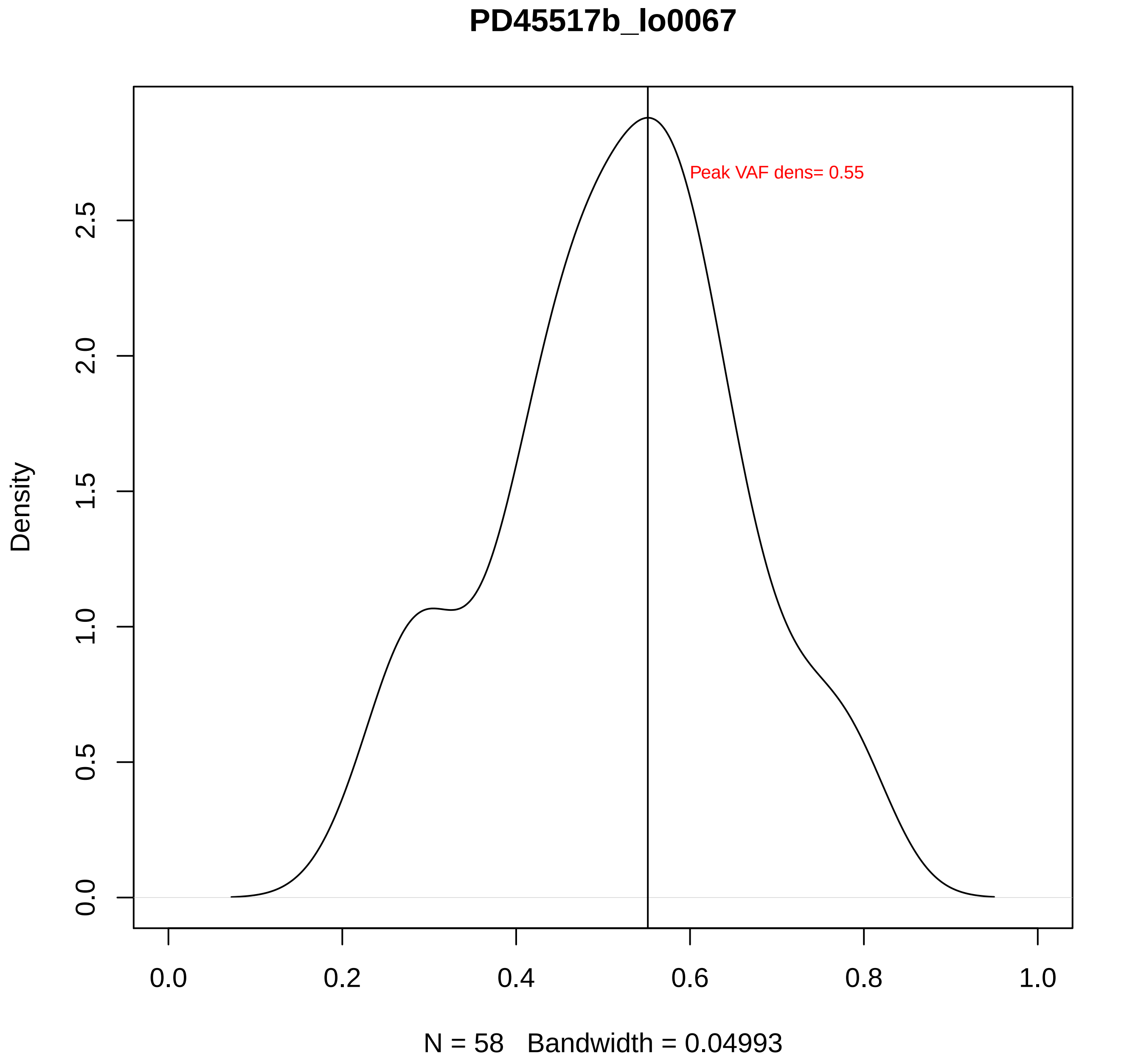
PD45517b_lo0067
Peak VAF dens= 0.55
Density
0.0
0.5
1.0
1.5
2.0
2.5
0.0
0.2
0.4
0.6
0.8
1.0
N = 58   Bandwidth = 0.04993

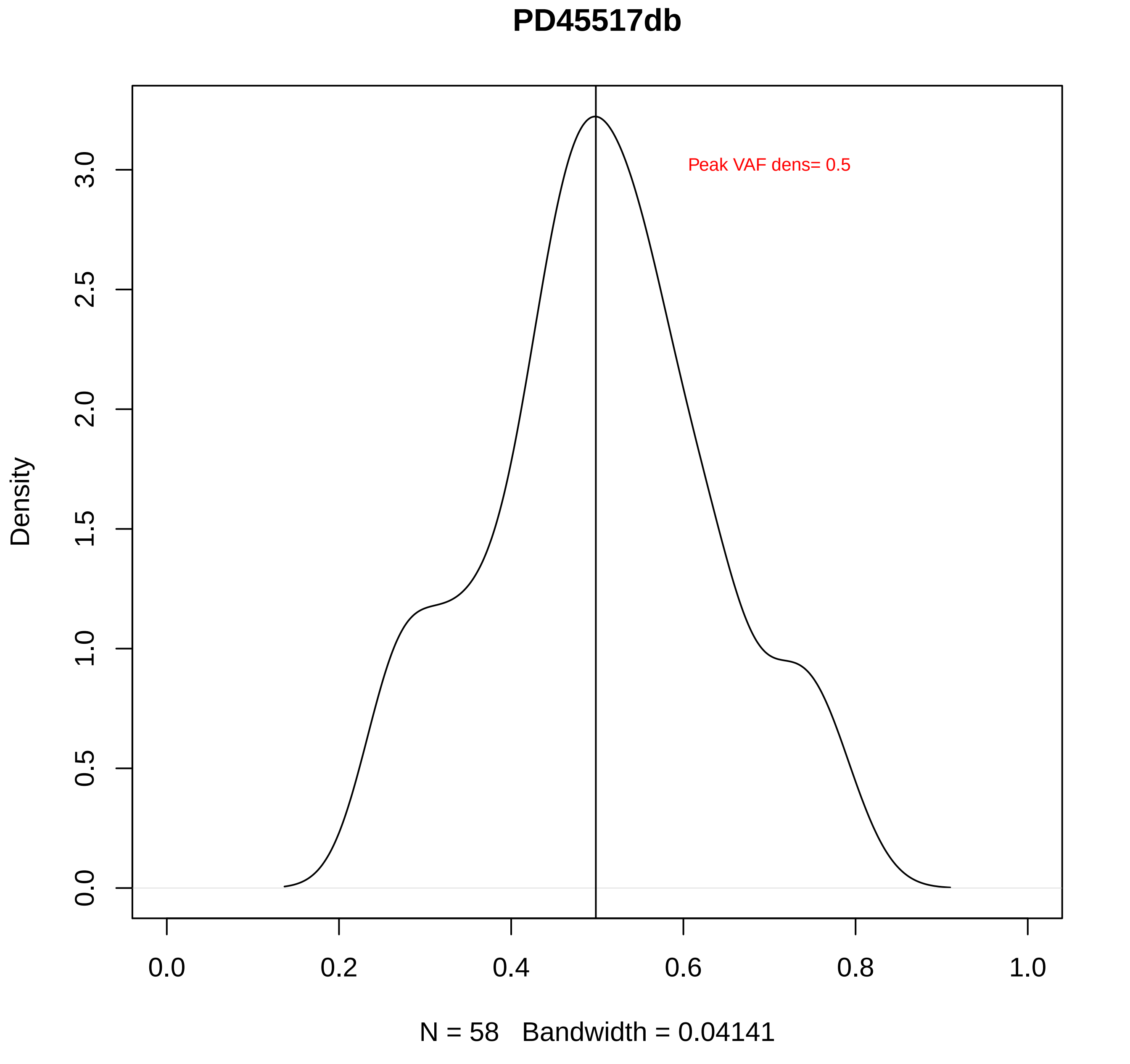
PD45517db
Peak VAF dens= 0.5
Density
0.0
0.5
1.0
1.5
2.0
2.5
3.0
0.0
0.2
0.4
0.6
0.8
1.0
N = 58   Bandwidth = 0.04141

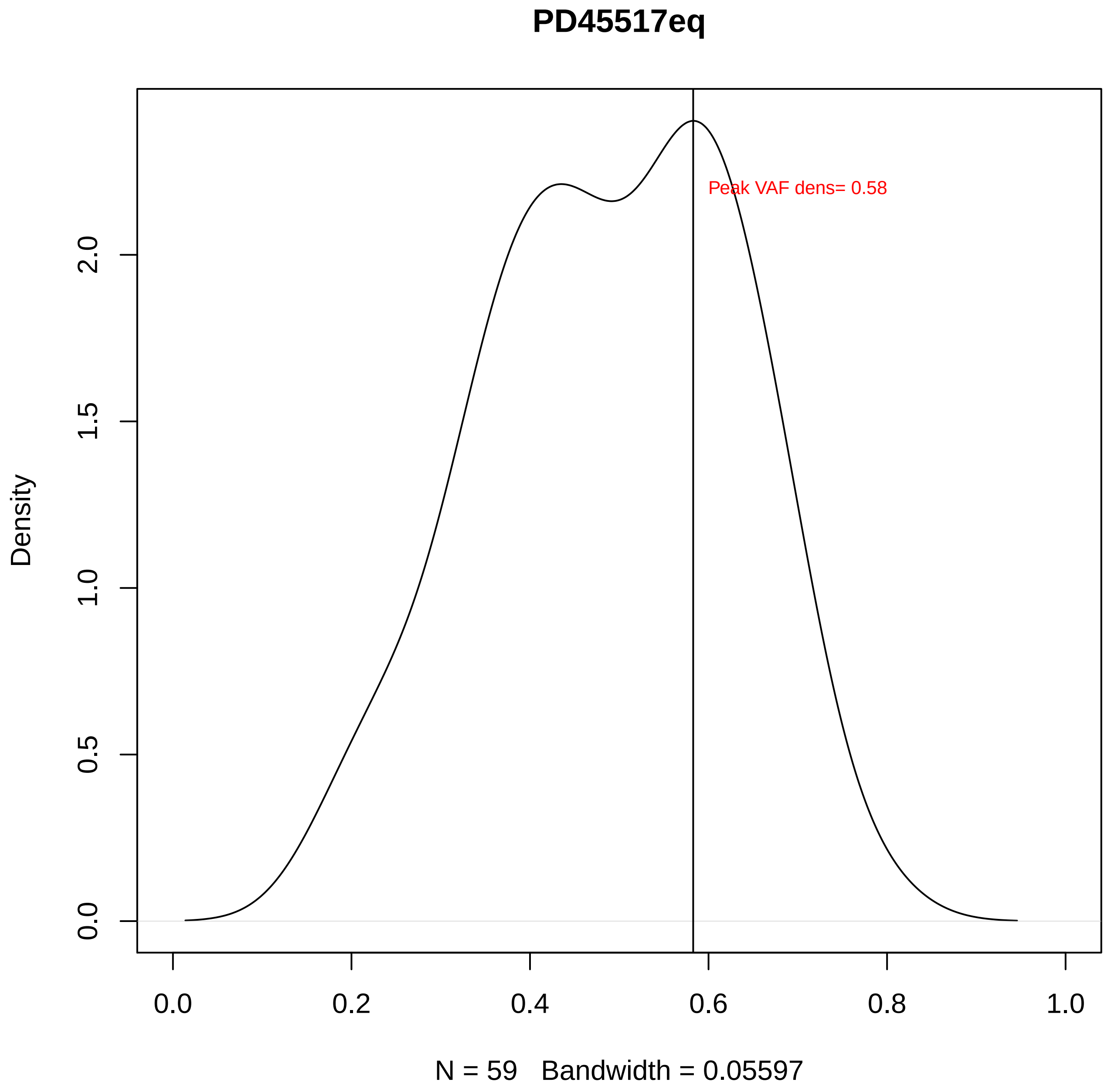
PD45517eq
Peak VAF dens= 0.58
Density
0.0
0.5
1.0
1.5
2.0
0.0
0.2
0.4
0.6
0.8
1.0
N = 59   Bandwidth = 0.05597

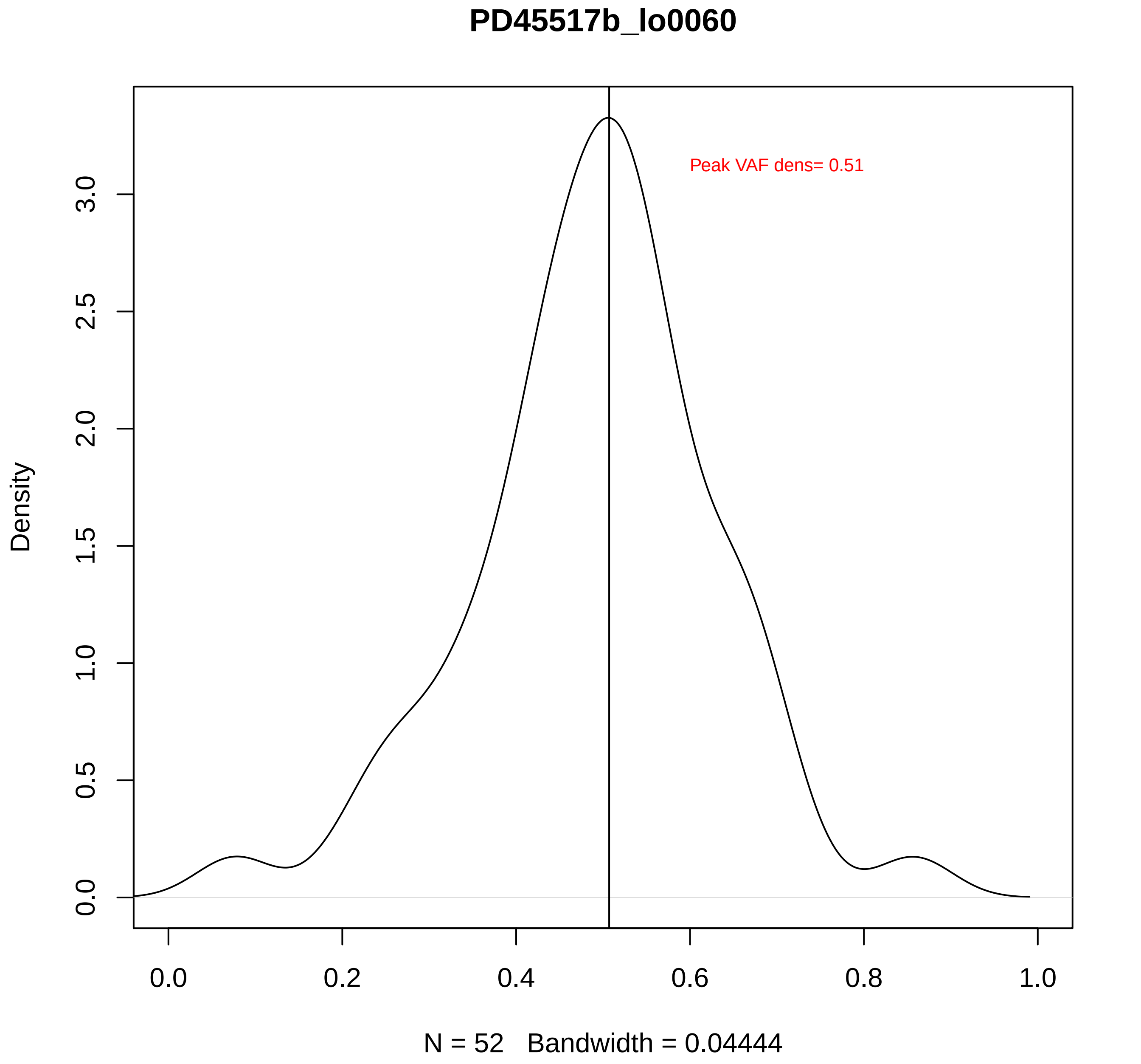

PD45517b_lo0060
Peak VAF dens= 0.51
Density
0.0
0.5
1.0
1.5
2.0
2.5
3.0
0.0
0.2
0.4
0.6
0.8
1.0
N = 52   Bandwidth = 0.04444

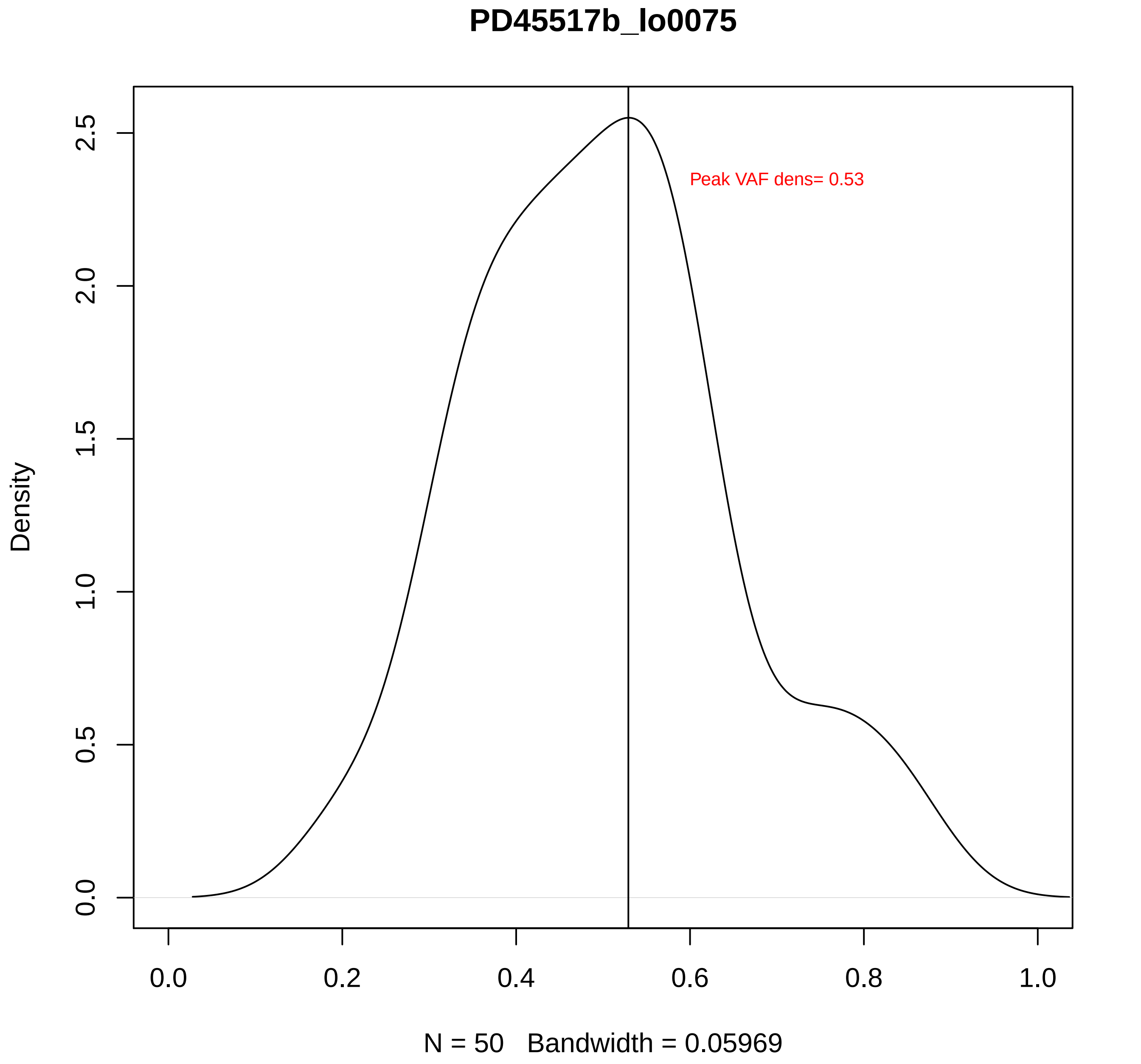
PD45517b_lo0075
Peak VAF dens= 0.53
Density
0.0
0.5
1.0
1.5
2.0
2.5
0.0
0.2
0.4
0.6
0.8
1.0
N = 50 Bandwidth = 0.05969

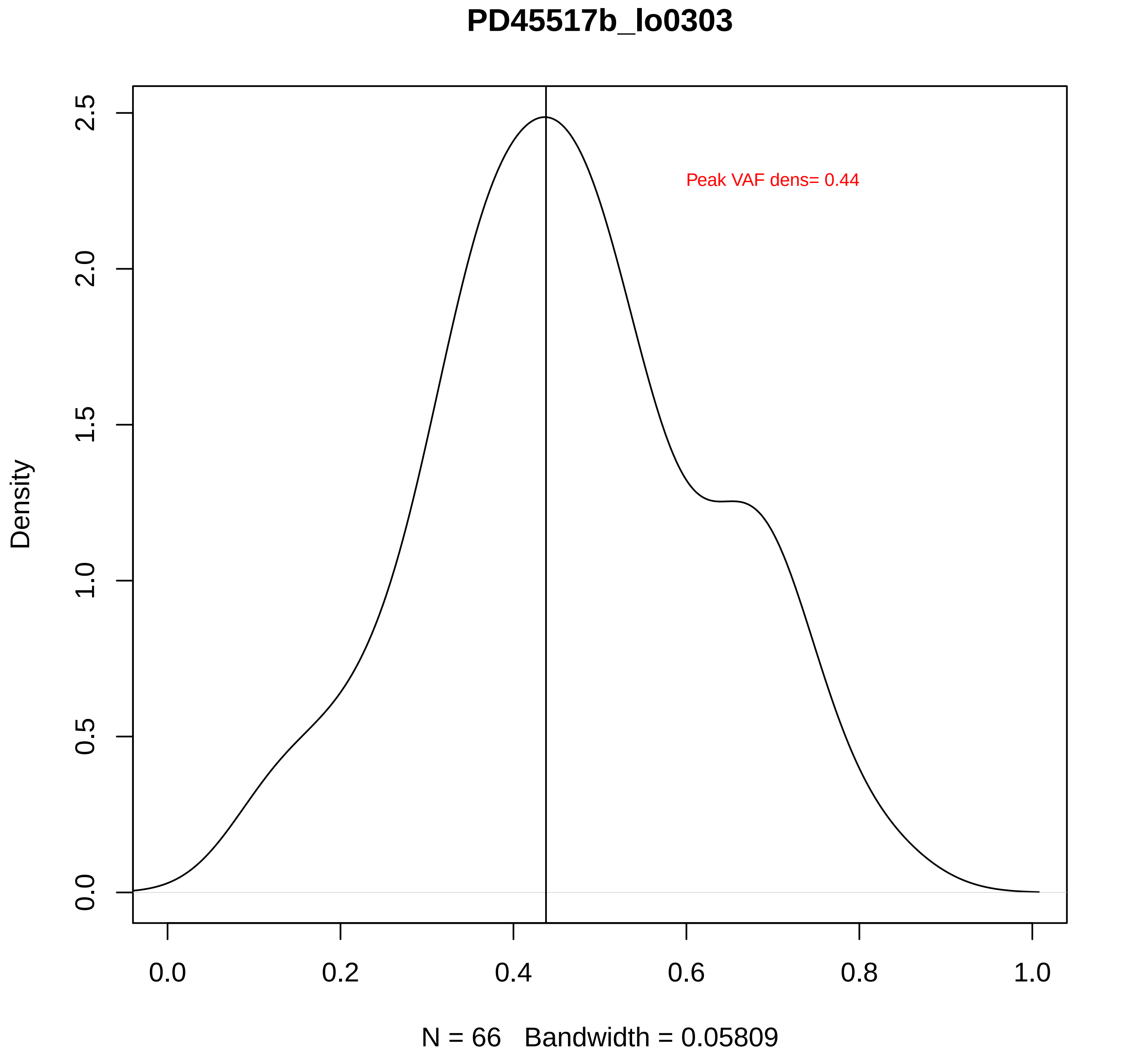
PD45517b_lo0303
Peak VAF dens= 0.44
Density
0.0
0.5
1.0
1.5
2.0
2.5
0.0
0.2
0.4
0.6
0.8
1.0
N = 66   Bandwidth = 0.05809

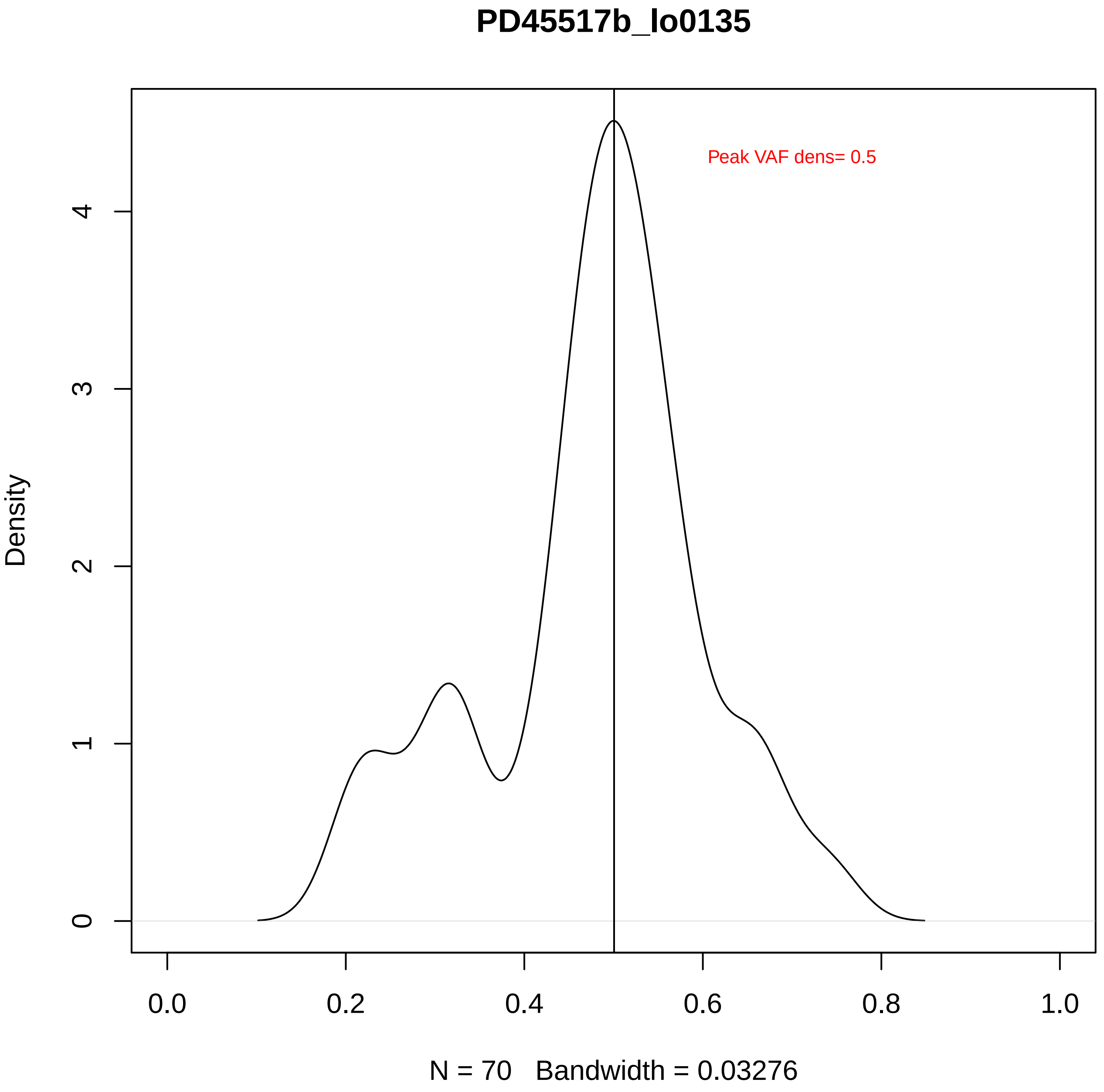
PD45517b_lo0135
Peak VAF dens= 0.5
Density
0
1
2
3
4
0.0
0.2
0.4
0.6
0.8
1.0
N = 70 Bandwidth = 0.03276

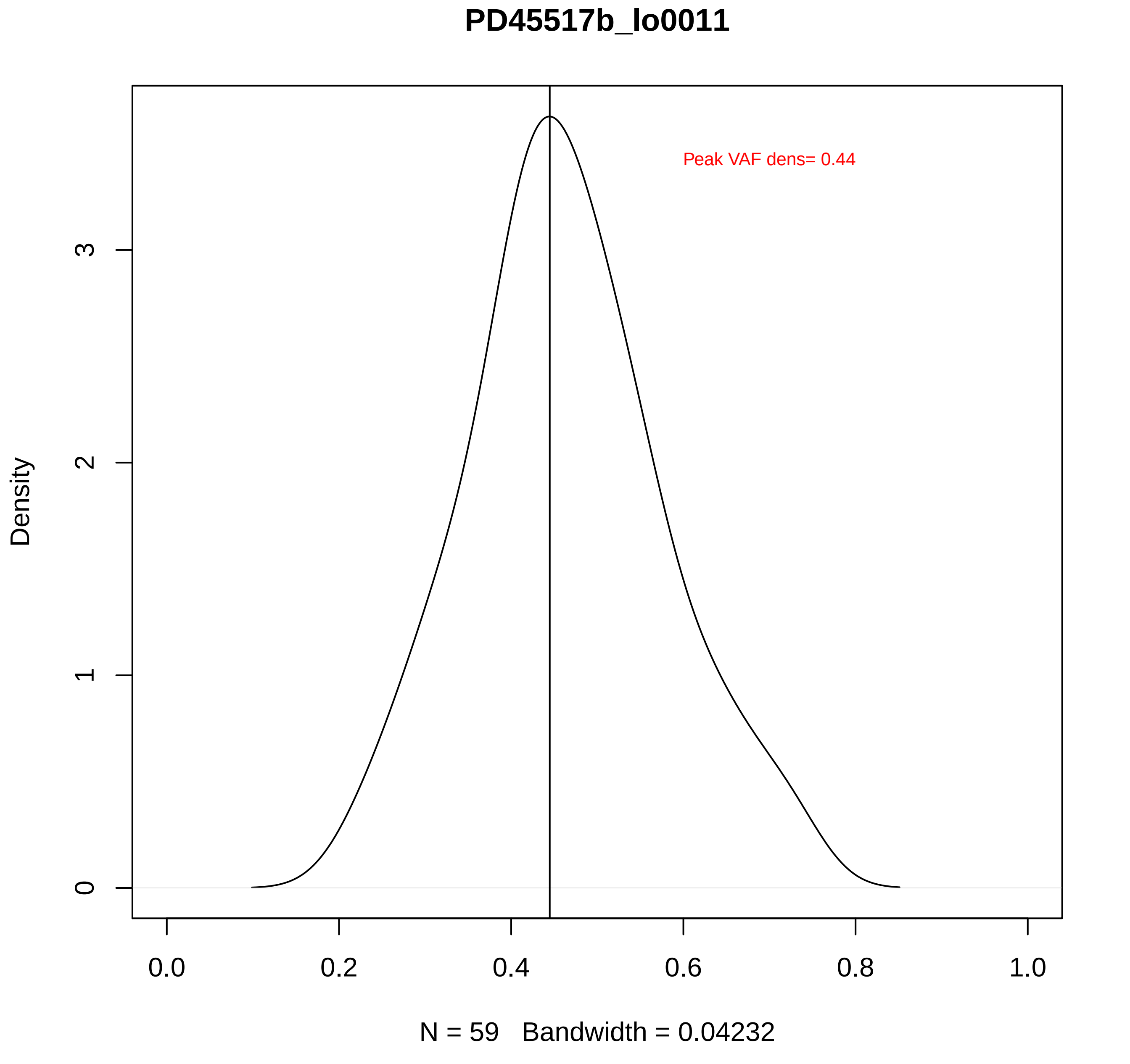
PD45517b_lo0011
Peak VAF dens= 0.44
Density
0
1
2
3
0.0
0.2
0.4
0.6
0.8
1.0
N = 59   Bandwidth = 0.04232

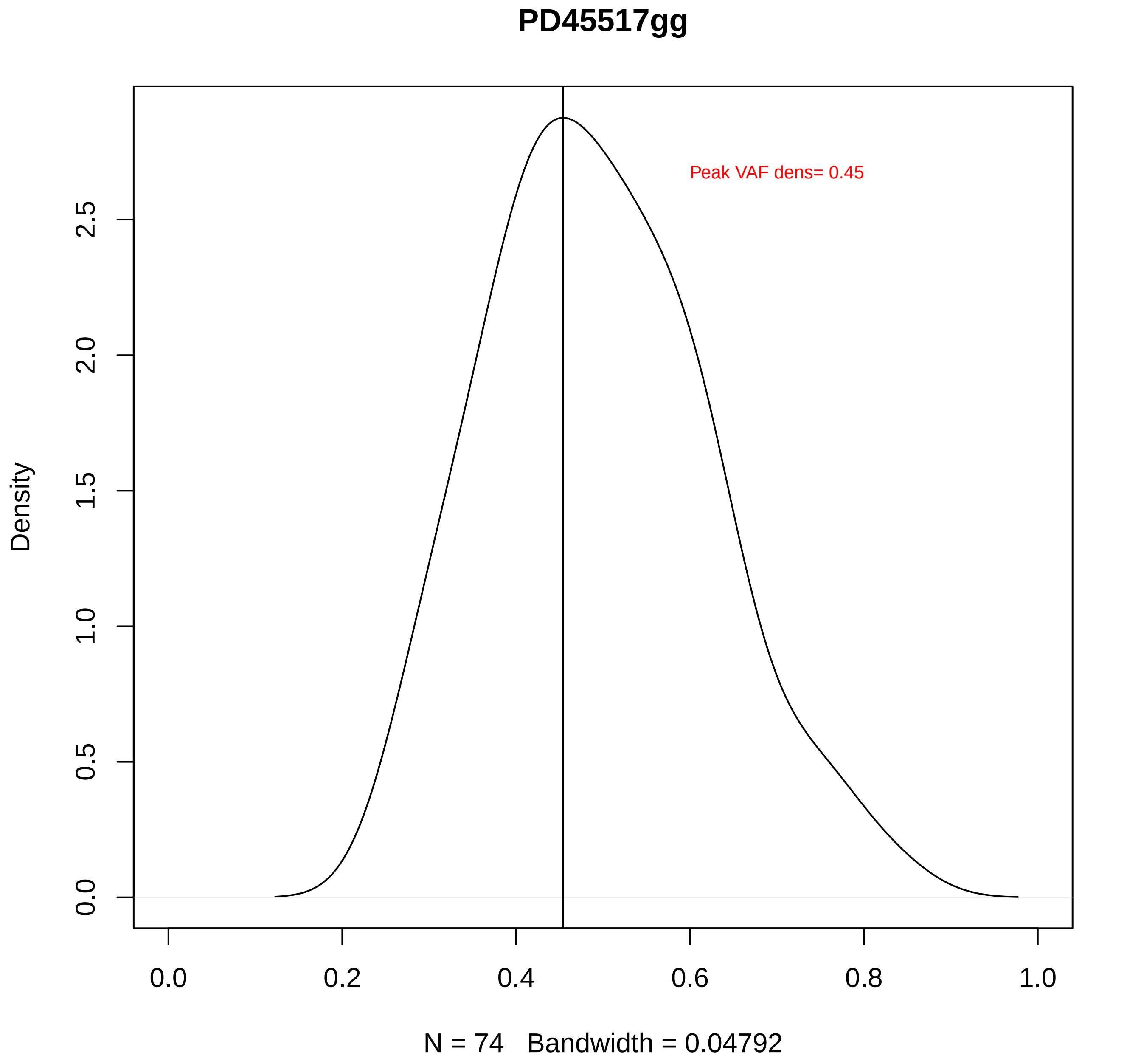
PD45517gg
Peak VAF dens= 0.45
Density
0.0
0.5
1.0
1.5
2.0
2.5
0.0
0.2
0.4
0.6
0.8
1.0
N = 74   Bandwidth = 0.04792

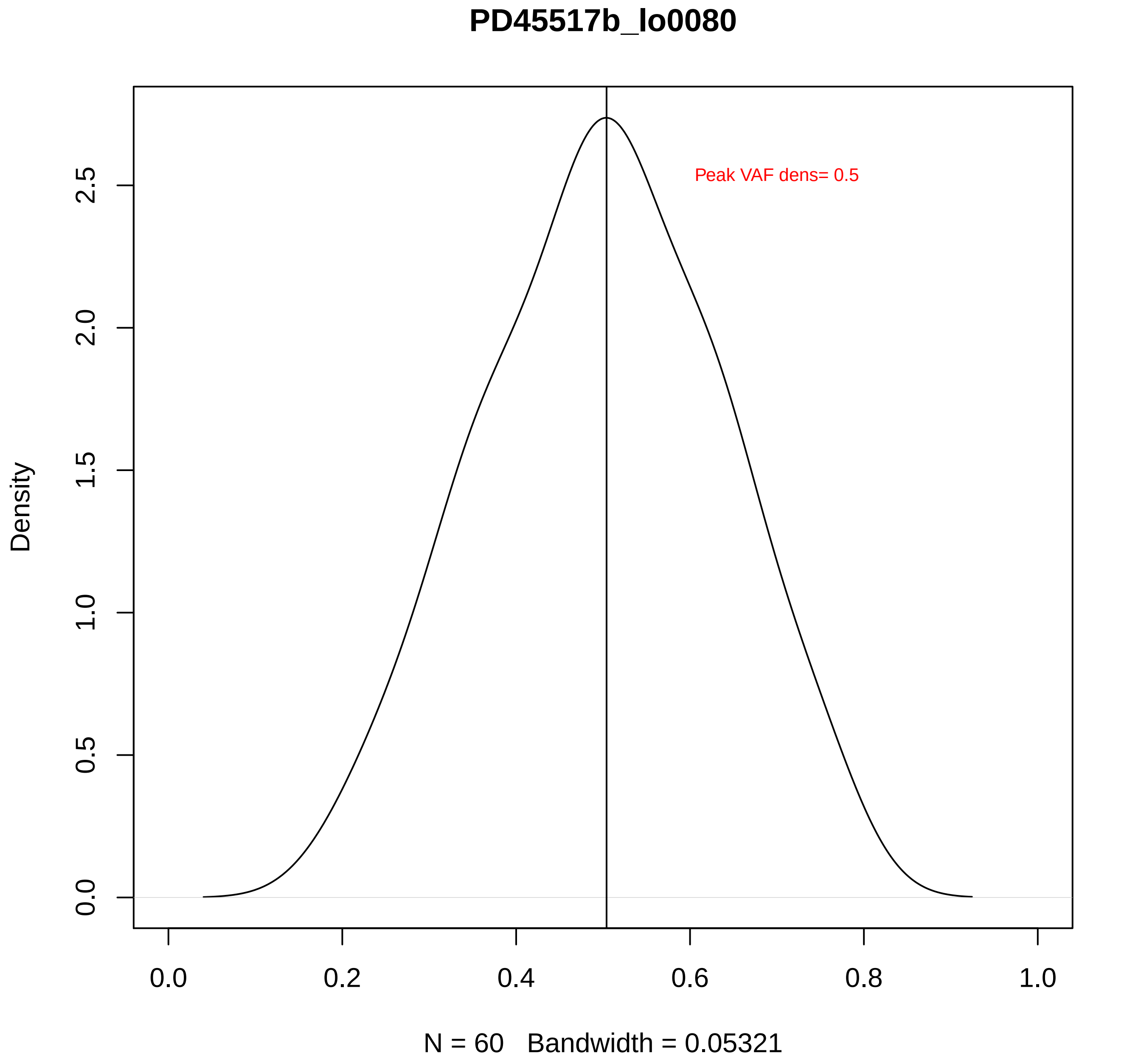
PD45517b_lo0080
Peak VAF dens= 0.5
Density
0.0
0.5
1.0
1.5
2.0
2.5
0.0
0.2
0.4
0.6
0.8
1.0
N = 60   Bandwidth = 0.05321

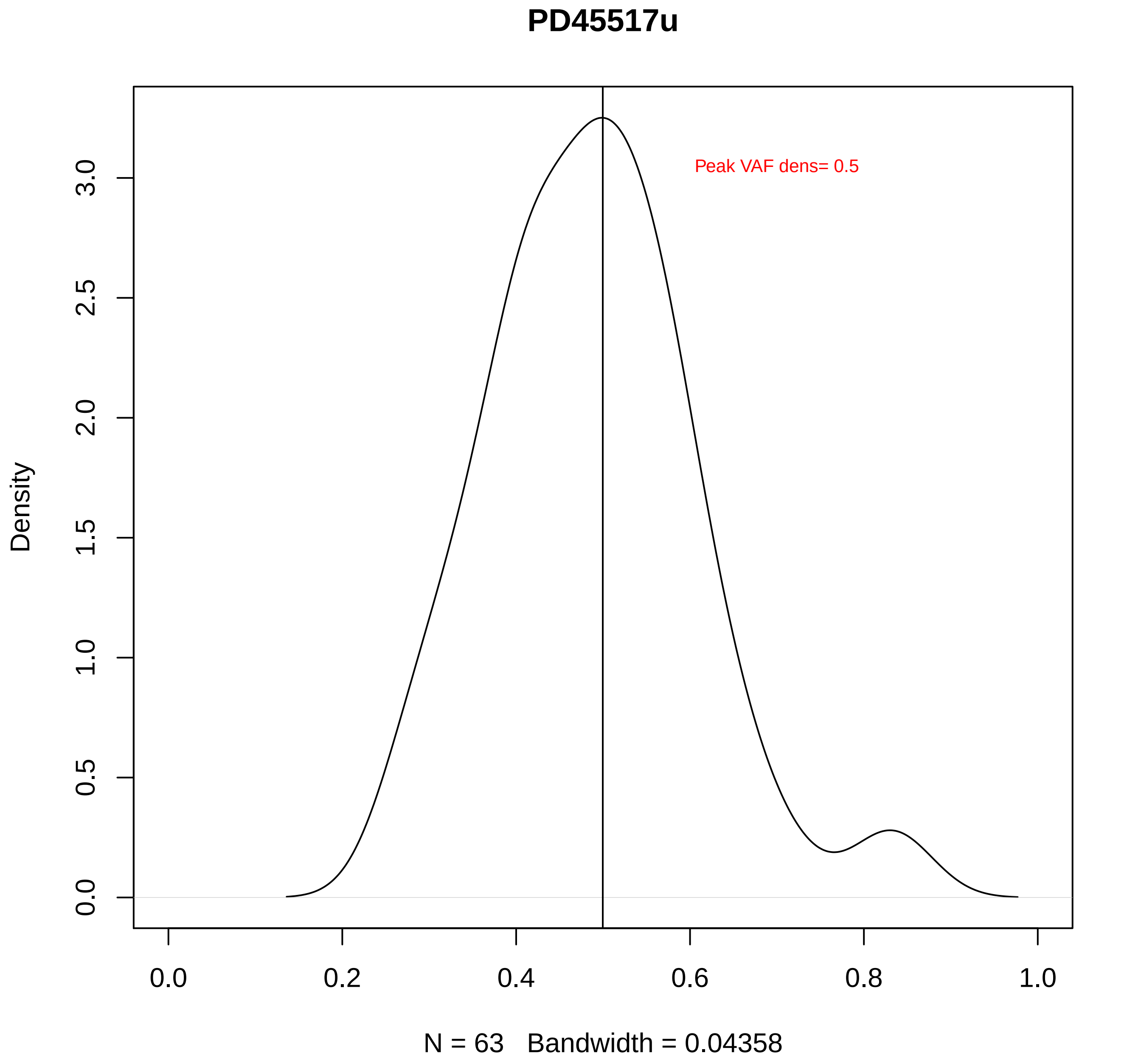
PD45517u
Peak VAF dens= 0.5
Density
0.0
0.5
1.0
1.5
2.0
2.5
3.0
0.0
0.2
0.4
0.6
0.8
1.0
N = 63   Bandwidth = 0.04358

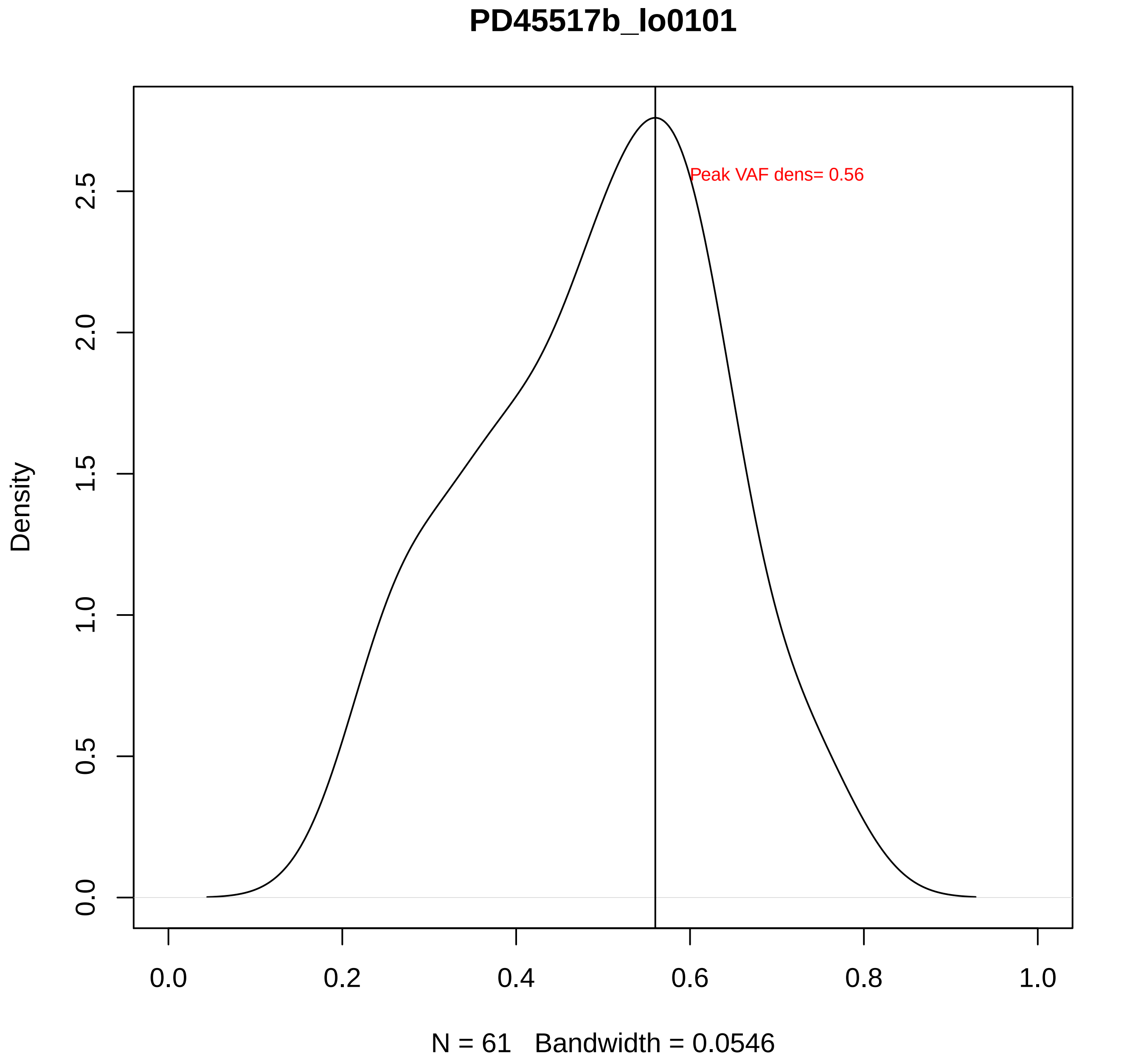
PD45517b_lo0101
Peak VAF dens= 0.56
Density
0.0
0.5
1.0
1.5
2.0
2.5
0.0
0.2
0.4
0.6
0.8
1.0
N = 61   Bandwidth = 0.0546

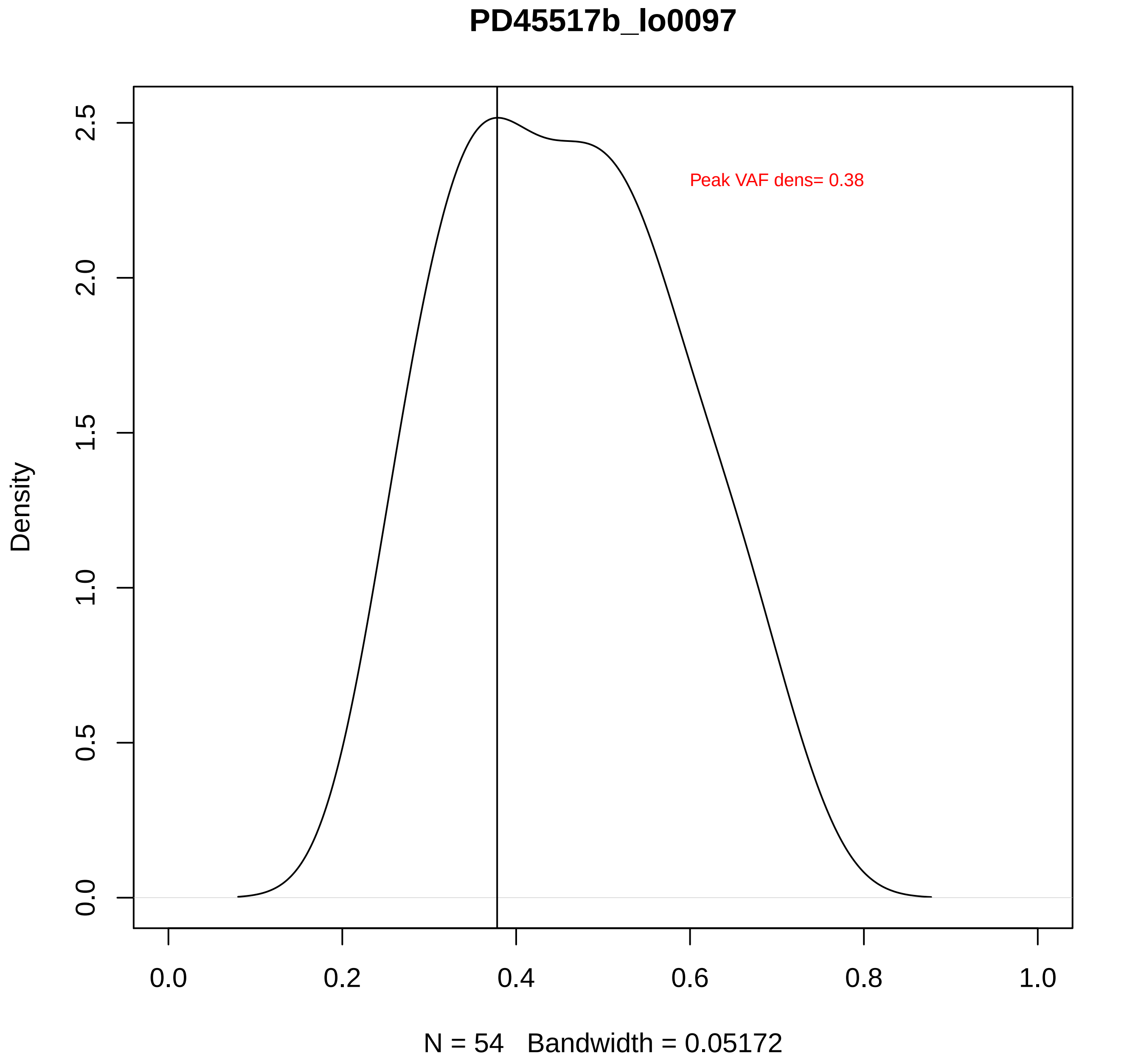

PD45517b_lo0097
Peak VAF dens= 0.38
Density
0.0
0.5
1.0
1.5
2.0
2.5
0.0
0.2
0.4
0.6
0.8
1.0
N = 54 Bandwidth = 0.05172

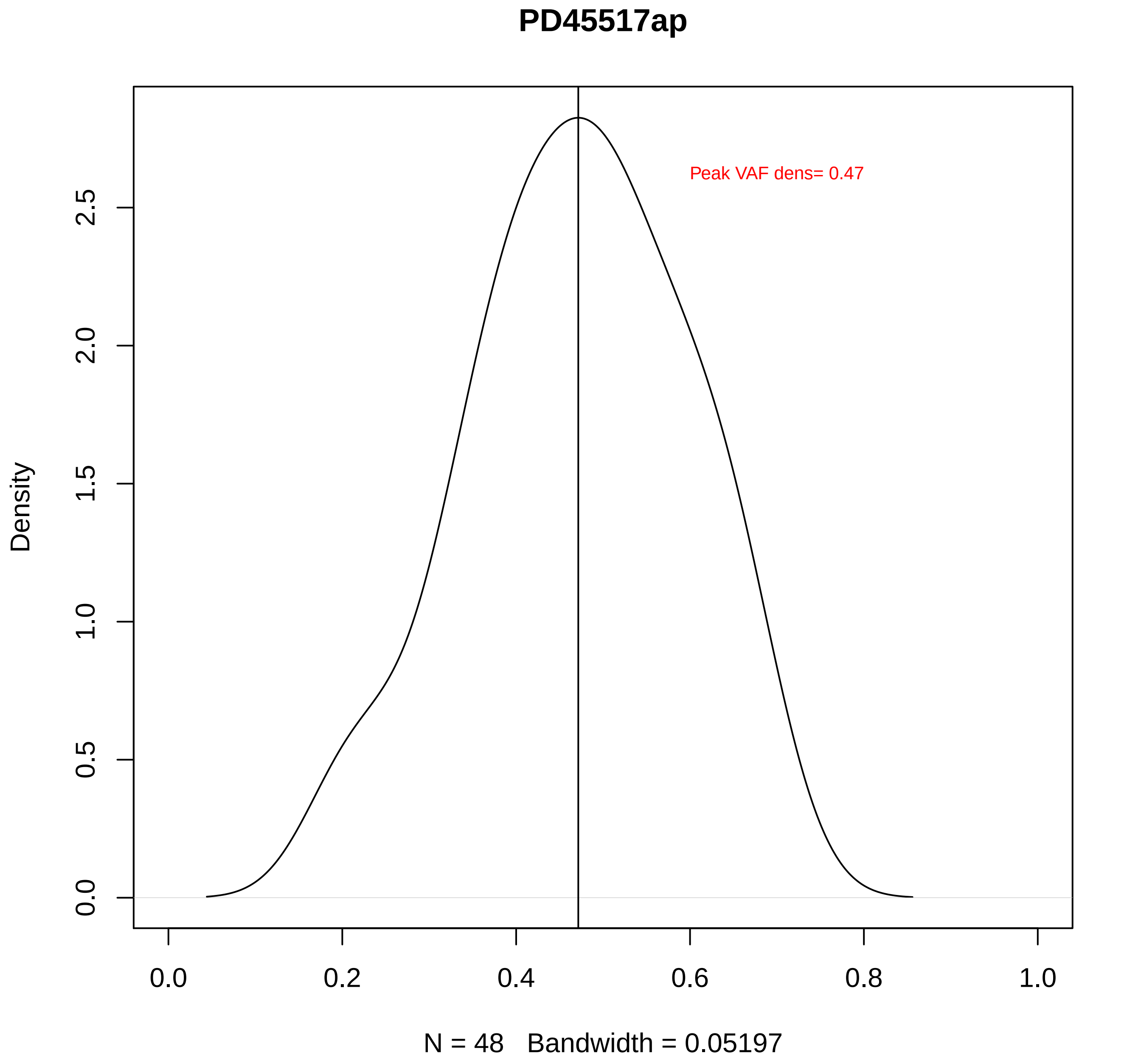
PD45517ap
Peak VAF dens= 0.47
Density
0.0
0.5
1.0
1.5
2.0
2.5
0.0
0.2
0.4
0.6
0.8
1.0
N = 48   Bandwidth = 0.05197

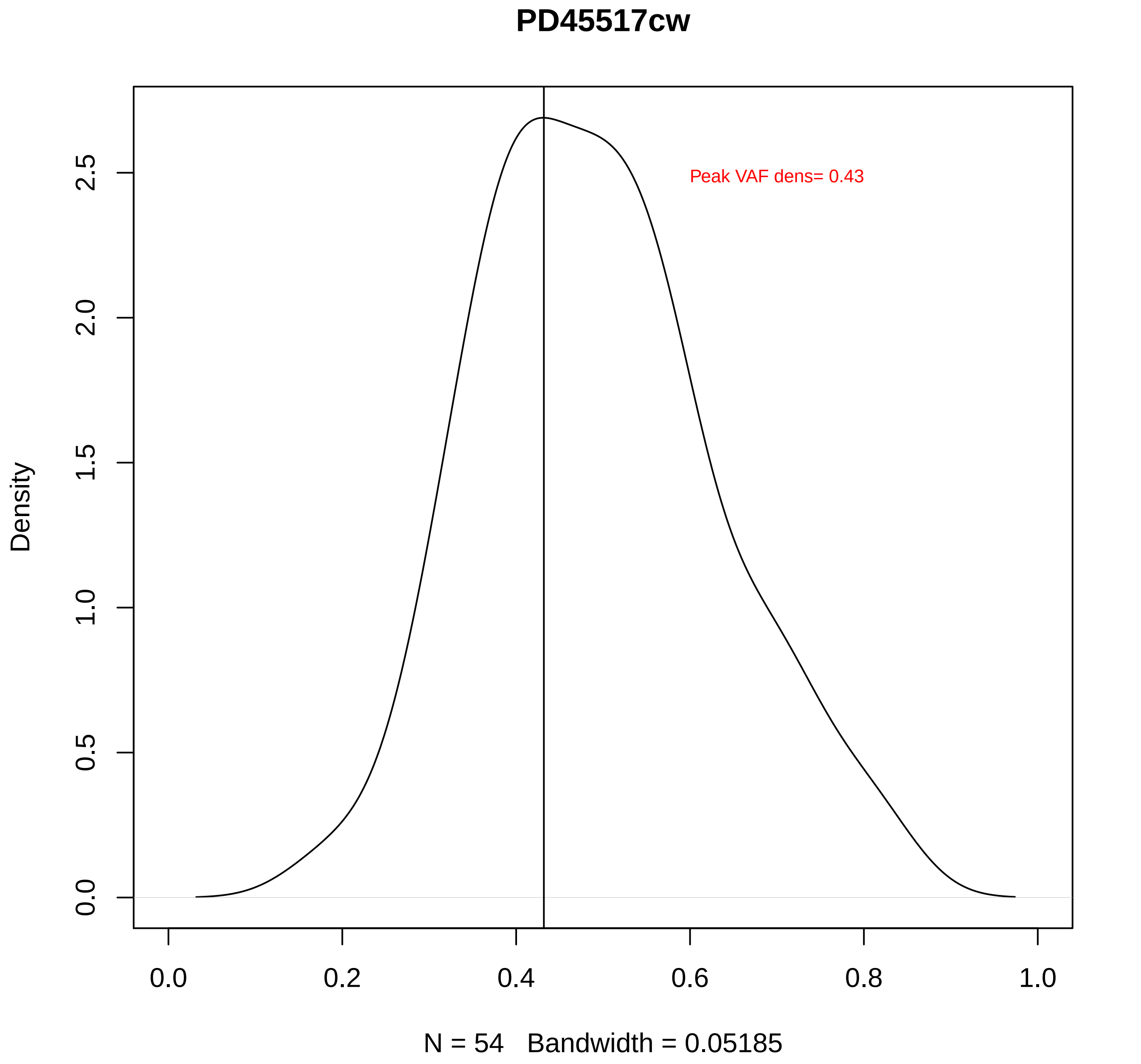

PD45517cw
Peak VAF dens= 0.43
Density
0.0
0.5
1.0
1.5
2.0
2.5
0.0
0.2
0.4
0.6
0.8
1.0
N = 54   Bandwidth = 0.05185

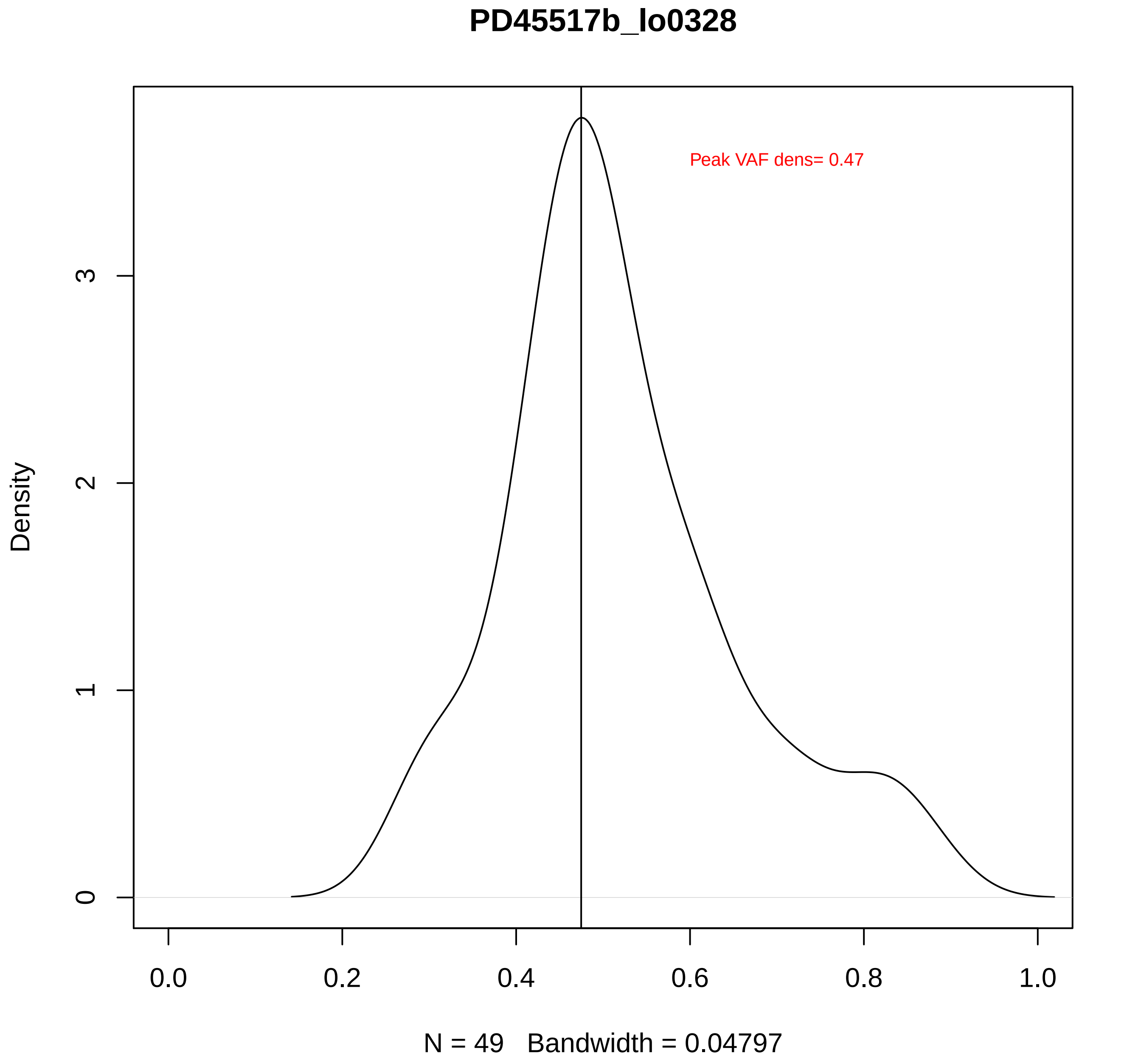
PD45517b_lo0328
Peak VAF dens= 0.47
Density
0
1
2
3
0.0
0.2
0.4
0.6
0.8
1.0
N = 49   Bandwidth = 0.04797

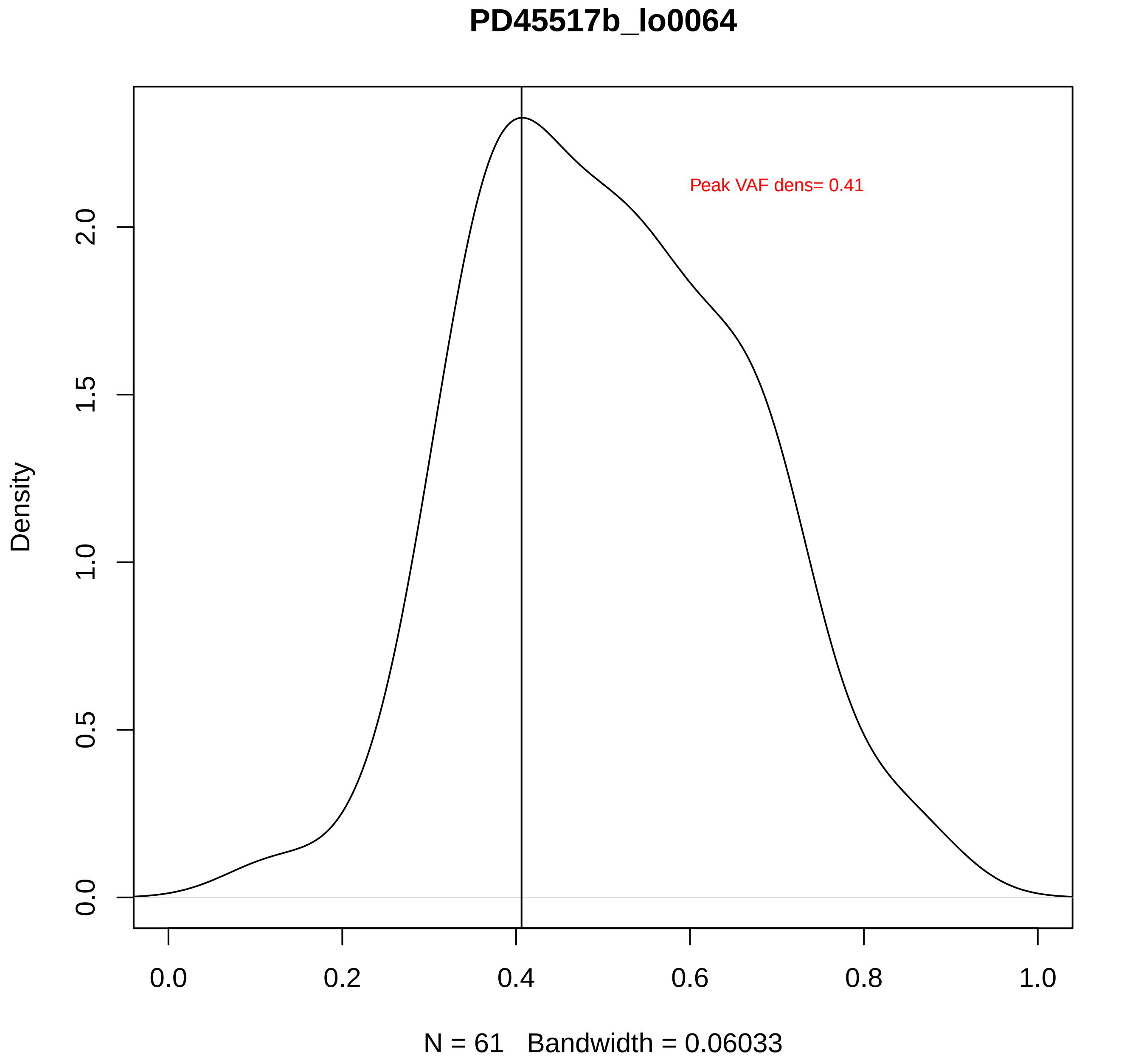
PD45517b_lo0064
Peak VAF dens= 0.41
Density
0.0
0.5
1.0
1.5
2.0
0.0
0.2
0.4
0.6
0.8
1.0
N = 61   Bandwidth = 0.06033

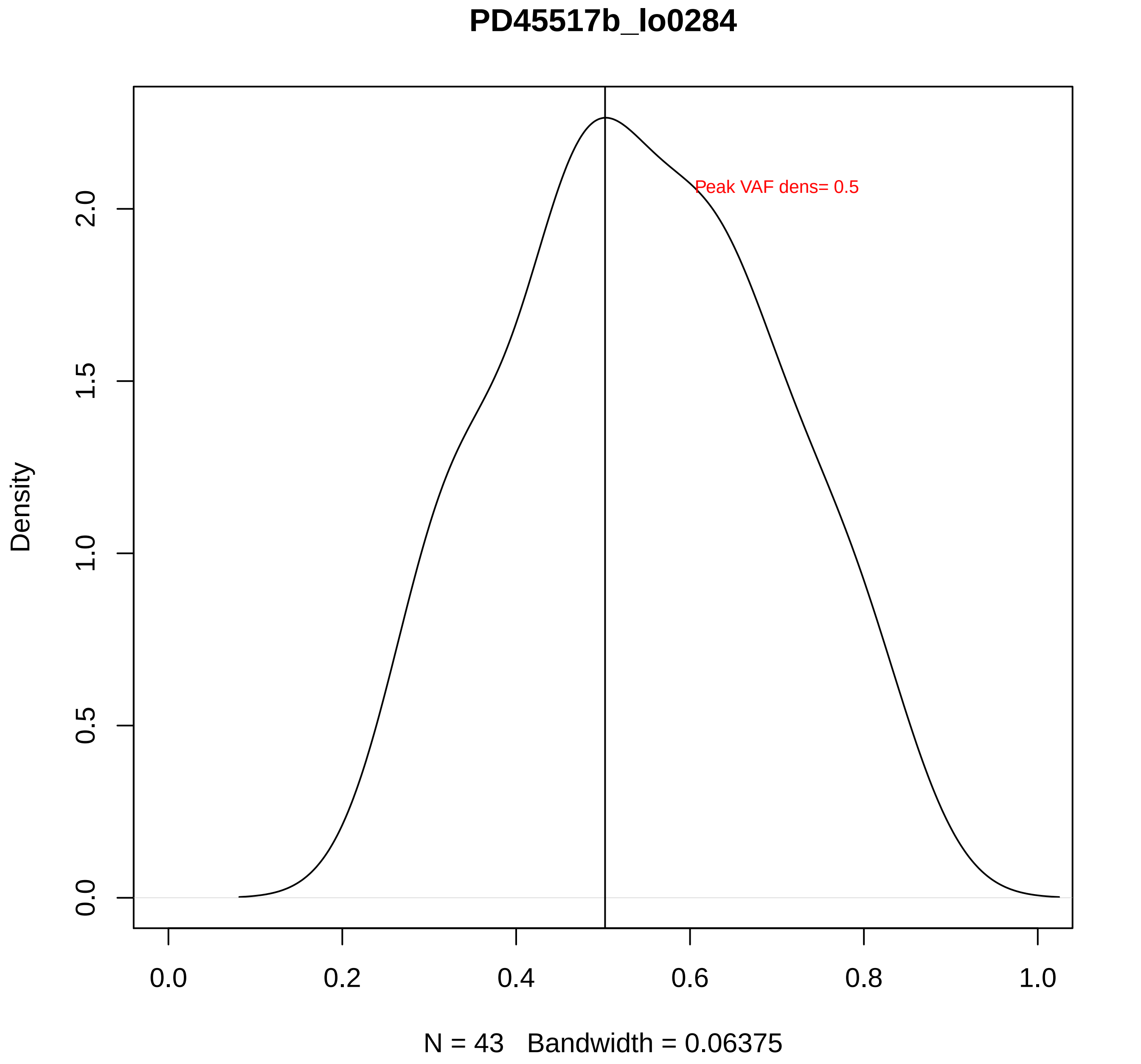
PD45517b_lo0284
Peak VAF dens= 0.5
Density
0.0
0.5
1.0
1.5
2.0
0.0
0.2
0.4
0.6
0.8
1.0
N = 43   Bandwidth = 0.06375

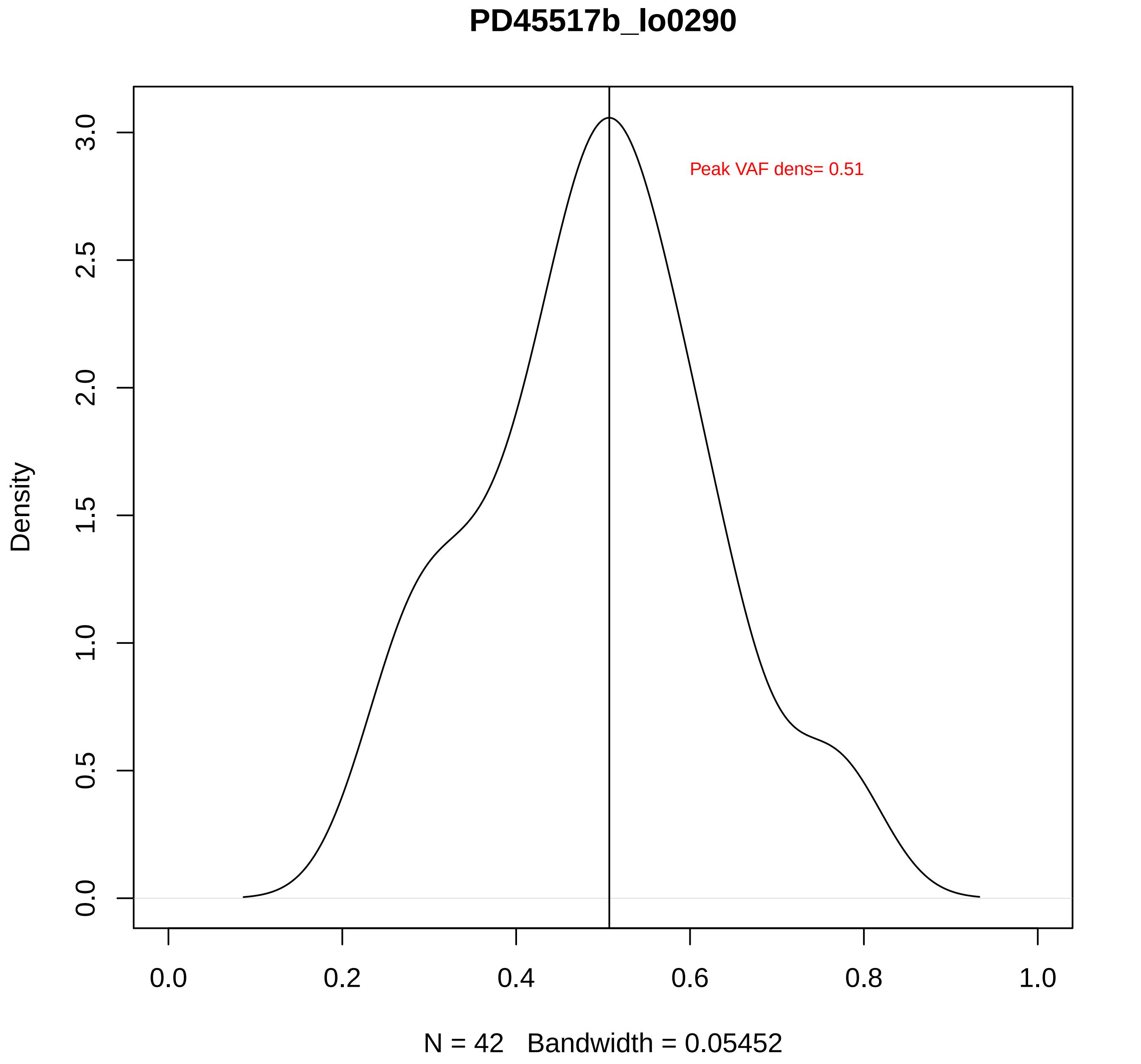
PD45517b_lo0290
Peak VAF dens= 0.51
Density
0.0
0.5
1.0
1.5
2.0
2.5
3.0
0.0
0.2
0.4
0.6
0.8
1.0
N = 42   Bandwidth = 0.05452

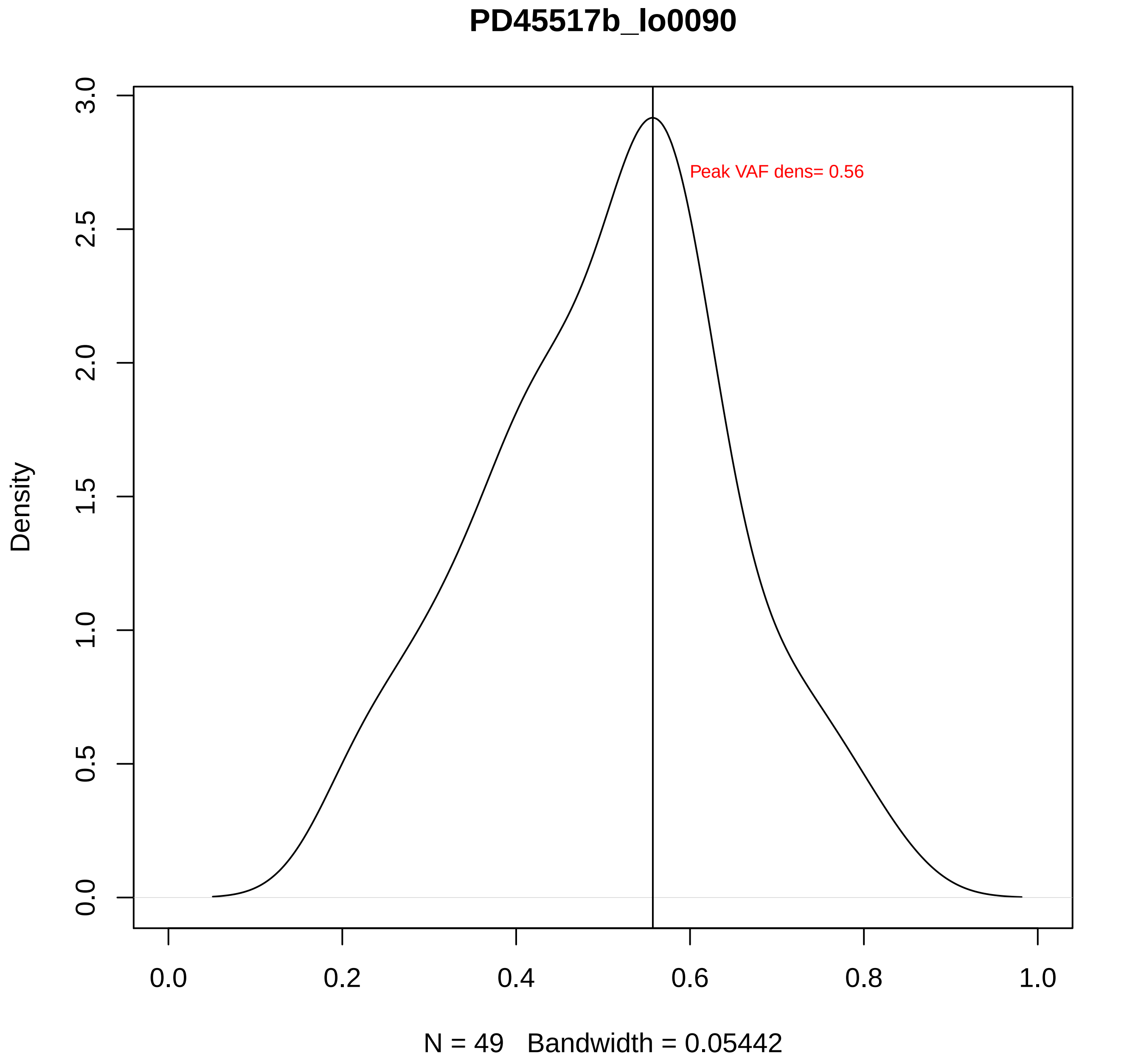
PD45517b_lo0090
Peak VAF dens= 0.56
Density
3.0
2.5
2.0
1.5
1.0
0.5
0.0
0.0
0.2
0.4
0.6
0.8
1.0
N = 49 Bandwidth = 0.05442

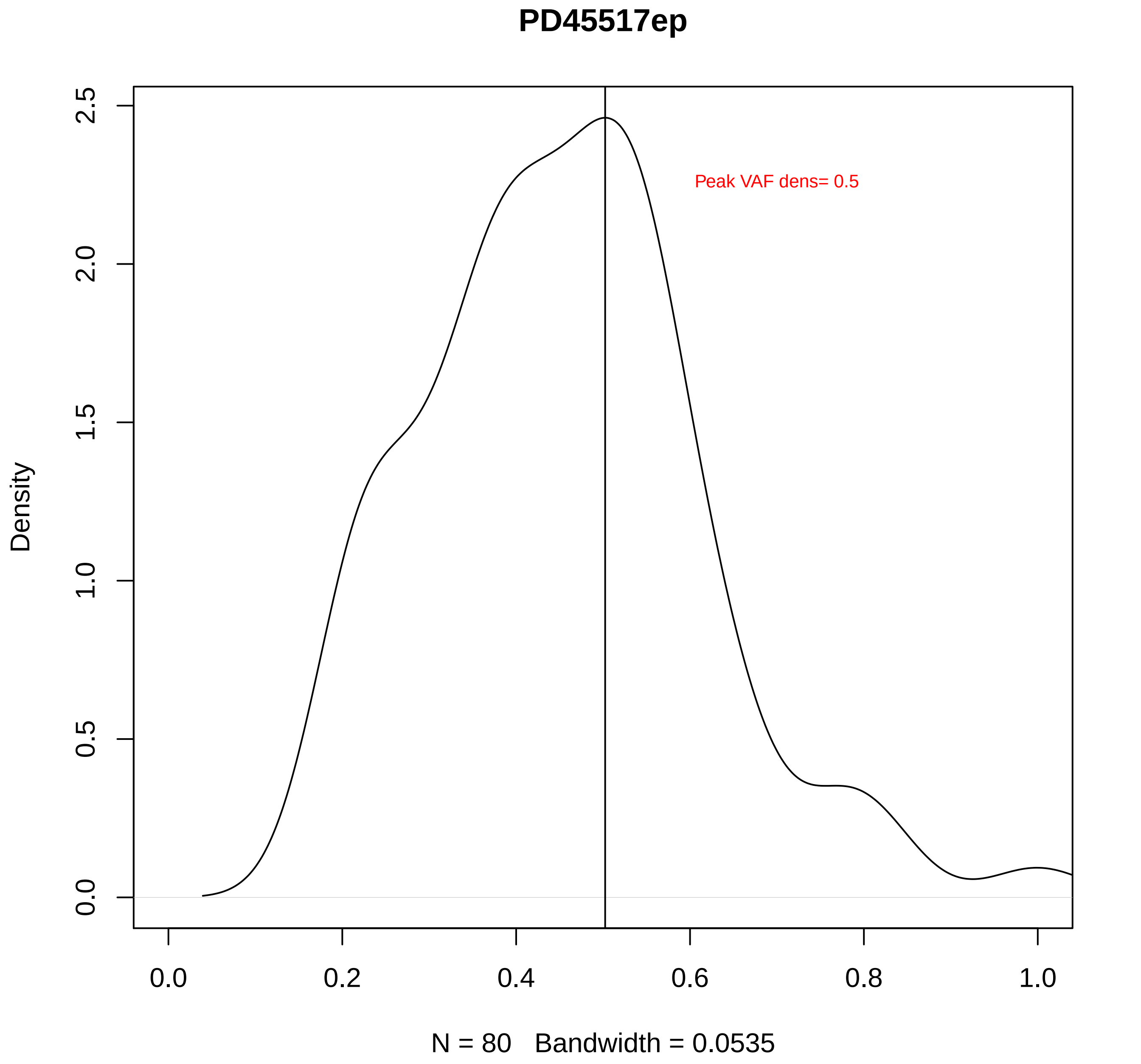
PD45517ep
Peak VAF dens= 0.5
Density
0.0
0.5
1.0
1.5
2.0
2.5
0.0
0.2
0.4
0.6
0.8
1.0
N = 80 Bandwidth = 0.0535

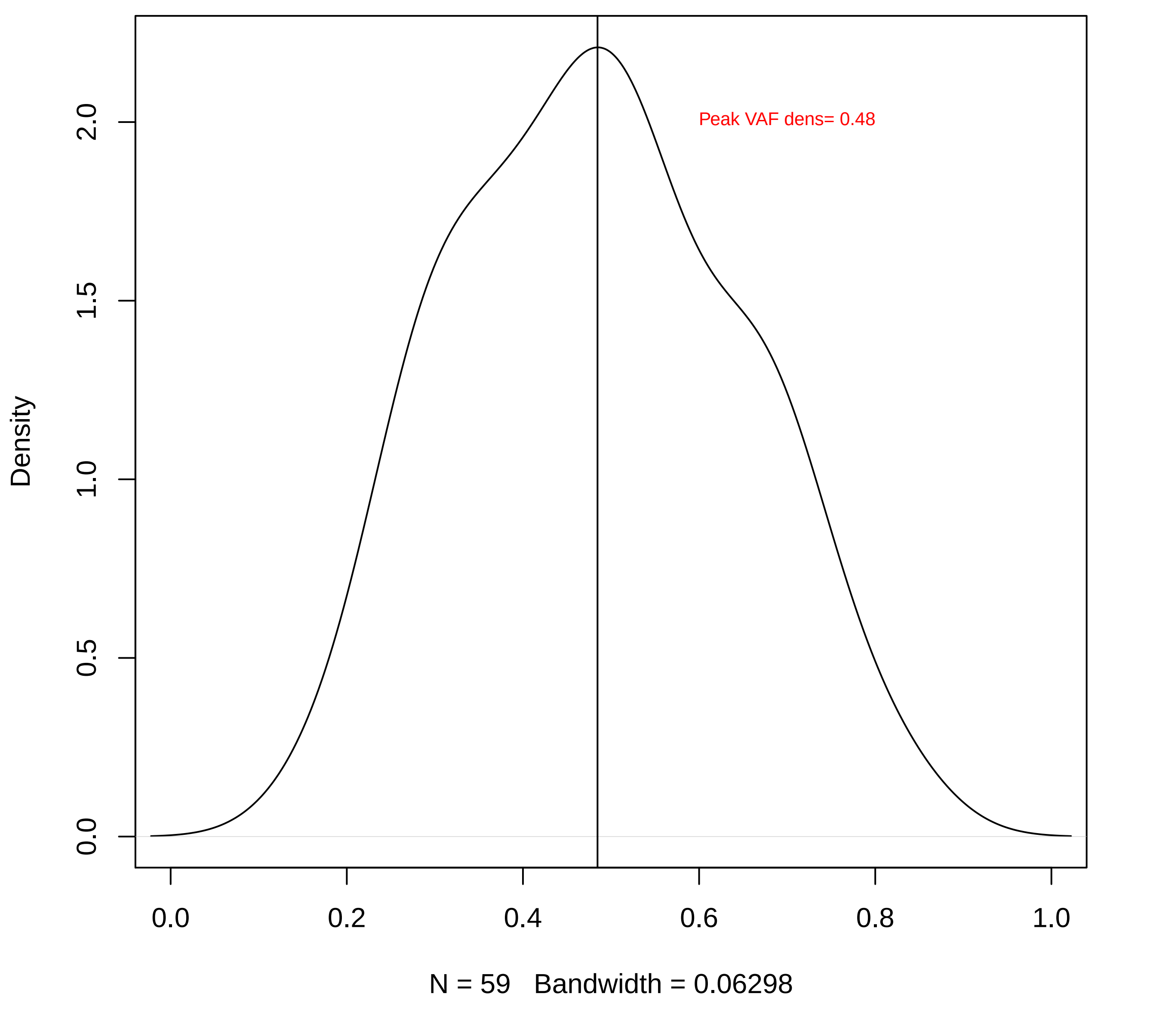
PD45517b_lo0061
Peak VAF dens= 0.48
Density
0.0
0.5
1.0
1.5
2.0
0.0
0.2
0.4
0.6
0.8
1.0
N = 59   Bandwidth = 0.06298

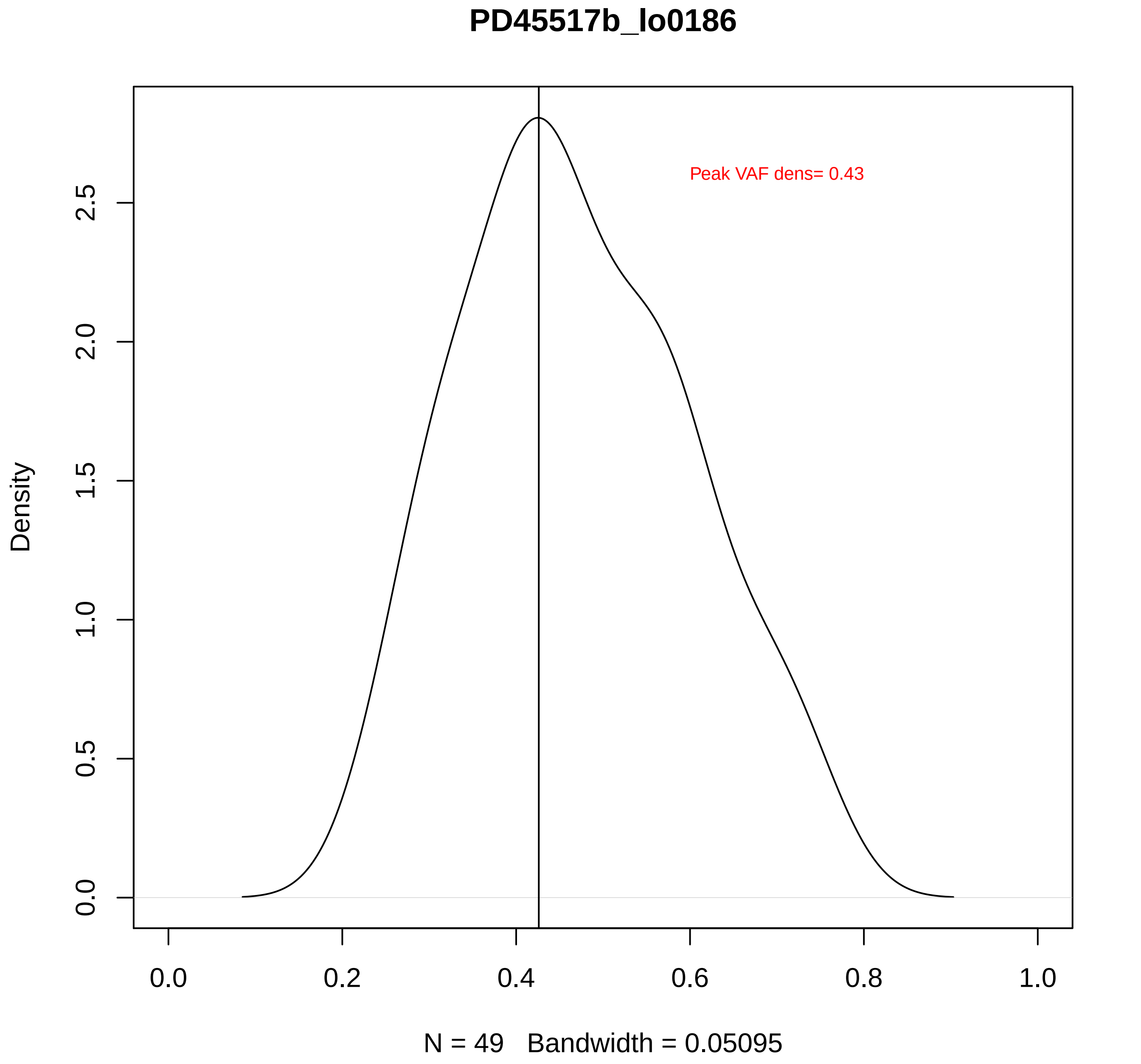
PD45517b_lo0186
Peak VAF dens= 0.43
Density
0.0
0.5
1.0
1.5
2.0
2.5
0.0
0.2
0.4
0.6
0.8
1.0
N = 49   Bandwidth = 0.05095

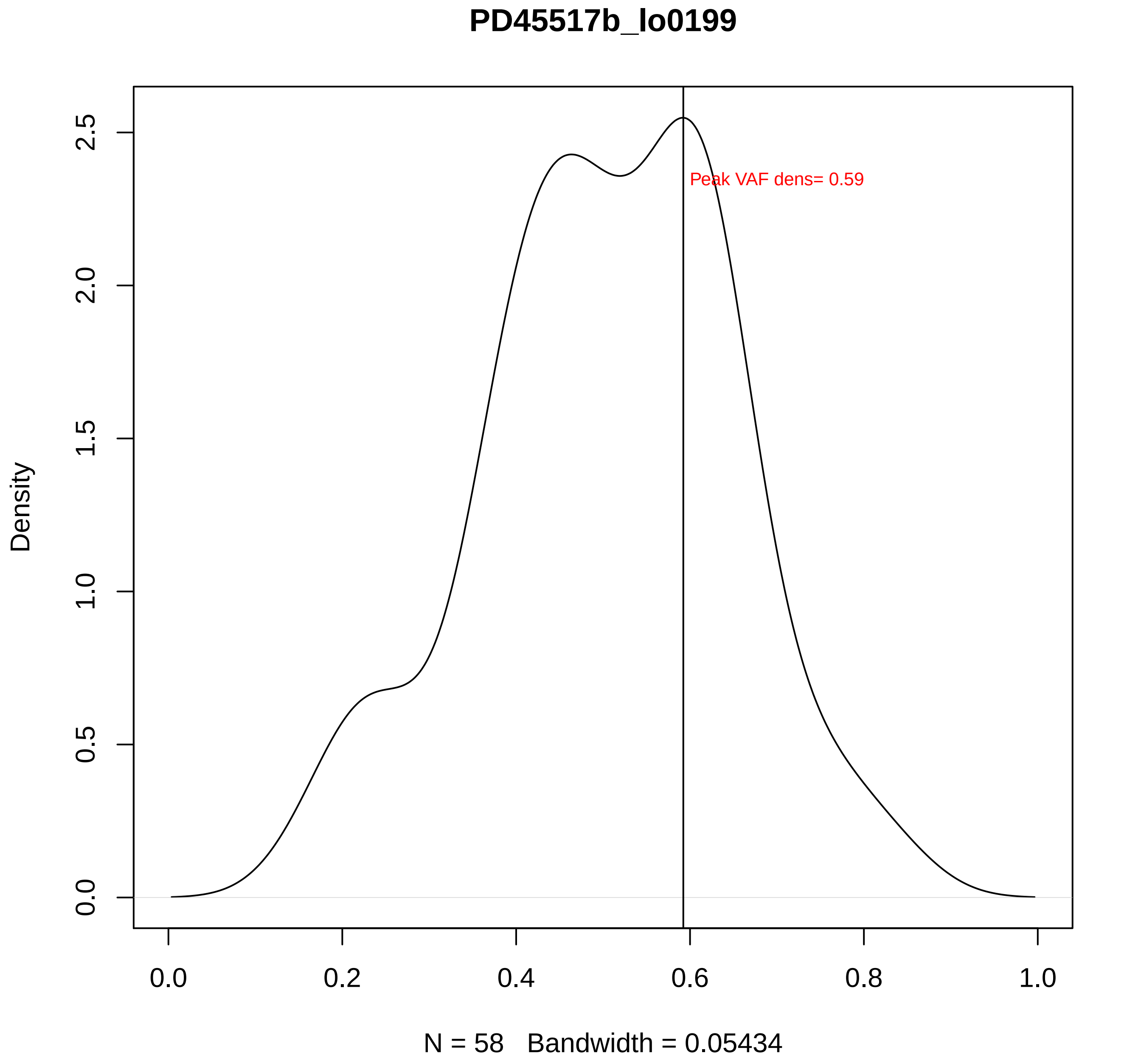
PD45517b_lo0199
Peak VAF dens= 0.59
Density
0.0
0.5
1.0
1.5
2.0
2.5
0.0
0.2
0.4
0.6
0.8
1.0
N = 58   Bandwidth = 0.05434

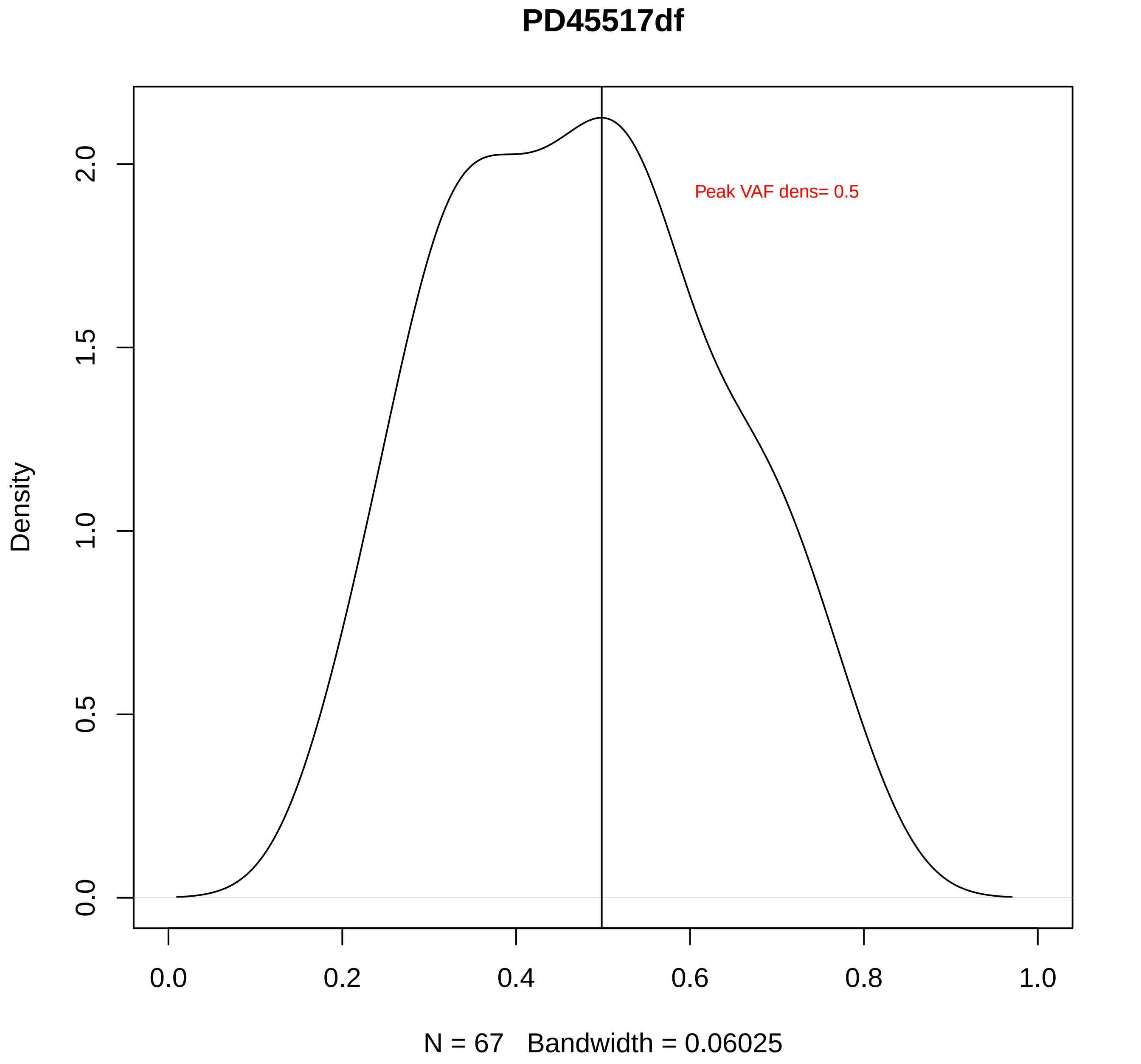
PD45517df
Peak VAF dens= 0.5
Density
0.0
0.5
1.0
1.5
2.0
0.0
0.2
0.4
0.6
0.8
1.0
N = 67   Bandwidth = 0.06025

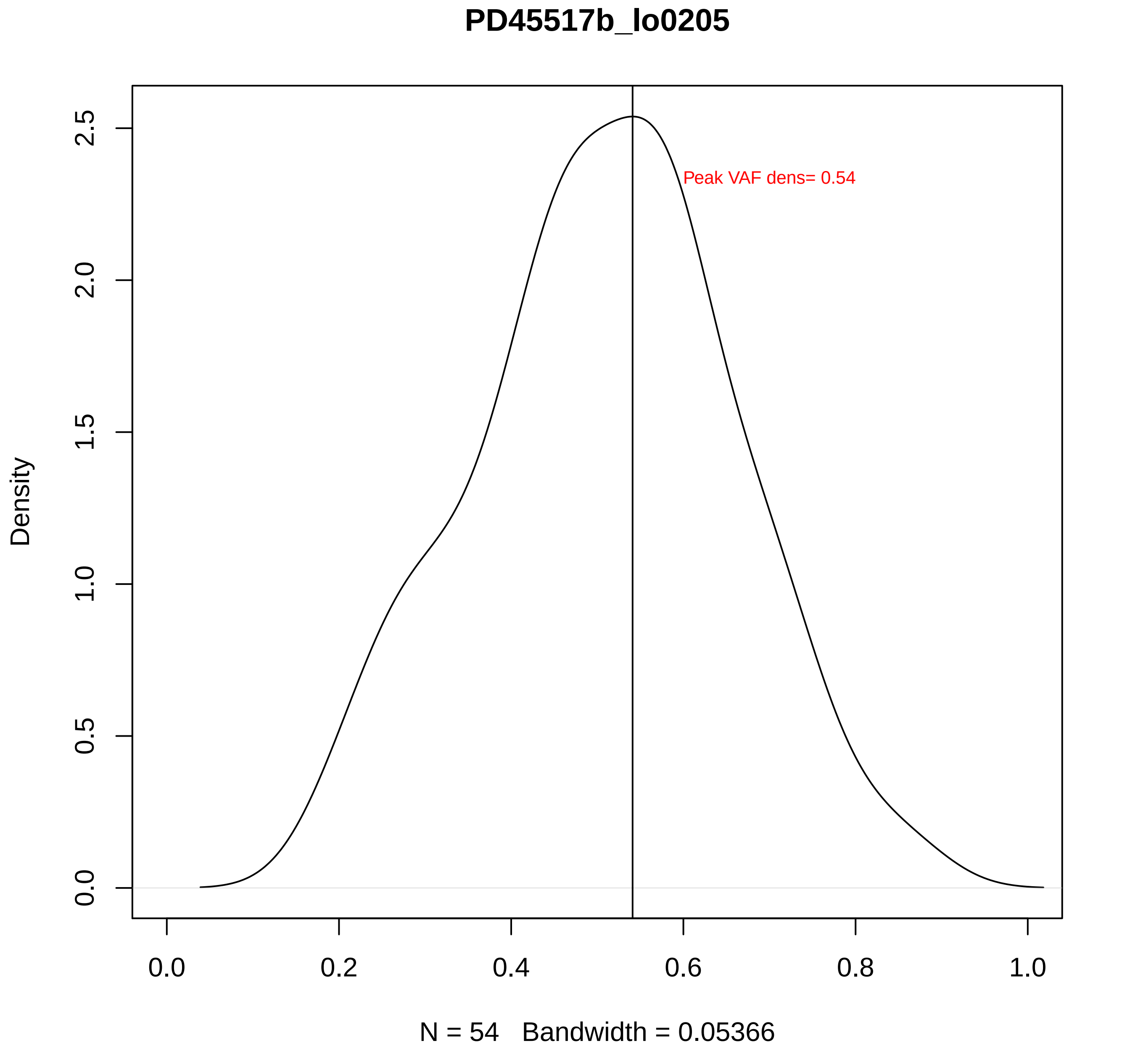
PD45517b_lo0205
Peak VAF dens= 0.54
Density
0.0
0.5
1.0
1.5
2.0
2.5
0.0
0.2
0.4
0.6
0.8
1.0
N = 54 Bandwidth = 0.05366

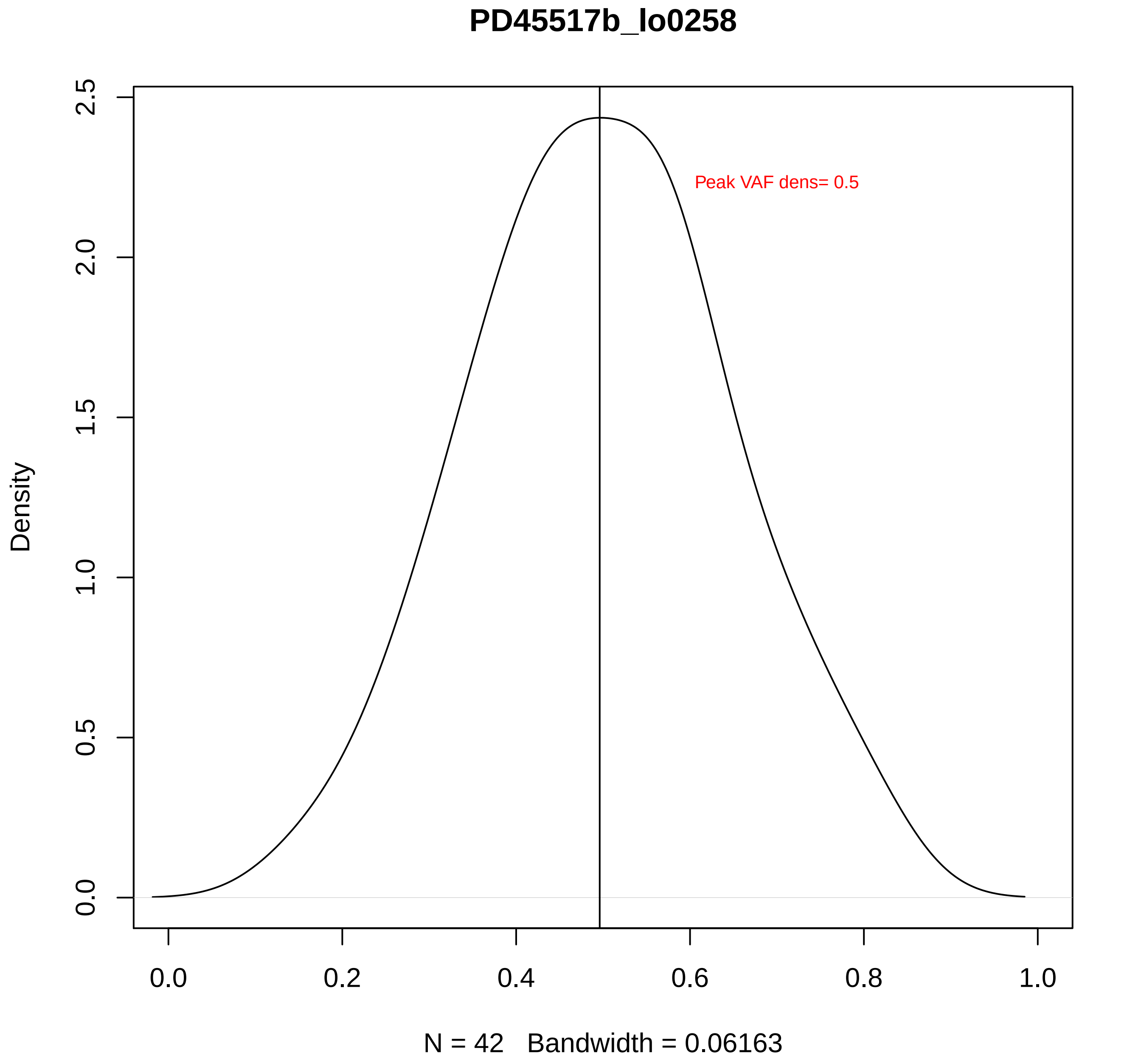

PD45517b_lo0258
Peak VAF dens= 0.5
Density
0.0
0.5
1.0
1.5
2.0
2.5
0.0
0.2
0.4
0.6
0.8
1.0
N = 42   Bandwidth = 0.06163

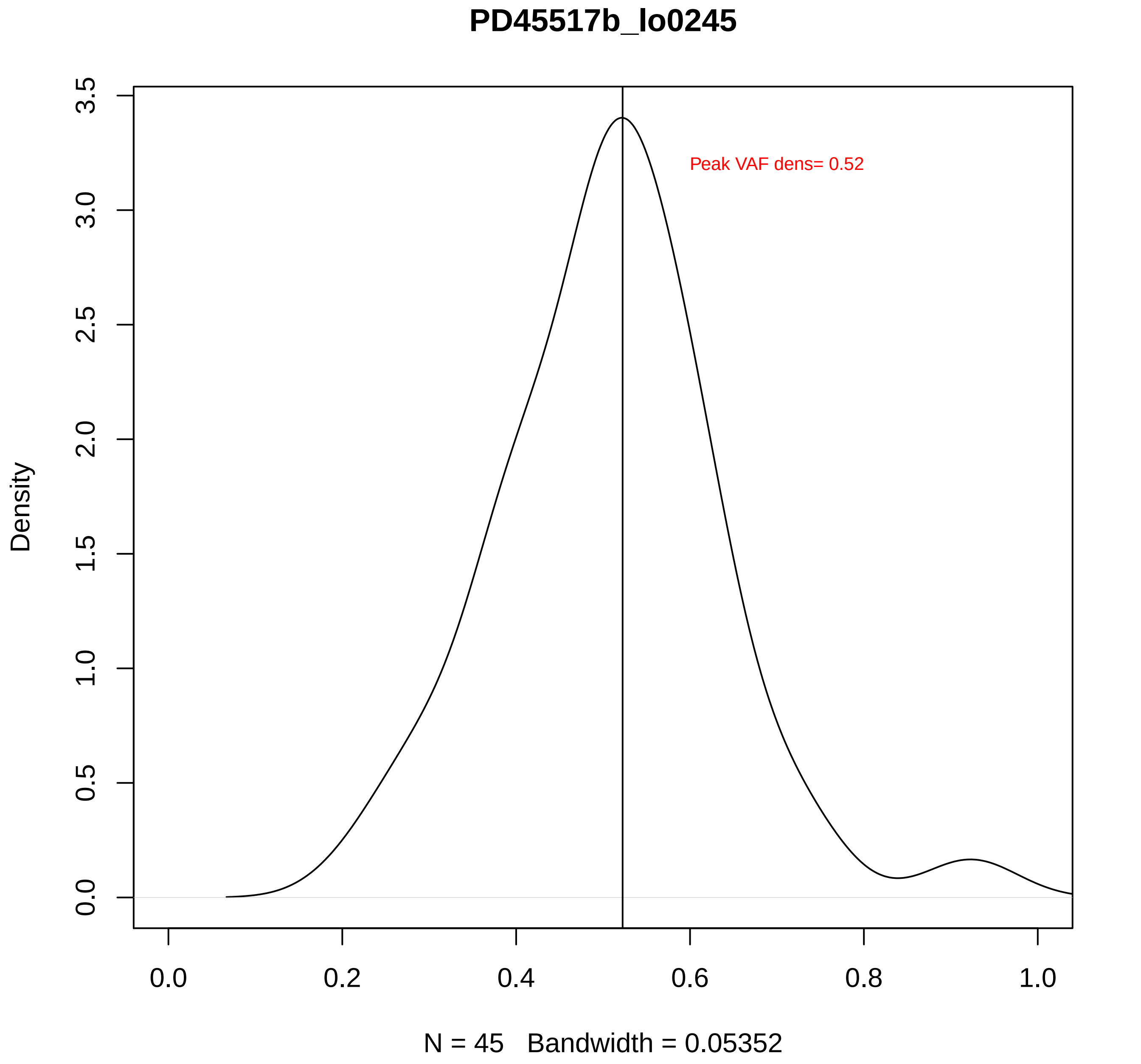
PD45517b_lo0245
Peak VAF dens= 0.52
Density
0.0
0.5
1.0
1.5
2.0
2.5
3.0
3.5
0.0
0.2
0.4
0.6
0.8
1.0
N = 45   Bandwidth = 0.05352

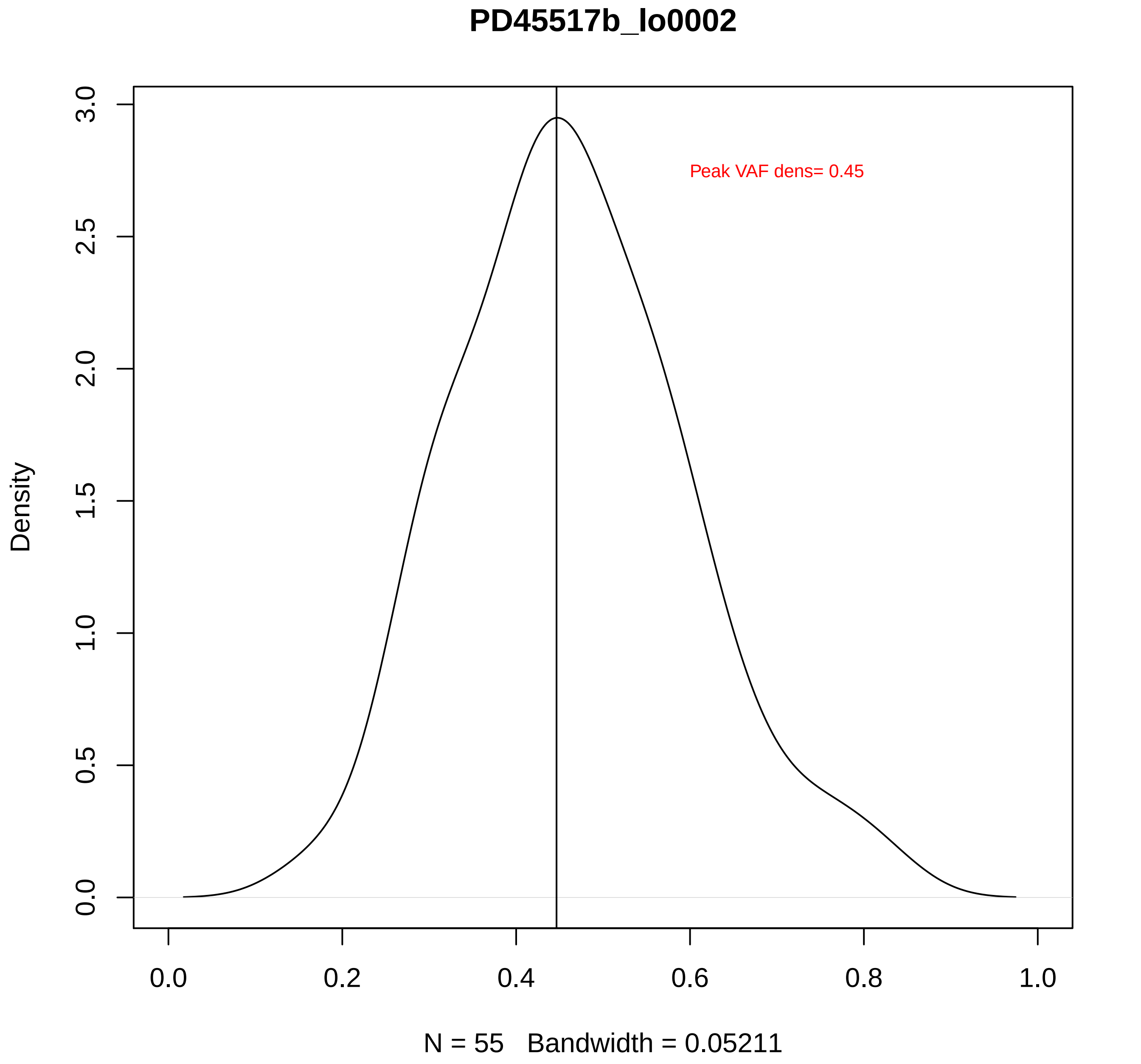
PD45517b_lo0002
Peak VAF dens= 0.45
Density
0.0
0.5
1.0
1.5
2.0
2.5
3.0
0.0
0.2
0.4
0.6
0.8
1.0
N = 55 Bandwidth = 0.05211

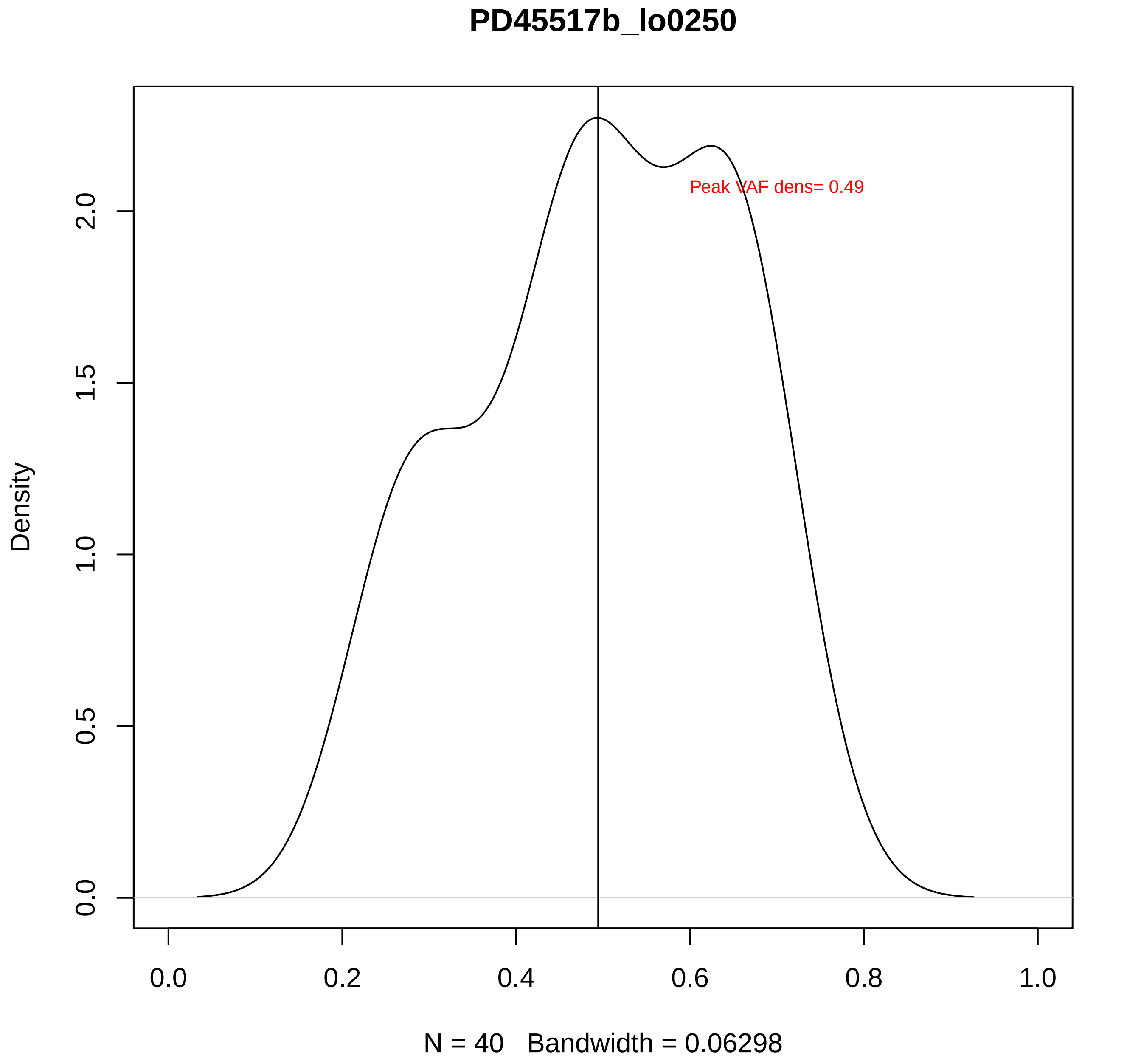
PD45517b_lo0250
Peak VAF dens= 0.49
Density
2.0
1.5
1.0
0.5
0.0
0.0
0.2
0.4
0.6
0.8
1.0
N = 40   Bandwidth = 0.06298

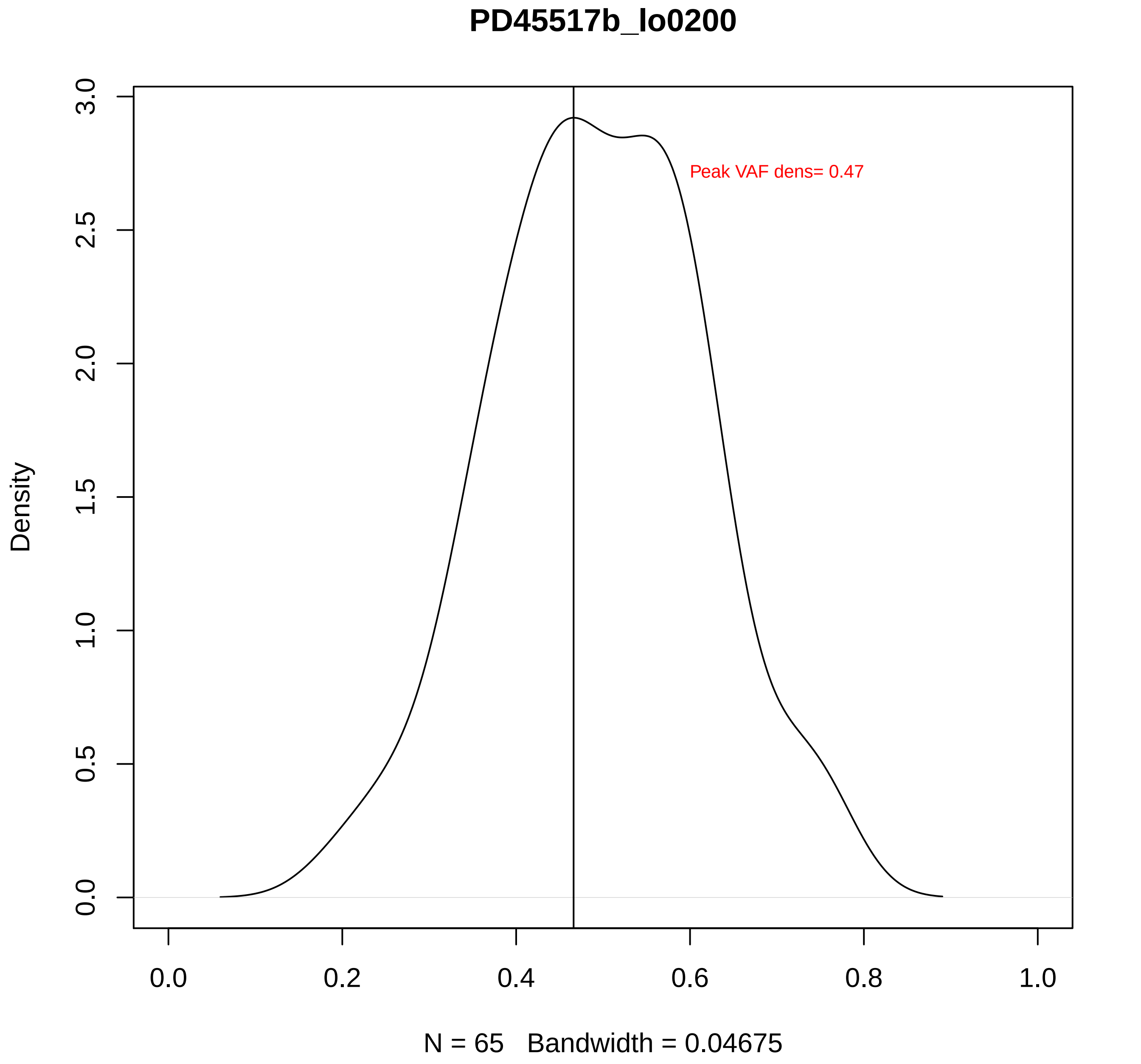
PD45517b_lo0200
Peak VAF dens= 0.47
Density
0.0
0.5
1.0
1.5
2.0
2.5
3.0
0.0
0.2
0.4
0.6
0.8
1.0
N = 65   Bandwidth = 0.04675

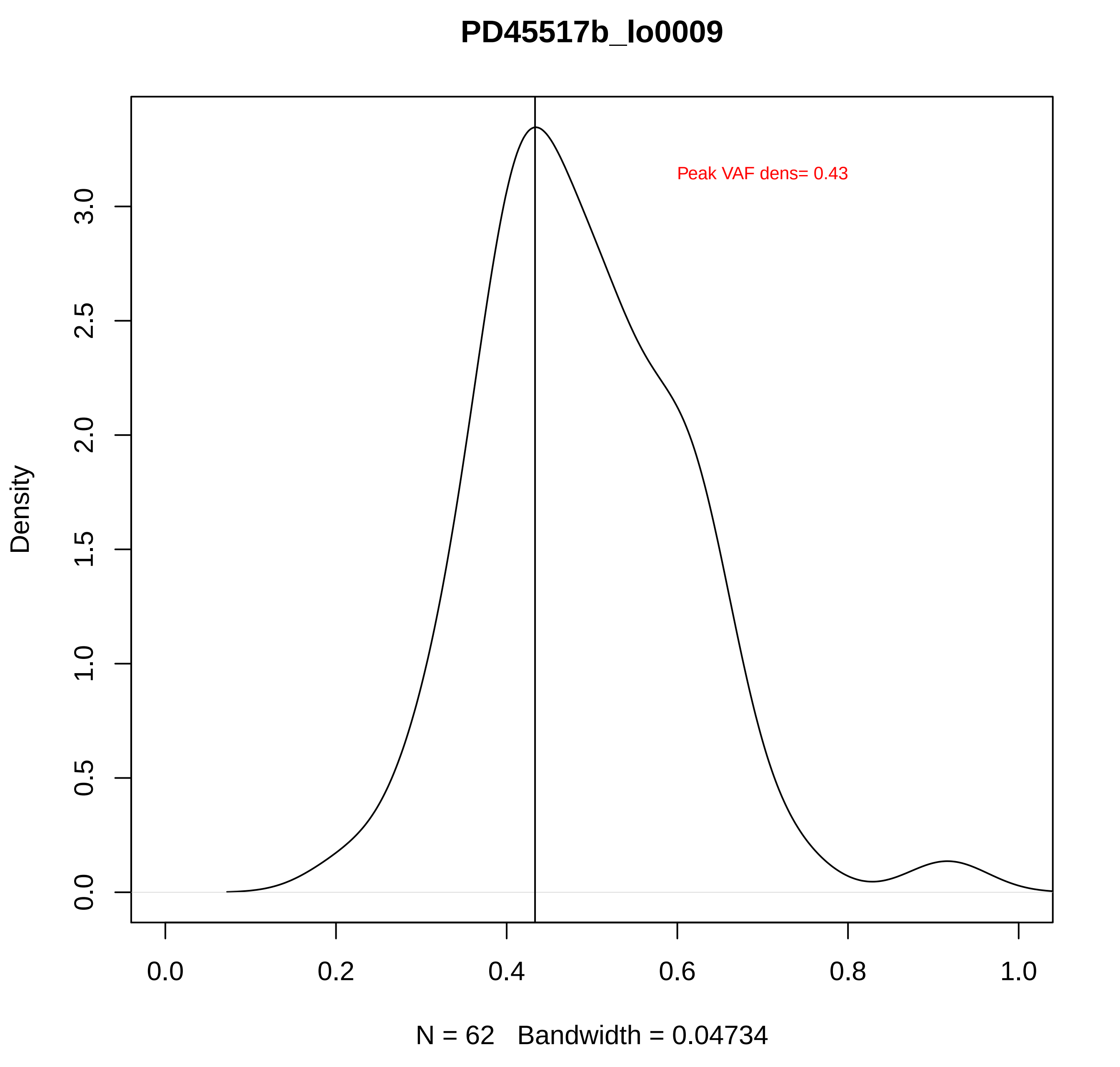

PD45517b_lo0009
Peak VAF dens= 0.43
Density
0.0
0.5
1.0
1.5
2.0
2.5
3.0
0.0
0.2
0.4
0.6
0.8
1.0
N = 62   Bandwidth = 0.04734

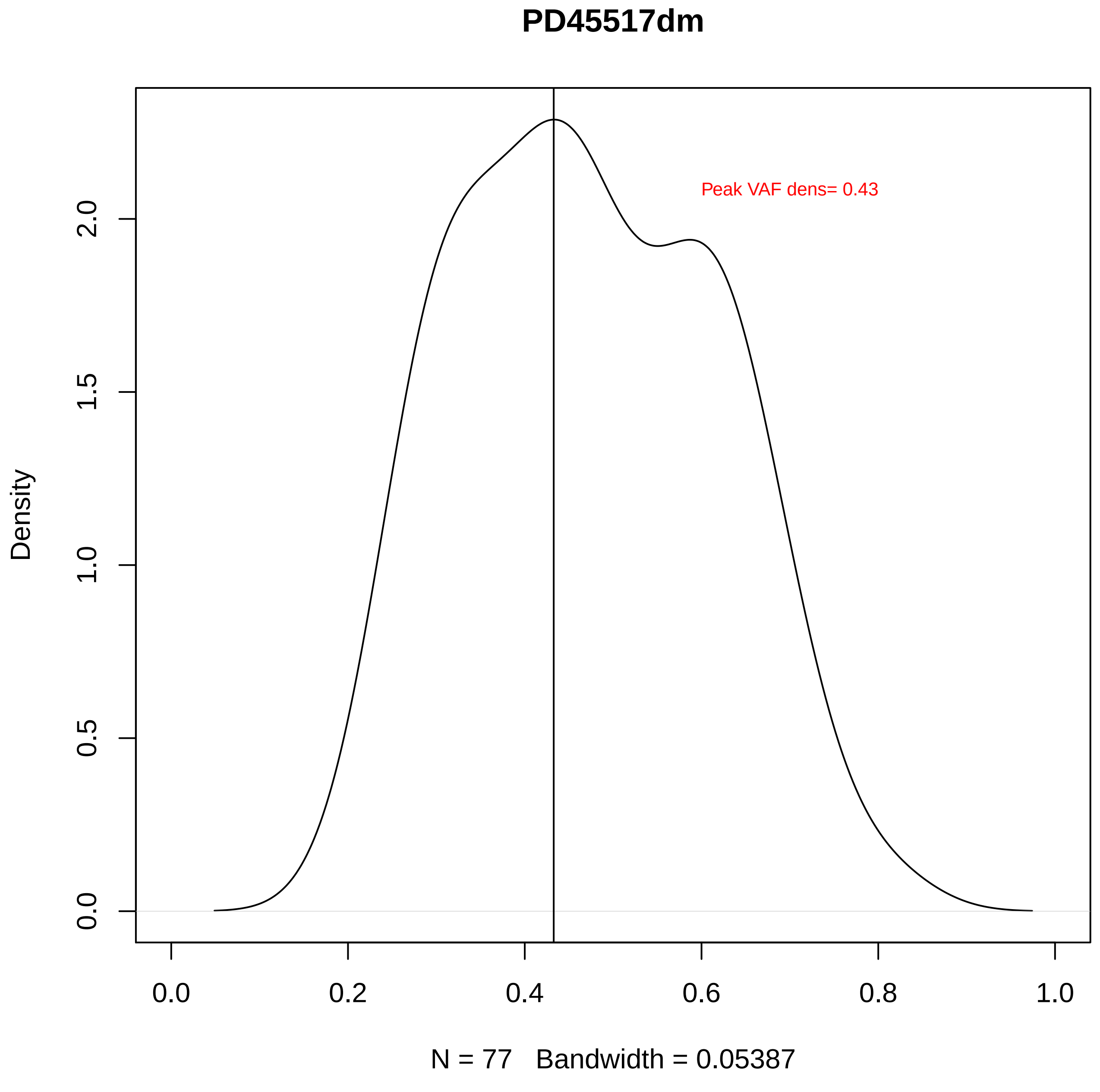

PD45517dm
Peak VAF dens= 0.43
Density
0.0
0.5
1.0
1.5
2.0
0.0
0.2
0.4
0.6
0.8
1.0
N = 77   Bandwidth = 0.05387

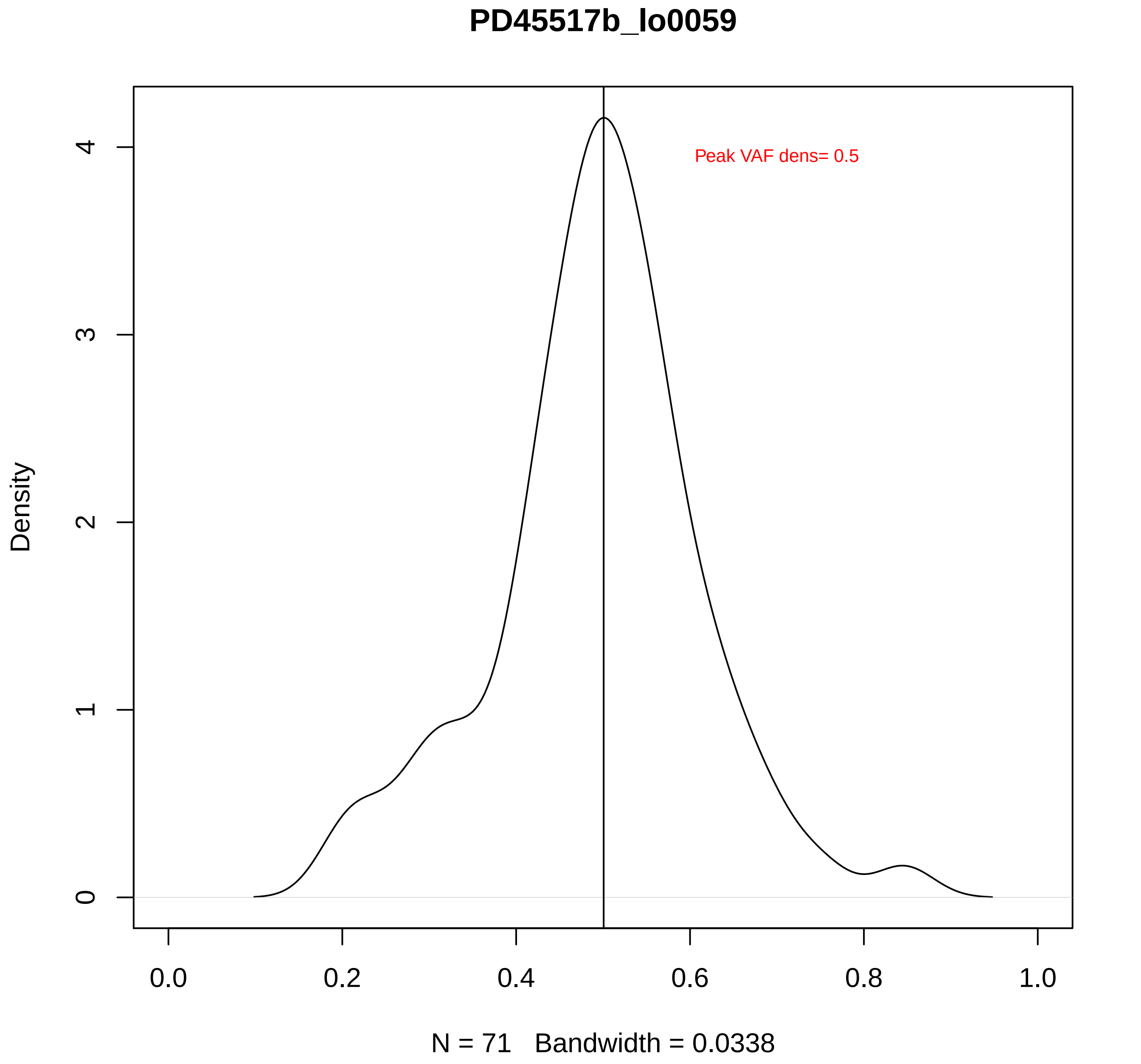

PD45517b_lo0059
Peak VAF dens= 0.5
Density
0
1
2
3
4
0.0
0.2
0.4
0.6
0.8
1.0
N = 71 Bandwidth = 0.0338

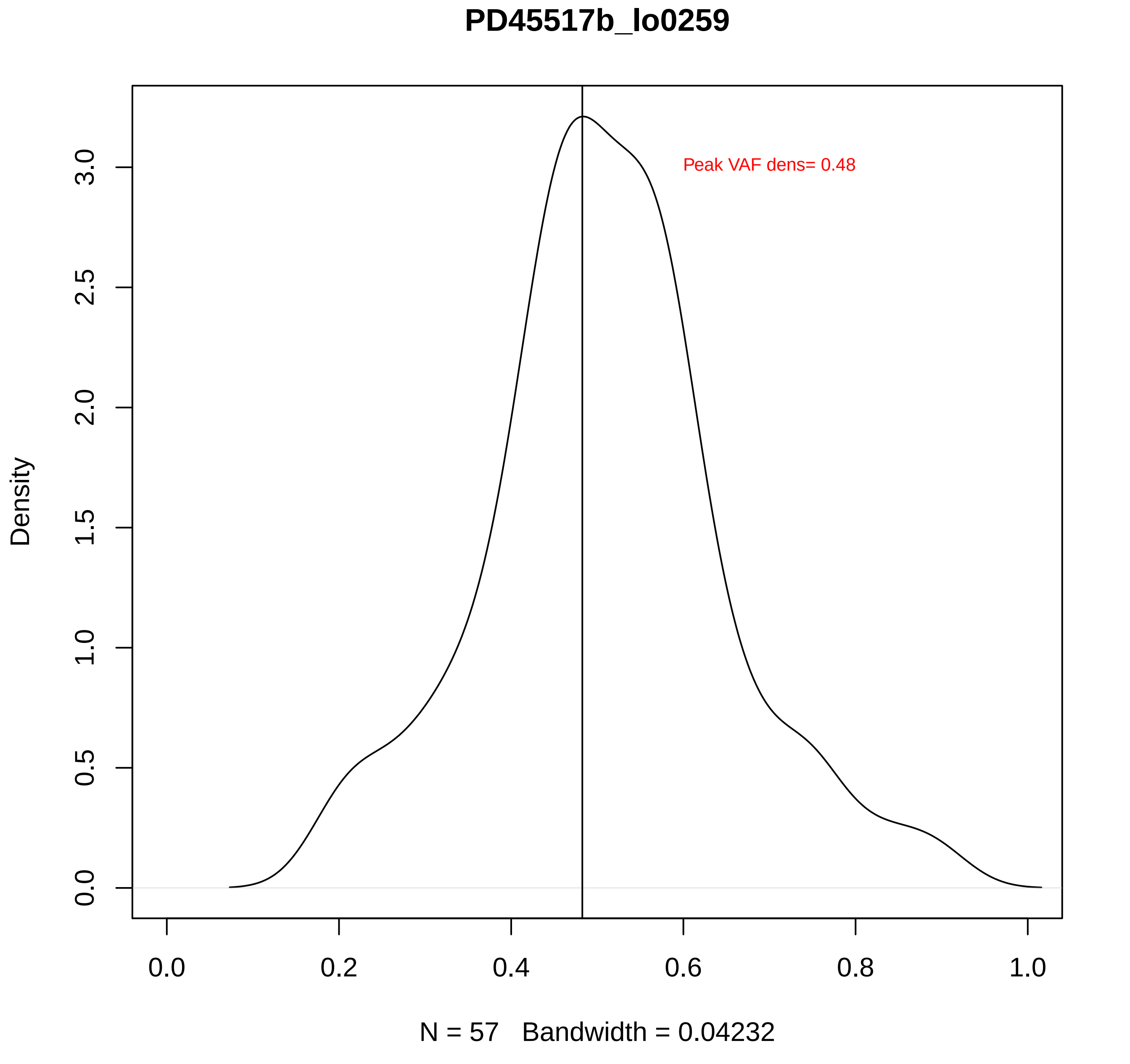

PD45517b_lo0259
Peak VAF dens= 0.48
Density
0.0
0.5
1.0
1.5
2.0
2.5
3.0
0.0
0.2
0.4
0.6
0.8
1.0
N = 57 Bandwidth = 0.04232

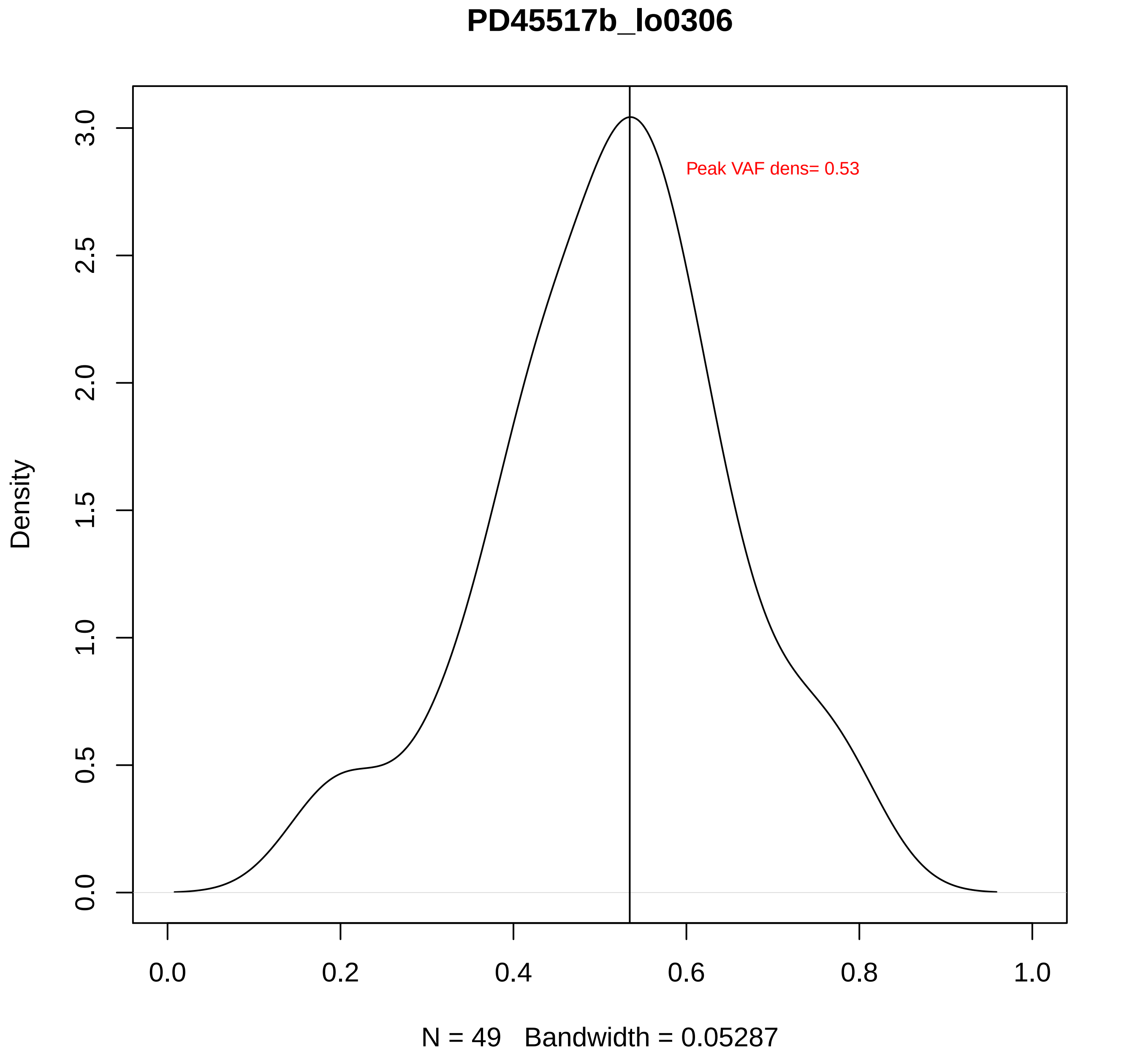
PD45517b_lo0306
Peak VAF dens= 0.53
Density
0.0
0.5
1.0
1.5
2.0
2.5
3.0
0.0
0.2
0.4
0.6
0.8
1.0
N = 49   Bandwidth = 0.05287

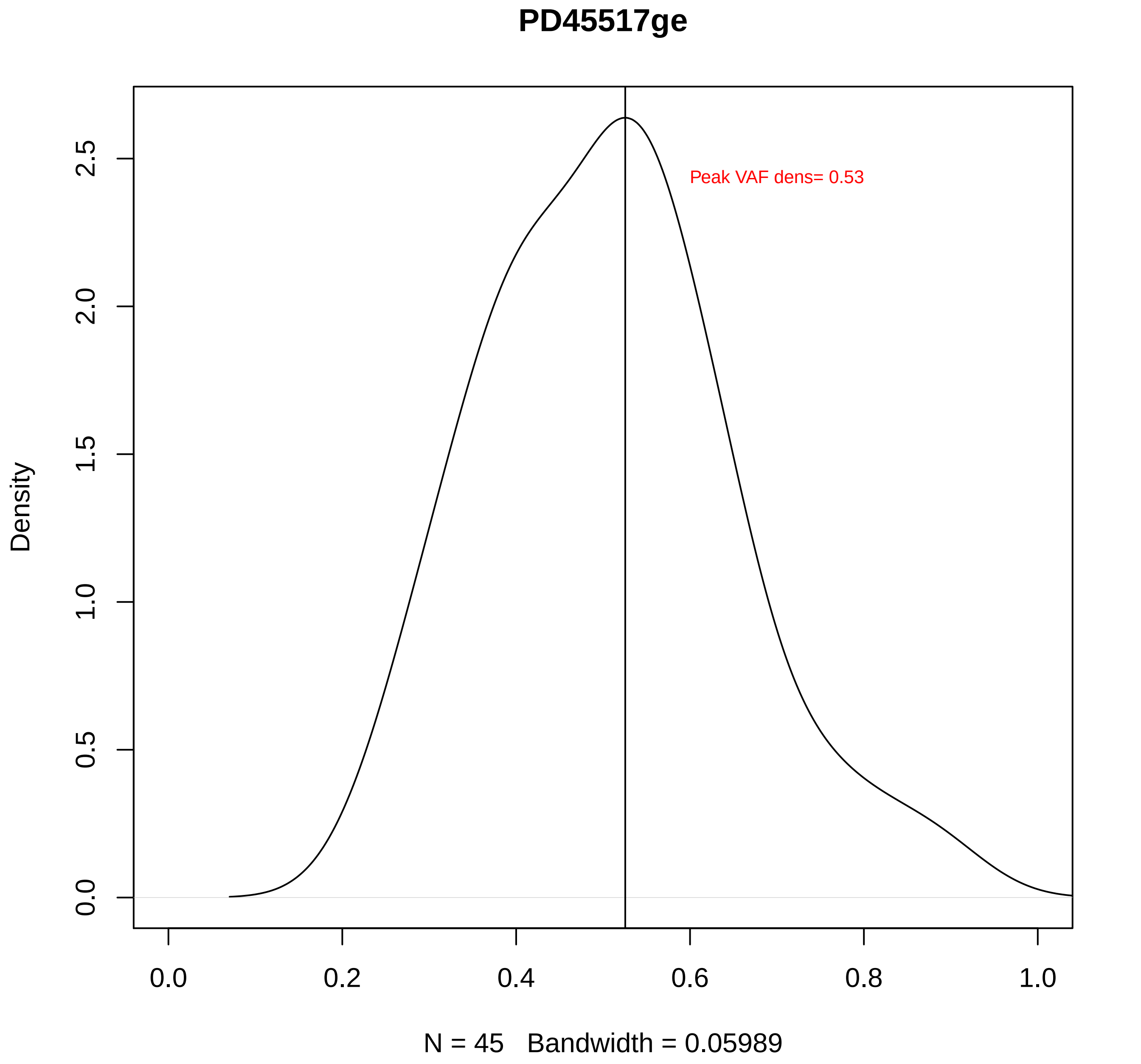

PD45517ge
Peak VAF dens= 0.53
Density
0.0
0.5
1.0
1.5
2.0
2.5
0.0
0.2
0.4
0.6
0.8
1.0
N = 45   Bandwidth = 0.05989

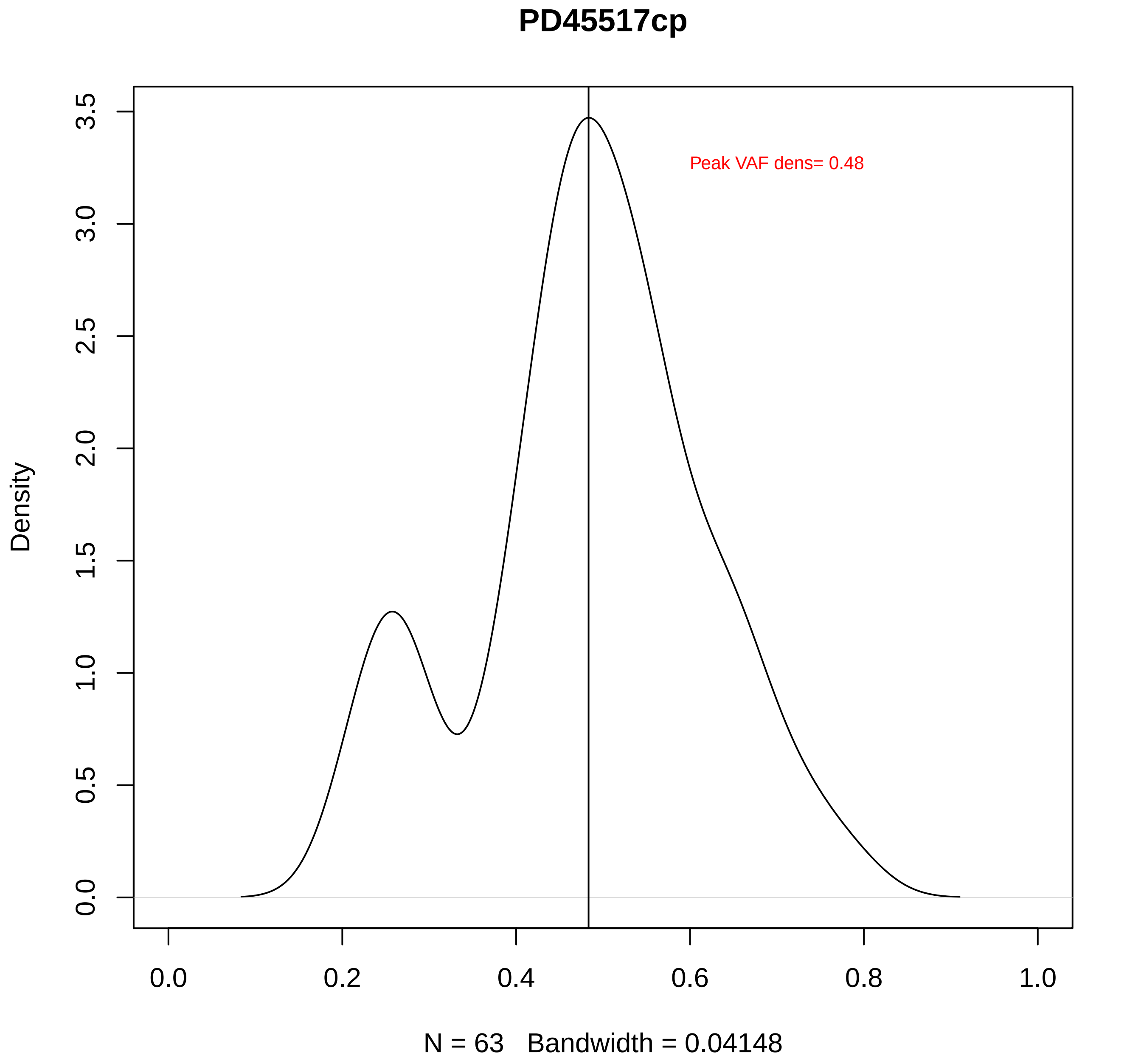
PD45517cp
Peak VAF dens= 0.48
Density
0.0
0.5
1.0
1.5
2.0
2.5
3.0
3.5
0.0
0.2
0.4
0.6
0.8
1.0
N = 63   Bandwidth = 0.04148

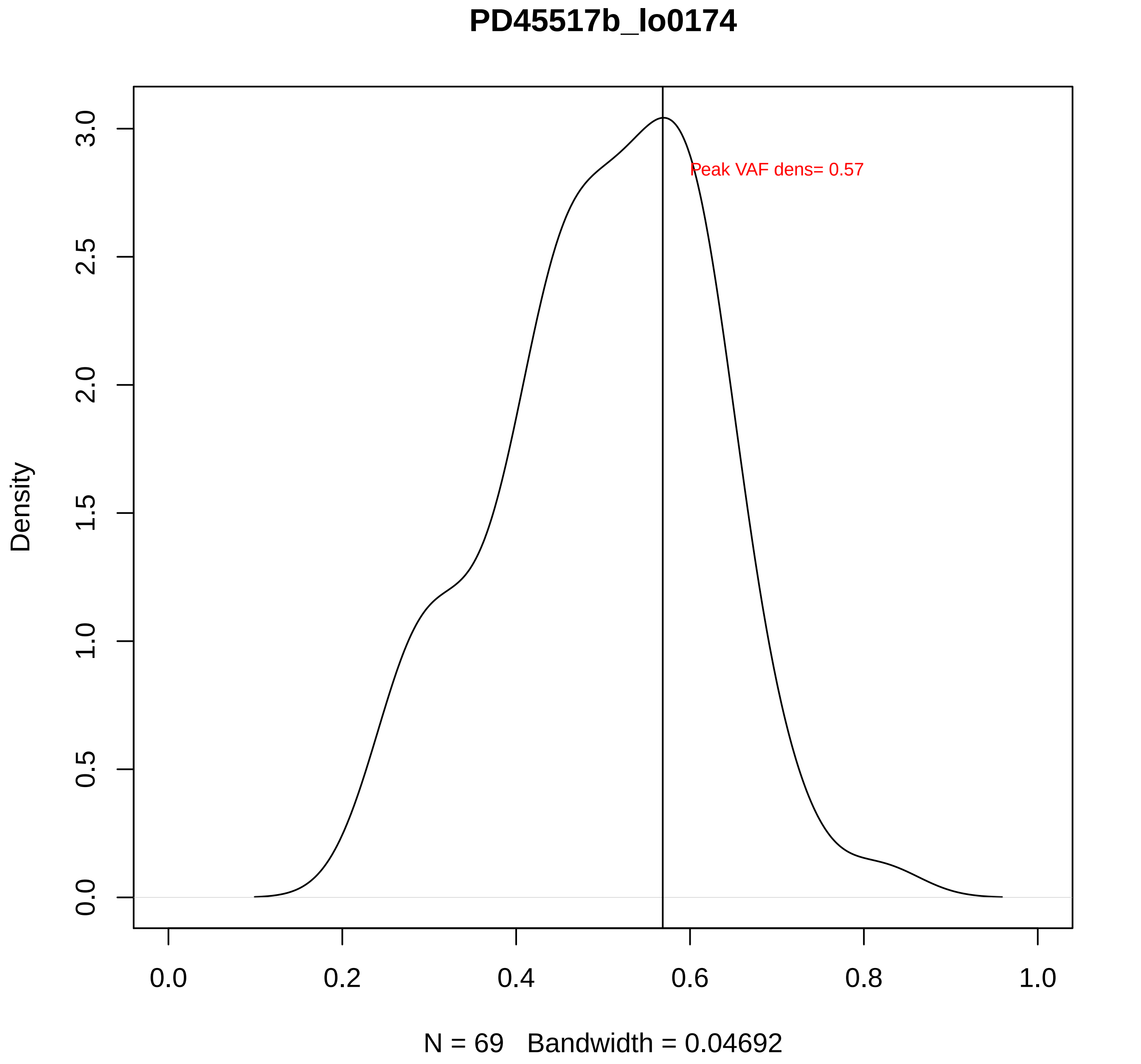

PD45517b_lo0174
Peak VAF dens= 0.57
Density
0.0
0.5
1.0
1.5
2.0
2.5
3.0
0.0
0.2
0.4
0.6
0.8
1.0
N = 69 Bandwidth = 0.04692

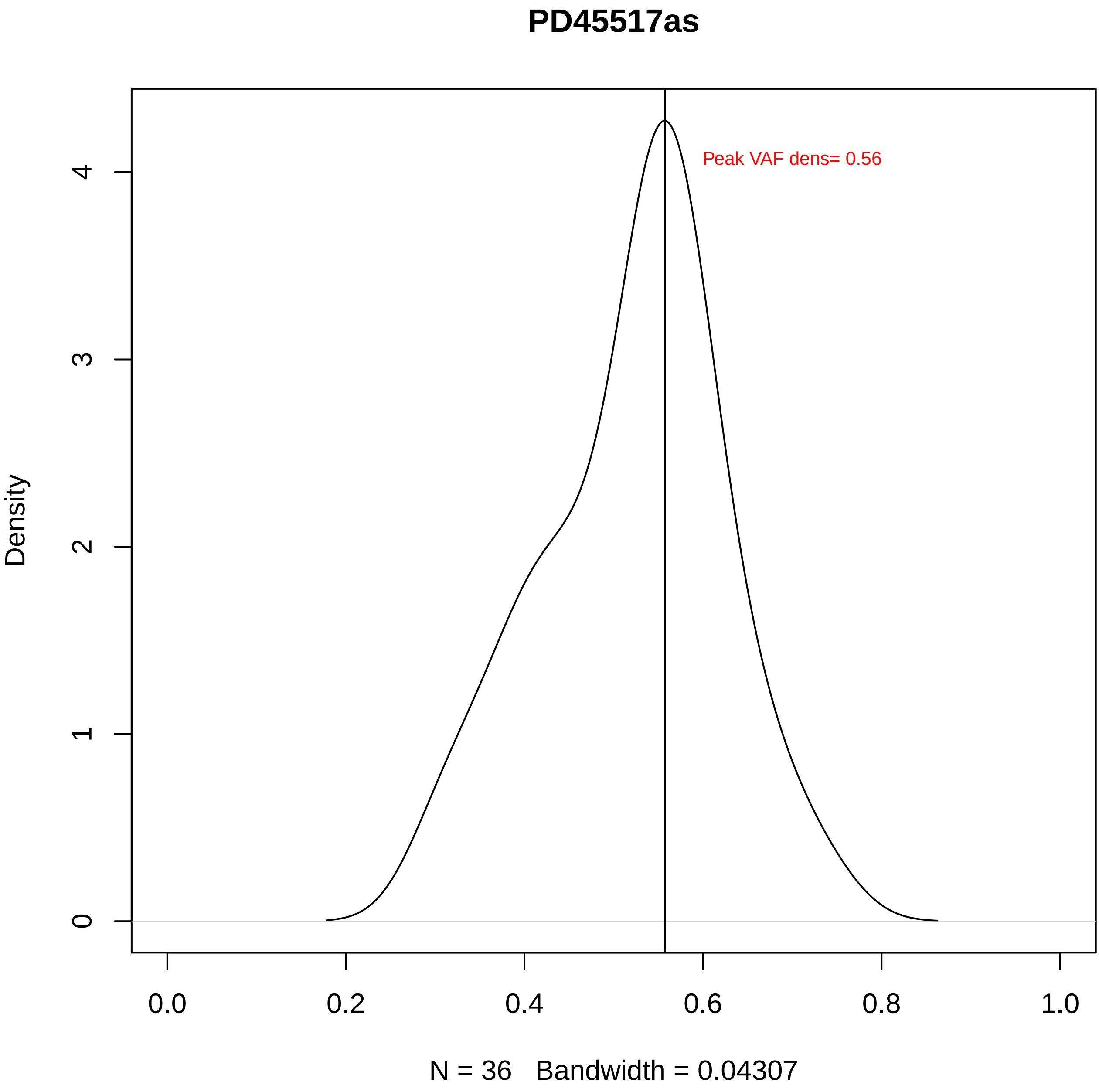

PD45517as
Peak VAF dens= 0.56
Density
0
1
2
3
4
0.0
0.2
0.4
0.6
0.8
1.0
N = 36   Bandwidth = 0.04307

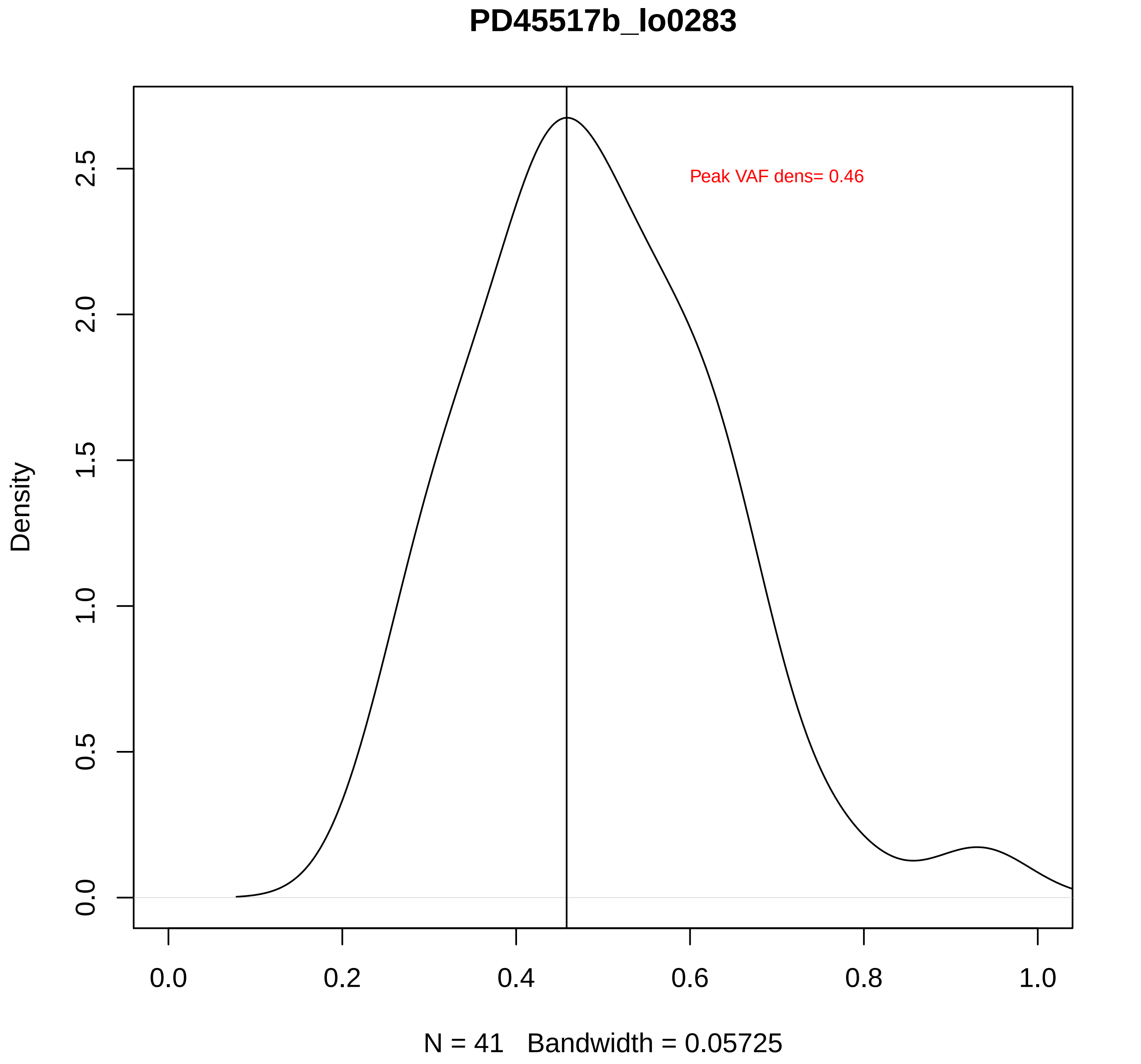
PD45517b_lo0283
Peak VAF dens= 0.46
Density
0.0
0.5
1.0
1.5
2.0
2.5
0.0
0.2
0.4
0.6
0.8
1.0
N = 41   Bandwidth = 0.05725

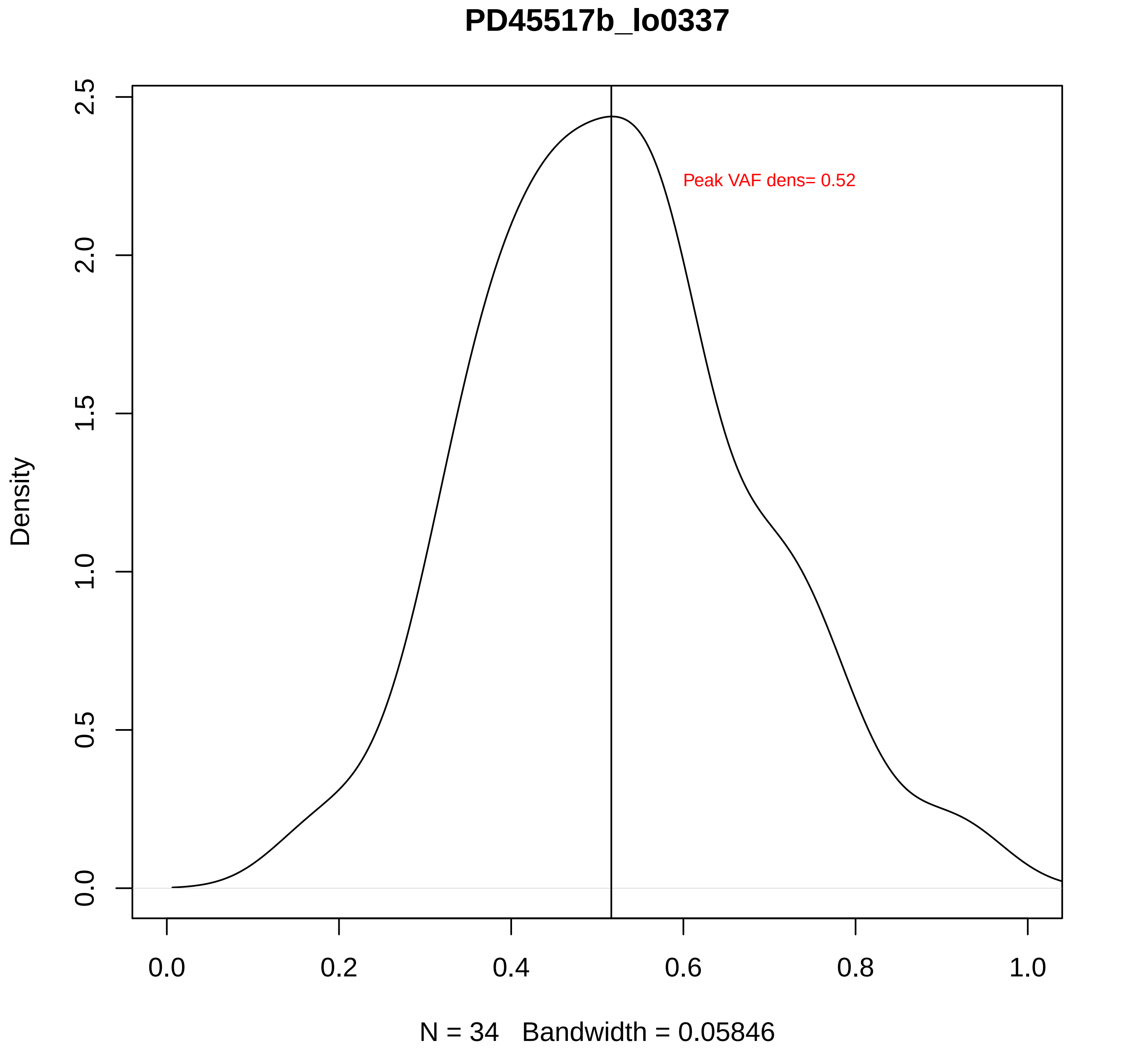
PD45517b_lo0337
Density
Peak VAF dens= 0.52
0.0
0.5
1.0
1.5
2.0
2.5
0.0
0.2
0.4
0.6
0.8
1.0
N = 34   Bandwidth = 0.05846

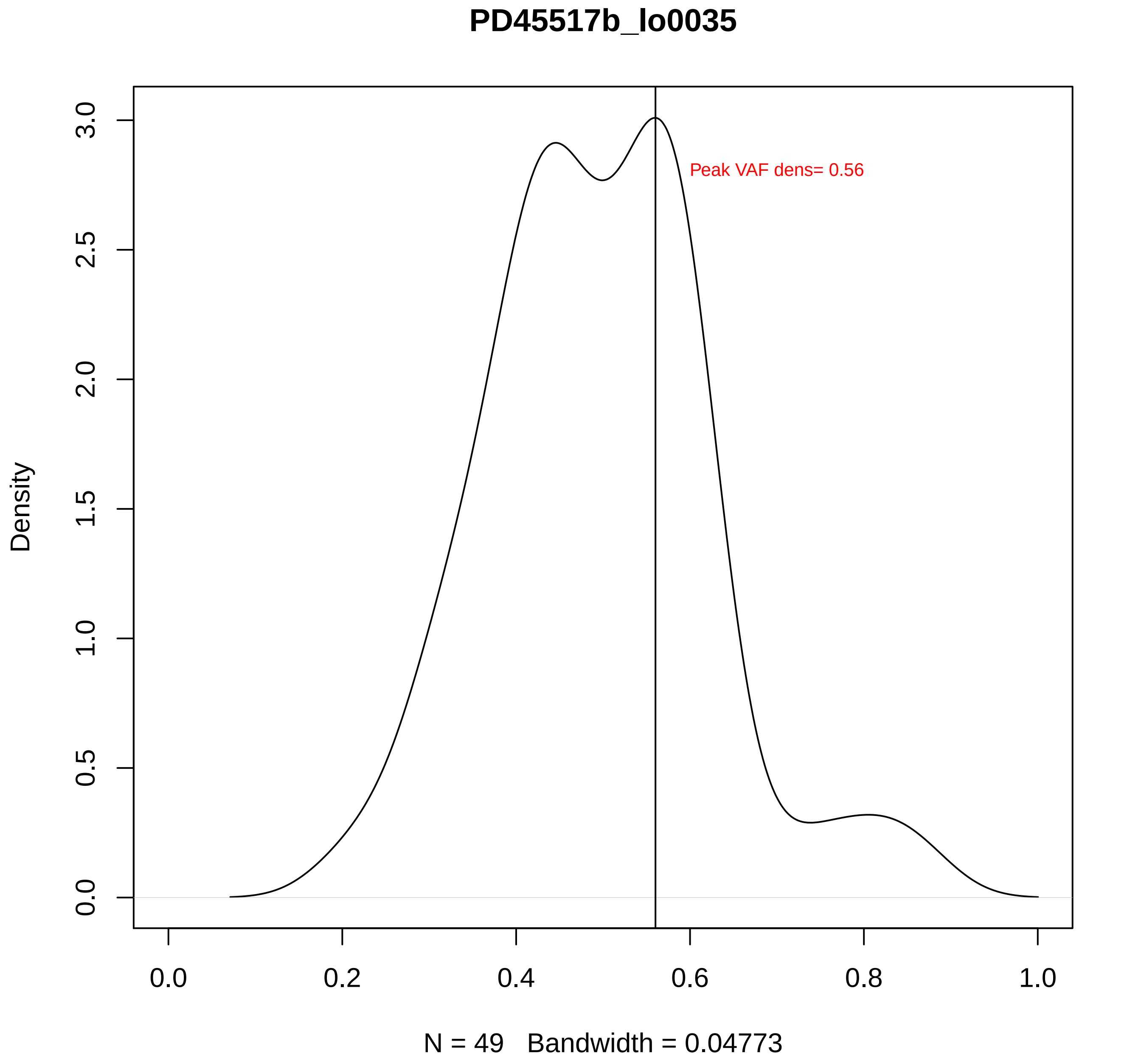
PD45517b_lo0035
Peak VAF dens= 0.56
Density
0.0
0.5
1.0
1.5
2.0
2.5
3.0
0.0
0.2
0.4
0.6
0.8
1.0
N = 49 Bandwidth = 0.04773

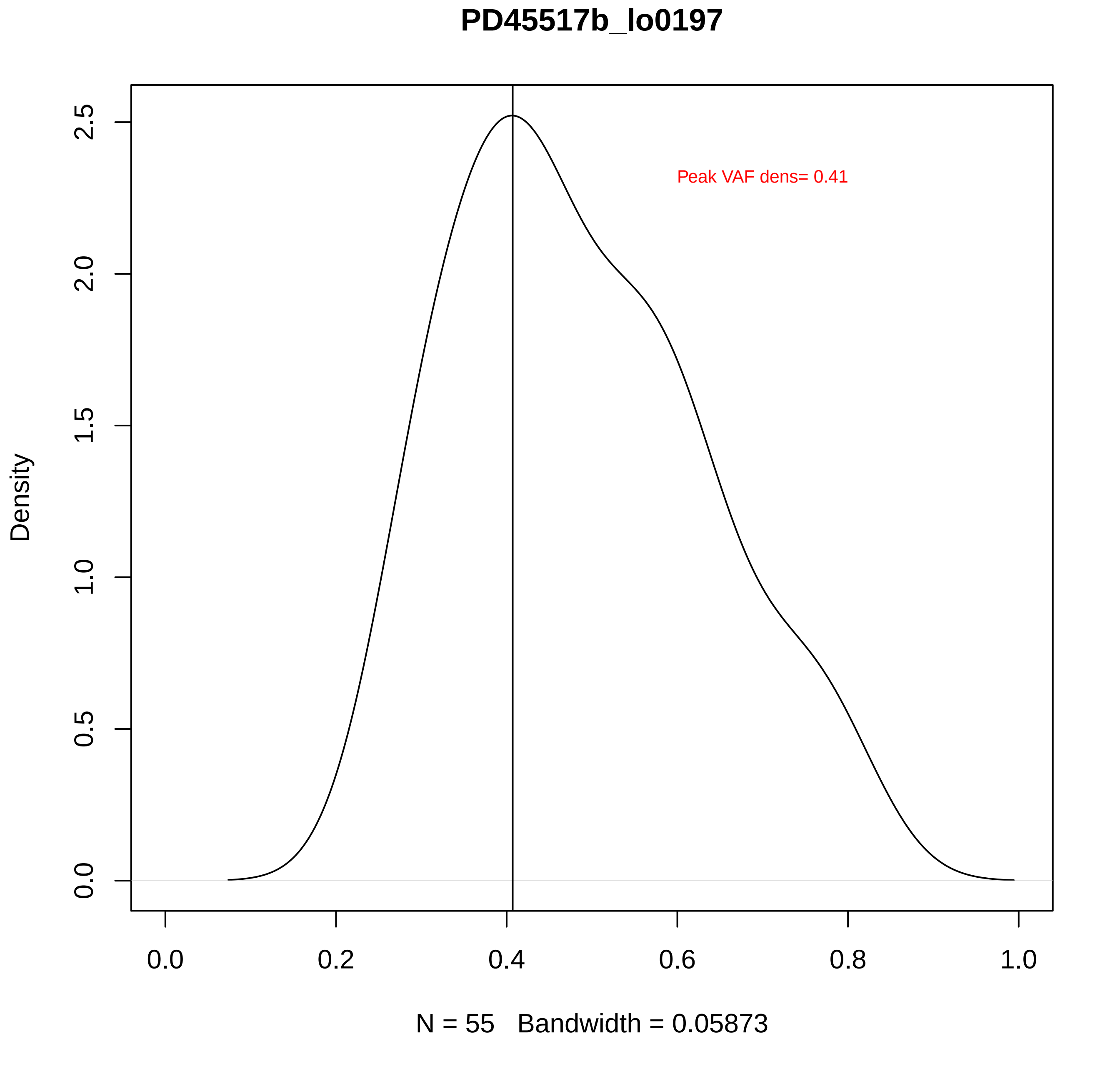
PD45517b_lo0197
Peak VAF dens= 0.41
Density
0.0
0.5
1.0
1.5
2.0
2.5
0.0
0.2
0.4
0.6
0.8
1.0
N = 55 Bandwidth = 0.05873

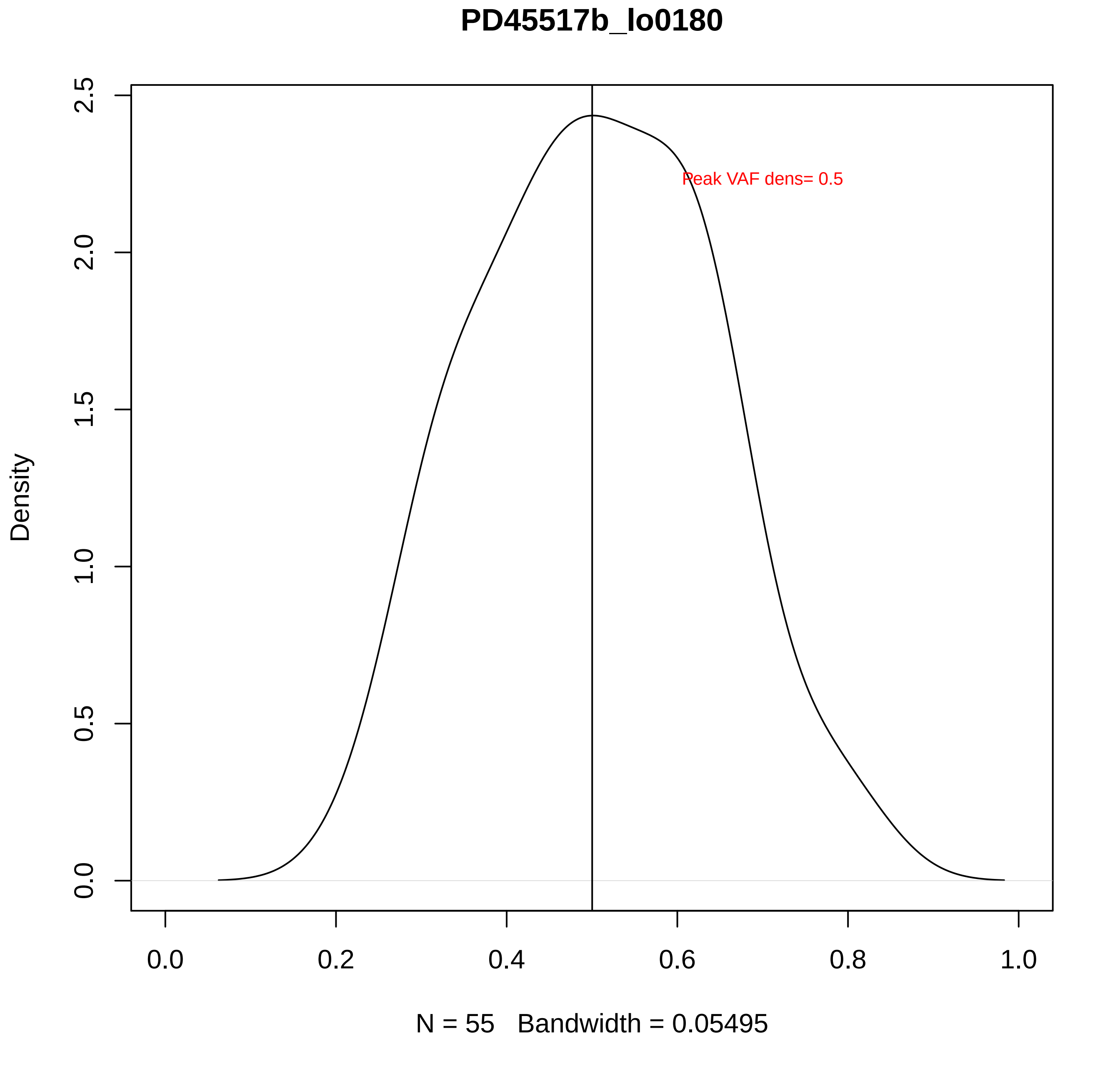
PD45517b_lo0180
Peak VAF dens= 0.5
Density
0.0
0.5
1.0
1.5
2.0
2.5
0.0
0.2
0.4
0.6
0.8
1.0
N = 55   Bandwidth = 0.05495

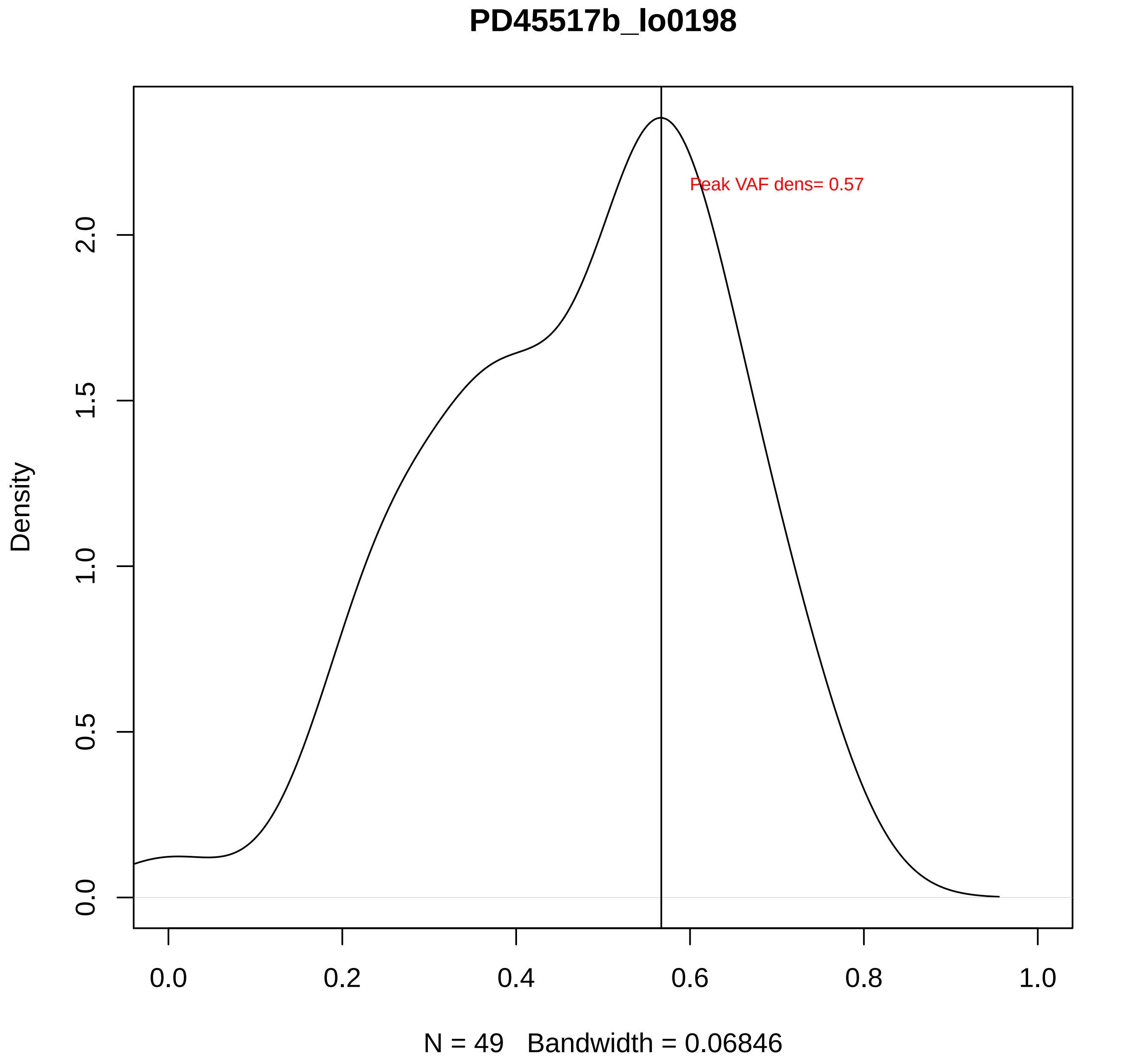
PD45517b_lo0198
Peak VAF dens= 0.57
Density
0.0
0.5
1.0
1.5
2.0
0.0
0.2
0.4
0.6
0.8
1.0
N = 49   Bandwidth = 0.06846

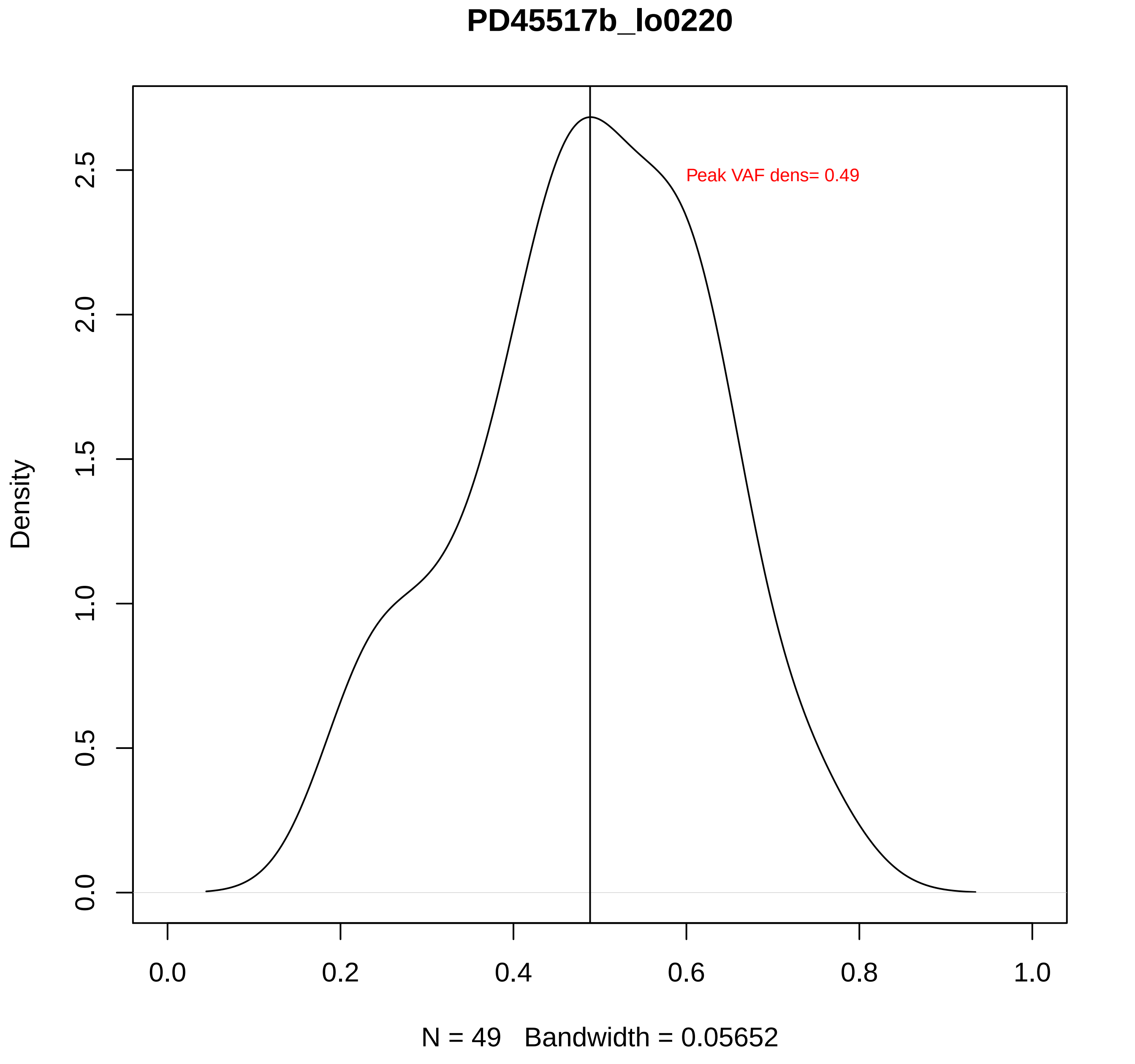
PD45517b_lo0220
Peak VAF dens= 0.49
Density
0.0
0.5
1.0
1.5
2.0
2.5
0.0
0.2
0.4
0.6
0.8
1.0
N = 49   Bandwidth = 0.05652

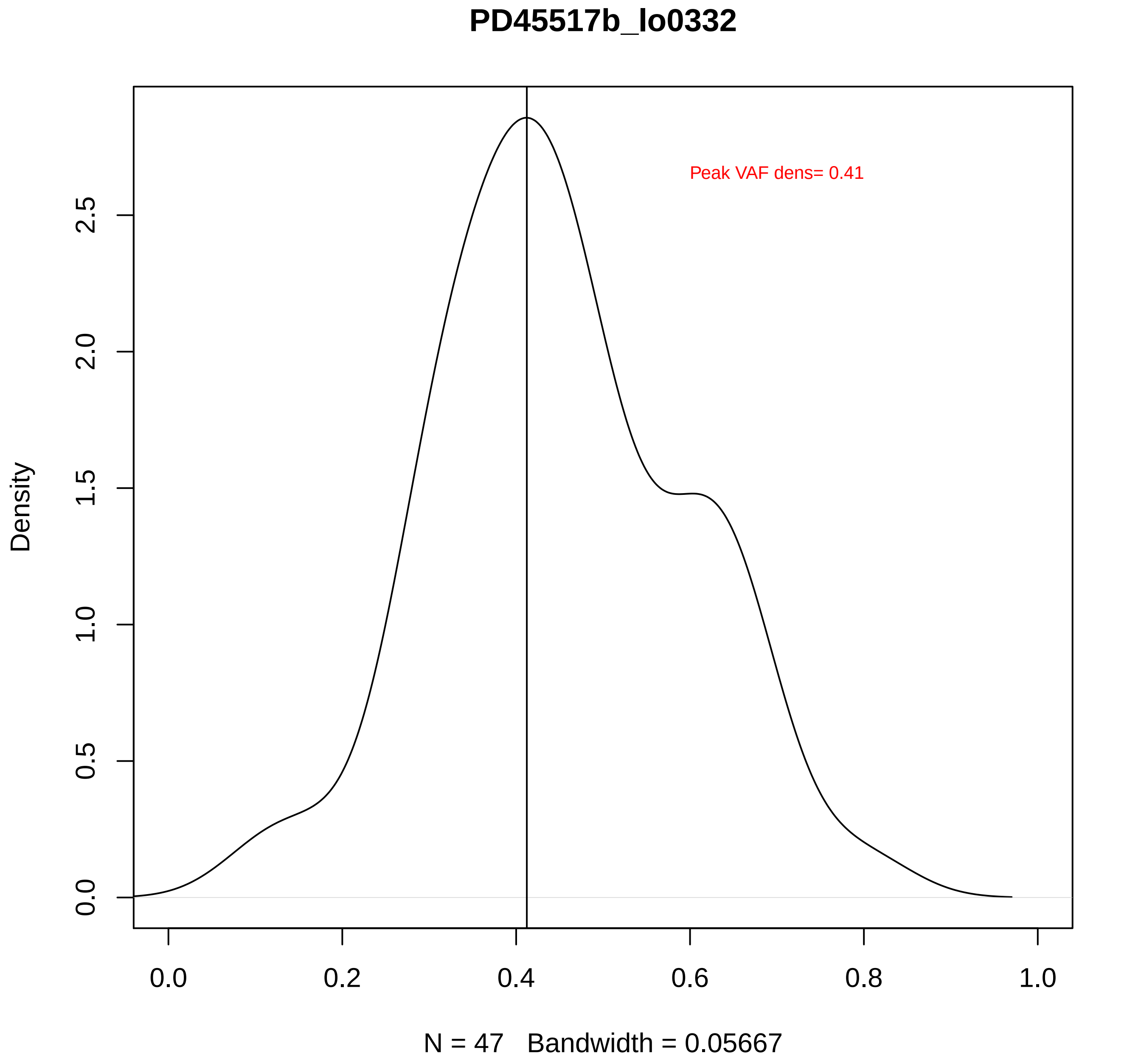

PD45517b_lo0332
Peak VAF dens= 0.41
Density
0.0
0.5
1.0
1.5
2.0
2.5
0.0
0.2
0.4
0.6
0.8
1.0
N = 47 Bandwidth = 0.05667

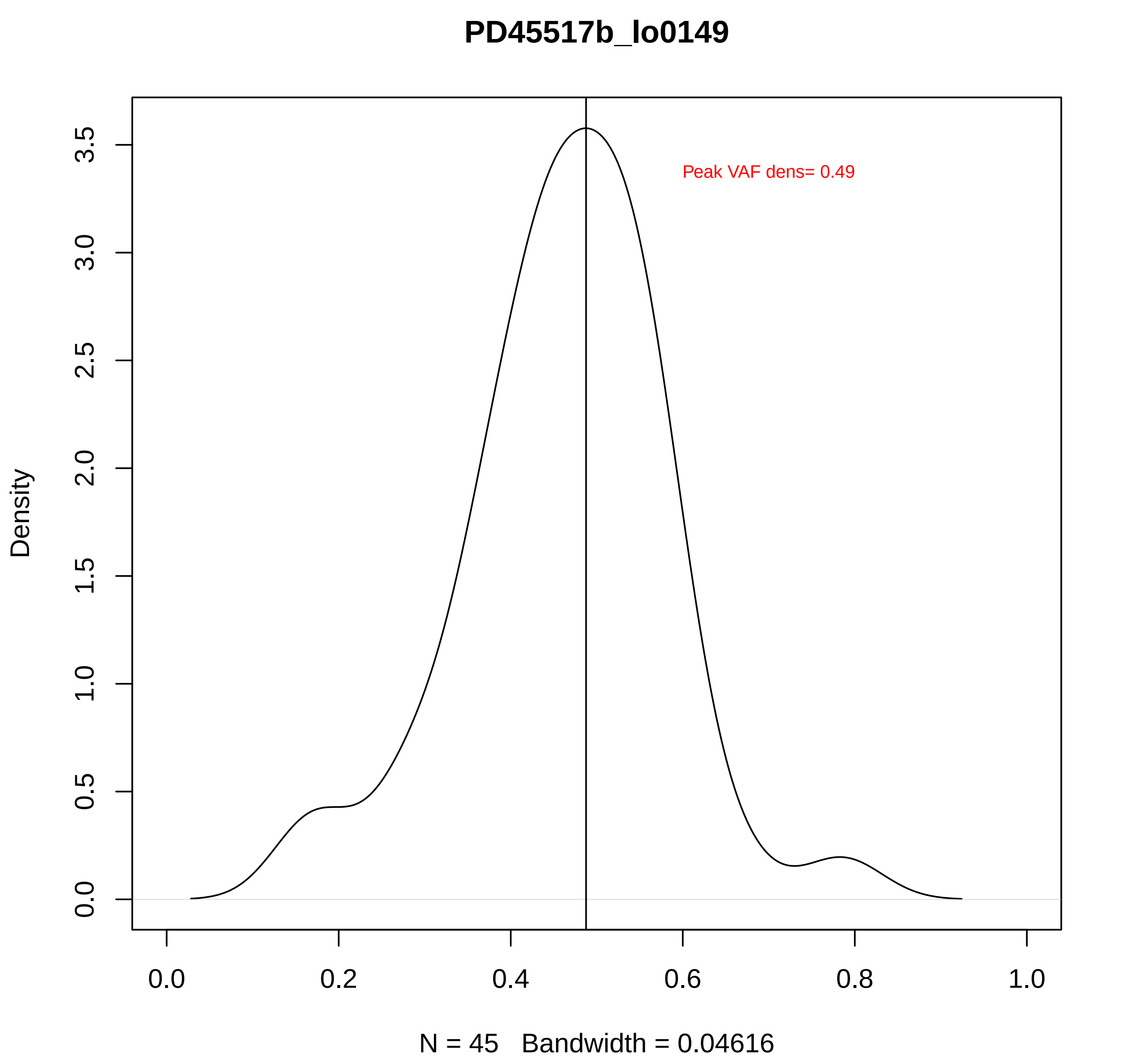

PD45517b_lo0149
Peak VAF dens= 0.49
Density
0.0
0.5
1.0
1.5
2.0
2.5
3.0
3.5
0.0
0.2
0.4
0.6
0.8
1.0
N = 45 Bandwidth = 0.04616

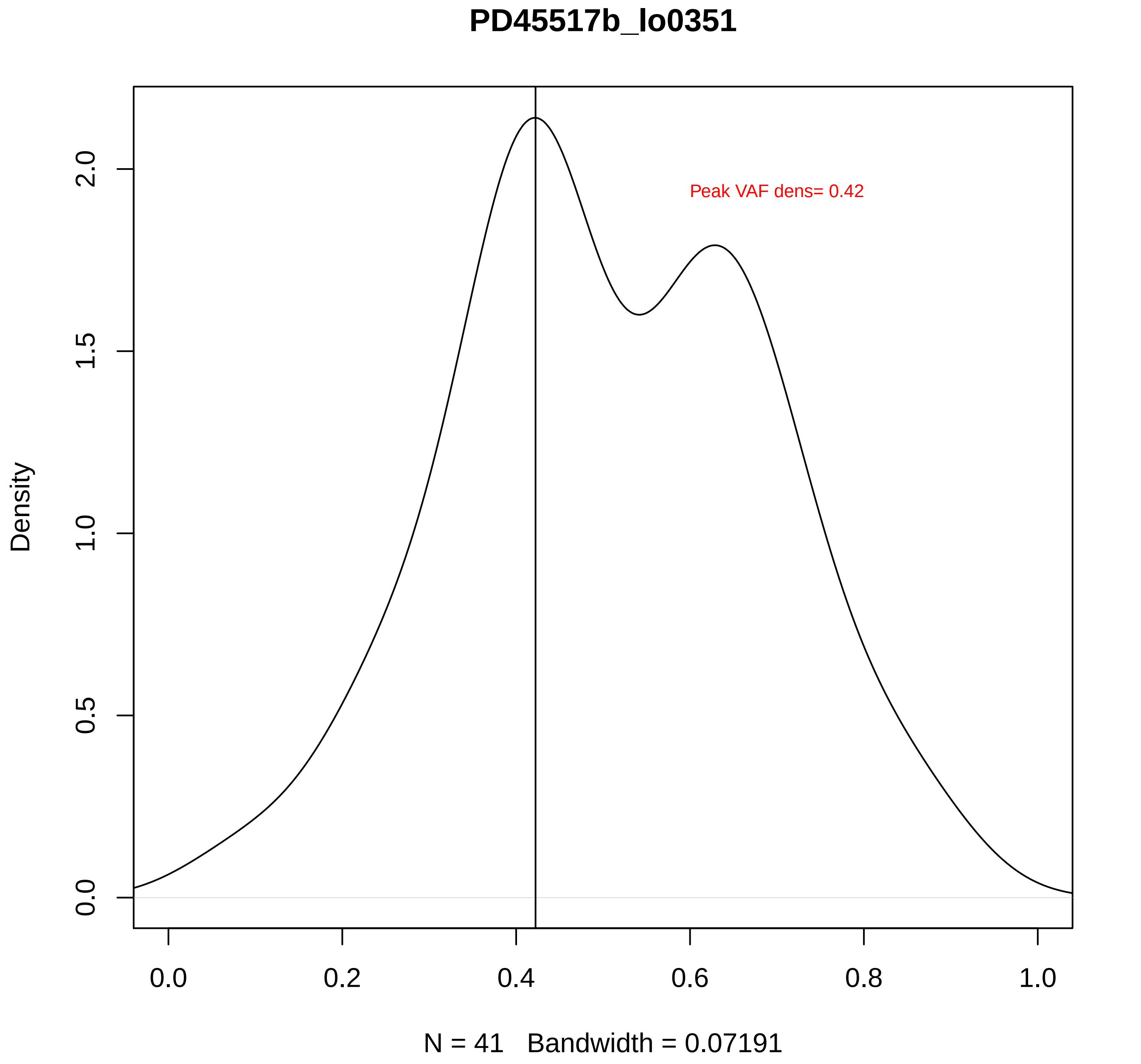

PD45517b_lo0351
Peak VAF dens= 0.42
Density
0.0
0.5
1.0
1.5
2.0
0.0
0.2
0.4
0.6
0.8
1.0
N = 41   Bandwidth = 0.07191

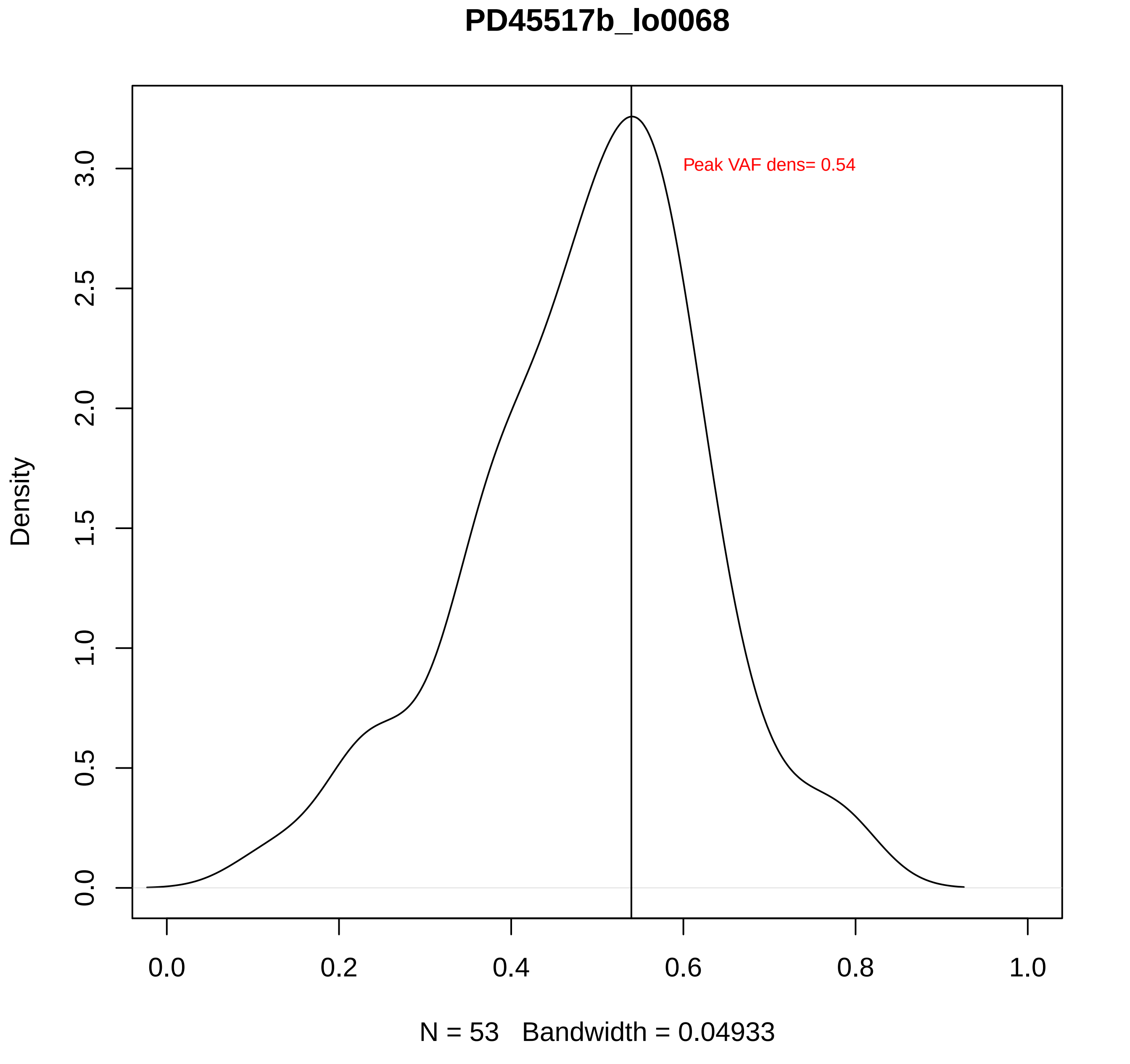
PD45517b_lo0068
Peak VAF dens= 0.54
Density
0.0
0.5
1.0
1.5
2.0
2.5
3.0
0.0
0.2
0.4
0.6
0.8
1.0
N = 53 Bandwidth = 0.04933

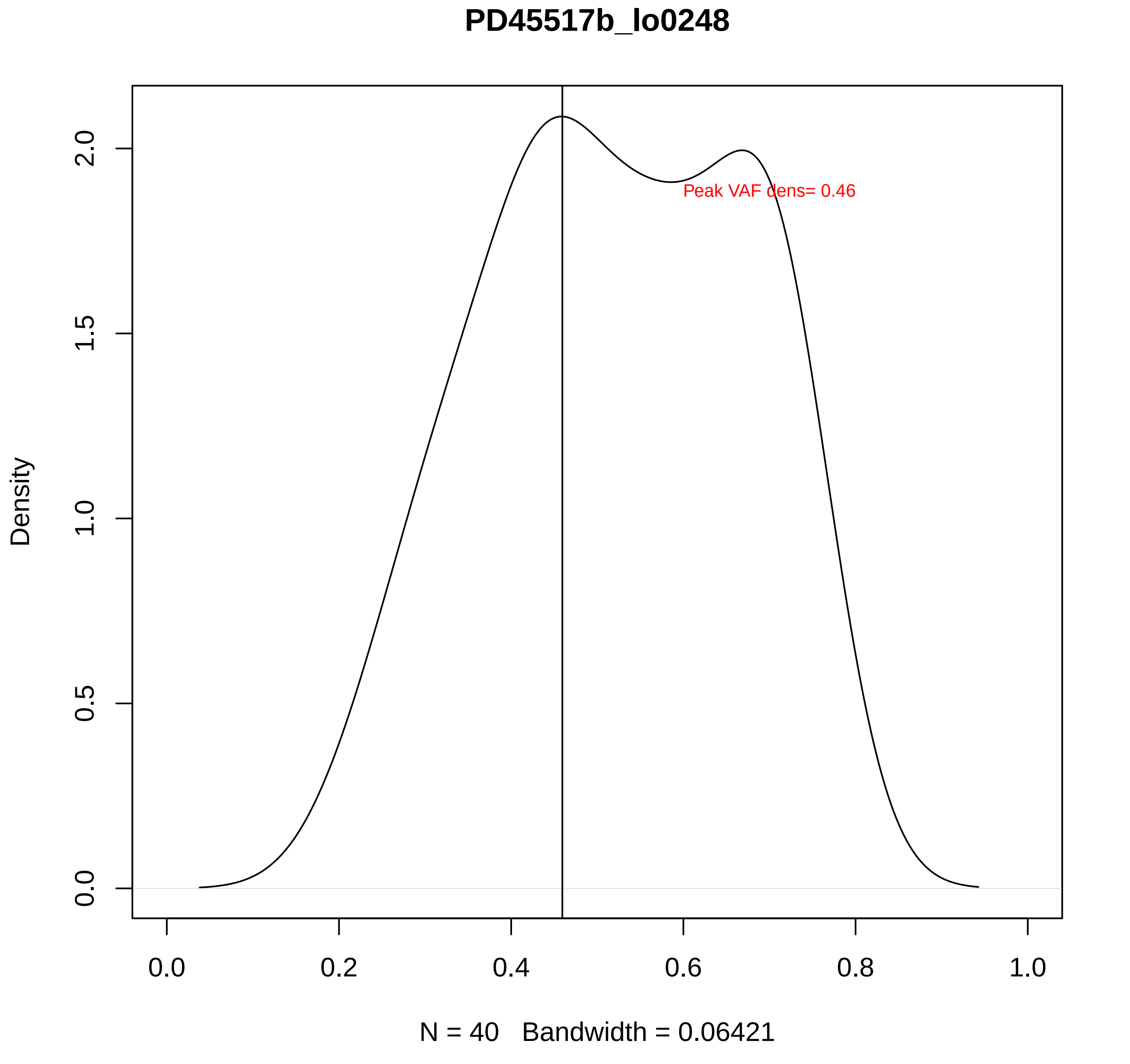
PD45517b_lo0248
Peak VAF dens= 0.46
Density
0.0
0.5
1.0
1.5
2.0
0.0
0.2
0.4
0.6
0.8
1.0
N = 40   Bandwidth = 0.06421

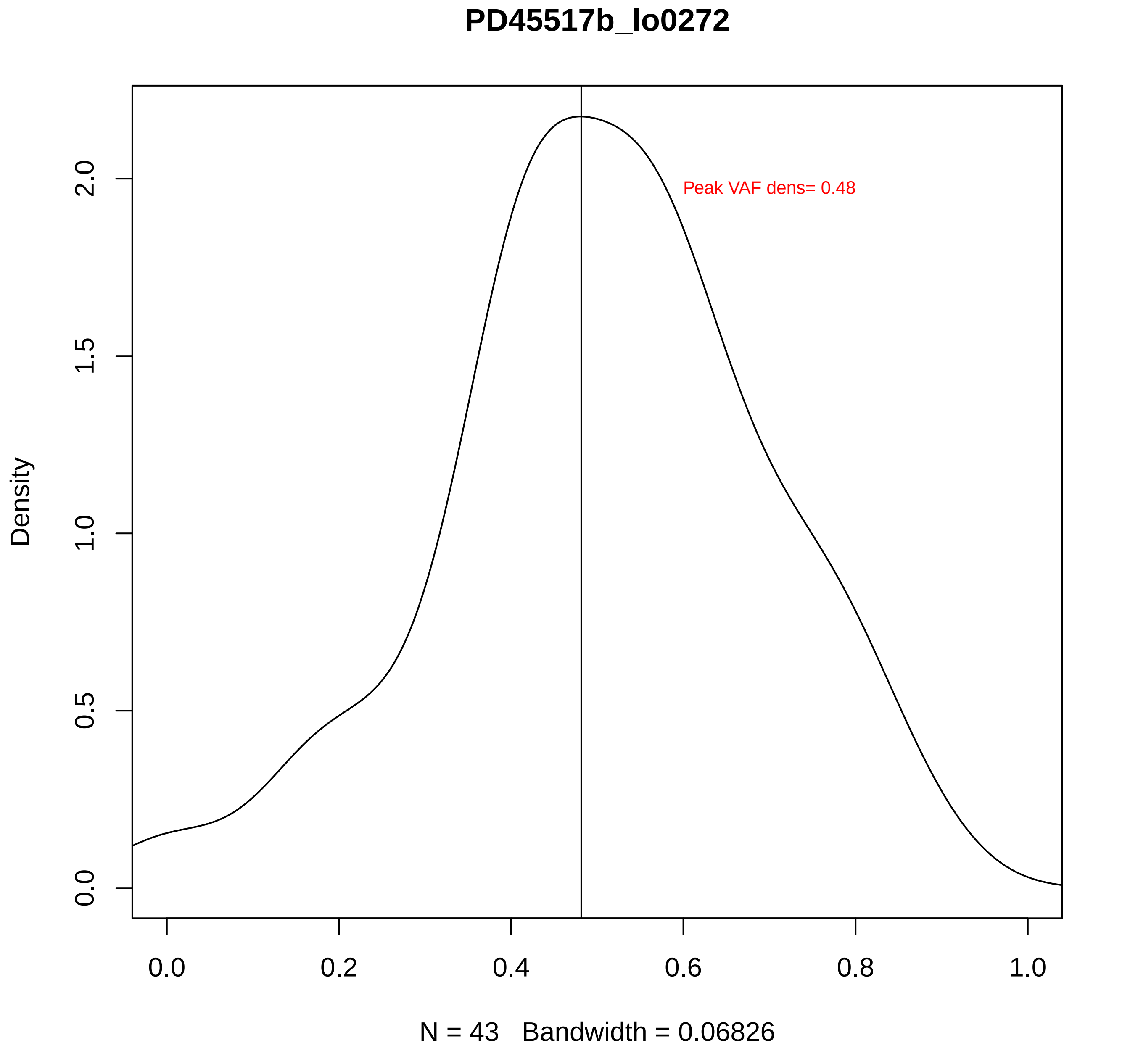
PD45517b_lo0272
Peak VAF dens= 0.48
Density
0.0
0.5
1.0
1.5
2.0
0.0
0.2
0.4
0.6
0.8
1.0
N = 43   Bandwidth = 0.06826

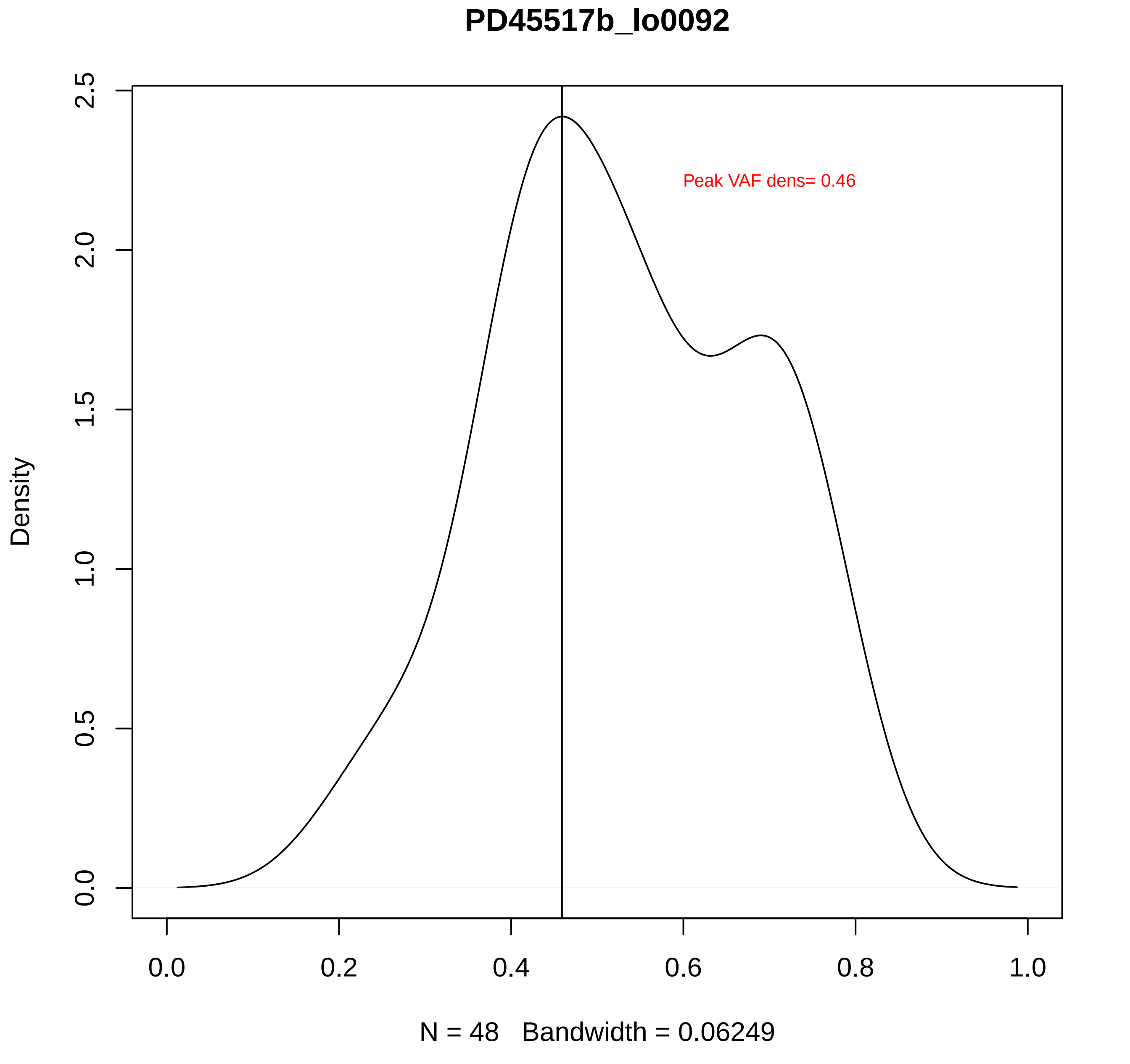

PD45517b_lo0092
Peak VAF dens= 0.46
Density
0.0
0.5
1.0
1.5
2.0
2.5
0.0
0.2
0.4
0.6
0.8
1.0
N = 48 Bandwidth = 0.06249

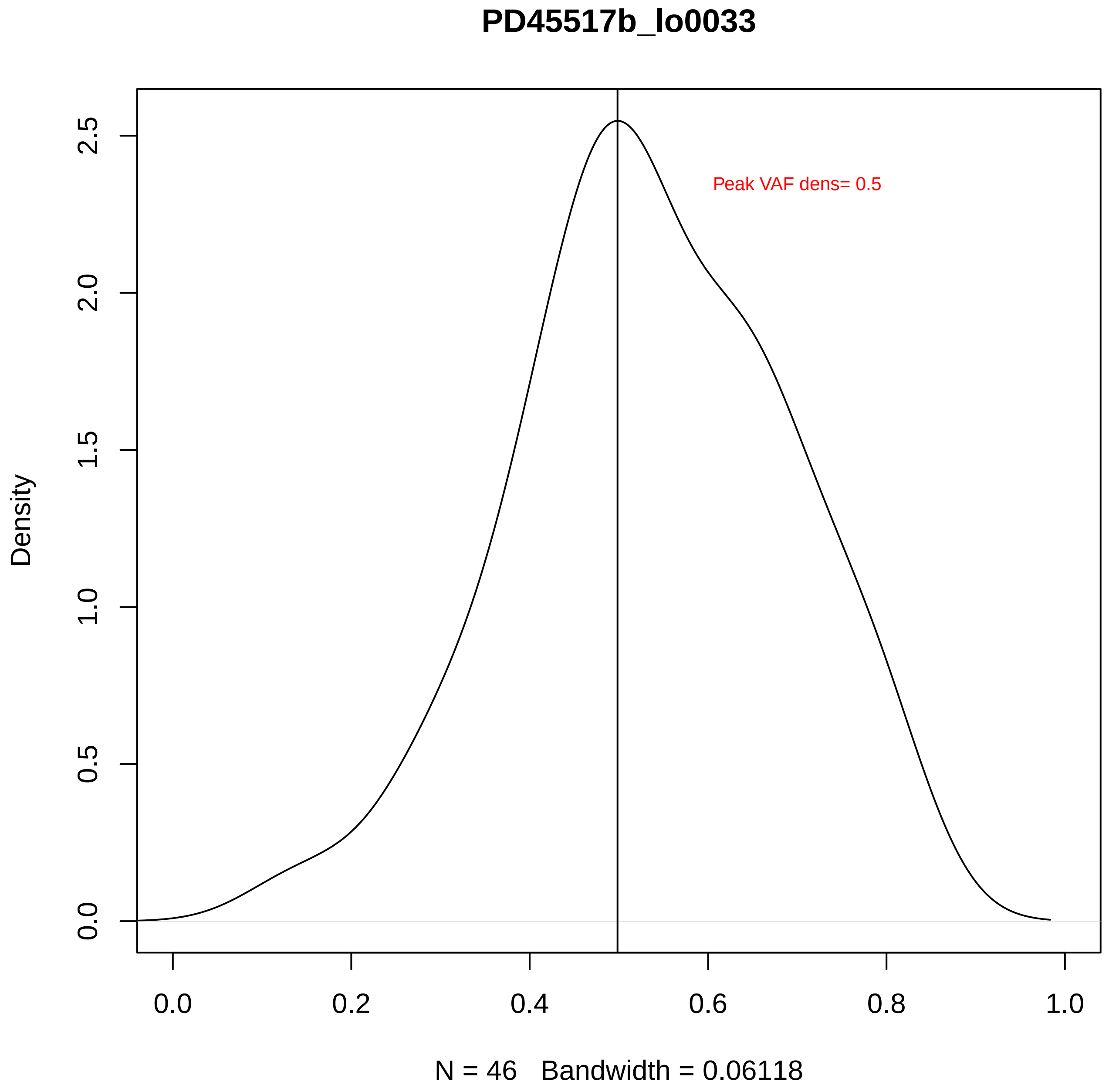
PD45517b_lo0033
Peak VAF dens= 0.5
Density
0.0
0.5
1.0
1.5
2.0
2.5
0.0
0.2
0.4
0.6
0.8
1.0
N = 46   Bandwidth = 0.06118

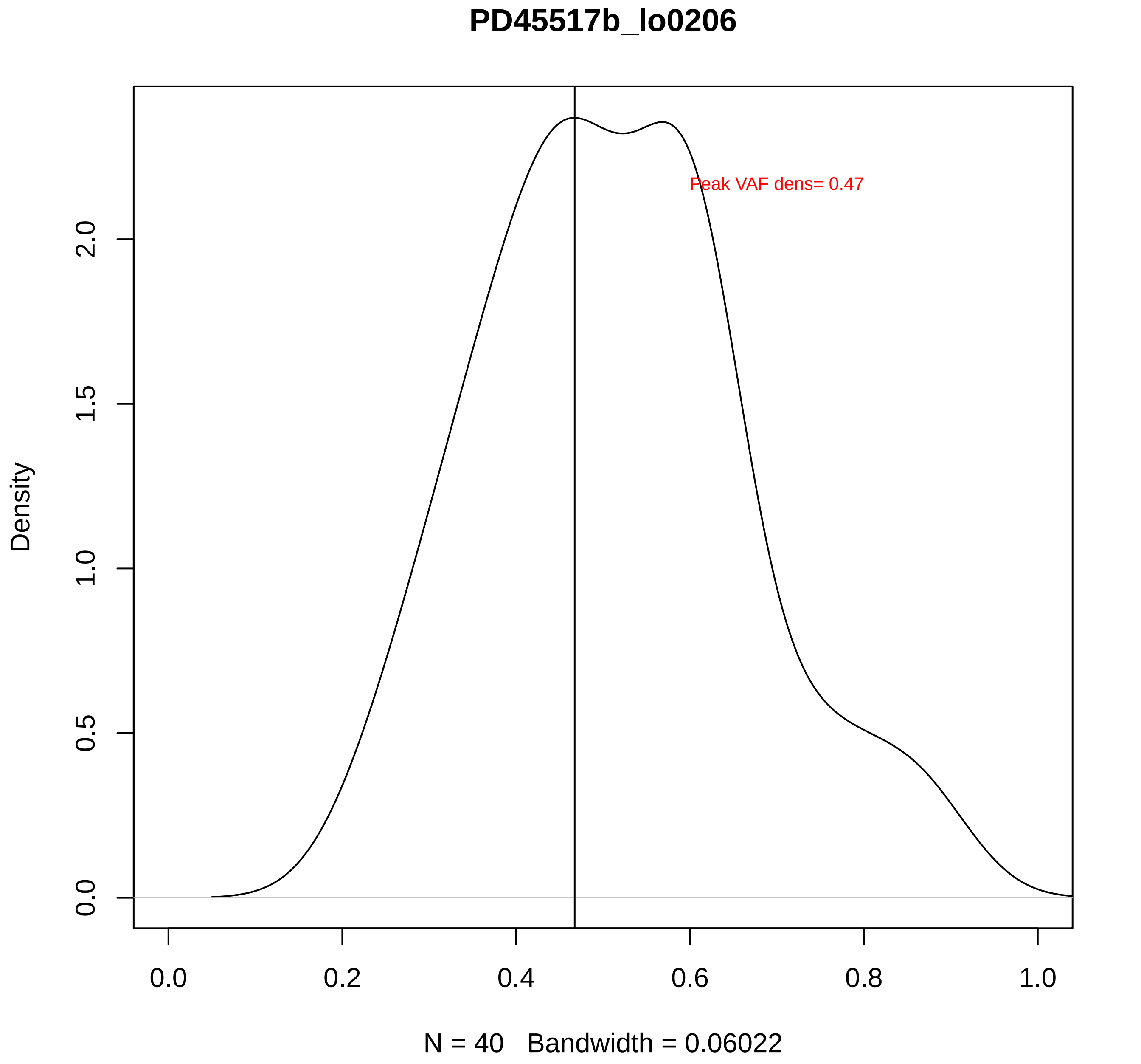

PD45517b_lo0206
Peak VAF dens= 0.47
Density
0.0
0.5
1.0
1.5
2.0
0.0
0.2
0.4
0.6
0.8
1.0
N = 40 Bandwidth = 0.06022

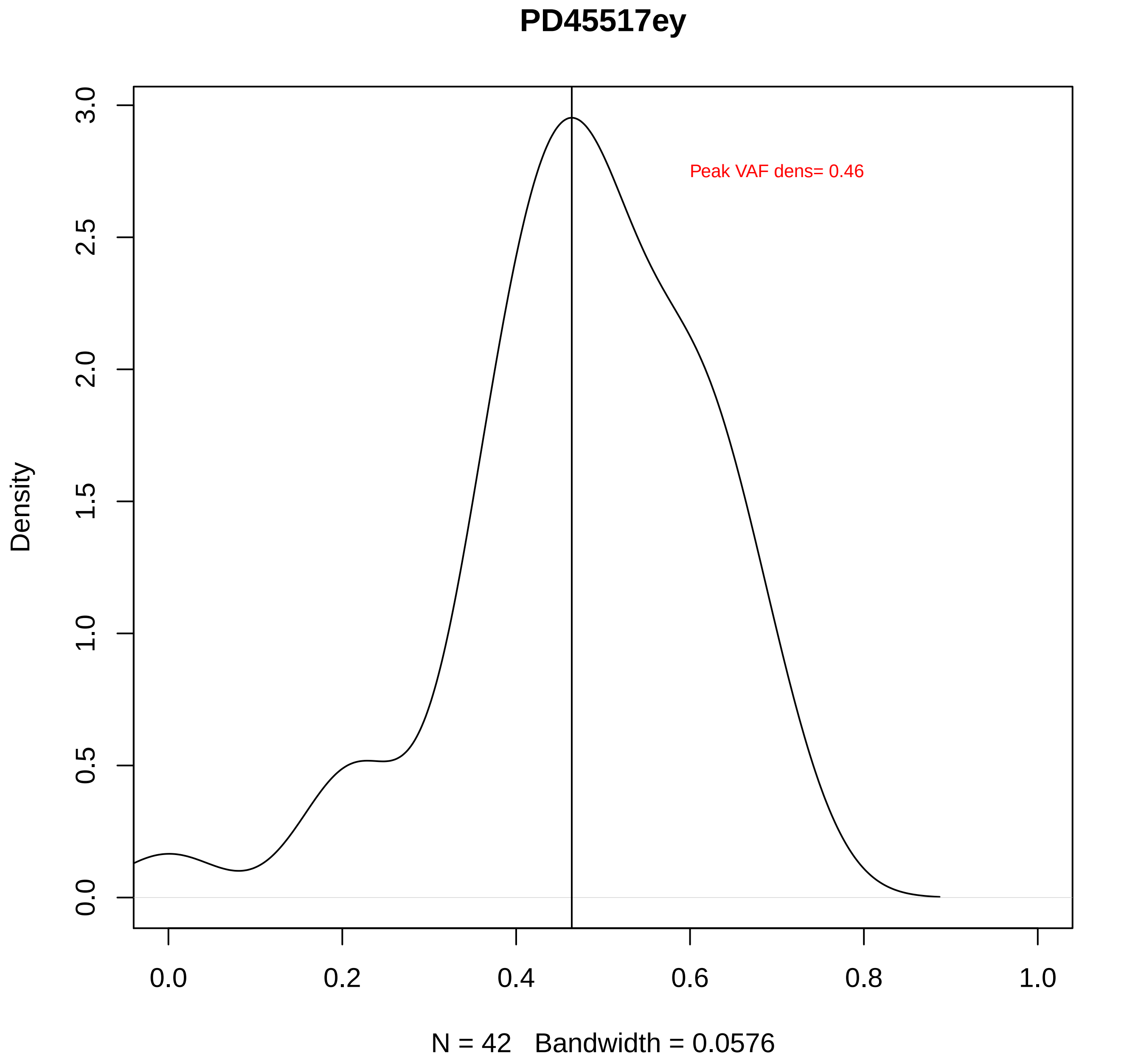

PD45517ey
Peak VAF dens= 0.46
Density
0.0
0.5
1.0
1.5
2.0
2.5
3.0
0.0
0.2
0.4
0.6
0.8
1.0
N = 42   Bandwidth = 0.0576

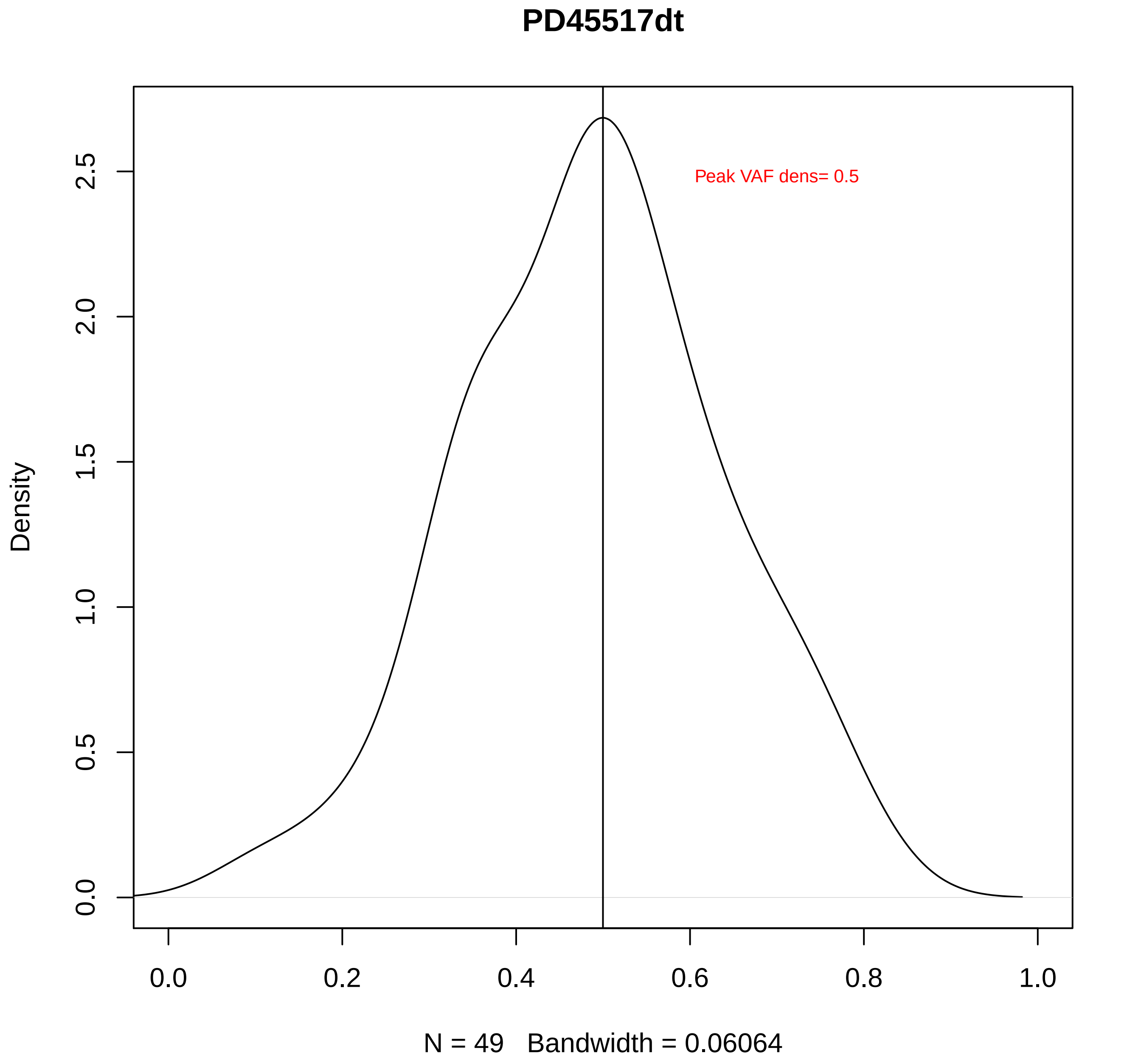

PD45517dt
Peak VAF dens= 0.5
Density
0.0
0.5
1.0
1.5
2.0
2.5
0.0
0.2
0.4
0.6
0.8
1.0
N = 49   Bandwidth = 0.06064

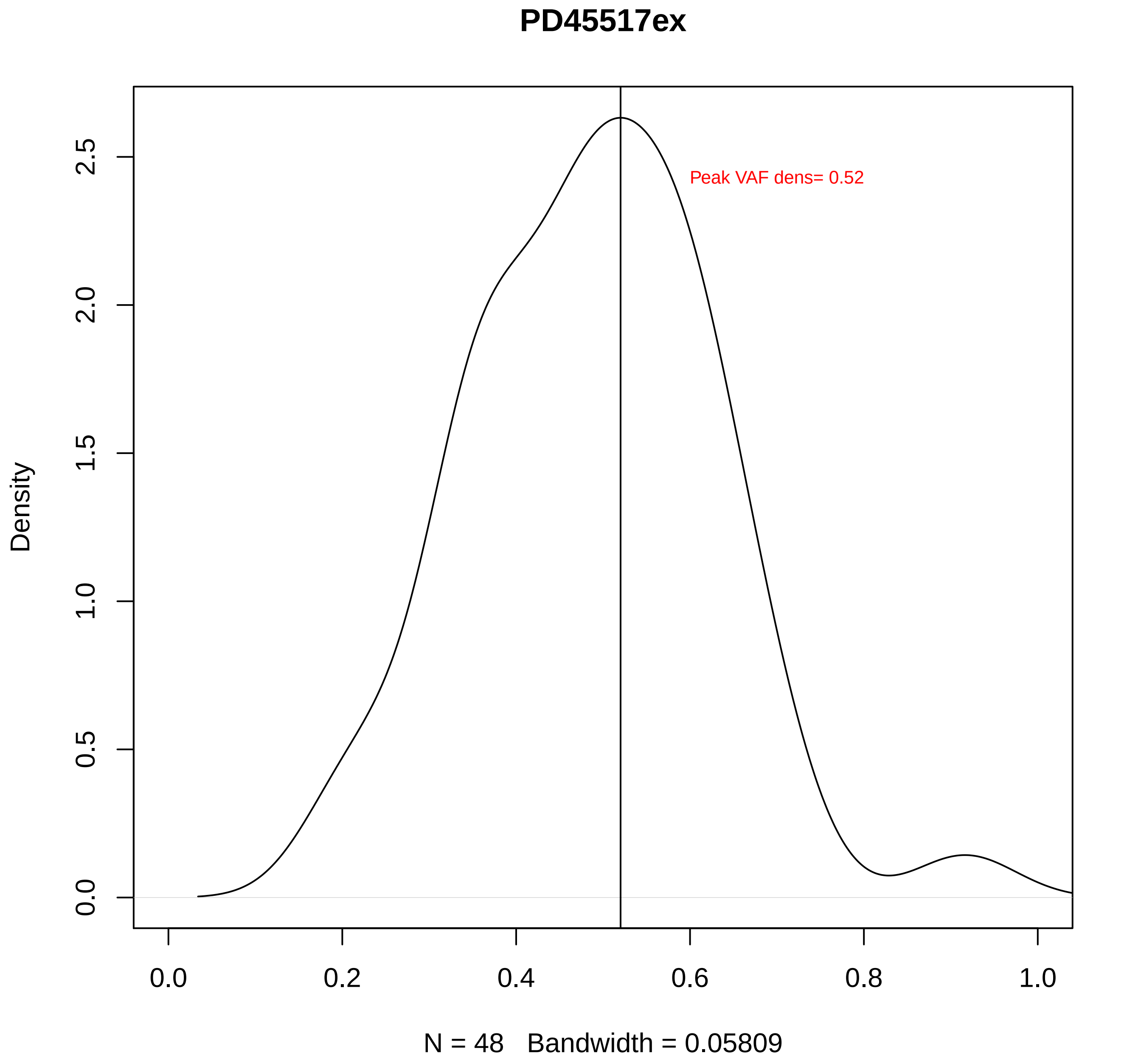
PD45517ex
Peak VAF dens= 0.52
Density
0.0
0.5
1.0
1.5
2.0
2.5
0.0
0.2
0.4
0.6
0.8
1.0
N = 48   Bandwidth = 0.05809

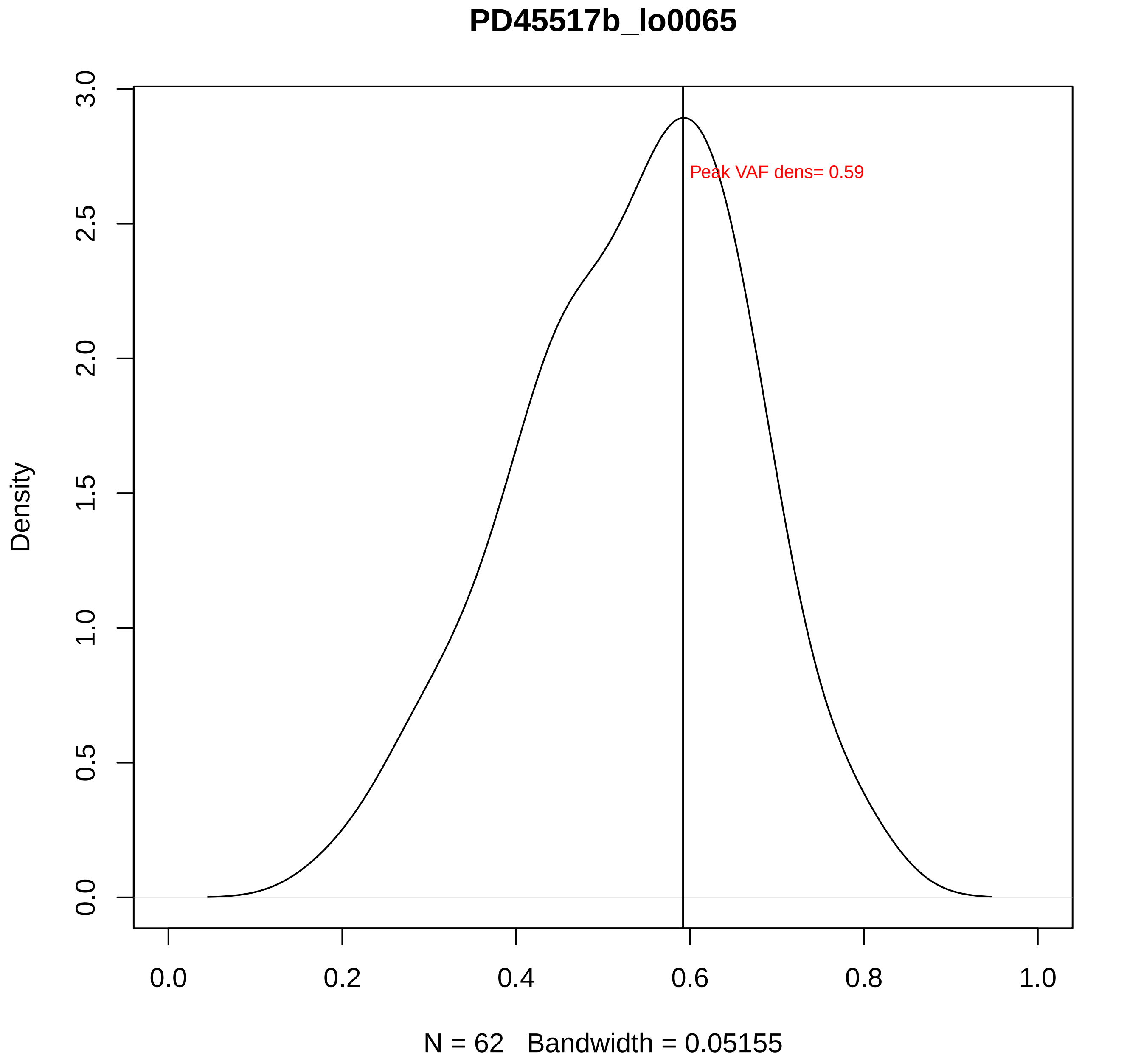

PD45517b_lo0065
Density
3.0
2.5
2.0
1.5
1.0
0.5
0.0
Peak VAF dens= 0.59
0.0
0.2
0.4
0.6
0.8
1.0
N = 62 Bandwidth = 0.05155

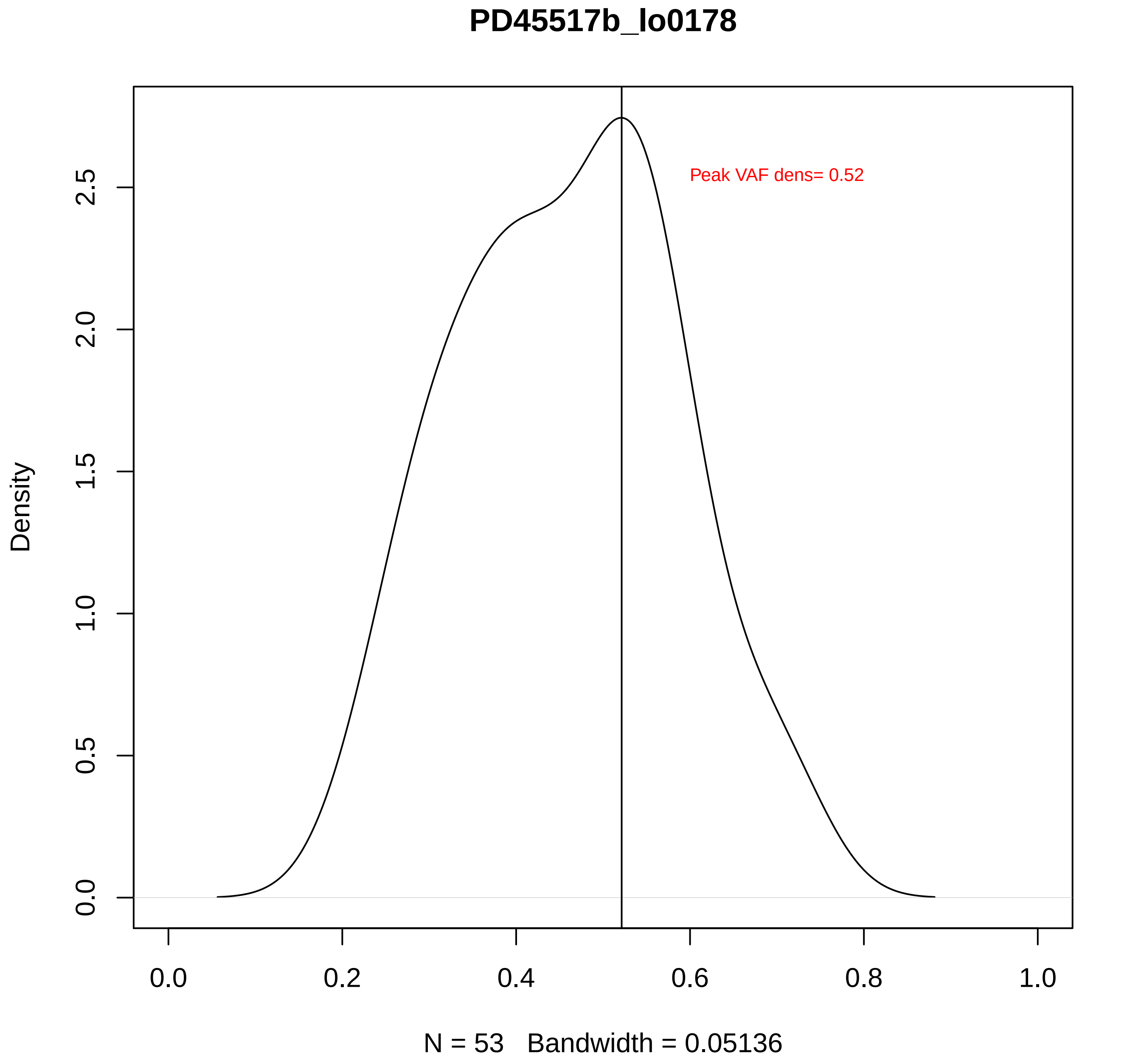

PD45517b_lo0178
Peak VAF dens= 0.52
Density
0.0
0.5
1.0
1.5
2.0
2.5
0.0
0.2
0.4
0.6
0.8
1.0
N = 53 Bandwidth = 0.05136

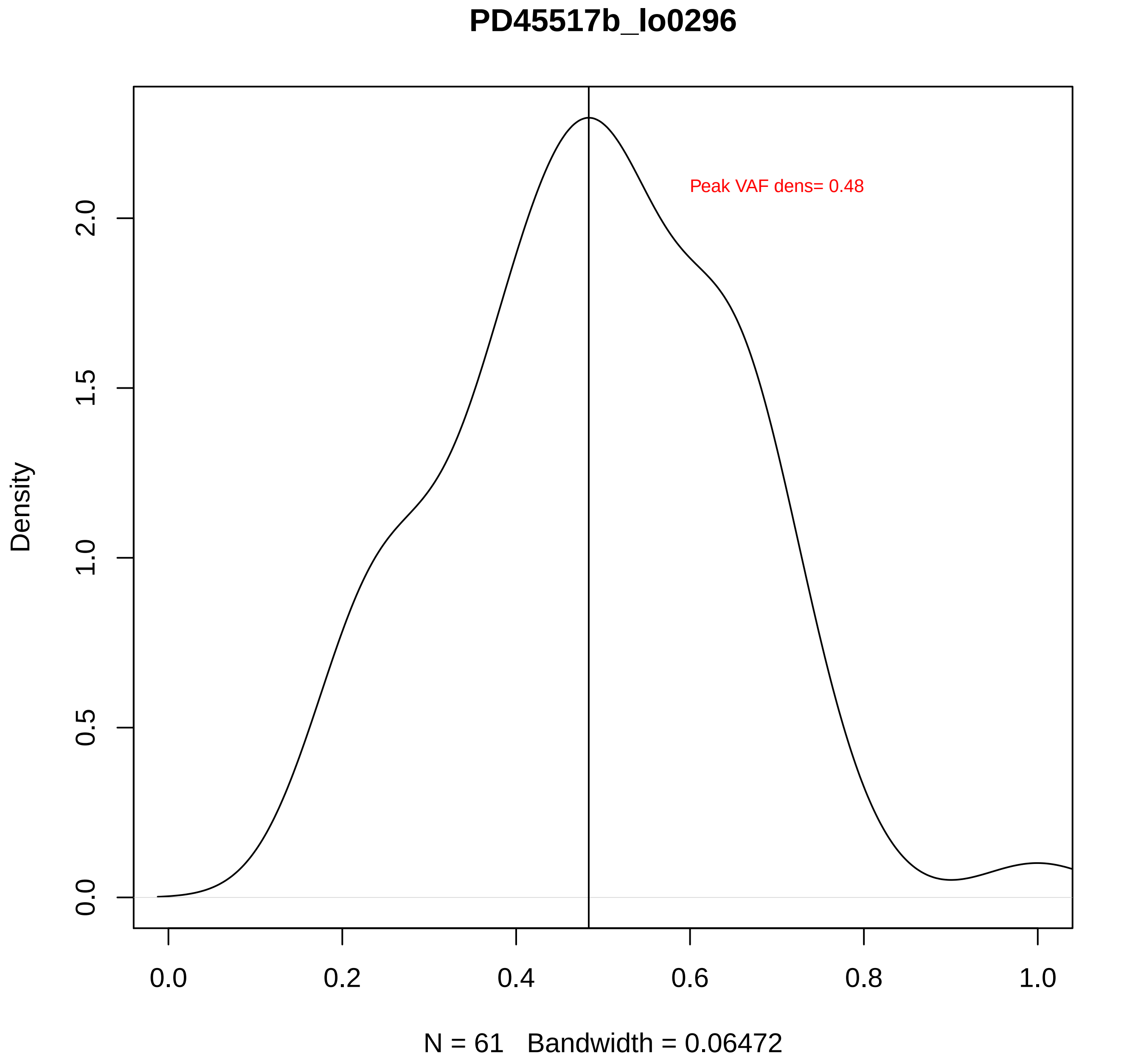
PD45517b_lo0296
Peak VAF dens= 0.48
Density
0.0
0.5
1.0
1.5
2.0
0.0
0.2
0.4
0.6
0.8
1.0
N = 61   Bandwidth = 0.06472

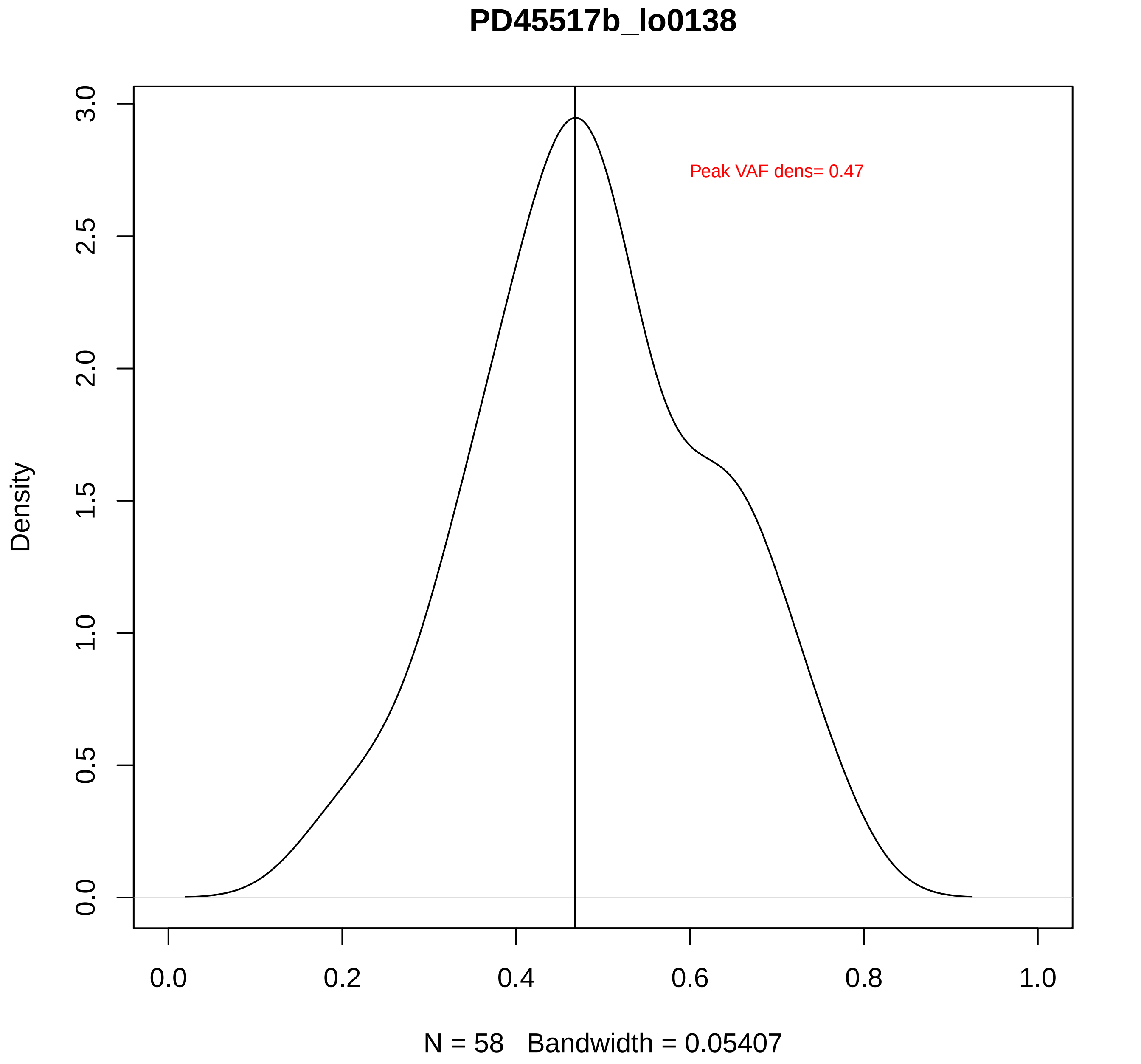
PD45517b_lo0138
Peak VAF dens= 0.47
Density
0.0
0.5
1.0
1.5
2.0
2.5
3.0
0.0
0.2
0.4
0.6
0.8
1.0
N = 58   Bandwidth = 0.05407

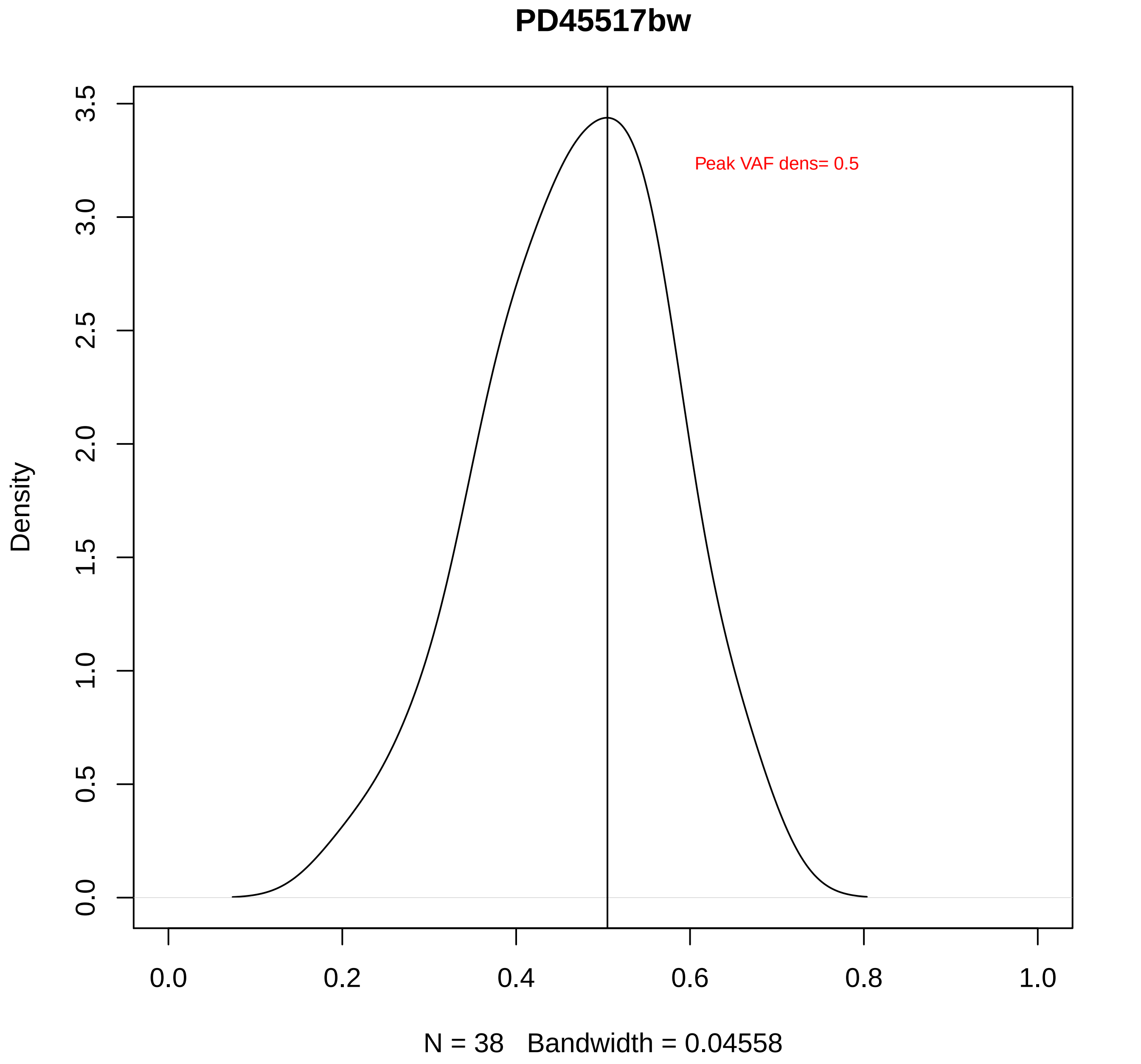
PD45517bw
Peak VAF dens= 0.5
Density
0.0
0.5
1.0
1.5
2.0
2.5
3.0
3.5
0.0
0.2
0.4
0.6
0.8
1.0
N = 38   Bandwidth = 0.04558

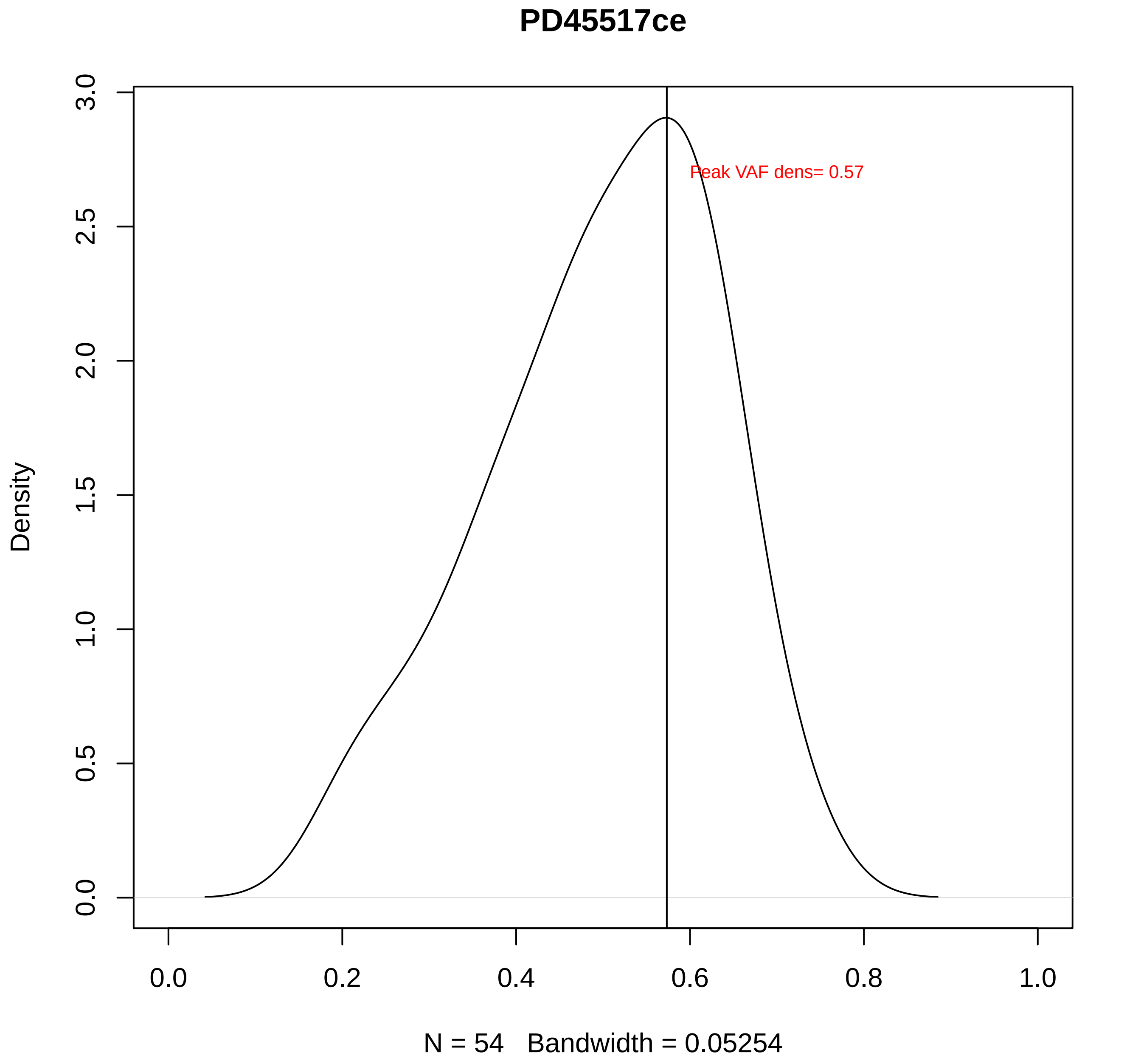
PD45517ce
Peak VAF dens= 0.57
Density
0.0
0.5
1.0
1.5
2.0
2.5
3.0
0.0
0.2
0.4
0.6
0.8
1.0
N = 54   Bandwidth = 0.05254

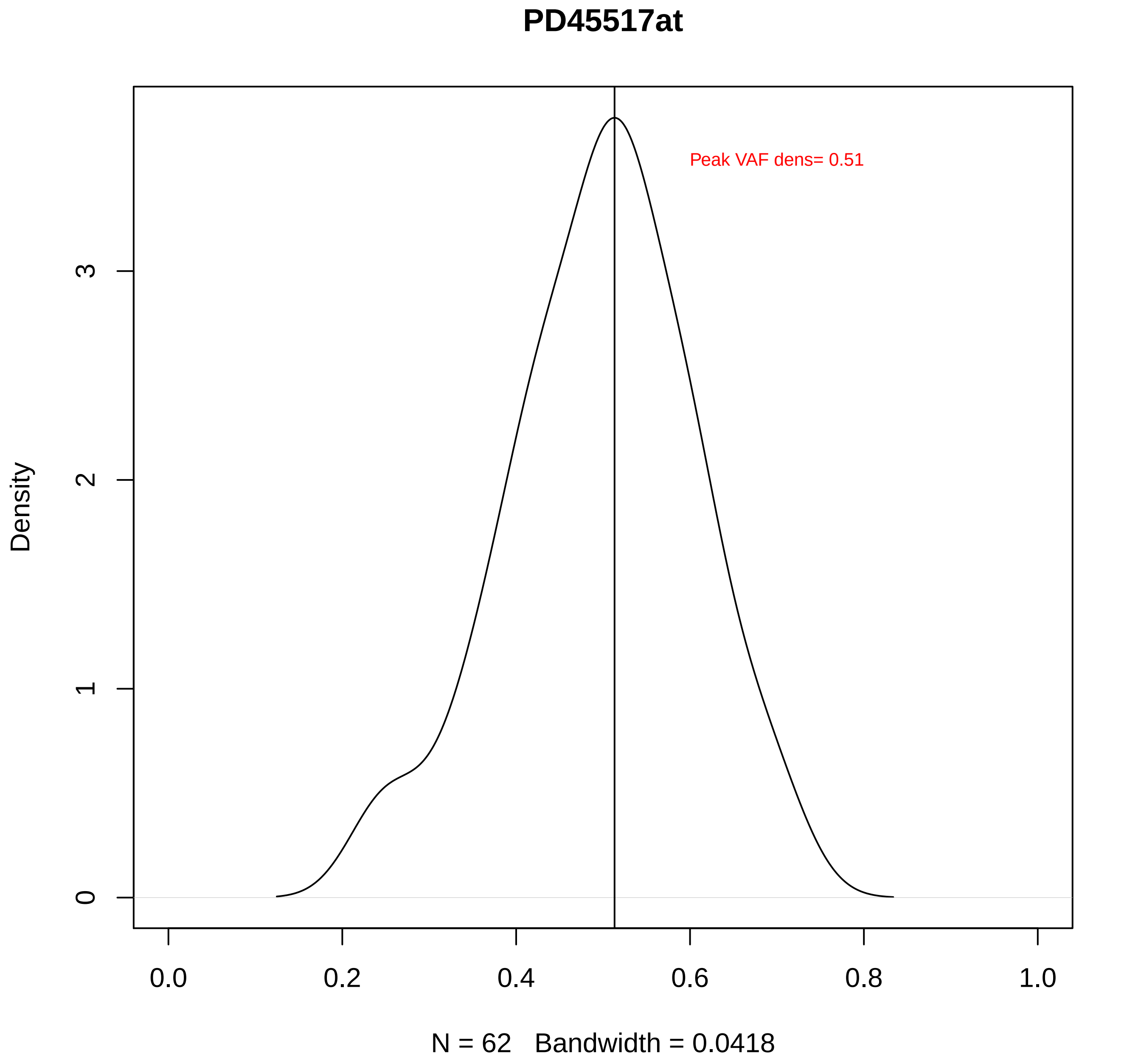

PD45517at
Peak VAF dens= 0.51
Density
0
1
2
3
0.0
0.2
0.4
0.6
0.8
1.0
N = 62 Bandwidth = 0.0418

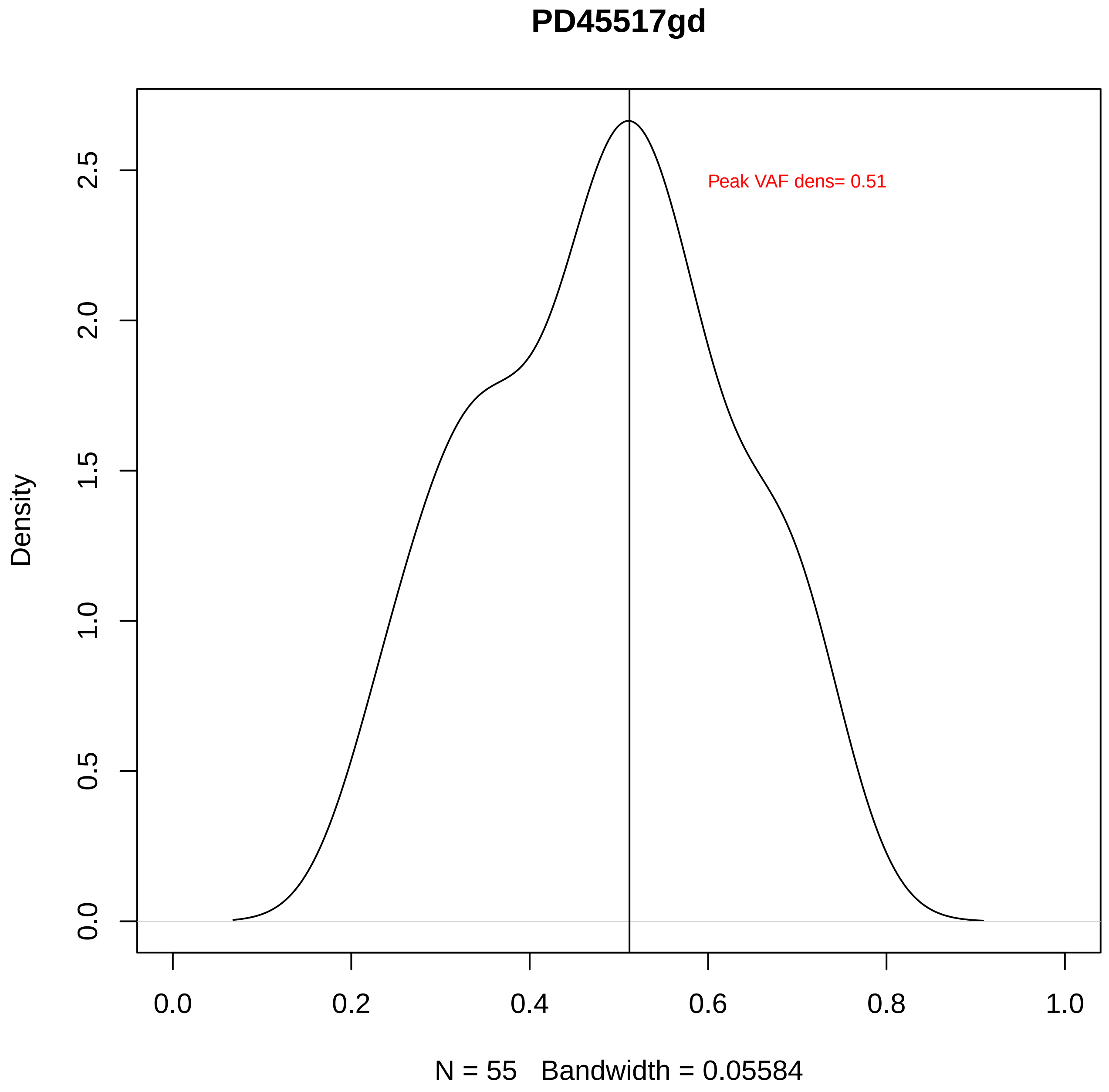
PD45517gd
Peak VAF dens= 0.51
Density
0.0
0.5
1.0
1.5
2.0
2.5
0.0
0.2
0.4
0.6
0.8
1.0
N = 55 Bandwidth = 0.05584

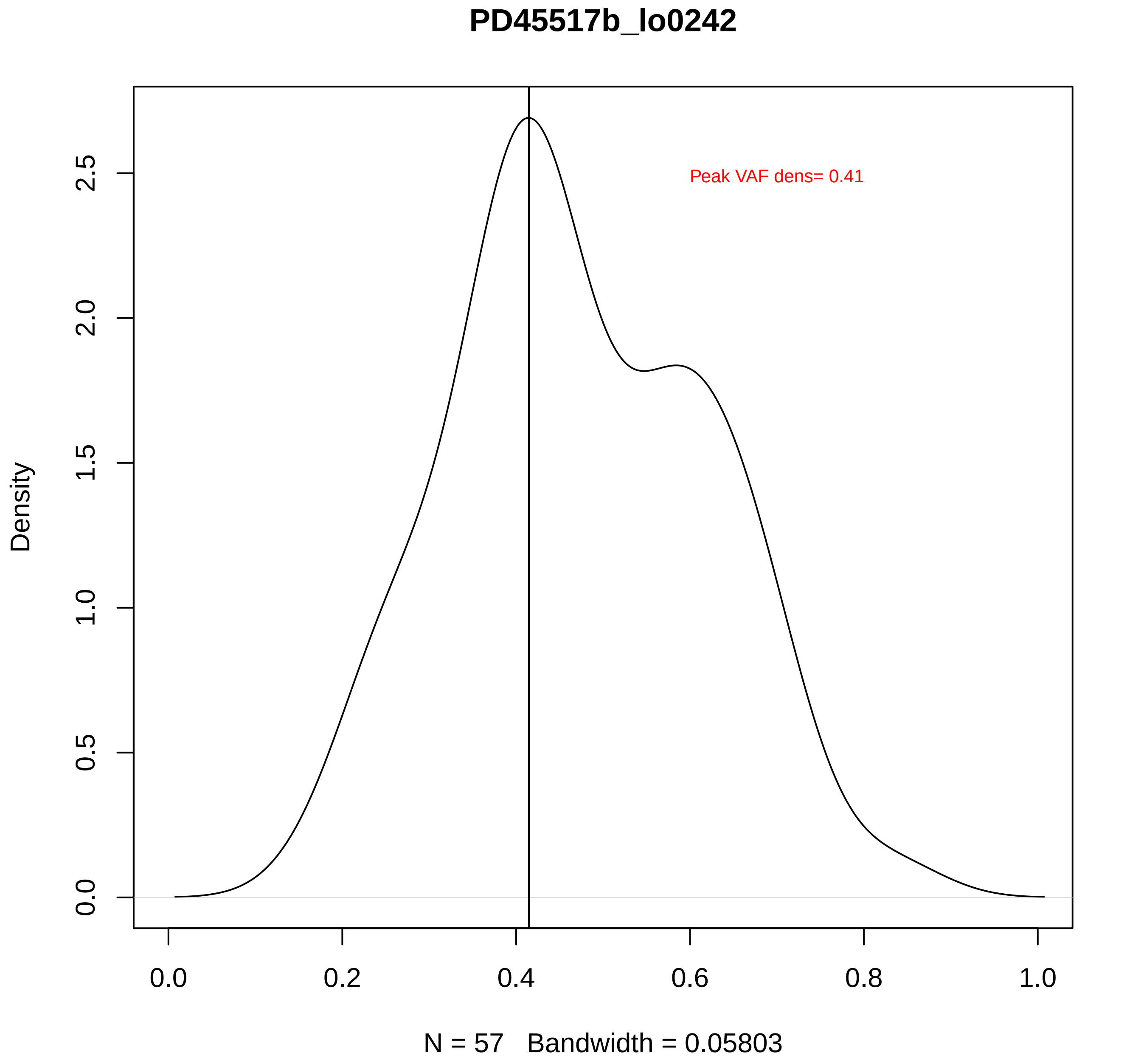
PD45517b_lo0242
Peak VAF dens= 0.41
Density
0.0
0.5
1.0
1.5
2.0
2.5
0.0
0.2
0.4
0.6
0.8
1.0
N = 57 Bandwidth = 0.05803

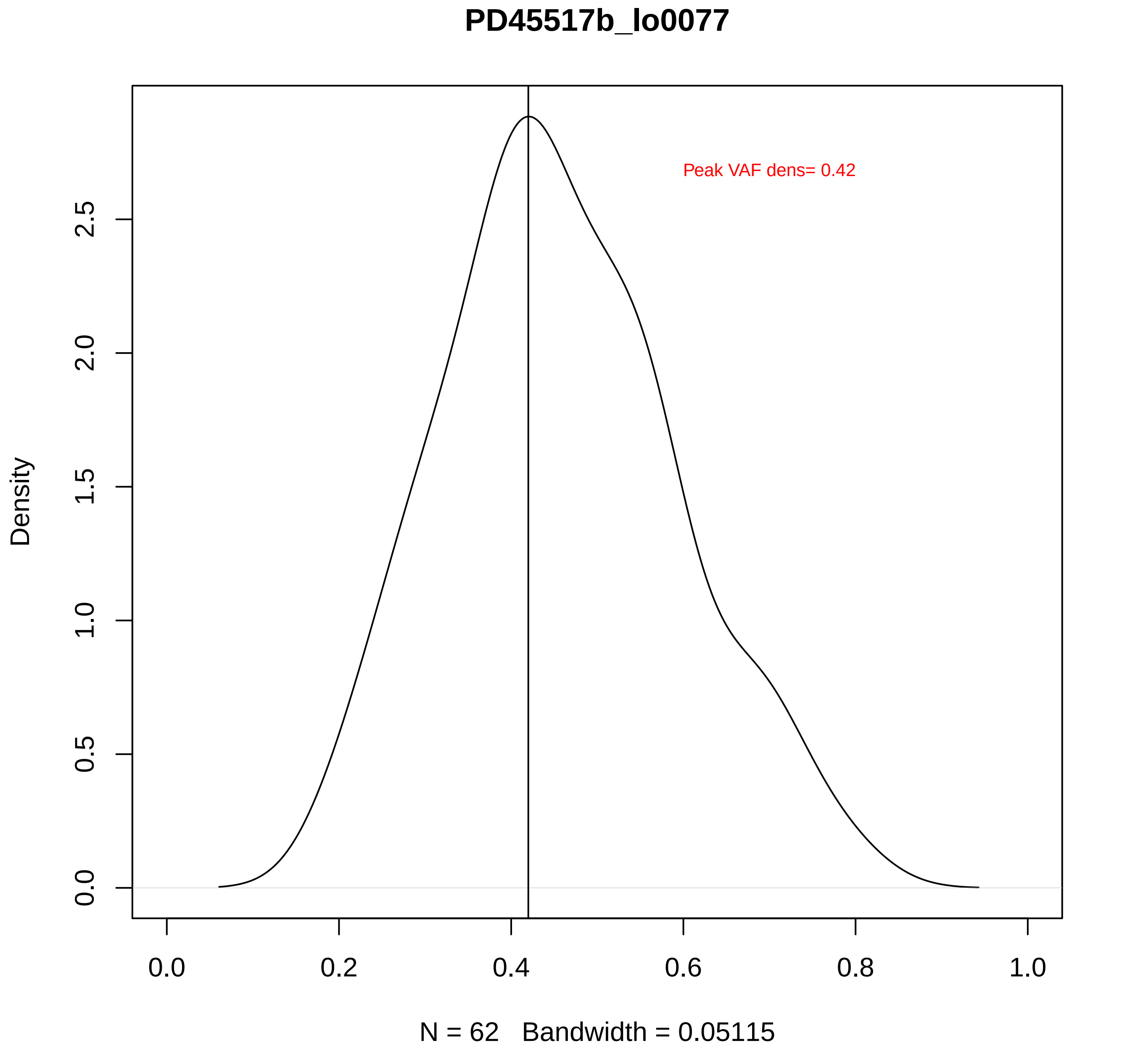
PD45517b_lo0077
Peak VAF dens= 0.42
Density
0.0
0.5
1.0
1.5
2.0
2.5
0.0
0.2
0.4
0.6
0.8
1.0
N = 62 Bandwidth = 0.05115

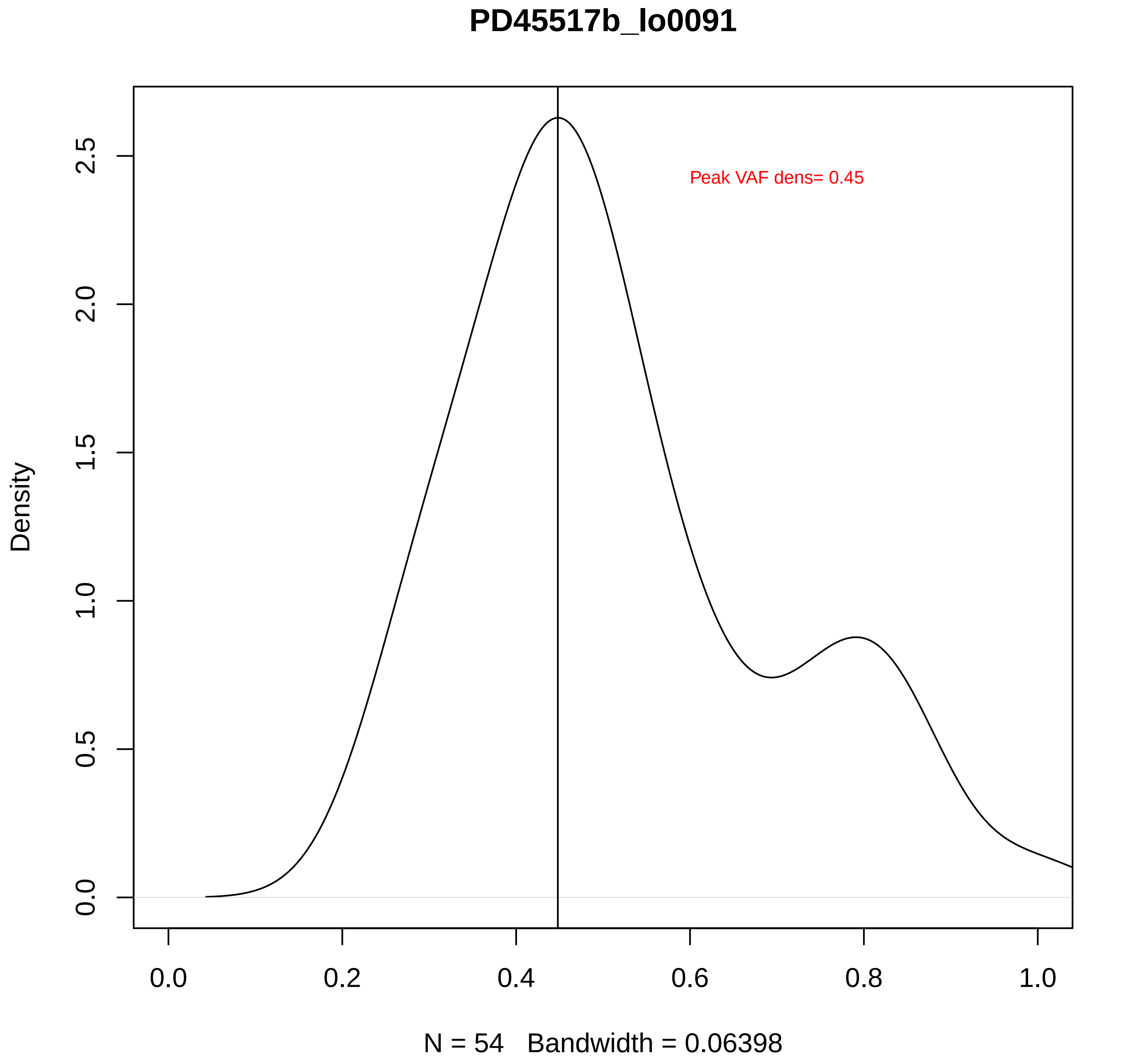
PD45517b_lo0091
Peak VAF dens= 0.45
Density
0.0
0.5
1.0
1.5
2.0
2.5
0.0
0.2
0.4
0.6
0.8
1.0
N = 54   Bandwidth = 0.06398

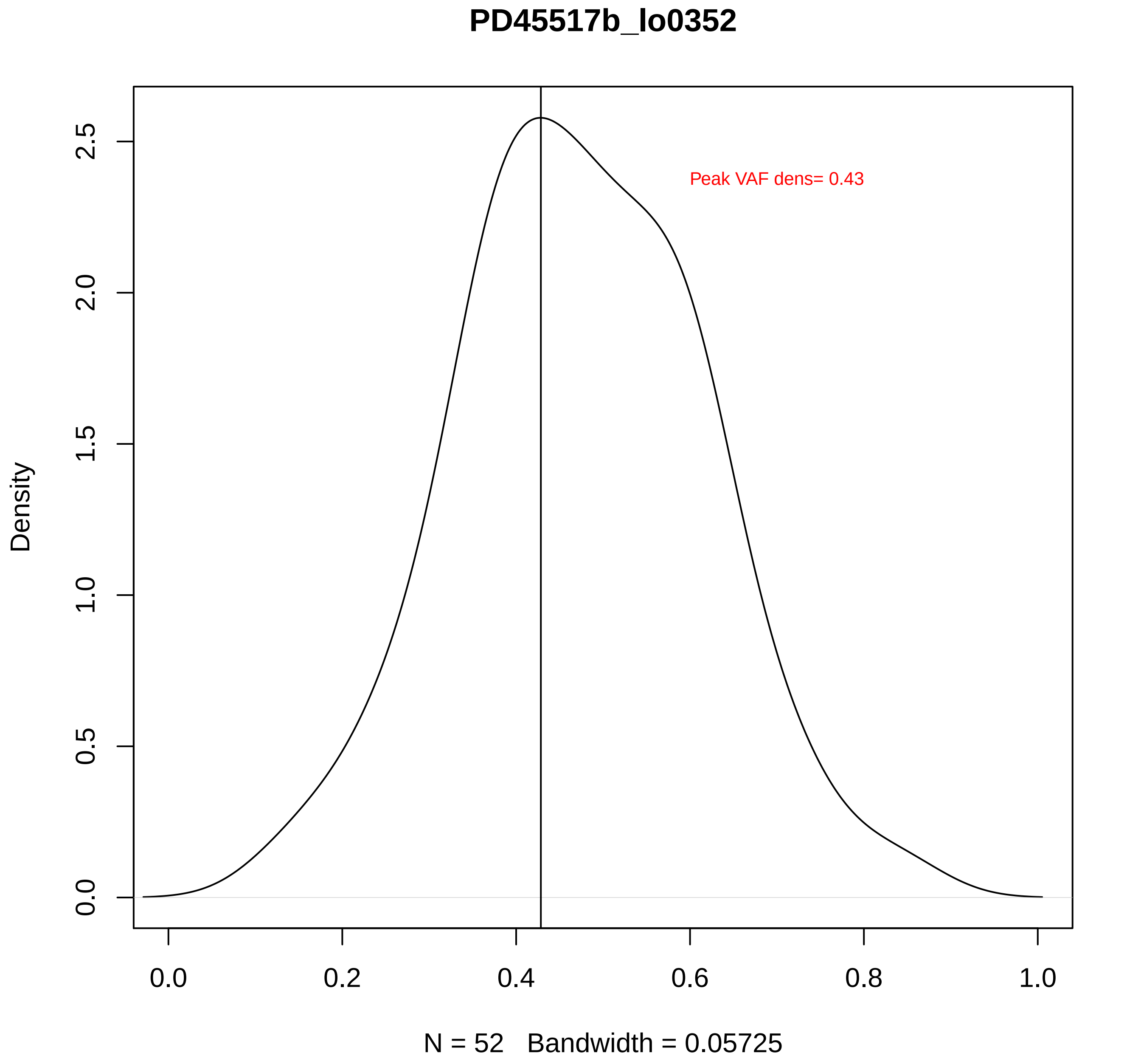

PD45517b_lo0352
Peak VAF dens= 0.43
Density
0.0
0.5
1.0
1.5
2.0
2.5
0.0
0.2
0.4
0.6
0.8
1.0
N = 52 Bandwidth = 0.05725

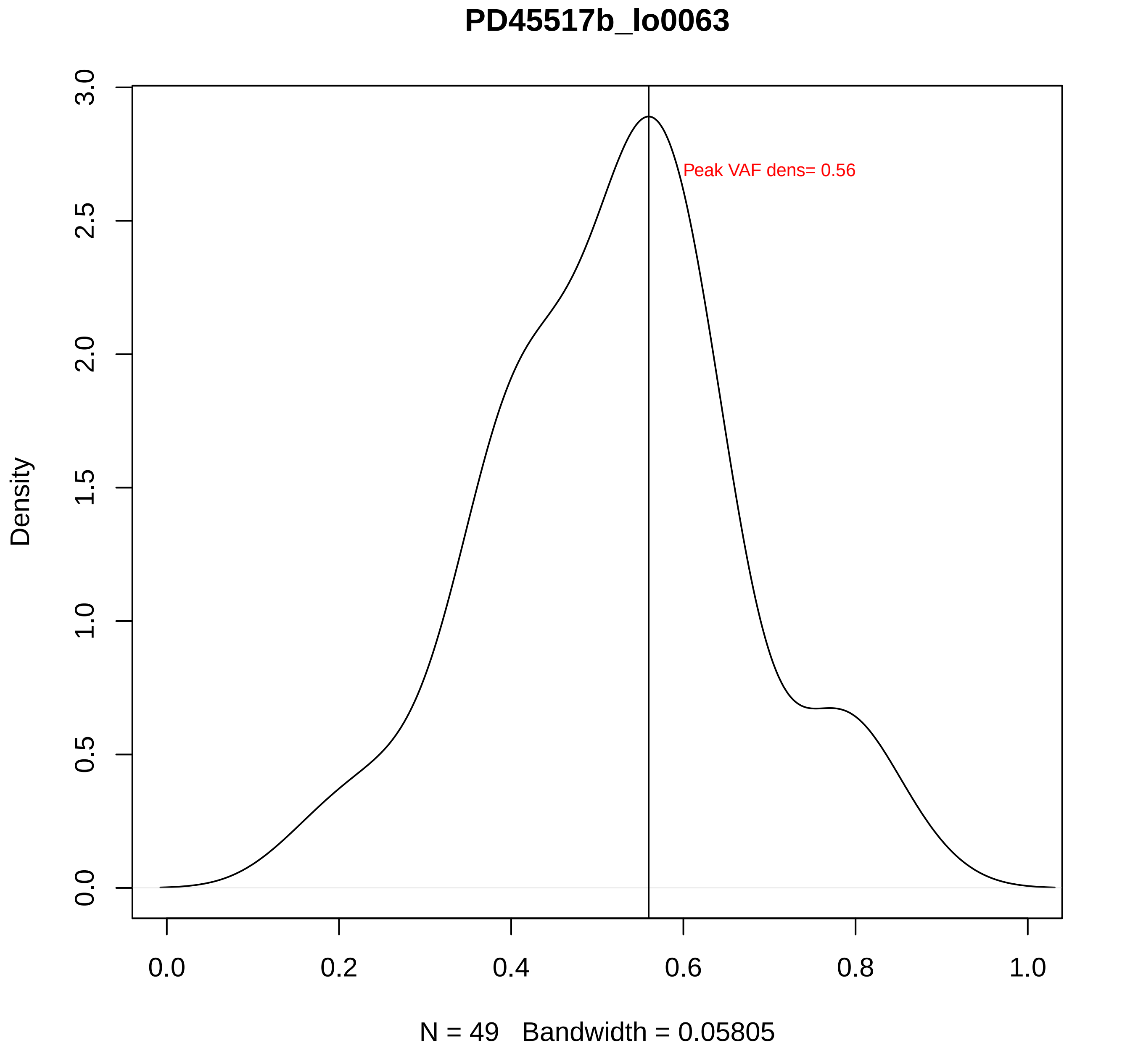
PD45517b_lo0063
Peak VAF dens= 0.56
Density
0.0
0.5
1.0
1.5
2.0
2.5
3.0
0.0
0.2
0.4
0.6
0.8
1.0
N = 49 Bandwidth = 0.05805

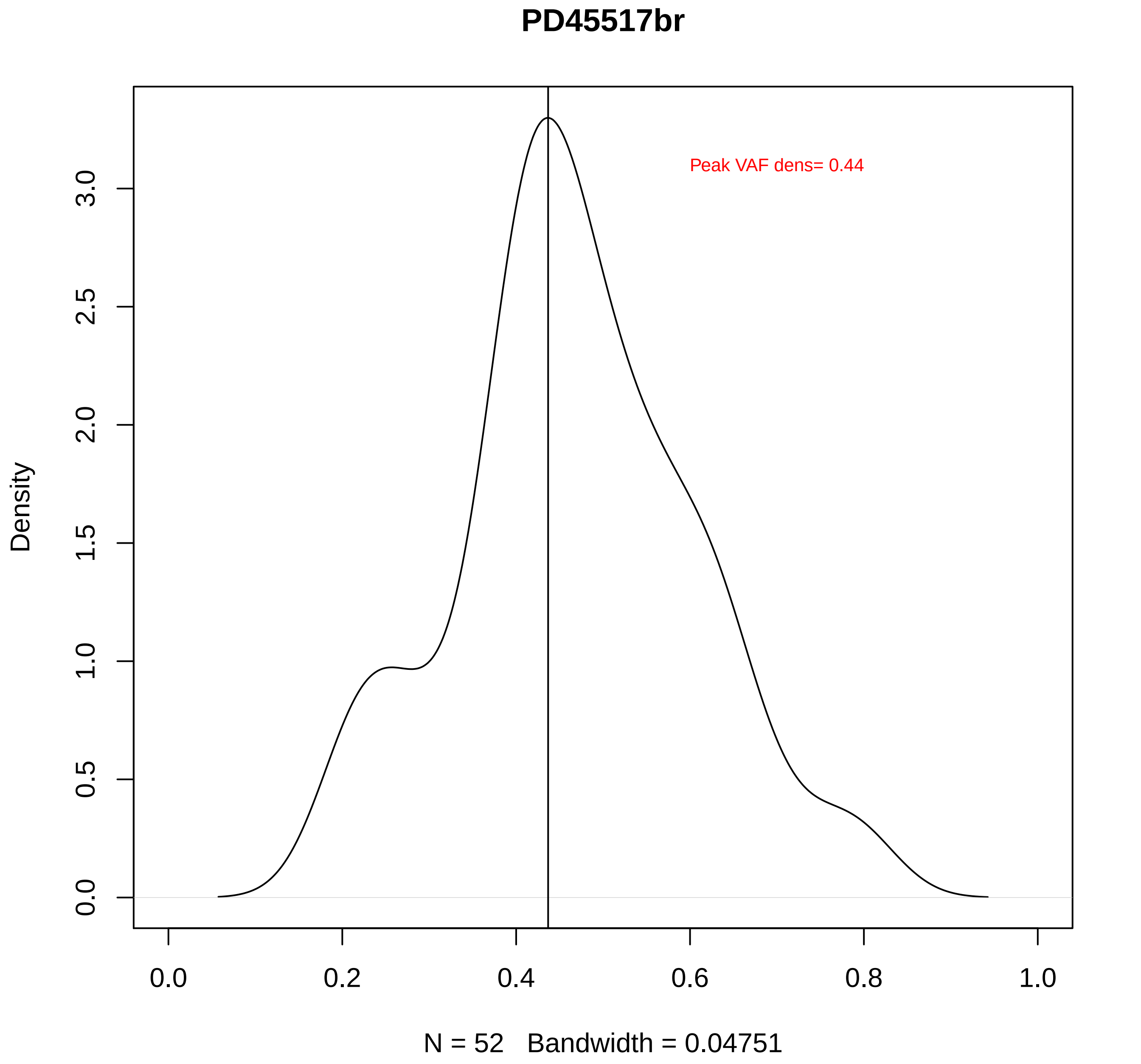
PD45517br
Peak VAF dens= 0.44
Density
0.0
0.5
1.0
1.5
2.0
2.5
3.0
0.0
0.2
0.4
0.6
0.8
1.0
N = 52   Bandwidth = 0.04751

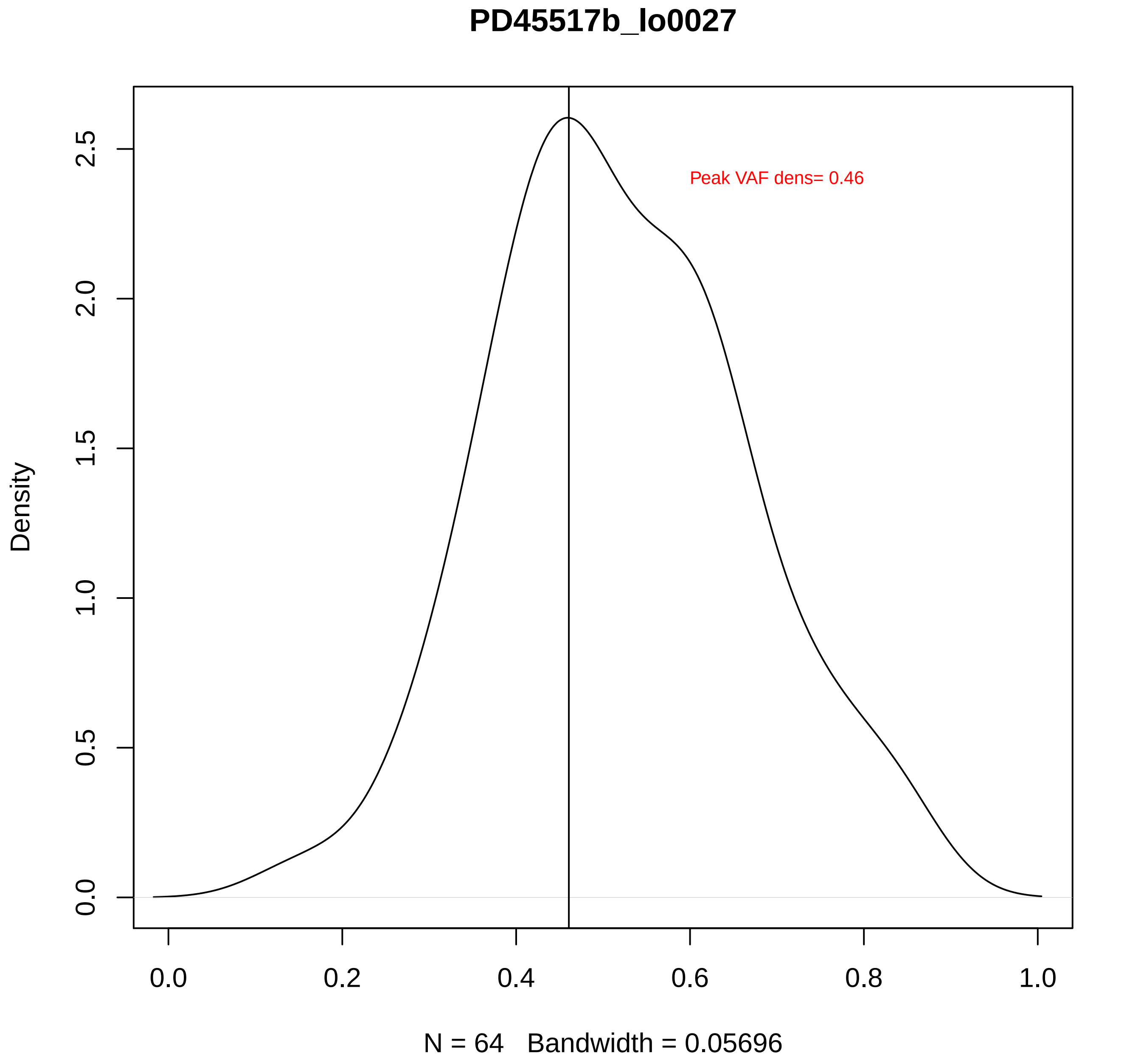
PD45517b_lo0027
Peak VAF dens= 0.46
Density
0.0
0.5
1.0
1.5
2.0
2.5
0.0
0.2
0.4
0.6
0.8
1.0
N = 64   Bandwidth = 0.05696

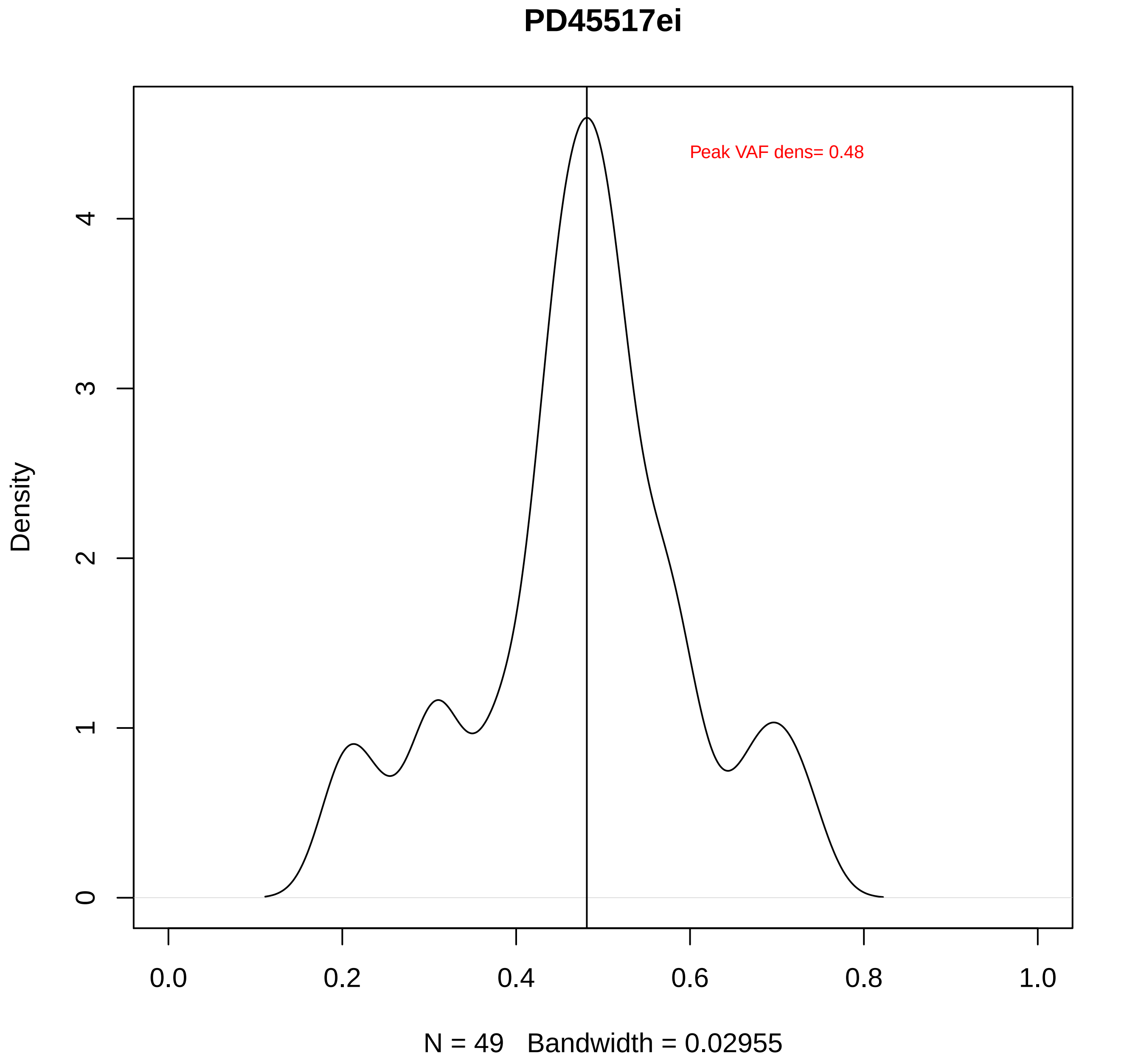
PD45517ei
Peak VAF dens= 0.48
Density
0
1
2
3
4
0.0
0.2
0.4
0.6
0.8
1.0
N = 49   Bandwidth = 0.02955

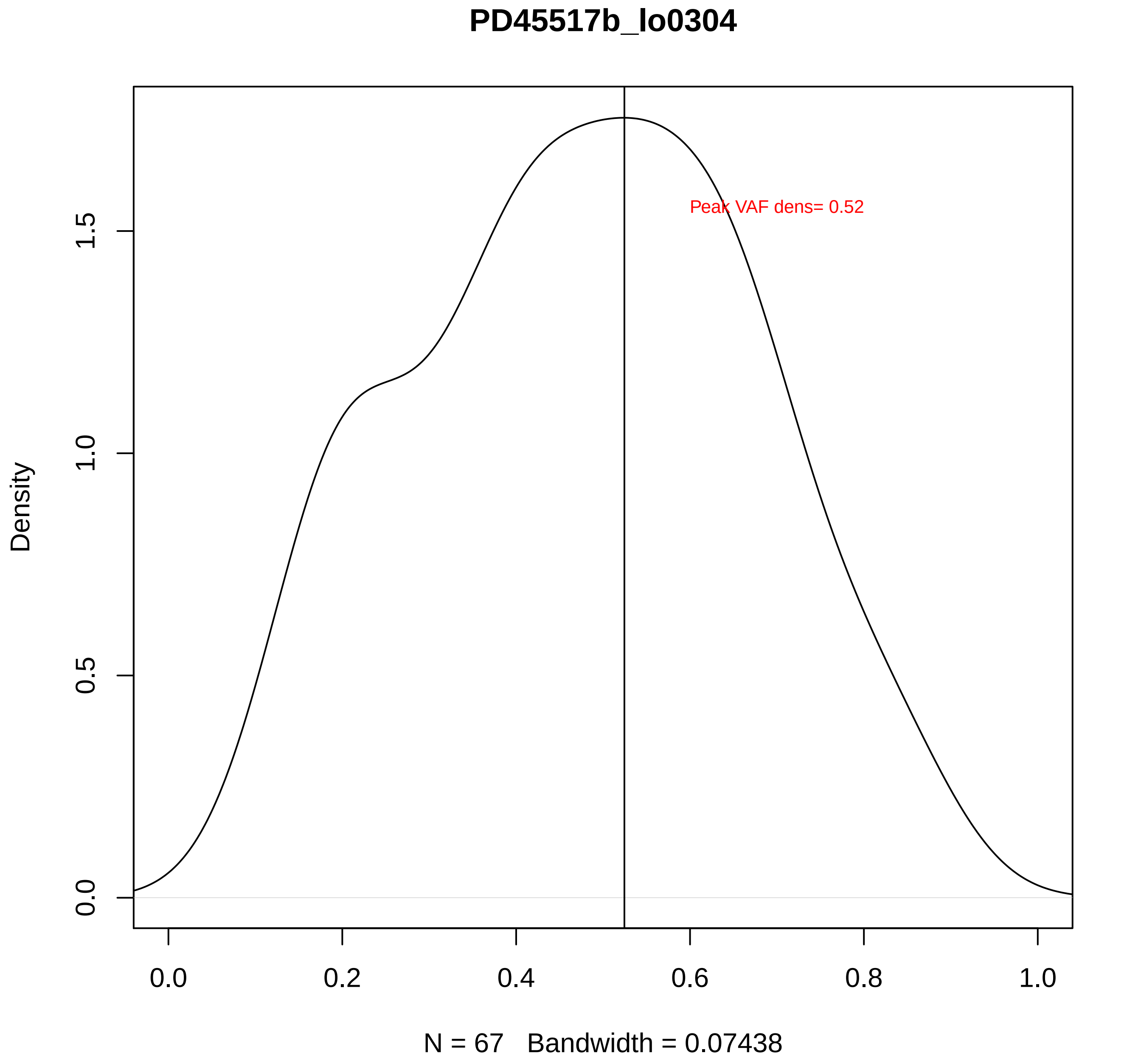

PD45517b_lo0304
Density
0.0
0.5
1.0
1.5
Peak VAF dens= 0.52
0.0
0.2
0.4
0.6
0.8
1.0
N = 67 Bandwidth = 0.07438

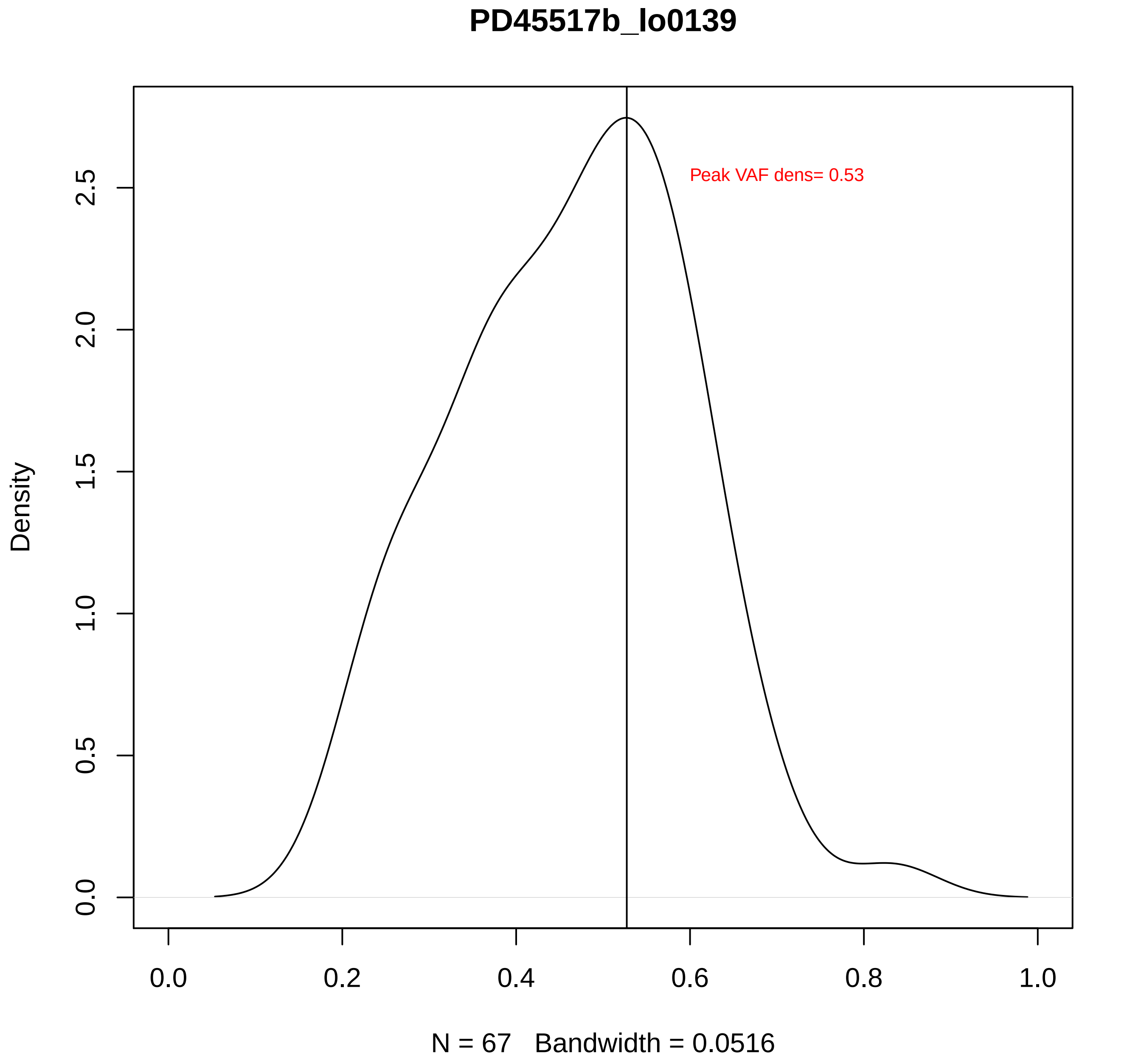
PD45517b_lo0139
Peak VAF dens= 0.53
Density
0.0
0.5
1.0
1.5
2.0
2.5
0.0
0.2
0.4
0.6
0.8
1.0
N = 67   Bandwidth = 0.0516

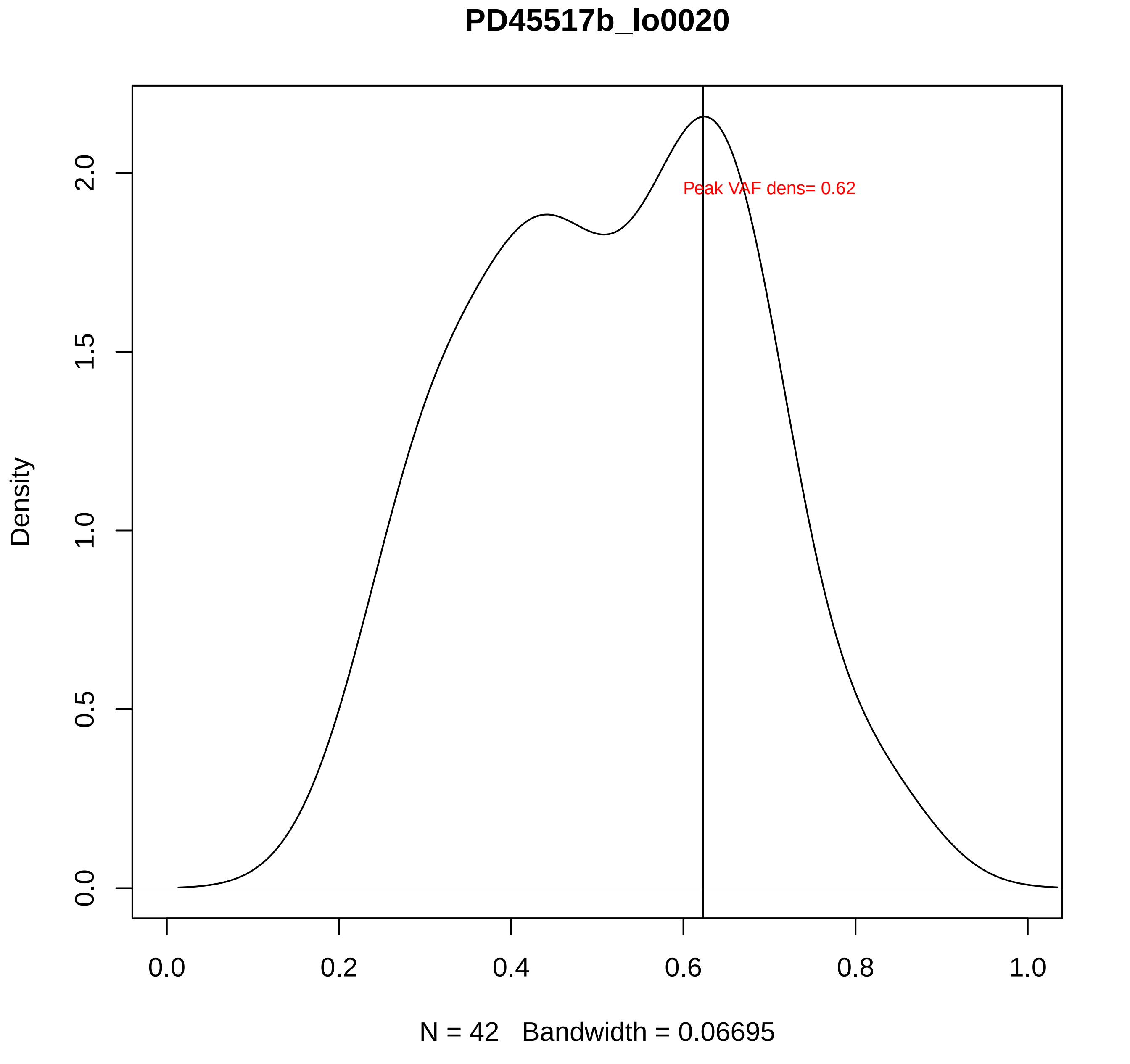

PD45517b_lo0020
Peak VAF dens= 0.62
Density
0.0
0.5
1.0
1.5
2.0
0.0
0.2
0.4
0.6
0.8
1.0
N = 42   Bandwidth = 0.06695

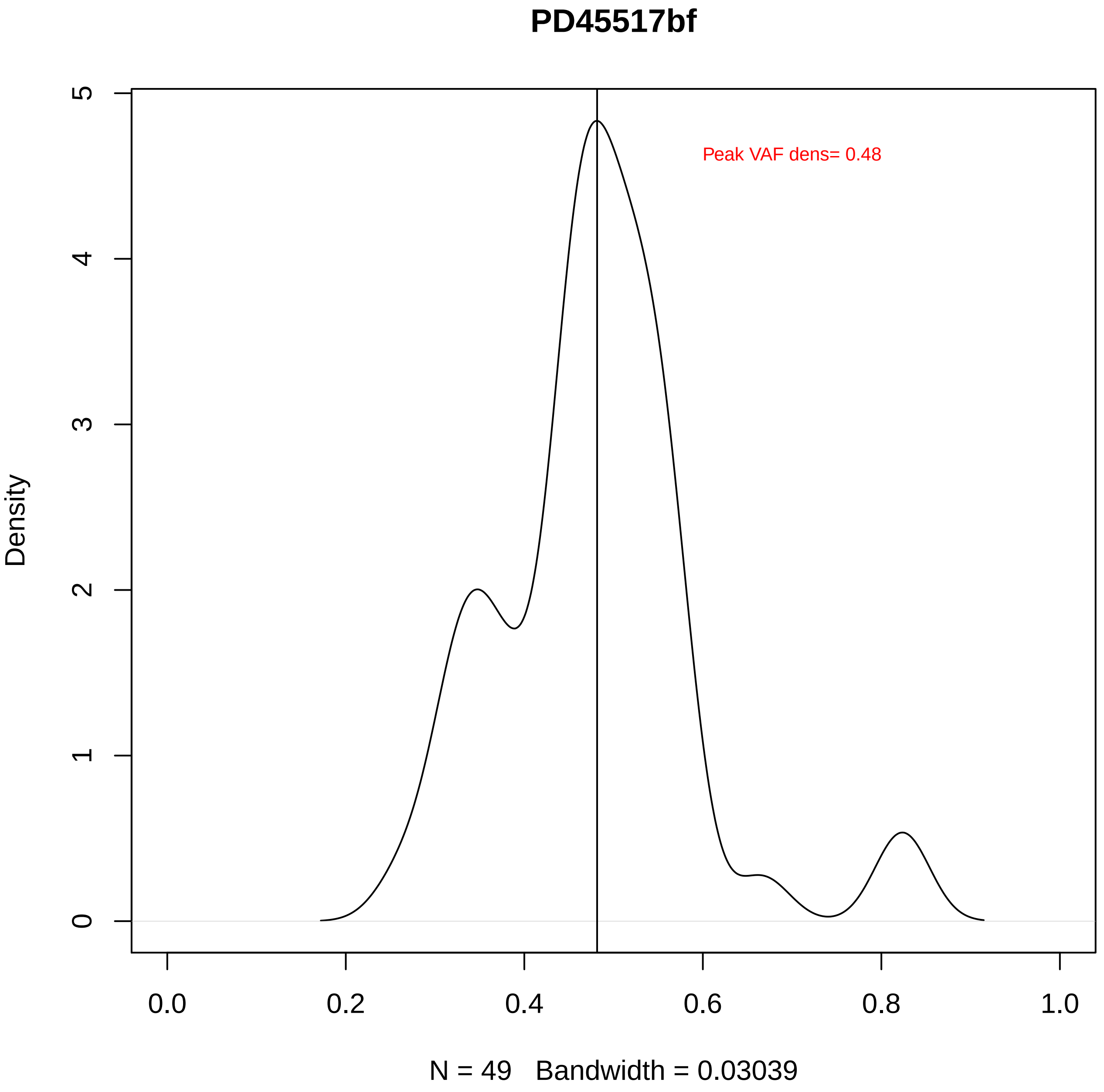

PD45517bf
Peak VAF dens= 0.48
Density
0
1
2
3
4
5
0.0
0.2
0.4
0.6
0.8
1.0
N = 49 Bandwidth = 0.03039

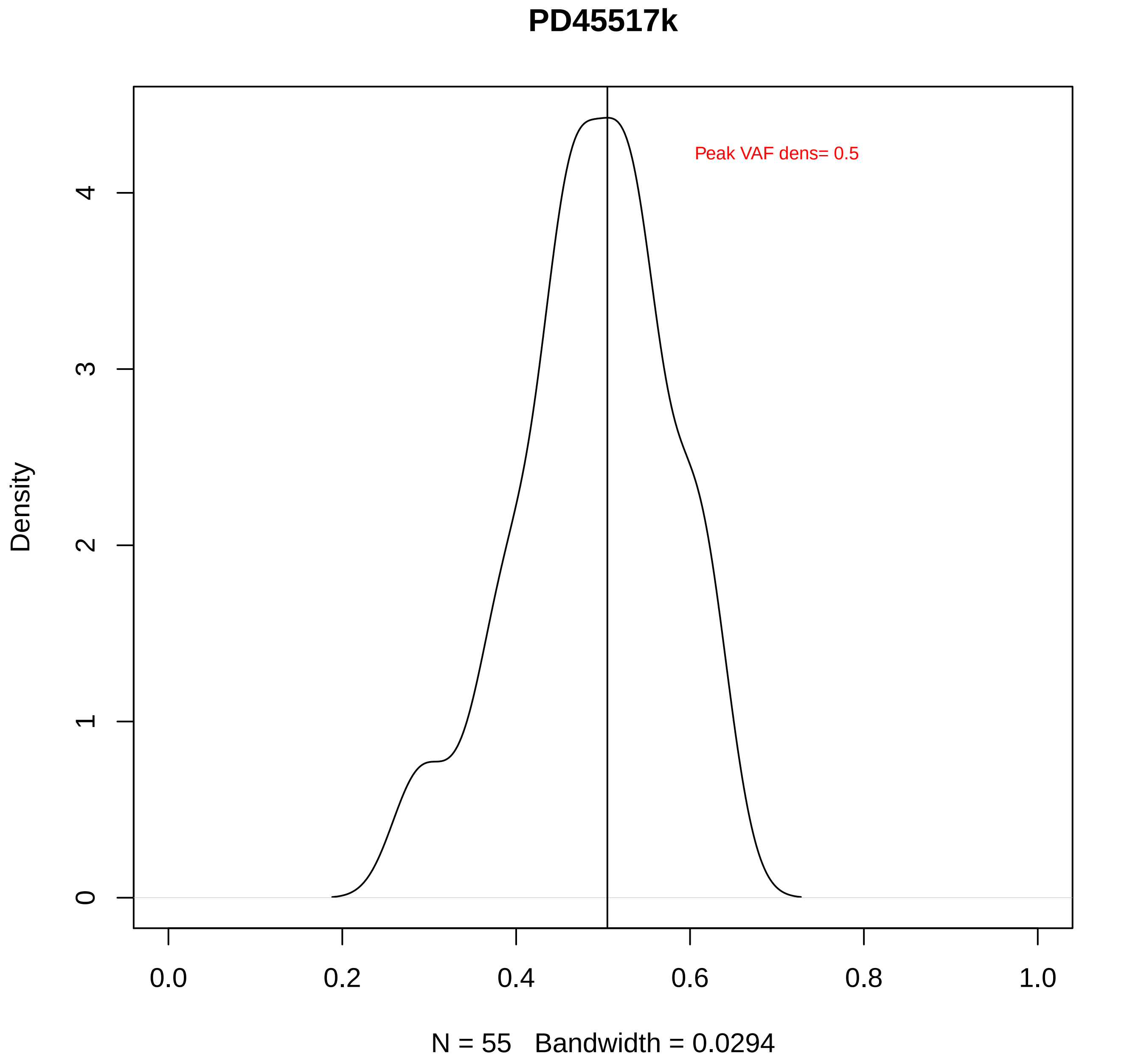
PD45517k
Peak VAF dens= 0.5
Density
0
1
2
3
4
0.0
0.2
0.4
0.6
0.8
1.0
N = 55   Bandwidth = 0.0294

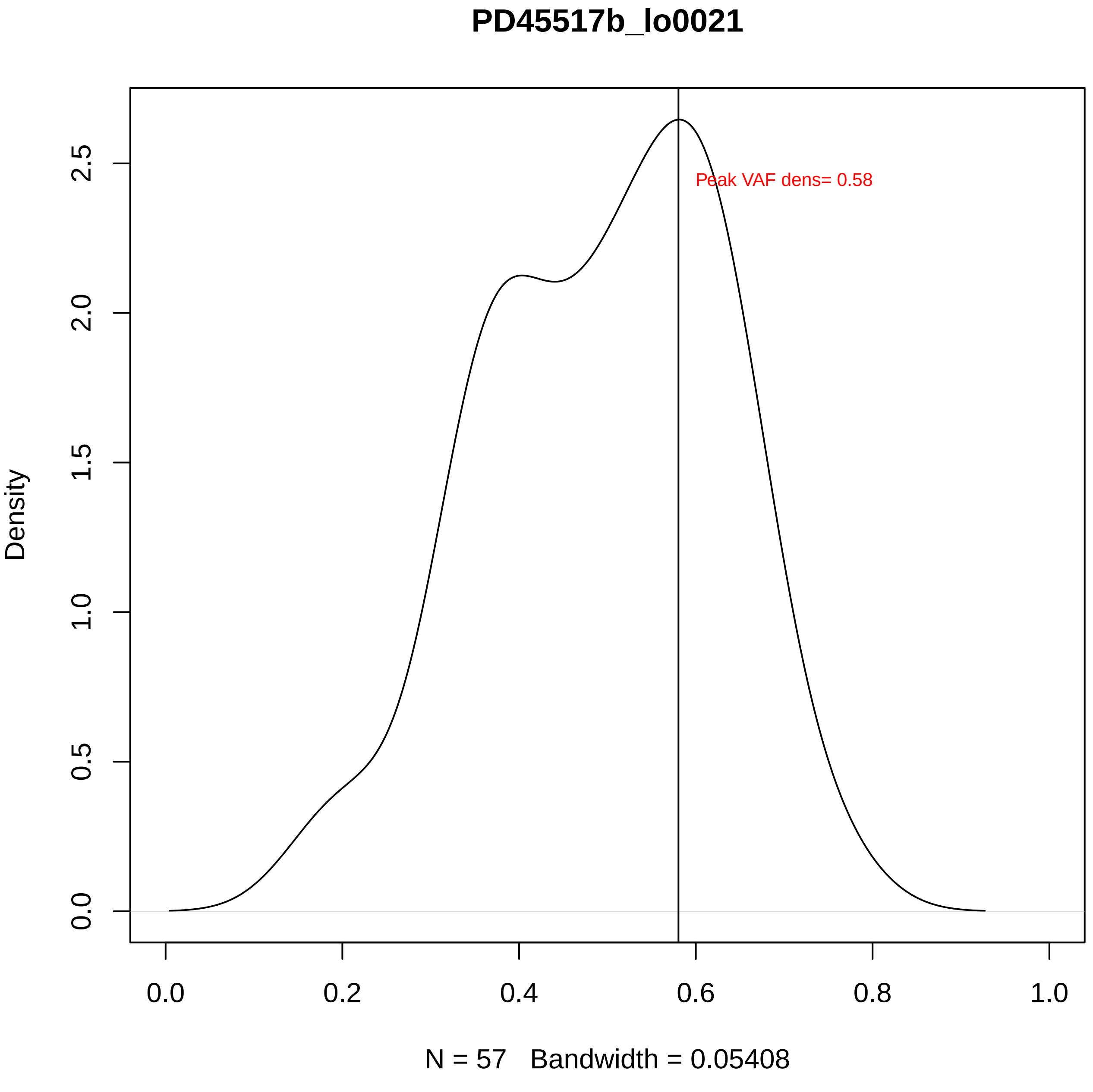
PD45517b_lo0021
Peak VAF dens= 0.58
Density
0.0
0.5
1.0
1.5
2.0
2.5
0.0
0.2
0.4
0.6
0.8
1.0
N = 57   Bandwidth = 0.05408

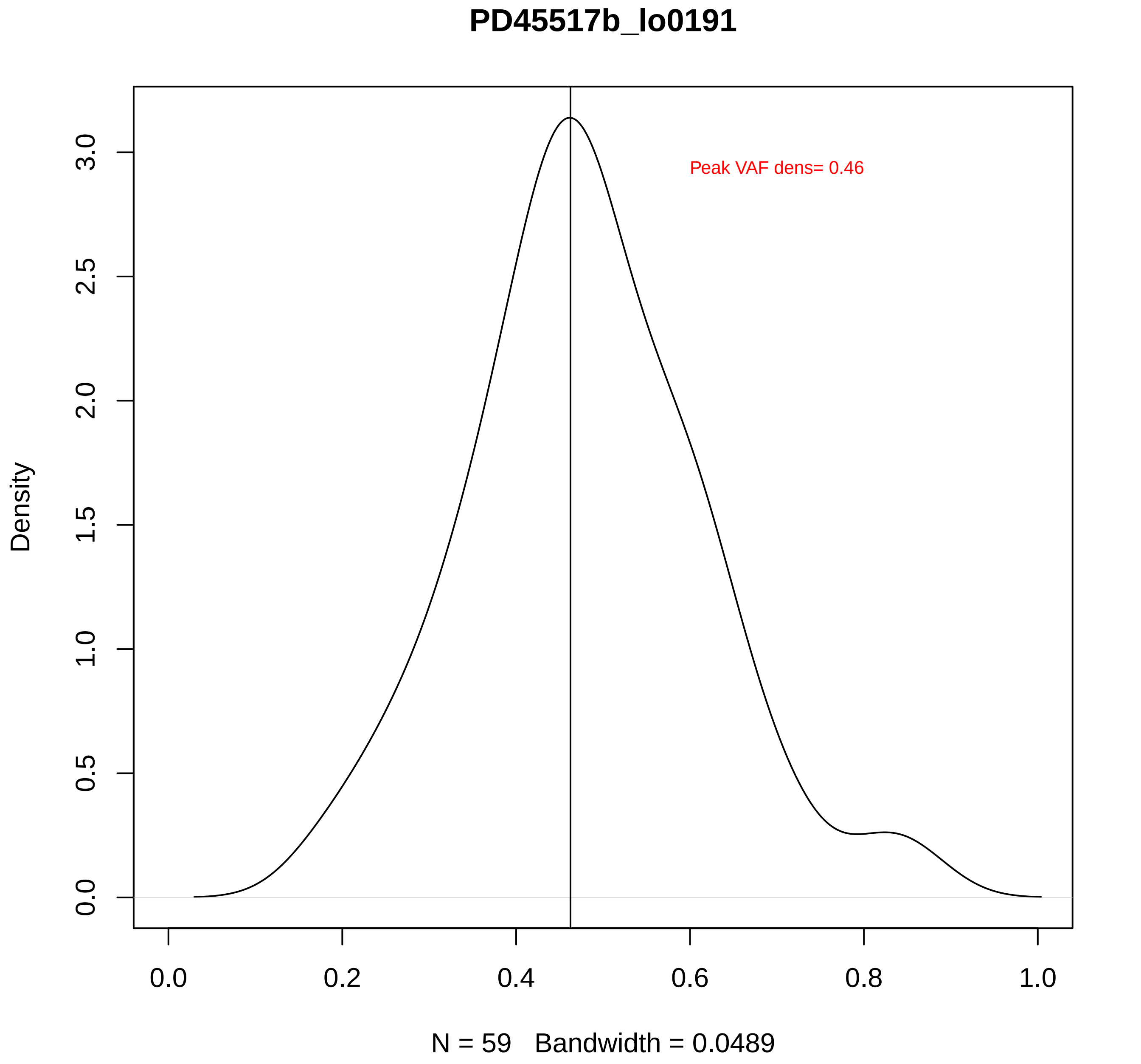

PD45517b_lo0191
Peak VAF dens= 0.46
Density
0.0
0.5
1.0
1.5
2.0
2.5
3.0
0.0
0.2
0.4
0.6
0.8
1.0
N = 59 Bandwidth = 0.0489

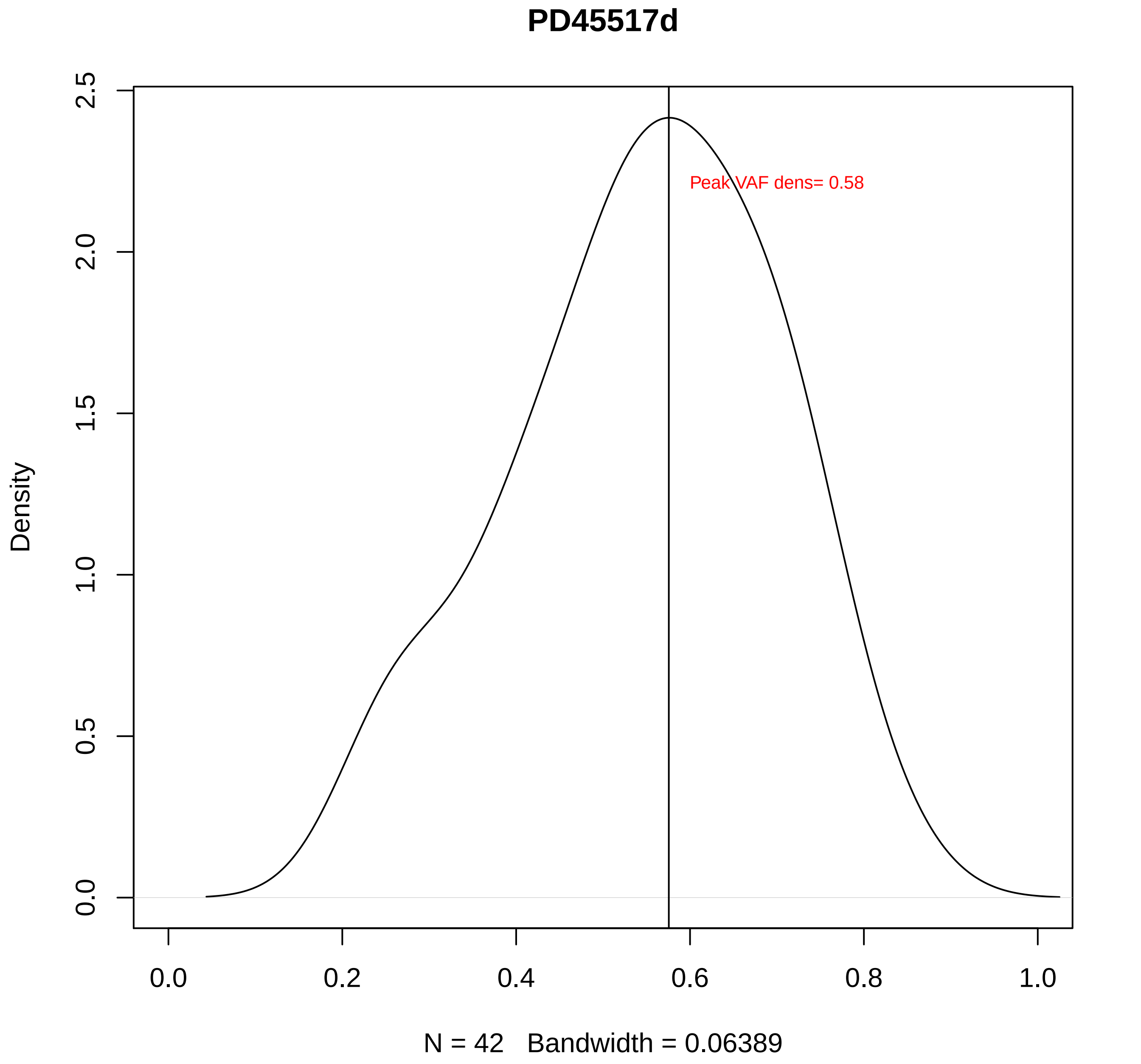

PD45517d
Density
Peak VAF dens= 0.58
0.0
0.5
1.0
1.5
2.0
2.5
0.0
0.2
0.4
0.6
0.8
1.0
N = 42   Bandwidth = 0.06389

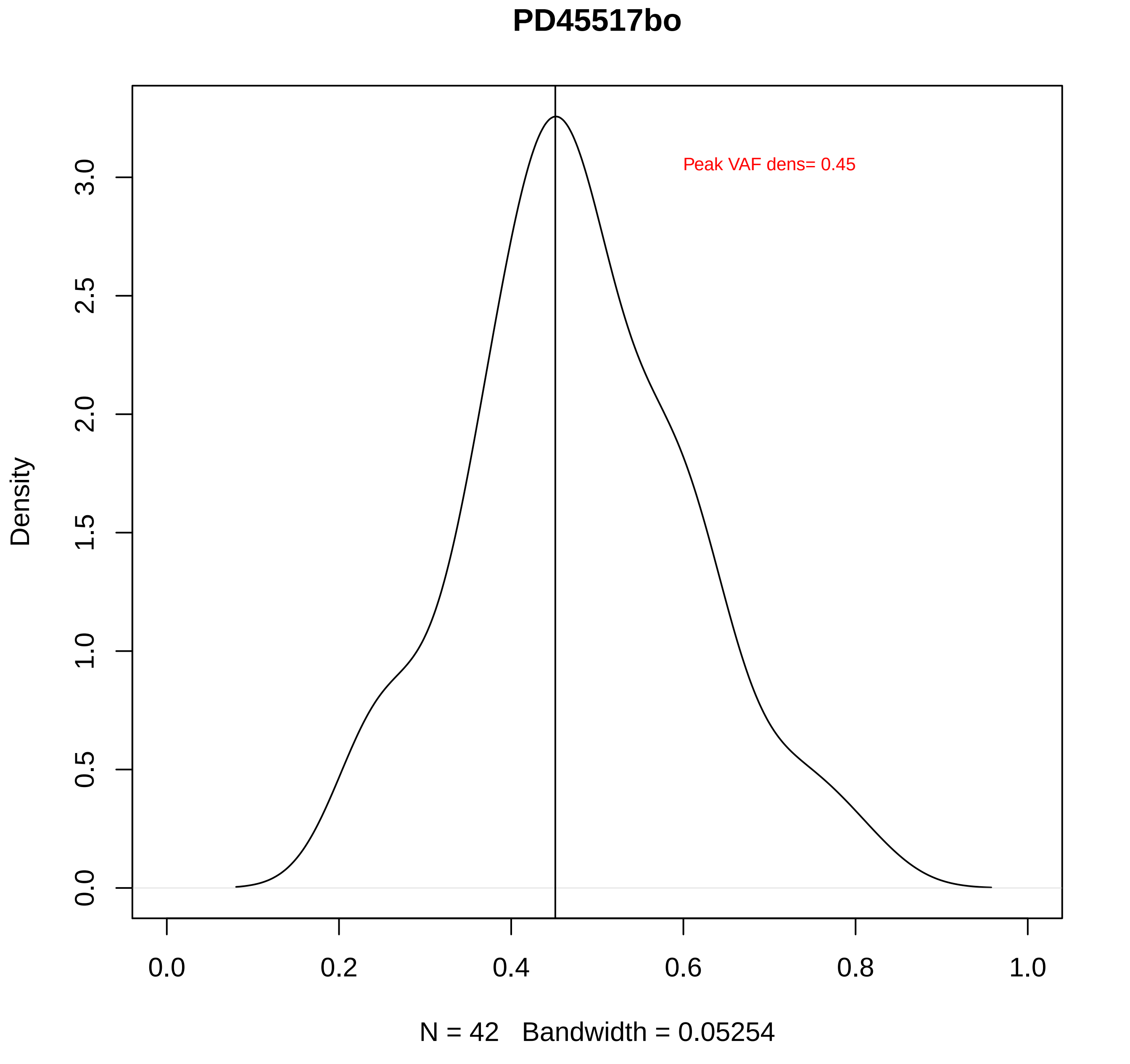
PD45517bo
Peak VAF dens= 0.45
Density
0.0
0.5
1.0
1.5
2.0
2.5
3.0
0.0
0.2
0.4
0.6
0.8
1.0
N = 42   Bandwidth = 0.05254

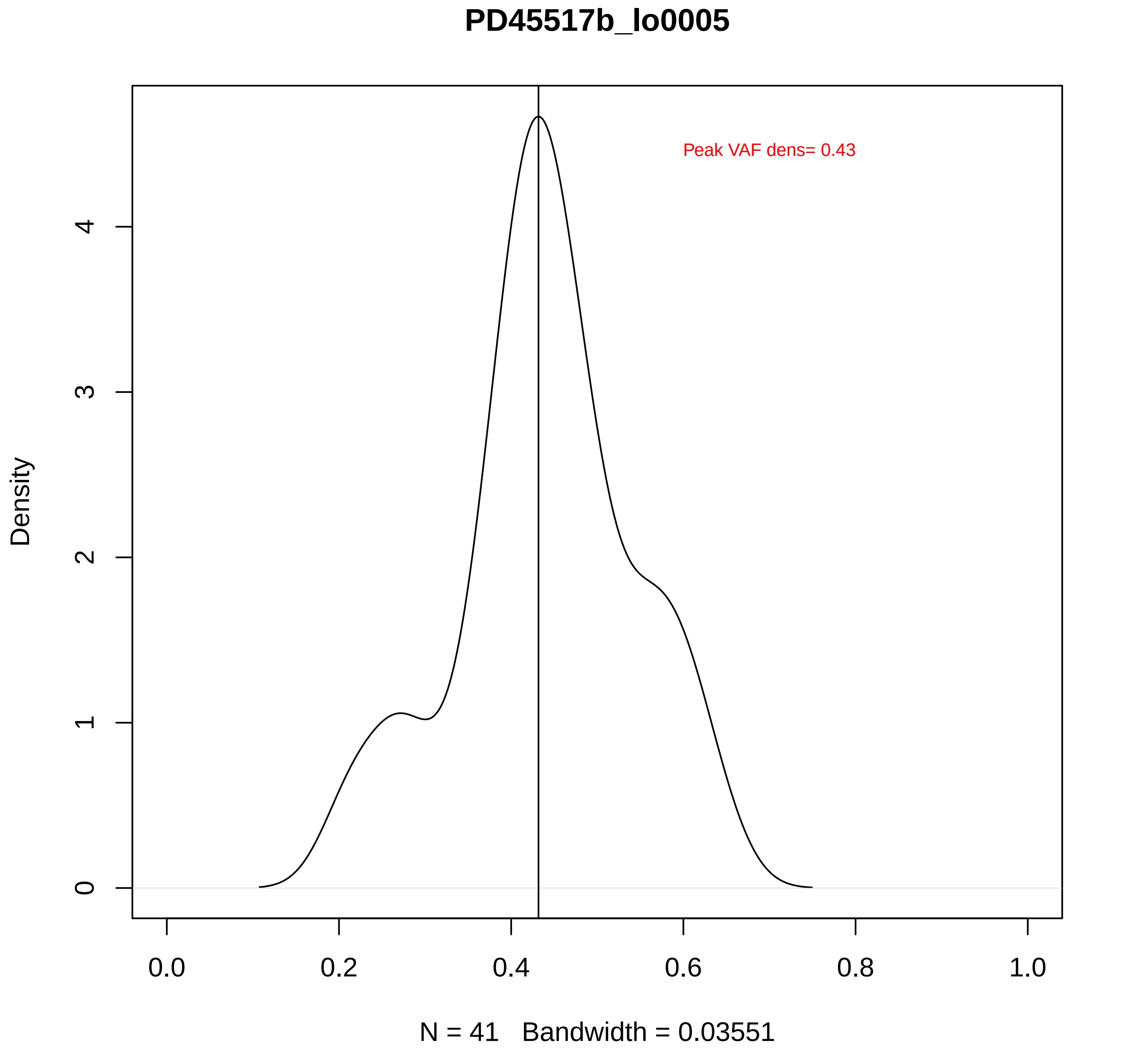
PD45517b_lo0005
Peak VAF dens= 0.43
Density
0
1
2
3
4
0.0
0.2
0.4
0.6
0.8
1.0
N = 41 Bandwidth = 0.03551

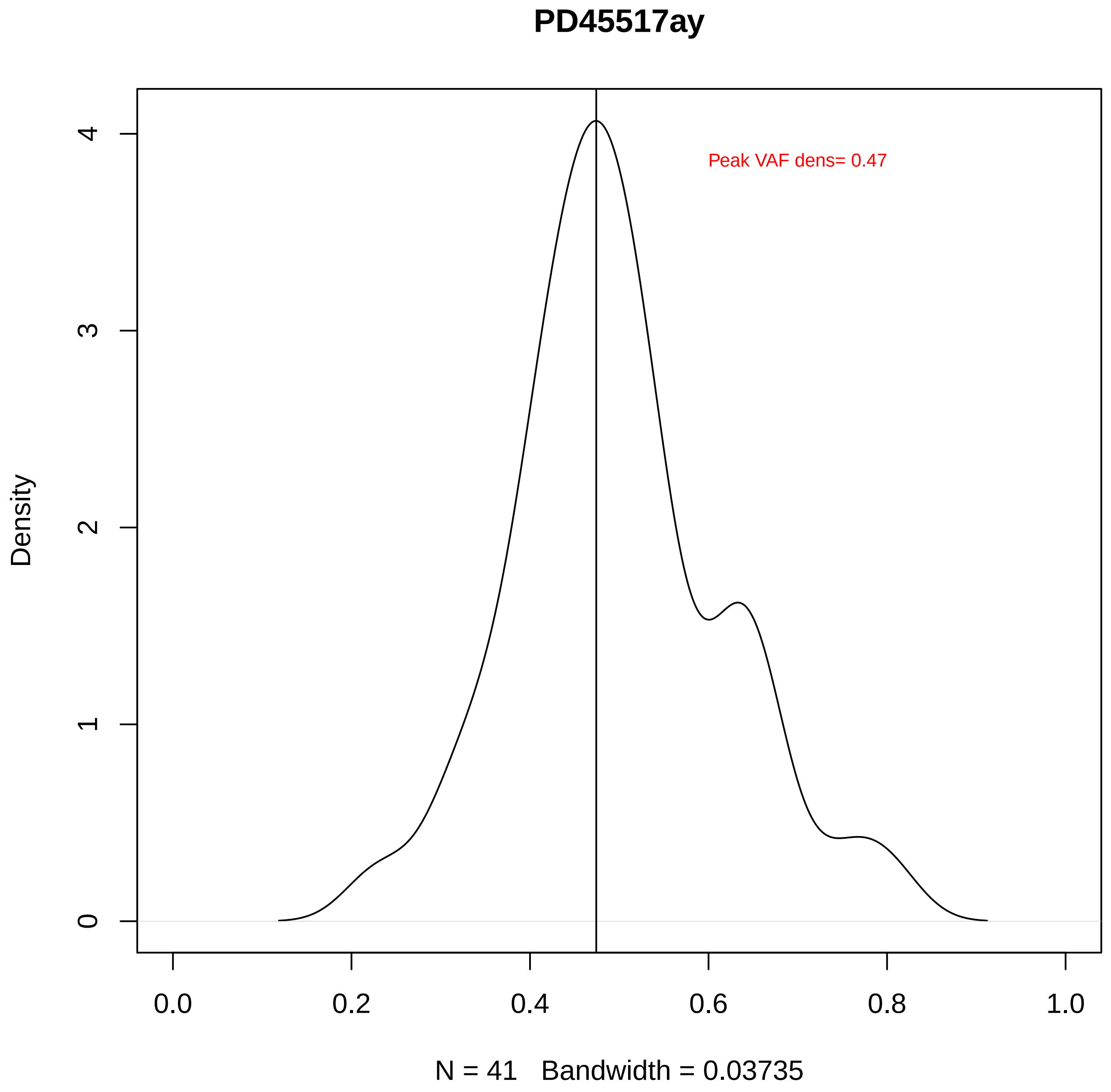
PD45517ay
Peak VAF dens= 0.47
Density
0
1
2
3
4
0.0
0.2
0.4
0.6
0.8
1.0
N = 41   Bandwidth = 0.03735

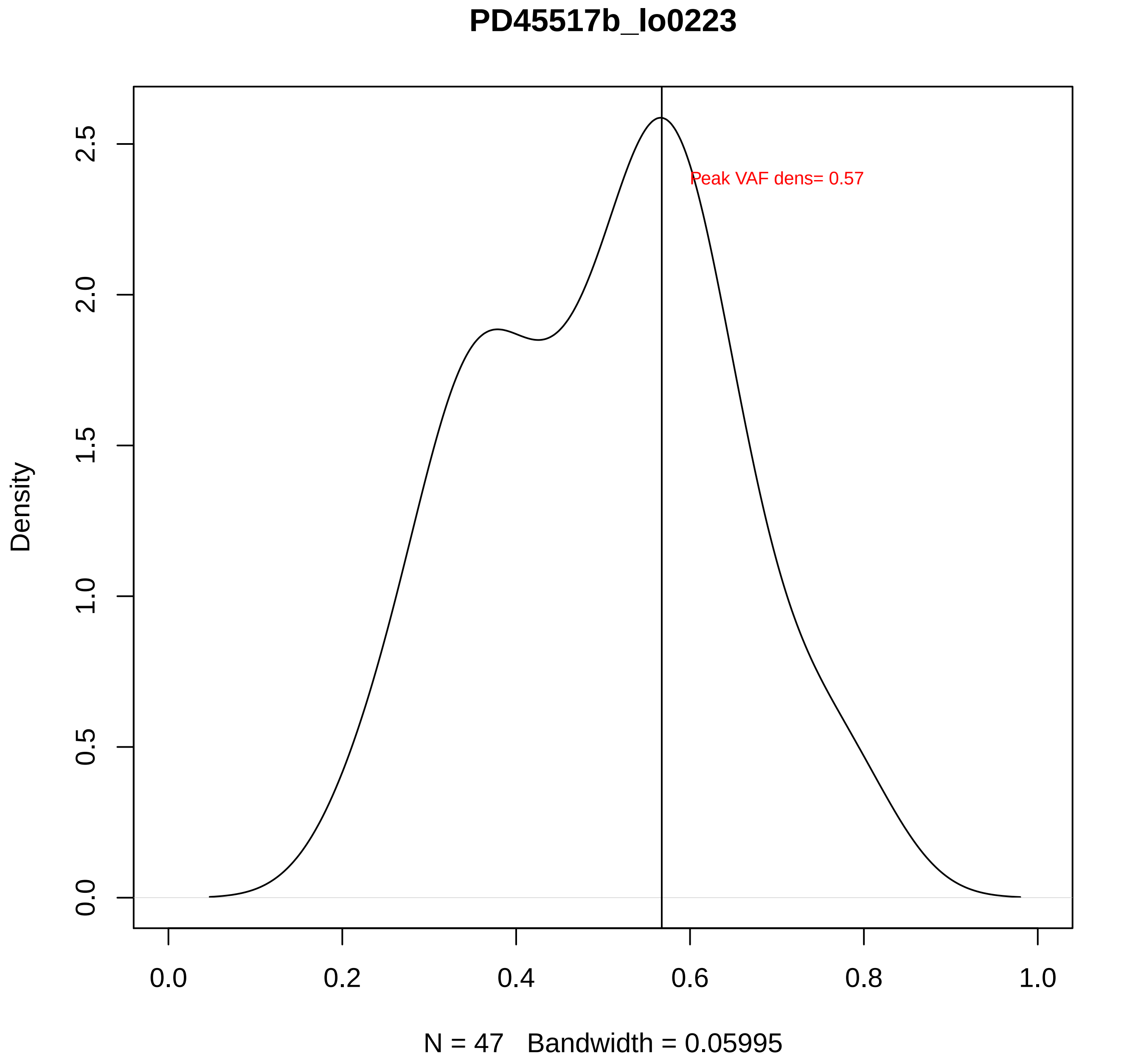
PD45517b_lo0223
Peak VAF dens= 0.57
Density
0.0
0.5
1.0
1.5
2.0
2.5
0.0
0.2
0.4
0.6
0.8
1.0
N = 47   Bandwidth = 0.05995

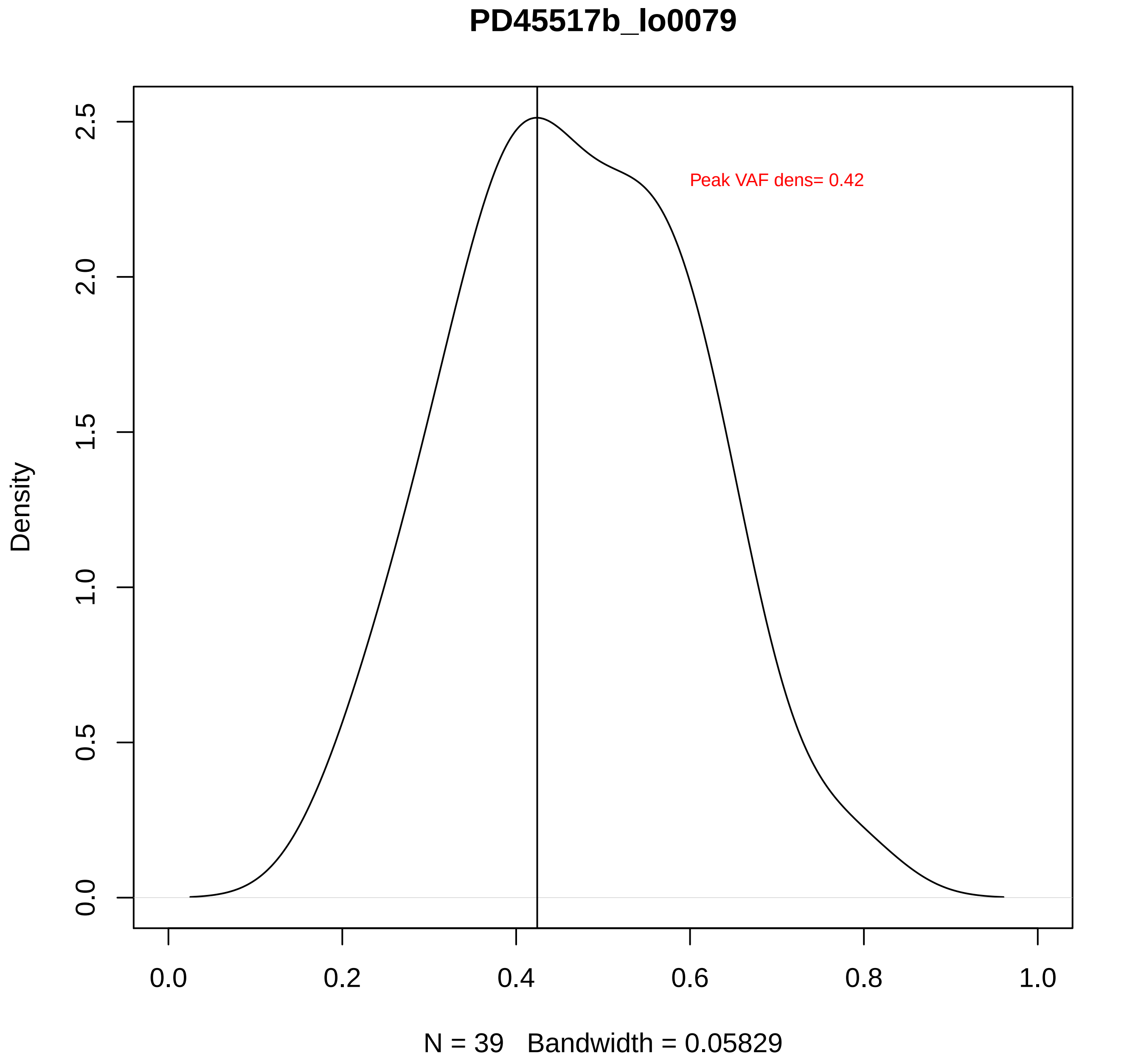
PD45517b_lo0079
Peak VAF dens= 0.42
Density
0.0
0.5
1.0
1.5
2.0
2.5
0.0
0.2
0.4
0.6
0.8
1.0
N = 39   Bandwidth = 0.05829

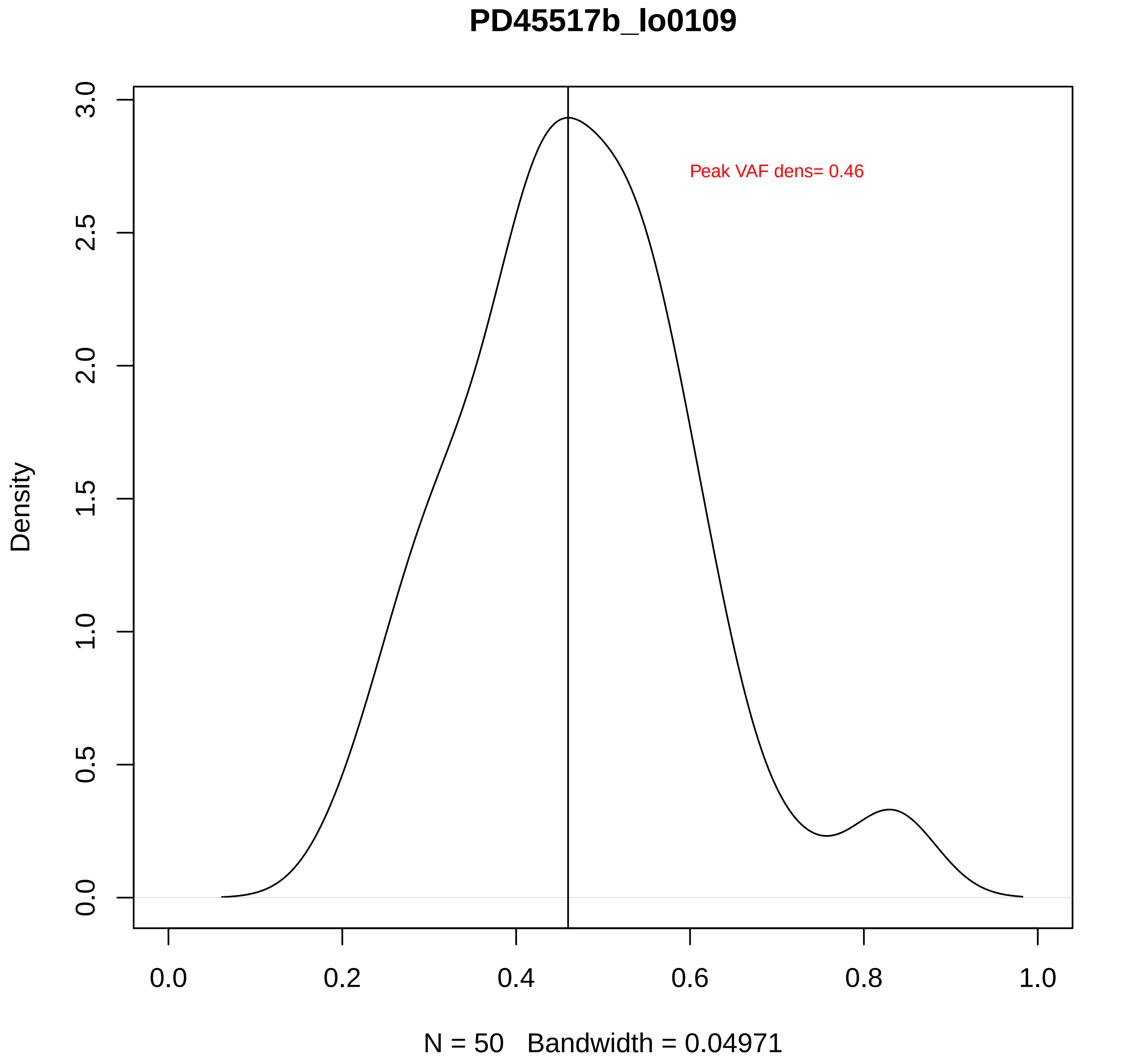
PD45517b_lo0109
Peak VAF dens= 0.46
Density
0.0
0.5
1.0
1.5
2.0
2.5
3.0
0.0
0.2
0.4
0.6
0.8
1.0
N = 50   Bandwidth = 0.04971

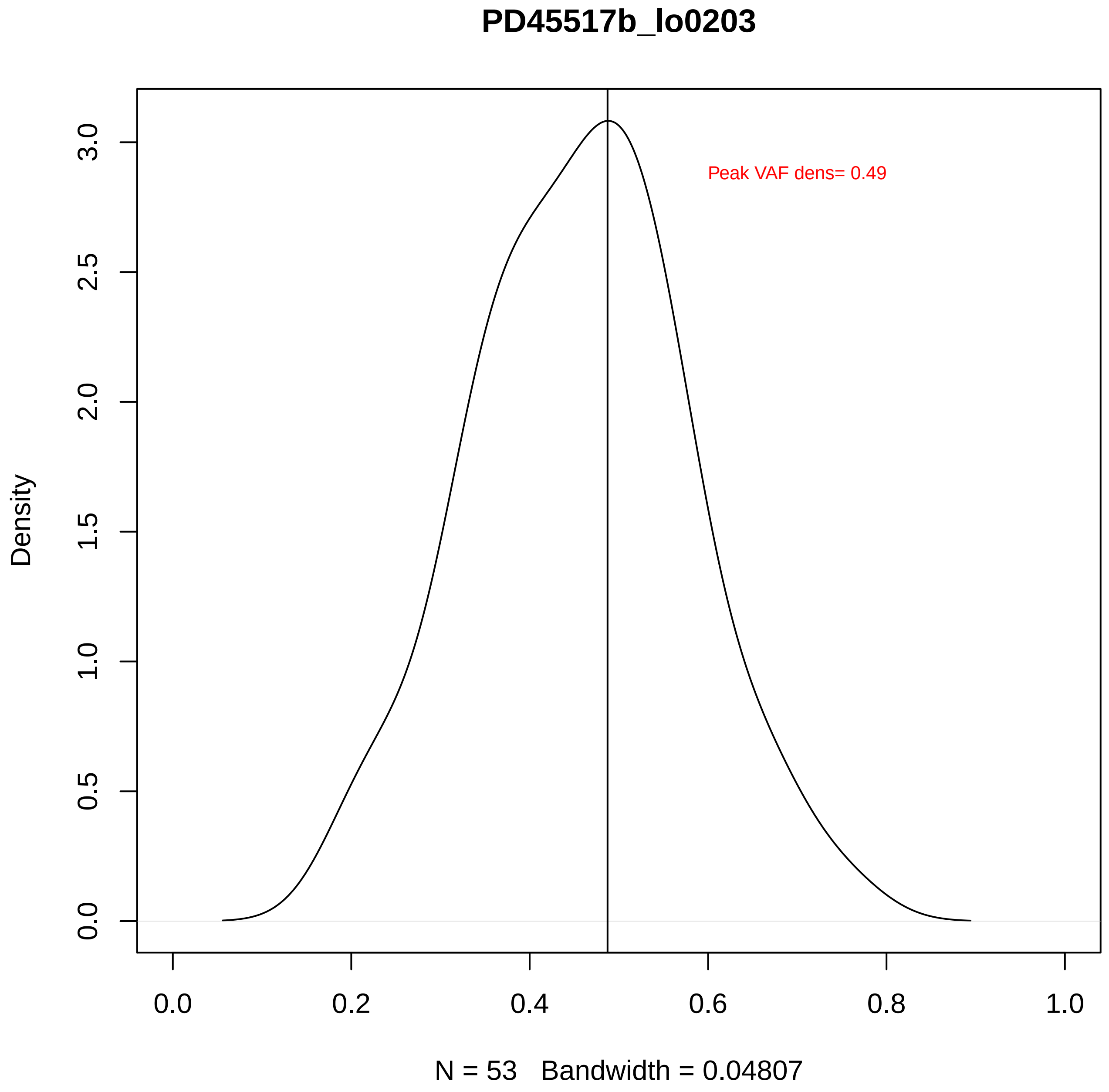

PD45517b_lo0203
Peak VAF dens= 0.49
Density
0.0
0.5
1.0
1.5
2.0
2.5
3.0
0.0
0.2
0.4
0.6
0.8
1.0
N = 53   Bandwidth = 0.04807

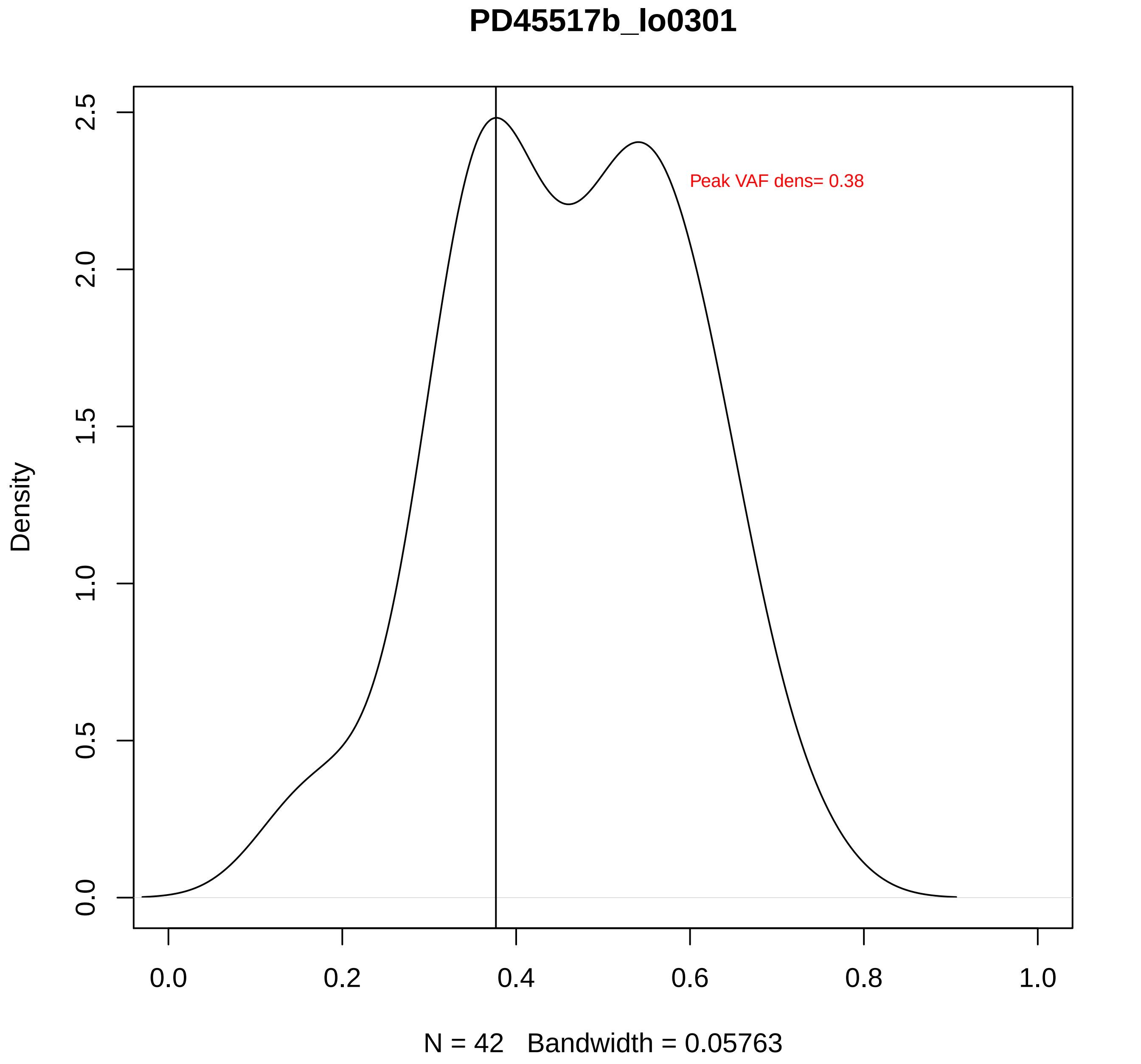
PD45517b_lo0301
Peak VAF dens= 0.38
Density
0.0
0.5
1.0
1.5
2.0
2.5
0.0
0.2
0.4
0.6
0.8
1.0
N = 42   Bandwidth = 0.05763

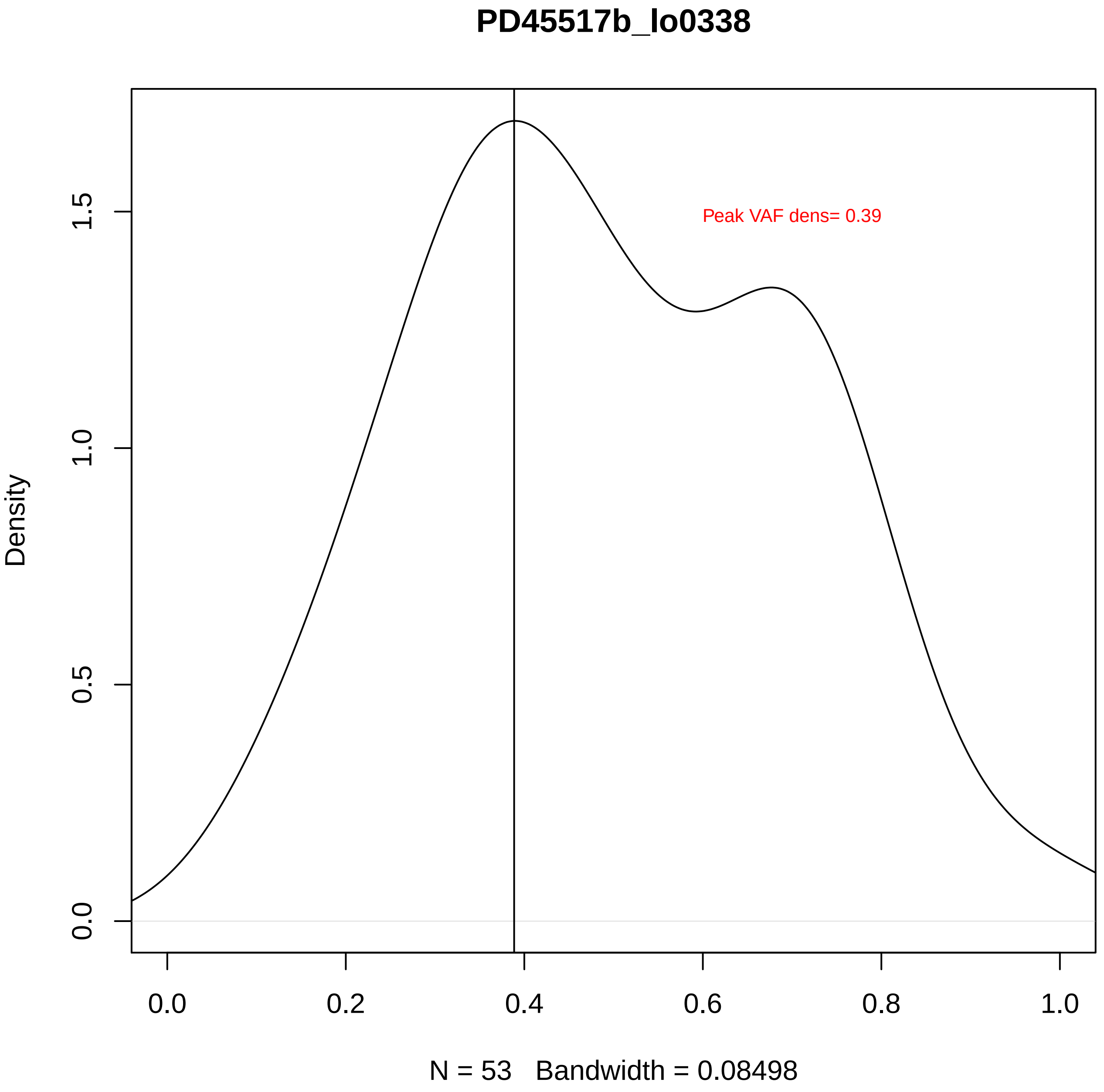
PD45517b_lo0338
Peak VAF dens= 0.39
Density
0.0
0.5
1.0
1.5
0.0
0.2
0.4
0.6
0.8
1.0
N = 53   Bandwidth = 0.08498

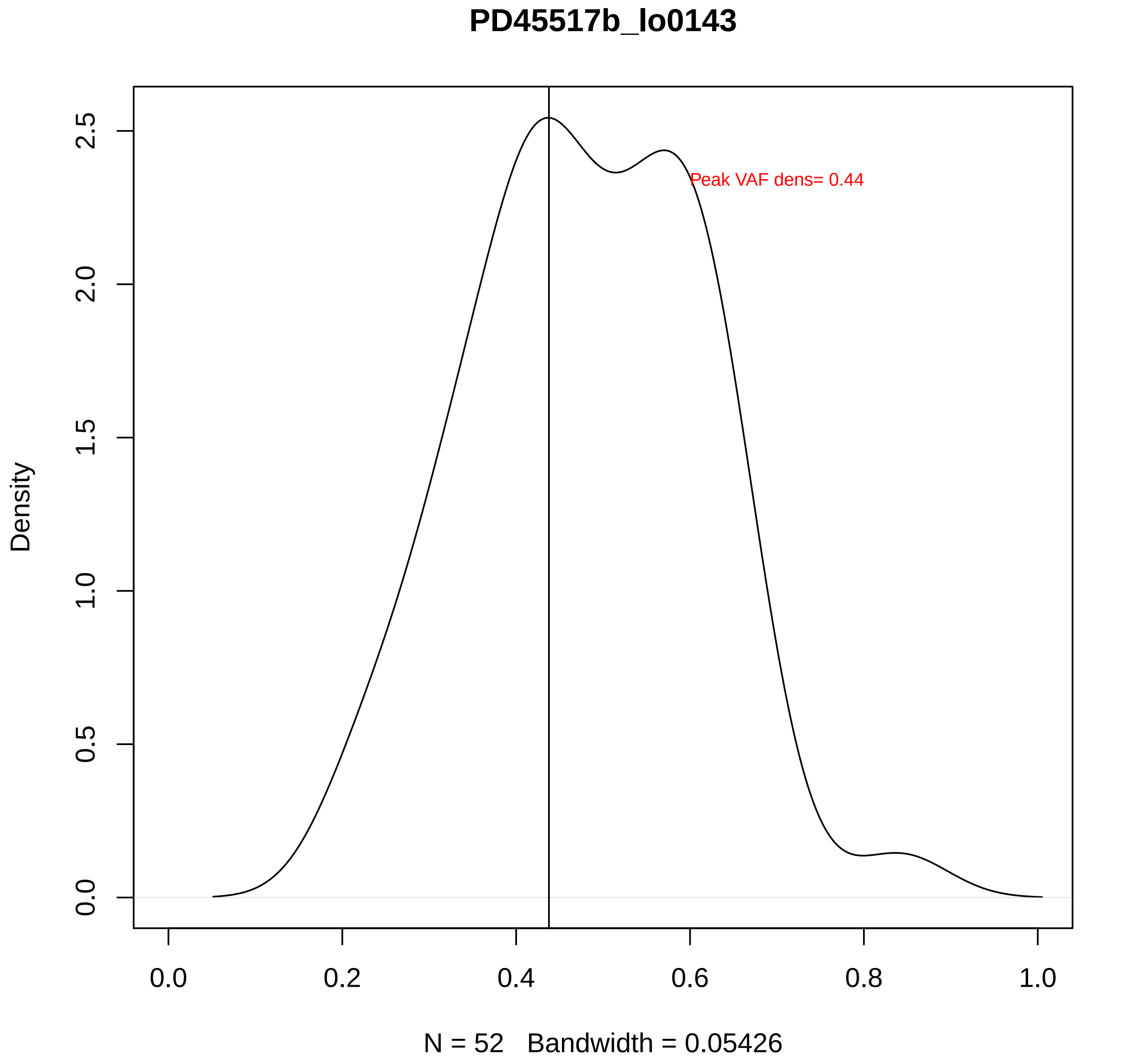
PD45517b_lo0143
Peak VAF dens= 0.44
Density
0.0
0.5
1.0
1.5
2.0
2.5
0.0
0.2
0.4
0.6
0.8
1.0
N = 52   Bandwidth = 0.05426

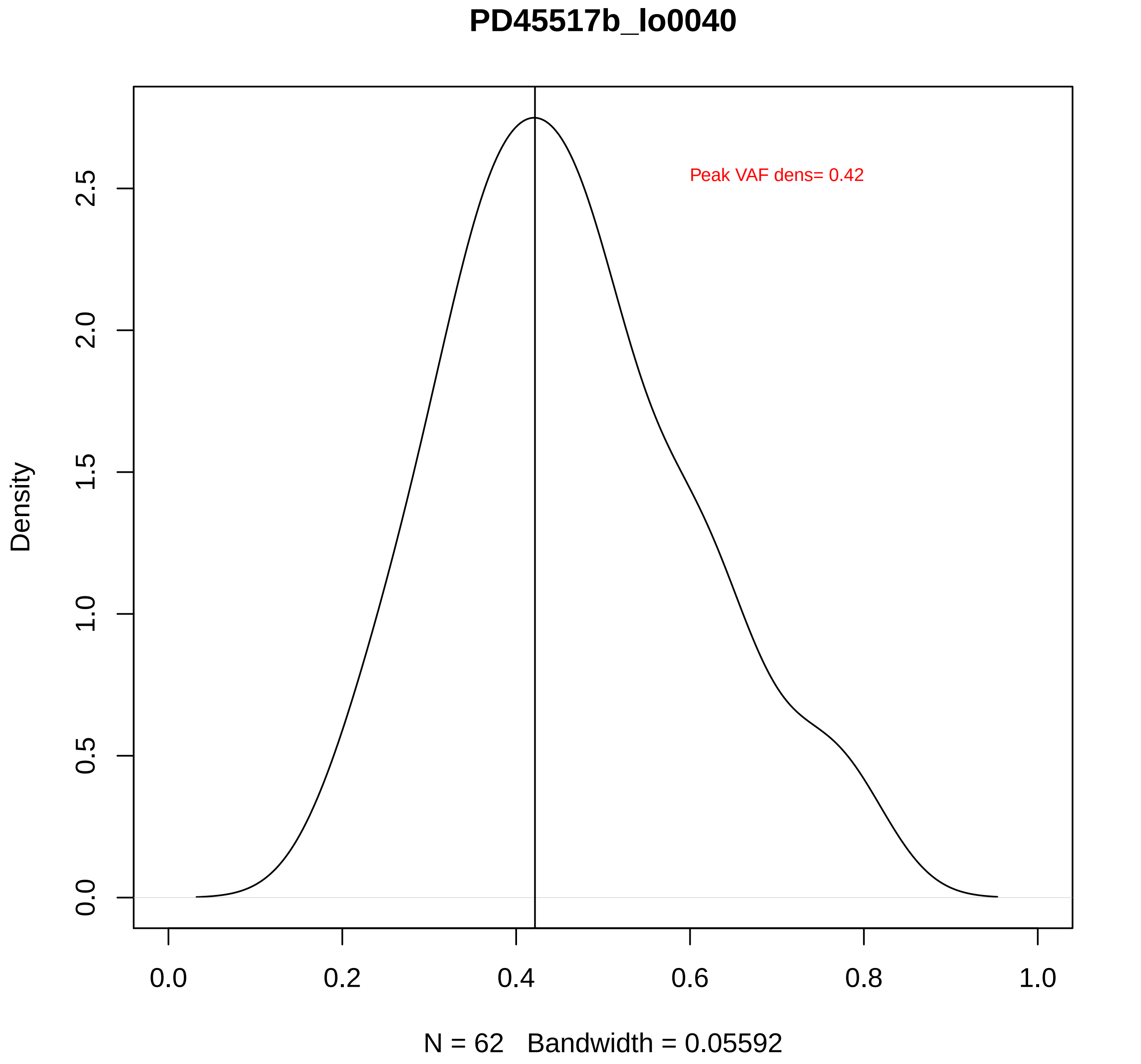
PD45517b_lo0040
Peak VAF dens= 0.42
Density
0.0
0.5
1.0
1.5
2.0
2.5
0.0
0.2
0.4
0.6
0.8
1.0
N = 62   Bandwidth = 0.05592

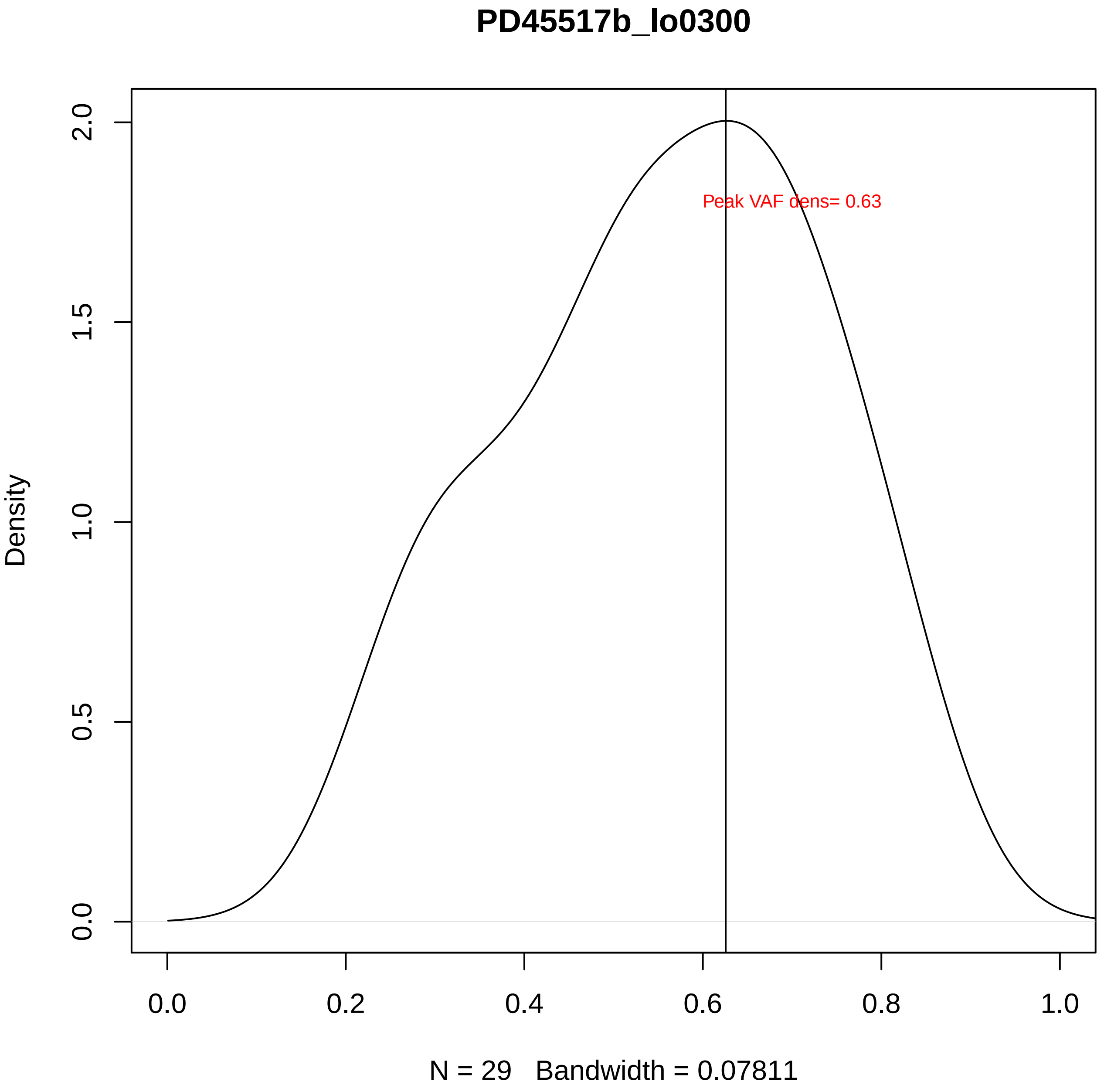

PD45517b_lo0300
Density
2.0
1.5
1.0
0.5
0.0
Peak VAF dens= 0.63
0.0
0.2
0.4
0.6
0.8
1.0
N = 29 Bandwidth = 0.07811

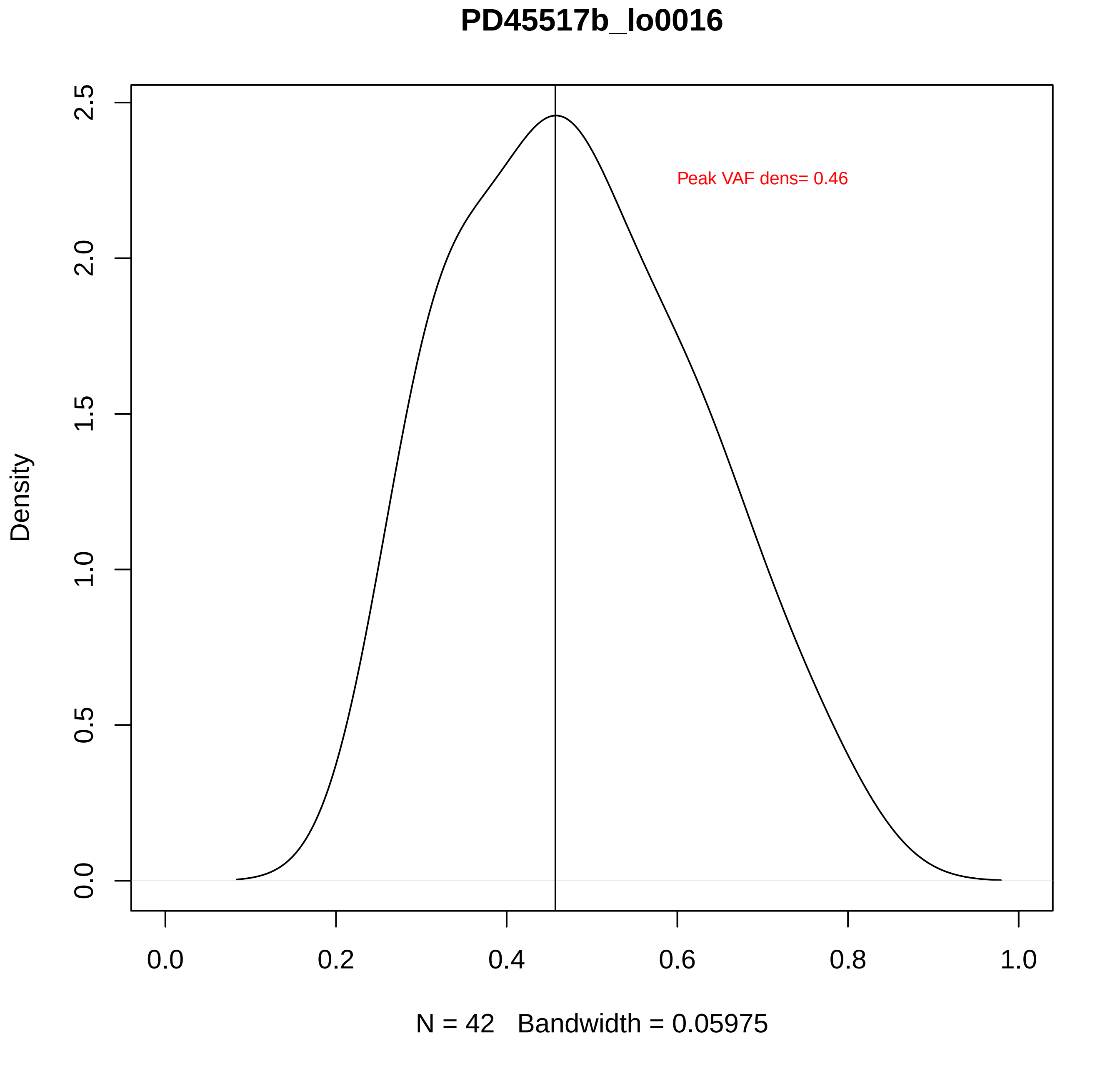

PD45517b_lo0016
Peak VAF dens= 0.46
Density
0.0
0.5
1.0
1.5
2.0
2.5
0.0
0.2
0.4
0.6
0.8
1.0
N = 42 Bandwidth = 0.05975

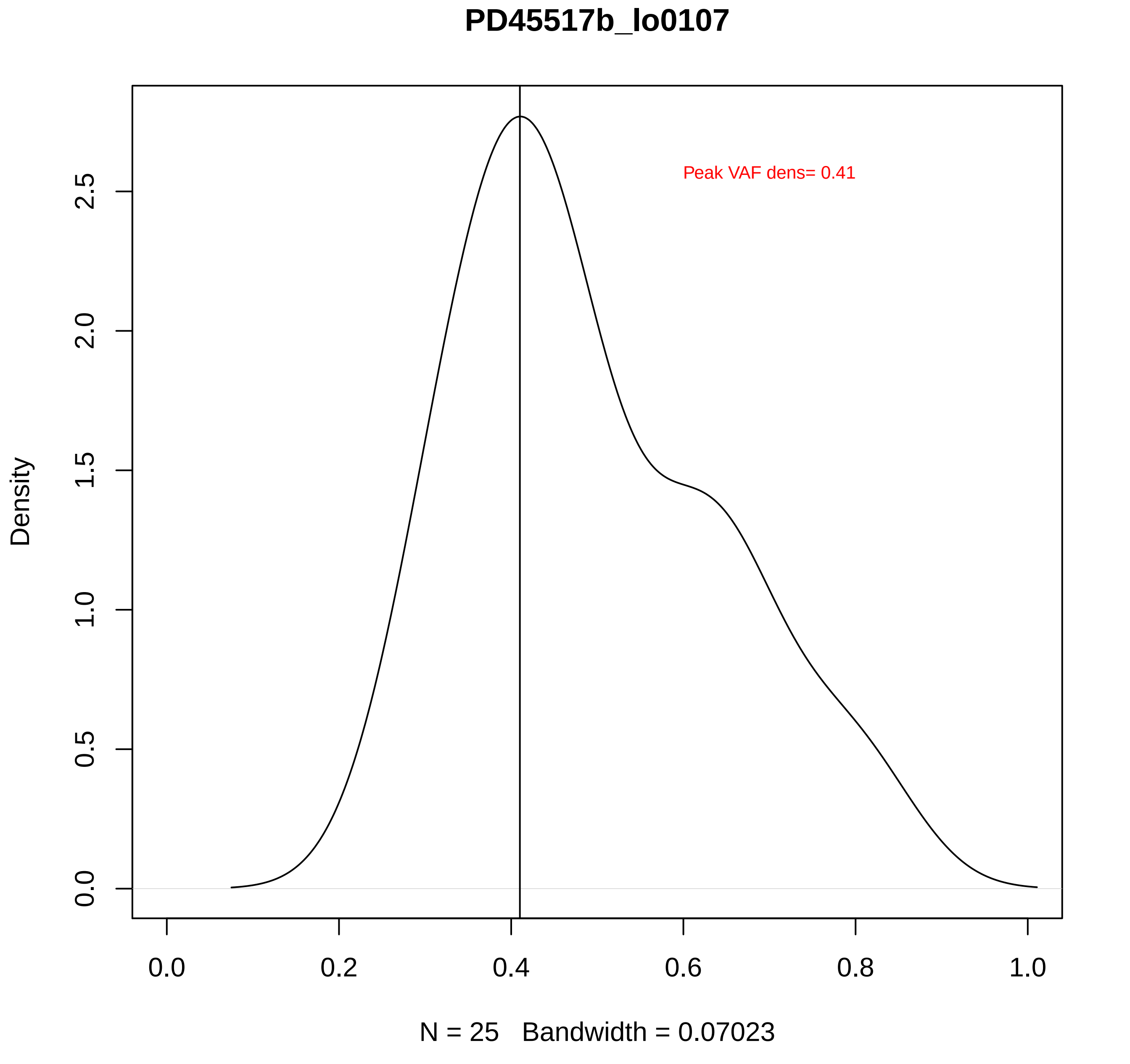
PD45517b_lo0107
Peak VAF dens= 0.41
Density
0.0
0.5
1.0
1.5
2.0
2.5
0.0
0.2
0.4
0.6
0.8
1.0
N = 25 Bandwidth = 0.07023

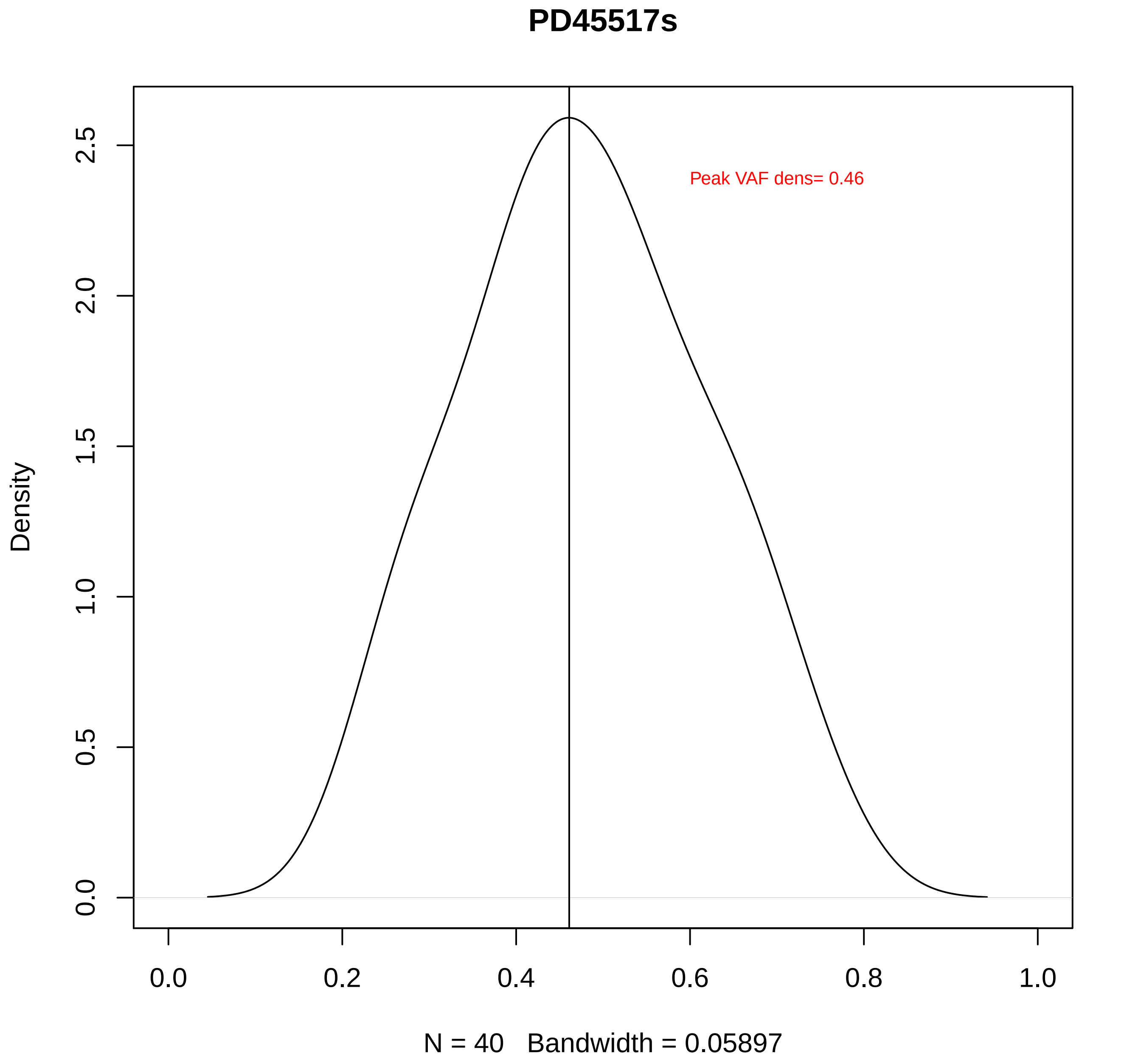
PD45517s
Peak VAF dens= 0.46
Density
0.0
0.5
1.0
1.5
2.0
2.5
0.0
0.2
0.4
0.6
0.8
1.0
N = 40   Bandwidth = 0.05897

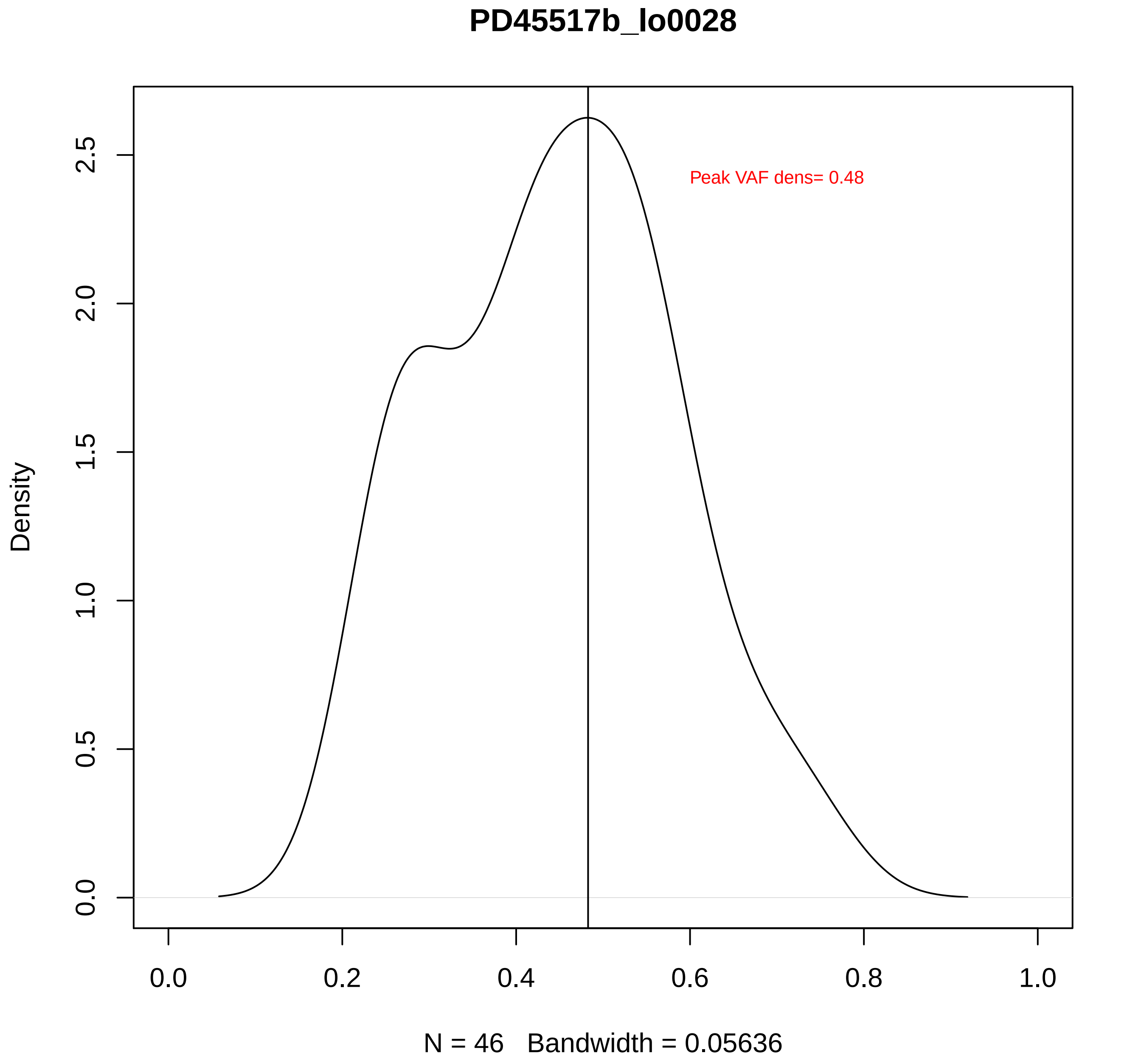
PD45517b_lo0028
Peak VAF dens= 0.48
Density
0.0
0.5
1.0
1.5
2.0
2.5
0.0
0.2
0.4
0.6
0.8
1.0
N = 46 Bandwidth = 0.05636

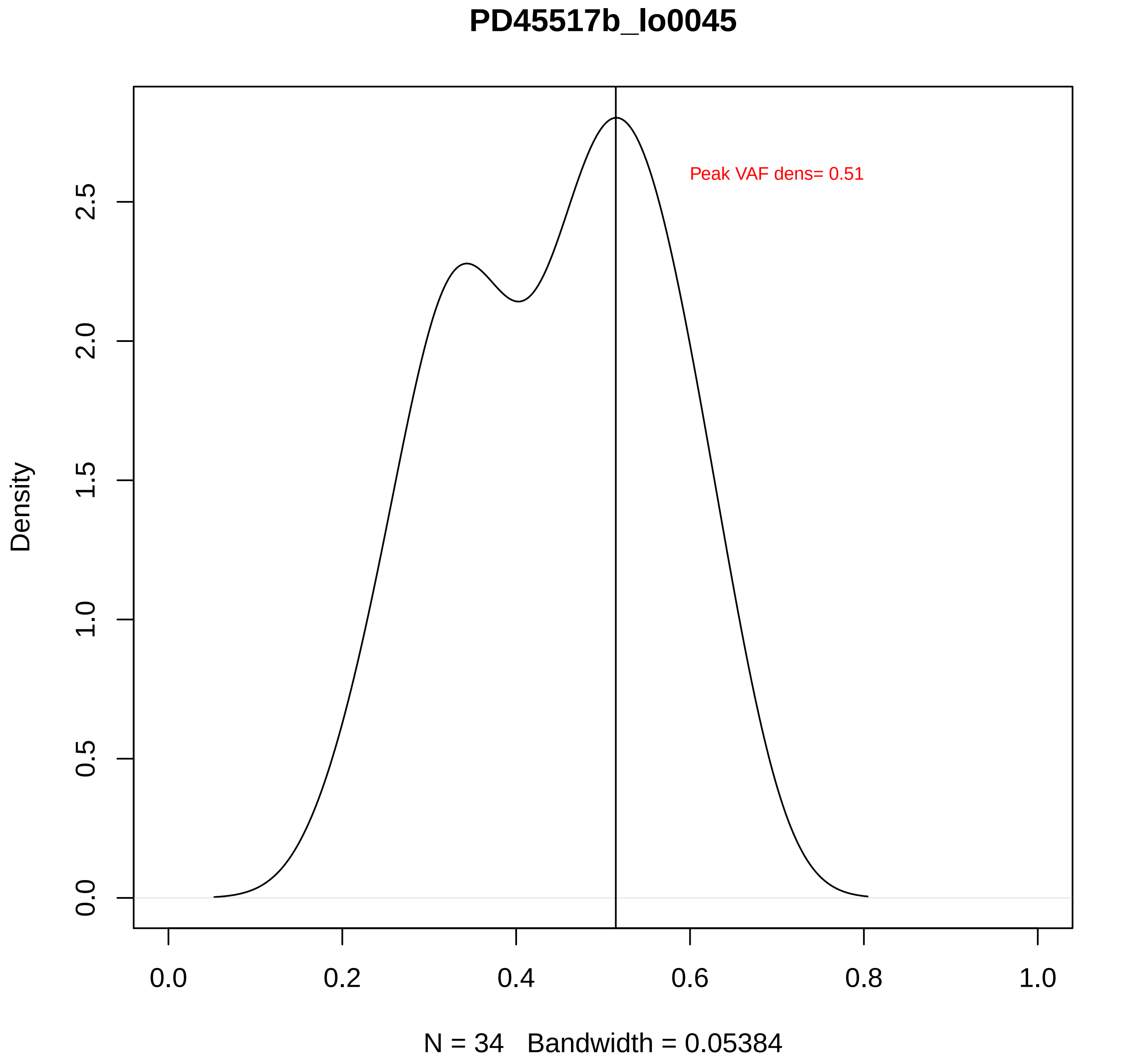
PD45517b_lo0045
Peak VAF dens= 0.51
Density
0.0
0.5
1.0
1.5
2.0
2.5
0.0
0.2
0.4
0.6
0.8
1.0
N = 34   Bandwidth = 0.05384

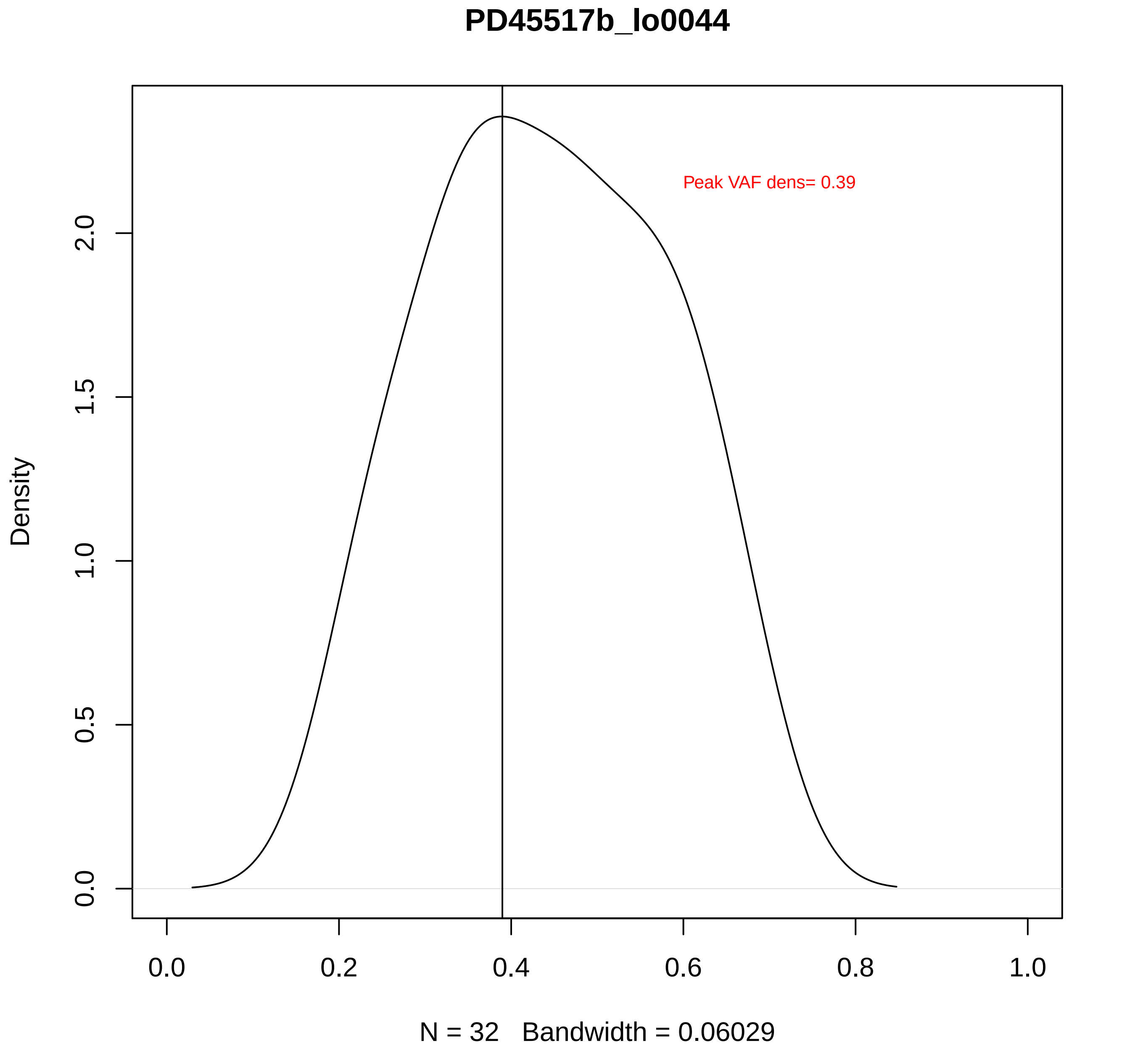
PD45517b_lo0044
Peak VAF dens= 0.39
Density
0.0
0.5
1.0
1.5
2.0
0.0
0.2
0.4
0.6
0.8
1.0
N = 32   Bandwidth = 0.06029

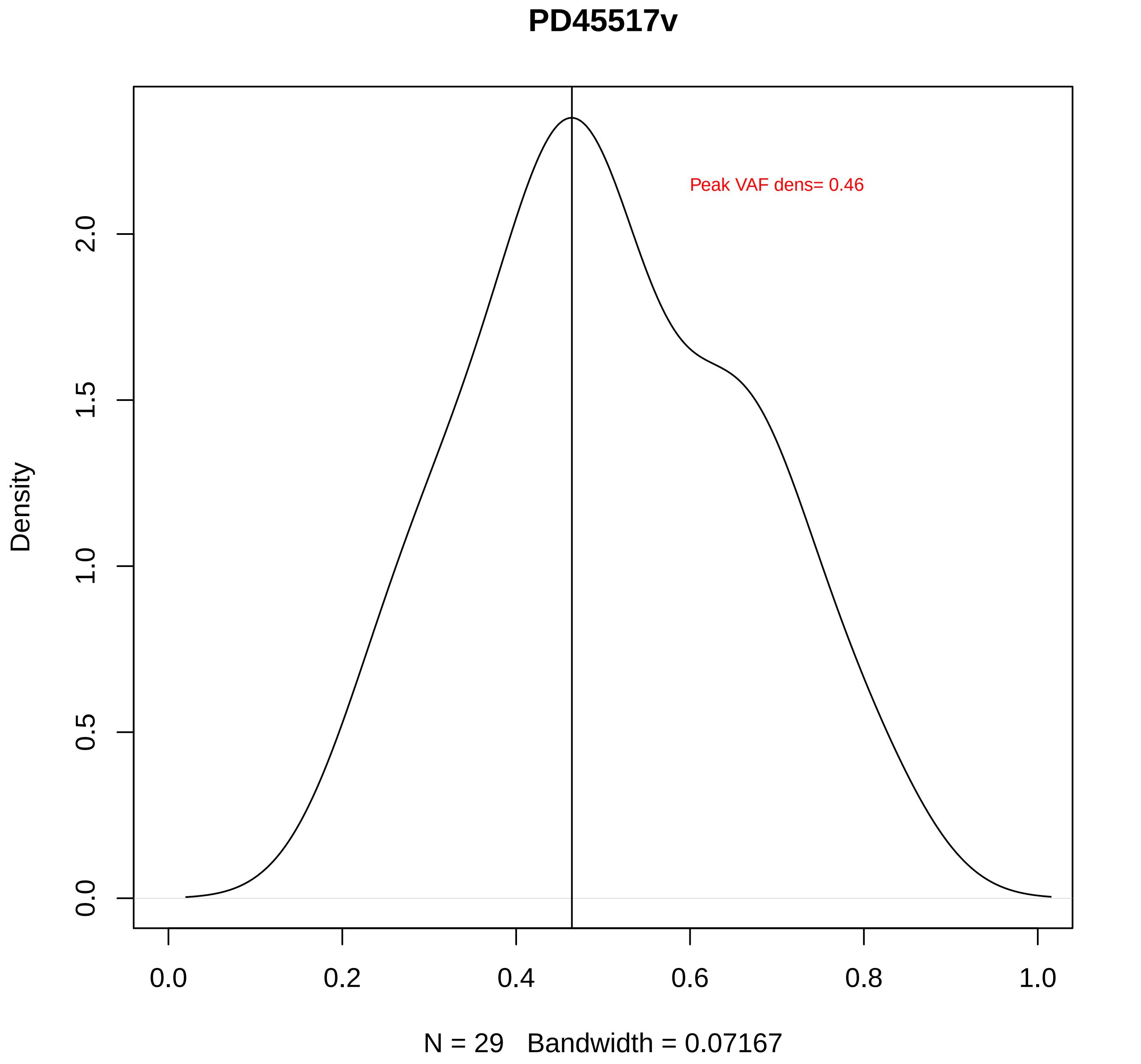

PD45517v
Peak VAF dens= 0.46
Density
0.0
0.5
1.0
1.5
2.0
0.0
0.2
0.4
0.6
0.8
1.0
N = 29   Bandwidth = 0.07167

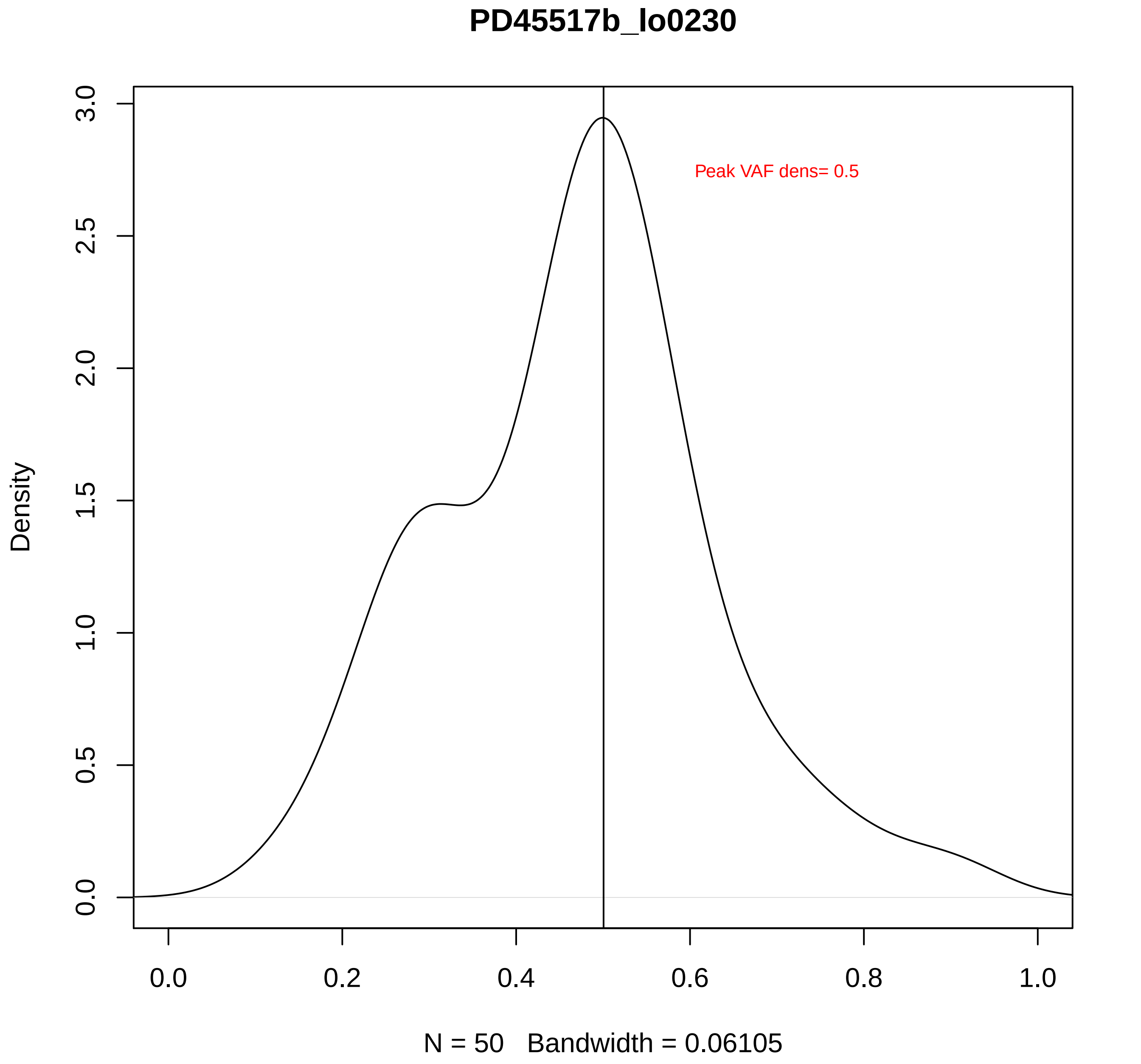
PD45517b_lo0230
Peak VAF dens= 0.5
Density
0.0
0.5
1.0
1.5
2.0
2.5
3.0
0.0
0.2
0.4
0.6
0.8
1.0
N = 50   Bandwidth = 0.06105

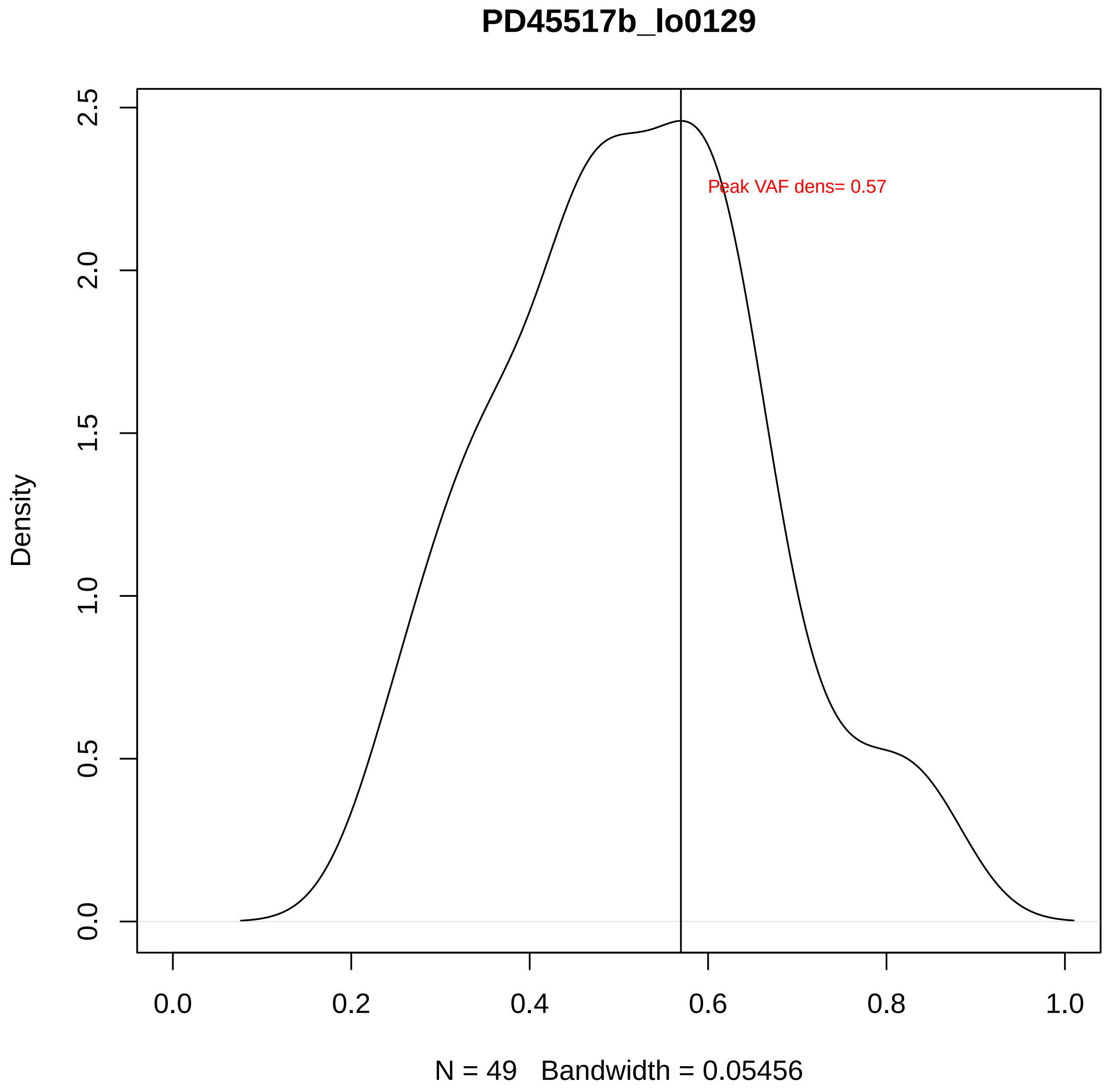
PD45517b_lo0129
Peak VAF dens= 0.57
Density
0.0
0.5
1.0
1.5
2.0
2.5
0.0
0.2
0.4
0.6
0.8
1.0
N = 49 Bandwidth = 0.05456

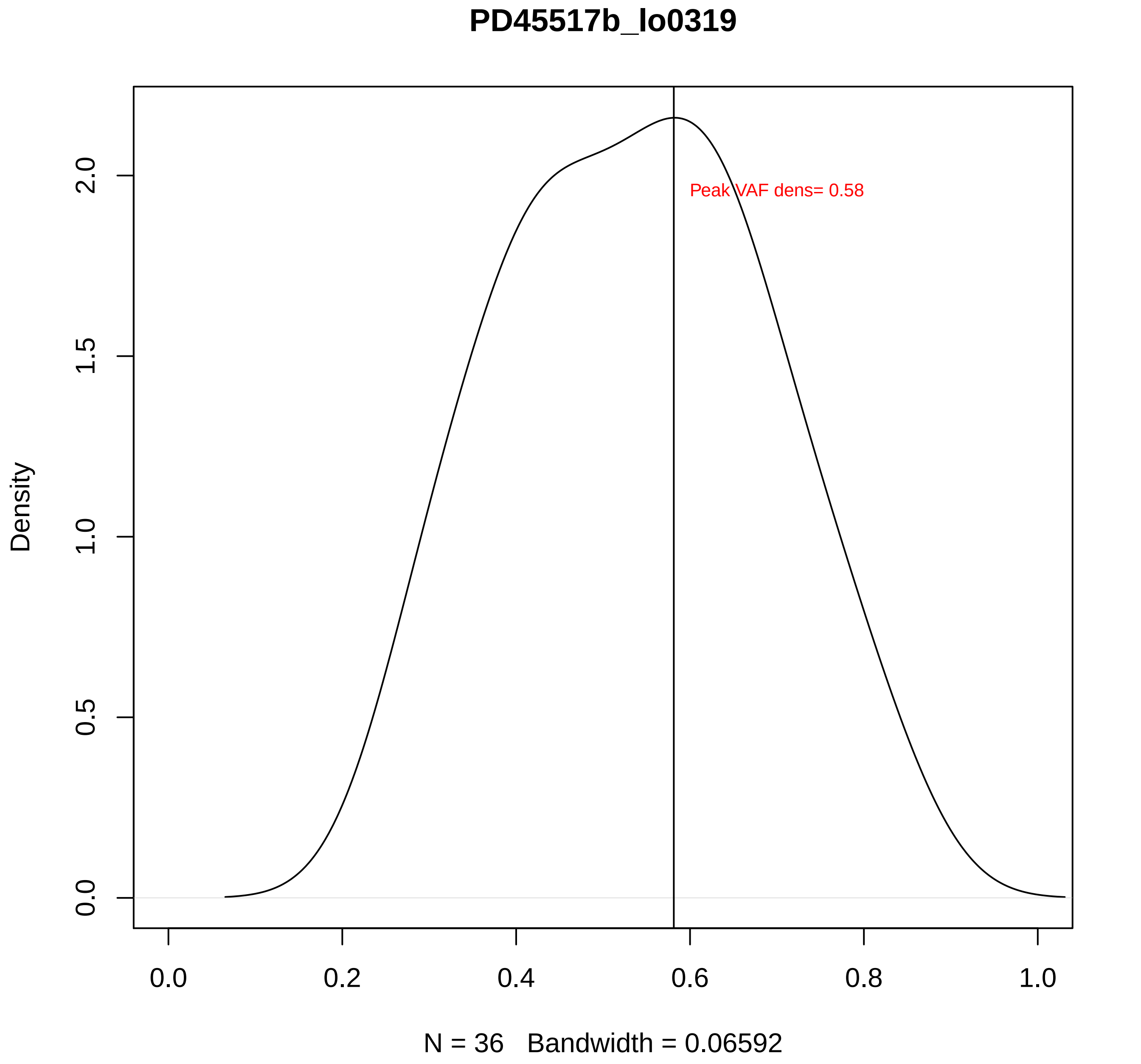

PD45517b_lo0319
Peak VAF dens= 0.58
Density
0.0
0.5
1.0
1.5
2.0
0.0
0.2
0.4
0.6
0.8
1.0
N = 36 Bandwidth = 0.06592

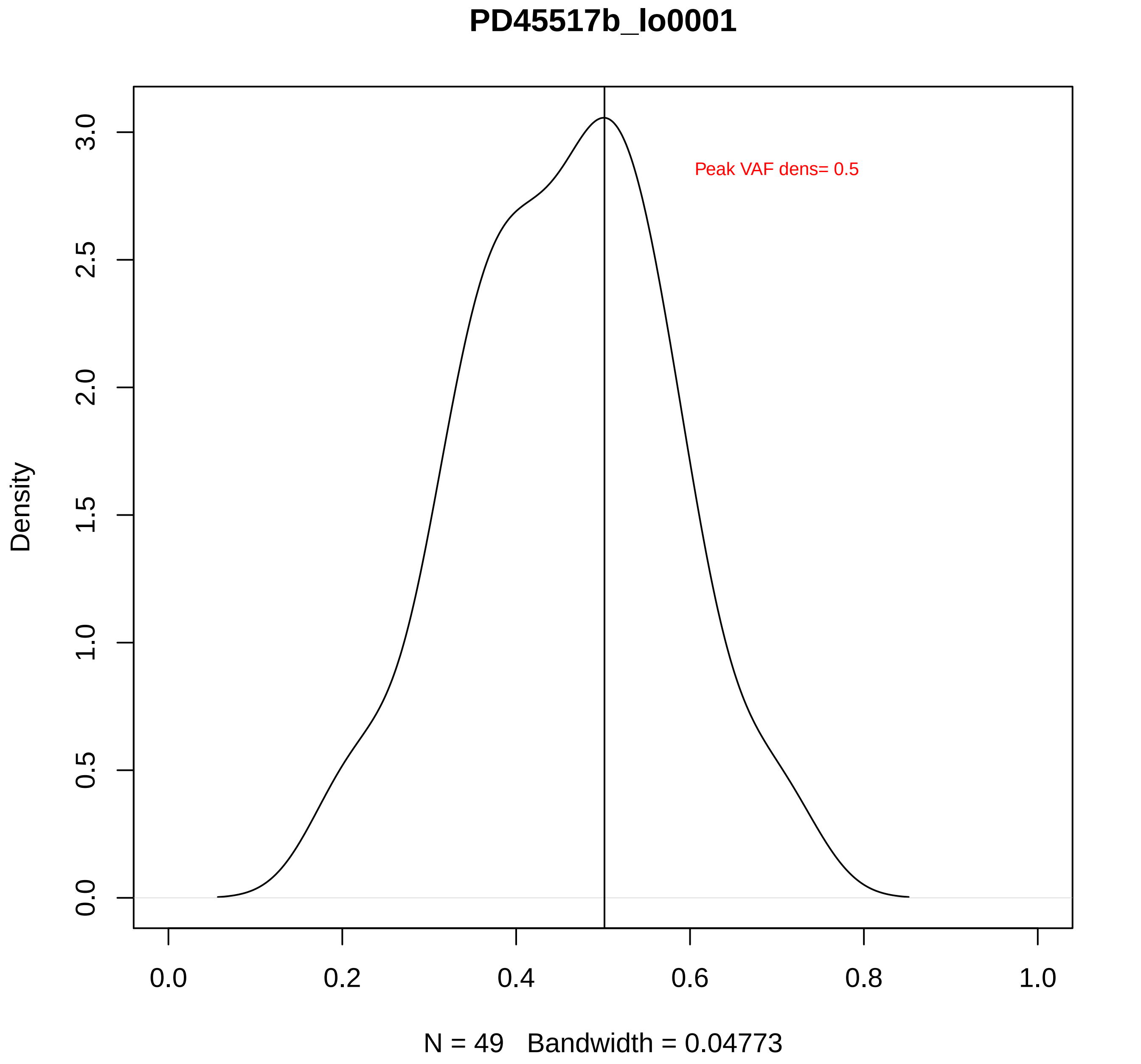

PD45517b_lo0001
Peak VAF dens= 0.5
Density
0.0
0.5
1.0
1.5
2.0
2.5
3.0
0.0
0.2
0.4
0.6
0.8
1.0
N = 49   Bandwidth = 0.04773

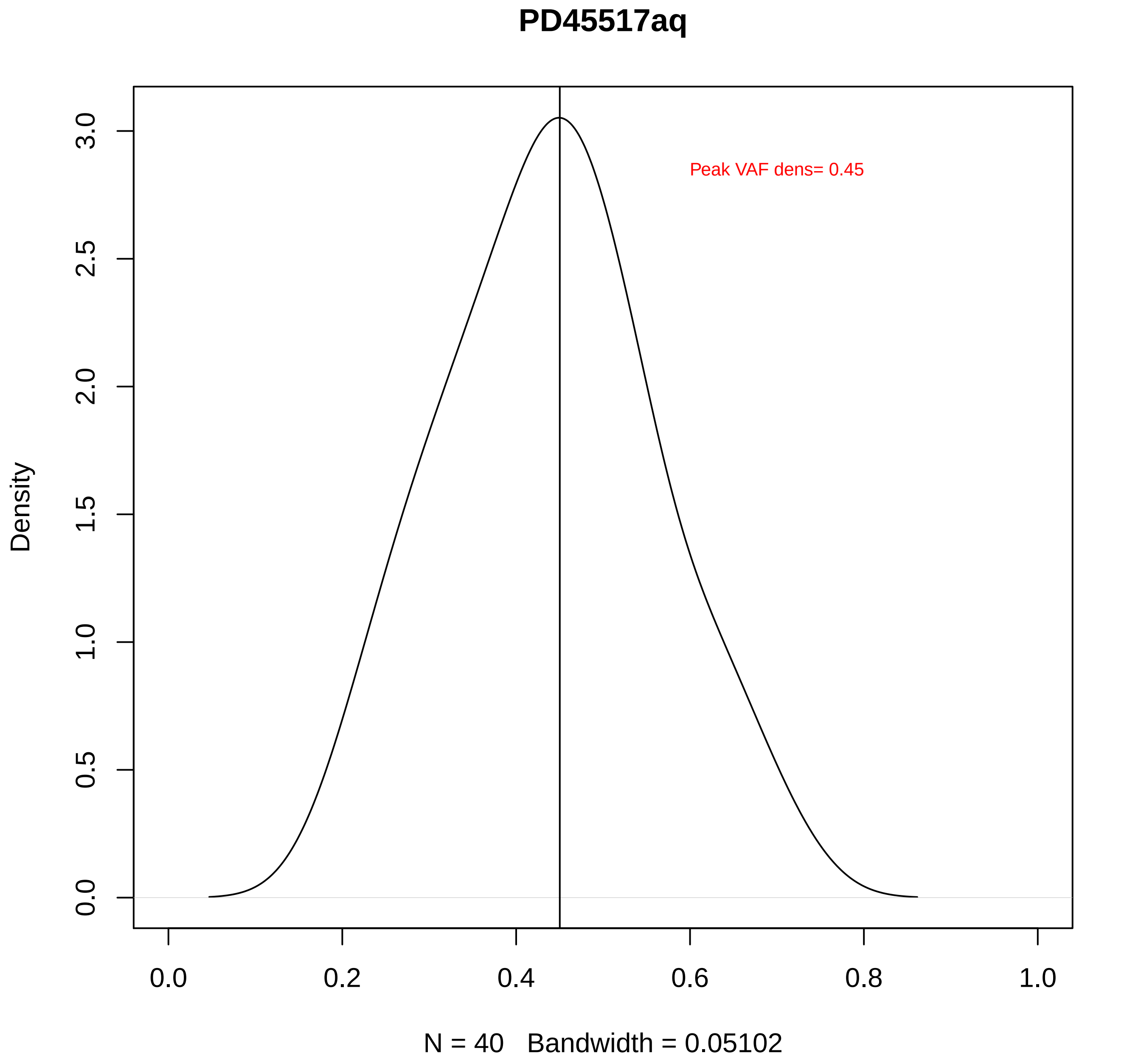
PD45517aq
Peak VAF dens= 0.45
Density
0.0
0.5
1.0
1.5
2.0
2.5
3.0
0.0
0.2
0.4
0.6
0.8
1.0
N = 40   Bandwidth = 0.05102

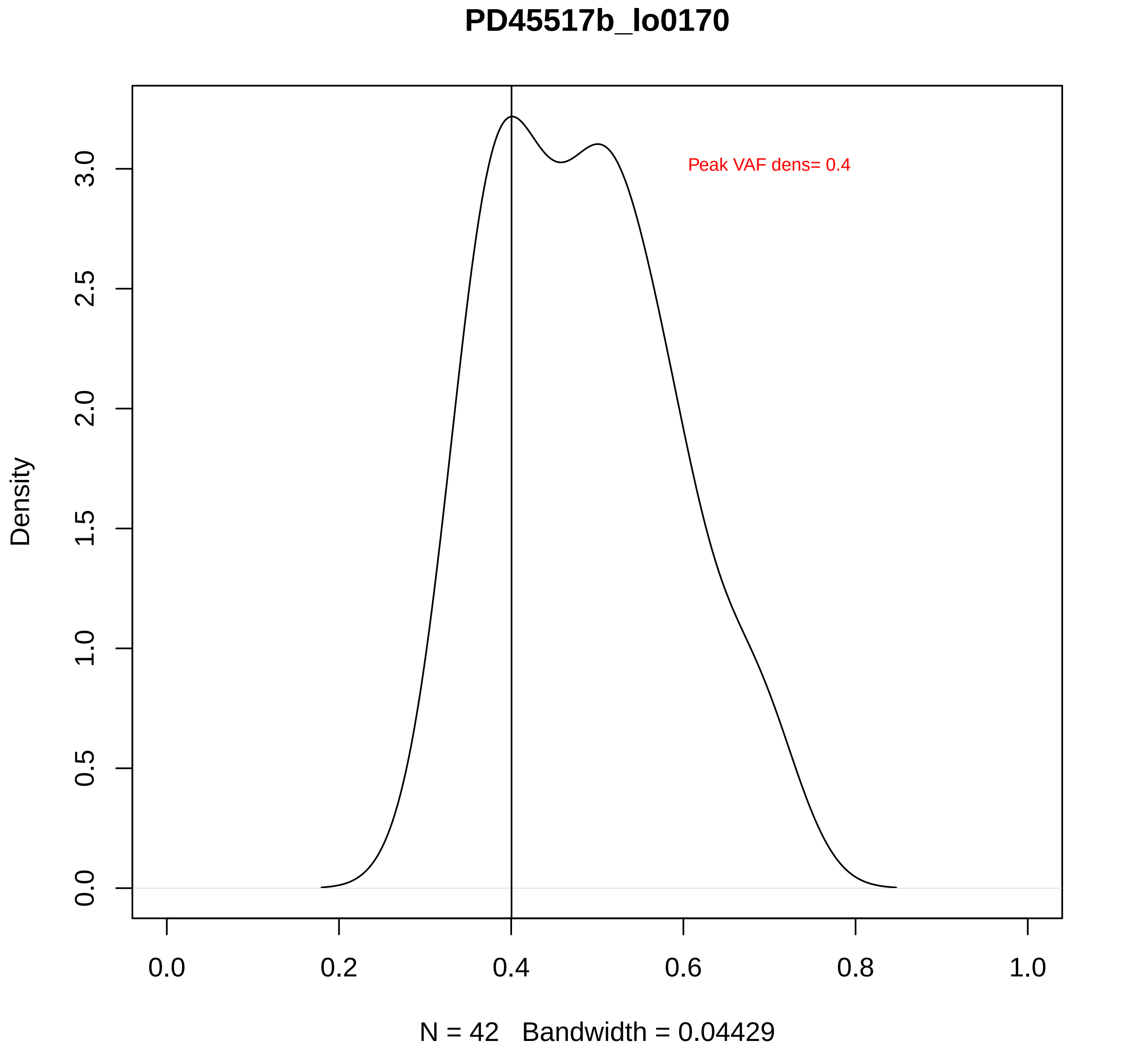
PD45517b_lo0170
Peak VAF dens= 0.4
Density
0.0
0.5
1.0
1.5
2.0
2.5
3.0
0.0
0.2
0.4
0.6
0.8
1.0
N = 42   Bandwidth = 0.04429

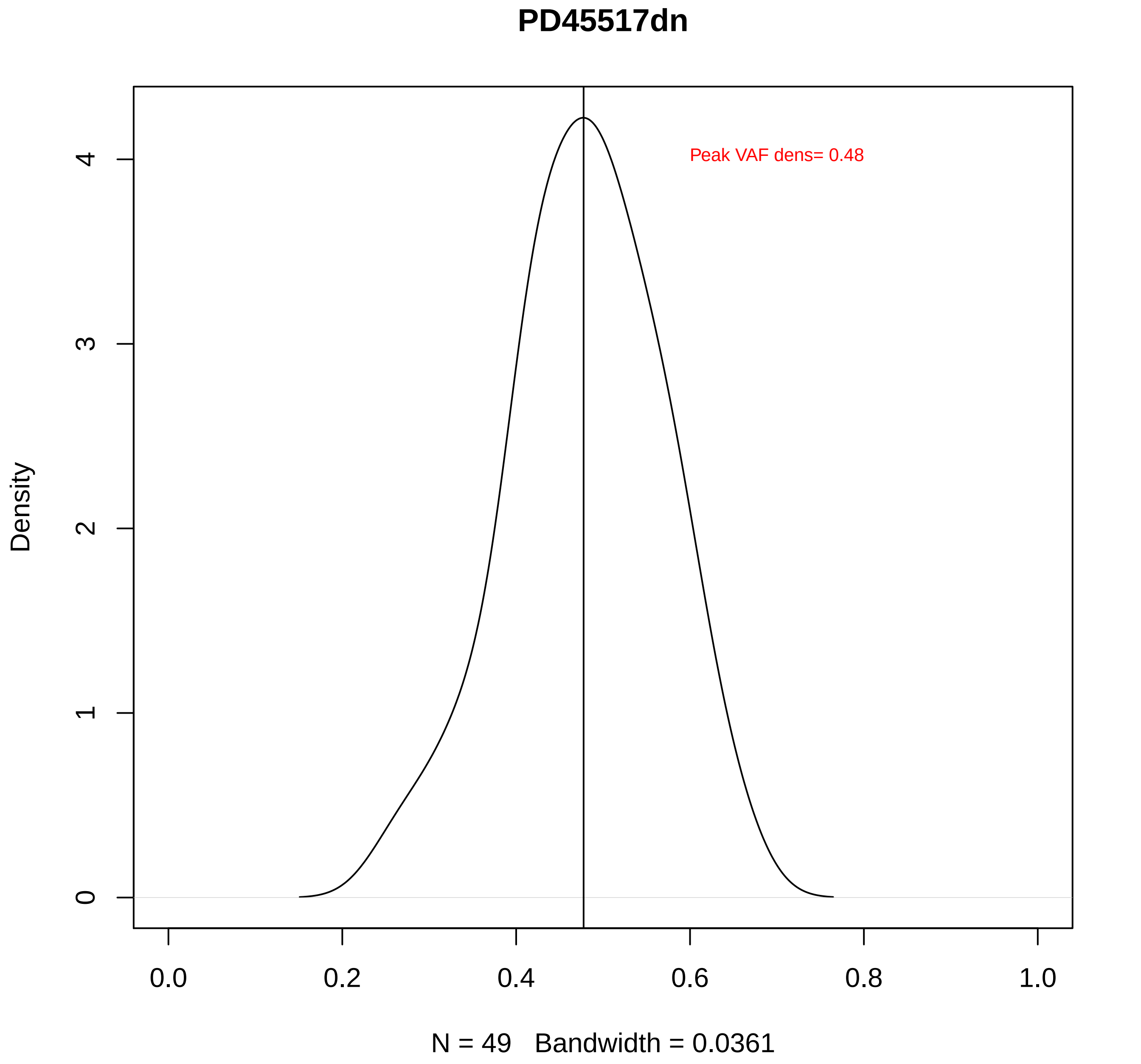

PD45517dn
Peak VAF dens= 0.48
Density
0
1
2
3
4
0.0
0.2
0.4
0.6
0.8
1.0
N = 49   Bandwidth = 0.0361

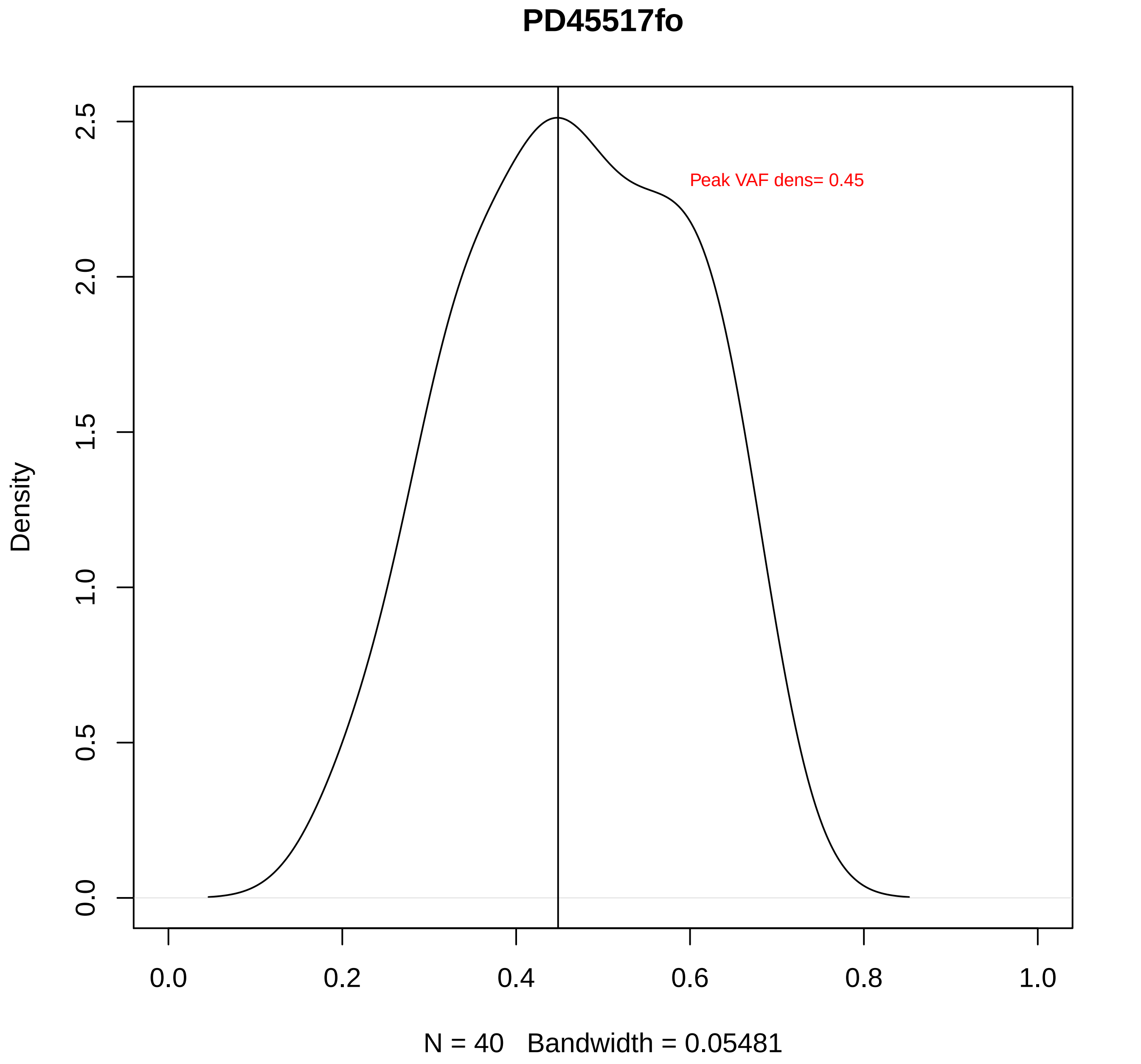
PD45517fo
Peak VAF dens= 0.45
Density
0.0
0.5
1.0
1.5
2.0
2.5
0.0
0.2
0.4
0.6
0.8
1.0
N = 40 Bandwidth = 0.05481

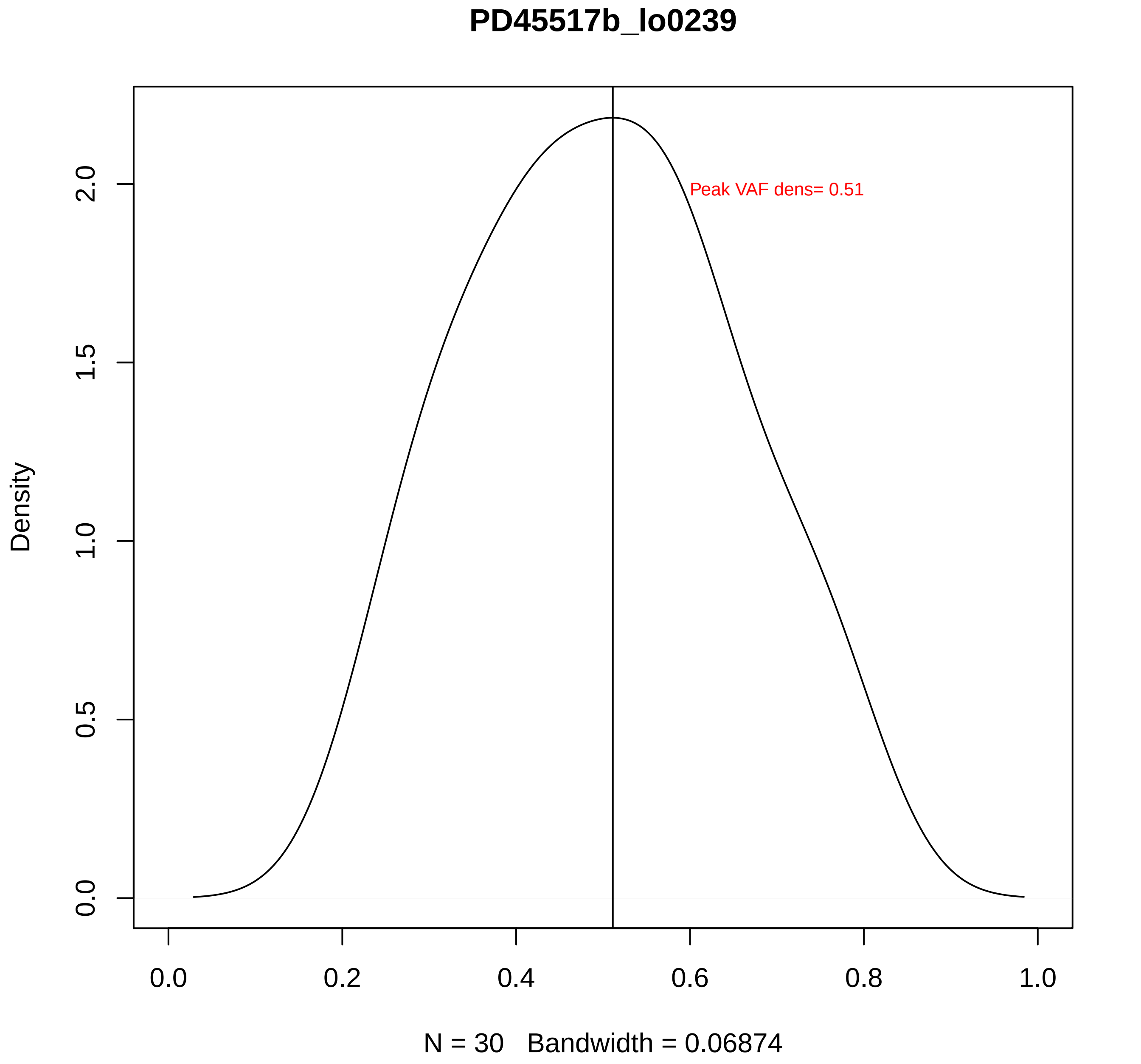

PD45517b_lo0239
Peak VAF dens= 0.51
Density
0.0
0.5
1.0
1.5
2.0
0.0
0.2
0.4
0.6
0.8
1.0
N = 30   Bandwidth = 0.06874

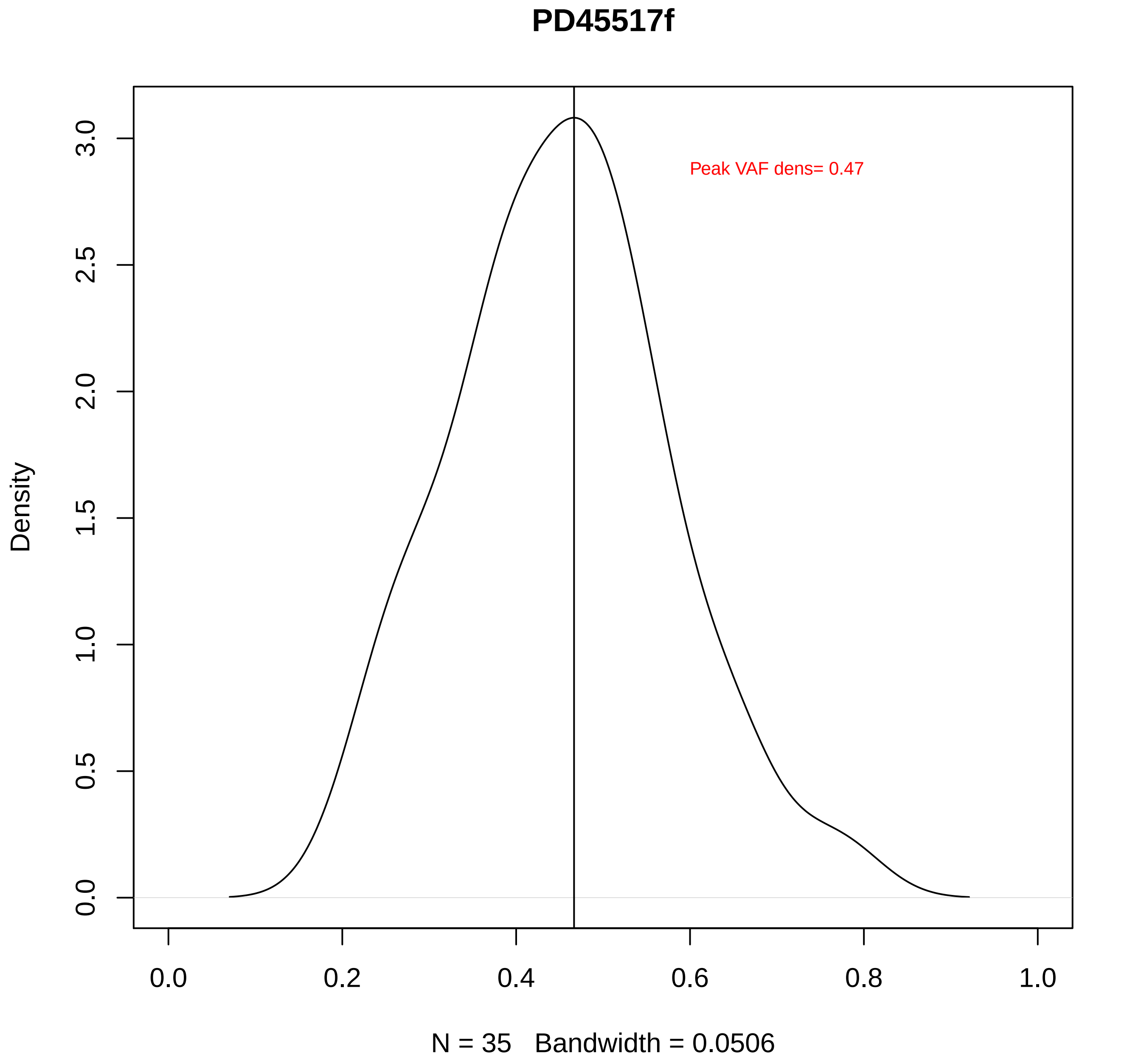
PD45517f
Peak VAF dens= 0.47
Density
0.0
0.5
1.0
1.5
2.0
2.5
3.0
0.0
0.2
0.4
0.6
0.8
1.0
N = 35 Bandwidth = 0.0506

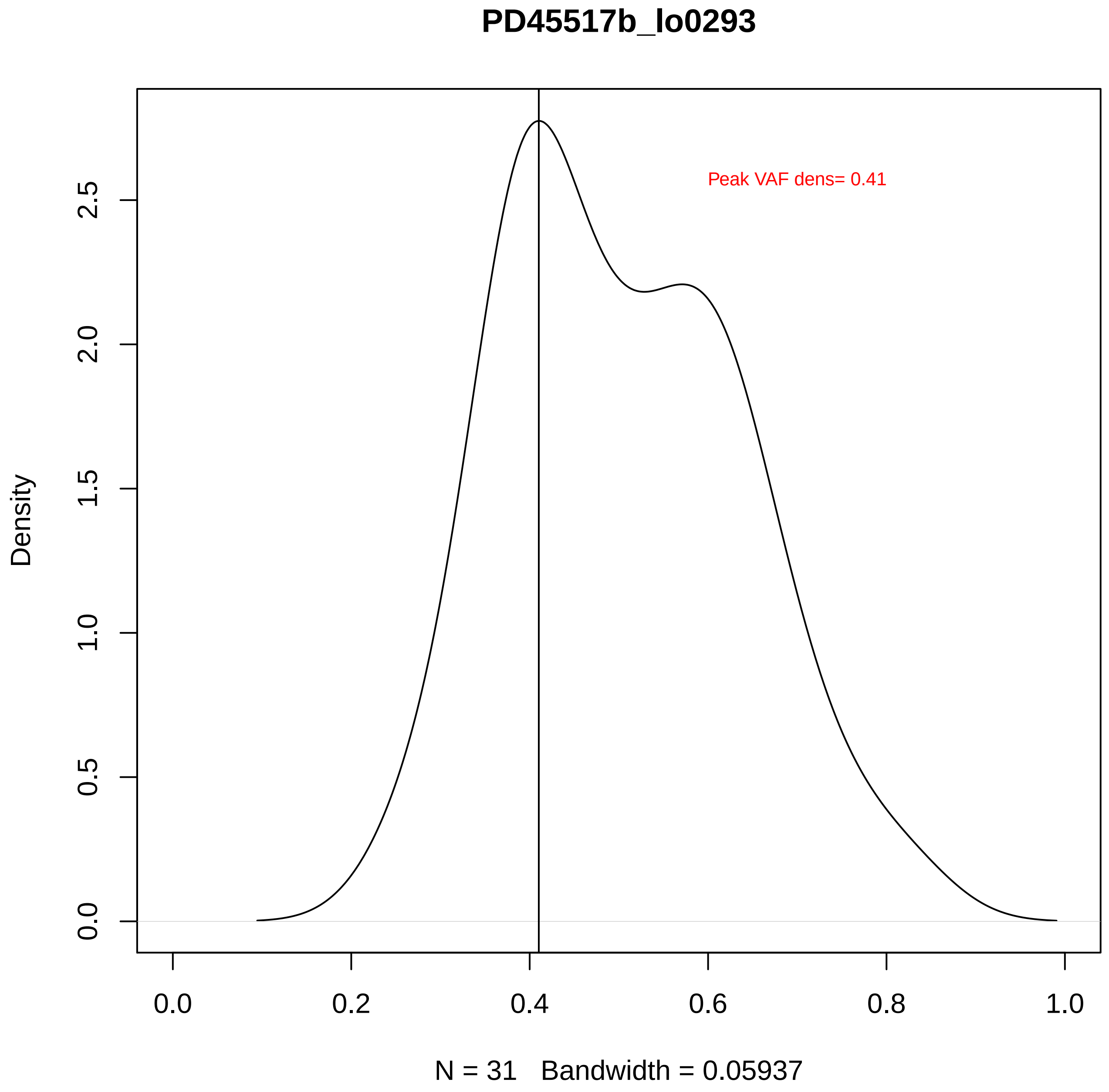
PD45517b_lo0293
Peak VAF dens= 0.41
Density
0.0
0.5
1.0
1.5
2.0
2.5
0.0
0.2
0.4
0.6
0.8
1.0
N = 31   Bandwidth = 0.05937

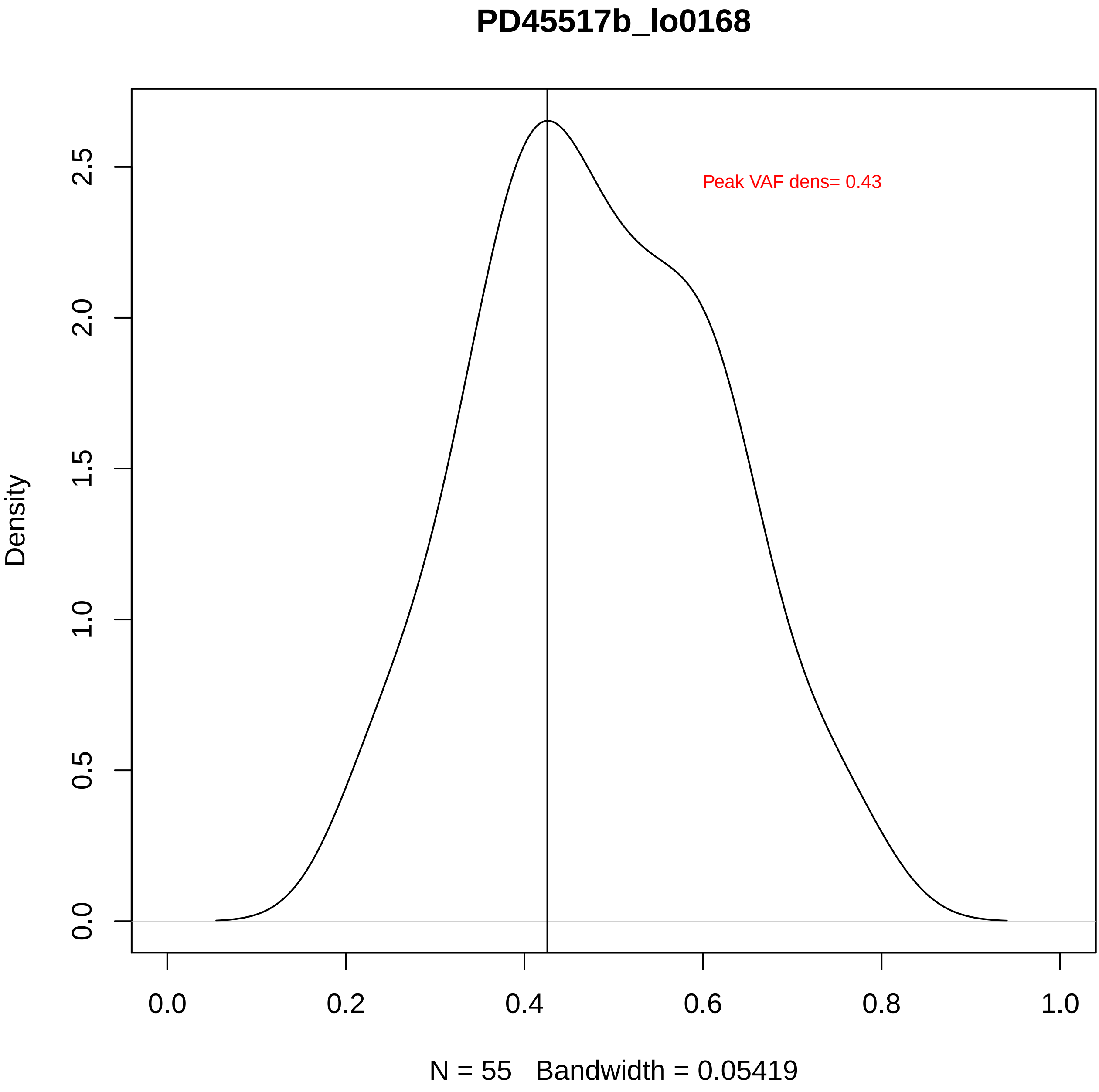

PD45517b_lo0168
Peak VAF dens= 0.43
Density
0.0
0.5
1.0
1.5
2.0
2.5
0.0
0.2
0.4
0.6
0.8
1.0
N = 55   Bandwidth = 0.05419

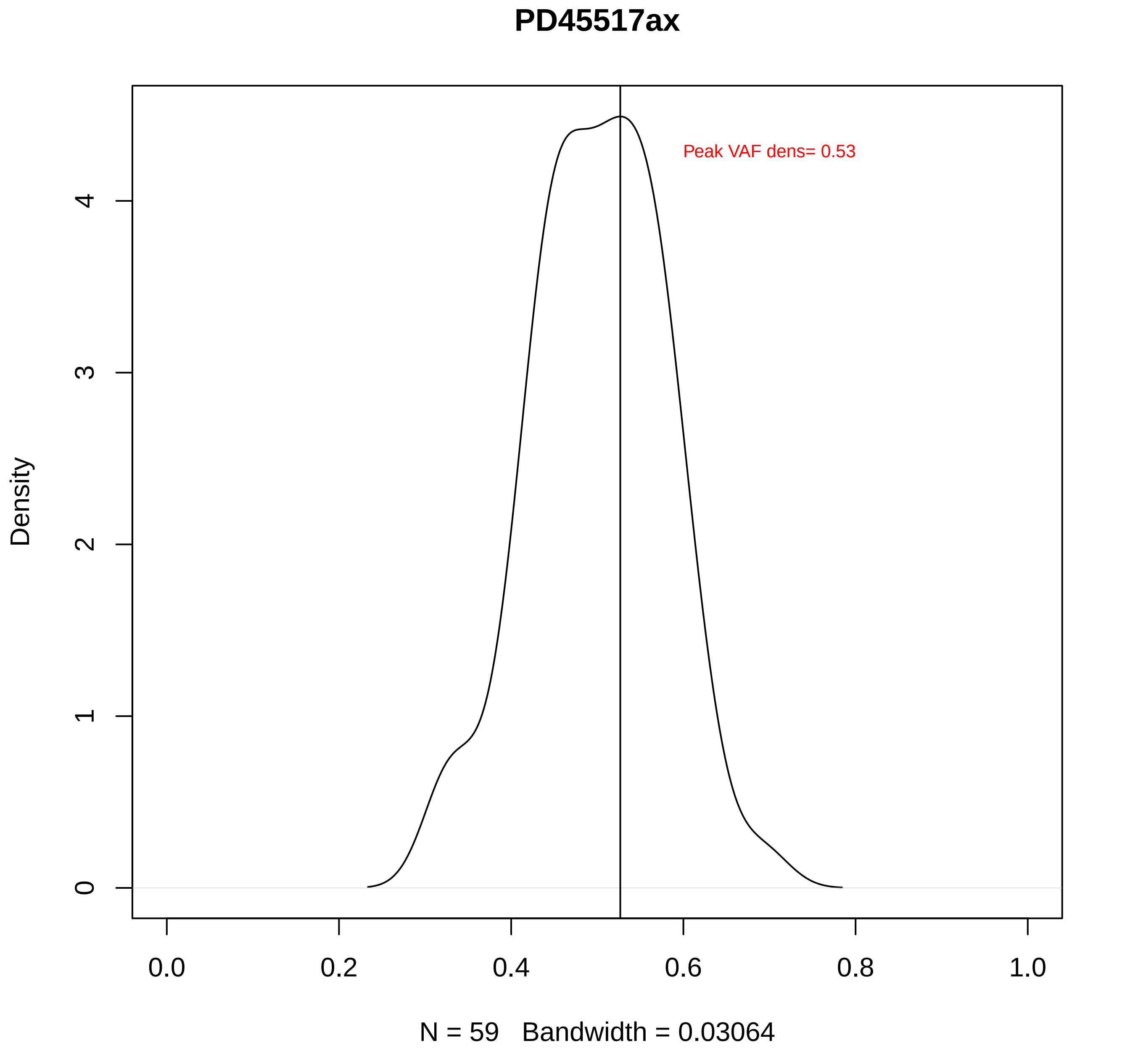

PD45517ax
Peak VAF dens= 0.53
Density
0
1
2
3
4
0.0
0.2
0.4
0.6
0.8
1.0
N = 59 Bandwidth = 0.03064

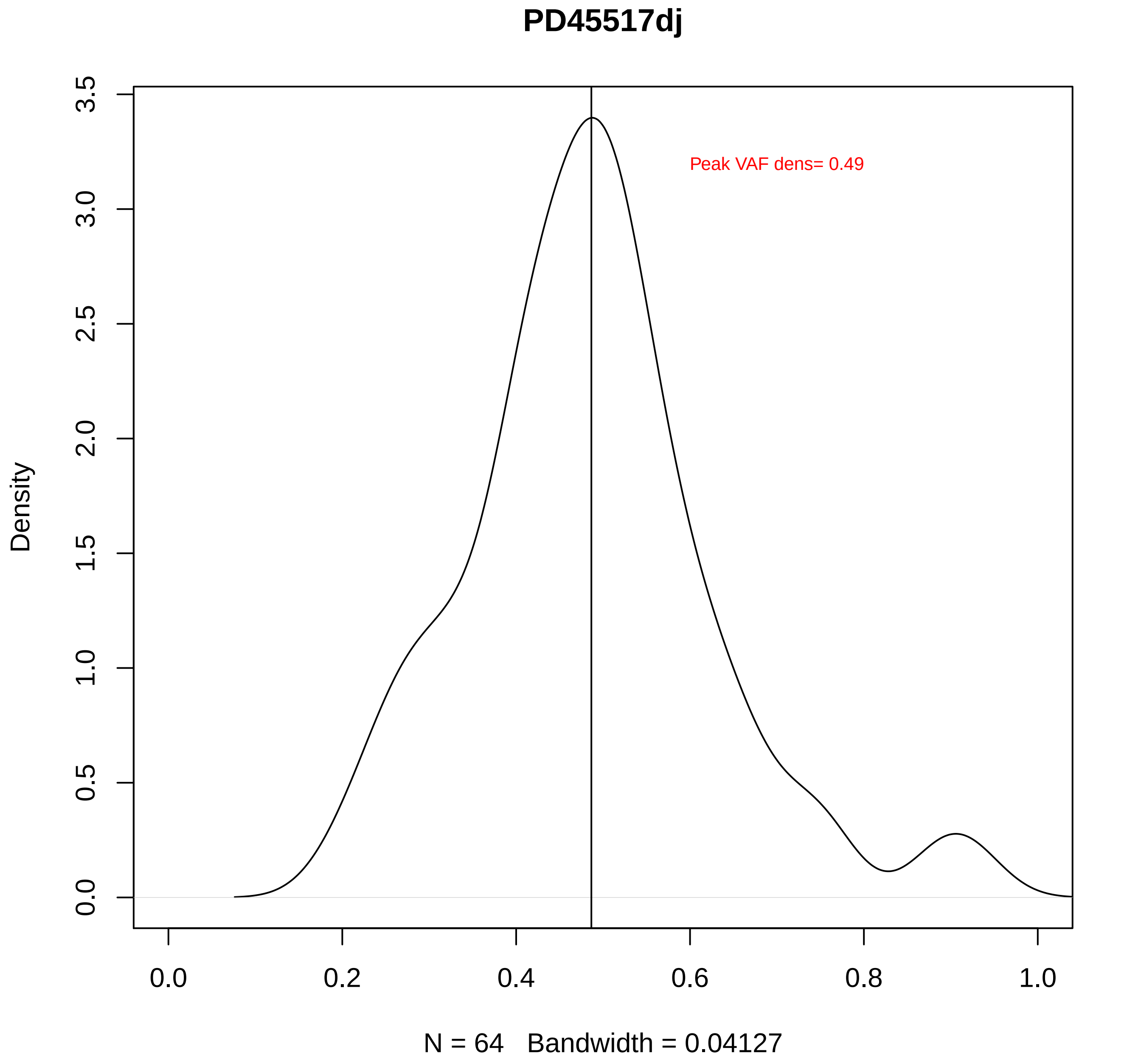

PD45517dj
Peak VAF dens= 0.49
Density
0.0
0.5
1.0
1.5
2.0
2.5
3.0
3.5
0.0
0.2
0.4
0.6
0.8
1.0
N = 64 Bandwidth = 0.04127

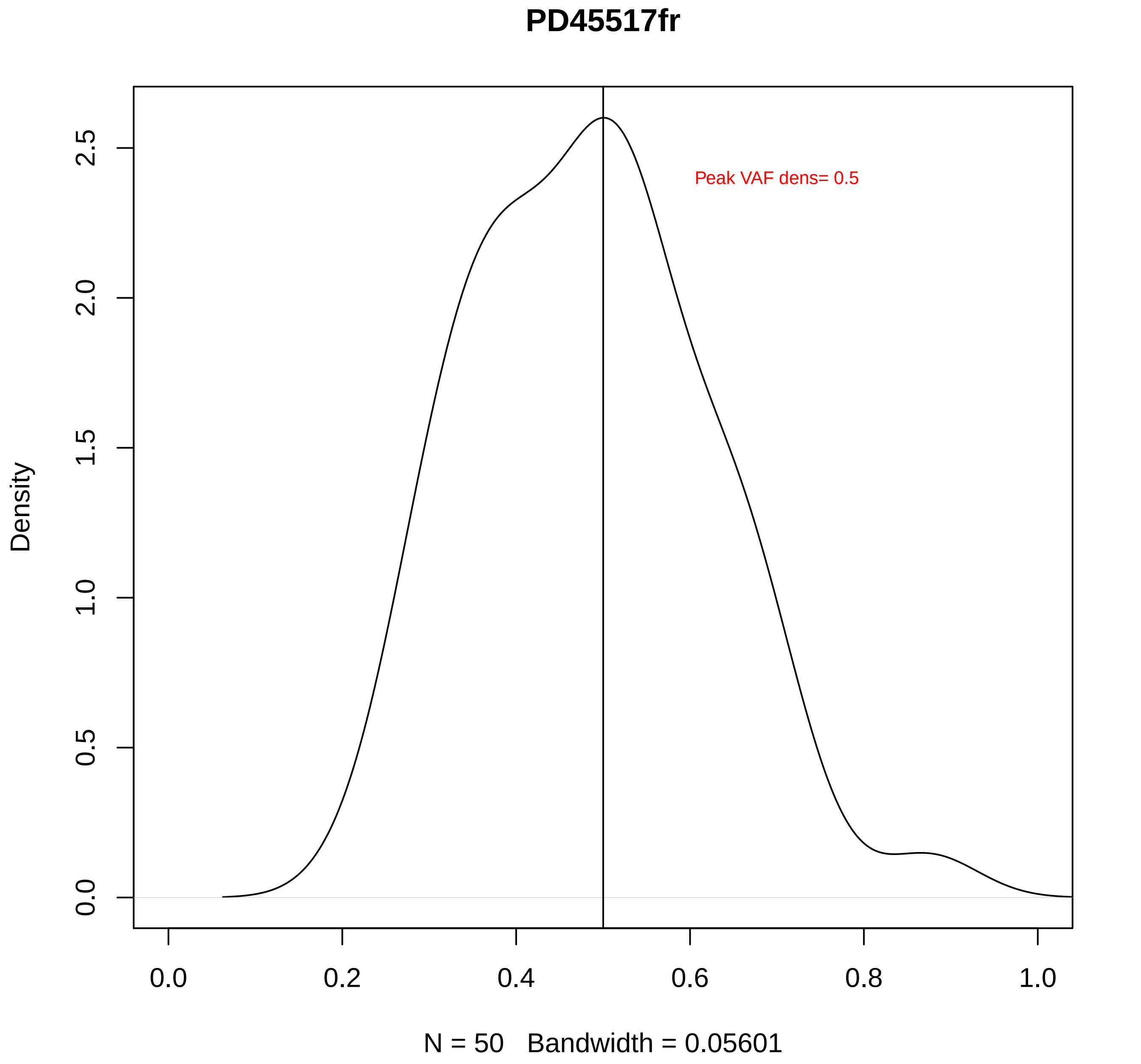
PD45517fr
Peak VAF dens= 0.5
Density
0.0
0.5
1.0
1.5
2.0
2.5
0.0
0.2
0.4
0.6
0.8
1.0
N = 50   Bandwidth = 0.05601

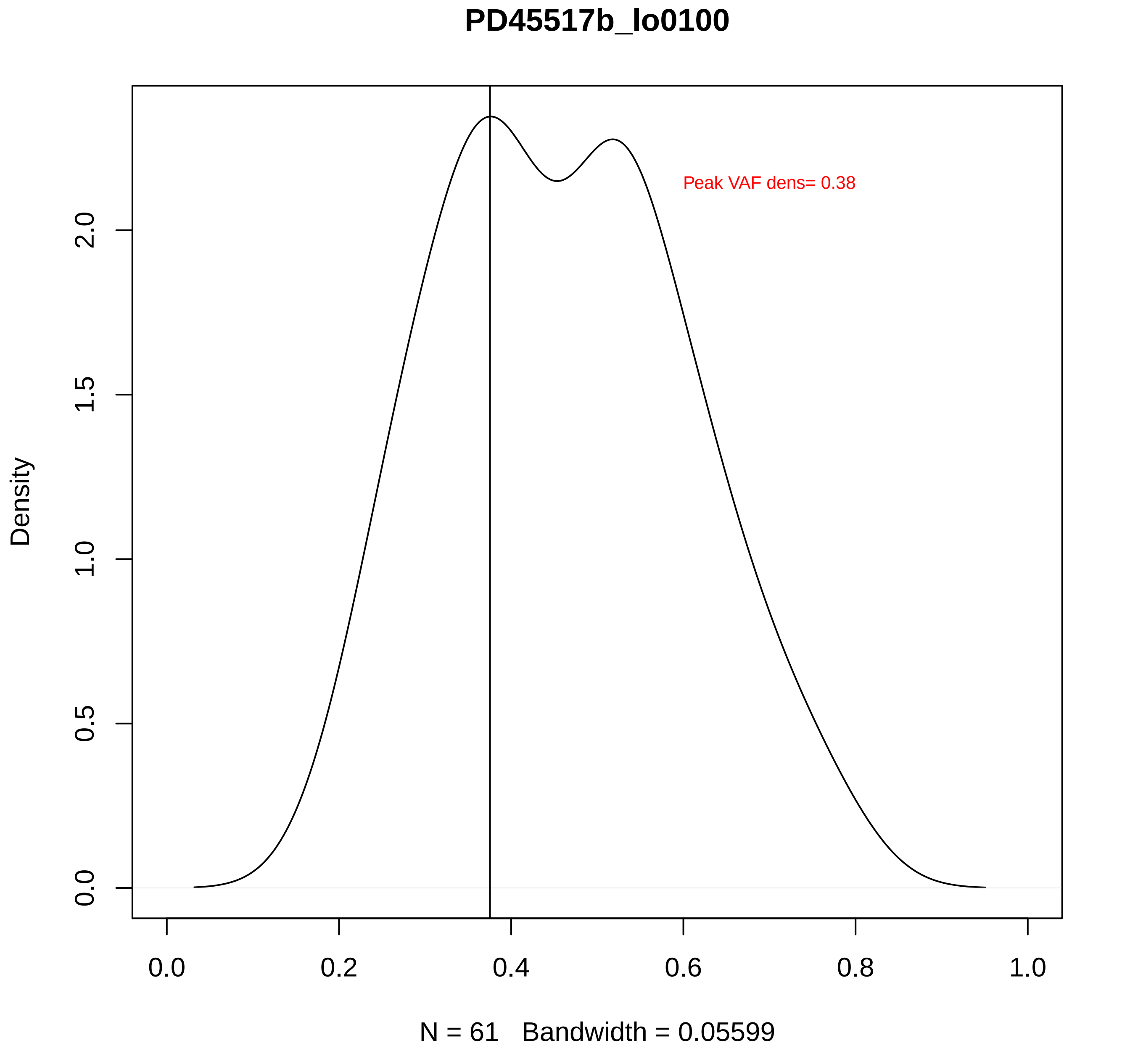
PD45517b_lo0100
Peak VAF dens= 0.38
Density
0.0
0.5
1.0
1.5
2.0
0.0
0.2
0.4
0.6
0.8
1.0
N = 61   Bandwidth = 0.05599

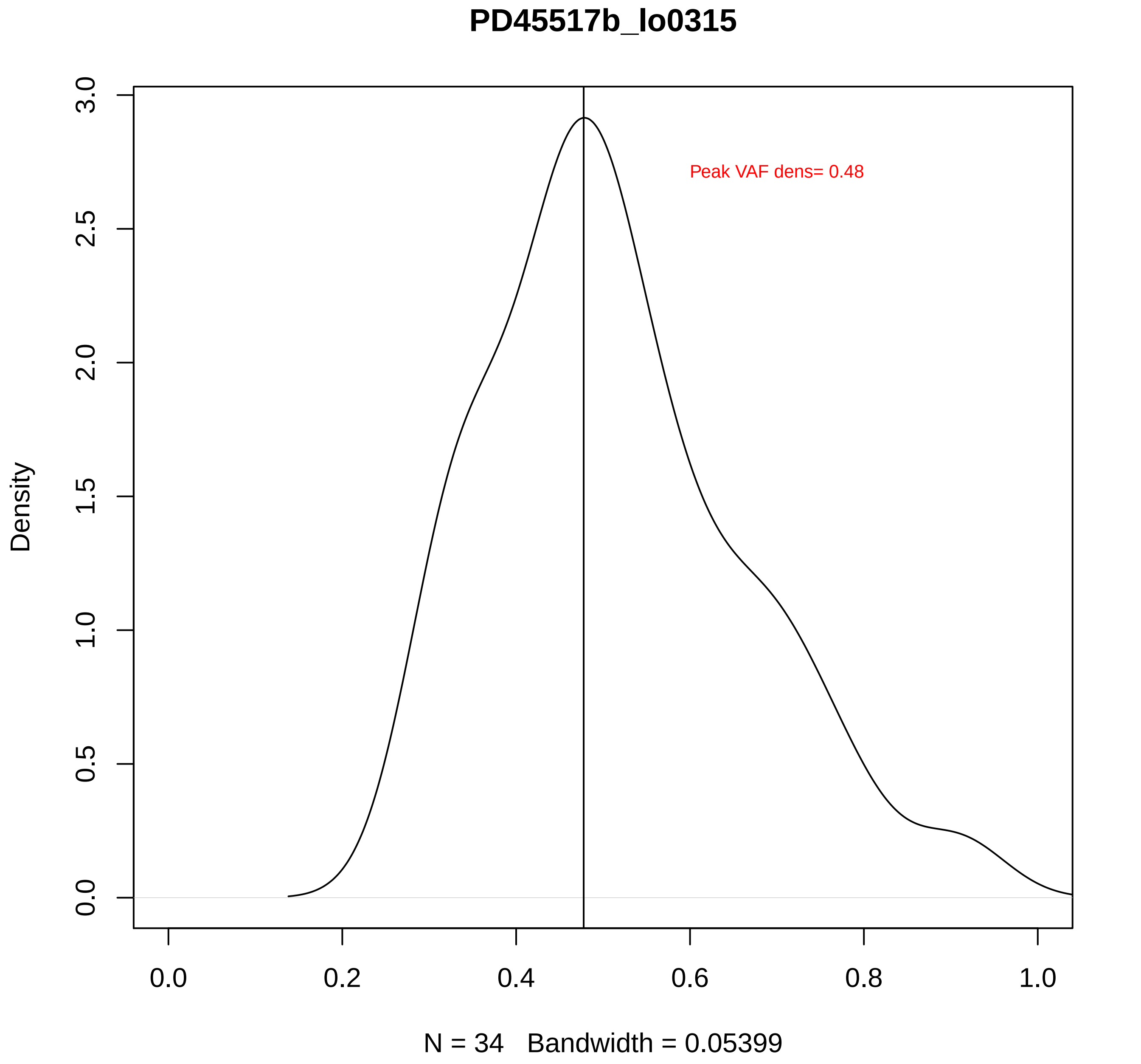

PD45517b_lo0315
Peak VAF dens= 0.48
Density
0.0
0.5
1.0
1.5
2.0
2.5
3.0
0.0
0.2
0.4
0.6
0.8
1.0
N = 34   Bandwidth = 0.05399

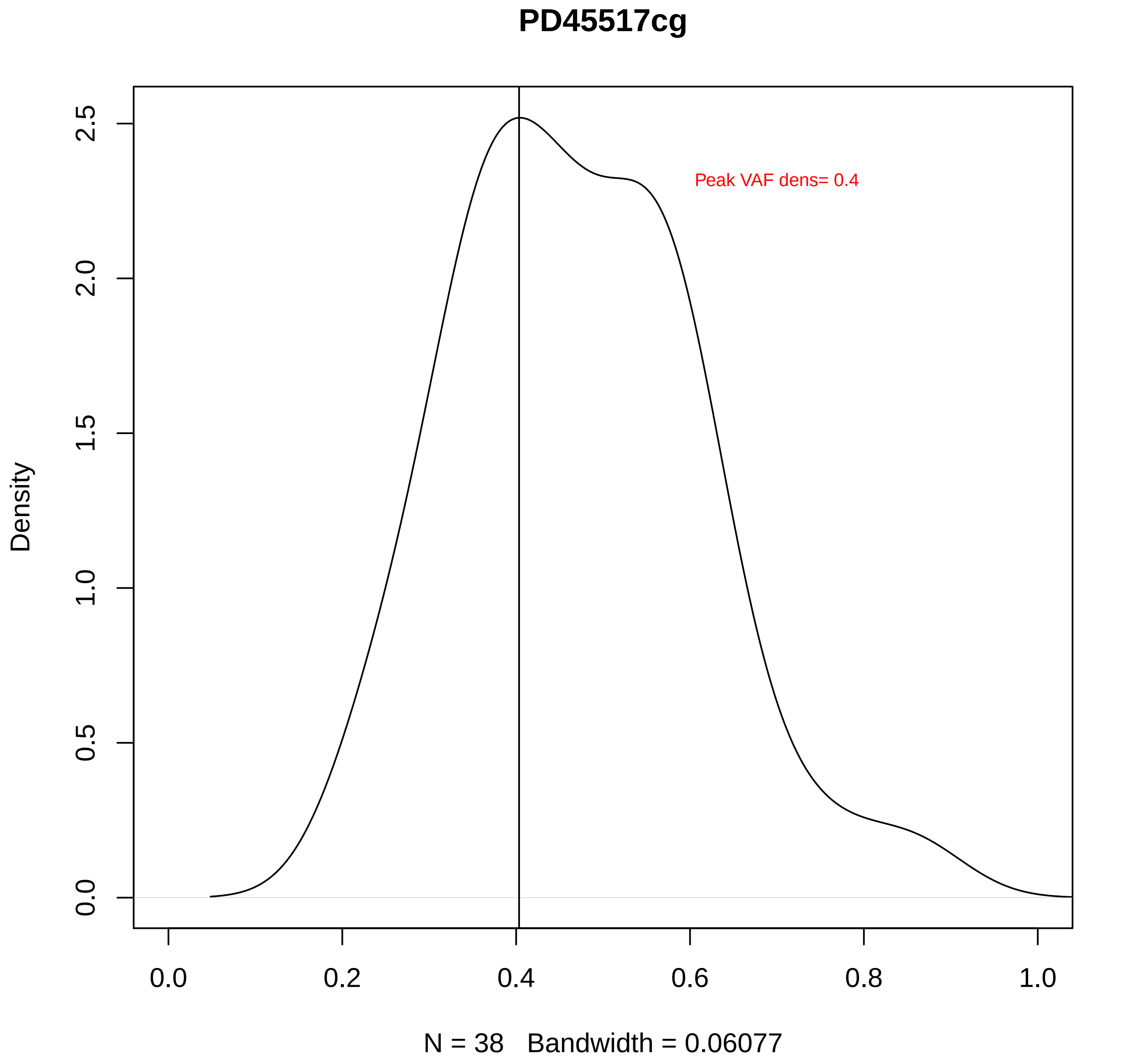
PD45517cg
Peak VAF dens= 0.4
Density
0.0
0.5
1.0
1.5
2.0
2.5
0.0
0.2
0.4
0.6
0.8
1.0
N = 38 Bandwidth = 0.06077

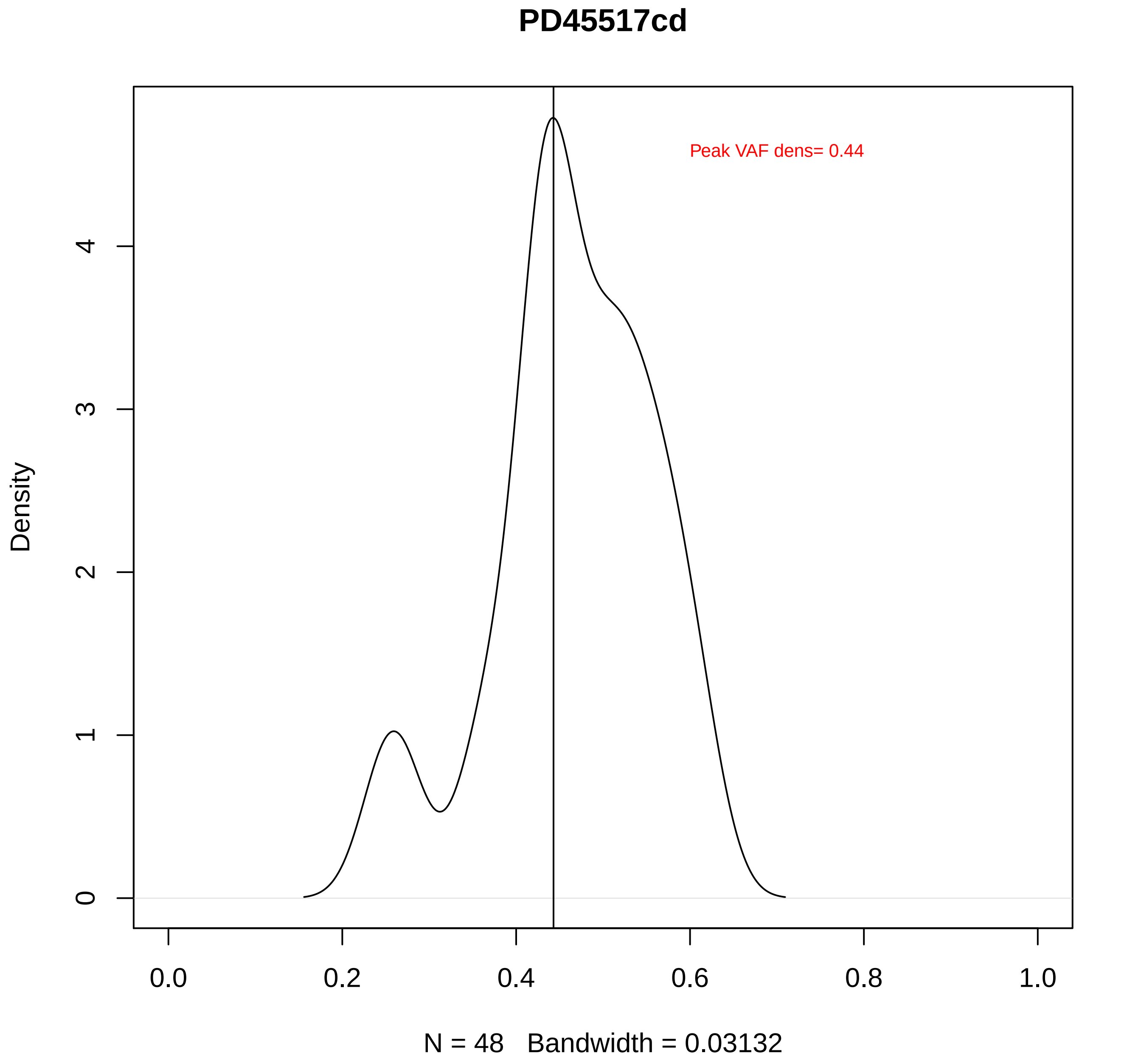
PD45517cd
Peak VAF dens= 0.44
Density
0
1
2
3
4
0.0
0.2
0.4
0.6
0.8
1.0
N = 48   Bandwidth = 0.03132

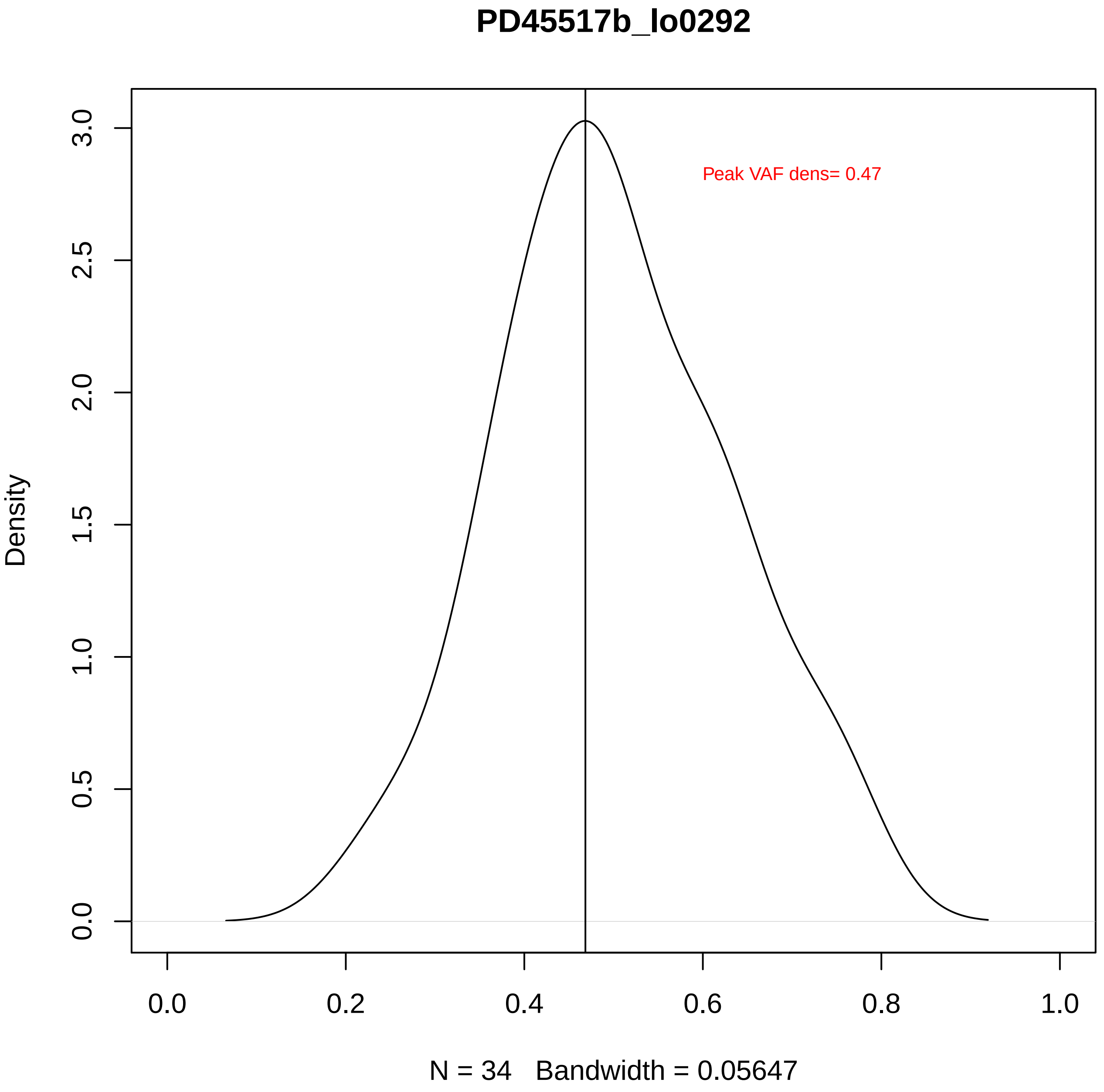
PD45517b_lo0292
Peak VAF dens= 0.47
Density
0.0
0.5
1.0
1.5
2.0
2.5
3.0
0.0
0.2
0.4
0.6
0.8
1.0
N = 34 Bandwidth = 0.05647

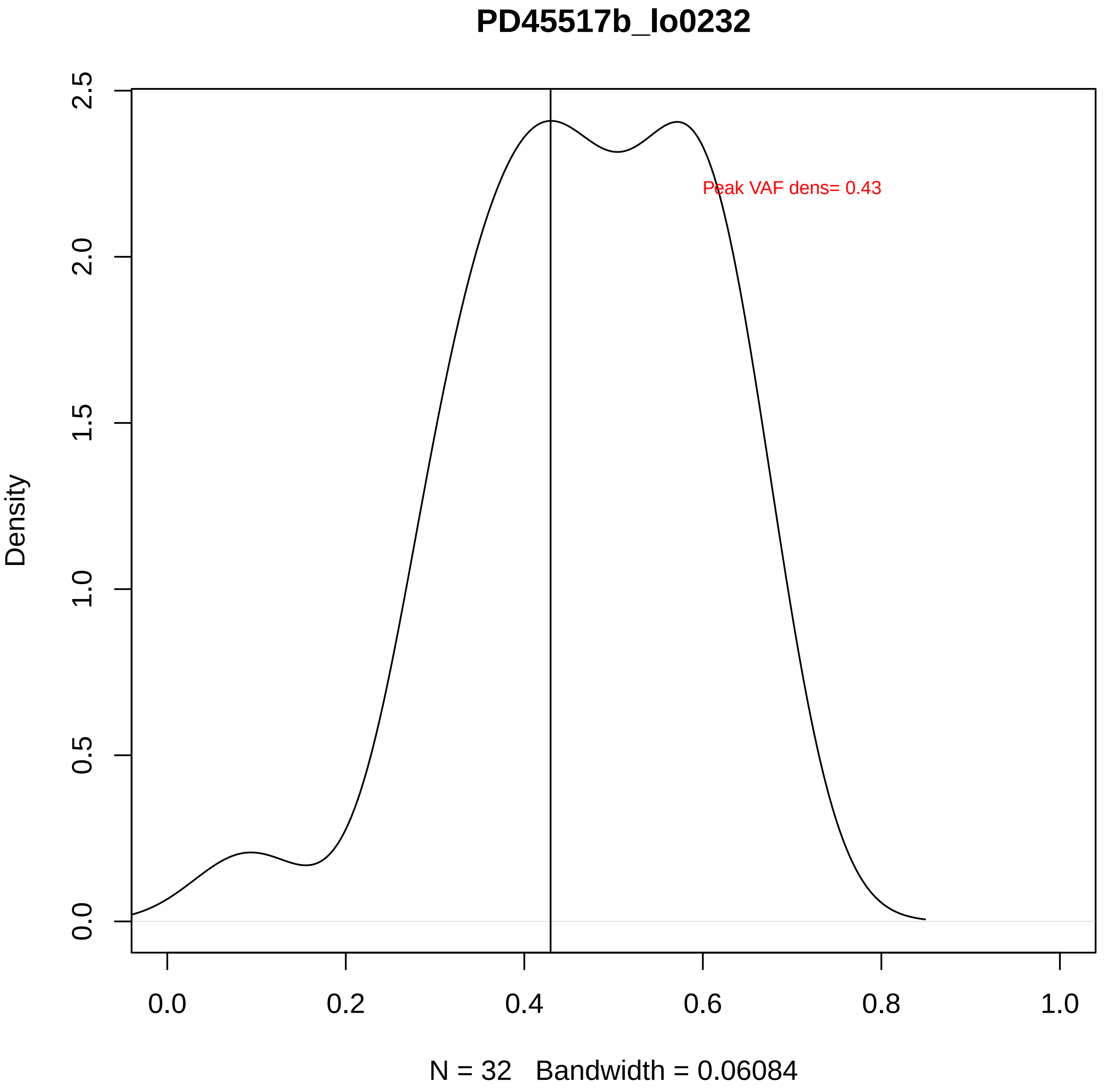
PD45517b_lo0232
Peak VAF dens= 0.43
Density
N = 32   Bandwidth = 0.06084
0.0
0.5
1.0
1.5
2.0
2.5
0.0
0.2
0.4
0.6
0.8
1.0

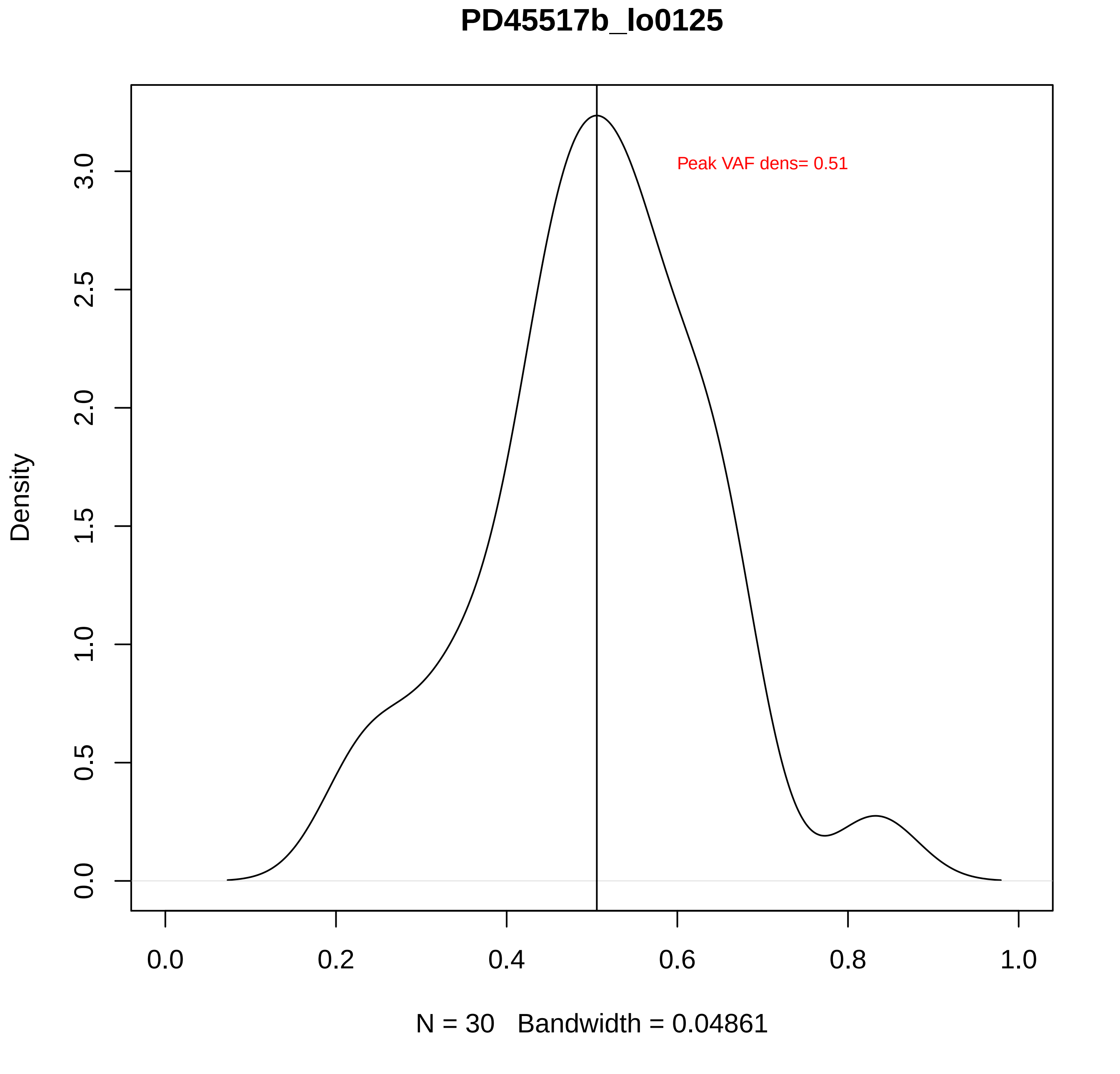
PD45517b_lo0125
Peak VAF dens= 0.51
Density
0.0
0.5
1.0
1.5
2.0
2.5
3.0
0.0
0.2
0.4
0.6
0.8
1.0
N = 30 Bandwidth = 0.04861

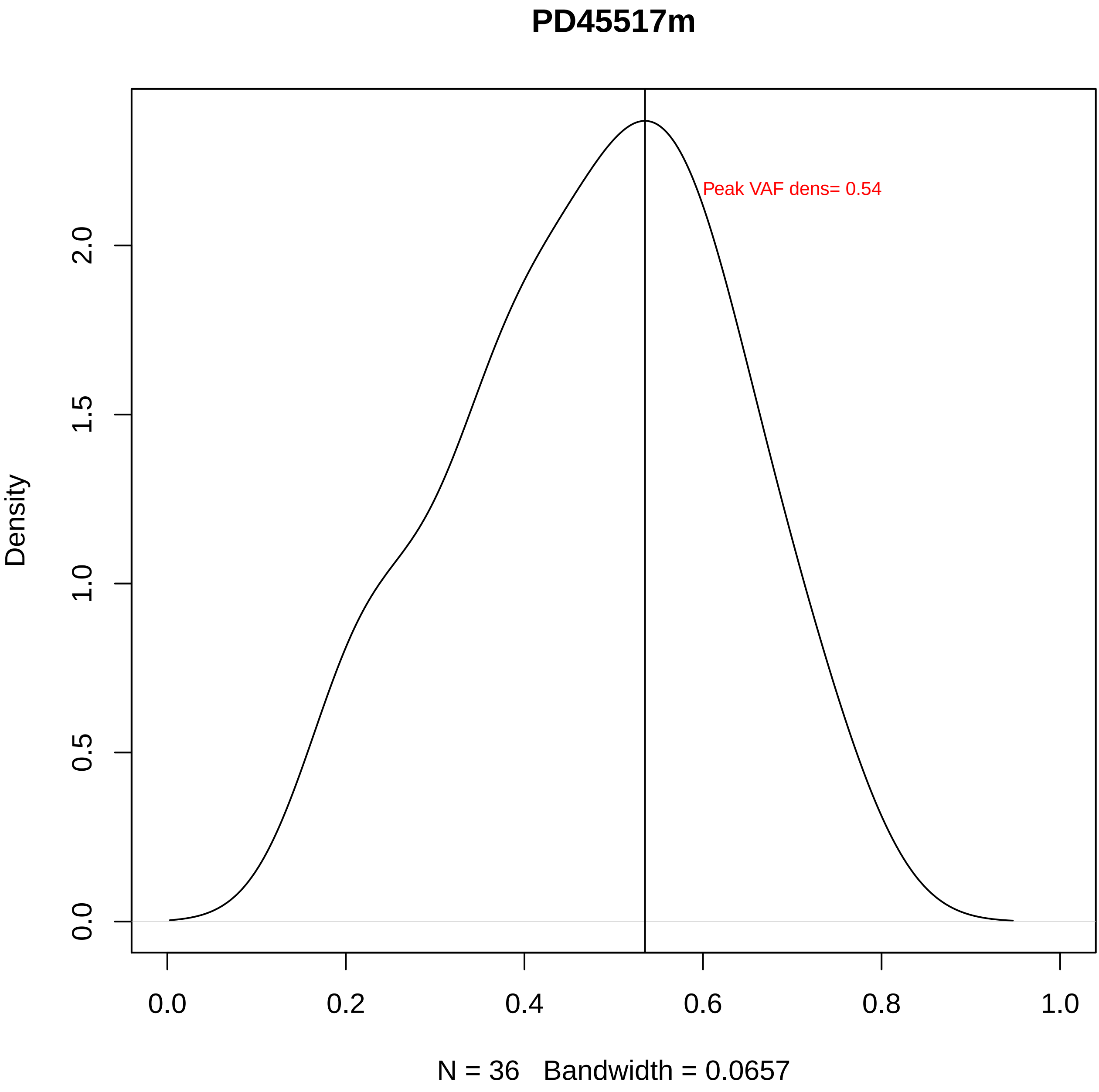
PD45517m
Peak VAF dens= 0.54
Density
0.0
0.5
1.0
1.5
2.0
0.0
0.2
0.4
0.6
0.8
1.0
N = 36 Bandwidth = 0.0657

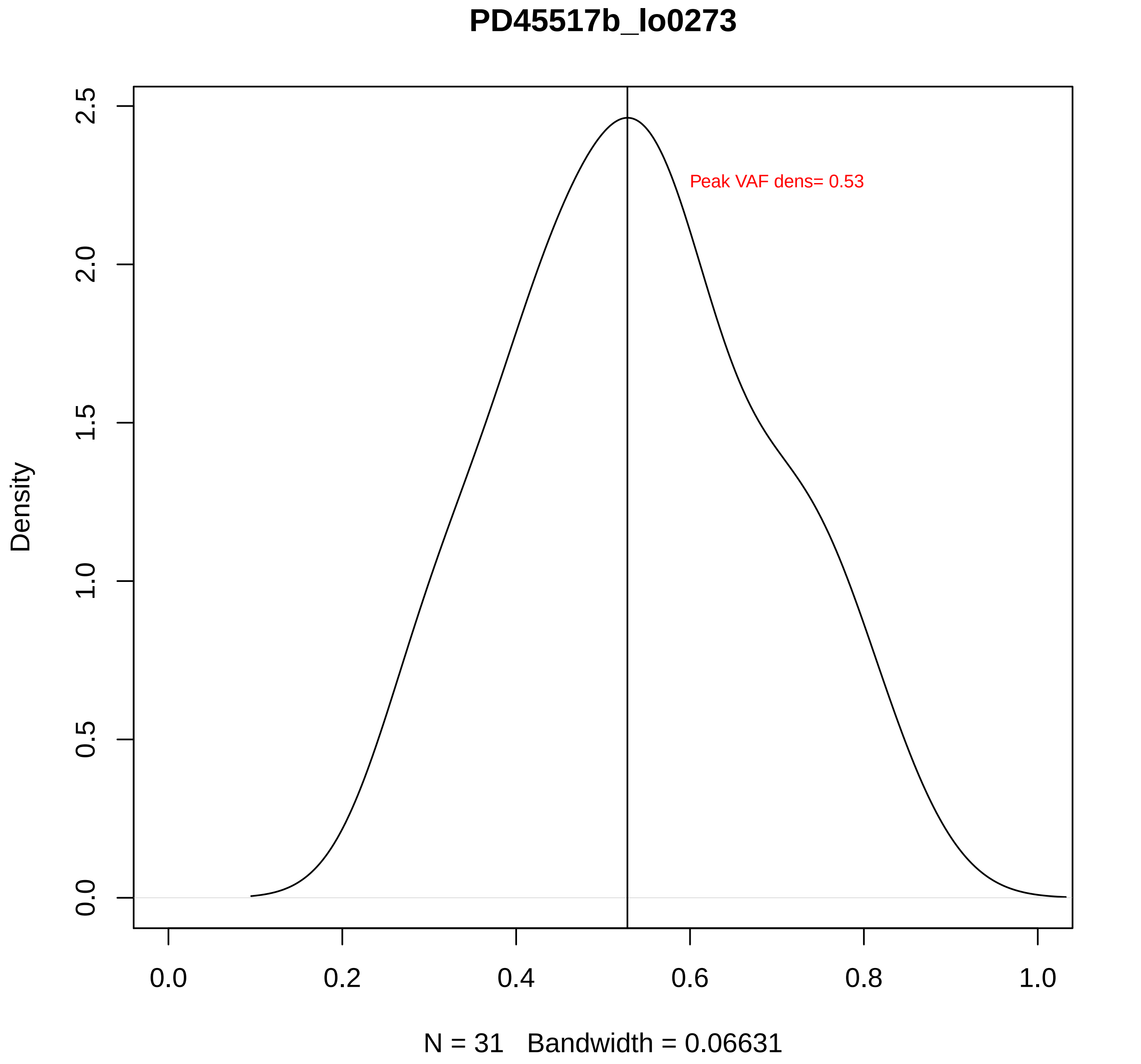
PD45517b_lo0273
Peak VAF dens= 0.53
Density
0.0
0.5
1.0
1.5
2.0
2.5
0.0
0.2
0.4
0.6
0.8
1.0
N = 31 Bandwidth = 0.06631

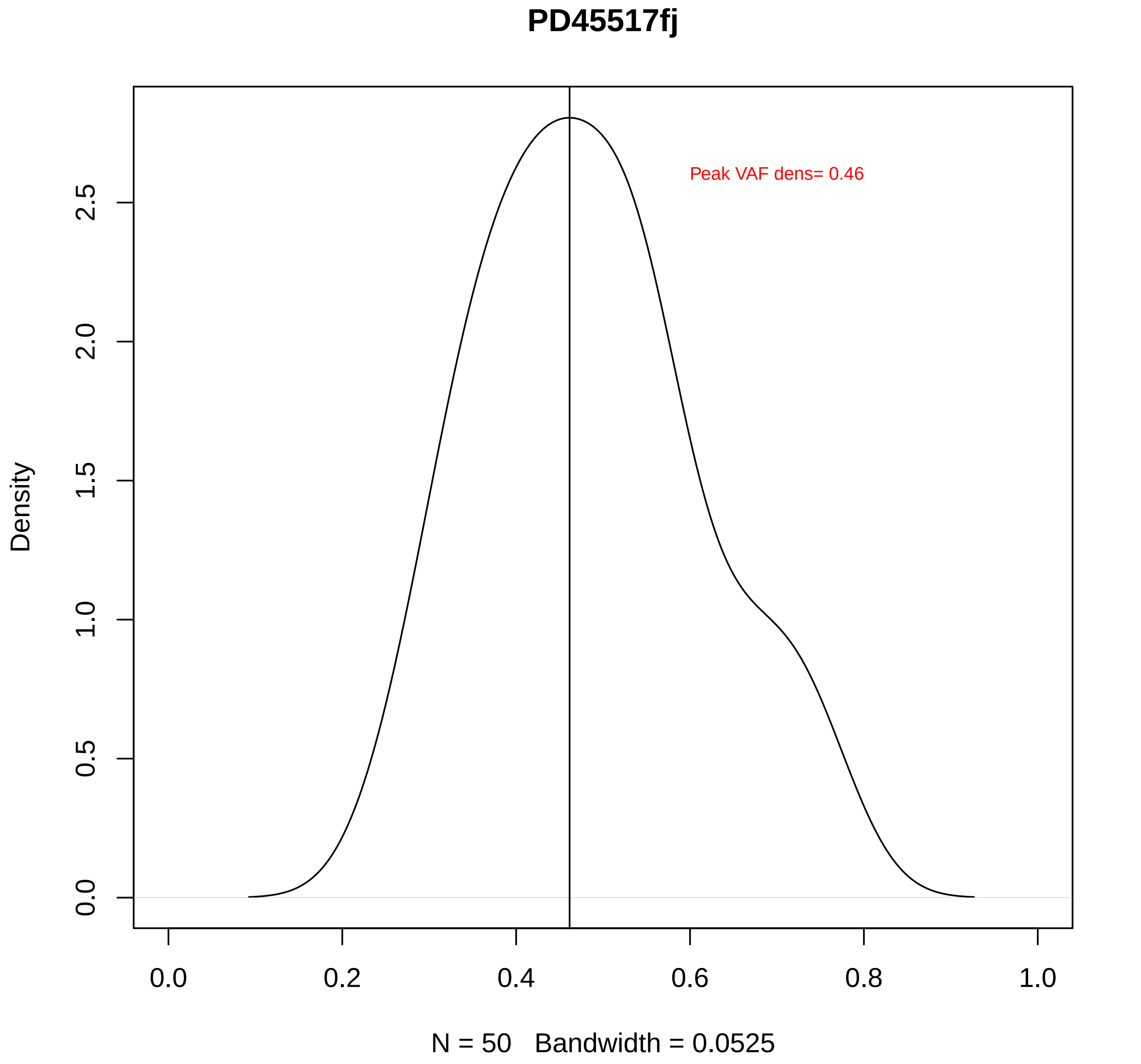
PD45517fj
Peak VAF dens= 0.46
Density
0.0
0.5
1.0
1.5
2.0
2.5
0.0
0.2
0.4
0.6
0.8
1.0
N = 50 Bandwidth = 0.0525

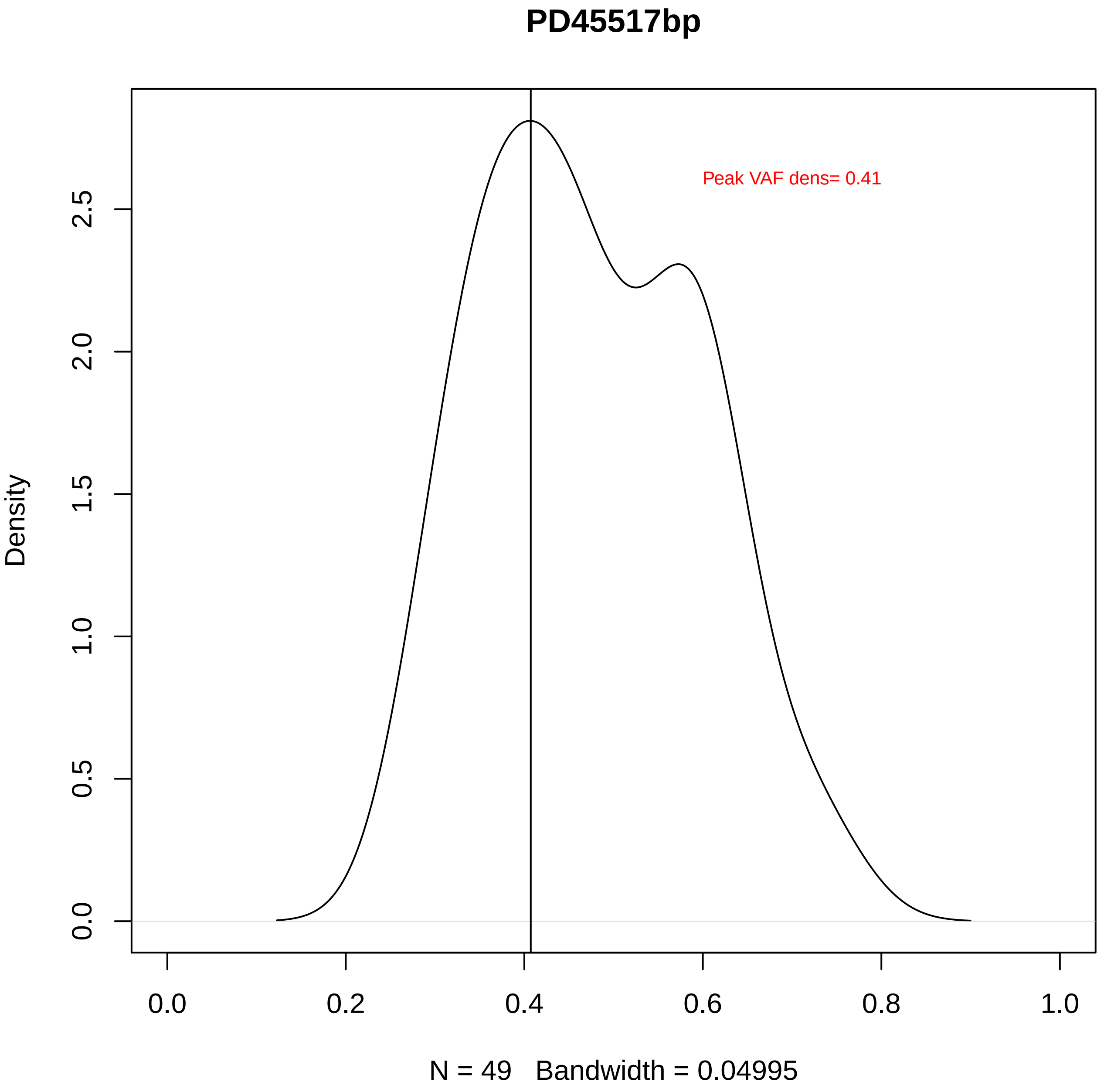
PD45517bp
Peak VAF dens= 0.41
Density
0.0
0.5
1.0
1.5
2.0
2.5
0.0
0.2
0.4
0.6
0.8
1.0
N = 49   Bandwidth = 0.04995

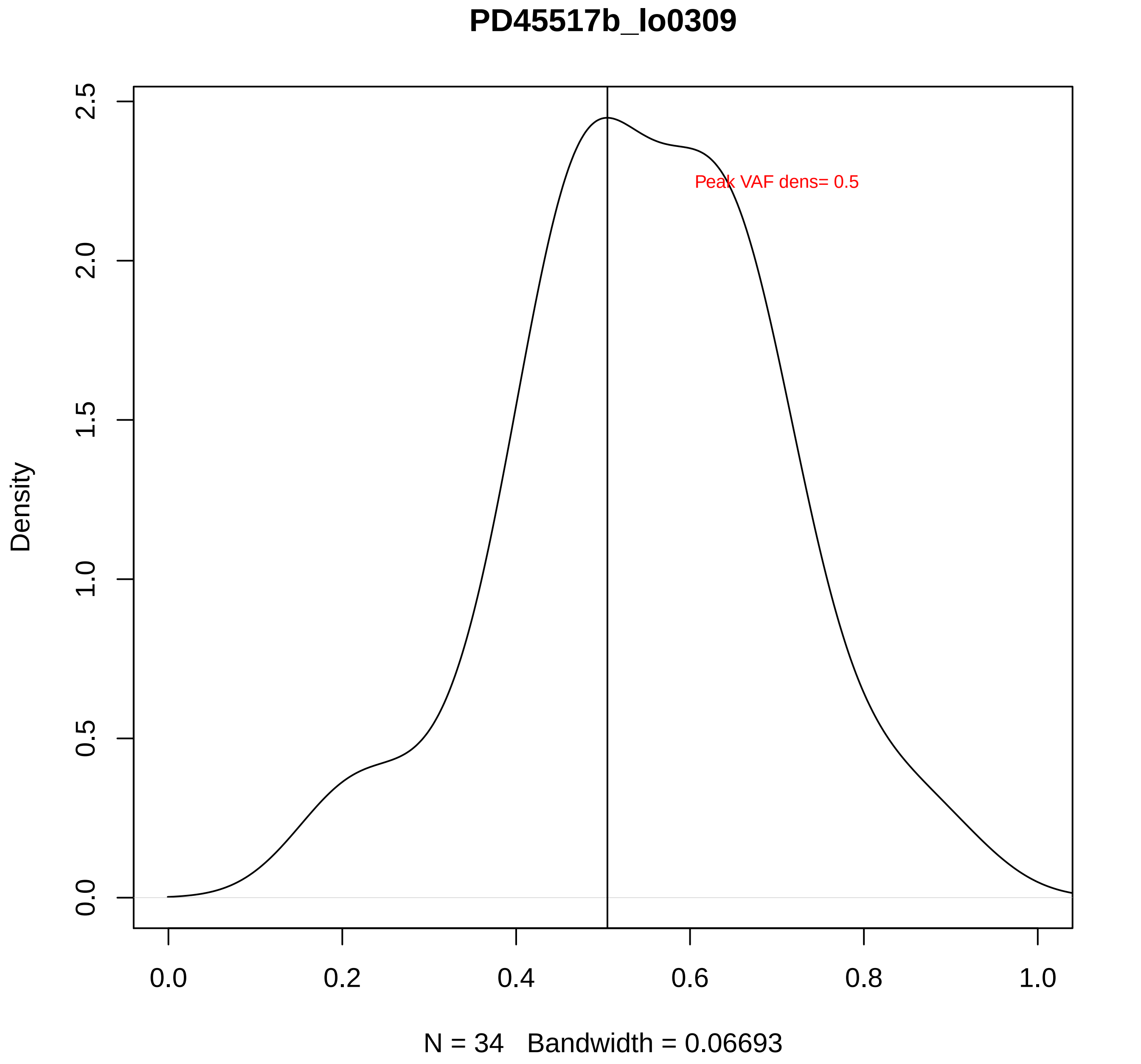
PD45517b_lo0309
Peak VAF dens= 0.5
Density
0.0
0.5
1.0
1.5
2.0
2.5
0.0
0.2
0.4
0.6
0.8
1.0
N = 34 Bandwidth = 0.06693

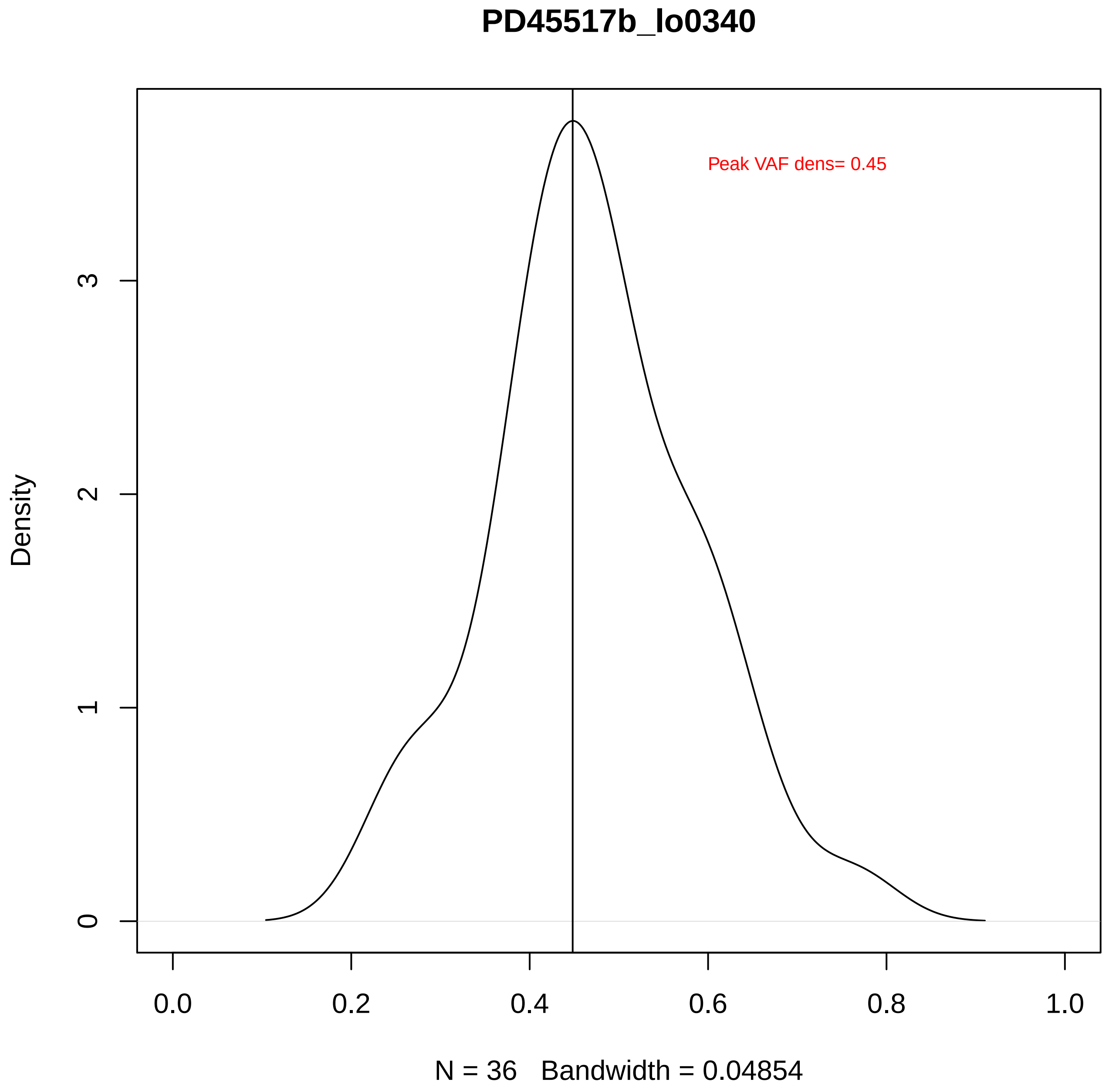
PD45517b_lo0340
Peak VAF dens= 0.45
Density
0
1
2
3
0.0
0.2
0.4
0.6
0.8
1.0
N = 36 Bandwidth = 0.04854

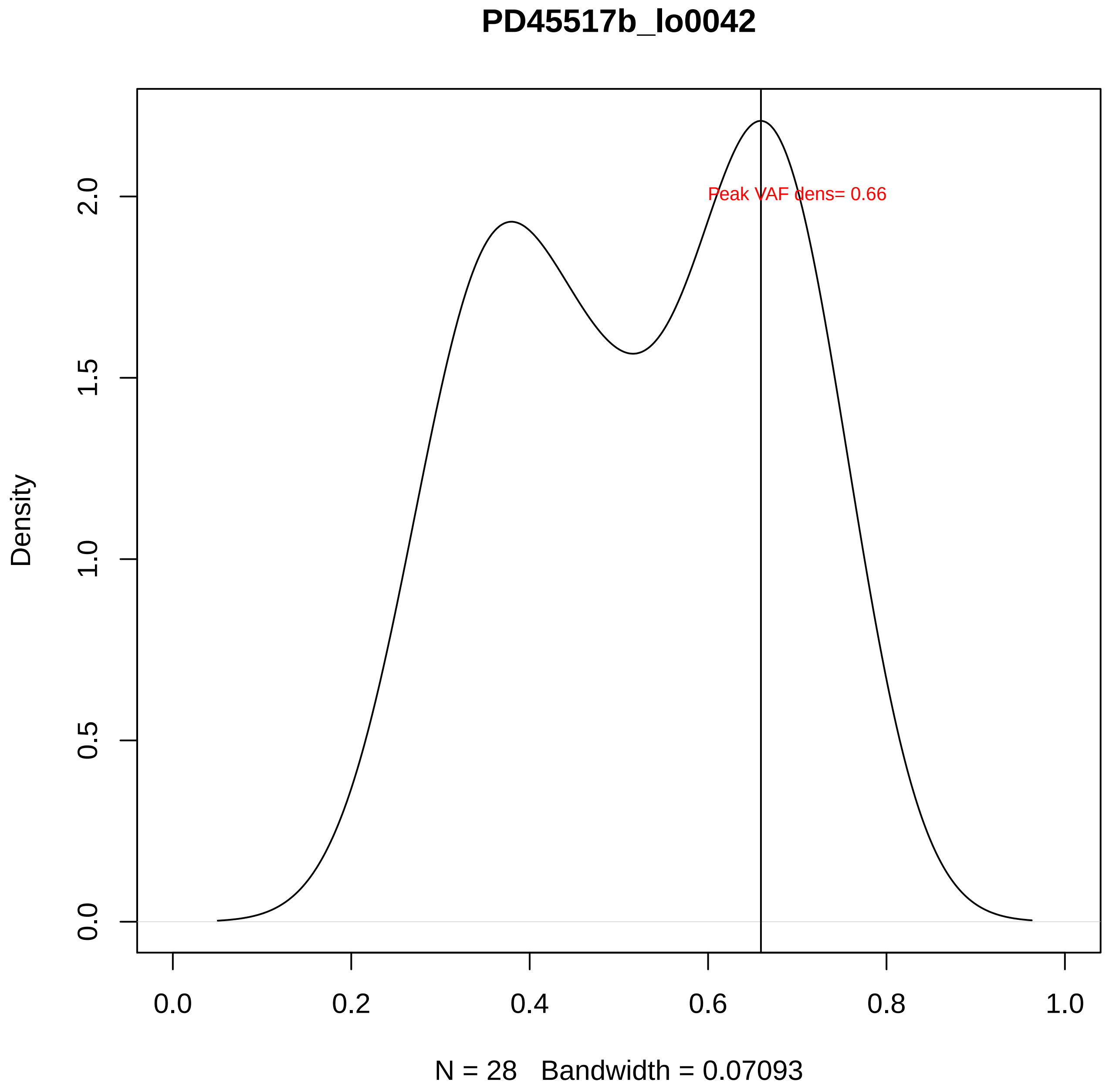
PD45517b_lo0042
Peak VAF dens= 0.66
Density
0.0
0.5
1.0
1.5
2.0
0.0
0.2
0.4
0.6
0.8
1.0
N = 28 Bandwidth = 0.07093

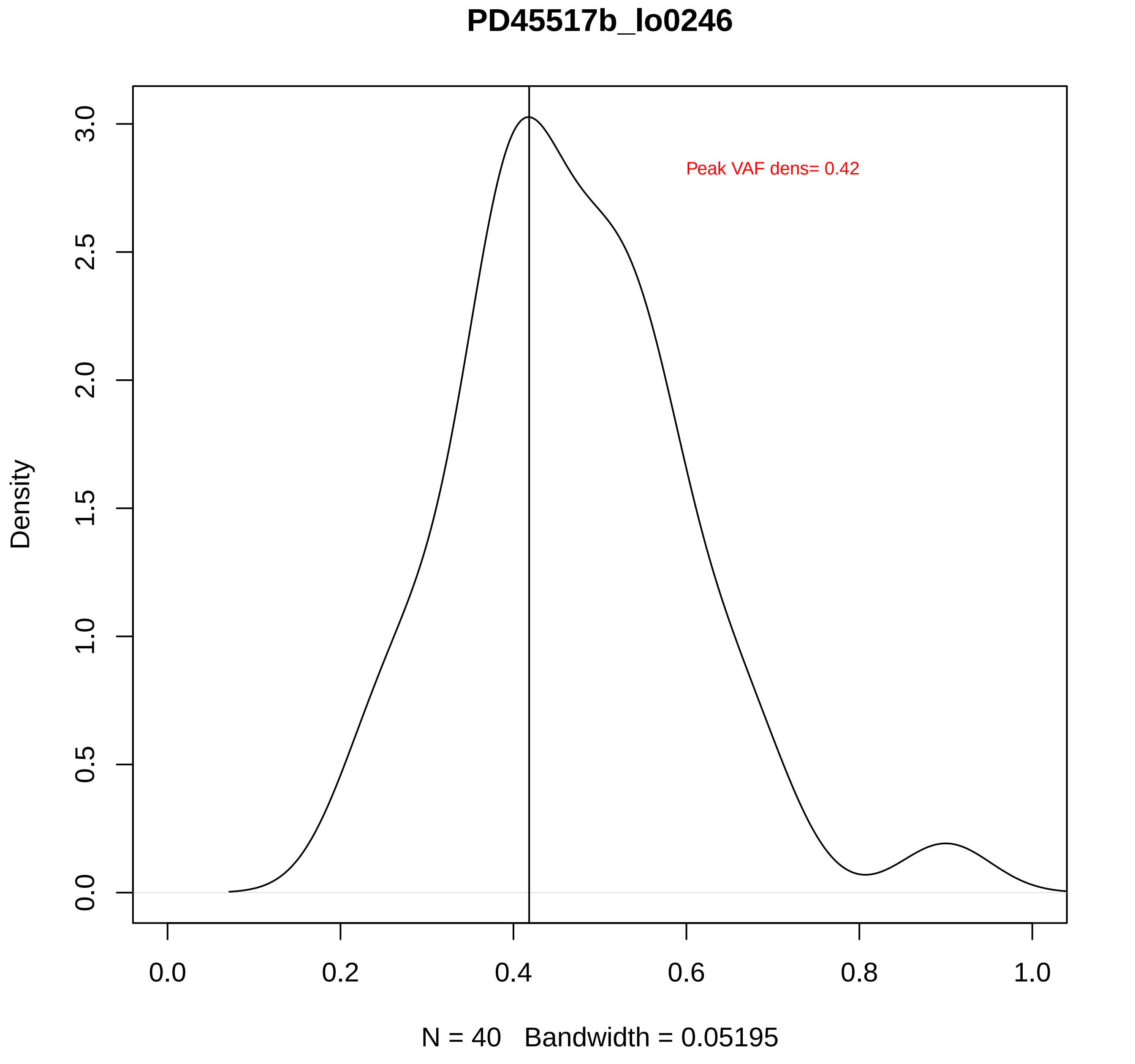

PD45517b_lo0246
Peak VAF dens= 0.42
Density
0.0
0.5
1.0
1.5
2.0
2.5
3.0
0.0
0.2
0.4
0.6
0.8
1.0
N = 40   Bandwidth = 0.05195

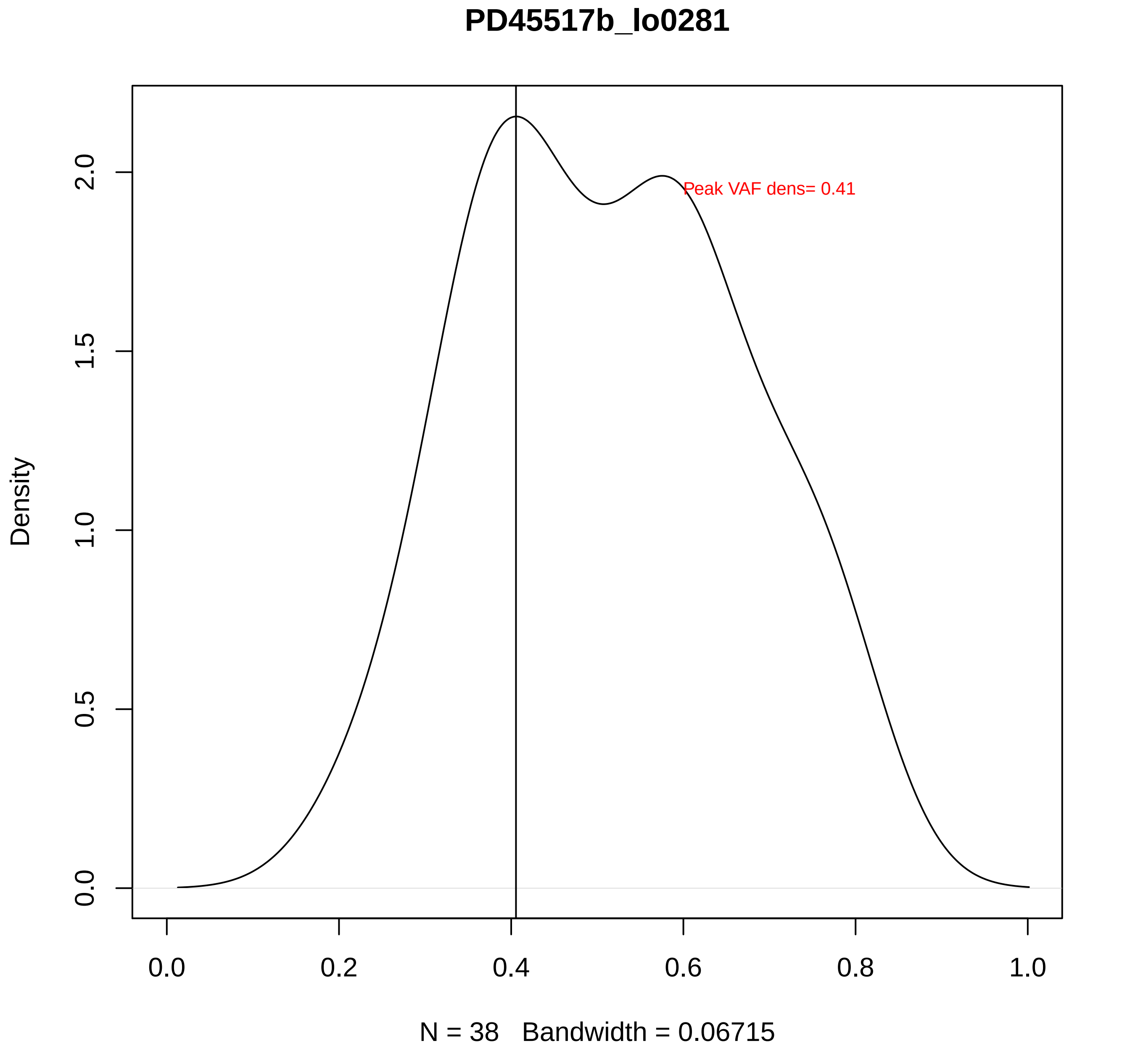
PD45517b_lo0281
Peak VAF dens= 0.41
Density
0.0
0.5
1.0
1.5
2.0
0.0
0.2
0.4
0.6
0.8
1.0
N = 38 Bandwidth = 0.06715

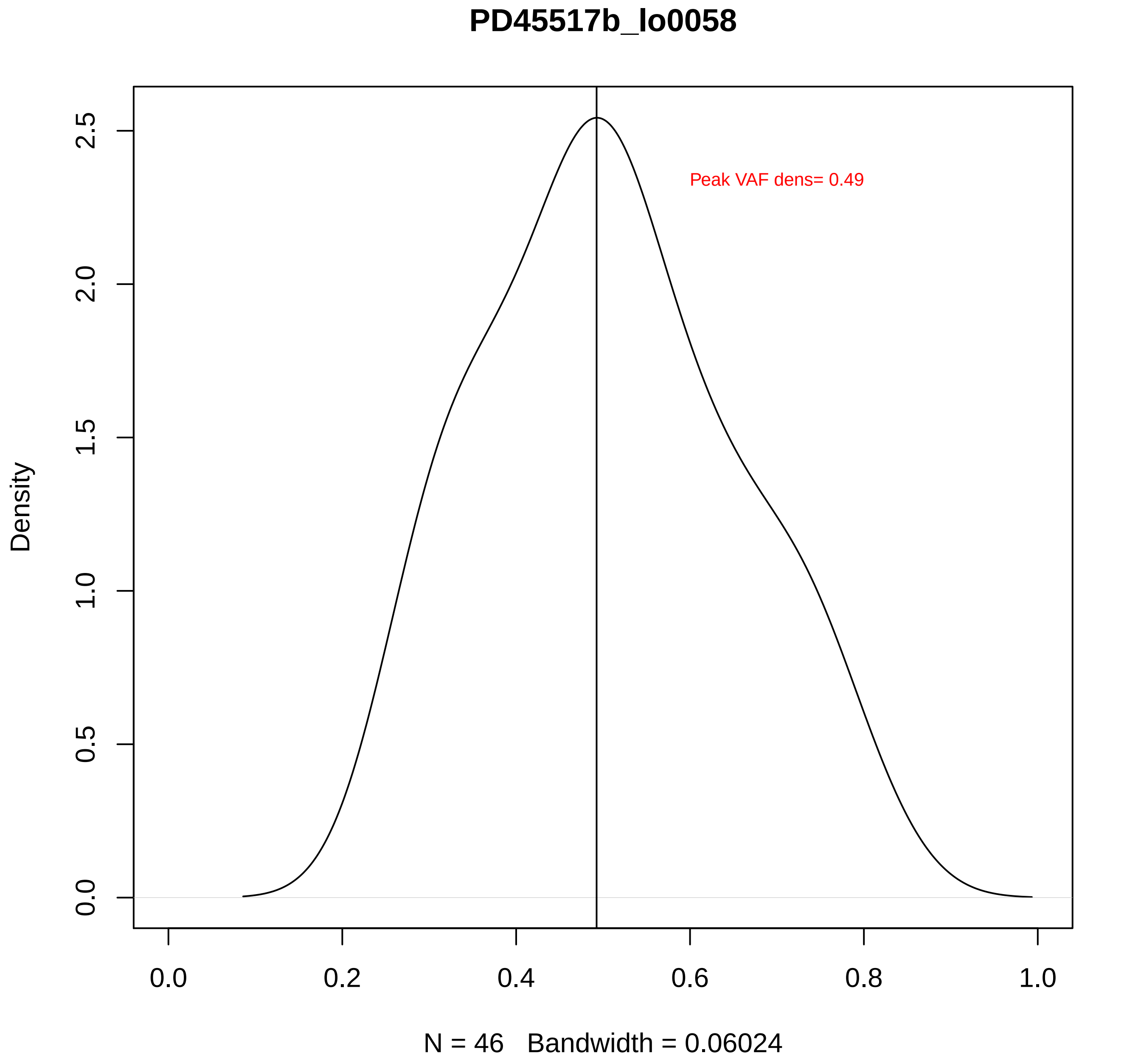
PD45517b_lo0058
Peak VAF dens= 0.49
Density
0.0
0.5
1.0
1.5
2.0
2.5
0.0
0.2
0.4
0.6
0.8
1.0
N = 46   Bandwidth = 0.06024

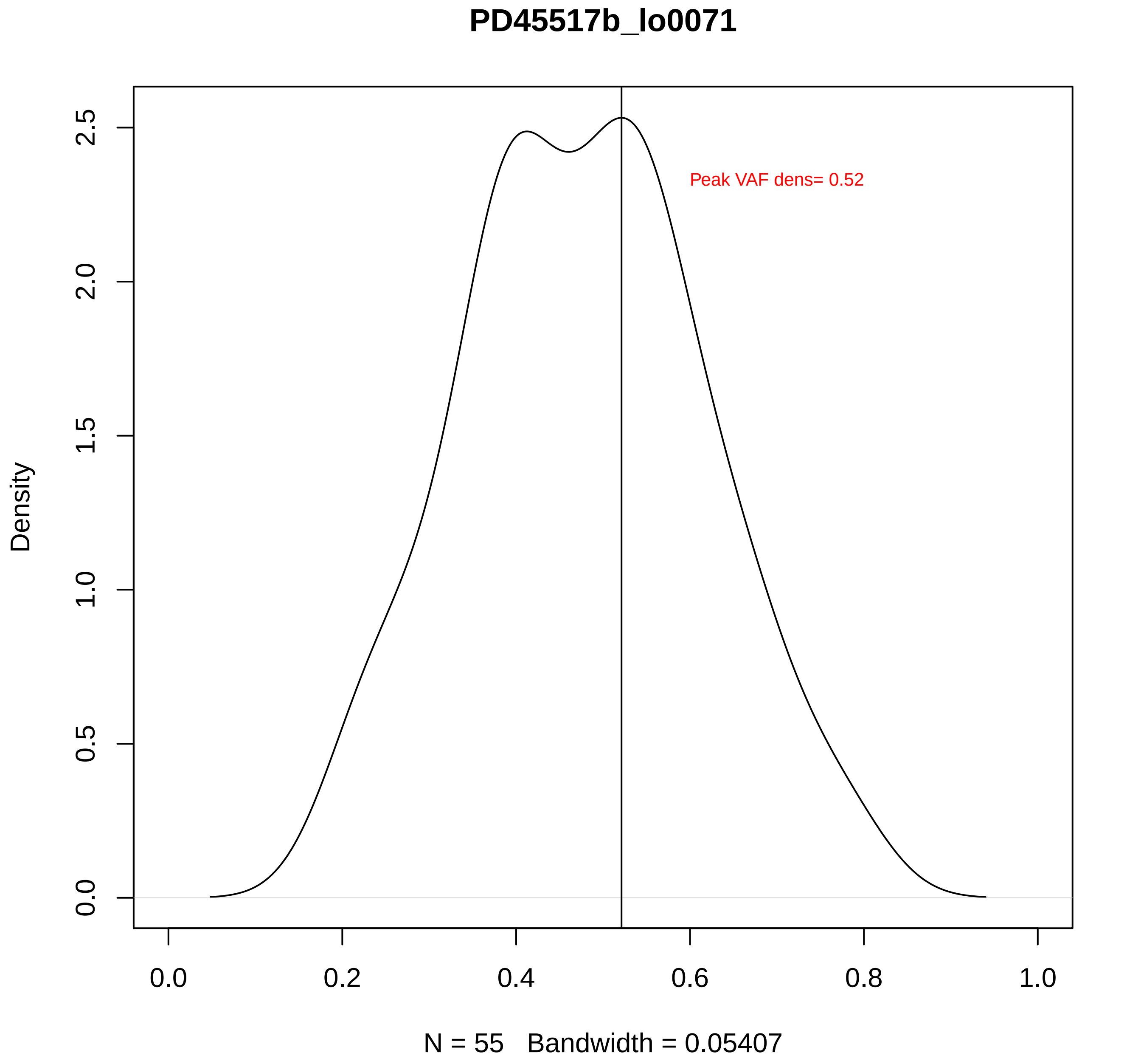
PD45517b_lo0071
Peak VAF dens= 0.52
Density
0.0
0.5
1.0
1.5
2.0
2.5
0.0
0.2
0.4
0.6
0.8
1.0
N = 55 Bandwidth = 0.05407

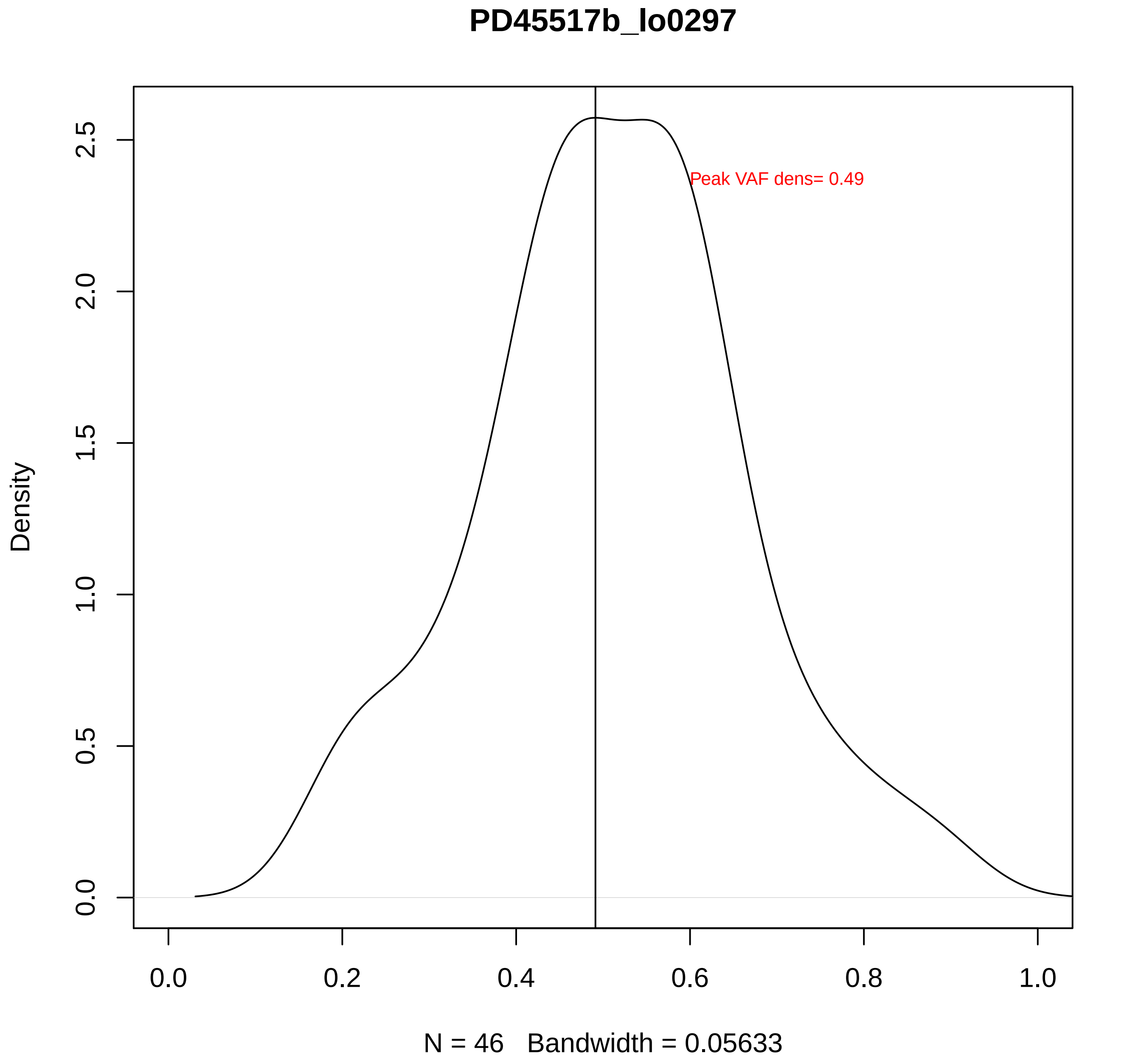
PD45517b_lo0297
Peak VAF dens= 0.49
Density
0.0
0.5
1.0
1.5
2.0
2.5
0.0
0.2
0.4
0.6
0.8
1.0
N = 46   Bandwidth = 0.05633

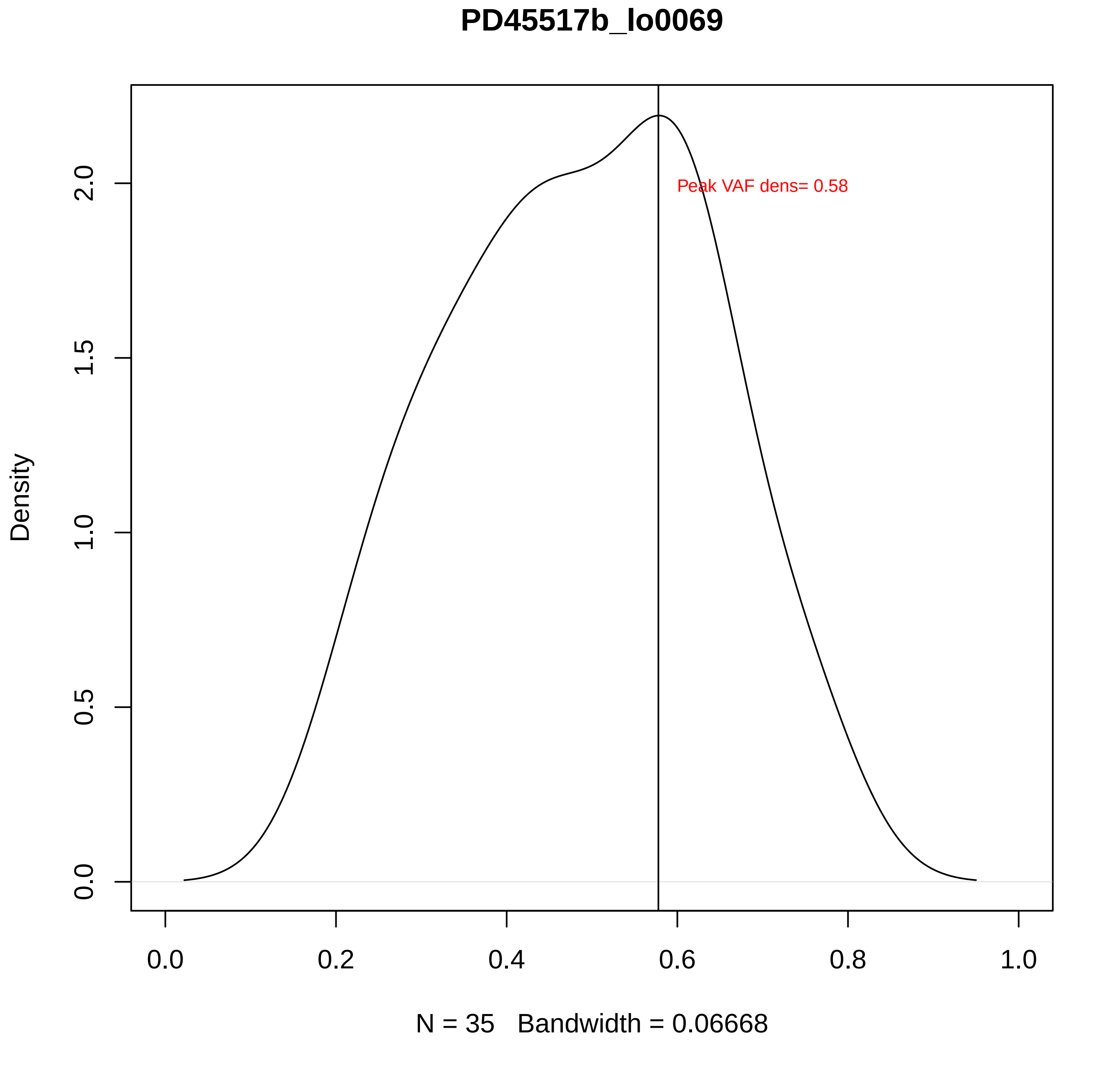
PD45517b_lo0069
Peak VAF dens= 0.58
Density
0.0
0.5
1.0
1.5
2.0
0.0
0.2
0.4
0.6
0.8
1.0
N = 35   Bandwidth = 0.06668

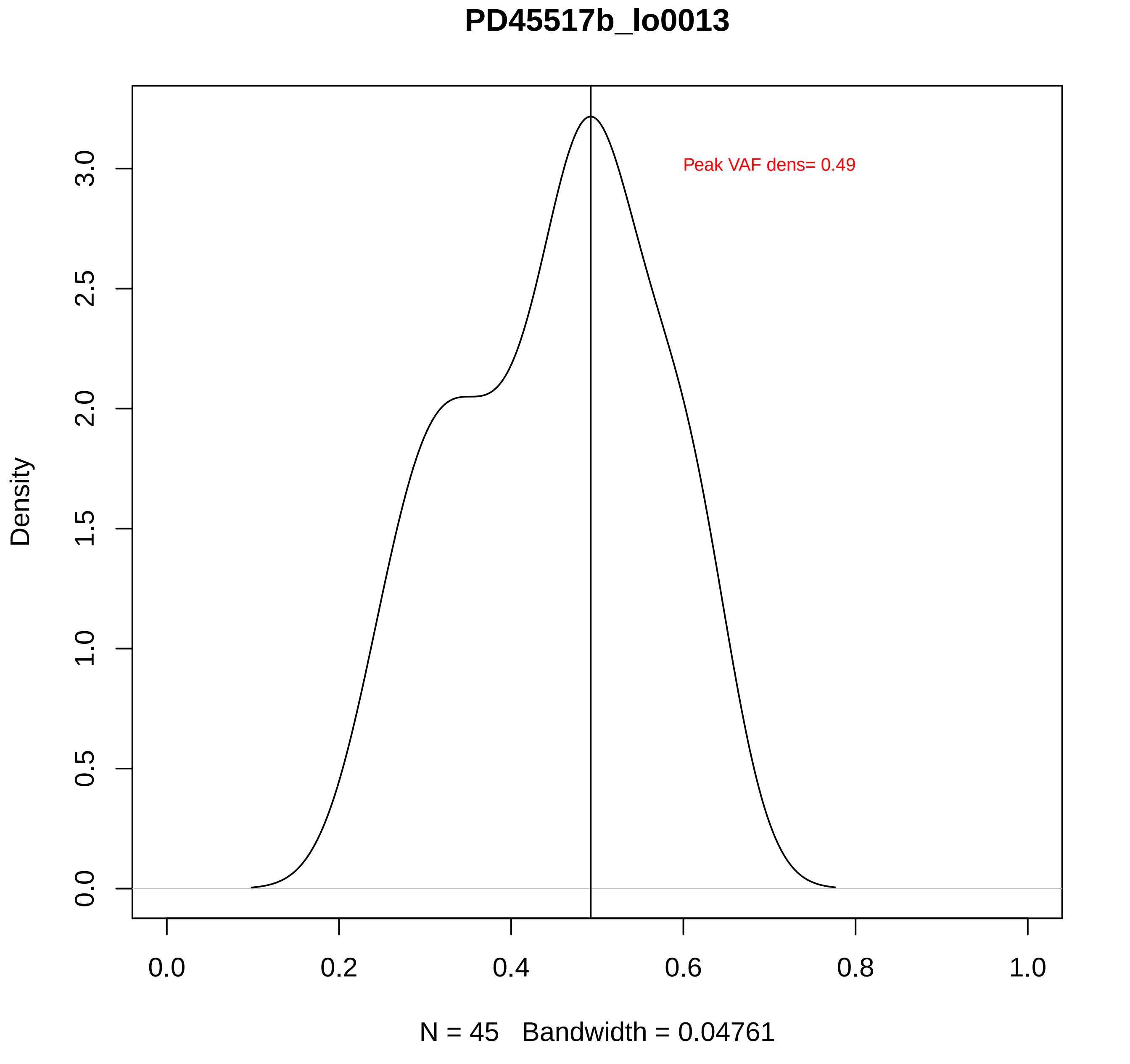
PD45517b_lo0013
Peak VAF dens= 0.49
Density
0.0
0.5
1.0
1.5
2.0
2.5
3.0
0.0
0.2
0.4
0.6
0.8
1.0
N = 45 Bandwidth = 0.04761

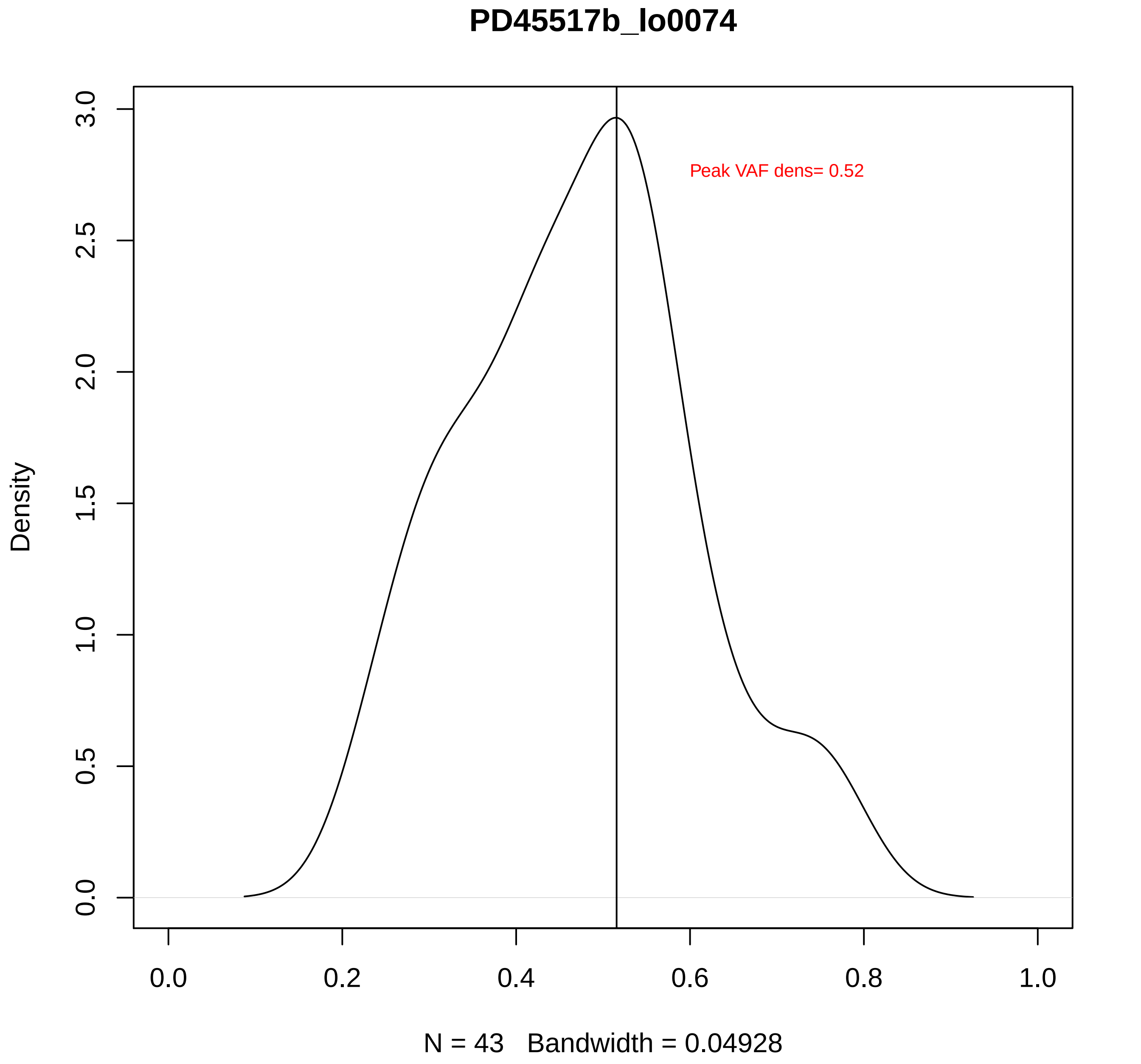
PD45517b_lo0074
Peak VAF dens= 0.52
Density
0.0
0.5
1.0
1.5
2.0
2.5
3.0
0.0
0.2
0.4
0.6
0.8
1.0
N = 43   Bandwidth = 0.04928

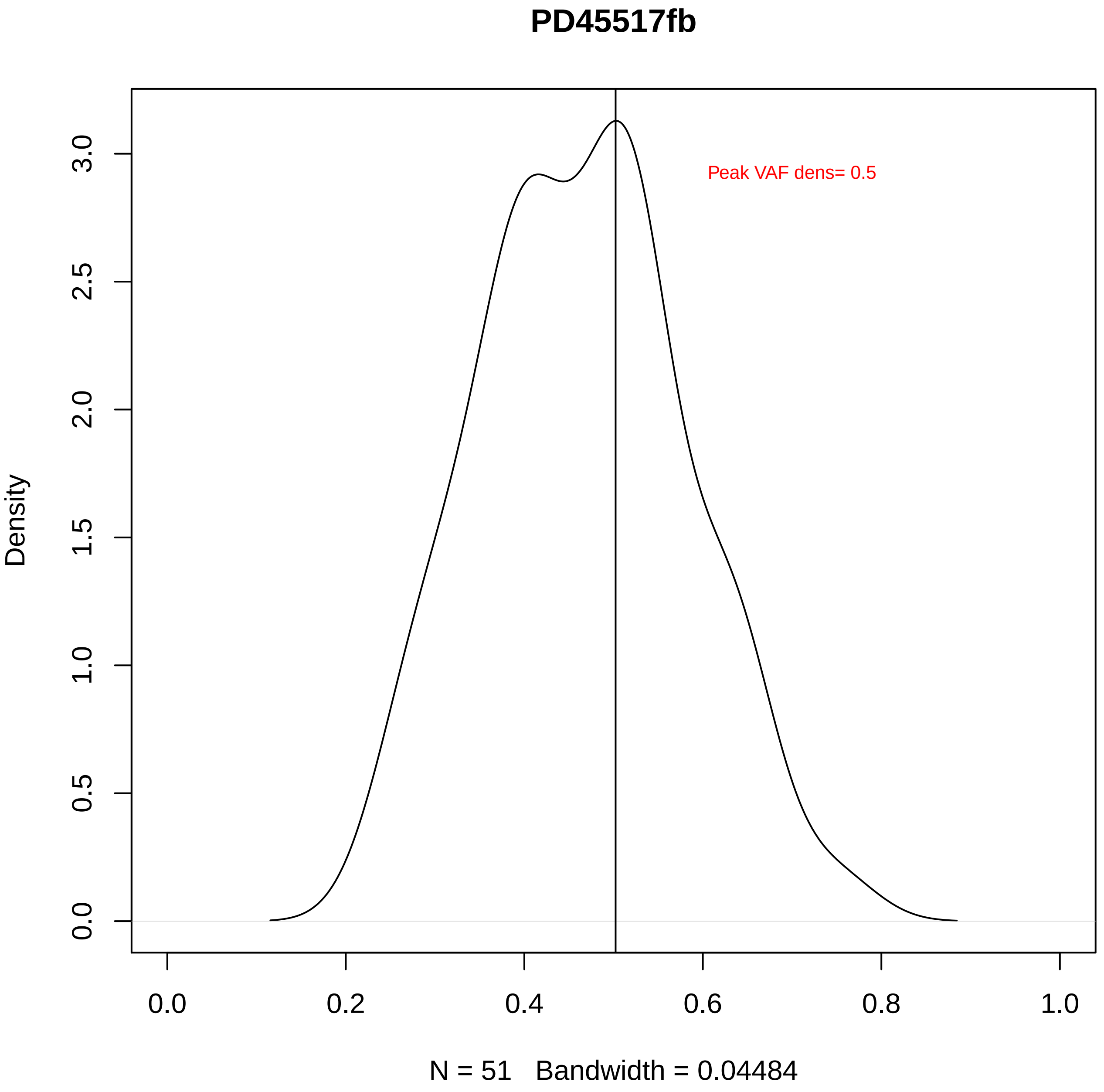
PD45517fb
Peak VAF dens= 0.5
Density
0.0
0.5
1.0
1.5
2.0
2.5
3.0
0.0
0.2
0.4
0.6
0.8
1.0
N = 51 Bandwidth = 0.04484

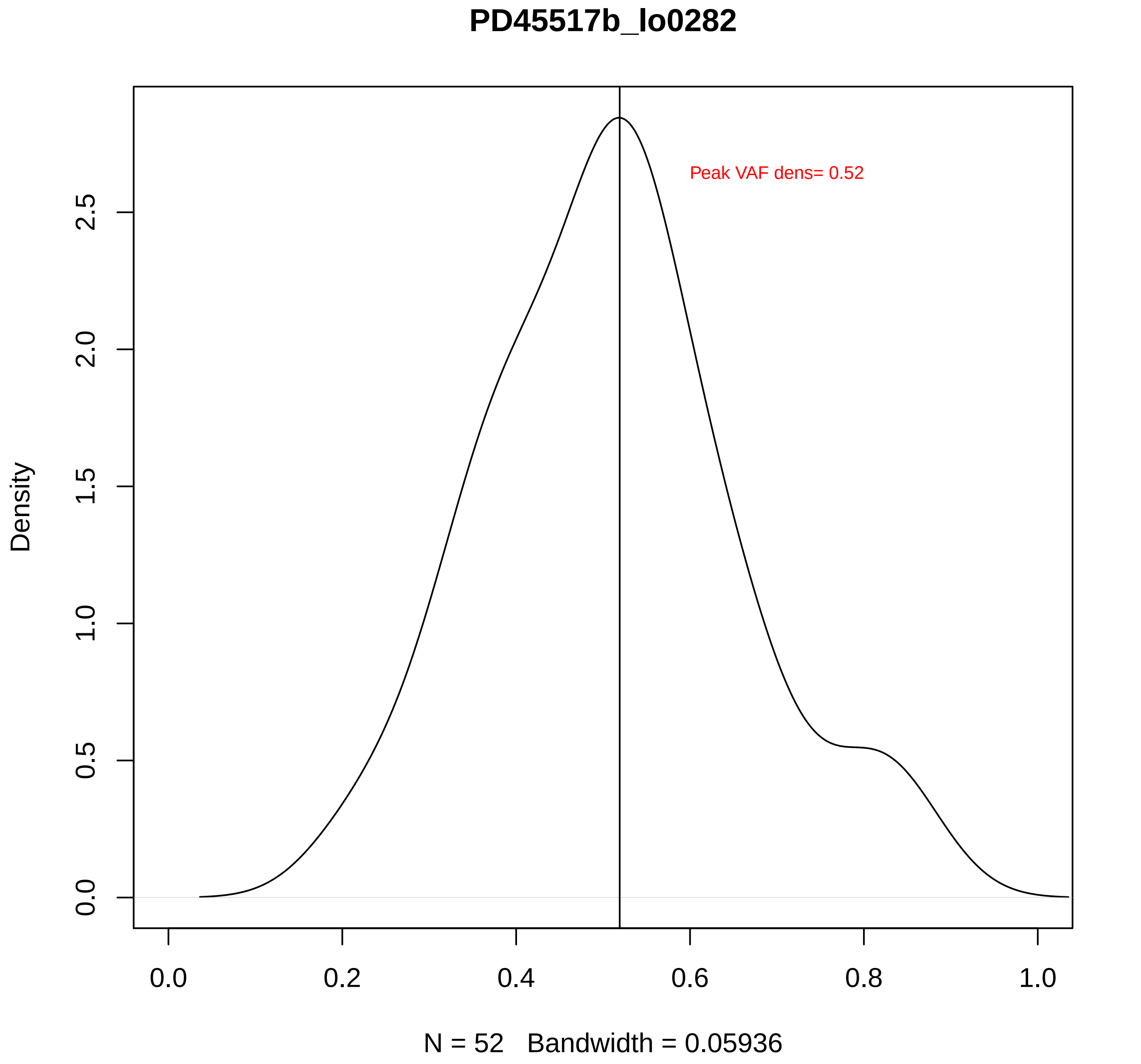

PD45517b_lo0282
Peak VAF dens= 0.52
Density
0.0
0.5
1.0
1.5
2.0
2.5
0.0
0.2
0.4
0.6
0.8
1.0
N = 52 Bandwidth = 0.05936

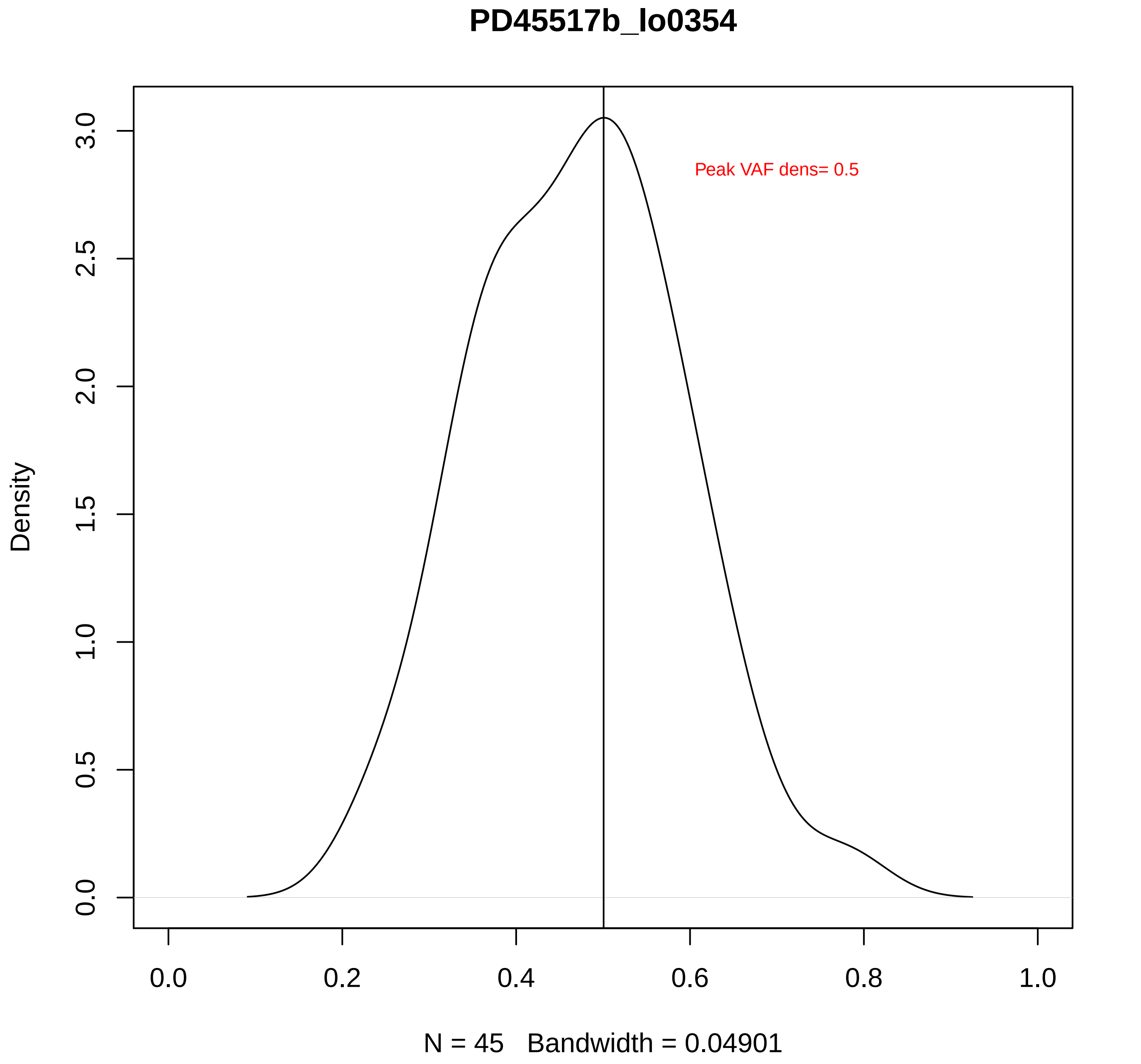

PD45517b_lo0354
Peak VAF dens= 0.5
Density
0.0
0.5
1.0
1.5
2.0
2.5
3.0
0.0
0.2
0.4
0.6
0.8
1.0
N = 45 Bandwidth = 0.04901

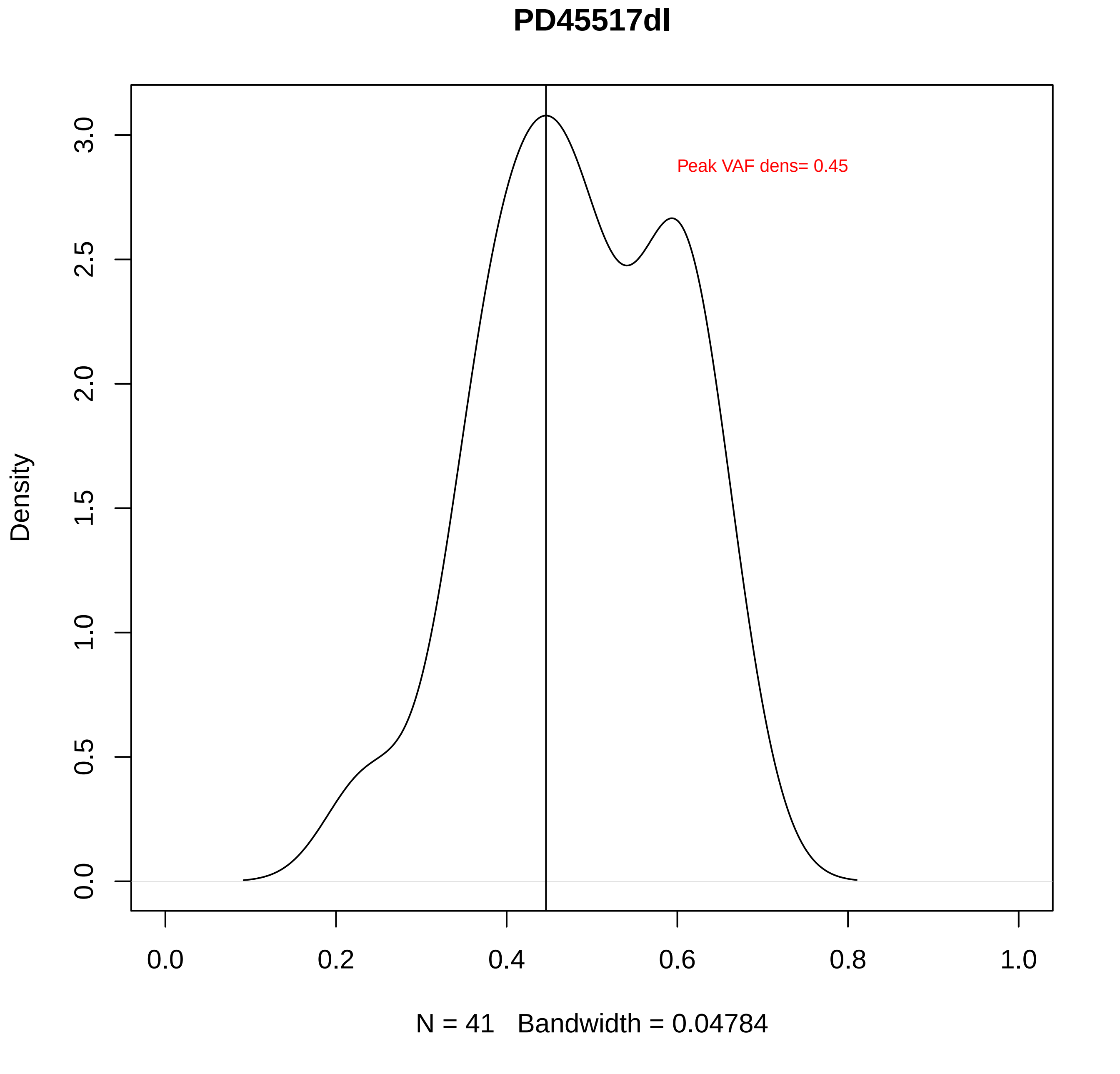
PD45517dl
Peak VAF dens= 0.45
Density
0.0
0.5
1.0
1.5
2.0
2.5
3.0
0.0
0.2
0.4
0.6
0.8
1.0
N = 41   Bandwidth = 0.04784

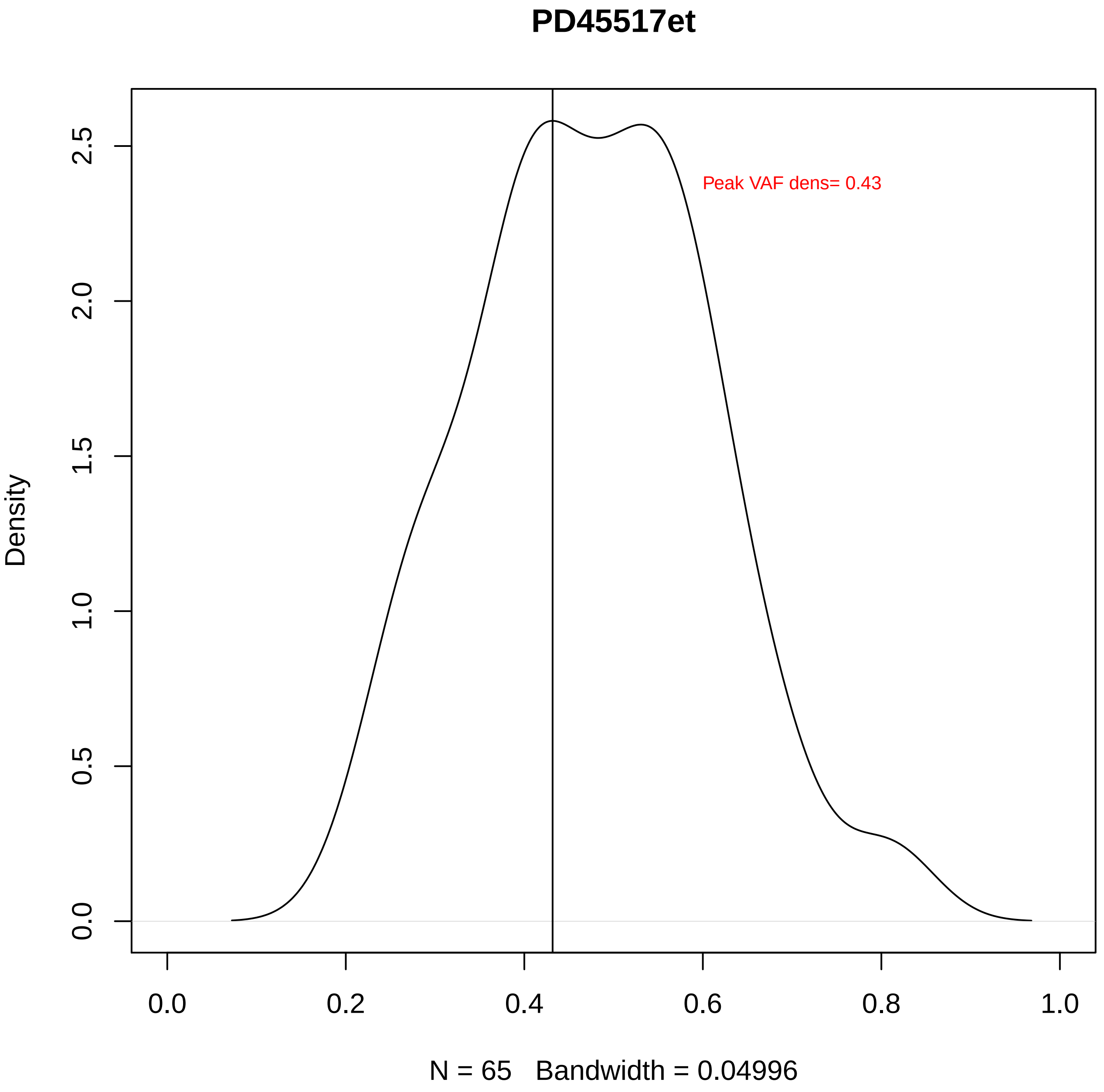
PD45517et
Peak VAF dens= 0.43
Density
0.0
0.5
1.0
1.5
2.0
2.5
0.0
0.2
0.4
0.6
0.8
1.0
N = 65 Bandwidth = 0.04996

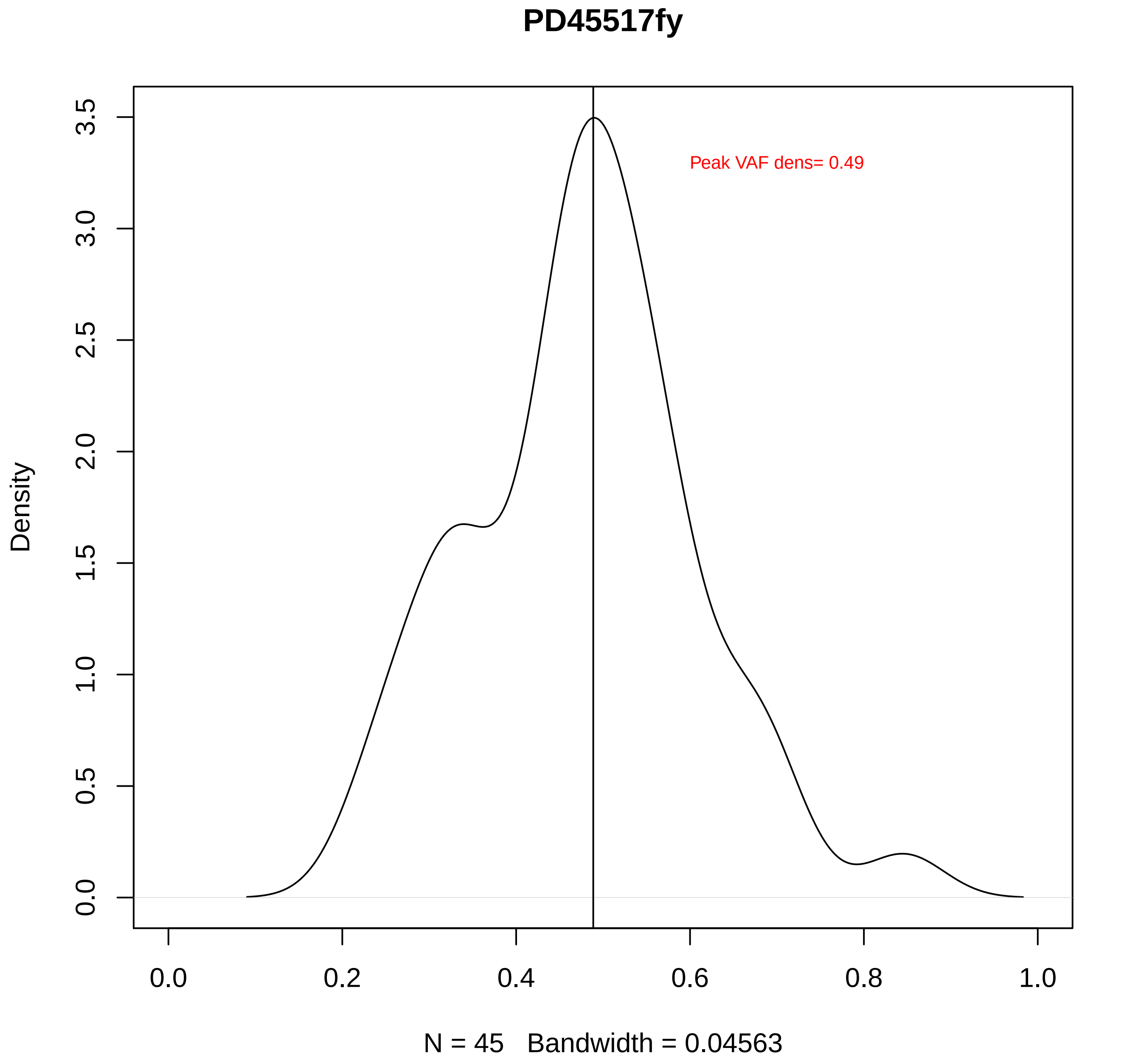

PD45517fy
Peak VAF dens= 0.49
Density
0.0
0.5
1.0
1.5
2.0
2.5
3.0
3.5
0.0
0.2
0.4
0.6
0.8
1.0
N = 45 Bandwidth = 0.04563

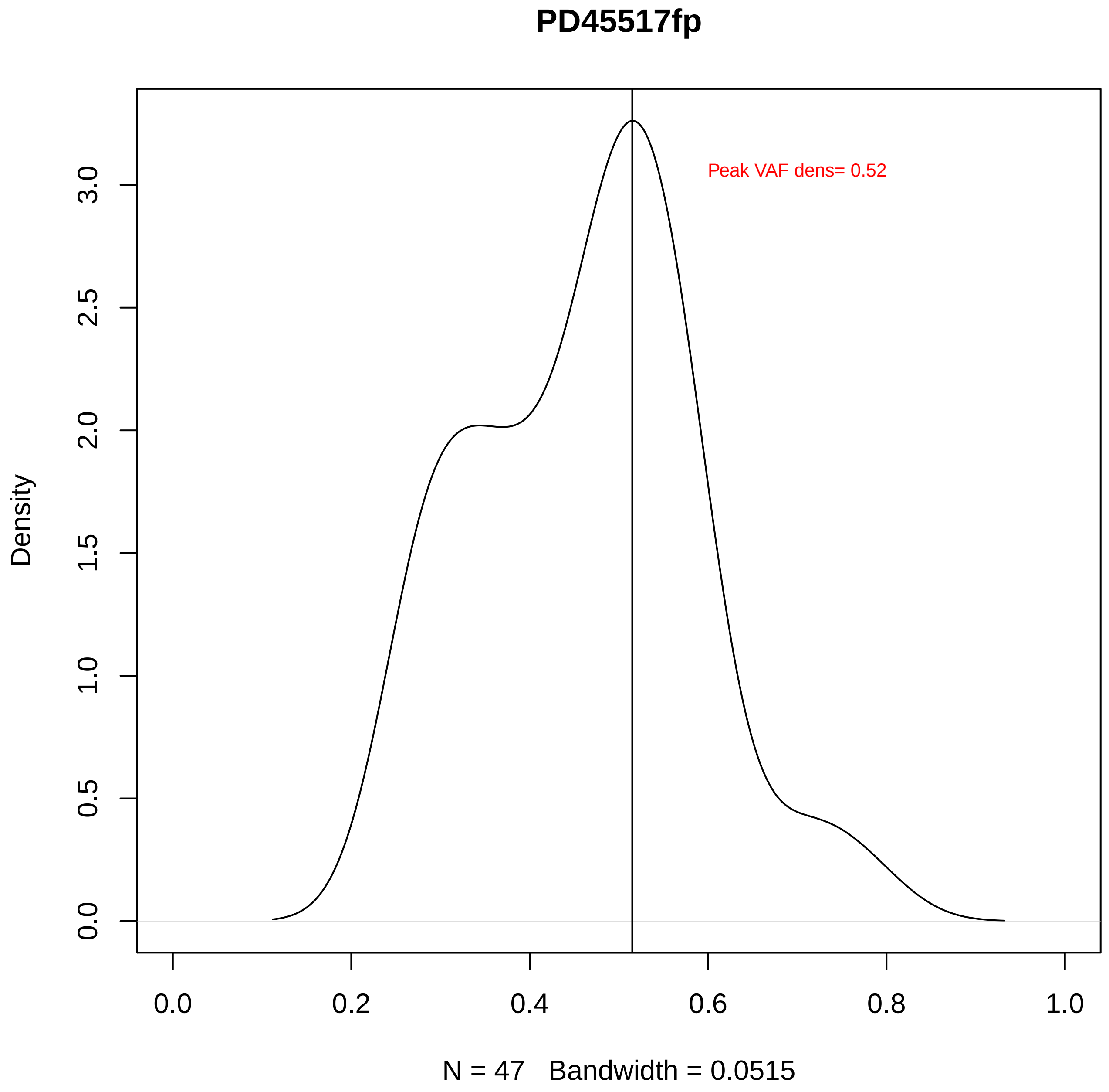
PD45517fp
Peak VAF dens= 0.52
Density
0.0
0.5
1.0
1.5
2.0
2.5
3.0
0.0
0.2
0.4
0.6
0.8
1.0
N = 47   Bandwidth = 0.0515

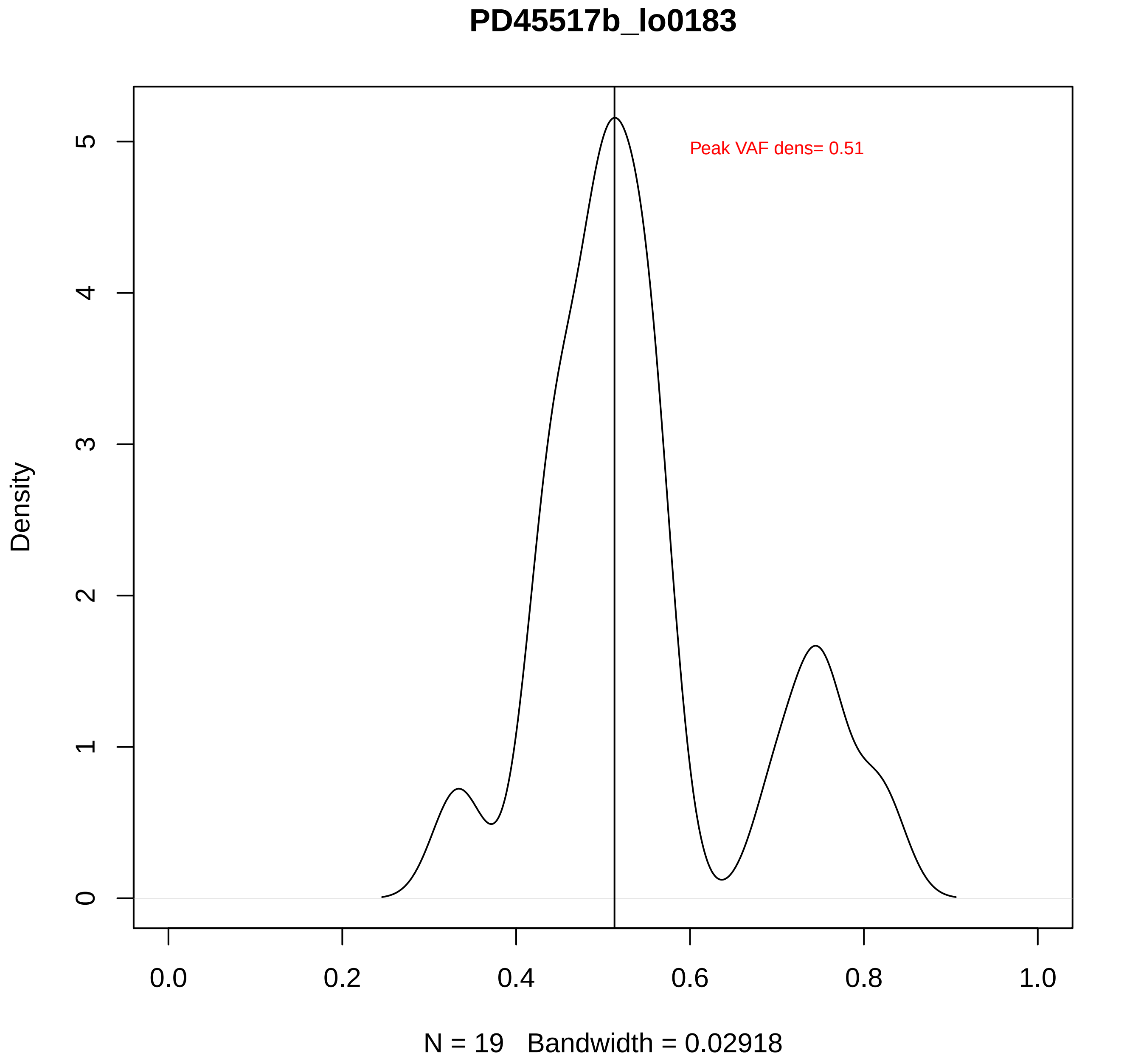
PD45517b_lo0183
Peak VAF dens= 0.51
Density
0
1
2
3
4
5
0.0
0.2
0.4
0.6
0.8
1.0
N = 19 Bandwidth = 0.02918

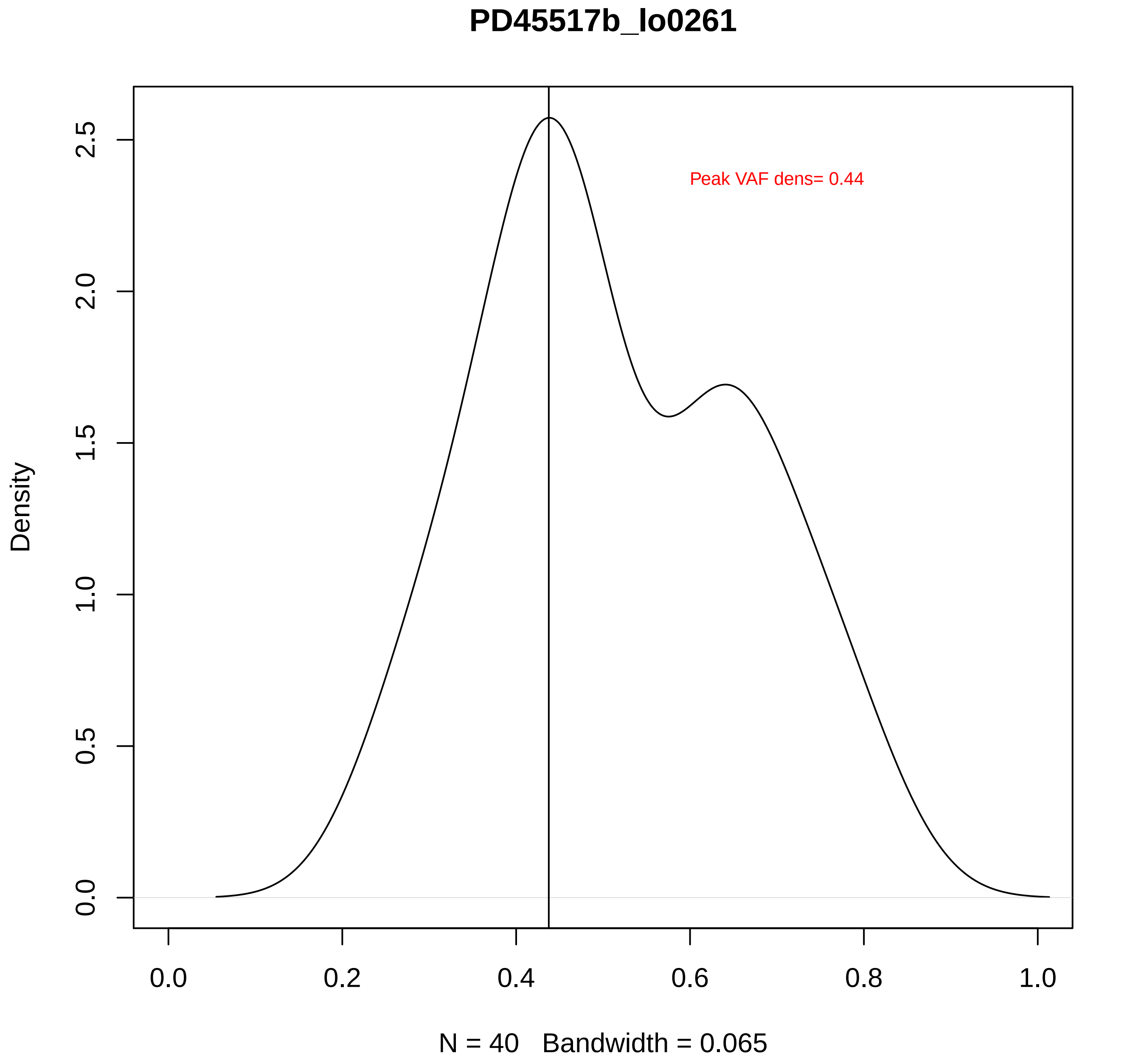
PD45517b_lo0261
Peak VAF dens= 0.44
Density
0.0
0.5
1.0
1.5
2.0
2.5
0.0
0.2
0.4
0.6
0.8
1.0
N = 40   Bandwidth = 0.065

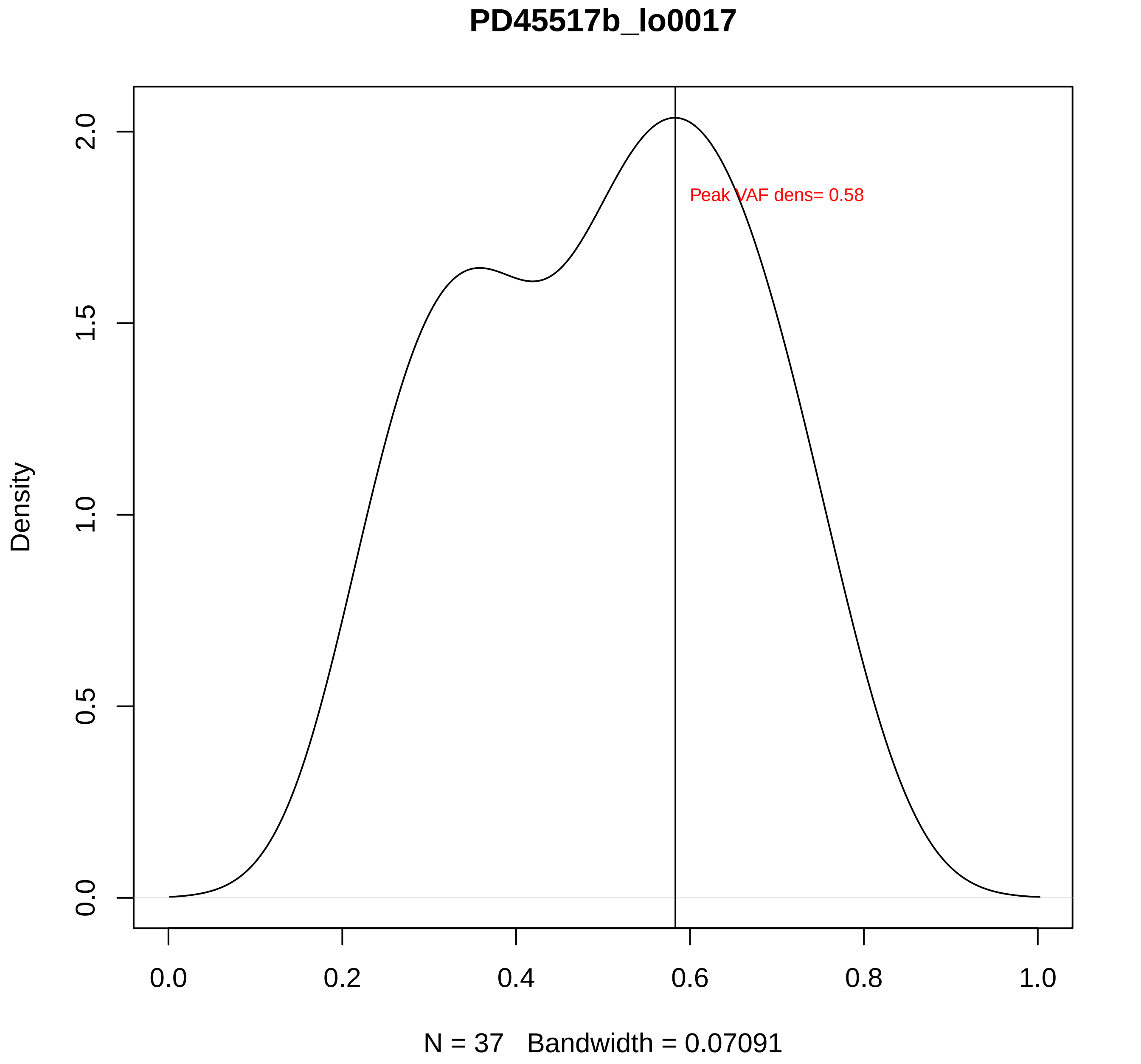

PD45517b_lo0017
Peak VAF dens= 0.58
Density
0.0
0.5
1.0
1.5
2.0
0.0
0.2
0.4
0.6
0.8
1.0
N = 37 Bandwidth = 0.07091

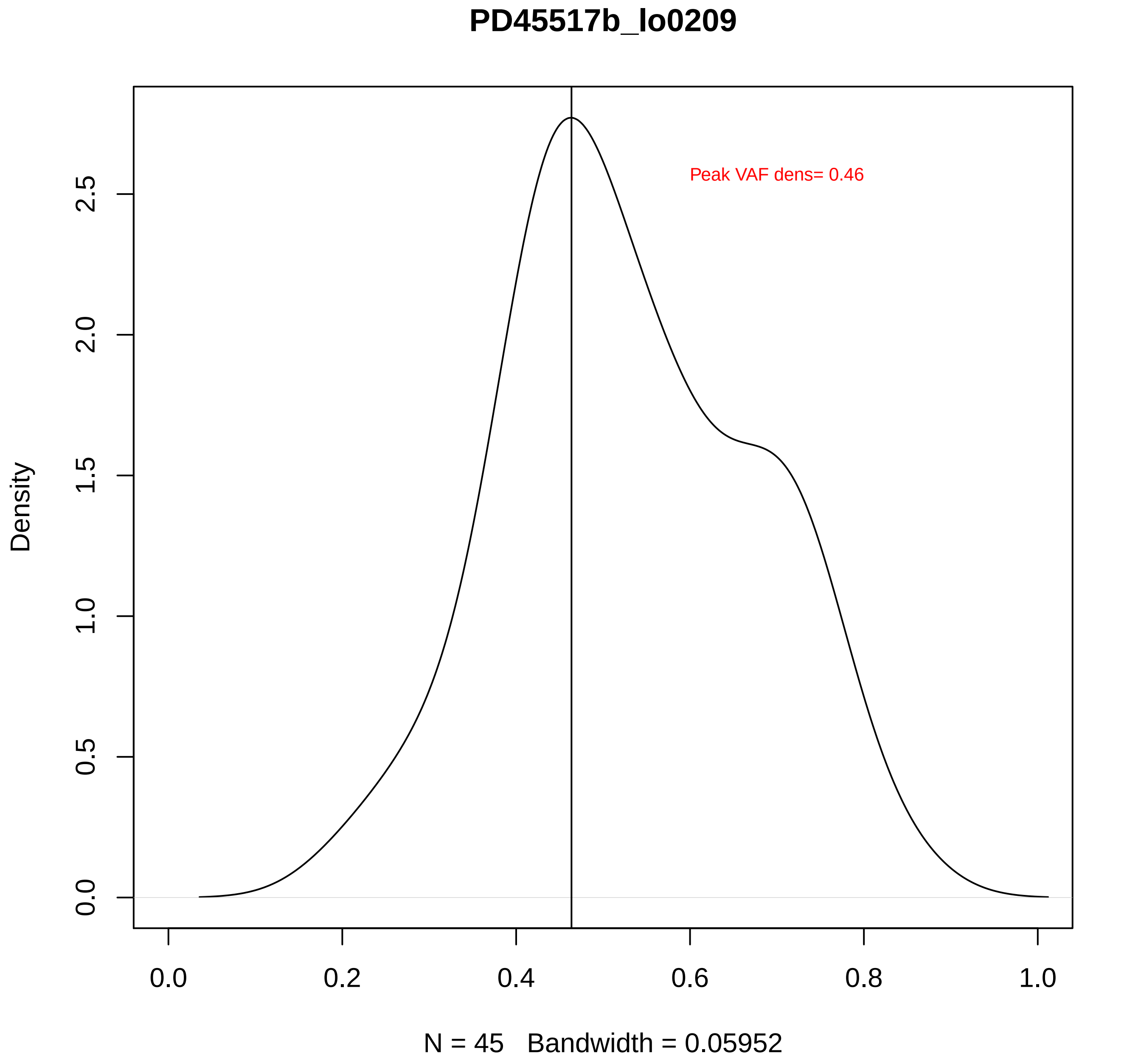

PD45517b_lo0209
Peak VAF dens= 0.46
Density
0.0
0.5
1.0
1.5
2.0
2.5
0.0
0.2
0.4
0.6
0.8
1.0
N = 45 Bandwidth = 0.05952

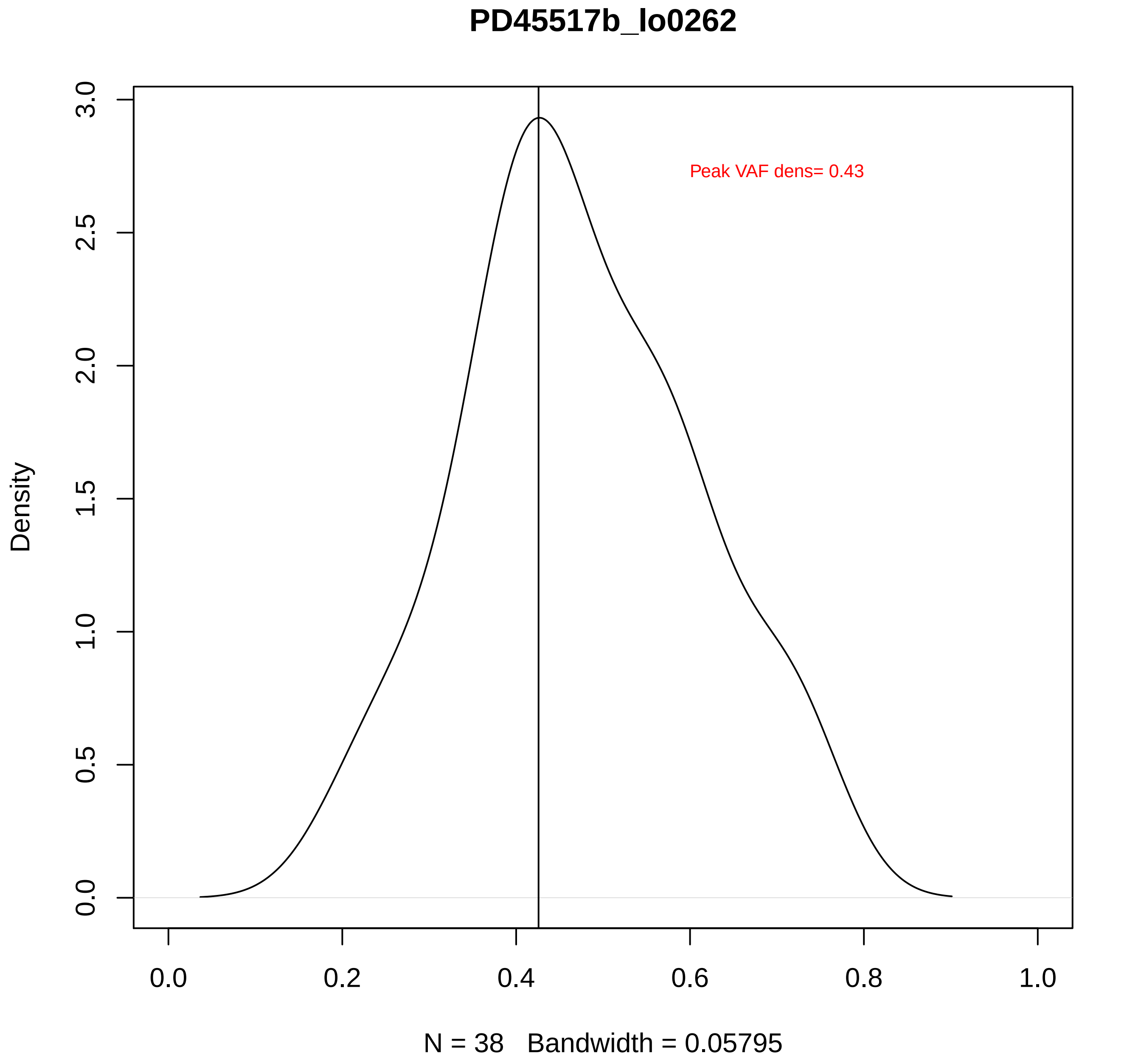
PD45517b_lo0262
Peak VAF dens= 0.43
Density
0.0
0.5
1.0
1.5
2.0
2.5
3.0
0.0
0.2
0.4
0.6
0.8
1.0
N = 38 Bandwidth = 0.05795

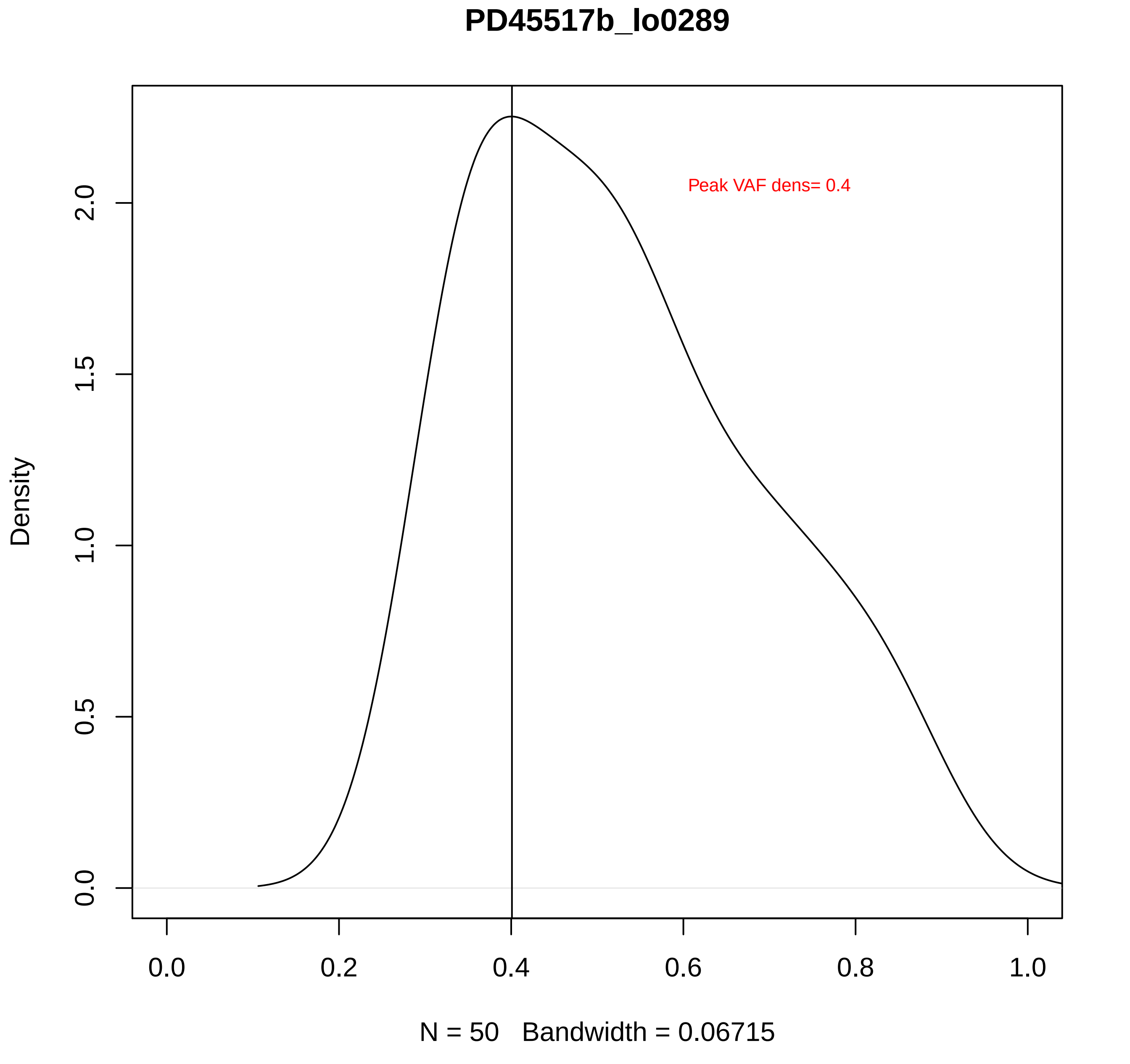

PD45517b_lo0289
Peak VAF dens= 0.4
Density
0.0
0.5
1.0
1.5
2.0
0.0
0.2
0.4
0.6
0.8
1.0
N = 50 Bandwidth = 0.06715

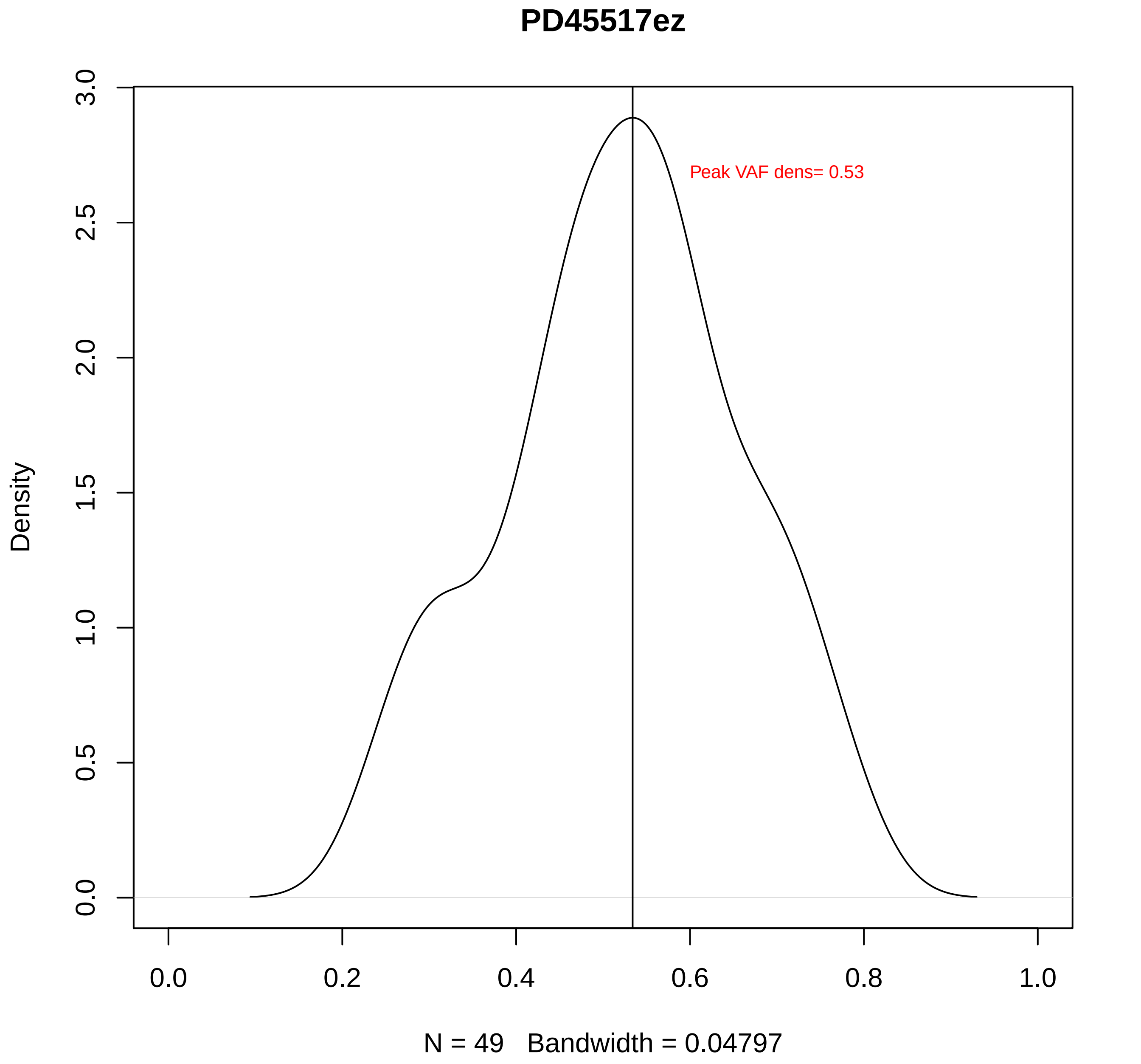
PD45517ez
Peak VAF dens= 0.53
Density
0.0
0.5
1.0
1.5
2.0
2.5
3.0
0.0
0.2
0.4
0.6
0.8
1.0
N = 49   Bandwidth = 0.04797

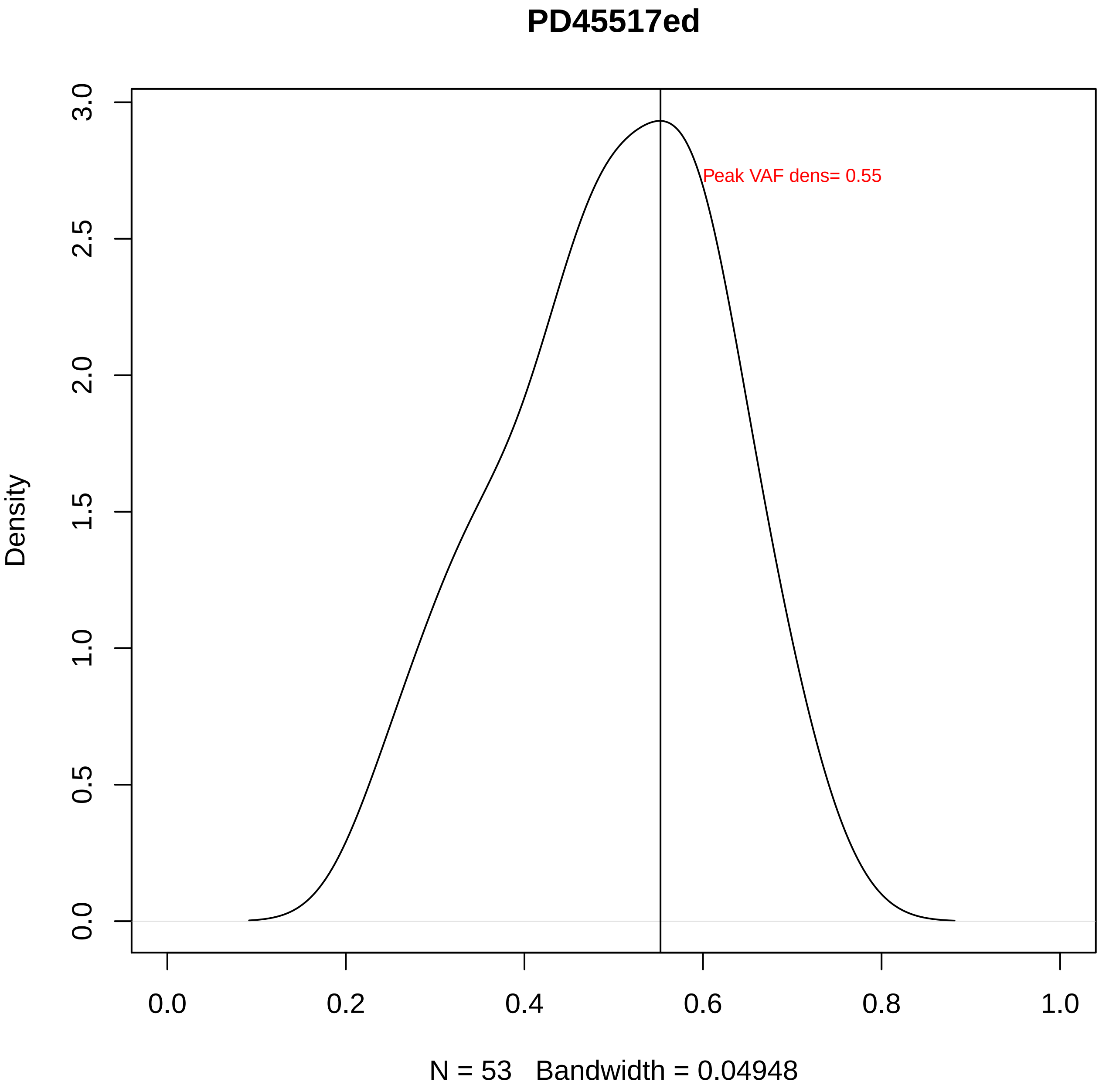
PD45517ed
Peak VAF dens= 0.55
Density
0.0
0.5
1.0
1.5
2.0
2.5
3.0
0.0
0.2
0.4
0.6
0.8
1.0
N = 53   Bandwidth = 0.04948

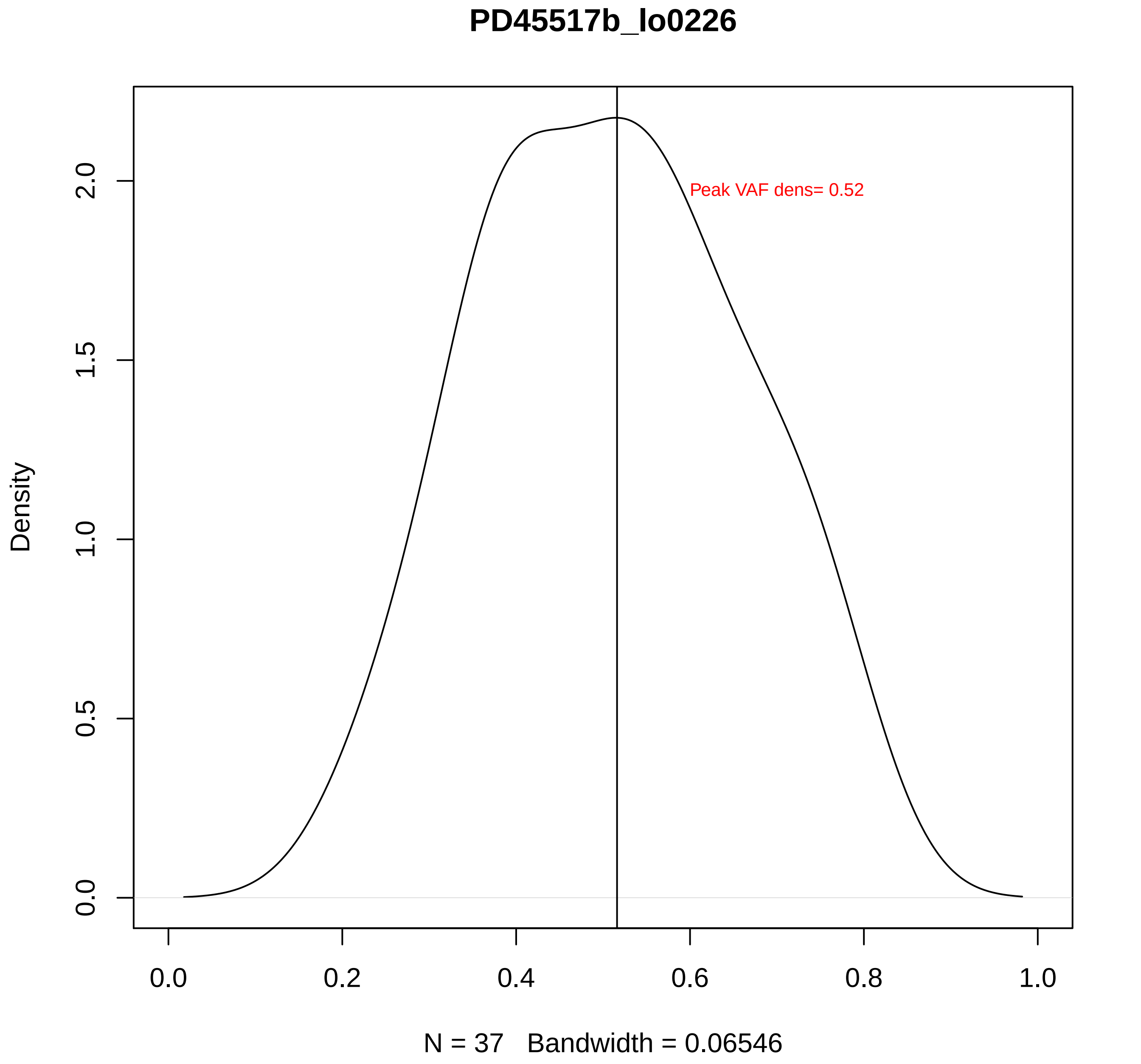
PD45517b_lo0226
Density
2.0
1.5
1.0
0.5
0.0
Peak VAF dens= 0.52
0.0
0.2
0.4
0.6
0.8
1.0
N = 37 Bandwidth = 0.06546

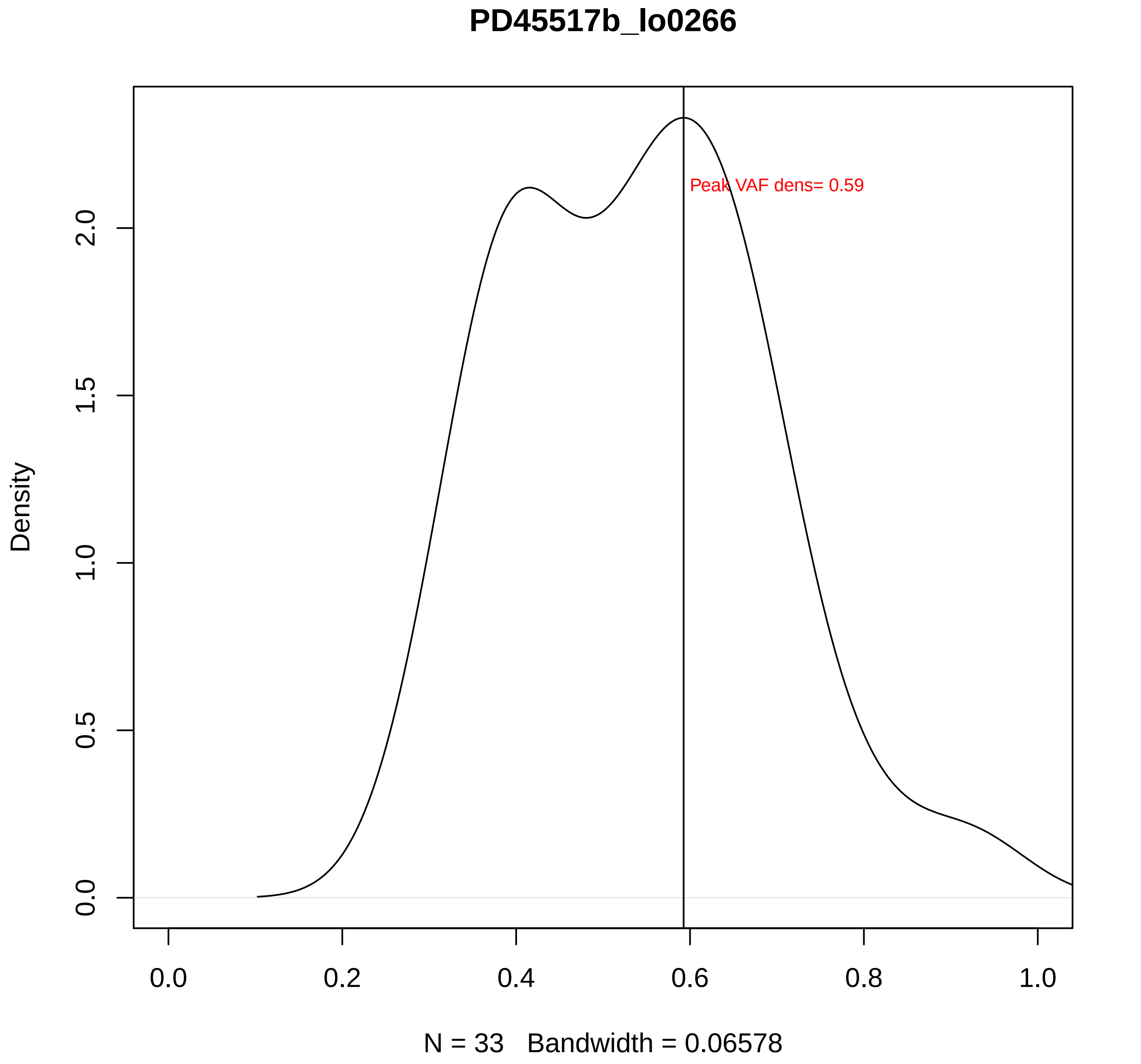
PD45517b_lo0266
Peak VAF dens= 0.59
Density
0.0
0.5
1.0
1.5
2.0
0.0
0.2
0.4
0.6
0.8
1.0
N = 33   Bandwidth = 0.06578

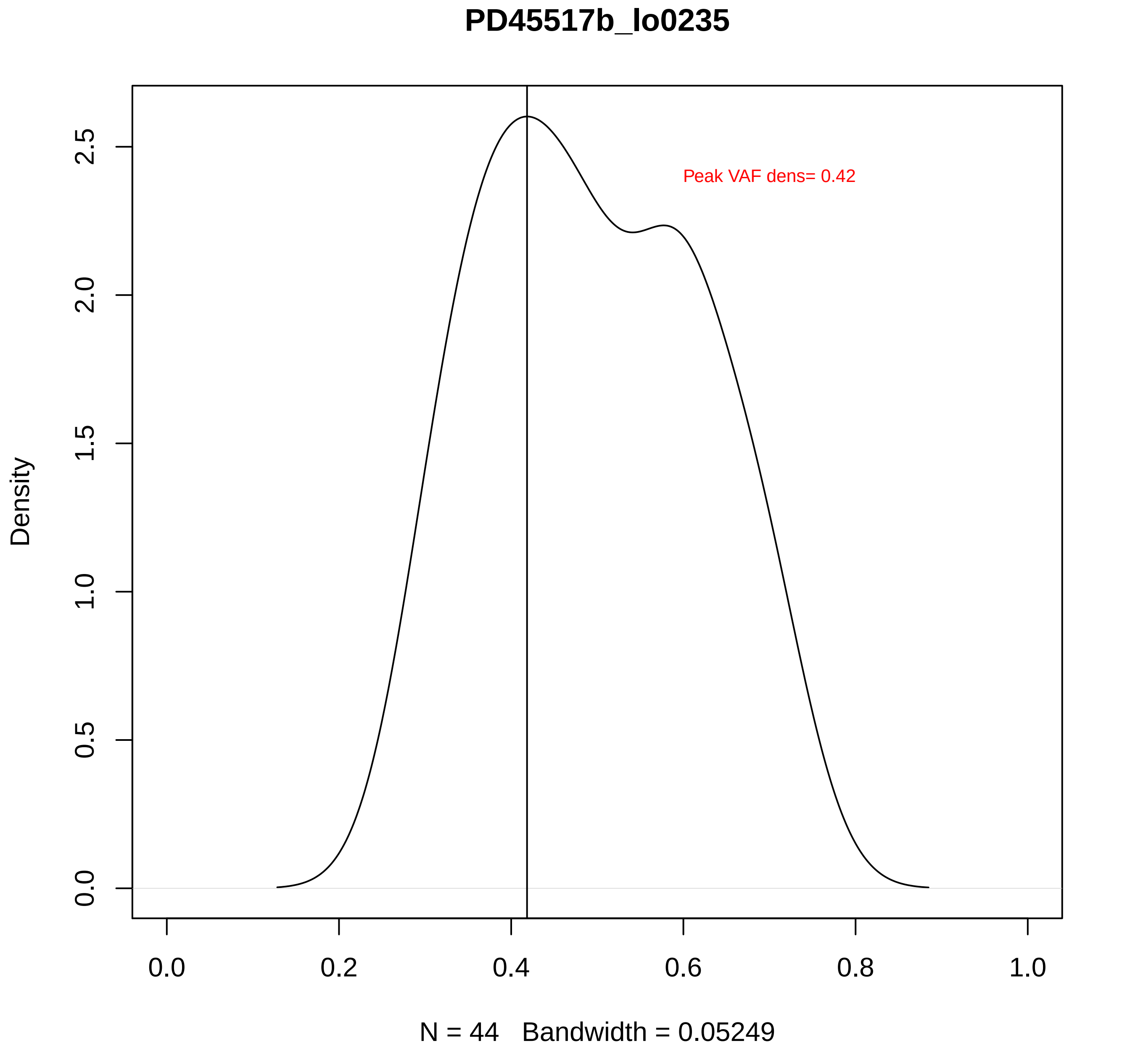
PD45517b_lo0235
Peak VAF dens= 0.42
Density
0.0
0.5
1.0
1.5
2.0
2.5
0.0
0.2
0.4
0.6
0.8
1.0
N = 44   Bandwidth = 0.05249

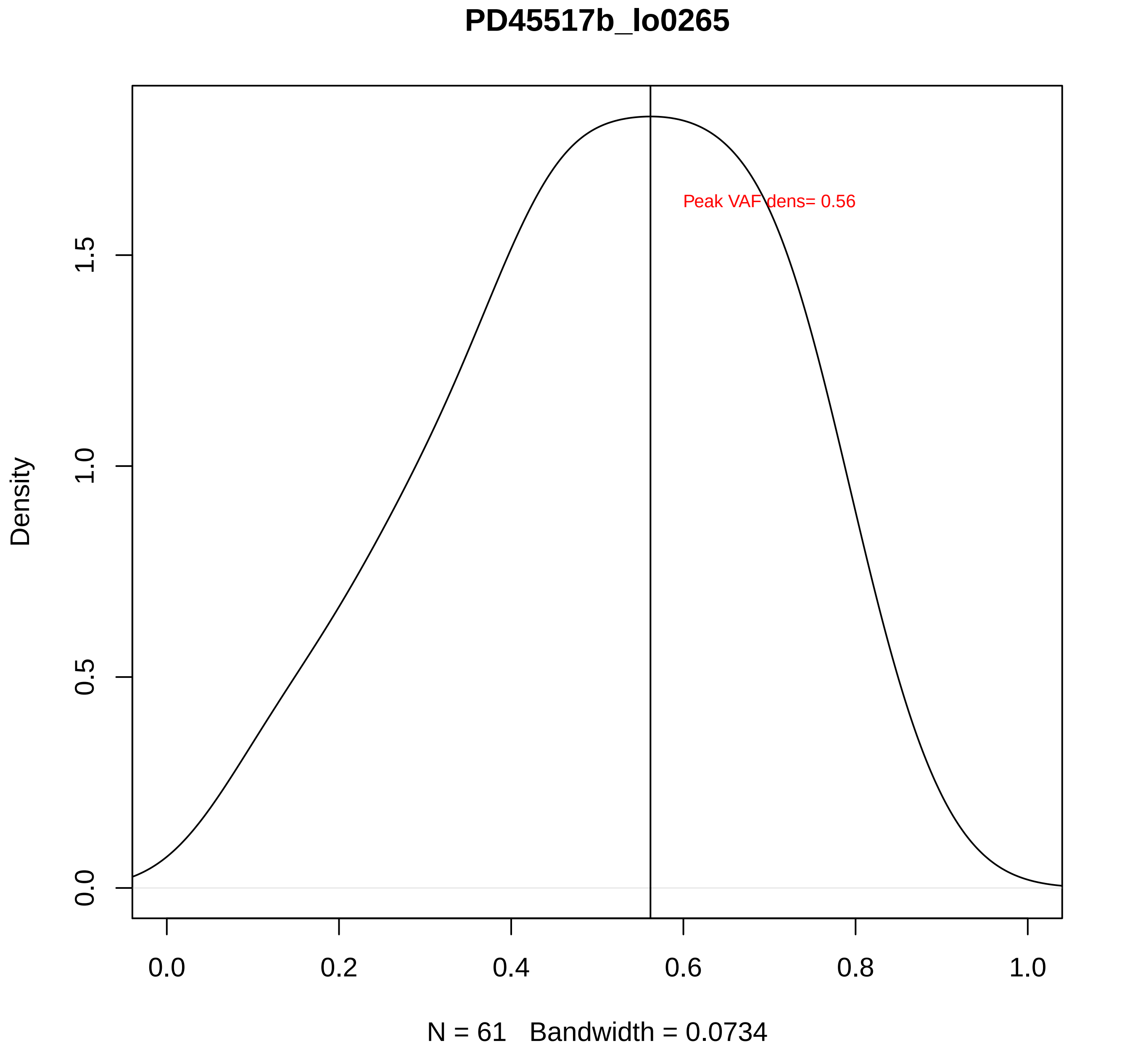

PD45517b_lo0265
Peak VAF dens= 0.56
Density
0.0
0.5
1.0
1.5
0.0
0.2
0.4
0.6
0.8
1.0
N = 61   Bandwidth = 0.0734

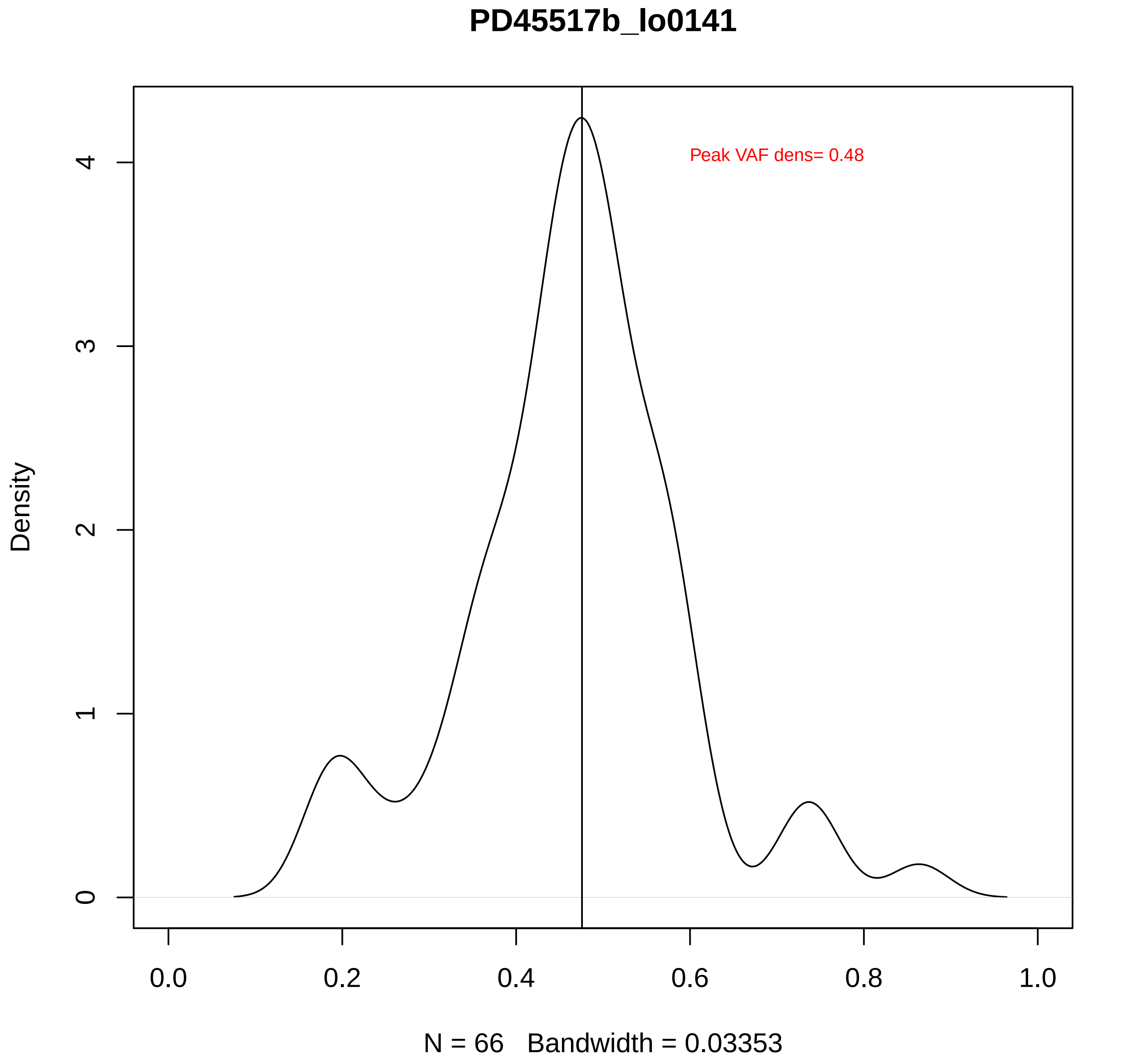
PD45517b_lo0141
Peak VAF dens= 0.48
Density
0
1
2
3
4
0.0
0.2
0.4
0.6
0.8
1.0
N = 66   Bandwidth = 0.03353

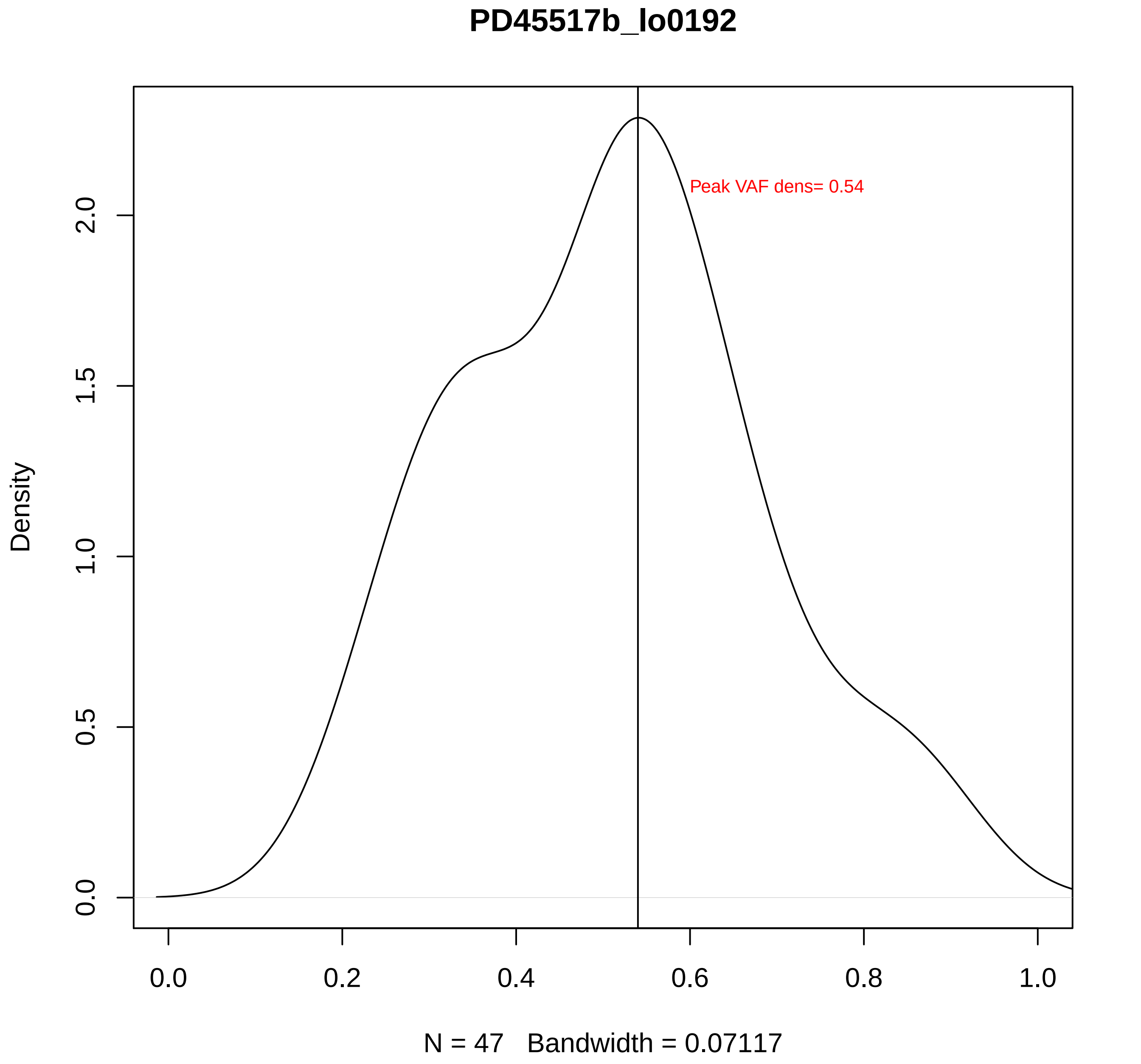

PD45517b_lo0192
Peak VAF dens= 0.54
Density
0.0
0.5
1.0
1.5
2.0
0.0
0.2
0.4
0.6
0.8
1.0
N = 47 Bandwidth = 0.07117

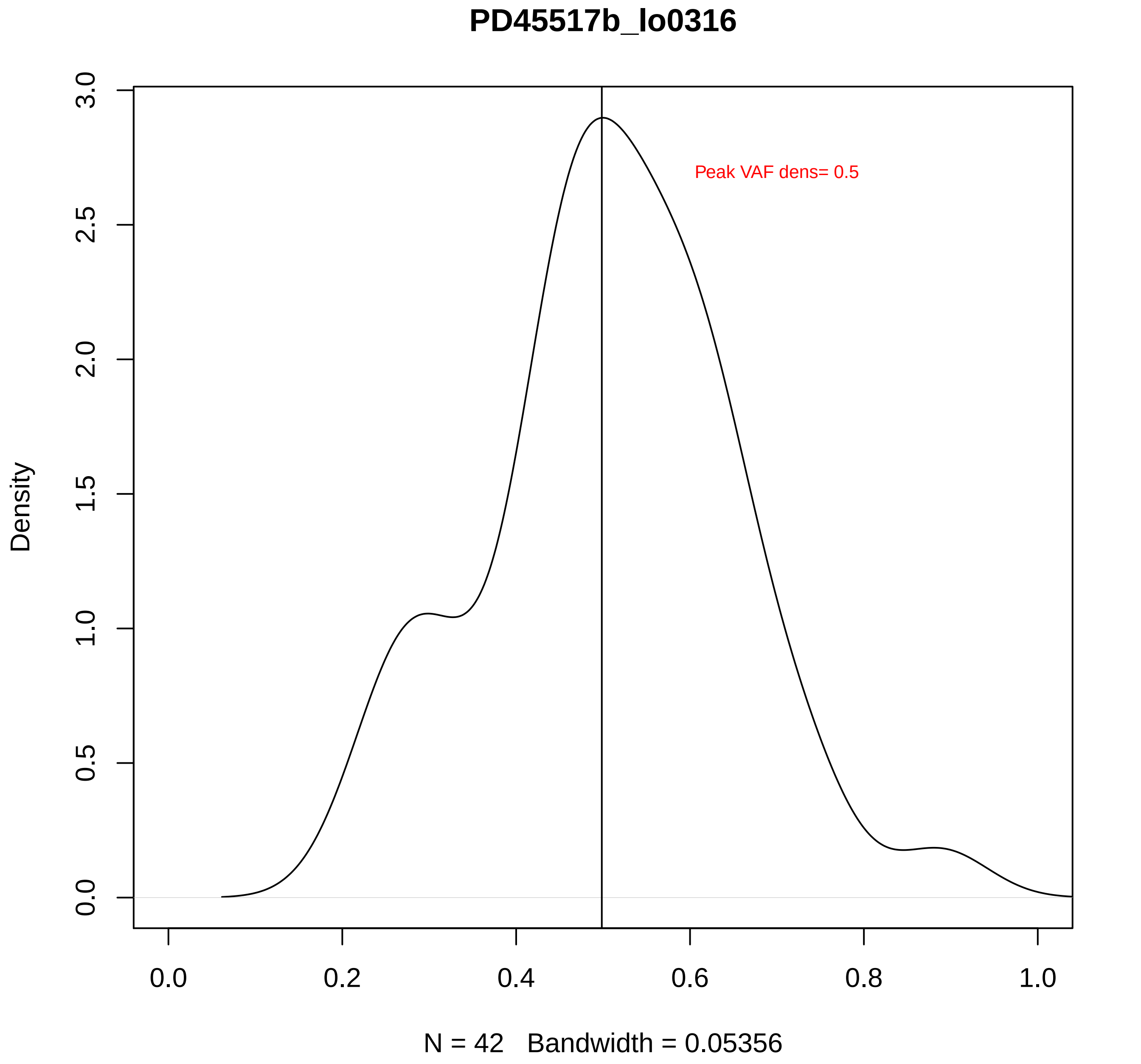
PD45517b_lo0316
Peak VAF dens= 0.5
Density
0.0
0.5
1.0
1.5
2.0
2.5
3.0
0.0
0.2
0.4
0.6
0.8
1.0
N = 42   Bandwidth = 0.05356

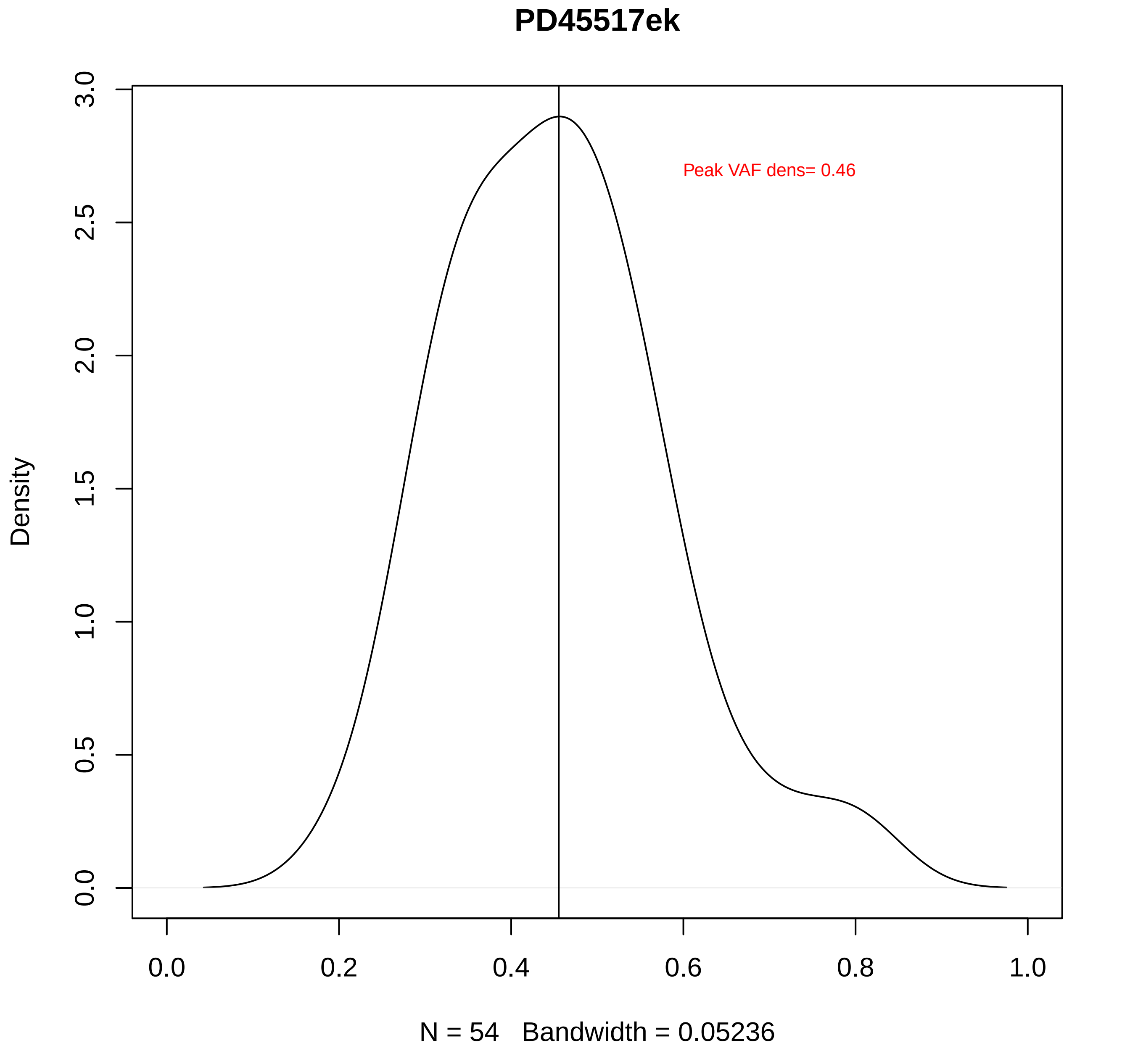

PD45517ek
Peak VAF dens= 0.46
Density
0.0
0.5
1.0
1.5
2.0
2.5
3.0
0.0
0.2
0.4
0.6
0.8
1.0
N = 54 Bandwidth = 0.05236

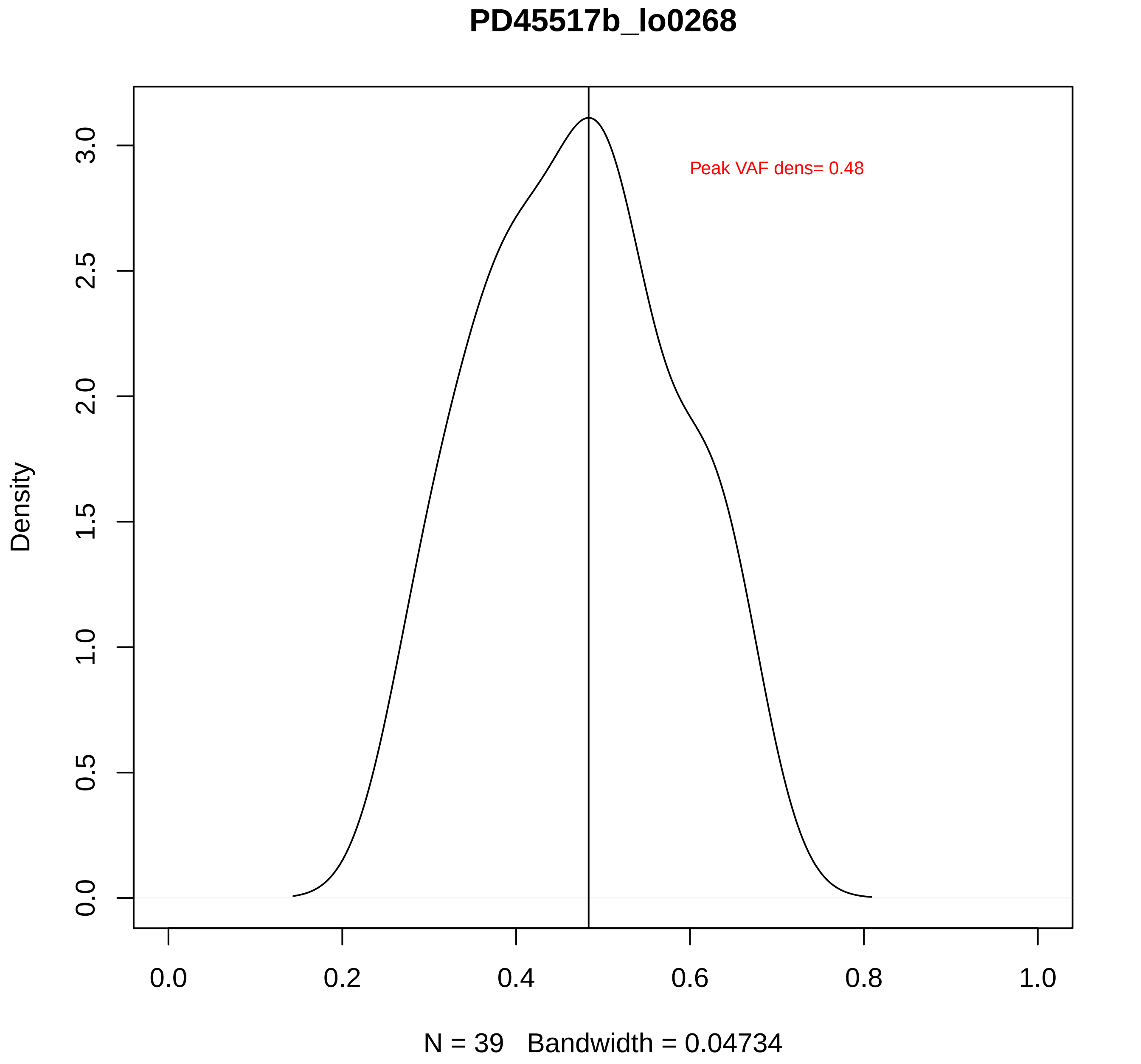
PD45517b_lo0268
Peak VAF dens= 0.48
Density
0.0
0.5
1.0
1.5
2.0
2.5
3.0
0.0
0.2
0.4
0.6
0.8
1.0
N = 39 Bandwidth = 0.04734

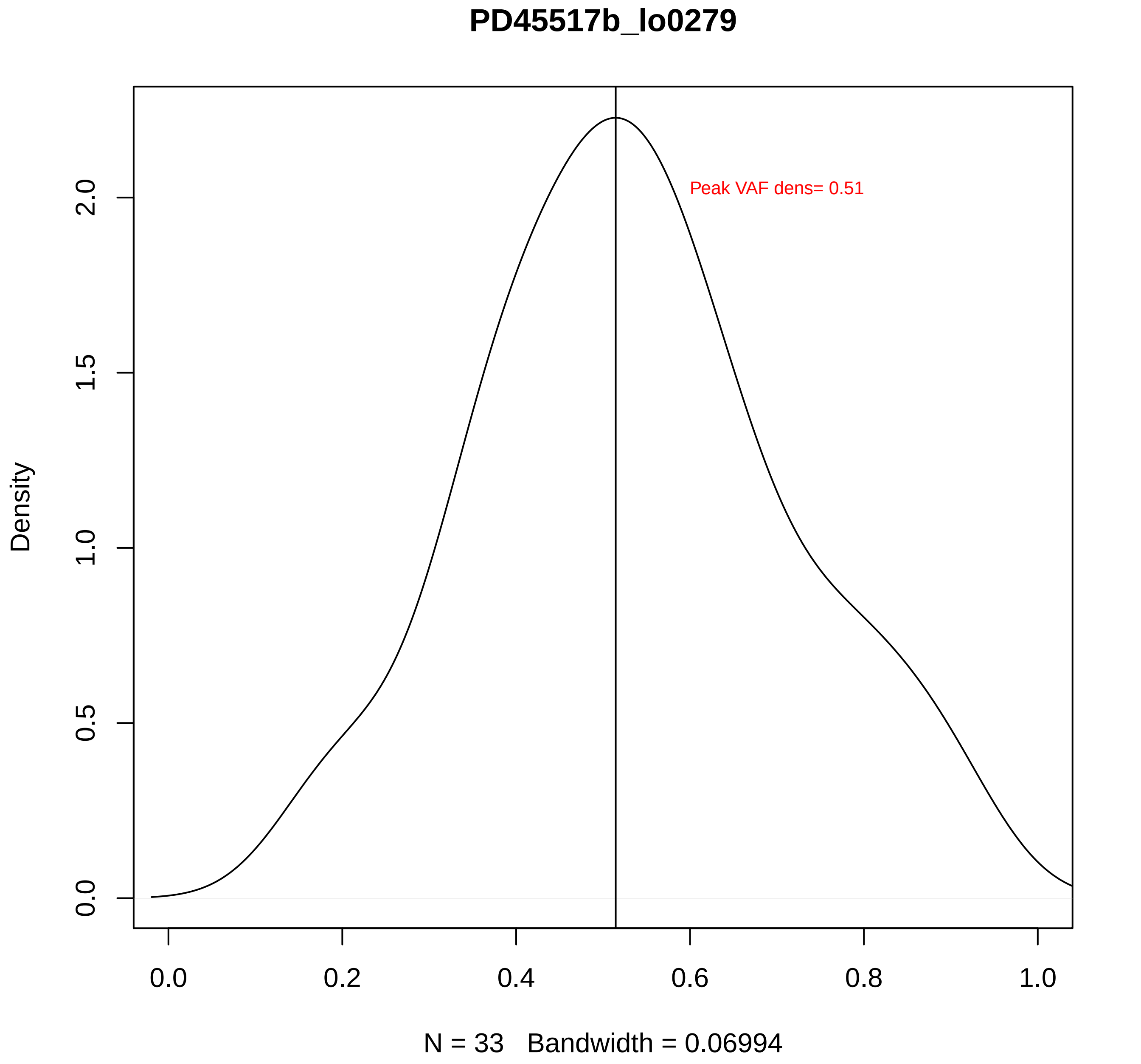

PD45517b_lo0279
Peak VAF dens= 0.51
Density
0.0
0.5
1.0
1.5
2.0
0.0
0.2
0.4
0.6
0.8
1.0
N = 33 Bandwidth = 0.06994

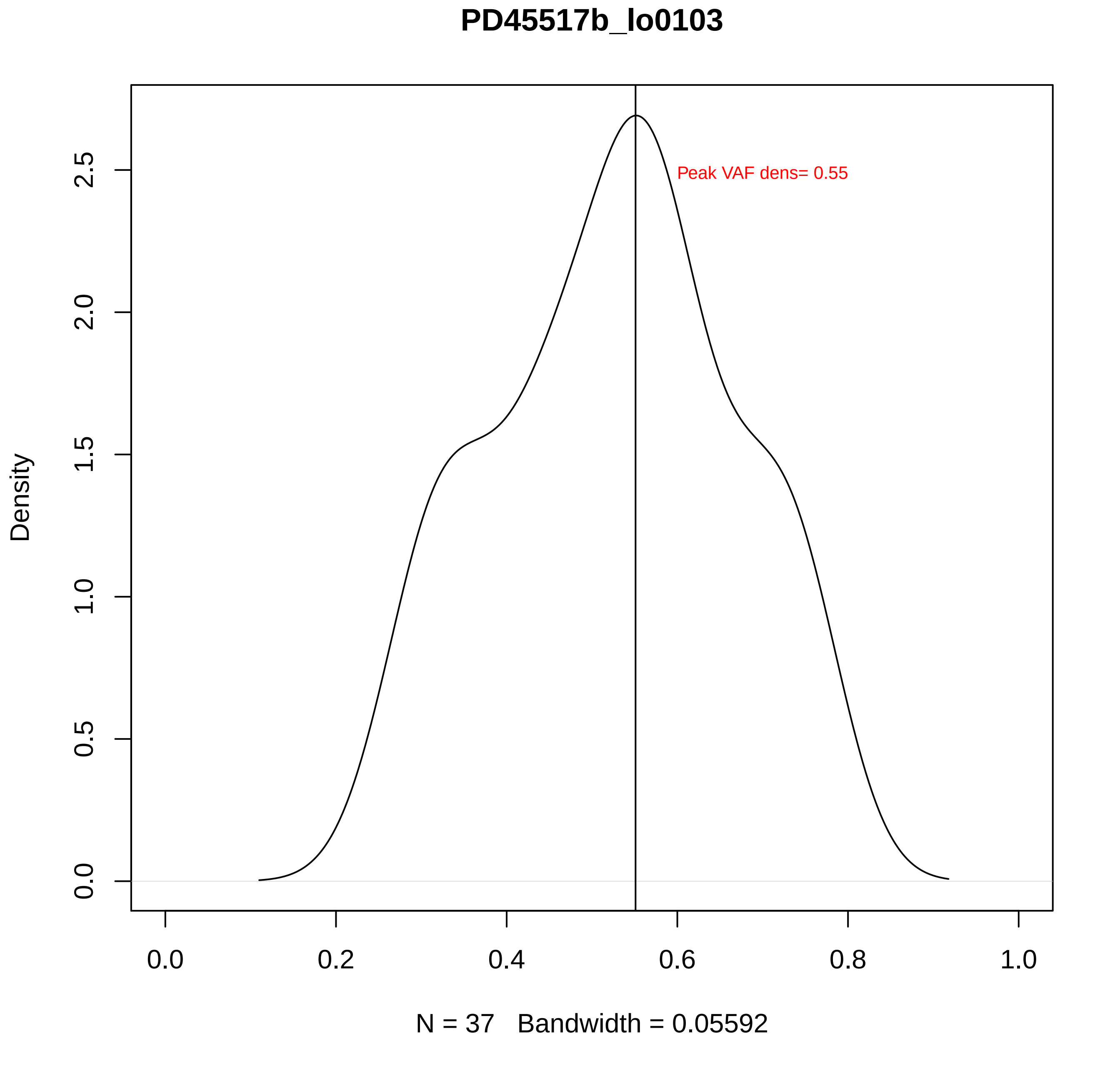
PD45517b_lo0103
Peak VAF dens= 0.55
Density
0.0
0.5
1.0
1.5
2.0
2.5
0.0
0.2
0.4
0.6
0.8
1.0
N = 37   Bandwidth = 0.05592

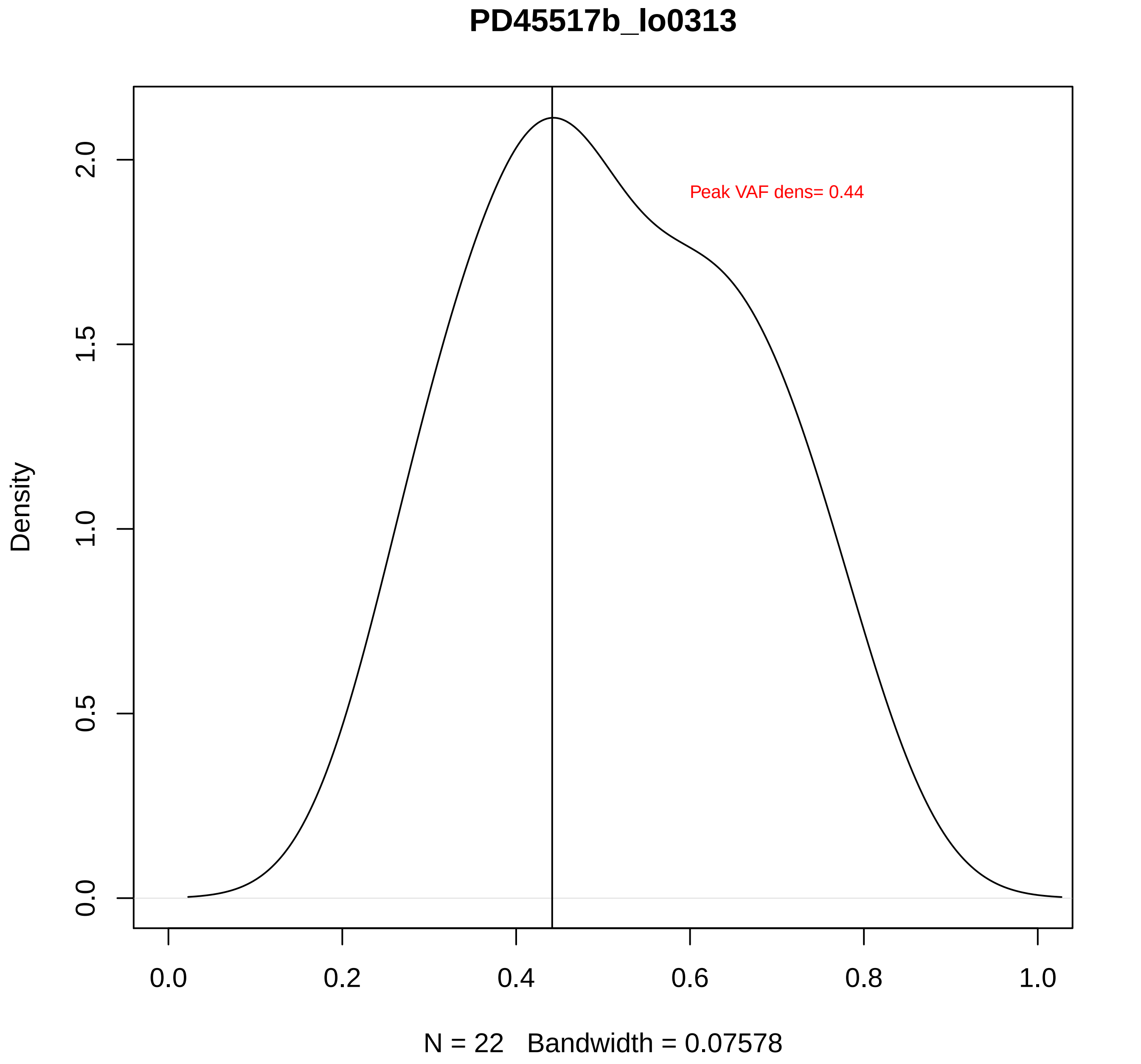
PD45517b_lo0313
Peak VAF dens= 0.44
Density
0.0
0.5
1.0
1.5
2.0
0.0
0.2
0.4
0.6
0.8
1.0
N = 22   Bandwidth = 0.07578

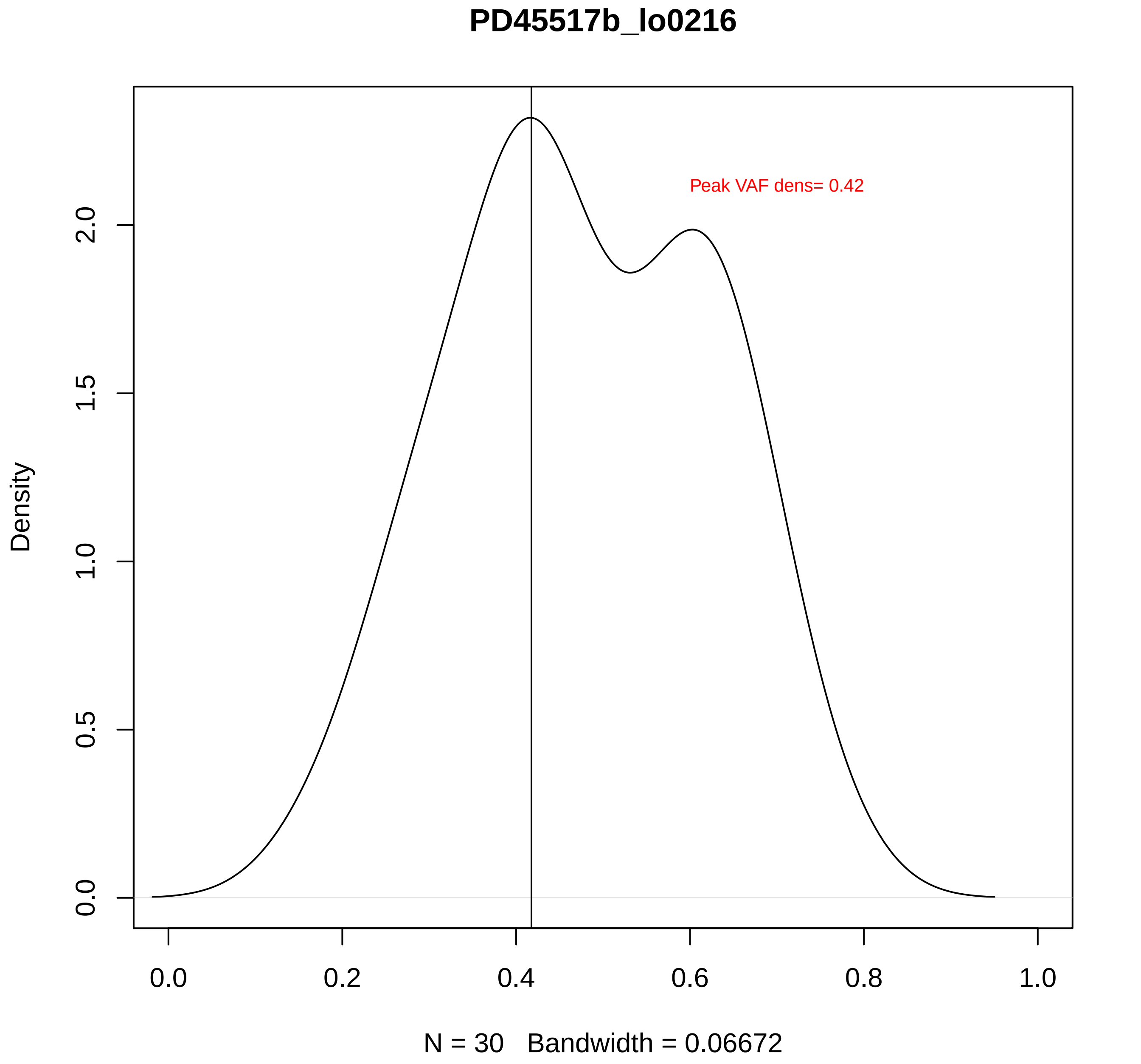
PD45517b_lo0216
Peak VAF dens= 0.42
Density
0.0
0.5
1.0
1.5
2.0
0.0
0.2
0.4
0.6
0.8
1.0
N = 30   Bandwidth = 0.06672

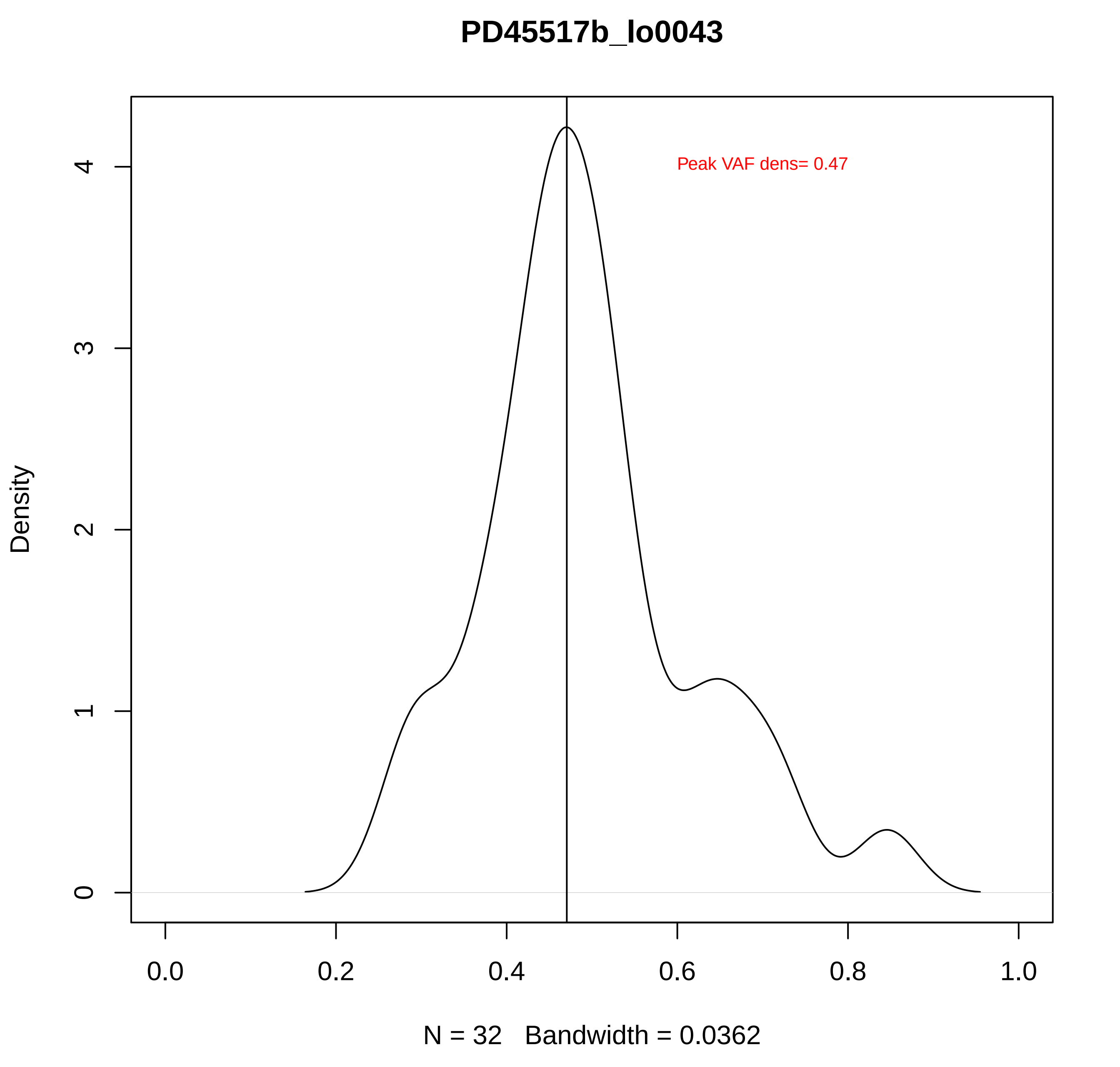

PD45517b_lo0043
Peak VAF dens= 0.47
Density
0
1
2
3
4
0.0
0.2
0.4
0.6
0.8
1.0
N = 32 Bandwidth = 0.0362

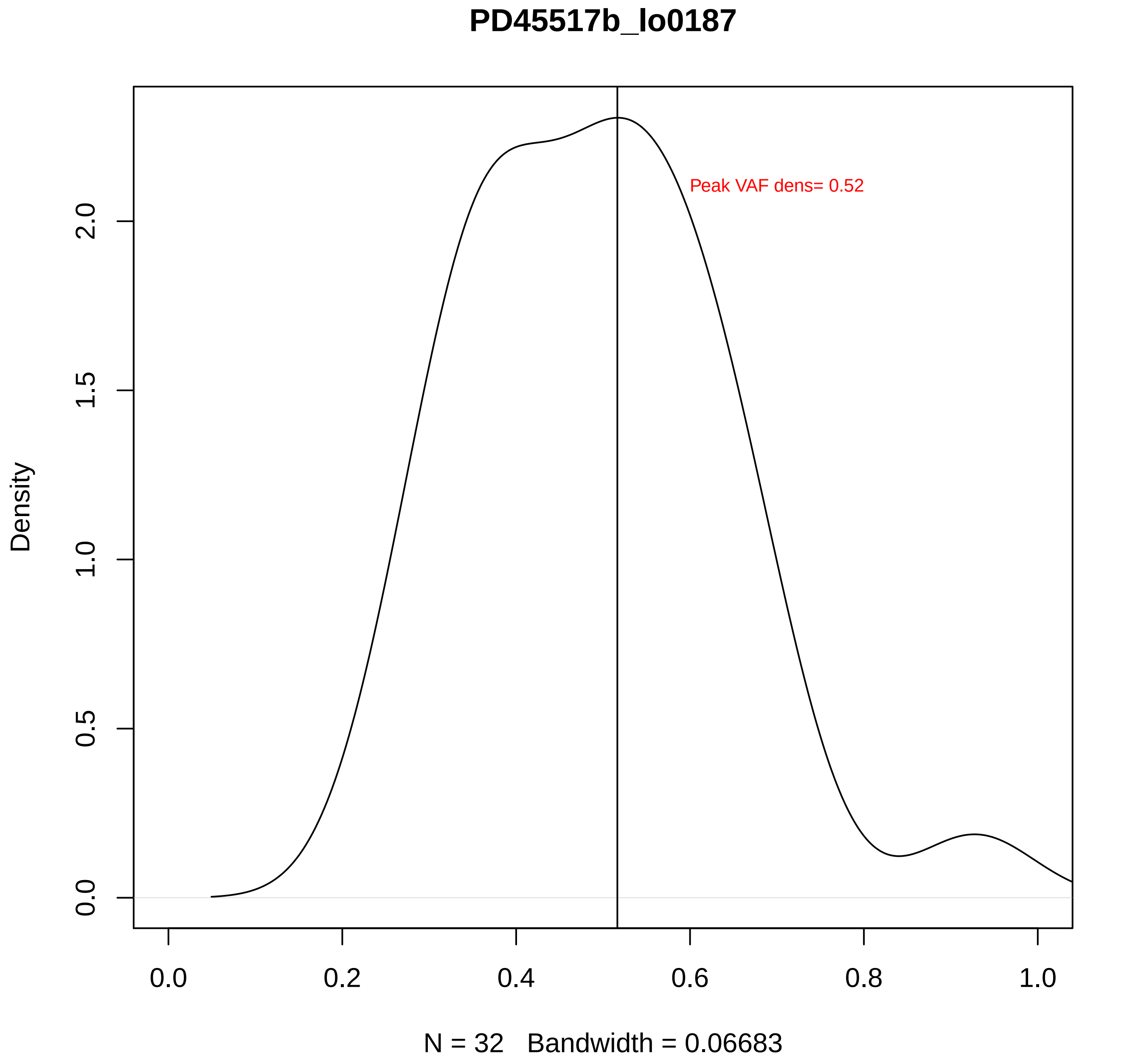
PD45517b_lo0187
Peak VAF dens= 0.52
Density
0.0
0.5
1.0
1.5
2.0
0.0
0.2
0.4
0.6
0.8
1.0
N = 32   Bandwidth = 0.06683

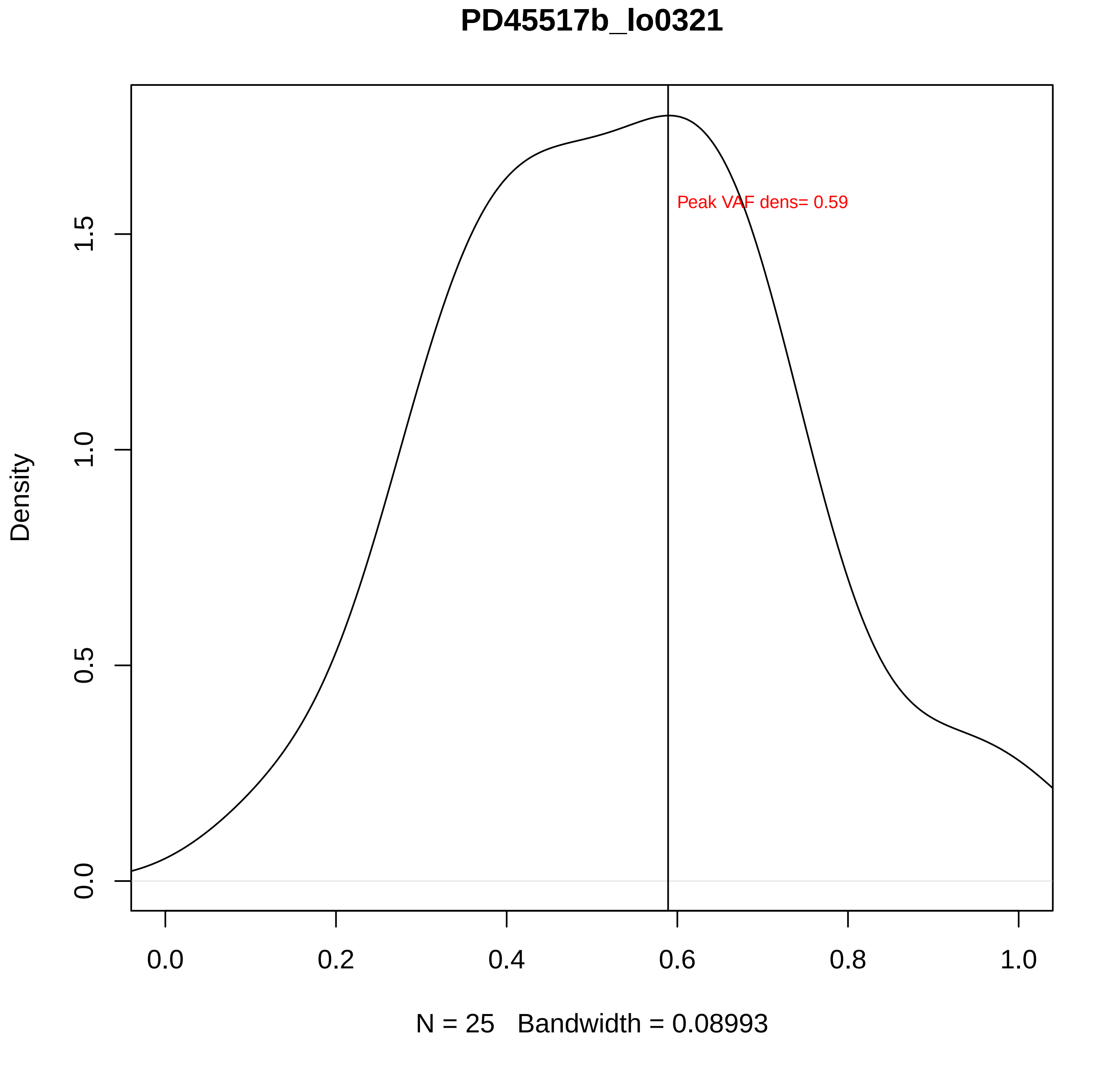
PD45517b_lo0321
Peak VAF dens= 0.59
Density
0.0
0.5
1.0
1.5
0.0
0.2
0.4
0.6
0.8
1.0
N = 25   Bandwidth = 0.08993

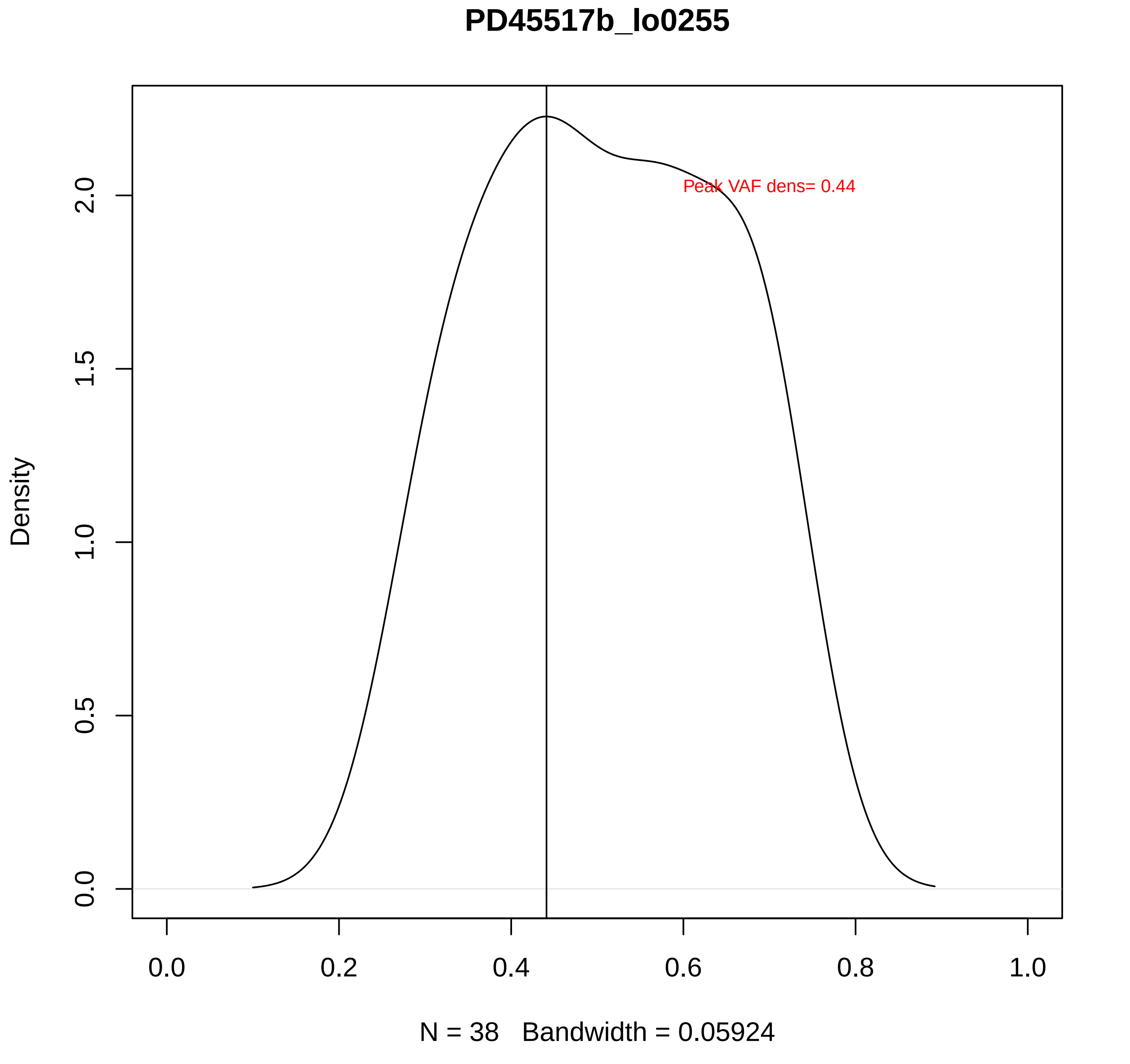
PD45517b_lo0255
Peak VAF dens= 0.44
Density
0.0
0.5
1.0
1.5
2.0
0.0
0.2
0.4
0.6
0.8
1.0
N = 38   Bandwidth = 0.05924

PD45517b_lo0053

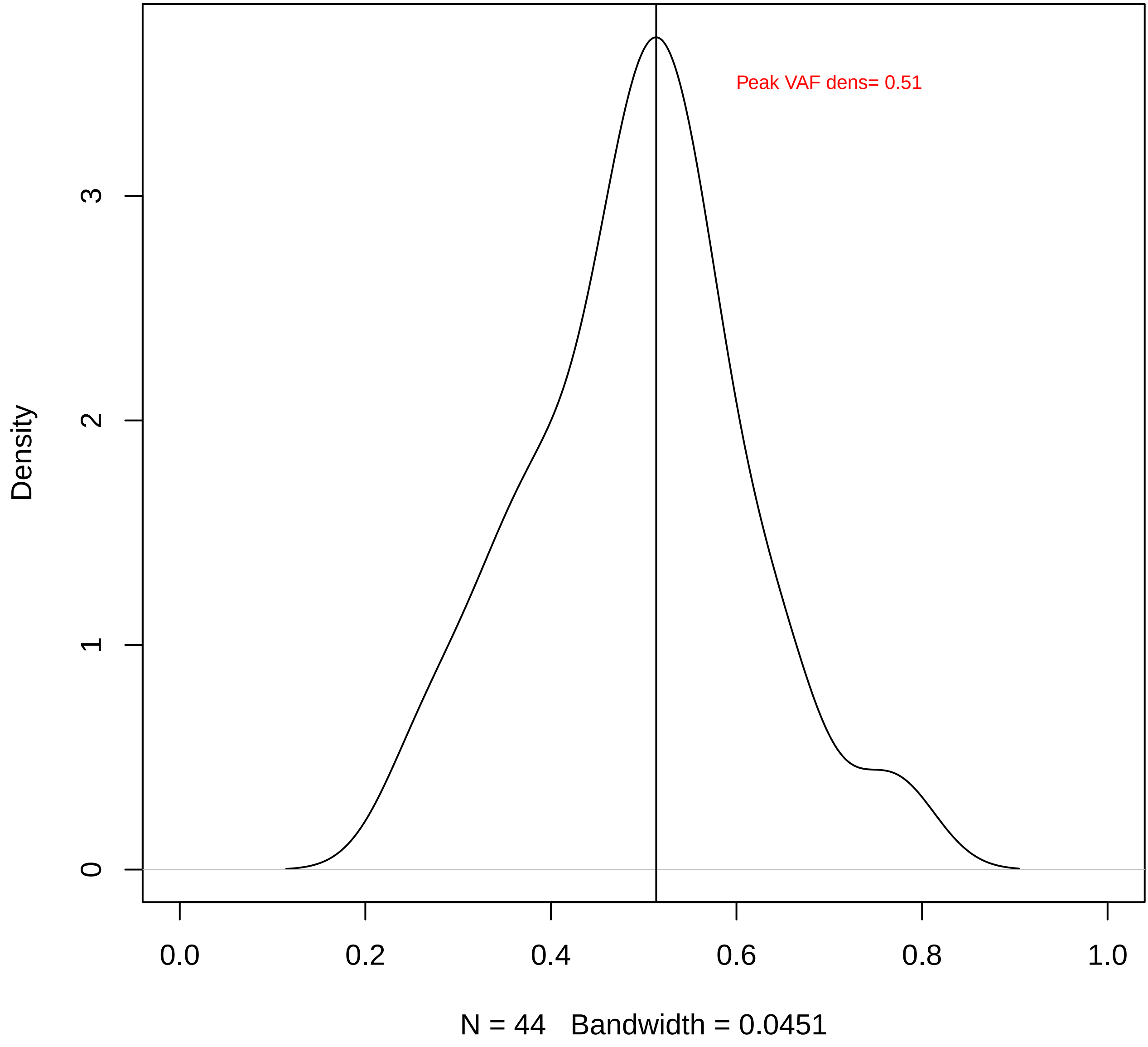

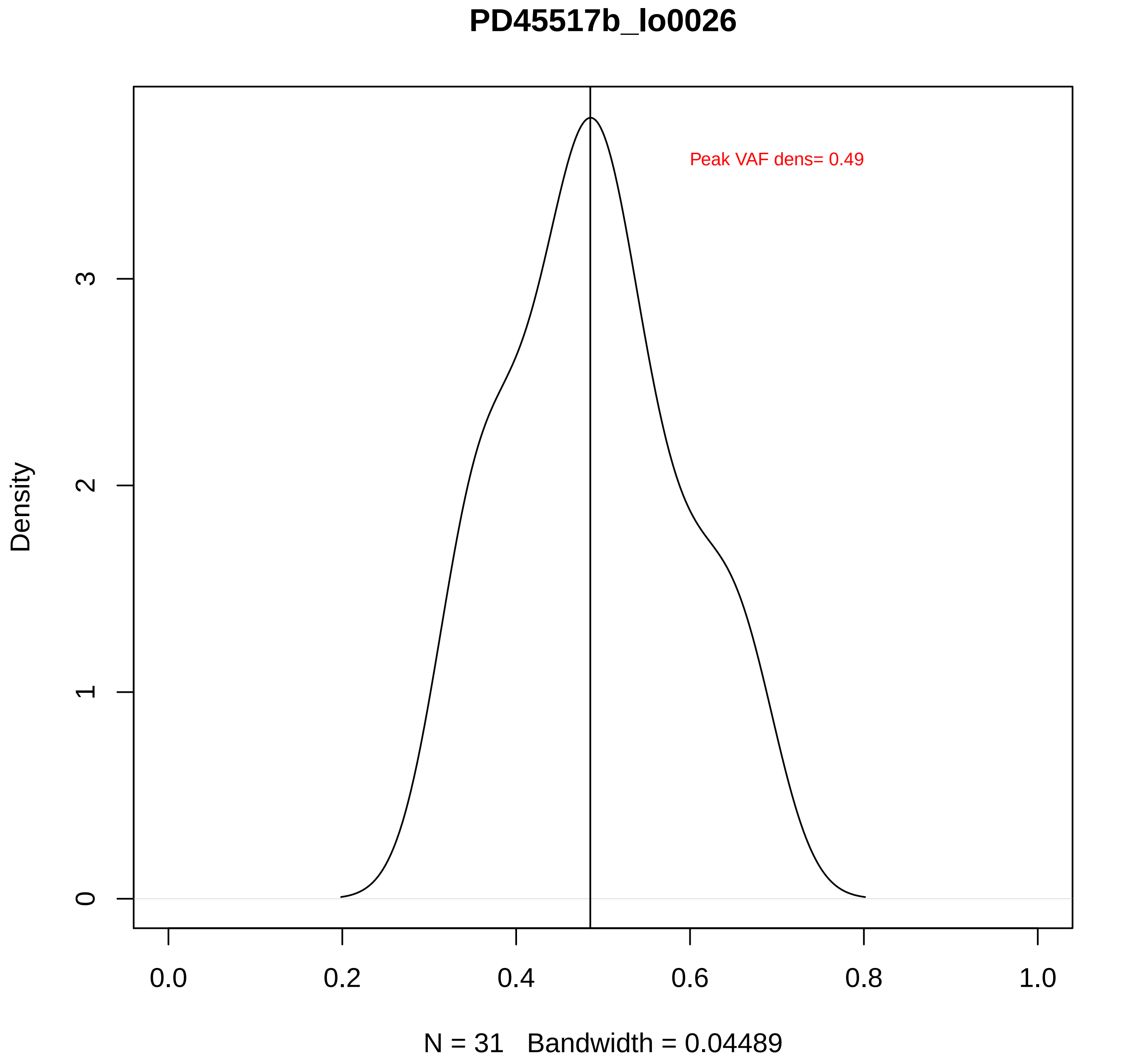
PD45517b_lo0026
Peak VAF dens= 0.49
Density
0
1
2
3
0.0
0.2
0.4
0.6
0.8
1.0
N = 31   Bandwidth = 0.04489

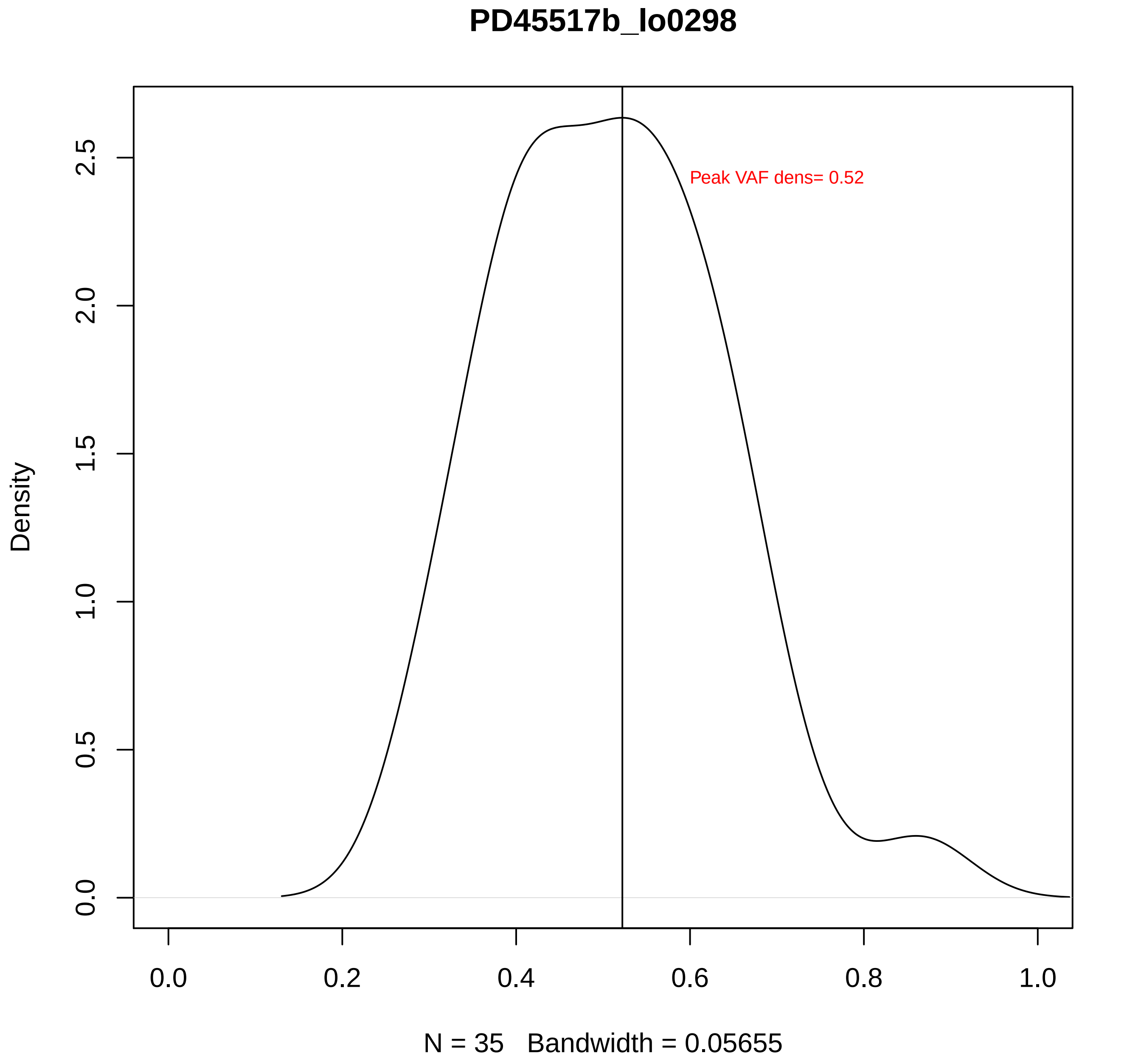
PD45517b_lo0298
Density
Peak VAF dens= 0.52
0.0
0.5
1.0
1.5
2.0
2.5
0.0
0.2
0.4
0.6
0.8
1.0
N = 35   Bandwidth = 0.05655

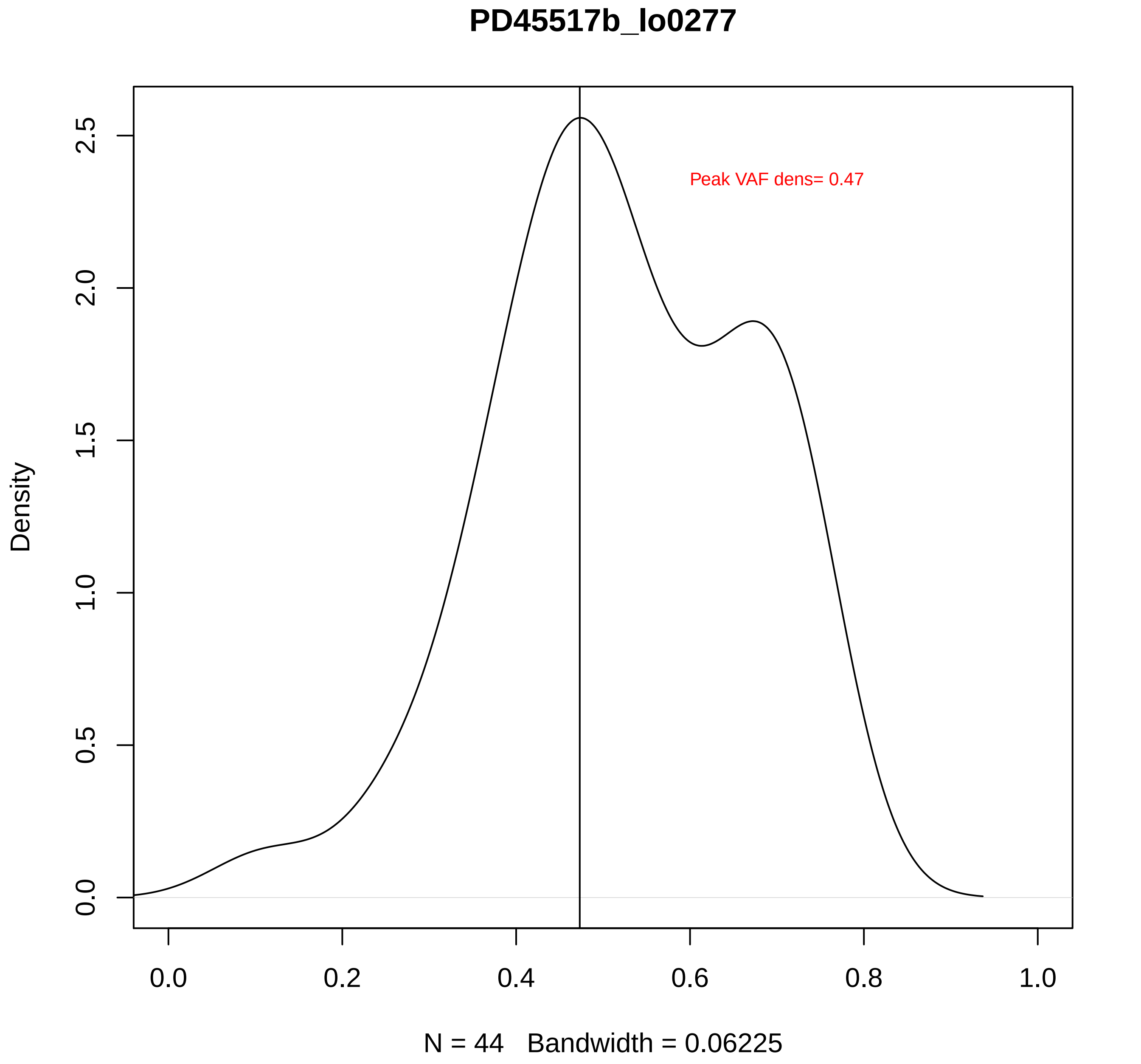
PD45517b_lo0277
Peak VAF dens= 0.47
Density
0.0
0.5
1.0
1.5
2.0
2.5
0.0
0.2
0.4
0.6
0.8
1.0
N = 44 Bandwidth = 0.06225

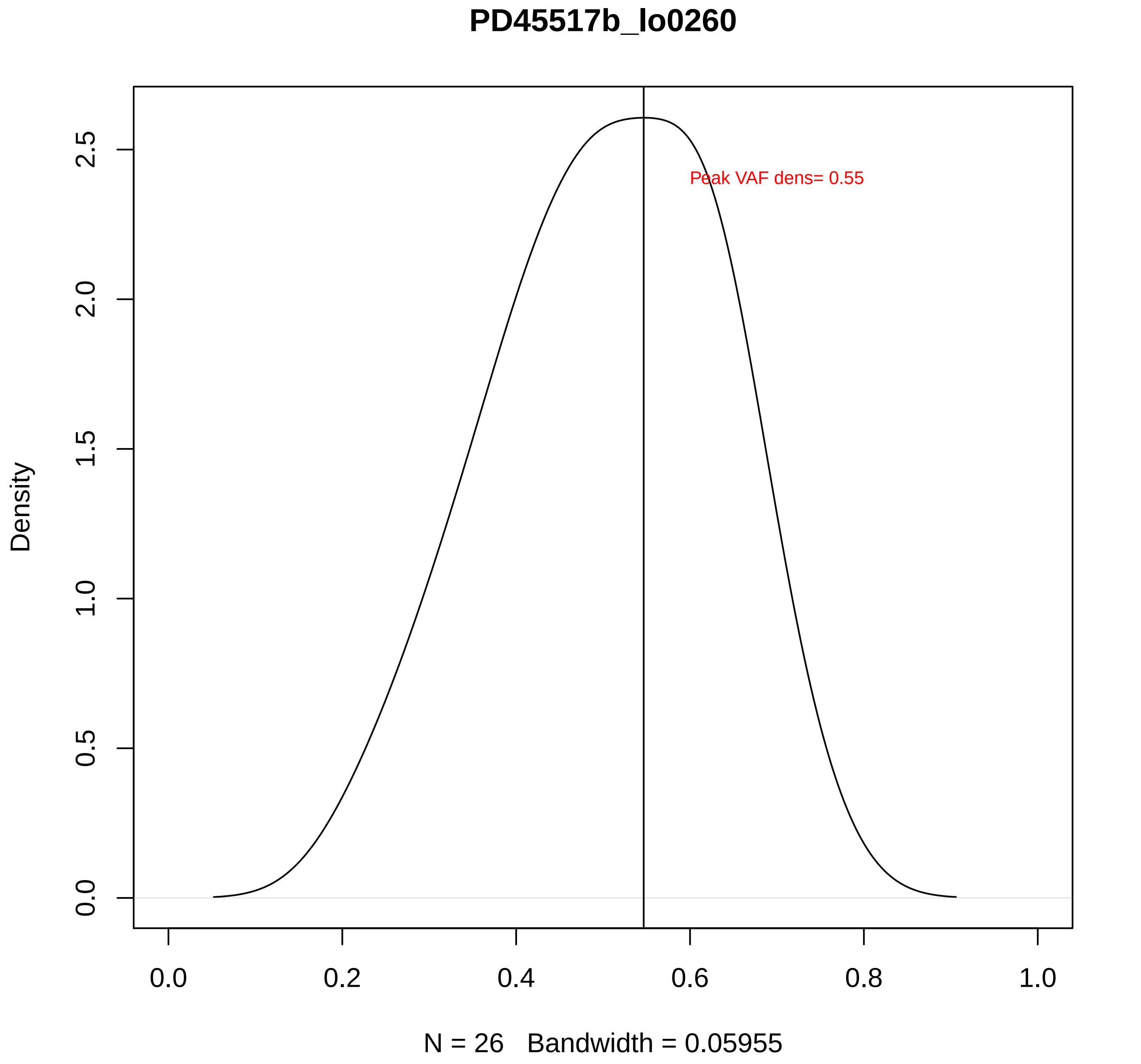
PD45517b_lo0260
Peak VAF dens= 0.55
Density
0.0
0.5
1.0
1.5
2.0
2.5
0.0
0.2
0.4
0.6
0.8
1.0
N = 26   Bandwidth = 0.05955

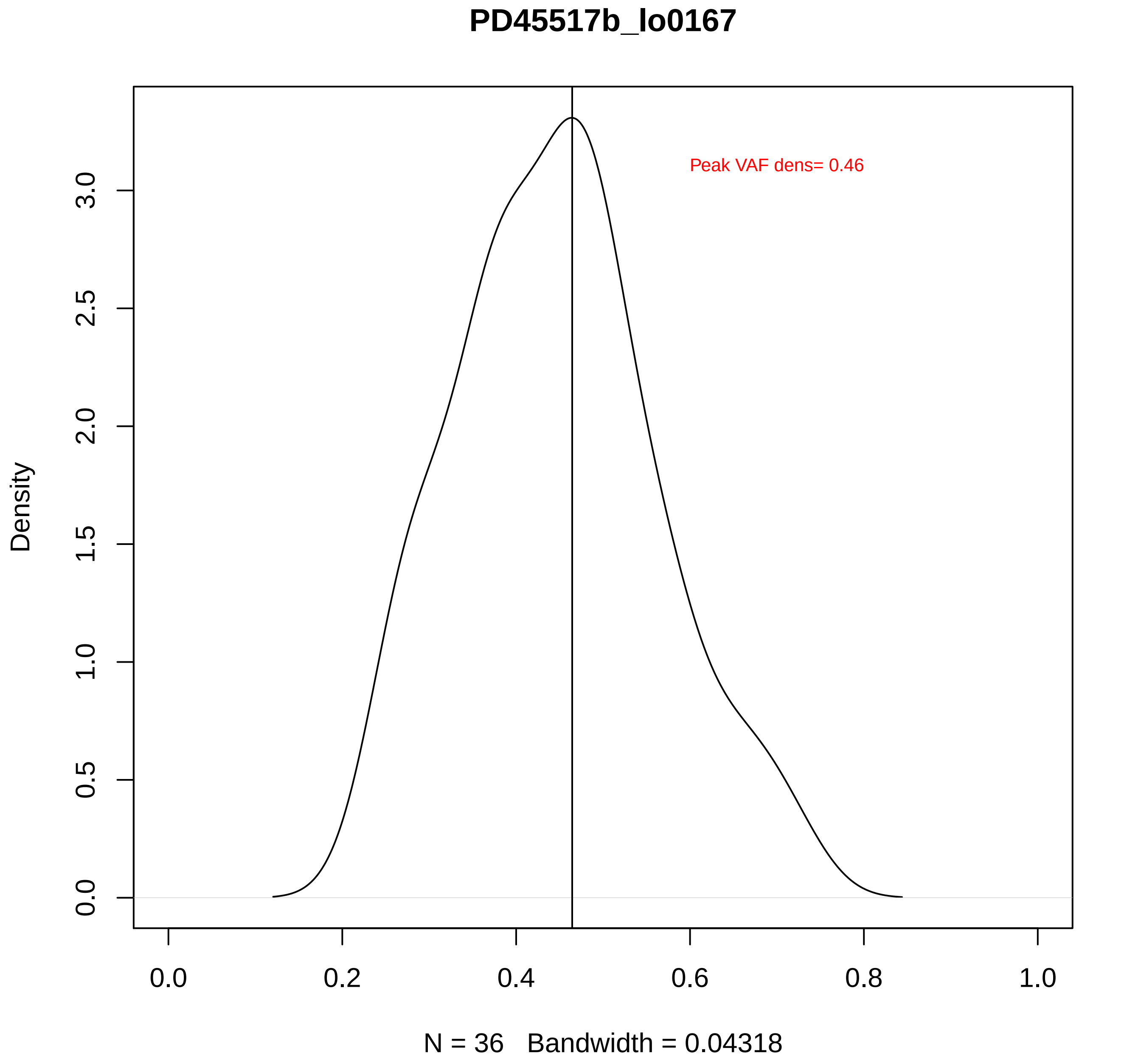

PD45517b_lo0167
Peak VAF dens= 0.46
Density
0.0
0.5
1.0
1.5
2.0
2.5
3.0
0.0
0.2
0.4
0.6
0.8
1.0
N = 36 Bandwidth = 0.04318

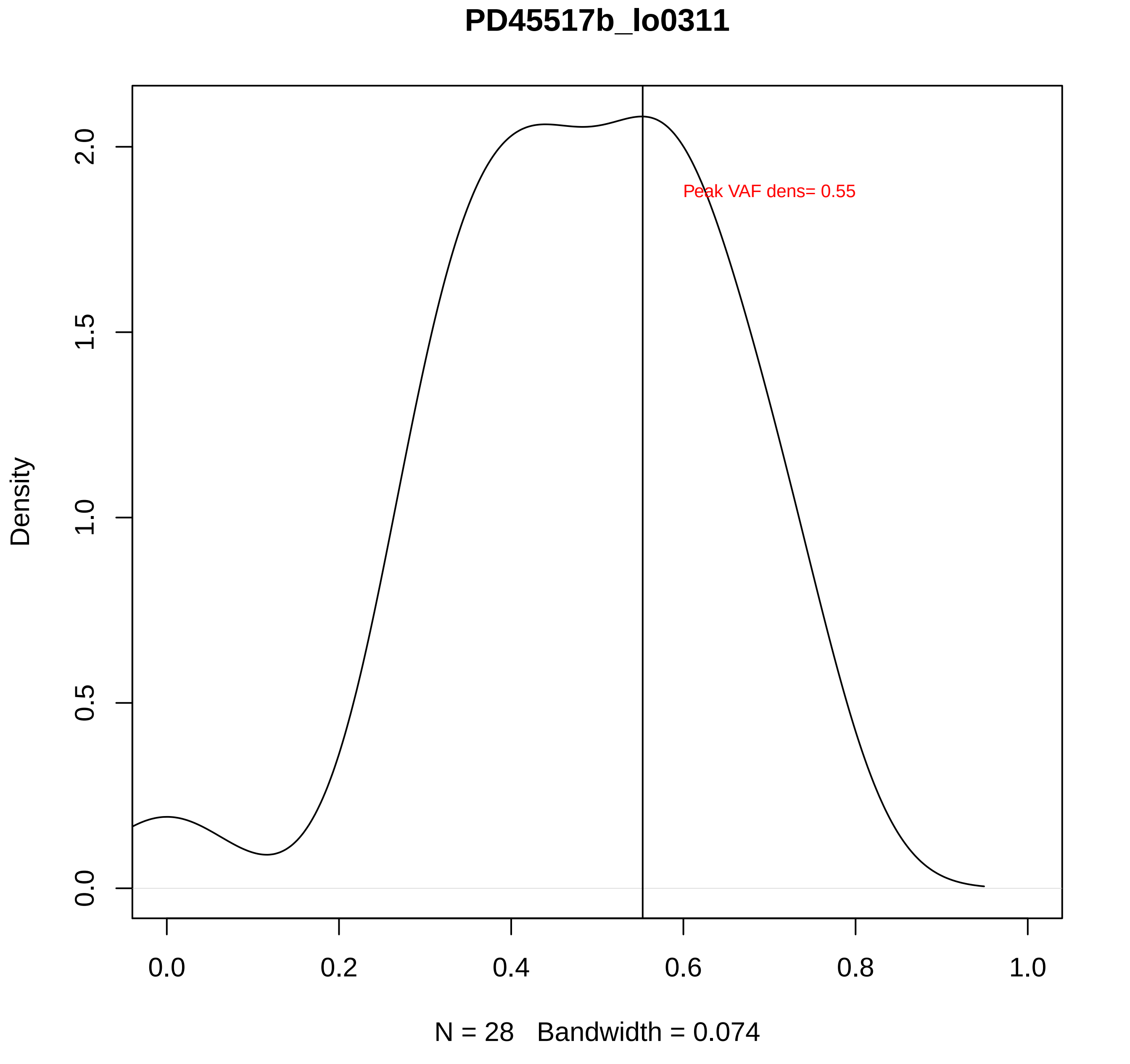
PD45517b_lo0311
Density
2.0
1.5
1.0
0.5
0.0
Peak VAF dens= 0.55
0.0
0.2
0.4
0.6
0.8
1.0
N = 28   Bandwidth = 0.074

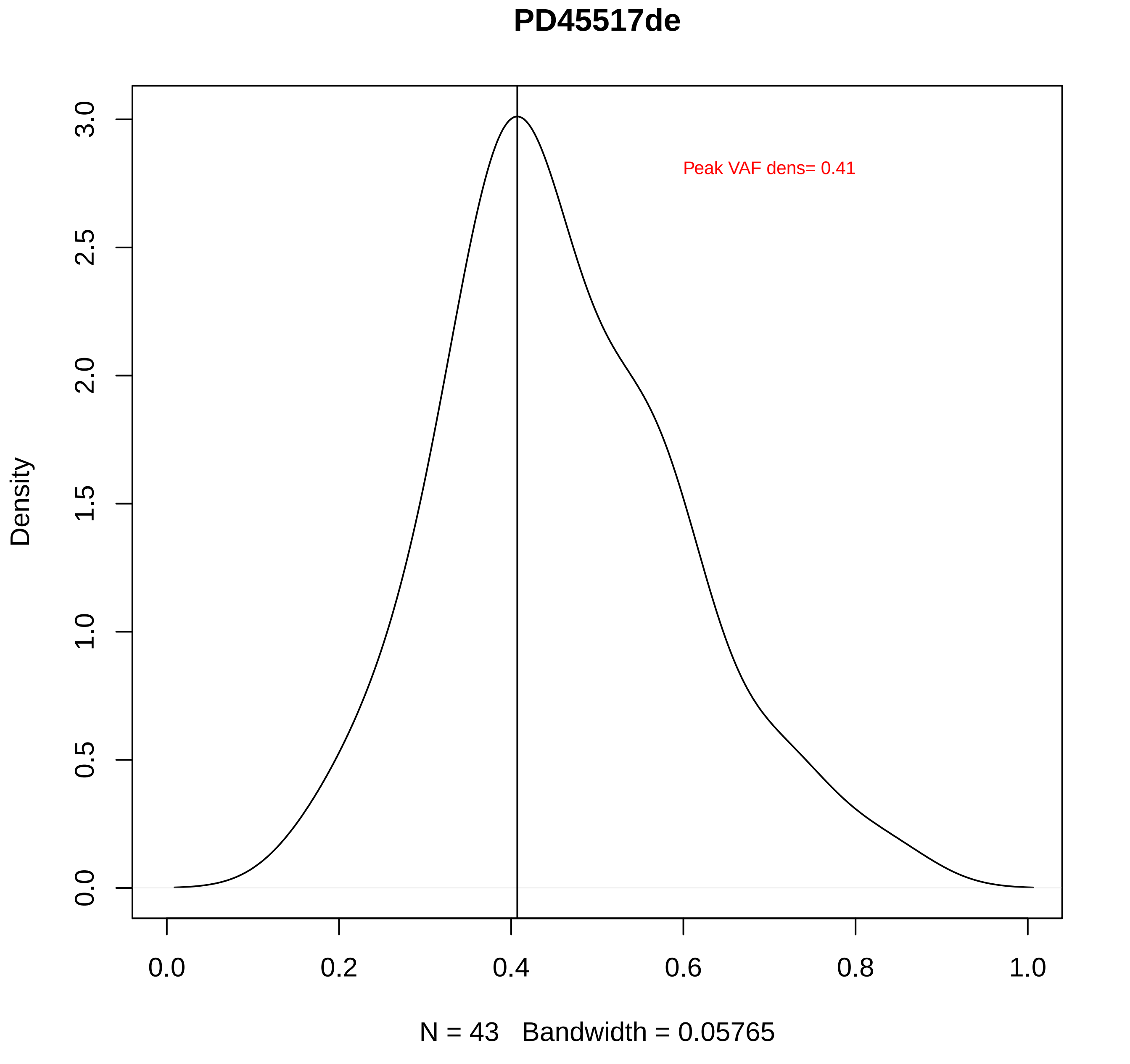
PD45517de
Peak VAF dens= 0.41
Density
3.0
2.5
2.0
1.5
1.0
0.5
0.0
0.0
0.2
0.4
0.6
0.8
1.0
N = 43 Bandwidth = 0.05765

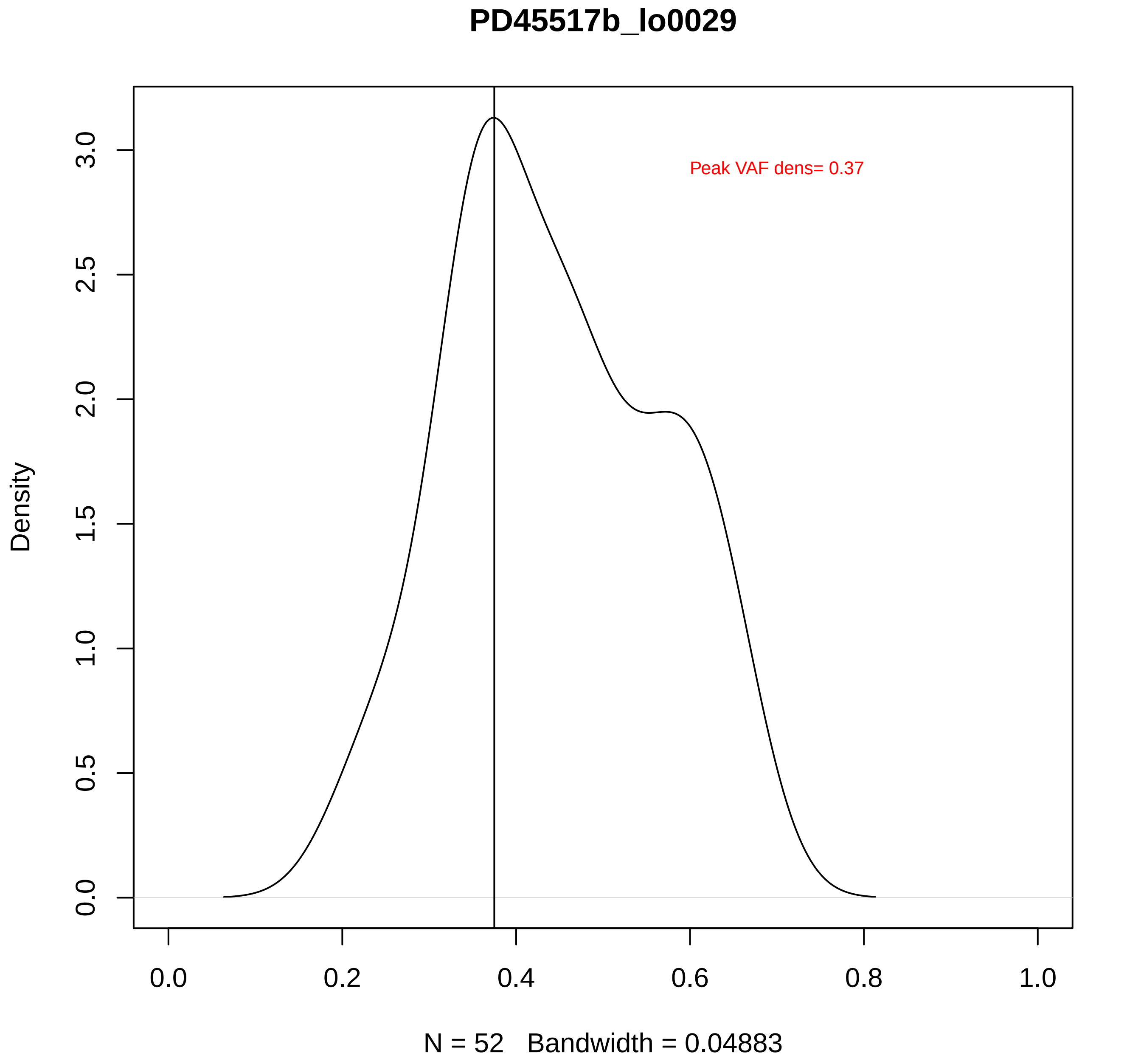

PD45517b_lo0029
Peak VAF dens= 0.37
Density
0.0
0.5
1.0
1.5
2.0
2.5
3.0
0.0
0.2
0.4
0.6
0.8
1.0
N = 52   Bandwidth = 0.04883

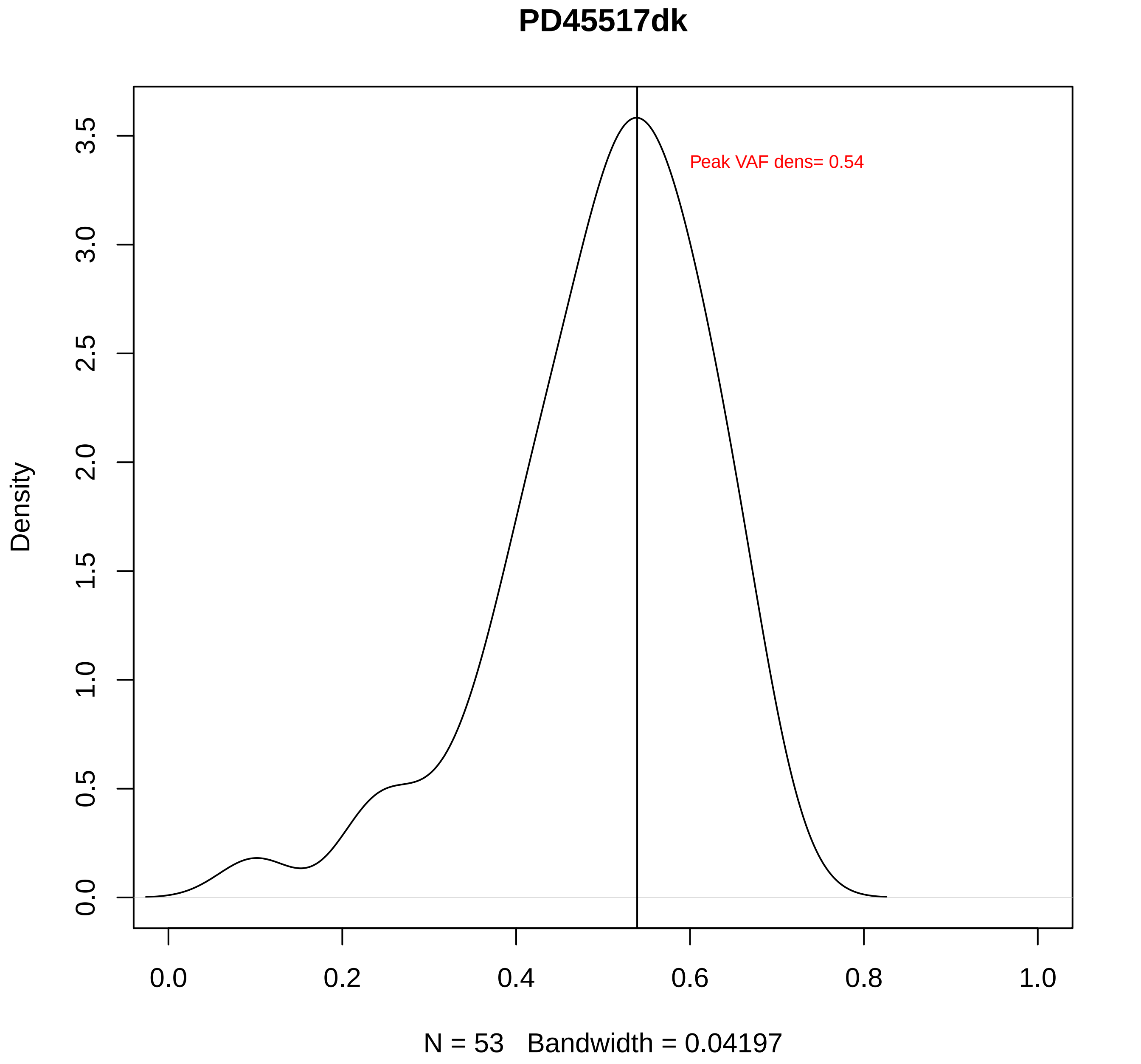

PD45517dk
Peak VAF dens= 0.54
Density
0.0
0.5
1.0
1.5
2.0
2.5
3.0
3.5
0.0
0.2
0.4
0.6
0.8
1.0
N = 53 Bandwidth = 0.04197

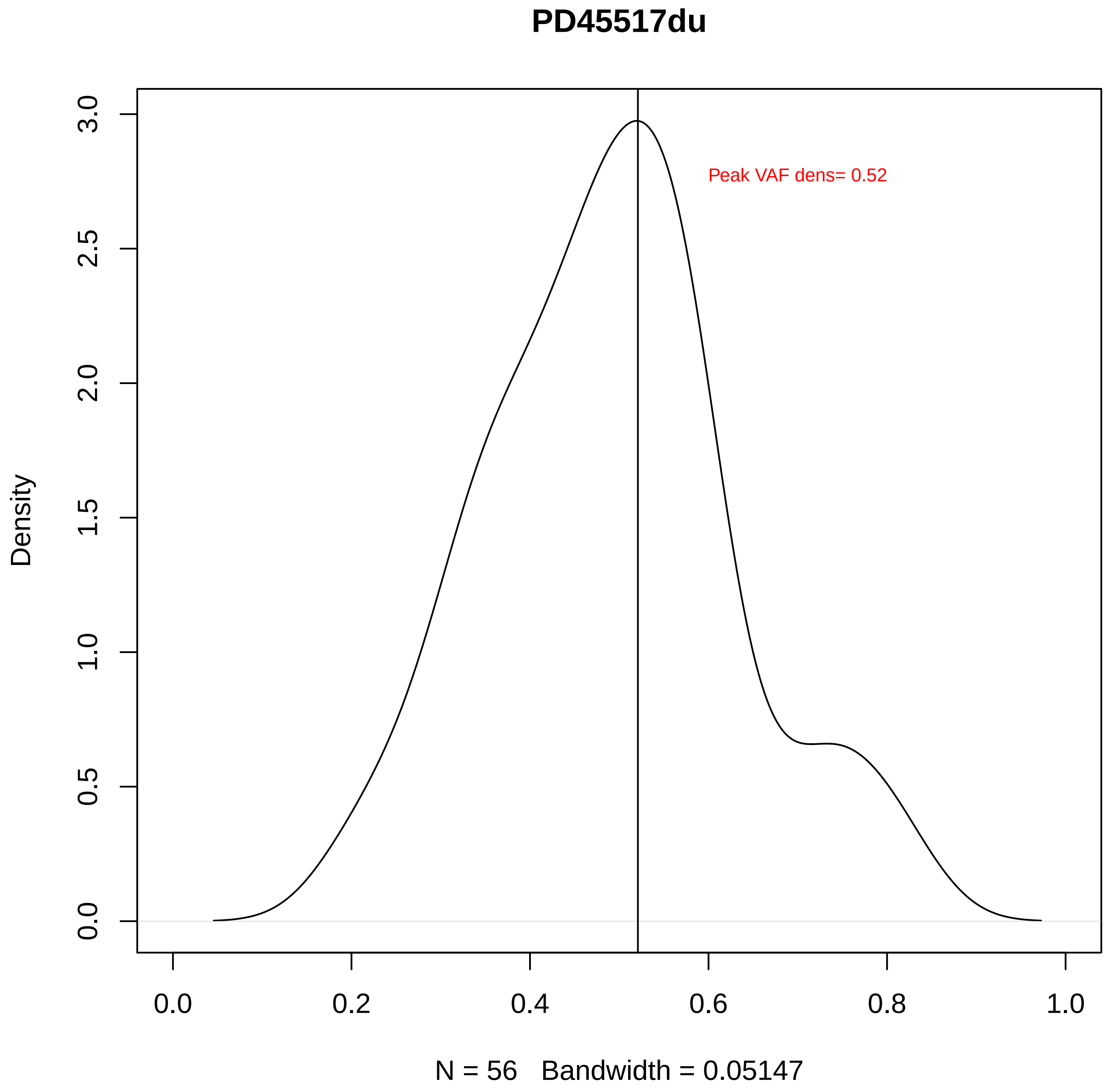
PD45517du
Peak VAF dens= 0.52
Density
3.0
2.5
2.0
1.5
1.0
0.5
0.0
0.0
0.2
0.4
0.6
0.8
1.0
N = 56   Bandwidth = 0.05147

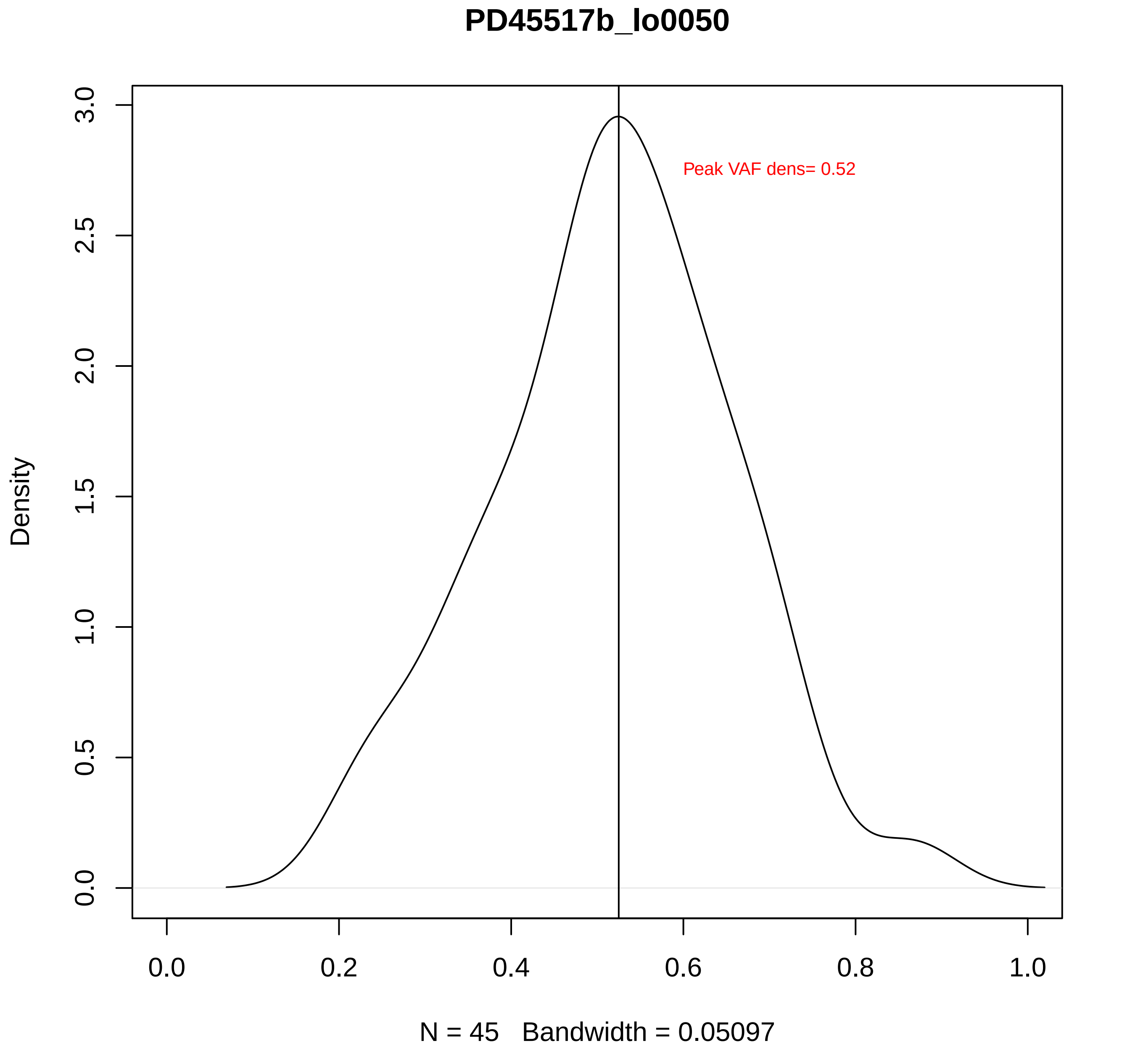
PD45517b_lo0050
Peak VAF dens= 0.52
Density
0.0
0.5
1.0
1.5
2.0
2.5
3.0
0.0
0.2
0.4
0.6
0.8
1.0
N = 45 Bandwidth = 0.05097

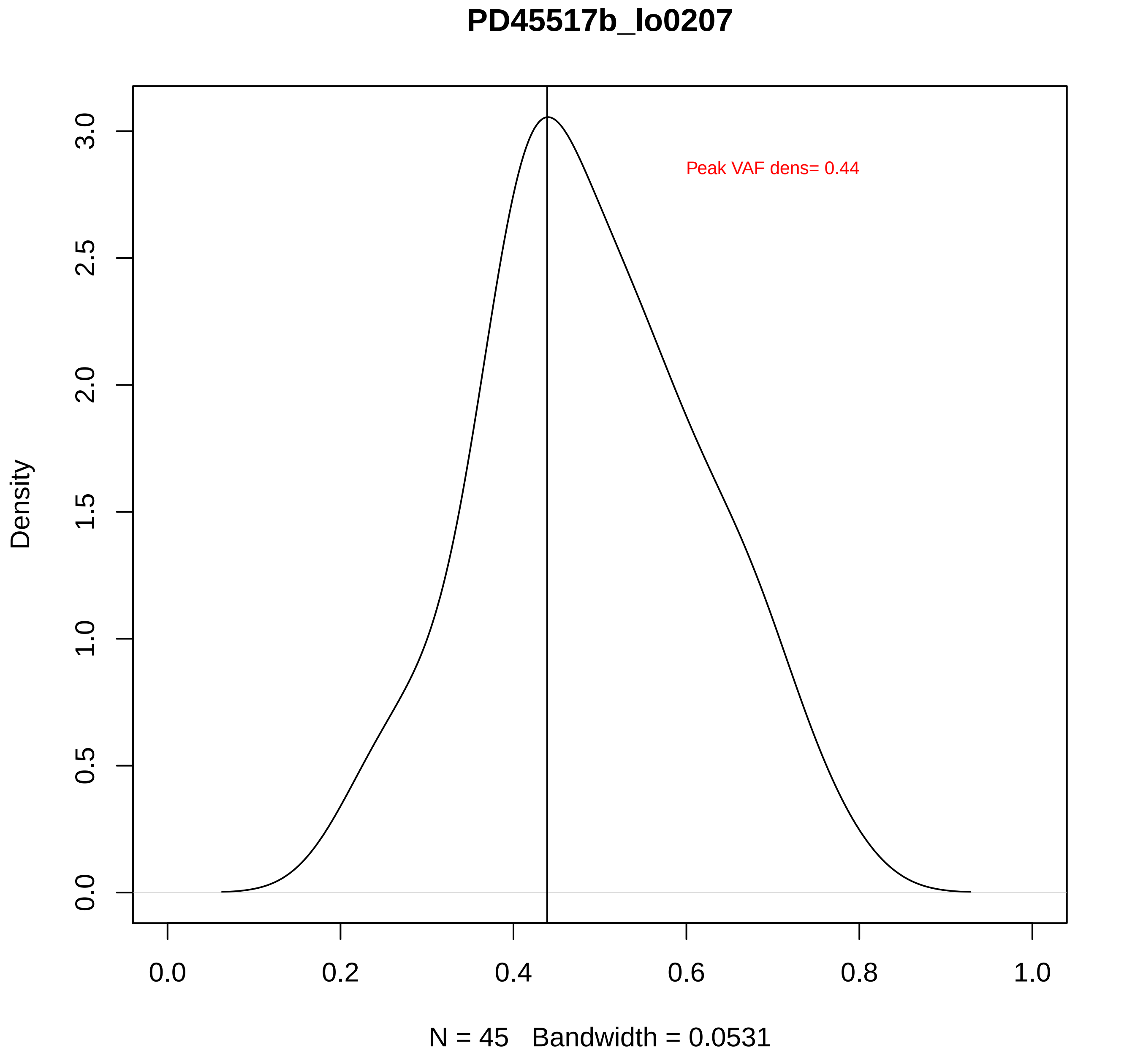
PD45517b_lo0207
Peak VAF dens= 0.44
Density
0.0
0.5
1.0
1.5
2.0
2.5
3.0
0.0
0.2
0.4
0.6
0.8
1.0
N = 45 Bandwidth = 0.0531

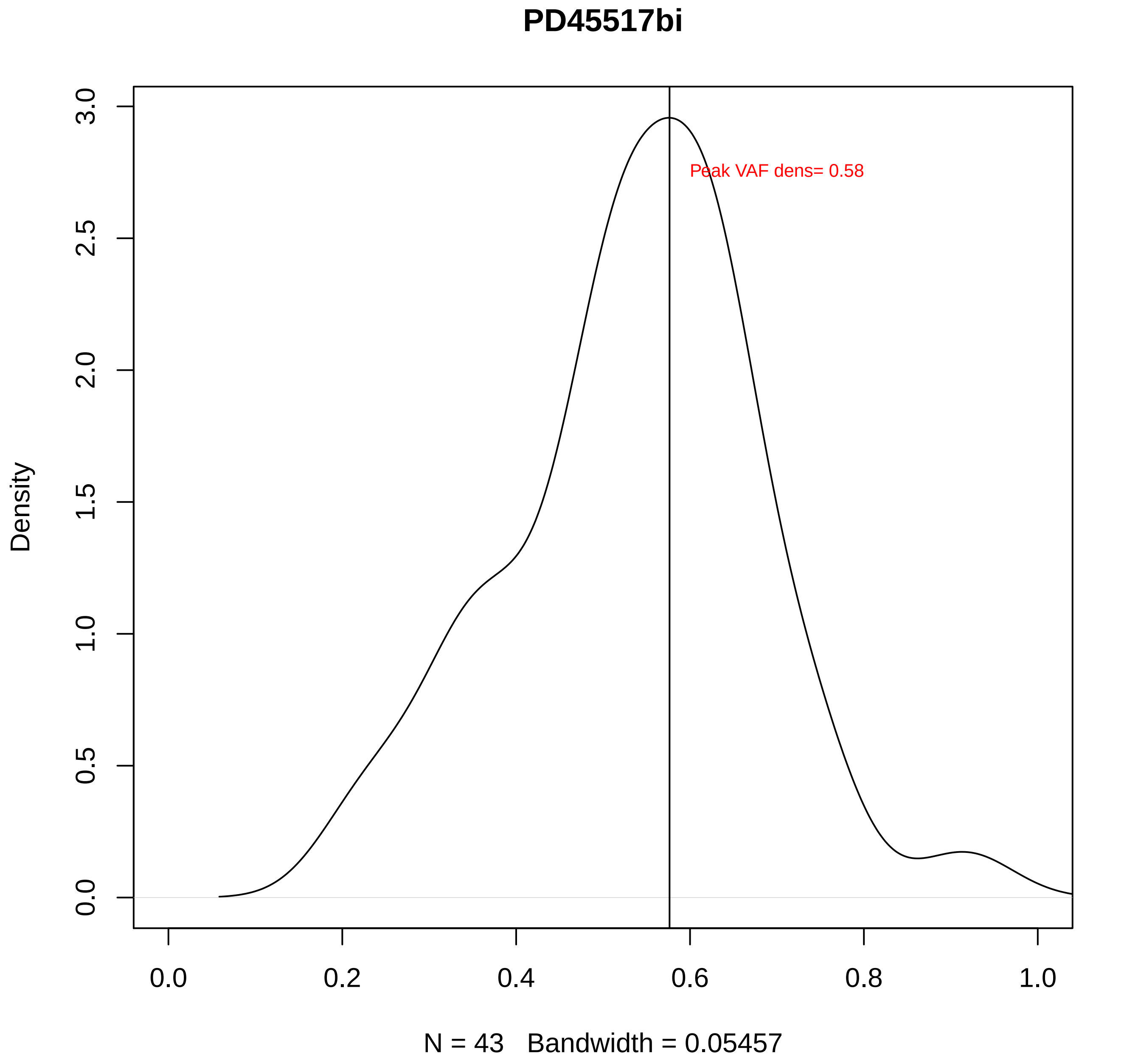

PD45517bi
Peak VAF dens= 0.58
Density
3.0
2.5
2.0
1.5
1.0
0.5
0.0
0.0
0.2
0.4
0.6
0.8
1.0
N = 43   Bandwidth = 0.05457

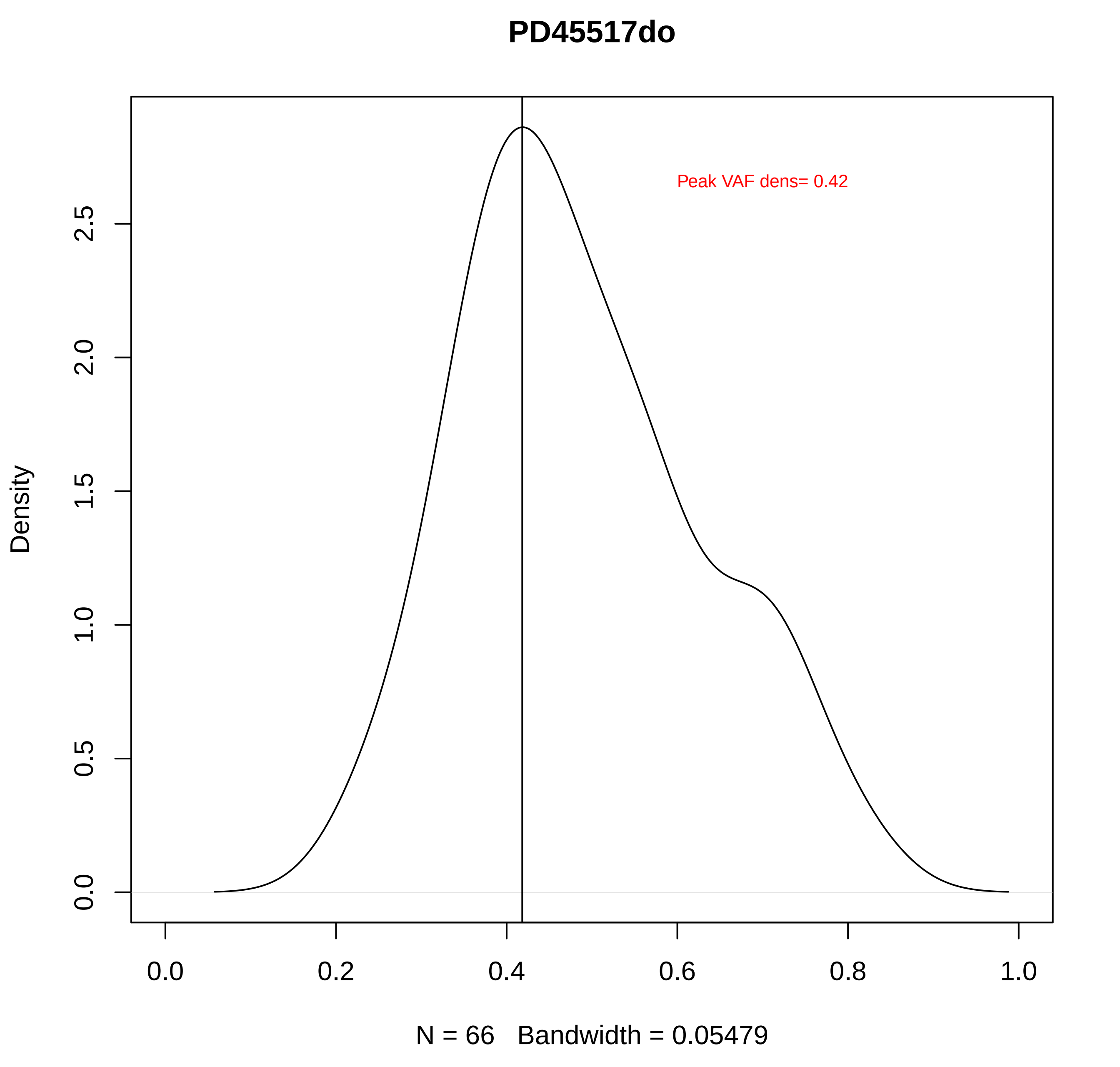
PD45517do
Peak VAF dens= 0.42
Density
0.0
0.5
1.0
1.5
2.0
2.5
0.0
0.2
0.4
0.6
0.8
1.0
N = 66   Bandwidth = 0.05479

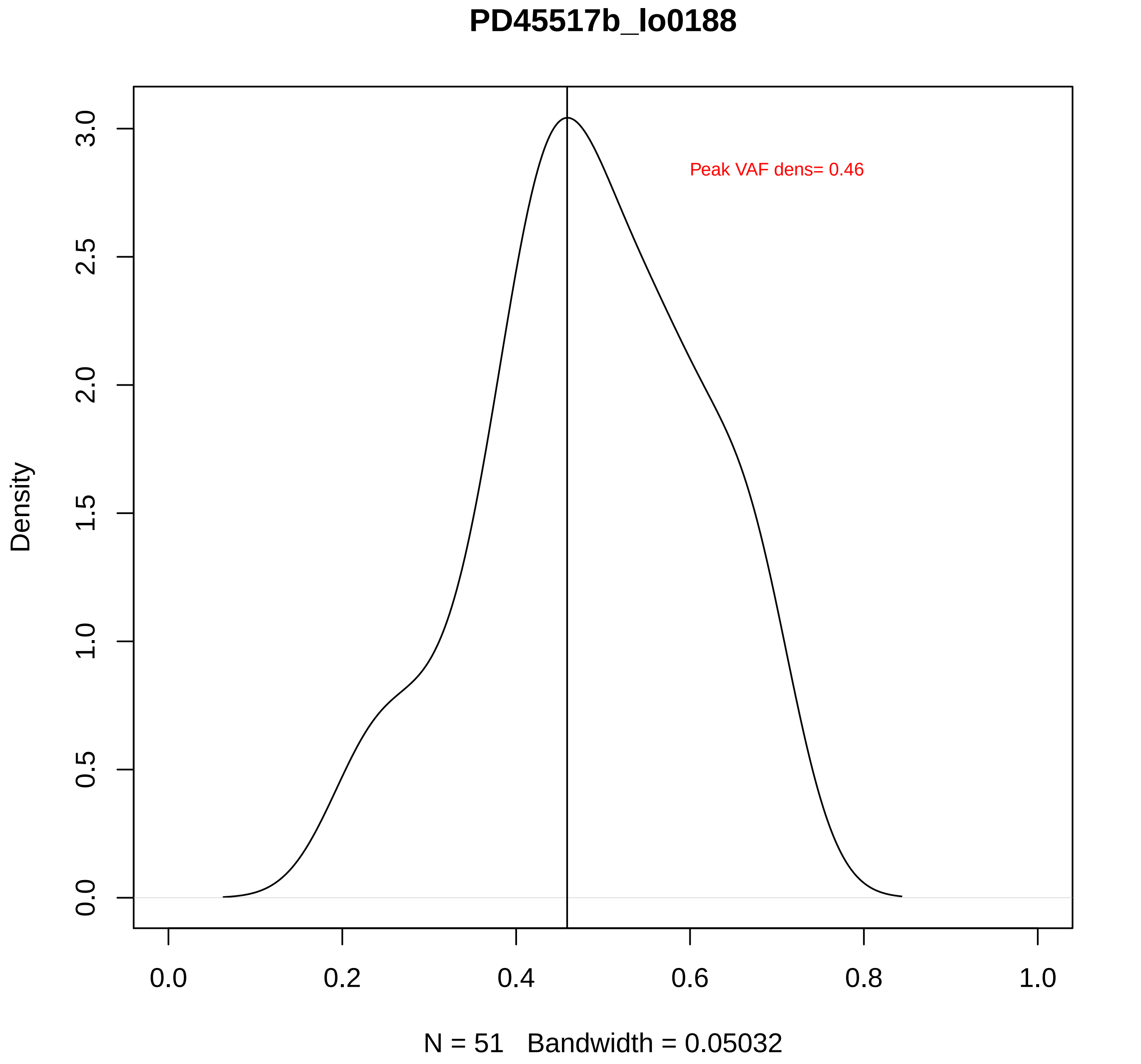
PD45517b_lo0188
Peak VAF dens= 0.46
Density
0.0
0.5
1.0
1.5
2.0
2.5
3.0
0.0
0.2
0.4
0.6
0.8
1.0
N = 51   Bandwidth = 0.05032

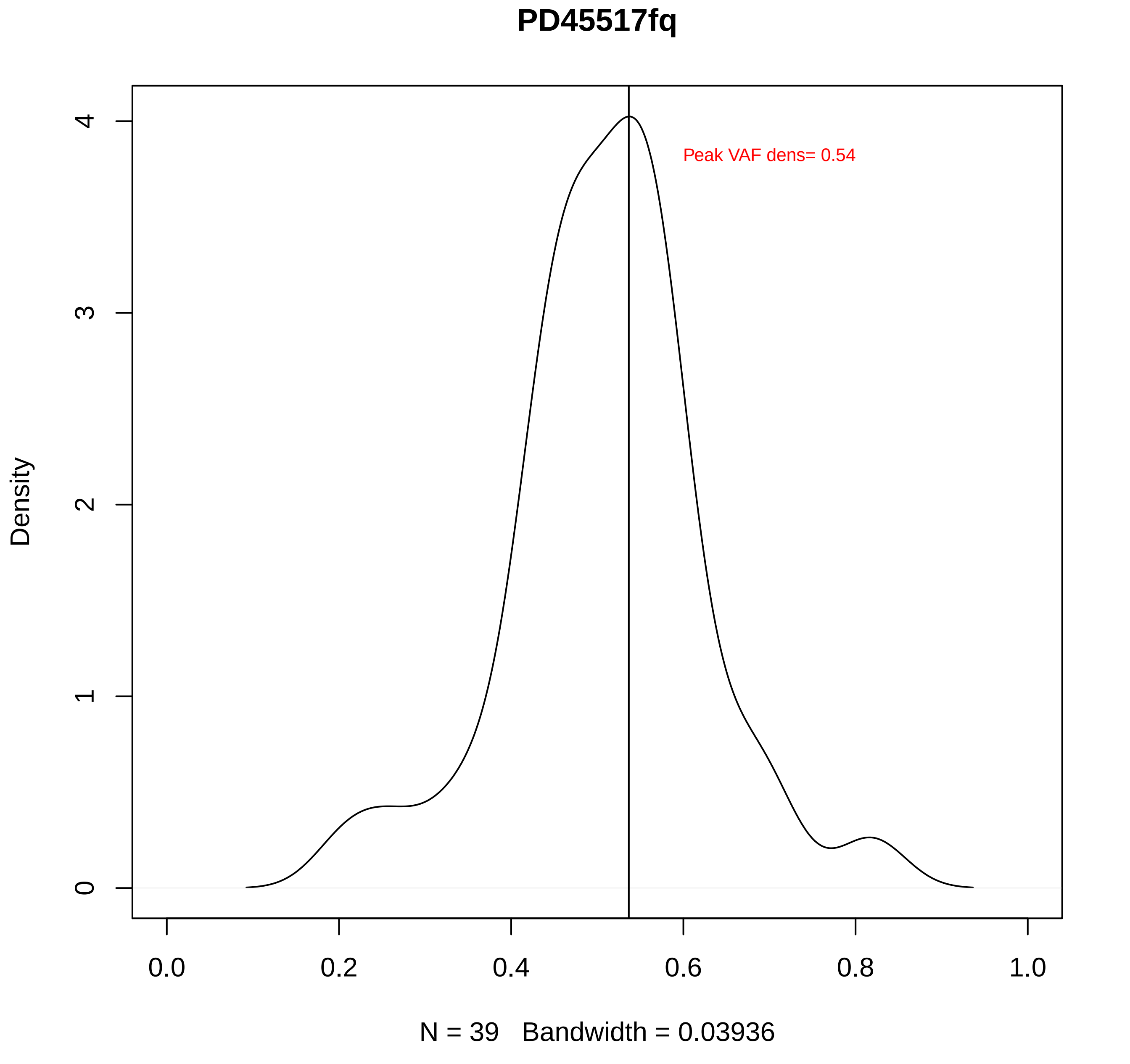
PD45517fq
Peak VAF dens= 0.54
Density
0
1
2
3
4
0.0
0.2
0.4
0.6
0.8
1.0
N = 39   Bandwidth = 0.03936

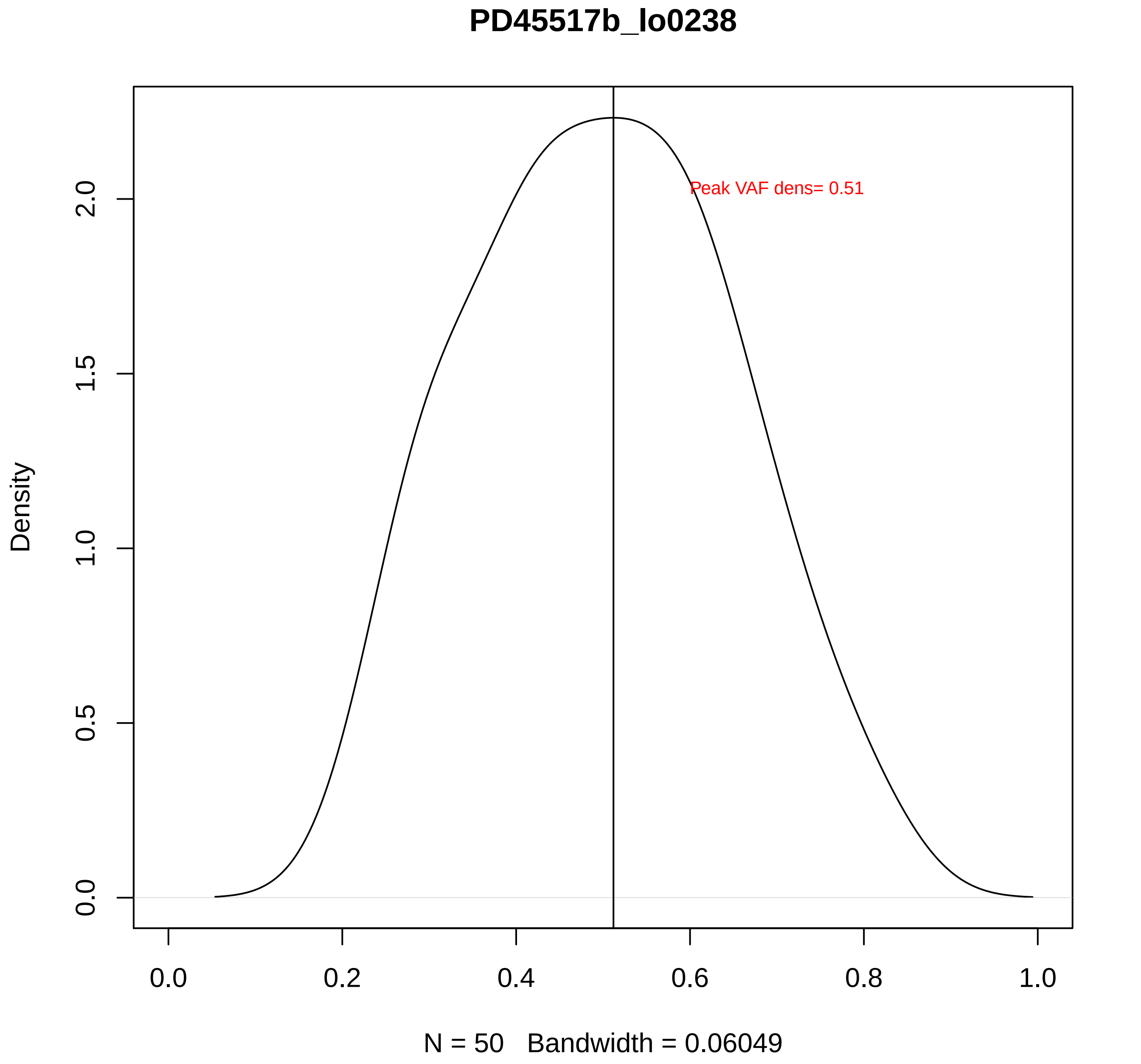
PD45517b_lo0238
Peak VAF dens= 0.51
Density
0.0
0.5
1.0
1.5
2.0
0.0
0.2
0.4
0.6
0.8
1.0
N = 50   Bandwidth = 0.06049

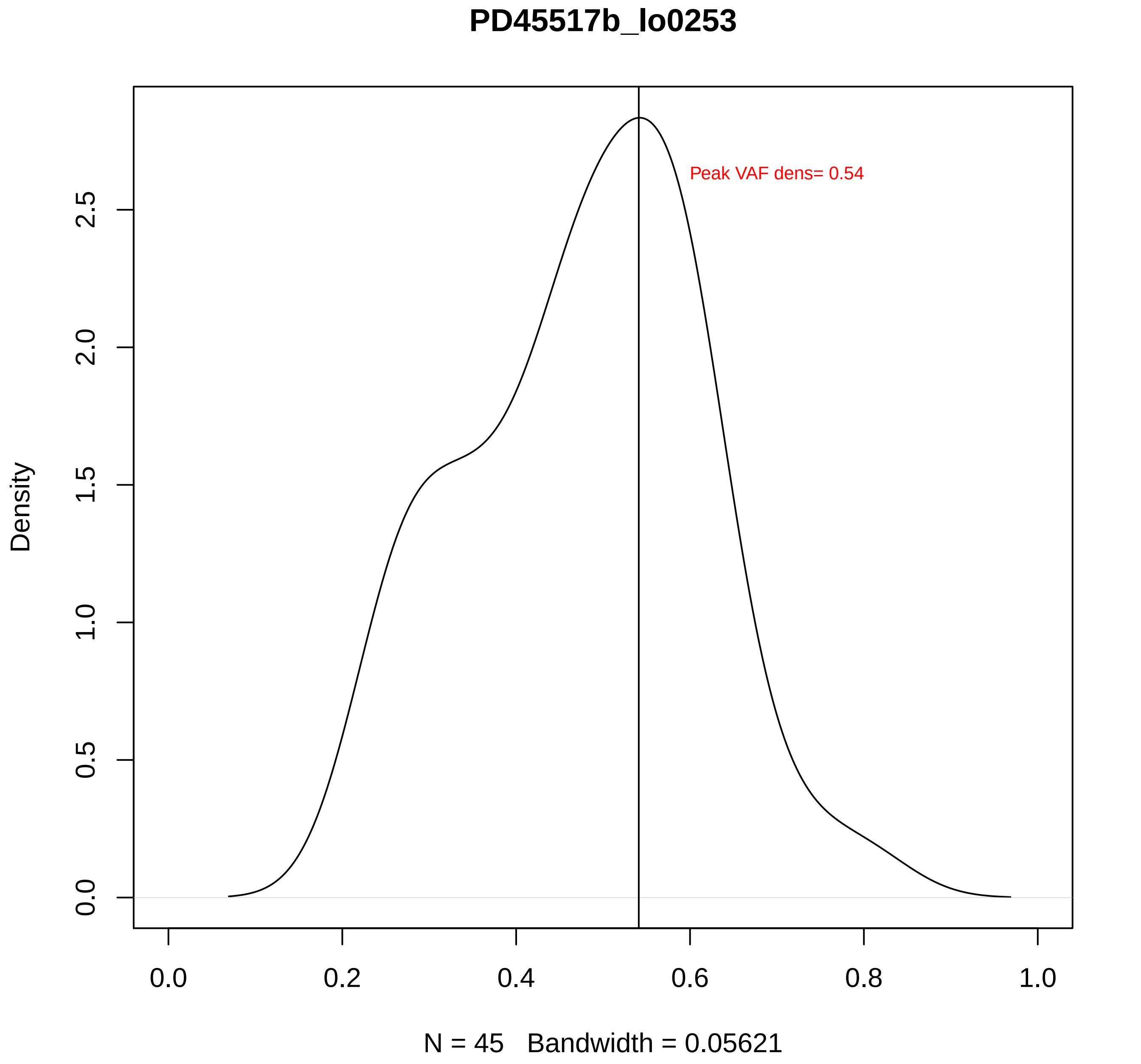
PD45517b_lo0253
Peak VAF dens= 0.54
Density
0.0
0.5
1.0
1.5
2.0
2.5
0.0
0.2
0.4
0.6
0.8
1.0
N = 45   Bandwidth = 0.05621

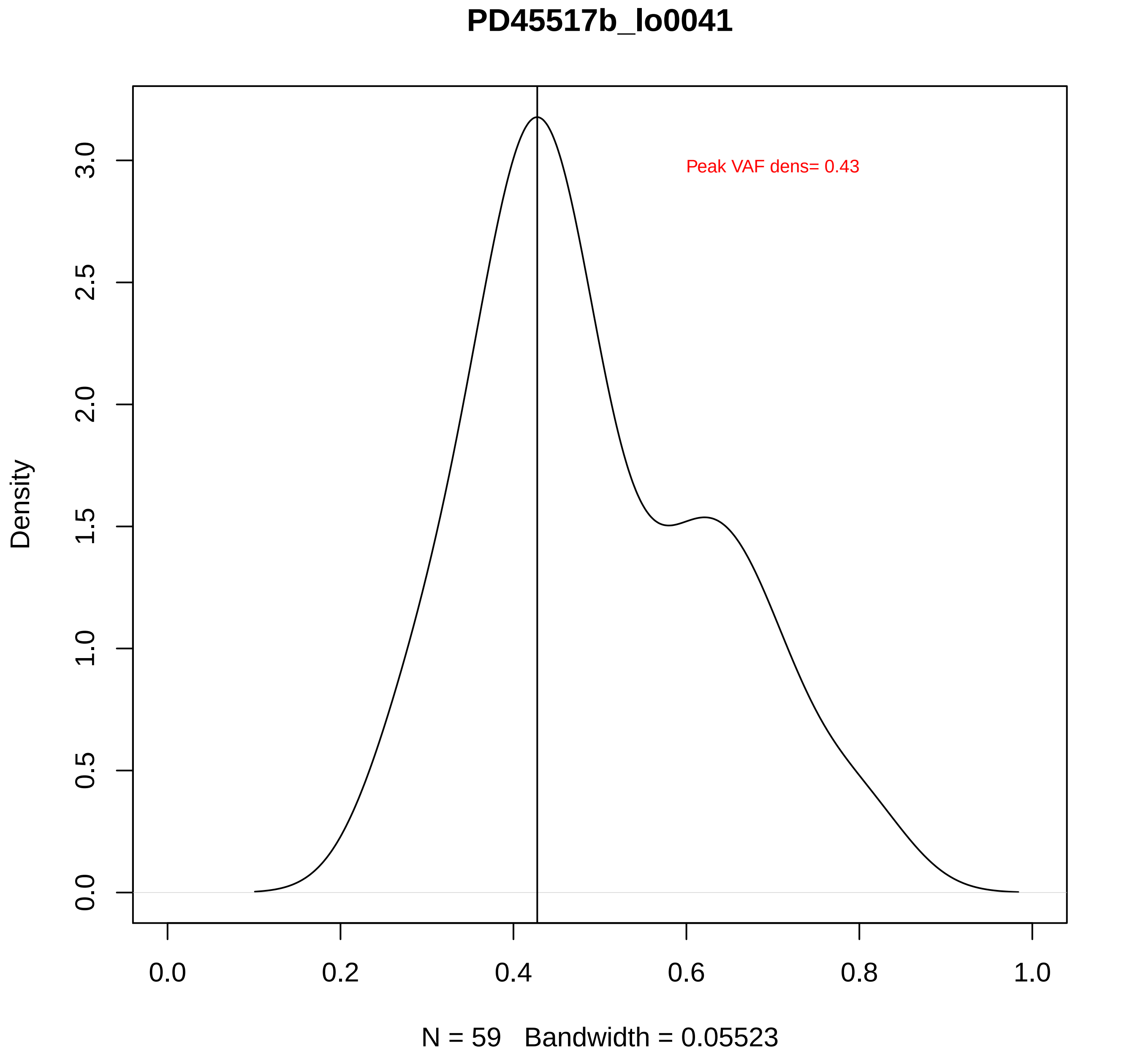
PD45517b_lo0041
Peak VAF dens= 0.43
Density
0.0
0.5
1.0
1.5
2.0
2.5
3.0
0.0
0.2
0.4
0.6
0.8
1.0
N = 59 Bandwidth = 0.05523

PD45517b_lo0324

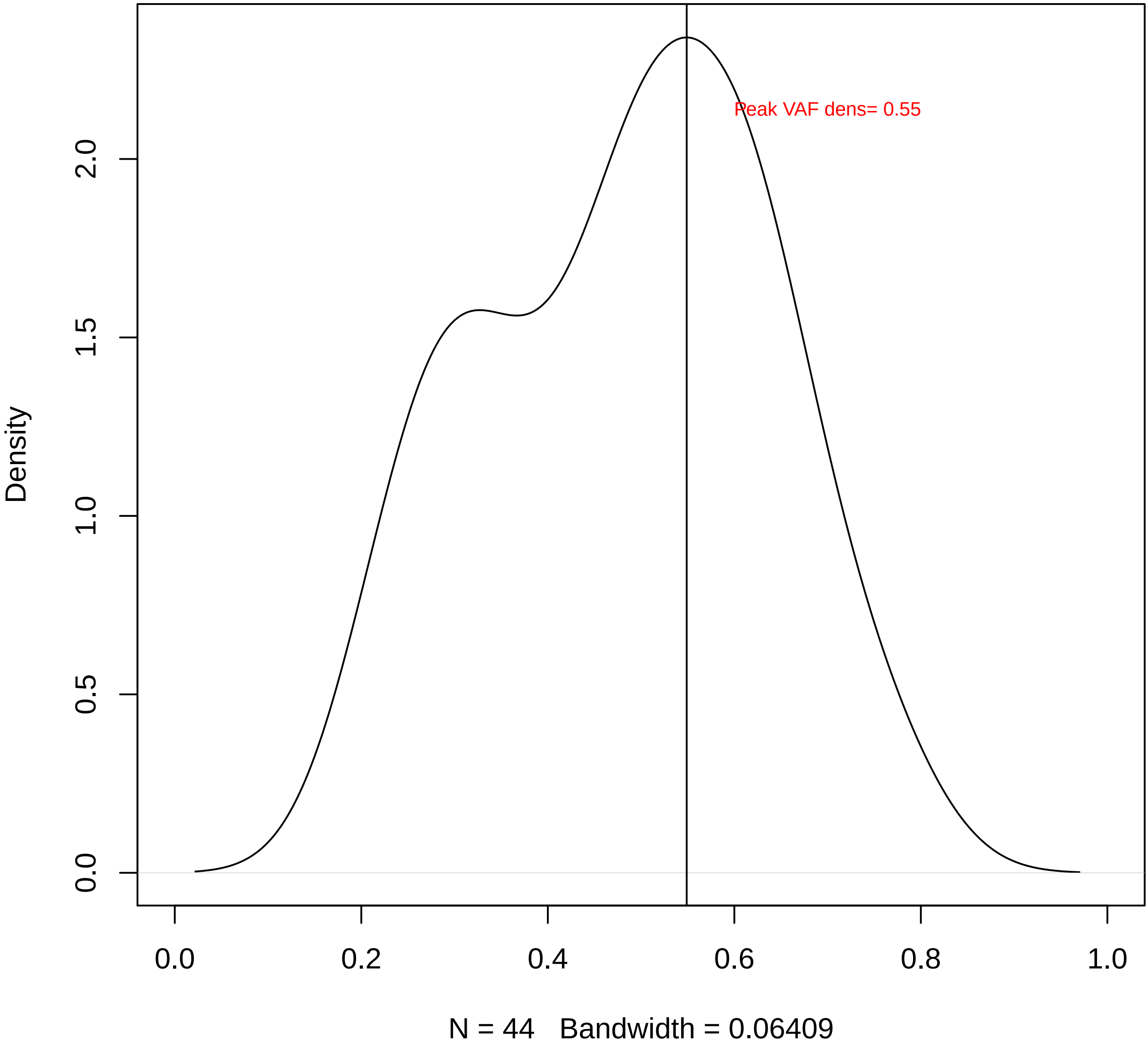

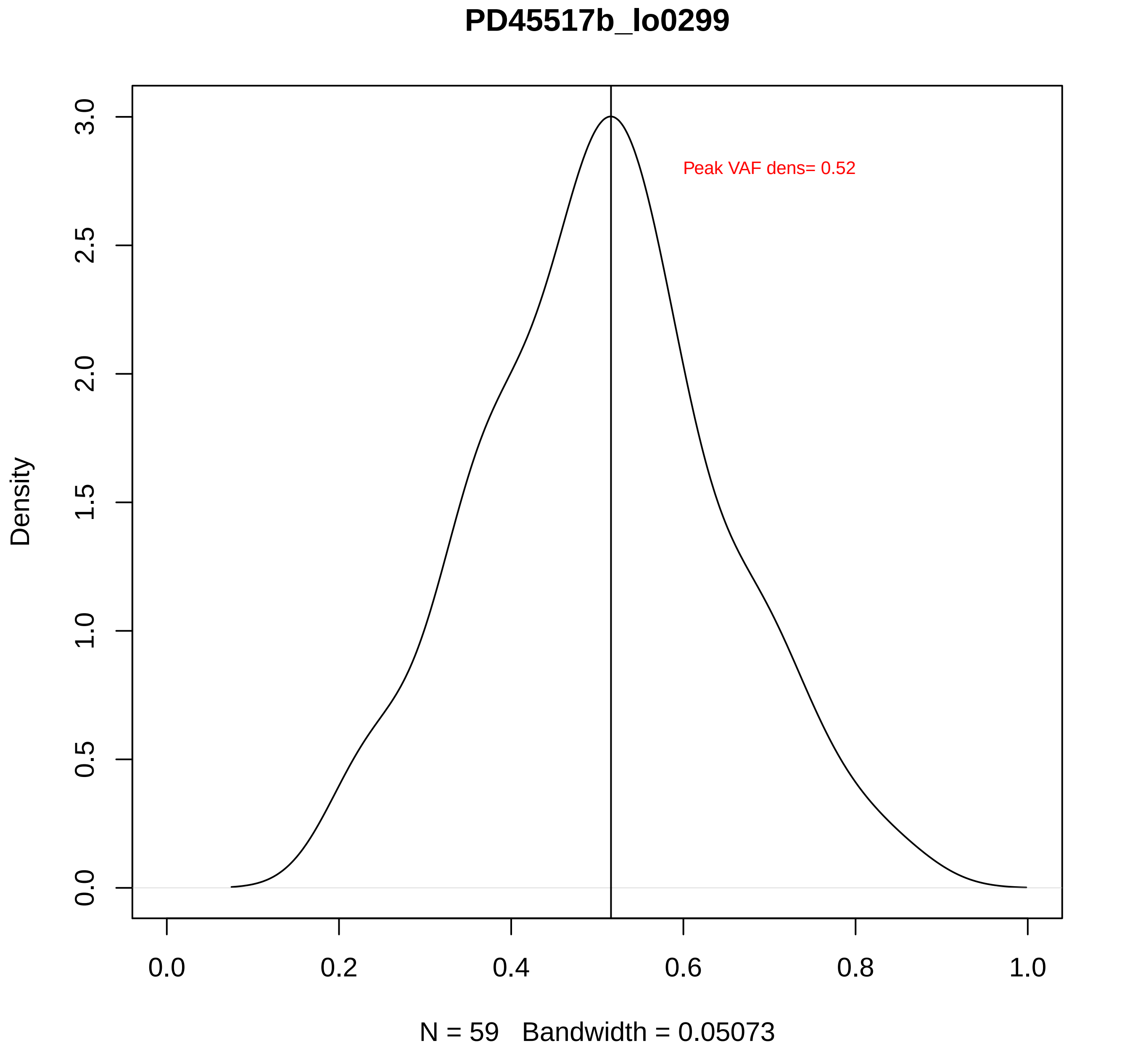
PD45517b_lo0299
Peak VAF dens= 0.52
Density
0.0
0.5
1.0
1.5
2.0
2.5
3.0
0.0
0.2
0.4
0.6
0.8
1.0
N = 59 Bandwidth = 0.05073

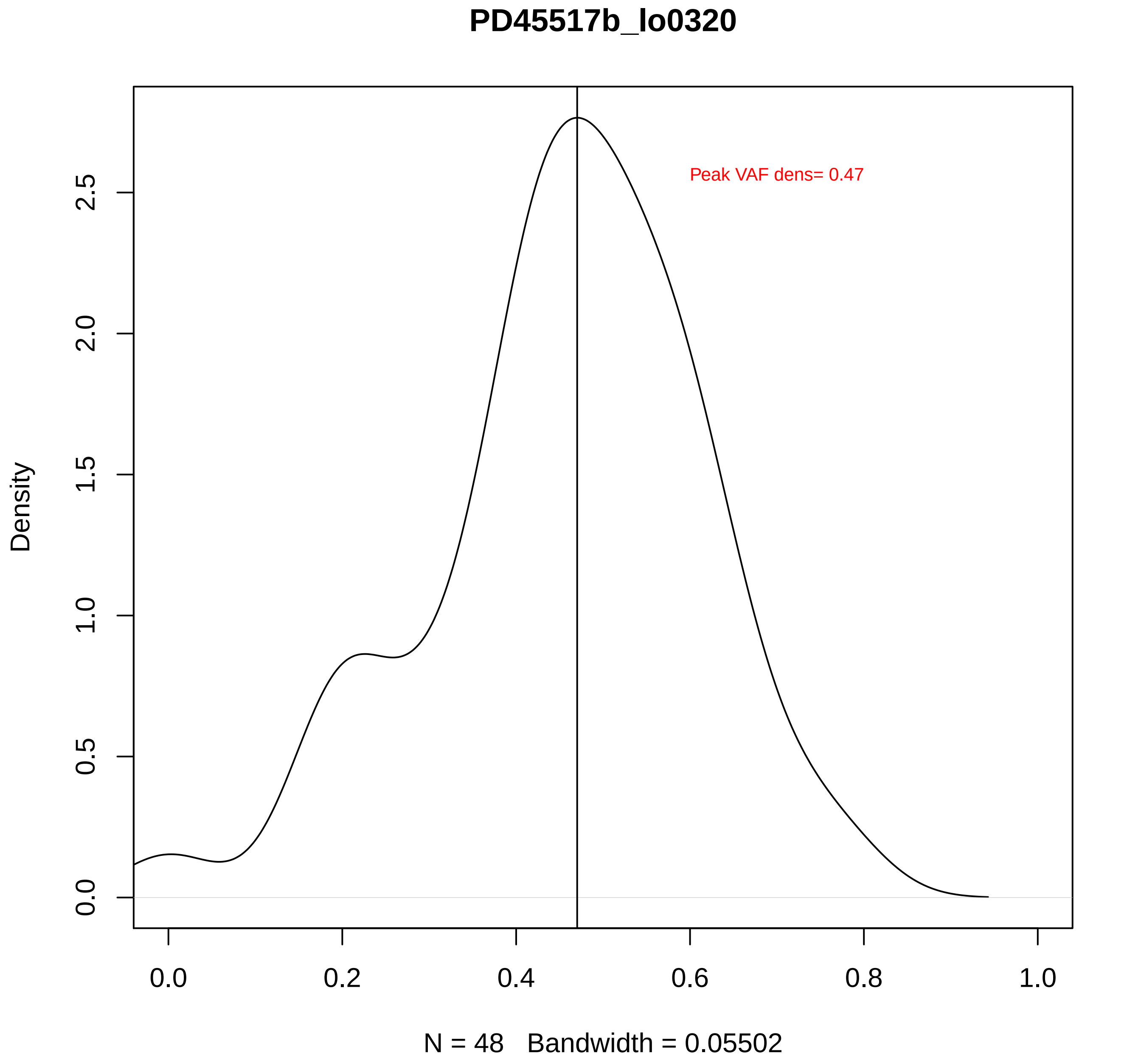

PD45517b_lo0320
Peak VAF dens= 0.47
Density
0.0
0.5
1.0
1.5
2.0
2.5
0.0
0.2
0.4
0.6
0.8
1.0
N = 48   Bandwidth = 0.05502

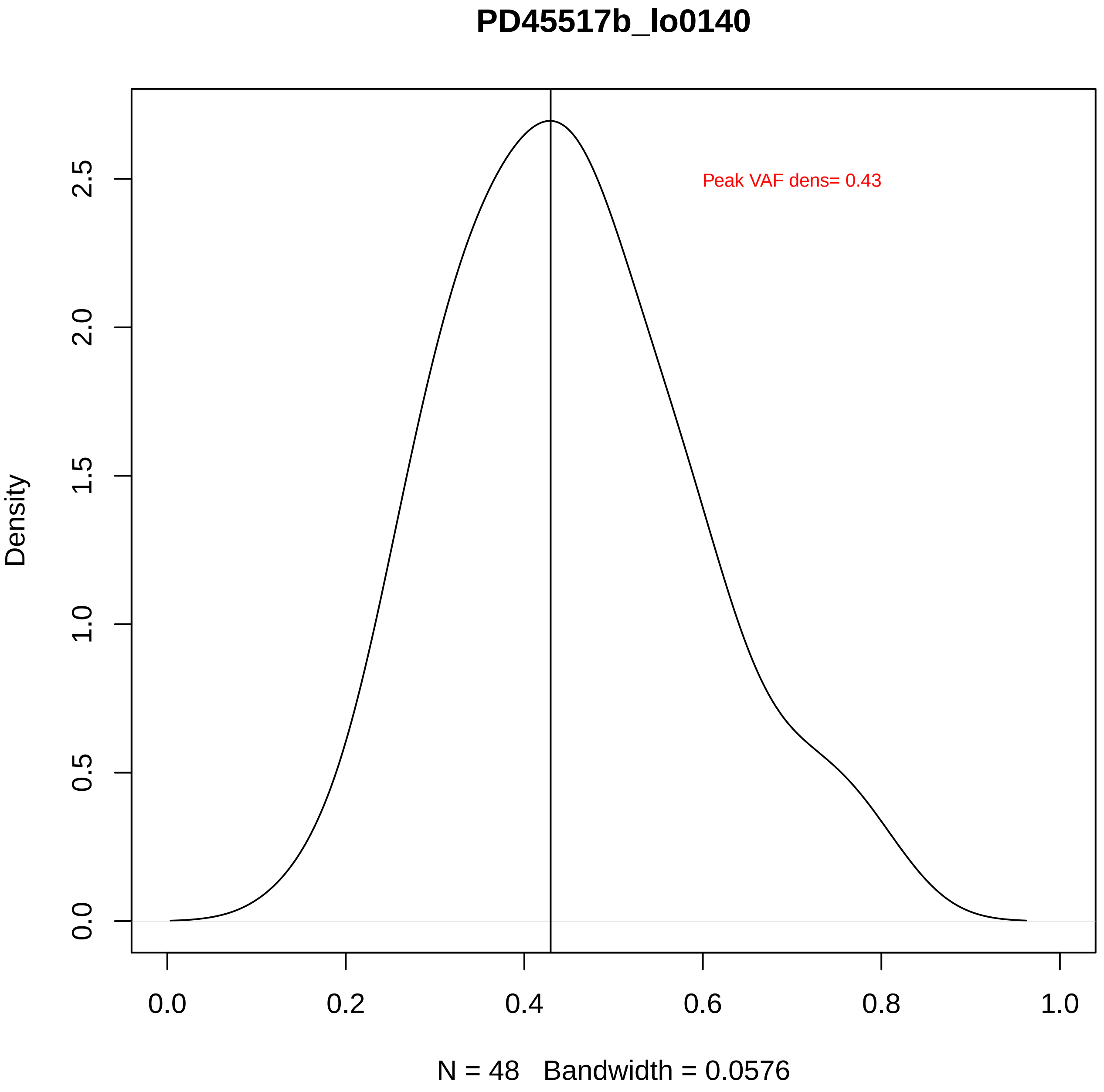
PD45517b_lo0140
Peak VAF dens= 0.43
Density
0.0
0.5
1.0
1.5
2.0
2.5
0.0
0.2
0.4
0.6
0.8
1.0
N = 48 Bandwidth = 0.0576

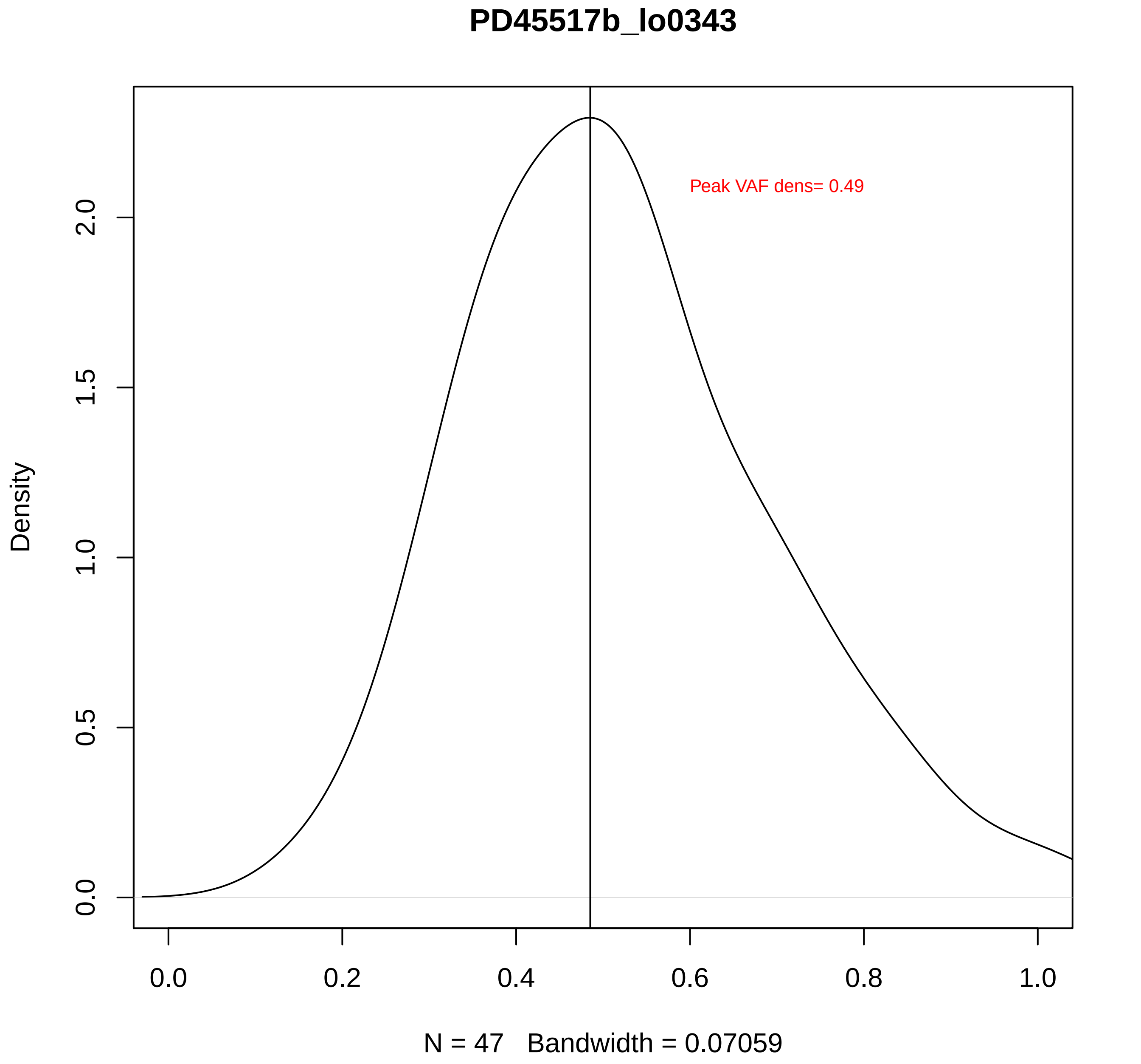
PD45517b_lo0343
Peak VAF dens= 0.49
Density
0.0
0.5
1.0
1.5
2.0
0.0
0.2
0.4
0.6
0.8
1.0
N = 47   Bandwidth = 0.07059

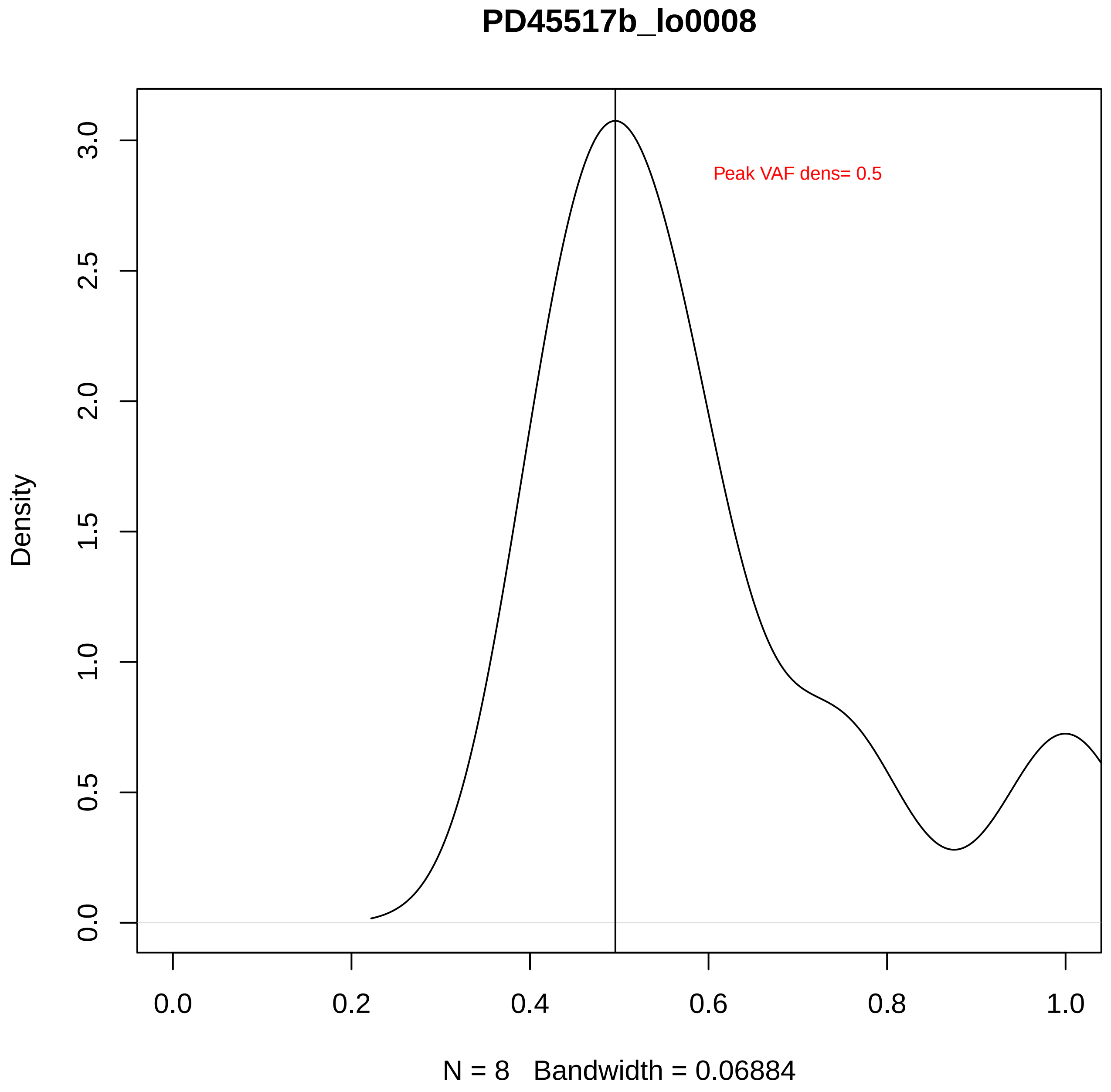
PD45517b_lo0008
Peak VAF dens= 0.5
Density
0.0
0.5
1.0
1.5
2.0
2.5
3.0
0.0
0.2
0.4
0.6
0.8
1.0
N = 8   Bandwidth = 0.06884

PD45517b_lo0249

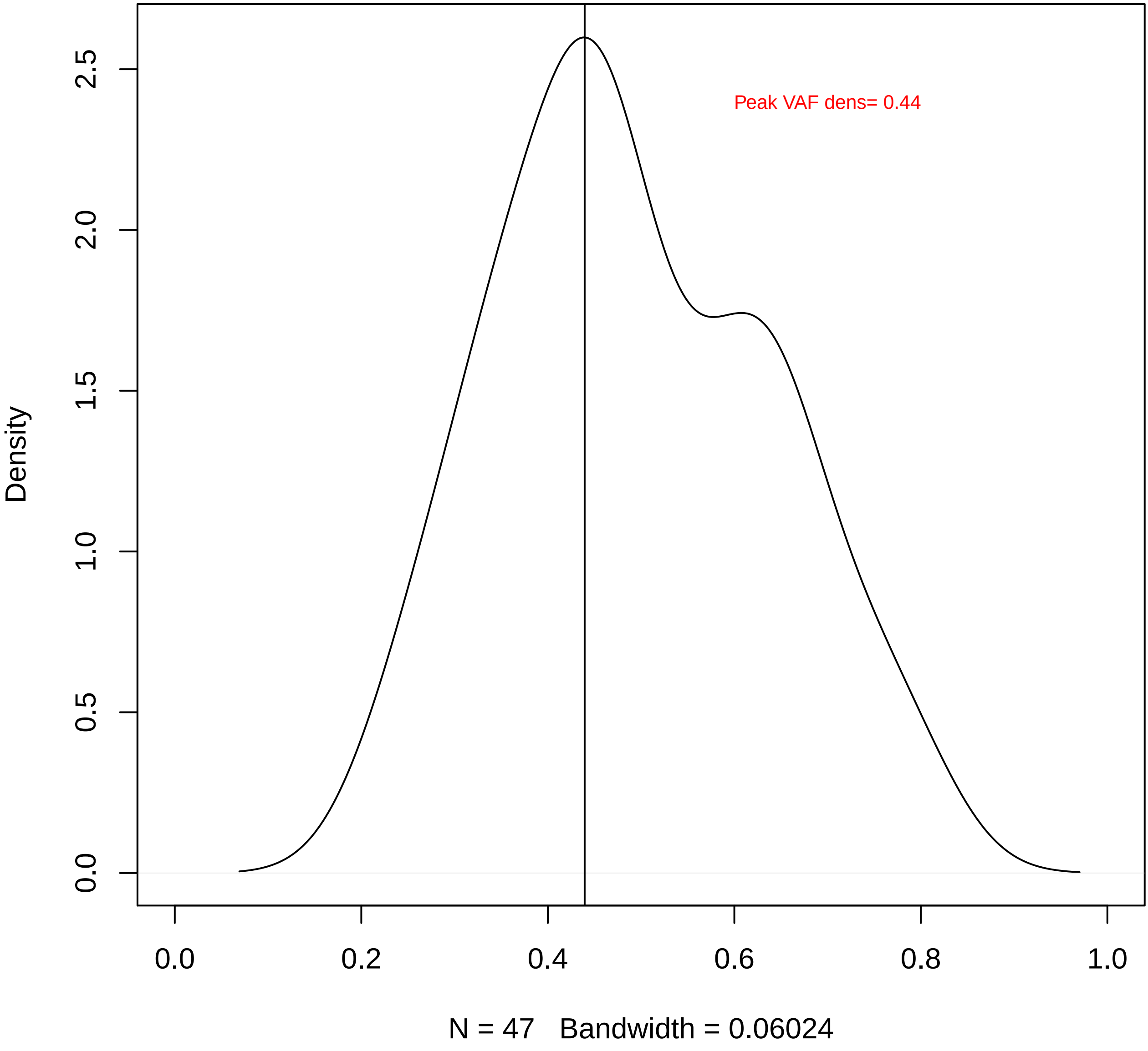

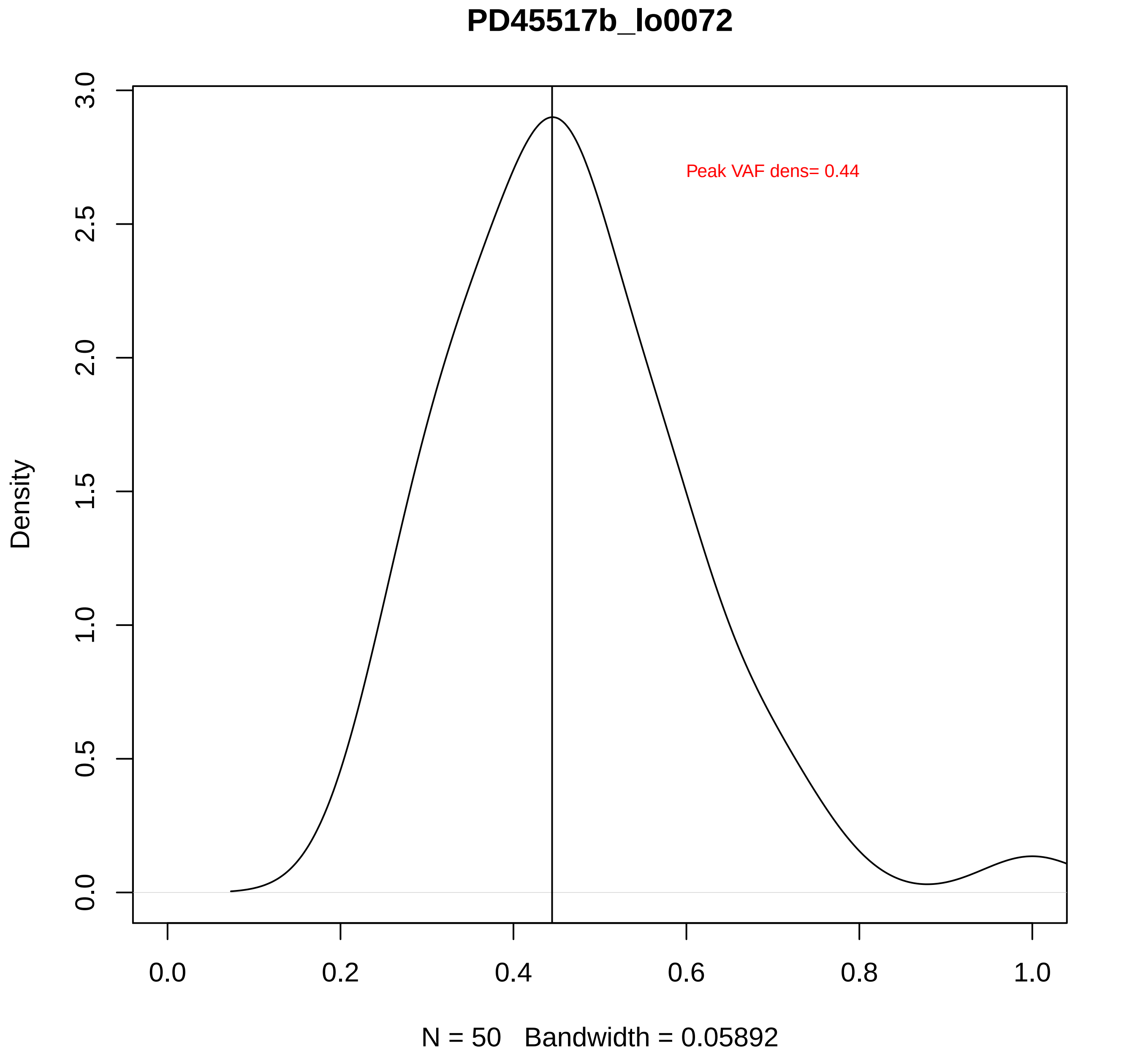
PD45517b_lo0072
Peak VAF dens= 0.44
Density
0.0
0.5
1.0
1.5
2.0
2.5
3.0
0.0
0.2
0.4
0.6
0.8
1.0
N = 50 Bandwidth = 0.05892

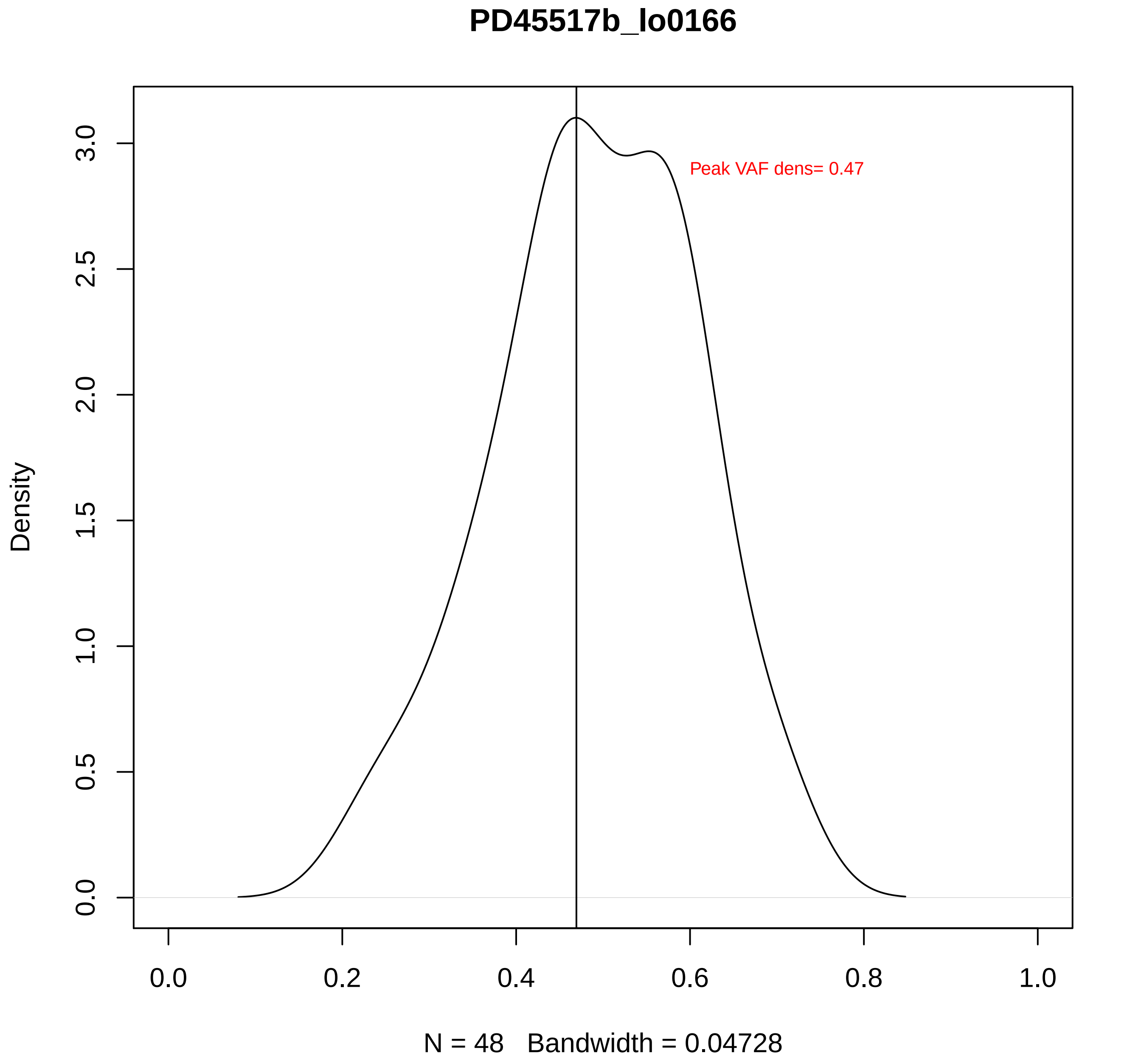
PD45517b_lo0166
Peak VAF dens= 0.47
Density
0.0
0.5
1.0
1.5
2.0
2.5
3.0
0.0
0.2
0.4
0.6
0.8
1.0
N = 48 Bandwidth = 0.04728
