# Supplementary material for: Clonal dynamics of haematopoiesis across the human lifespan
Source: Nature. 2022 Jun 1;606(7913):343–50. doi: 10.1038/s41586-022-04786-y (PMC9177428; doi:10.1038/s41586-022-04786-y)
Supplement: Supplementary file 4 — HTMLs of notebooks outlining key statistical analyses presented in the manuscript, including analysis of phylogenetic trees. [file 41586_2022_4786_MOESM4_ESM.zip › Supplementary_code/SNV_indel_analysis/KX007_sample_vaf_plots.pdf]

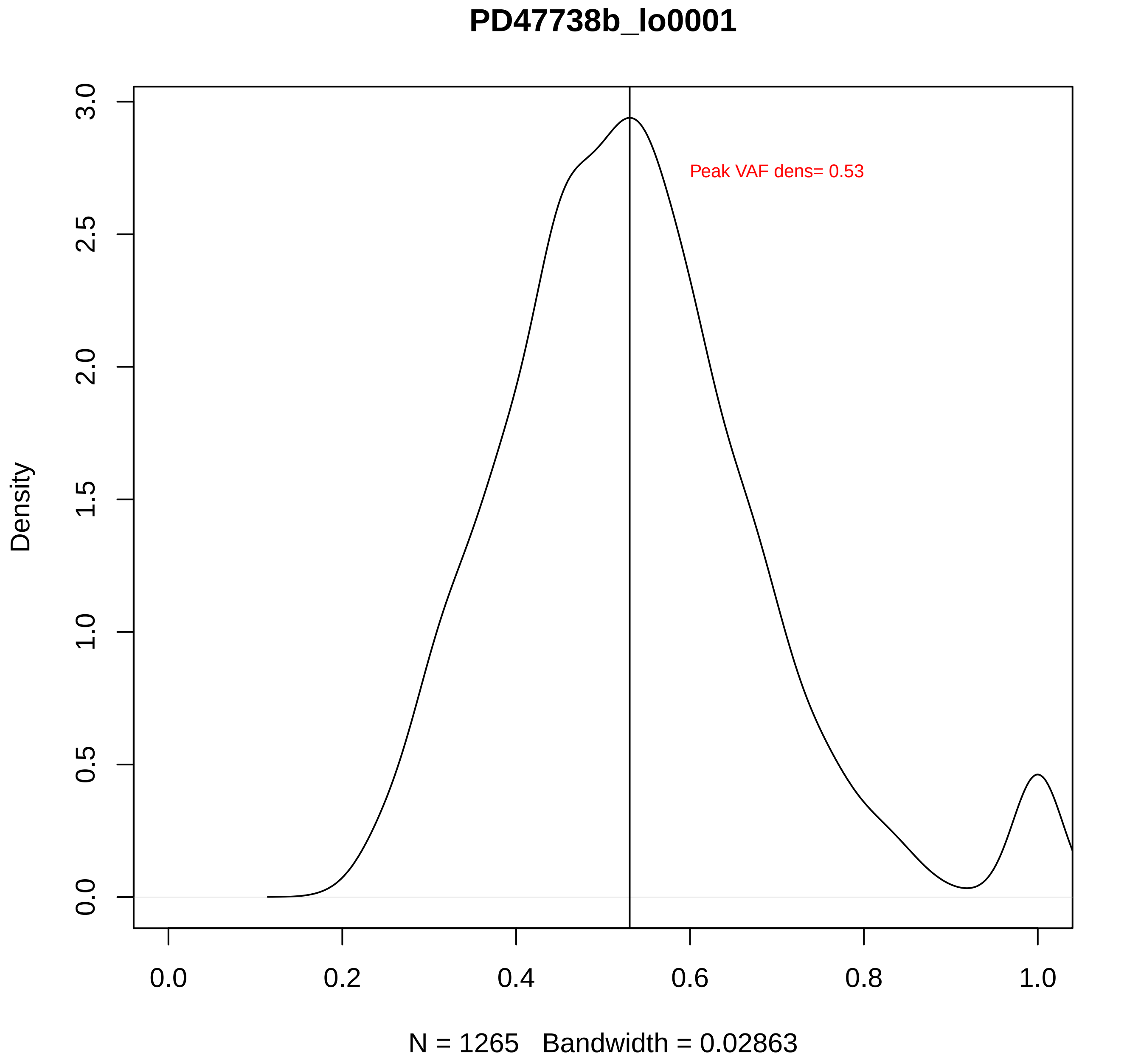
PD47738b_lo0001
Peak VAF dens= 0.53
Density
0.0
0.5
1.0
1.5
2.0
2.5
3.0
0.0
0.2
0.4
0.6
0.8
1.0
N = 1265   Bandwidth = 0.02863

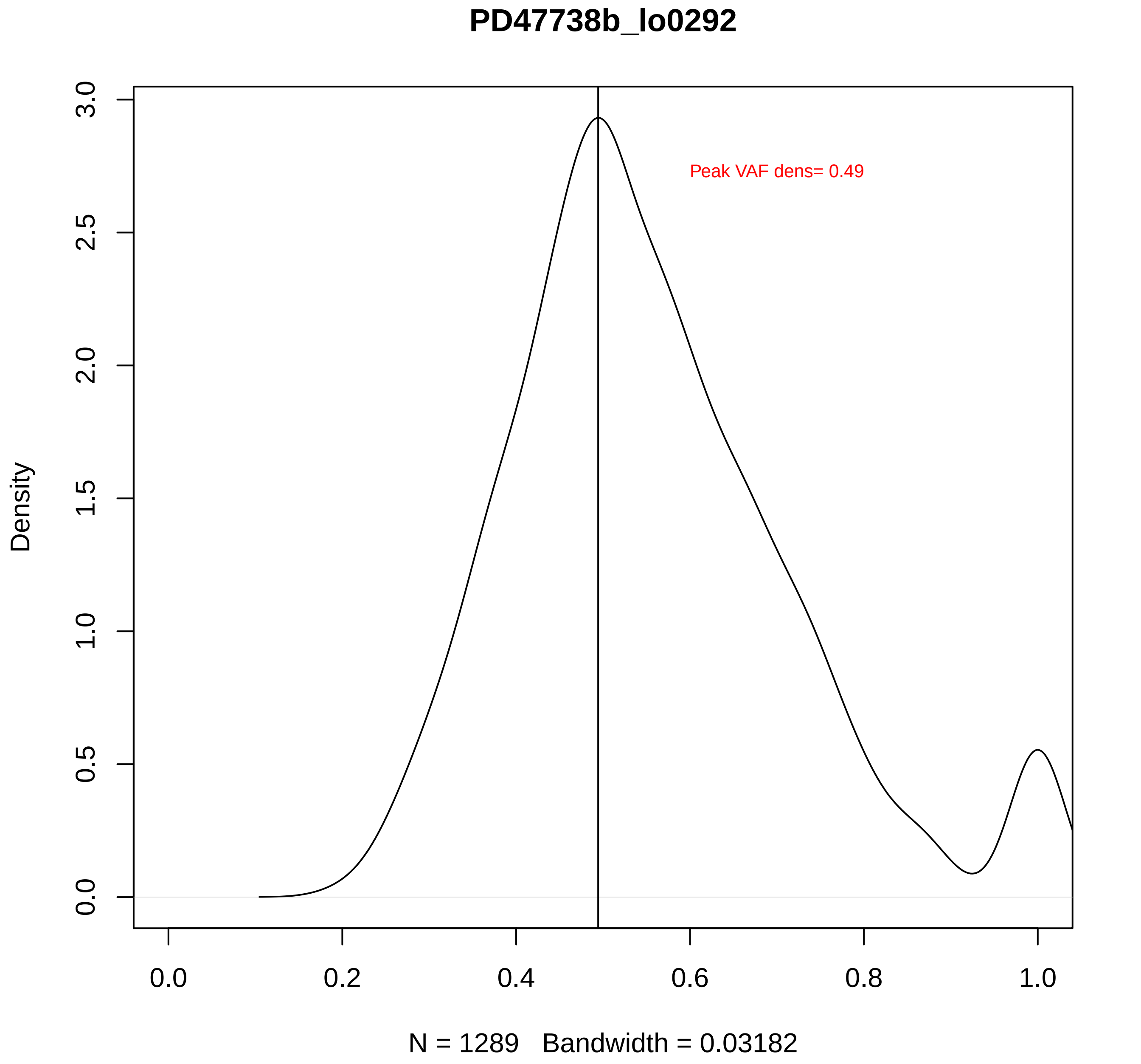
PD47738b_lo0292
Peak VAF dens= 0.49
Density
0.0
0.5
1.0
1.5
2.0
2.5
3.0
0.0
0.2
0.4
0.6
0.8
1.0
N = 1289   Bandwidth = 0.03182

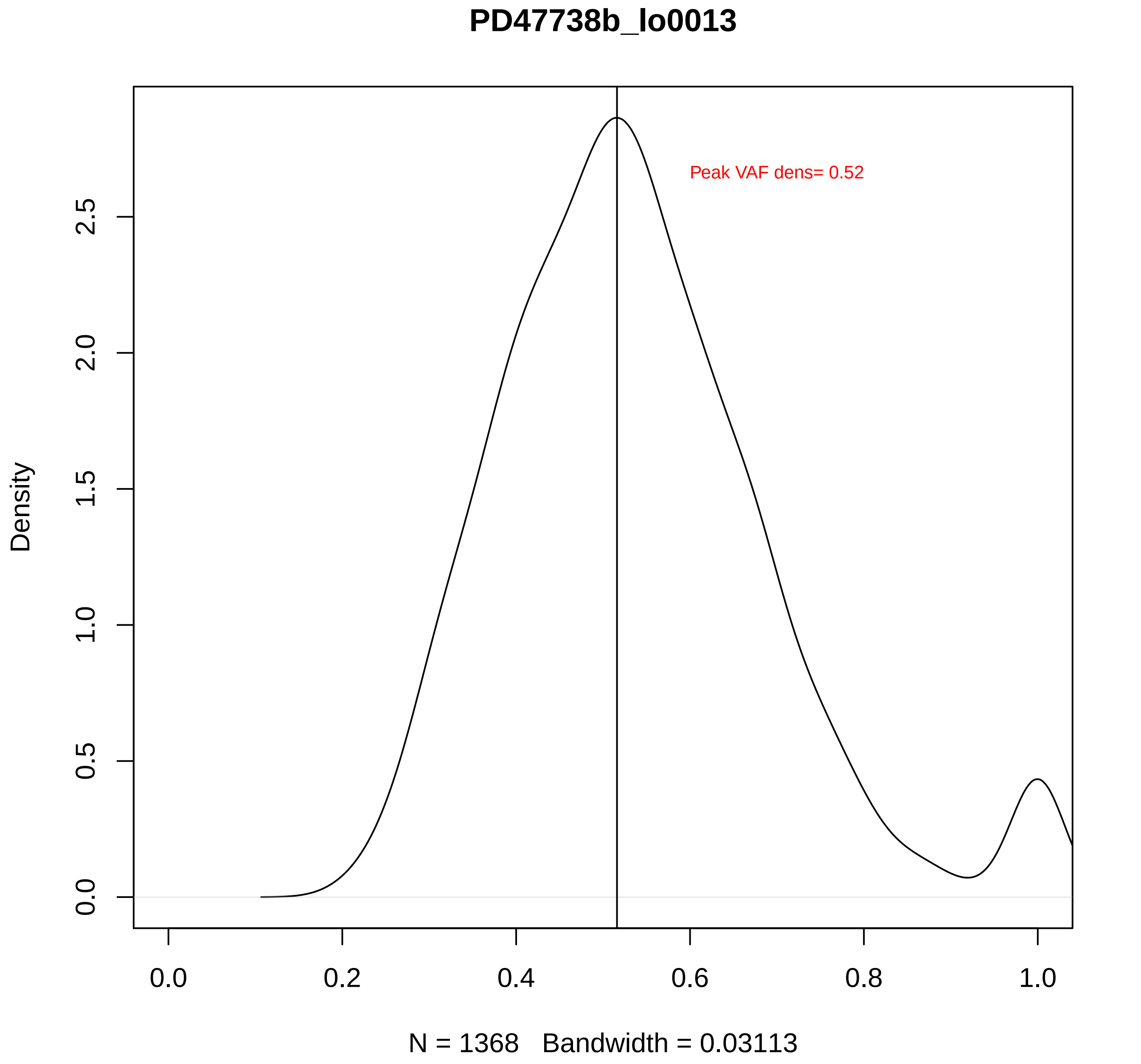
PD47738b_lo0013
Peak VAF dens= 0.52
Density
0.0
0.5
1.0
1.5
2.0
2.5
0.0
0.2
0.4
0.6
0.8
1.0
N = 1368   Bandwidth = 0.03113

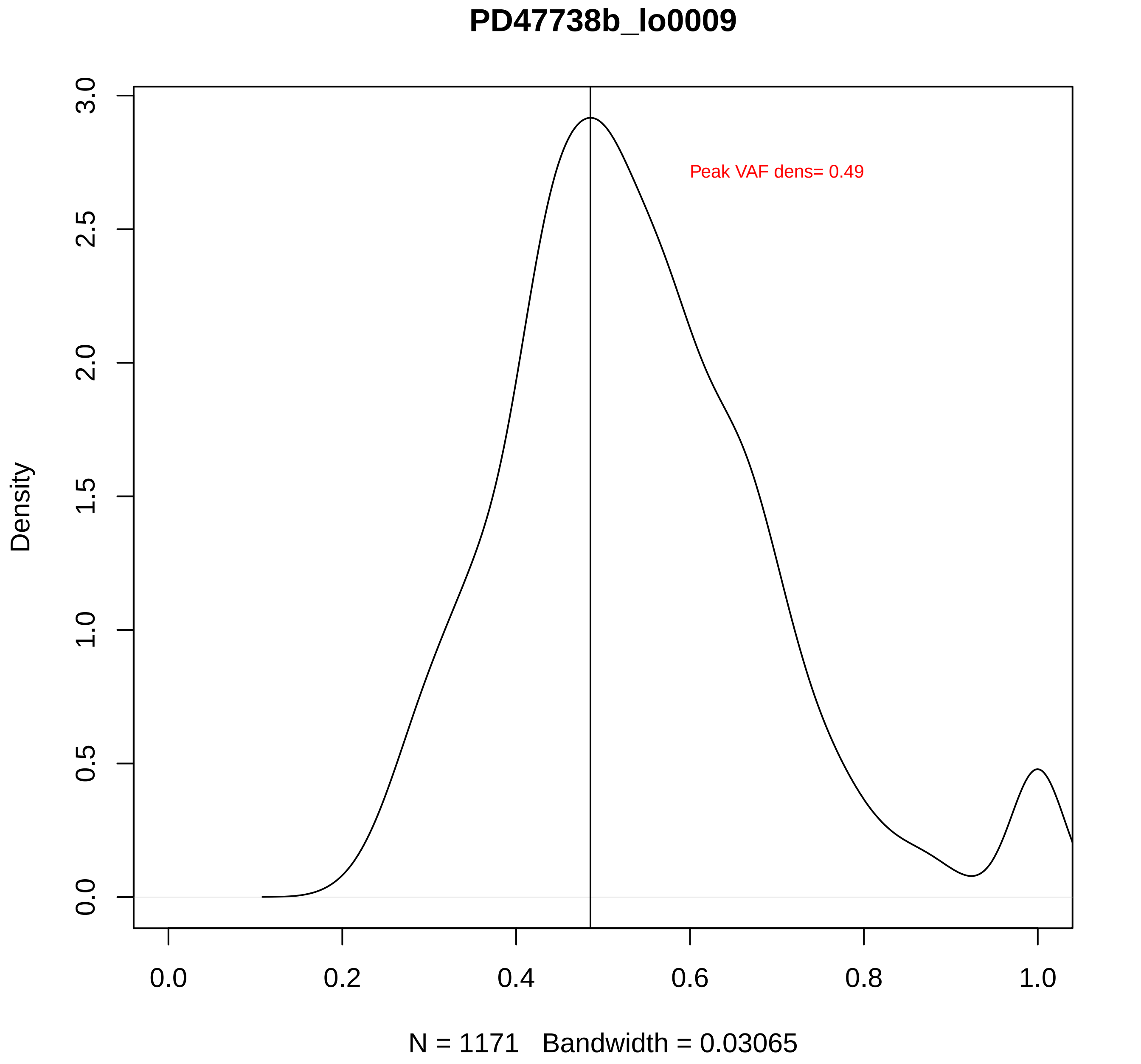
PD47738b_lo0009
Peak VAF dens= 0.49
Density
0.0
0.5
1.0
1.5
2.0
2.5
3.0
0.0
0.2
0.4
0.6
0.8
1.0
N = 1171   Bandwidth = 0.03065

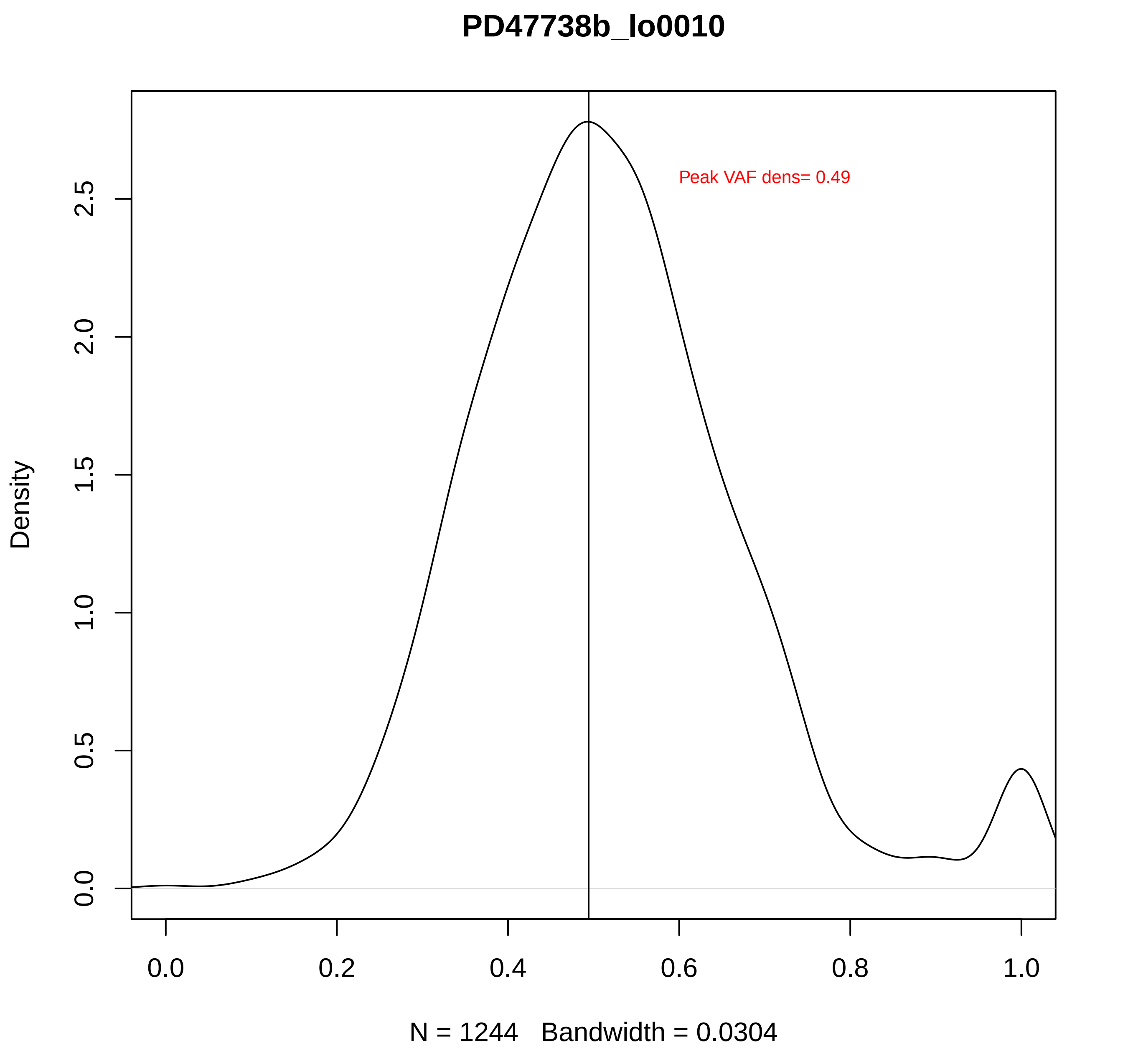
PD47738b_lo0010
Peak VAF dens= 0.49
Density
0.0
0.5
1.0
1.5
2.0
2.5
0.0
0.2
0.4
0.6
0.8
1.0
N = 1244 Bandwidth = 0.0304

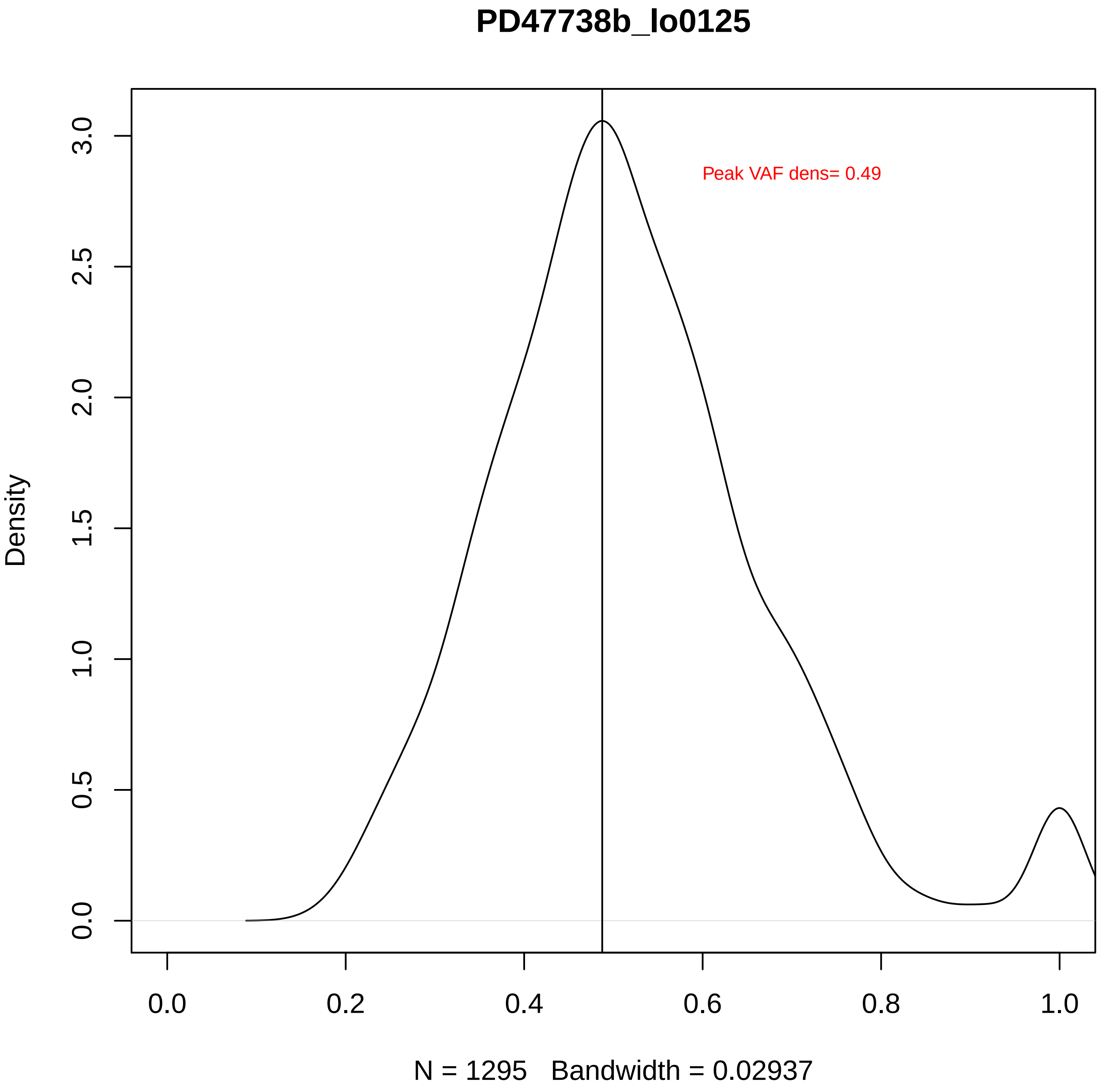
PD47738b_lo0125
Peak VAF dens= 0.49
Density
0.0
0.5
1.0
1.5
2.0
2.5
3.0
0.0
0.2
0.4
0.6
0.8
1.0
N = 1295   Bandwidth = 0.02937

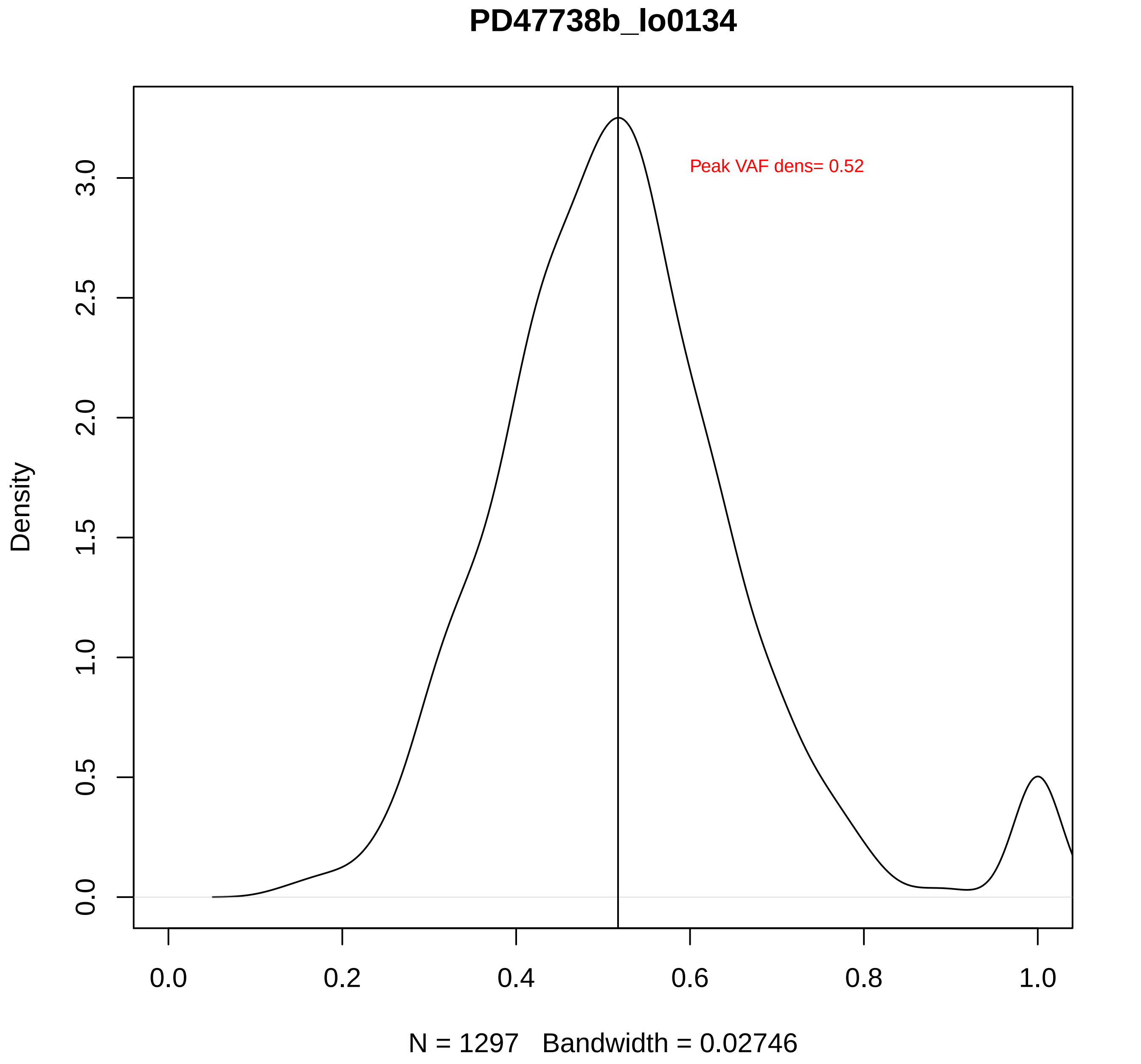

PD47738b_lo0134
Peak VAF dens= 0.52
Density
0.0
0.5
1.0
1.5
2.0
2.5
3.0
0.0
0.2
0.4
0.6
0.8
1.0
N = 1297   Bandwidth = 0.02746

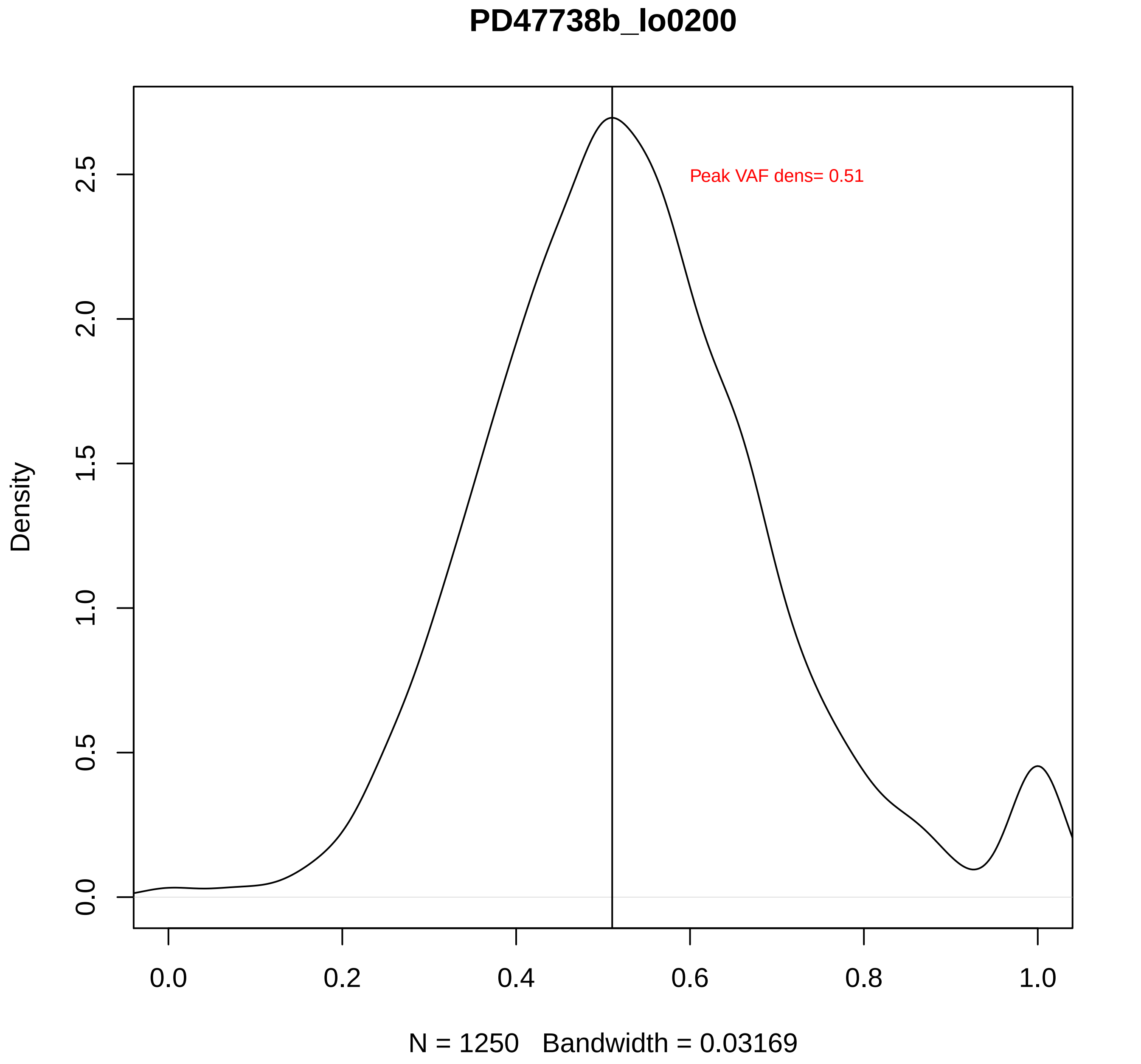

PD47738b_lo0200
Peak VAF dens= 0.51
Density
0.0
0.5
1.0
1.5
2.0
2.5
0.0
0.2
0.4
0.6
0.8
1.0
N = 1250   Bandwidth = 0.03169

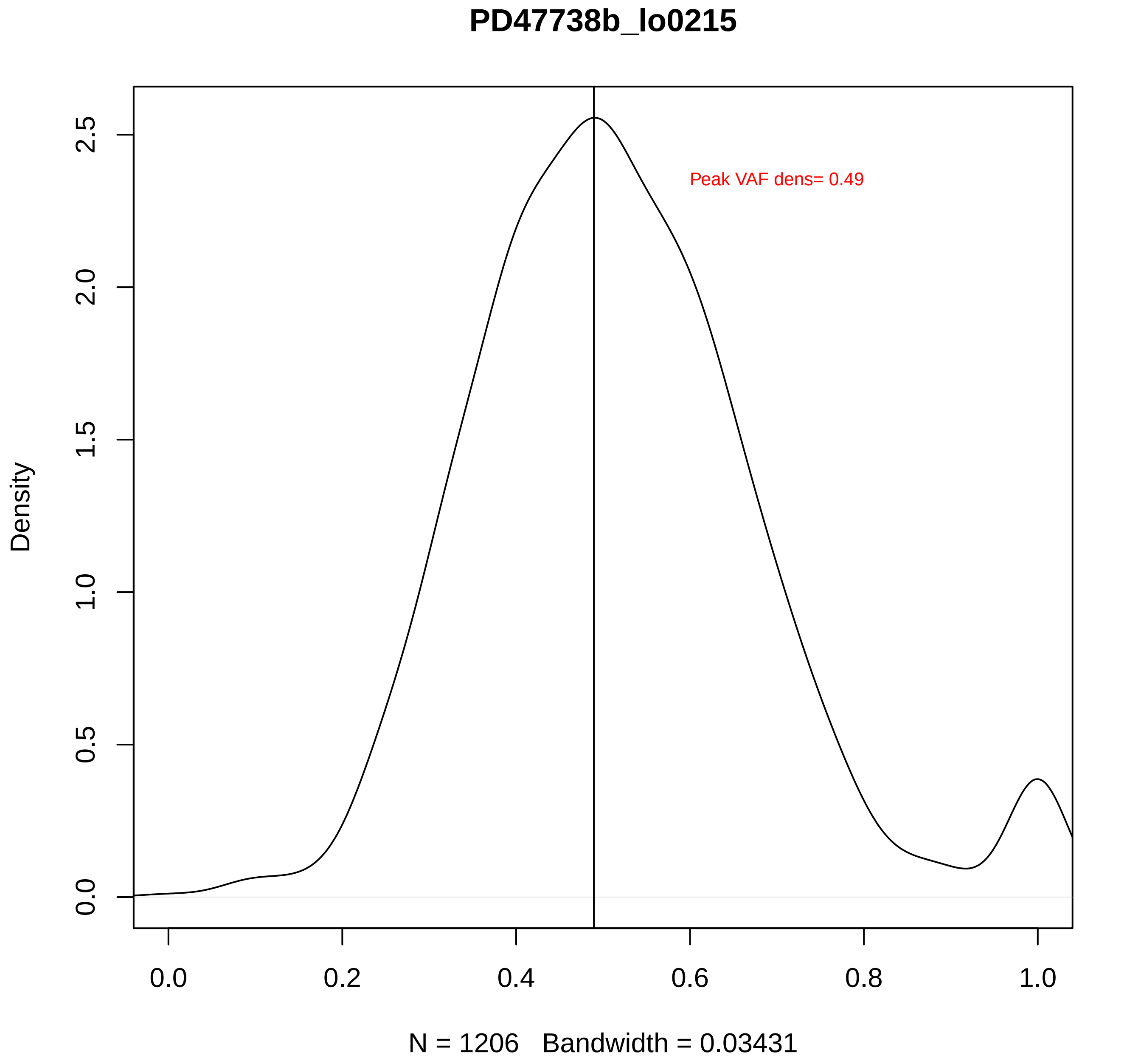
PD47738b_lo0215
Peak VAF dens= 0.49
Density
0.0
0.5
1.0
1.5
2.0
2.5
0.0
0.2
0.4
0.6
0.8
1.0
N = 1206 Bandwidth = 0.03431

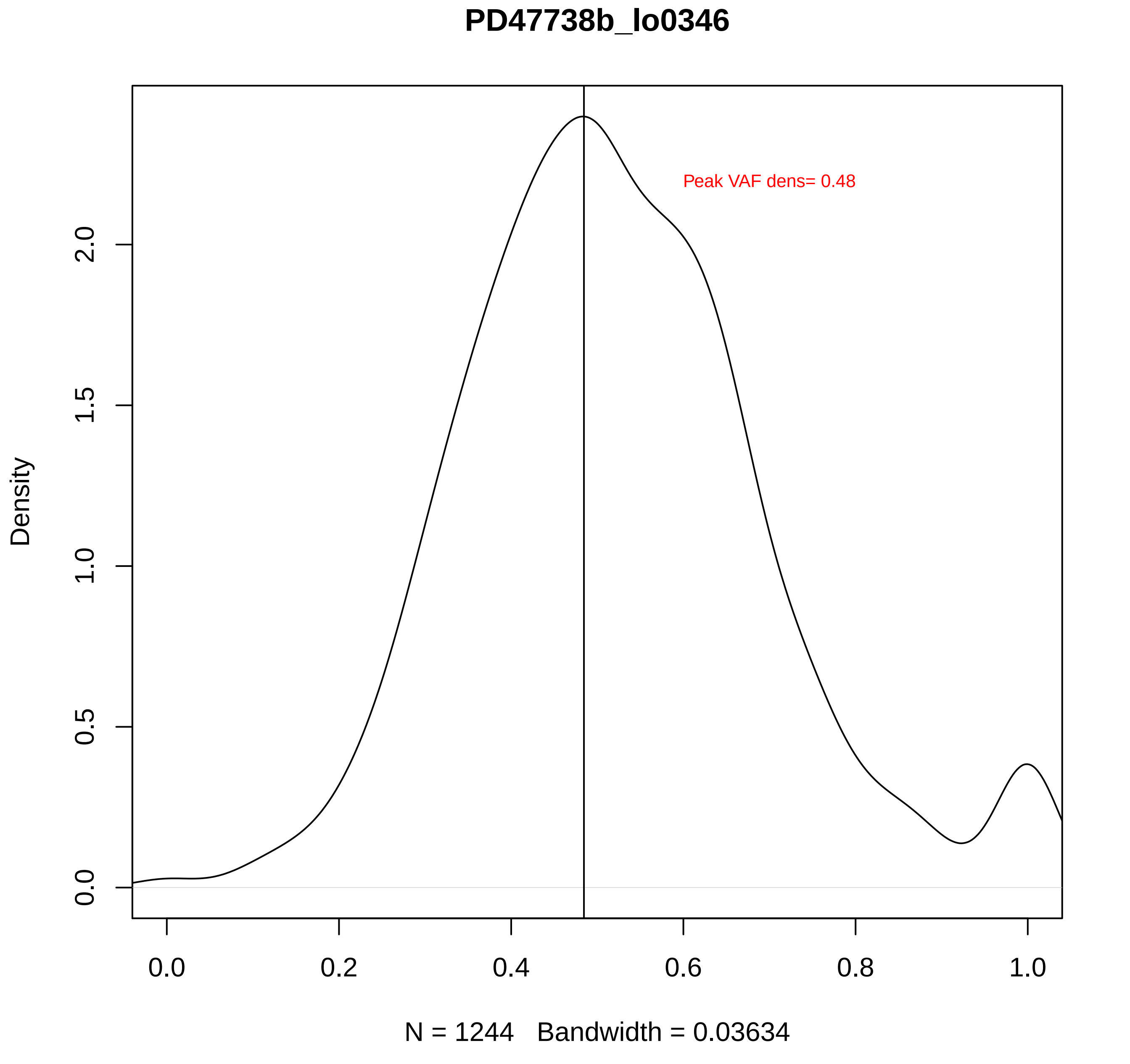
PD47738b_lo0346
Peak VAF dens= 0.48
Density
0.0
0.5
1.0
1.5
2.0
0.0
0.2
0.4
0.6
0.8
1.0
N = 1244   Bandwidth = 0.03634

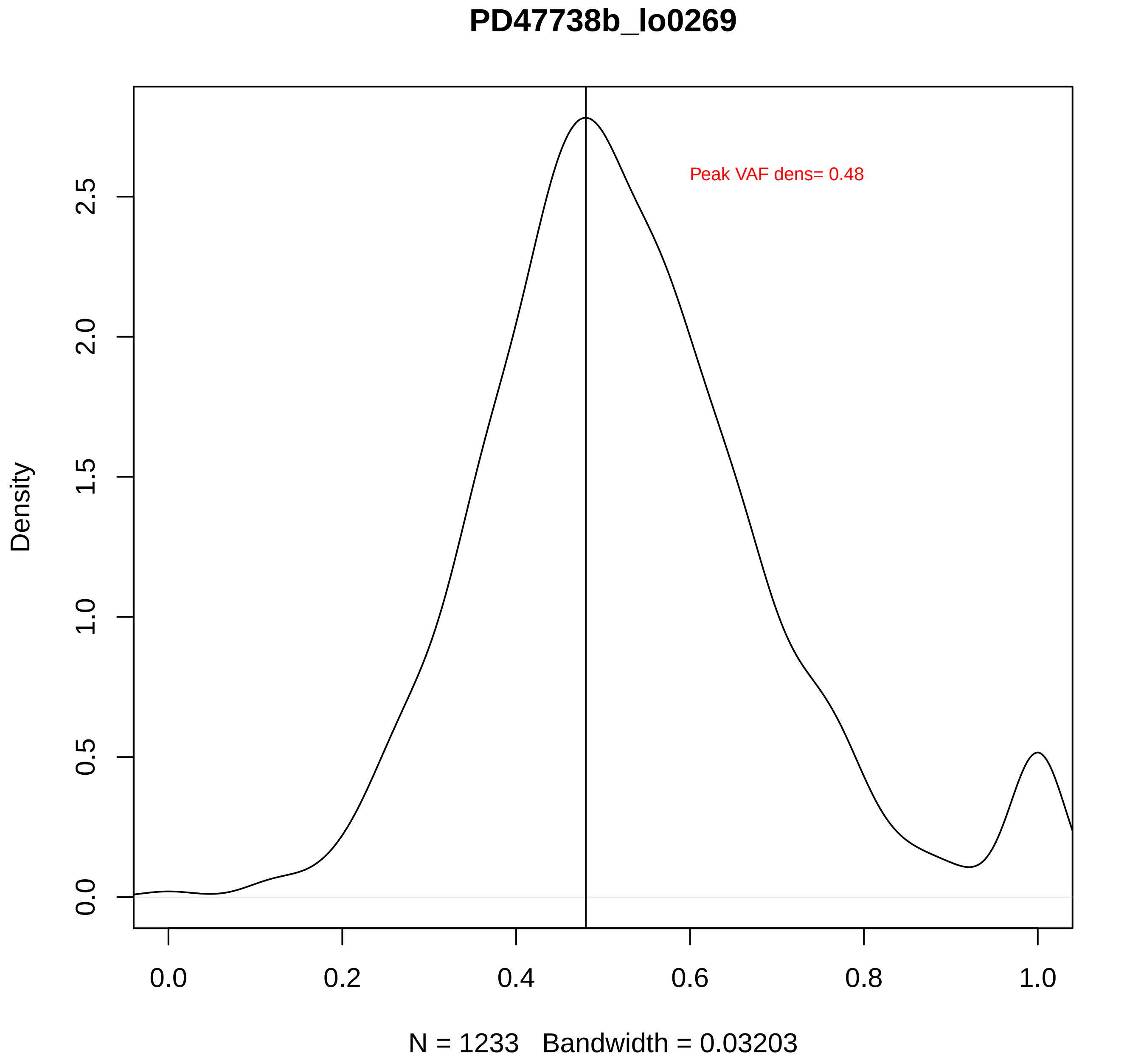
PD47738b_lo0269
Peak VAF dens= 0.48
Density
0.0
0.5
1.0
1.5
2.0
2.5
0.0
0.2
0.4
0.6
0.8
1.0
N = 1233   Bandwidth = 0.03203

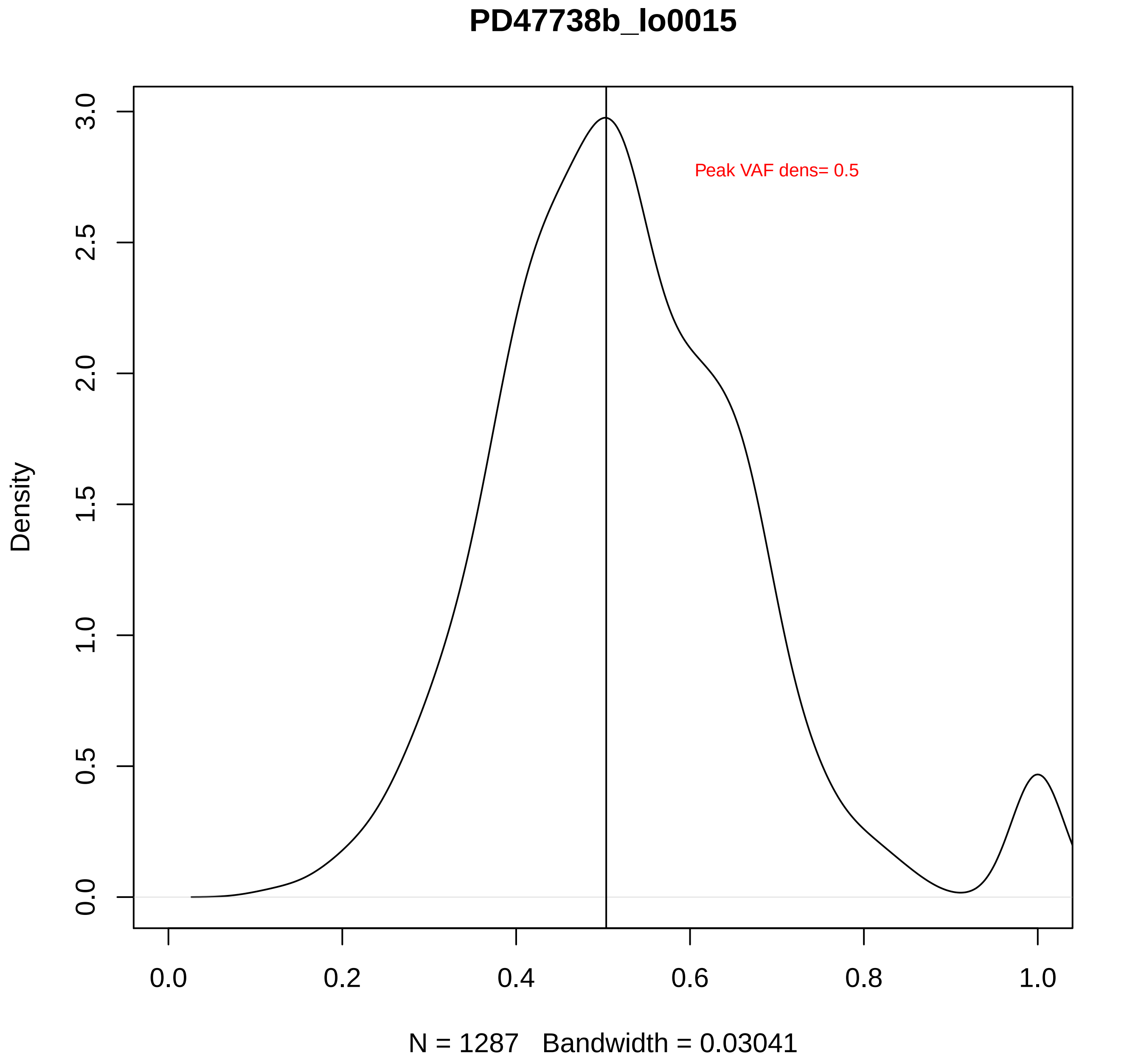
PD47738b_lo0015
Peak VAF dens= 0.5
Density
0.0
0.5
1.0
1.5
2.0
2.5
3.0
0.0
0.2
0.4
0.6
0.8
1.0
N = 1287   Bandwidth = 0.03041

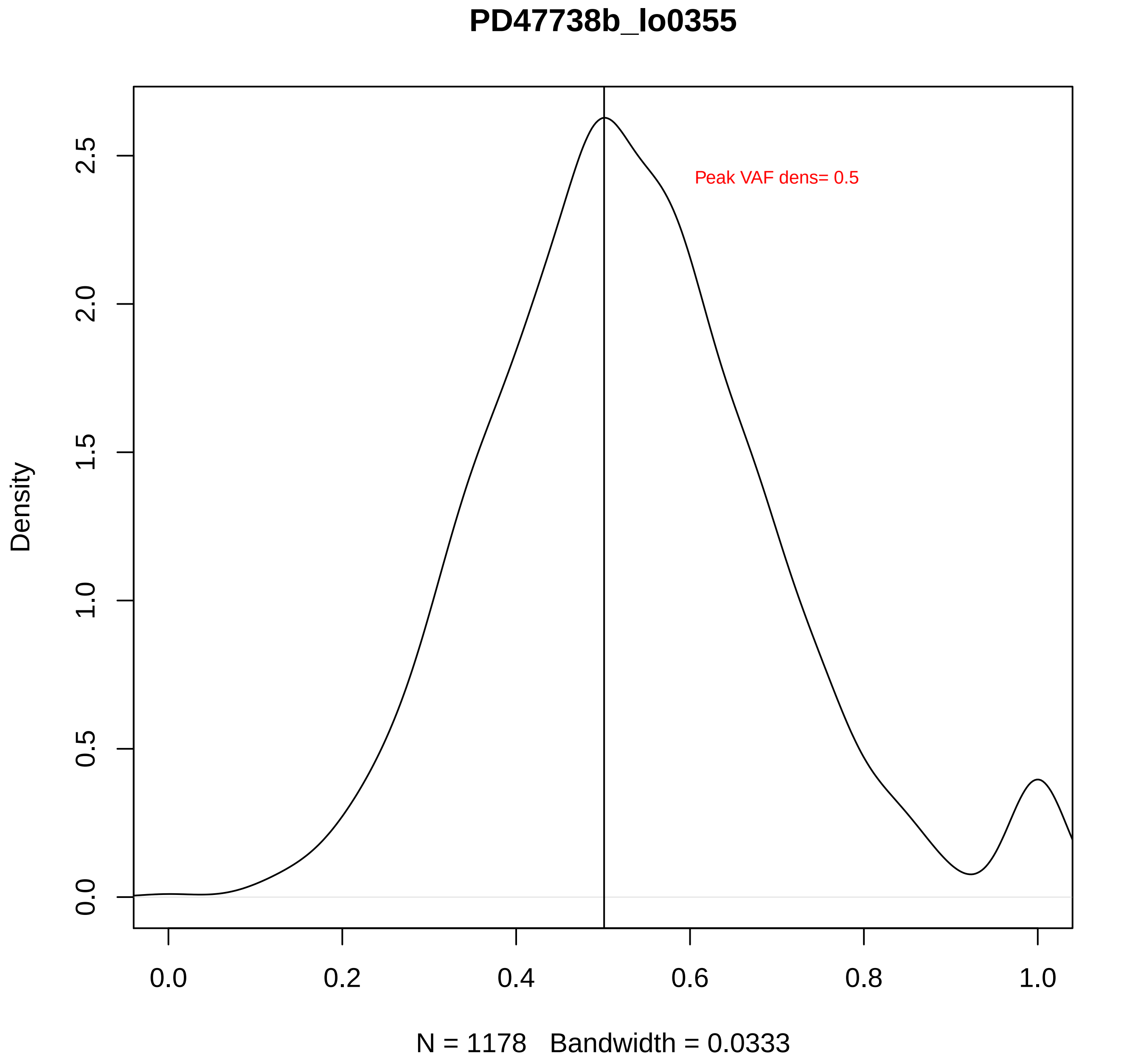
PD47738b_lo0355
Peak VAF dens= 0.5
Density
0.0
0.5
1.0
1.5
2.0
2.5
0.0
0.2
0.4
0.6
0.8
1.0
N = 1178   Bandwidth = 0.0333

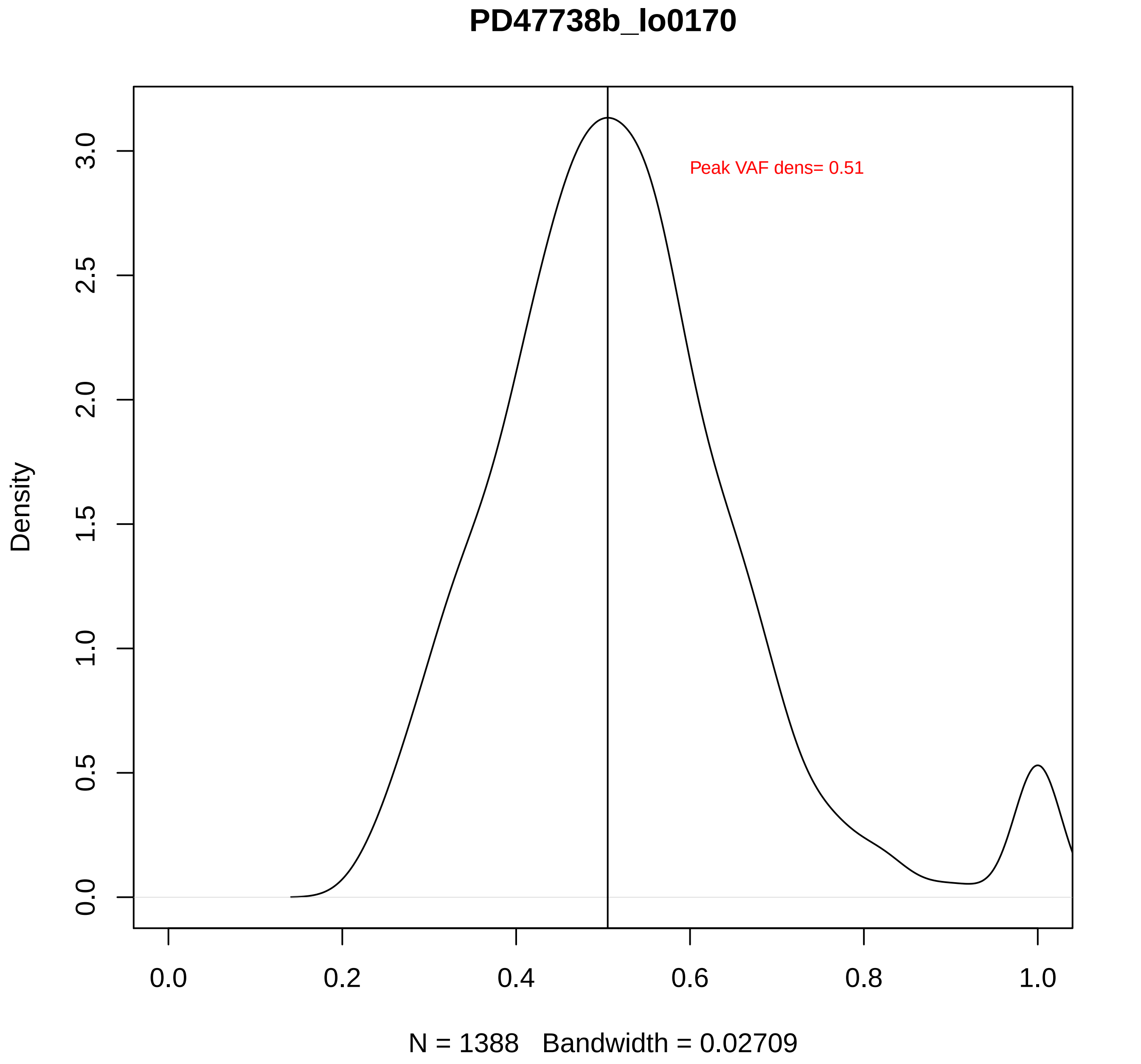
PD47738b_lo0170
Peak VAF dens= 0.51
Density
0.0
0.5
1.0
1.5
2.0
2.5
3.0
0.0
0.2
0.4
0.6
0.8
1.0
N = 1388   Bandwidth = 0.02709

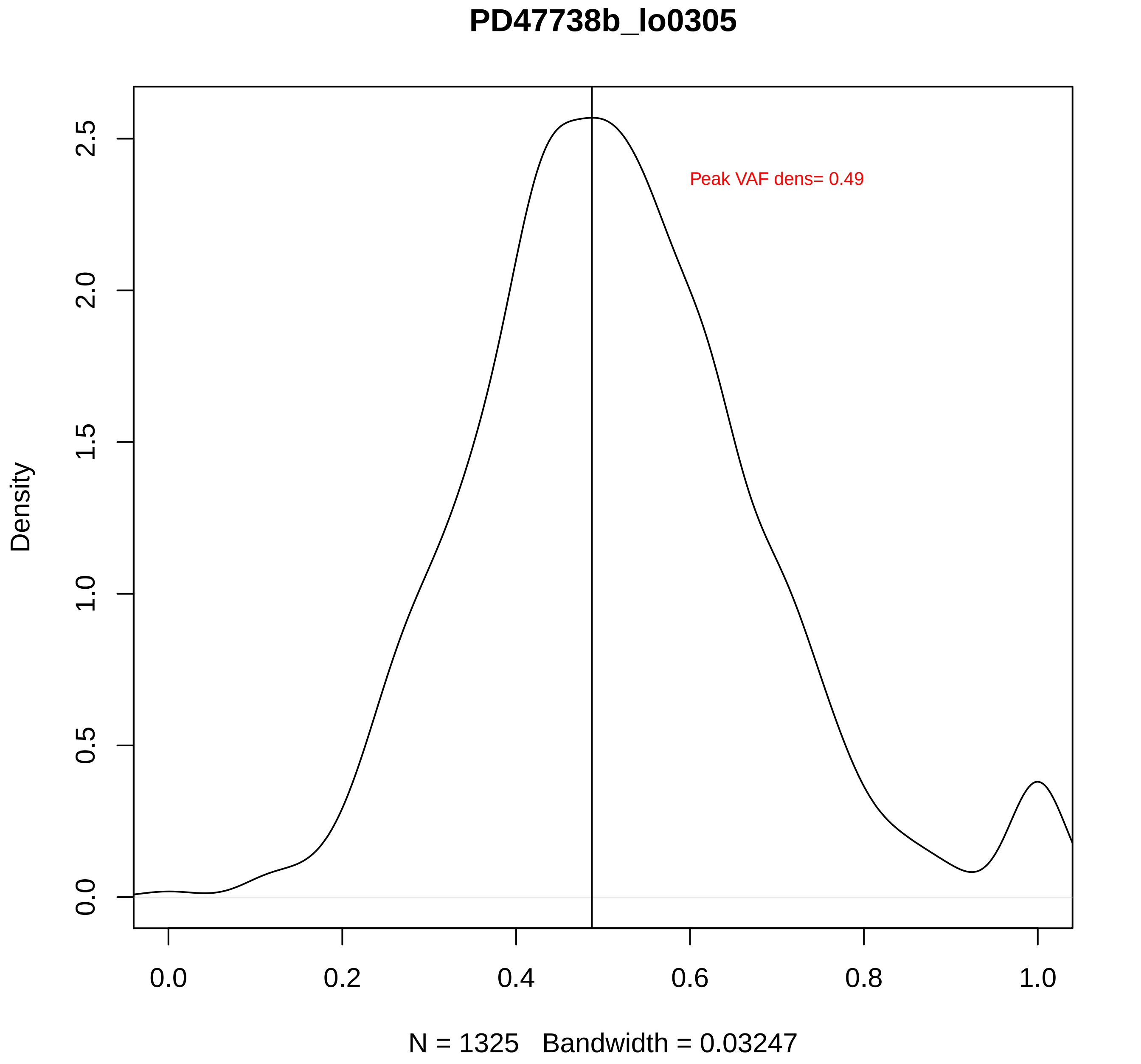

PD47738b_lo0305
Peak VAF dens= 0.49
Density
0.0
0.5
1.0
1.5
2.0
2.5
0.0
0.2
0.4
0.6
0.8
1.0
N = 1325   Bandwidth = 0.03247

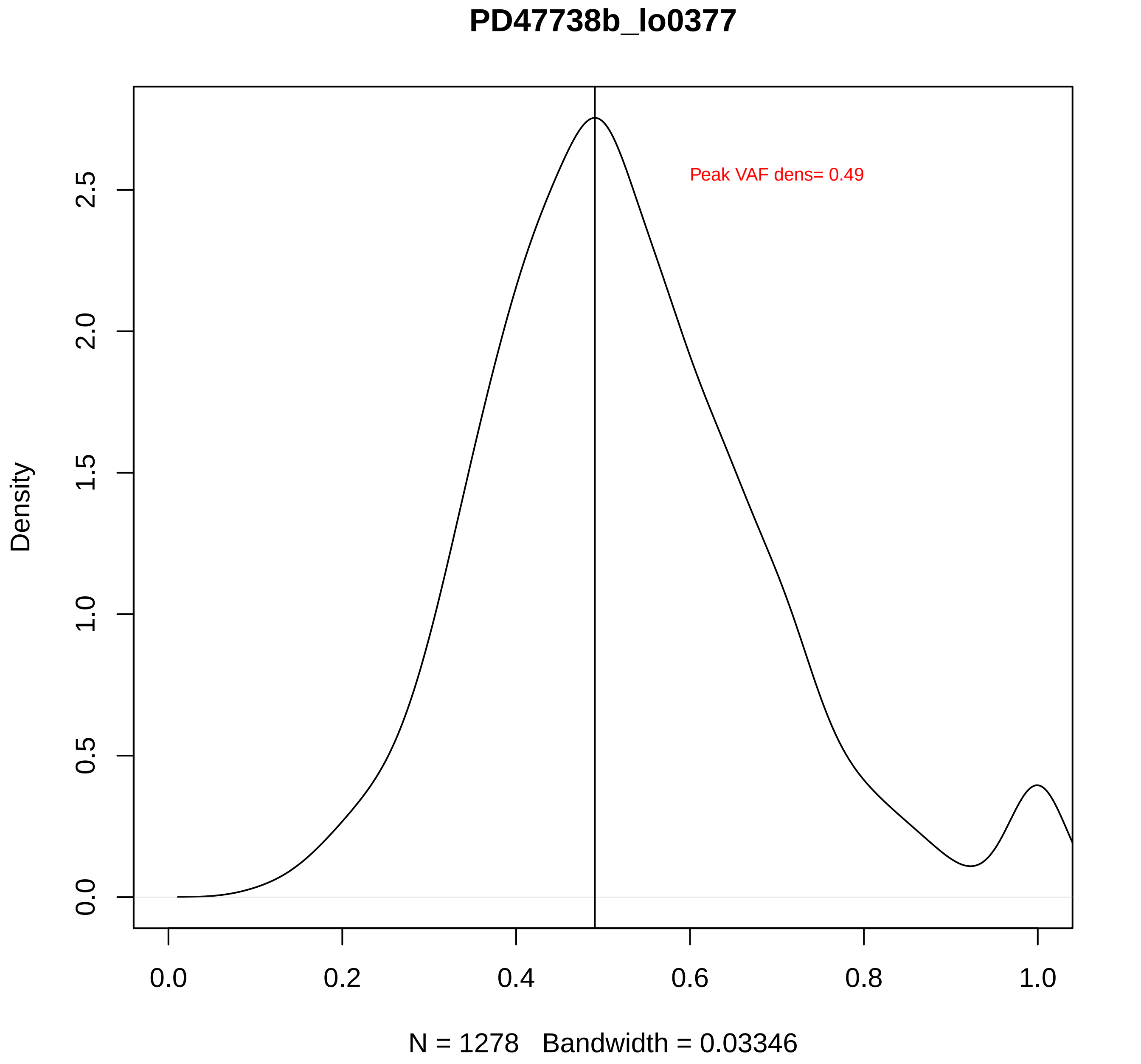

PD47738b_lo0377
Peak VAF dens= 0.49
Density
0.0
0.5
1.0
1.5
2.0
2.5
0.0
0.2
0.4
0.6
0.8
1.0
N = 1278   Bandwidth = 0.03346

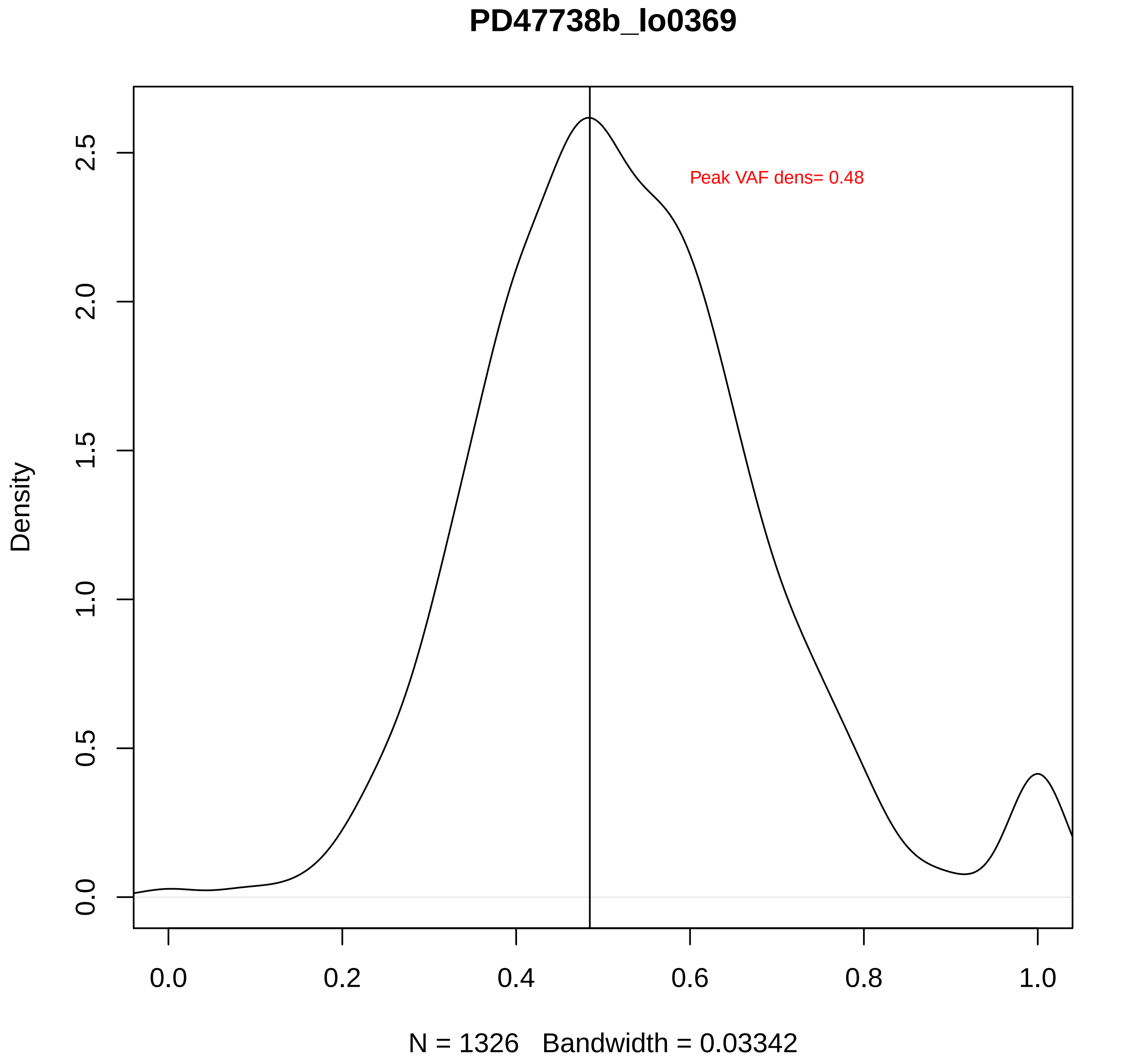
PD47738b_lo0369
Peak VAF dens= 0.48
Density
0.0
0.5
1.0
1.5
2.0
2.5
0.0
0.2
0.4
0.6
0.8
1.0
N = 1326   Bandwidth = 0.03342

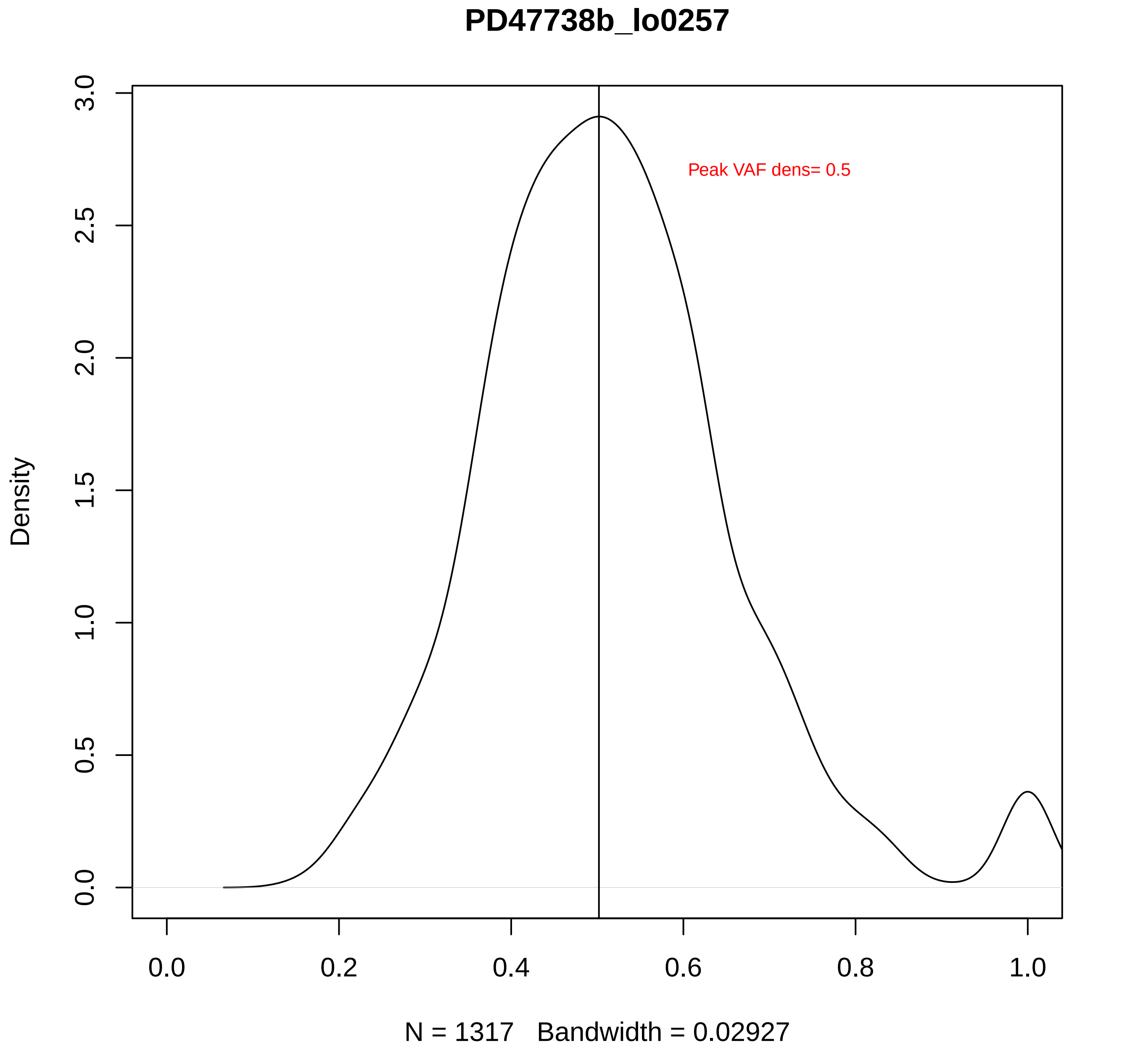

PD47738b_lo0257
Peak VAF dens= 0.5
Density
0.0
0.5
1.0
1.5
2.0
2.5
3.0
0.0
0.2
0.4
0.6
0.8
1.0
N = 1317 Bandwidth = 0.02927

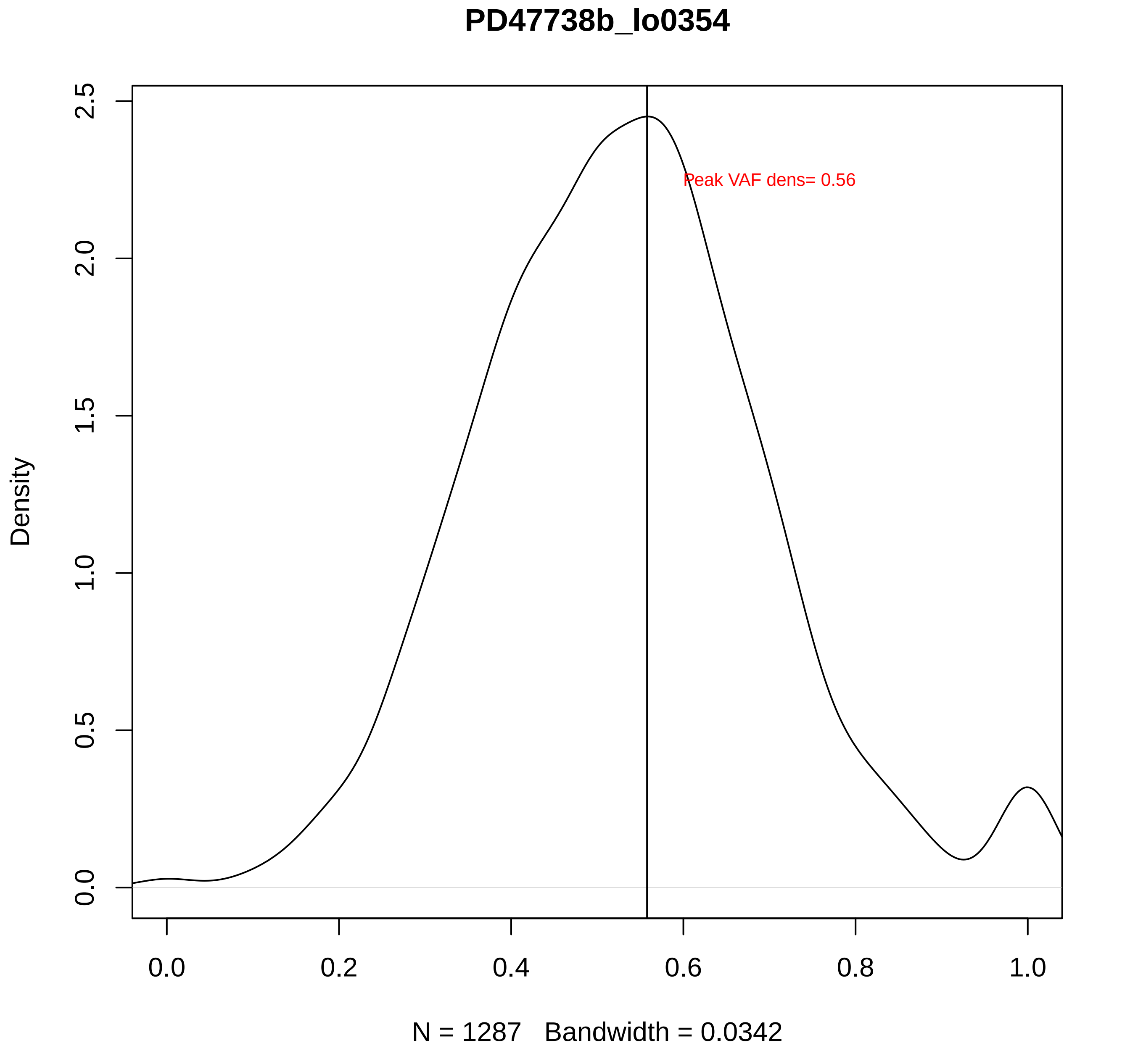
PD47738b_lo0354
Peak VAF dens= 0.56
Density
0.0
0.5
1.0
1.5
2.0
2.5
0.0
0.2
0.4
0.6
0.8
1.0
N = 1287   Bandwidth = 0.0342

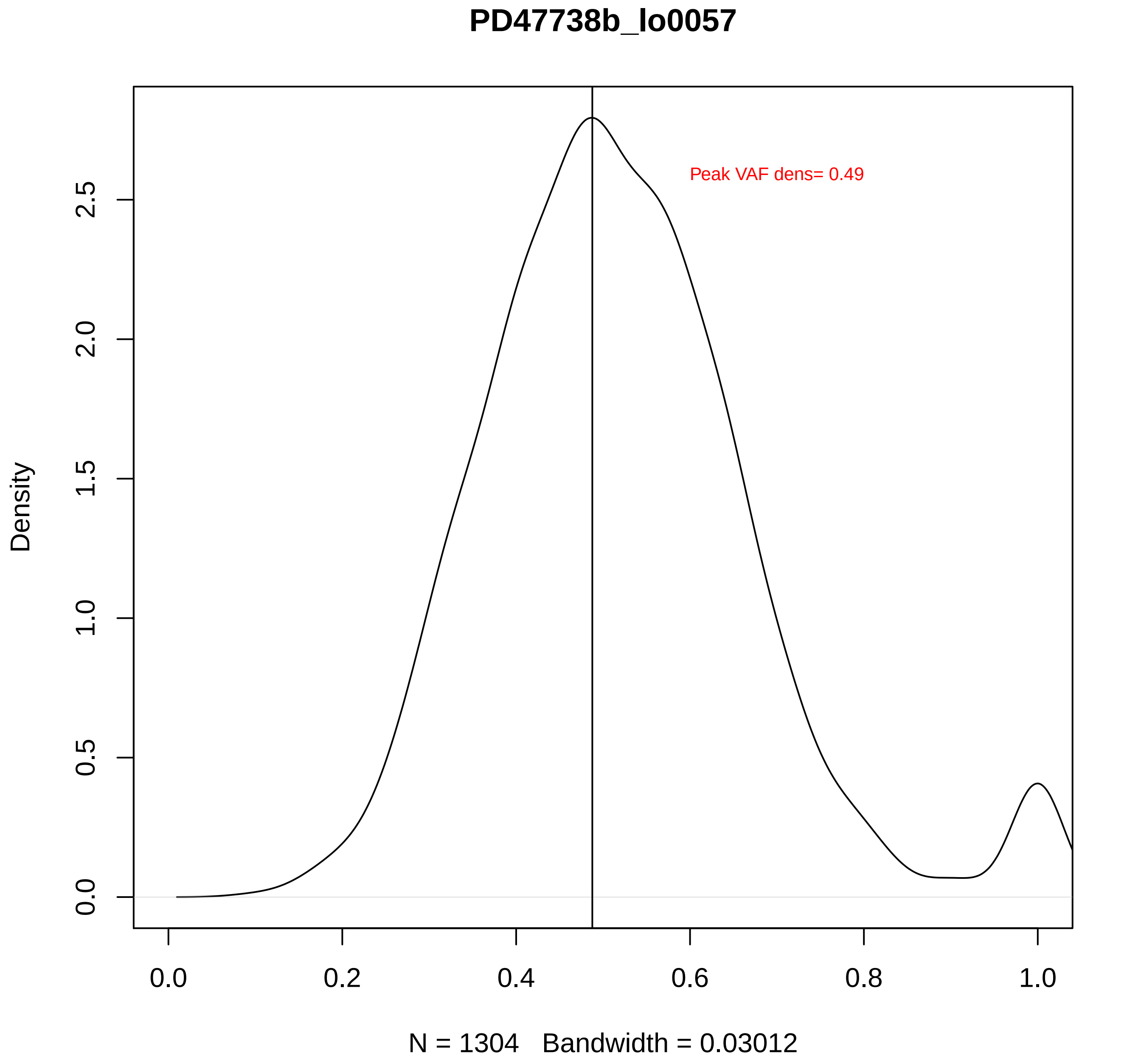
PD47738b_lo0057
Peak VAF dens= 0.49
Density
0.0
0.5
1.0
1.5
2.0
2.5
0.0
0.2
0.4
0.6
0.8
1.0
N = 1304   Bandwidth = 0.03012

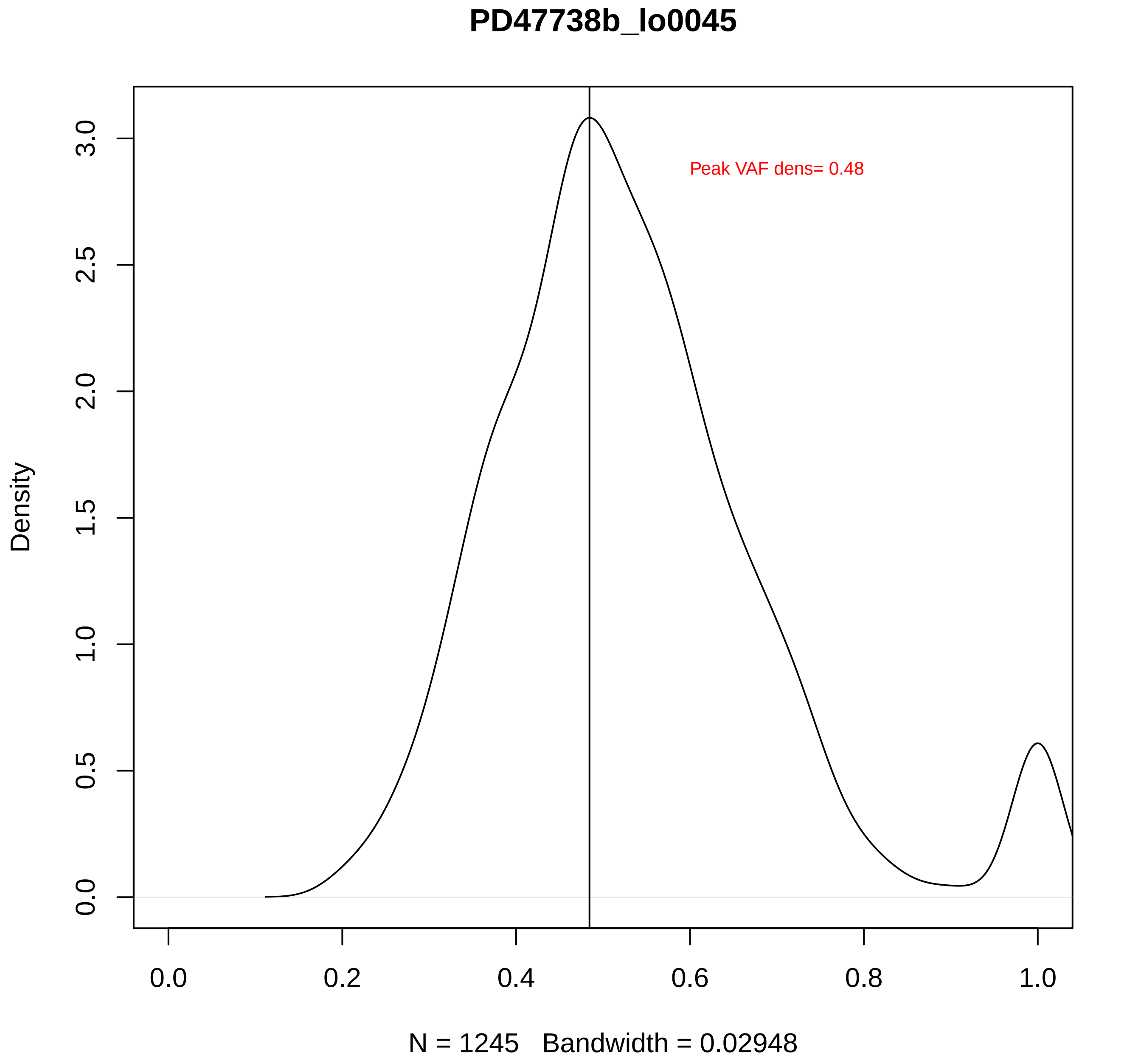
PD47738b_lo0045
Peak VAF dens= 0.48
Density
0.0
0.5
1.0
1.5
2.0
2.5
3.0
0.0
0.2
0.4
0.6
0.8
1.0
N = 1245   Bandwidth = 0.02948

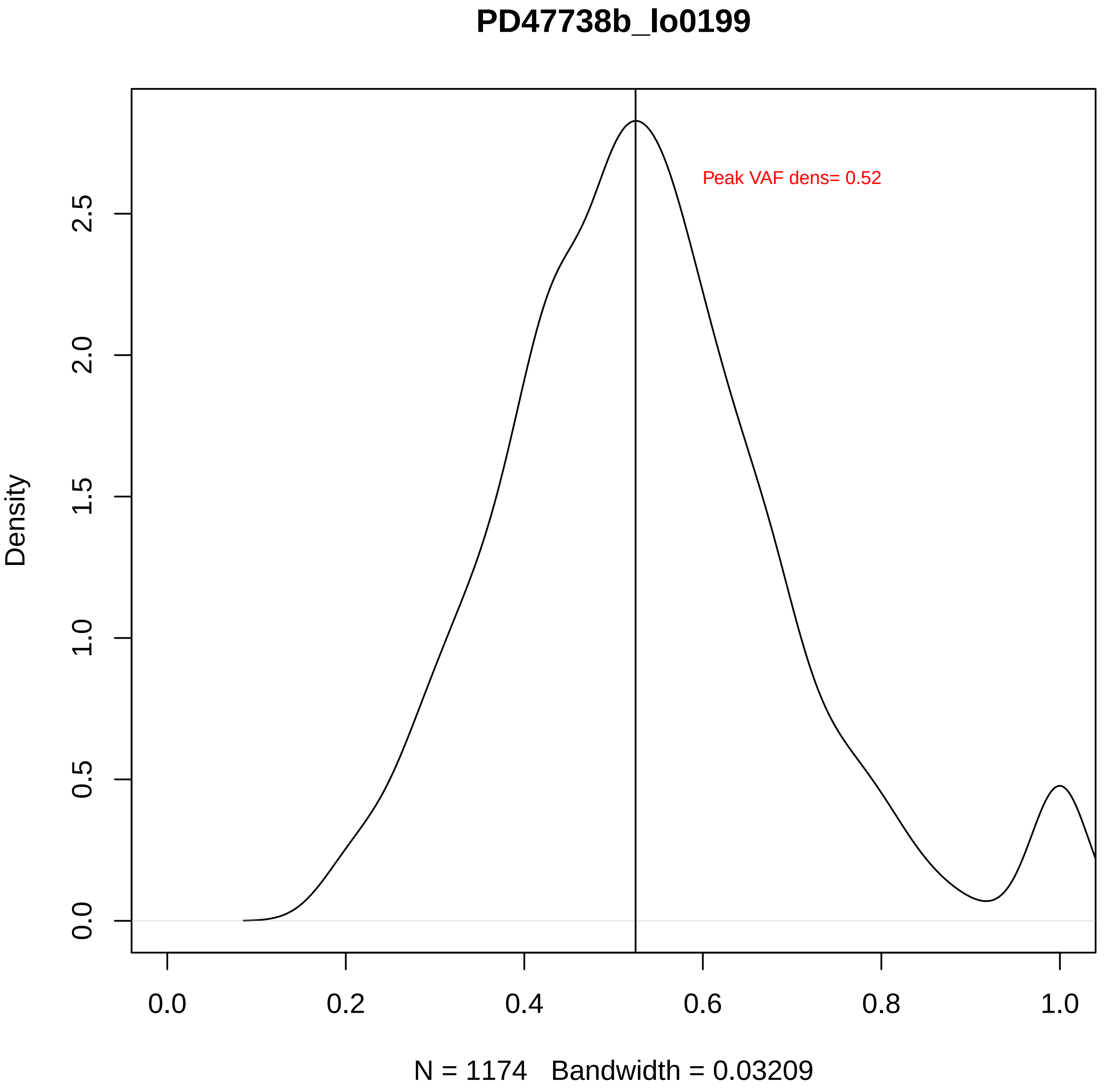
PD47738b_lo0199
Peak VAF dens= 0.52
Density
0.0
0.5
1.0
1.5
2.0
2.5
0.0
0.2
0.4
0.6
0.8
1.0
N = 1174 Bandwidth = 0.03209

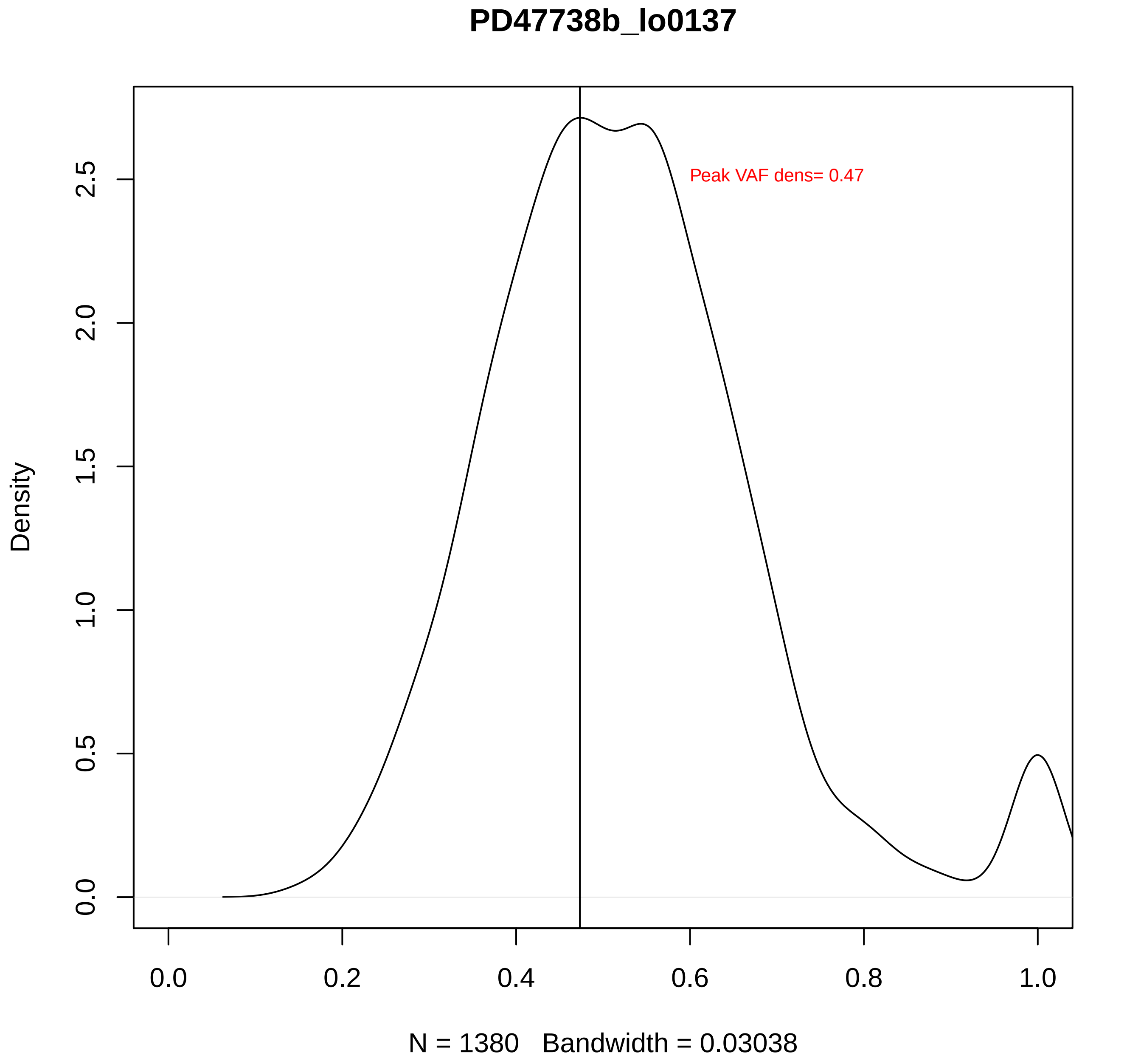

PD47738b_lo0137
Peak VAF dens= 0.47
Density
0.0
0.5
1.0
1.5
2.0
2.5
0.0
0.2
0.4
0.6
0.8
1.0
N = 1380   Bandwidth = 0.03038

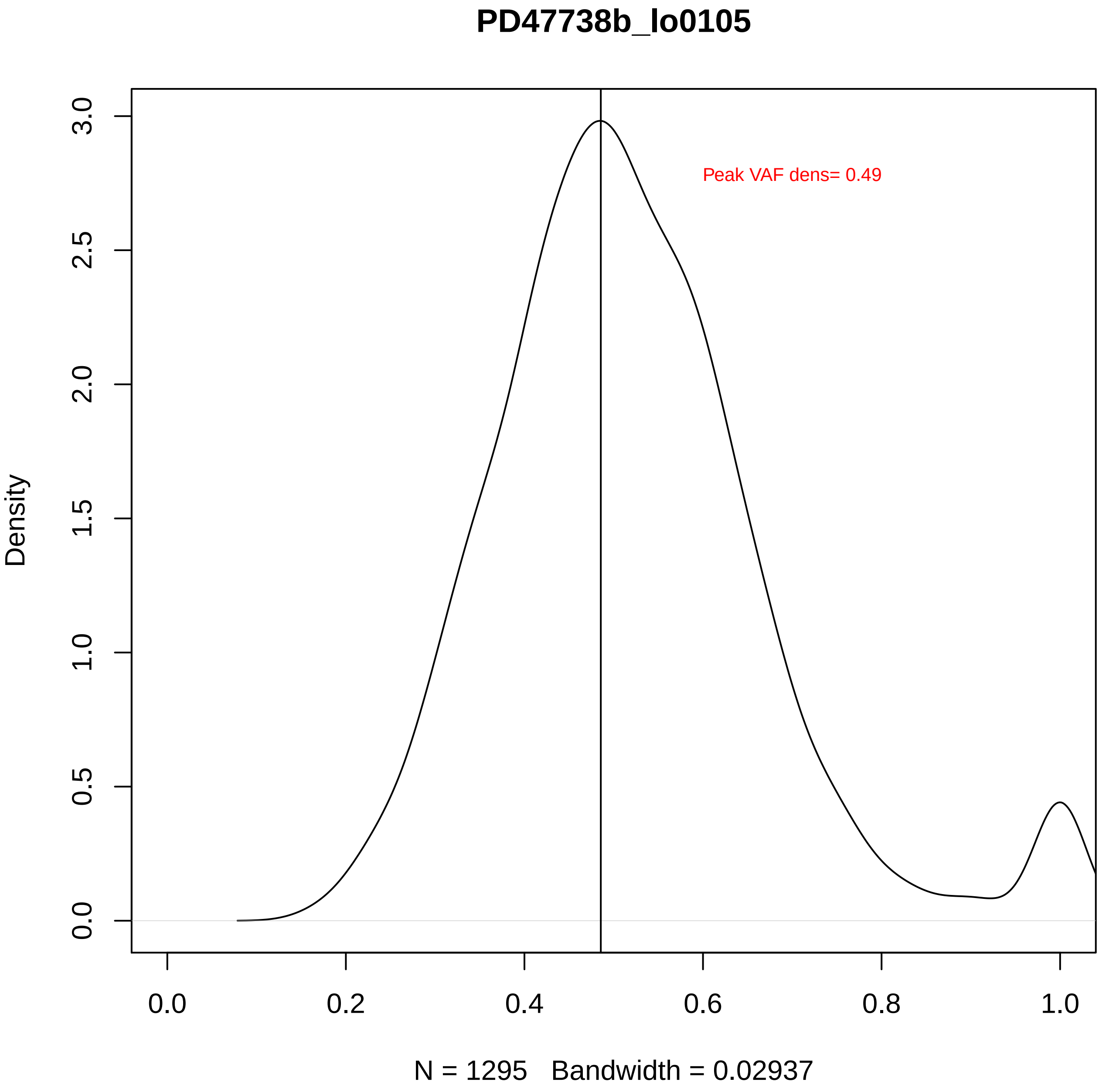

PD47738b_lo0105
Peak VAF dens= 0.49
Density
3.0
2.5
2.0
1.5
1.0
0.5
0.0
0.0
0.2
0.4
0.6
0.8
1.0
N = 1295   Bandwidth = 0.02937

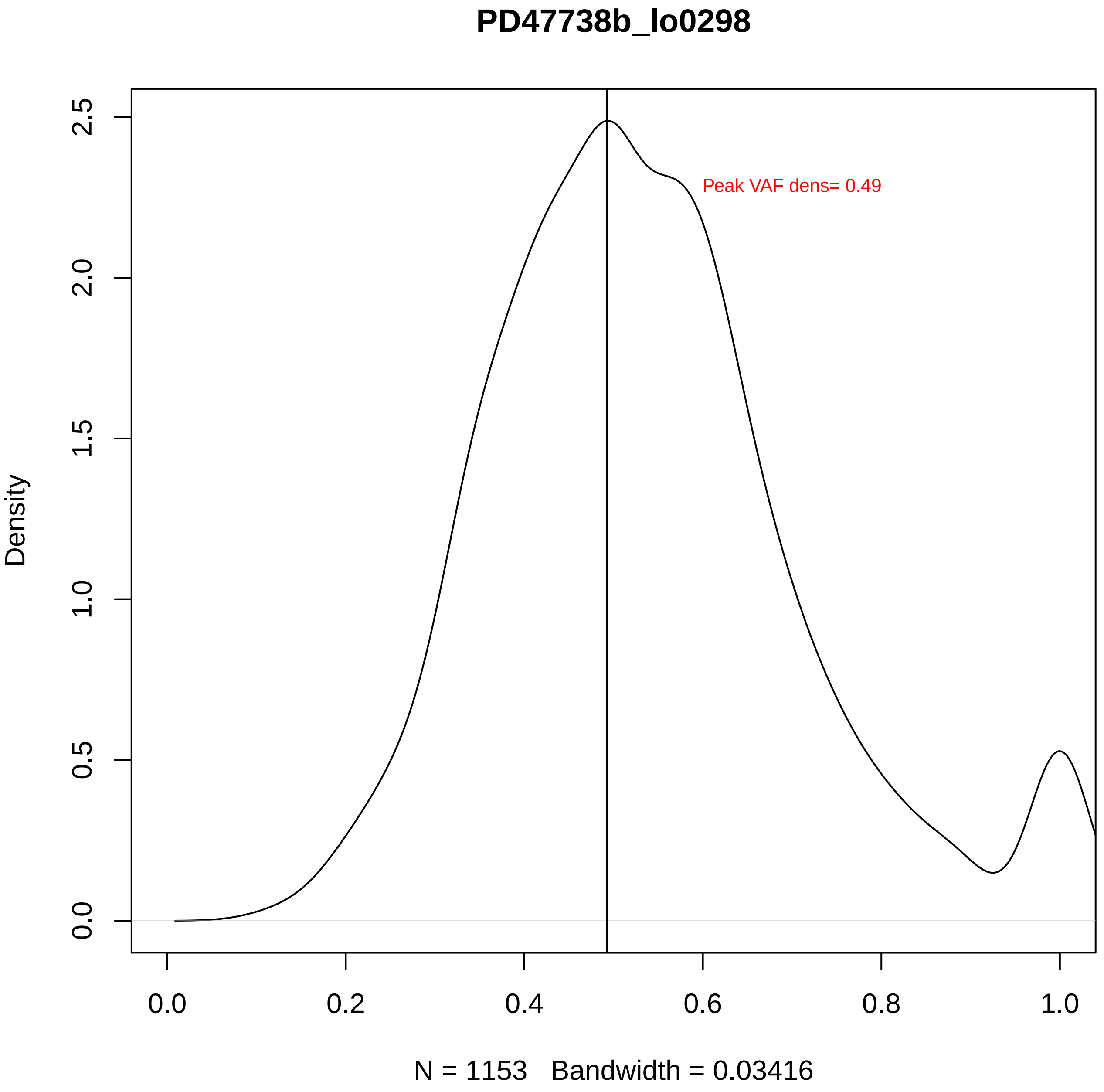

PD47738b_lo0298
Peak VAF dens= 0.49
Density
2.5
2.0
1.5
1.0
0.5
0.0
0.0
0.2
0.4
0.6
0.8
1.0
N = 1153   Bandwidth = 0.03416

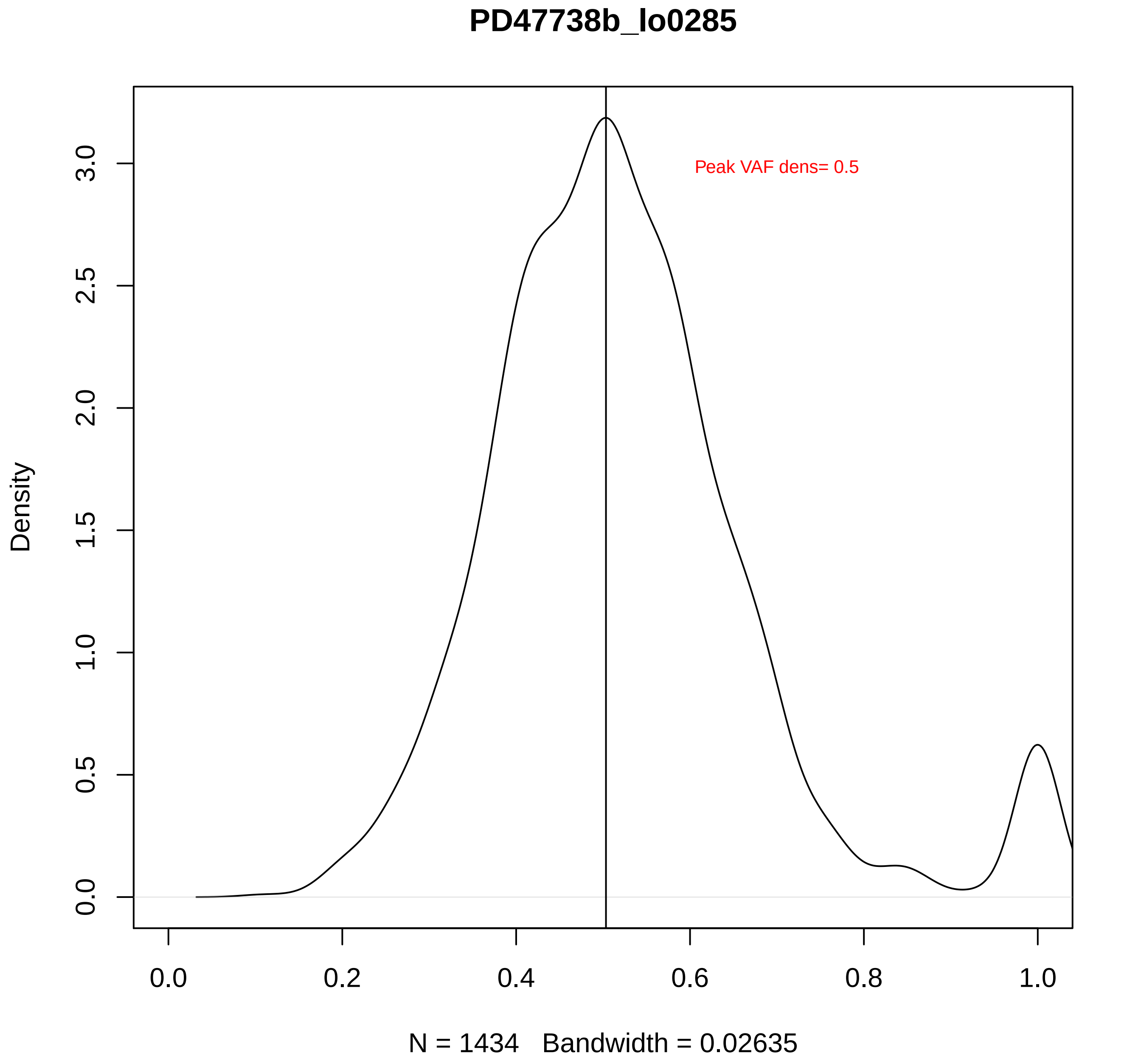
PD47738b_lo0285
Peak VAF dens= 0.5
Density
0.0
0.5
1.0
1.5
2.0
2.5
3.0
0.0
0.2
0.4
0.6
0.8
1.0
N = 1434   Bandwidth = 0.02635

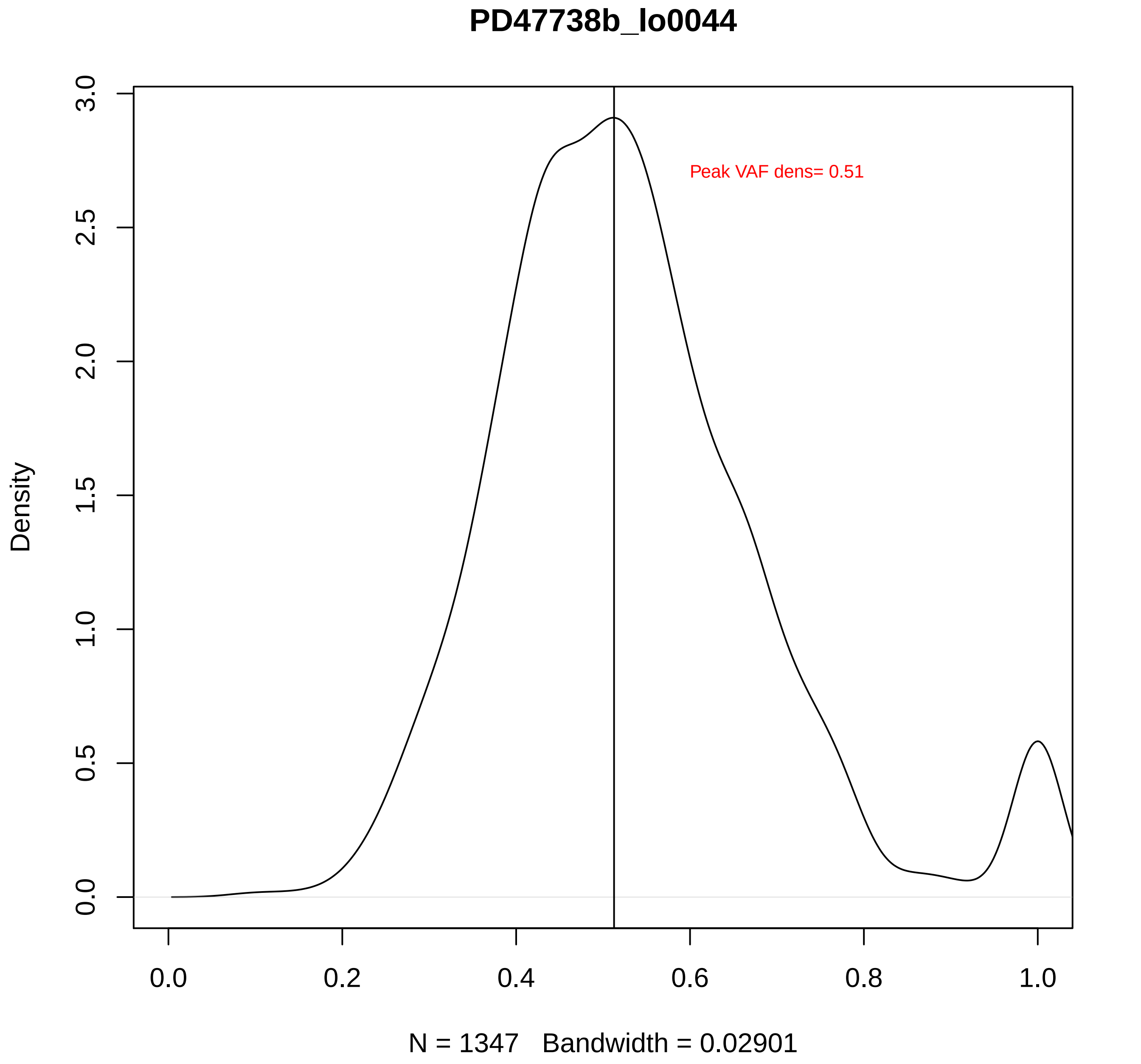

PD47738b_lo0044
Peak VAF dens= 0.51
Density
0.0
0.5
1.0
1.5
2.0
2.5
3.0
0.0
0.2
0.4
0.6
0.8
1.0
N = 1347   Bandwidth = 0.02901

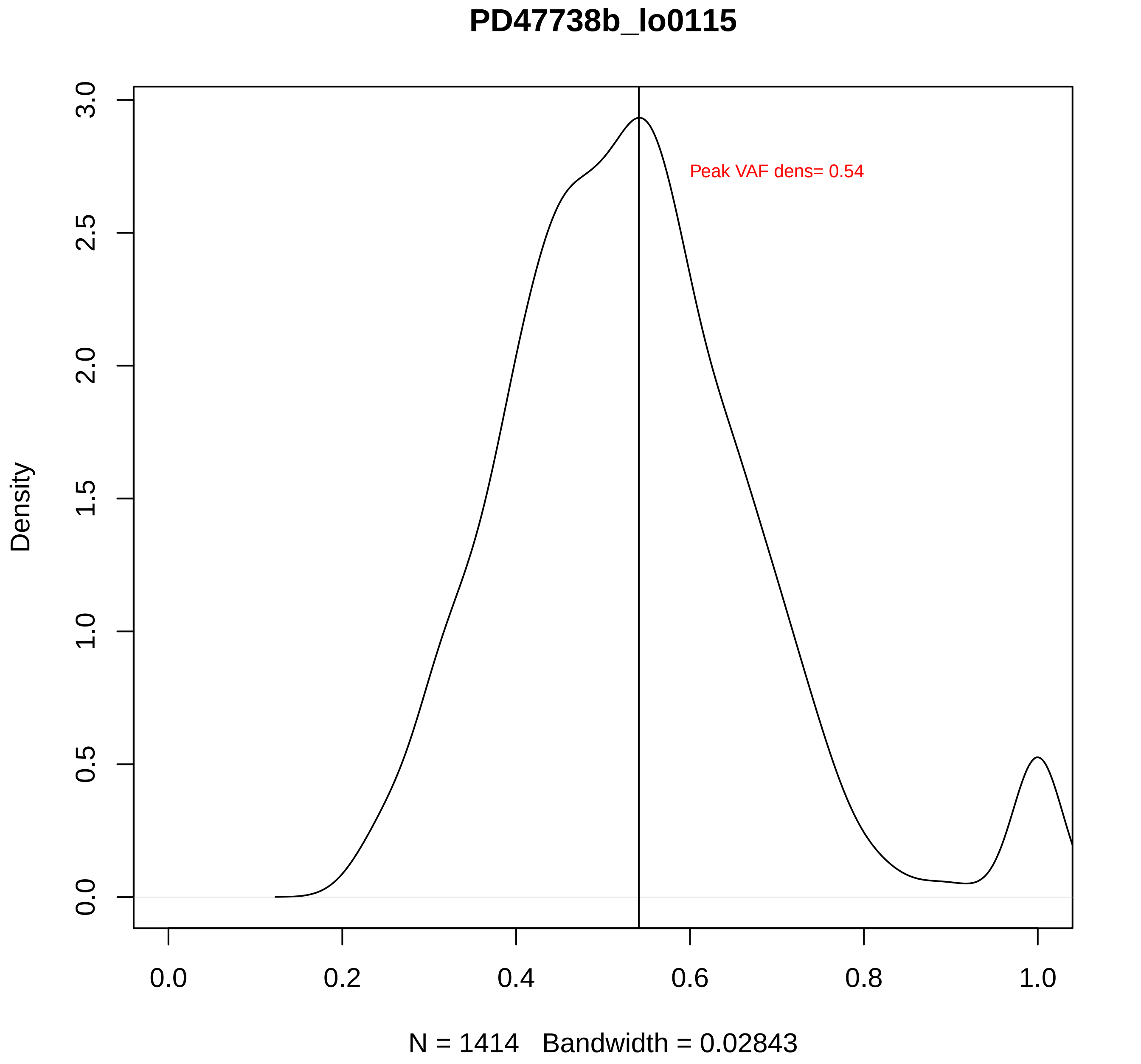
PD47738b_lo0115
Peak VAF dens= 0.54
Density
0.0
0.5
1.0
1.5
2.0
2.5
3.0
0.0
0.2
0.4
0.6
0.8
1.0
N = 1414 Bandwidth = 0.02843

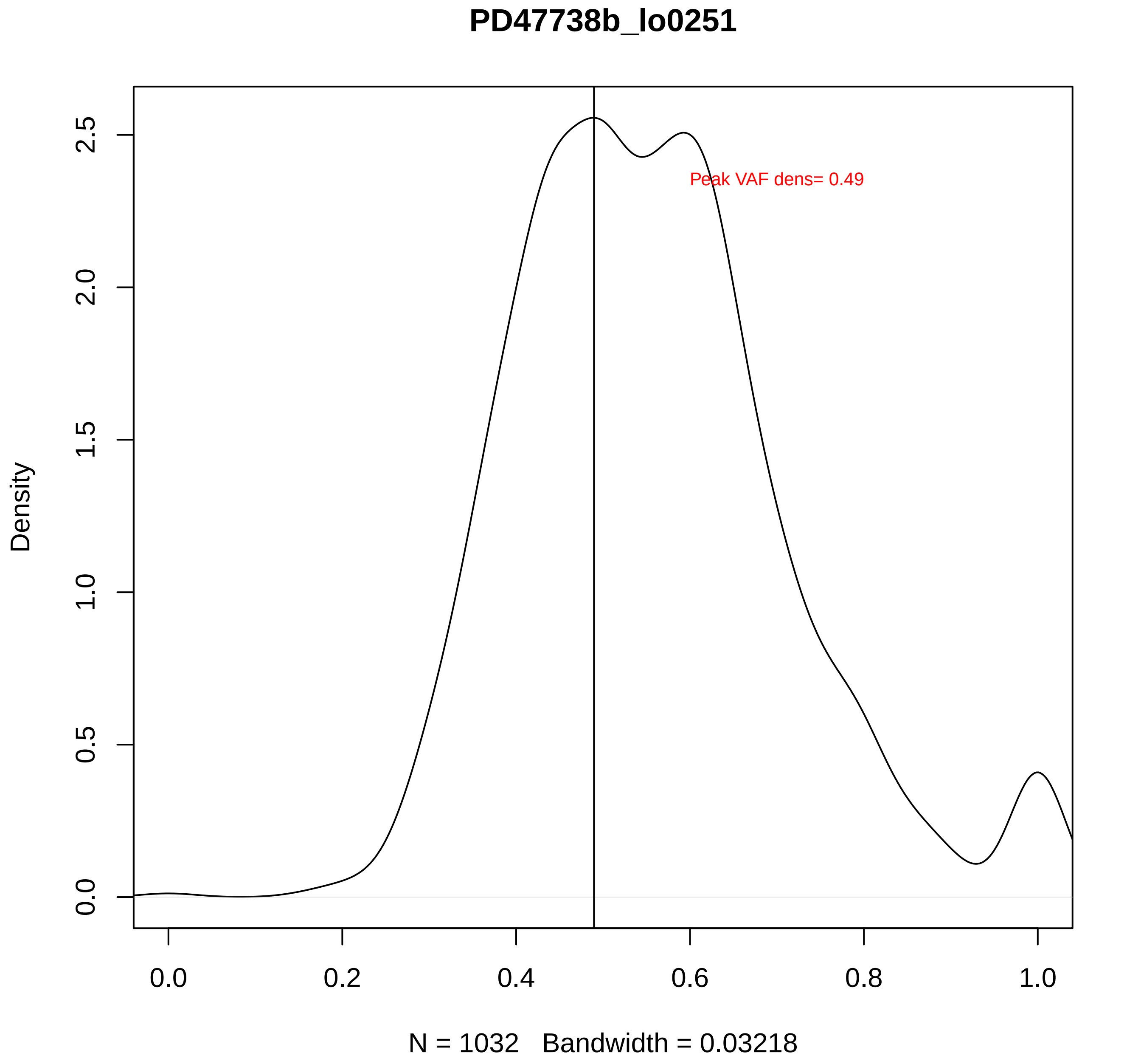
PD47738b_lo0251
Peak VAF dens= 0.49
Density
0.0
0.5
1.0
1.5
2.0
2.5
0.0
0.2
0.4
0.6
0.8
1.0
N = 1032 Bandwidth = 0.03218

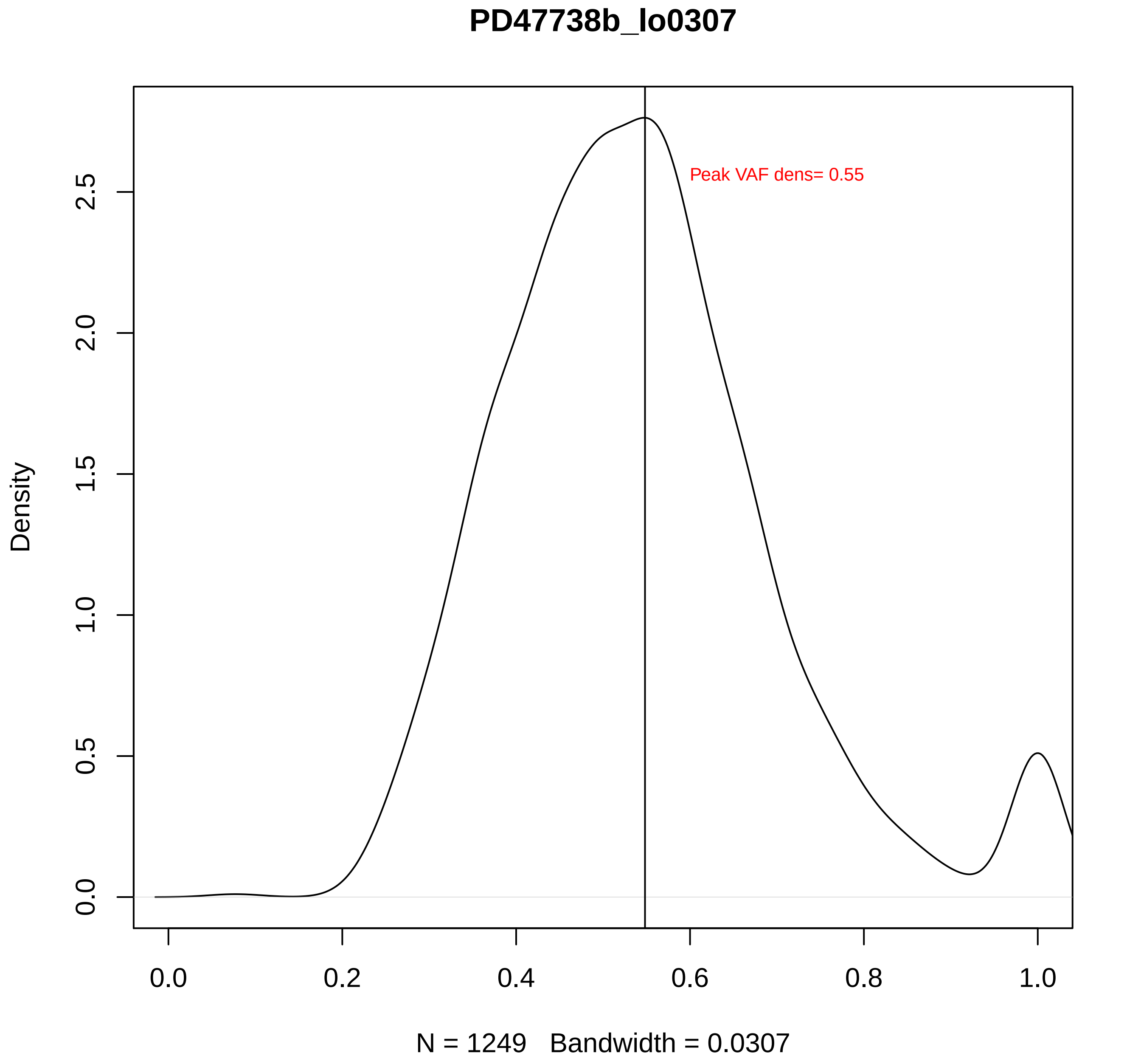
PD47738b_lo0307
Peak VAF dens= 0.55
Density
0.0
0.5
1.0
1.5
2.0
2.5
0.0
0.2
0.4
0.6
0.8
1.0
N = 1249   Bandwidth = 0.0307

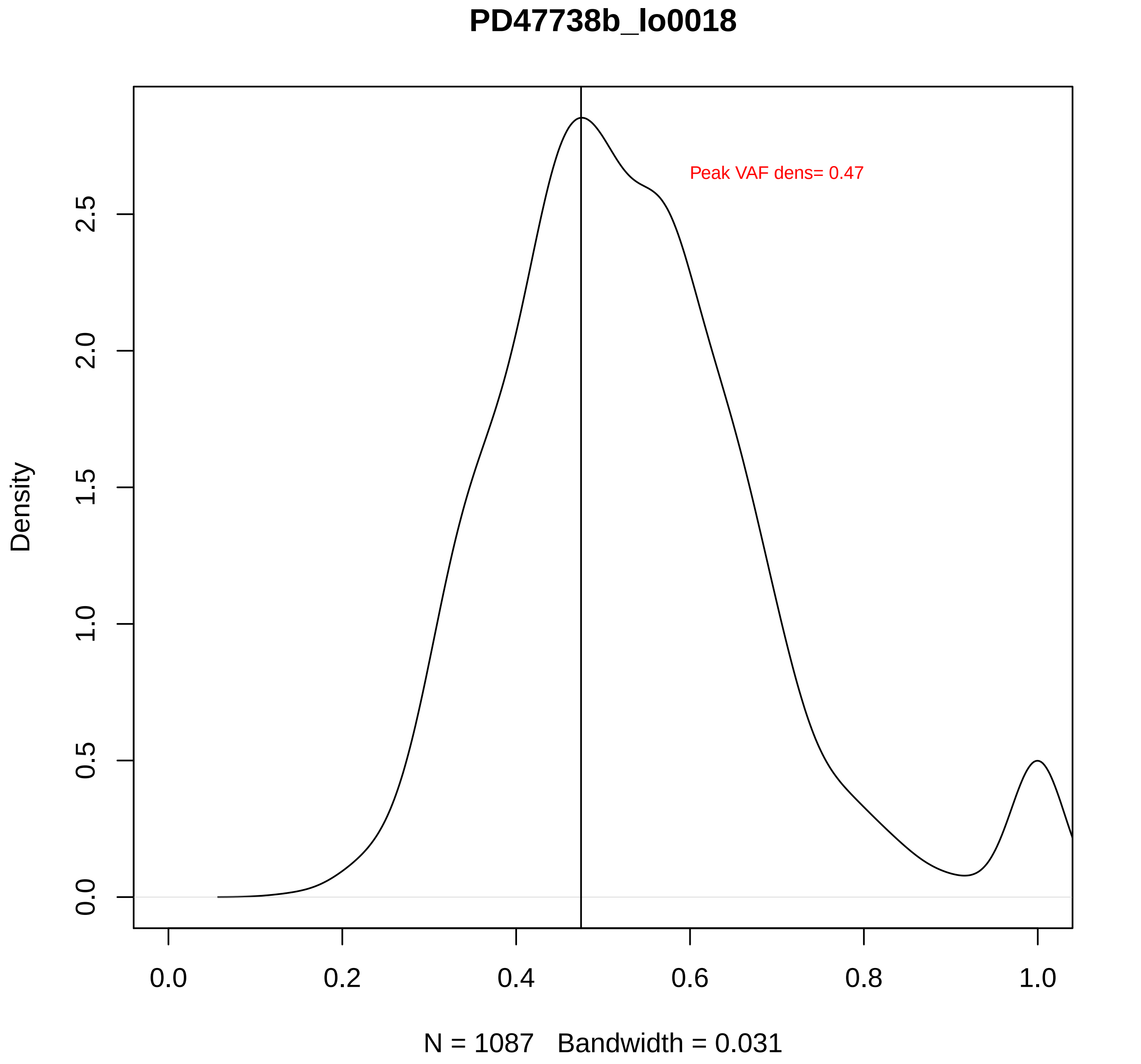
PD47738b_lo0018
Peak VAF dens= 0.47
Density
0.0
0.5
1.0
1.5
2.0
2.5
0.0
0.2
0.4
0.6
0.8
1.0
N = 1087   Bandwidth = 0.031

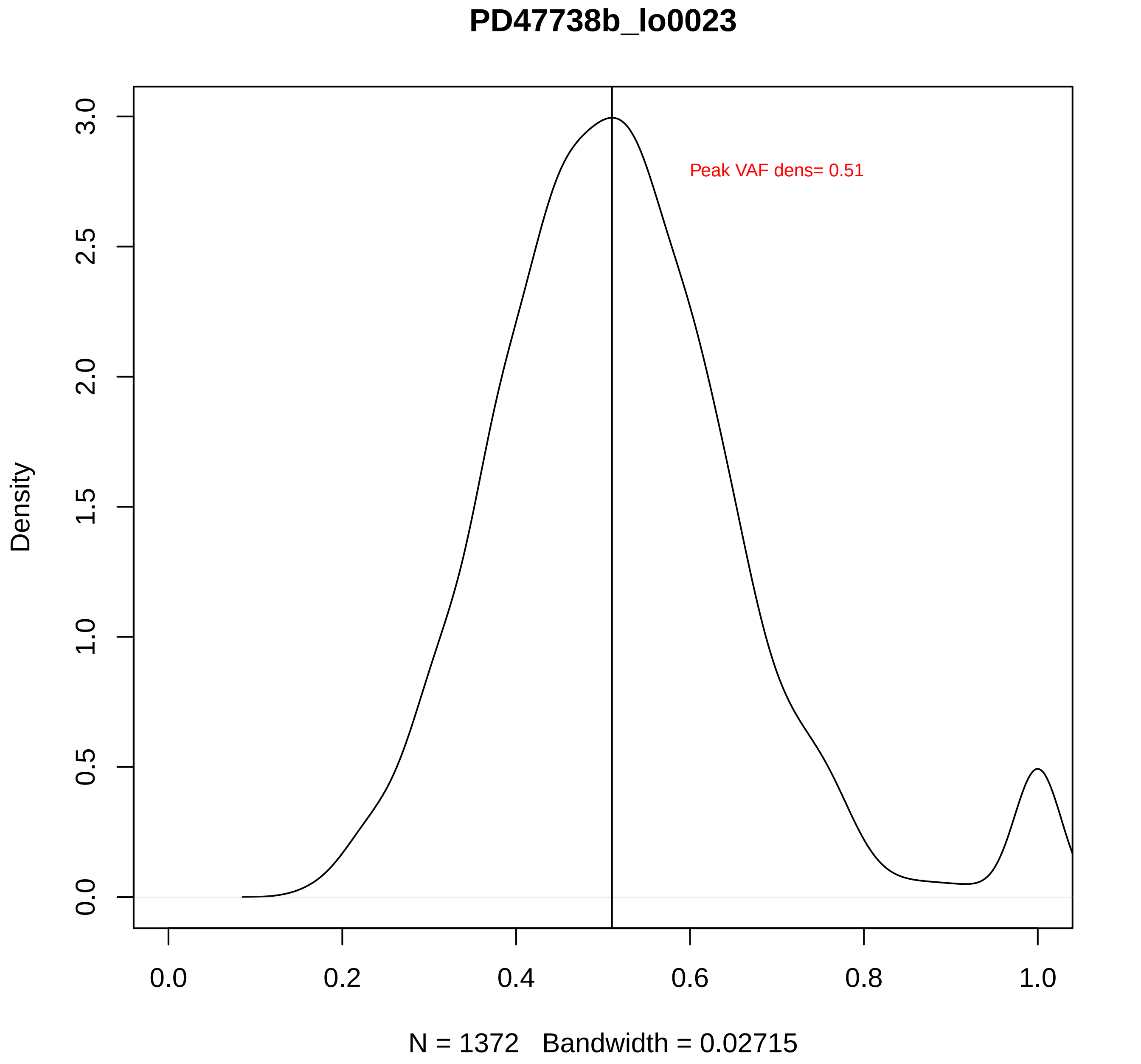

PD47738b_lo0023
Peak VAF dens= 0.51
Density
0.0
0.5
1.0
1.5
2.0
2.5
3.0
0.0
0.2
0.4
0.6
0.8
1.0
N = 1372 Bandwidth = 0.02715

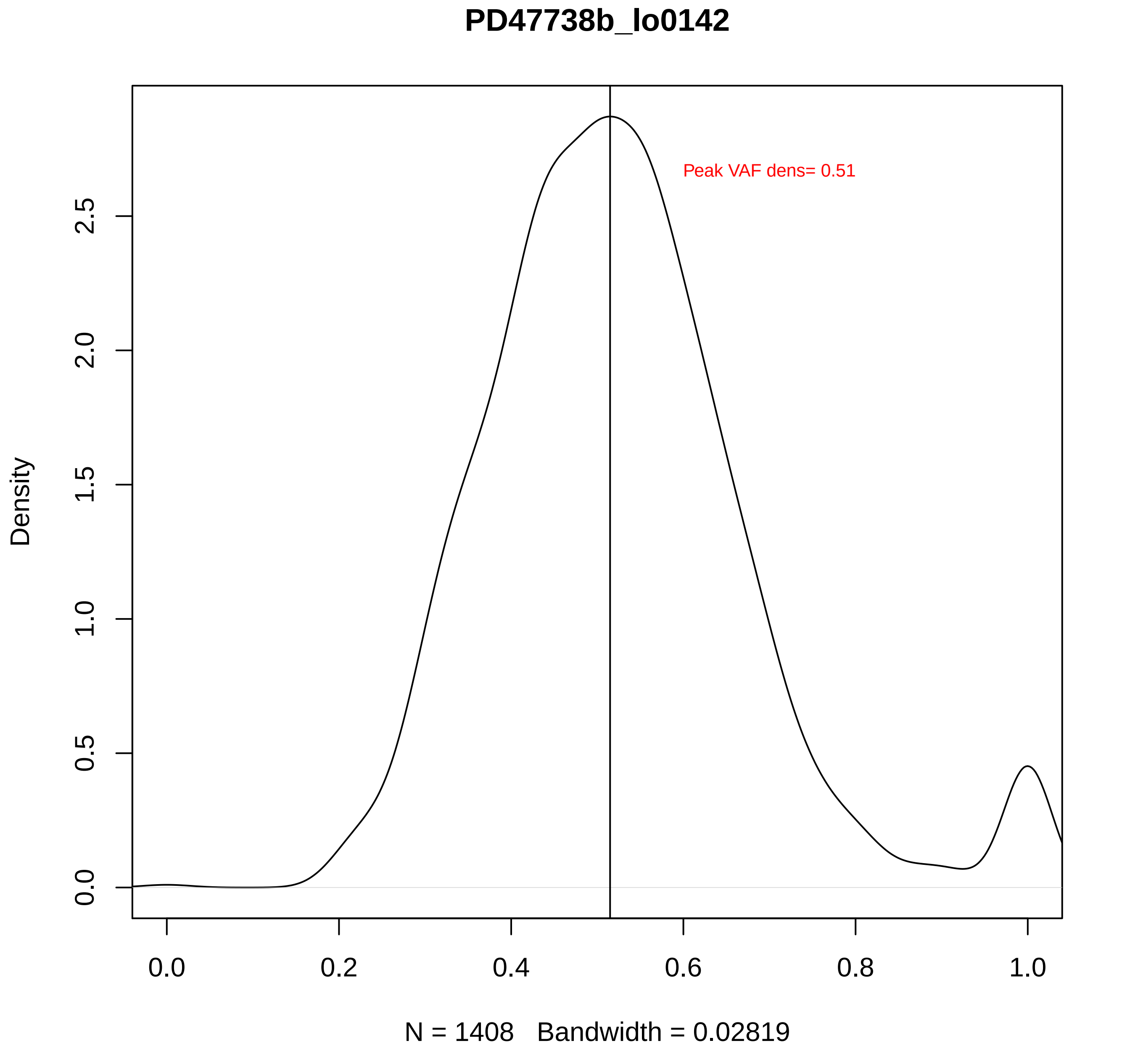
PD47738b_lo0142
Peak VAF dens= 0.51
Density
0.0
0.5
1.0
1.5
2.0
2.5
0.0
0.2
0.4
0.6
0.8
1.0
N = 1408   Bandwidth = 0.02819

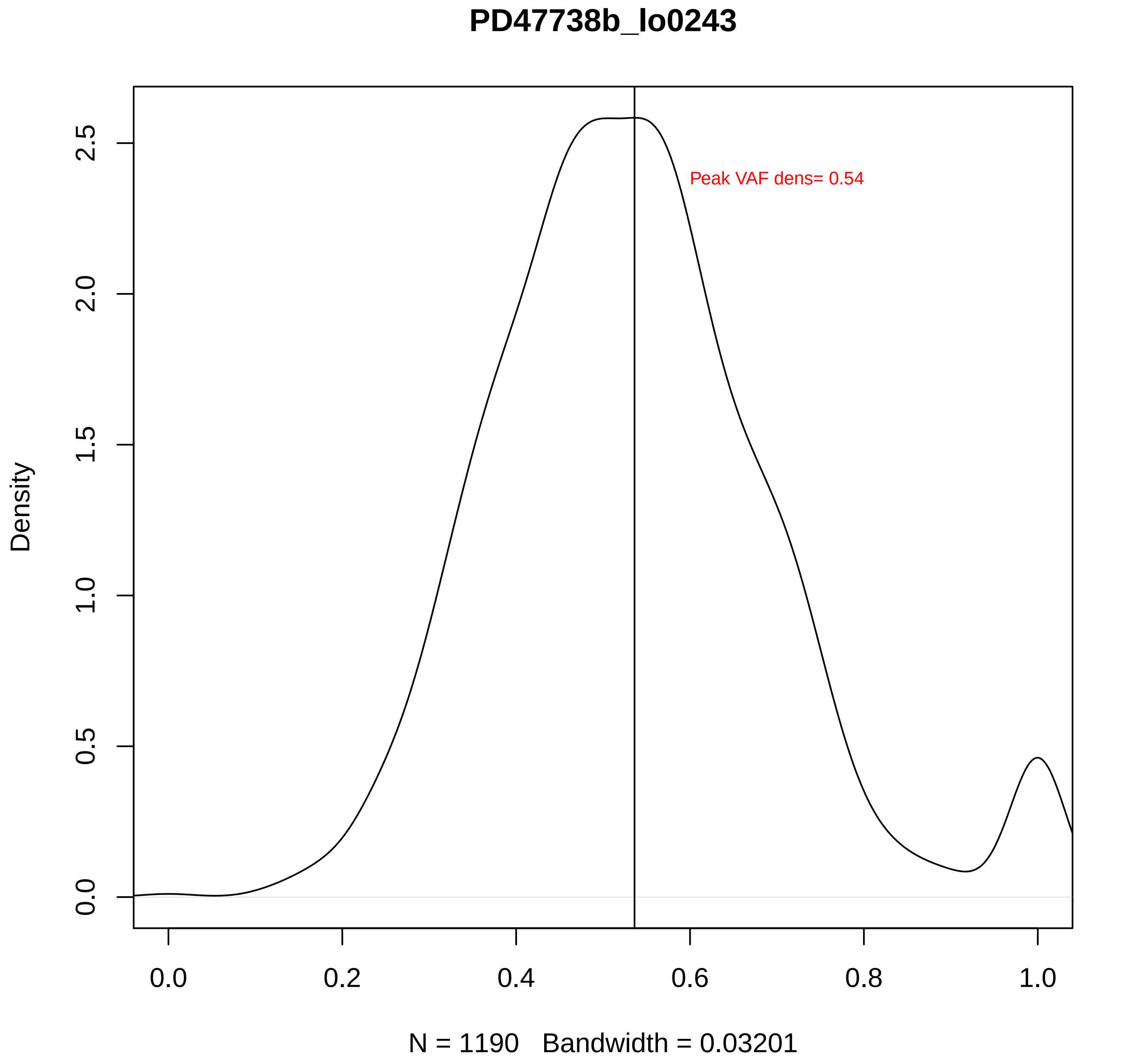
PD47738b_lo0243
Peak VAF dens= 0.54
Density
2.5
2.0
1.5
1.0
0.5
0.0
0.0
0.2
0.4
0.6
0.8
1.0
N = 1190   Bandwidth = 0.03201

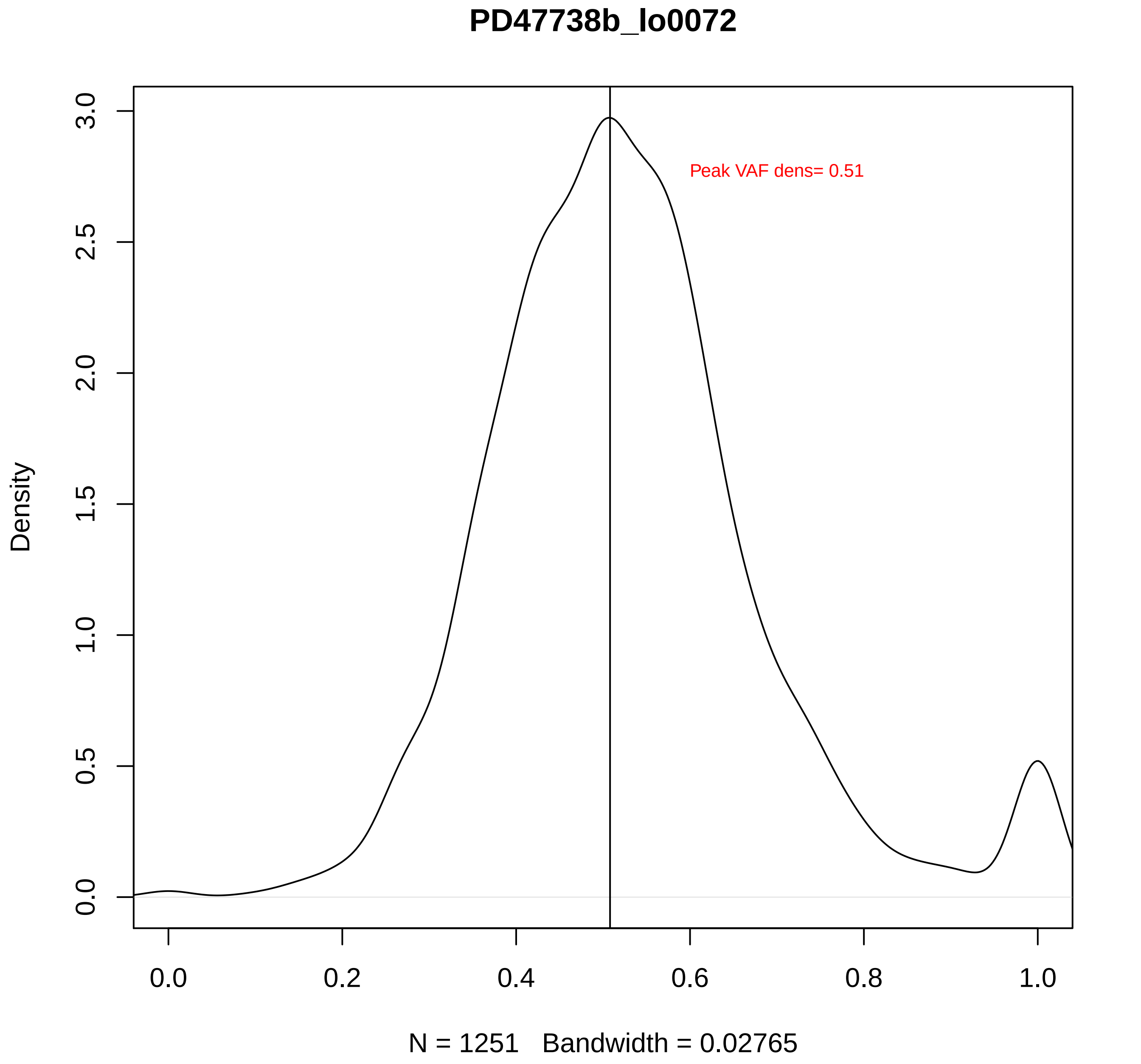
PD47738b_lo0072
Peak VAF dens= 0.51
Density
0.0
0.5
1.0
1.5
2.0
2.5
3.0
0.0
0.2
0.4
0.6
0.8
1.0
N = 1251   Bandwidth = 0.02765

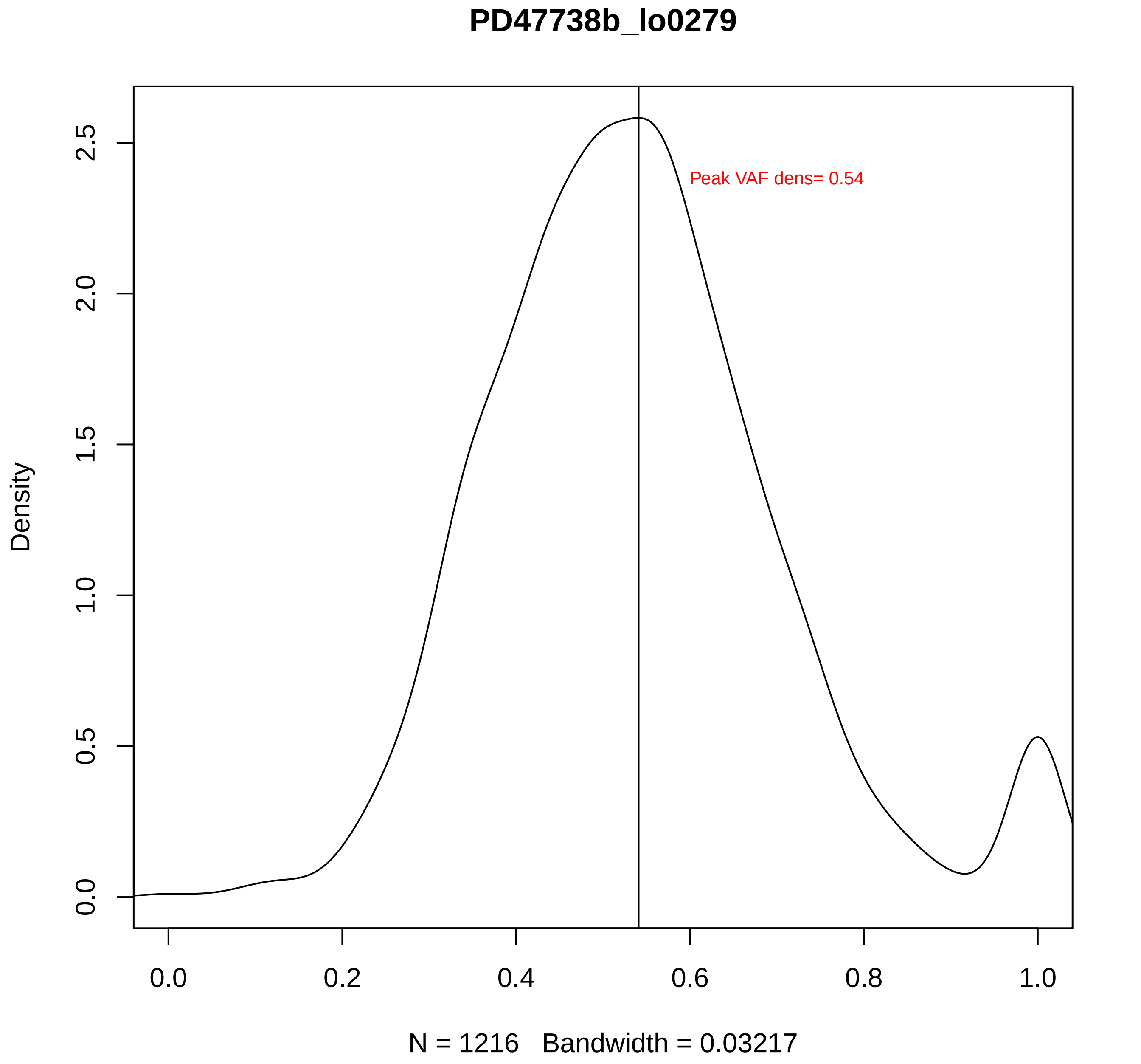
PD47738b_lo0279
Peak VAF dens= 0.54
Density
0.0
0.5
1.0
1.5
2.0
2.5
0.0
0.2
0.4
0.6
0.8
1.0
N = 1216 Bandwidth = 0.03217

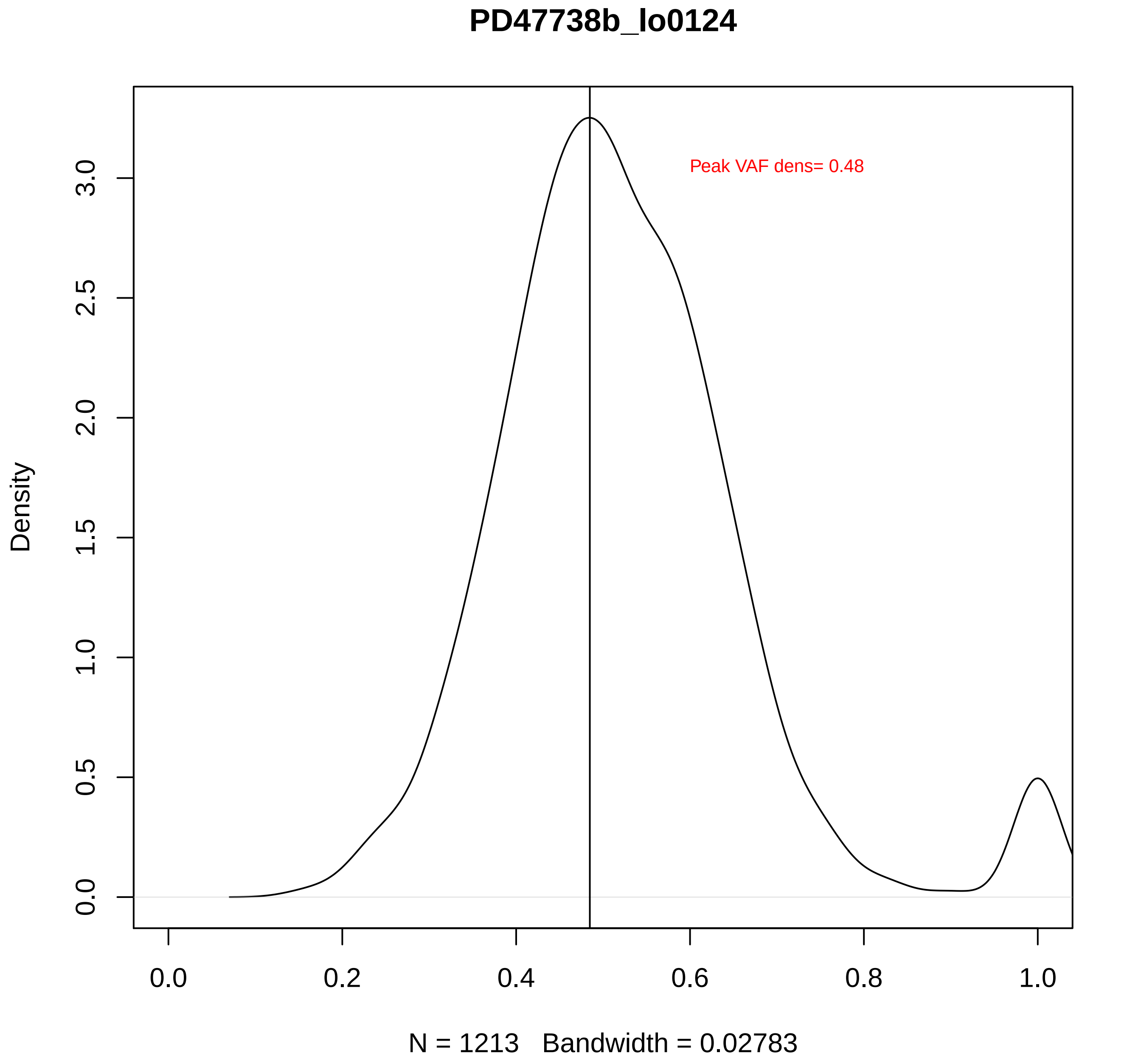

PD47738b_lo0124
Peak VAF dens= 0.48
Density
0.0
0.5
1.0
1.5
2.0
2.5
3.0
0.0
0.2
0.4
0.6
0.8
1.0
N = 1213   Bandwidth = 0.02783

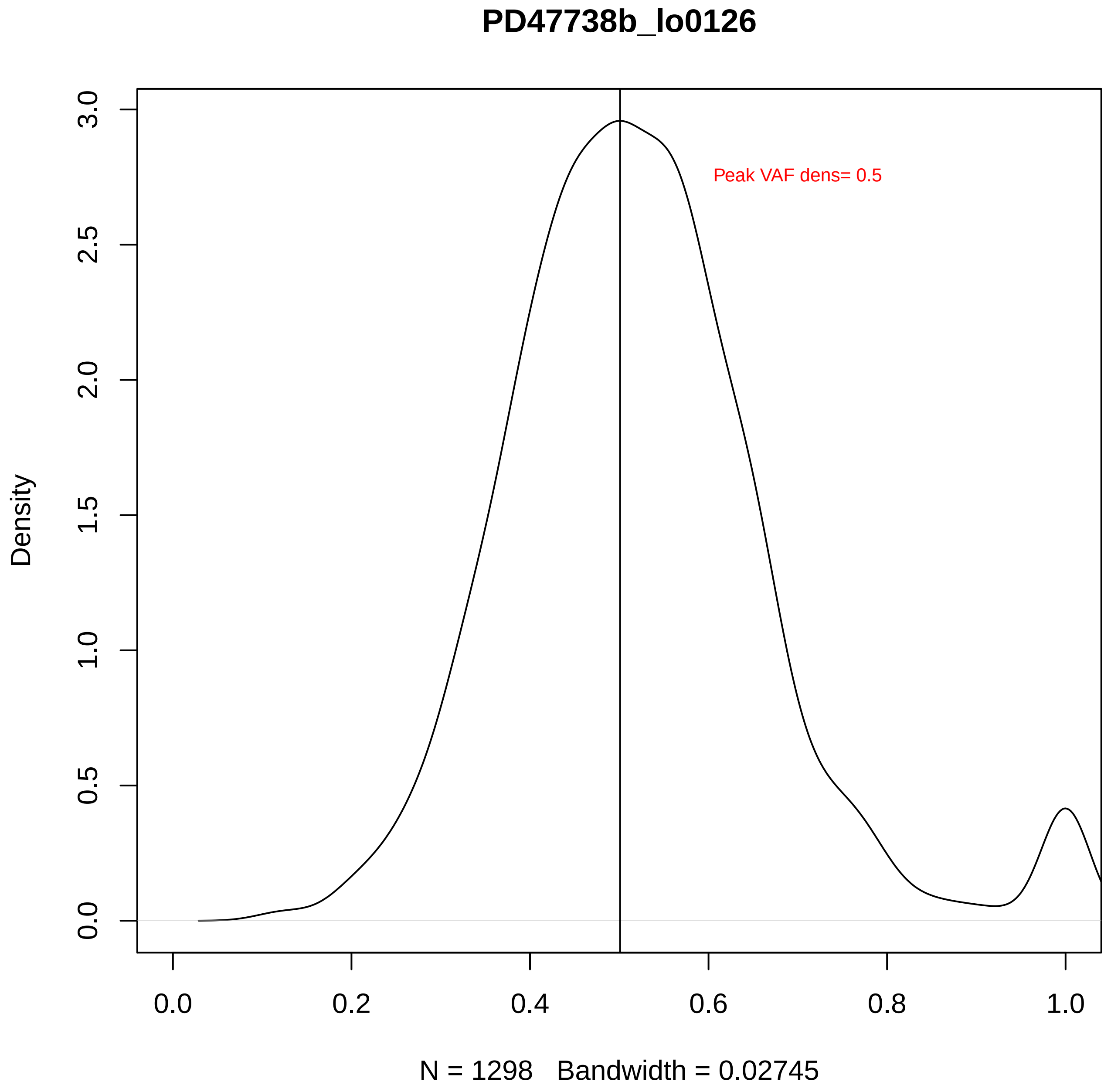
PD47738b_lo0126
Peak VAF dens= 0.5
Density
3.0
2.5
2.0
1.5
1.0
0.5
0.0
0.0
0.2
0.4
0.6
0.8
1.0
N = 1298   Bandwidth = 0.02745

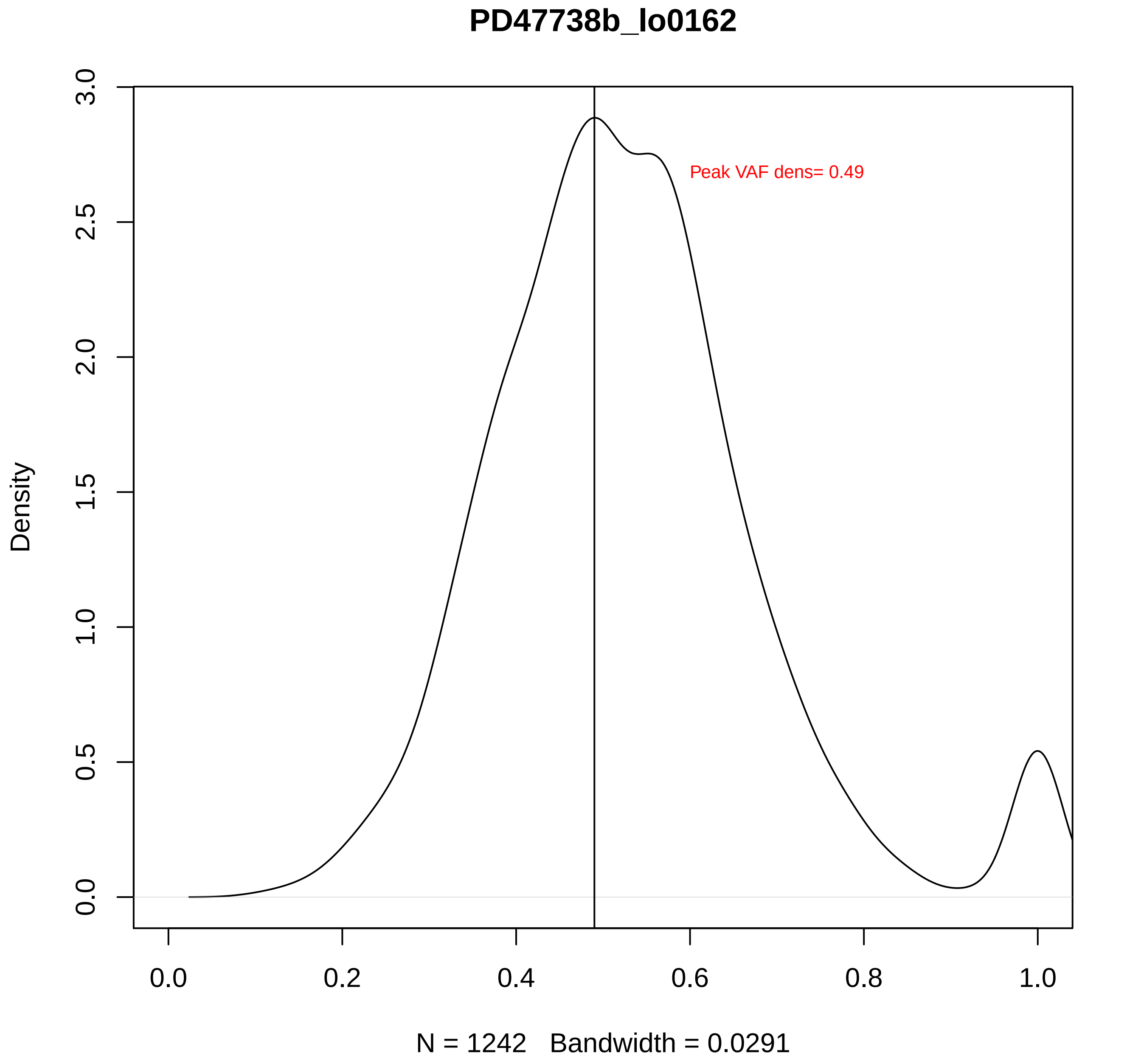
PD47738b_lo0162
Peak VAF dens= 0.49
Density
3.0
2.5
2.0
1.5
1.0
0.5
0.0
0.0
0.2
0.4
0.6
0.8
1.0
N = 1242   Bandwidth = 0.0291

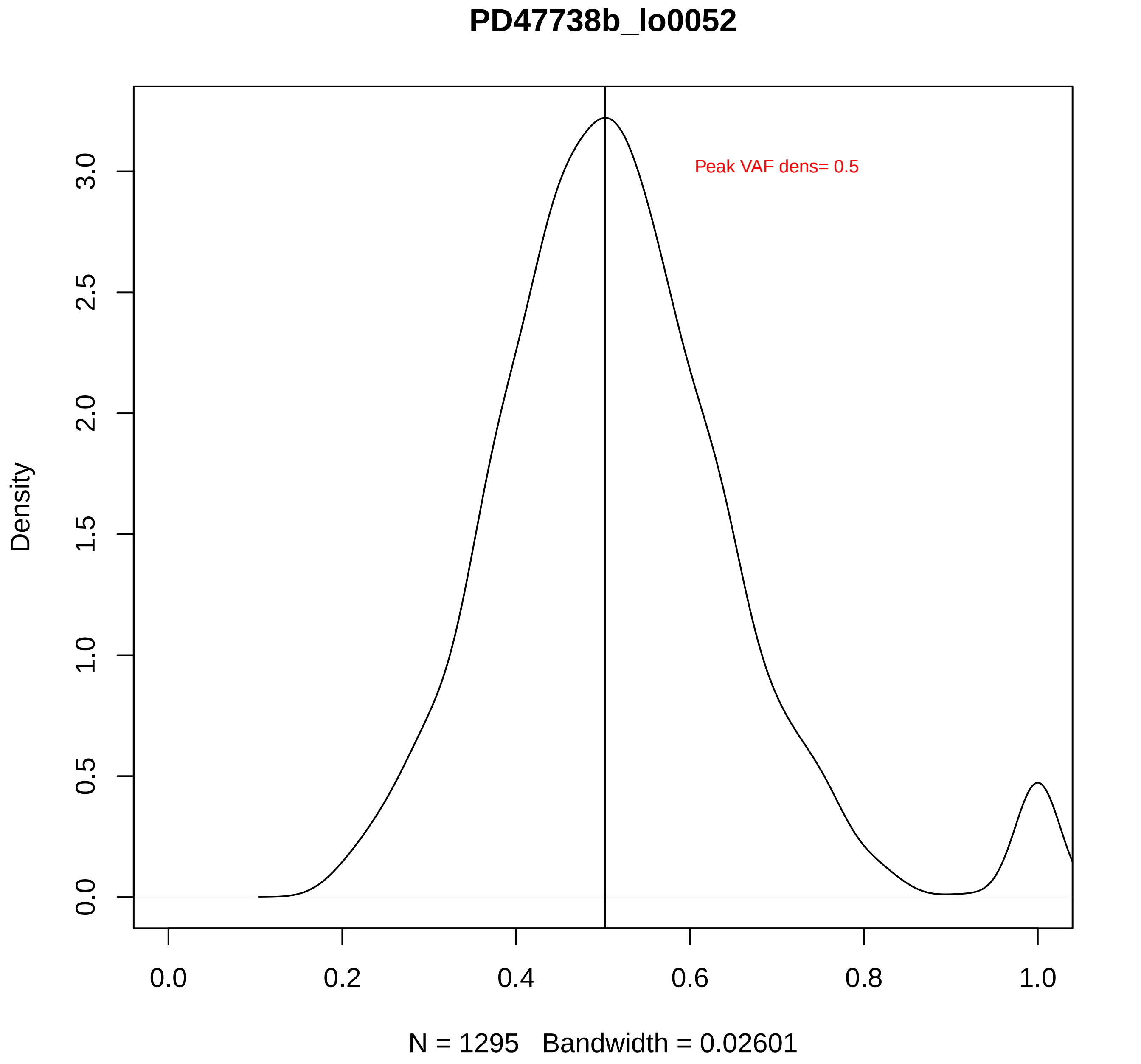
PD47738b_lo0052
Peak VAF dens= 0.5
Density
0.0
0.5
1.0
1.5
2.0
2.5
3.0
0.0
0.2
0.4
0.6
0.8
1.0
N = 1295   Bandwidth = 0.02601

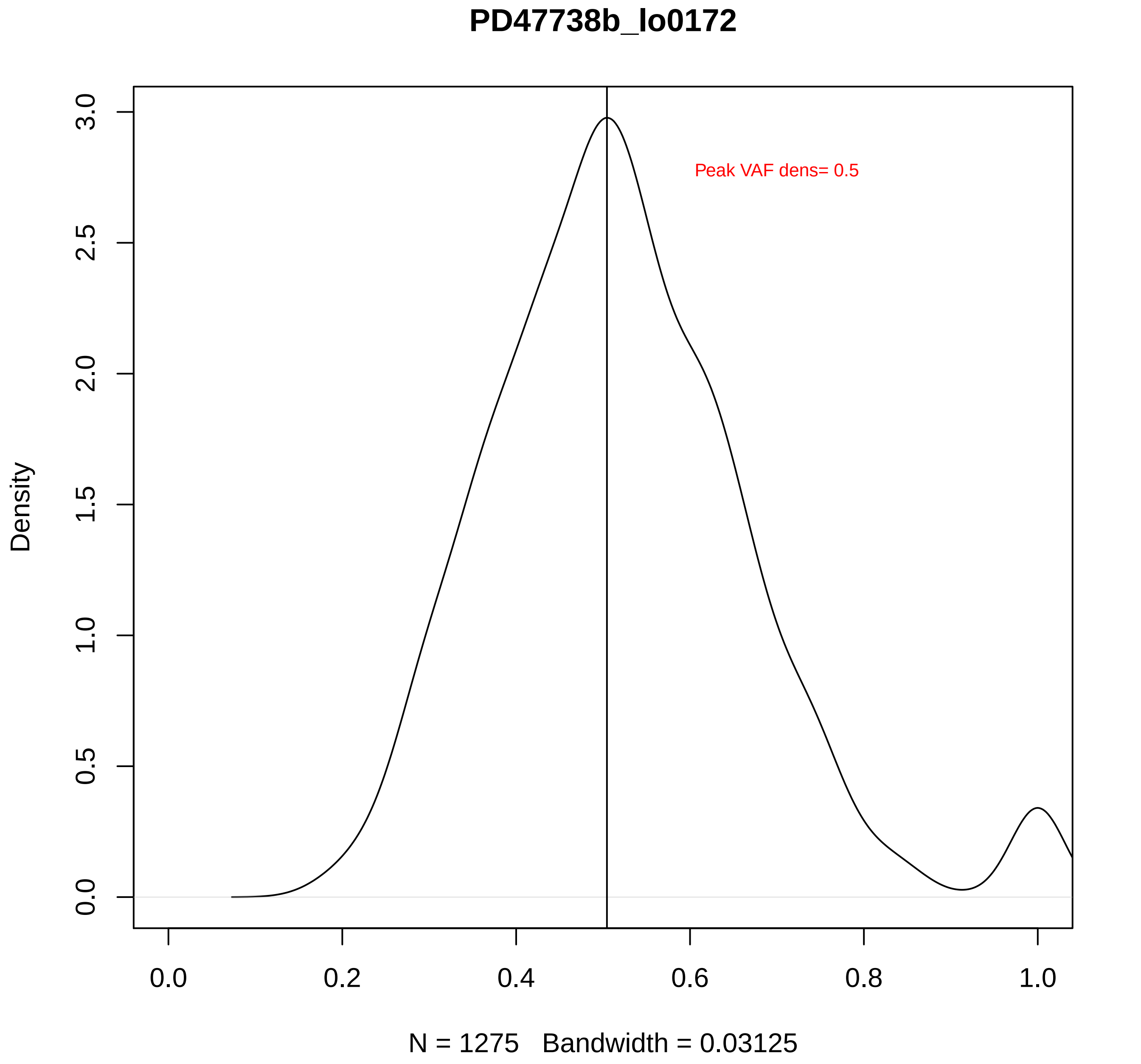
PD47738b_lo0172
Peak VAF dens= 0.5
Density
0.0
0.5
1.0
1.5
2.0
2.5
3.0
0.0
0.2
0.4
0.6
0.8
1.0
N = 1275   Bandwidth = 0.03125

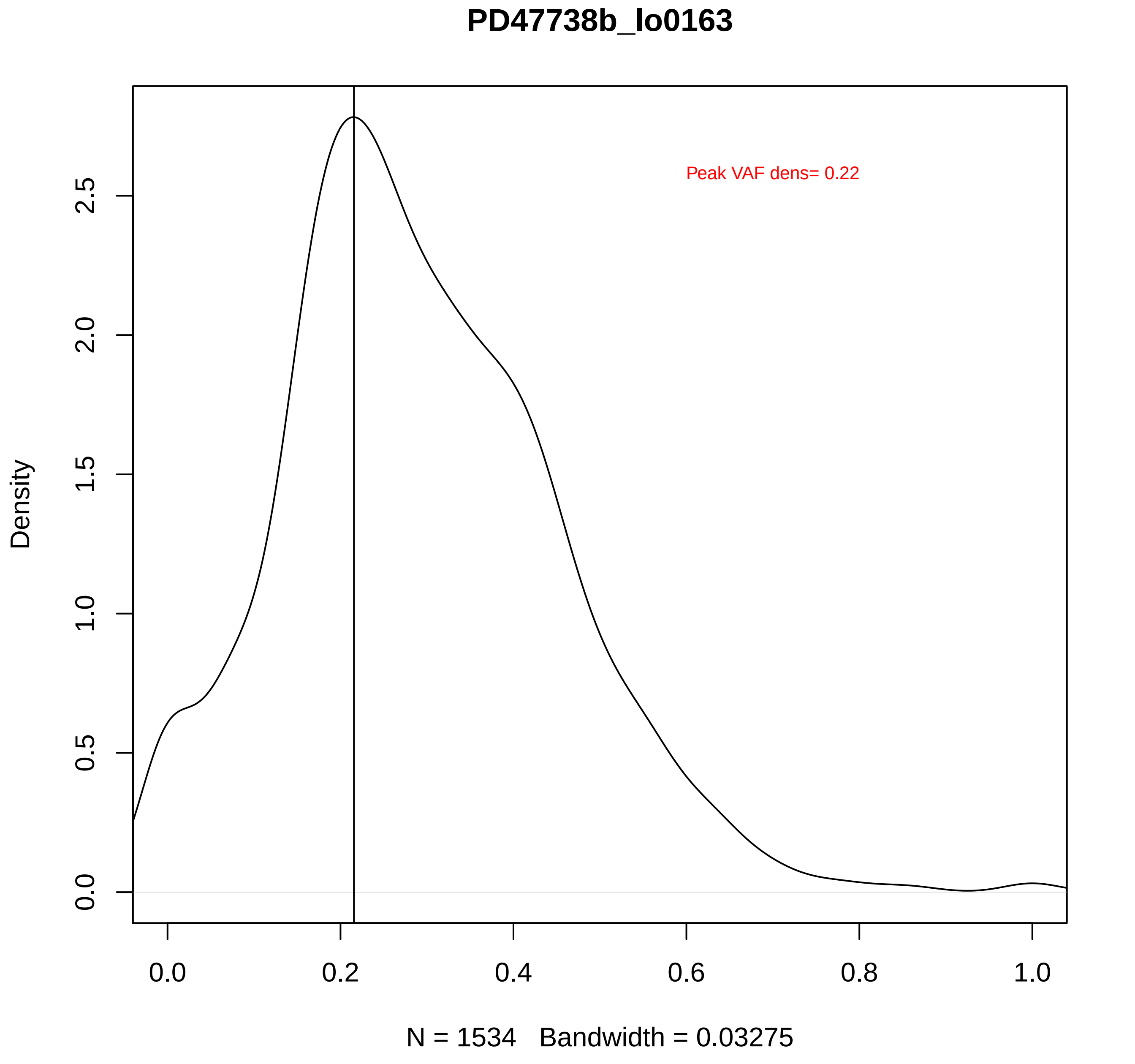
PD47738b_lo0163
Peak VAF dens= 0.22
Density
0.0
0.5
1.0
1.5
2.0
2.5
0.0
0.2
0.4
0.6
0.8
1.0
N = 1534 Bandwidth = 0.03275

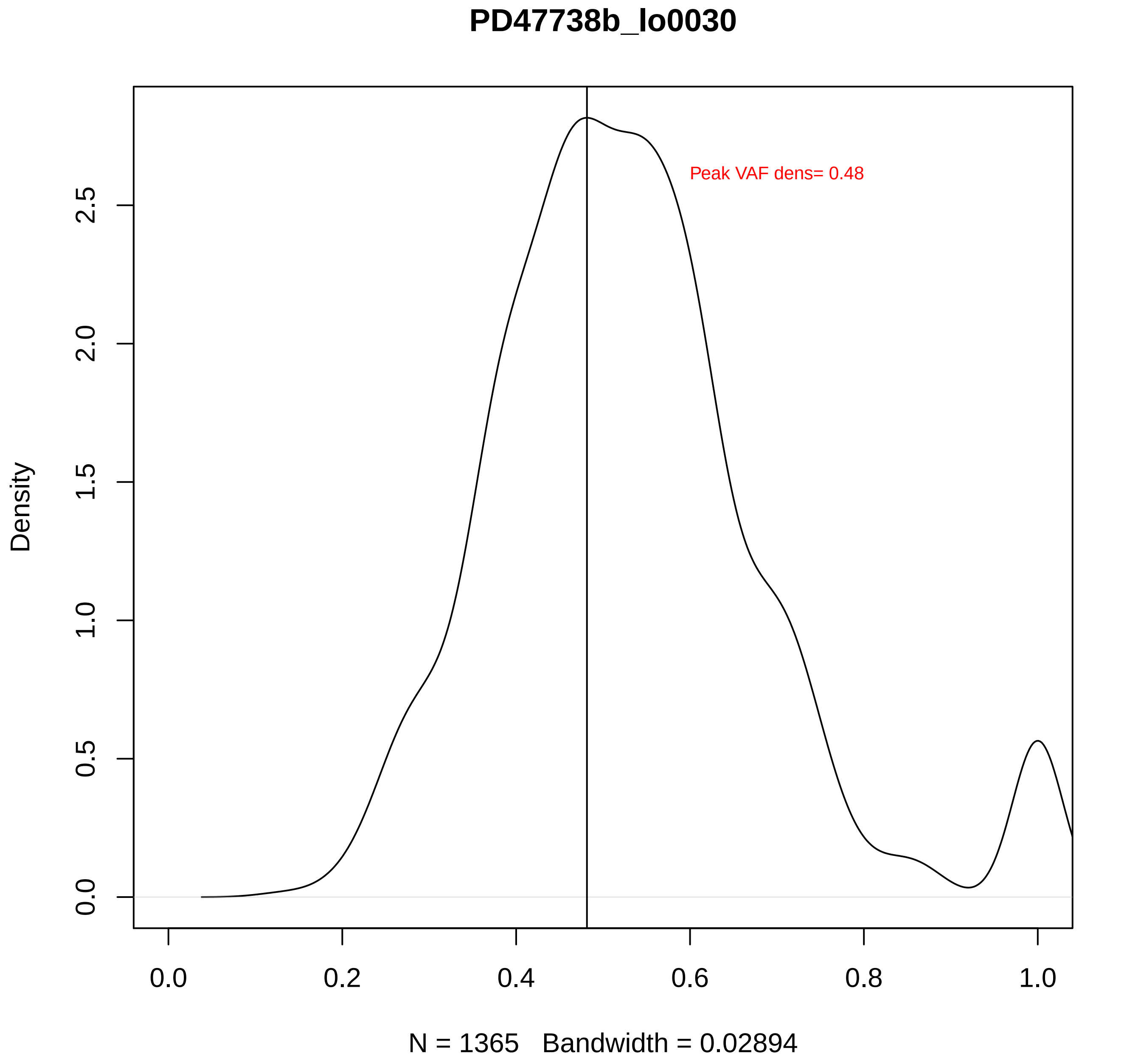
PD47738b_lo0030
Peak VAF dens= 0.48
Density
0.0
0.5
1.0
1.5
2.0
2.5
0.0
0.2
0.4
0.6
0.8
1.0
N = 1365 Bandwidth = 0.02894

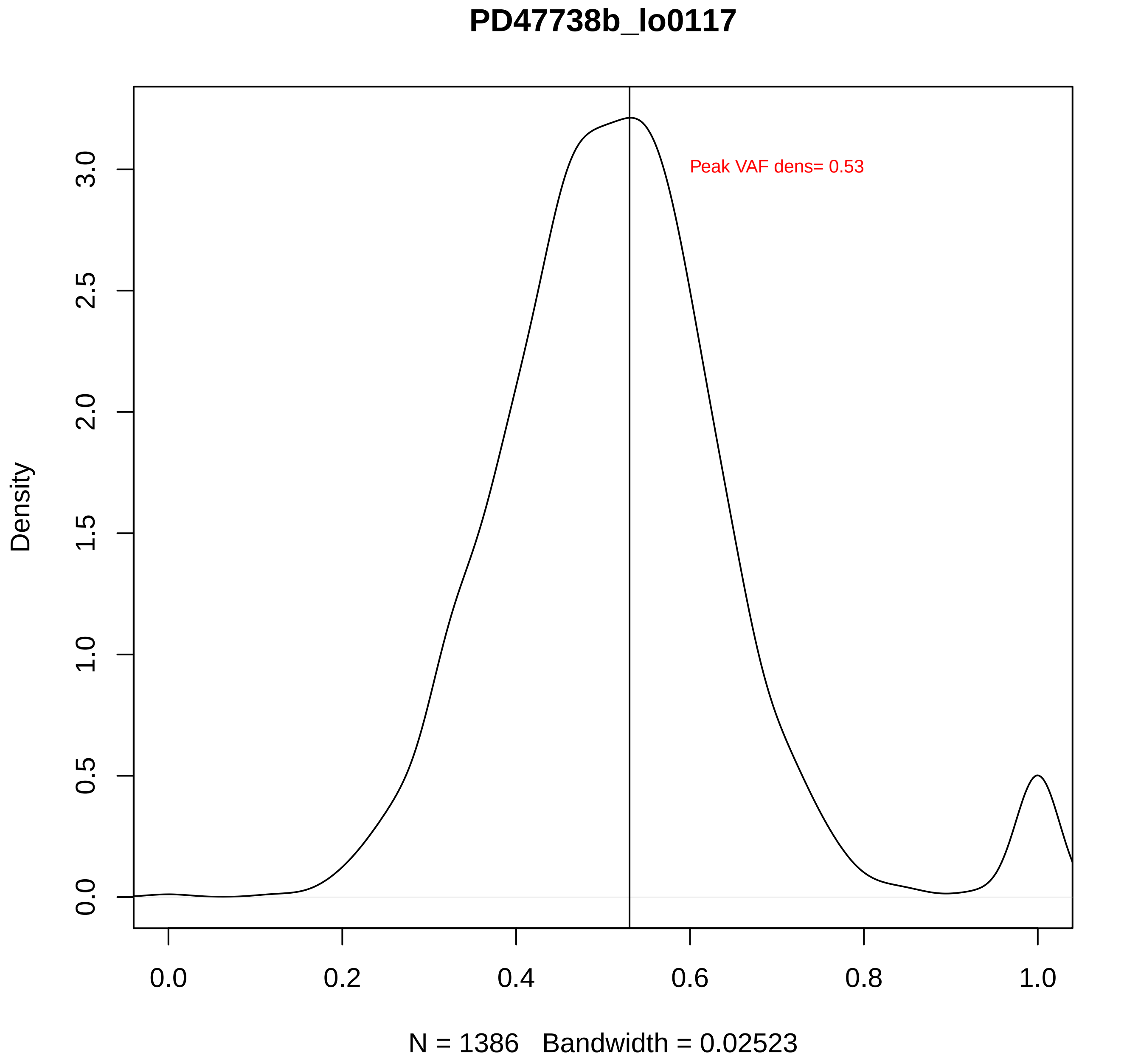
PD47738b_lo0117
Peak VAF dens= 0.53
Density
0.0
0.5
1.0
1.5
2.0
2.5
3.0
0.0
0.2
0.4
0.6
0.8
1.0
N = 1386   Bandwidth = 0.02523

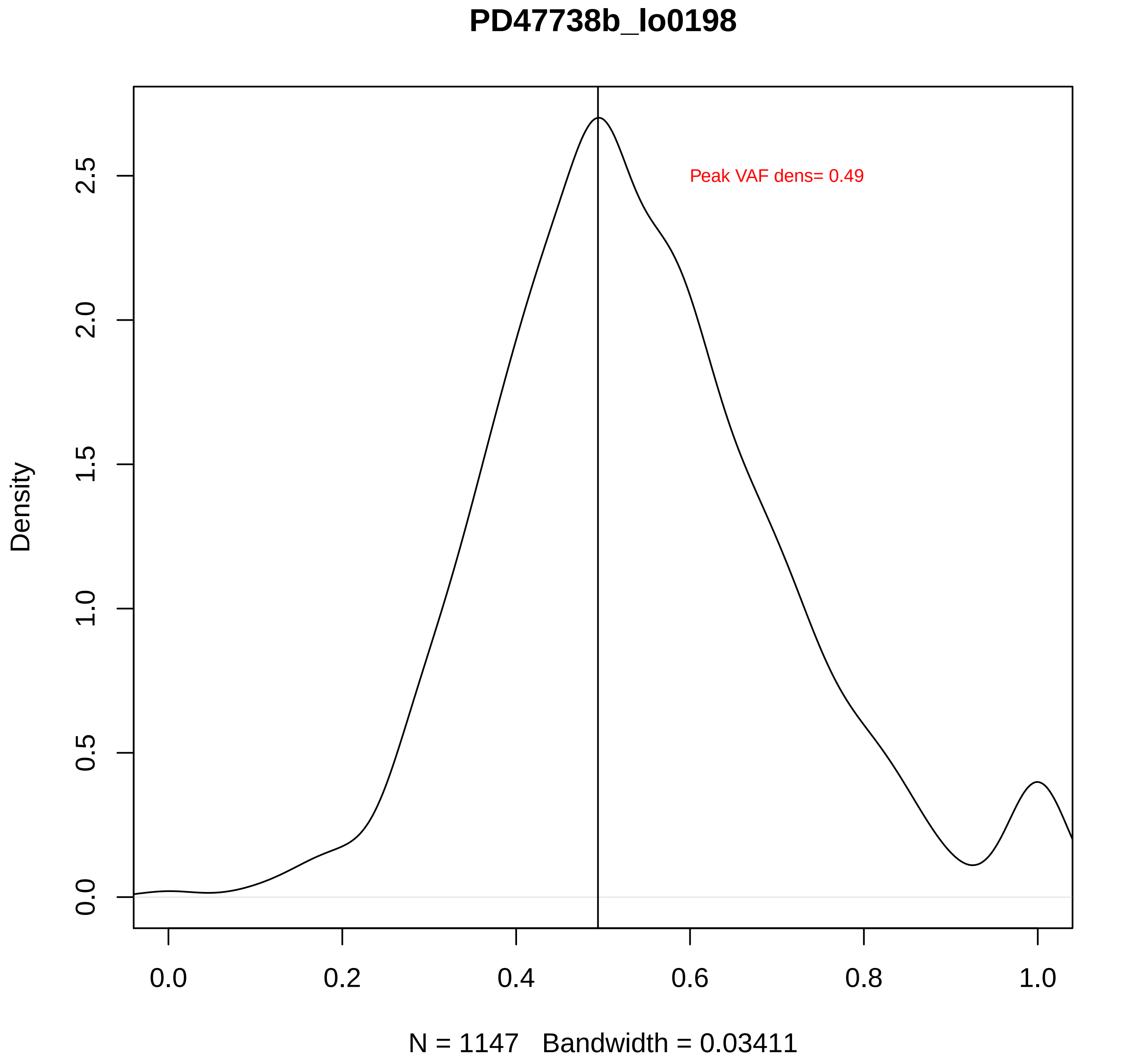
PD47738b_lo0198
Peak VAF dens= 0.49
Density
0.0
0.5
1.0
1.5
2.0
2.5
0.0
0.2
0.4
0.6
0.8
1.0
N = 1147   Bandwidth = 0.03411

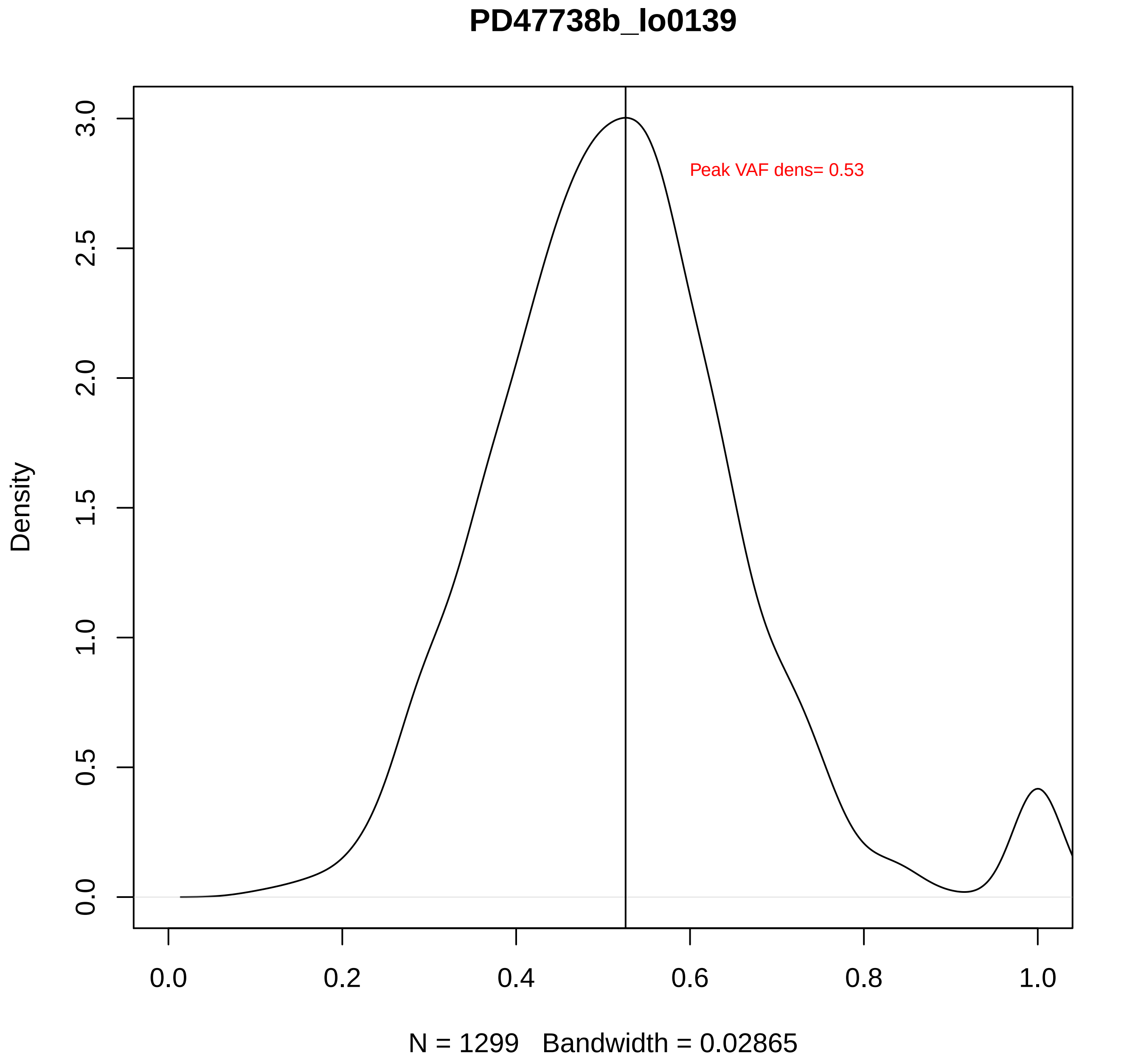
PD47738b_lo0139
Peak VAF dens= 0.53
Density
0.0
0.5
1.0
1.5
2.0
2.5
3.0
0.0
0.2
0.4
0.6
0.8
1.0
N = 1299   Bandwidth = 0.02865

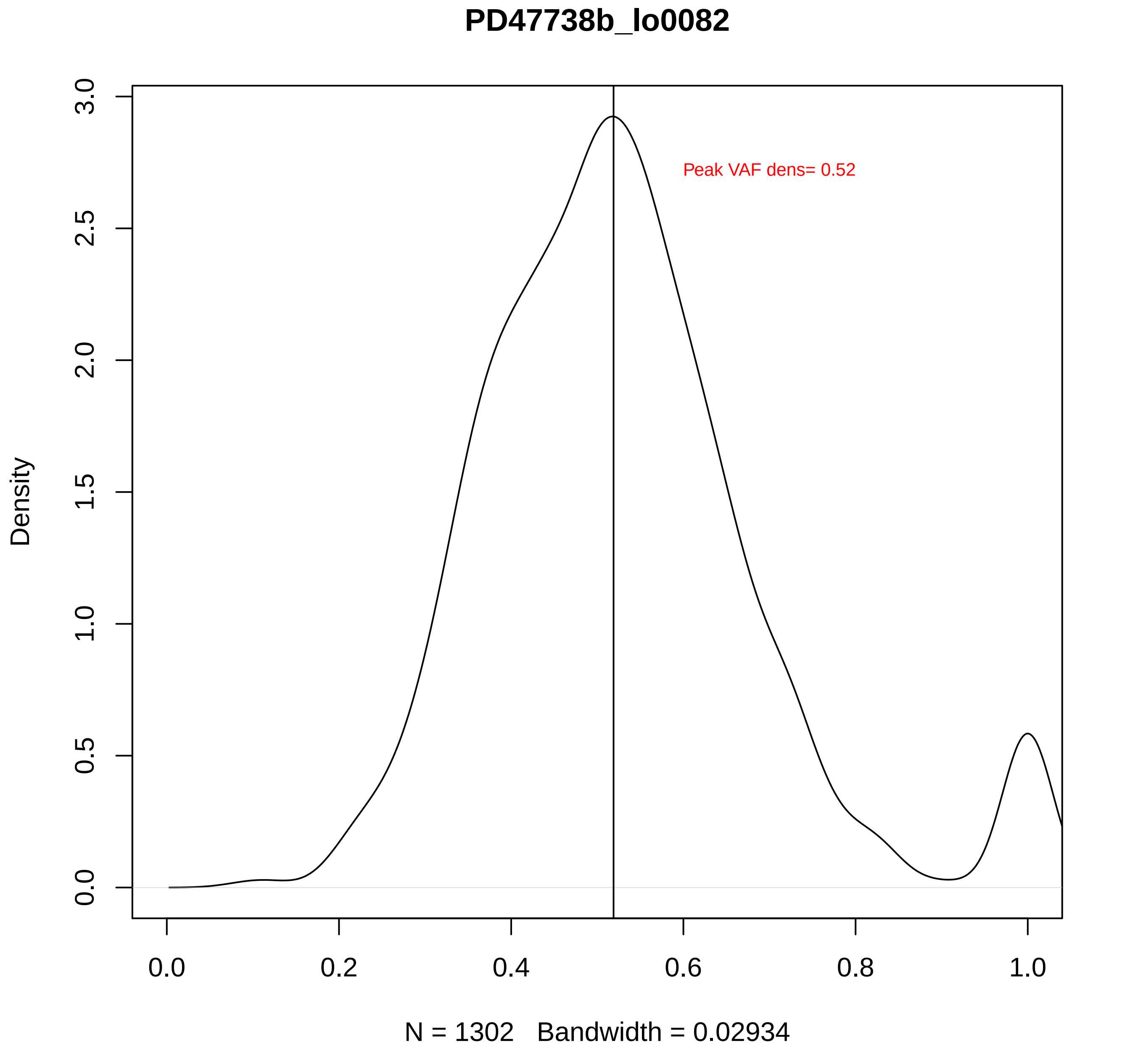

PD47738b_lo0082
Peak VAF dens= 0.52
Density
0.0
0.5
1.0
1.5
2.0
2.5
3.0
0.0
0.2
0.4
0.6
0.8
1.0
N = 1302   Bandwidth = 0.02934

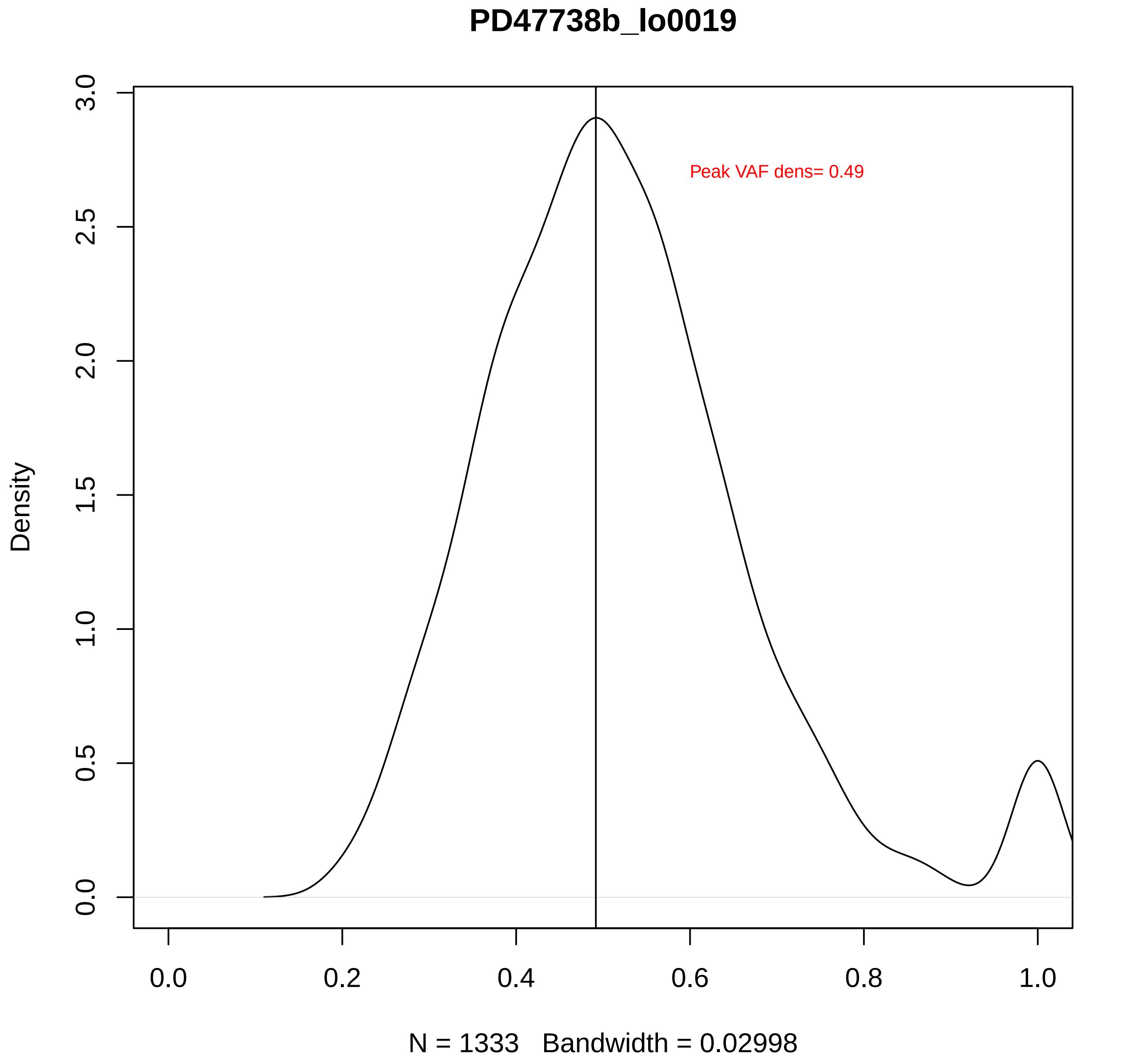
PD47738b_lo0019
Peak VAF dens= 0.49
Density
0.0
0.5
1.0
1.5
2.0
2.5
3.0
0.0
0.2
0.4
0.6
0.8
1.0
N = 1333   Bandwidth = 0.02998

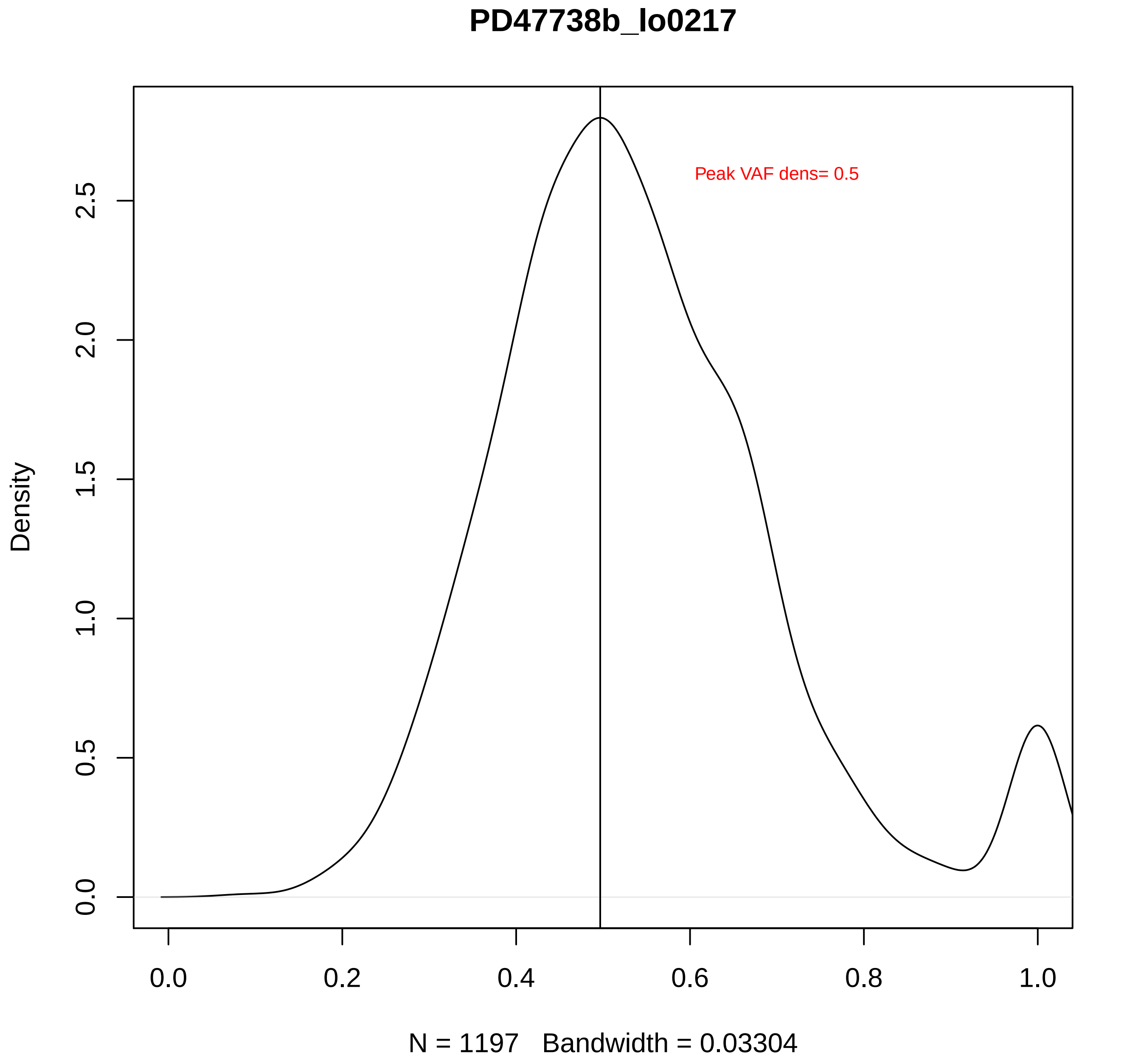
PD47738b_lo0217
Peak VAF dens= 0.5
Density
0.0
0.5
1.0
1.5
2.0
2.5
0.0
0.2
0.4
0.6
0.8
1.0
N = 1197   Bandwidth = 0.03304

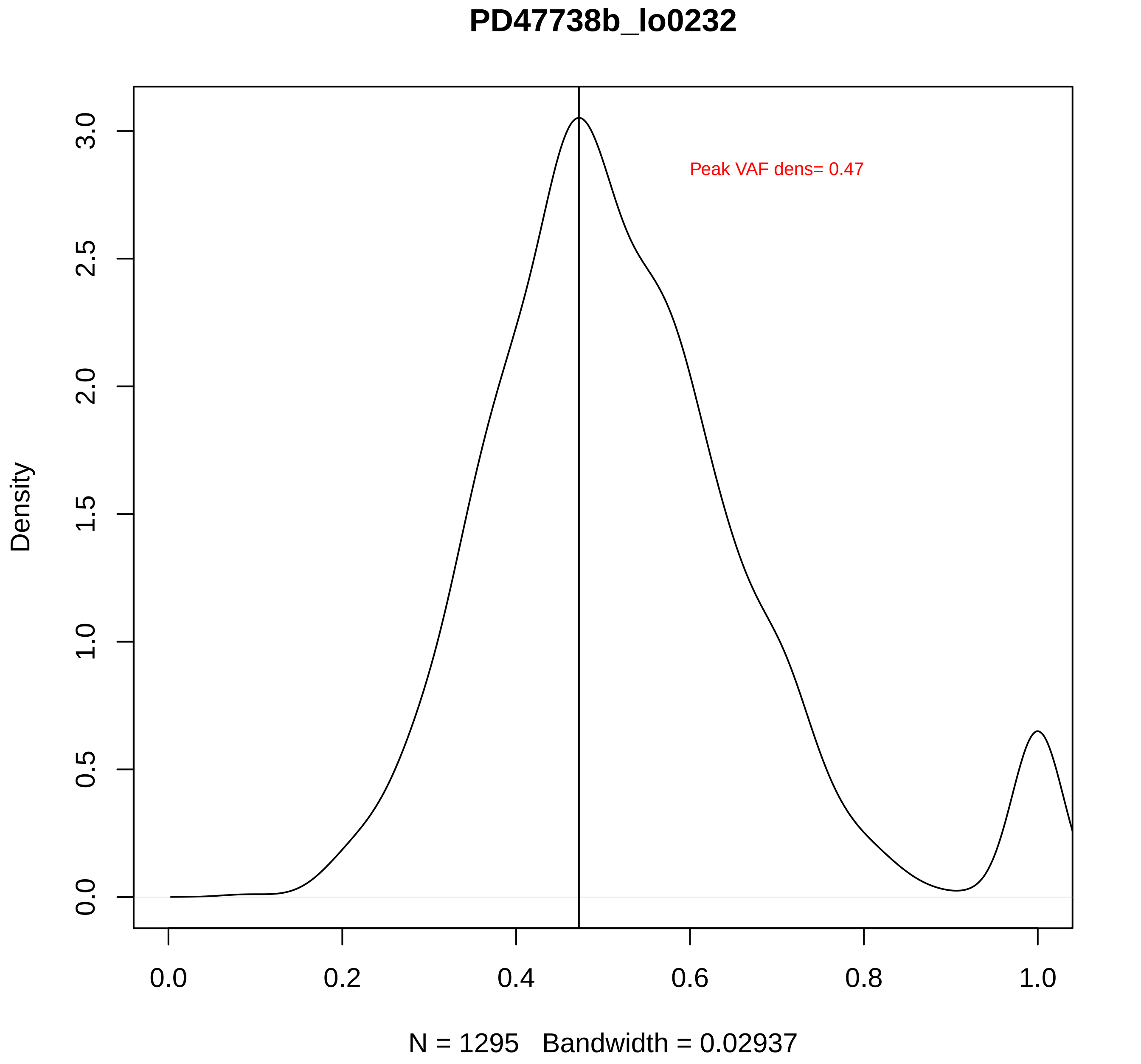
PD47738b_lo0232
Peak VAF dens= 0.47
Density
0.0
0.5
1.0
1.5
2.0
2.5
3.0
0.0
0.2
0.4
0.6
0.8
1.0
N = 1295   Bandwidth = 0.02937

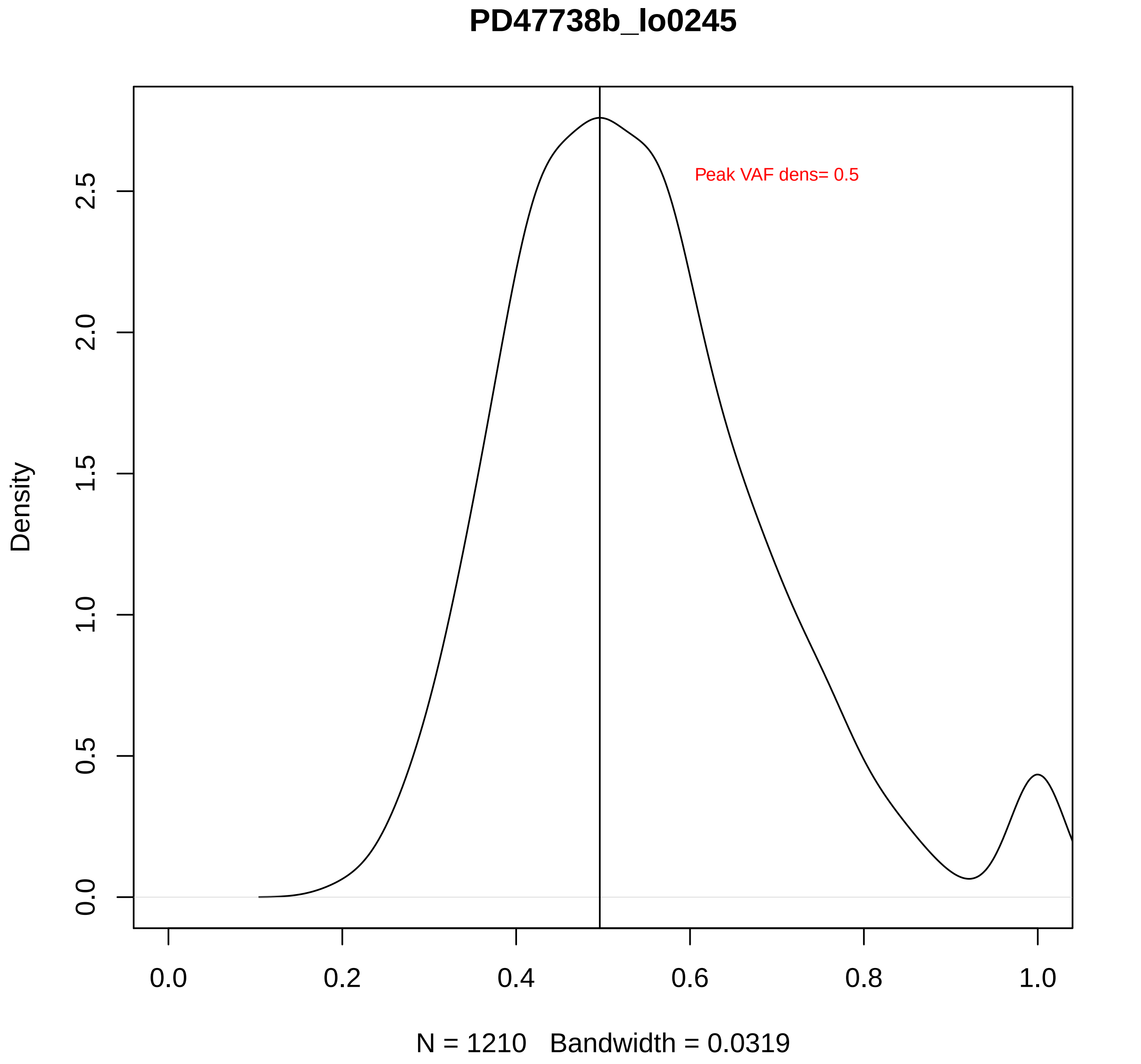

PD47738b_lo0245
Peak VAF dens= 0.5
Density
0.0
0.5
1.0
1.5
2.0
2.5
0.0
0.2
0.4
0.6
0.8
1.0
N = 1210   Bandwidth = 0.0319

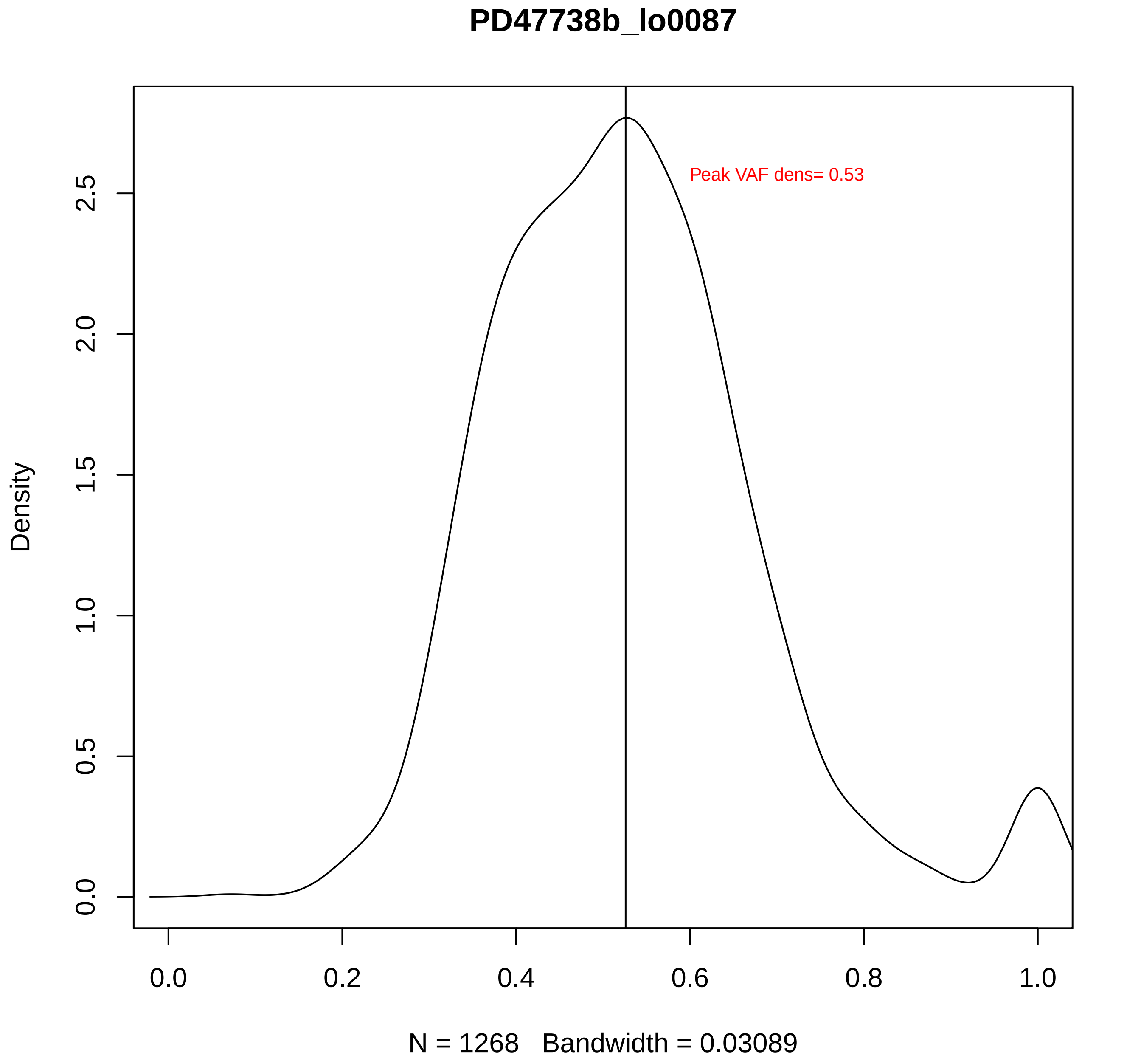
PD47738b_lo0087
Peak VAF dens= 0.53
Density
0.0
0.5
1.0
1.5
2.0
2.5
0.0
0.2
0.4
0.6
0.8
1.0
N = 1268   Bandwidth = 0.03089

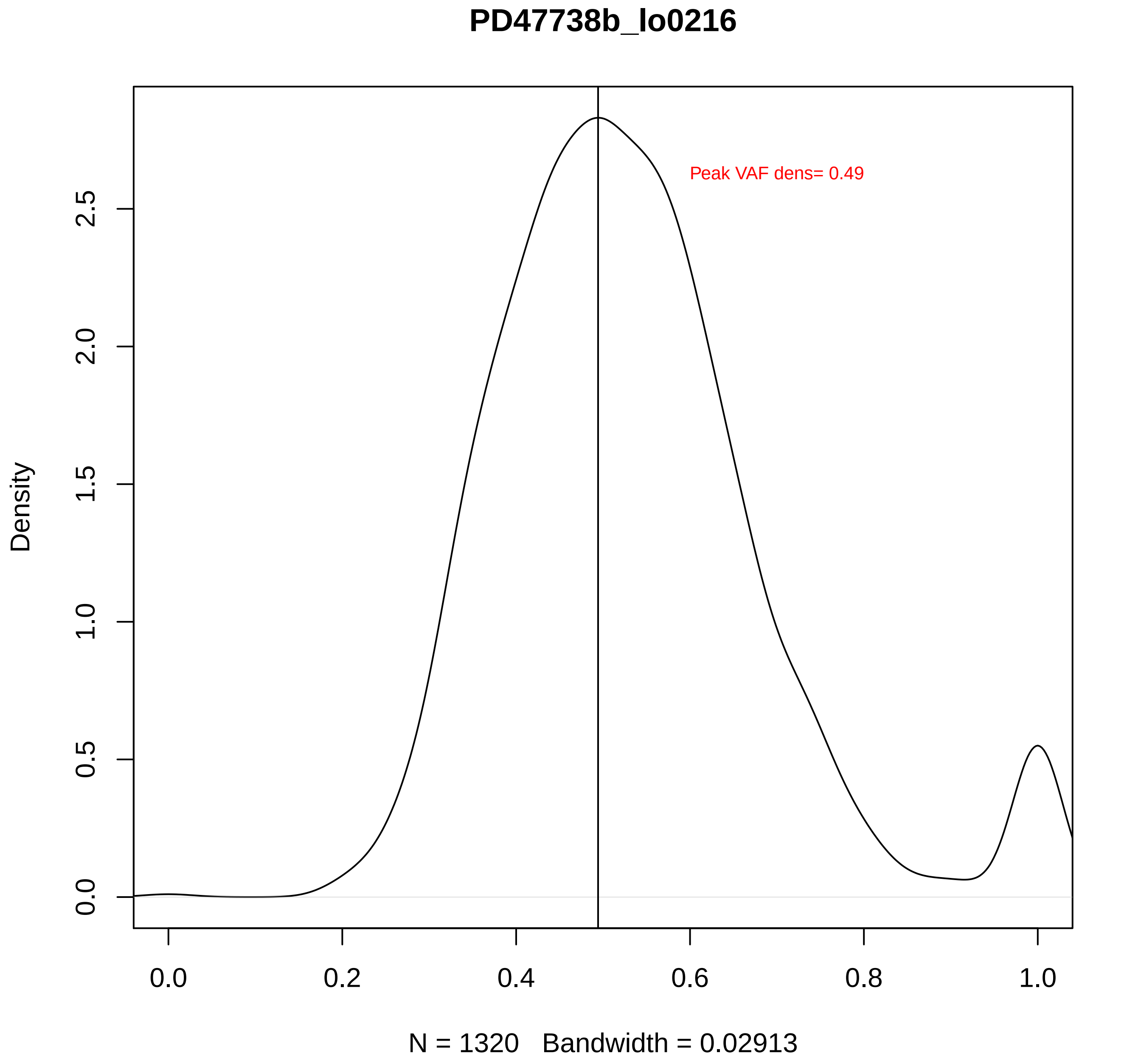
PD47738b_lo0216
Peak VAF dens= 0.49
Density
0.0
0.5
1.0
1.5
2.0
2.5
0.0
0.2
0.4
0.6
0.8
1.0
N = 1320   Bandwidth = 0.02913

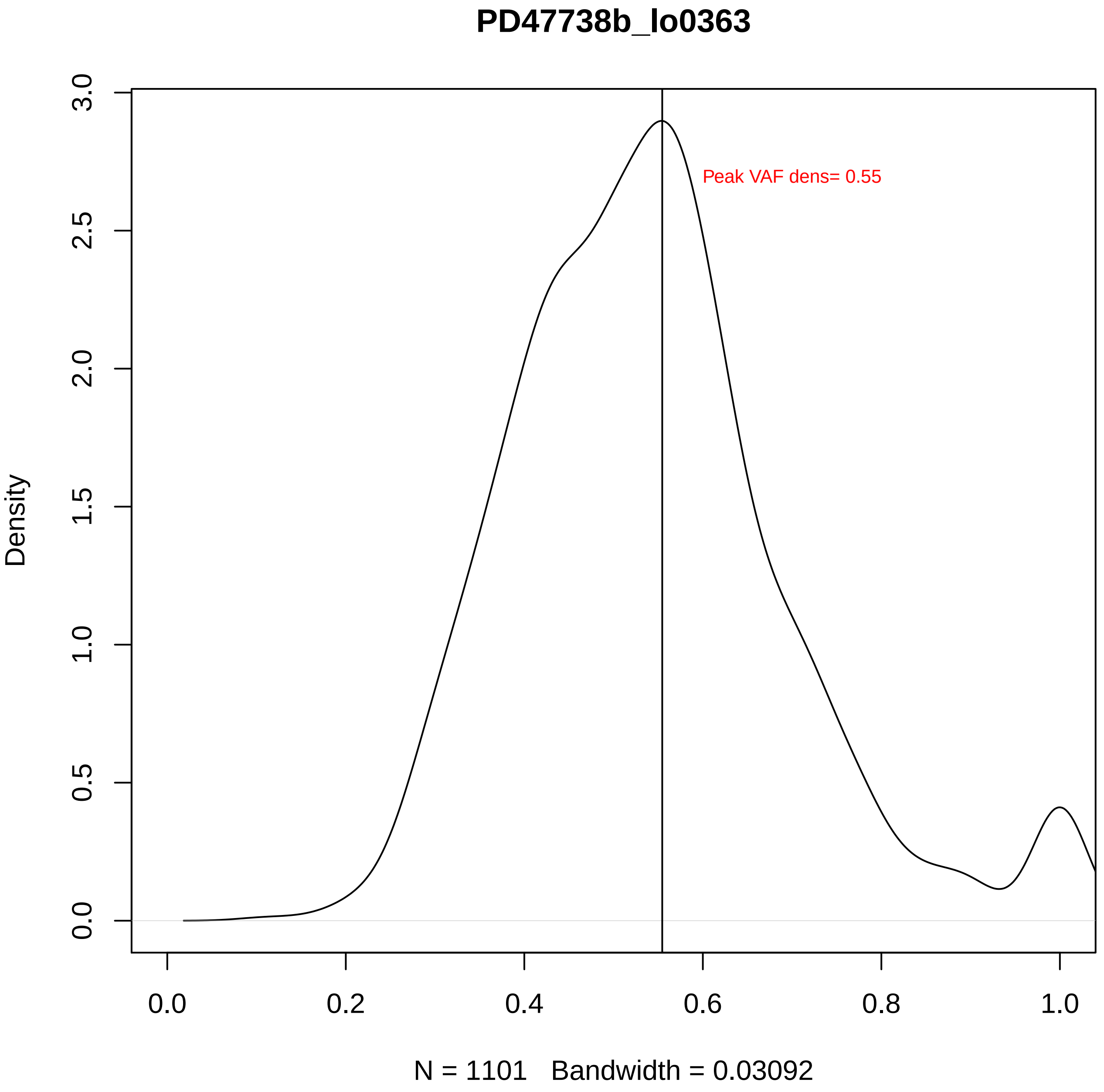
PD47738b_lo0363
Peak VAF dens= 0.55
Density
0.0
0.5
1.0
1.5
2.0
2.5
3.0
0.0
0.2
0.4
0.6
0.8
1.0
N = 1101   Bandwidth = 0.03092

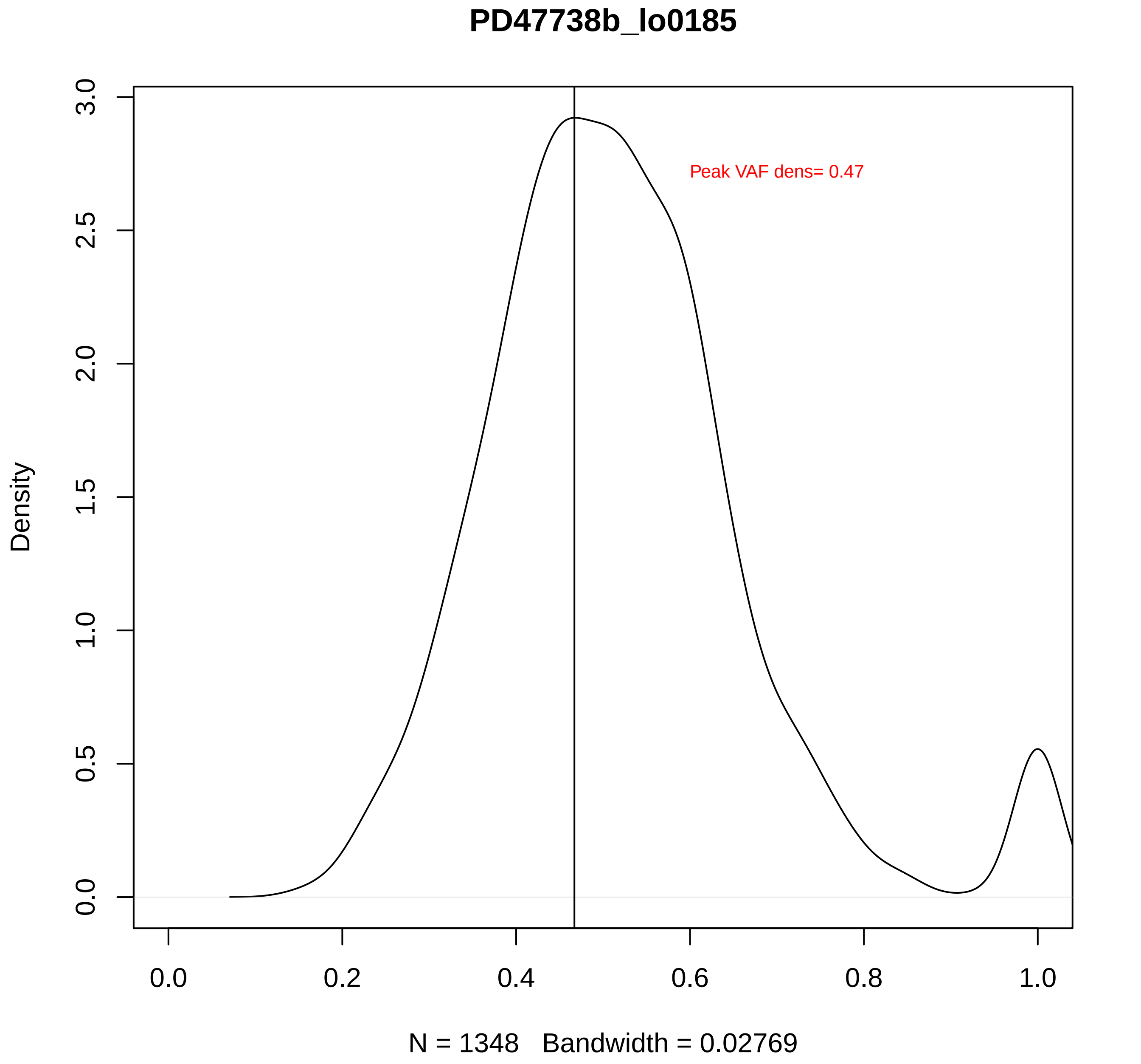
PD47738b_lo0185
Peak VAF dens= 0.47
Density
0.0
0.5
1.0
1.5
2.0
2.5
3.0
0.0
0.2
0.4
0.6
0.8
1.0
N = 1348   Bandwidth = 0.02769

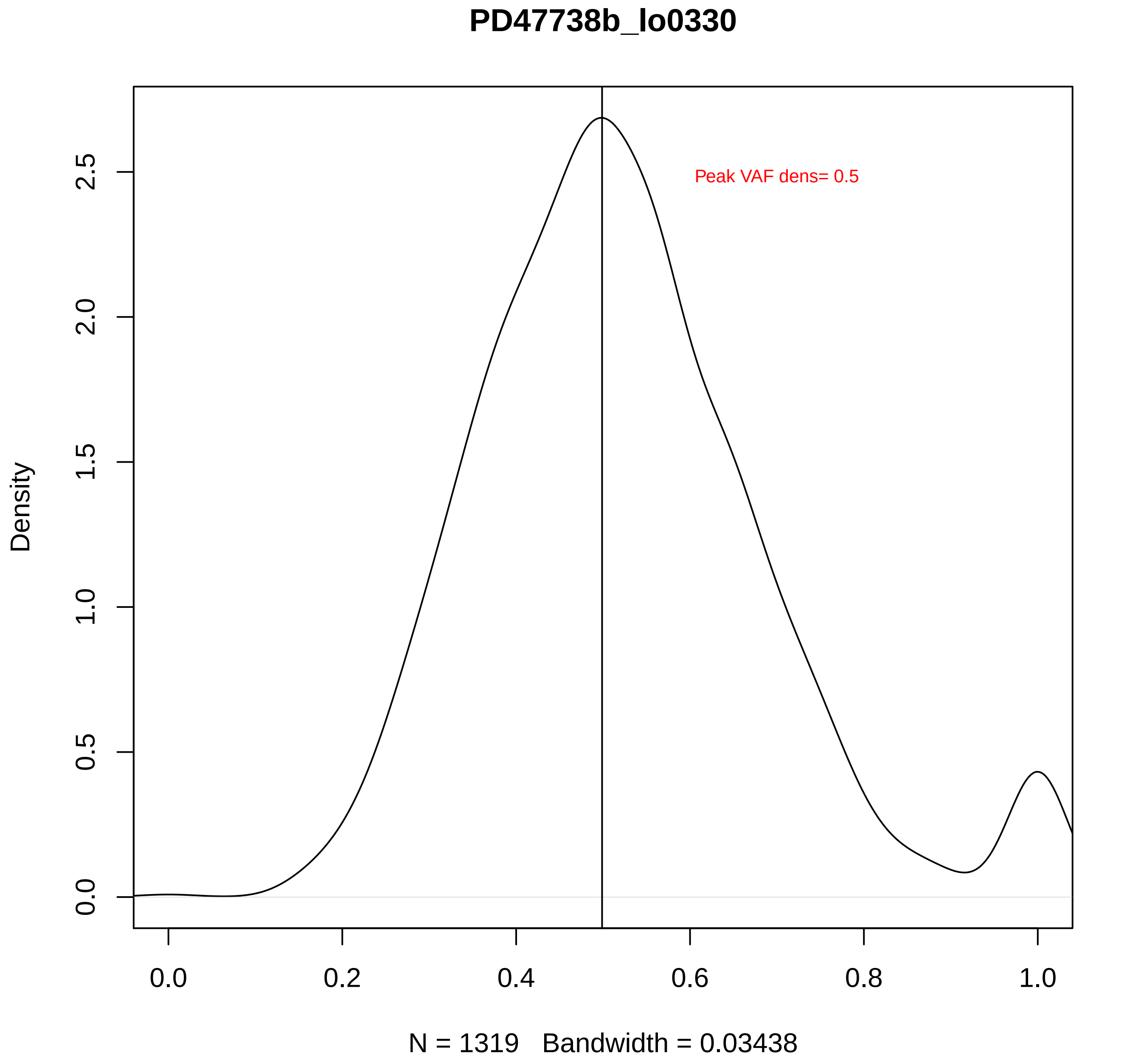
PD47738b_lo0330
Peak VAF dens= 0.5
Density
0.0
0.5
1.0
1.5
2.0
2.5
0.0
0.2
0.4
0.6
0.8
1.0
N = 1319 Bandwidth = 0.03438

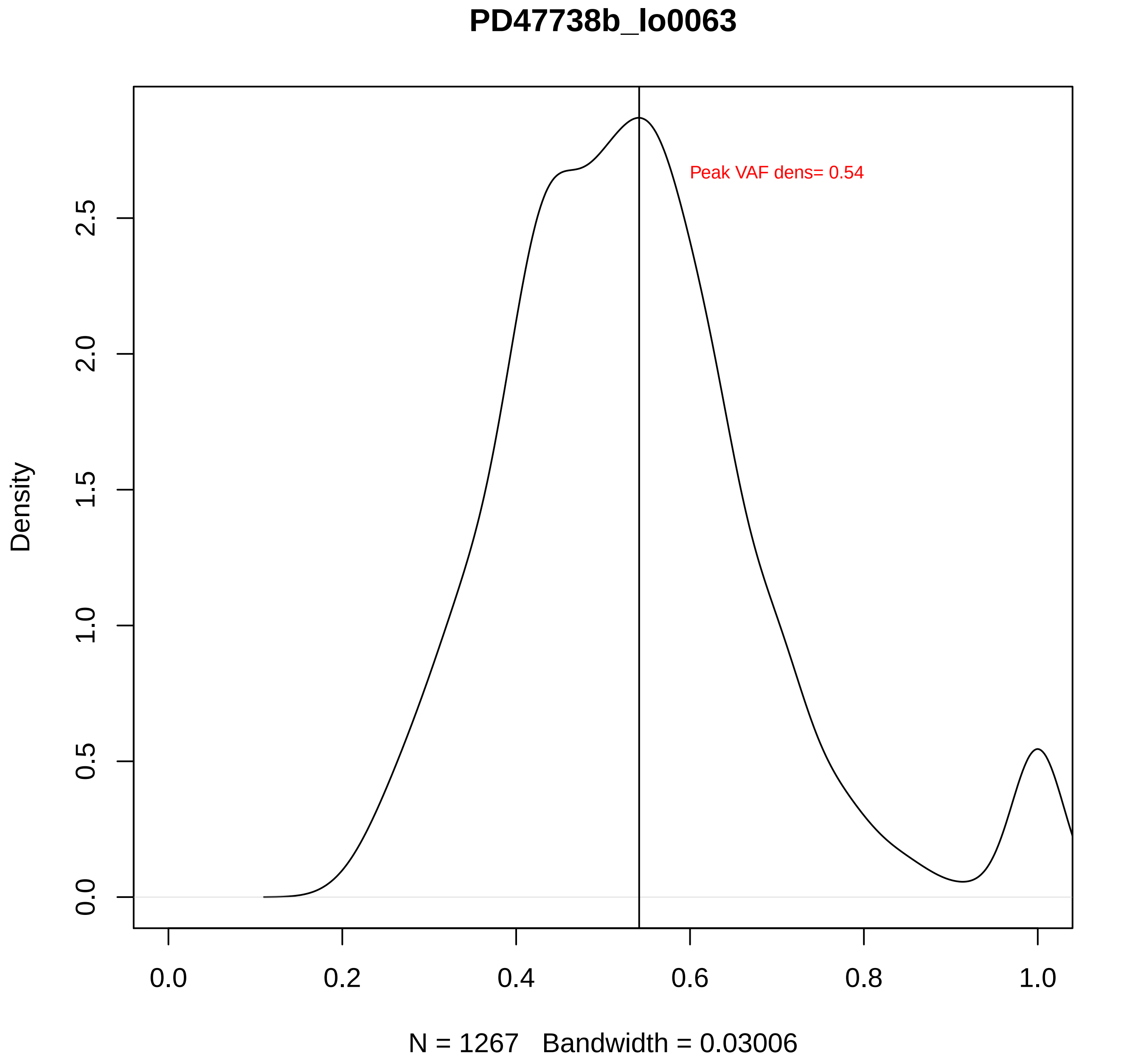

PD47738b_lo0063
Peak VAF dens= 0.54
Density
0.0
0.5
1.0
1.5
2.0
2.5
0.0
0.2
0.4
0.6
0.8
1.0
N = 1267   Bandwidth = 0.03006

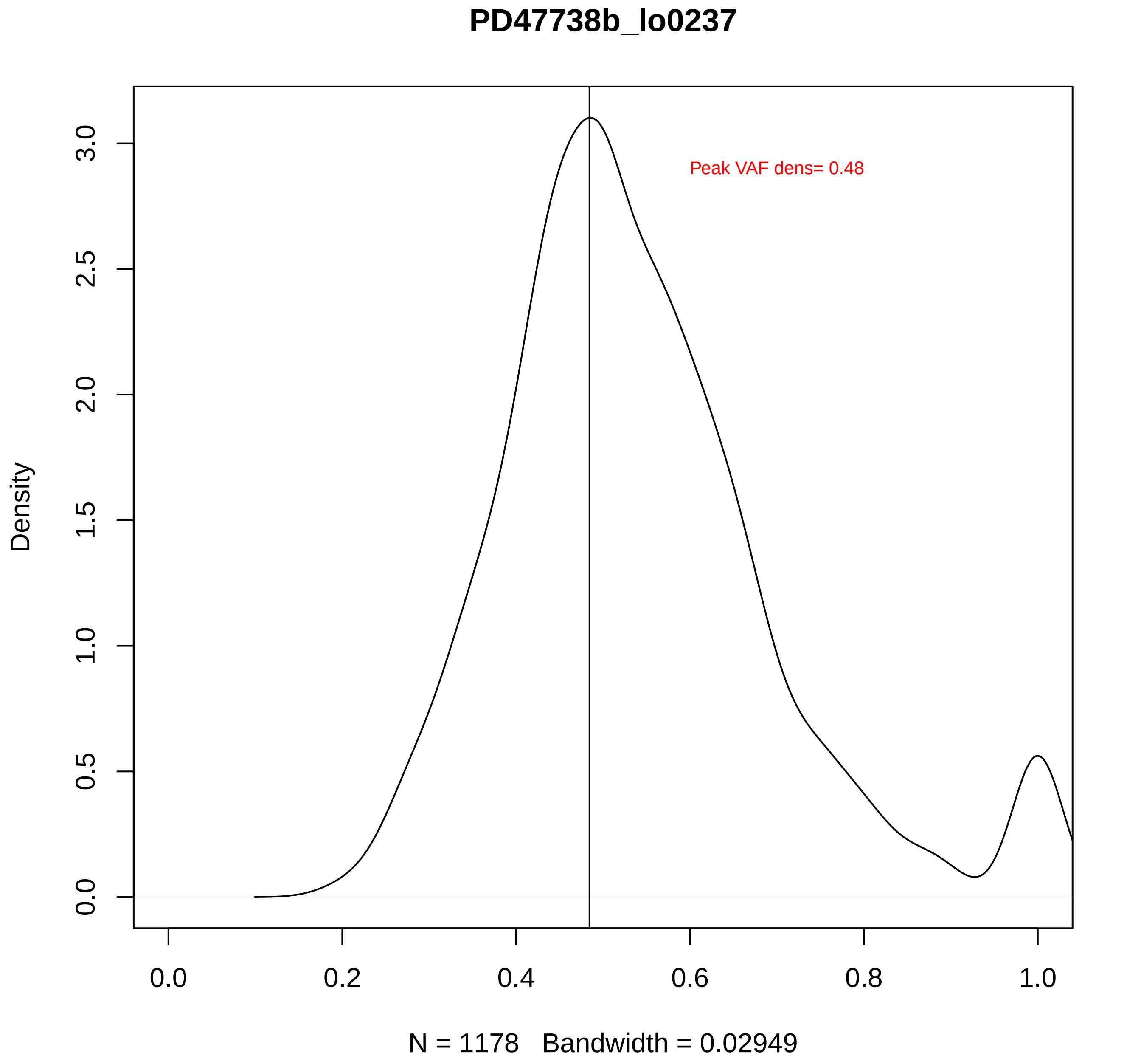
PD47738b_lo0237
Peak VAF dens= 0.48
Density
0.0
0.5
1.0
1.5
2.0
2.5
3.0
0.0
0.2
0.4
0.6
0.8
1.0
N = 1178   Bandwidth = 0.02949

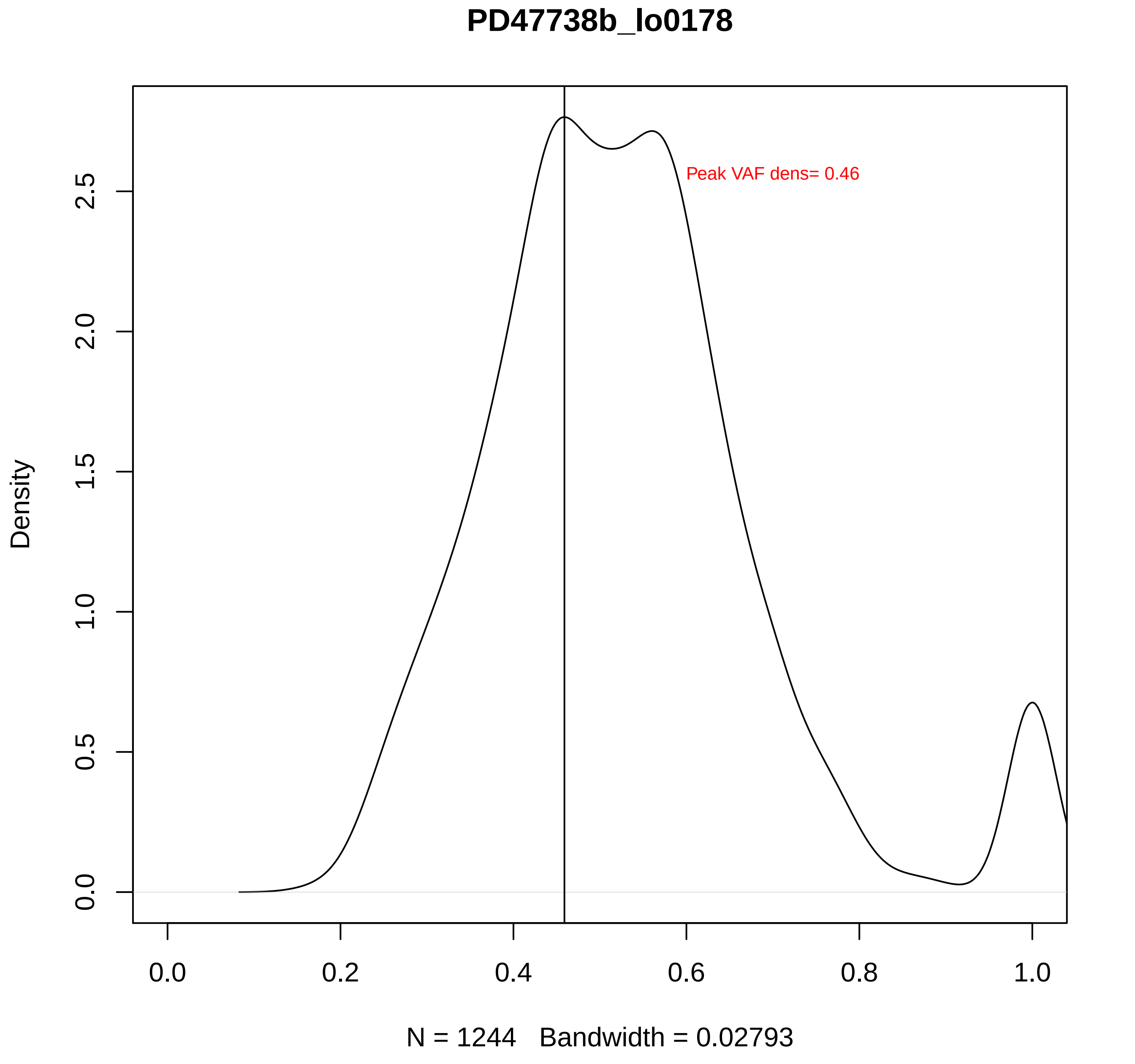
PD47738b_lo0178
Peak VAF dens= 0.46
Density
0.0
0.5
1.0
1.5
2.0
2.5
0.0
0.2
0.4
0.6
0.8
1.0
N = 1244 Bandwidth = 0.02793

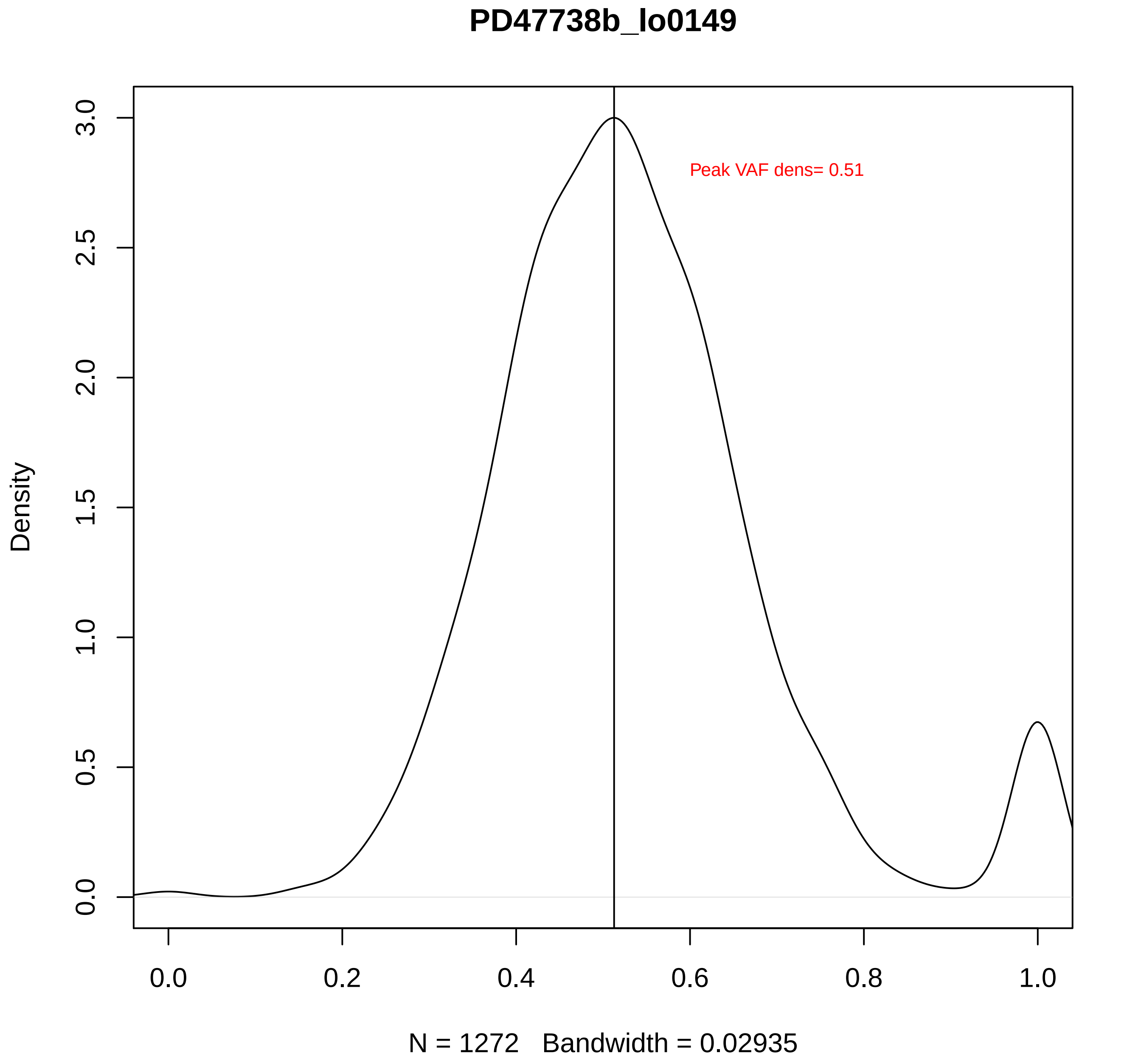

PD47738b_lo0149
Peak VAF dens= 0.51
Density
0.0
0.5
1.0
1.5
2.0
2.5
3.0
0.0
0.2
0.4
0.6
0.8
1.0
N = 1272   Bandwidth = 0.02935

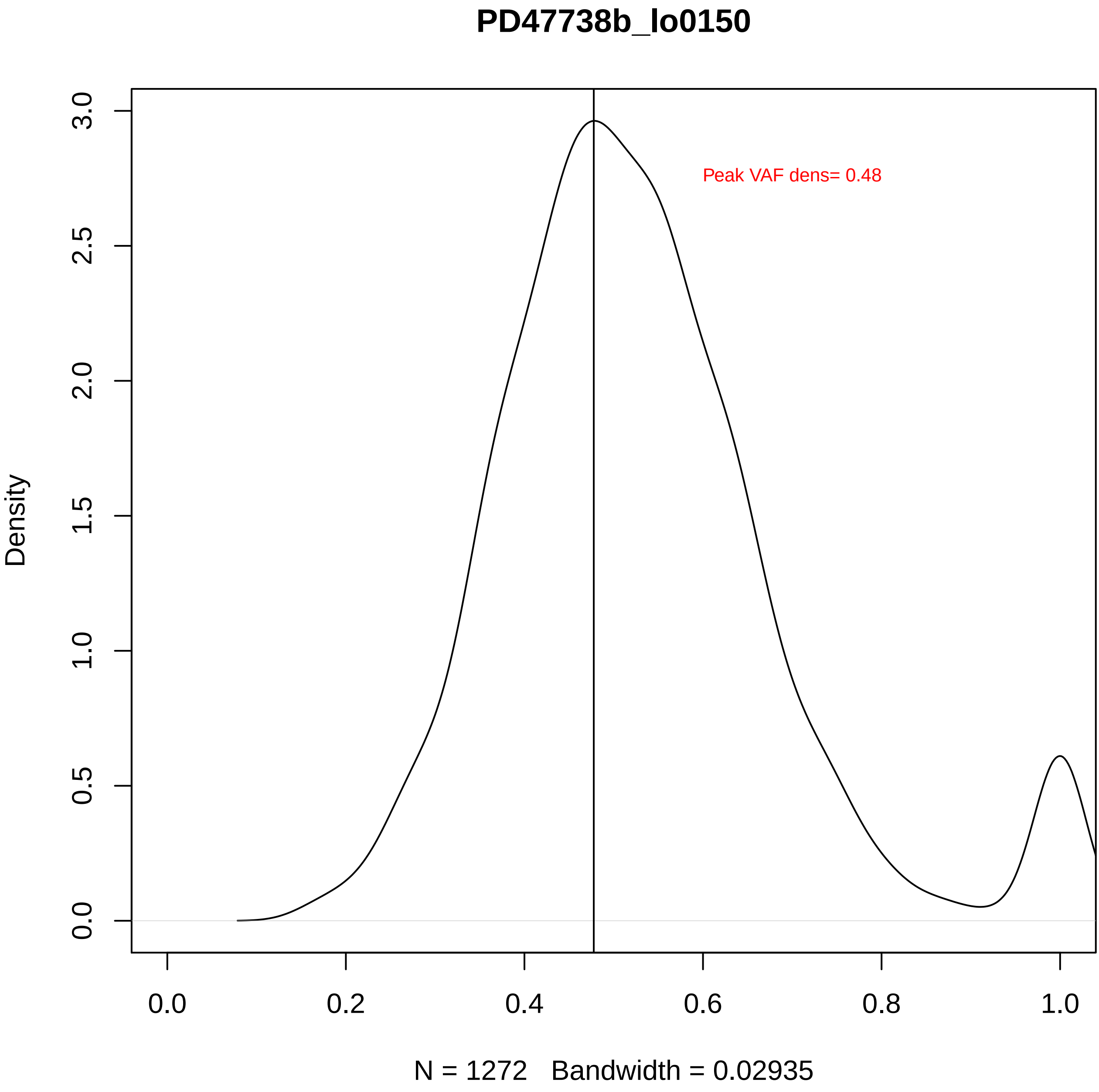
PD47738b_lo0150
Peak VAF dens= 0.48
Density
0.0
0.5
1.0
1.5
2.0
2.5
3.0
0.0
0.2
0.4
0.6
0.8
1.0
N = 1272   Bandwidth = 0.02935

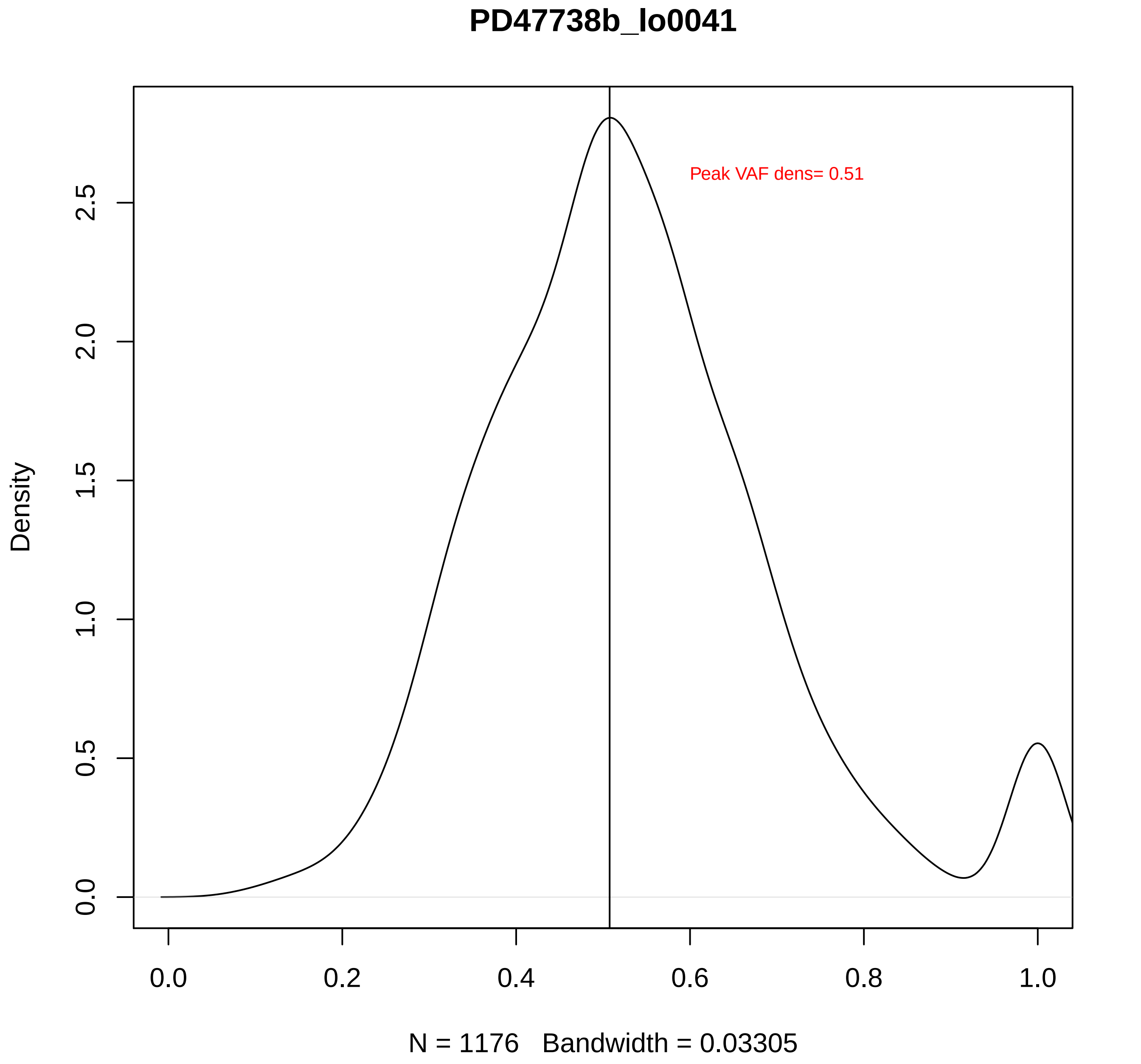
PD47738b_lo0041
Peak VAF dens= 0.51
Density
0.0
0.5
1.0
1.5
2.0
2.5
0.0
0.2
0.4
0.6
0.8
1.0
N = 1176   Bandwidth = 0.03305

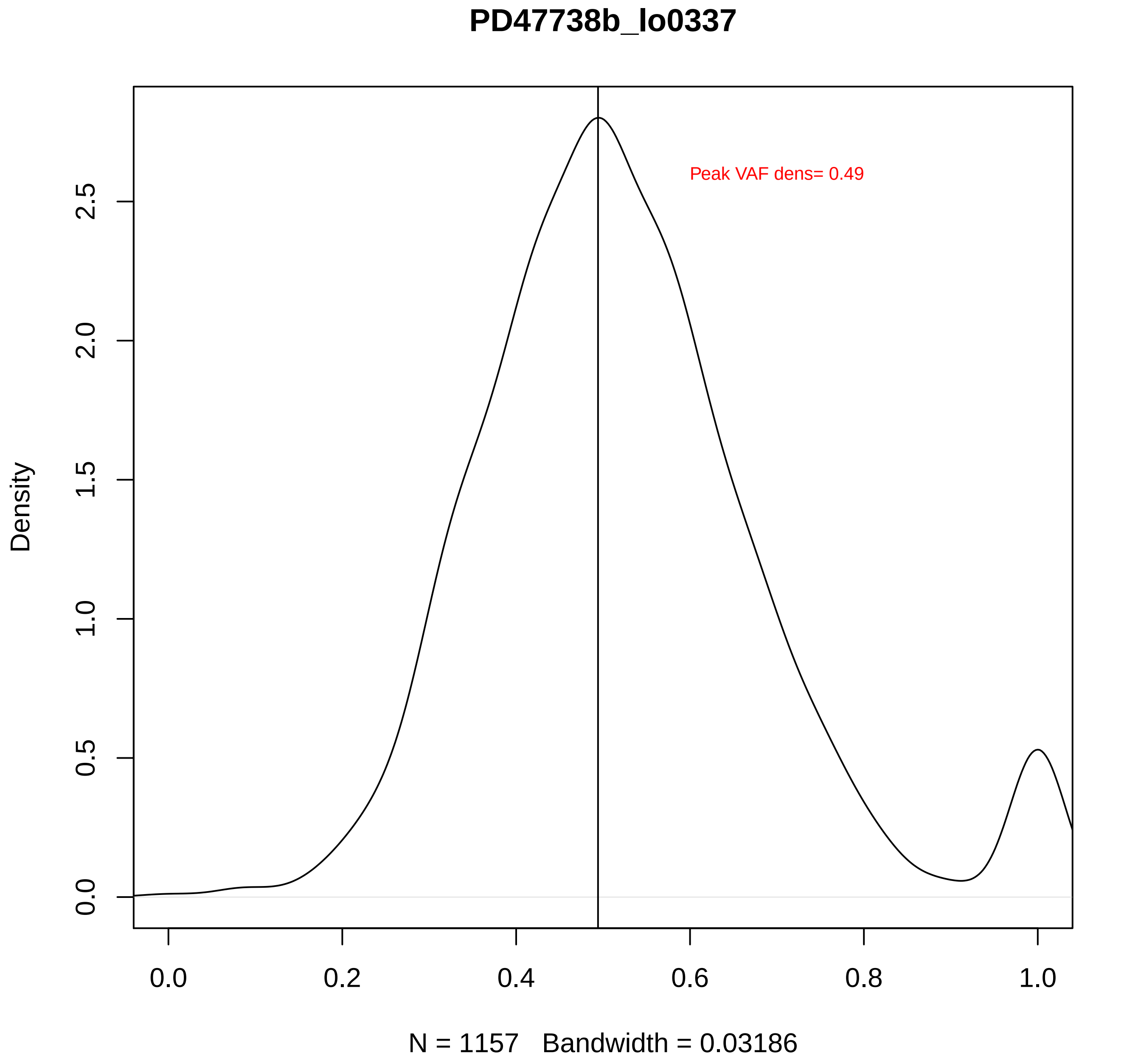

PD47738b_lo0337
Peak VAF dens= 0.49
Density
0.0
0.5
1.0
1.5
2.0
2.5
0.0
0.2
0.4
0.6
0.8
1.0
N = 1157   Bandwidth = 0.03186

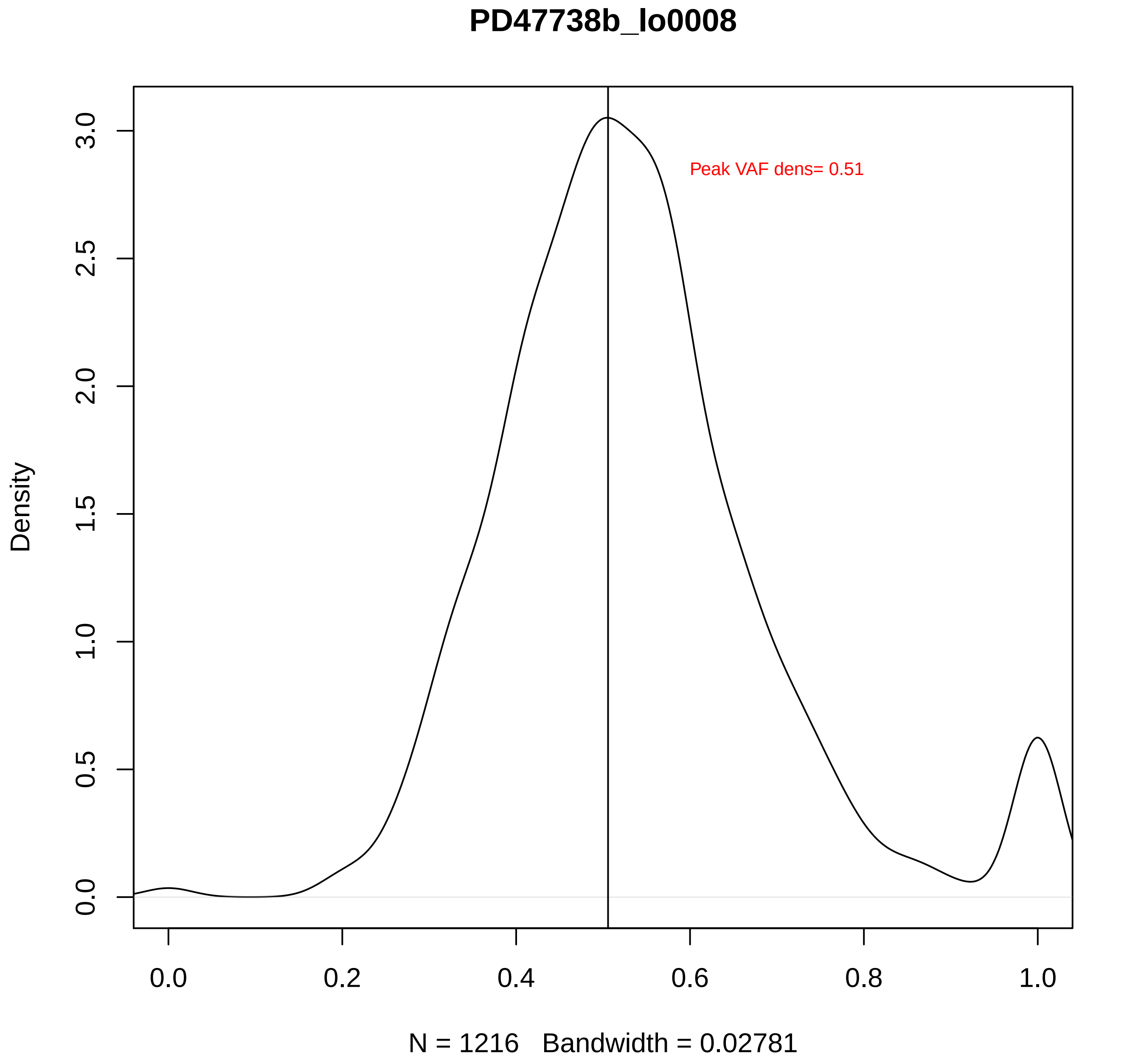
PD47738b_lo0008
Peak VAF dens= 0.51
Density
0.0
0.5
1.0
1.5
2.0
2.5
3.0
0.0
0.2
0.4
0.6
0.8
1.0
N = 1216   Bandwidth = 0.02781

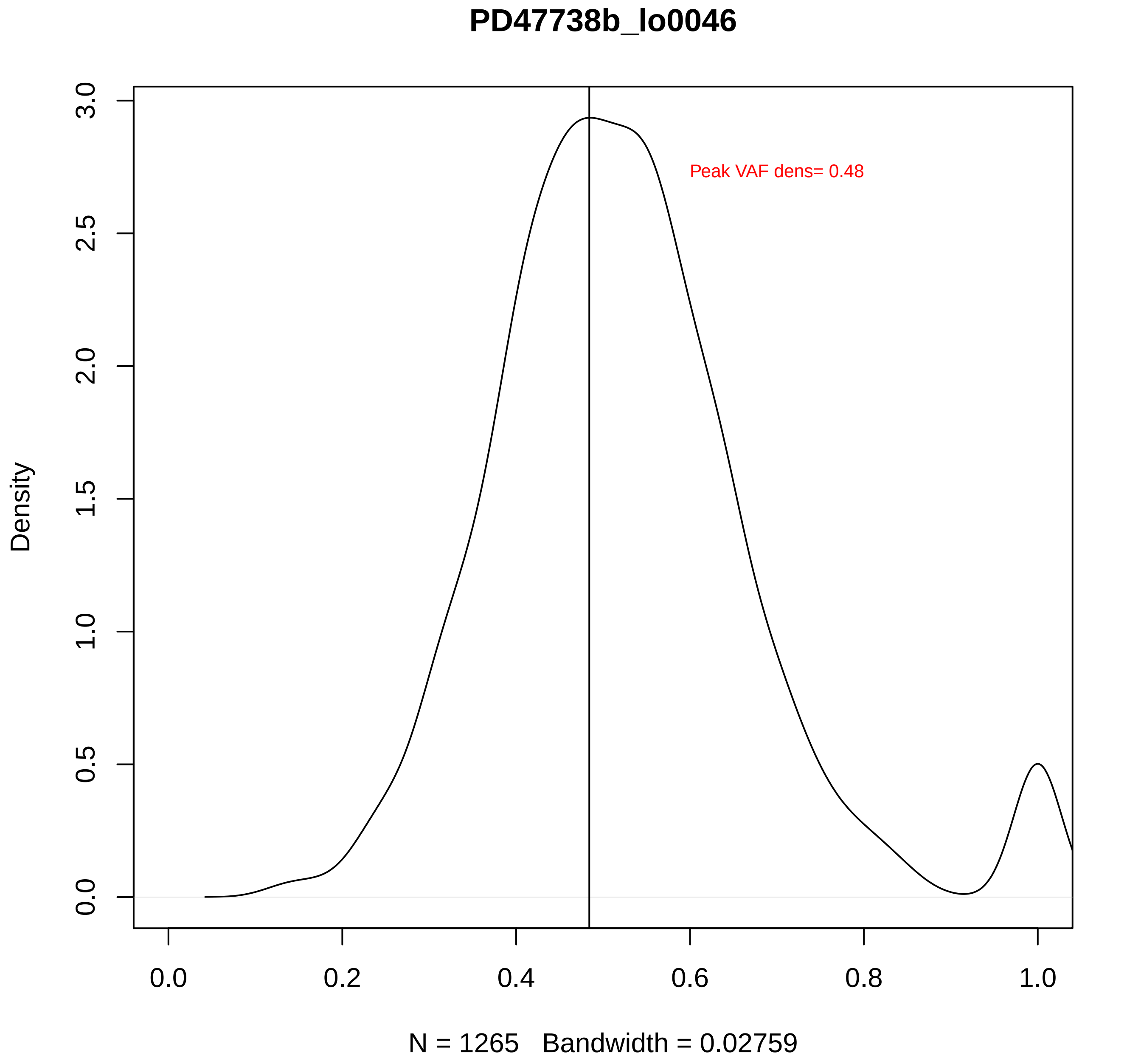

PD47738b_lo0046
Peak VAF dens= 0.48
Density
0.0
0.5
1.0
1.5
2.0
2.5
3.0
0.0
0.2
0.4
0.6
0.8
1.0
N = 1265   Bandwidth = 0.02759

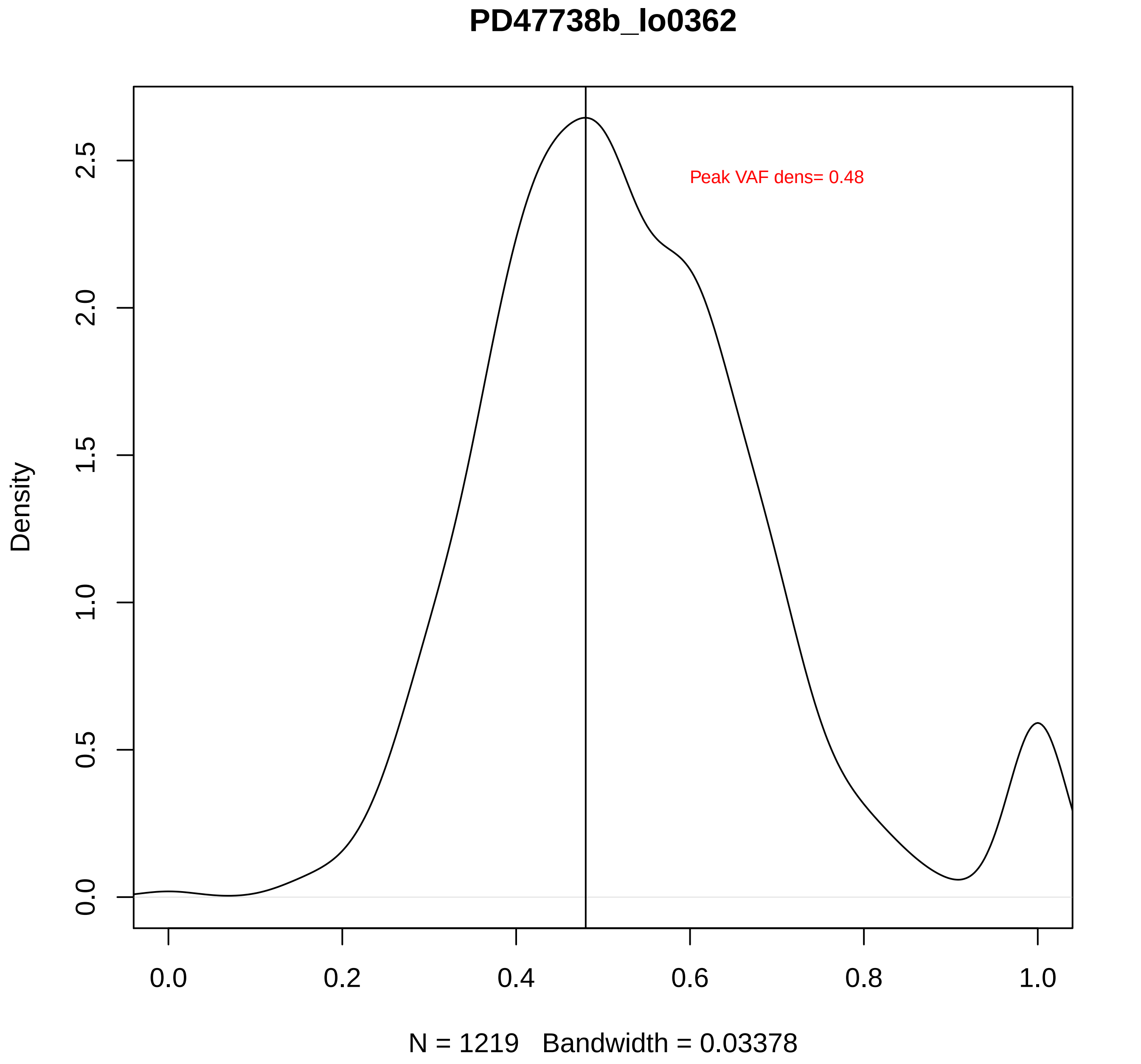

PD47738b_lo0362
Peak VAF dens= 0.48
Density
0.0
0.5
1.0
1.5
2.0
2.5
0.0
0.2
0.4
0.6
0.8
1.0
N = 1219   Bandwidth = 0.03378

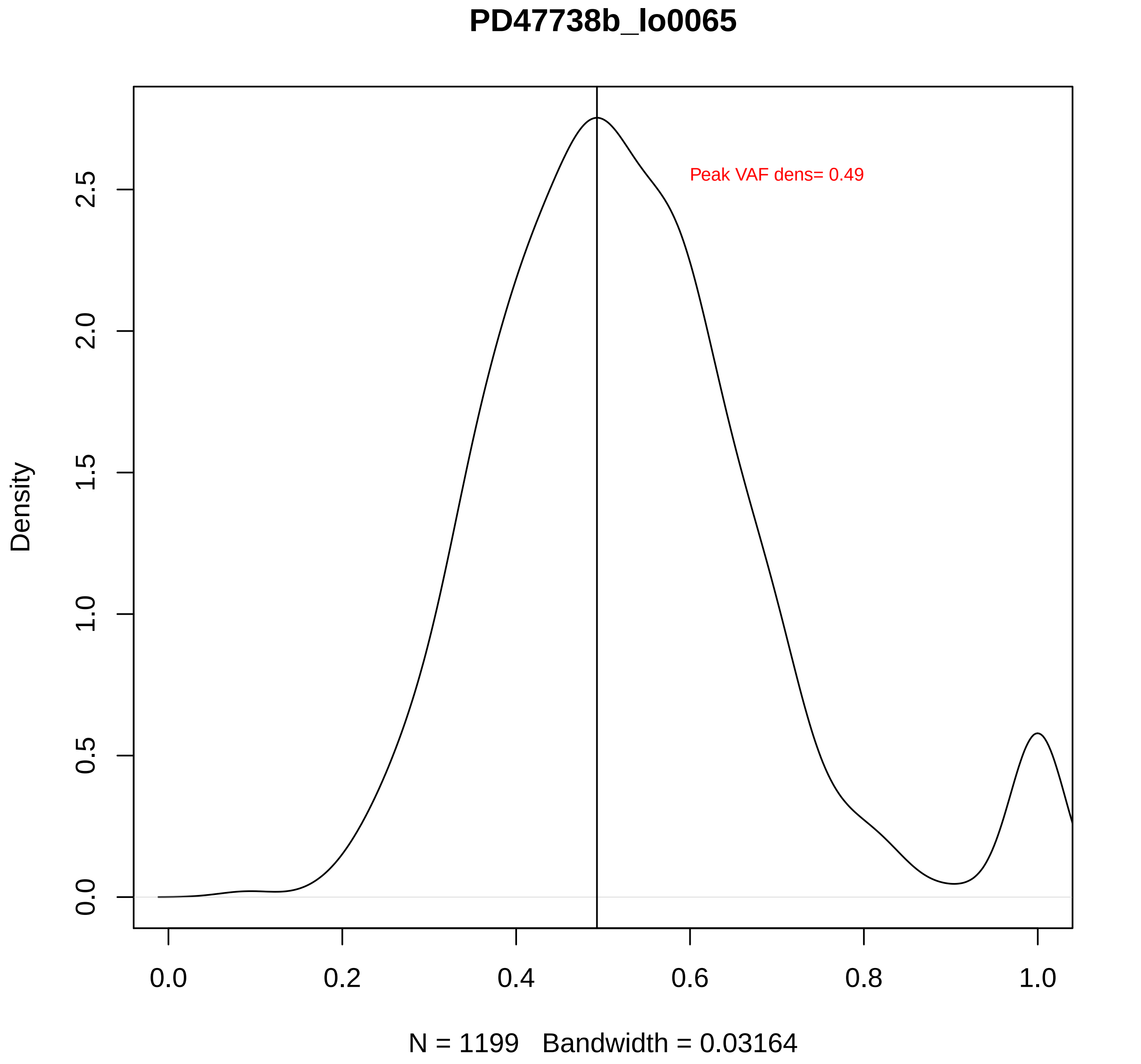

PD47738b_lo0065
Peak VAF dens= 0.49
Density
0.0
0.5
1.0
1.5
2.0
2.5
0.0
0.2
0.4
0.6
0.8
1.0
N = 1199 Bandwidth = 0.03164

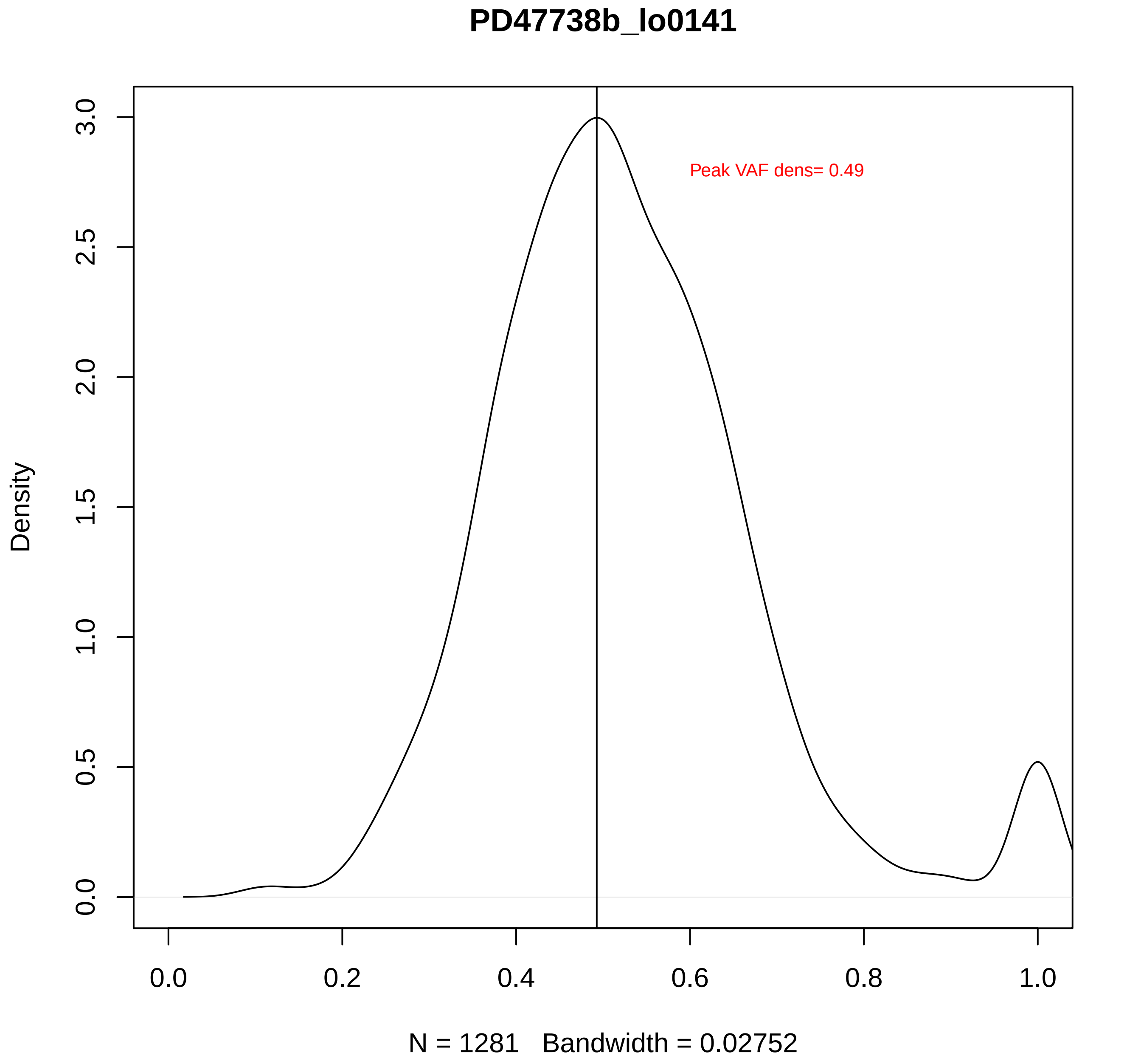

PD47738b_lo0141
Peak VAF dens= 0.49
Density
0.0
0.5
1.0
1.5
2.0
2.5
3.0
0.0
0.2
0.4
0.6
0.8
1.0
N = 1281   Bandwidth = 0.02752

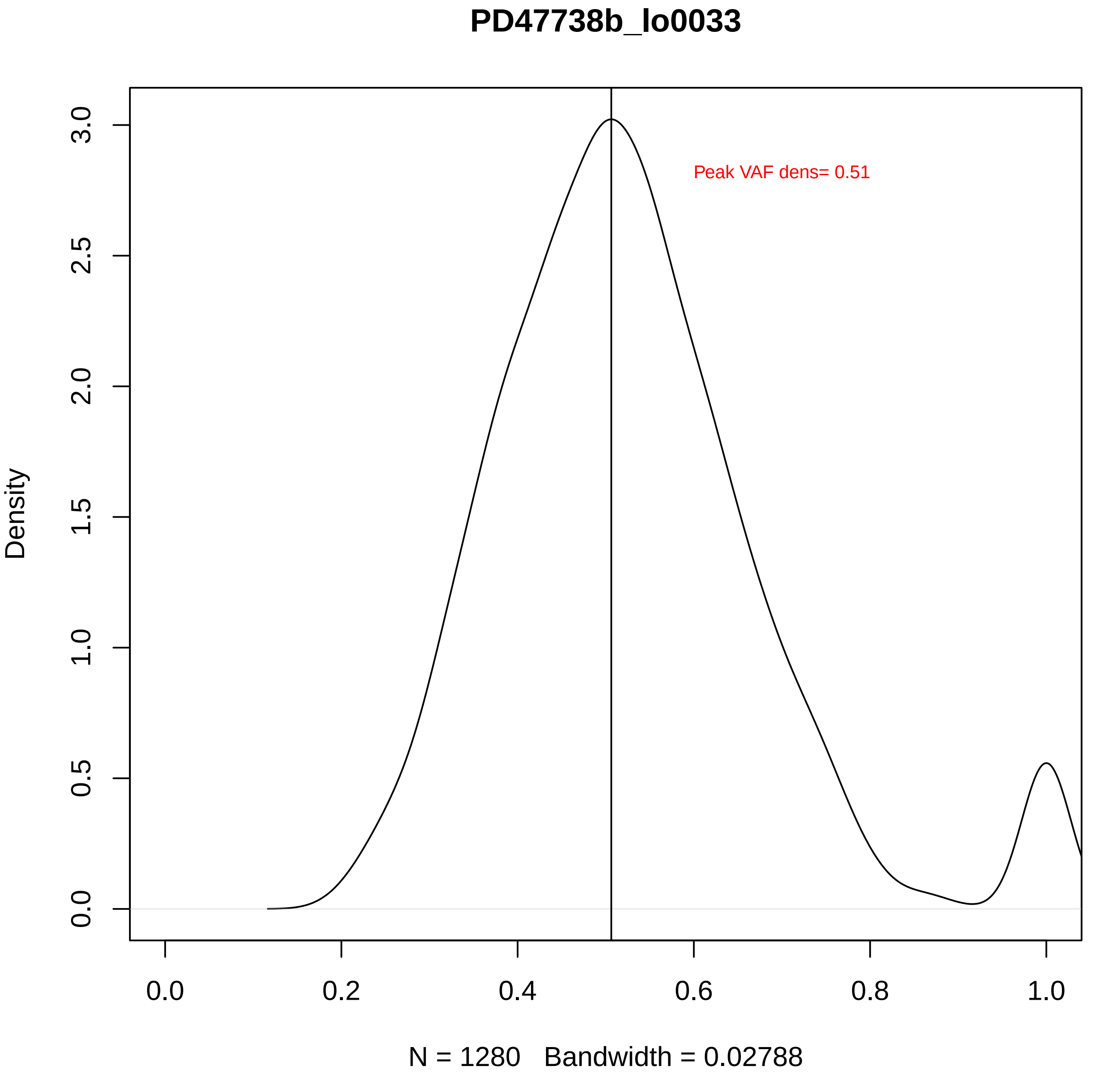
PD47738b_lo0033
Peak VAF dens= 0.51
Density
0.0
0.5
1.0
1.5
2.0
2.5
3.0
0.0
0.2
0.4
0.6
0.8
1.0
N = 1280 Bandwidth = 0.02788

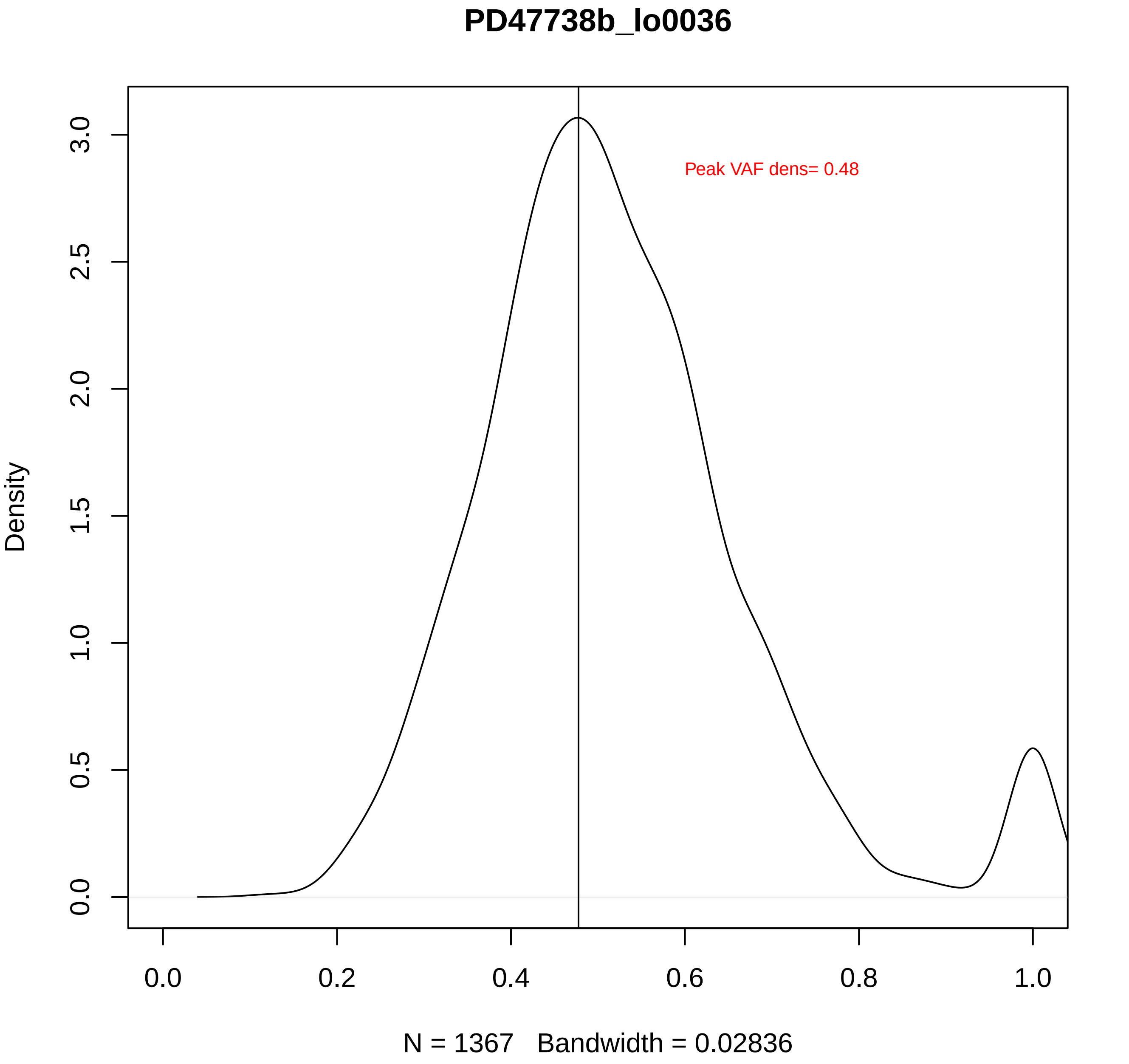

PD47738b_lo0036
Peak VAF dens= 0.48
Density
0.0
0.5
1.0
1.5
2.0
2.5
3.0
0.0
0.2
0.4
0.6
0.8
1.0
N = 1367 Bandwidth = 0.02836

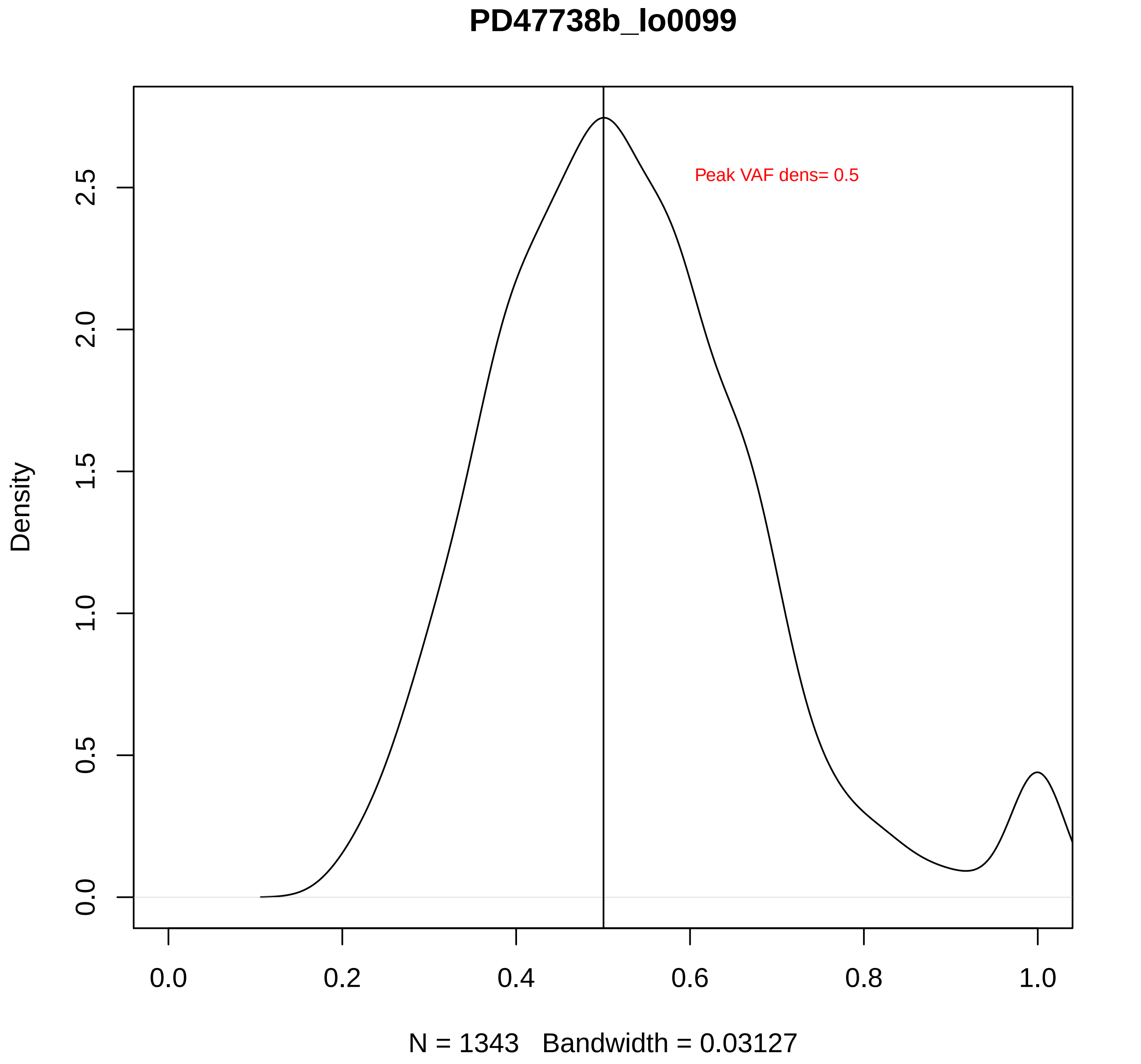
PD47738b_lo0099
Peak VAF dens= 0.5
Density
0.0
0.5
1.0
1.5
2.0
2.5
0.0
0.2
0.4
0.6
0.8
1.0
N = 1343   Bandwidth = 0.03127

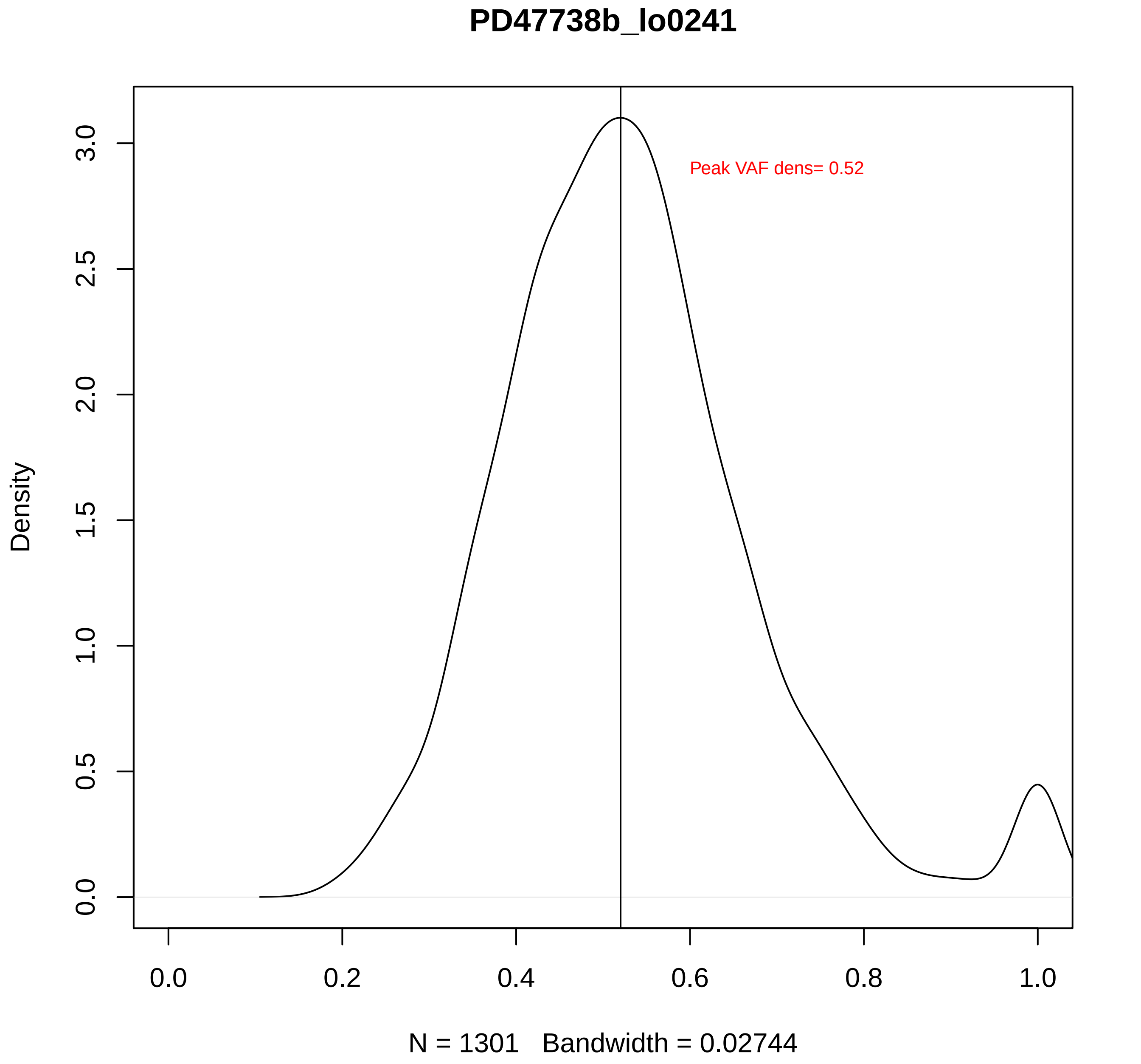

PD47738b_lo0241
Peak VAF dens= 0.52
Density
3.0
2.5
2.0
1.5
1.0
0.5
0.0
0.2
0.4
0.6
0.8
1.0
N = 1301   Bandwidth = 0.02744

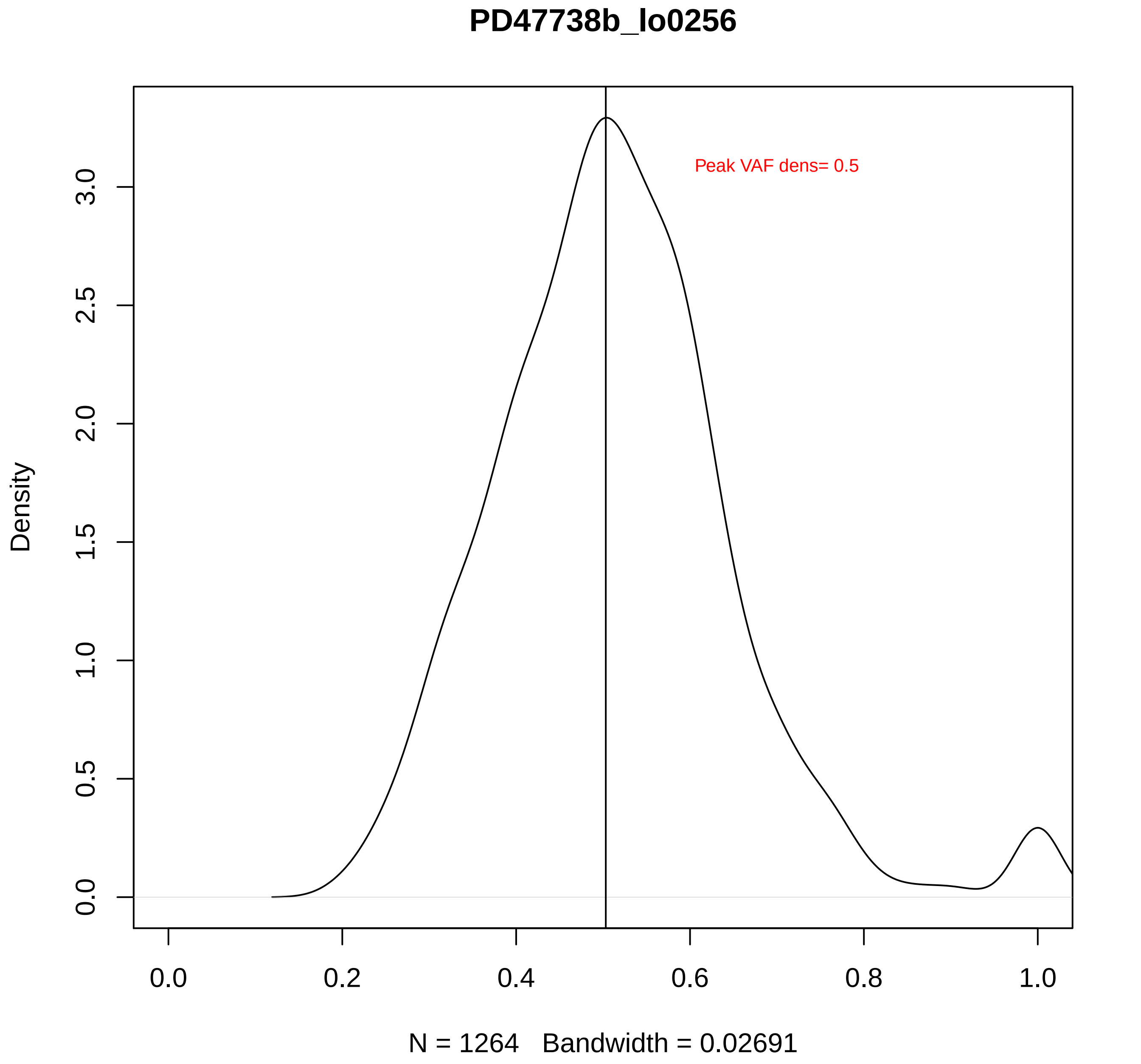
PD47738b_lo0256
Peak VAF dens= 0.5
Density
0.0
0.5
1.0
1.5
2.0
2.5
3.0
0.0
0.2
0.4
0.6
0.8
1.0
N = 1264   Bandwidth = 0.02691

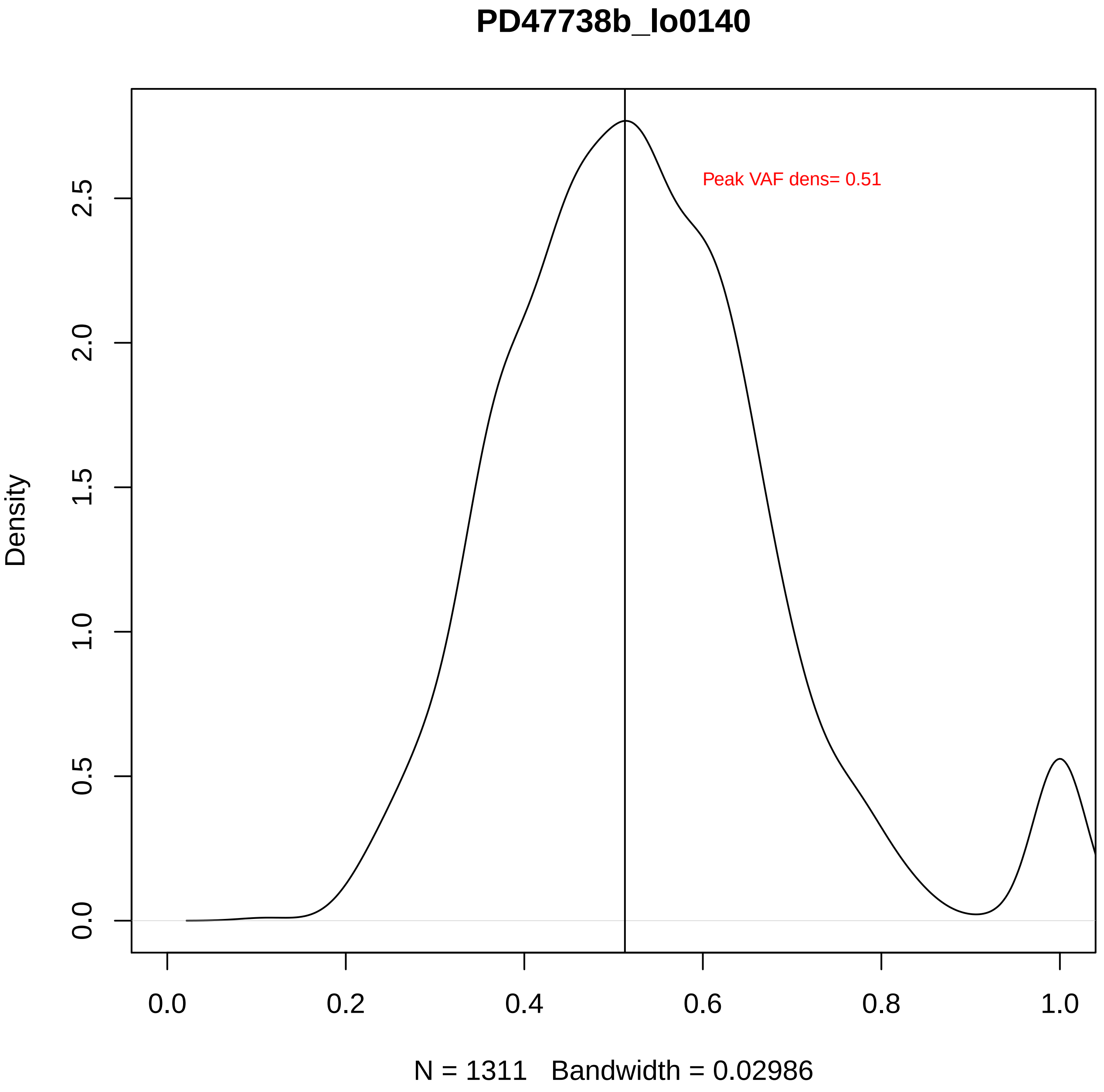
PD47738b_lo0140
Peak VAF dens= 0.51
Density
0.0
0.5
1.0
1.5
2.0
2.5
0.0
0.2
0.4
0.6
0.8
1.0
N = 1311   Bandwidth = 0.02986

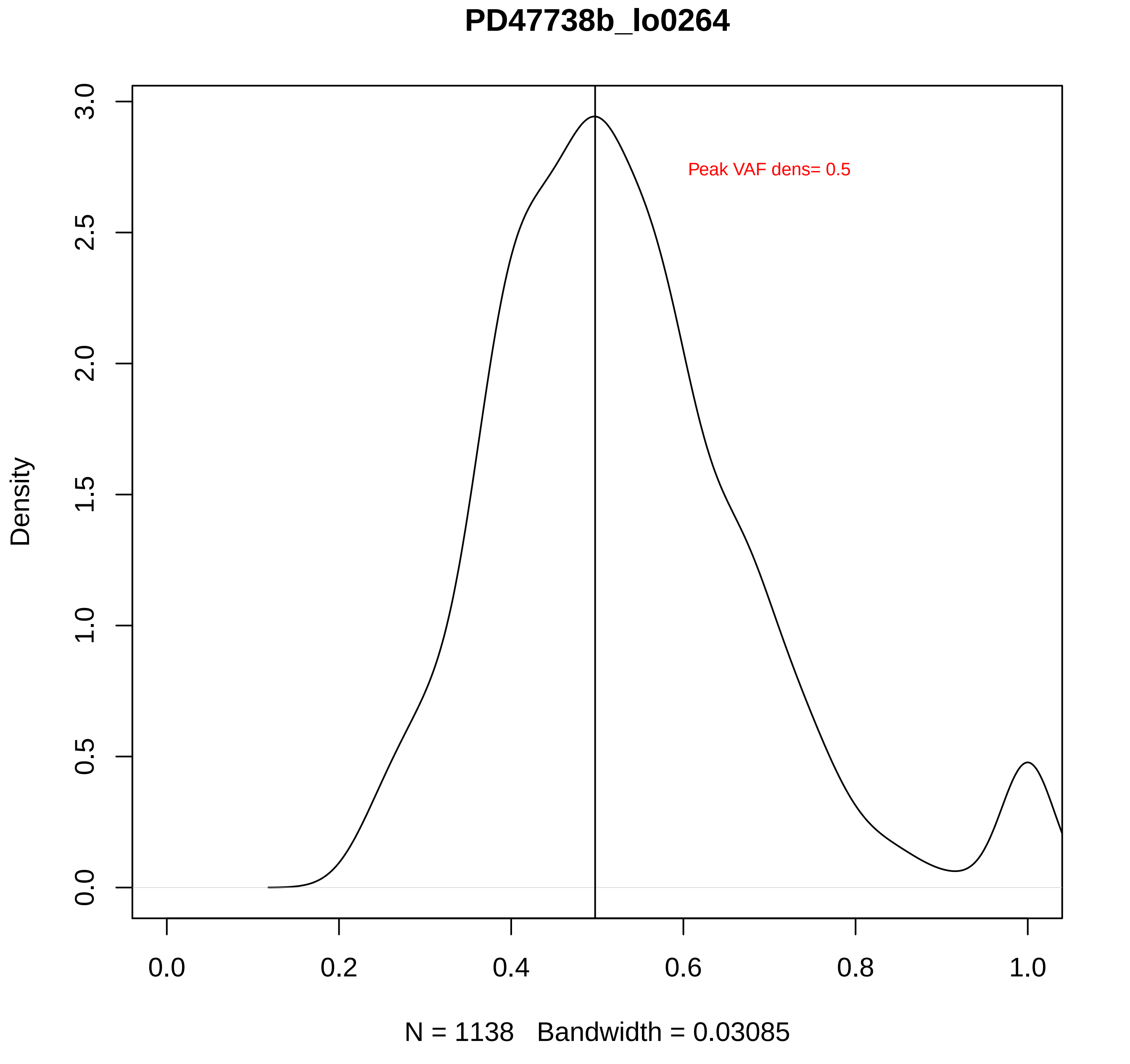

PD47738b_lo0264
Peak VAF dens= 0.5
Density
0.0
0.5
1.0
1.5
2.0
2.5
3.0
0.0
0.2
0.4
0.6
0.8
1.0
N = 1138   Bandwidth = 0.03085

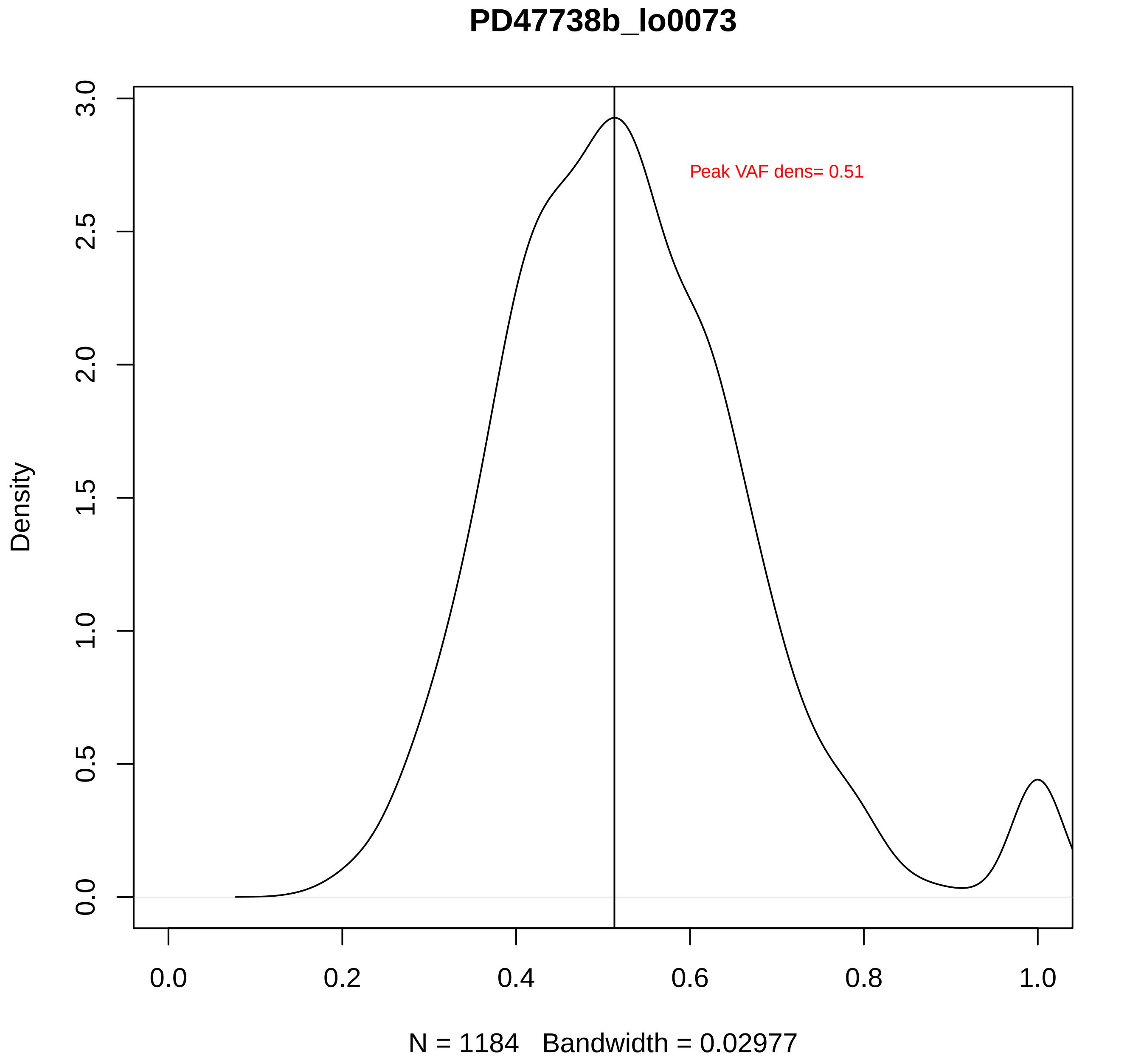

PD47738b_lo0073
Peak VAF dens= 0.51
Density
0.0
0.5
1.0
1.5
2.0
2.5
3.0
0.0
0.2
0.4
0.6
0.8
1.0
N = 1184 Bandwidth = 0.02977

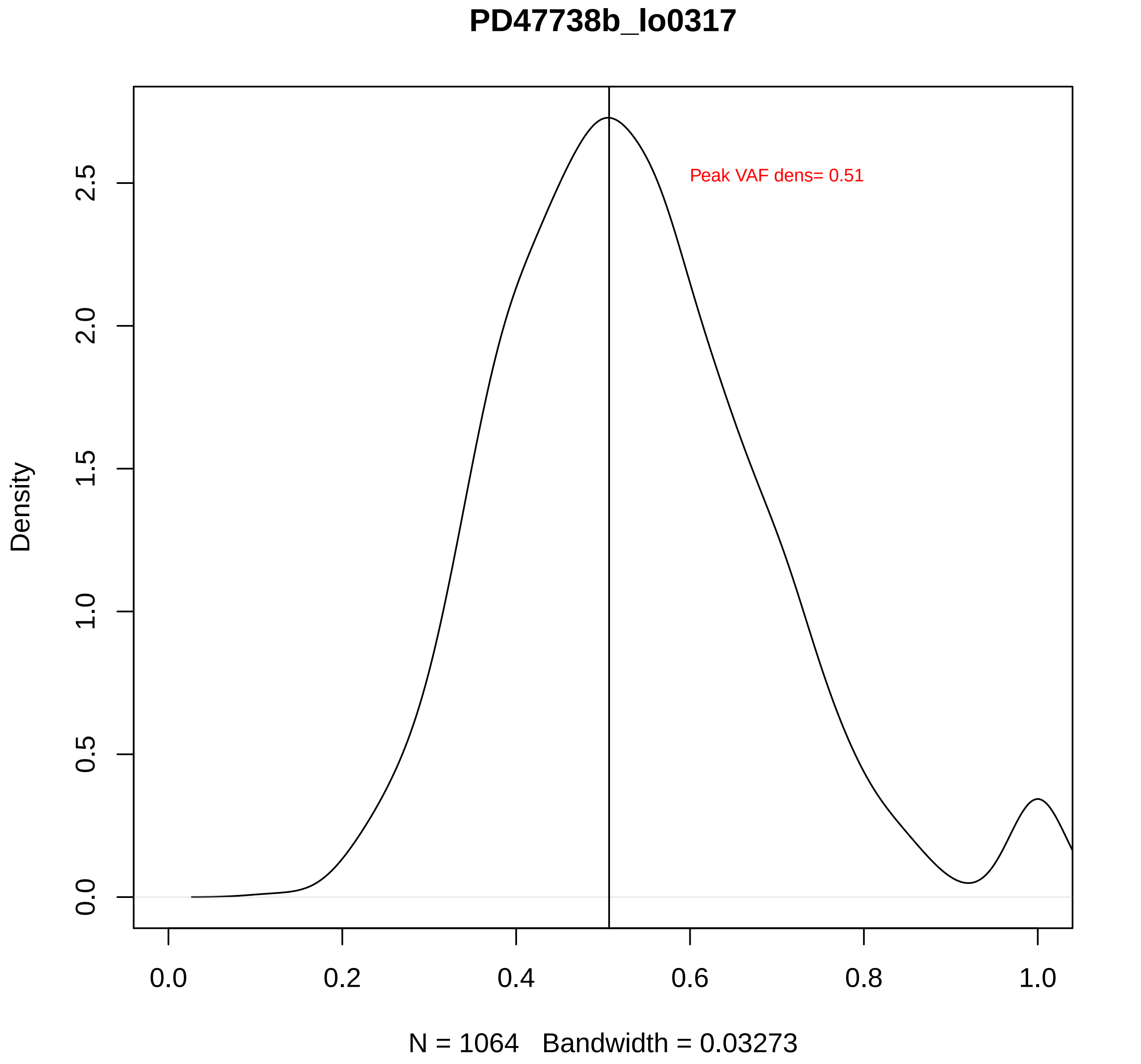
PD47738b_lo0317
Peak VAF dens= 0.51
Density
0.0
0.5
1.0
1.5
2.0
2.5
0.0
0.2
0.4
0.6
0.8
1.0
N = 1064   Bandwidth = 0.03273

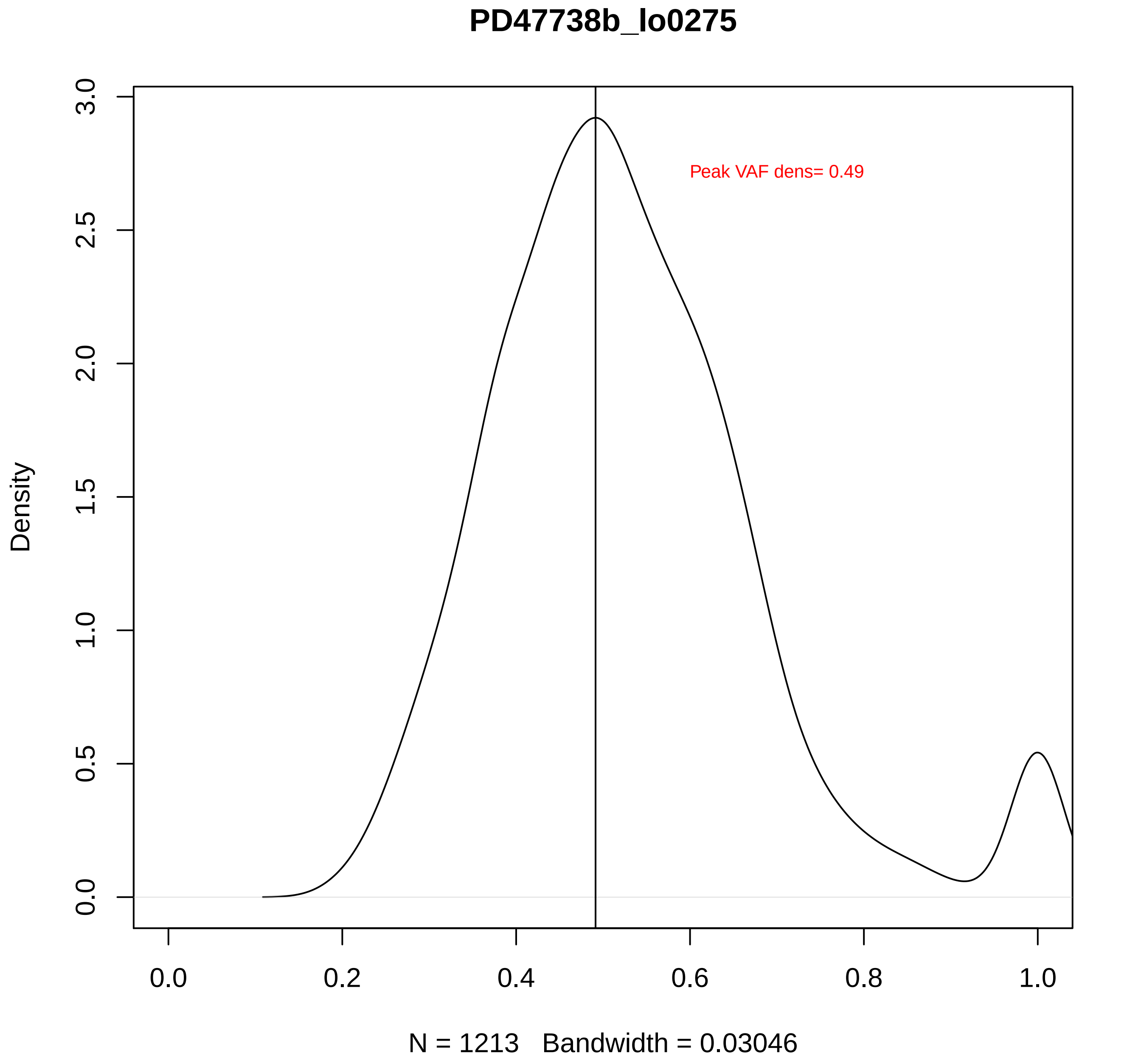
PD47738b_lo0275
Peak VAF dens= 0.49
Density
3.0
2.5
2.0
1.5
1.0
0.5
0.0
0.0
0.2
0.4
0.6
0.8
1.0
N = 1213   Bandwidth = 0.03046

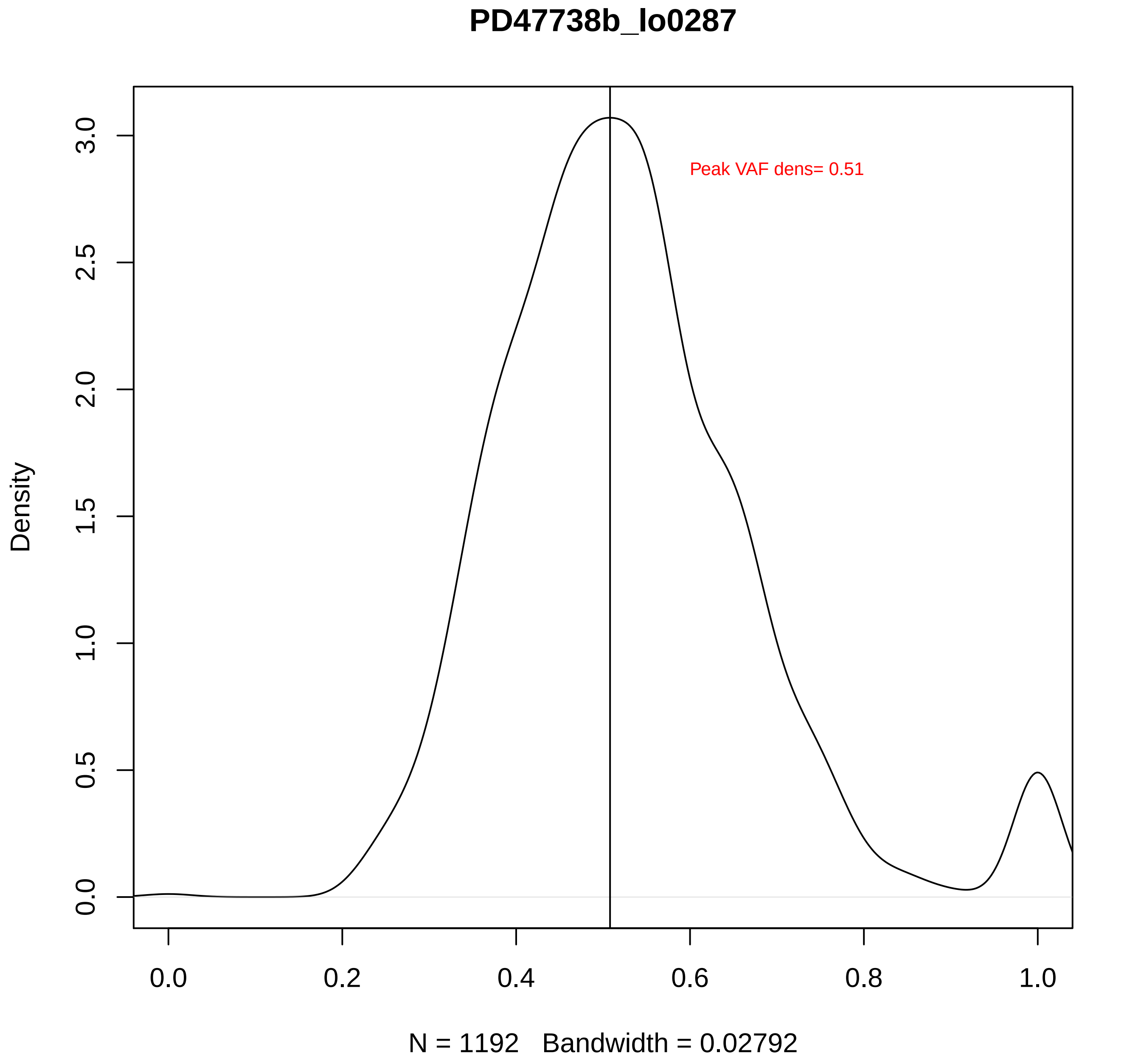

PD47738b_lo0287
Peak VAF dens= 0.51
Density
0.0
0.5
1.0
1.5
2.0
2.5
3.0
0.0
0.2
0.4
0.6
0.8
1.0
N = 1192   Bandwidth = 0.02792

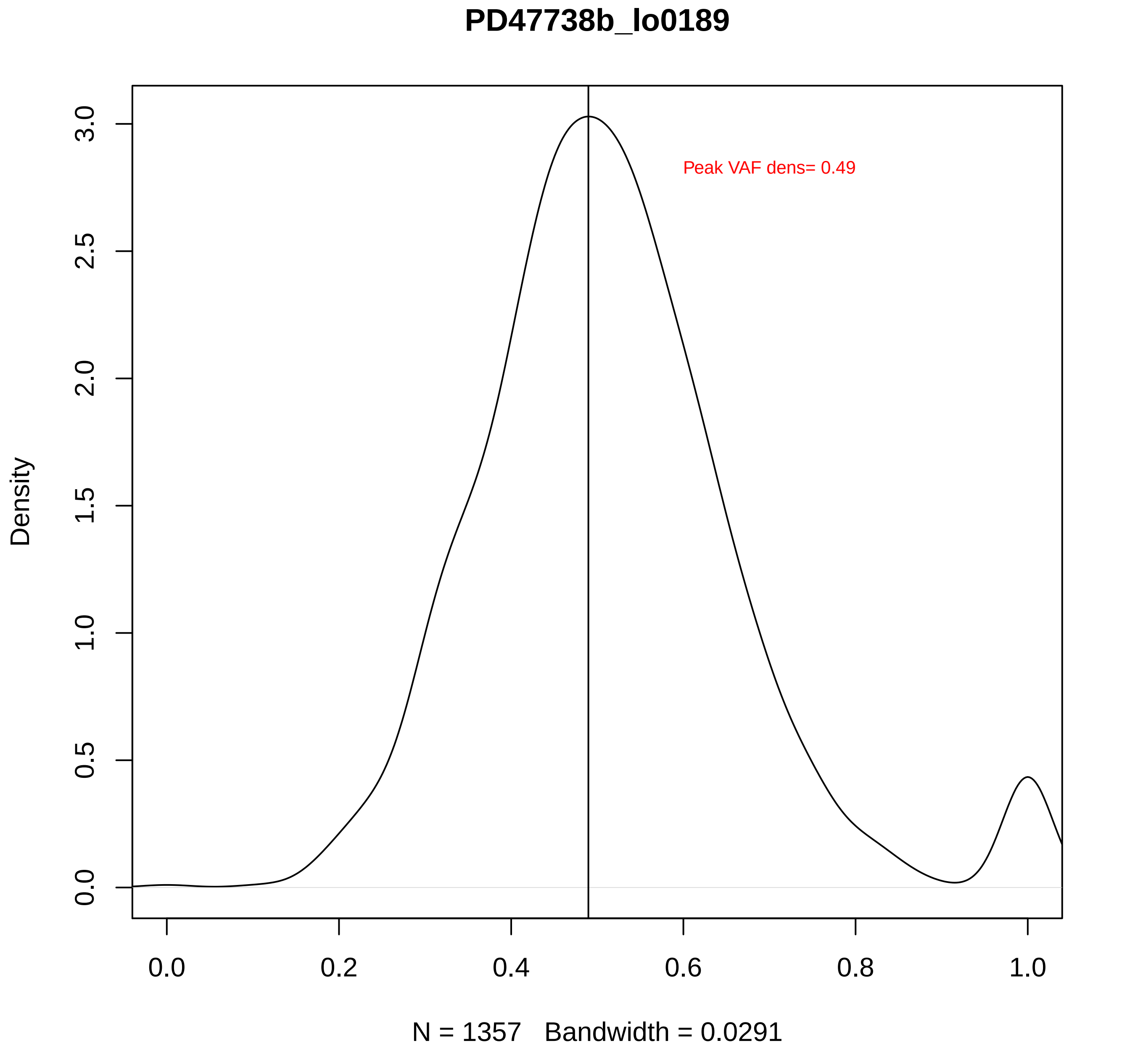

PD47738b_lo0189
Peak VAF dens= 0.49
Density
0.0
0.5
1.0
1.5
2.0
2.5
3.0
0.0
0.2
0.4
0.6
0.8
1.0
N = 1357 Bandwidth = 0.0291

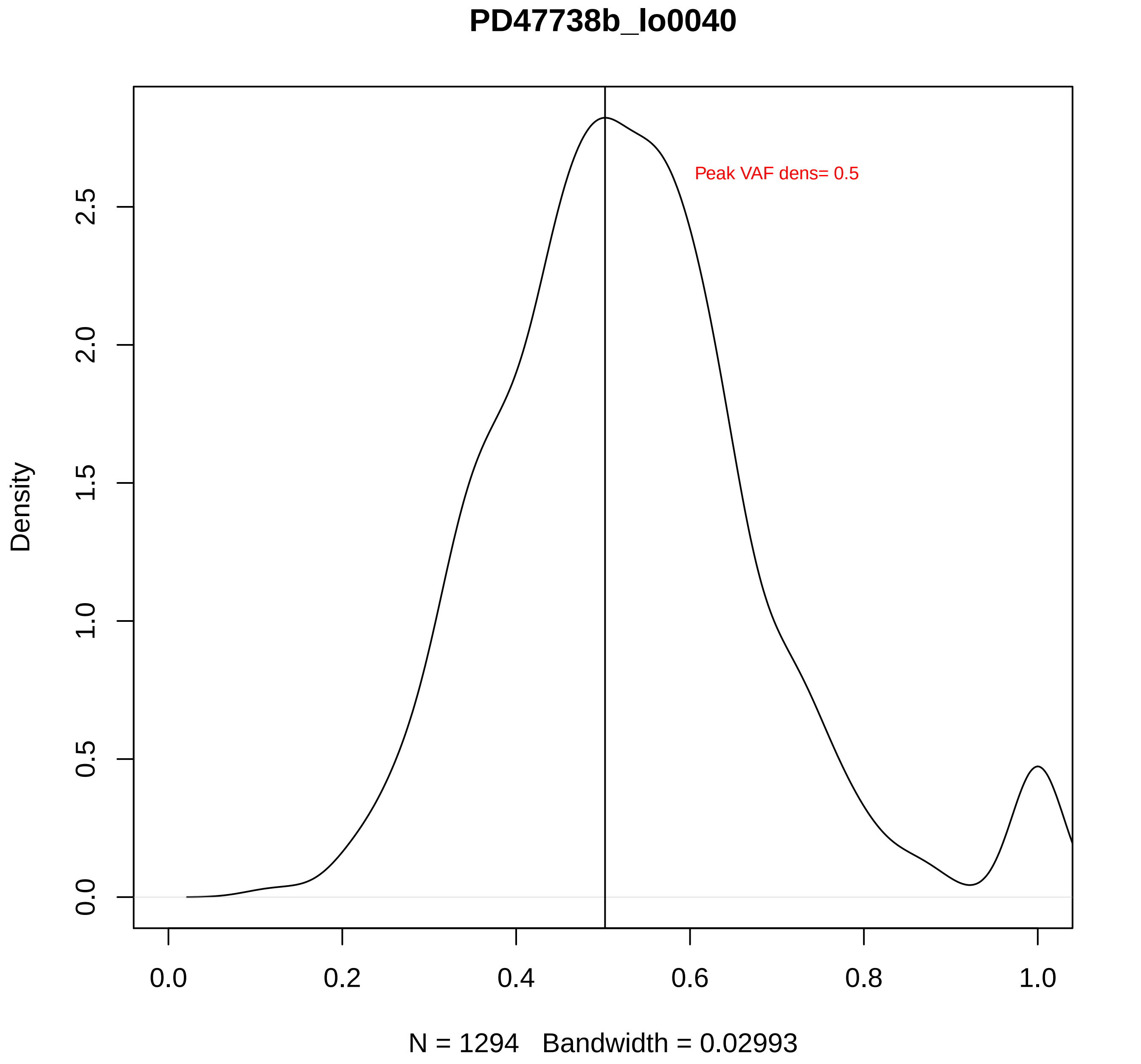
PD47738b_lo0040
Density
Peak VAF dens= 0.5
0.0
0.5
1.0
1.5
2.0
2.5
0.0
0.2
0.4
0.6
0.8
1.0
N = 1294   Bandwidth = 0.02993

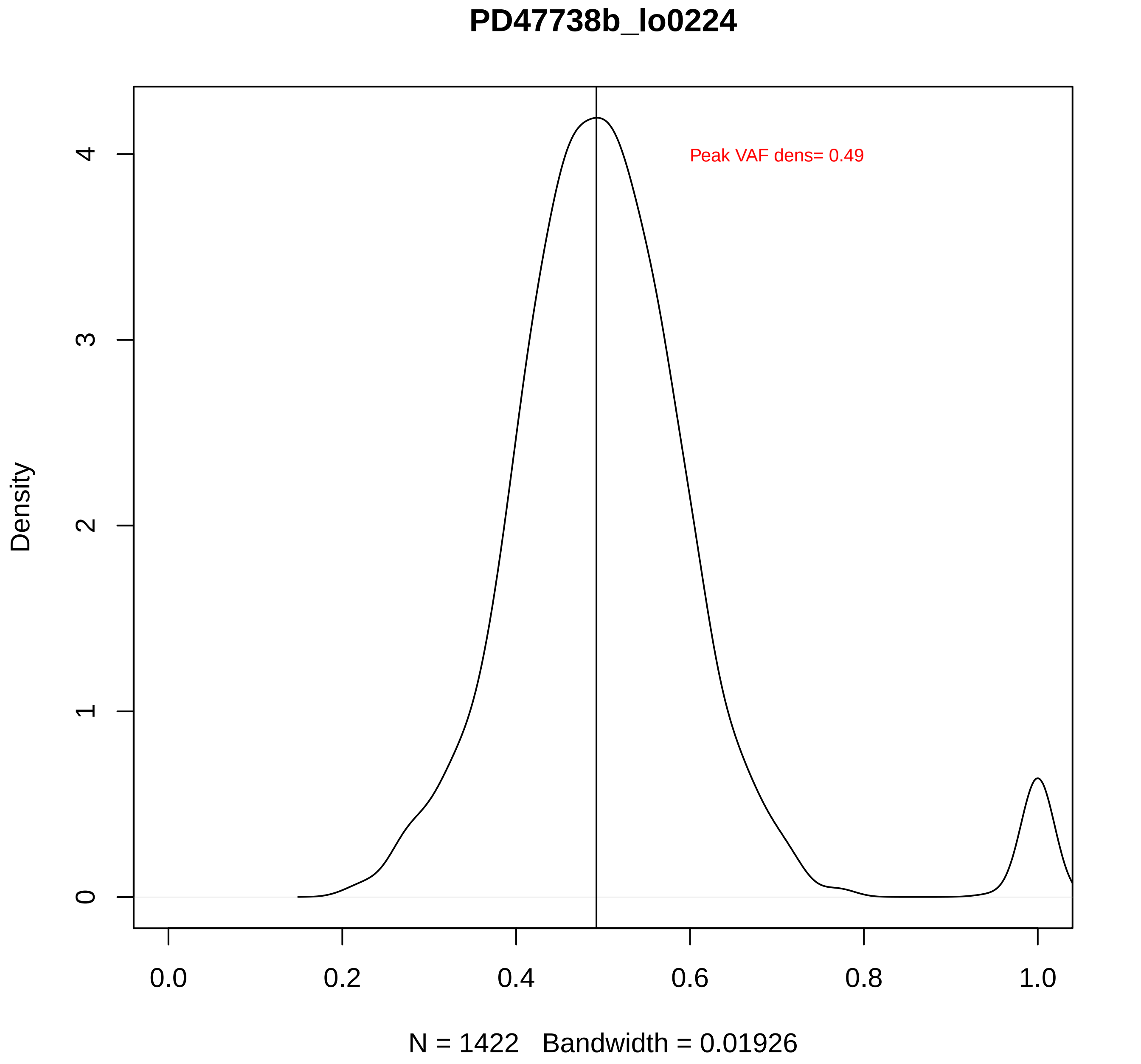
PD47738b_lo0224
Peak VAF dens= 0.49
Density
0
1
2
3
4
0.0
0.2
0.4
0.6
0.8
1.0
N = 1422 Bandwidth = 0.01926

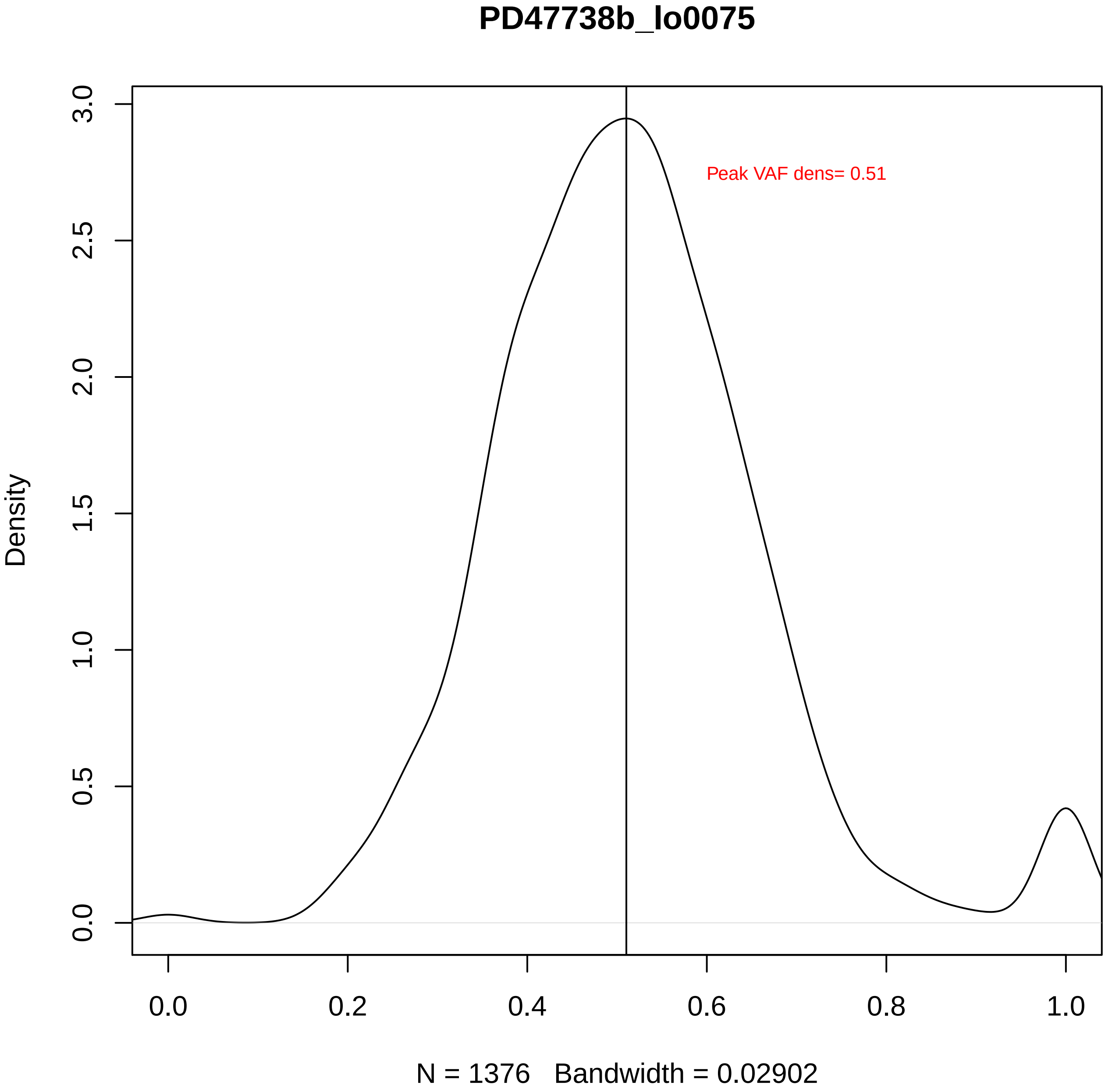
PD47738b_lo0075
Peak VAF dens= 0.51
Density
0.0
0.5
1.0
1.5
2.0
2.5
3.0
0.0
0.2
0.4
0.6
0.8
1.0
N = 1376   Bandwidth = 0.02902

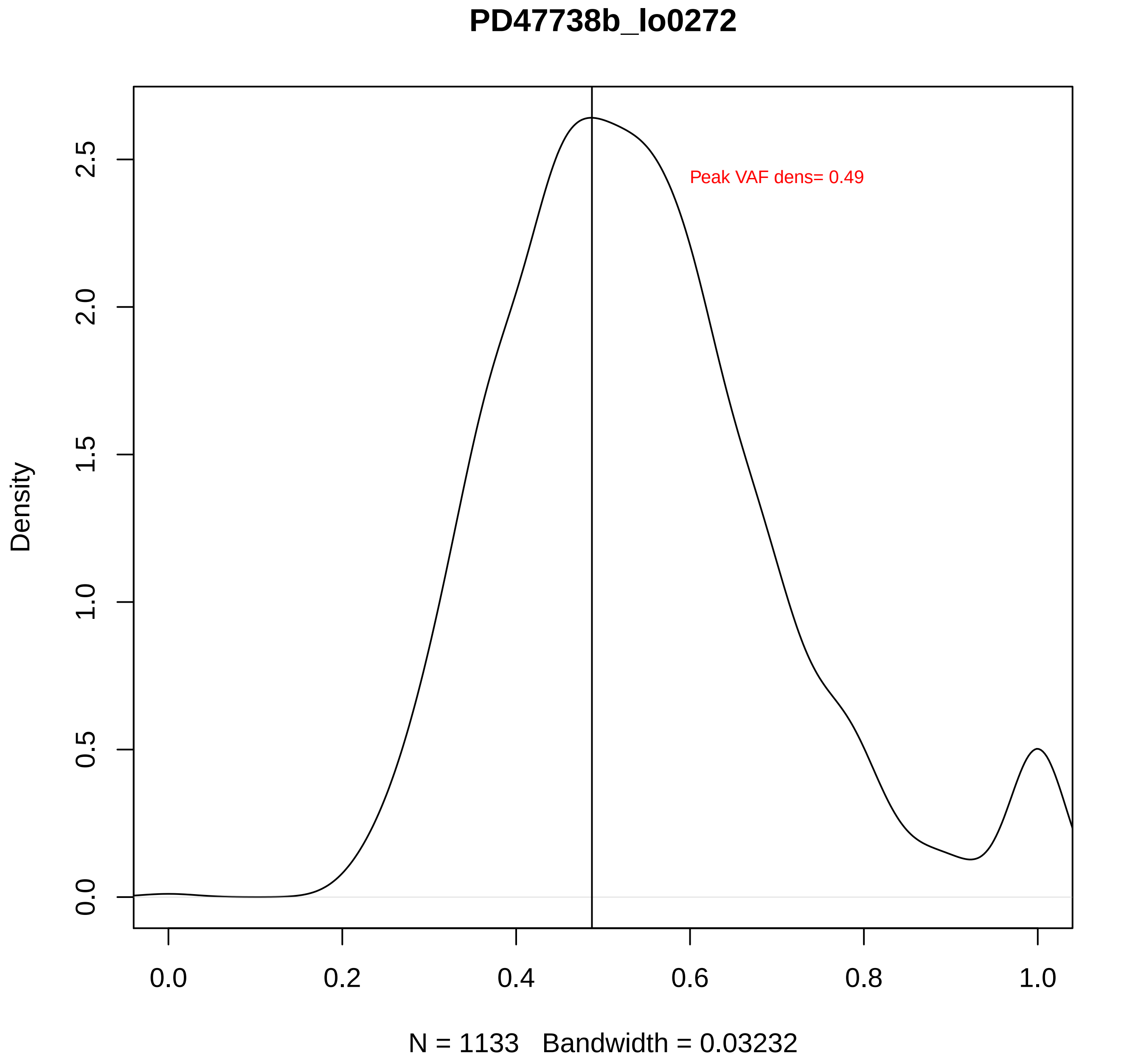
PD47738b_lo0272
Peak VAF dens= 0.49
Density
0.0
0.5
1.0
1.5
2.0
2.5
0.0
0.2
0.4
0.6
0.8
1.0
N = 1133   Bandwidth = 0.03232

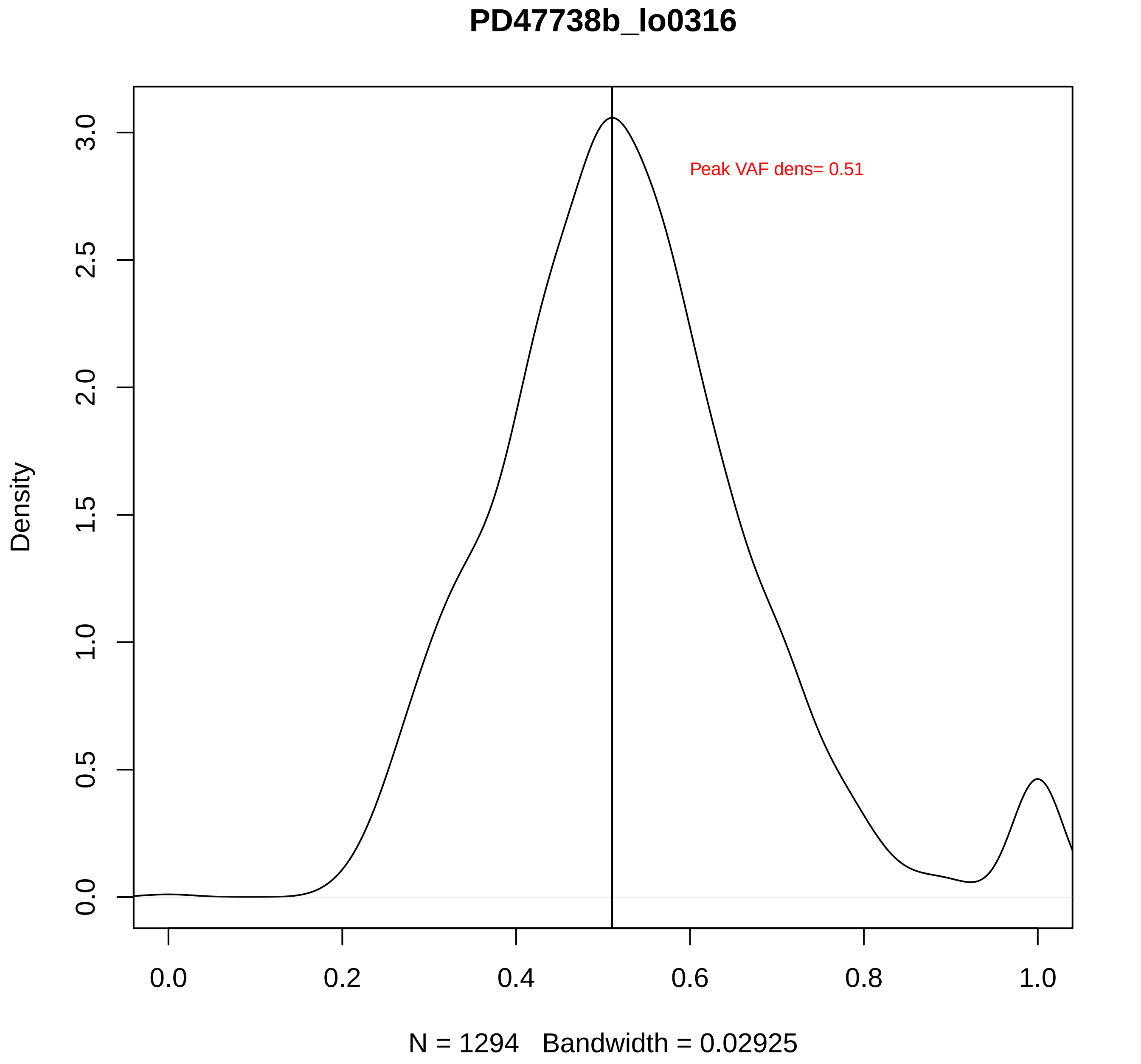
PD47738b_lo0316
Peak VAF dens= 0.51
Density
0.0
0.5
1.0
1.5
2.0
2.5
3.0
0.0
0.2
0.4
0.6
0.8
1.0
N = 1294   Bandwidth = 0.02925

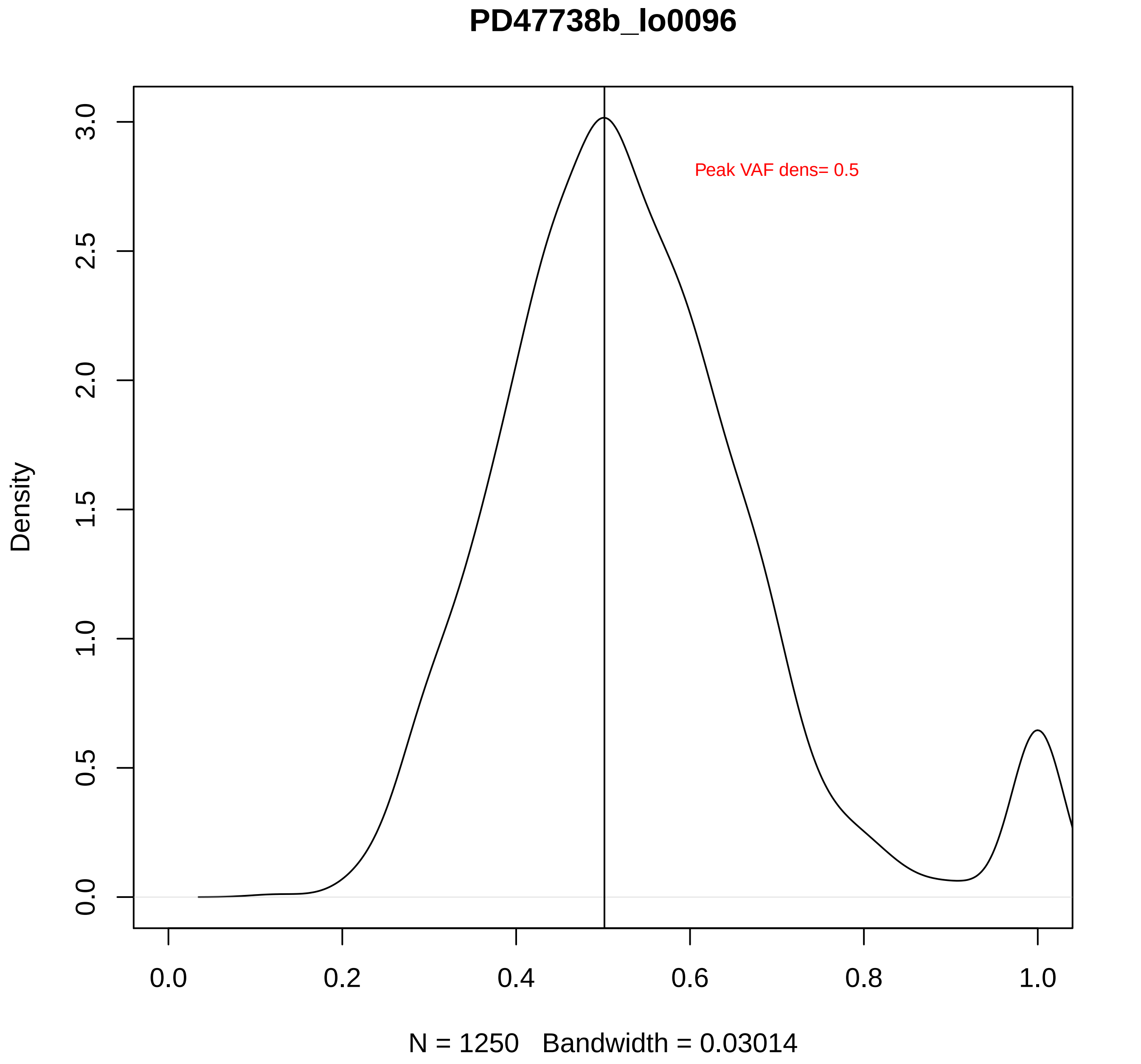
PD47738b_lo0096
Peak VAF dens= 0.5
Density
0.0
0.5
1.0
1.5
2.0
2.5
3.0
0.0
0.2
0.4
0.6
0.8
1.0
N = 1250   Bandwidth = 0.03014

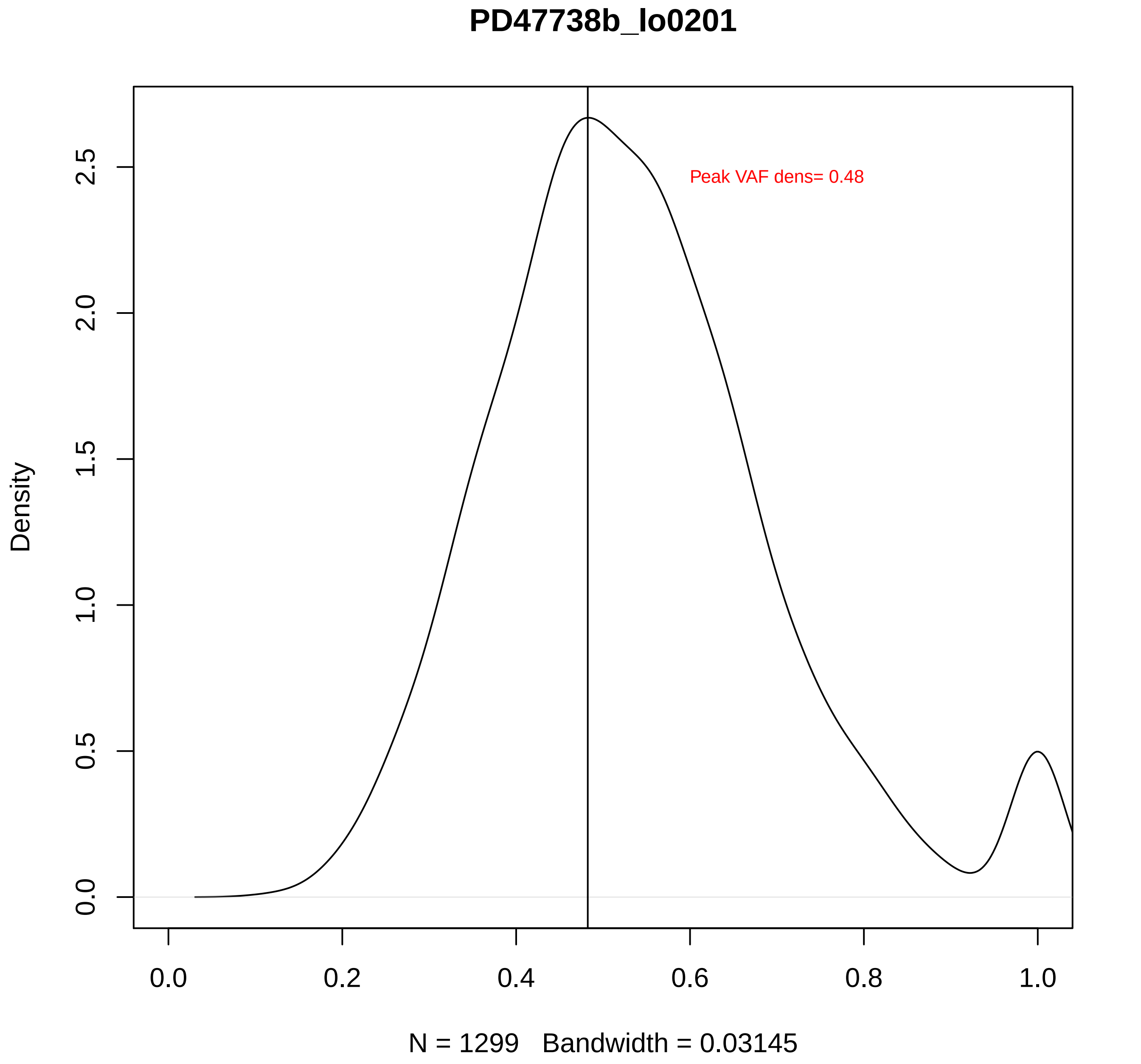

PD47738b_lo0201
Peak VAF dens= 0.48
Density
0.0
0.5
1.0
1.5
2.0
2.5
0.0
0.2
0.4
0.6
0.8
1.0
N = 1299   Bandwidth = 0.03145

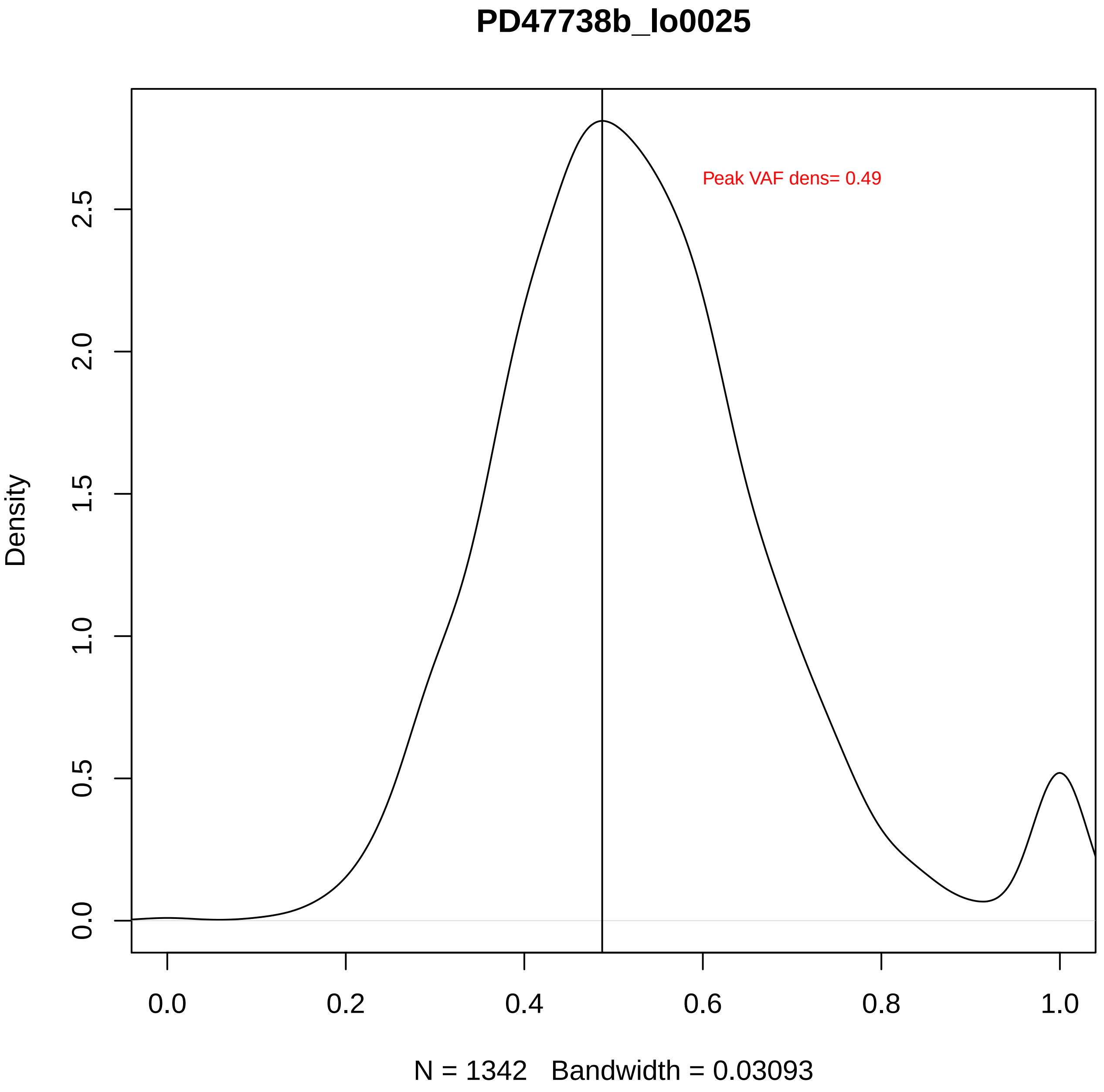
PD47738b_lo0025
Peak VAF dens= 0.49
Density
0.0
0.5
1.0
1.5
2.0
2.5
0.0
0.2
0.4
0.6
0.8
1.0
N = 1342   Bandwidth = 0.03093

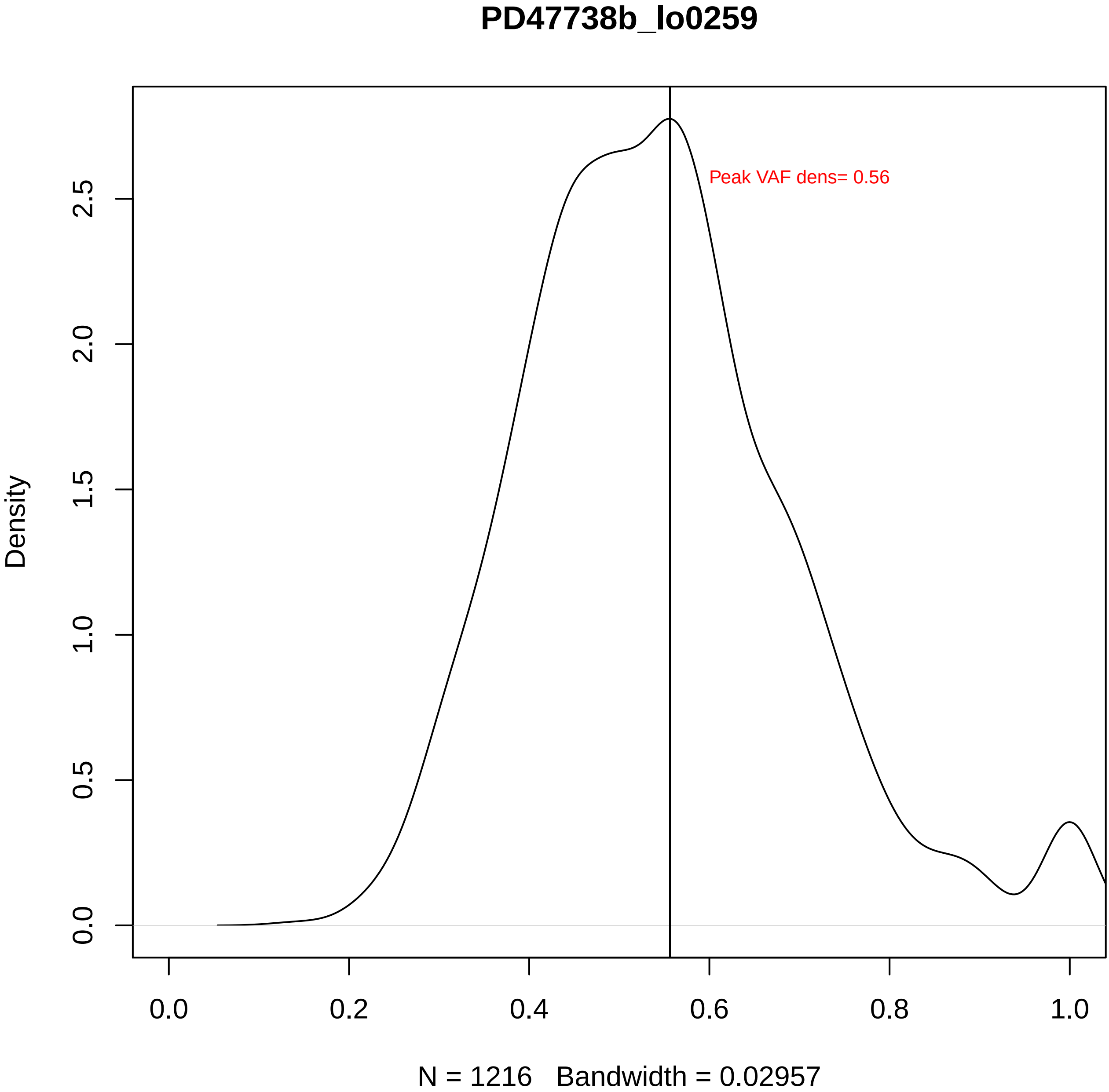
PD47738b_lo0259
Peak VAF dens= 0.56
Density
0.0
0.5
1.0
1.5
2.0
2.5
0.0
0.2
0.4
0.6
0.8
1.0
N = 1216   Bandwidth = 0.02957

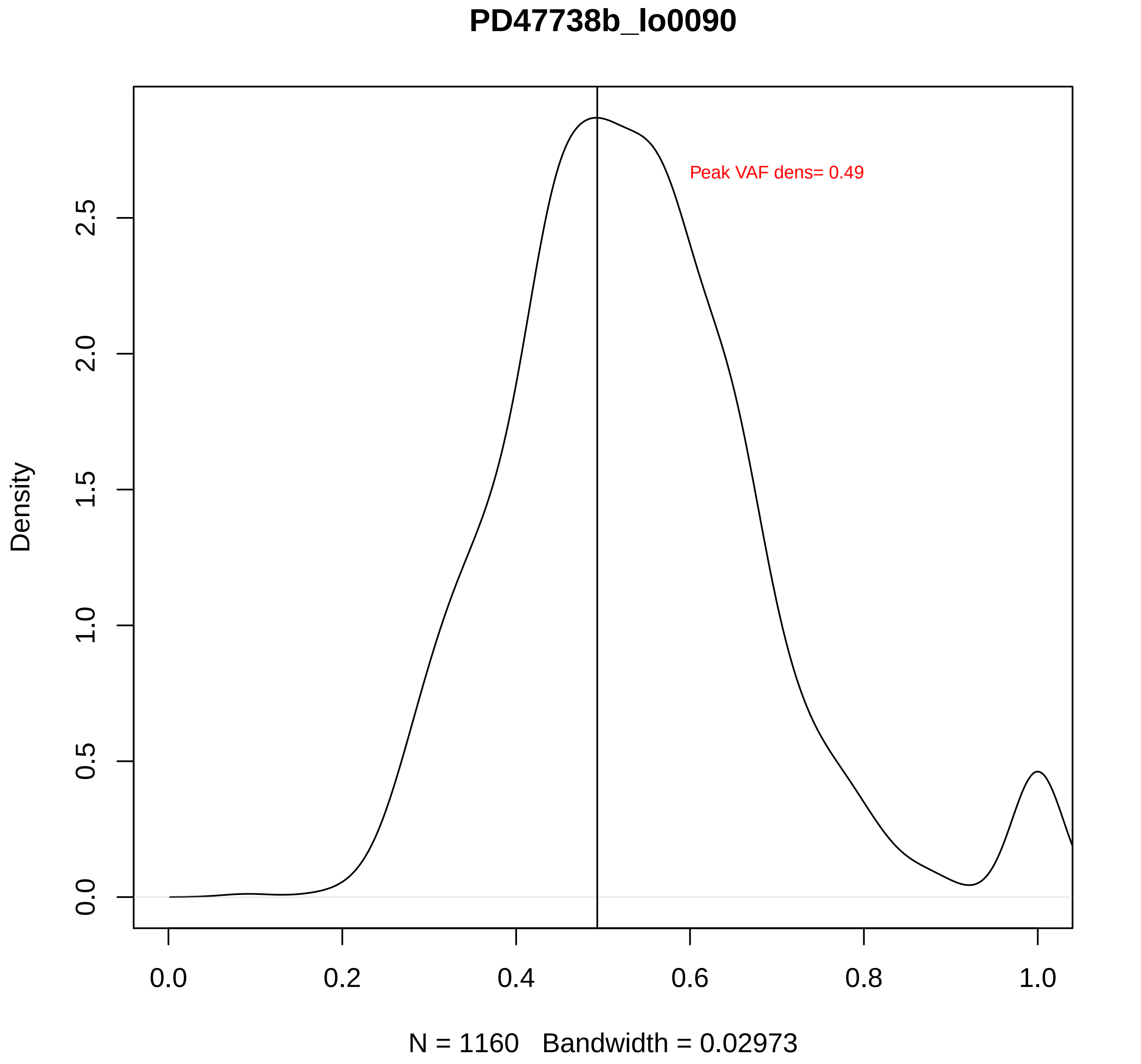
PD47738b_lo0090
Peak VAF dens= 0.49
Density
0.0
0.5
1.0
1.5
2.0
2.5
0.0
0.2
0.4
0.6
0.8
1.0
N = 1160   Bandwidth = 0.02973

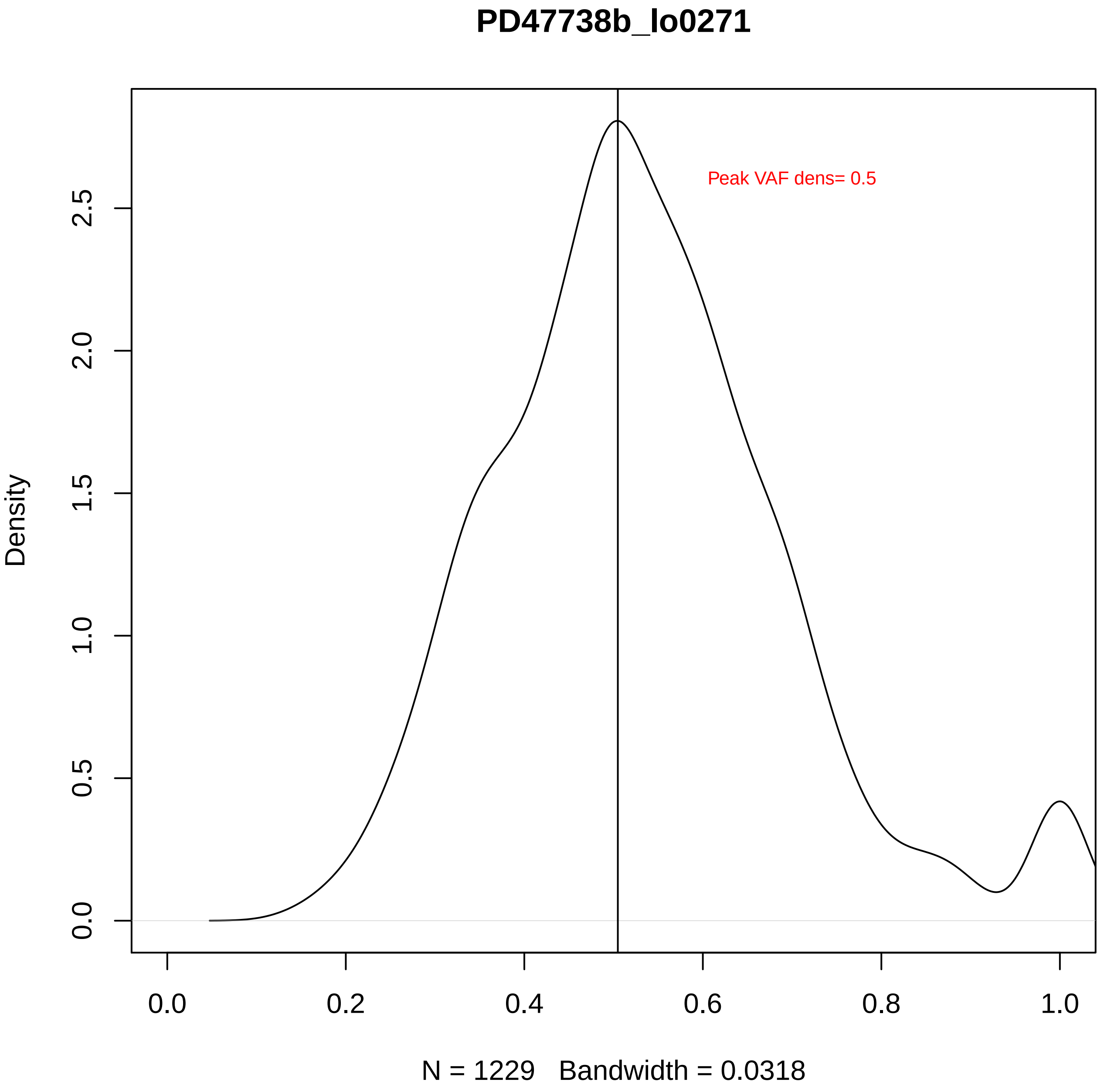

PD47738b_lo0271
Peak VAF dens= 0.5
Density
0.0
0.5
1.0
1.5
2.0
2.5
0.0
0.2
0.4
0.6
0.8
1.0
N = 1229   Bandwidth = 0.0318

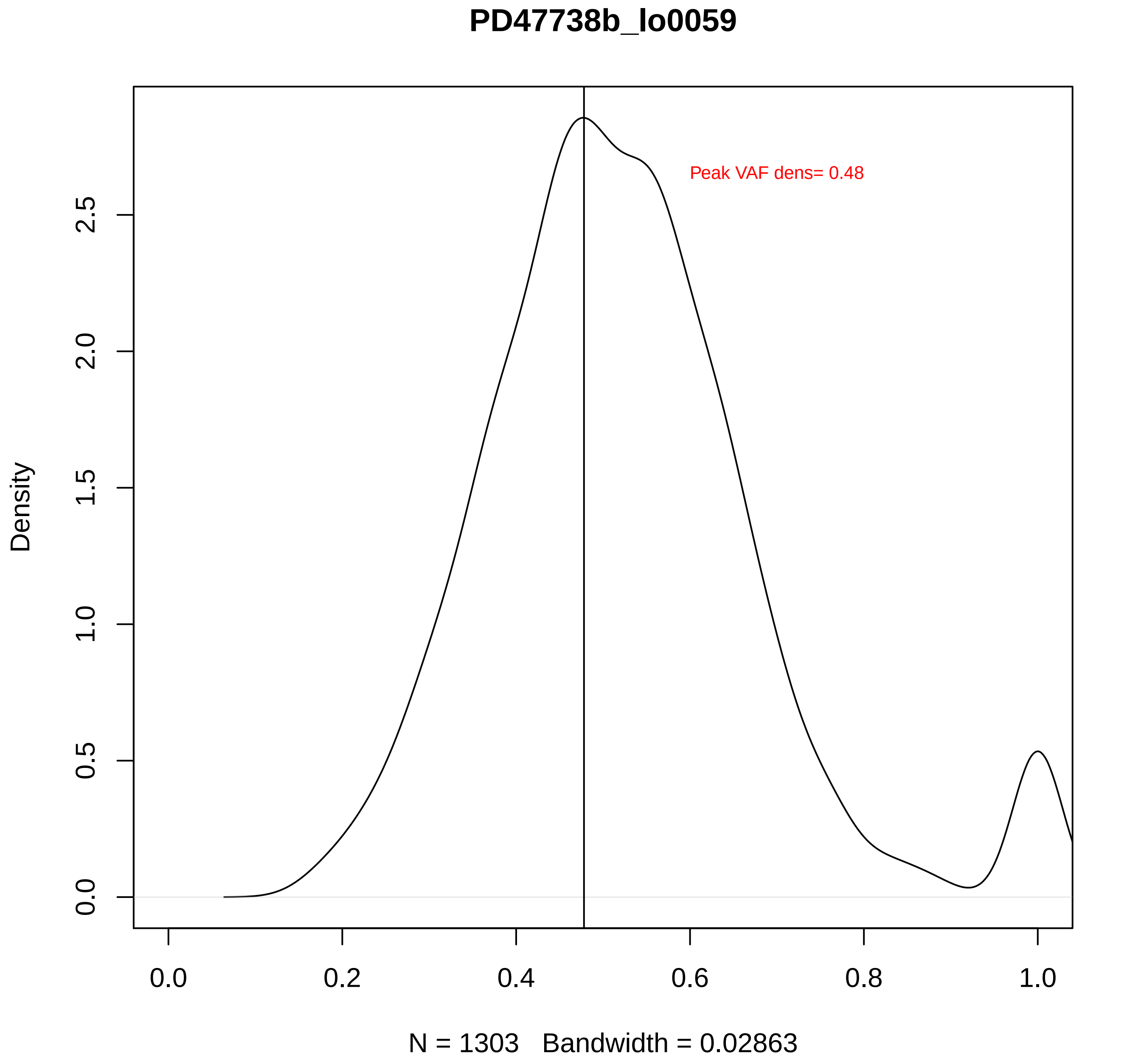
PD47738b_lo0059
Peak VAF dens= 0.48
Density
0.0
0.5
1.0
1.5
2.0
2.5
0.0
0.2
0.4
0.6
0.8
1.0
N = 1303   Bandwidth = 0.02863

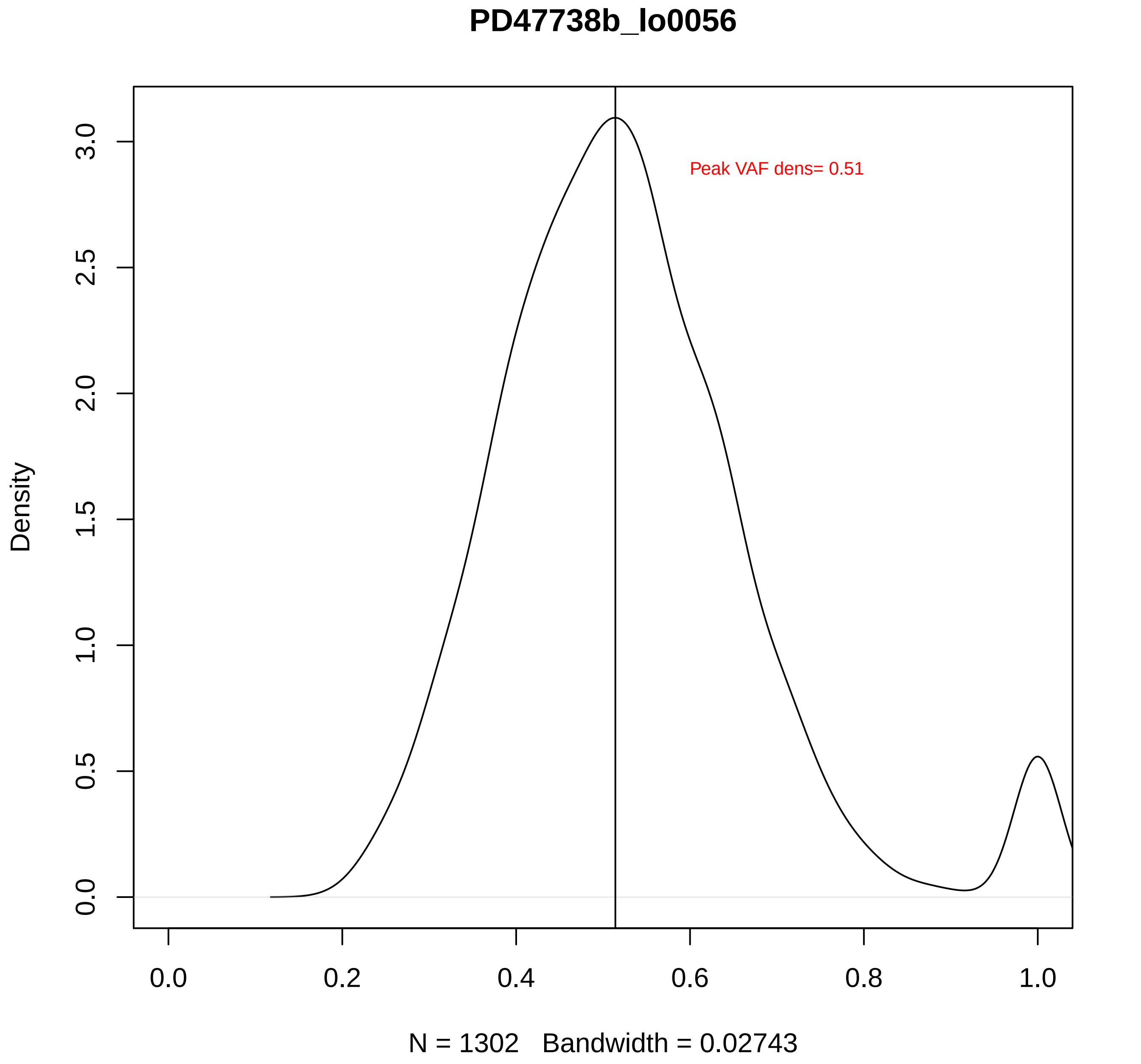
PD47738b_lo0056
Peak VAF dens= 0.51
Density
0.0
0.5
1.0
1.5
2.0
2.5
3.0
0.0
0.2
0.4
0.6
0.8
1.0
N = 1302 Bandwidth = 0.02743

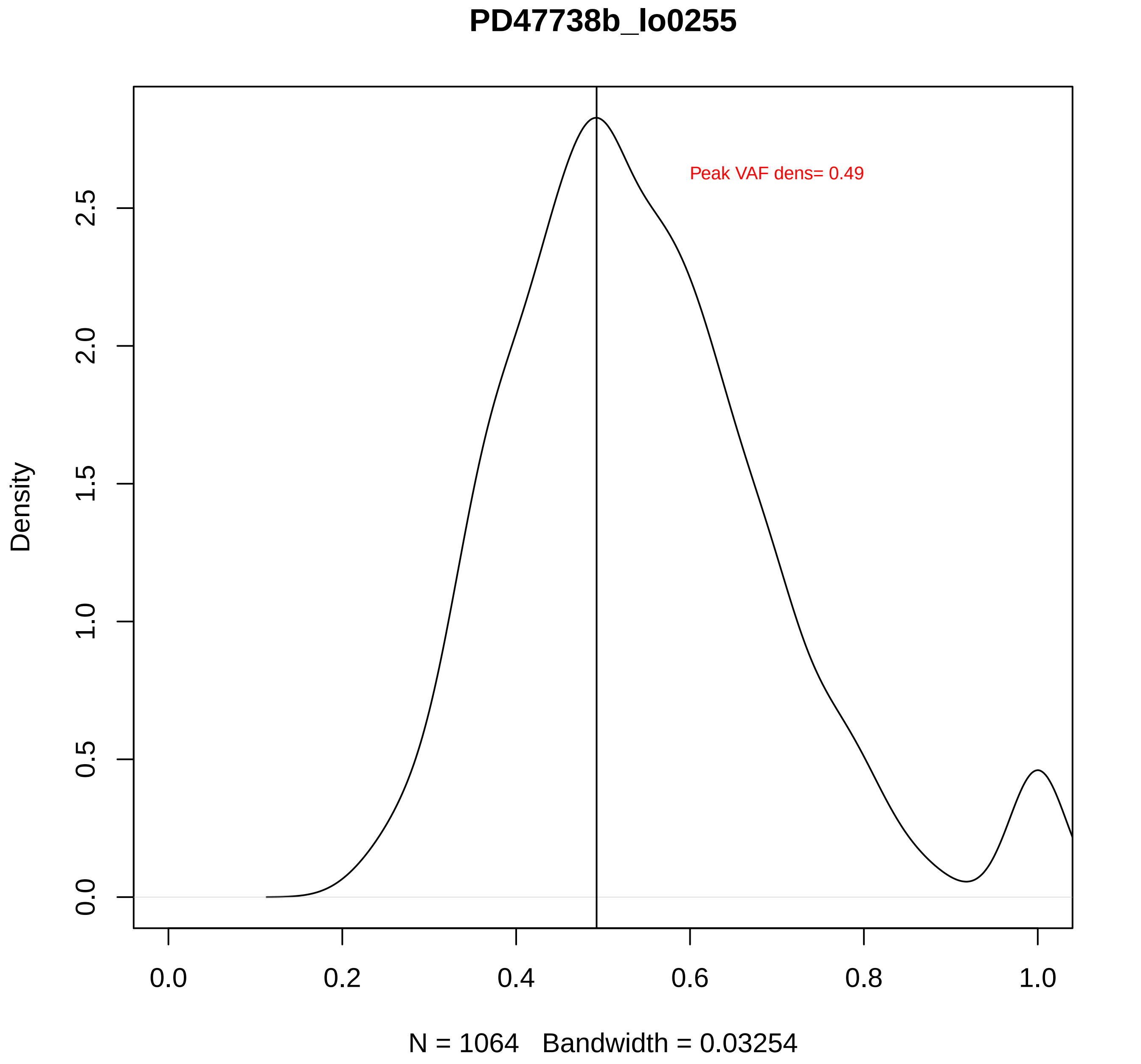

PD47738b_lo0255
Peak VAF dens= 0.49
Density
0.0
0.5
1.0
1.5
2.0
2.5
0.0
0.2
0.4
0.6
0.8
1.0
N = 1064 Bandwidth = 0.03254

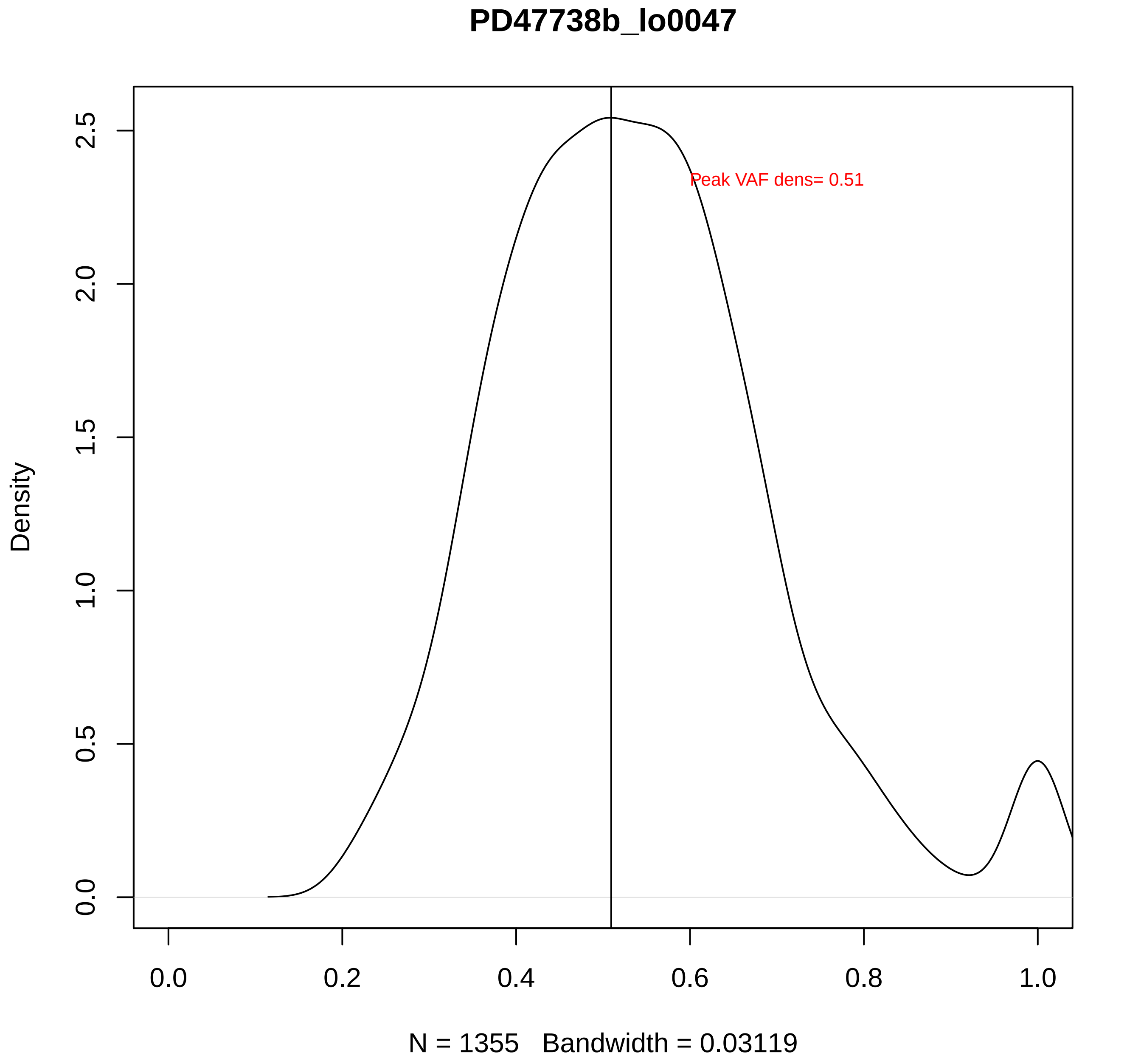
PD47738b_lo0047
Peak VAF dens= 0.51
Density
0.0
0.5
1.0
1.5
2.0
2.5
0.0
0.2
0.4
0.6
0.8
1.0
N = 1355   Bandwidth = 0.03119

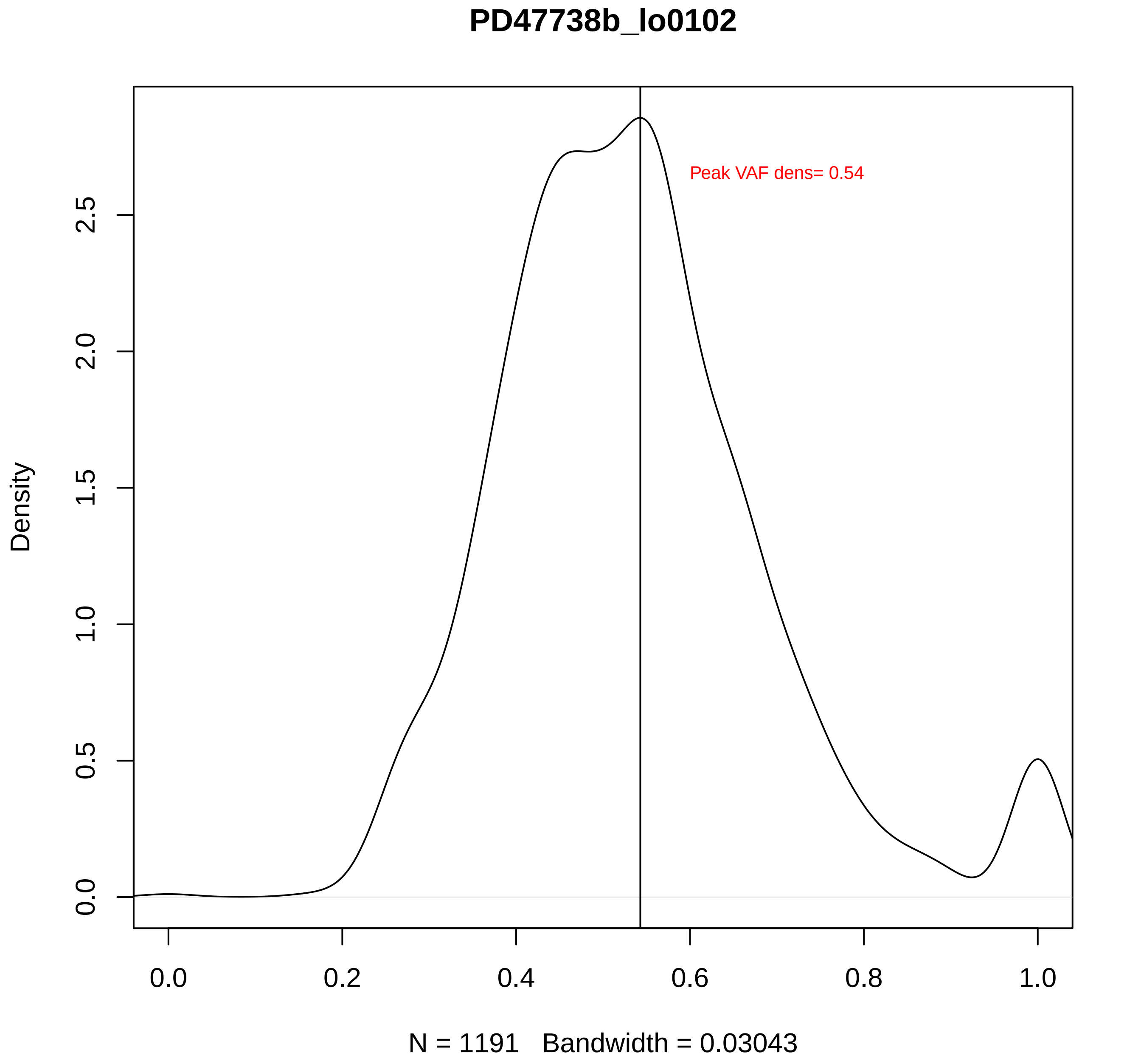
PD47738b_lo0102
Peak VAF dens= 0.54
Density
0.0
0.5
1.0
1.5
2.0
2.5
0.0
0.2
0.4
0.6
0.8
1.0
N = 1191   Bandwidth = 0.03043

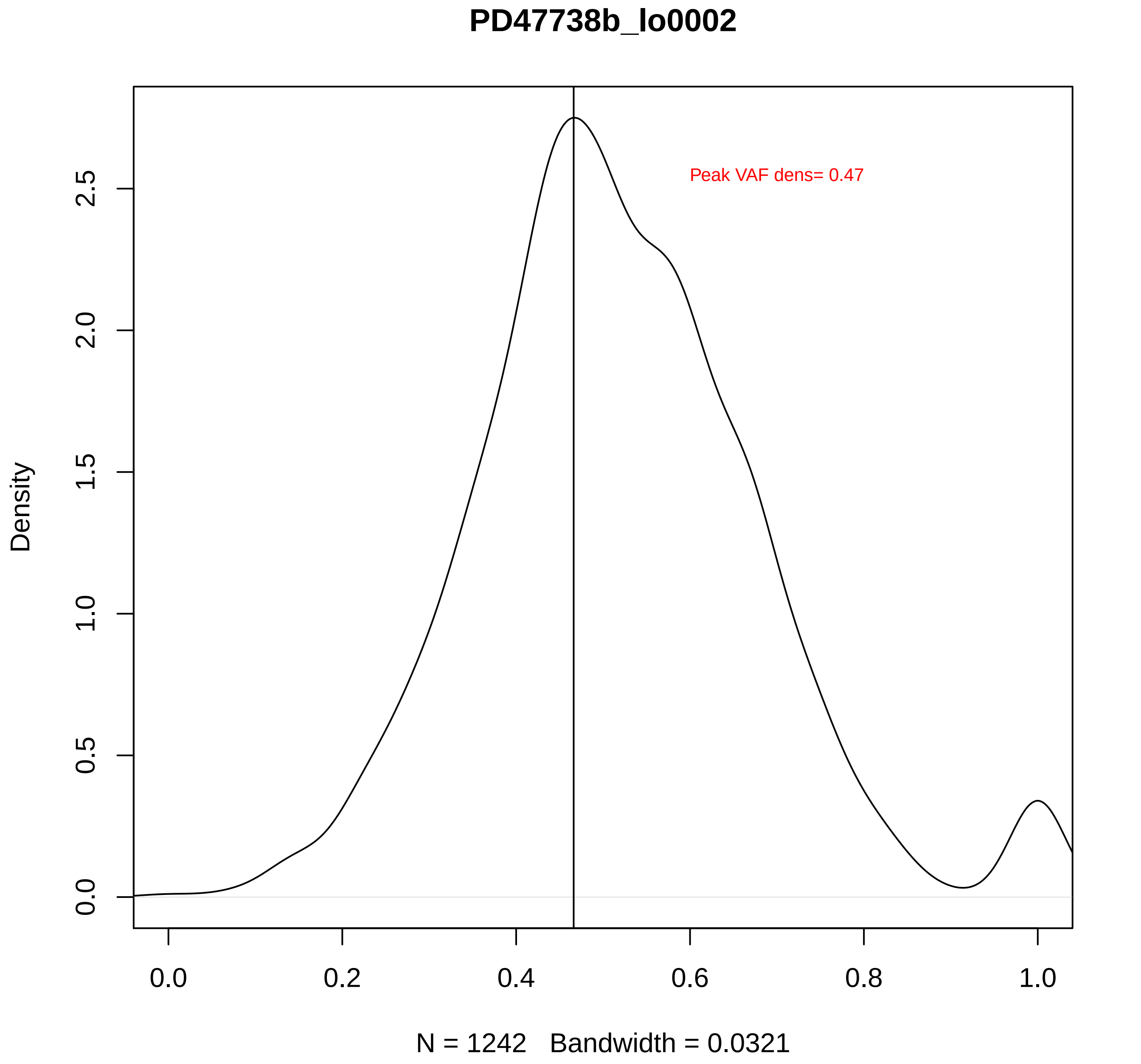

PD47738b_lo0002
Peak VAF dens= 0.47
Density
0.0
0.5
1.0
1.5
2.0
2.5
0.0
0.2
0.4
0.6
0.8
1.0
N = 1242 Bandwidth = 0.0321

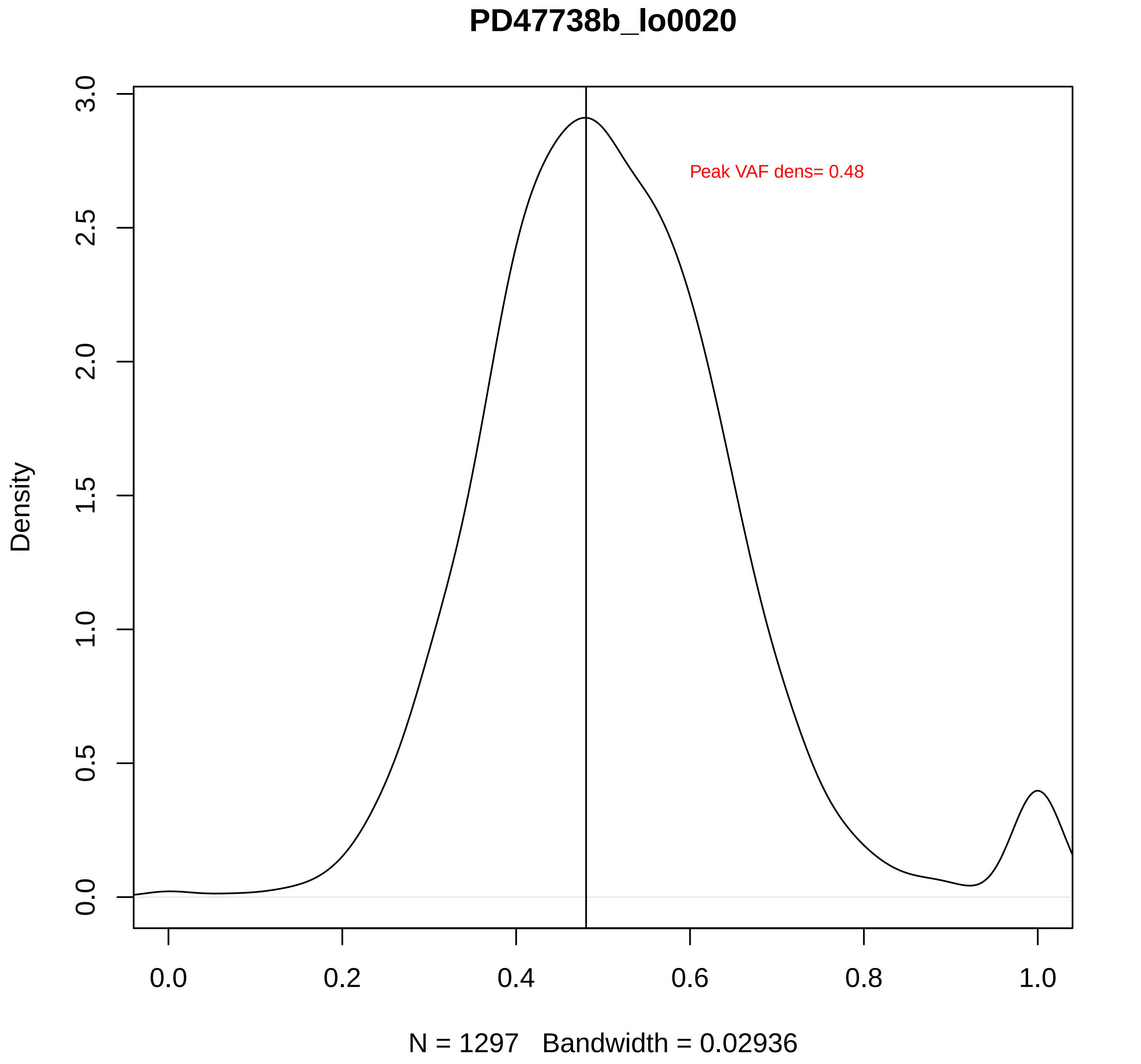
PD47738b_lo0020
Peak VAF dens= 0.48
Density
0.0
0.5
1.0
1.5
2.0
2.5
3.0
0.0
0.2
0.4
0.6
0.8
1.0
N = 1297   Bandwidth = 0.02936

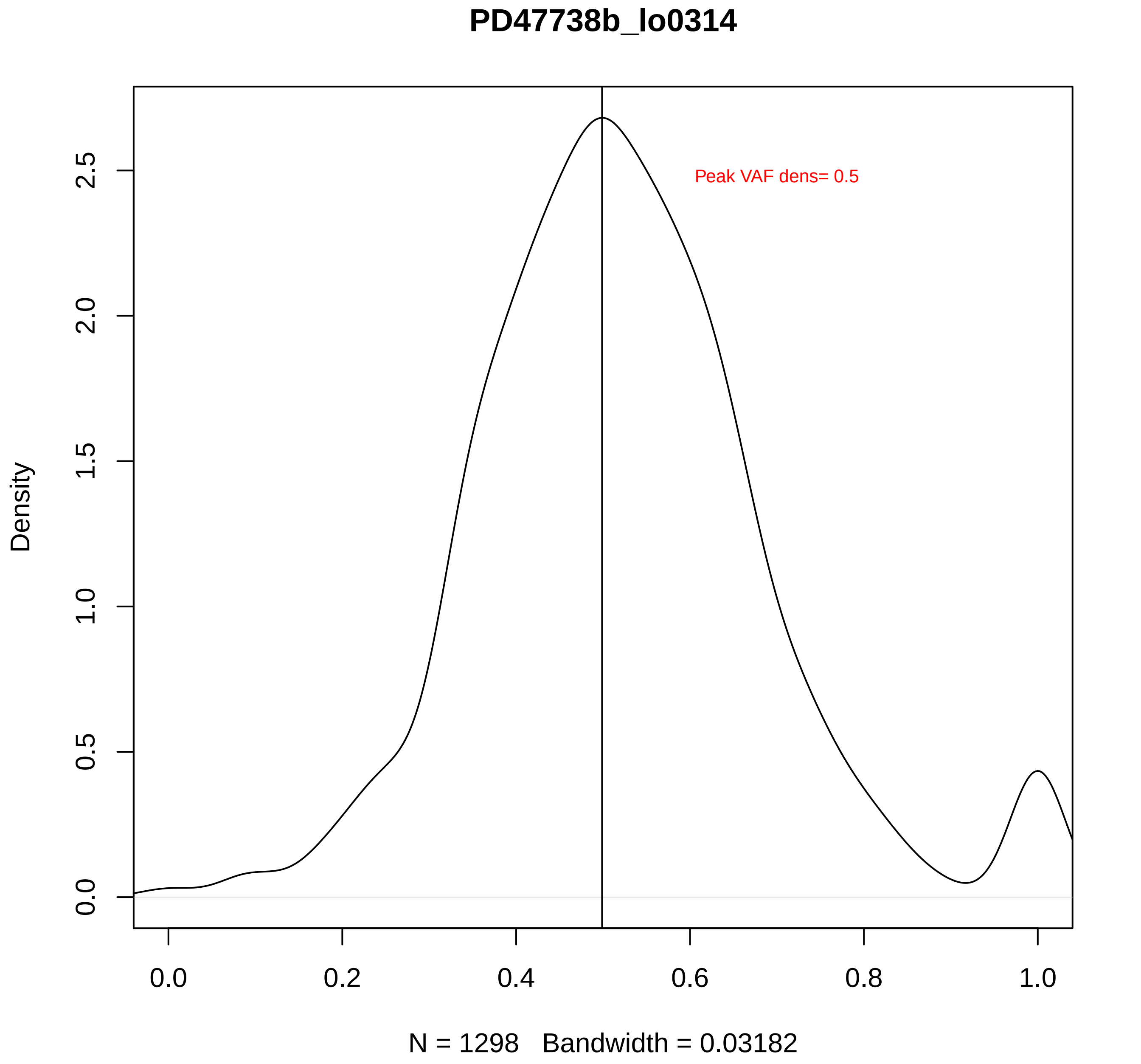

PD47738b_lo0314
Peak VAF dens= 0.5
Density
0.0
0.5
1.0
1.5
2.0
2.5
0.0
0.2
0.4
0.6
0.8
1.0
N = 1298 Bandwidth = 0.03182

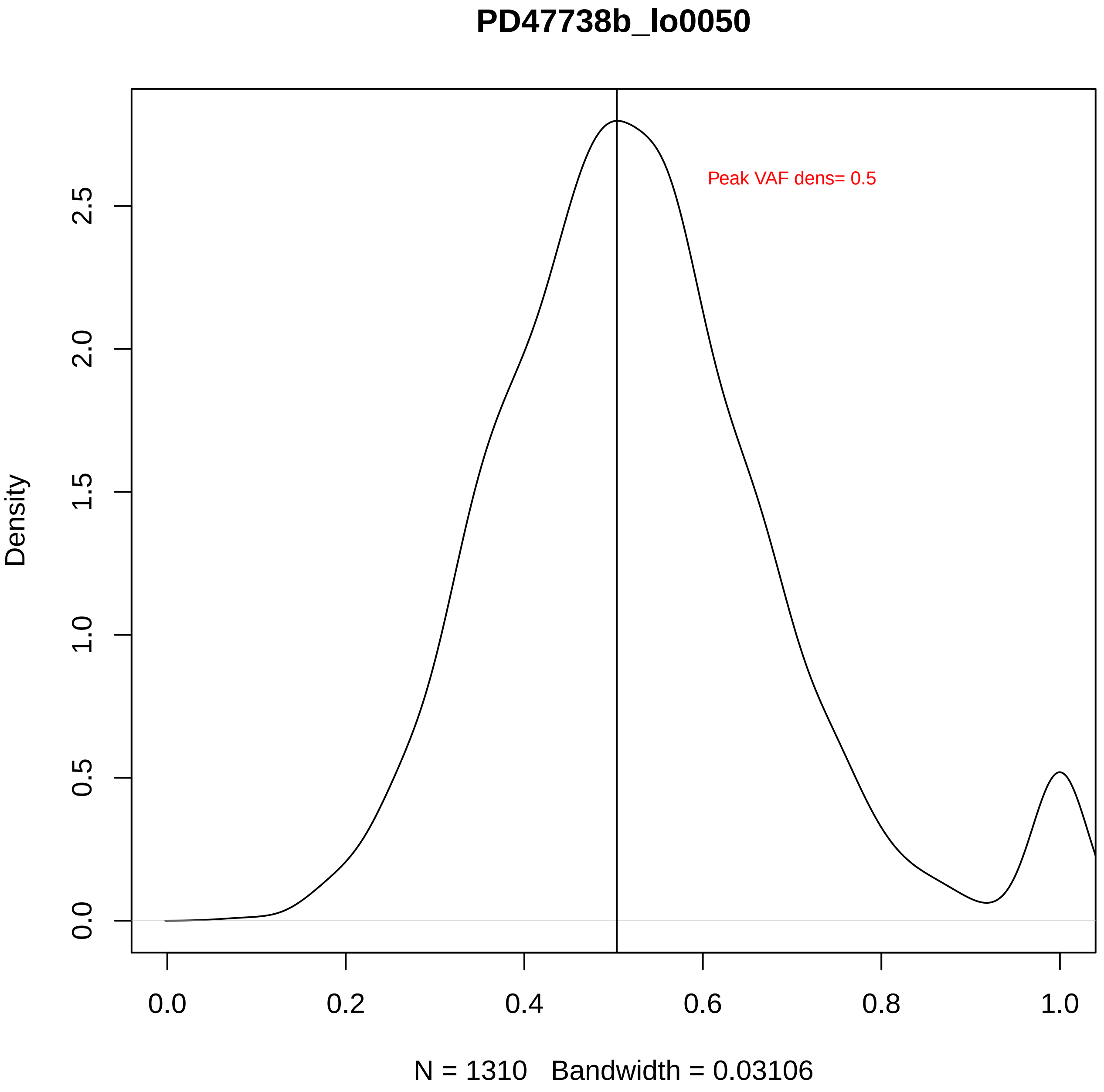
PD47738b_lo0050
Peak VAF dens= 0.5
Density
0.0
0.5
1.0
1.5
2.0
2.5
0.0
0.2
0.4
0.6
0.8
1.0
N = 1310 Bandwidth = 0.03106

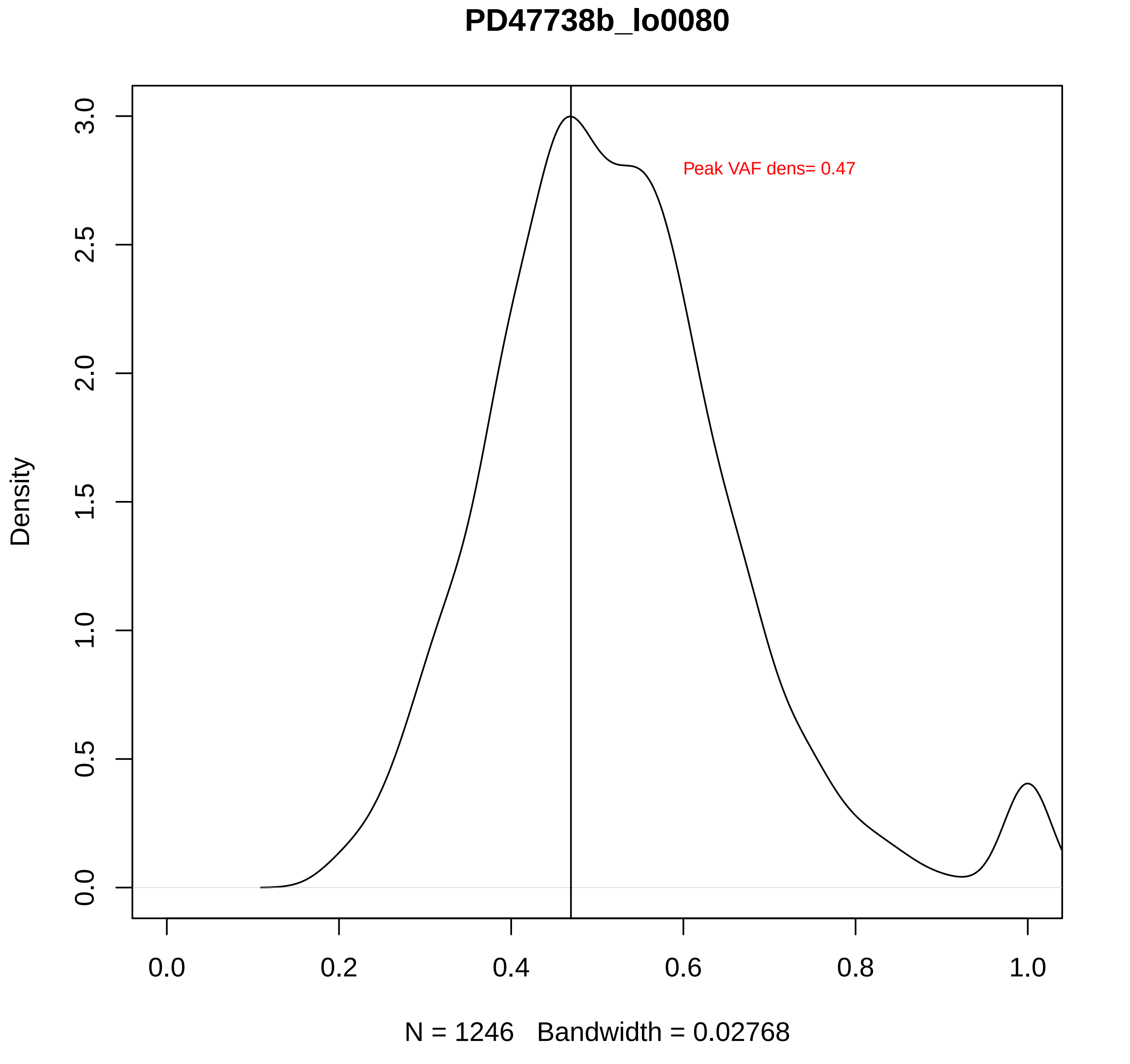

PD47738b_lo0080
Peak VAF dens= 0.47
Density
0.0
0.5
1.0
1.5
2.0
2.5
3.0
0.0
0.2
0.4
0.6
0.8
1.0
N = 1246   Bandwidth = 0.02768

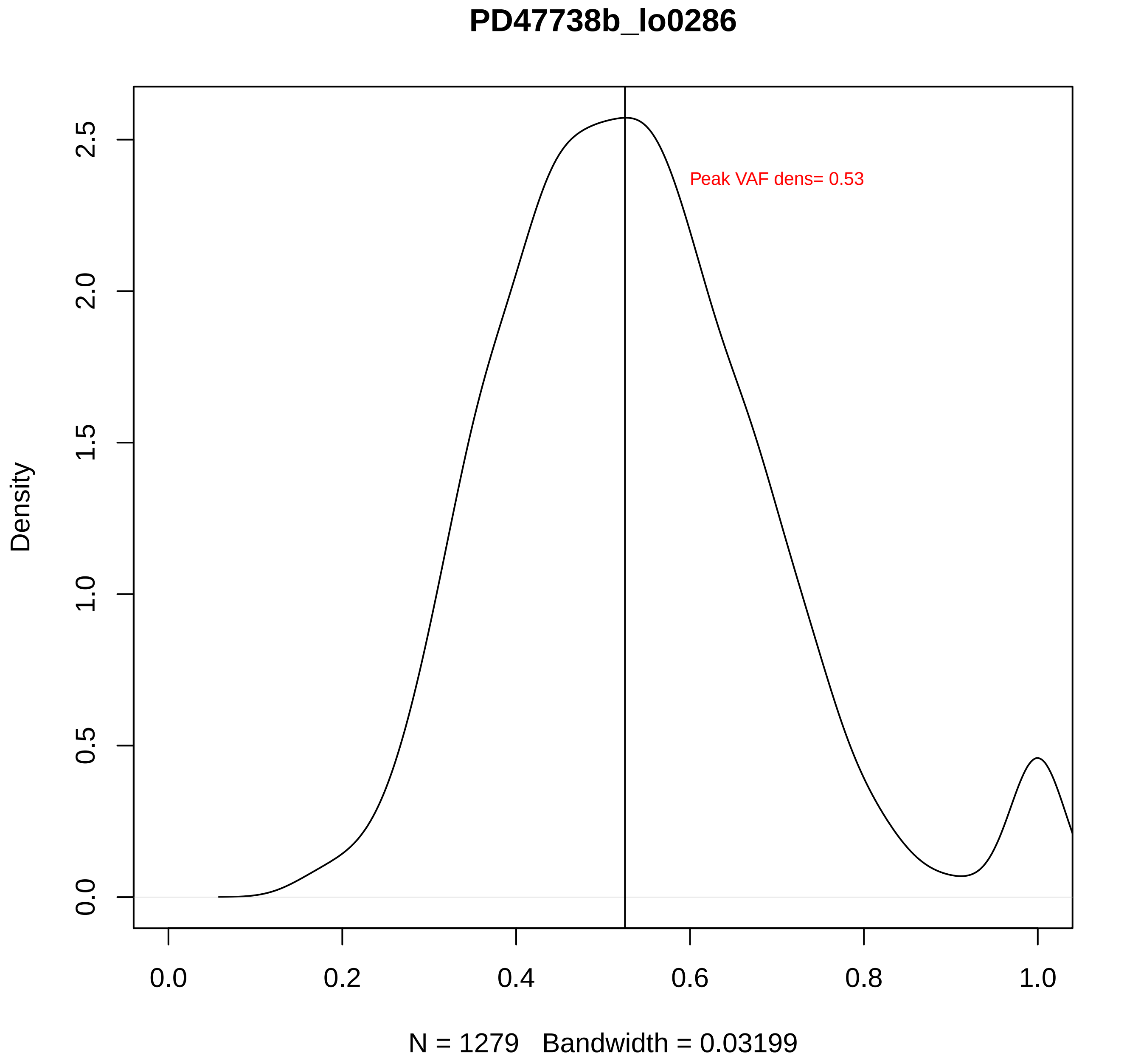
PD47738b_lo0286
Density
2.5
2.0
1.5
1.0
0.5
0.0
Peak VAF dens= 0.53
0.0
0.2
0.4
0.6
0.8
1.0
N = 1279   Bandwidth = 0.03199

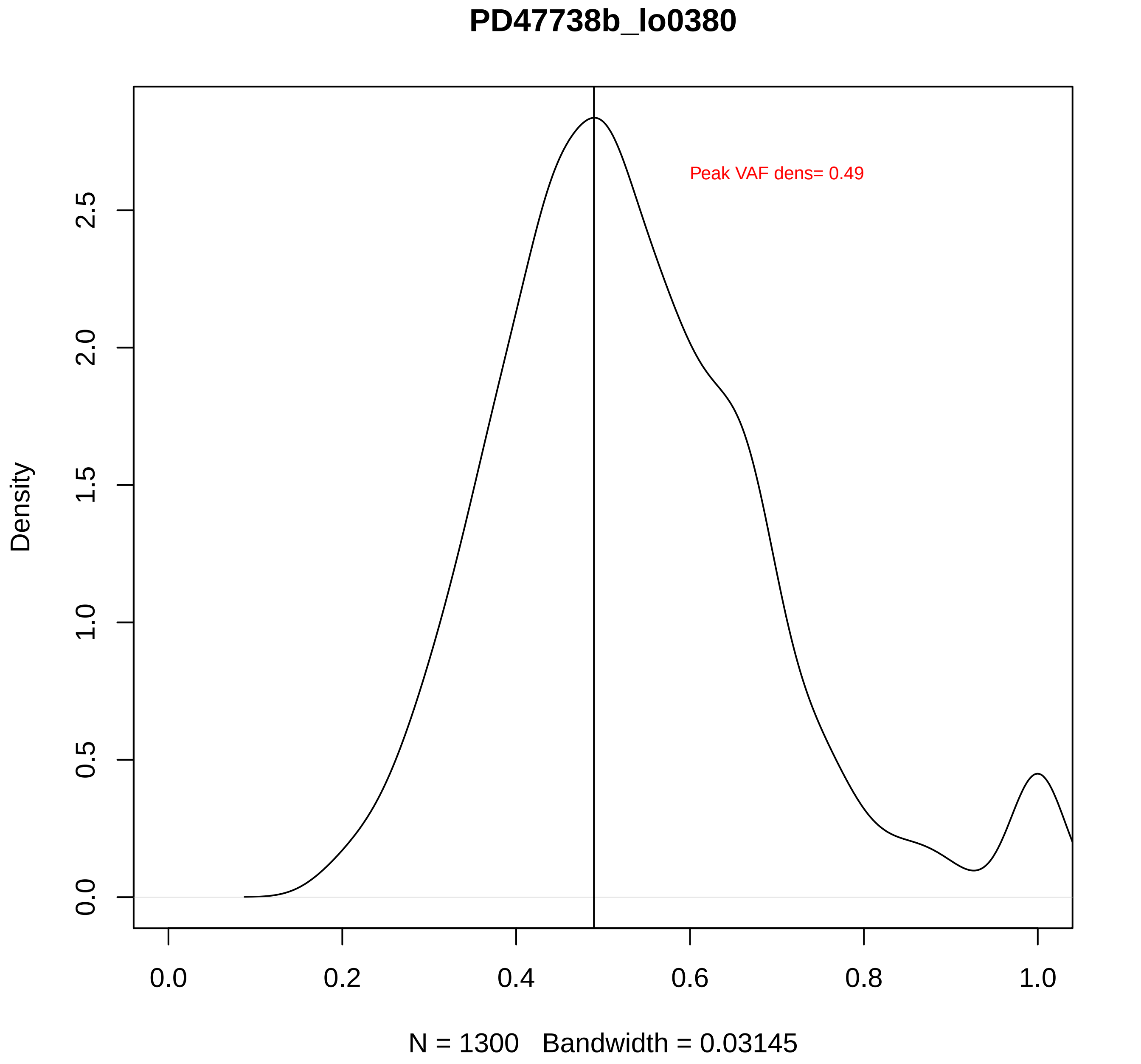

PD47738b_lo0380
Peak VAF dens= 0.49
Density
0.0
0.5
1.0
1.5
2.0
2.5
0.0
0.2
0.4
0.6
0.8
1.0
N = 1300   Bandwidth = 0.03145

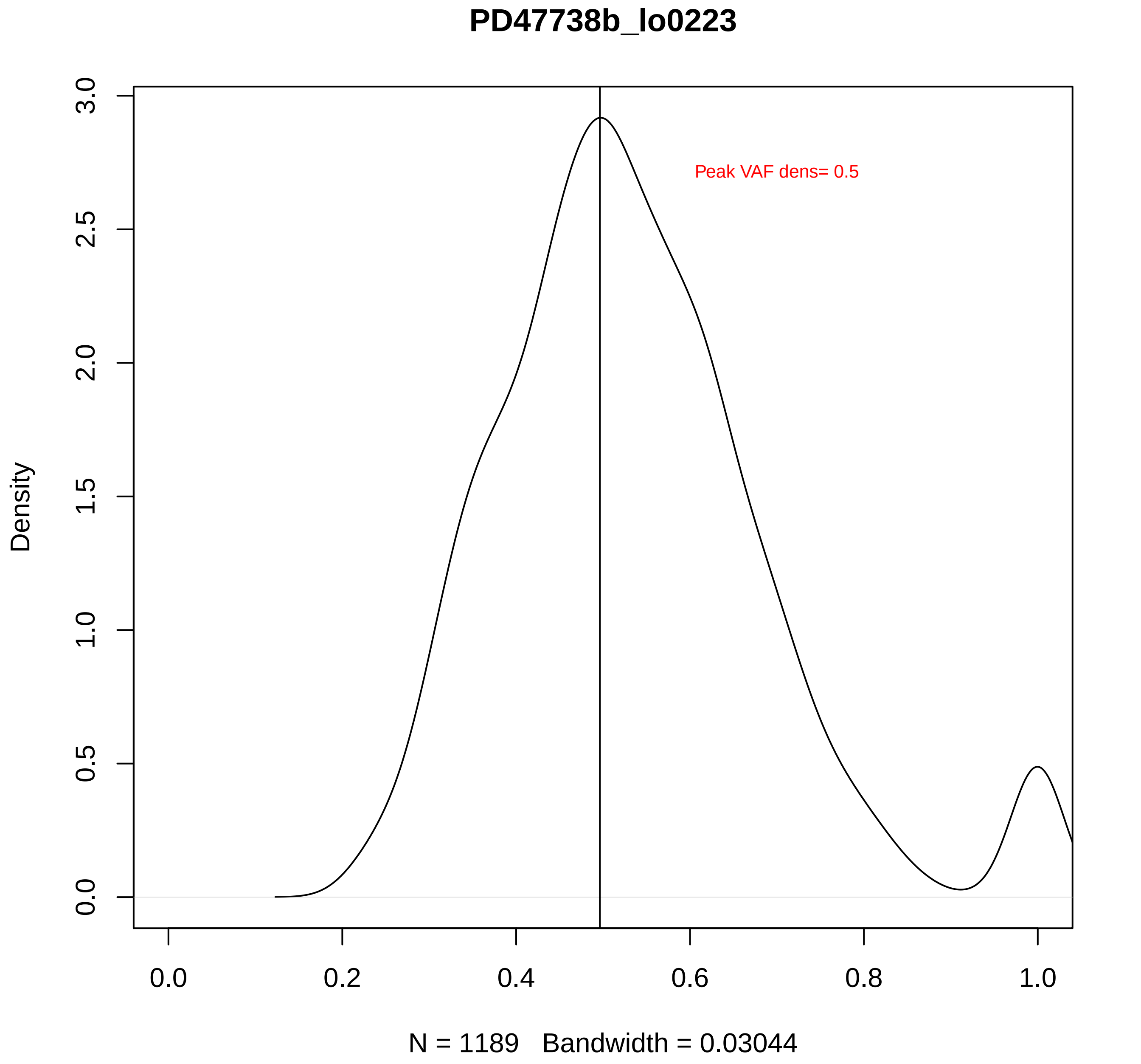
PD47738b_lo0223
Peak VAF dens= 0.5
Density
0.0
0.5
1.0
1.5
2.0
2.5
3.0
0.0
0.2
0.4
0.6
0.8
1.0
N = 1189   Bandwidth = 0.03044

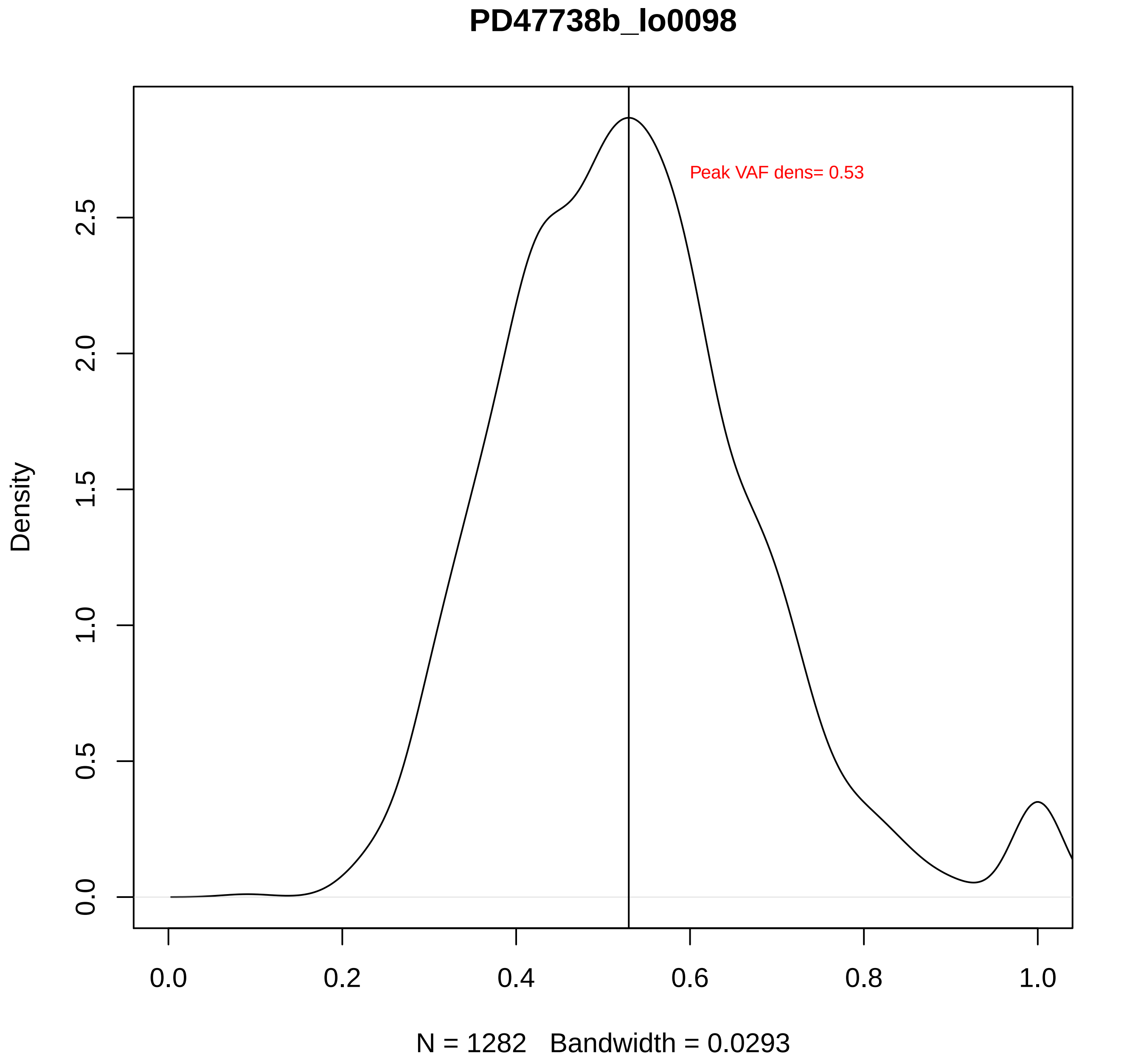
PD47738b_lo0098
Peak VAF dens= 0.53
Density
0.0
0.5
1.0
1.5
2.0
2.5
0.0
0.2
0.4
0.6
0.8
1.0
N = 1282   Bandwidth = 0.0293

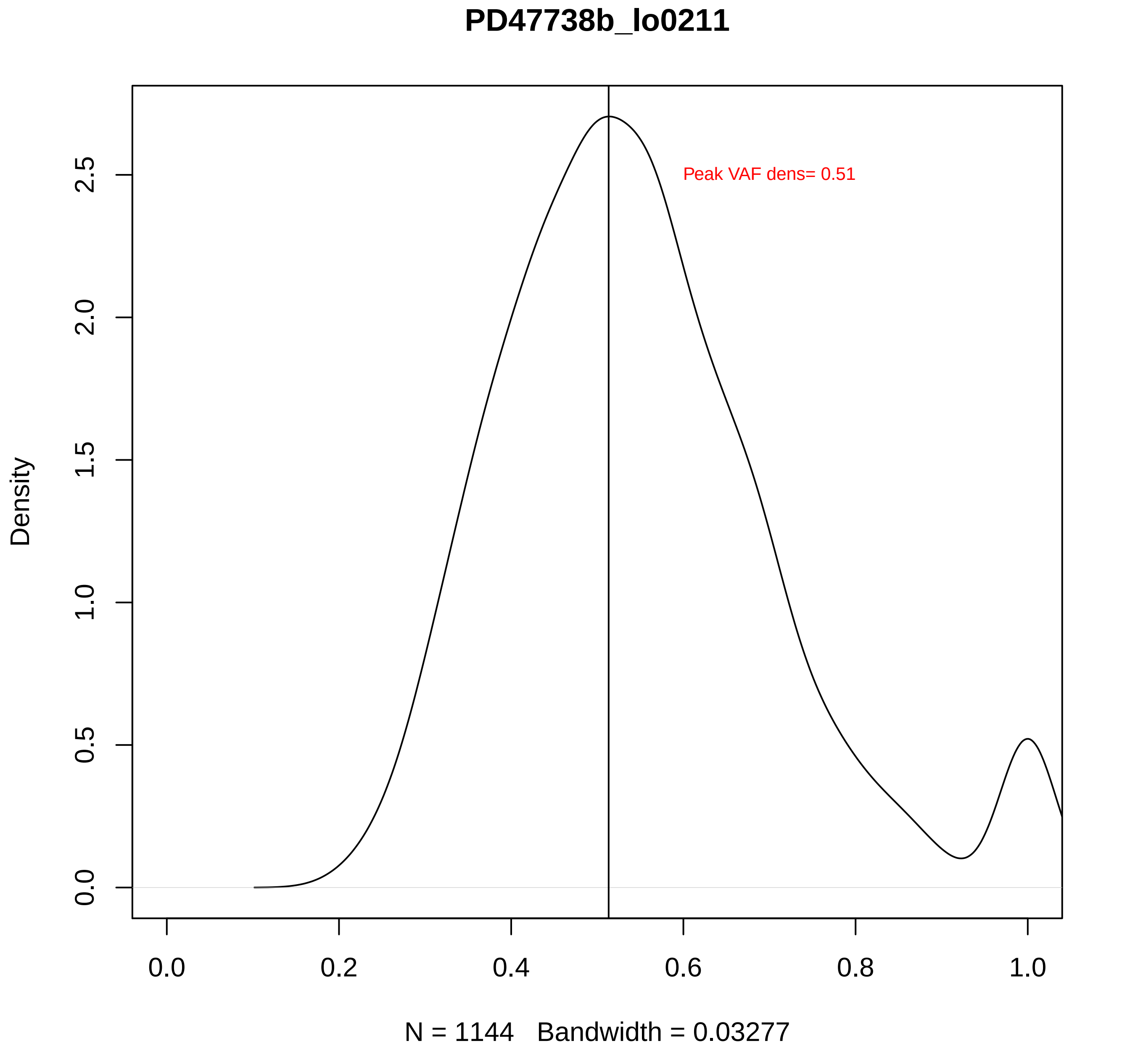

PD47738b_lo0211
Peak VAF dens= 0.51
Density
0.0
0.5
1.0
1.5
2.0
2.5
0.0
0.2
0.4
0.6
0.8
1.0
N = 1144   Bandwidth = 0.03277

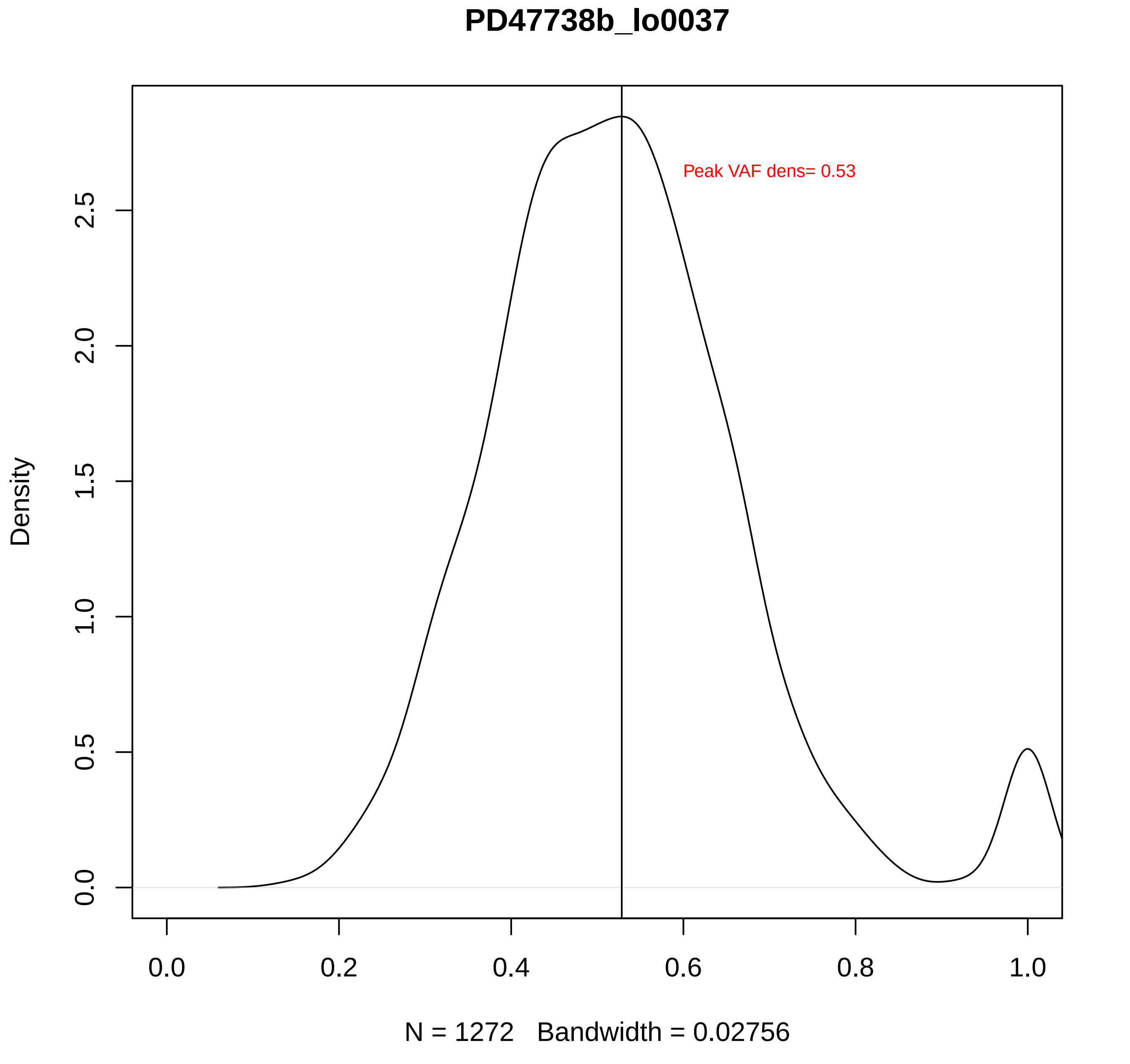

PD47738b_lo0037
Peak VAF dens= 0.53
Density
0.0
0.5
1.0
1.5
2.0
2.5
0.0
0.2
0.4
0.6
0.8
1.0
N = 1272   Bandwidth = 0.02756

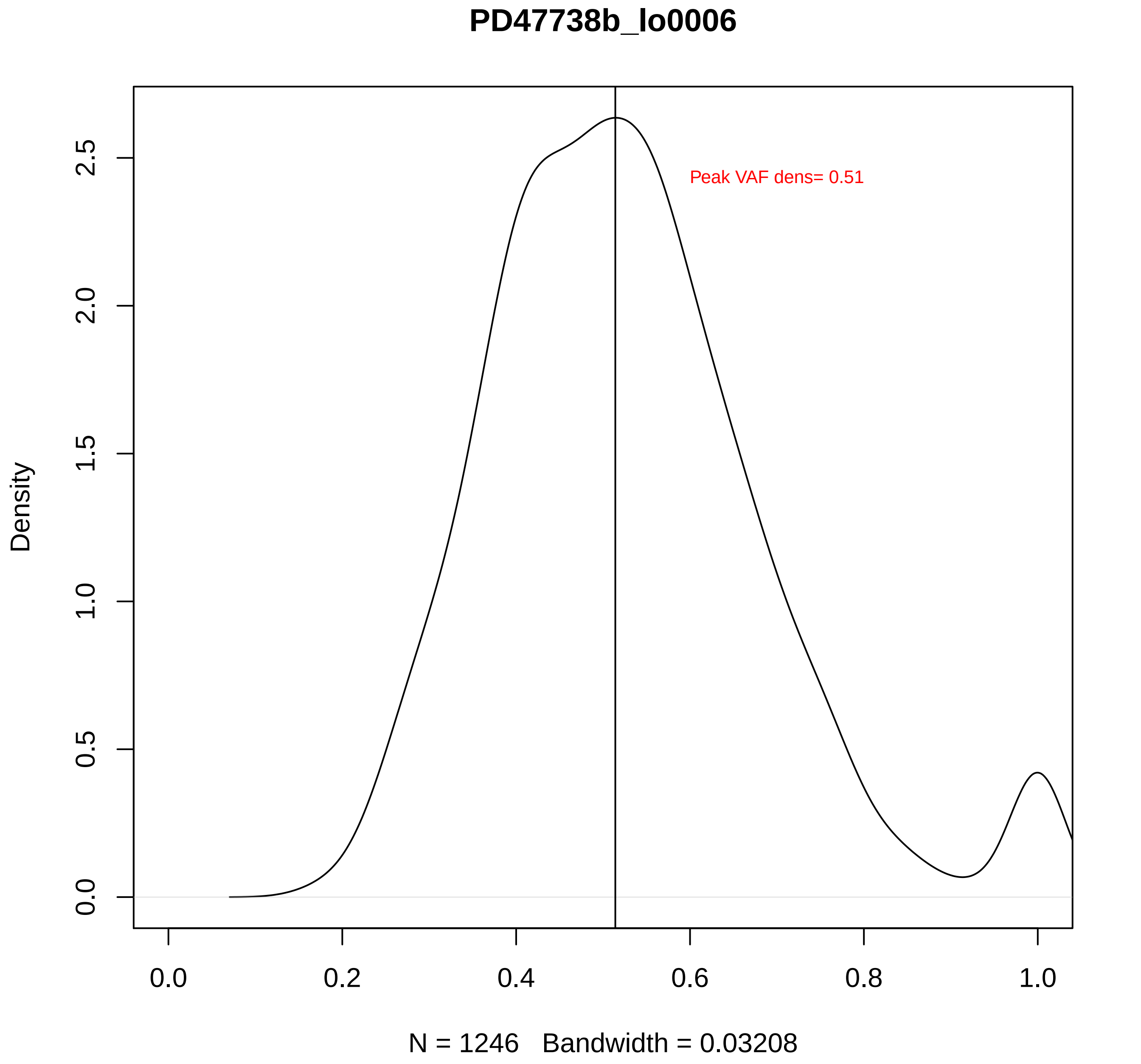

PD47738b_lo0006
Peak VAF dens= 0.51
Density
0.0
0.5
1.0
1.5
2.0
2.5
0.0
0.2
0.4
0.6
0.8
1.0
N = 1246   Bandwidth = 0.03208

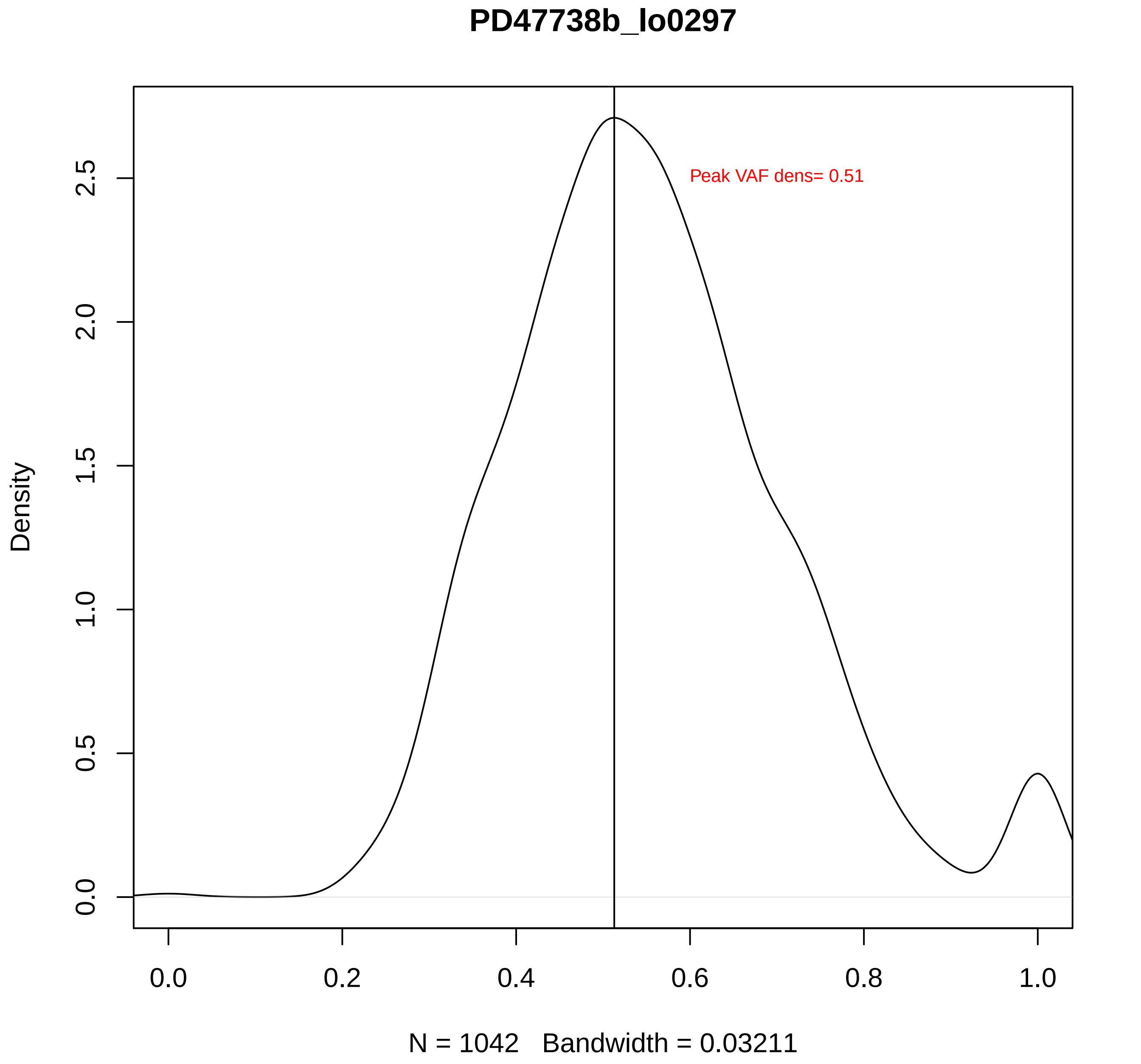
PD47738b_lo0297
Peak VAF dens= 0.51
Density
0.0
0.5
1.0
1.5
2.0
2.5
0.0
0.2
0.4
0.6
0.8
1.0
N = 1042   Bandwidth = 0.03211

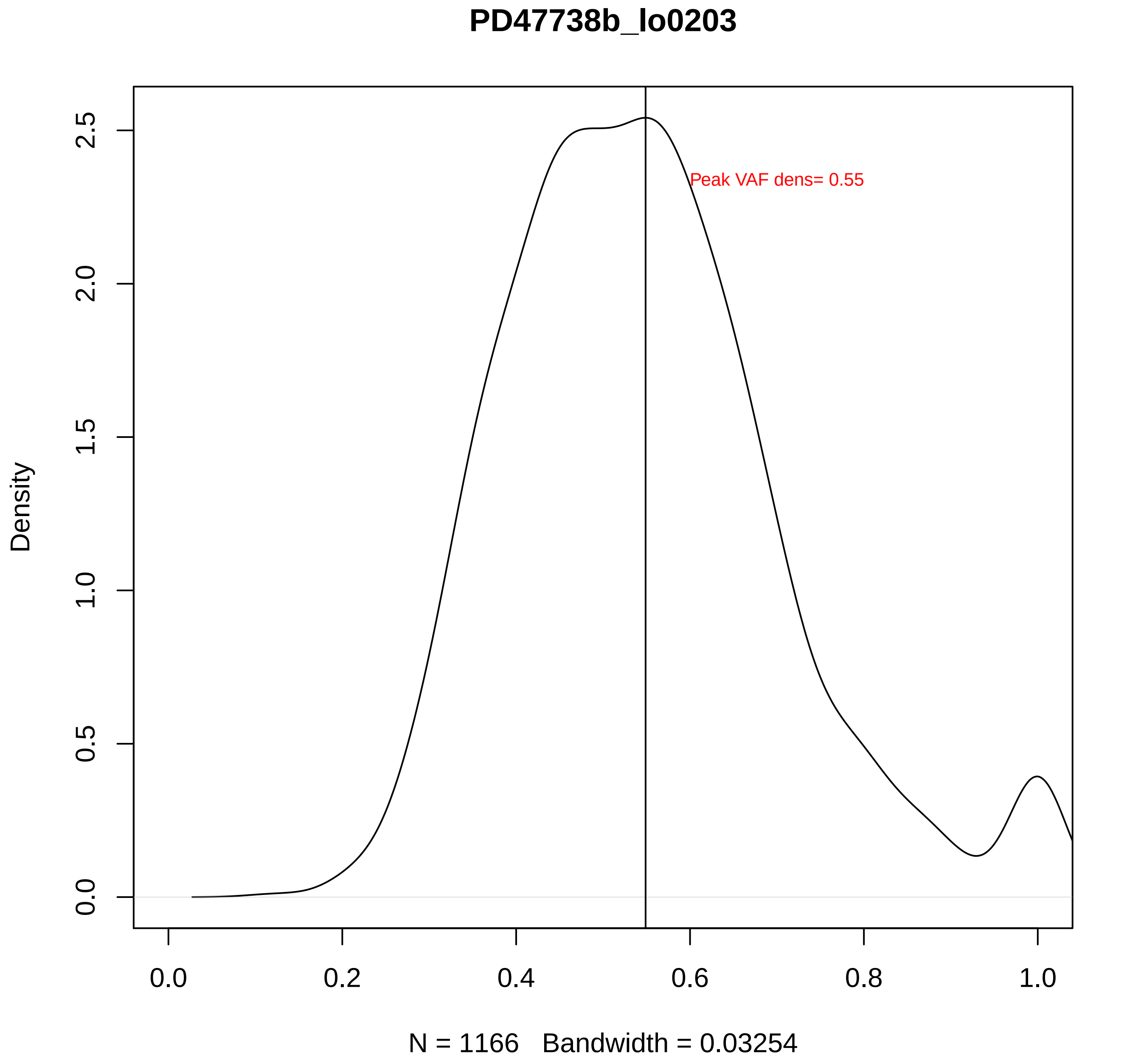
PD47738b_lo0203
Peak VAF dens= 0.55
Density
2.5
2.0
1.5
1.0
0.5
0.0
0.0
0.2
0.4
0.6
0.8
1.0
N = 1166   Bandwidth = 0.03254

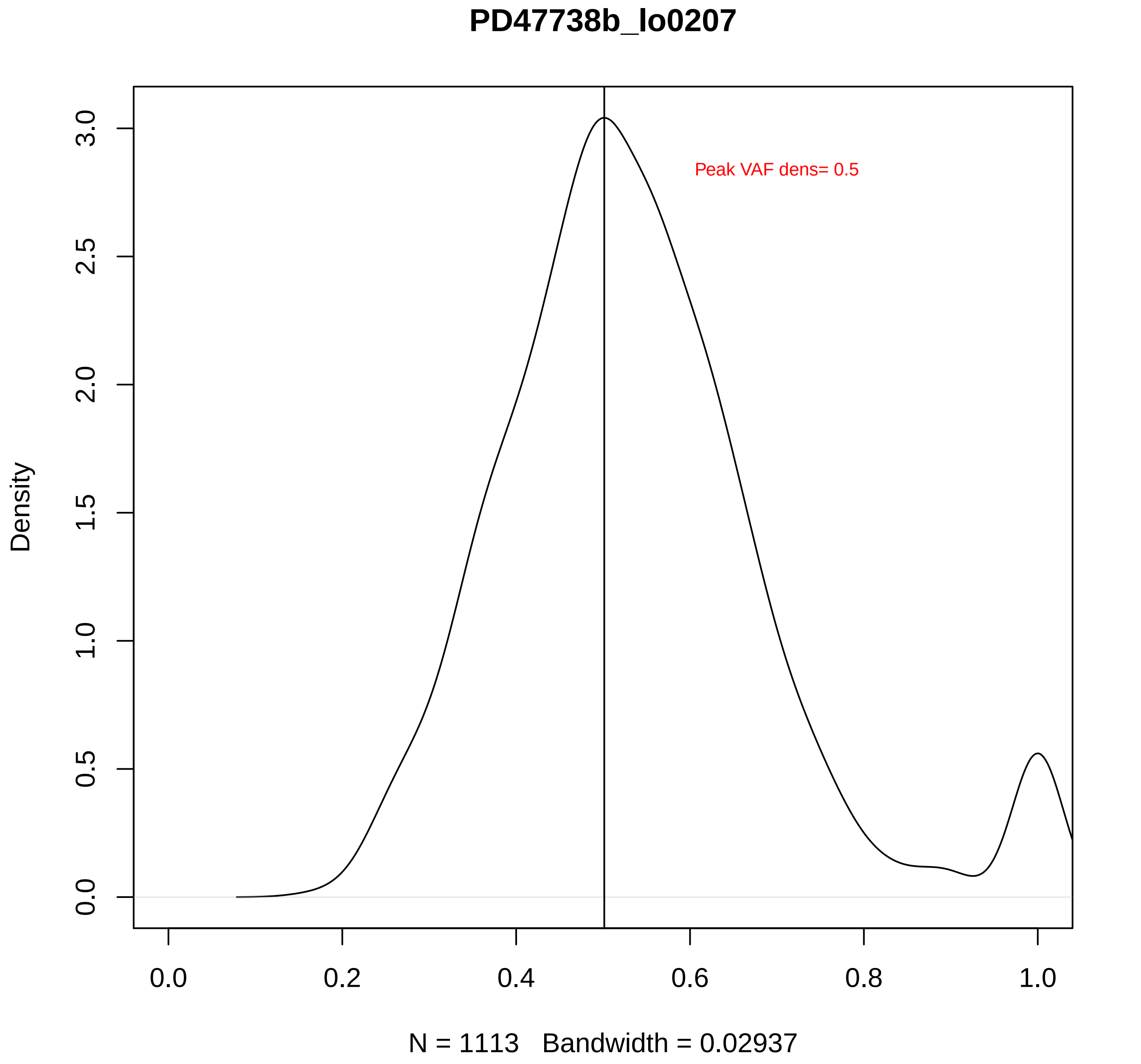

PD47738b_lo0207
Peak VAF dens= 0.5
Density
0.0
0.5
1.0
1.5
2.0
2.5
3.0
0.0
0.2
0.4
0.6
0.8
1.0
N = 1113   Bandwidth = 0.02937

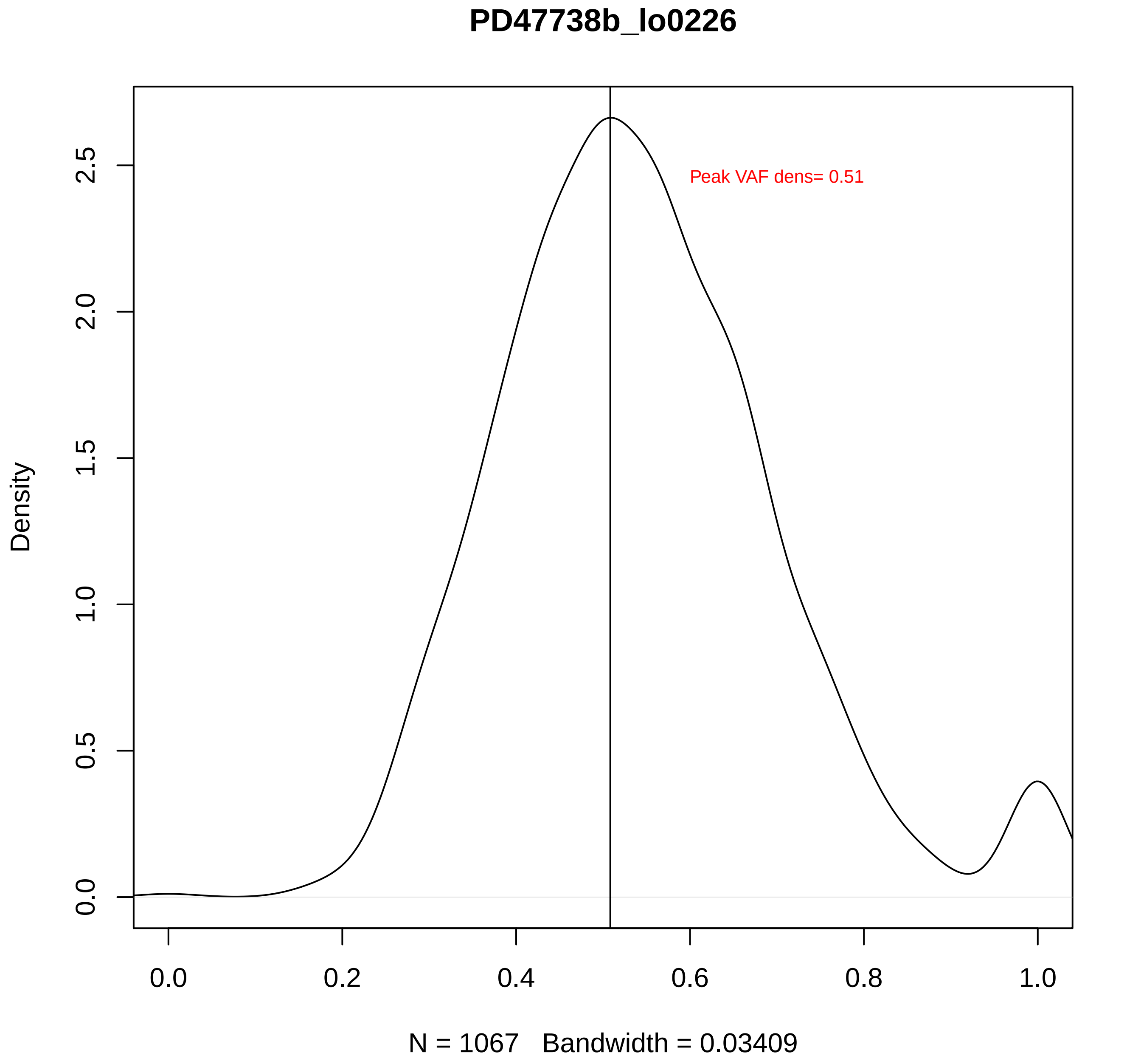
PD47738b_lo0226
Peak VAF dens= 0.51
Density
0.0
0.5
1.0
1.5
2.0
2.5
0.0
0.2
0.4
0.6
0.8
1.0
N = 1067 Bandwidth = 0.03409

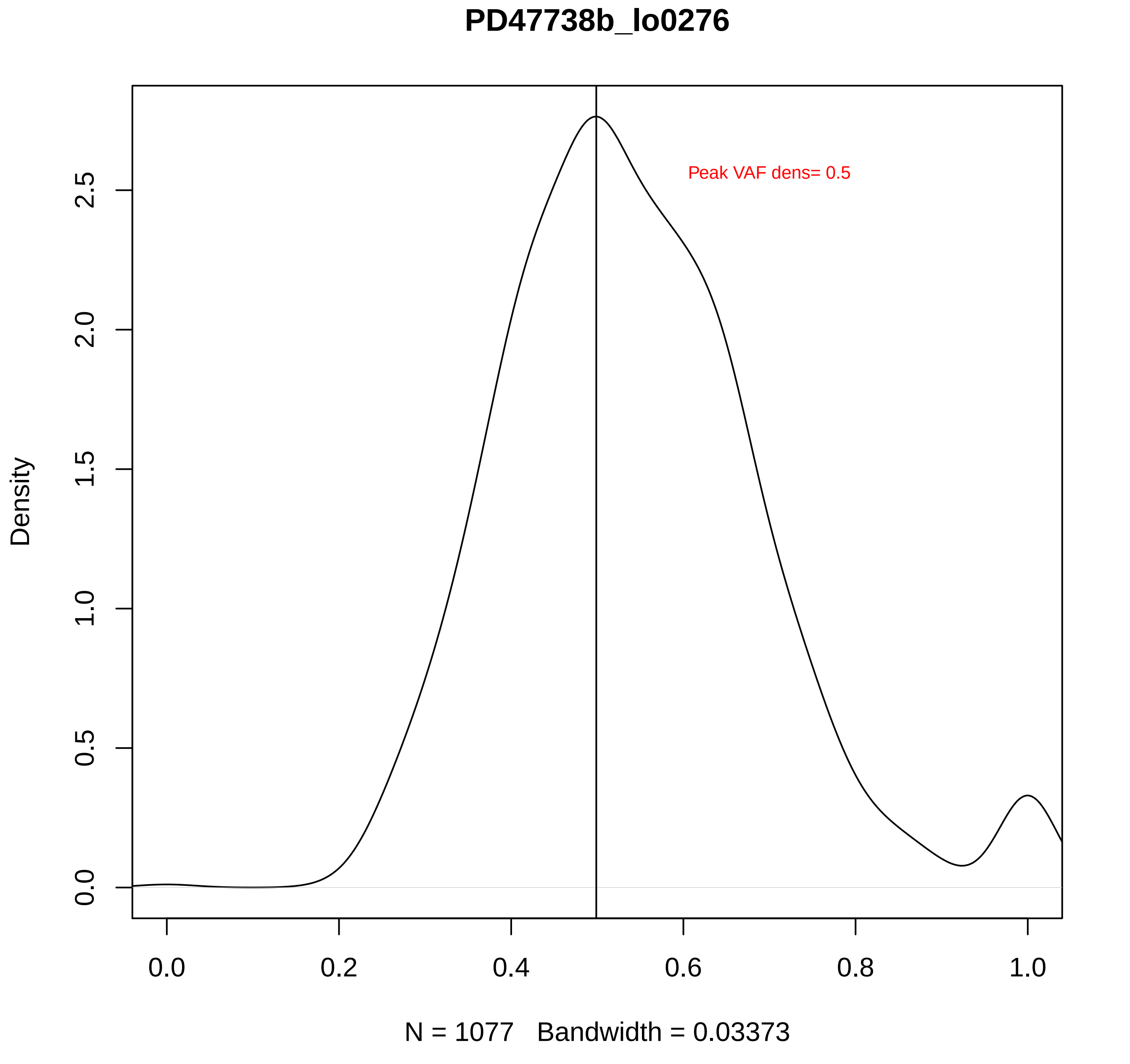
PD47738b_lo0276
Peak VAF dens= 0.5
Density
0.0
0.5
1.0
1.5
2.0
2.5
0.0
0.2
0.4
0.6
0.8
1.0
N = 1077   Bandwidth = 0.03373

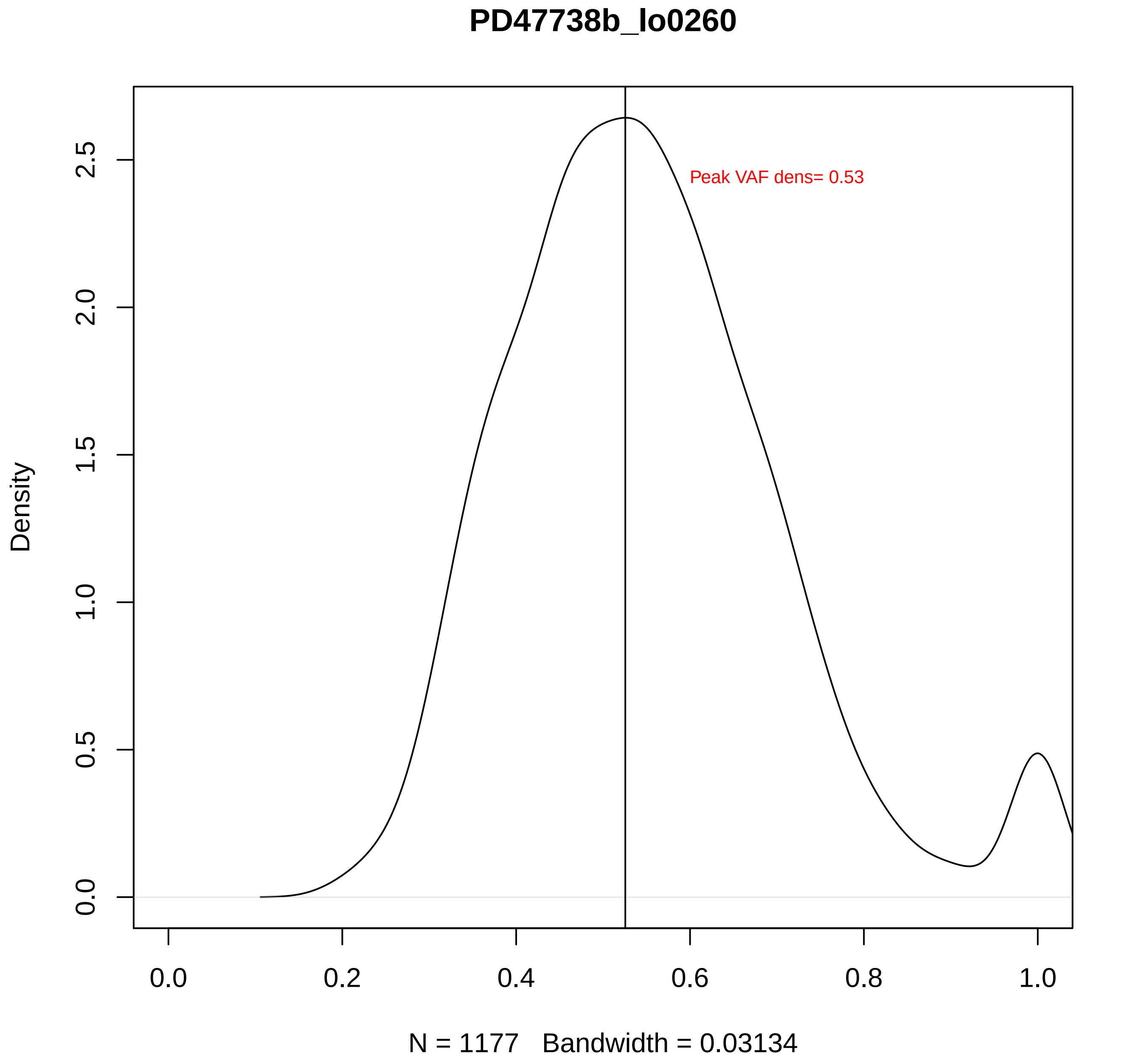
PD47738b_lo0260
Peak VAF dens= 0.53
Density
0.0
0.5
1.0
1.5
2.0
2.5
0.0
0.2
0.4
0.6
0.8
1.0
N = 1177 Bandwidth = 0.03134

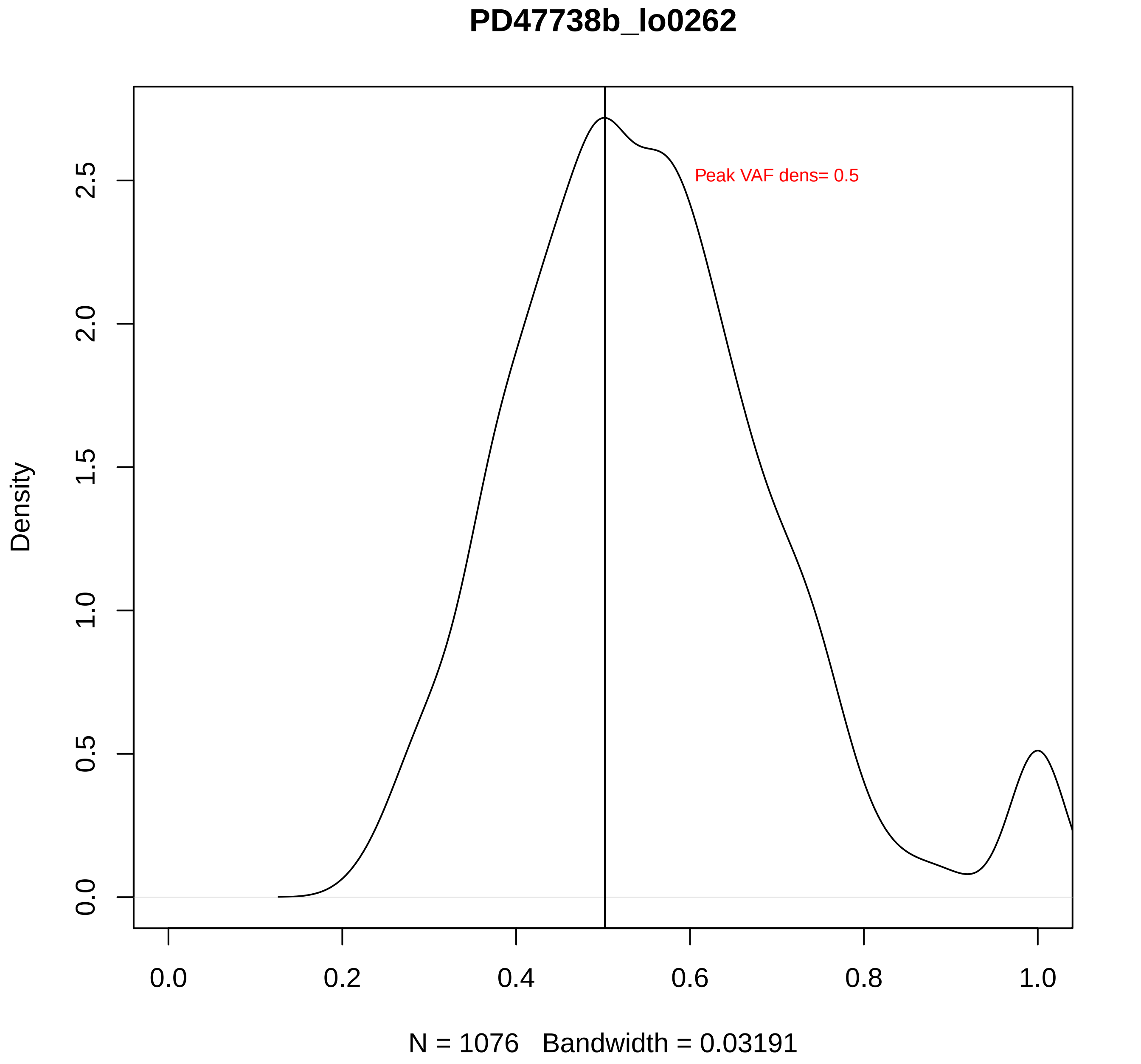

PD47738b_lo0262
Peak VAF dens= 0.5
Density
0.0
0.5
1.0
1.5
2.0
2.5
0.0
0.2
0.4
0.6
0.8
1.0
N = 1076   Bandwidth = 0.03191

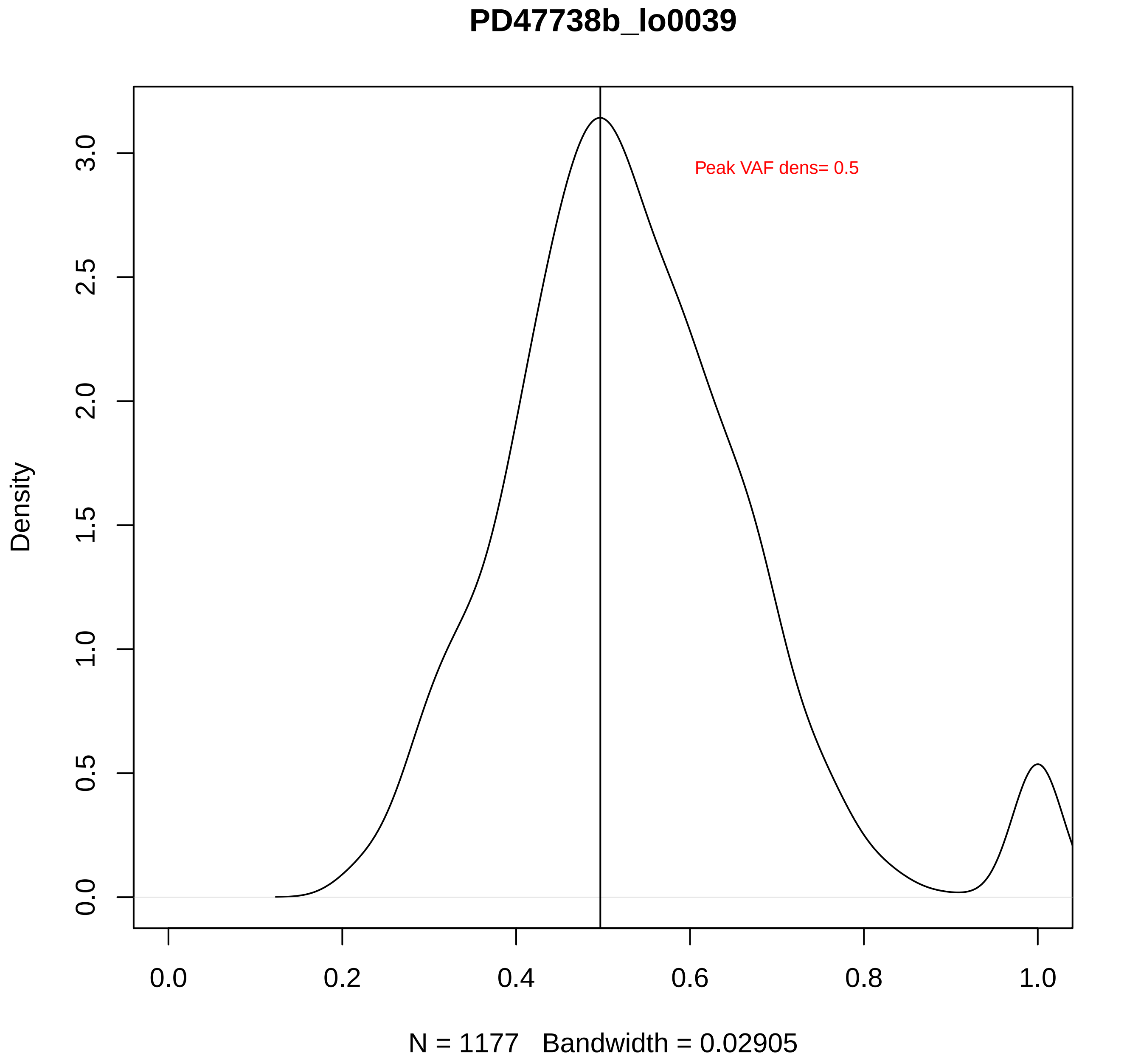
PD47738b_lo0039
Peak VAF dens= 0.5
Density
0.0
0.5
1.0
1.5
2.0
2.5
3.0
0.0
0.2
0.4
0.6
0.8
1.0
N = 1177   Bandwidth = 0.02905

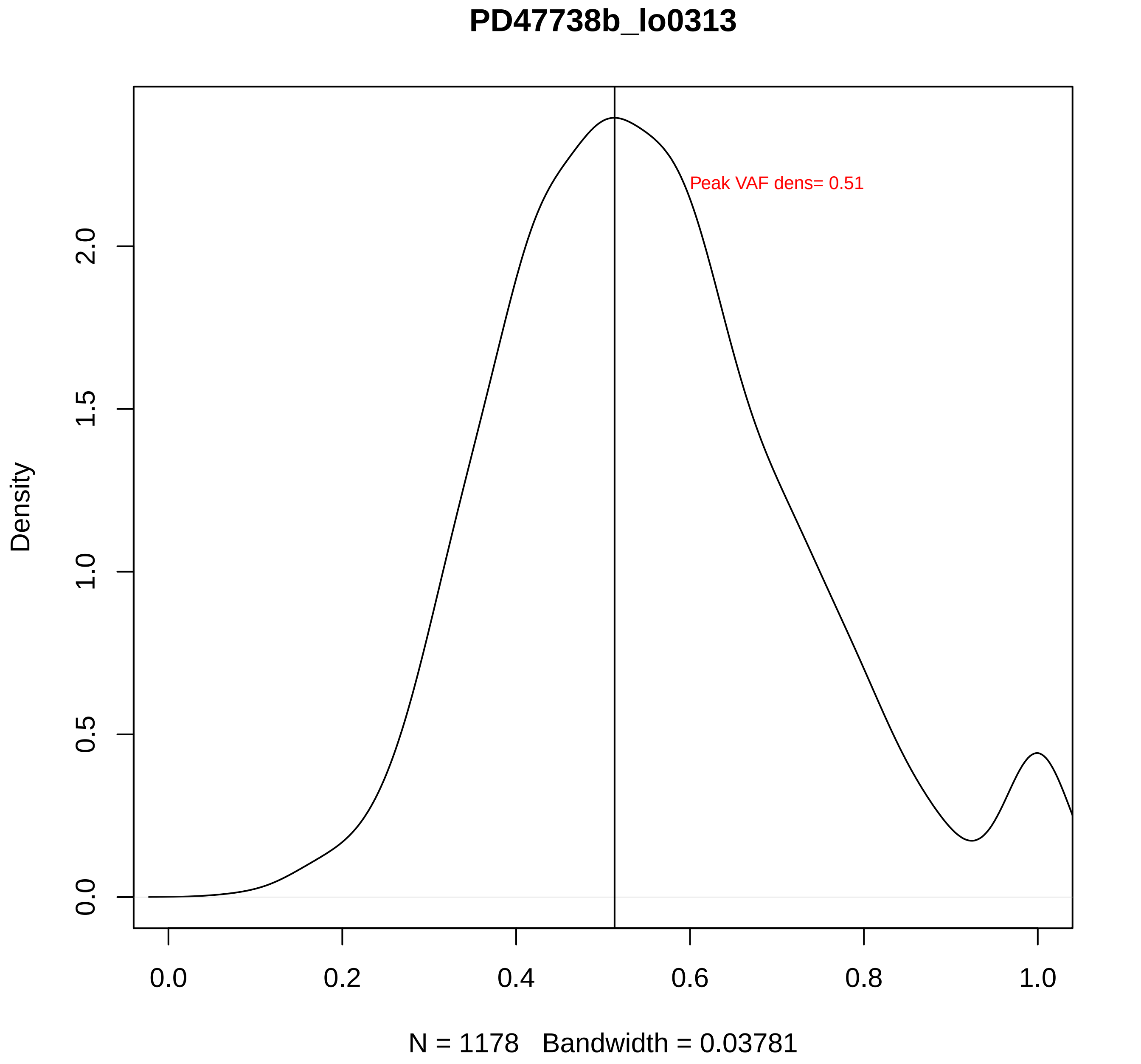
PD47738b_lo0313
Peak VAF dens= 0.51
Density
0.0
0.5
1.0
1.5
2.0
0.0
0.2
0.4
0.6
0.8
1.0
N = 1178   Bandwidth = 0.03781

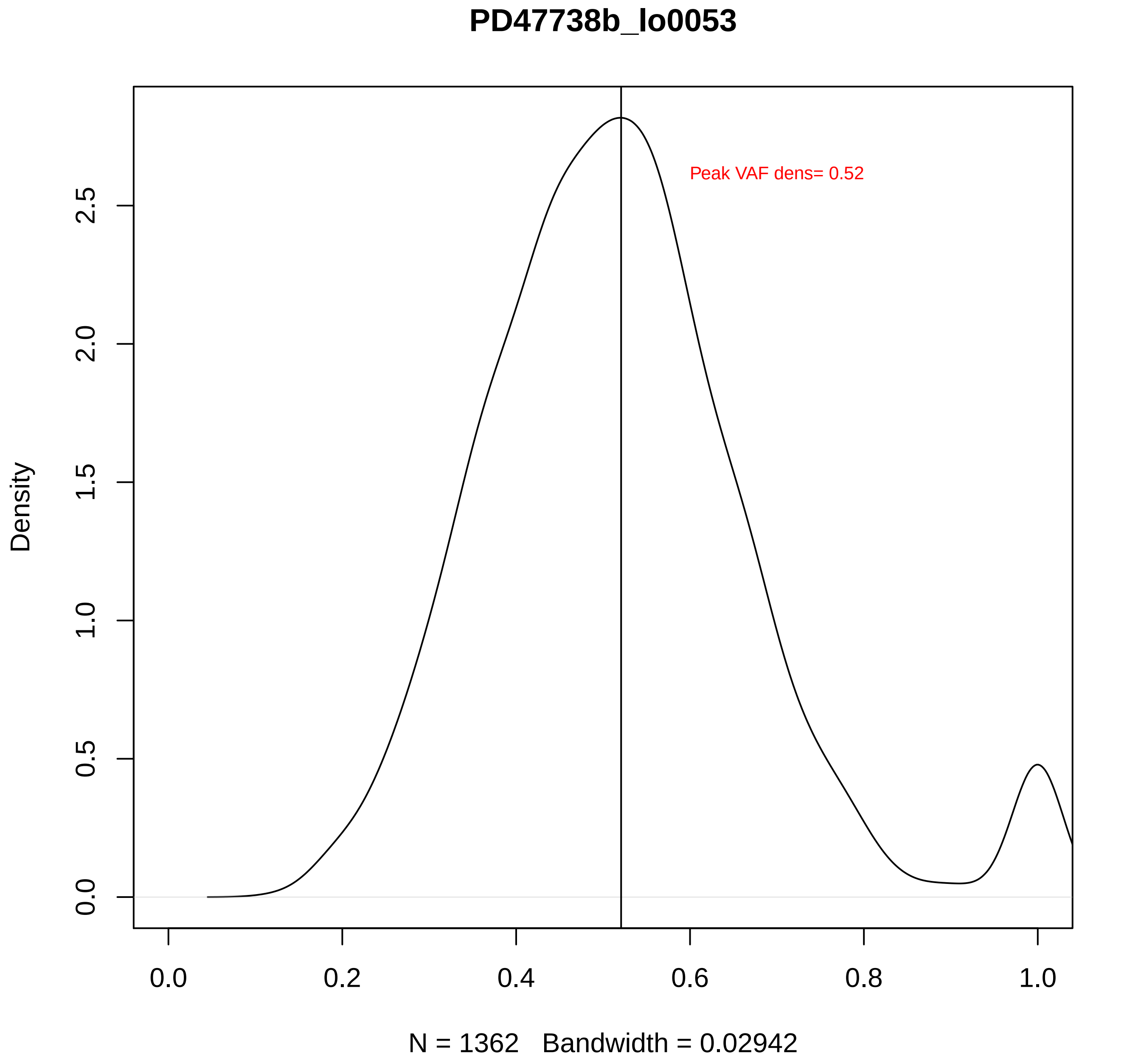
PD47738b_lo0053
Peak VAF dens= 0.52
Density
0.0
0.5
1.0
1.5
2.0
2.5
0.0
0.2
0.4
0.6
0.8
1.0
N = 1362   Bandwidth = 0.02942

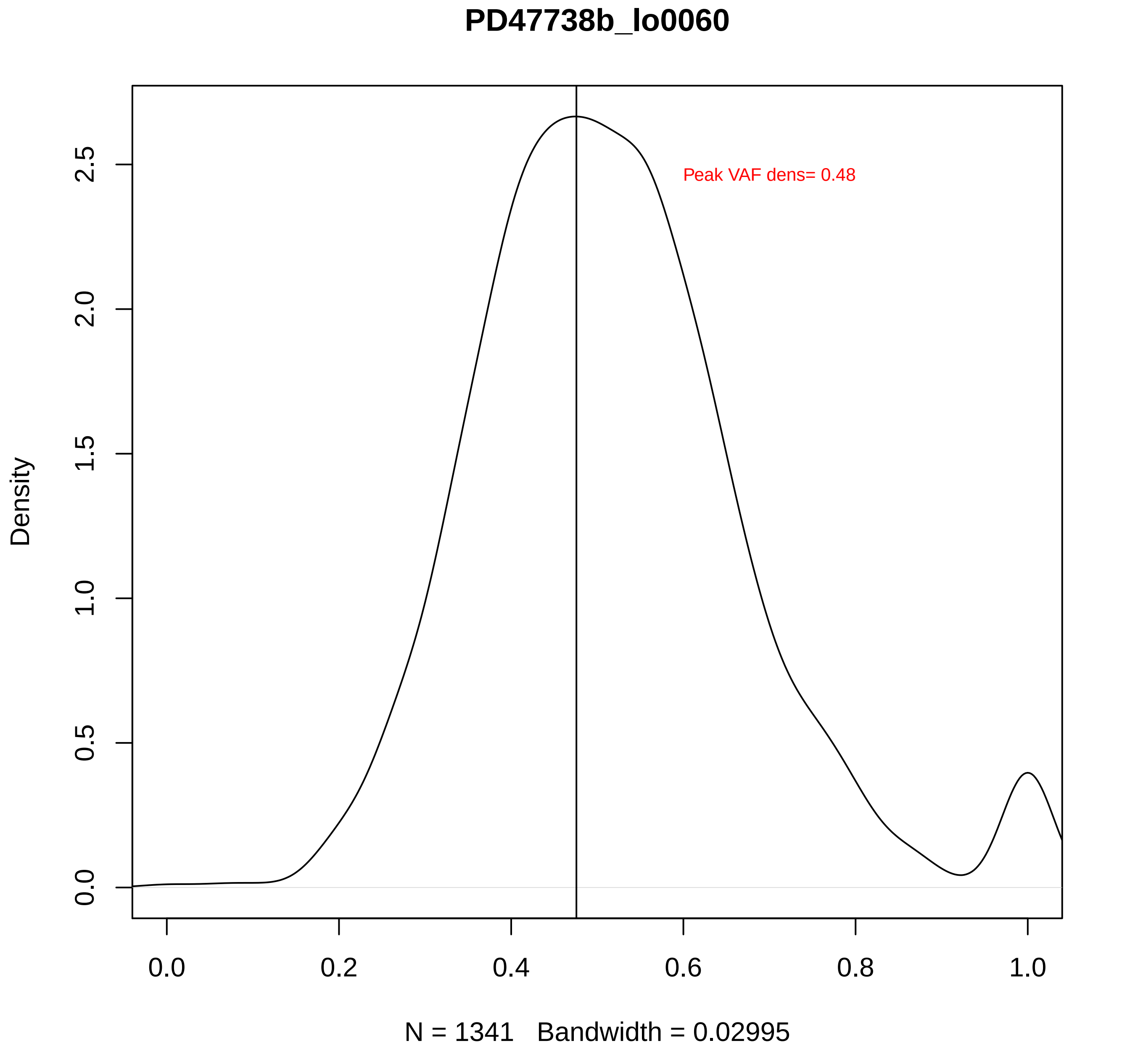

PD47738b_lo0060
Peak VAF dens= 0.48
Density
0.0
0.5
1.0
1.5
2.0
2.5
0.0
0.2
0.4
0.6
0.8
1.0
N = 1341   Bandwidth = 0.02995

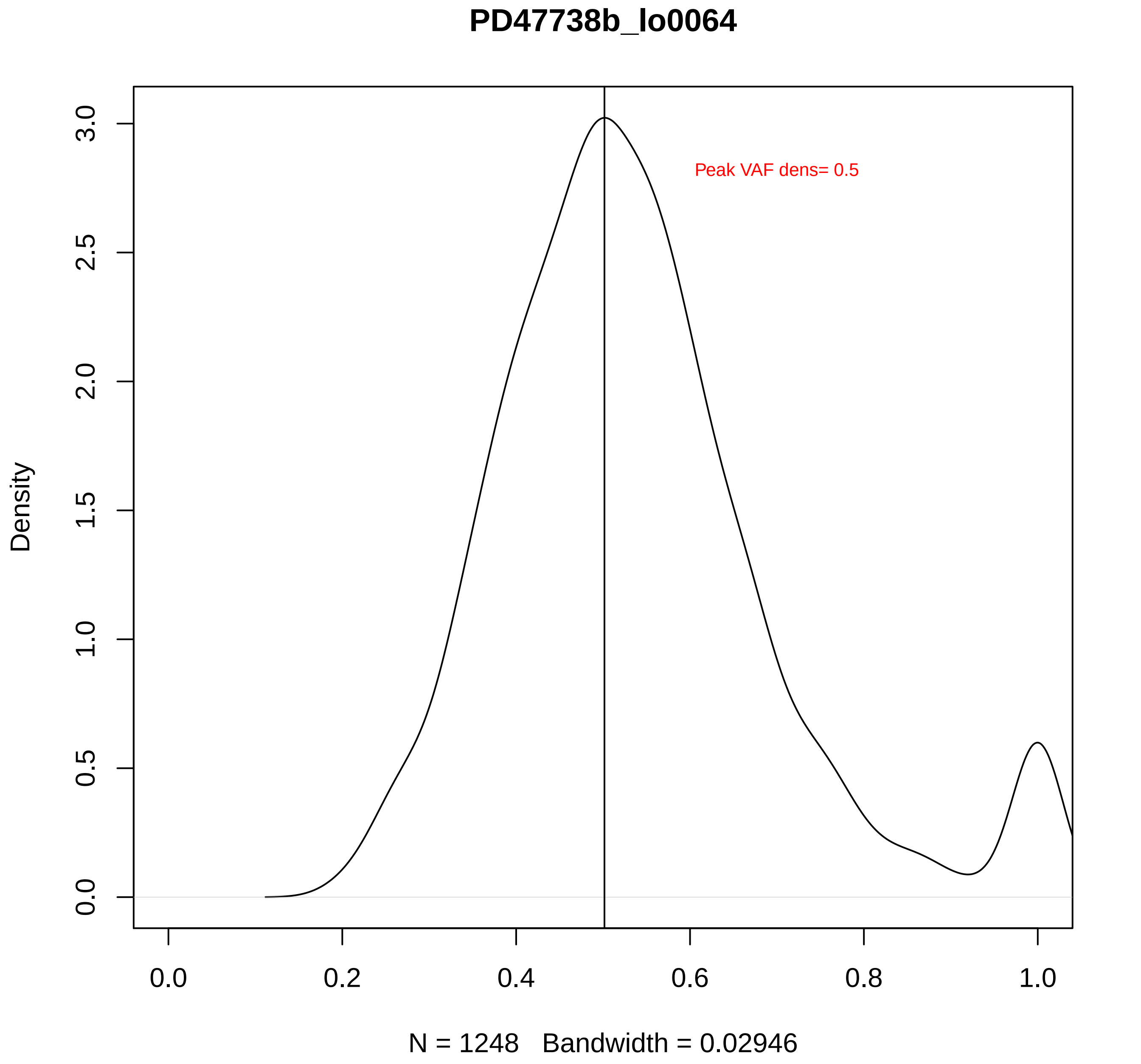
PD47738b_lo0064
Peak VAF dens= 0.5
Density
0.0
0.5
1.0
1.5
2.0
2.5
3.0
0.0
0.2
0.4
0.6
0.8
1.0
N = 1248   Bandwidth = 0.02946

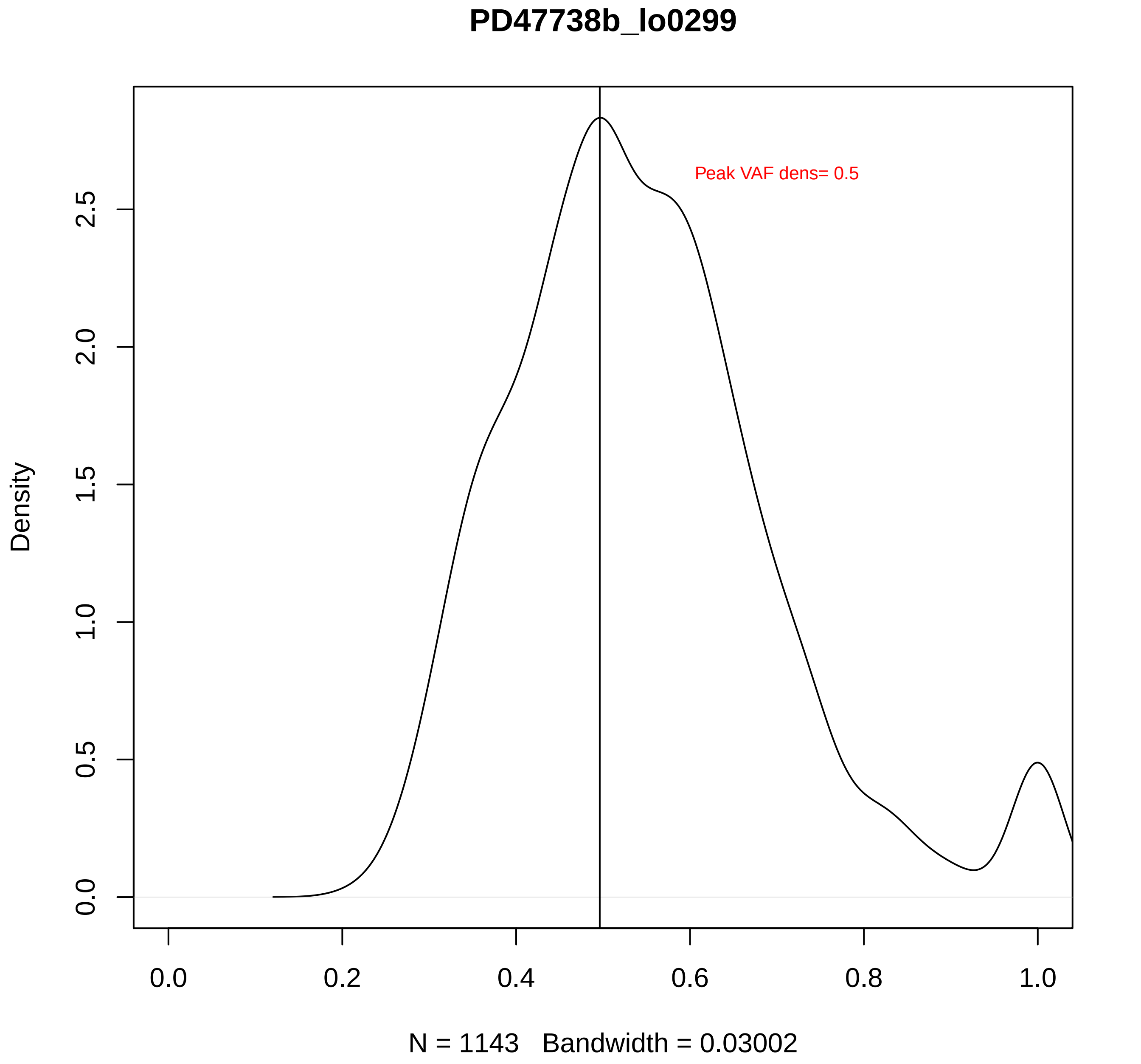
PD47738b_lo0299
Peak VAF dens= 0.5
Density
0.0
0.5
1.0
1.5
2.0
2.5
0.0
0.2
0.4
0.6
0.8
1.0
N = 1143   Bandwidth = 0.03002

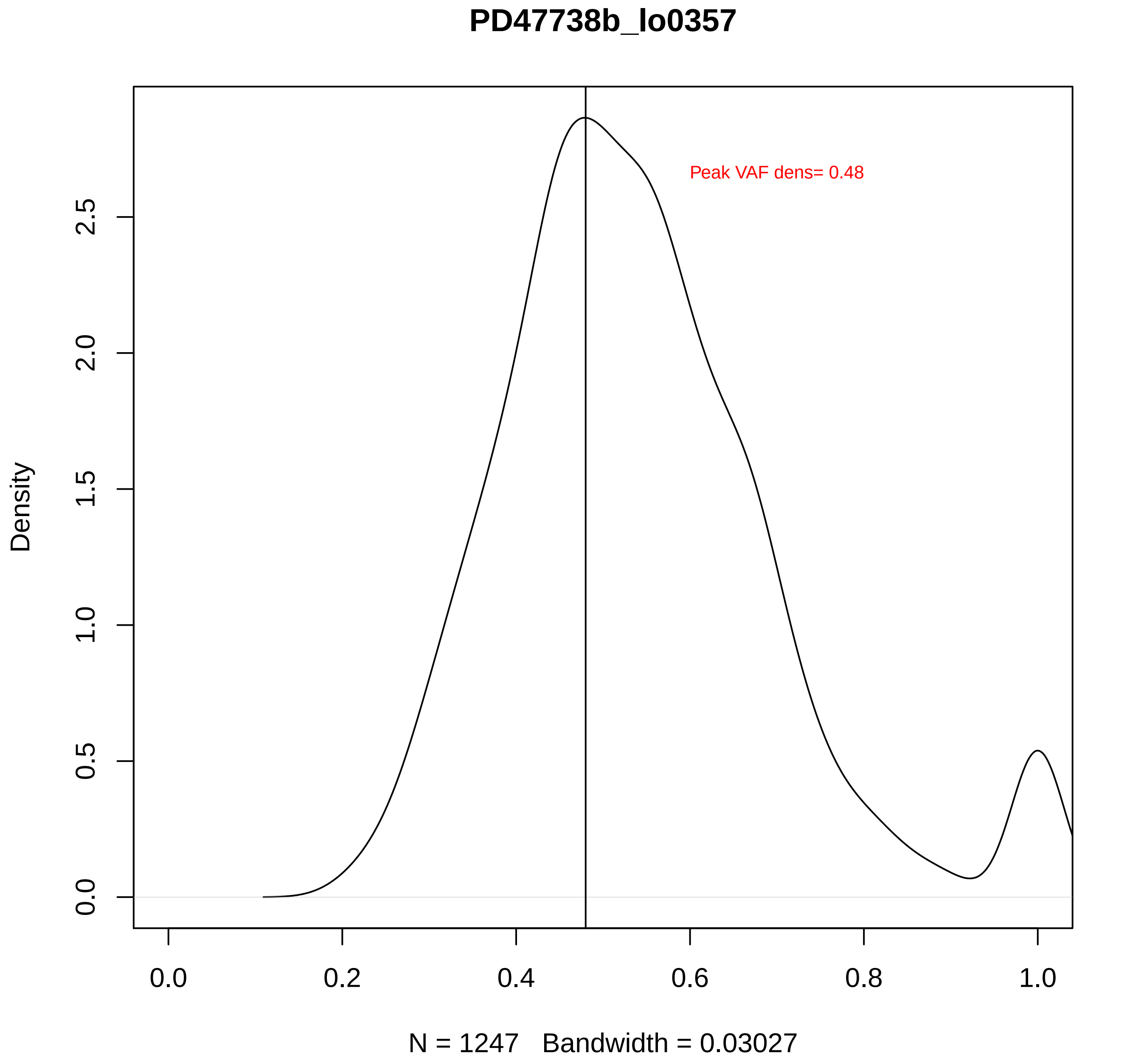
PD47738b_lo0357
Peak VAF dens= 0.48
Density
0.0
0.5
1.0
1.5
2.0
2.5
0.0
0.2
0.4
0.6
0.8
1.0
N = 1247 Bandwidth = 0.03027

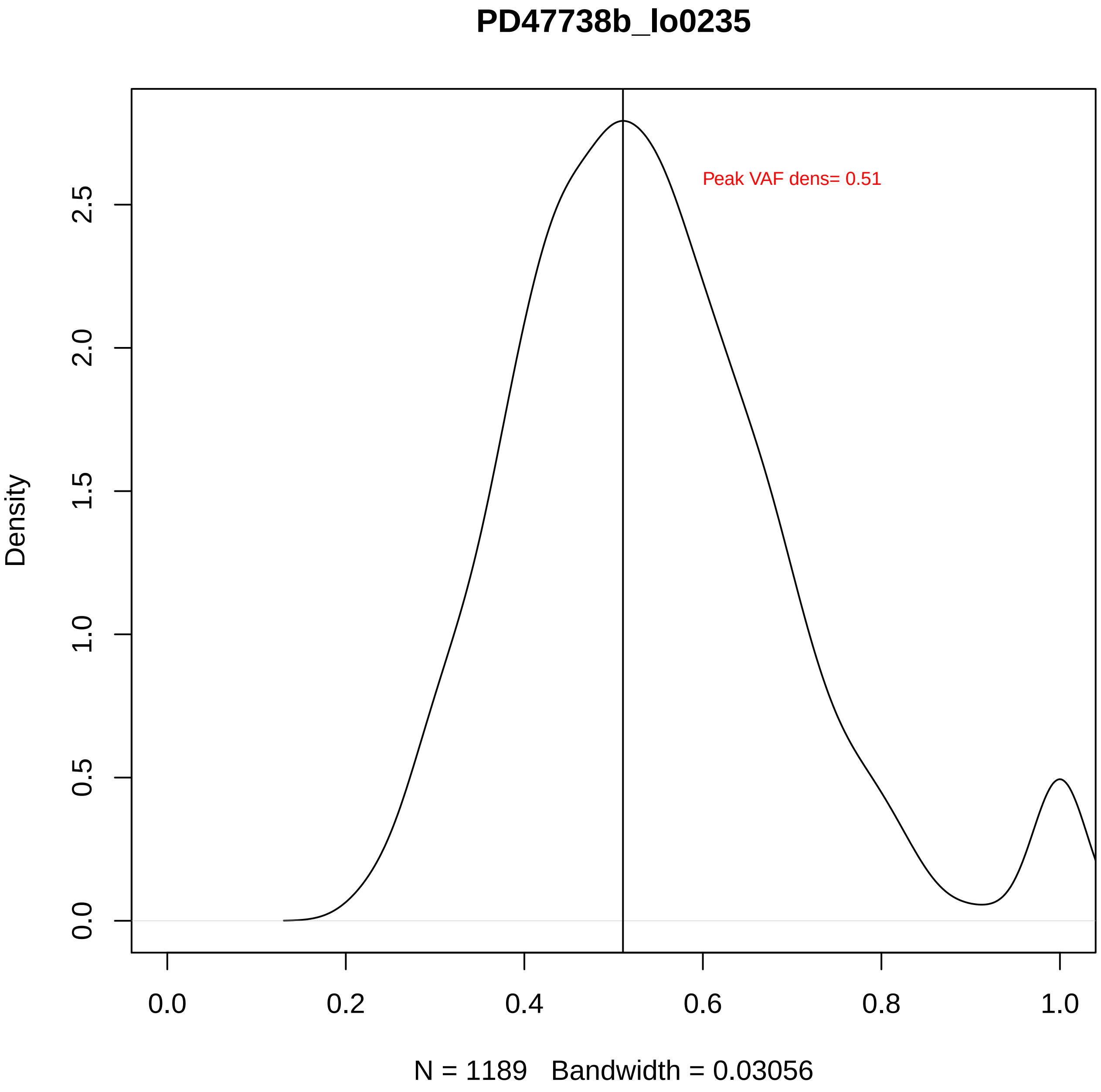

PD47738b_lo0235
Peak VAF dens= 0.51
Density
0.0
0.5
1.0
1.5
2.0
2.5
0.0
0.2
0.4
0.6
0.8
1.0
N = 1189 Bandwidth = 0.03056

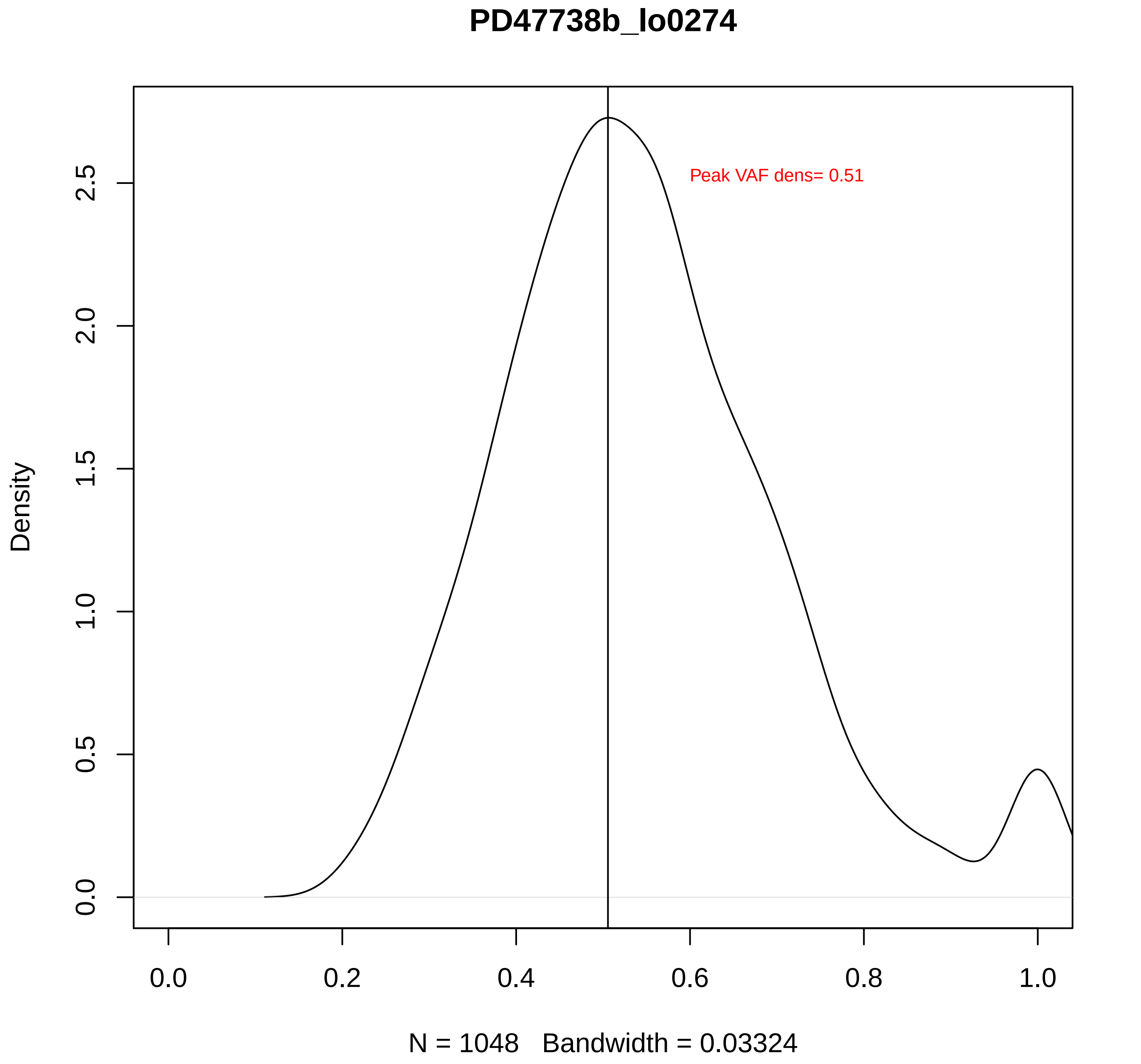
PD47738b_lo0274
Peak VAF dens= 0.51
Density
0.0
0.5
1.0
1.5
2.0
2.5
0.0
0.2
0.4
0.6
0.8
1.0
N = 1048   Bandwidth = 0.03324

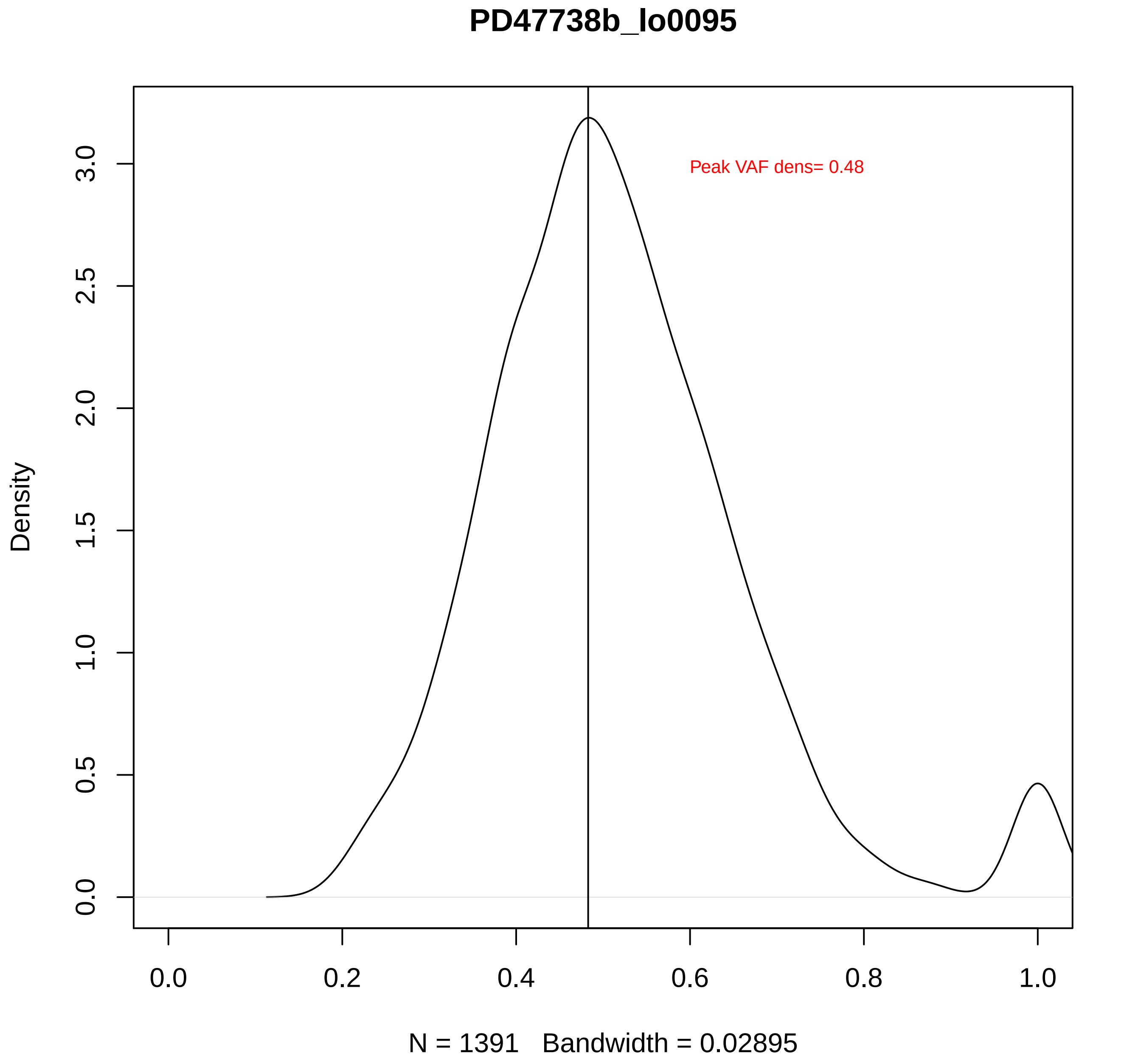
PD47738b_lo0095
Density
Peak VAF dens= 0.48
0.0
0.5
1.0
1.5
2.0
2.5
3.0
0.0
0.2
0.4
0.6
0.8
1.0
N = 1391   Bandwidth = 0.02895

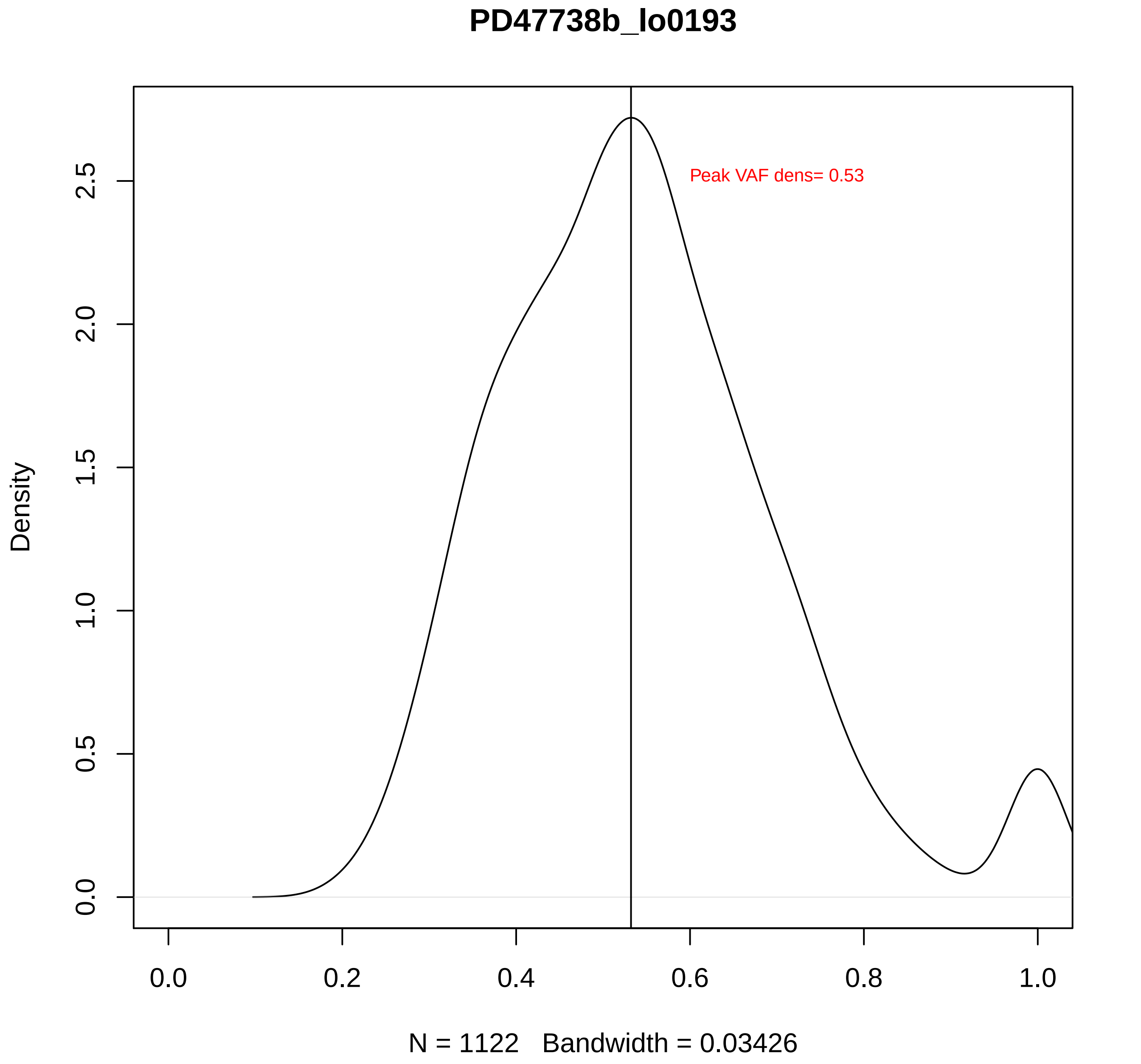
PD47738b_lo0193
Peak VAF dens= 0.53
Density
0.0
0.5
1.0
1.5
2.0
2.5
0.0
0.2
0.4
0.6
0.8
1.0
N = 1122   Bandwidth = 0.03426

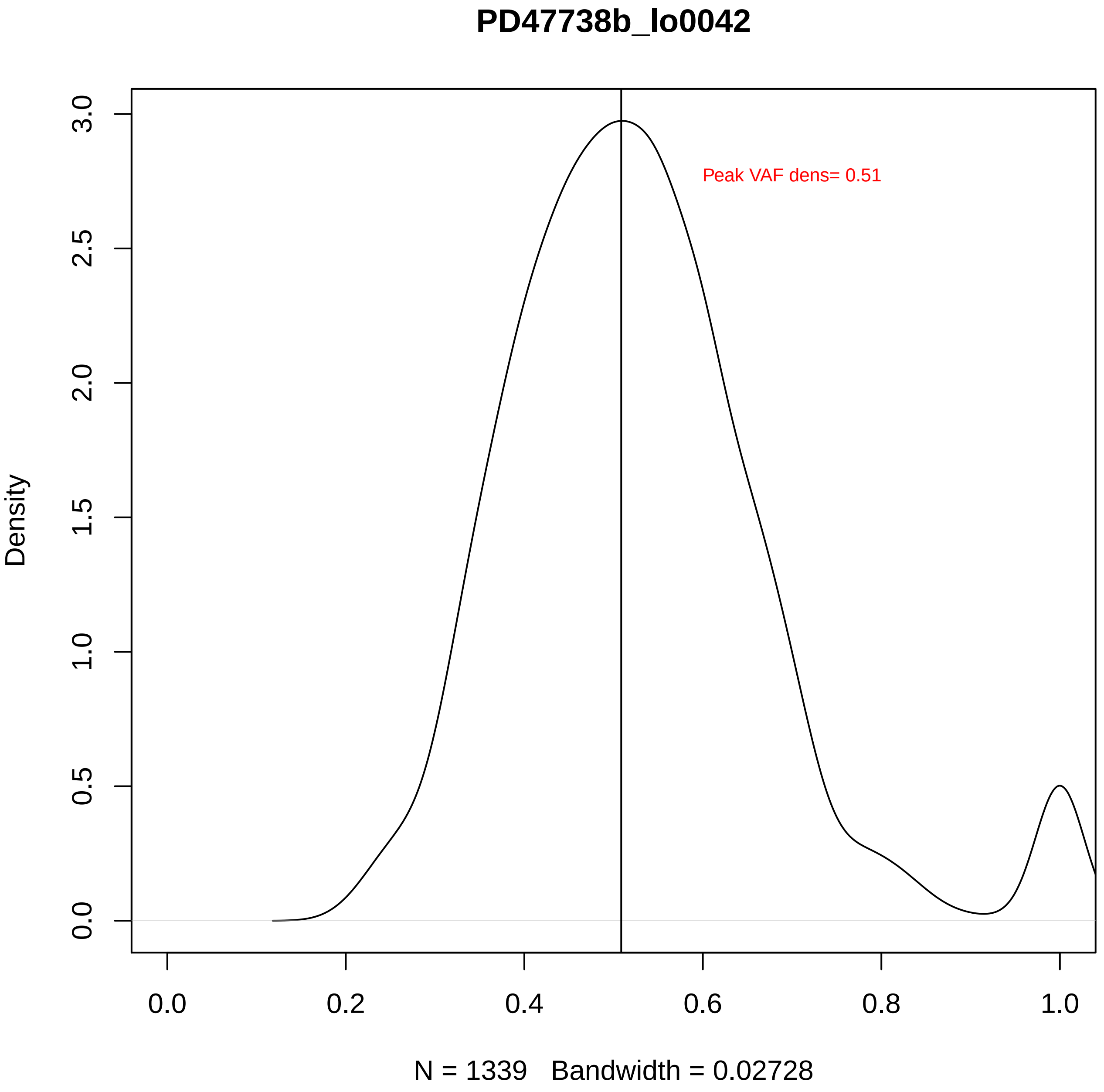
PD47738b_lo0042
Peak VAF dens= 0.51
Density
0.0
0.5
1.0
1.5
2.0
2.5
3.0
0.0
0.2
0.4
0.6
0.8
1.0
N = 1339   Bandwidth = 0.02728

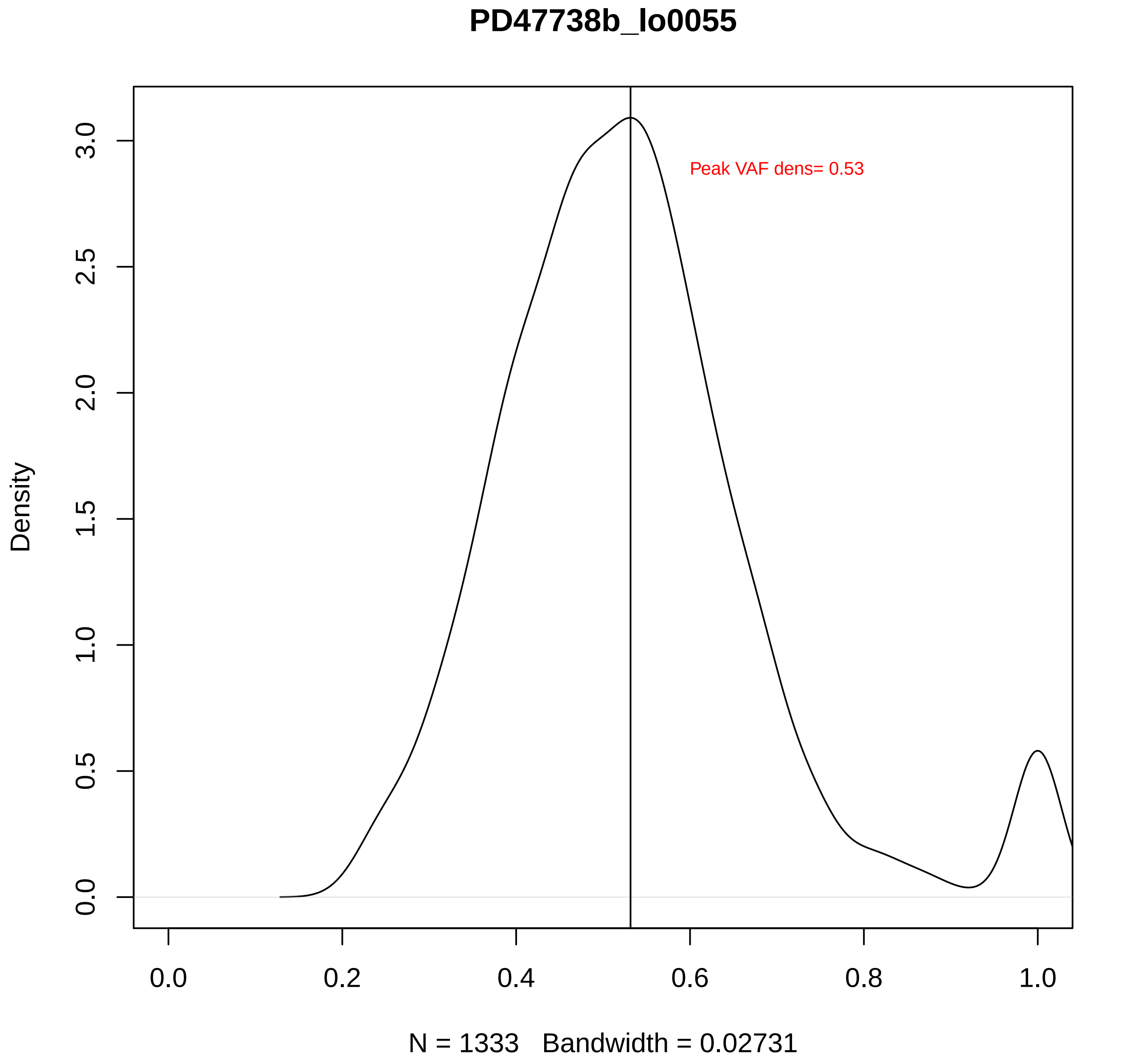
PD47738b_lo0055
Peak VAF dens= 0.53
Density
0.0
0.5
1.0
1.5
2.0
2.5
3.0
0.0
0.2
0.4
0.6
0.8
1.0
N = 1333   Bandwidth = 0.02731

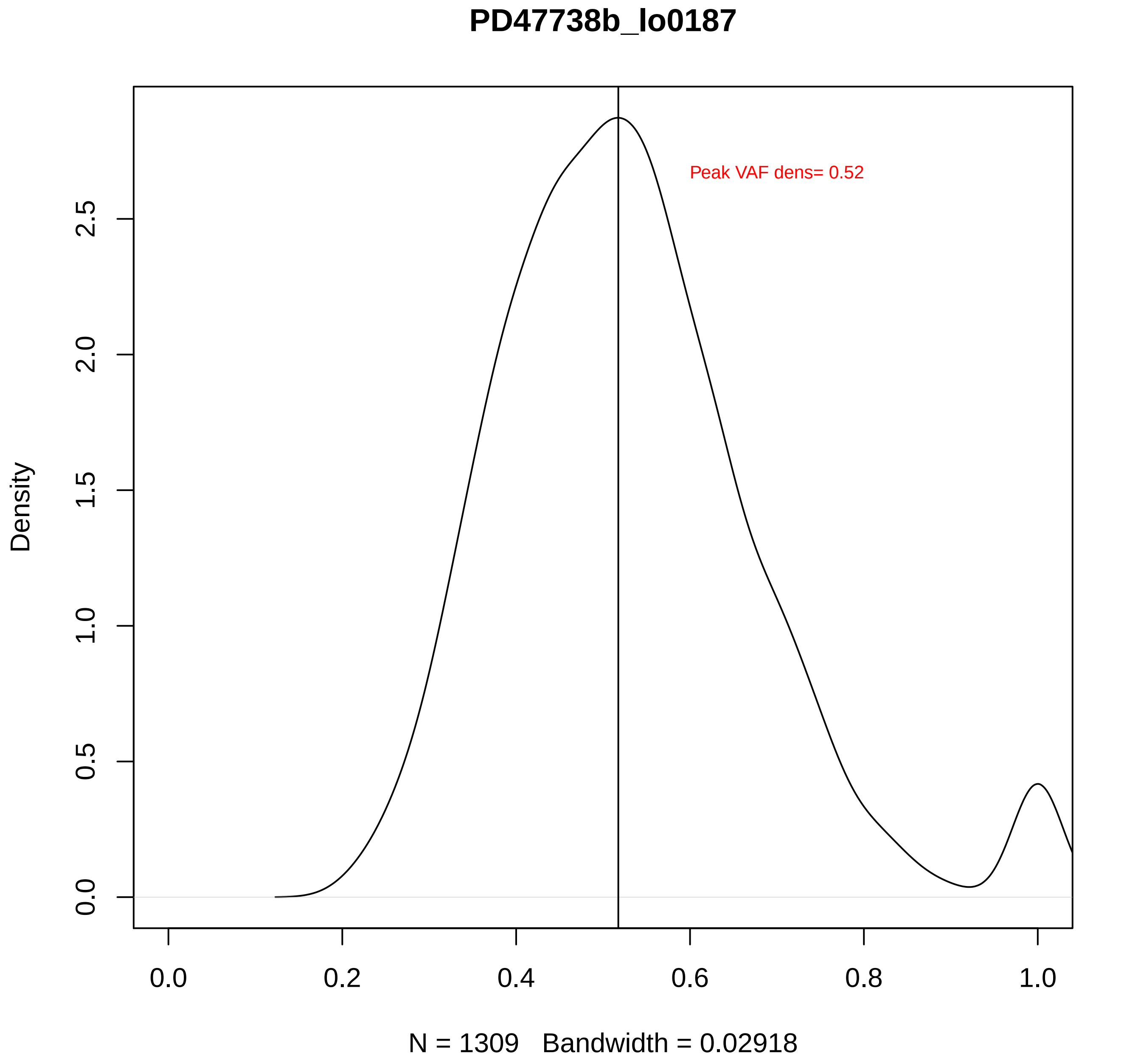
PD47738b_lo0187
Peak VAF dens= 0.52
Density
0.0
0.5
1.0
1.5
2.0
2.5
0.0
0.2
0.4
0.6
0.8
1.0
N = 1309   Bandwidth = 0.02918

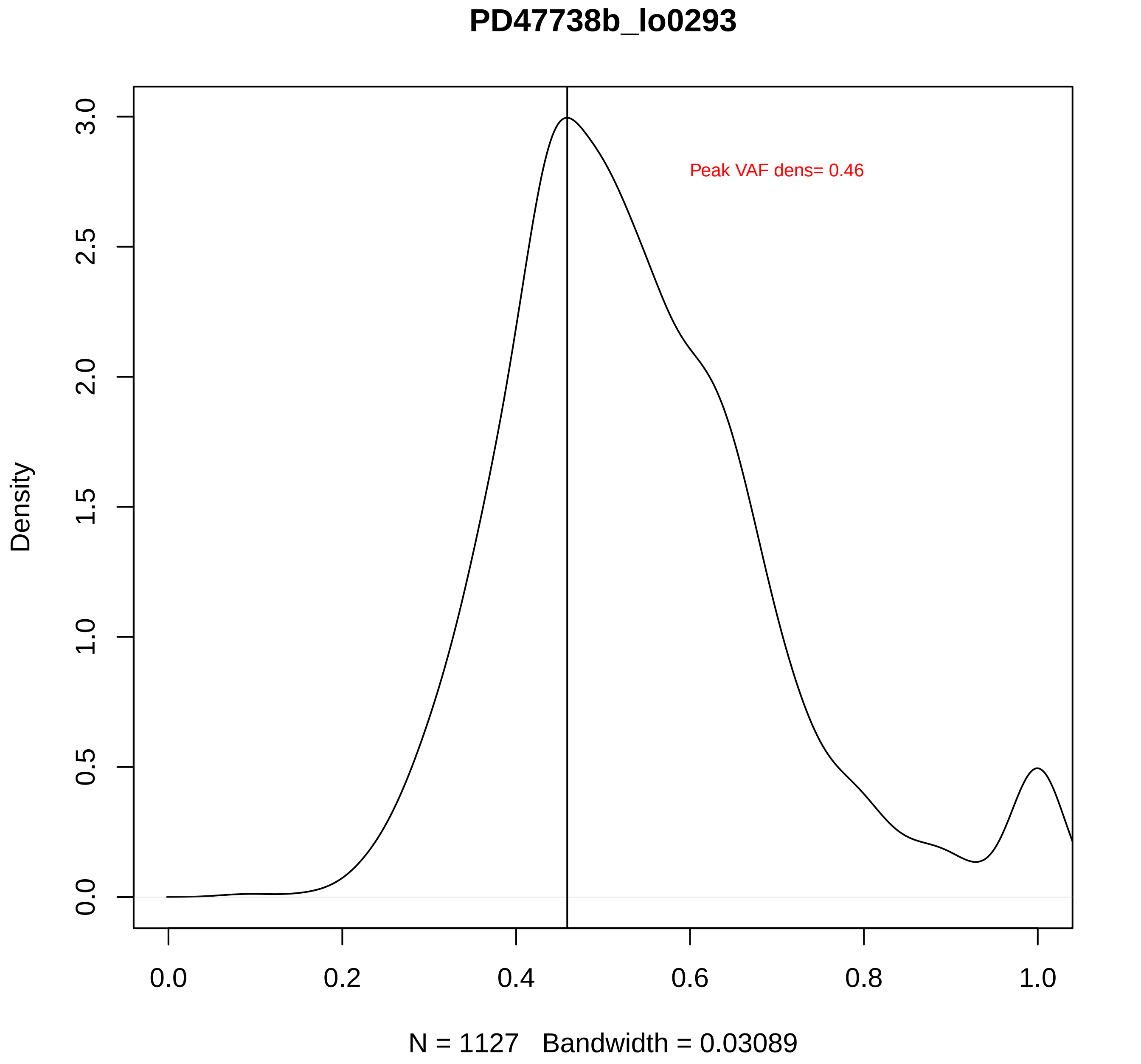
PD47738b_lo0293
Peak VAF dens= 0.46
Density
0.0
0.5
1.0
1.5
2.0
2.5
3.0
0.0
0.2
0.4
0.6
0.8
1.0
N = 1127   Bandwidth = 0.03089

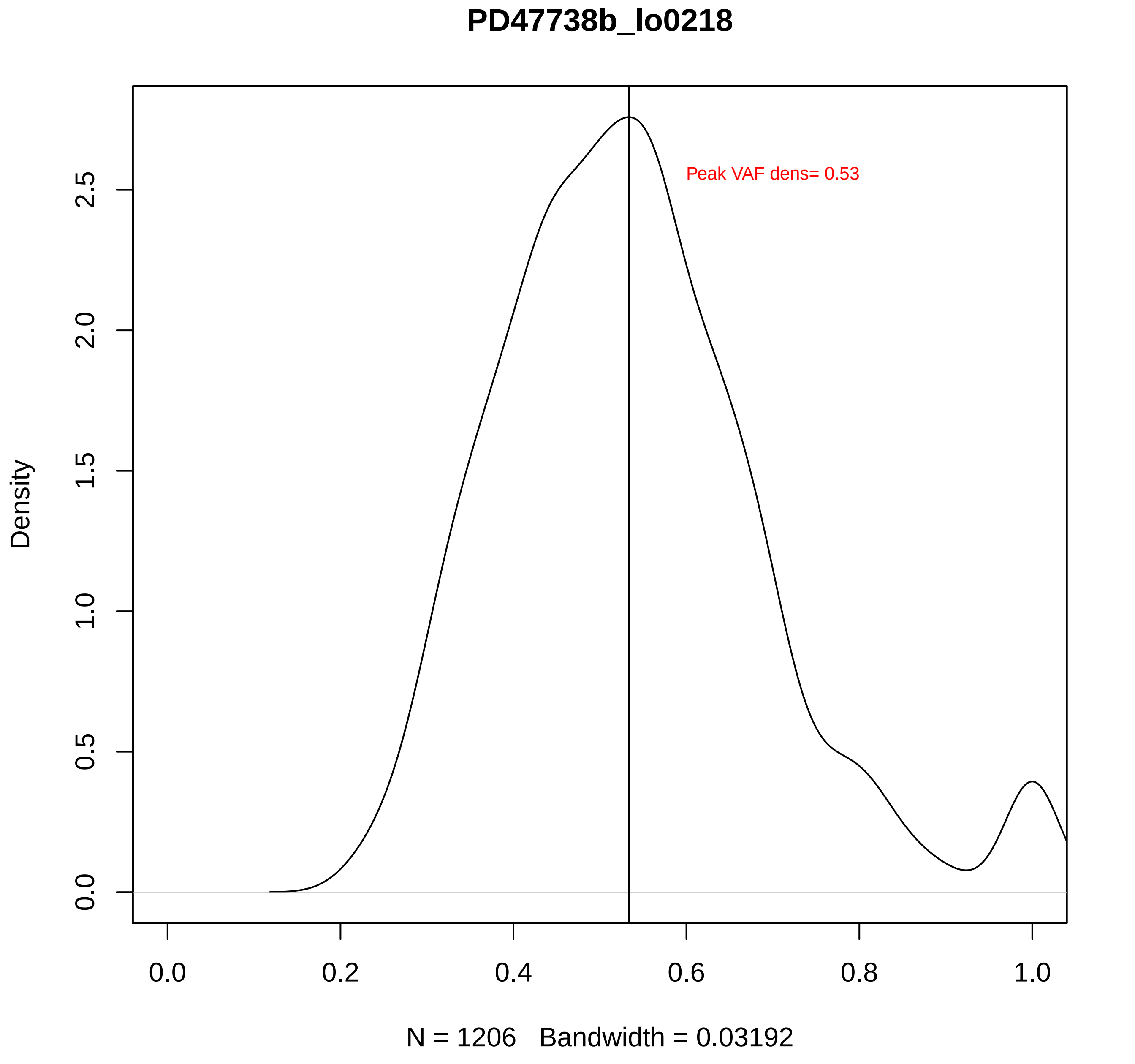
PD47738b_lo0218
Peak VAF dens= 0.53
Density
0.0
0.5
1.0
1.5
2.0
2.5
0.0
0.2
0.4
0.6
0.8
1.0
N = 1206   Bandwidth = 0.03192

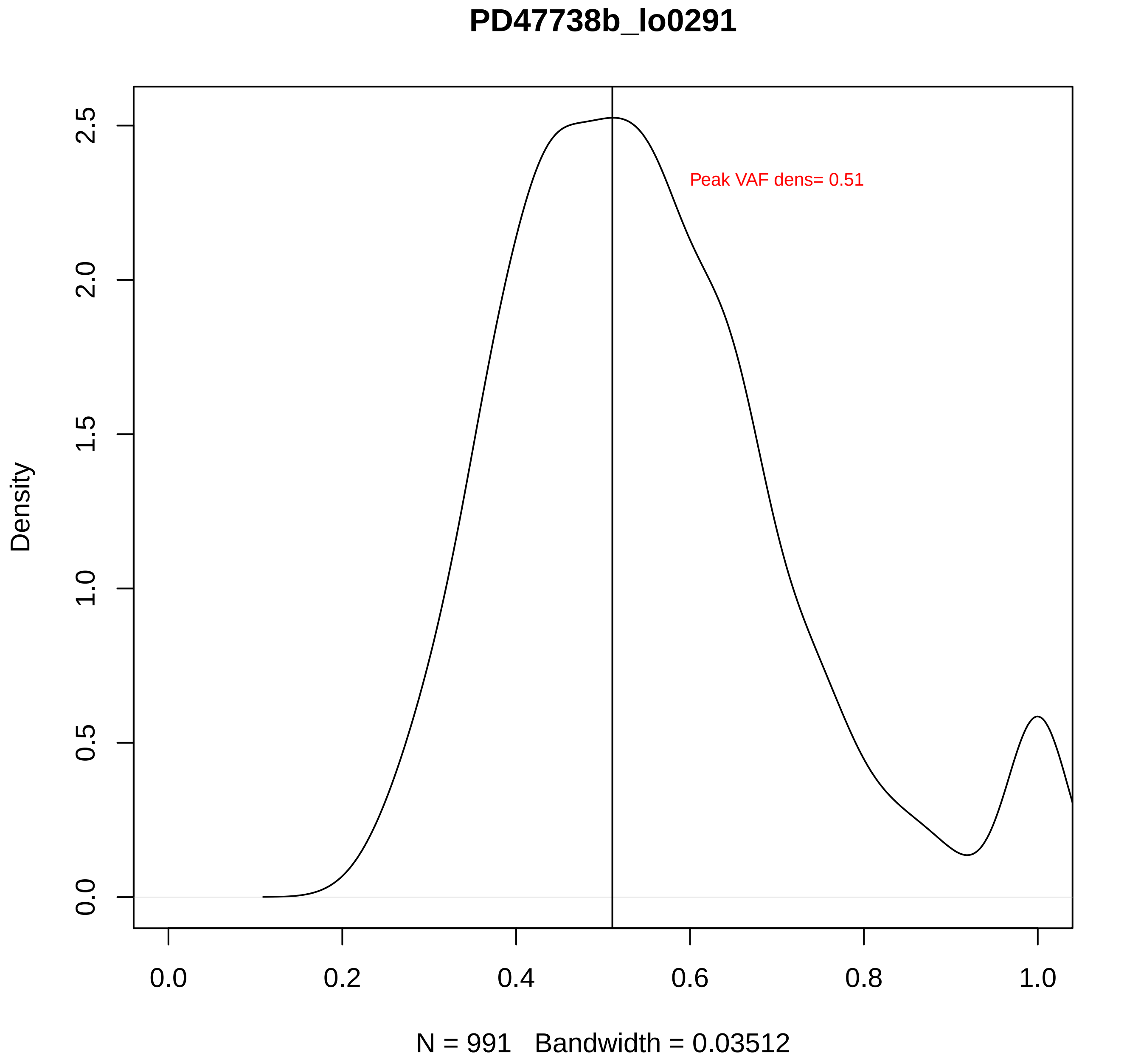
PD47738b_lo0291
Peak VAF dens= 0.51
Density
0.0
0.5
1.0
1.5
2.0
2.5
0.0
0.2
0.4
0.6
0.8
1.0
N = 991 Bandwidth = 0.03512

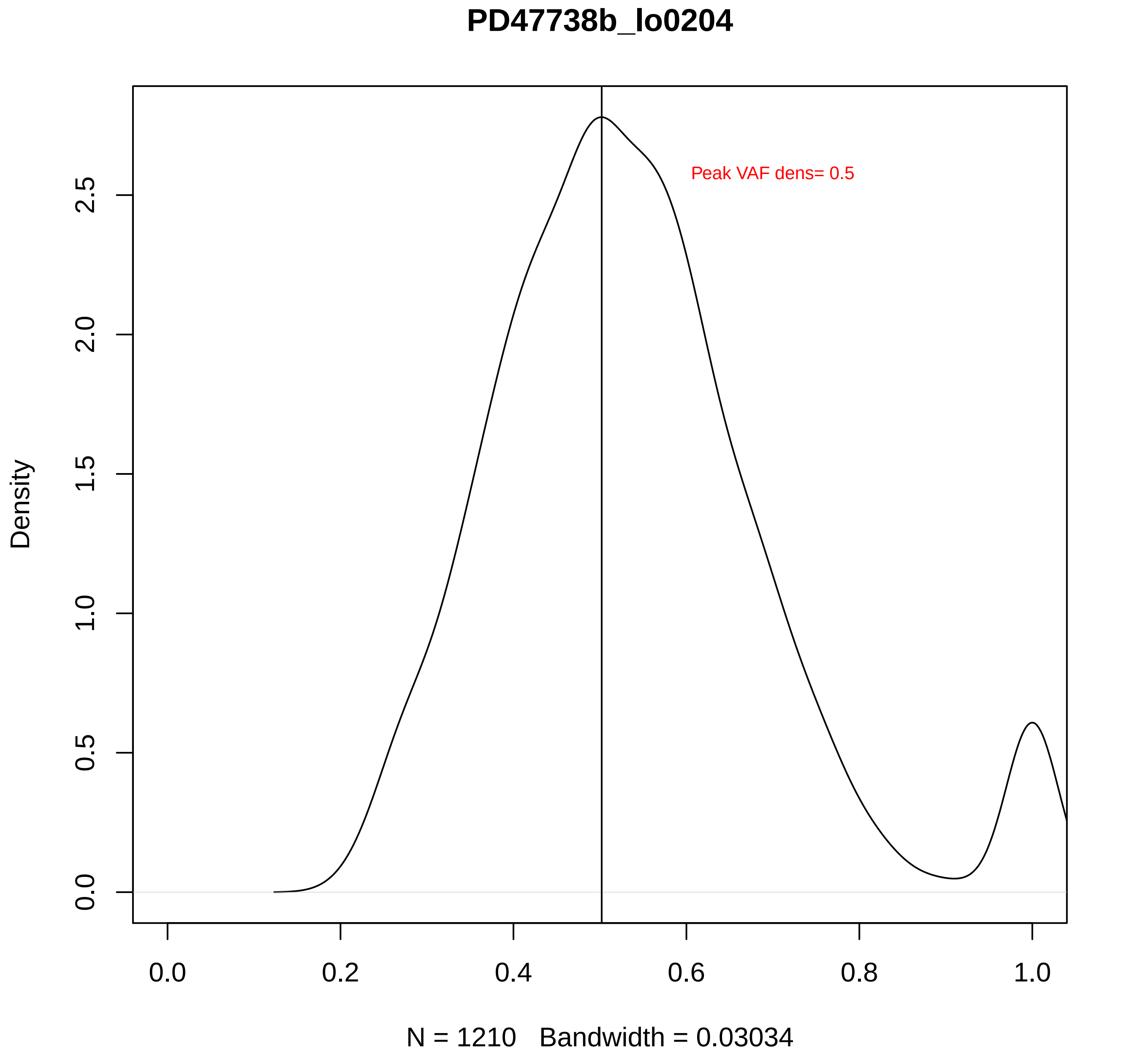

PD47738b_lo0204
Peak VAF dens= 0.5
Density
0.0
0.5
1.0
1.5
2.0
2.5
0.0
0.2
0.4
0.6
0.8
1.0
N = 1210 Bandwidth = 0.03034

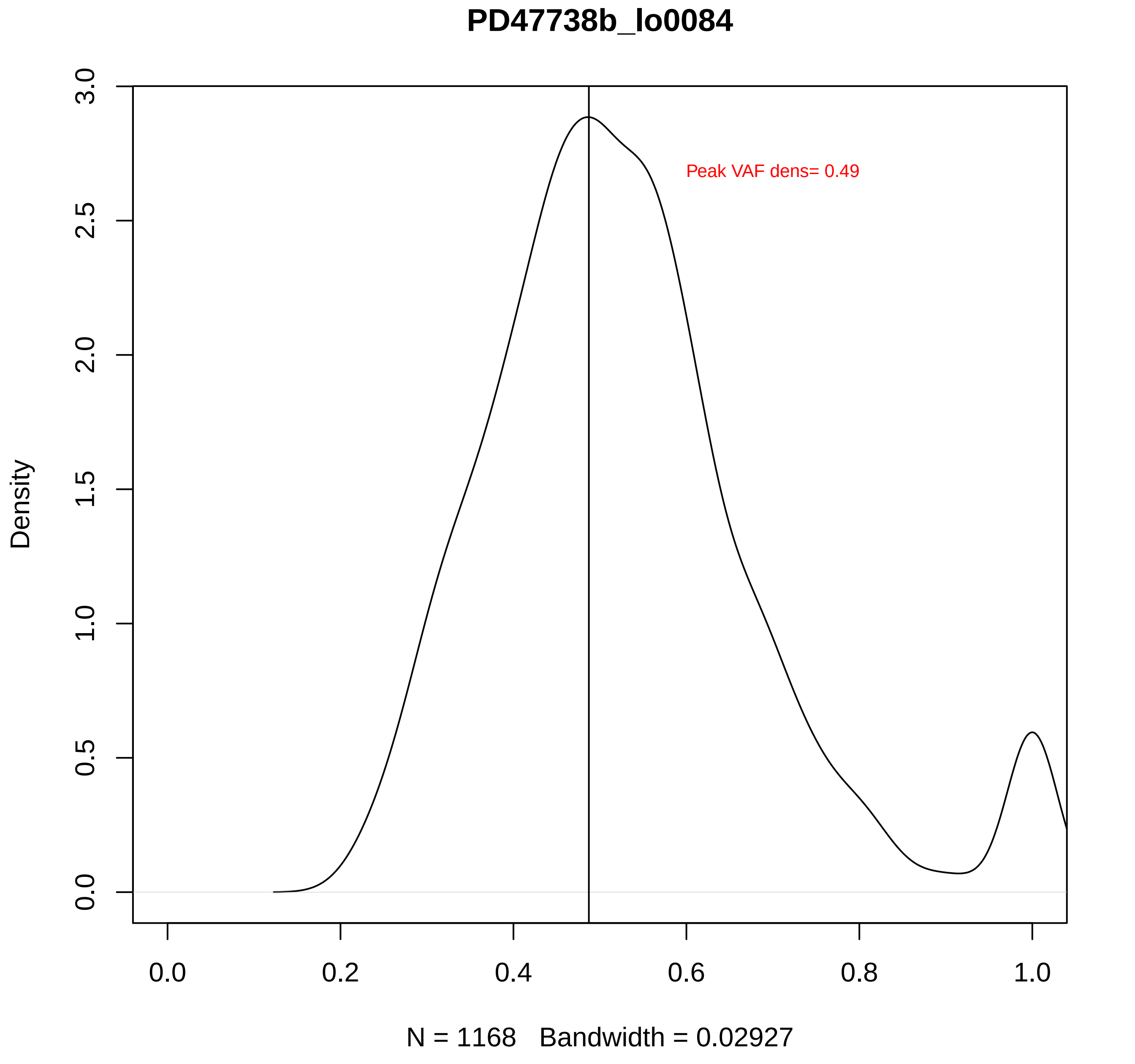
PD47738b_lo0084
Peak VAF dens= 0.49
Density
0.0
0.5
1.0
1.5
2.0
2.5
3.0
0.0
0.2
0.4
0.6
0.8
1.0
N = 1168   Bandwidth = 0.02927

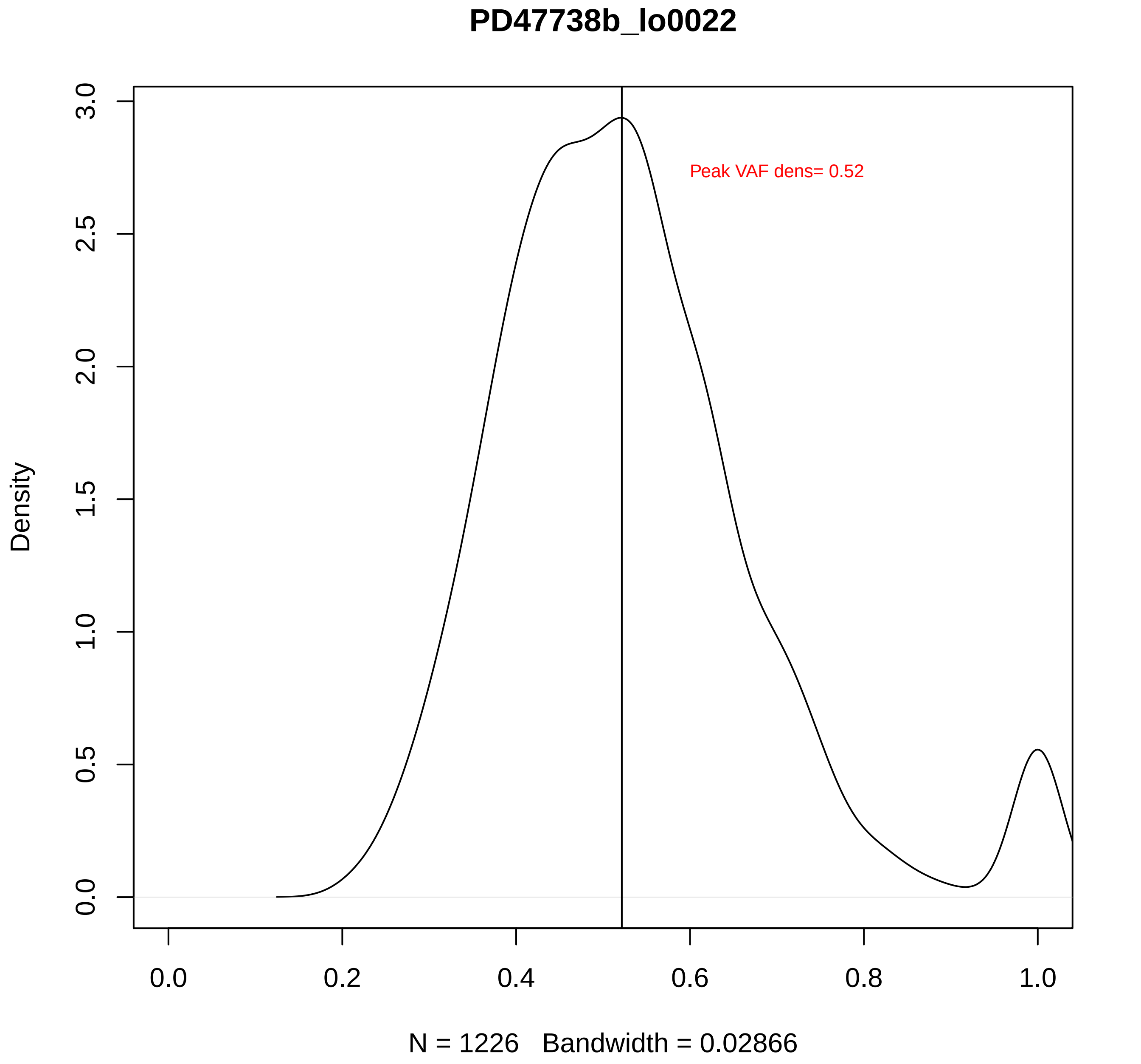

PD47738b_lo0022
Peak VAF dens= 0.52
Density
0.0
0.5
1.0
1.5
2.0
2.5
3.0
0.0
0.2
0.4
0.6
0.8
1.0
N = 1226 Bandwidth = 0.02866

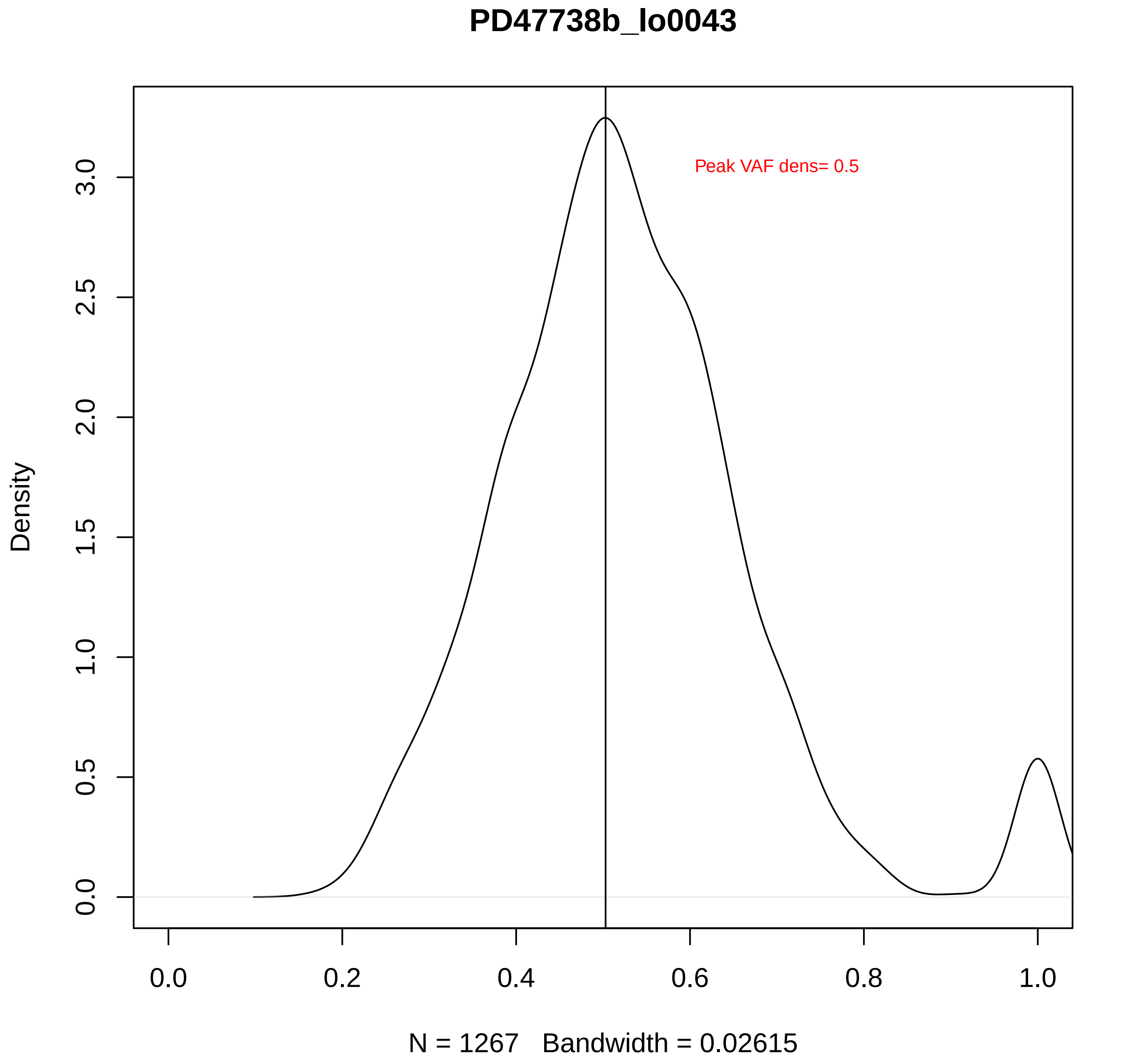
PD47738b_lo0043
Peak VAF dens= 0.5
Density
0.0
0.5
1.0
1.5
2.0
2.5
3.0
0.0
0.2
0.4
0.6
0.8
1.0
N = 1267   Bandwidth = 0.02615

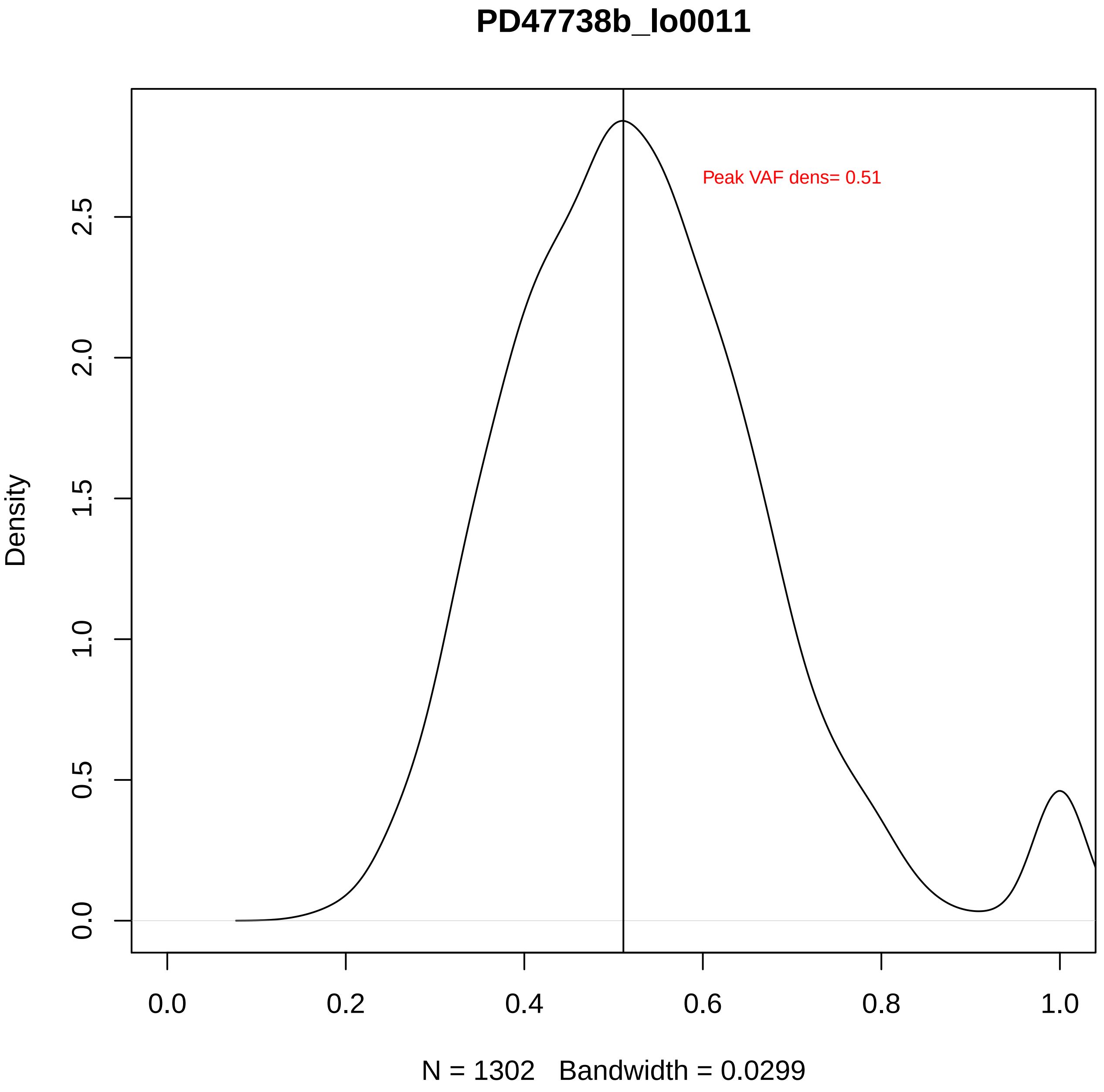
PD47738b_lo0011
Peak VAF dens= 0.51
Density
0.0
0.5
1.0
1.5
2.0
2.5
0.0
0.2
0.4
0.6
0.8
1.0
N = 1302   Bandwidth = 0.0299

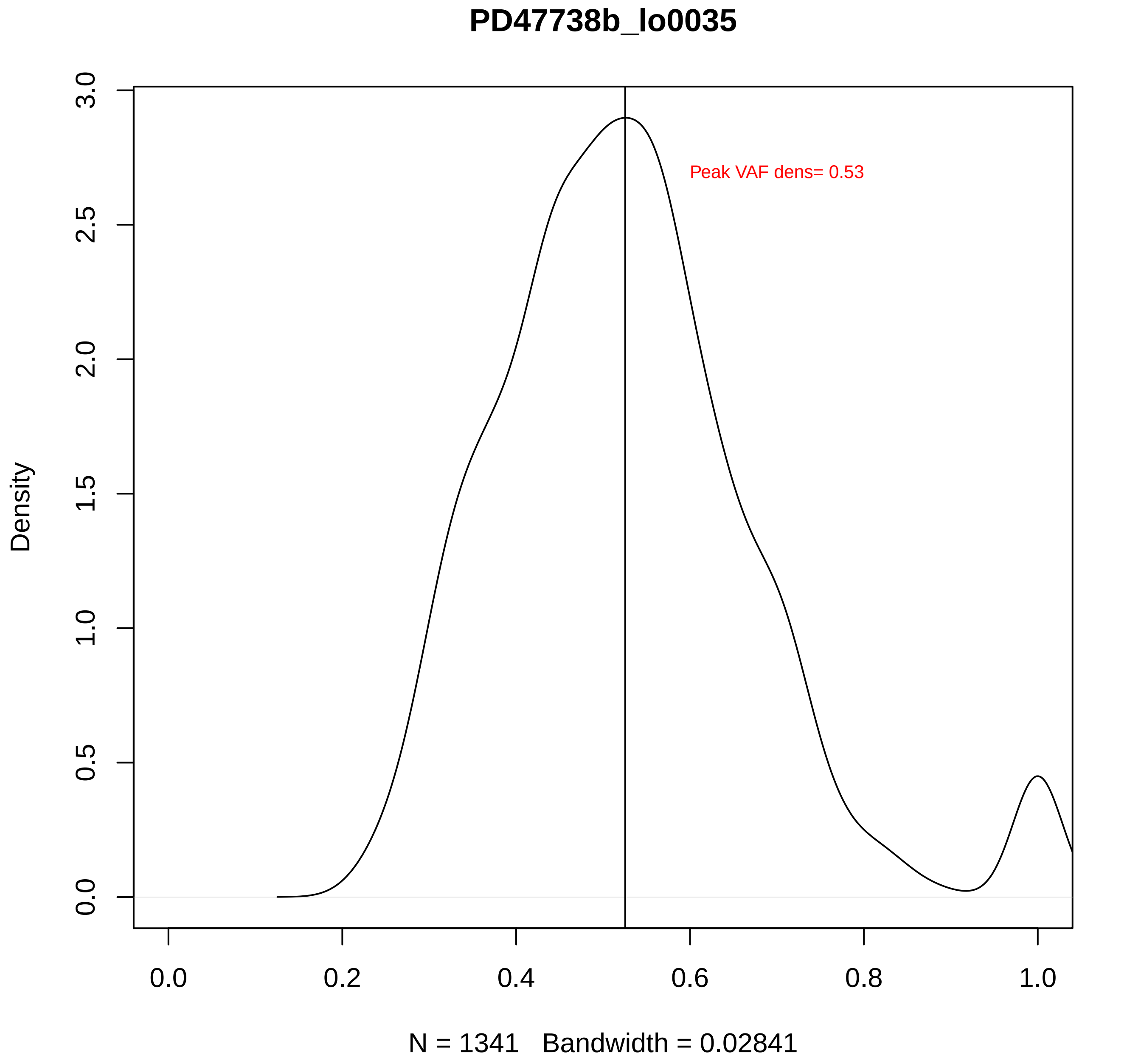
PD47738b_lo0035
Peak VAF dens= 0.53
Density
3.0
2.5
2.0
1.5
1.0
0.5
0.0
0.0
0.2
0.4
0.6
0.8
1.0
N = 1341   Bandwidth = 0.02841

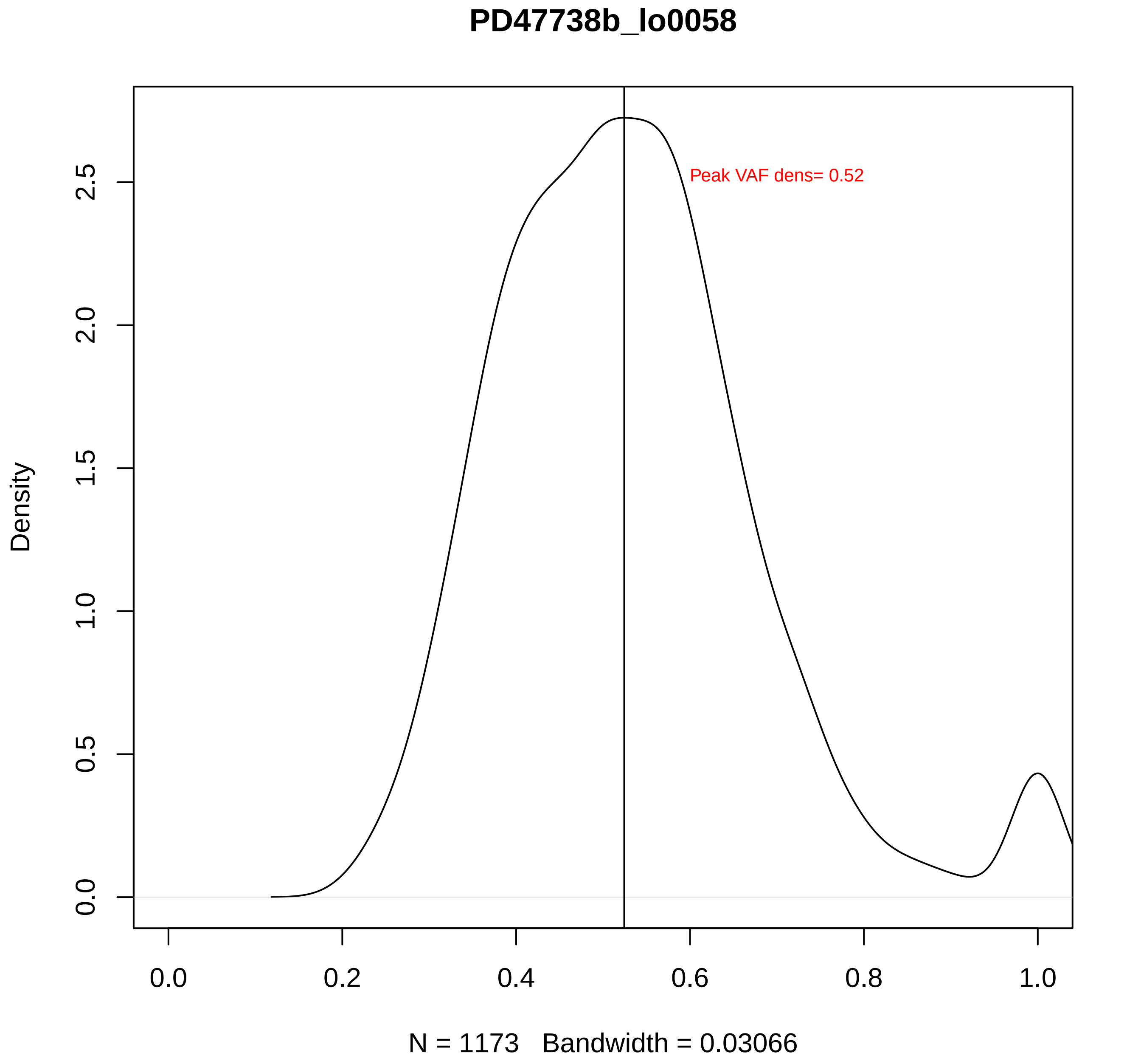

PD47738b_lo0058
Peak VAF dens= 0.52
Density
0.0
0.5
1.0
1.5
2.0
2.5
0.0
0.2
0.4
0.6
0.8
1.0
N = 1173   Bandwidth = 0.03066

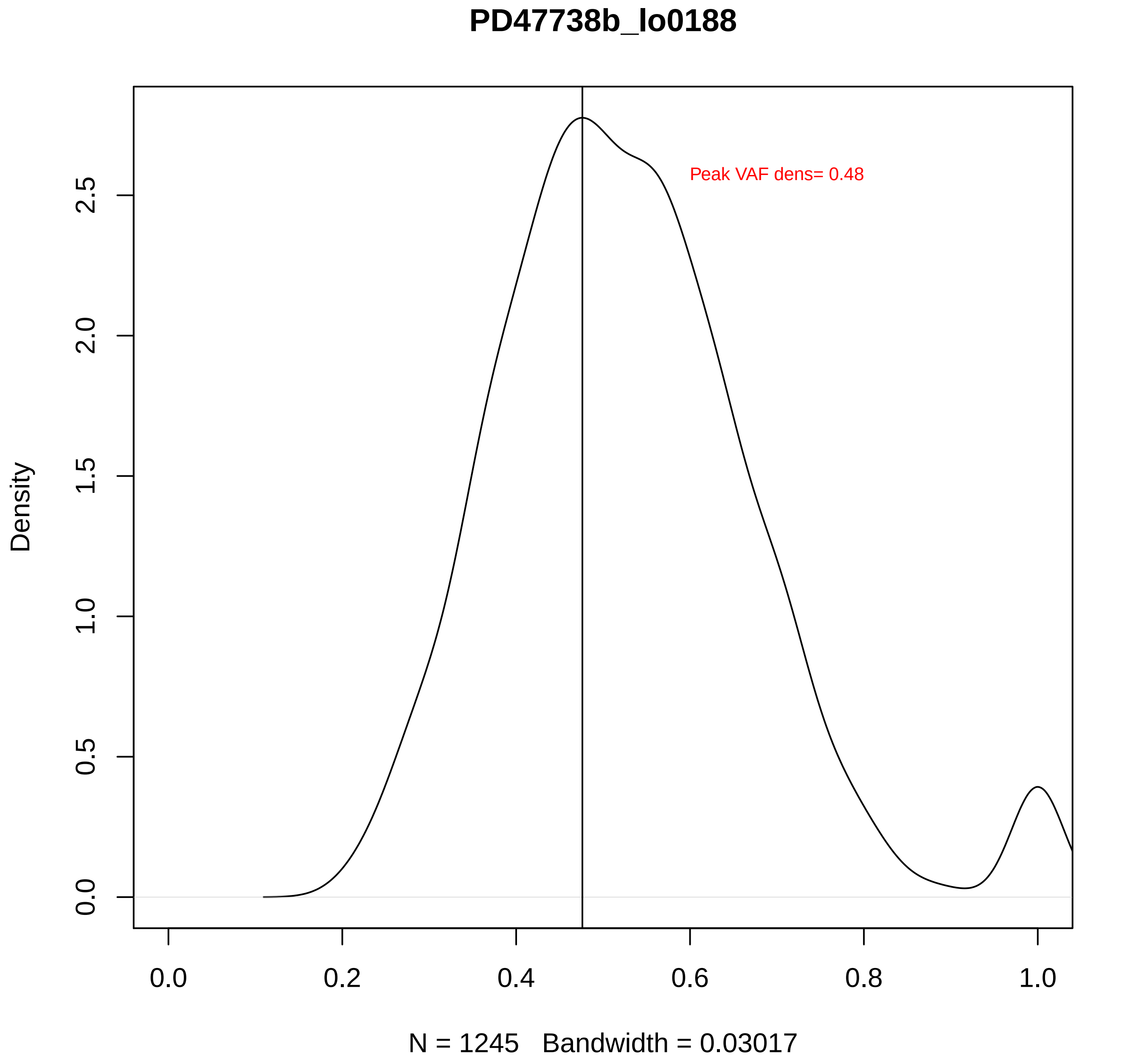
PD47738b_lo0188
Peak VAF dens= 0.48
Density
0.0
0.5
1.0
1.5
2.0
2.5
0.0
0.2
0.4
0.6
0.8
1.0
N = 1245   Bandwidth = 0.03017

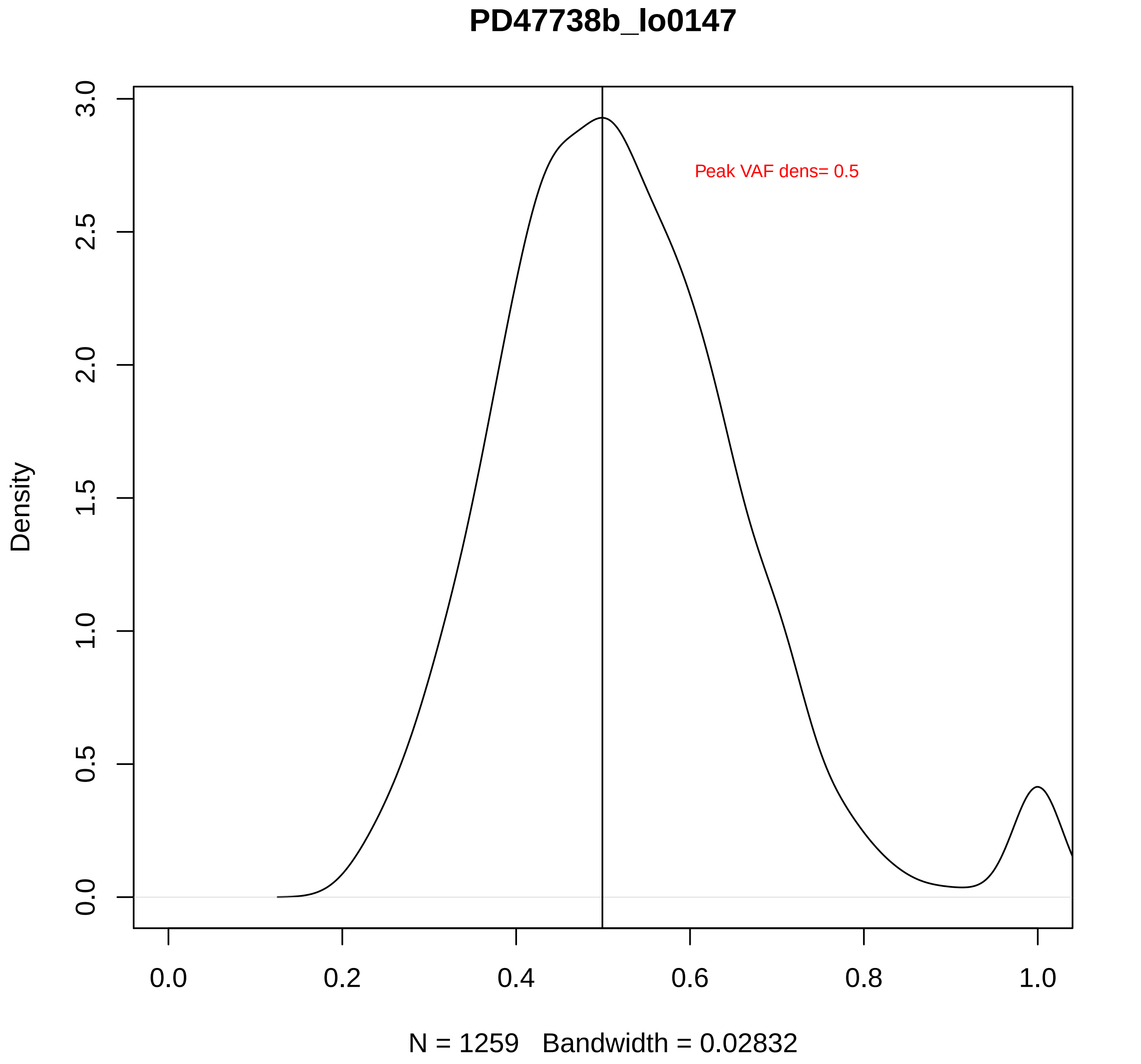

PD47738b_lo0147
Peak VAF dens= 0.5
Density
3.0
2.5
2.0
1.5
1.0
0.5
0.0
0.0
0.2
0.4
0.6
0.8
1.0
N = 1259   Bandwidth = 0.02832

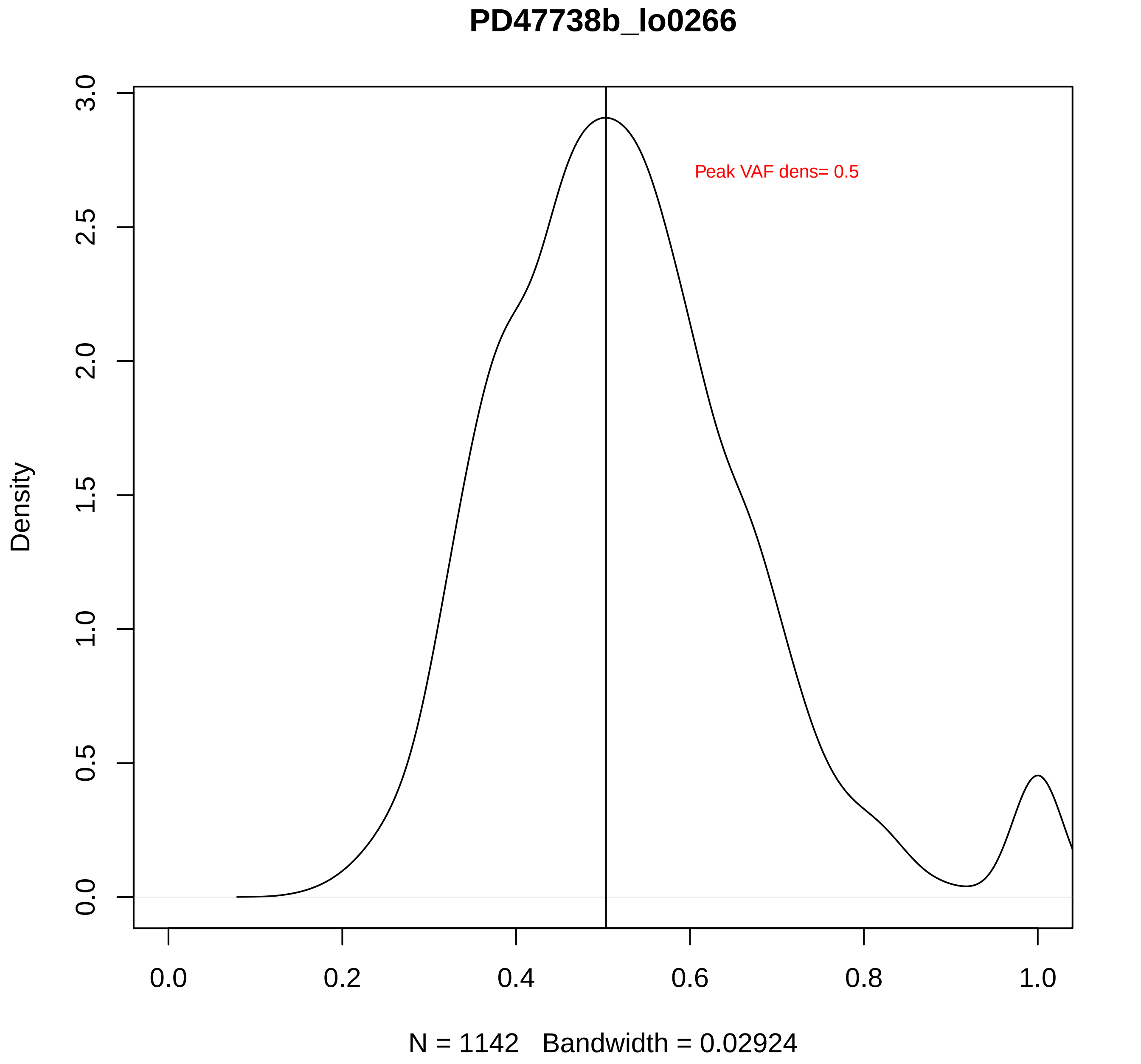

PD47738b_lo0266
Peak VAF dens= 0.5
Density
3.0
2.5
2.0
1.5
1.0
0.5
0.0
0.0
0.2
0.4
0.6
0.8
1.0
N = 1142   Bandwidth = 0.02924

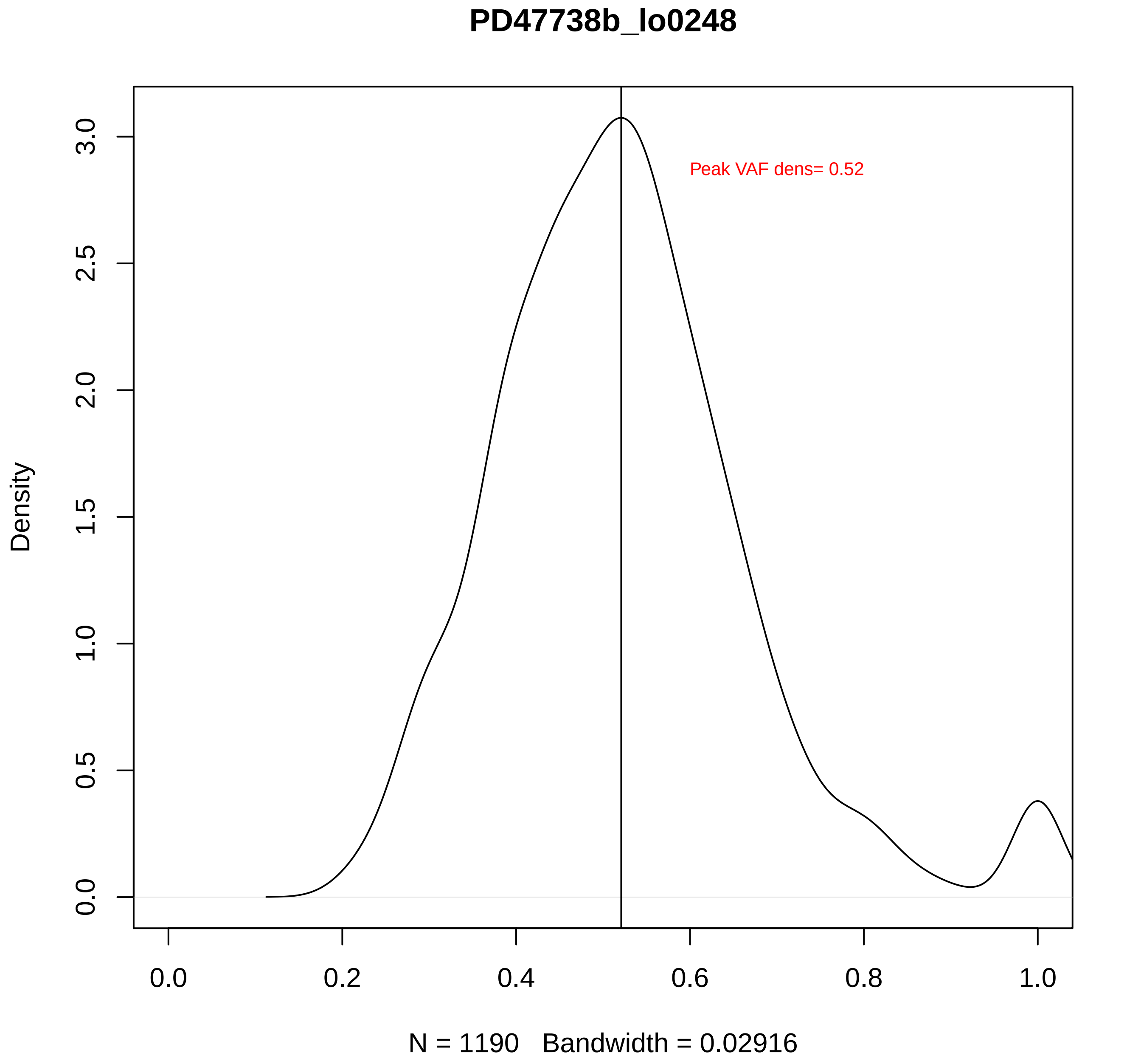
PD47738b_lo0248
Peak VAF dens= 0.52
Density
0.0
0.5
1.0
1.5
2.0
2.5
3.0
0.0
0.2
0.4
0.6
0.8
1.0
N = 1190   Bandwidth = 0.02916

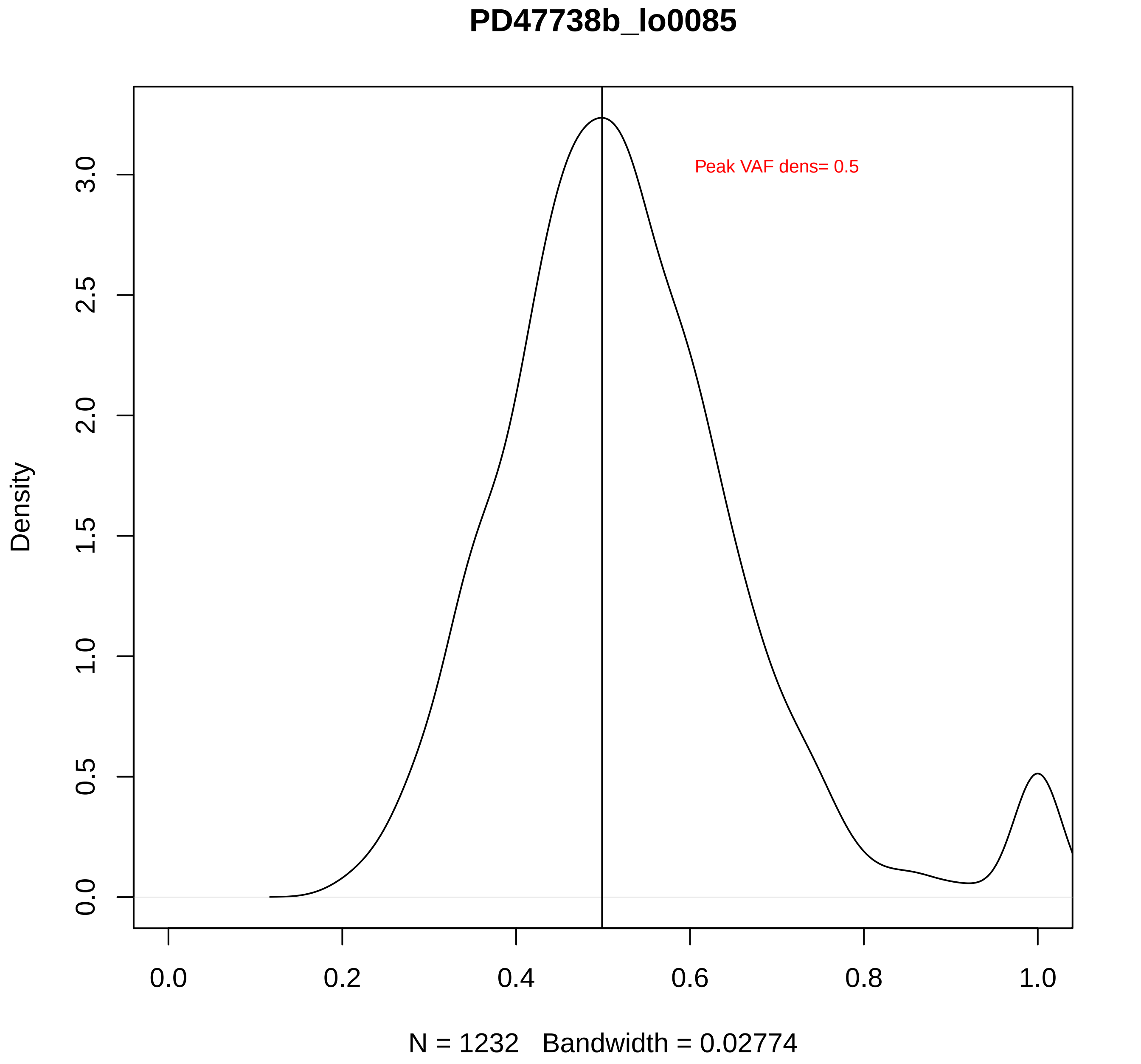
PD47738b_lo0085
Peak VAF dens= 0.5
Density
0.0
0.5
1.0
1.5
2.0
2.5
3.0
0.0
0.2
0.4
0.6
0.8
1.0
N = 1232 Bandwidth = 0.02774

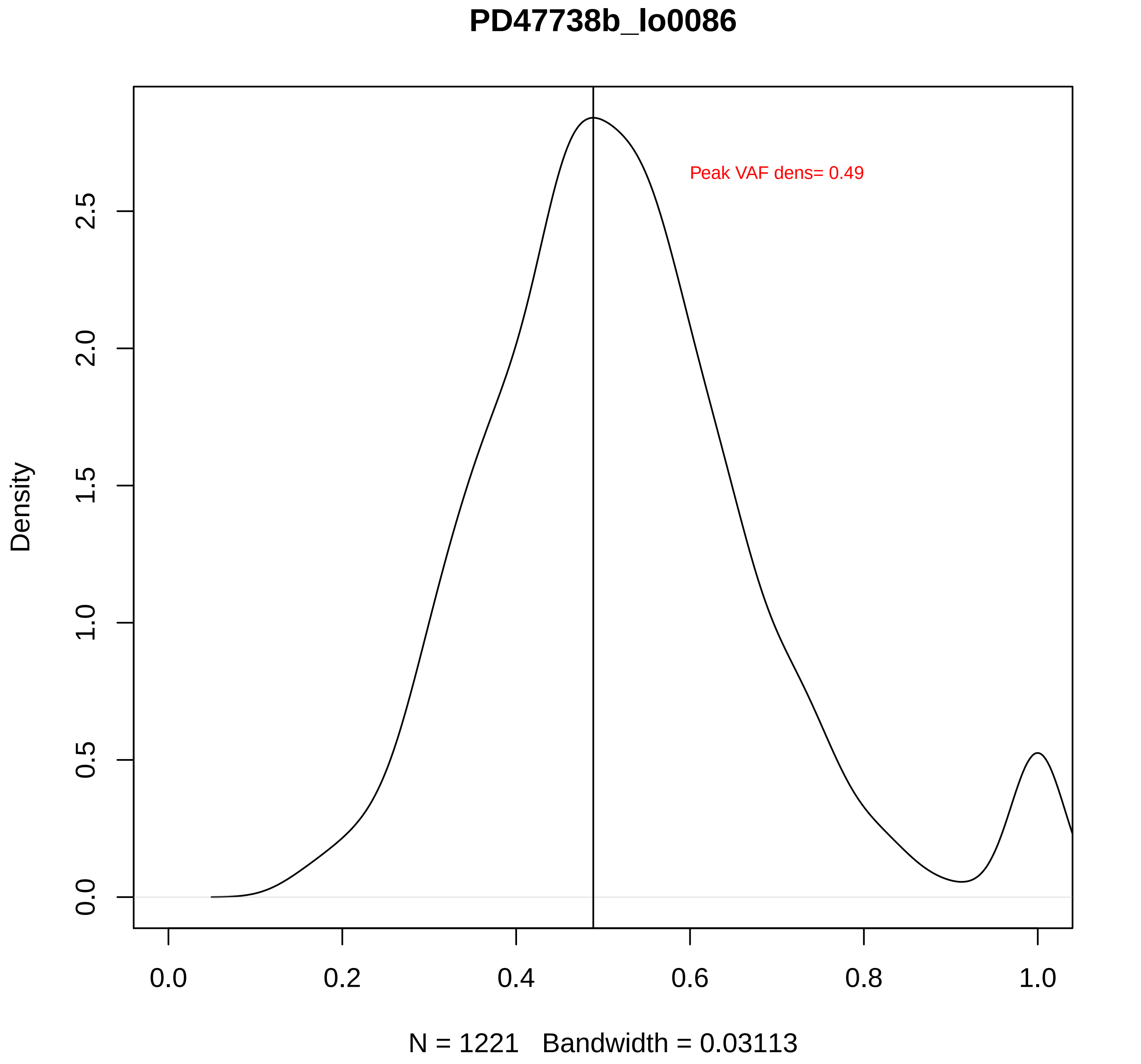
PD47738b_lo0086
Peak VAF dens= 0.49
Density
0.0
0.5
1.0
1.5
2.0
2.5
0.0
0.2
0.4
0.6
0.8
1.0
N = 1221   Bandwidth = 0.03113

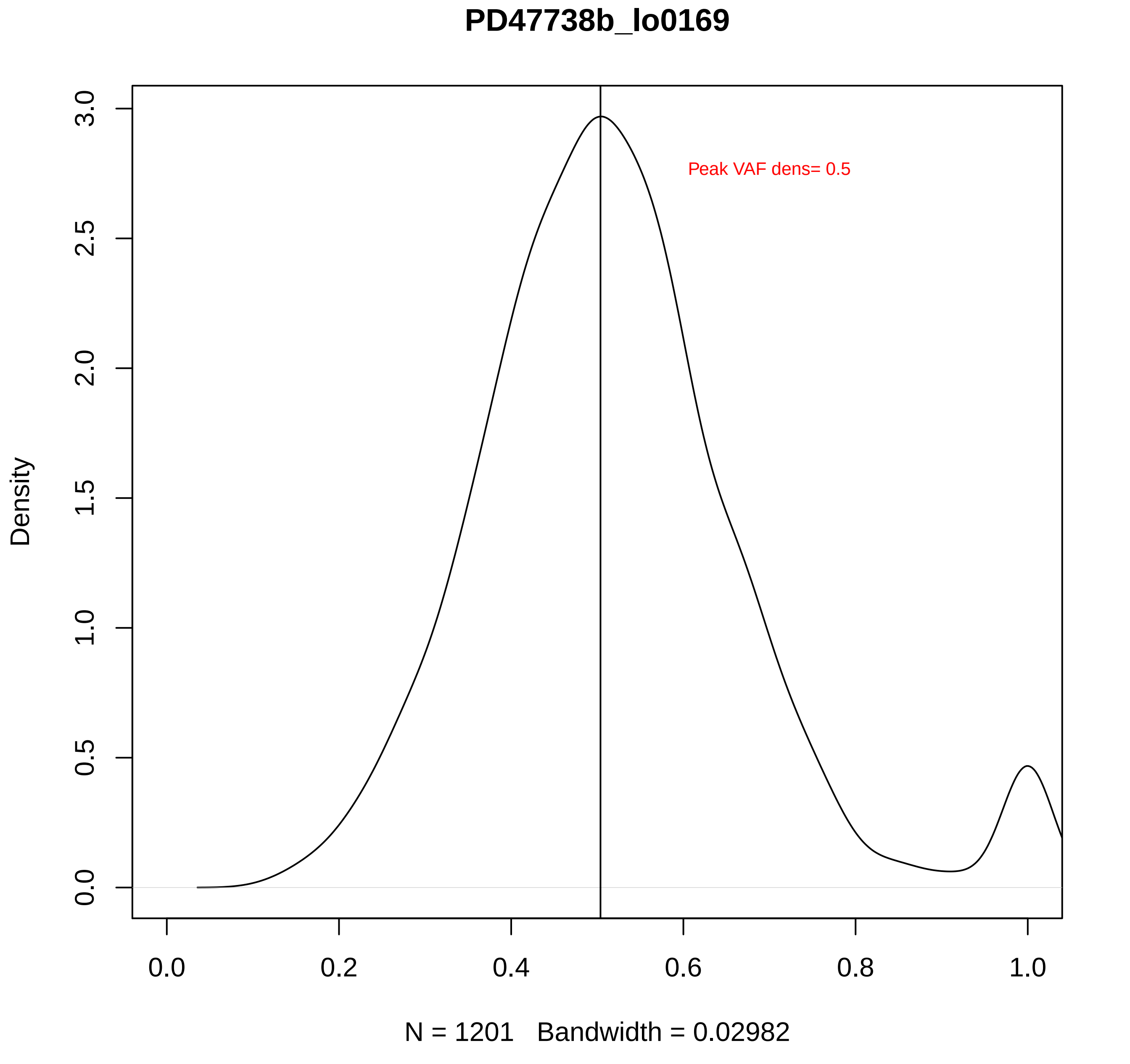

PD47738b_lo0169
Peak VAF dens= 0.5
Density
0.0
0.5
1.0
1.5
2.0
2.5
3.0
0.0
0.2
0.4
0.6
0.8
1.0
N = 1201 Bandwidth = 0.02982

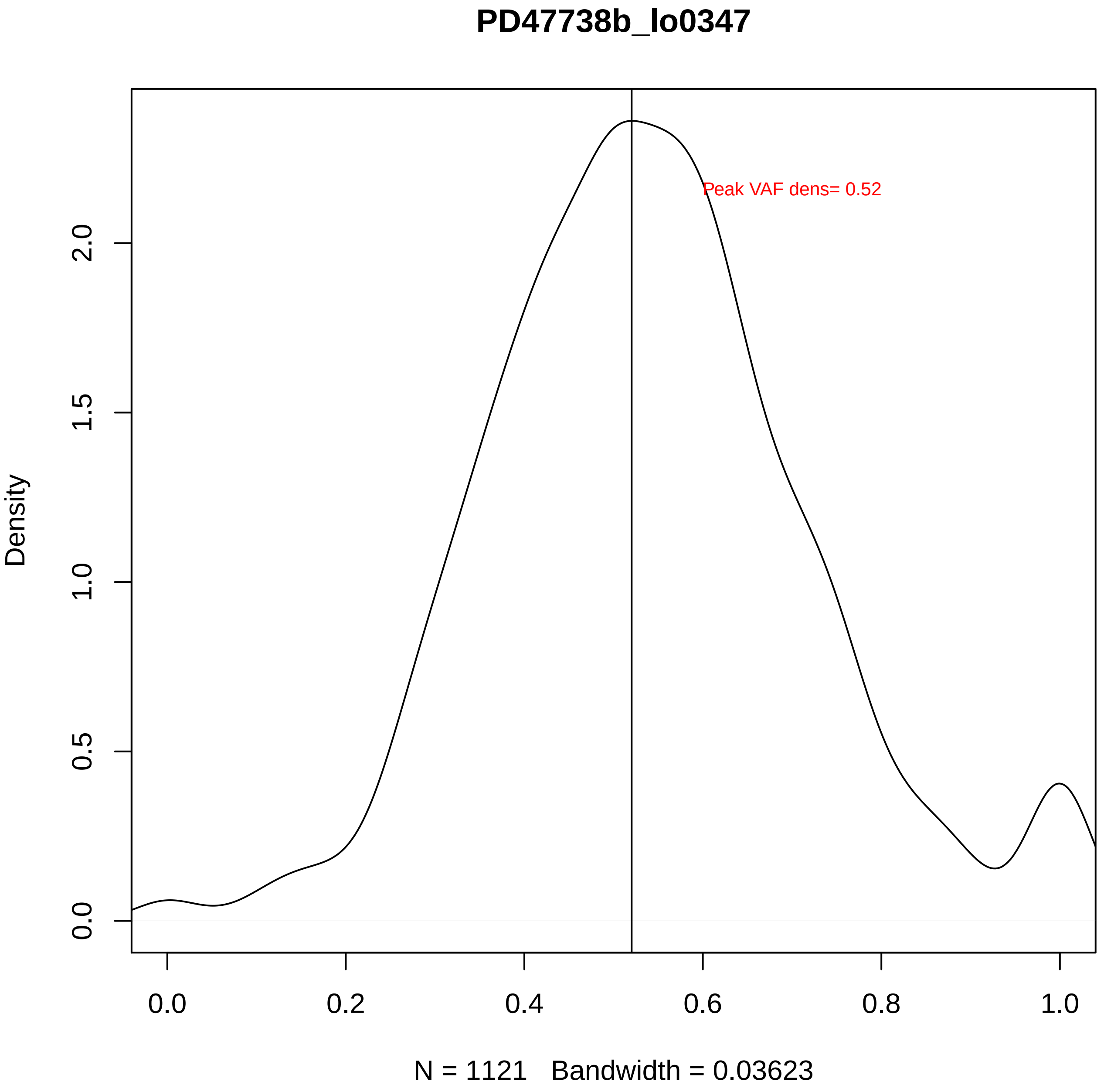
PD47738b_lo0347
Peak VAF dens= 0.52
Density
0.0
0.5
1.0
1.5
2.0
0.0
0.2
0.4
0.6
0.8
1.0
N = 1121   Bandwidth = 0.03623

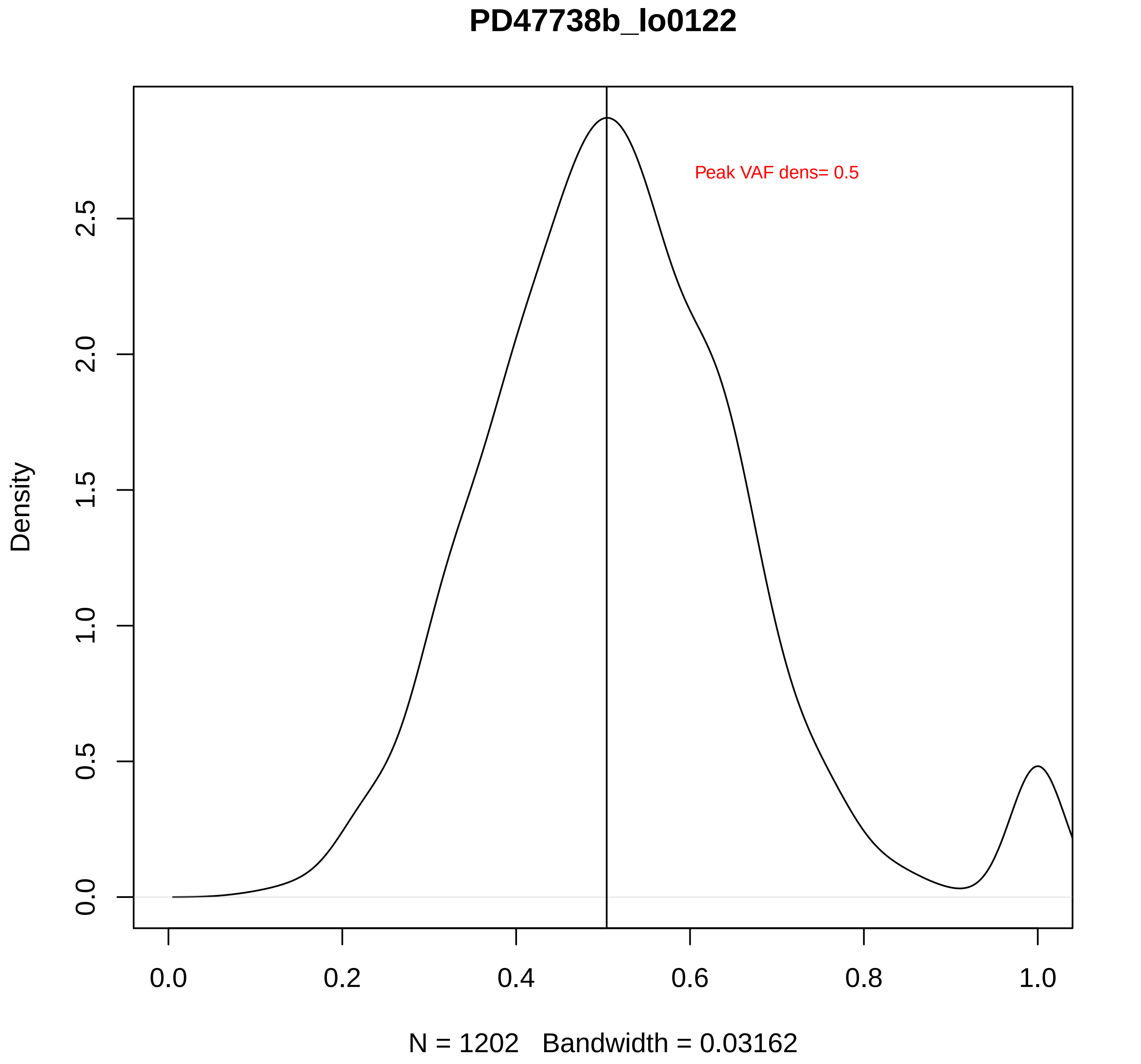

PD47738b_lo0122
Peak VAF dens= 0.5
Density
0.0
0.5
1.0
1.5
2.0
2.5
0.0
0.2
0.4
0.6
0.8
1.0
N = 1202   Bandwidth = 0.03162

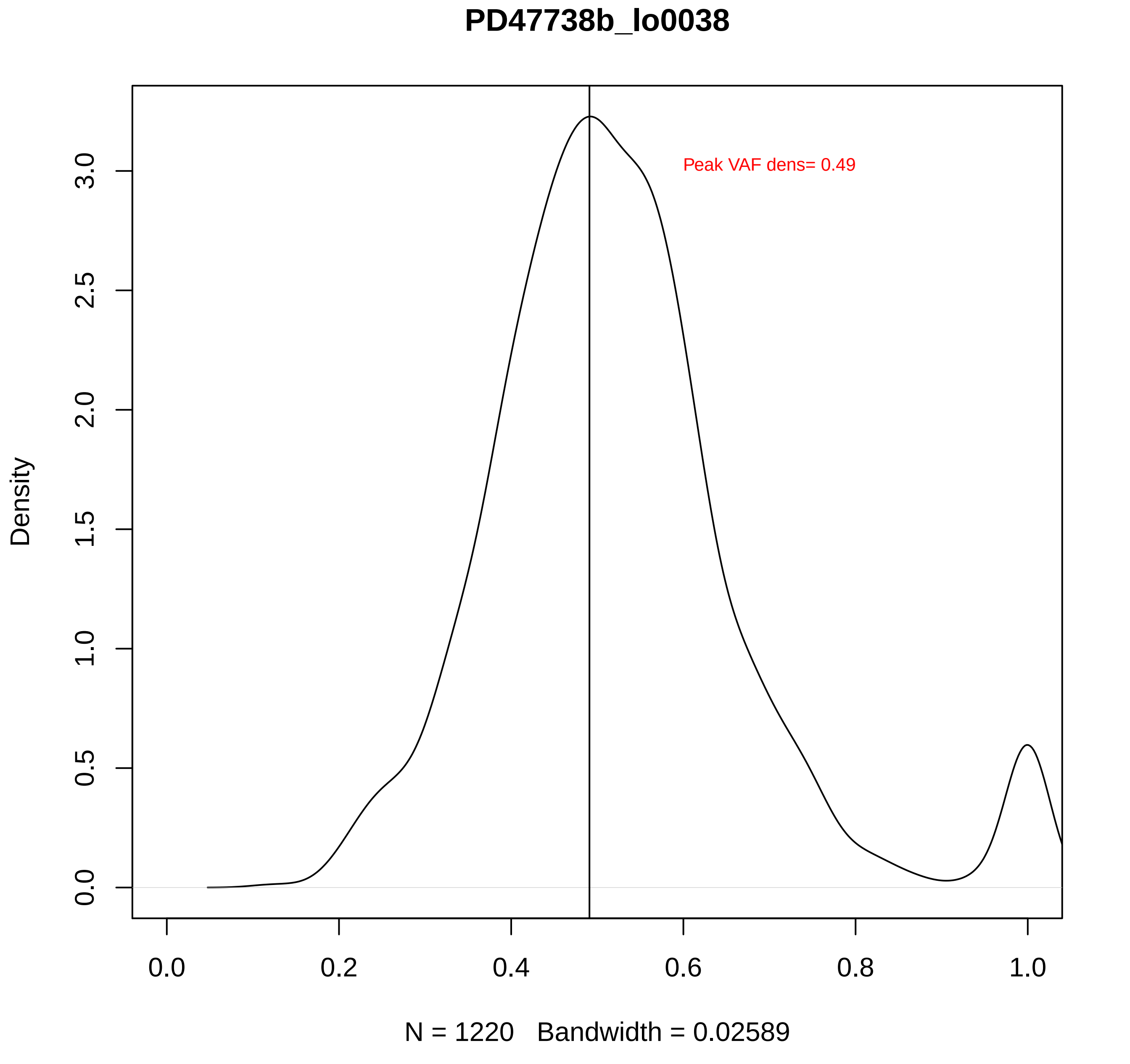
PD47738b_lo0038
Peak VAF dens= 0.49
Density
0.0
0.5
1.0
1.5
2.0
2.5
3.0
0.0
0.2
0.4
0.6
0.8
1.0
N = 1220   Bandwidth = 0.02589

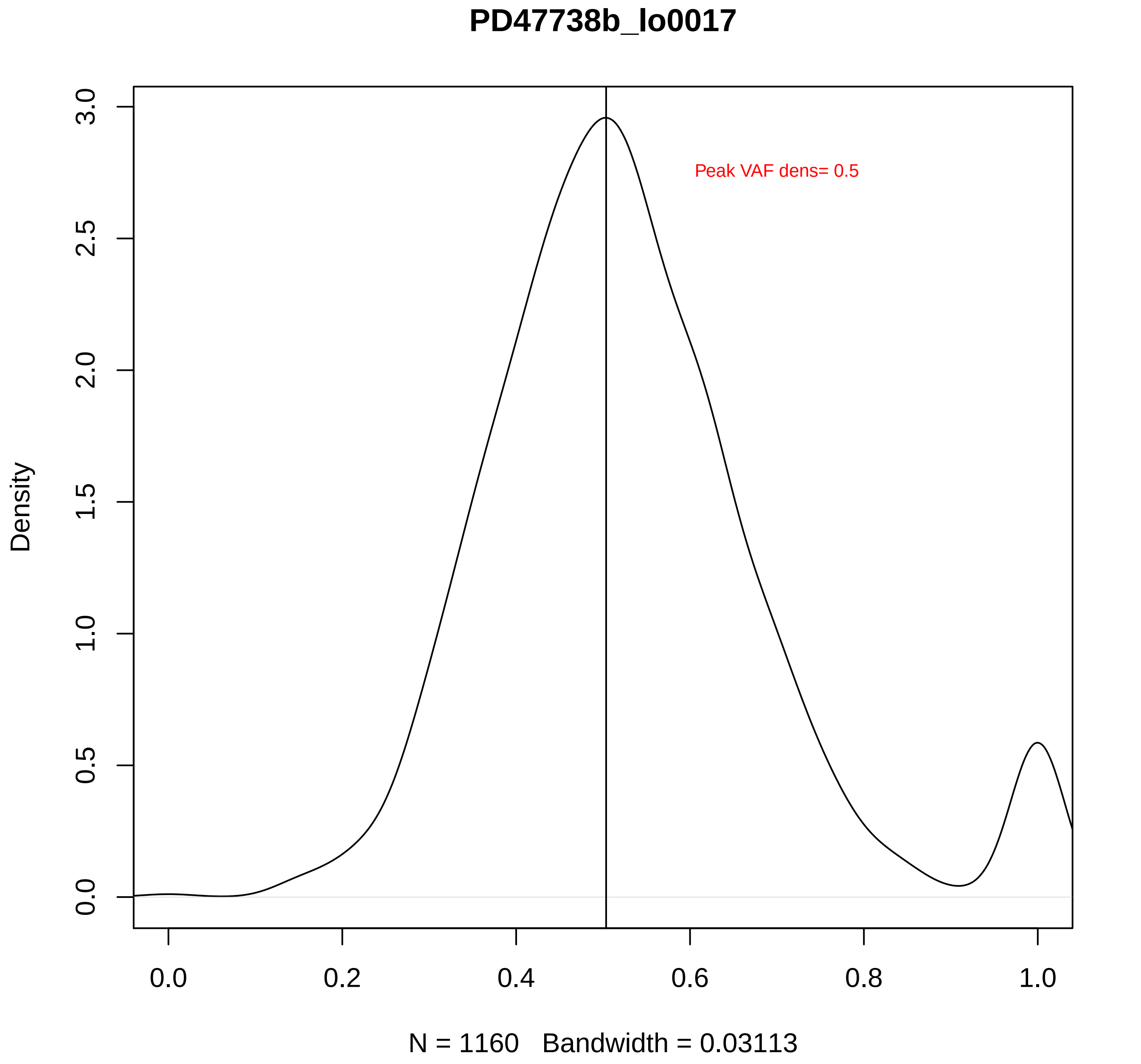
PD47738b_lo0017
Peak VAF dens= 0.5
Density
0.0
0.5
1.0
1.5
2.0
2.5
3.0
0.0
0.2
0.4
0.6
0.8
1.0
N = 1160   Bandwidth = 0.03113

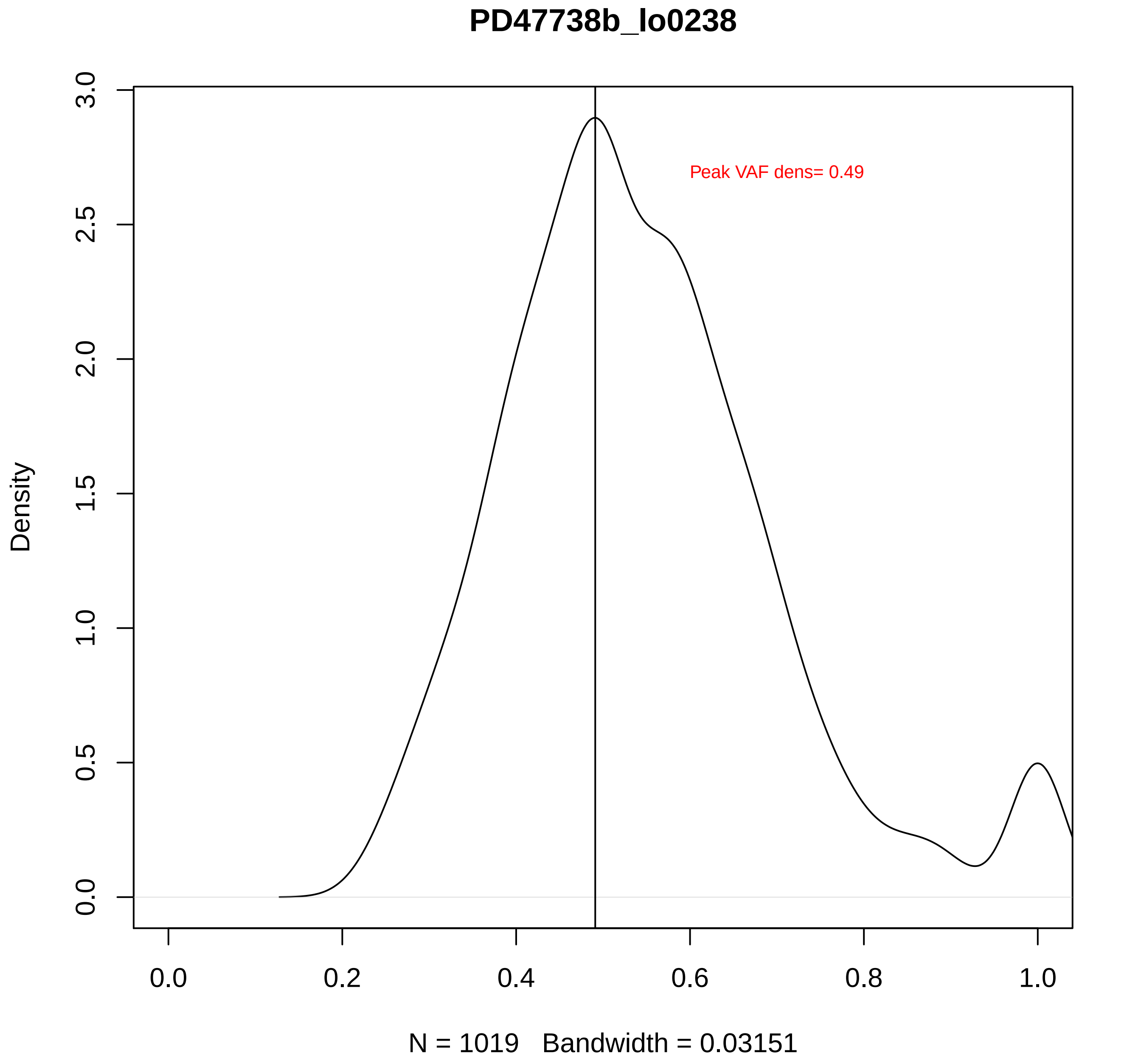
PD47738b_lo0238
Peak VAF dens= 0.49
Density
3.0
2.5
2.0
1.5
1.0
0.5
0.0
0.0
0.2
0.4
0.6
0.8
1.0
N = 1019   Bandwidth = 0.03151

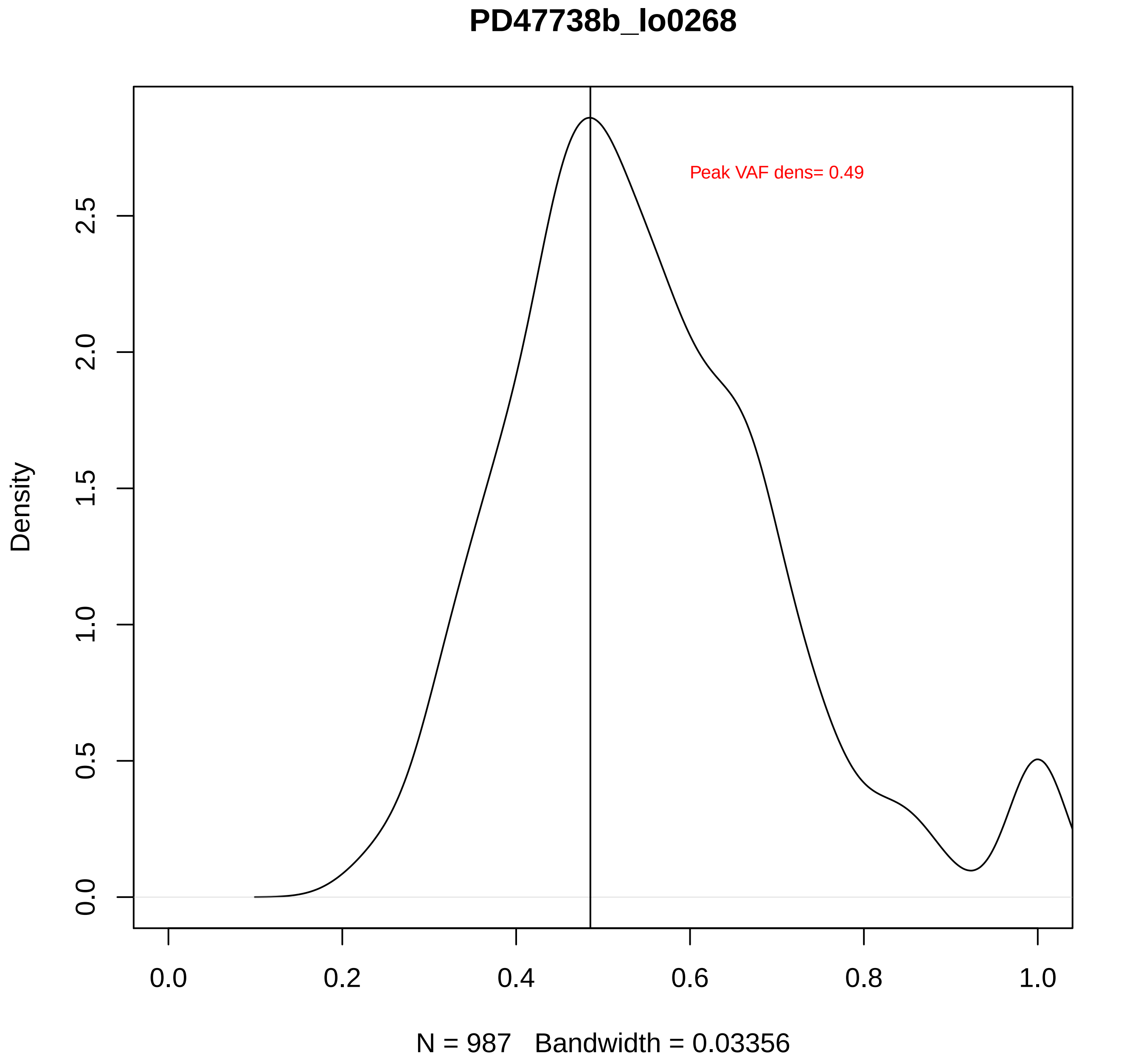
PD47738b_lo0268
Peak VAF dens= 0.49
Density
0.0
0.5
1.0
1.5
2.0
2.5
0.0
0.2
0.4
0.6
0.8
1.0
N = 987   Bandwidth = 0.03356

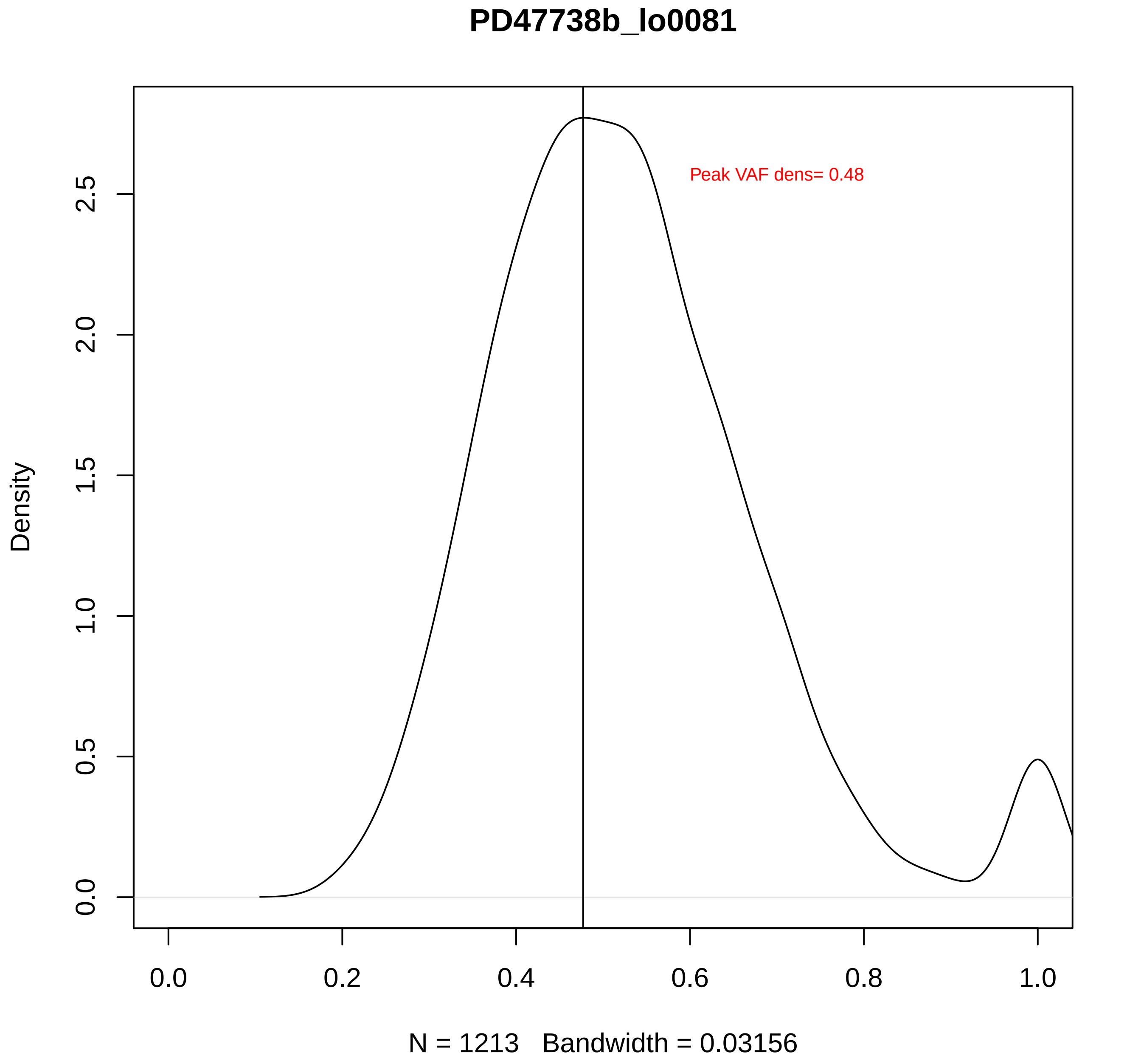

PD47738b_lo0081
Peak VAF dens= 0.48
Density
0.0
0.5
1.0
1.5
2.0
2.5
0.0
0.2
0.4
0.6
0.8
1.0
N = 1213   Bandwidth = 0.03156

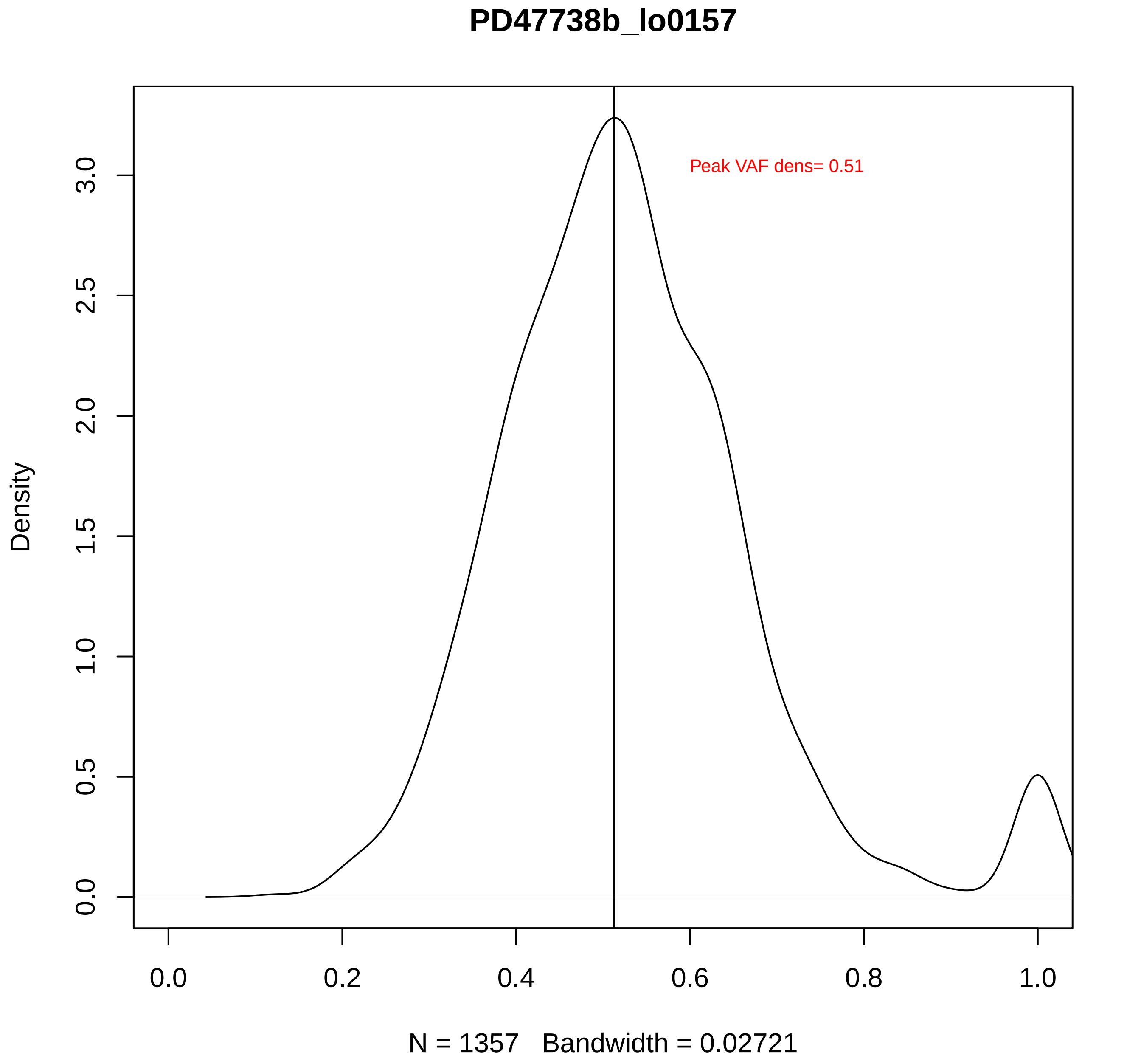
PD47738b_lo0157
Peak VAF dens= 0.51
Density
0.0
0.5
1.0
1.5
2.0
2.5
3.0
0.0
0.2
0.4
0.6
0.8
1.0
N = 1357   Bandwidth = 0.02721

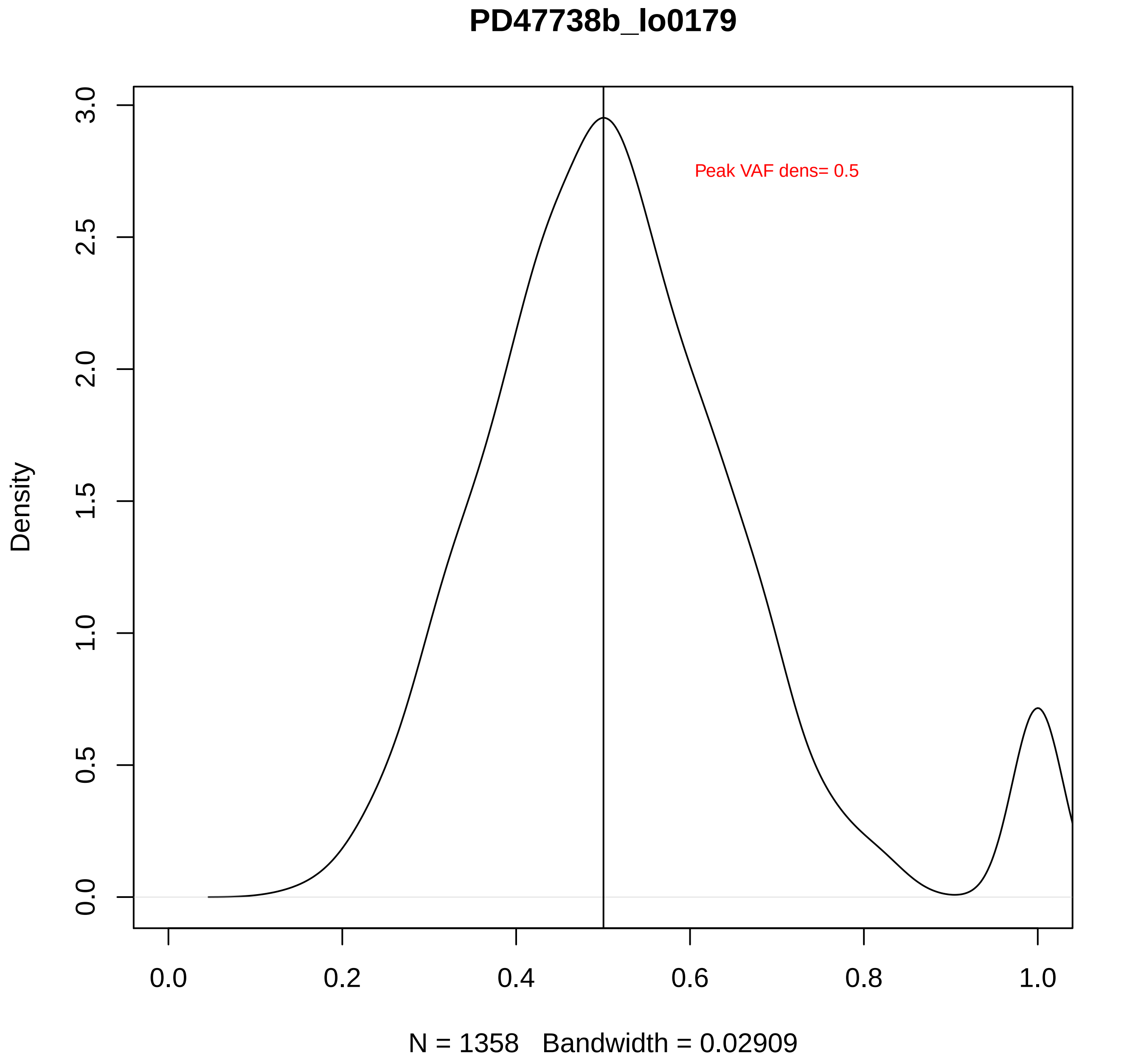

PD47738b_lo0179
Peak VAF dens= 0.5
Density
3.0
2.5
2.0
1.5
1.0
0.5
0.0
0.0
0.2
0.4
0.6
0.8
1.0
N = 1358   Bandwidth = 0.02909

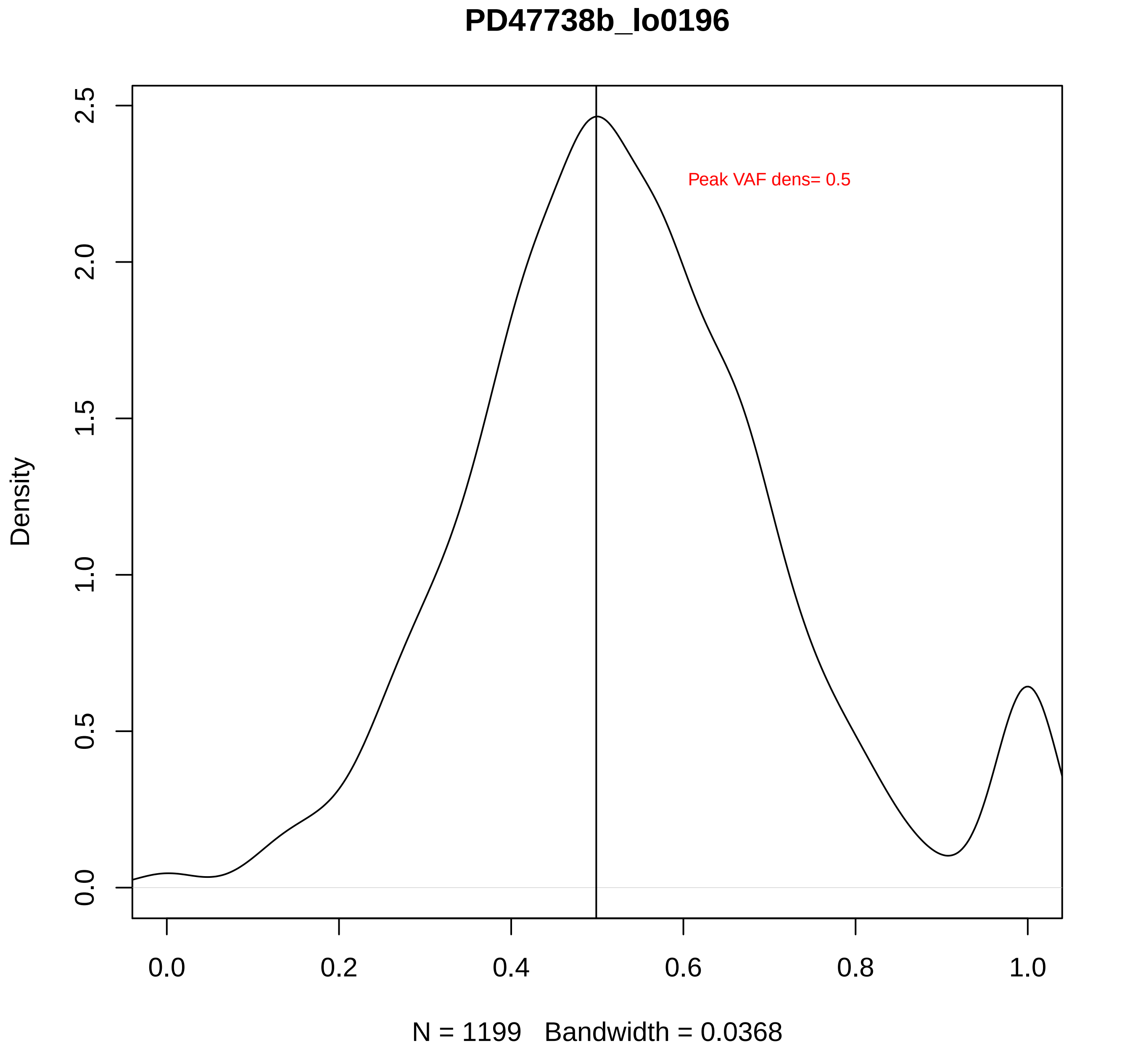
PD47738b_lo0196
Density
Peak VAF dens= 0.5
0.0
0.5
1.0
1.5
2.0
2.5
0.0
0.2
0.4
0.6
0.8
1.0
N = 1199   Bandwidth = 0.0368

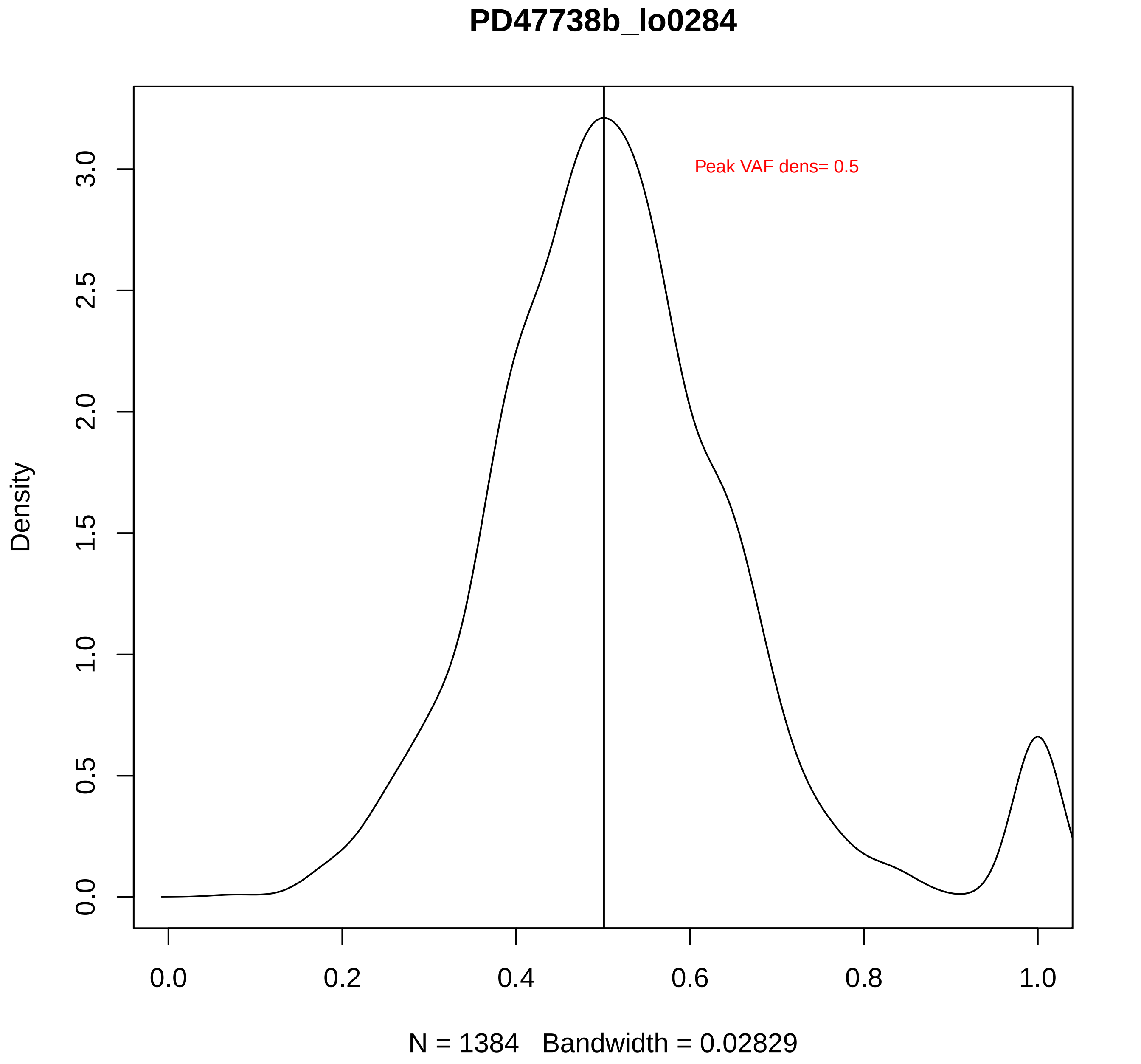
PD47738b_lo0284
Peak VAF dens= 0.5
Density
0.0
0.5
1.0
1.5
2.0
2.5
3.0
0.0
0.2
0.4
0.6
0.8
1.0
N = 1384   Bandwidth = 0.02829

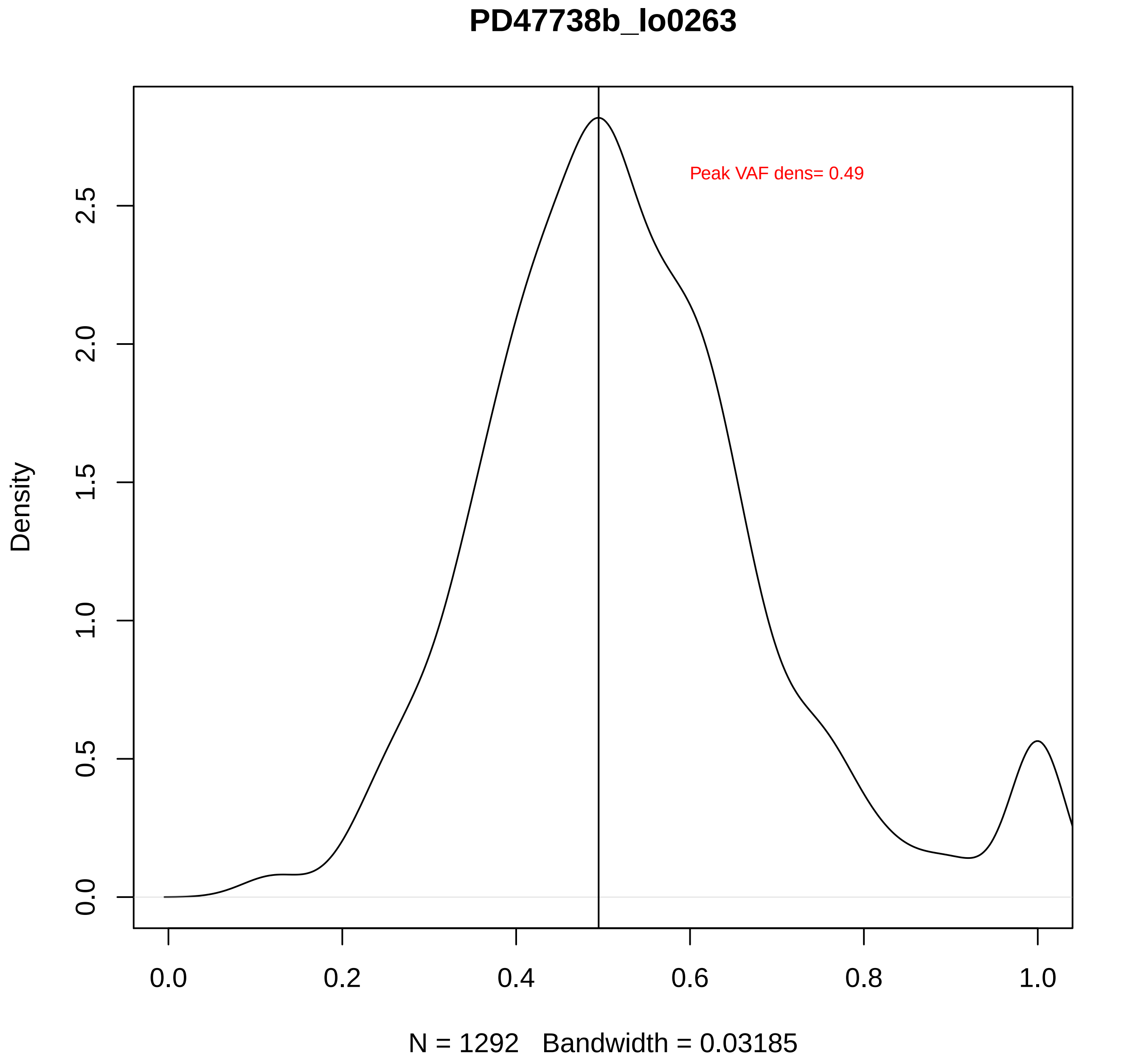
PD47738b_lo0263
Peak VAF dens= 0.49
Density
0.0
0.5
1.0
1.5
2.0
2.5
0.0
0.2
0.4
0.6
0.8
1.0
N = 1292 Bandwidth = 0.03185

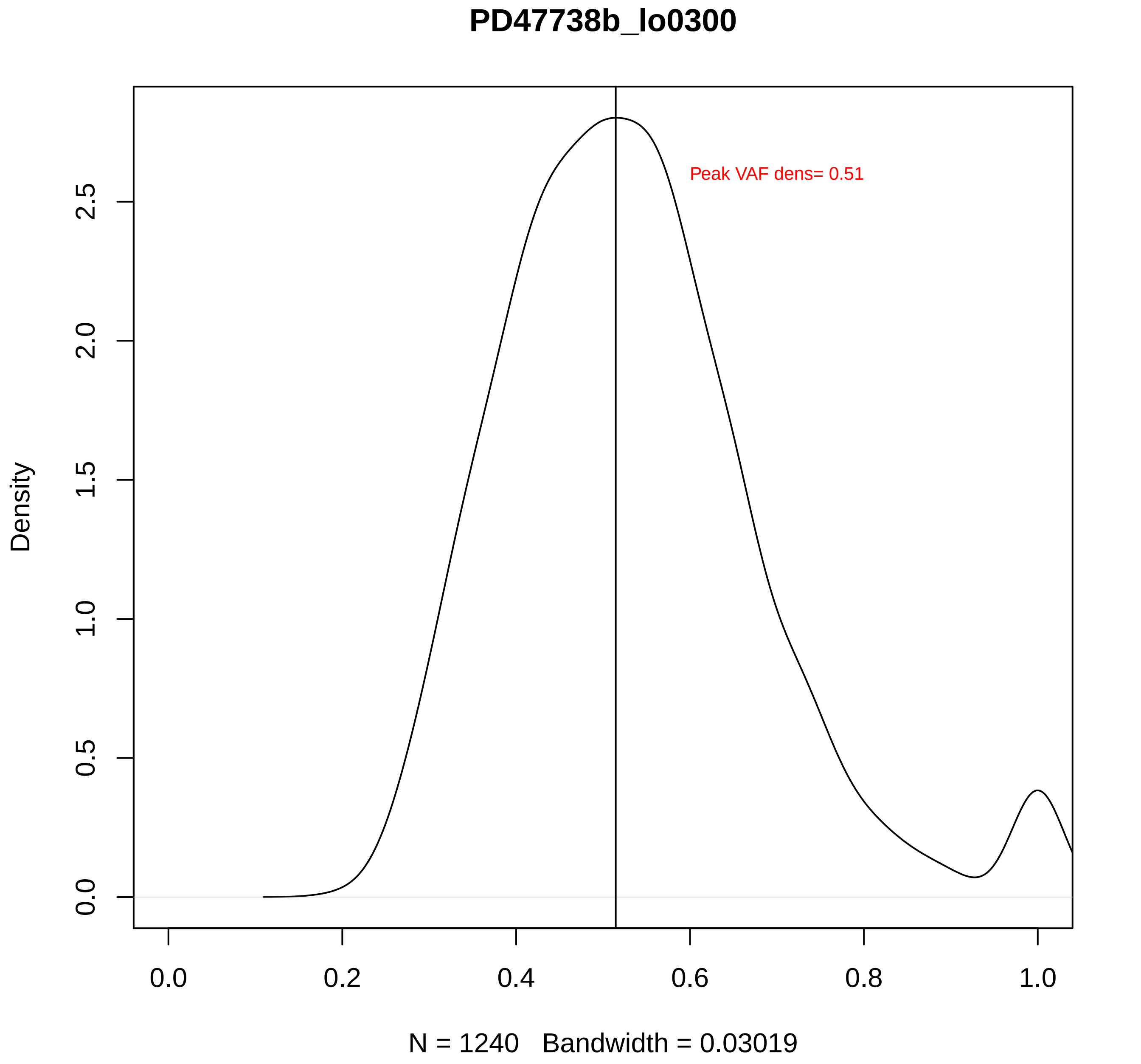
PD47738b_lo0300
Peak VAF dens= 0.51
Density
0.0
0.5
1.0
1.5
2.0
2.5
0.0
0.2
0.4
0.6
0.8
1.0
N = 1240   Bandwidth = 0.03019

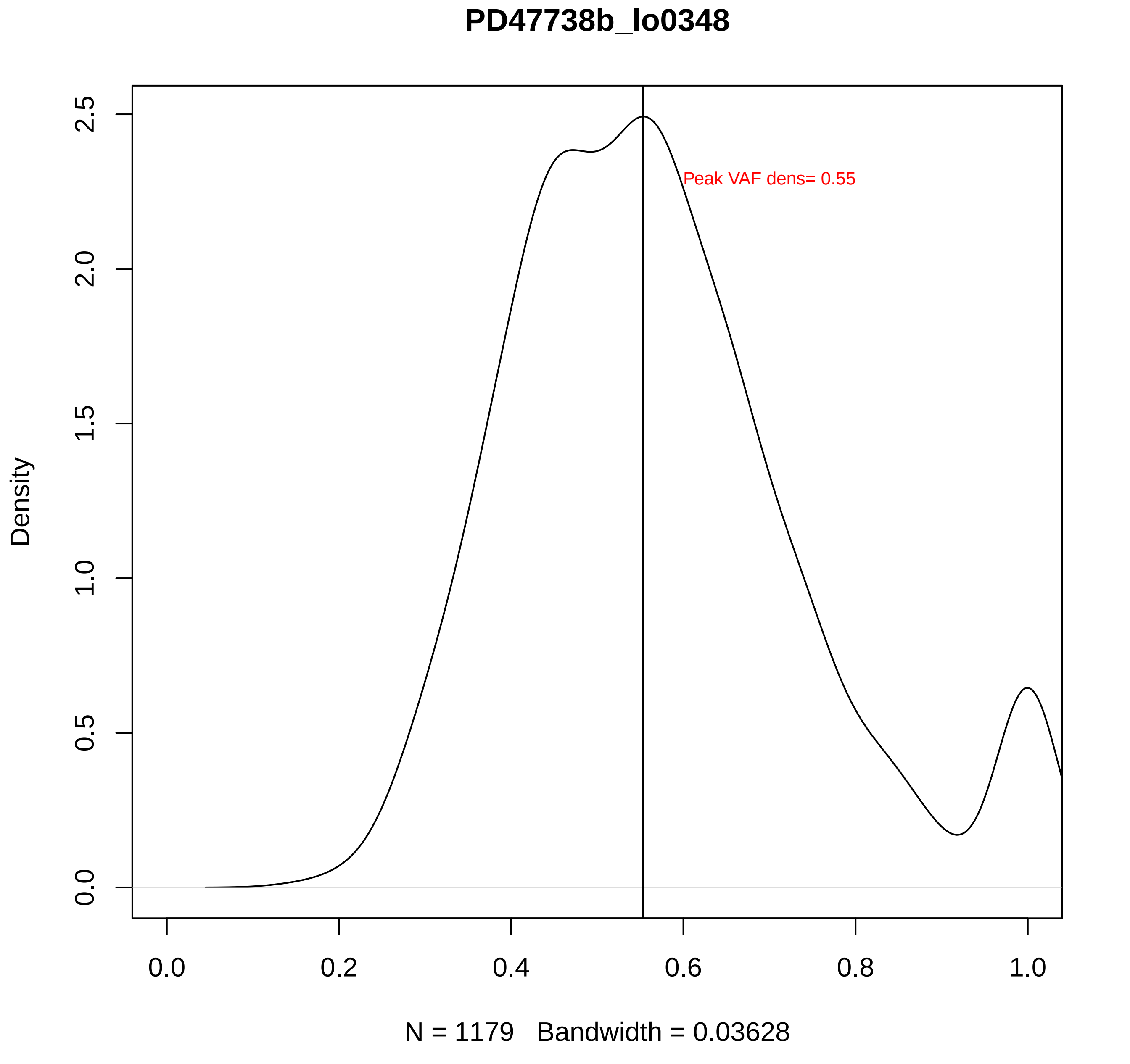

PD47738b_lo0348
Peak VAF dens= 0.55
Density
0.0
0.5
1.0
1.5
2.0
2.5
0.0
0.2
0.4
0.6
0.8
1.0
N = 1179 Bandwidth = 0.03628

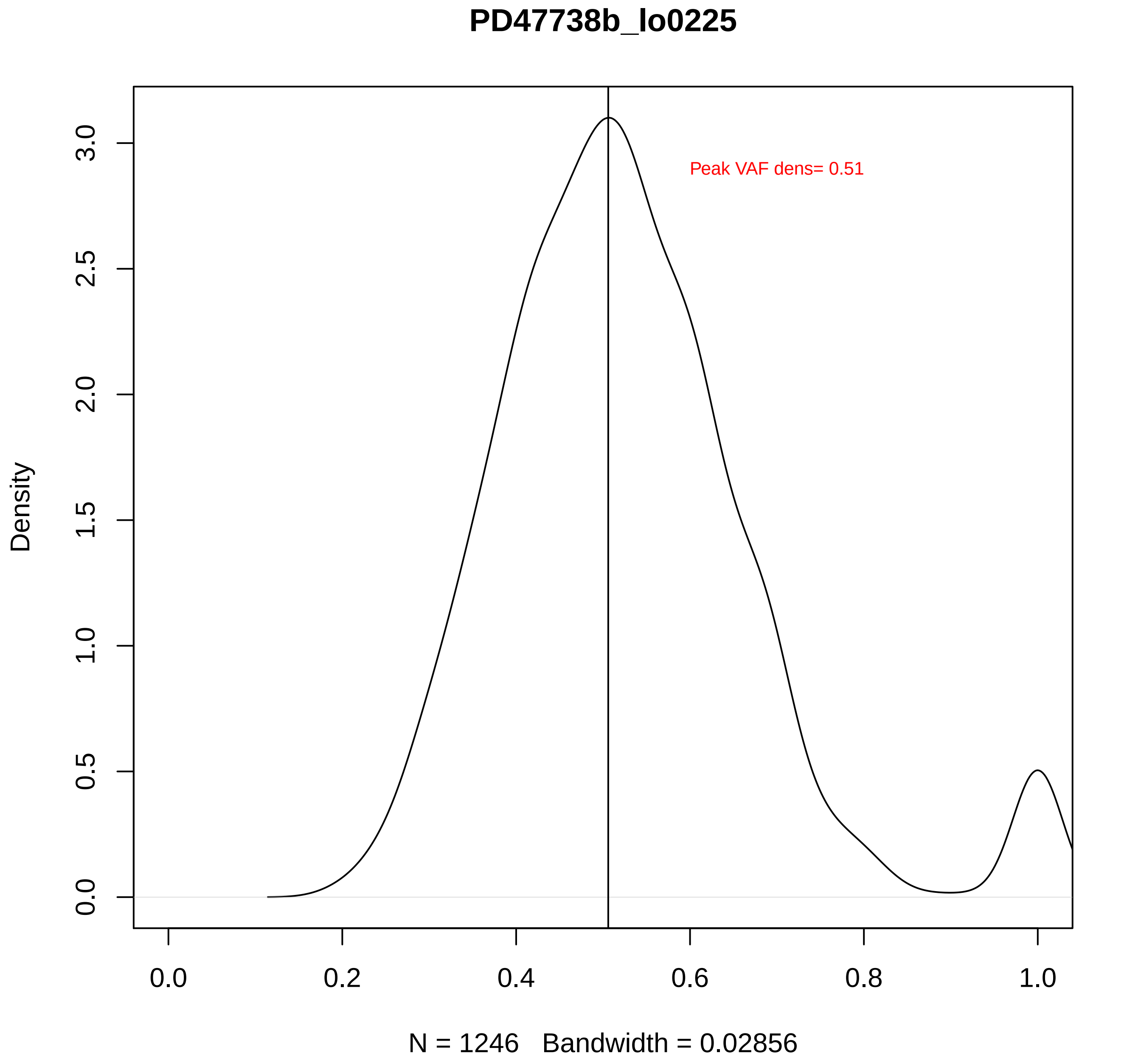
PD47738b_lo0225
Peak VAF dens= 0.51
Density
0.0
0.5
1.0
1.5
2.0
2.5
3.0
0.0
0.2
0.4
0.6
0.8
1.0
N = 1246 Bandwidth = 0.02856

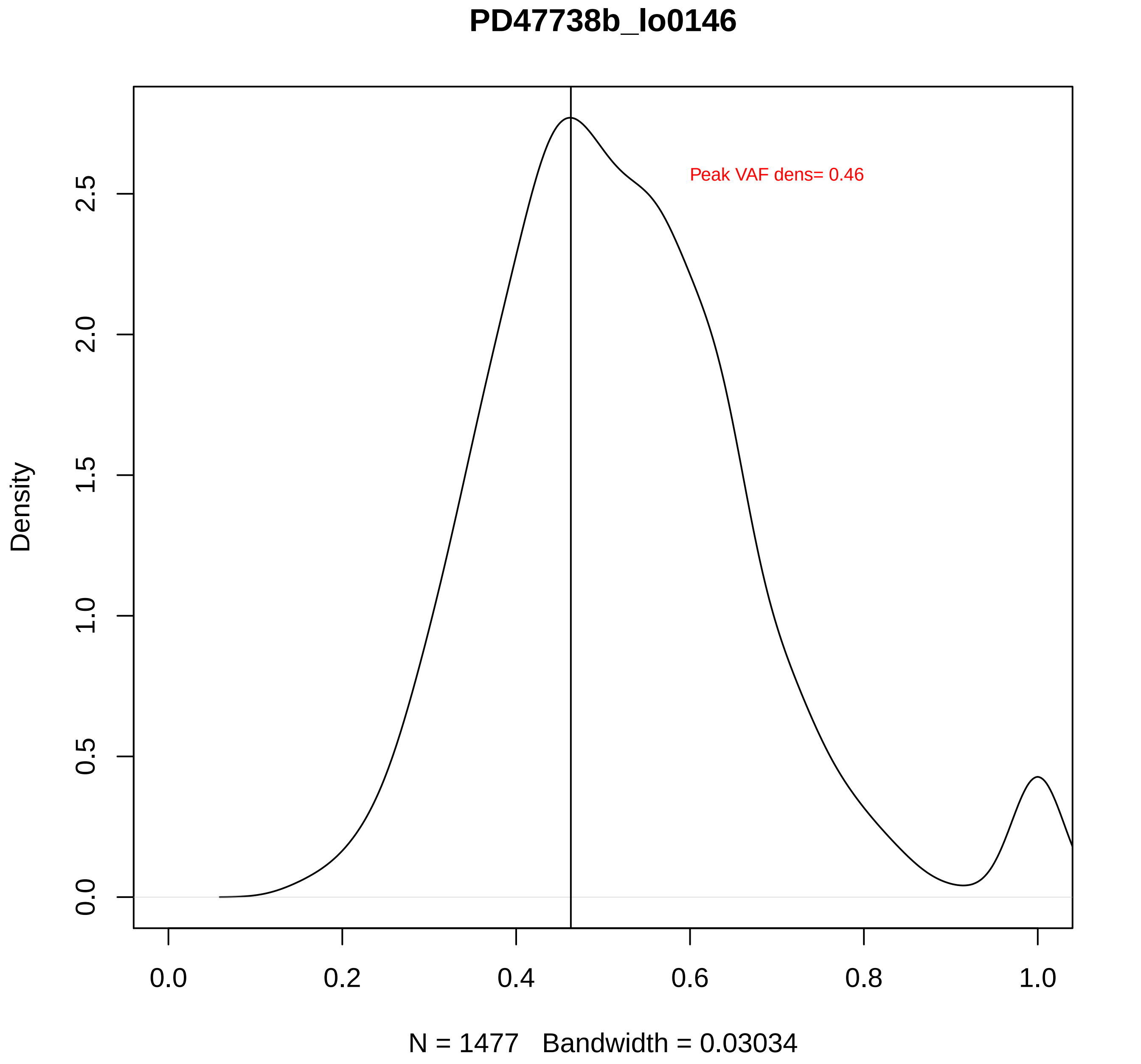
PD47738b_lo0146
Peak VAF dens= 0.46
Density
0.0
0.5
1.0
1.5
2.0
2.5
0.0
0.2
0.4
0.6
0.8
1.0
N = 1477   Bandwidth = 0.03034

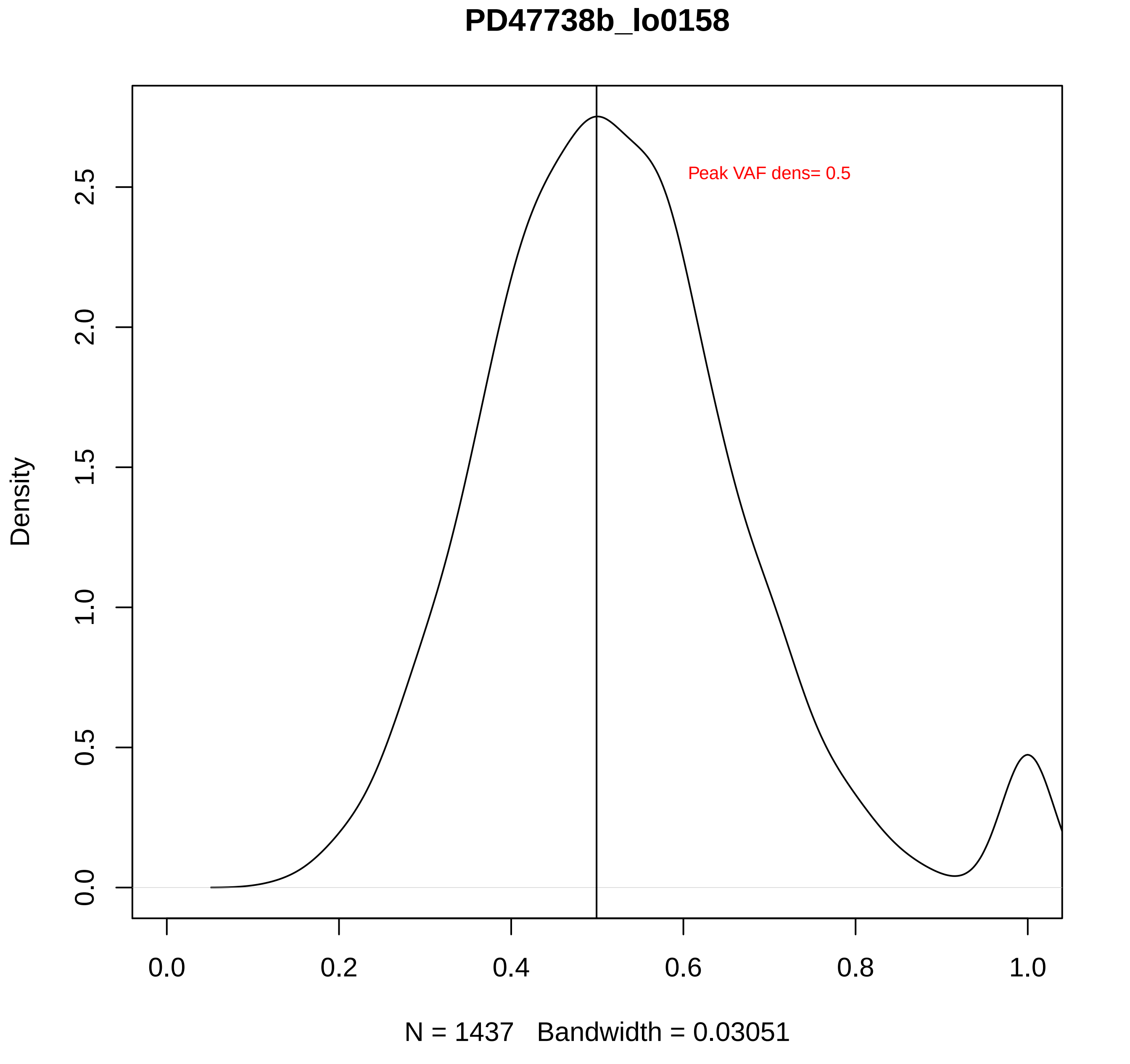
PD47738b_lo0158
Peak VAF dens= 0.5
Density
0.0
0.5
1.0
1.5
2.0
2.5
0.0
0.2
0.4
0.6
0.8
1.0
N = 1437   Bandwidth = 0.03051

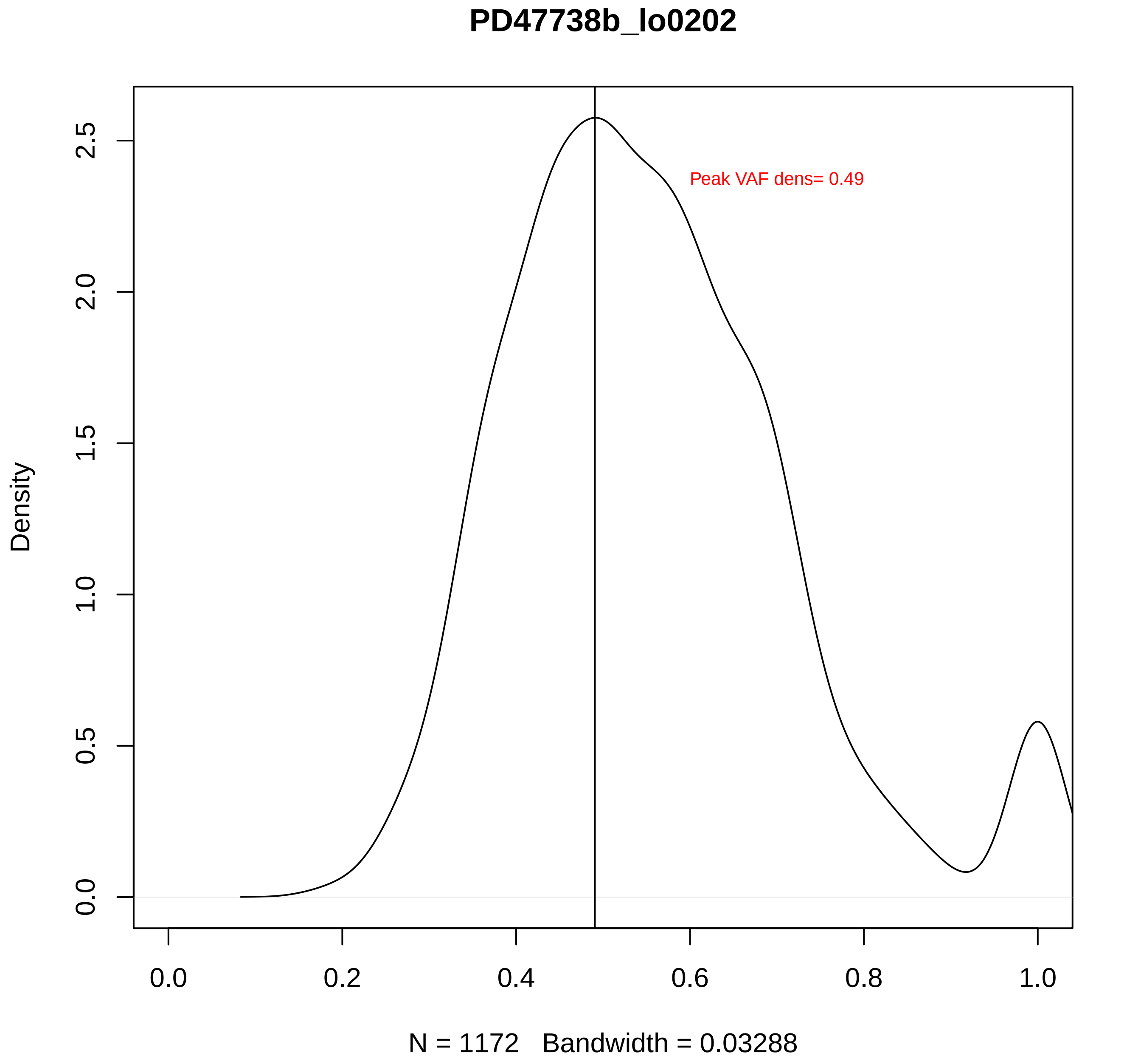

PD47738b_lo0202
Peak VAF dens= 0.49
Density
0.0
0.5
1.0
1.5
2.0
2.5
0.0
0.2
0.4
0.6
0.8
1.0
N = 1172 Bandwidth = 0.03288

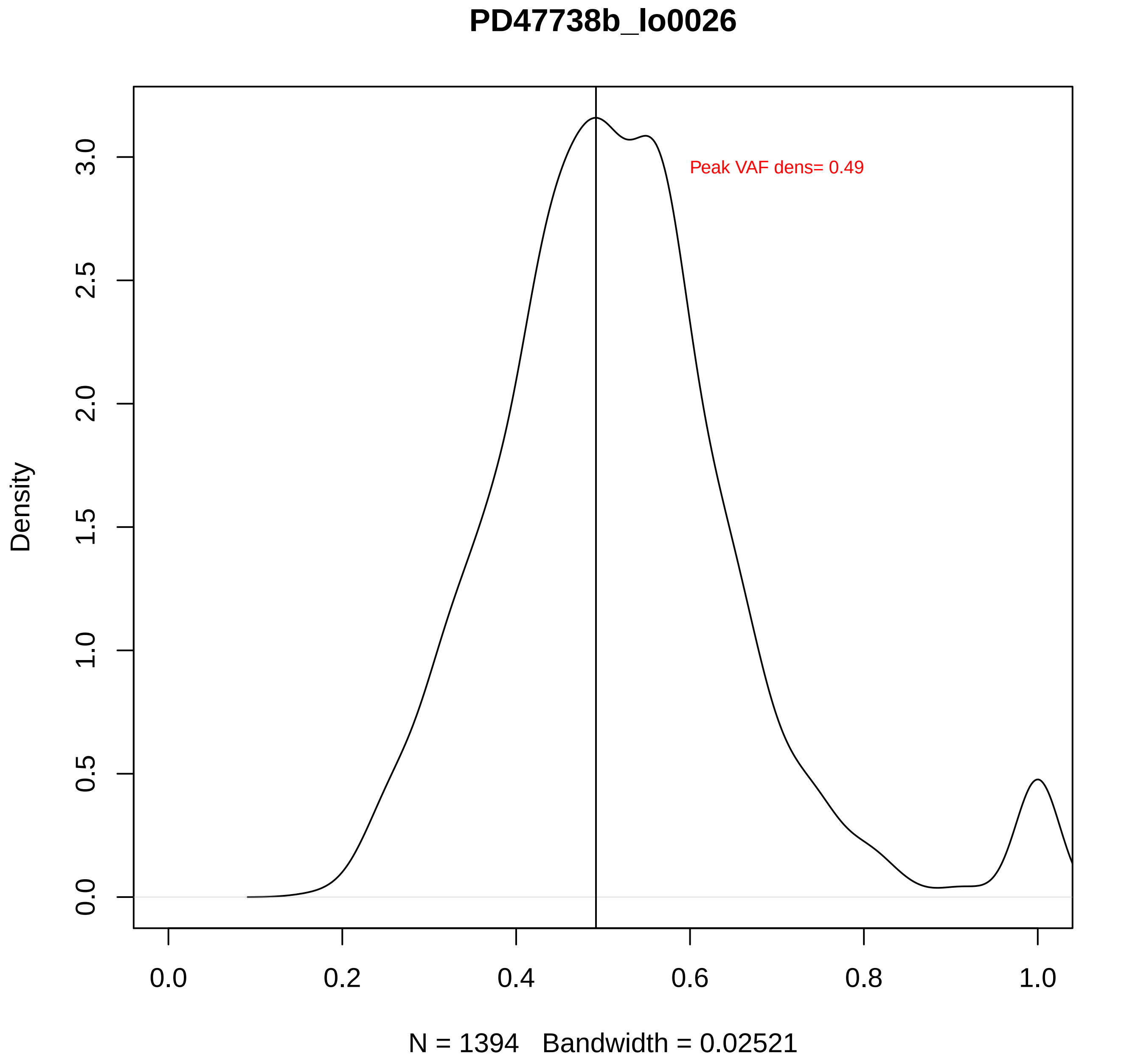
PD47738b_lo0026
Peak VAF dens= 0.49
Density
0.0
0.5
1.0
1.5
2.0
2.5
3.0
0.0
0.2
0.4
0.6
0.8
1.0
N = 1394   Bandwidth = 0.02521

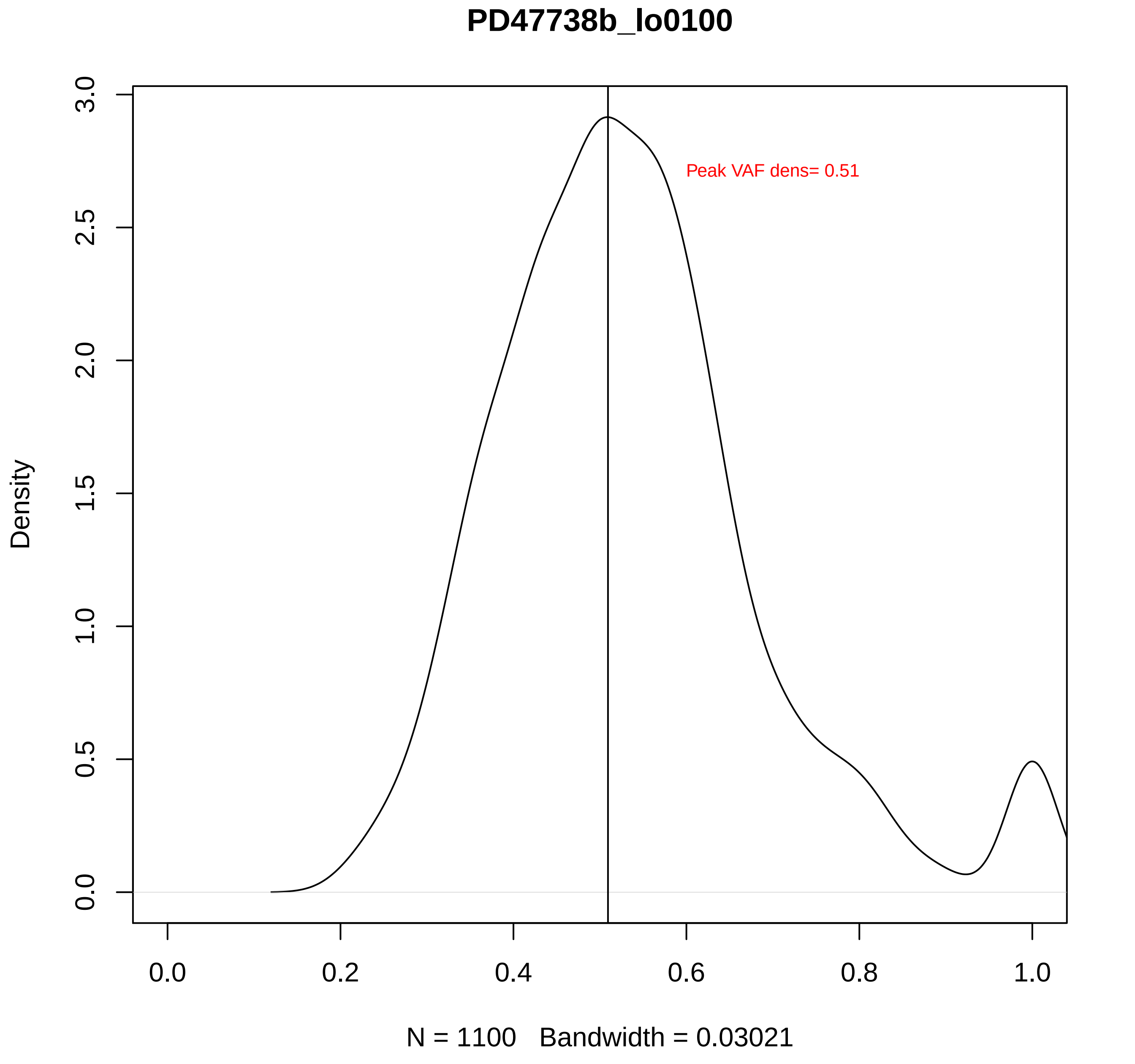
PD47738b_lo0100
Peak VAF dens= 0.51
Density
0.0
0.5
1.0
1.5
2.0
2.5
3.0
0.0
0.2
0.4
0.6
0.8
1.0
N = 1100   Bandwidth = 0.03021

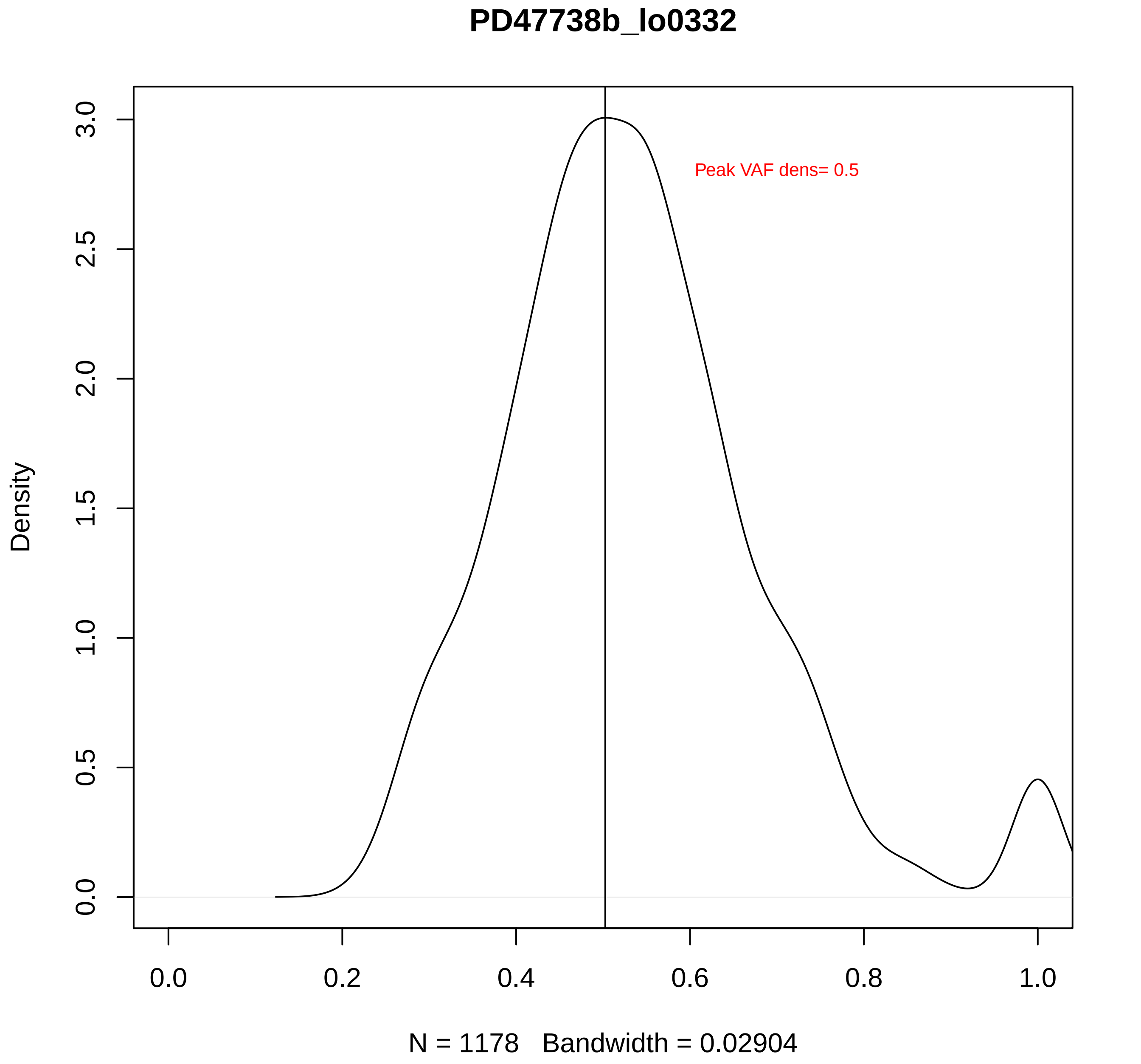
PD47738b_lo0332
Peak VAF dens= 0.5
Density
0.0
0.5
1.0
1.5
2.0
2.5
3.0
0.0
0.2
0.4
0.6
0.8
1.0
N = 1178   Bandwidth = 0.02904

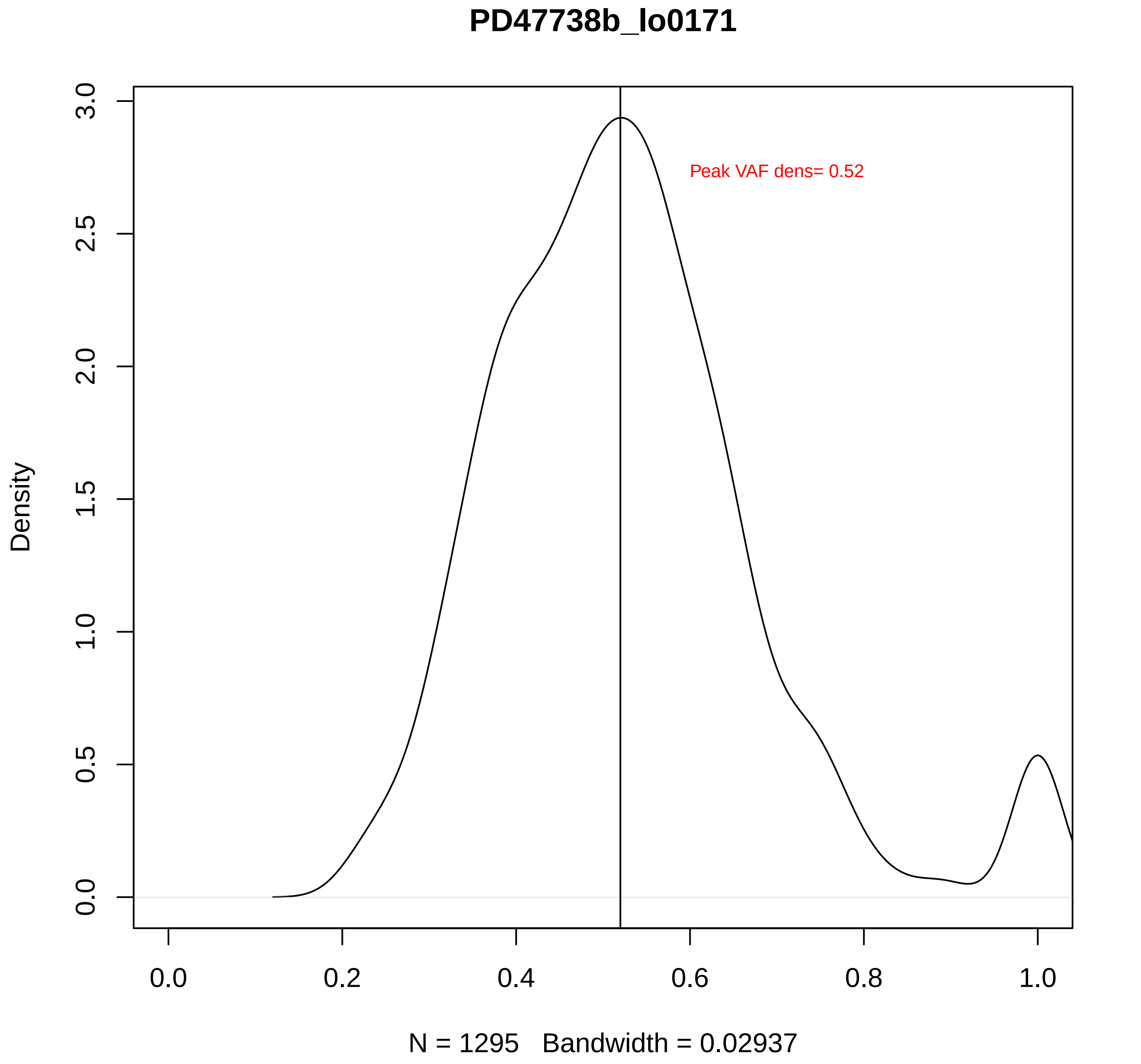
PD47738b_lo0171
Peak VAF dens= 0.52
Density
0.0
0.5
1.0
1.5
2.0
2.5
3.0
0.0
0.2
0.4
0.6
0.8
1.0
N = 1295   Bandwidth = 0.02937

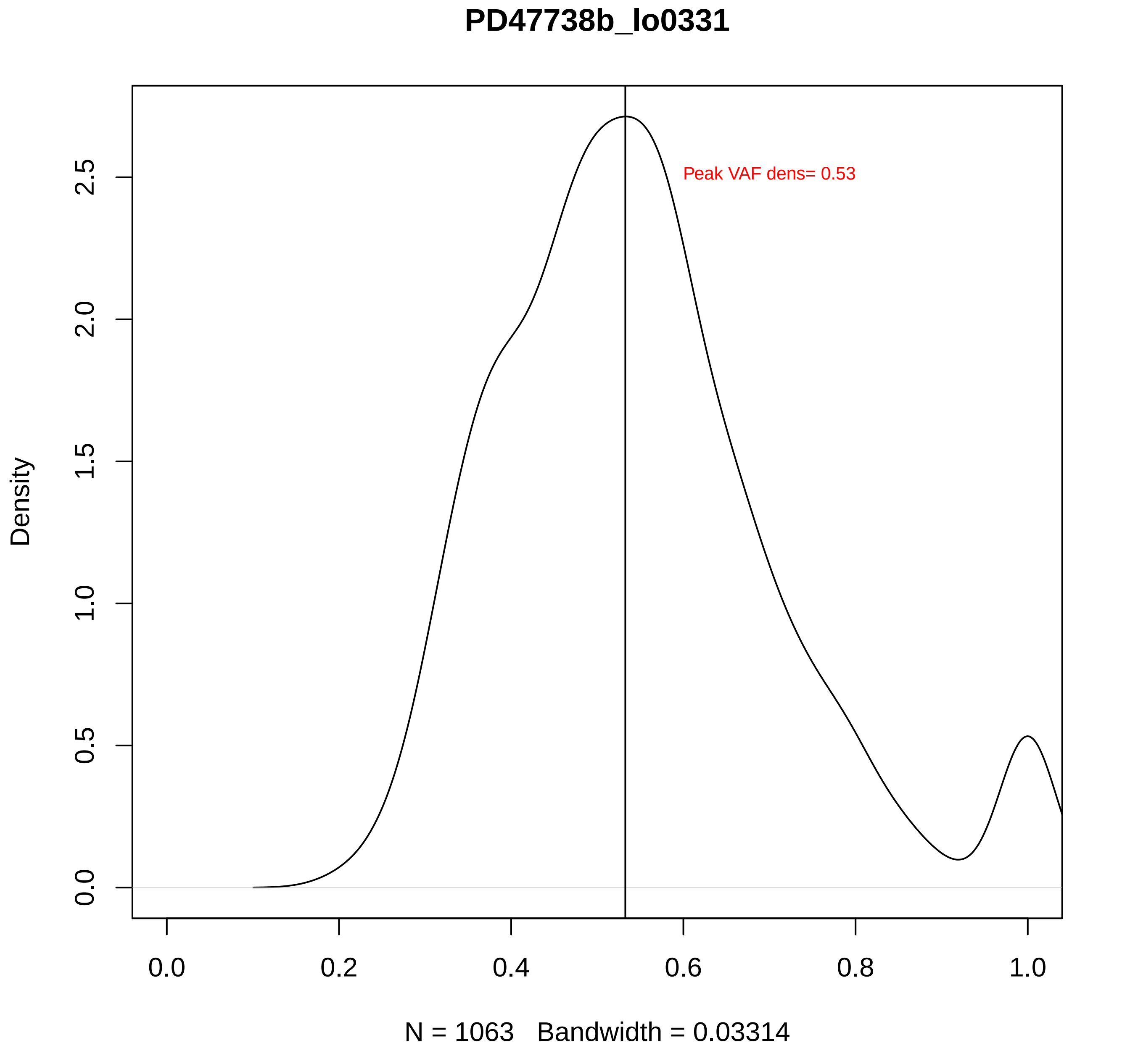
PD47738b_lo0331
Peak VAF dens= 0.53
Density
0.0
0.5
1.0
1.5
2.0
2.5
0.0
0.2
0.4
0.6
0.8
1.0
N = 1063   Bandwidth = 0.03314

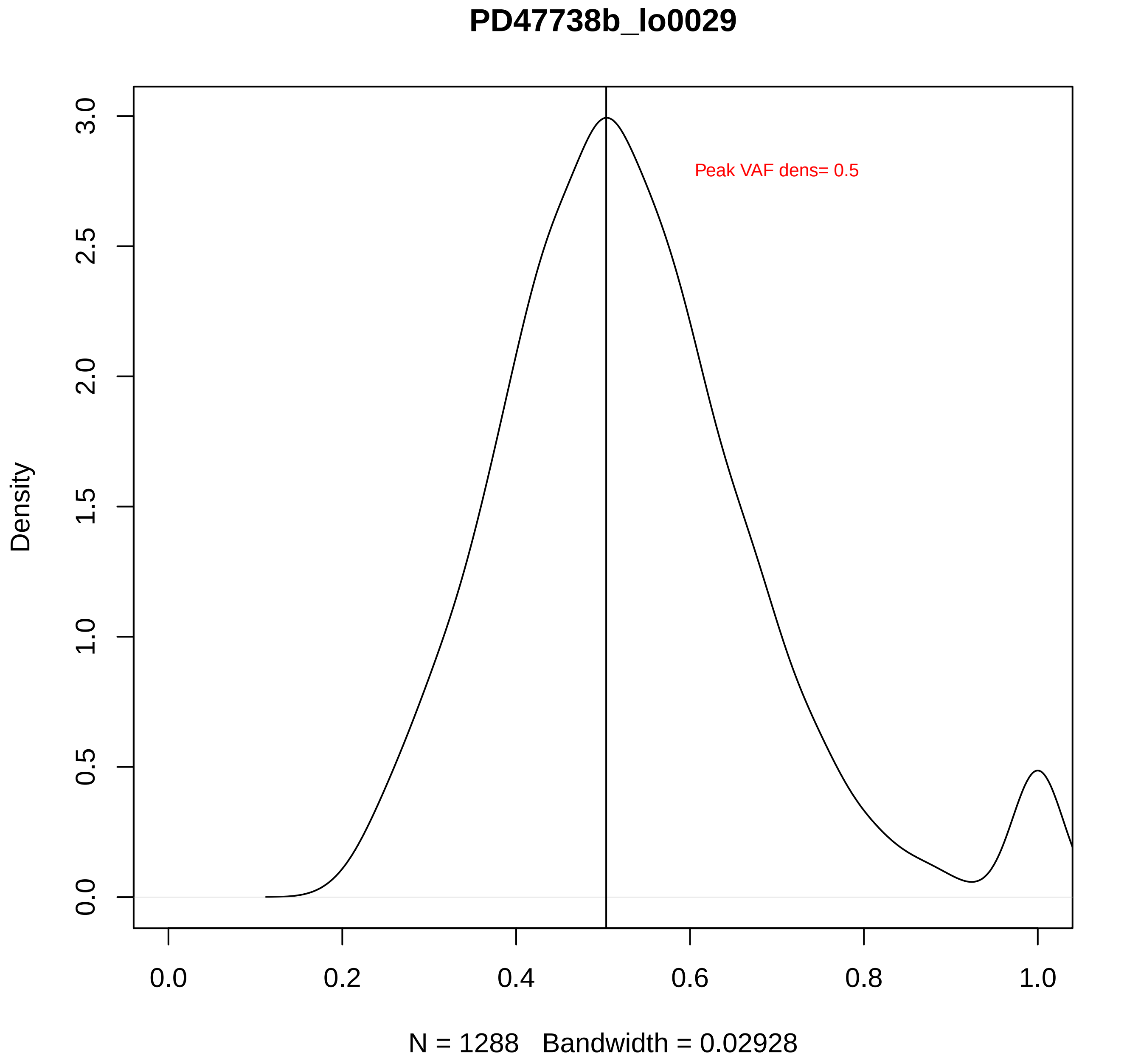

PD47738b_lo0029
Peak VAF dens= 0.5
Density
0.0
0.5
1.0
1.5
2.0
2.5
3.0
0.0
0.2
0.4
0.6
0.8
1.0
N = 1288 Bandwidth = 0.02928

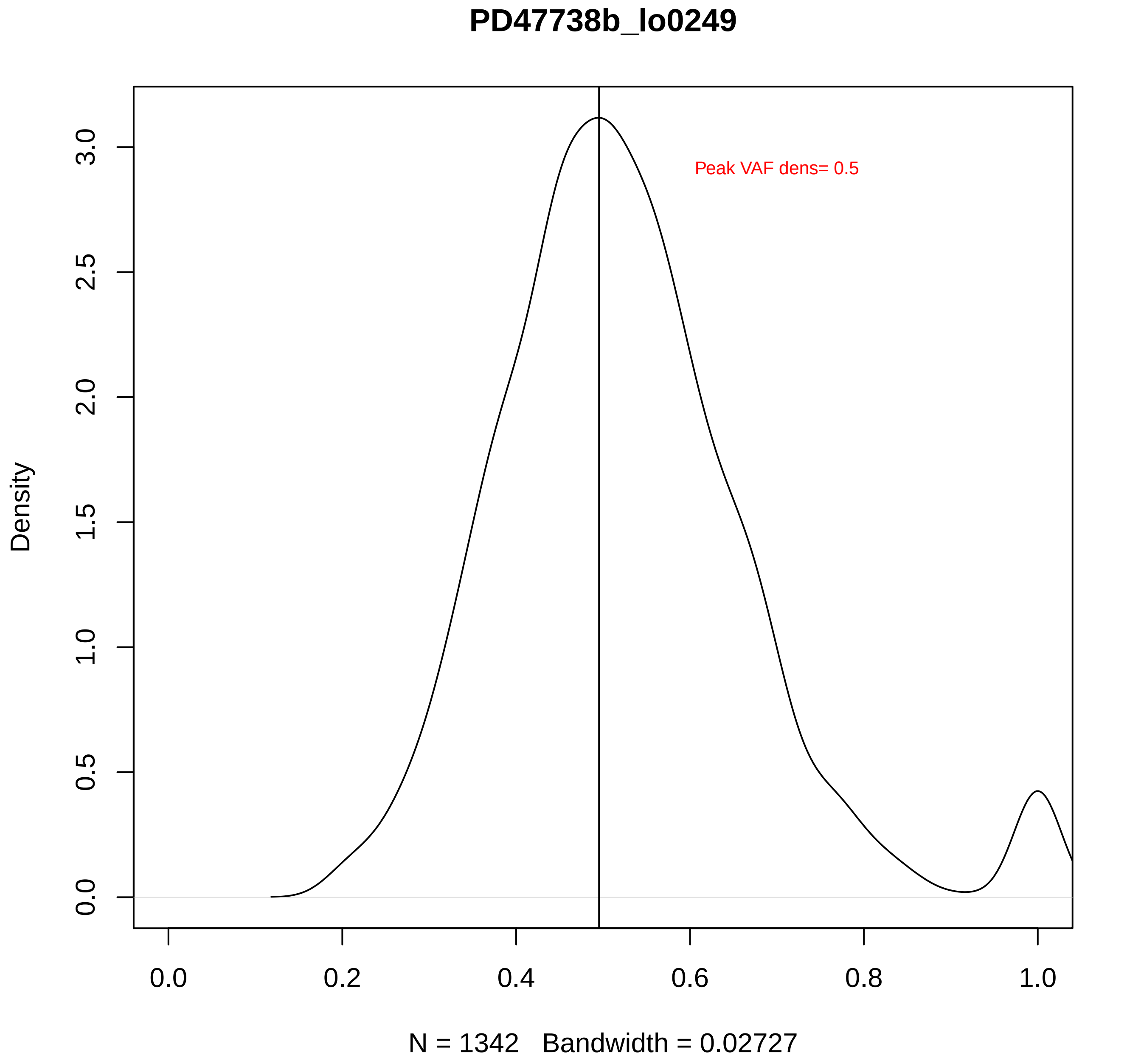
PD47738b_lo0249
Peak VAF dens= 0.5
Density
0.0
0.5
1.0
1.5
2.0
2.5
3.0
0.0
0.2
0.4
0.6
0.8
1.0
N = 1342   Bandwidth = 0.02727

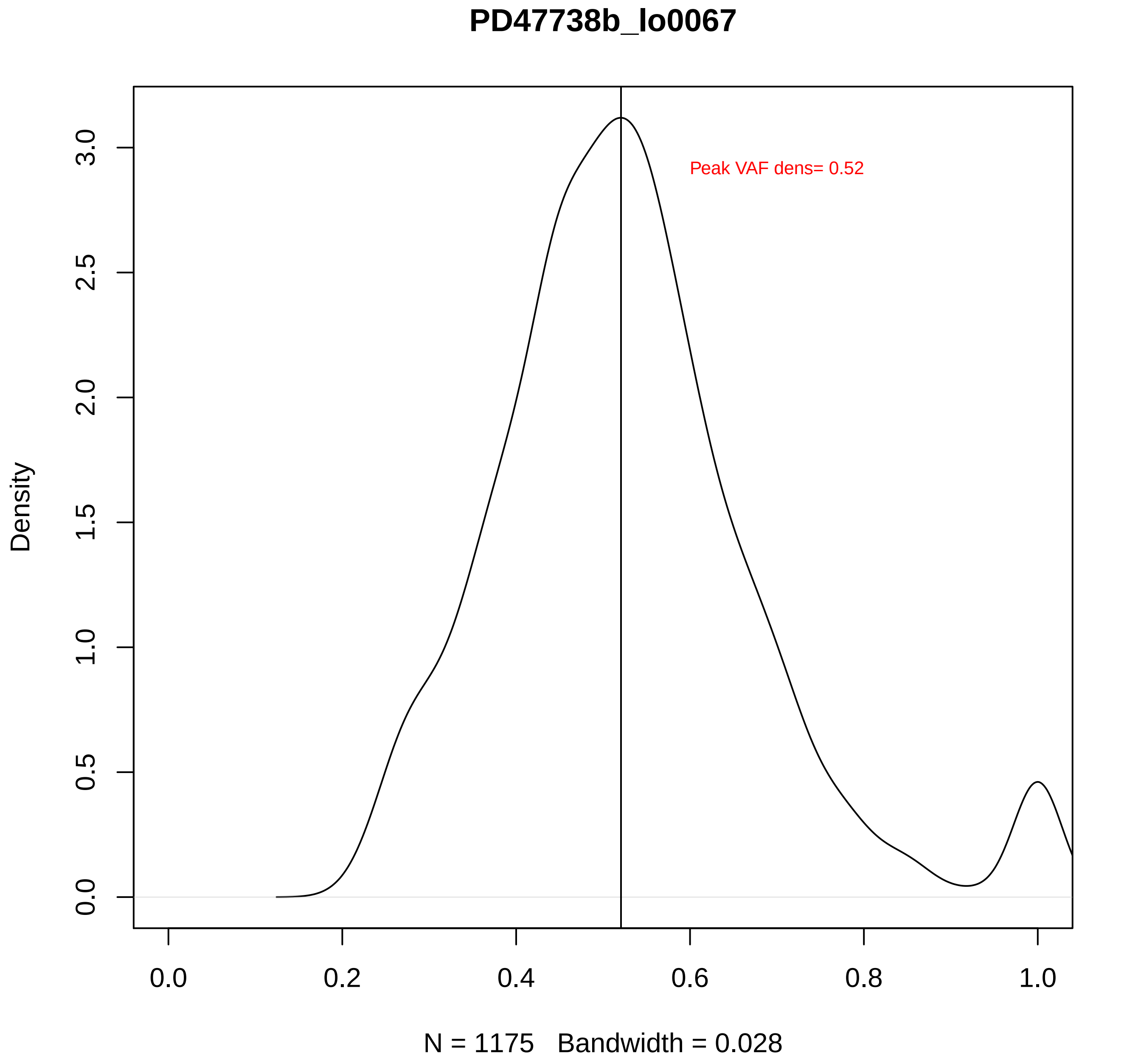

PD47738b_lo0067
Peak VAF dens= 0.52
Density
0.0
0.5
1.0
1.5
2.0
2.5
3.0
0.0
0.2
0.4
0.6
0.8
1.0
N = 1175 Bandwidth = 0.028

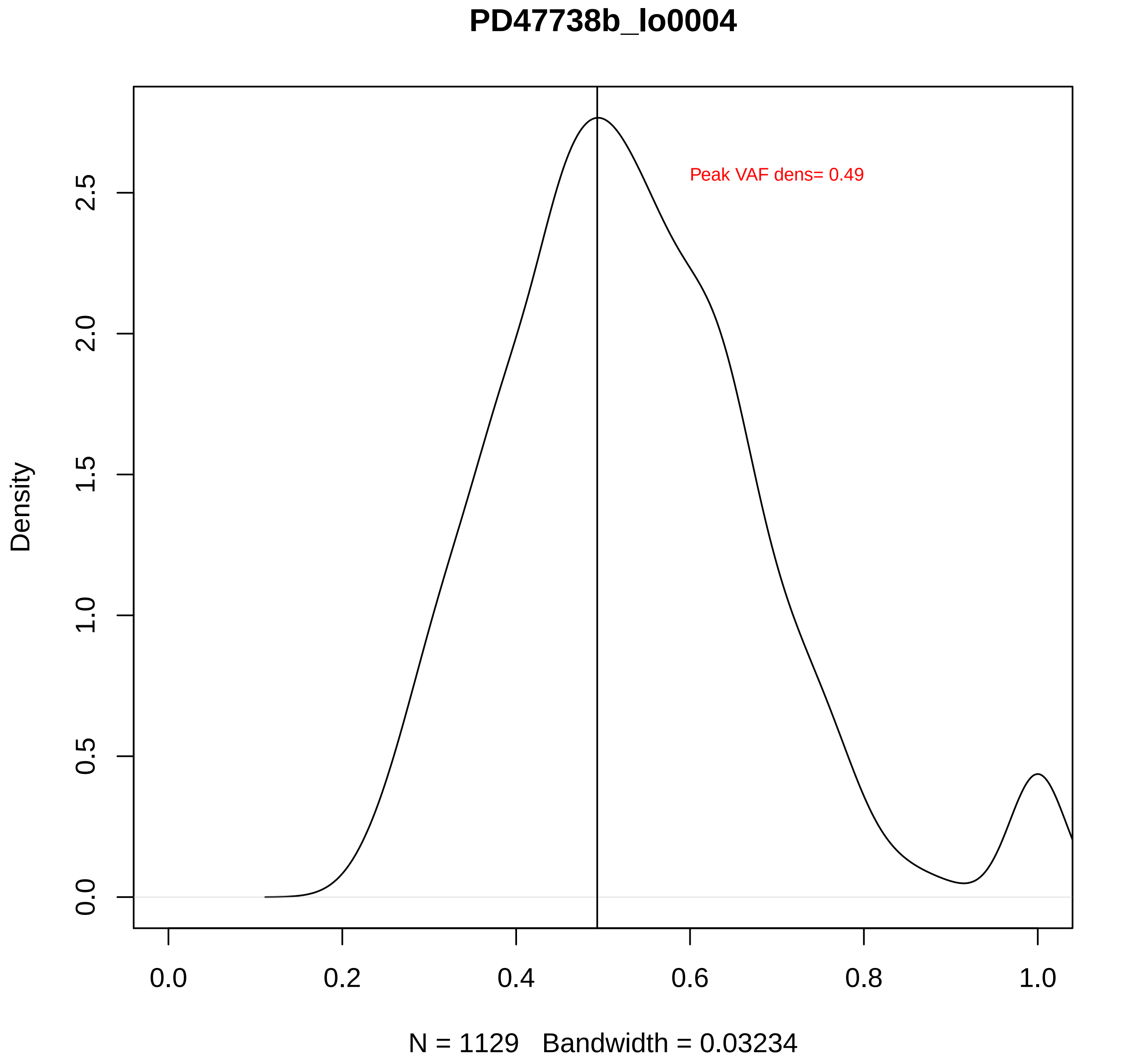

PD47738b_lo0004
Peak VAF dens= 0.49
Density
0.0
0.5
1.0
1.5
2.0
2.5
0.0
0.2
0.4
0.6
0.8
1.0
N = 1129   Bandwidth = 0.03234

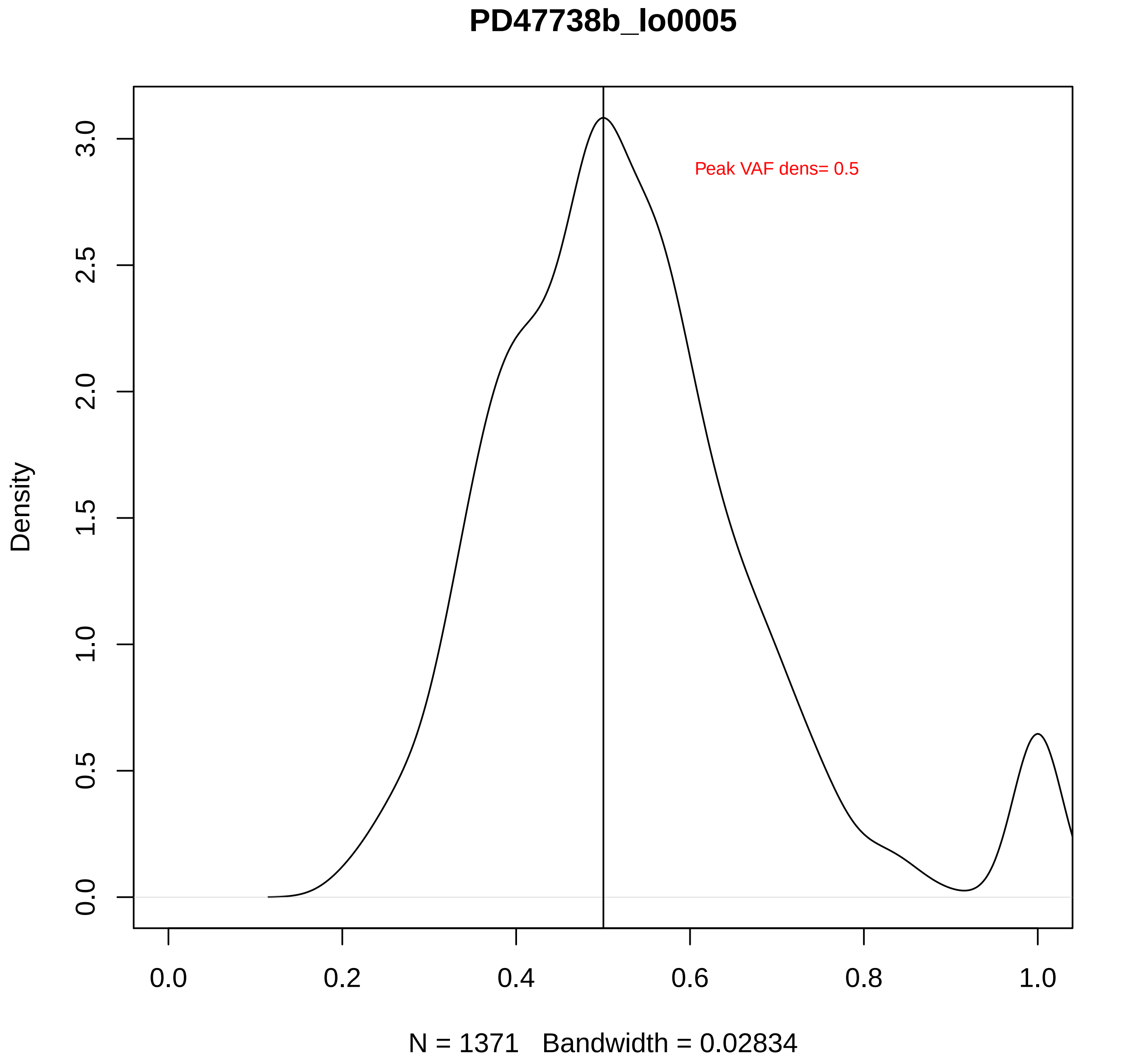
PD47738b_lo0005
Peak VAF dens= 0.5
Density
0.0
0.5
1.0
1.5
2.0
2.5
3.0
0.0
0.2
0.4
0.6
0.8
1.0
N = 1371   Bandwidth = 0.02834

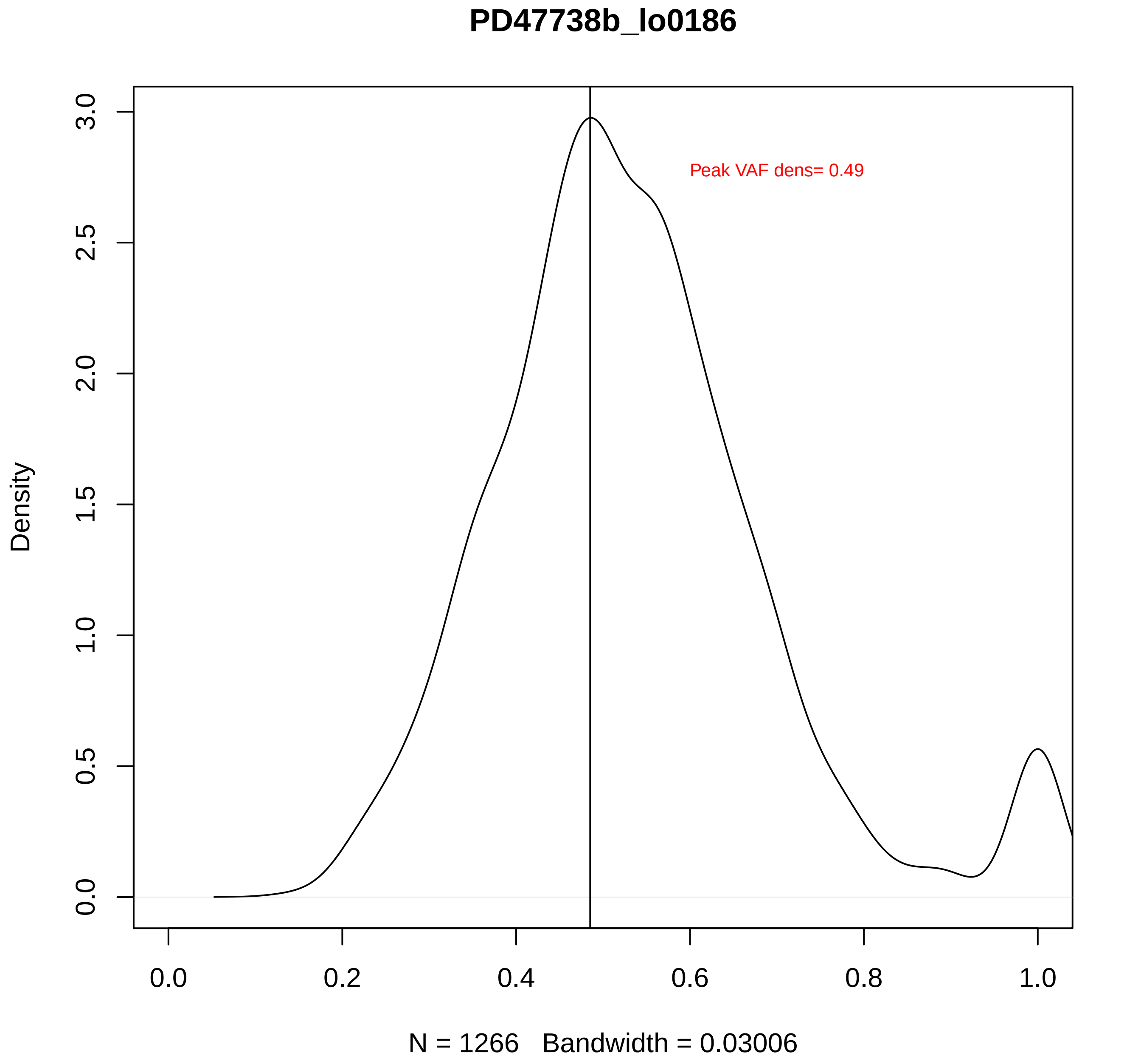
PD47738b_lo0186
Peak VAF dens= 0.49
Density
0.0
0.5
1.0
1.5
2.0
2.5
3.0
0.0
0.2
0.4
0.6
0.8
1.0
N = 1266   Bandwidth = 0.03006

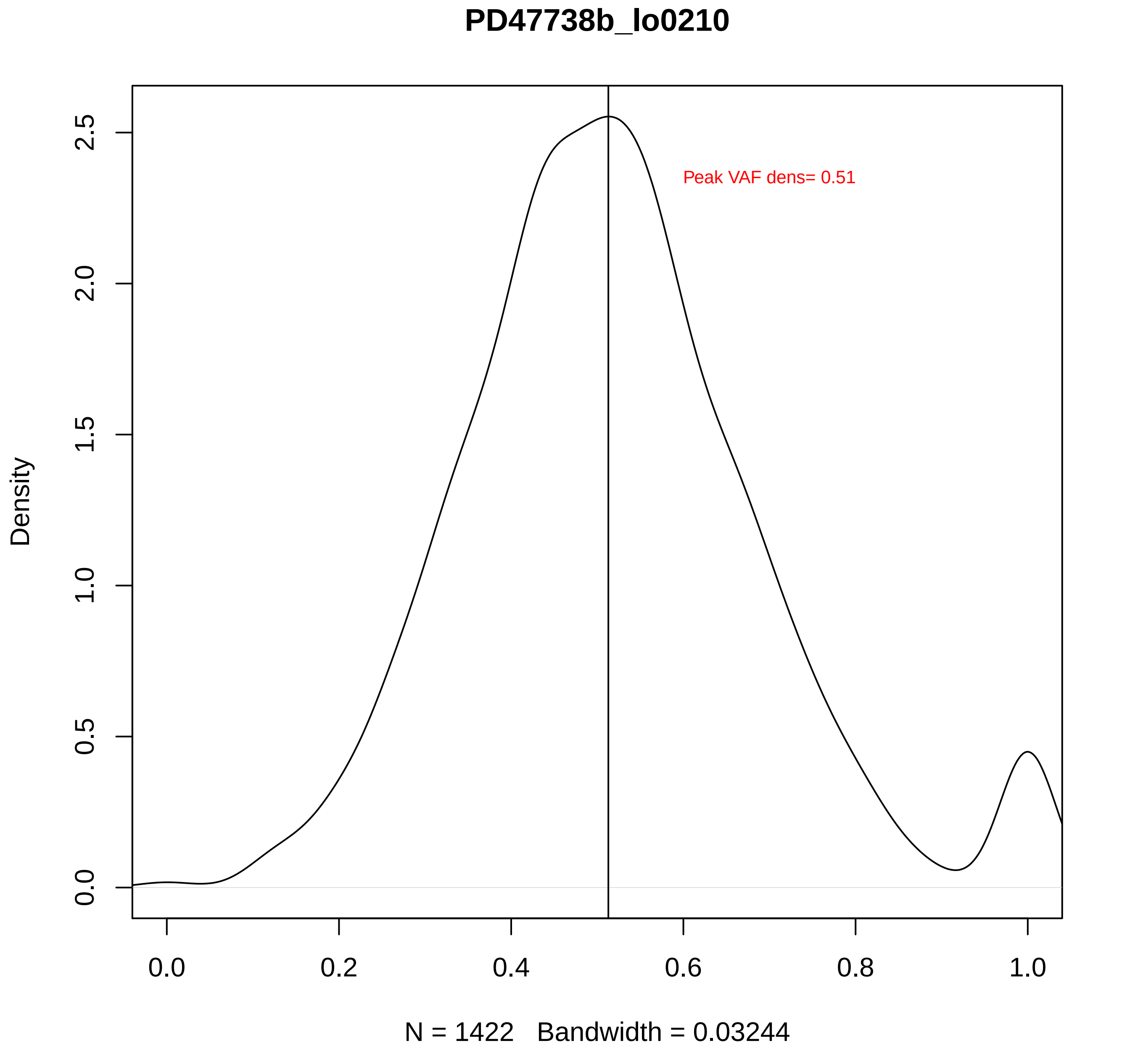

PD47738b_lo0210
Peak VAF dens= 0.51
Density
0.0
0.5
1.0
1.5
2.0
2.5
0.0
0.2
0.4
0.6
0.8
1.0
N = 1422   Bandwidth = 0.03244

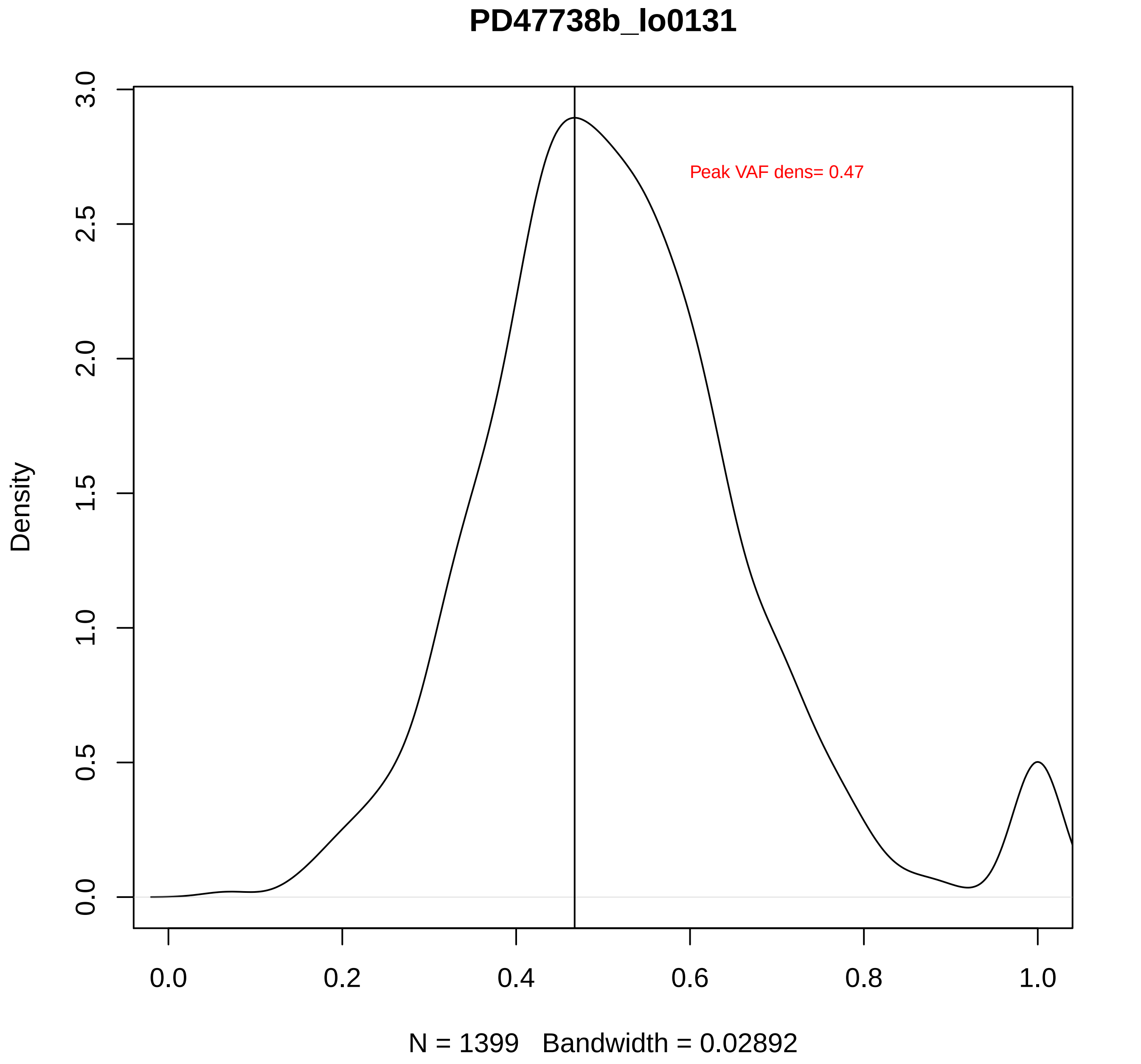

PD47738b_lo0131
Peak VAF dens= 0.47
Density
3.0
2.5
2.0
1.5
1.0
0.5
0.0
0.0
0.2
0.4
0.6
0.8
1.0
N = 1399 Bandwidth = 0.02892

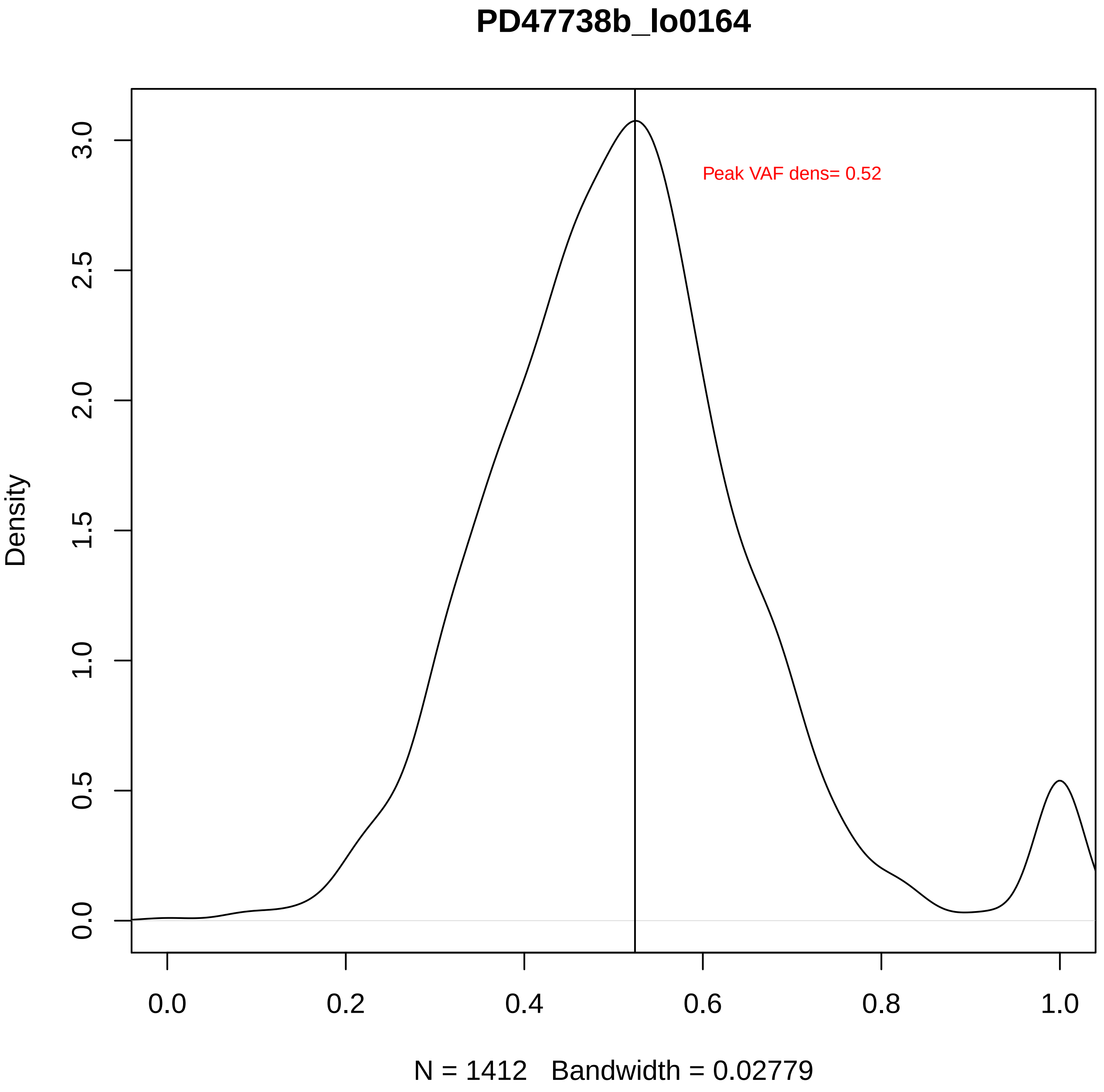
PD47738b_lo0164
Peak VAF dens= 0.52
Density
0.0
0.5
1.0
1.5
2.0
2.5
3.0
0.0
0.2
0.4
0.6
0.8
1.0
N = 1412   Bandwidth = 0.02779

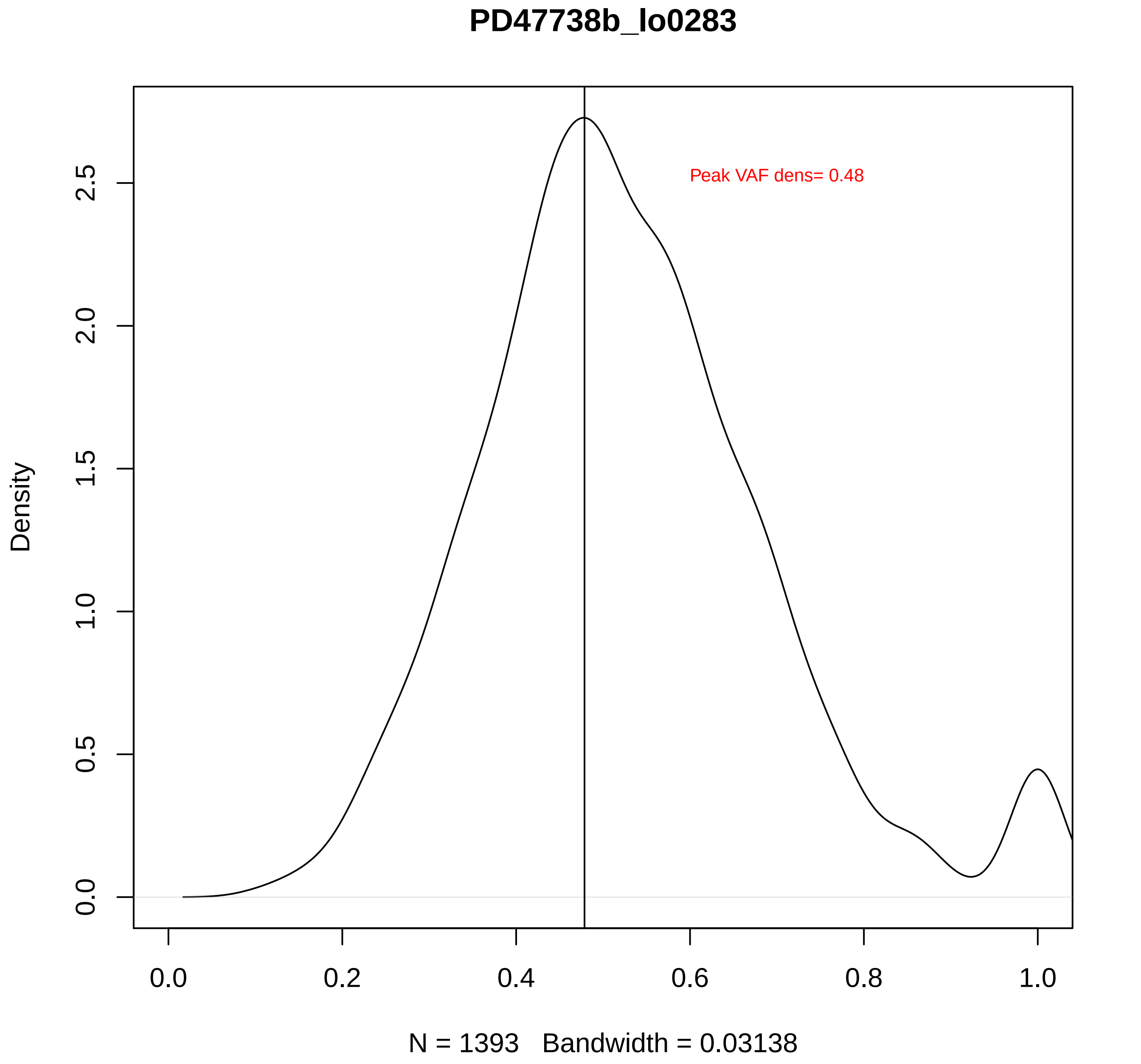
PD47738b_lo0283
Peak VAF dens= 0.48
Density
0.0
0.5
1.0
1.5
2.0
2.5
0.0
0.2
0.4
0.6
0.8
1.0
N = 1393   Bandwidth = 0.03138

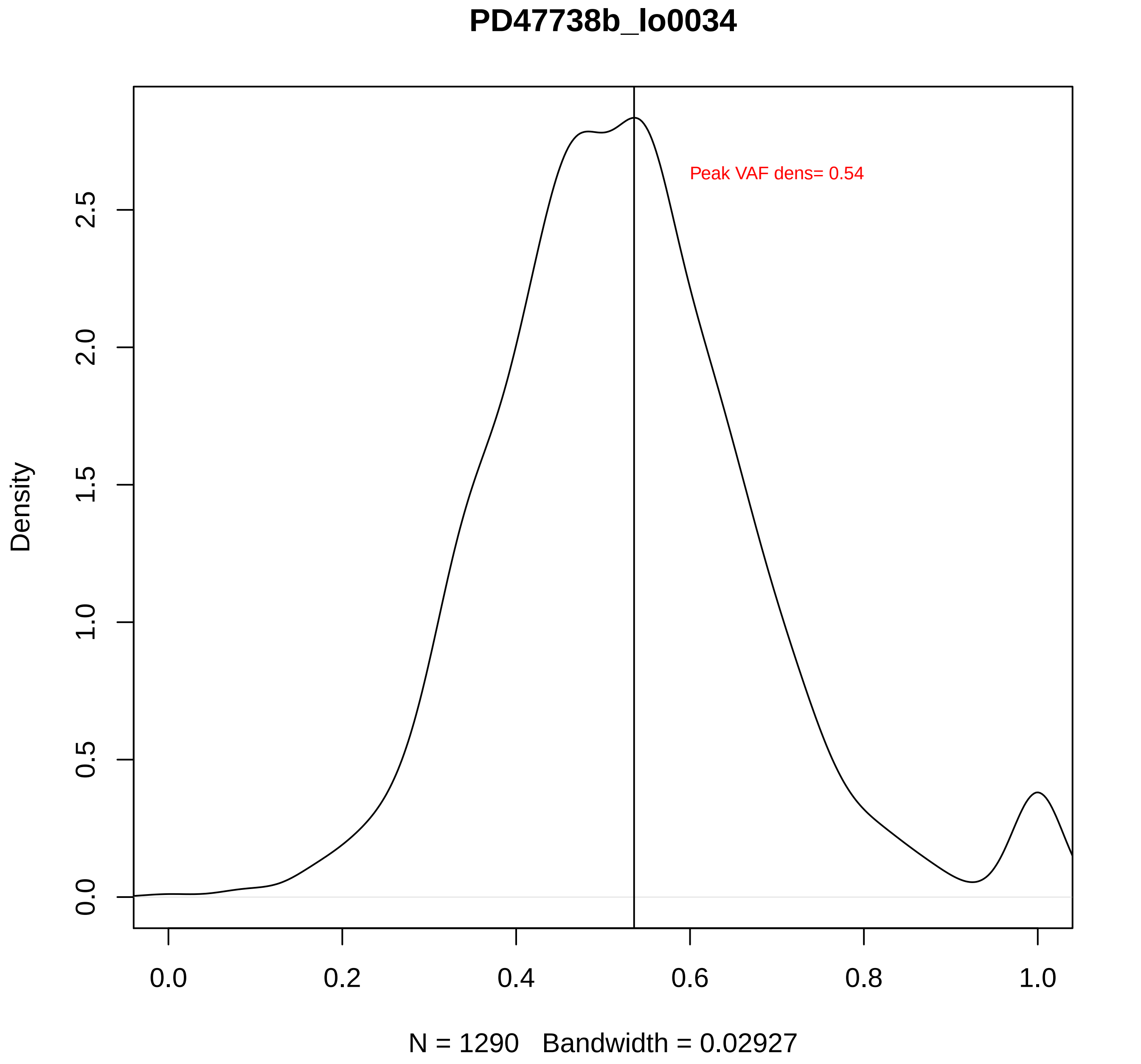
PD47738b_lo0034
Peak VAF dens= 0.54
Density
0.0
0.5
1.0
1.5
2.0
2.5
0.0
0.2
0.4
0.6
0.8
1.0
N = 1290   Bandwidth = 0.02927

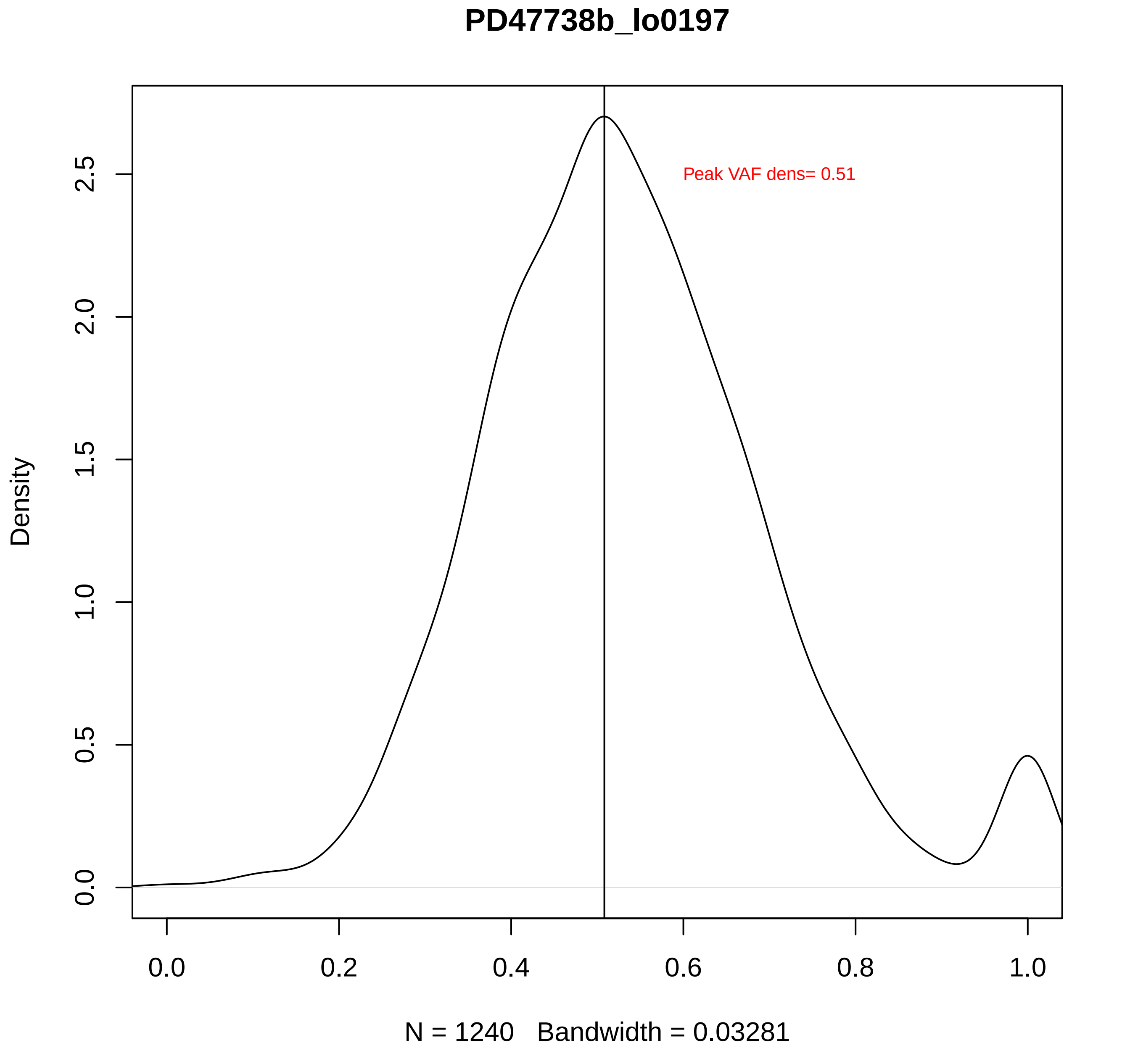

PD47738b_lo0197
Peak VAF dens= 0.51
Density
0.0
0.5
1.0
1.5
2.0
2.5
0.0
0.2
0.4
0.6
0.8
1.0
N = 1240   Bandwidth = 0.03281

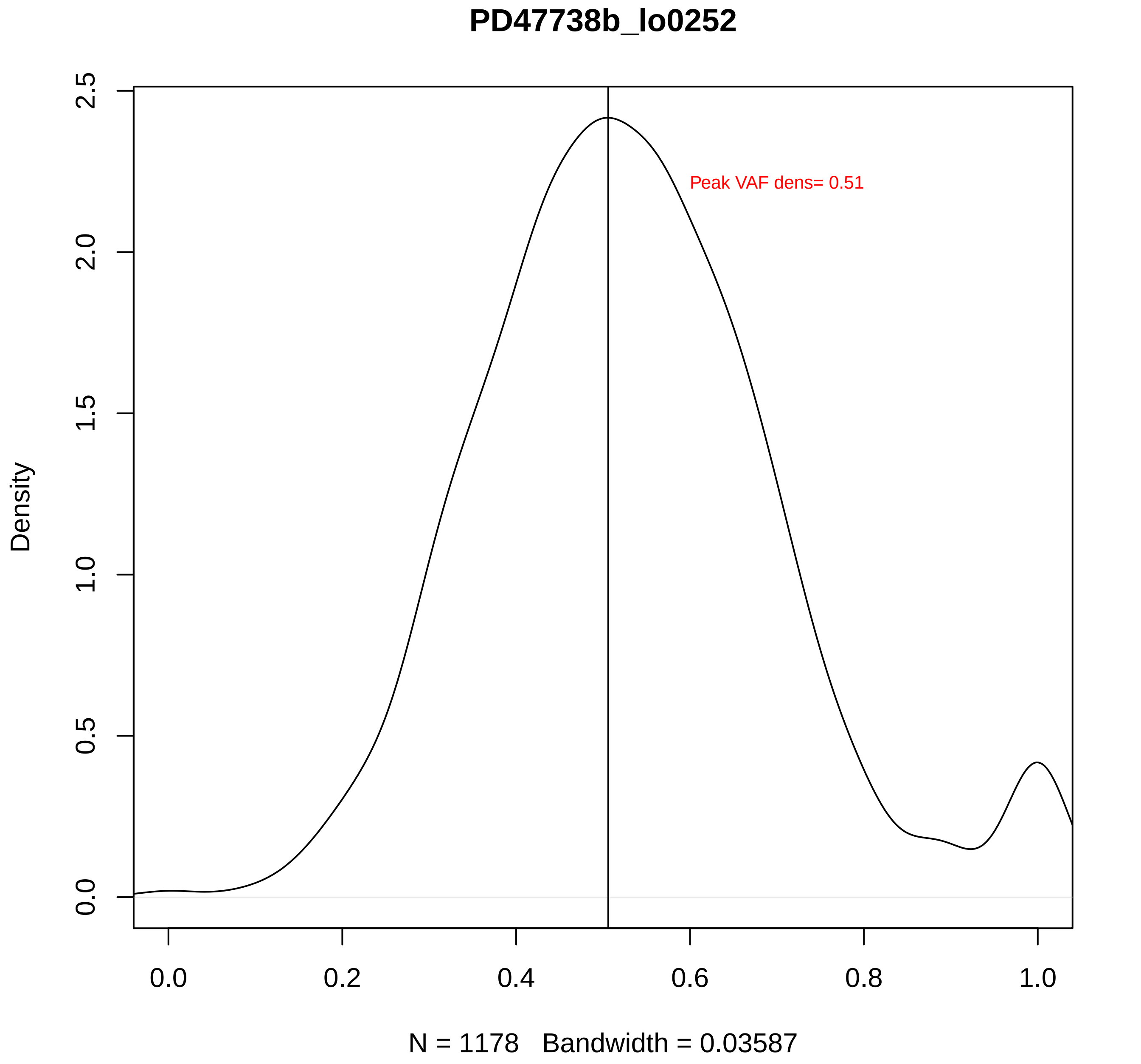
PD47738b_lo0252
Peak VAF dens= 0.51
Density
0.0
0.5
1.0
1.5
2.0
2.5
0.0
0.2
0.4
0.6
0.8
1.0
N = 1178   Bandwidth = 0.03587

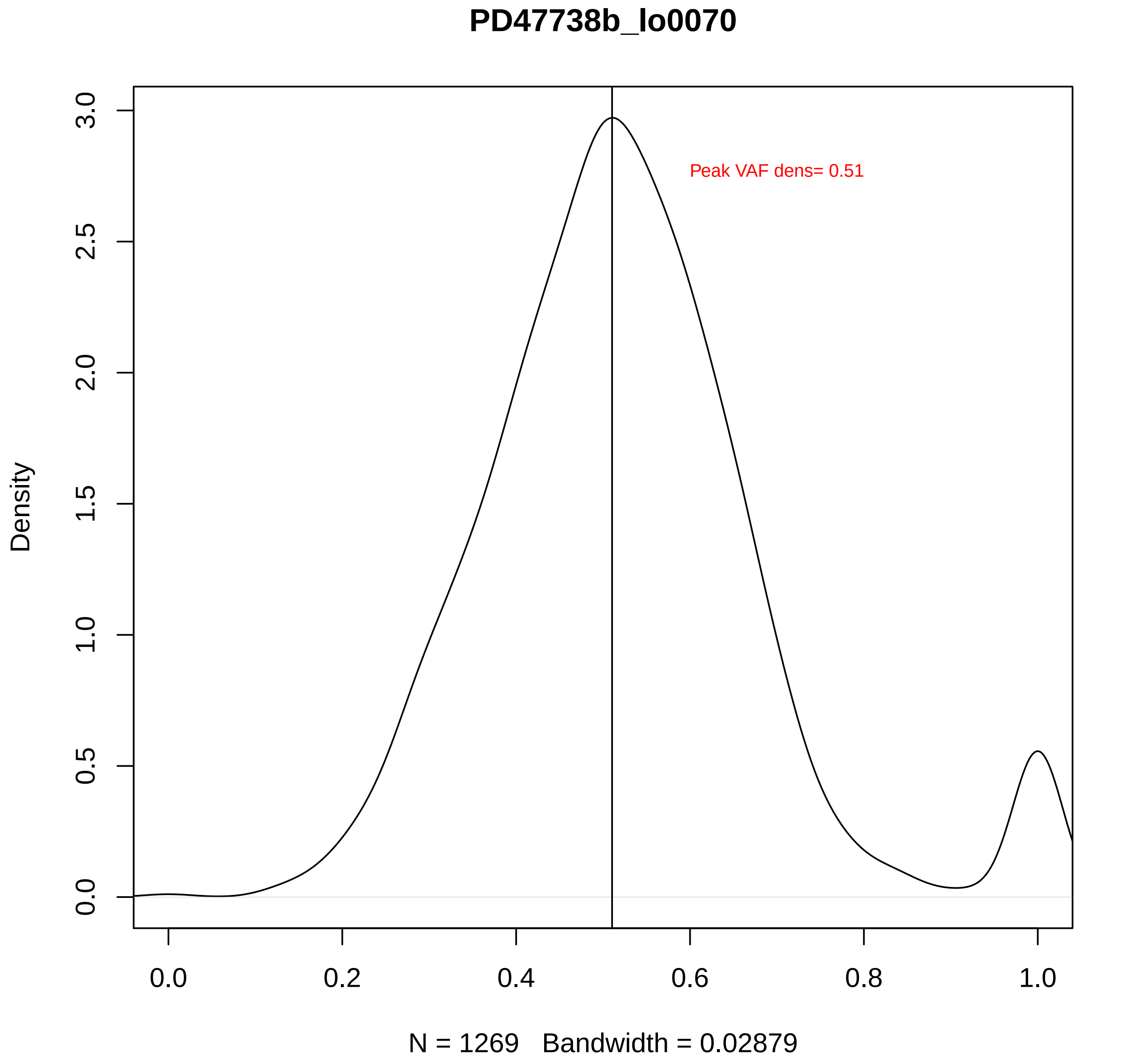
PD47738b_lo0070
Peak VAF dens= 0.51
Density
0.0
0.5
1.0
1.5
2.0
2.5
3.0
0.0
0.2
0.4
0.6
0.8
1.0
N = 1269   Bandwidth = 0.02879

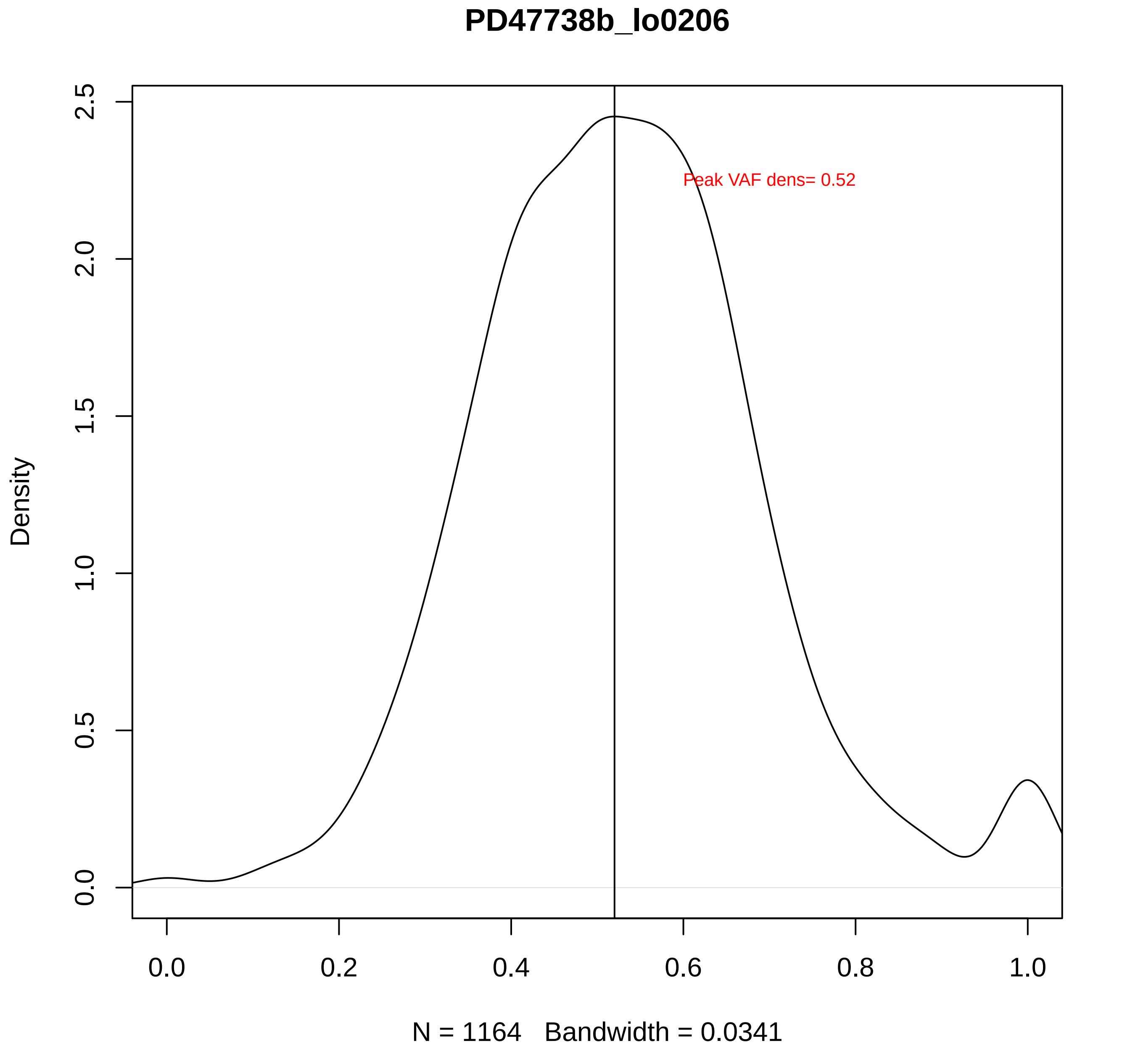

PD47738b_lo0206
Peak VAF dens= 0.52
Density
0.0
0.5
1.0
1.5
2.0
2.5
0.0
0.2
0.4
0.6
0.8
1.0
N = 1164   Bandwidth = 0.0341

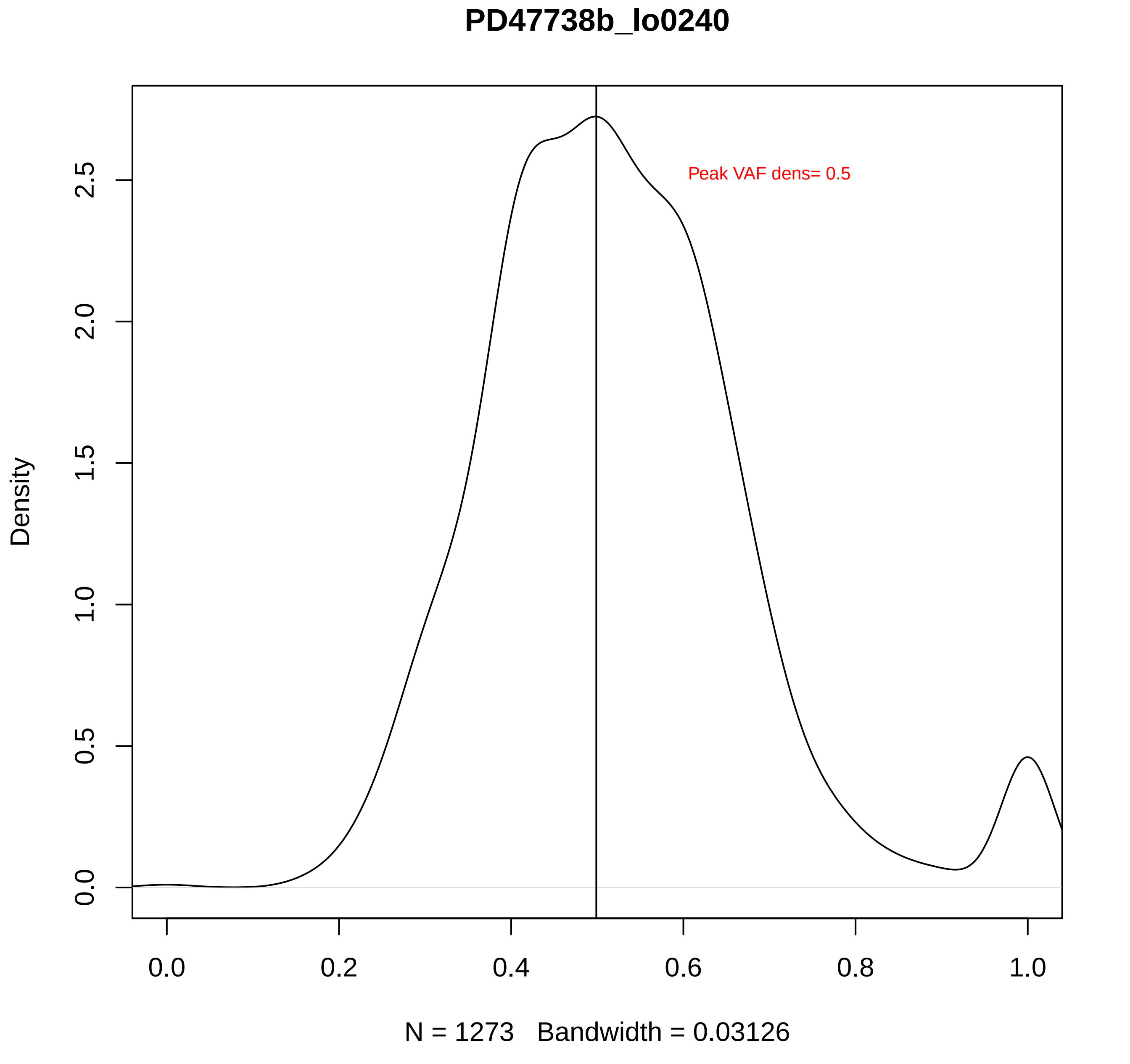
PD47738b_lo0240
Peak VAF dens= 0.5
Density
0.0
0.5
1.0
1.5
2.0
2.5
0.0
0.2
0.4
0.6
0.8
1.0
N = 1273   Bandwidth = 0.03126

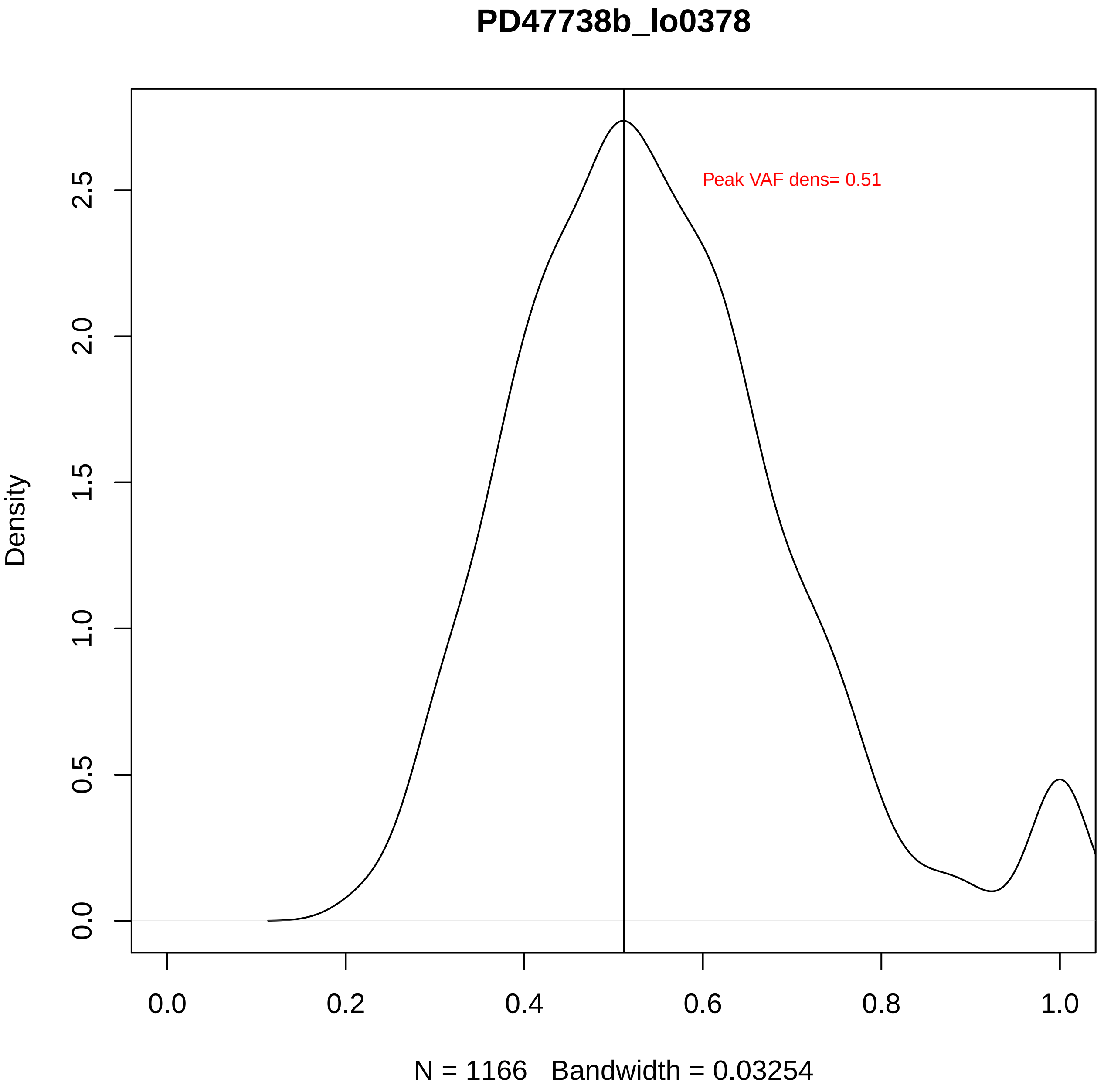
PD47738b_lo0378
Peak VAF dens= 0.51
Density
0.0
0.5
1.0
1.5
2.0
2.5
0.0
0.2
0.4
0.6
0.8
1.0
N = 1166   Bandwidth = 0.03254

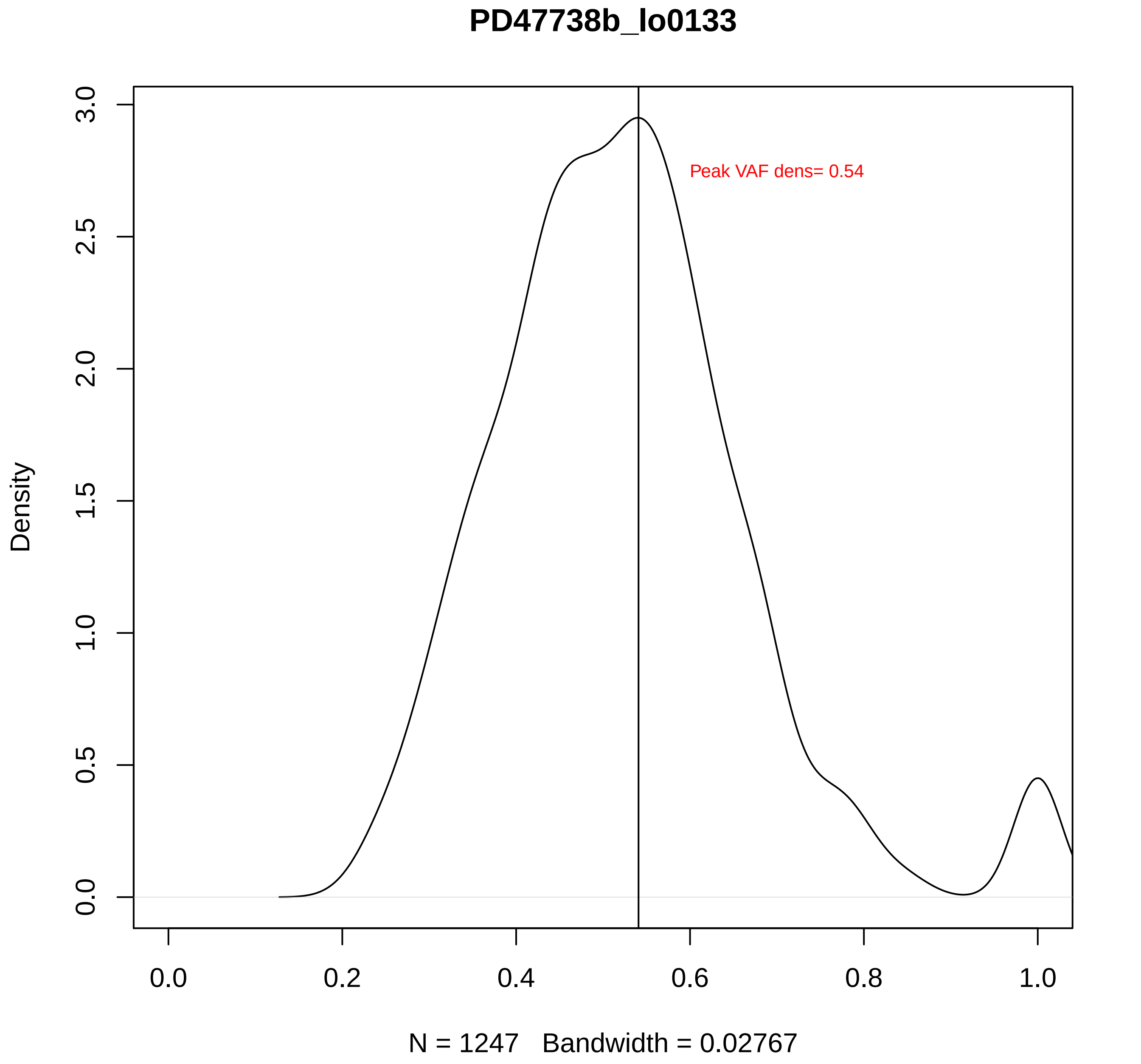
PD47738b_lo0133
Peak VAF dens= 0.54
Density
0.0
0.5
1.0
1.5
2.0
2.5
3.0
0.0
0.2
0.4
0.6
0.8
1.0
N = 1247   Bandwidth = 0.02767

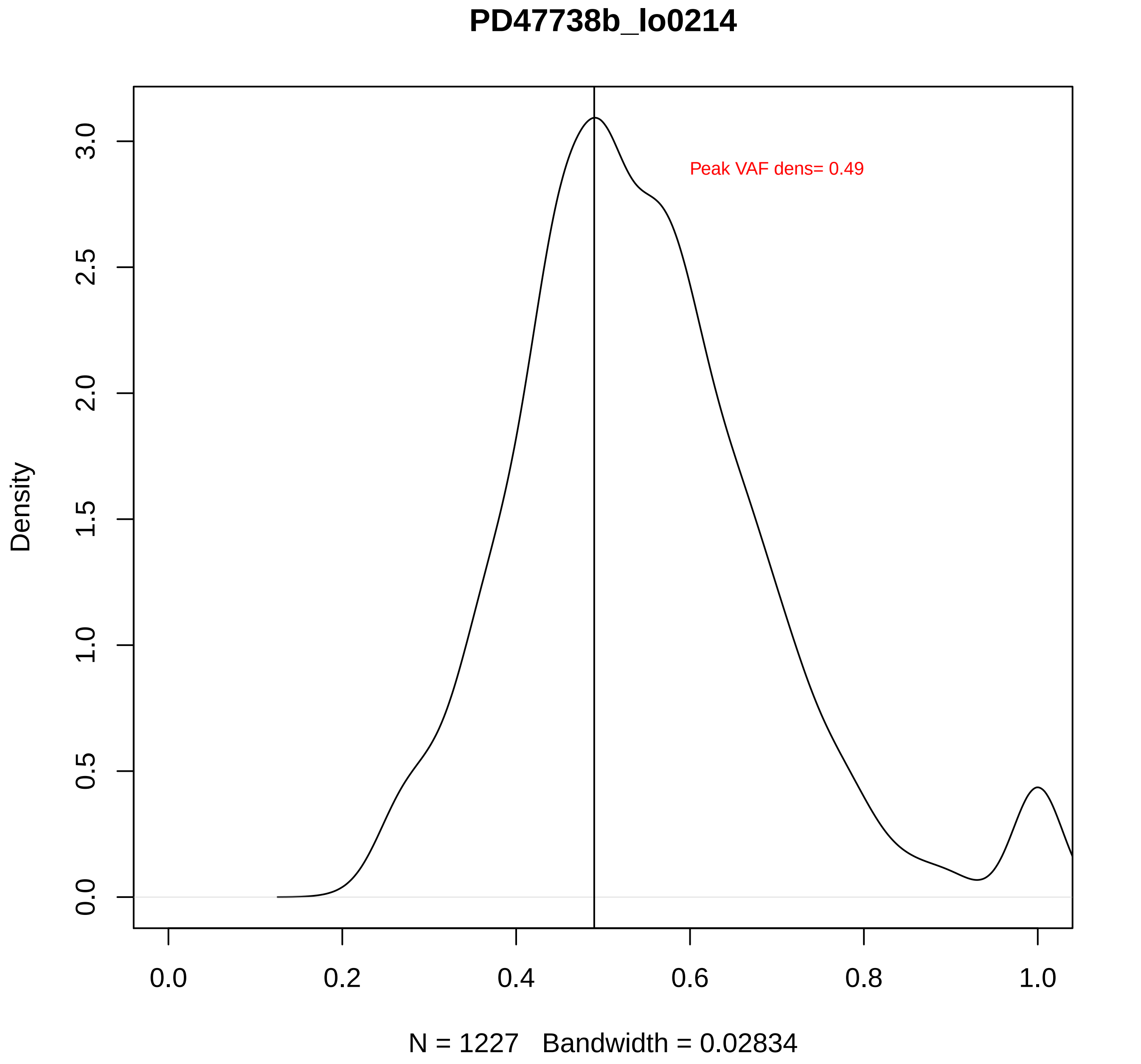
PD47738b_lo0214
Peak VAF dens= 0.49
Density
0.0
0.5
1.0
1.5
2.0
2.5
3.0
0.0
0.2
0.4
0.6
0.8
1.0
N = 1227   Bandwidth = 0.02834

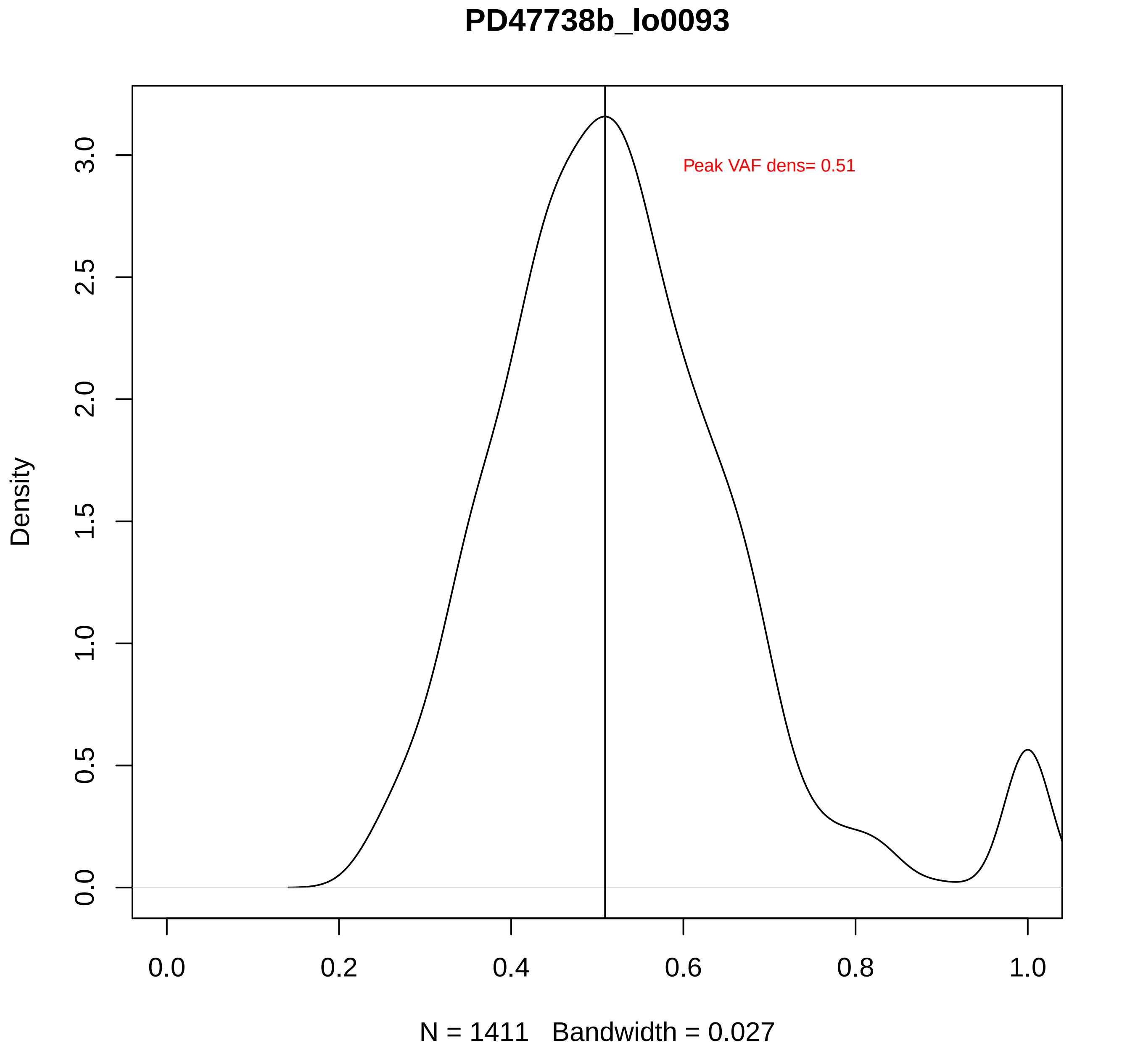
PD47738b_lo0093
Peak VAF dens= 0.51
Density
0.0
0.5
1.0
1.5
2.0
2.5
3.0
0.0
0.2
0.4
0.6
0.8
1.0
N = 1411 Bandwidth = 0.027

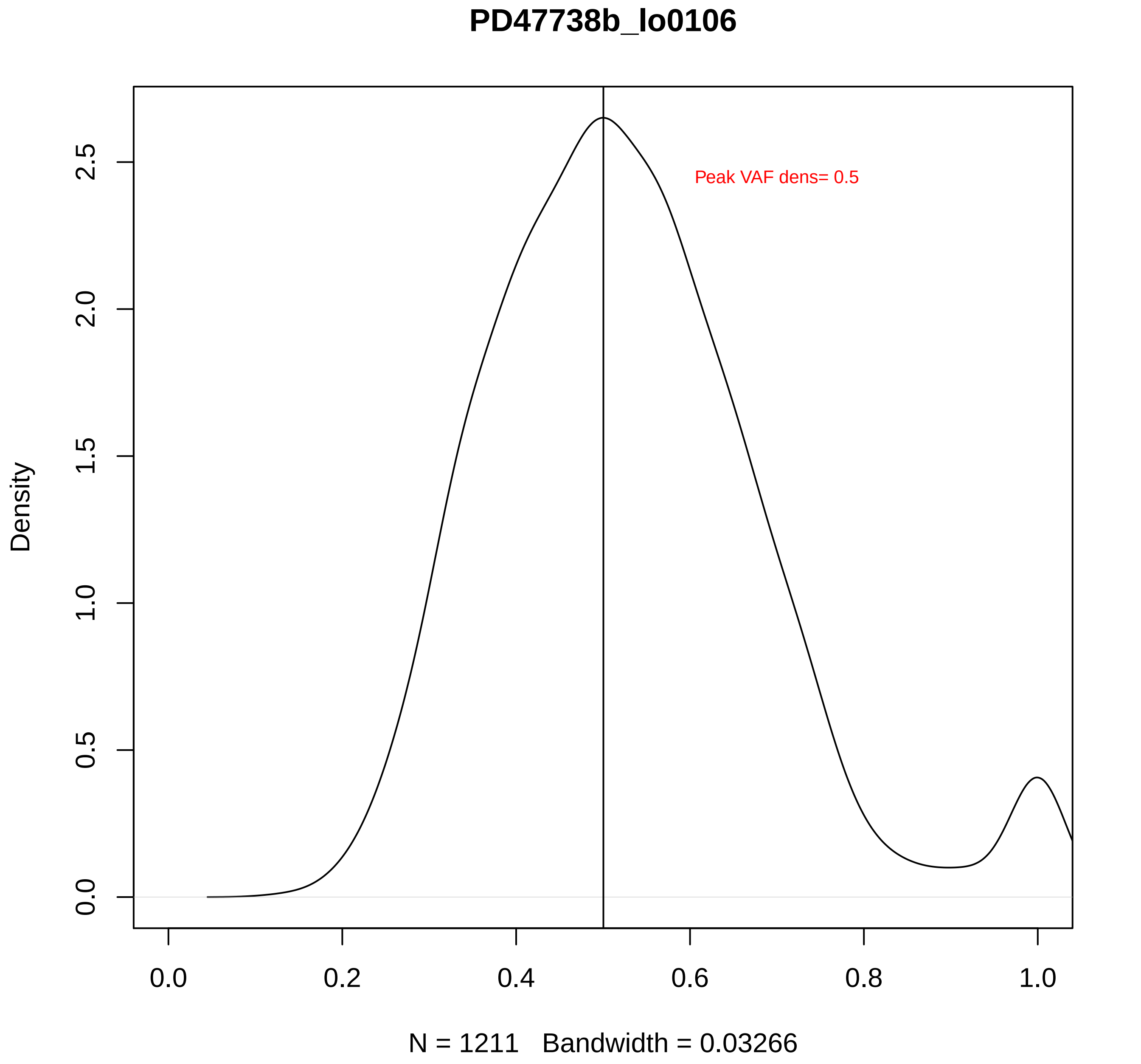
PD47738b_lo0106
Peak VAF dens= 0.5
Density
0.0
0.5
1.0
1.5
2.0
2.5
0.0
0.2
0.4
0.6
0.8
1.0
N = 1211 Bandwidth = 0.03266

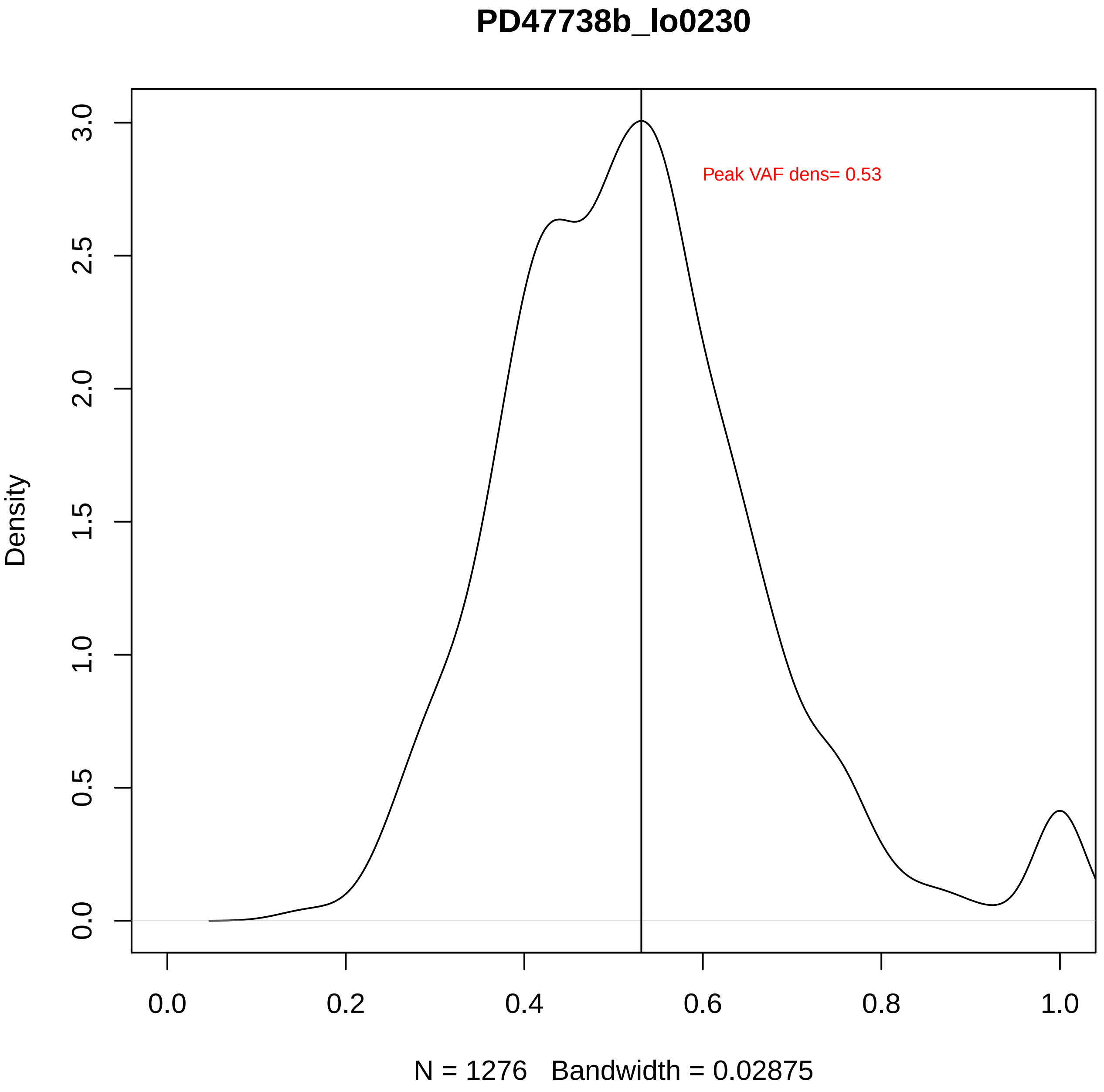
PD47738b_lo0230
Peak VAF dens= 0.53
Density
0.0
0.5
1.0
1.5
2.0
2.5
3.0
0.0
0.2
0.4
0.6
0.8
1.0
N = 1276   Bandwidth = 0.02875

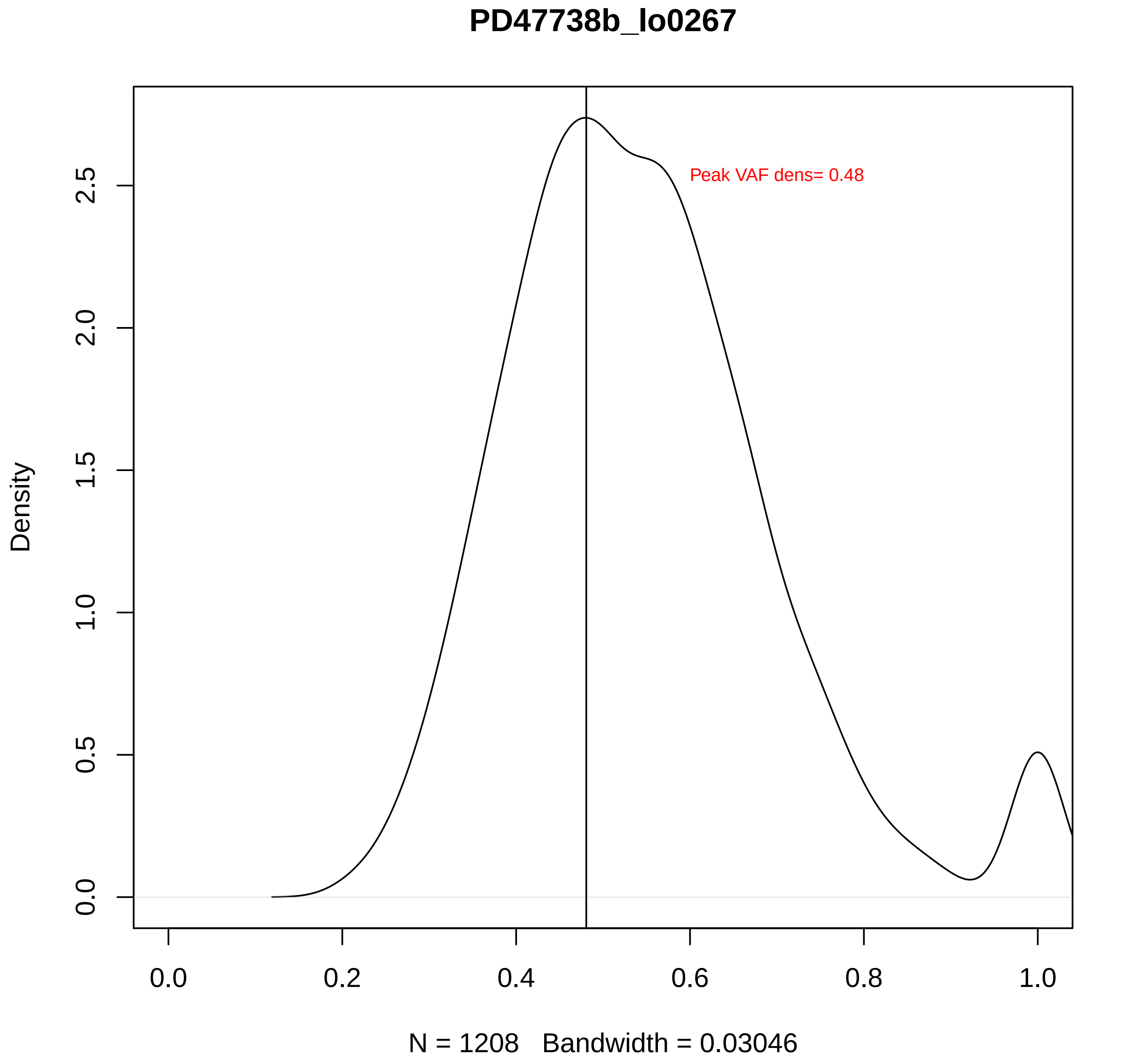
PD47738b_lo0267
Density
Peak VAF dens= 0.48
0.0
0.5
1.0
1.5
2.0
2.5
0.0
0.2
0.4
0.6
0.8
1.0
N = 1208 Bandwidth = 0.03046

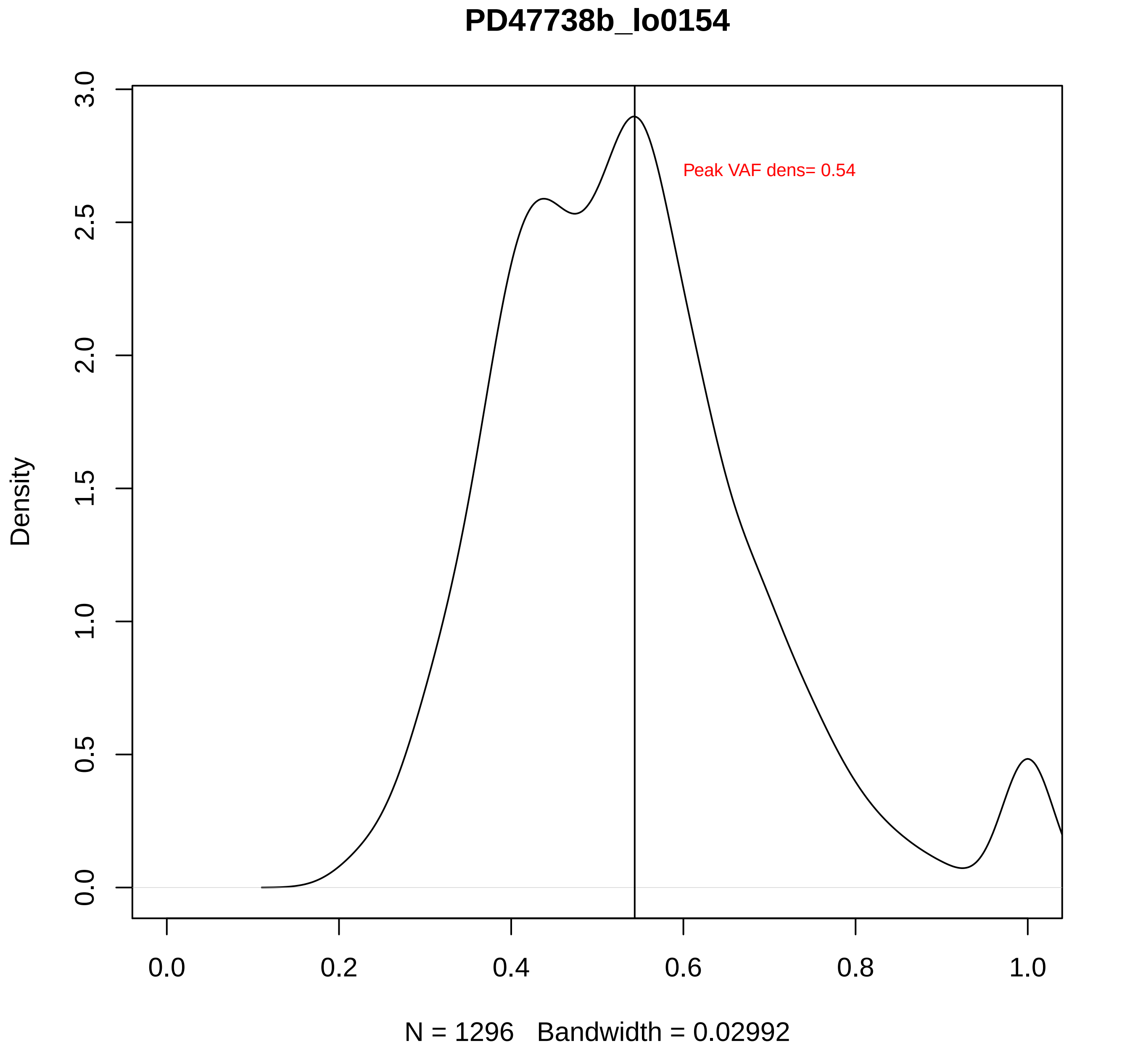
PD47738b_lo0154
Peak VAF dens= 0.54
Density
3.0
2.5
2.0
1.5
1.0
0.5
0.0
0.0
0.2
0.4
0.6
0.8
1.0
N = 1296 Bandwidth = 0.02992

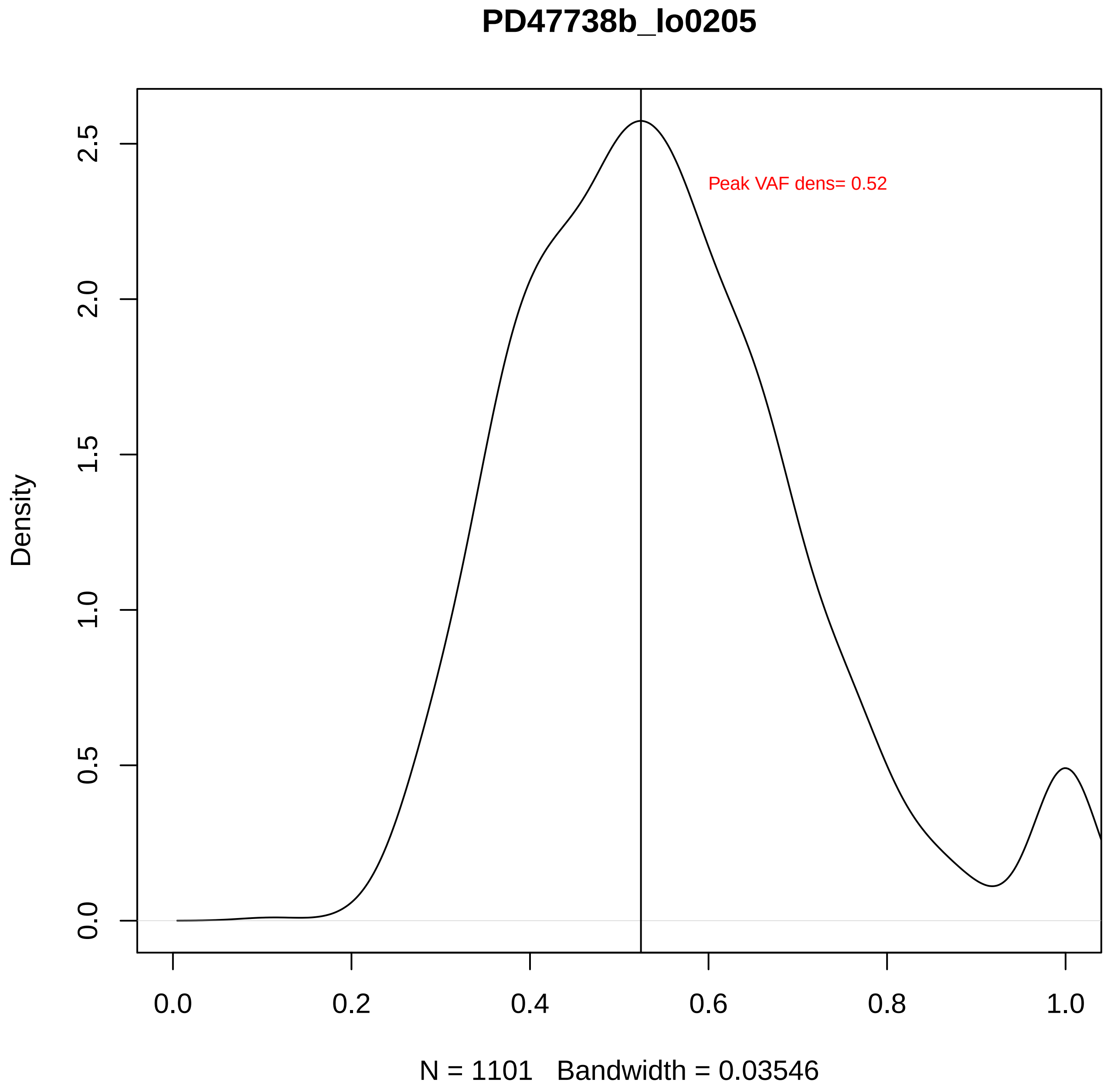
PD47738b_lo0205
Peak VAF dens= 0.52
Density
0.0
0.5
1.0
1.5
2.0
2.5
0.0
0.2
0.4
0.6
0.8
1.0
N = 1101   Bandwidth = 0.03546

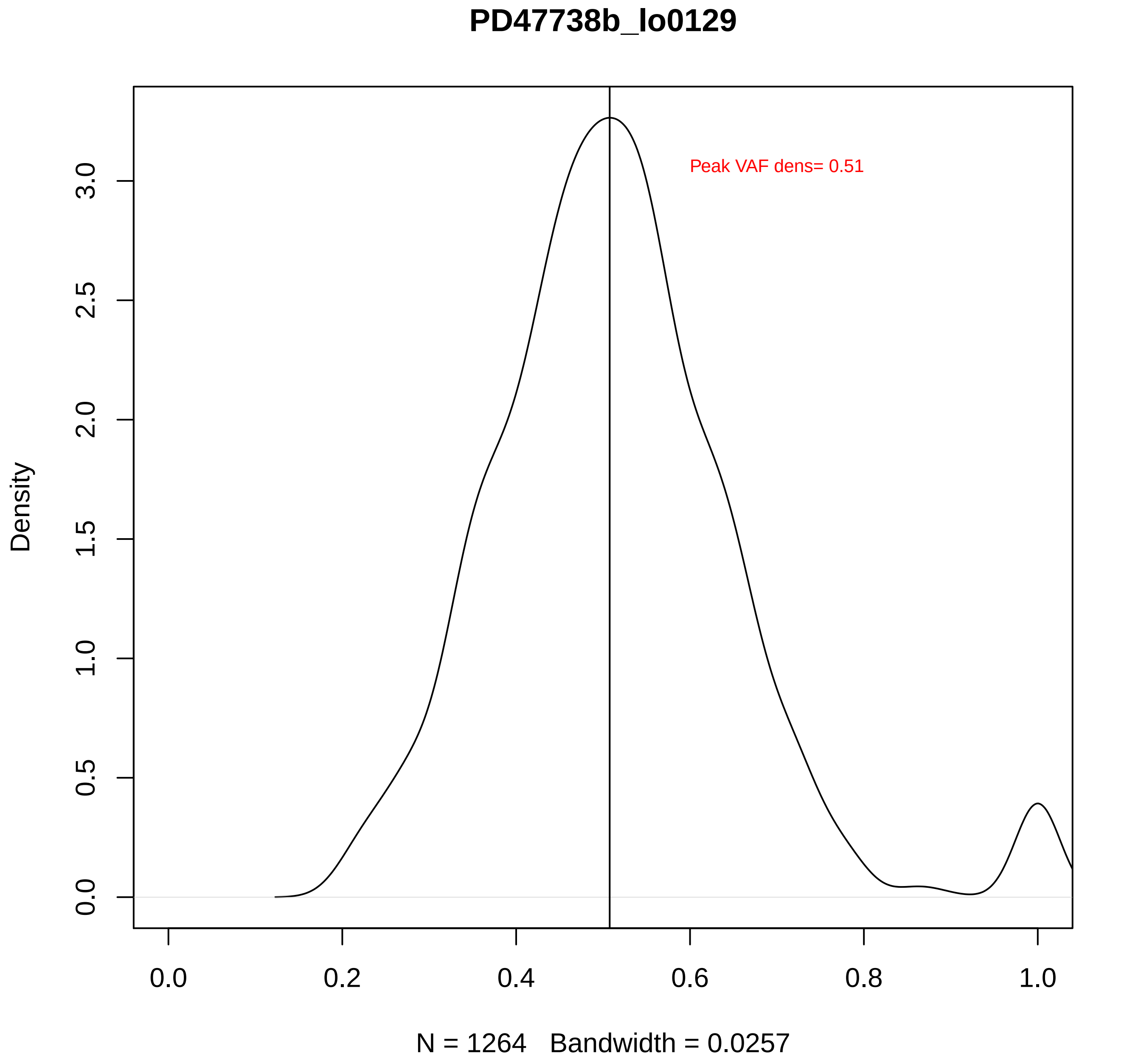
PD47738b_lo0129
Peak VAF dens= 0.51
Density
0.0
0.5
1.0
1.5
2.0
2.5
3.0
0.0
0.2
0.4
0.6
0.8
1.0
N = 1264   Bandwidth = 0.0257

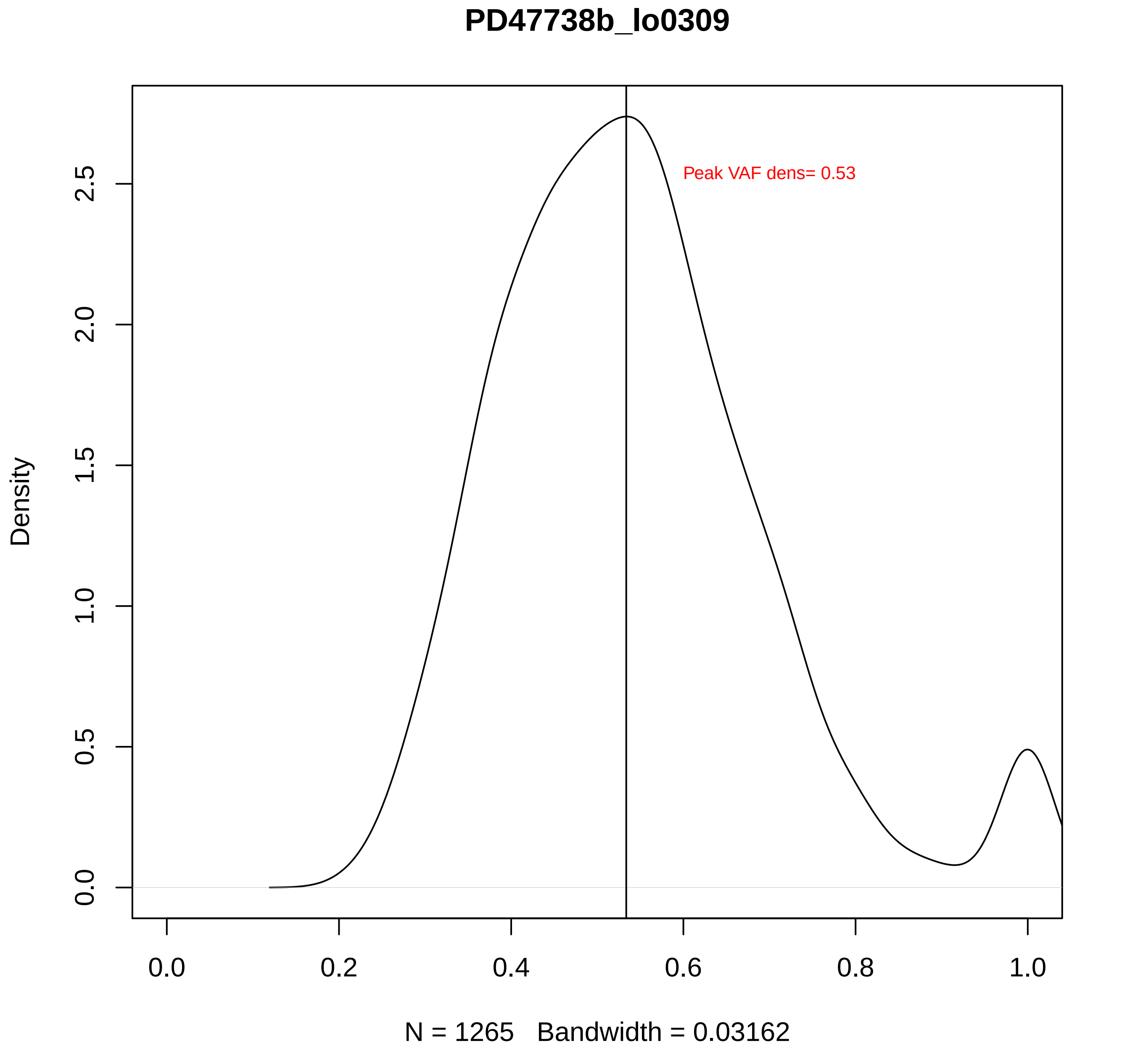
PD47738b_lo0309
Peak VAF dens= 0.53
Density
0.0
0.5
1.0
1.5
2.0
2.5
0.0
0.2
0.4
0.6
0.8
1.0
N = 1265   Bandwidth = 0.03162

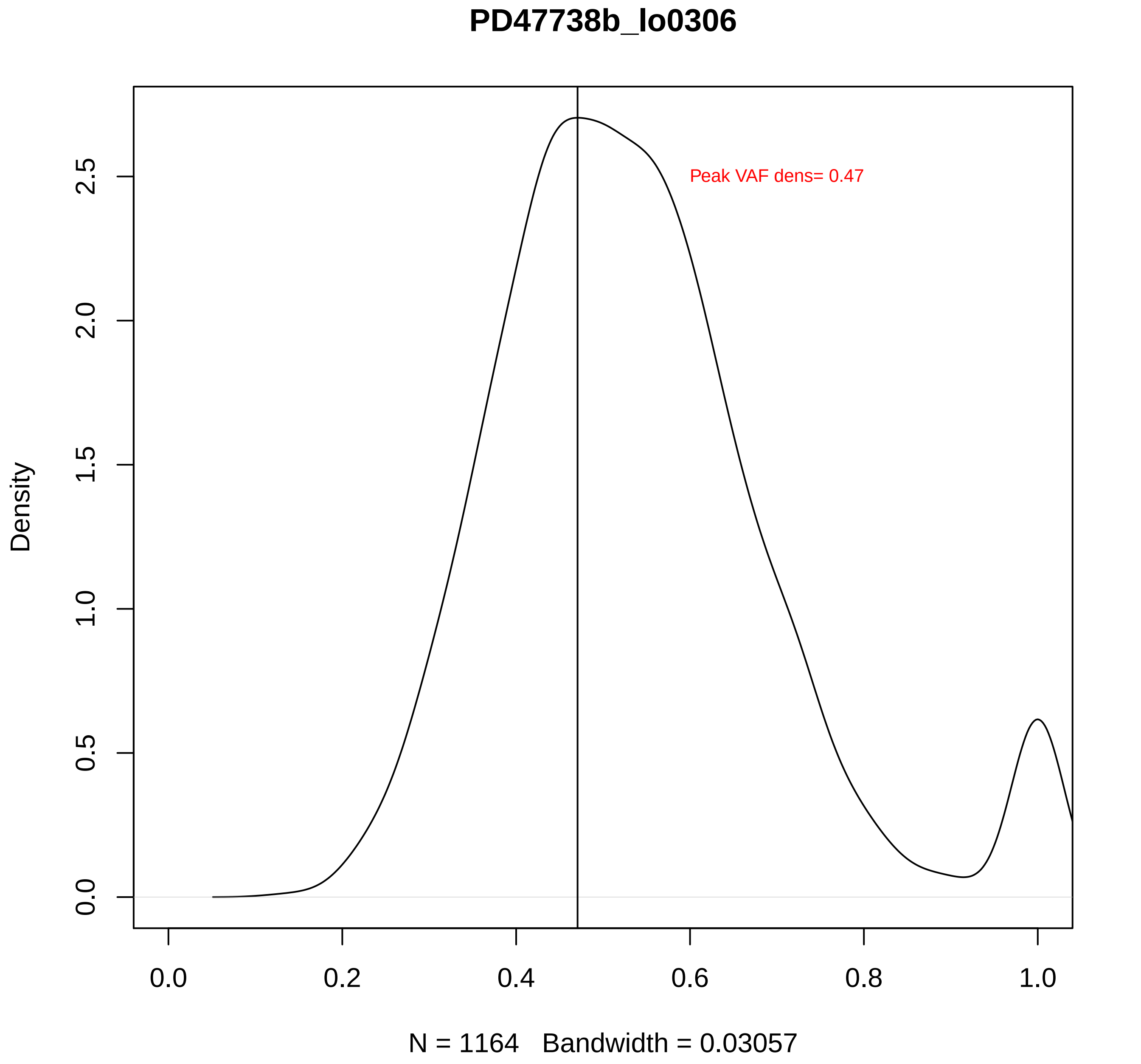
PD47738b_lo0306
Peak VAF dens= 0.47
Density
0.0
0.5
1.0
1.5
2.0
2.5
0.0
0.2
0.4
0.6
0.8
1.0
N = 1164   Bandwidth = 0.03057

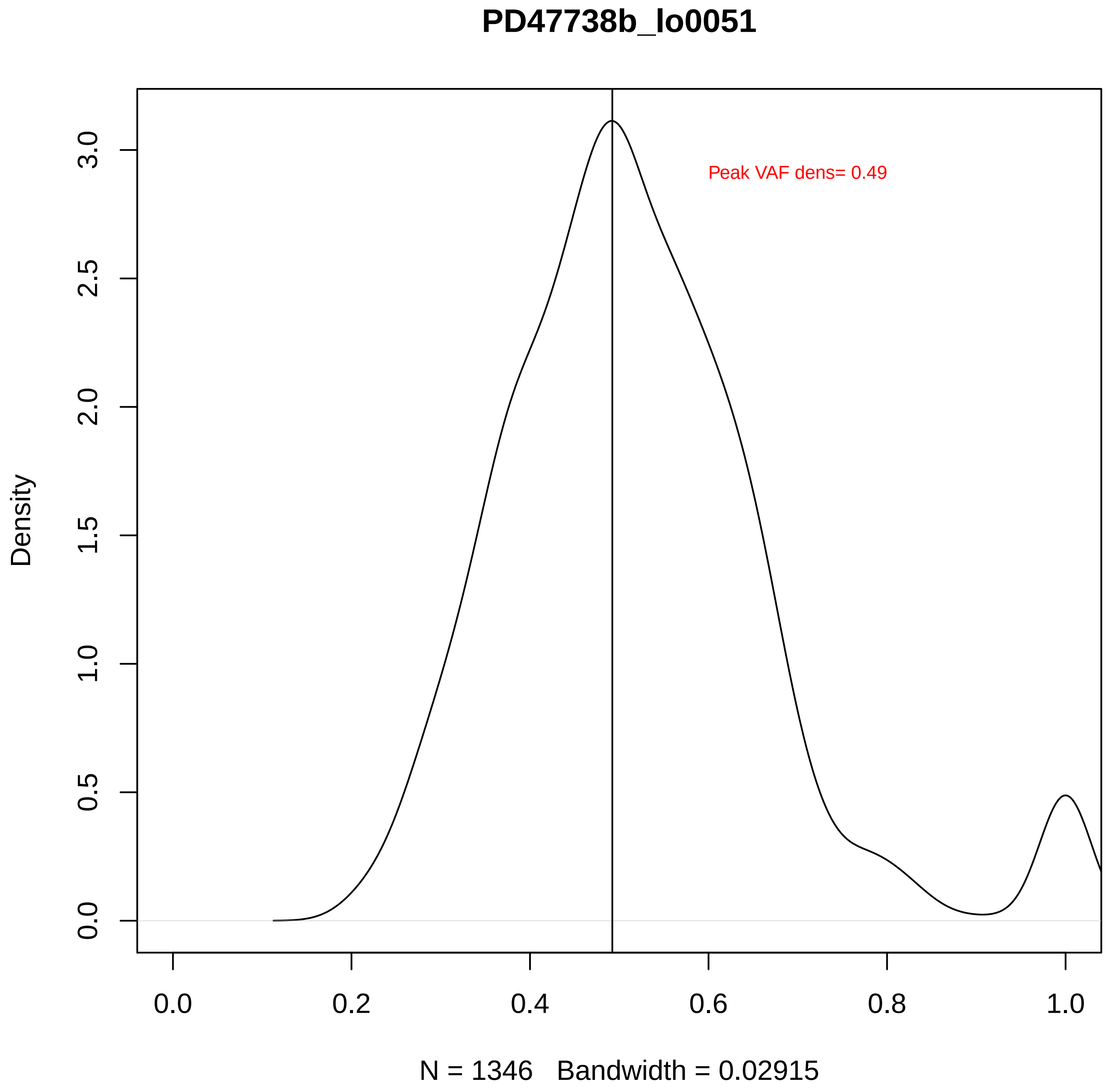
PD47738b_lo0051
Peak VAF dens= 0.49
Density
0.0
0.5
1.0
1.5
2.0
2.5
3.0
0.0
0.2
0.4
0.6
0.8
1.0
N = 1346   Bandwidth = 0.02915

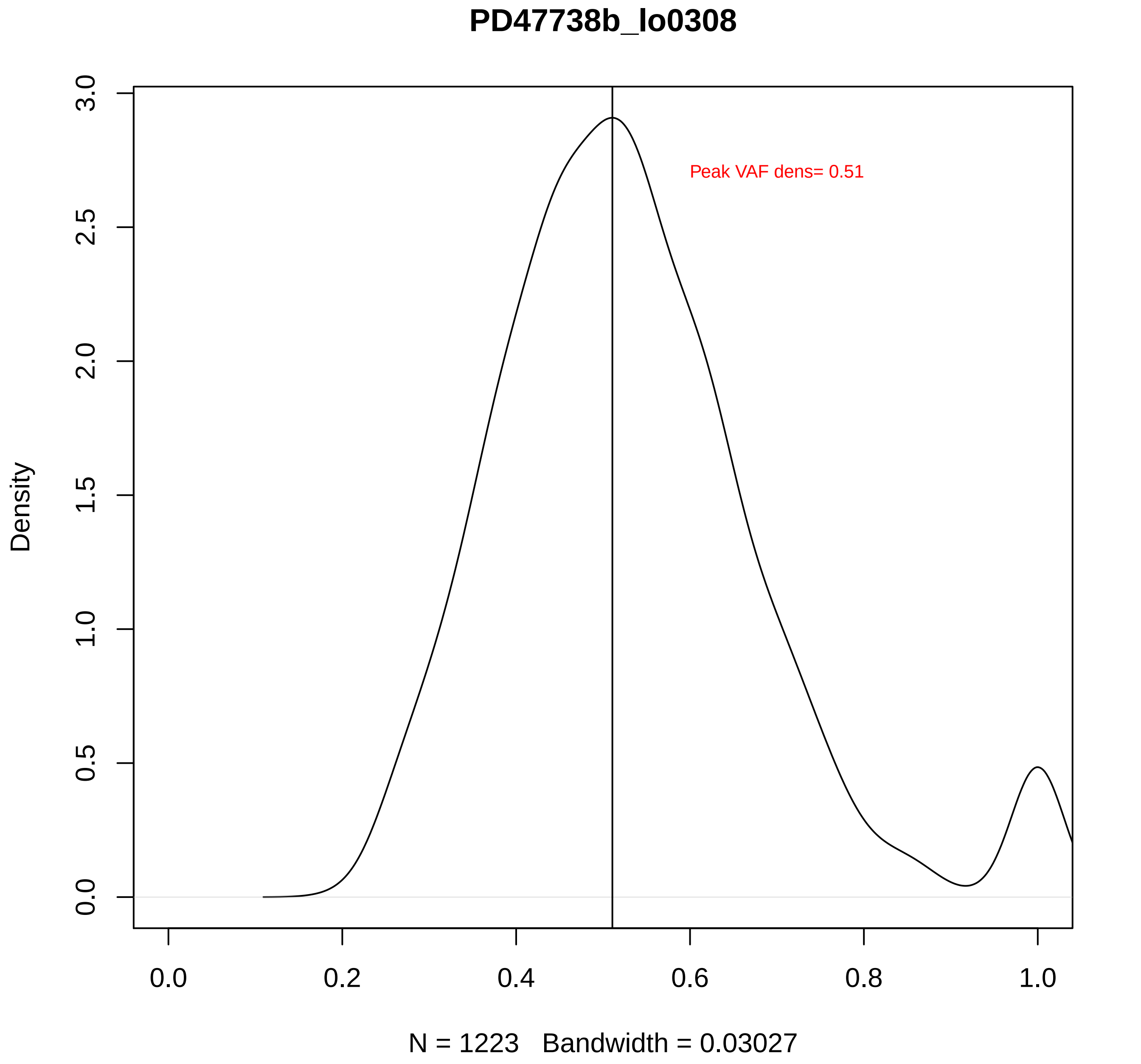

PD47738b_lo0308
Peak VAF dens= 0.51
Density
0.0
0.5
1.0
1.5
2.0
2.5
3.0
0.0
0.2
0.4
0.6
0.8
1.0
N = 1223   Bandwidth = 0.03027

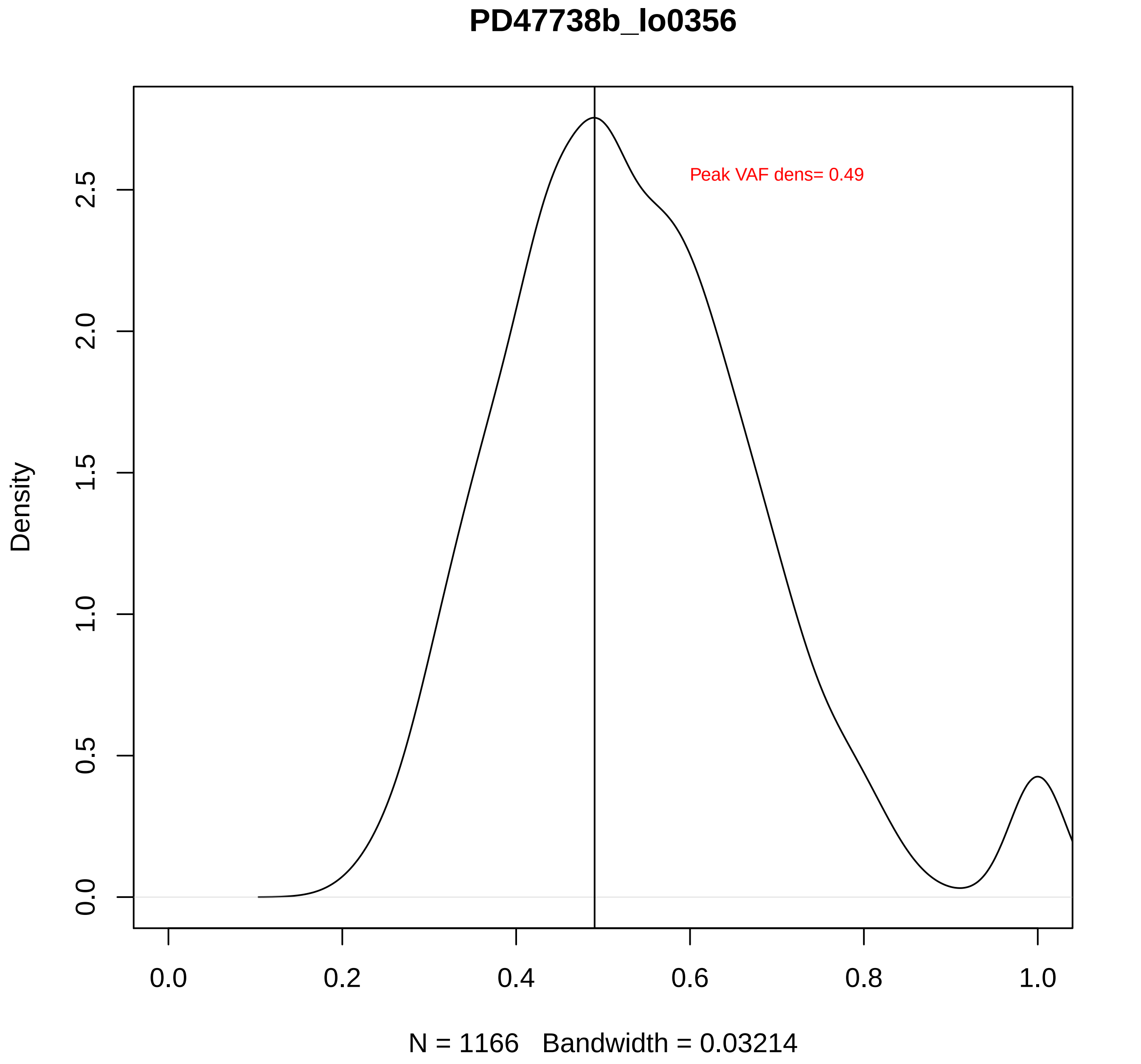

PD47738b_lo0356
Peak VAF dens= 0.49
Density
0.0
0.5
1.0
1.5
2.0
2.5
0.0
0.2
0.4
0.6
0.8
1.0
N = 1166 Bandwidth = 0.03214

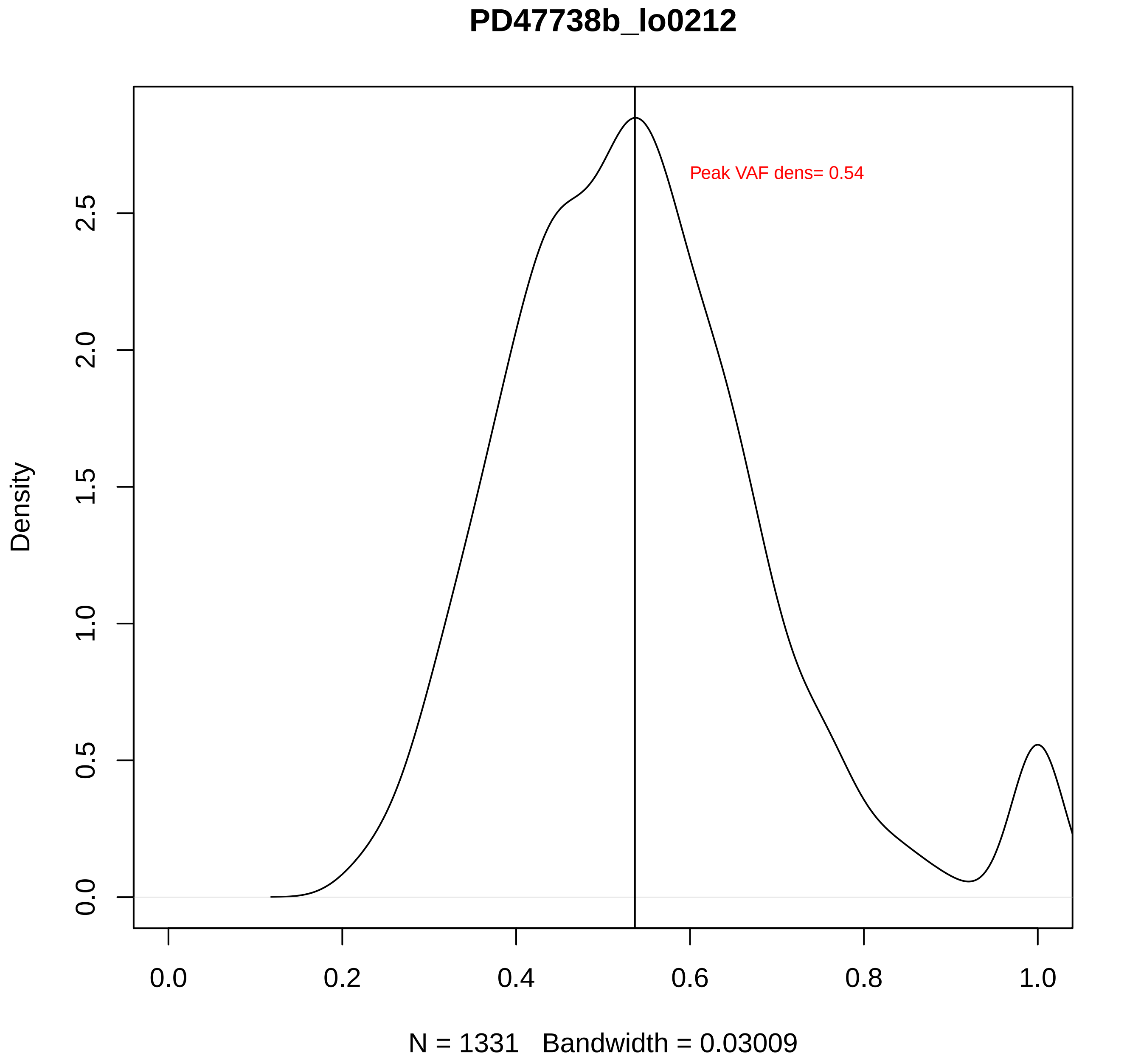

PD47738b_lo0212
Peak VAF dens= 0.54
Density
0.0
0.5
1.0
1.5
2.0
2.5
0.0
0.2
0.4
0.6
0.8
1.0
N = 1331 Bandwidth = 0.03009

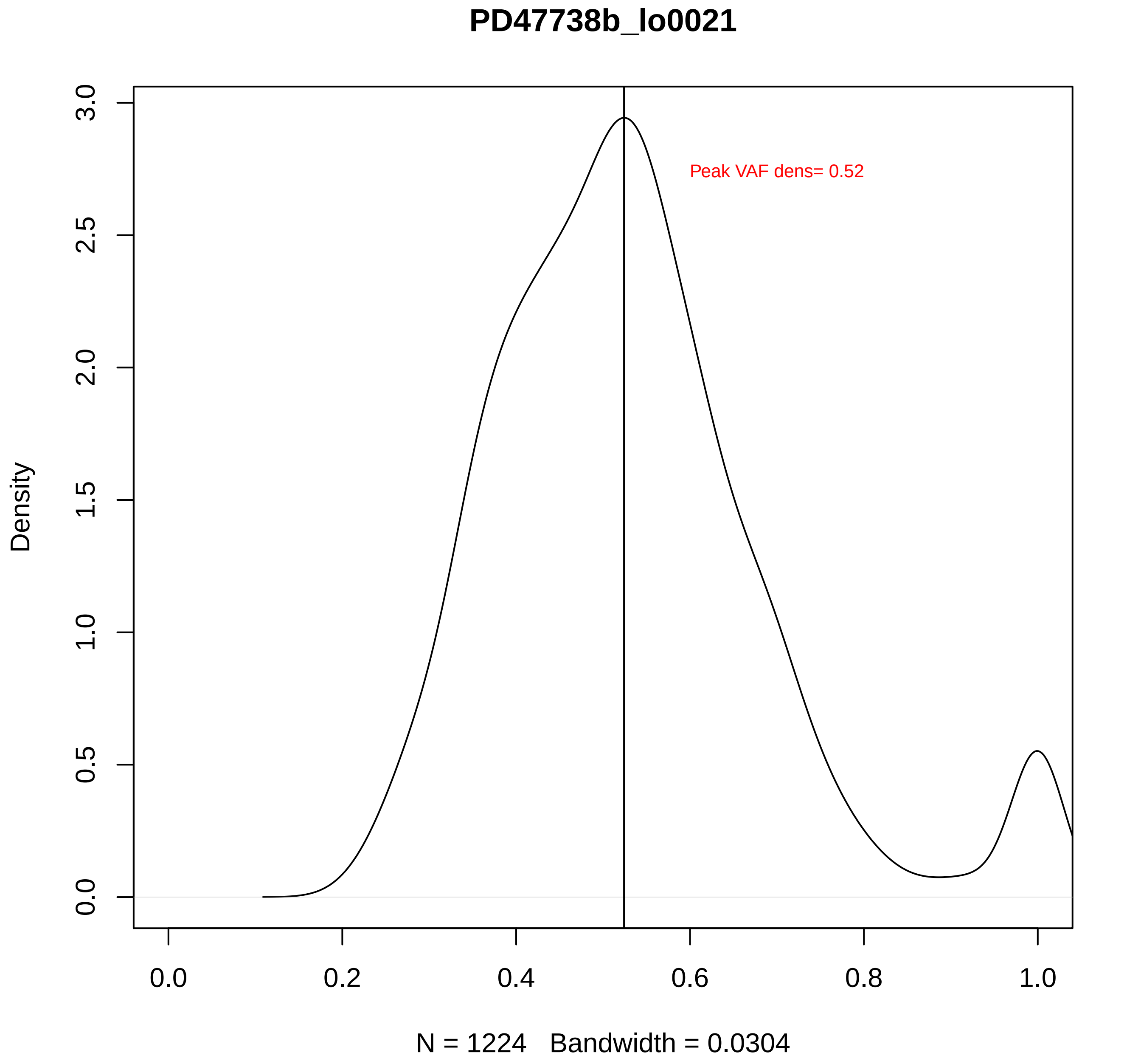

PD47738b_lo0021
Peak VAF dens= 0.52
Density
3.0
2.5
2.0
1.5
1.0
0.5
0.0
0.0
0.2
0.4
0.6
0.8
1.0
N = 1224   Bandwidth = 0.0304

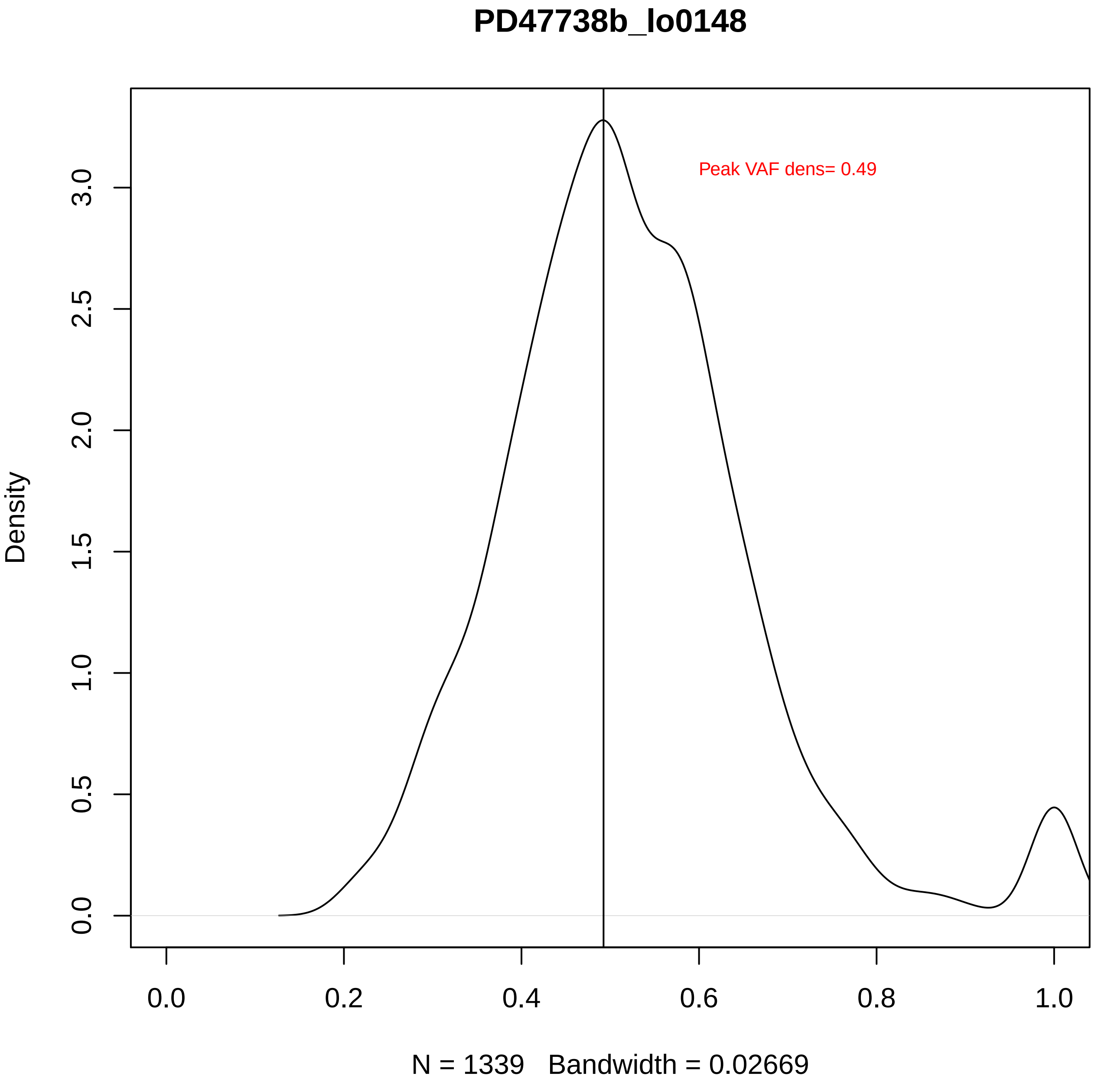

PD47738b_lo0148
Peak VAF dens= 0.49
Density
0.0
0.5
1.0
1.5
2.0
2.5
3.0
0.0
0.2
0.4
0.6
0.8
1.0
N = 1339   Bandwidth = 0.02669

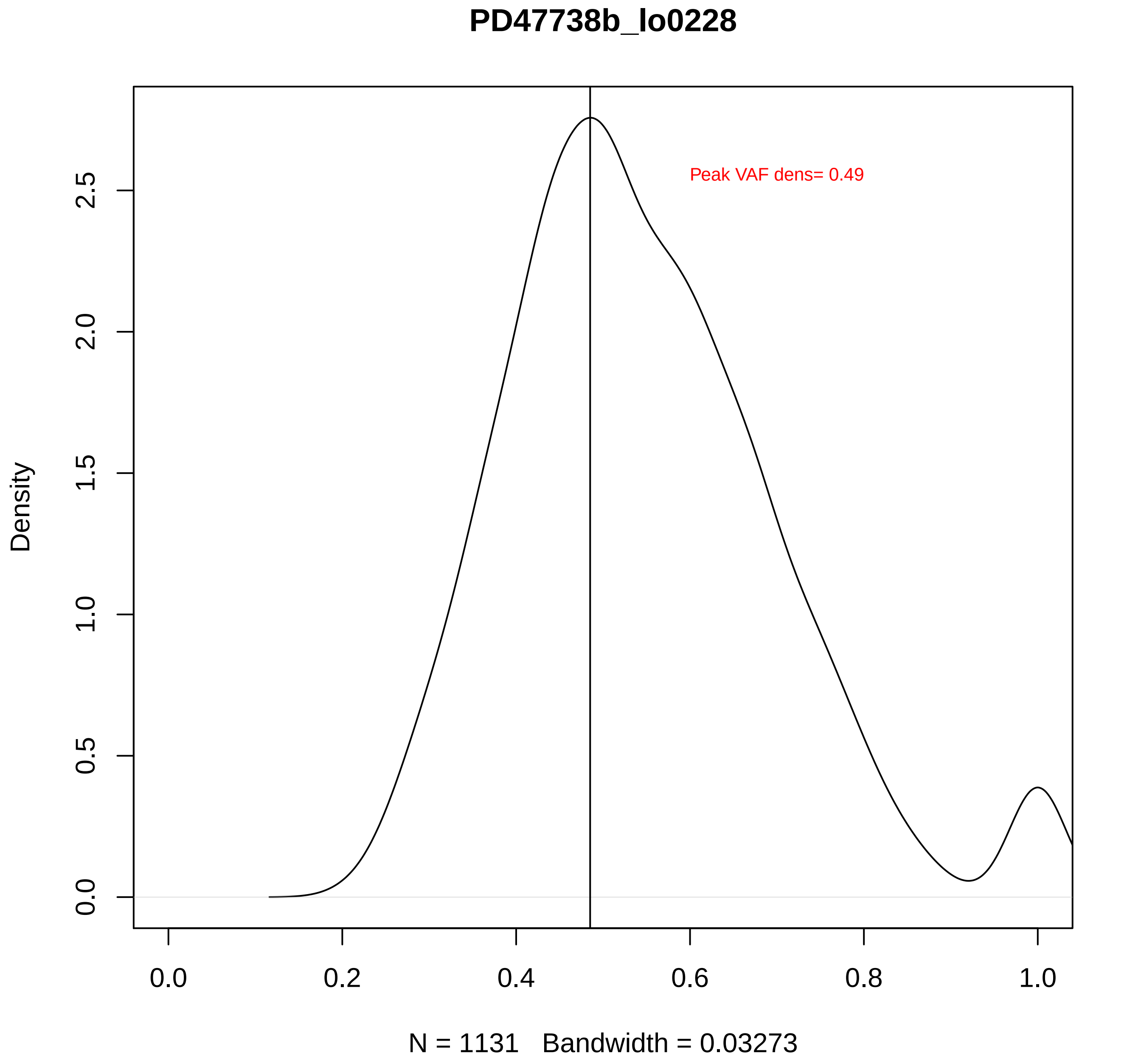
PD47738b_lo0228
Peak VAF dens= 0.49
Density
0.0
0.5
1.0
1.5
2.0
2.5
0.0
0.2
0.4
0.6
0.8
1.0
N = 1131   Bandwidth = 0.03273

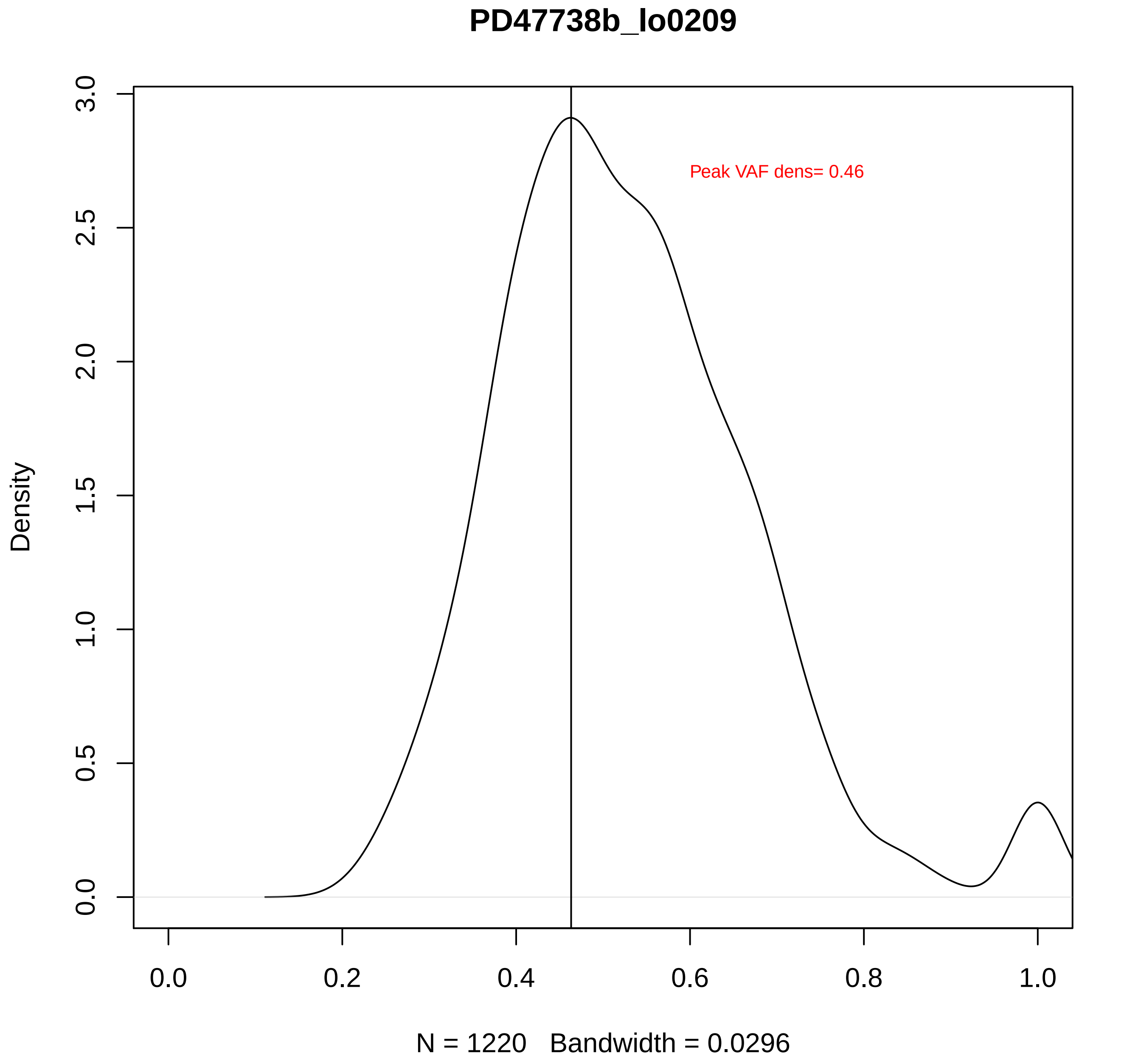
PD47738b_lo0209
Peak VAF dens= 0.46
Density
0.0
0.5
1.0
1.5
2.0
2.5
3.0
0.0
0.2
0.4
0.6
0.8
1.0
N = 1220 Bandwidth = 0.0296

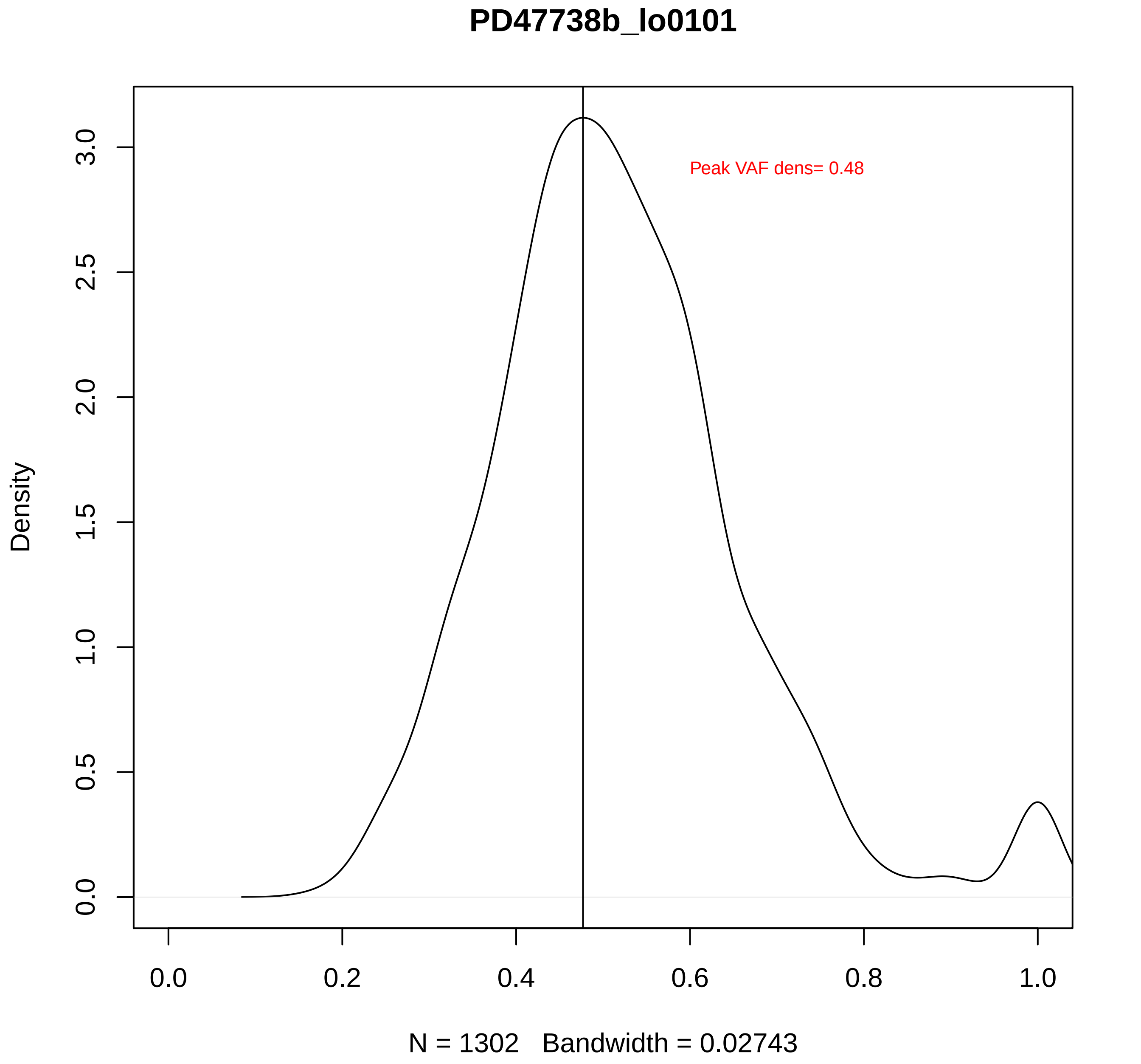
PD47738b_lo0101
Peak VAF dens= 0.48
Density
0.0
0.5
1.0
1.5
2.0
2.5
3.0
0.0
0.2
0.4
0.6
0.8
1.0
N = 1302   Bandwidth = 0.02743

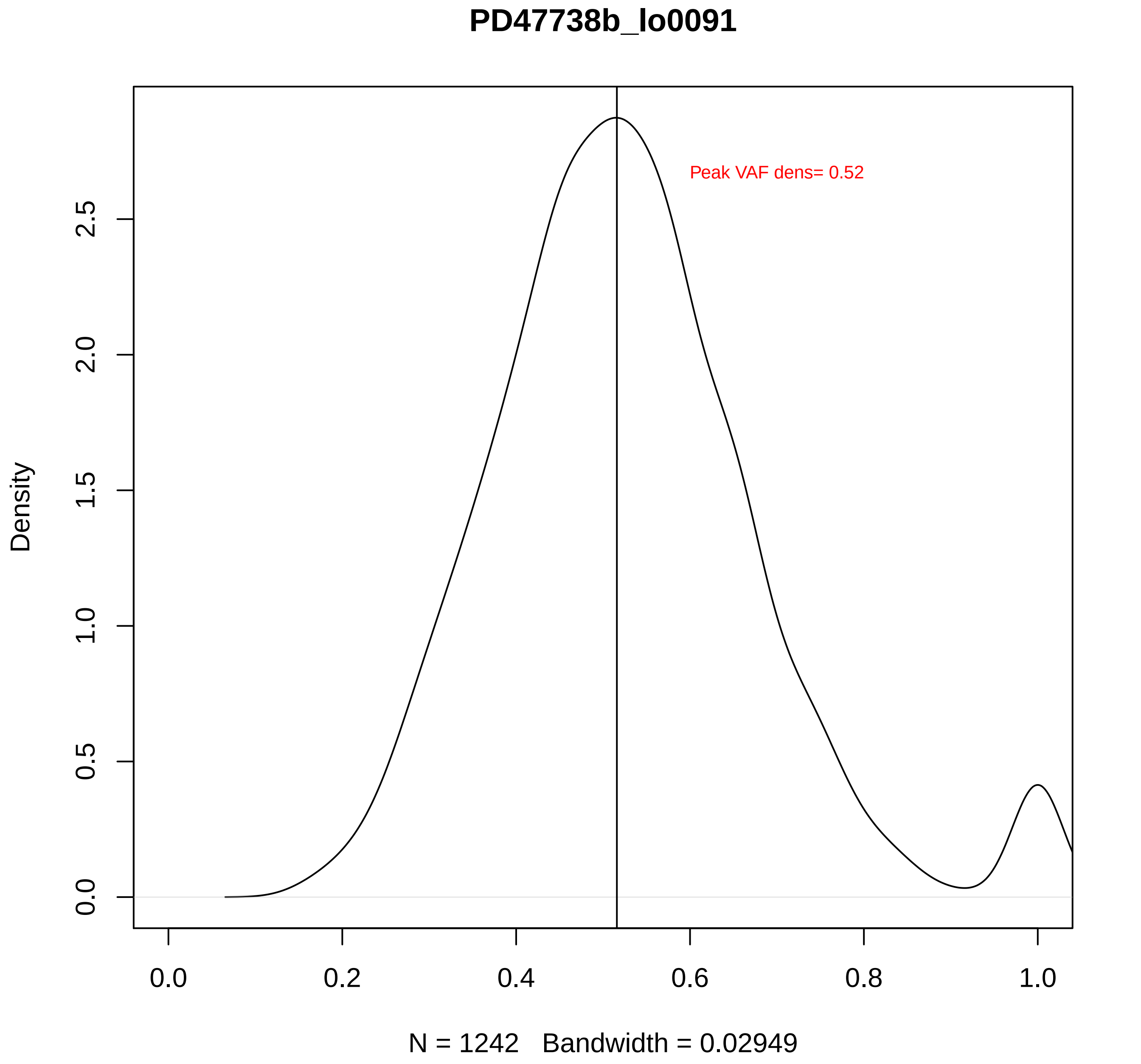
PD47738b_lo0091
Peak VAF dens= 0.52
Density
0.0
0.5
1.0
1.5
2.0
2.5
0.0
0.2
0.4
0.6
0.8
1.0
N = 1242 Bandwidth = 0.02949

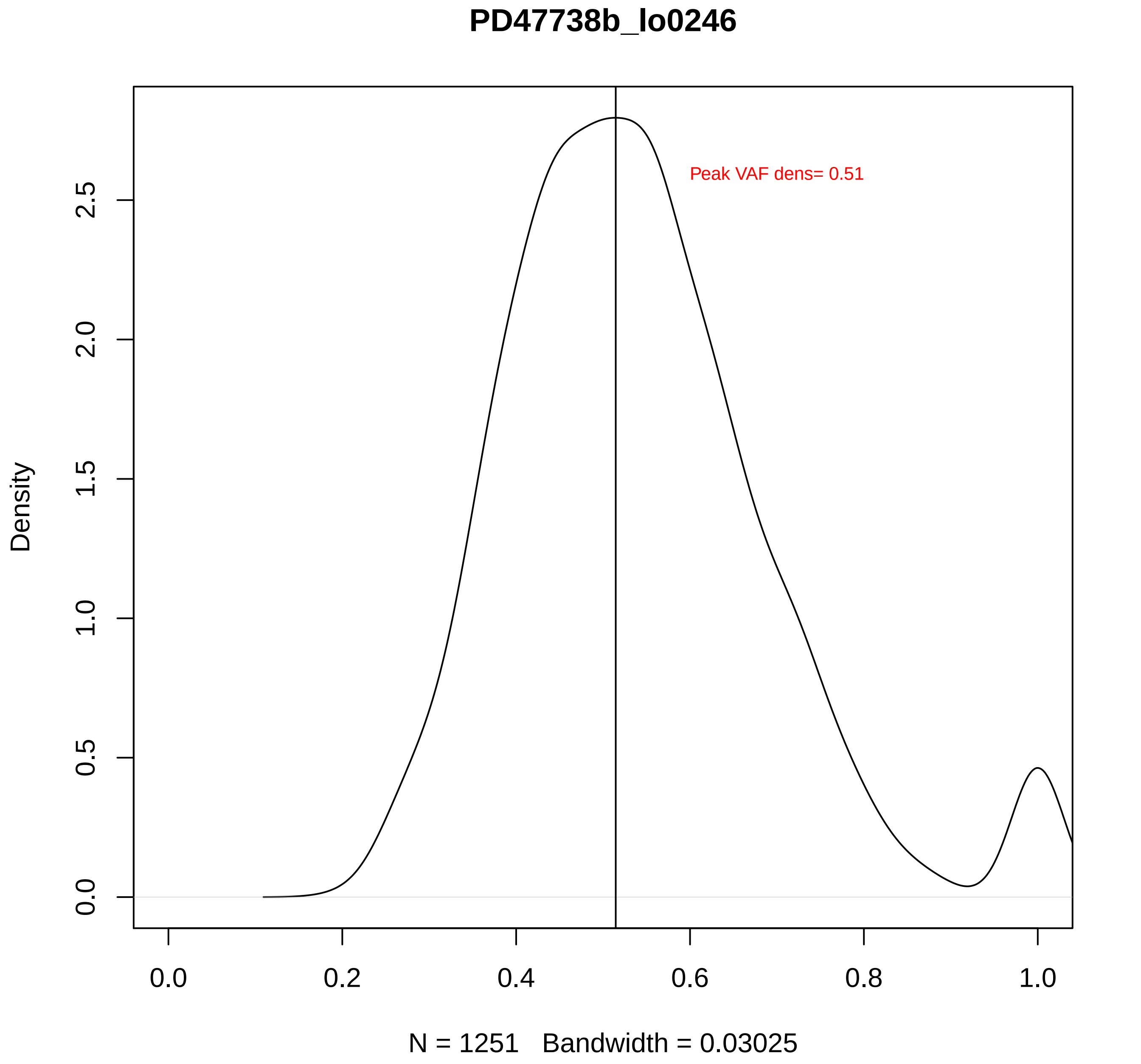

PD47738b_lo0246
Peak VAF dens= 0.51
Density
0.0
0.5
1.0
1.5
2.0
2.5
0.0
0.2
0.4
0.6
0.8
1.0
N = 1251   Bandwidth = 0.03025

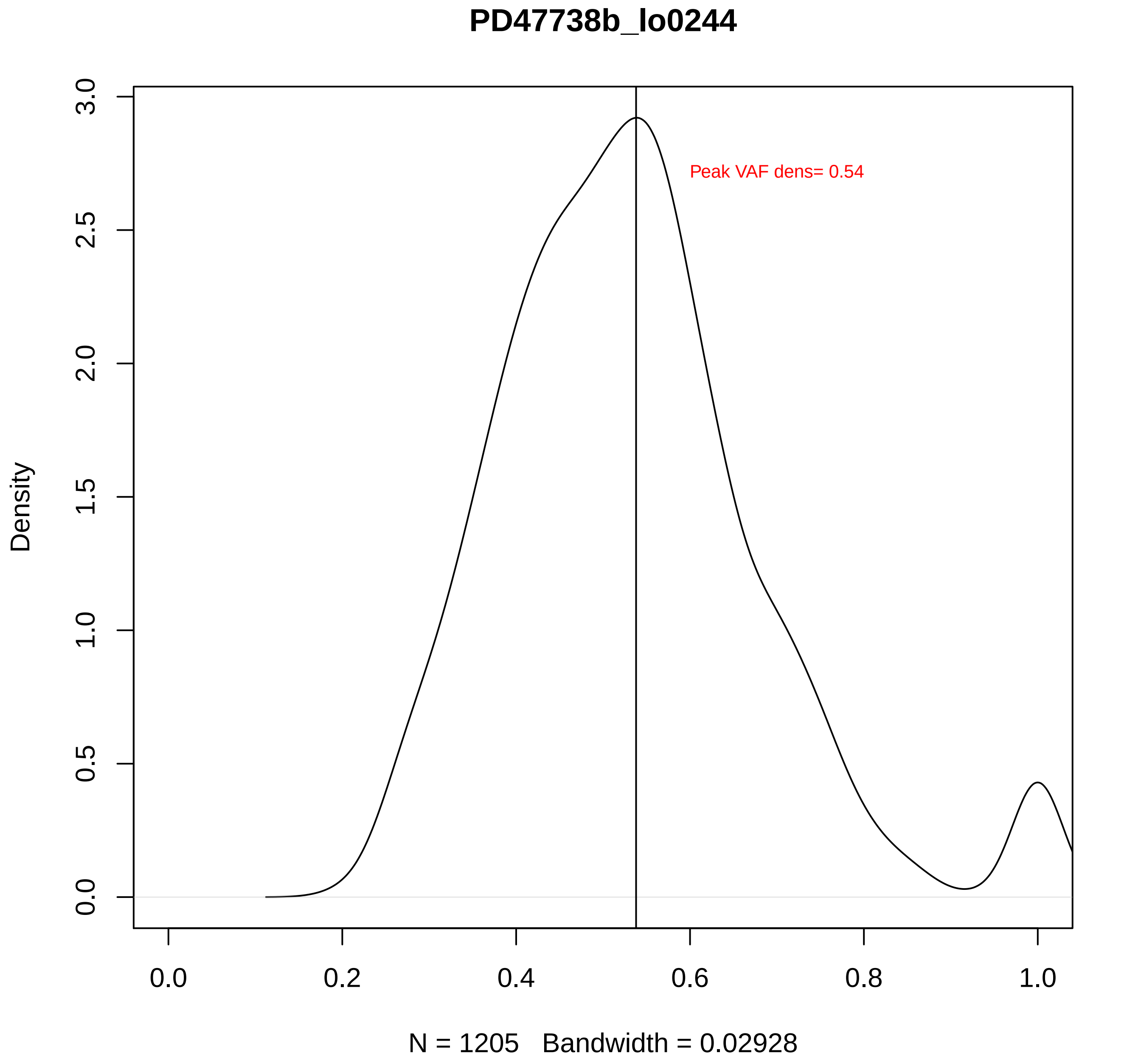
PD47738b_lo0244
Peak VAF dens= 0.54
Density
0.0
0.5
1.0
1.5
2.0
2.5
3.0
0.0
0.2
0.4
0.6
0.8
1.0
N = 1205   Bandwidth = 0.02928

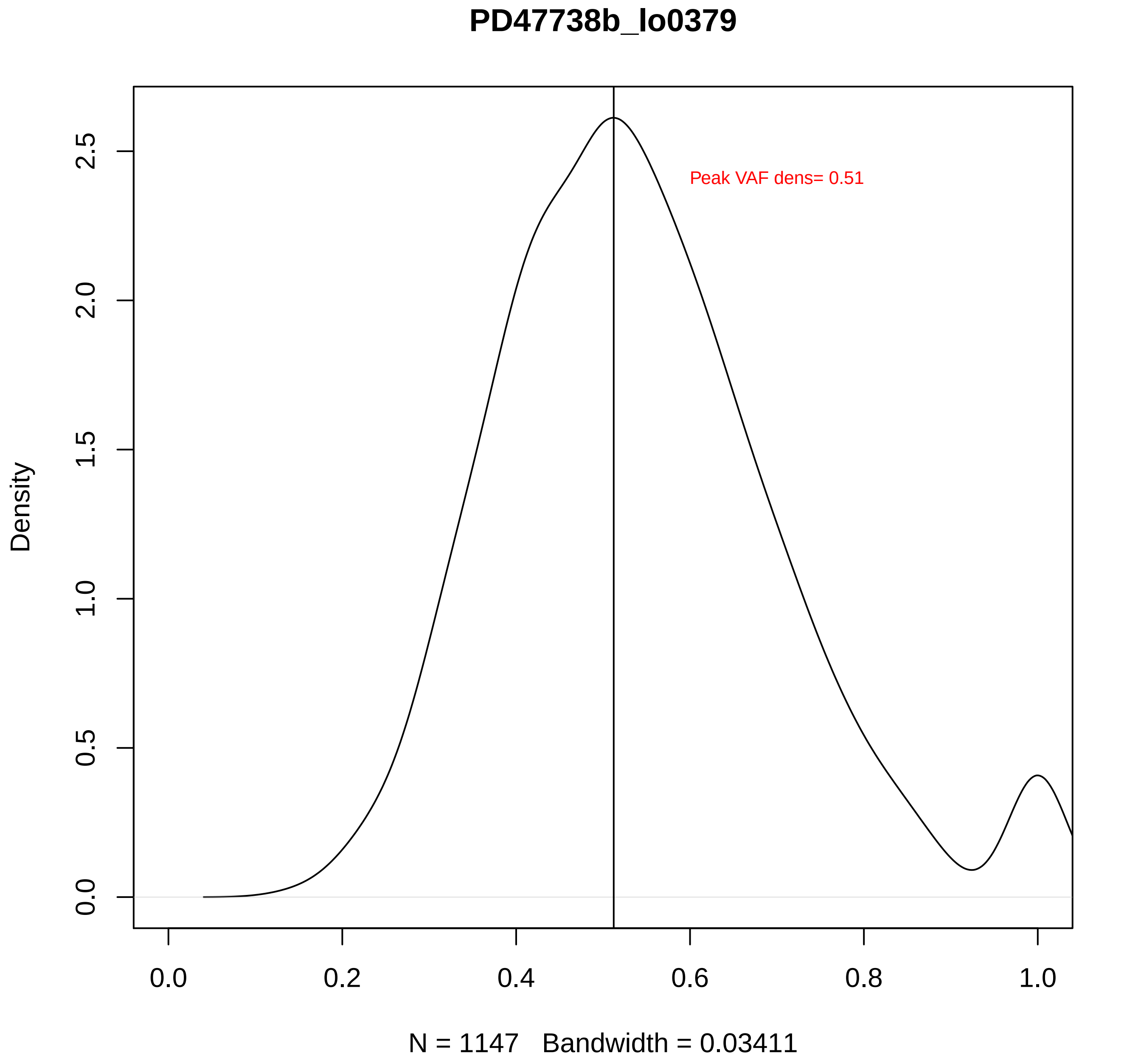
PD47738b_lo0379
Peak VAF dens= 0.51
Density
0.0
0.5
1.0
1.5
2.0
2.5
0.0
0.2
0.4
0.6
0.8
1.0
N = 1147   Bandwidth = 0.03411

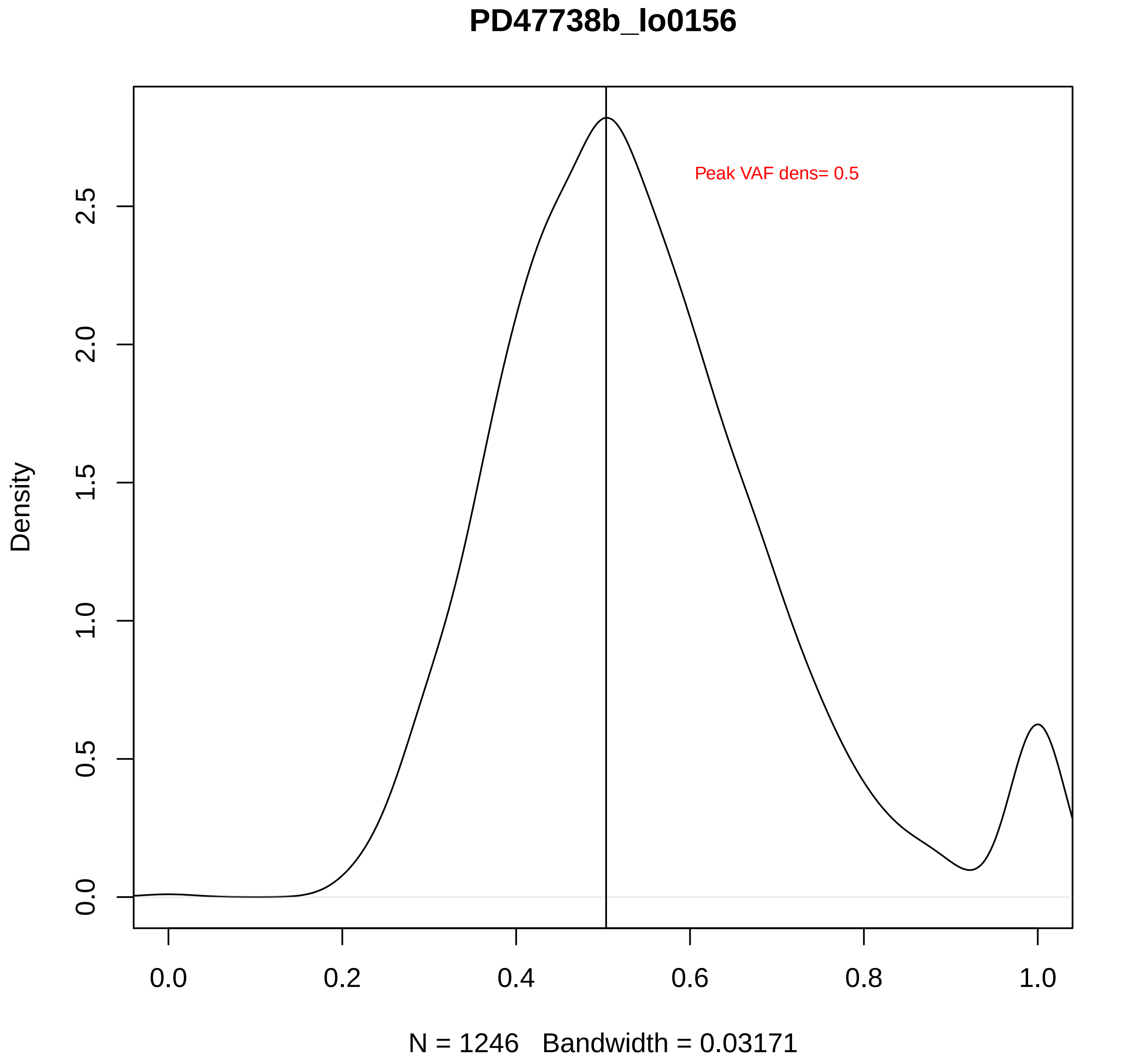

PD47738b_lo0156
Peak VAF dens= 0.5
Density
0.0
0.5
1.0
1.5
2.0
2.5
0.0
0.2
0.4
0.6
0.8
1.0
N = 1246   Bandwidth = 0.03171

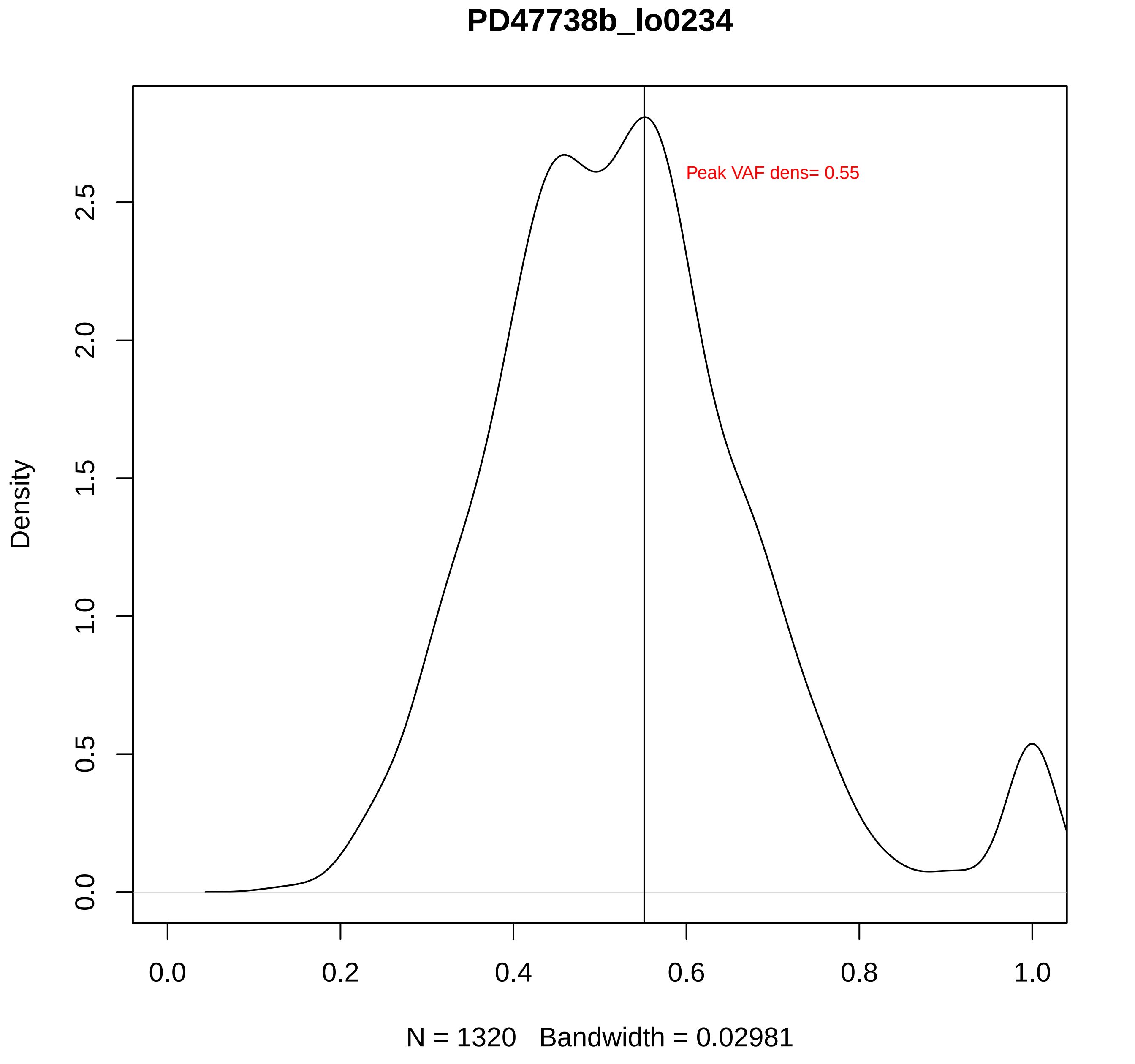
PD47738b_lo0234
Peak VAF dens= 0.55
Density
0.0
0.5
1.0
1.5
2.0
2.5
0.0
0.2
0.4
0.6
0.8
1.0
N = 1320 Bandwidth = 0.02981

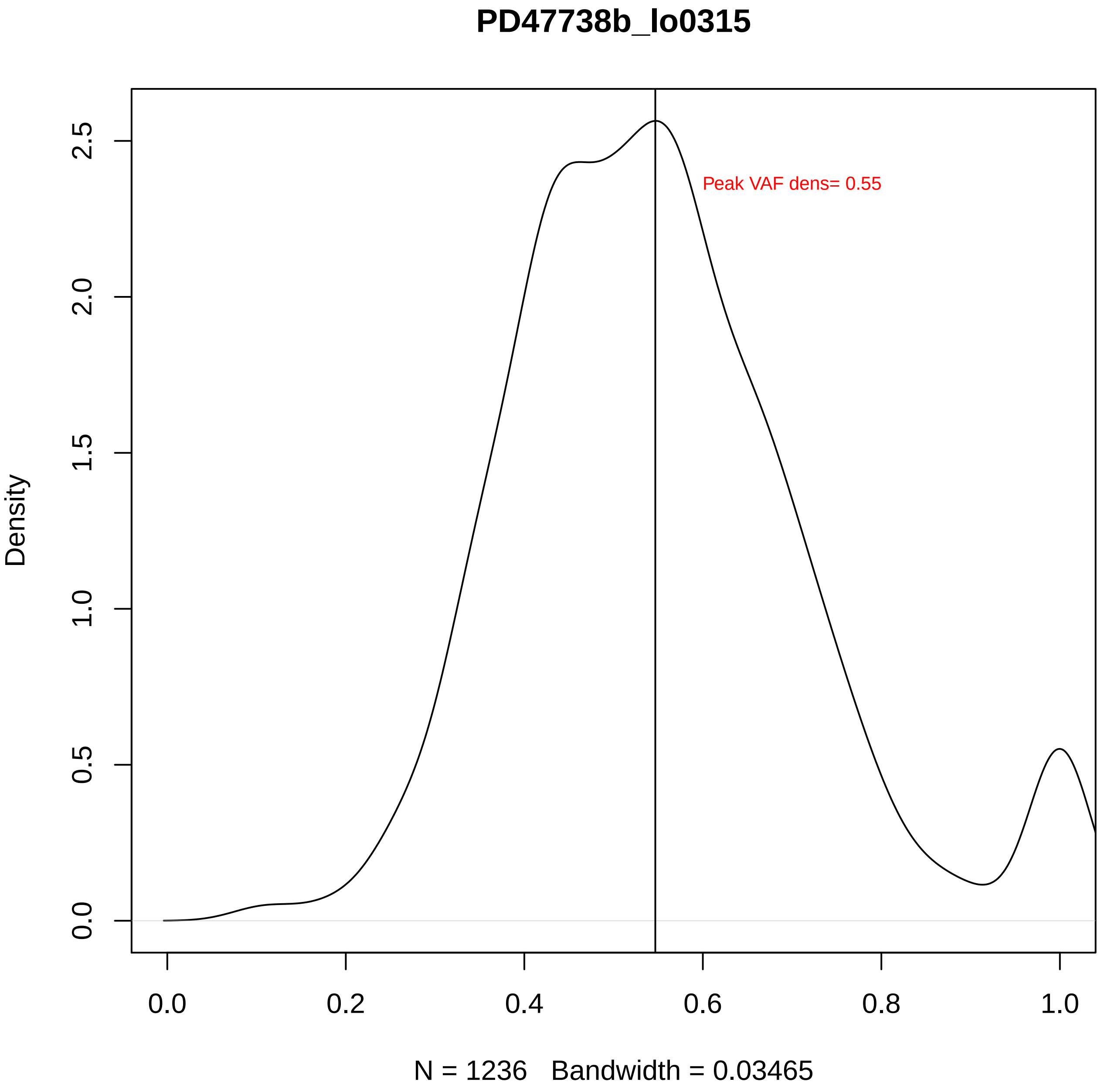
PD47738b_lo0315
Peak VAF dens= 0.55
Density
0.0
0.5
1.0
1.5
2.0
2.5
0.0
0.2
0.4
0.6
0.8
1.0
N = 1236   Bandwidth = 0.03465

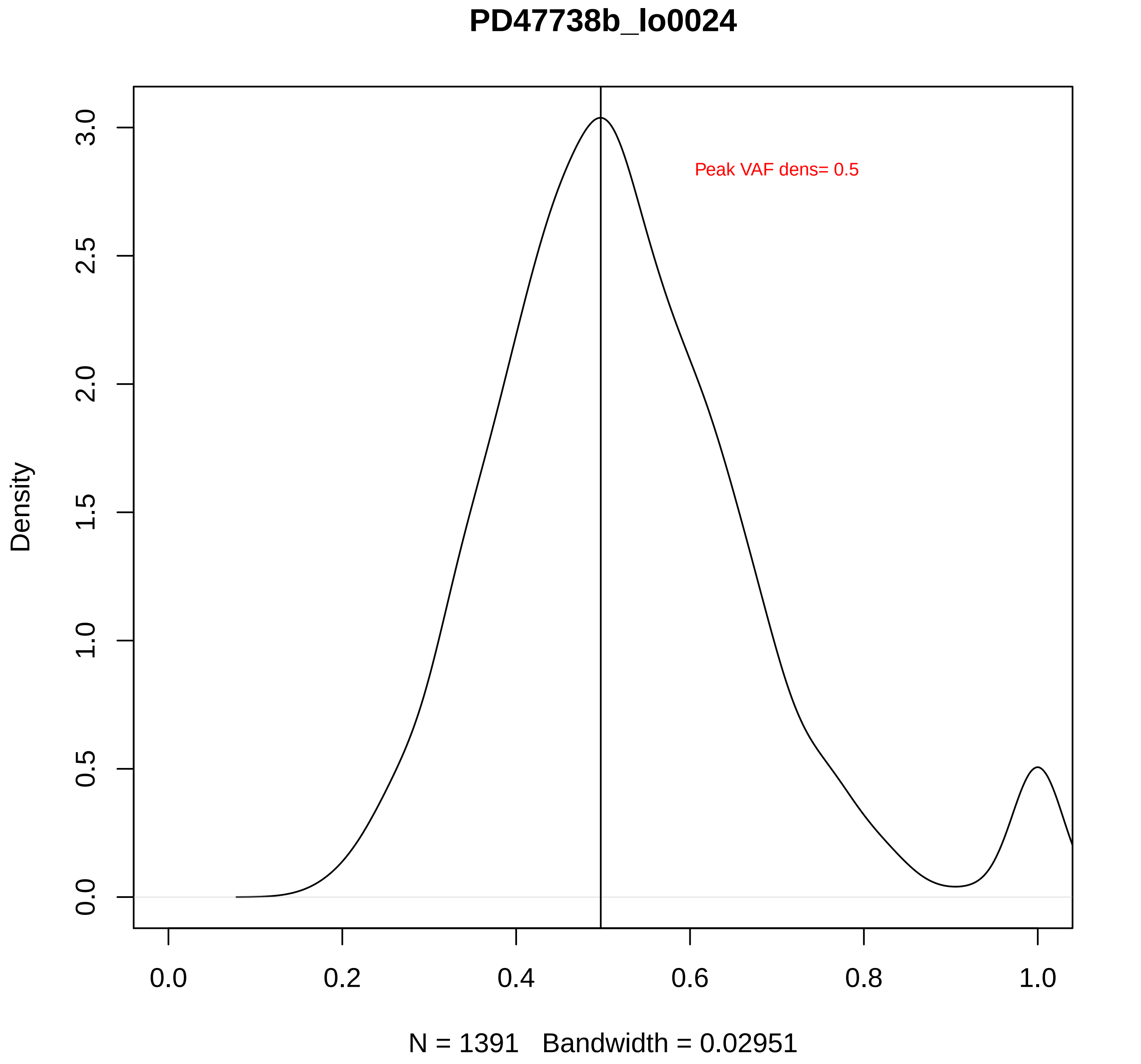
PD47738b_lo0024
Peak VAF dens= 0.5
Density
0.0
0.5
1.0
1.5
2.0
2.5
3.0
0.0
0.2
0.4
0.6
0.8
1.0
N = 1391   Bandwidth = 0.02951

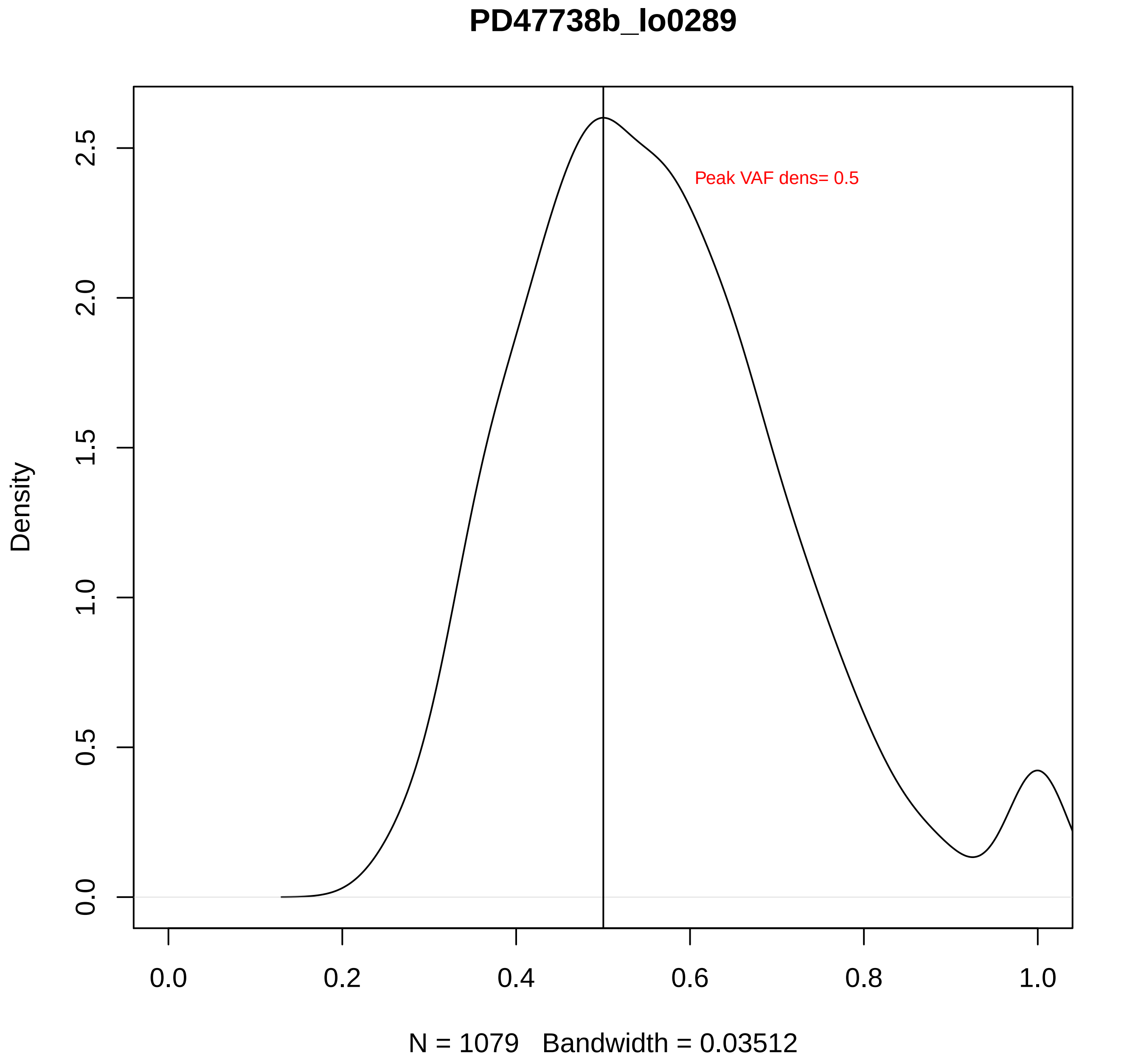

PD47738b_lo0289
Peak VAF dens= 0.5
Density
0.0
0.5
1.0
1.5
2.0
2.5
0.0
0.2
0.4
0.6
0.8
1.0
N = 1079   Bandwidth = 0.03512

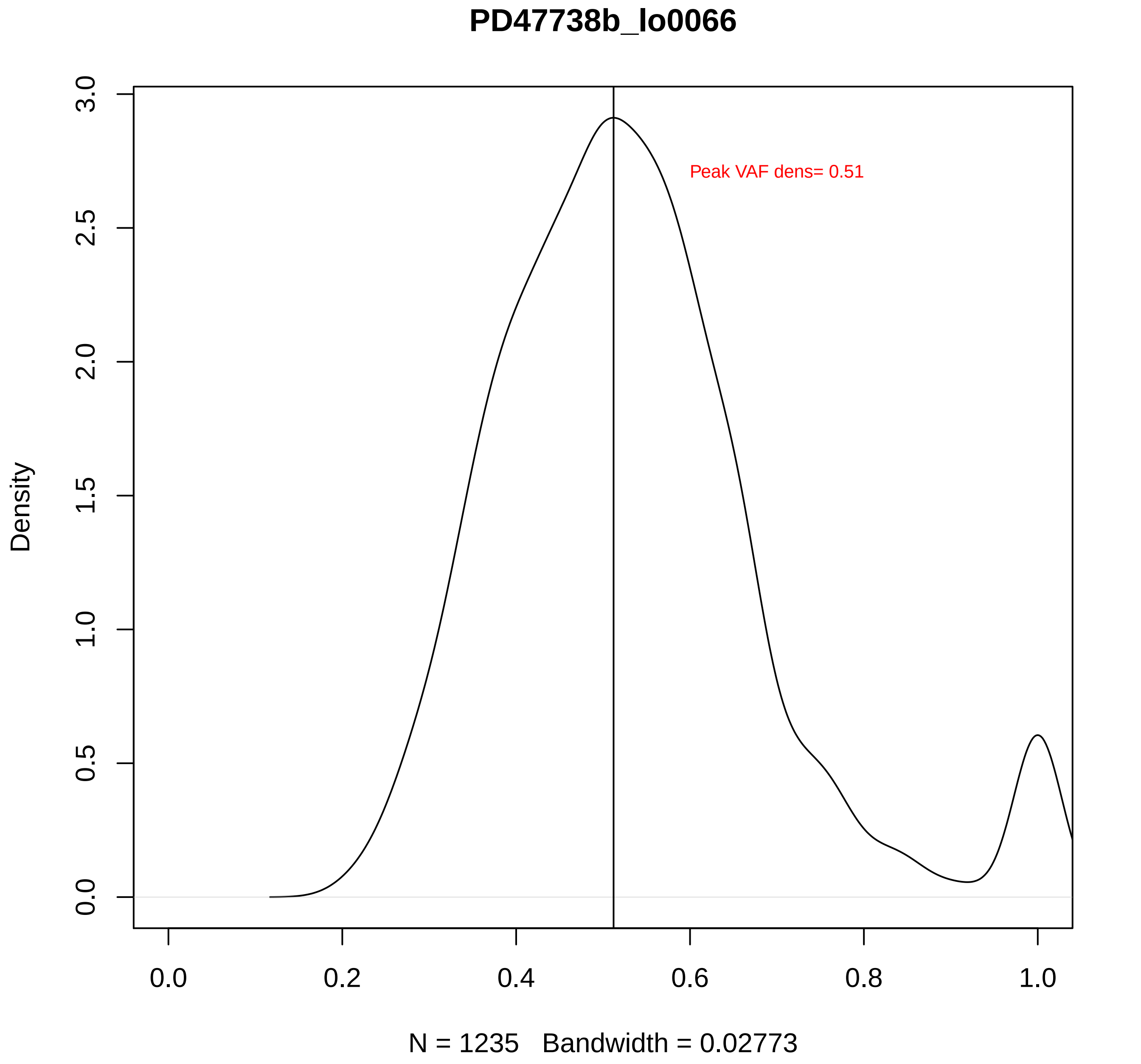
PD47738b_lo0066
Peak VAF dens= 0.51
Density
0.0
0.5
1.0
1.5
2.0
2.5
3.0
0.0
0.2
0.4
0.6
0.8
1.0
N = 1235   Bandwidth = 0.02773

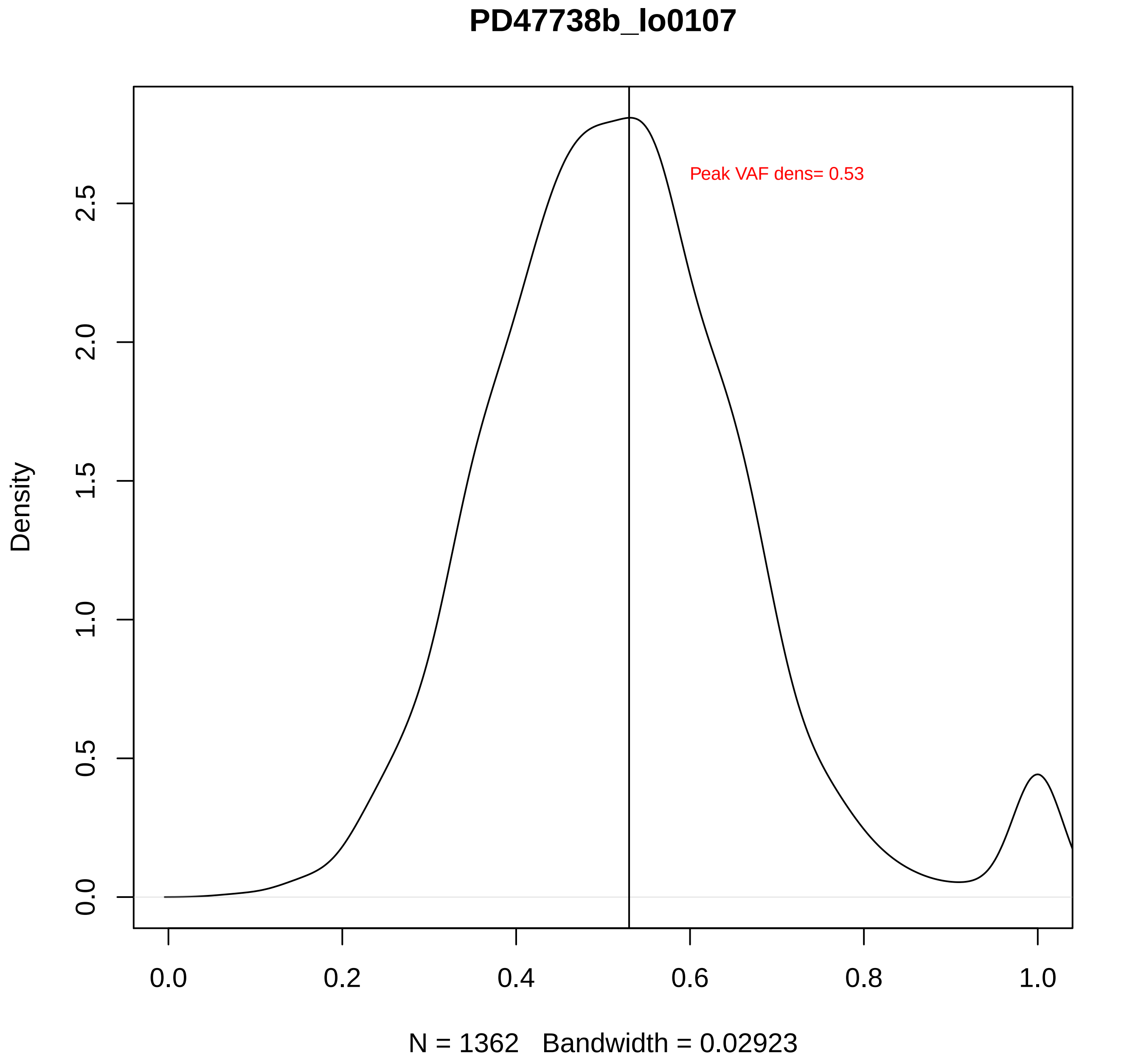
PD47738b_lo0107
Peak VAF dens= 0.53
Density
0.0
0.5
1.0
1.5
2.0
2.5
0.0
0.2
0.4
0.6
0.8
1.0
N = 1362 Bandwidth = 0.02923

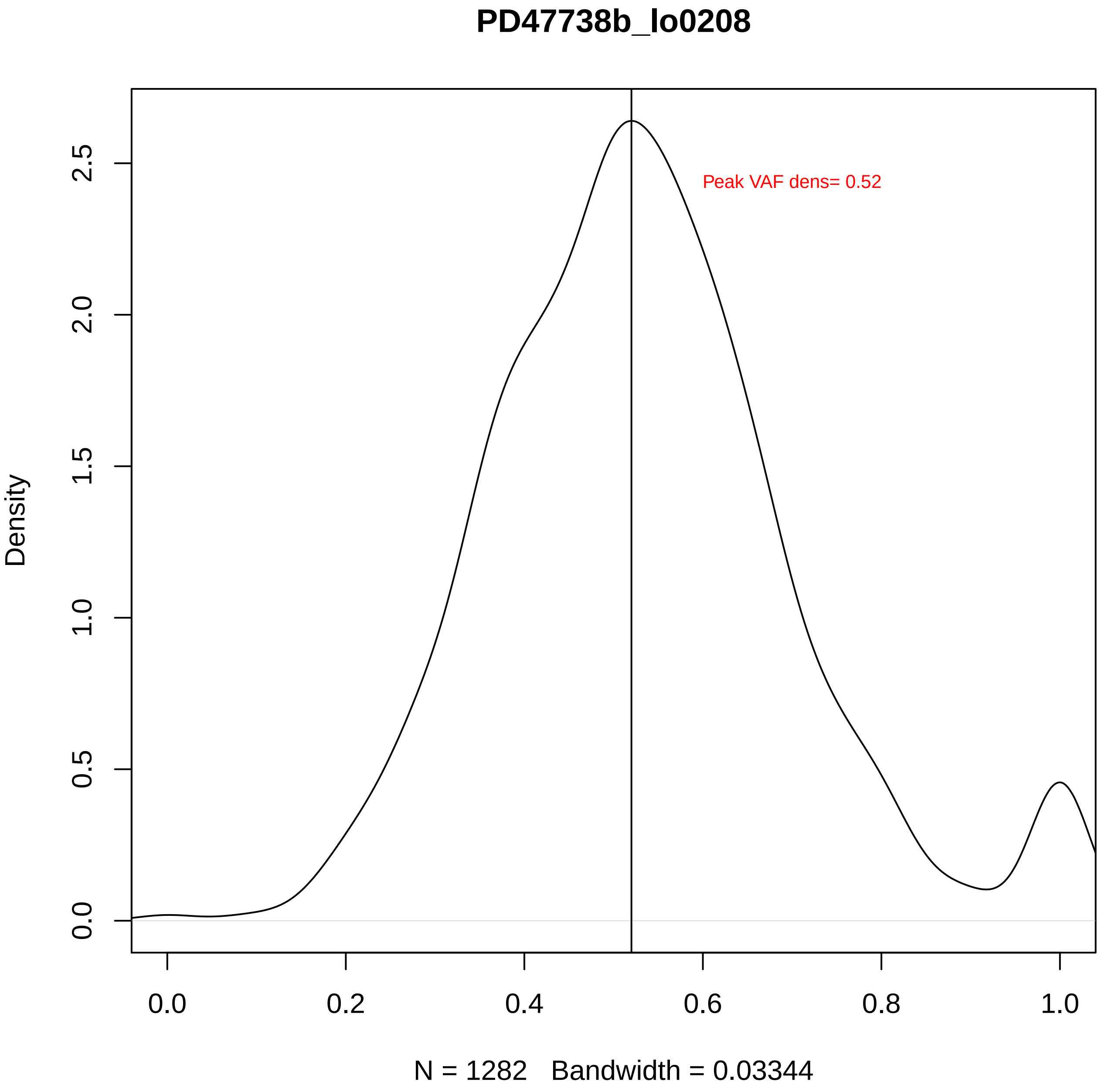
PD47738b_lo0208
Peak VAF dens= 0.52
Density
0.0
0.5
1.0
1.5
2.0
2.5
0.0
0.2
0.4
0.6
0.8
1.0
N = 1282   Bandwidth = 0.03344

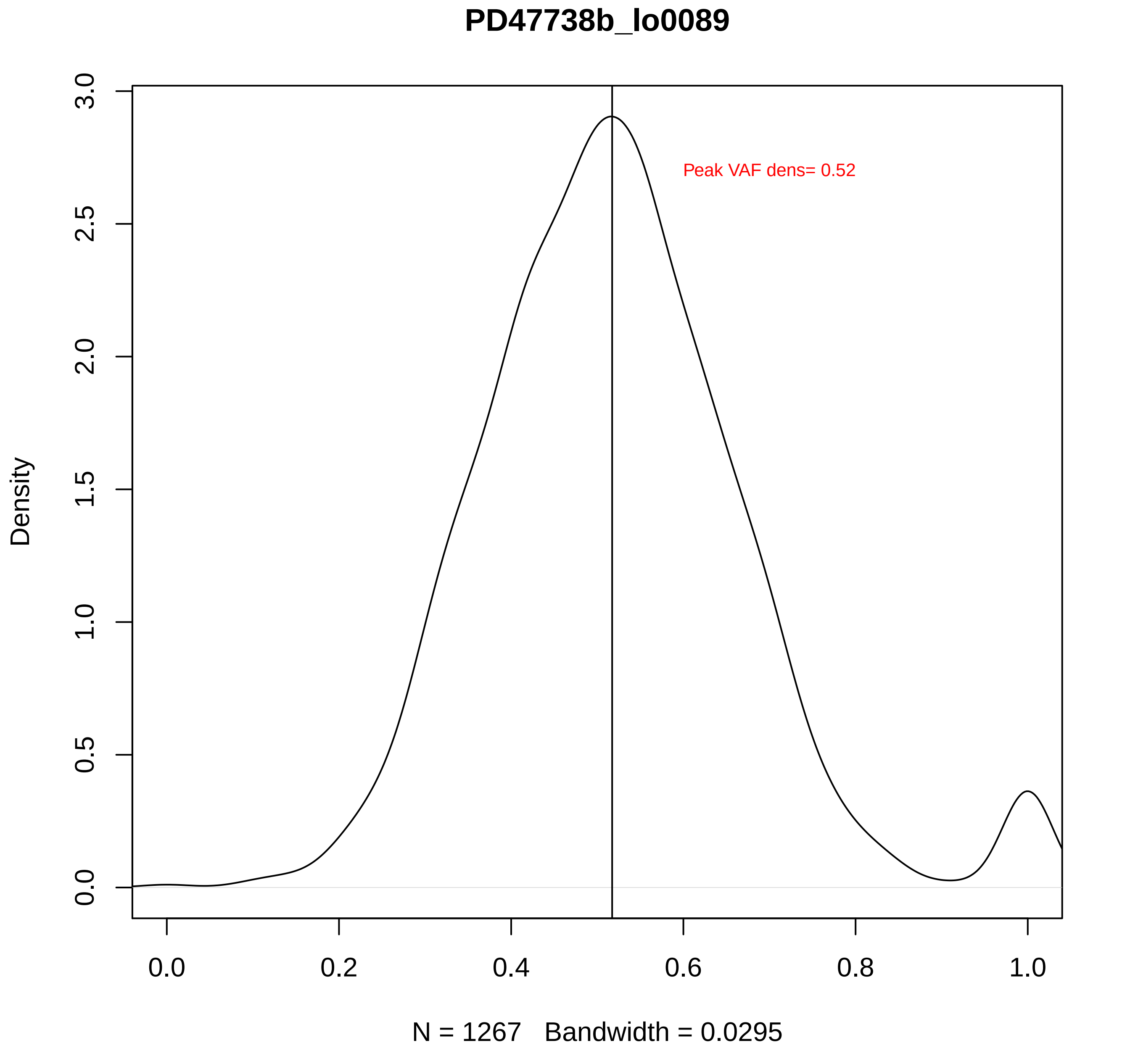

PD47738b_lo0089
Peak VAF dens= 0.52
Density
3.0
2.5
2.0
1.5
1.0
0.5
0.0
0.0
0.2
0.4
0.6
0.8
1.0
N = 1267 Bandwidth = 0.0295

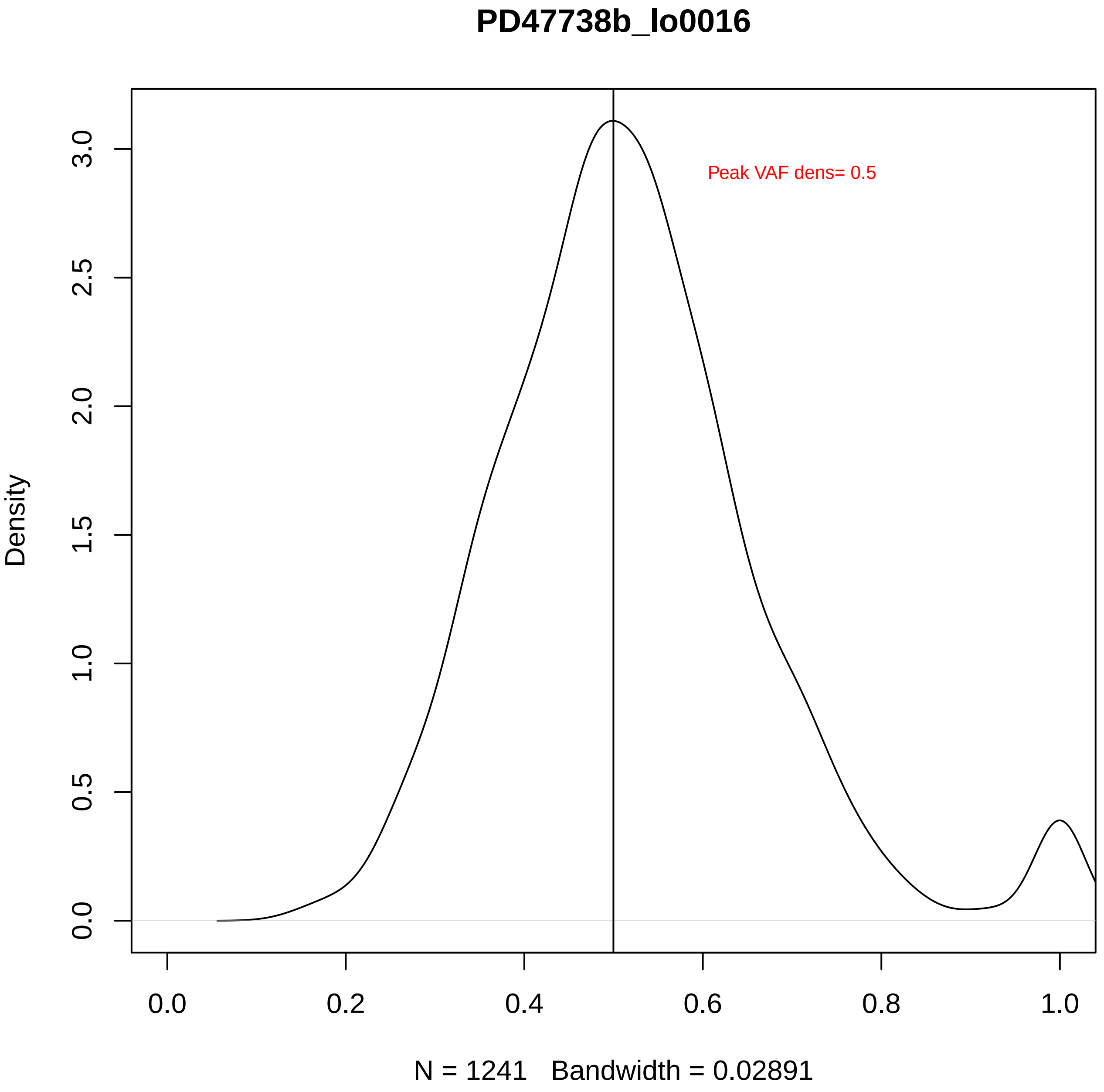
PD47738b_lo0016
Peak VAF dens= 0.5
Density
0.0
0.5
1.0
1.5
2.0
2.5
3.0
0.0
0.2
0.4
0.6
0.8
1.0
N = 1241   Bandwidth = 0.02891

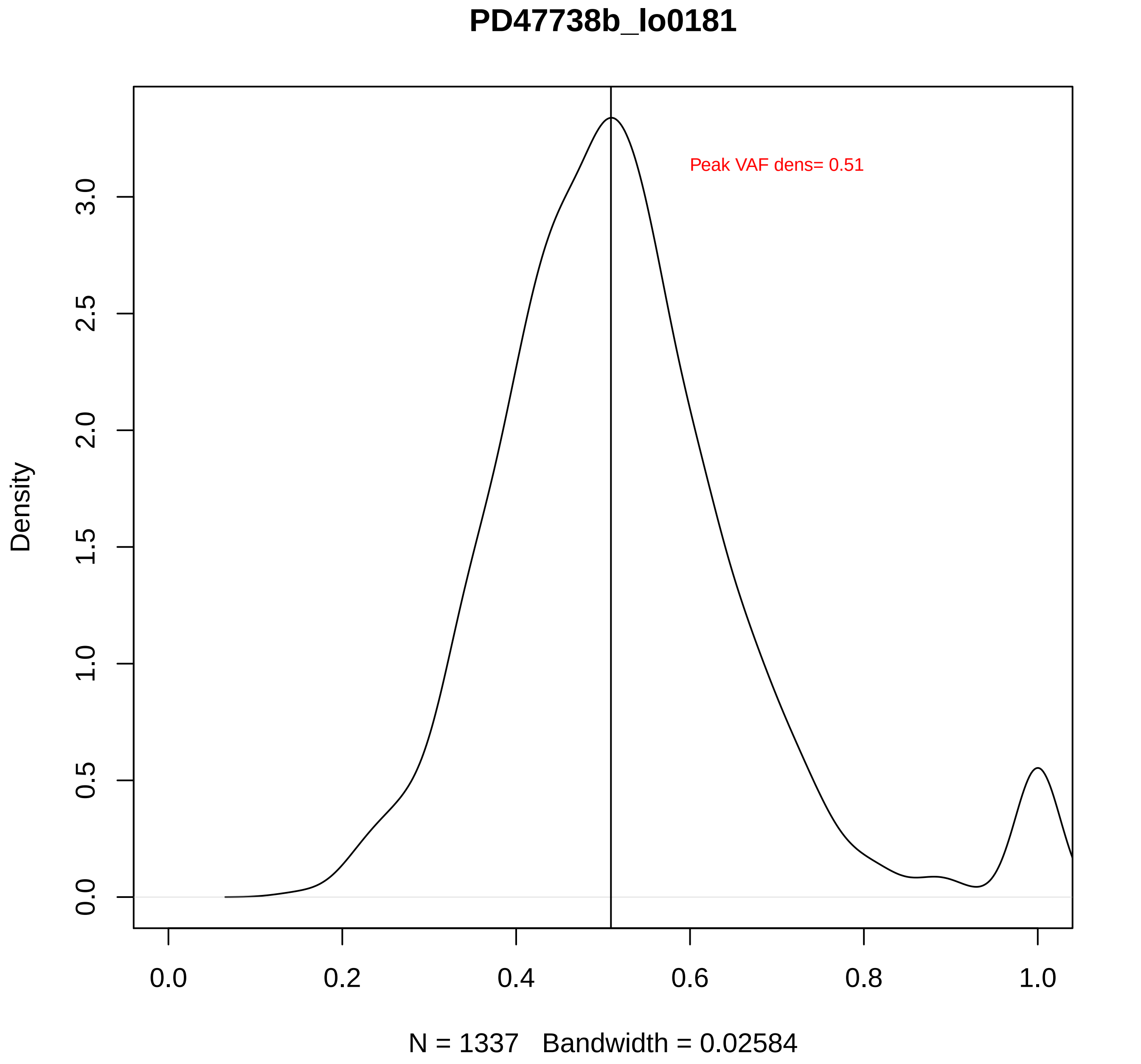
PD47738b_lo0181
Peak VAF dens= 0.51
Density
0.0
0.5
1.0
1.5
2.0
2.5
3.0
0.0
0.2
0.4
0.6
0.8
1.0
N = 1337   Bandwidth = 0.02584

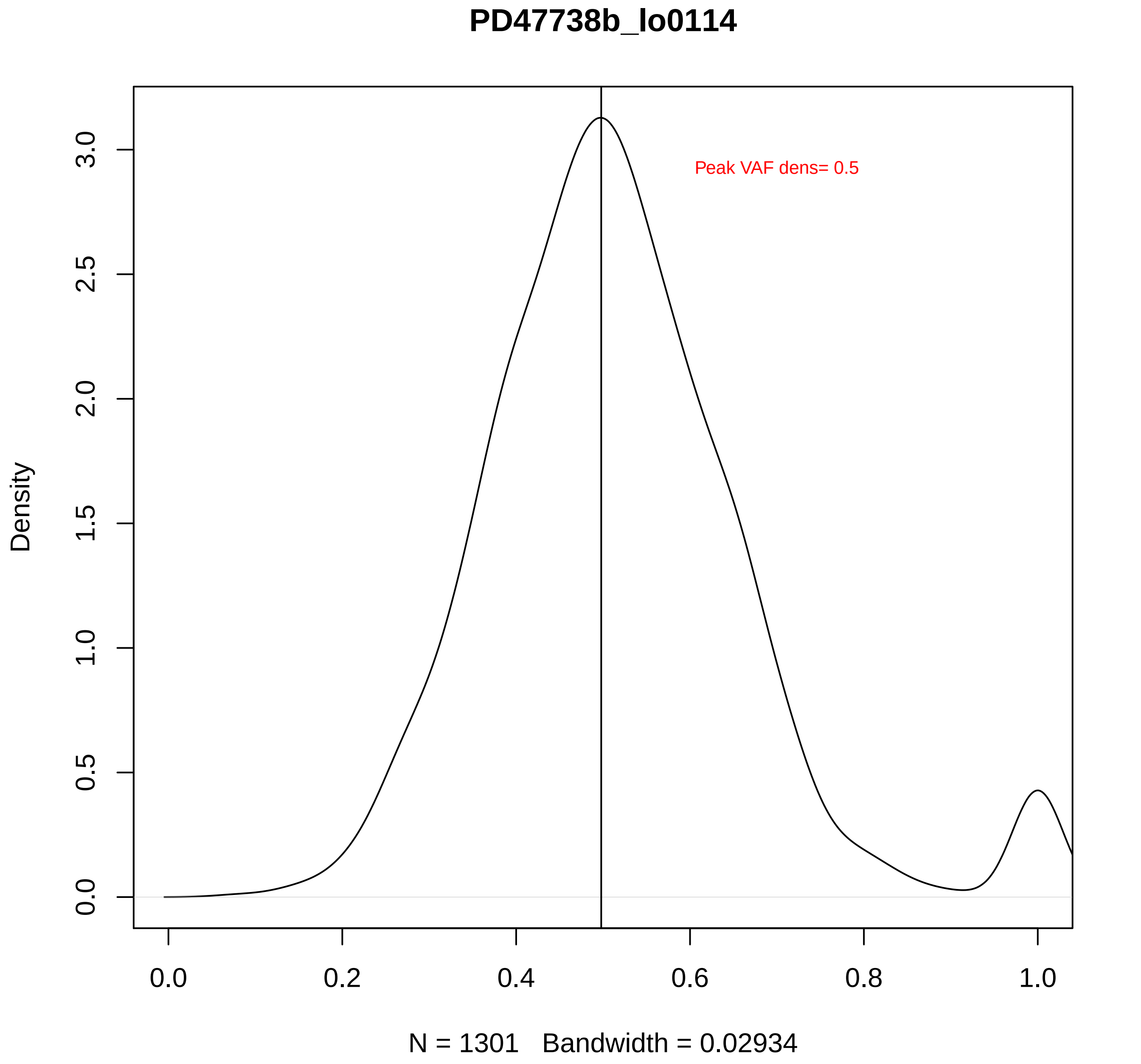
PD47738b_lo0114
Peak VAF dens= 0.5
Density
0.0
0.5
1.0
1.5
2.0
2.5
3.0
0.0
0.2
0.4
0.6
0.8
1.0
N = 1301   Bandwidth = 0.02934

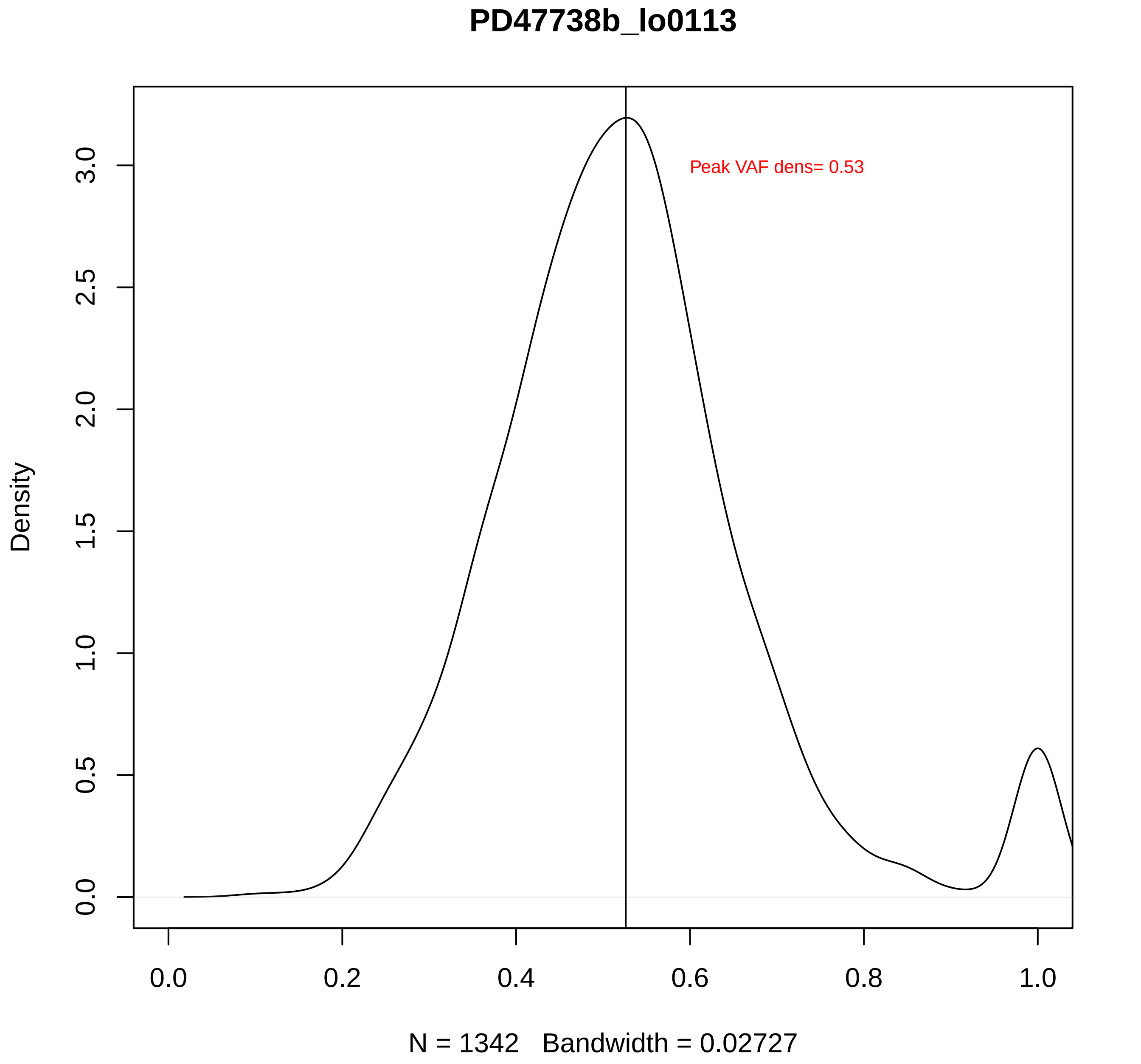
PD47738b_lo0113
Peak VAF dens= 0.53
Density
0.0
0.5
1.0
1.5
2.0
2.5
3.0
0.0
0.2
0.4
0.6
0.8
1.0
N = 1342   Bandwidth = 0.02727

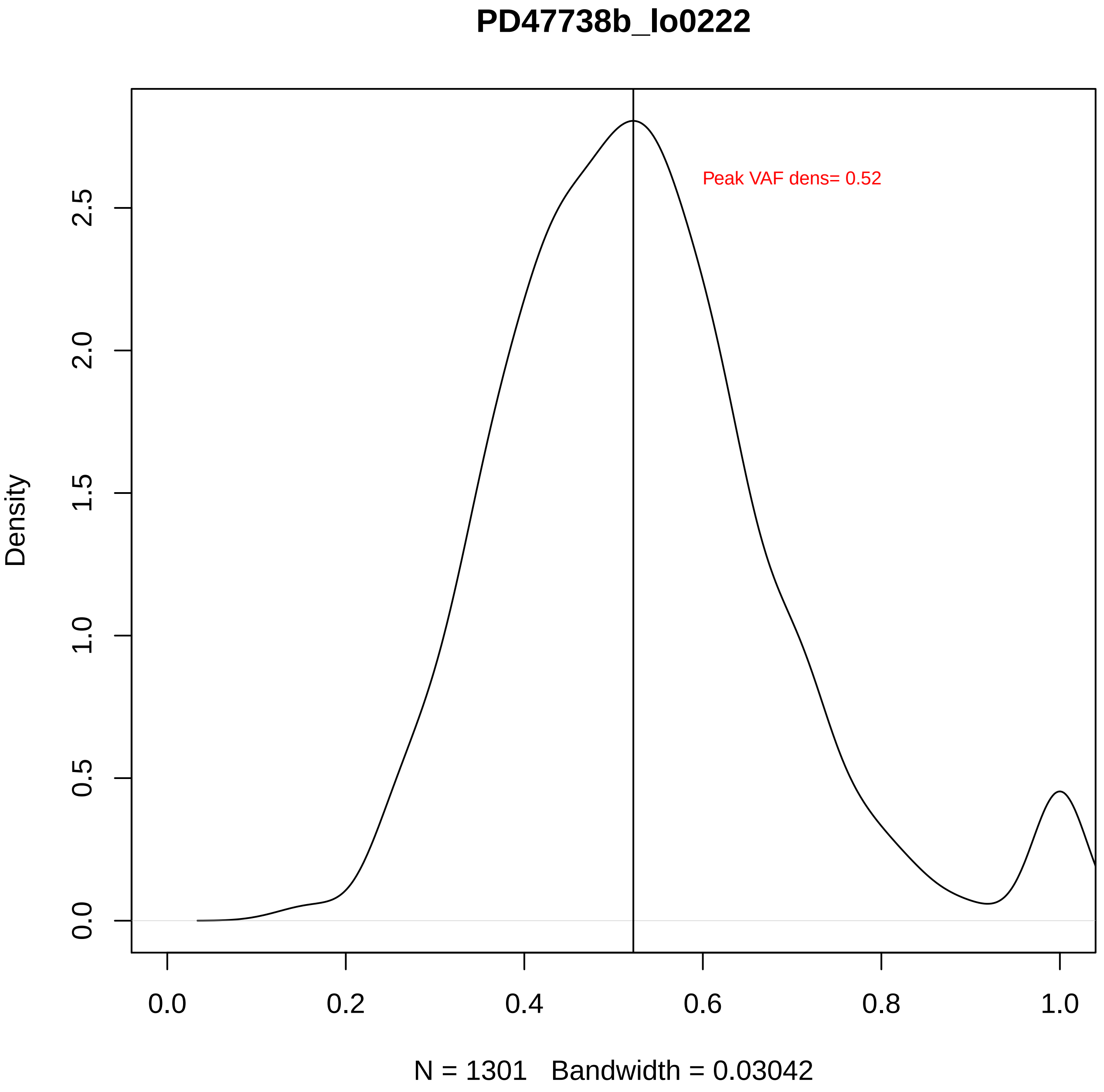
PD47738b_lo0222
Peak VAF dens= 0.52
Density
0.0
0.5
1.0
1.5
2.0
2.5
0.0
0.2
0.4
0.6
0.8
1.0
N = 1301 Bandwidth = 0.03042

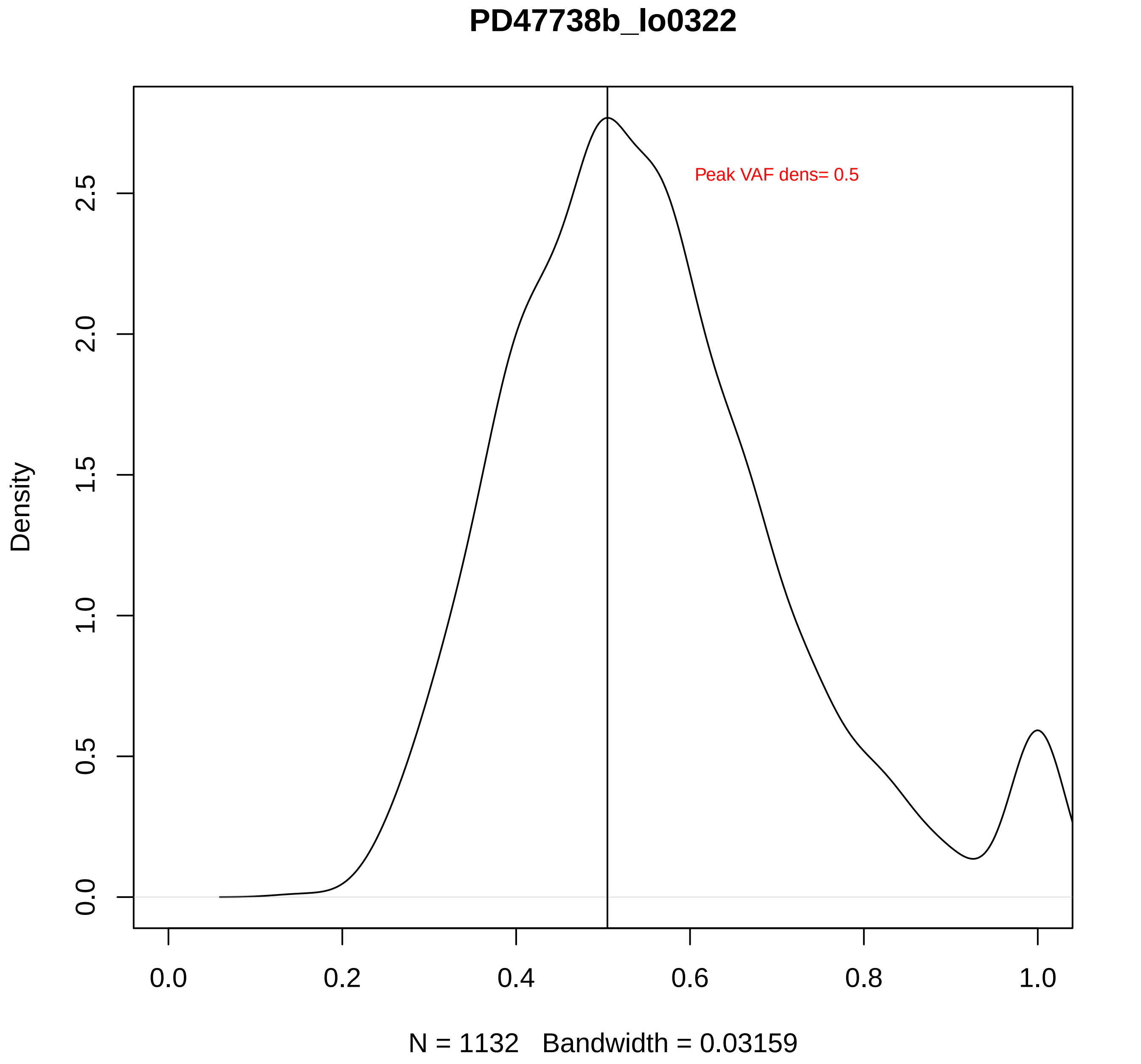

PD47738b_lo0322
Peak VAF dens= 0.5
Density
0.0
0.5
1.0
1.5
2.0
2.5
0.0
0.2
0.4
0.6
0.8
1.0
N = 1132   Bandwidth = 0.03159

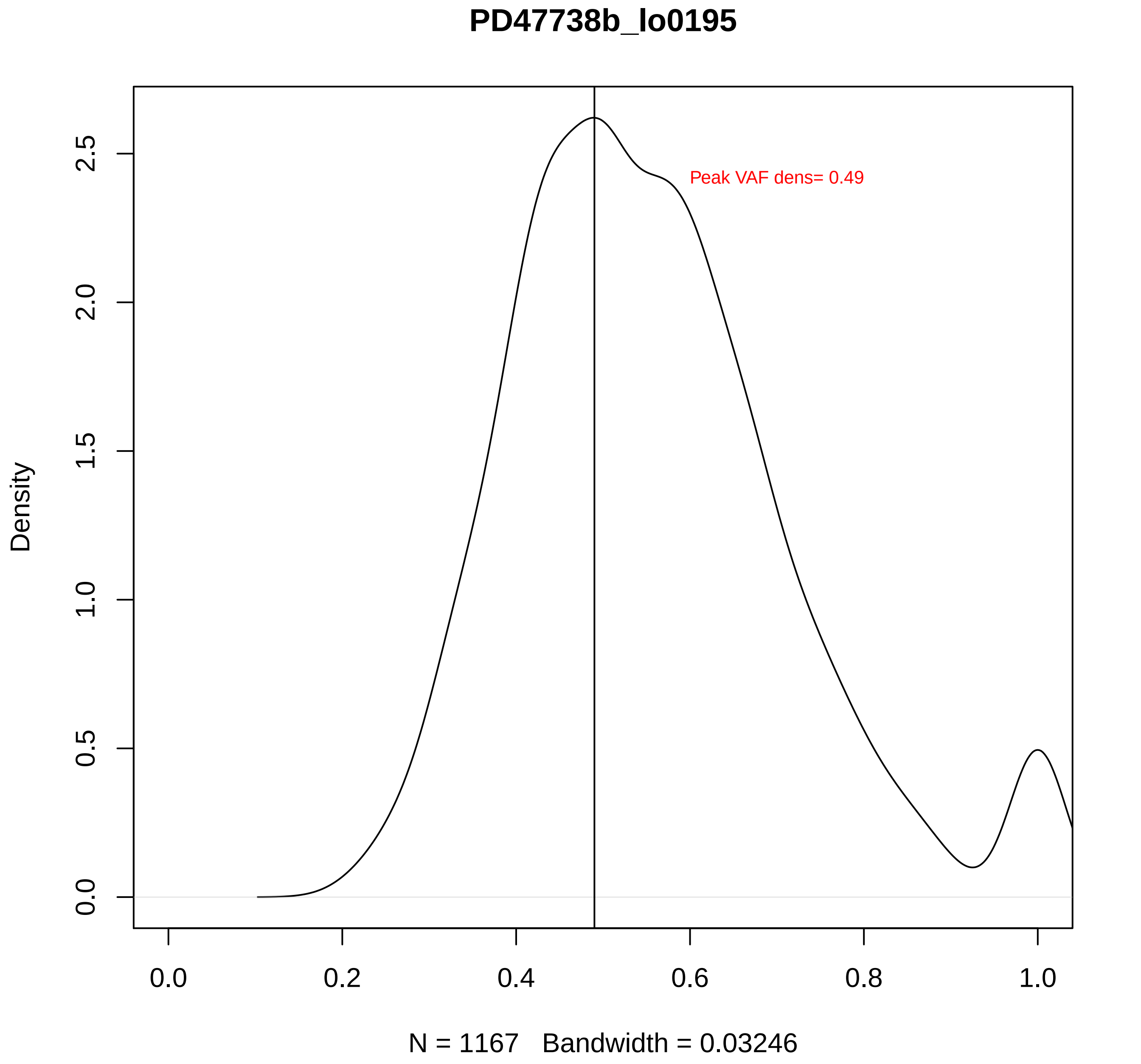

PD47738b_lo0195
Peak VAF dens= 0.49
Density
0.0
0.5
1.0
1.5
2.0
2.5
0.0
0.2
0.4
0.6
0.8
1.0
N = 1167   Bandwidth = 0.03246

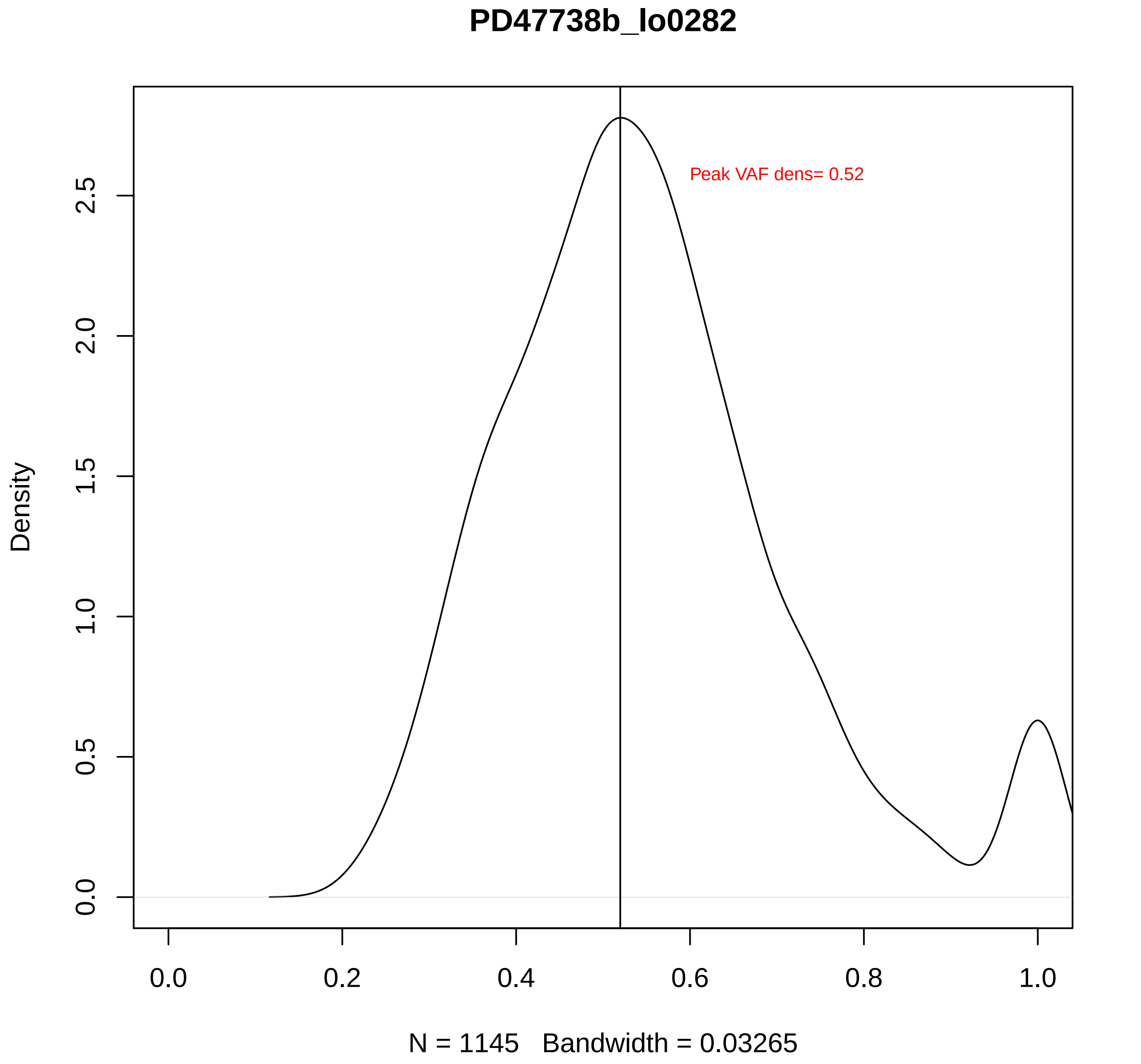
PD47738b_lo0282
Peak VAF dens= 0.52
Density
0.0
0.5
1.0
1.5
2.0
2.5
0.0
0.2
0.4
0.6
0.8
1.0
N = 1145   Bandwidth = 0.03265

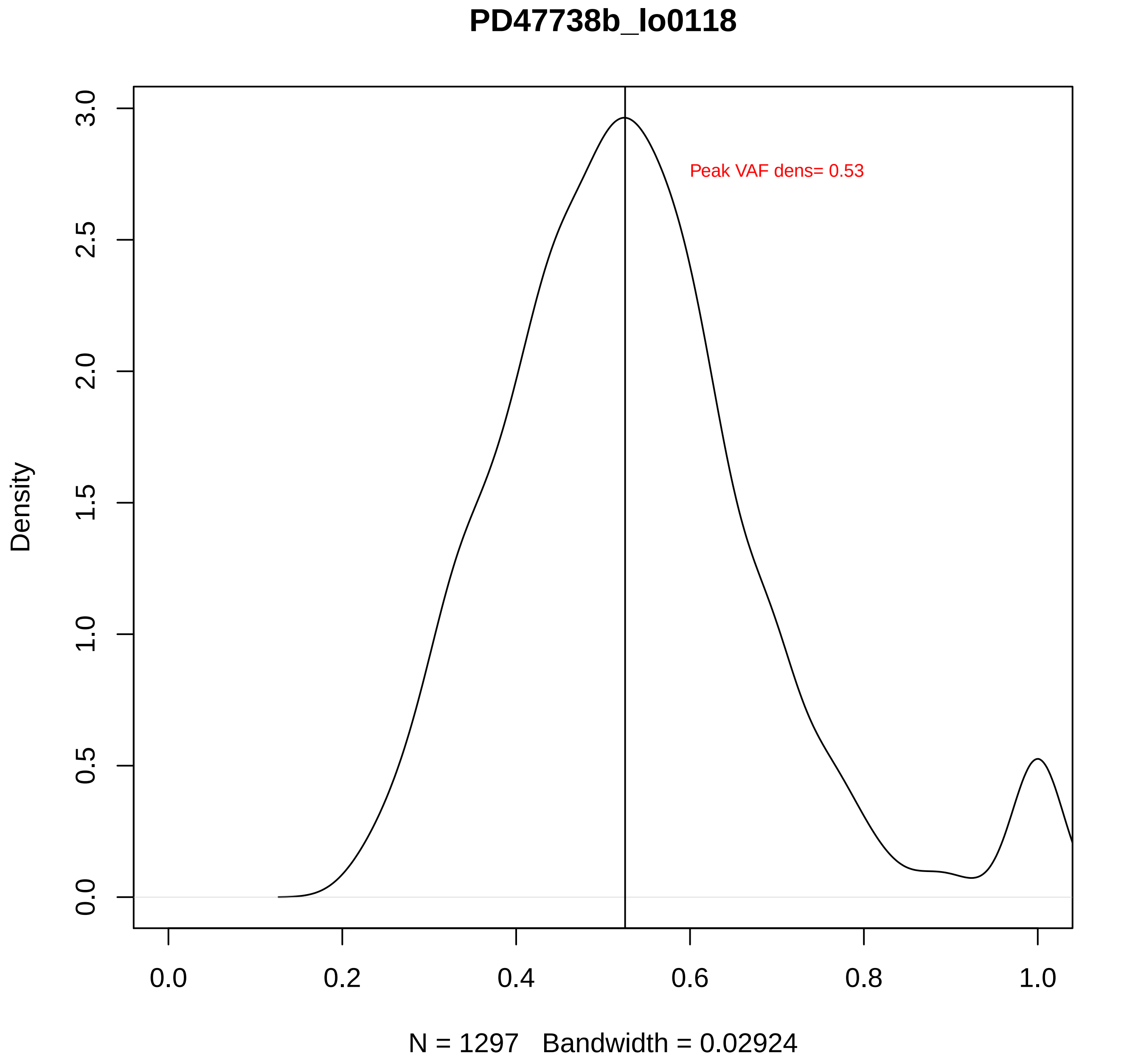
PD47738b_lo0118
Peak VAF dens= 0.53
Density
3.0
2.5
2.0
1.5
1.0
0.5
0.0
0.2
0.4
0.6
0.8
1.0
N = 1297   Bandwidth = 0.02924

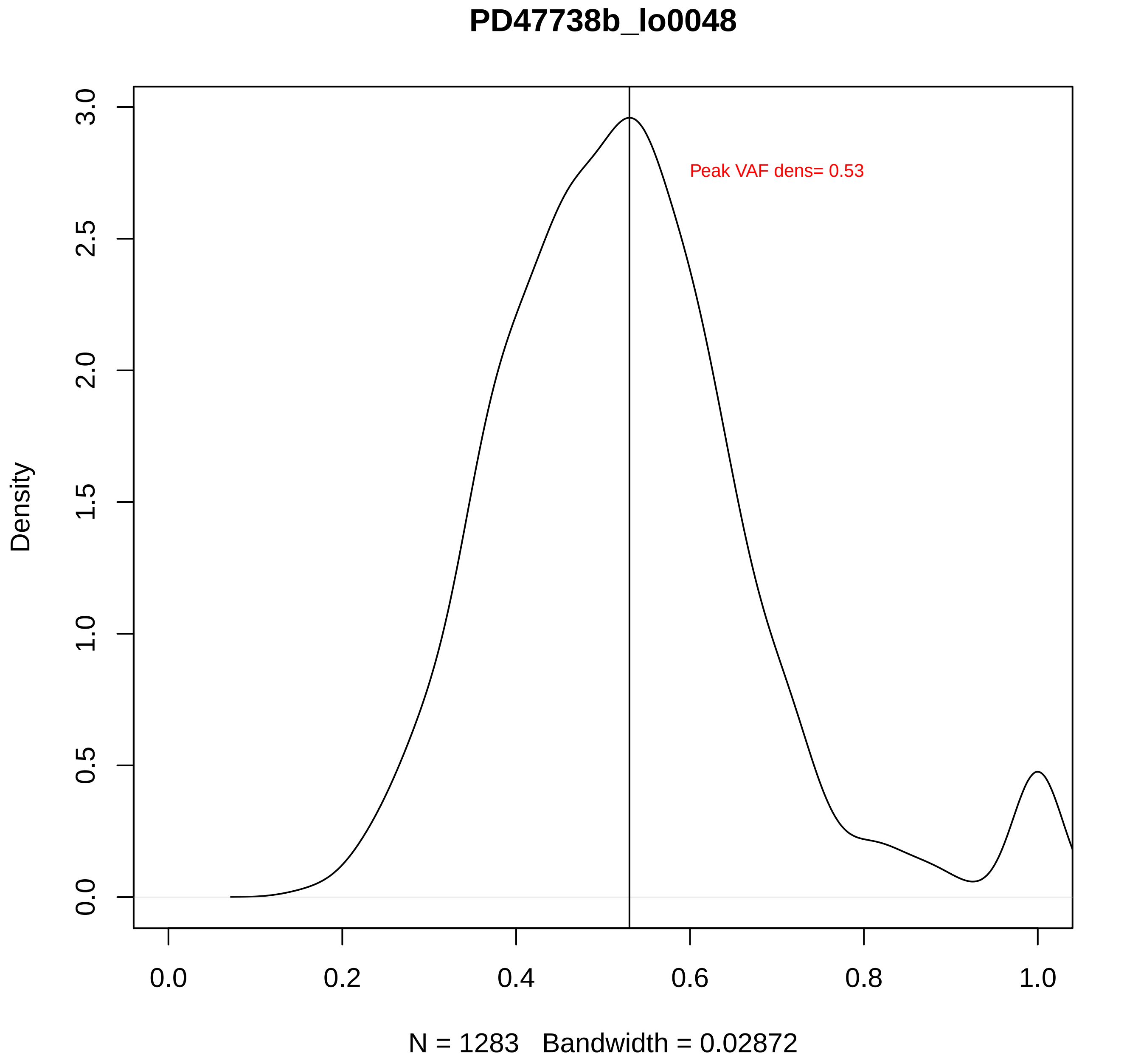
PD47738b_lo0048
Peak VAF dens= 0.53
Density
3.0
2.5
2.0
1.5
1.0
0.5
0.0
0.0
0.2
0.4
0.6
0.8
1.0
N = 1283 Bandwidth = 0.02872

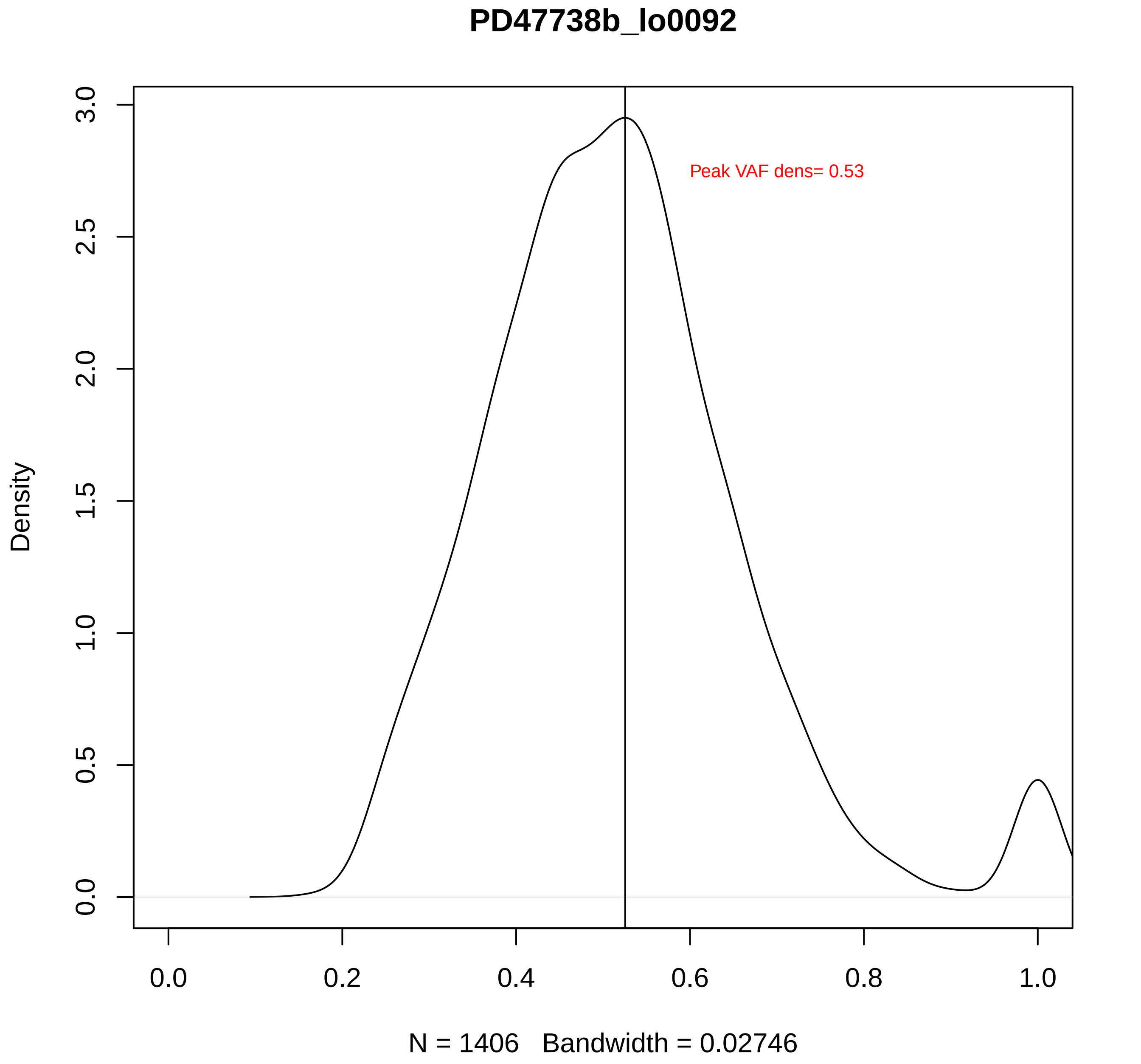

PD47738b_lo0092
Peak VAF dens= 0.53
Density
0.0
0.5
1.0
1.5
2.0
2.5
3.0
0.0
0.2
0.4
0.6
0.8
1.0
N = 1406 Bandwidth = 0.02746

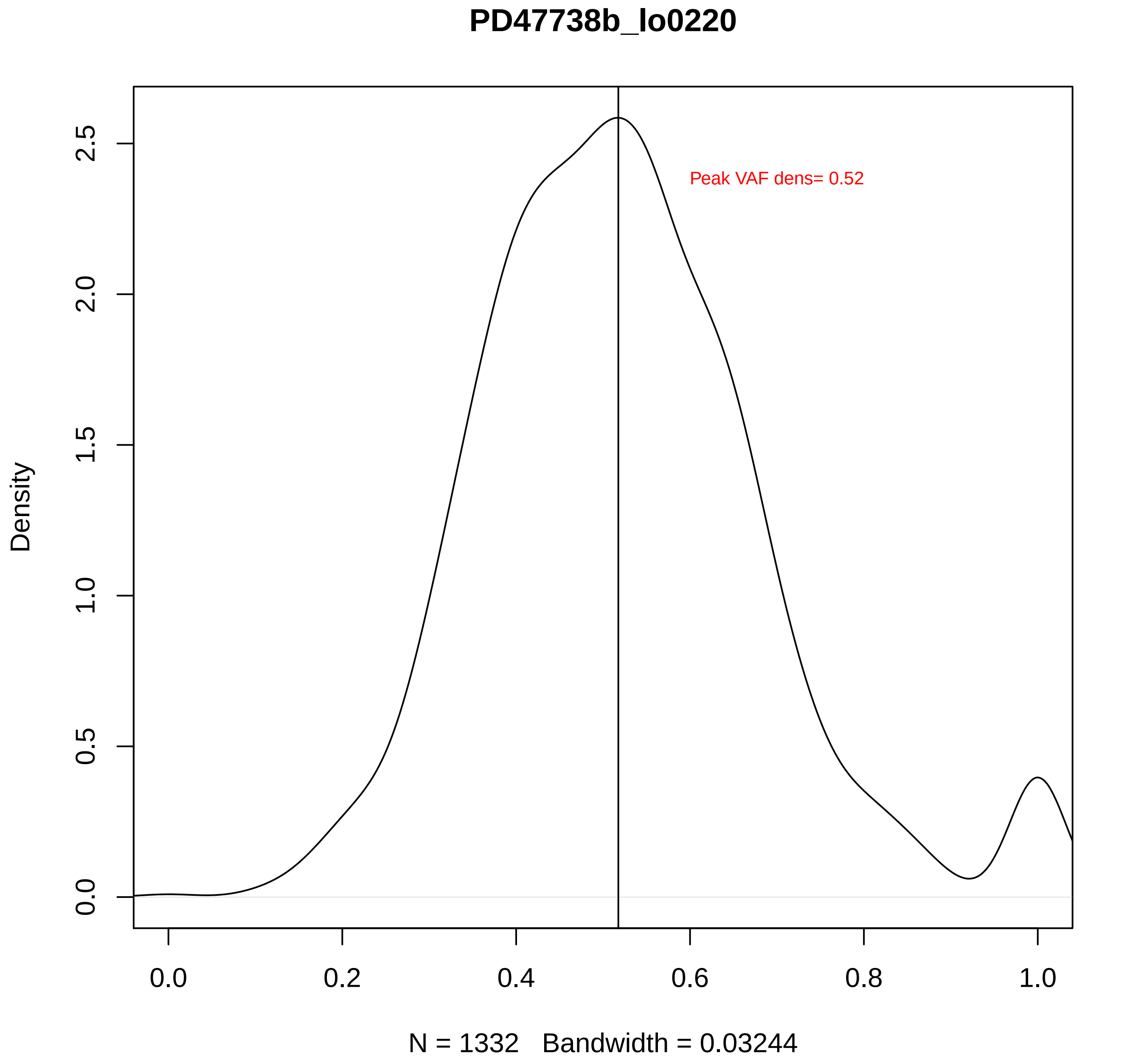

PD47738b_lo0220
Peak VAF dens= 0.52
Density
0.0
0.5
1.0
1.5
2.0
2.5
0.0
0.2
0.4
0.6
0.8
1.0
N = 1332 Bandwidth = 0.03244

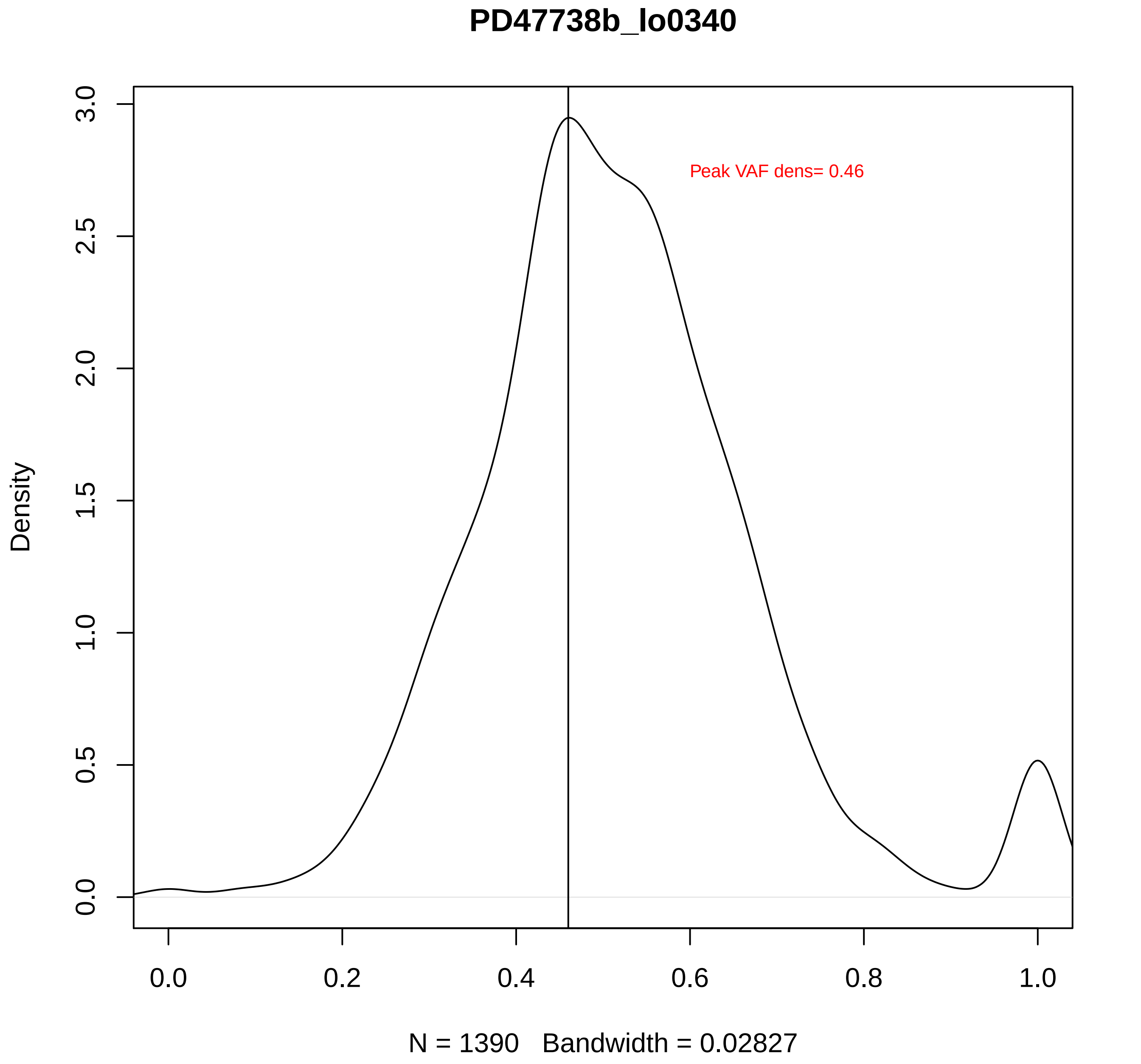
PD47738b_lo0340
Peak VAF dens= 0.46
Density
3.0
2.5
2.0
1.5
1.0
0.5
0.0
0.0
0.2
0.4
0.6
0.8
1.0
N = 1390   Bandwidth = 0.02827

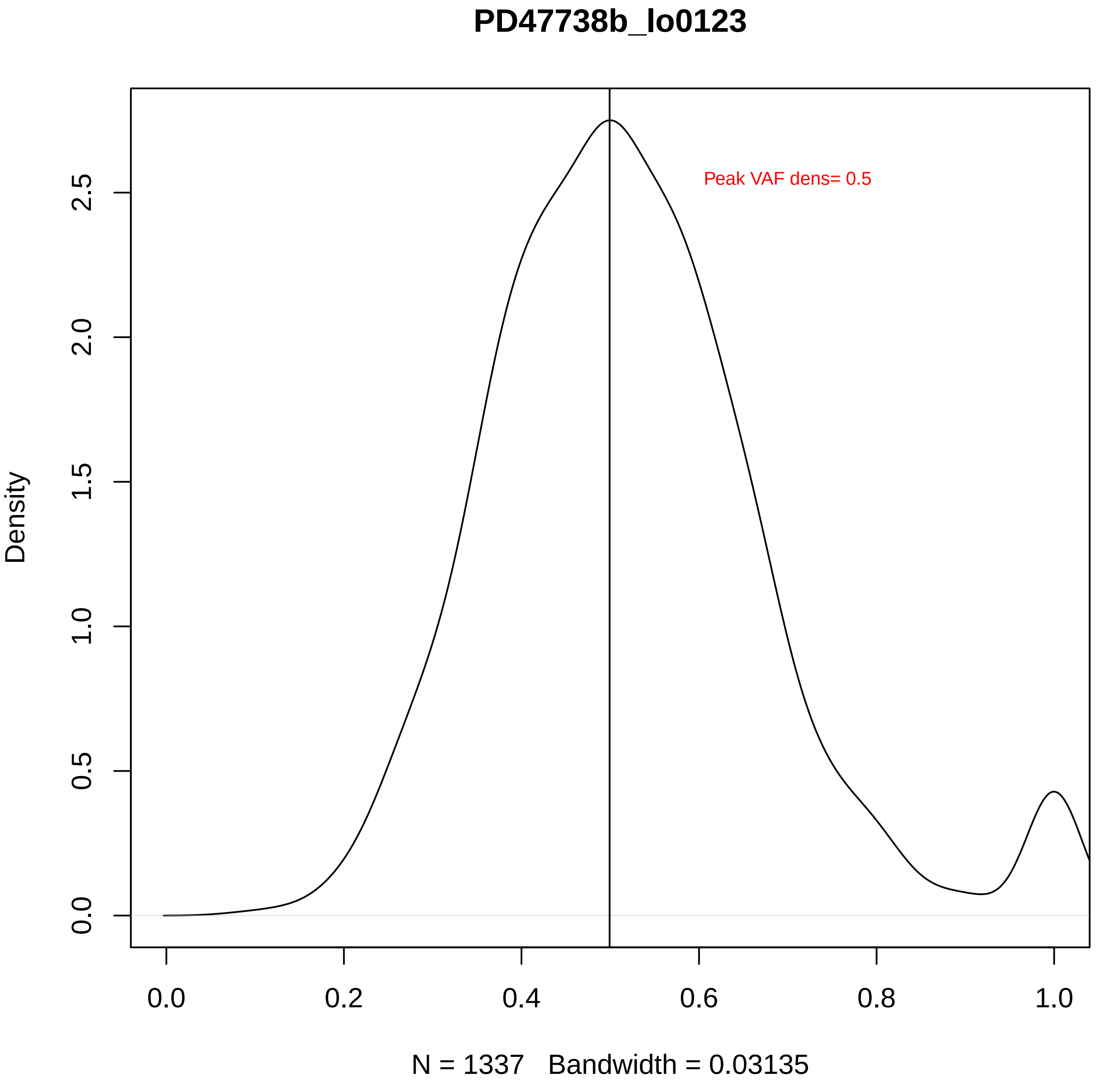
PD47738b_lo0123
Peak VAF dens= 0.5
Density
0.0
0.5
1.0
1.5
2.0
2.5
0.0
0.2
0.4
0.6
0.8
1.0
N = 1337   Bandwidth = 0.03135

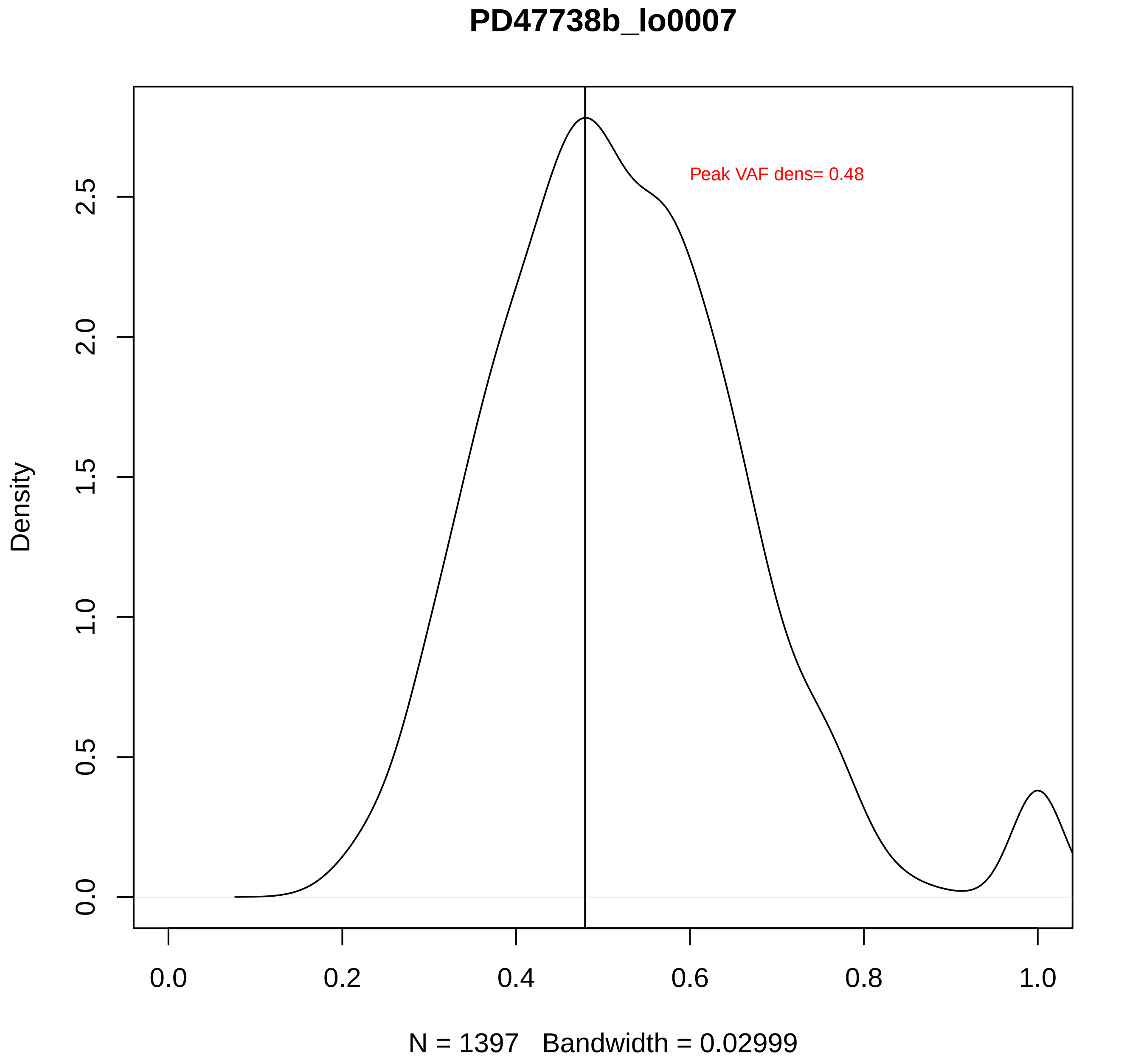

PD47738b_lo0007
Peak VAF dens= 0.48
Density
0.0
0.5
1.0
1.5
2.0
2.5
0.0
0.2
0.4
0.6
0.8
1.0
N = 1397   Bandwidth = 0.02999

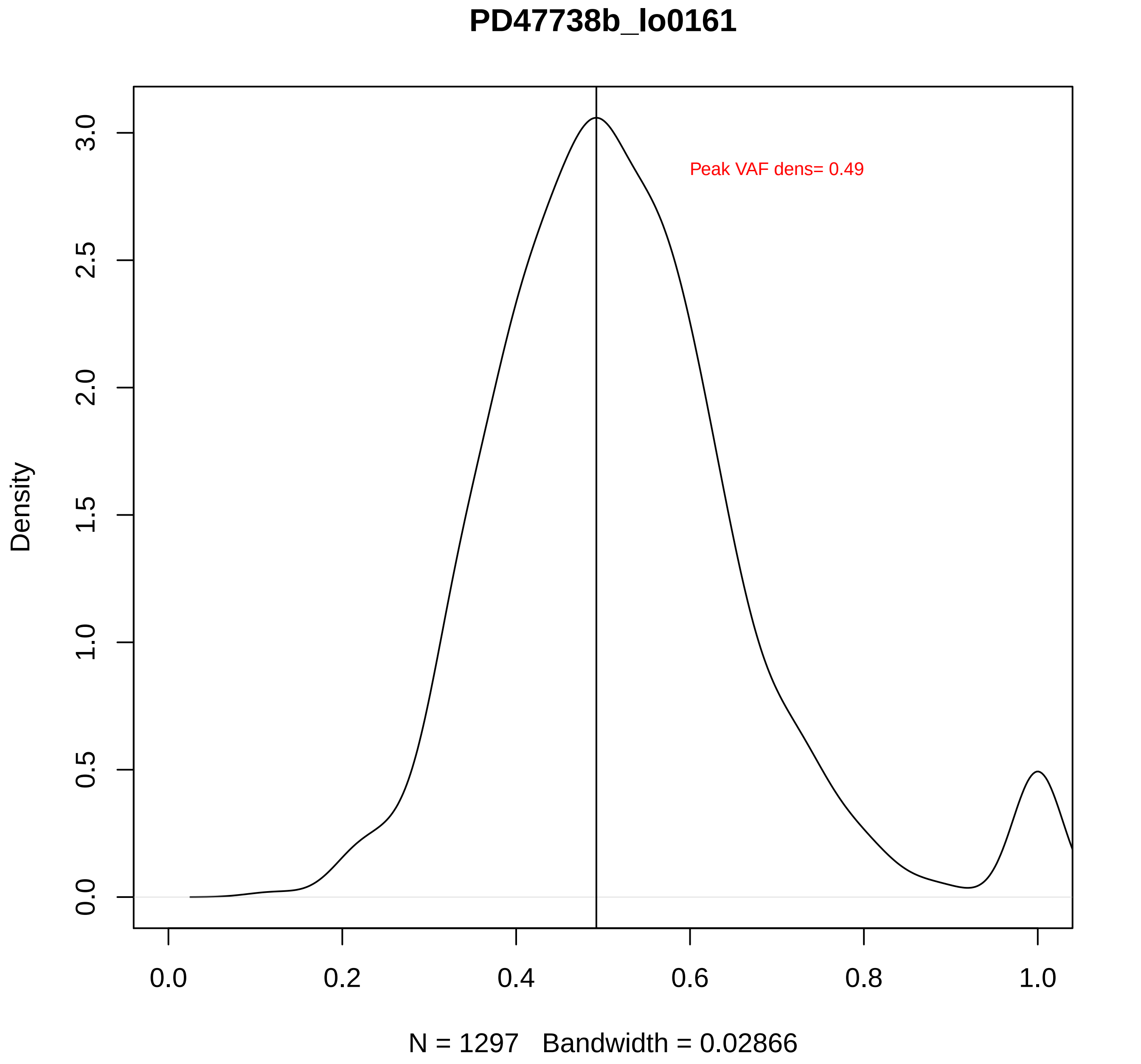
PD47738b_lo0161
Peak VAF dens= 0.49
Density
3.0
2.5
2.0
1.5
1.0
0.5
0.0
0.0
0.2
0.4
0.6
0.8
1.0
N = 1297   Bandwidth = 0.02866

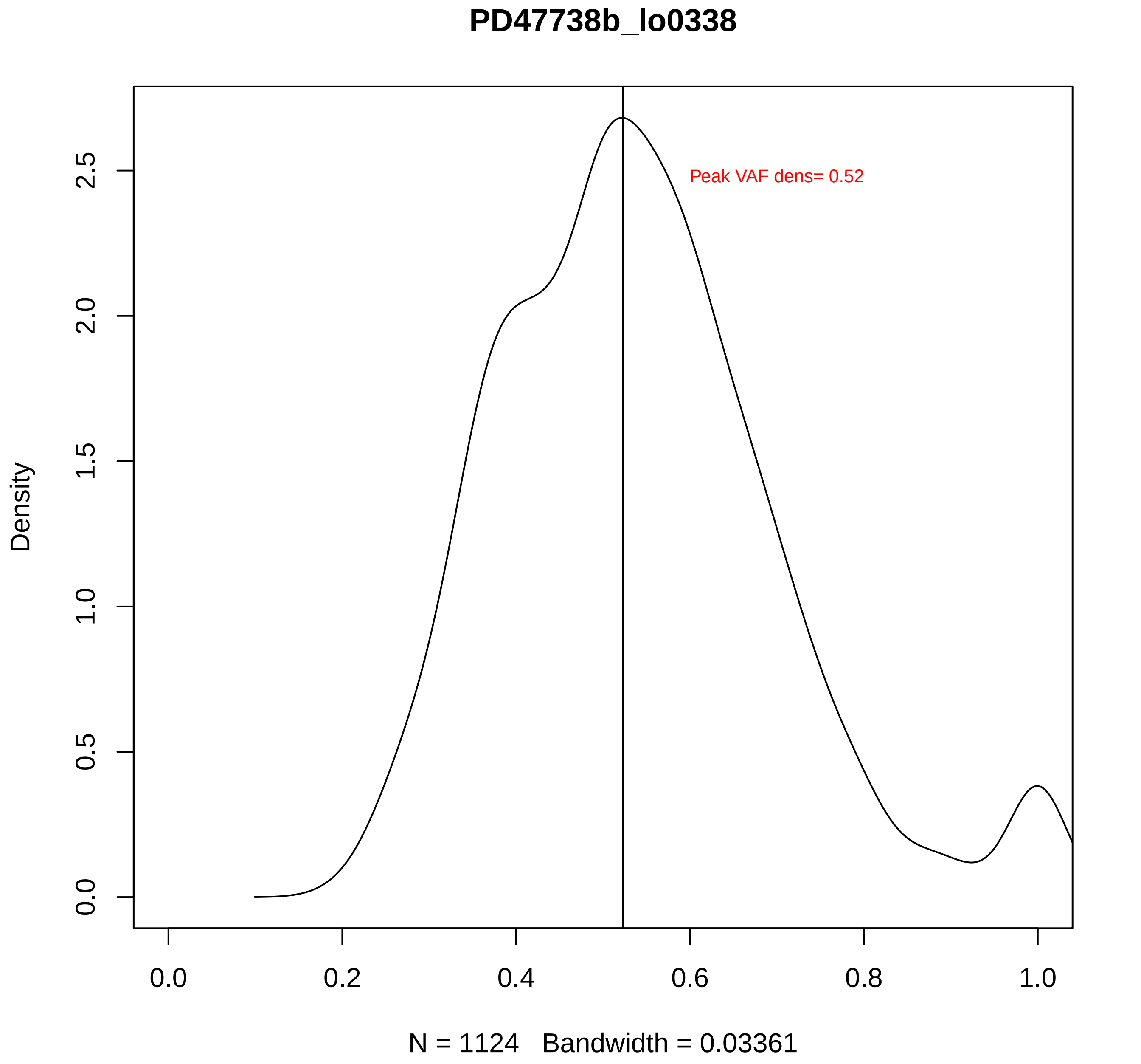

PD47738b_lo0338
Peak VAF dens= 0.52
Density
0.0
0.5
1.0
1.5
2.0
2.5
0.0
0.2
0.4
0.6
0.8
1.0
N = 1124   Bandwidth = 0.03361

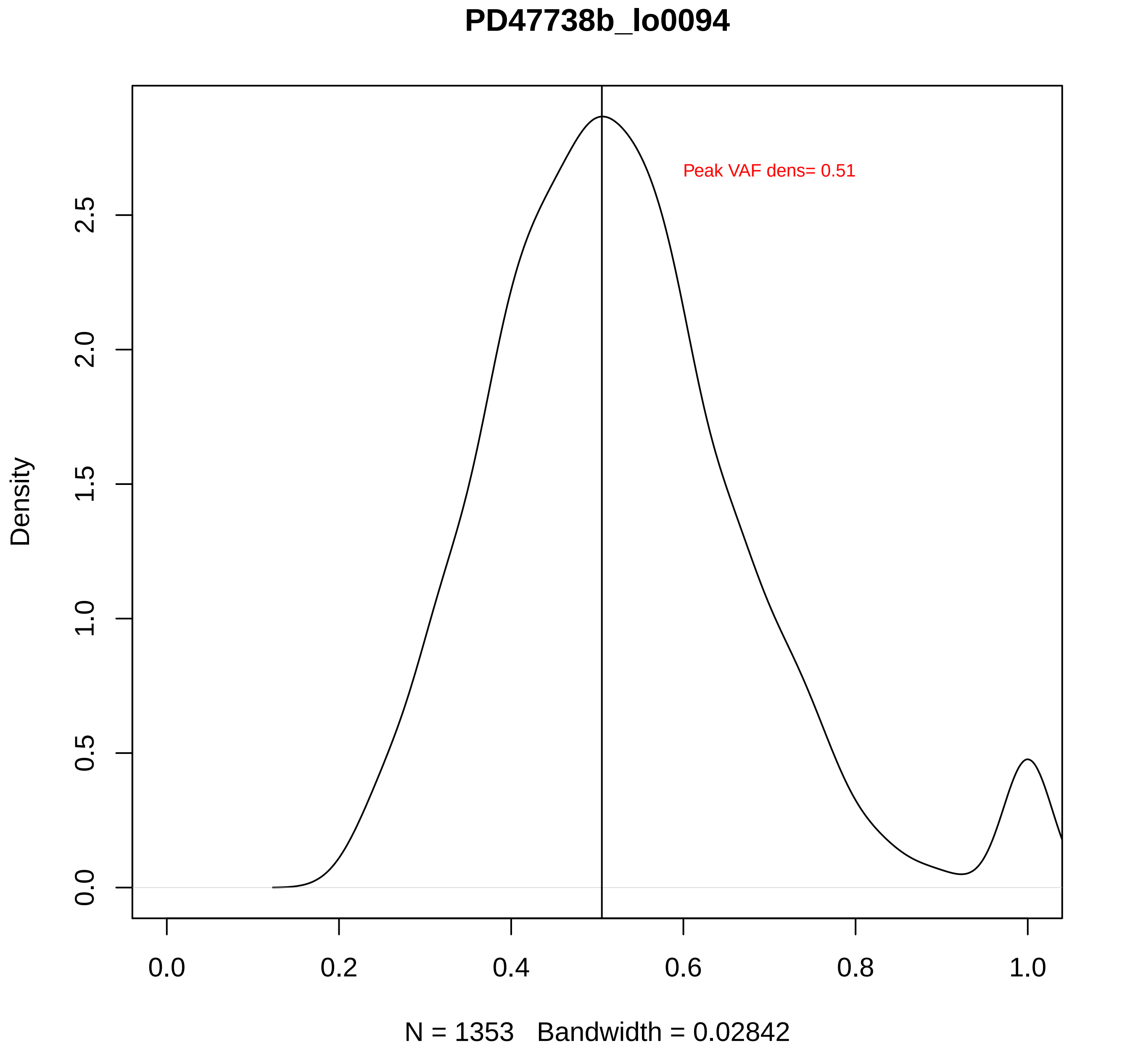

PD47738b_lo0094
Peak VAF dens= 0.51
Density
0.0
0.5
1.0
1.5
2.0
2.5
0.0
0.2
0.4
0.6
0.8
1.0
N = 1353   Bandwidth = 0.02842

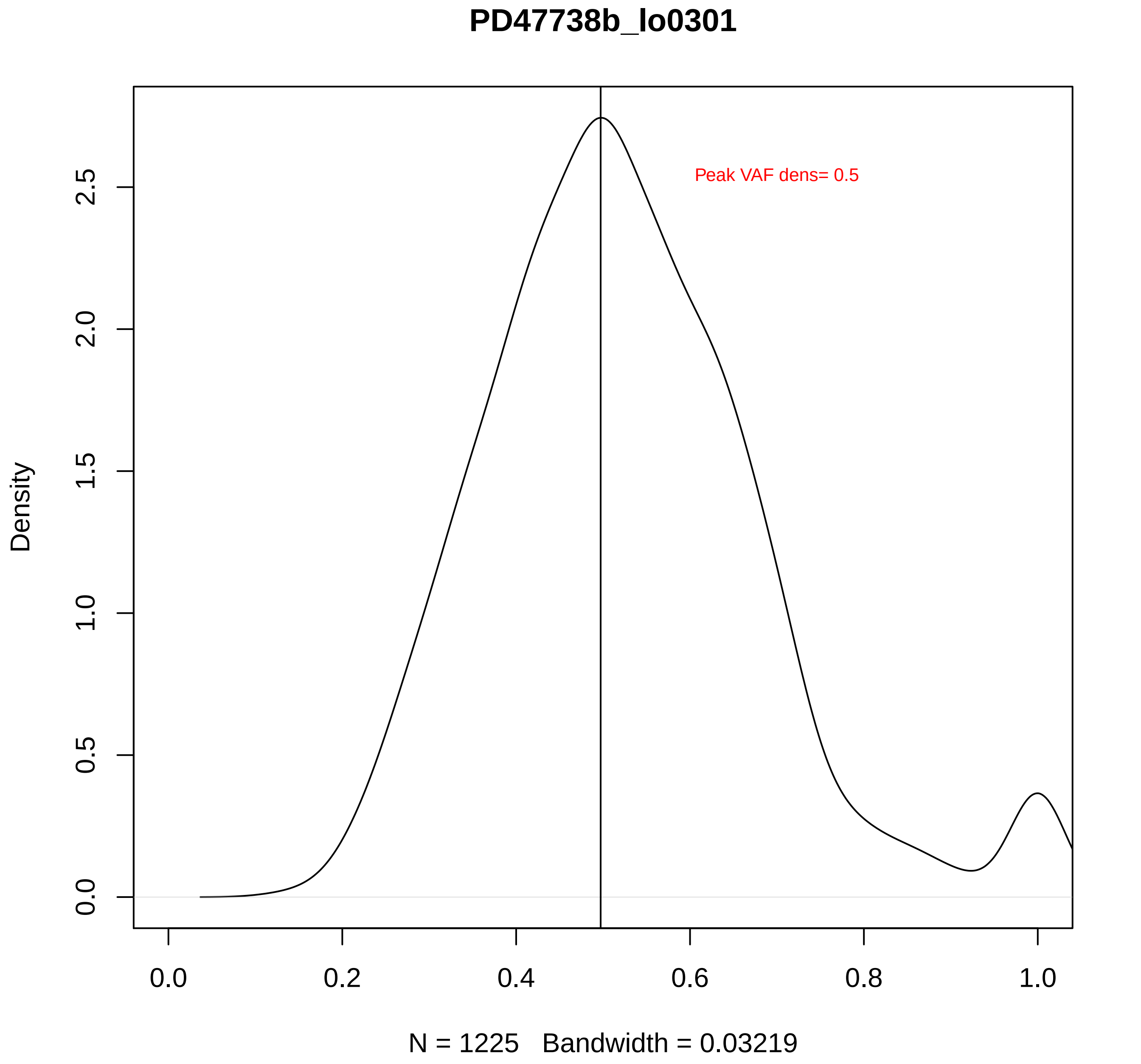
PD47738b_lo0301
Peak VAF dens= 0.5
Density
0.0
0.5
1.0
1.5
2.0
2.5
0.0
0.2
0.4
0.6
0.8
1.0
N = 1225   Bandwidth = 0.03219

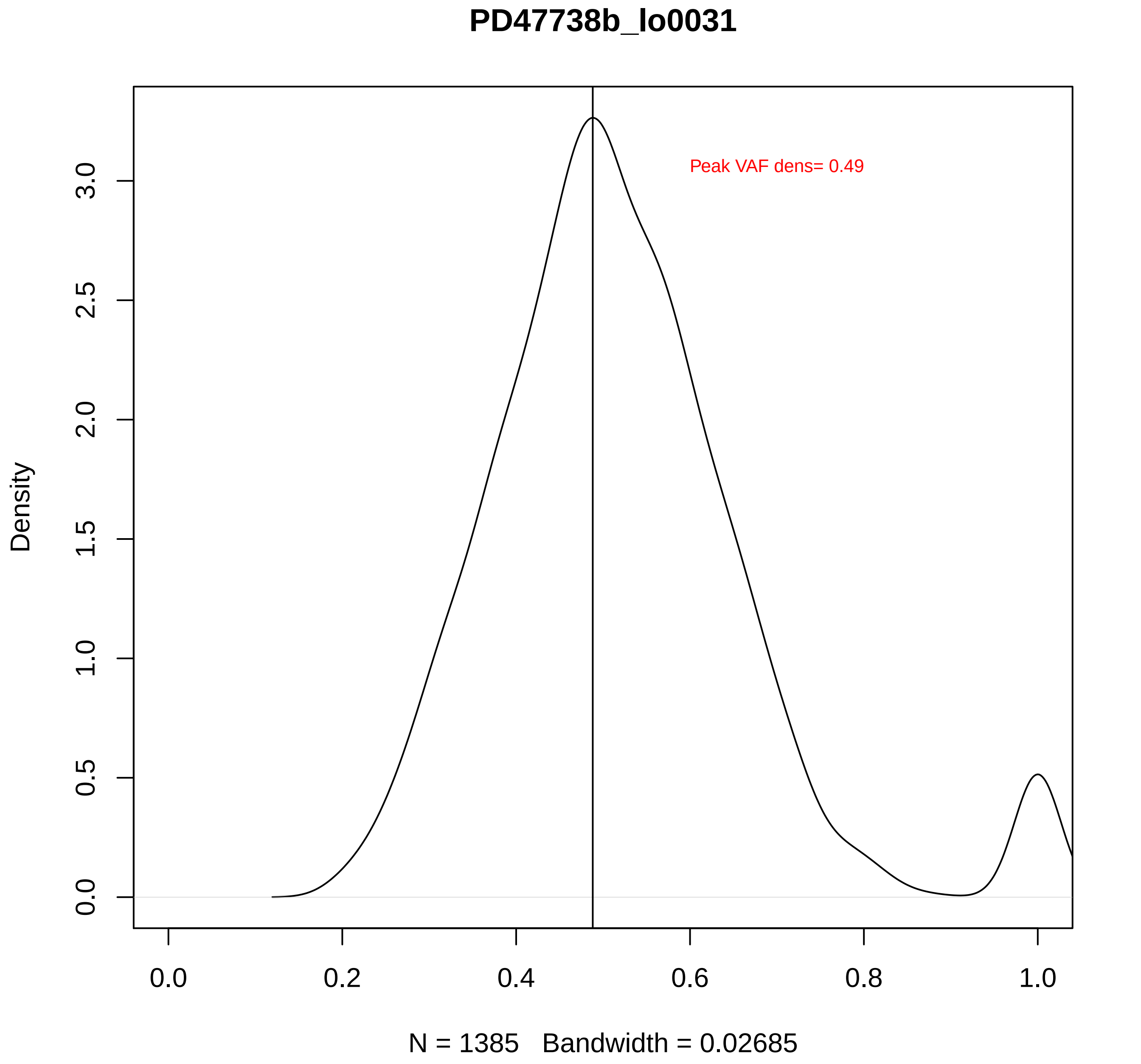
PD47738b_lo0031
Peak VAF dens= 0.49
Density
0.0
0.5
1.0
1.5
2.0
2.5
3.0
0.0
0.2
0.4
0.6
0.8
1.0
N = 1385   Bandwidth = 0.02685

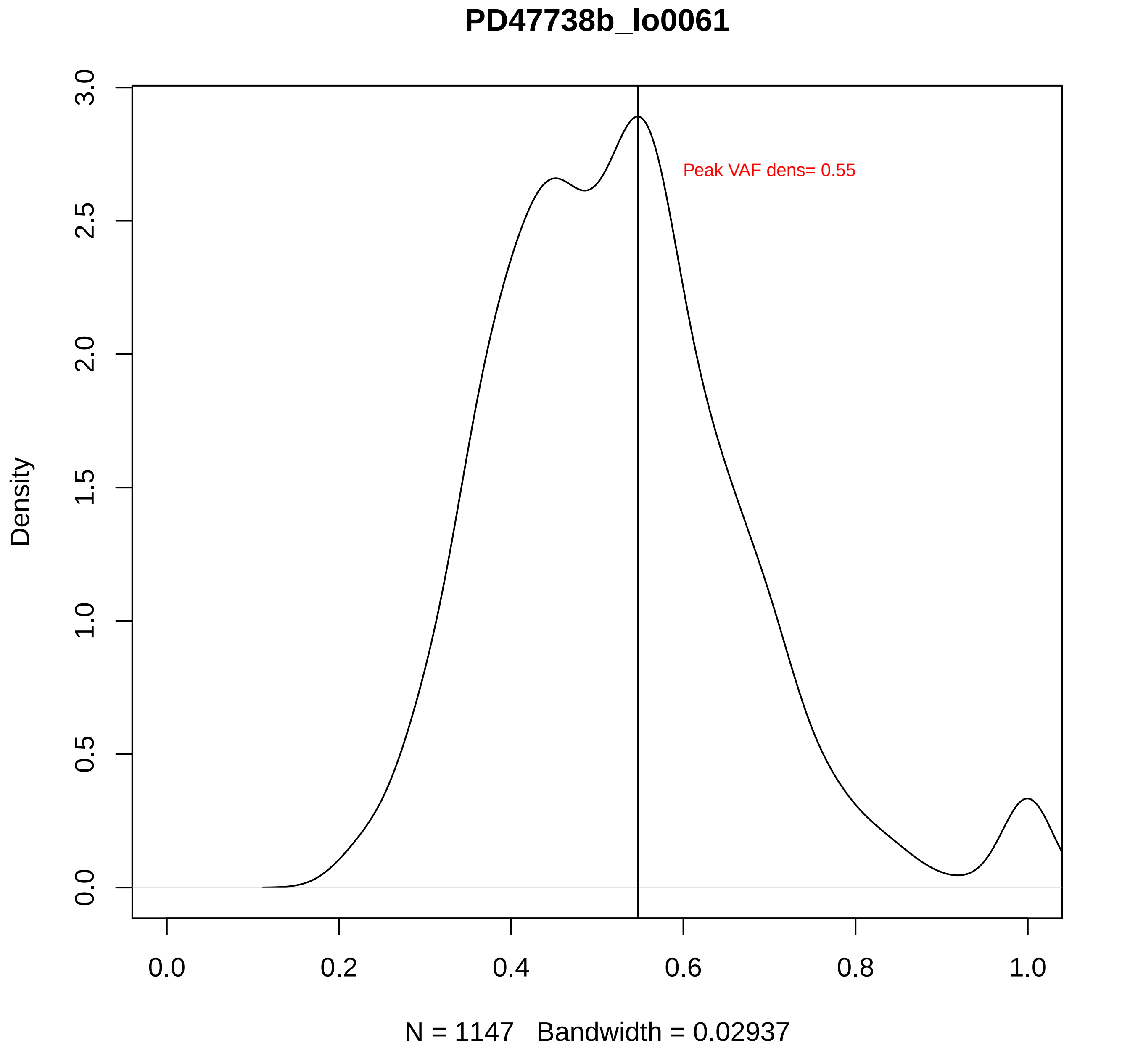

PD47738b_lo0061
Peak VAF dens= 0.55
Density
0.0
0.5
1.0
1.5
2.0
2.5
3.0
0.0
0.2
0.4
0.6
0.8
1.0
N = 1147   Bandwidth = 0.02937

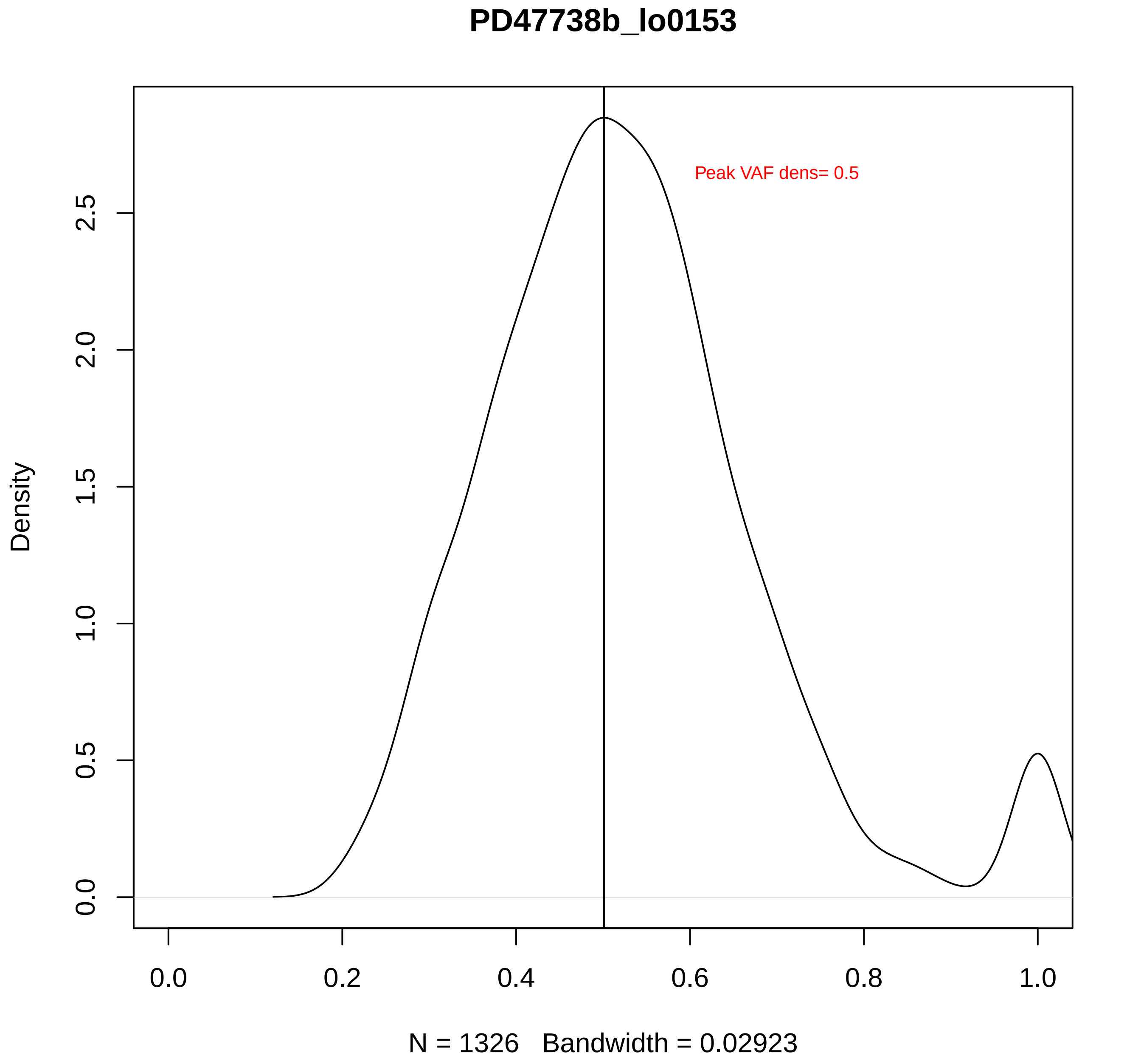
PD47738b_lo0153
Peak VAF dens= 0.5
Density
0.0
0.5
1.0
1.5
2.0
2.5
0.0
0.2
0.4
0.6
0.8
1.0
N = 1326 Bandwidth = 0.02923

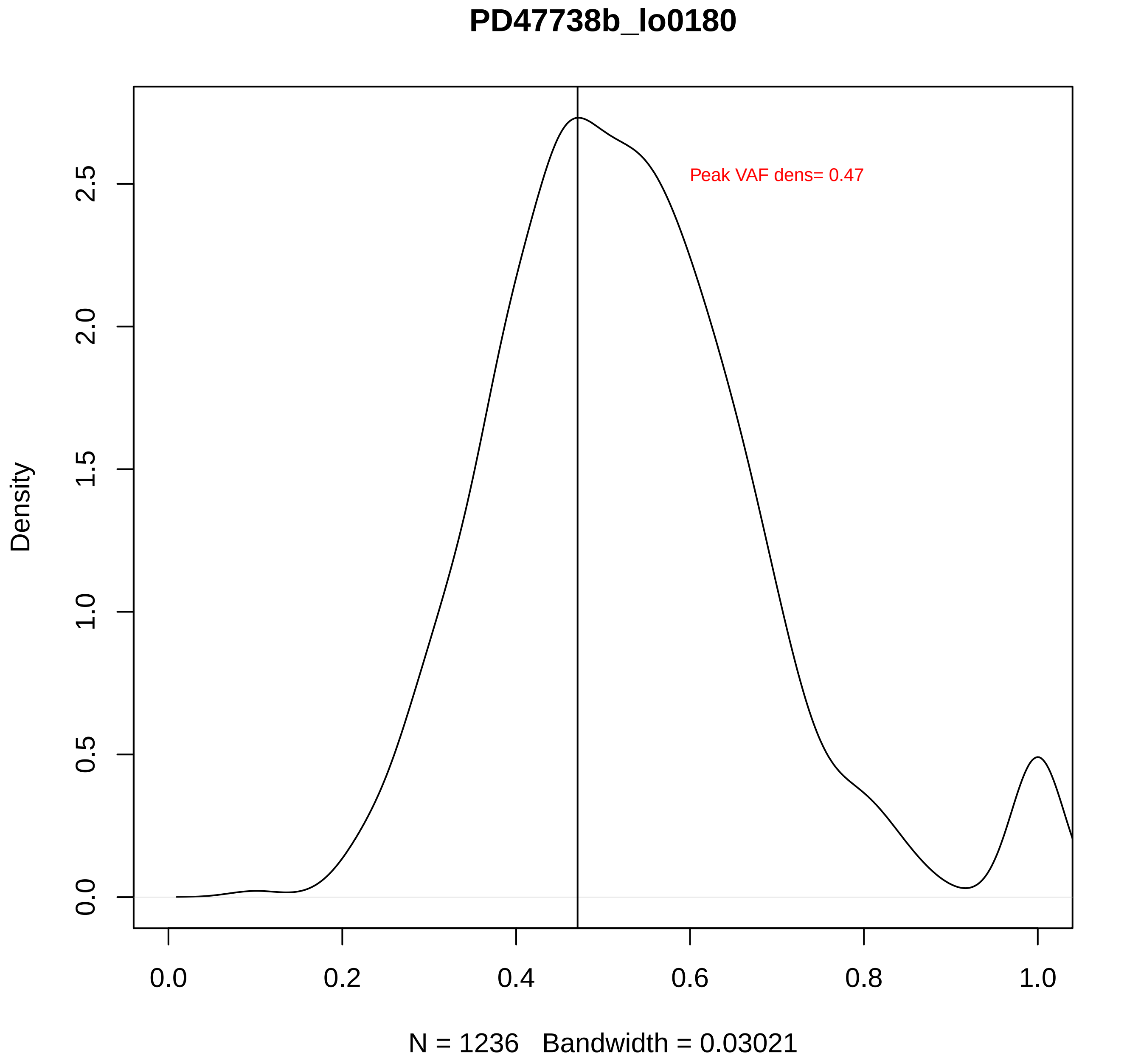
PD47738b_lo0180
Peak VAF dens= 0.47
Density
0.0
0.5
1.0
1.5
2.0
2.5
0.0
0.2
0.4
0.6
0.8
1.0
N = 1236 Bandwidth = 0.03021

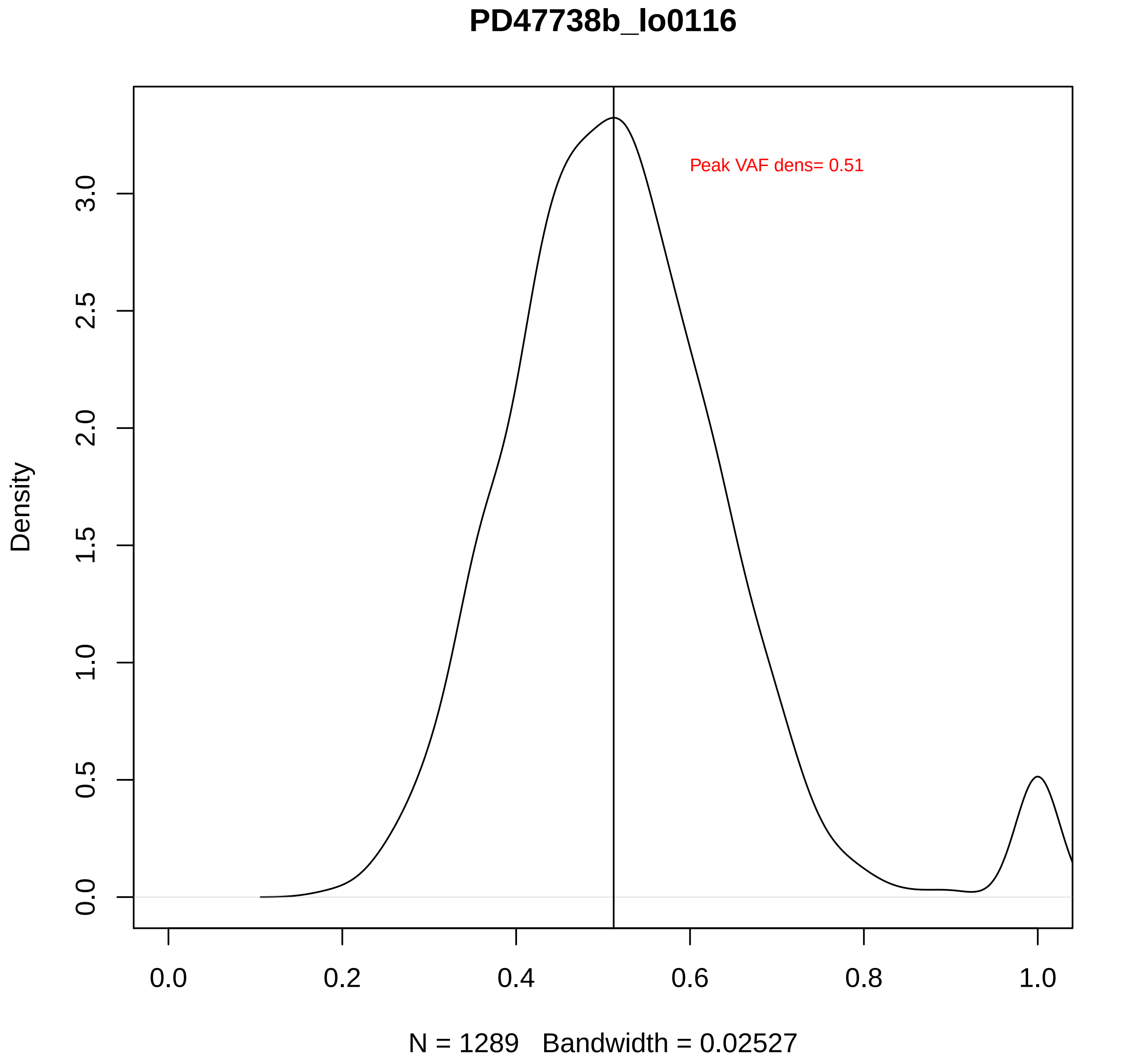
PD47738b_lo0116
Peak VAF dens= 0.51
Density
0.0
0.5
1.0
1.5
2.0
2.5
3.0
0.0
0.2
0.4
0.6
0.8
1.0
N = 1289   Bandwidth = 0.02527

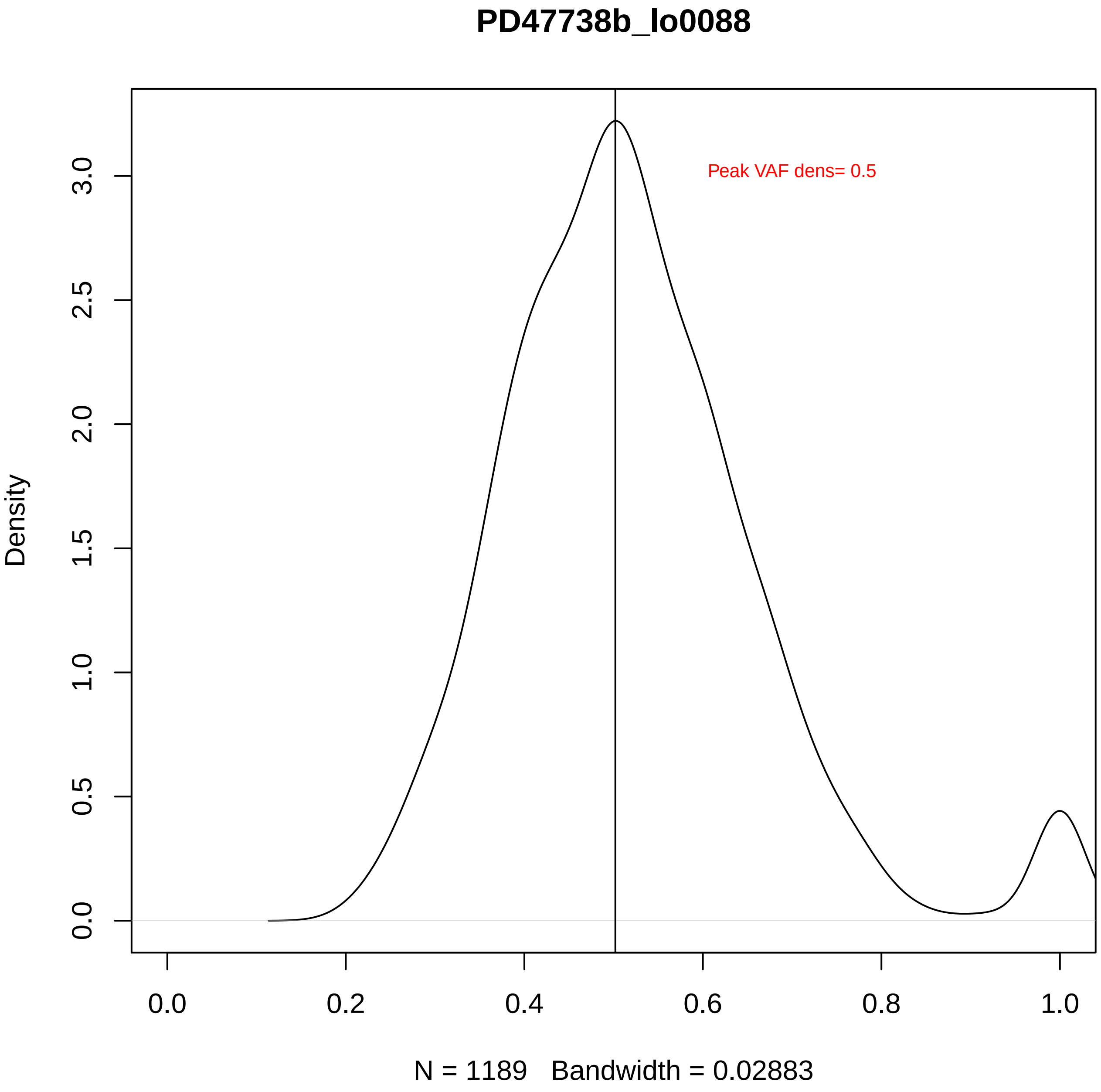
PD47738b_lo0088
Peak VAF dens= 0.5
Density
0.0
0.5
1.0
1.5
2.0
2.5
3.0
0.0
0.2
0.4
0.6
0.8
1.0
N = 1189   Bandwidth = 0.02883

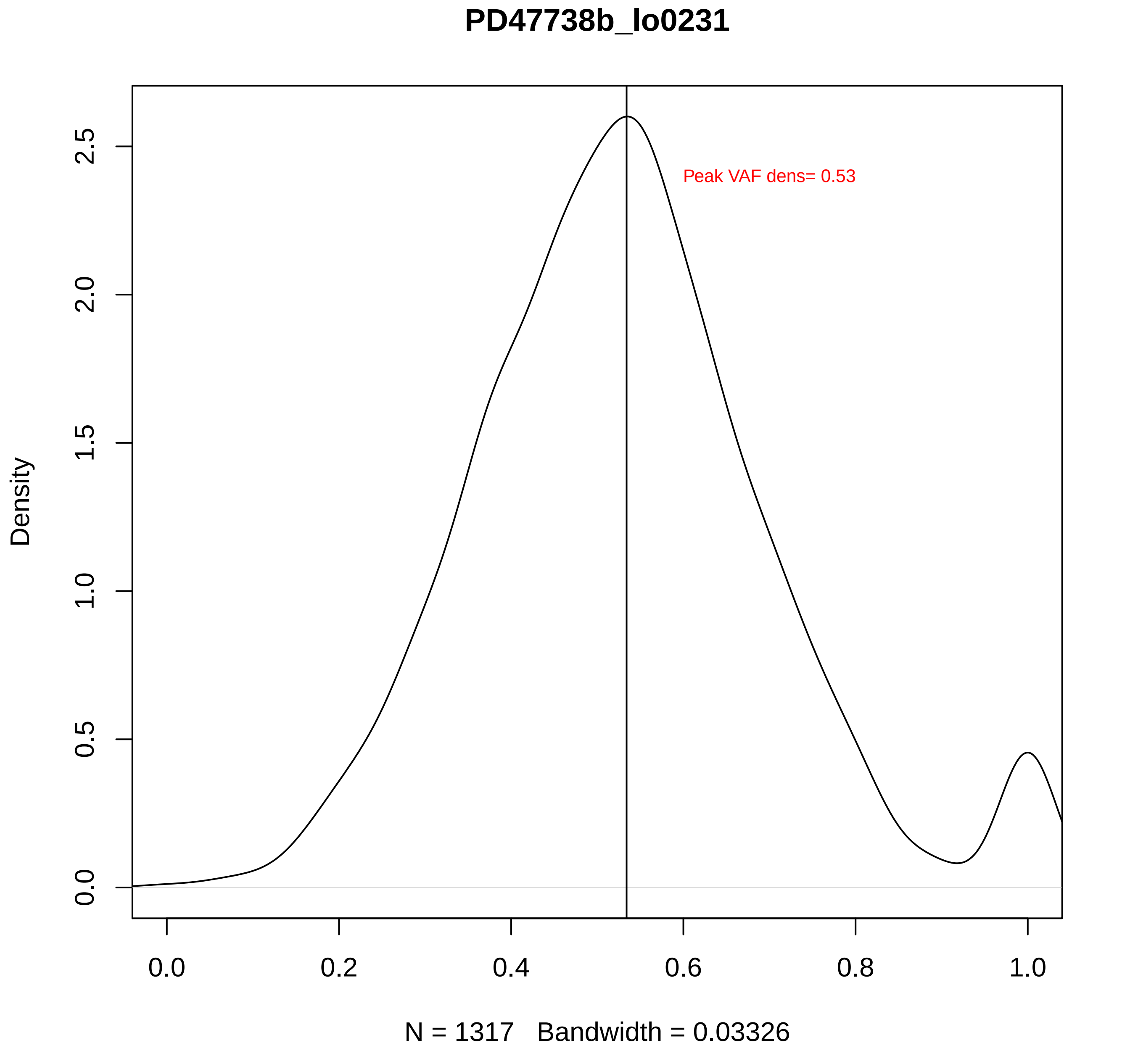

PD47738b_lo0231
Peak VAF dens= 0.53
Density
0.0
0.5
1.0
1.5
2.0
2.5
0.0
0.2
0.4
0.6
0.8
1.0
N = 1317   Bandwidth = 0.03326

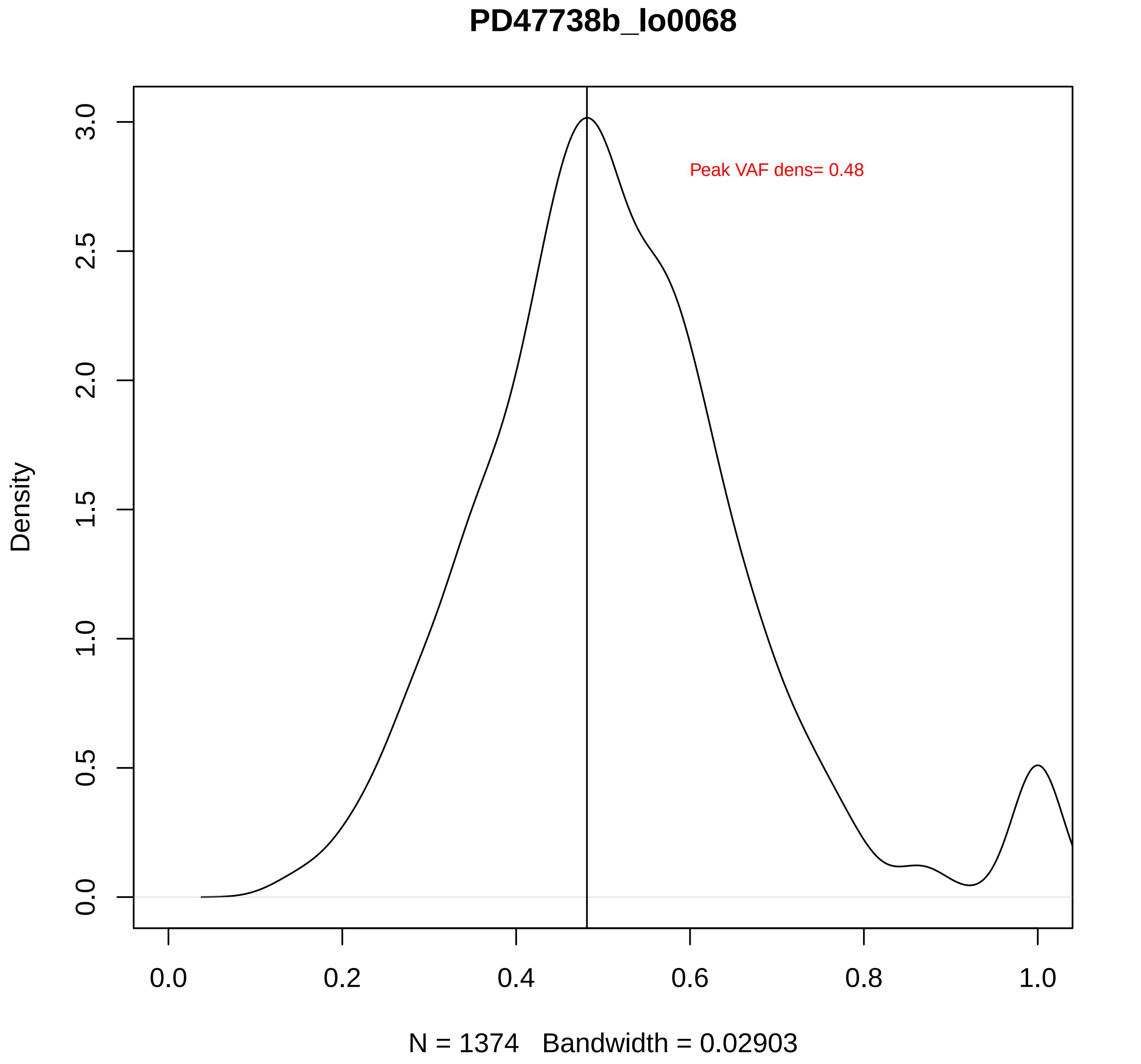
PD47738b_lo0068
Peak VAF dens= 0.48
Density
3.0
2.5
2.0
1.5
1.0
0.5
0.0
0.2
0.4
0.6
0.8
1.0
N = 1374   Bandwidth = 0.02903

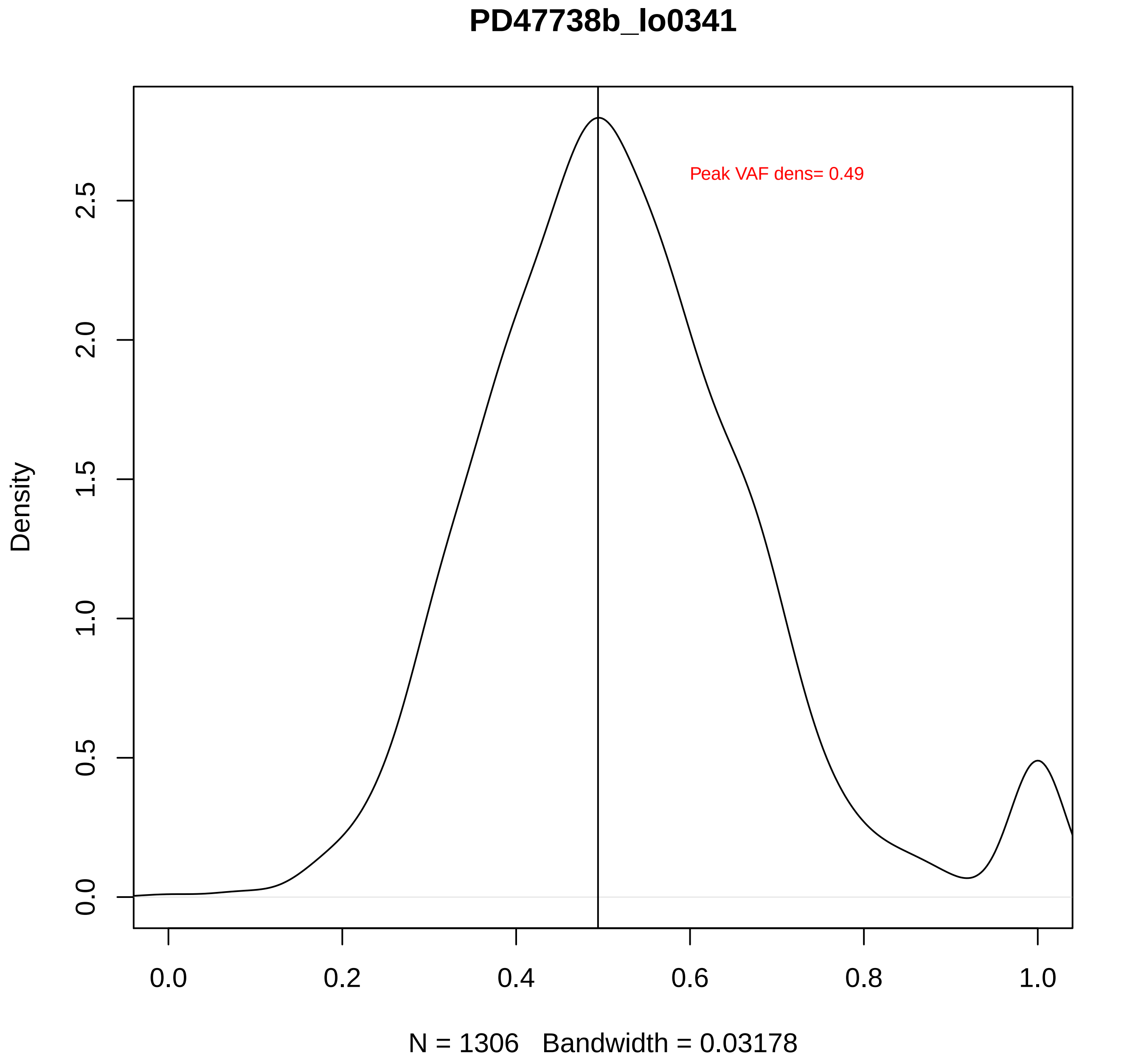
PD47738b_lo0341
Peak VAF dens= 0.49
Density
0.0
0.5
1.0
1.5
2.0
2.5
0.0
0.2
0.4
0.6
0.8
1.0
N = 1306   Bandwidth = 0.03178

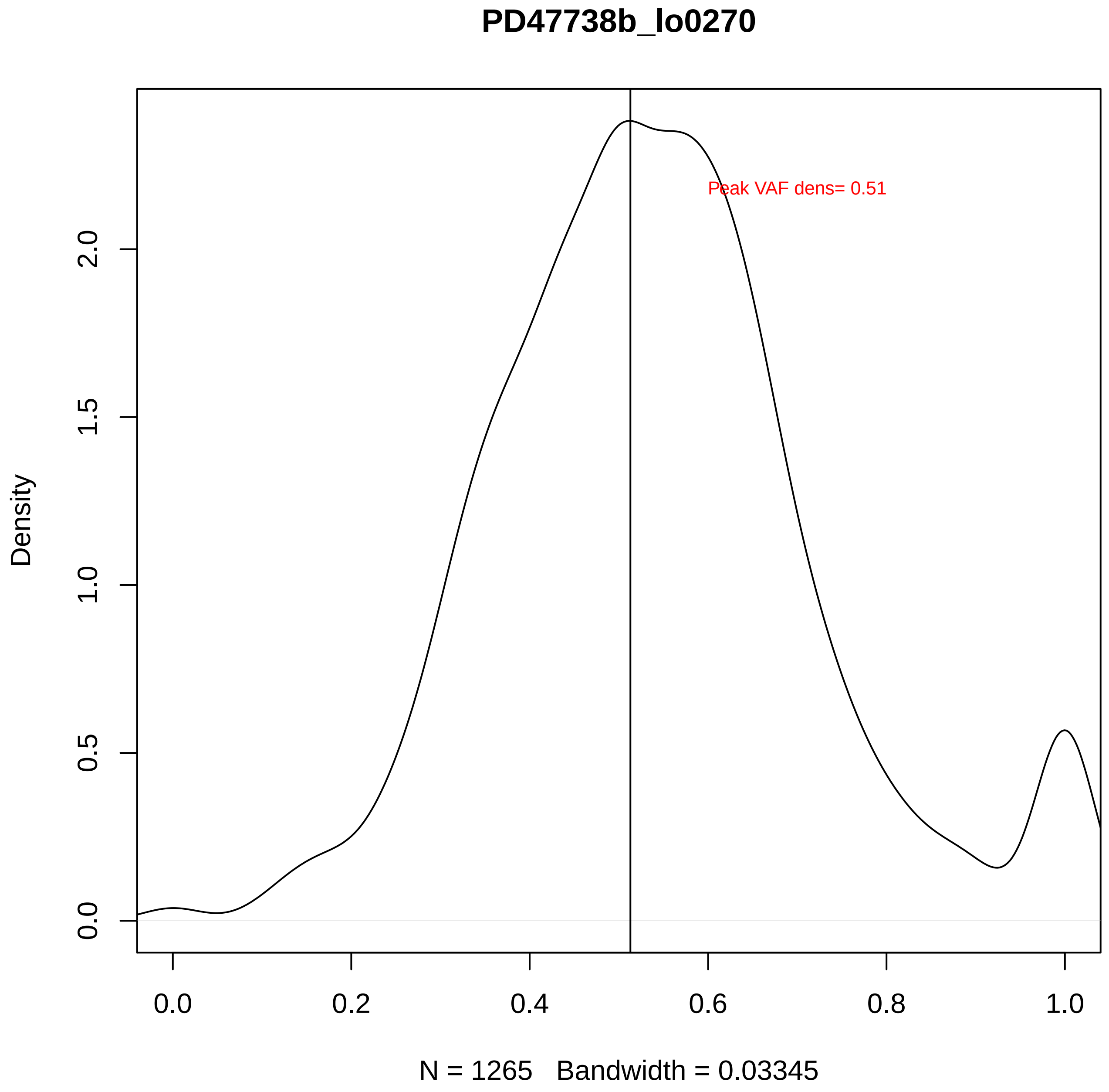
PD47738b_lo0270
Peak VAF dens= 0.51
Density
0.0
0.5
1.0
1.5
2.0
0.0
0.2
0.4
0.6
0.8
1.0
N = 1265   Bandwidth = 0.03345

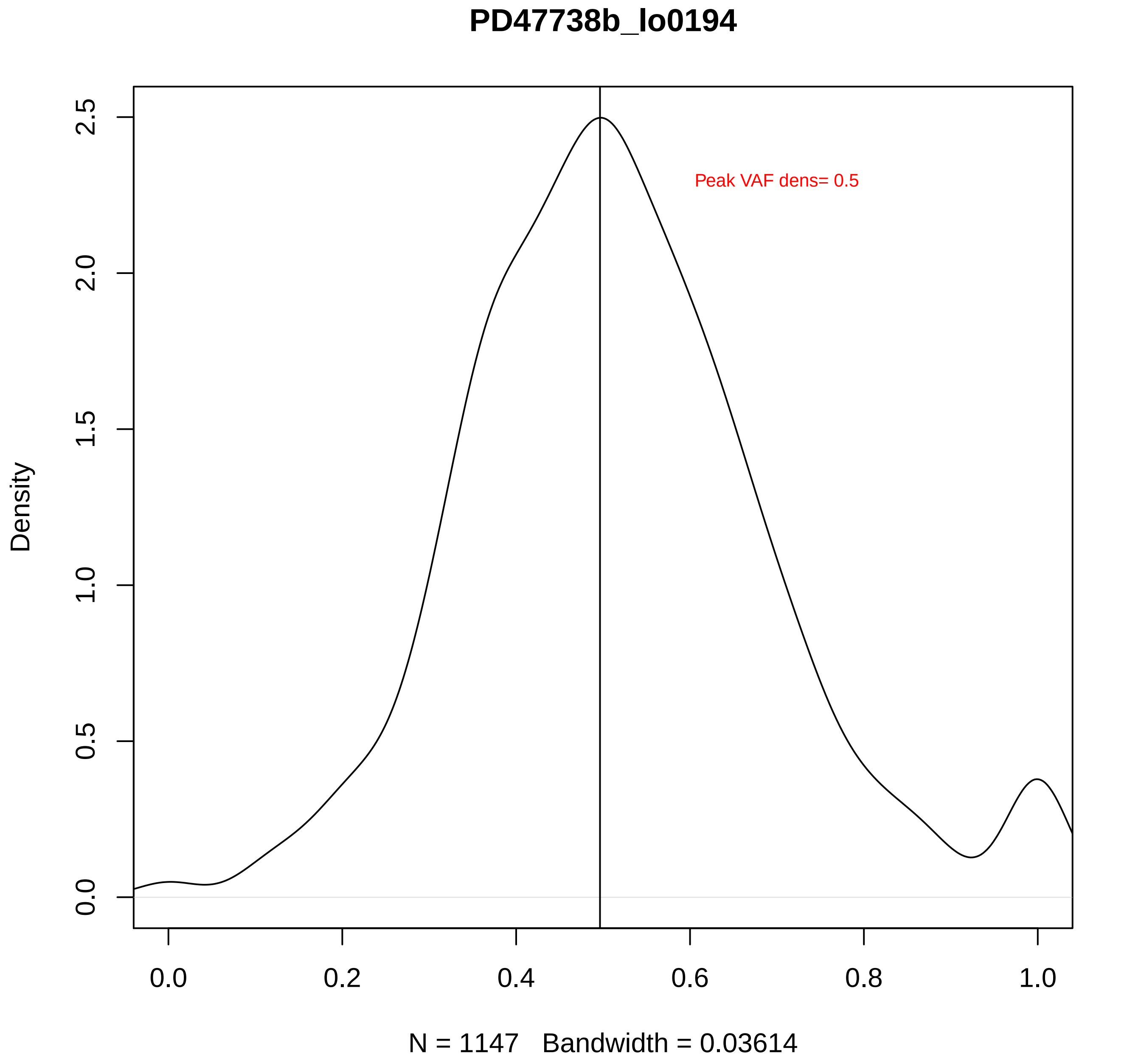
PD47738b_lo0194
Peak VAF dens= 0.5
Density
0.0
0.5
1.0
1.5
2.0
2.5
0.0
0.2
0.4
0.6
0.8
1.0
N = 1147   Bandwidth = 0.03614

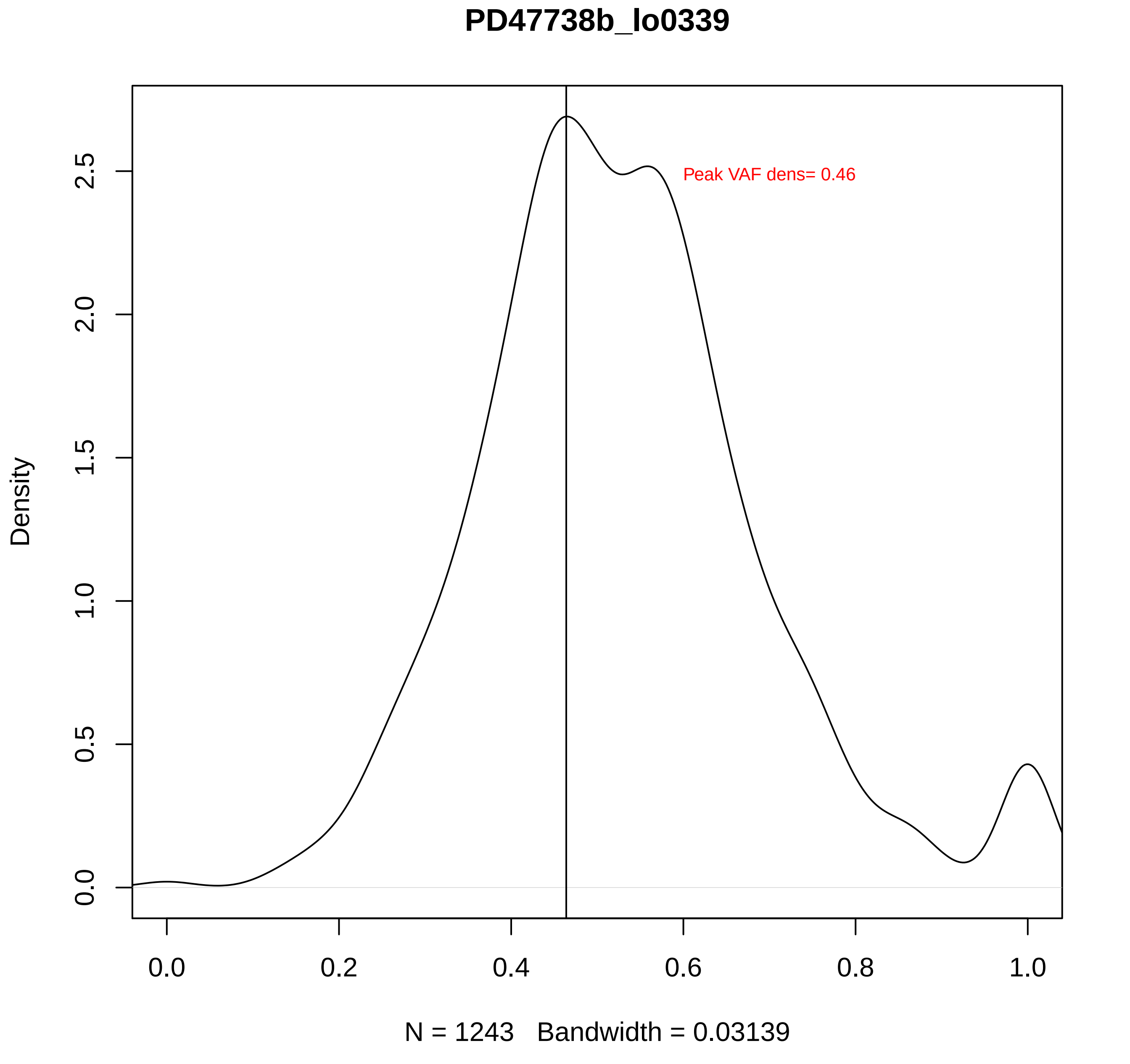

PD47738b_lo0339
Peak VAF dens= 0.46
Density
0.0
0.5
1.0
1.5
2.0
2.5
0.0
0.2
0.4
0.6
0.8
1.0
N = 1243   Bandwidth = 0.03139

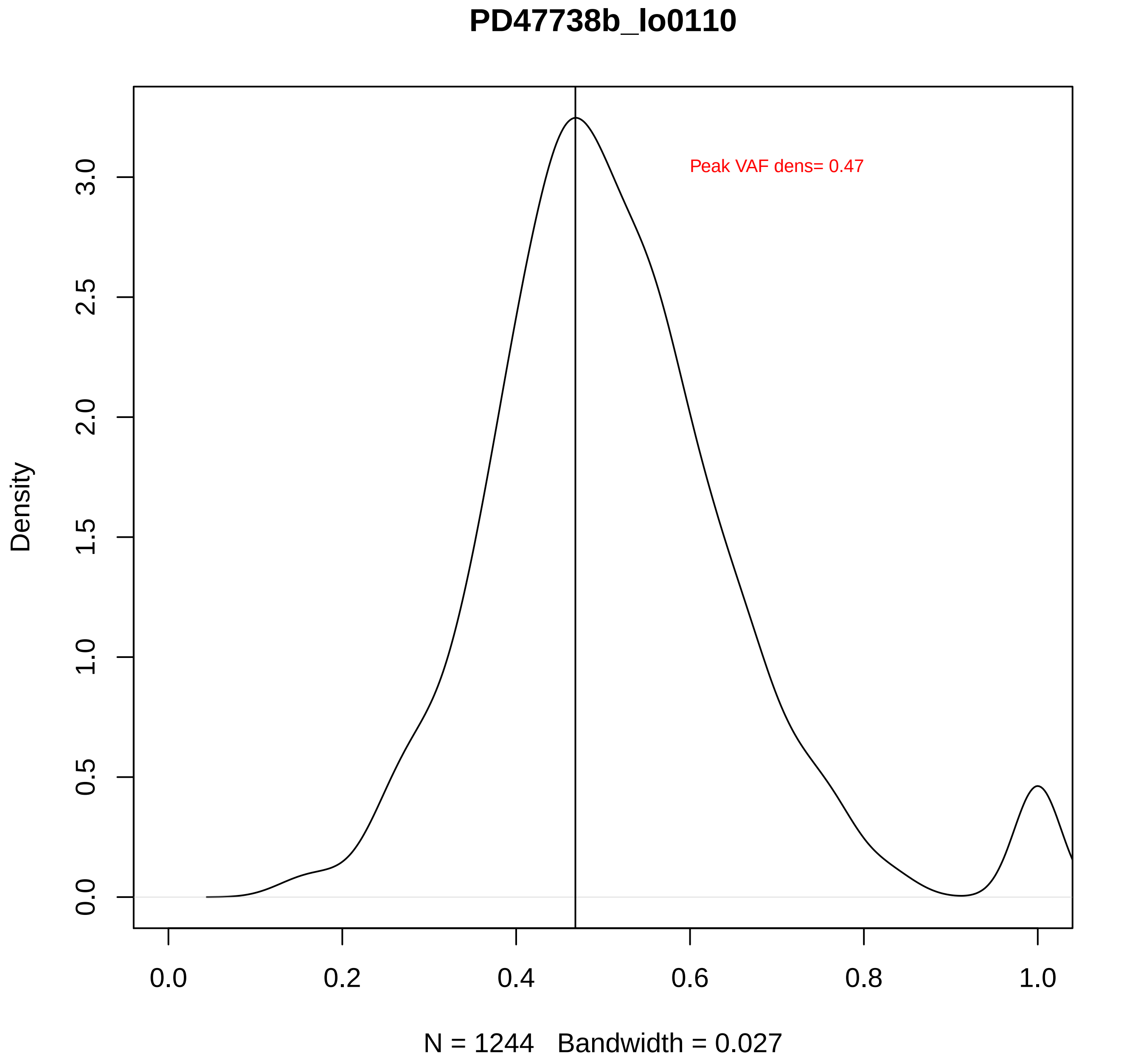
PD47738b_lo0110
Peak VAF dens= 0.47
Density
0.0
0.5
1.0
1.5
2.0
2.5
3.0
0.0
0.2
0.4
0.6
0.8
1.0
N = 1244   Bandwidth = 0.027

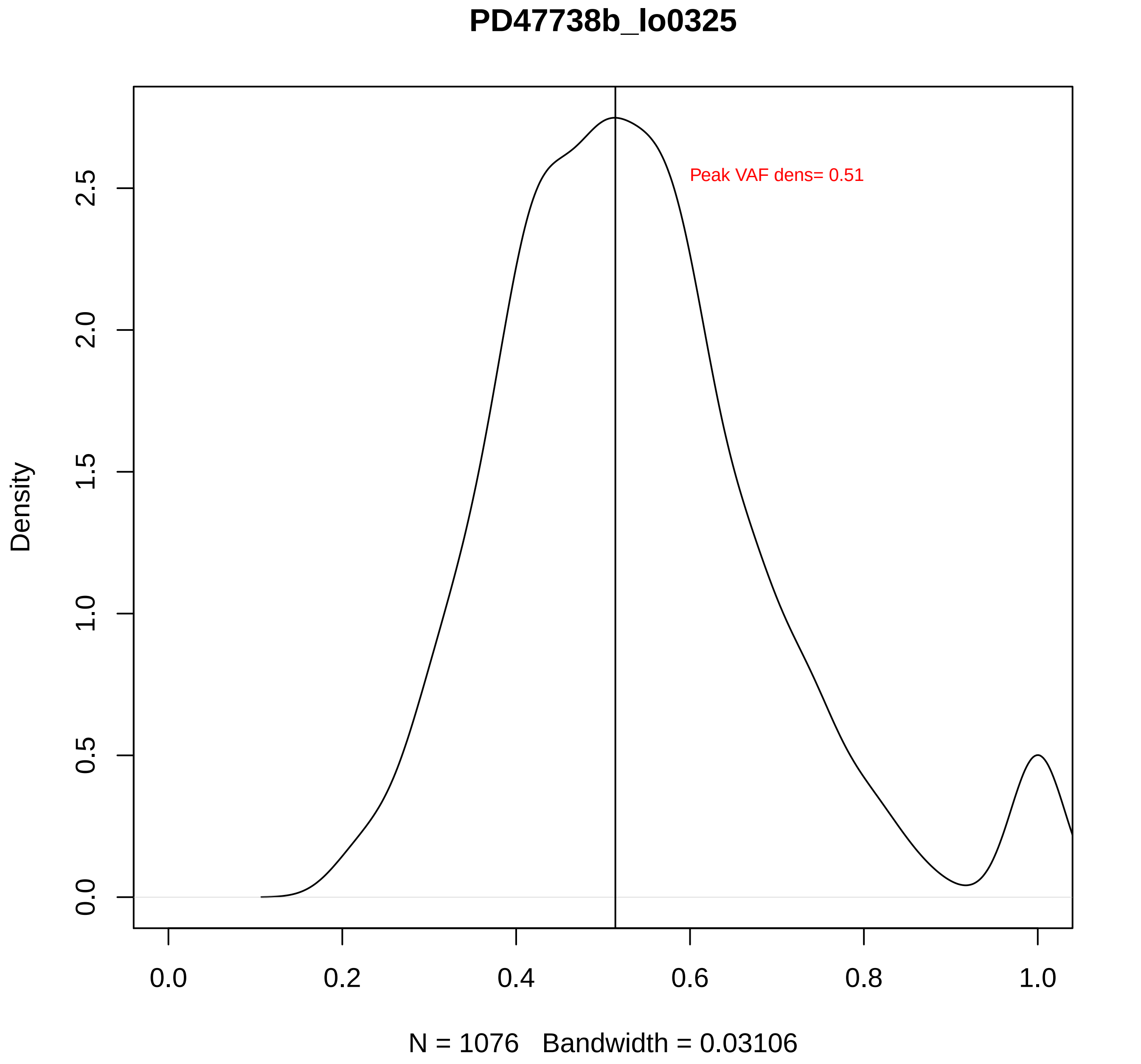

PD47738b_lo0325
Peak VAF dens= 0.51
Density
0.0
0.5
1.0
1.5
2.0
2.5
0.0
0.2
0.4
0.6
0.8
1.0
N = 1076   Bandwidth = 0.03106

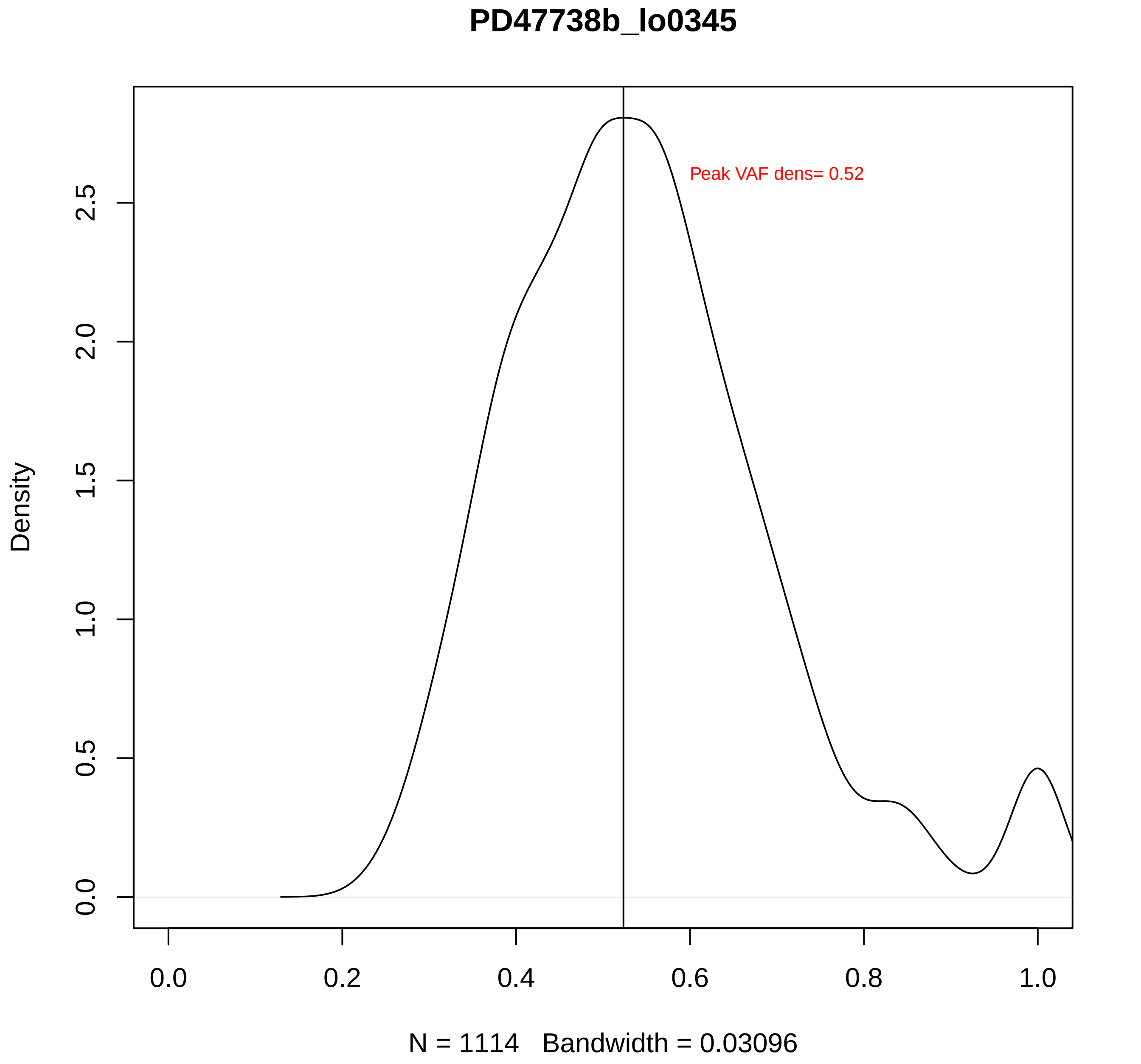
PD47738b_lo0345
Peak VAF dens= 0.52
Density
0.0
0.5
1.0
1.5
2.0
2.5
0.0
0.2
0.4
0.6
0.8
1.0
N = 1114   Bandwidth = 0.03096

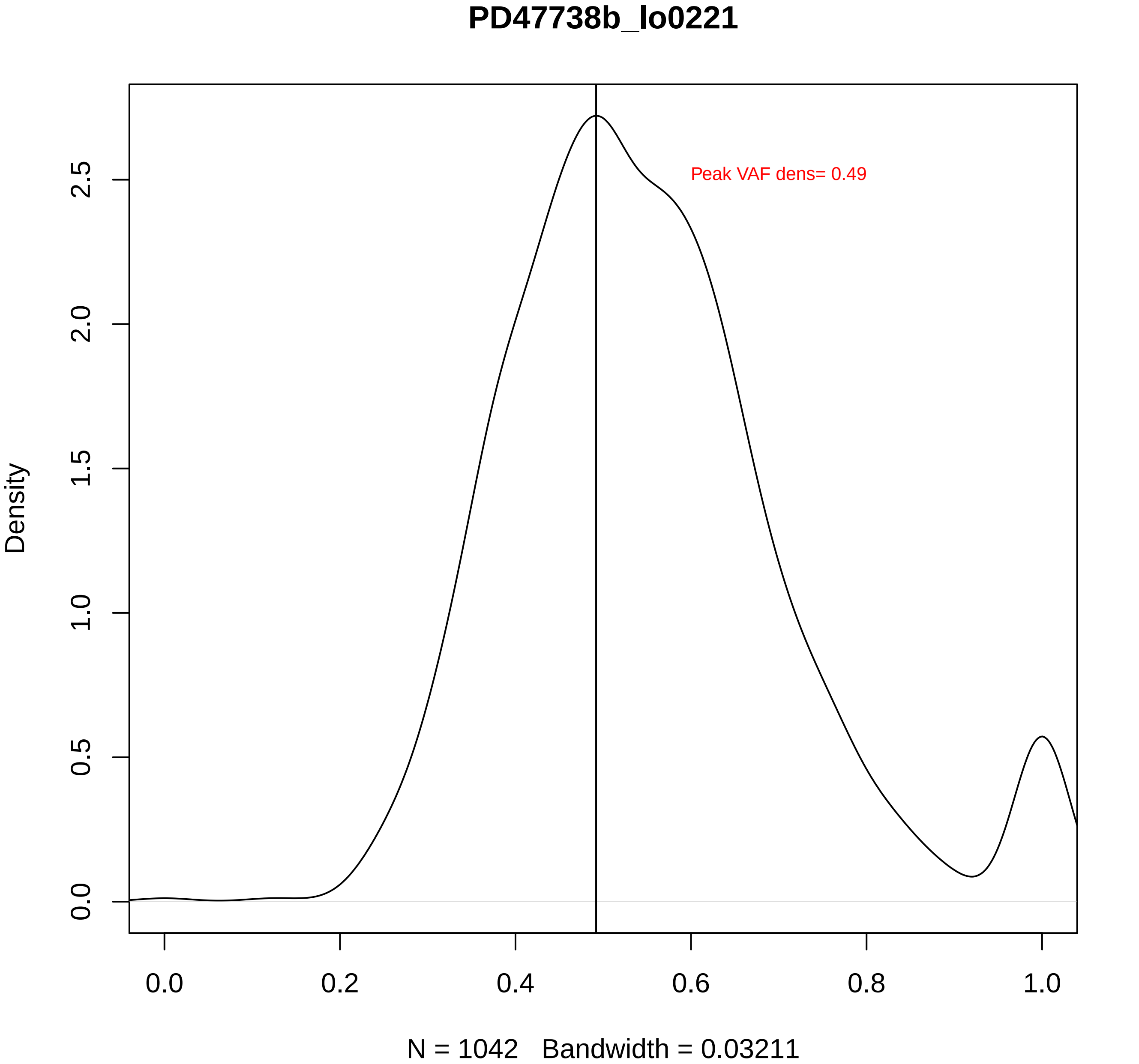
PD47738b_lo0221
Peak VAF dens= 0.49
Density
0.0
0.5
1.0
1.5
2.0
2.5
0.0
0.2
0.4
0.6
0.8
1.0
N = 1042   Bandwidth = 0.03211

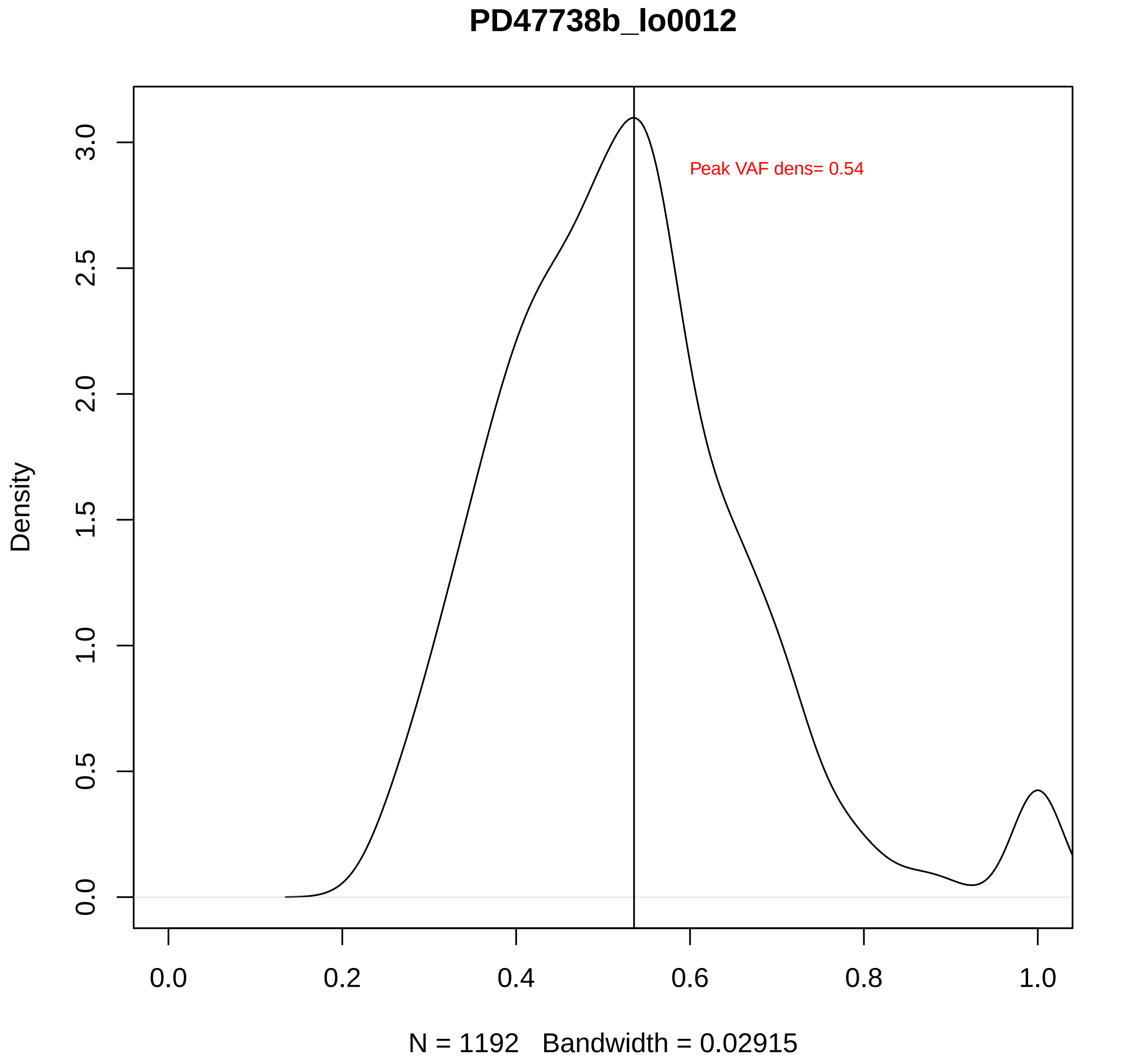

PD47738b_lo0012
Peak VAF dens= 0.54
Density
0.0
0.5
1.0
1.5
2.0
2.5
3.0
0.0
0.2
0.4
0.6
0.8
1.0
N = 1192 Bandwidth = 0.02915

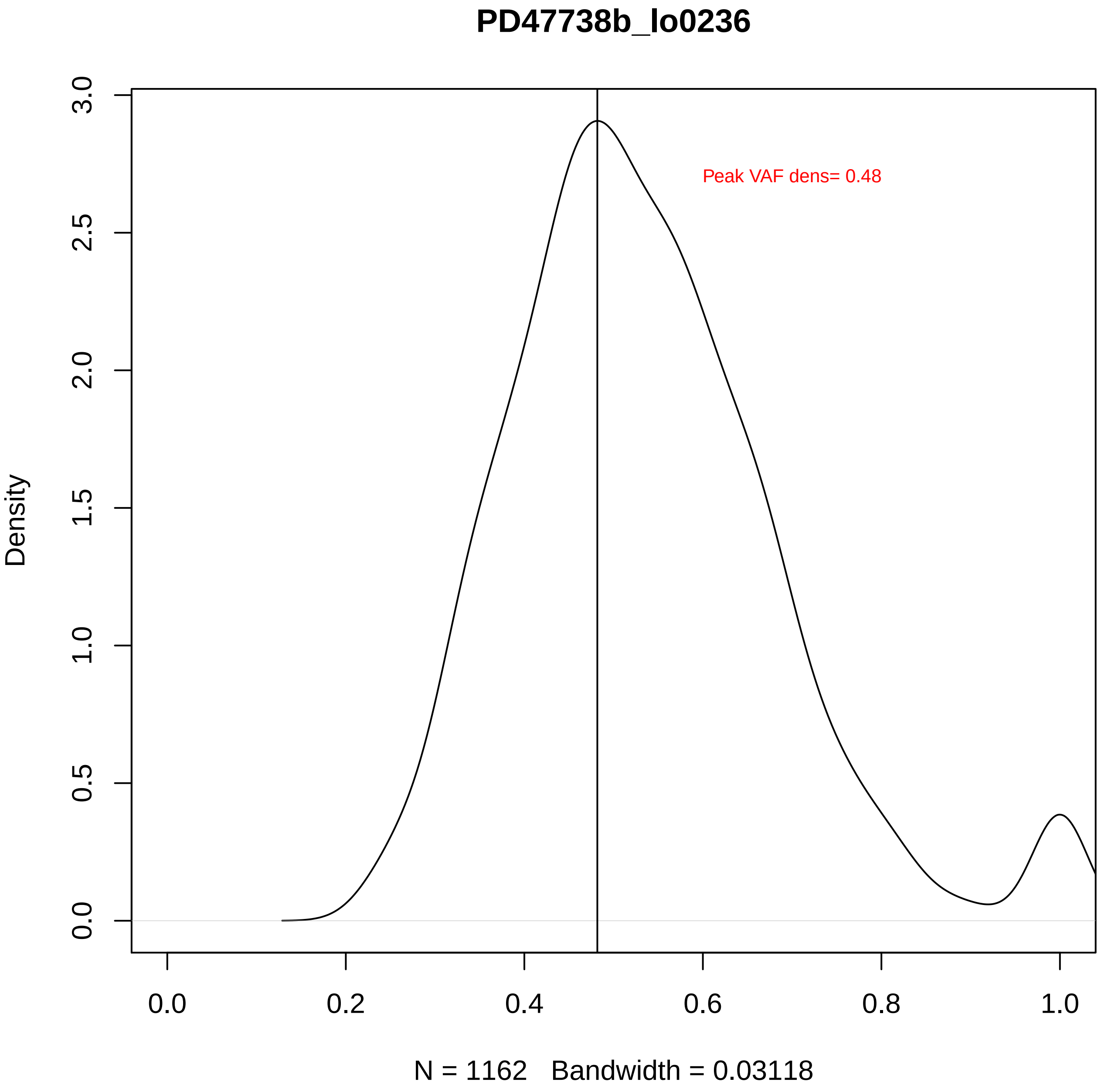
PD47738b_lo0236
Peak VAF dens= 0.48
Density
0.0
0.5
1.0
1.5
2.0
2.5
3.0
0.0
0.2
0.4
0.6
0.8
1.0
N = 1162   Bandwidth = 0.03118

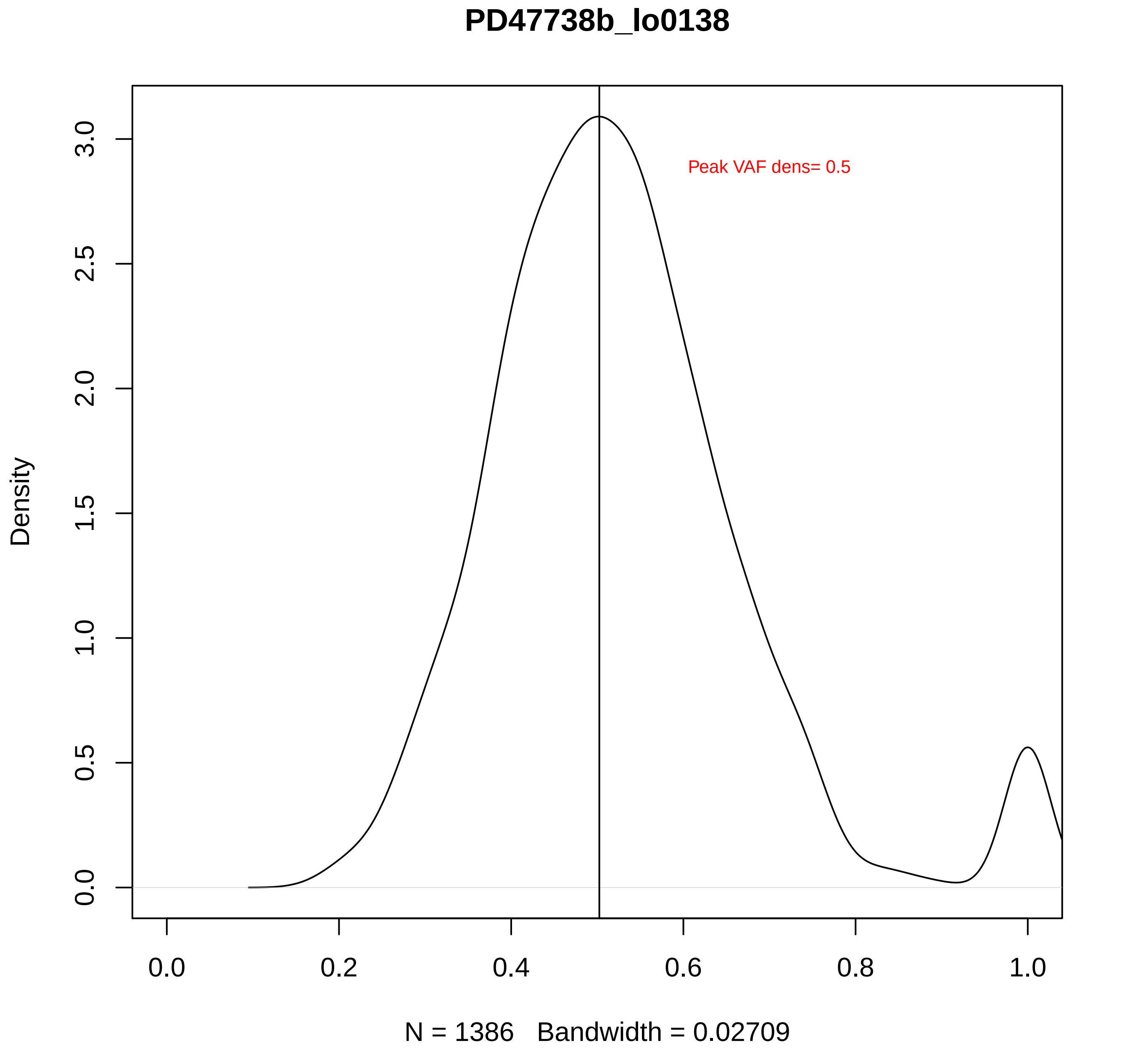
PD47738b_lo0138
Peak VAF dens= 0.5
Density
0.0
0.5
1.0
1.5
2.0
2.5
3.0
0.0
0.2
0.4
0.6
0.8
1.0
N = 1386   Bandwidth = 0.02709

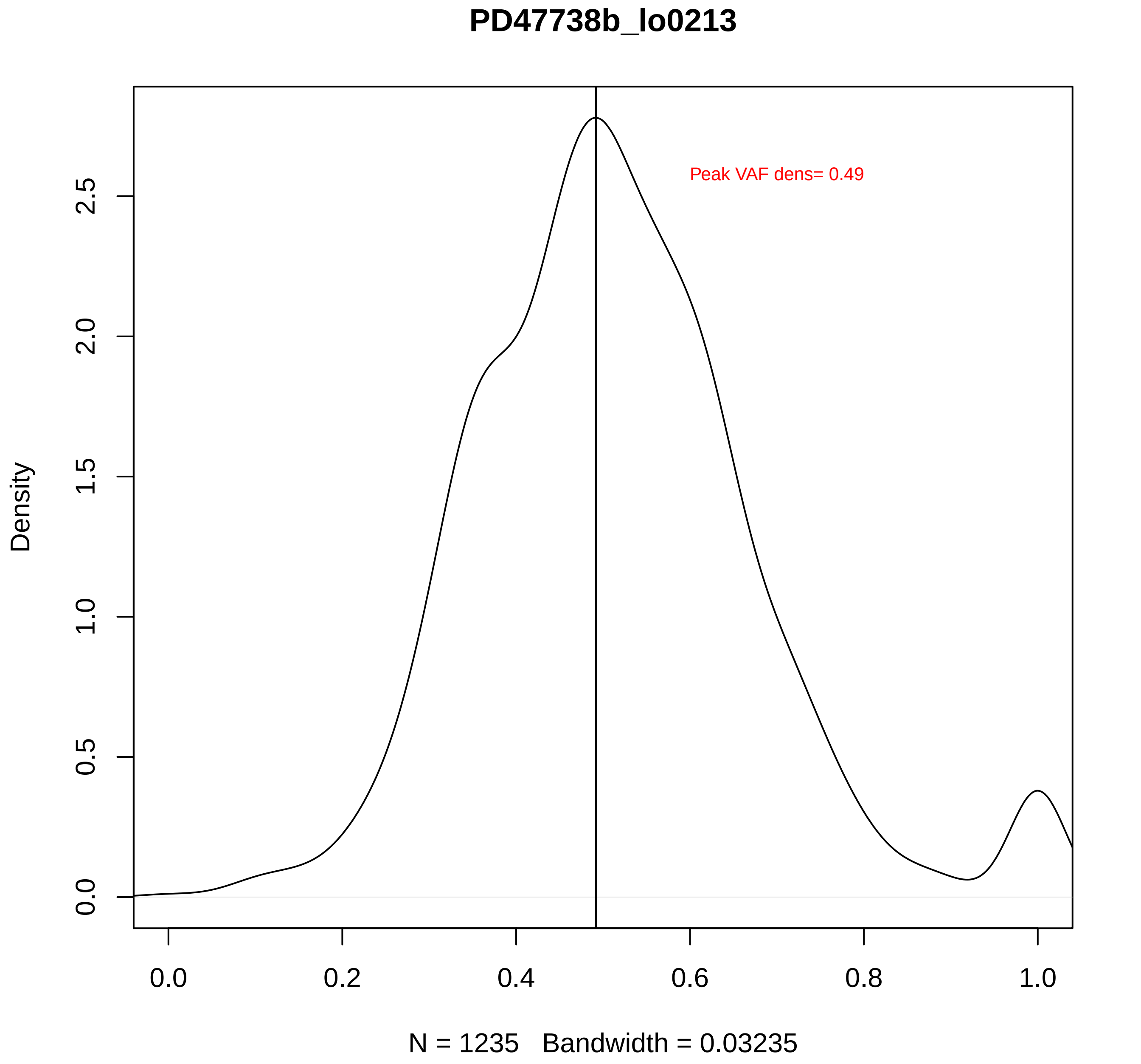
PD47738b_lo0213
Peak VAF dens= 0.49
Density
0.0
0.5
1.0
1.5
2.0
2.5
0.0
0.2
0.4
0.6
0.8
1.0
N = 1235   Bandwidth = 0.03235

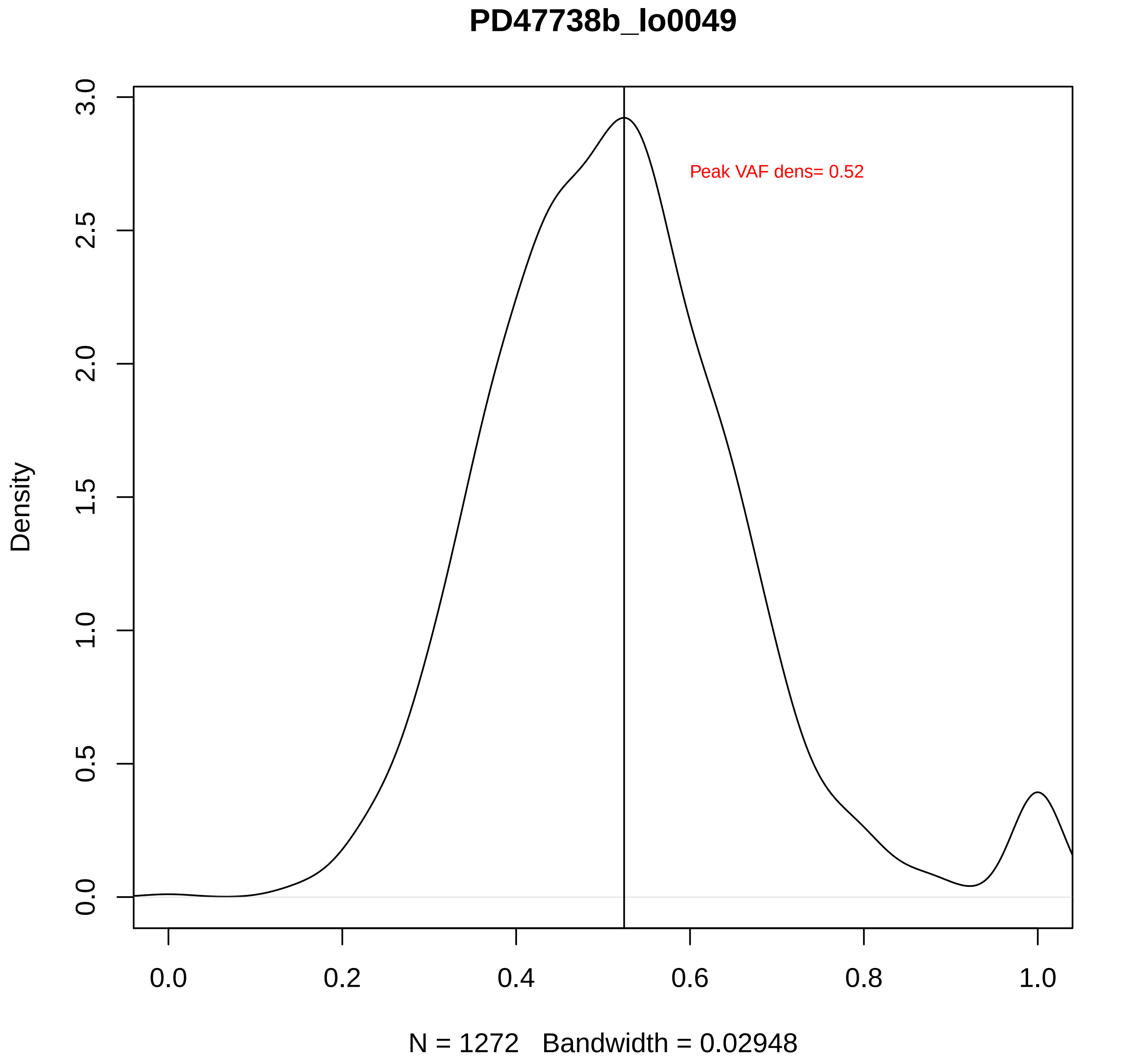

PD47738b_lo0049
Peak VAF dens= 0.52
Density
3.0
2.5
2.0
1.5
1.0
0.5
0.0
0.0
0.2
0.4
0.6
0.8
1.0
N = 1272   Bandwidth = 0.02948

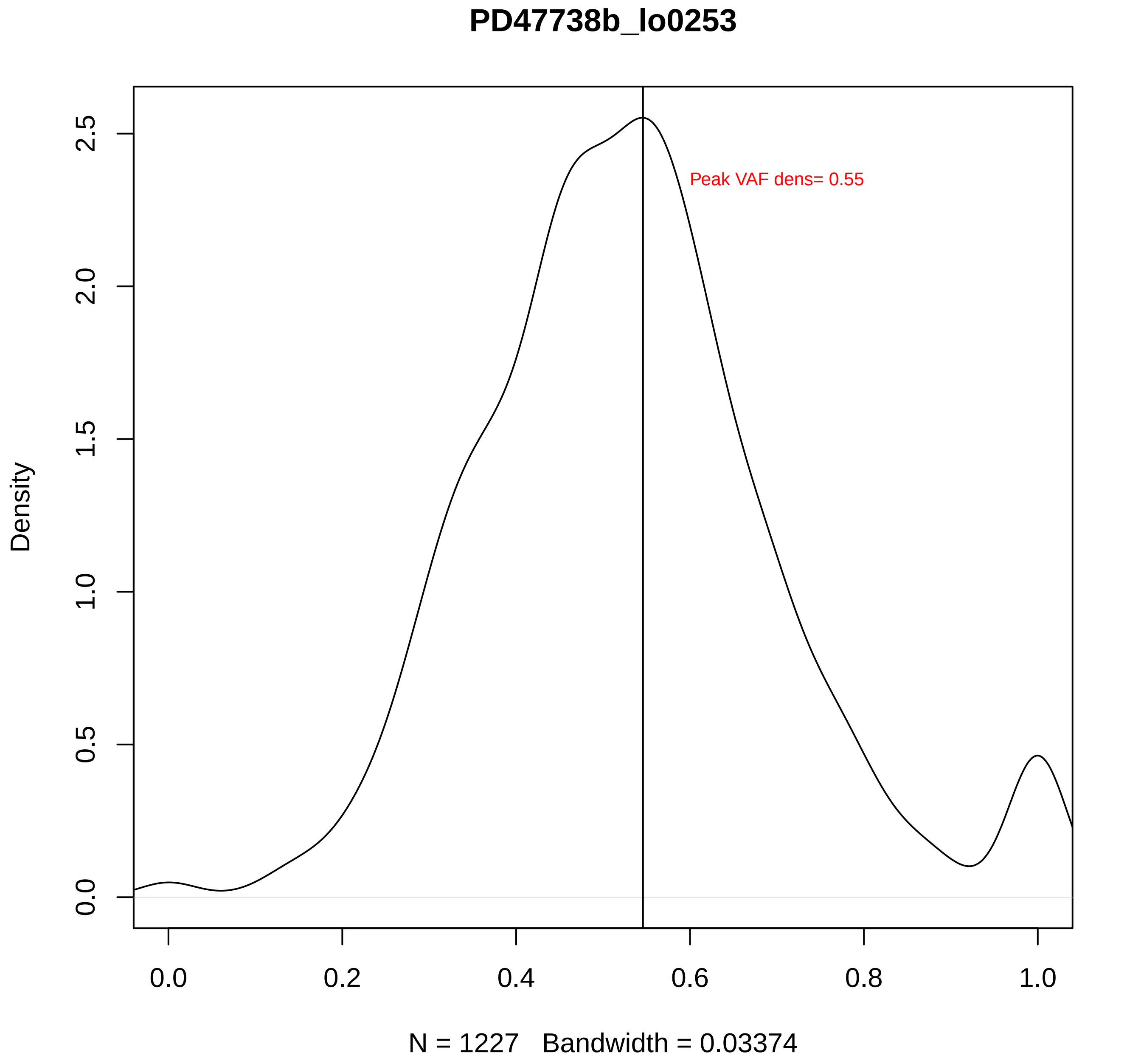
PD47738b_lo0253
Peak VAF dens= 0.55
Density
0.0
0.5
1.0
1.5
2.0
2.5
0.0
0.2
0.4
0.6
0.8
1.0
N = 1227   Bandwidth = 0.03374

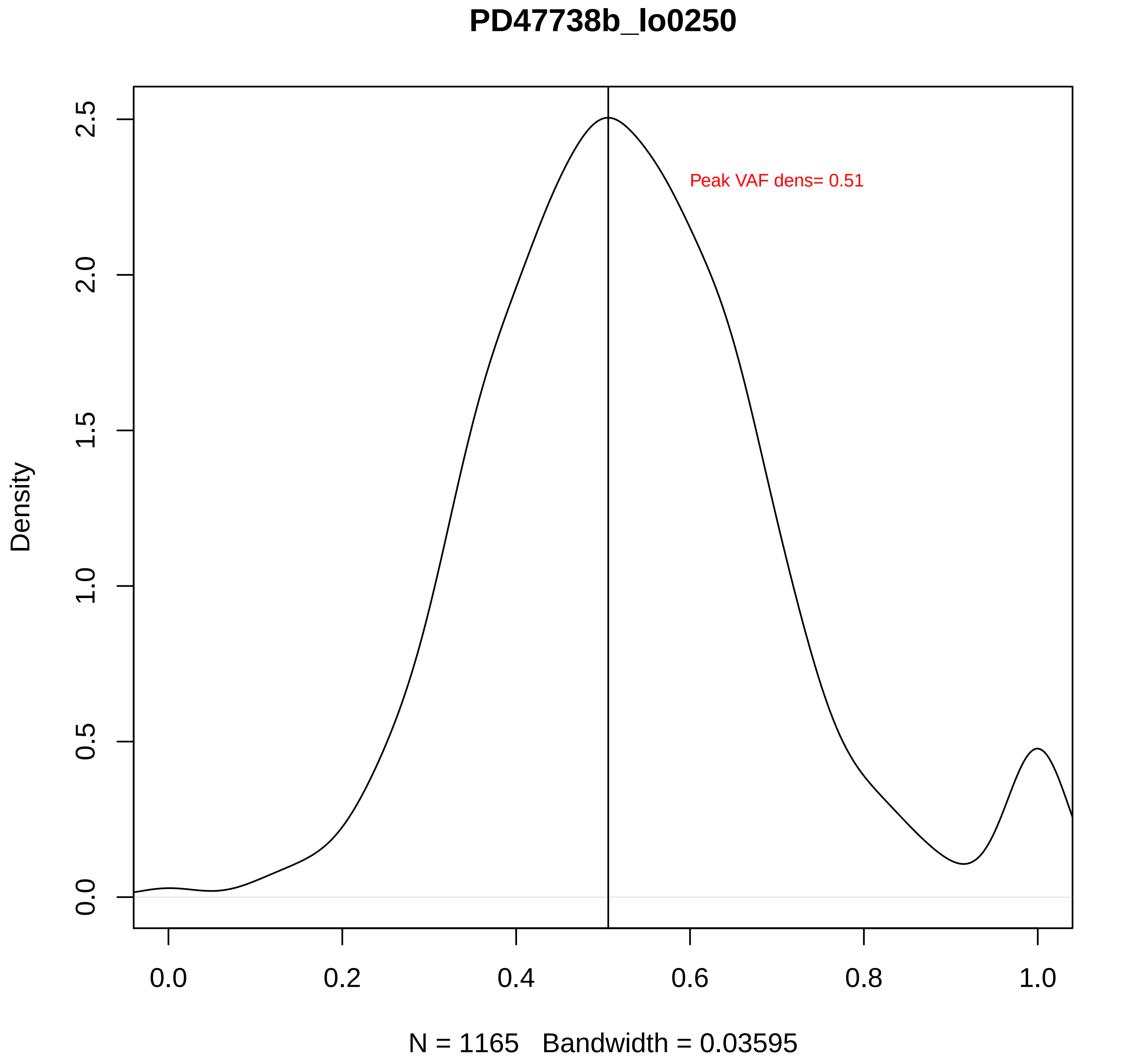

PD47738b_lo0250
Peak VAF dens= 0.51
Density
0.0
0.5
1.0
1.5
2.0
2.5
0.0
0.2
0.4
0.6
0.8
1.0
N = 1165   Bandwidth = 0.03595

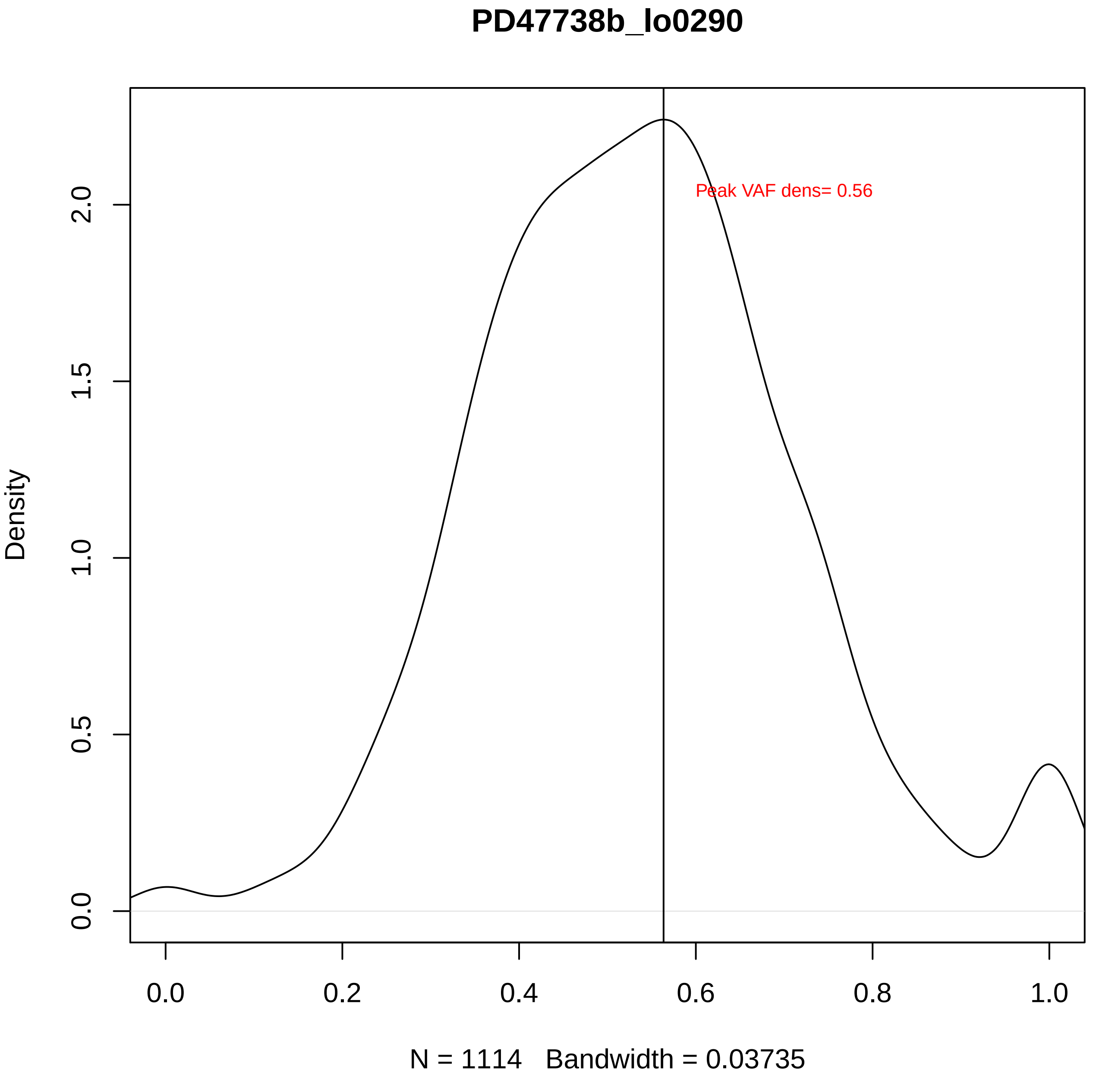

PD47738b_lo0290
Peak VAF dens= 0.56
Density
0.0
0.5
1.0
1.5
2.0
0.0
0.2
0.4
0.6
0.8
1.0
N = 1114   Bandwidth = 0.03735

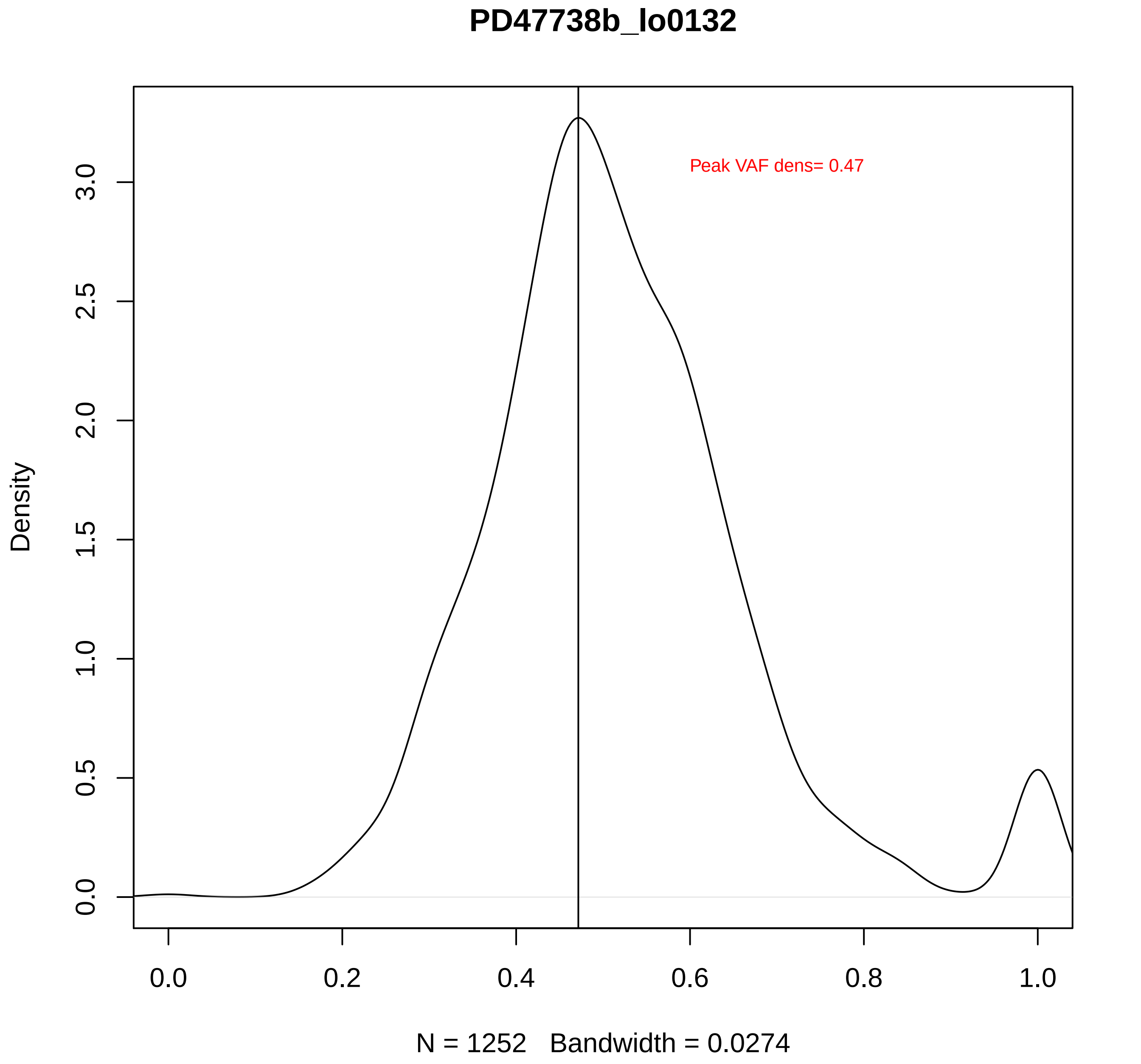

PD47738b_lo0132
Peak VAF dens= 0.47
Density
0.0
0.5
1.0
1.5
2.0
2.5
3.0
0.0
0.2
0.4
0.6
0.8
1.0
N = 1252   Bandwidth = 0.0274

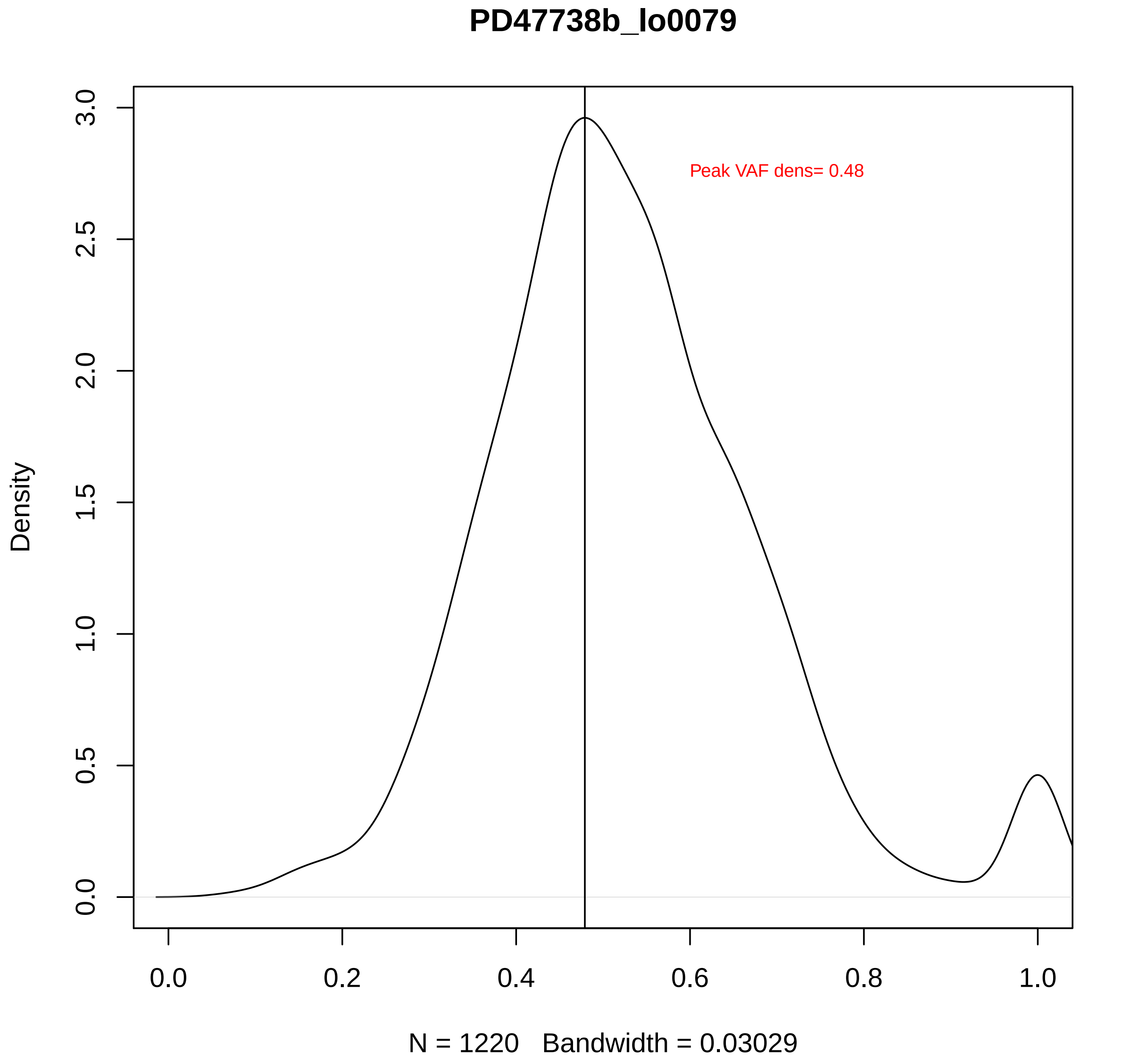
PD47738b_lo0079
Peak VAF dens= 0.48
Density
3.0
2.5
2.0
1.5
1.0
0.5
0.0
0.0
0.2
0.4
0.6
0.8
1.0
N = 1220   Bandwidth = 0.03029

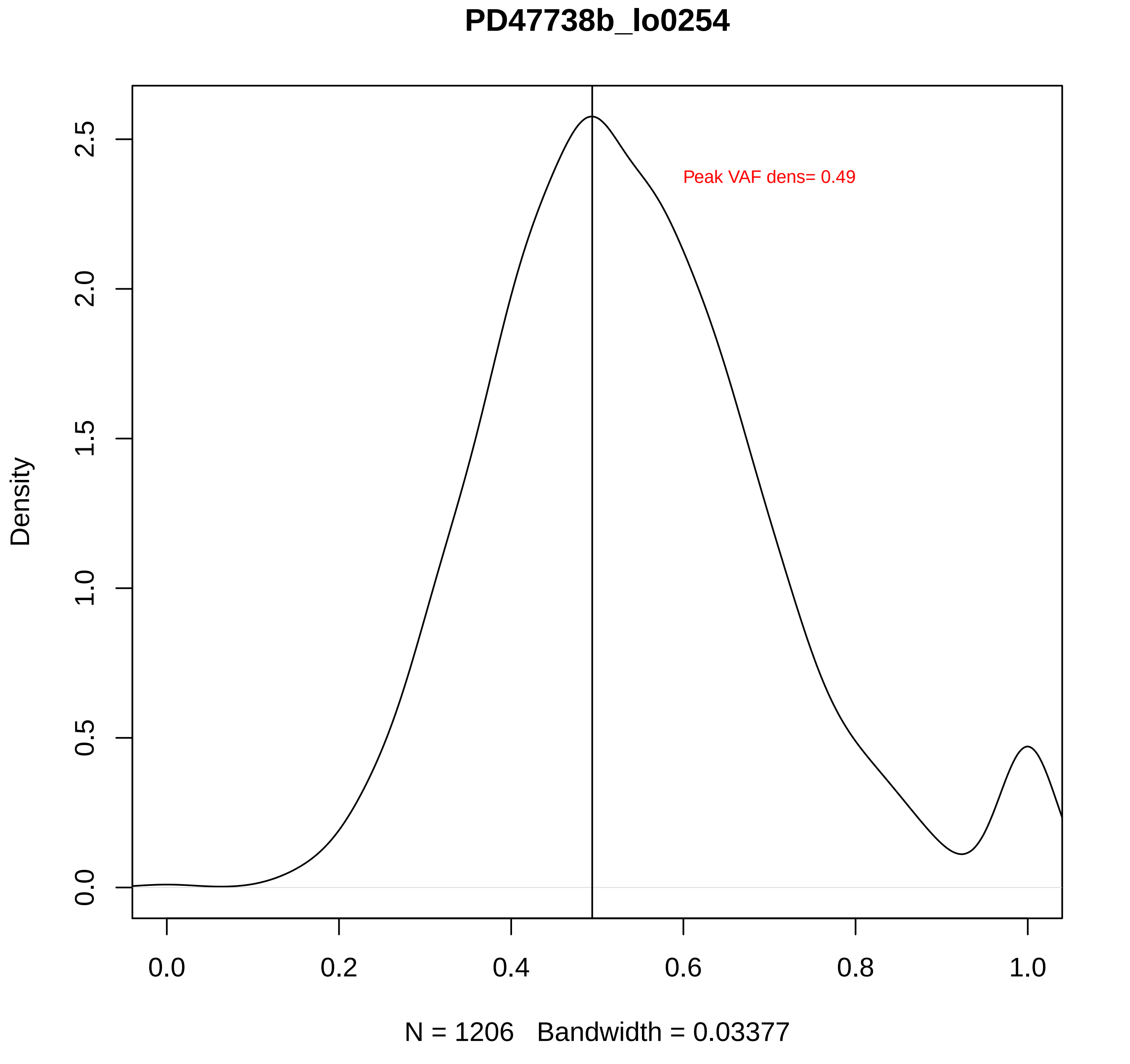
PD47738b_lo0254
Peak VAF dens= 0.49
Density
0.0
0.5
1.0
1.5
2.0
2.5
0.0
0.2
0.4
0.6
0.8
1.0
N = 1206 Bandwidth = 0.03377

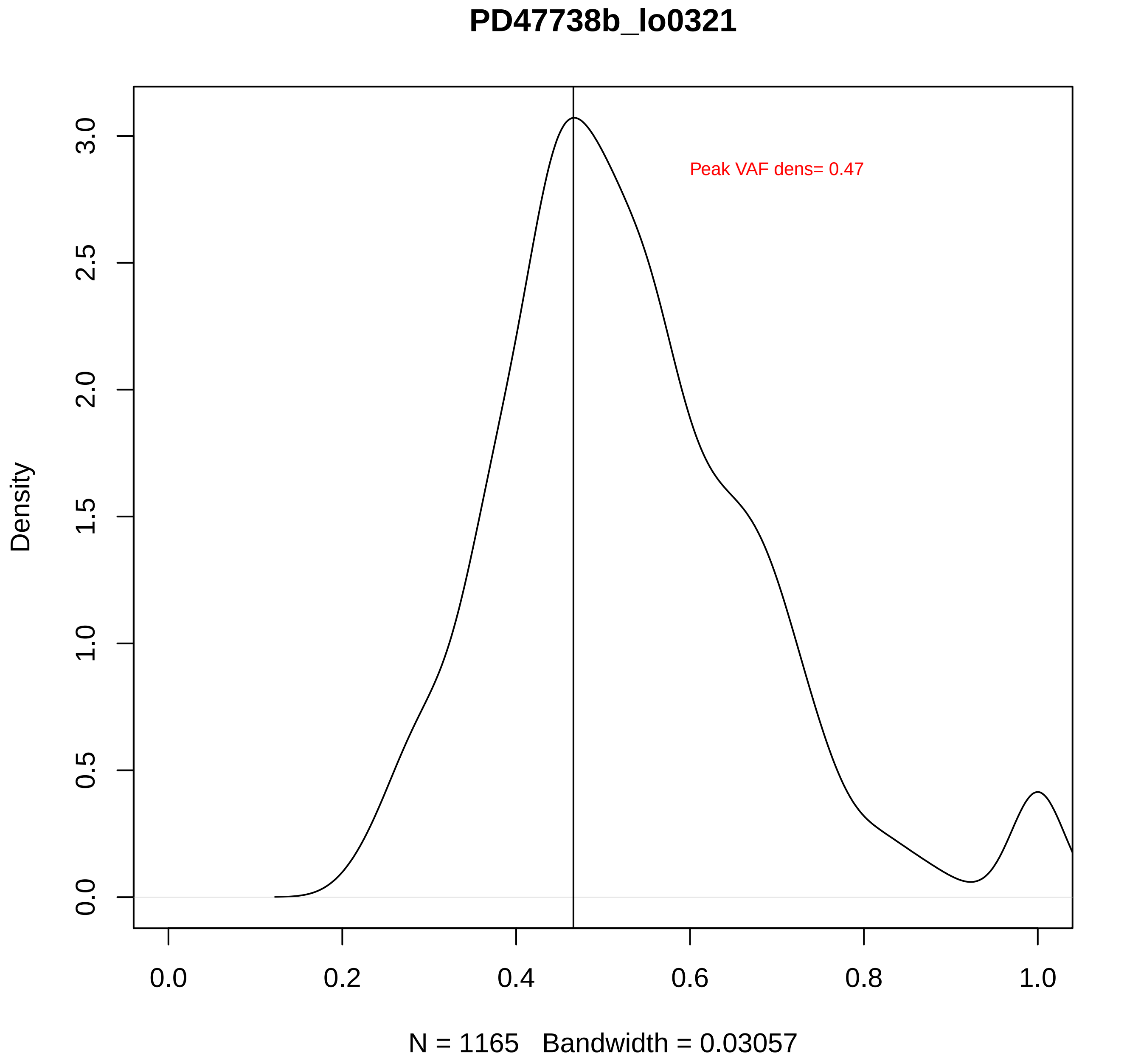
PD47738b_lo0321
Peak VAF dens= 0.47
Density
0.0
0.5
1.0
1.5
2.0
2.5
3.0
0.0
0.2
0.4
0.6
0.8
1.0
N = 1165   Bandwidth = 0.03057

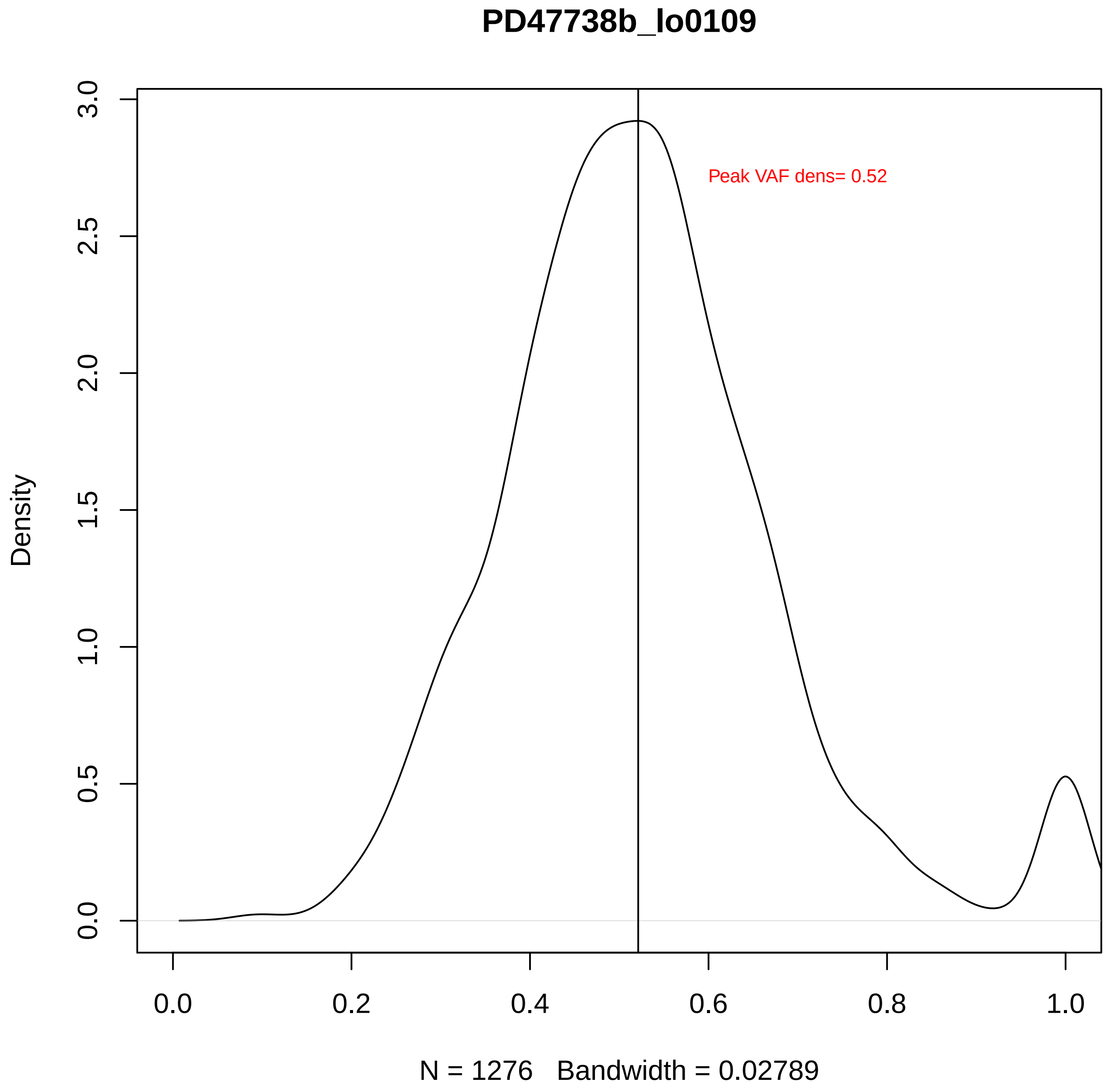

PD47738b_lo0109
Density
0.0
0.5
1.0
1.5
2.0
2.5
3.0
0.0
0.2
0.4
0.6
0.8
1.0
Peak VAF dens= 0.52
N = 1276   Bandwidth = 0.02789

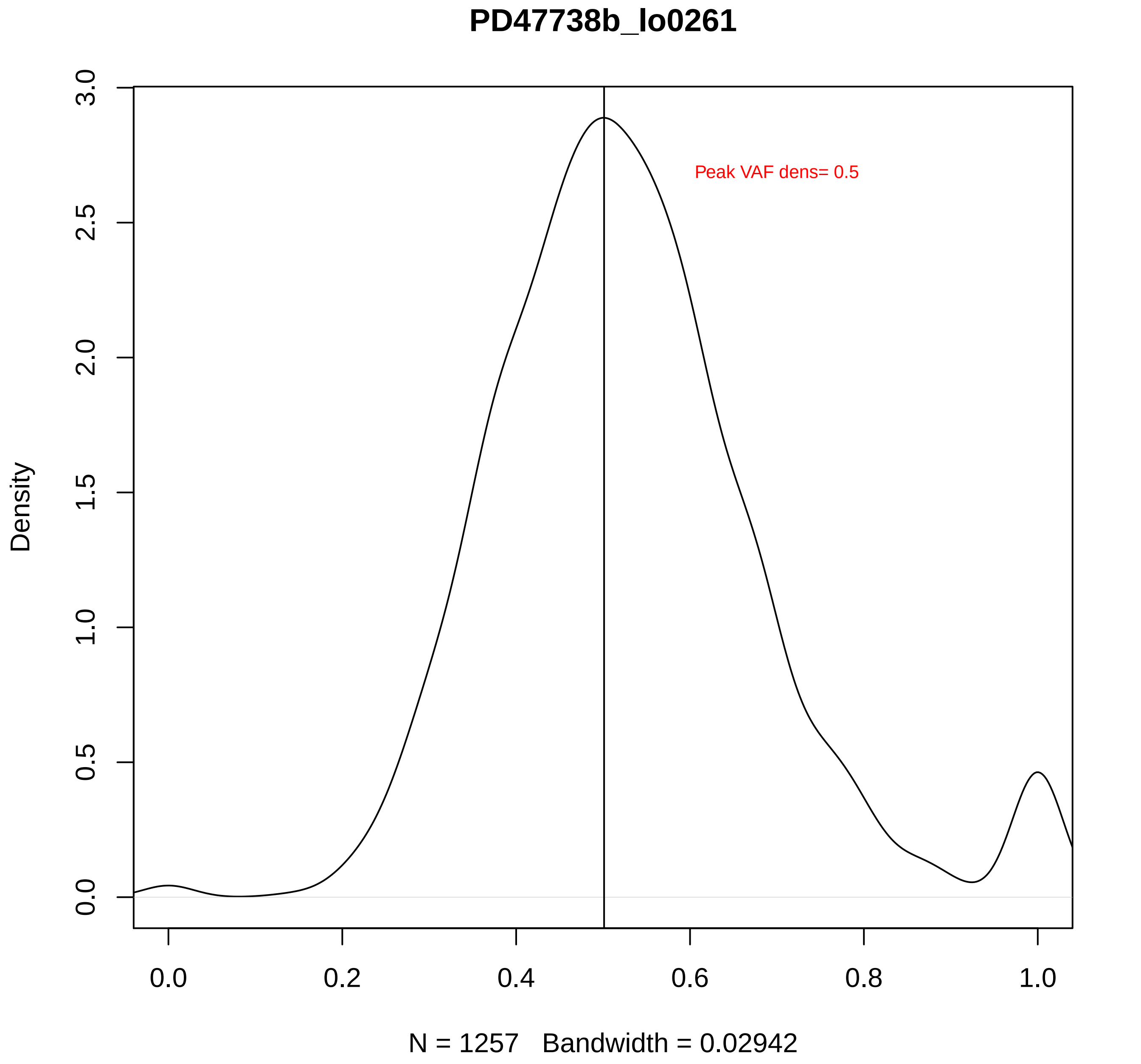

PD47738b_lo0261
Peak VAF dens= 0.5
Density
3.0
2.5
2.0
1.5
1.0
0.5
0.0
0.0
0.2
0.4
0.6
0.8
1.0
N = 1257   Bandwidth = 0.02942

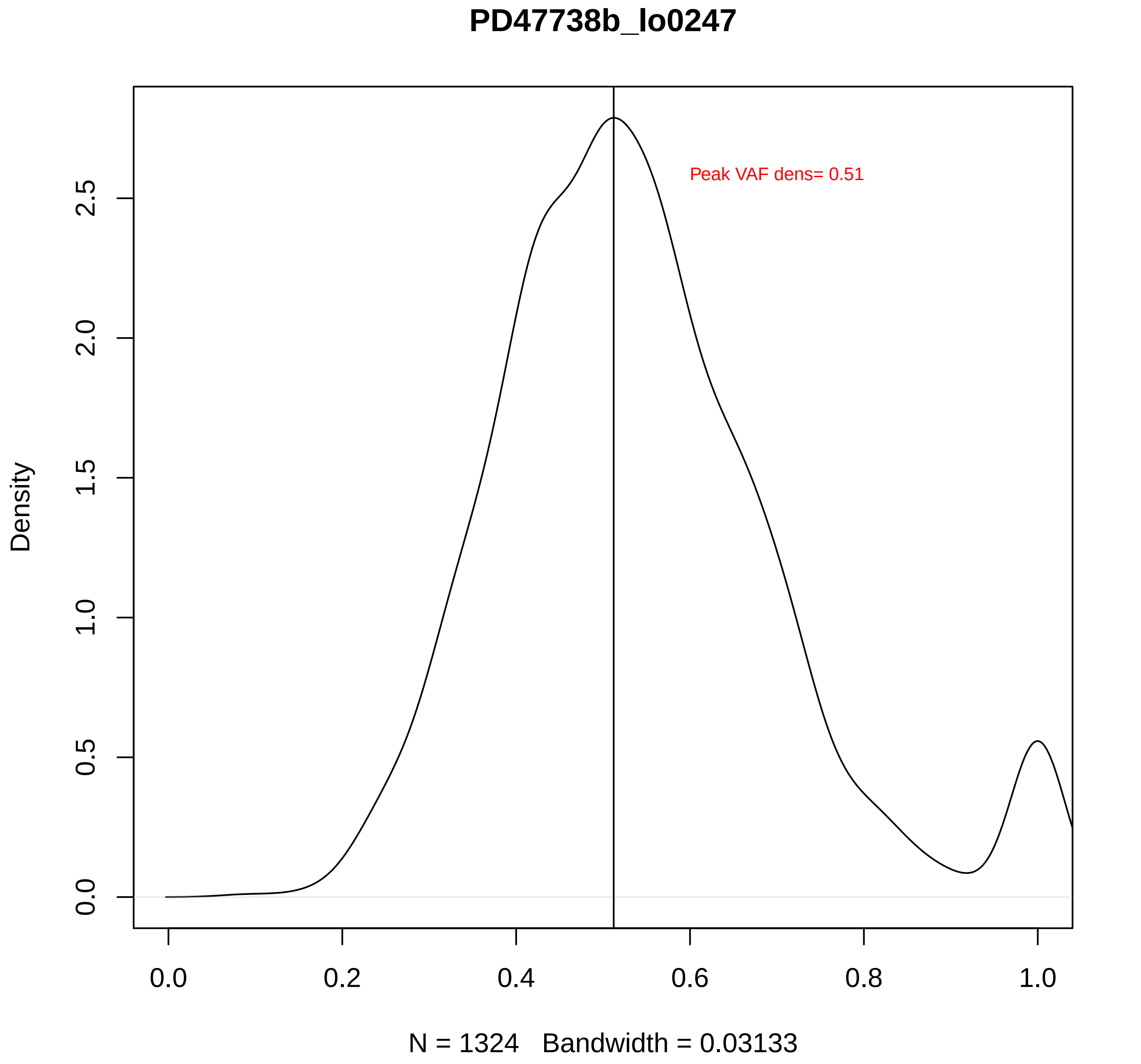

PD47738b_lo0247
Peak VAF dens= 0.51
Density
0.0
0.5
1.0
1.5
2.0
2.5
0.0
0.2
0.4
0.6
0.8
1.0
N = 1324   Bandwidth = 0.03133

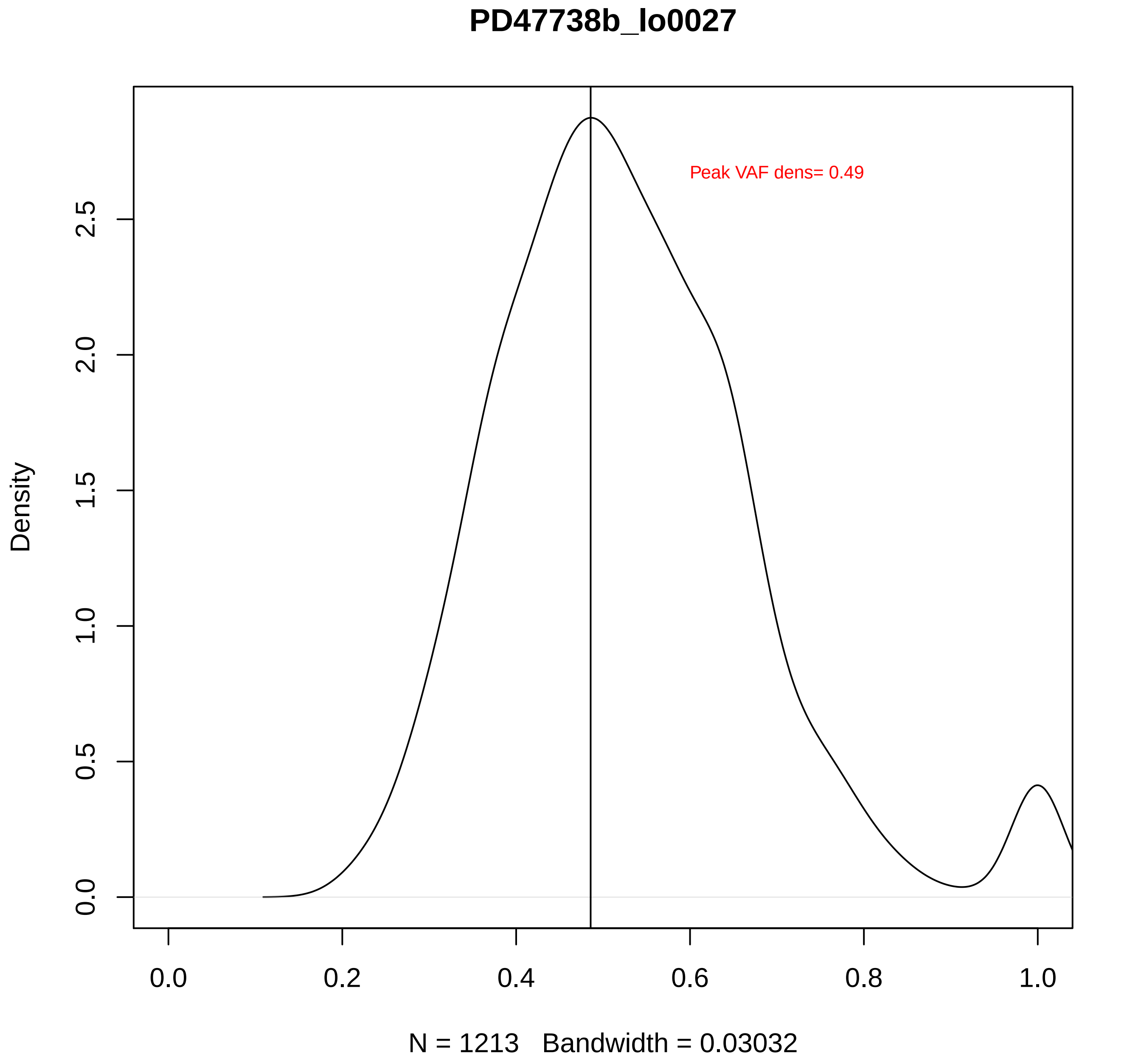

PD47738b_lo0027
Peak VAF dens= 0.49
Density
0.0
0.5
1.0
1.5
2.0
2.5
0.0
0.2
0.4
0.6
0.8
1.0
N = 1213   Bandwidth = 0.03032

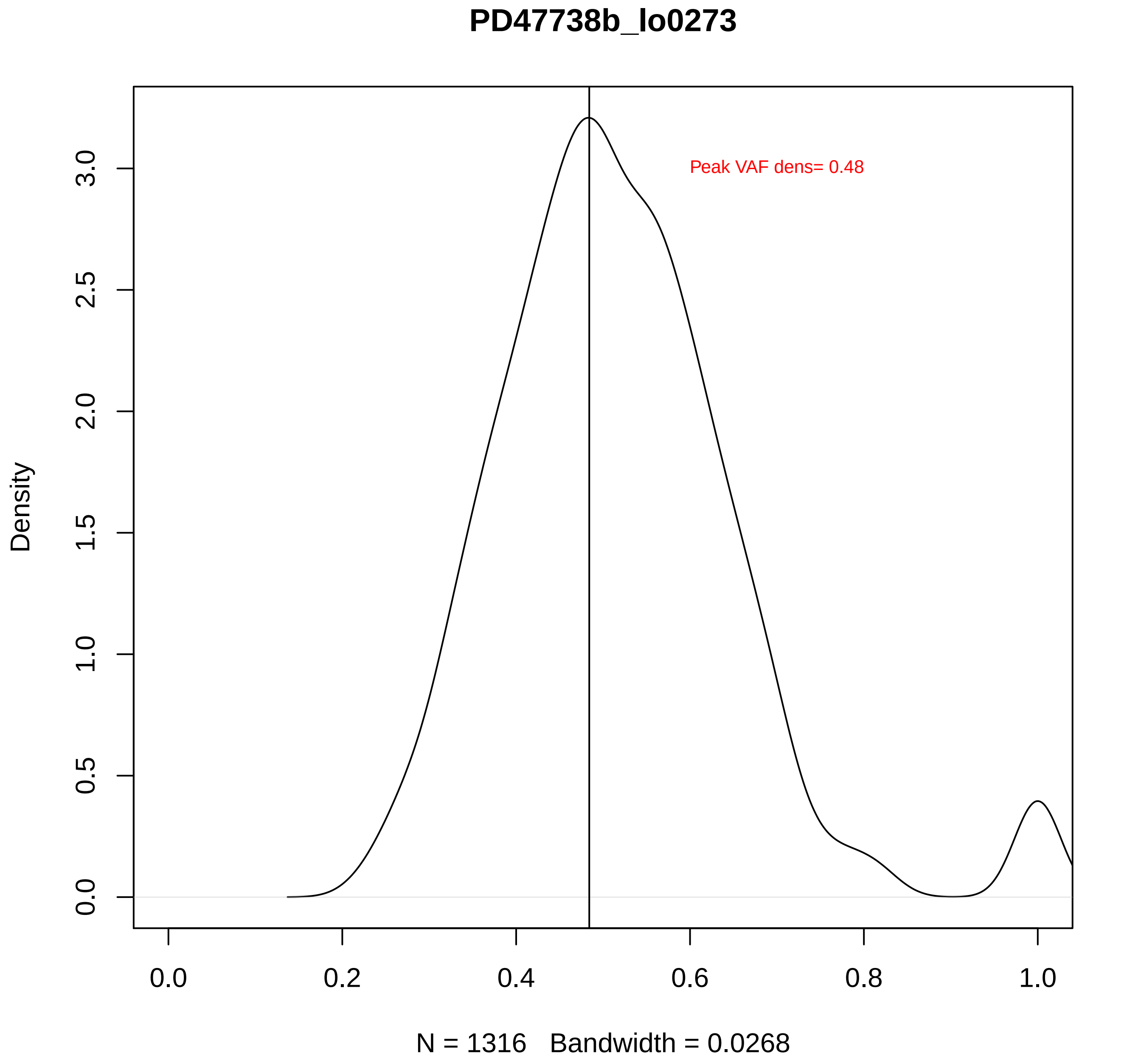
PD47738b_lo0273
Peak VAF dens= 0.48
Density
0.0
0.5
1.0
1.5
2.0
2.5
3.0
0.0
0.2
0.4
0.6
0.8
1.0
N = 1316   Bandwidth = 0.0268

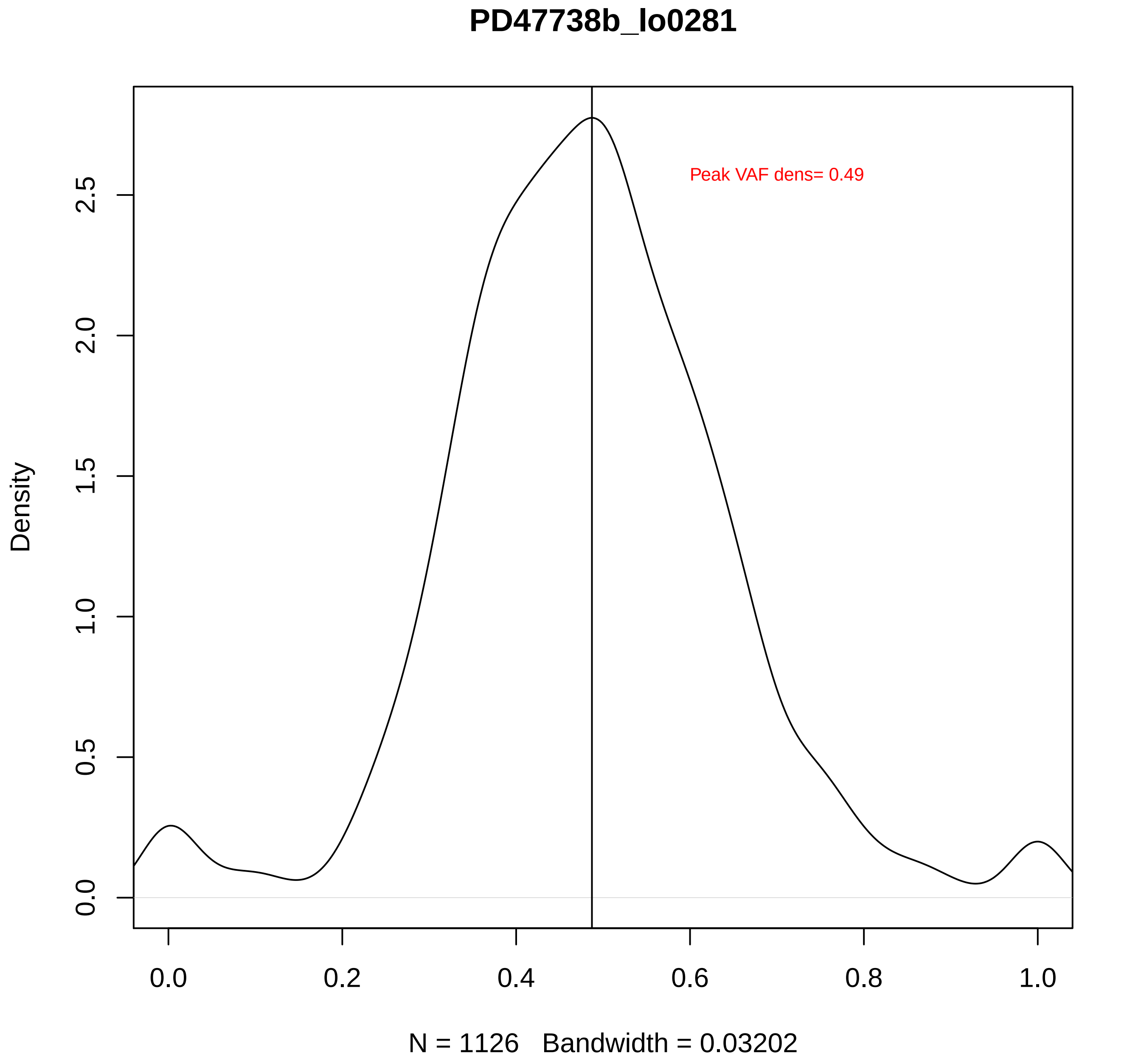

PD47738b_lo0281
Peak VAF dens= 0.49
Density
0.0
0.5
1.0
1.5
2.0
2.5
0.0
0.2
0.4
0.6
0.8
1.0
N = 1126 Bandwidth = 0.03202

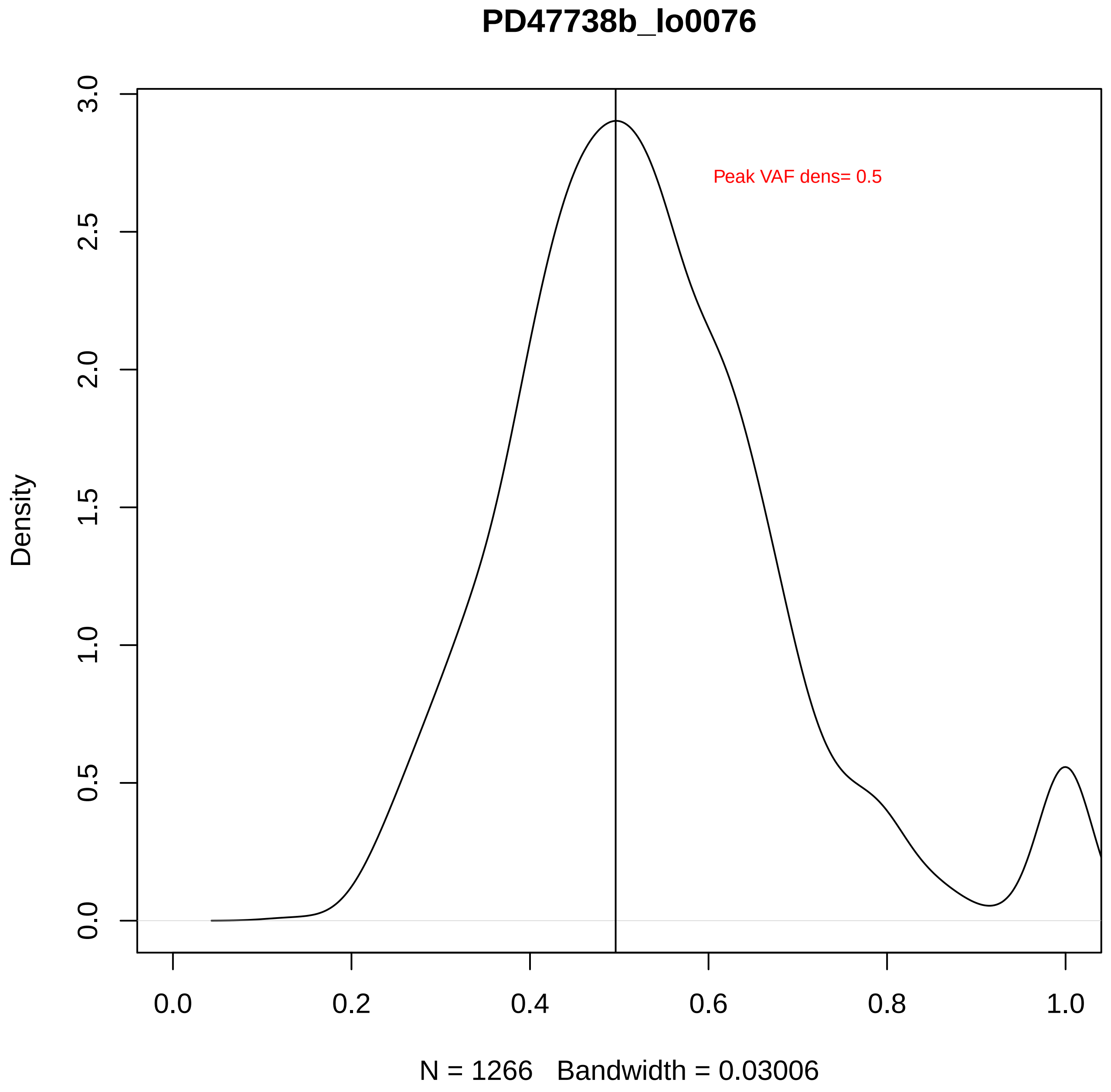
PD47738b_lo0076
Peak VAF dens= 0.5
Density
0.0
0.5
1.0
1.5
2.0
2.5
3.0
0.0
0.2
0.4
0.6
0.8
1.0
N = 1266   Bandwidth = 0.03006

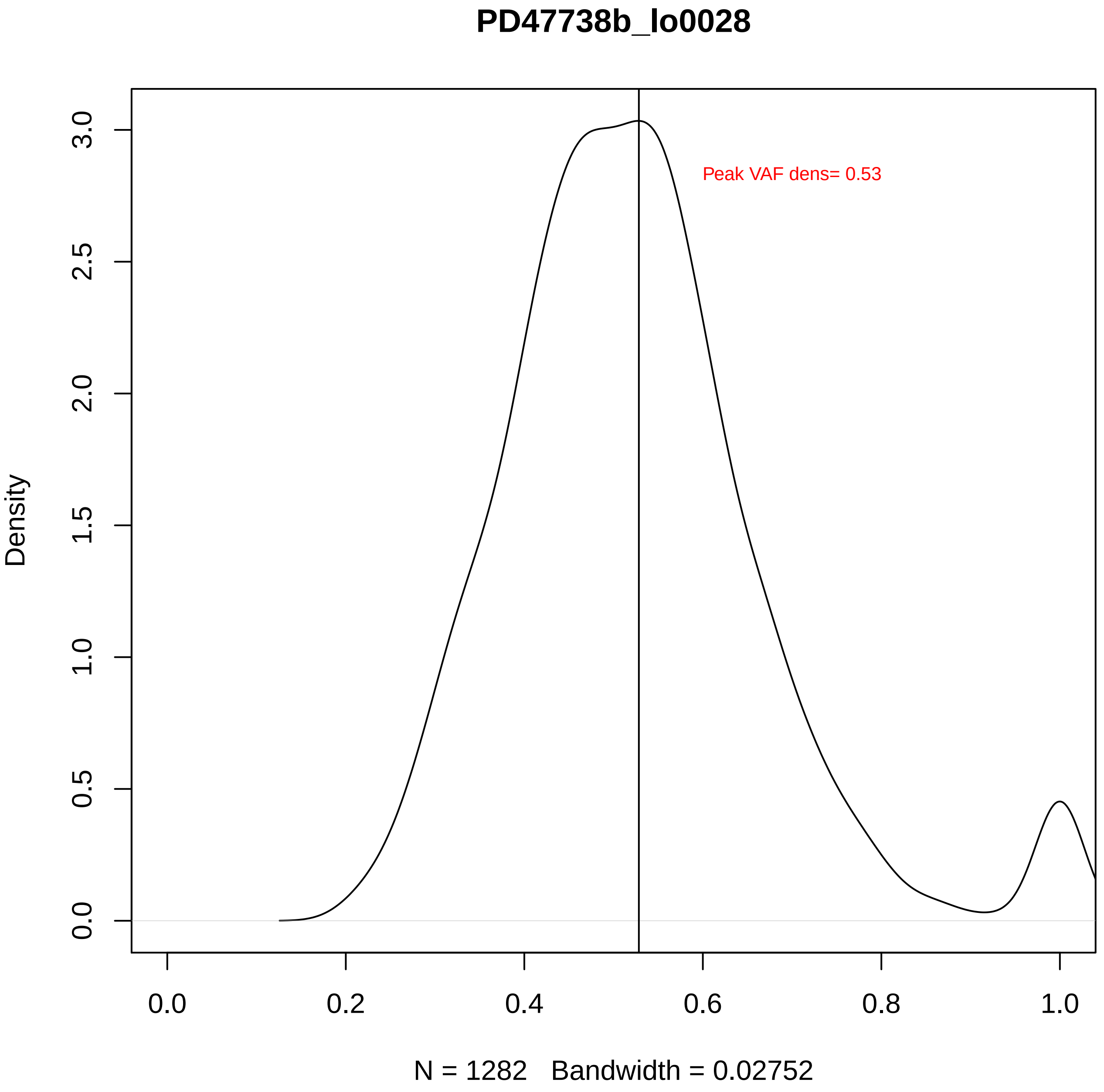
PD47738b_lo0028
Peak VAF dens= 0.53
Density
0.0
0.5
1.0
1.5
2.0
2.5
3.0
0.0
0.2
0.4
0.6
0.8
1.0
N = 1282 Bandwidth = 0.02752

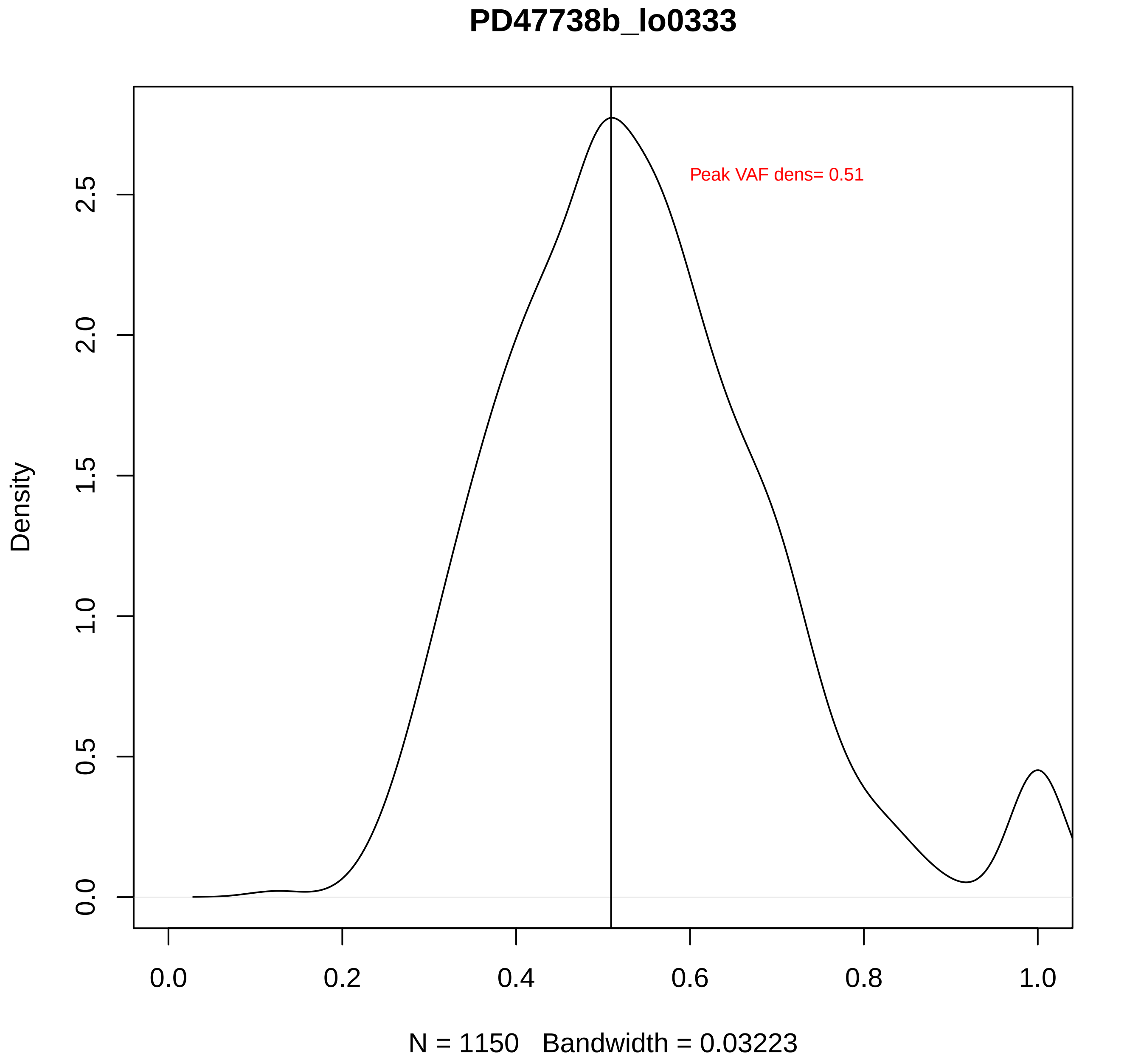
PD47738b_lo0333
Peak VAF dens= 0.51
Density
0.0
0.5
1.0
1.5
2.0
2.5
0.0
0.2
0.4
0.6
0.8
1.0
N = 1150   Bandwidth = 0.03223

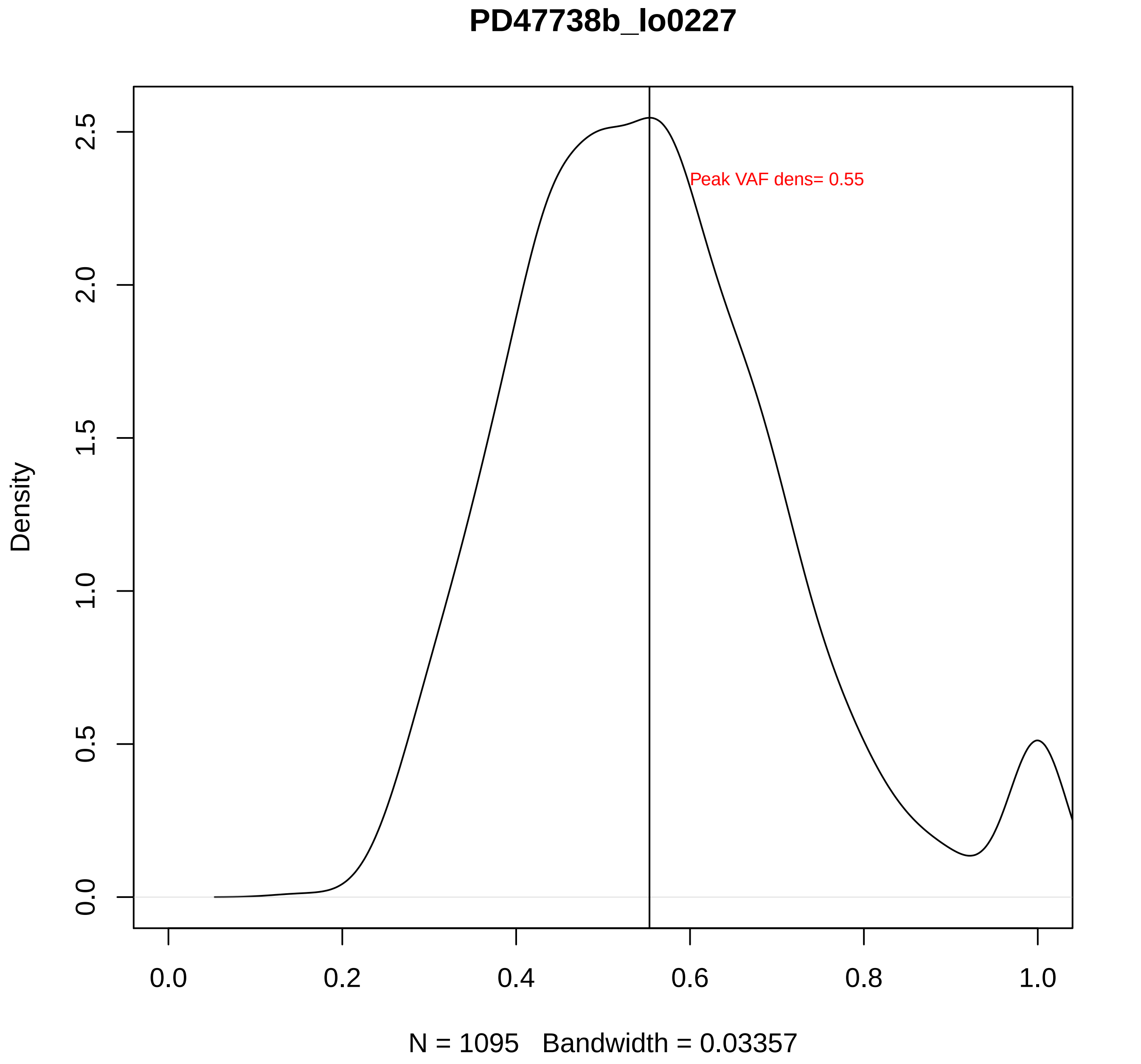
PD47738b_lo0227
Peak VAF dens= 0.55
Density
2.5
2.0
1.5
1.0
0.5
0.0
0.0
0.2
0.4
0.6
0.8
1.0
N = 1095   Bandwidth = 0.03357

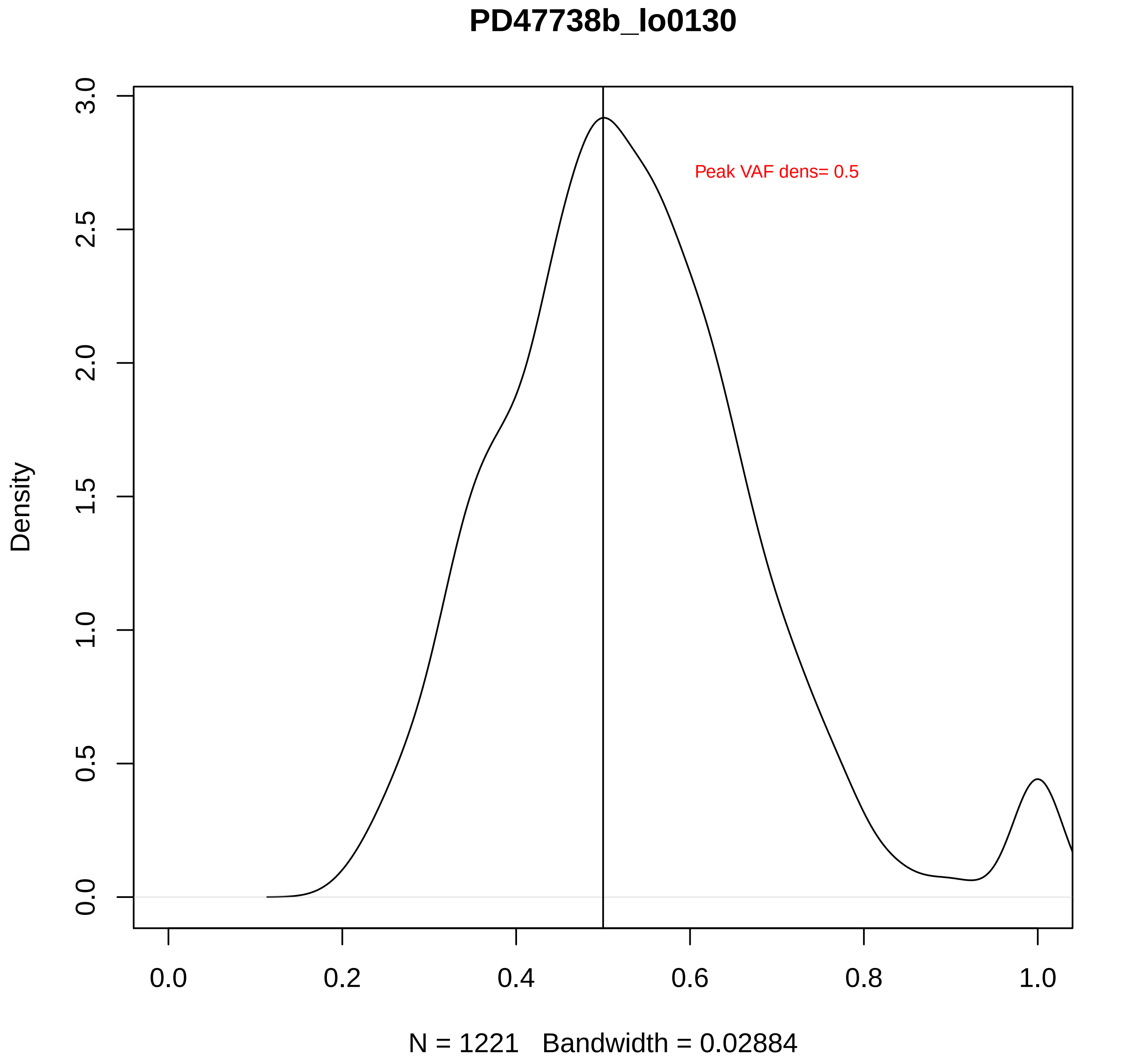
PD47738b_lo0130
Peak VAF dens= 0.5
Density
0.0
0.5
1.0
1.5
2.0
2.5
3.0
0.0
0.2
0.4
0.6
0.8
1.0
N = 1221   Bandwidth = 0.02884

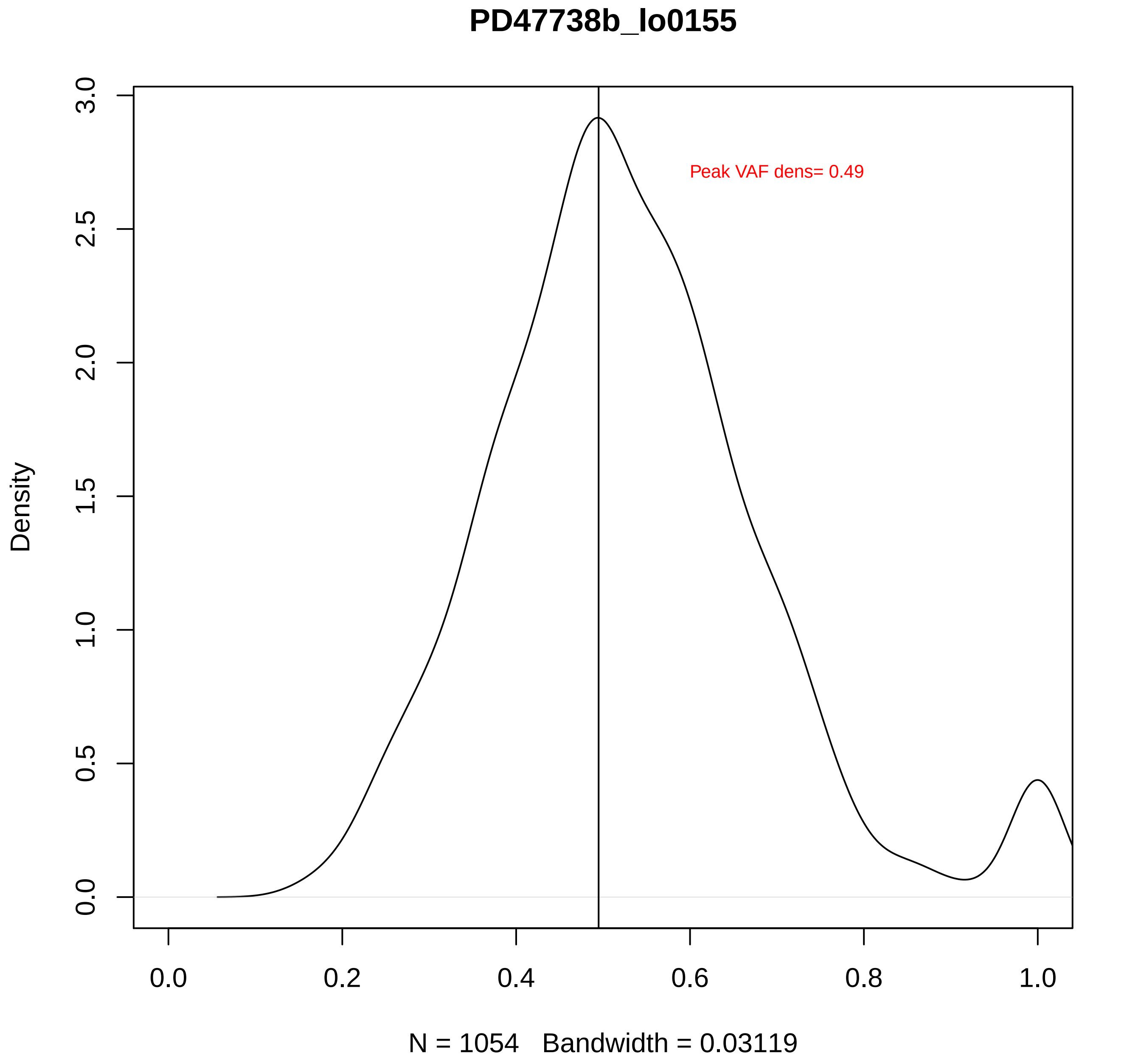

PD47738b_lo0155
Peak VAF dens= 0.49
Density
0.0
0.5
1.0
1.5
2.0
2.5
3.0
0.0
0.2
0.4
0.6
0.8
1.0
N = 1054   Bandwidth = 0.03119

PD47738b_lo0165

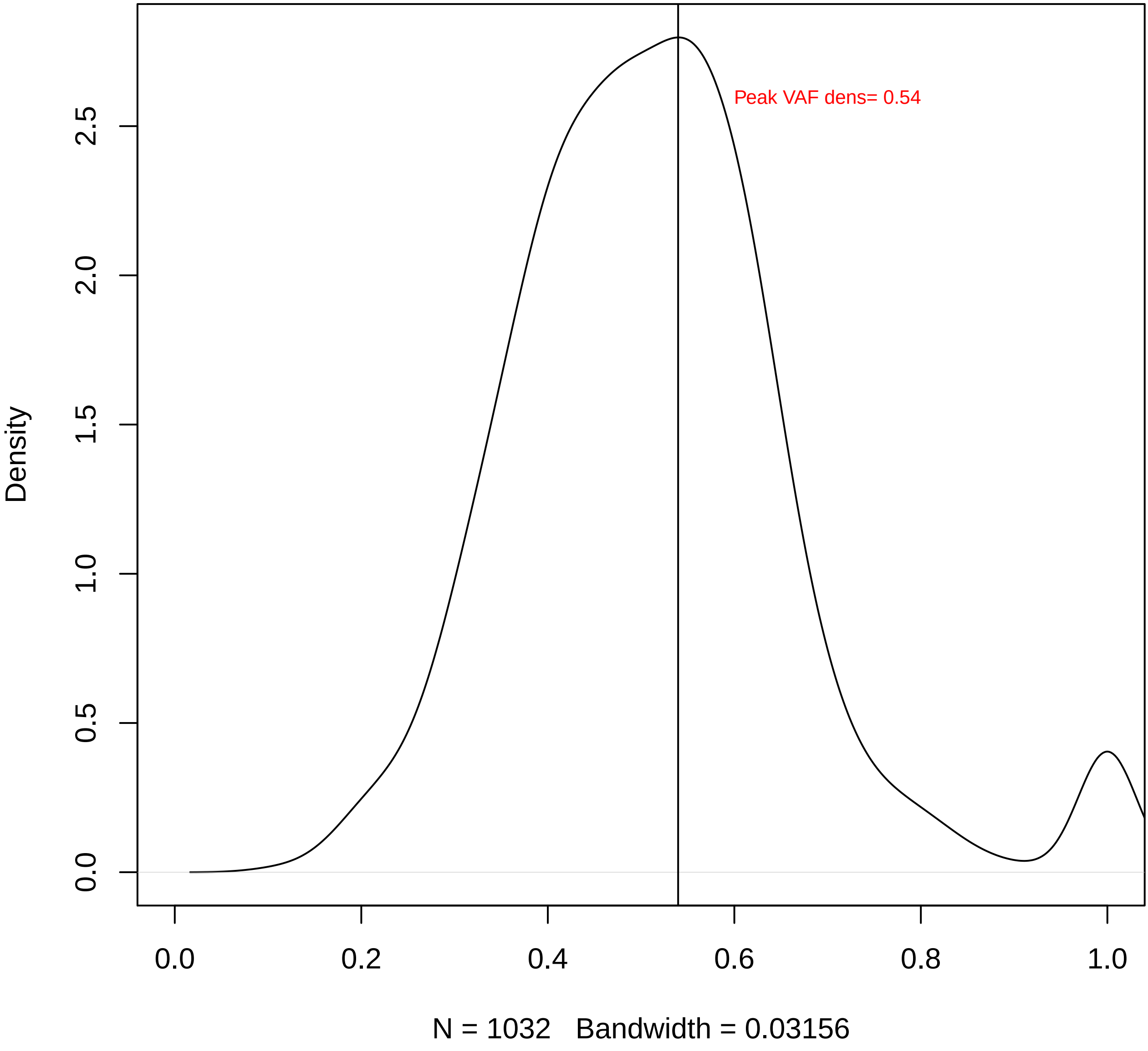

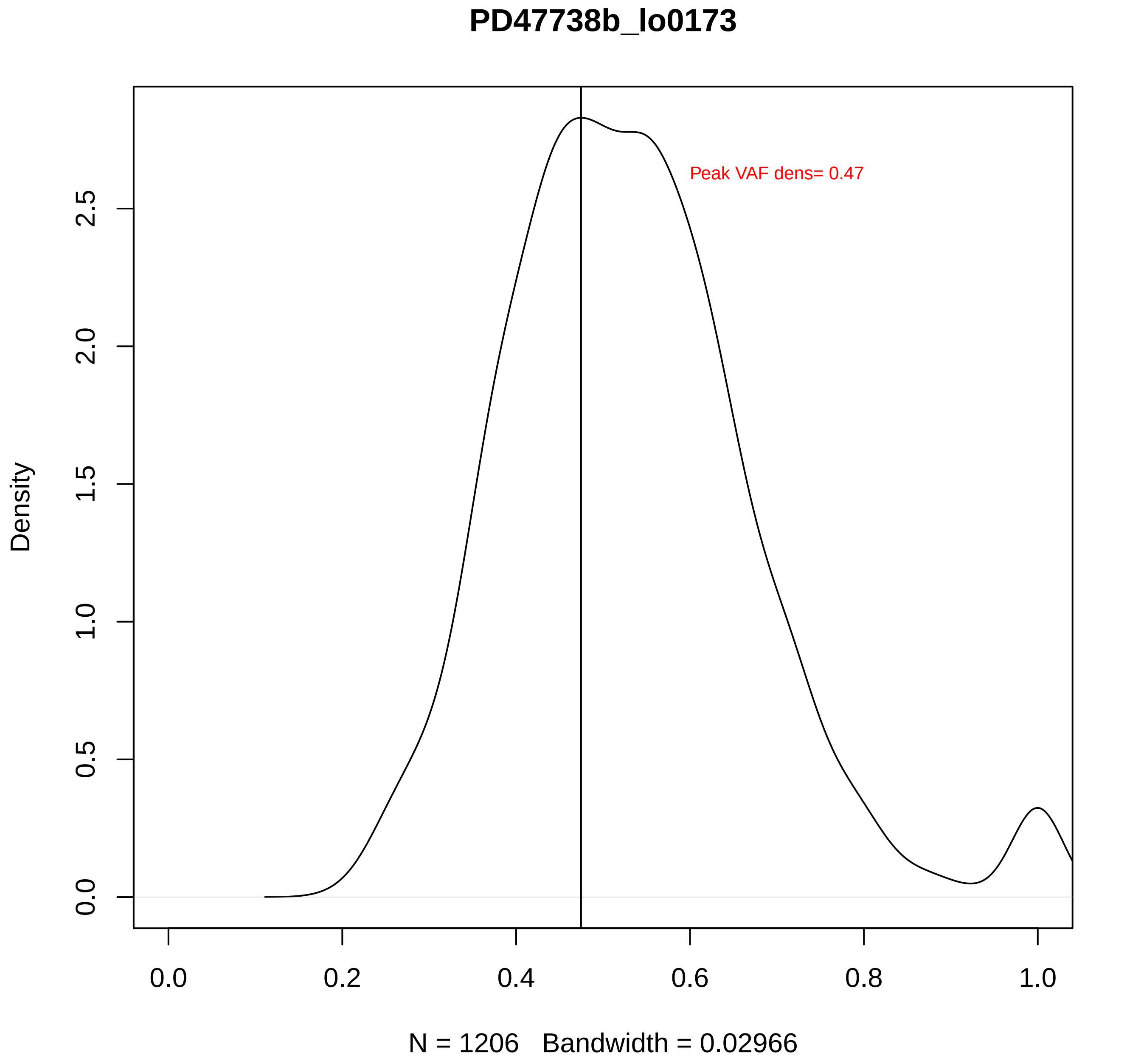

PD47738b_lo0173
Peak VAF dens= 0.47
Density
0.0
0.5
1.0
1.5
2.0
2.5
0.0
0.2
0.4
0.6
0.8
1.0
N = 1206   Bandwidth = 0.02966

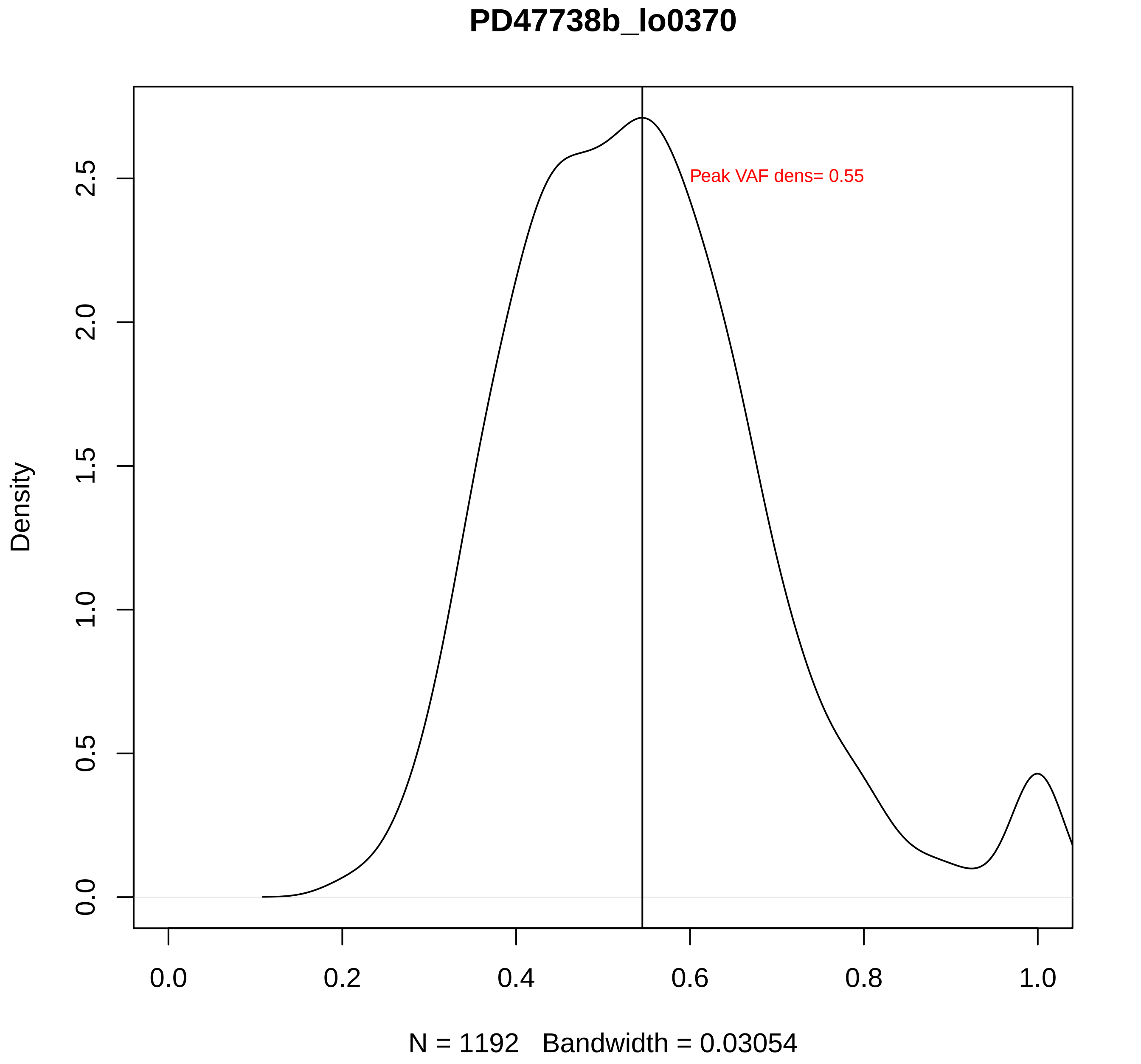

PD47738b_lo0370
Peak VAF dens= 0.55
Density
0.0
0.5
1.0
1.5
2.0
2.5
0.0
0.2
0.4
0.6
0.8
1.0
N = 1192   Bandwidth = 0.03054

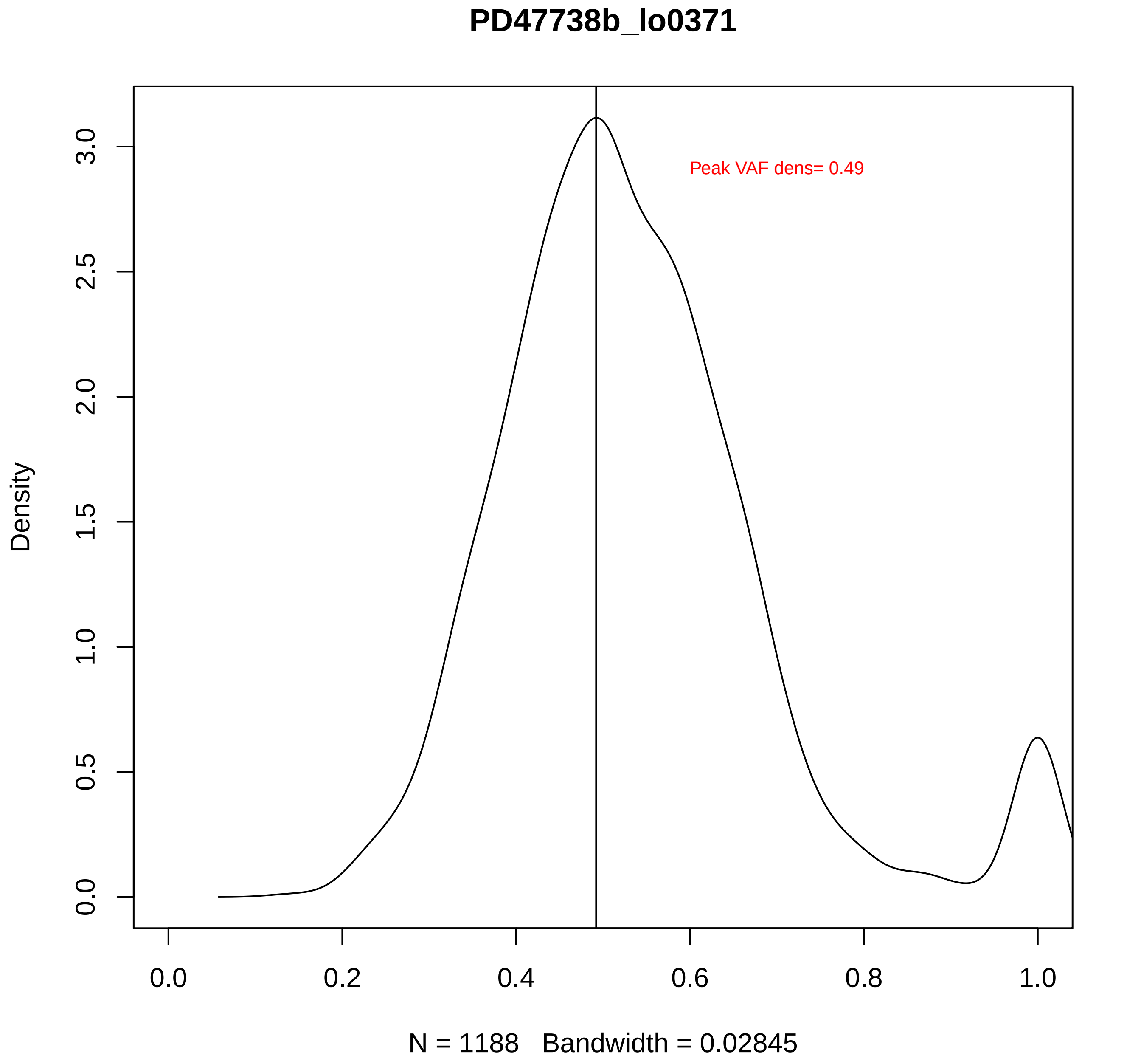

PD47738b_lo0371
Peak VAF dens= 0.49
Density
0.0
0.5
1.0
1.5
2.0
2.5
3.0
0.0
0.2
0.4
0.6
0.8
1.0
N = 1188 Bandwidth = 0.02845

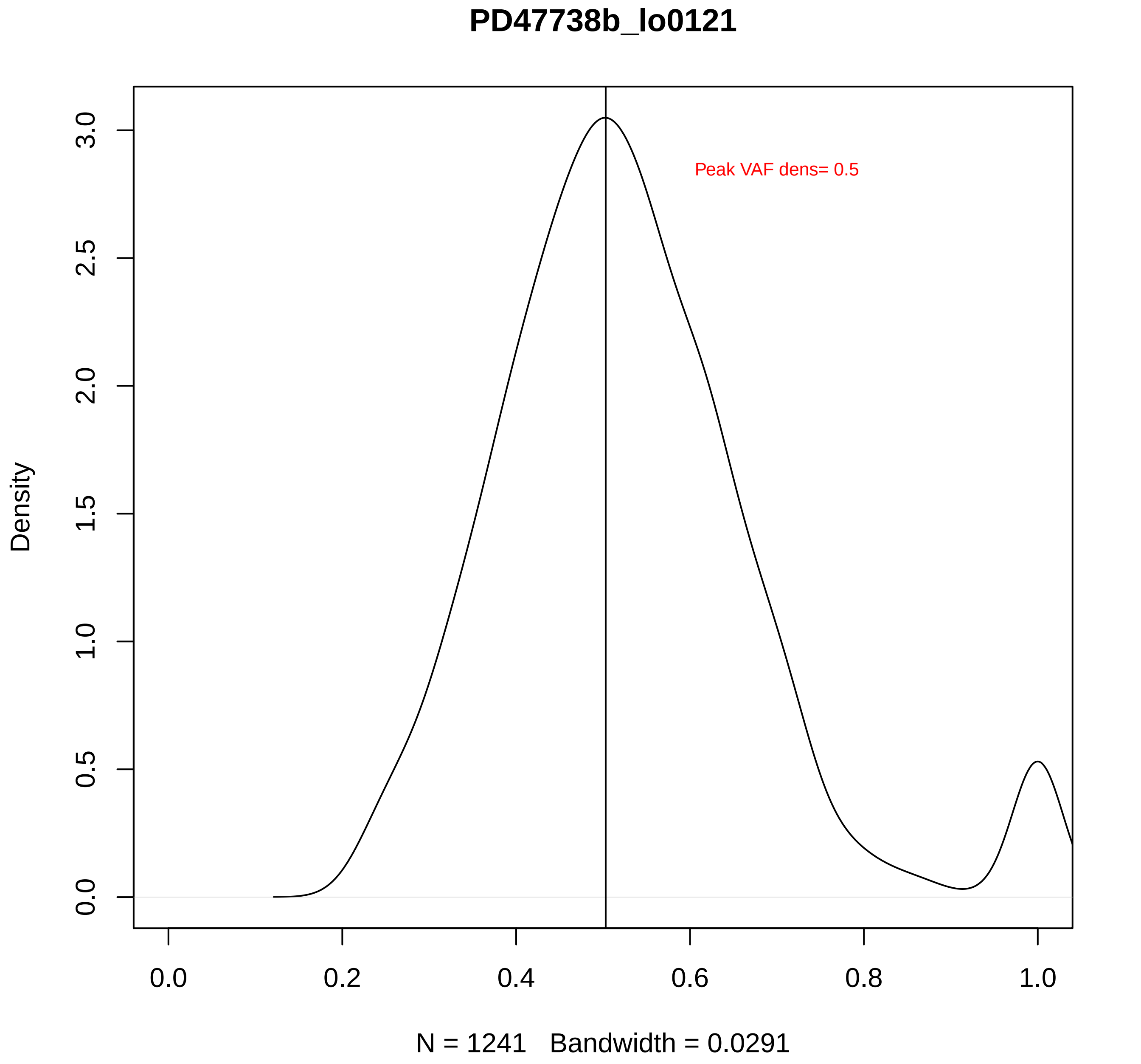
PD47738b_lo0121
Peak VAF dens= 0.5
Density
0.0
0.5
1.0
1.5
2.0
2.5
3.0
0.0
0.2
0.4
0.6
0.8
1.0
N = 1241   Bandwidth = 0.0291

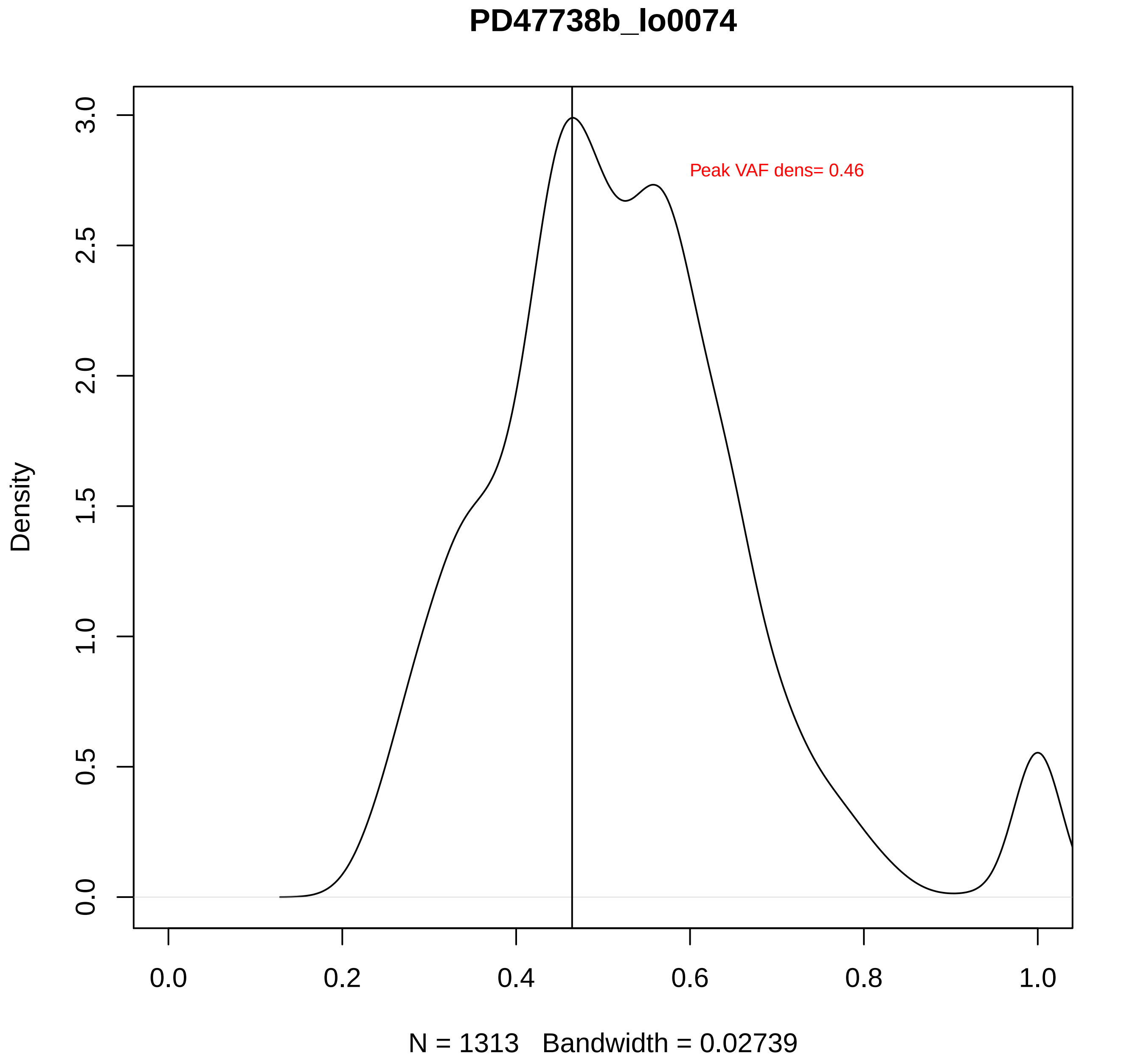
PD47738b_lo0074
Peak VAF dens= 0.46
Density
0.0
0.5
1.0
1.5
2.0
2.5
3.0
0.0
0.2
0.4
0.6
0.8
1.0
N = 1313 Bandwidth = 0.02739

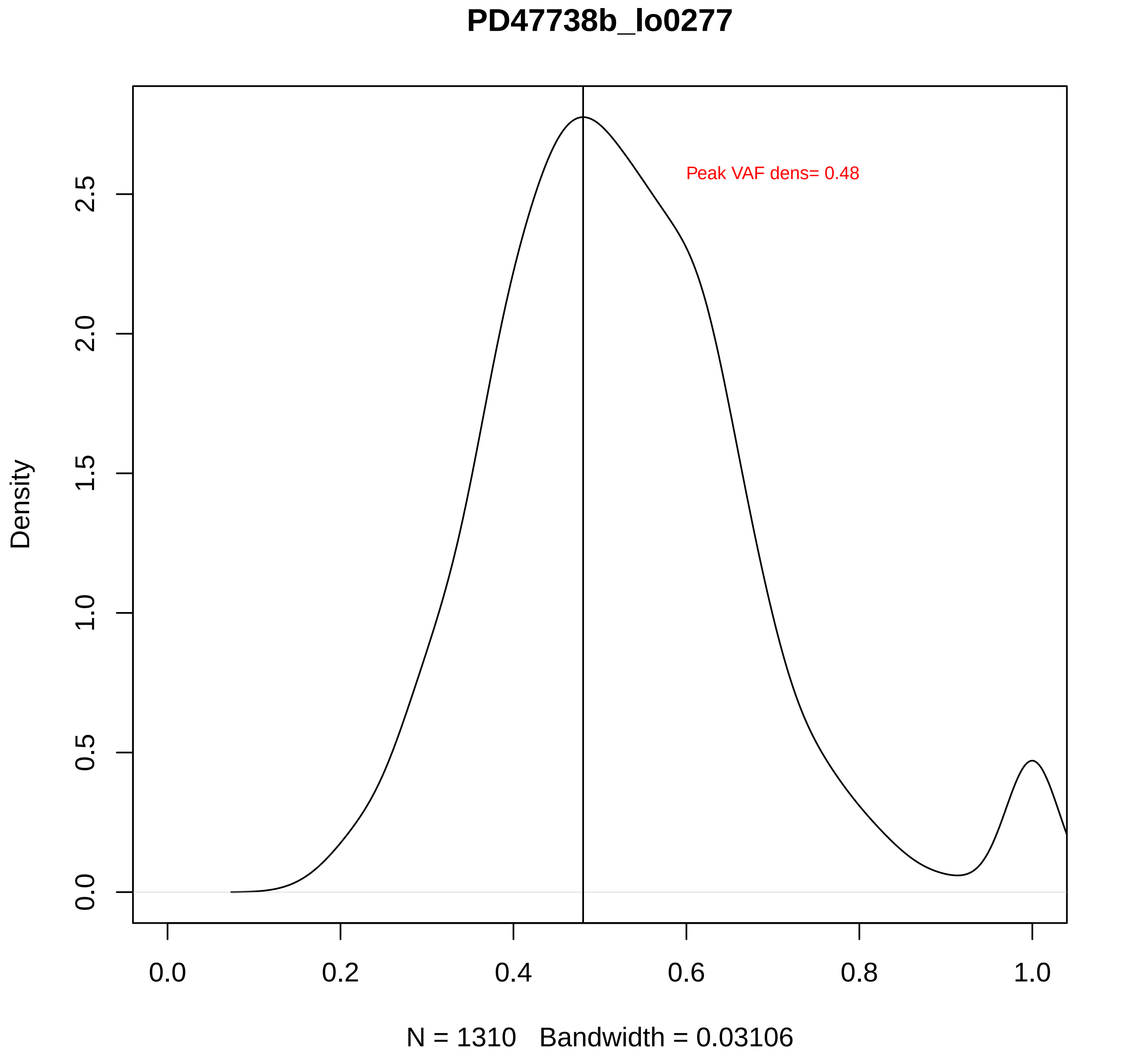

PD47738b_lo0277
Peak VAF dens= 0.48
Density
0.0
0.5
1.0
1.5
2.0
2.5
0.0
0.2
0.4
0.6
0.8
1.0
N = 1310   Bandwidth = 0.03106

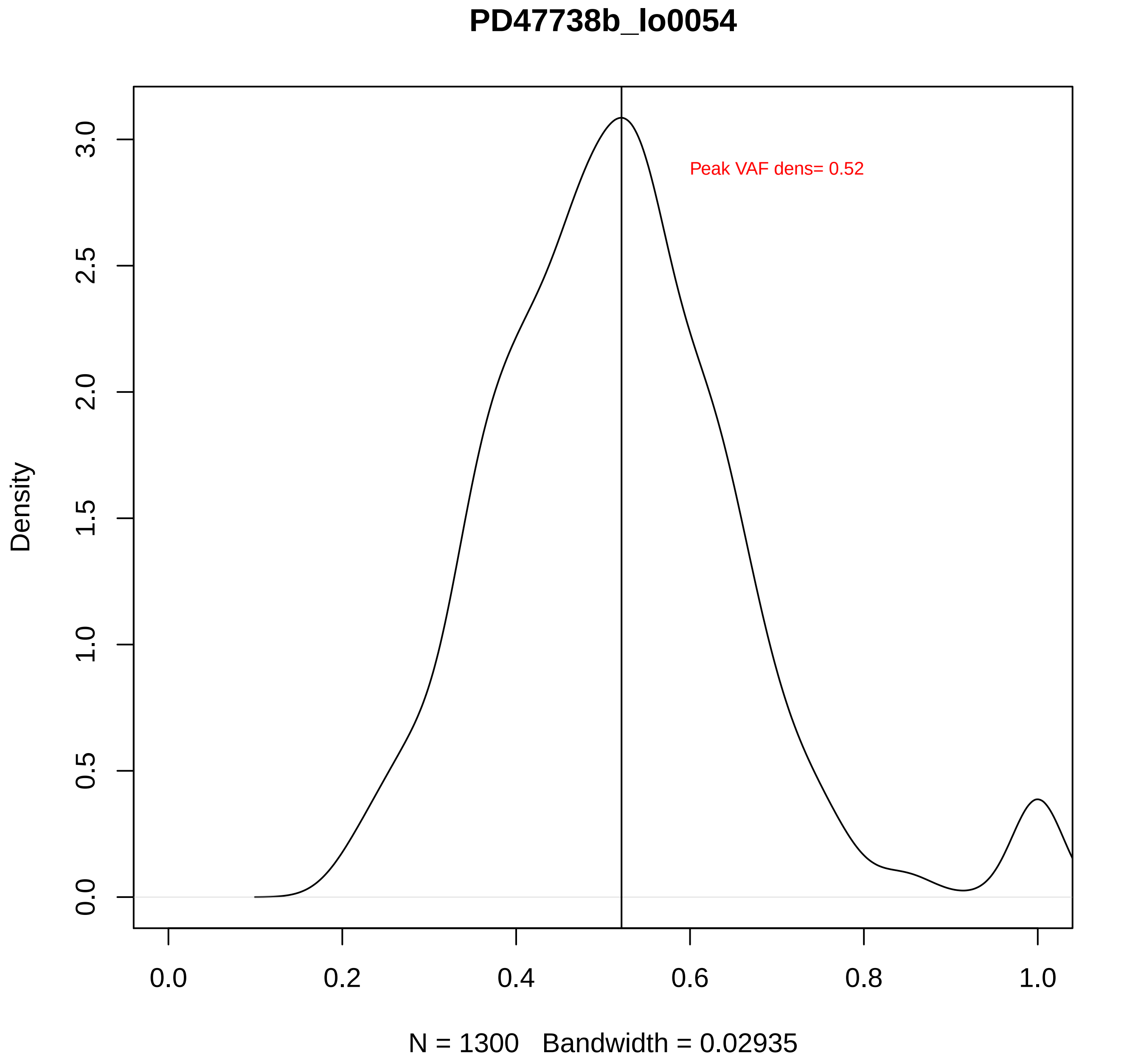
PD47738b_lo0054
Peak VAF dens= 0.52
Density
0.0
0.5
1.0
1.5
2.0
2.5
3.0
0.0
0.2
0.4
0.6
0.8
1.0
N = 1300 Bandwidth = 0.02935

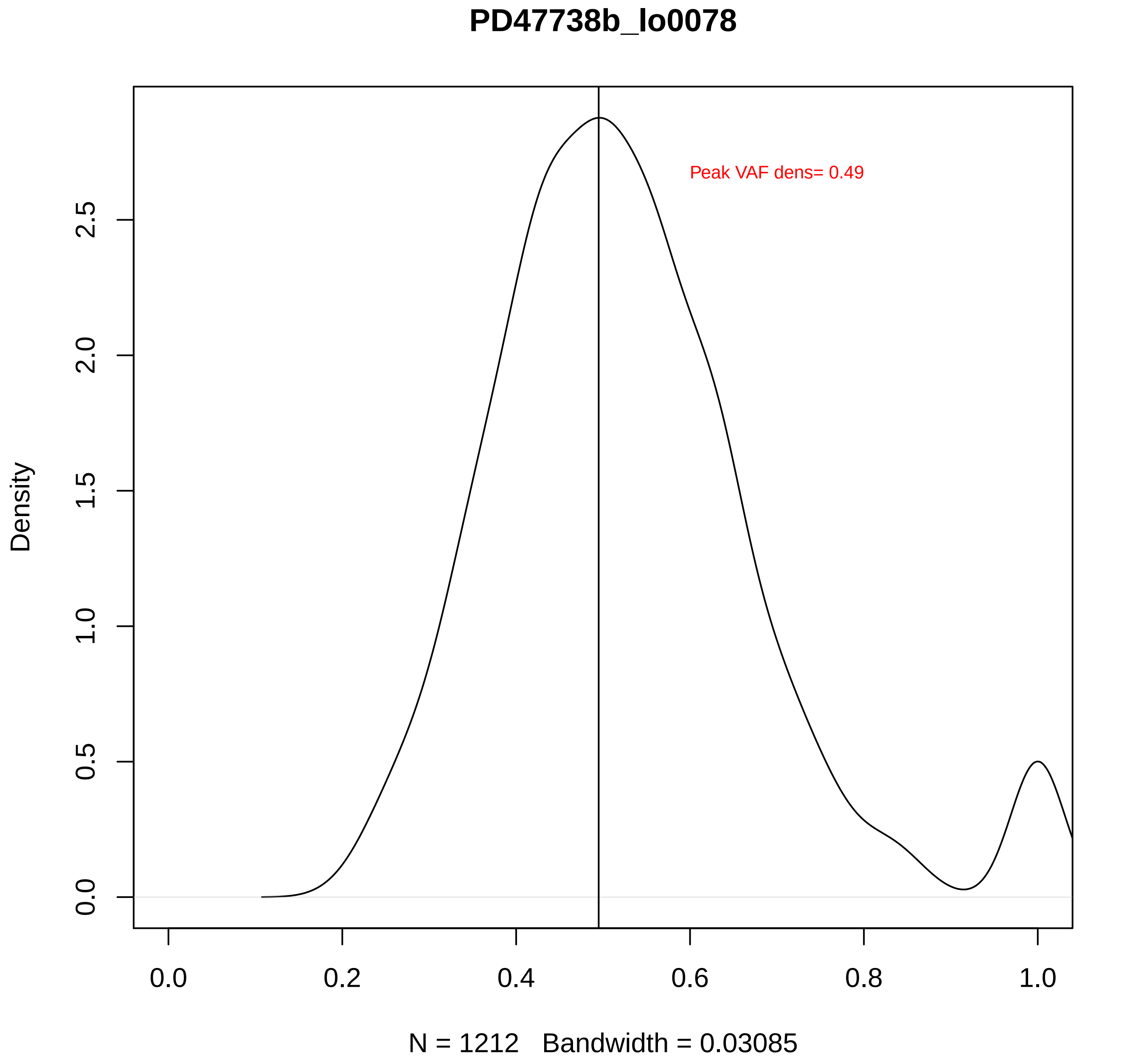
PD47738b_lo0078
Peak VAF dens= 0.49
Density
0.0
0.5
1.0
1.5
2.0
2.5
0.0
0.2
0.4
0.6
0.8
1.0
N = 1212   Bandwidth = 0.03085

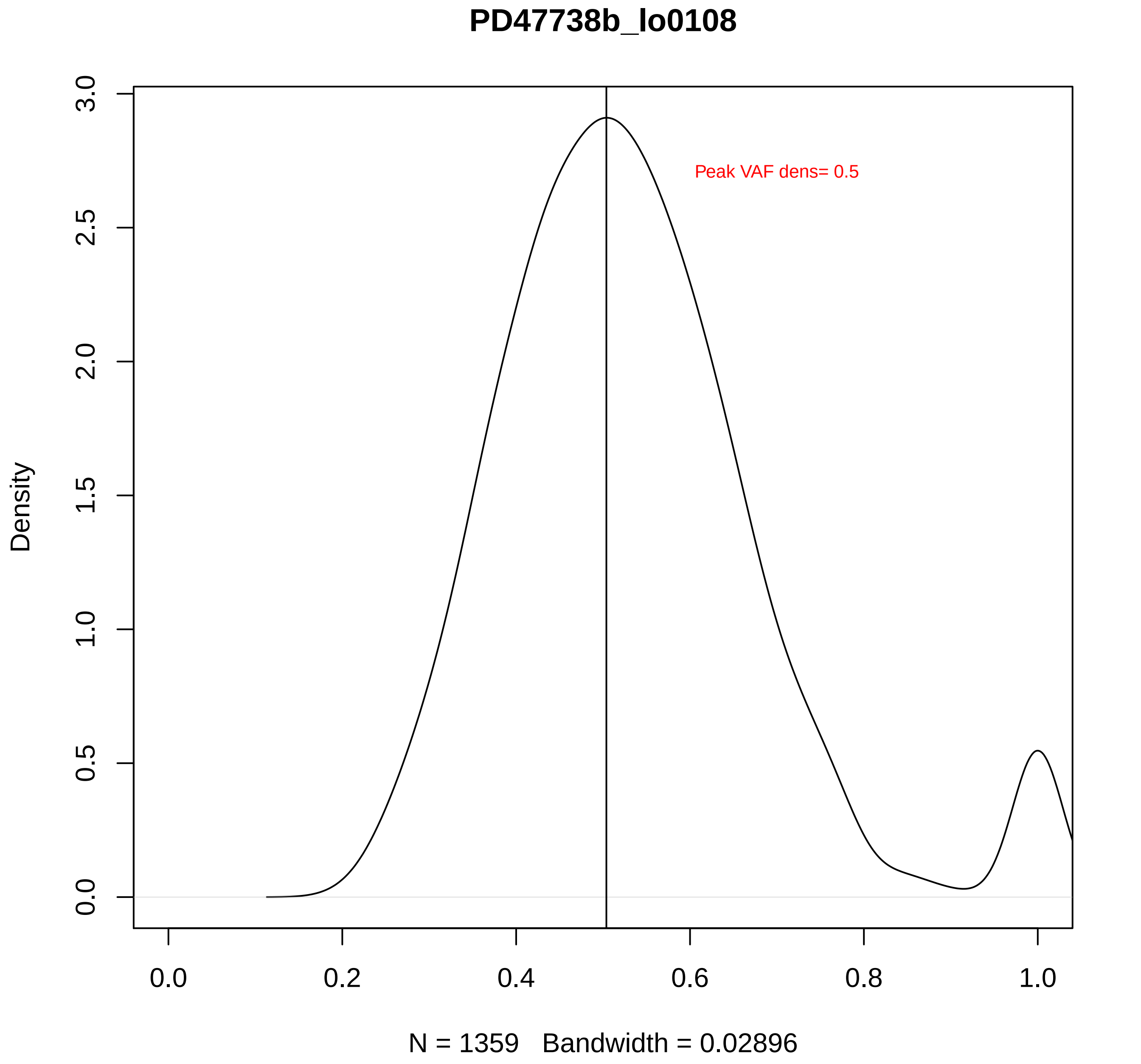
PD47738b_lo0108
Peak VAF dens= 0.5
Density
0.0
0.5
1.0
1.5
2.0
2.5
3.0
0.0
0.2
0.4
0.6
0.8
1.0
N = 1359   Bandwidth = 0.02896

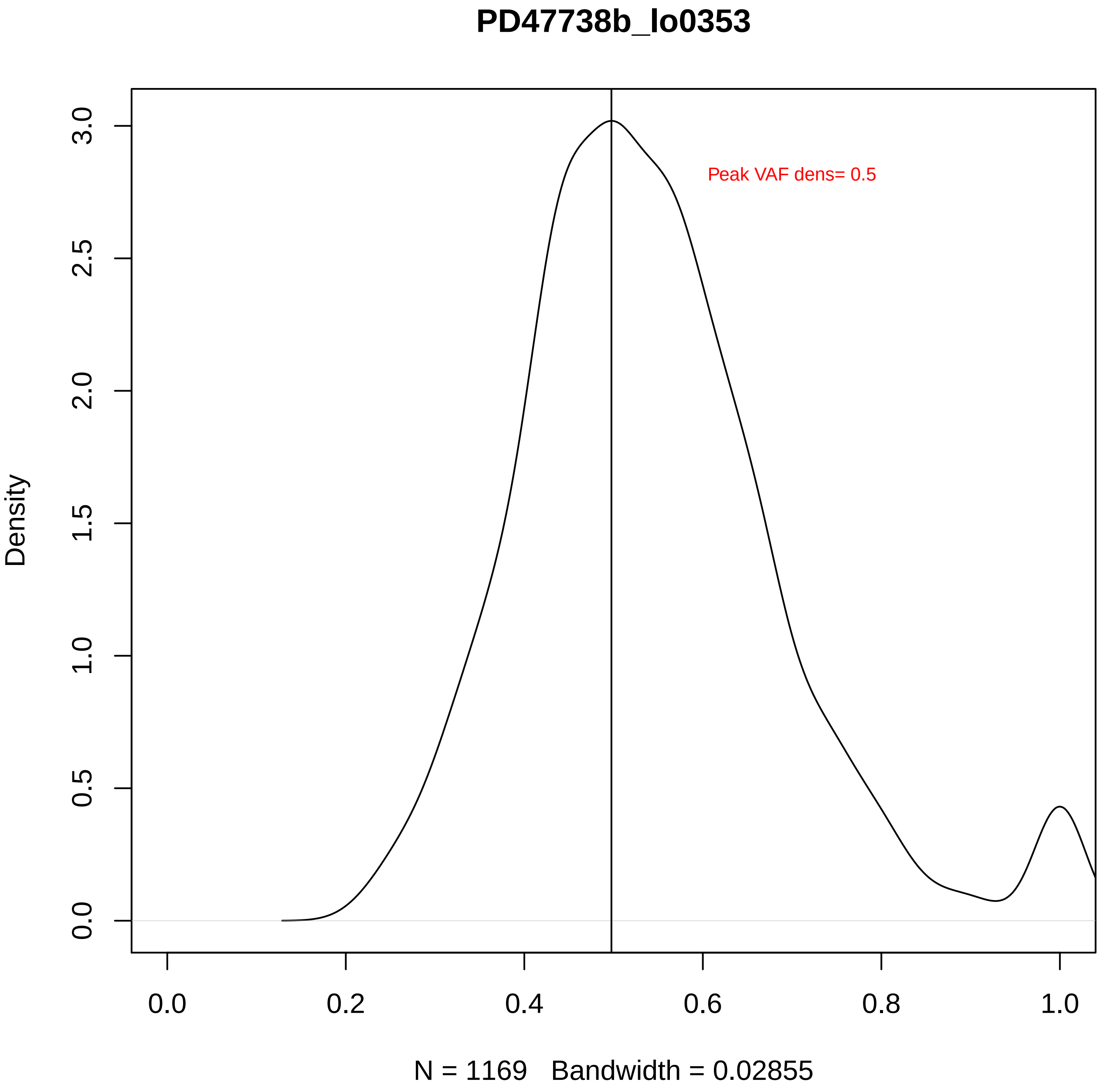
PD47738b_lo0353
Peak VAF dens= 0.5
Density
0.0
0.5
1.0
1.5
2.0
2.5
3.0
0.0
0.2
0.4
0.6
0.8
1.0
N = 1169   Bandwidth = 0.02855

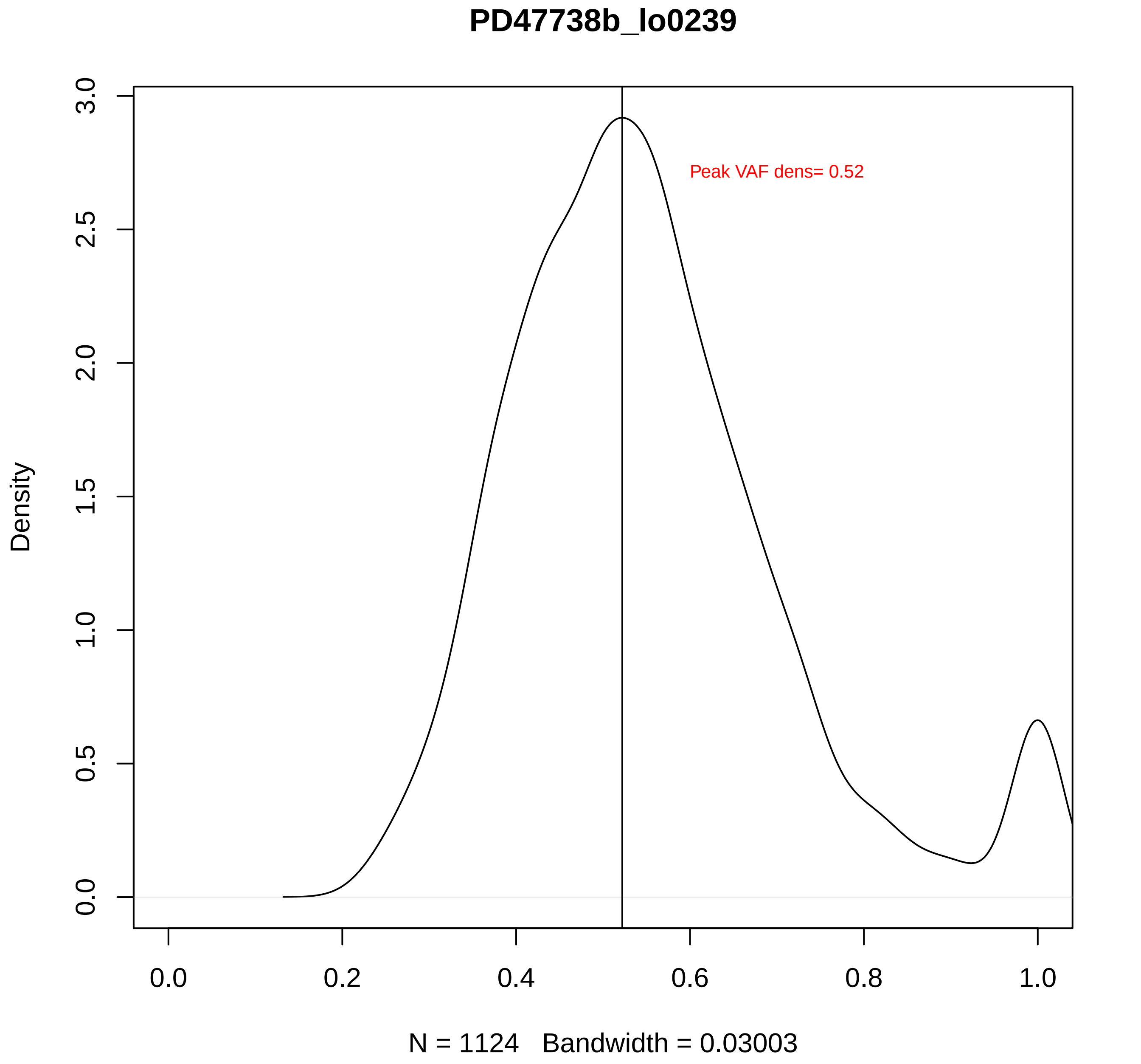
PD47738b_lo0239
Peak VAF dens= 0.52
Density
0.0
0.5
1.0
1.5
2.0
2.5
3.0
0.0
0.2
0.4
0.6
0.8
1.0
N = 1124   Bandwidth = 0.03003

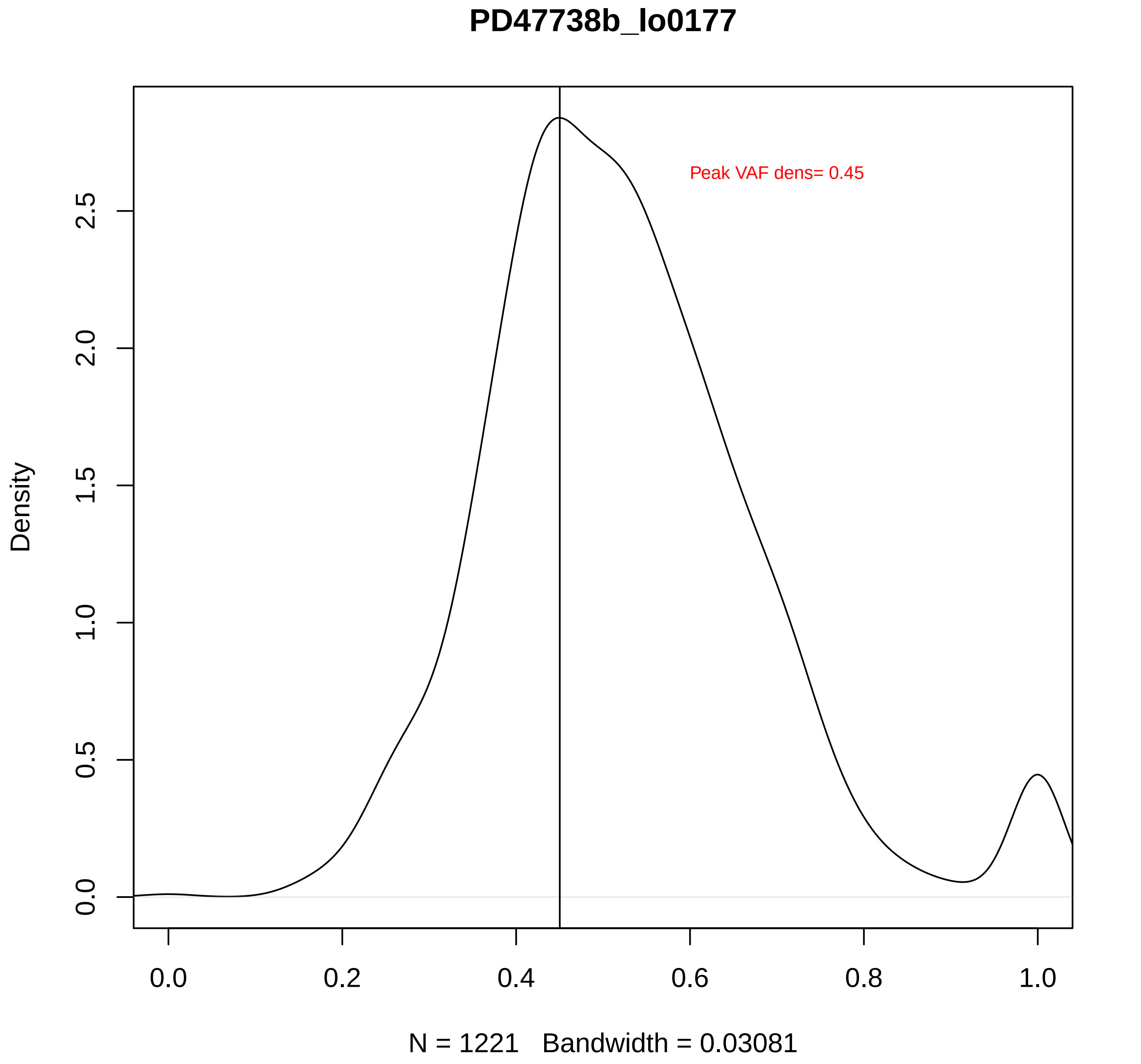

PD47738b_lo0177
Peak VAF dens= 0.45
Density
0.0
0.5
1.0
1.5
2.0
2.5
0.0
0.2
0.4
0.6
0.8
1.0
N = 1221   Bandwidth = 0.03081

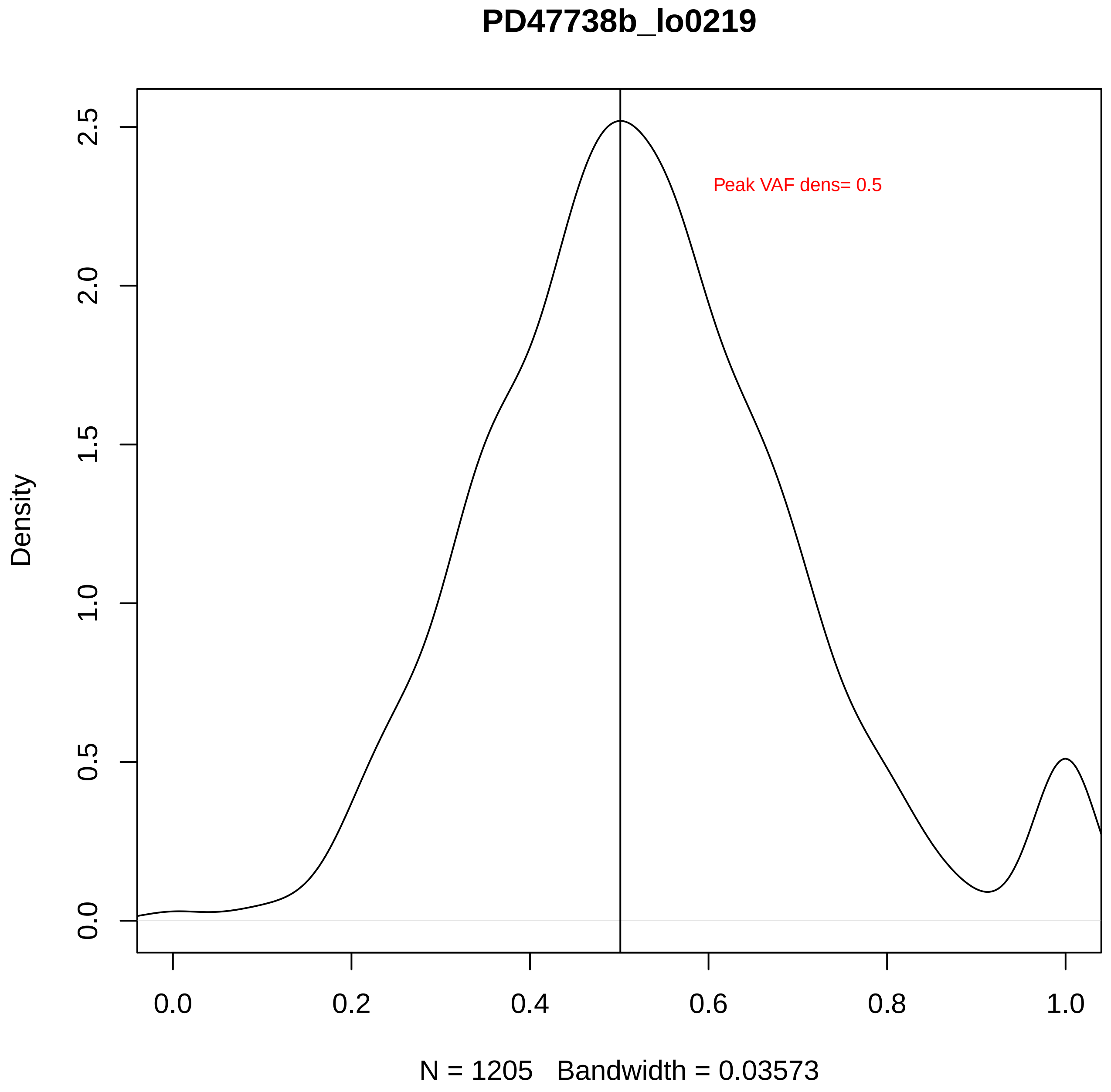
PD47738b_lo0219
Peak VAF dens= 0.5
Density
0.0
0.5
1.0
1.5
2.0
2.5
0.0
0.2
0.4
0.6
0.8
1.0
N = 1205   Bandwidth = 0.03573

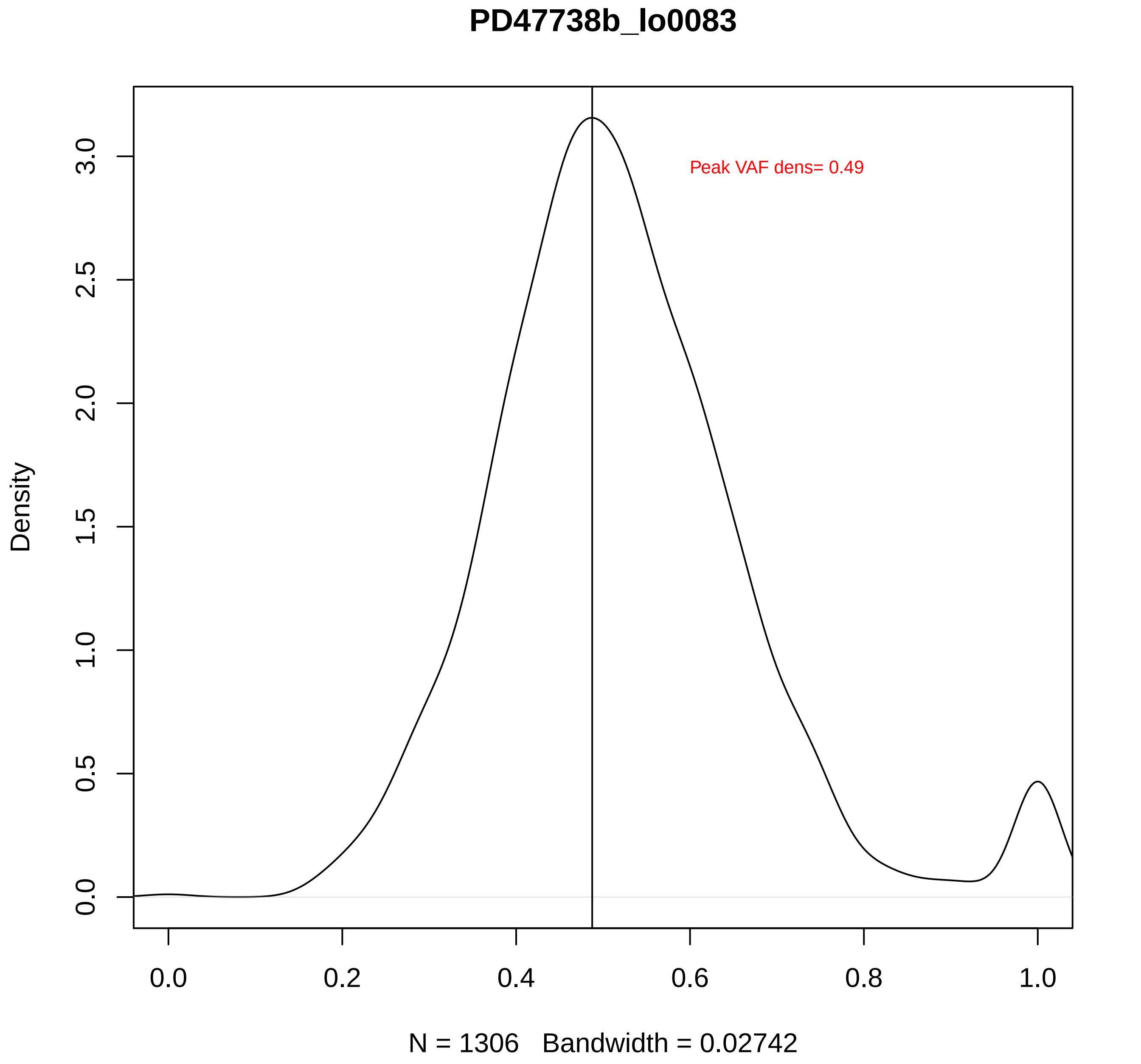

PD47738b_lo0083
Peak VAF dens= 0.49
Density
0.0
0.5
1.0
1.5
2.0
2.5
3.0
0.0
0.2
0.4
0.6
0.8
1.0
N = 1306   Bandwidth = 0.02742

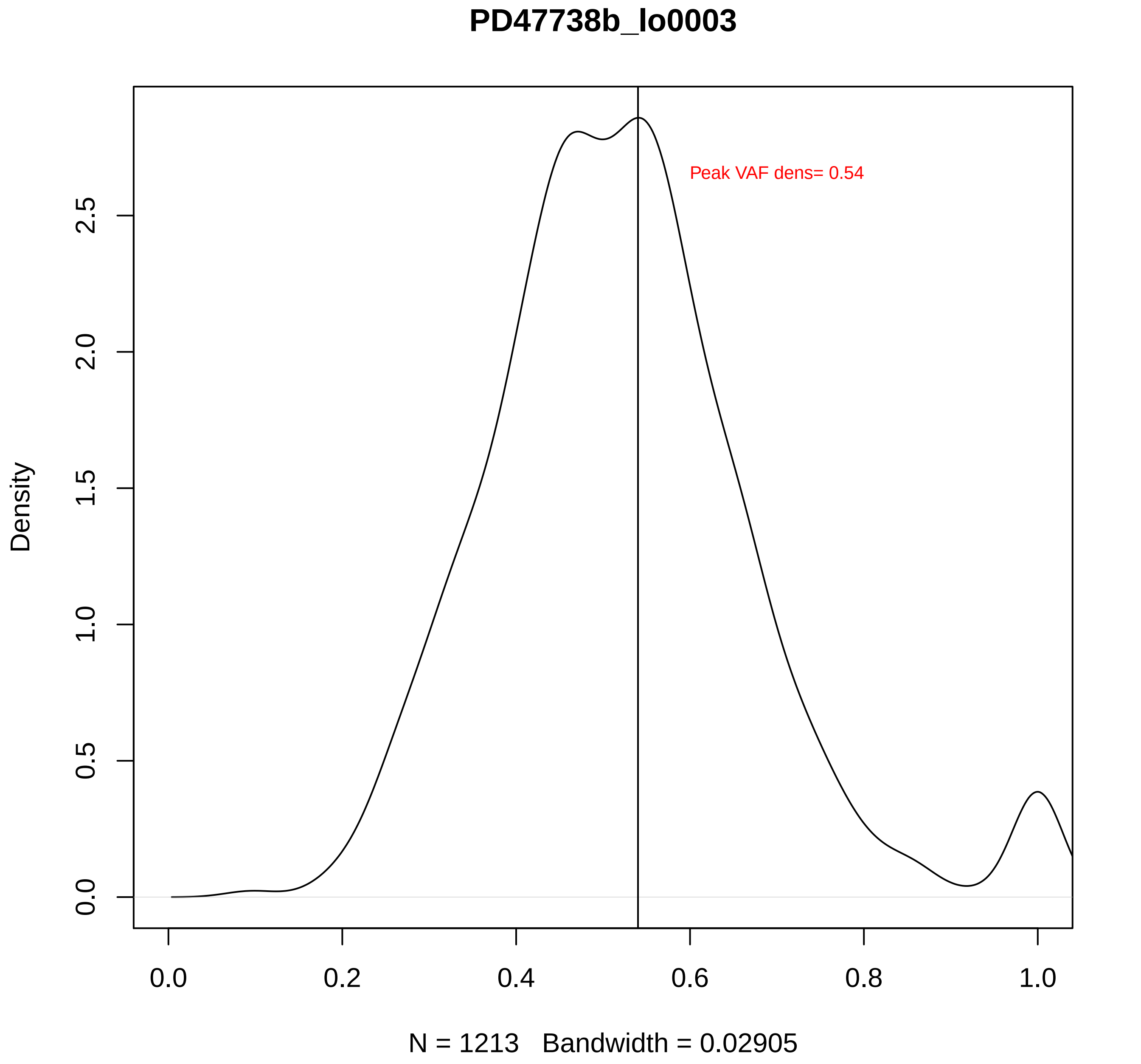
PD47738b_lo0003
Peak VAF dens= 0.54
Density
0.0
0.5
1.0
1.5
2.0
2.5
0.0
0.2
0.4
0.6
0.8
1.0
N = 1213   Bandwidth = 0.02905

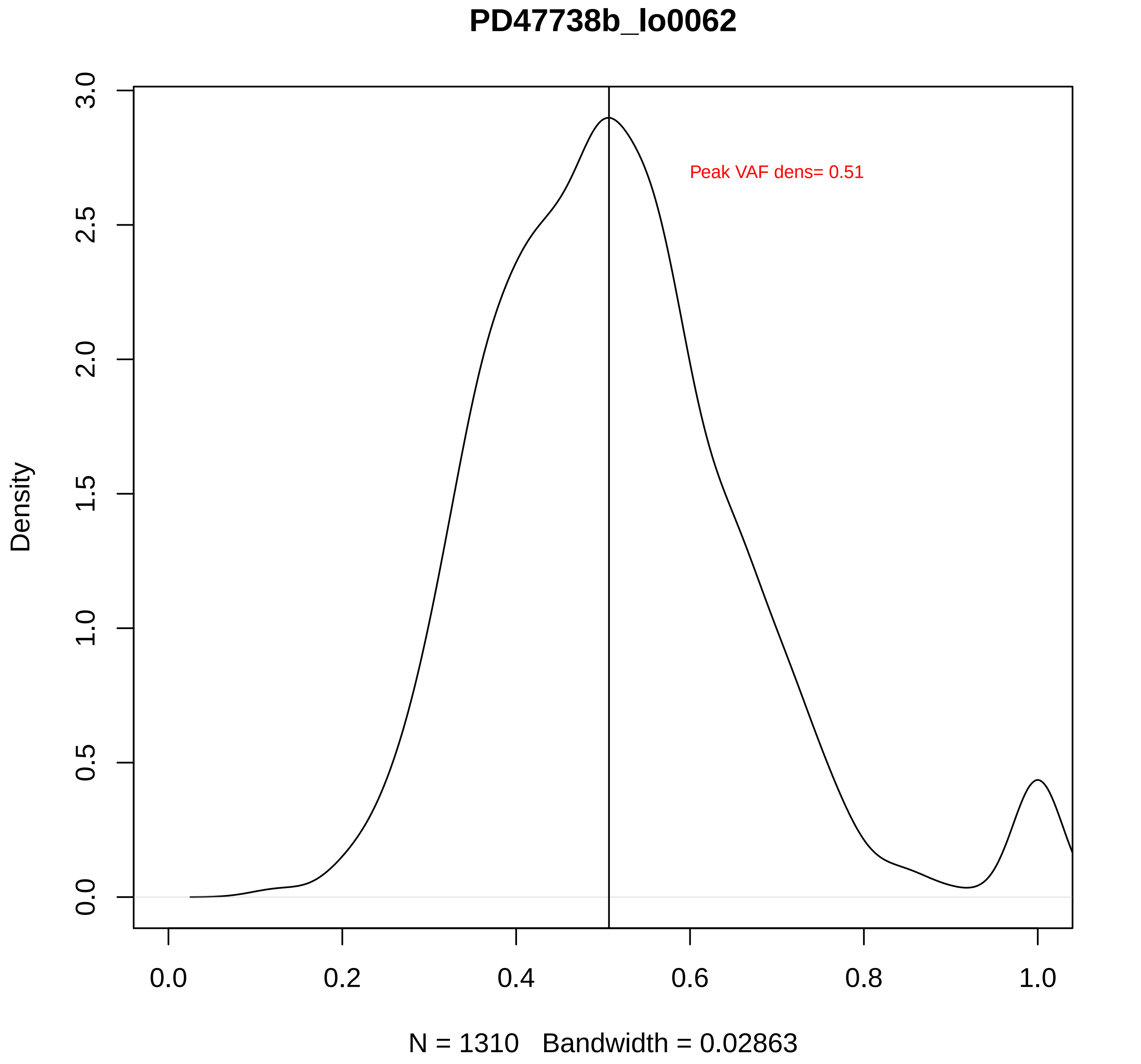
PD47738b_lo0062
Peak VAF dens= 0.51
Density
0.0
0.5
1.0
1.5
2.0
2.5
3.0
0.0
0.2
0.4
0.6
0.8
1.0
N = 1310   Bandwidth = 0.02863

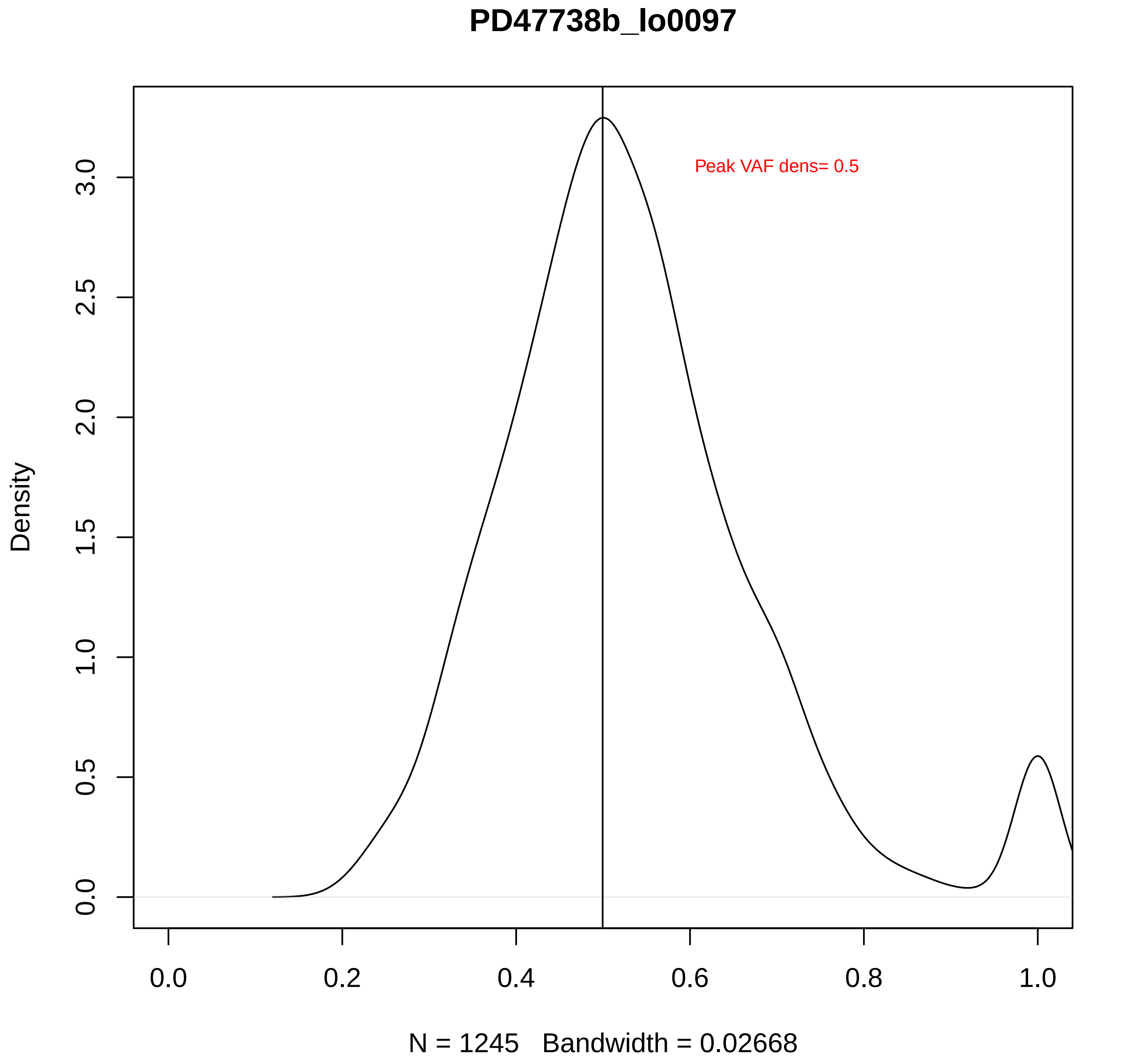
PD47738b_lo0097
Peak VAF dens= 0.5
Density
0.0
0.5
1.0
1.5
2.0
2.5
3.0
0.0
0.2
0.4
0.6
0.8
1.0
N = 1245 Bandwidth = 0.02668

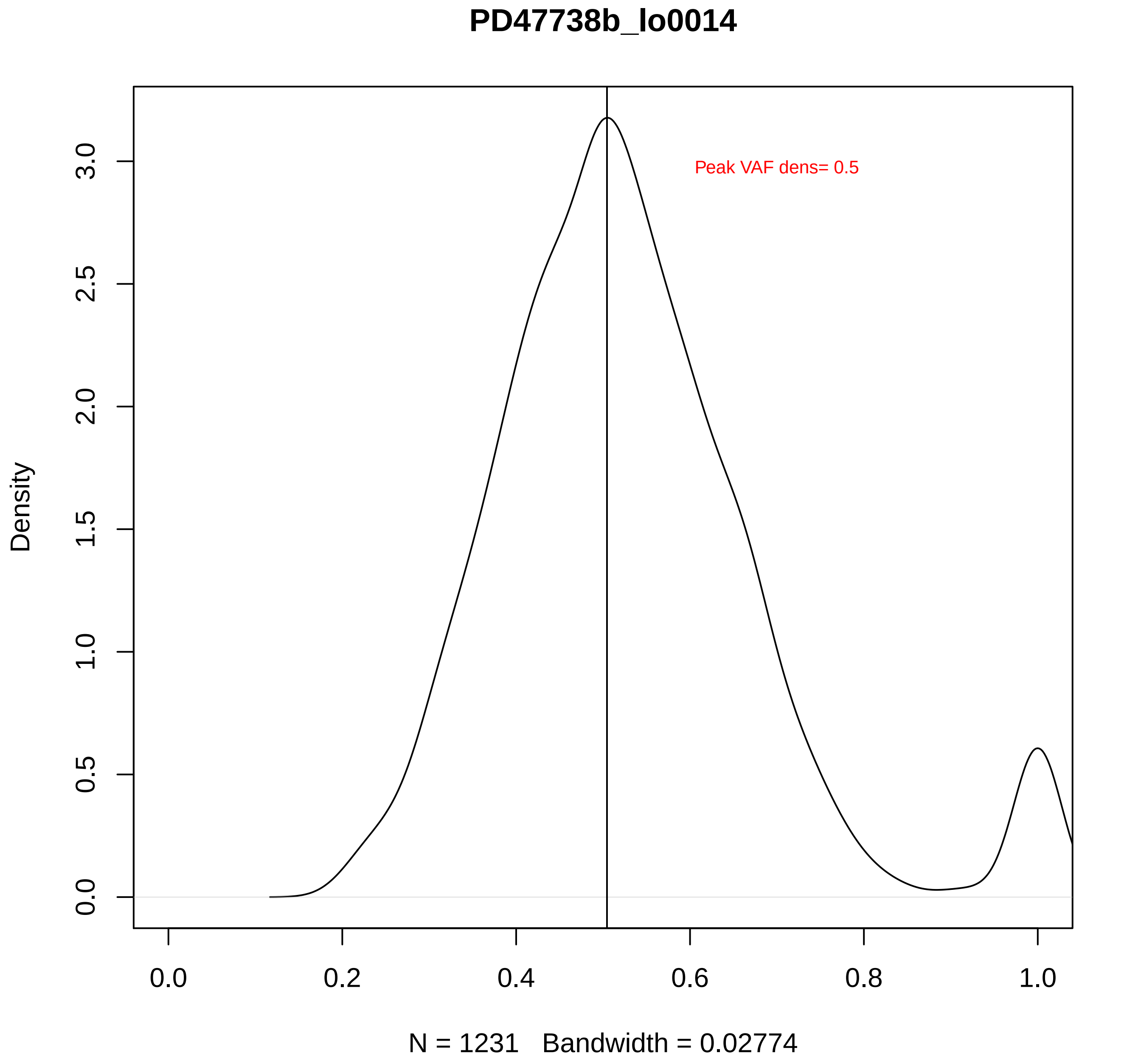
PD47738b_lo0014
Peak VAF dens= 0.5
Density
0.0
0.5
1.0
1.5
2.0
2.5
3.0
0.0
0.2
0.4
0.6
0.8
1.0
N = 1231   Bandwidth = 0.02774

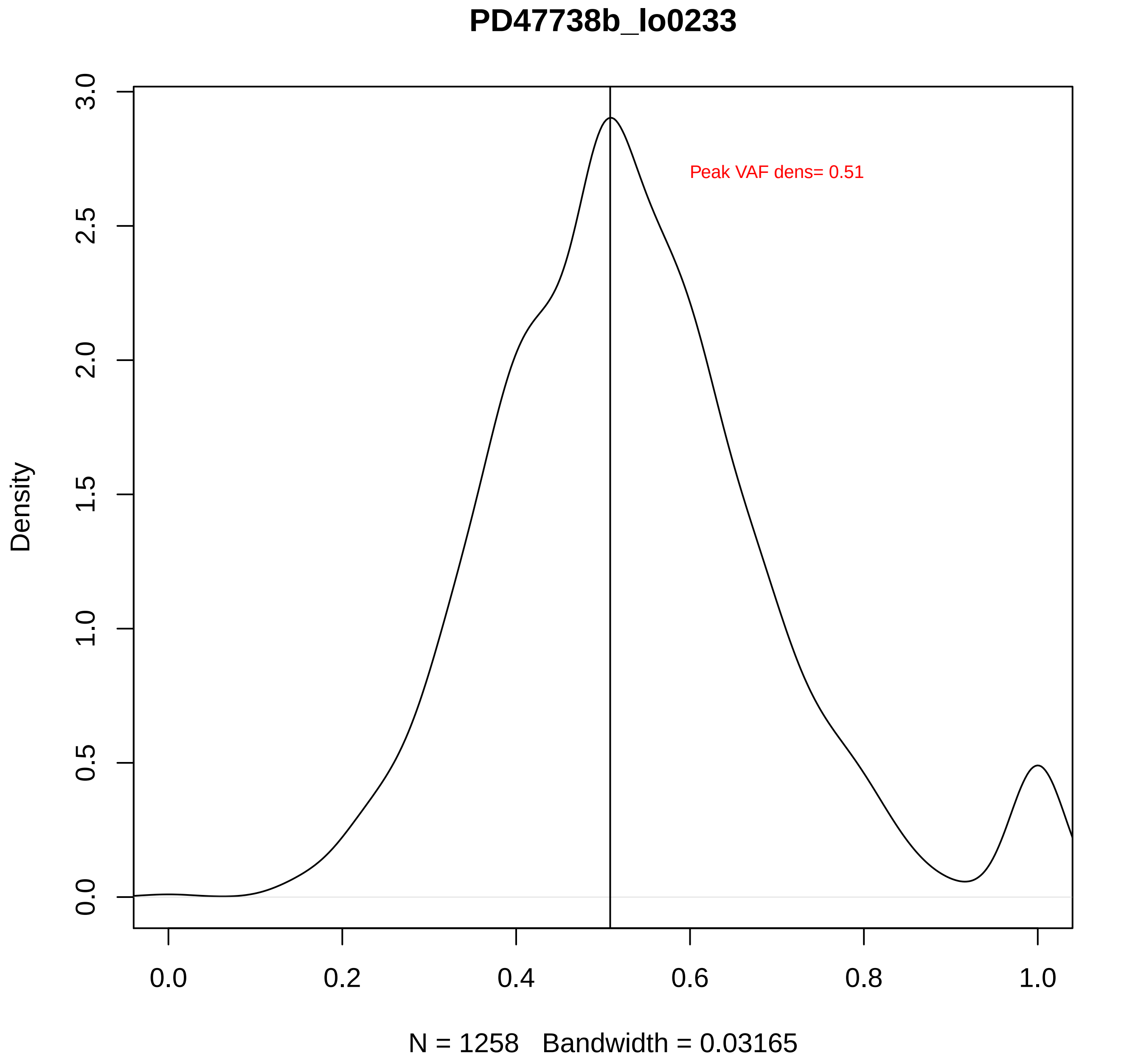

PD47738b_lo0233
Peak VAF dens= 0.51
Density
0.0
0.5
1.0
1.5
2.0
2.5
3.0
0.0
0.2
0.4
0.6
0.8
1.0
N = 1258 Bandwidth = 0.03165

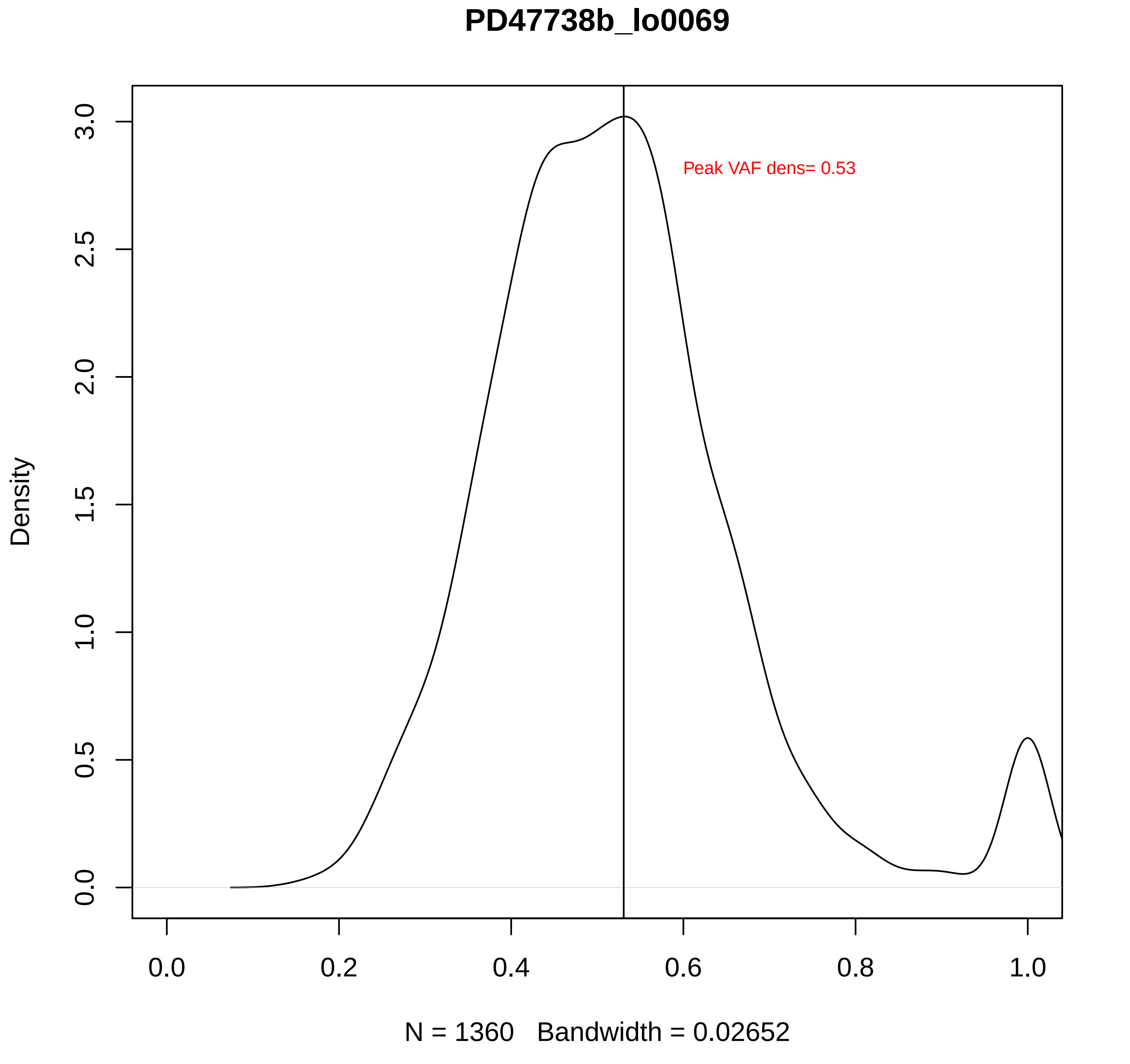
PD47738b_lo0069
Peak VAF dens= 0.53
Density
0.0
0.5
1.0
1.5
2.0
2.5
3.0
0.0
0.2
0.4
0.6
0.8
1.0
N = 1360   Bandwidth = 0.02652

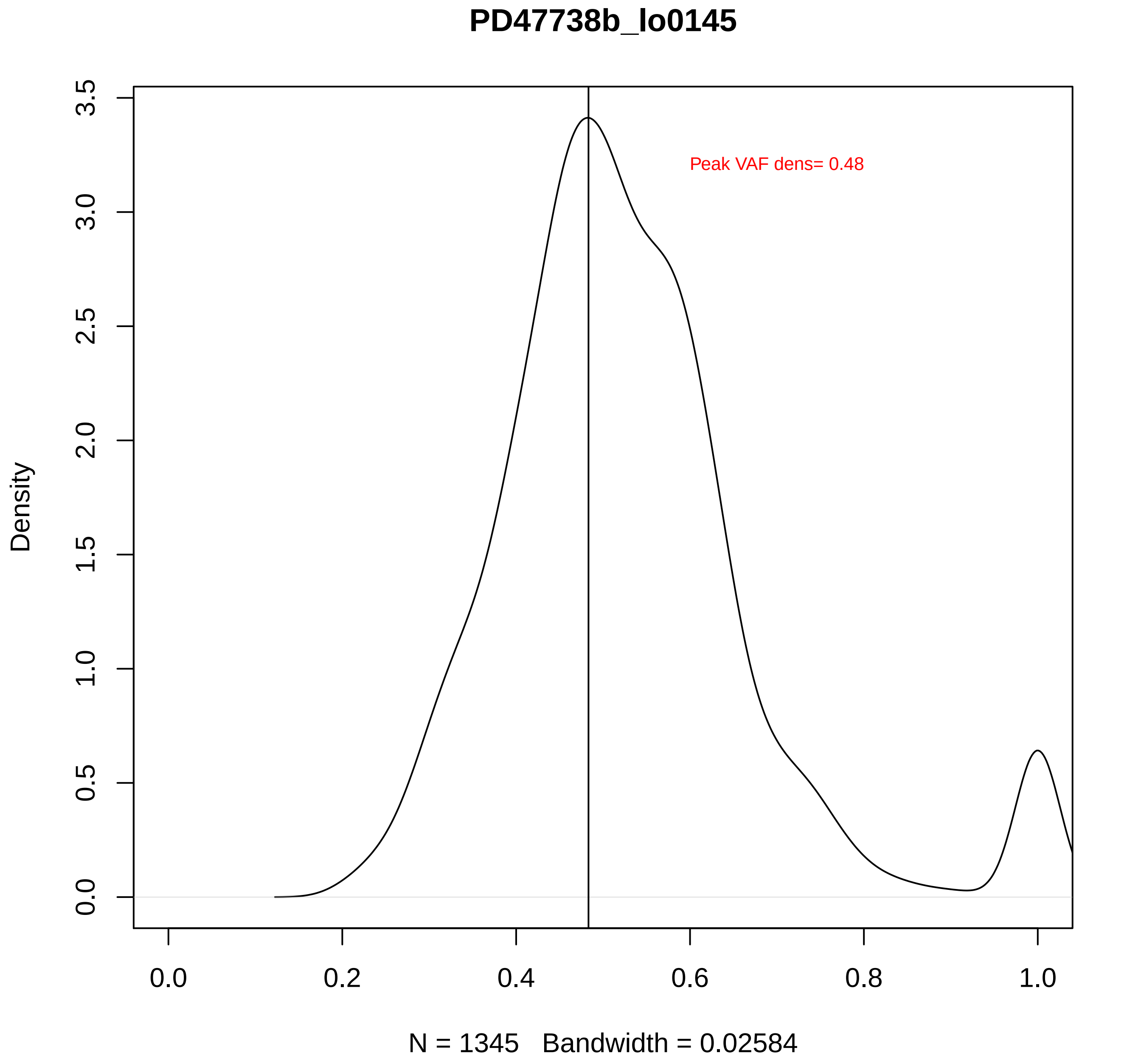

PD47738b_lo0145
Peak VAF dens= 0.48
Density
0.0
0.5
1.0
1.5
2.0
2.5
3.0
3.5
0.0
0.2
0.4
0.6
0.8
1.0
N = 1345   Bandwidth = 0.02584

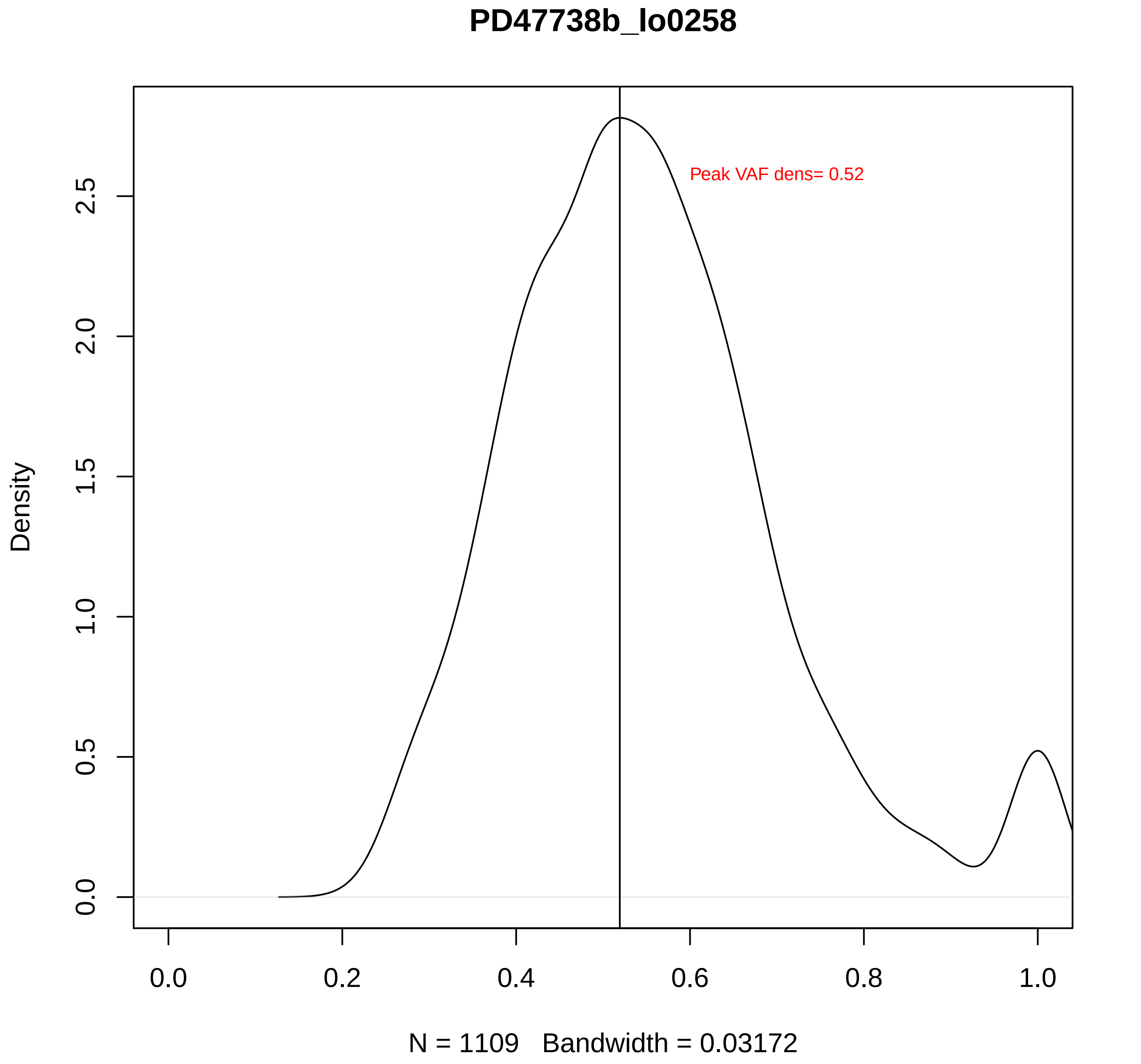

PD47738b_lo0258
Peak VAF dens= 0.52
Density
0.0
0.5
1.0
1.5
2.0
2.5
0.0
0.2
0.4
0.6
0.8
1.0
N = 1109   Bandwidth = 0.03172

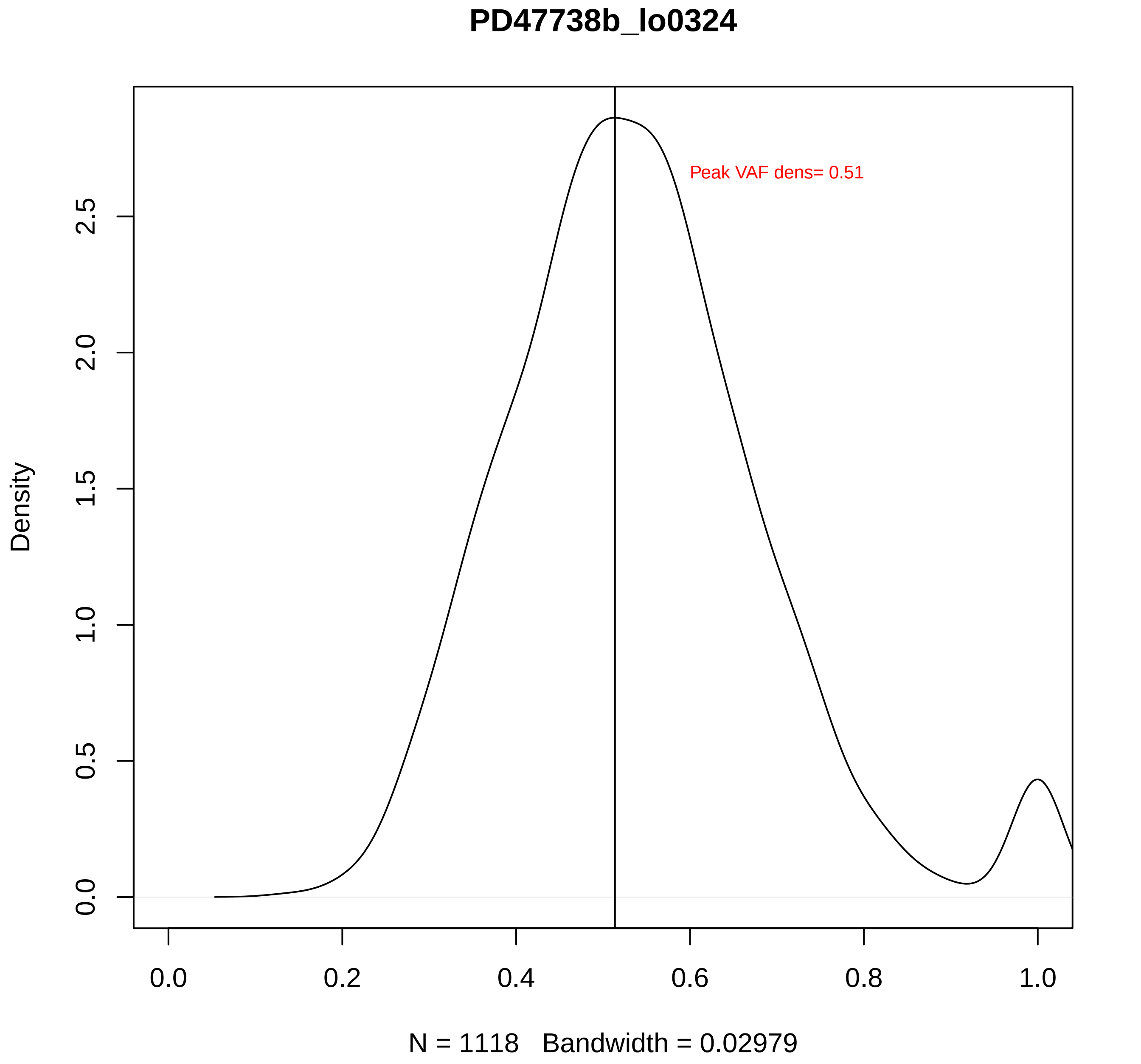

PD47738b_lo0324
Peak VAF dens= 0.51
Density
0.0
0.5
1.0
1.5
2.0
2.5
0.0
0.2
0.4
0.6
0.8
1.0
N = 1118   Bandwidth = 0.02979

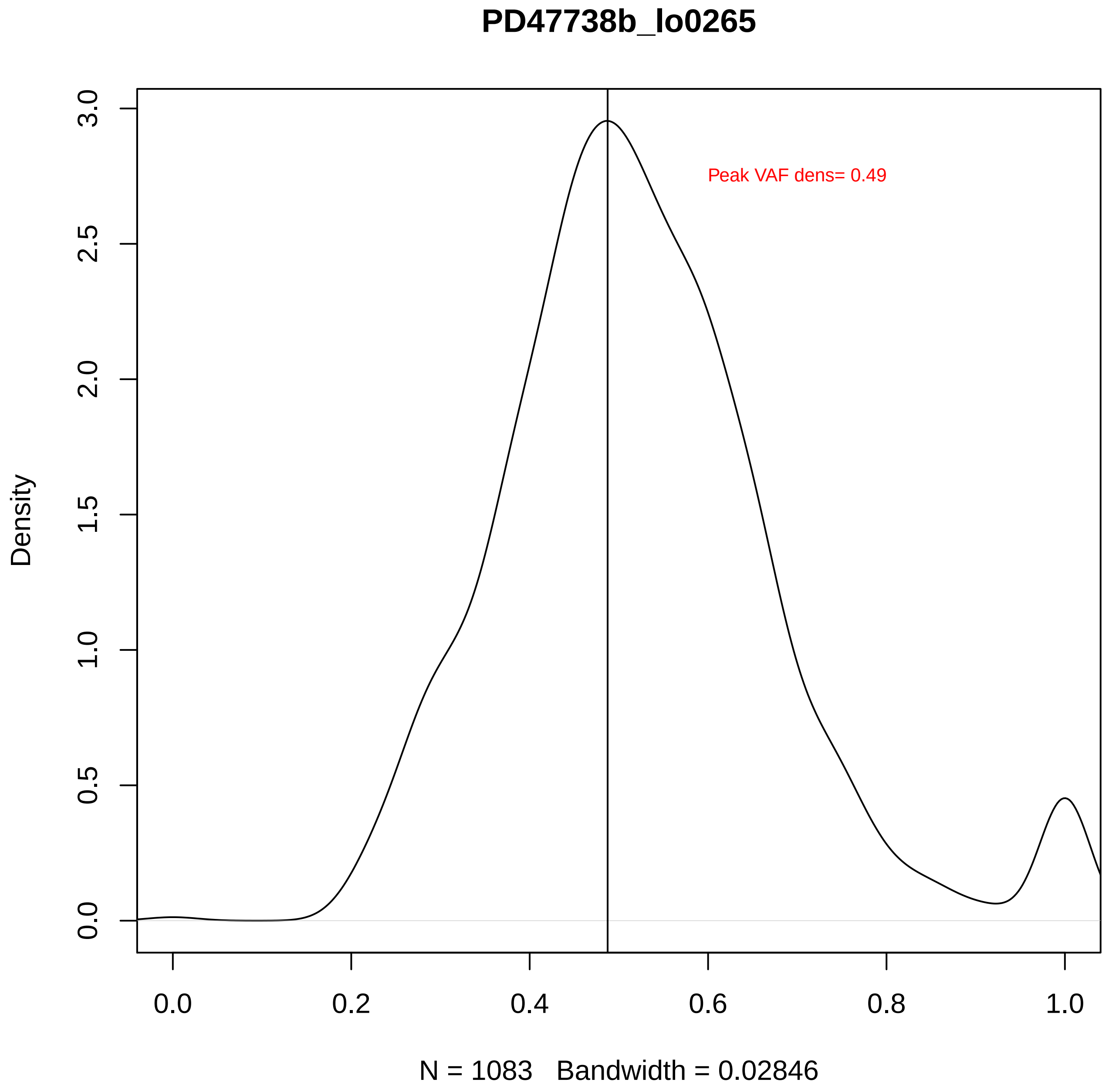
PD47738b_lo0265
Peak VAF dens= 0.49
Density
3.0
2.5
2.0
1.5
1.0
0.5
0.0
0.0
0.2
0.4
0.6
0.8
1.0
N = 1083   Bandwidth = 0.02846

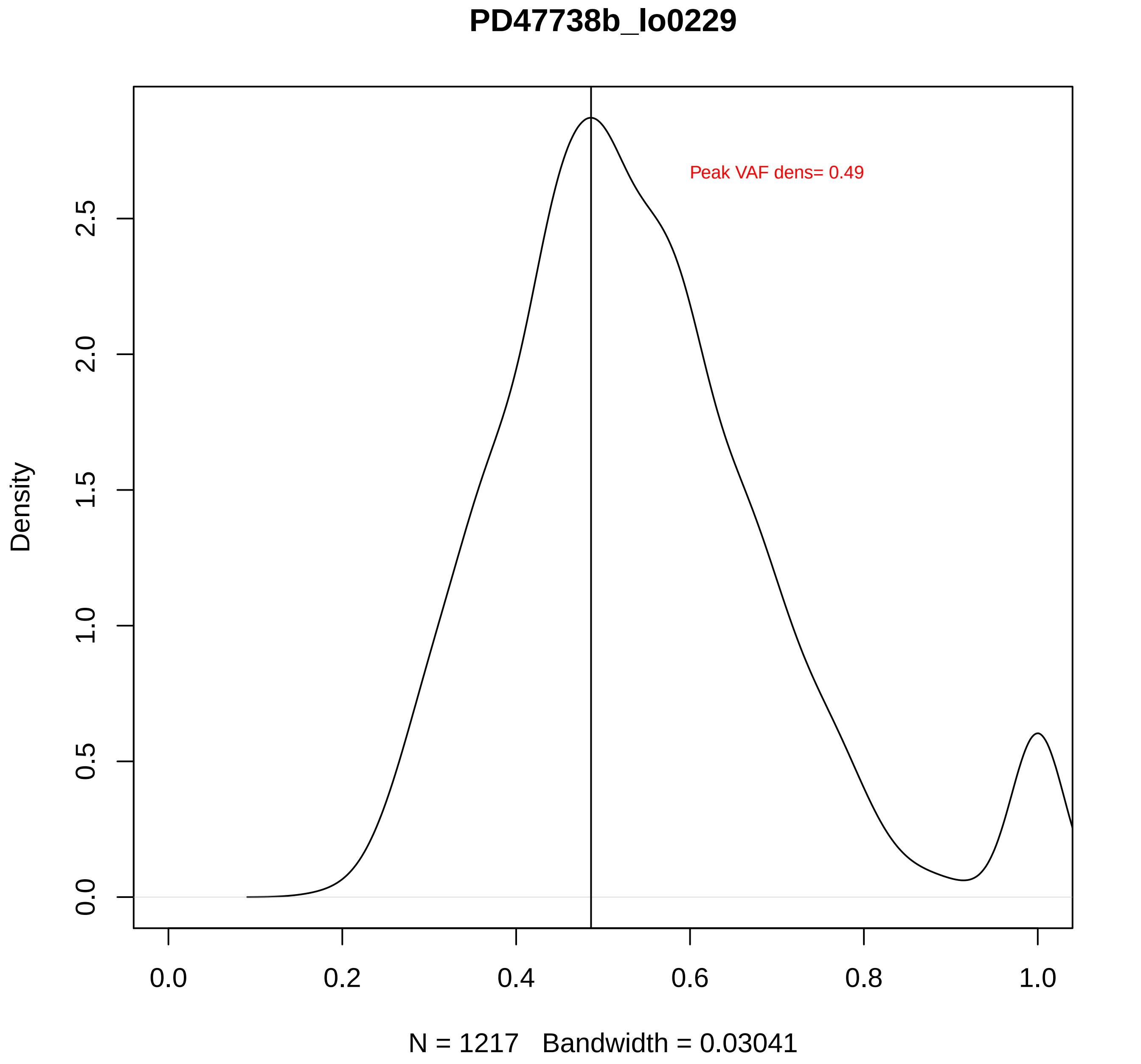

PD47738b_lo0229
Peak VAF dens= 0.49
Density
0.0
0.5
1.0
1.5
2.0
2.5
0.0
0.2
0.4
0.6
0.8
1.0
N = 1217   Bandwidth = 0.03041

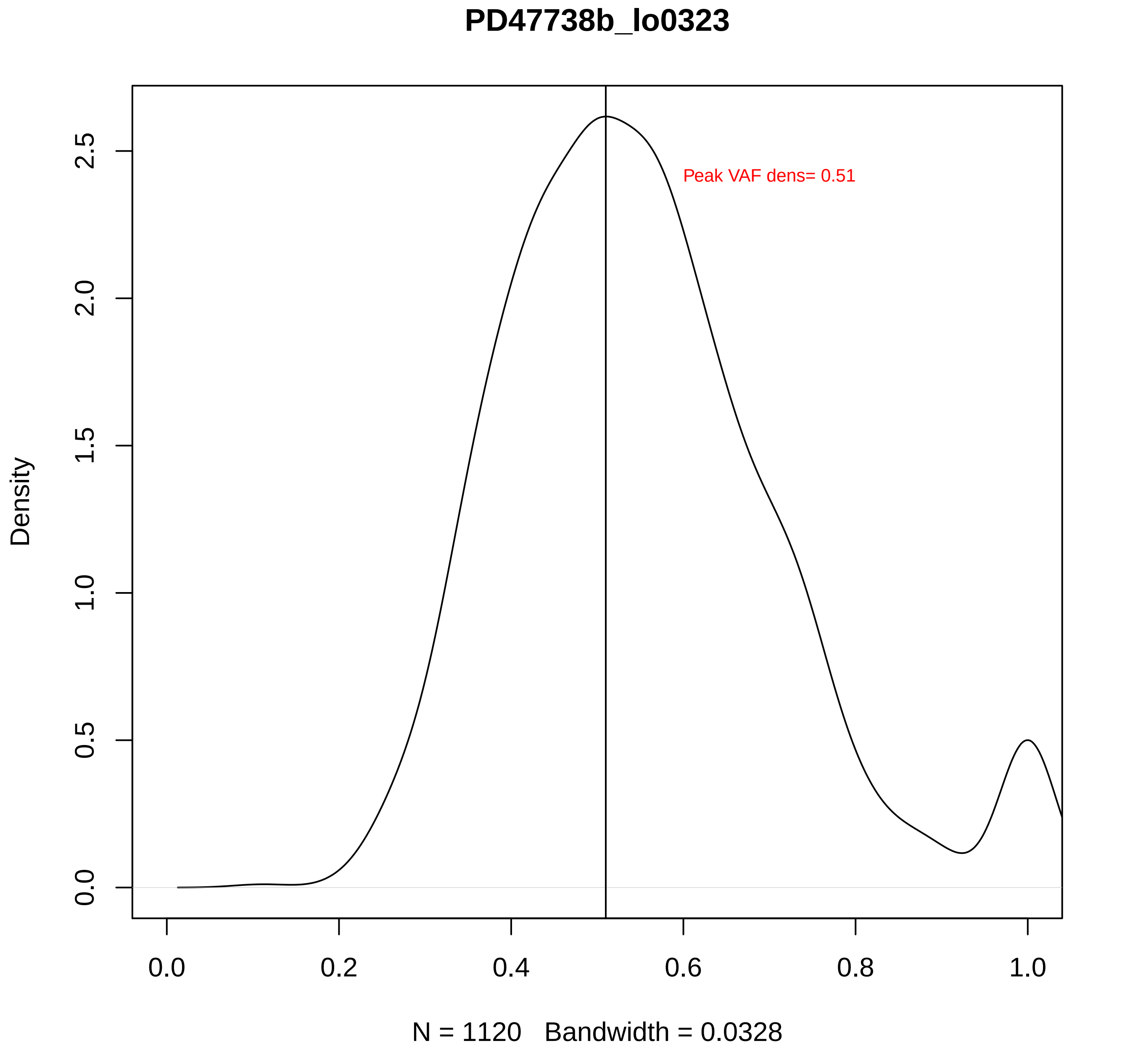
PD47738b_lo0323
Peak VAF dens= 0.51
Density
0.0
0.5
1.0
1.5
2.0
2.5
0.0
0.2
0.4
0.6
0.8
1.0
N = 1120   Bandwidth = 0.0328

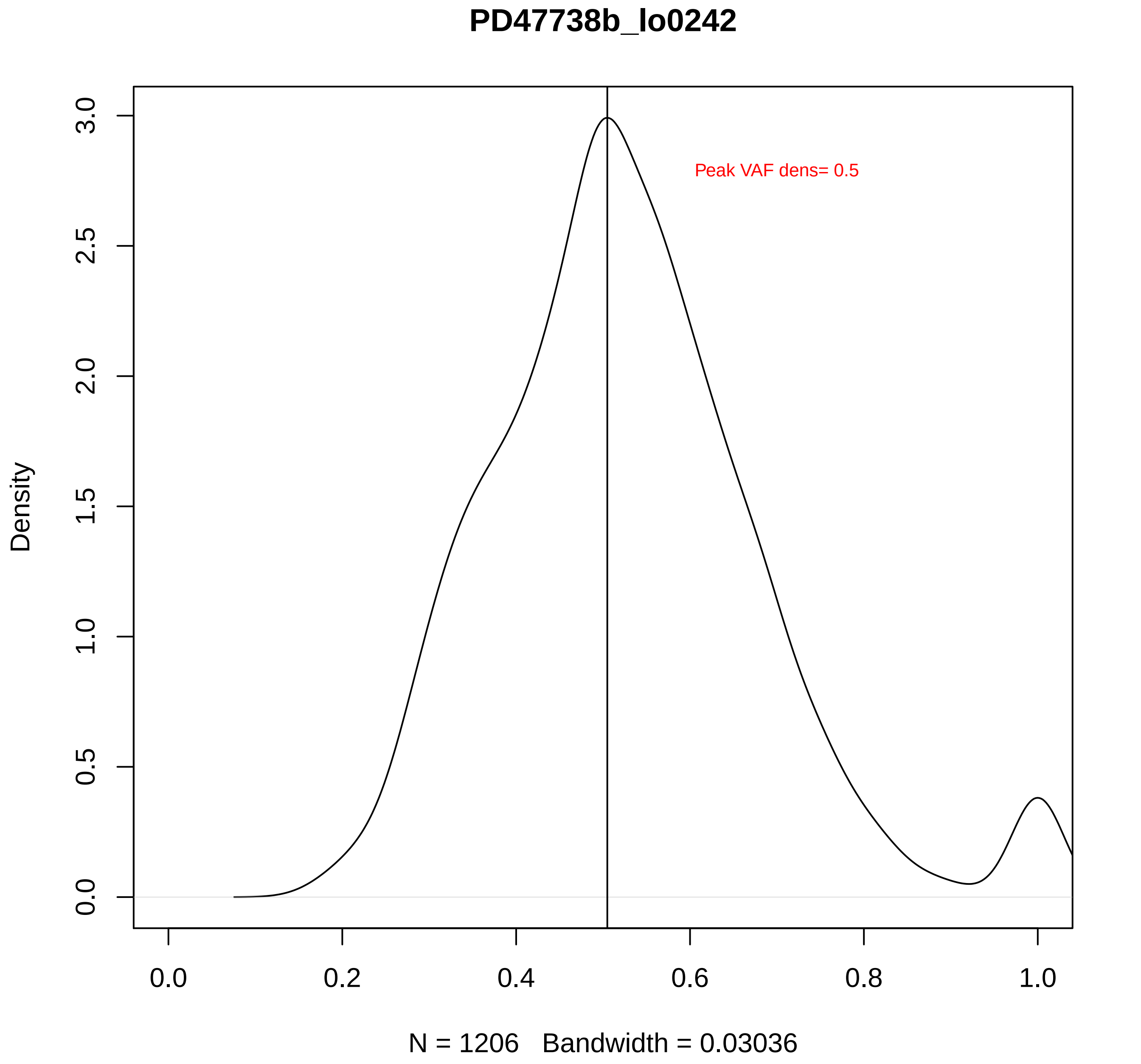

PD47738b_lo0242
Peak VAF dens= 0.5
Density
3.0
2.5
2.0
1.5
1.0
0.5
0.0
0.0
0.2
0.4
0.6
0.8
1.0
N = 1206   Bandwidth = 0.03036

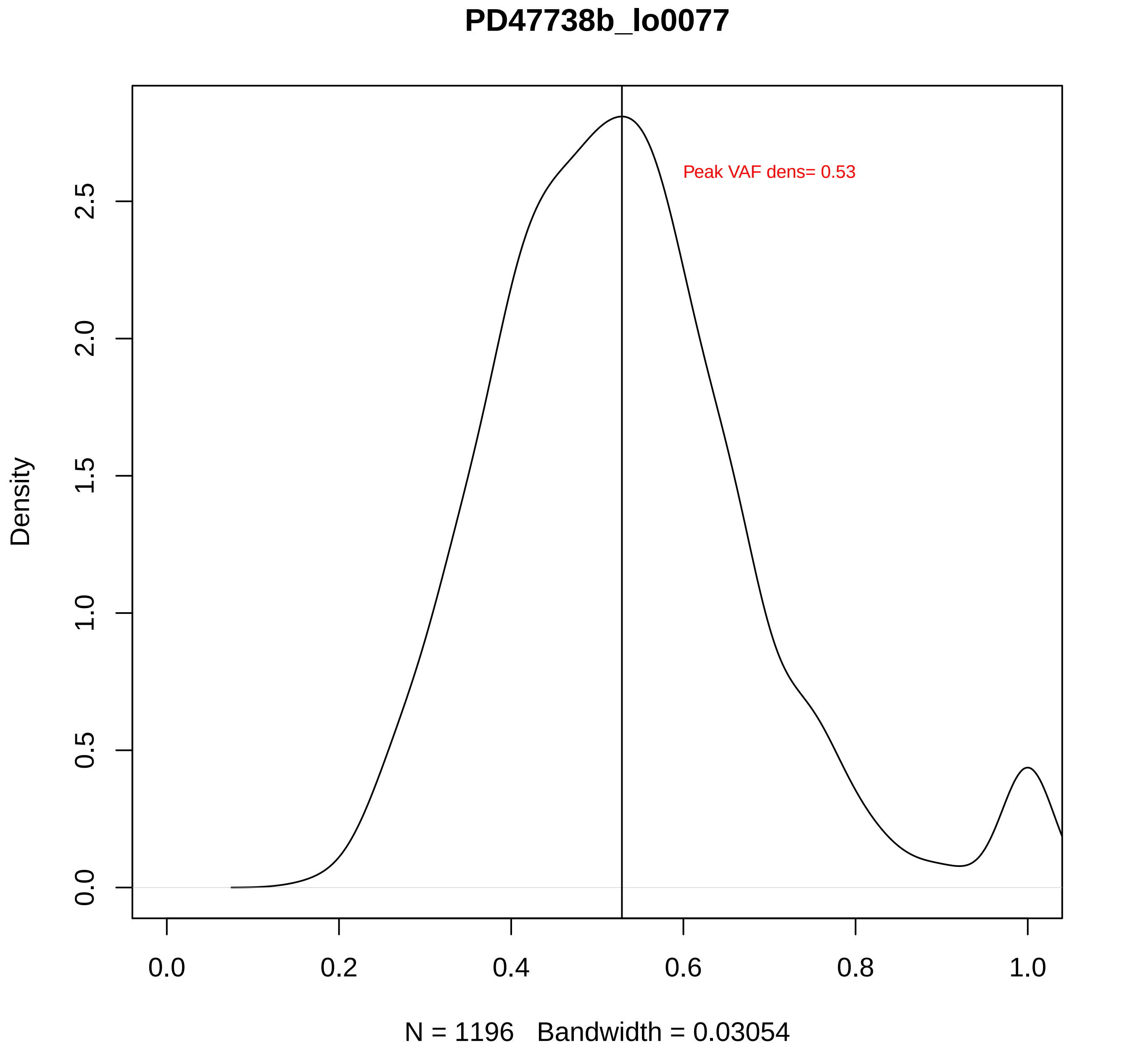
PD47738b_lo0077
Peak VAF dens= 0.53
Density
0.0
0.5
1.0
1.5
2.0
2.5
0.0
0.2
0.4
0.6
0.8
1.0
N = 1196   Bandwidth = 0.03054

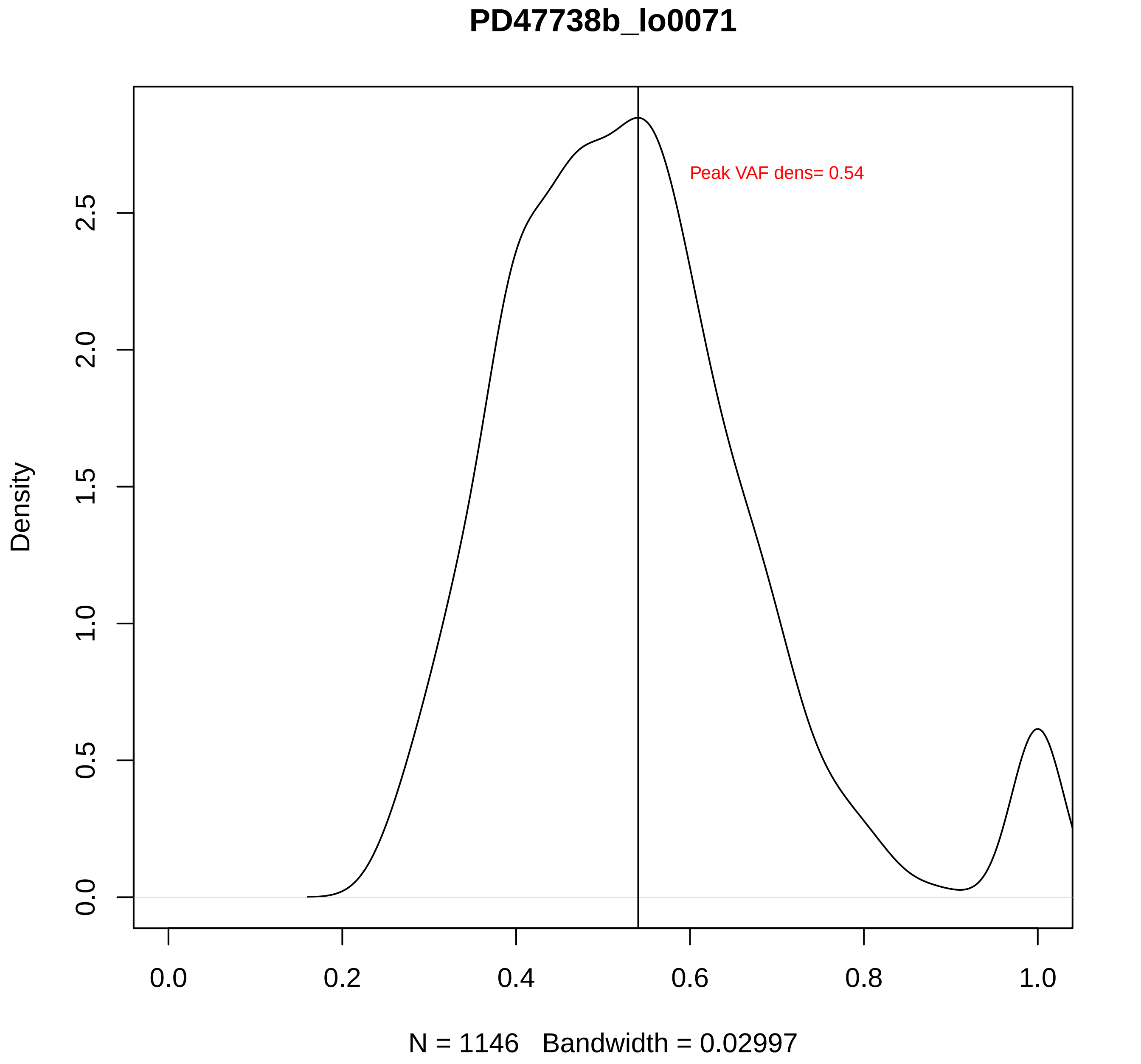

PD47738b_lo0071
Peak VAF dens= 0.54
Density
0.0
0.5
1.0
1.5
2.0
2.5
0.0
0.2
0.4
0.6
0.8
1.0
N = 1146   Bandwidth = 0.02997

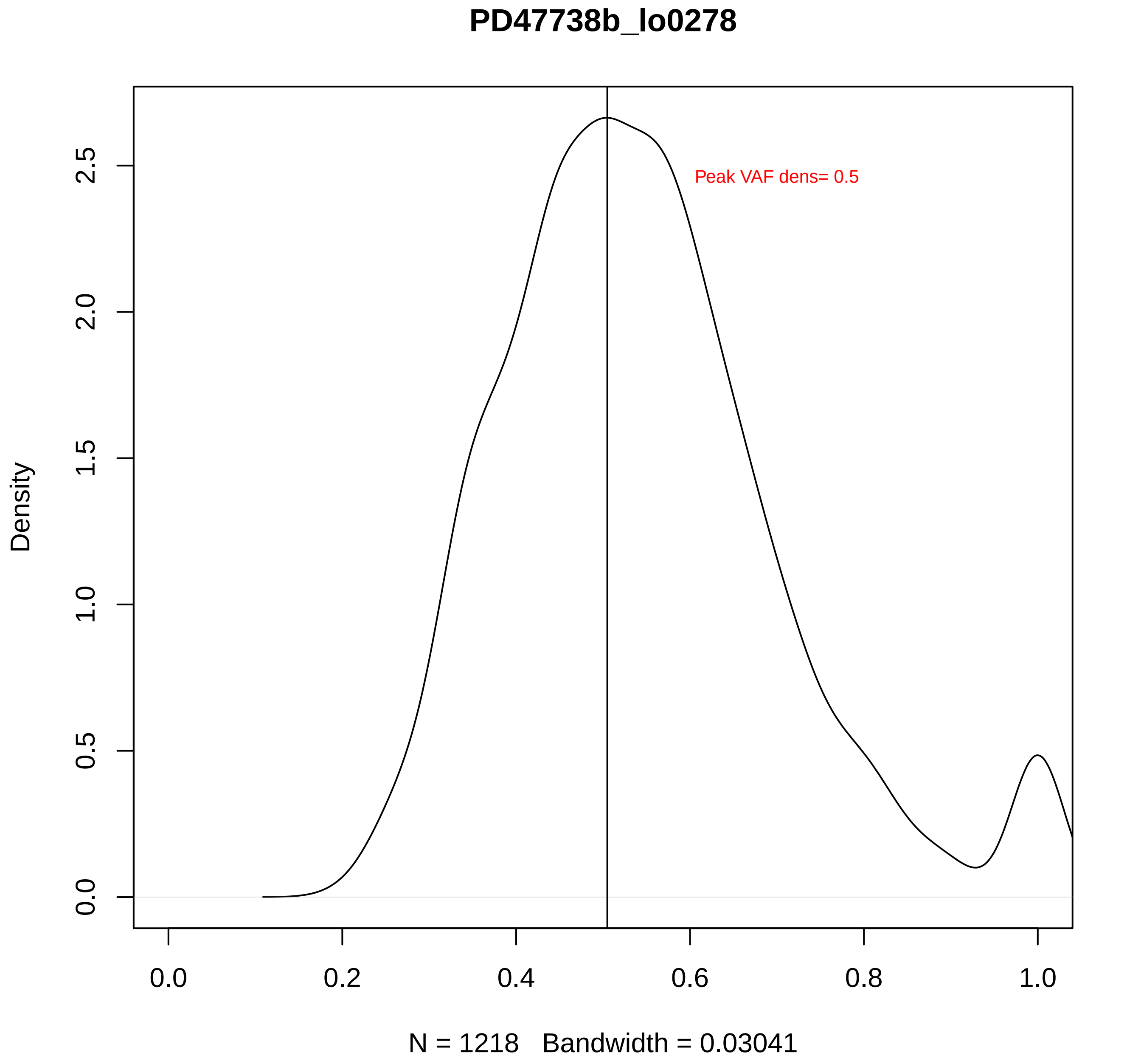
PD47738b_lo0278
Peak VAF dens= 0.5
Density
0.0
0.5
1.0
1.5
2.0
2.5
0.0
0.2
0.4
0.6
0.8
1.0
N = 1218 Bandwidth = 0.03041

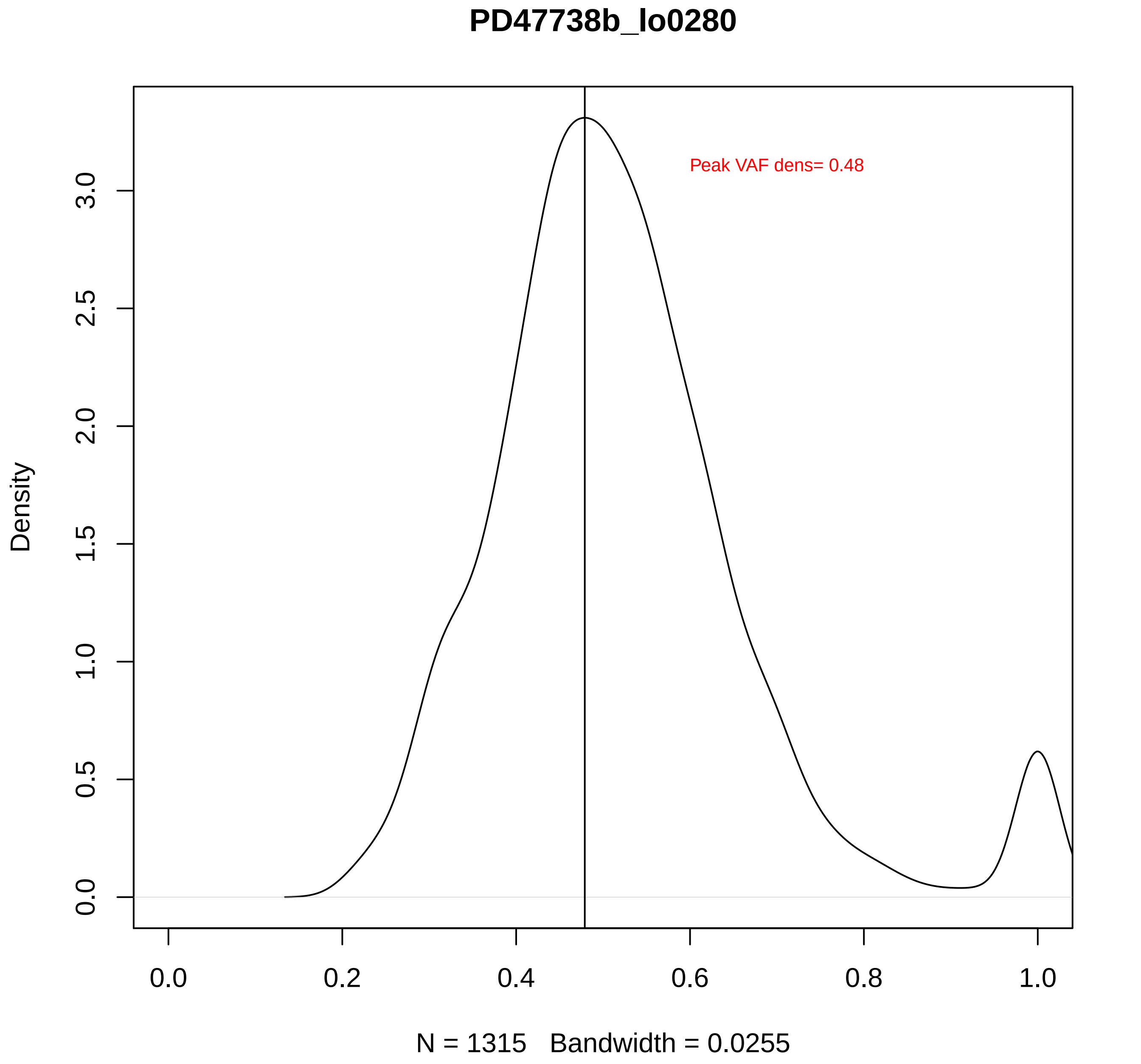
PD47738b_lo0280
Peak VAF dens= 0.48
Density
0.0
0.5
1.0
1.5
2.0
2.5
3.0
0.0
0.2
0.4
0.6
0.8
1.0
N = 1315   Bandwidth = 0.0255

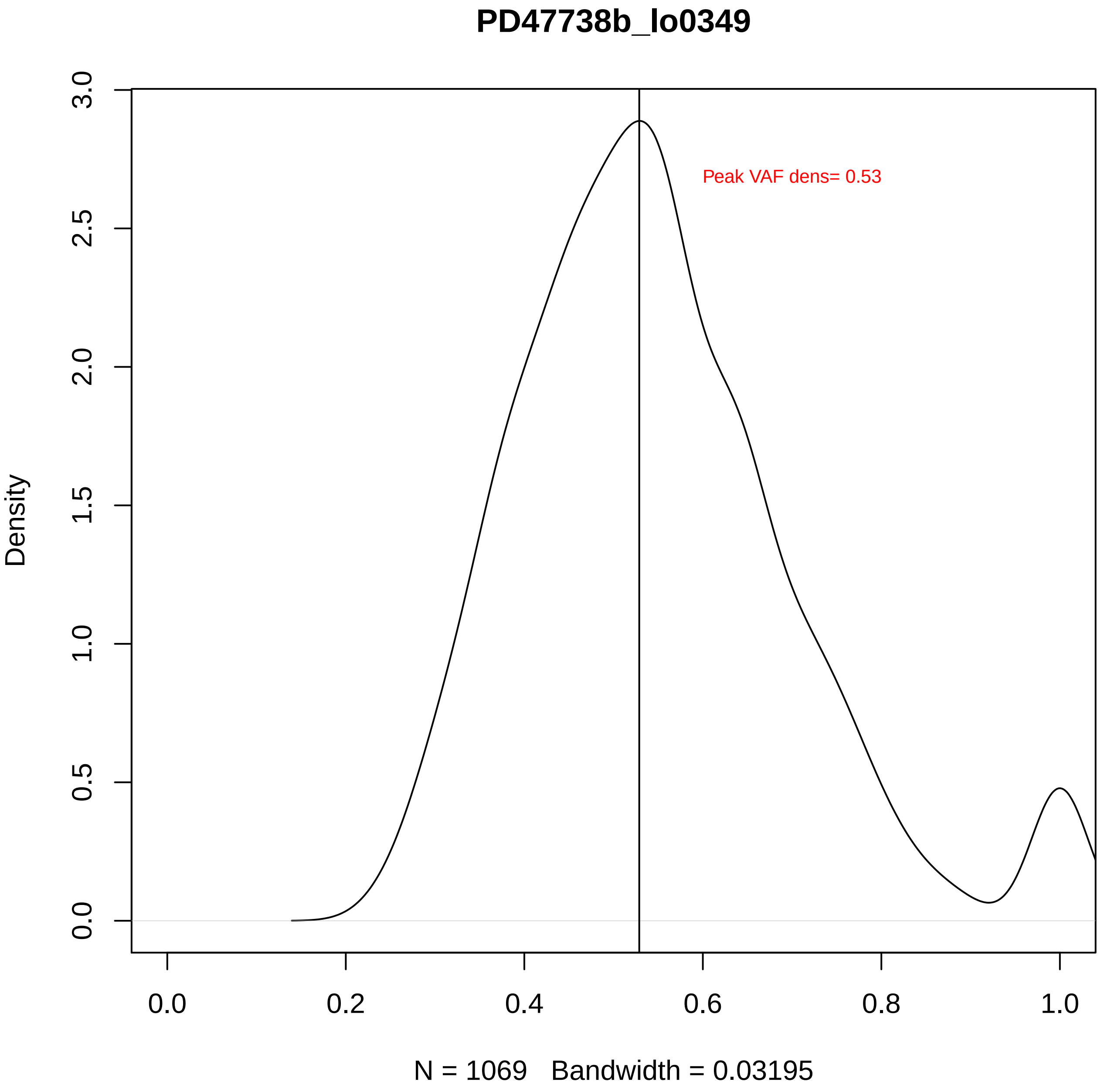
PD47738b_lo0349
Peak VAF dens= 0.53
Density
3.0
2.5
2.0
1.5
1.0
0.5
0.0
0.0
0.2
0.4
0.6
0.8
1.0
N = 1069   Bandwidth = 0.03195

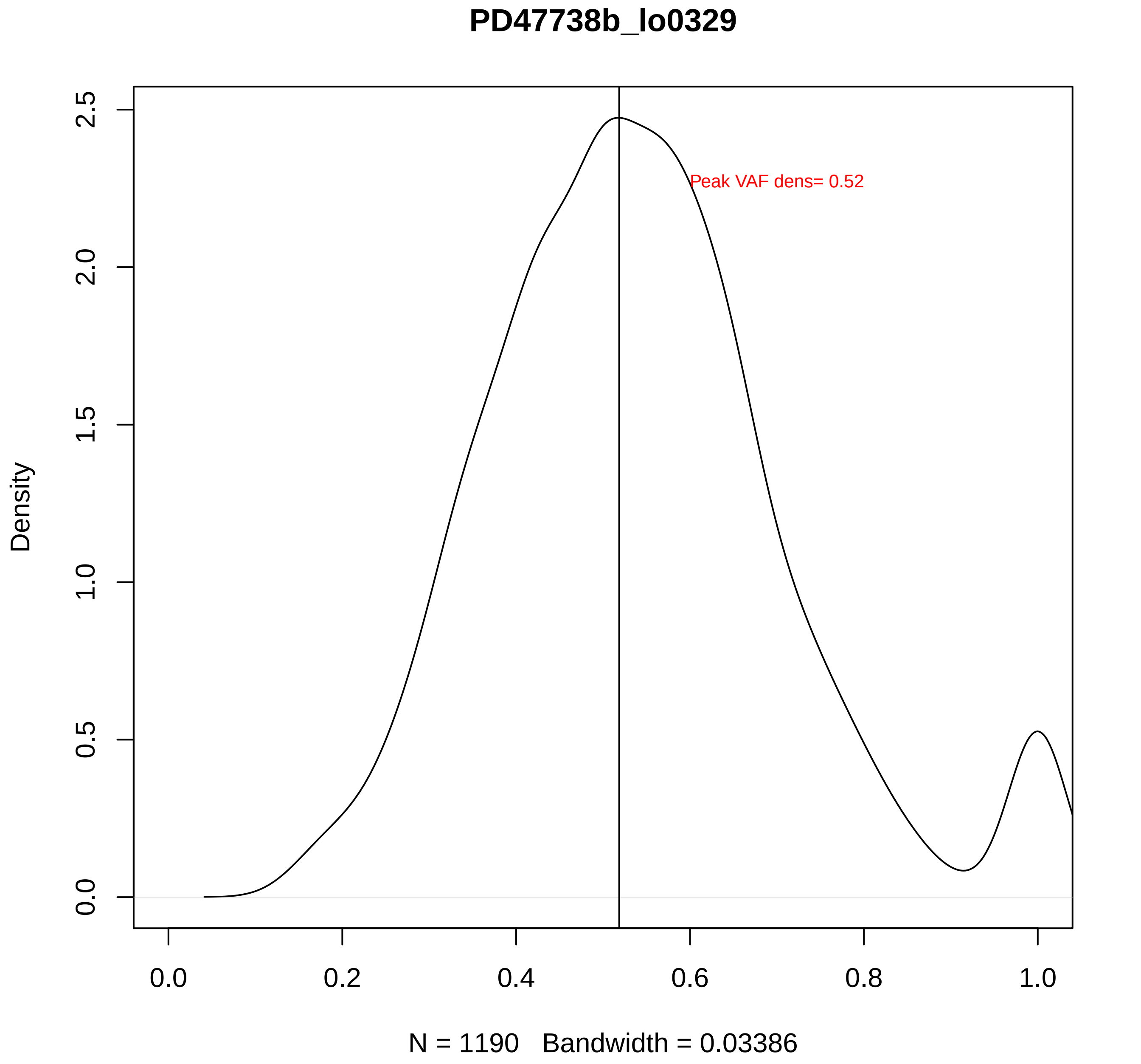
PD47738b_lo0329
Peak VAF dens= 0.52
Density
0.0
0.5
1.0
1.5
2.0
2.5
0.0
0.2
0.4
0.6
0.8
1.0
N = 1190   Bandwidth = 0.03386

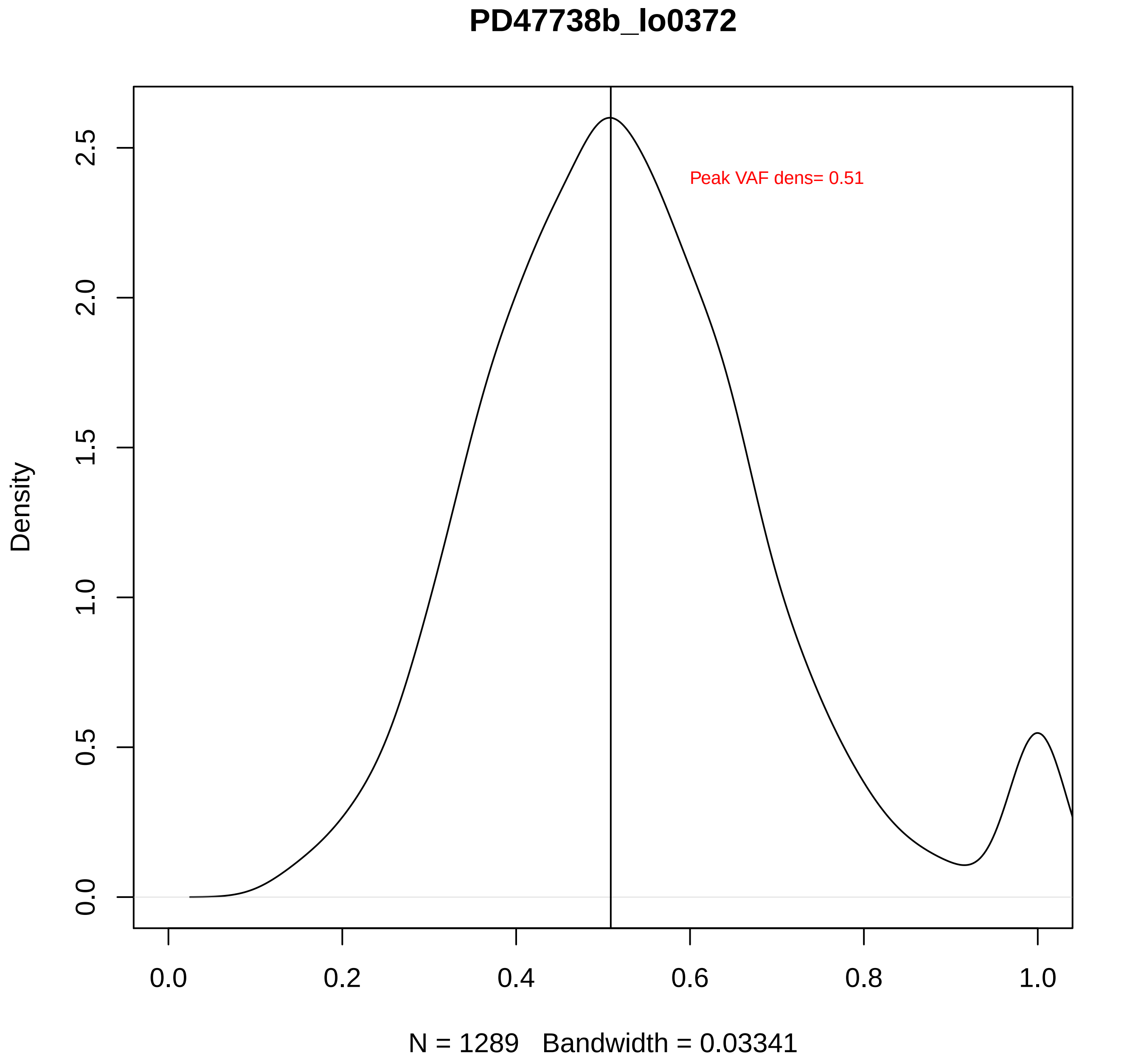
PD47738b_lo0372
Peak VAF dens= 0.51
Density
0.0
0.5
1.0
1.5
2.0
2.5
0.0
0.2
0.4
0.6
0.8
1.0
N = 1289   Bandwidth = 0.03341

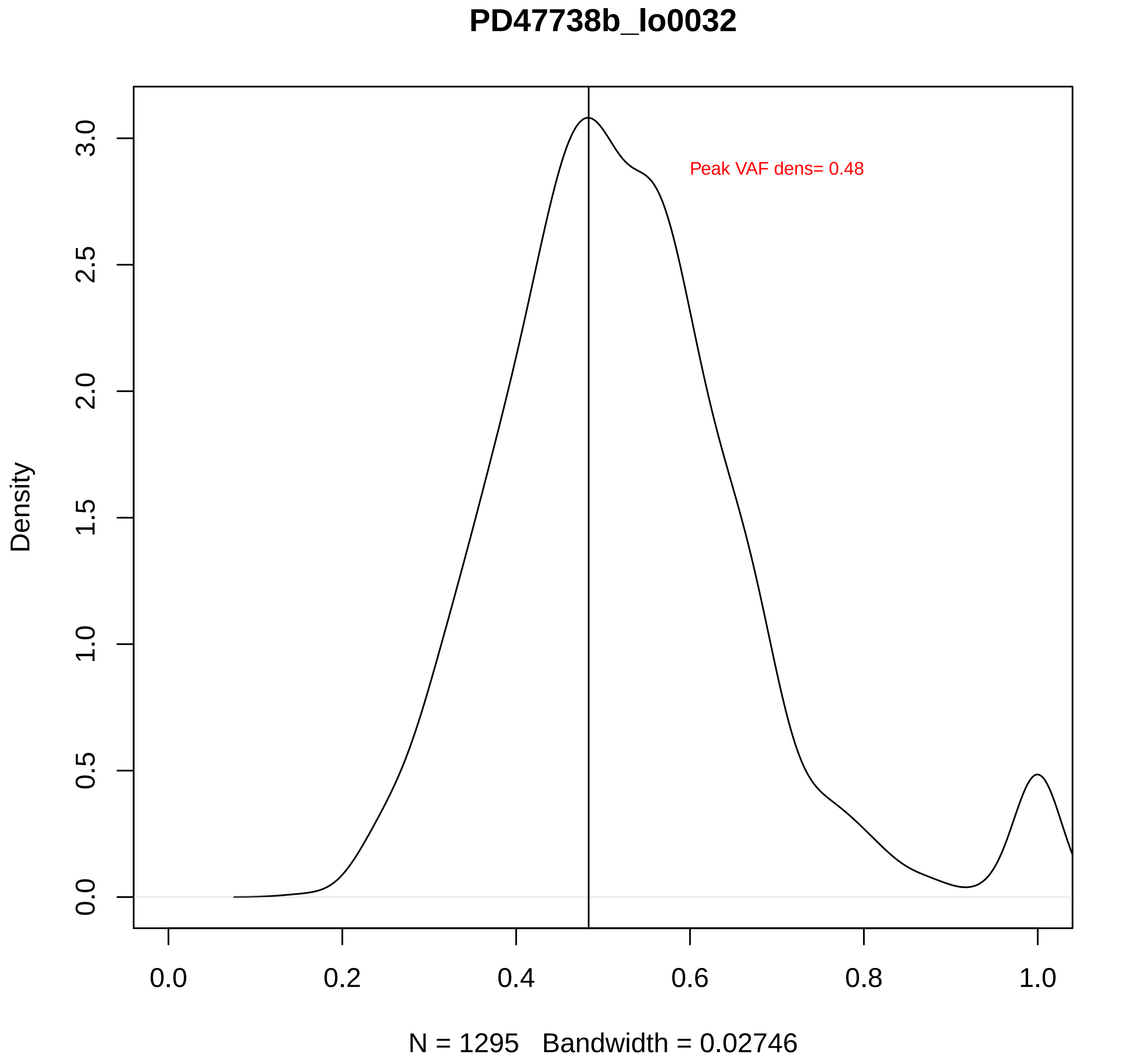
PD47738b_lo0032
Peak VAF dens= 0.48
Density
3.0
2.5
2.0
1.5
1.0
0.5
0.0
0.0
0.2
0.4
0.6
0.8
1.0
N = 1295   Bandwidth = 0.02746

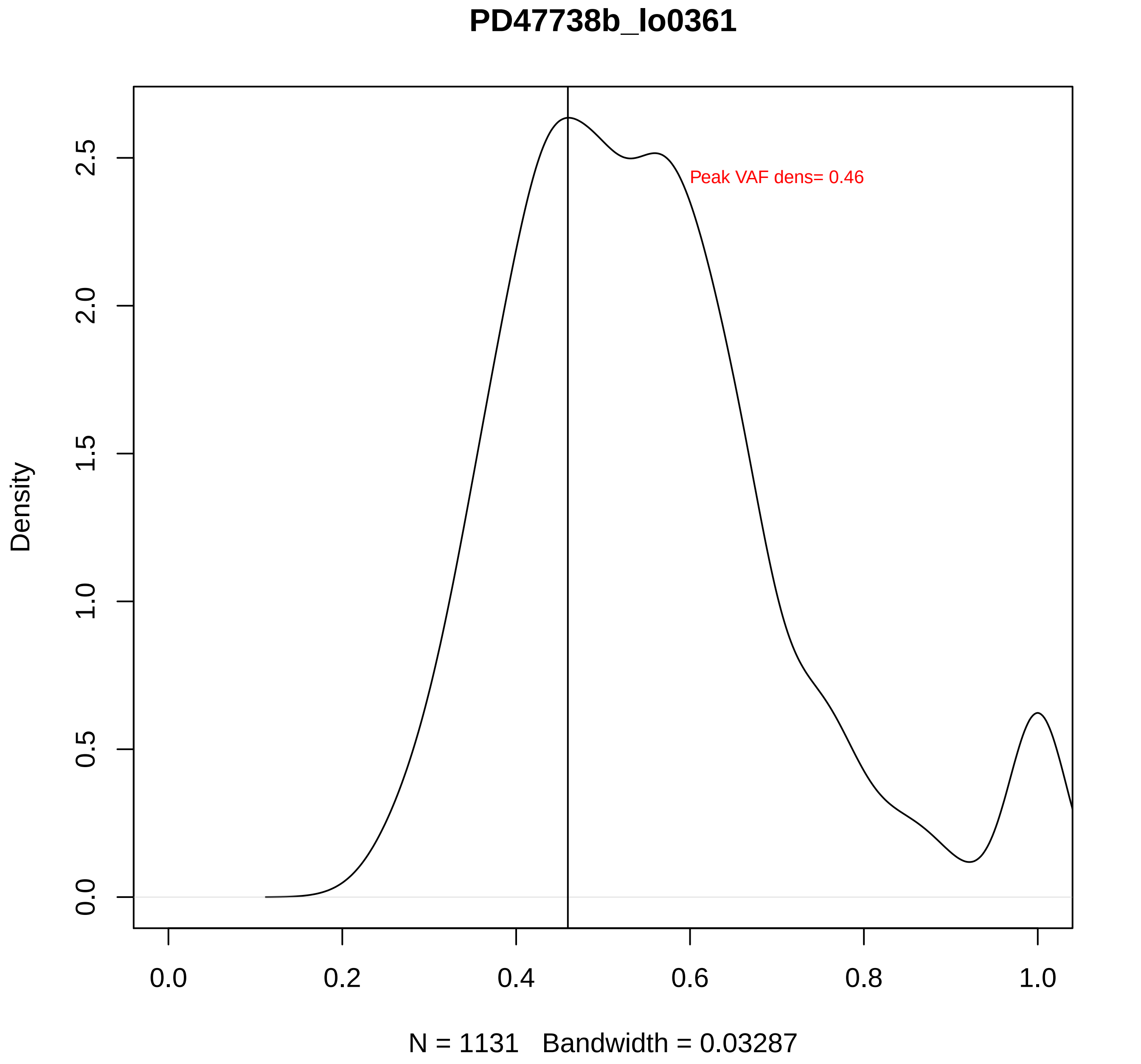
PD47738b_lo0361
Peak VAF dens= 0.46
Density
0.0
0.5
1.0
1.5
2.0
2.5
0.0
0.2
0.4
0.6
0.8
1.0
N = 1131   Bandwidth = 0.03287

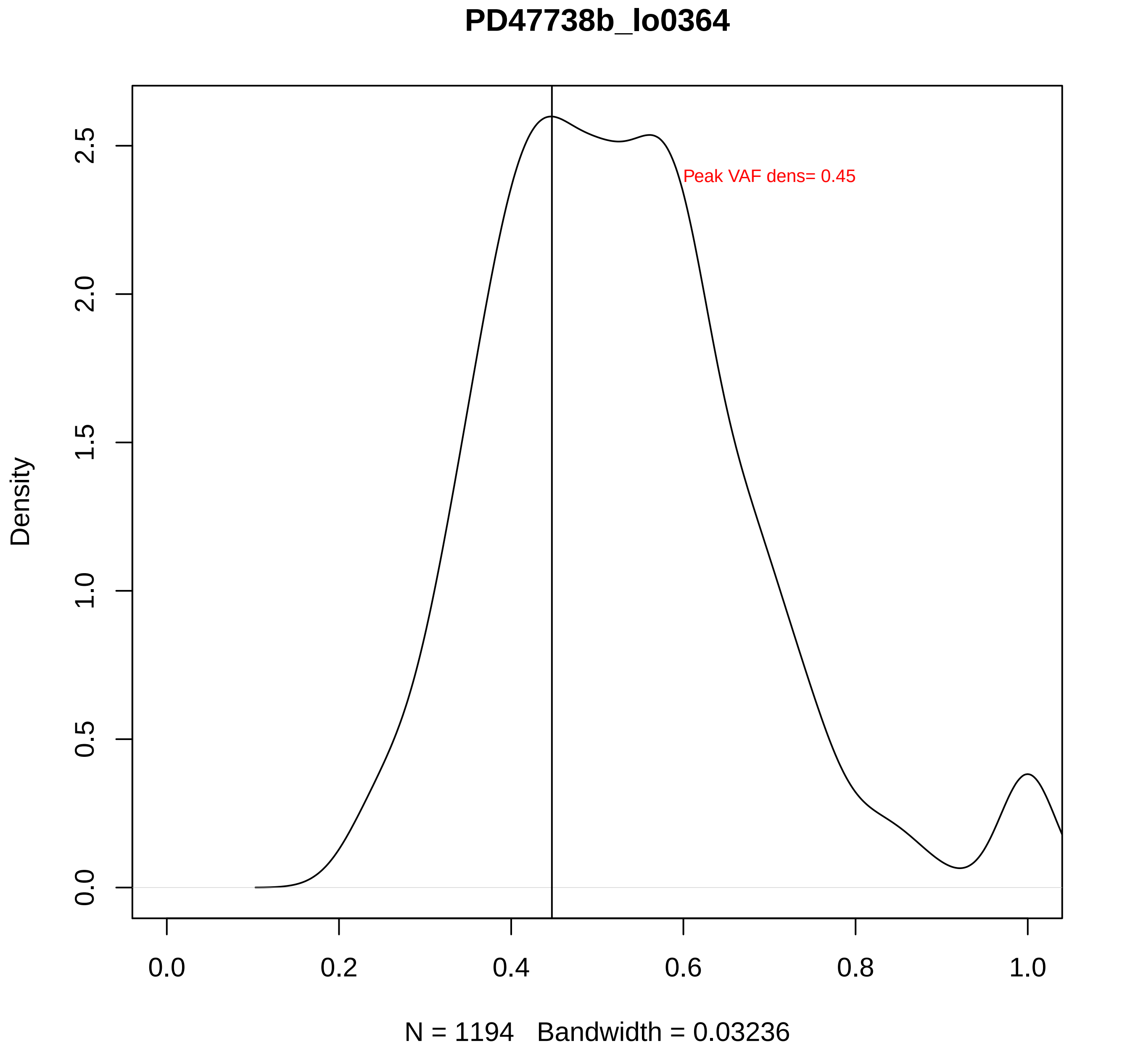
PD47738b_lo0364
Peak VAF dens= 0.45
Density
0.0
0.5
1.0
1.5
2.0
2.5
0.0
0.2
0.4
0.6
0.8
1.0
N = 1194 Bandwidth = 0.03236
