# Supplementary material for: Clonal dynamics of haematopoiesis across the human lifespan
Source: Nature. 2022 Jun 1;606(7913):343–50. doi: 10.1038/s41586-022-04786-y (PMC9177428; doi:10.1038/s41586-022-04786-y)
Supplement: Supplementary file 4 — HTMLs of notebooks outlining key statistical analyses presented in the manuscript, including analysis of phylogenetic trees. [file 41586_2022_4786_MOESM4_ESM.zip › Supplementary_code/SNV_indel_analysis/SX001_vaf_plots.pdf]

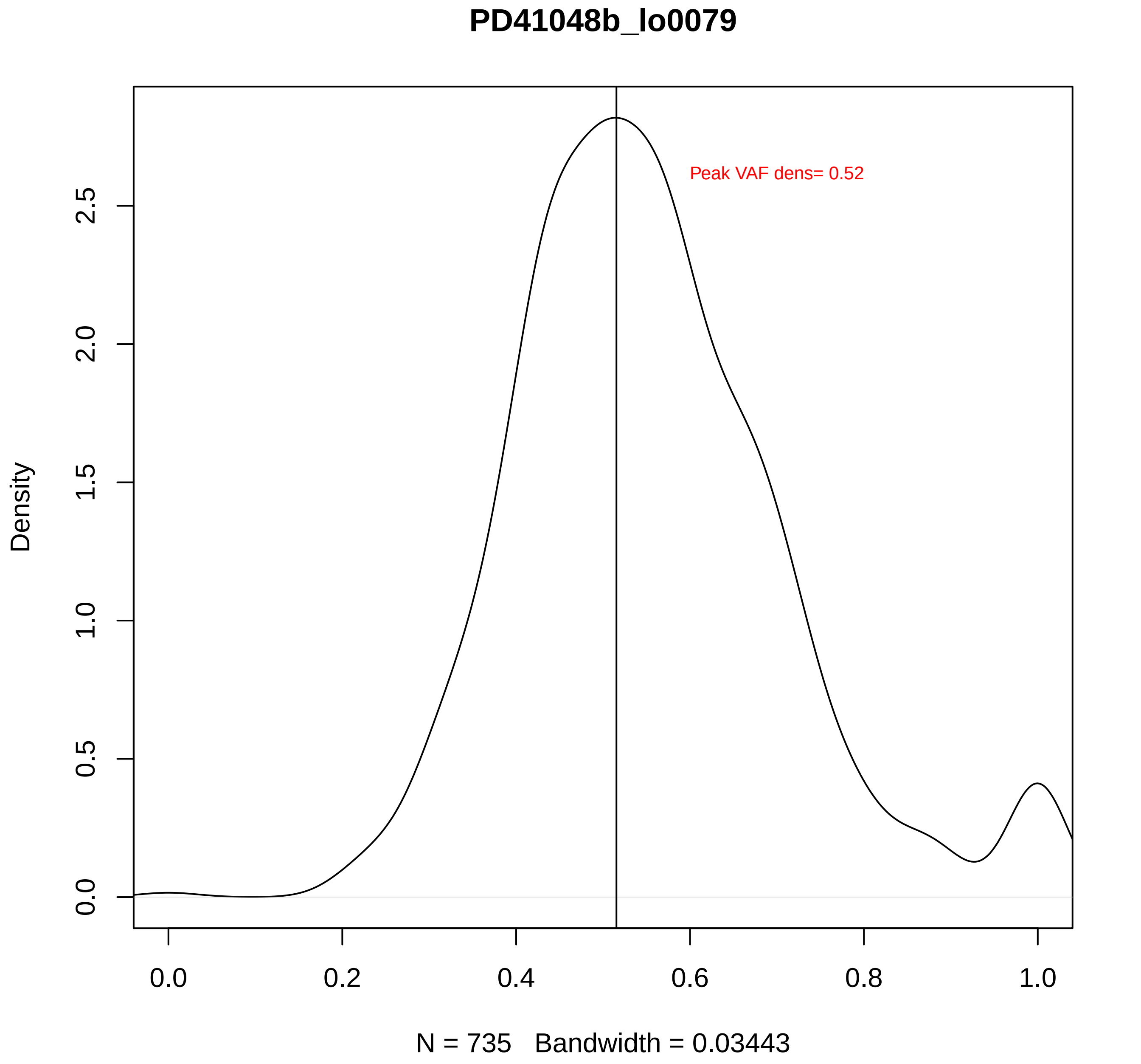
PD41048b_lo0079
Peak VAF dens= 0.52
Density
0.0
0.5
1.0
1.5
2.0
2.5
0.0
0.2
0.4
0.6
0.8
1.0
N = 735   Bandwidth = 0.03443

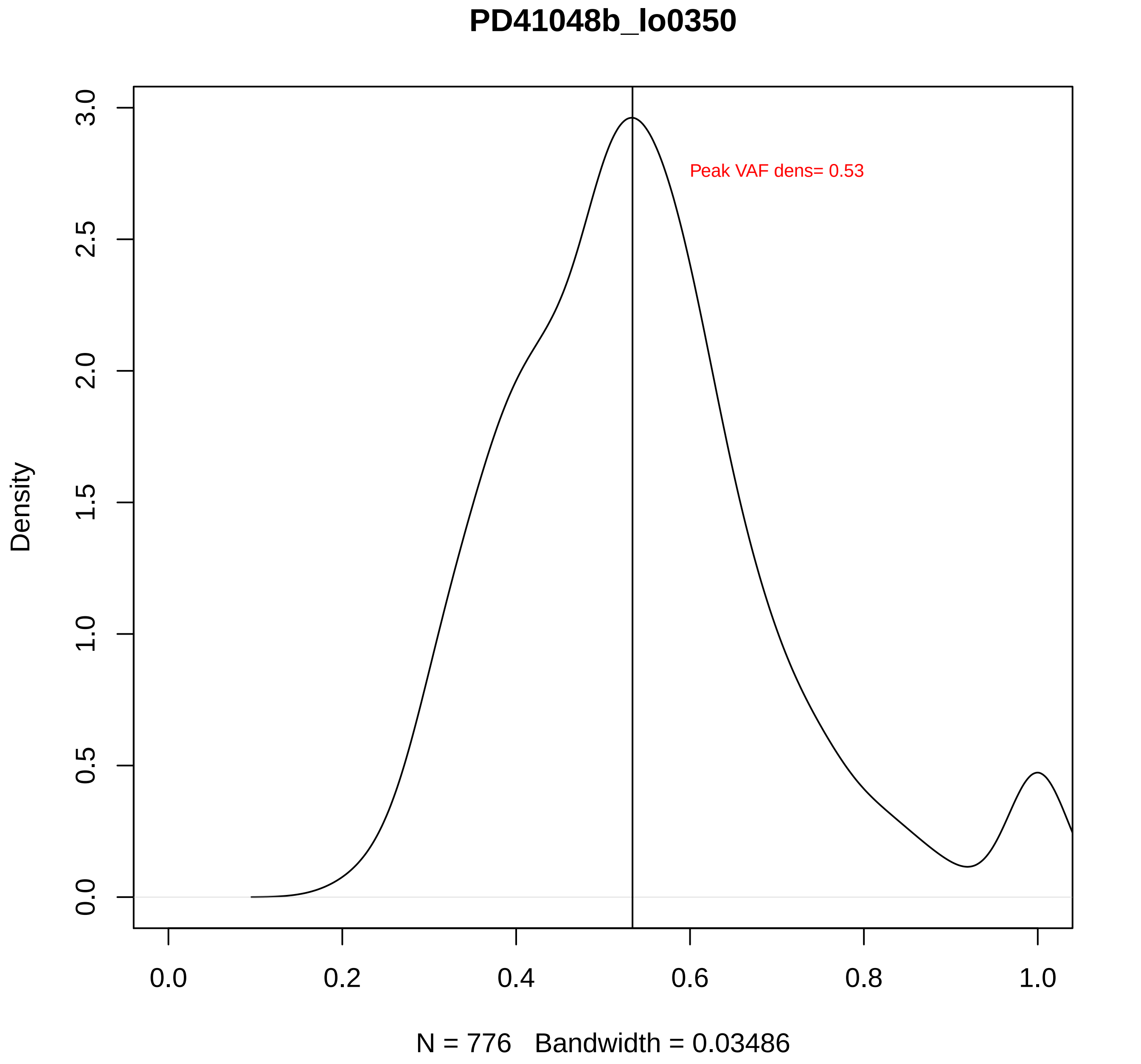
PD41048b_lo0350
Peak VAF dens= 0.53
Density
0.0
0.5
1.0
1.5
2.0
2.5
3.0
0.0
0.2
0.4
0.6
0.8
1.0
N = 776   Bandwidth = 0.03486

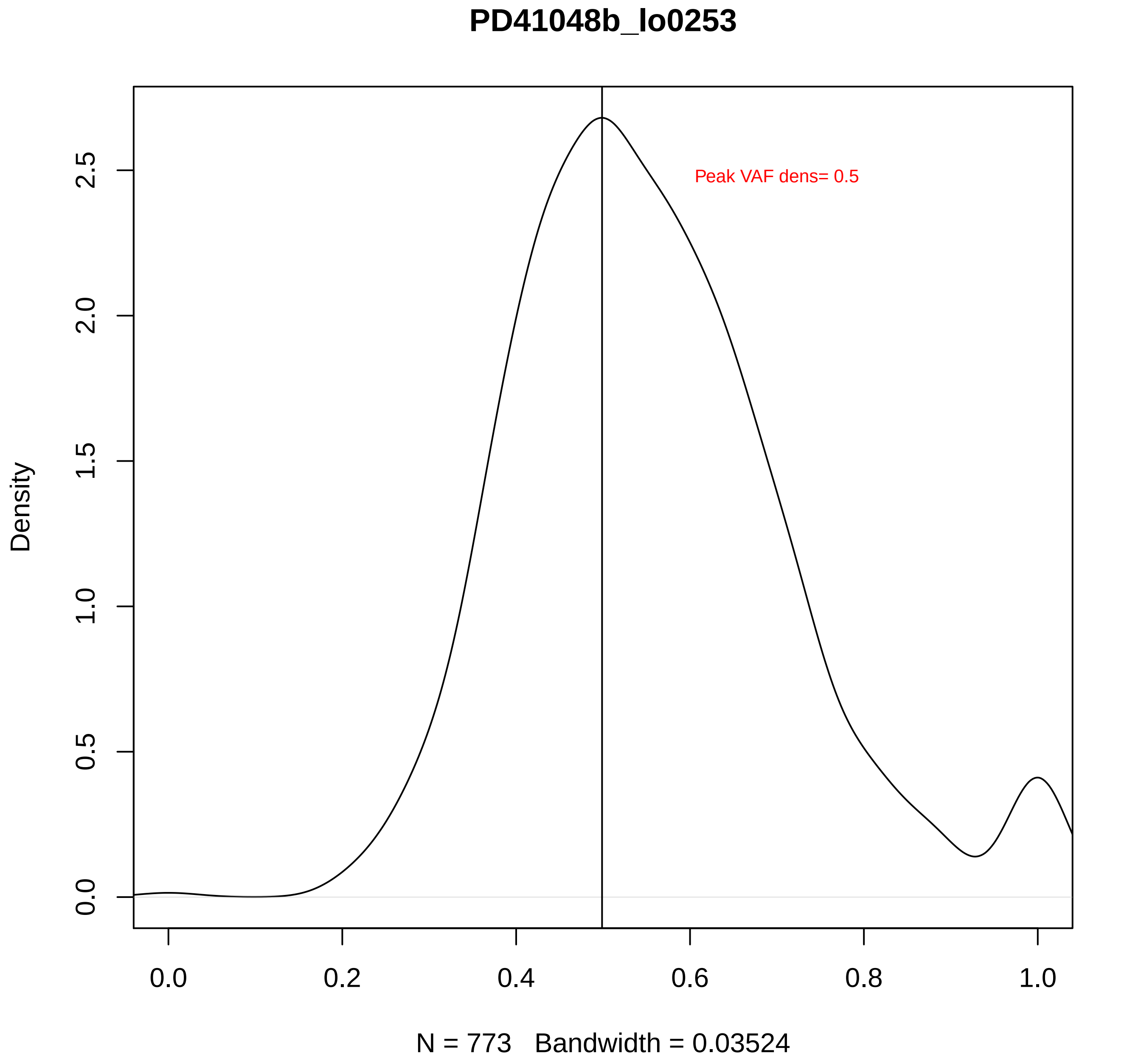
PD41048b_lo0253
Peak VAF dens= 0.5
Density
0.0
0.5
1.0
1.5
2.0
2.5
0.0
0.2
0.4
0.6
0.8
1.0
N = 773   Bandwidth = 0.03524

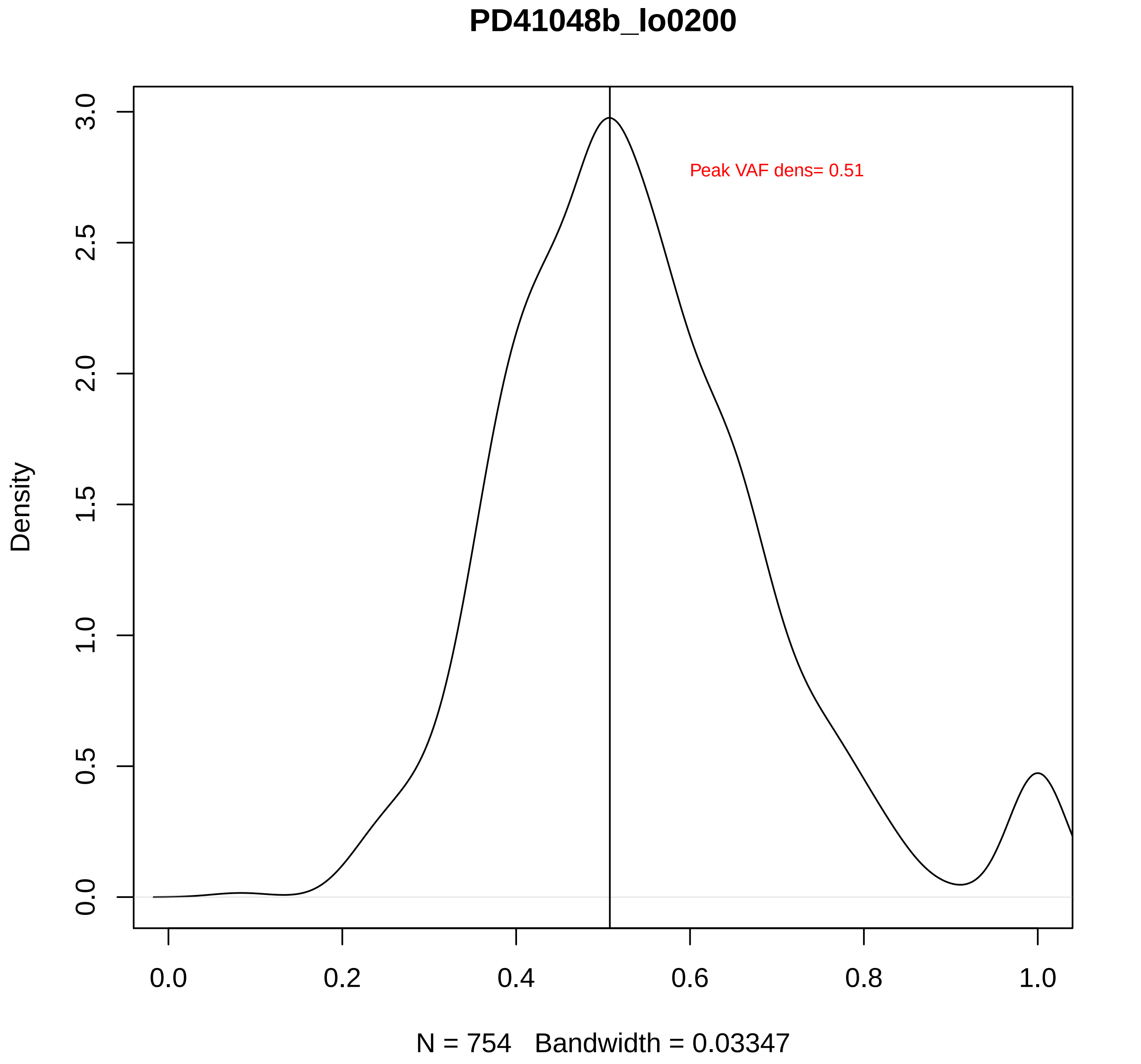
PD41048b_lo0200
Peak VAF dens= 0.51
Density
3.0
2.5
2.0
1.5
1.0
0.5
0.0
0.0
0.2
0.4
0.6
0.8
1.0
N = 754   Bandwidth = 0.03347

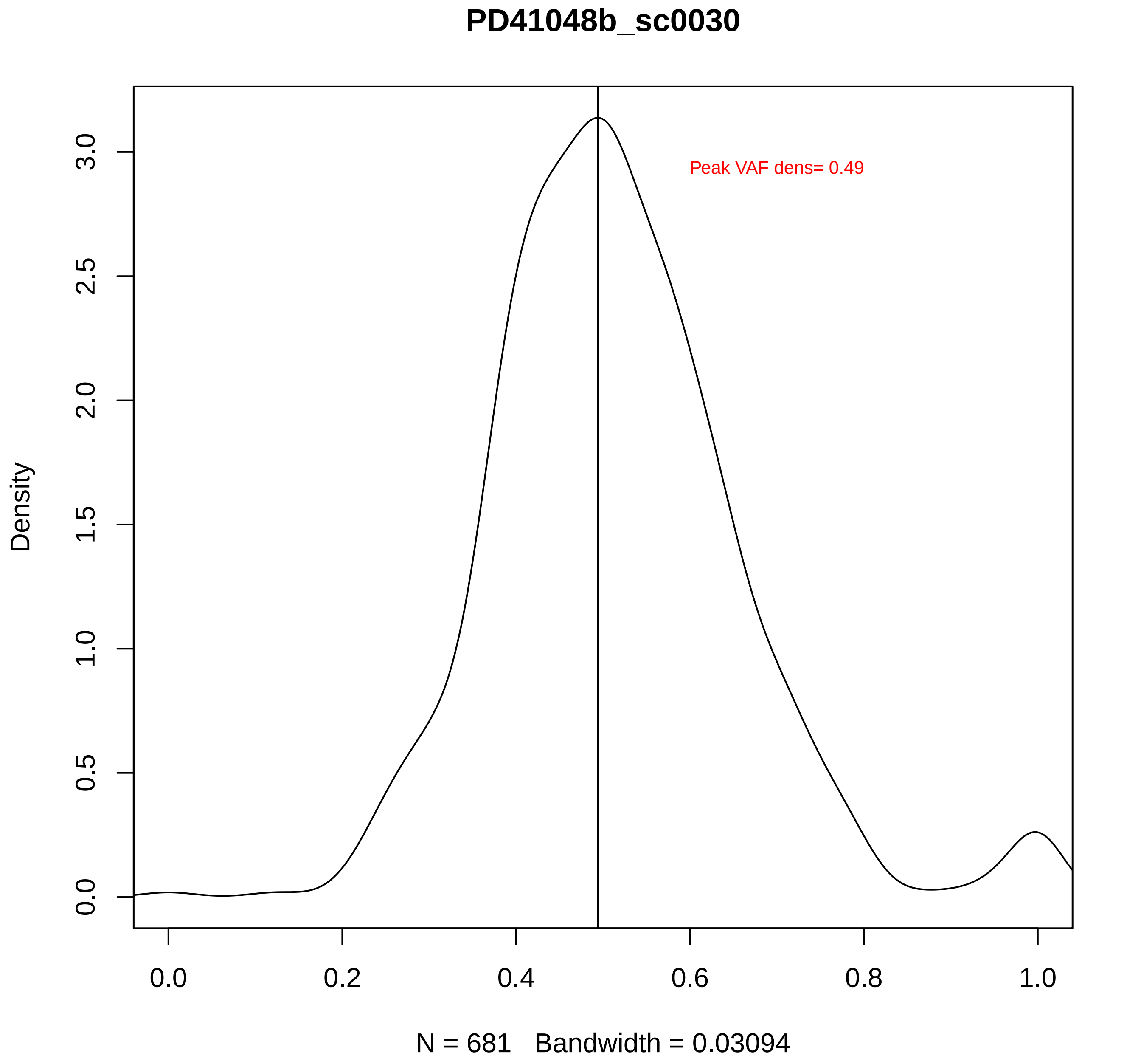
PD41048b_sc0030
Peak VAF dens= 0.49
Density
0.0
0.5
1.0
1.5
2.0
2.5
3.0
0.0
0.2
0.4
0.6
0.8
1.0
N = 681   Bandwidth = 0.03094

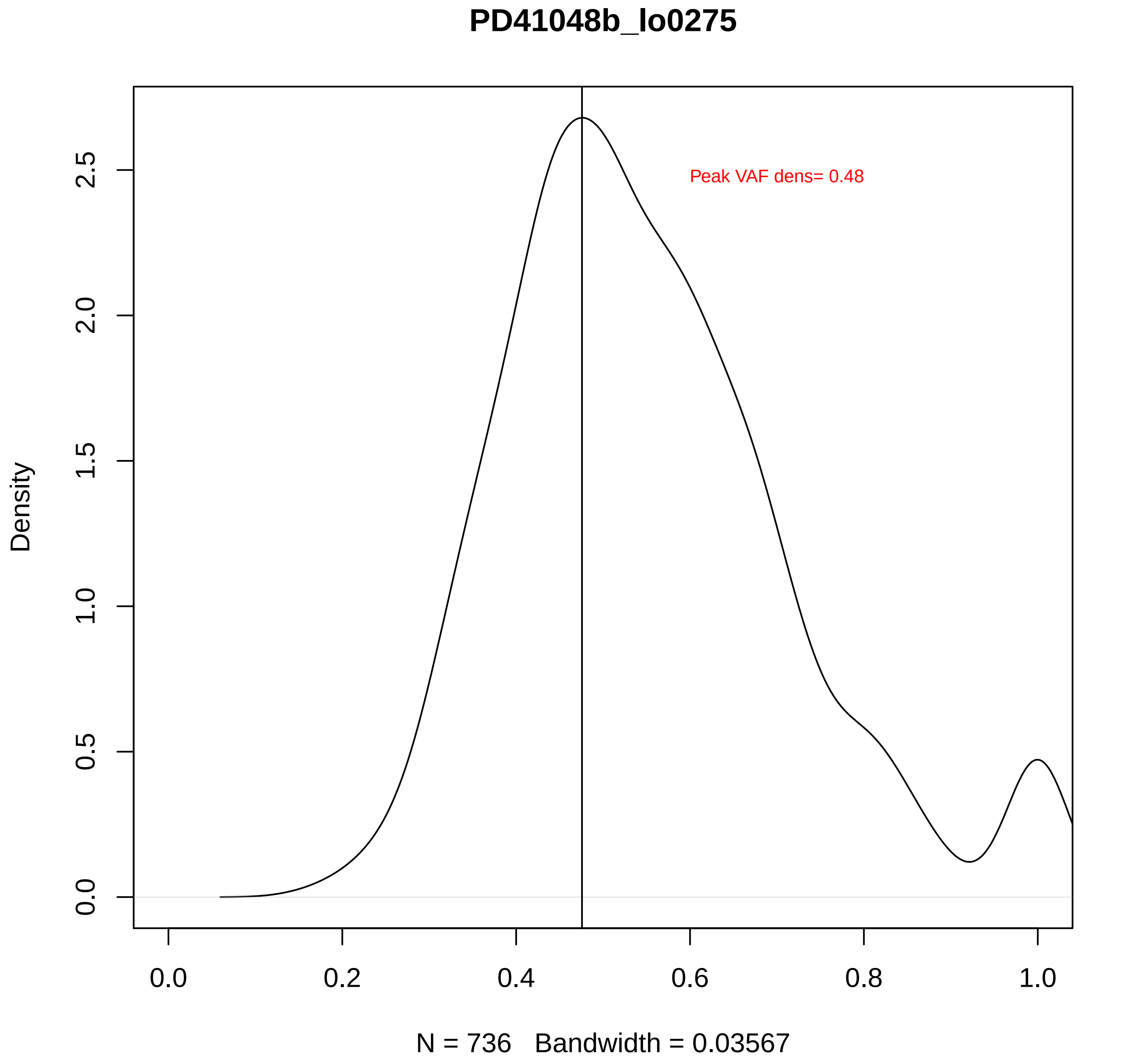
PD41048b_lo0275
Peak VAF dens= 0.48
Density
0.0
0.5
1.0
1.5
2.0
2.5
0.0
0.2
0.4
0.6
0.8
1.0
N = 736   Bandwidth = 0.03567

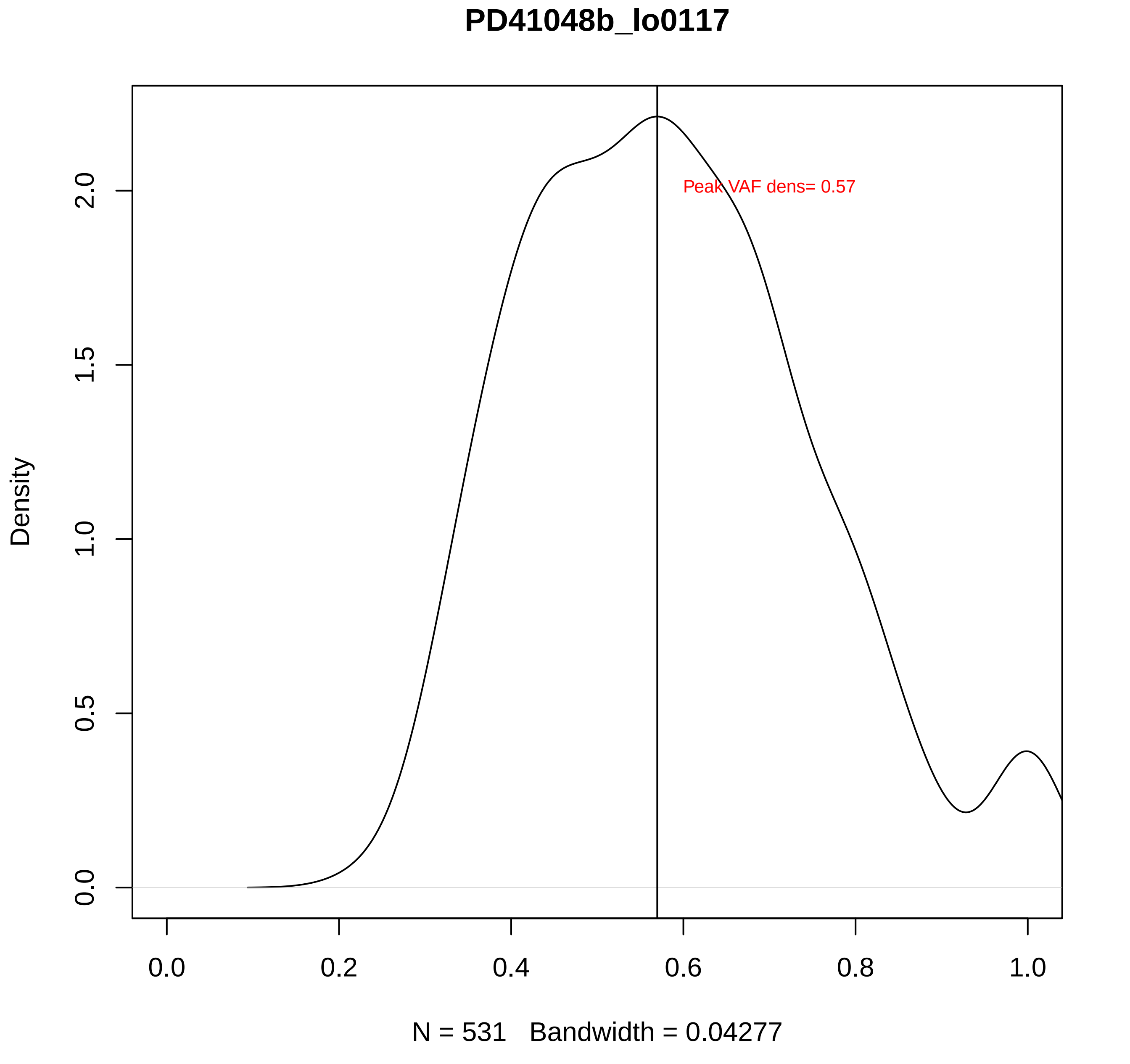

PD41048b_lo0117
Peak VAF dens= 0.57
Density
0.0
0.5
1.0
1.5
2.0
0.0
0.2
0.4
0.6
0.8
1.0
N = 531 Bandwidth = 0.04277

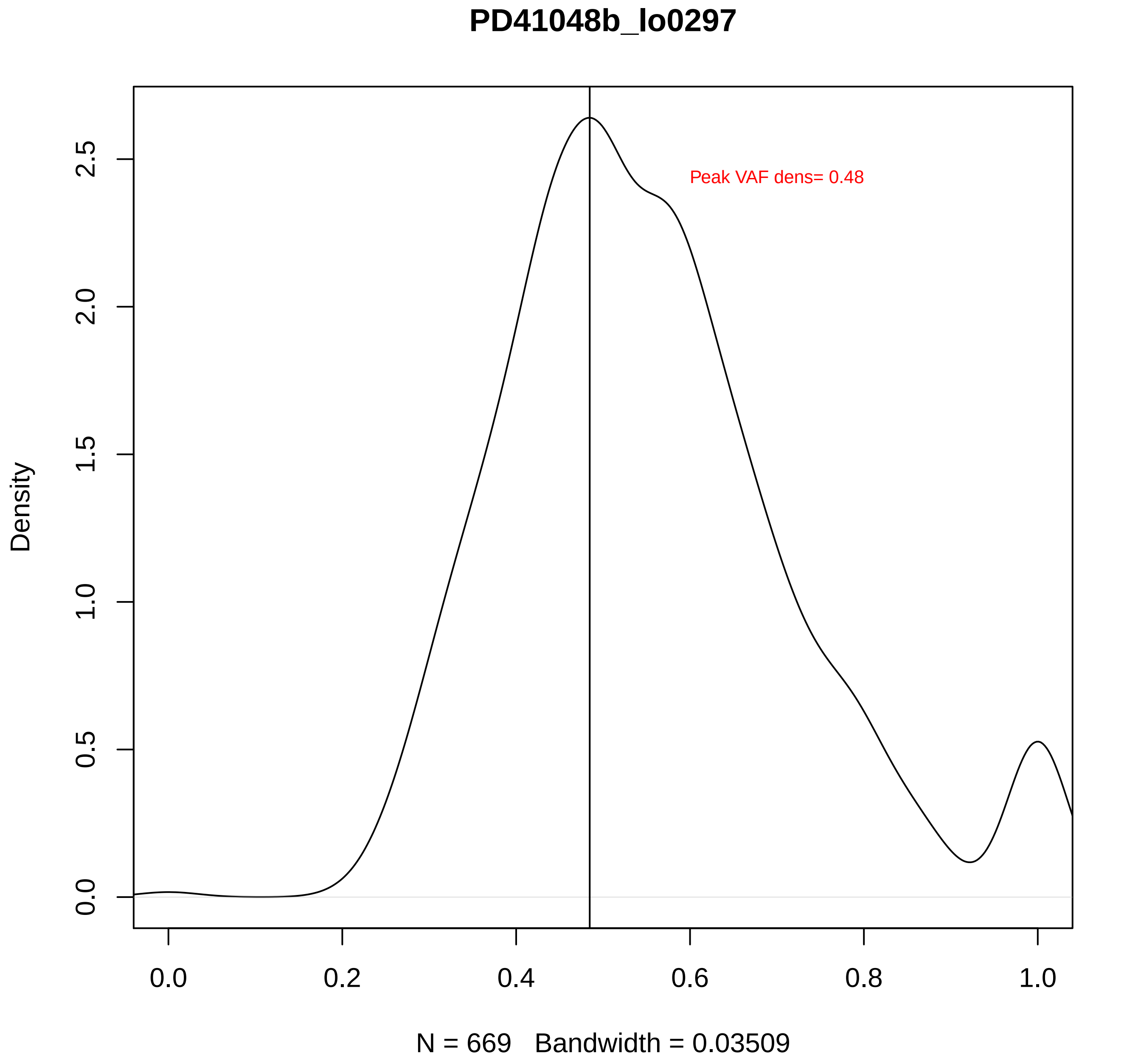
PD41048b_lo0297
Peak VAF dens= 0.48
Density
0.0
0.5
1.0
1.5
2.0
2.5
0.0
0.2
0.4
0.6
0.8
1.0
N = 669 Bandwidth = 0.03509

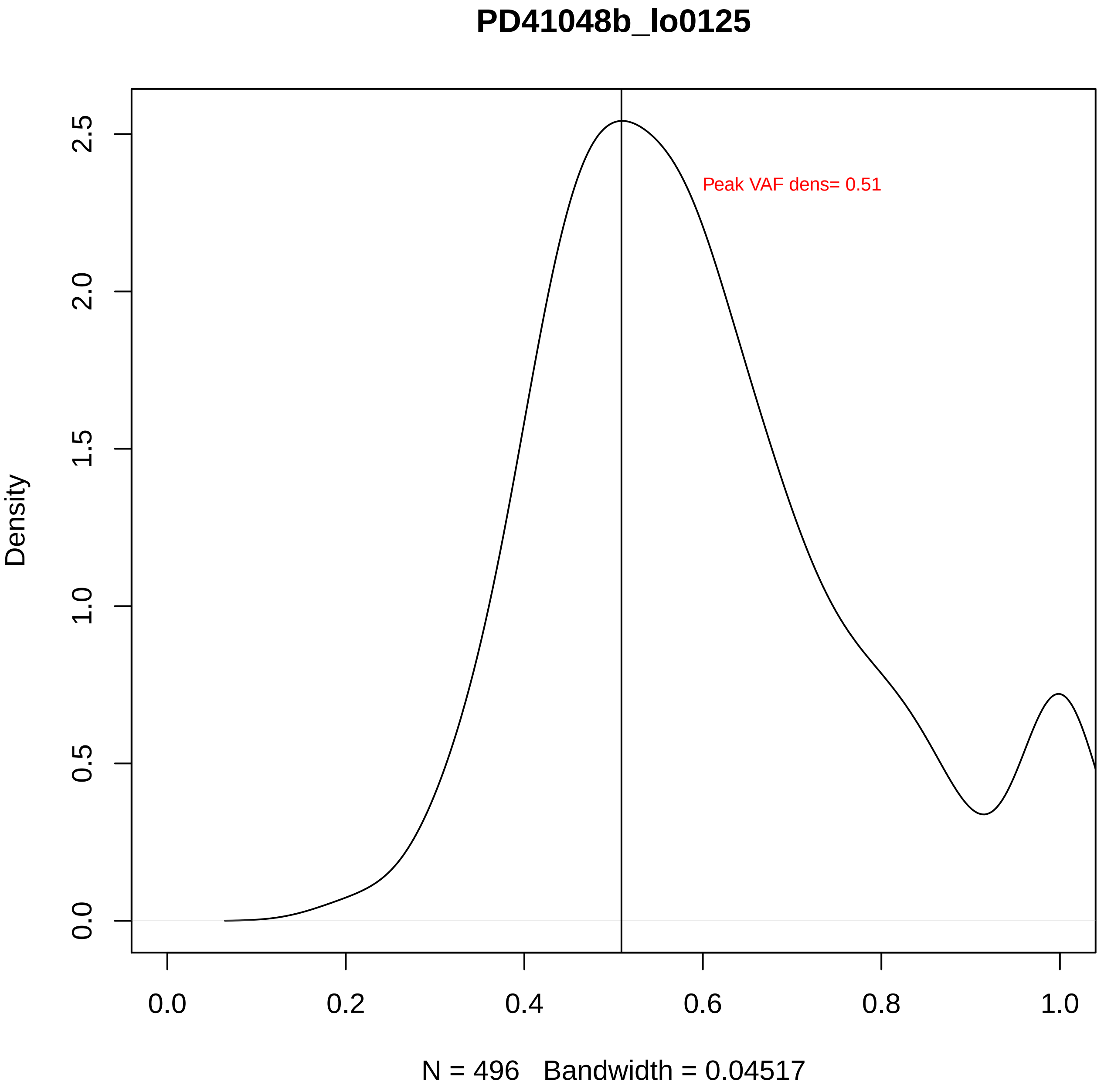
PD41048b_lo0125
Peak VAF dens= 0.51
Density
0.0
0.5
1.0
1.5
2.0
2.5
0.0
0.2
0.4
0.6
0.8
1.0
N = 496   Bandwidth = 0.04517

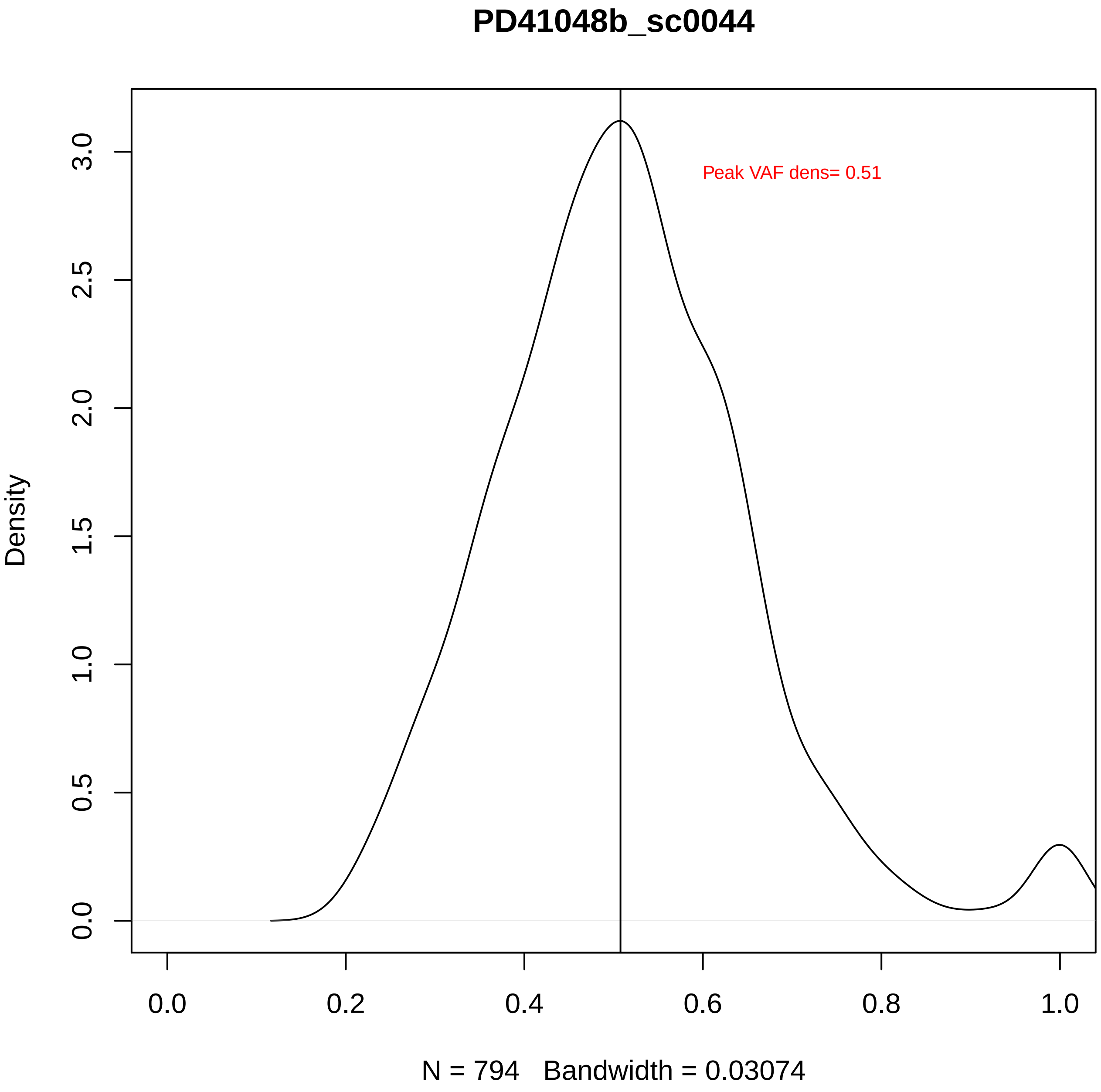
PD41048b_sc0044
Peak VAF dens= 0.51
Density
0.0
0.5
1.0
1.5
2.0
2.5
3.0
0.0
0.2
0.4
0.6
0.8
1.0
N = 794   Bandwidth = 0.03074

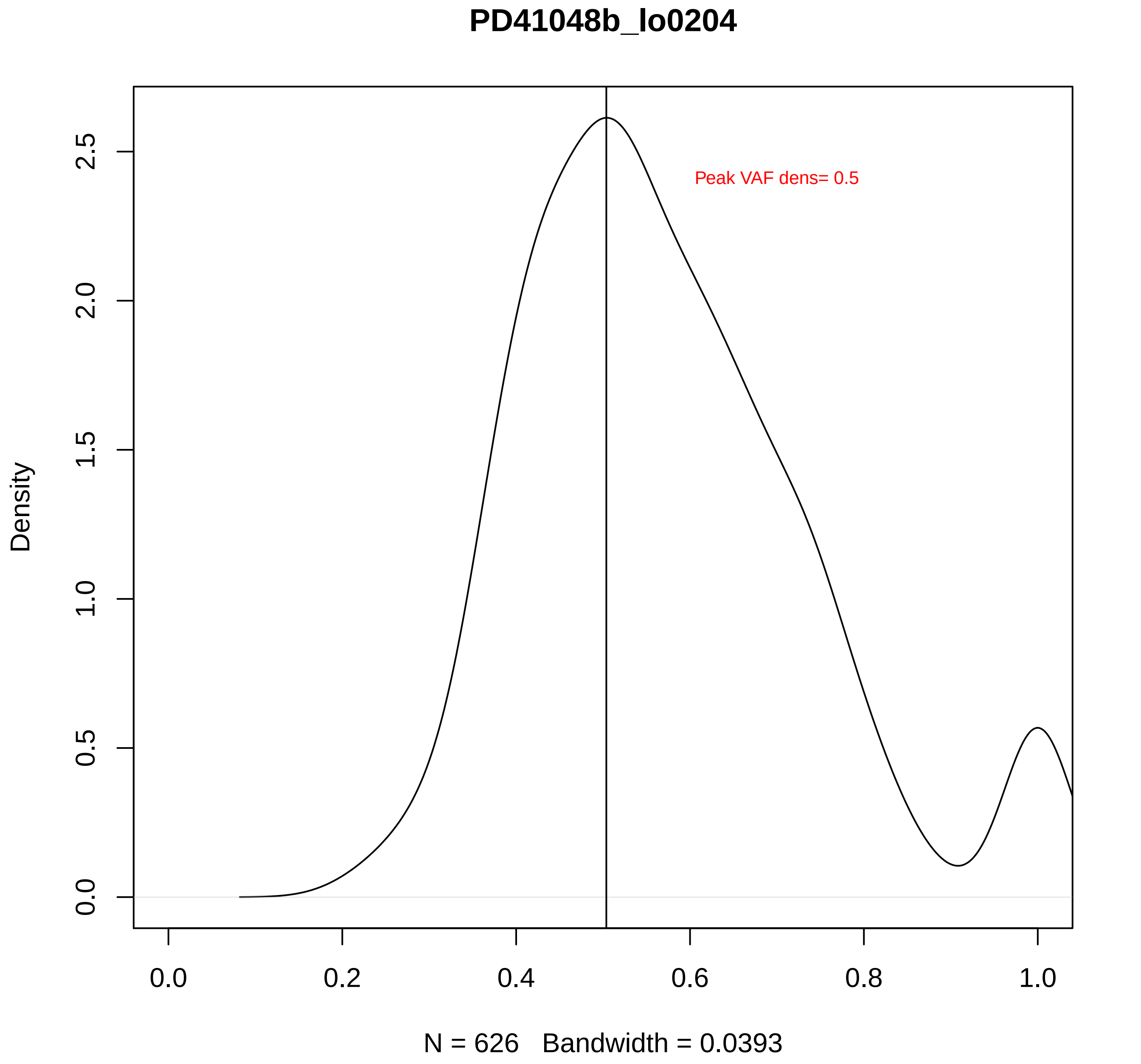

PD41048b_lo0204
Peak VAF dens= 0.5
Density
0.0
0.5
1.0
1.5
2.0
2.5
0.0
0.2
0.4
0.6
0.8
1.0
N = 626   Bandwidth = 0.0393

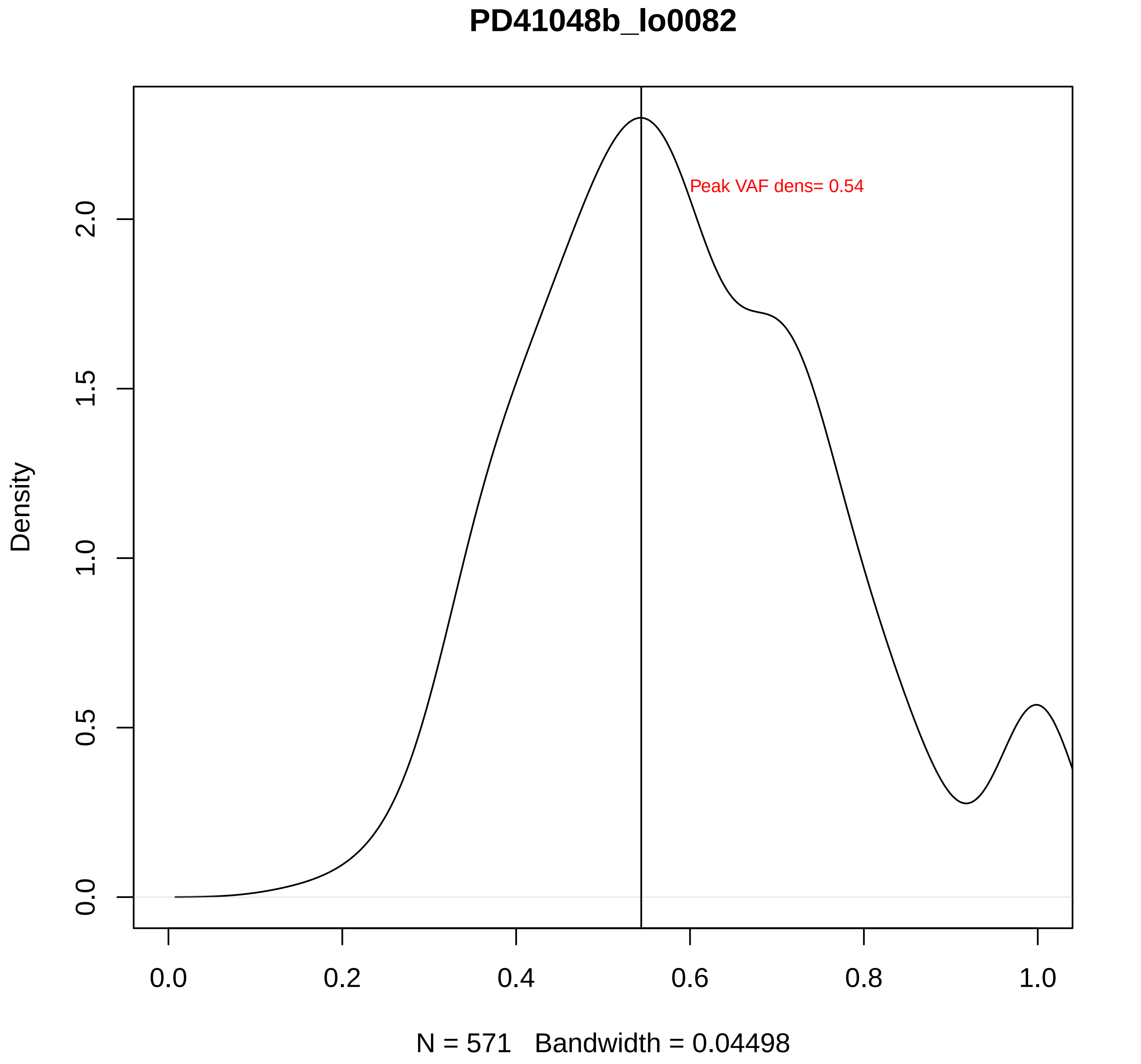
PD41048b_lo0082
Peak VAF dens= 0.54
Density
0.0
0.5
1.0
1.5
2.0
0.0
0.2
0.4
0.6
0.8
1.0
N = 571   Bandwidth = 0.04498

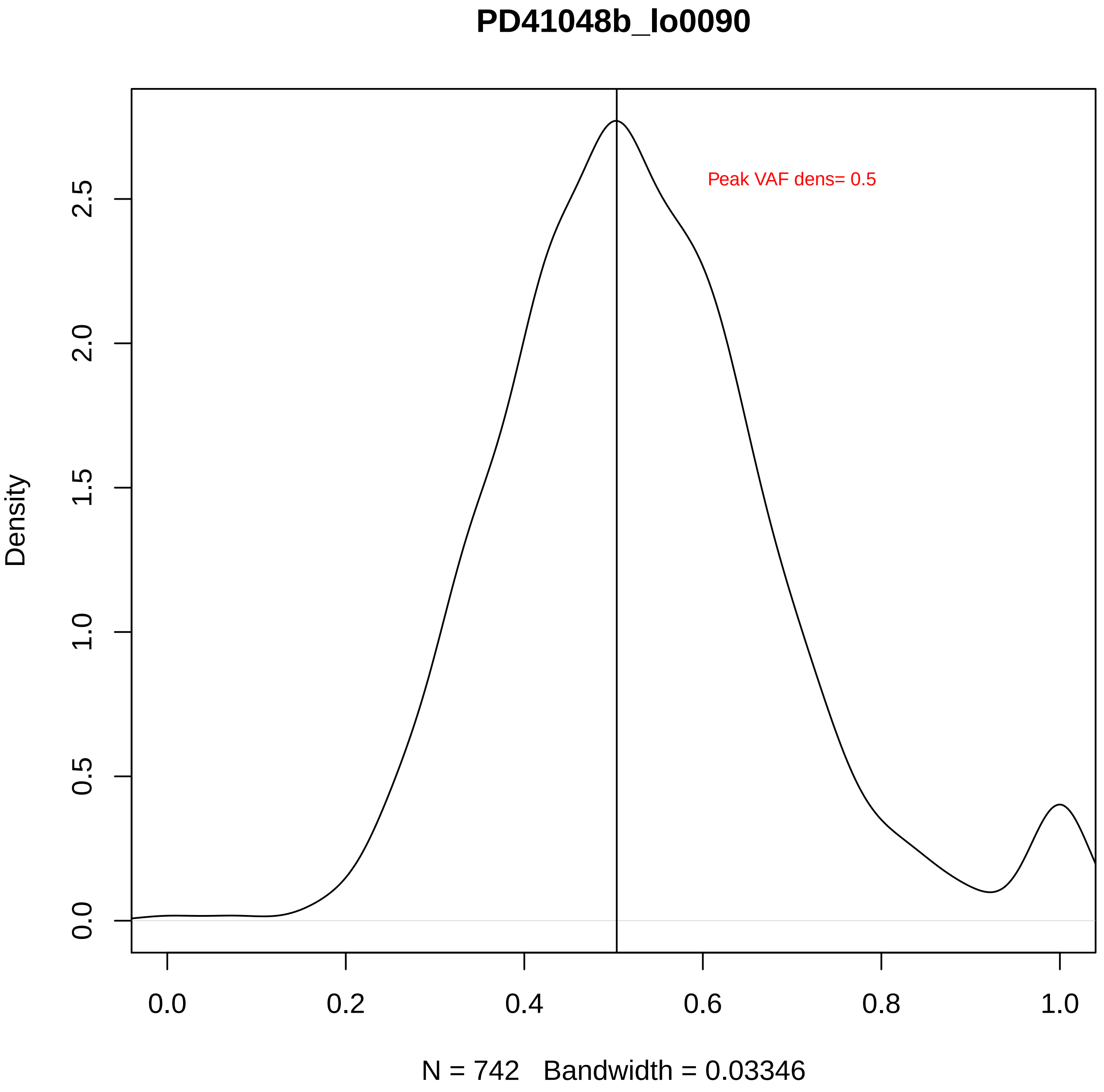
PD41048b_lo0090
Peak VAF dens= 0.5
Density
0.0
0.5
1.0
1.5
2.0
2.5
0.0
0.2
0.4
0.6
0.8
1.0
N = 742 Bandwidth = 0.03346

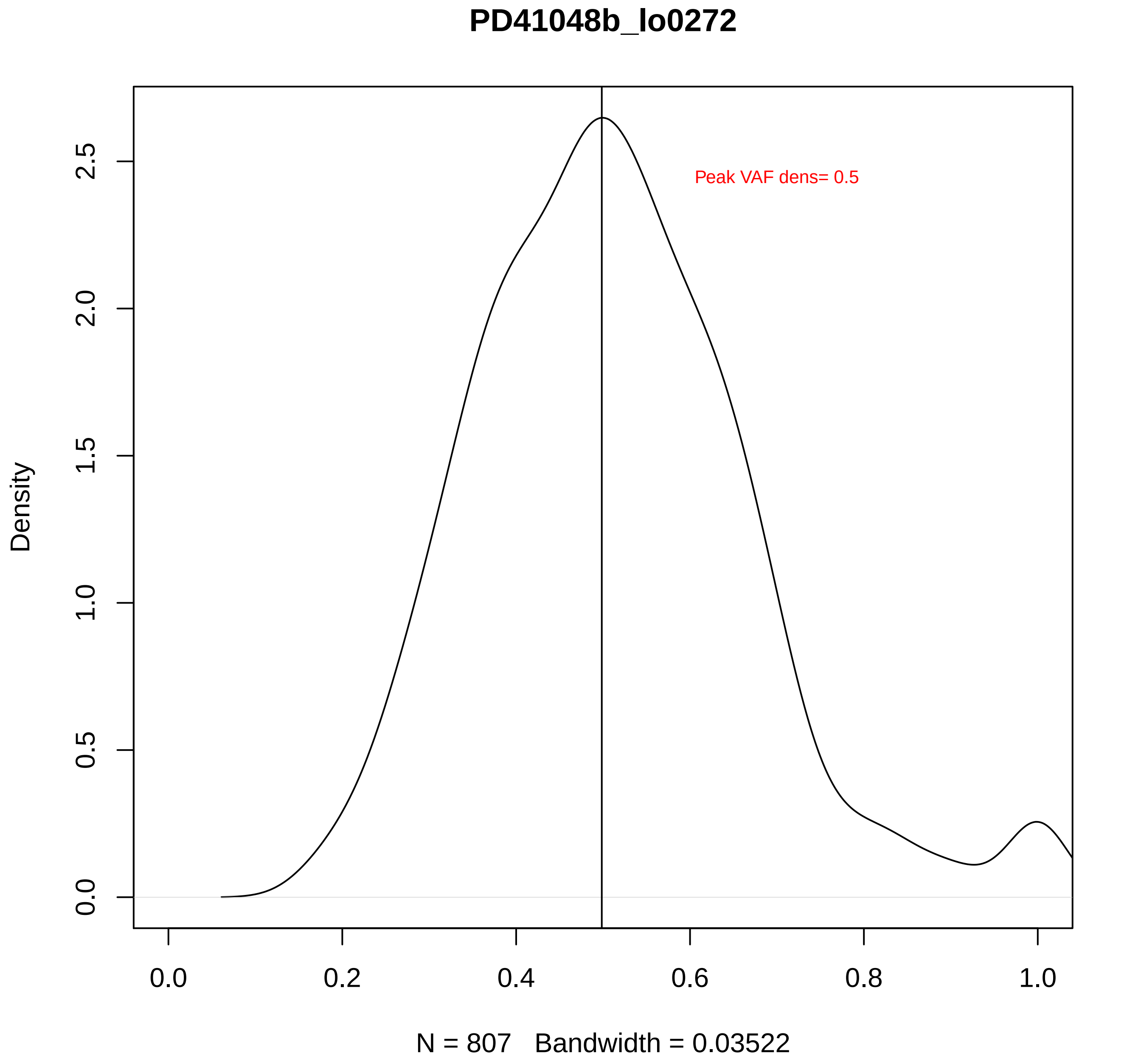
PD41048b_lo0272
Peak VAF dens= 0.5
Density
0.0
0.5
1.0
1.5
2.0
2.5
0.0
0.2
0.4
0.6
0.8
1.0
N = 807   Bandwidth = 0.03522

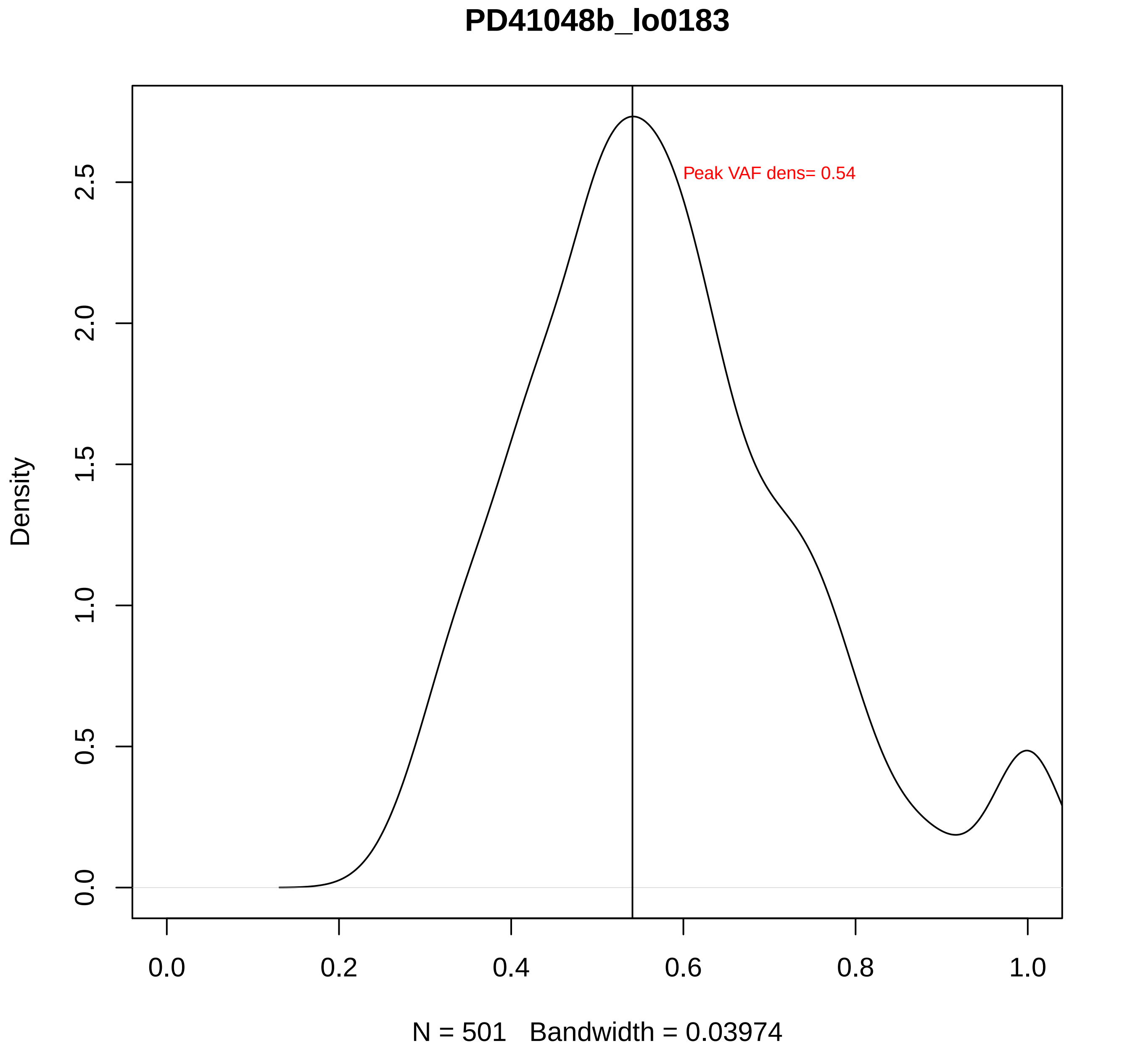
PD41048b_lo0183
Peak VAF dens= 0.54
Density
0.0
0.5
1.0
1.5
2.0
2.5
0.0
0.2
0.4
0.6
0.8
1.0
N = 501   Bandwidth = 0.03974

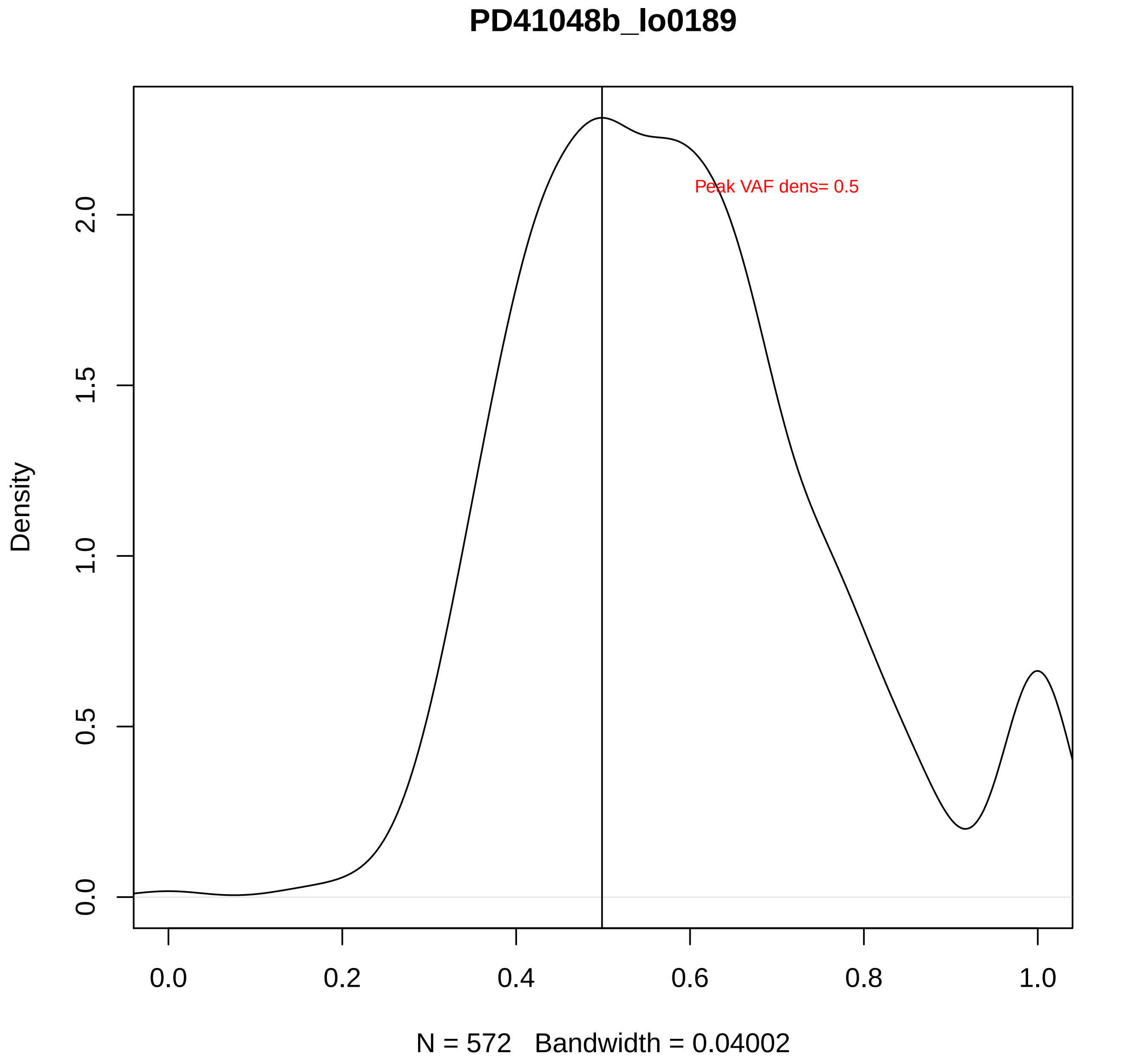
PD41048b_lo0189
Peak VAF dens= 0.5
Density
0.0
0.5
1.0
1.5
2.0
0.0
0.2
0.4
0.6
0.8
1.0
N = 572   Bandwidth = 0.04002

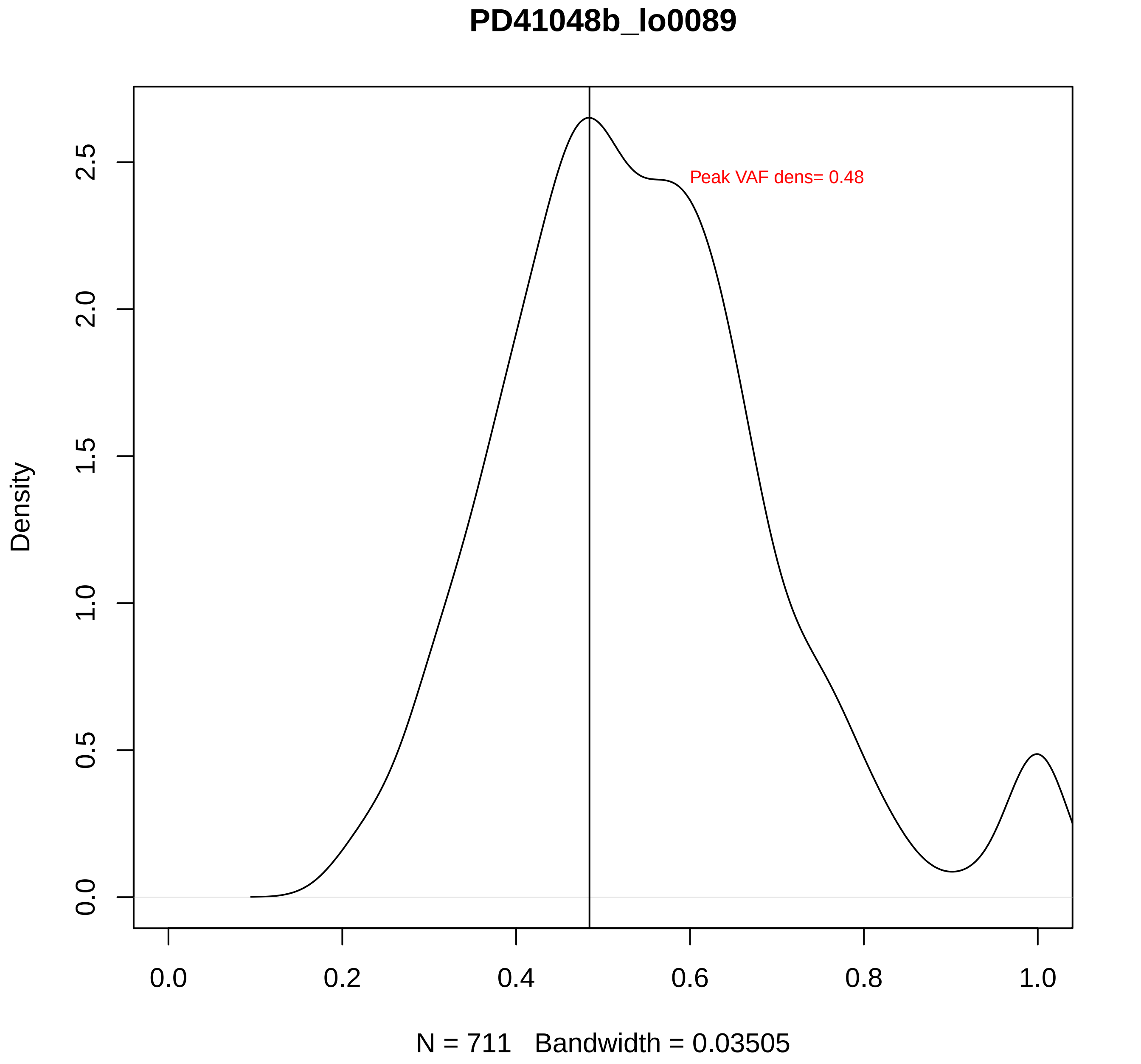
PD41048b_lo0089
Density
2.5
2.0
1.5
1.0
0.5
0.0
Peak VAF dens= 0.48
0.0
0.2
0.4
0.6
0.8
1.0
N = 711 Bandwidth = 0.03505

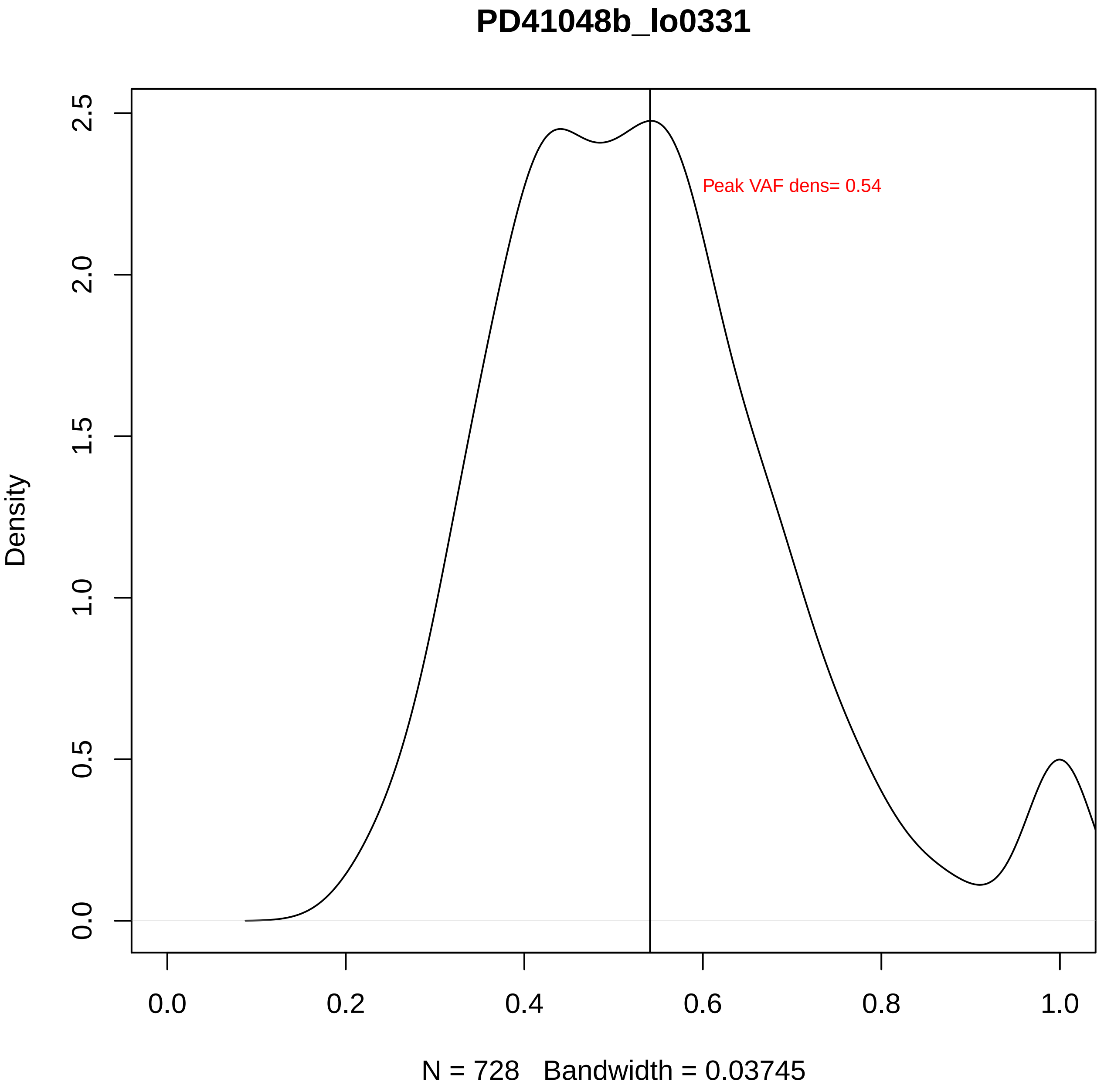
PD41048b_lo0331
Peak VAF dens= 0.54
Density
0.0
0.5
1.0
1.5
2.0
2.5
0.0
0.2
0.4
0.6
0.8
1.0
N = 728 Bandwidth = 0.03745

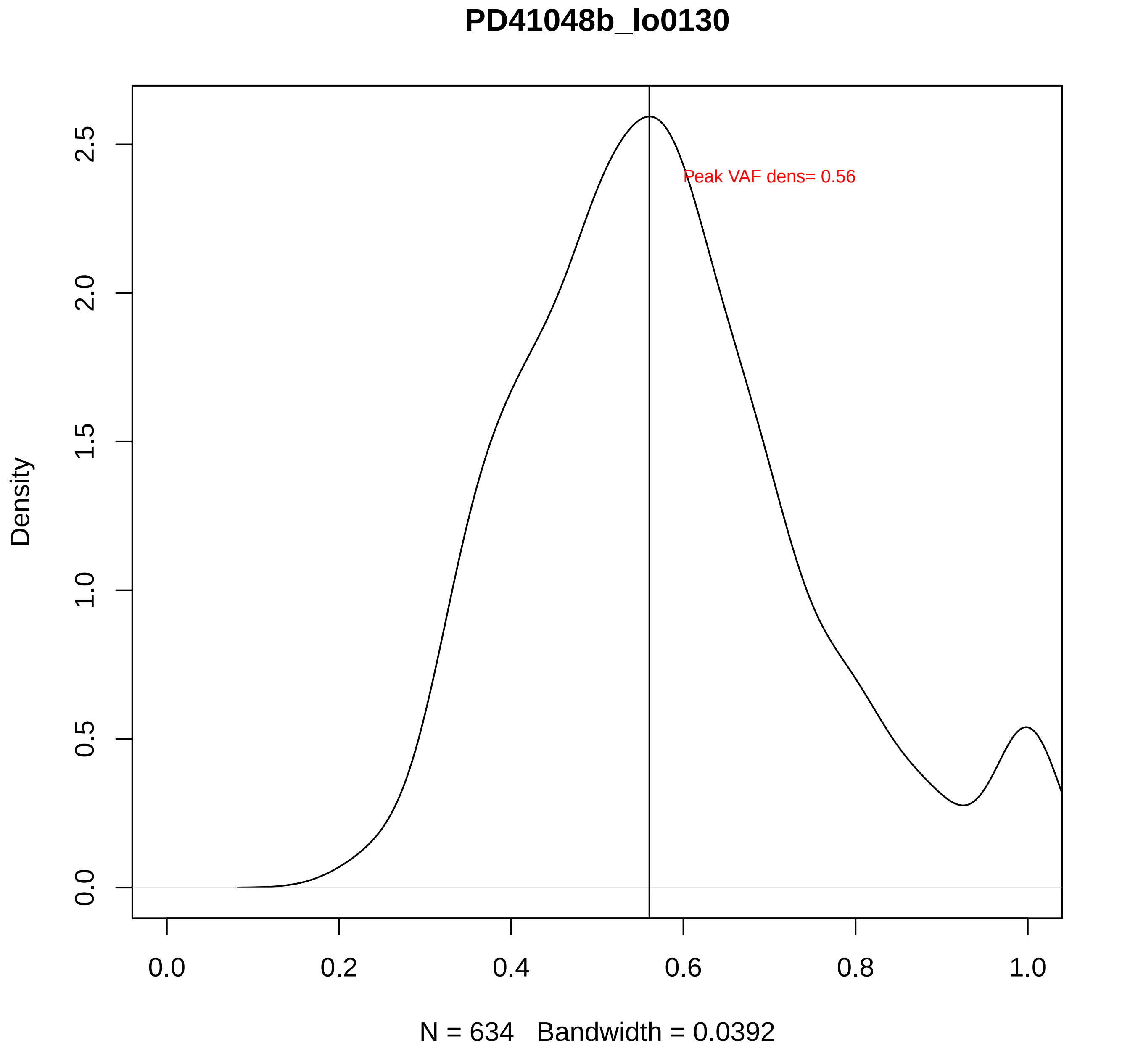
PD41048b_lo0130
Peak VAF dens= 0.56
Density
0.0
0.5
1.0
1.5
2.0
2.5
0.0
0.2
0.4
0.6
0.8
1.0
N = 634   Bandwidth = 0.0392

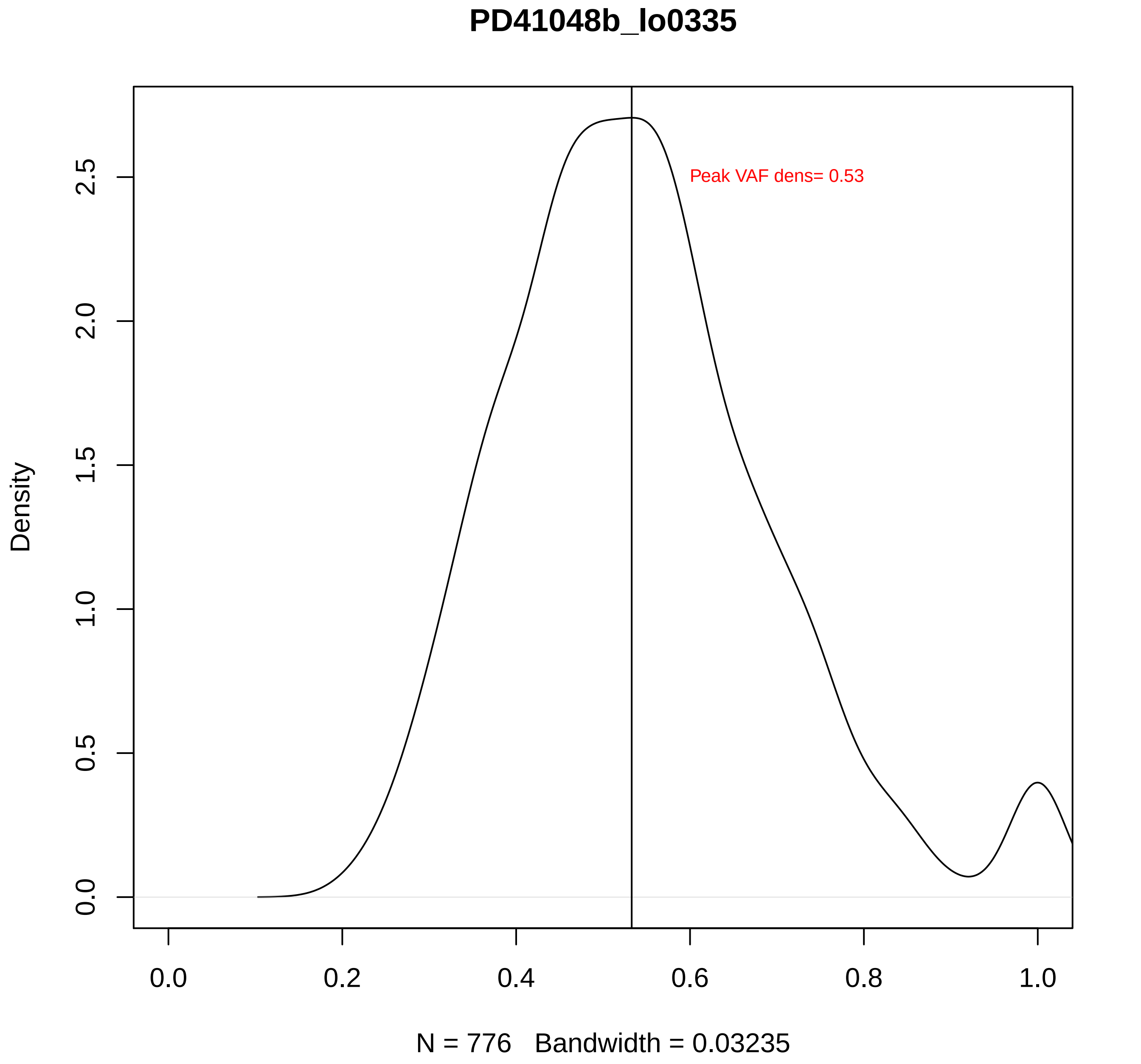

PD41048b_lo0335
Peak VAF dens= 0.53
Density
0.0
0.5
1.0
1.5
2.0
2.5
0.0
0.2
0.4
0.6
0.8
1.0
N = 776 Bandwidth = 0.03235

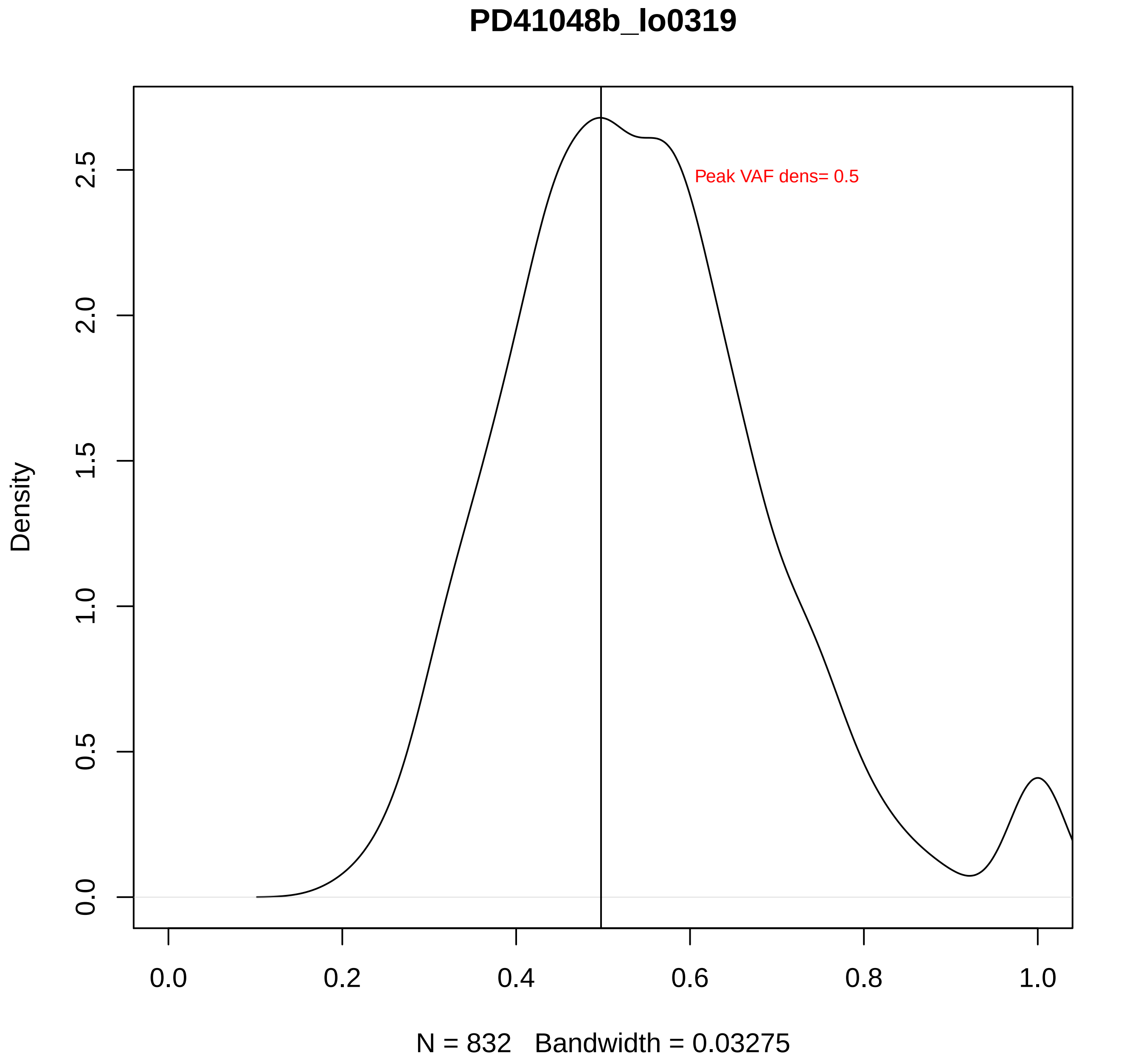
PD41048b_lo0319
Peak VAF dens= 0.5
Density
0.0
0.5
1.0
1.5
2.0
2.5
0.0
0.2
0.4
0.6
0.8
1.0
N = 832 Bandwidth = 0.03275

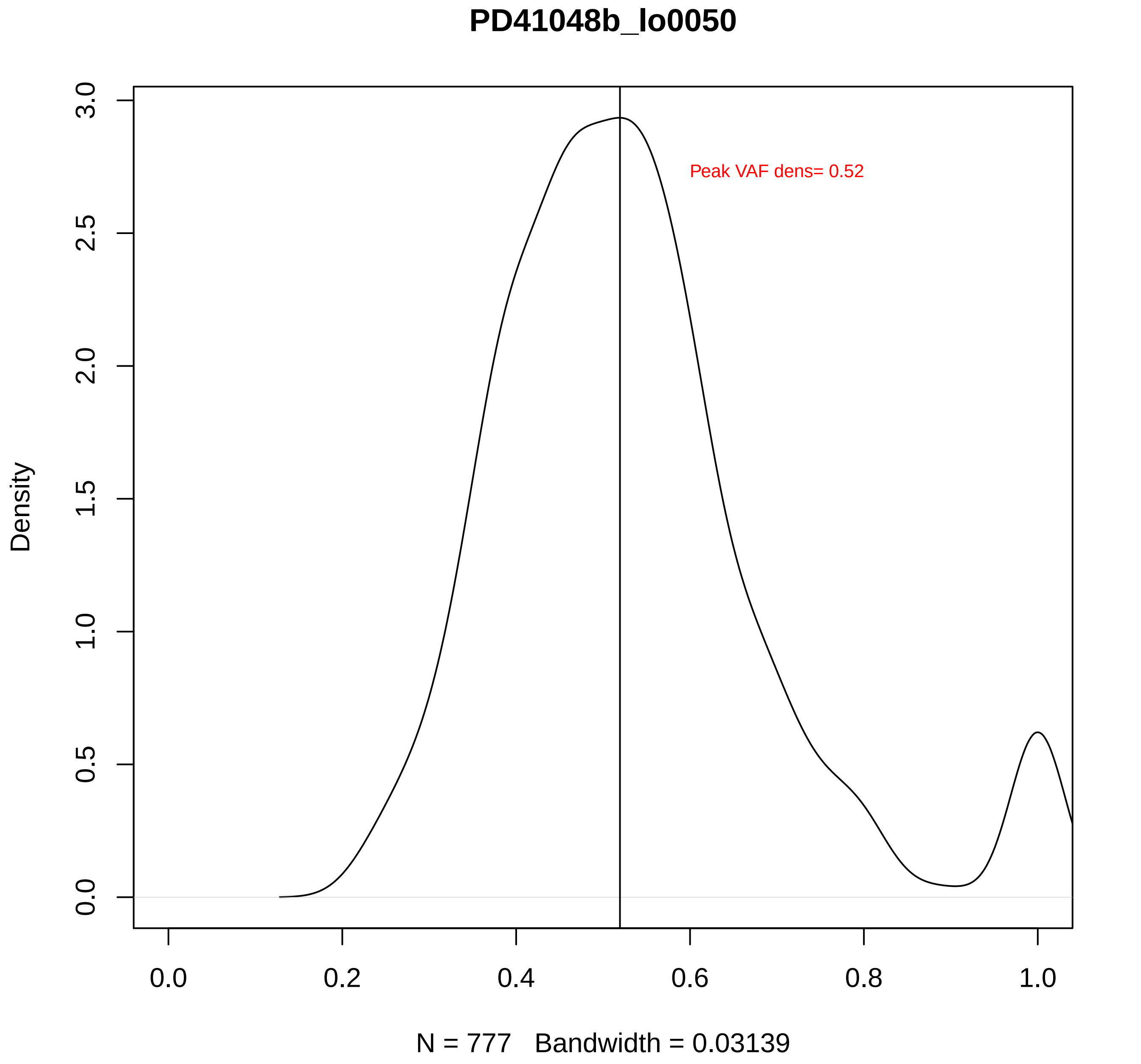
PD41048b_lo0050
Peak VAF dens= 0.52
Density
0.0
0.5
1.0
1.5
2.0
2.5
3.0
0.0
0.2
0.4
0.6
0.8
1.0
N = 777   Bandwidth = 0.03139

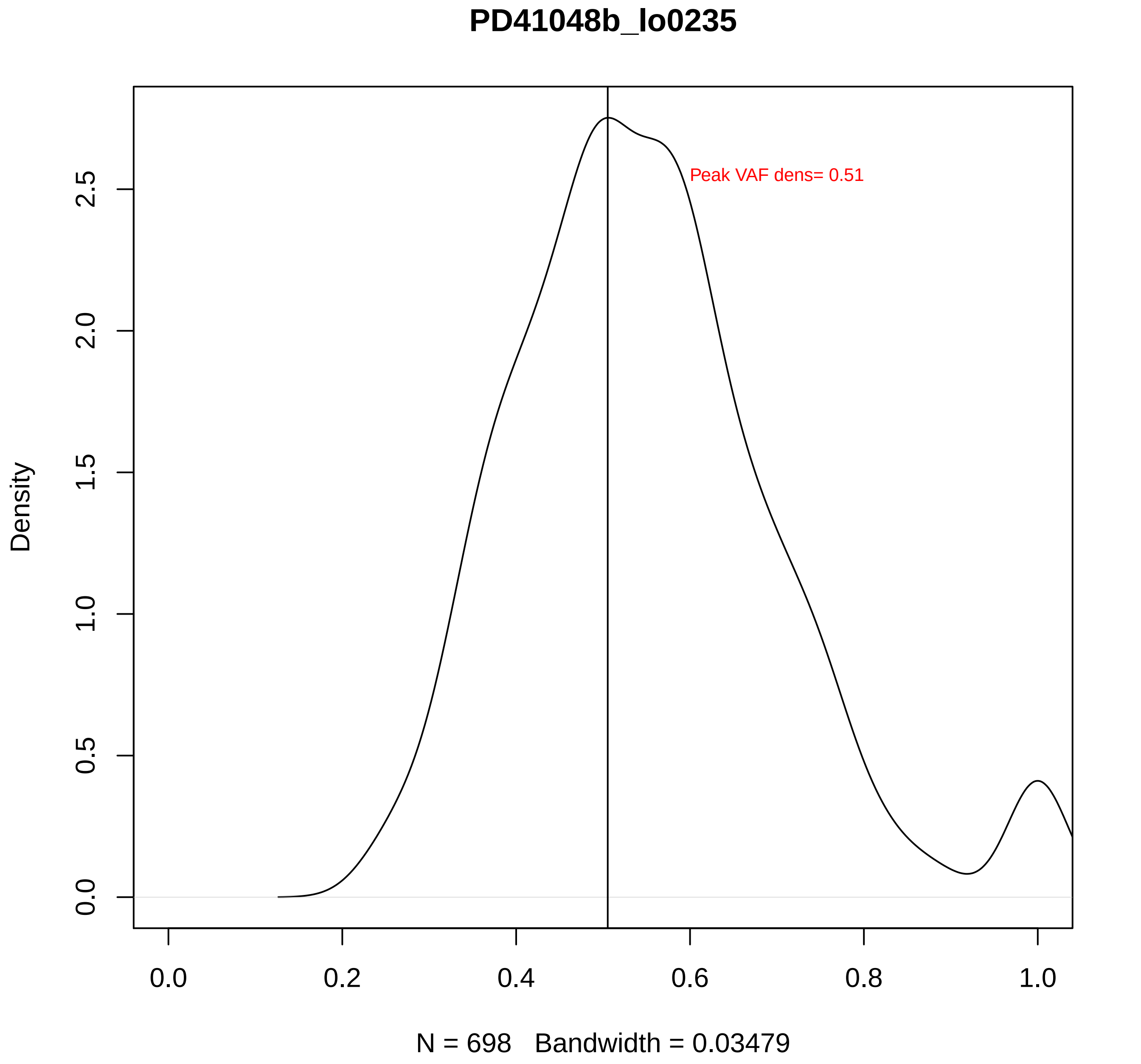

PD41048b_lo0235
Peak VAF dens= 0.51
Density
0.0
0.5
1.0
1.5
2.0
2.5
0.0
0.2
0.4
0.6
0.8
1.0
N = 698 Bandwidth = 0.03479

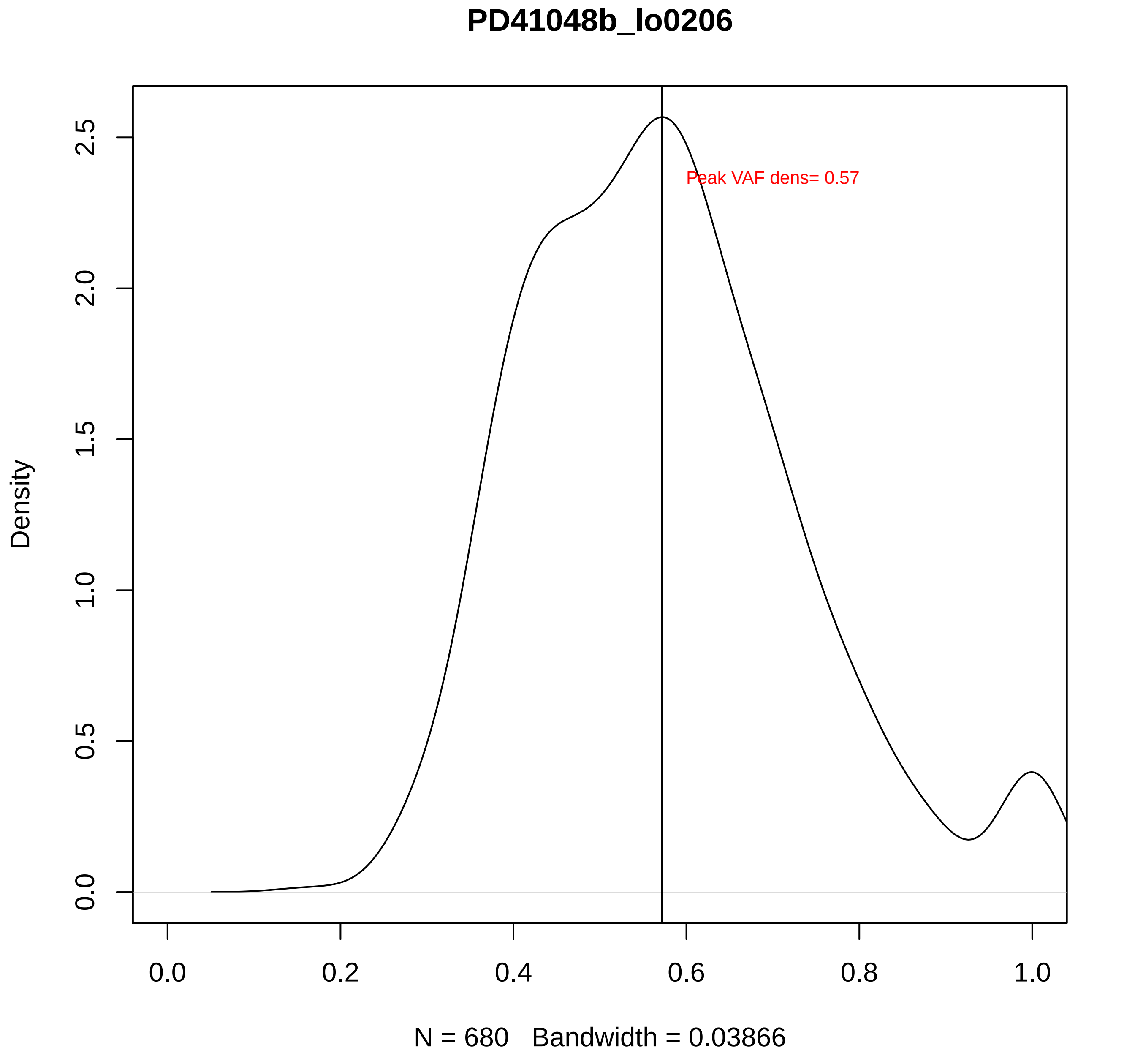

PD41048b_lo0206
Peak VAF dens= 0.57
Density
0.0
0.5
1.0
1.5
2.0
2.5
0.0
0.2
0.4
0.6
0.8
1.0
N = 680 Bandwidth = 0.03866

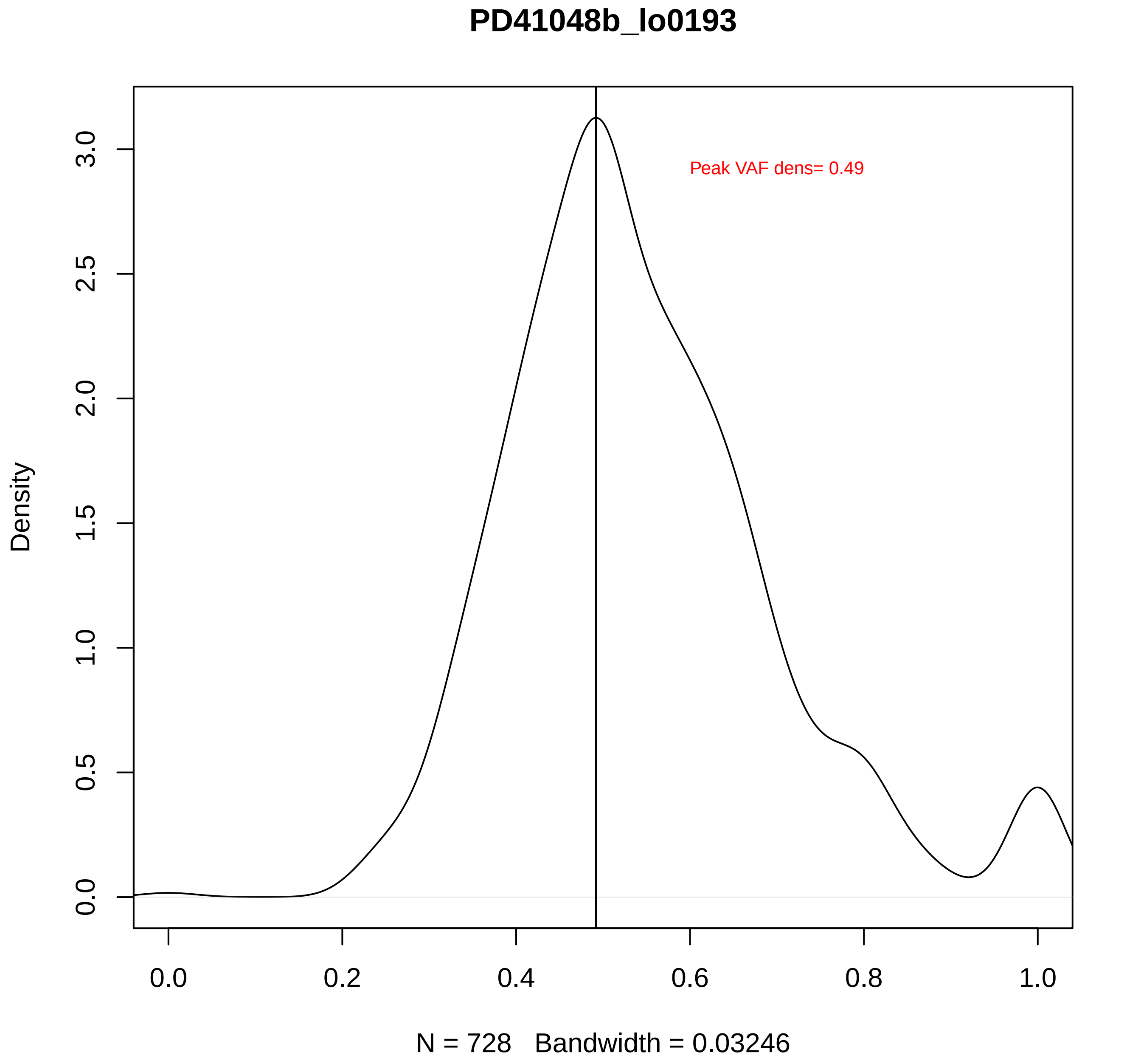

PD41048b_lo0193
Peak VAF dens= 0.49
Density
0.0
0.5
1.0
1.5
2.0
2.5
3.0
0.0
0.2
0.4
0.6
0.8
1.0
N = 728   Bandwidth = 0.03246

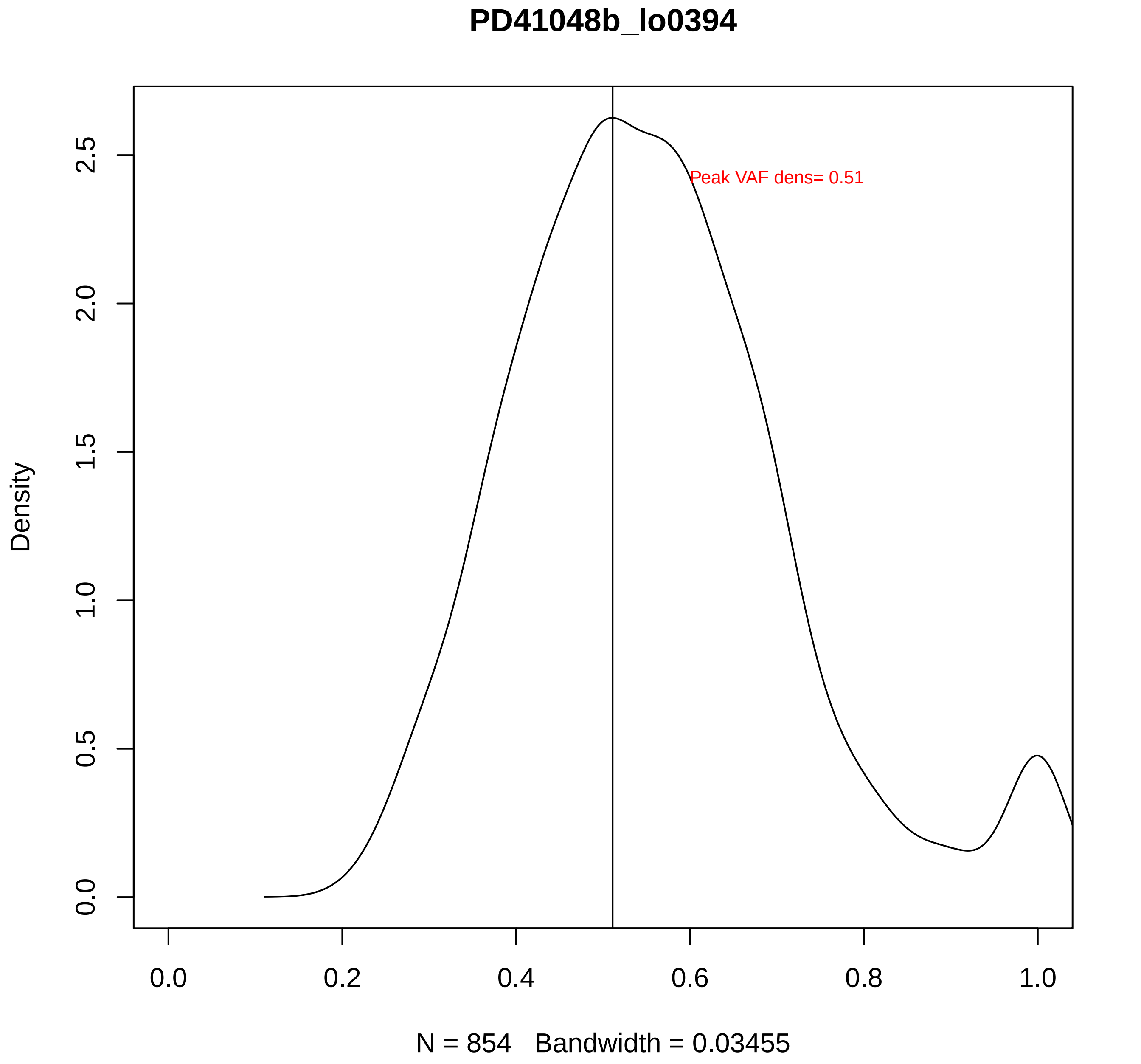

PD41048b_lo0394
Peak VAF dens= 0.51
Density
0.0
0.5
1.0
1.5
2.0
2.5
0.0
0.2
0.4
0.6
0.8
1.0
N = 854   Bandwidth = 0.03455

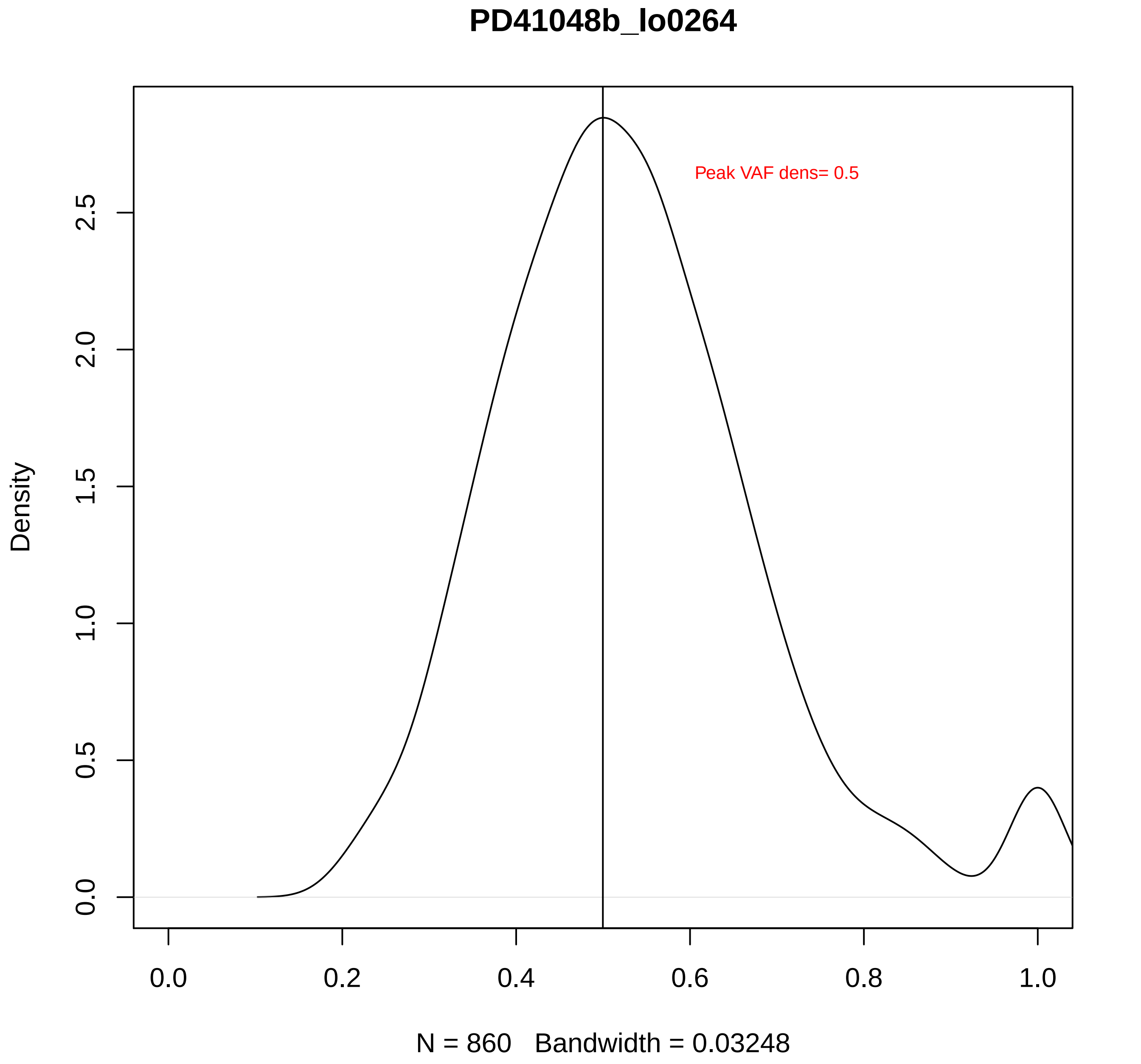

PD41048b_lo0264
Peak VAF dens= 0.5
Density
0.0
0.5
1.0
1.5
2.0
2.5
0.0
0.2
0.4
0.6
0.8
1.0
N = 860   Bandwidth = 0.03248

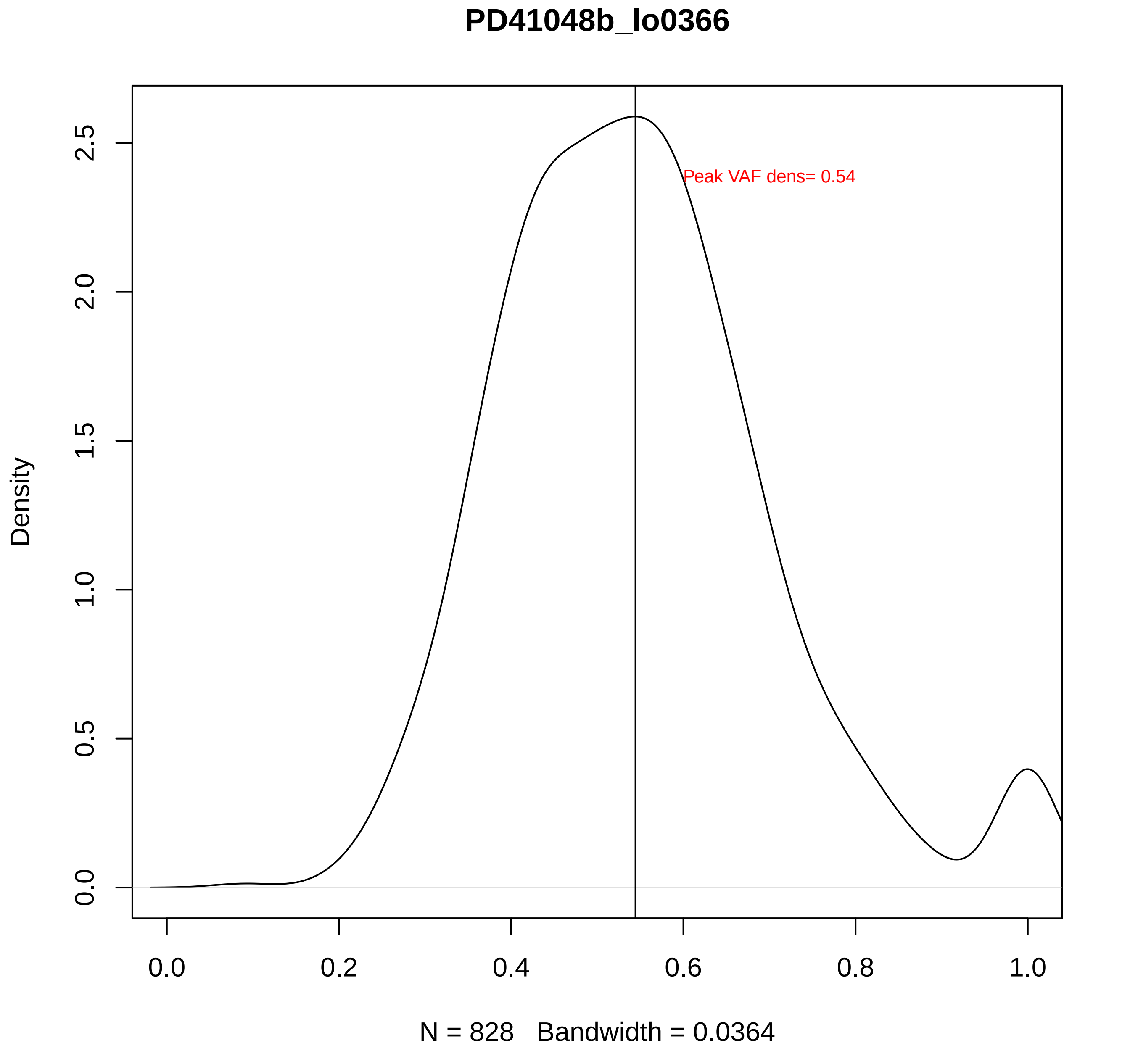
PD41048b_lo0366
Density
2.5
2.0
1.5
1.0
0.5
0.0
Peak VAF dens= 0.54
0.0
0.2
0.4
0.6
0.8
1.0
N = 828   Bandwidth = 0.0364

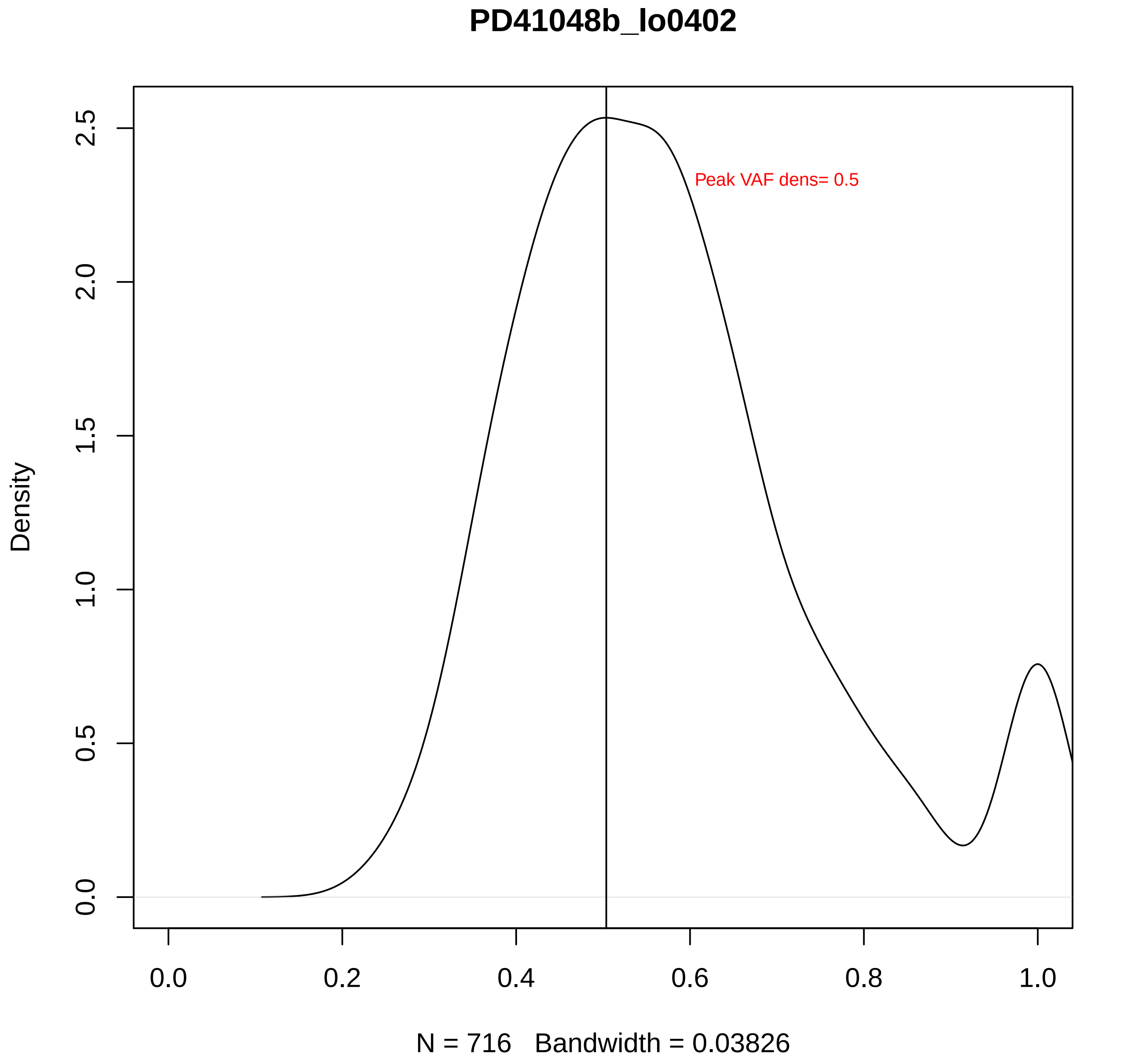
PD41048b_lo0402
Peak VAF dens= 0.5
Density
0.0
0.5
1.0
1.5
2.0
2.5
0.0
0.2
0.4
0.6
0.8
1.0
N = 716   Bandwidth = 0.03826

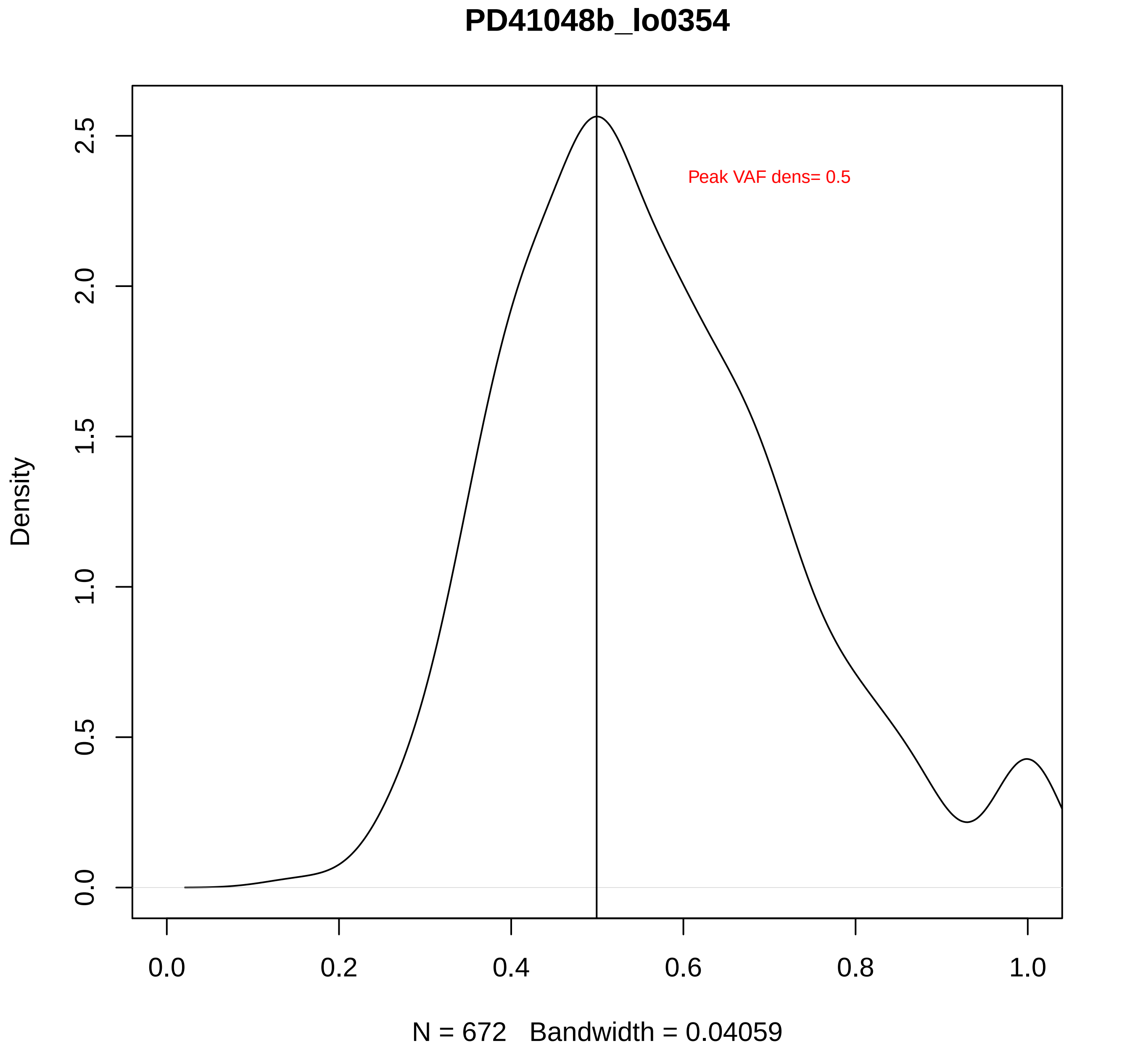

PD41048b_lo0354
Peak VAF dens= 0.5
Density
0.0
0.5
1.0
1.5
2.0
2.5
0.0
0.2
0.4
0.6
0.8
1.0
N = 672 Bandwidth = 0.04059

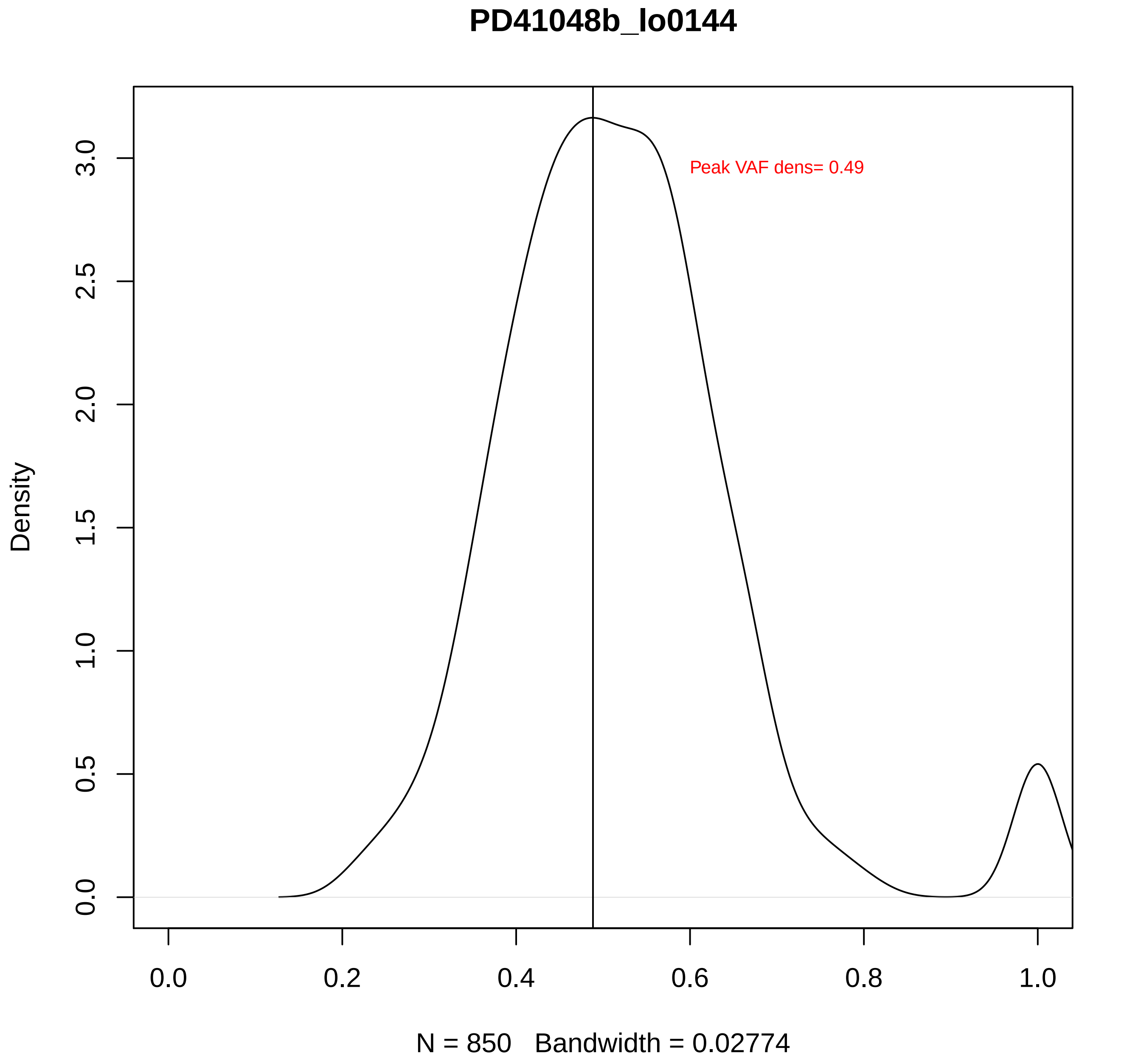
PD41048b_lo0144
Peak VAF dens= 0.49
Density
0.0
0.5
1.0
1.5
2.0
2.5
3.0
0.0
0.2
0.4
0.6
0.8
1.0
N = 850 Bandwidth = 0.02774

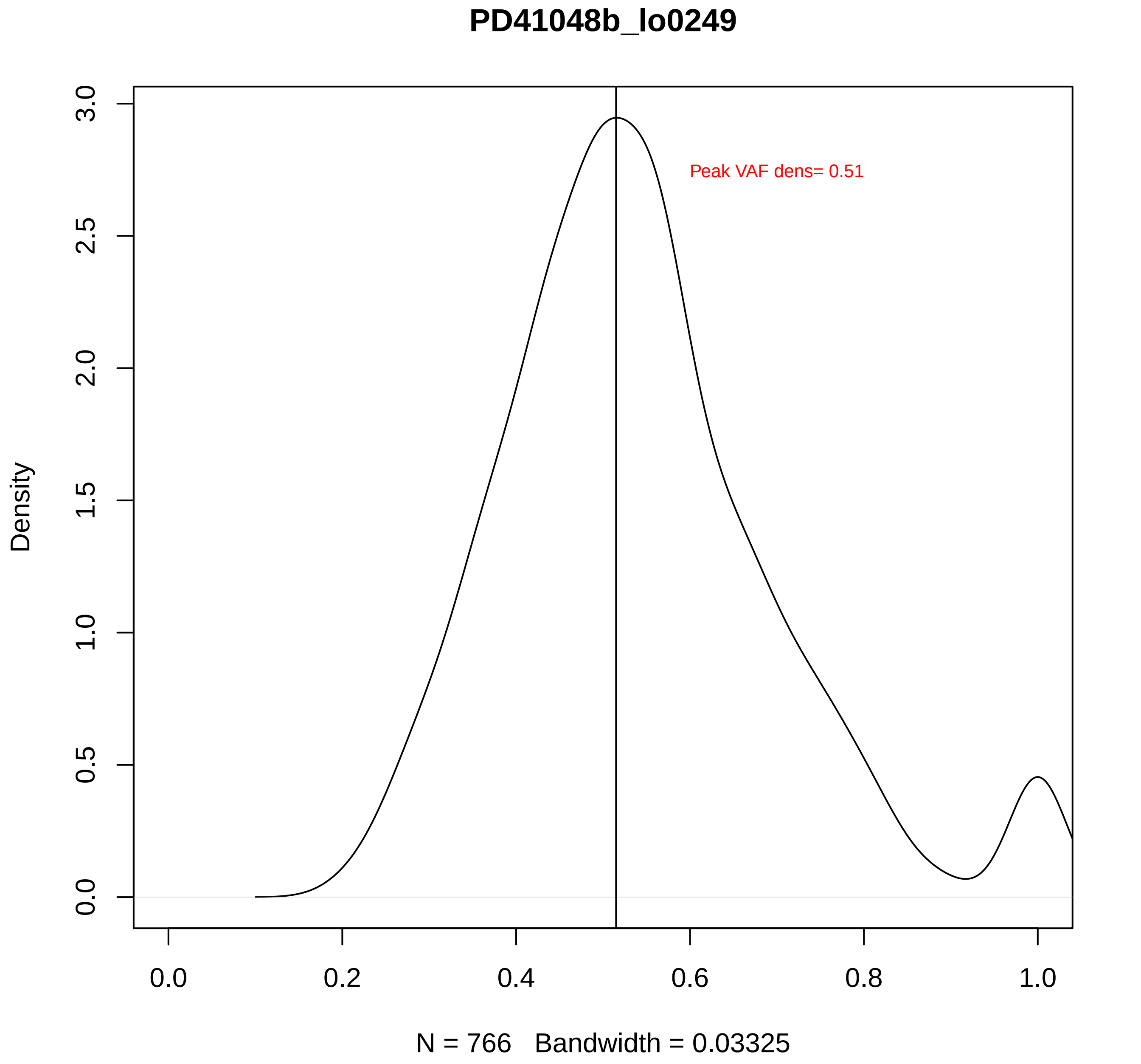

PD41048b_lo0249
Peak VAF dens= 0.51
Density
0.0
0.5
1.0
1.5
2.0
2.5
3.0
0.0
0.2
0.4
0.6
0.8
1.0
N = 766 Bandwidth = 0.03325

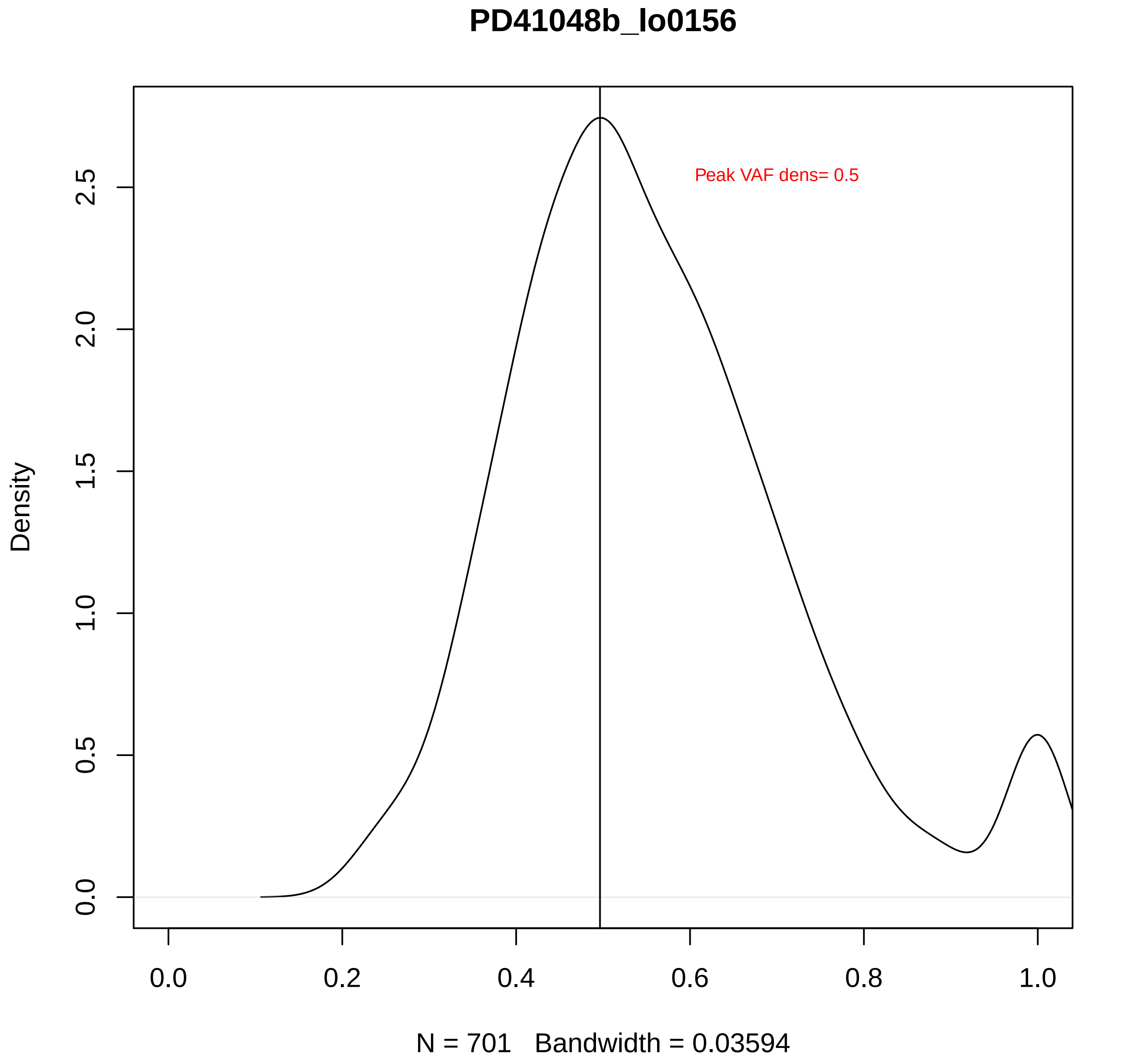
PD41048b_lo0156
Peak VAF dens= 0.5
Density
0.0
0.5
1.0
1.5
2.0
2.5
0.0
0.2
0.4
0.6
0.8
1.0
N = 701 Bandwidth = 0.03594

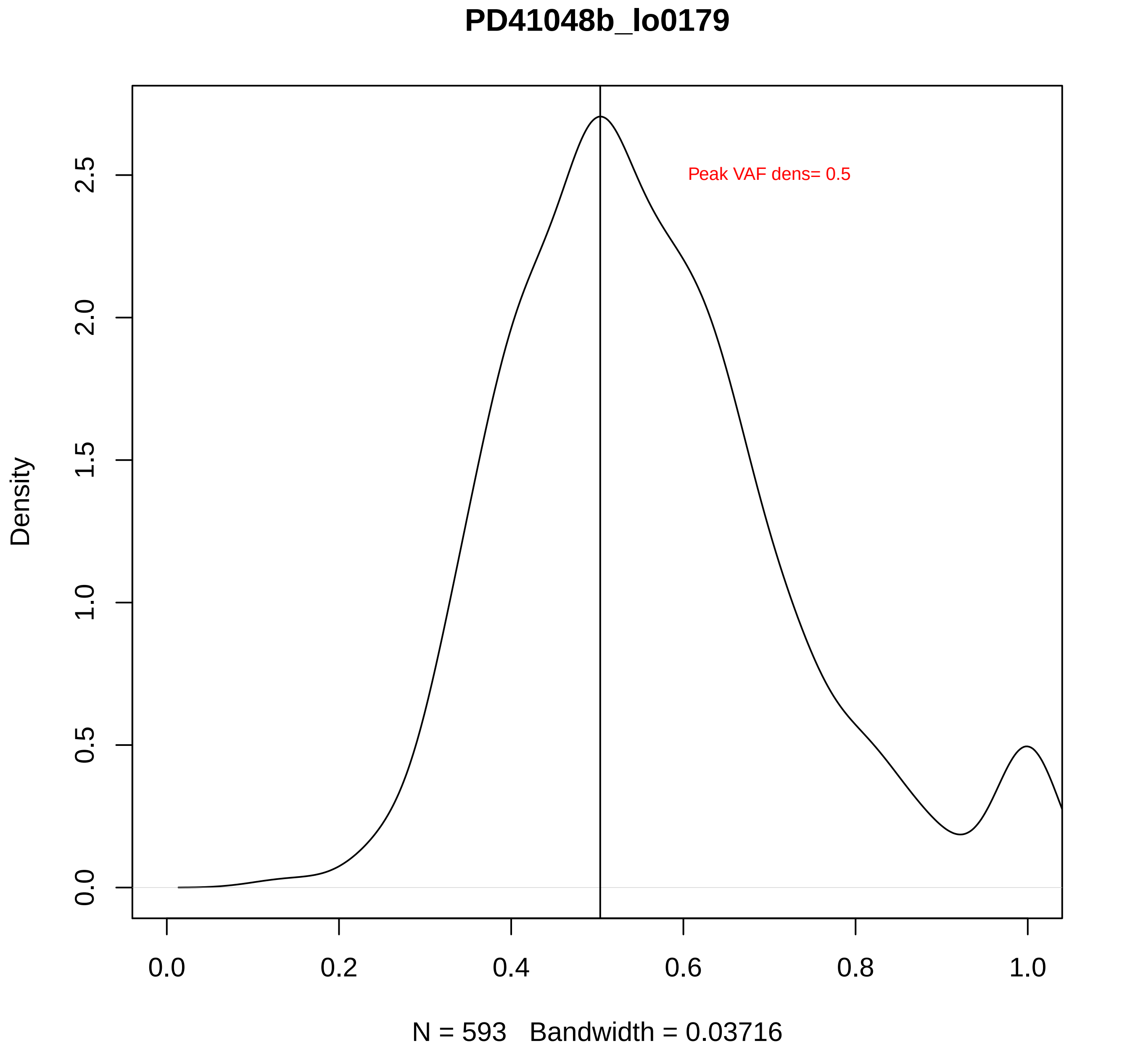
PD41048b_lo0179
Peak VAF dens= 0.5
Density
0.0
0.5
1.0
1.5
2.0
2.5
0.0
0.2
0.4
0.6
0.8
1.0
N = 593 Bandwidth = 0.03716

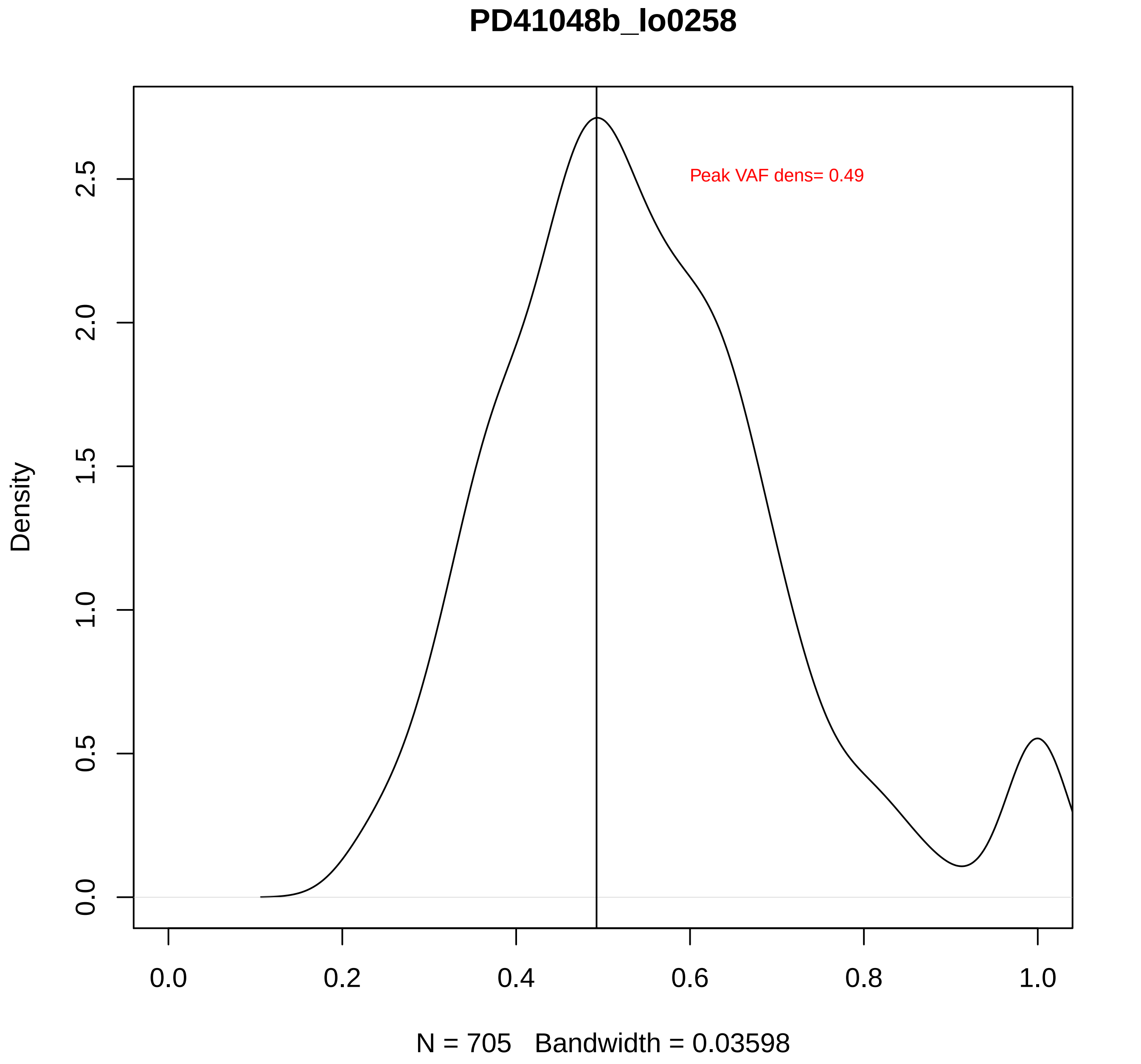

PD41048b_lo0258
Peak VAF dens= 0.49
Density
0.0
0.5
1.0
1.5
2.0
2.5
0.0
0.2
0.4
0.6
0.8
1.0
N = 705 Bandwidth = 0.03598

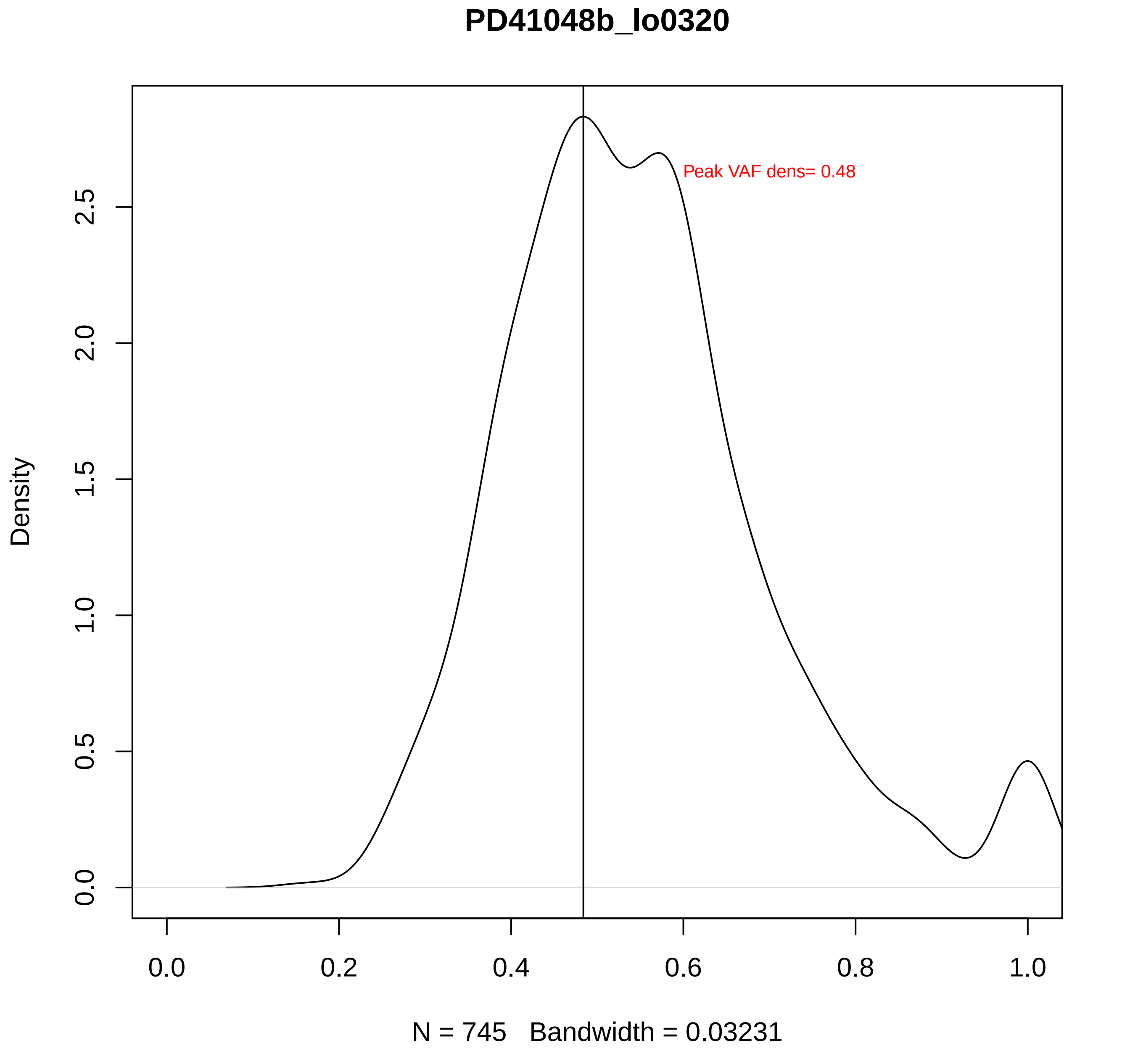

PD41048b_lo0320
Peak VAF dens= 0.48
Density
0.0
0.5
1.0
1.5
2.0
2.5
0.0
0.2
0.4
0.6
0.8
1.0
N = 745   Bandwidth = 0.03231

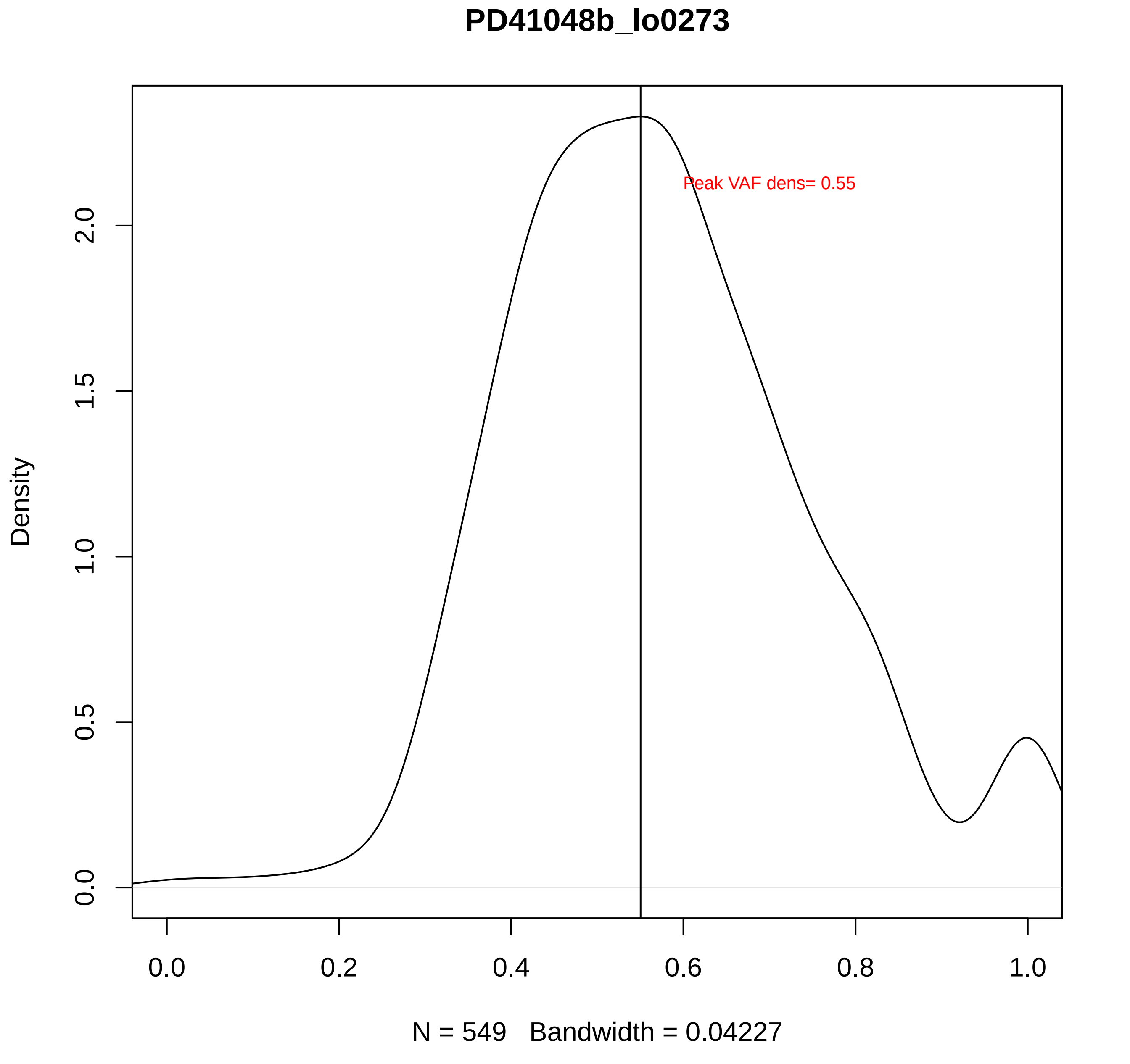

PD41048b_lo0273
Peak VAF dens= 0.55
Density
0.0
0.5
1.0
1.5
2.0
0.0
0.2
0.4
0.6
0.8
1.0
N = 549   Bandwidth = 0.04227

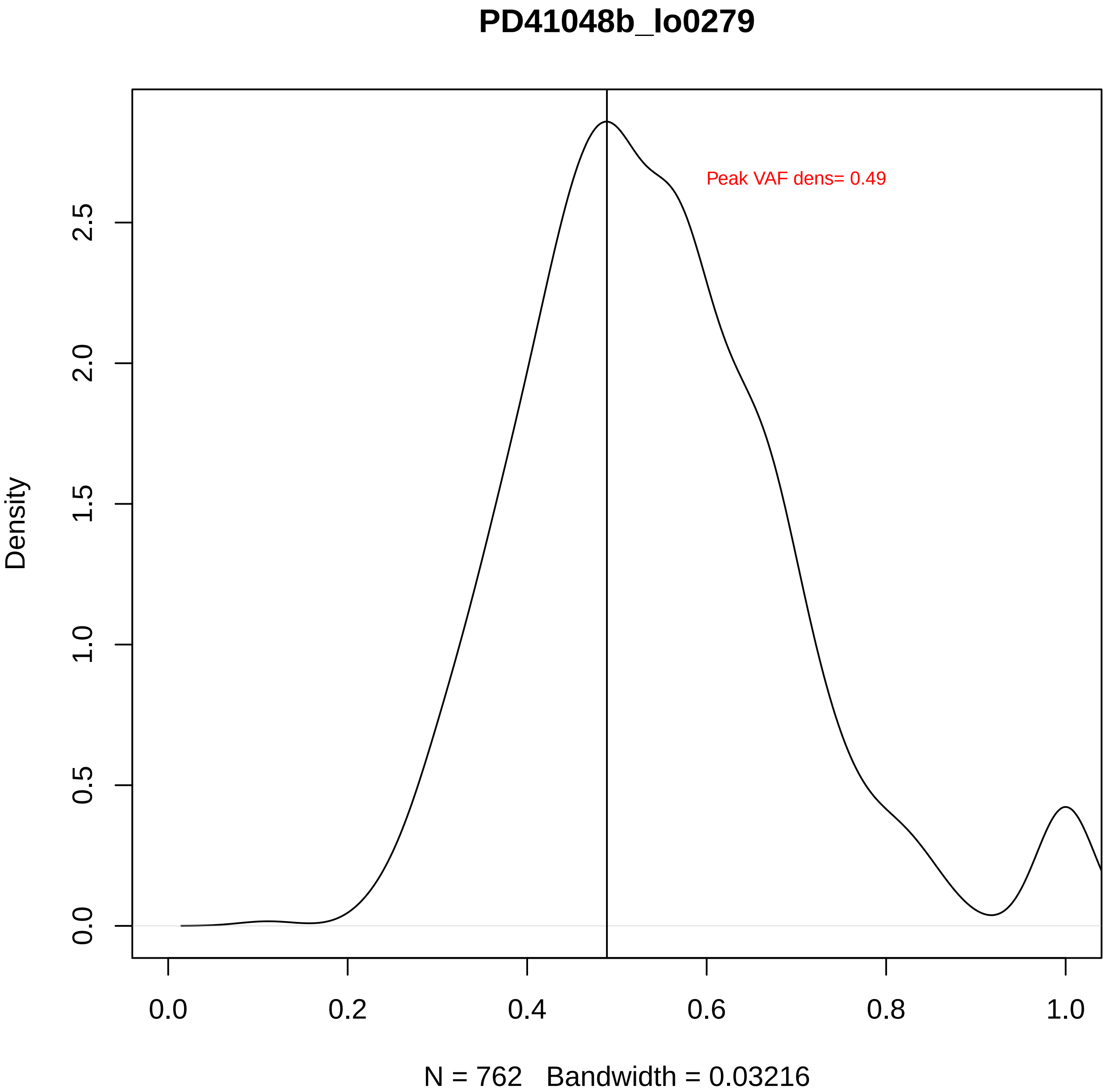

PD41048b_lo0279
Peak VAF dens= 0.49
Density
0.0
0.5
1.0
1.5
2.0
2.5
0.0
0.2
0.4
0.6
0.8
1.0
N = 762   Bandwidth = 0.03216

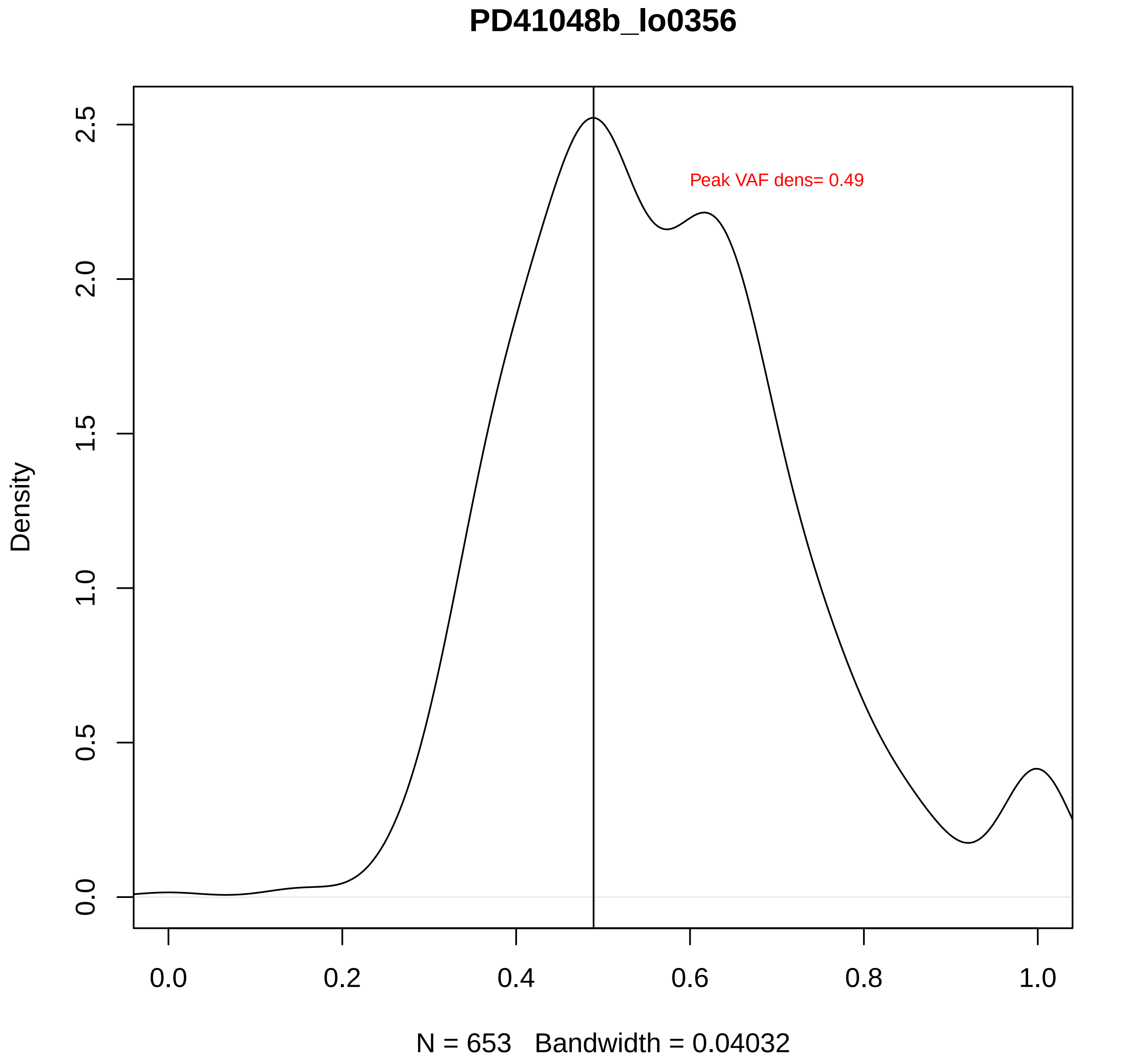
PD41048b_lo0356
Peak VAF dens= 0.49
Density
0.0
0.5
1.0
1.5
2.0
2.5
0.0
0.2
0.4
0.6
0.8
1.0
N = 653 Bandwidth = 0.04032

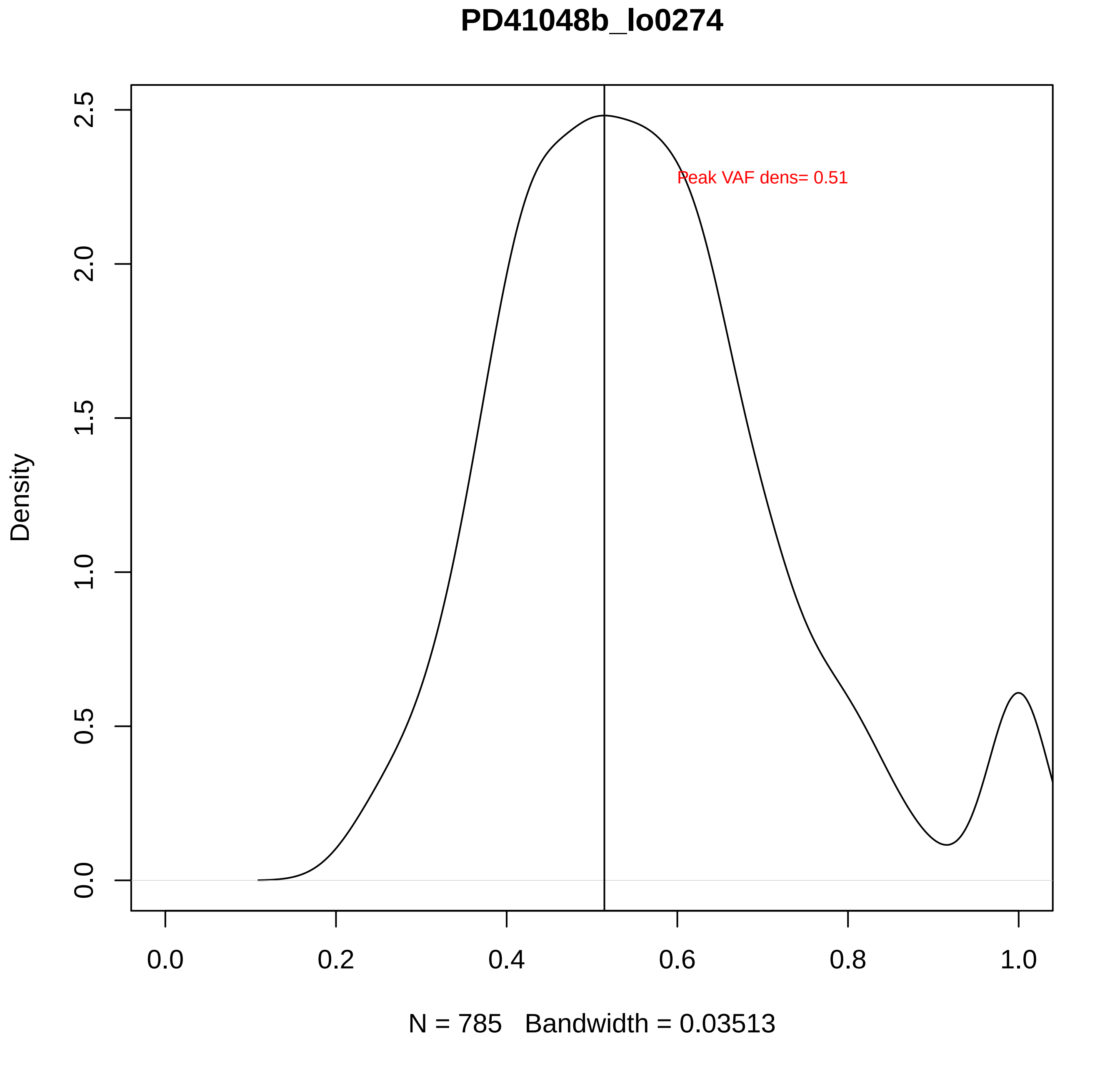
PD41048b_lo0274
Peak VAF dens= 0.51
Density
2.5
2.0
1.5
1.0
0.5
0.0
0.0
0.2
0.4
0.6
0.8
1.0
N = 785   Bandwidth = 0.03513

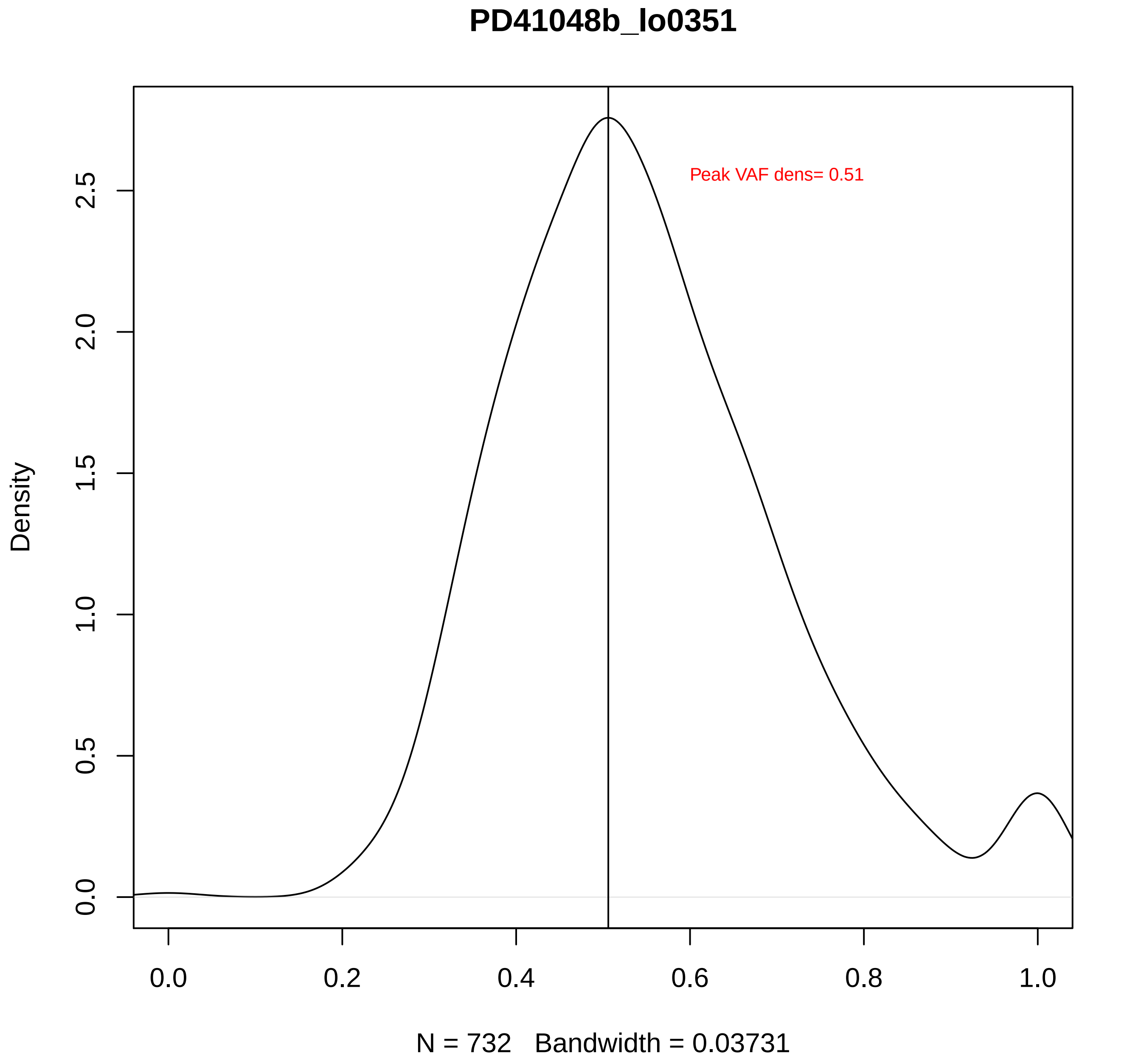
PD41048b_lo0351
Peak VAF dens= 0.51
Density
0.0
0.5
1.0
1.5
2.0
2.5
0.0
0.2
0.4
0.6
0.8
1.0
N = 732   Bandwidth = 0.03731

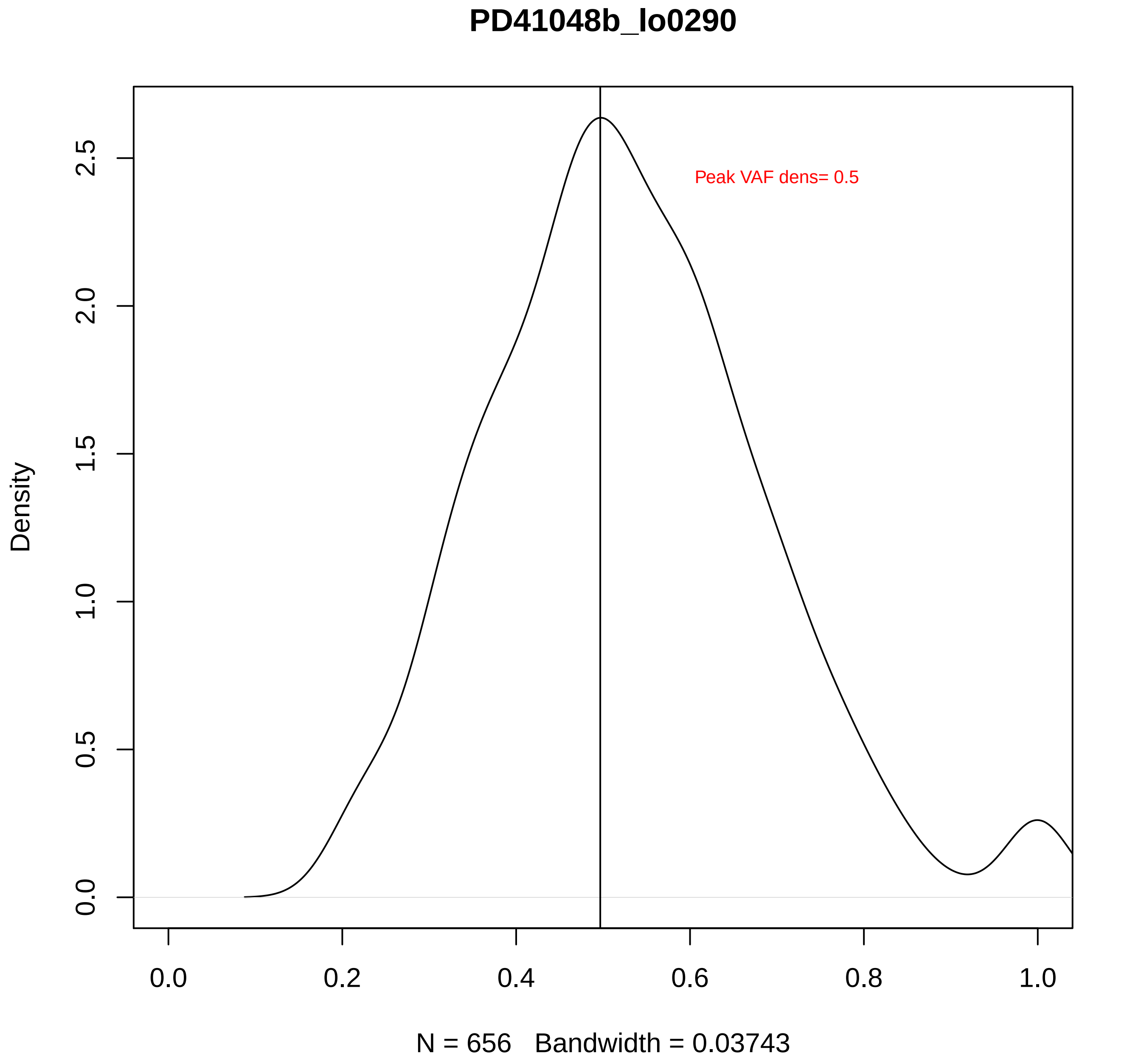

PD41048b_lo0290
Peak VAF dens= 0.5
Density
0.0
0.5
1.0
1.5
2.0
2.5
0.0
0.2
0.4
0.6
0.8
1.0
N = 656 Bandwidth = 0.03743

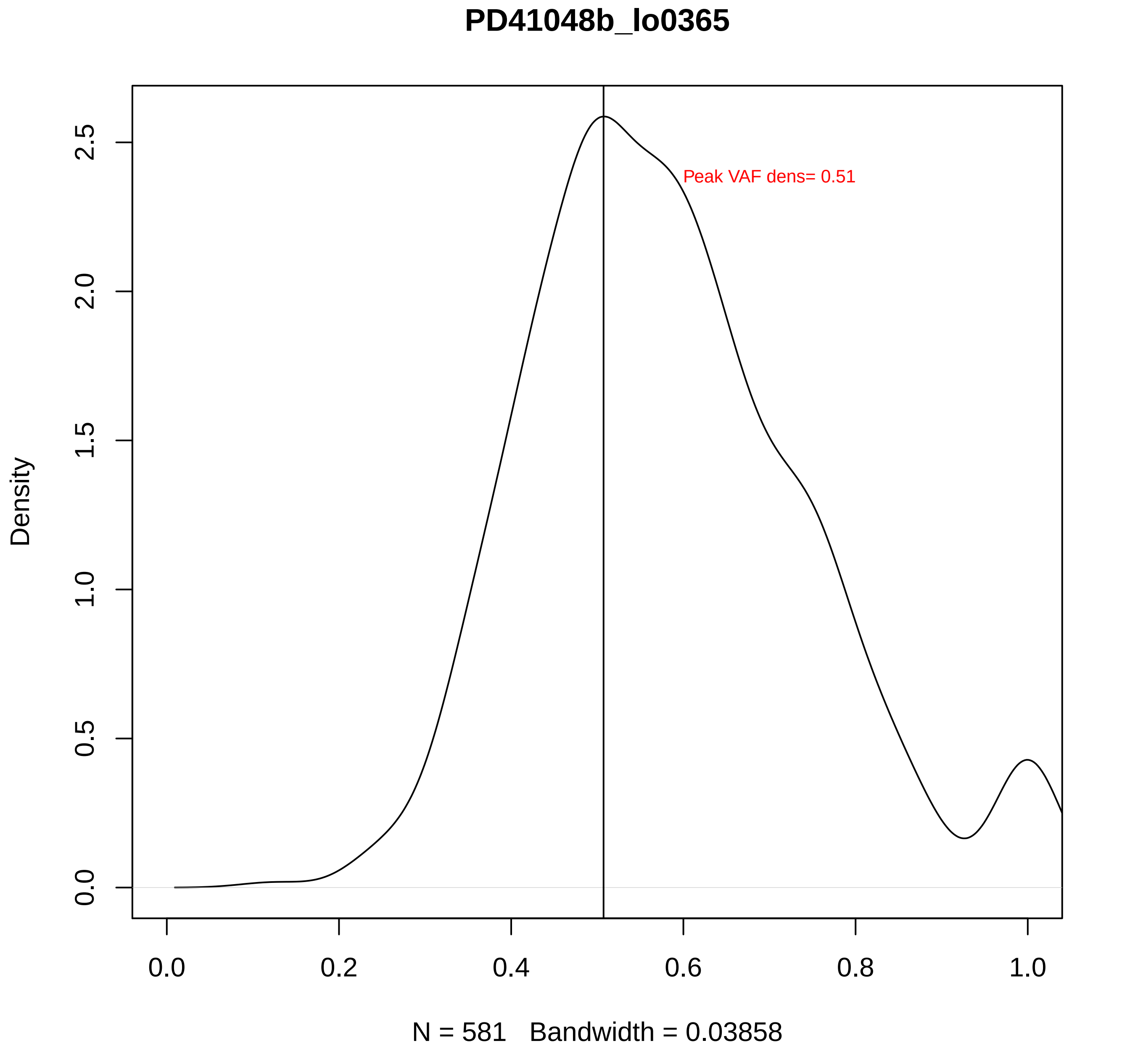

PD41048b_lo0365
Peak VAF dens= 0.51
Density
0.0
0.5
1.0
1.5
2.0
2.5
0.0
0.2
0.4
0.6
0.8
1.0
N = 581   Bandwidth = 0.03858

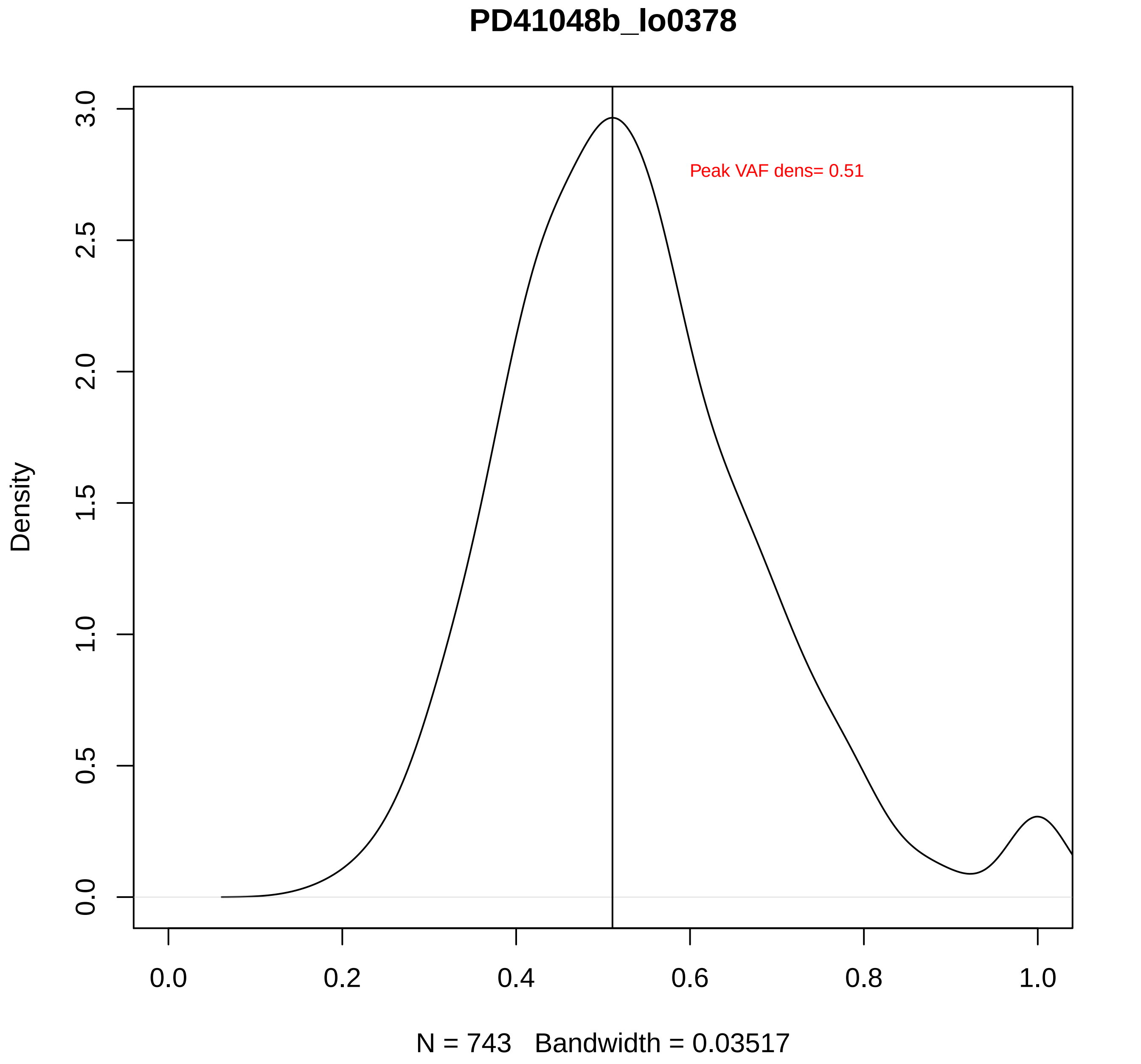

PD41048b_lo0378
Peak VAF dens= 0.51
Density
3.0
2.5
2.0
1.5
1.0
0.5
0.0
0.0
0.2
0.4
0.6
0.8
1.0
N = 743 Bandwidth = 0.03517

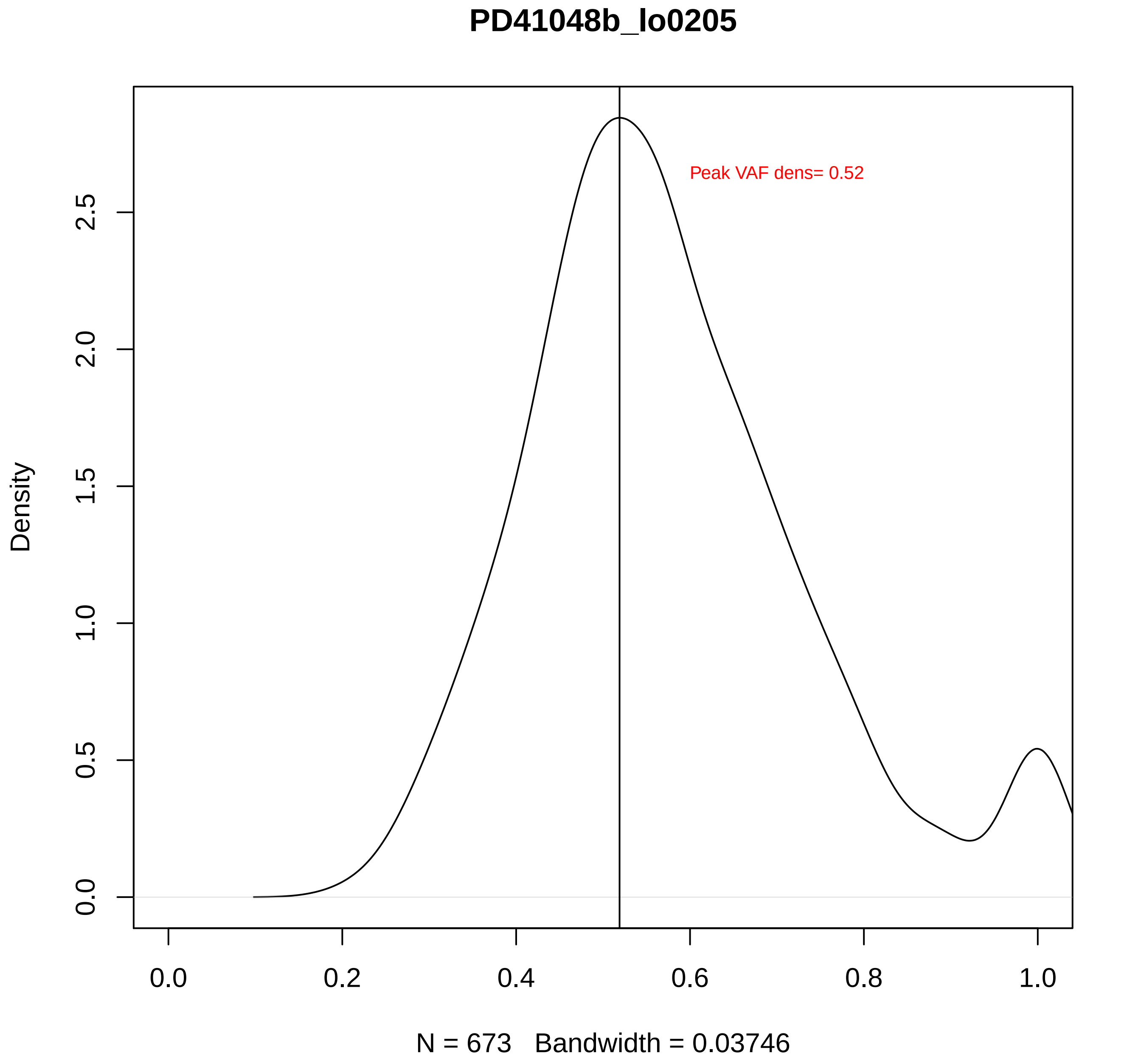
PD41048b_lo0205
Peak VAF dens= 0.52
Density
0.0
0.5
1.0
1.5
2.0
2.5
0.0
0.2
0.4
0.6
0.8
1.0
N = 673   Bandwidth = 0.03746

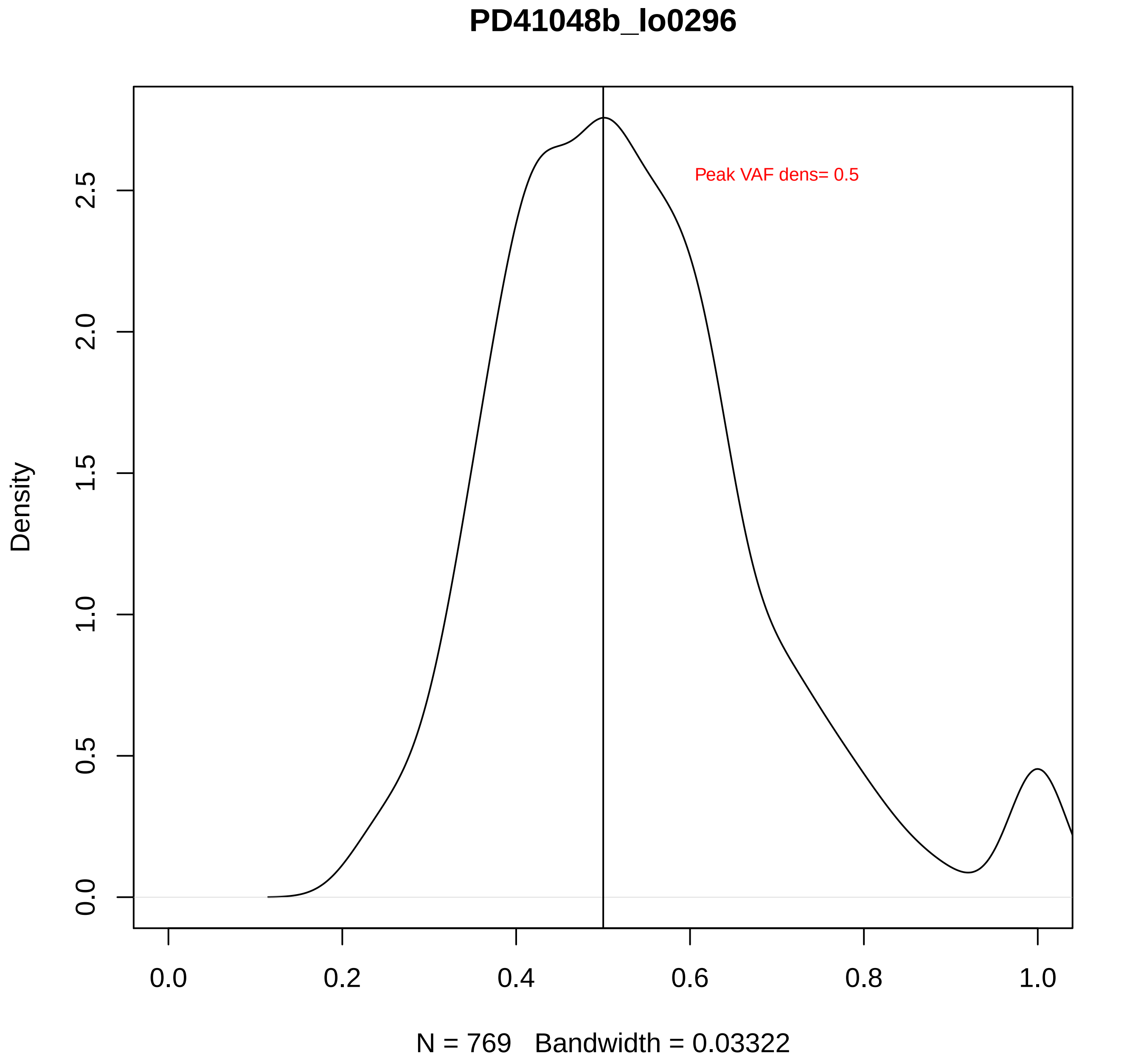

PD41048b_lo0296
Peak VAF dens= 0.5
Density
0.0
0.5
1.0
1.5
2.0
2.5
0.0
0.2
0.4
0.6
0.8
1.0
N = 769   Bandwidth = 0.03322

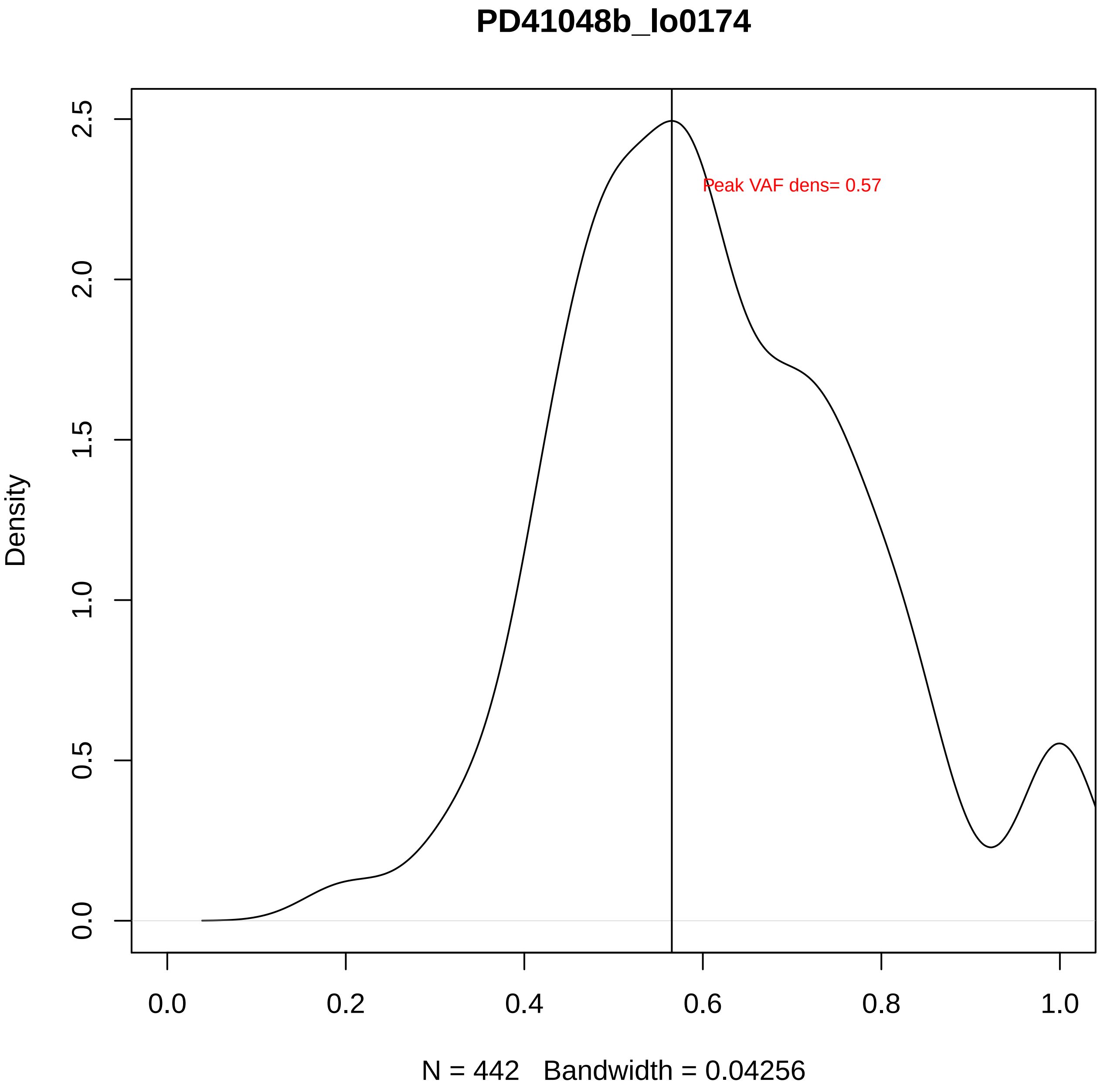
PD41048b_lo0174
Density
Peak VAF dens= 0.57
0.0
0.5
1.0
1.5
2.0
2.5
0.0
0.2
0.4
0.6
0.8
1.0
N = 442 Bandwidth = 0.04256

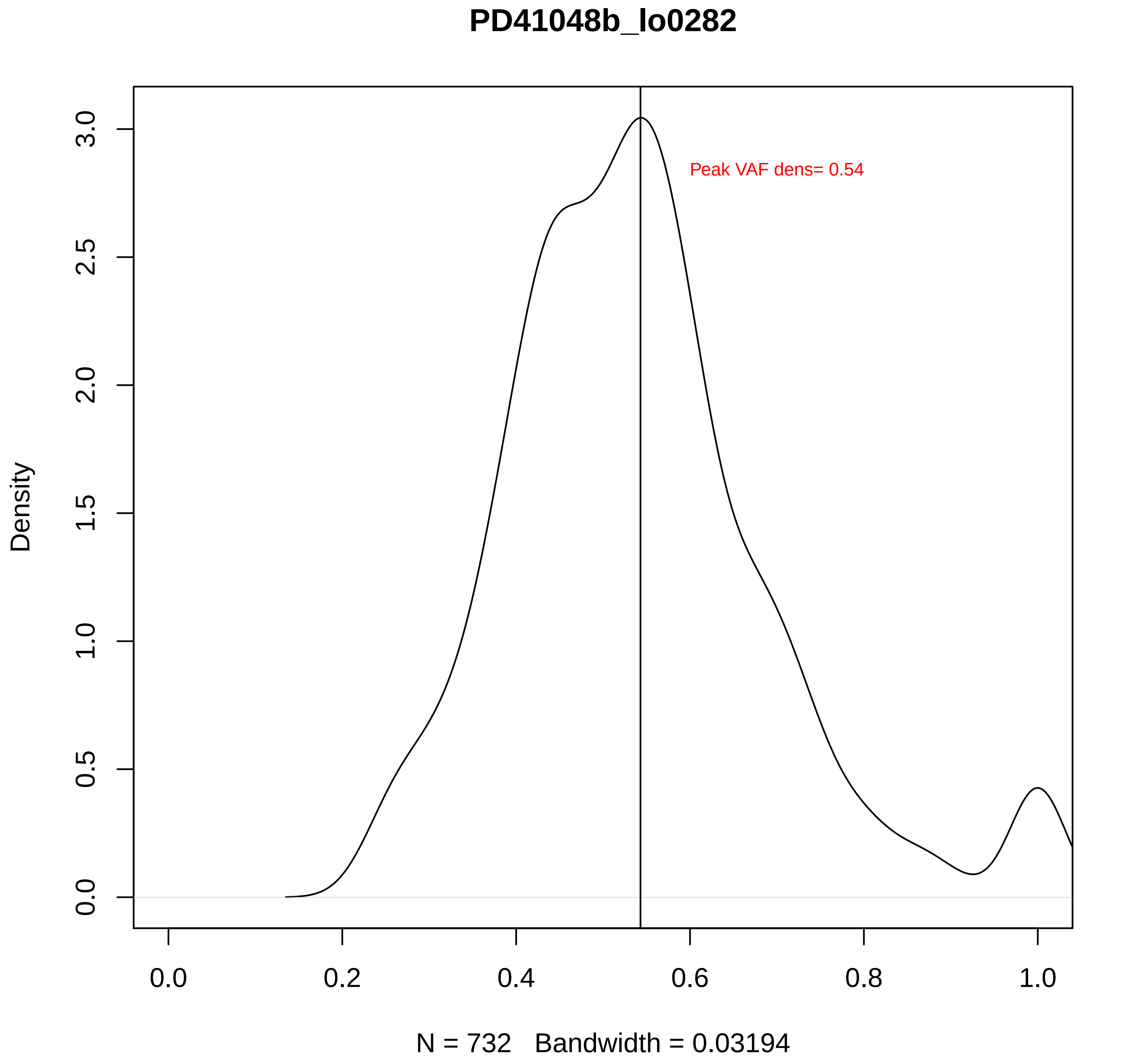
PD41048b_lo0282
Peak VAF dens= 0.54
Density
0.0
0.5
1.0
1.5
2.0
2.5
3.0
0.0
0.2
0.4
0.6
0.8
1.0
N = 732 Bandwidth = 0.03194

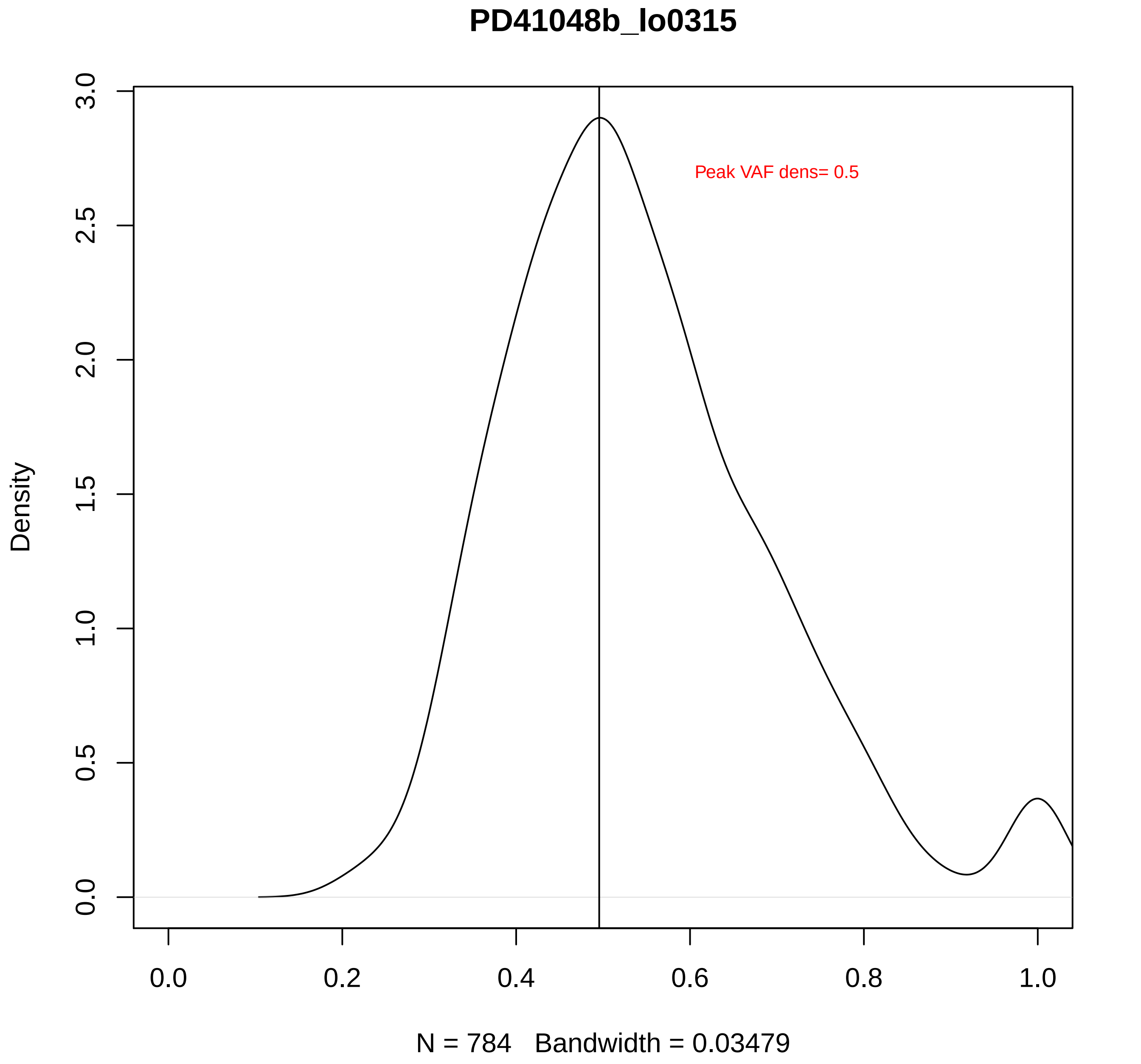
PD41048b_lo0315
Peak VAF dens= 0.5
Density
0.0
0.5
1.0
1.5
2.0
2.5
3.0
0.0
0.2
0.4
0.6
0.8
1.0
N = 784 Bandwidth = 0.03479

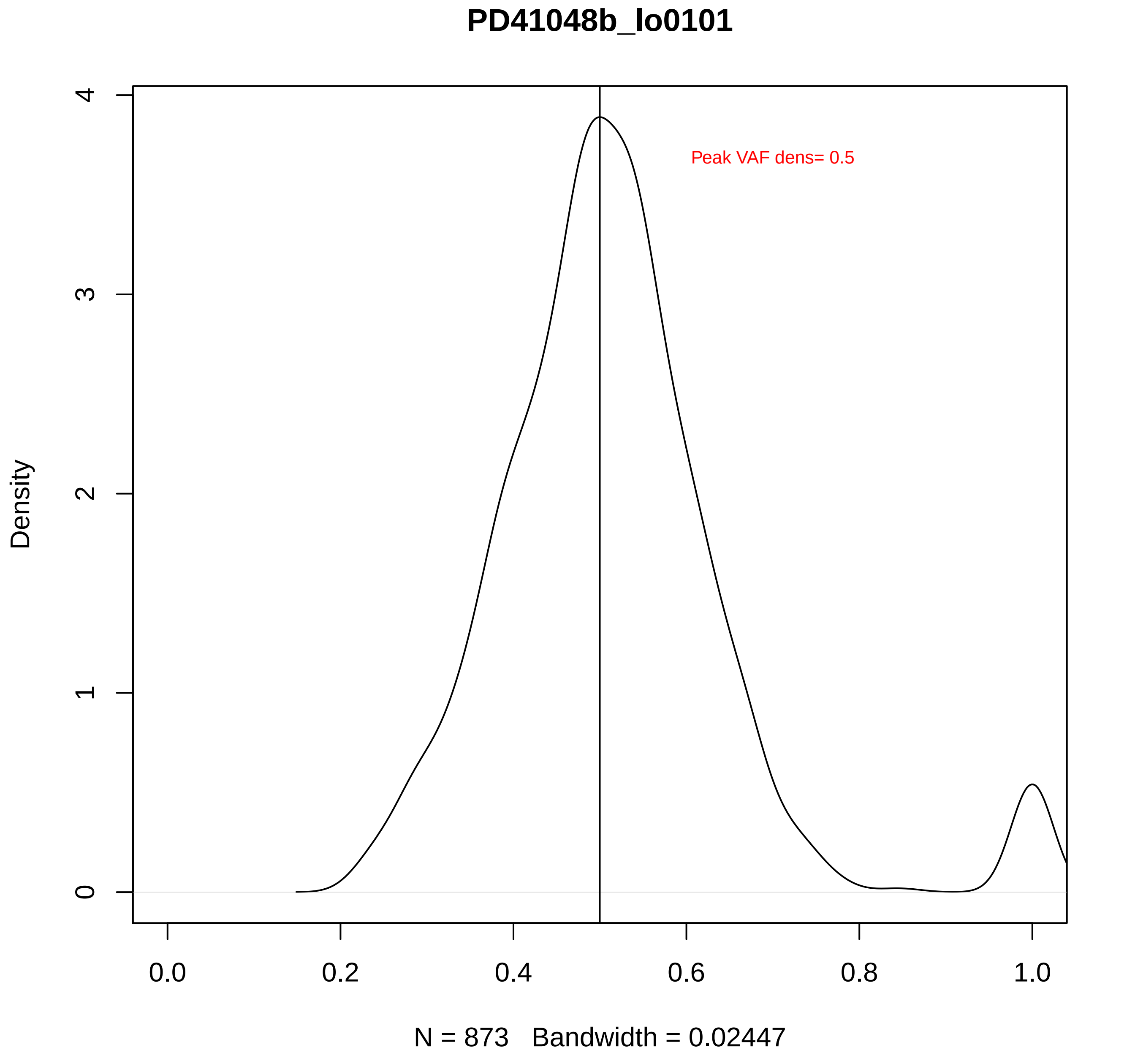

PD41048b_lo0101
Peak VAF dens= 0.5
Density
0
1
2
3
4
0.0
0.2
0.4
0.6
0.8
1.0
N = 873   Bandwidth = 0.02447

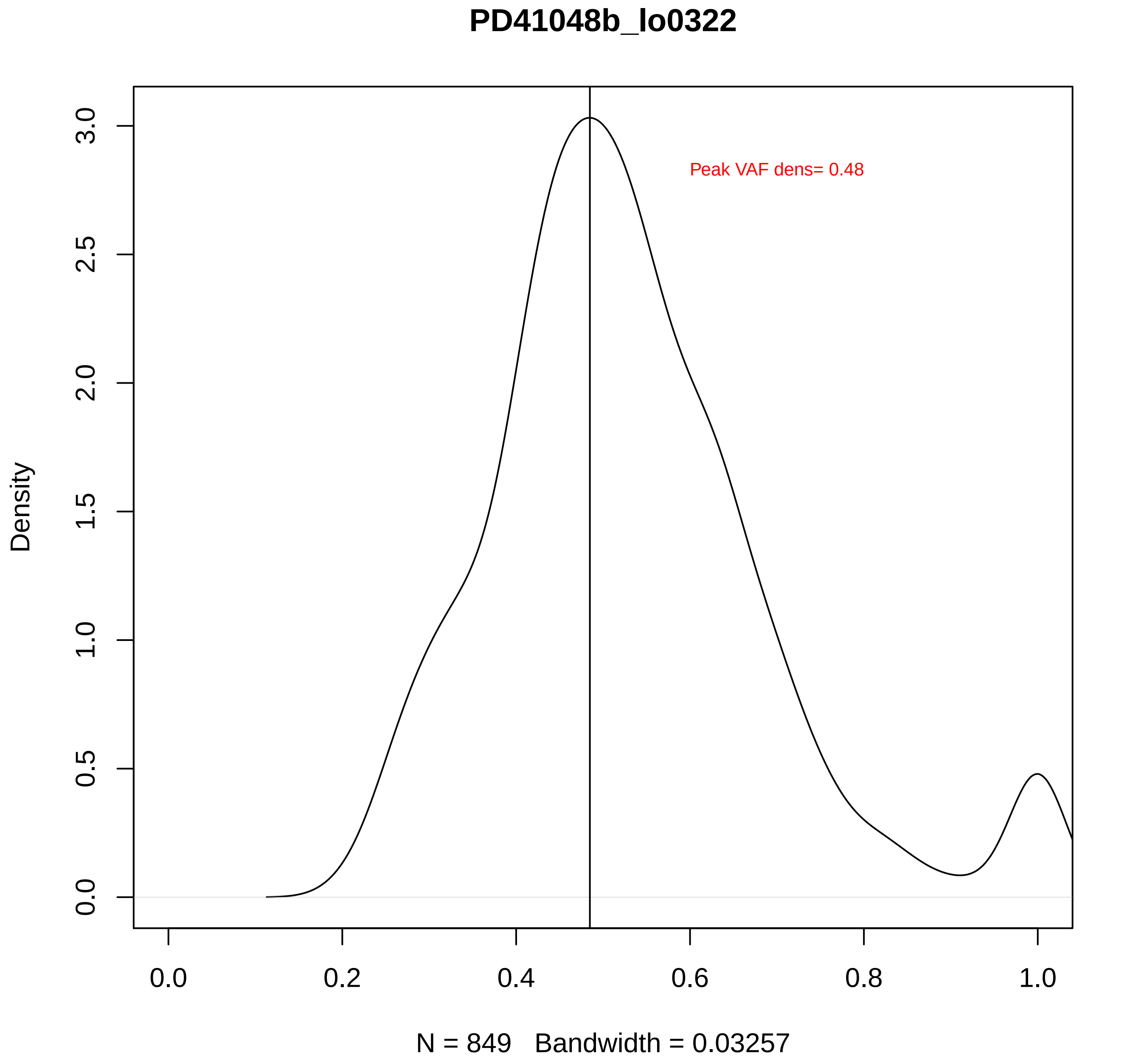

PD41048b_lo0322
Peak VAF dens= 0.48
Density
3.0
2.5
2.0
1.5
1.0
0.5
0.0
0.0
0.2
0.4
0.6
0.8
1.0
N = 849   Bandwidth = 0.03257

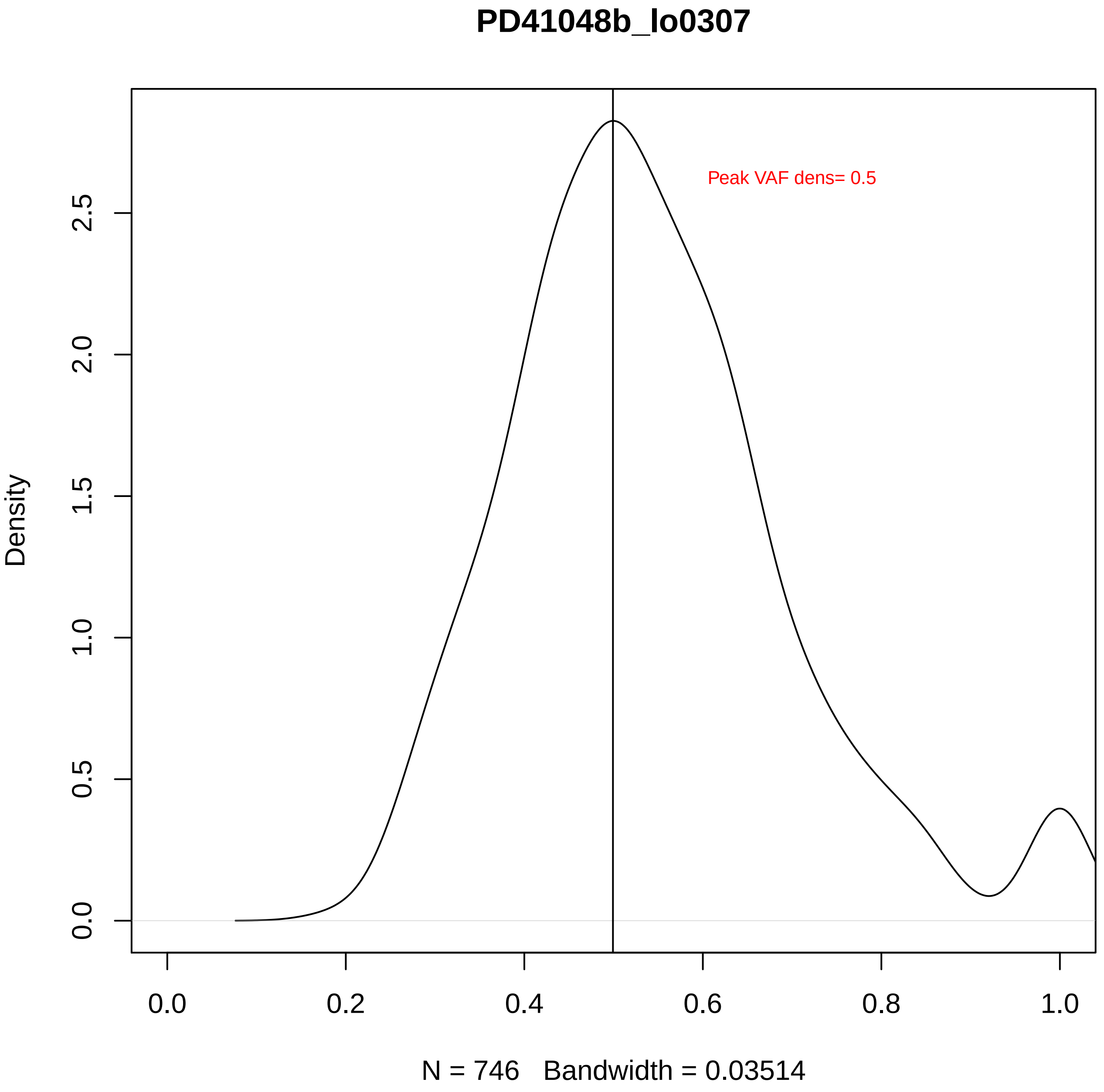
PD41048b_lo0307
Peak VAF dens= 0.5
Density
0.0
0.5
1.0
1.5
2.0
2.5
0.0
0.2
0.4
0.6
0.8
1.0
N = 746   Bandwidth = 0.03514

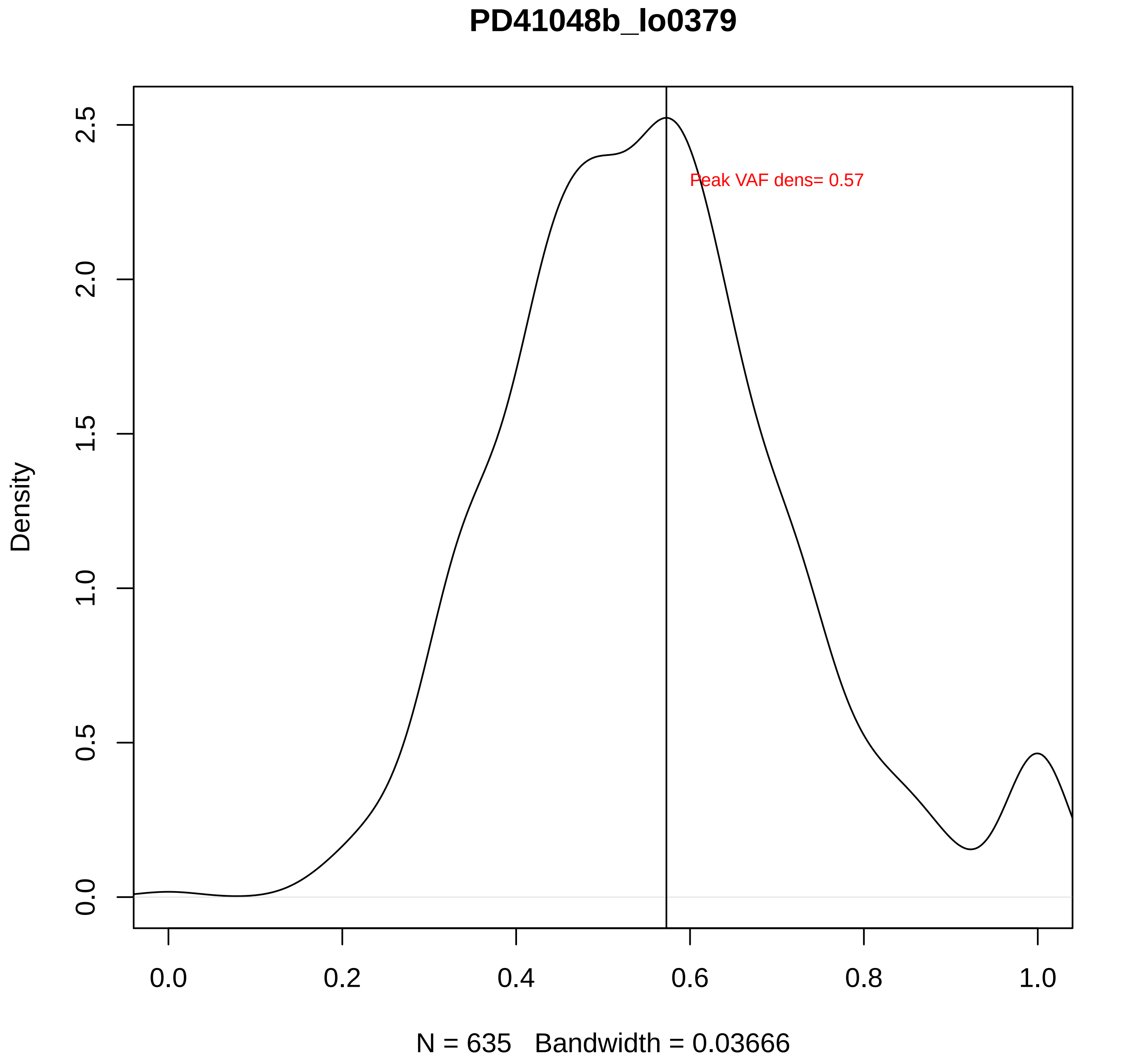
PD41048b_lo0379
Peak VAF dens= 0.57
Density
0.0
0.5
1.0
1.5
2.0
2.5
0.0
0.2
0.4
0.6
0.8
1.0
N = 635   Bandwidth = 0.03666

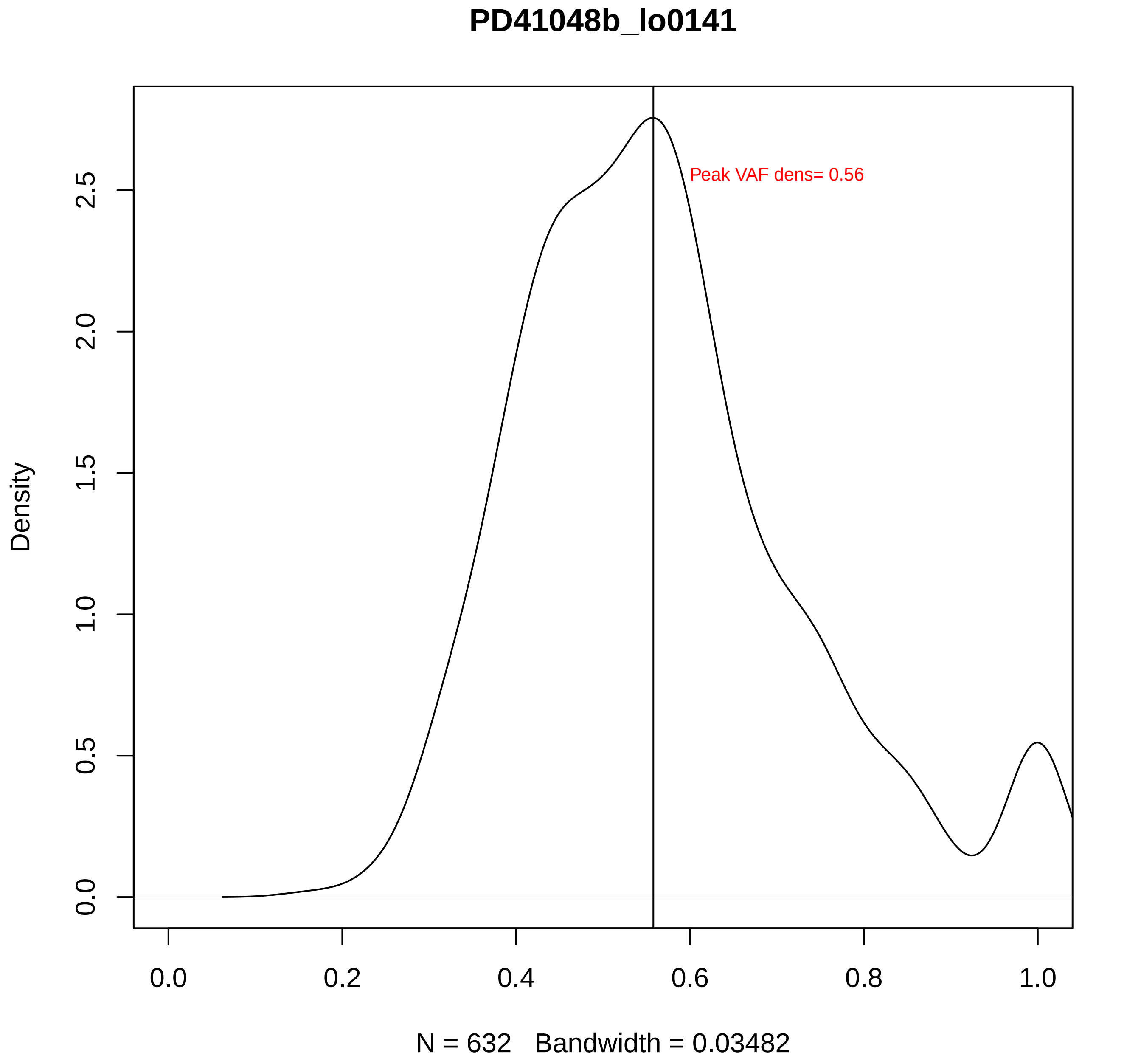
PD41048b_lo0141
Peak VAF dens= 0.56
Density
0.0
0.5
1.0
1.5
2.0
2.5
0.0
0.2
0.4
0.6
0.8
1.0
N = 632   Bandwidth = 0.03482

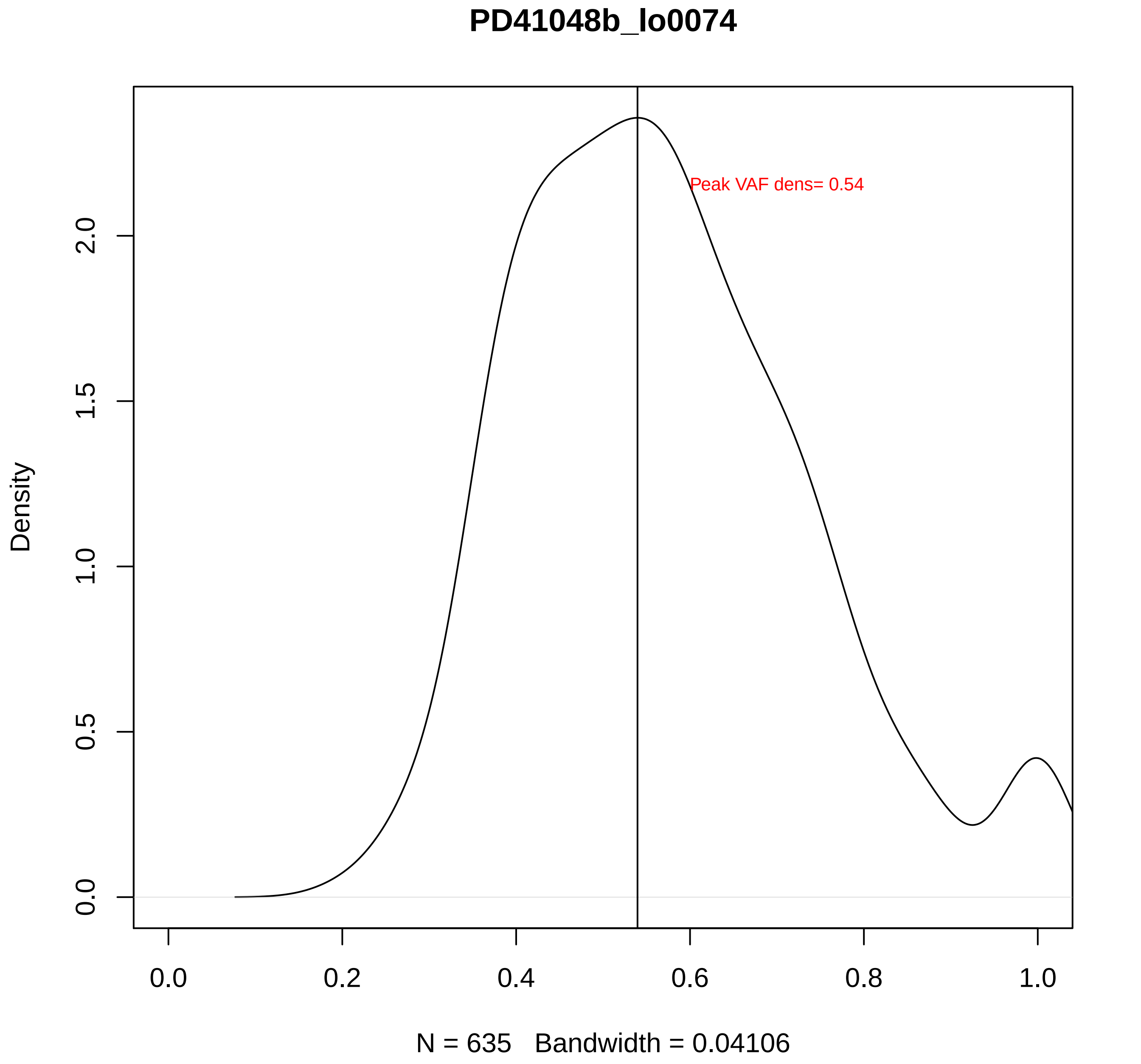
PD41048b_lo0074
Peak VAF dens= 0.54
Density
0.0
0.5
1.0
1.5
2.0
0.0
0.2
0.4
0.6
0.8
1.0
N = 635 Bandwidth = 0.04106

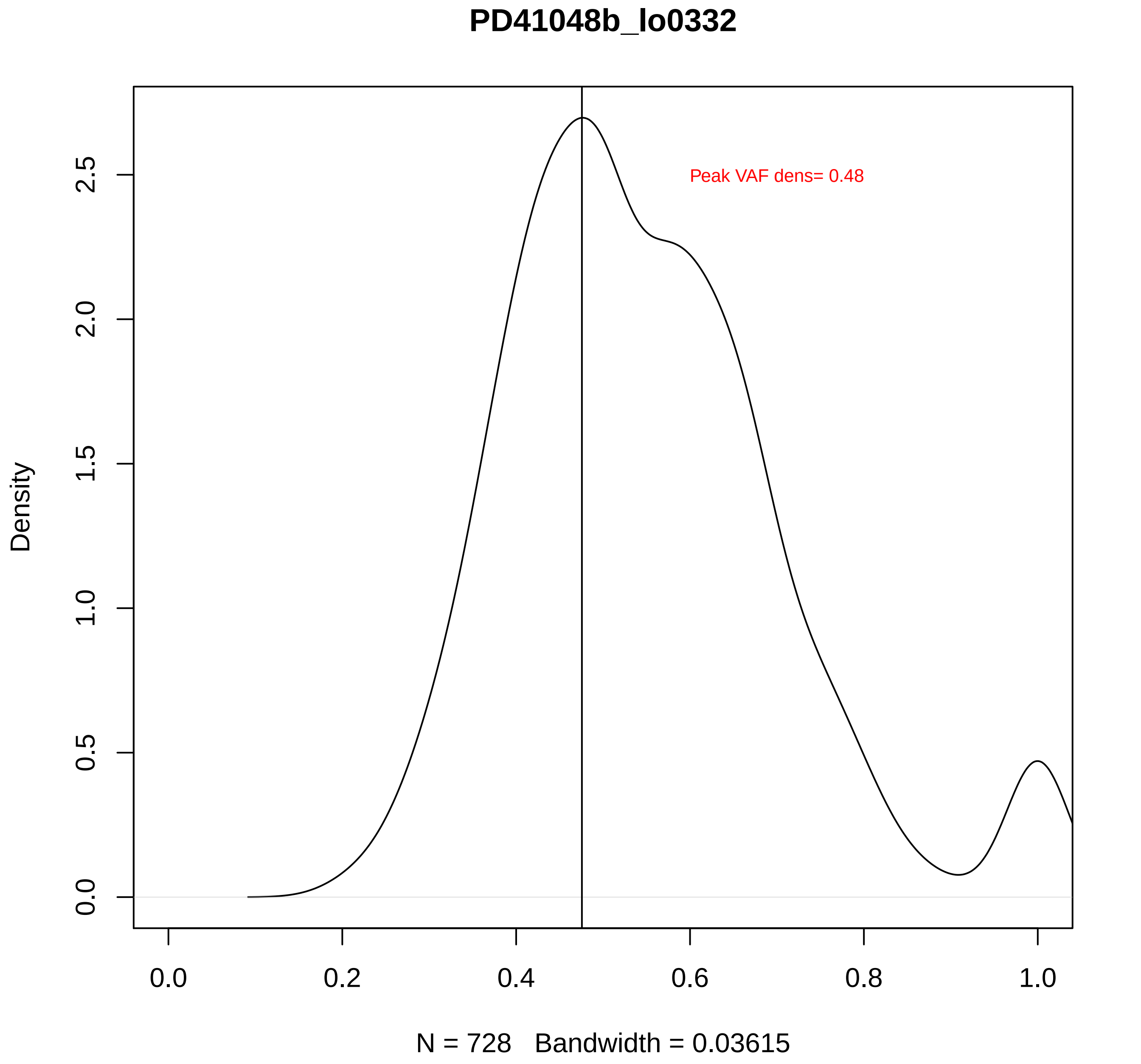
PD41048b_lo0332
Peak VAF dens= 0.48
Density
0.0
0.5
1.0
1.5
2.0
2.5
0.0
0.2
0.4
0.6
0.8
1.0
N = 728 Bandwidth = 0.03615

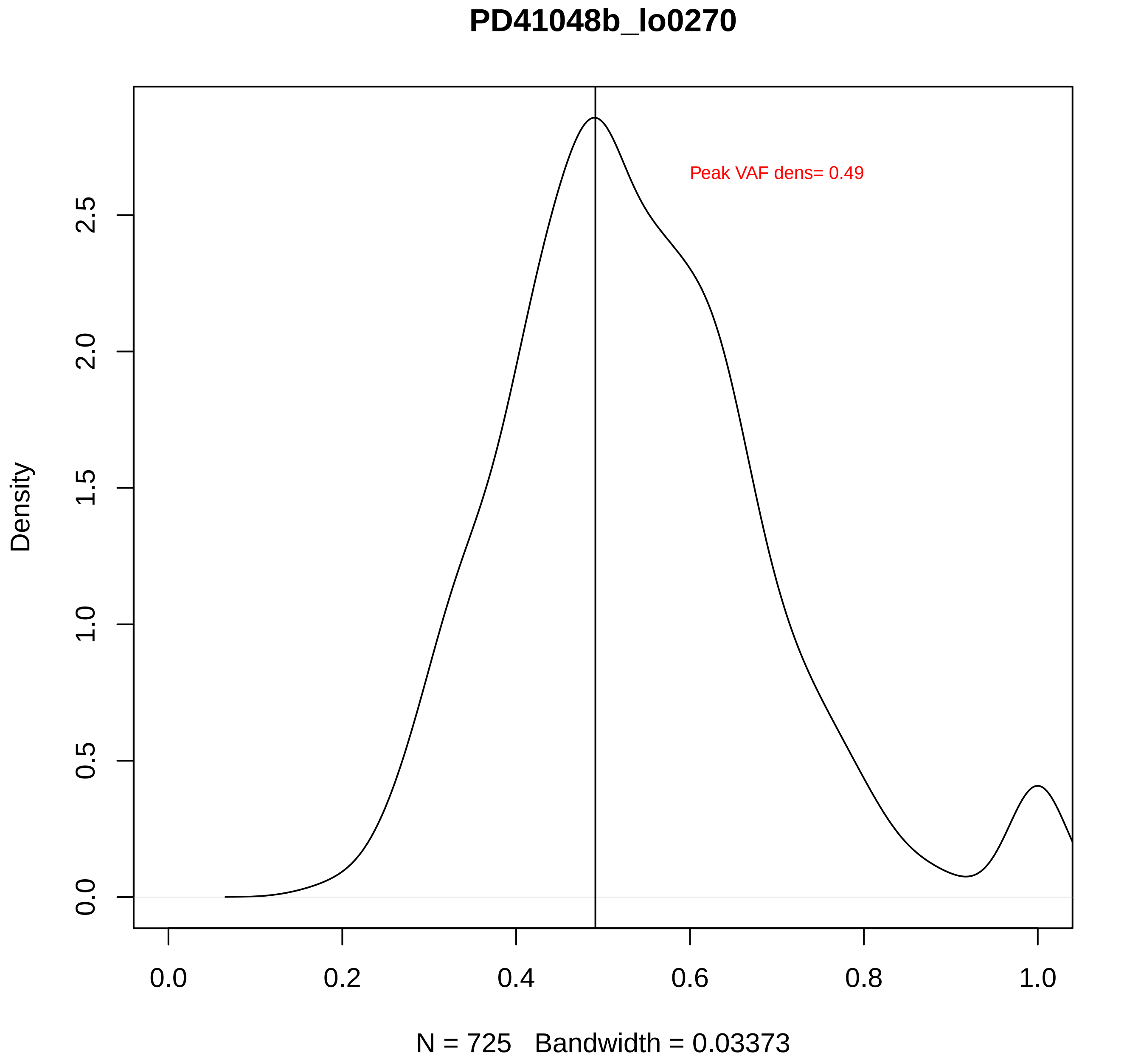
PD41048b_lo0270
Peak VAF dens= 0.49
Density
0.0
0.5
1.0
1.5
2.0
2.5
0.0
0.2
0.4
0.6
0.8
1.0
N = 725   Bandwidth = 0.03373

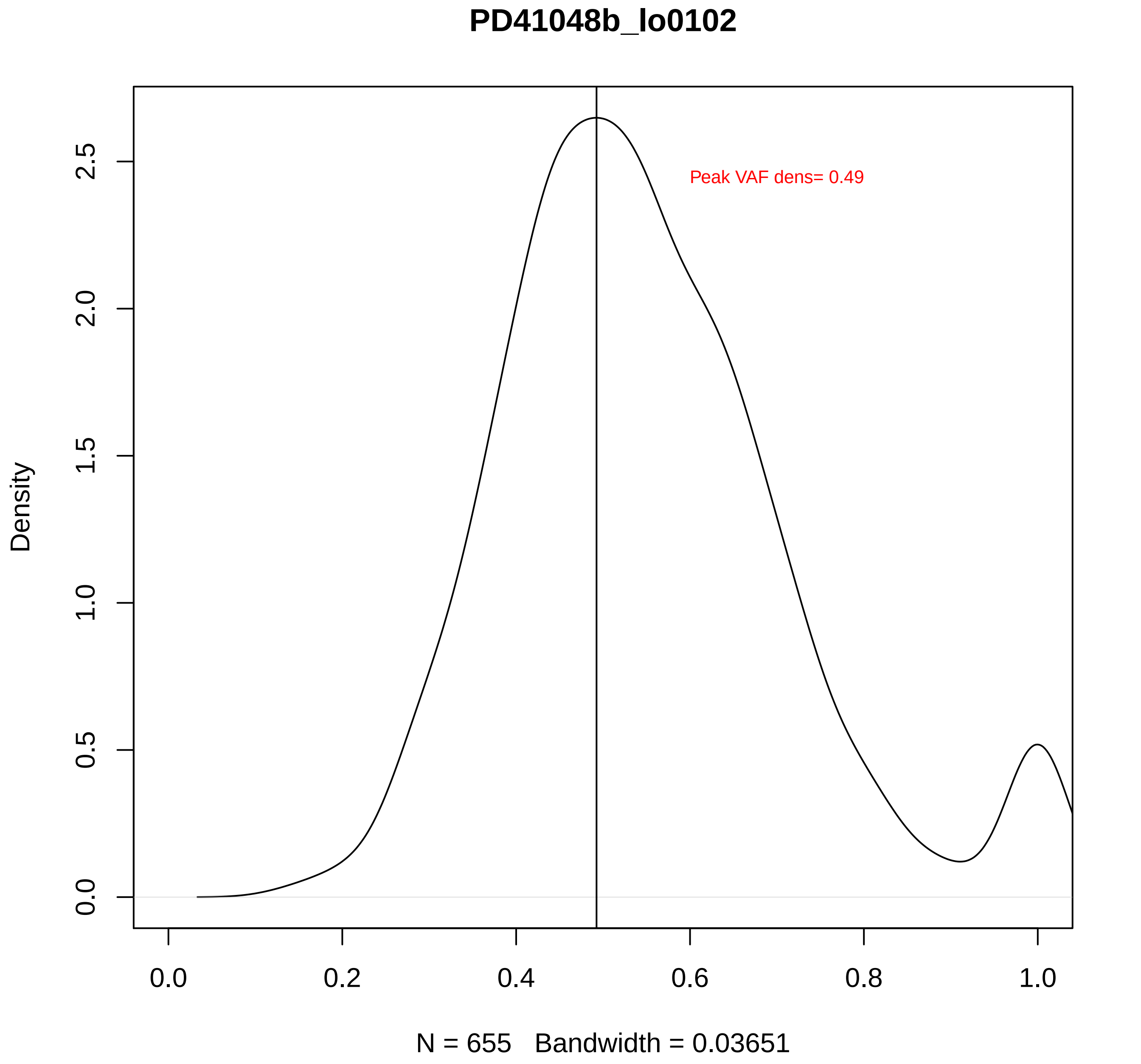

PD41048b_lo0102
Peak VAF dens= 0.49
Density
0.0
0.5
1.0
1.5
2.0
2.5
0.0
0.2
0.4
0.6
0.8
1.0
N = 655   Bandwidth = 0.03651

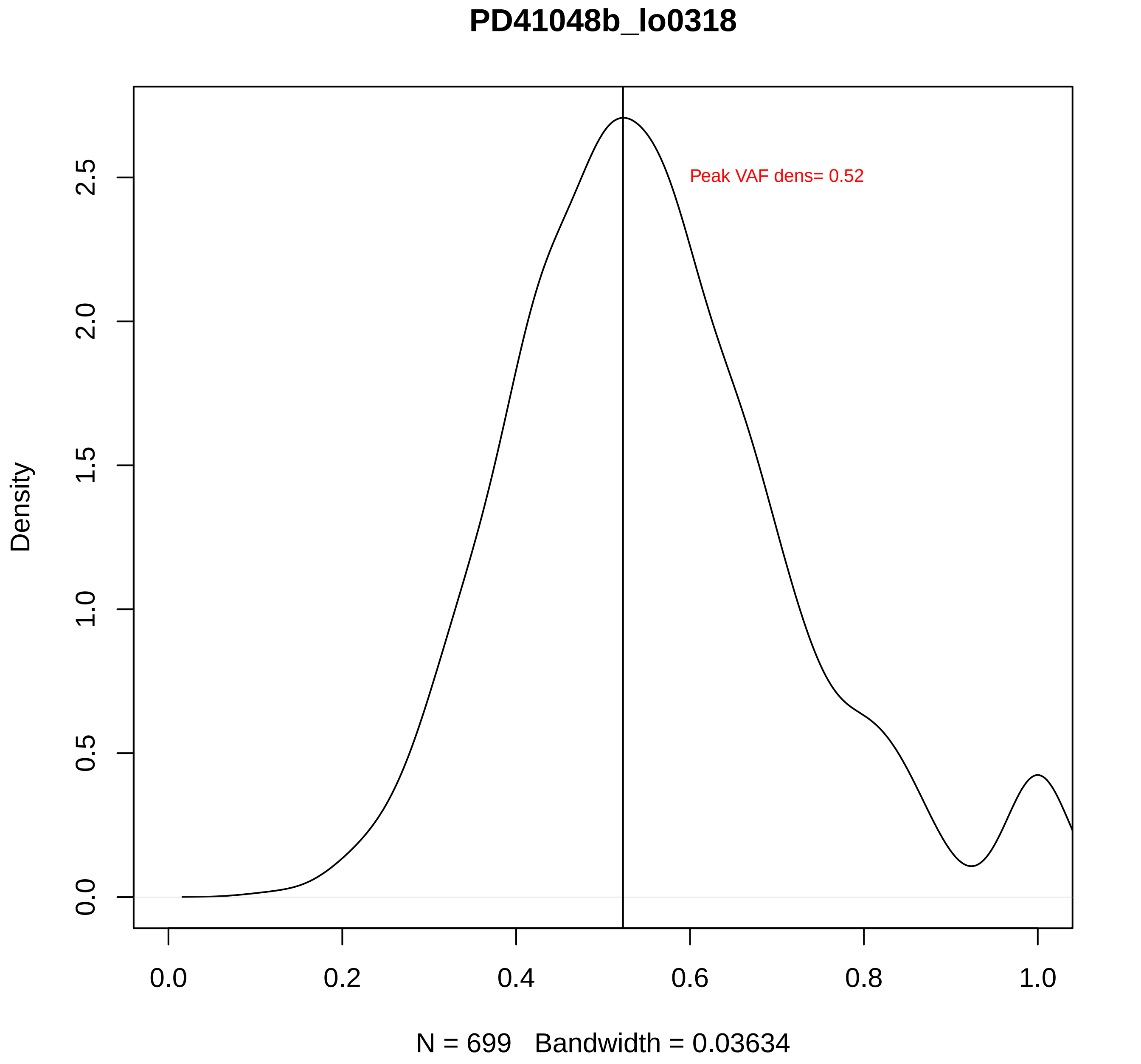
PD41048b_lo0318
Peak VAF dens= 0.52
Density
0.0
0.5
1.0
1.5
2.0
2.5
0.0
0.2
0.4
0.6
0.8
1.0
N = 699 Bandwidth = 0.03634

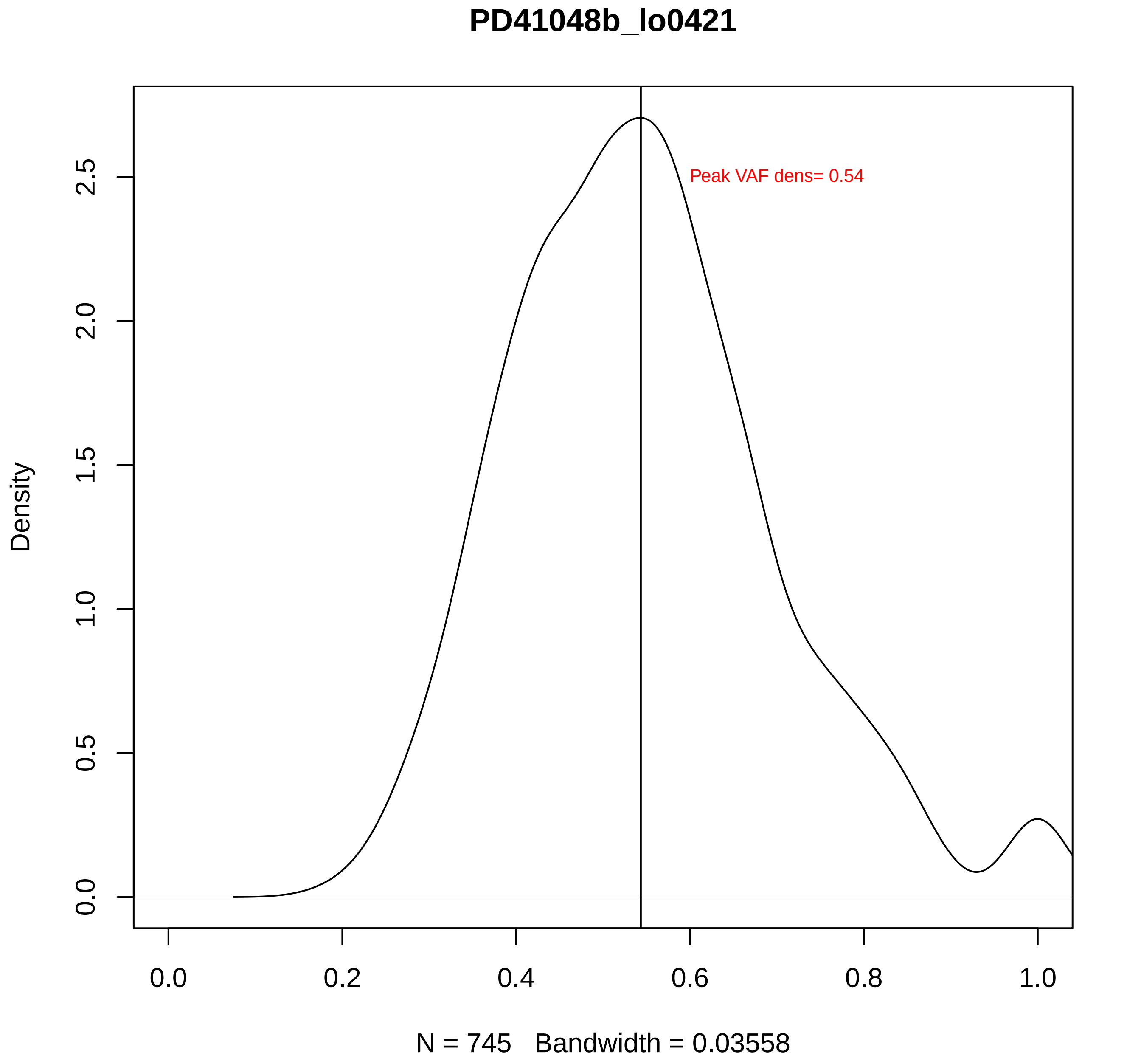
PD41048b_lo0421
Peak VAF dens= 0.54
Density
0.0
0.5
1.0
1.5
2.0
2.5
0.0
0.2
0.4
0.6
0.8
1.0
N = 745 Bandwidth = 0.03558

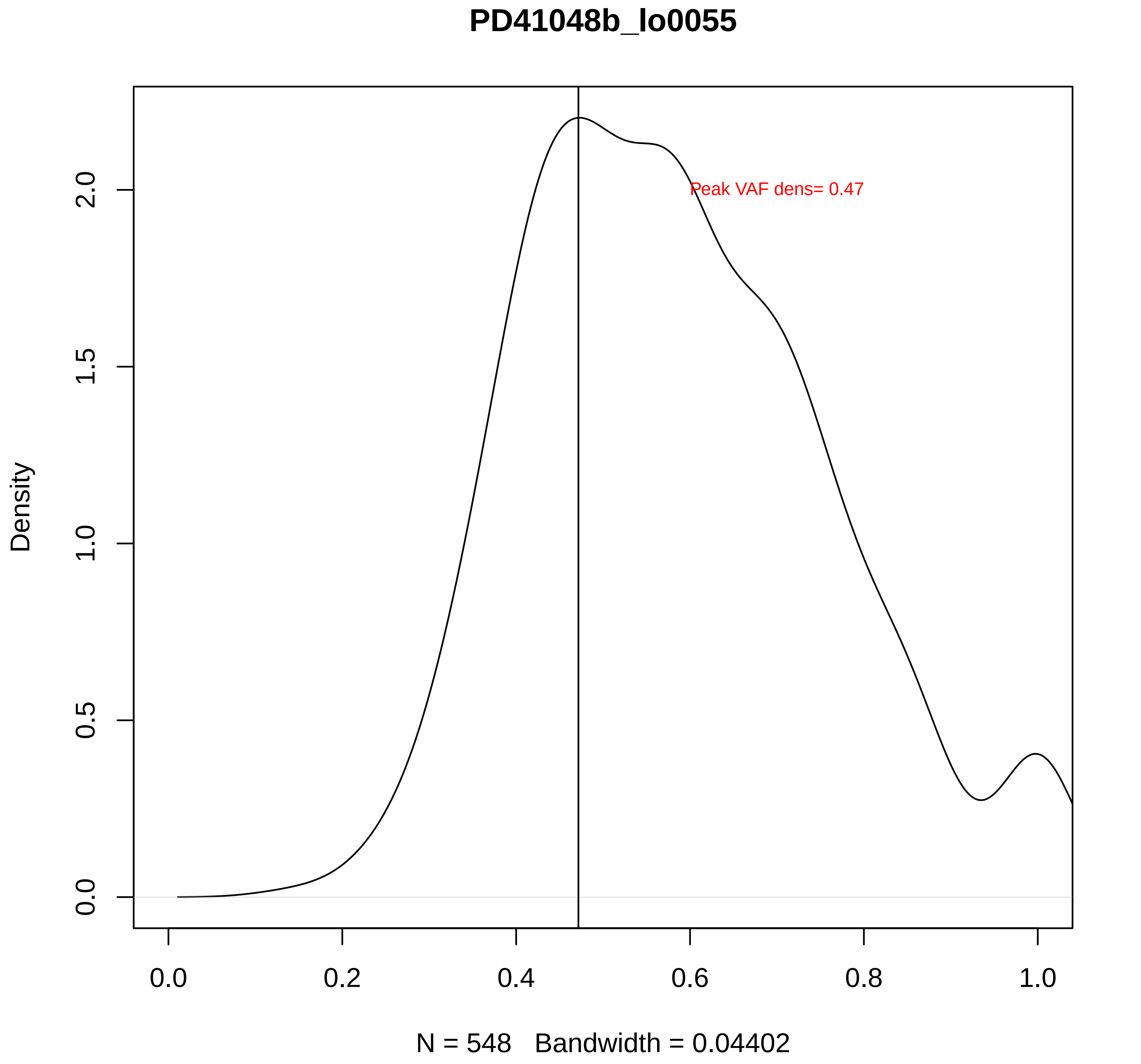

PD41048b_lo0055
Density
Peak VAF dens= 0.47
0.0
0.5
1.0
1.5
2.0
0.0
0.2
0.4
0.6
0.8
1.0
N = 548 Bandwidth = 0.04402

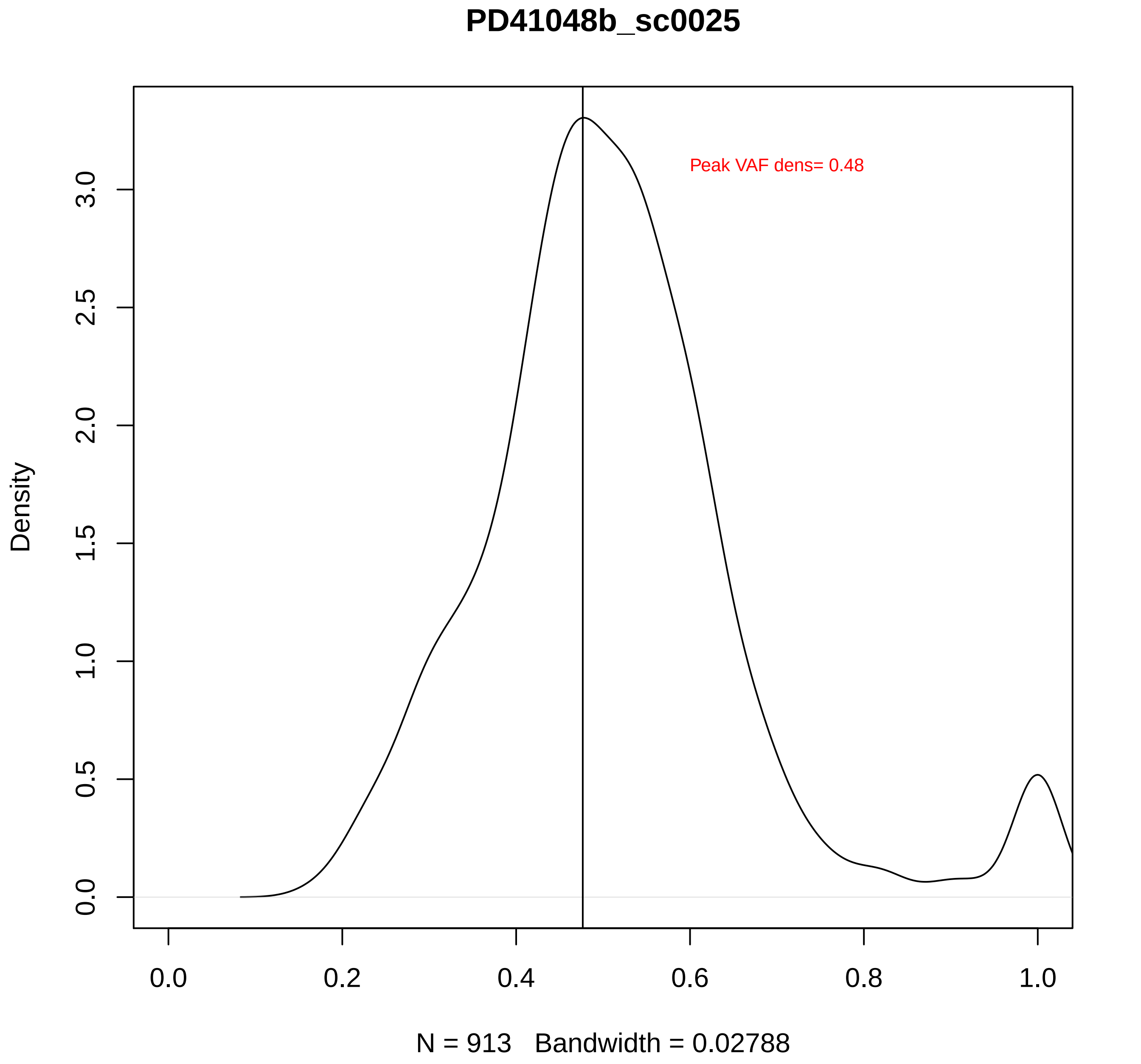
PD41048b_sc0025
Peak VAF dens= 0.48
Density
0.0
0.5
1.0
1.5
2.0
2.5
3.0
0.0
0.2
0.4
0.6
0.8
1.0
N = 913   Bandwidth = 0.02788

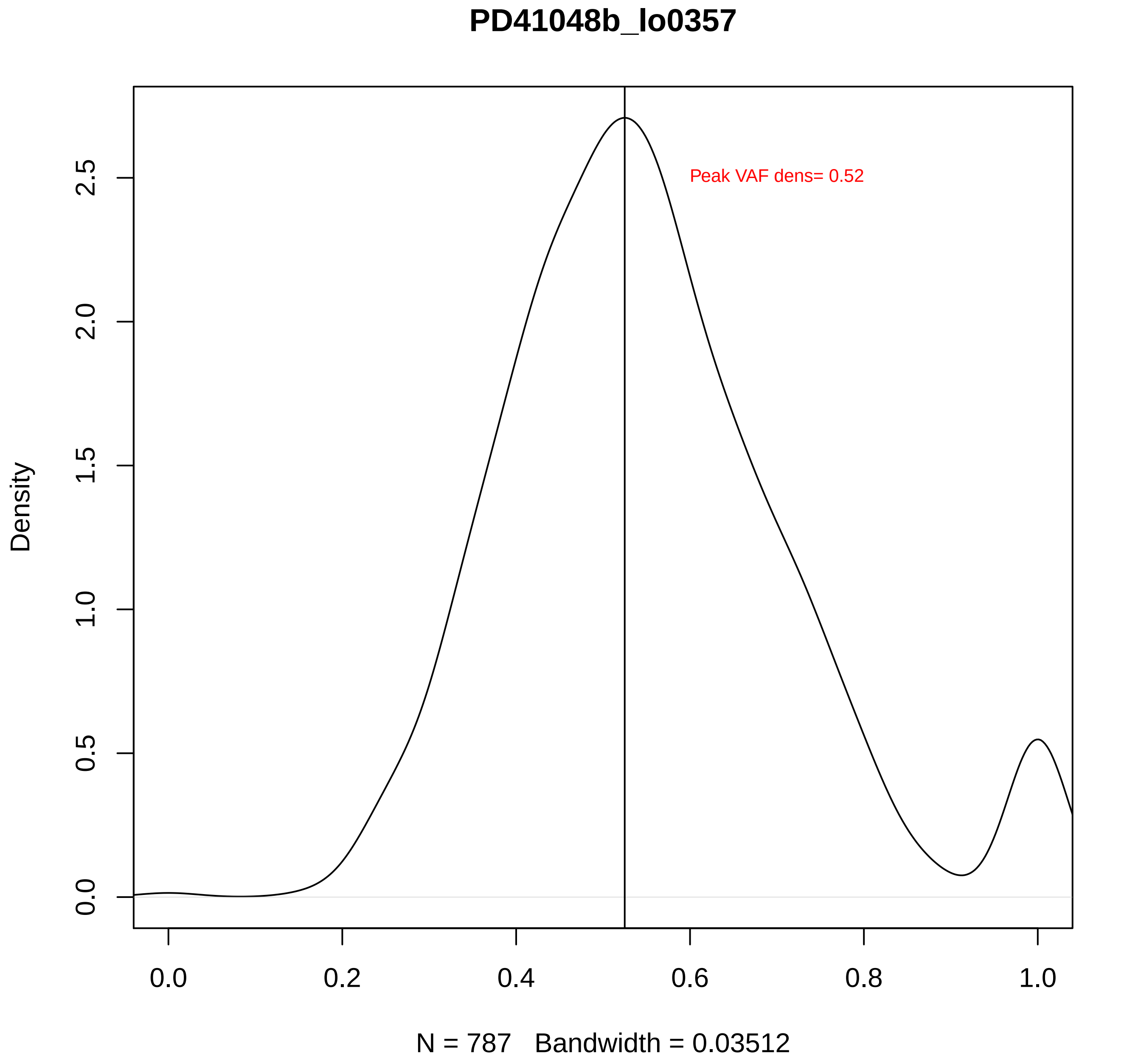
PD41048b_lo0357
Peak VAF dens= 0.52
Density
0.0
0.5
1.0
1.5
2.0
2.5
0.0
0.2
0.4
0.6
0.8
1.0
N = 787   Bandwidth = 0.03512

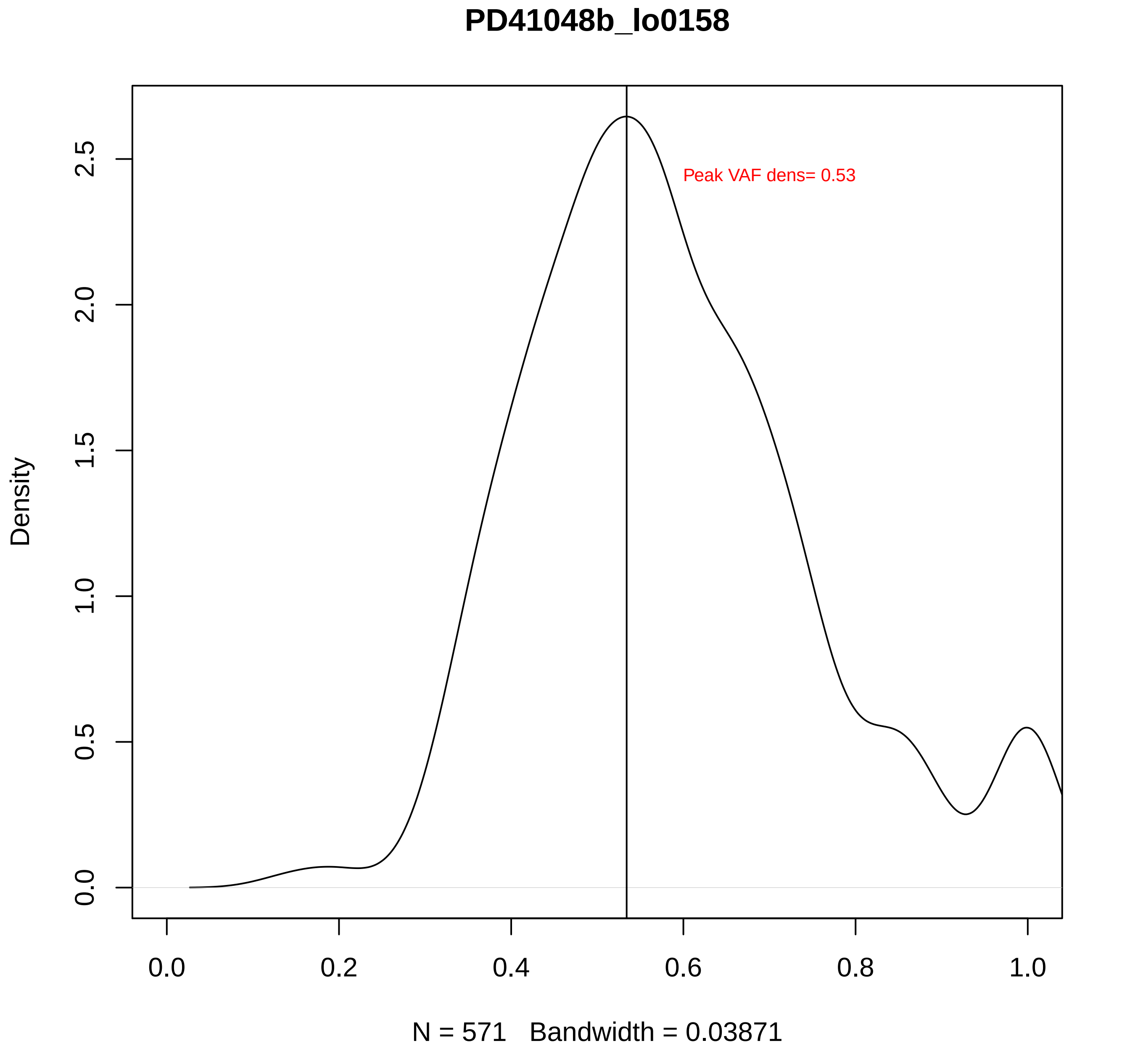
PD41048b_lo0158
Peak VAF dens= 0.53
Density
0.0
0.5
1.0
1.5
2.0
2.5
0.0
0.2
0.4
0.6
0.8
1.0
N = 571 Bandwidth = 0.03871

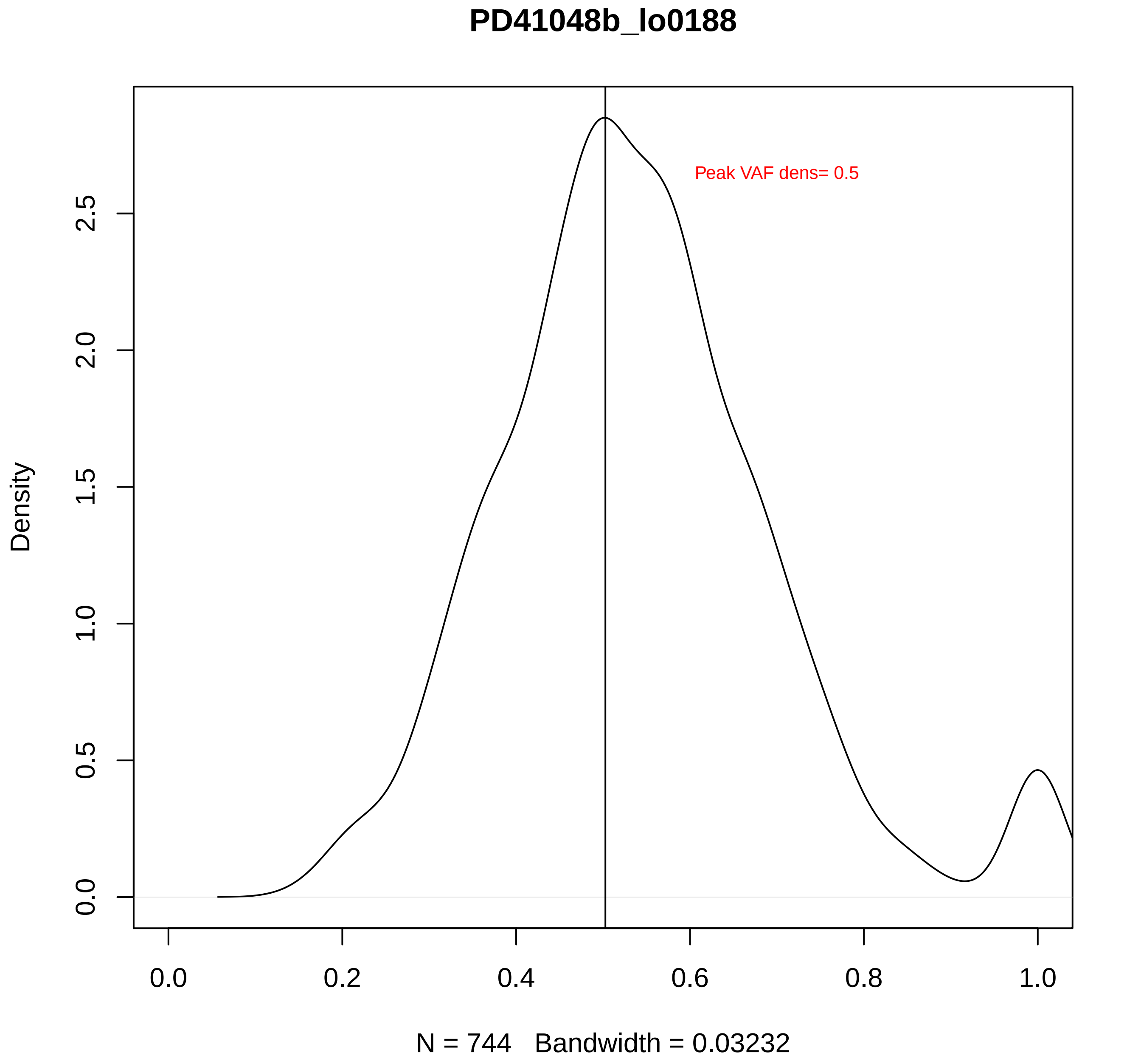
PD41048b_lo0188
Peak VAF dens= 0.5
Density
0.0
0.5
1.0
1.5
2.0
2.5
0.0
0.2
0.4
0.6
0.8
1.0
N = 744   Bandwidth = 0.03232

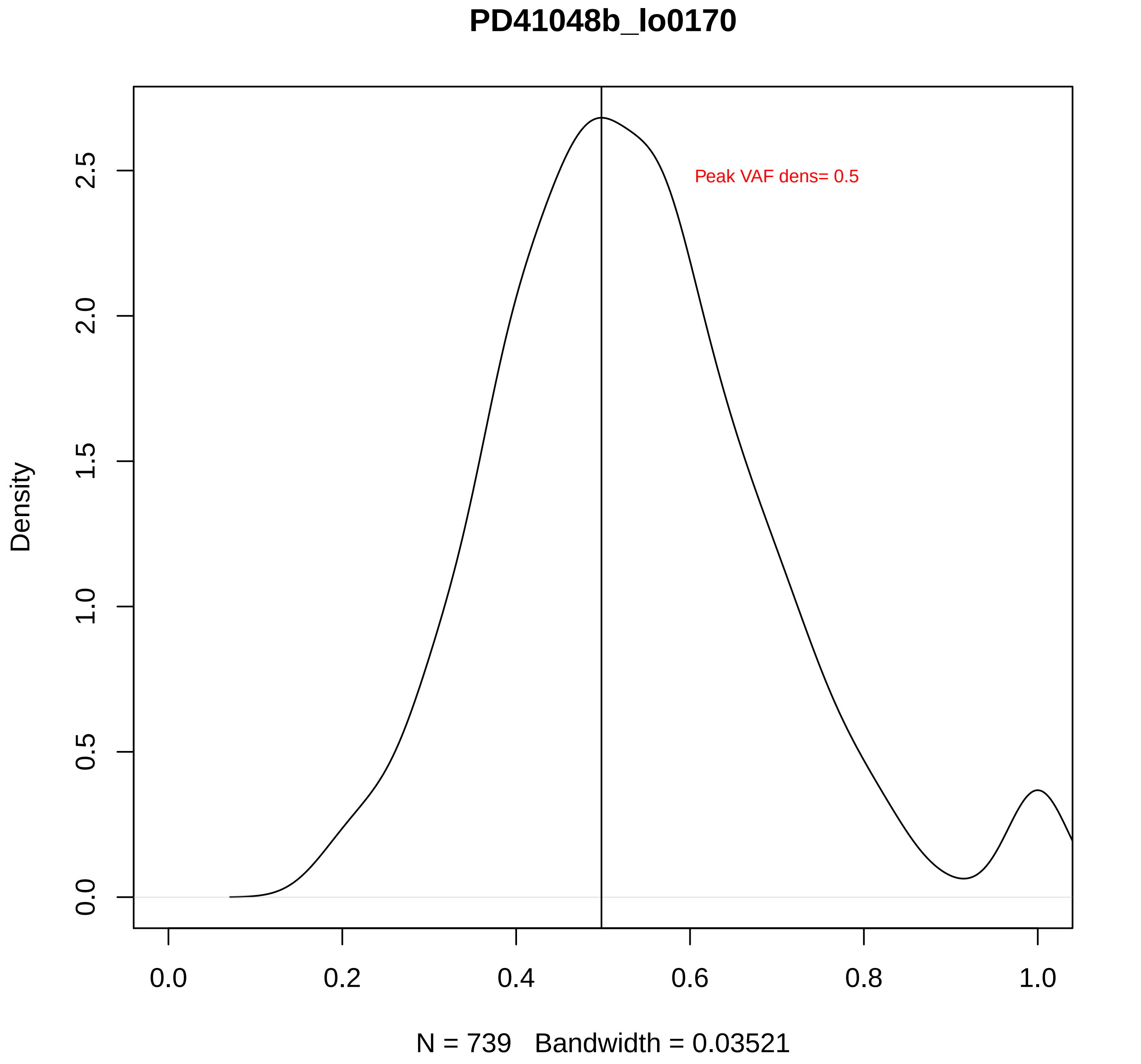
PD41048b_lo0170
Peak VAF dens= 0.5
Density
0.0
0.5
1.0
1.5
2.0
2.5
0.0
0.2
0.4
0.6
0.8
1.0
N = 739 Bandwidth = 0.03521

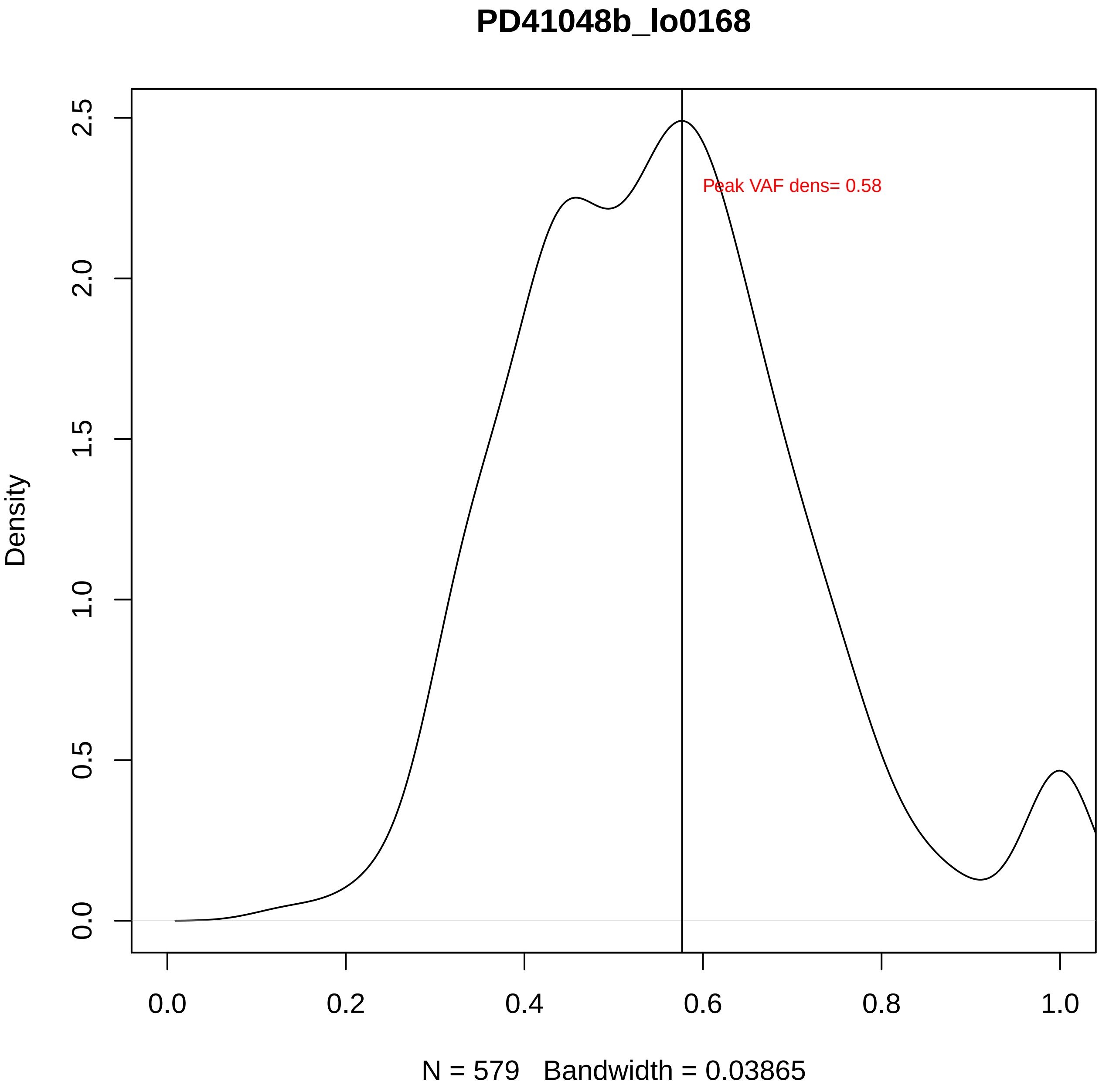

PD41048b_lo0168
Peak VAF dens= 0.58
Density
0.0
0.5
1.0
1.5
2.0
2.5
0.0
0.2
0.4
0.6
0.8
1.0
N = 579   Bandwidth = 0.03865

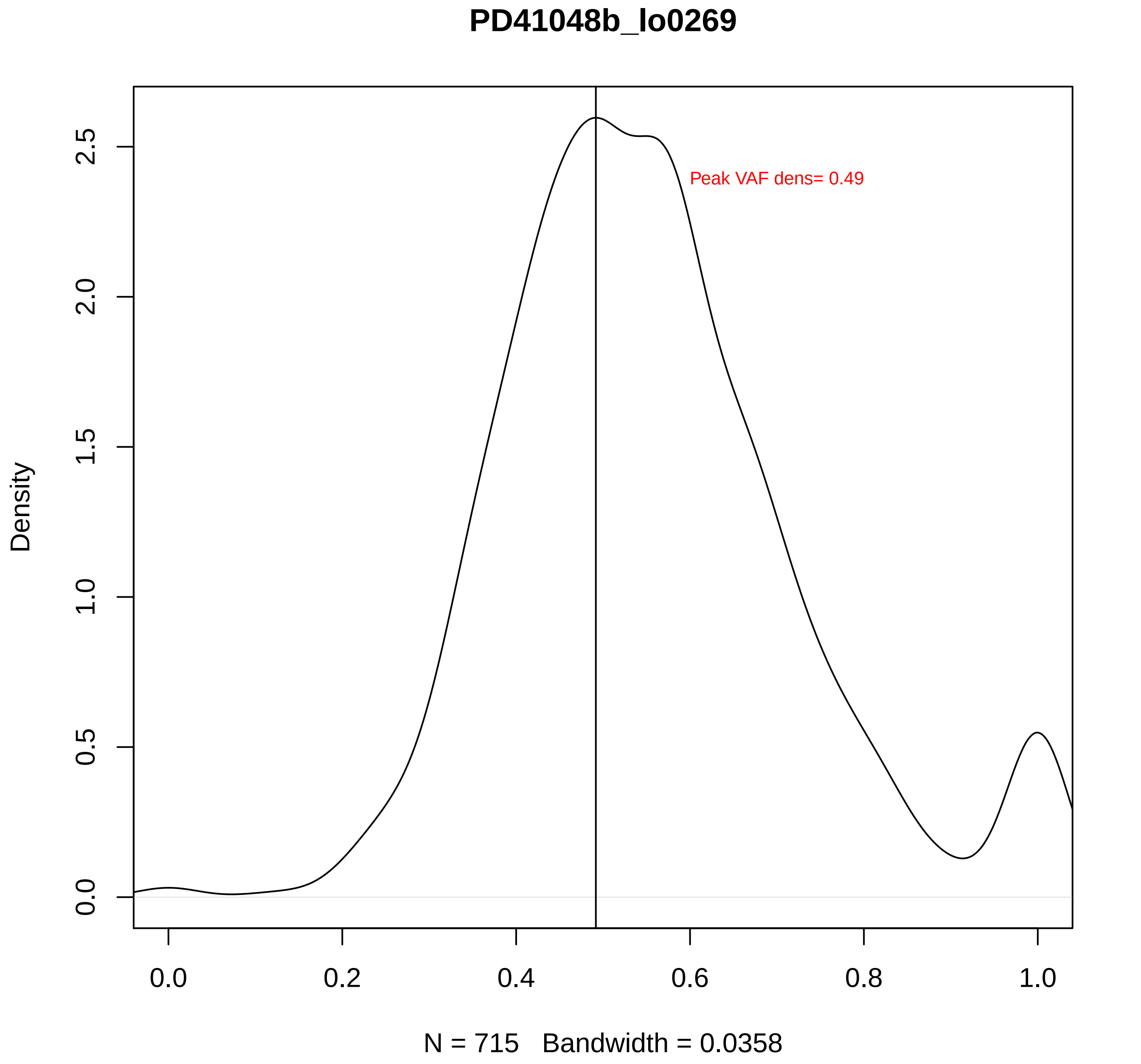
PD41048b_lo0269
Peak VAF dens= 0.49
Density
0.0
0.5
1.0
1.5
2.0
2.5
0.0
0.2
0.4
0.6
0.8
1.0
N = 715   Bandwidth = 0.0358

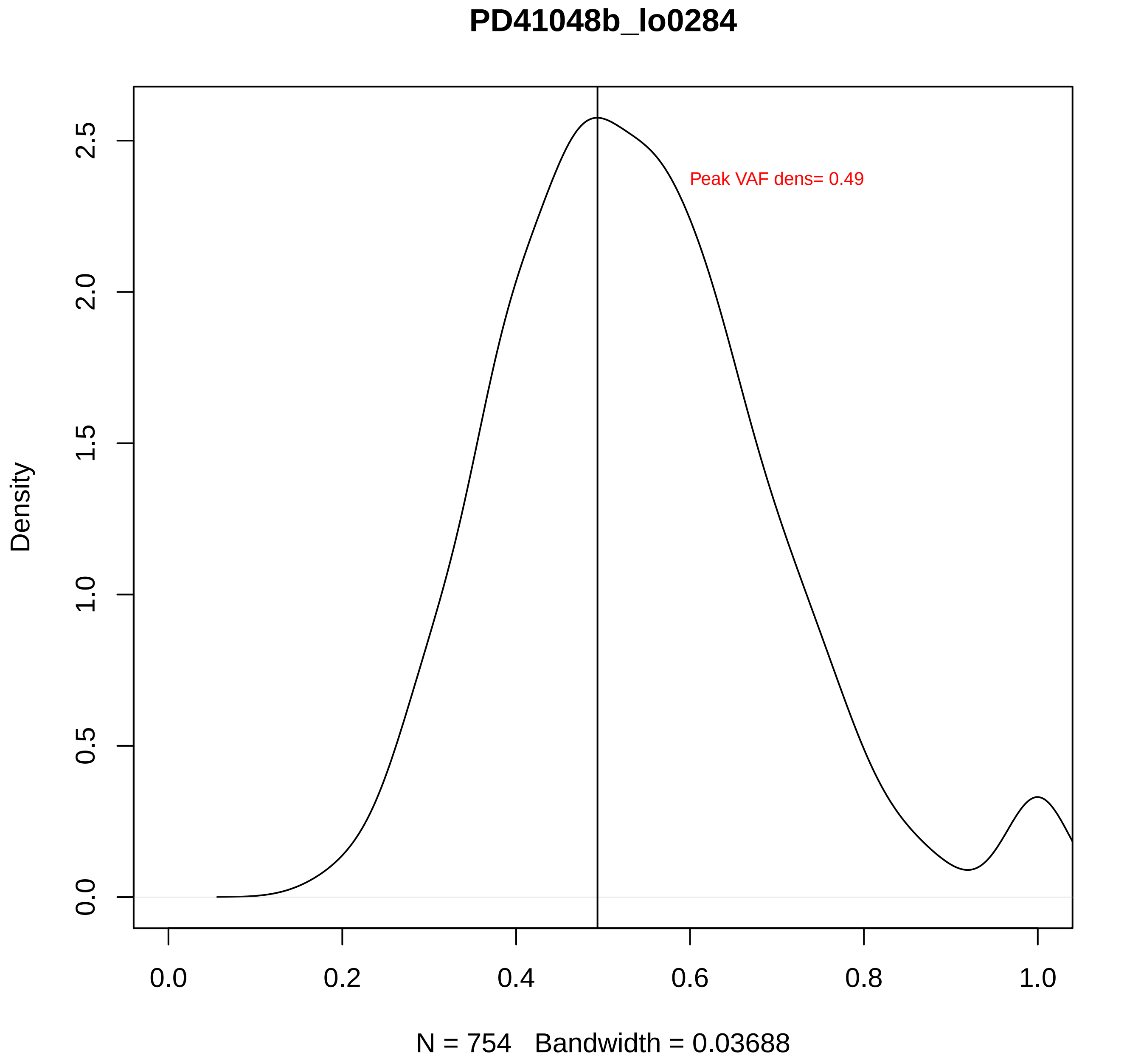

PD41048b_lo0284
Density
Peak VAF dens= 0.49
0.0
0.5
1.0
1.5
2.0
2.5
0.0
0.2
0.4
0.6
0.8
1.0
N = 754 Bandwidth = 0.03688

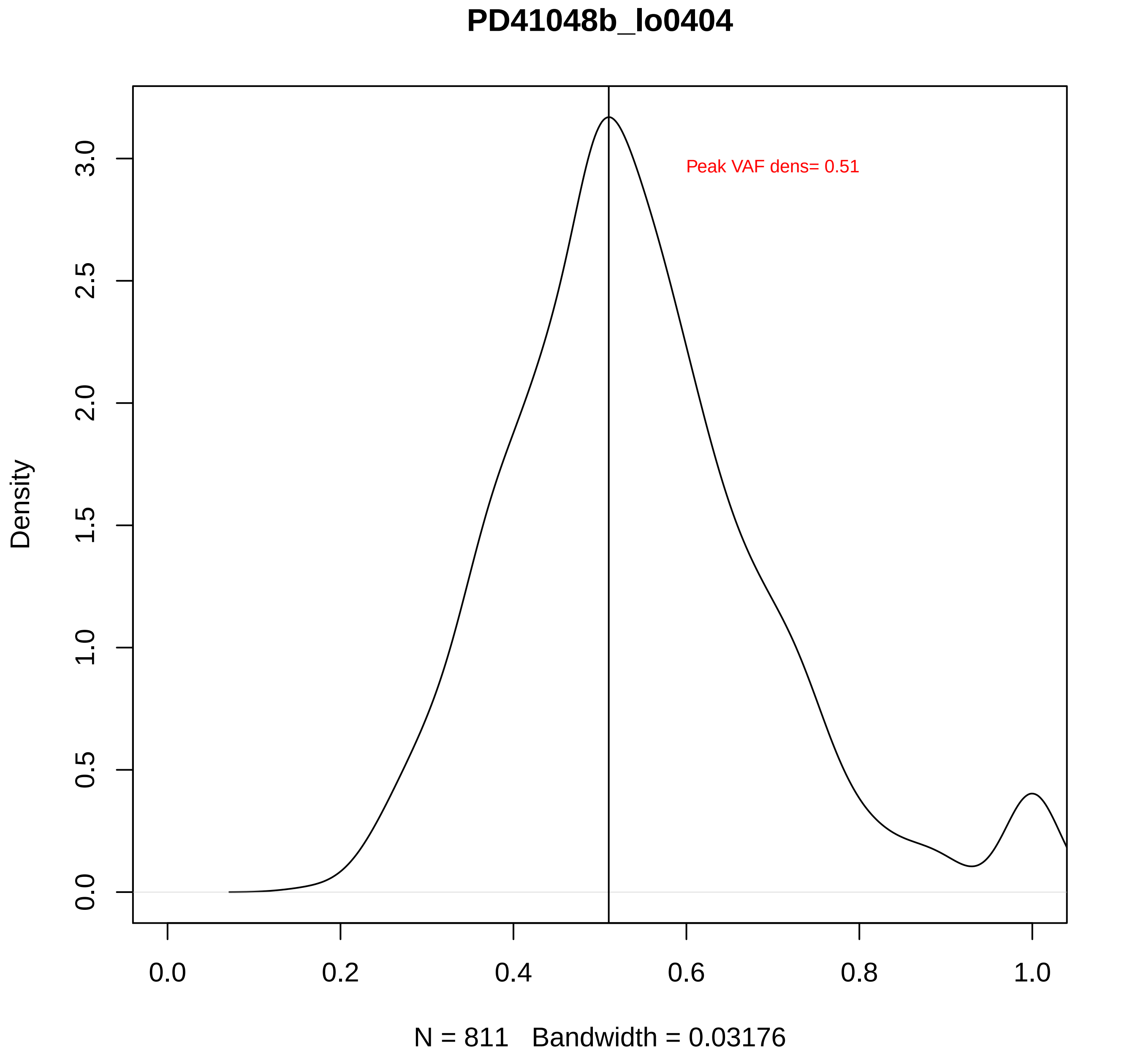
PD41048b_lo0404
Peak VAF dens= 0.51
Density
0.0
0.5
1.0
1.5
2.0
2.5
3.0
0.0
0.2
0.4
0.6
0.8
1.0
N = 811 Bandwidth = 0.03176

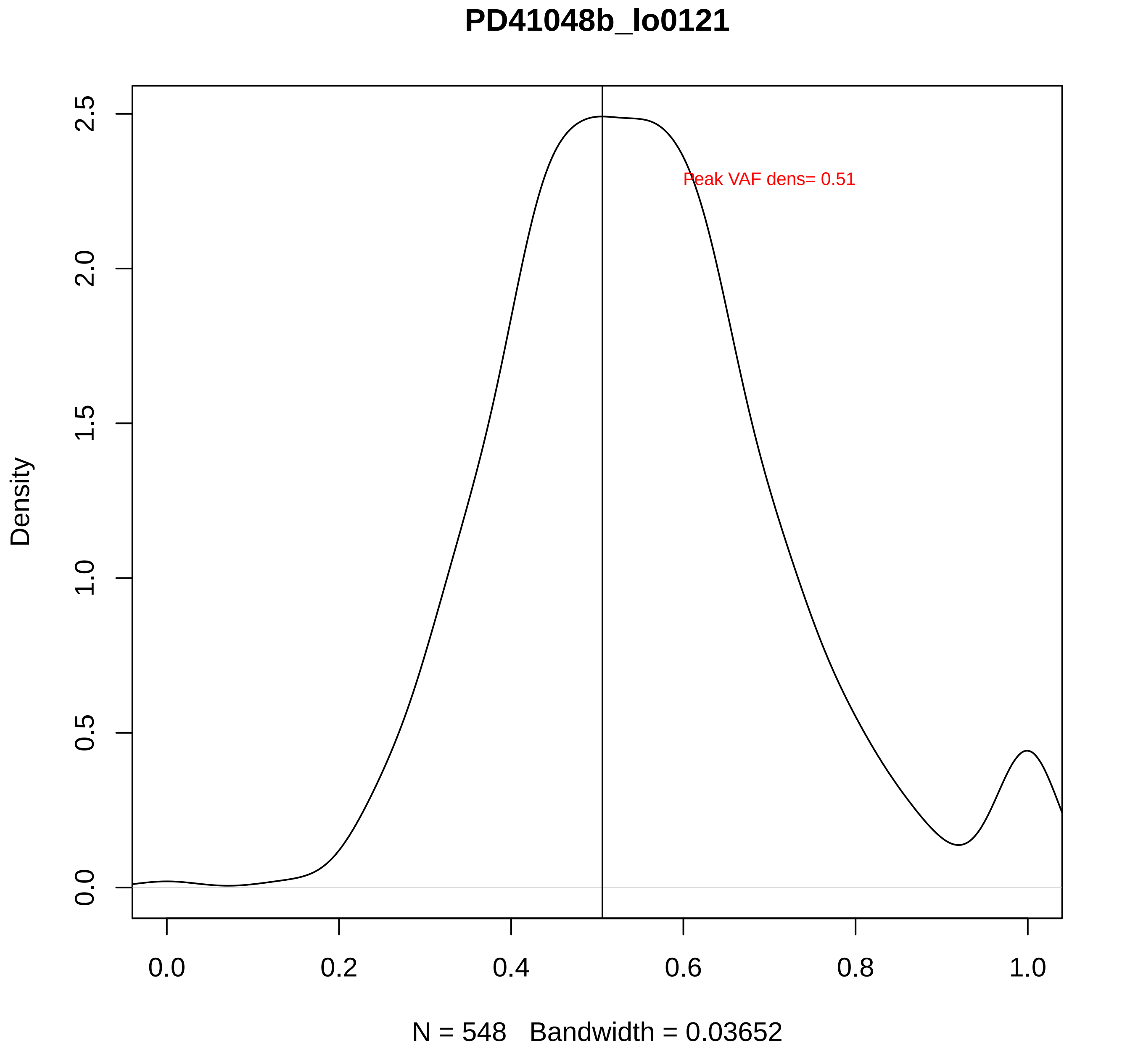

PD41048b_lo0121
Peak VAF dens= 0.51
Density
0.0
0.5
1.0
1.5
2.0
2.5
0.0
0.2
0.4
0.6
0.8
1.0
N = 548 Bandwidth = 0.03652

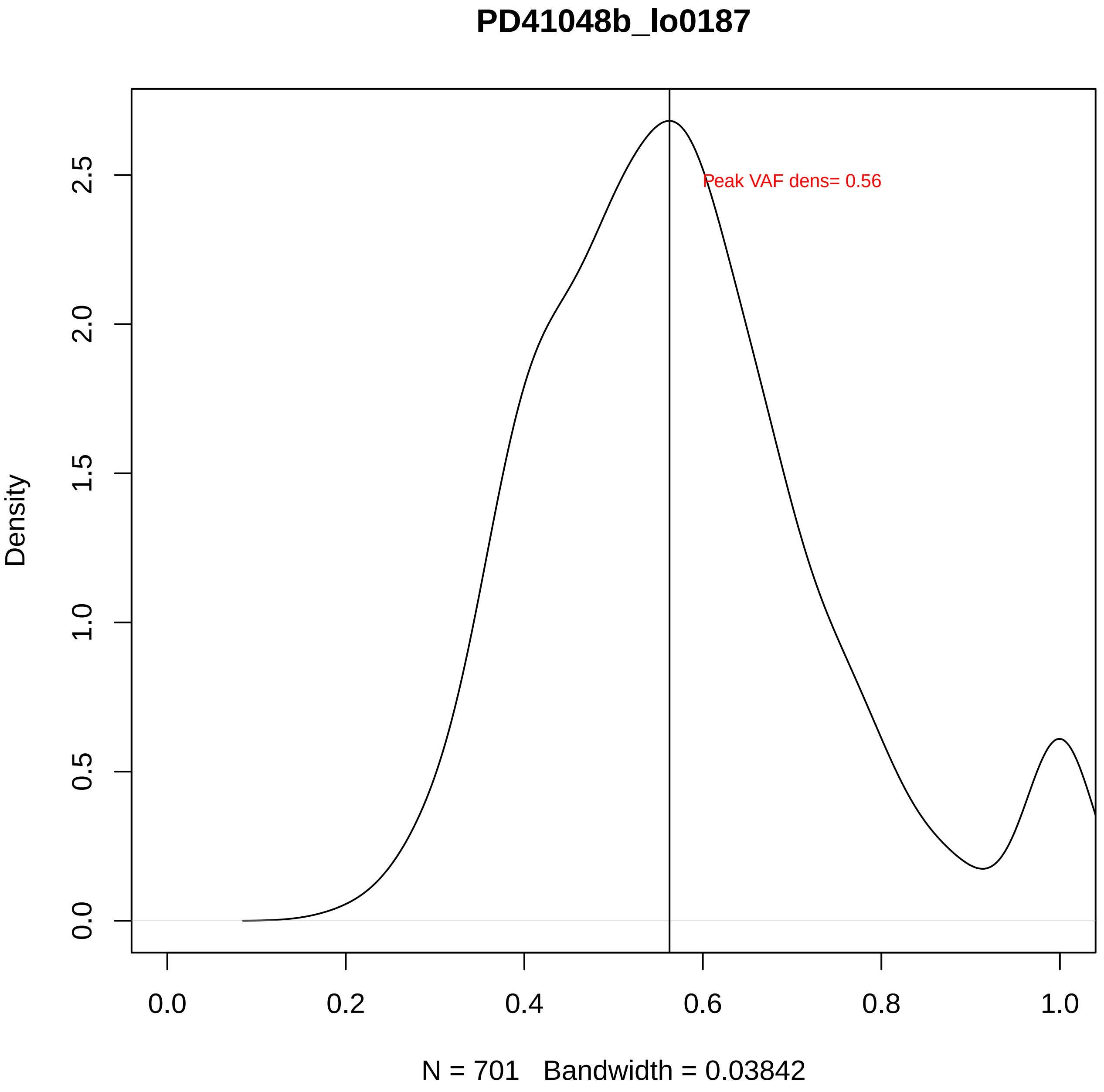
PD41048b_lo0187
Peak VAF dens= 0.56
Density
0.0
0.5
1.0
1.5
2.0
2.5
0.0
0.2
0.4
0.6
0.8
1.0
N = 701 Bandwidth = 0.03842

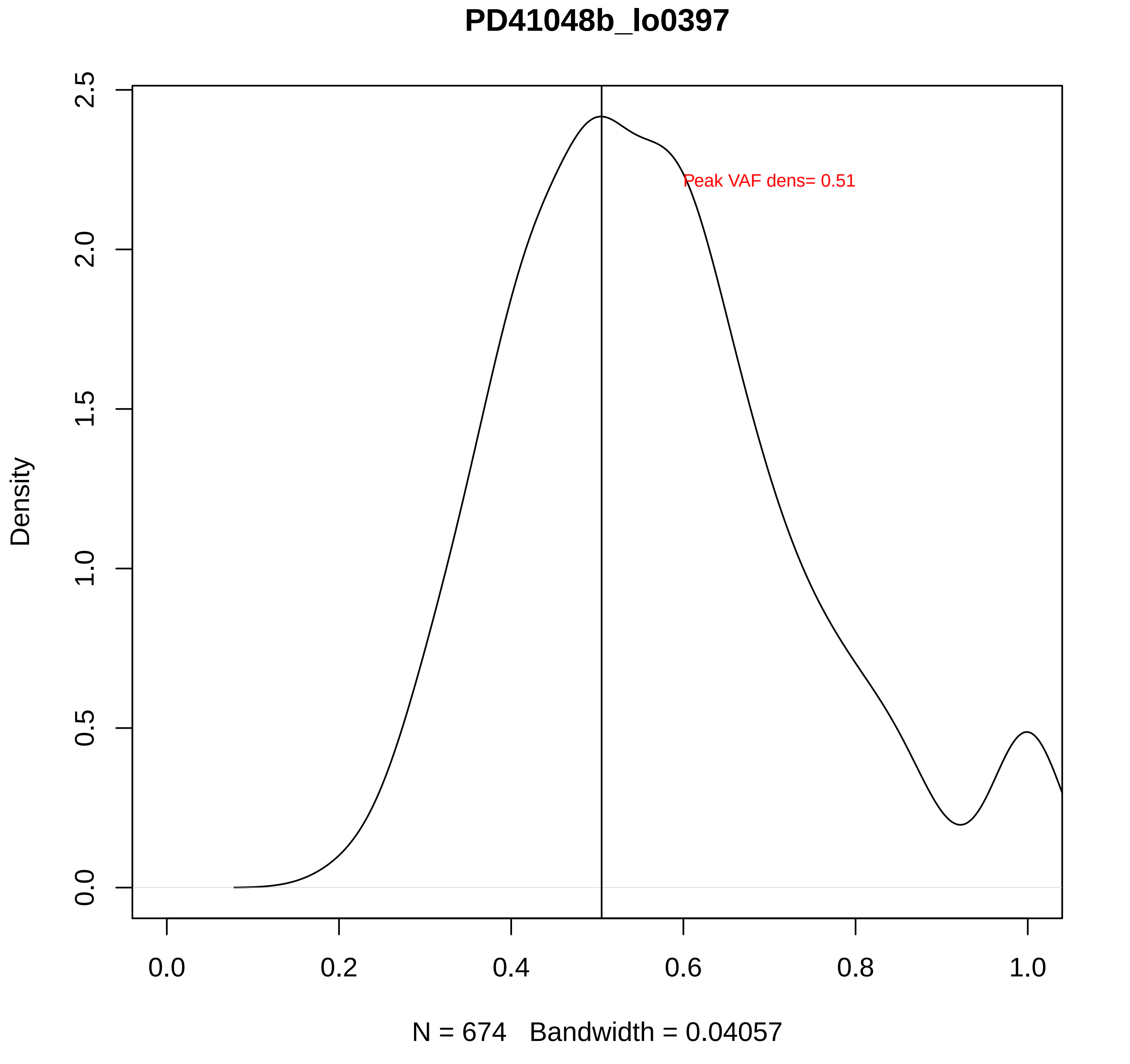
PD41048b_lo0397
Density
Peak VAF dens= 0.51
0.0
0.5
1.0
1.5
2.0
2.5
0.0
0.2
0.4
0.6
0.8
1.0
N = 674   Bandwidth = 0.04057

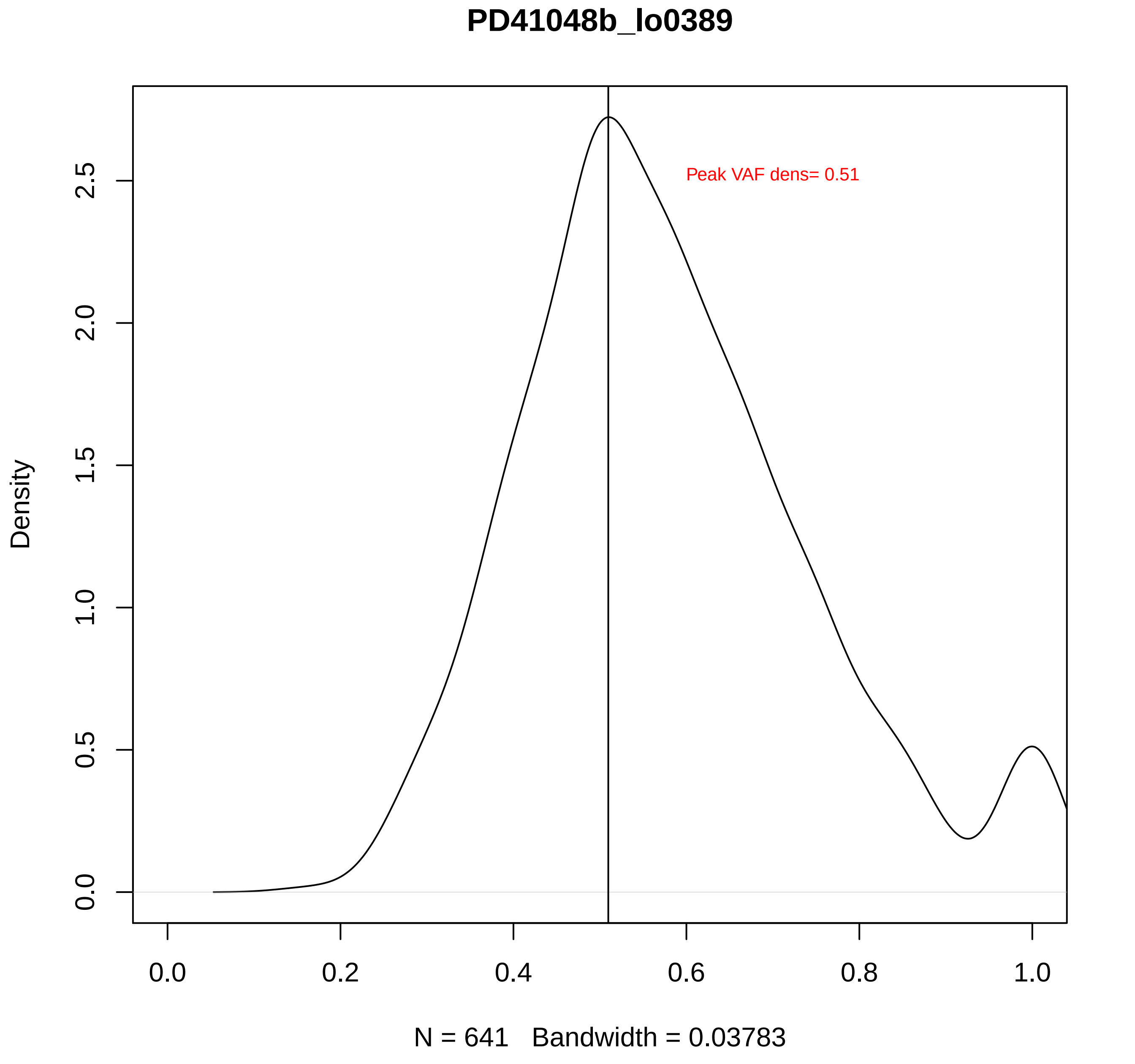

PD41048b_lo0389
Peak VAF dens= 0.51
Density
0.0
0.5
1.0
1.5
2.0
2.5
0.0
0.2
0.4
0.6
0.8
1.0
N = 641 Bandwidth = 0.03783

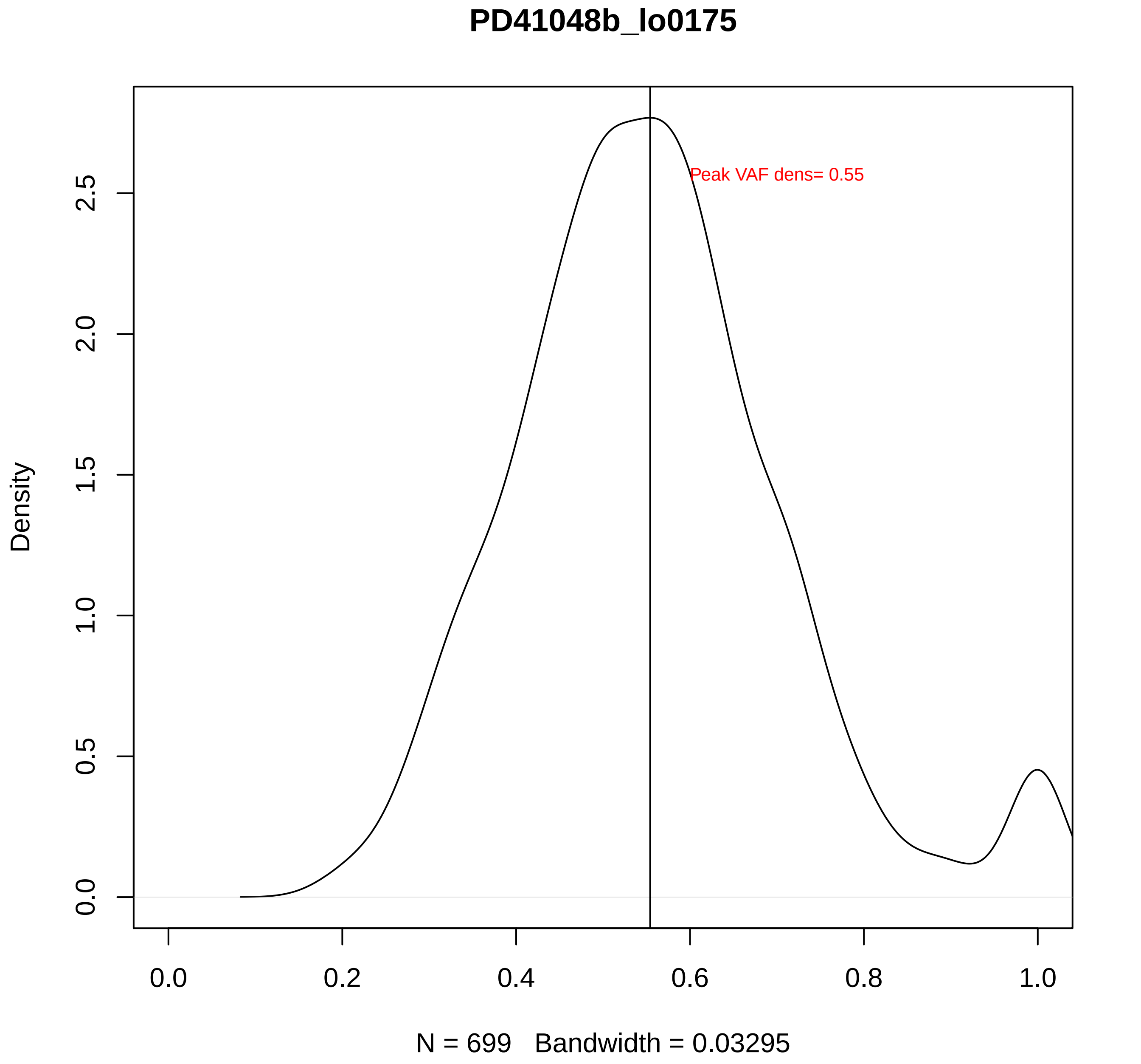

PD41048b_lo0175
Peak VAF dens= 0.55
Density
0.0
0.5
1.0
1.5
2.0
2.5
0.0
0.2
0.4
0.6
0.8
1.0
N = 699 Bandwidth = 0.03295

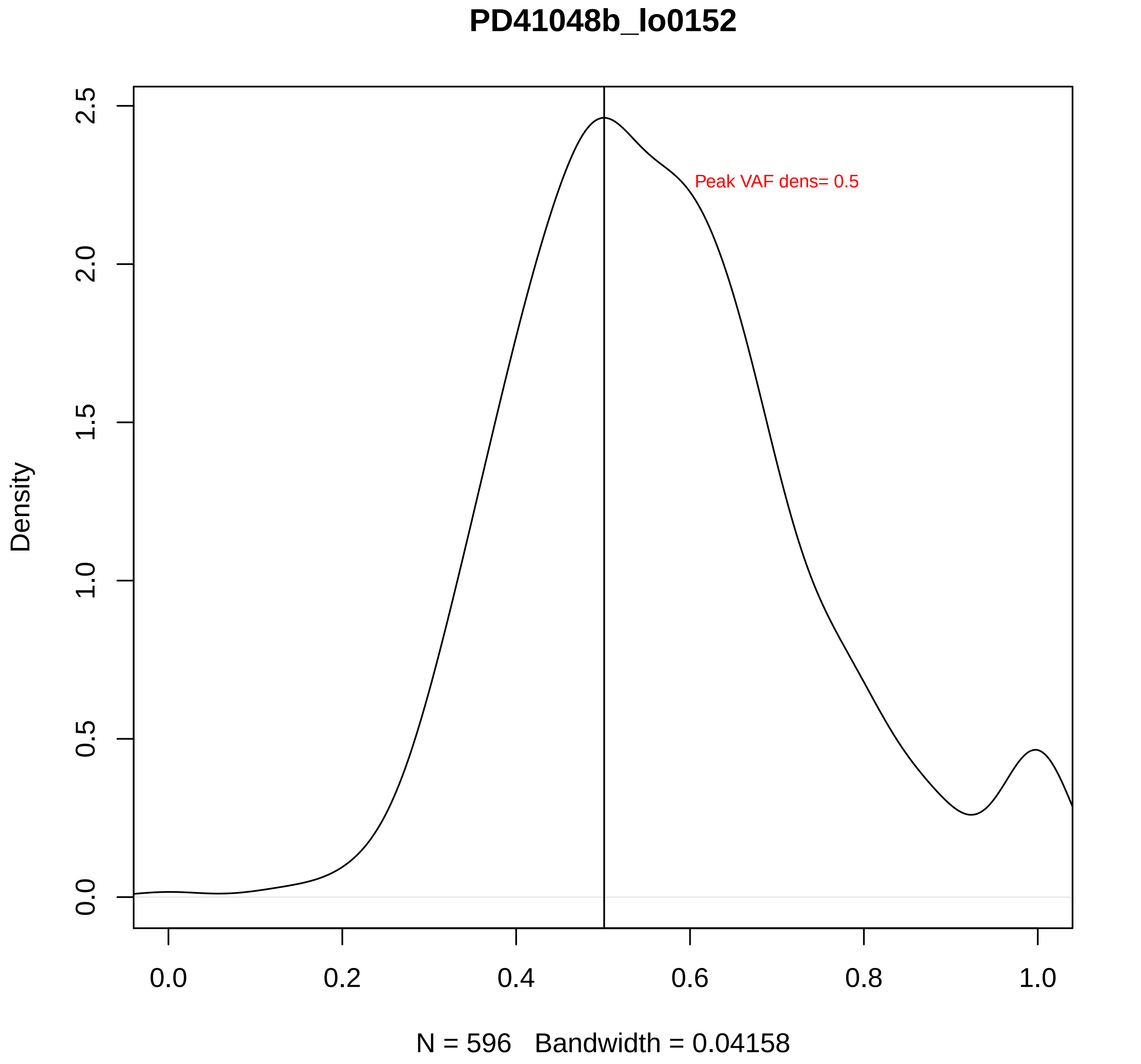
PD41048b_lo0152
Density
Peak VAF dens= 0.5
0.0
0.5
1.0
1.5
2.0
2.5
0.0
0.2
0.4
0.6
0.8
1.0
N = 596 Bandwidth = 0.04158

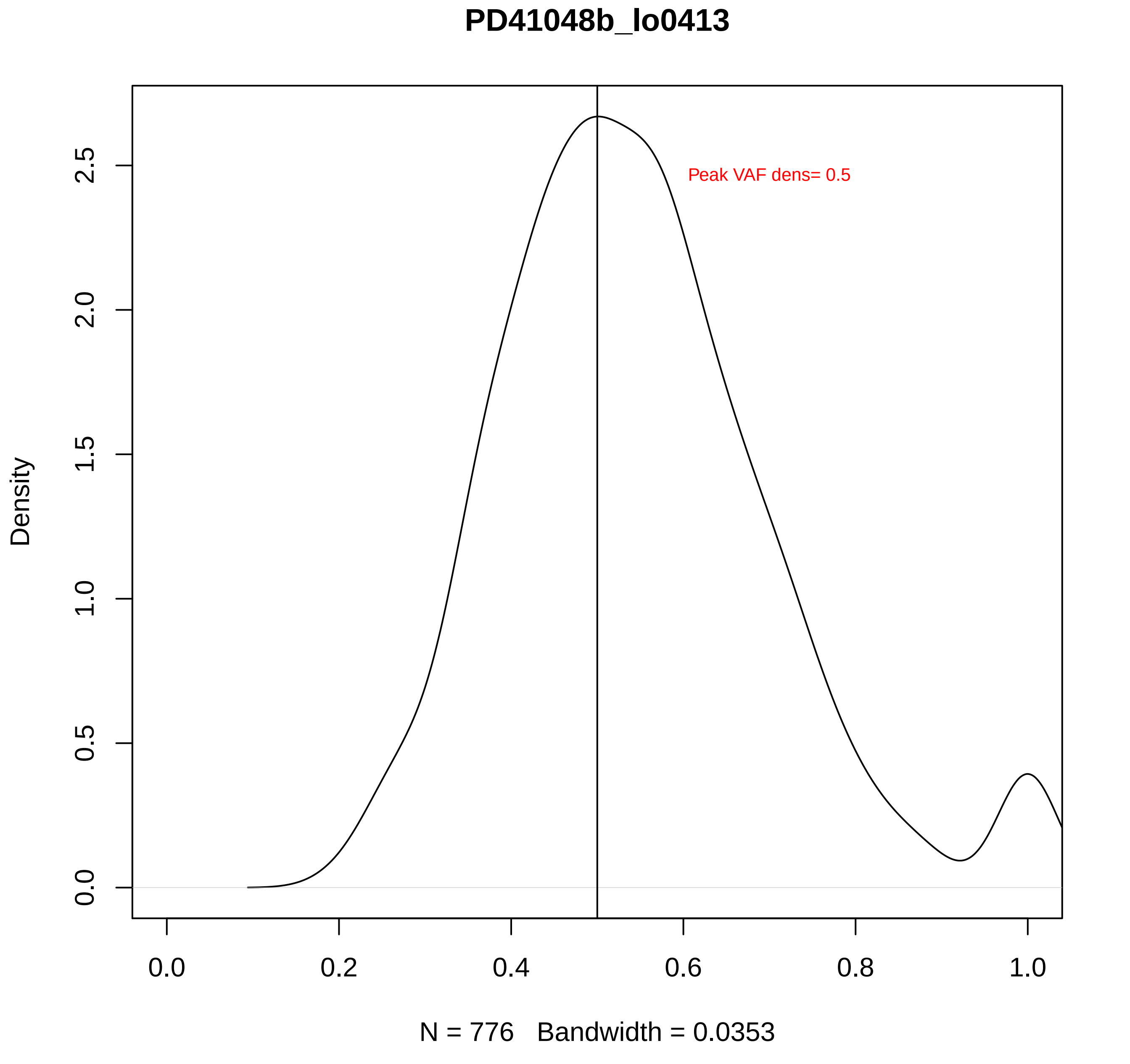
PD41048b_lo0413
Peak VAF dens= 0.5
Density
0.0
0.5
1.0
1.5
2.0
2.5
0.0
0.2
0.4
0.6
0.8
1.0
N = 776   Bandwidth = 0.0353

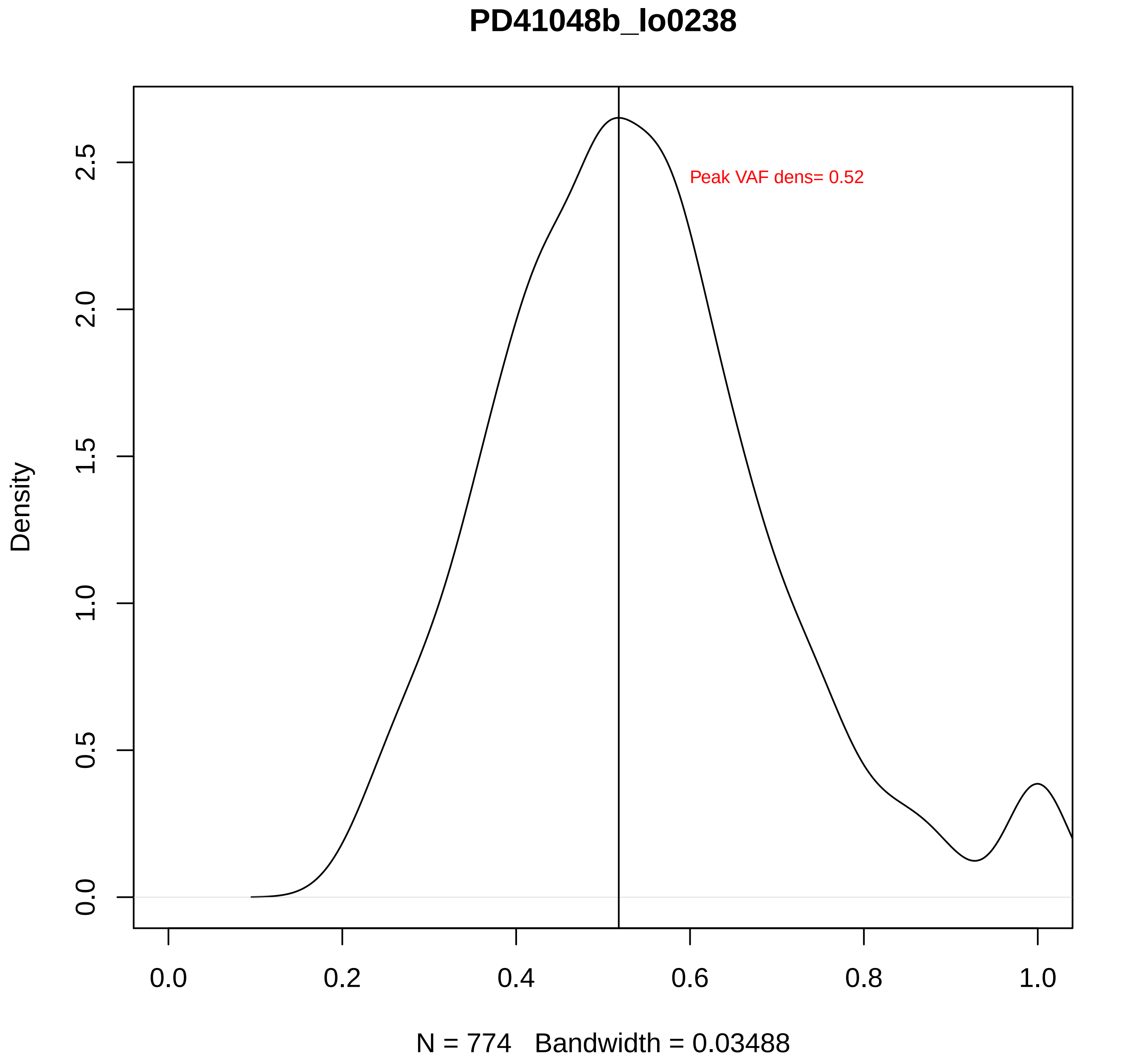

PD41048b_lo0238
Peak VAF dens= 0.52
Density
0.0
0.5
1.0
1.5
2.0
2.5
0.0
0.2
0.4
0.6
0.8
1.0
N = 774   Bandwidth = 0.03488

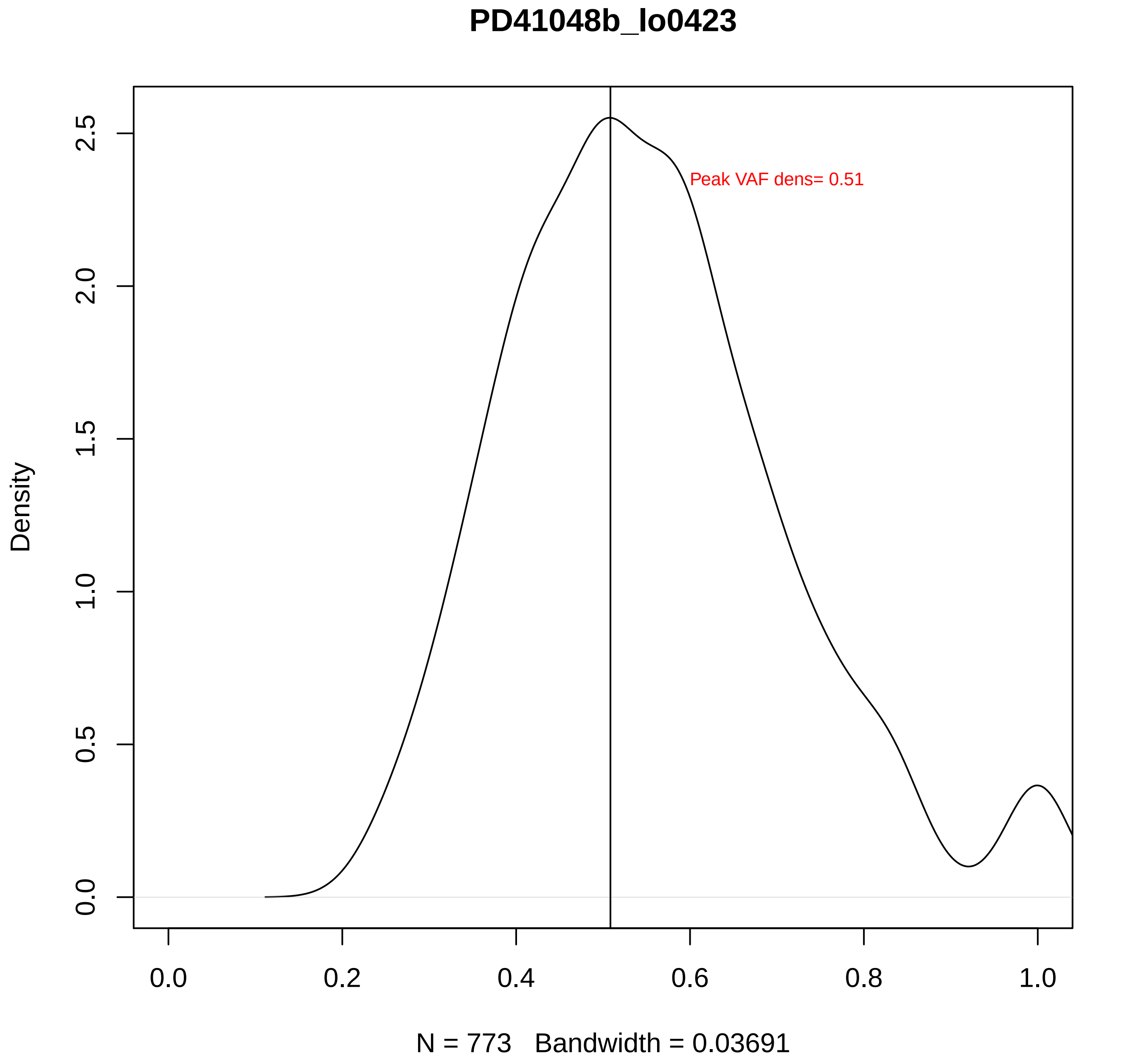

PD41048b_lo0423
Peak VAF dens= 0.51
Density
0.0
0.5
1.0
1.5
2.0
2.5
0.0
0.2
0.4
0.6
0.8
1.0
N = 773   Bandwidth = 0.03691

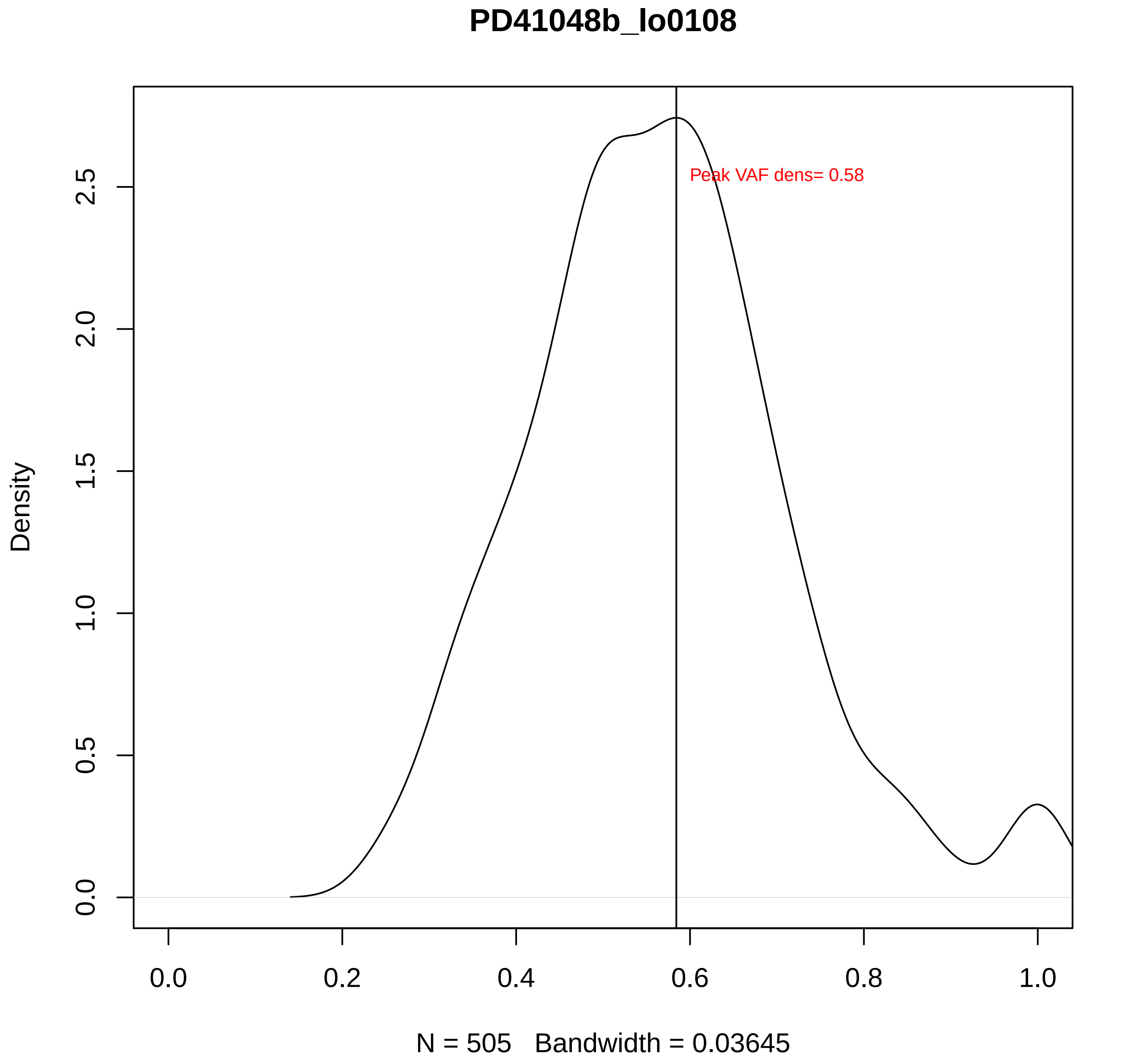
PD41048b_lo0108
Peak VAF dens= 0.58
Density
0.0
0.5
1.0
1.5
2.0
2.5
0.0
0.2
0.4
0.6
0.8
1.0
N = 505   Bandwidth = 0.03645

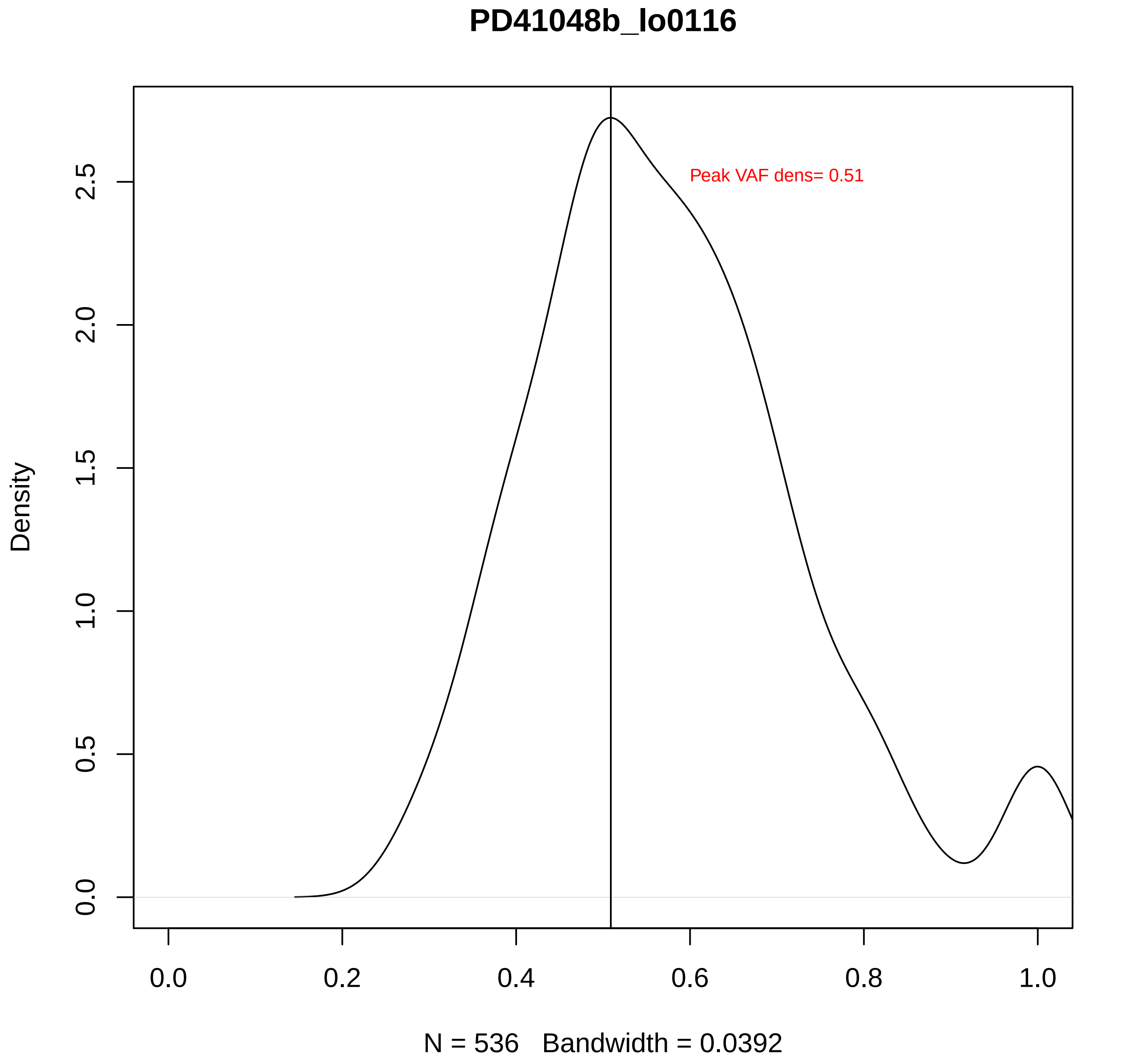

PD41048b_lo0116
Peak VAF dens= 0.51
Density
0.0
0.5
1.0
1.5
2.0
2.5
0.0
0.2
0.4
0.6
0.8
1.0
N = 536 Bandwidth = 0.0392

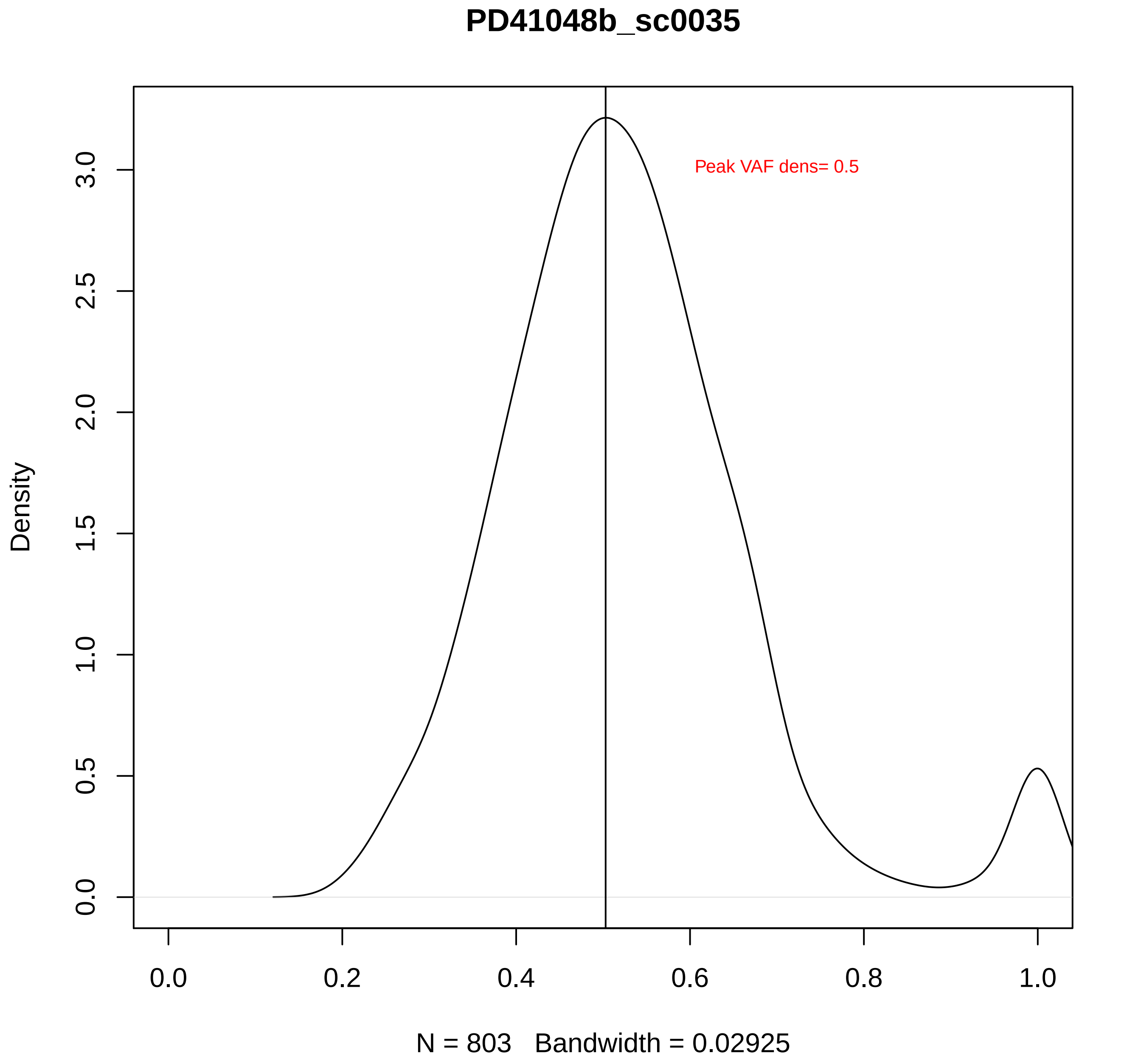
PD41048b_sc0035
Peak VAF dens= 0.5
Density
0.0
0.5
1.0
1.5
2.0
2.5
3.0
0.0
0.2
0.4
0.6
0.8
1.0
N = 803   Bandwidth = 0.02925

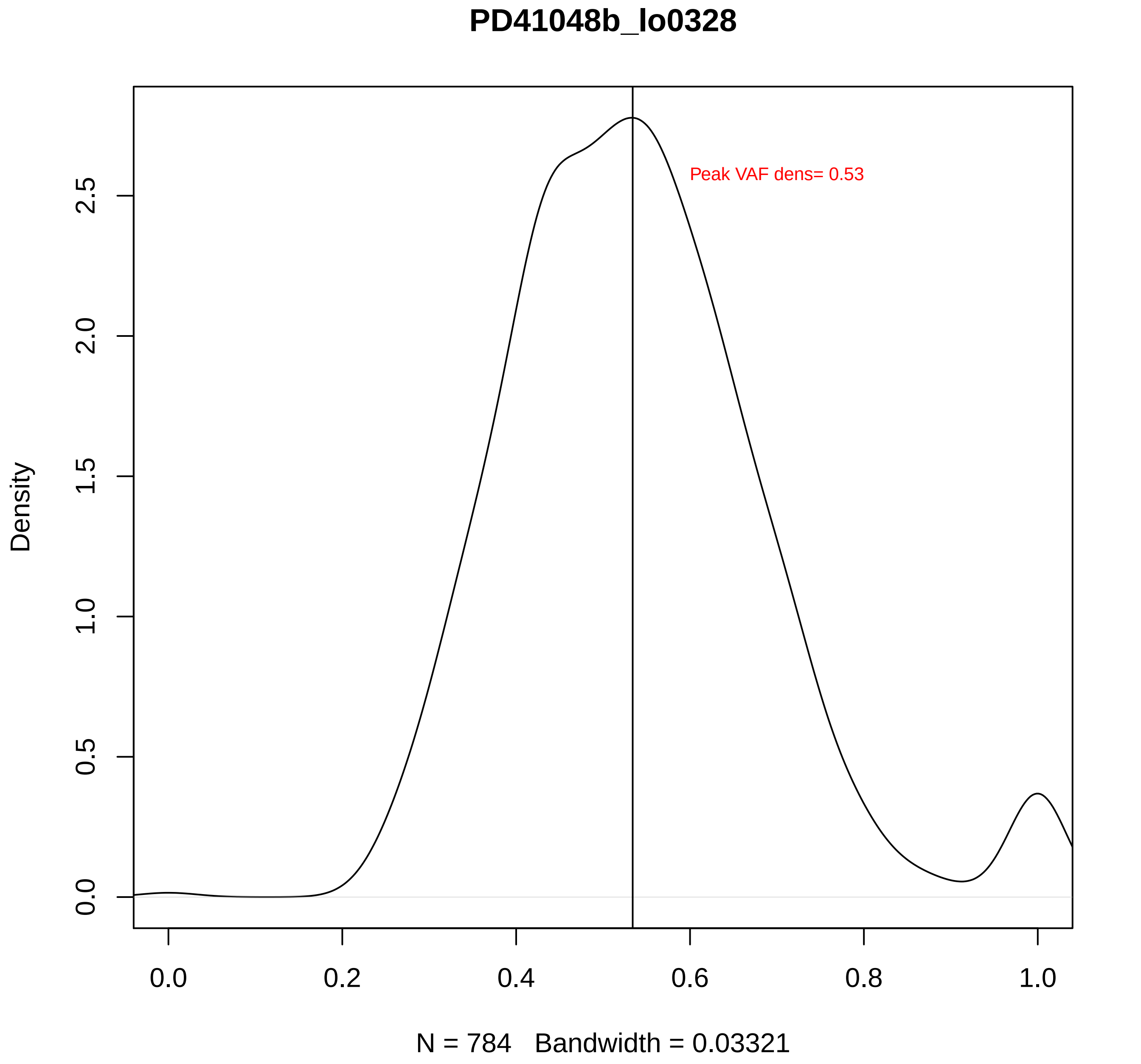
PD41048b_lo0328
Peak VAF dens= 0.53
Density
0.0
0.5
1.0
1.5
2.0
2.5
0.0
0.2
0.4
0.6
0.8
1.0
N = 784   Bandwidth = 0.03321

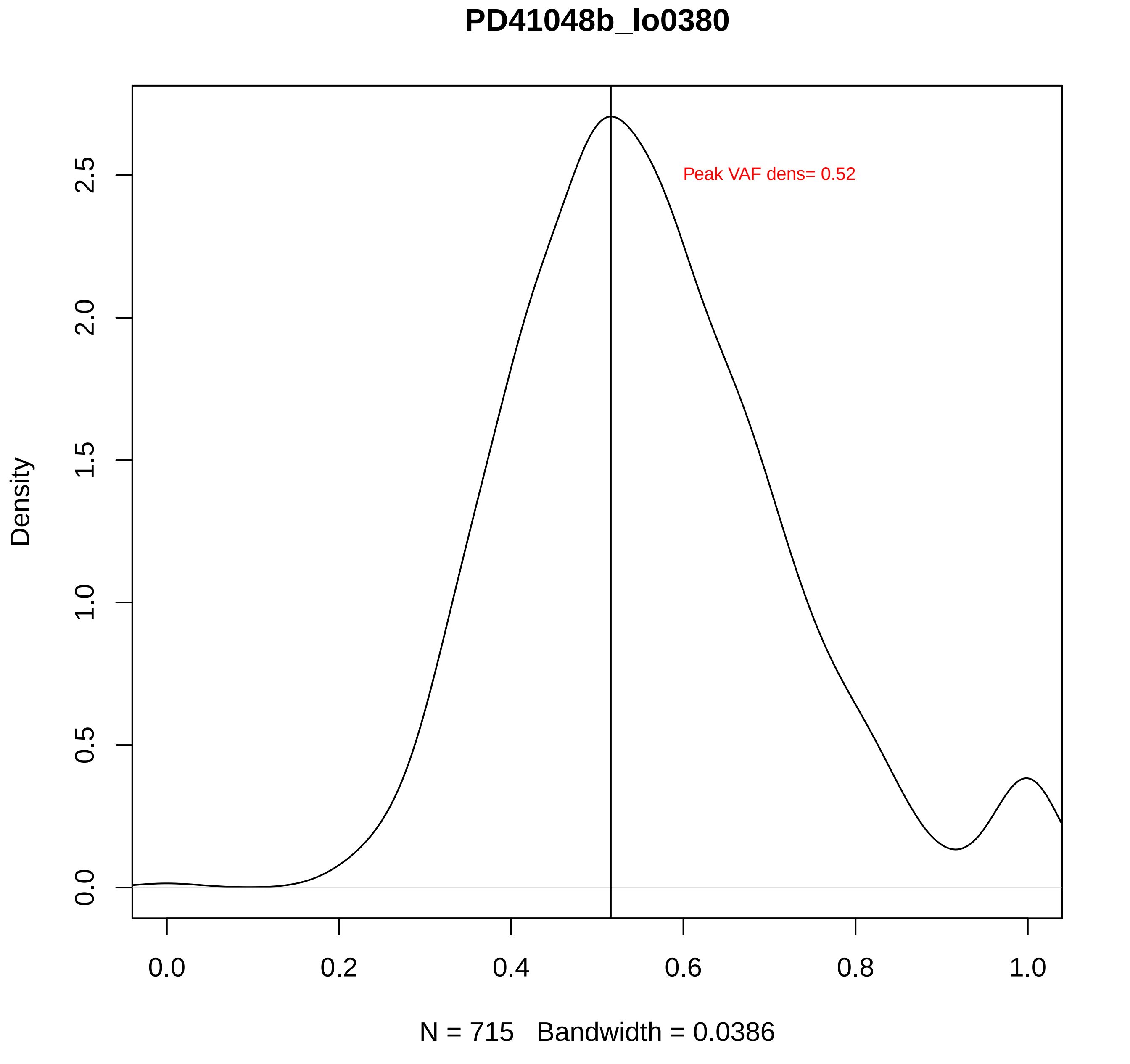
PD41048b_lo0380
Peak VAF dens= 0.52
Density
0.0
0.5
1.0
1.5
2.0
2.5
0.0
0.2
0.4
0.6
0.8
1.0
N = 715   Bandwidth = 0.0386

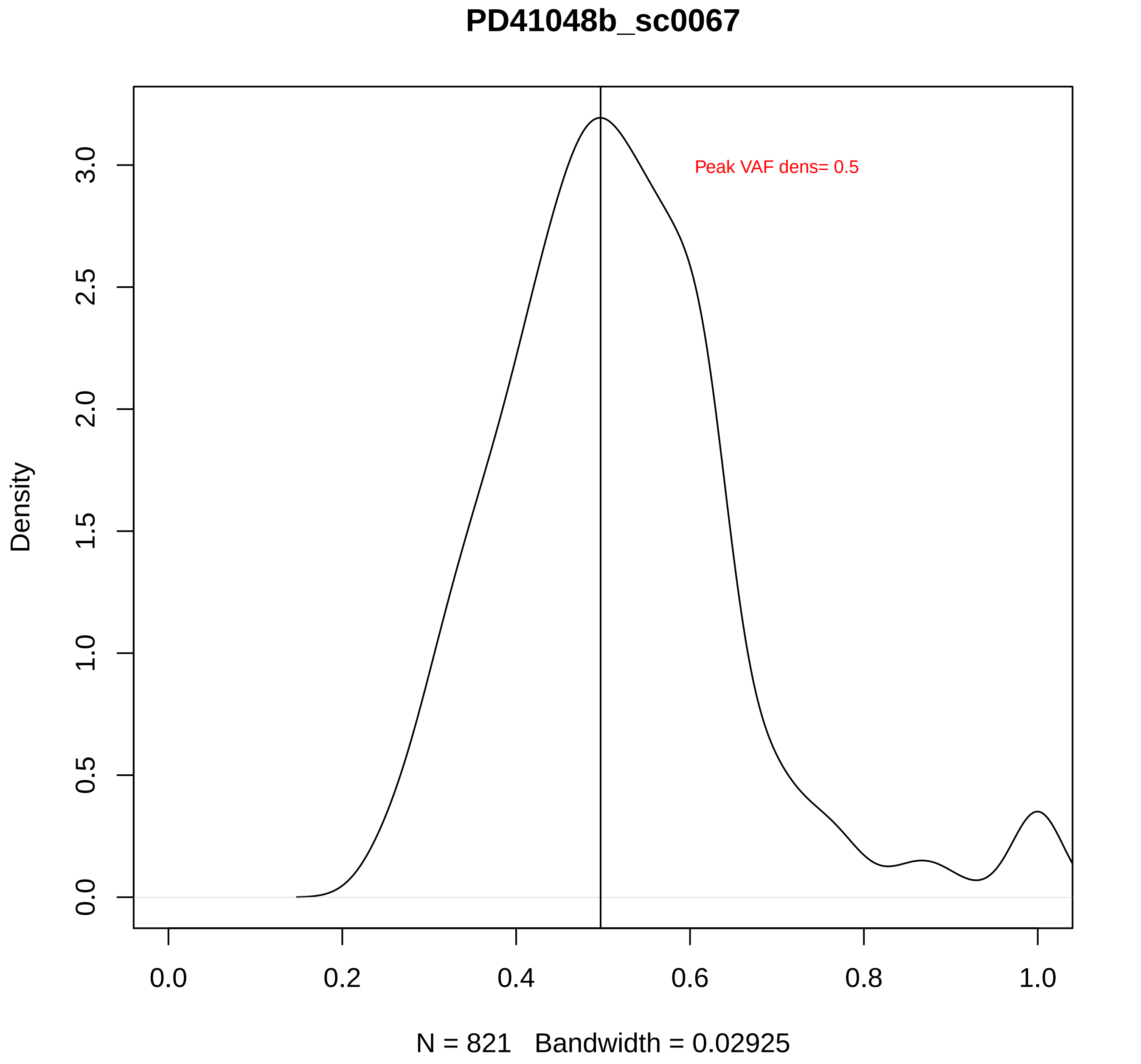

PD41048b_sc0067
Peak VAF dens= 0.5
Density
0.0
0.5
1.0
1.5
2.0
2.5
3.0
0.0
0.2
0.4
0.6
0.8
1.0
N = 821   Bandwidth = 0.02925

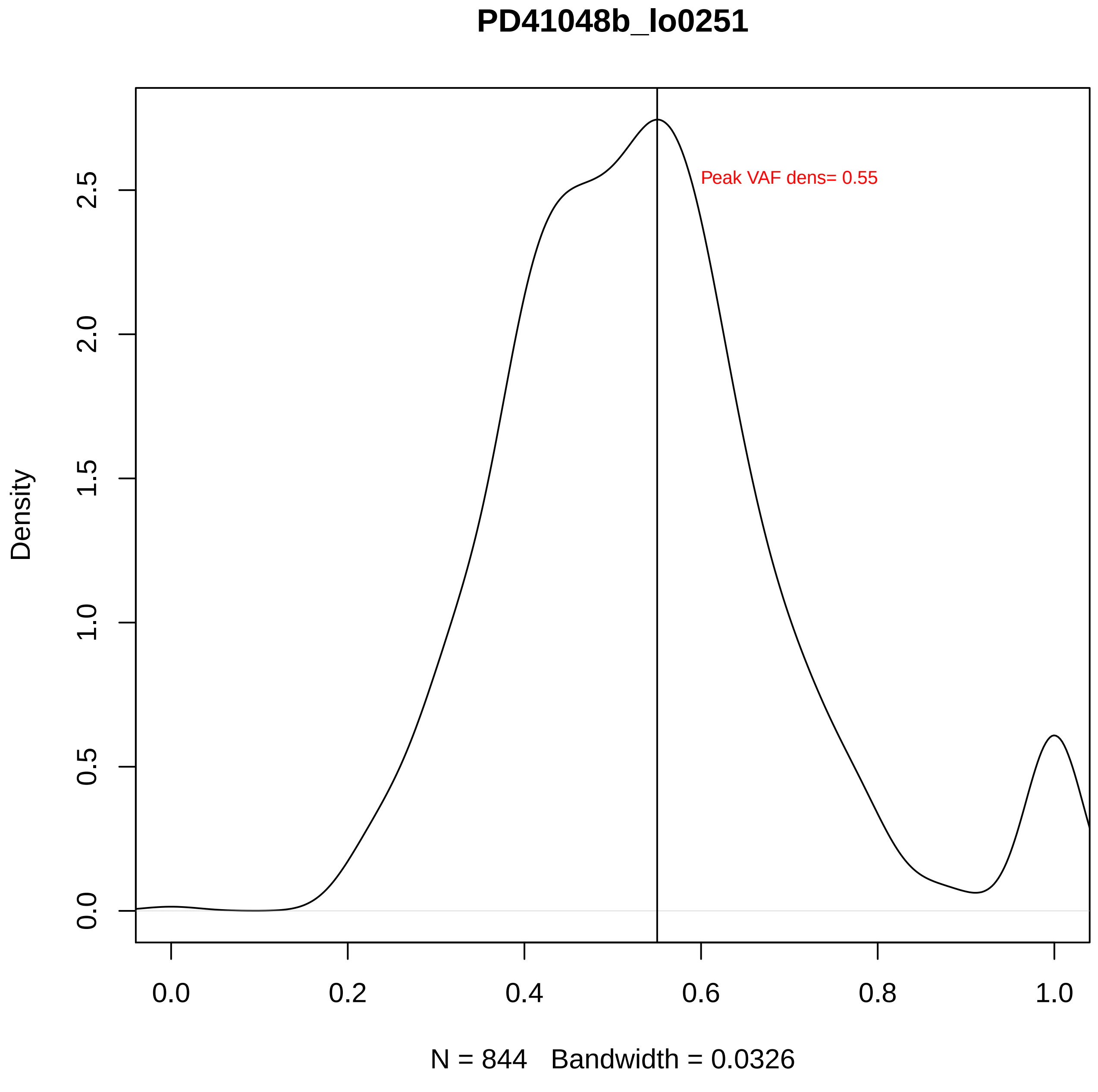

PD41048b_lo0251
Peak VAF dens= 0.55
Density
0.0
0.5
1.0
1.5
2.0
2.5
0.0
0.2
0.4
0.6
0.8
1.0
N = 844   Bandwidth = 0.0326

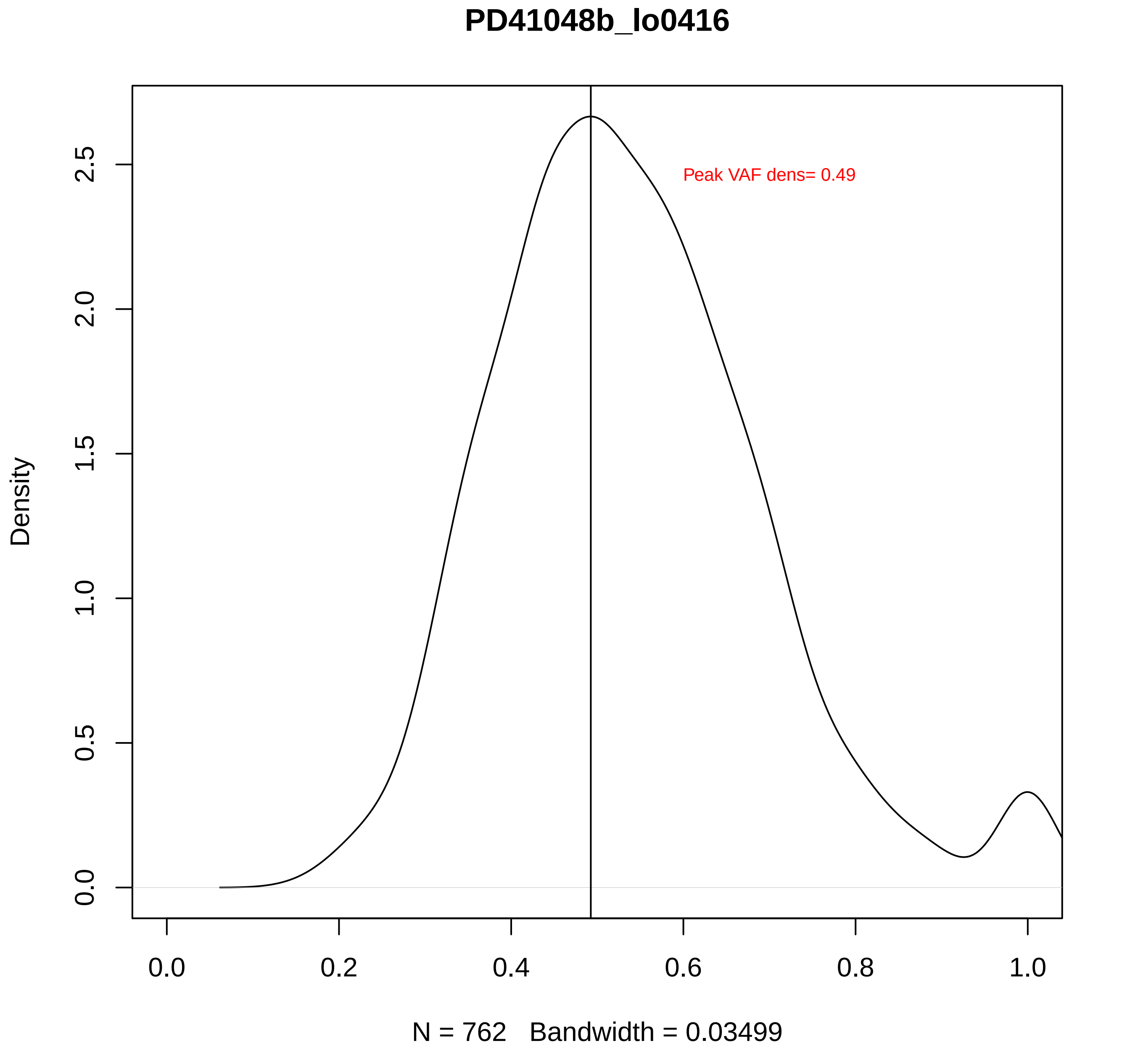

PD41048b_lo0416
Peak VAF dens= 0.49
Density
0.0
0.5
1.0
1.5
2.0
2.5
0.0
0.2
0.4
0.6
0.8
1.0
N = 762   Bandwidth = 0.03499

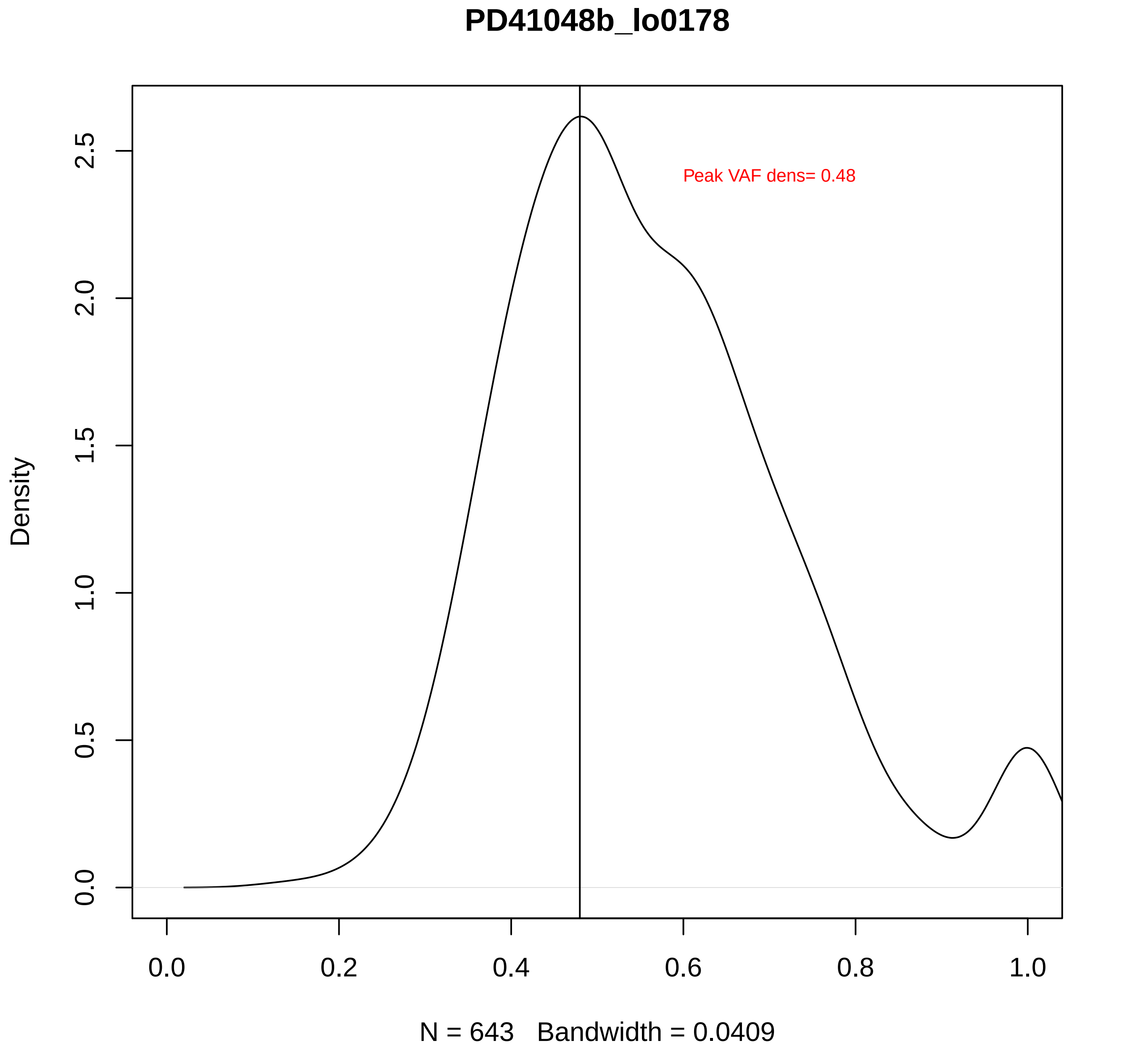

PD41048b_lo0178
Peak VAF dens= 0.48
Density
0.0
0.5
1.0
1.5
2.0
2.5
0.0
0.2
0.4
0.6
0.8
1.0
N = 643 Bandwidth = 0.0409

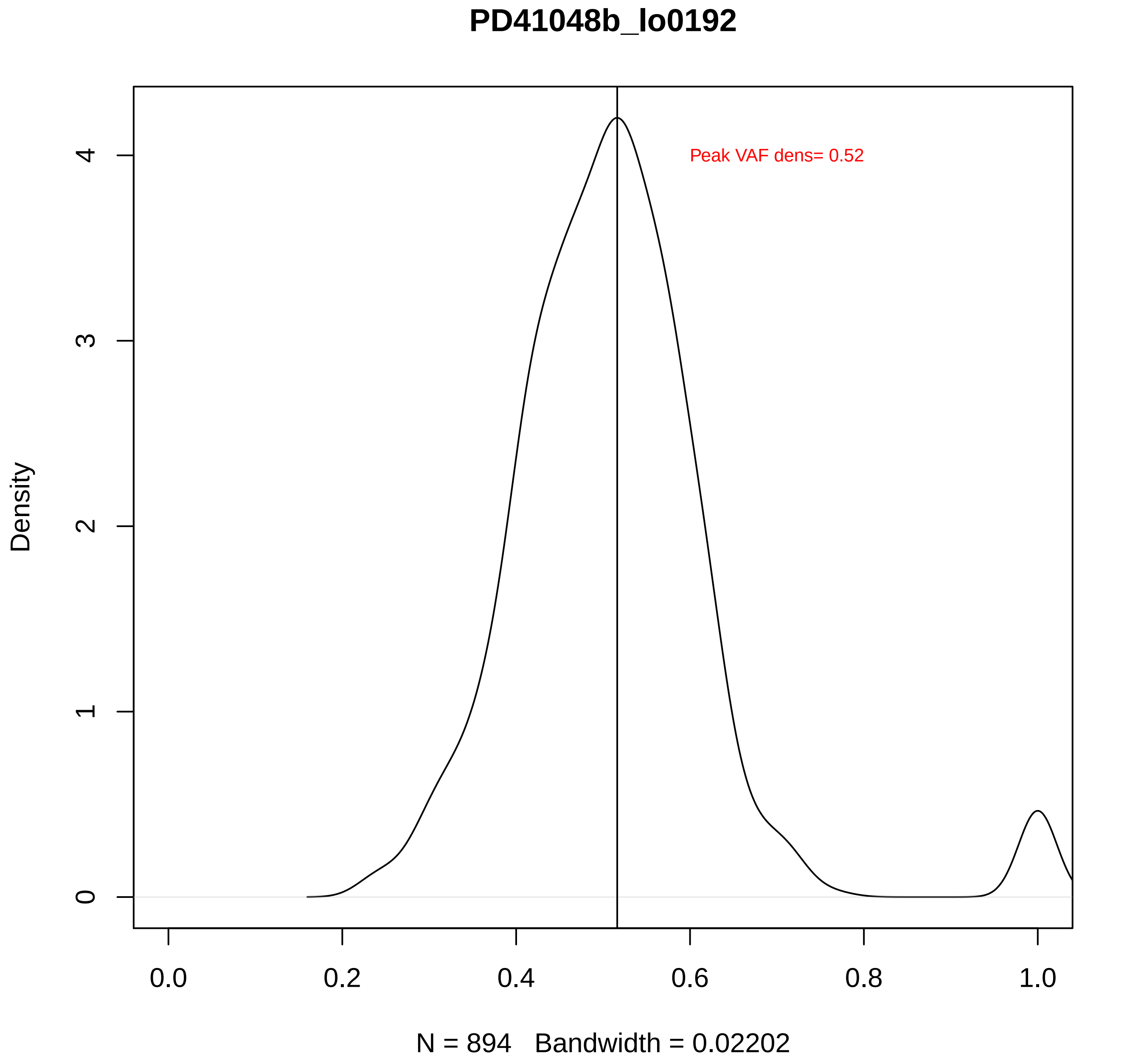

PD41048b_lo0192
Peak VAF dens= 0.52
Density
0
1
2
3
4
0.0
0.2
0.4
0.6
0.8
1.0
N = 894 Bandwidth = 0.02202

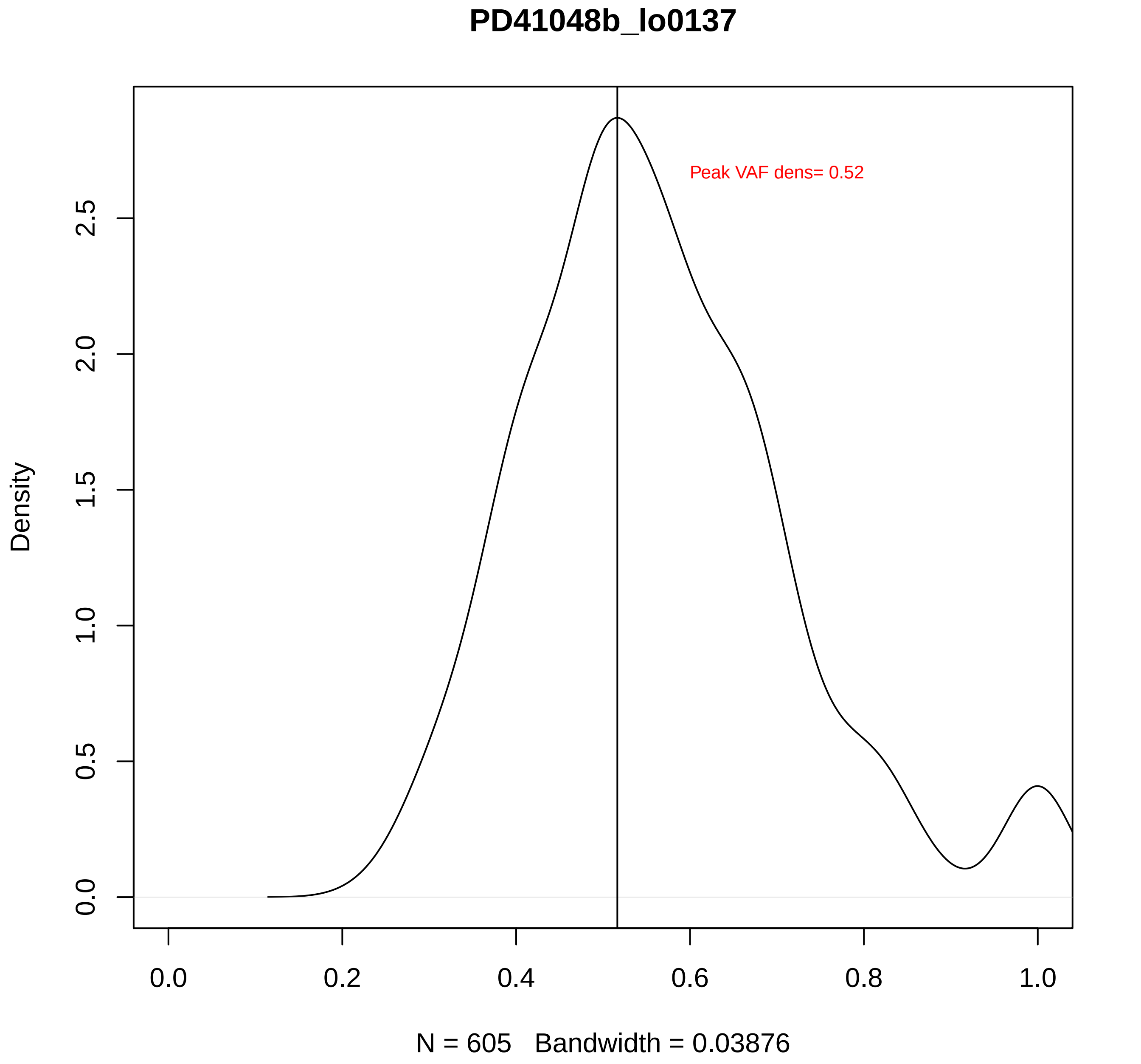
PD41048b_lo0137
Peak VAF dens= 0.52
Density
0.0
0.5
1.0
1.5
2.0
2.5
0.0
0.2
0.4
0.6
0.8
1.0
N = 605   Bandwidth = 0.03876

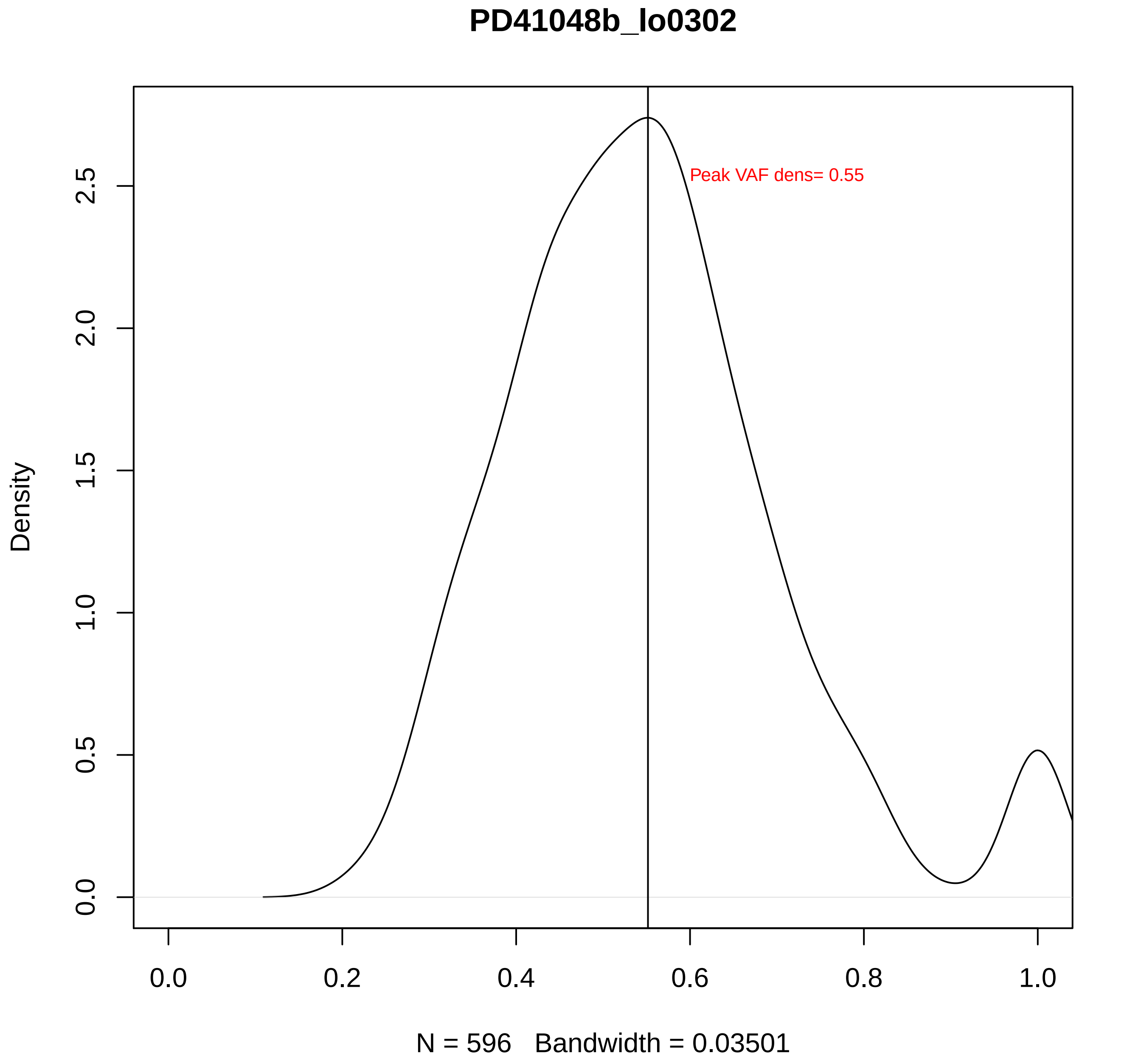

PD41048b_lo0302
Peak VAF dens= 0.55
Density
0.0
0.5
1.0
1.5
2.0
2.5
0.0
0.2
0.4
0.6
0.8
1.0
N = 596   Bandwidth = 0.03501

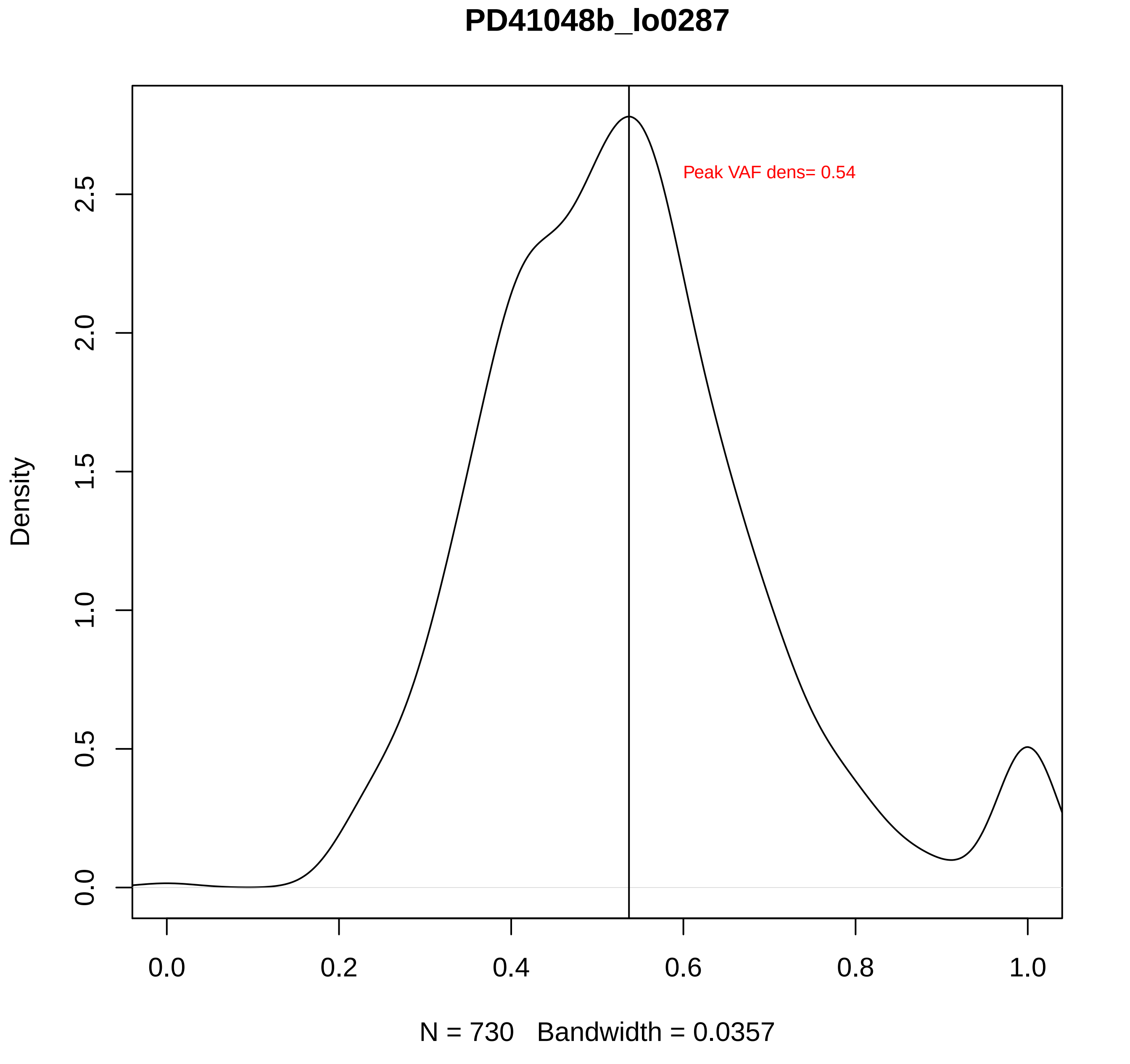
PD41048b_lo0287
Peak VAF dens= 0.54
Density
0.0
0.5
1.0
1.5
2.0
2.5
0.0
0.2
0.4
0.6
0.8
1.0
N = 730 Bandwidth = 0.0357

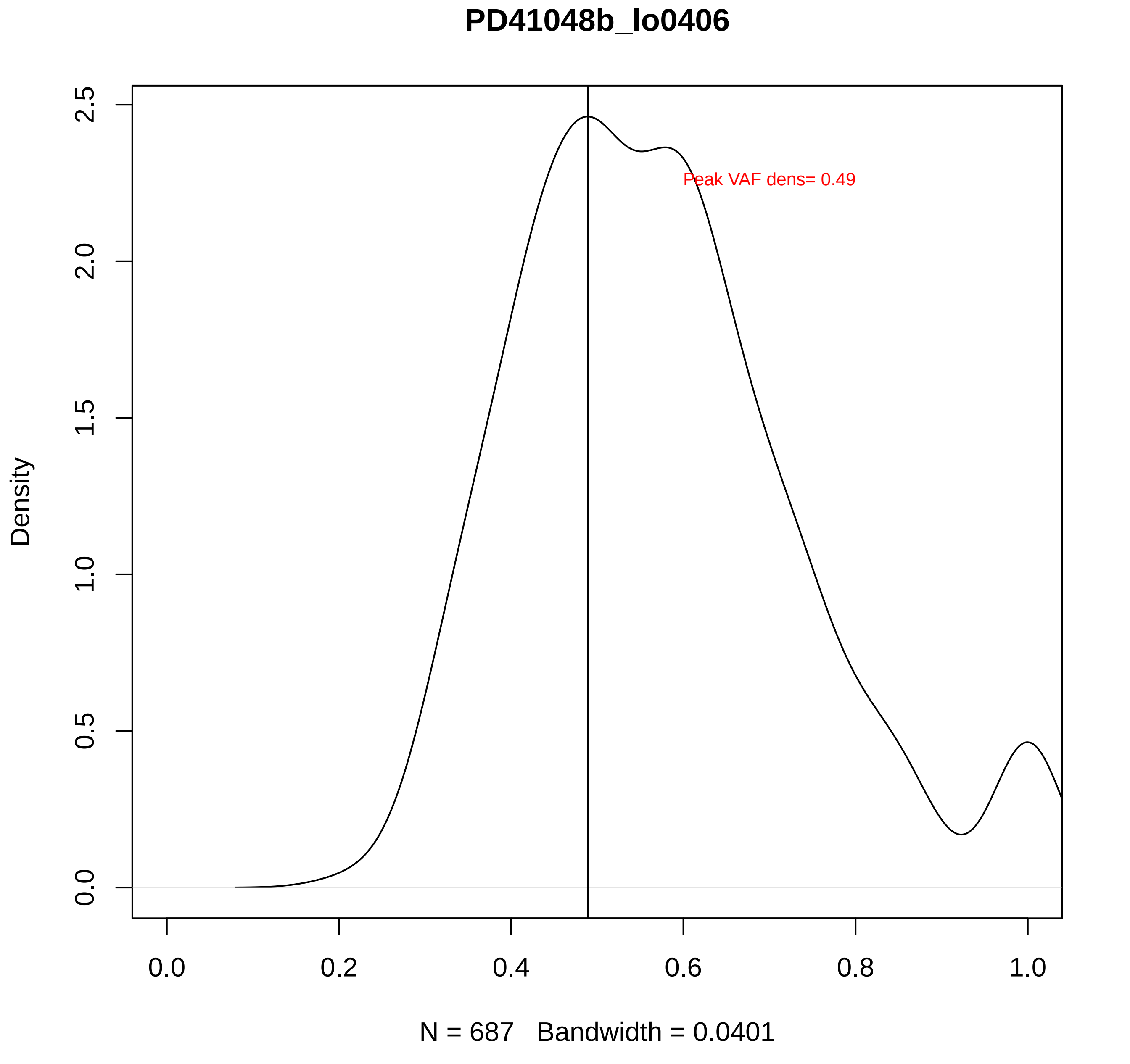
PD41048b_lo0406
Peak VAF dens= 0.49
Density
0.0
0.5
1.0
1.5
2.0
2.5
0.0
0.2
0.4
0.6
0.8
1.0
N = 687   Bandwidth = 0.0401

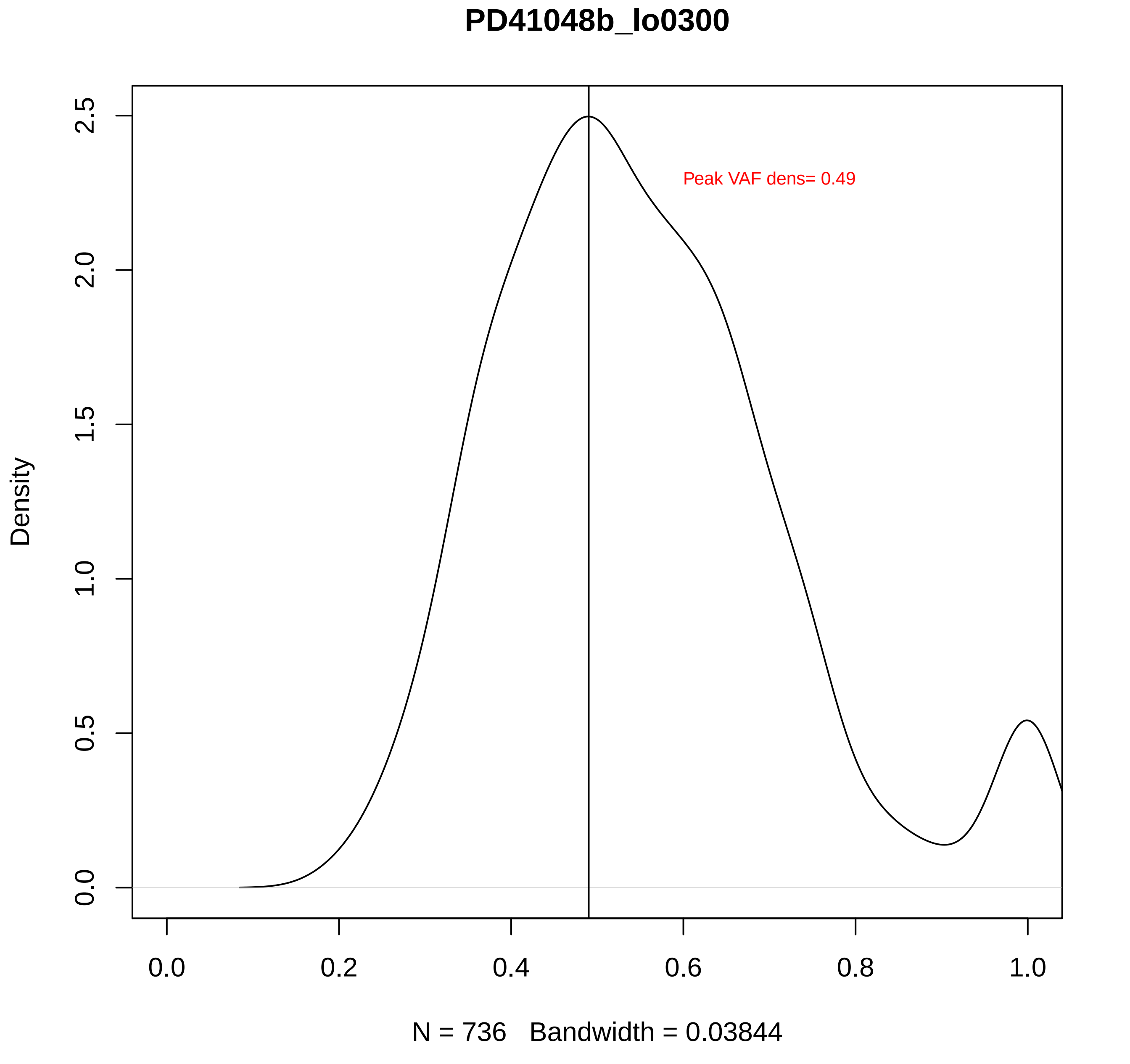
PD41048b_lo0300
Peak VAF dens= 0.49
Density
0.0
0.5
1.0
1.5
2.0
2.5
0.0
0.2
0.4
0.6
0.8
1.0
N = 736   Bandwidth = 0.03844

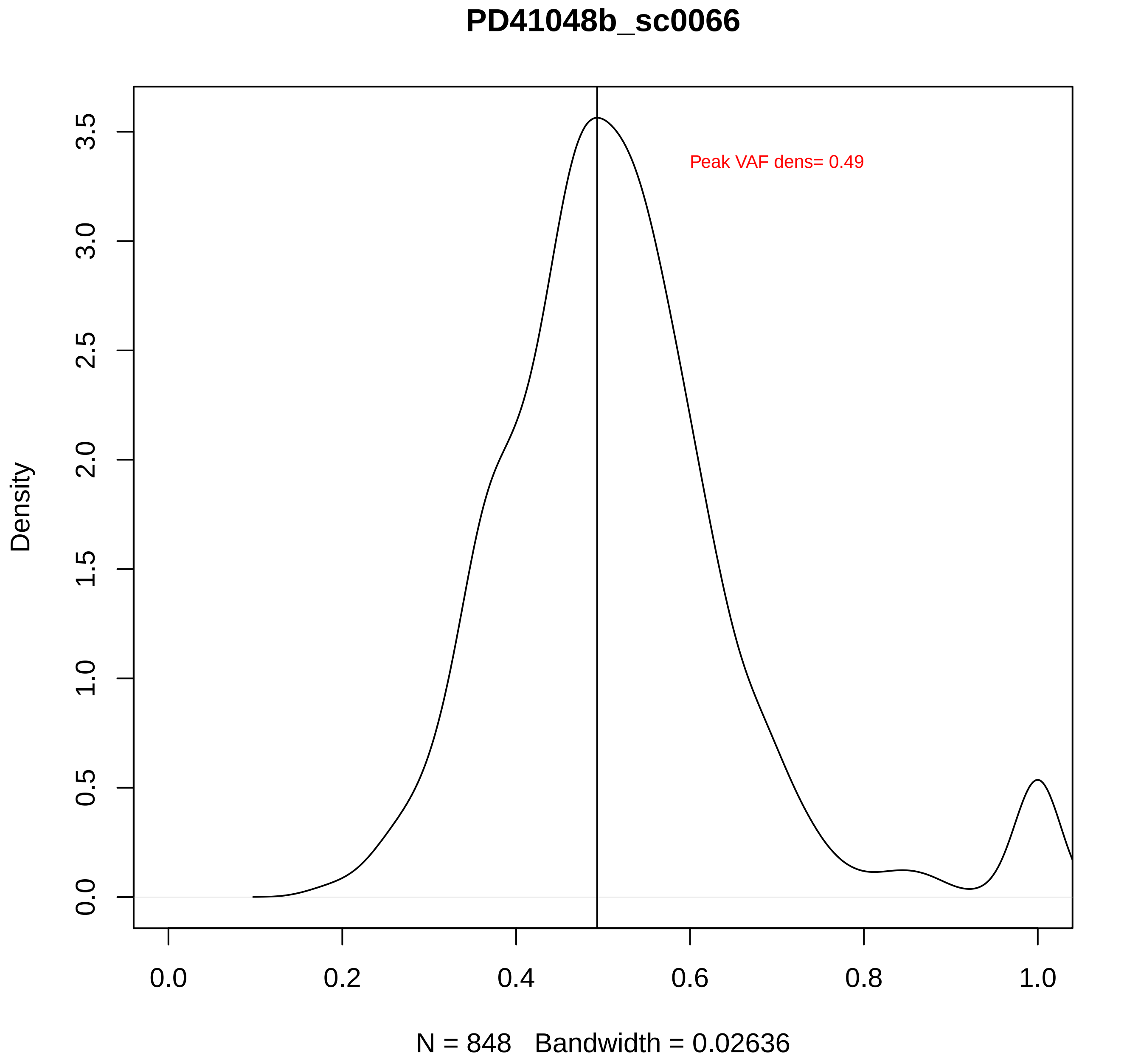
PD41048b_sc0066
Peak VAF dens= 0.49
Density
0.0
0.5
1.0
1.5
2.0
2.5
3.0
3.5
0.0
0.2
0.4
0.6
0.8
1.0
N = 848   Bandwidth = 0.02636

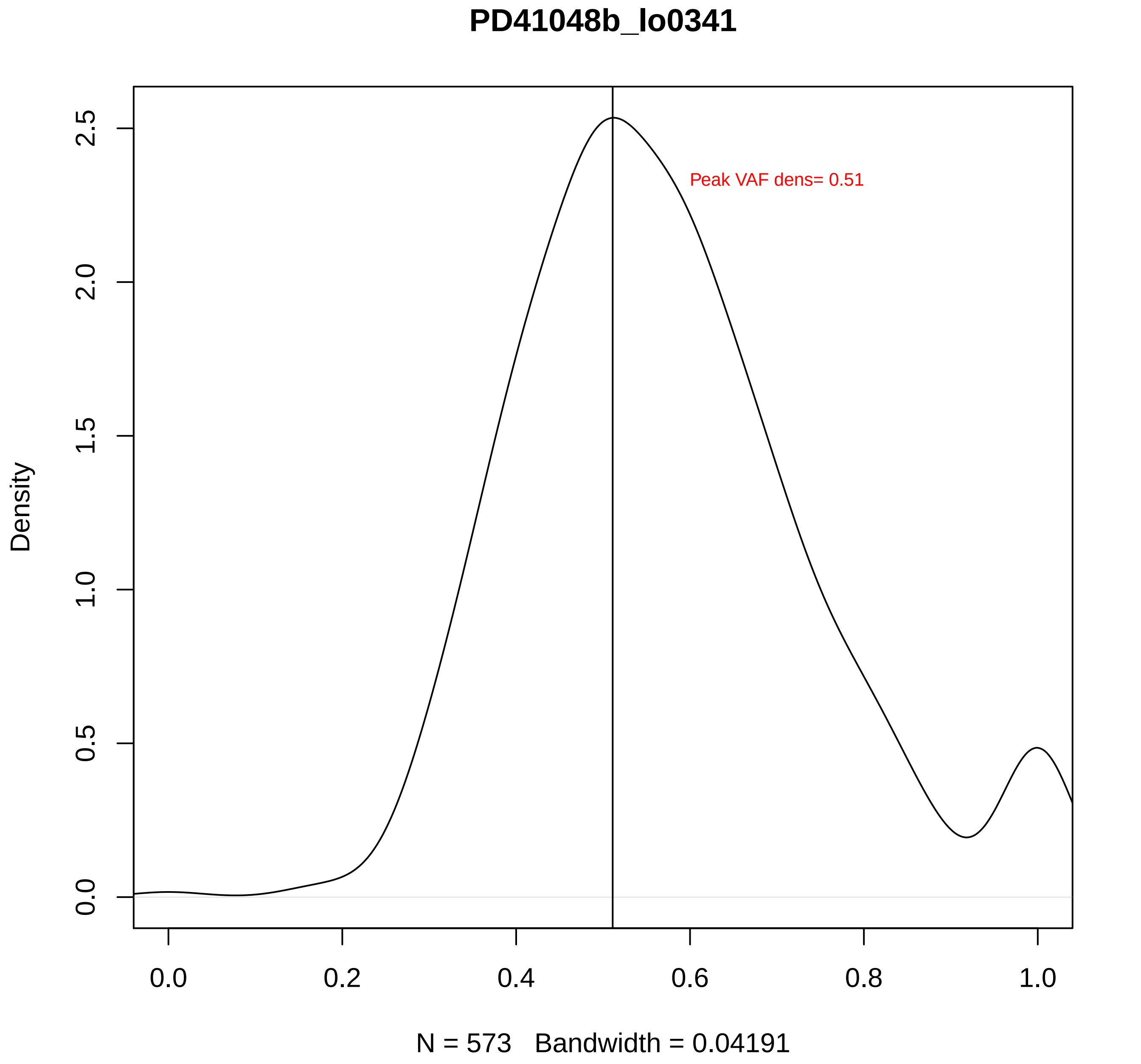
PD41048b_lo0341
Peak VAF dens= 0.51
Density
0.0
0.5
1.0
1.5
2.0
2.5
0.0
0.2
0.4
0.6
0.8
1.0
N = 573 Bandwidth = 0.04191

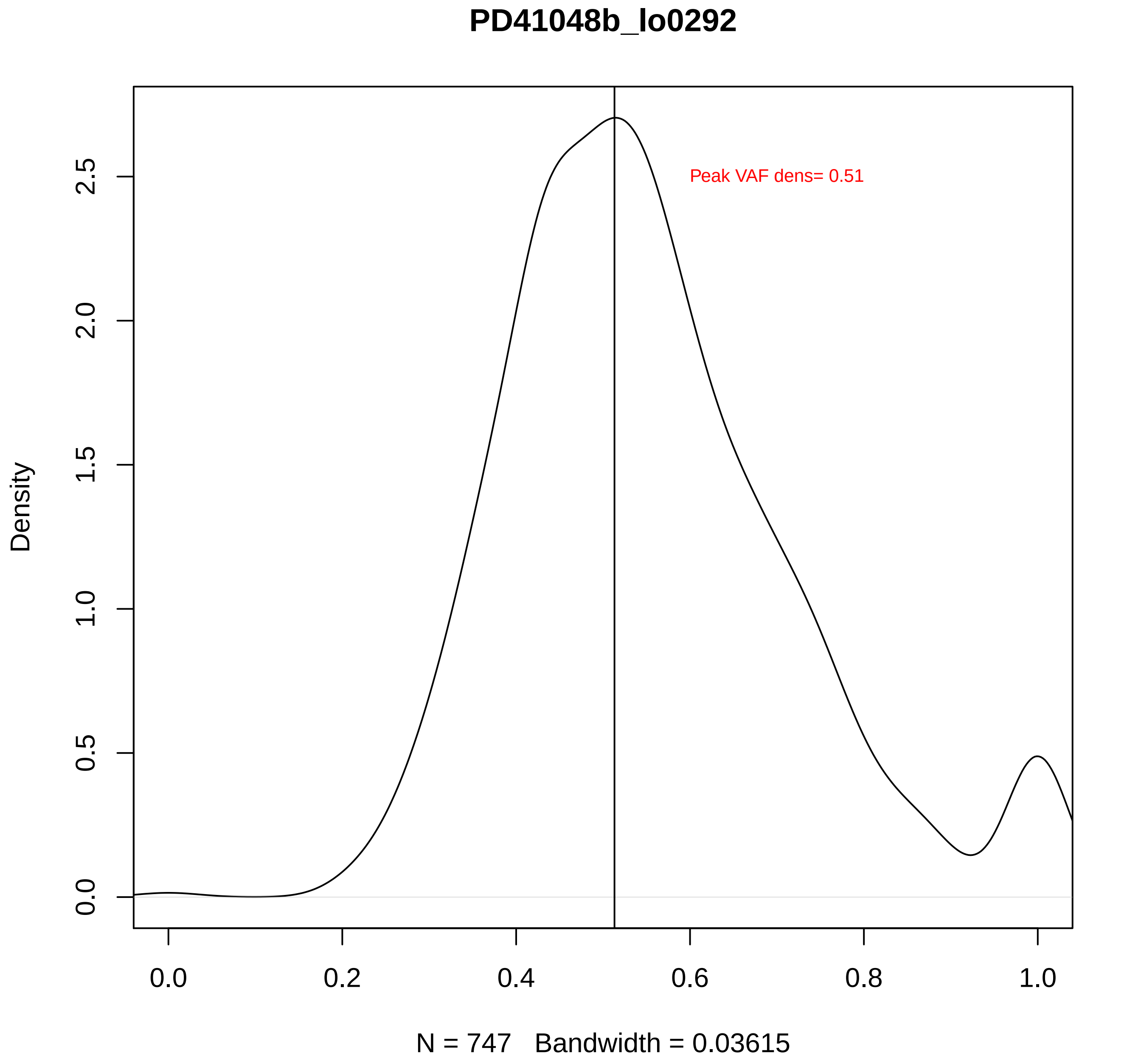

PD41048b_lo0292
Peak VAF dens= 0.51
Density
0.0
0.5
1.0
1.5
2.0
2.5
0.0
0.2
0.4
0.6
0.8
1.0
N = 747   Bandwidth = 0.03615

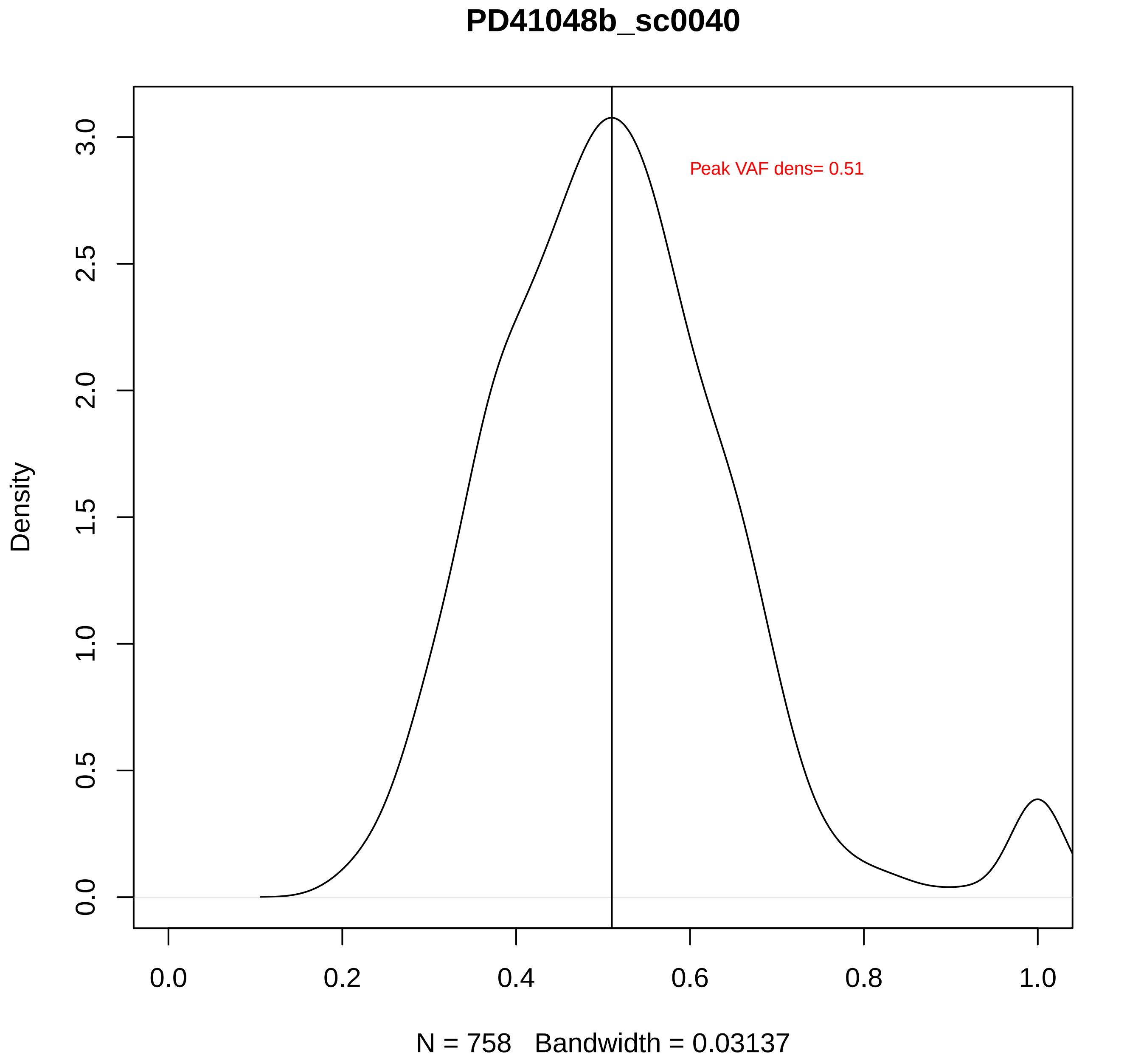
PD41048b_sc0040
Peak VAF dens= 0.51
Density
0.0
0.5
1.0
1.5
2.0
2.5
3.0
0.0
0.2
0.4
0.6
0.8
1.0
N = 758   Bandwidth = 0.03137

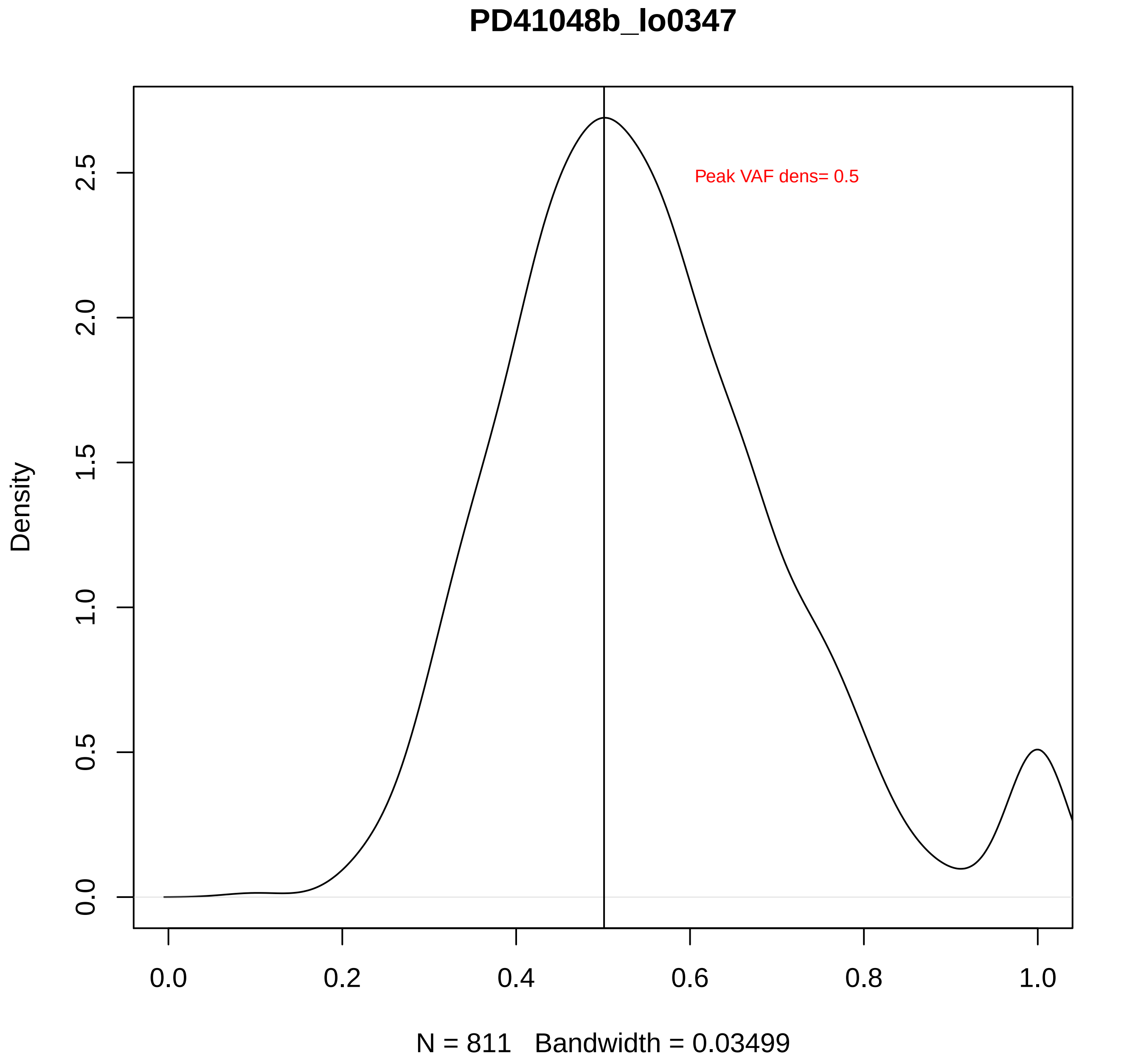

PD41048b_lo0347
Peak VAF dens= 0.5
Density
0.0
0.5
1.0
1.5
2.0
2.5
0.0
0.2
0.4
0.6
0.8
1.0
N = 811 Bandwidth = 0.03499

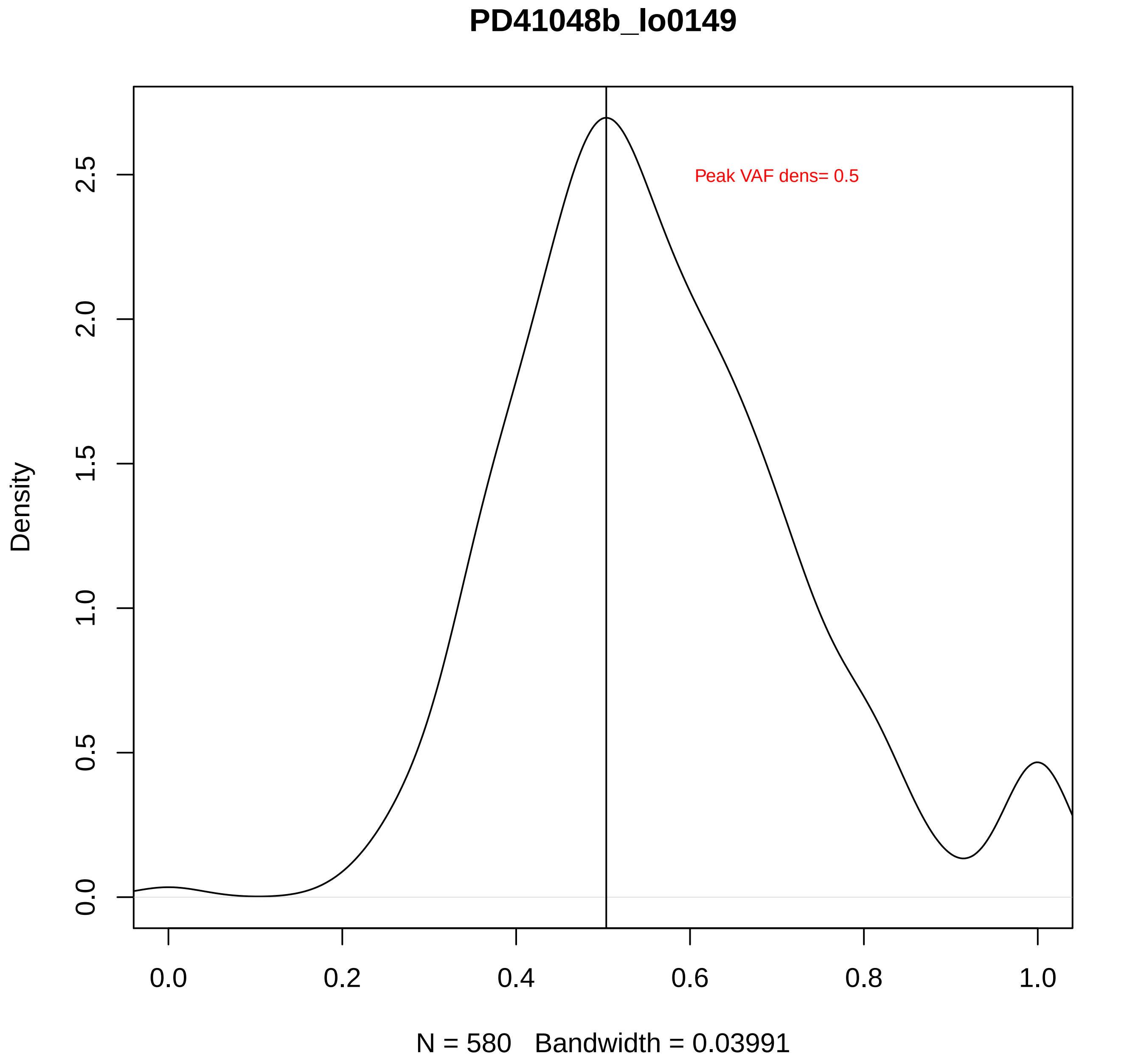
PD41048b_lo0149
Peak VAF dens= 0.5
Density
0.0
0.5
1.0
1.5
2.0
2.5
0.0
0.2
0.4
0.6
0.8
1.0
N = 580   Bandwidth = 0.03991

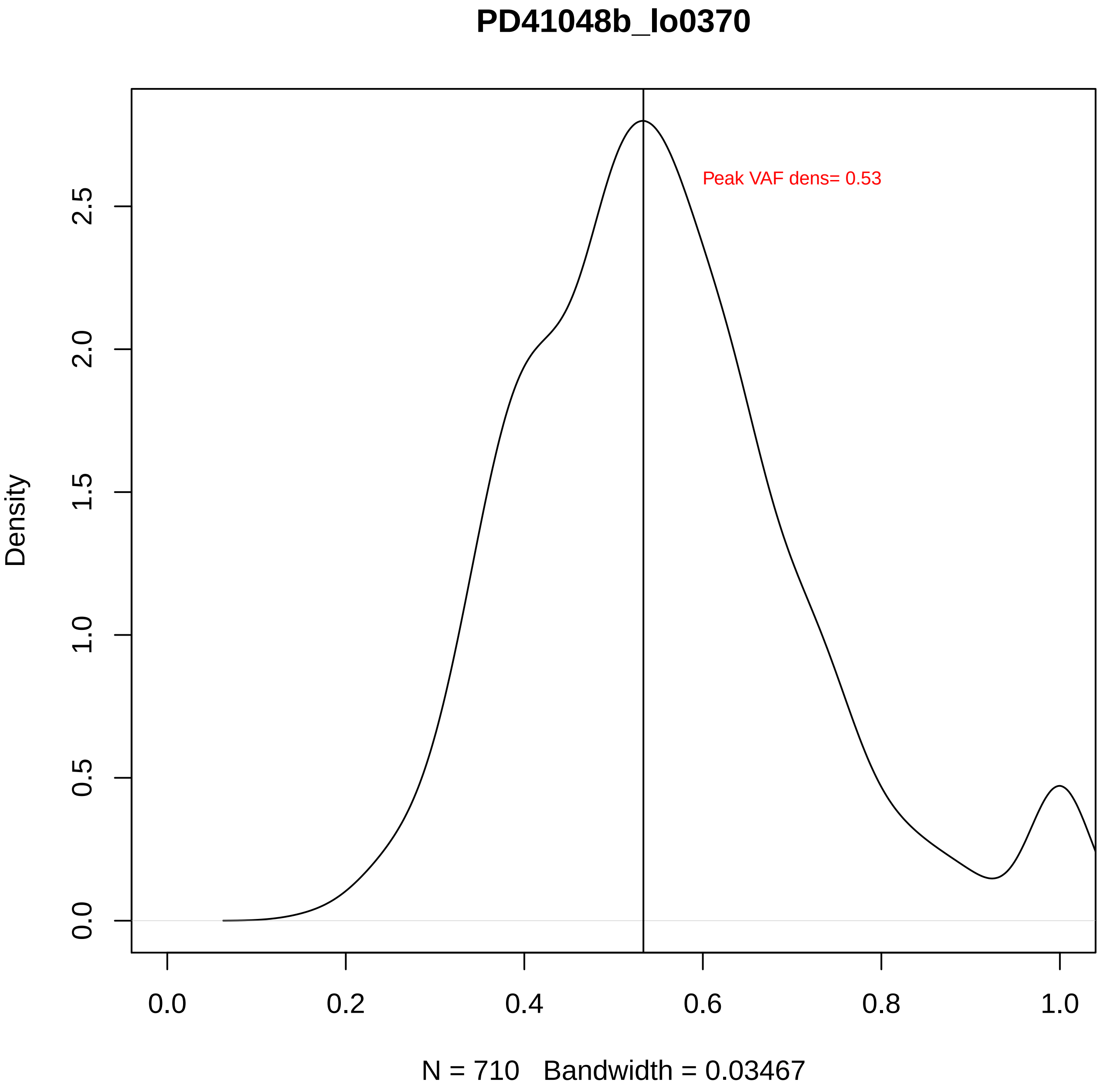

PD41048b_lo0370
Peak VAF dens= 0.53
Density
0.0
0.5
1.0
1.5
2.0
2.5
0.0
0.2
0.4
0.6
0.8
1.0
N = 710 Bandwidth = 0.03467

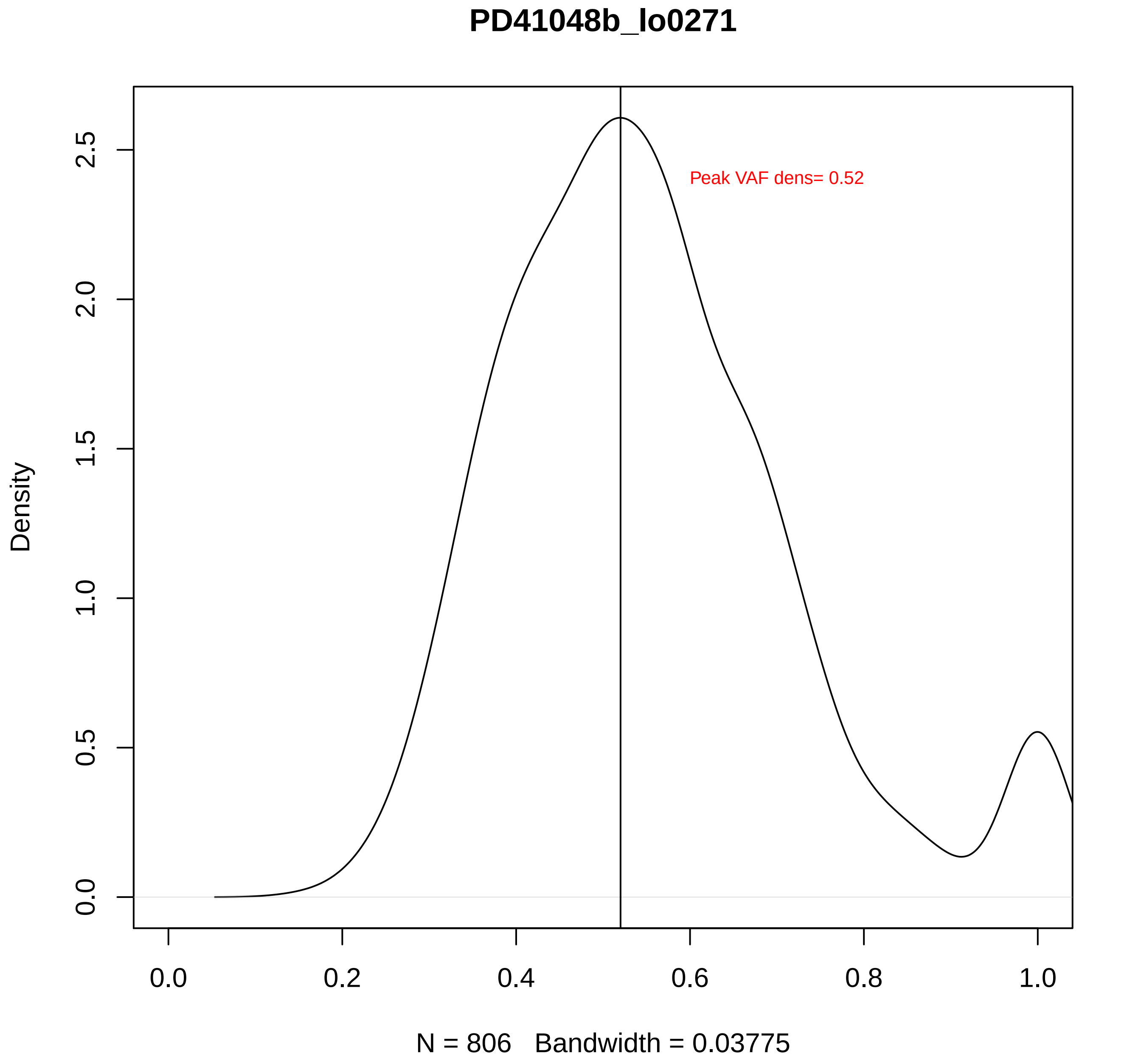

PD41048b_lo0271
Peak VAF dens= 0.52
Density
0.0
0.5
1.0
1.5
2.0
2.5
0.0
0.2
0.4
0.6
0.8
1.0
N = 806 Bandwidth = 0.03775

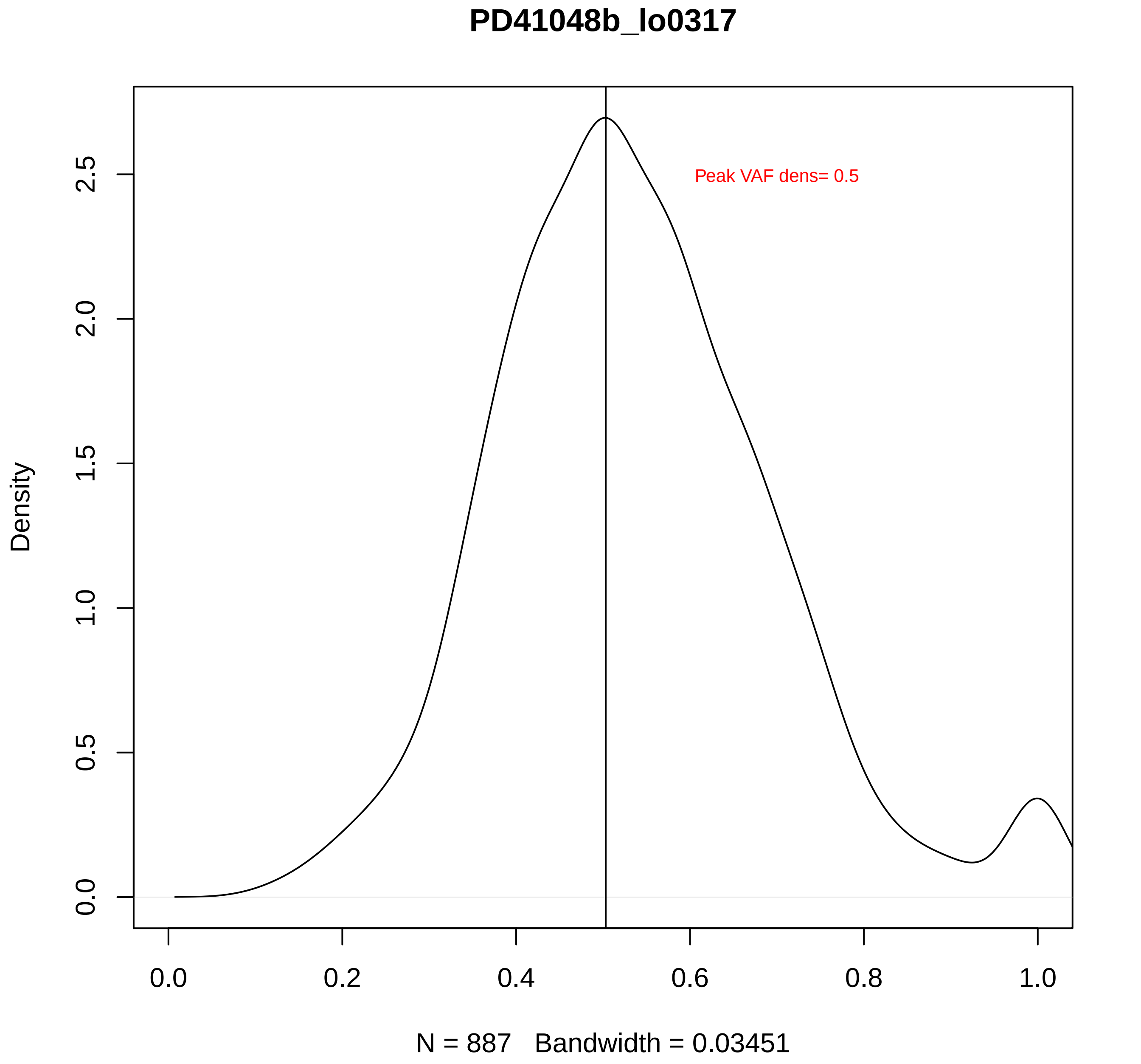

PD41048b_lo0317
Peak VAF dens= 0.5
Density
0.0
0.5
1.0
1.5
2.0
2.5
0.0
0.2
0.4
0.6
0.8
1.0
N = 887 Bandwidth = 0.03451

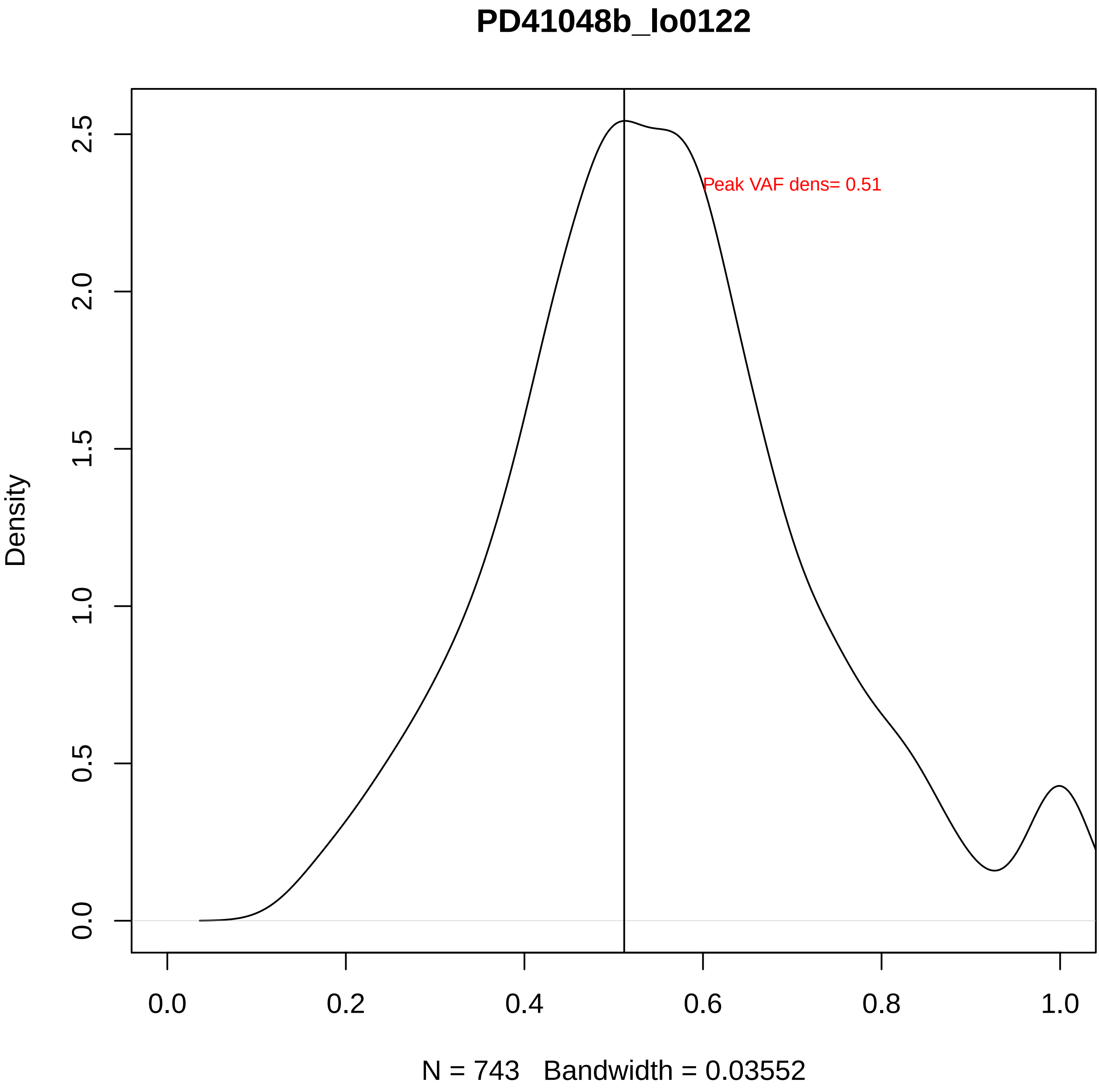
PD41048b_lo0122
Peak VAF dens= 0.51
Density
0.0
0.5
1.0
1.5
2.0
2.5
0.0
0.2
0.4
0.6
0.8
1.0
N = 743   Bandwidth = 0.03552

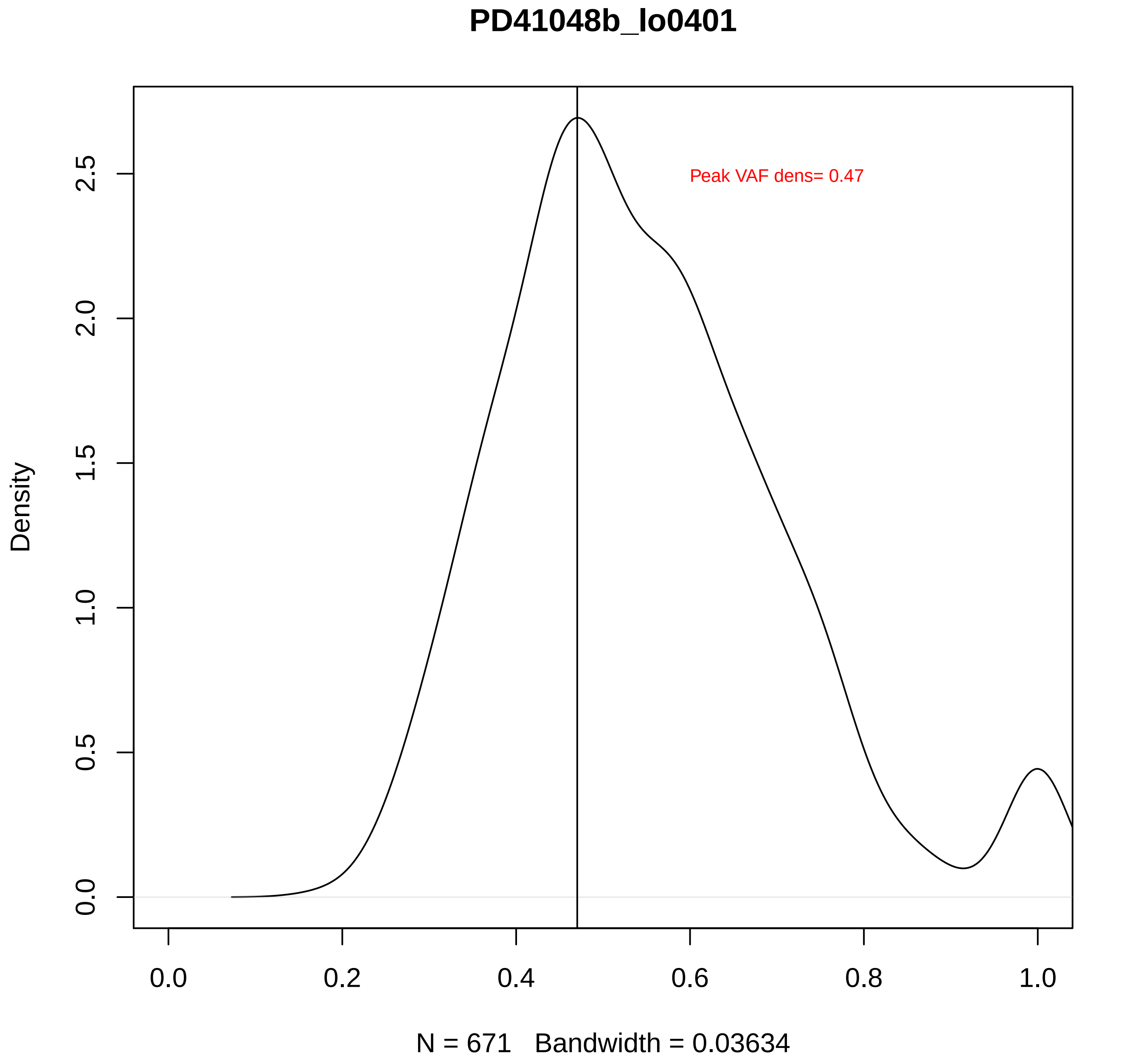
PD41048b_lo0401
Peak VAF dens= 0.47
Density
0.0
0.5
1.0
1.5
2.0
2.5
0.0
0.2
0.4
0.6
0.8
1.0
N = 671 Bandwidth = 0.03634

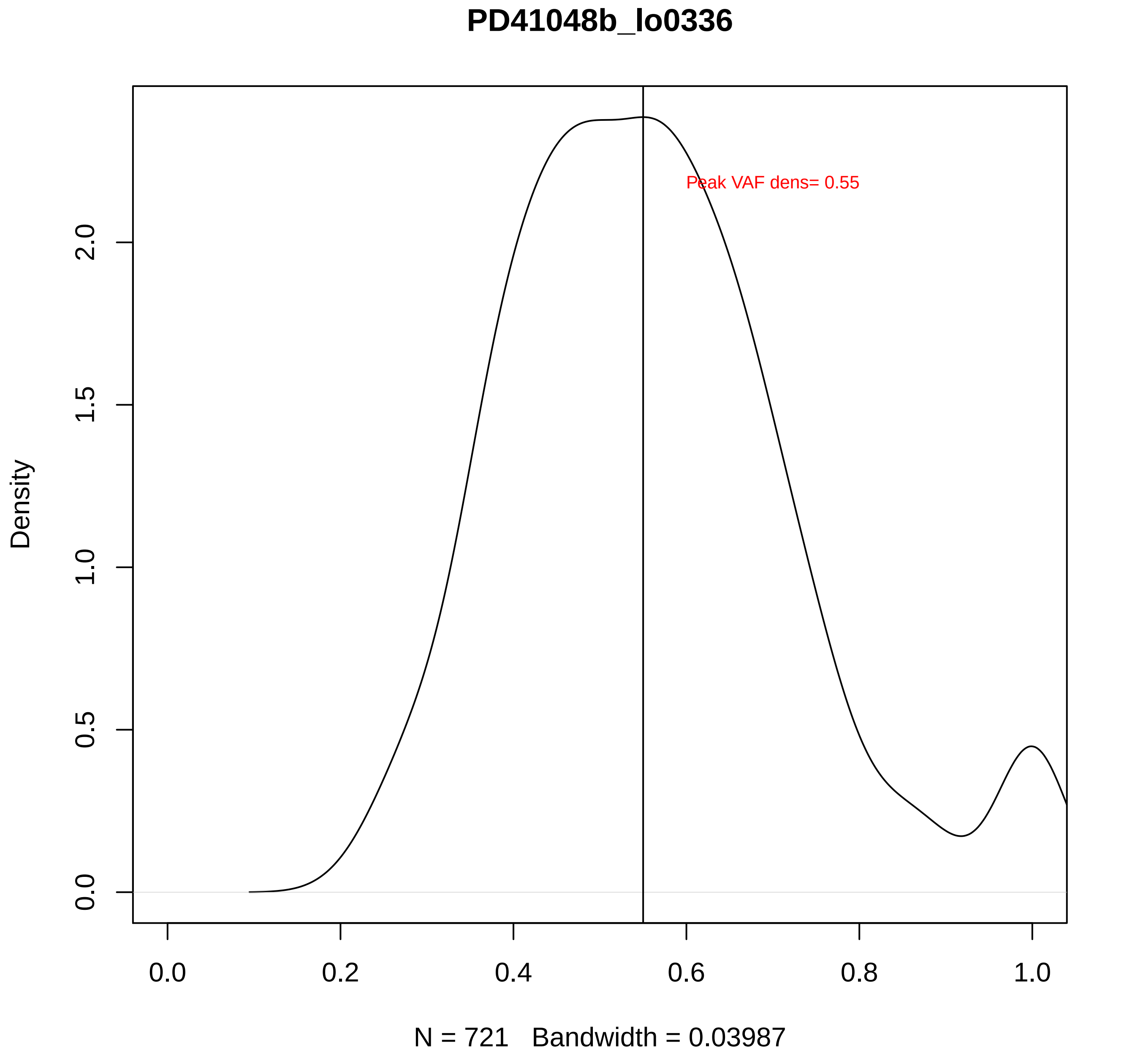
PD41048b_lo0336
Peak VAF dens= 0.55
Density
0.0
0.5
1.0
1.5
2.0
0.0
0.2
0.4
0.6
0.8
1.0
N = 721   Bandwidth = 0.03987

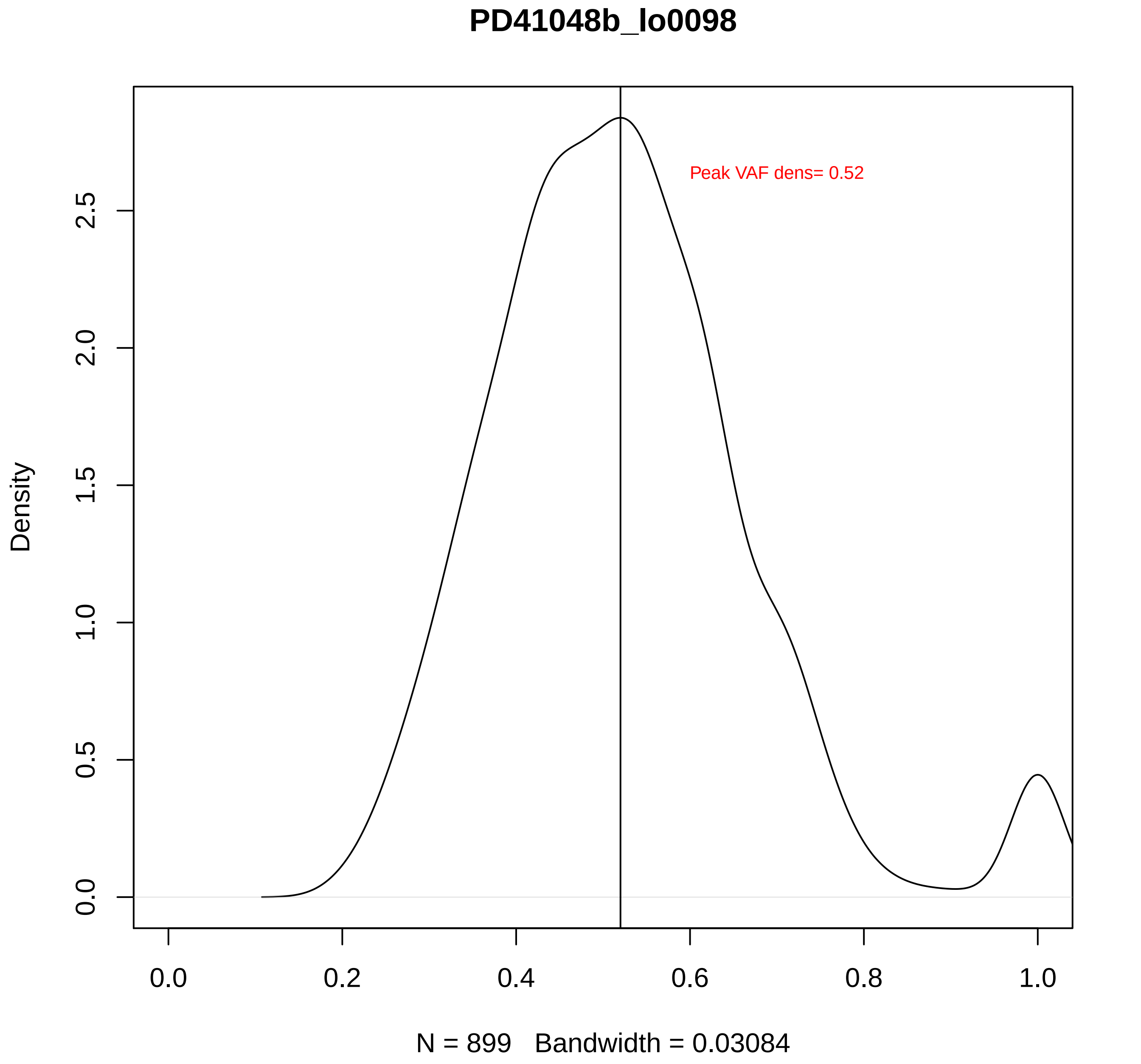
PD41048b_lo0098
Peak VAF dens= 0.52
Density
0.0
0.5
1.0
1.5
2.0
2.5
0.0
0.2
0.4
0.6
0.8
1.0
N = 899   Bandwidth = 0.03084

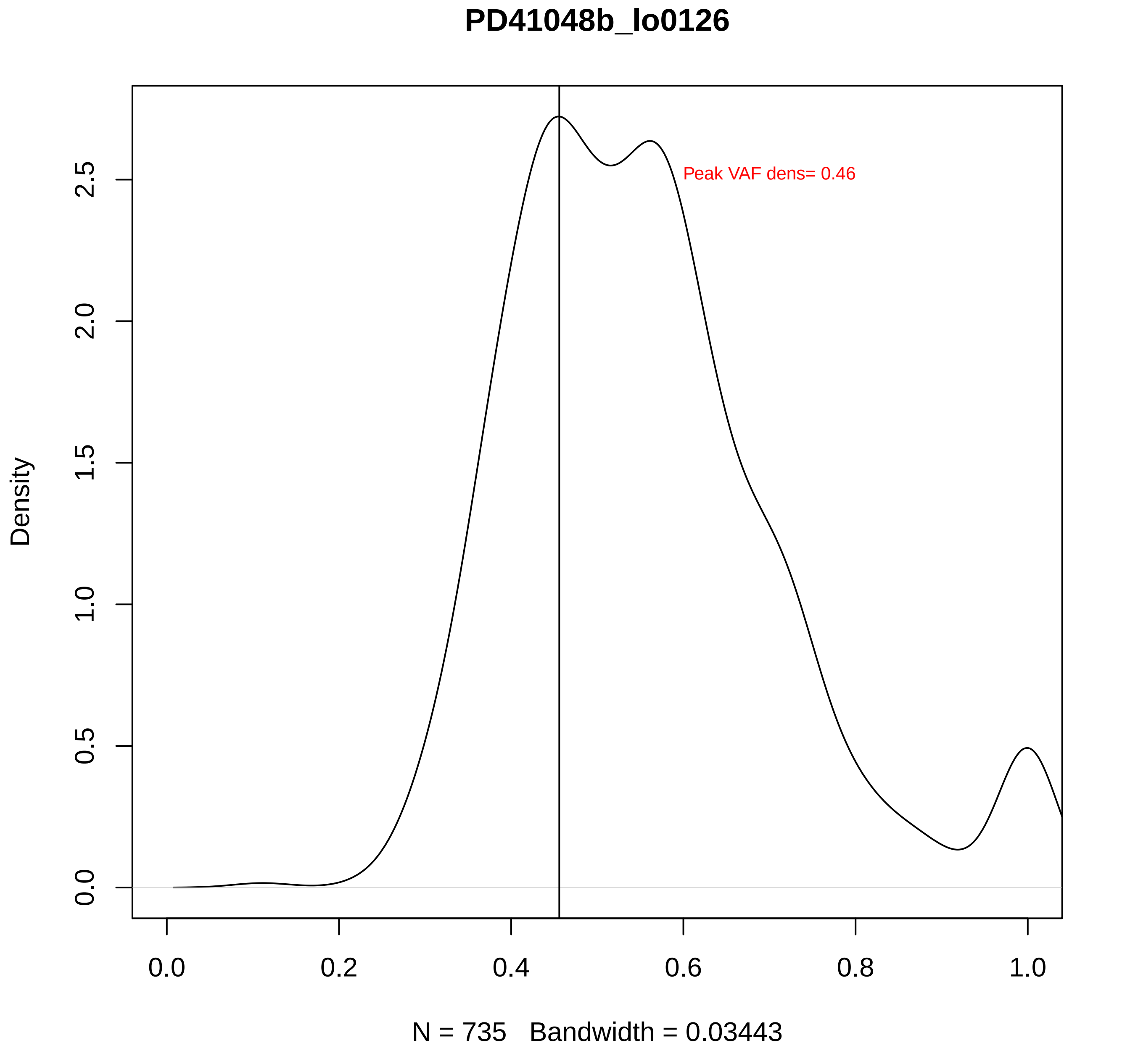
PD41048b_lo0126
Peak VAF dens= 0.46
Density
0.0
0.5
1.0
1.5
2.0
2.5
0.0
0.2
0.4
0.6
0.8
1.0
N = 735   Bandwidth = 0.03443

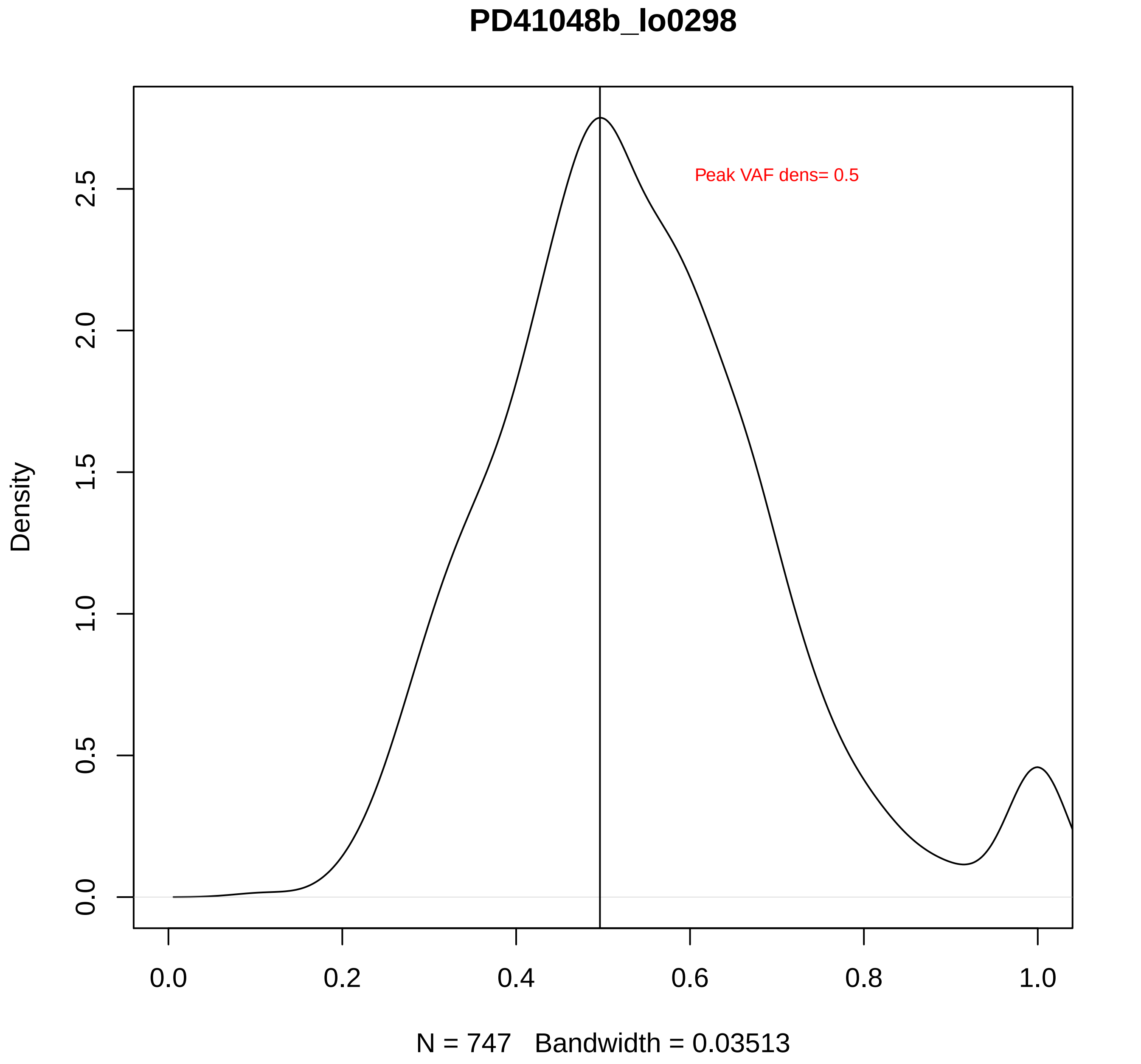

PD41048b_lo0298
Peak VAF dens= 0.5
Density
0.0
0.5
1.0
1.5
2.0
2.5
0.0
0.2
0.4
0.6
0.8
1.0
N = 747   Bandwidth = 0.03513

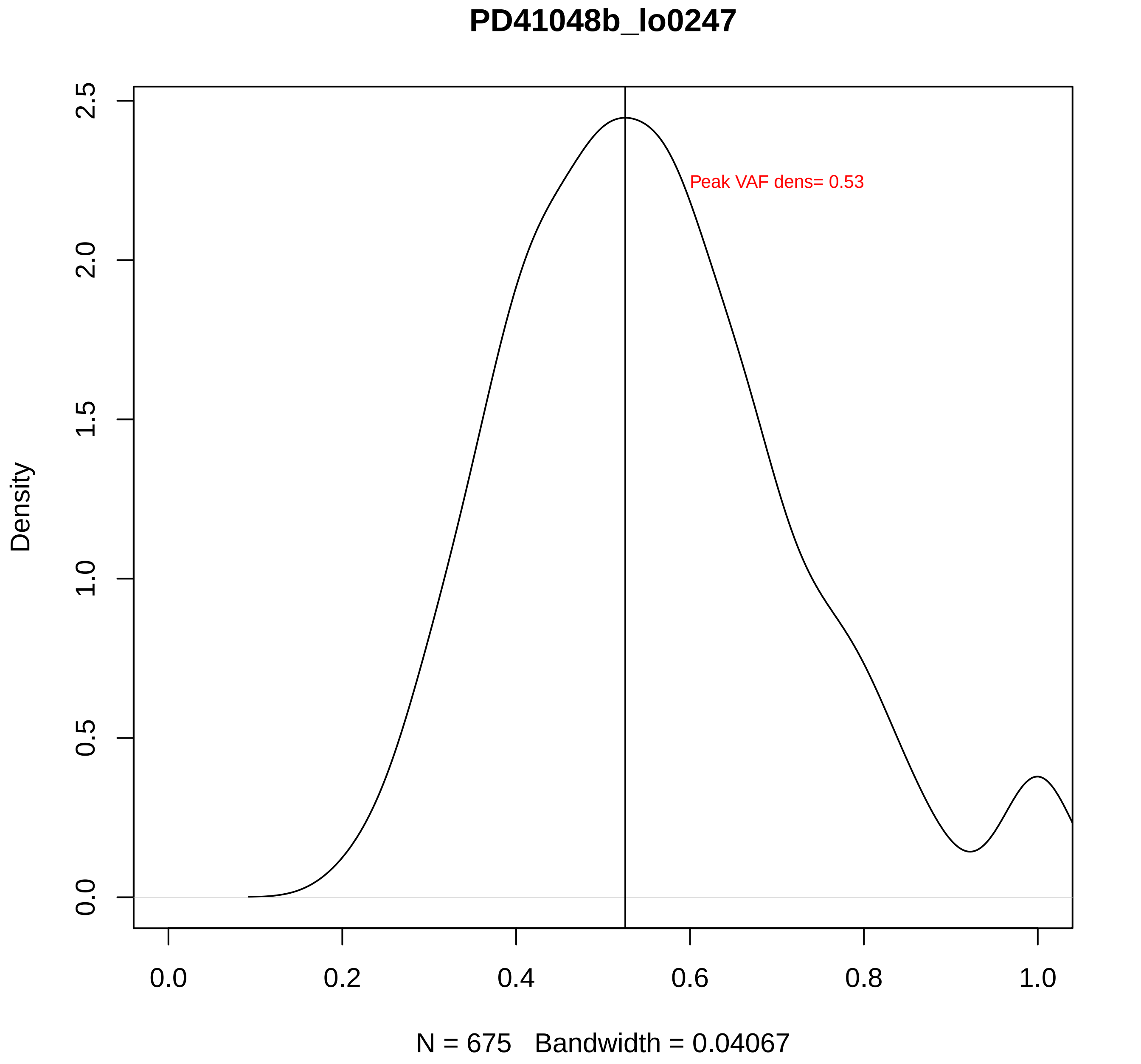
PD41048b_lo0247
Peak VAF dens= 0.53
Density
0.0
0.5
1.0
1.5
2.0
2.5
0.0
0.2
0.4
0.6
0.8
1.0
N = 675   Bandwidth = 0.04067

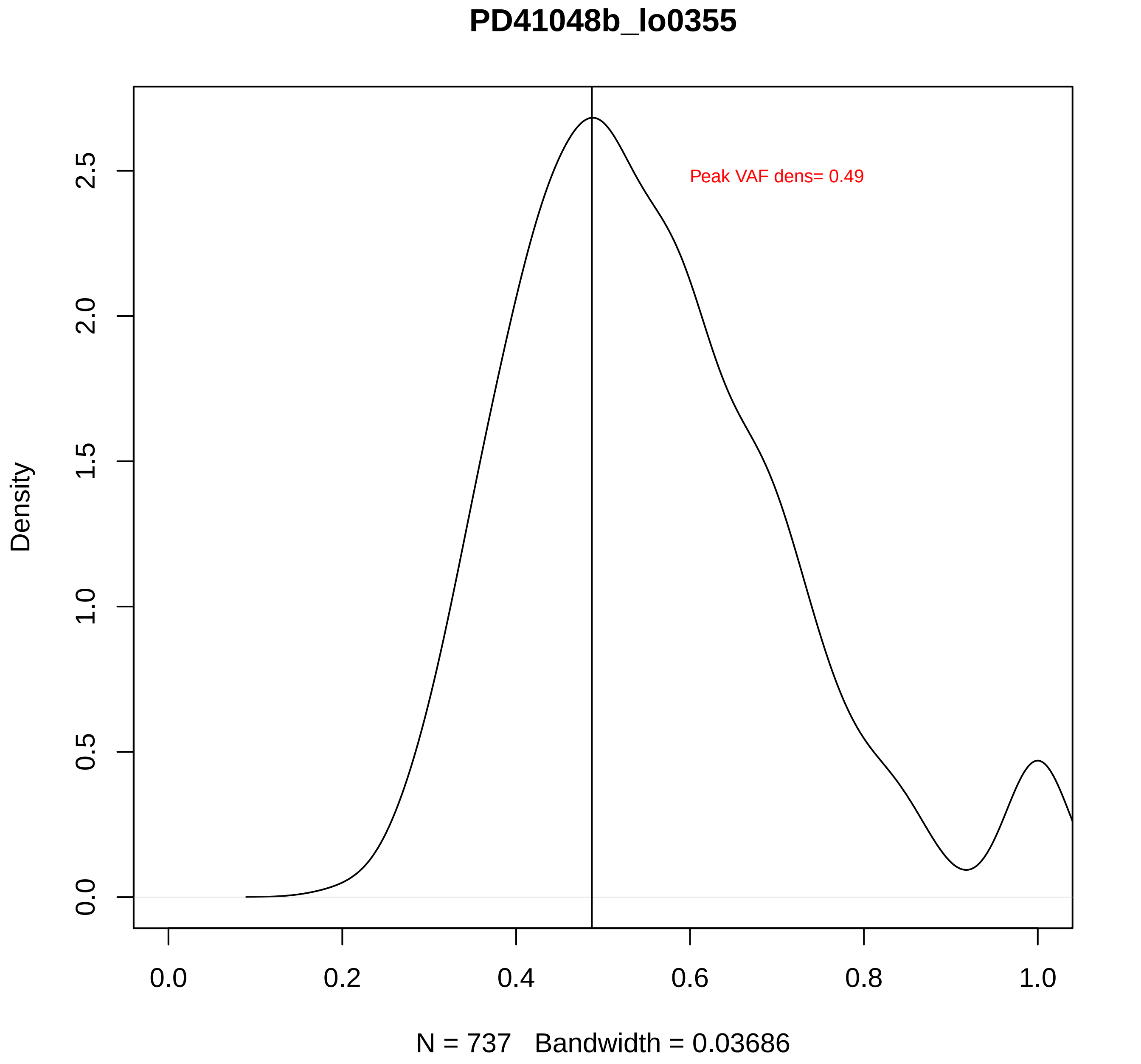

PD41048b_lo0355
Peak VAF dens= 0.49
Density
0.0
0.5
1.0
1.5
2.0
2.5
0.0
0.2
0.4
0.6
0.8
1.0
N = 737   Bandwidth = 0.03686

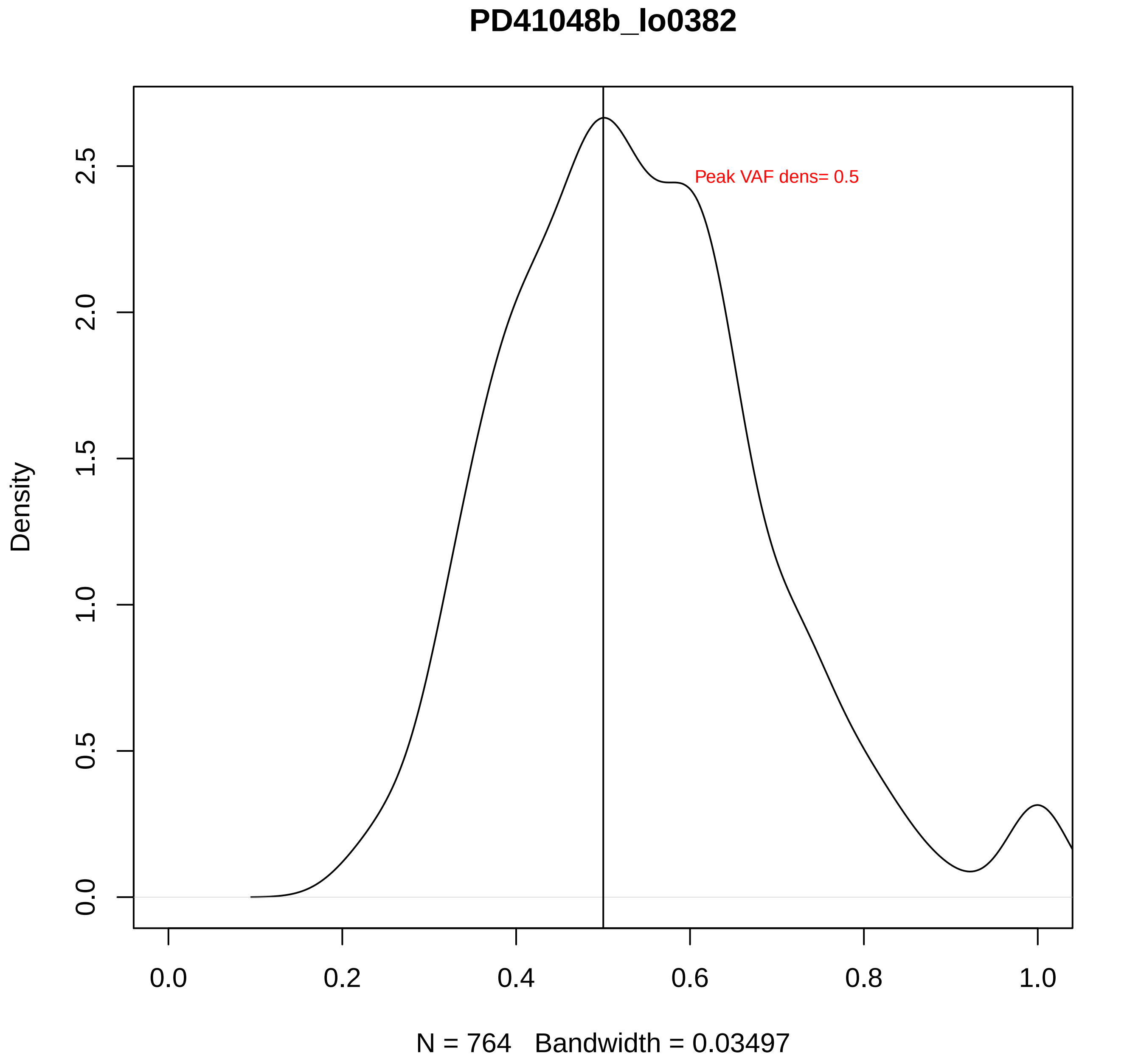
PD41048b_lo0382
Peak VAF dens= 0.5
Density
0.0
0.5
1.0
1.5
2.0
2.5
0.0
0.2
0.4
0.6
0.8
1.0
N = 764   Bandwidth = 0.03497

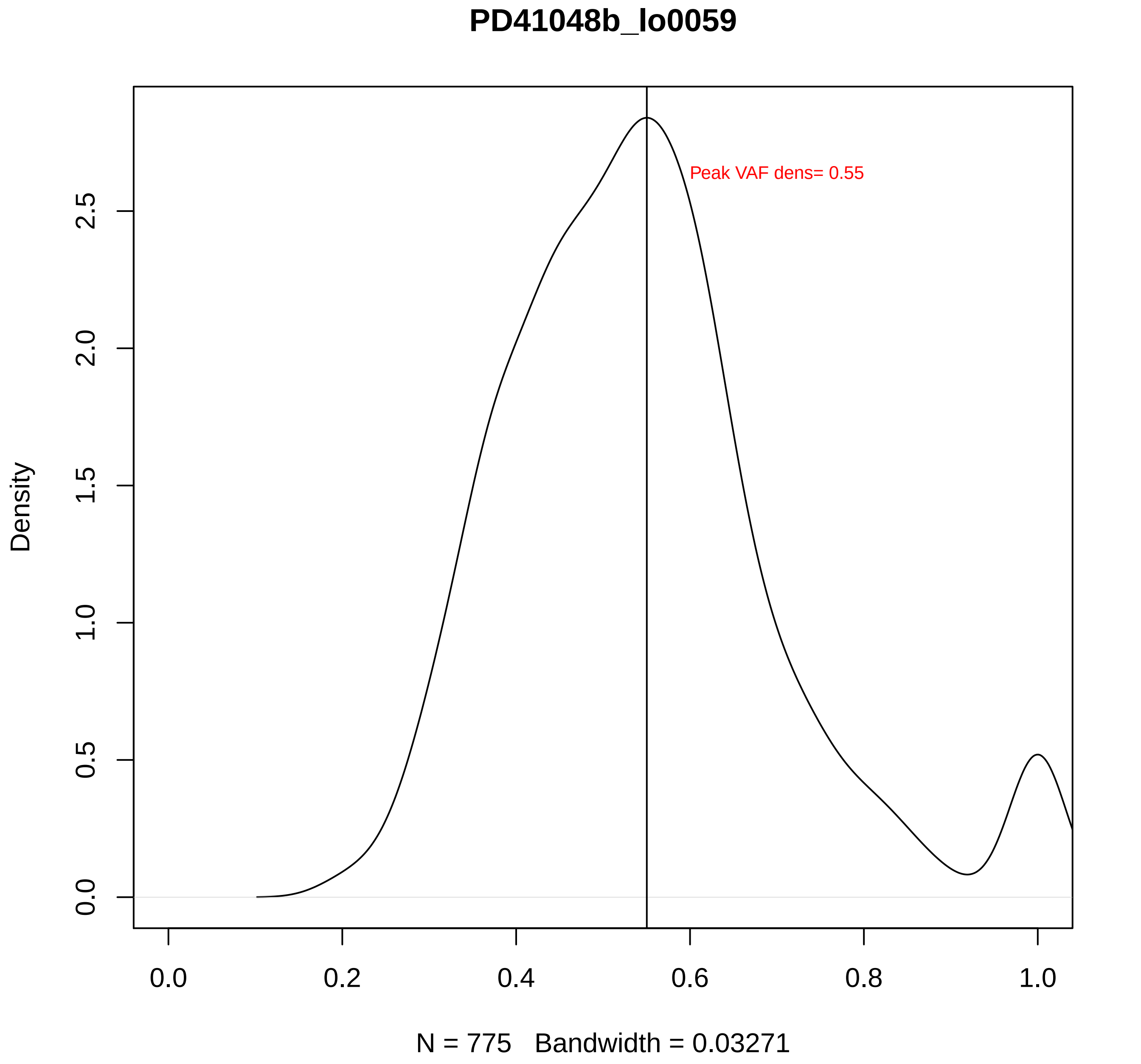
PD41048b_lo0059
Peak VAF dens= 0.55
Density
0.0
0.5
1.0
1.5
2.0
2.5
0.0
0.2
0.4
0.6
0.8
1.0
N = 775 Bandwidth = 0.03271

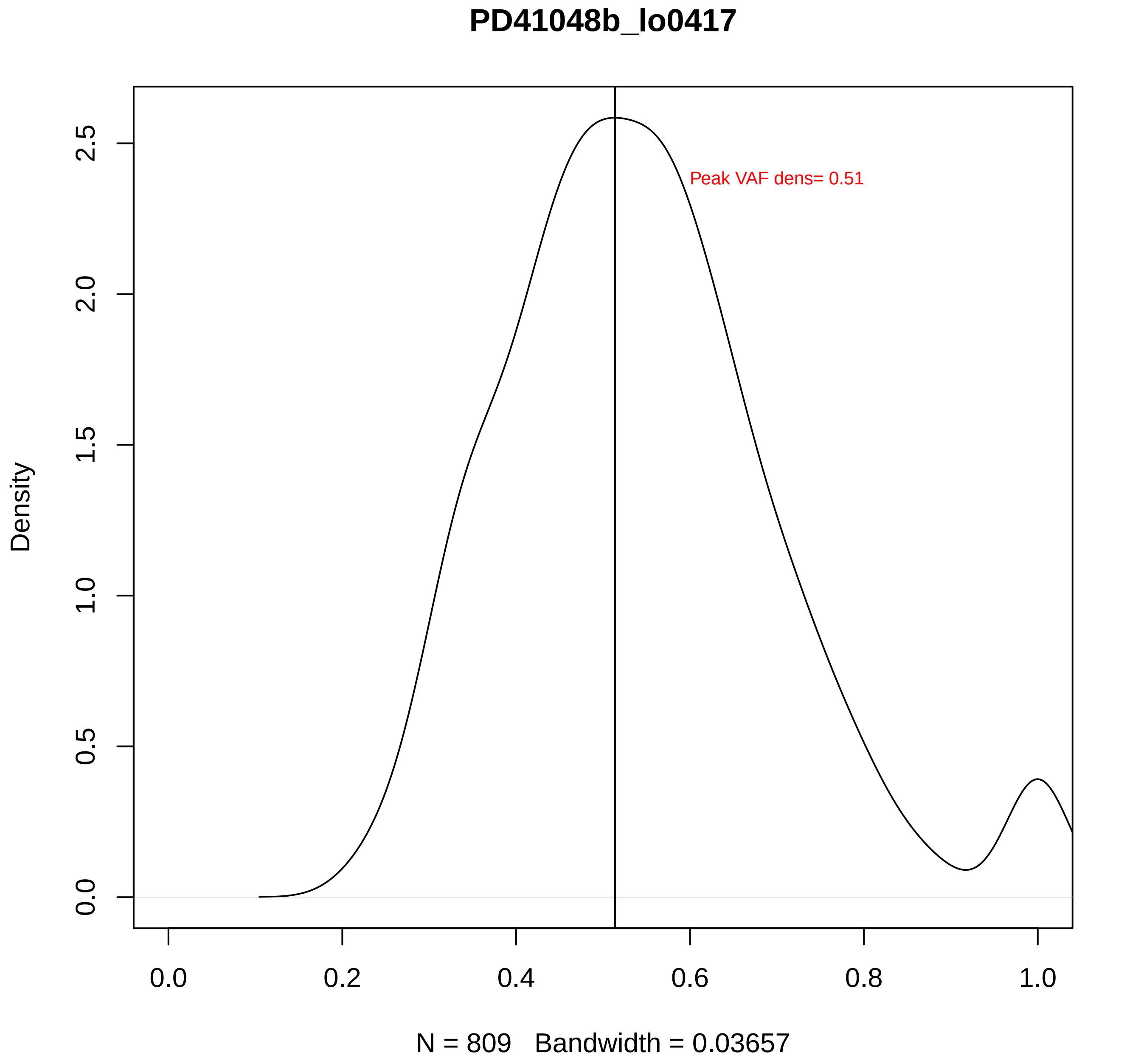
PD41048b_lo0417
Peak VAF dens= 0.51
Density
0.0
0.5
1.0
1.5
2.0
2.5
0.0
0.2
0.4
0.6
0.8
1.0
N = 809   Bandwidth = 0.03657

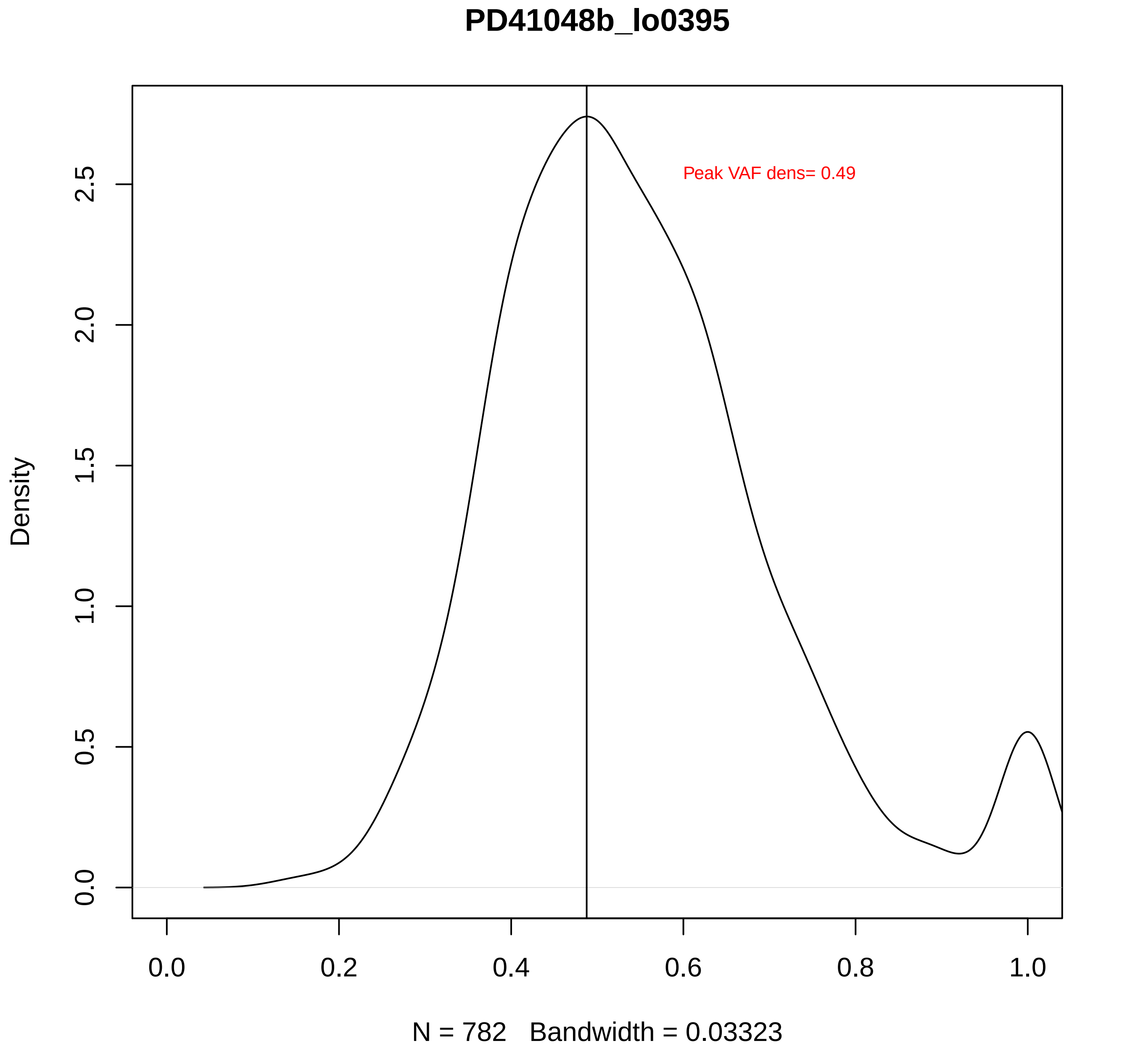
PD41048b_lo0395
Peak VAF dens= 0.49
Density
0.0
0.5
1.0
1.5
2.0
2.5
0.0
0.2
0.4
0.6
0.8
1.0
N = 782   Bandwidth = 0.03323

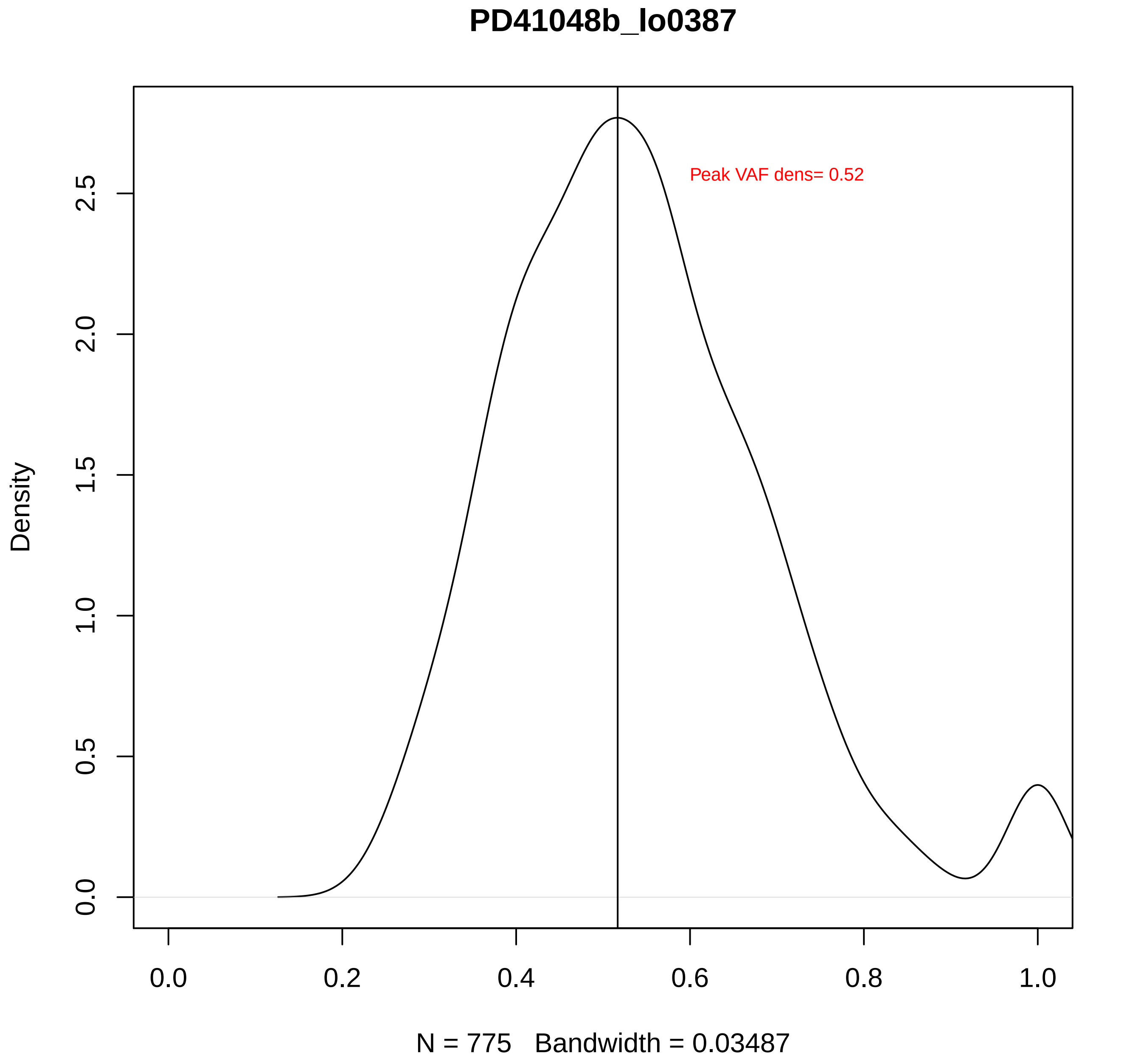
PD41048b_lo0387
Peak VAF dens= 0.52
Density
0.0
0.5
1.0
1.5
2.0
2.5
0.0
0.2
0.4
0.6
0.8
1.0
N = 775   Bandwidth = 0.03487

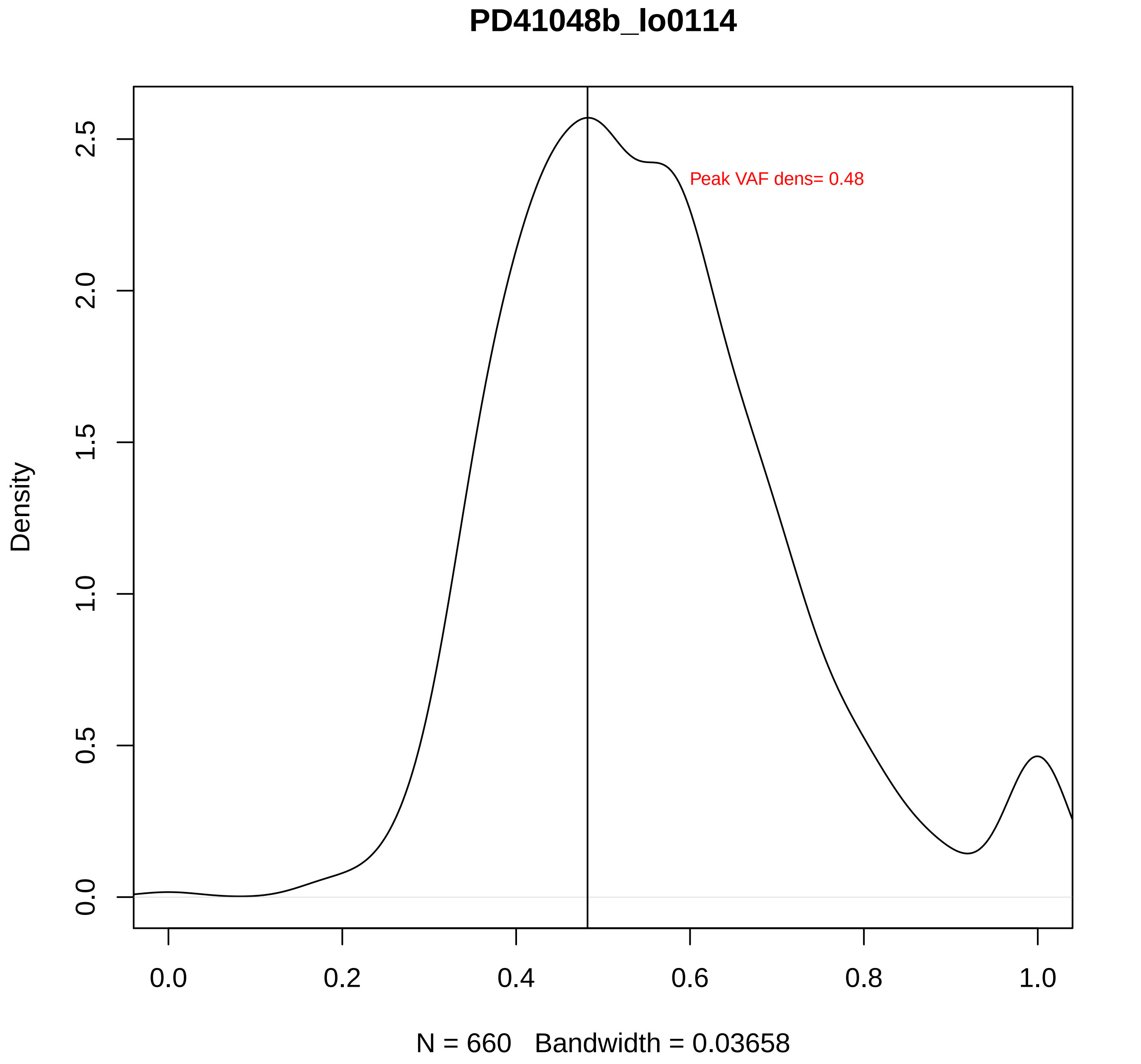
PD41048b_lo0114
Peak VAF dens= 0.48
Density
0.0
0.5
1.0
1.5
2.0
2.5
0.0
0.2
0.4
0.6
0.8
1.0
N = 660   Bandwidth = 0.03658

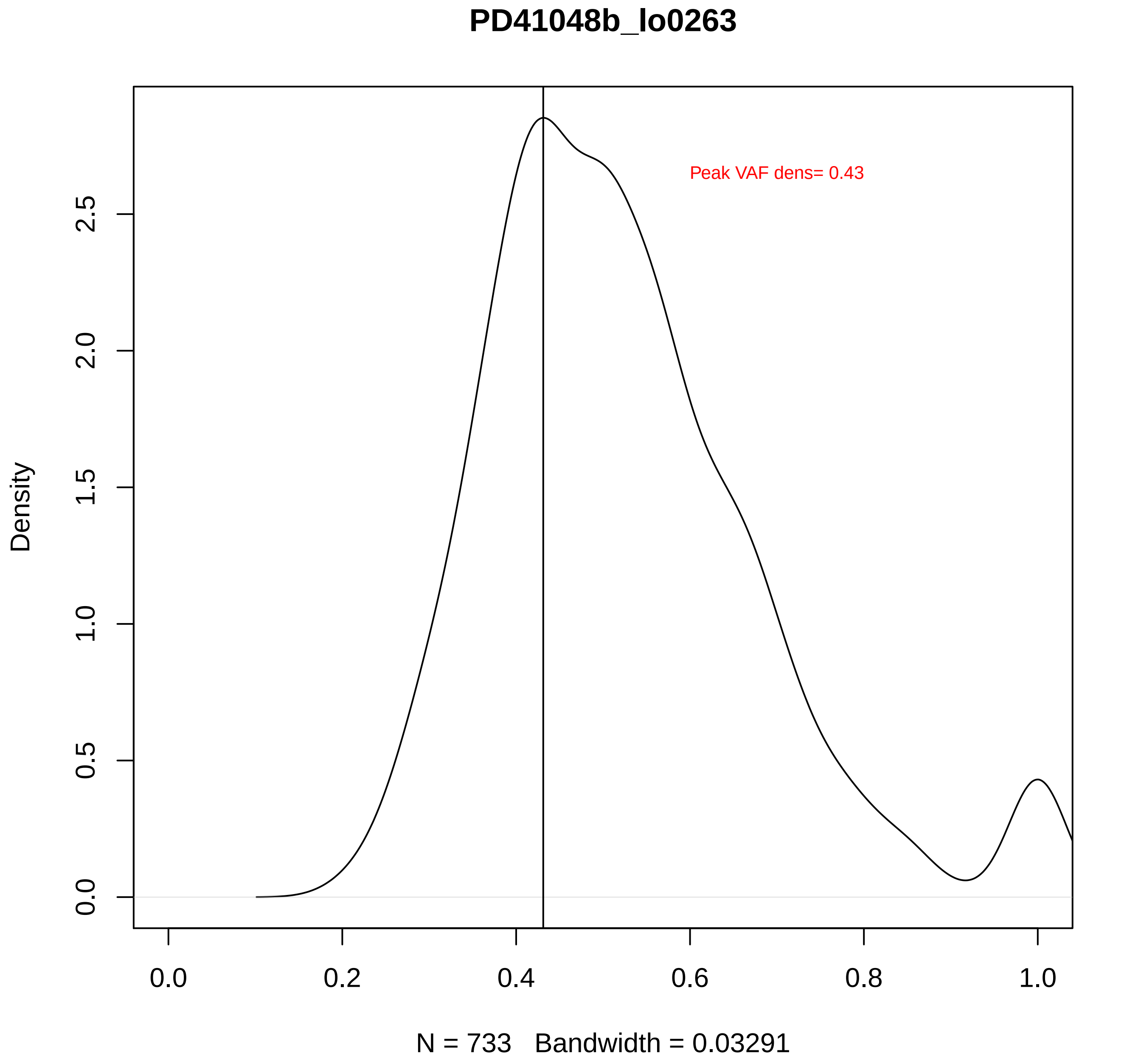
PD41048b_lo0263
Peak VAF dens= 0.43
Density
0.0
0.5
1.0
1.5
2.0
2.5
0.0
0.2
0.4
0.6
0.8
1.0
N = 733   Bandwidth = 0.03291

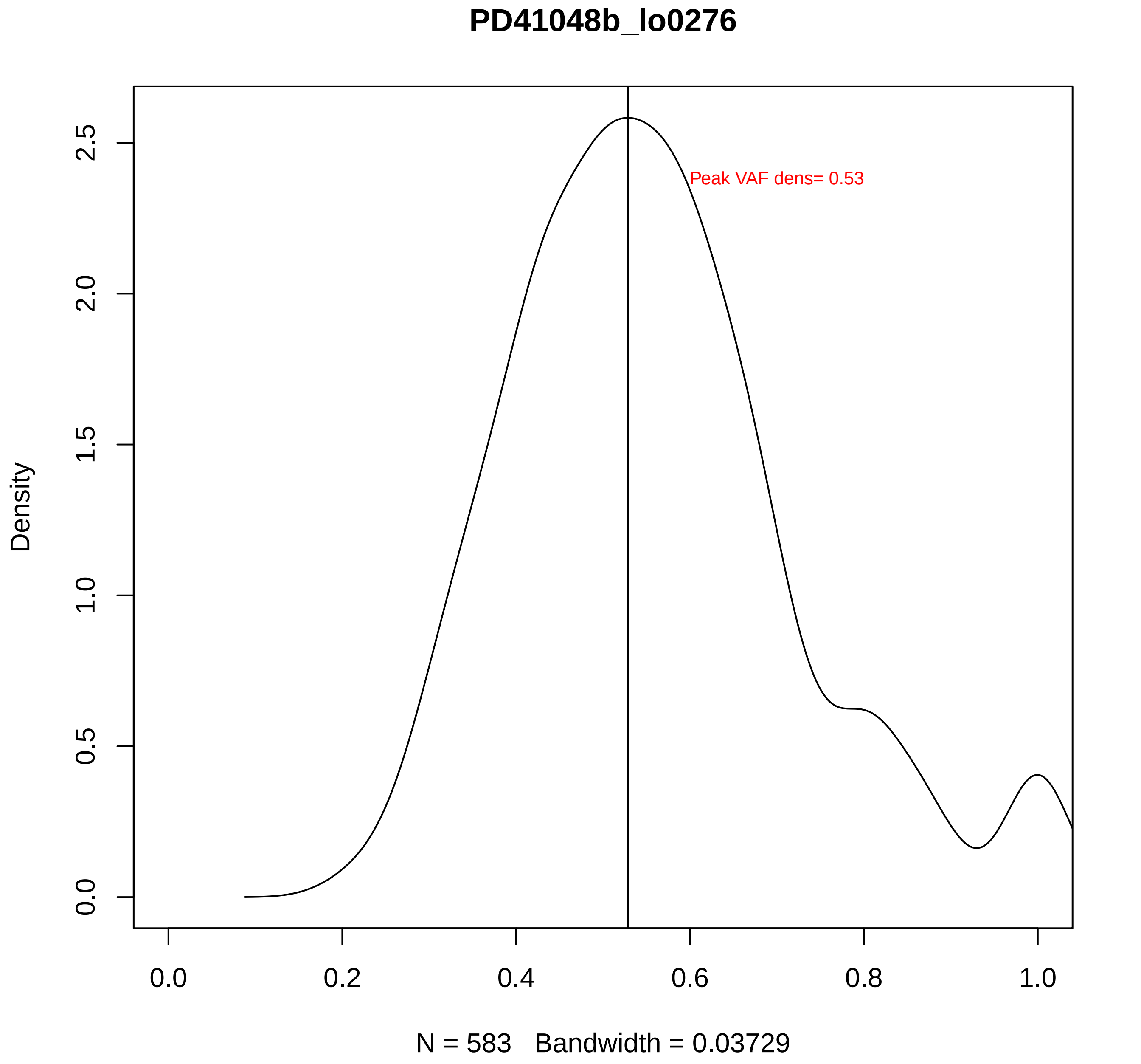
PD41048b_lo0276
Peak VAF dens= 0.53
Density
0.0
0.5
1.0
1.5
2.0
2.5
0.0
0.2
0.4
0.6
0.8
1.0
N = 583   Bandwidth = 0.03729

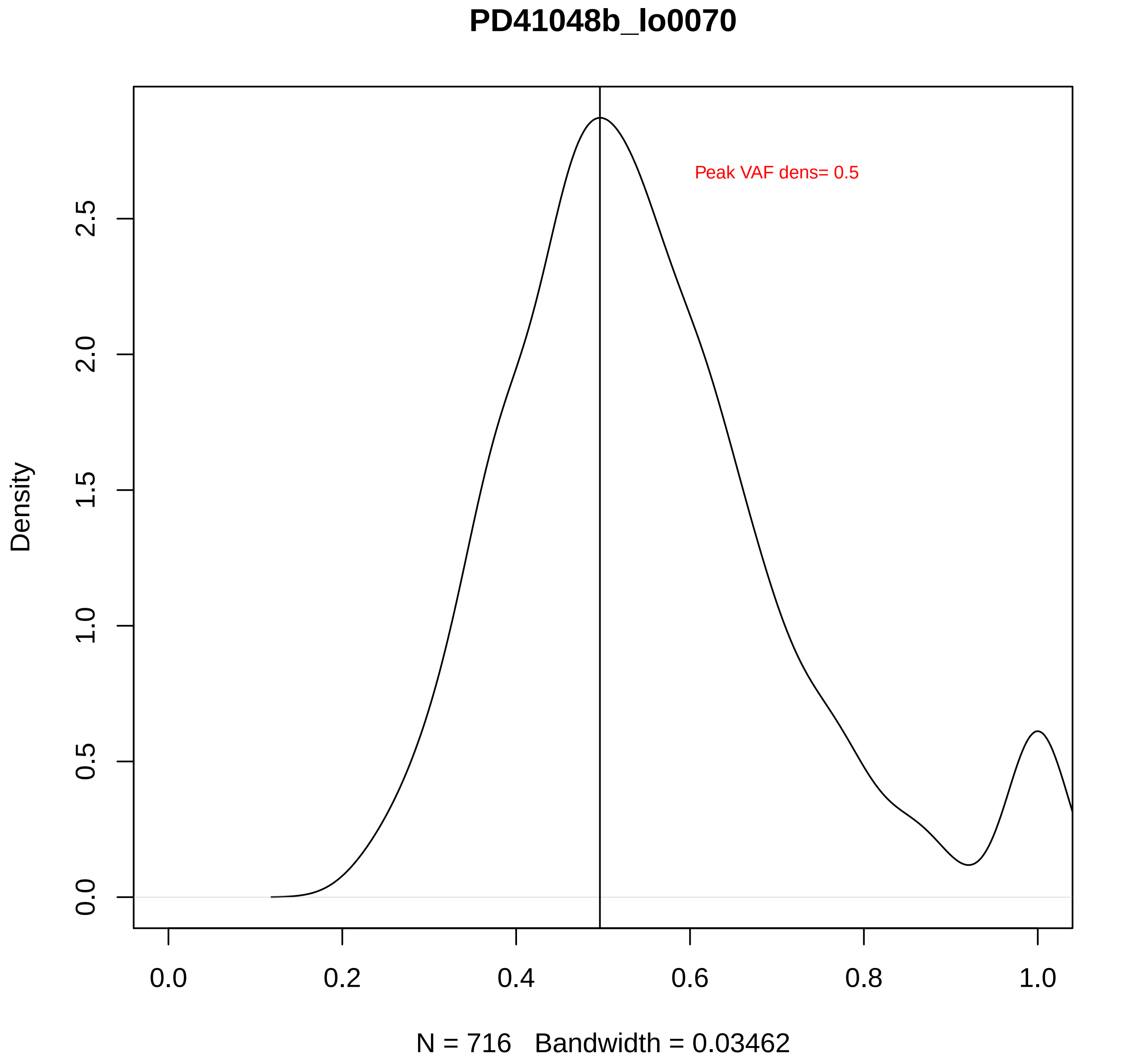
PD41048b_lo0070
Peak VAF dens= 0.5
Density
0.0
0.5
1.0
1.5
2.0
2.5
0.0
0.2
0.4
0.6
0.8
1.0
N = 716   Bandwidth = 0.03462

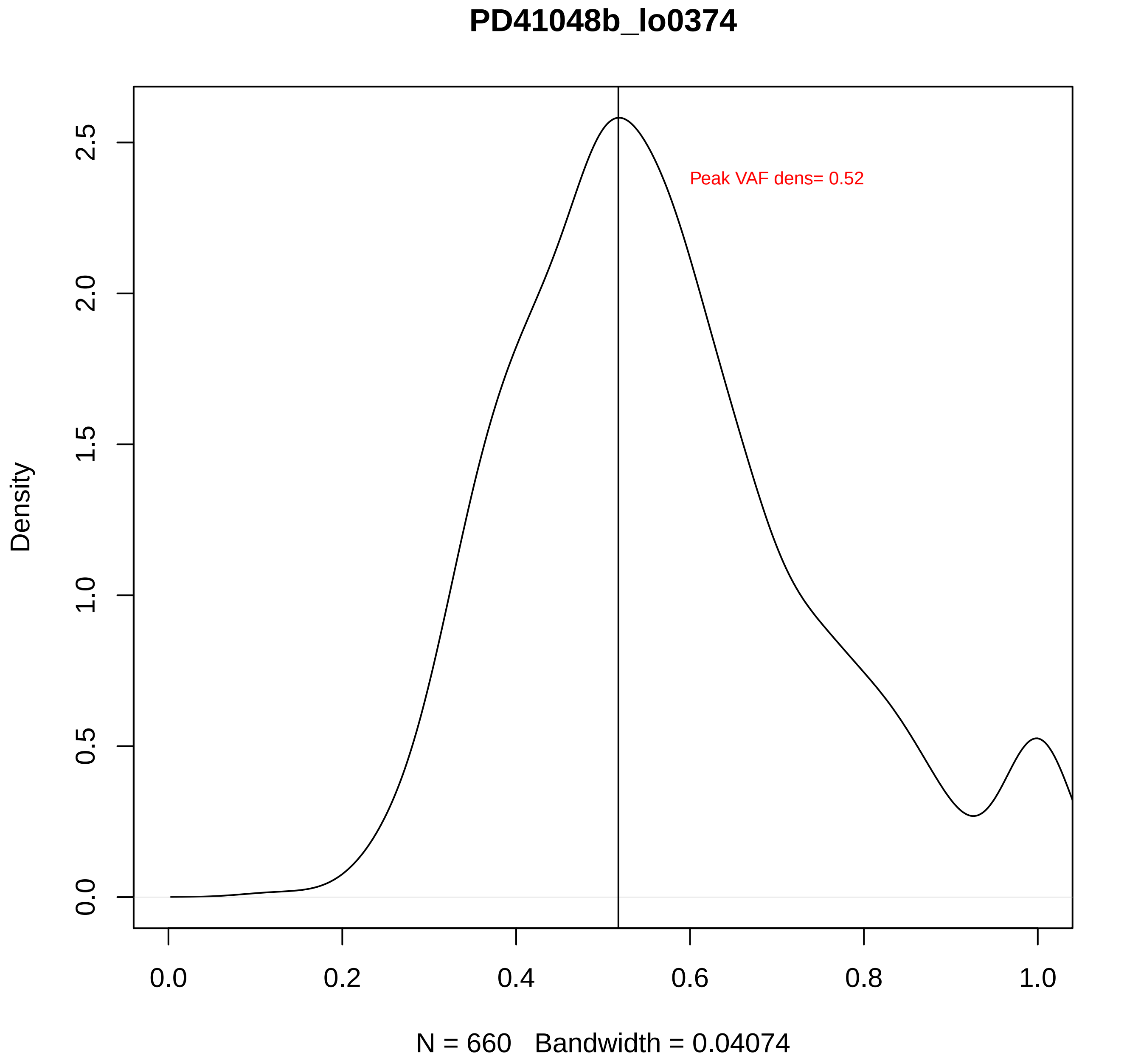
PD41048b_lo0374
Density
Peak VAF dens= 0.52
0.0
0.5
1.0
1.5
2.0
2.5
0.0
0.2
0.4
0.6
0.8
1.0
N = 660   Bandwidth = 0.04074

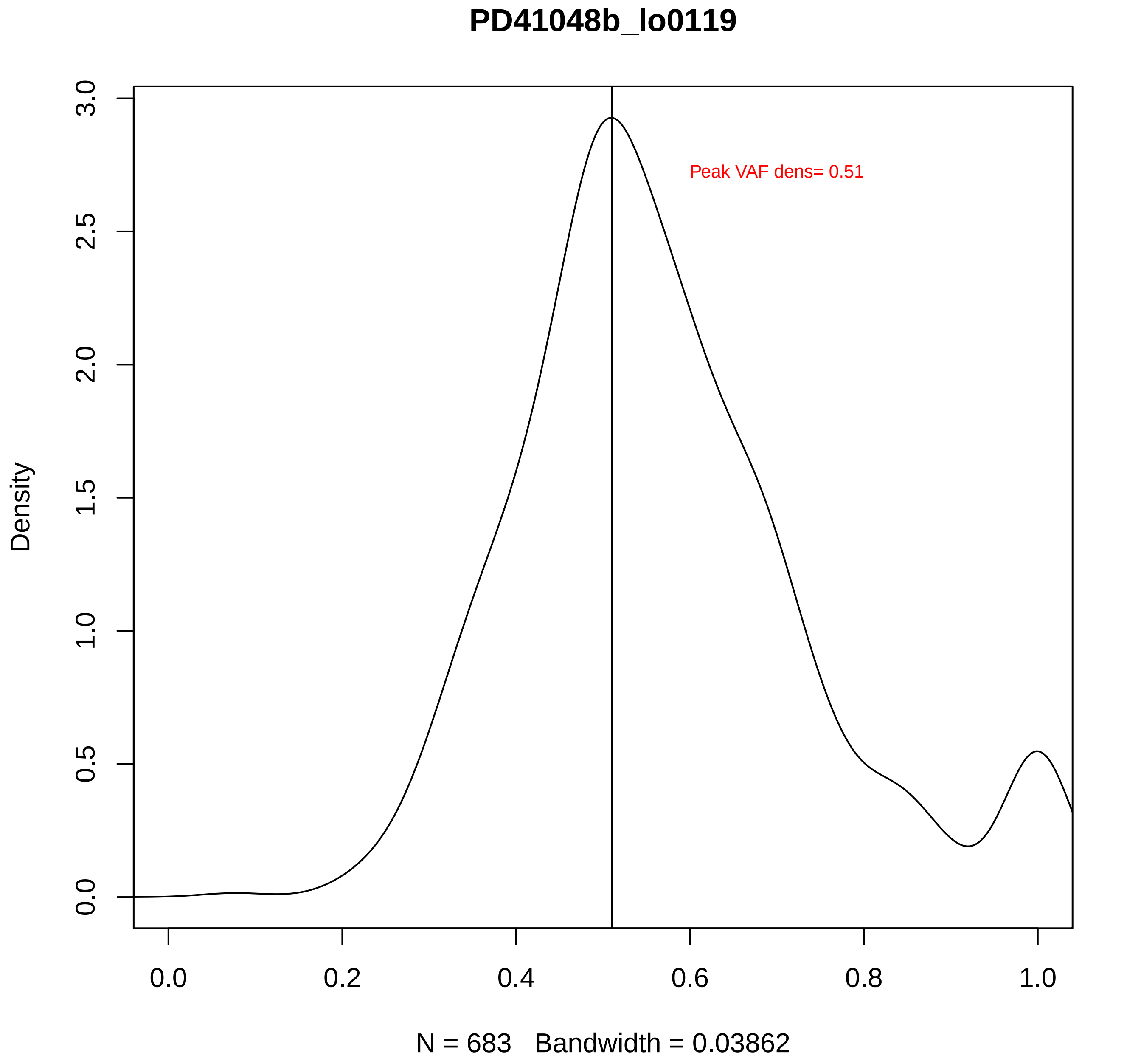
PD41048b_lo0119
Peak VAF dens= 0.51
Density
0.0
0.5
1.0
1.5
2.0
2.5
3.0
0.0
0.2
0.4
0.6
0.8
1.0
N = 683   Bandwidth = 0.03862

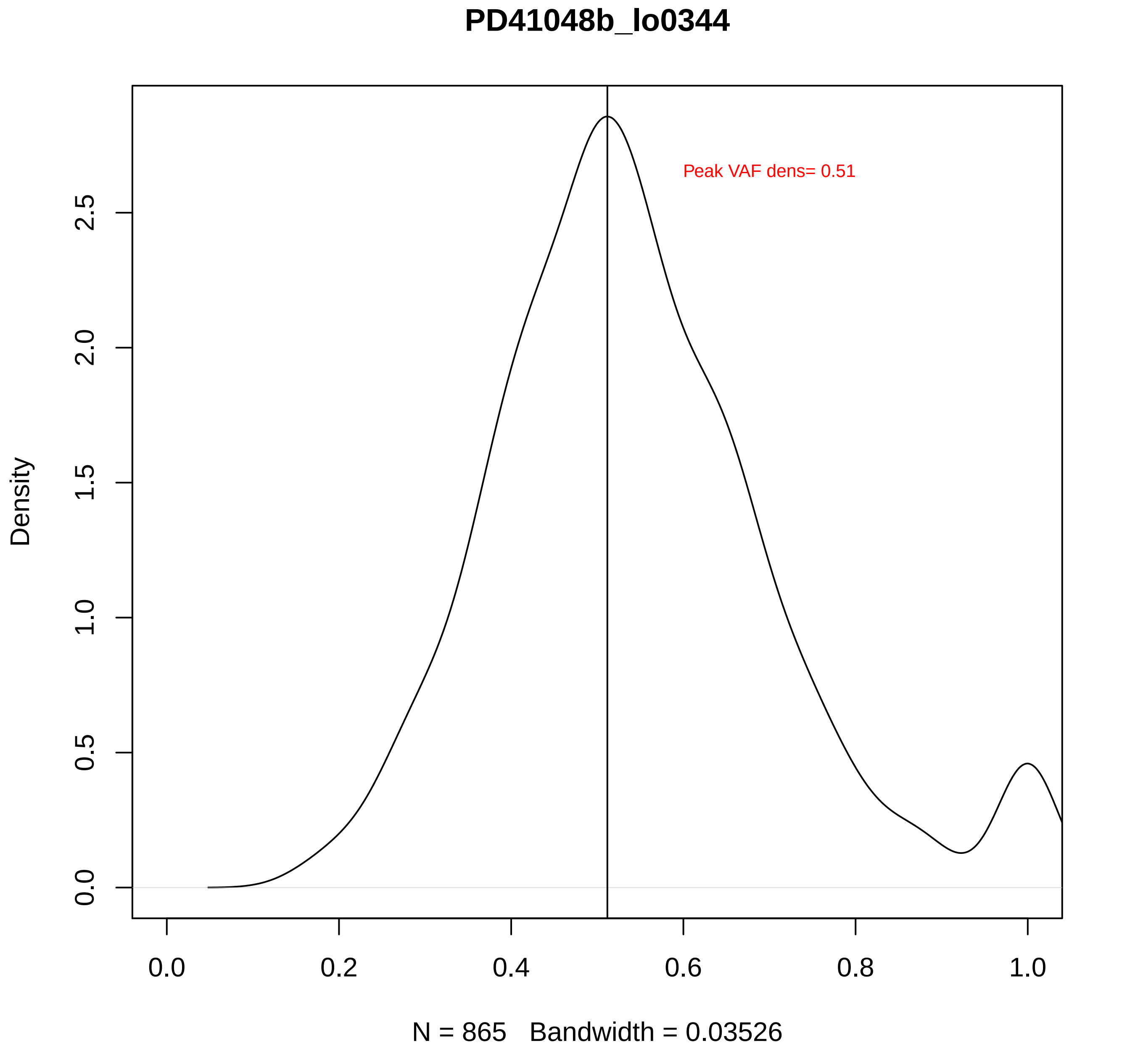

PD41048b_lo0344
Peak VAF dens= 0.51
Density
0.0
0.5
1.0
1.5
2.0
2.5
0.0
0.2
0.4
0.6
0.8
1.0
N = 865 Bandwidth = 0.03526

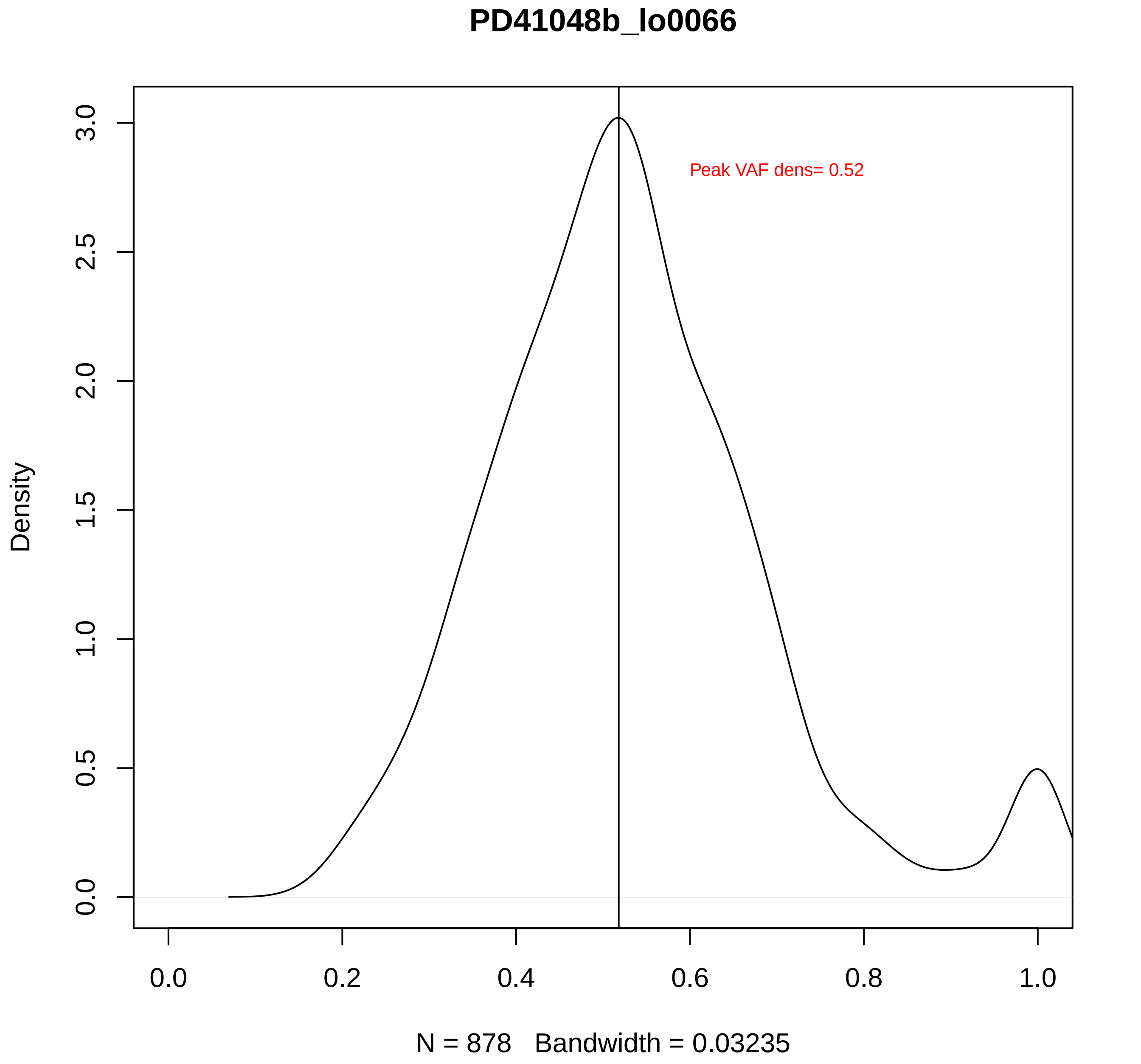

PD41048b_lo0066
Peak VAF dens= 0.52
Density
0.0
0.5
1.0
1.5
2.0
2.5
3.0
0.0
0.2
0.4
0.6
0.8
1.0
N = 878   Bandwidth = 0.03235

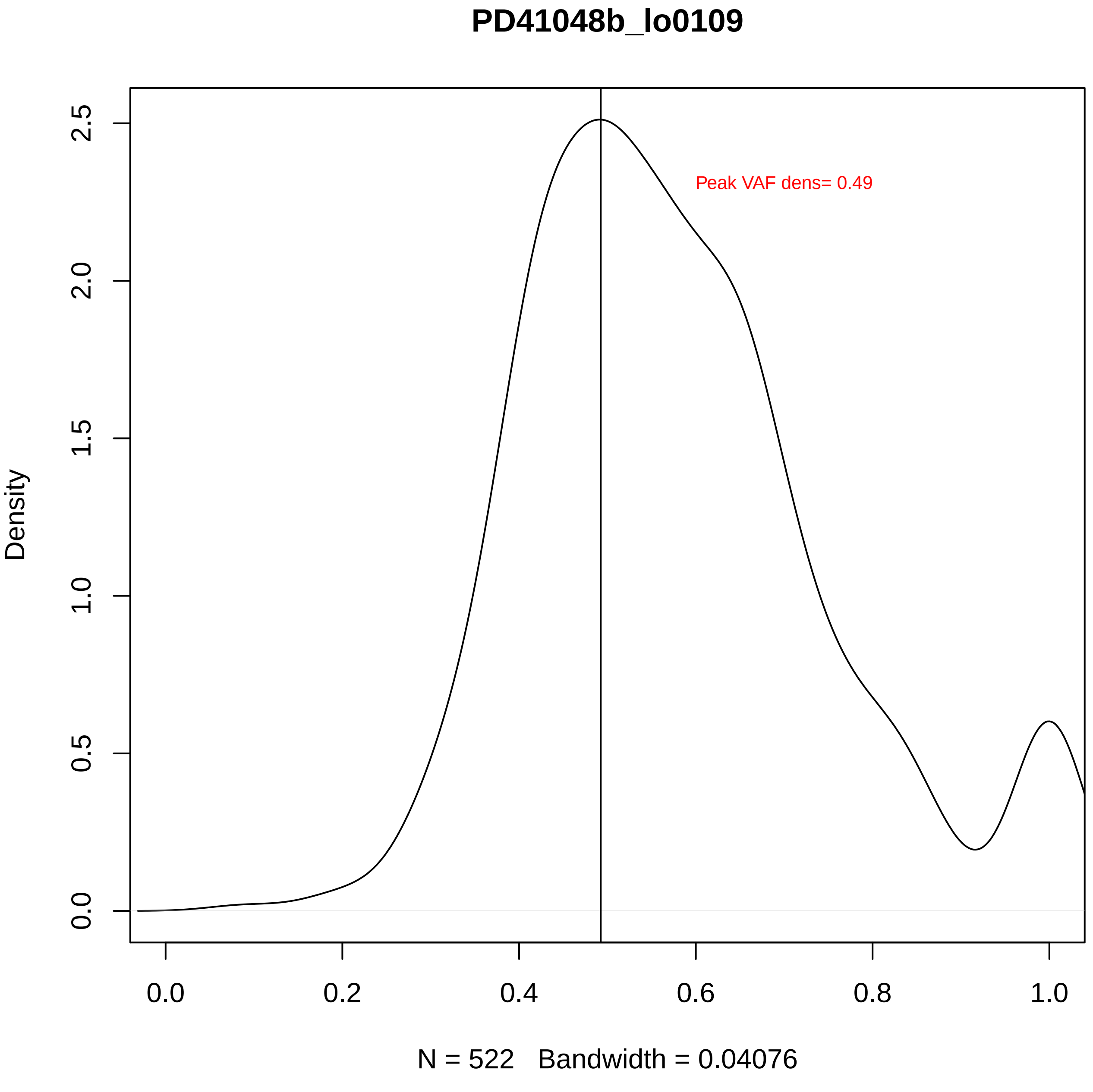
PD41048b_lo0109
Peak VAF dens= 0.49
Density
0.0
0.5
1.0
1.5
2.0
2.5
0.0
0.2
0.4
0.6
0.8
1.0
N = 522   Bandwidth = 0.04076

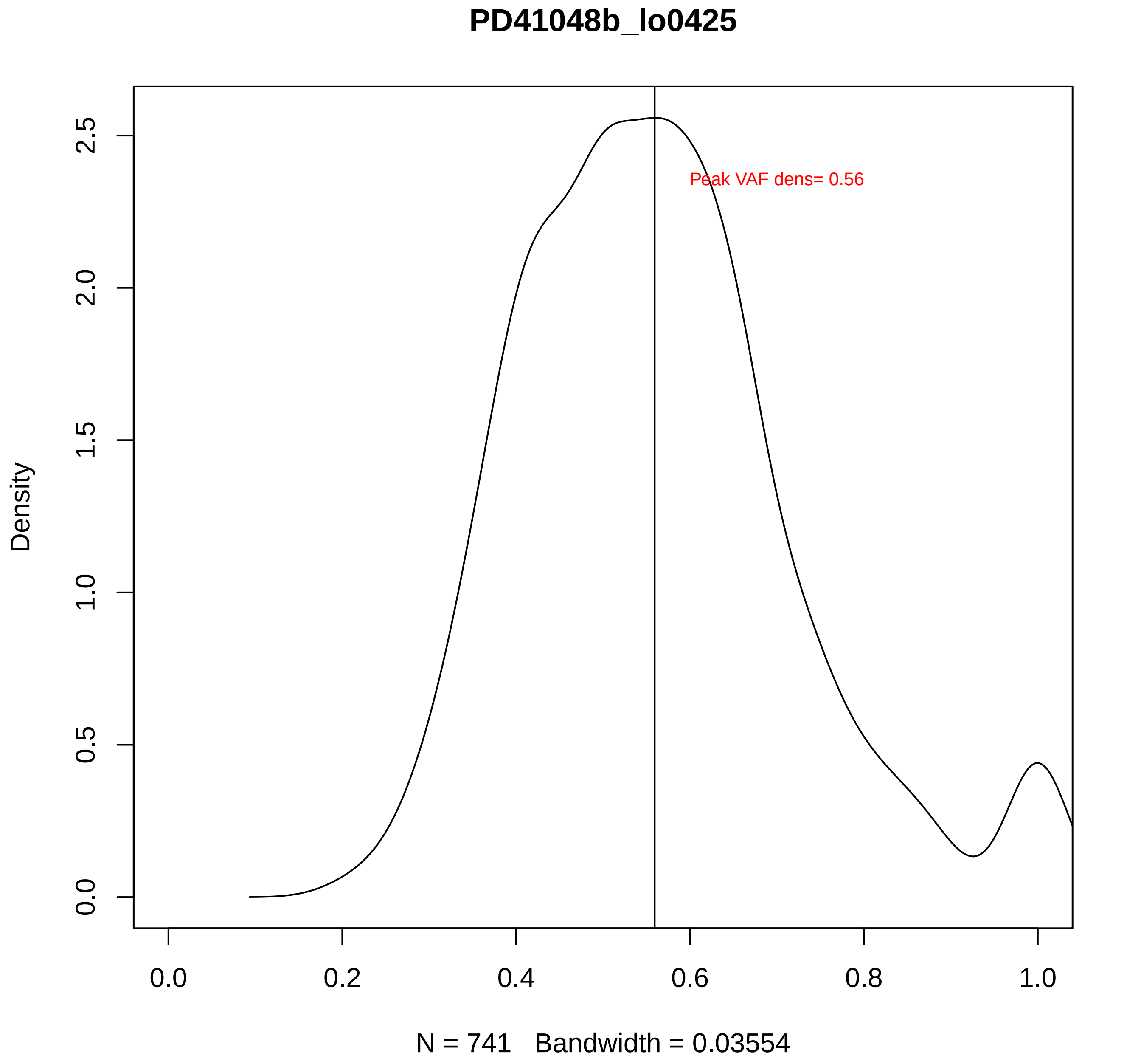

PD41048b_lo0425
Peak VAF dens= 0.56
Density
0.0
0.5
1.0
1.5
2.0
2.5
0.0
0.2
0.4
0.6
0.8
1.0
N = 741   Bandwidth = 0.03554

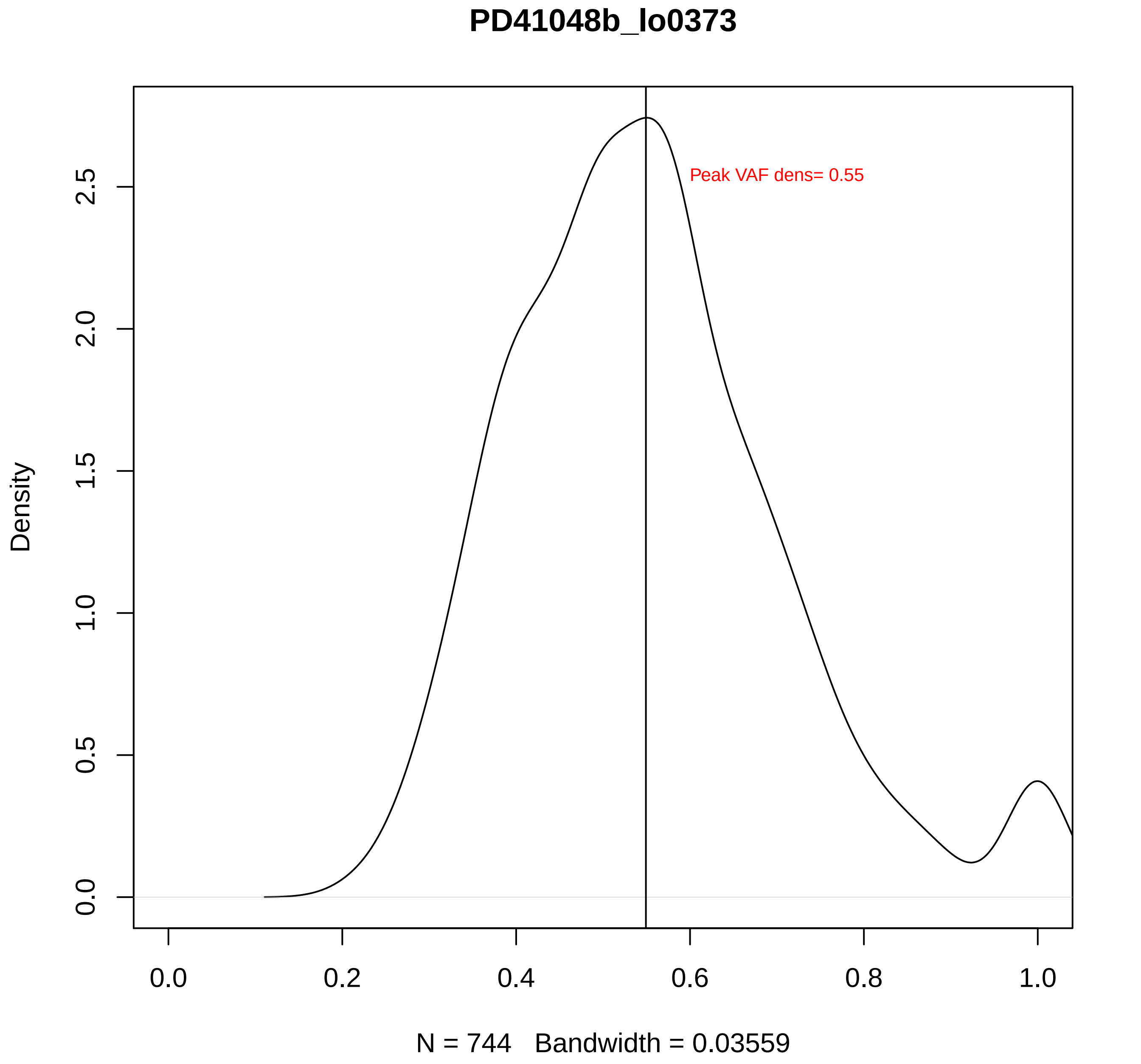
PD41048b_lo0373
Peak VAF dens= 0.55
Density
0.0
0.5
1.0
1.5
2.0
2.5
0.0
0.2
0.4
0.6
0.8
1.0
N = 744   Bandwidth = 0.03559

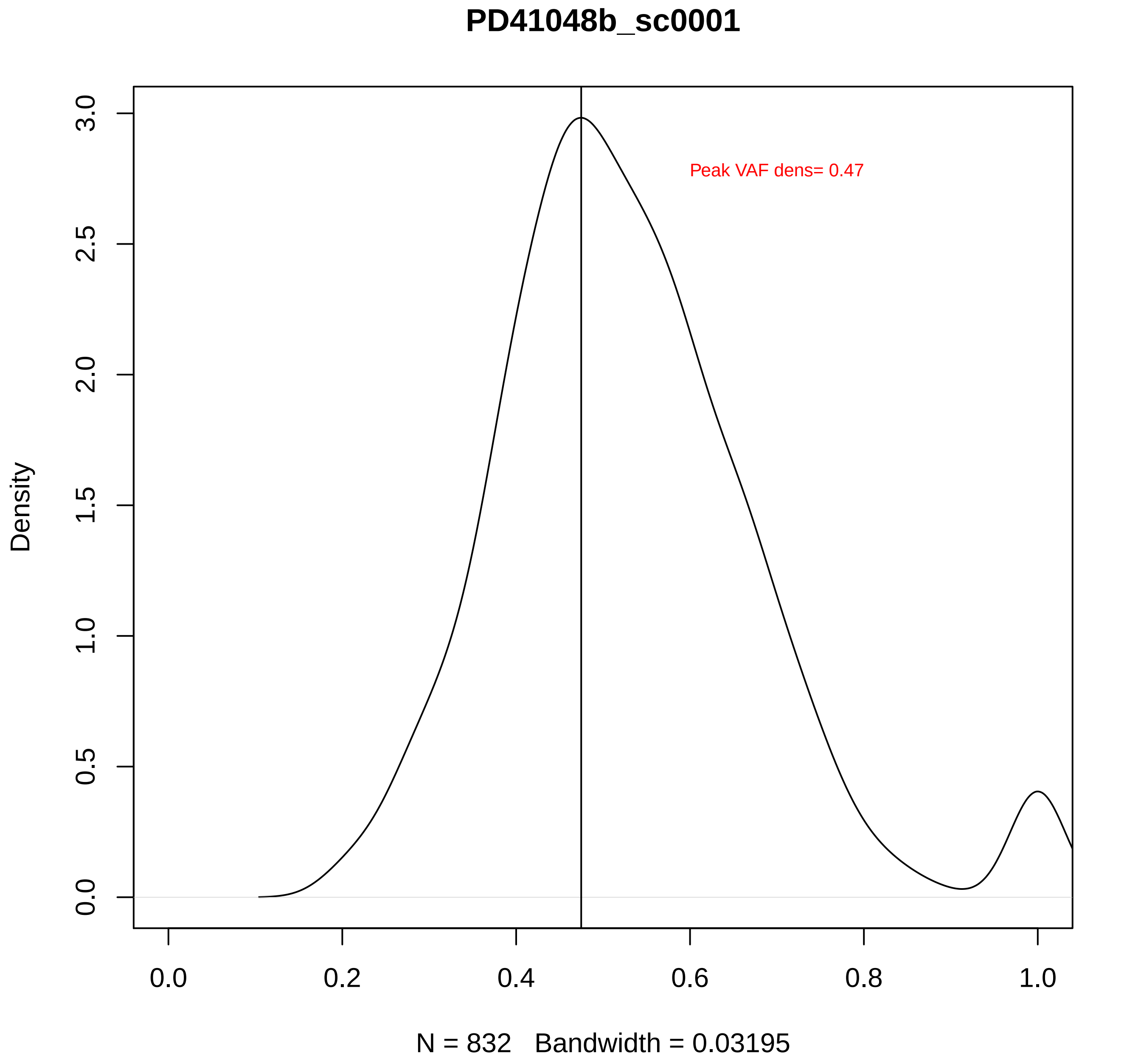

PD41048b_sc0001
Peak VAF dens= 0.47
Density
3.0
2.5
2.0
1.5
1.0
0.5
0.0
0.0
0.2
0.4
0.6
0.8
1.0
N = 832   Bandwidth = 0.03195

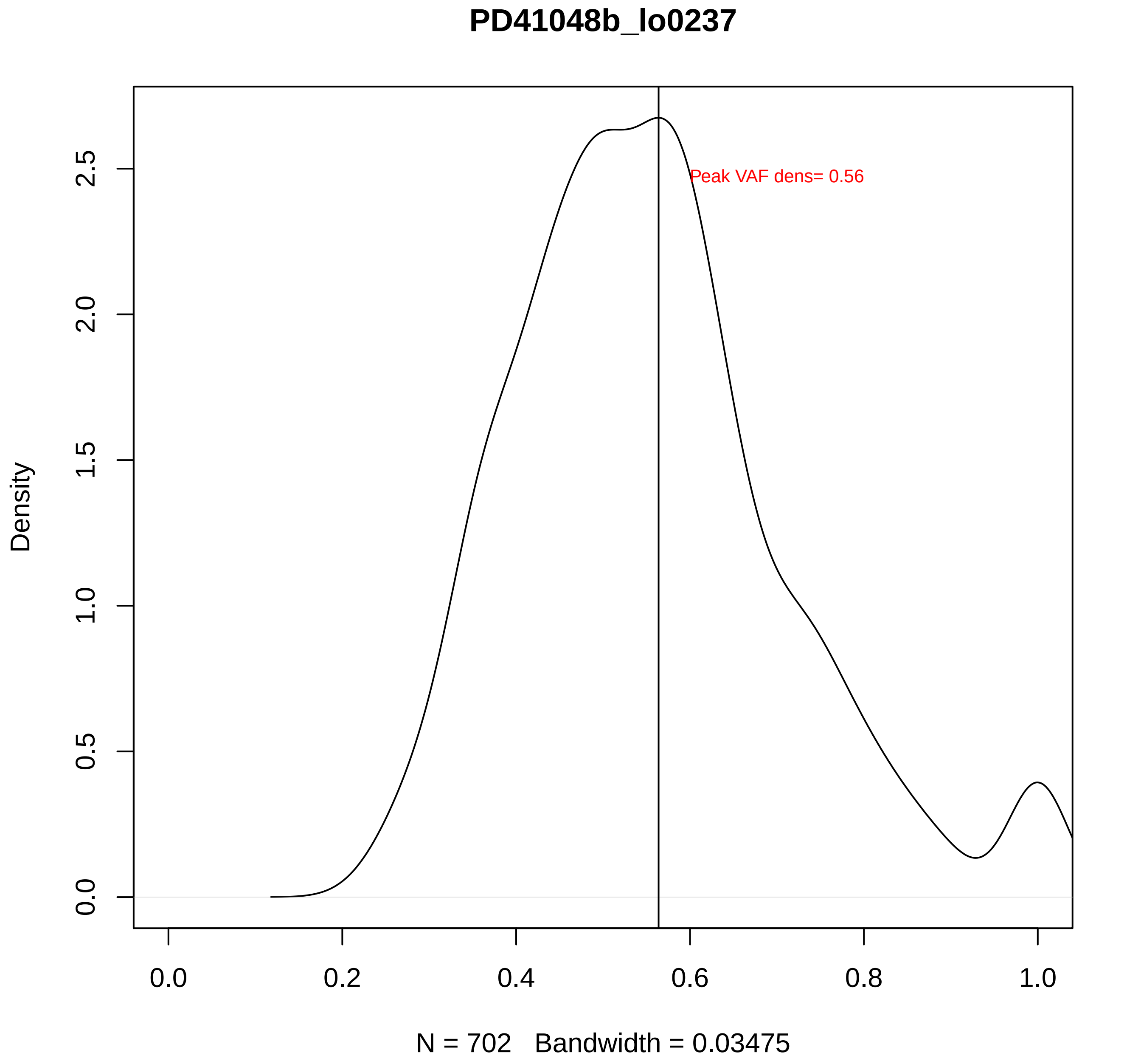

PD41048b_lo0237
Peak VAF dens= 0.56
Density
0.0
0.5
1.0
1.5
2.0
2.5
0.0
0.2
0.4
0.6
0.8
1.0
N = 702 Bandwidth = 0.03475

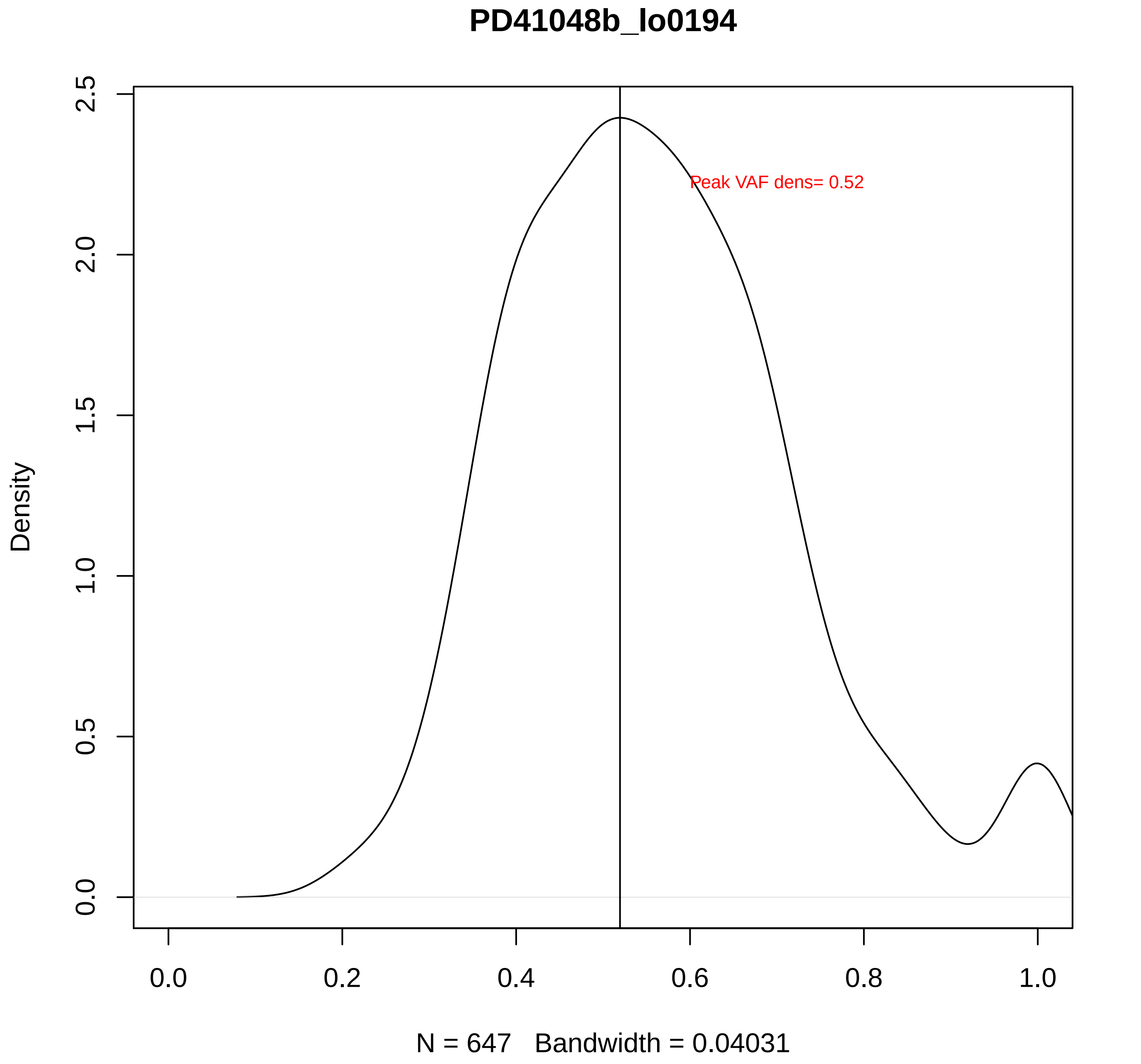

PD41048b_lo0194
Density
Peak VAF dens= 0.52
0.0
0.5
1.0
1.5
2.0
2.5
0.0
0.2
0.4
0.6
0.8
1.0
N = 647 Bandwidth = 0.04031

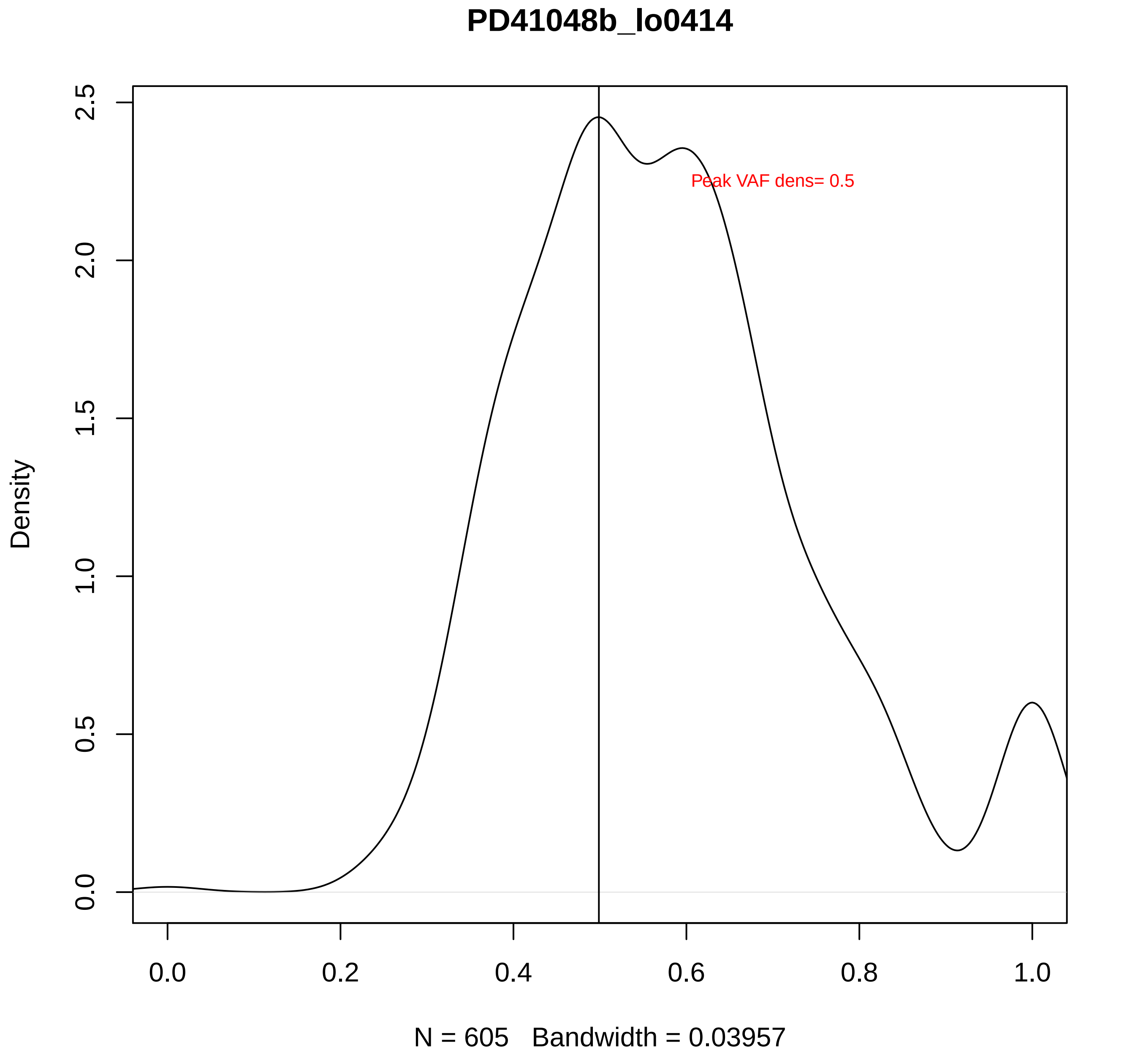
PD41048b_lo0414
Density
2.5
2.0
1.5
1.0
0.5
0.0
Peak VAF dens= 0.5
0.0
0.2
0.4
0.6
0.8
1.0
N = 605 Bandwidth = 0.03957

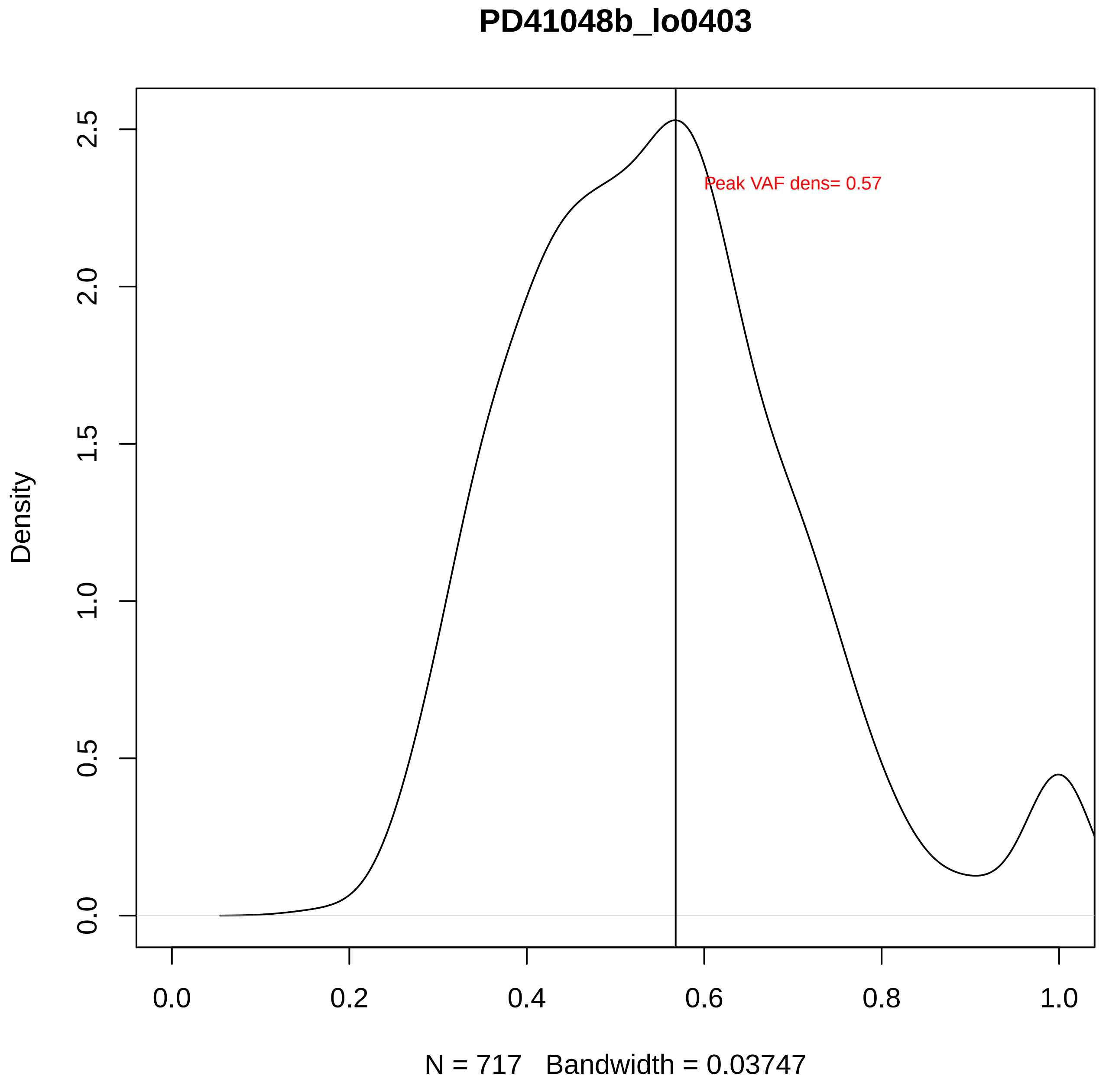

PD41048b_lo0403
Density
Peak VAF dens= 0.57
0.0
0.5
1.0
1.5
2.0
2.5
0.0
0.2
0.4
0.6
0.8
1.0
N = 717 Bandwidth = 0.03747

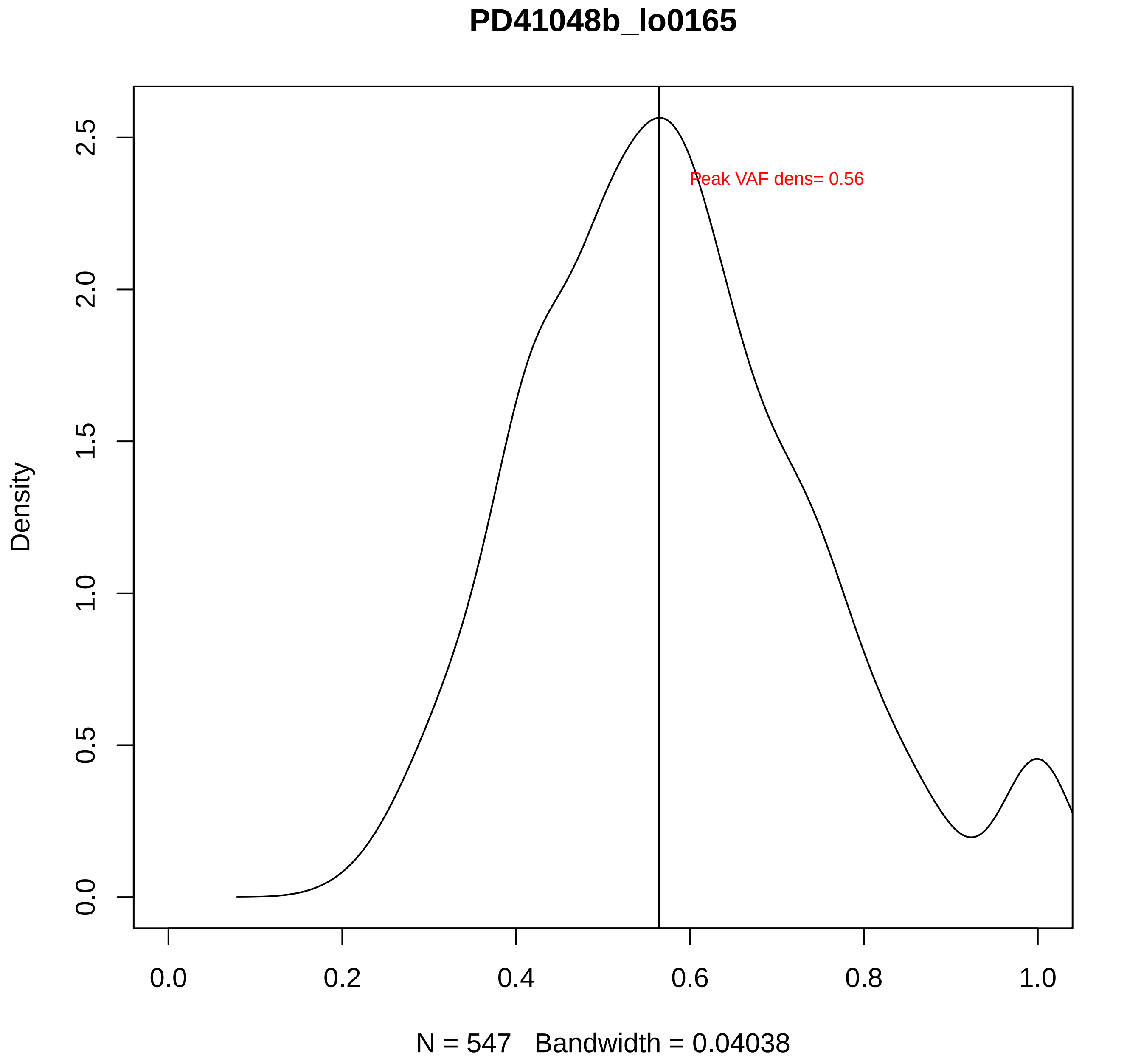
PD41048b_lo0165
Peak VAF dens= 0.56
Density
0.0
0.5
1.0
1.5
2.0
2.5
0.0
0.2
0.4
0.6
0.8
1.0
N = 547   Bandwidth = 0.04038

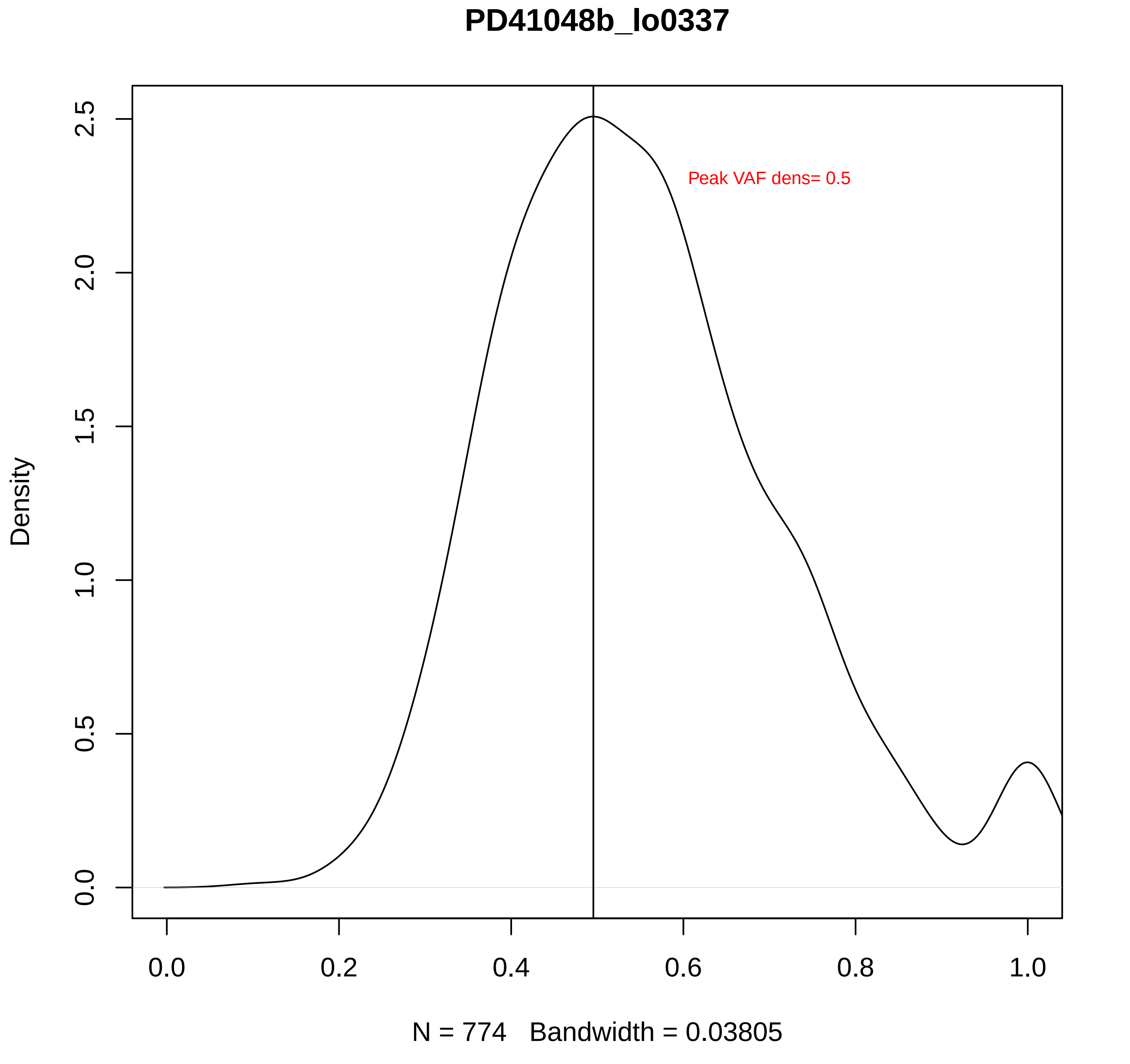
PD41048b_lo0337
Density
Peak VAF dens= 0.5
0.0
0.5
1.0
1.5
2.0
2.5
0.0
0.2
0.4
0.6
0.8
1.0
N = 774   Bandwidth = 0.03805

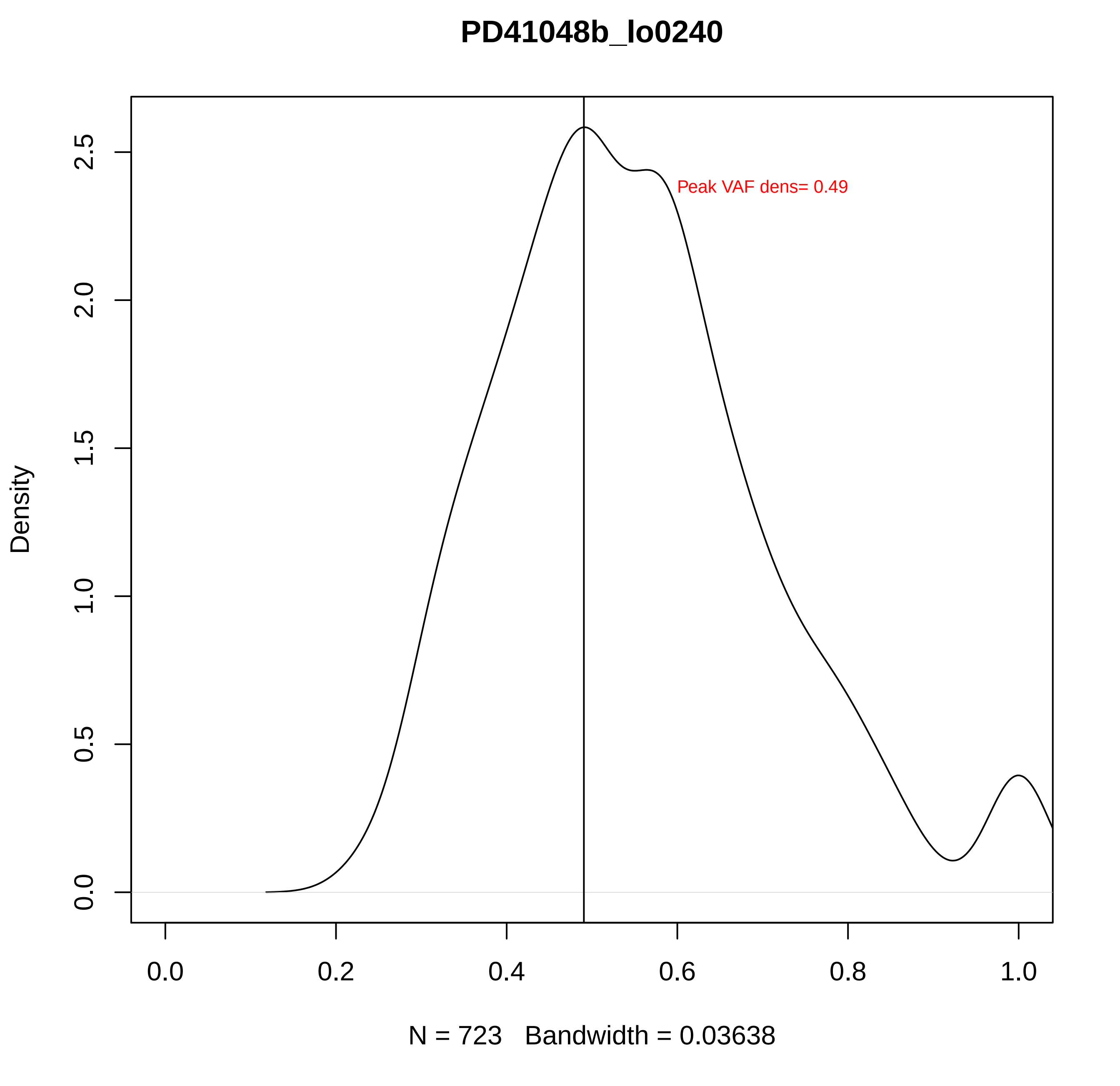
PD41048b_lo0240
Peak VAF dens= 0.49
Density
0.0
0.5
1.0
1.5
2.0
2.5
0.0
0.2
0.4
0.6
0.8
1.0
N = 723 Bandwidth = 0.03638

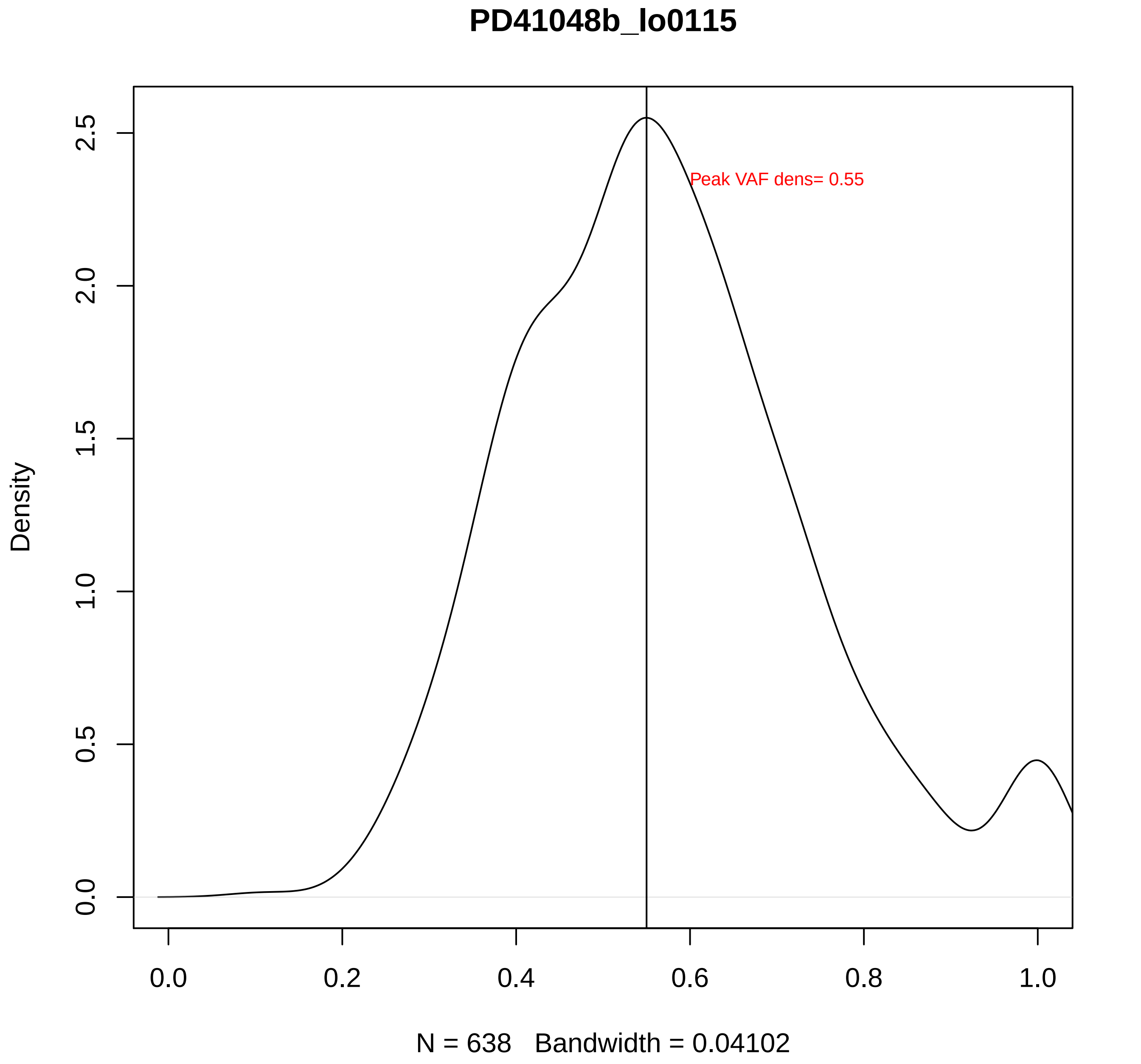

PD41048b_lo0115
Peak VAF dens= 0.55
Density
0.0
0.5
1.0
1.5
2.0
2.5
0.0
0.2
0.4
0.6
0.8
1.0
N = 638   Bandwidth = 0.04102

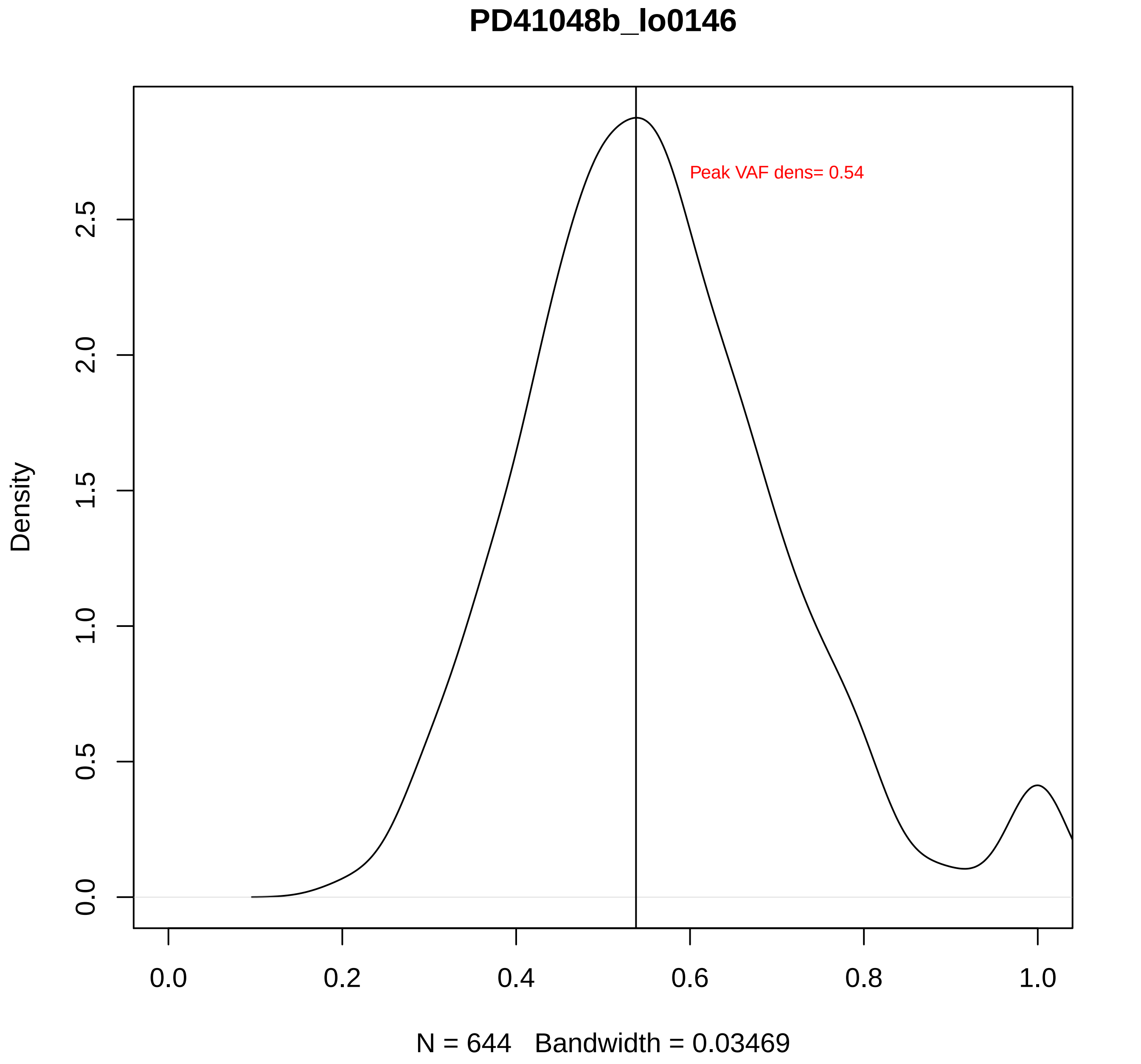
PD41048b_lo0146
Peak VAF dens= 0.54
Density
0.0
0.5
1.0
1.5
2.0
2.5
0.0
0.2
0.4
0.6
0.8
1.0
N = 644   Bandwidth = 0.03469

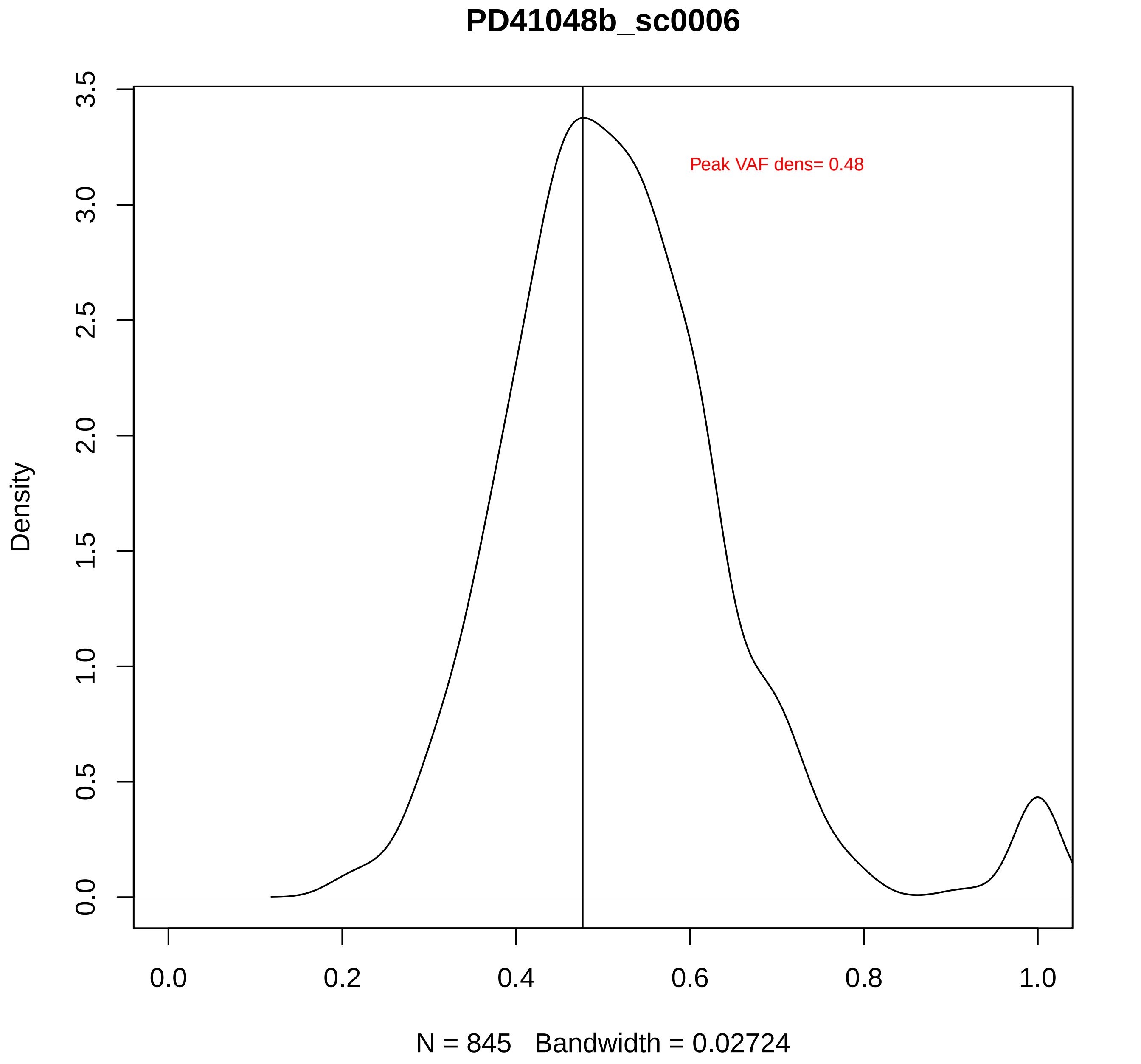
PD41048b_sc0006
Peak VAF dens= 0.48
Density
0.0
0.5
1.0
1.5
2.0
2.5
3.0
3.5
0.0
0.2
0.4
0.6
0.8
1.0
N = 845   Bandwidth = 0.02724

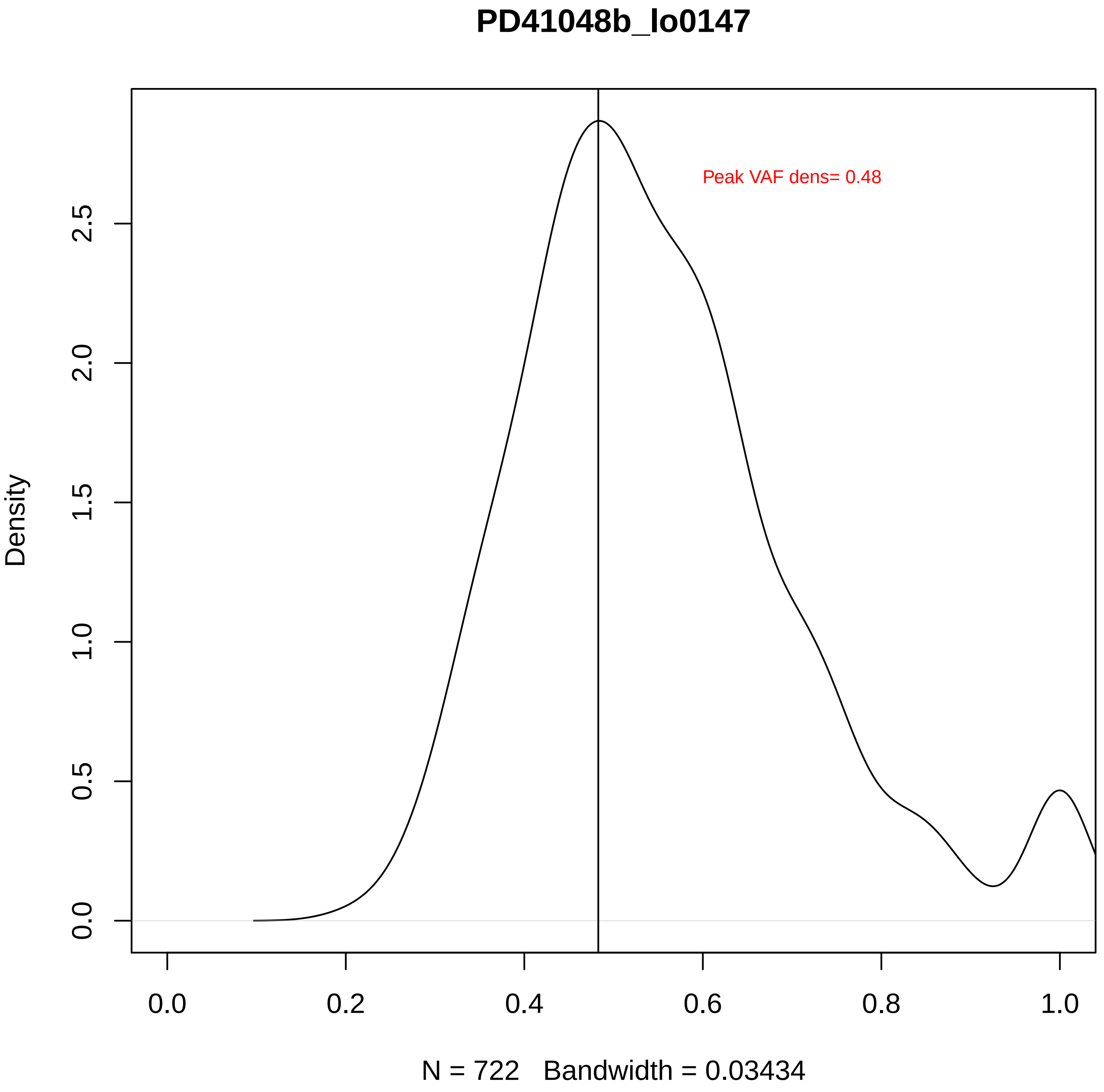
PD41048b_lo0147
Peak VAF dens= 0.48
Density
0.0
0.5
1.0
1.5
2.0
2.5
0.0
0.2
0.4
0.6
0.8
1.0
N = 722   Bandwidth = 0.03434

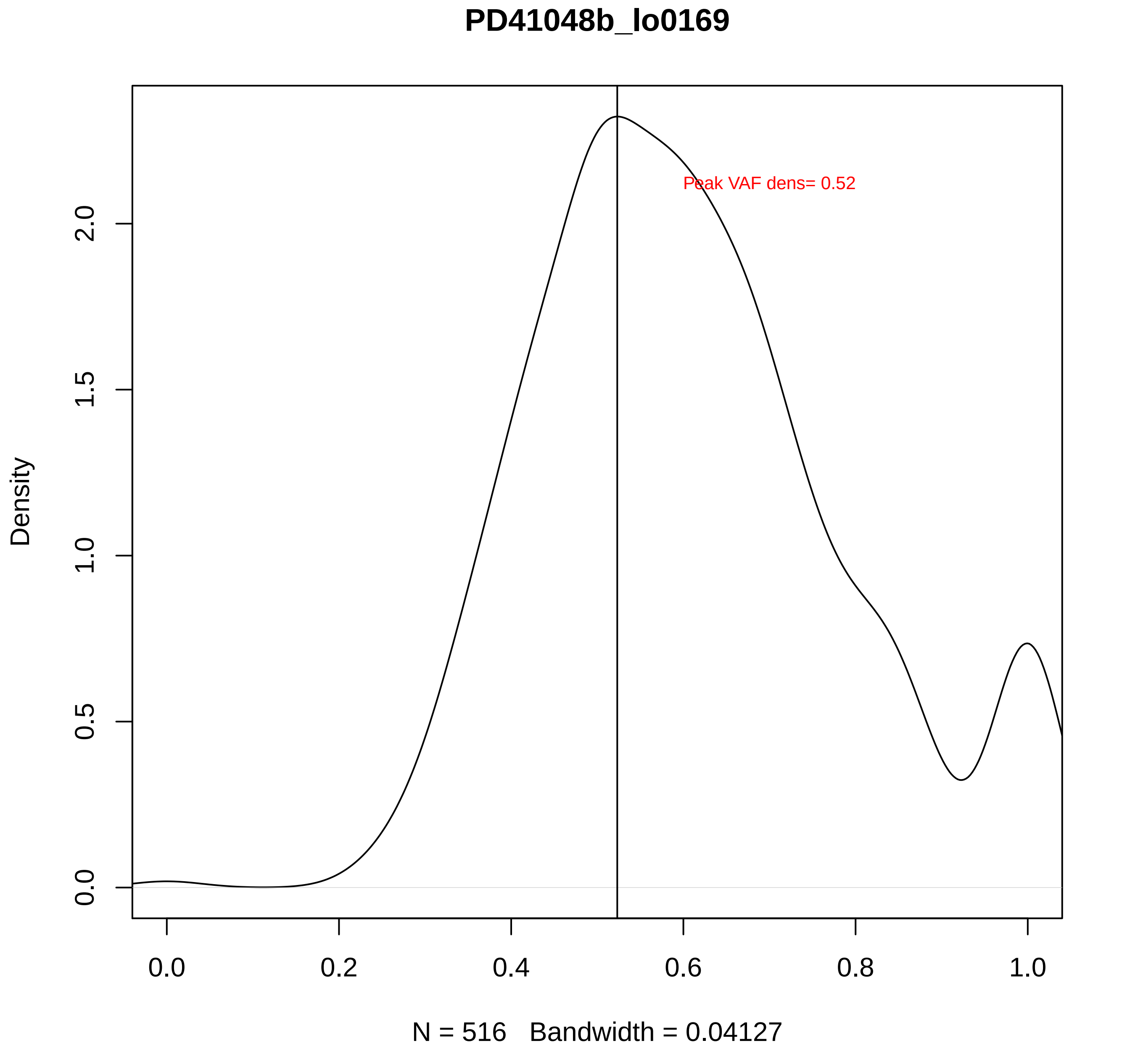
PD41048b_lo0169
Peak VAF dens= 0.52
Density
0.0
0.5
1.0
1.5
2.0
0.0
0.2
0.4
0.6
0.8
1.0
N = 516   Bandwidth = 0.04127

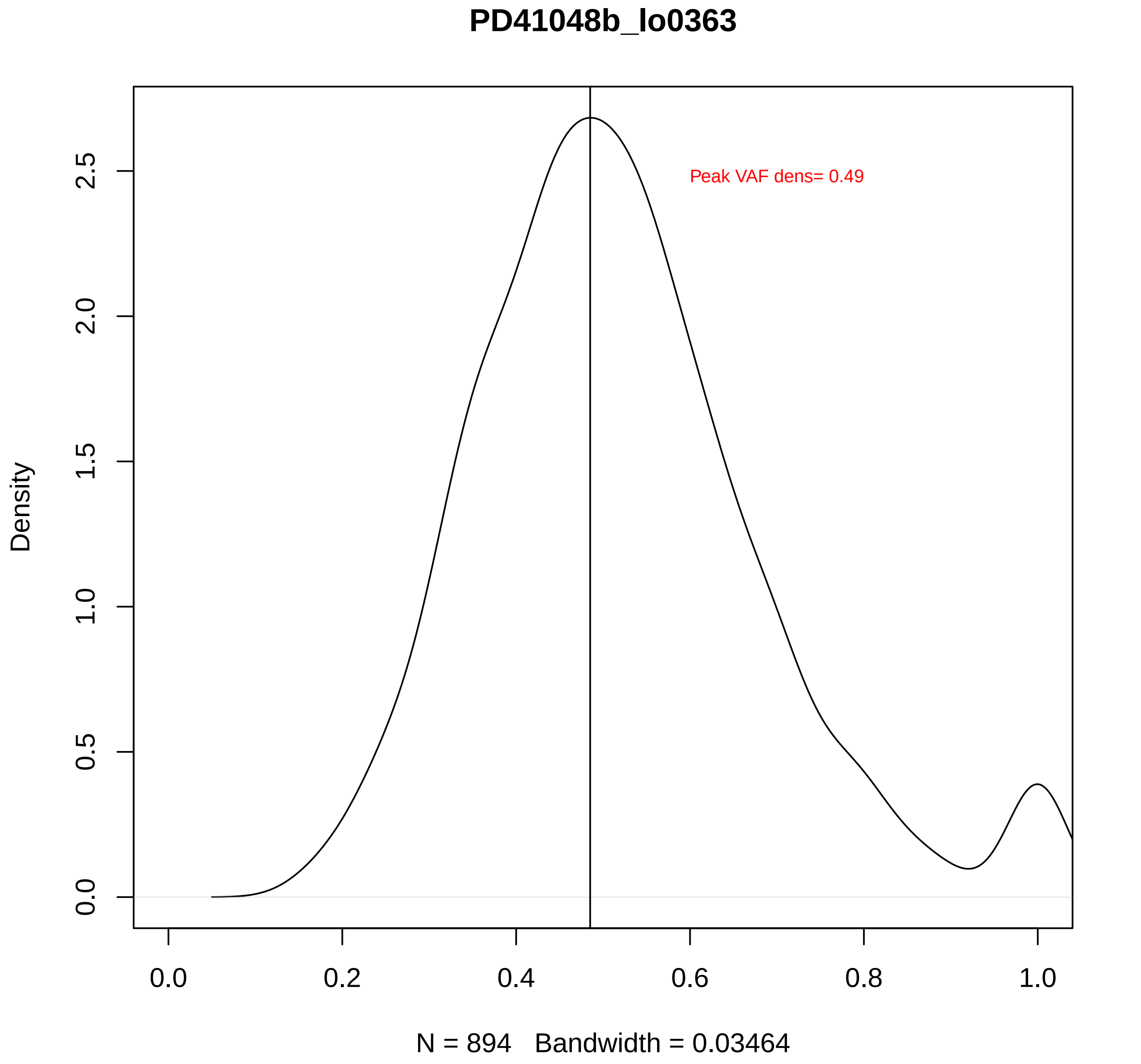
PD41048b_lo0363
Density
0.0
0.5
1.0
1.5
2.0
2.5
0.0
0.2
0.4
0.6
0.8
1.0
Peak VAF dens= 0.49
N = 894   Bandwidth = 0.03464

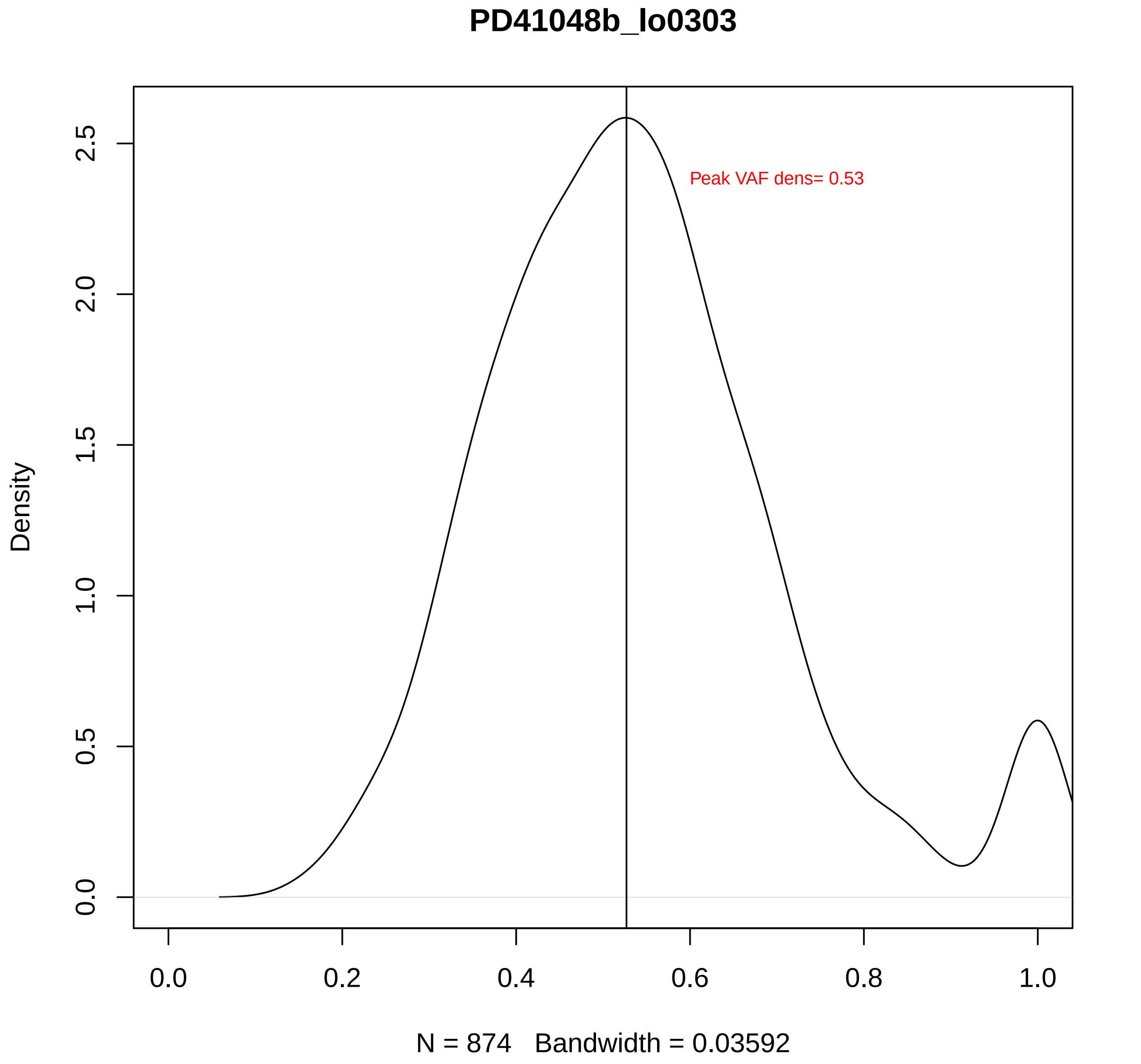
PD41048b_lo0303
Peak VAF dens= 0.53
Density
0.0
0.5
1.0
1.5
2.0
2.5
0.0
0.2
0.4
0.6
0.8
1.0
N = 874   Bandwidth = 0.03592

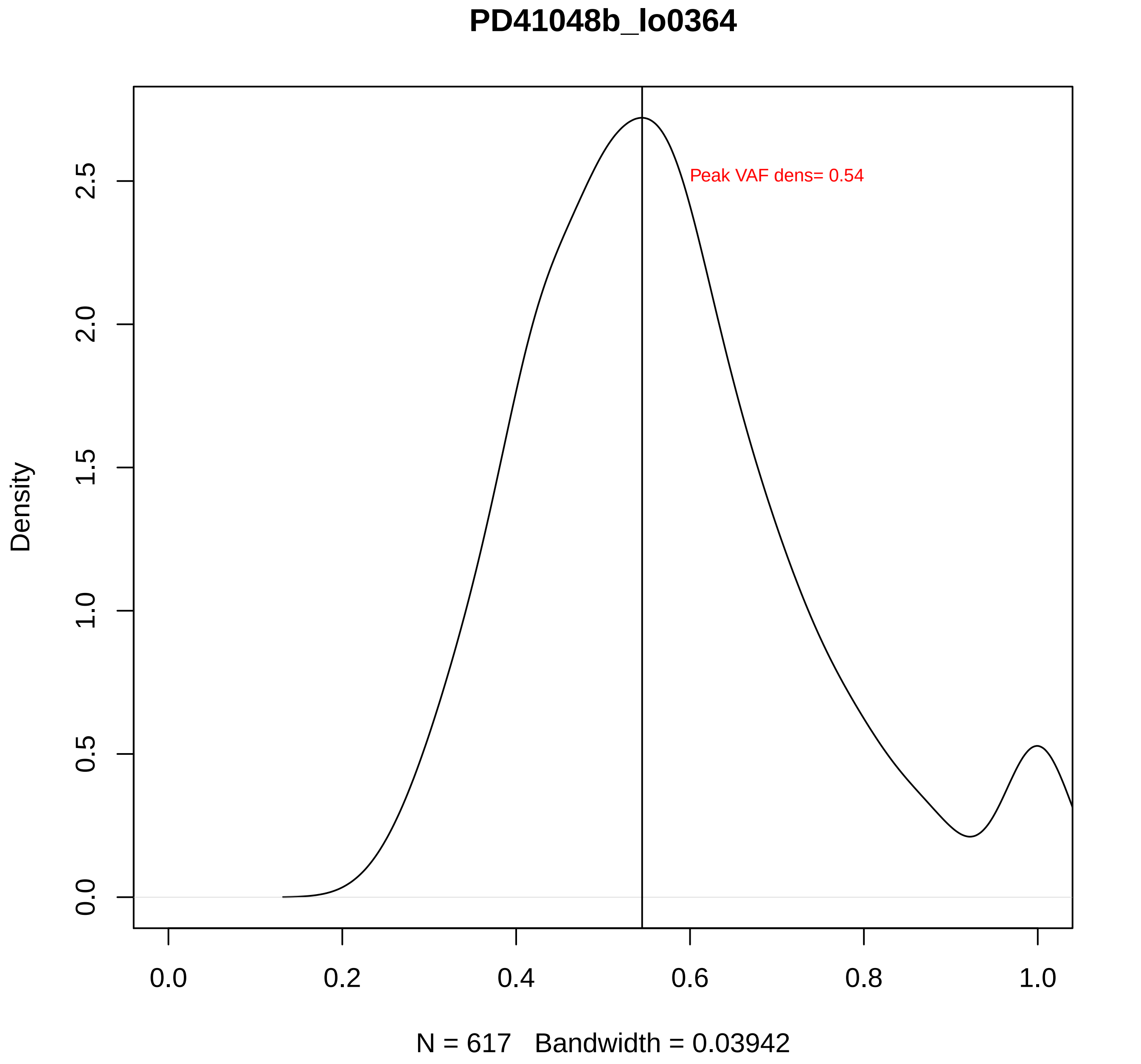
PD41048b_lo0364
Peak VAF dens= 0.54
Density
0.0
0.5
1.0
1.5
2.0
2.5
0.0
0.2
0.4
0.6
0.8
1.0
N = 617   Bandwidth = 0.03942

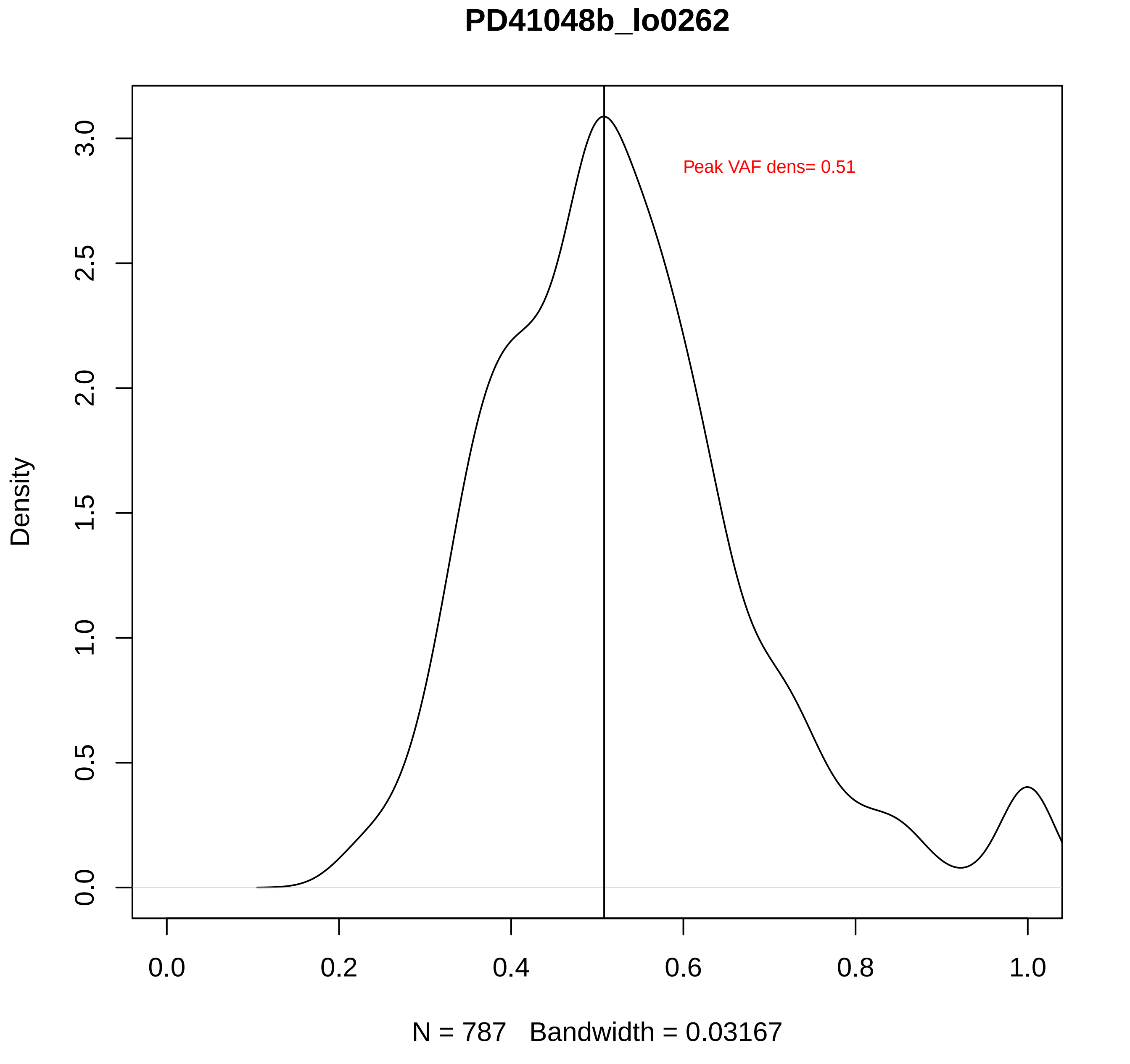
PD41048b_lo0262
Peak VAF dens= 0.51
Density
0.0
0.5
1.0
1.5
2.0
2.5
3.0
0.0
0.2
0.4
0.6
0.8
1.0
N = 787   Bandwidth = 0.03167

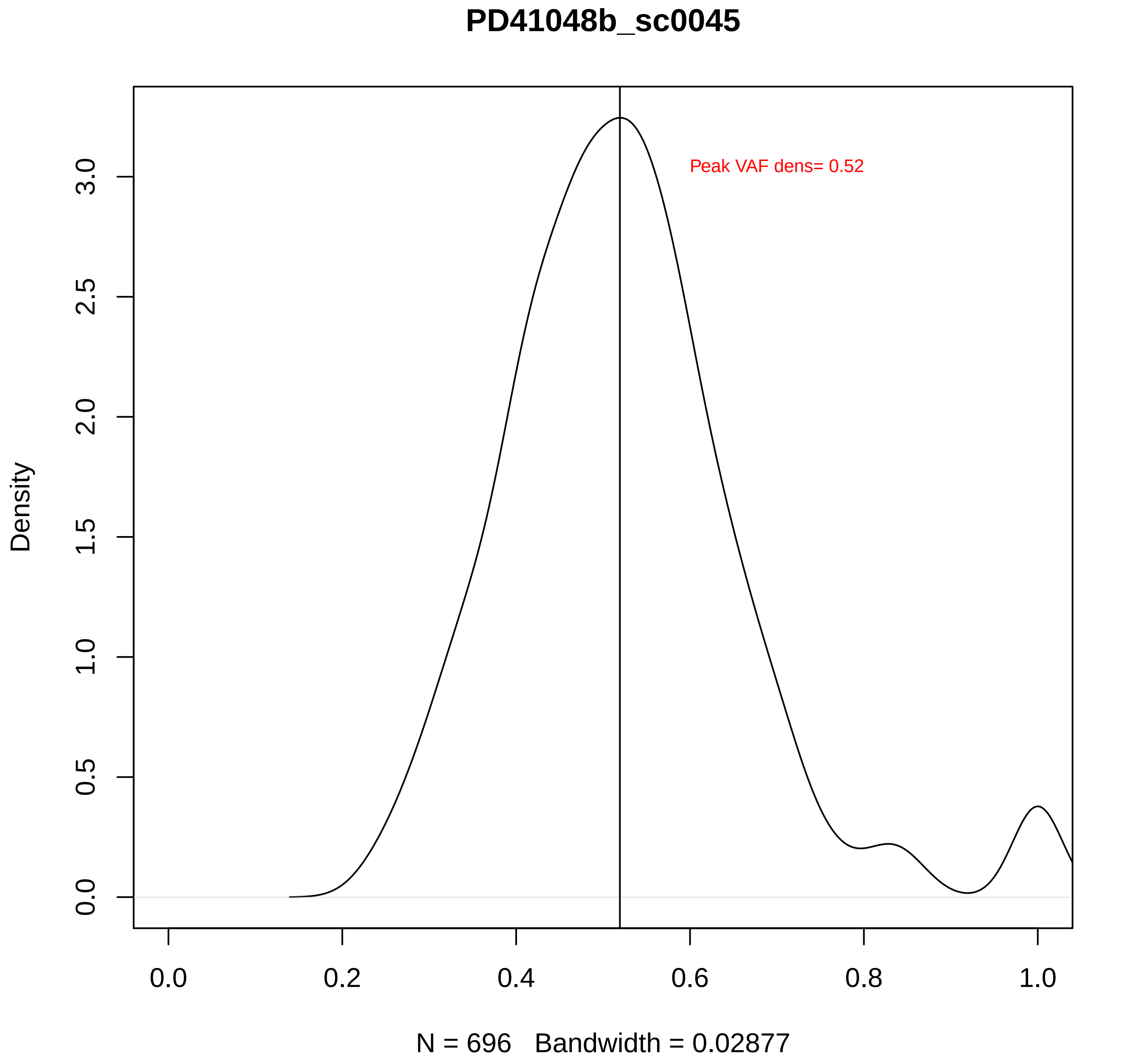

PD41048b_sc0045
Peak VAF dens= 0.52
Density
0.0
0.5
1.0
1.5
2.0
2.5
3.0
0.0
0.2
0.4
0.6
0.8
1.0
N = 696 Bandwidth = 0.02877

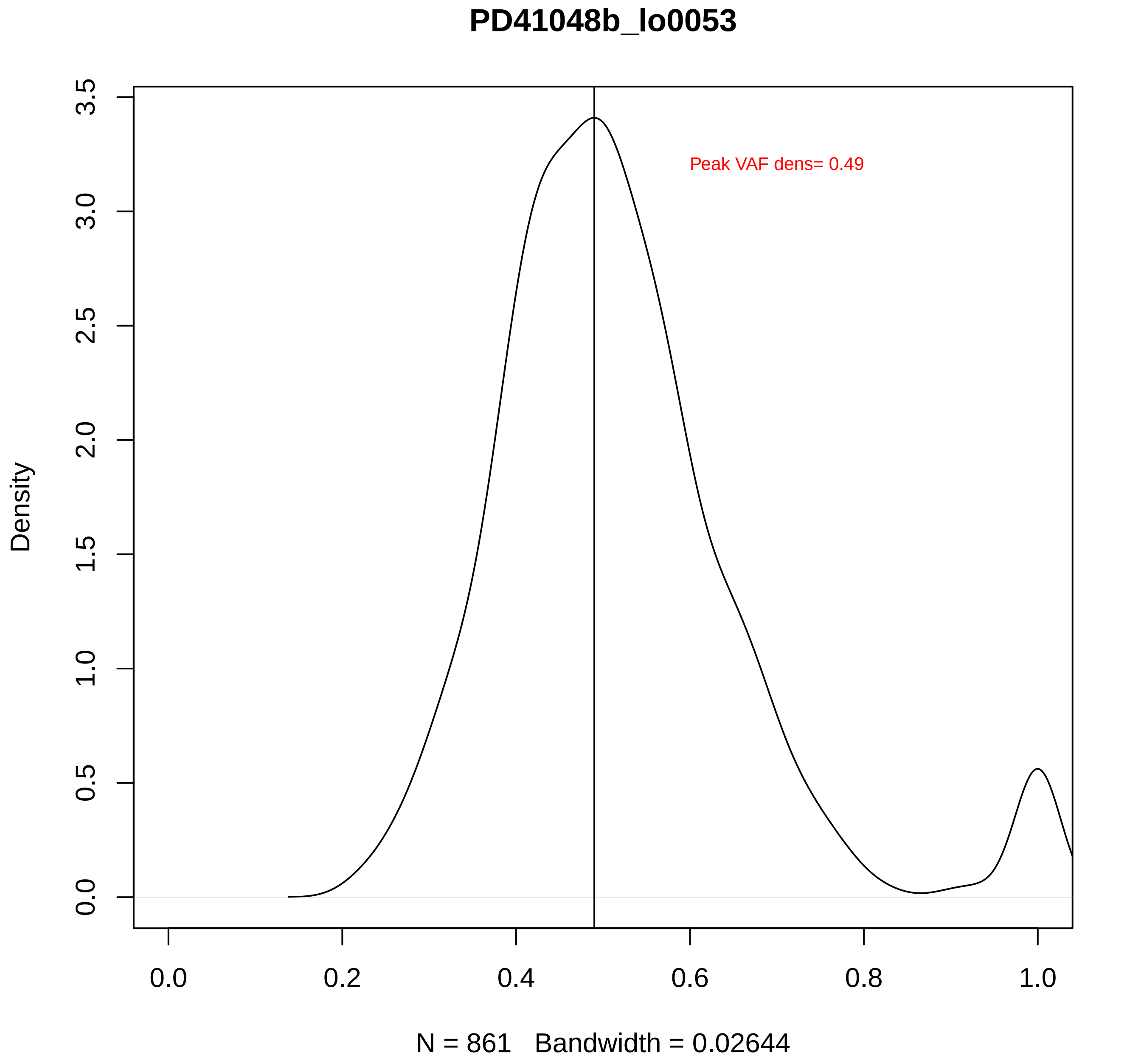
PD41048b_lo0053
Peak VAF dens= 0.49
Density
0.0
0.5
1.0
1.5
2.0
2.5
3.0
3.5
0.0
0.2
0.4
0.6
0.8
1.0
N = 861   Bandwidth = 0.02644

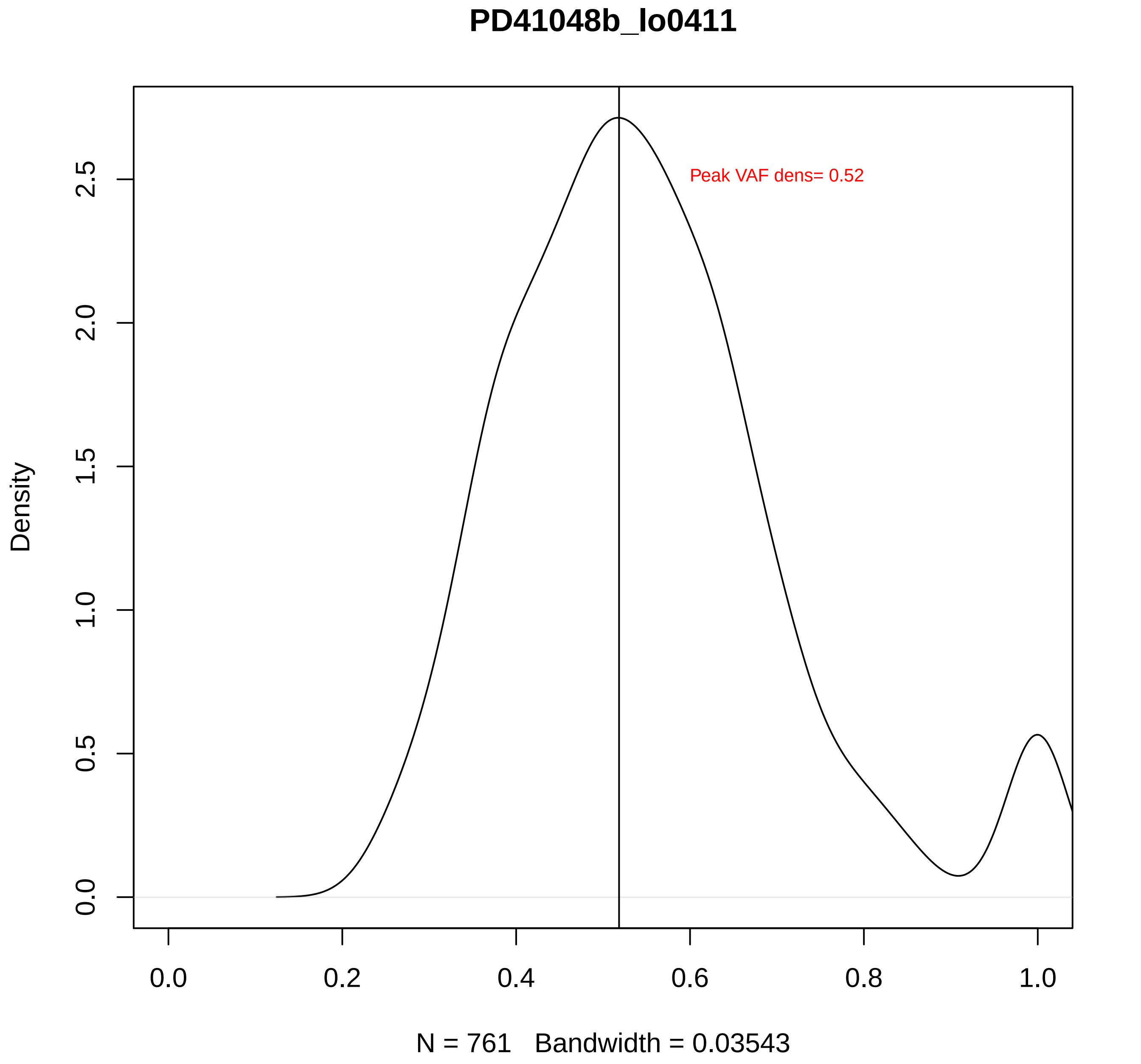
PD41048b_lo0411
Peak VAF dens= 0.52
Density
0.0
0.5
1.0
1.5
2.0
2.5
0.0
0.2
0.4
0.6
0.8
1.0
N = 761   Bandwidth = 0.03543

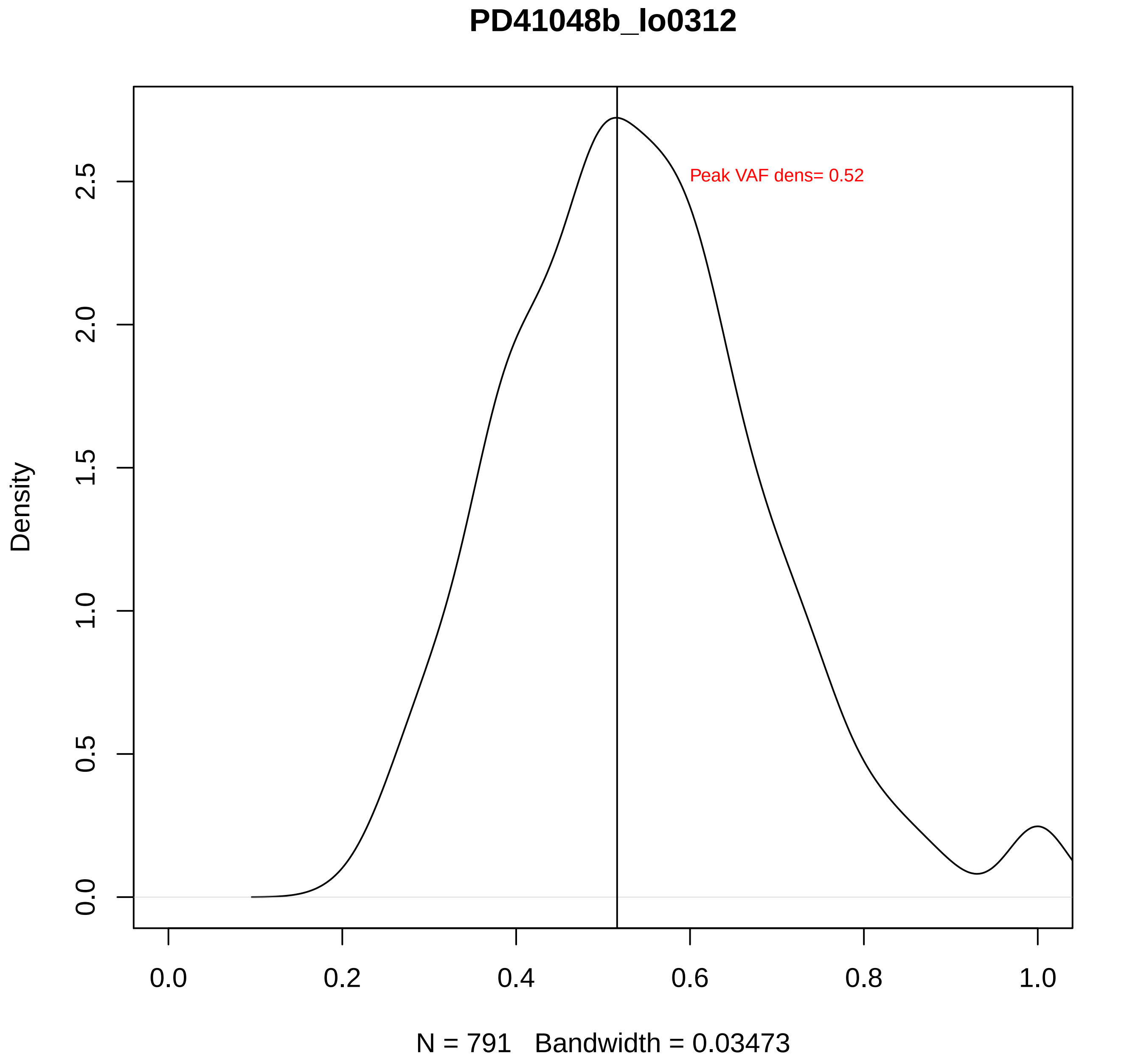
PD41048b_lo0312
Peak VAF dens= 0.52
Density
0.0
0.5
1.0
1.5
2.0
2.5
0.0
0.2
0.4
0.6
0.8
1.0
N = 791 Bandwidth = 0.03473

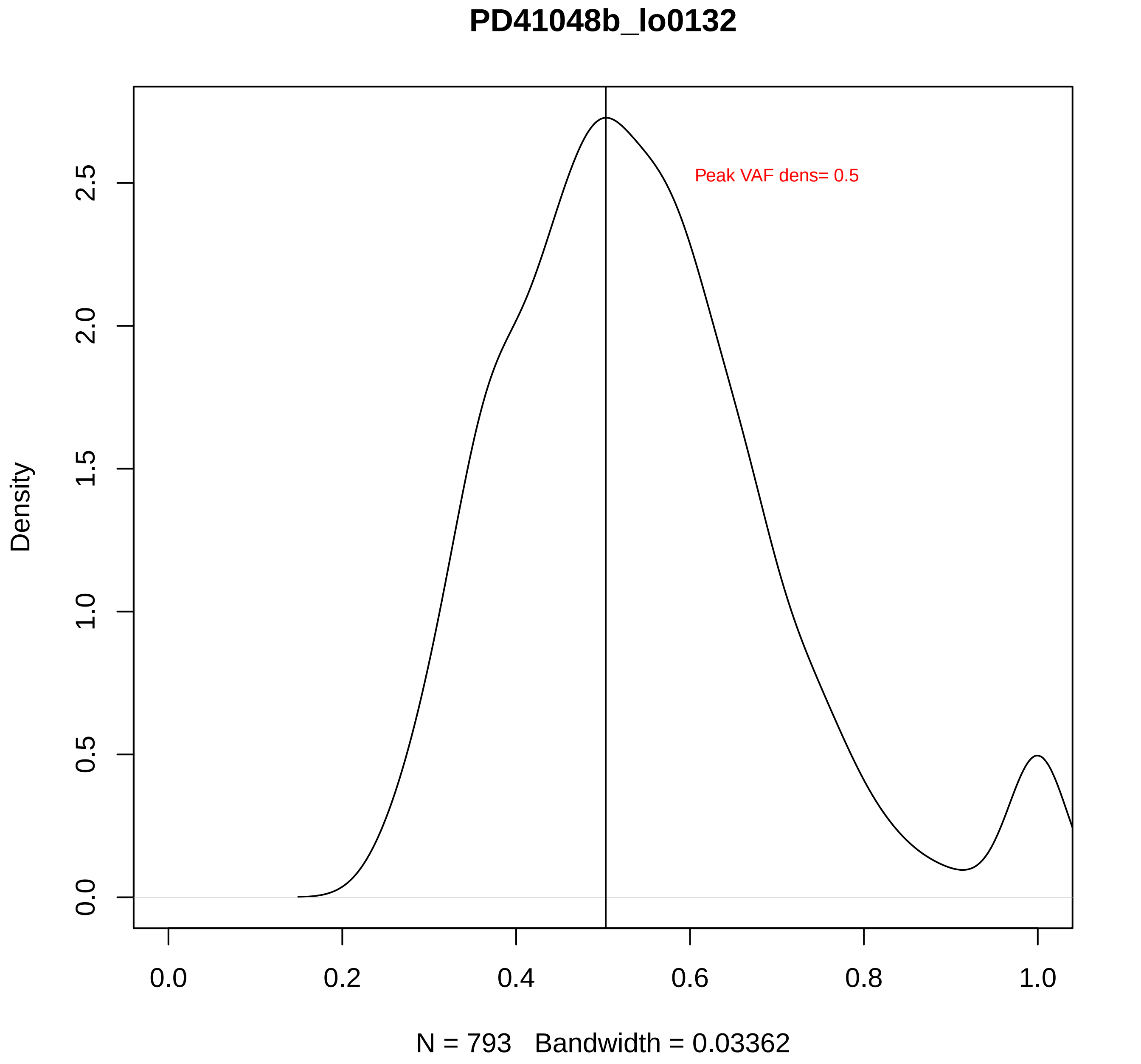

PD41048b_lo0132
Peak VAF dens= 0.5
Density
0.0
0.5
1.0
1.5
2.0
2.5
0.0
0.2
0.4
0.6
0.8
1.0
N = 793 Bandwidth = 0.03362

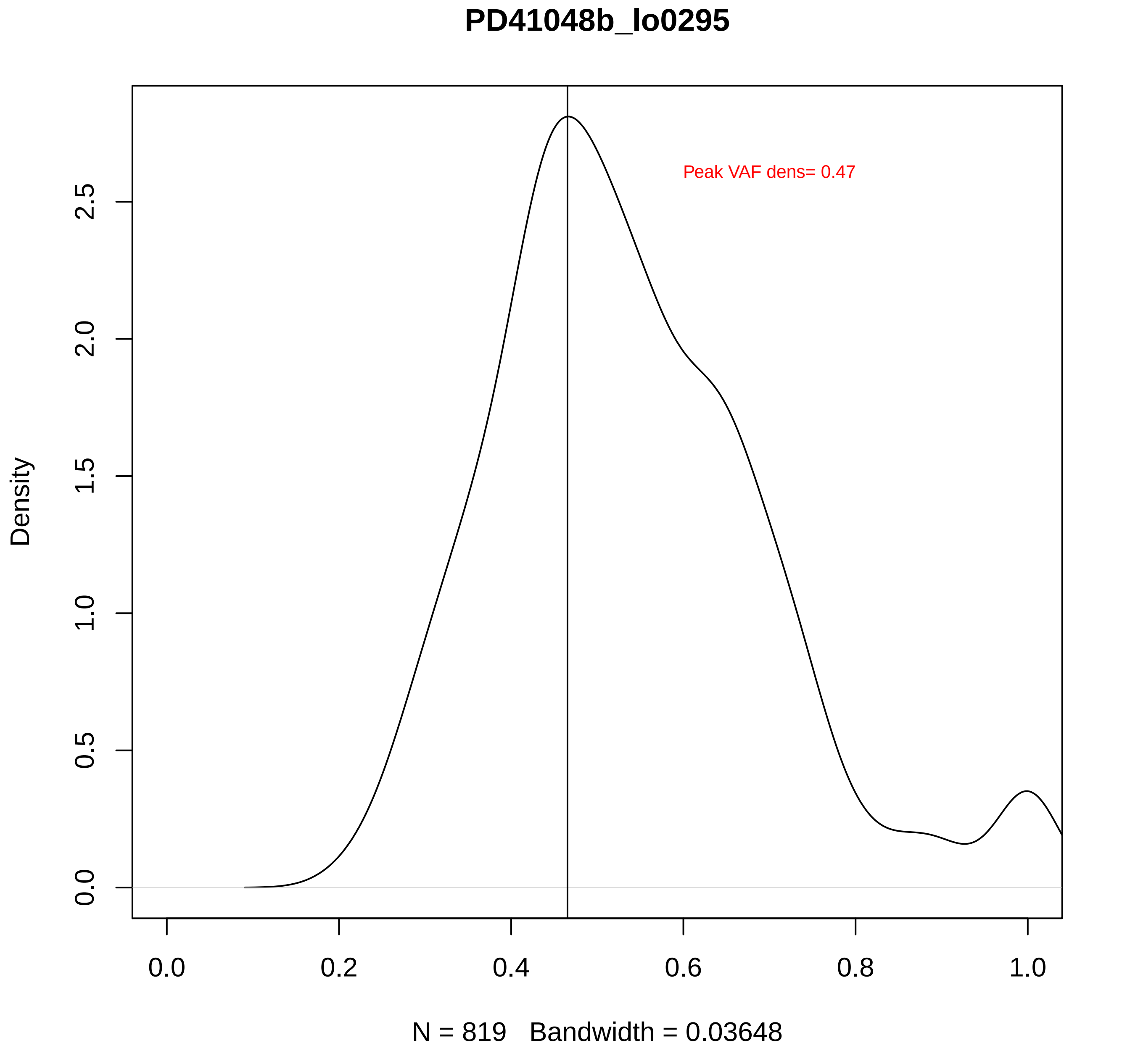

PD41048b_lo0295
Peak VAF dens= 0.47
Density
0.0
0.5
1.0
1.5
2.0
2.5
0.0
0.2
0.4
0.6
0.8
1.0
N = 819 Bandwidth = 0.03648

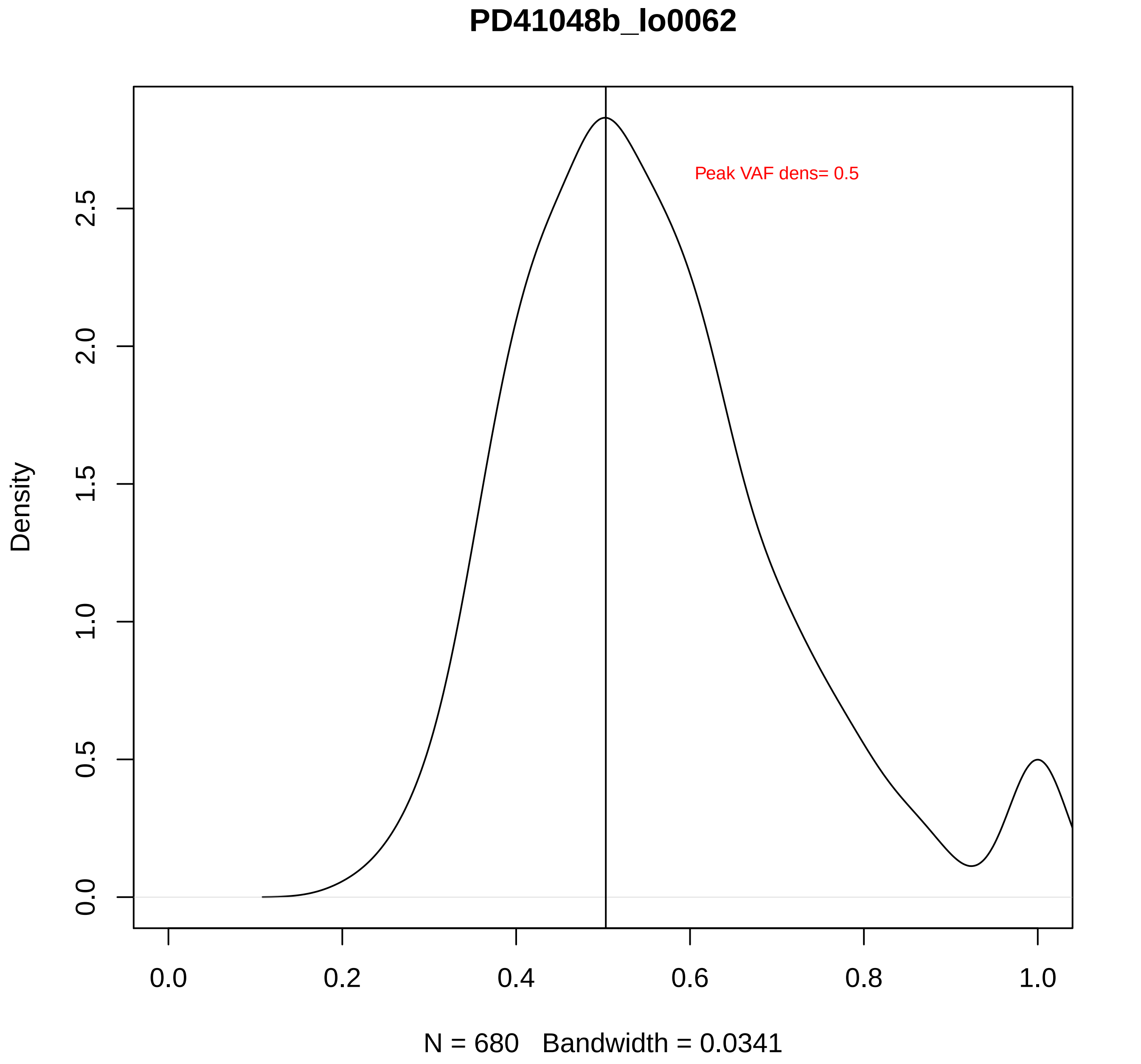

PD41048b_lo0062
Peak VAF dens= 0.5
Density
0.0
0.5
1.0
1.5
2.0
2.5
0.0
0.2
0.4
0.6
0.8
1.0
N = 680 Bandwidth = 0.0341

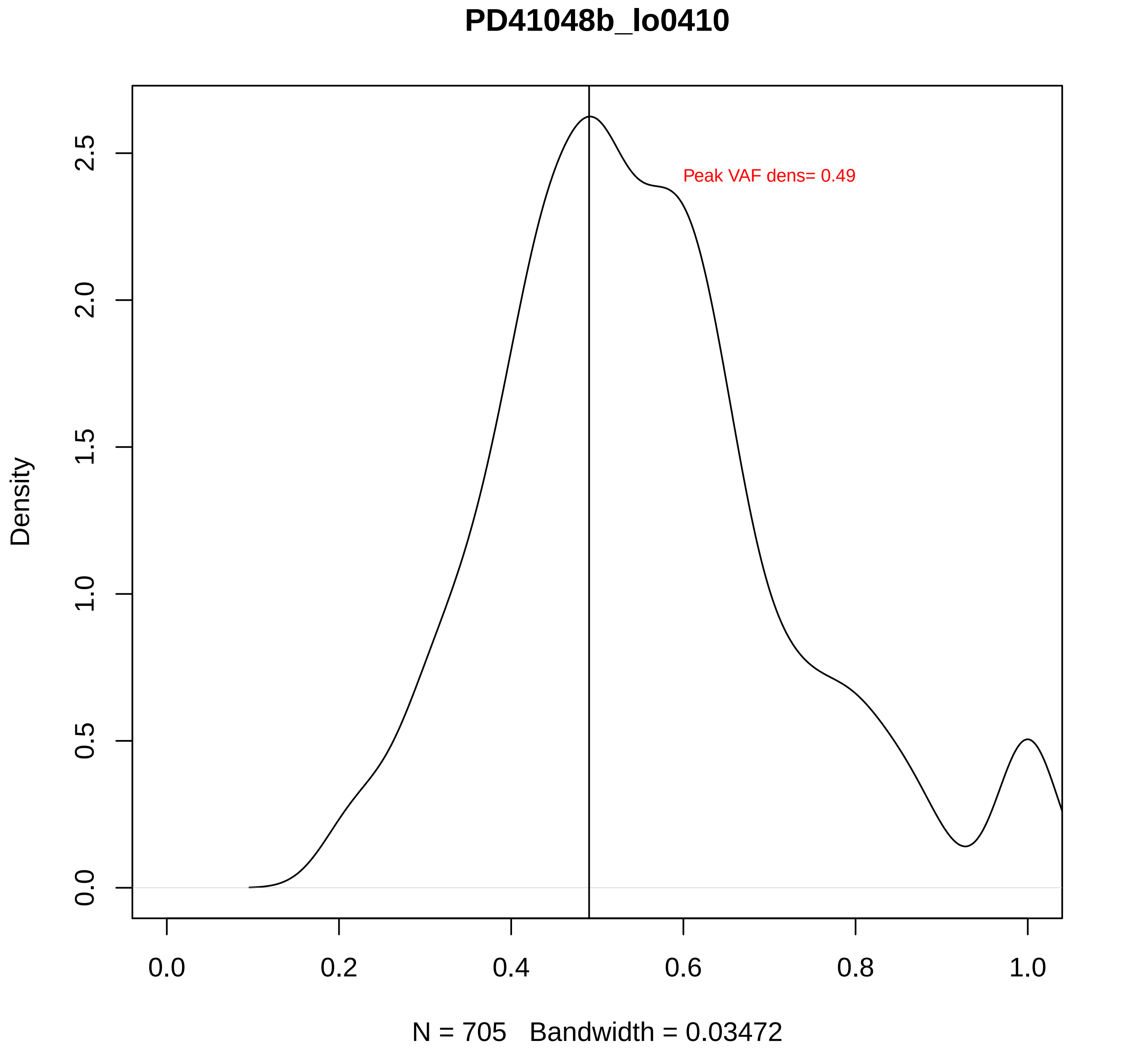
PD41048b_lo0410
Peak VAF dens= 0.49
Density
2.5
2.0
1.5
1.0
0.5
0.0
0.0
0.2
0.4
0.6
0.8
1.0
N = 705 Bandwidth = 0.03472

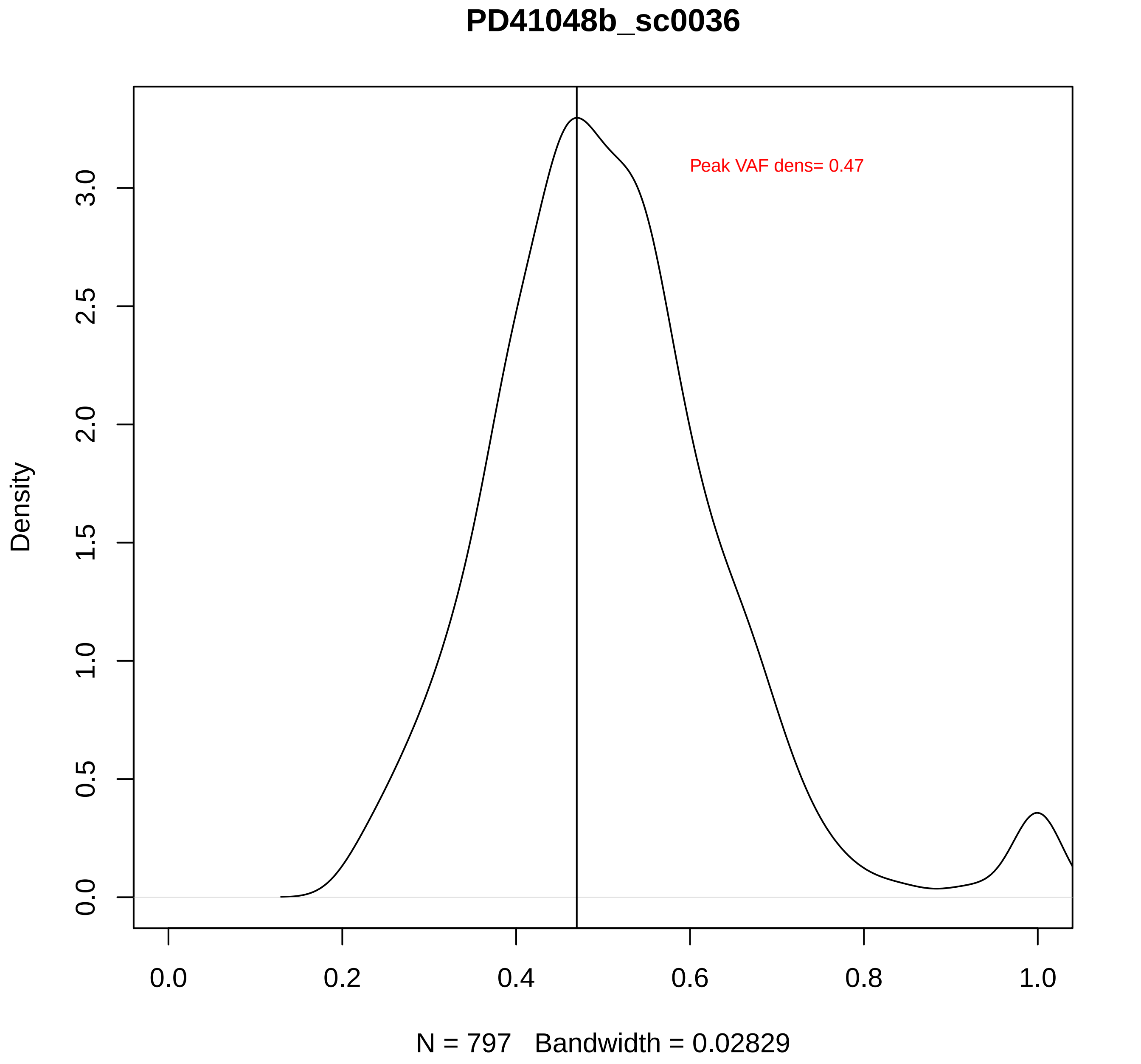

PD41048b_sc0036
Peak VAF dens= 0.47
Density
0.0
0.5
1.0
1.5
2.0
2.5
3.0
0.0
0.2
0.4
0.6
0.8
1.0
N = 797   Bandwidth = 0.02829

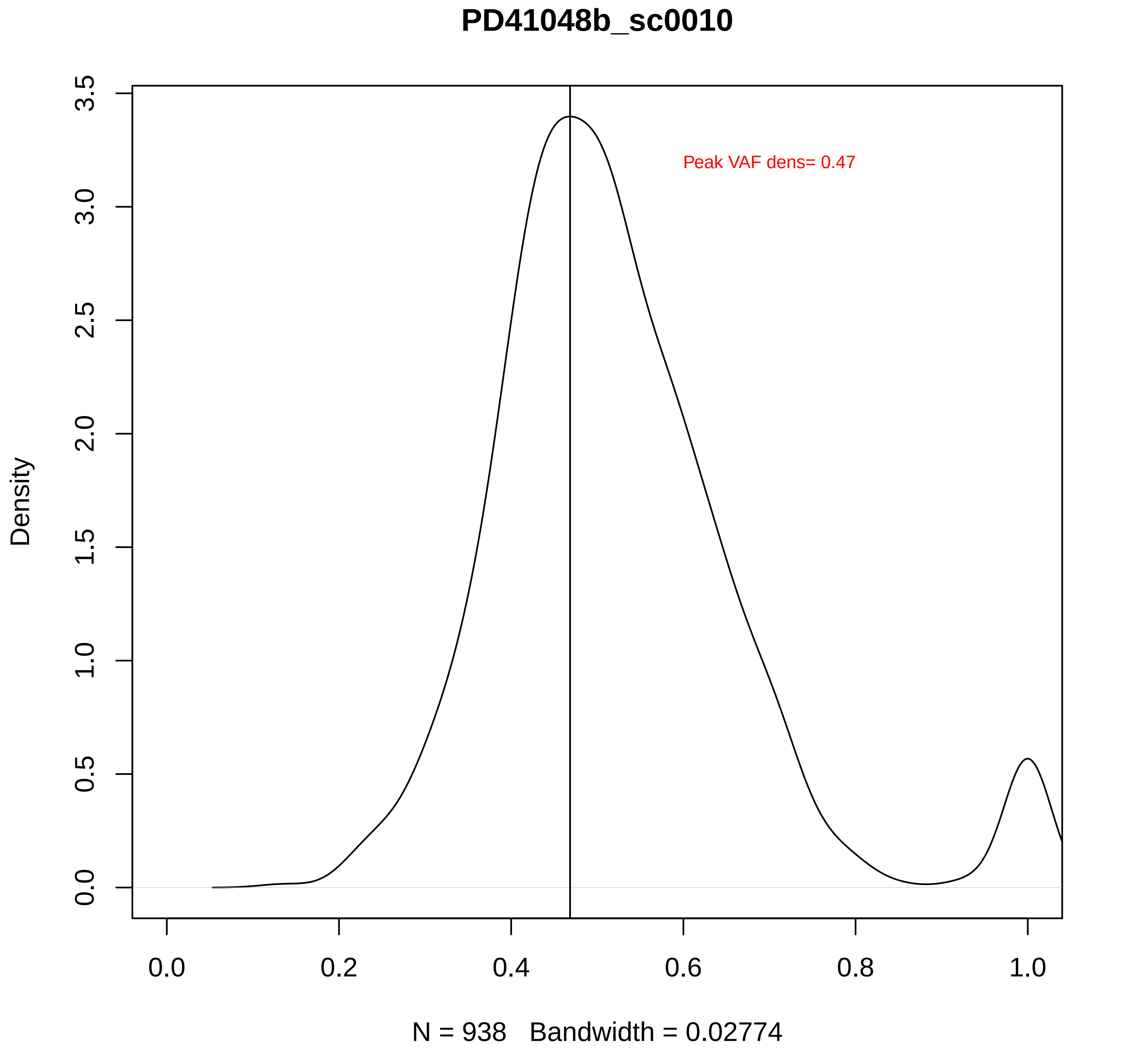

PD41048b_sc0010
Peak VAF dens= 0.47
Density
0.0
0.5
1.0
1.5
2.0
2.5
3.0
3.5
0.0
0.2
0.4
0.6
0.8
1.0
N = 938   Bandwidth = 0.02774

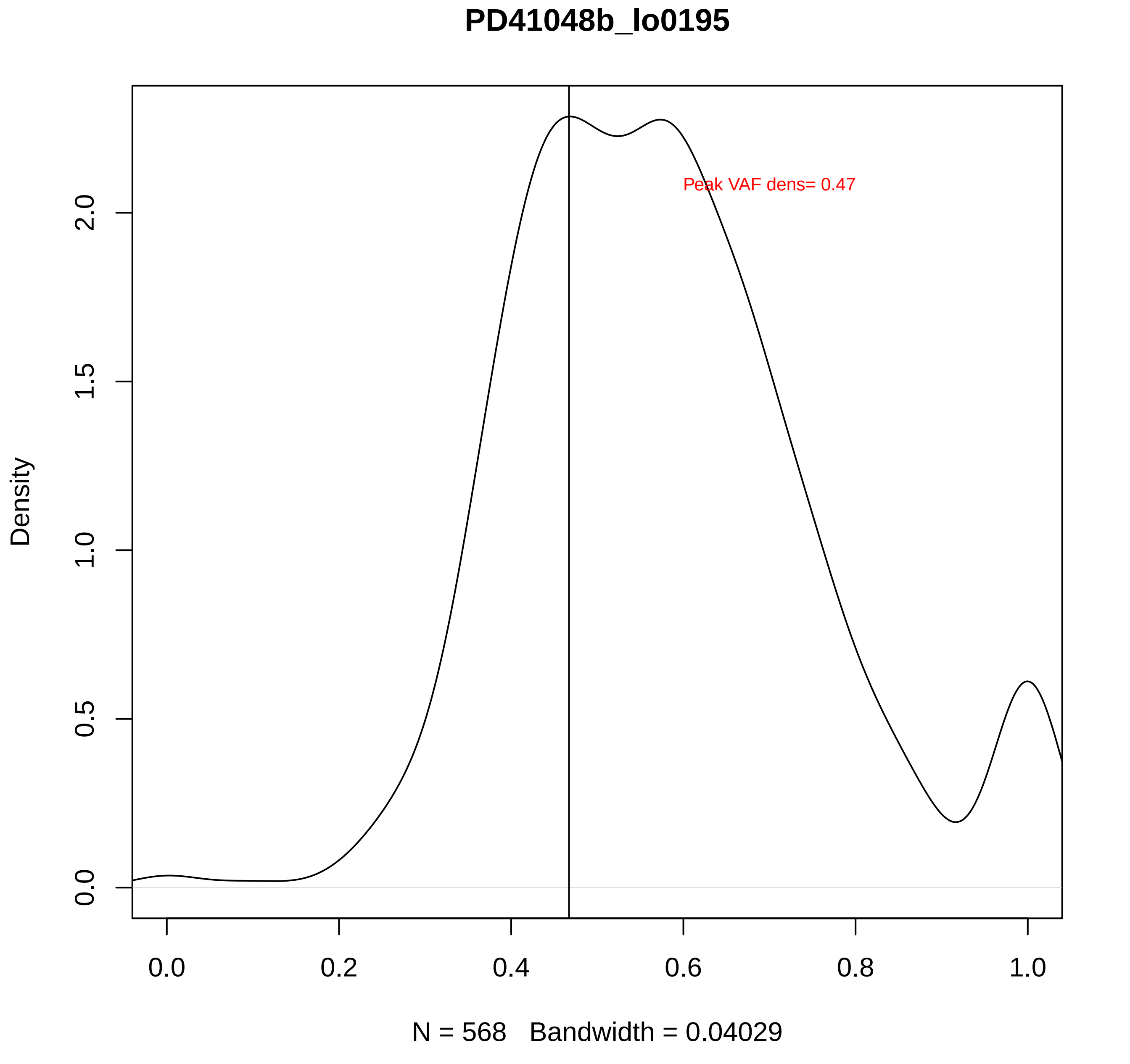
PD41048b_lo0195
Peak VAF dens= 0.47
Density
0.0
0.5
1.0
1.5
2.0
0.0
0.2
0.4
0.6
0.8
1.0
N = 568 Bandwidth = 0.04029

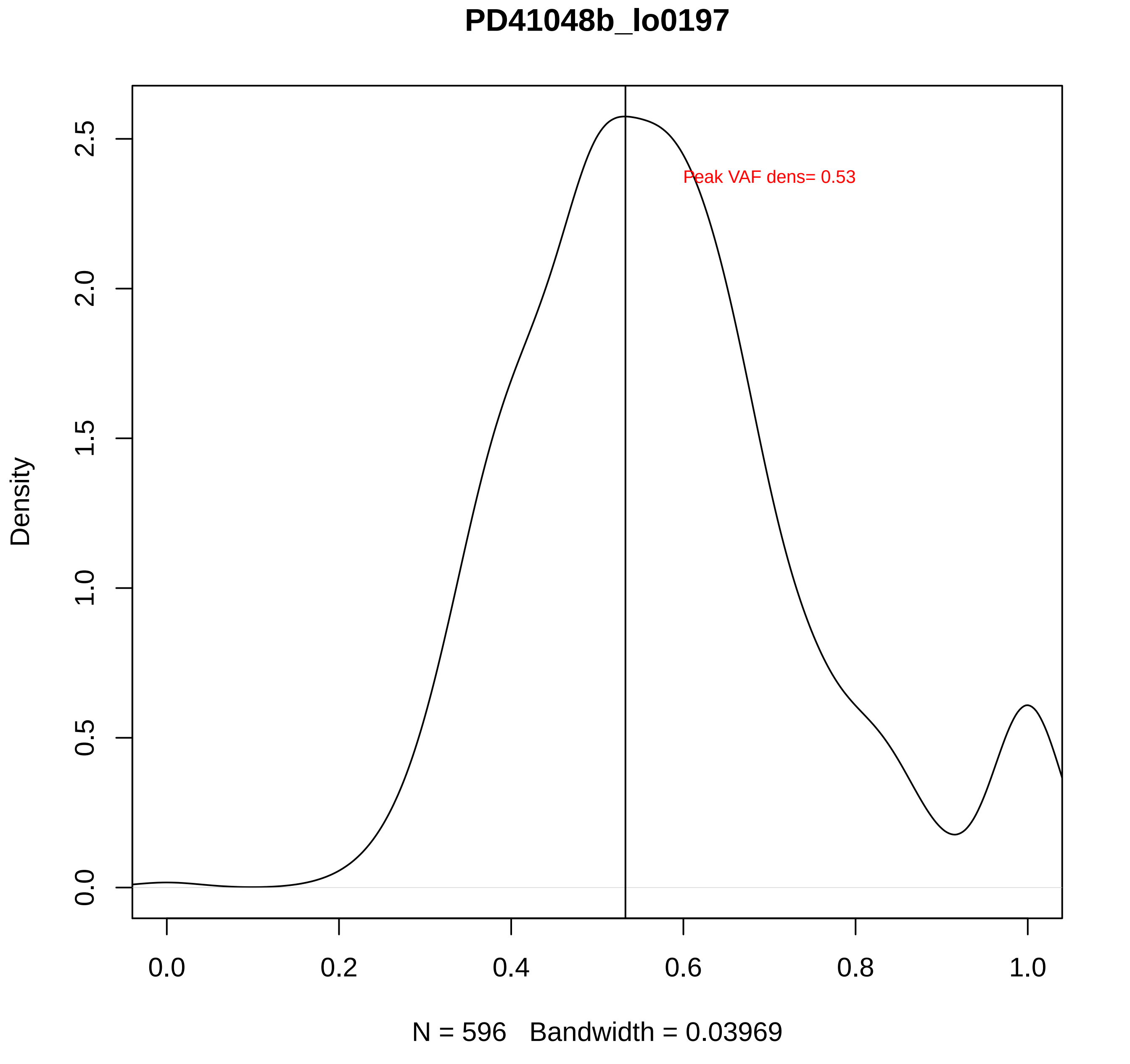
PD41048b_lo0197
Peak VAF dens= 0.53
Density
0.0
0.5
1.0
1.5
2.0
2.5
0.0
0.2
0.4
0.6
0.8
1.0
N = 596   Bandwidth = 0.03969

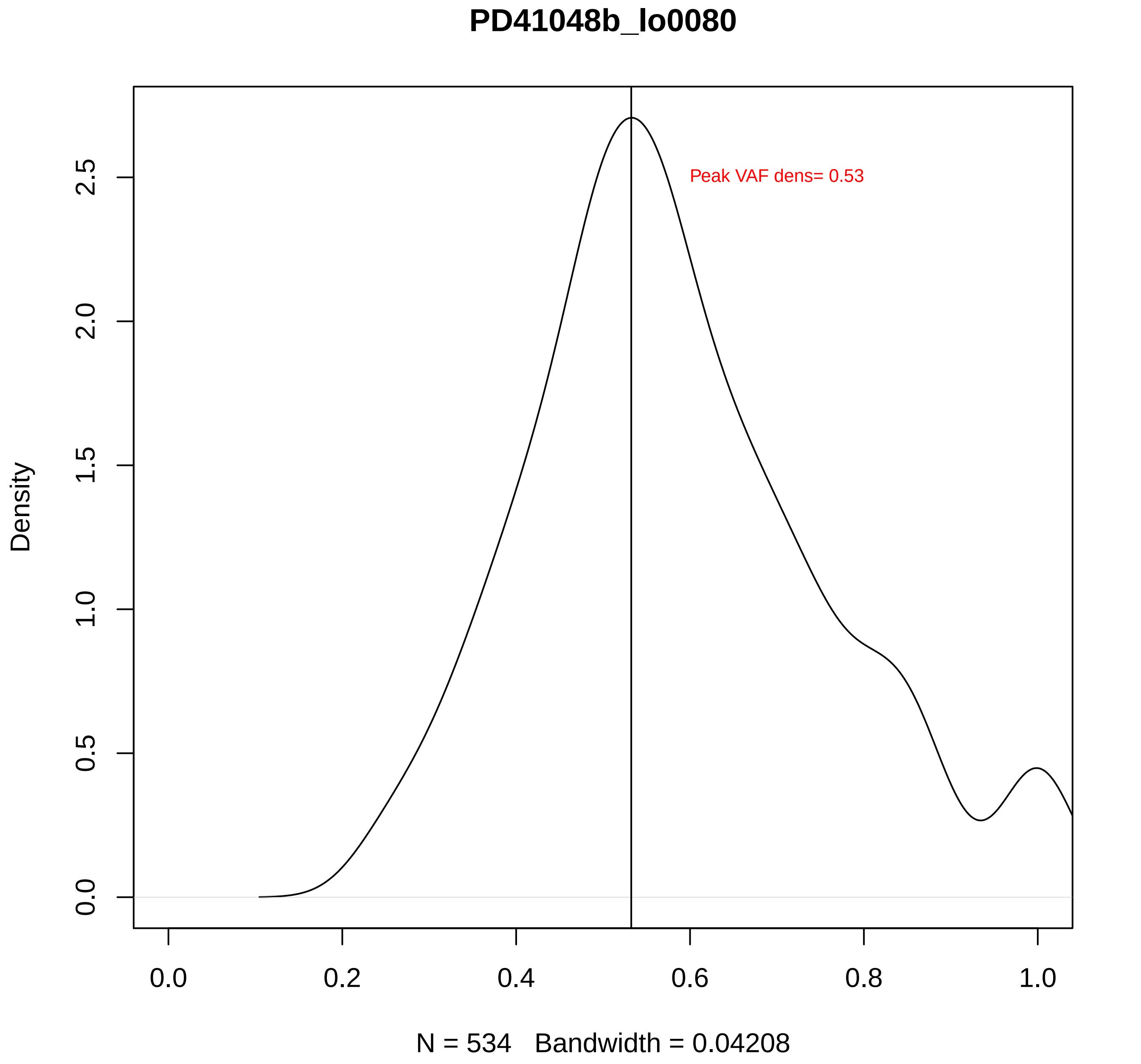

PD41048b_lo0080
Peak VAF dens= 0.53
Density
0.0
0.5
1.0
1.5
2.0
2.5
0.0
0.2
0.4
0.6
0.8
1.0
N = 534   Bandwidth = 0.04208

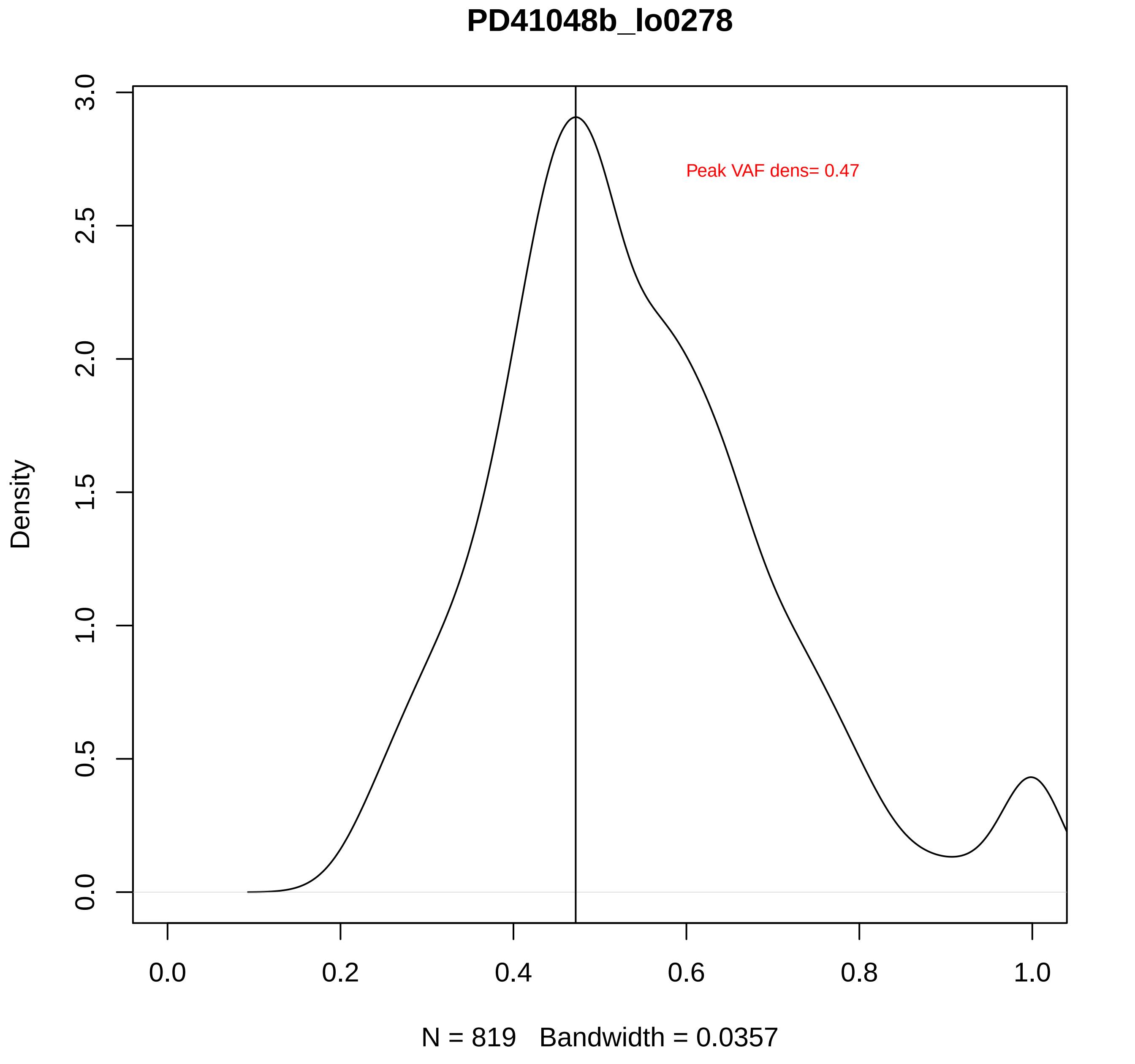

PD41048b_lo0278
Peak VAF dens= 0.47
Density
0.0
0.5
1.0
1.5
2.0
2.5
3.0
0.0
0.2
0.4
0.6
0.8
1.0
N = 819 Bandwidth = 0.0357

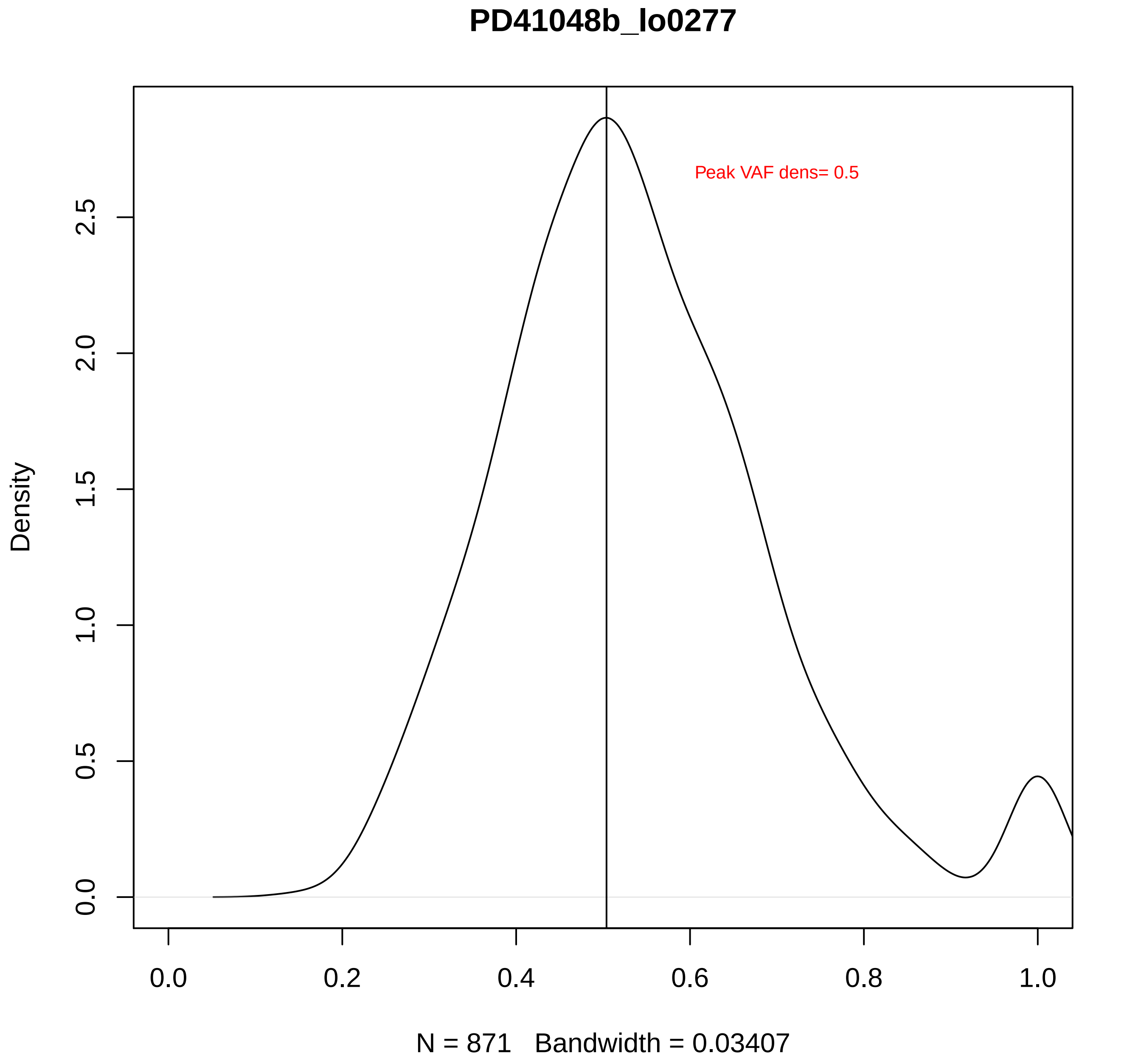

PD41048b_lo0277
Peak VAF dens= 0.5
Density
0.0
0.5
1.0
1.5
2.0
2.5
0.0
0.2
0.4
0.6
0.8
1.0
N = 871   Bandwidth = 0.03407

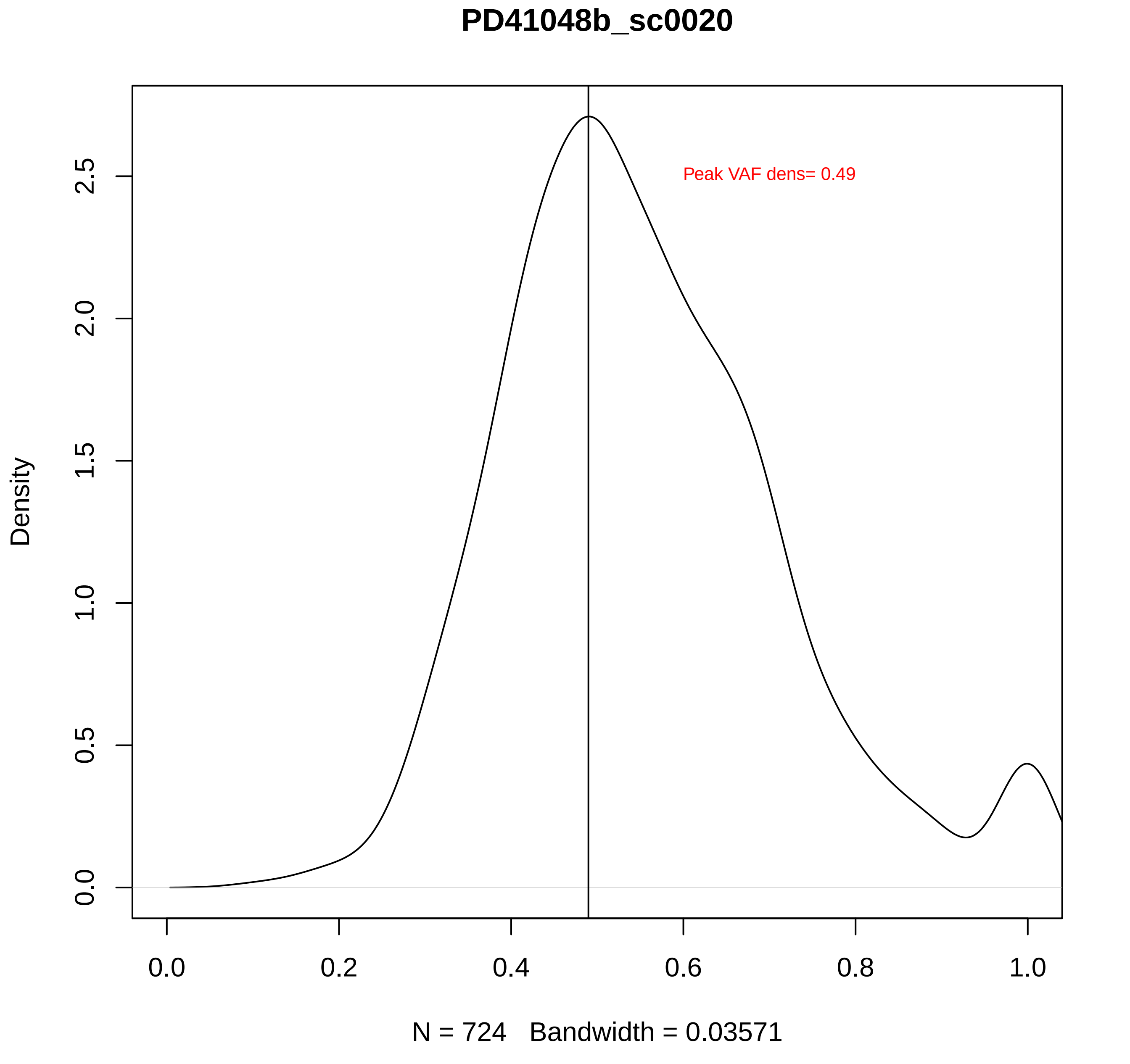

PD41048b_sc0020
Peak VAF dens= 0.49
Density
0.0
0.5
1.0
1.5
2.0
2.5
0.0
0.2
0.4
0.6
0.8
1.0
N = 724 Bandwidth = 0.03571

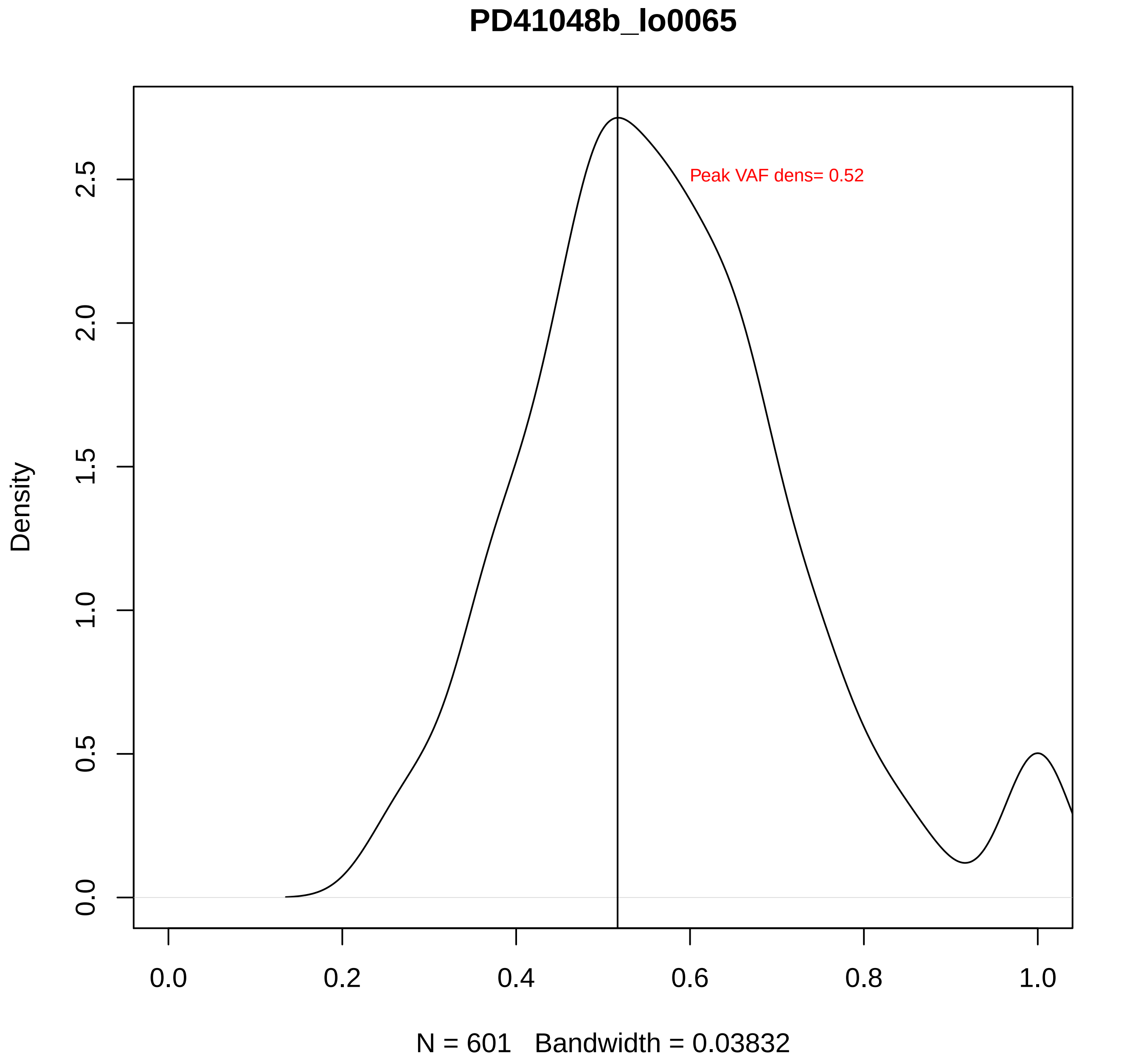
PD41048b_lo0065
Peak VAF dens= 0.52
Density
0.0
0.5
1.0
1.5
2.0
2.5
0.0
0.2
0.4
0.6
0.8
1.0
N = 601   Bandwidth = 0.03832

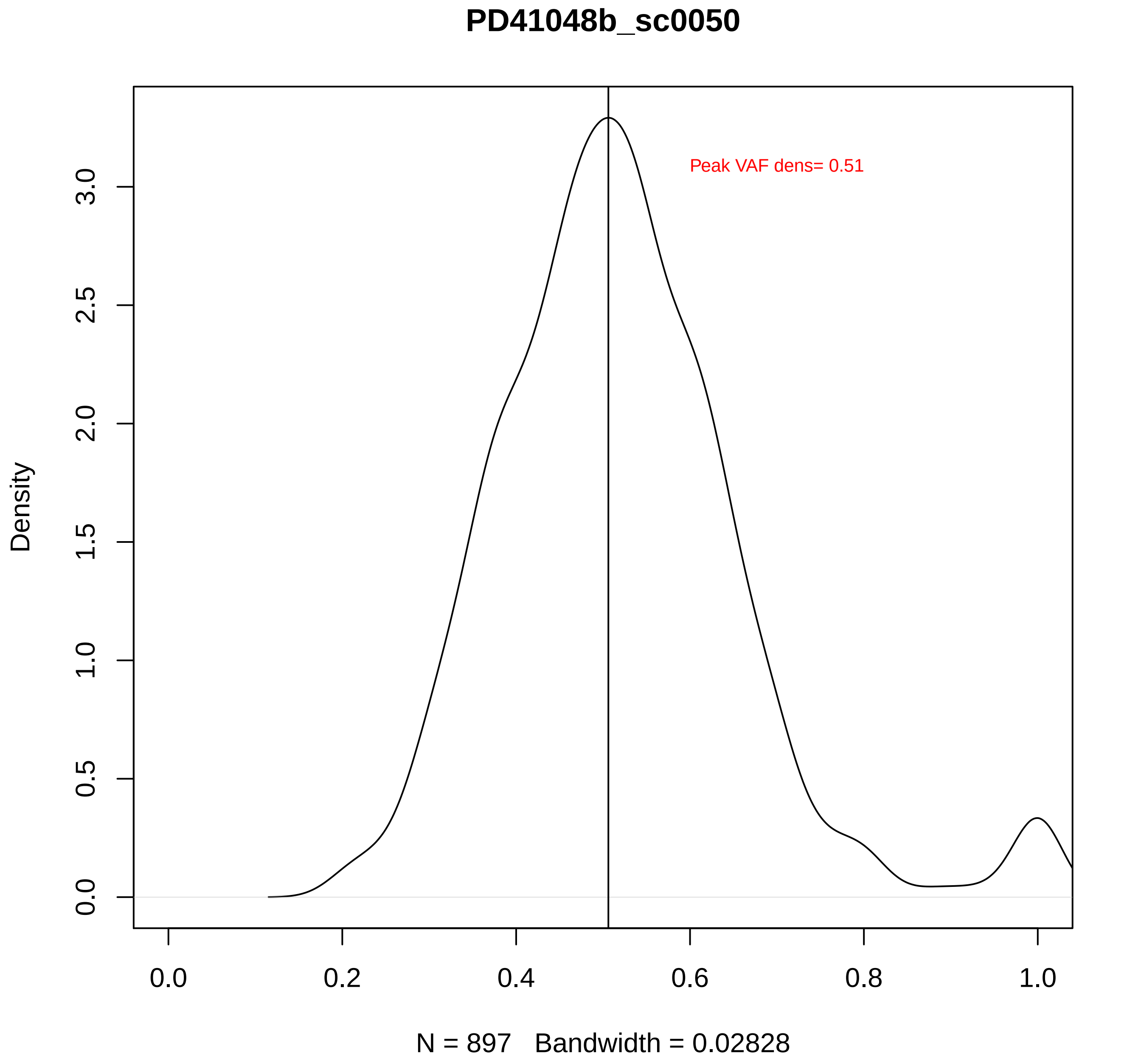
PD41048b_sc0050
Peak VAF dens= 0.51
Density
0.0
0.5
1.0
1.5
2.0
2.5
3.0
0.0
0.2
0.4
0.6
0.8
1.0
N = 897   Bandwidth = 0.02828

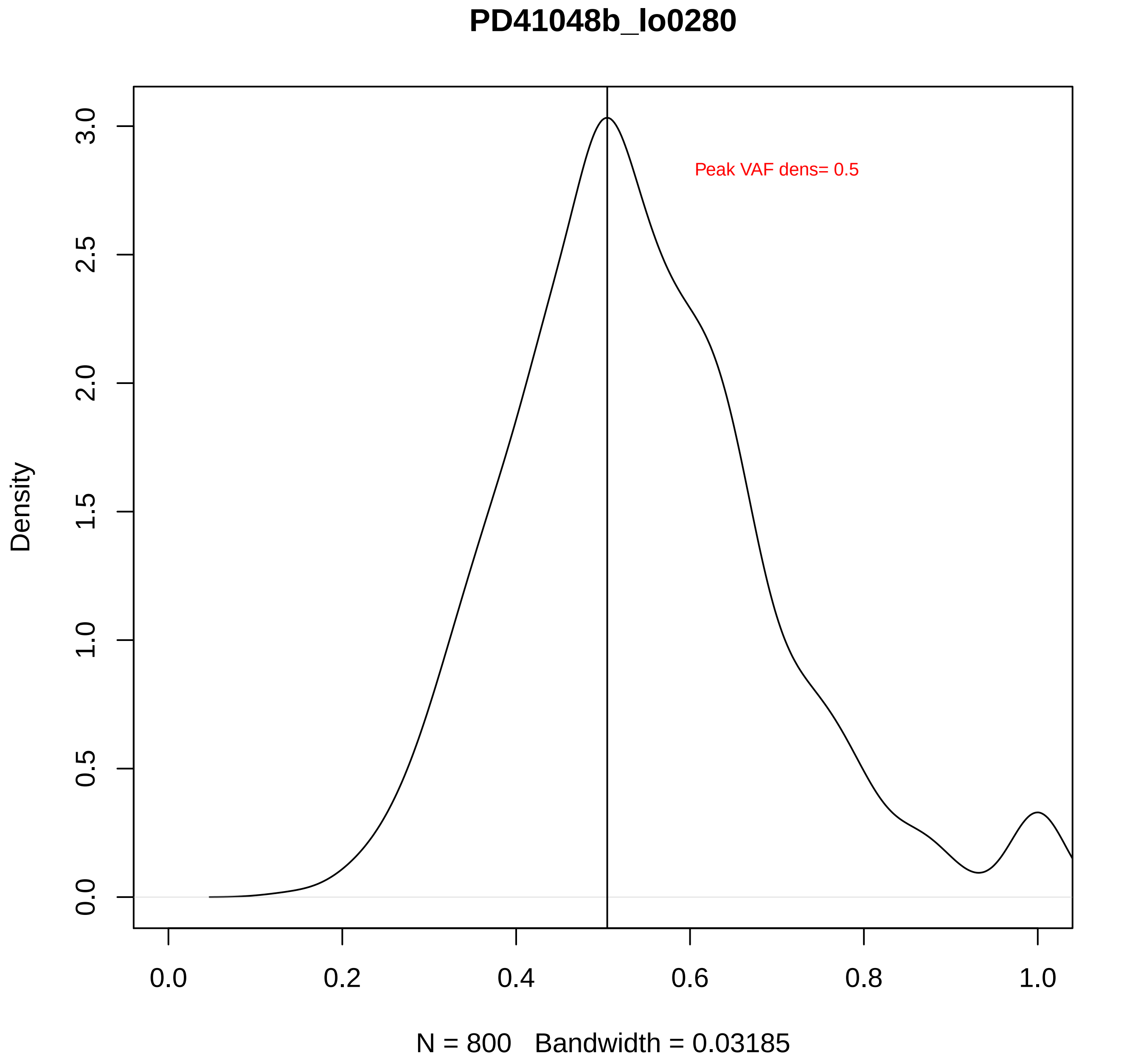

PD41048b_lo0280
Peak VAF dens= 0.5
Density
0.0
0.5
1.0
1.5
2.0
2.5
3.0
0.0
0.2
0.4
0.6
0.8
1.0
N = 800   Bandwidth = 0.03185

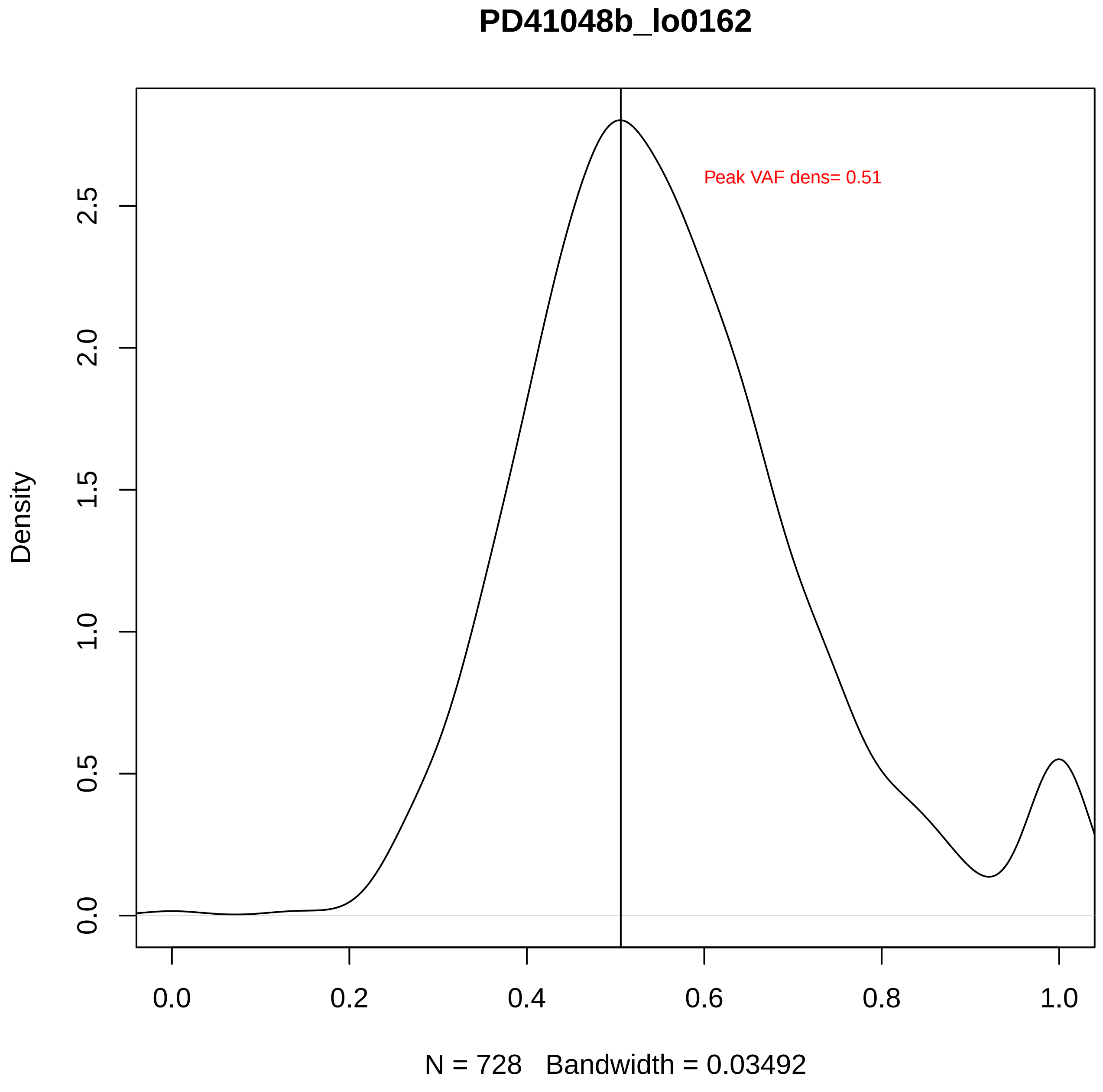

PD41048b_lo0162
Peak VAF dens= 0.51
Density
0.0
0.5
1.0
1.5
2.0
2.5
0.0
0.2
0.4
0.6
0.8
1.0
N = 728   Bandwidth = 0.03492

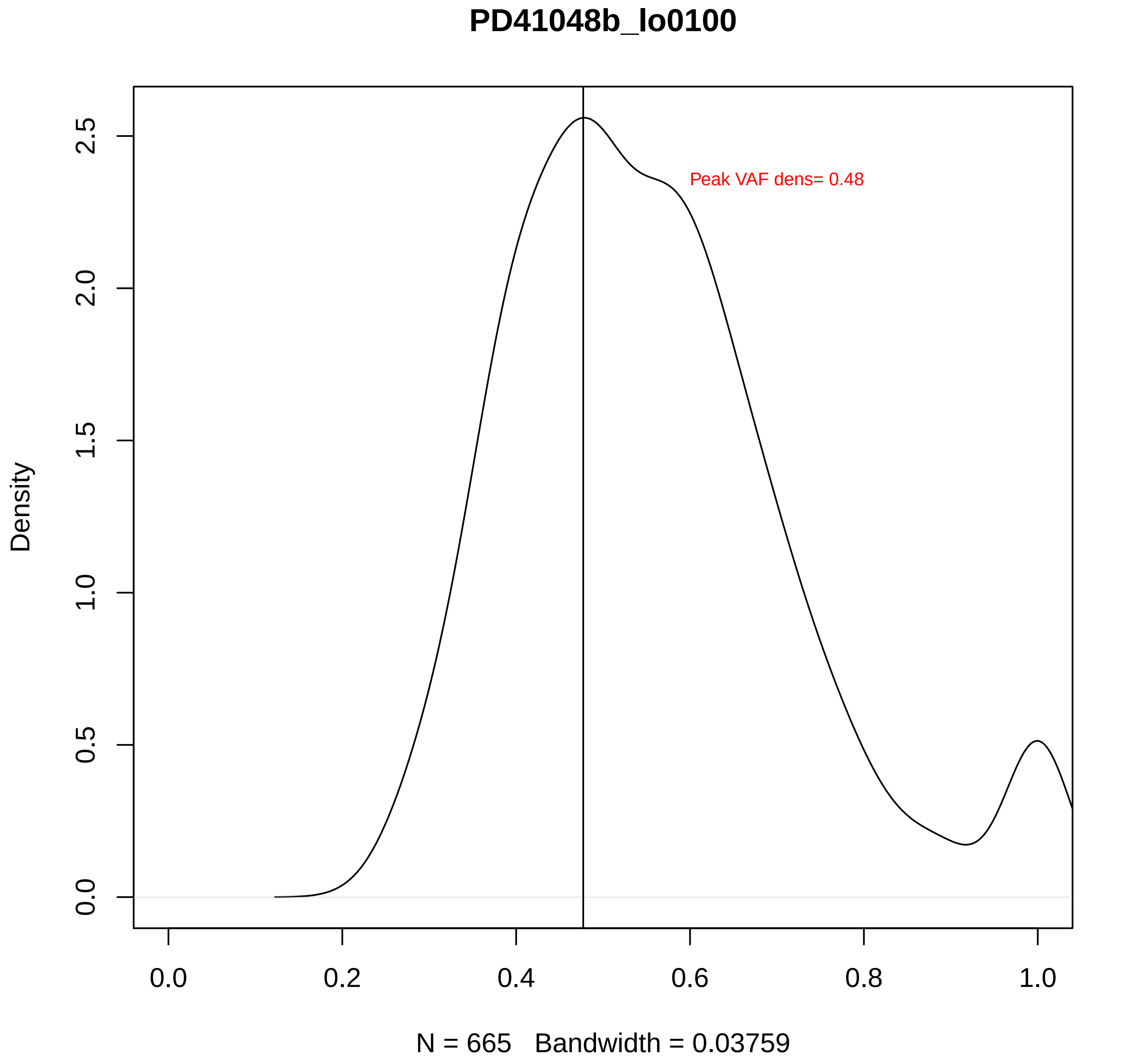
PD41048b_lo0100
Density
0.0
0.5
1.0
1.5
2.0
2.5
0.0
0.2
0.4
0.6
0.8
1.0
Peak VAF dens= 0.48
N = 665   Bandwidth = 0.03759

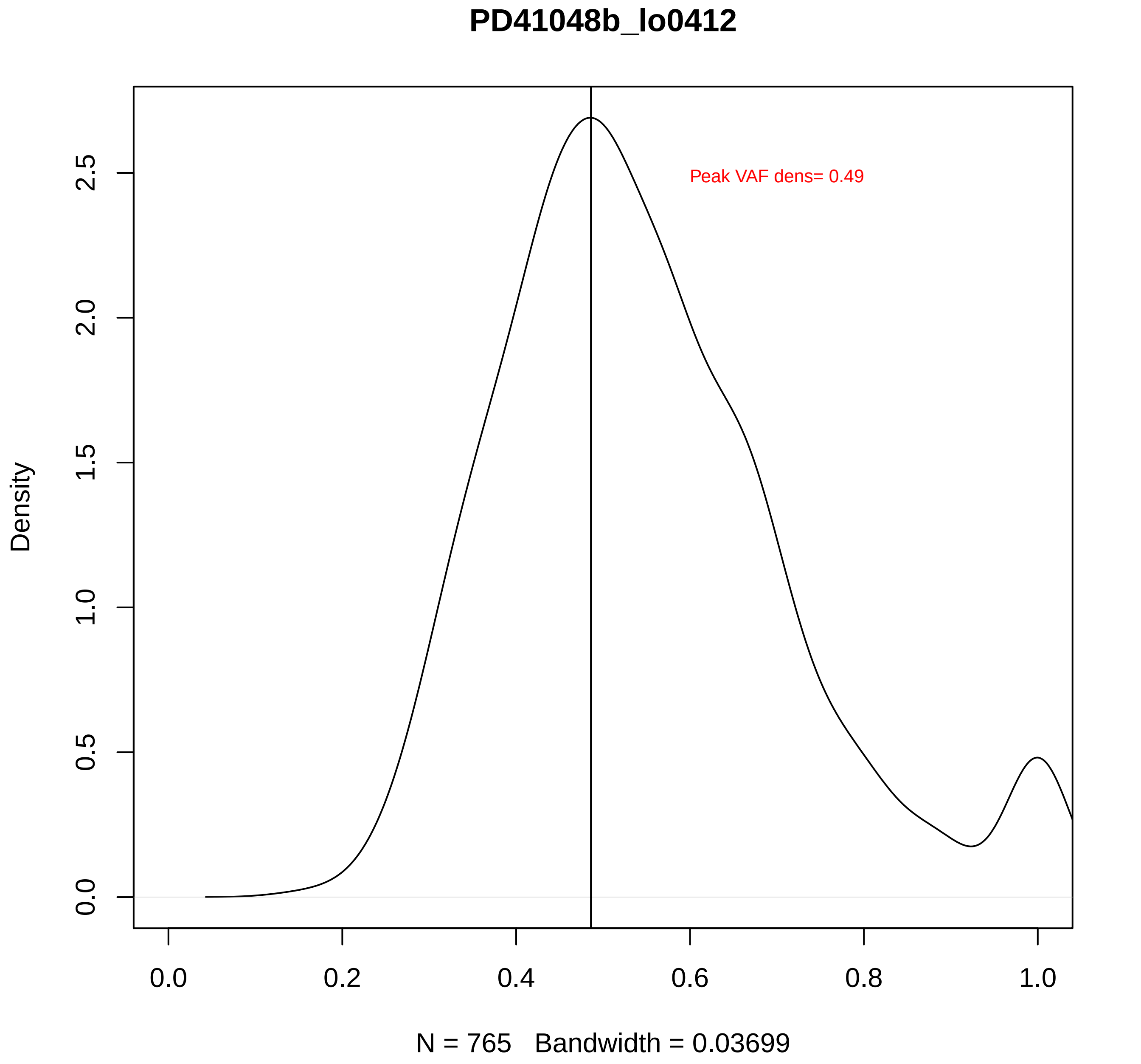
PD41048b_lo0412
Peak VAF dens= 0.49
Density
0.0
0.5
1.0
1.5
2.0
2.5
0.0
0.2
0.4
0.6
0.8
1.0
N = 765   Bandwidth = 0.03699

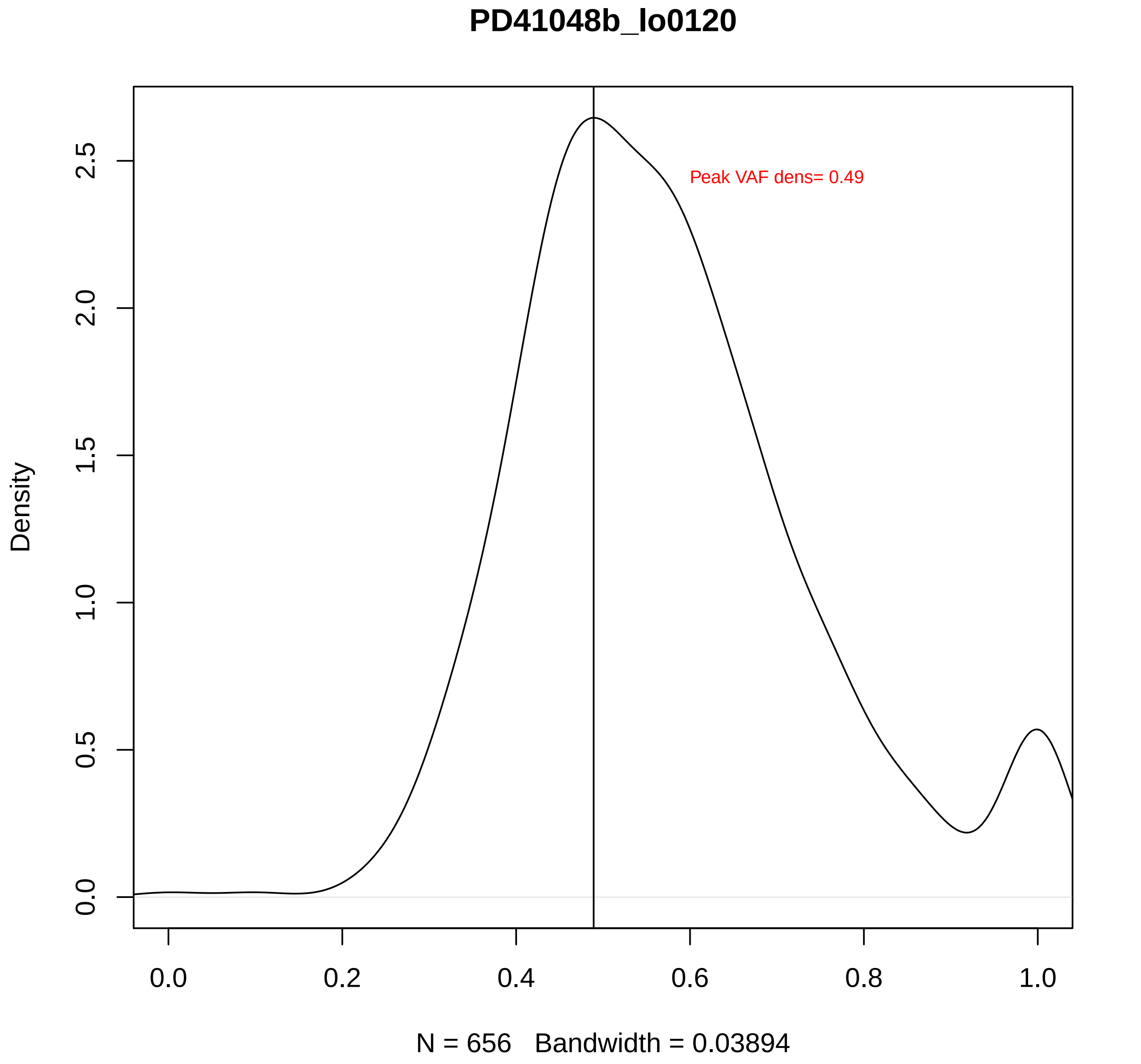
PD41048b_lo0120
Peak VAF dens= 0.49
Density
0.0
0.5
1.0
1.5
2.0
2.5
0.0
0.2
0.4
0.6
0.8
1.0
N = 656 Bandwidth = 0.03894

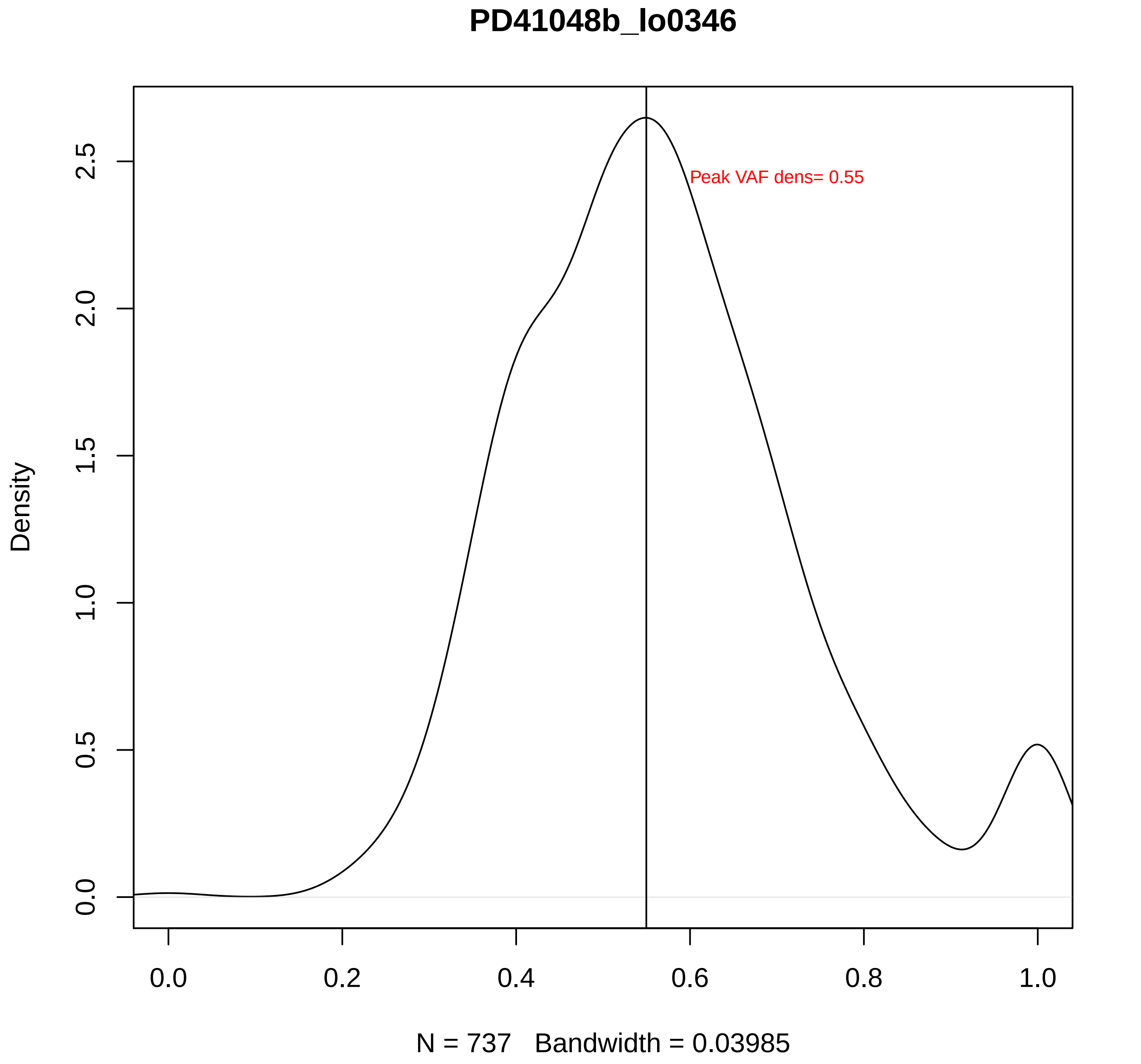
PD41048b_lo0346
Peak VAF dens= 0.55
Density
0.0
0.5
1.0
1.5
2.0
2.5
0.0
0.2
0.4
0.6
0.8
1.0
N = 737   Bandwidth = 0.03985

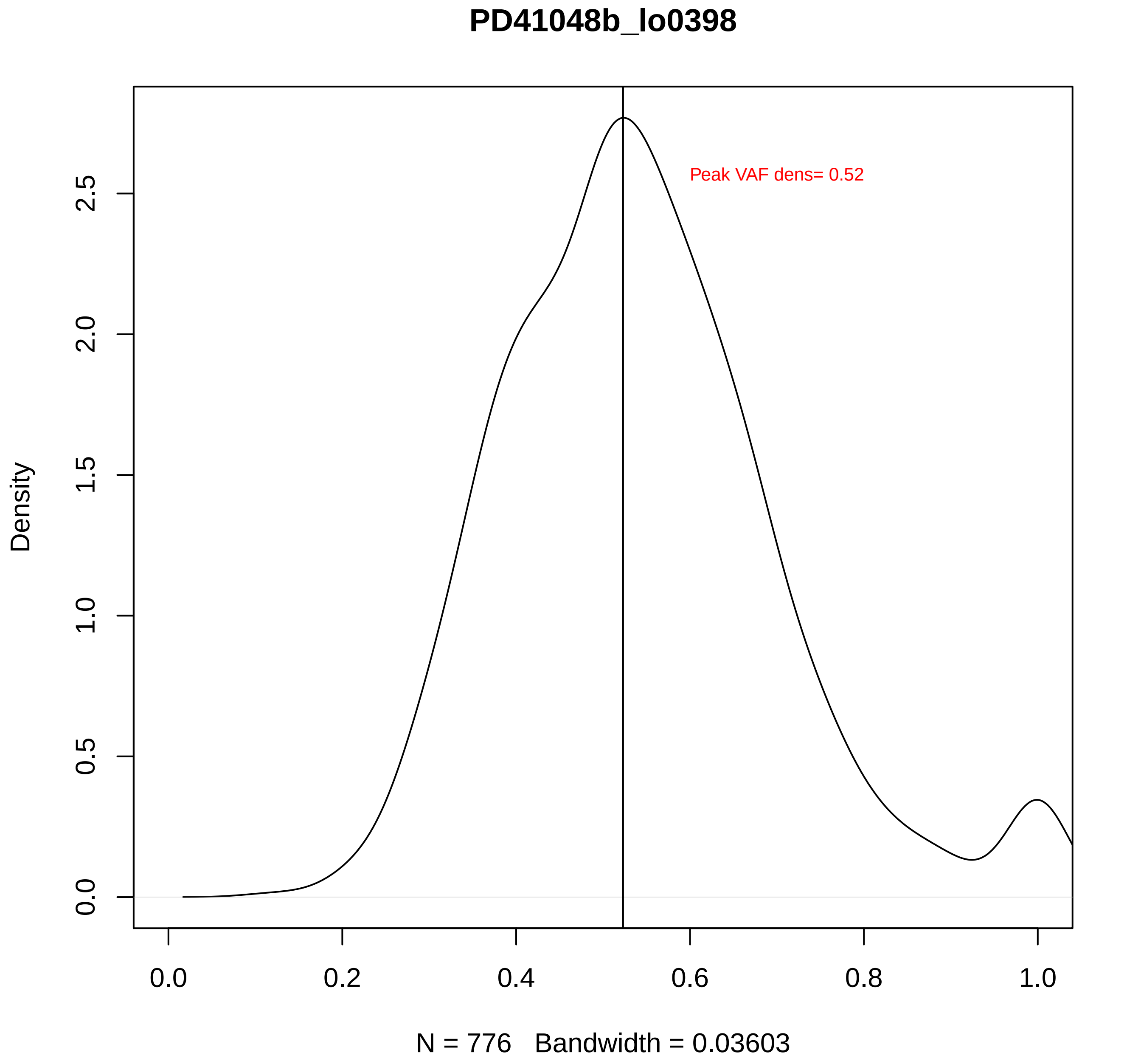
PD41048b_lo0398
Peak VAF dens= 0.52
Density
0.0
0.5
1.0
1.5
2.0
2.5
0.0
0.2
0.4
0.6
0.8
1.0
N = 776   Bandwidth = 0.03603

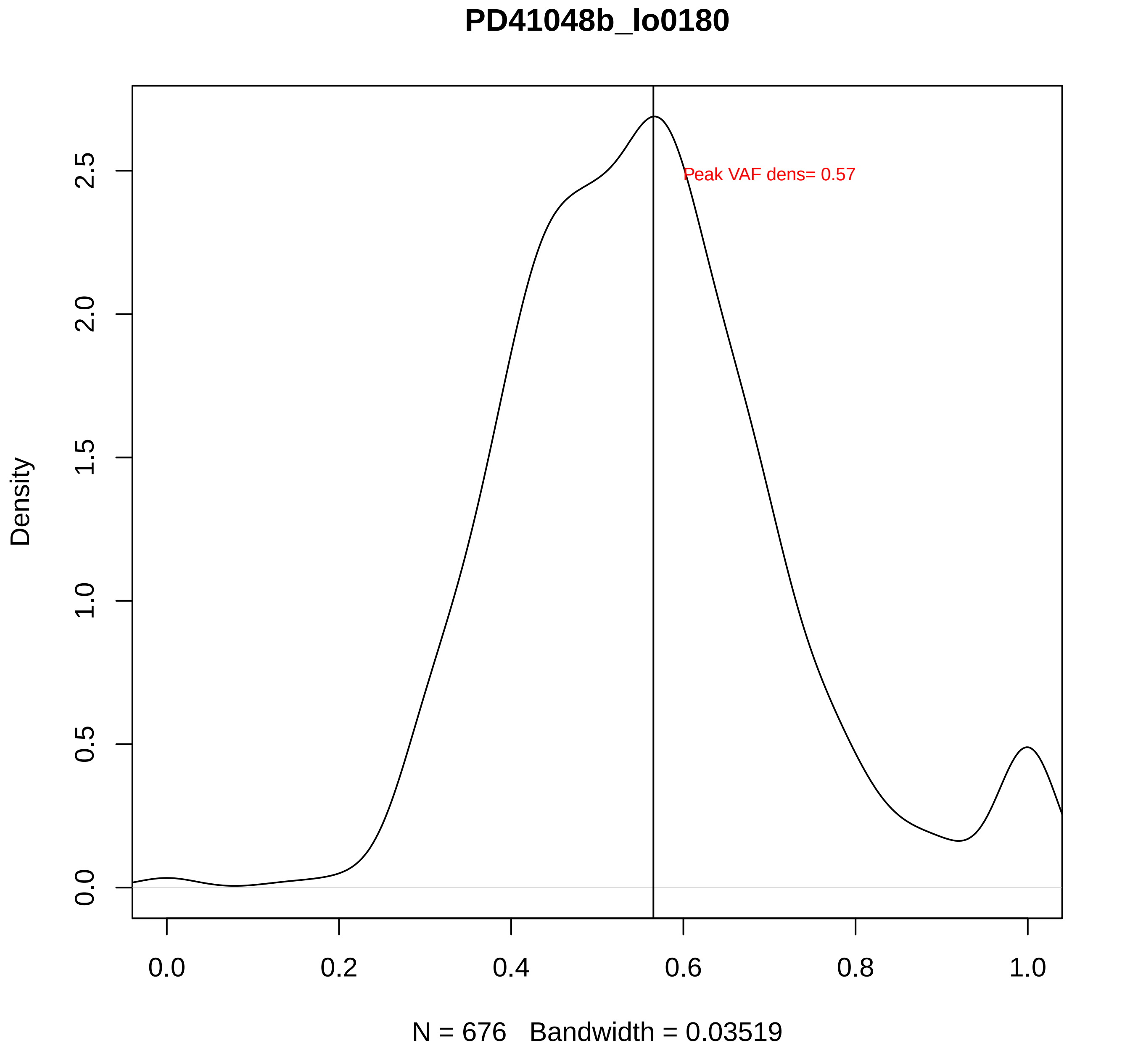

PD41048b_lo0180
Peak VAF dens= 0.57
Density
0.0
0.5
1.0
1.5
2.0
2.5
0.0
0.2
0.4
0.6
0.8
1.0
N = 676   Bandwidth = 0.03519

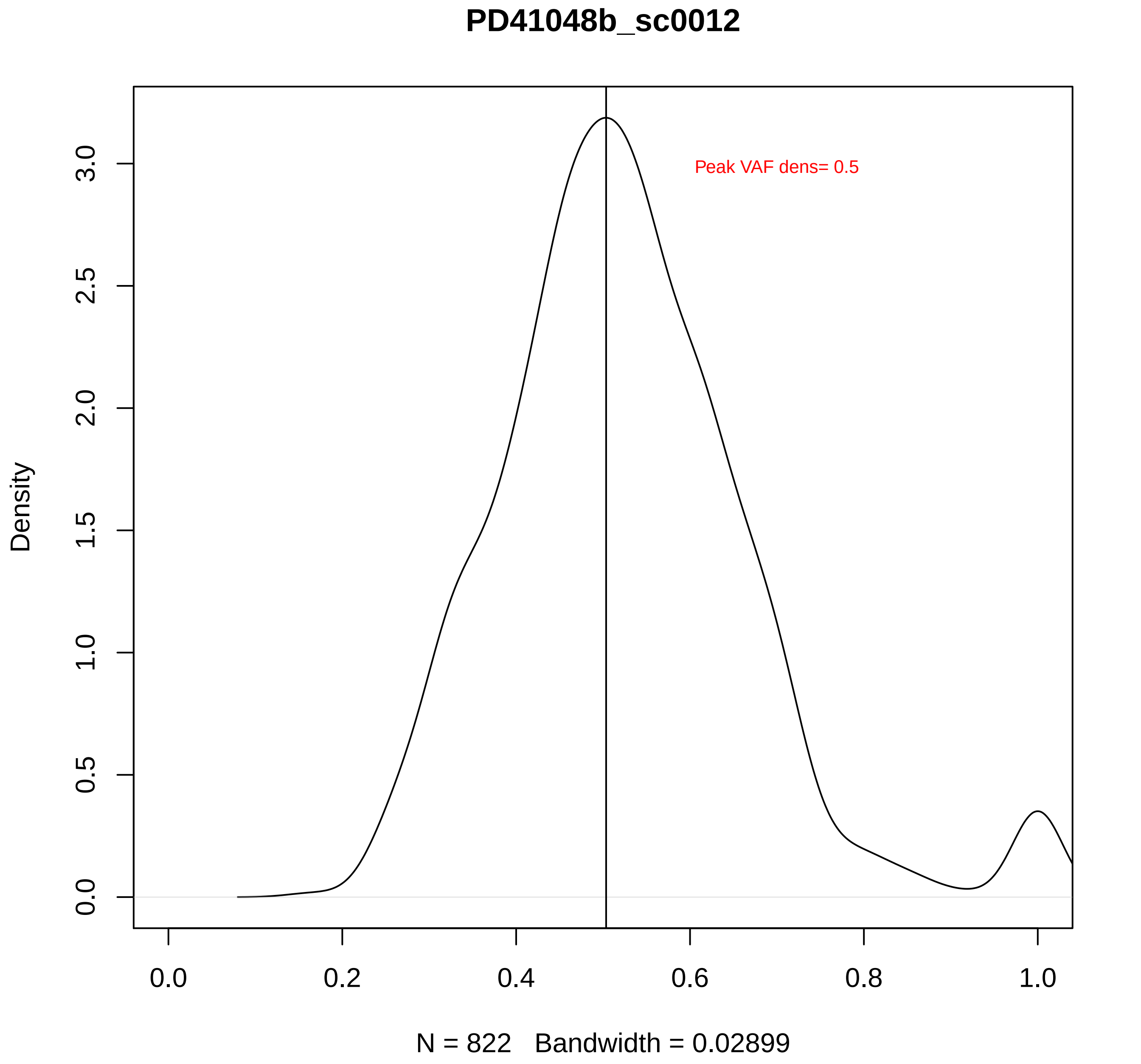
PD41048b_sc0012
Peak VAF dens= 0.5
Density
0.0
0.5
1.0
1.5
2.0
2.5
3.0
0.0
0.2
0.4
0.6
0.8
1.0
N = 822   Bandwidth = 0.02899

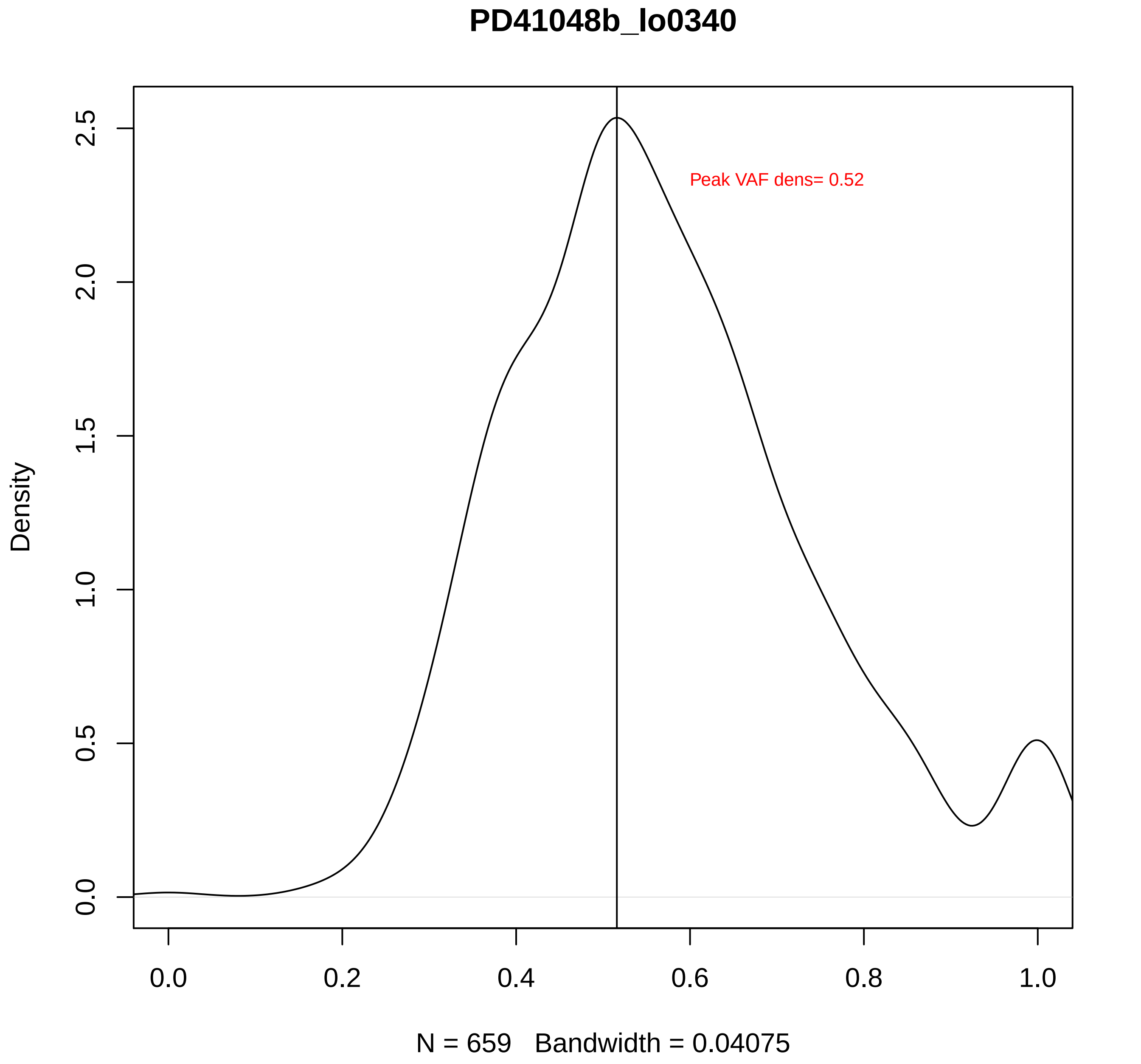
PD41048b_lo0340
Peak VAF dens= 0.52
Density
0.0
0.5
1.0
1.5
2.0
2.5
0.0
0.2
0.4
0.6
0.8
1.0
N = 659 Bandwidth = 0.04075

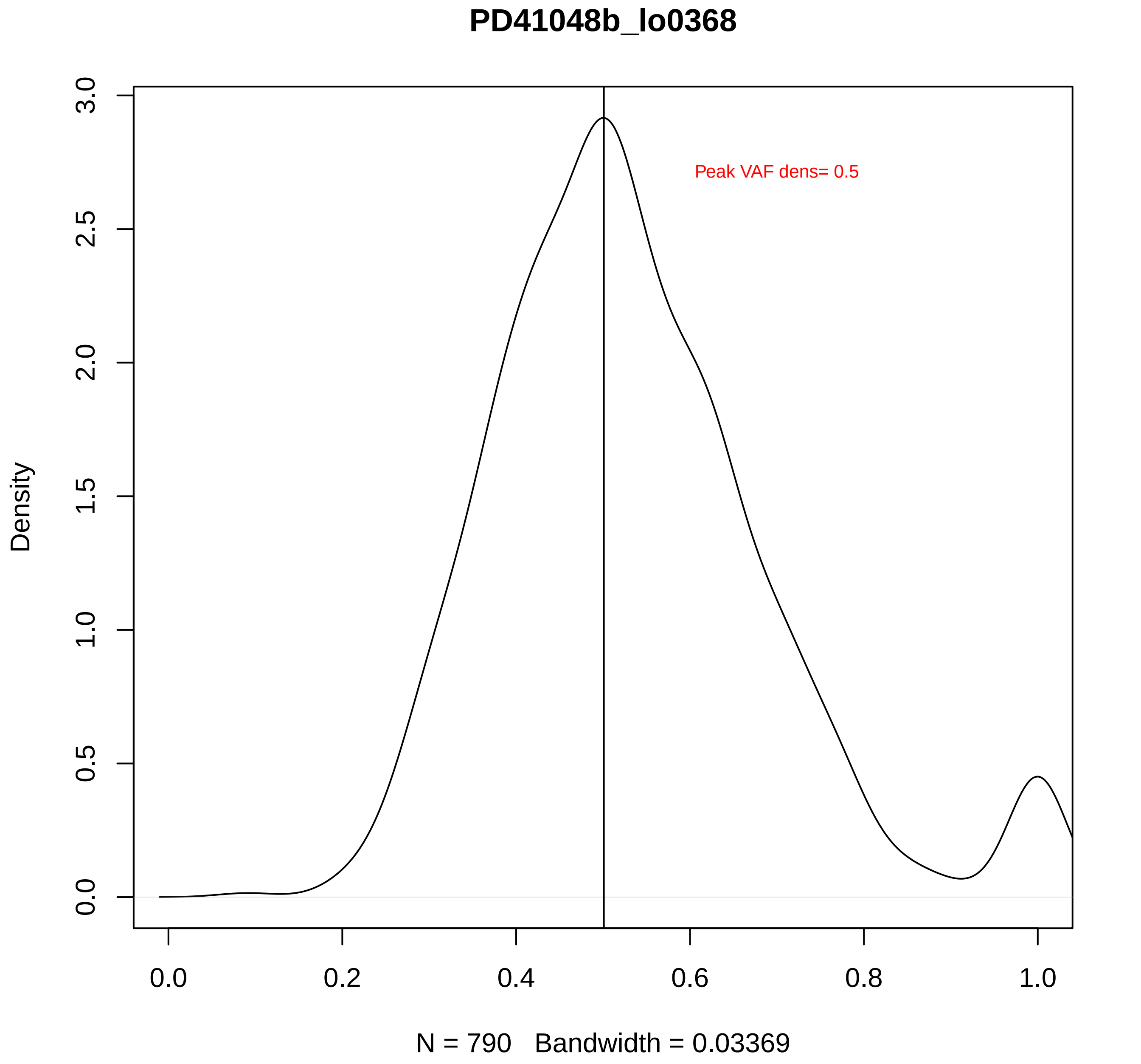
PD41048b_lo0368
Peak VAF dens= 0.5
Density
0.0
0.5
1.0
1.5
2.0
2.5
3.0
0.0
0.2
0.4
0.6
0.8
1.0
N = 790   Bandwidth = 0.03369

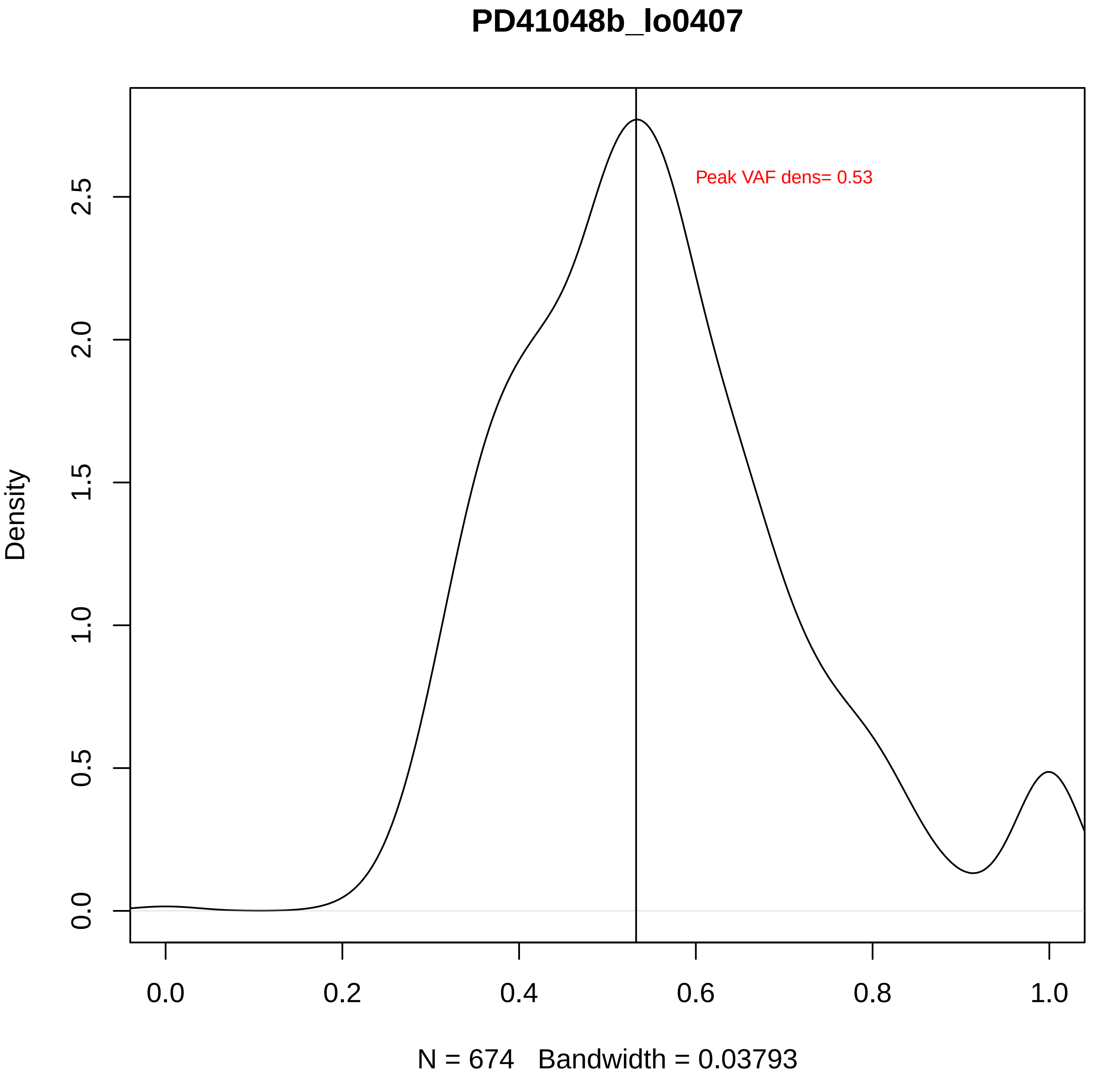
PD41048b_lo0407
Peak VAF dens= 0.53
Density
0.0
0.5
1.0
1.5
2.0
2.5
0.0
0.2
0.4
0.6
0.8
1.0
N = 674 Bandwidth = 0.03793

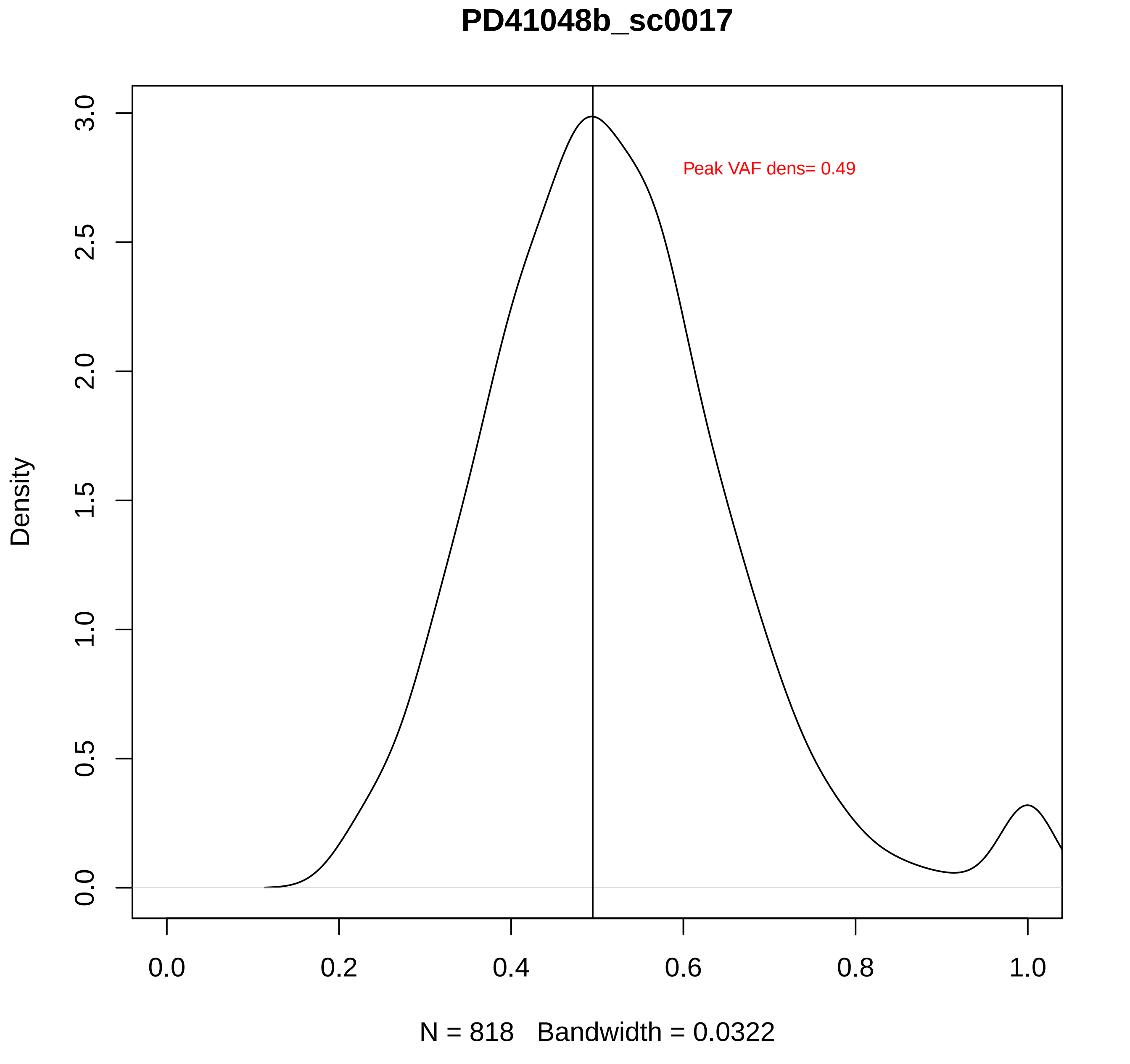

PD41048b_sc0017
Peak VAF dens= 0.49
Density
0.0
0.5
1.0
1.5
2.0
2.5
3.0
0.0
0.2
0.4
0.6
0.8
1.0
N = 818   Bandwidth = 0.0322

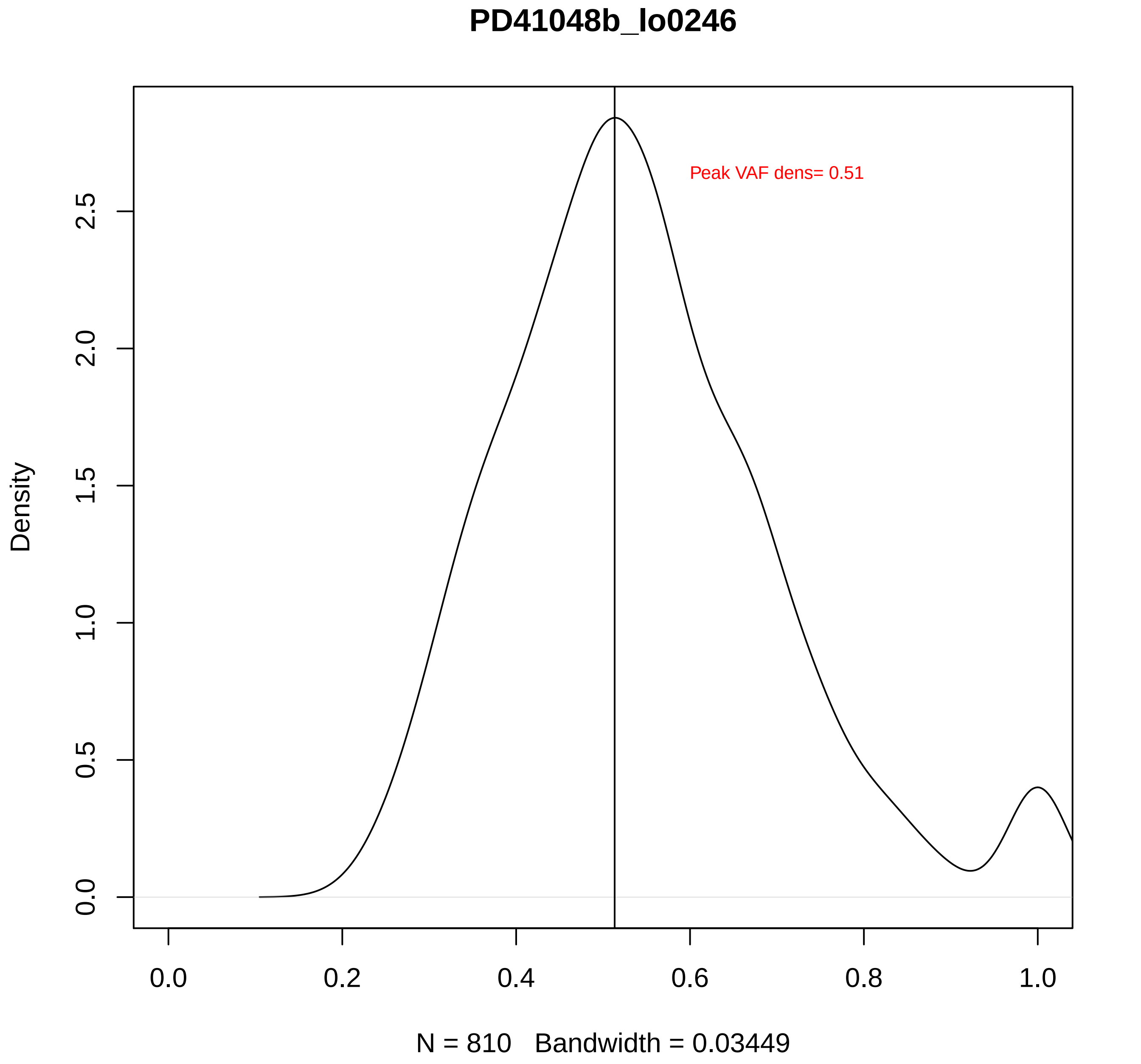
PD41048b_lo0246
Peak VAF dens= 0.51
Density
0.0
0.5
1.0
1.5
2.0
2.5
0.0
0.2
0.4
0.6
0.8
1.0
N = 810   Bandwidth = 0.03449

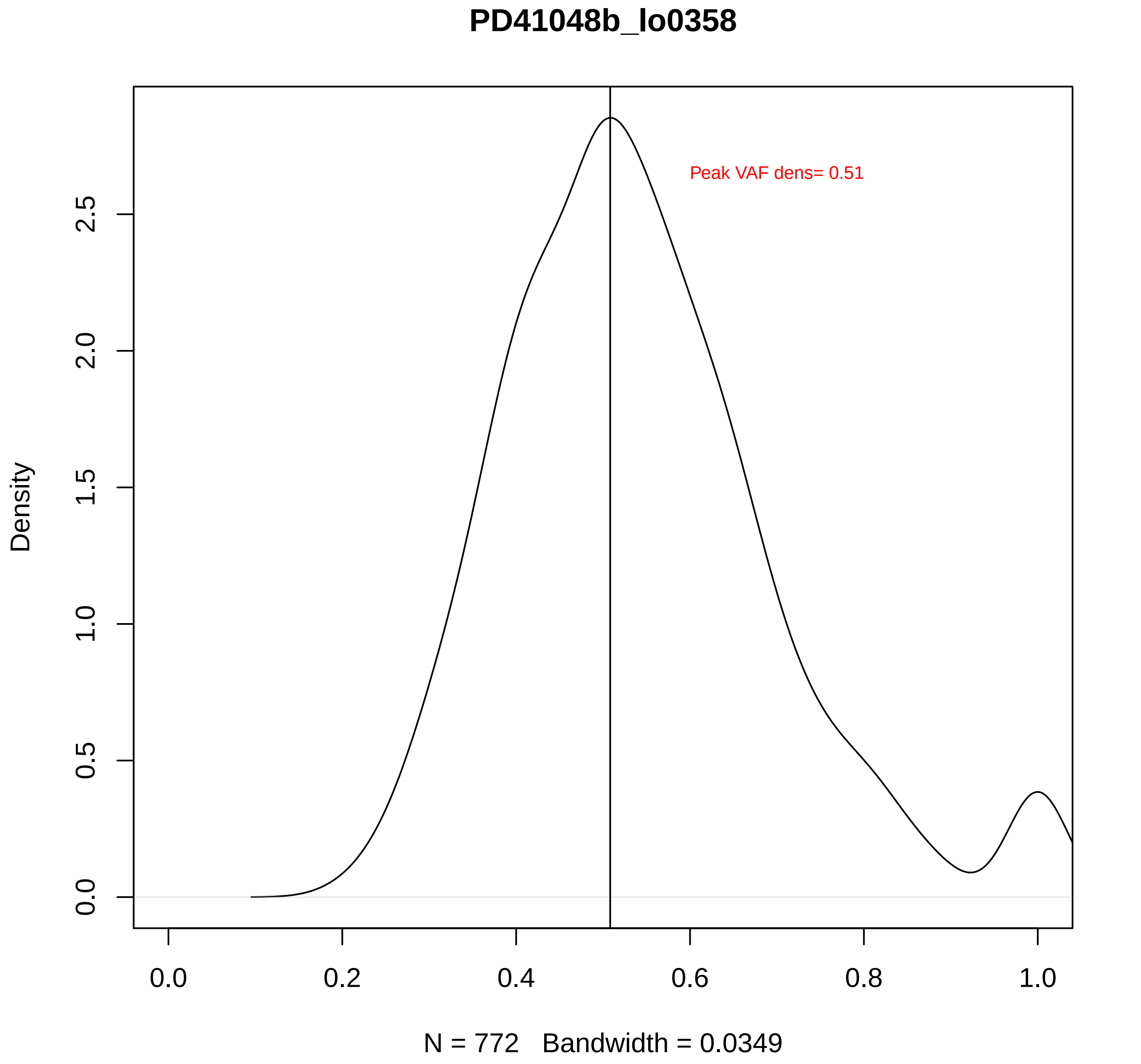
PD41048b_lo0358
Peak VAF dens= 0.51
Density
0.0
0.5
1.0
1.5
2.0
2.5
0.0
0.2
0.4
0.6
0.8
1.0
N = 772   Bandwidth = 0.0349

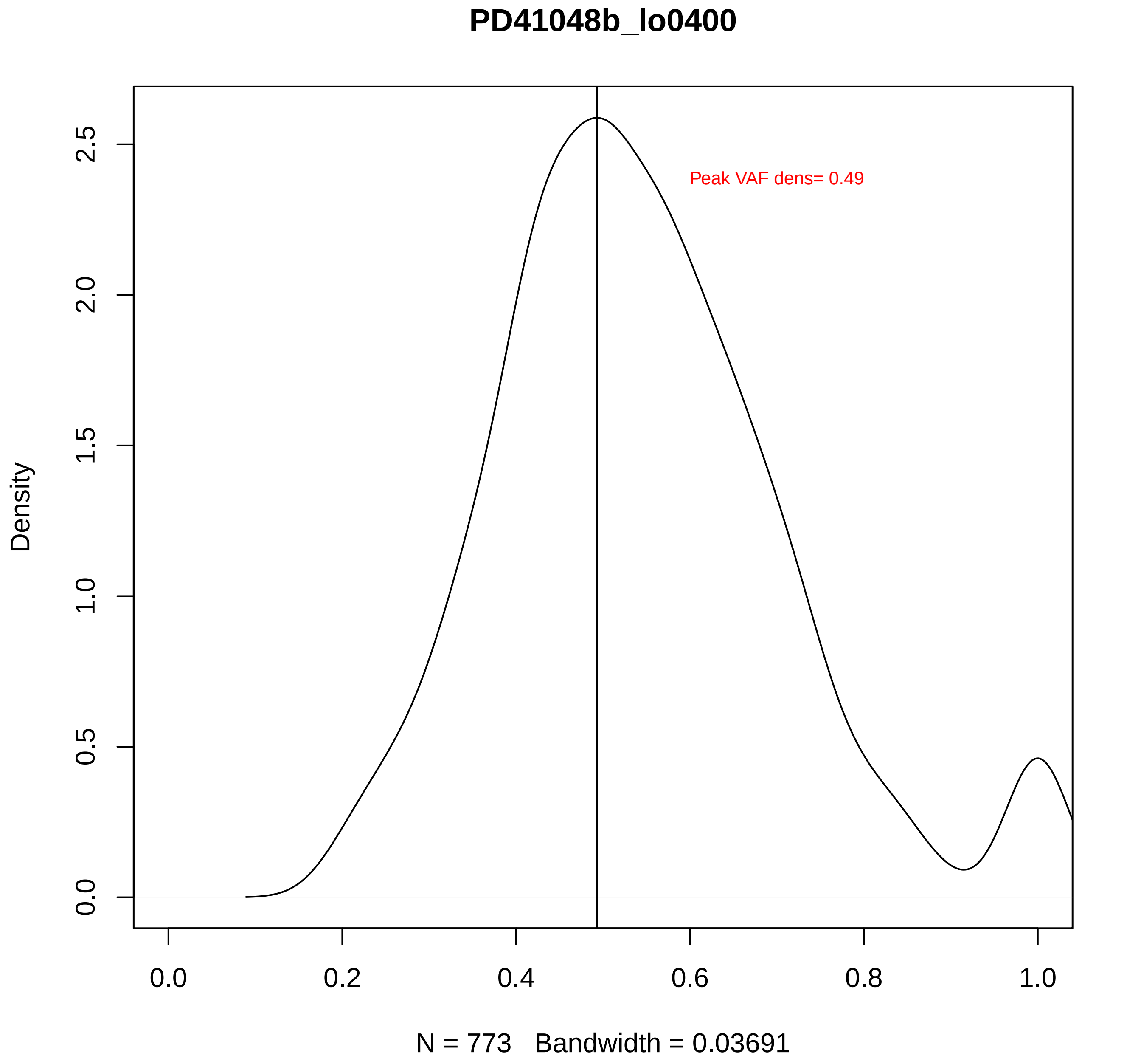

PD41048b_lo0400
Peak VAF dens= 0.49
Density
0.0
0.5
1.0
1.5
2.0
2.5
0.0
0.2
0.4
0.6
0.8
1.0
N = 773   Bandwidth = 0.03691

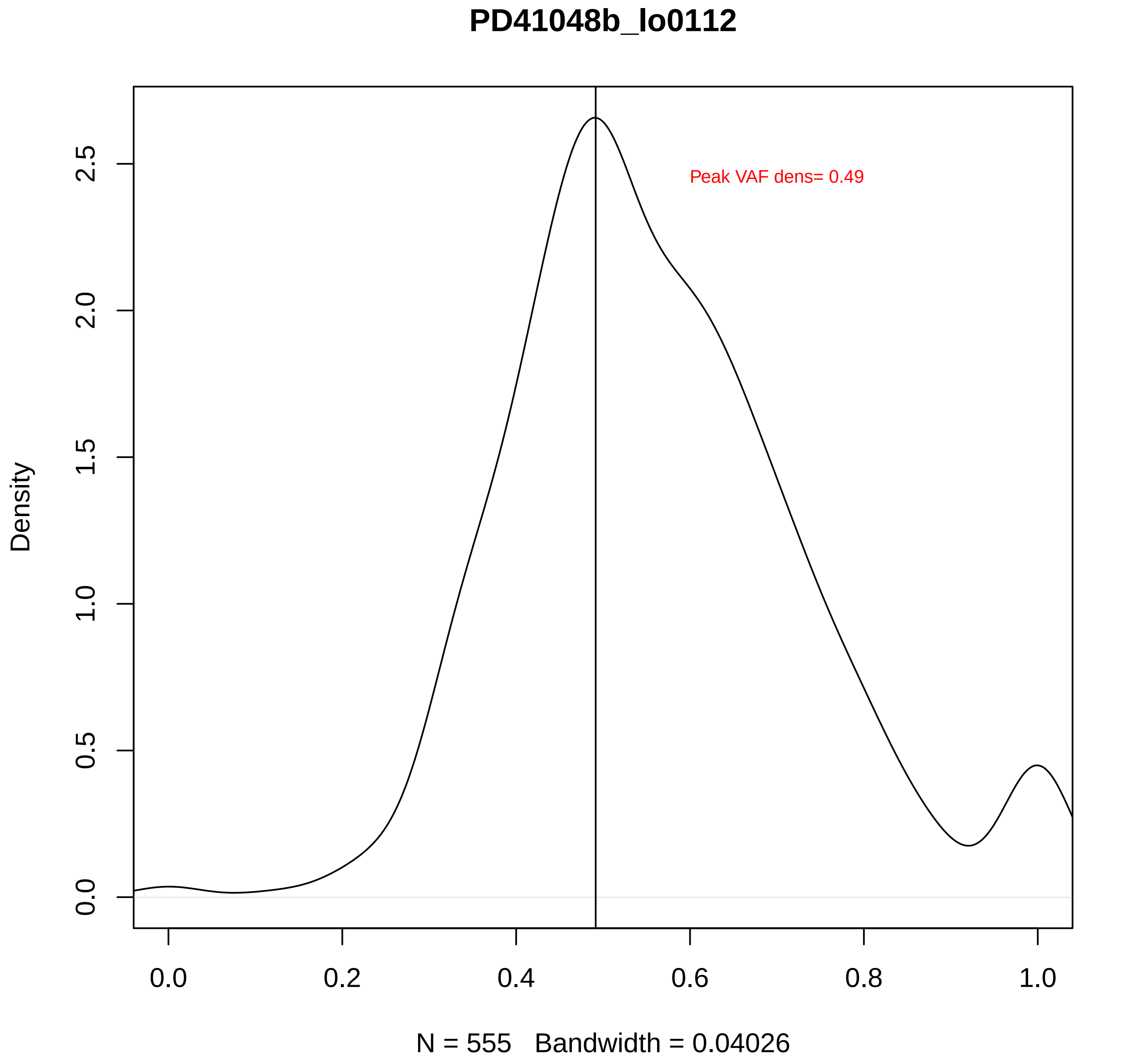
PD41048b_lo0112
Peak VAF dens= 0.49
Density
0.0
0.5
1.0
1.5
2.0
2.5
0.0
0.2
0.4
0.6
0.8
1.0
N = 555   Bandwidth = 0.04026

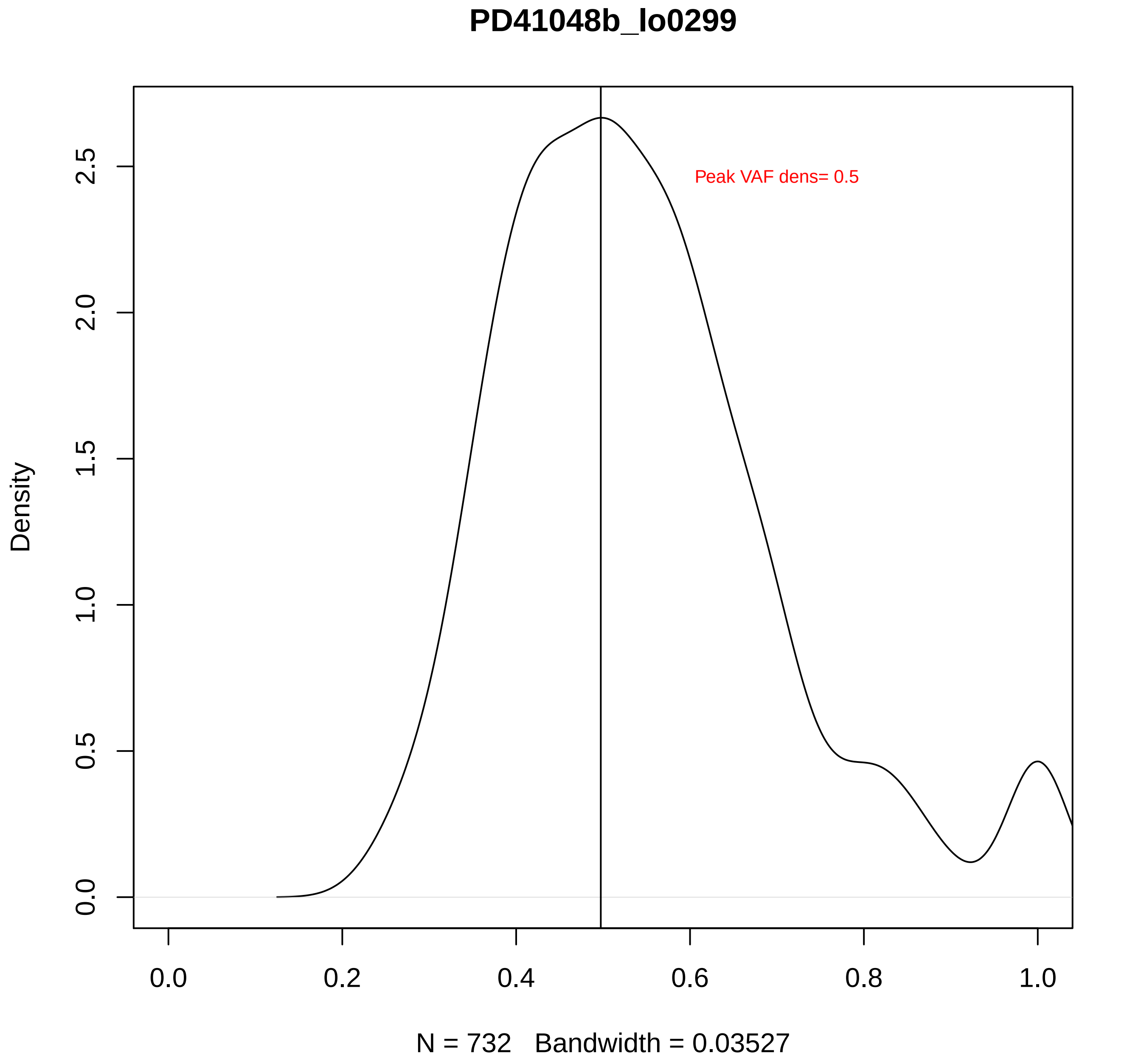

PD41048b_lo0299
Peak VAF dens= 0.5
Density
0.0
0.5
1.0
1.5
2.0
2.5
0.0
0.2
0.4
0.6
0.8
1.0
N = 732   Bandwidth = 0.03527

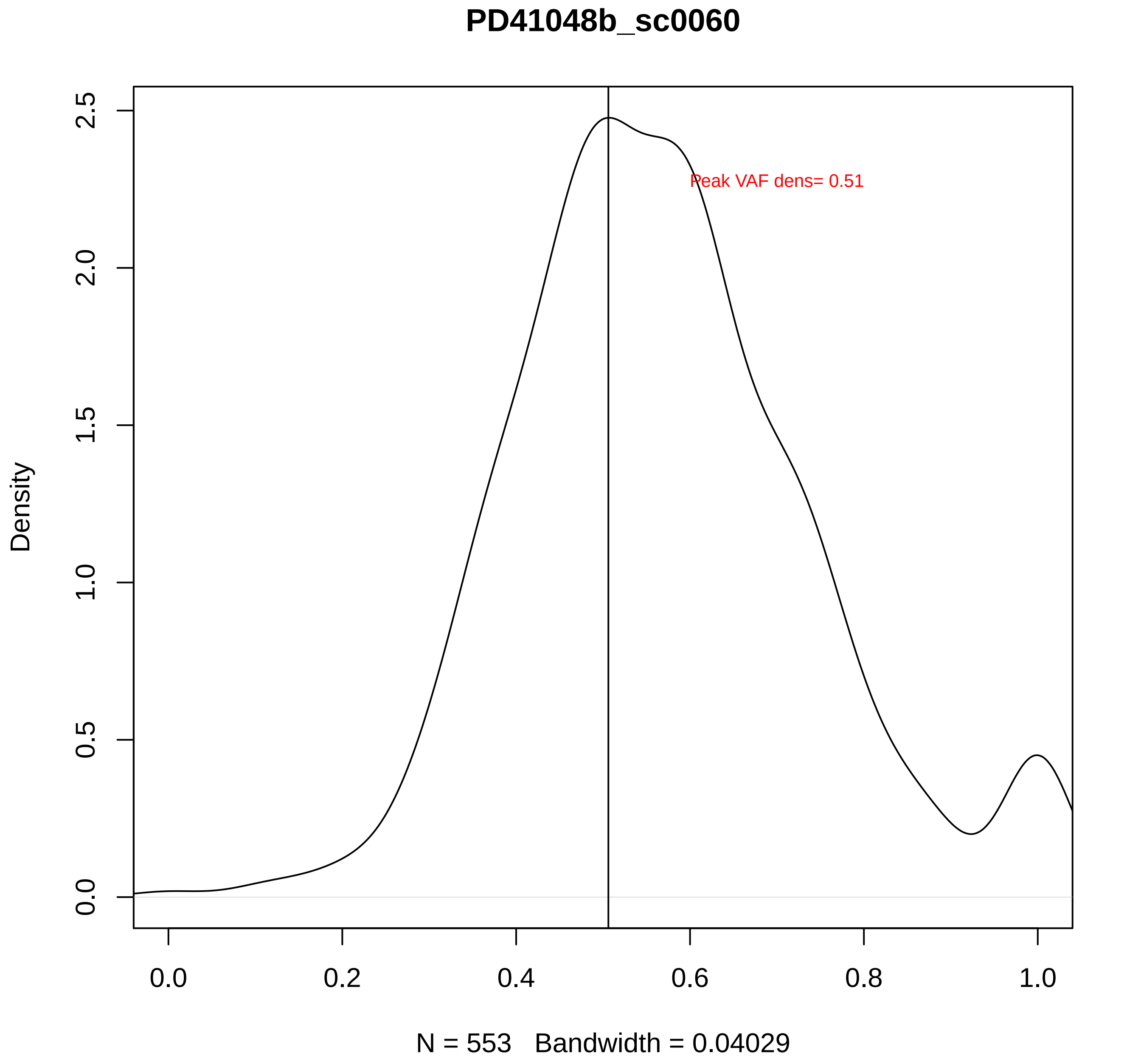
PD41048b_sc0060
Density
2.5
2.0
1.5
1.0
0.5
0.0
Peak VAF dens= 0.51
0.0
0.2
0.4
0.6
0.8
1.0
N = 553   Bandwidth = 0.04029

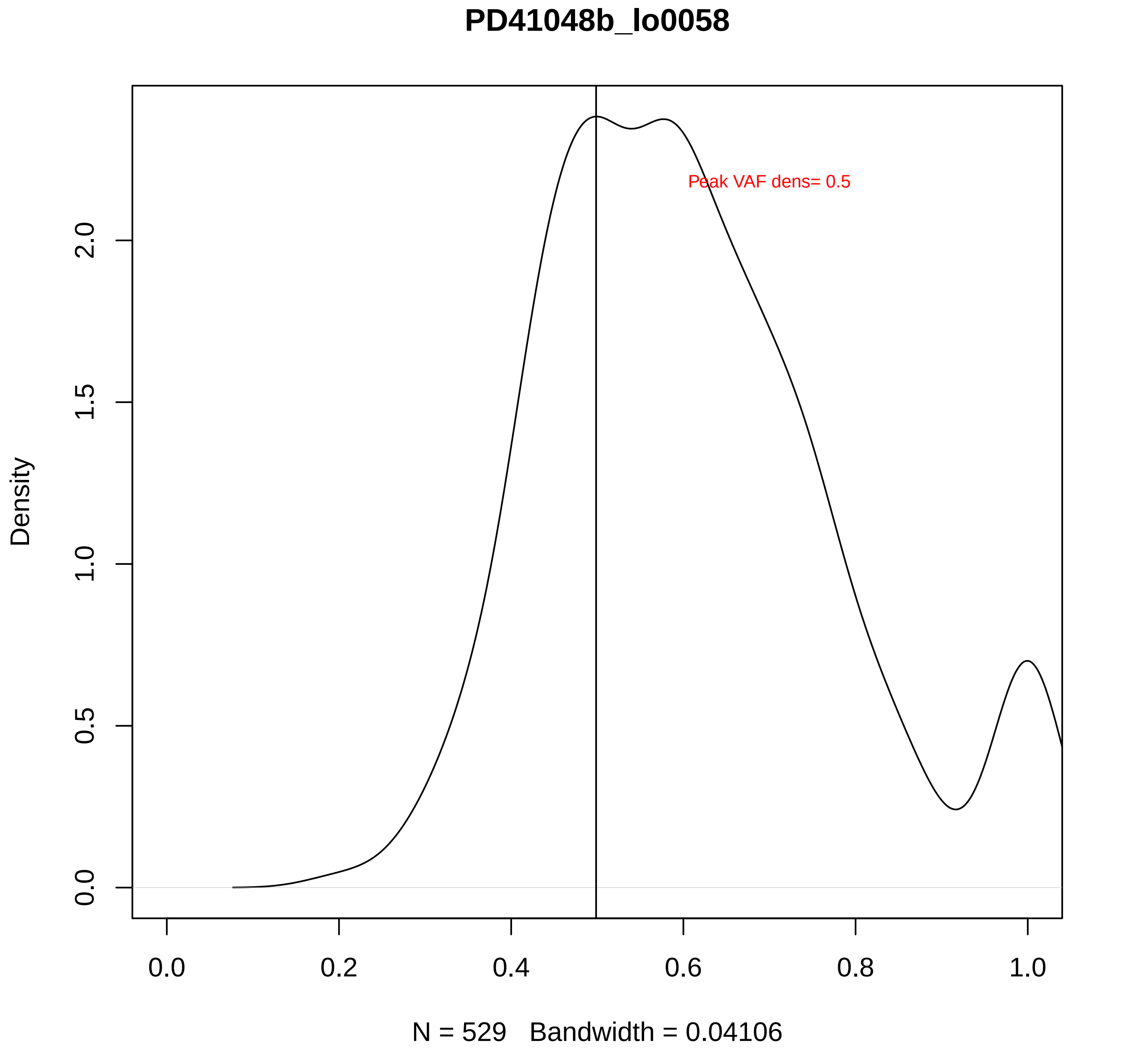
PD41048b_lo0058
Peak VAF dens= 0.5
Density
0.0
0.5
1.0
1.5
2.0
0.0
0.2
0.4
0.6
0.8
1.0
N = 529   Bandwidth = 0.04106

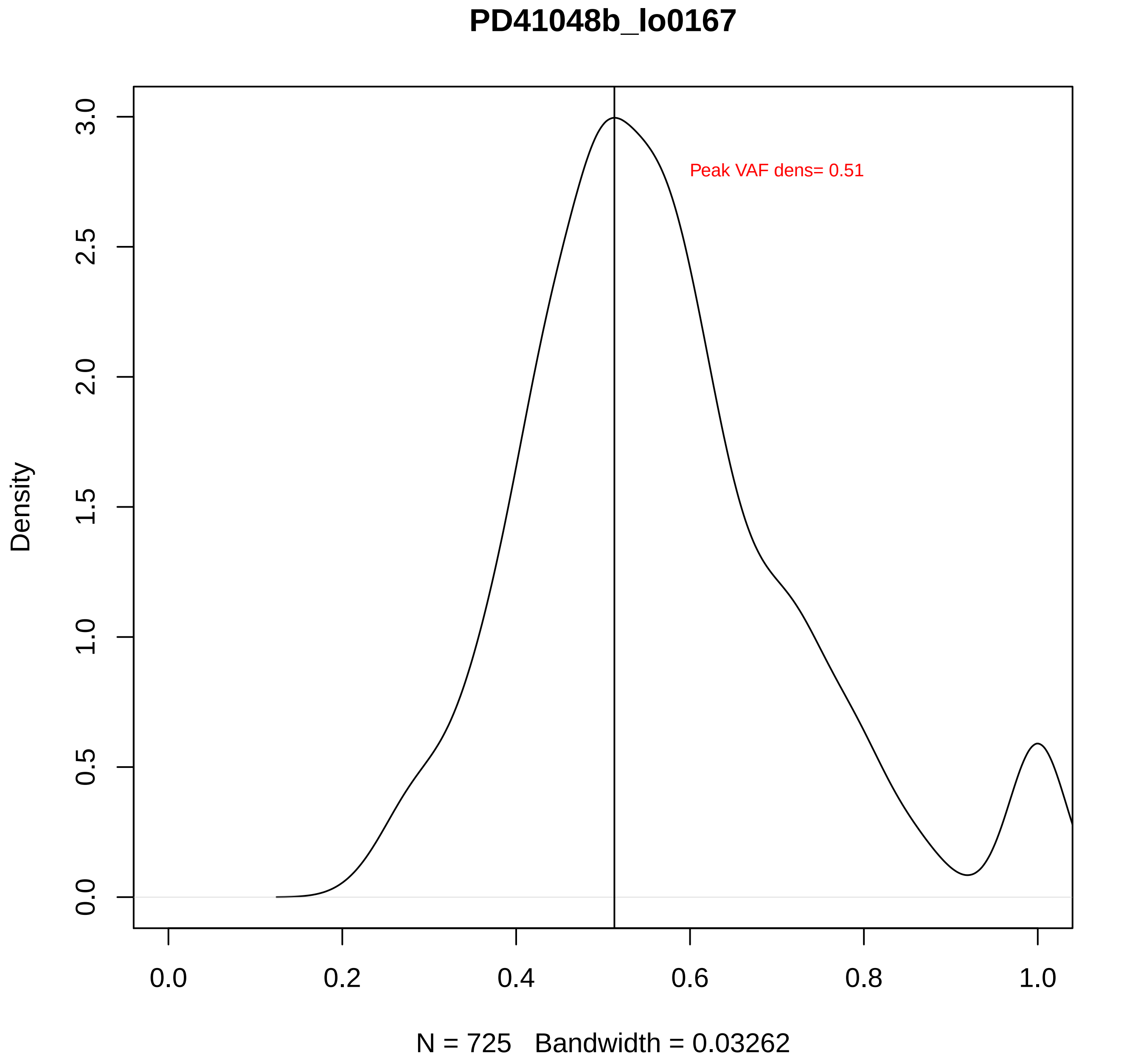

PD41048b_lo0167
Peak VAF dens= 0.51
Density
0.0
0.5
1.0
1.5
2.0
2.5
3.0
0.0
0.2
0.4
0.6
0.8
1.0
N = 725 Bandwidth = 0.03262

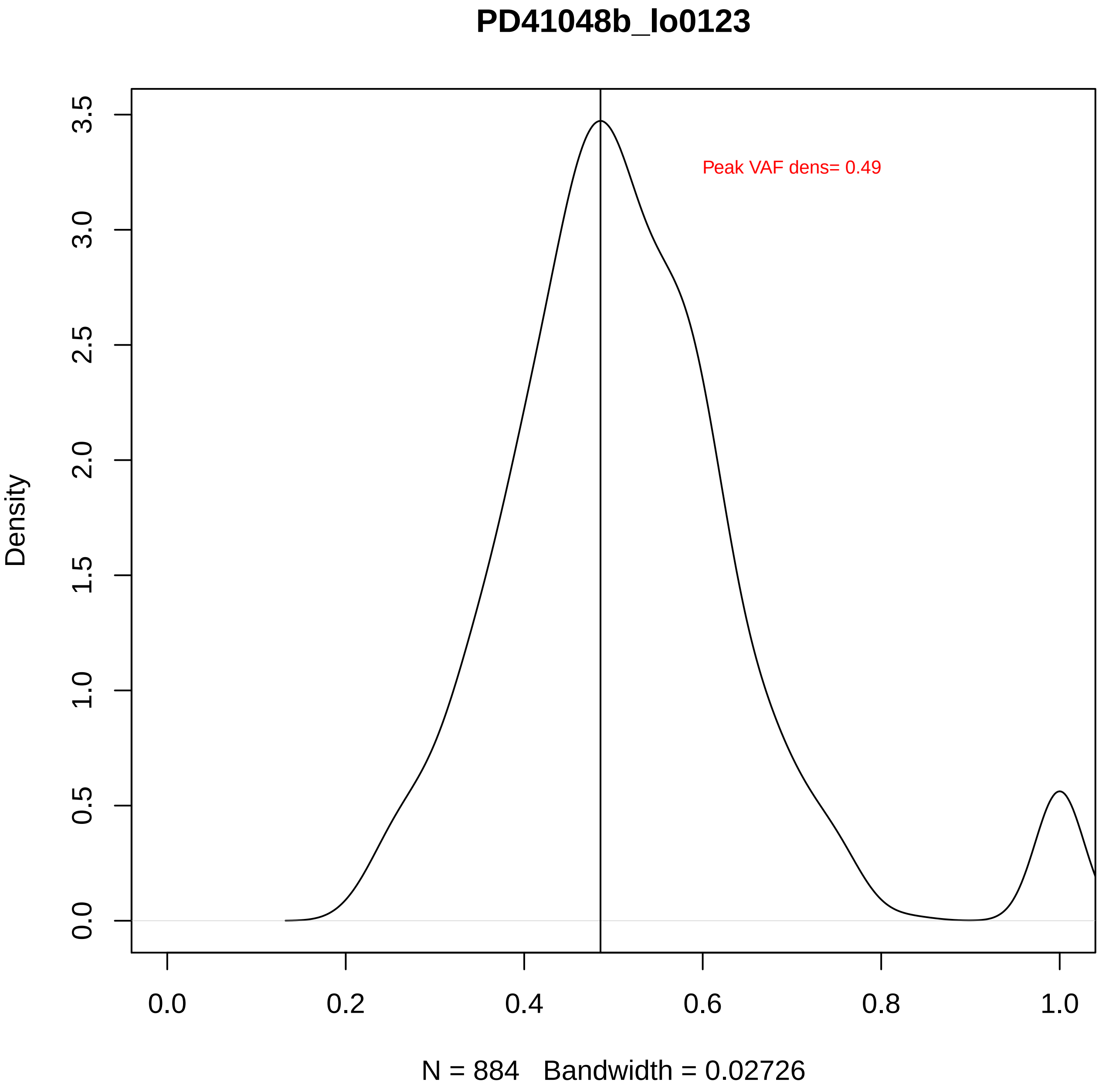
PD41048b_lo0123
Peak VAF dens= 0.49
Density
0.0
0.5
1.0
1.5
2.0
2.5
3.0
3.5
0.0
0.2
0.4
0.6
0.8
1.0
N = 884   Bandwidth = 0.02726

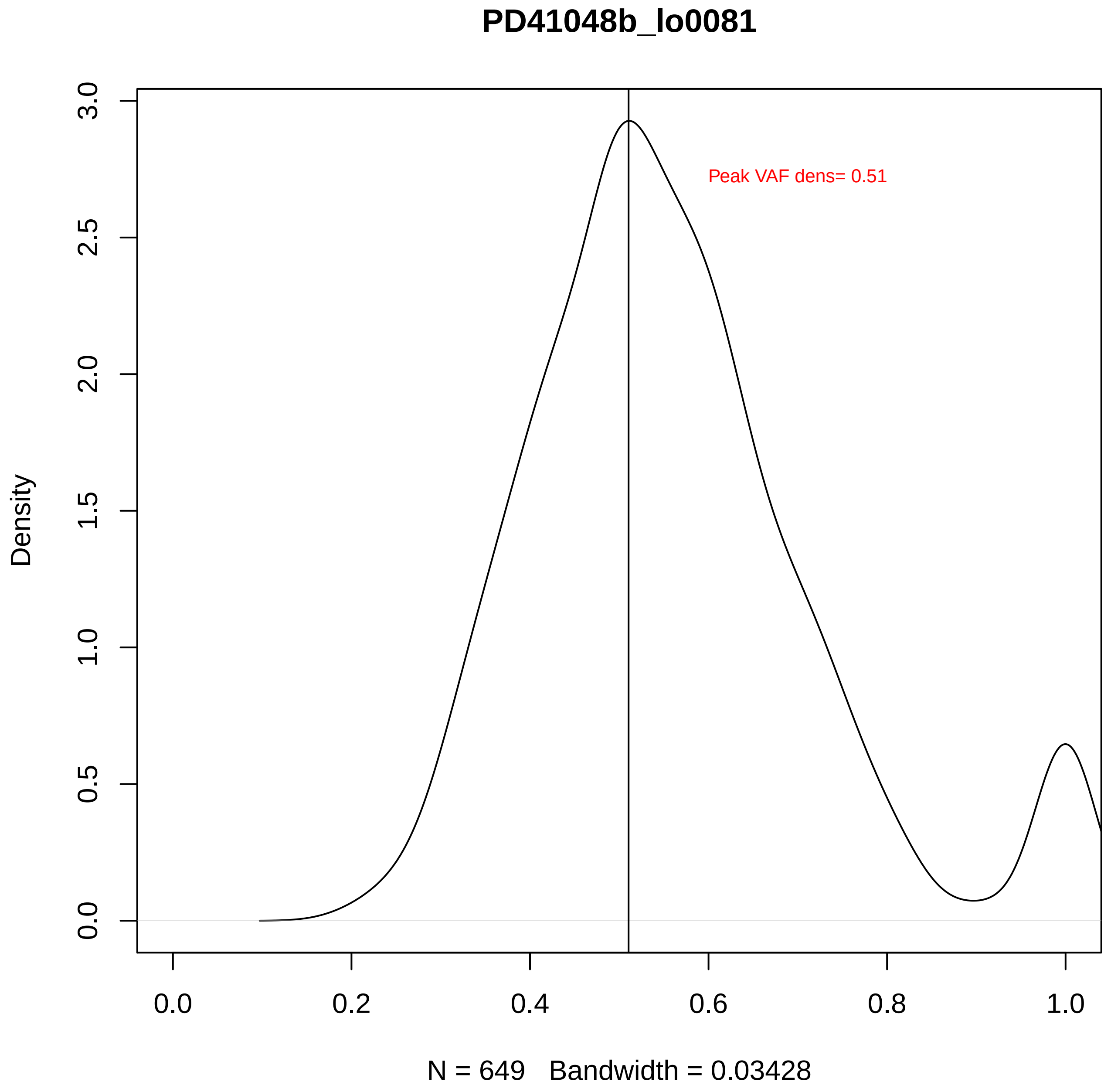
PD41048b_lo0081
Peak VAF dens= 0.51
Density
0.0
0.5
1.0
1.5
2.0
2.5
3.0
0.0
0.2
0.4
0.6
0.8
1.0
N = 649   Bandwidth = 0.03428

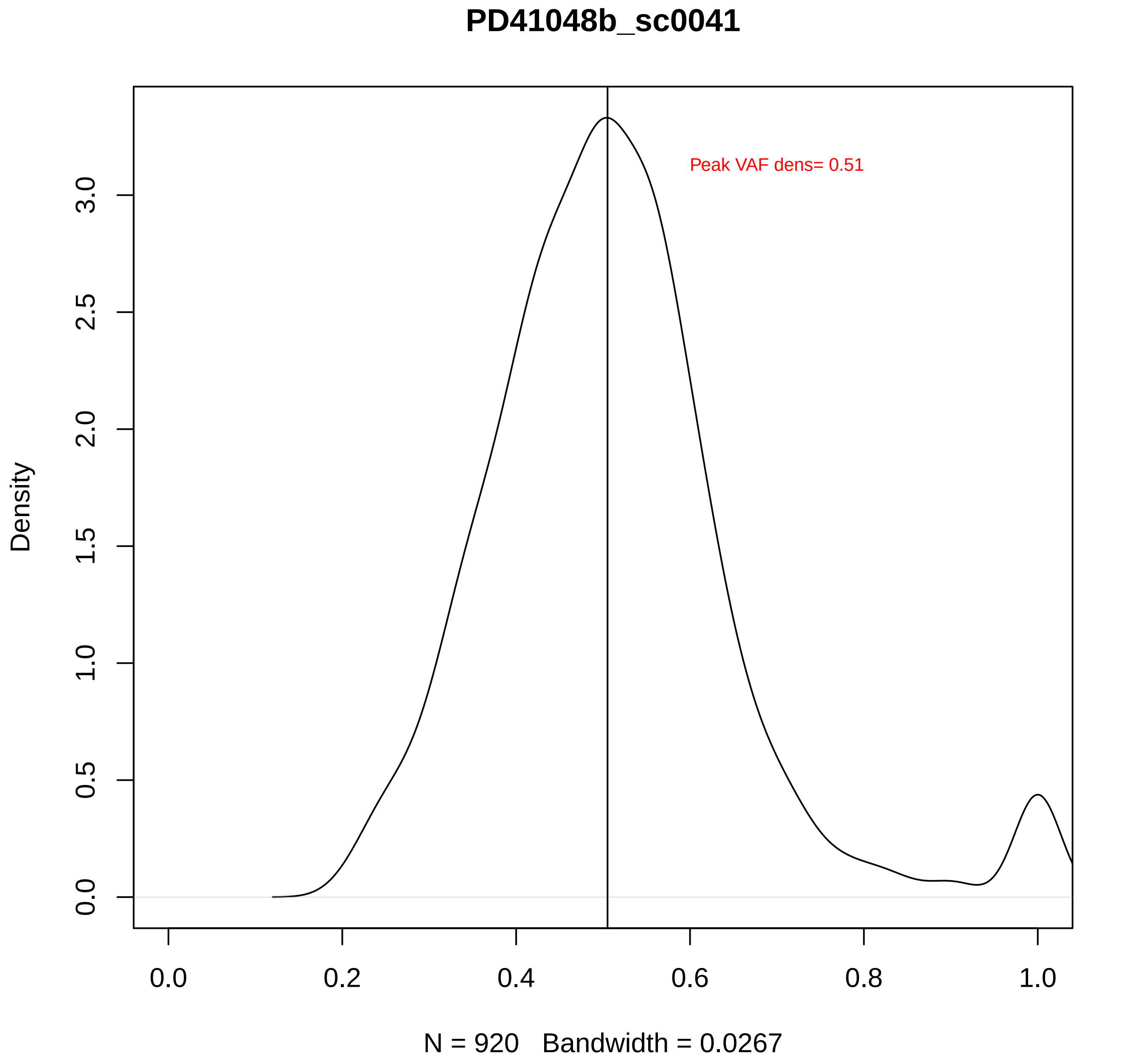

PD41048b_sc0041
Peak VAF dens= 0.51
Density
0.0
0.5
1.0
1.5
2.0
2.5
3.0
0.0
0.2
0.4
0.6
0.8
1.0
N = 920 Bandwidth = 0.0267

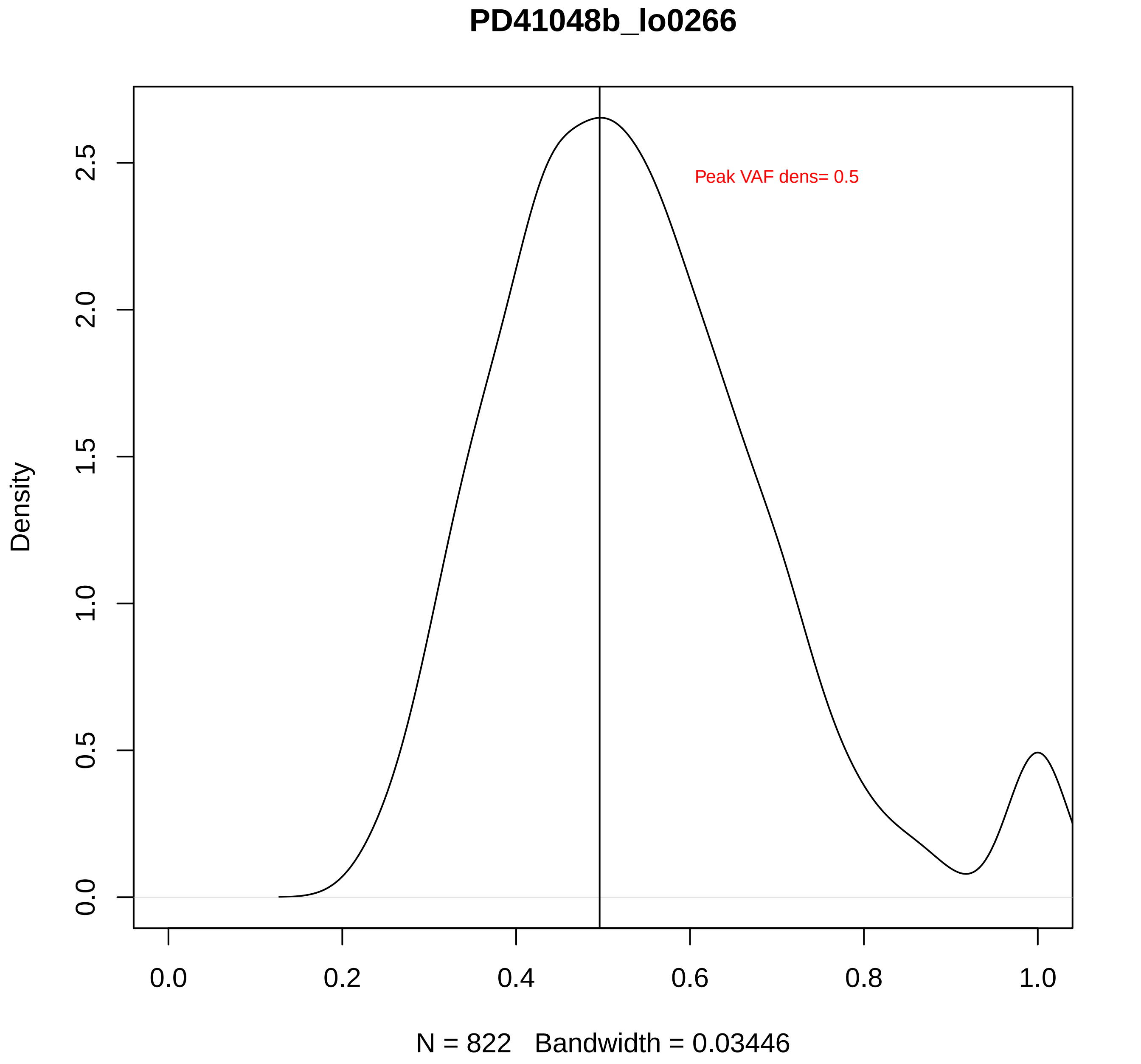
PD41048b_lo0266
Peak VAF dens= 0.5
Density
0.0
0.5
1.0
1.5
2.0
2.5
0.0
0.2
0.4
0.6
0.8
1.0
N = 822 Bandwidth = 0.03446

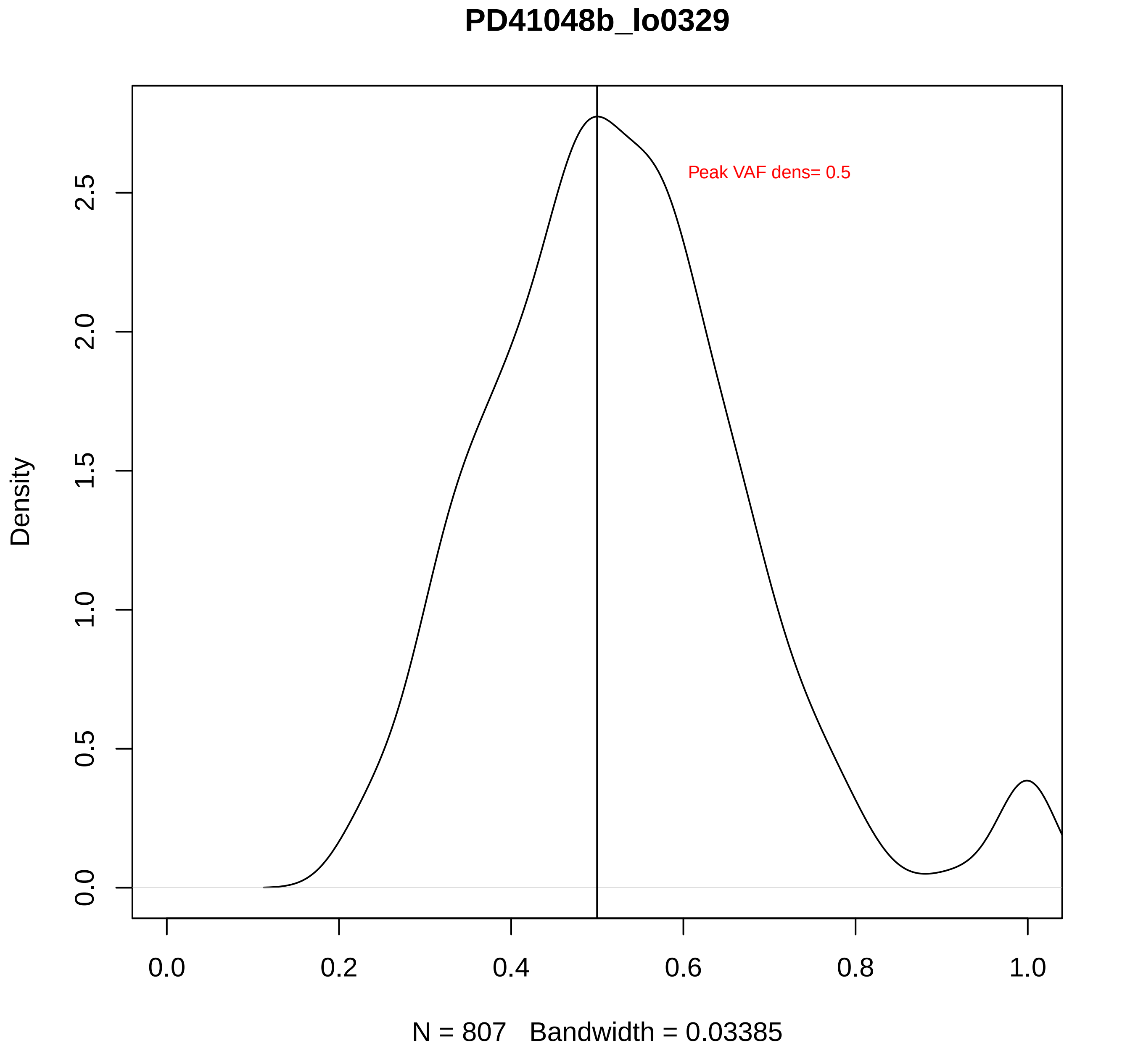
PD41048b_lo0329
Peak VAF dens= 0.5
Density
0.0
0.5
1.0
1.5
2.0
2.5
0.0
0.2
0.4
0.6
0.8
1.0
N = 807 Bandwidth = 0.03385

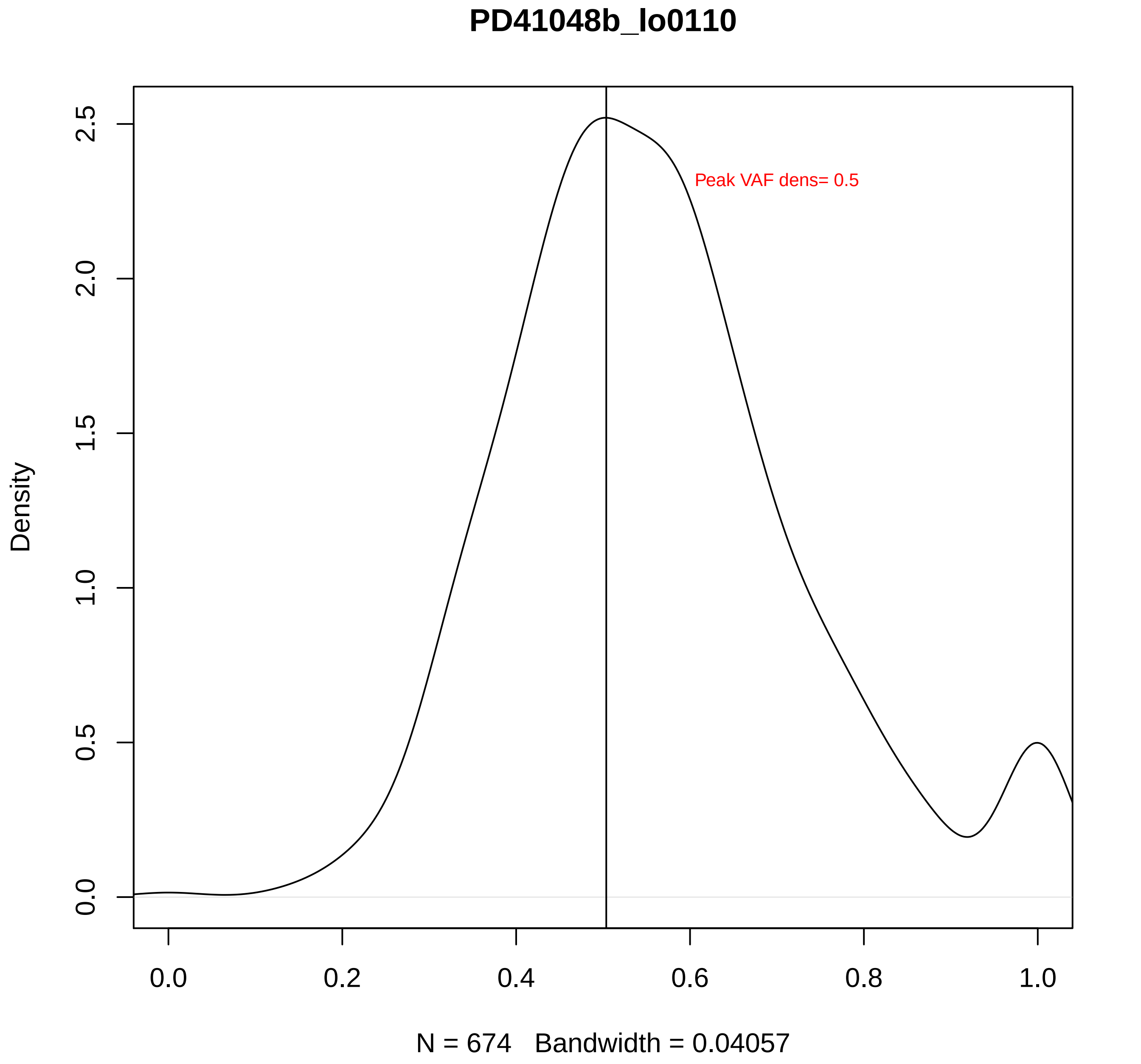
PD41048b_lo0110
Peak VAF dens= 0.5
Density
0.0
0.5
1.0
1.5
2.0
2.5
0.0
0.2
0.4
0.6
0.8
1.0
N = 674   Bandwidth = 0.04057

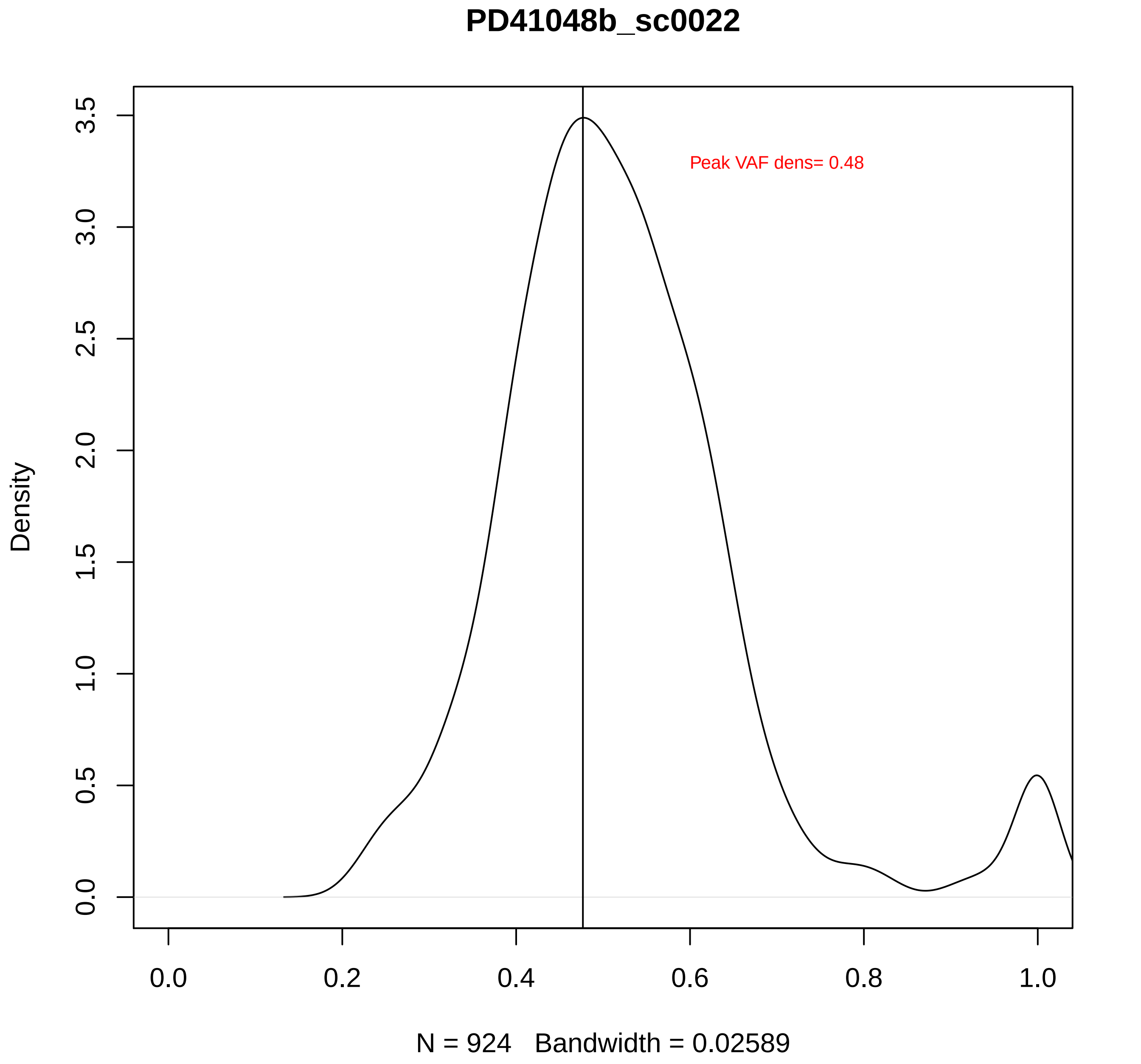

PD41048b_sc0022
Peak VAF dens= 0.48
Density
0.0
0.5
1.0
1.5
2.0
2.5
3.0
3.5
0.0
0.2
0.4
0.6
0.8
1.0
N = 924   Bandwidth = 0.02589

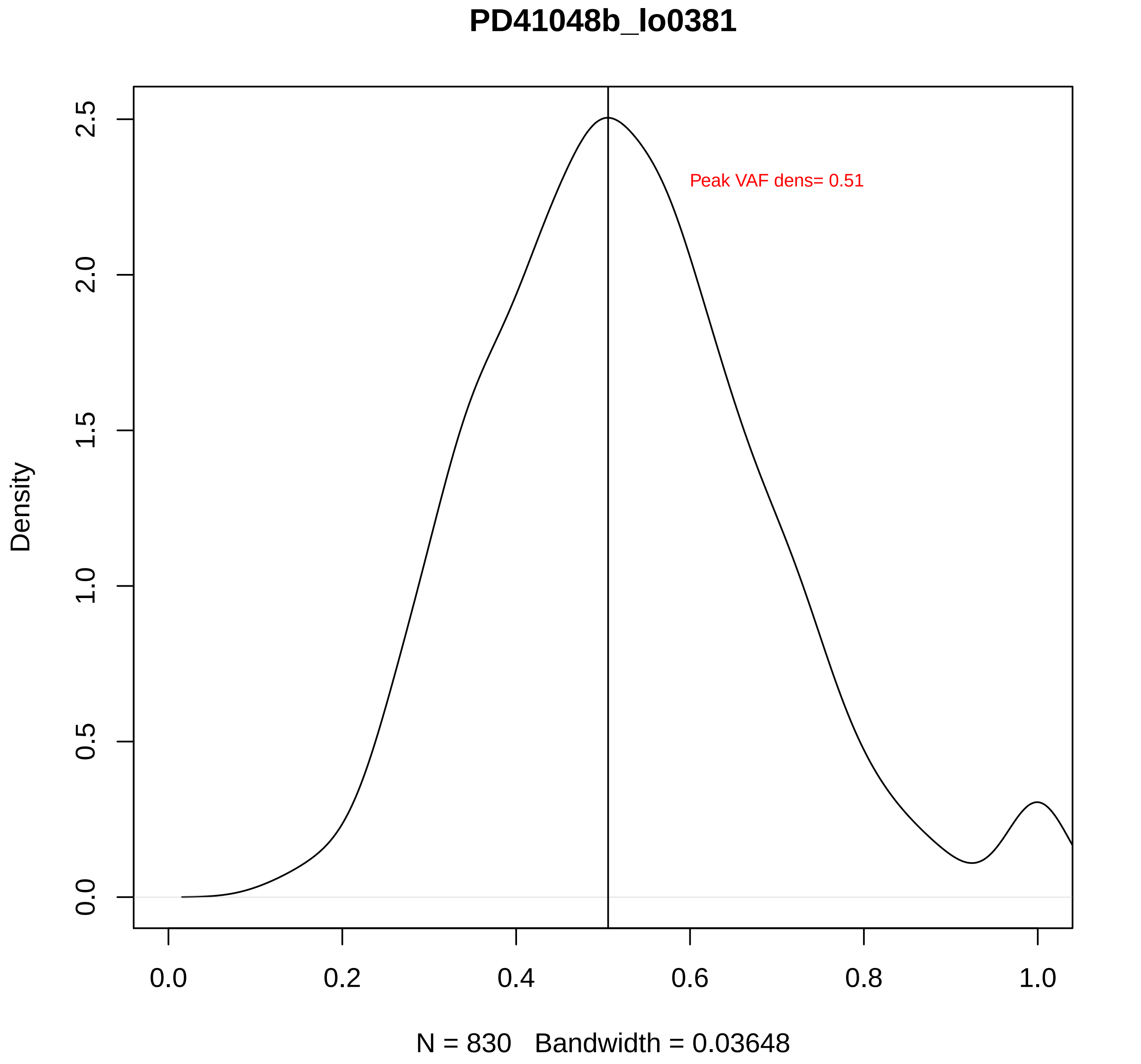
PD41048b_lo0381
Peak VAF dens= 0.51
Density
0.0
0.5
1.0
1.5
2.0
2.5
0.0
0.2
0.4
0.6
0.8
1.0
N = 830   Bandwidth = 0.03648

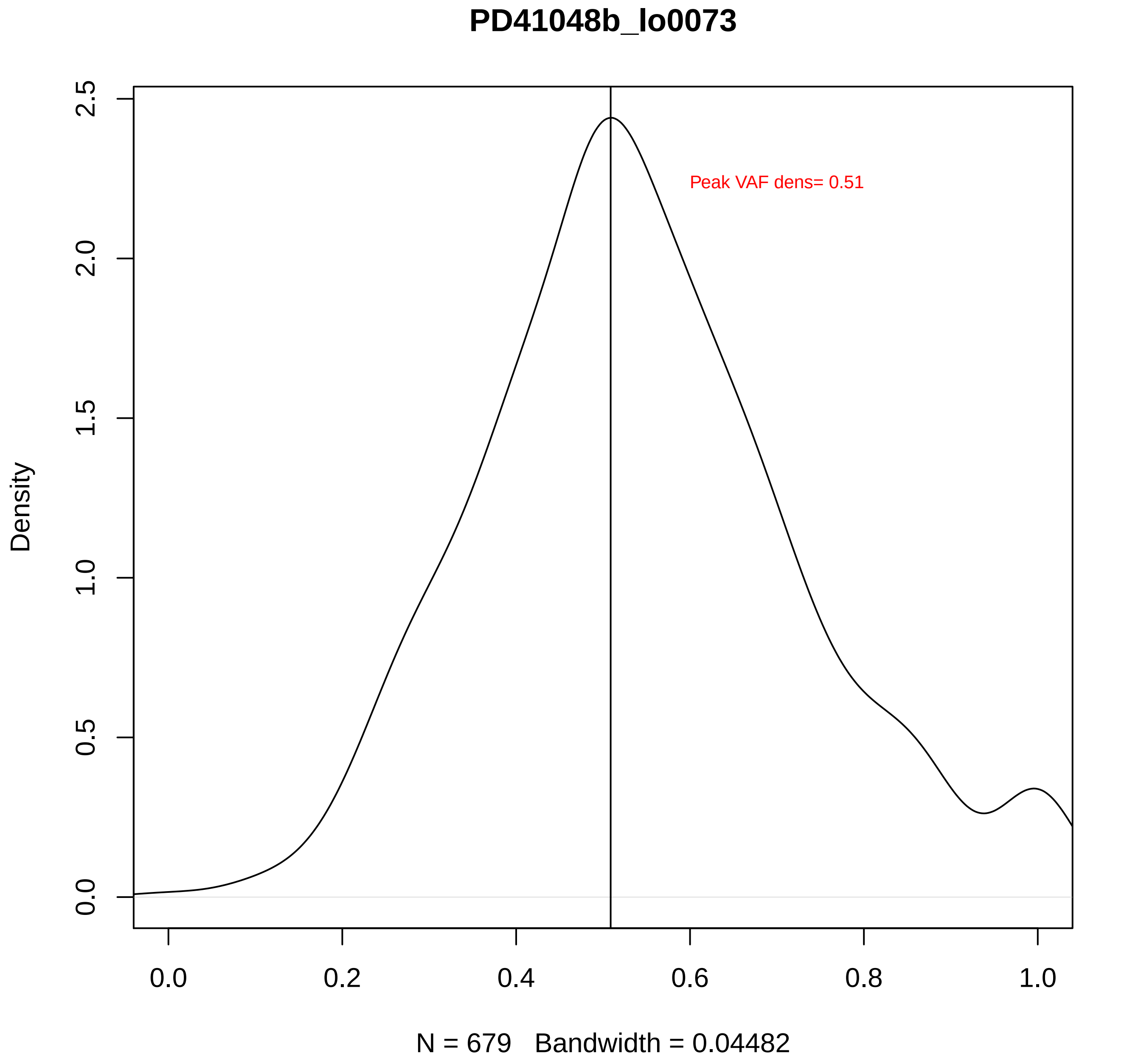

PD41048b_lo0073
Peak VAF dens= 0.51
Density
0.0
0.5
1.0
1.5
2.0
2.5
0.0
0.2
0.4
0.6
0.8
1.0
N = 679   Bandwidth = 0.04482

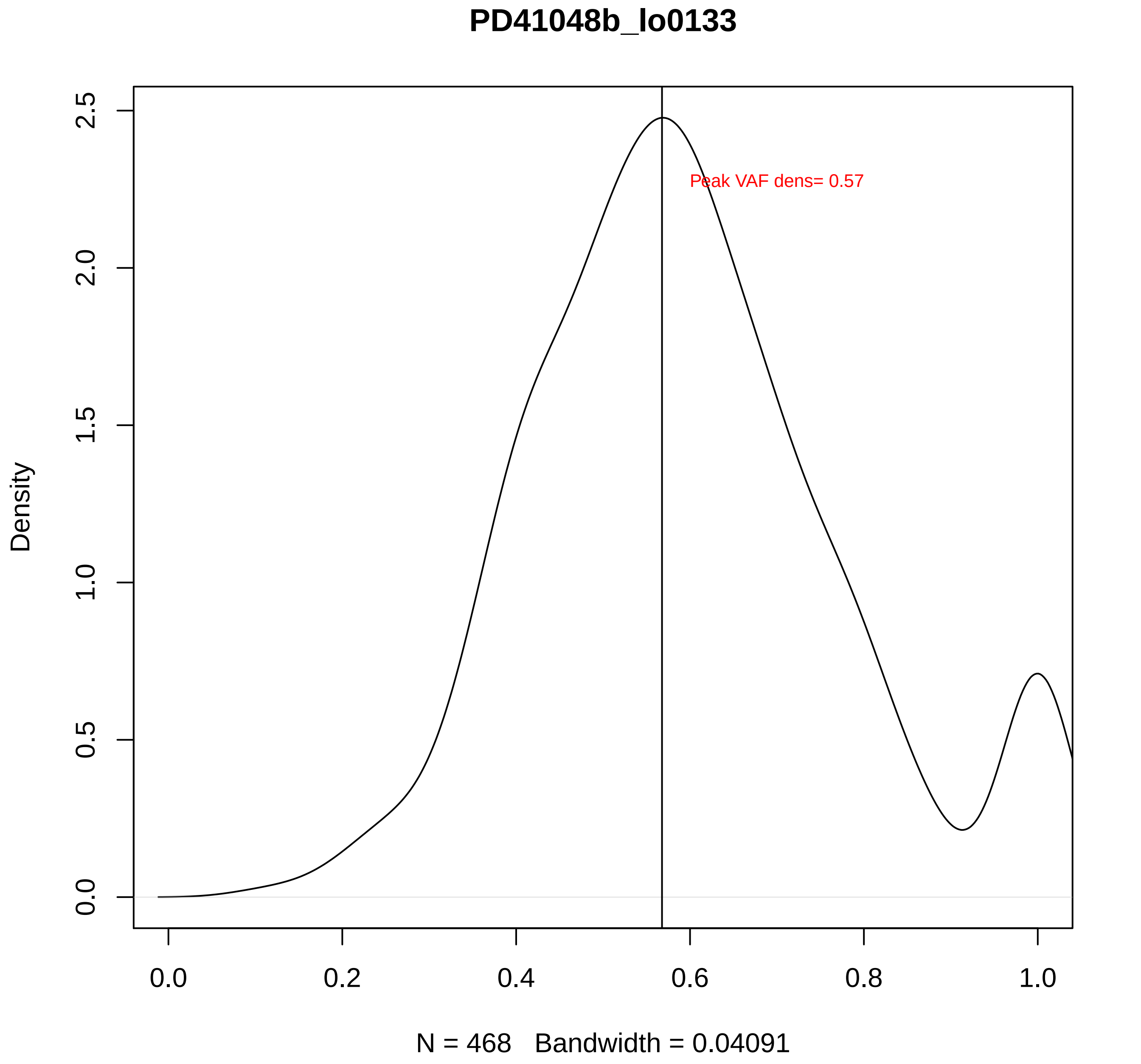
PD41048b_lo0133
Peak VAF dens= 0.57
Density
0.0
0.5
1.0
1.5
2.0
2.5
0.0
0.2
0.4
0.6
0.8
1.0
N = 468   Bandwidth = 0.04091

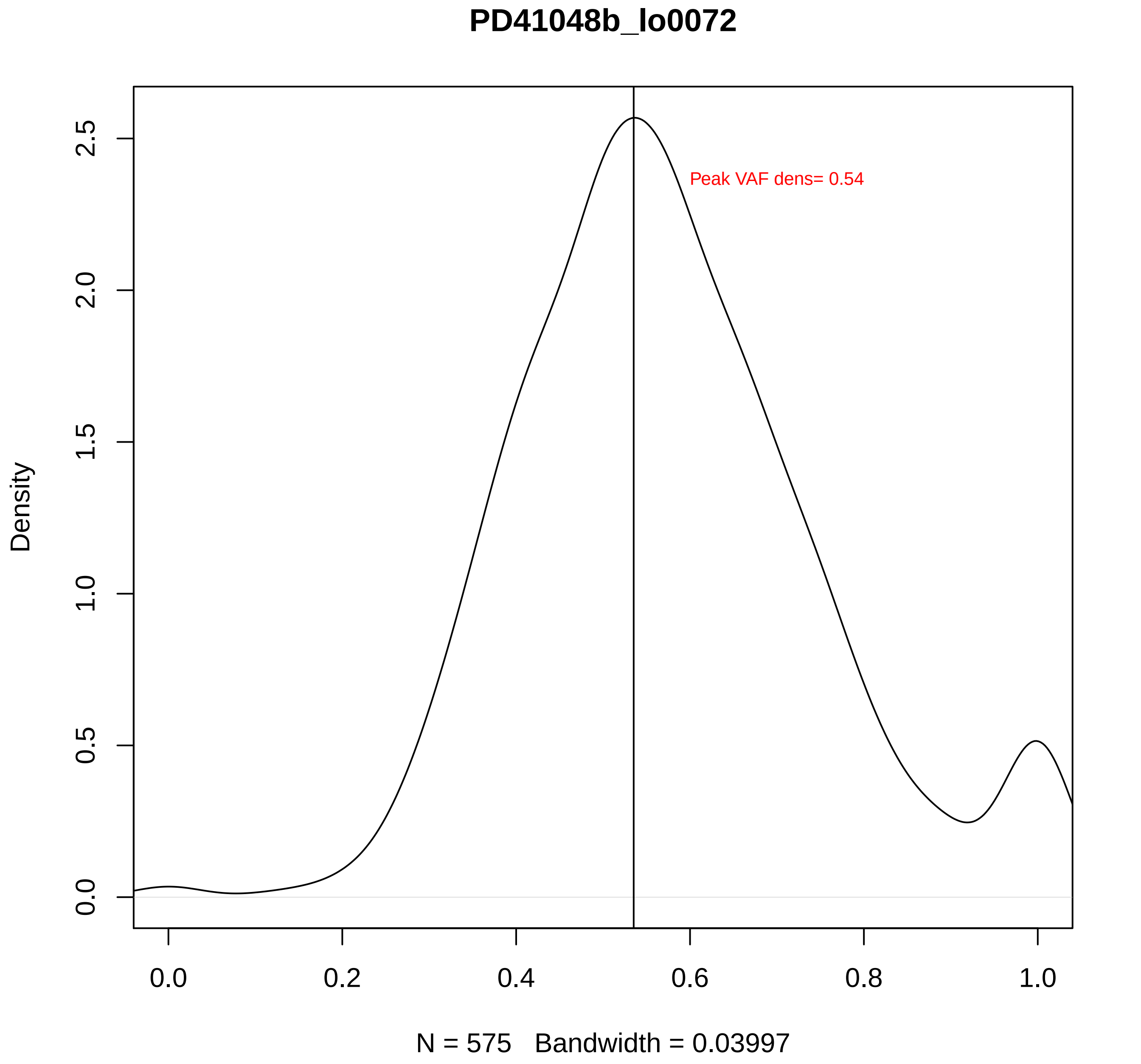

PD41048b_lo0072
Peak VAF dens= 0.54
Density
0.0
0.5
1.0
1.5
2.0
2.5
0.0
0.2
0.4
0.6
0.8
1.0
N = 575 Bandwidth = 0.03997

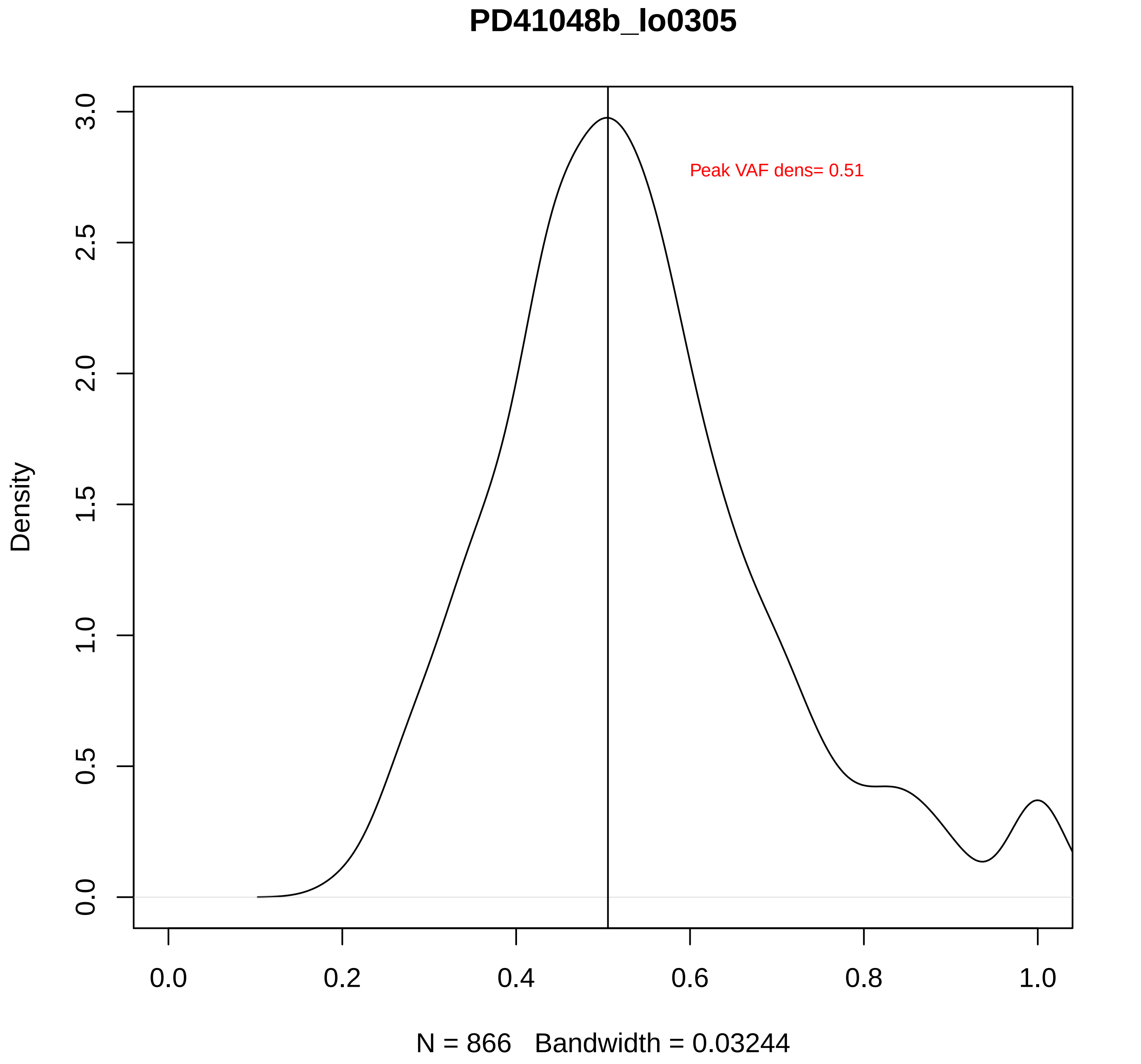
PD41048b_lo0305
Density
Peak VAF dens= 0.51
3.0
2.5
2.0
1.5
1.0
0.5
0.0
0.0
0.2
0.4
0.6
0.8
1.0
N = 866 Bandwidth = 0.03244

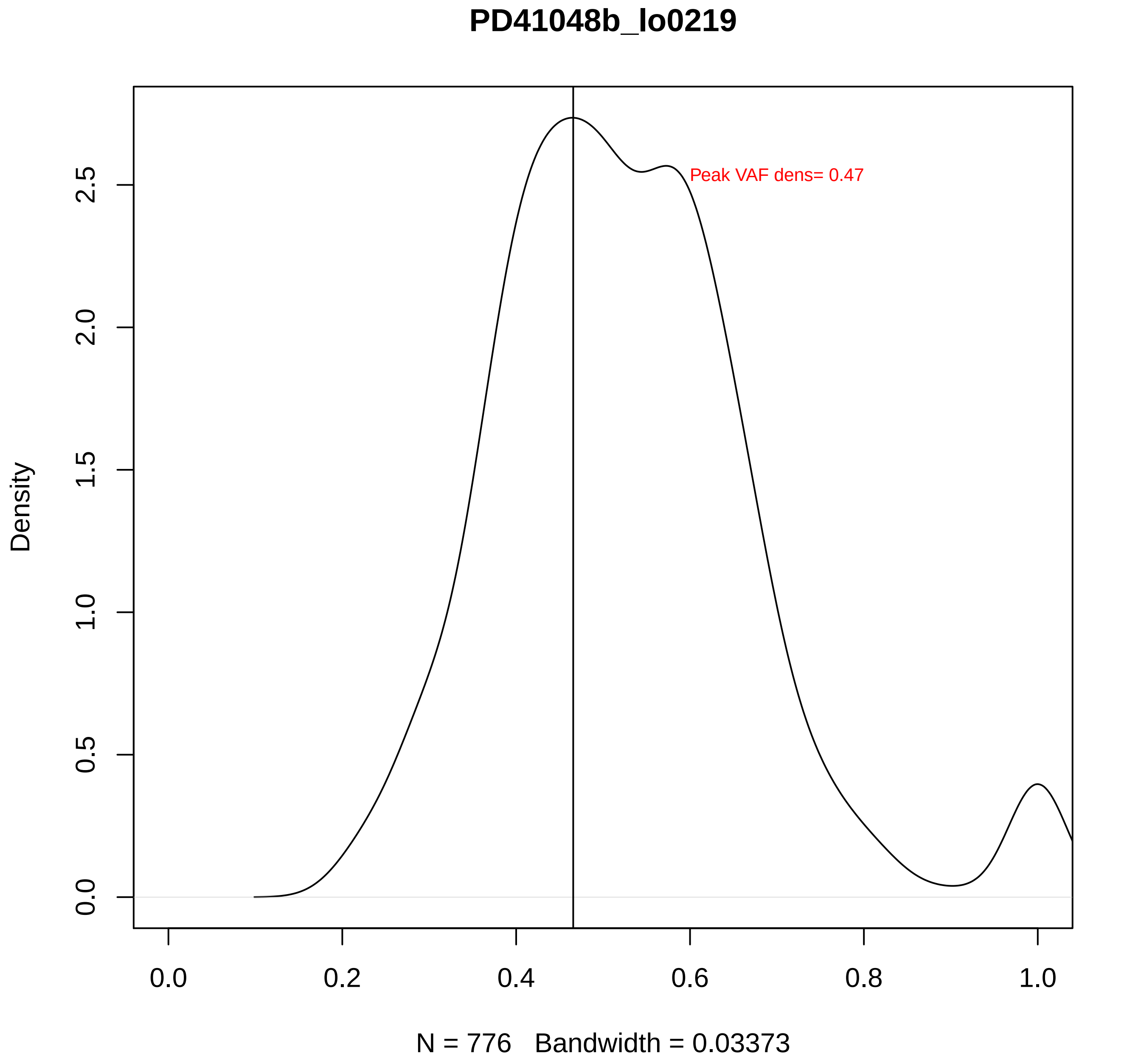
PD41048b_lo0219
Peak VAF dens= 0.47
Density
0.0
0.5
1.0
1.5
2.0
2.5
0.0
0.2
0.4
0.6
0.8
1.0
N = 776   Bandwidth = 0.03373

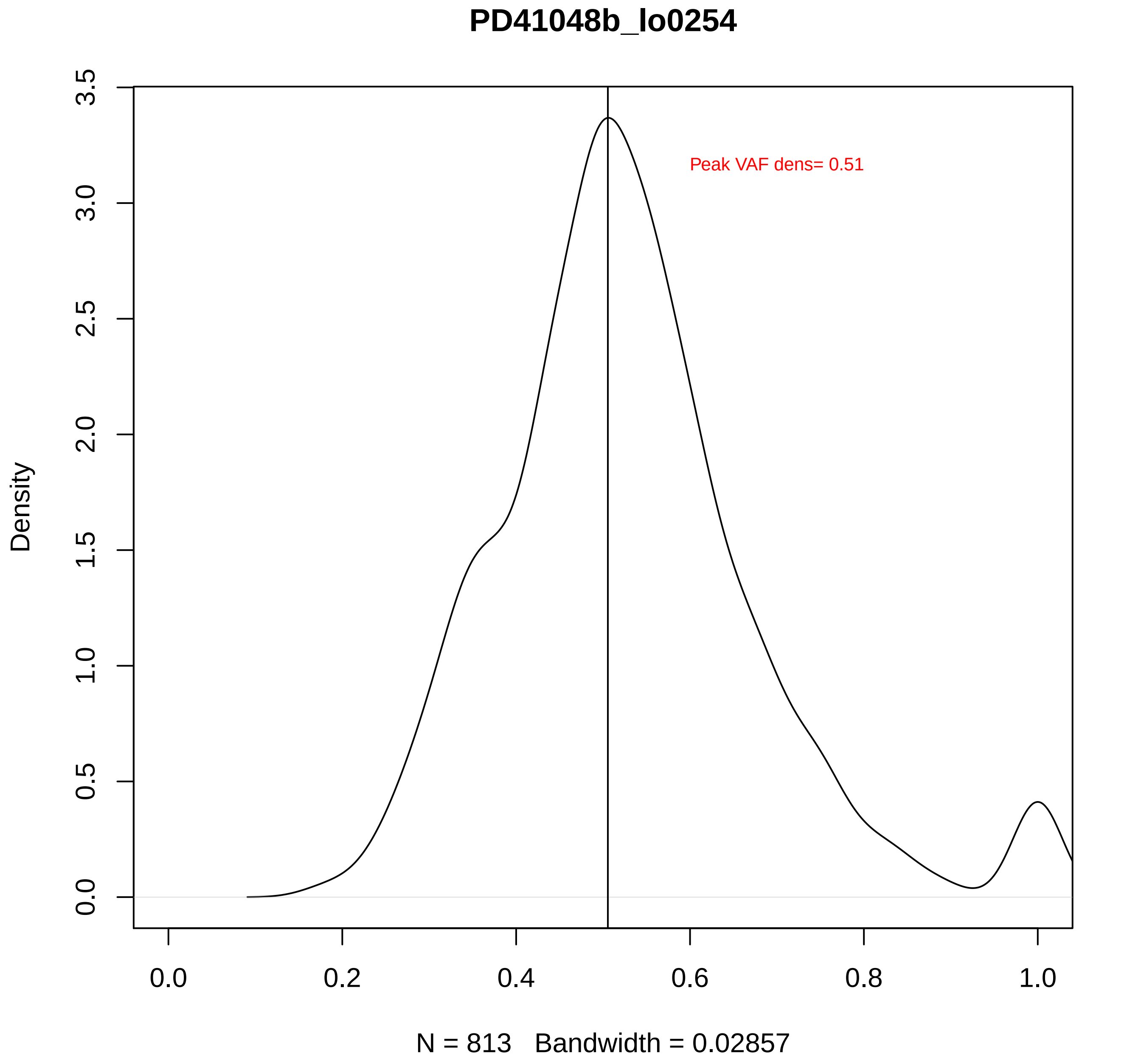

PD41048b_lo0254
Peak VAF dens= 0.51
Density
0.0
0.5
1.0
1.5
2.0
2.5
3.0
3.5
0.0
0.2
0.4
0.6
0.8
1.0
N = 813 Bandwidth = 0.02857

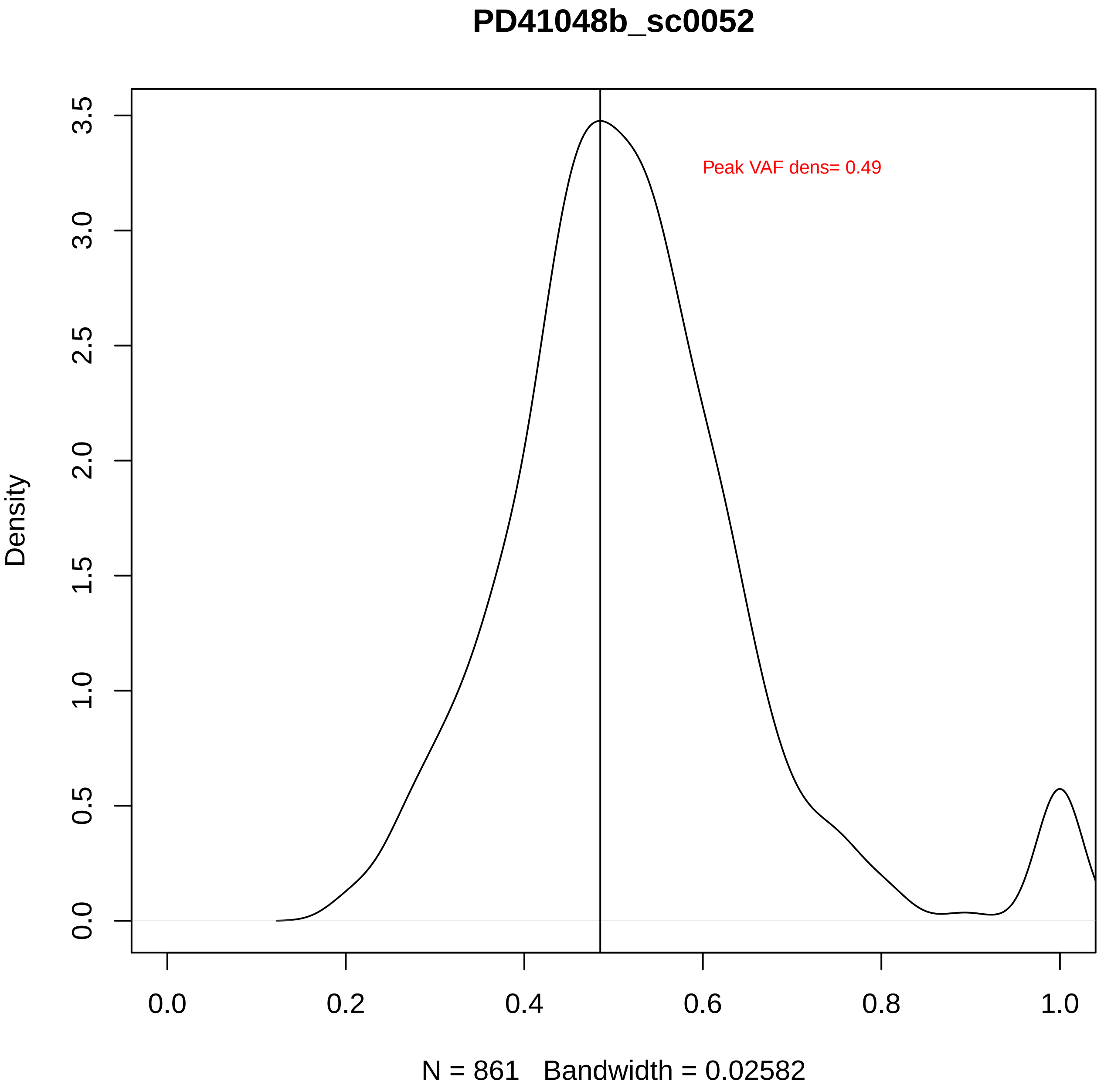
PD41048b_sc0052
Peak VAF dens= 0.49
Density
0.0
0.5
1.0
1.5
2.0
2.5
3.0
3.5
0.0
0.2
0.4
0.6
0.8
1.0
N = 861   Bandwidth = 0.02582

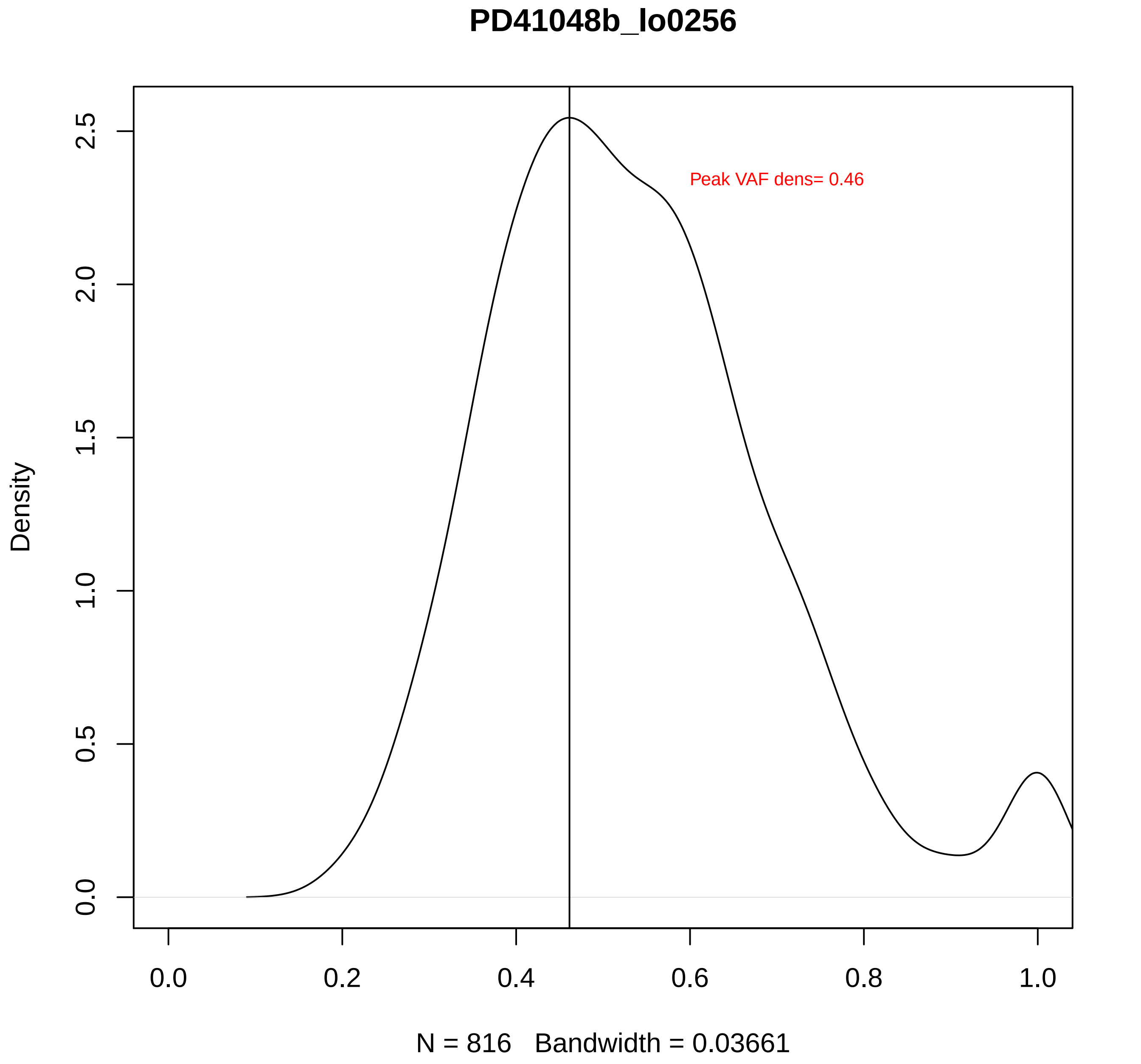

PD41048b_lo0256
Peak VAF dens= 0.46
Density
2.5
2.0
1.5
1.0
0.5
0.0
0.0
0.2
0.4
0.6
0.8
1.0
N = 816 Bandwidth = 0.03661

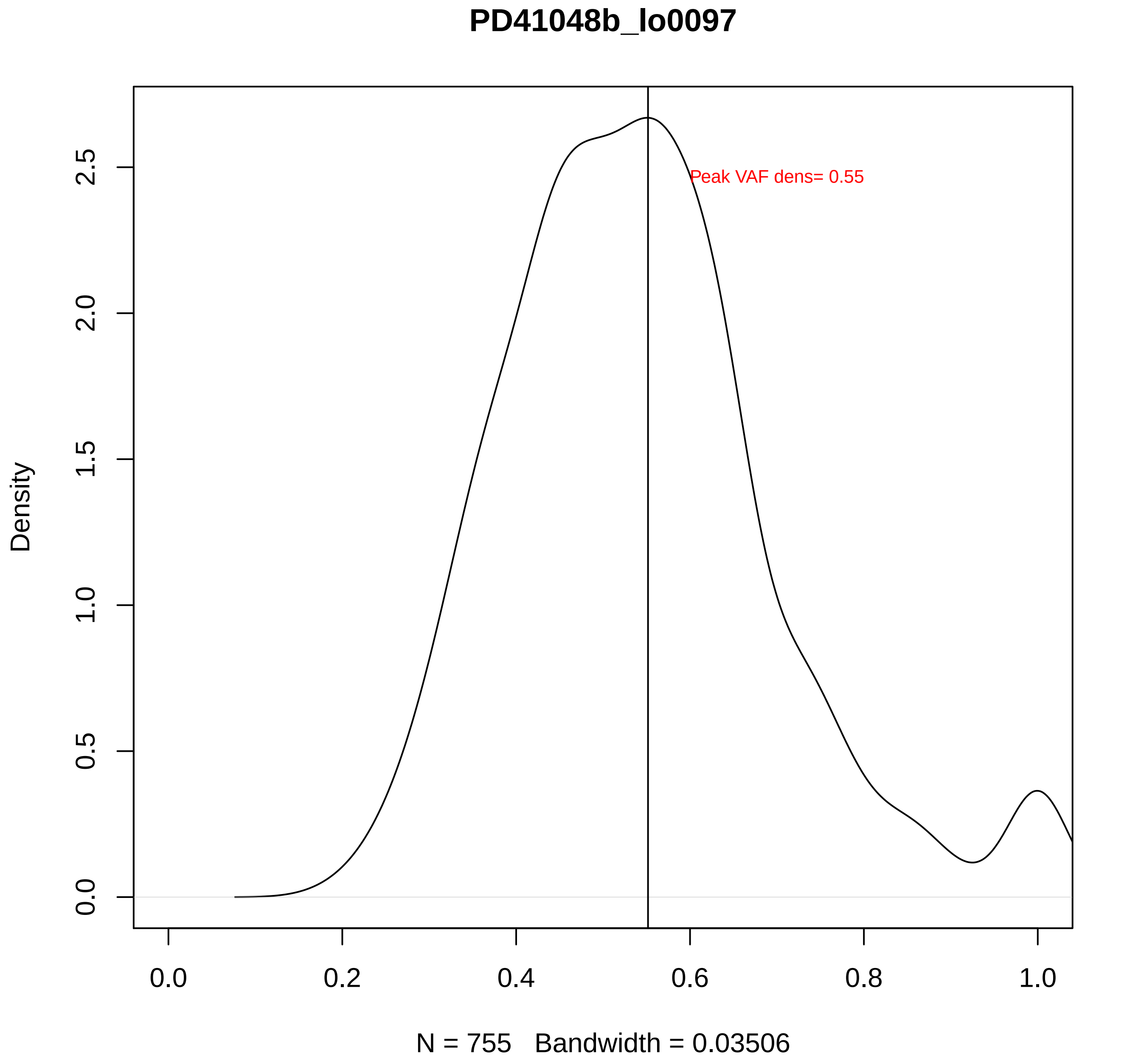

PD41048b_lo0097
Peak VAF dens= 0.55
Density
0.0
0.5
1.0
1.5
2.0
2.5
0.0
0.2
0.4
0.6
0.8
1.0
N = 755 Bandwidth = 0.03506

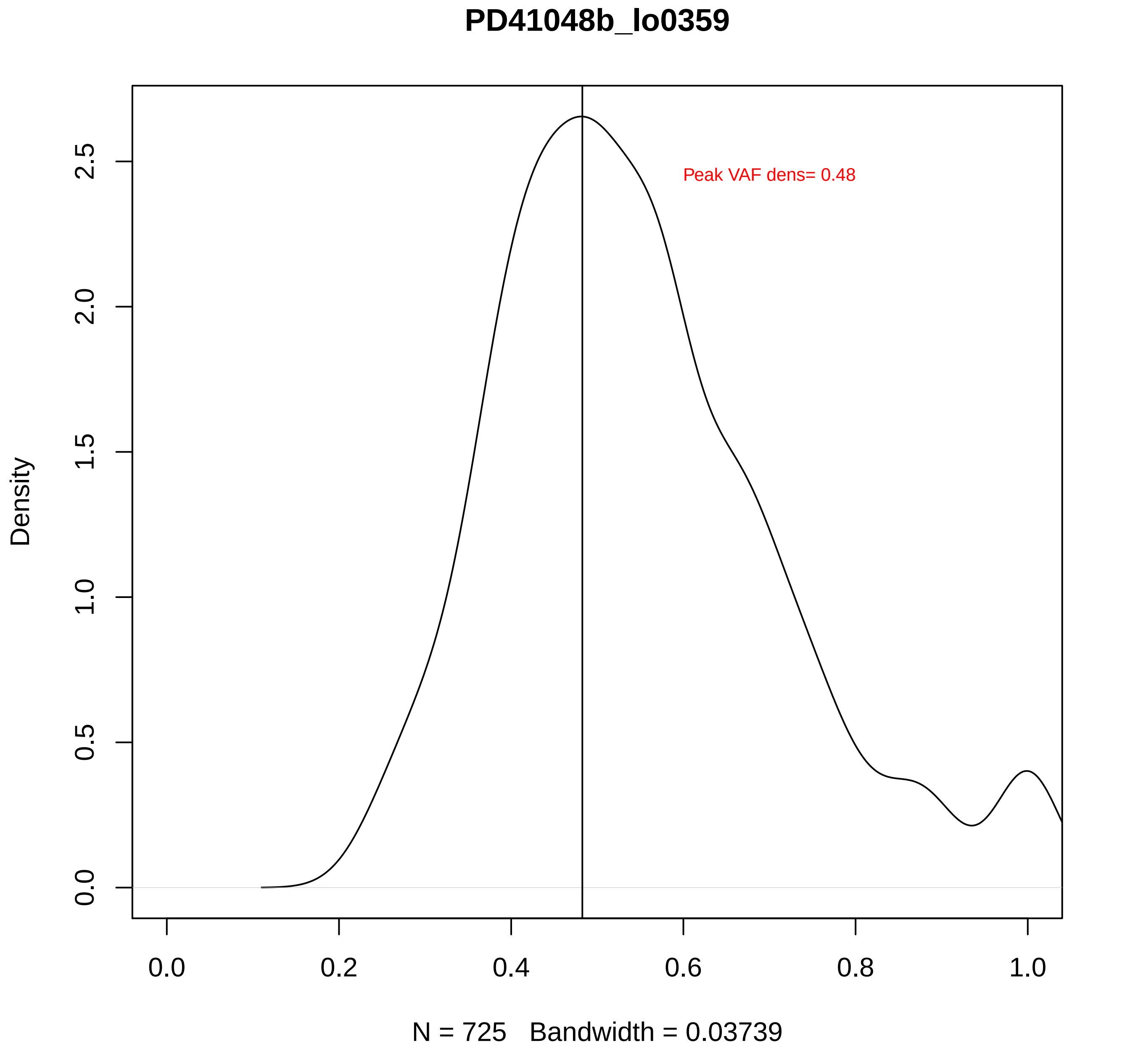

PD41048b_lo0359
Peak VAF dens= 0.48
Density
0.0
0.5
1.0
1.5
2.0
2.5
0.0
0.2
0.4
0.6
0.8
1.0
N = 725   Bandwidth = 0.03739

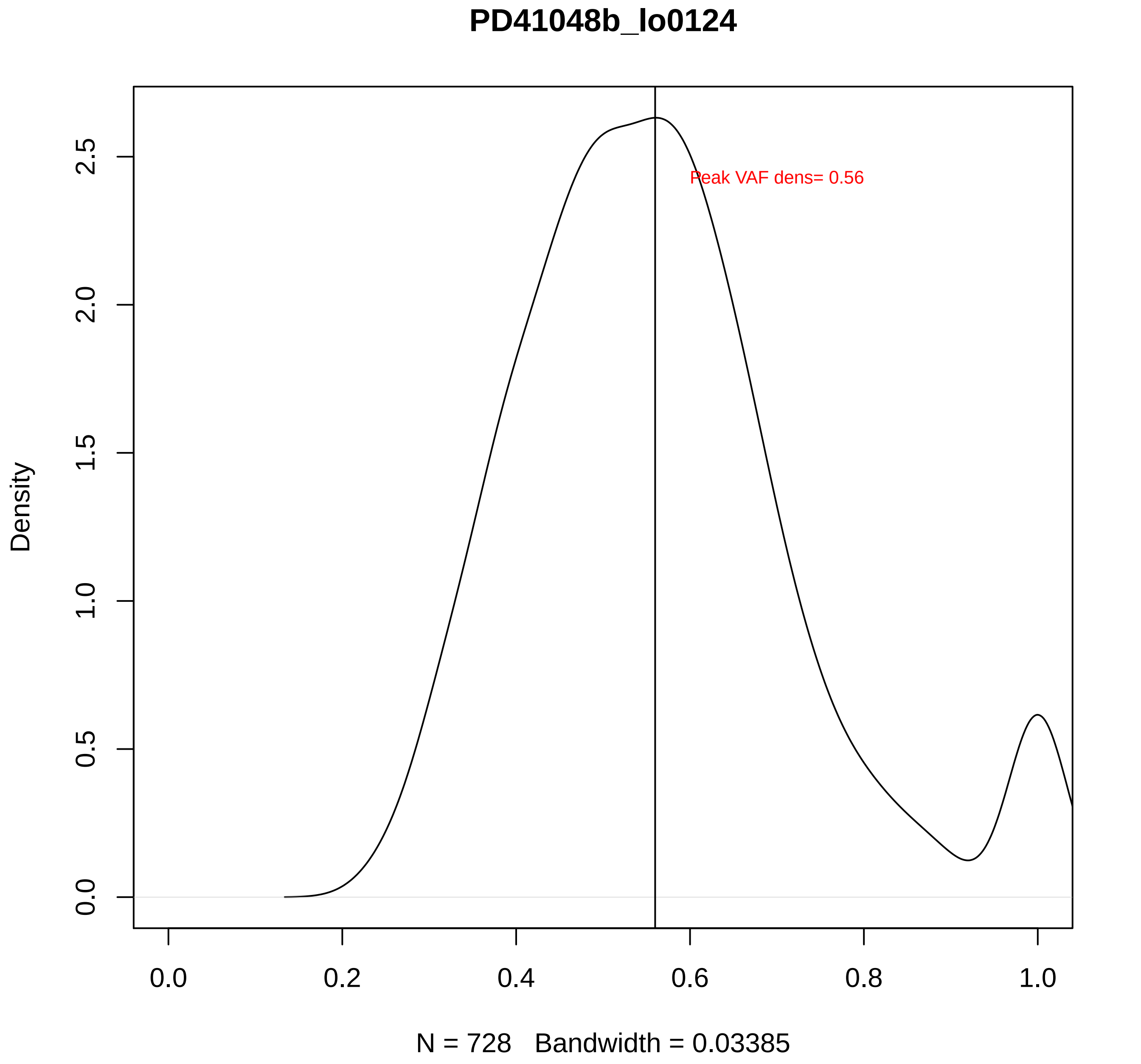
PD41048b_lo0124
Peak VAF dens= 0.56
Density
0.0
0.5
1.0
1.5
2.0
2.5
0.0
0.2
0.4
0.6
0.8
1.0
N = 728   Bandwidth = 0.03385

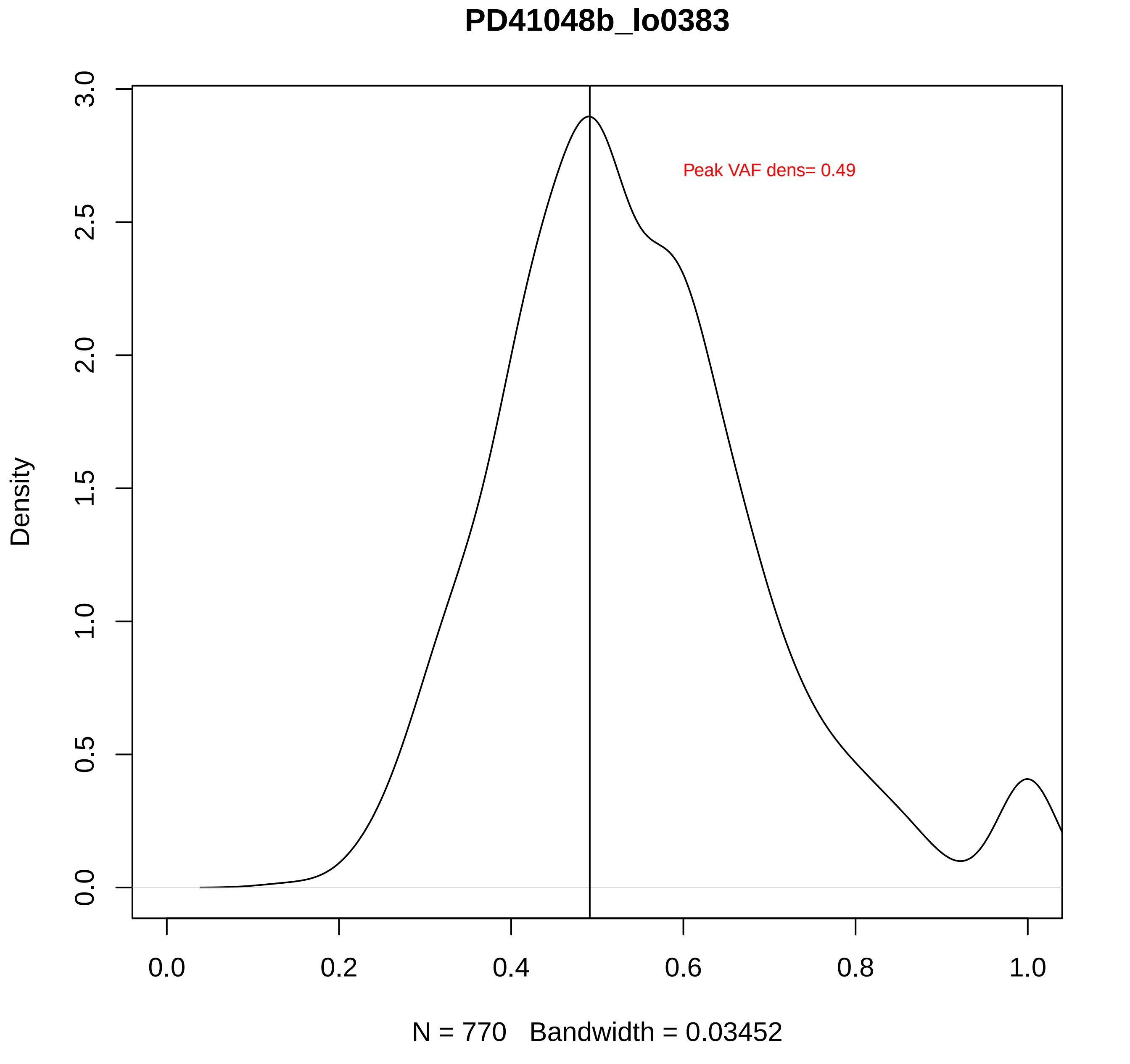
PD41048b_lo0383
Peak VAF dens= 0.49
Density
0.0
0.5
1.0
1.5
2.0
2.5
3.0
0.0
0.2
0.4
0.6
0.8
1.0
N = 770 Bandwidth = 0.03452

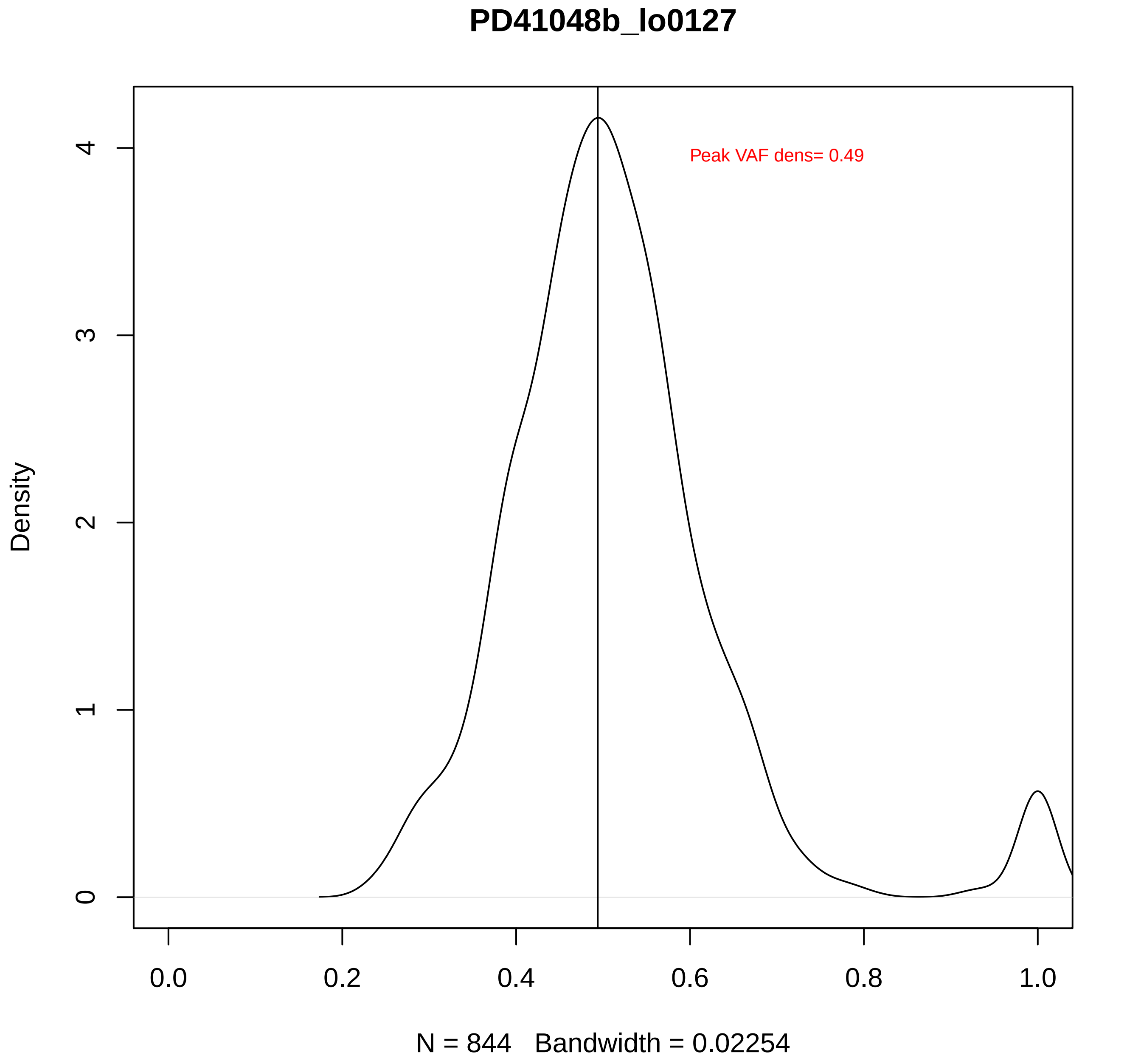
PD41048b_lo0127
Peak VAF dens= 0.49
Density
0
1
2
3
4
0.0
0.2
0.4
0.6
0.8
1.0
N = 844   Bandwidth = 0.02254

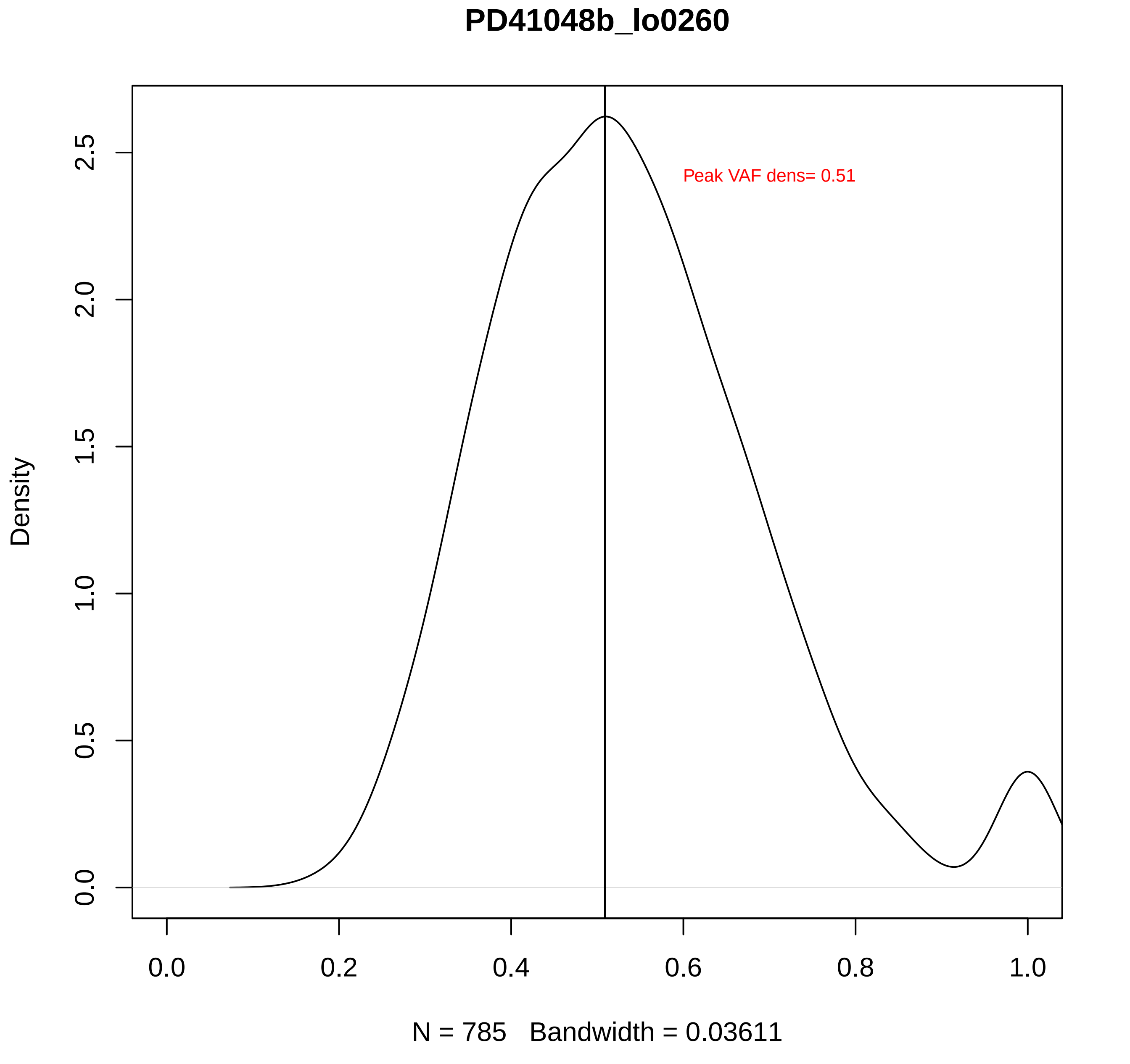
PD41048b_lo0260
Peak VAF dens= 0.51
Density
0.0
0.5
1.0
1.5
2.0
2.5
0.0
0.2
0.4
0.6
0.8
1.0
N = 785 Bandwidth = 0.03611

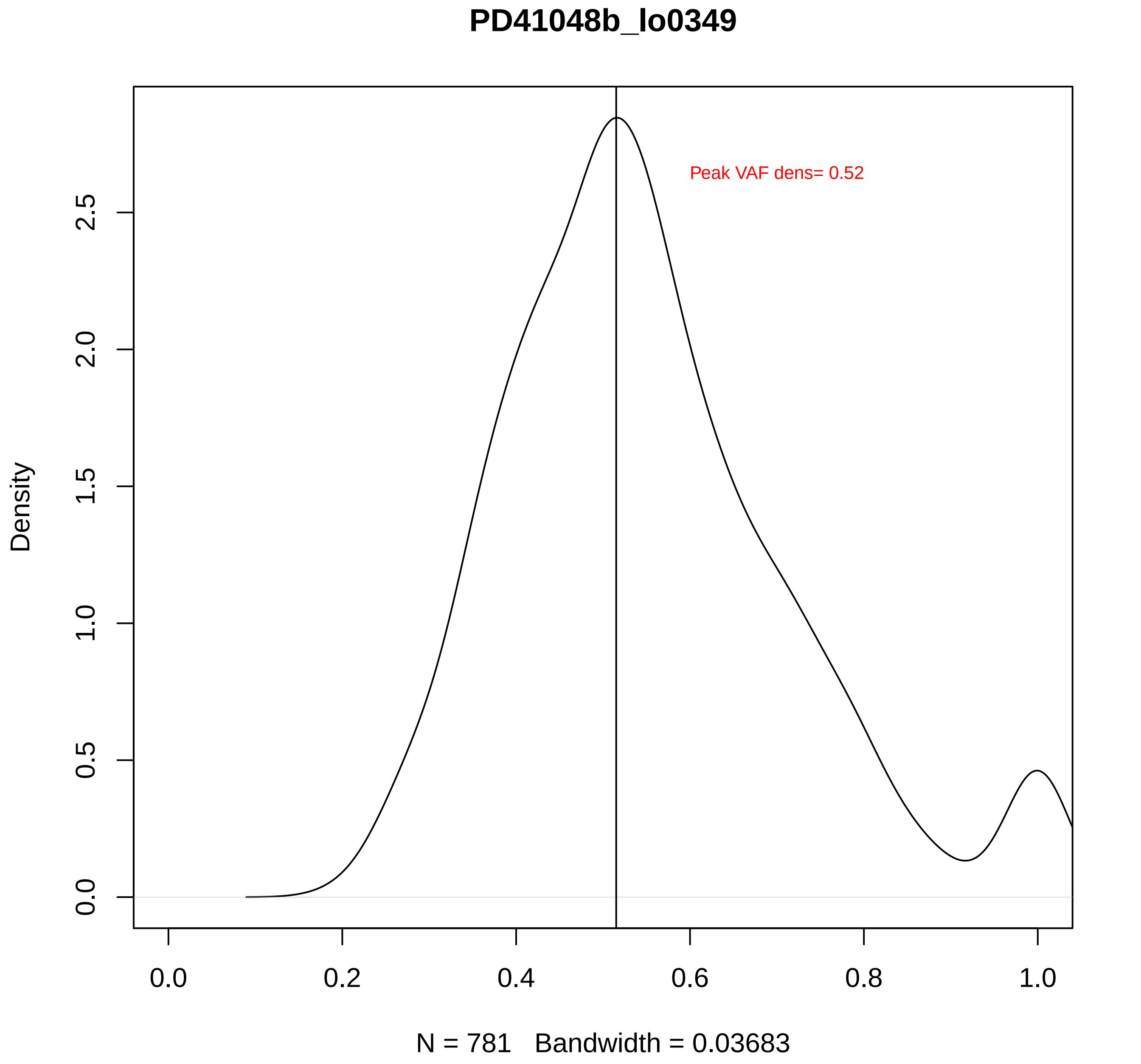

PD41048b_lo0349
Peak VAF dens= 0.52
Density
0.0
0.5
1.0
1.5
2.0
2.5
0.0
0.2
0.4
0.6
0.8
1.0
N = 781   Bandwidth = 0.03683

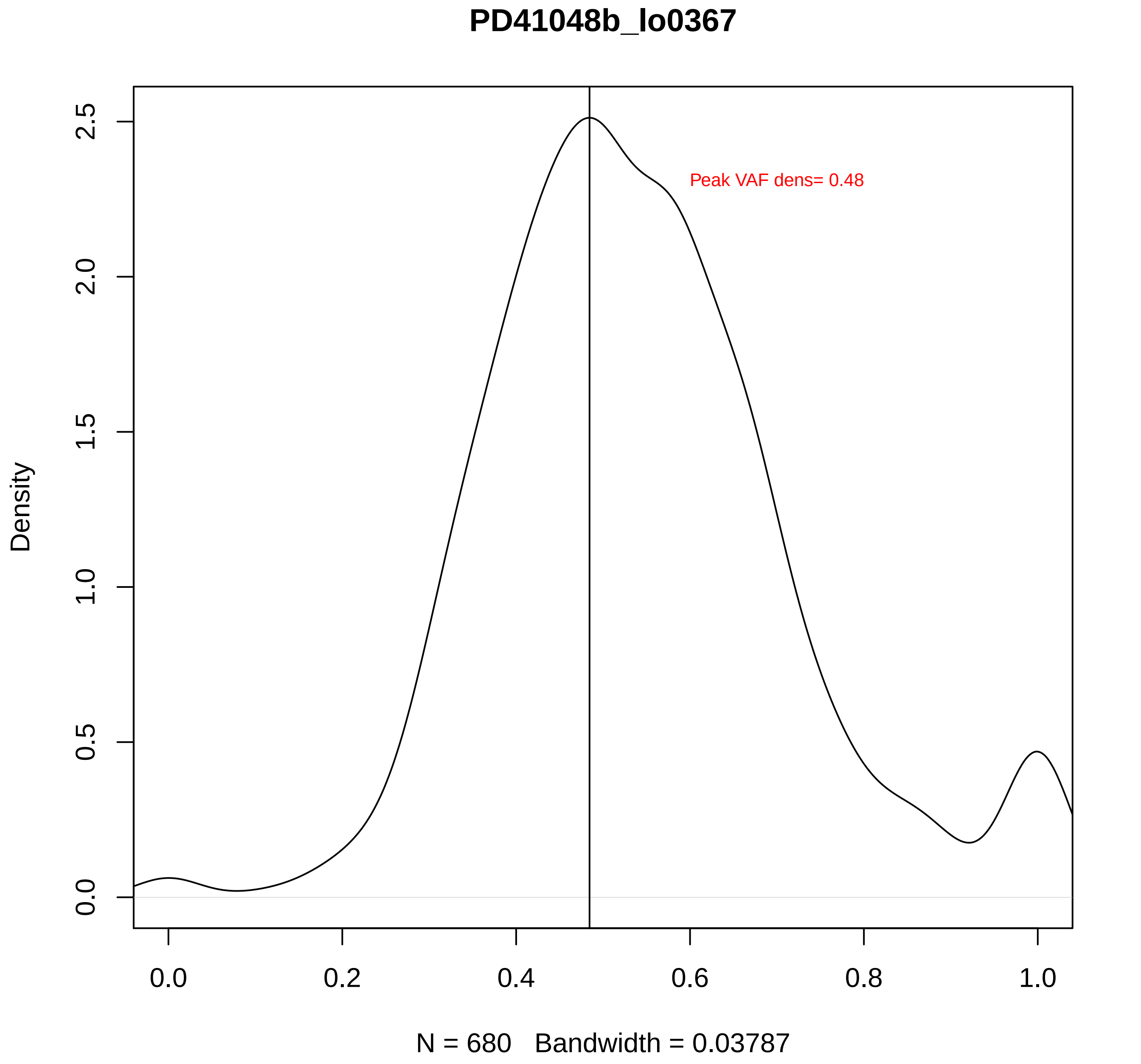

PD41048b_lo0367
Peak VAF dens= 0.48
Density
0.0
0.5
1.0
1.5
2.0
2.5
0.0
0.2
0.4
0.6
0.8
1.0
N = 680 Bandwidth = 0.03787

PD41048b_lo0111

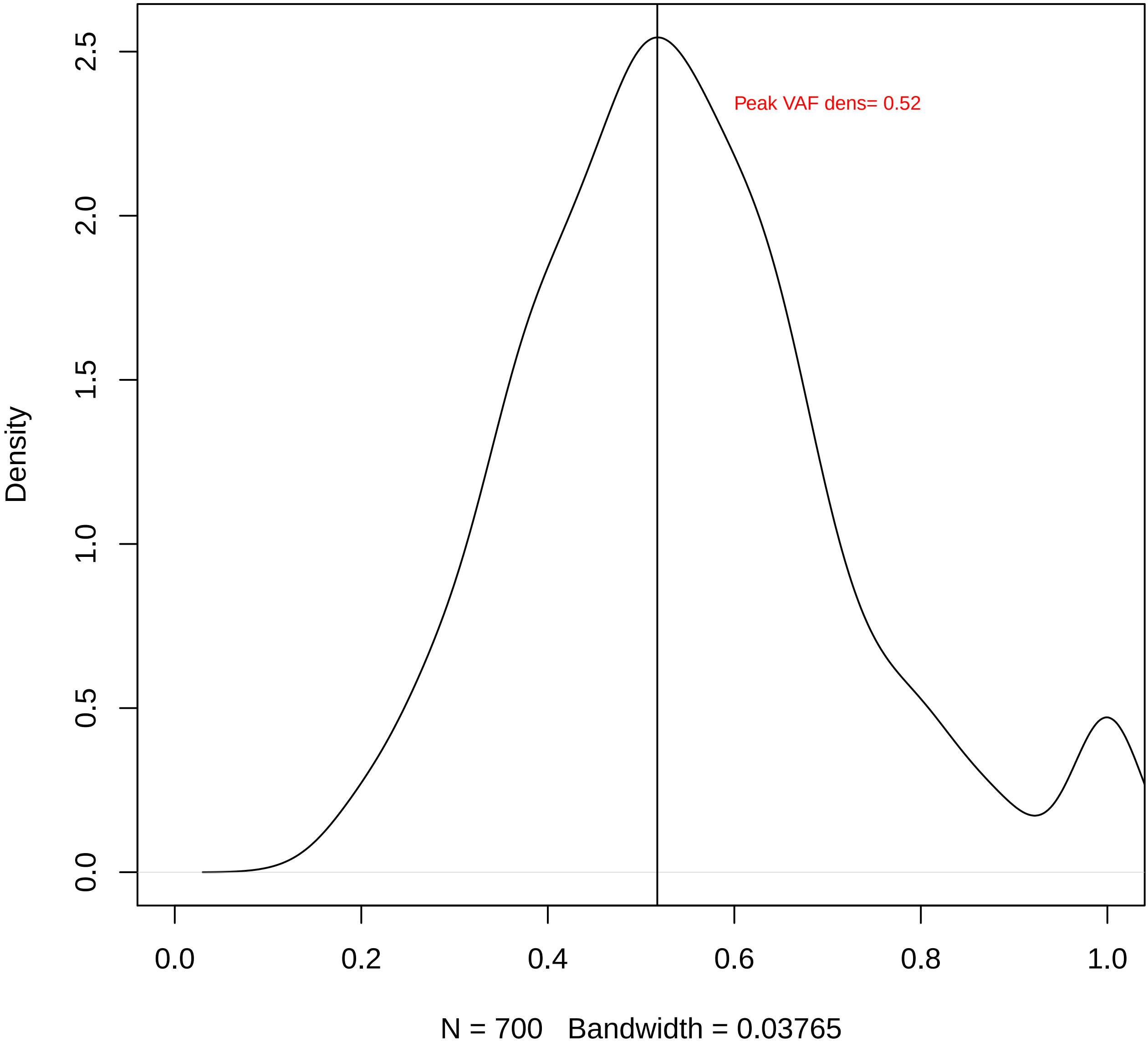

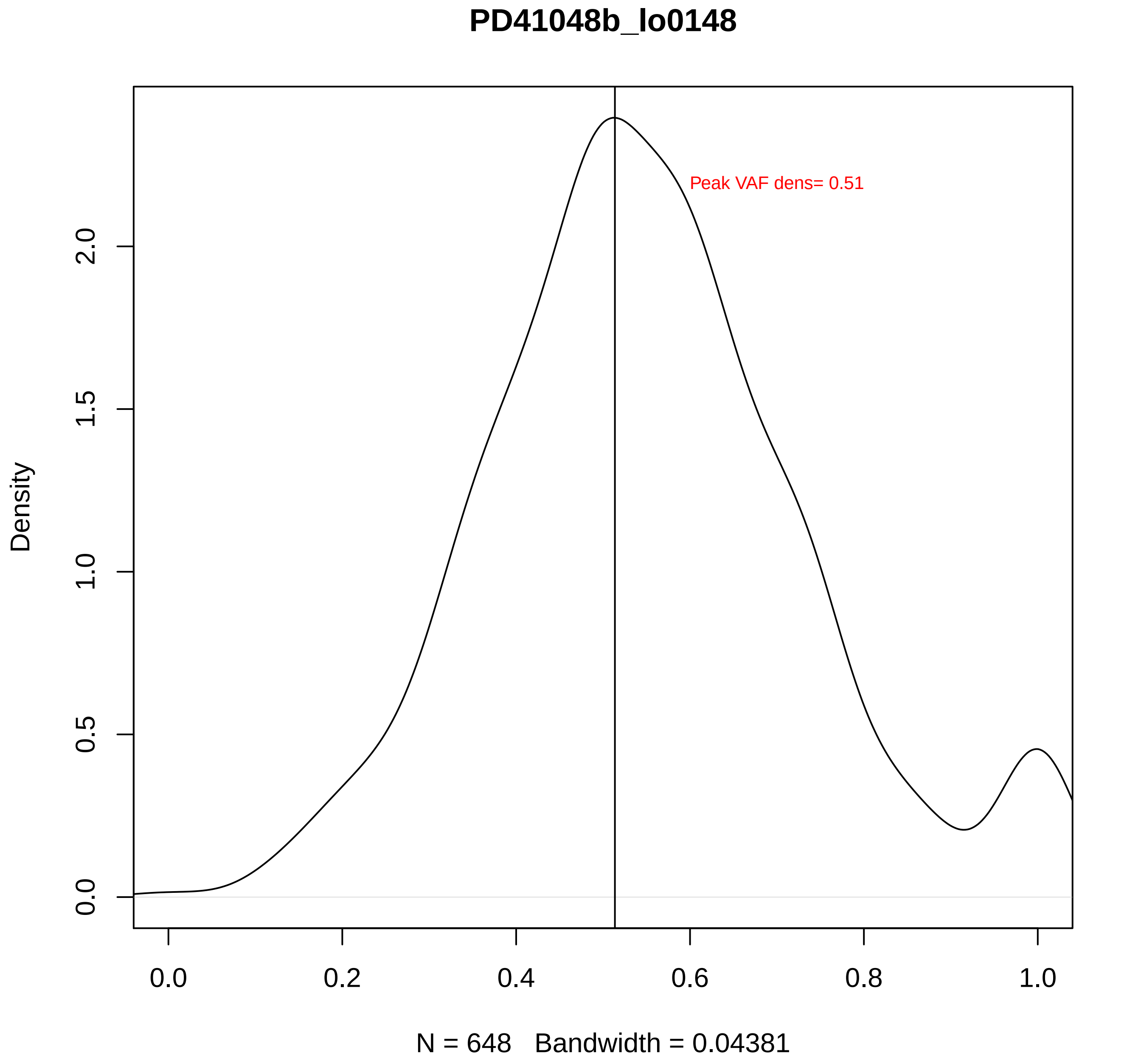
PD41048b_lo0148
Peak VAF dens= 0.51
Density
0.0
0.5
1.0
1.5
2.0
0.0
0.2
0.4
0.6
0.8
1.0
N = 648   Bandwidth = 0.04381

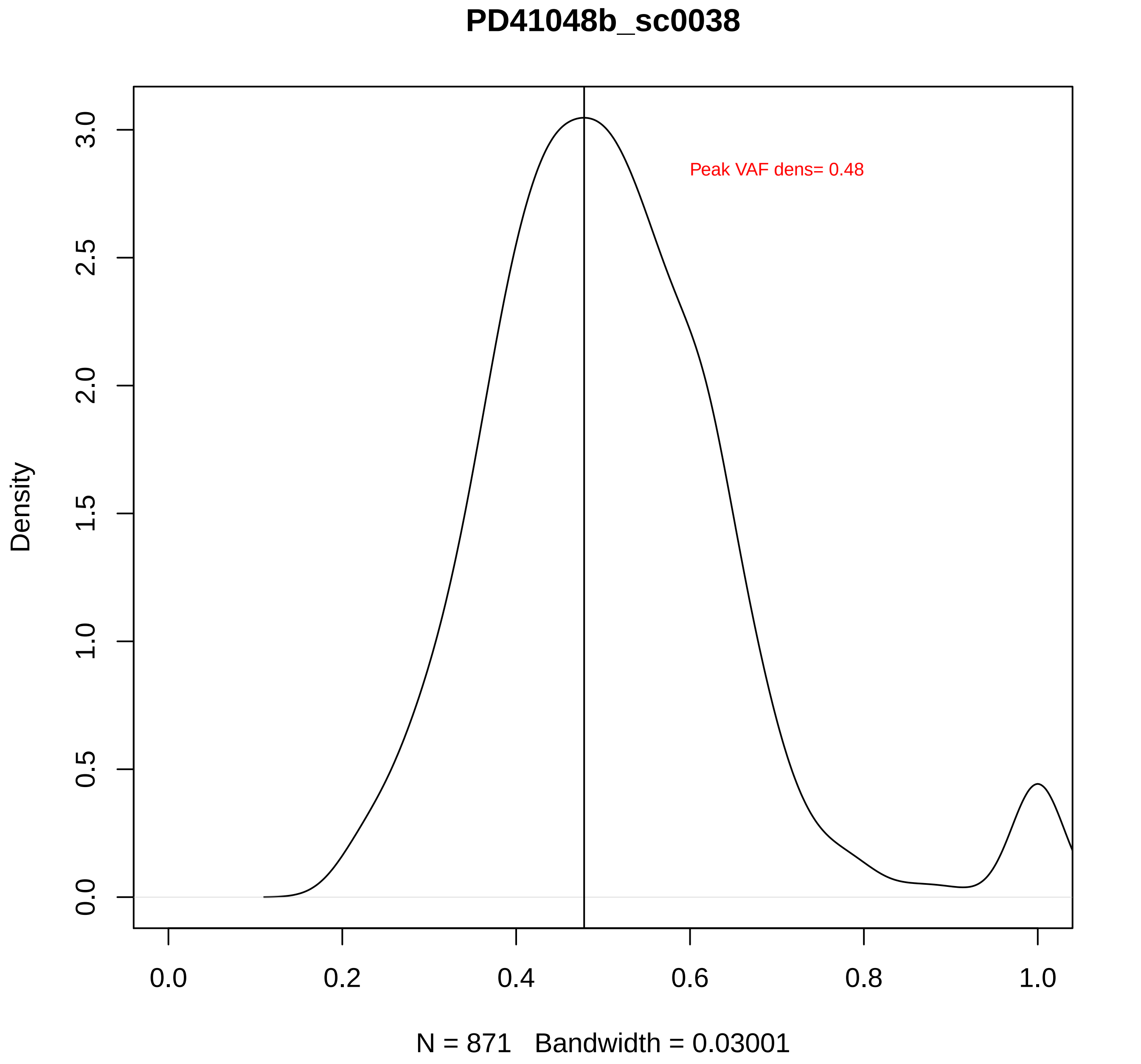

PD41048b_sc0038
Peak VAF dens= 0.48
Density
0.0
0.5
1.0
1.5
2.0
2.5
3.0
0.0
0.2
0.4
0.6
0.8
1.0
N = 871 Bandwidth = 0.03001

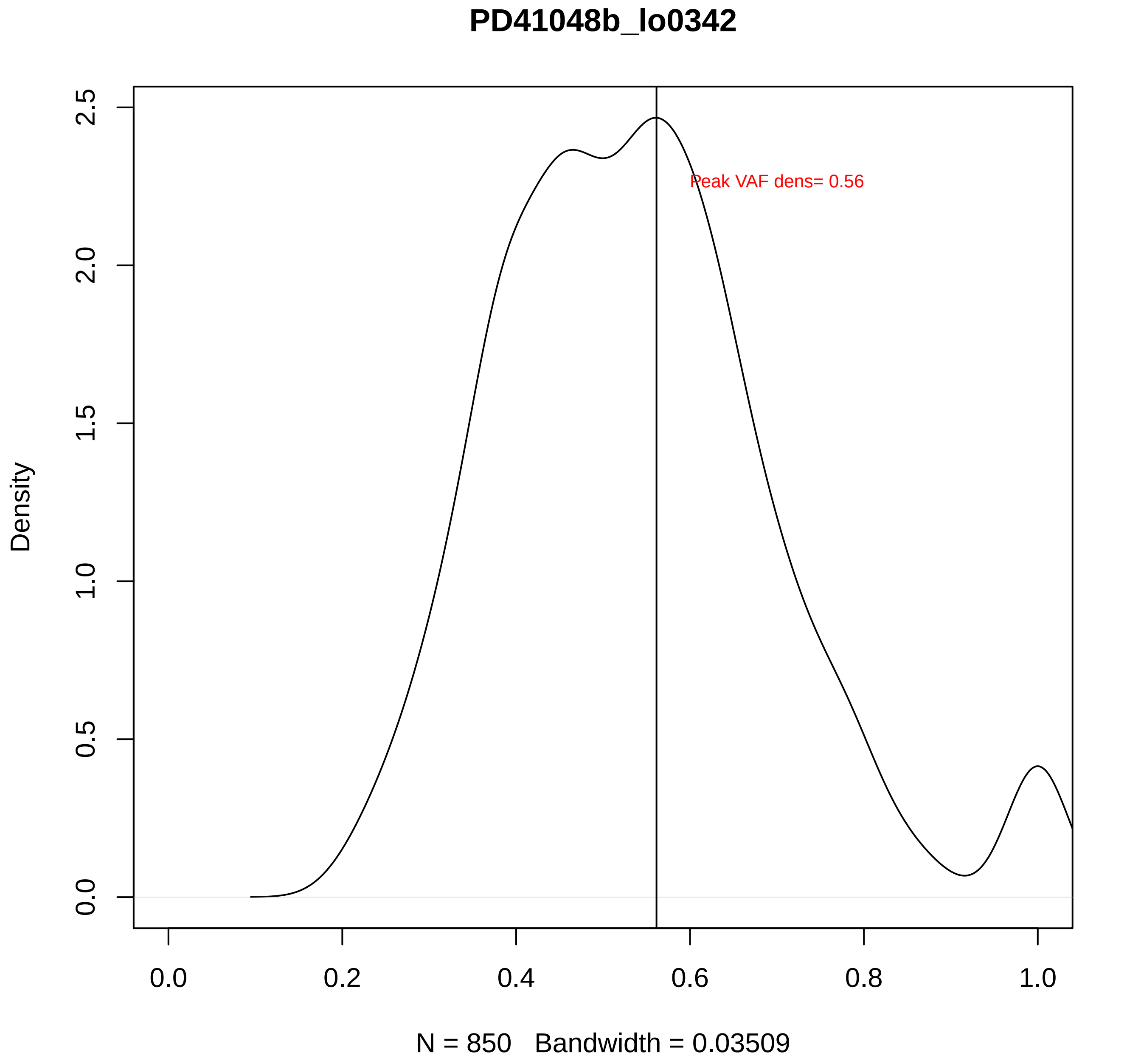
PD41048b_lo0342
Peak VAF dens= 0.56
Density
0.0
0.5
1.0
1.5
2.0
2.5
0.0
0.2
0.4
0.6
0.8
1.0
N = 850   Bandwidth = 0.03509

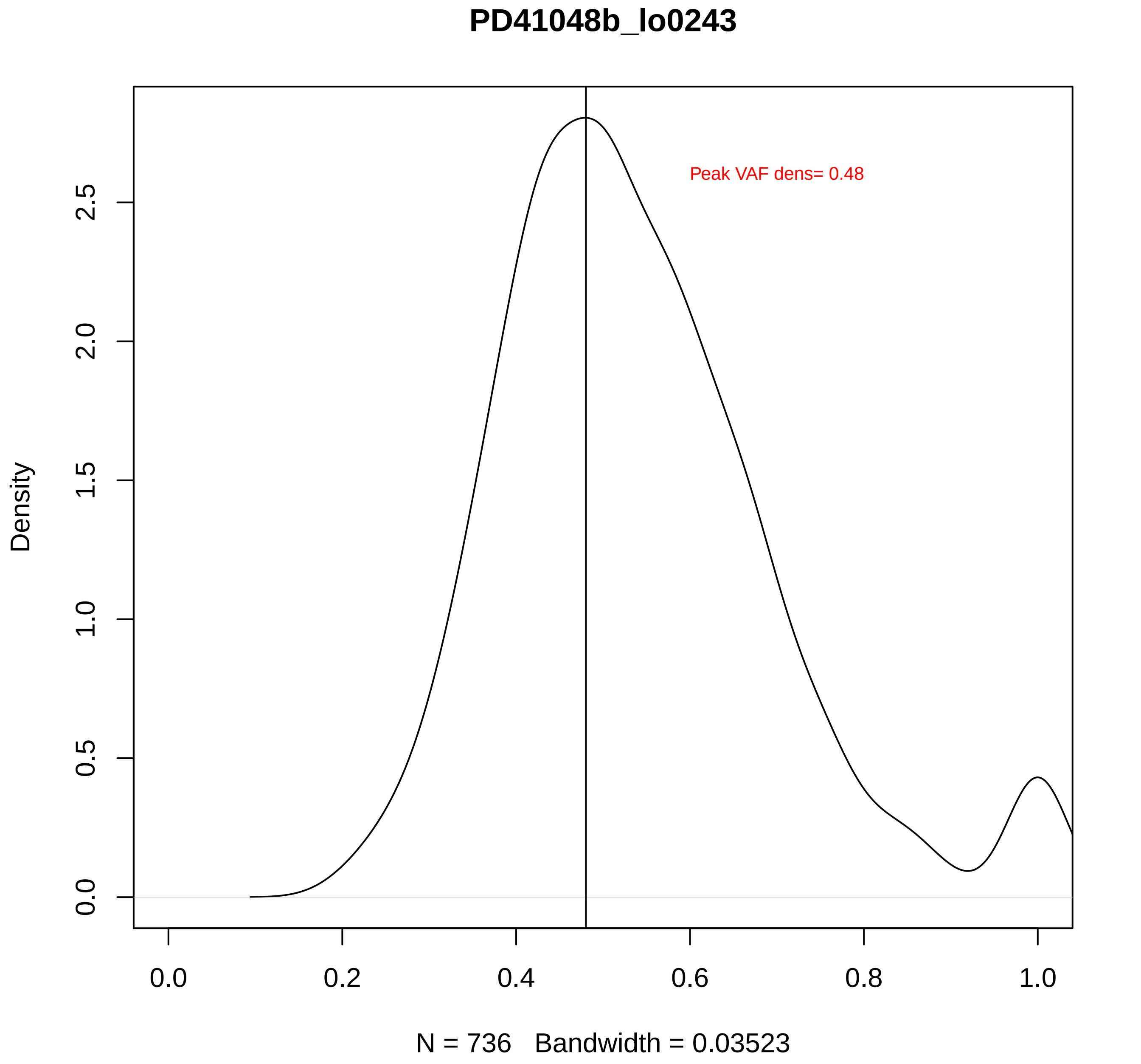
PD41048b_lo0243
Peak VAF dens= 0.48
Density
0.0
0.5
1.0
1.5
2.0
2.5
0.0
0.2
0.4
0.6
0.8
1.0
N = 736   Bandwidth = 0.03523

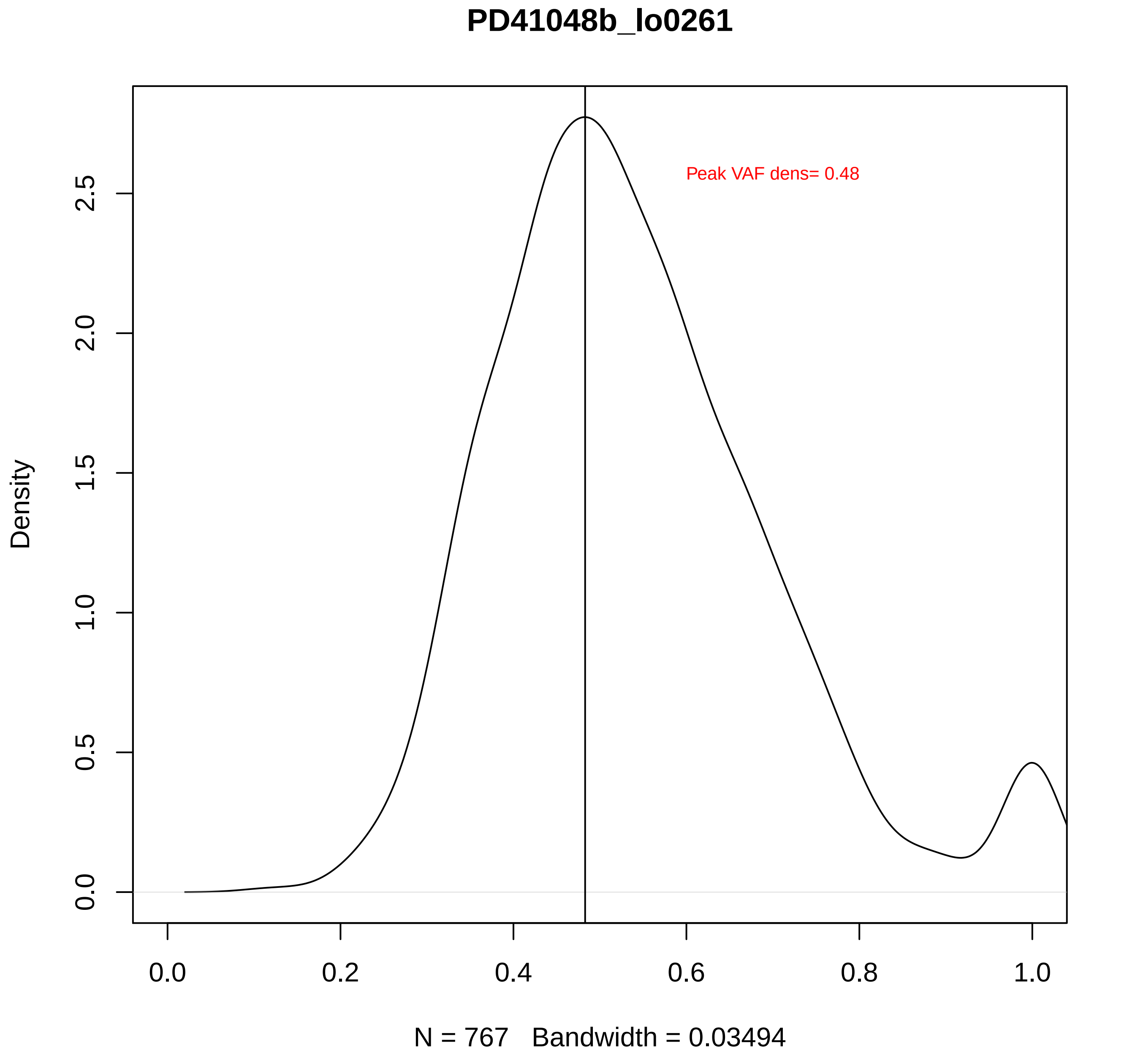
PD41048b_lo0261
Peak VAF dens= 0.48
Density
0.0
0.5
1.0
1.5
2.0
2.5
0.0
0.2
0.4
0.6
0.8
1.0
N = 767   Bandwidth = 0.03494

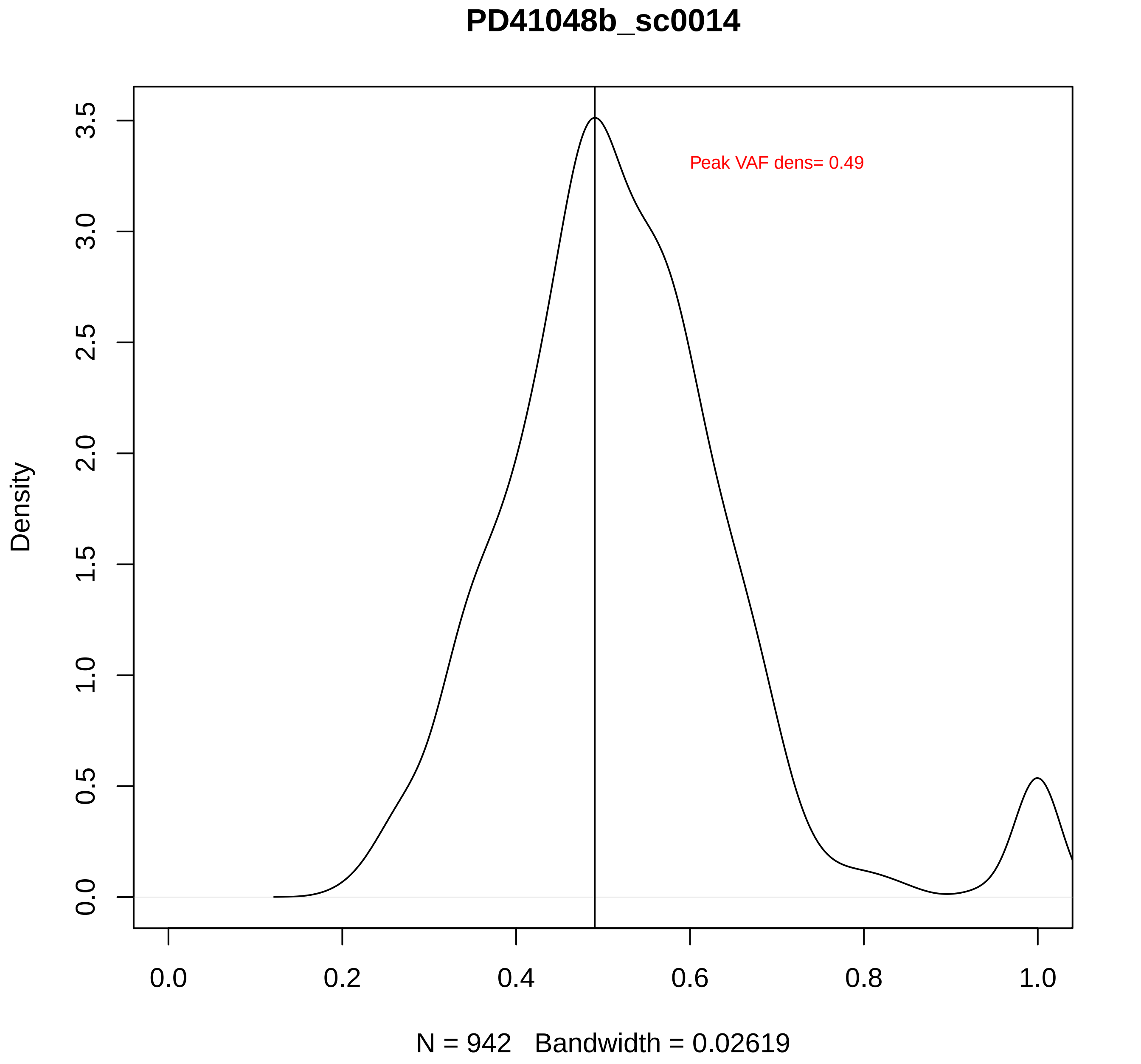

PD41048b_sc0014
Peak VAF dens= 0.49
Density
0.0
0.5
1.0
1.5
2.0
2.5
3.0
3.5
0.0
0.2
0.4
0.6
0.8
1.0
N = 942 Bandwidth = 0.02619

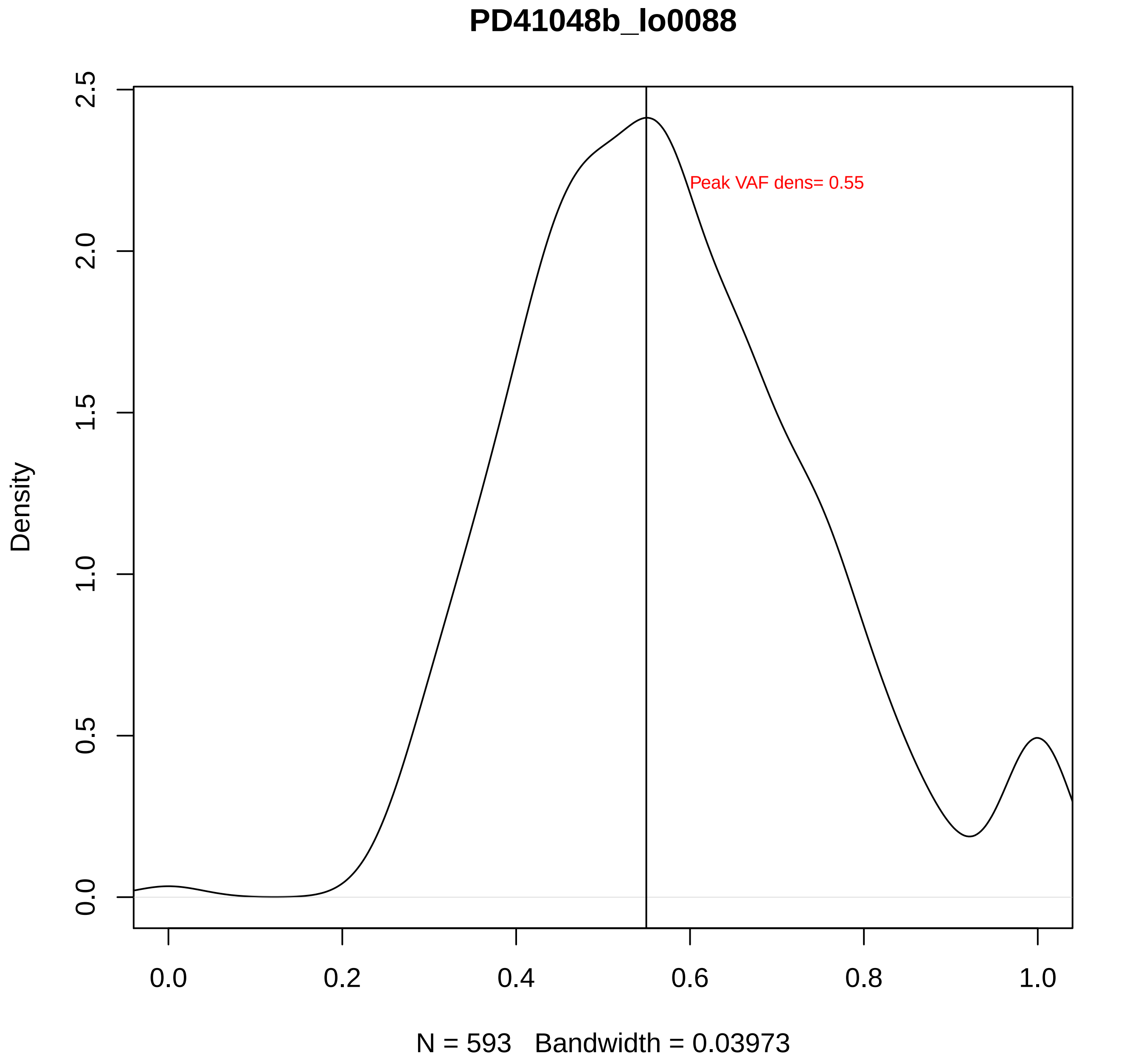
PD41048b_lo0088
Peak VAF dens= 0.55
Density
0.0
0.5
1.0
1.5
2.0
2.5
0.0
0.2
0.4
0.6
0.8
1.0
N = 593   Bandwidth = 0.03973

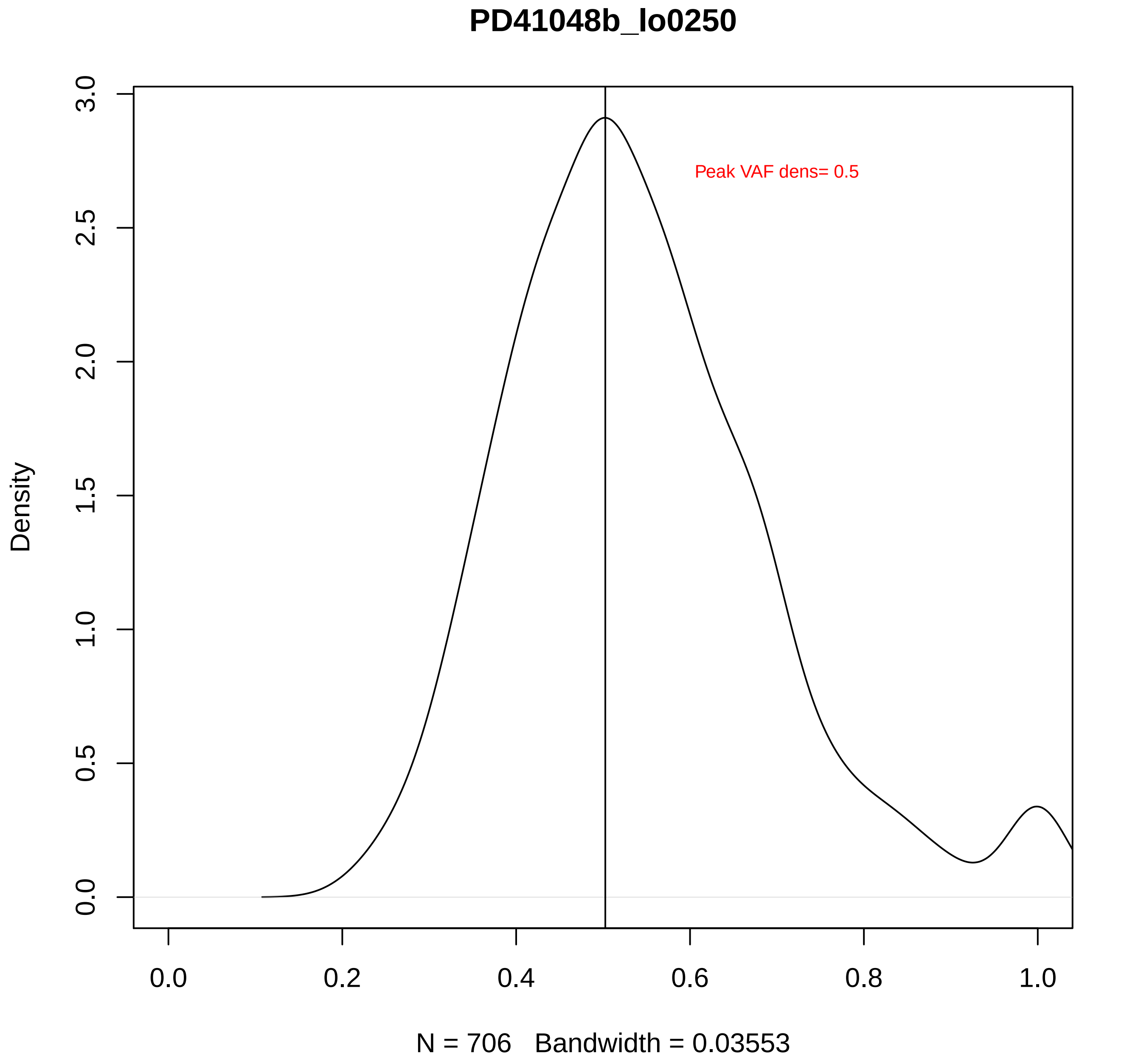
PD41048b_lo0250
Peak VAF dens= 0.5
Density
0.0
0.5
1.0
1.5
2.0
2.5
3.0
0.0
0.2
0.4
0.6
0.8
1.0
N = 706 Bandwidth = 0.03553

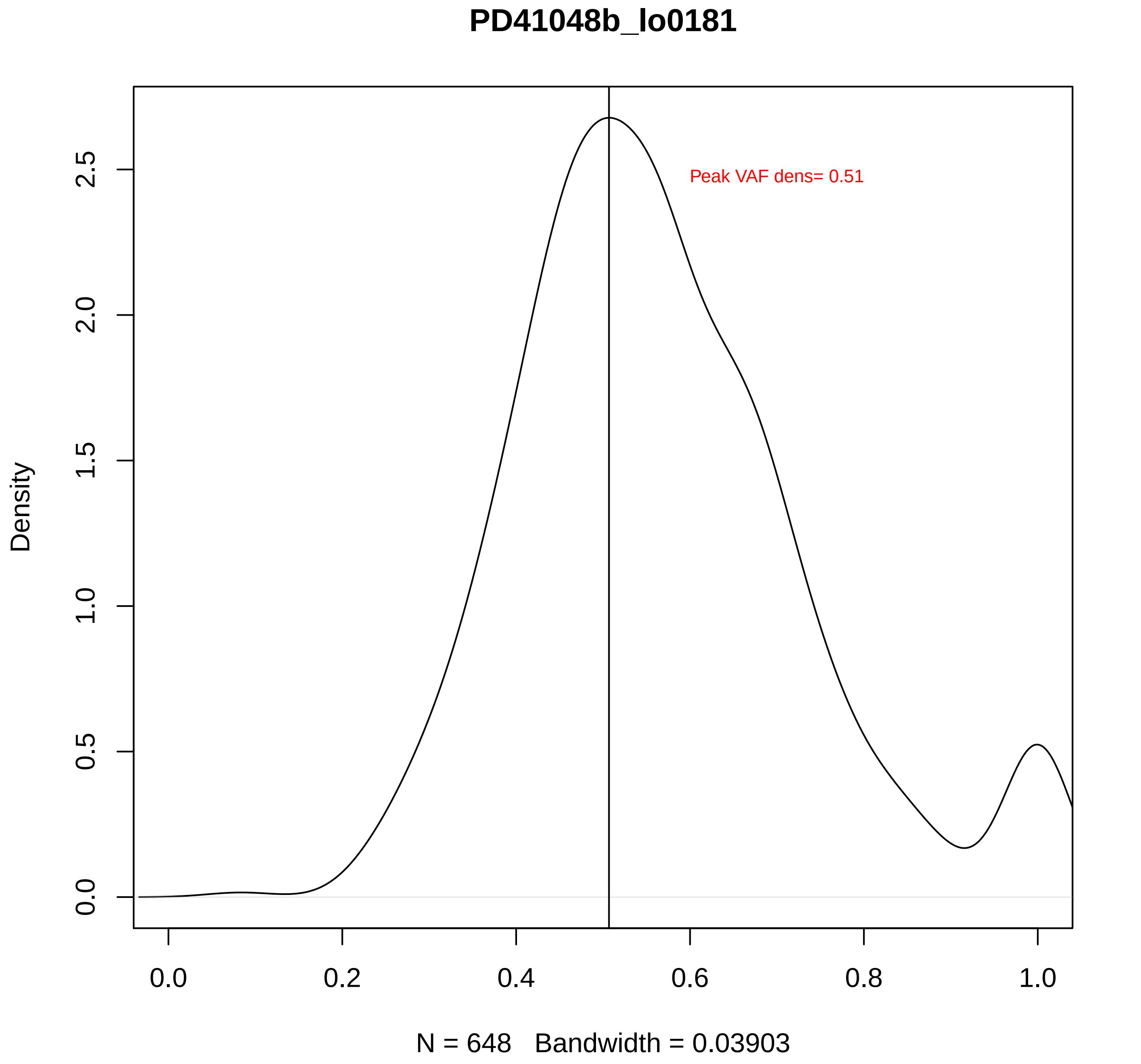
PD41048b_lo0181
Peak VAF dens= 0.51
Density
0.0
0.5
1.0
1.5
2.0
2.5
0.0
0.2
0.4
0.6
0.8
1.0
N = 648   Bandwidth = 0.03903

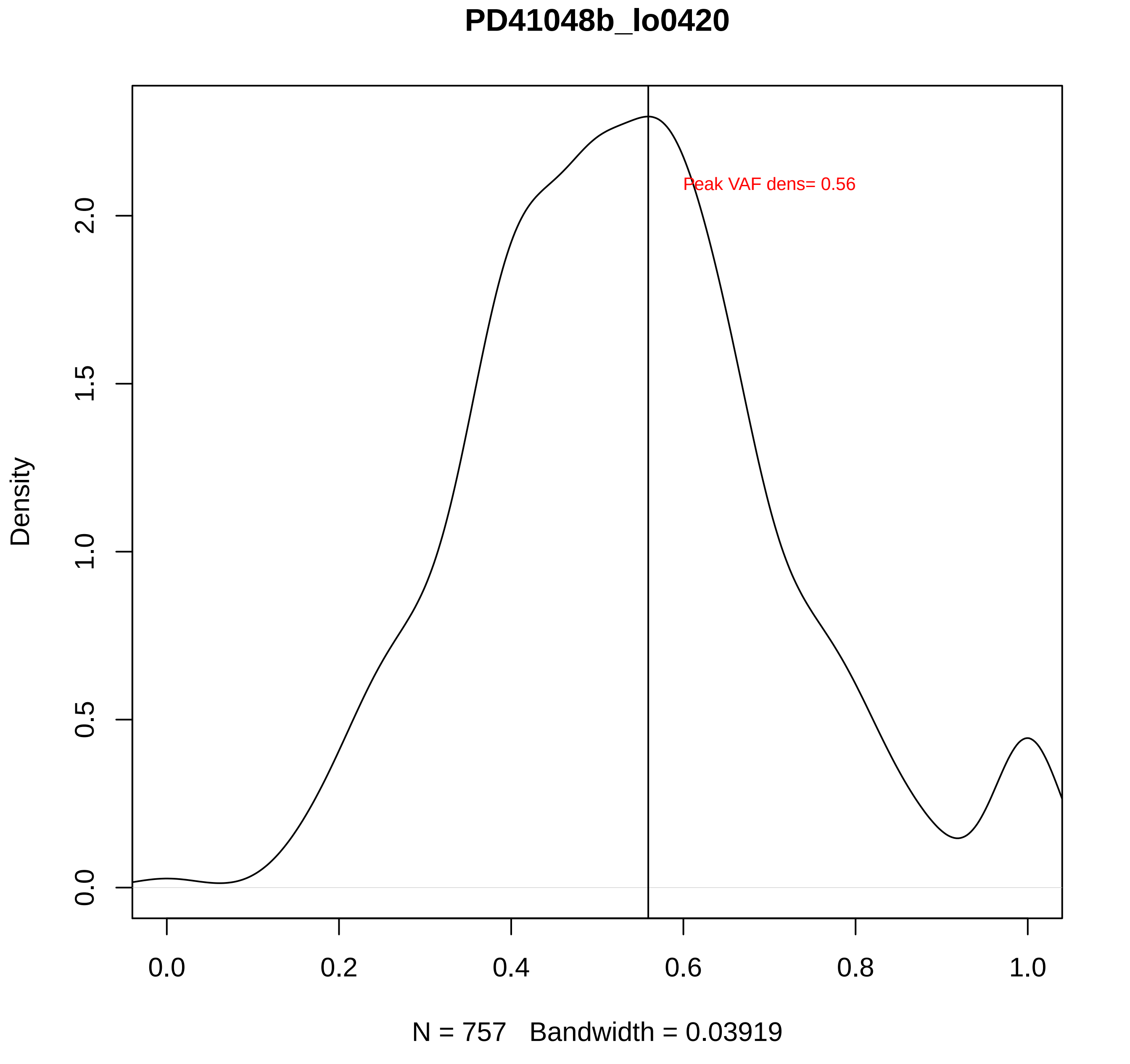

PD41048b_lo0420
Peak VAF dens= 0.56
Density
0.0
0.5
1.0
1.5
2.0
0.0
0.2
0.4
0.6
0.8
1.0
N = 757 Bandwidth = 0.03919

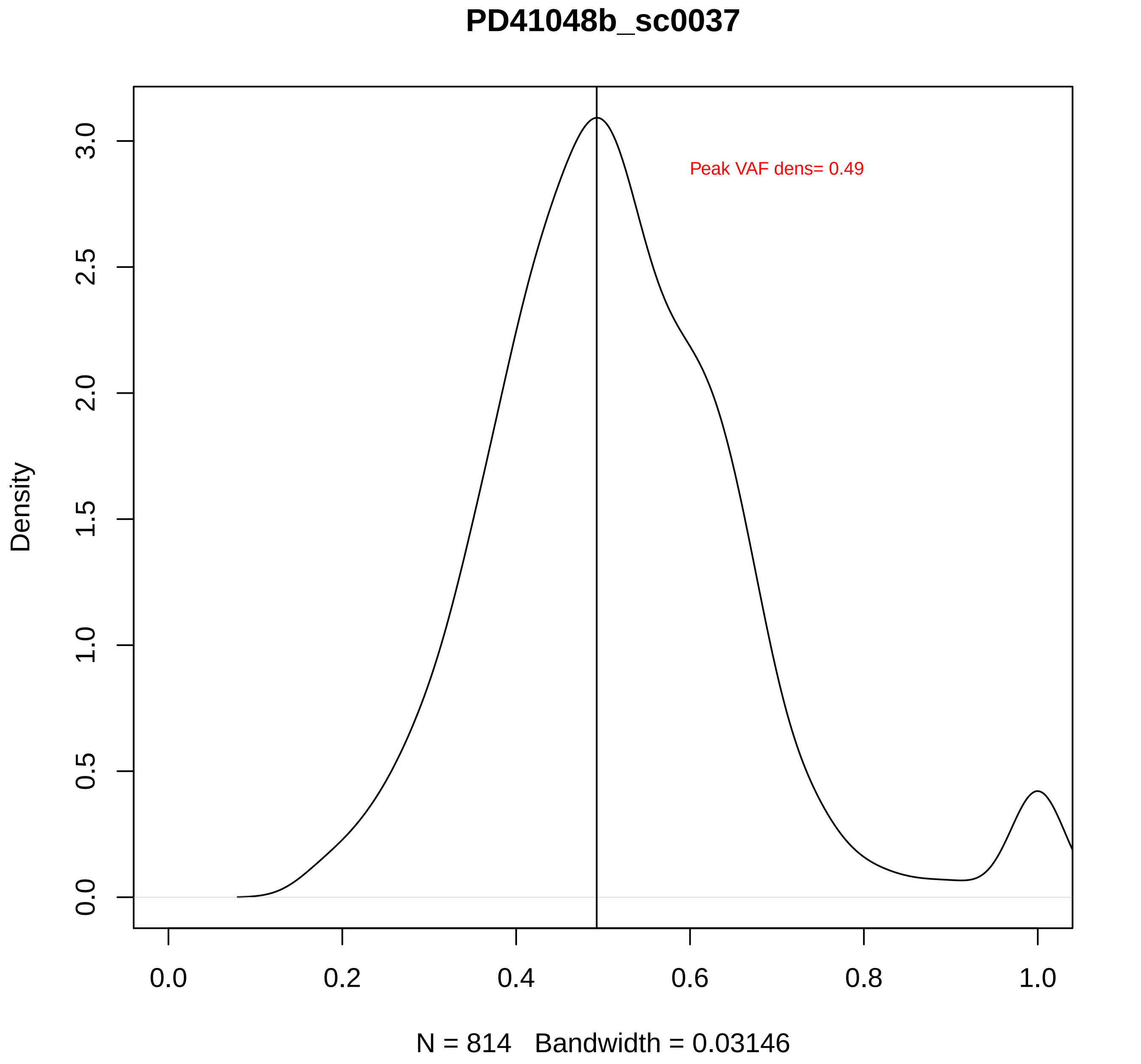
PD41048b_sc0037
Peak VAF dens= 0.49
Density
0.0
0.5
1.0
1.5
2.0
2.5
3.0
0.0
0.2
0.4
0.6
0.8
1.0
N = 814 Bandwidth = 0.03146

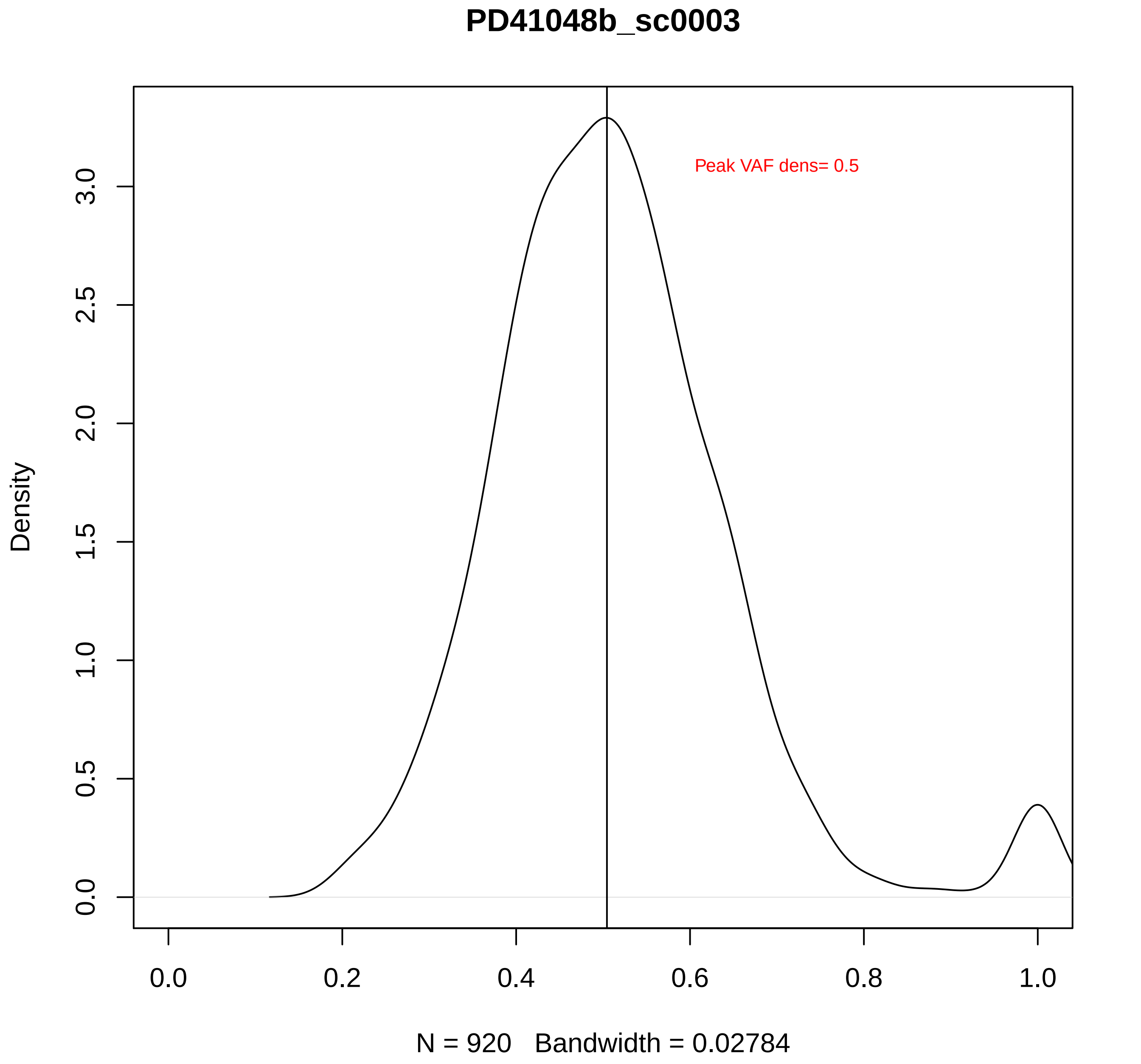
PD41048b_sc0003
Peak VAF dens= 0.5
Density
0.0
0.5
1.0
1.5
2.0
2.5
3.0
0.0
0.2
0.4
0.6
0.8
1.0
N = 920   Bandwidth = 0.02784

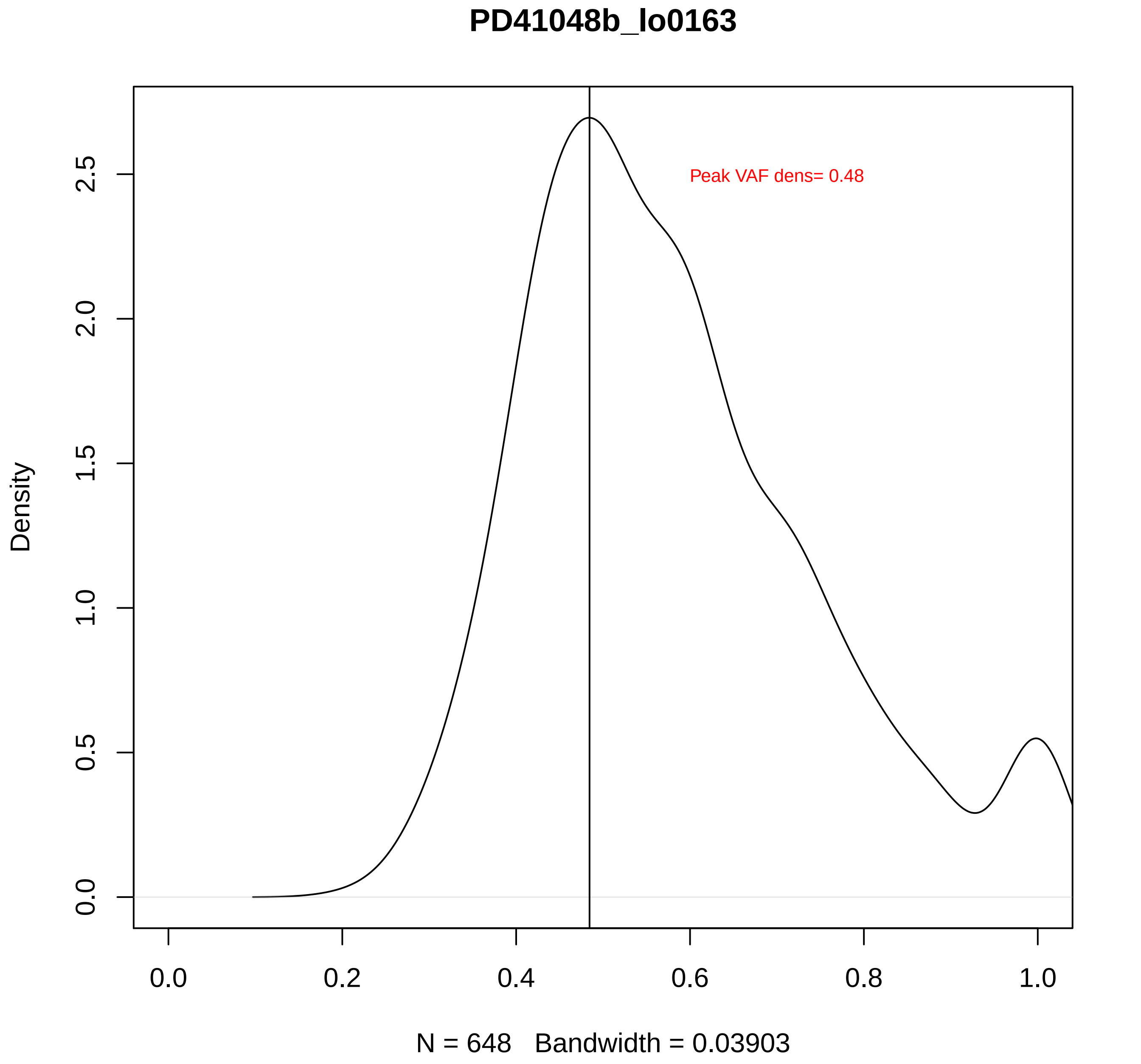
PD41048b_lo0163
Peak VAF dens= 0.48
Density
0.0
0.5
1.0
1.5
2.0
2.5
0.0
0.2
0.4
0.6
0.8
1.0
N = 648   Bandwidth = 0.03903

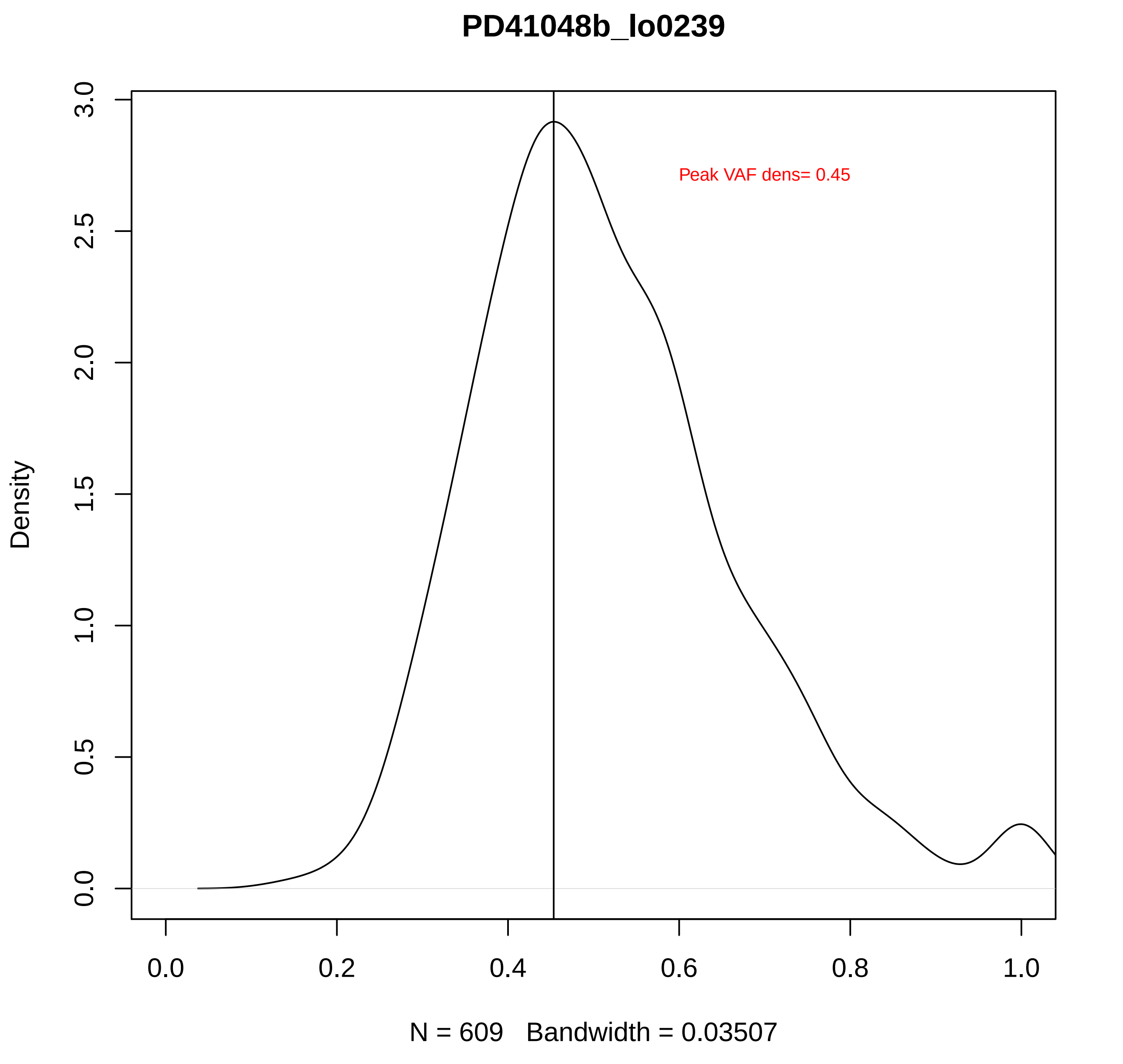

PD41048b_lo0239
Peak VAF dens= 0.45
Density
0.0
0.5
1.0
1.5
2.0
2.5
3.0
0.0
0.2
0.4
0.6
0.8
1.0
N = 609   Bandwidth = 0.03507

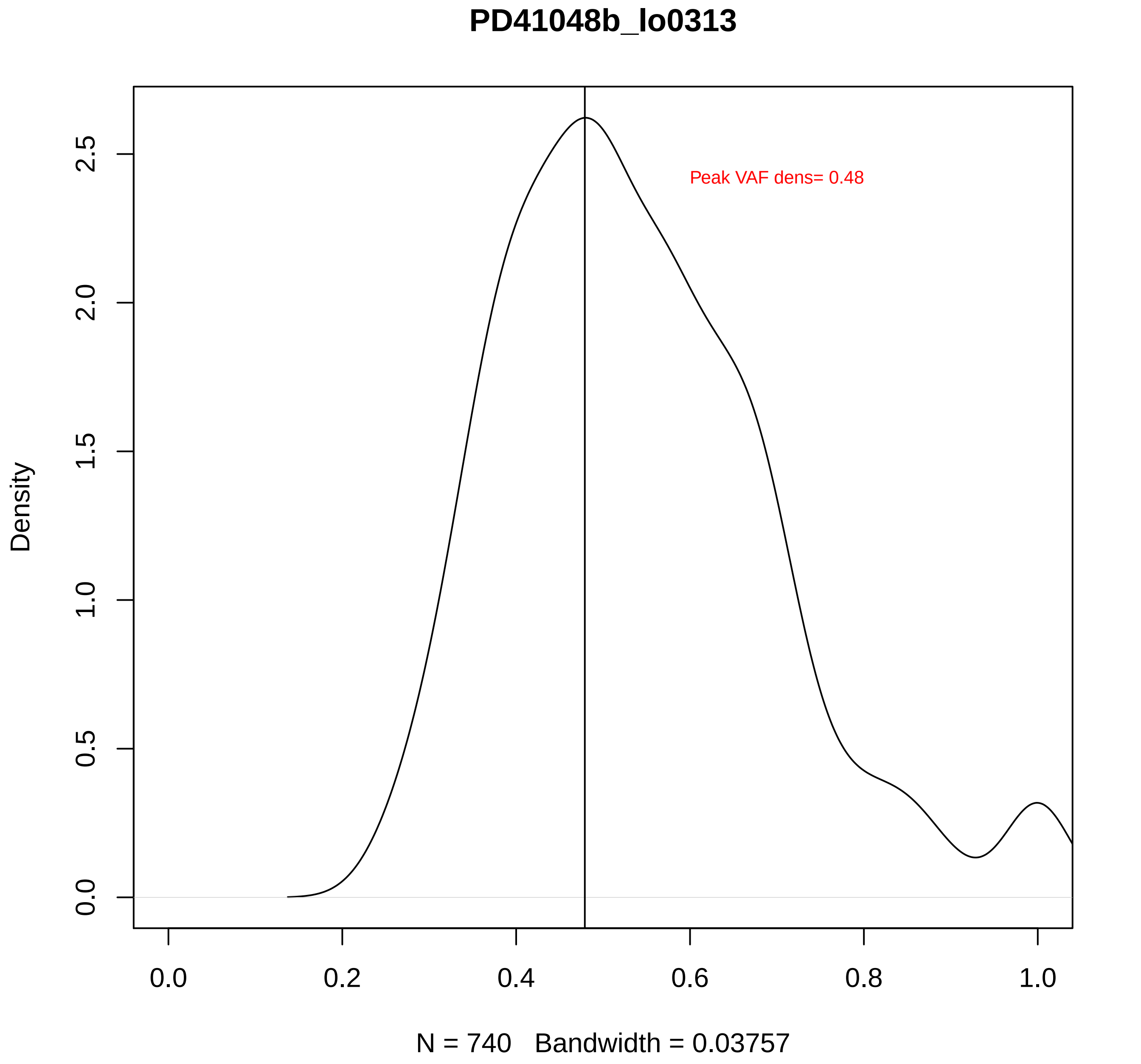
PD41048b_lo0313
Peak VAF dens= 0.48
Density
0.0
0.5
1.0
1.5
2.0
2.5
0.0
0.2
0.4
0.6
0.8
1.0
N = 740 Bandwidth = 0.03757

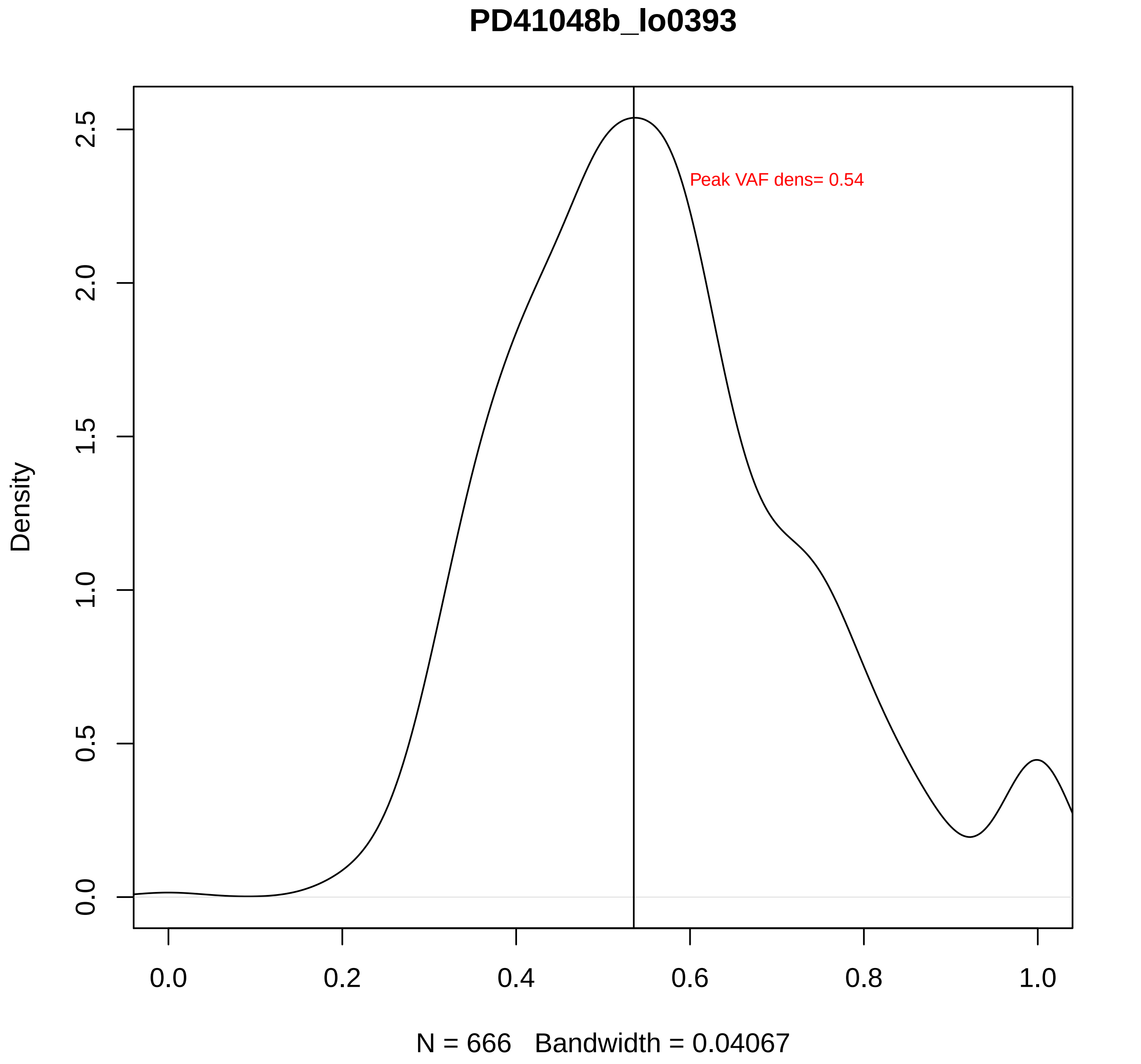
PD41048b_lo0393
Peak VAF dens= 0.54
Density
0.0
0.5
1.0
1.5
2.0
2.5
0.0
0.2
0.4
0.6
0.8
1.0
N = 666   Bandwidth = 0.04067

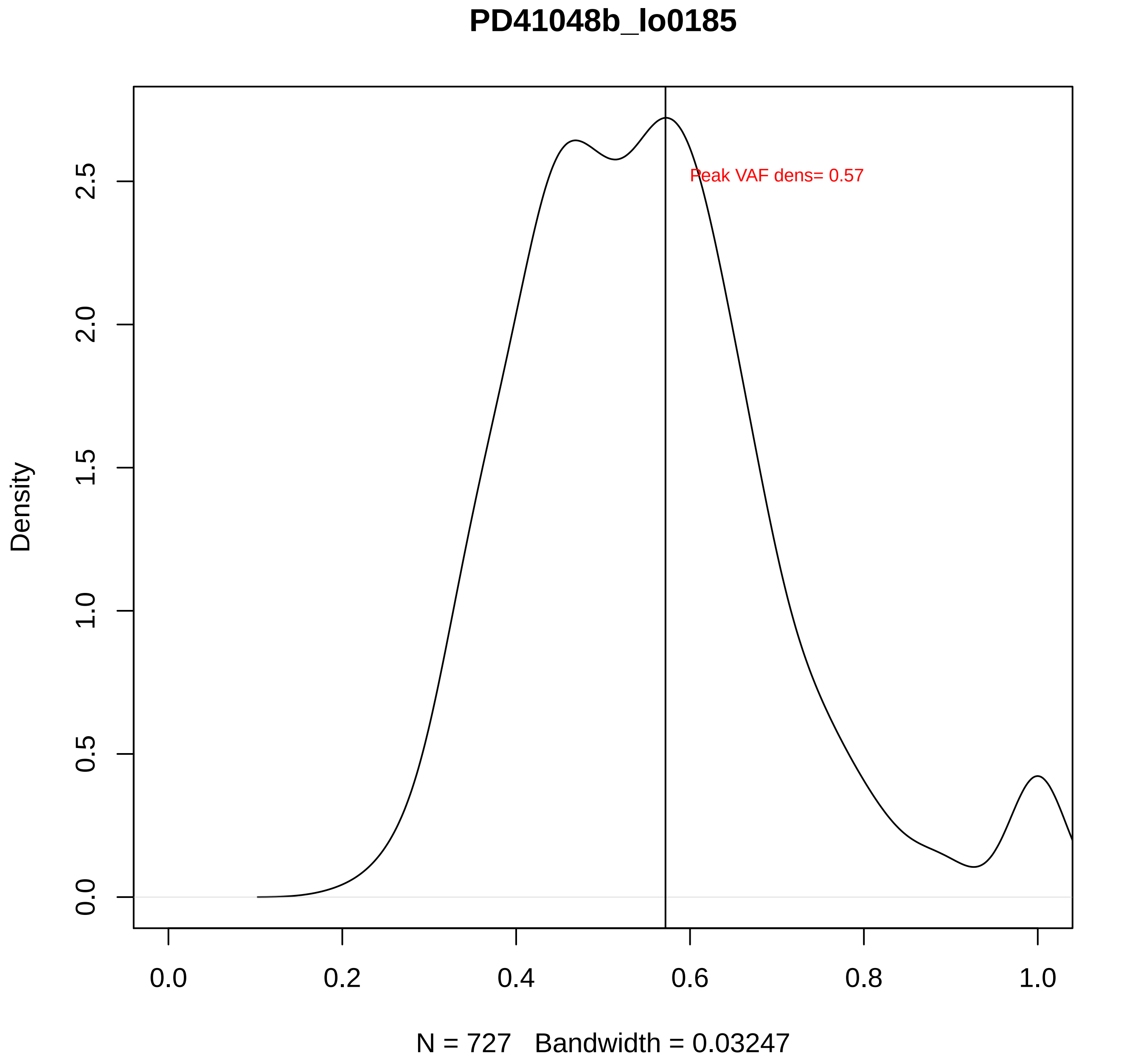

PD41048b_lo0185
Density
Peak VAF dens= 0.57
0.0
0.5
1.0
1.5
2.0
2.5
0.0
0.2
0.4
0.6
0.8
1.0
N = 727 Bandwidth = 0.03247

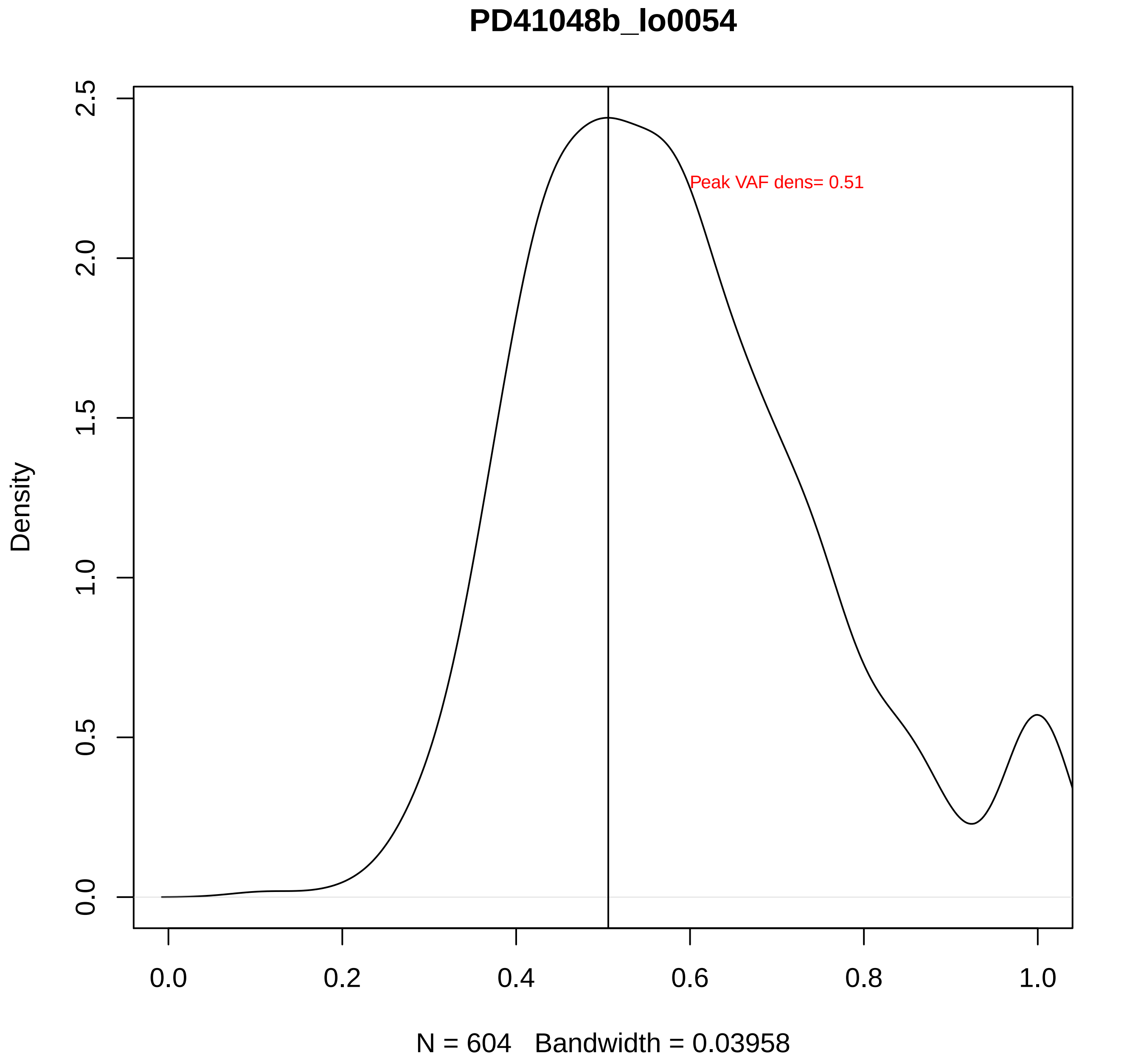

PD41048b_lo0054
Peak VAF dens= 0.51
Density
0.0
0.5
1.0
1.5
2.0
2.5
0.0
0.2
0.4
0.6
0.8
1.0
N = 604   Bandwidth = 0.03958

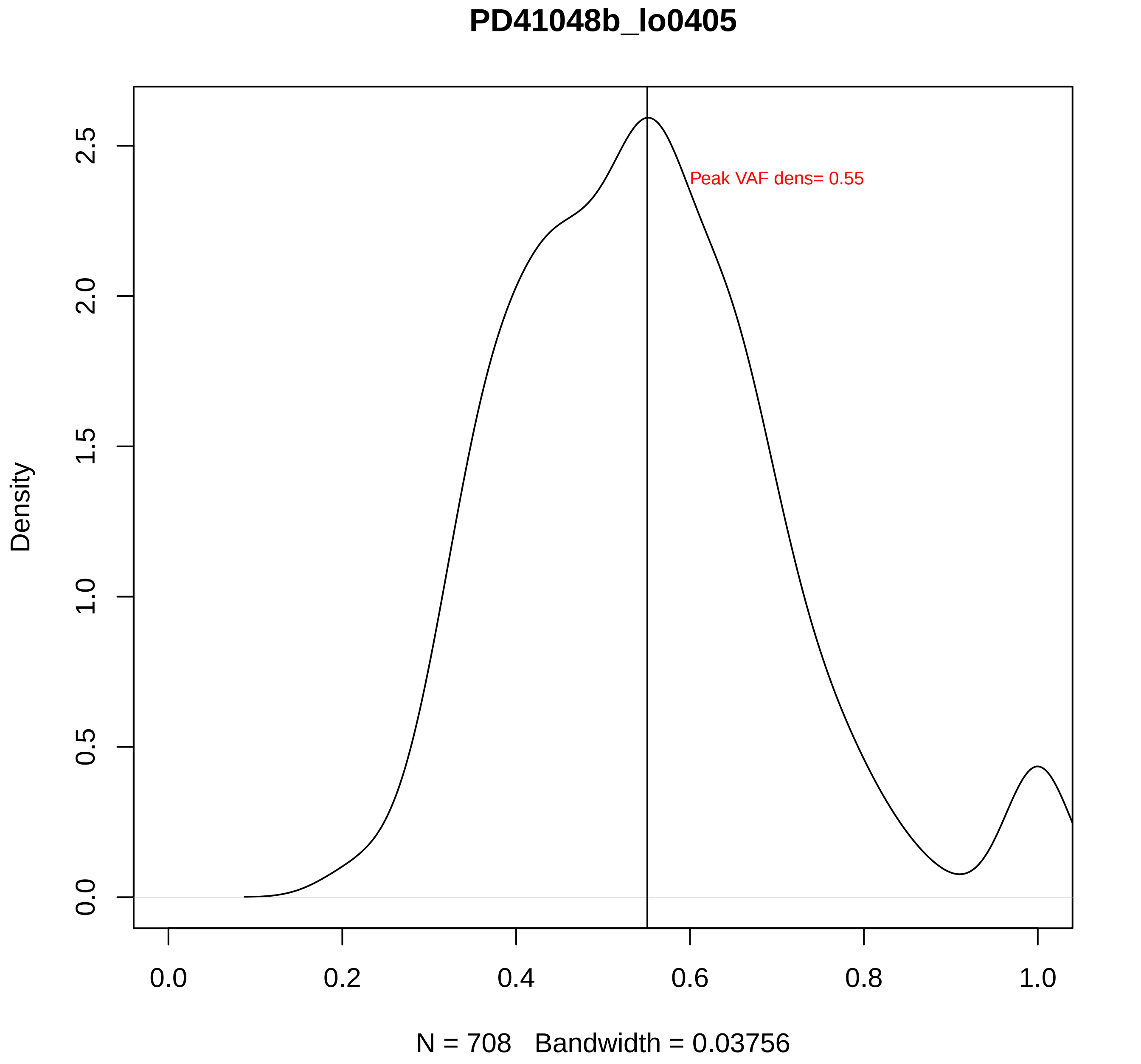
PD41048b_lo0405
Peak VAF dens= 0.55
Density
0.0
0.5
1.0
1.5
2.0
2.5
0.0
0.2
0.4
0.6
0.8
1.0
N = 708   Bandwidth = 0.03756

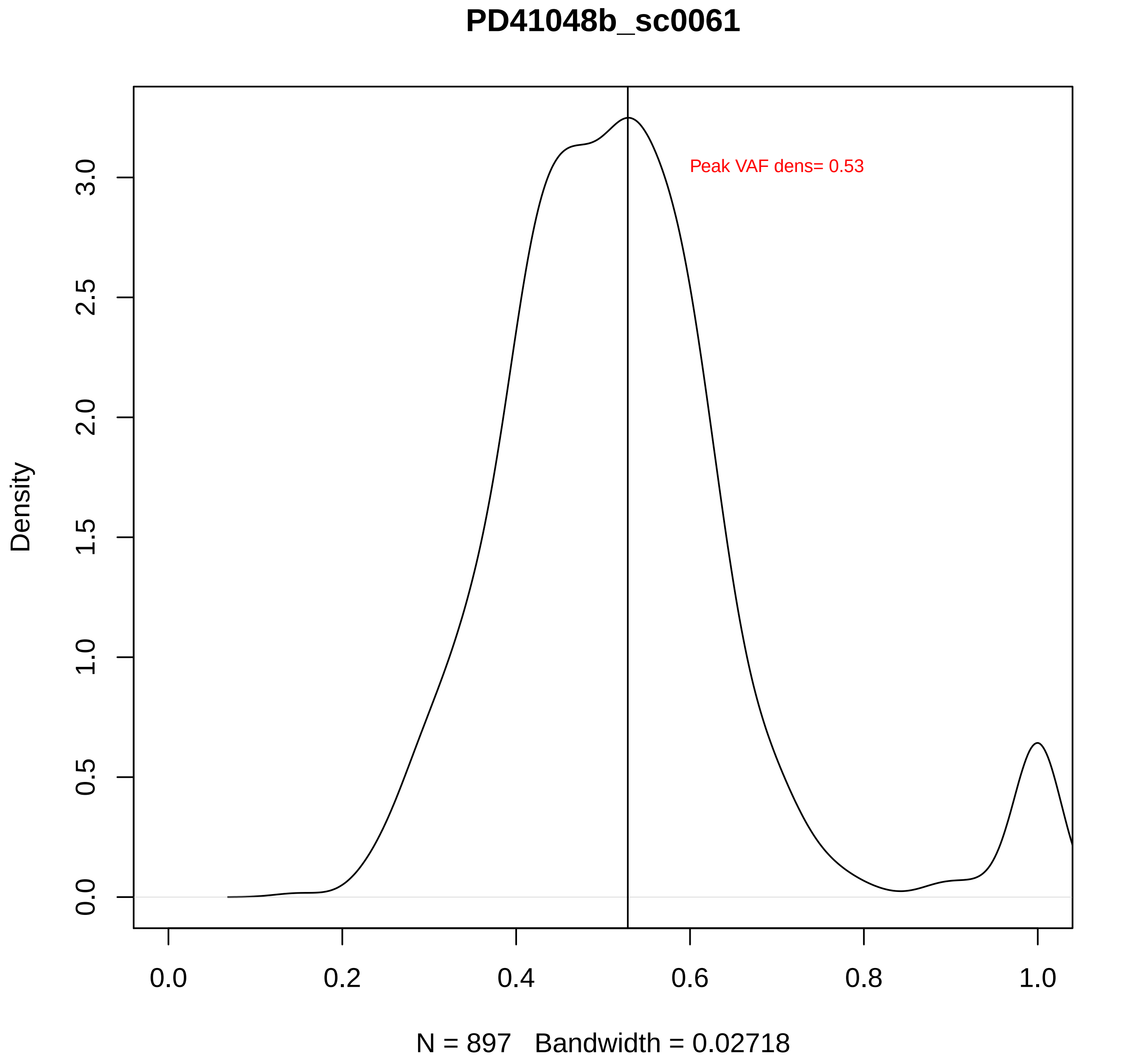

PD41048b_sc0061
Peak VAF dens= 0.53
Density
0.0
0.5
1.0
1.5
2.0
2.5
3.0
0.0
0.2
0.4
0.6
0.8
1.0
N = 897   Bandwidth = 0.02718

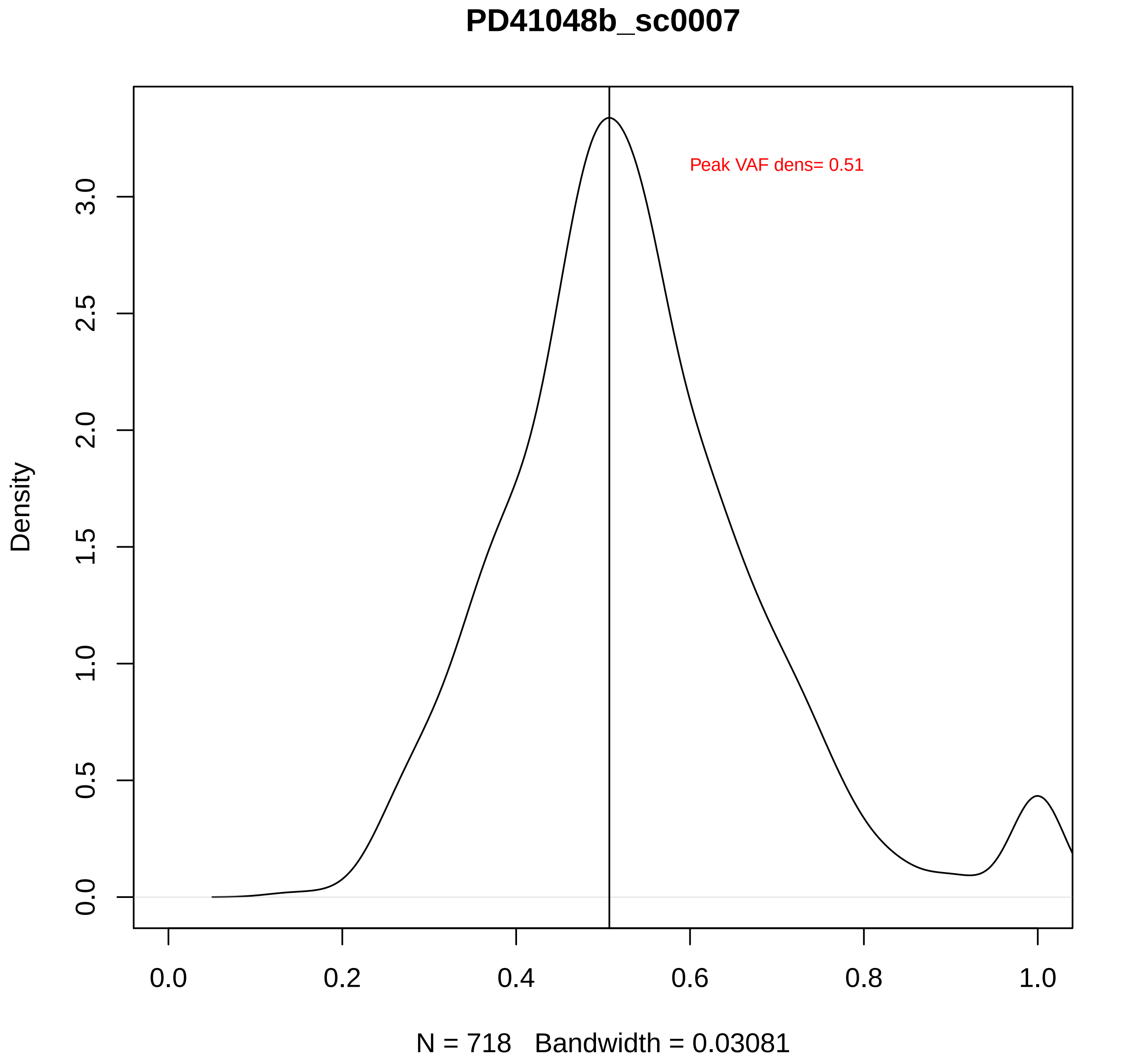
PD41048b_sc0007
Peak VAF dens= 0.51
Density
0.0
0.5
1.0
1.5
2.0
2.5
3.0
0.0
0.2
0.4
0.6
0.8
1.0
N = 718 Bandwidth = 0.03081

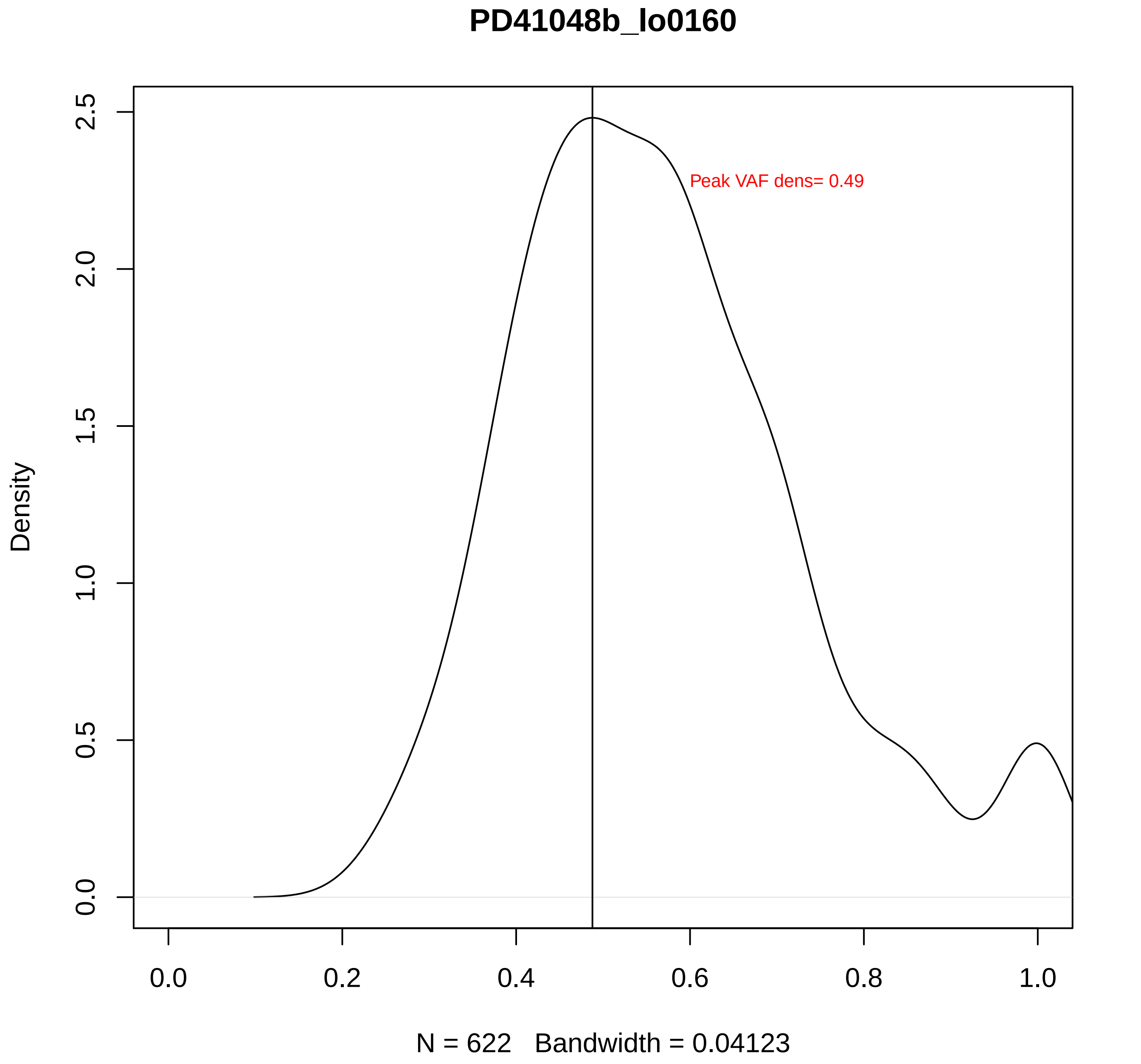
PD41048b_lo0160
Peak VAF dens= 0.49
Density
0.0
0.5
1.0
1.5
2.0
2.5
0.0
0.2
0.4
0.6
0.8
1.0
N = 622 Bandwidth = 0.04123

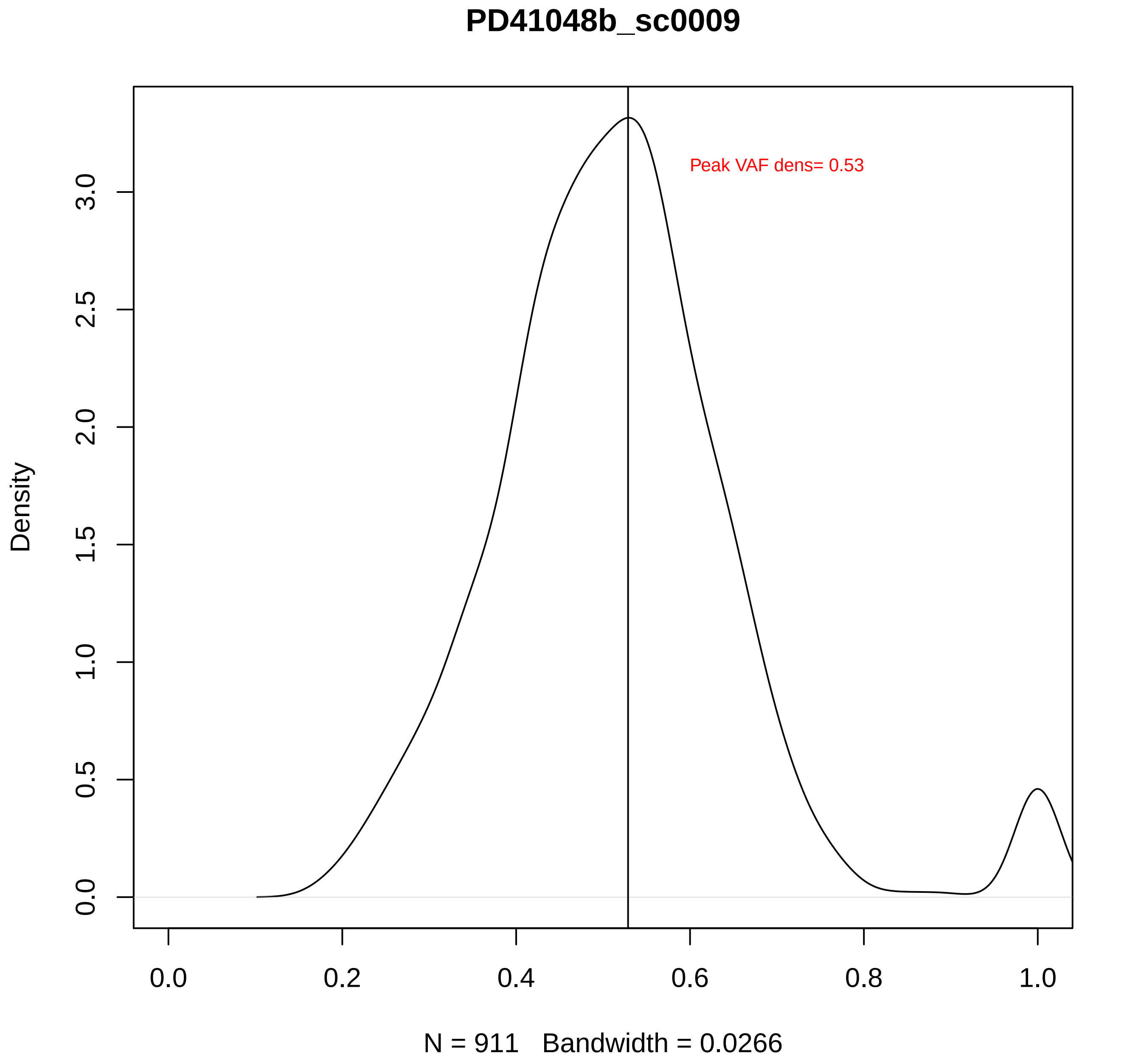
PD41048b_sc0009
Peak VAF dens= 0.53
Density
0.0
0.5
1.0
1.5
2.0
2.5
3.0
0.0
0.2
0.4
0.6
0.8
1.0
N = 911   Bandwidth = 0.0266

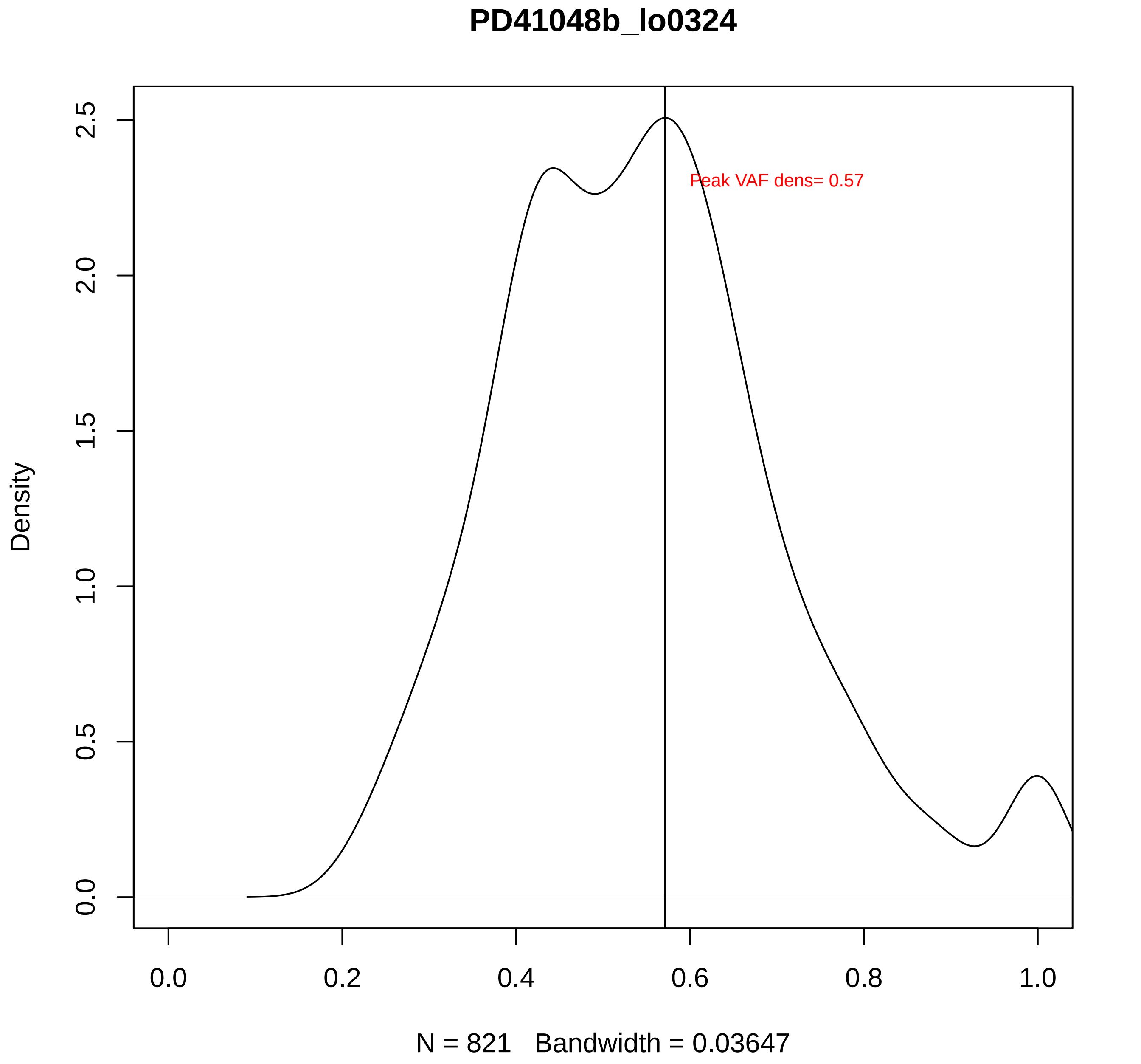

PD41048b_lo0324
Peak VAF dens= 0.57
Density
0.0
0.5
1.0
1.5
2.0
2.5
0.0
0.2
0.4
0.6
0.8
1.0
N = 821   Bandwidth = 0.03647

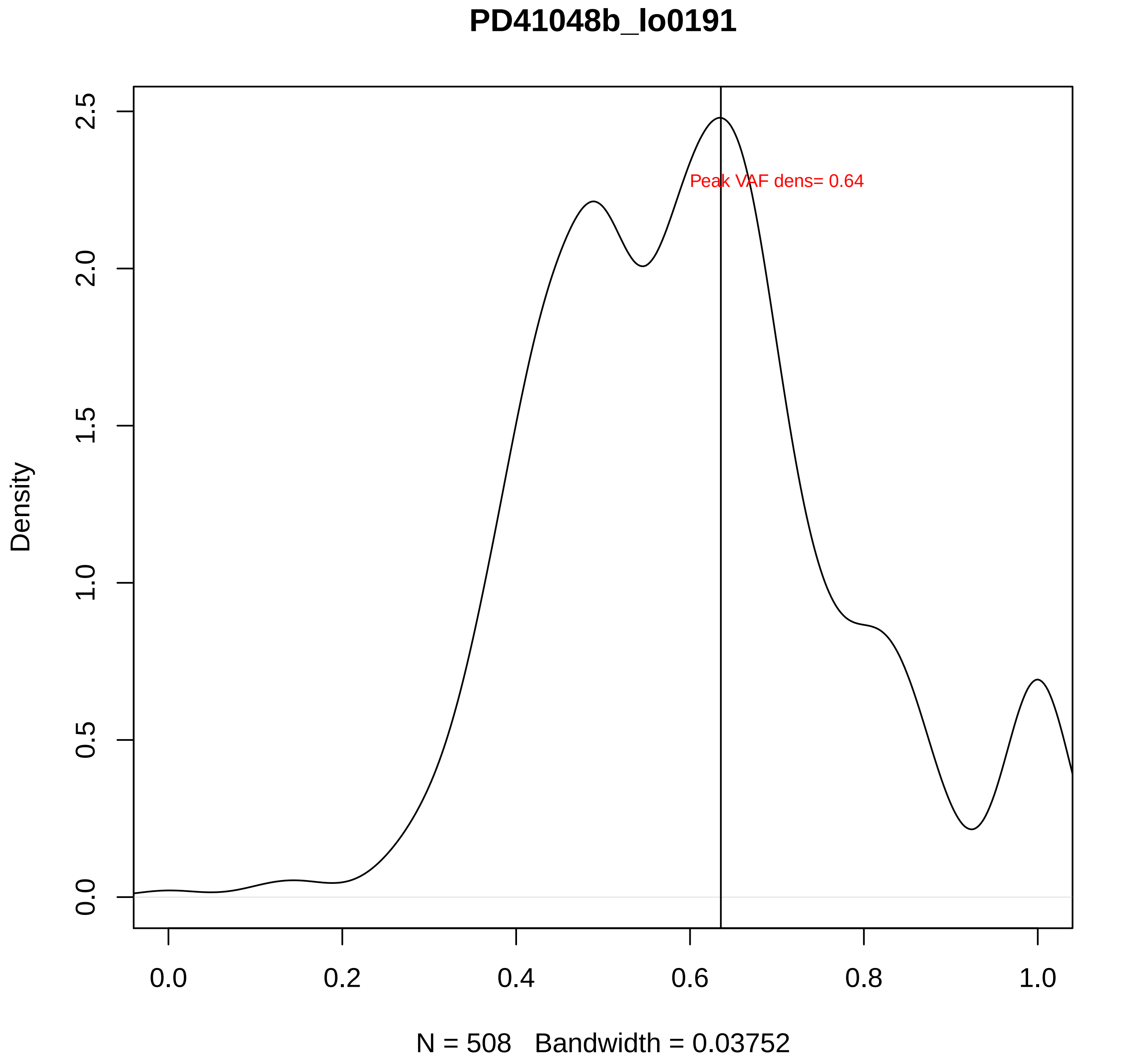
PD41048b_lo0191
Peak VAF dens= 0.64
Density
0.0
0.5
1.0
1.5
2.0
2.5
0.0
0.2
0.4
0.6
0.8
1.0
N = 508 Bandwidth = 0.03752

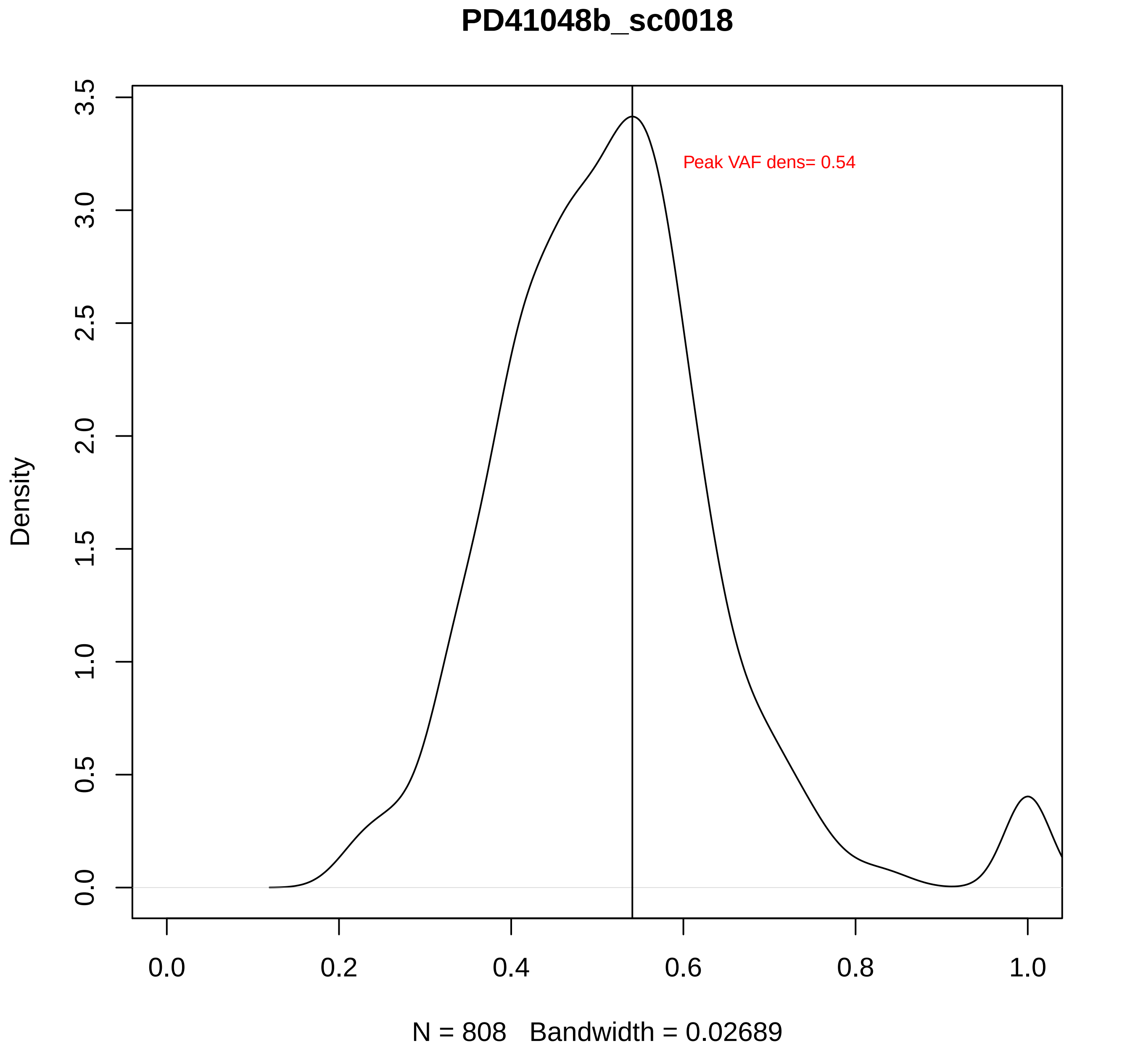
PD41048b_sc0018
Peak VAF dens= 0.54
Density
0.0
0.5
1.0
1.5
2.0
2.5
3.0
3.5
0.0
0.2
0.4
0.6
0.8
1.0
N = 808   Bandwidth = 0.02689

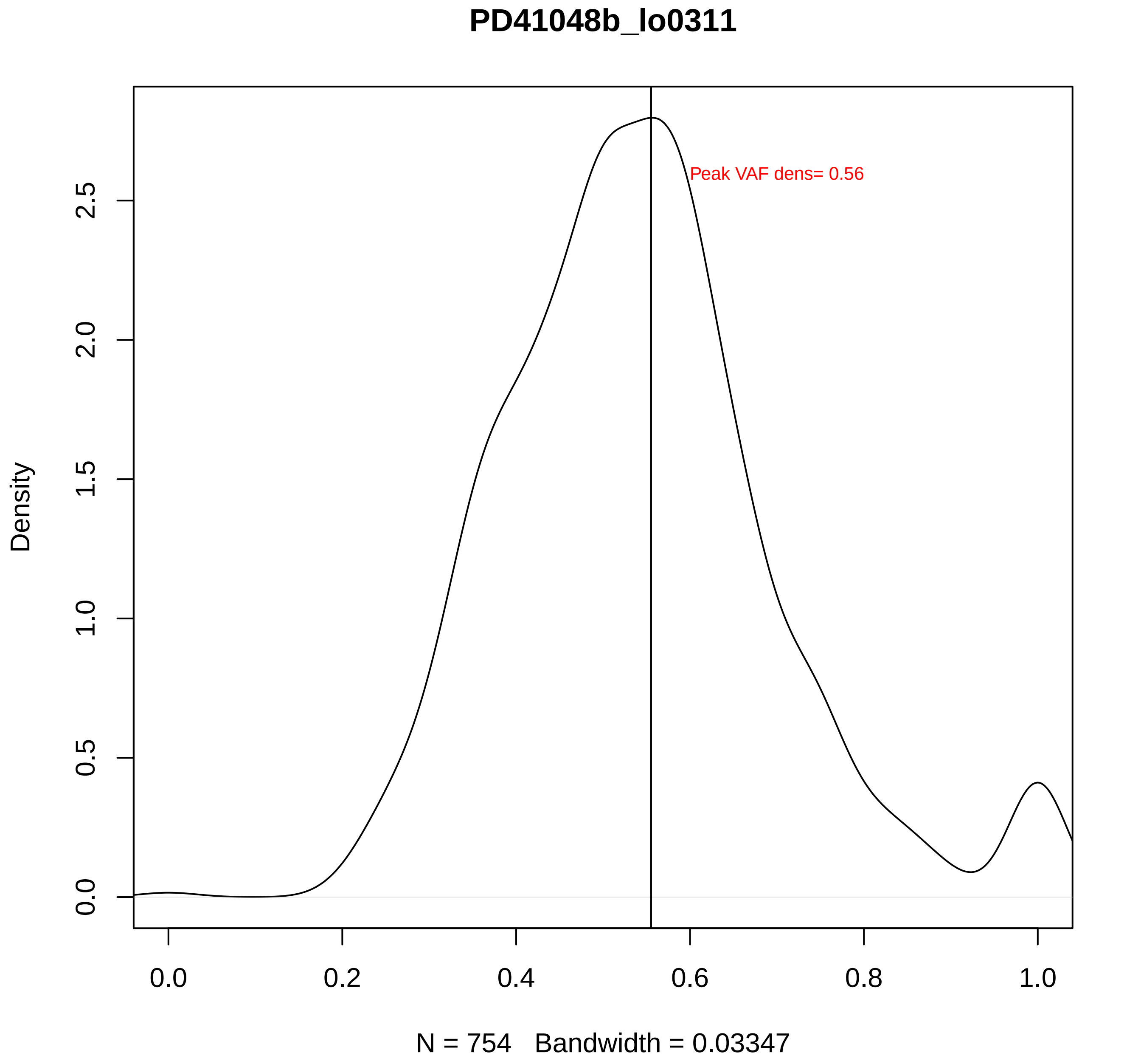

PD41048b_lo0311
Peak VAF dens= 0.56
Density
0.0
0.5
1.0
1.5
2.0
2.5
0.0
0.2
0.4
0.6
0.8
1.0
N = 754   Bandwidth = 0.03347

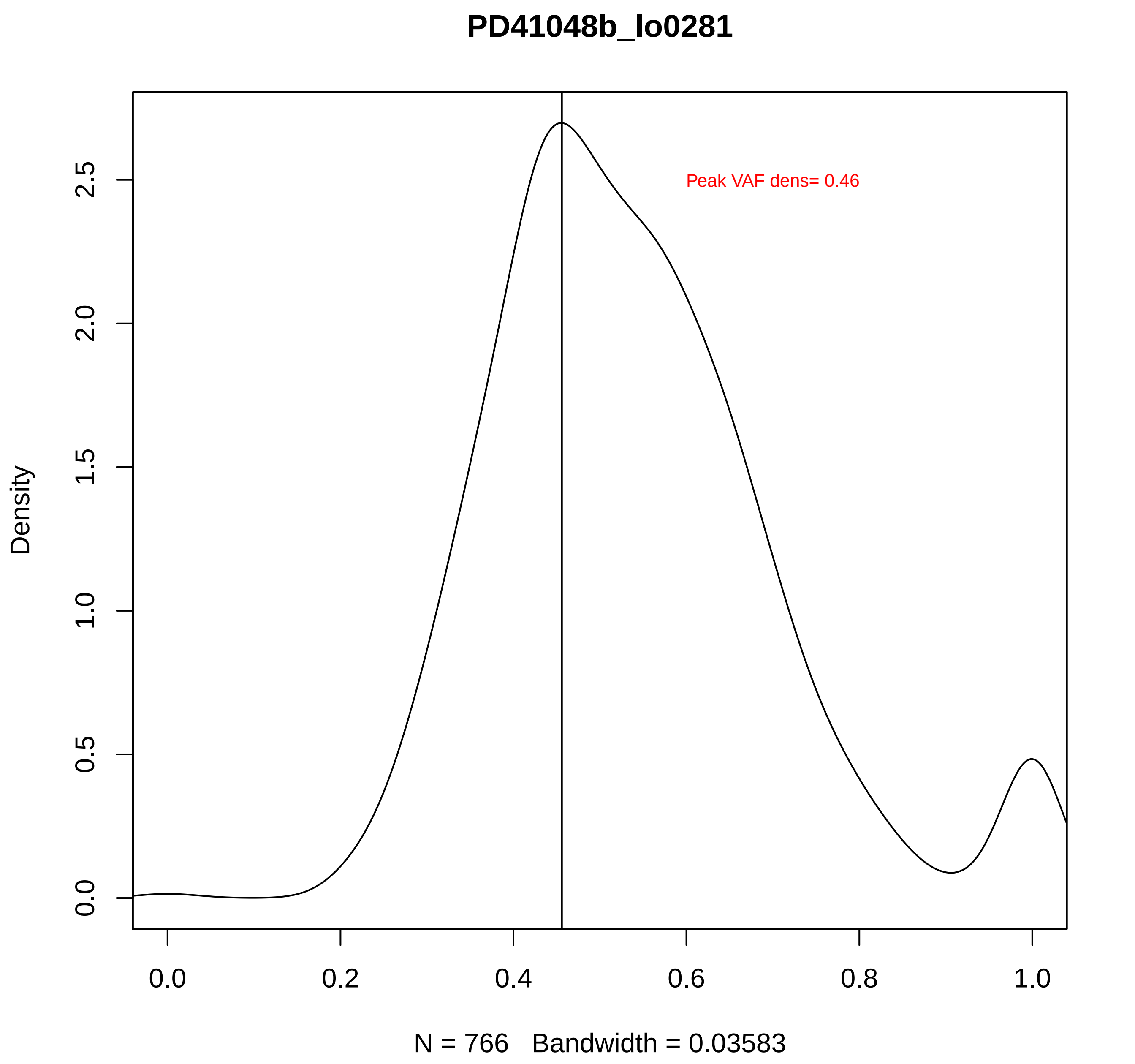
PD41048b_lo0281
Peak VAF dens= 0.46
Density
0.0
0.5
1.0
1.5
2.0
2.5
0.0
0.2
0.4
0.6
0.8
1.0
N = 766 Bandwidth = 0.03583

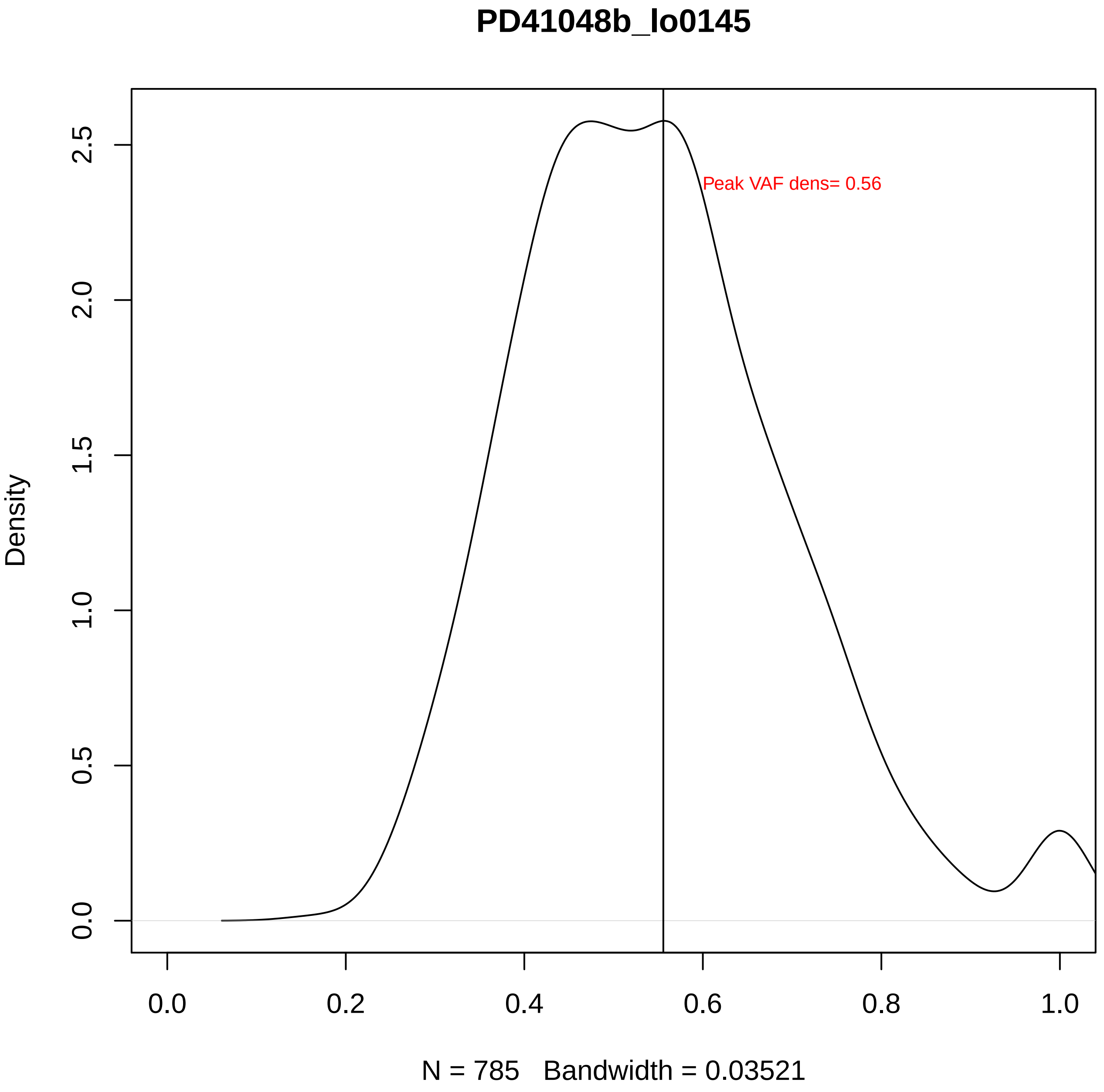

PD41048b_lo0145
Peak VAF dens= 0.56
Density
0.0
0.5
1.0
1.5
2.0
2.5
0.0
0.2
0.4
0.6
0.8
1.0
N = 785   Bandwidth = 0.03521

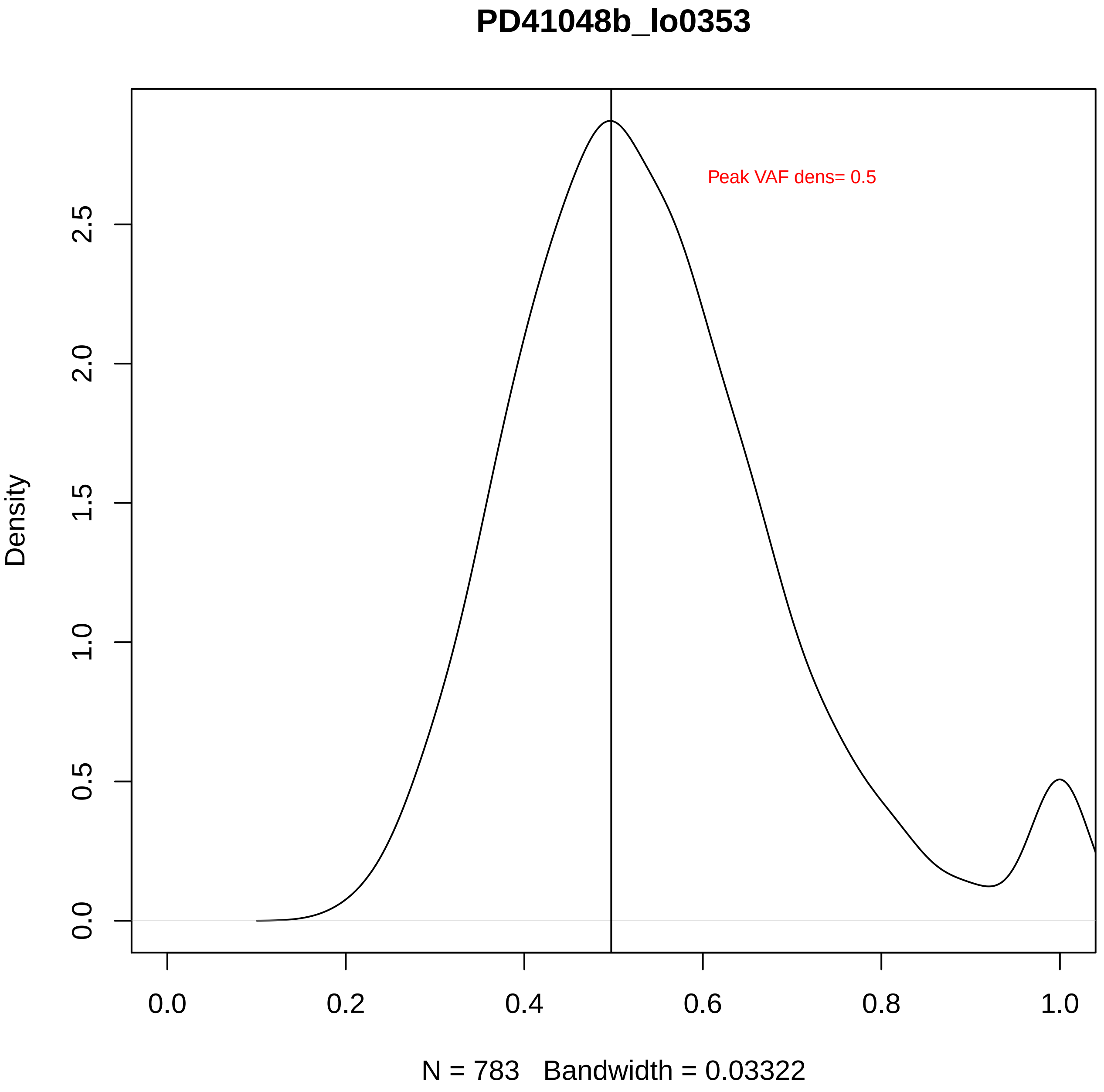
PD41048b_lo0353
Peak VAF dens= 0.5
Density
0.0
0.5
1.0
1.5
2.0
2.5
0.0
0.2
0.4
0.6
0.8
1.0
N = 783   Bandwidth = 0.03322

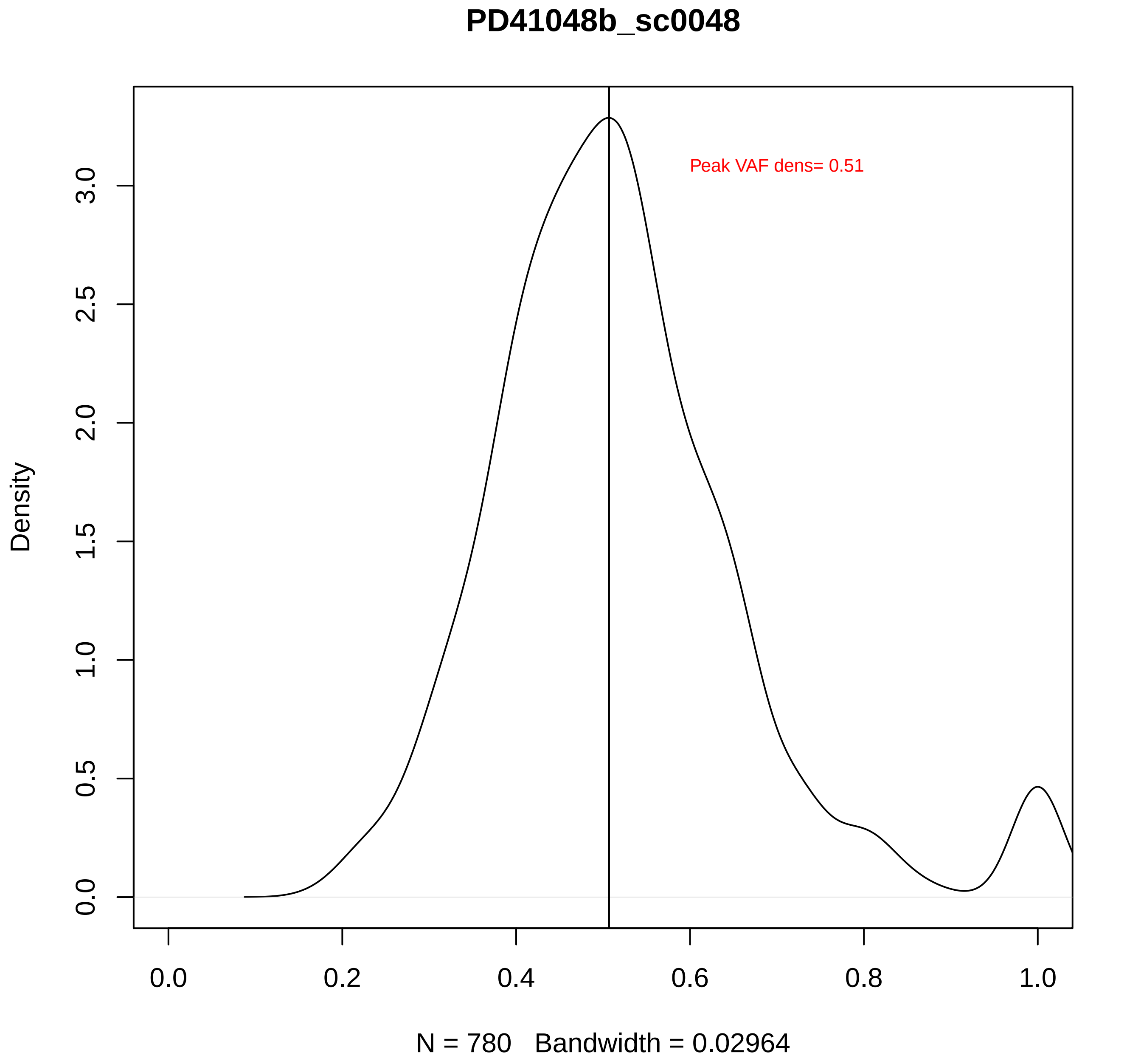
PD41048b_sc0048
Peak VAF dens= 0.51
Density
0.0
0.5
1.0
1.5
2.0
2.5
3.0
0.0
0.2
0.4
0.6
0.8
1.0
N = 780 Bandwidth = 0.02964

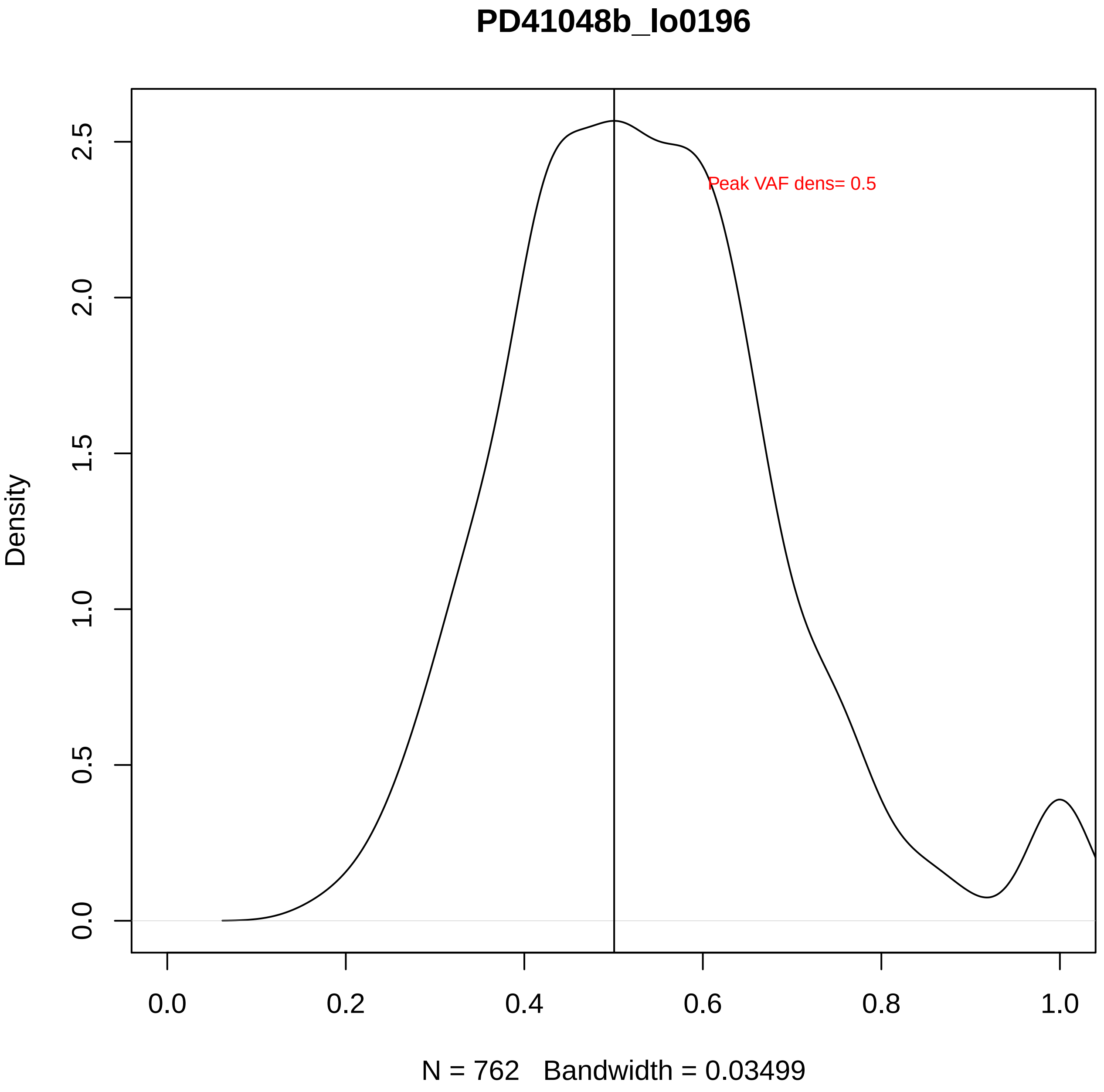
PD41048b_lo0196
Peak VAF dens= 0.5
Density
0.0
0.5
1.0
1.5
2.0
2.5
0.0
0.2
0.4
0.6
0.8
1.0
N = 762 Bandwidth = 0.03499

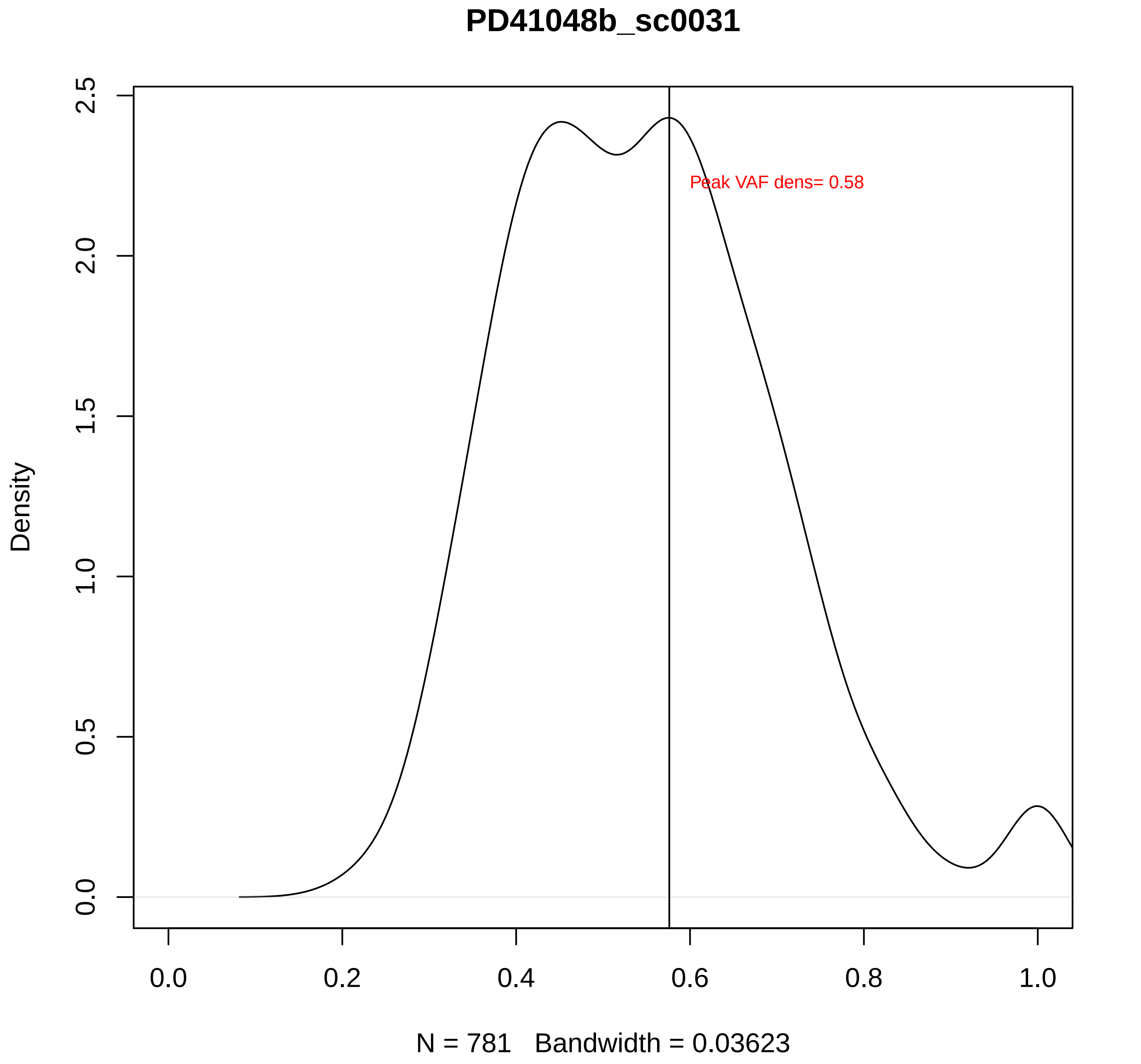
PD41048b_sc0031
Peak VAF dens= 0.58
Density
2.5
2.0
1.5
1.0
0.5
0.0
0.0
0.2
0.4
0.6
0.8
1.0
N = 781   Bandwidth = 0.03623

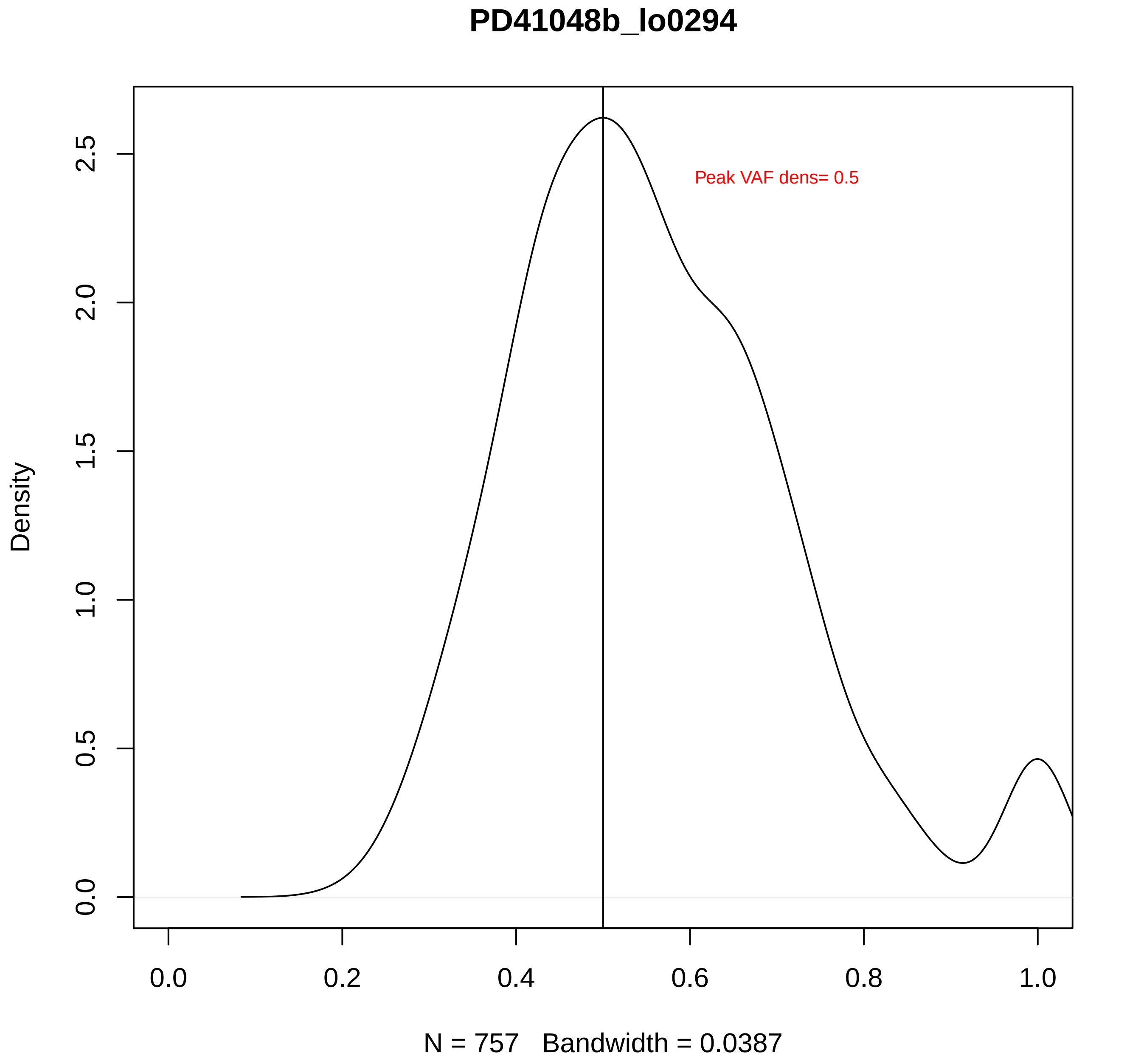
PD41048b_lo0294
Peak VAF dens= 0.5
Density
0.0
0.5
1.0
1.5
2.0
2.5
0.0
0.2
0.4
0.6
0.8
1.0
N = 757 Bandwidth = 0.0387

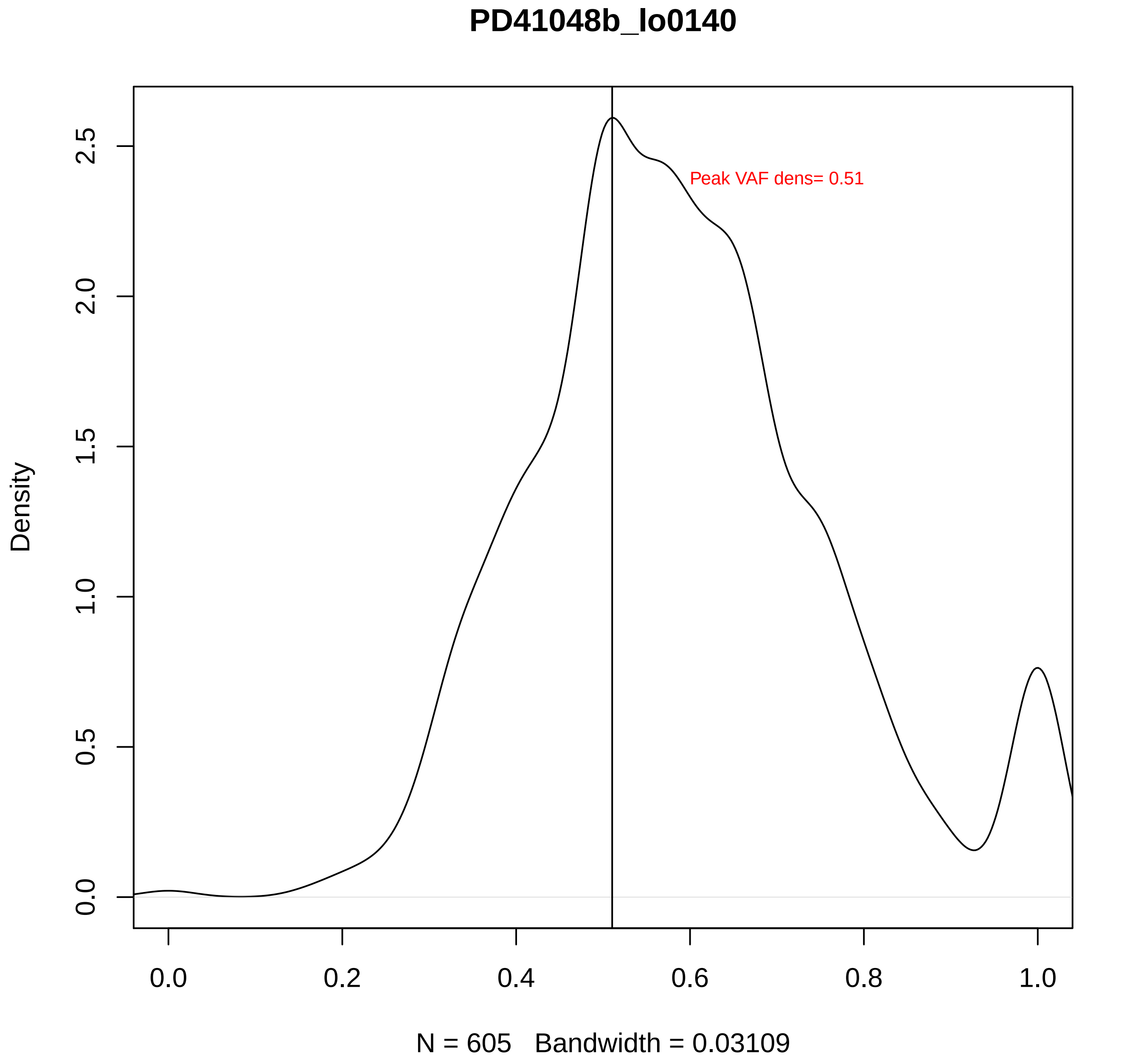
PD41048b_lo0140
Peak VAF dens= 0.51
Density
0.0
0.5
1.0
1.5
2.0
2.5
0.0
0.2
0.4
0.6
0.8
1.0
N = 605   Bandwidth = 0.03109

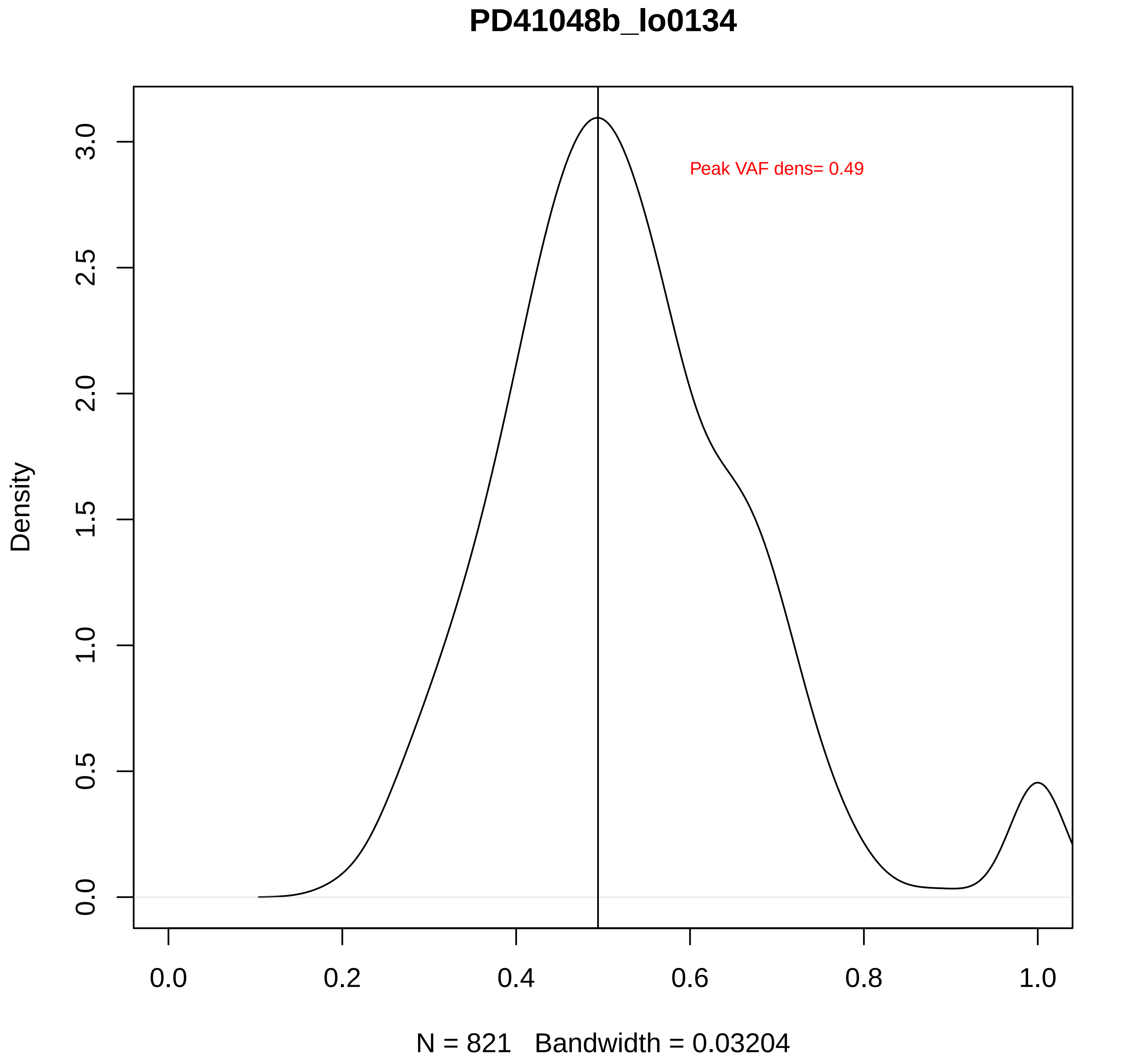
PD41048b_lo0134
Peak VAF dens= 0.49
Density
0.0
0.5
1.0
1.5
2.0
2.5
3.0
0.0
0.2
0.4
0.6
0.8
1.0
N = 821   Bandwidth = 0.03204

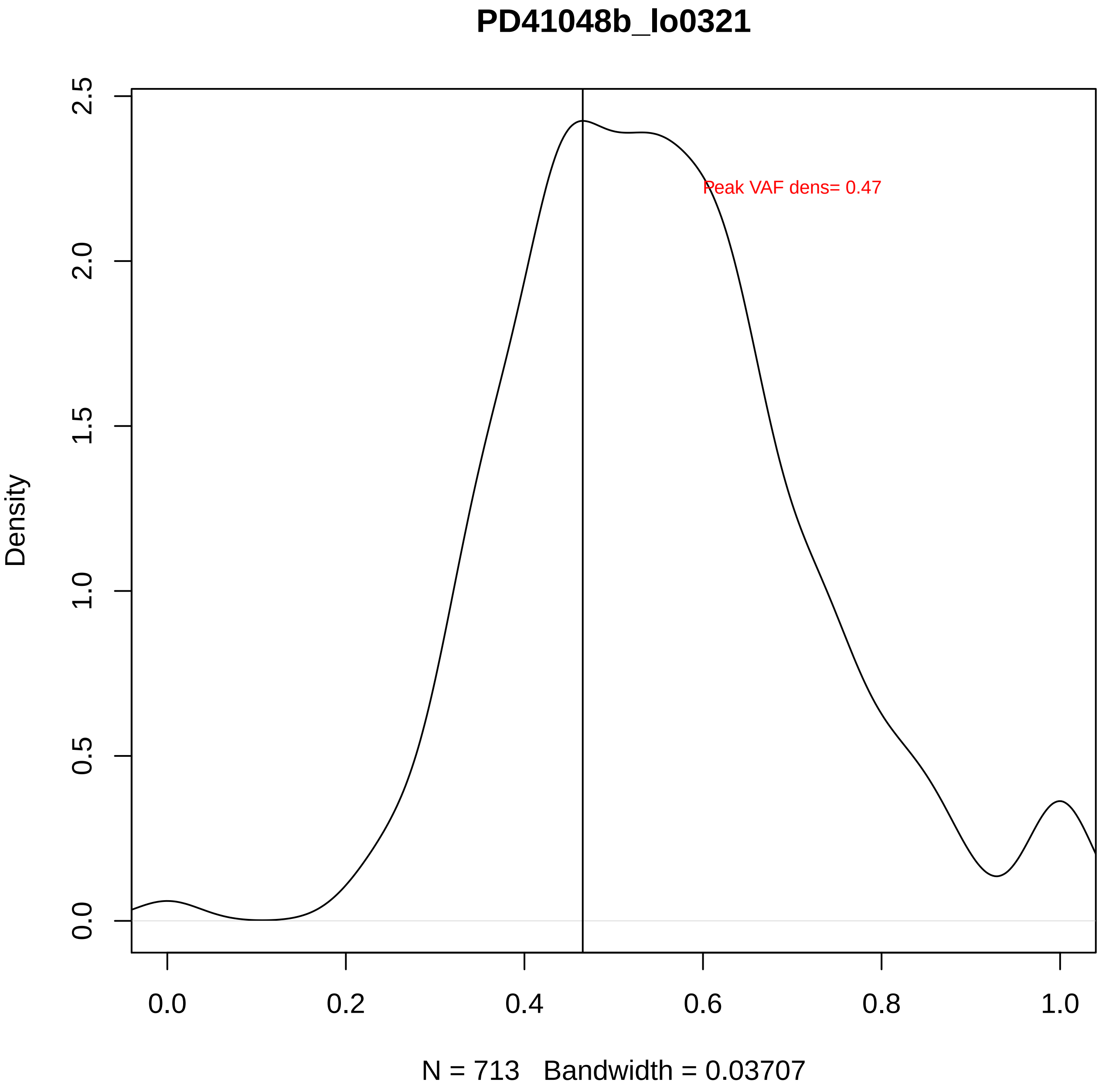

PD41048b_lo0321
Peak VAF dens= 0.47
Density
0.0
0.5
1.0
1.5
2.0
2.5
0.0
0.2
0.4
0.6
0.8
1.0
N = 713 Bandwidth = 0.03707

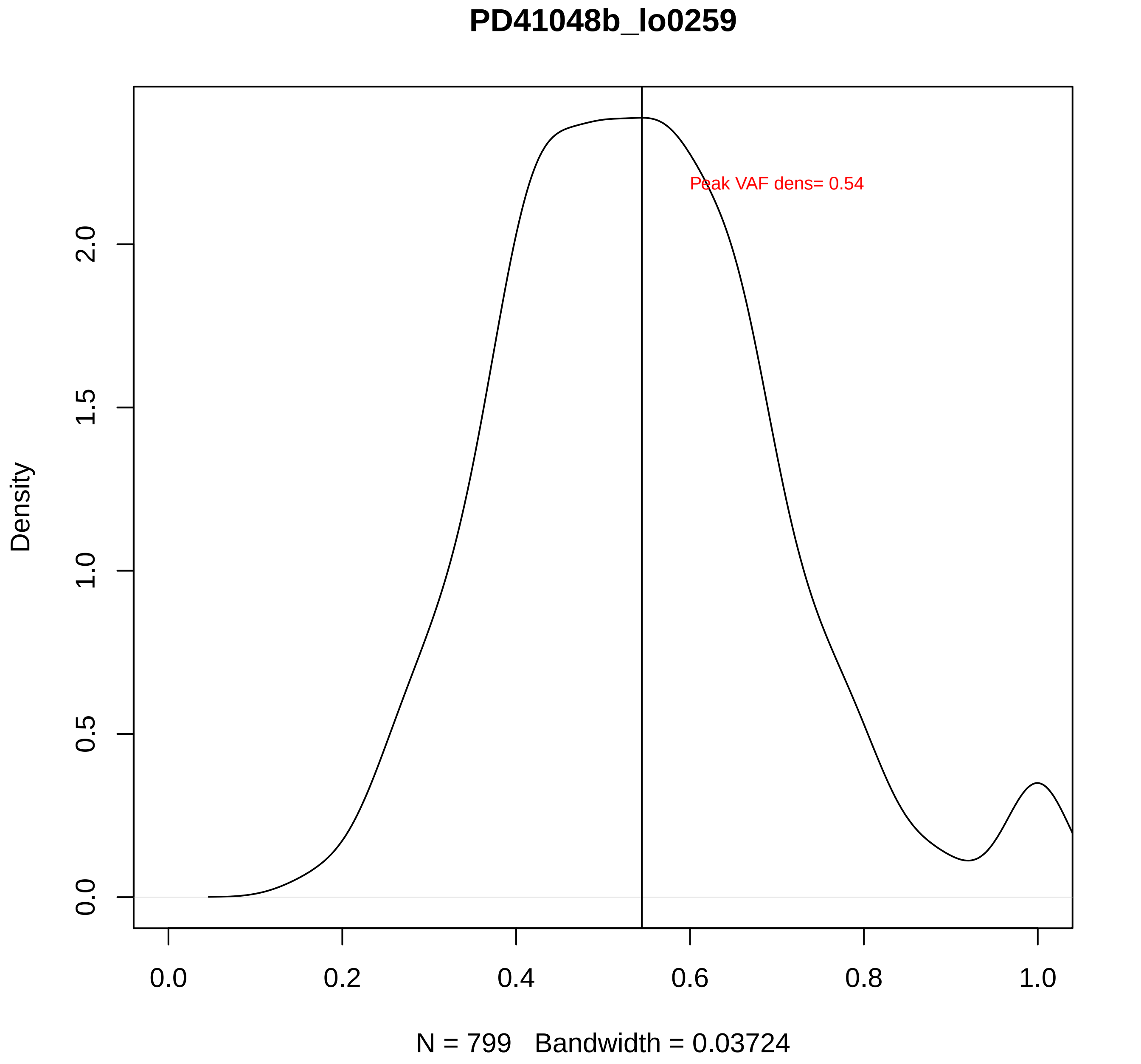

PD41048b_lo0259
Peak VAF dens= 0.54
Density
2.0
1.5
1.0
0.5
0.0
0.0
0.2
0.4
0.6
0.8
1.0
N = 799   Bandwidth = 0.03724

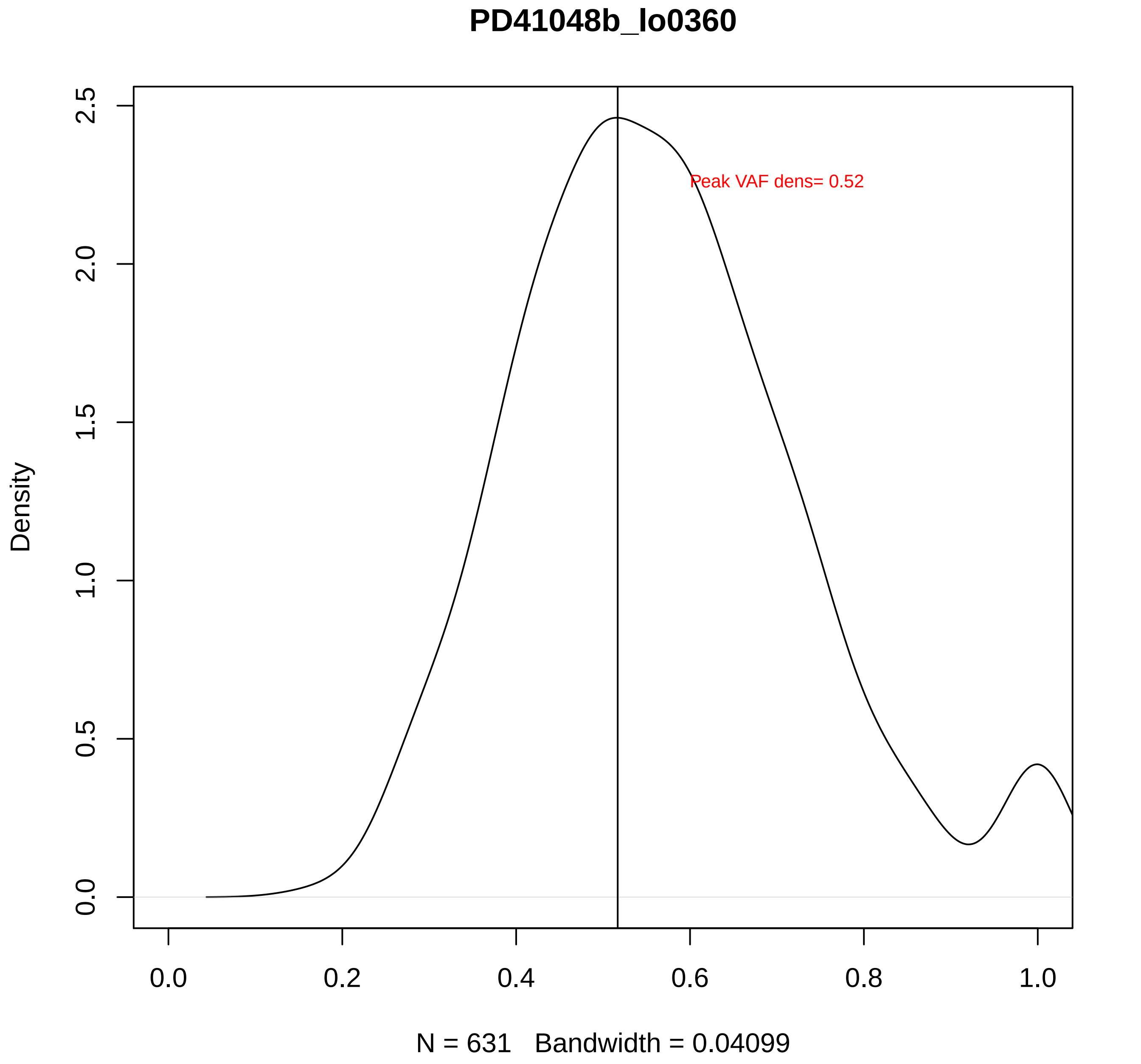
PD41048b_lo0360
Peak VAF dens= 0.52
Density
0.0
0.5
1.0
1.5
2.0
2.5
0.0
0.2
0.4
0.6
0.8
1.0
N = 631 Bandwidth = 0.04099

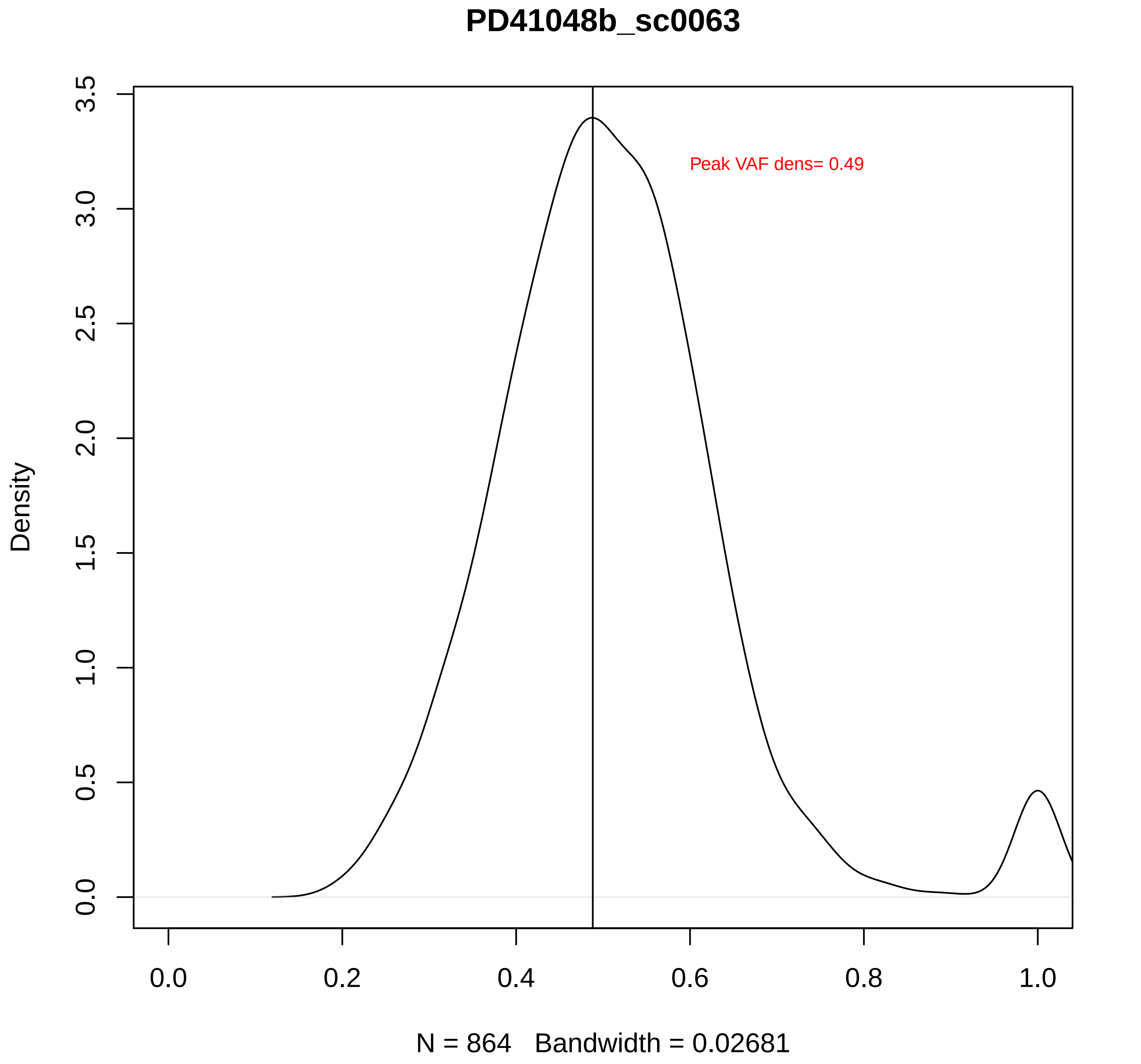
PD41048b_sc0063
Peak VAF dens= 0.49
Density
0.0
0.5
1.0
1.5
2.0
2.5
3.0
3.5
0.0
0.2
0.4
0.6
0.8
1.0
N = 864   Bandwidth = 0.02681

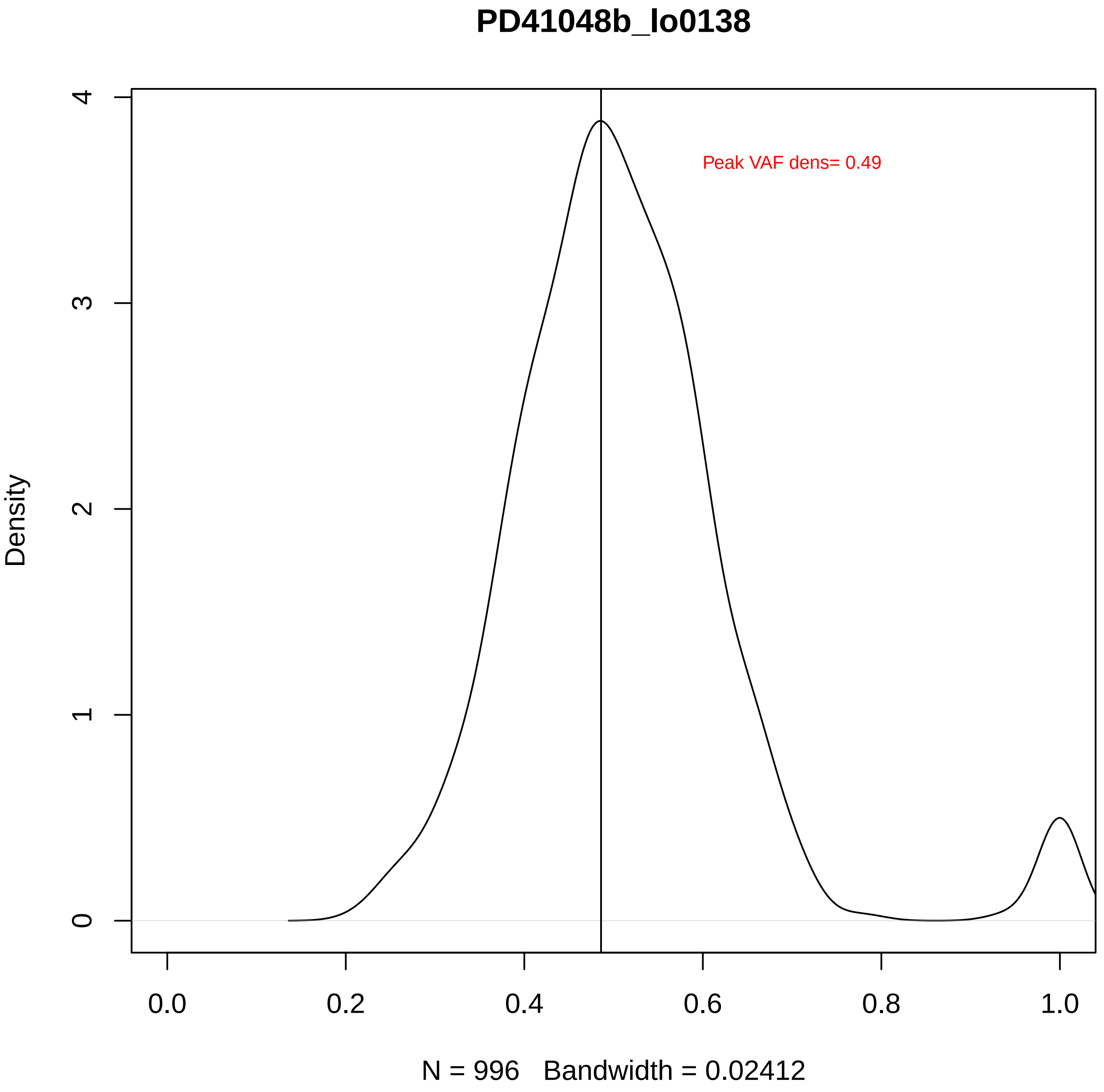
PD41048b_lo0138
Peak VAF dens= 0.49
Density
0
1
2
3
4
0.0
0.2
0.4
0.6
0.8
1.0
N = 996 Bandwidth = 0.02412

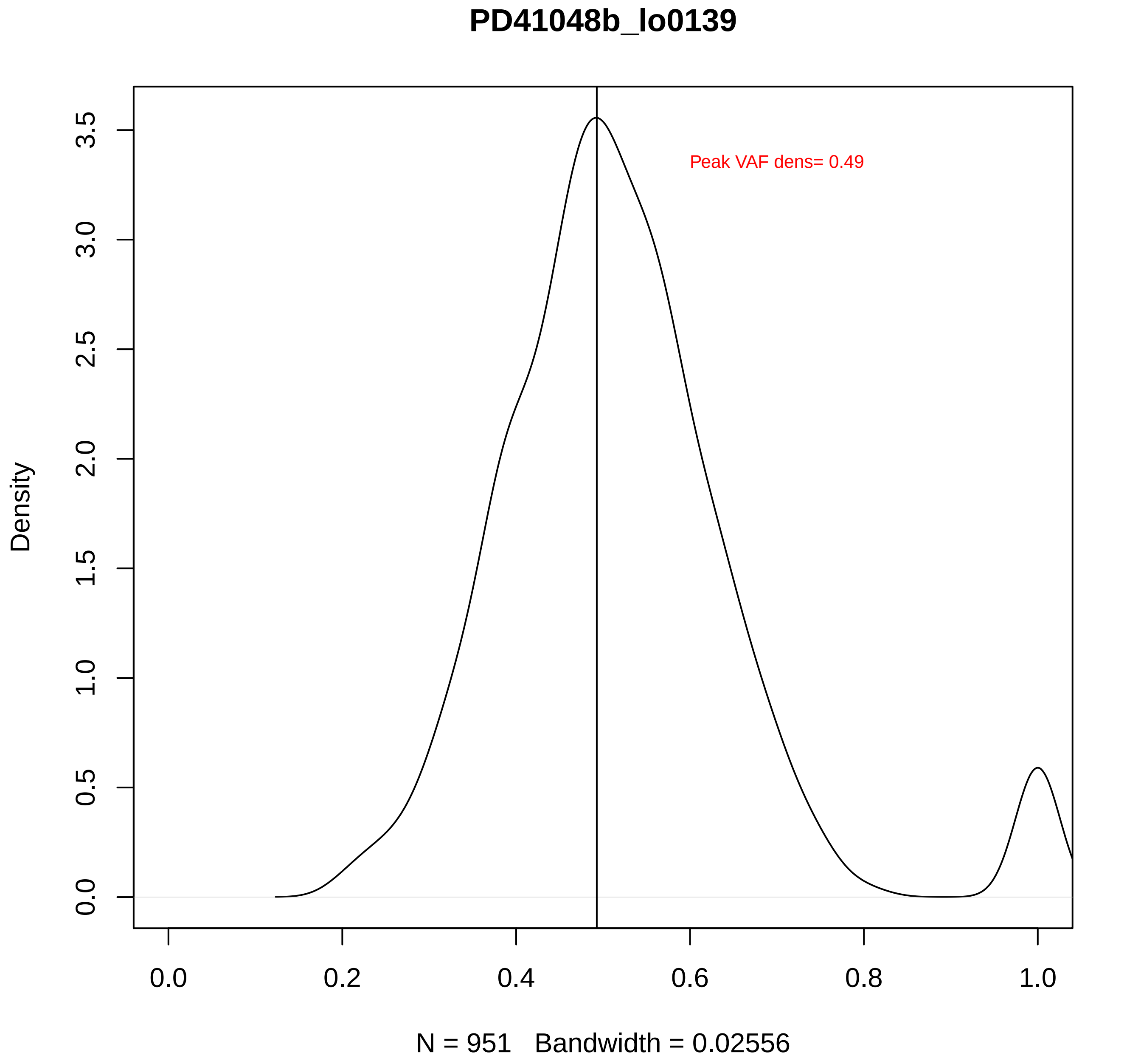
PD41048b_lo0139
Peak VAF dens= 0.49
Density
0.0
0.5
1.0
1.5
2.0
2.5
3.0
3.5
0.0
0.2
0.4
0.6
0.8
1.0
N = 951 Bandwidth = 0.02556

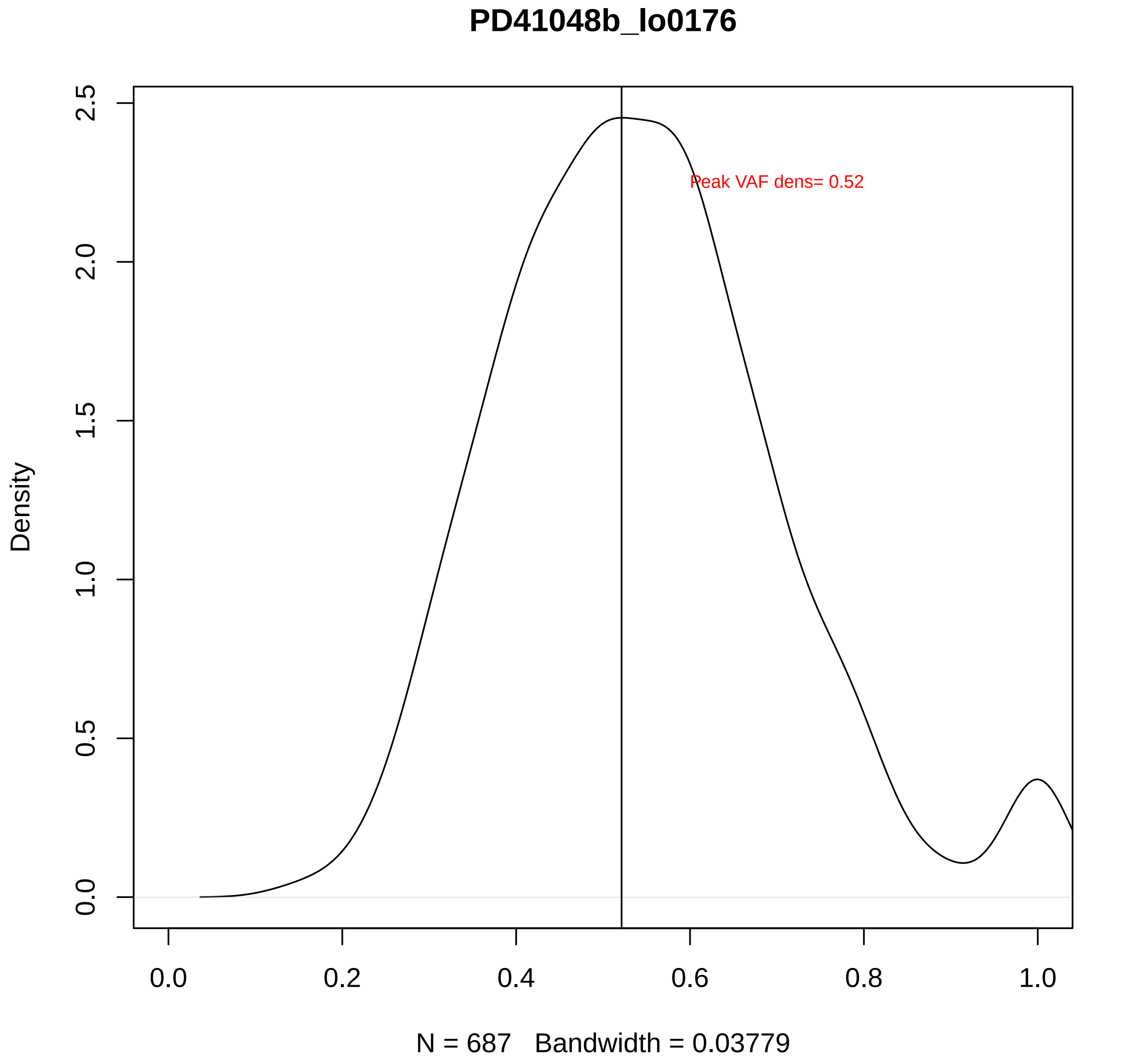
PD41048b_lo0176
Density
Peak VAF dens= 0.52
0.0
0.5
1.0
1.5
2.0
2.5
0.0
0.2
0.4
0.6
0.8
1.0
N = 687   Bandwidth = 0.03779

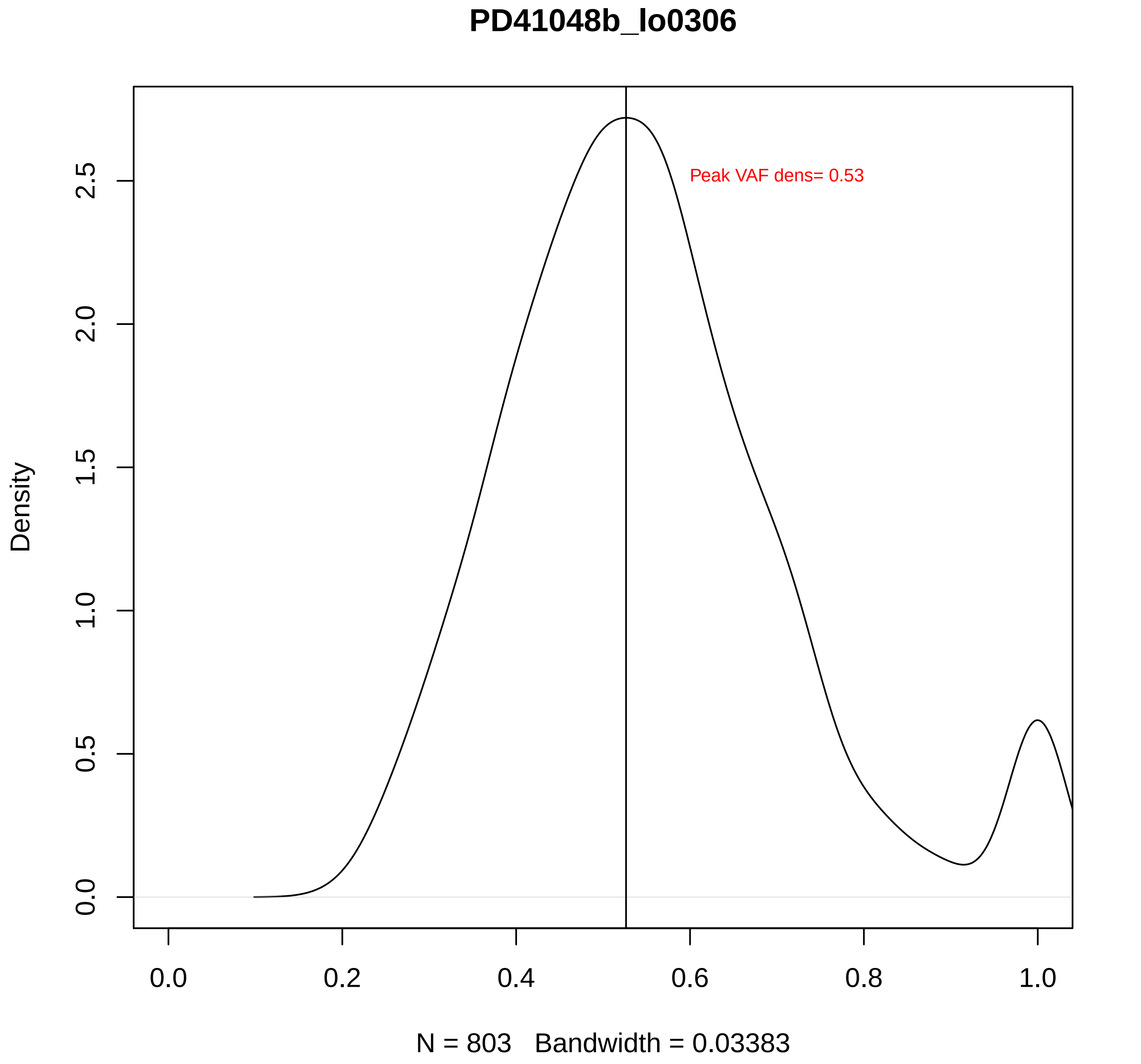
PD41048b_lo0306
Peak VAF dens= 0.53
Density
0.0
0.5
1.0
1.5
2.0
2.5
0.0
0.2
0.4
0.6
0.8
1.0
N = 803   Bandwidth = 0.03383

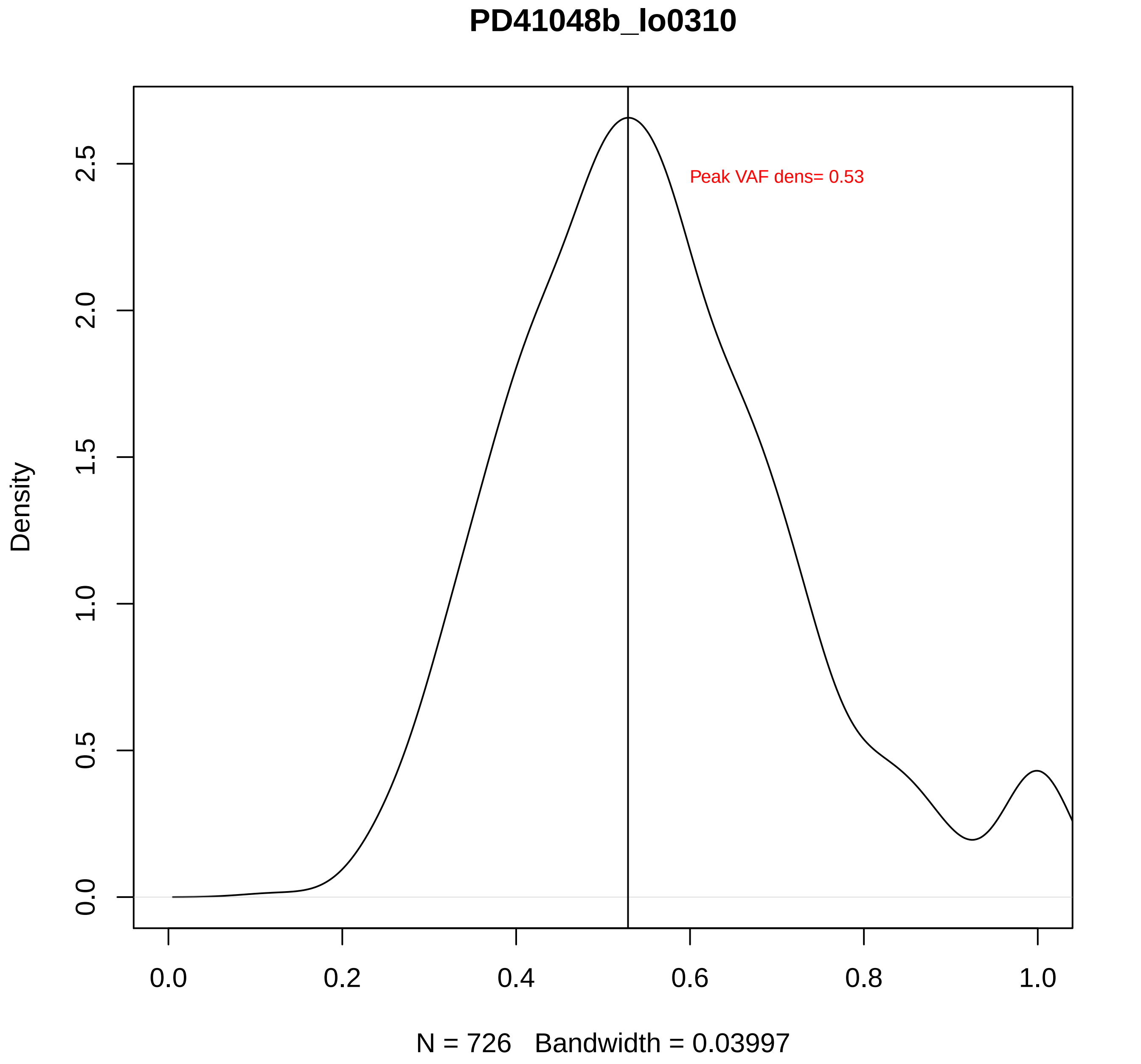
PD41048b_lo0310
Peak VAF dens= 0.53
Density
0.0
0.5
1.0
1.5
2.0
2.5
0.0
0.2
0.4
0.6
0.8
1.0
N = 726 Bandwidth = 0.03997

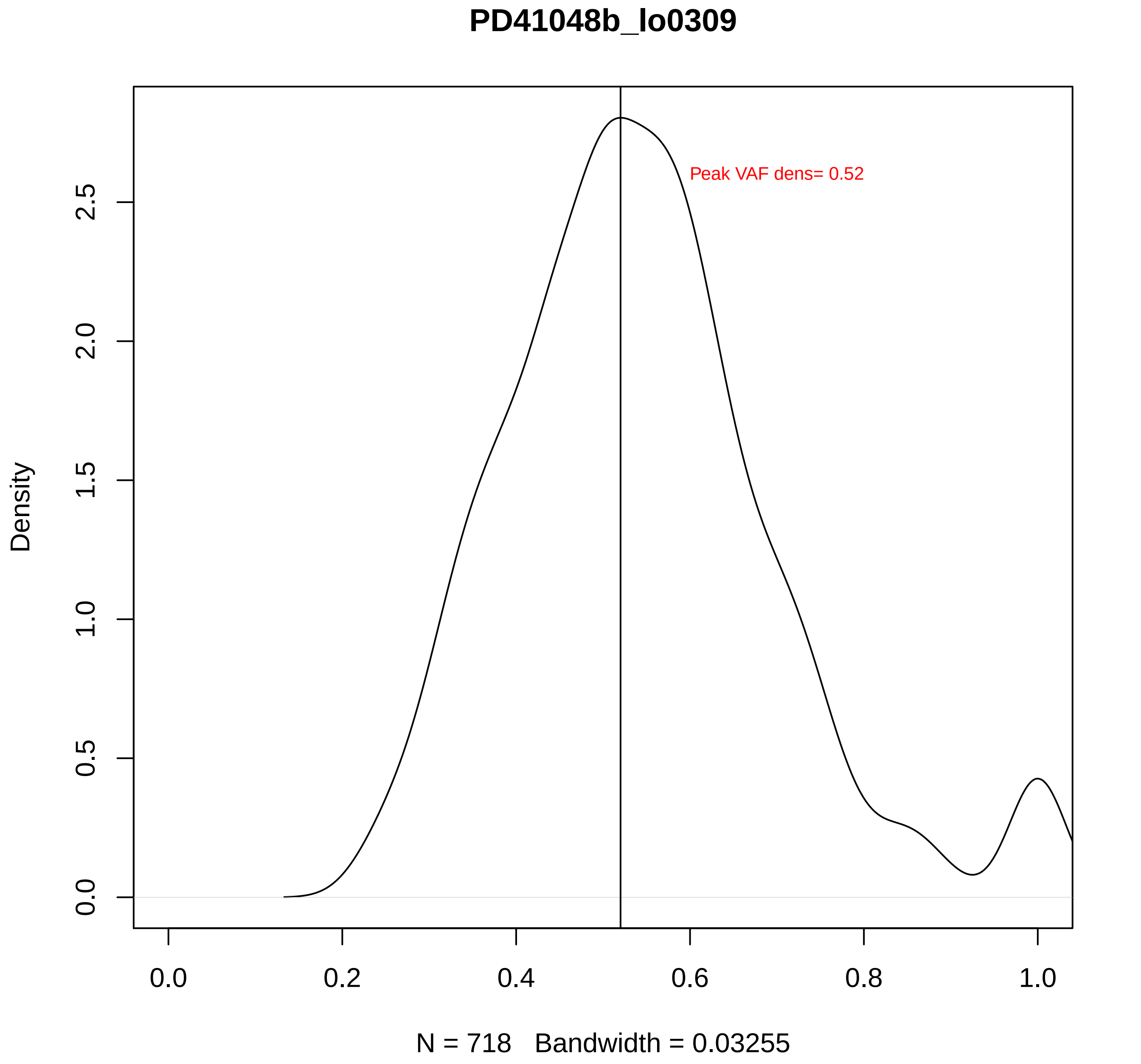
PD41048b_lo0309
Peak VAF dens= 0.52
Density
0.0
0.5
1.0
1.5
2.0
2.5
0.0
0.2
0.4
0.6
0.8
1.0
N = 718   Bandwidth = 0.03255

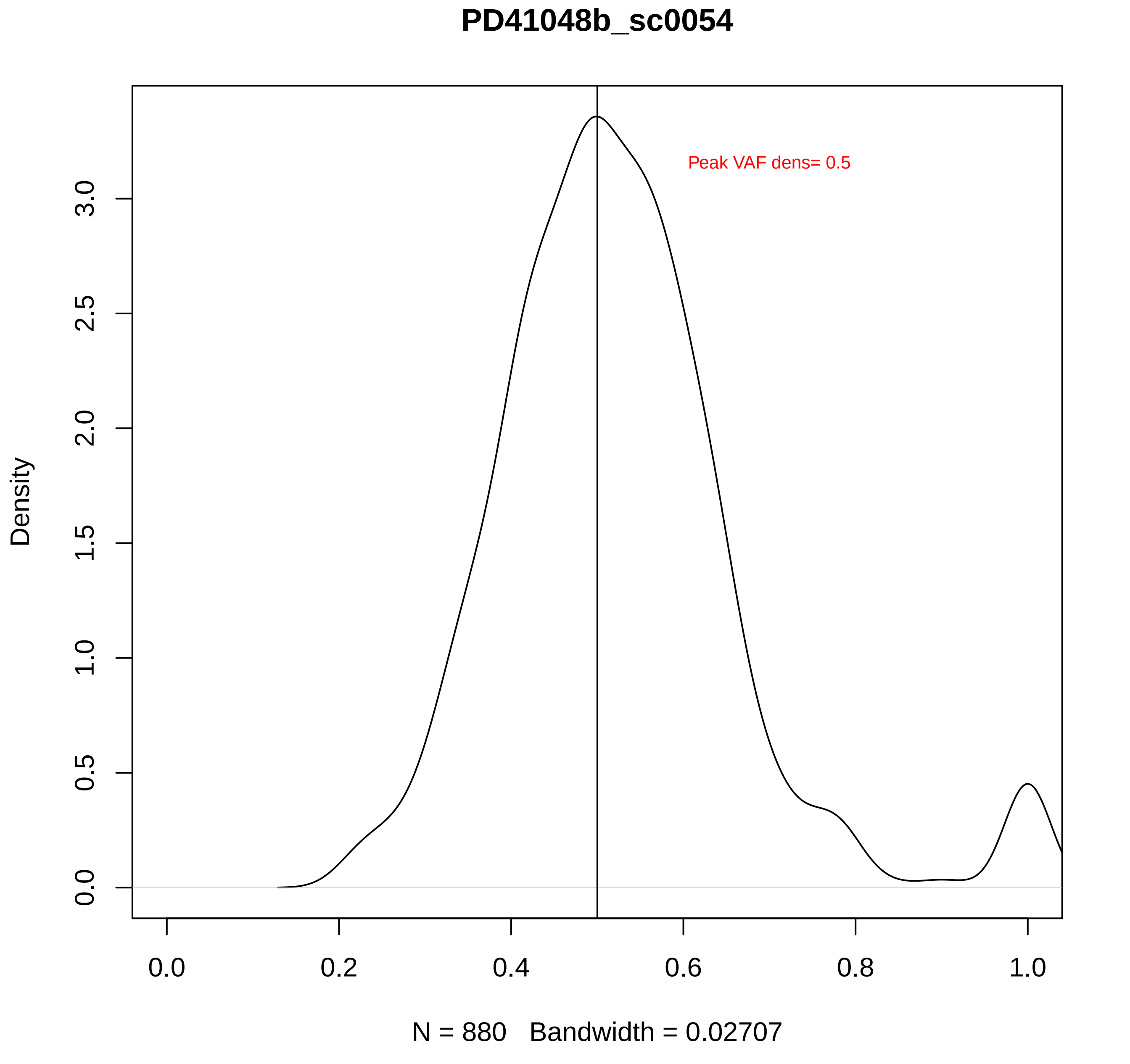
PD41048b_sc0054
Peak VAF dens= 0.5
Density
0.0
0.5
1.0
1.5
2.0
2.5
3.0
0.0
0.2
0.4
0.6
0.8
1.0
N = 880   Bandwidth = 0.02707

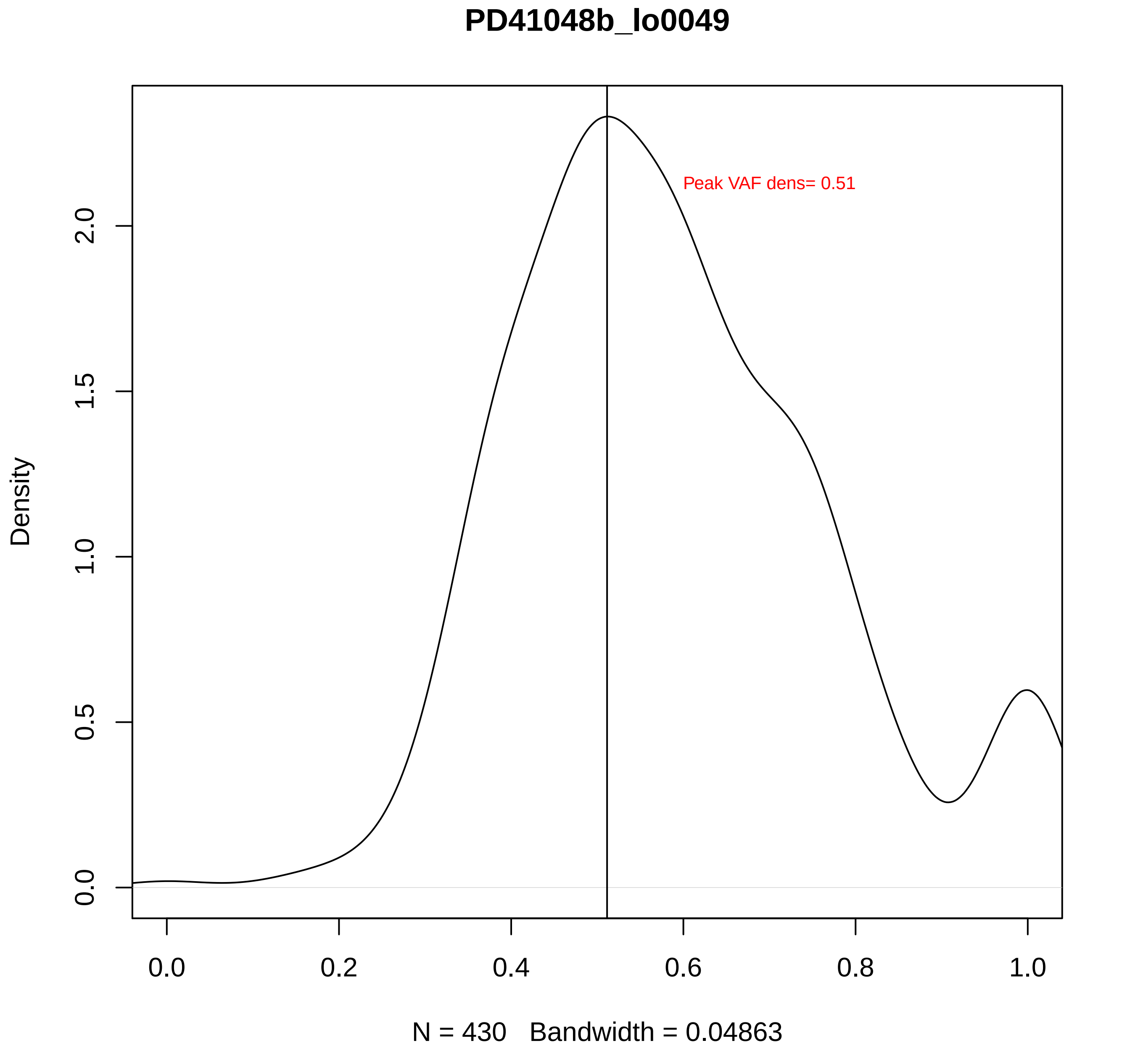
PD41048b_lo0049
Peak VAF dens= 0.51
Density
0.0
0.5
1.0
1.5
2.0
0.0
0.2
0.4
0.6
0.8
1.0
N = 430   Bandwidth = 0.04863

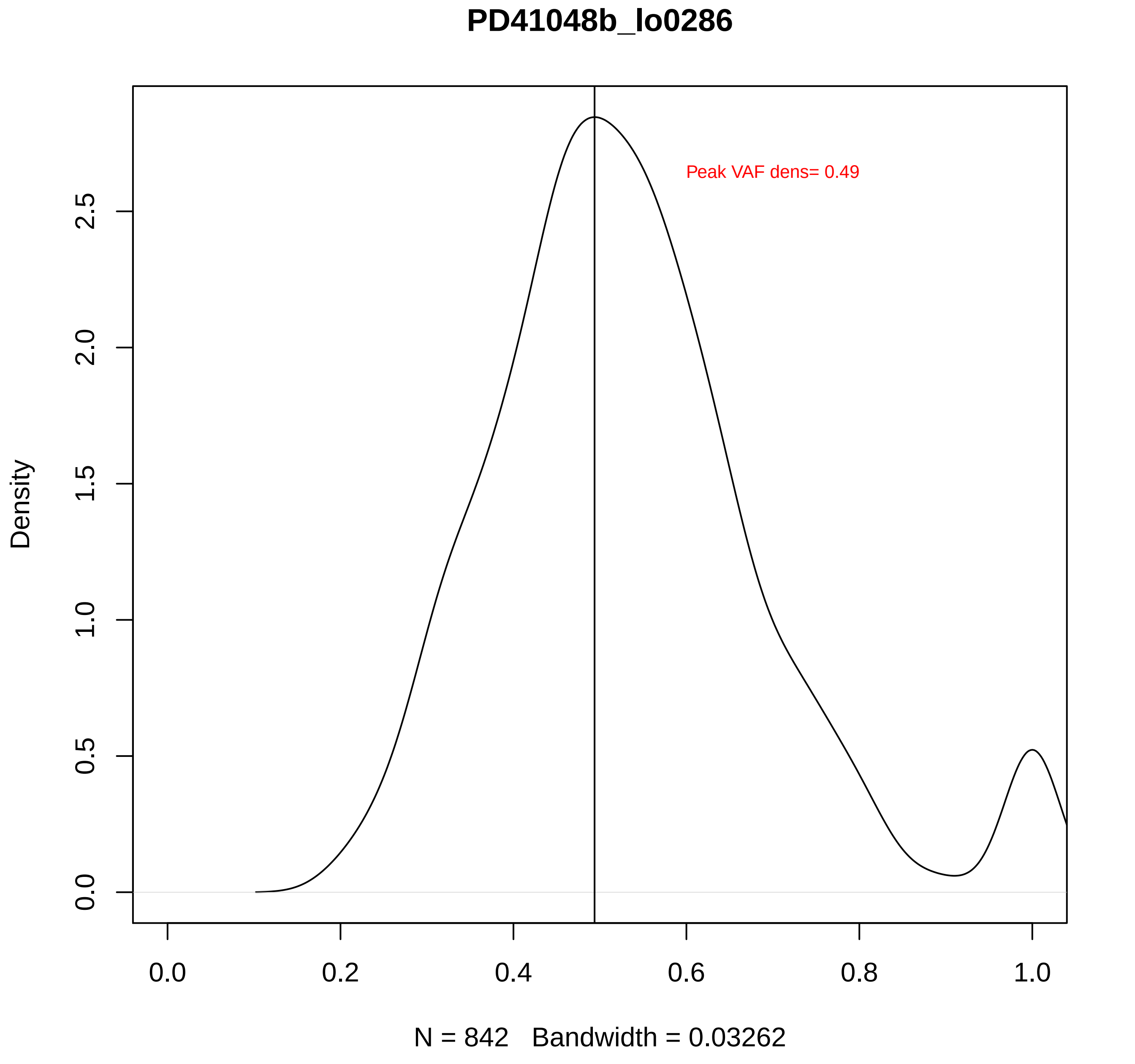
PD41048b_lo0286
Peak VAF dens= 0.49
Density
0.0
0.5
1.0
1.5
2.0
2.5
0.0
0.2
0.4
0.6
0.8
1.0
N = 842   Bandwidth = 0.03262

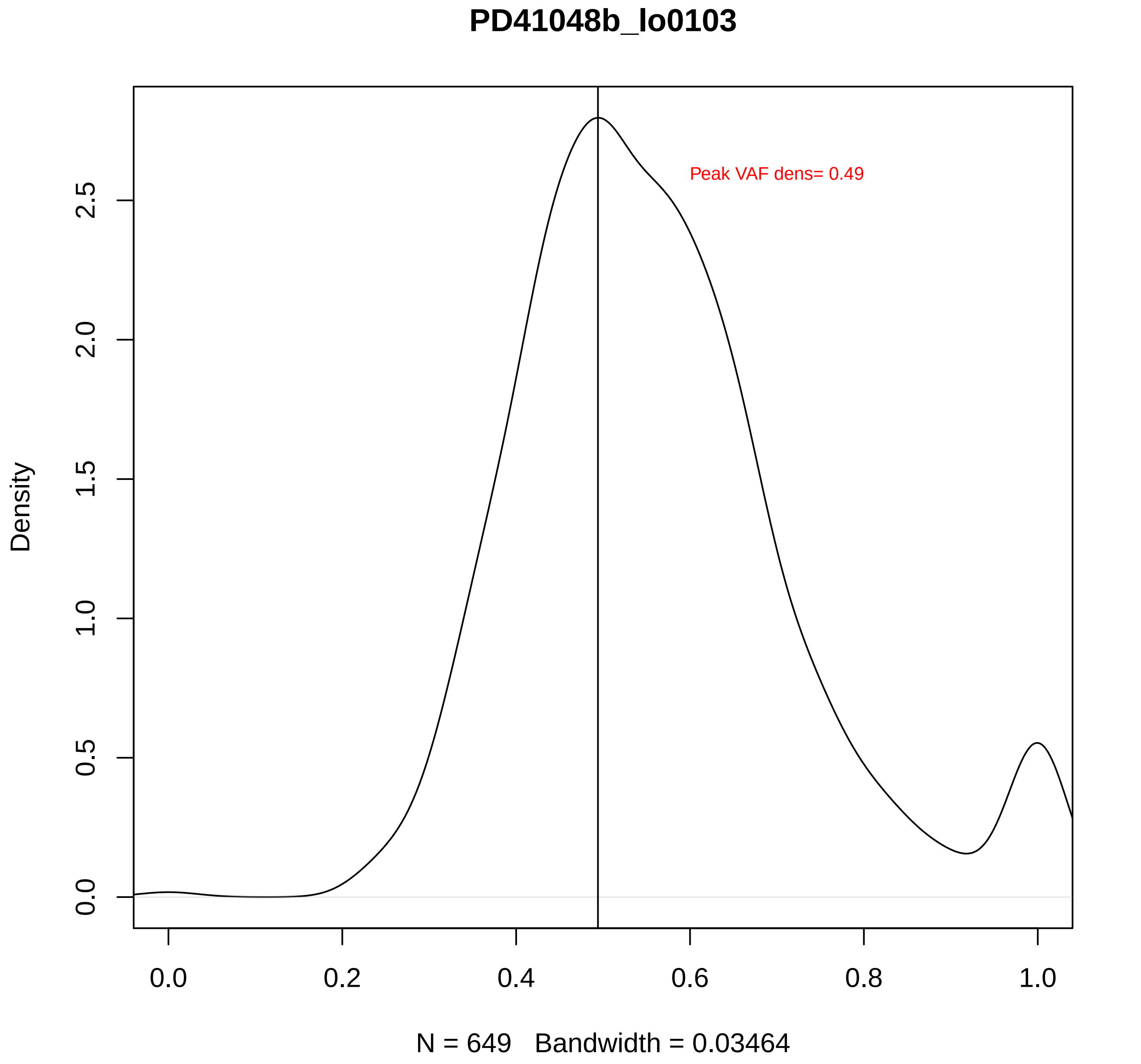
PD41048b_lo0103
Peak VAF dens= 0.49
Density
0.0
0.5
1.0
1.5
2.0
2.5
0.0
0.2
0.4
0.6
0.8
1.0
N = 649 Bandwidth = 0.03464

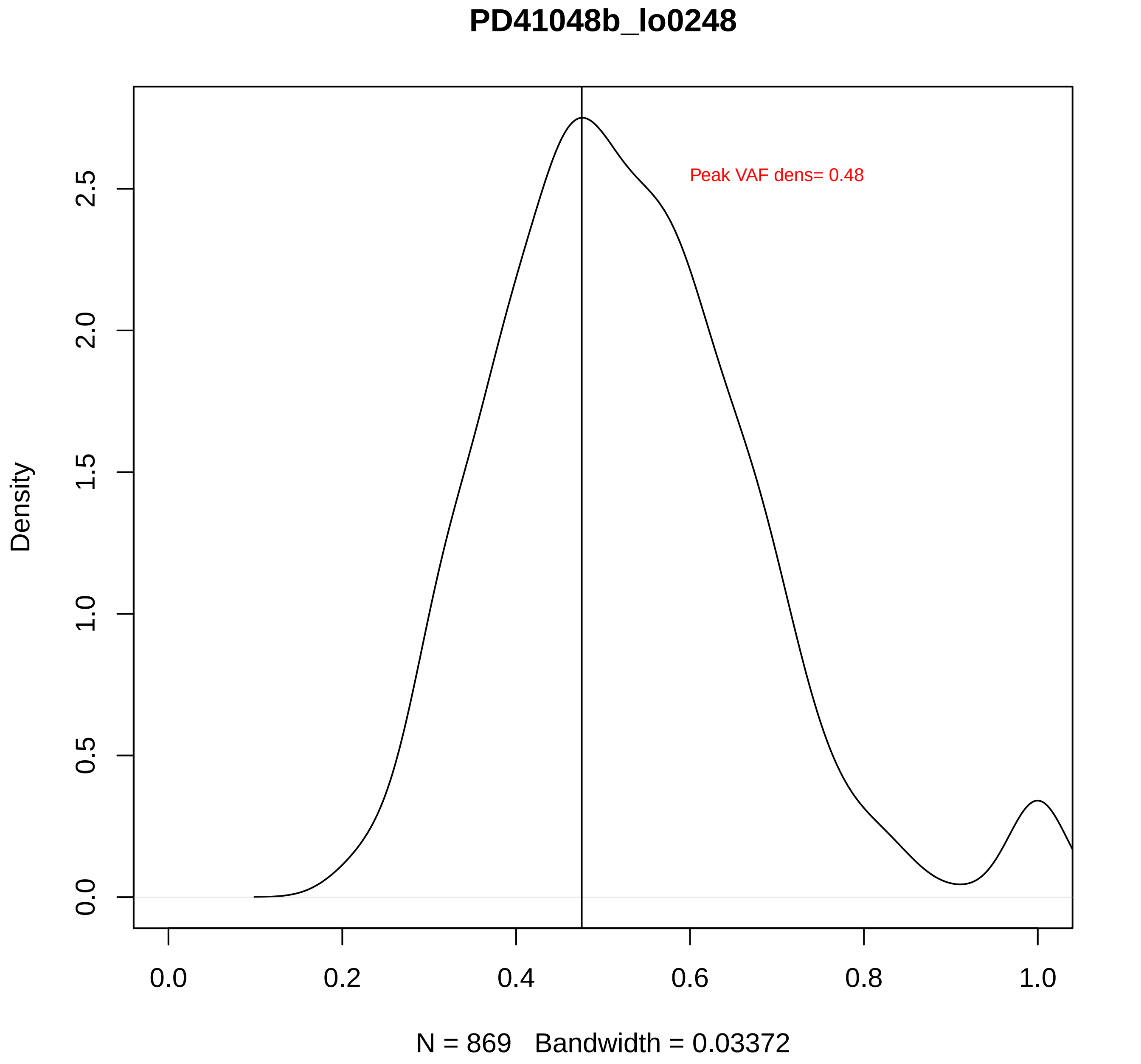
PD41048b_lo0248
Peak VAF dens= 0.48
Density
0.0
0.5
1.0
1.5
2.0
2.5
0.0
0.2
0.4
0.6
0.8
1.0
N = 869 Bandwidth = 0.03372

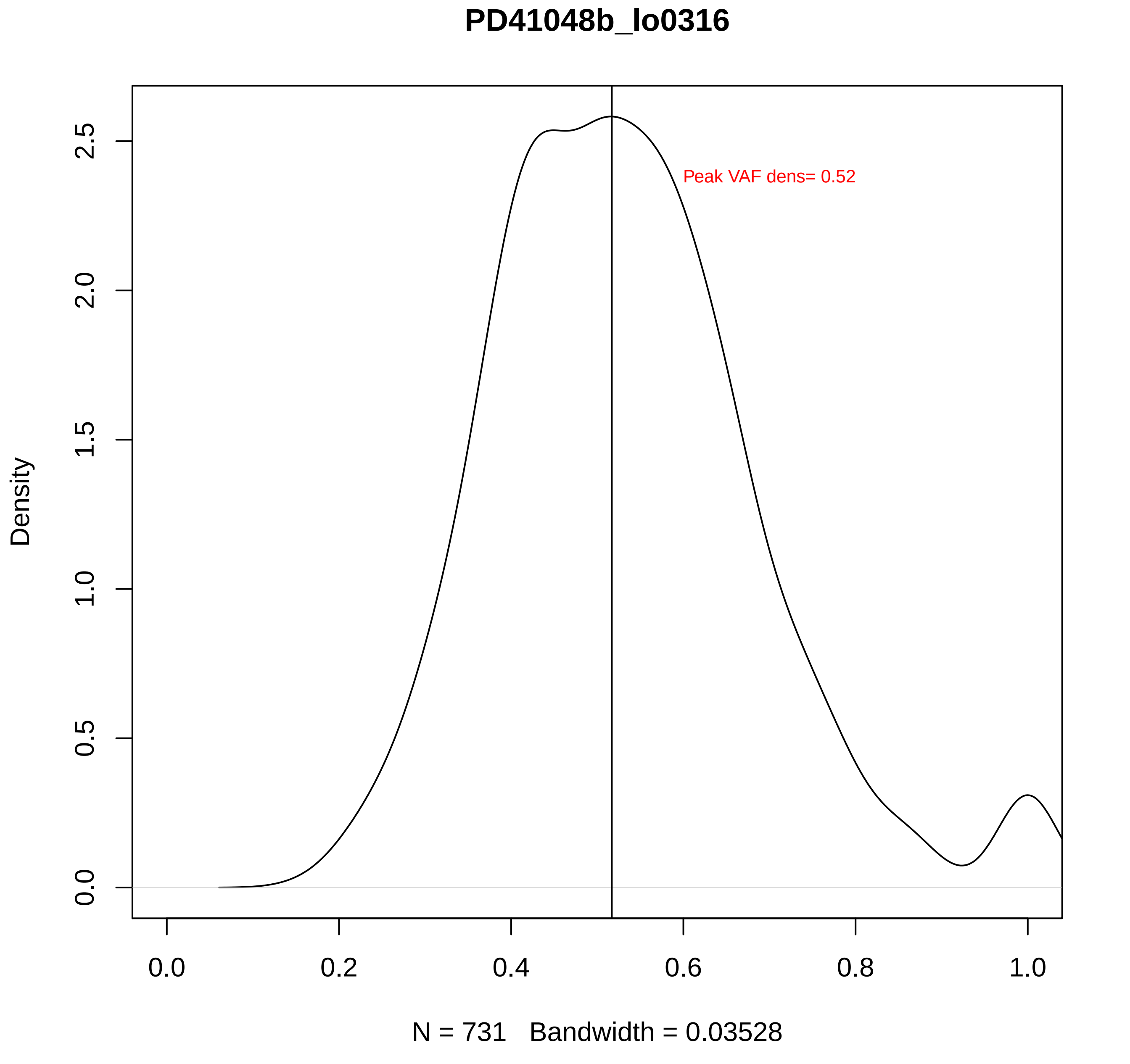

PD41048b_lo0316
Peak VAF dens= 0.52
Density
0.0
0.5
1.0
1.5
2.0
2.5
0.0
0.2
0.4
0.6
0.8
1.0
N = 731 Bandwidth = 0.03528

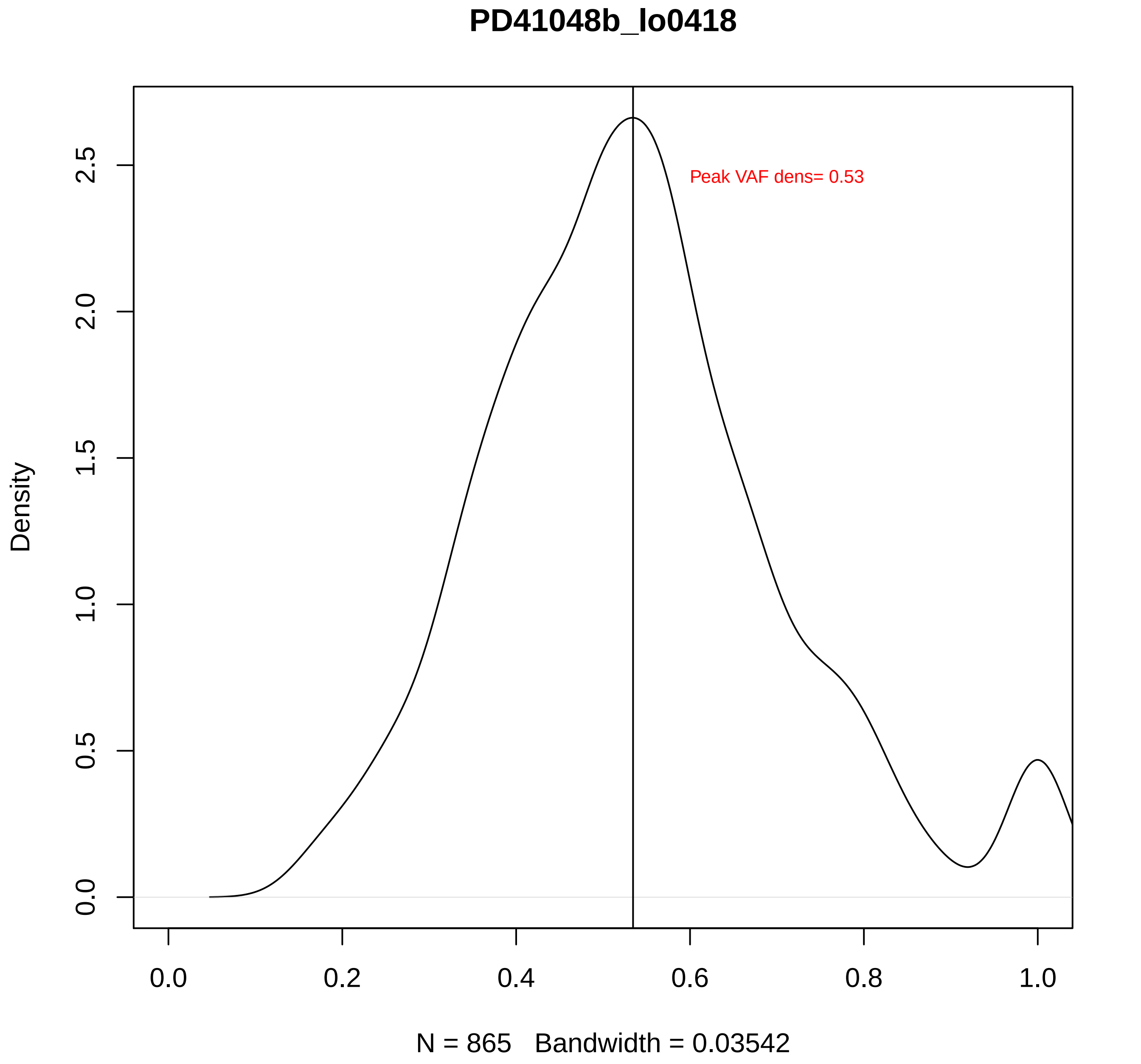
PD41048b_lo0418
Peak VAF dens= 0.53
Density
0.0
0.5
1.0
1.5
2.0
2.5
0.0
0.2
0.4
0.6
0.8
1.0
N = 865   Bandwidth = 0.03542

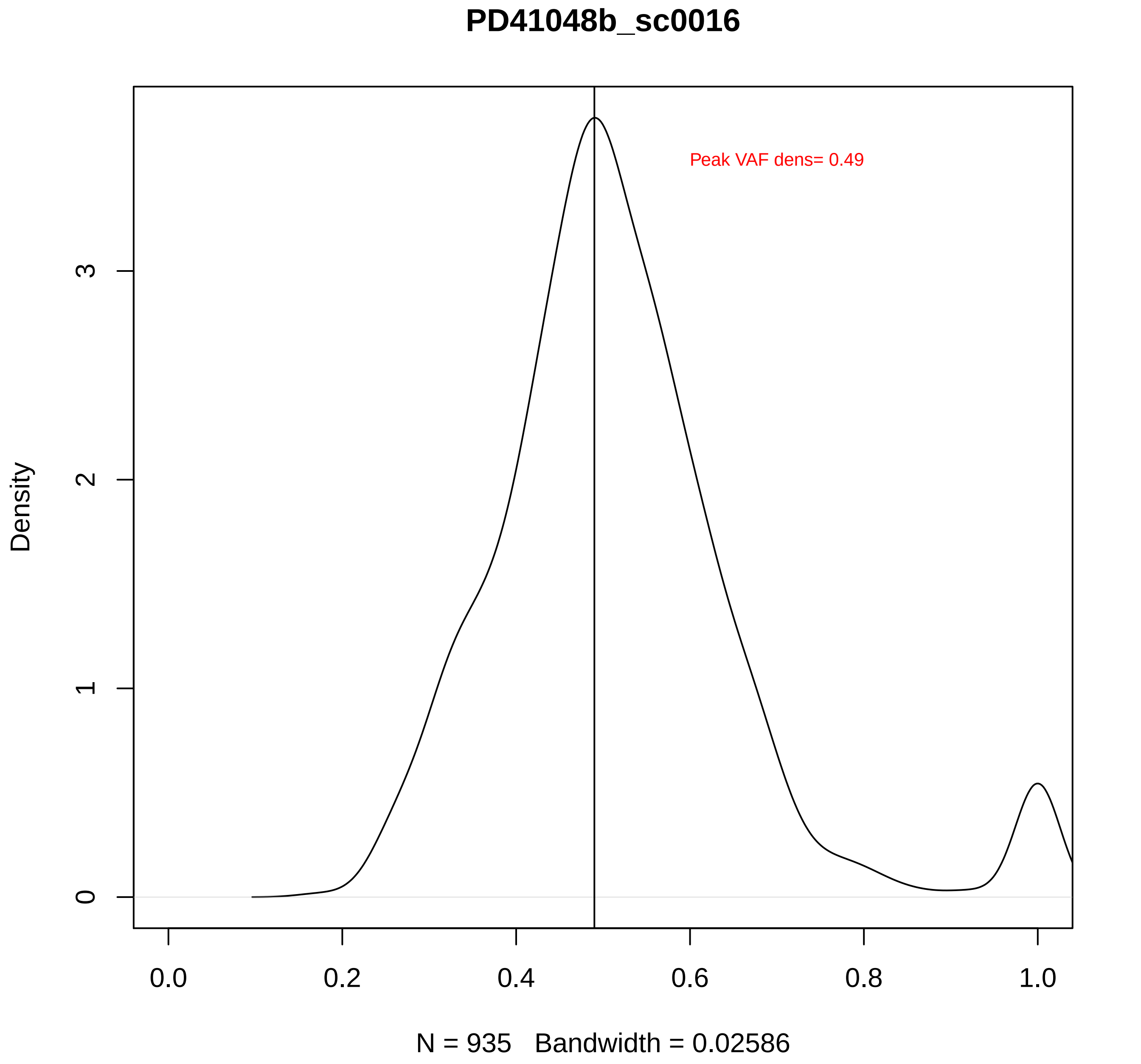

PD41048b_sc0016
Peak VAF dens= 0.49
Density
0
1
2
3
0.0
0.2
0.4
0.6
0.8
1.0
N = 935 Bandwidth = 0.02586

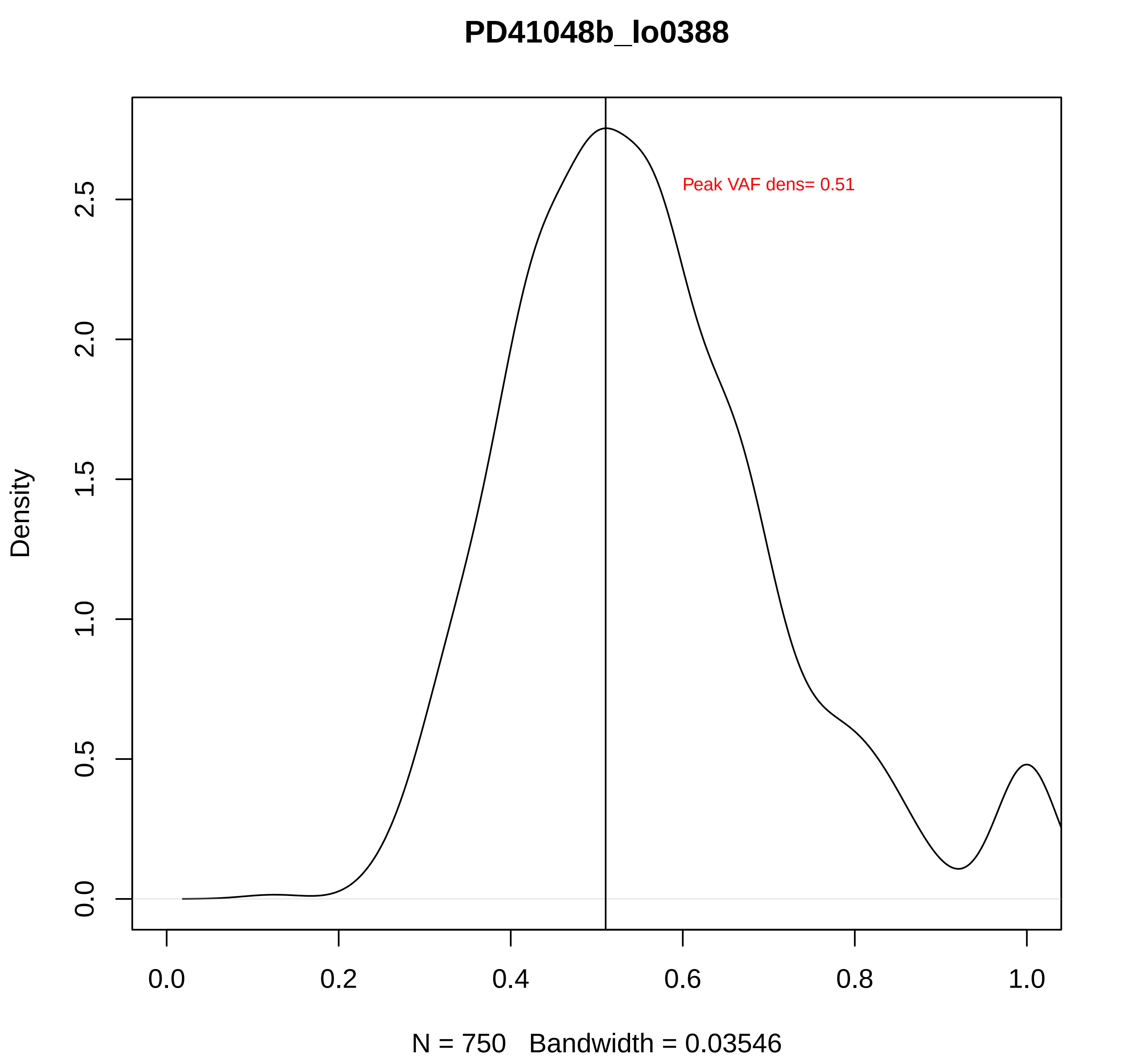
PD41048b_lo0388
Peak VAF dens= 0.51
Density
0.0
0.5
1.0
1.5
2.0
2.5
0.0
0.2
0.4
0.6
0.8
1.0
N = 750   Bandwidth = 0.03546

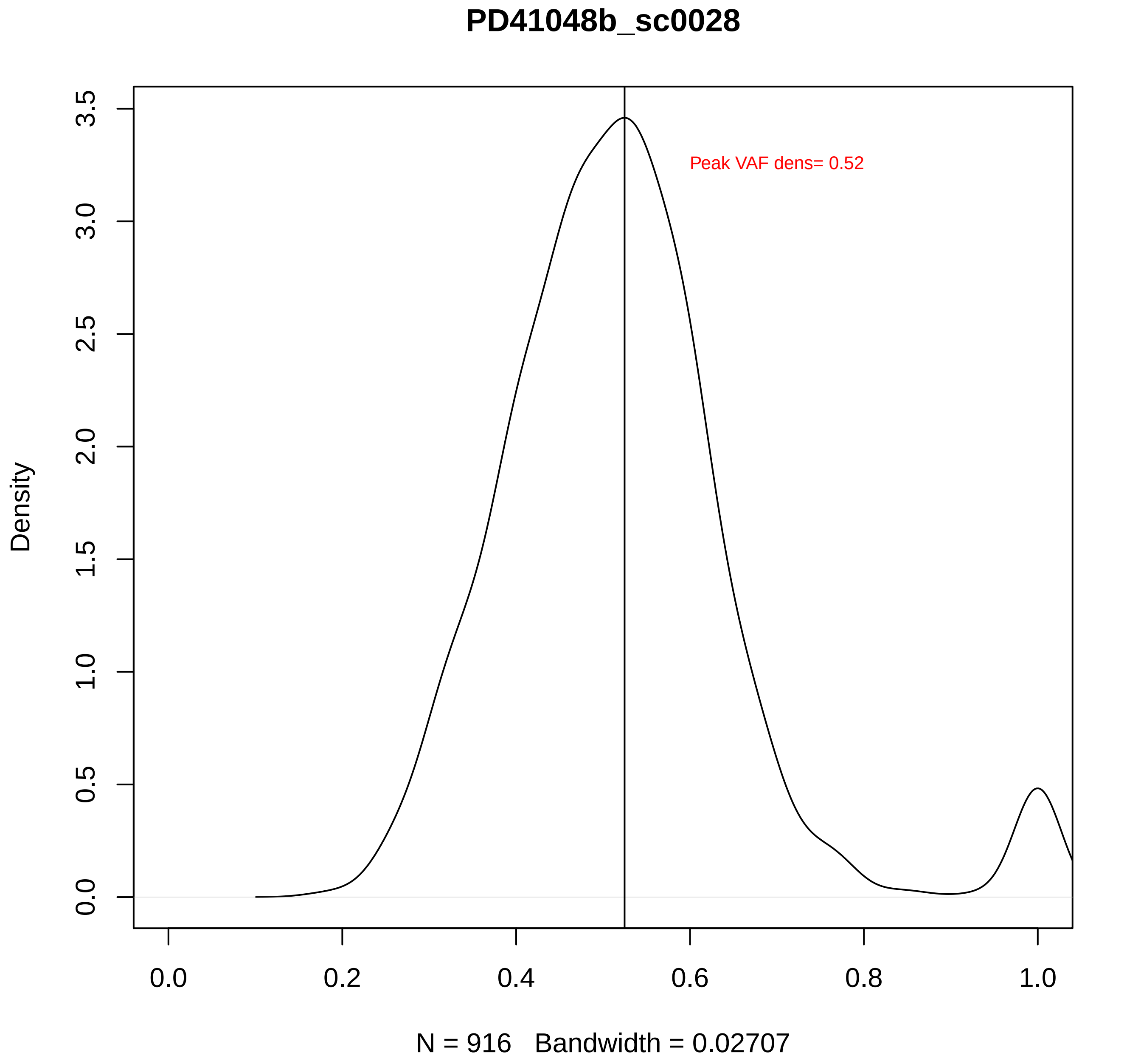
PD41048b_sc0028
Peak VAF dens= 0.52
Density
0.0
0.5
1.0
1.5
2.0
2.5
3.0
3.5
0.0
0.2
0.4
0.6
0.8
1.0
N = 916   Bandwidth = 0.02707

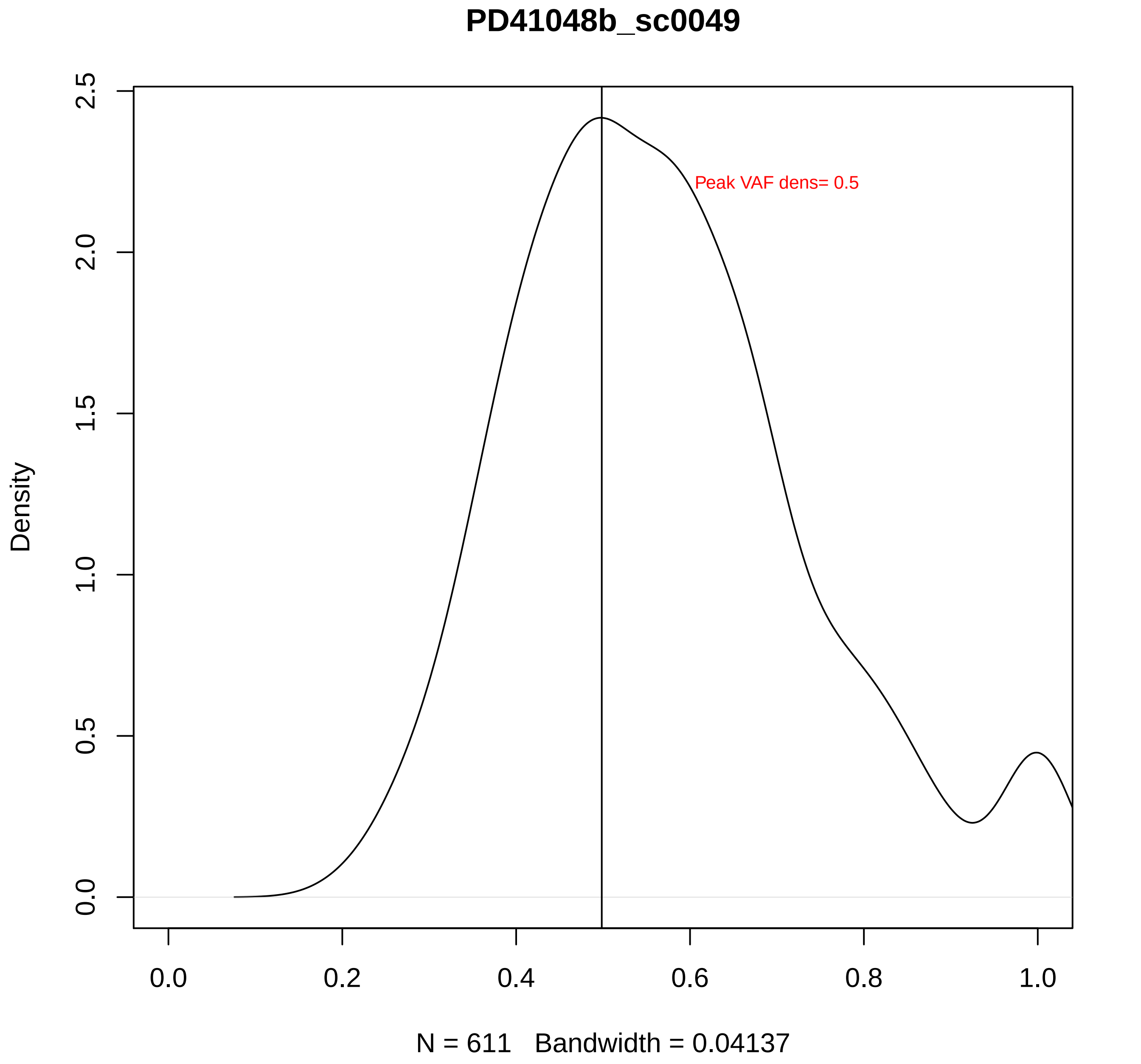

PD41048b_sc0049
Peak VAF dens= 0.5
Density
0.0
0.5
1.0
1.5
2.0
2.5
0.0
0.2
0.4
0.6
0.8
1.0
N = 611   Bandwidth = 0.04137

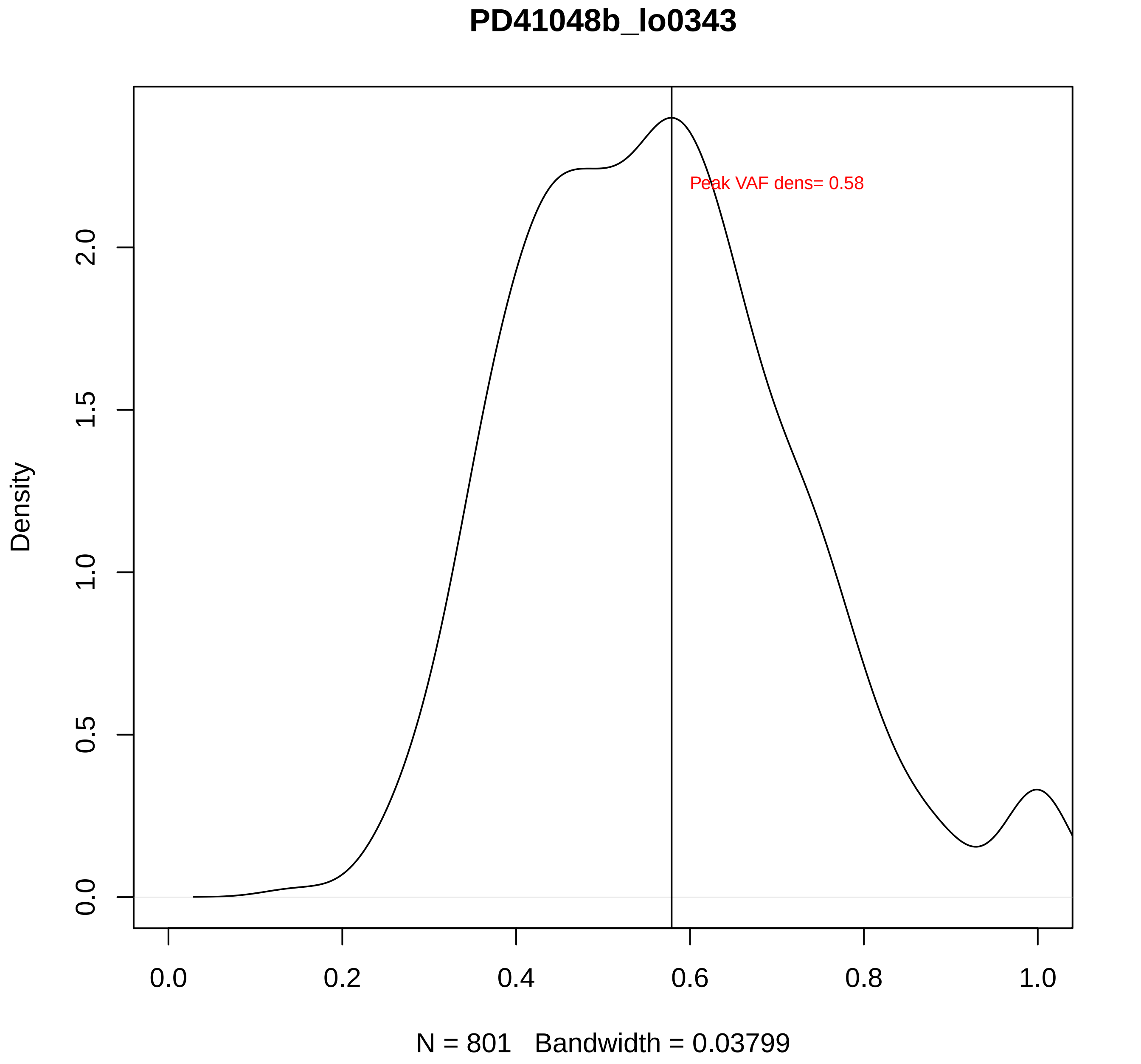

PD41048b_lo0343
Peak VAF dens= 0.58
Density
0.0
0.5
1.0
1.5
2.0
0.0
0.2
0.4
0.6
0.8
1.0
N = 801   Bandwidth = 0.03799

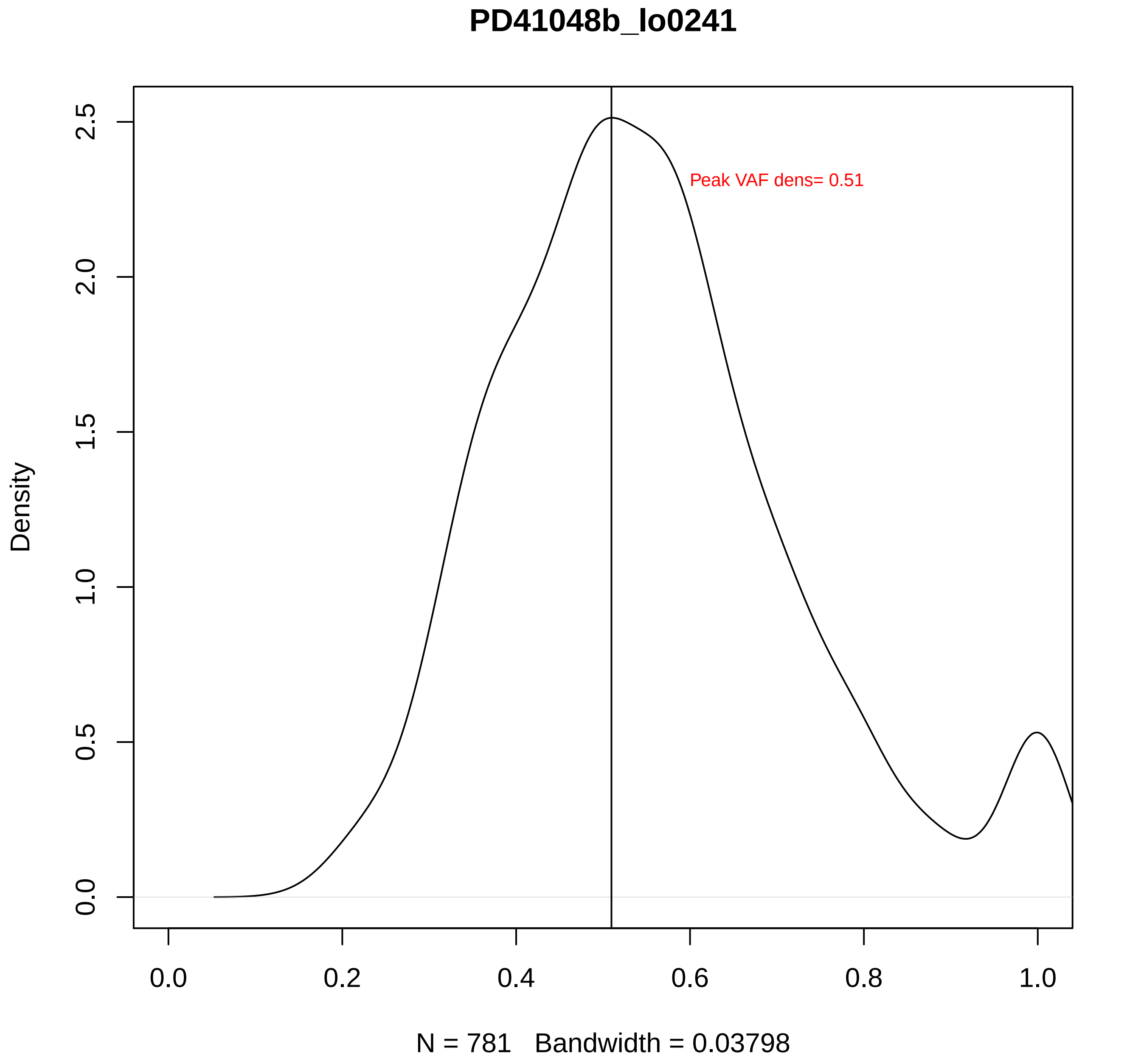
PD41048b_lo0241
Peak VAF dens= 0.51
Density
0.0
0.5
1.0
1.5
2.0
2.5
0.0
0.2
0.4
0.6
0.8
1.0
N = 781   Bandwidth = 0.03798

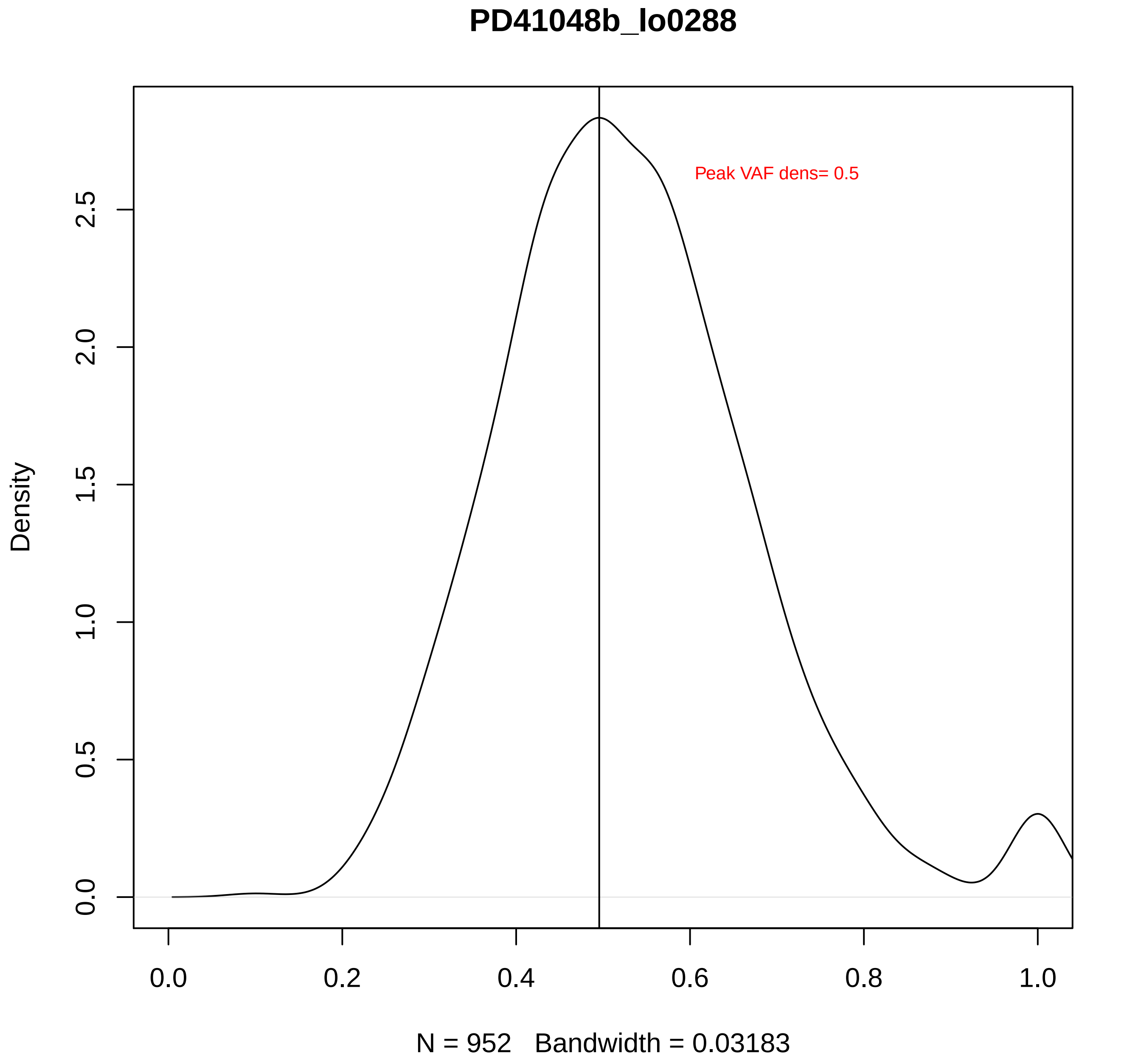

PD41048b_lo0288
Peak VAF dens= 0.5
Density
0.0
0.5
1.0
1.5
2.0
2.5
0.0
0.2
0.4
0.6
0.8
1.0
N = 952 Bandwidth = 0.03183

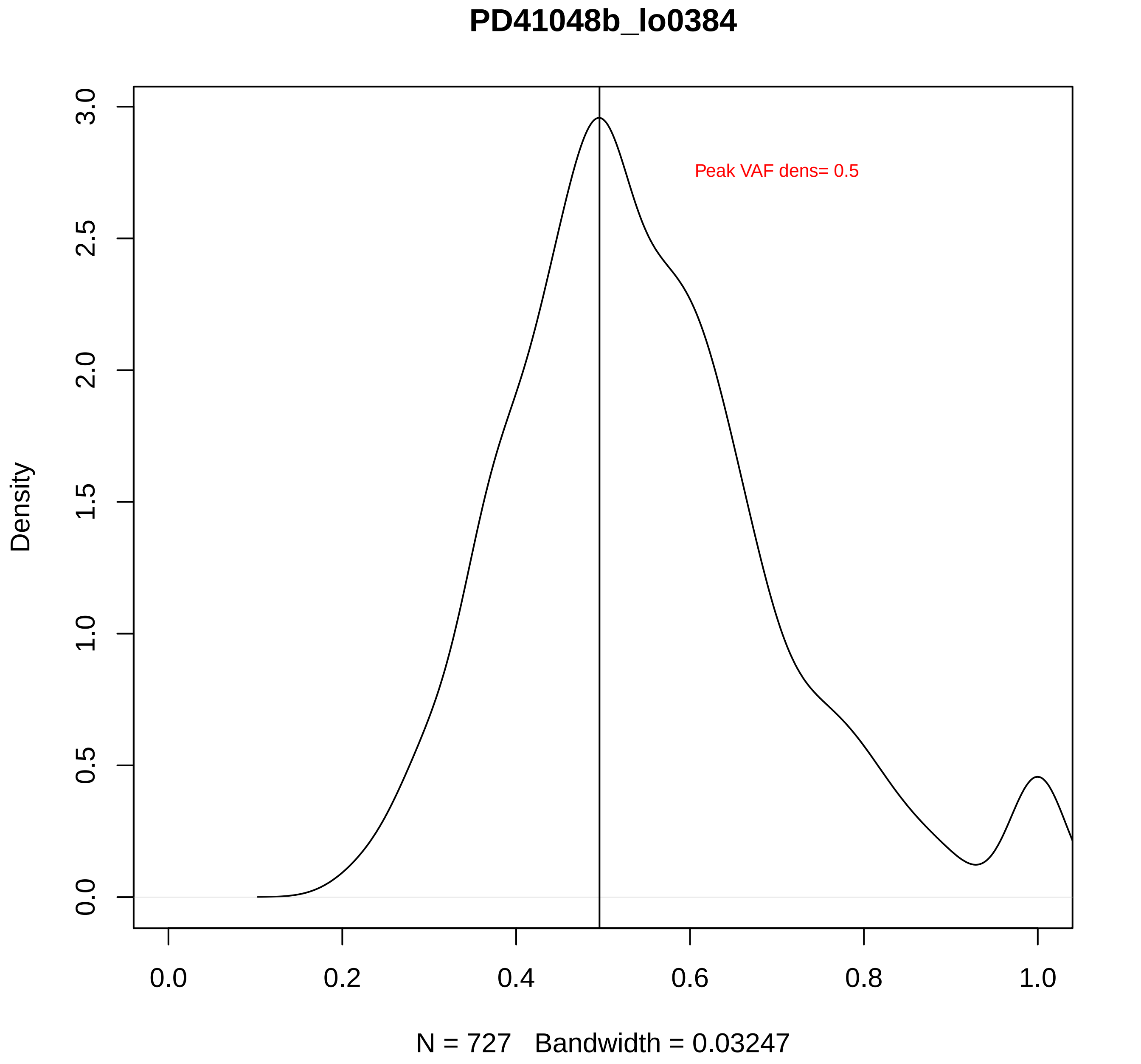
PD41048b_lo0384
Peak VAF dens= 0.5
Density
0.0
0.5
1.0
1.5
2.0
2.5
3.0
0.0
0.2
0.4
0.6
0.8
1.0
N = 727   Bandwidth = 0.03247

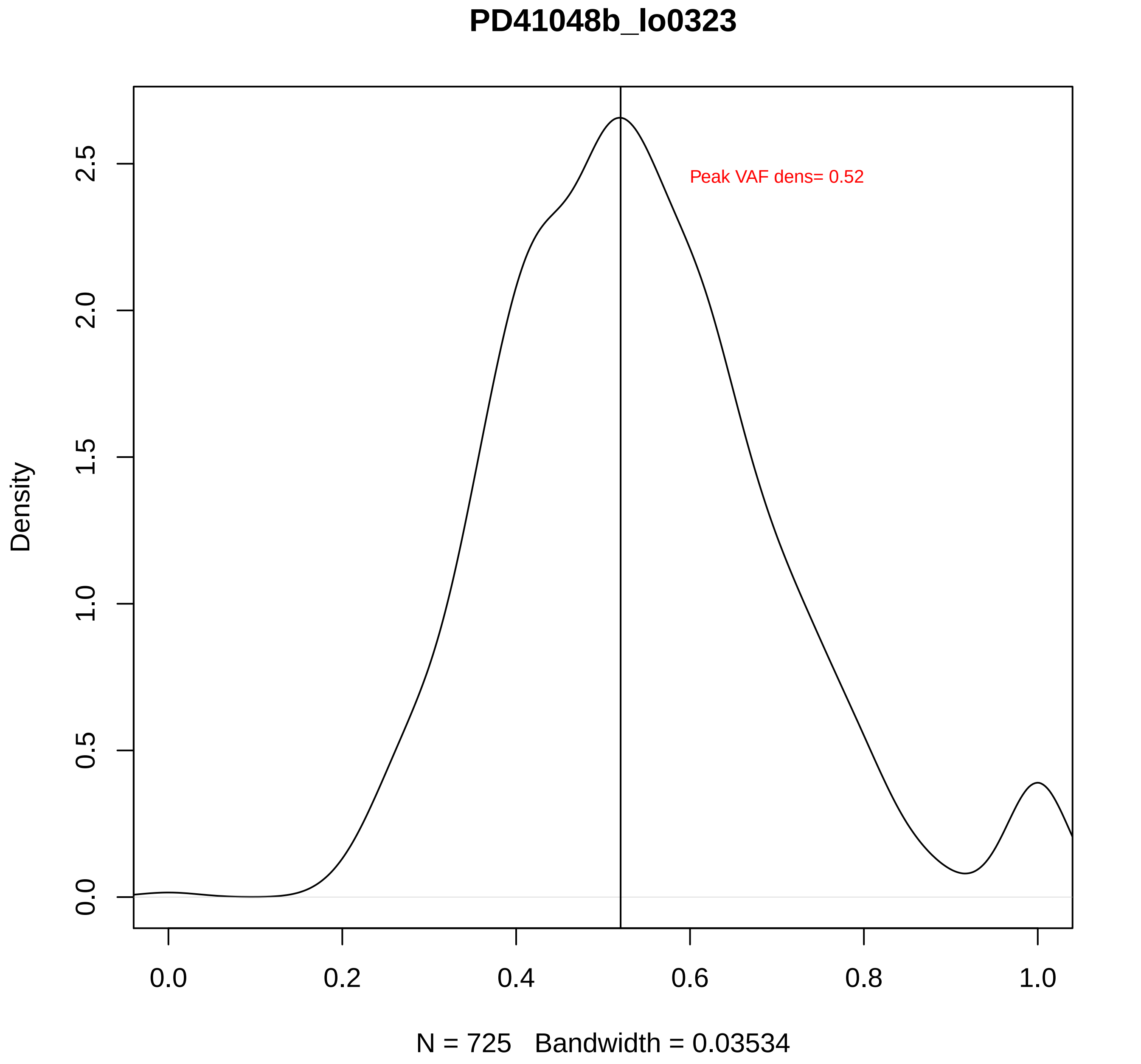
PD41048b_lo0323
Peak VAF dens= 0.52
Density
2.5
2.0
1.5
1.0
0.5
0.0
0.0
0.2
0.4
0.6
0.8
1.0
N = 725 Bandwidth = 0.03534

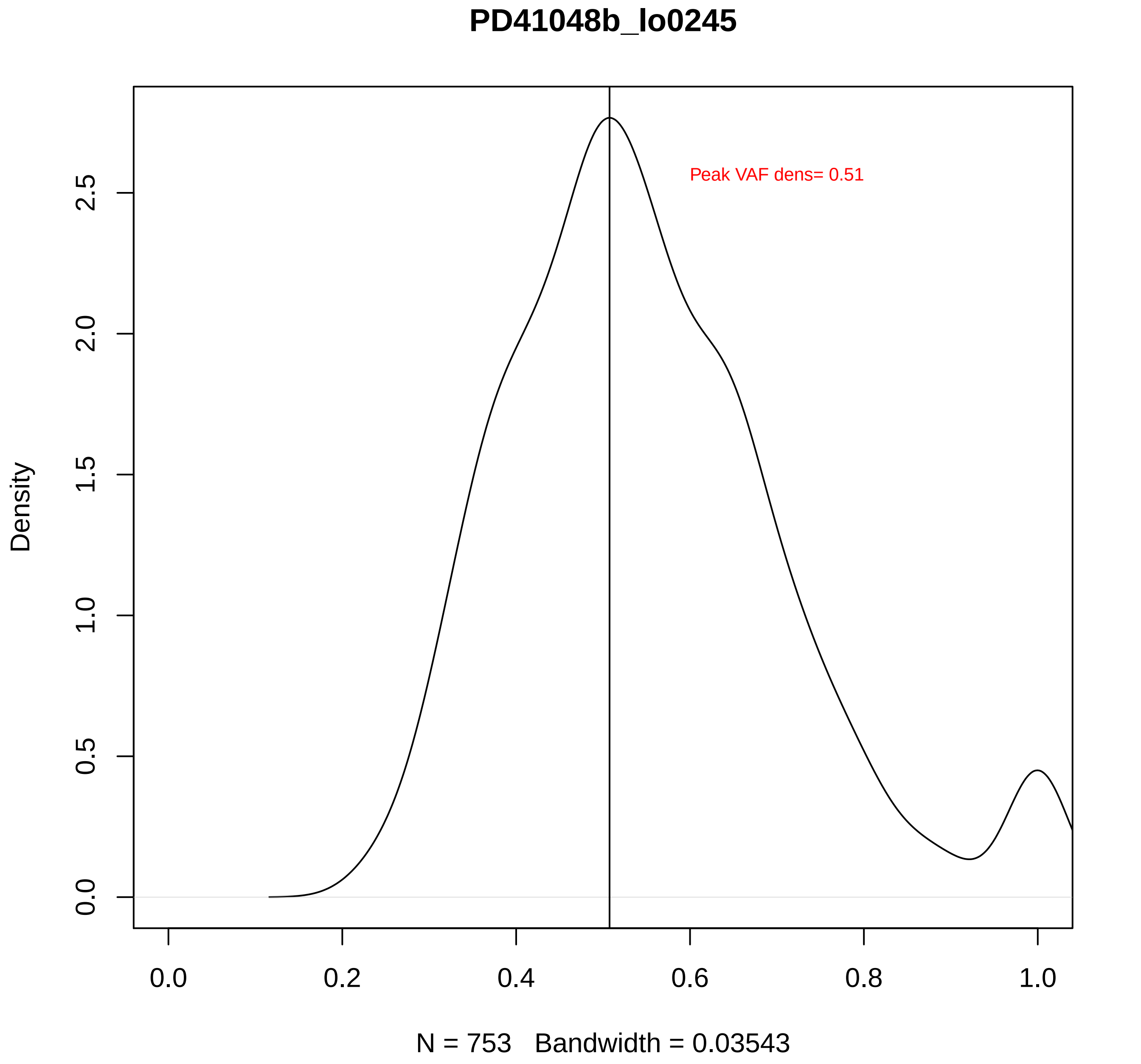

PD41048b_lo0245
Peak VAF dens= 0.51
Density
2.5
2.0
1.5
1.0
0.5
0.0
0.0
0.2
0.4
0.6
0.8
1.0
N = 753 Bandwidth = 0.03543

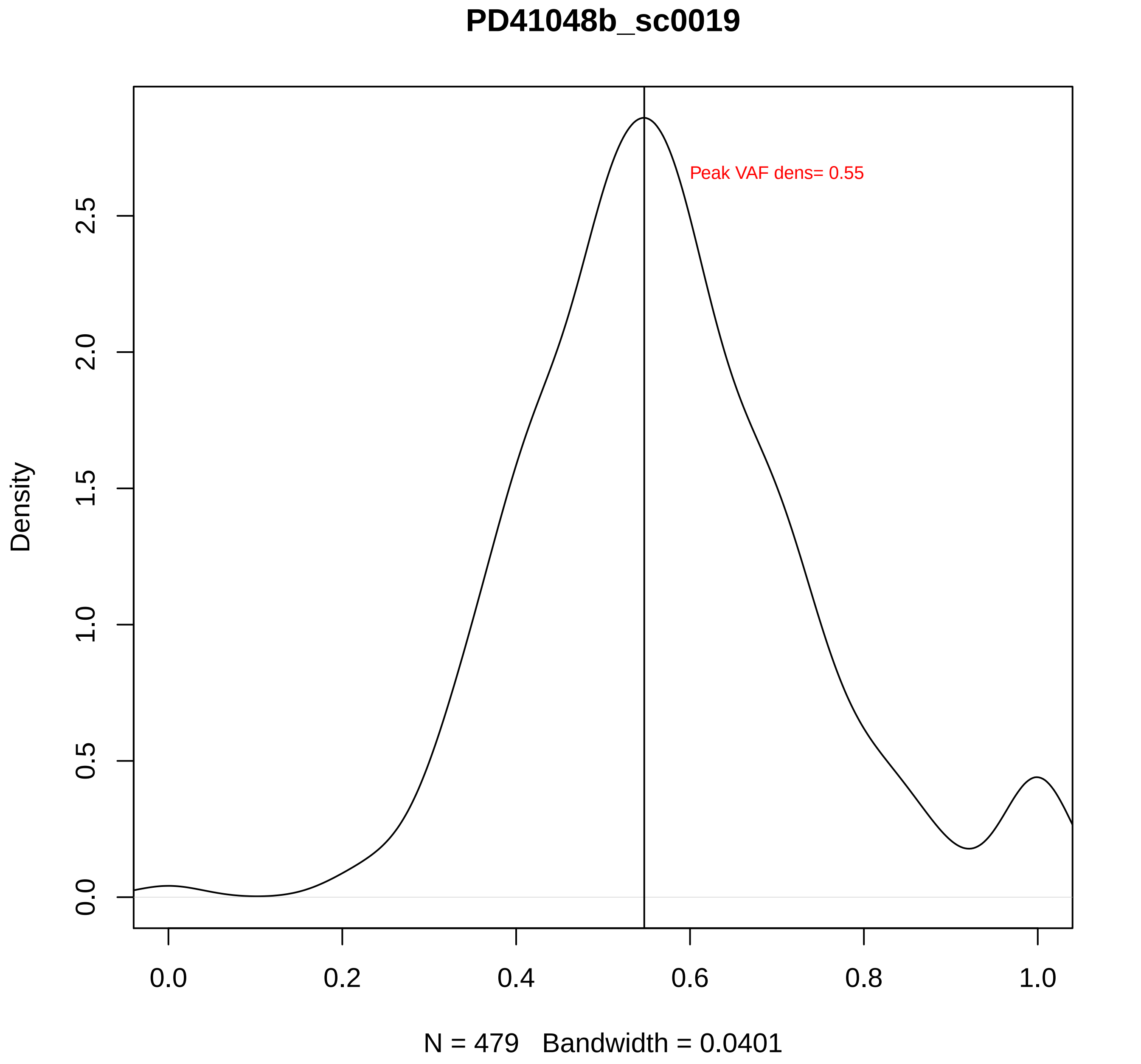
PD41048b_sc0019
Peak VAF dens= 0.55
Density
0.0
0.5
1.0
1.5
2.0
2.5
0.0
0.2
0.4
0.6
0.8
1.0
N = 479 Bandwidth = 0.0401

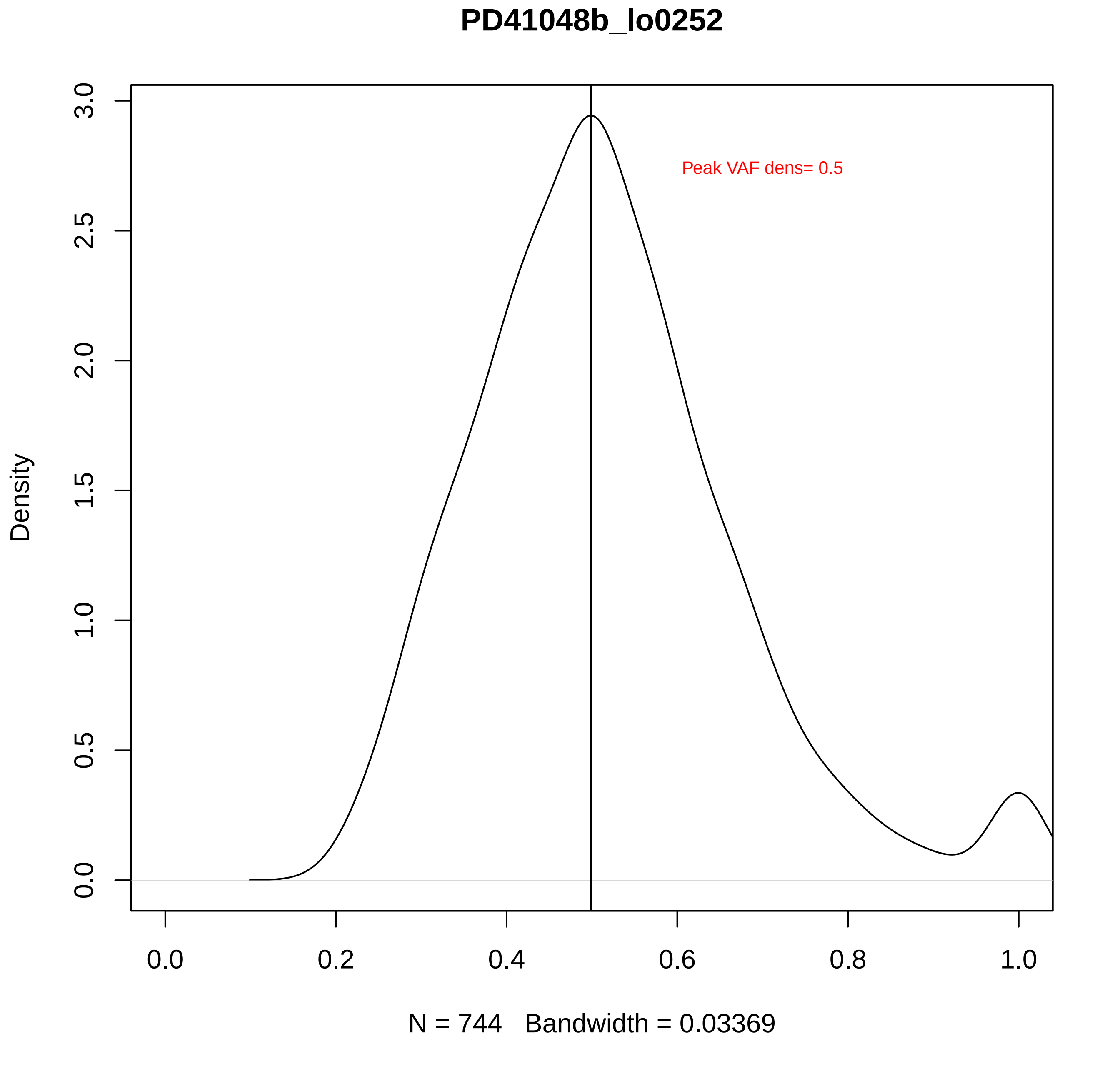
PD41048b_lo0252
Peak VAF dens= 0.5
Density
0.0
0.5
1.0
1.5
2.0
2.5
3.0
0.0
0.2
0.4
0.6
0.8
1.0
N = 744 Bandwidth = 0.03369

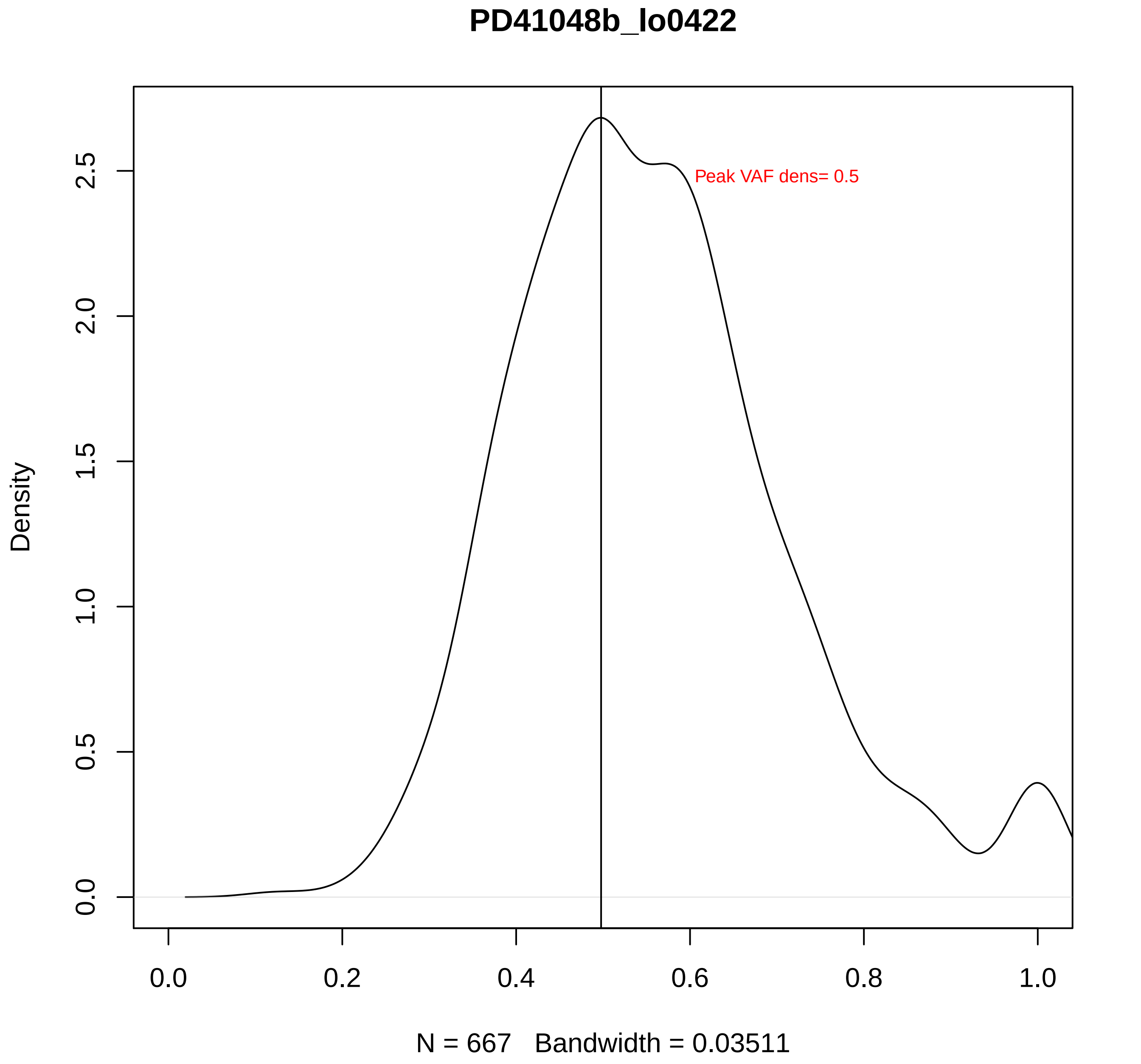

PD41048b_lo0422
Density
Peak VAF dens= 0.5
0.0
0.5
1.0
1.5
2.0
2.5
0.0
0.2
0.4
0.6
0.8
1.0
N = 667 Bandwidth = 0.03511

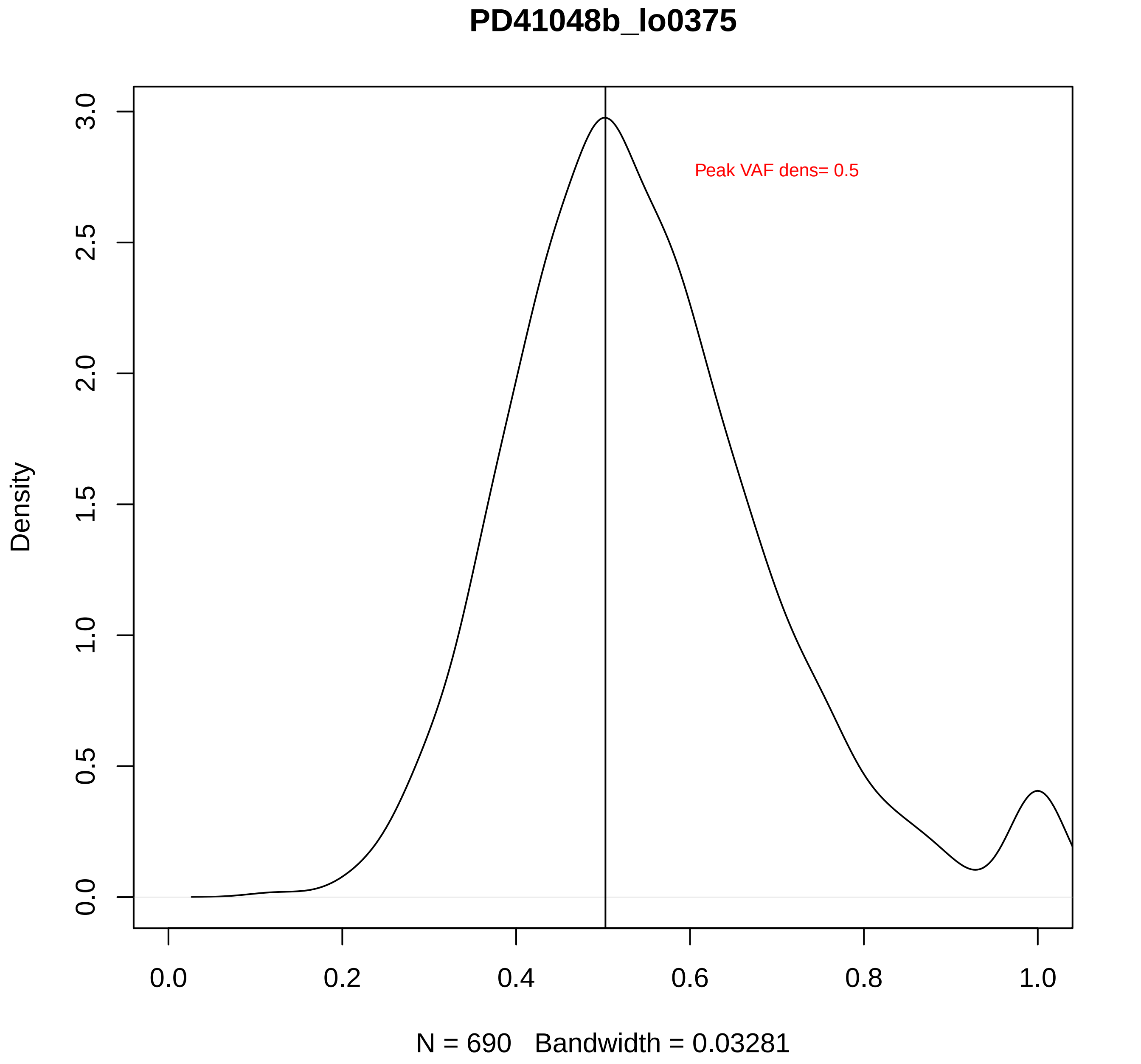
PD41048b_lo0375
Peak VAF dens= 0.5
Density
0.0
0.5
1.0
1.5
2.0
2.5
3.0
0.0
0.2
0.4
0.6
0.8
1.0
N = 690   Bandwidth = 0.03281

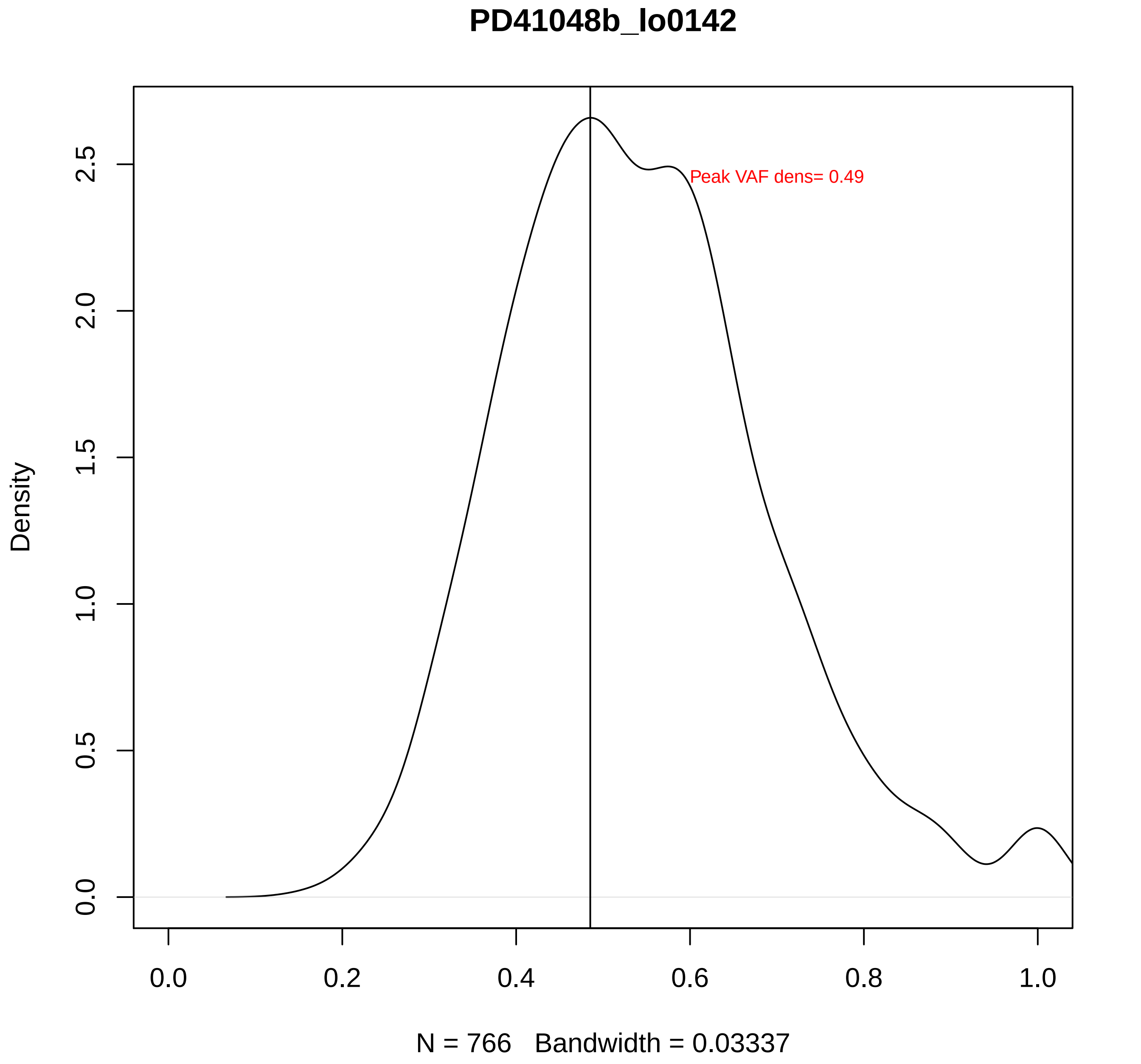

PD41048b_lo0142
Peak VAF dens= 0.49
Density
0.0
0.5
1.0
1.5
2.0
2.5
0.0
0.2
0.4
0.6
0.8
1.0
N = 766   Bandwidth = 0.03337

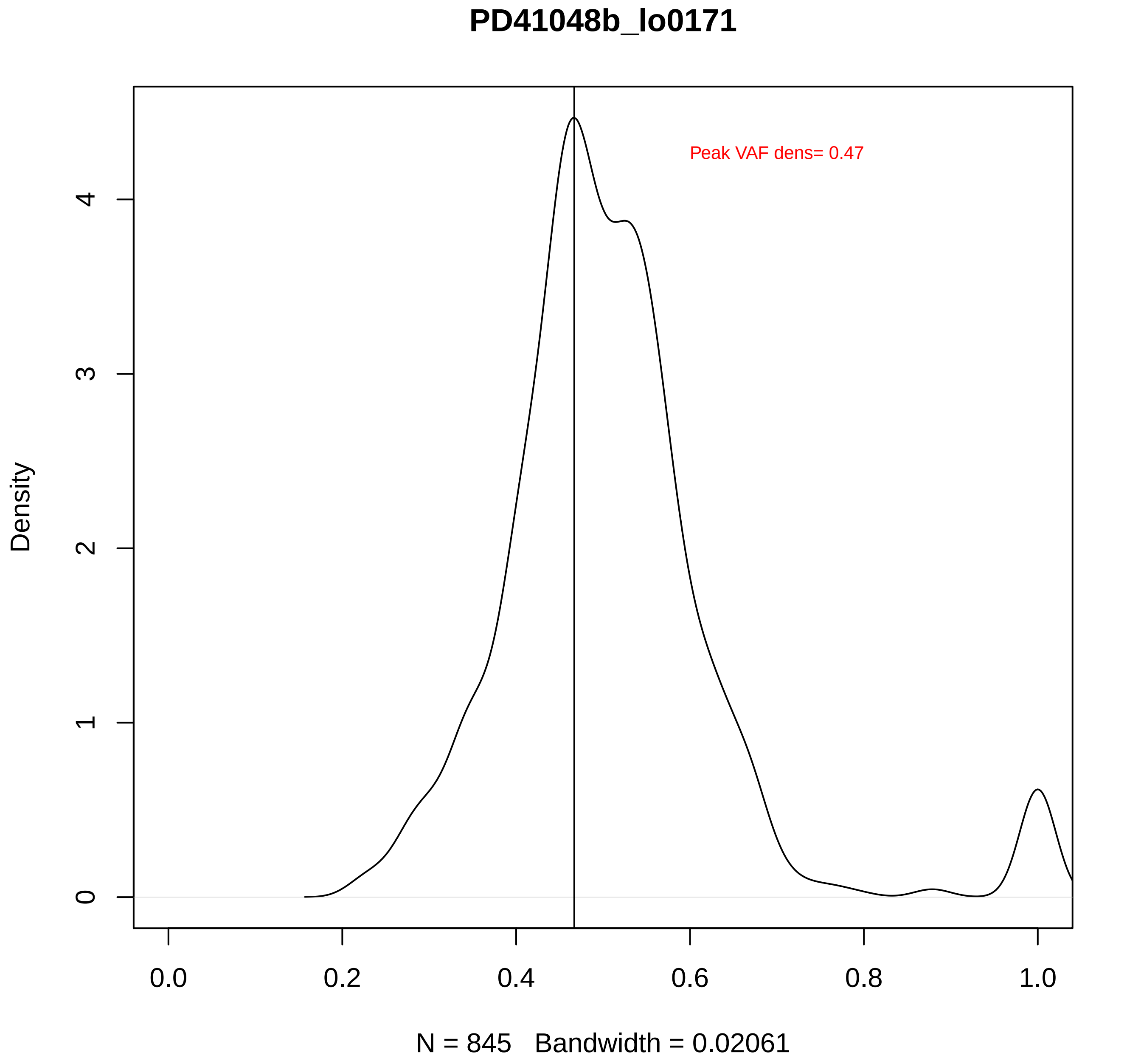
PD41048b_lo0171
Peak VAF dens= 0.47
Density
0
1
2
3
4
0.0
0.2
0.4
0.6
0.8
1.0
N = 845   Bandwidth = 0.02061

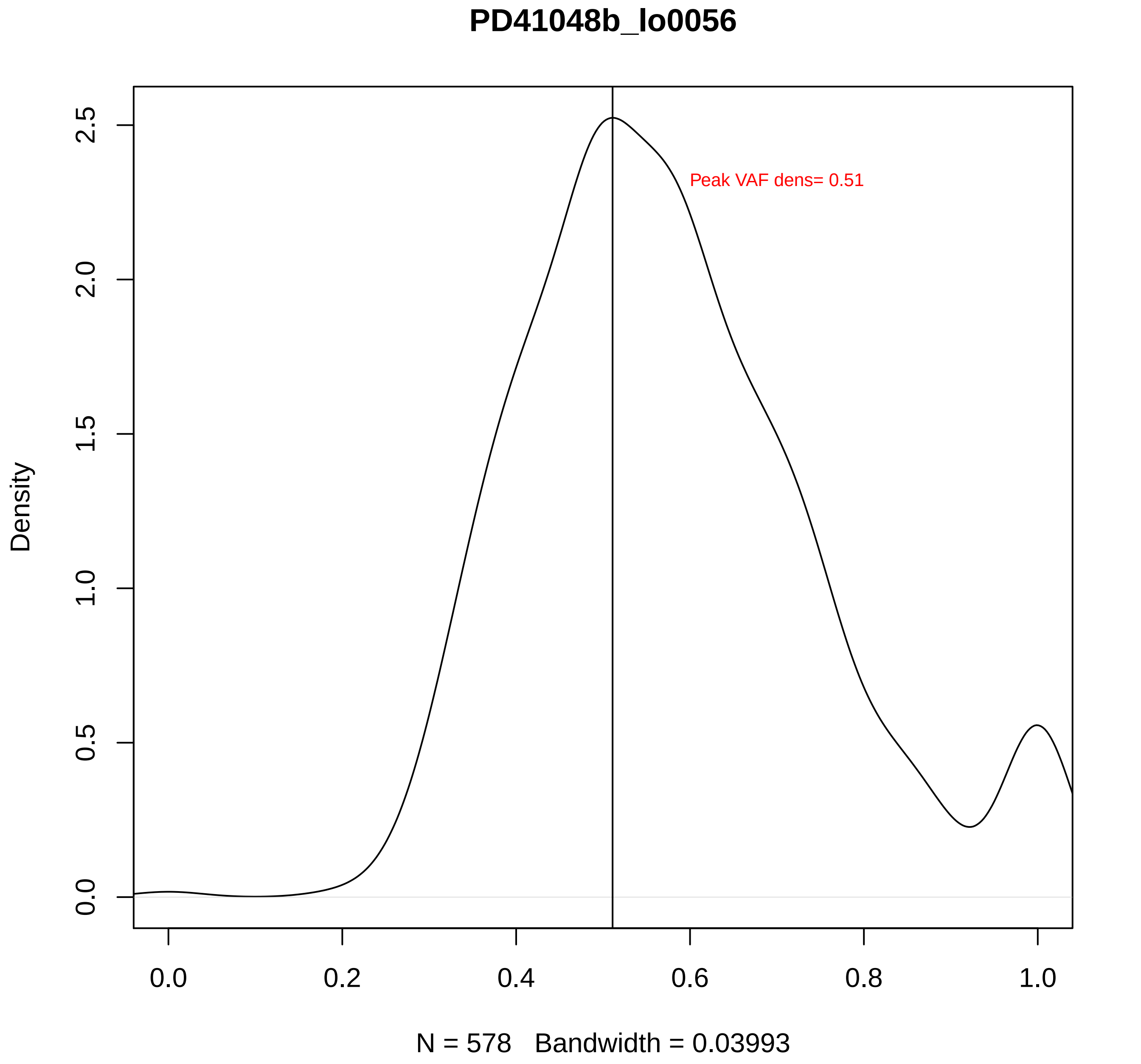
PD41048b_lo0056
Peak VAF dens= 0.51
Density
0.0
0.5
1.0
1.5
2.0
2.5
0.0
0.2
0.4
0.6
0.8
1.0
N = 578 Bandwidth = 0.03993

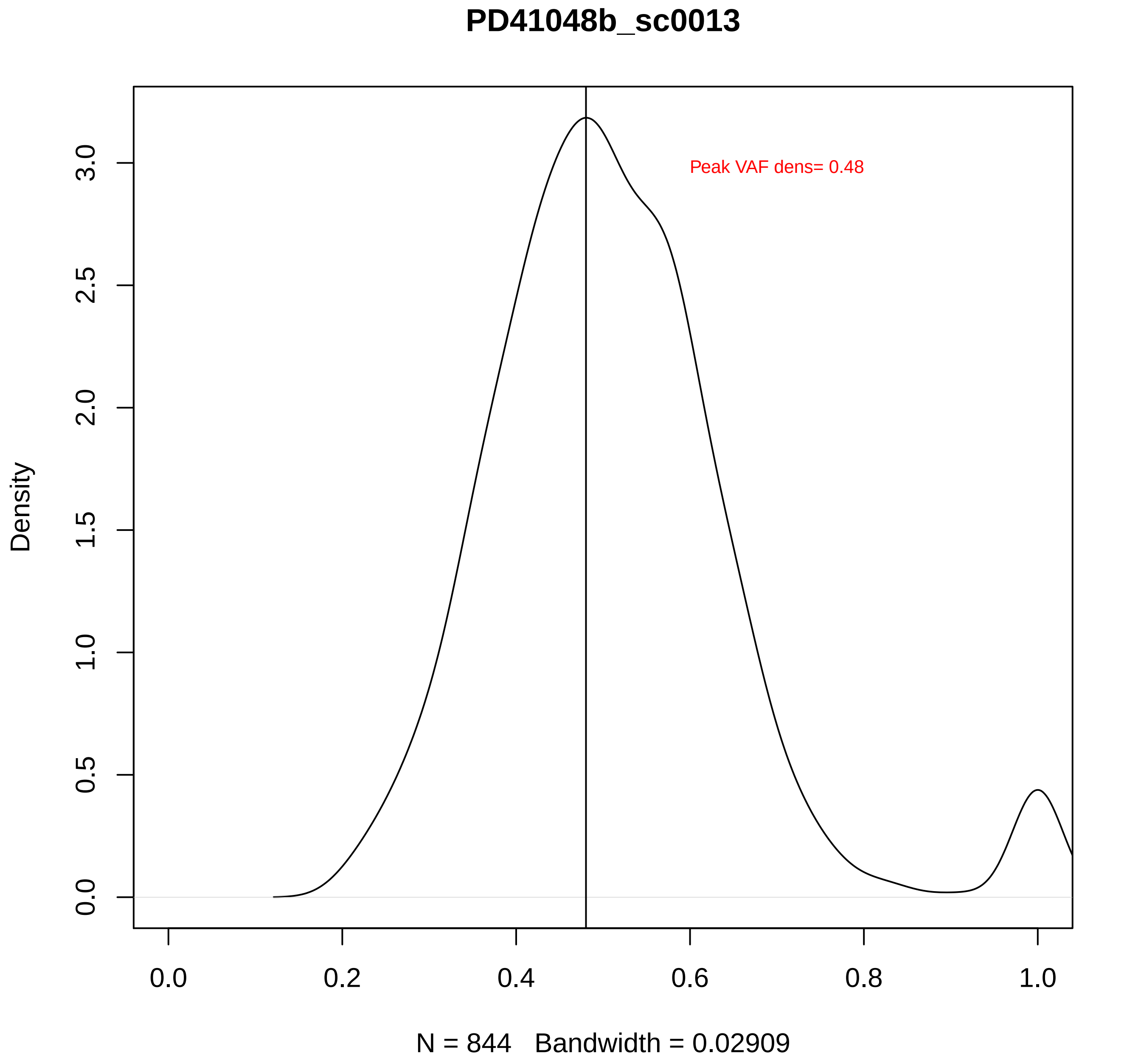

PD41048b_sc0013
Peak VAF dens= 0.48
Density
0.0
0.5
1.0
1.5
2.0
2.5
3.0
0.0
0.2
0.4
0.6
0.8
1.0
N = 844 Bandwidth = 0.02909

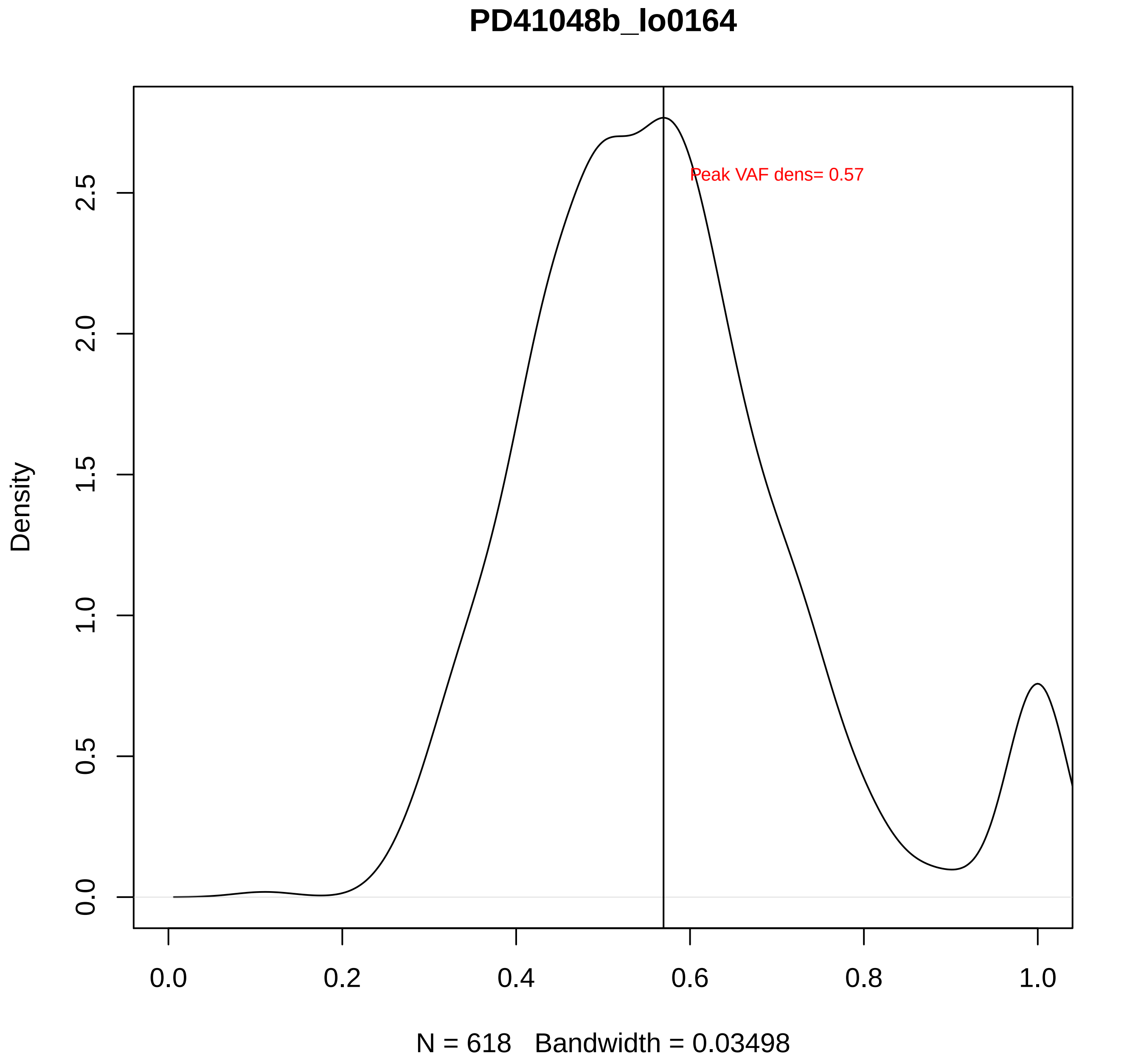

PD41048b_lo0164
Peak VAF dens= 0.57
Density
0.0
0.5
1.0
1.5
2.0
2.5
0.0
0.2
0.4
0.6
0.8
1.0
N = 618   Bandwidth = 0.03498

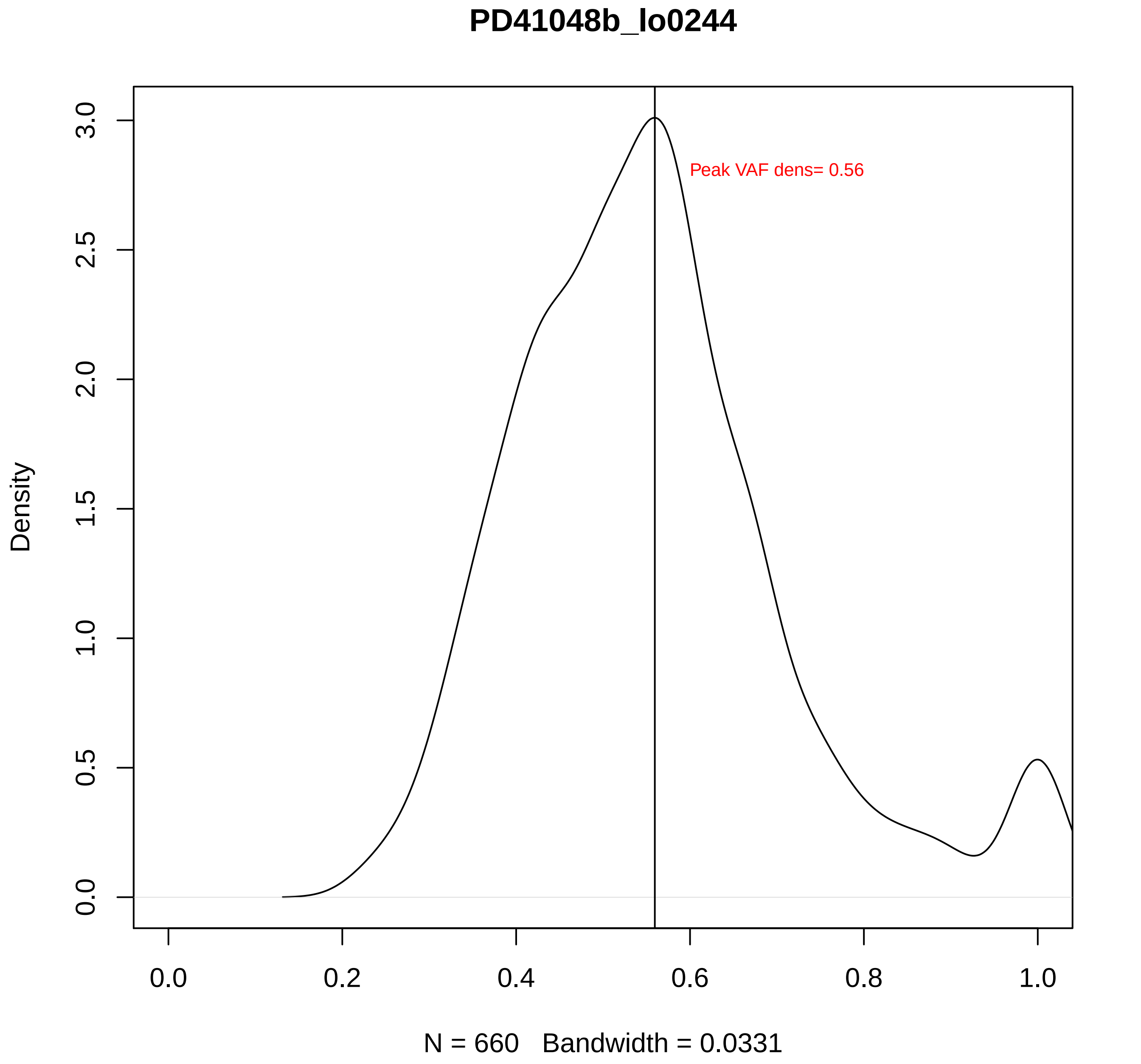
PD41048b_lo0244
Peak VAF dens= 0.56
Density
0.0
0.5
1.0
1.5
2.0
2.5
3.0
0.0
0.2
0.4
0.6
0.8
1.0
N = 660   Bandwidth = 0.0331

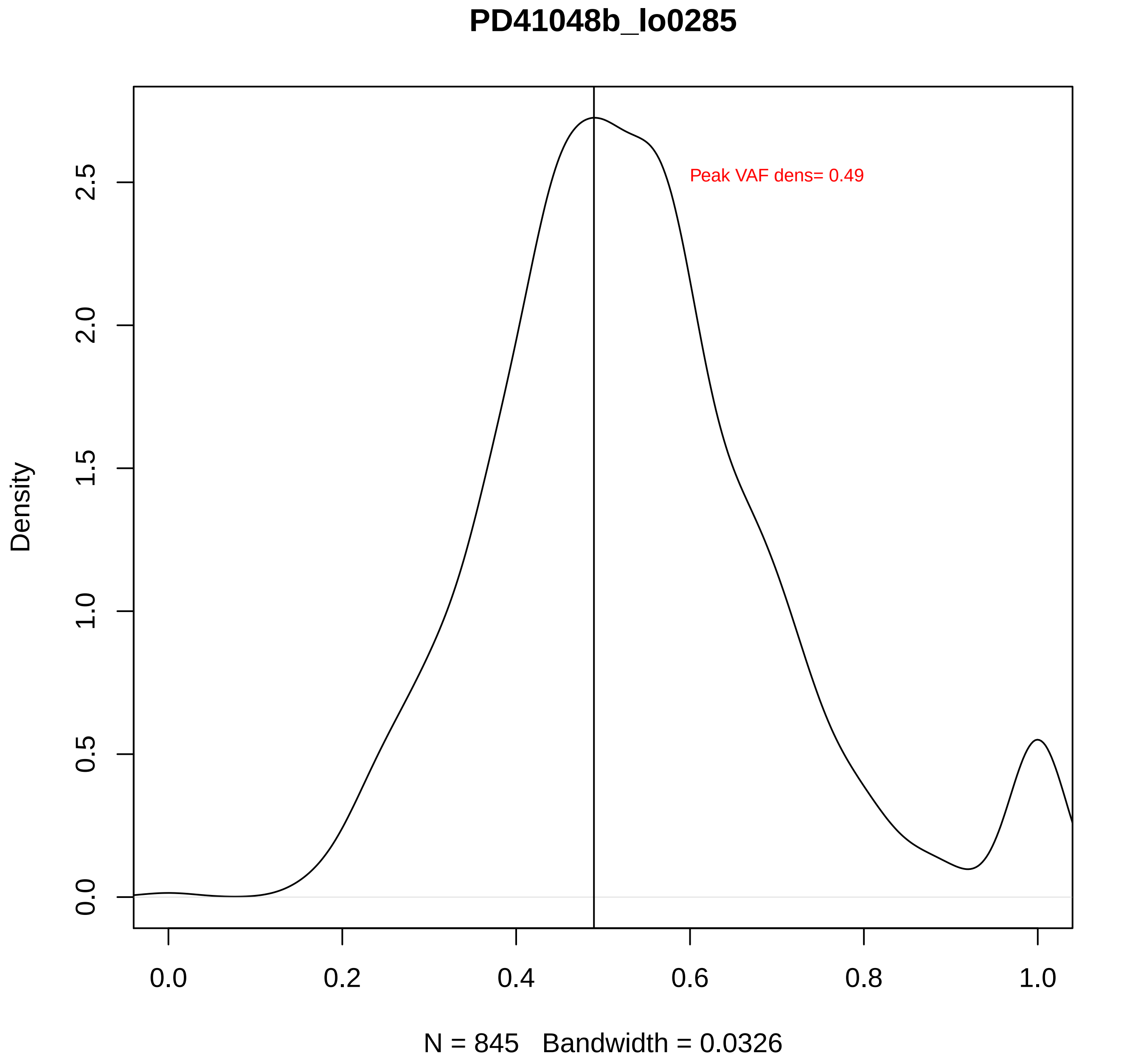

PD41048b_lo0285
Peak VAF dens= 0.49
Density
0.0
0.5
1.0
1.5
2.0
2.5
0.0
0.2
0.4
0.6
0.8
1.0
N = 845 Bandwidth = 0.0326

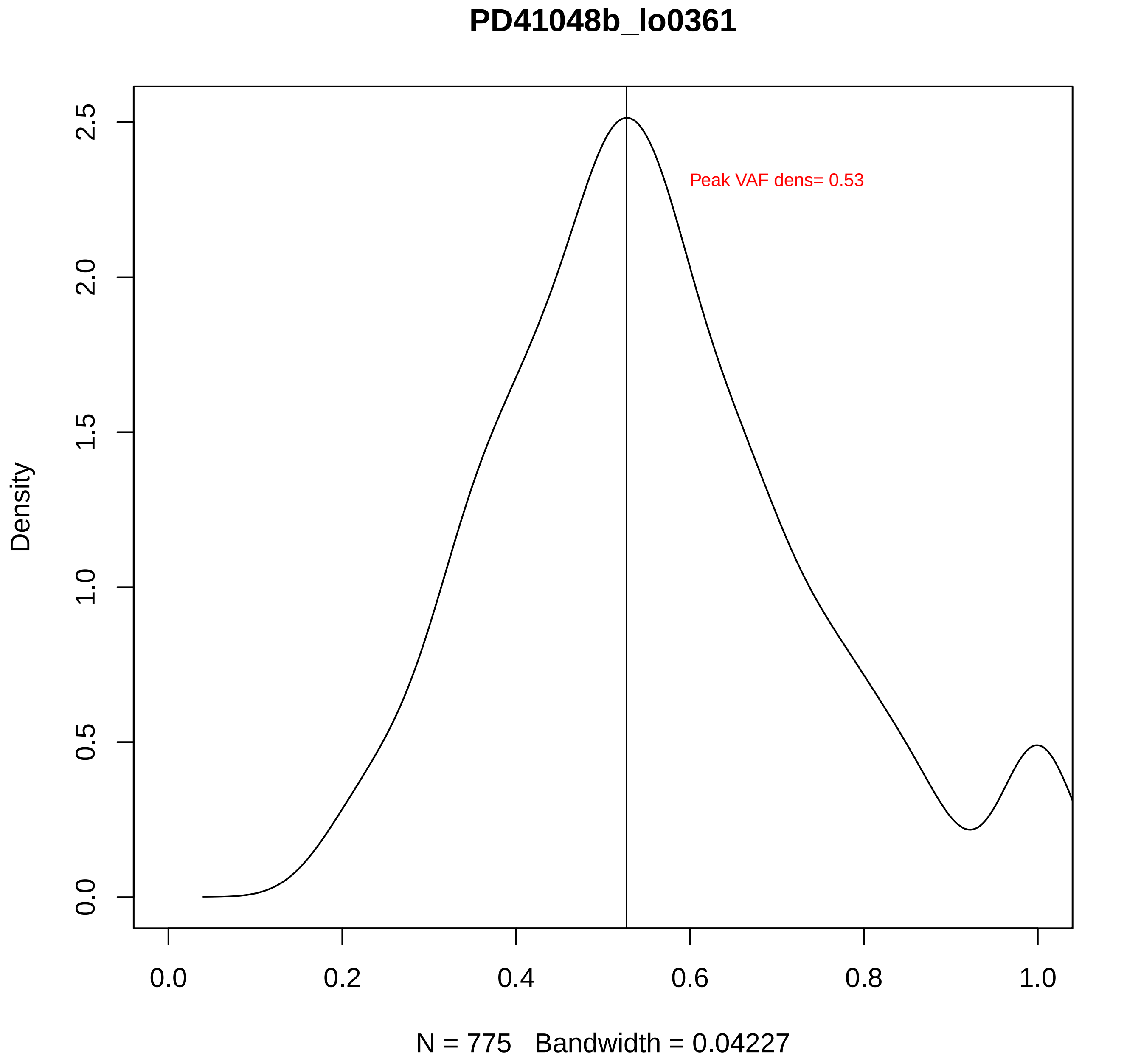
PD41048b_lo0361
Peak VAF dens= 0.53
Density
0.0
0.5
1.0
1.5
2.0
2.5
0.0
0.2
0.4
0.6
0.8
1.0
N = 775   Bandwidth = 0.04227

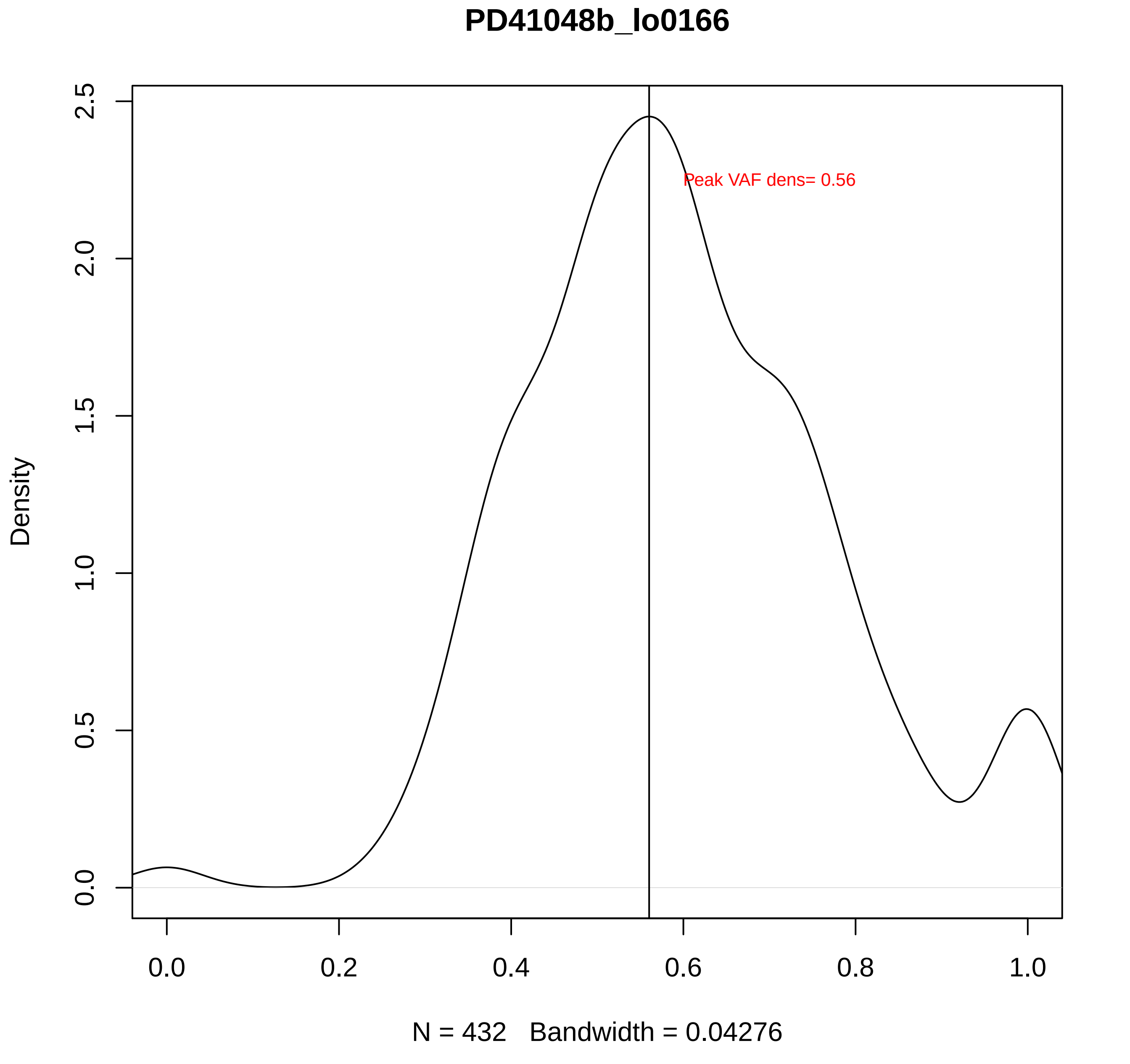

PD41048b_lo0166
Peak VAF dens= 0.56
Density
2.5
2.0
1.5
1.0
0.5
0.0
0.0
0.2
0.4
0.6
0.8
1.0
N = 432   Bandwidth = 0.04276

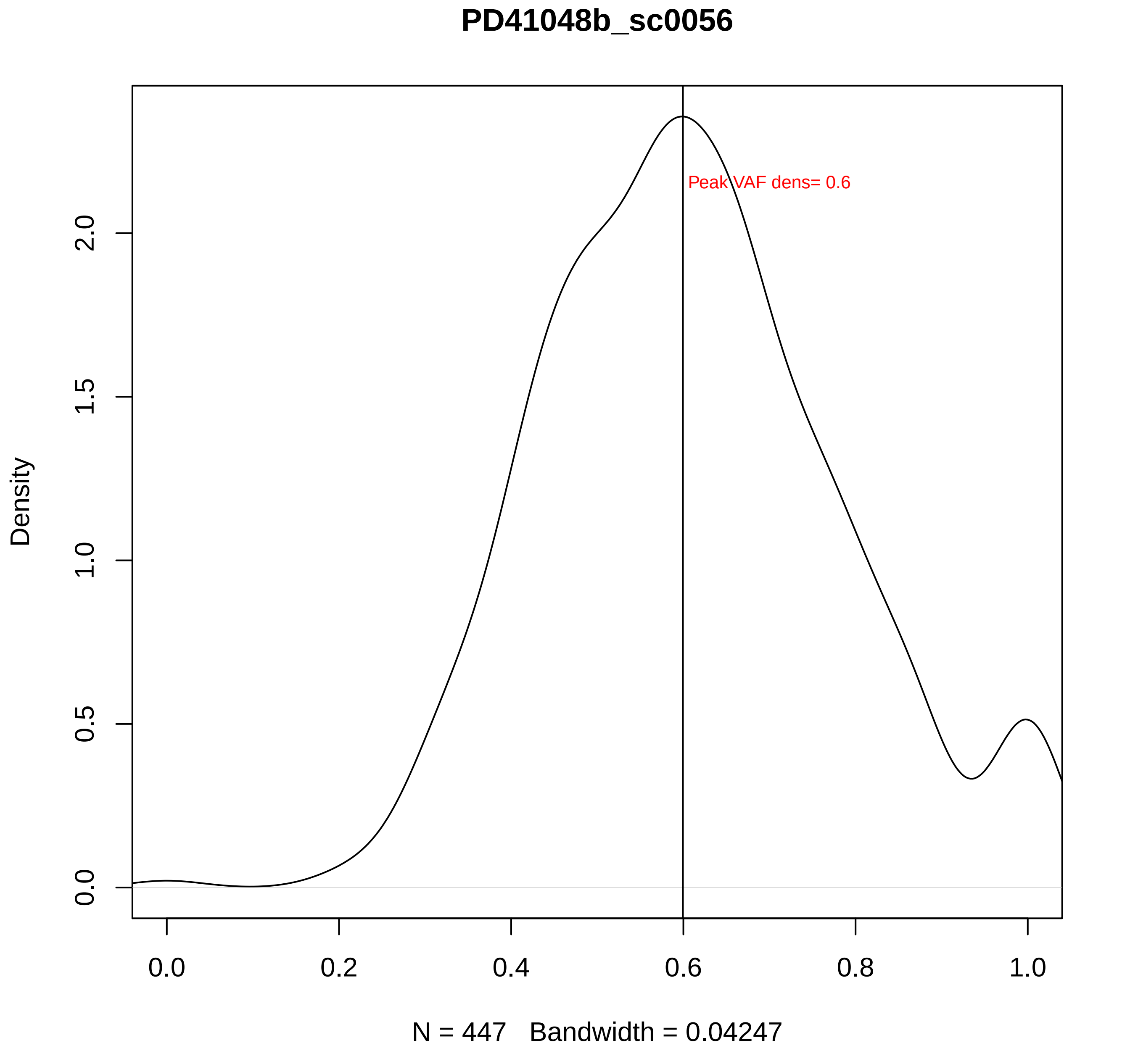
PD41048b_sc0056
Peak VAF dens= 0.6
Density
0.0
0.5
1.0
1.5
2.0
0.0
0.2
0.4
0.6
0.8
1.0
N = 447   Bandwidth = 0.04247

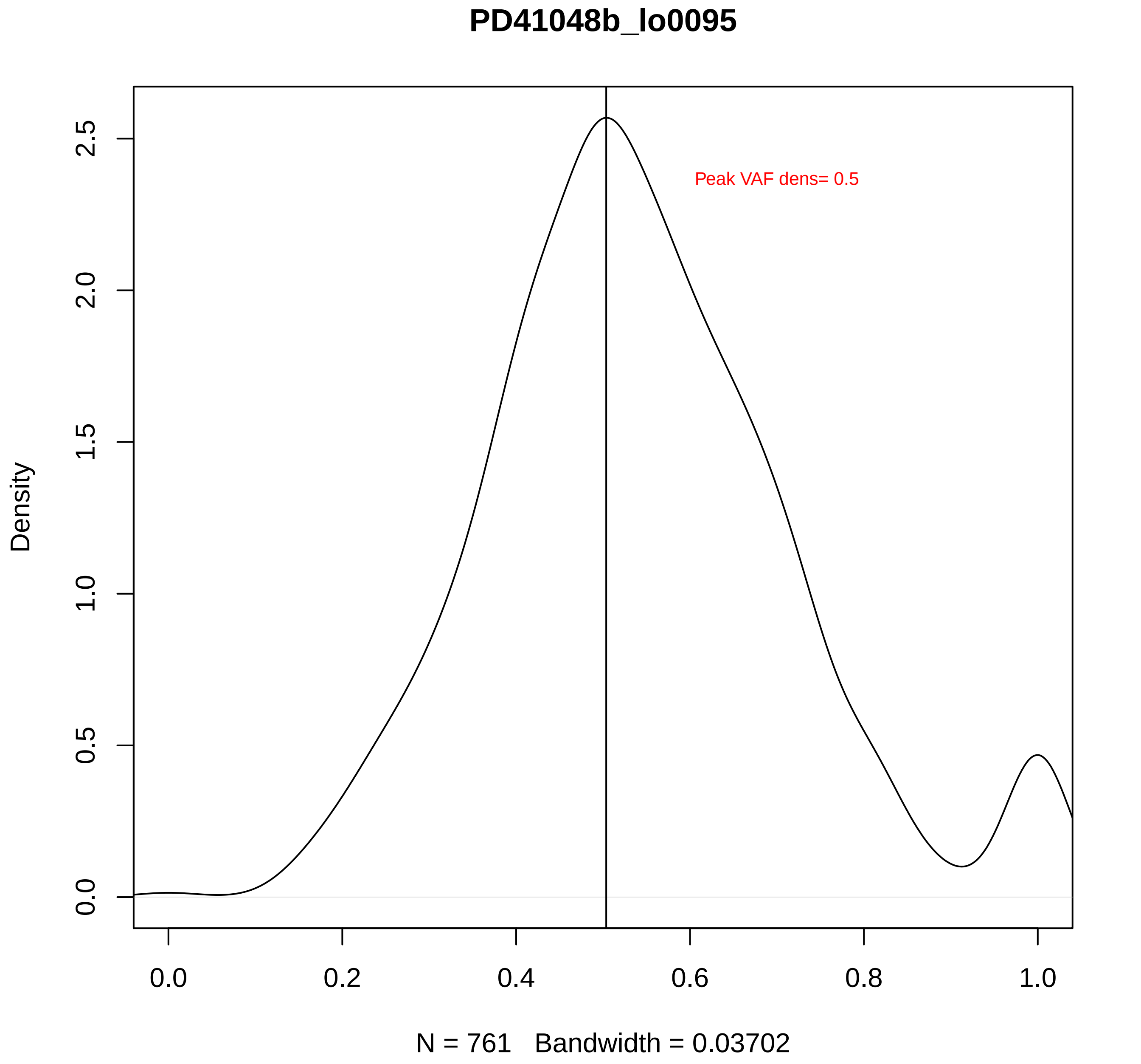
PD41048b_lo0095
Peak VAF dens= 0.5
Density
0.0
0.5
1.0
1.5
2.0
2.5
0.0
0.2
0.4
0.6
0.8
1.0
N = 761   Bandwidth = 0.03702

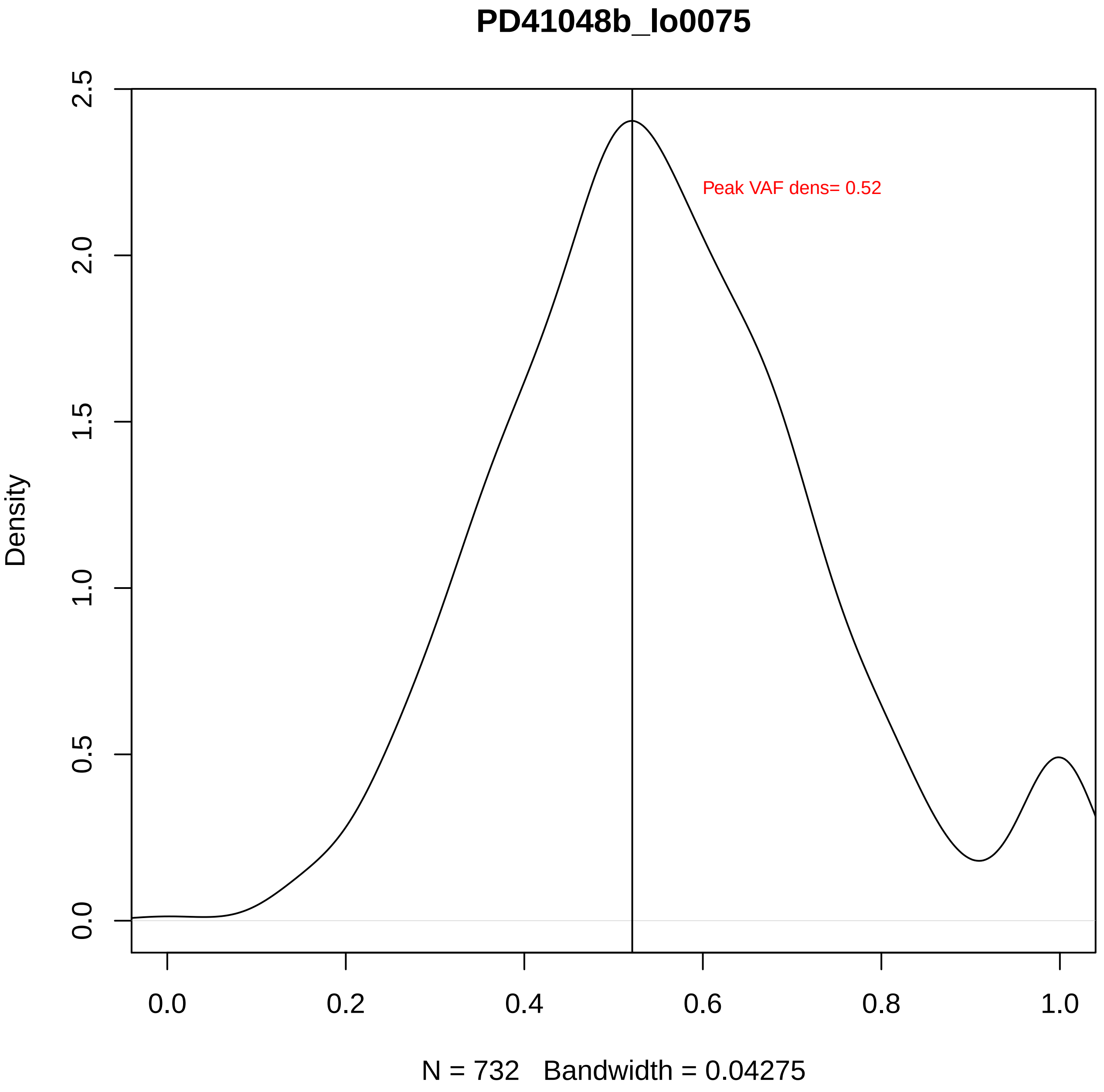
PD41048b_lo0075
Peak VAF dens= 0.52
Density
0.0
0.5
1.0
1.5
2.0
2.5
0.0
0.2
0.4
0.6
0.8
1.0
N = 732 Bandwidth = 0.04275

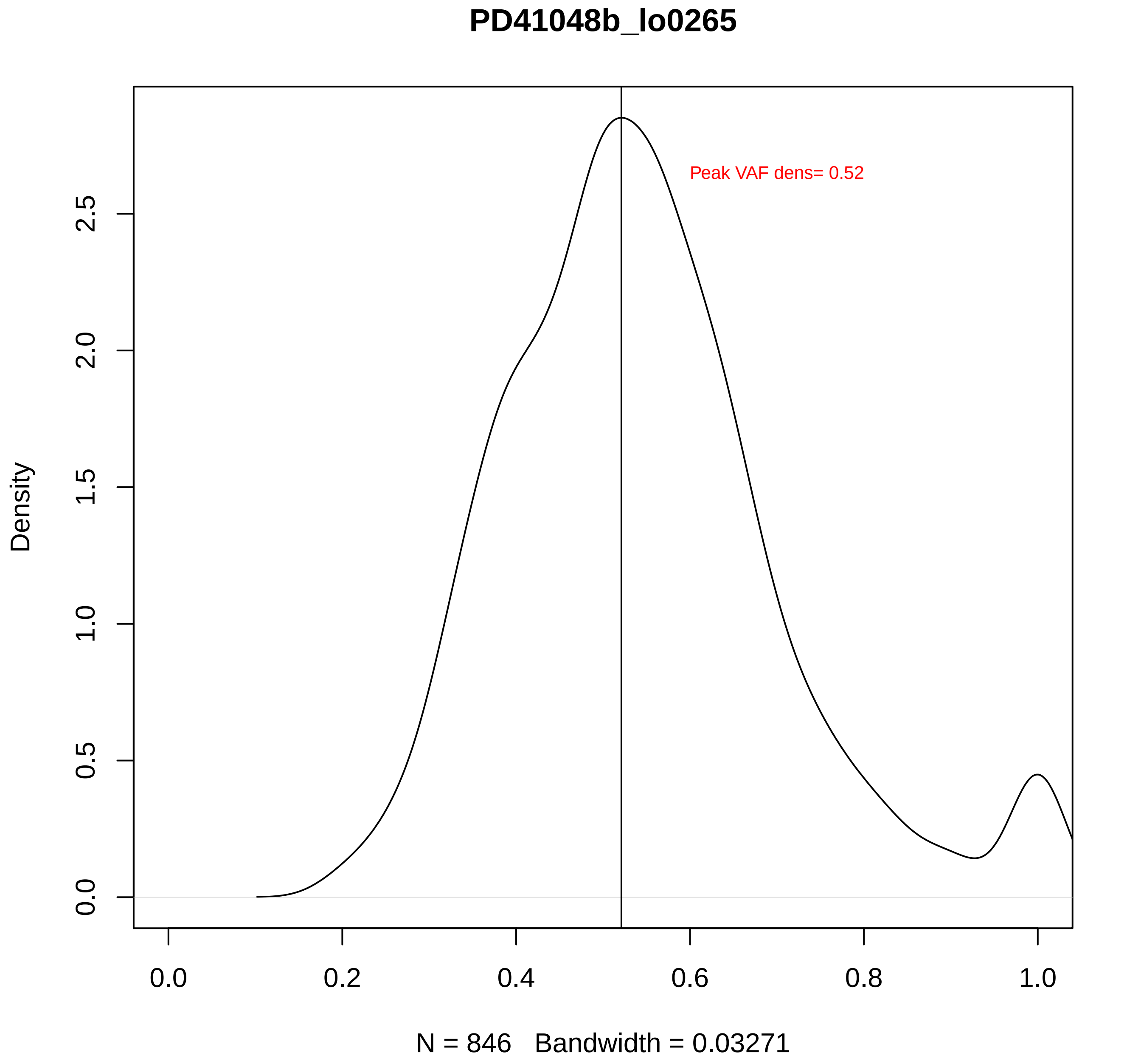

PD41048b_lo0265
Peak VAF dens= 0.52
Density
0.0
0.5
1.0
1.5
2.0
2.5
0.0
0.2
0.4
0.6
0.8
1.0
N = 846   Bandwidth = 0.03271

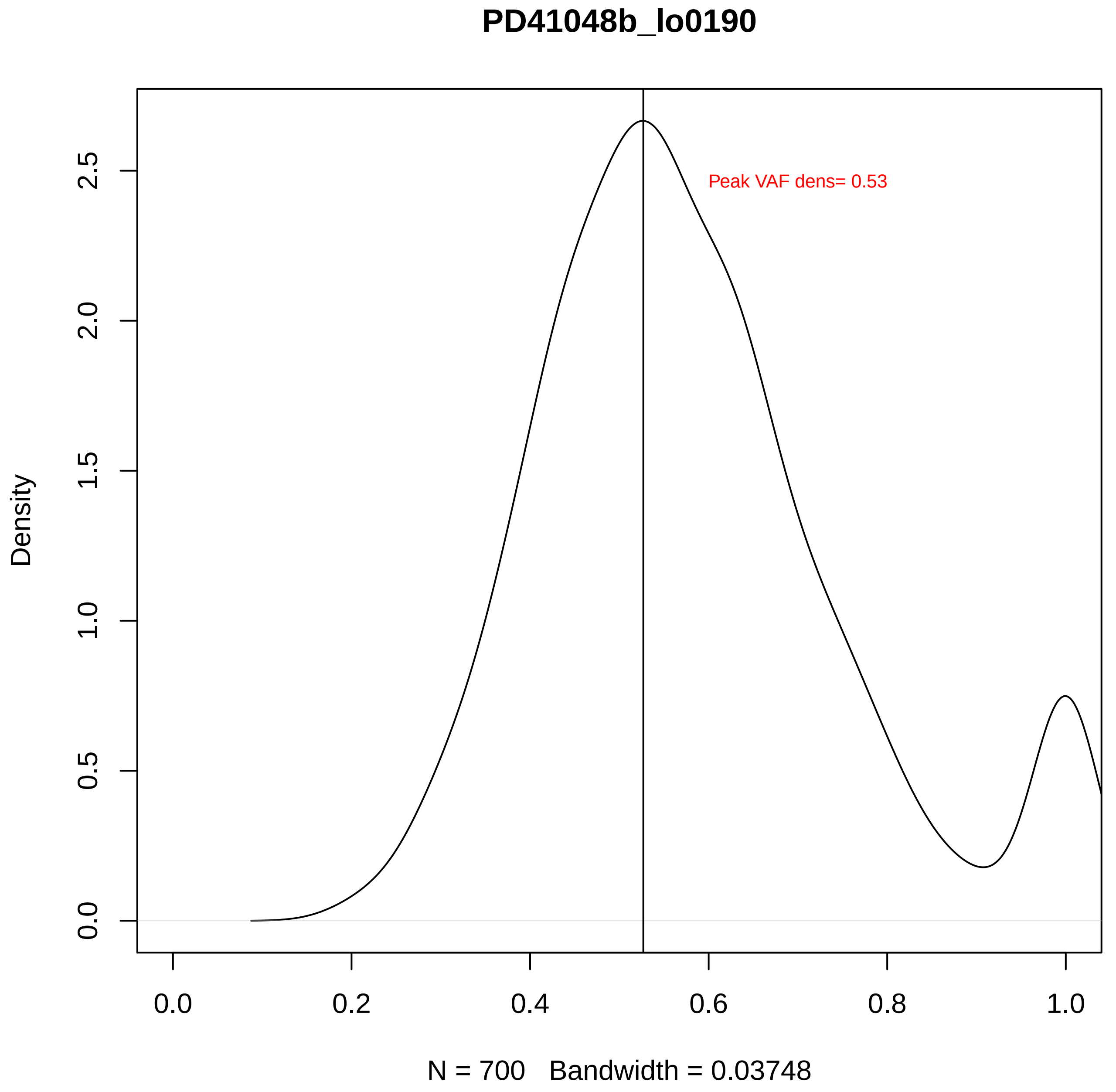

PD41048b_lo0190
Peak VAF dens= 0.53
Density
0.0
0.5
1.0
1.5
2.0
2.5
0.0
0.2
0.4
0.6
0.8
1.0
N = 700 Bandwidth = 0.03748

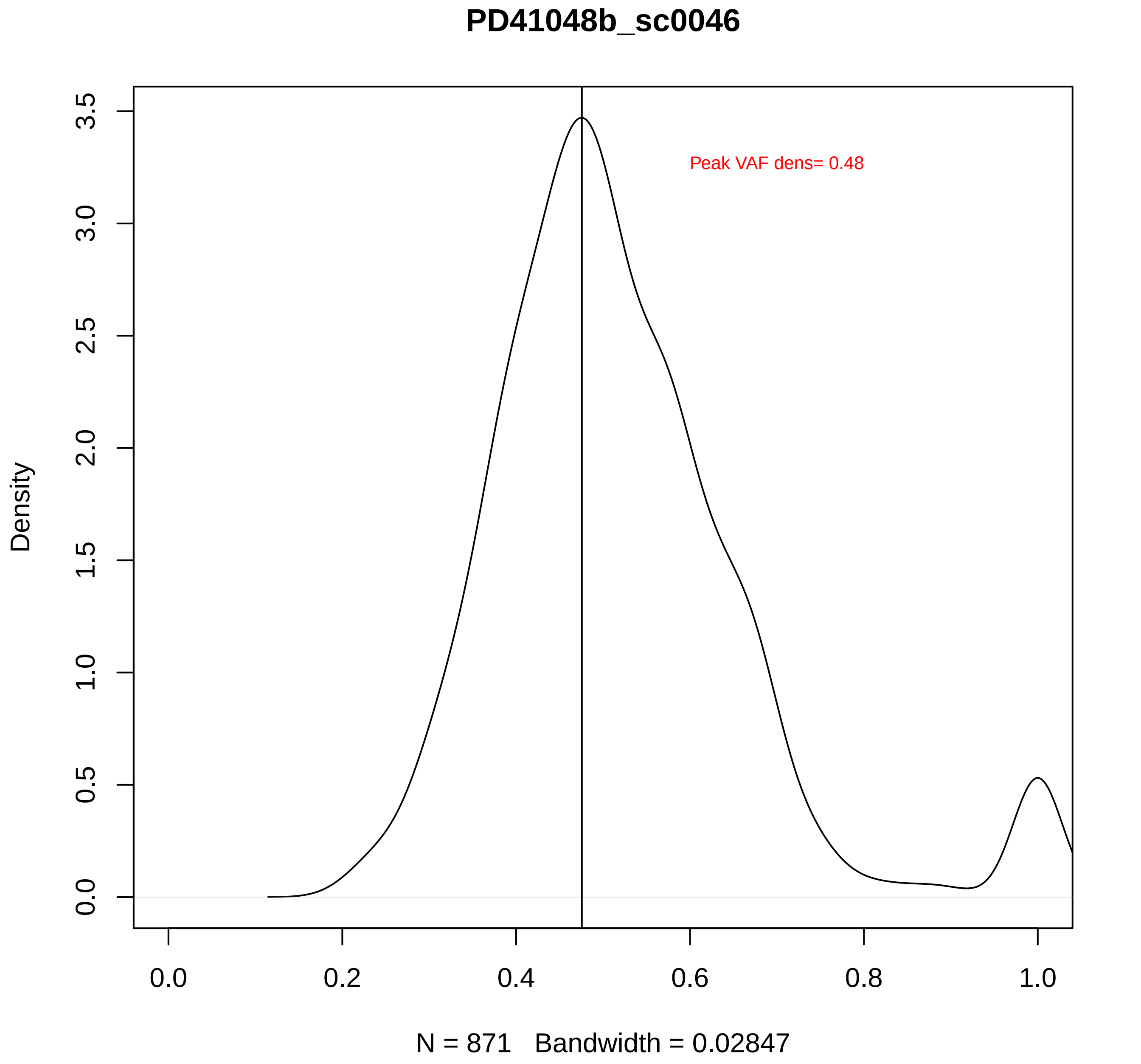
PD41048b_sc0046
Peak VAF dens= 0.48
Density
0.0
0.5
1.0
1.5
2.0
2.5
3.0
3.5
0.0
0.2
0.4
0.6
0.8
1.0
N = 871   Bandwidth = 0.02847

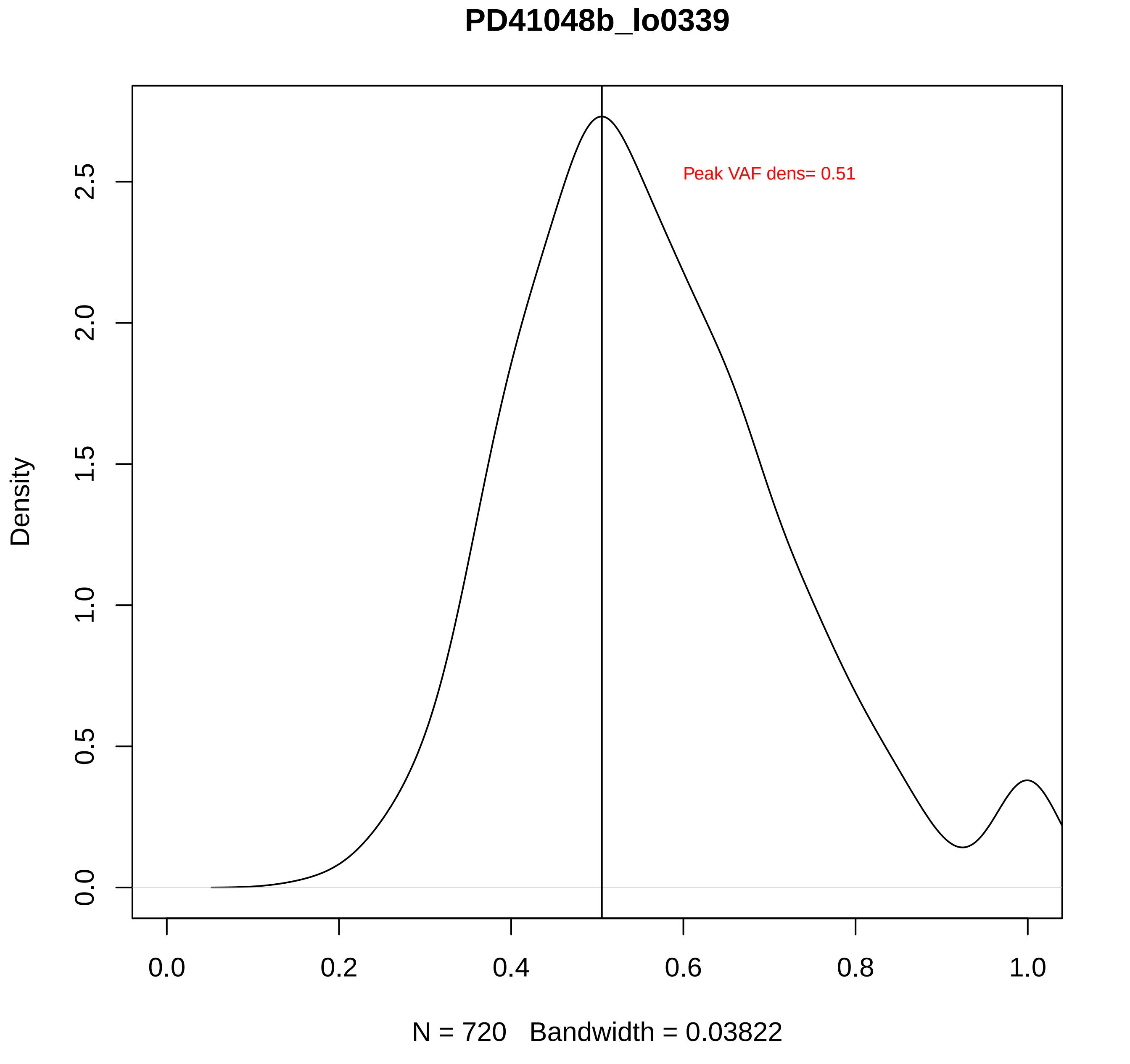
PD41048b_lo0339
Peak VAF dens= 0.51
Density
0.0
0.5
1.0
1.5
2.0
2.5
0.0
0.2
0.4
0.6
0.8
1.0
N = 720   Bandwidth = 0.03822

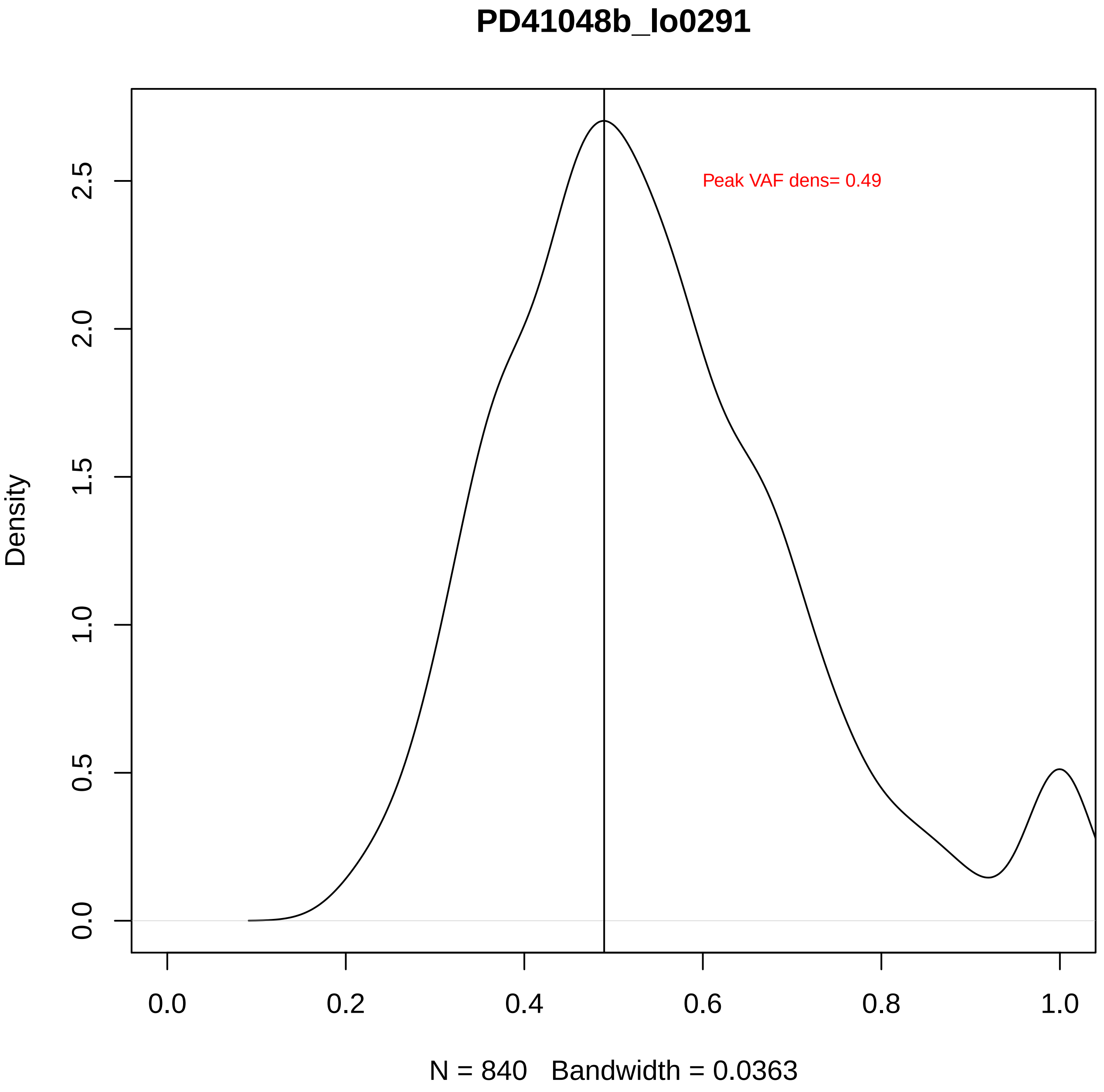
PD41048b_lo0291
Peak VAF dens= 0.49
Density
0.0
0.5
1.0
1.5
2.0
2.5
0.0
0.2
0.4
0.6
0.8
1.0
N = 840   Bandwidth = 0.0363

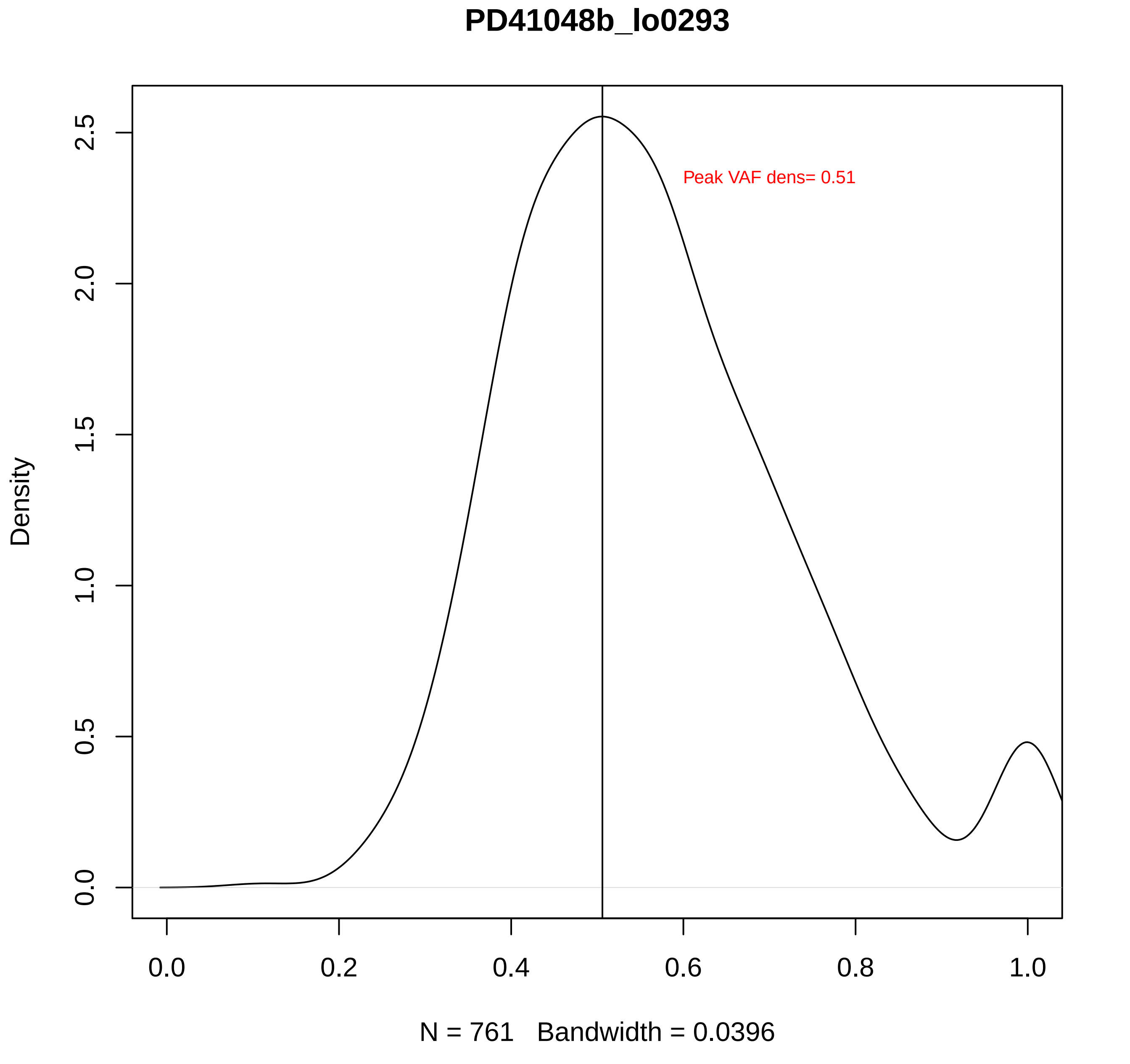
PD41048b_lo0293
Peak VAF dens= 0.51
Density
0.0
0.5
1.0
1.5
2.0
2.5
0.0
0.2
0.4
0.6
0.8
1.0
N = 761 Bandwidth = 0.0396

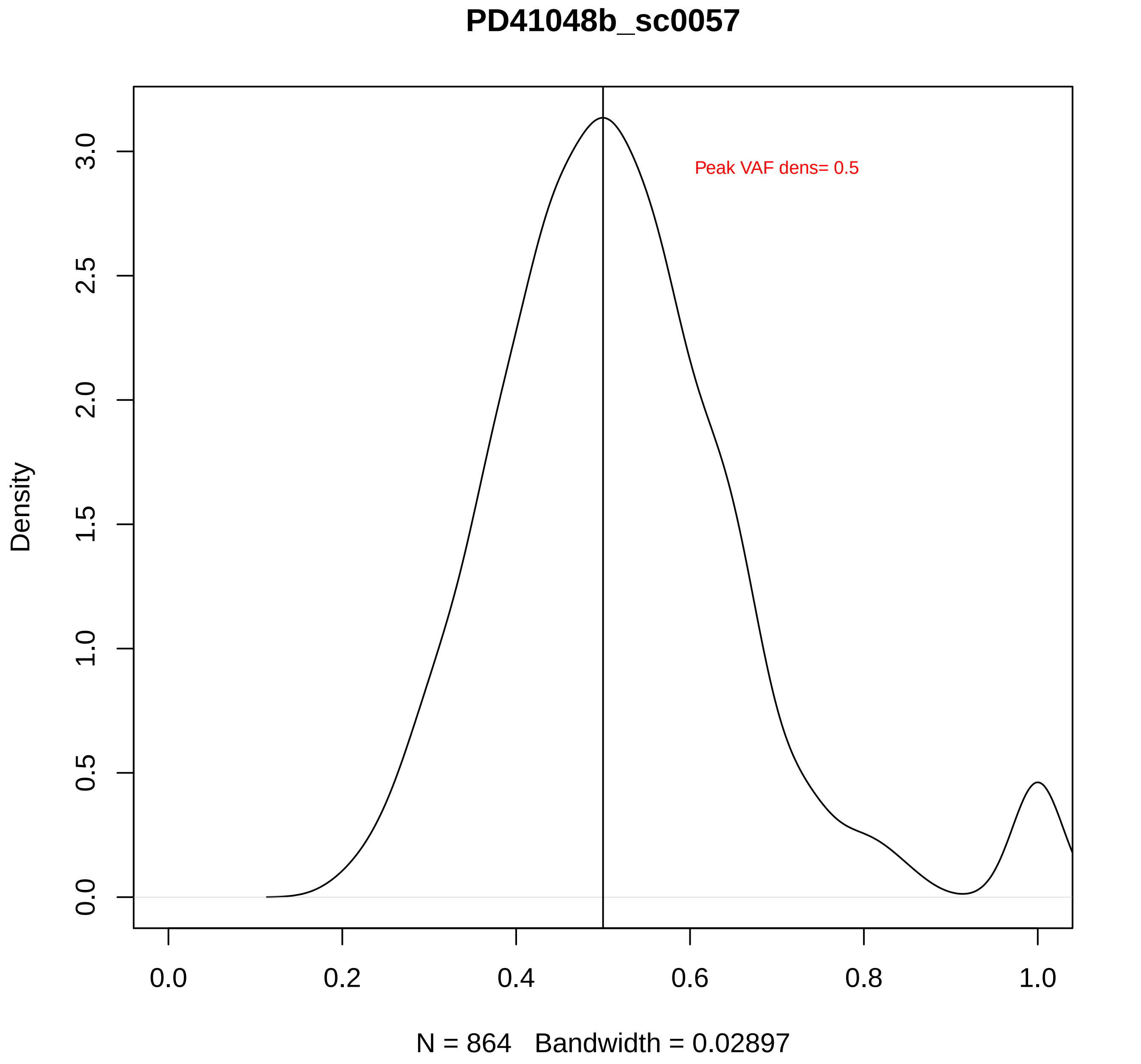

PD41048b_sc0057
Peak VAF dens= 0.5
Density
0.0
0.5
1.0
1.5
2.0
2.5
3.0
0.0
0.2
0.4
0.6
0.8
1.0
N = 864 Bandwidth = 0.02897

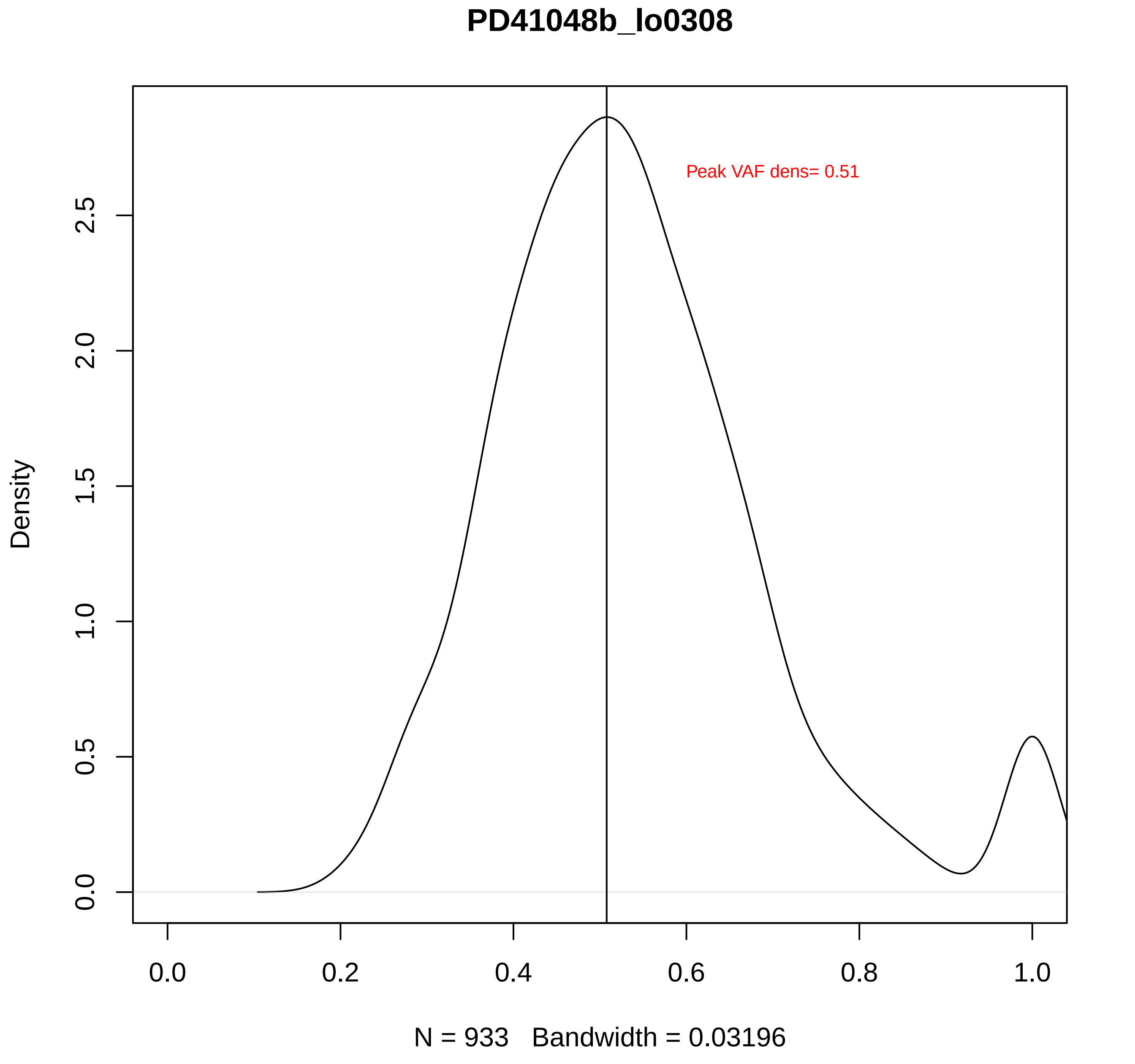
PD41048b_lo0308
Peak VAF dens= 0.51
Density
0.0
0.5
1.0
1.5
2.0
2.5
0.0
0.2
0.4
0.6
0.8
1.0
N = 933   Bandwidth = 0.03196

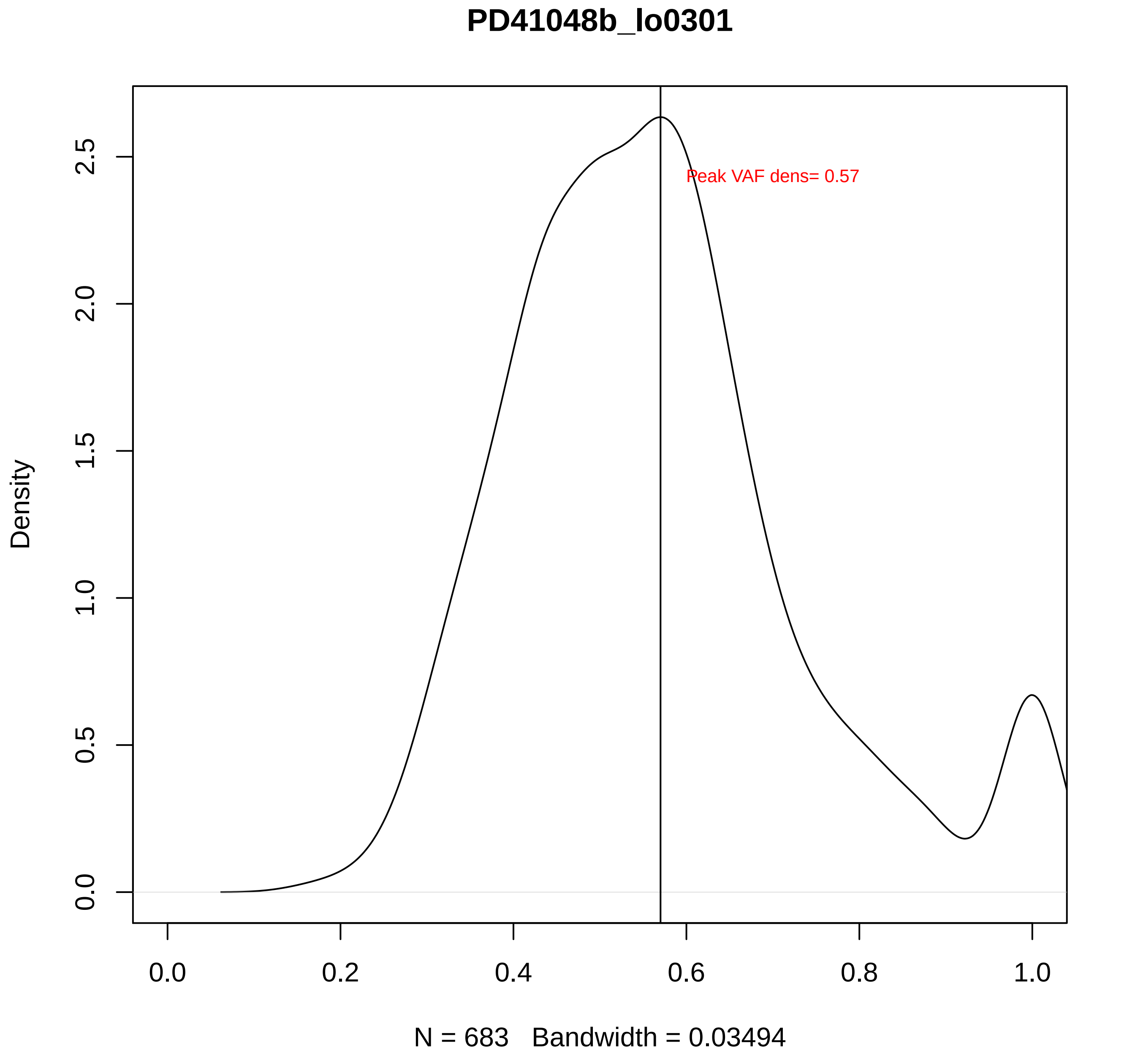
PD41048b_lo0301
Peak VAF dens= 0.57
Density
0.0
0.5
1.0
1.5
2.0
2.5
0.0
0.2
0.4
0.6
0.8
1.0
N = 683 Bandwidth = 0.03494

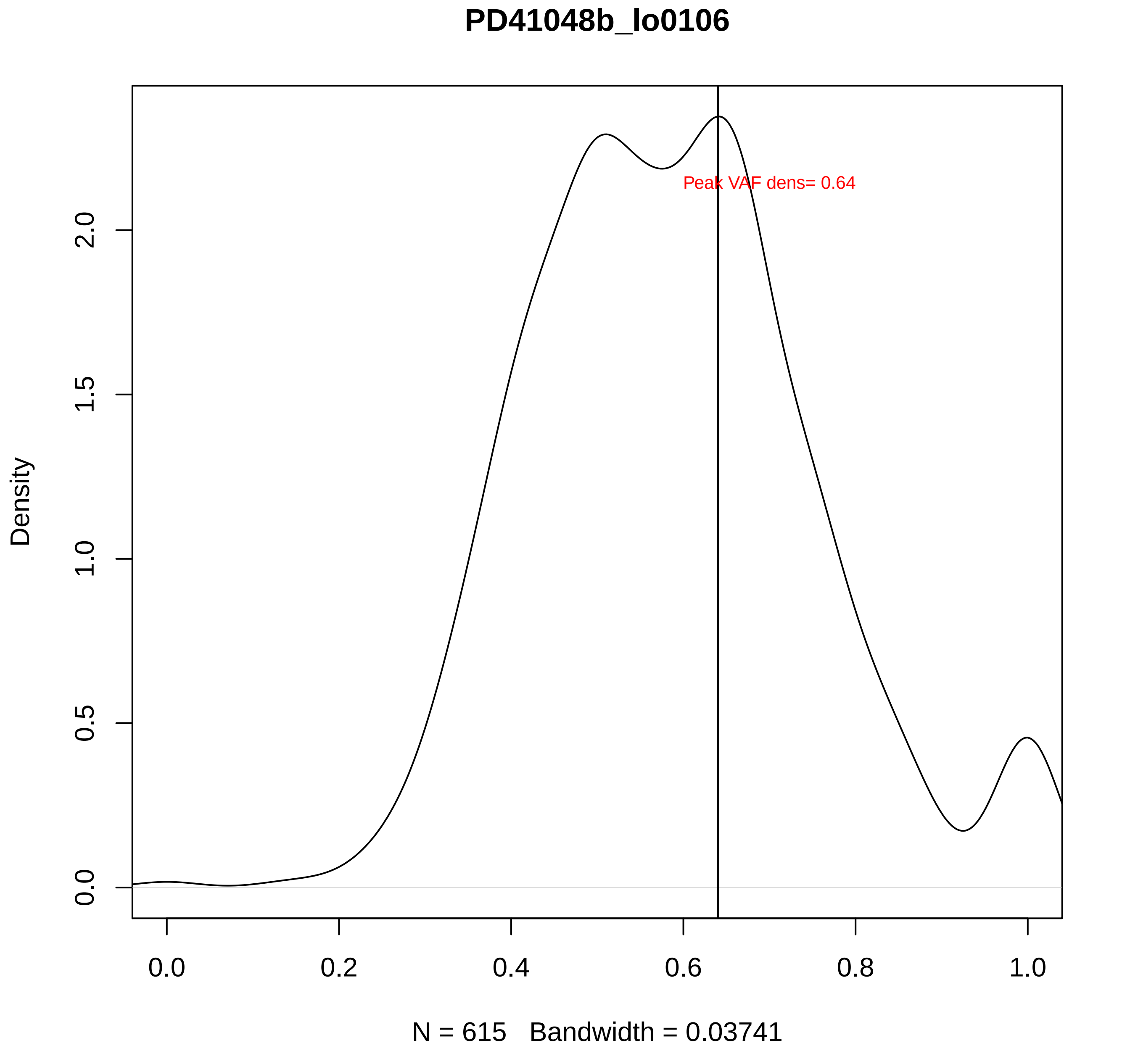
PD41048b_lo0106
Peak VAF dens= 0.64
Density
0.0
0.5
1.0
1.5
2.0
0.0
0.2
0.4
0.6
0.8
1.0
N = 615   Bandwidth = 0.03741

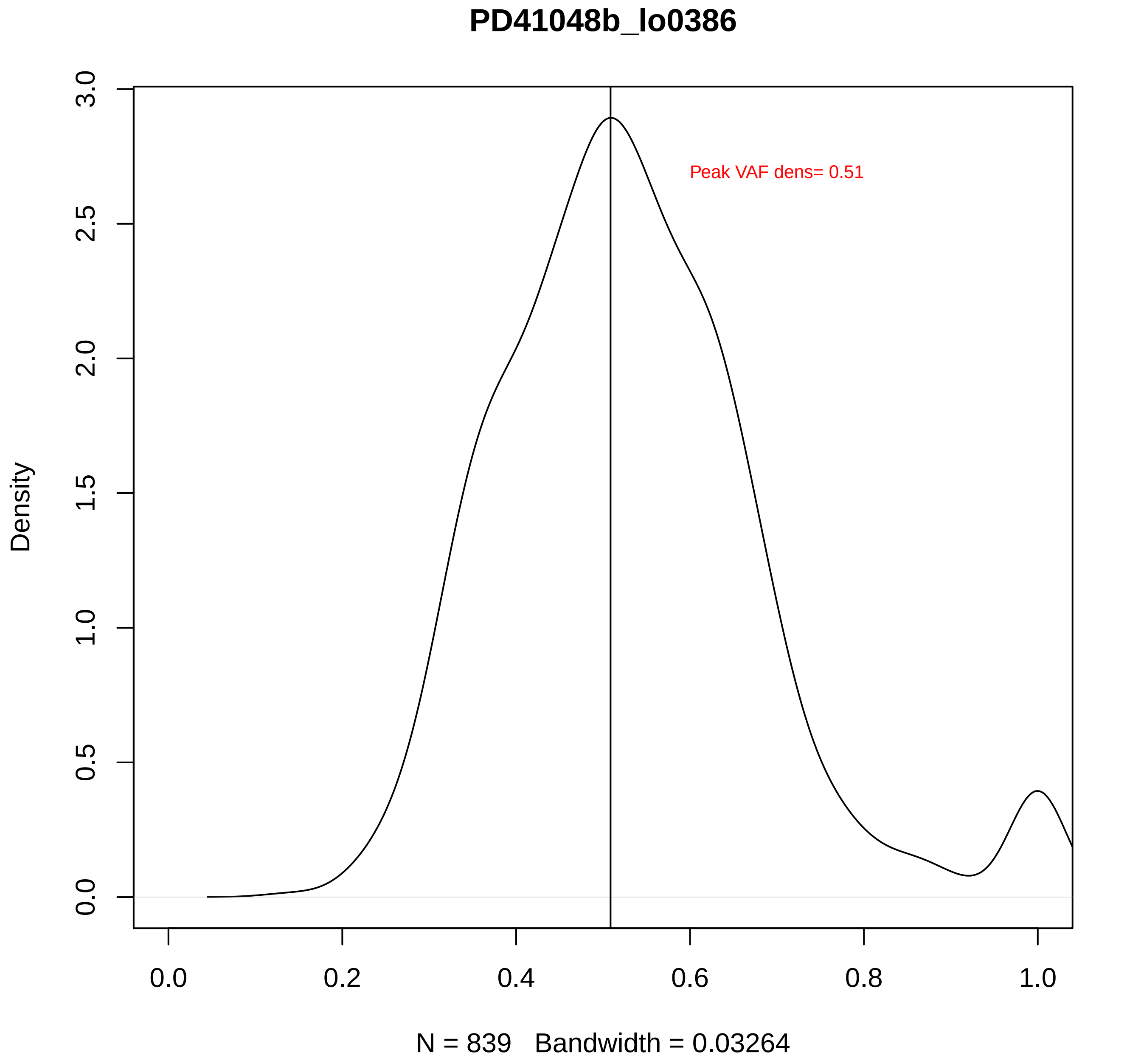
PD41048b_lo0386
Peak VAF dens= 0.51
Density
3.0
2.5
2.0
1.5
1.0
0.5
0.0
0.0
0.2
0.4
0.6
0.8
1.0
N = 839   Bandwidth = 0.03264

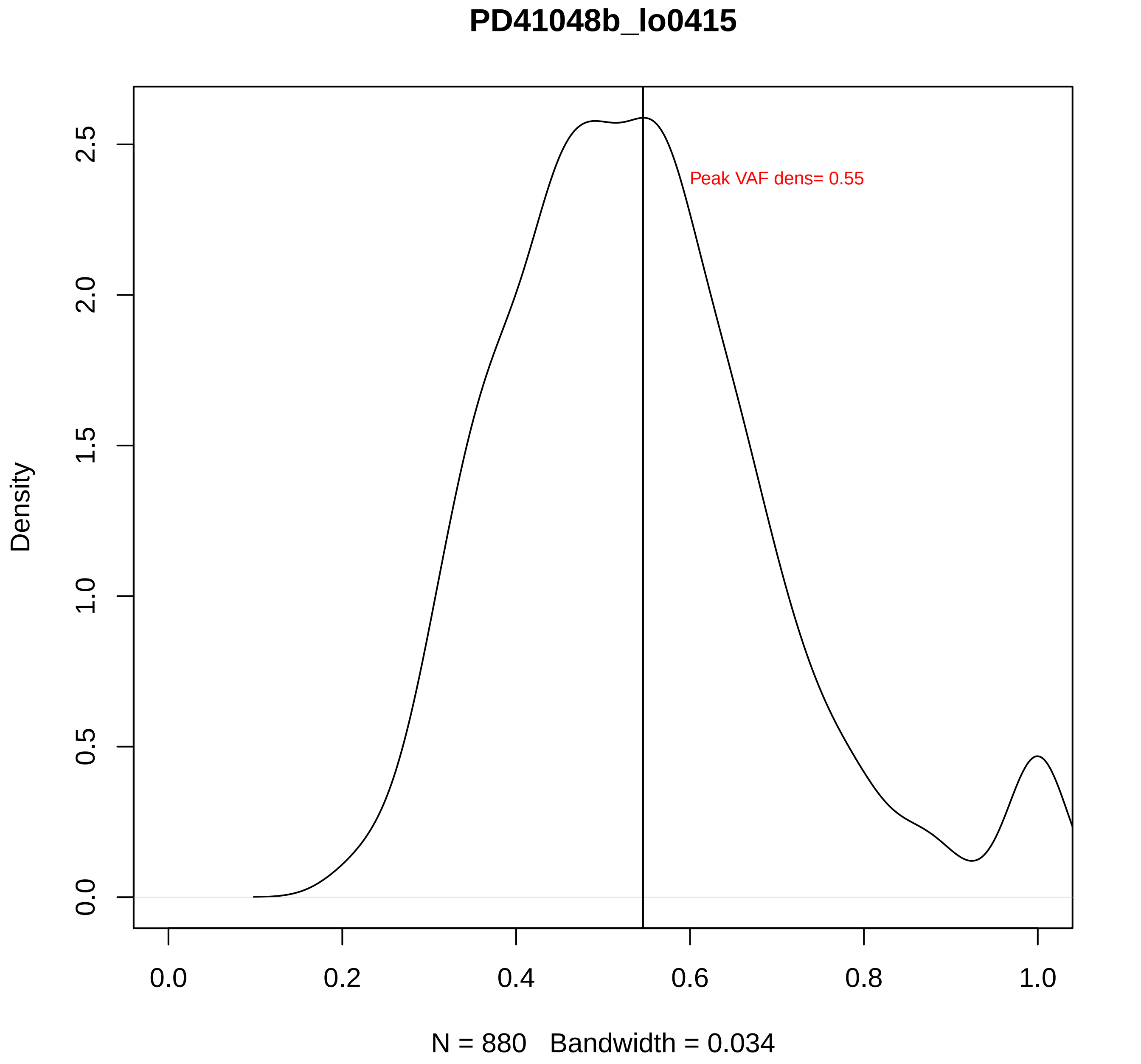
PD41048b_lo0415
Peak VAF dens= 0.55
Density
0.0
0.5
1.0
1.5
2.0
2.5
0.0
0.2
0.4
0.6
0.8
1.0
N = 880   Bandwidth = 0.034

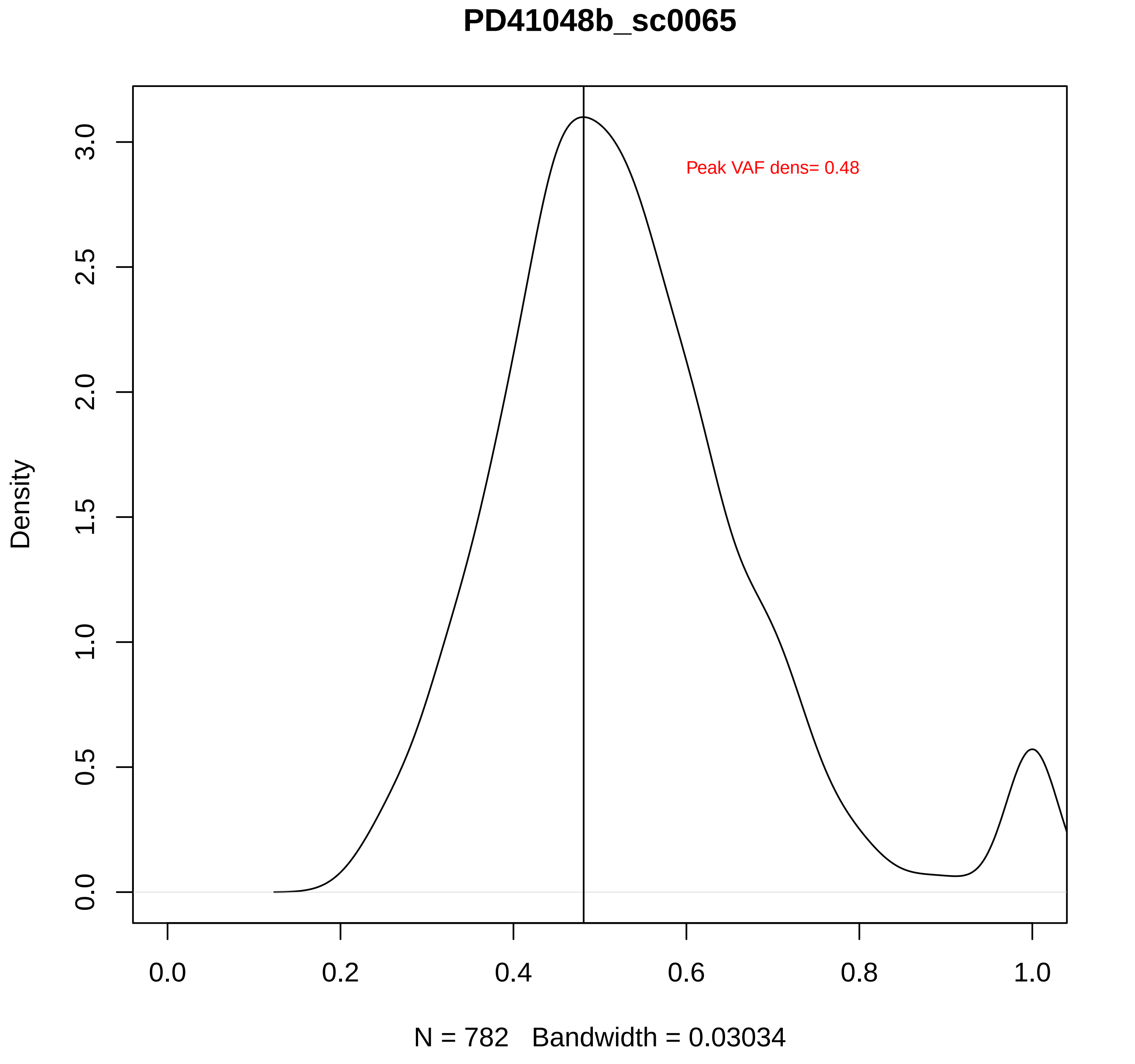
PD41048b_sc0065
Peak VAF dens= 0.48
Density
0.0
0.5
1.0
1.5
2.0
2.5
3.0
0.0
0.2
0.4
0.6
0.8
1.0
N = 782 Bandwidth = 0.03034

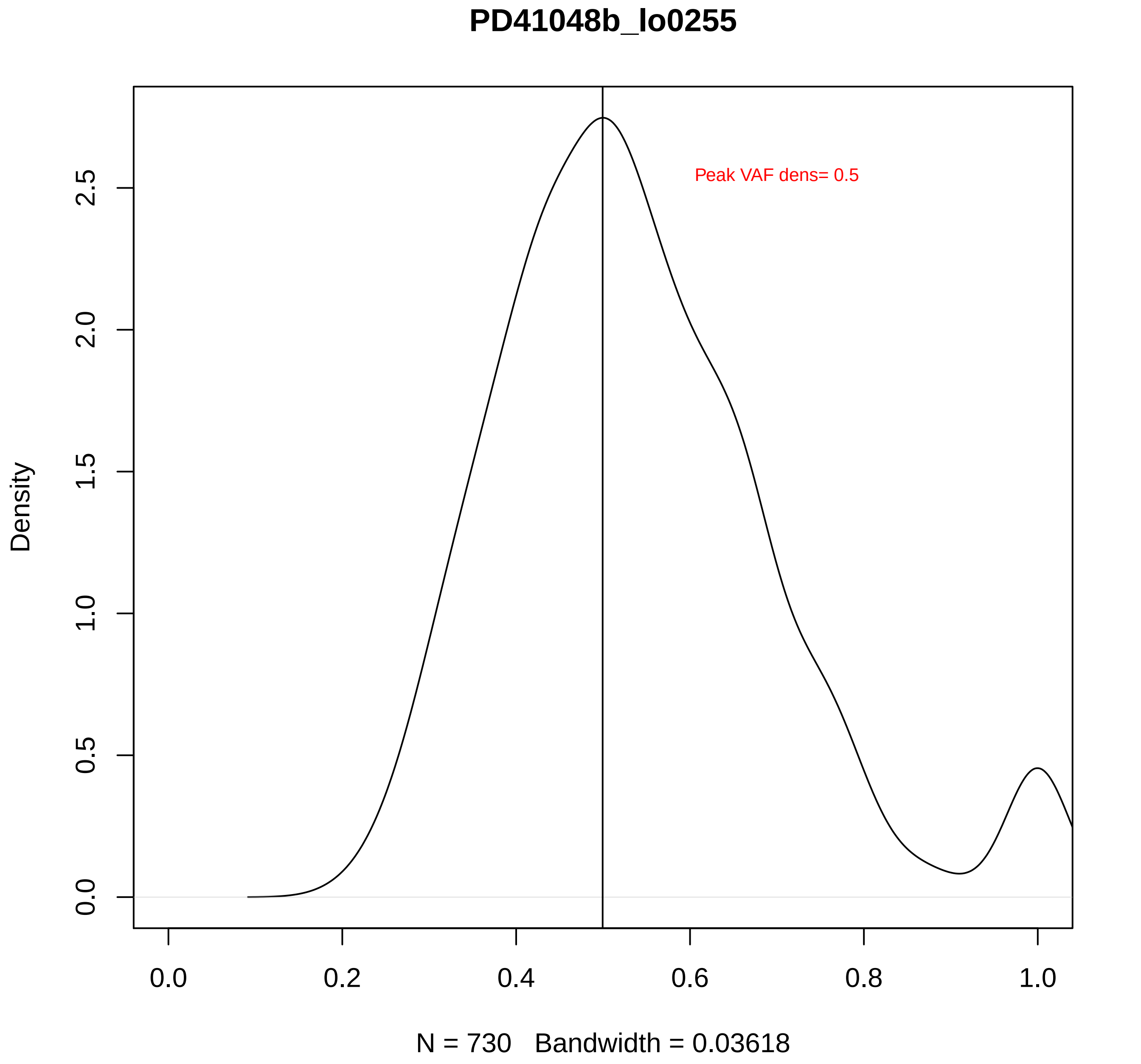
PD41048b_lo0255
Peak VAF dens= 0.5
Density
0.0
0.5
1.0
1.5
2.0
2.5
0.0
0.2
0.4
0.6
0.8
1.0
N = 730   Bandwidth = 0.03618

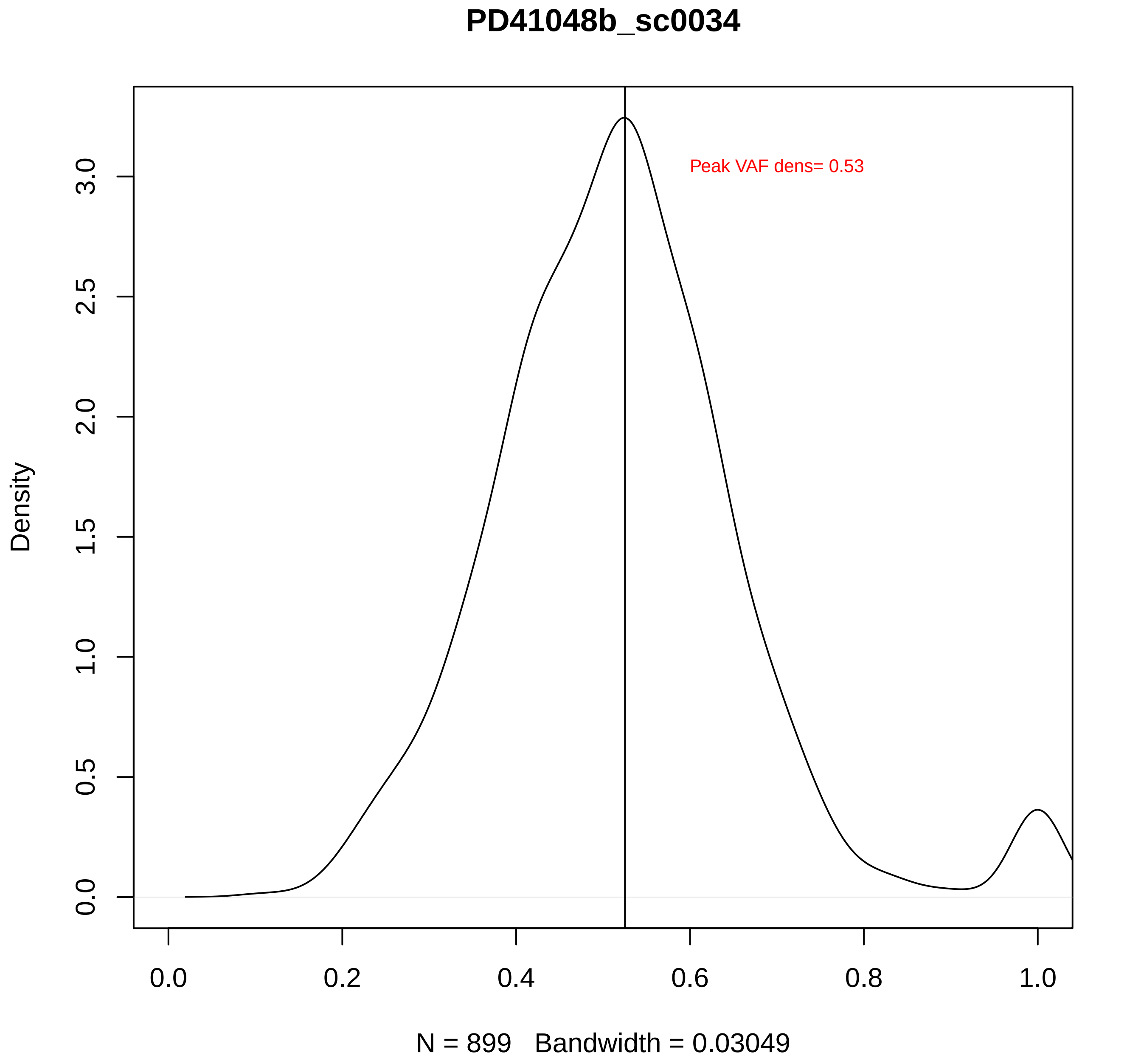
PD41048b_sc0034
Peak VAF dens= 0.53
Density
0.0
0.5
1.0
1.5
2.0
2.5
3.0
0.0
0.2
0.4
0.6
0.8
1.0
N = 899   Bandwidth = 0.03049

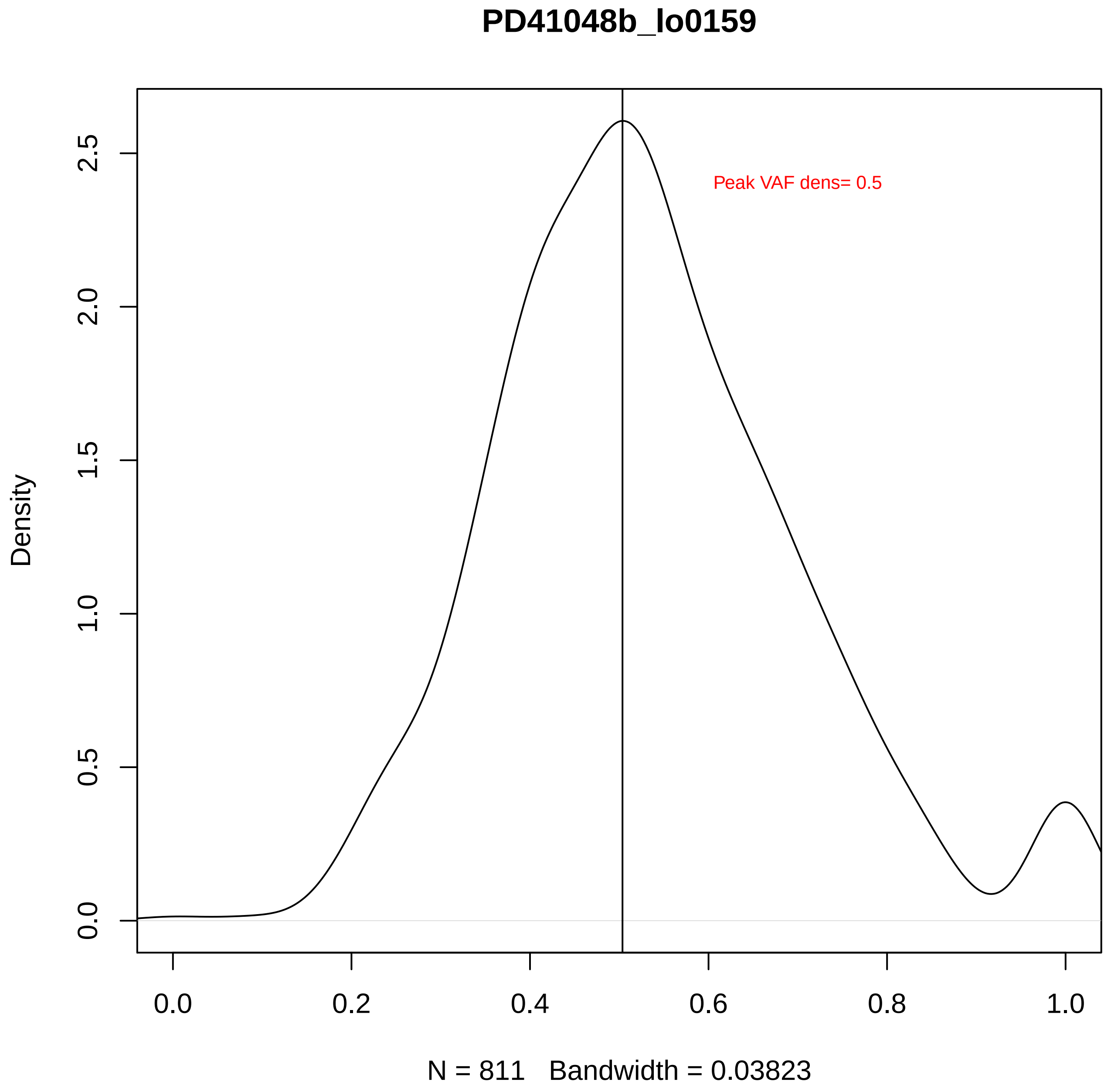

PD41048b_lo0159
Peak VAF dens= 0.5
Density
0.0
0.5
1.0
1.5
2.0
2.5
0.0
0.2
0.4
0.6
0.8
1.0
N = 811   Bandwidth = 0.03823

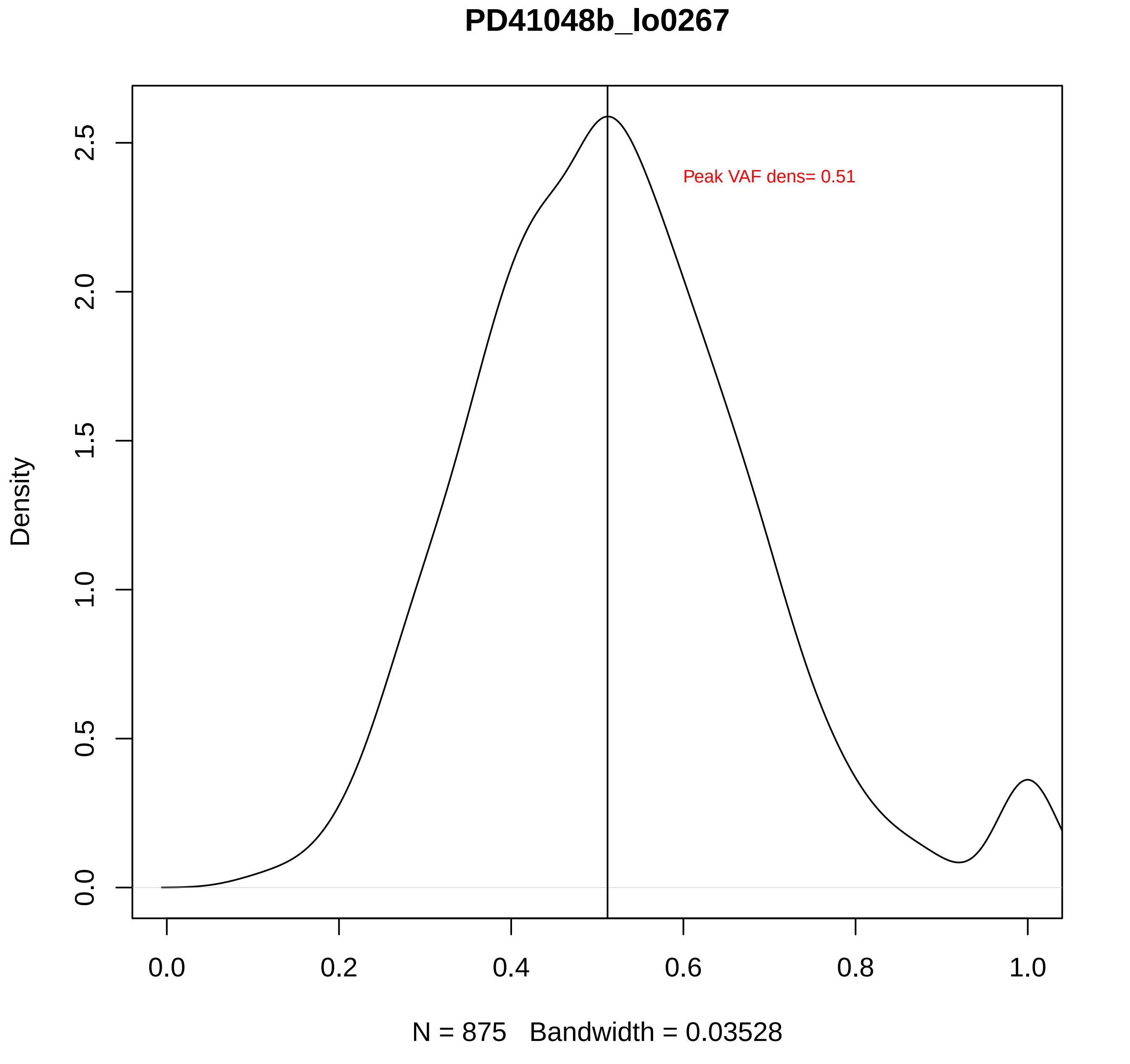
PD41048b_lo0267
Peak VAF dens= 0.51
Density
0.0
0.5
1.0
1.5
2.0
2.5
0.0
0.2
0.4
0.6
0.8
1.0
N = 875   Bandwidth = 0.03528

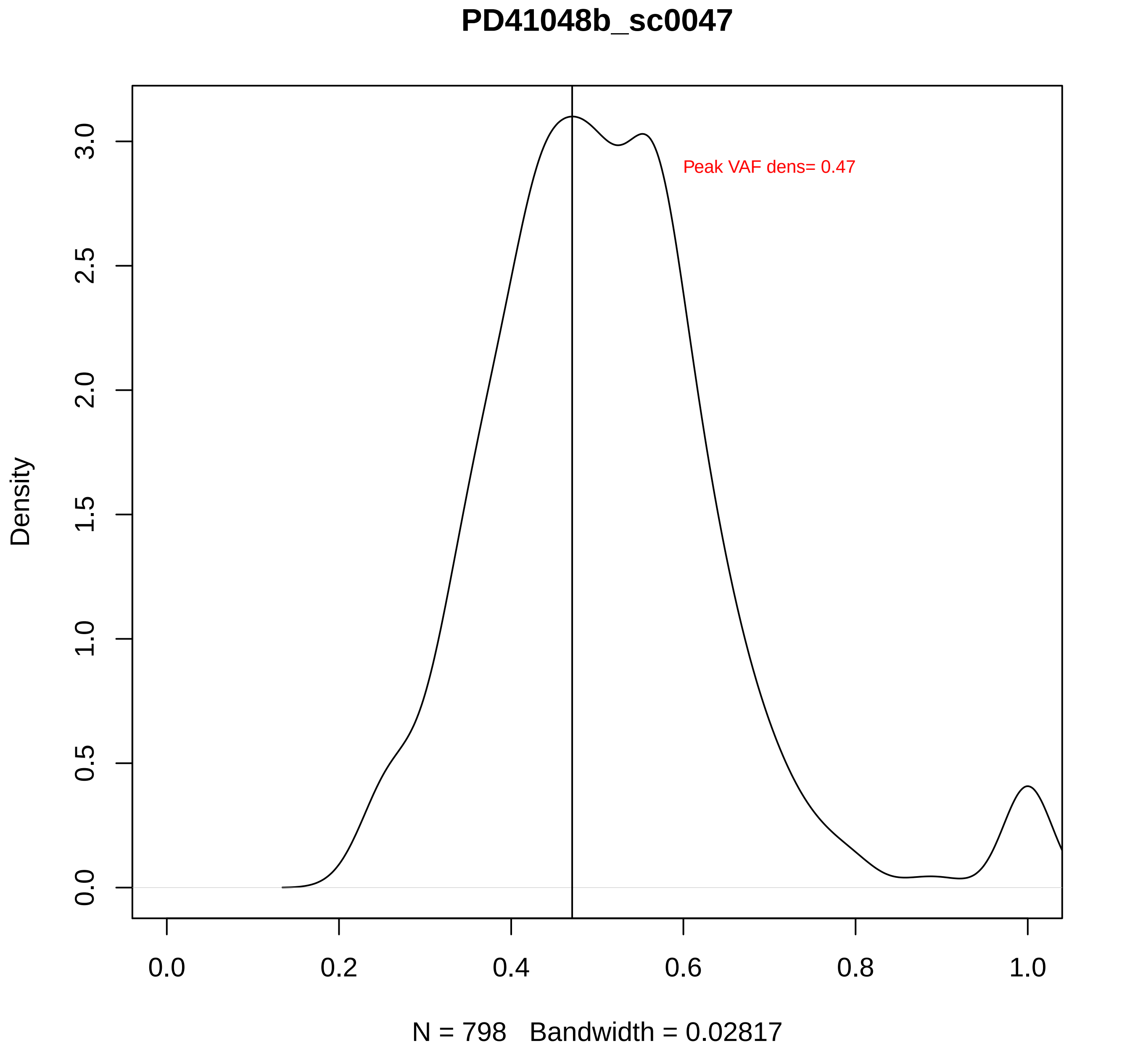
PD41048b_sc0047
Peak VAF dens= 0.47
Density
0.0
0.5
1.0
1.5
2.0
2.5
3.0
0.0
0.2
0.4
0.6
0.8
1.0
N = 798 Bandwidth = 0.02817

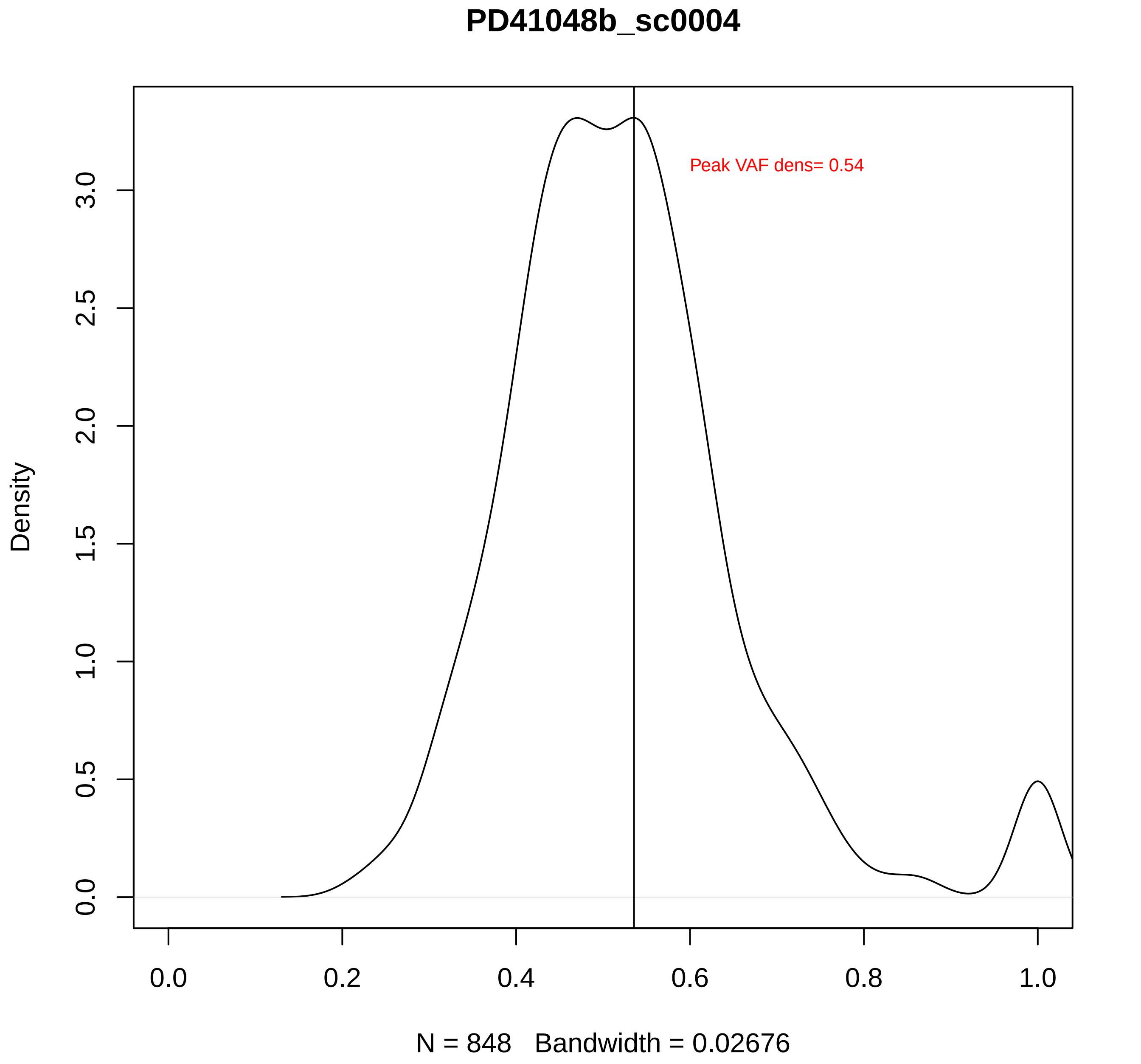

PD41048b_sc0004
Peak VAF dens= 0.54
Density
0.0
0.5
1.0
1.5
2.0
2.5
3.0
0.0
0.2
0.4
0.6
0.8
1.0
N = 848   Bandwidth = 0.02676

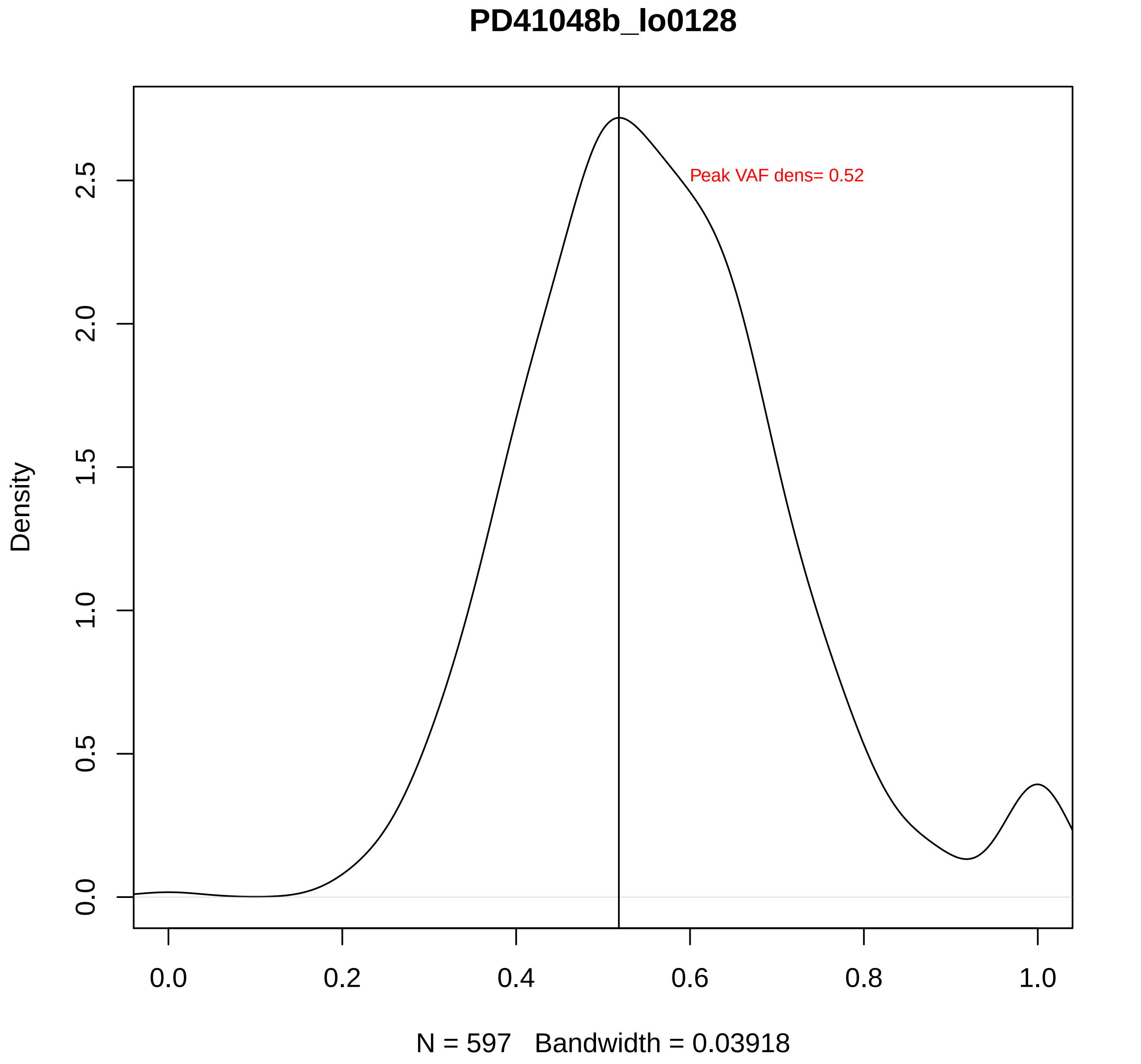

PD41048b_lo0128
Peak VAF dens= 0.52
Density
0.0
0.5
1.0
1.5
2.0
2.5
0.0
0.2
0.4
0.6
0.8
1.0
N = 597   Bandwidth = 0.03918

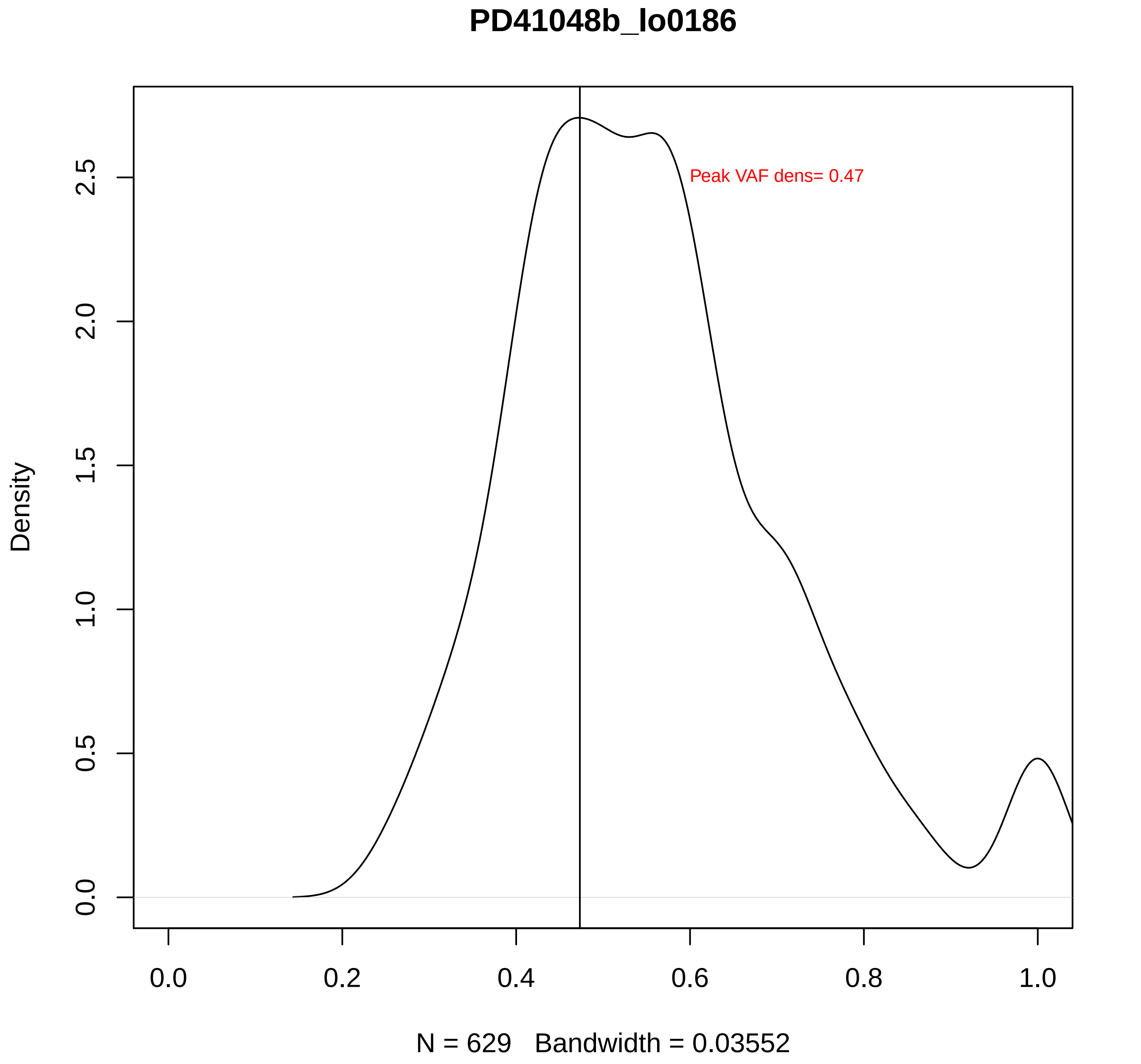

PD41048b_lo0186
Peak VAF dens= 0.47
Density
0.0
0.5
1.0
1.5
2.0
2.5
0.0
0.2
0.4
0.6
0.8
1.0
N = 629 Bandwidth = 0.03552

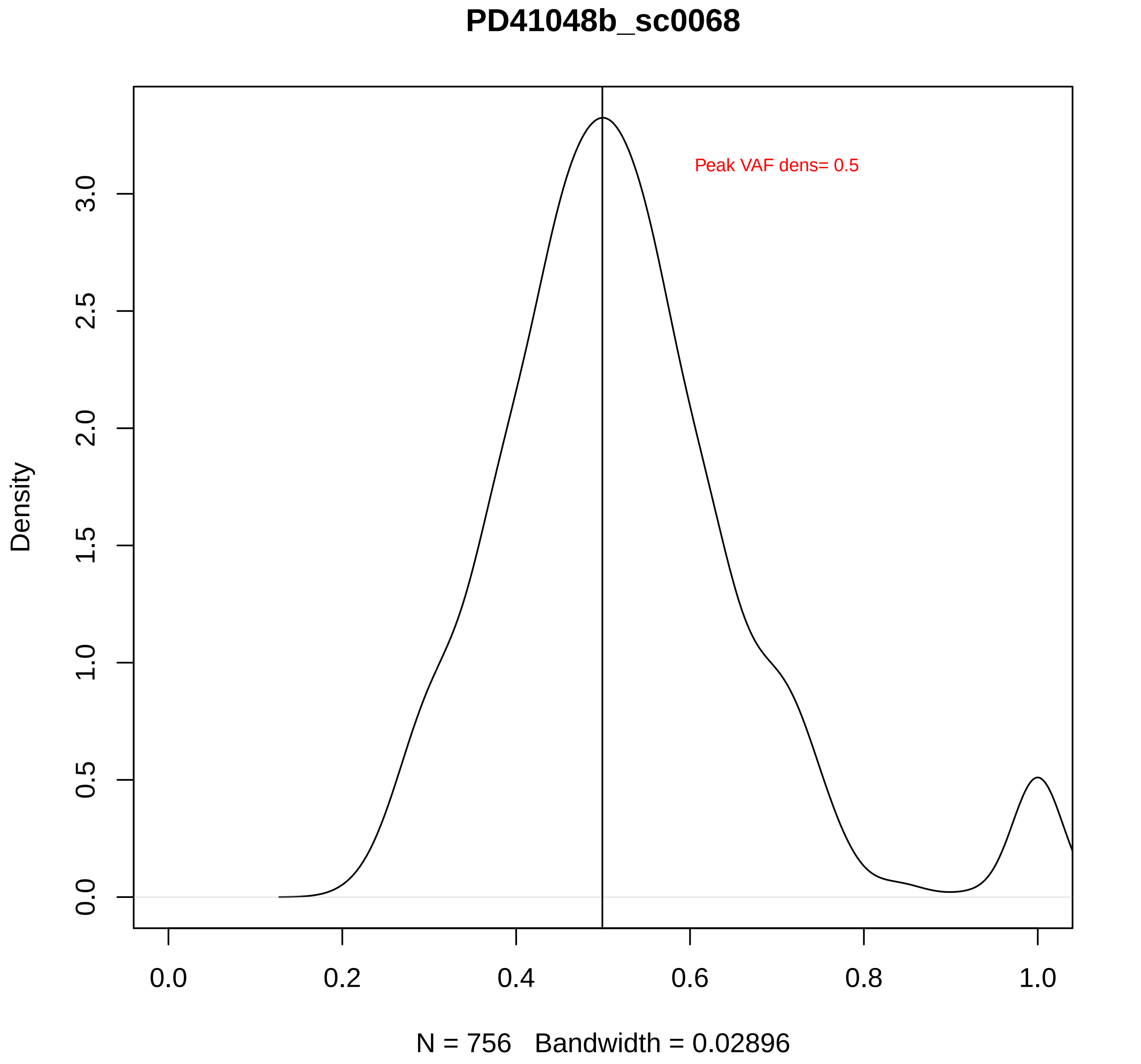
PD41048b_sc0068
Peak VAF dens= 0.5
Density
0.0
0.5
1.0
1.5
2.0
2.5
3.0
0.0
0.2
0.4
0.6
0.8
1.0
N = 756   Bandwidth = 0.02896

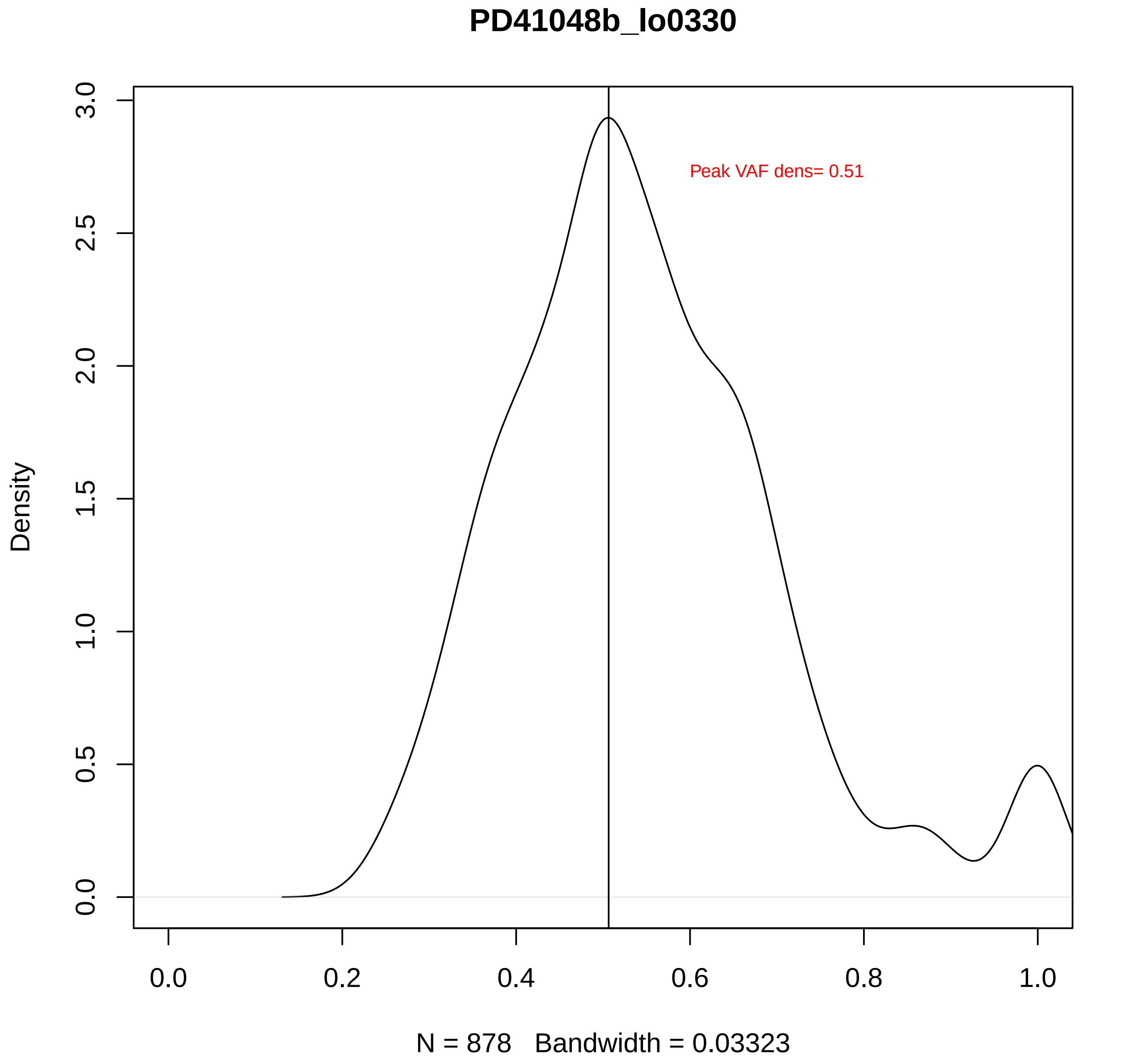
PD41048b_lo0330
Peak VAF dens= 0.51
Density
0.0
0.5
1.0
1.5
2.0
2.5
3.0
0.0
0.2
0.4
0.6
0.8
1.0
N = 878 Bandwidth = 0.03323

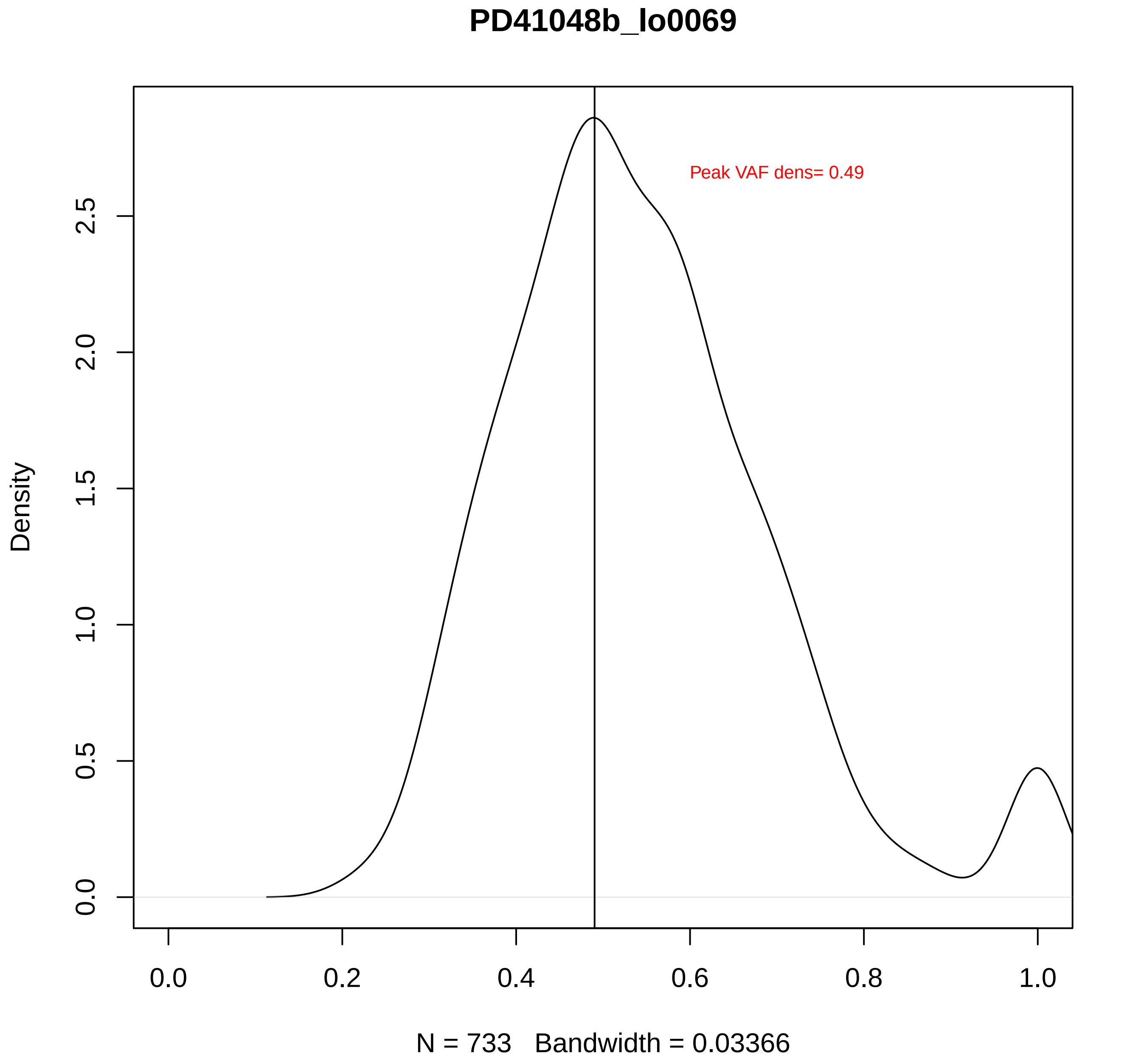

PD41048b_lo0069
Peak VAF dens= 0.49
Density
0.0
0.5
1.0
1.5
2.0
2.5
0.0
0.2
0.4
0.6
0.8
1.0
N = 733   Bandwidth = 0.03366

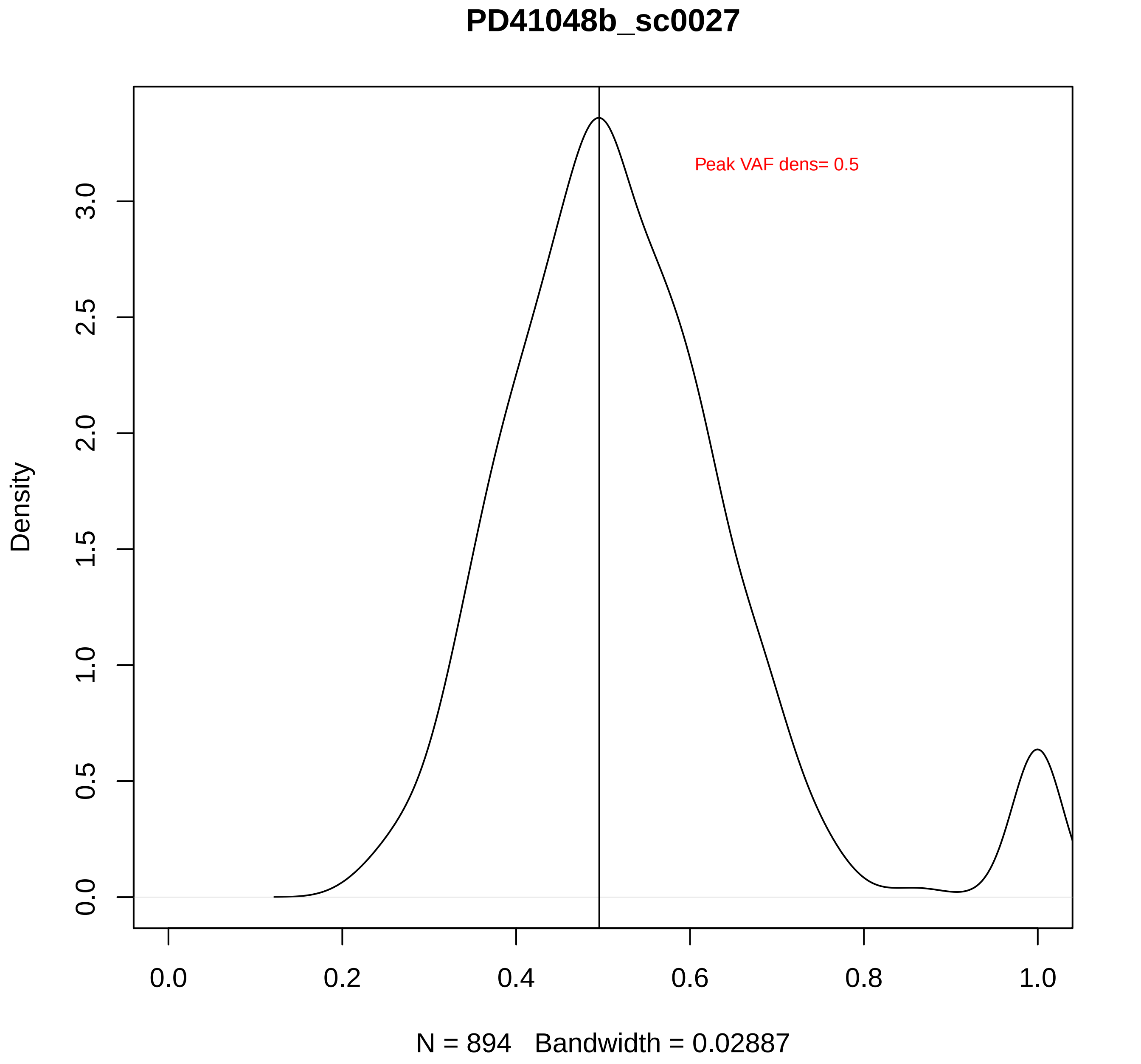

PD41048b_sc0027
Peak VAF dens= 0.5
Density
0.0
0.5
1.0
1.5
2.0
2.5
3.0
0.0
0.2
0.4
0.6
0.8
1.0
N = 894 Bandwidth = 0.02887

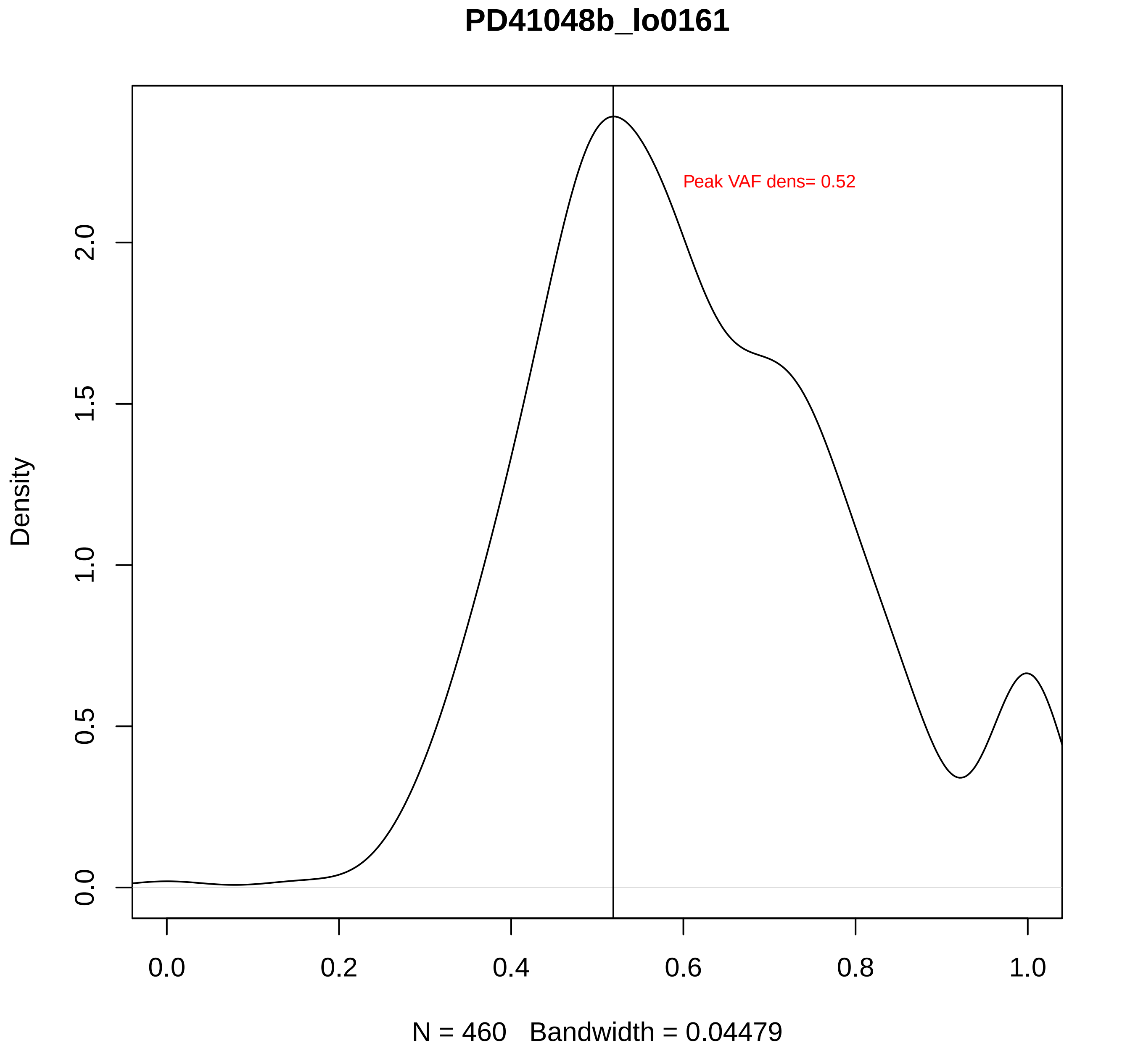
PD41048b_lo0161
Peak VAF dens= 0.52
Density
0.0
0.5
1.0
1.5
2.0
0.0
0.2
0.4
0.6
0.8
1.0
N = 460   Bandwidth = 0.04479

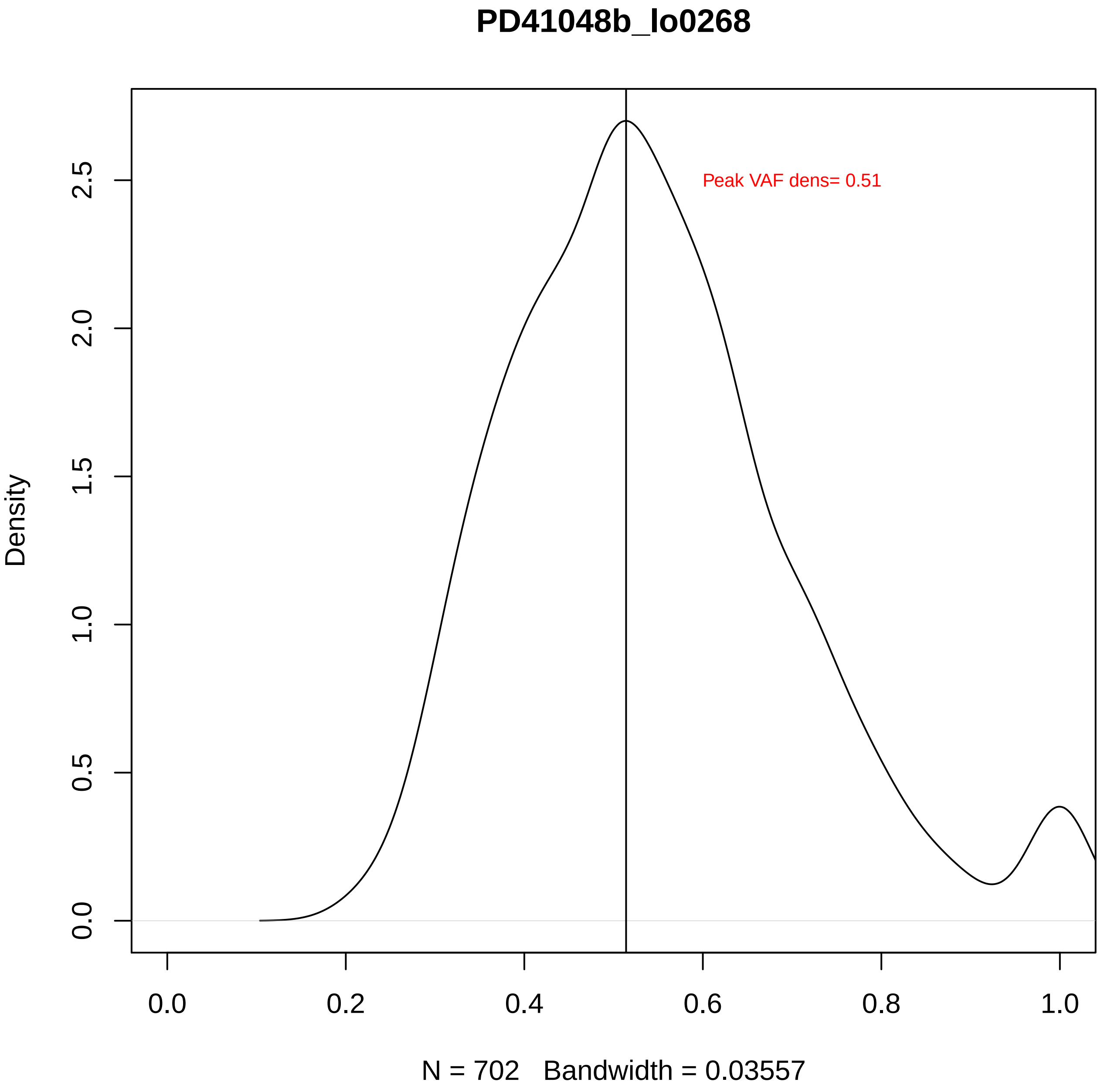

PD41048b_lo0268
Peak VAF dens= 0.51
Density
0.0
0.5
1.0
1.5
2.0
2.5
0.0
0.2
0.4
0.6
0.8
1.0
N = 702 Bandwidth = 0.03557

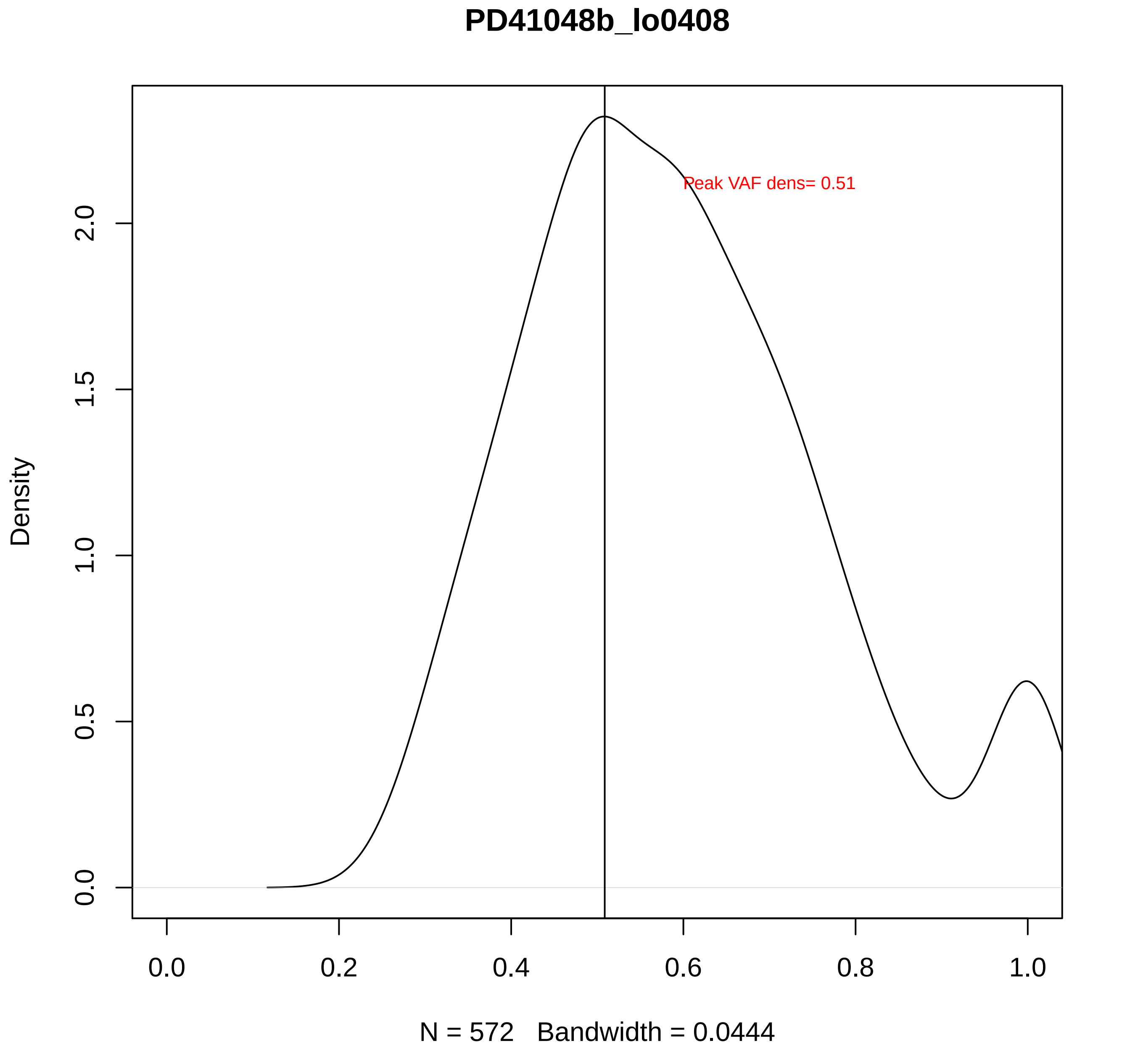

PD41048b_lo0408
Peak VAF dens= 0.51
Density
0.0
0.5
1.0
1.5
2.0
0.0
0.2
0.4
0.6
0.8
1.0
N = 572   Bandwidth = 0.0444

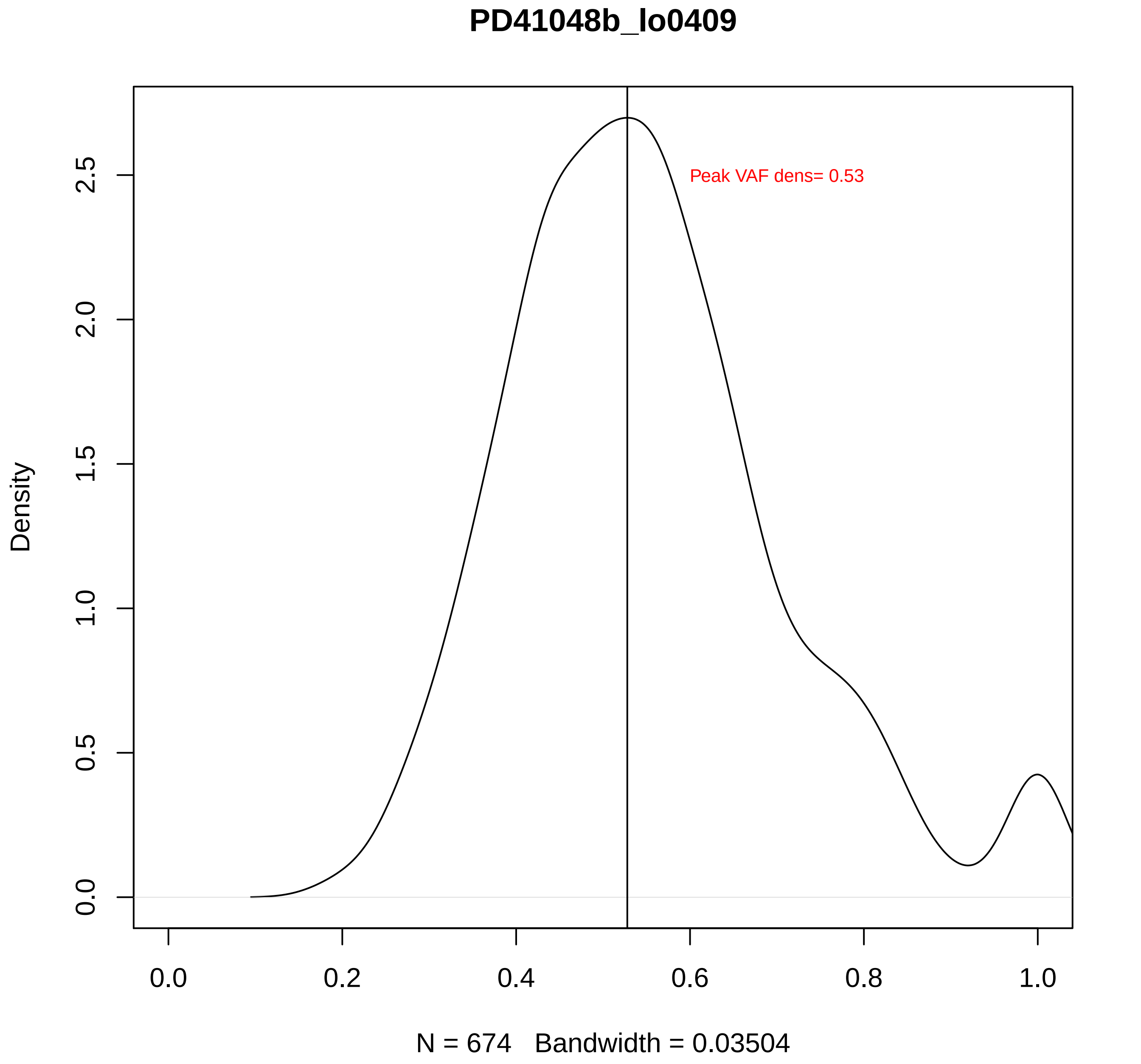
PD41048b_lo0409
Peak VAF dens= 0.53
Density
0.0
0.5
1.0
1.5
2.0
2.5
0.0
0.2
0.4
0.6
0.8
1.0
N = 674 Bandwidth = 0.03504

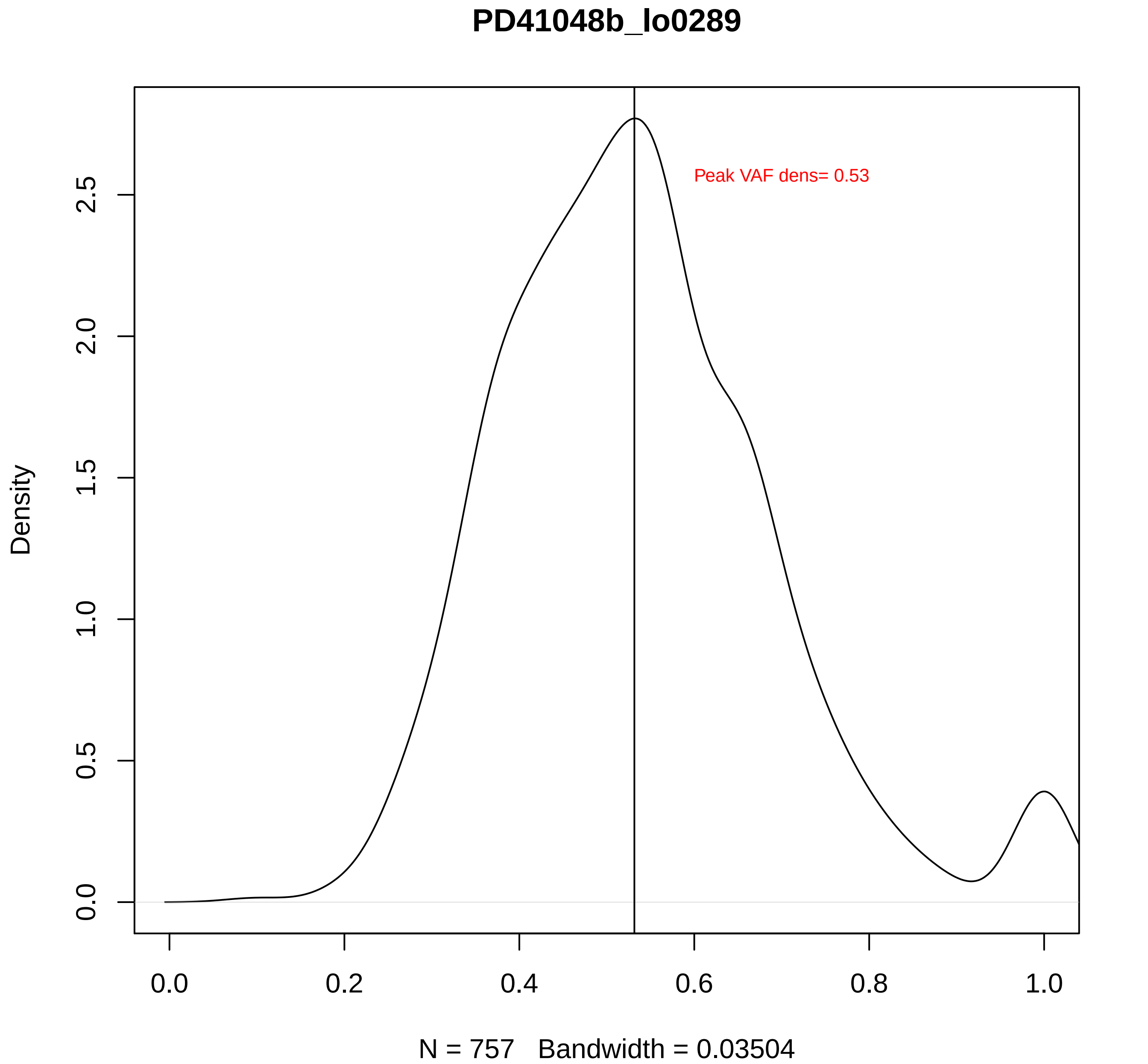

PD41048b_lo0289
Peak VAF dens= 0.53
Density
0.0
0.5
1.0
1.5
2.0
2.5
0.0
0.2
0.4
0.6
0.8
1.0
N = 757 Bandwidth = 0.03504

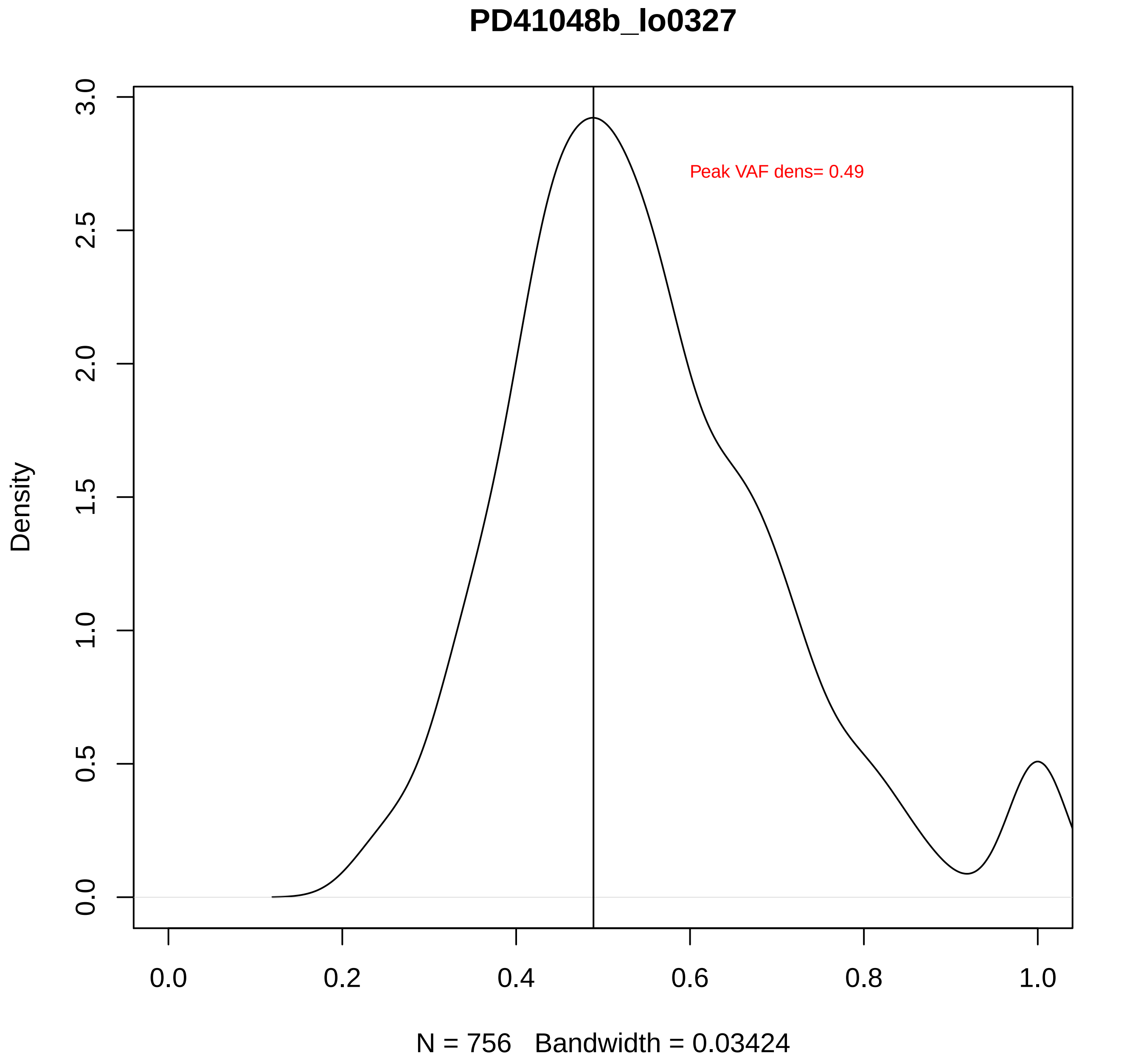
PD41048b_lo0327
Peak VAF dens= 0.49
Density
0.0
0.5
1.0
1.5
2.0
2.5
3.0
0.0
0.2
0.4
0.6
0.8
1.0
N = 756   Bandwidth = 0.03424

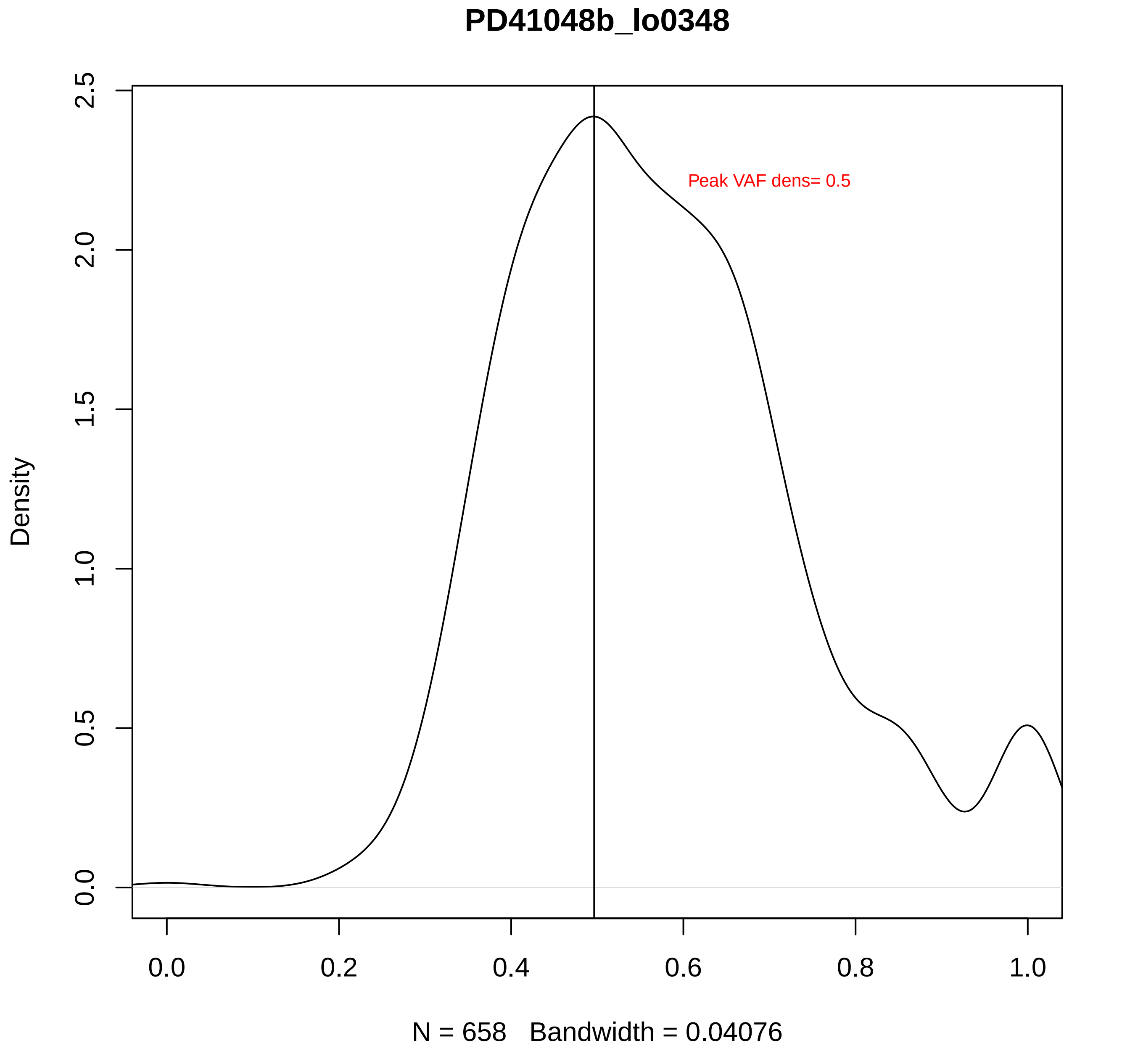

PD41048b_lo0348
Peak VAF dens= 0.5
Density
0.0
0.5
1.0
1.5
2.0
2.5
0.0
0.2
0.4
0.6
0.8
1.0
N = 658   Bandwidth = 0.04076

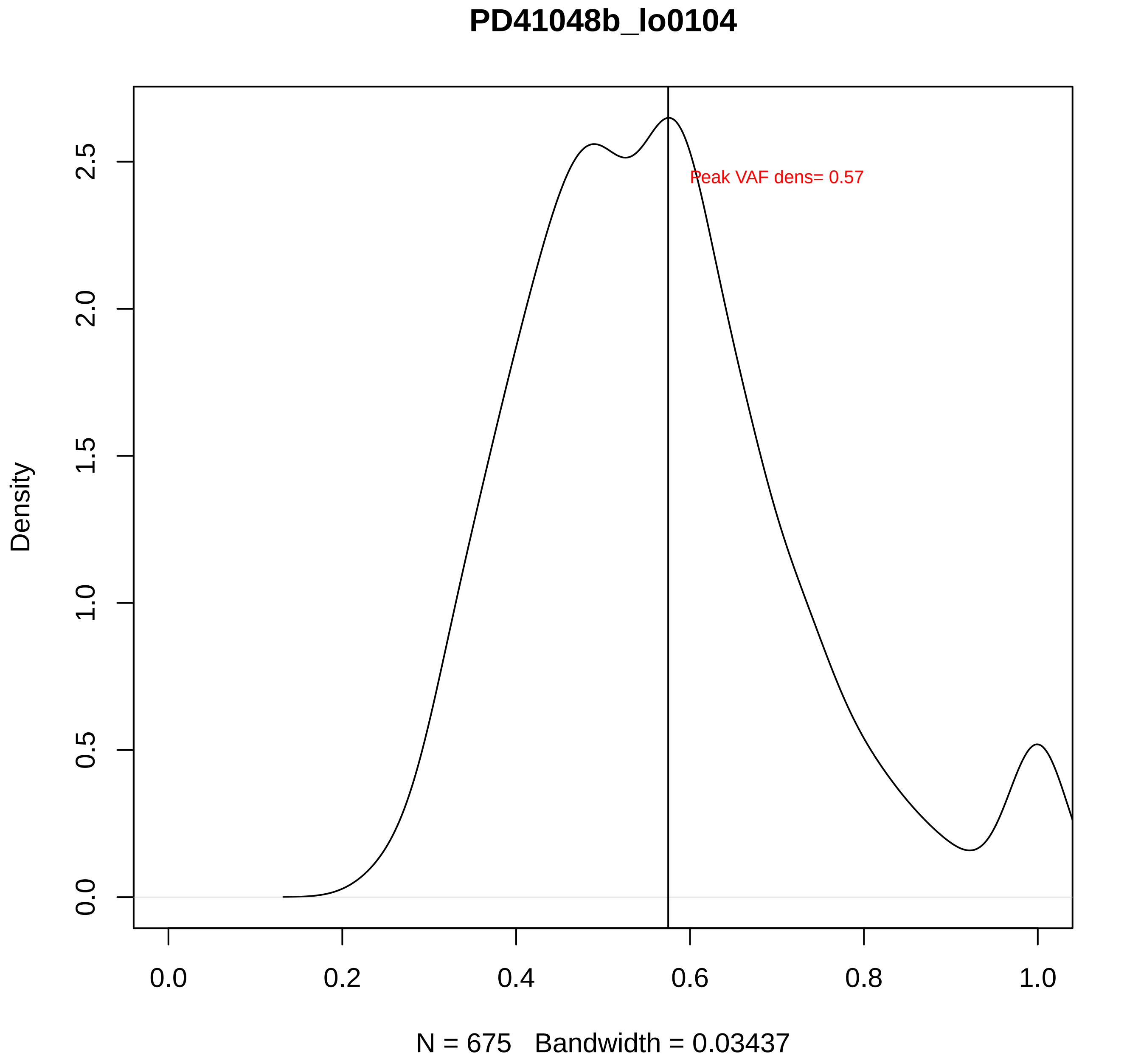
PD41048b_lo0104
Peak VAF dens= 0.57
Density
0.0
0.5
1.0
1.5
2.0
2.5
0.0
0.2
0.4
0.6
0.8
1.0
N = 675   Bandwidth = 0.03437

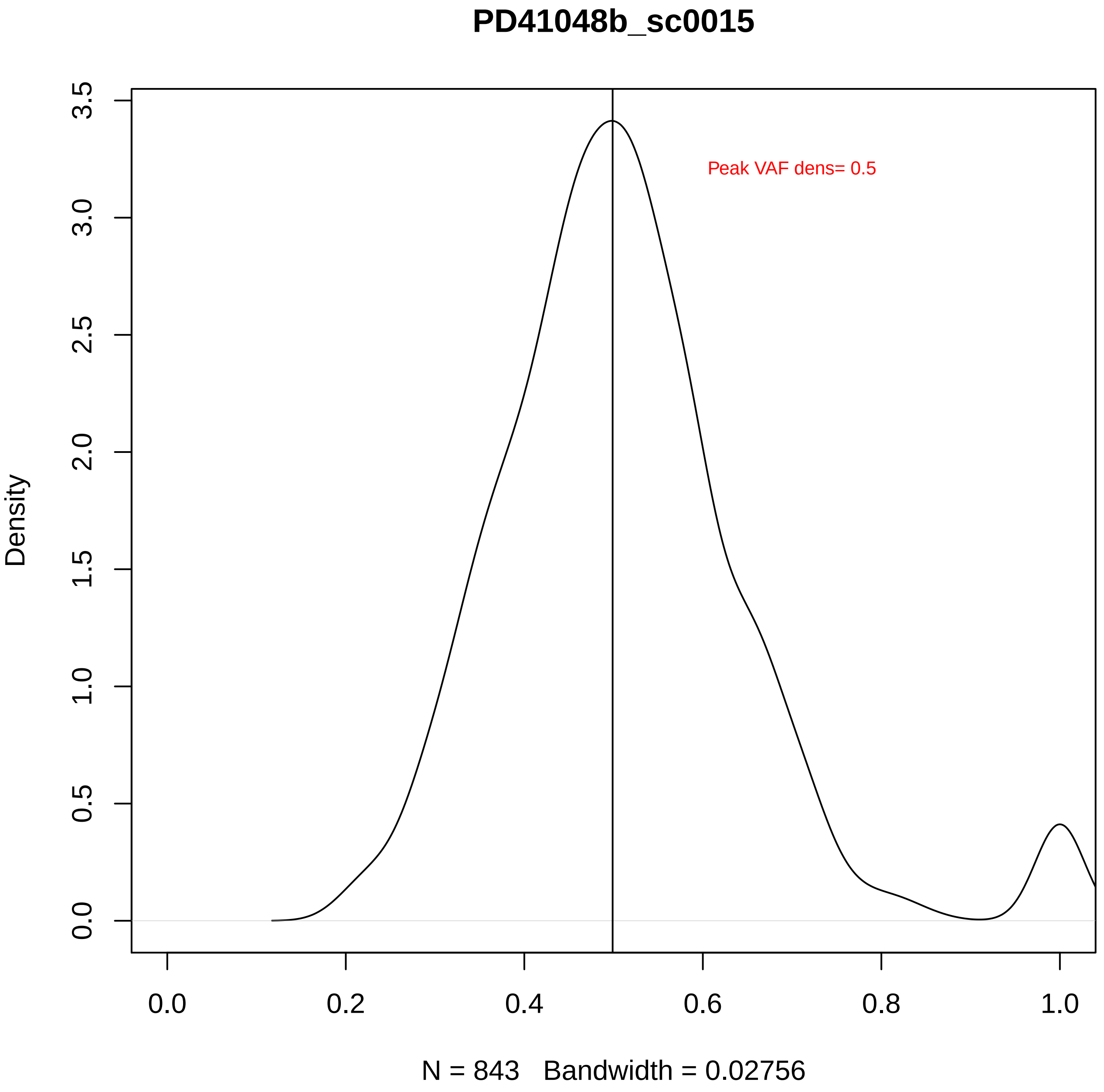
PD41048b_sc0015
Peak VAF dens= 0.5
Density
0.0
0.5
1.0
1.5
2.0
2.5
3.0
3.5
0.0
0.2
0.4
0.6
0.8
1.0
N = 843 Bandwidth = 0.02756

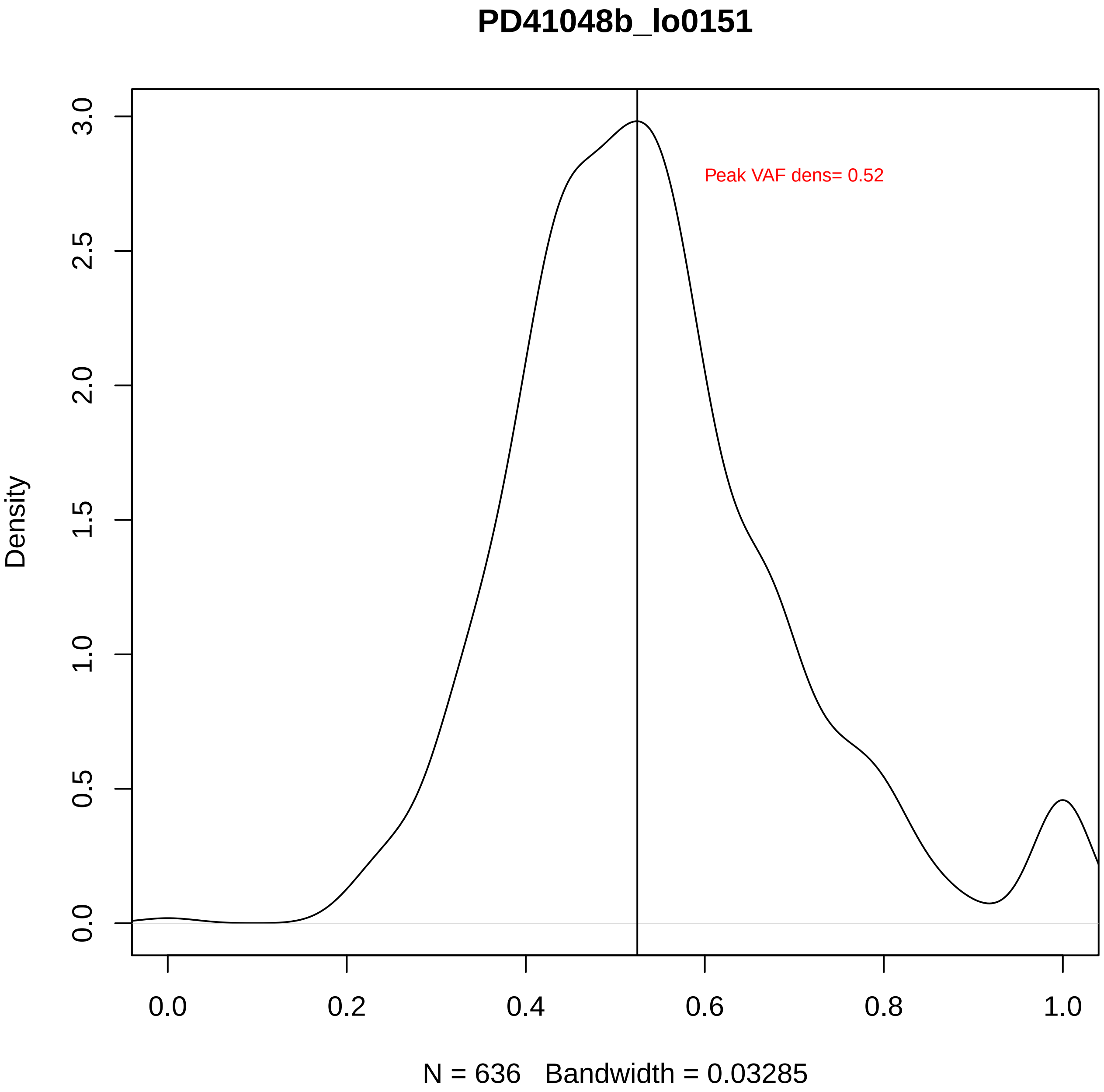
PD41048b_lo0151
Peak VAF dens= 0.52
Density
0.0
0.5
1.0
1.5
2.0
2.5
3.0
0.0
0.2
0.4
0.6
0.8
1.0
N = 636 Bandwidth = 0.03285

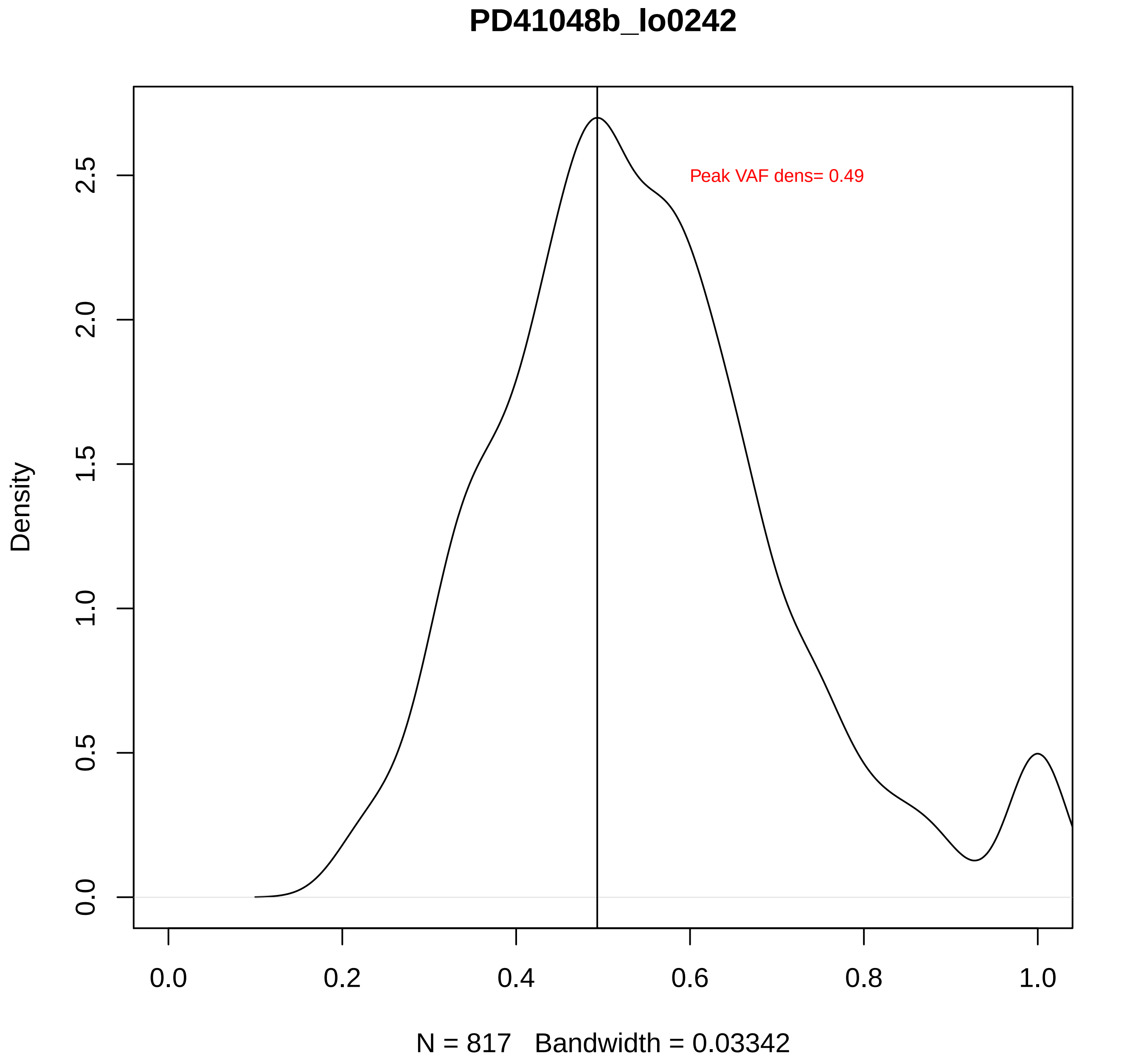
PD41048b_lo0242
Peak VAF dens= 0.49
Density
0.0
0.5
1.0
1.5
2.0
2.5
0.0
0.2
0.4
0.6
0.8
1.0
N = 817 Bandwidth = 0.03342

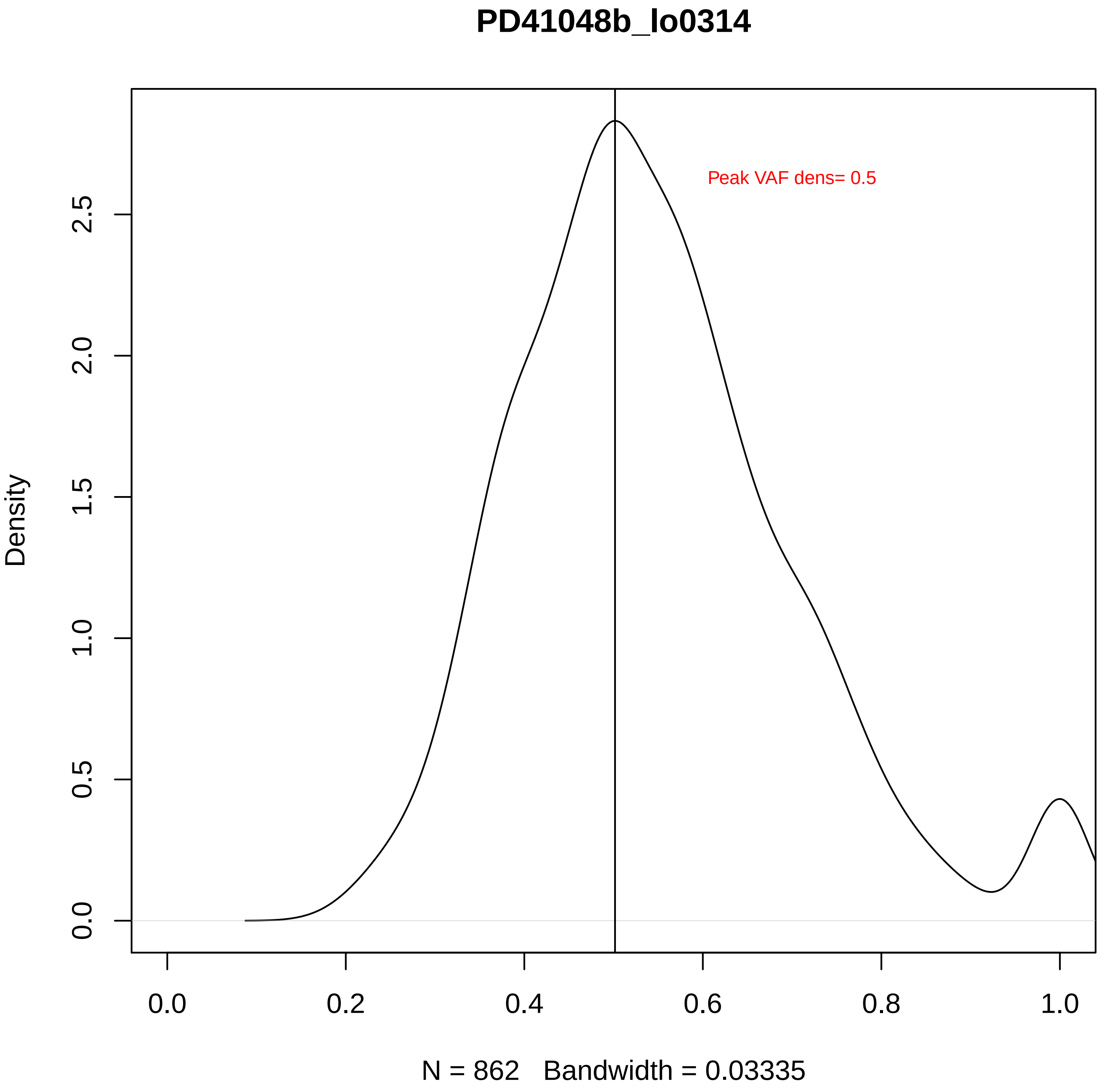
PD41048b_lo0314
Peak VAF dens= 0.5
Density
0.0
0.5
1.0
1.5
2.0
2.5
0.0
0.2
0.4
0.6
0.8
1.0
N = 862 Bandwidth = 0.03335

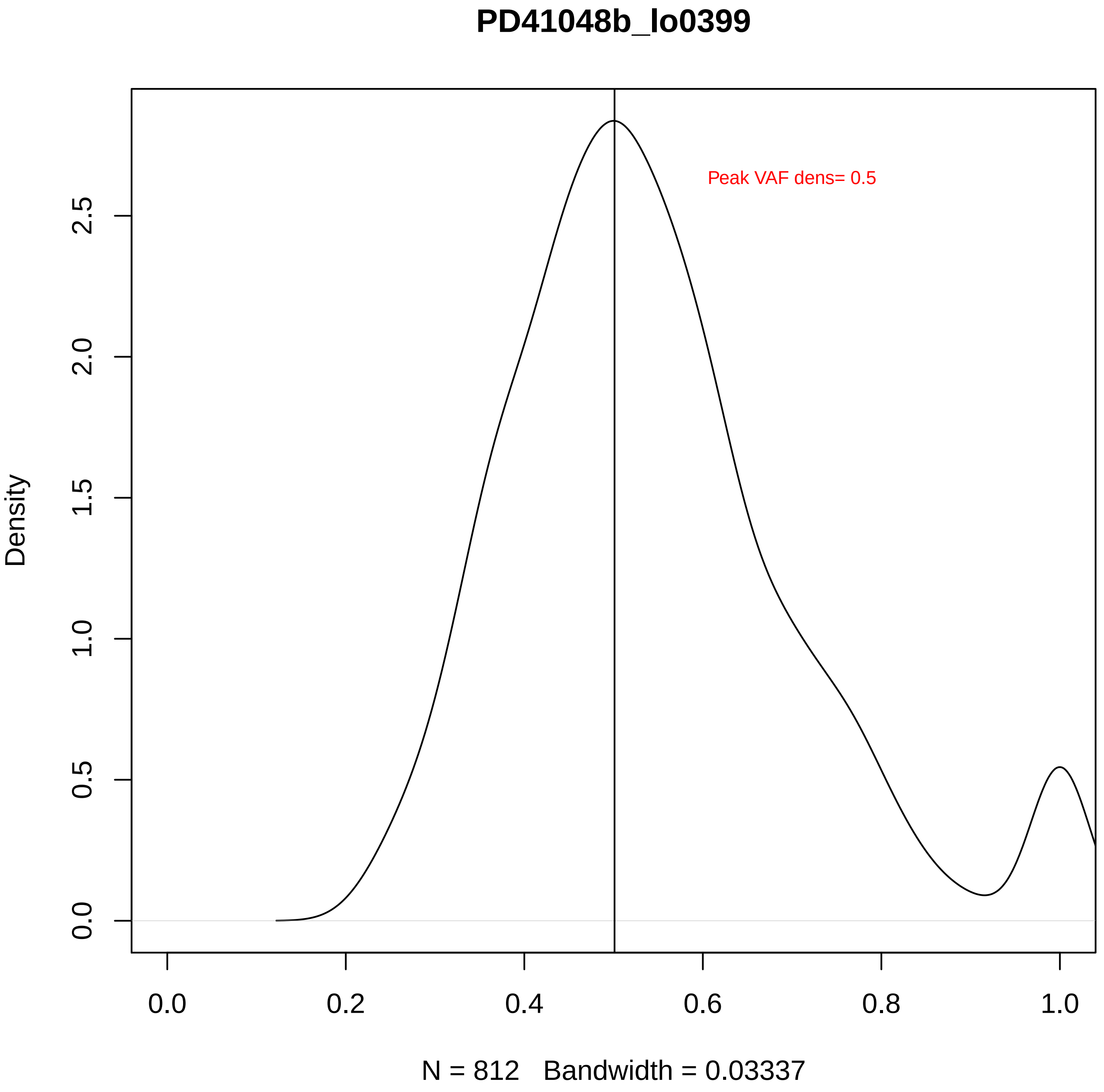
PD41048b_lo0399
Peak VAF dens= 0.5
Density
0.0
0.5
1.0
1.5
2.0
2.5
0.0
0.2
0.4
0.6
0.8
1.0
N = 812   Bandwidth = 0.03337

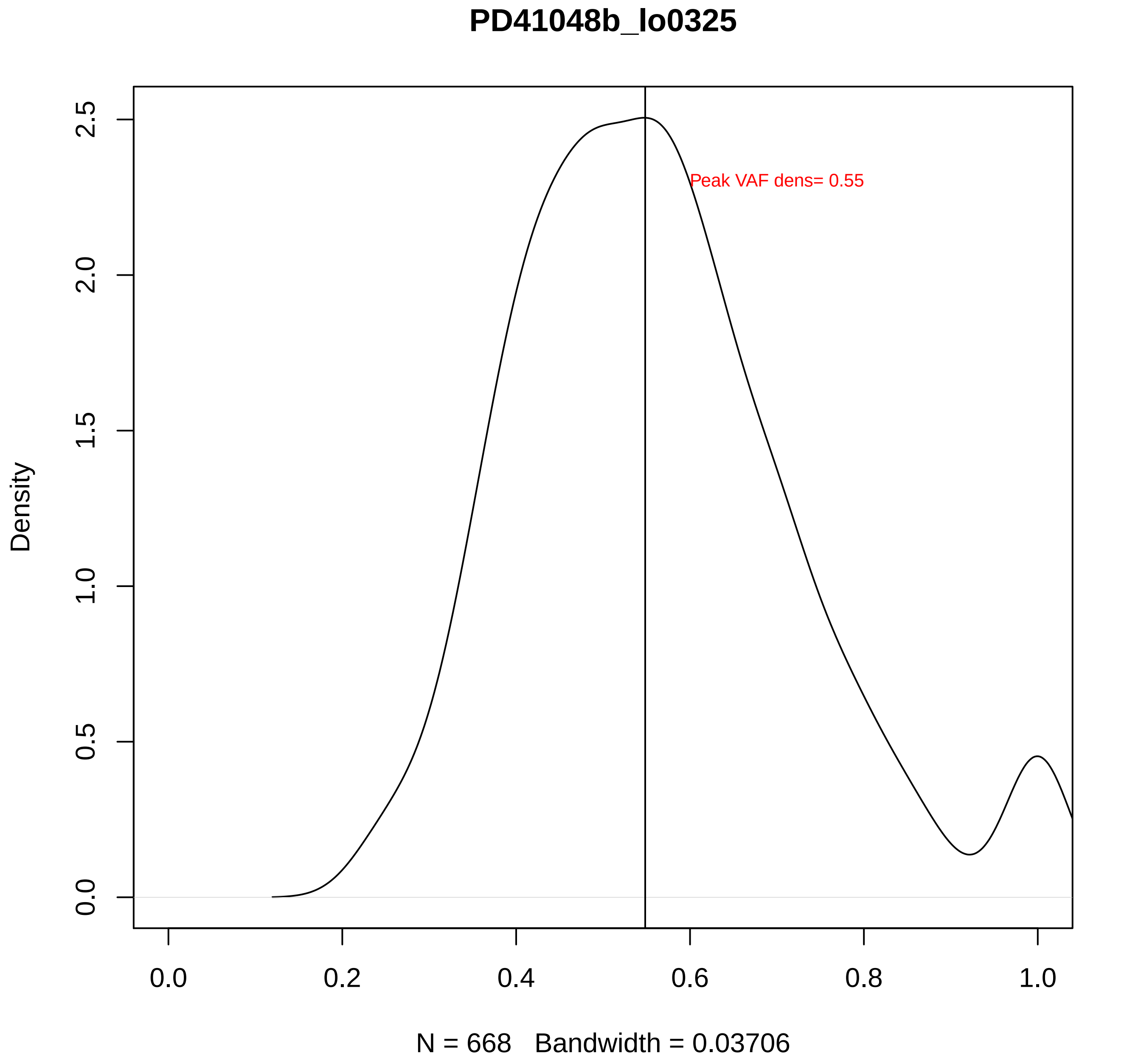
PD41048b_lo0325
Peak VAF dens= 0.55
Density
0.0
0.5
1.0
1.5
2.0
2.5
0.0
0.2
0.4
0.6
0.8
1.0
N = 668   Bandwidth = 0.03706

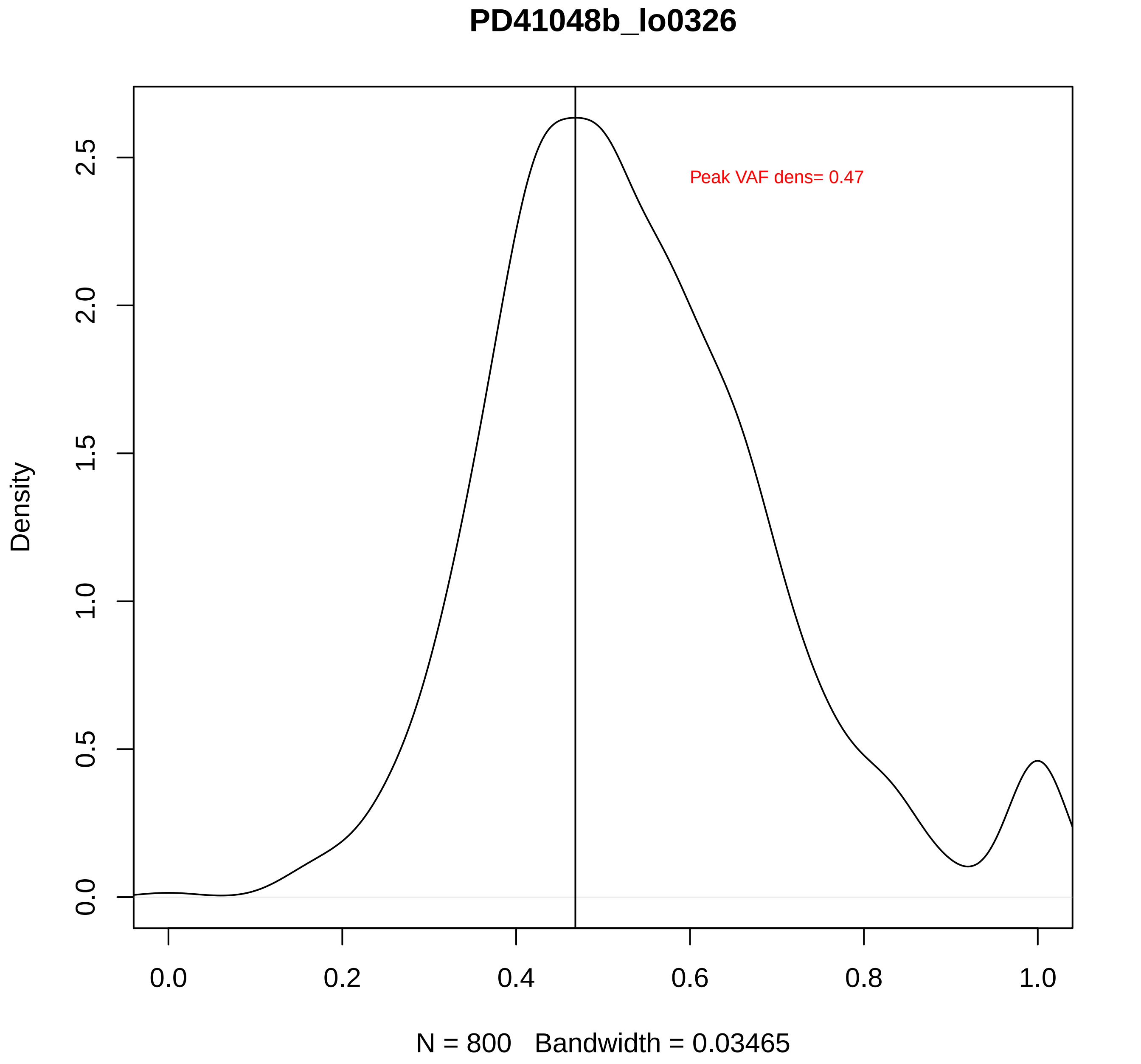

PD41048b_lo0326
Peak VAF dens= 0.47
Density
0.0
0.5
1.0
1.5
2.0
2.5
0.0
0.2
0.4
0.6
0.8
1.0
N = 800   Bandwidth = 0.03465

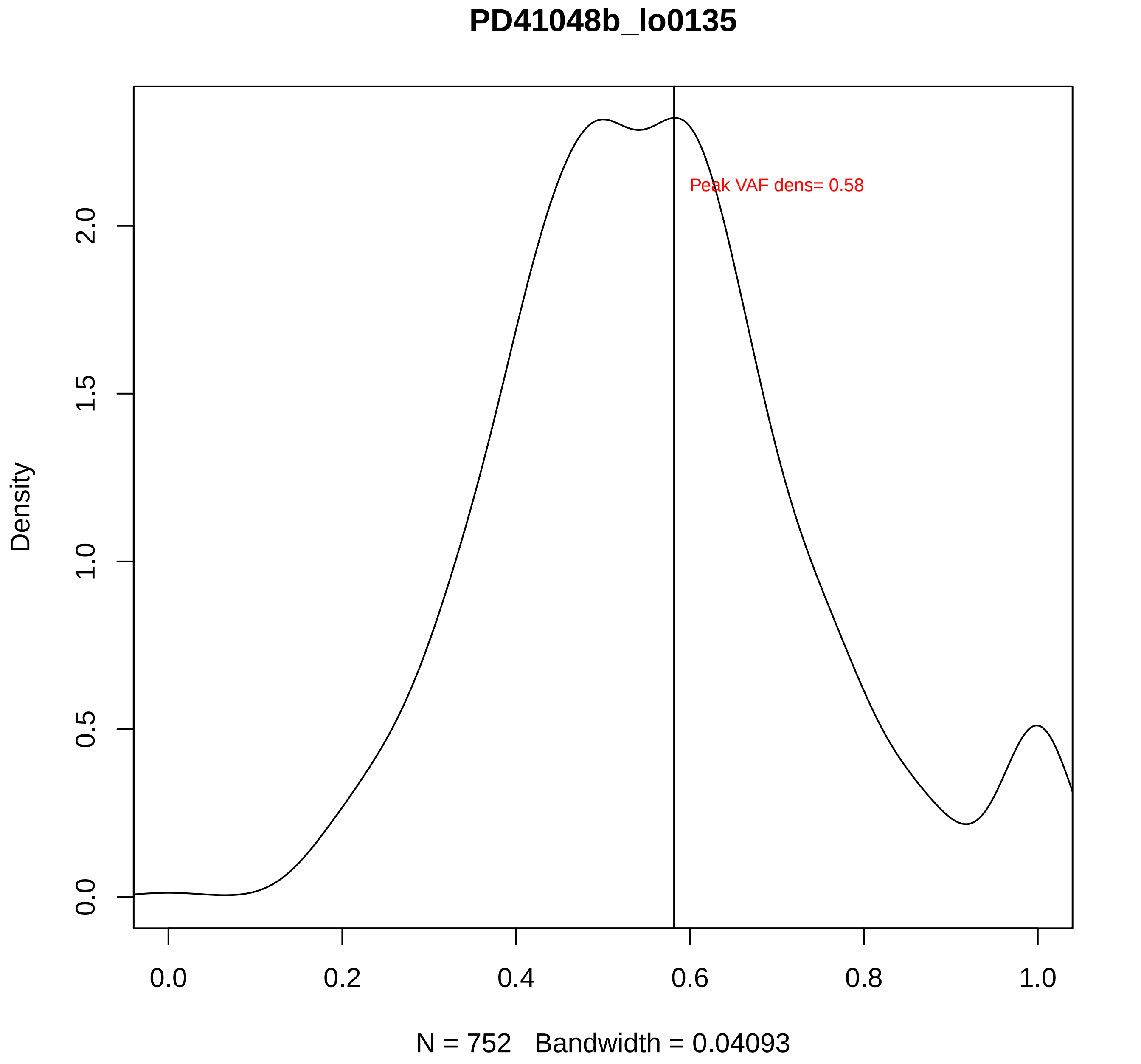
PD41048b_lo0135
Peak VAF dens= 0.58
Density
0.0
0.5
1.0
1.5
2.0
0.0
0.2
0.4
0.6
0.8
1.0
N = 752   Bandwidth = 0.04093

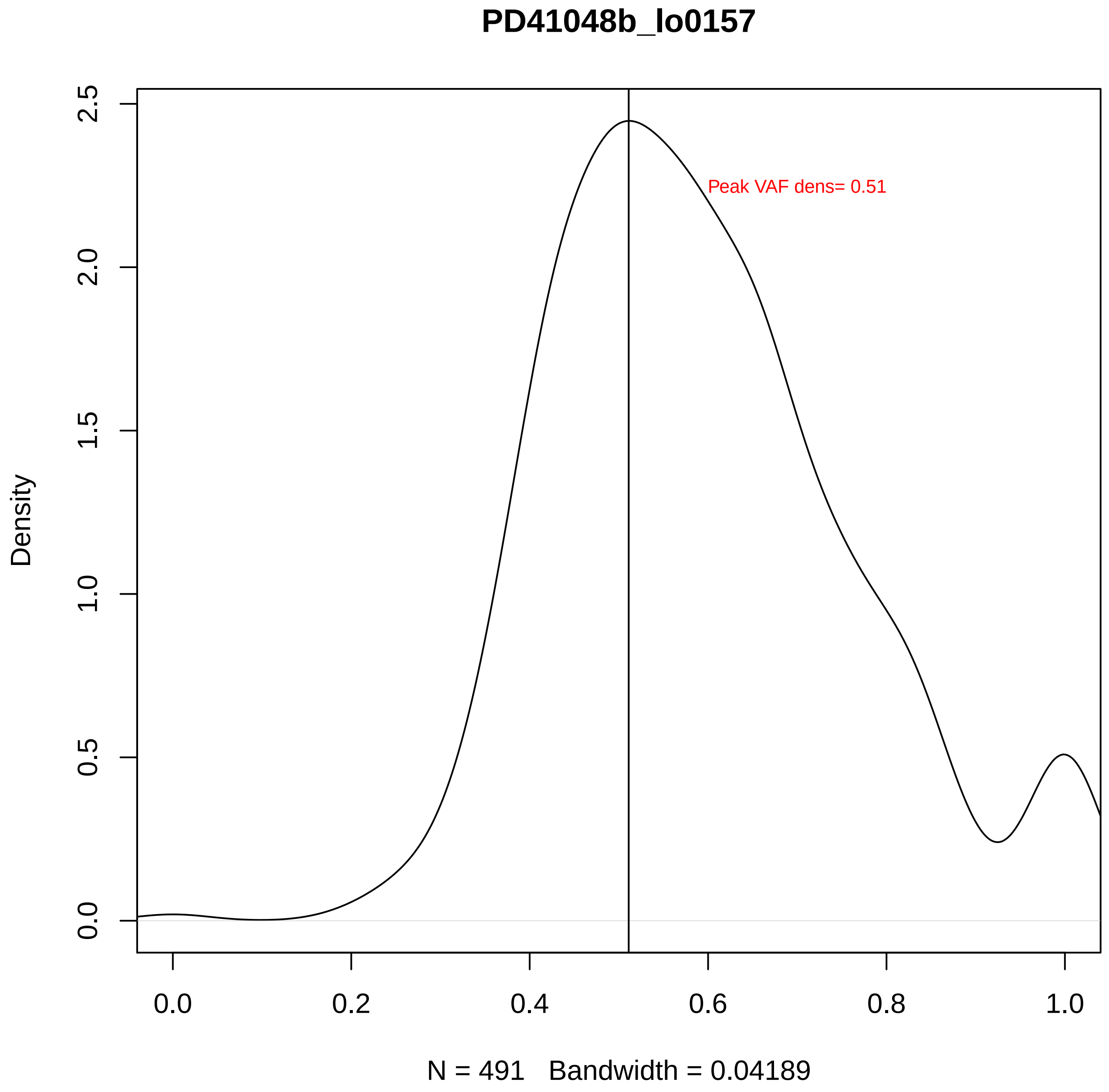
PD41048b_lo0157
Peak VAF dens= 0.51
Density
0.0
0.5
1.0
1.5
2.0
2.5
0.0
0.2
0.4
0.6
0.8
1.0
N = 491   Bandwidth = 0.04189

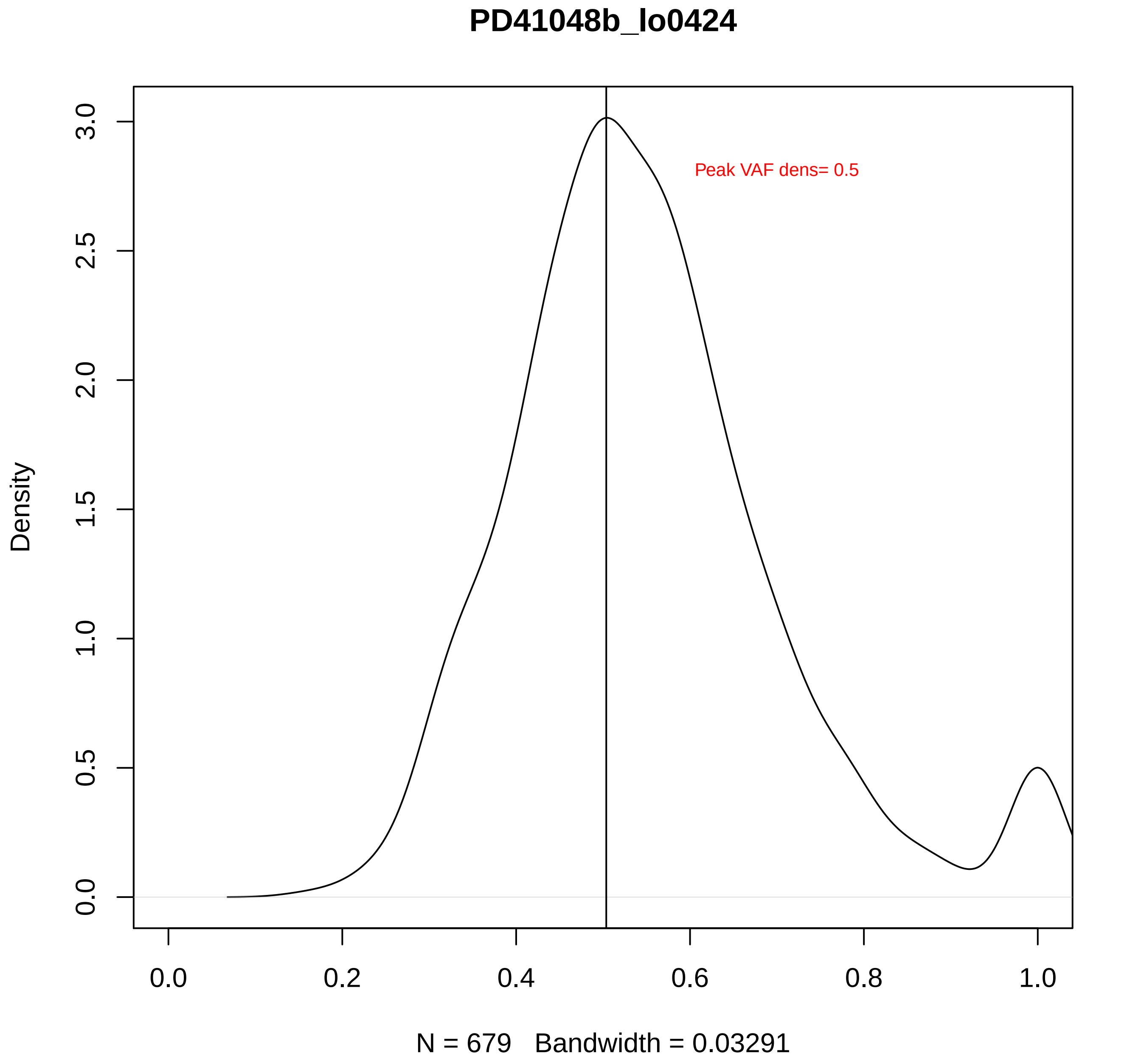
PD41048b_lo0424
Peak VAF dens= 0.5
Density
3.0
2.5
2.0
1.5
1.0
0.5
0.0
0.0
0.2
0.4
0.6
0.8
1.0
N = 679   Bandwidth = 0.03291

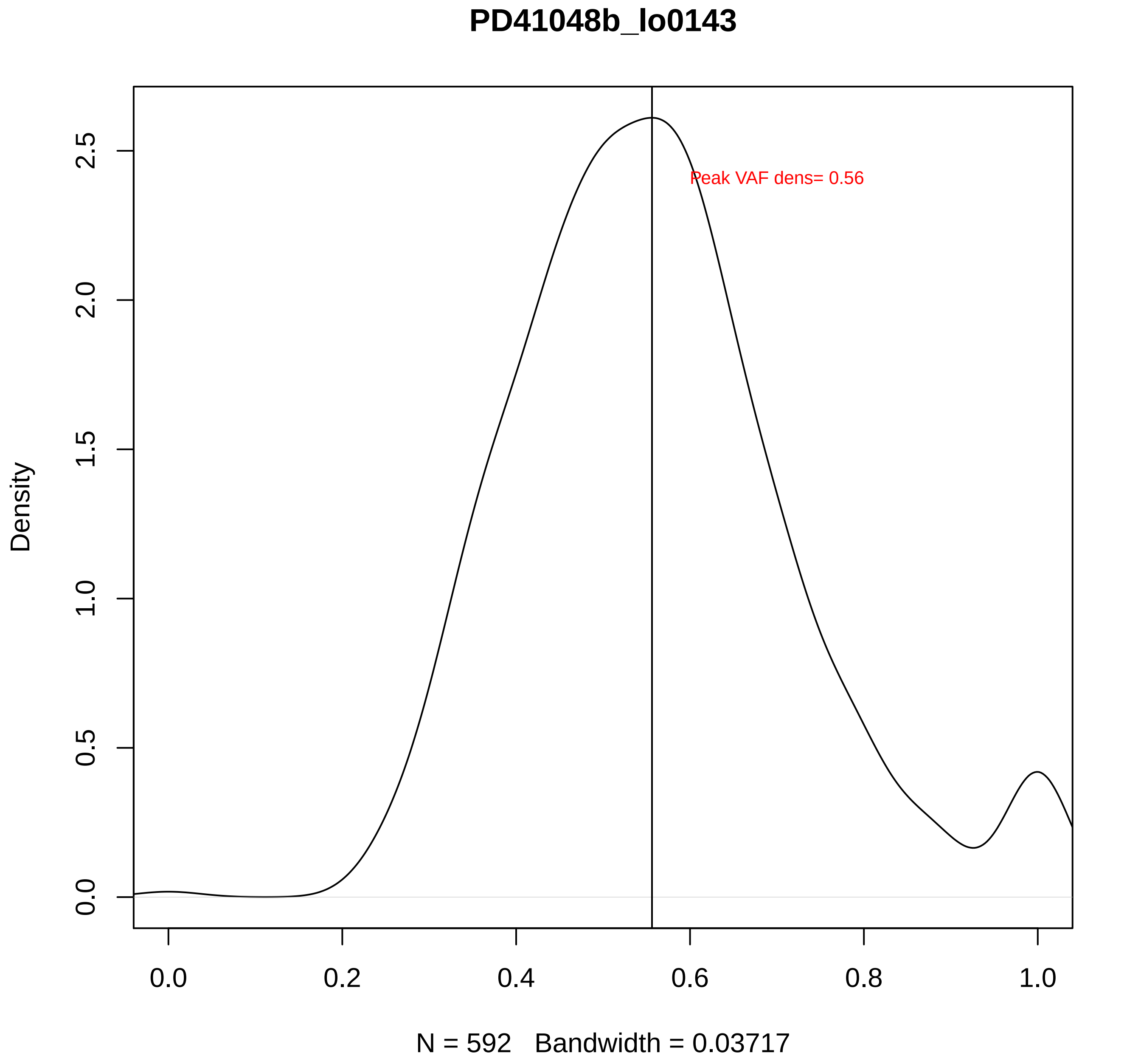
PD41048b_lo0143
Peak VAF dens= 0.56
Density
0.0
0.5
1.0
1.5
2.0
2.5
0.0
0.2
0.4
0.6
0.8
1.0
N = 592   Bandwidth = 0.03717

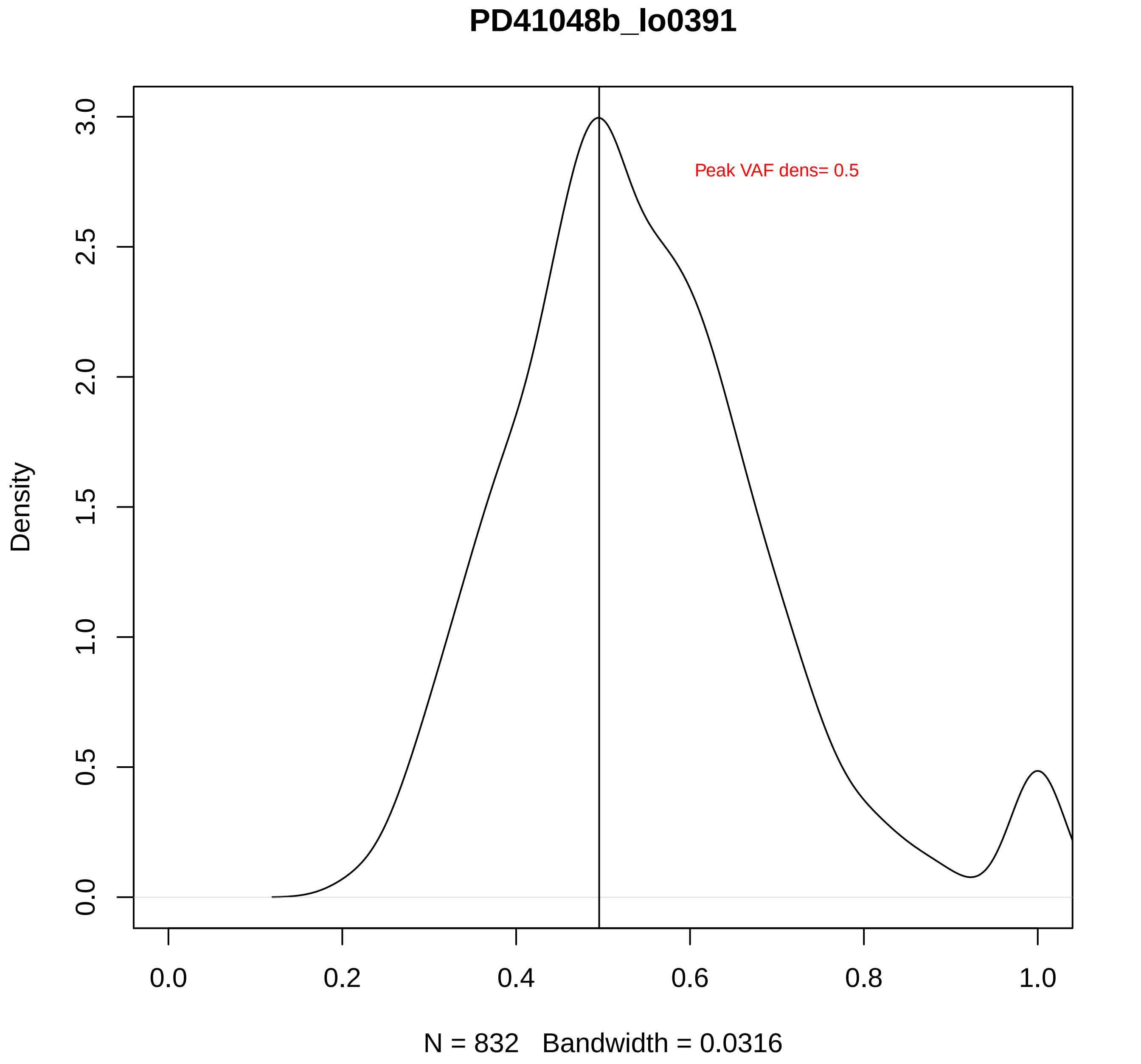
PD41048b_lo0391
Peak VAF dens= 0.5
Density
0.0
0.5
1.0
1.5
2.0
2.5
3.0
0.0
0.2
0.4
0.6
0.8
1.0
N = 832 Bandwidth = 0.0316

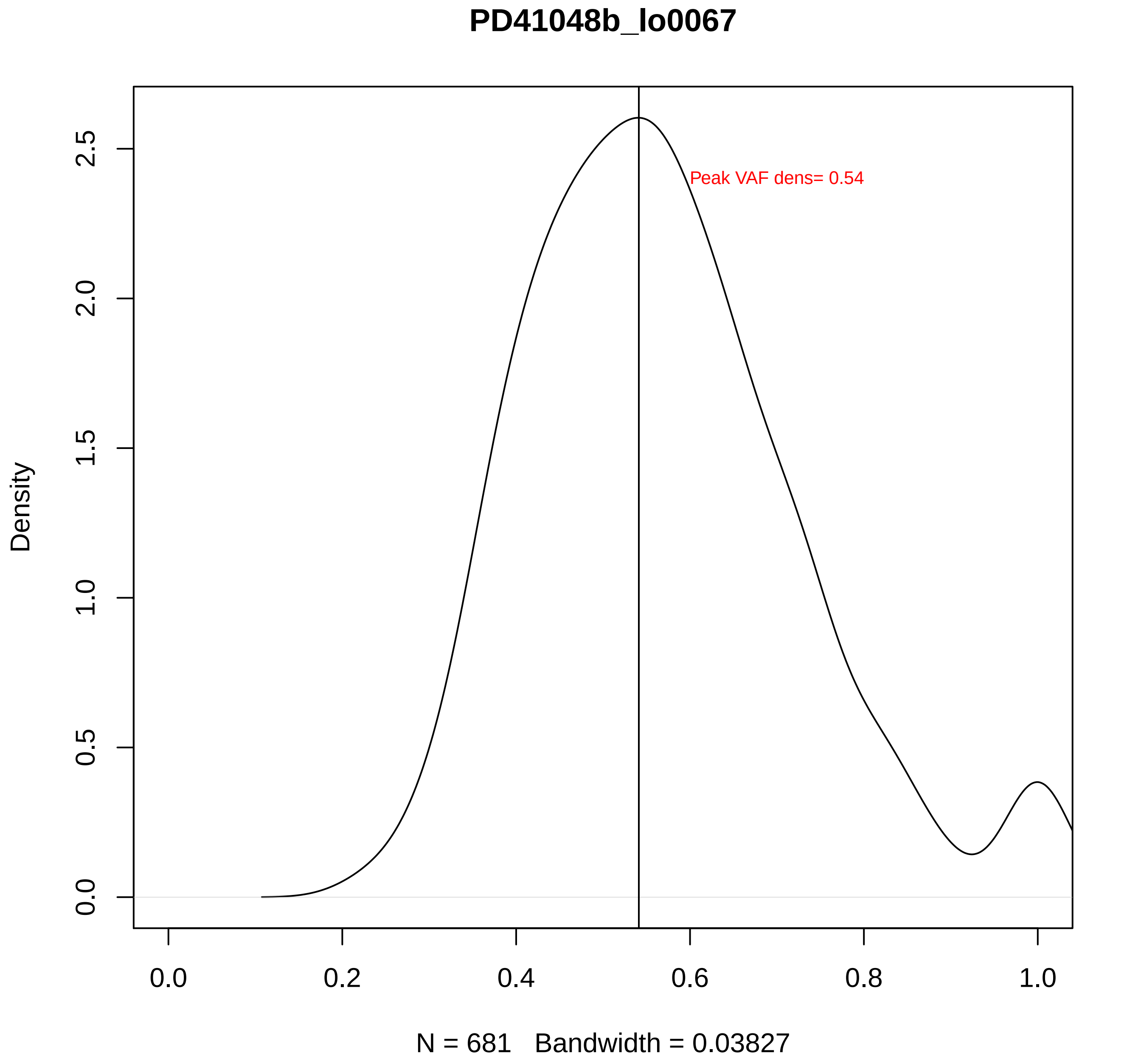
PD41048b_lo0067
Peak VAF dens= 0.54
Density
0.0
0.5
1.0
1.5
2.0
2.5
0.0
0.2
0.4
0.6
0.8
1.0
N = 681   Bandwidth = 0.03827

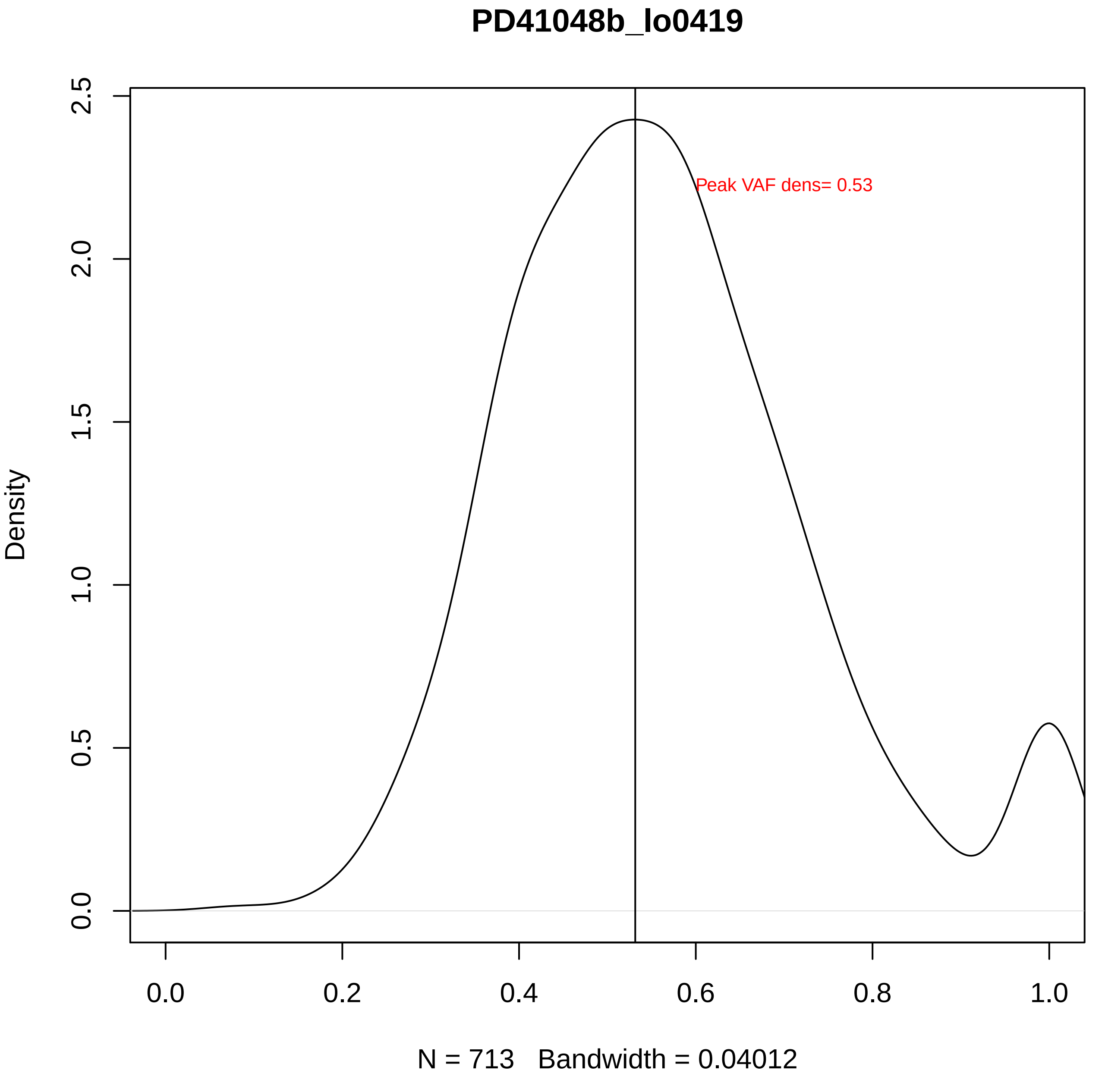
PD41048b_lo0419
Peak VAF dens= 0.53
Density
2.5
2.0
1.5
1.0
0.5
0.0
0.0
0.2
0.4
0.6
0.8
1.0
N = 713 Bandwidth = 0.04012

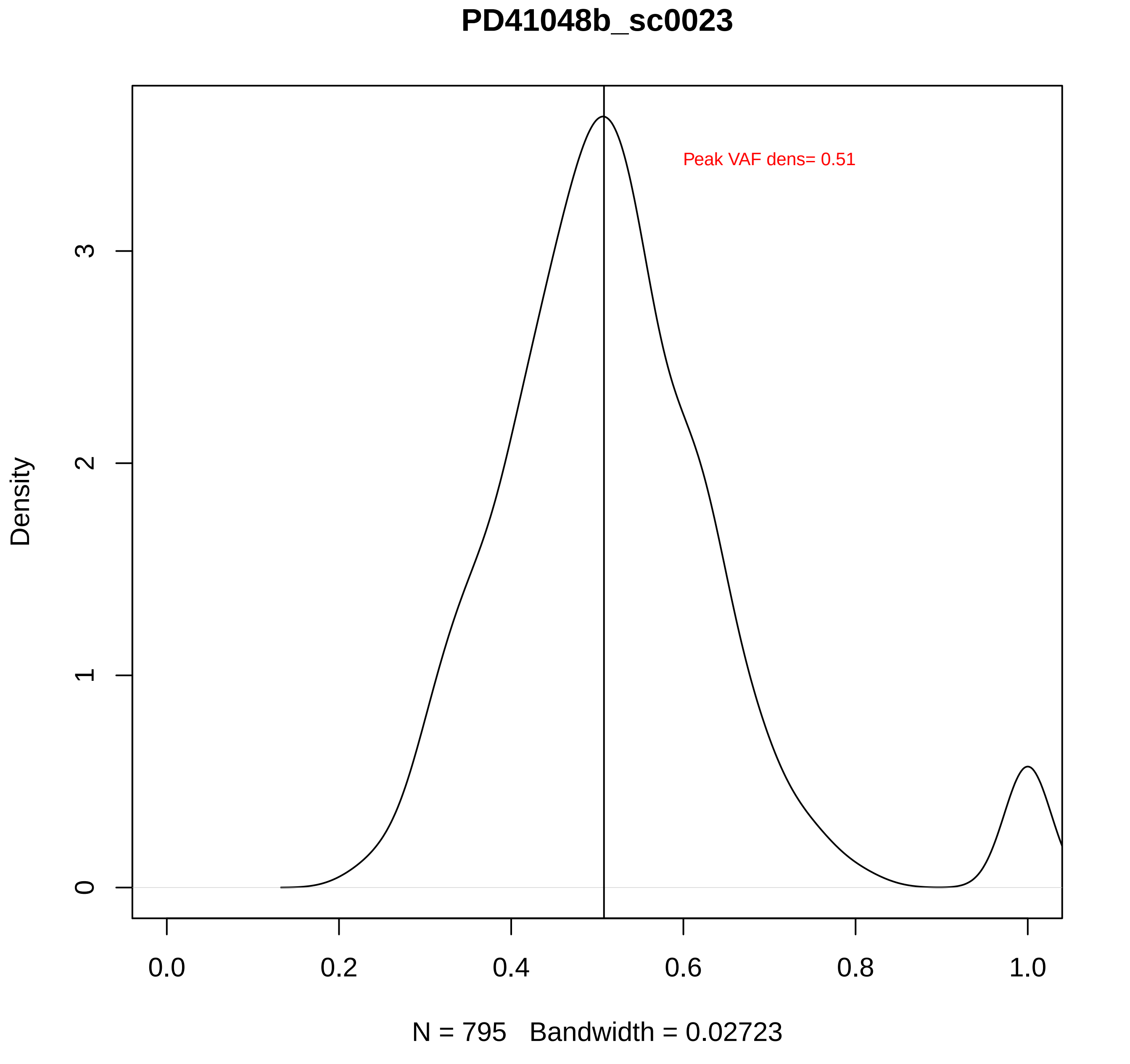

PD41048b_sc0023
Peak VAF dens= 0.51
Density
0
1
2
3
0.0
0.2
0.4
0.6
0.8
1.0
N = 795   Bandwidth = 0.02723

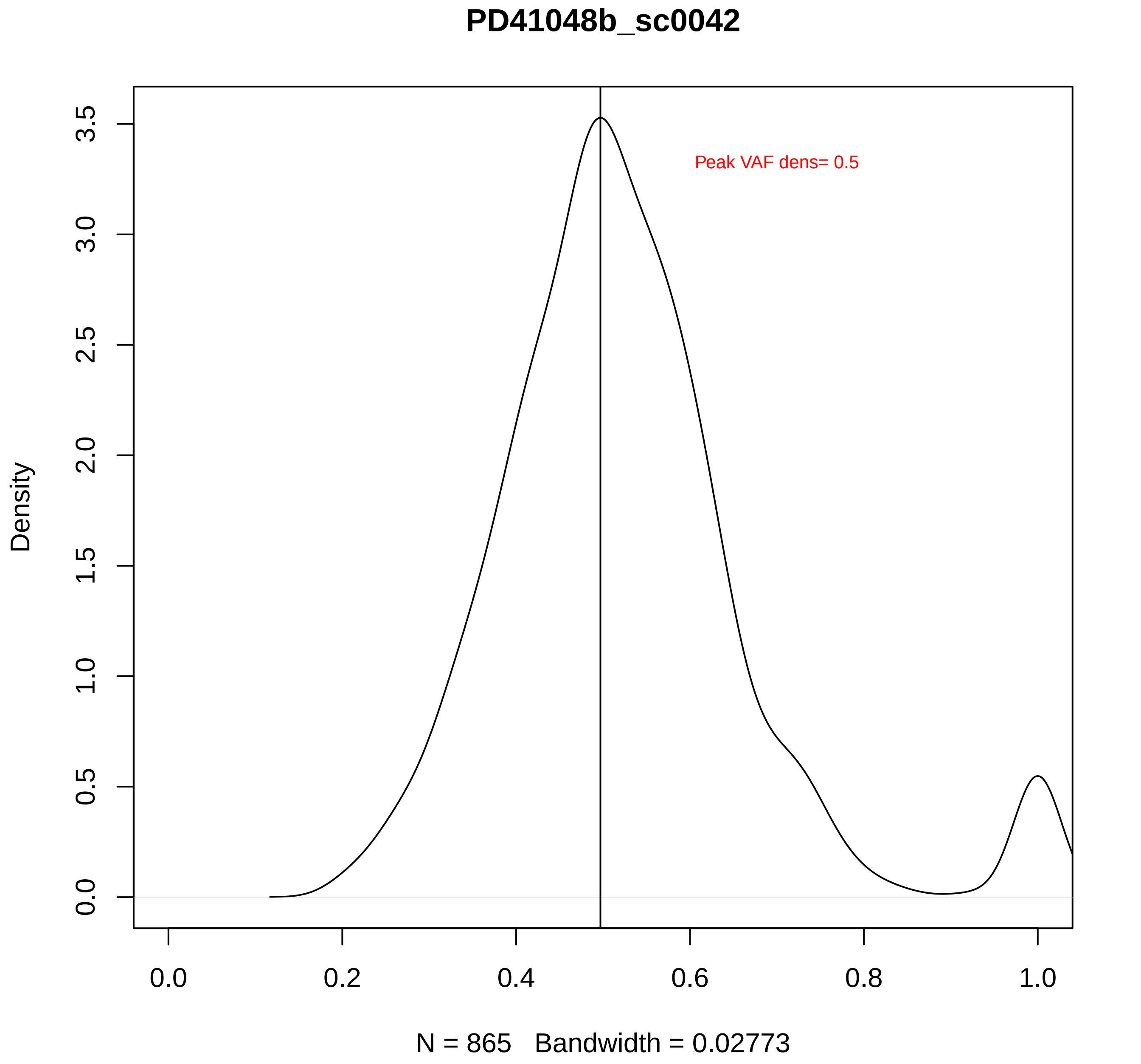
PD41048b_sc0042
Peak VAF dens= 0.5
Density
0.0
0.5
1.0
1.5
2.0
2.5
3.0
3.5
0.0
0.2
0.4
0.6
0.8
1.0
N = 865 Bandwidth = 0.02773

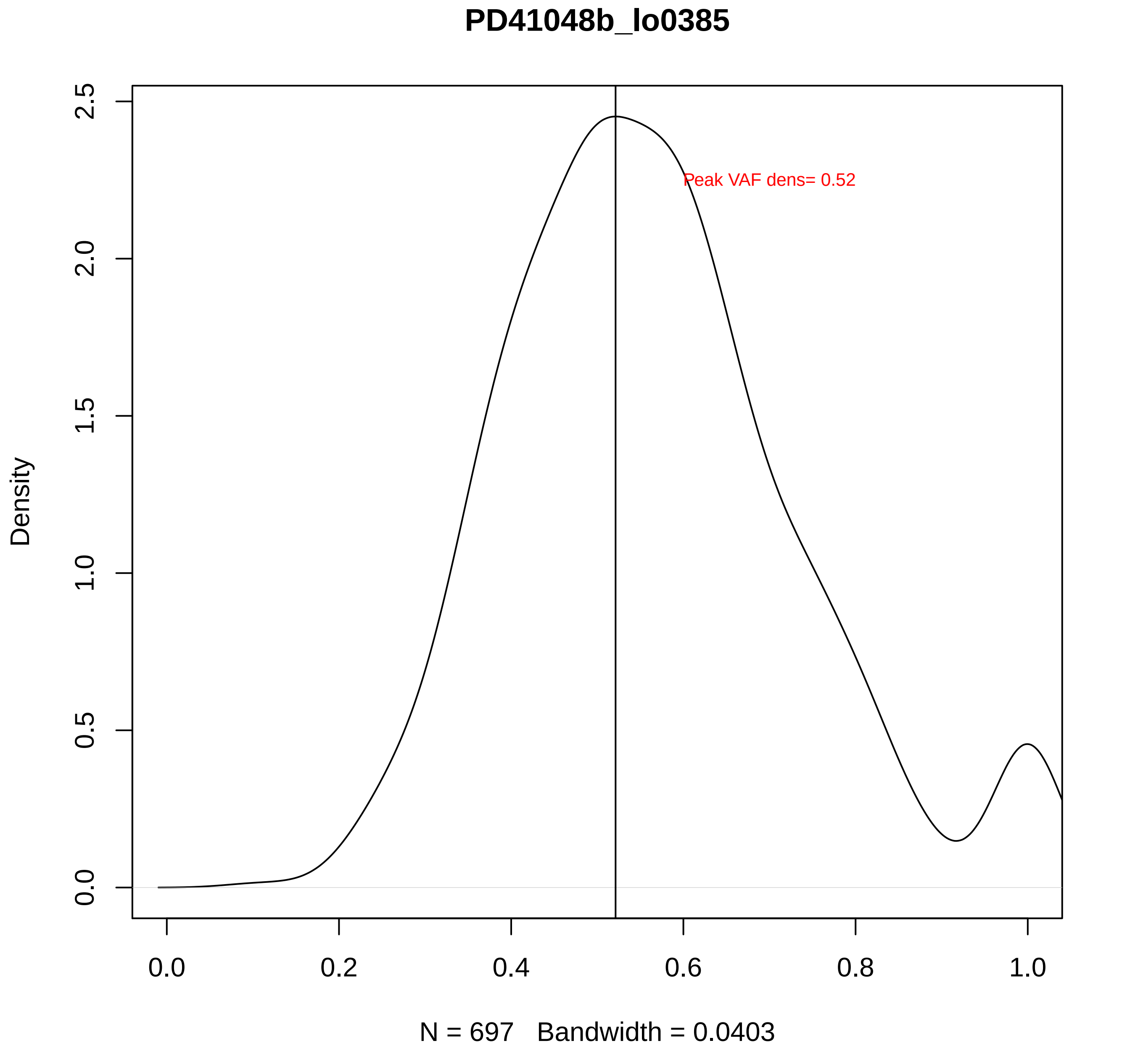
PD41048b_lo0385
Density
2.5
2.0
1.5
1.0
0.5
0.0
Peak VAF dens= 0.52
0.0
0.2
0.4
0.6
0.8
1.0
N = 697   Bandwidth = 0.0403

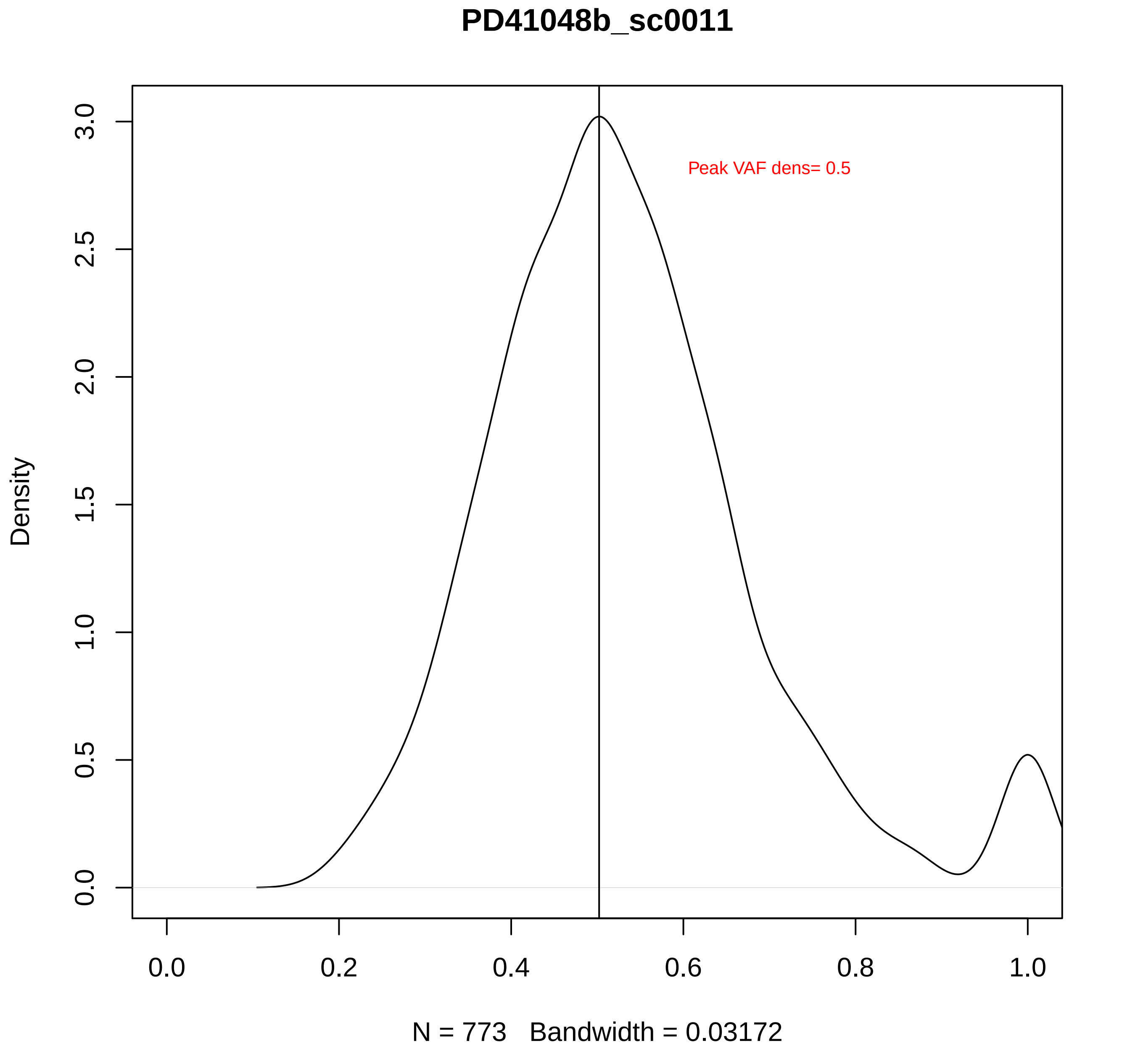

PD41048b_sc0011
Peak VAF dens= 0.5
Density
0.0
0.5
1.0
1.5
2.0
2.5
3.0
0.0
0.2
0.4
0.6
0.8
1.0
N = 773   Bandwidth = 0.03172

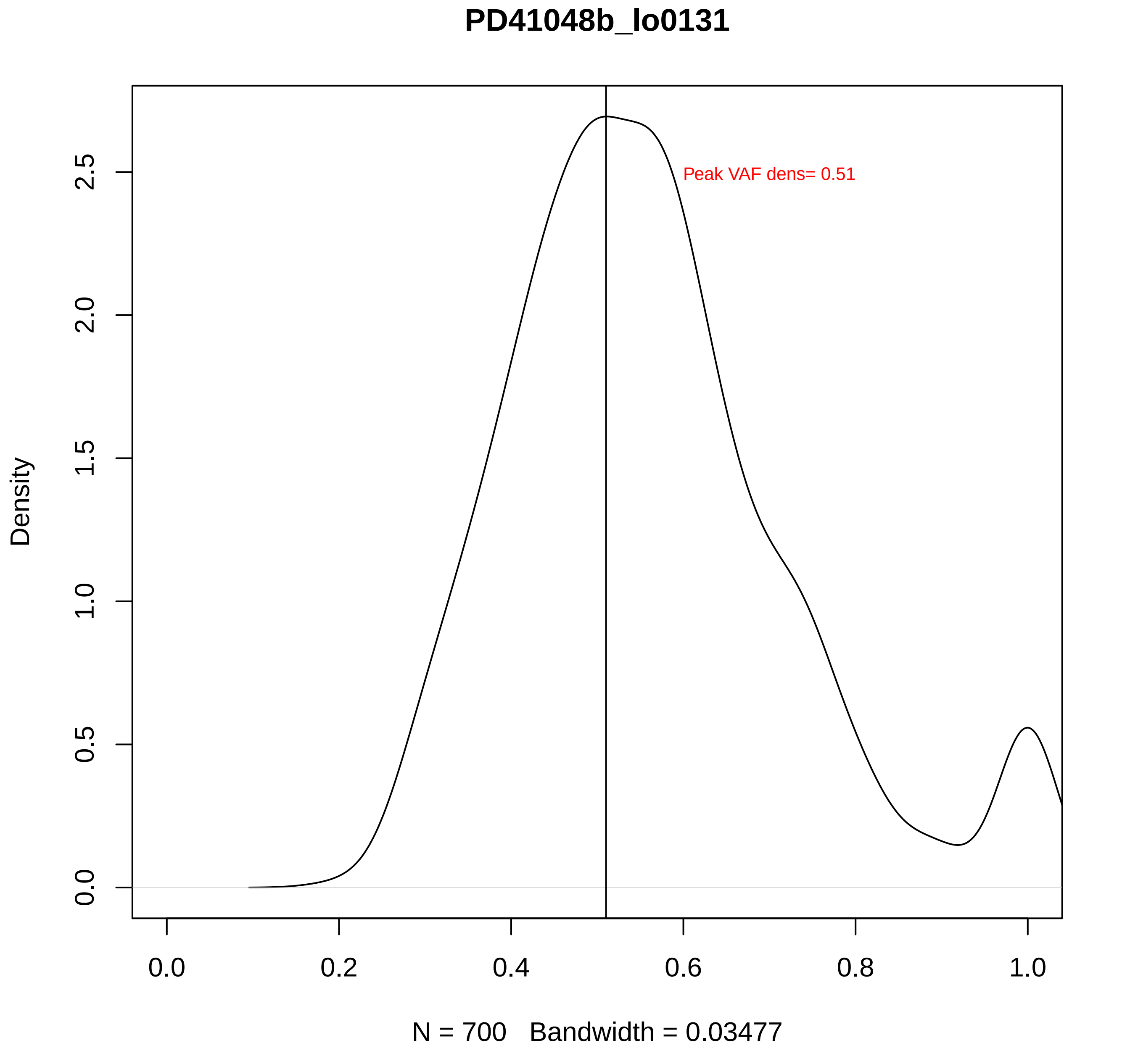

PD41048b_lo0131
Peak VAF dens= 0.51
Density
0.0
0.5
1.0
1.5
2.0
2.5
0.0
0.2
0.4
0.6
0.8
1.0
N = 700   Bandwidth = 0.03477

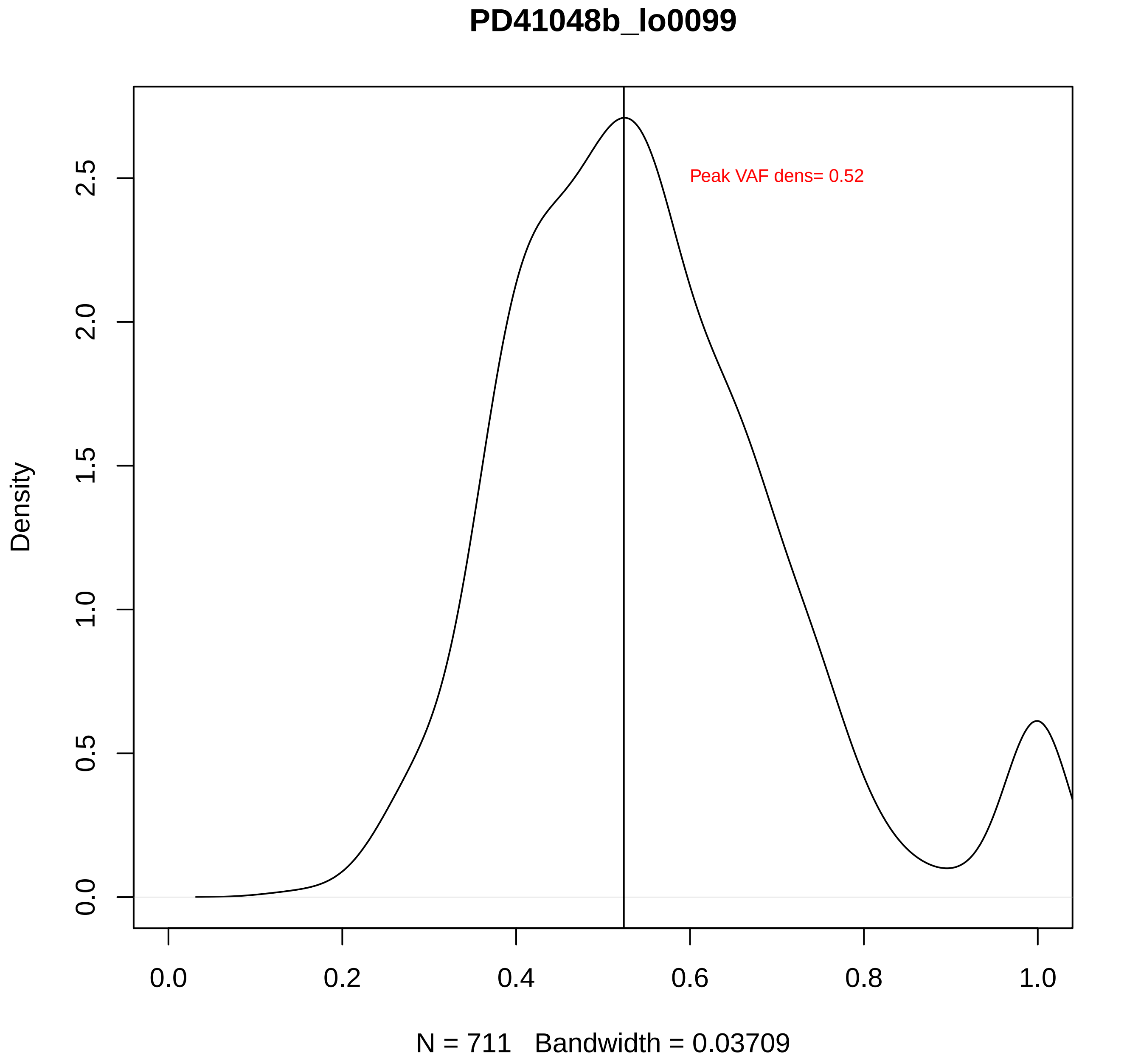
PD41048b_lo0099
Peak VAF dens= 0.52
Density
0.0
0.5
1.0
1.5
2.0
2.5
0.0
0.2
0.4
0.6
0.8
1.0
N = 711   Bandwidth = 0.03709

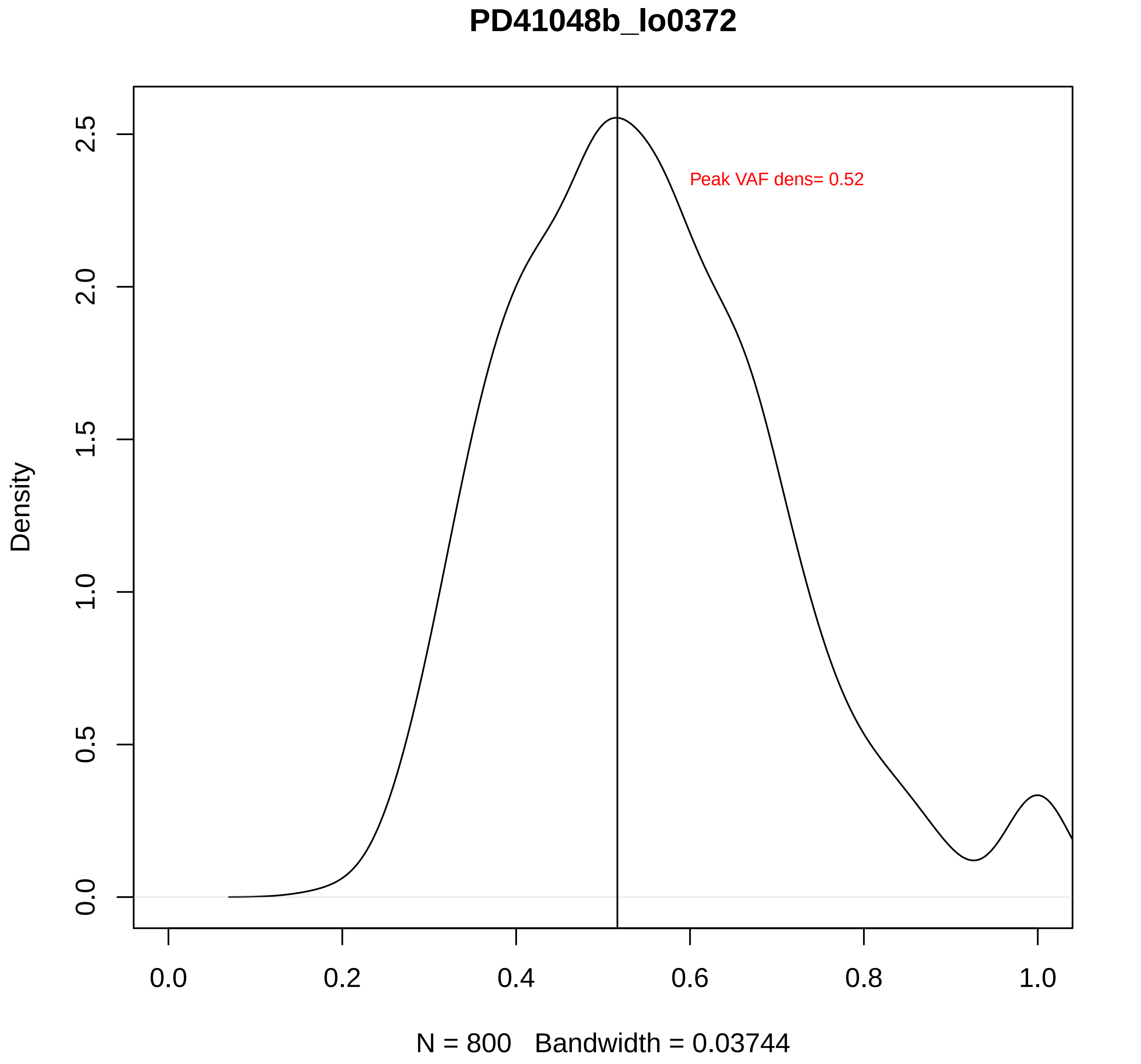
PD41048b_lo0372
Peak VAF dens= 0.52
Density
0.0
0.5
1.0
1.5
2.0
2.5
0.0
0.2
0.4
0.6
0.8
1.0
N = 800 Bandwidth = 0.03744

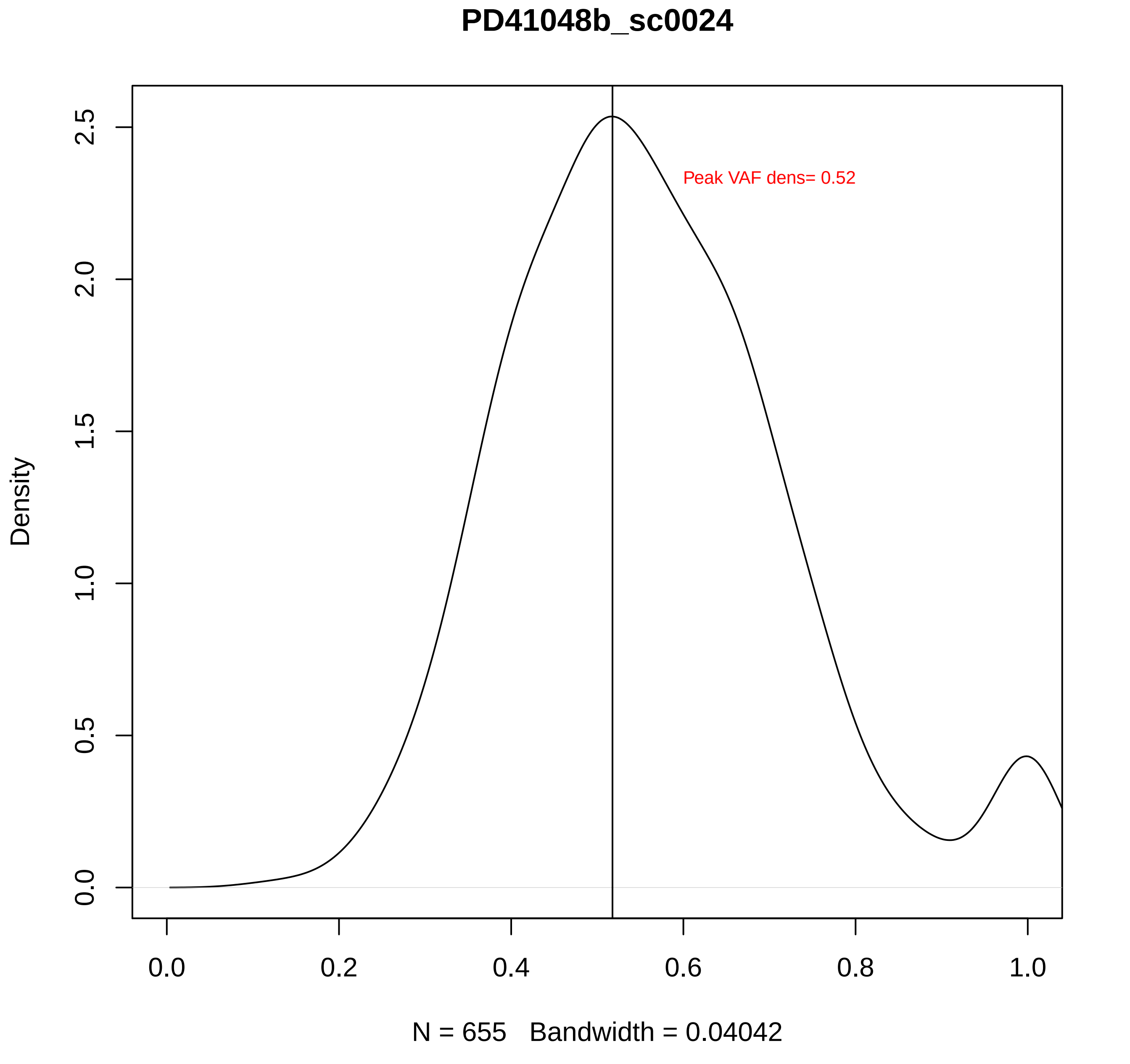

PD41048b_sc0024
Peak VAF dens= 0.52
Density
0.0
0.5
1.0
1.5
2.0
2.5
0.0
0.2
0.4
0.6
0.8
1.0
N = 655 Bandwidth = 0.04042

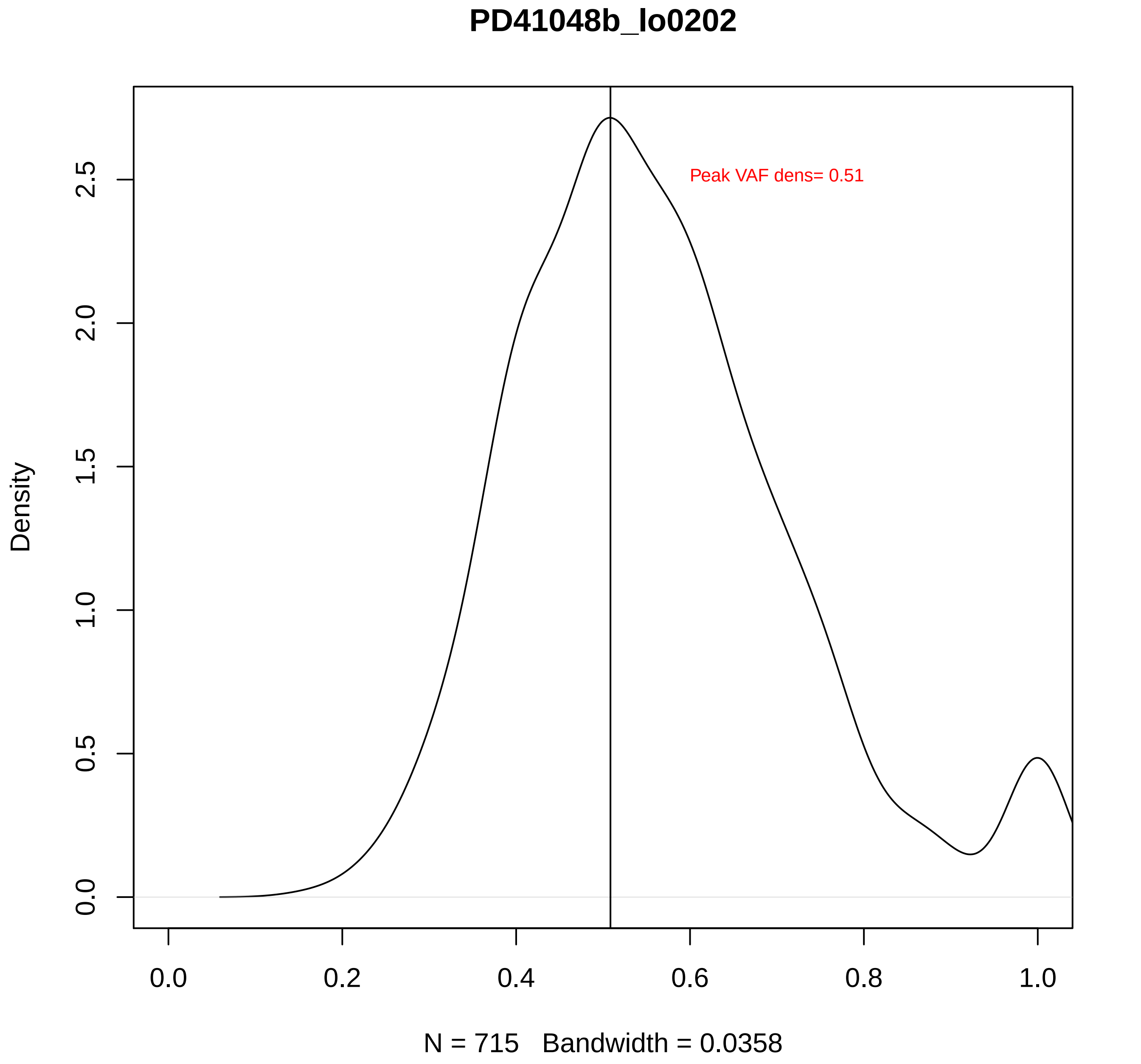

PD41048b_lo0202
Peak VAF dens= 0.51
Density
0.0
0.5
1.0
1.5
2.0
2.5
0.0
0.2
0.4
0.6
0.8
1.0
N = 715   Bandwidth = 0.0358

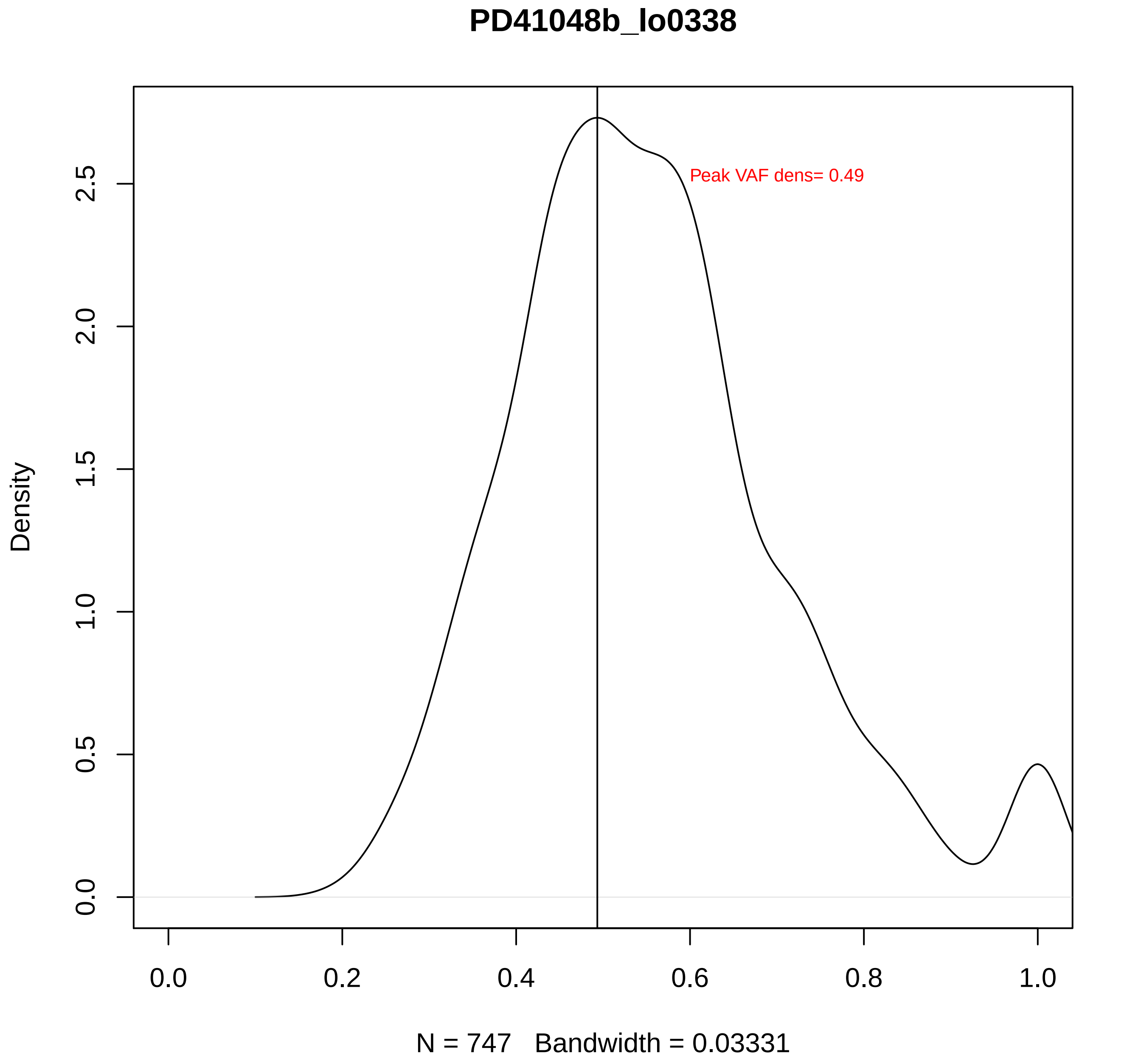
PD41048b_lo0338
Peak VAF dens= 0.49
Density
0.0
0.5
1.0
1.5
2.0
2.5
0.0
0.2
0.4
0.6
0.8
1.0
N = 747   Bandwidth = 0.03331

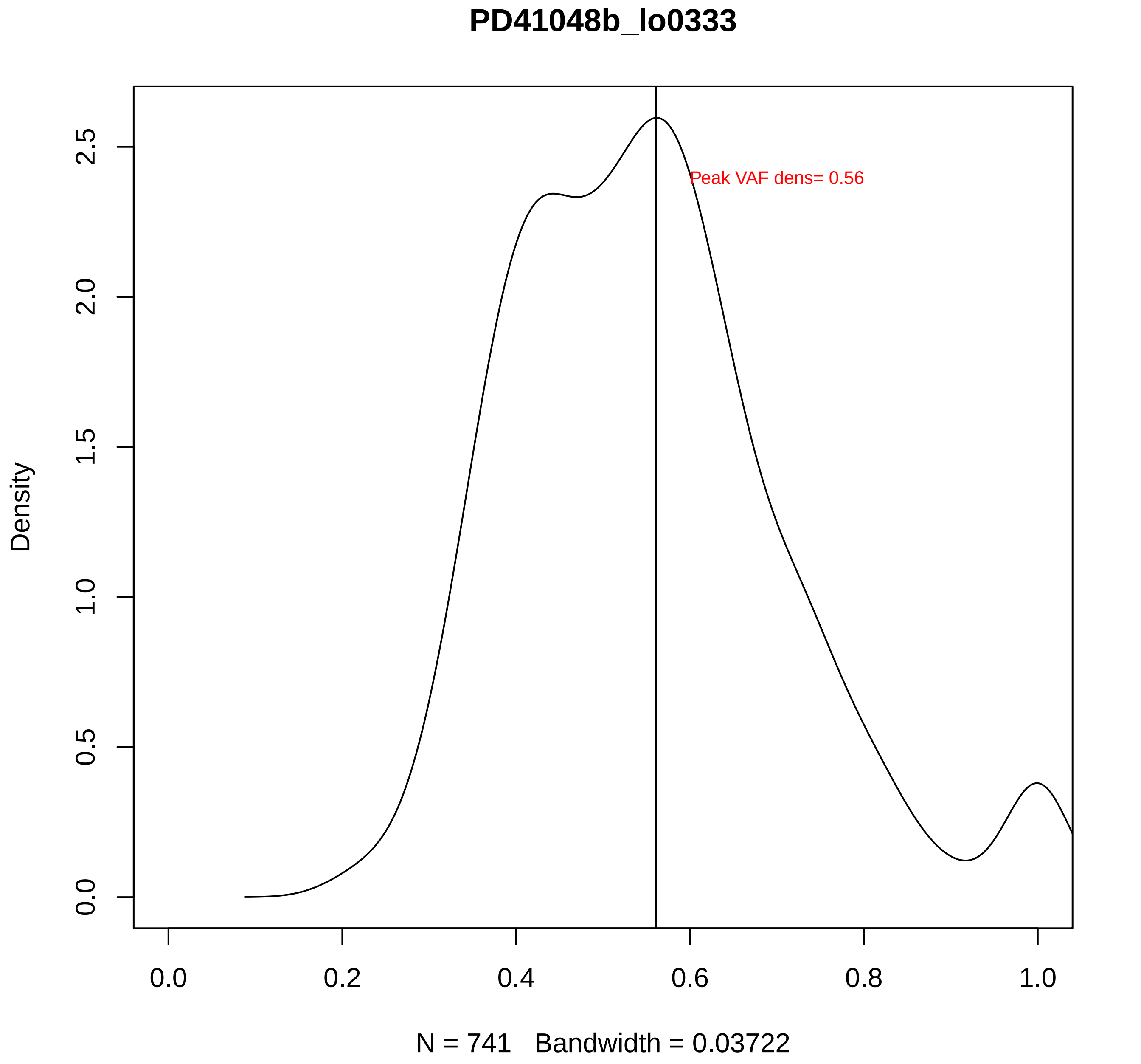
PD41048b_lo0333
Density
Peak VAF dens= 0.56
0.0
0.5
1.0
1.5
2.0
2.5
0.0
0.2
0.4
0.6
0.8
1.0
N = 741   Bandwidth = 0.03722

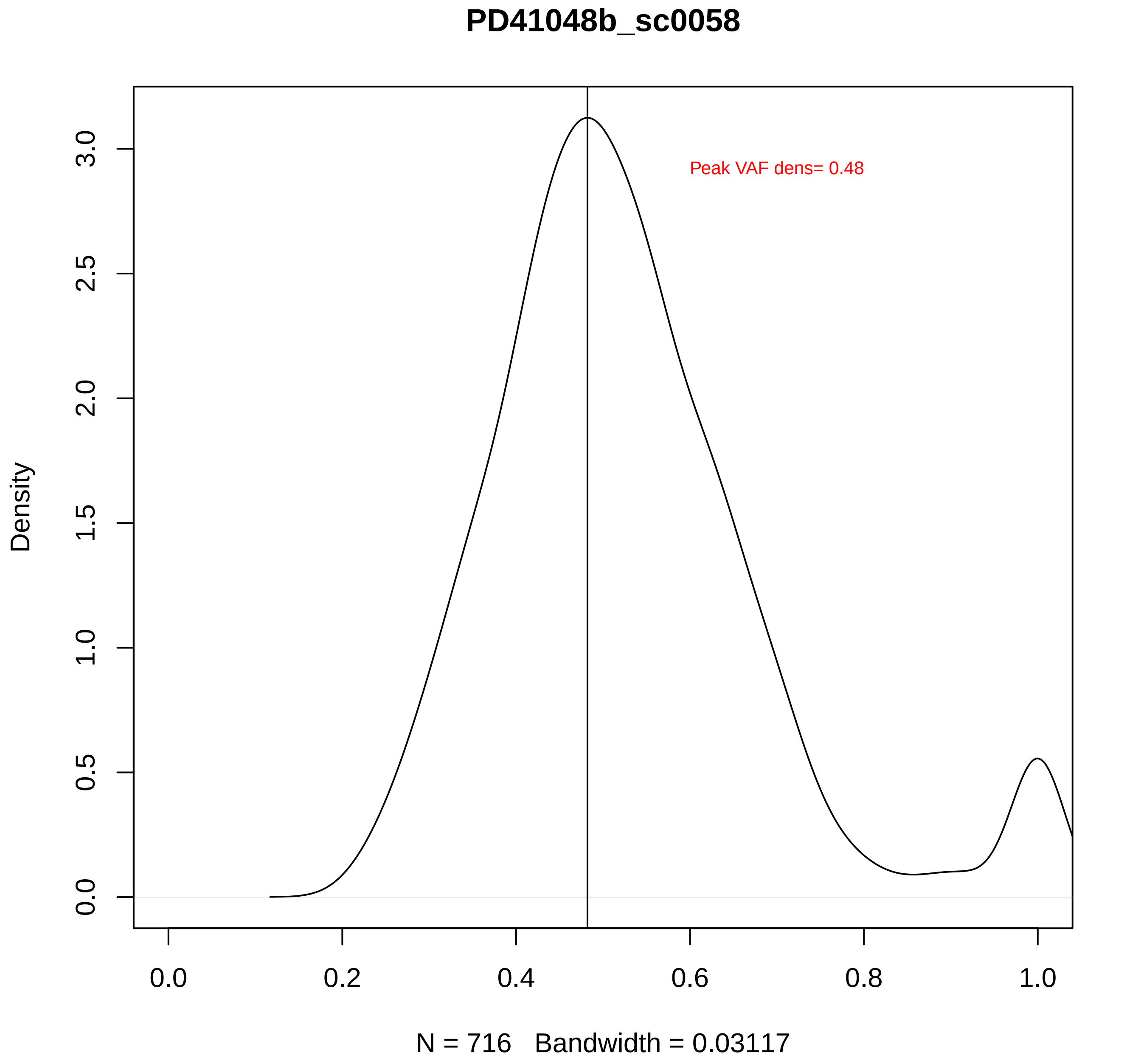
PD41048b_sc0058
Peak VAF dens= 0.48
Density
0.0
0.5
1.0
1.5
2.0
2.5
3.0
0.0
0.2
0.4
0.6
0.8
1.0
N = 716   Bandwidth = 0.03117

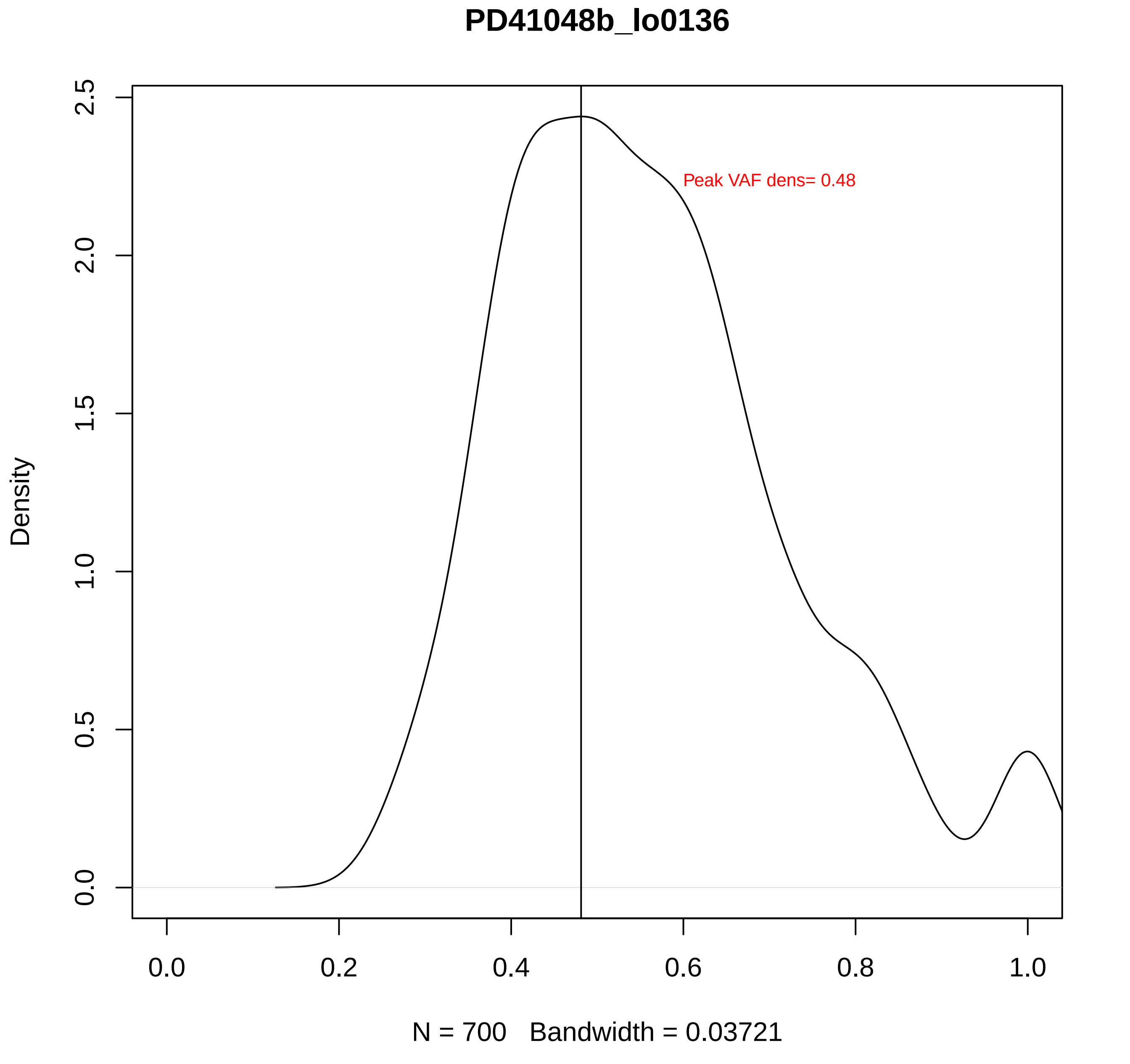
PD41048b_lo0136
Peak VAF dens= 0.48
Density
0.0
0.5
1.0
1.5
2.0
2.5
0.0
0.2
0.4
0.6
0.8
1.0
N = 700 Bandwidth = 0.03721

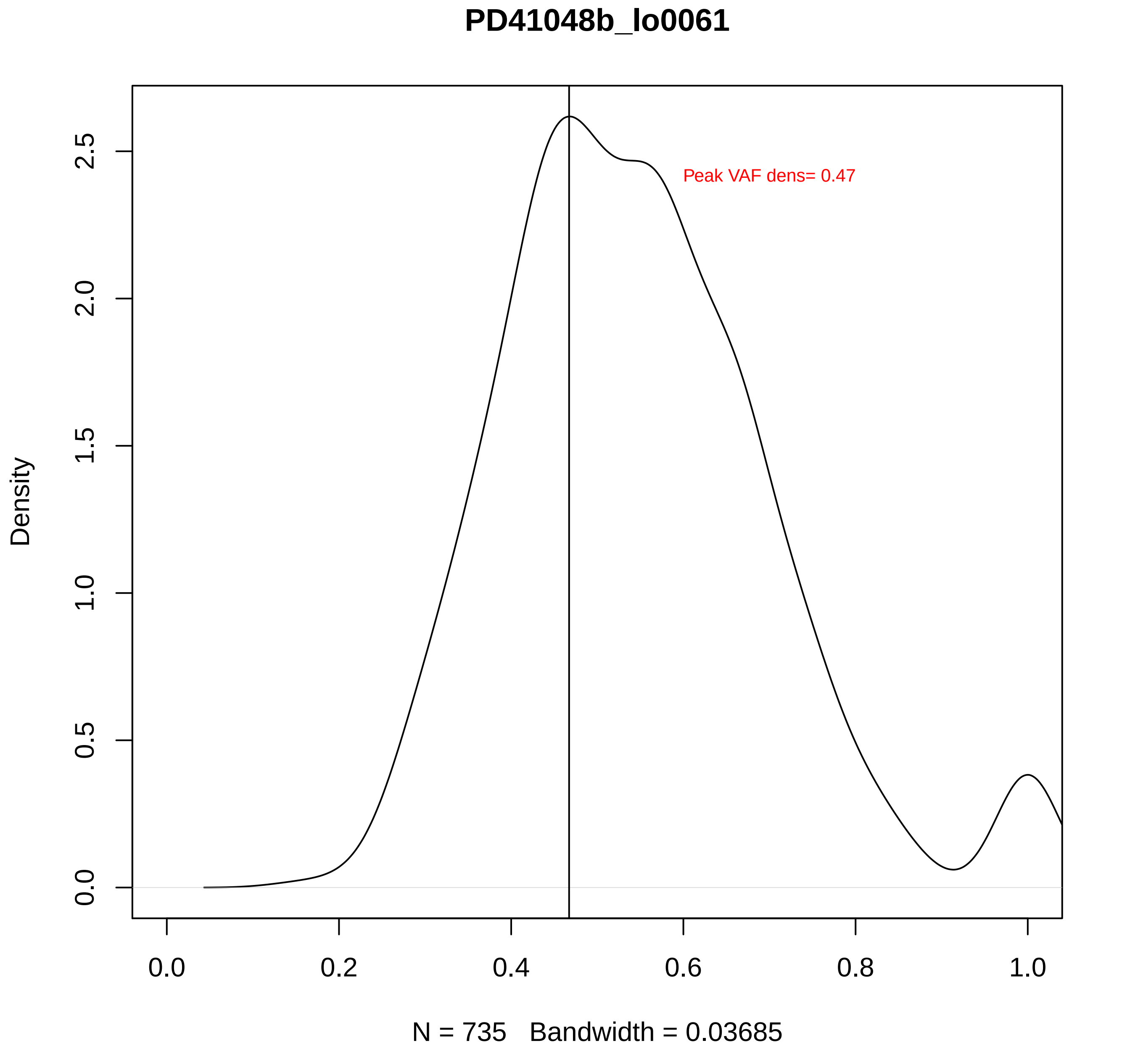
PD41048b_lo0061
Peak VAF dens= 0.47
Density
0.0
0.5
1.0
1.5
2.0
2.5
0.0
0.2
0.4
0.6
0.8
1.0
N = 735 Bandwidth = 0.03685

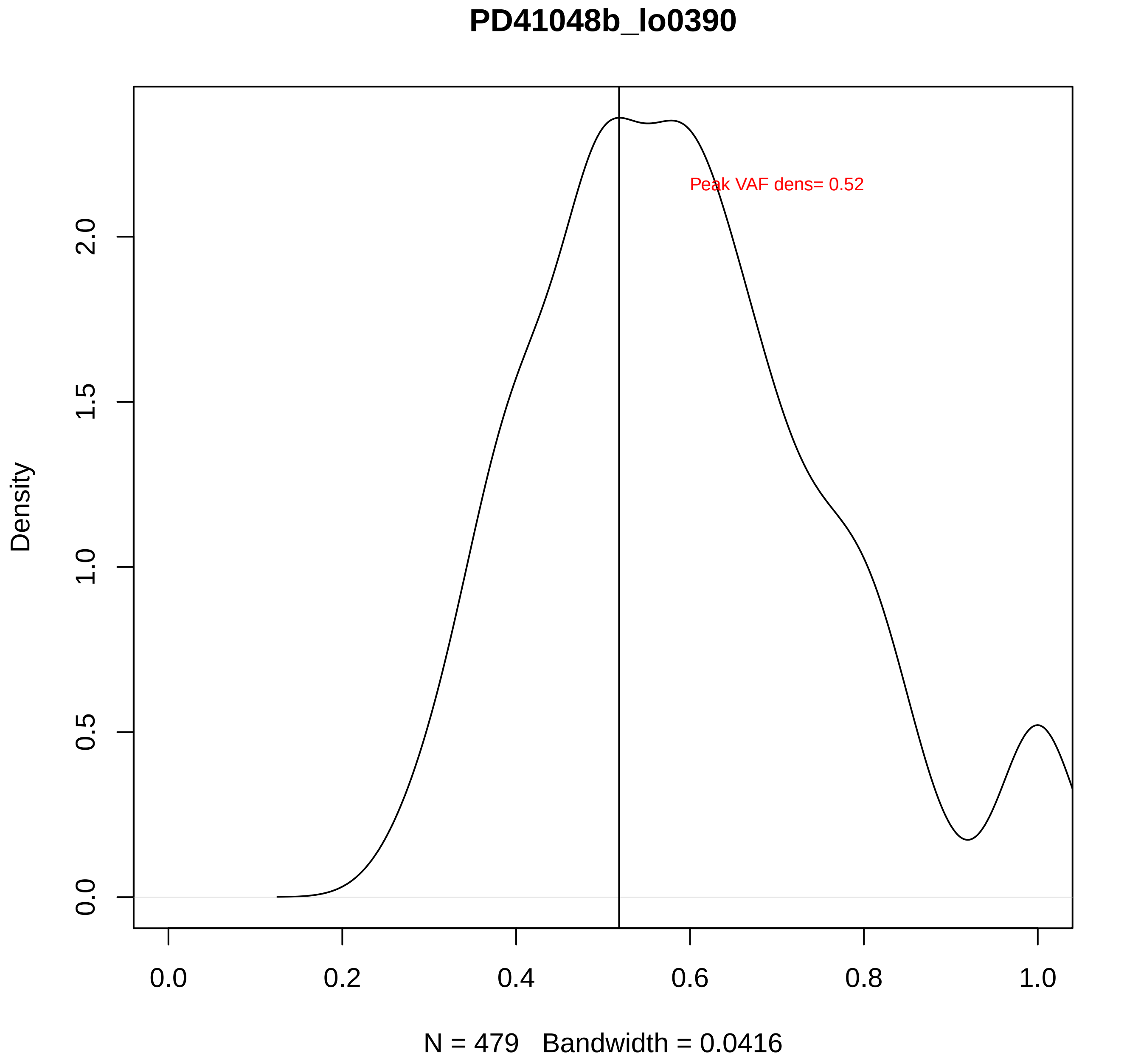

PD41048b_lo0390
Peak VAF dens= 0.52
Density
0.0
0.5
1.0
1.5
2.0
0.0
0.2
0.4
0.6
0.8
1.0
N = 479   Bandwidth = 0.0416

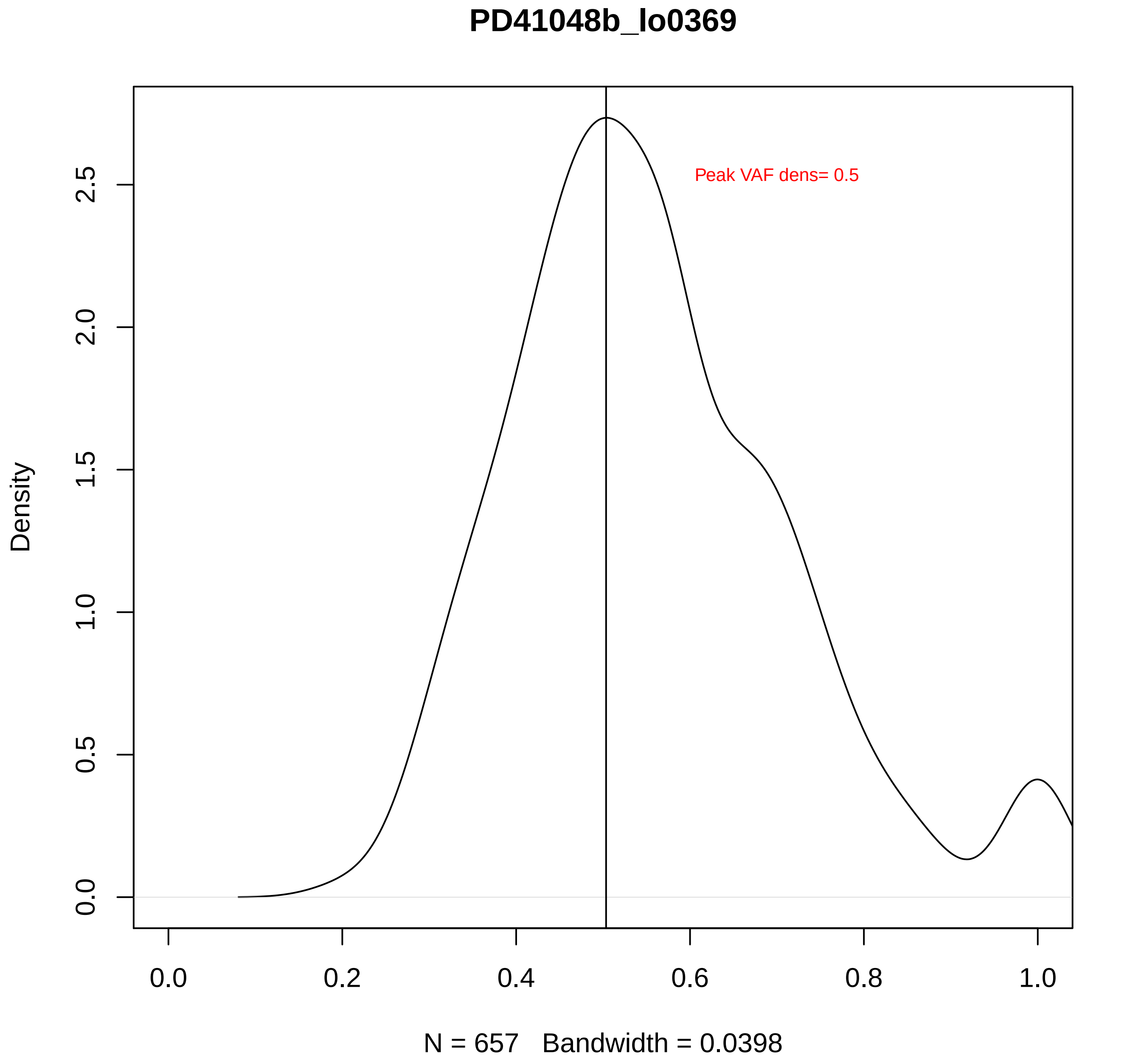
PD41048b_lo0369
Peak VAF dens= 0.5
Density
0.0
0.5
1.0
1.5
2.0
2.5
0.0
0.2
0.4
0.6
0.8
1.0
N = 657   Bandwidth = 0.0398

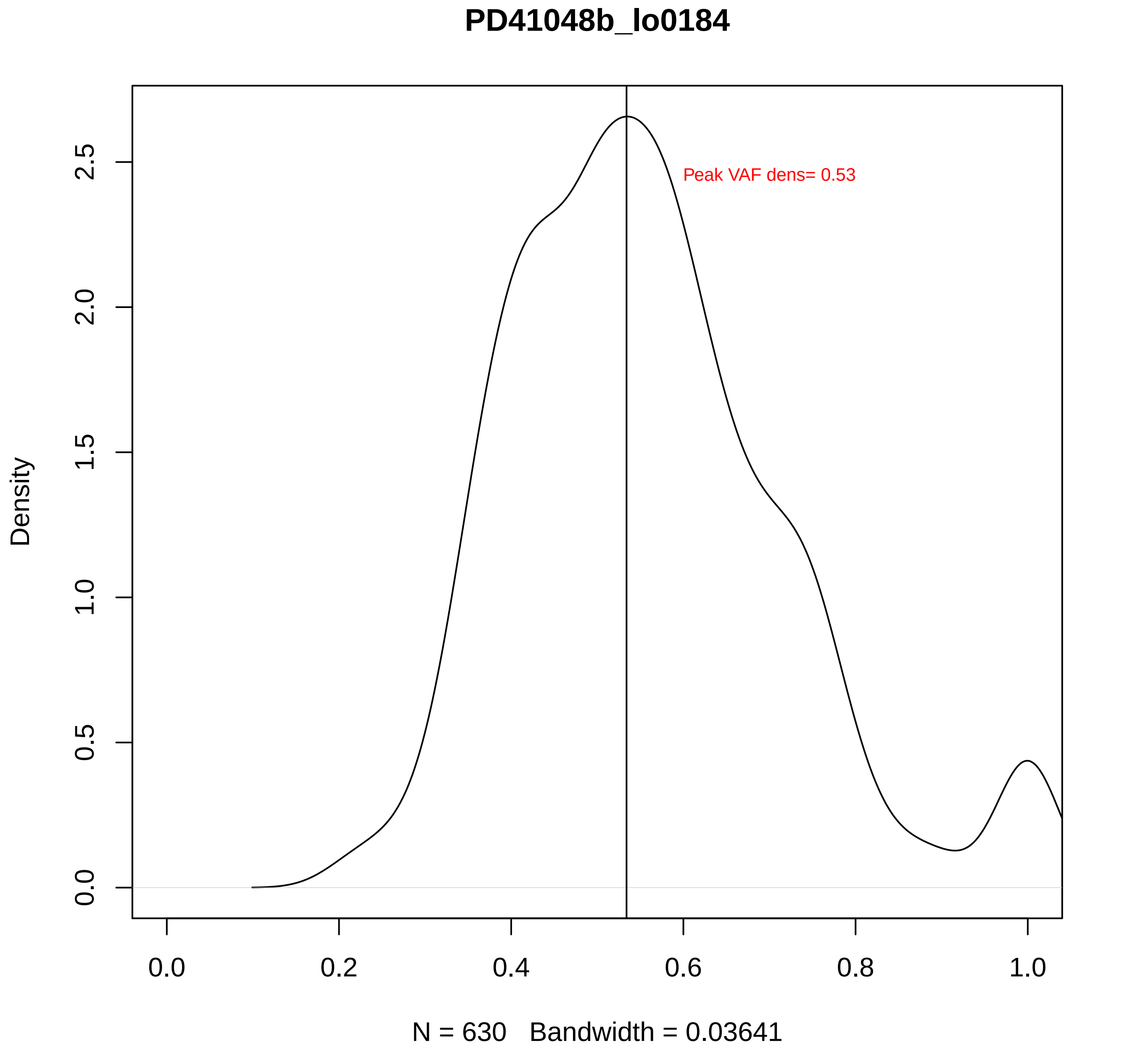
PD41048b_lo0184
Peak VAF dens= 0.53
Density
0.0
0.5
1.0
1.5
2.0
2.5
0.0
0.2
0.4
0.6
0.8
1.0
N = 630   Bandwidth = 0.03641

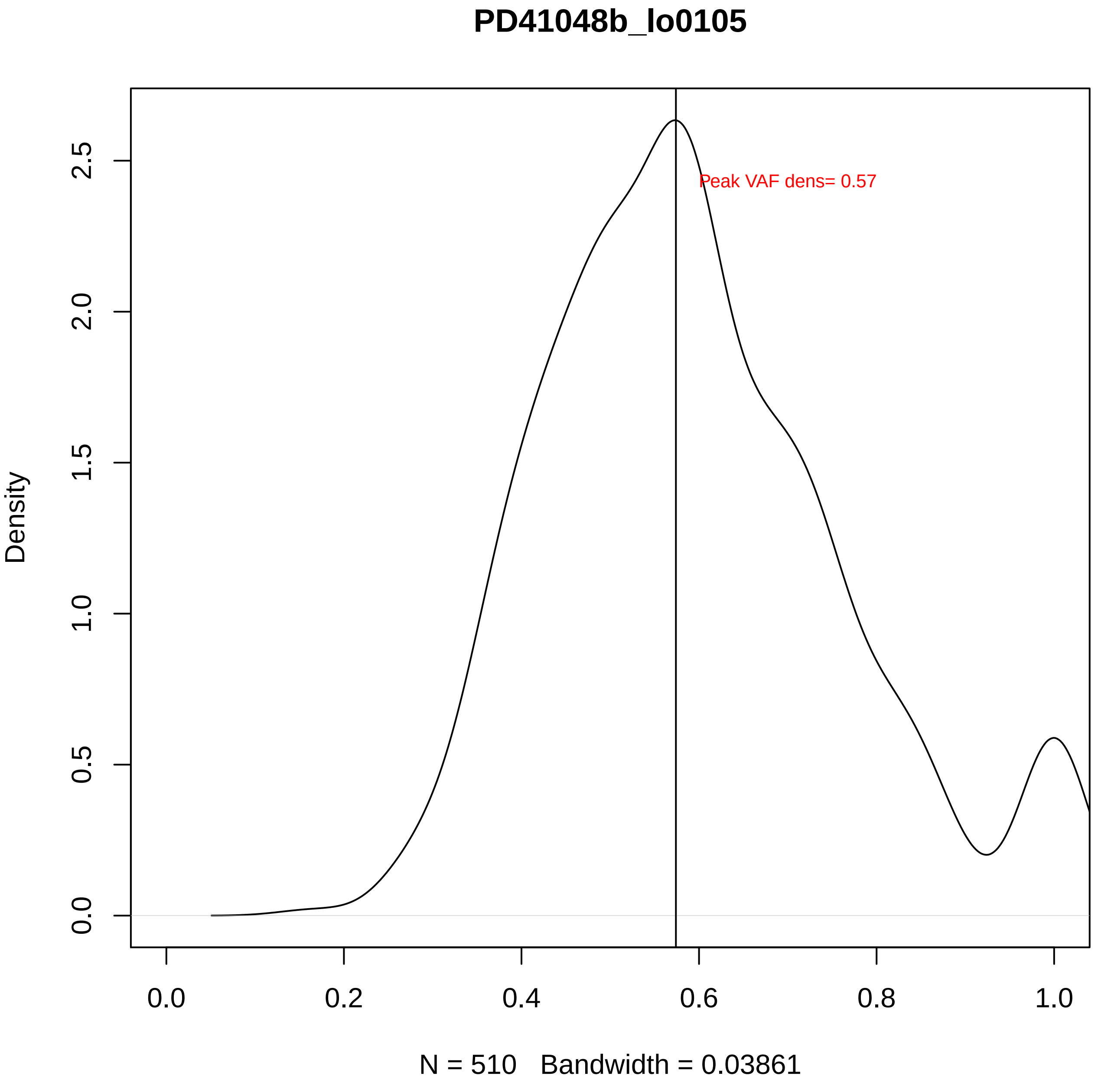

PD41048b_lo0105
Peak VAF dens= 0.57
Density
0.0
0.5
1.0
1.5
2.0
2.5
0.0
0.2
0.4
0.6
0.8
1.0
N = 510   Bandwidth = 0.03861

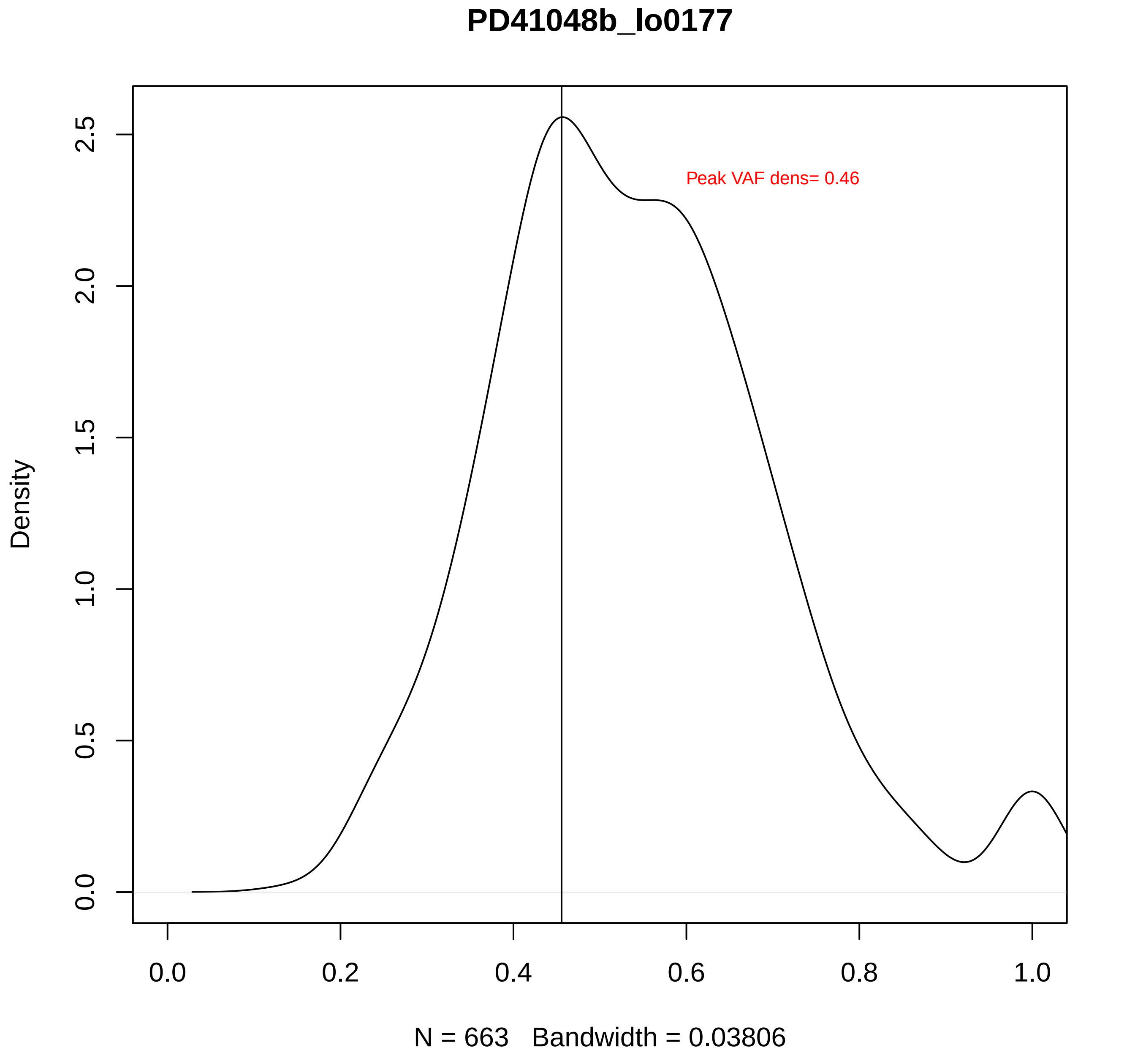

PD41048b_lo0177
Peak VAF dens= 0.46
Density
0.0
0.5
1.0
1.5
2.0
2.5
0.0
0.2
0.4
0.6
0.8
1.0
N = 663 Bandwidth = 0.03806

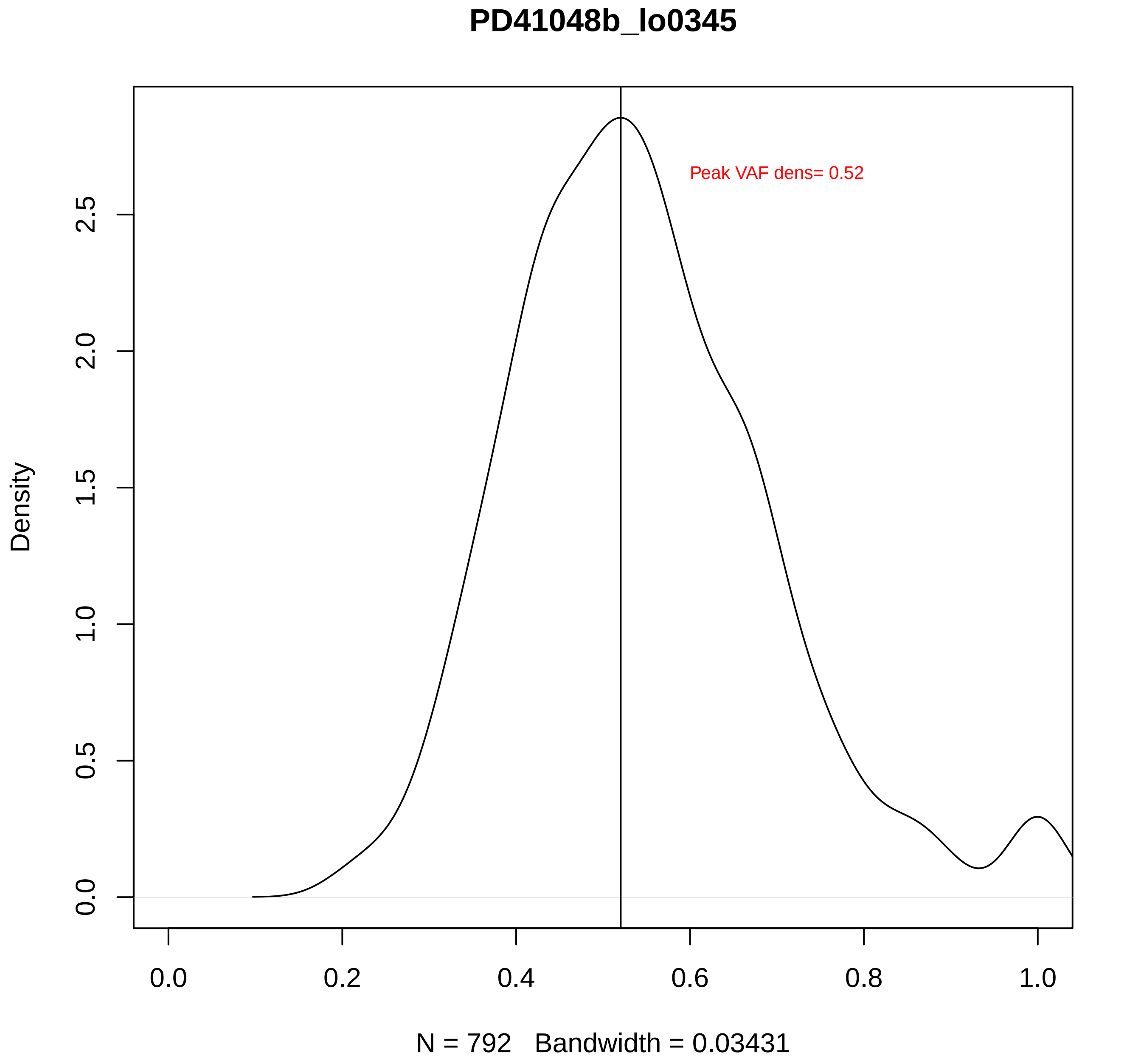
PD41048b_lo0345
Peak VAF dens= 0.52
Density
0.0
0.5
1.0
1.5
2.0
2.5
0.0
0.2
0.4
0.6
0.8
1.0
N = 792 Bandwidth = 0.03431

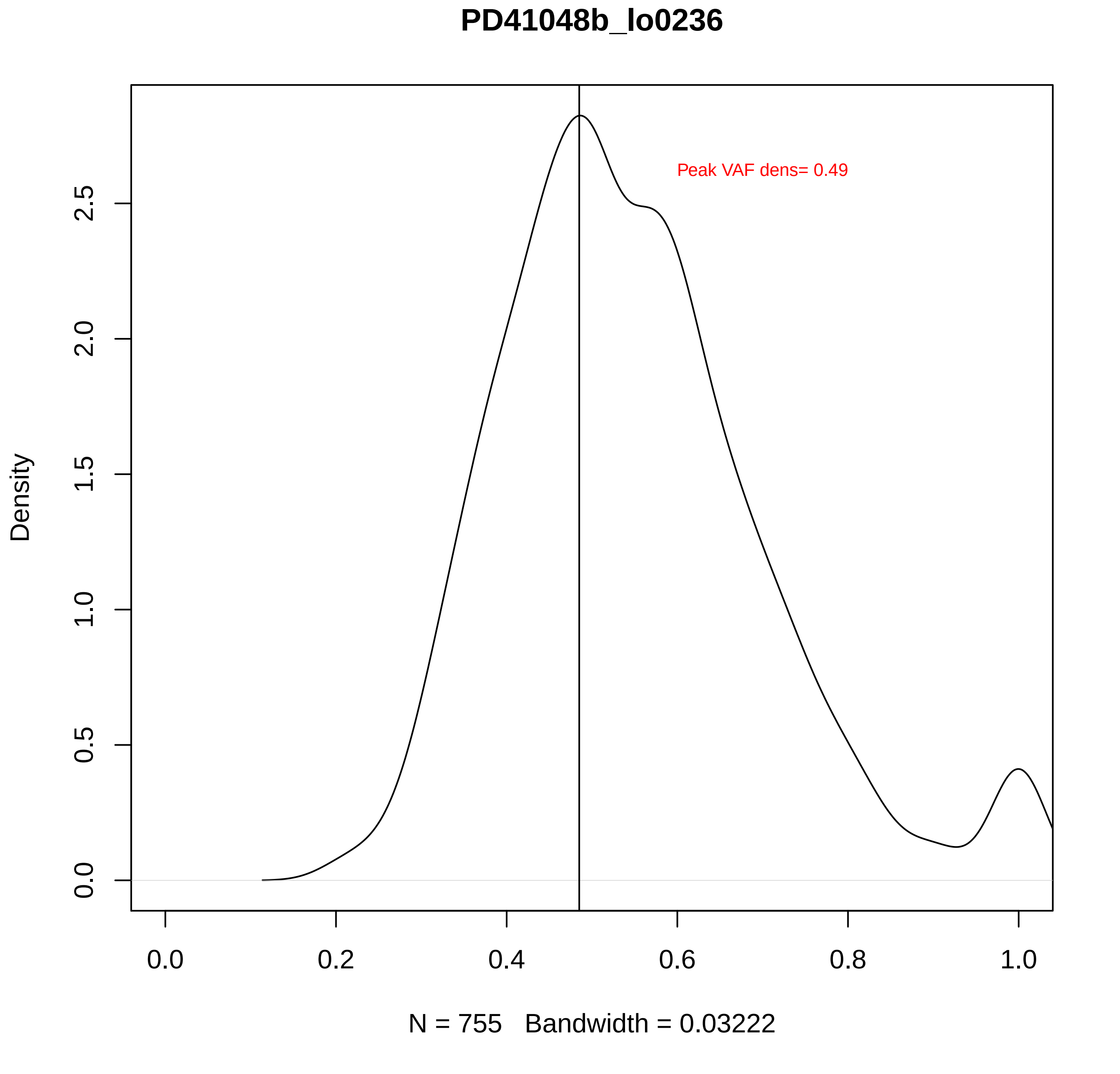
PD41048b_lo0236
Peak VAF dens= 0.49
Density
0.0
0.5
1.0
1.5
2.0
2.5
0.0
0.2
0.4
0.6
0.8
1.0
N = 755   Bandwidth = 0.03222

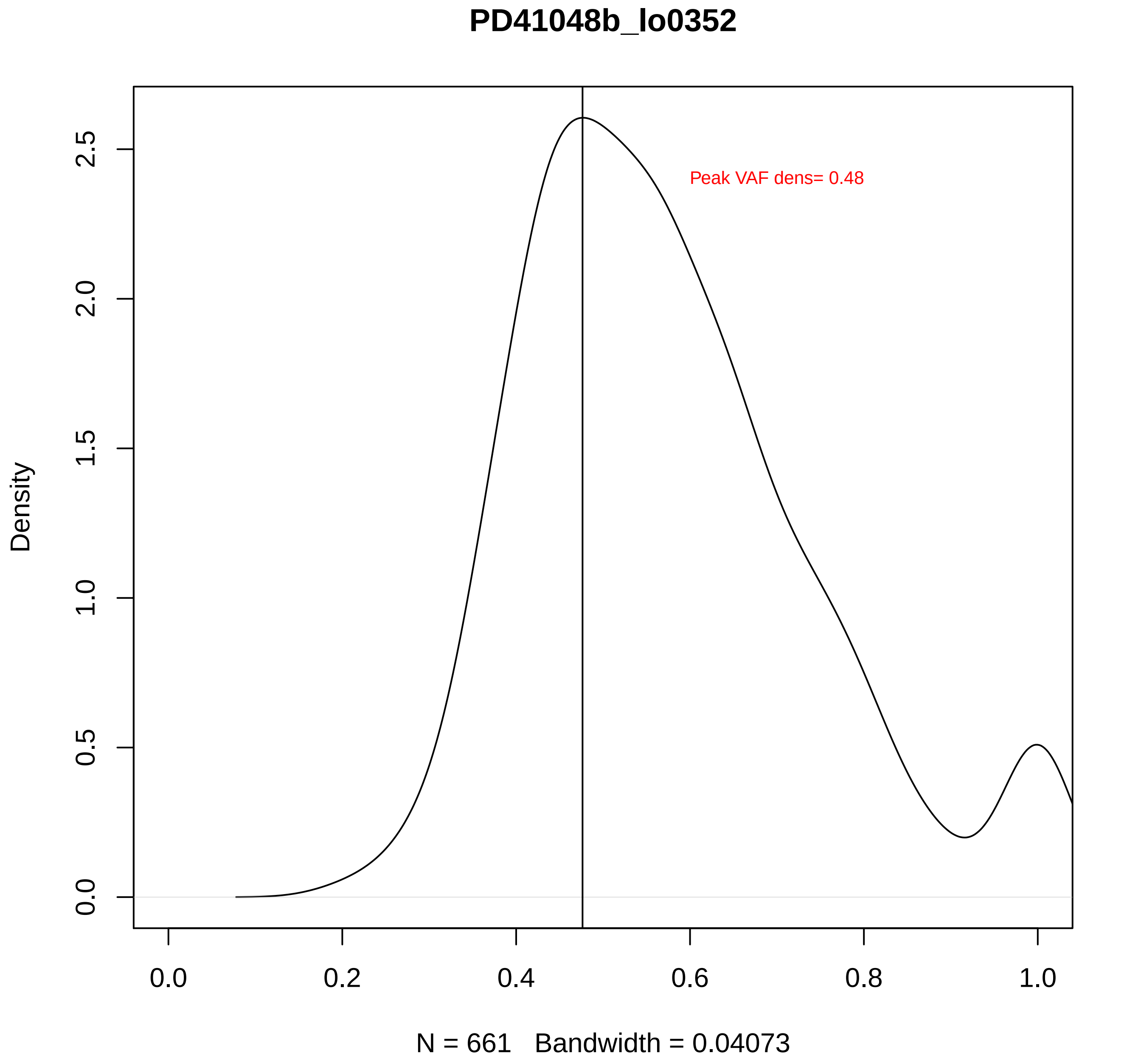
PD41048b_lo0352
Peak VAF dens= 0.48
Density
0.0
0.5
1.0
1.5
2.0
2.5
0.0
0.2
0.4
0.6
0.8
1.0
N = 661 Bandwidth = 0.04073

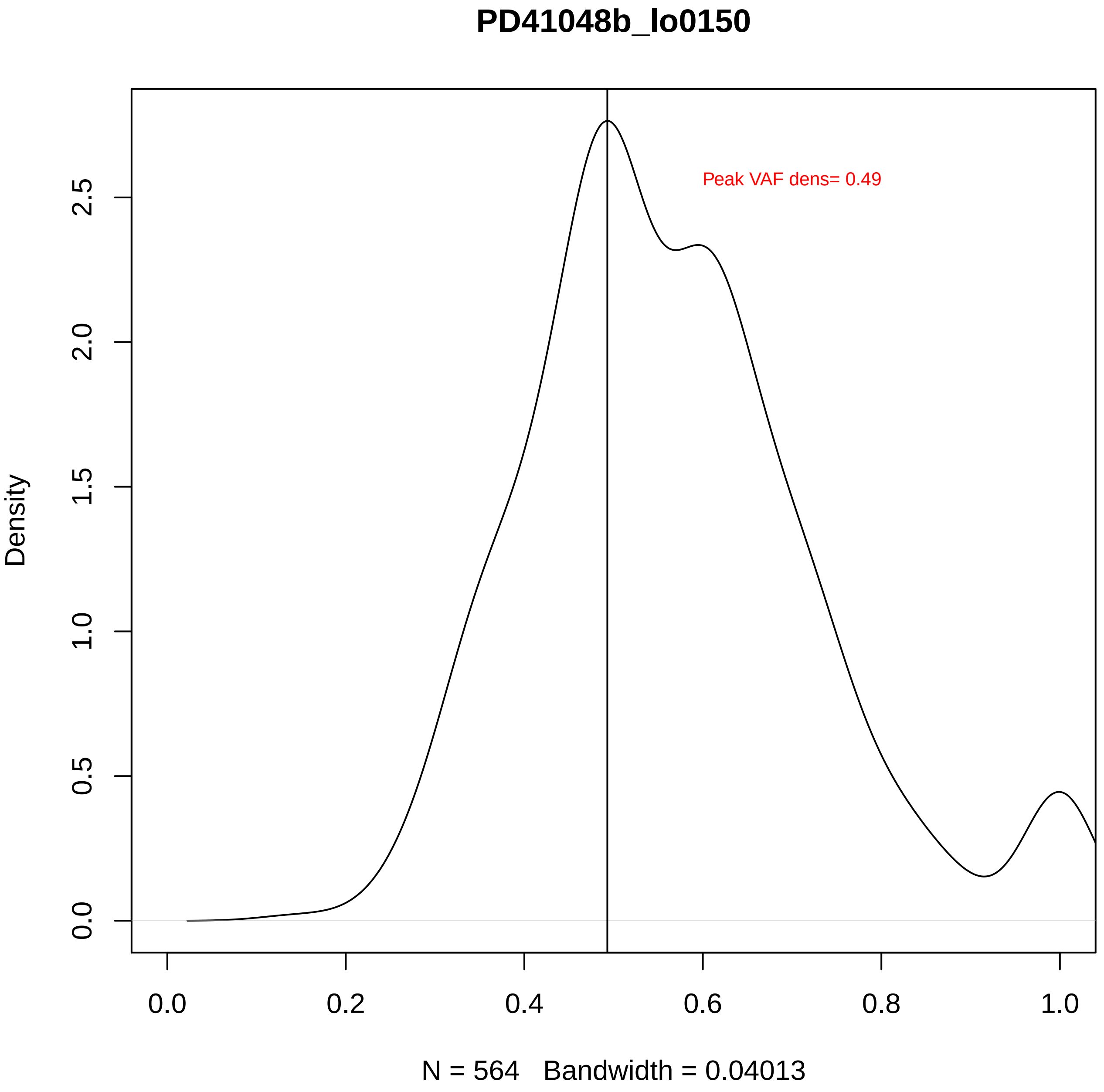

PD41048b_lo0150
Peak VAF dens= 0.49
Density
0.0
0.5
1.0
1.5
2.0
2.5
0.0
0.2
0.4
0.6
0.8
1.0
N = 564 Bandwidth = 0.04013

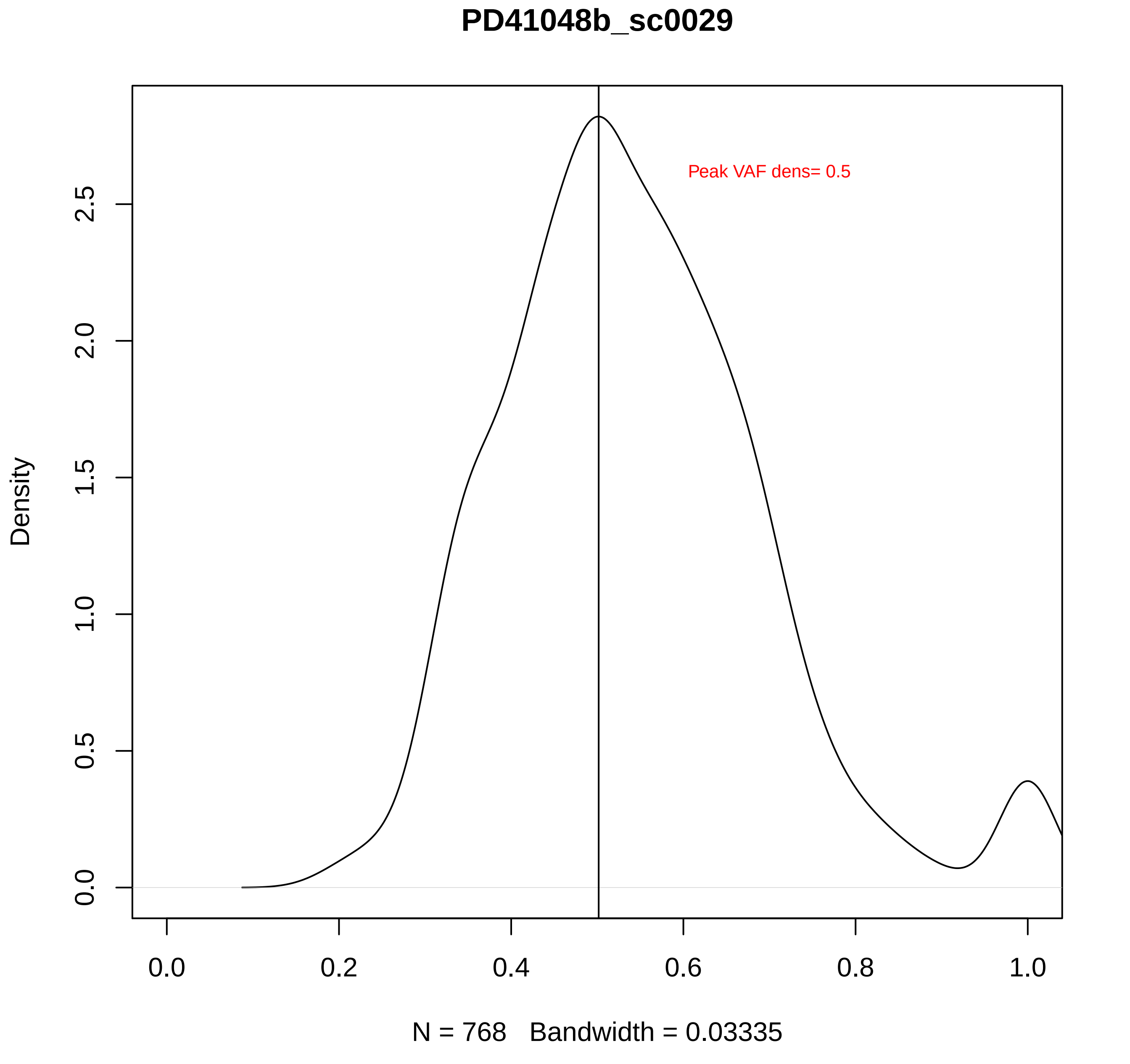
PD41048b_sc0029
Peak VAF dens= 0.5
Density
0.0
0.5
1.0
1.5
2.0
2.5
0.0
0.2
0.4
0.6
0.8
1.0
N = 768   Bandwidth = 0.03335

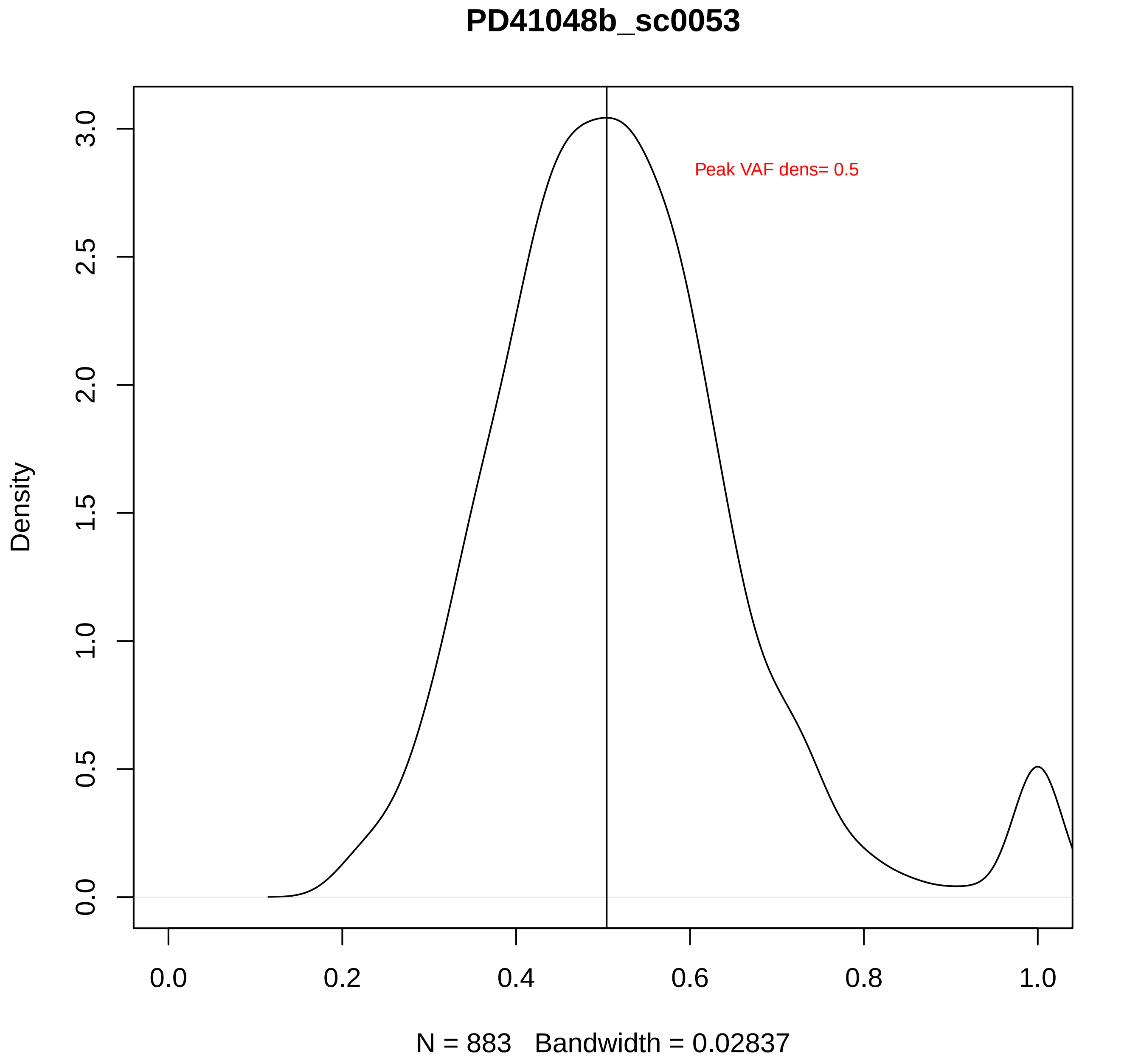
PD41048b_sc0053
Peak VAF dens= 0.5
Density
0.0
0.5
1.0
1.5
2.0
2.5
3.0
0.0
0.2
0.4
0.6
0.8
1.0
N = 883 Bandwidth = 0.02837

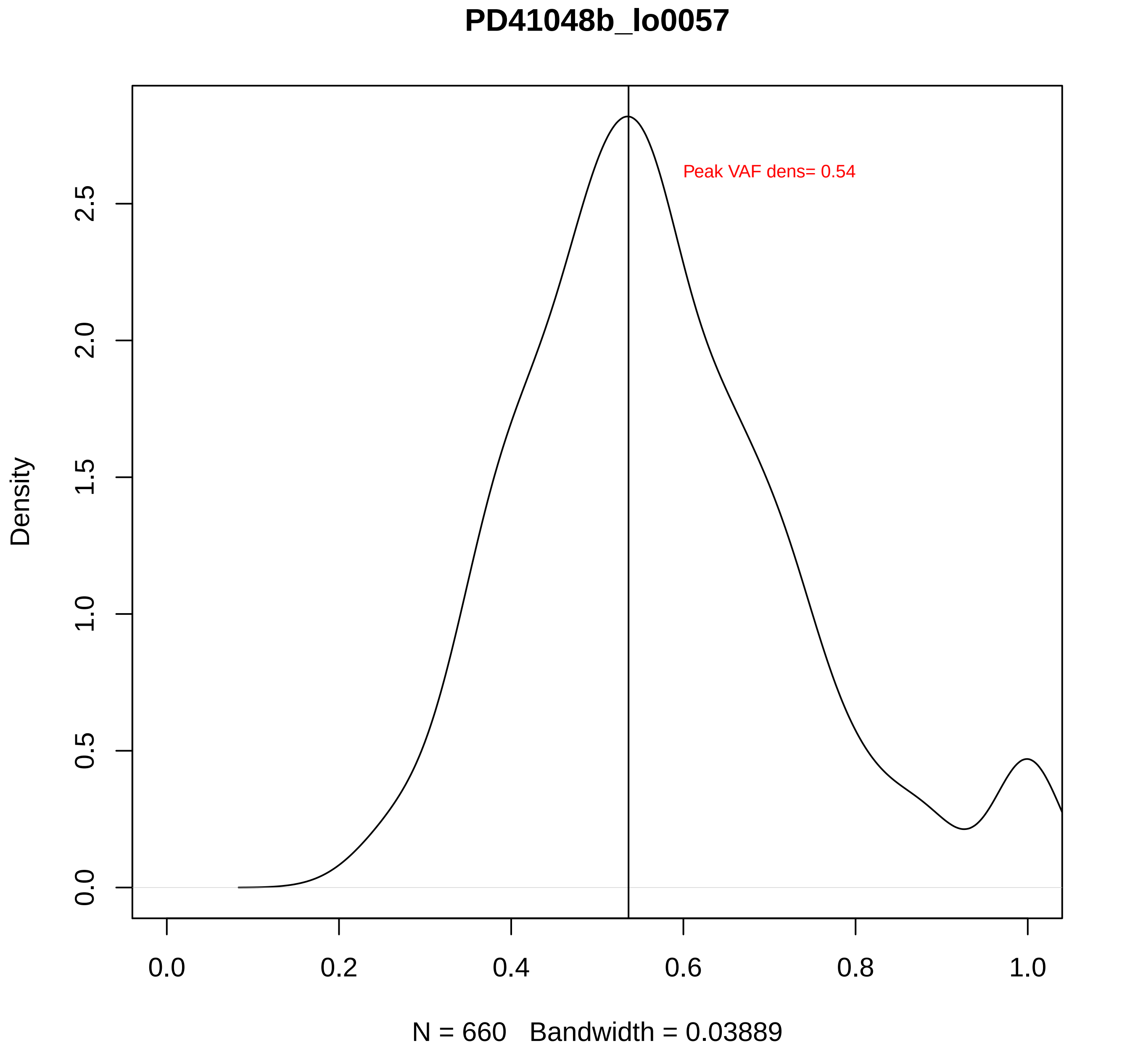

PD41048b_lo0057
Peak VAF dens= 0.54
Density
0.0
0.5
1.0
1.5
2.0
2.5
0.0
0.2
0.4
0.6
0.8
1.0
N = 660 Bandwidth = 0.03889

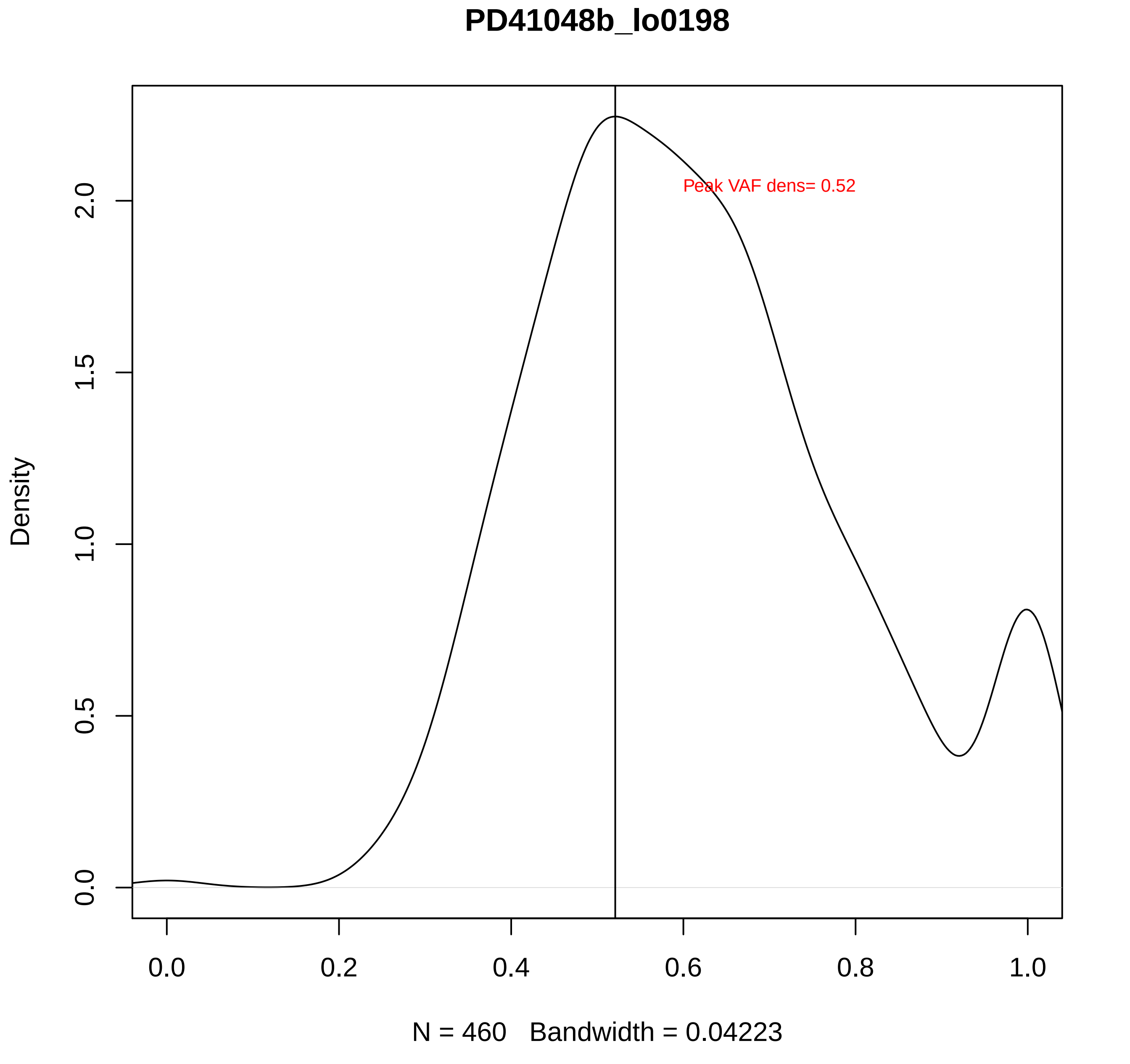

PD41048b_lo0198
Peak VAF dens= 0.52
Density
0.0
0.5
1.0
1.5
2.0
0.0
0.2
0.4
0.6
0.8
1.0
N = 460   Bandwidth = 0.04223

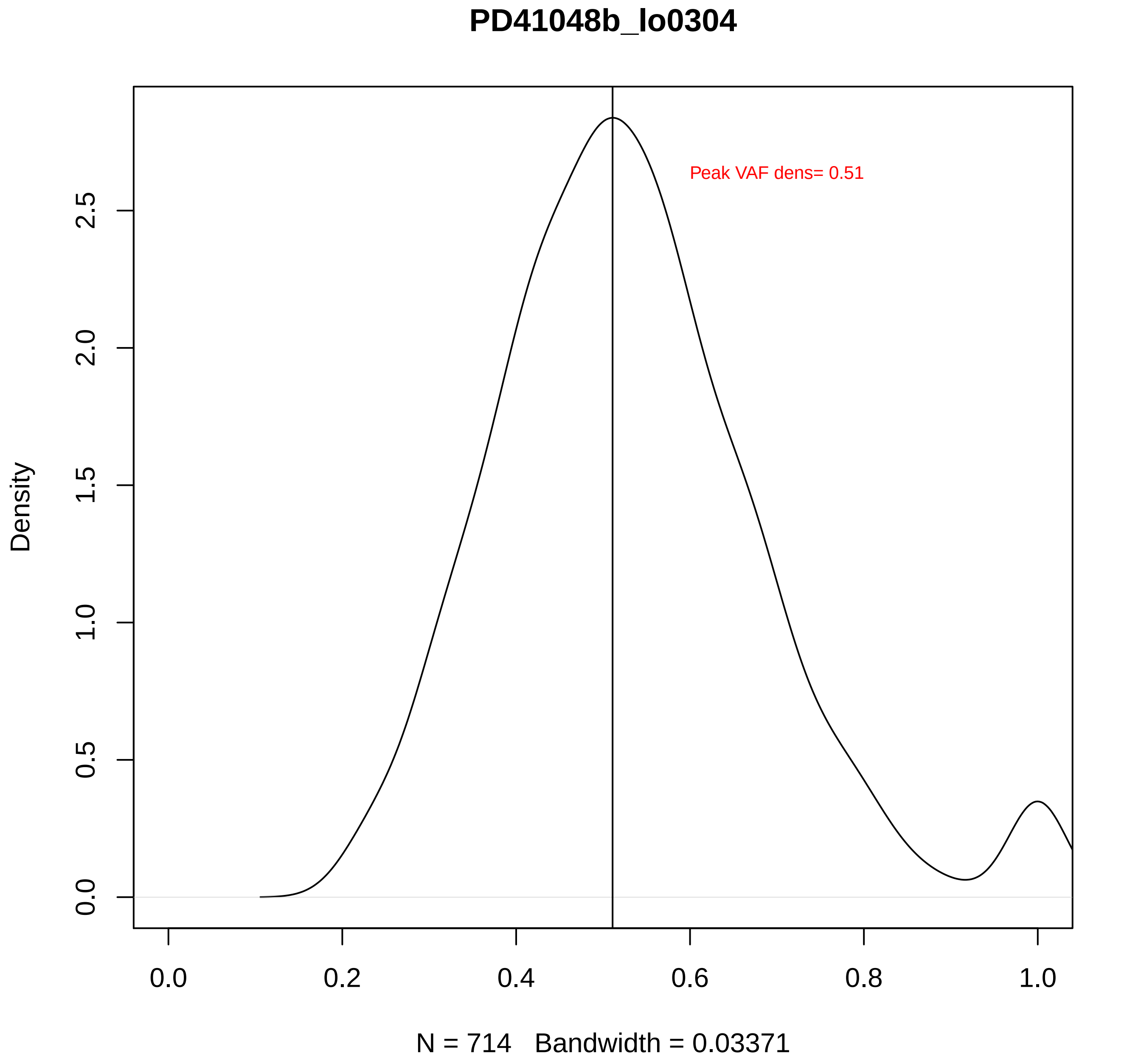

PD41048b_lo0304
Peak VAF dens= 0.51
Density
0.0
0.5
1.0
1.5
2.0
2.5
0.0
0.2
0.4
0.6
0.8
1.0
N = 714 Bandwidth = 0.03371

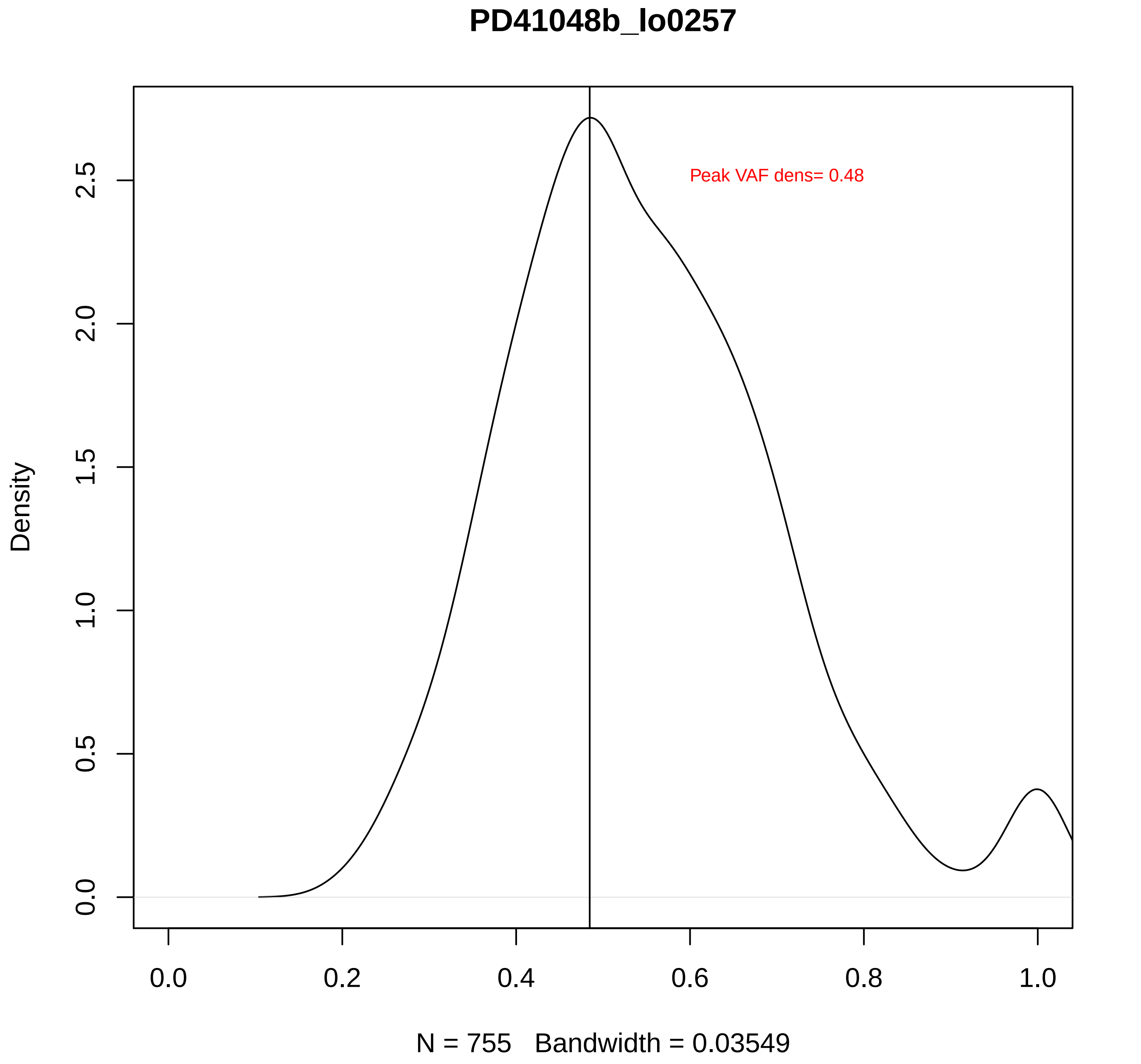
PD41048b_lo0257
Peak VAF dens= 0.48
Density
0.0
0.5
1.0
1.5
2.0
2.5
0.0
0.2
0.4
0.6
0.8
1.0
N = 755   Bandwidth = 0.03549
