# Supplementary material for: Clonal dynamics of haematopoiesis across the human lifespan
Source: Nature. 2022 Jun 1;606(7913):343–50. doi: 10.1038/s41586-022-04786-y (PMC9177428; doi:10.1038/s41586-022-04786-y)
Supplement: Supplementary file 4 — HTMLs of notebooks outlining key statistical analyses presented in the manuscript, including analysis of phylogenetic trees. [file 41586_2022_4786_MOESM4_ESM.zip › Supplementary_code/SNV_indel_analysis/KX002_sample_vaf_plots.pdf]

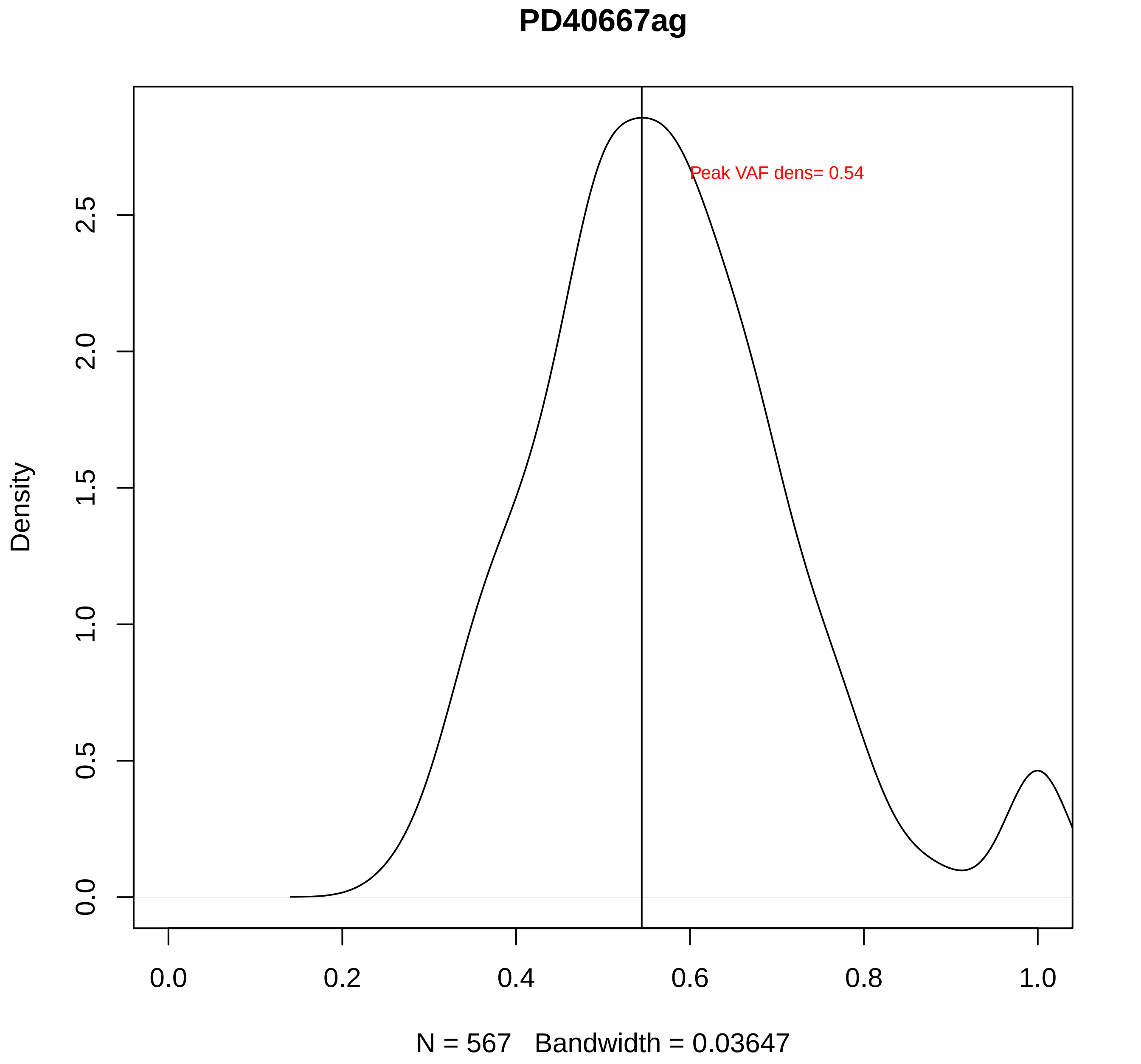
PD40667ag
Peak VAF dens= 0.54
Density
0.0
0.5
1.0
1.5
2.0
2.5
0.0
0.2
0.4
0.6
0.8
1.0
N = 567   Bandwidth = 0.03647

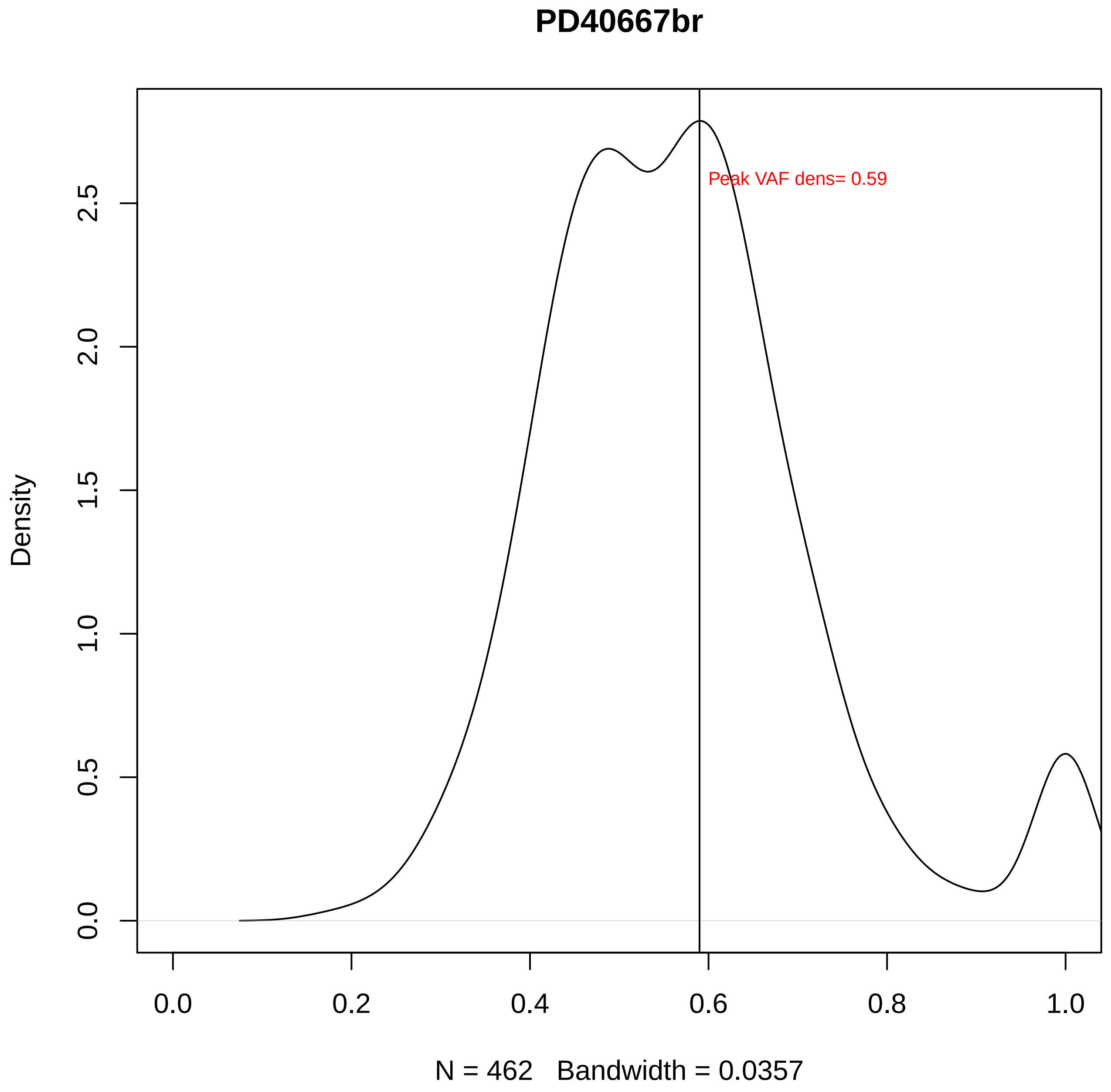

PD40667br
Peak VAF dens= 0.59
Density
0.0
0.5
1.0
1.5
2.0
2.5
0.0
0.2
0.4
0.6
0.8
1.0
N = 462 Bandwidth = 0.0357

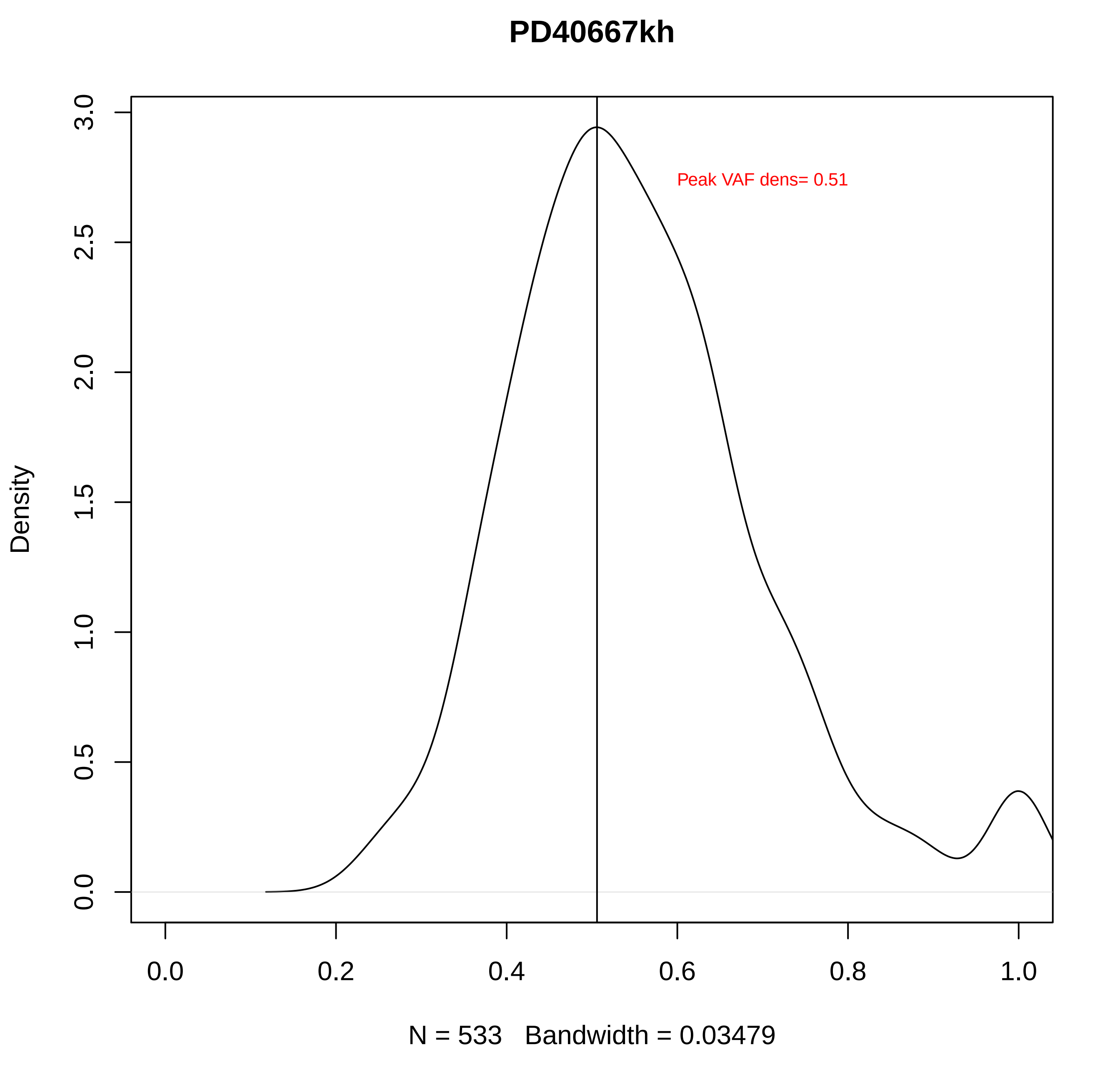

PD40667kh
Peak VAF dens= 0.51
Density
3.0
2.5
2.0
1.5
1.0
0.5
0.0
0.0
0.2
0.4
0.6
0.8
1.0
N = 533   Bandwidth = 0.03479

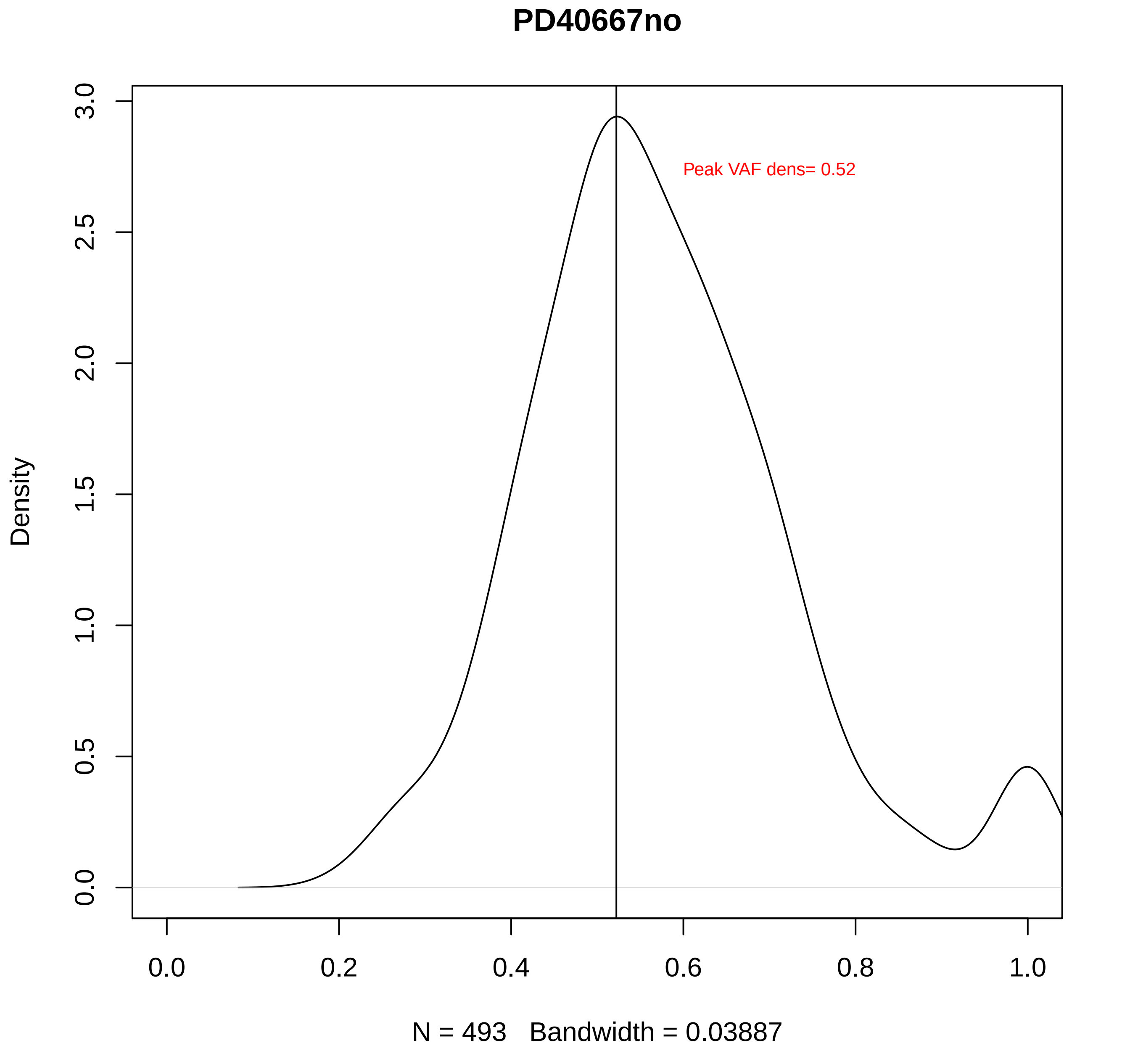
PD40667no
Peak VAF dens= 0.52
Density
0.0
0.5
1.0
1.5
2.0
2.5
3.0
0.0
0.2
0.4
0.6
0.8
1.0
N = 493 Bandwidth = 0.03887

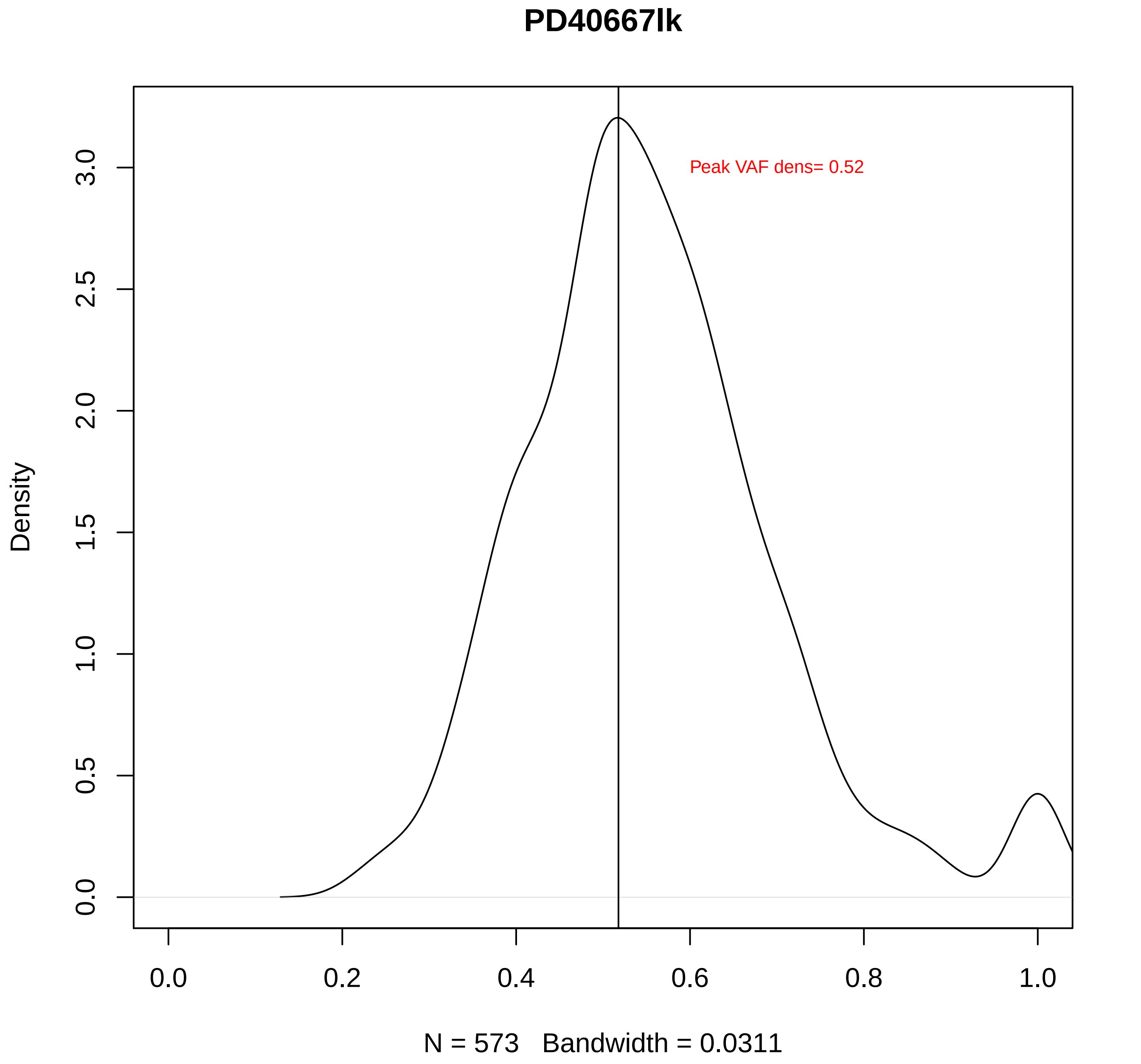

PD40667lk
Peak VAF dens= 0.52
Density
0.0
0.5
1.0
1.5
2.0
2.5
3.0
0.0
0.2
0.4
0.6
0.8
1.0
N = 573   Bandwidth = 0.0311

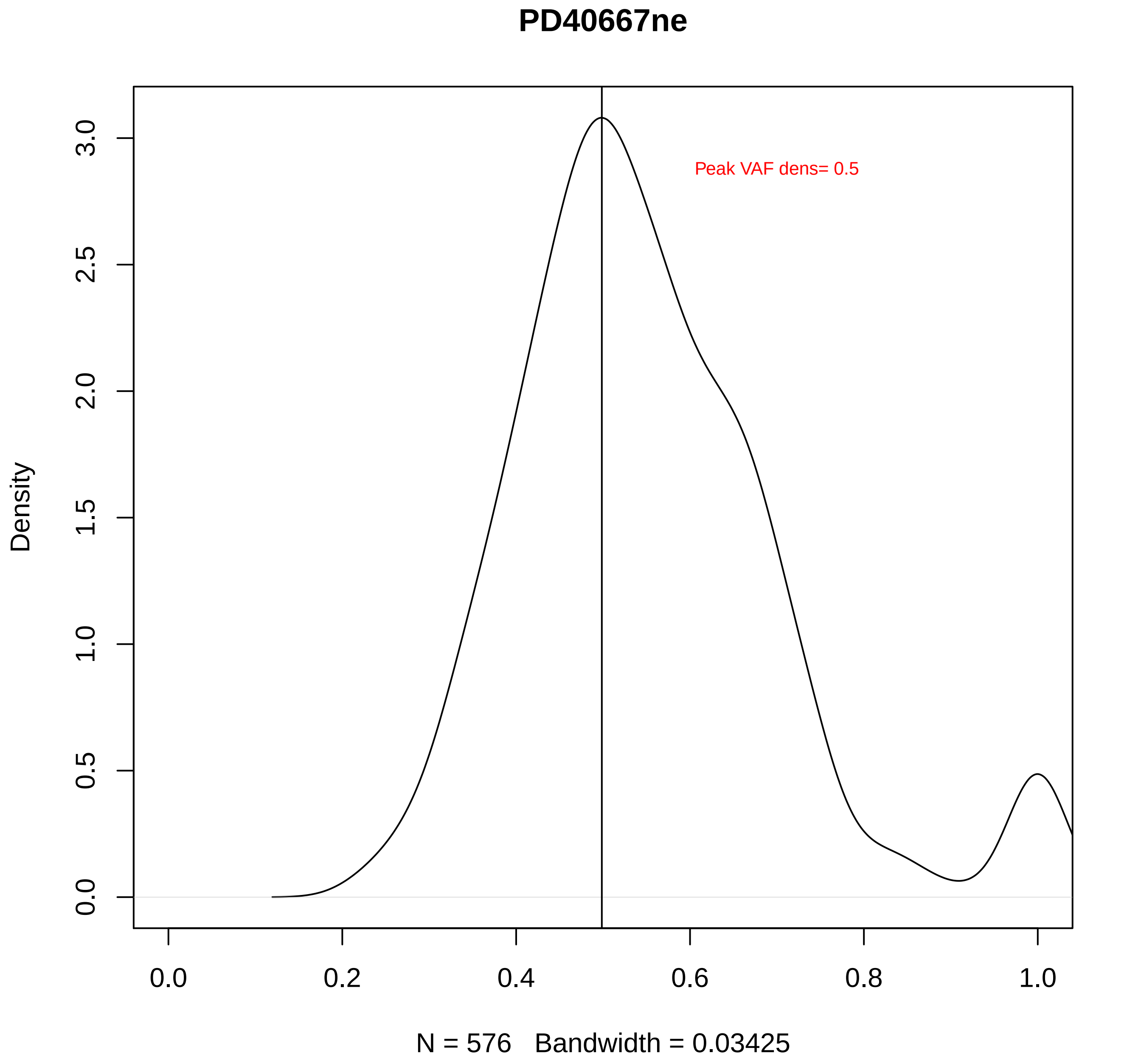
PD40667ne
Peak VAF dens= 0.5
Density
0.0
0.5
1.0
1.5
2.0
2.5
3.0
0.0
0.2
0.4
0.6
0.8
1.0
N = 576   Bandwidth = 0.03425

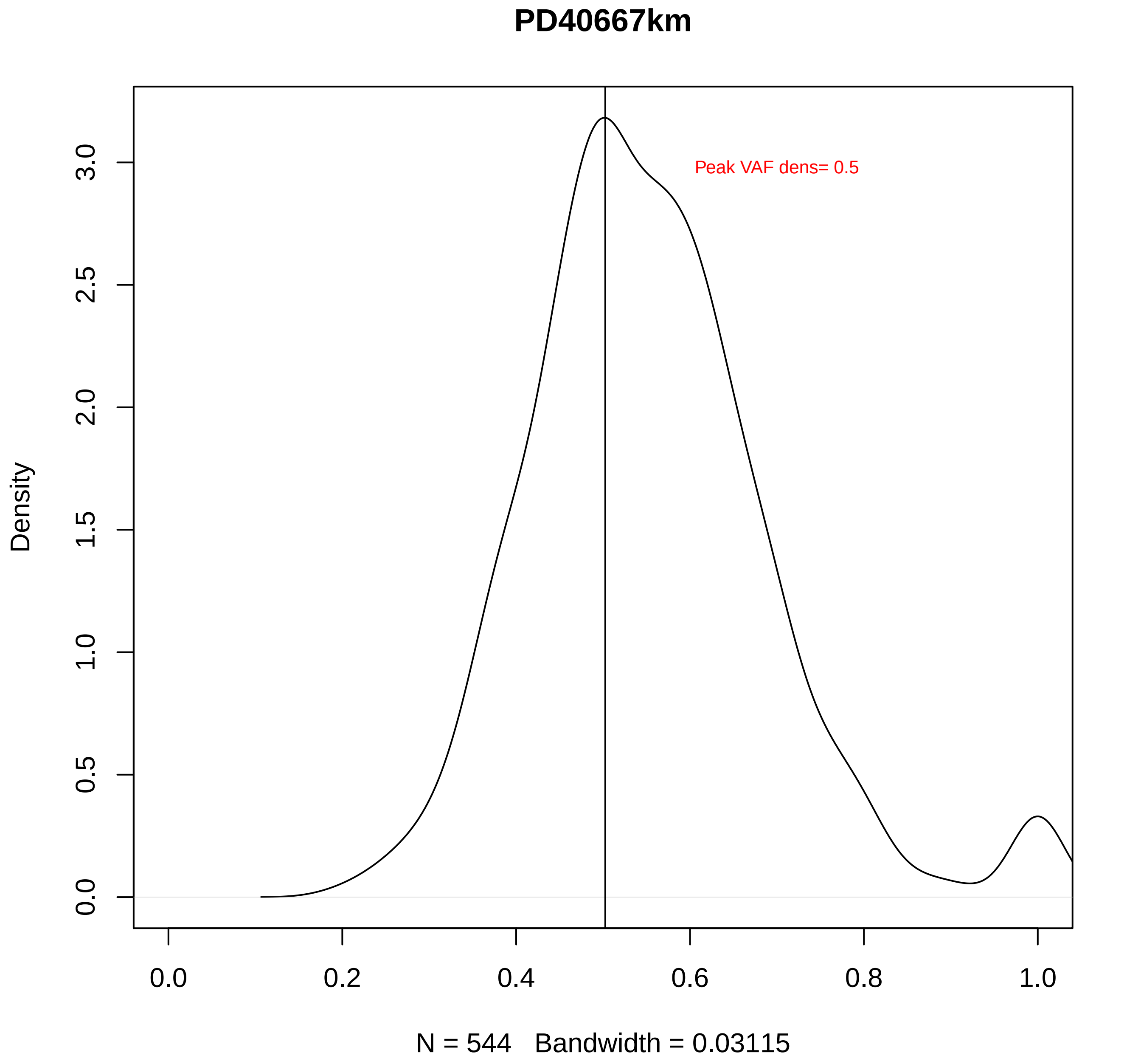
PD40667km
Peak VAF dens= 0.5
Density
0.0
0.5
1.0
1.5
2.0
2.5
3.0
0.0
0.2
0.4
0.6
0.8
1.0
N = 544   Bandwidth = 0.03115

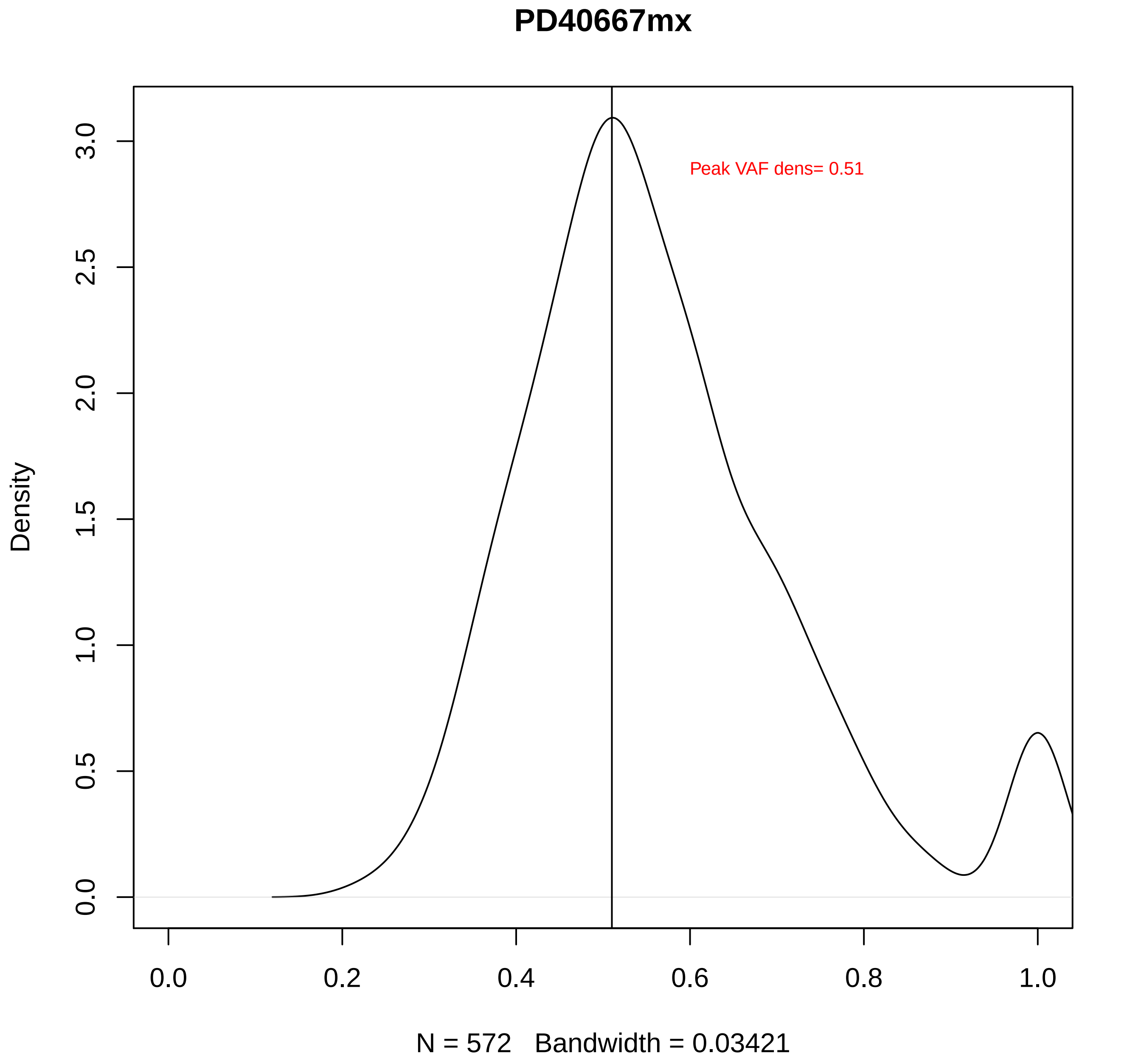
PD40667mx
Peak VAF dens= 0.51
Density
0.0
0.5
1.0
1.5
2.0
2.5
3.0
0.0
0.2
0.4
0.6
0.8
1.0
N = 572   Bandwidth = 0.03421

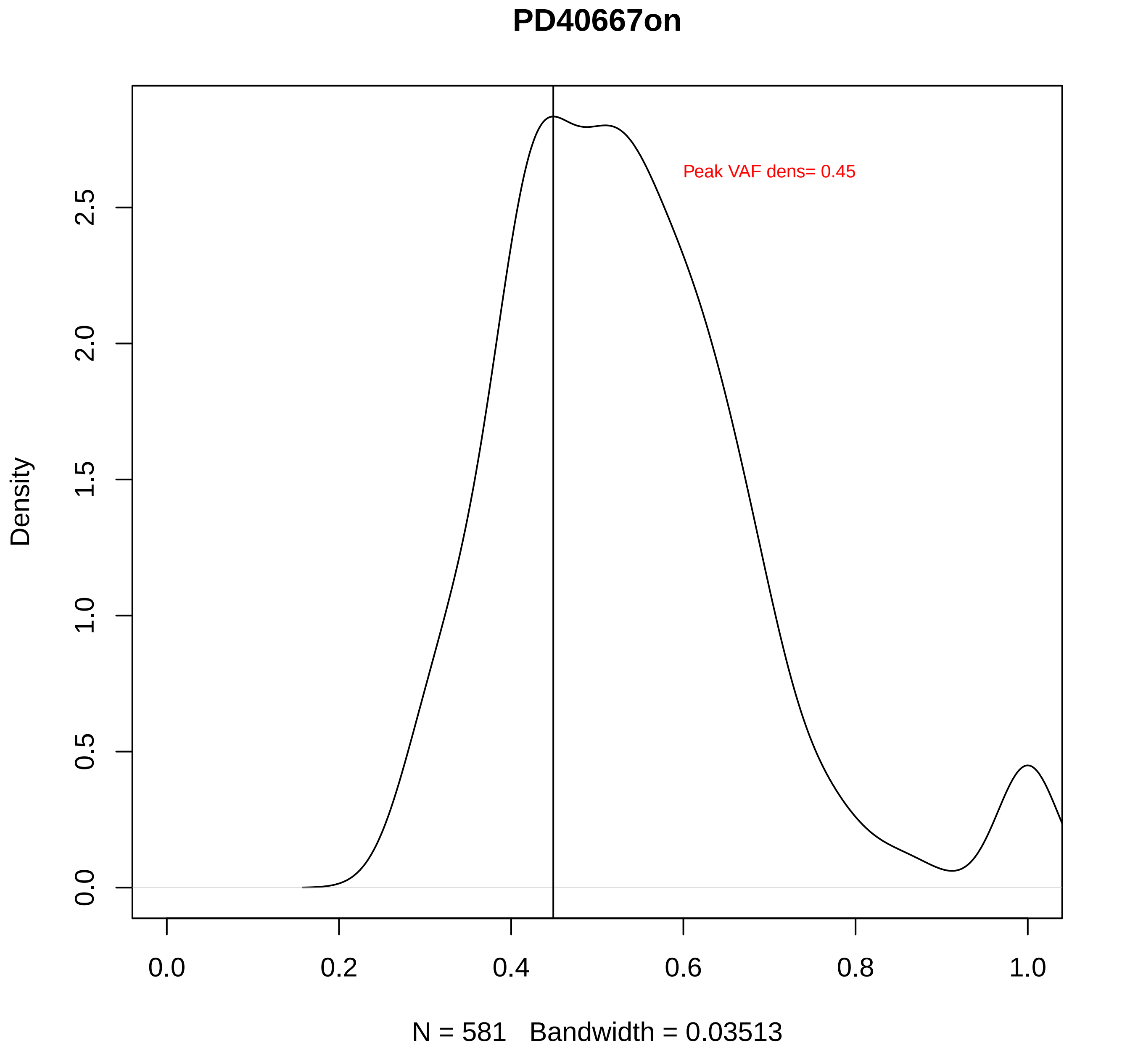
PD40667on
Peak VAF dens= 0.45
Density
0.0
0.5
1.0
1.5
2.0
2.5
0.0
0.2
0.4
0.6
0.8
1.0
N = 581   Bandwidth = 0.03513

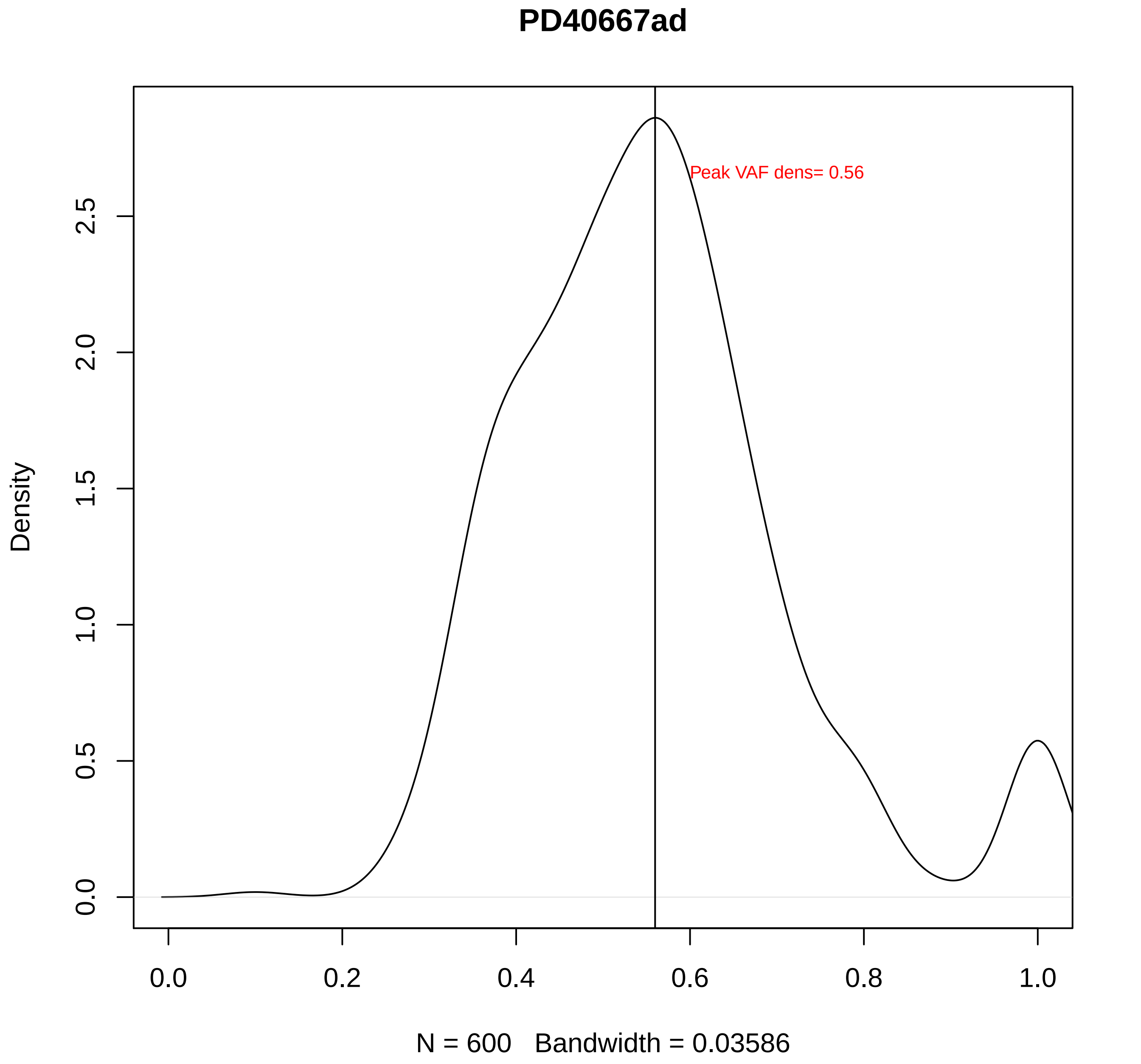
PD40667ad
Peak VAF dens= 0.56
Density
0.0
0.5
1.0
1.5
2.0
2.5
0.0
0.2
0.4
0.6
0.8
1.0
N = 600   Bandwidth = 0.03586

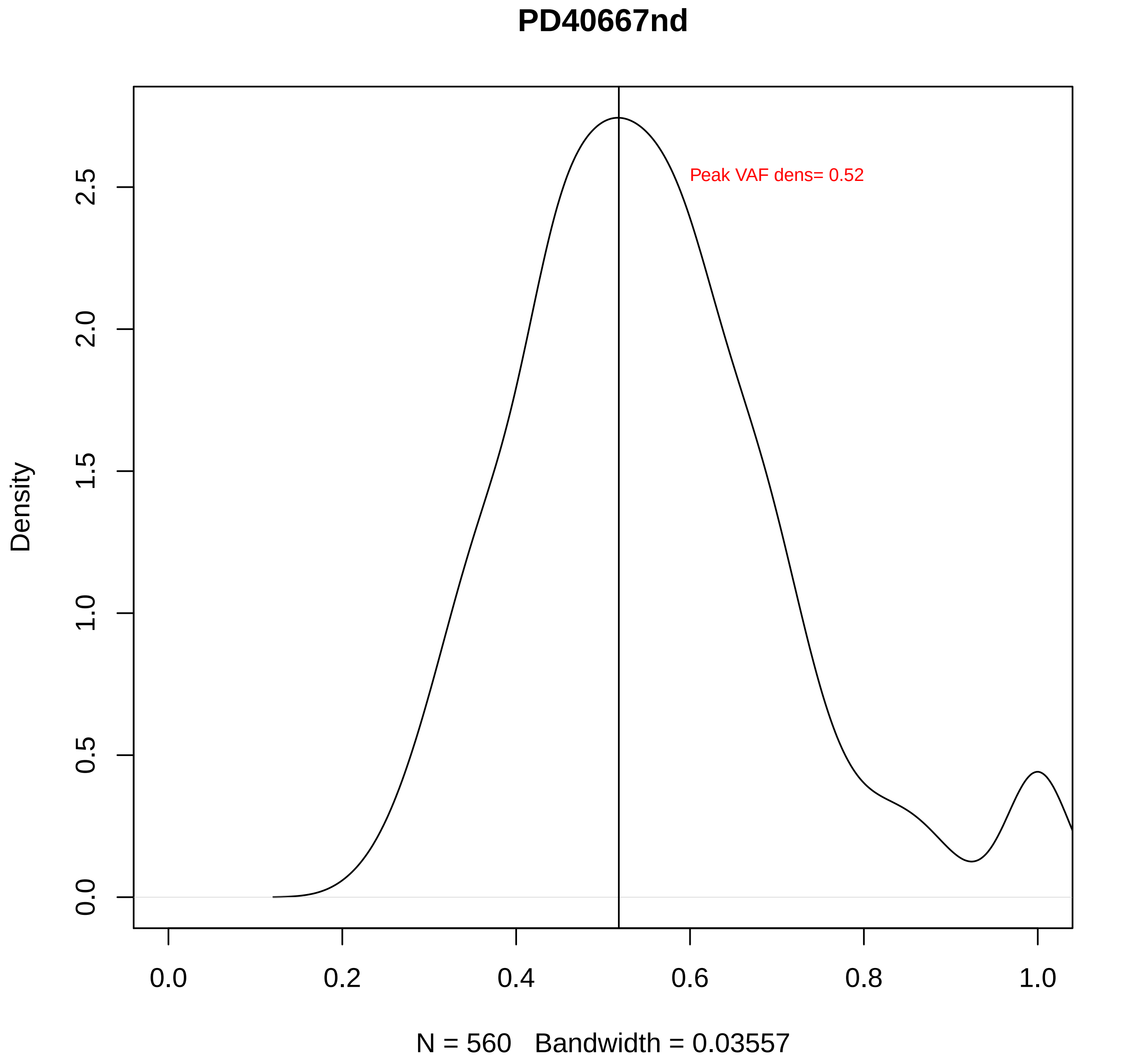
PD40667nd
Peak VAF dens= 0.52
Density
0.0
0.5
1.0
1.5
2.0
2.5
0.0
0.2
0.4
0.6
0.8
1.0
N = 560   Bandwidth = 0.03557

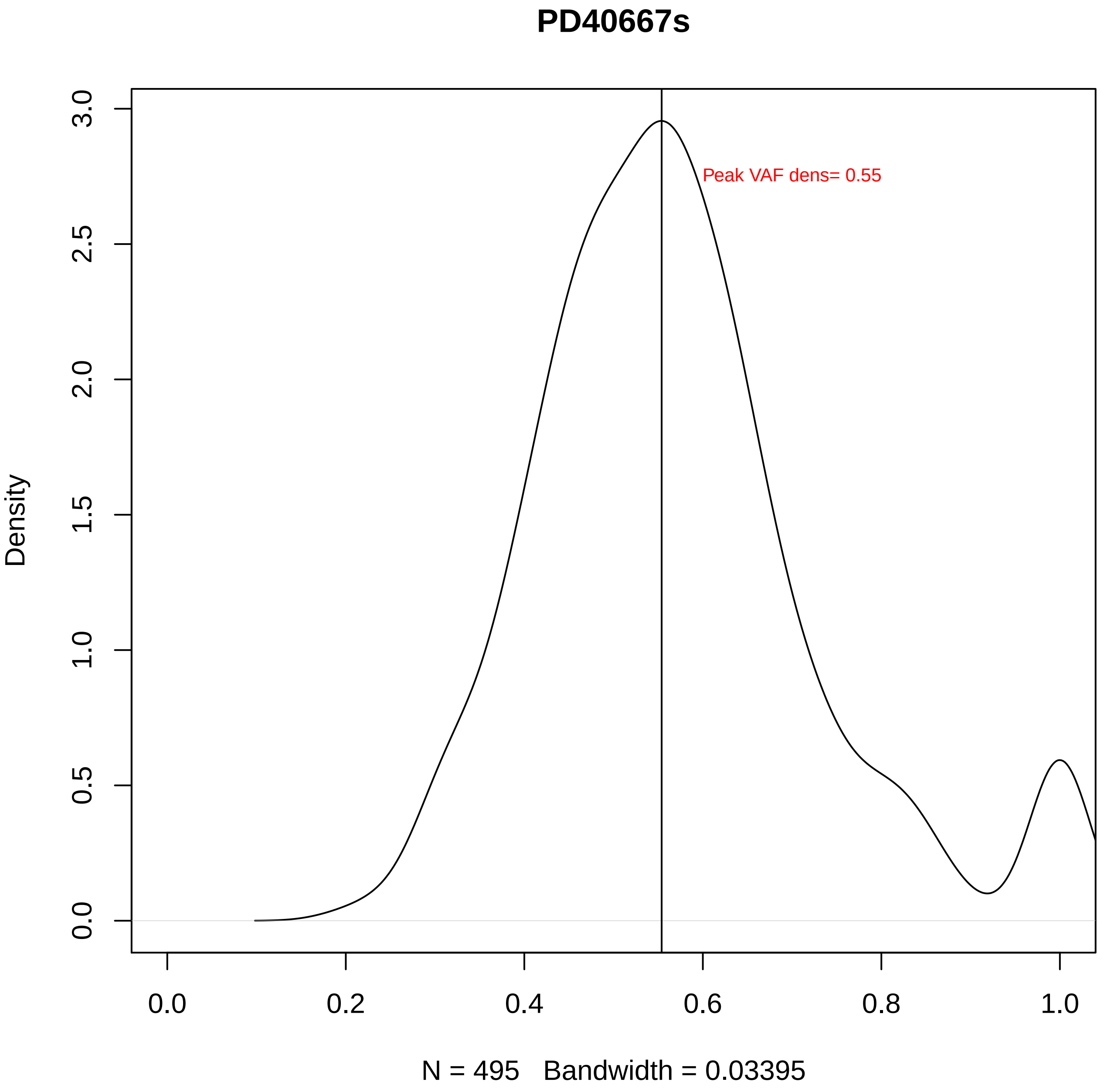
PD40667s
Peak VAF dens= 0.55
Density
0.0
0.5
1.0
1.5
2.0
2.5
3.0
0.0
0.2
0.4
0.6
0.8
1.0
N = 495   Bandwidth = 0.03395

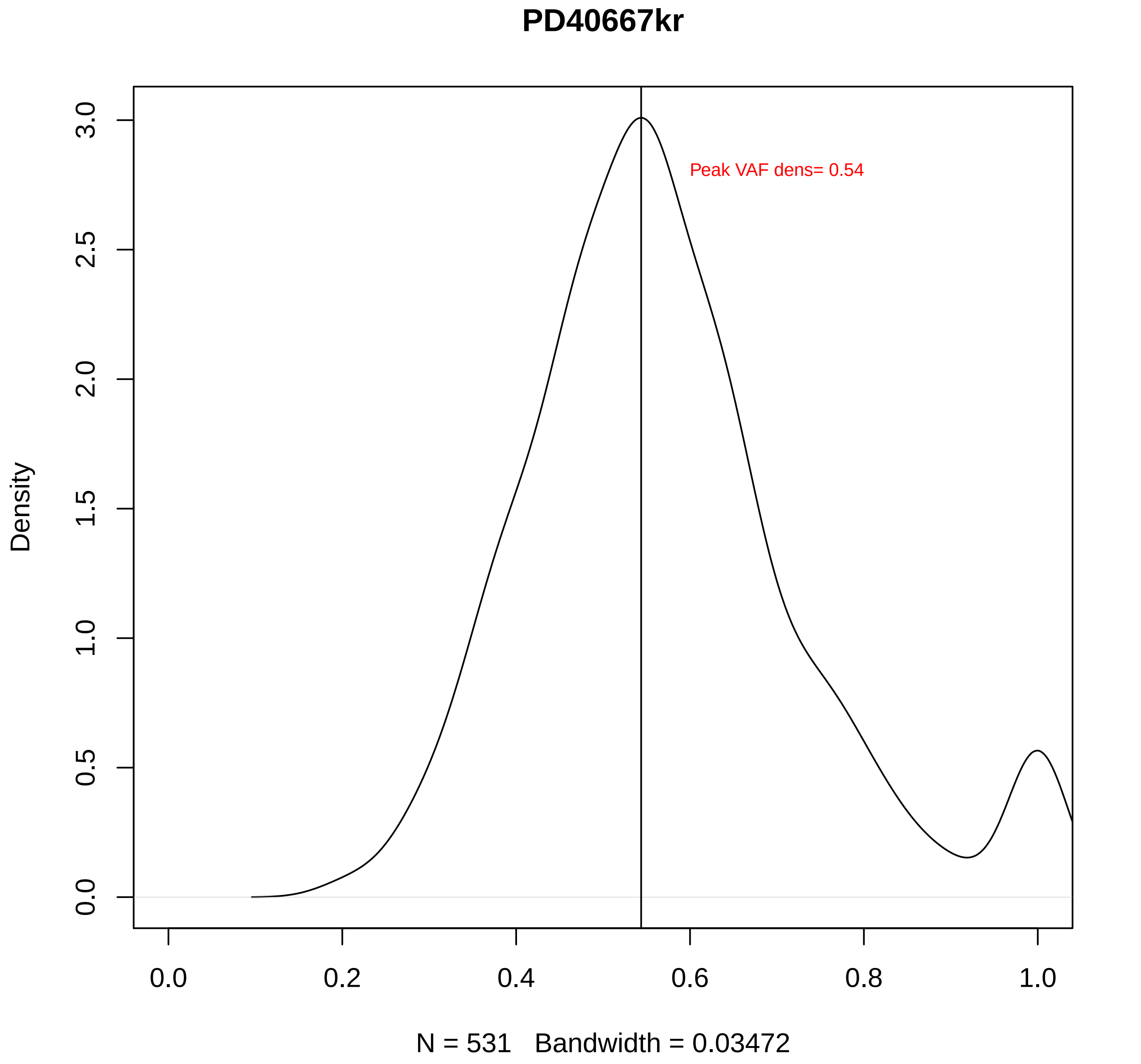

PD40667kr
Peak VAF dens= 0.54
Density
0.0
0.5
1.0
1.5
2.0
2.5
3.0
0.0
0.2
0.4
0.6
0.8
1.0
N = 531   Bandwidth = 0.03472

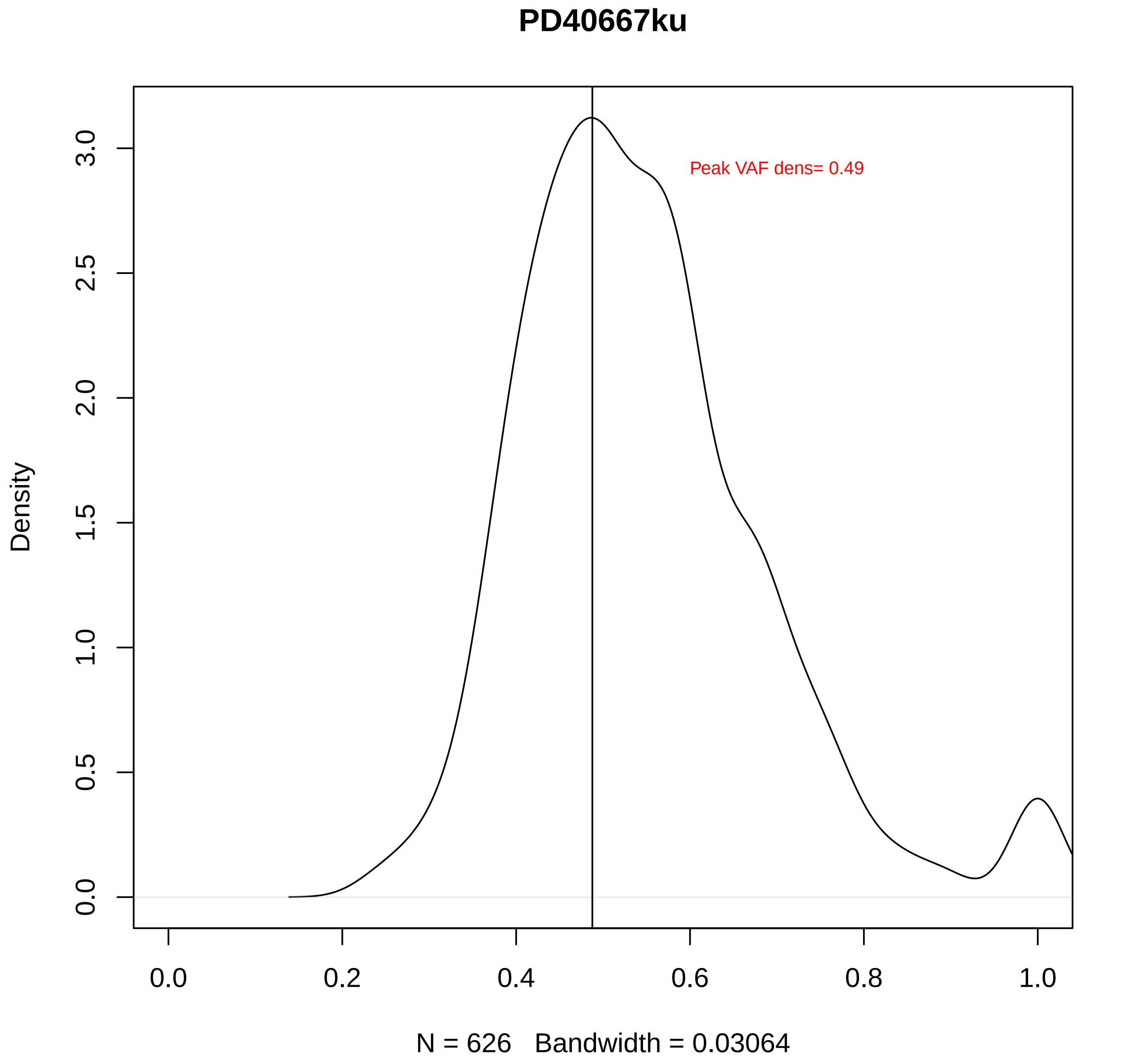
PD40667ku
Peak VAF dens= 0.49
Density
0.0
0.5
1.0
1.5
2.0
2.5
3.0
0.0
0.2
0.4
0.6
0.8
1.0
N = 626   Bandwidth = 0.03064

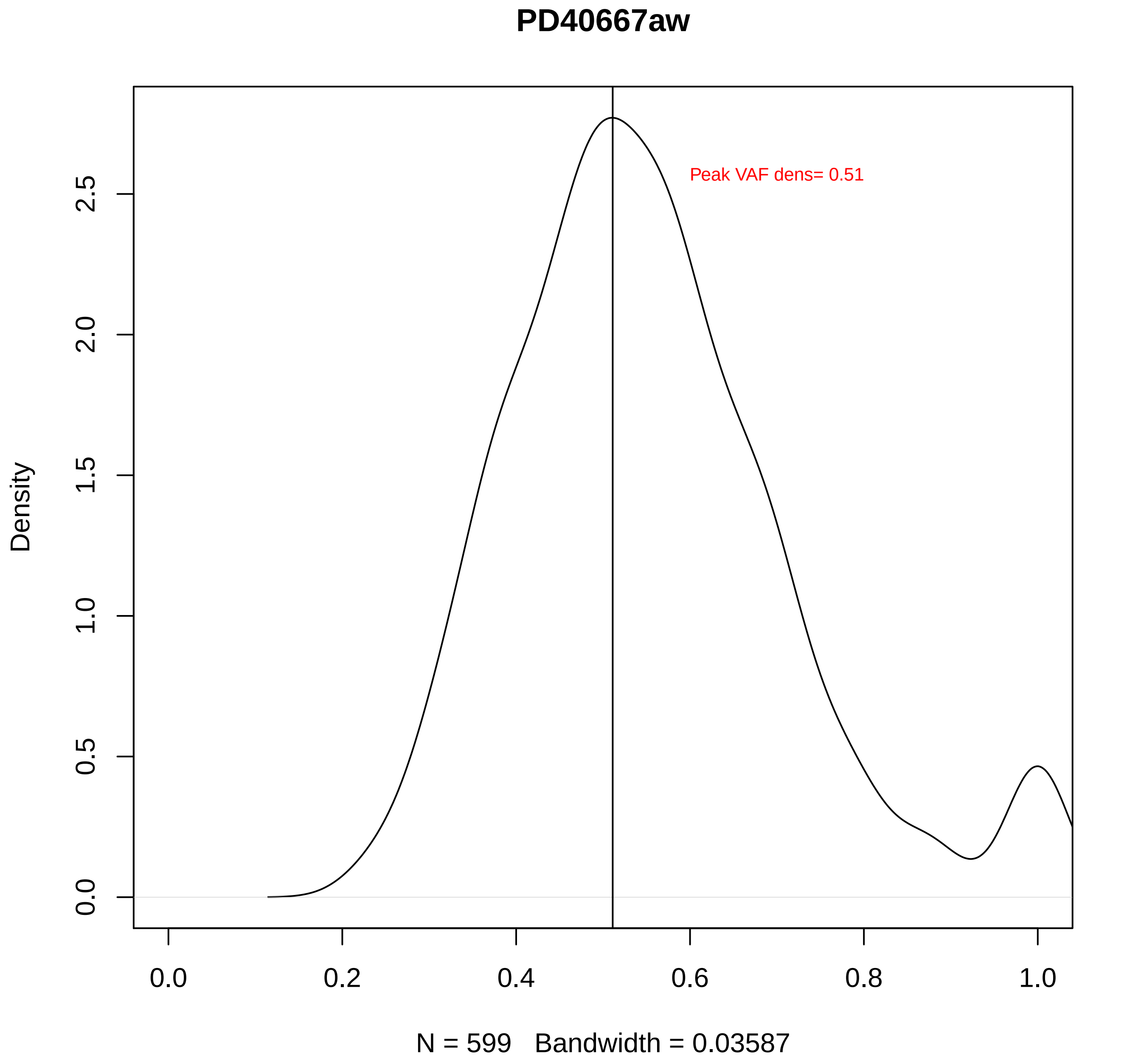

PD40667aw
Peak VAF dens= 0.51
Density
0.0
0.5
1.0
1.5
2.0
2.5
0.0
0.2
0.4
0.6
0.8
1.0
N = 599 Bandwidth = 0.03587

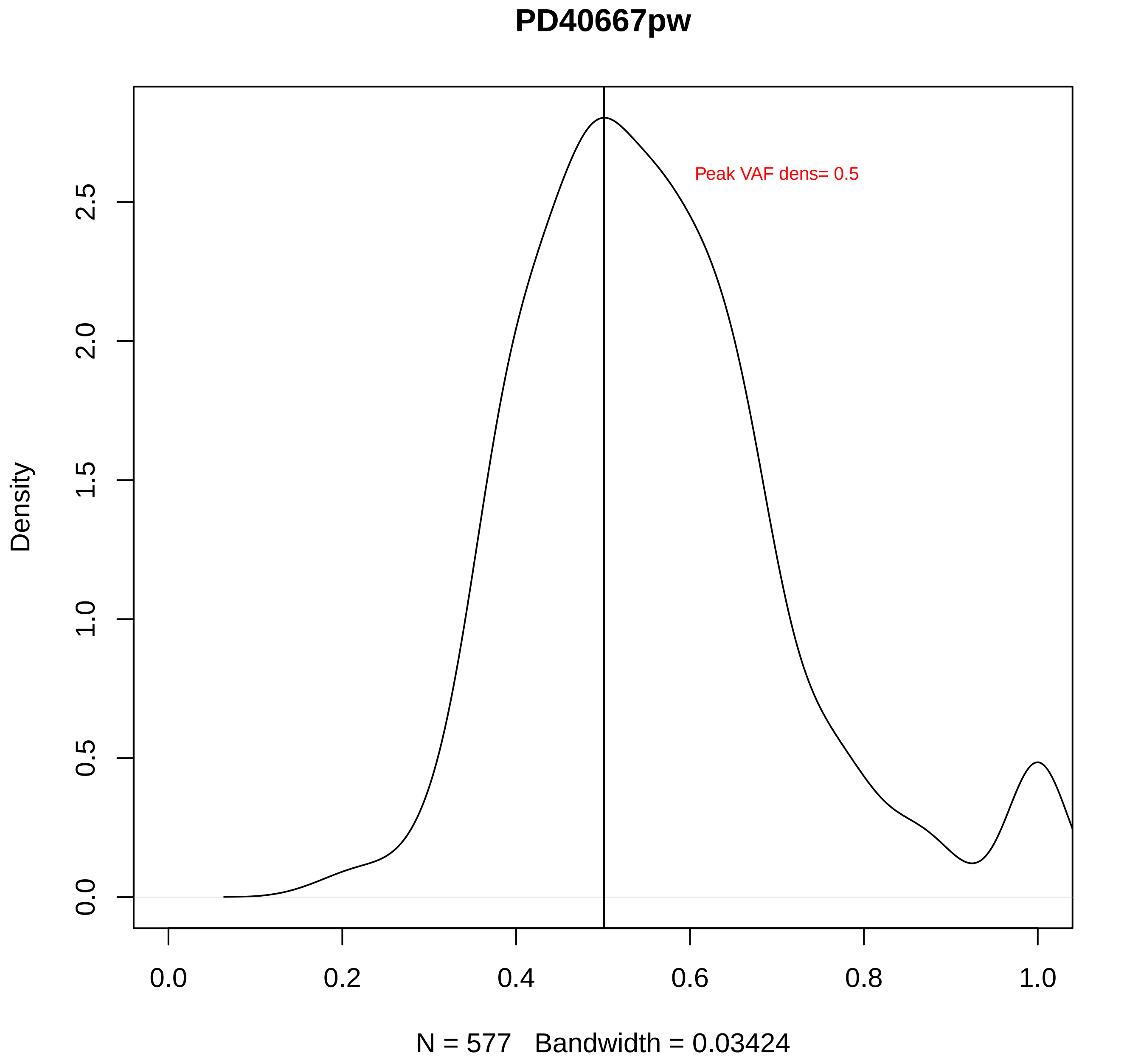
PD40667pw
Peak VAF dens= 0.5
Density
0.0
0.5
1.0
1.5
2.0
2.5
0.0
0.2
0.4
0.6
0.8
1.0
N = 577   Bandwidth = 0.03424

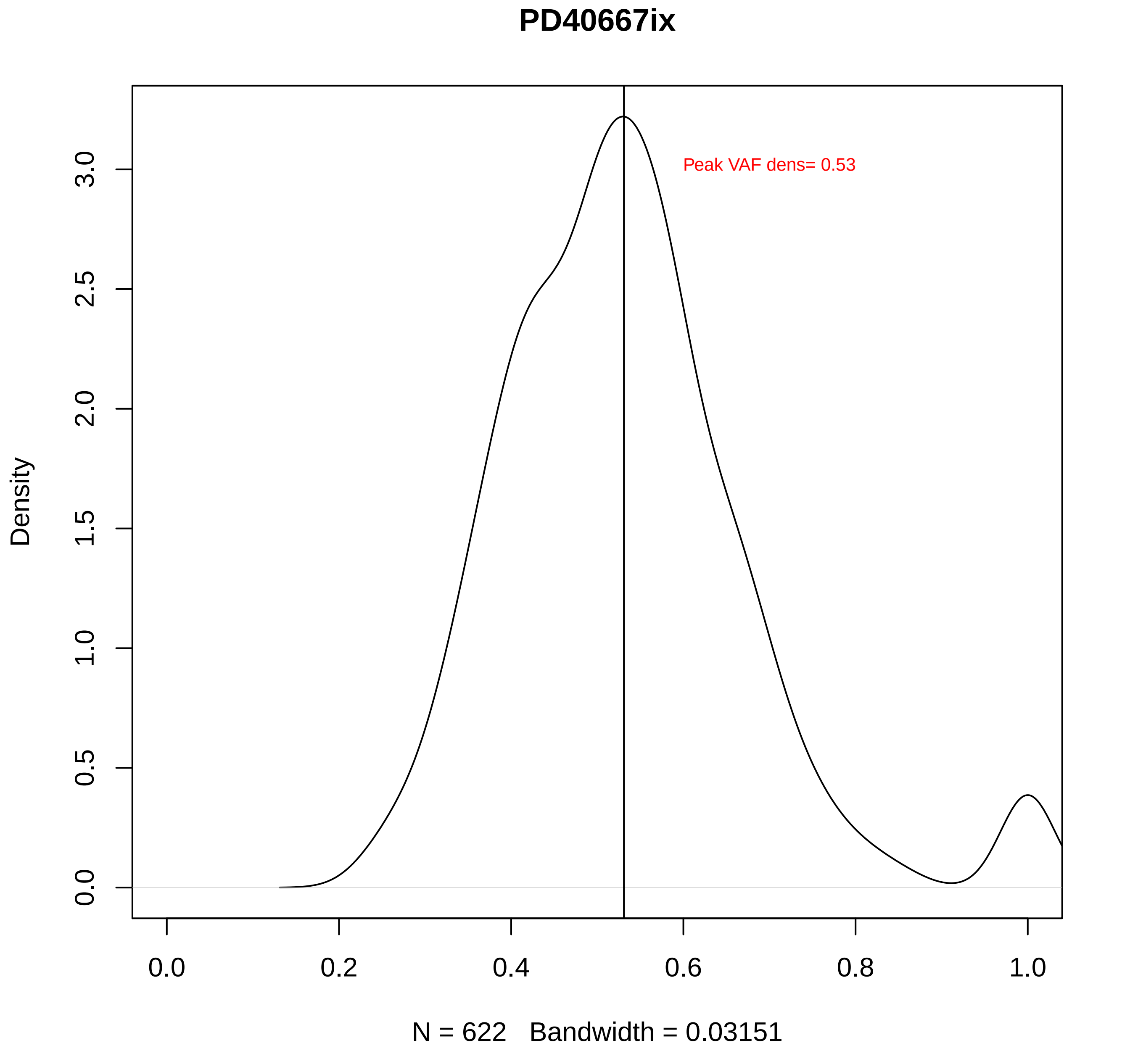
PD40667ix
Peak VAF dens= 0.53
Density
0.0
0.5
1.0
1.5
2.0
2.5
3.0
0.0
0.2
0.4
0.6
0.8
1.0
N = 622   Bandwidth = 0.03151

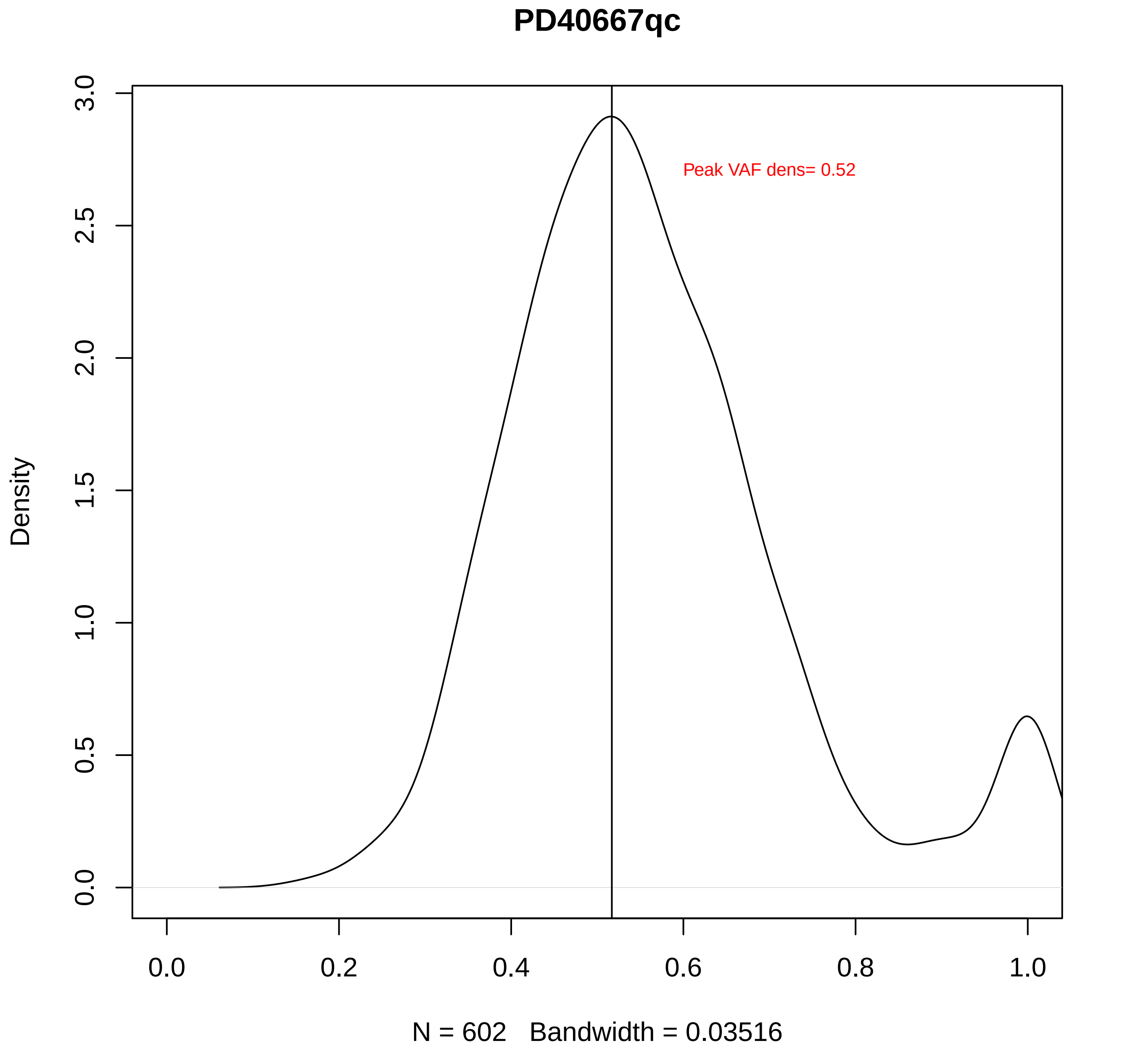

PD40667qc
Peak VAF dens= 0.52
Density
0.0
0.5
1.0
1.5
2.0
2.5
3.0
0.0
0.2
0.4
0.6
0.8
1.0
N = 602   Bandwidth = 0.03516

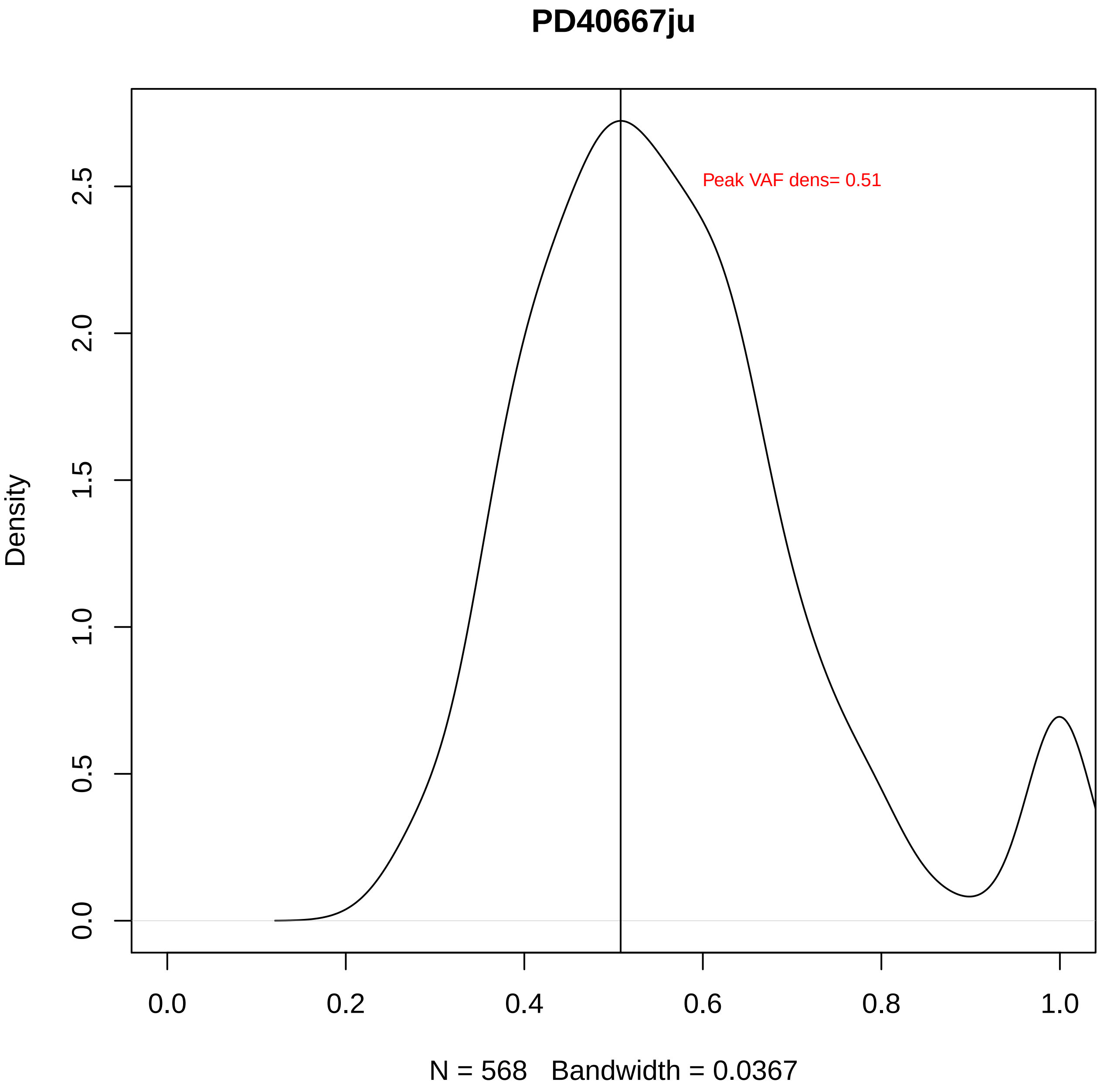

PD40667ju
Peak VAF dens= 0.51
Density
0.0
0.5
1.0
1.5
2.0
2.5
0.0
0.2
0.4
0.6
0.8
1.0
N = 568   Bandwidth = 0.0367

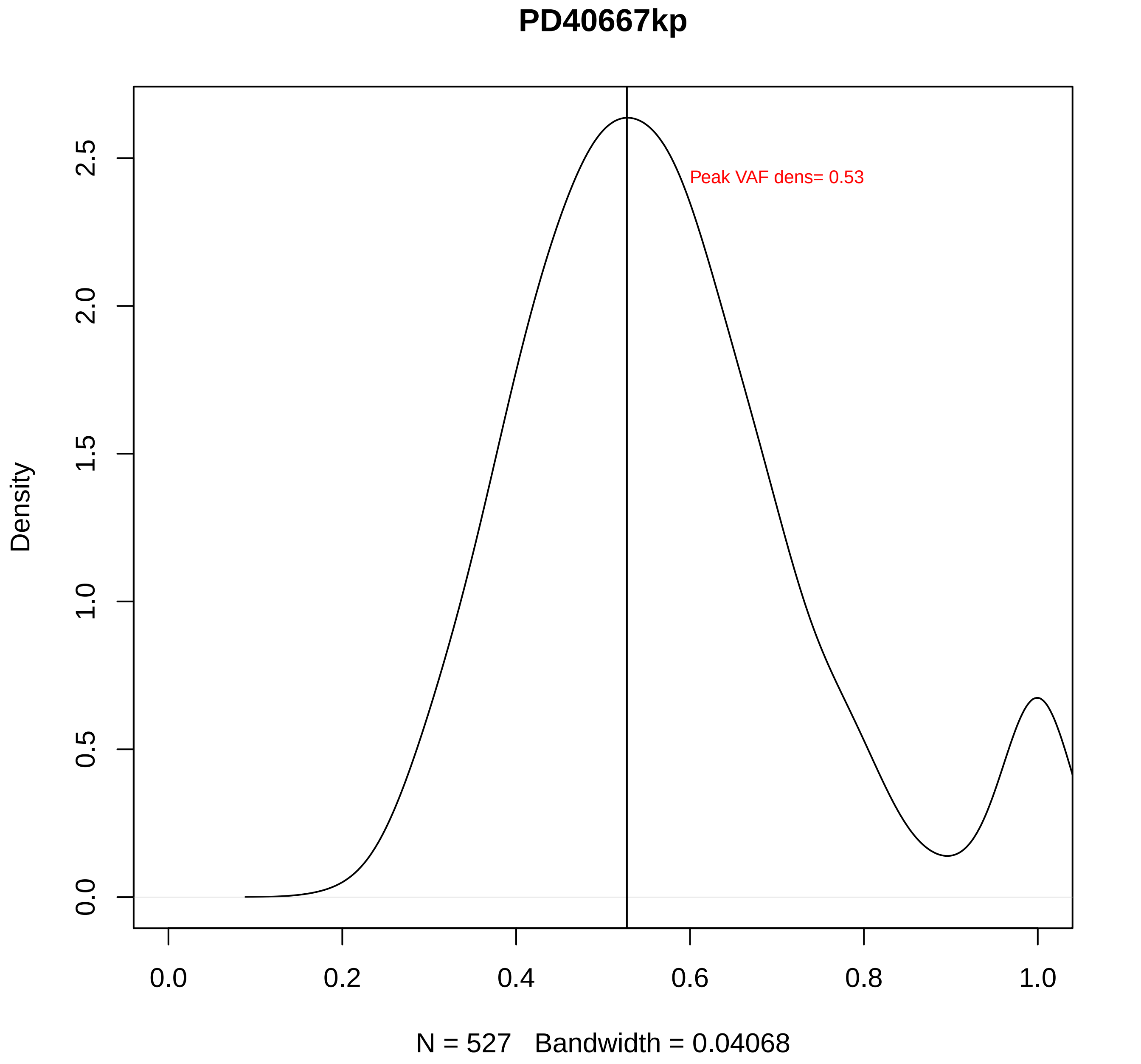
PD40667kp
Peak VAF dens= 0.53
Density
0.0
0.5
1.0
1.5
2.0
2.5
0.0
0.2
0.4
0.6
0.8
1.0
N = 527   Bandwidth = 0.04068

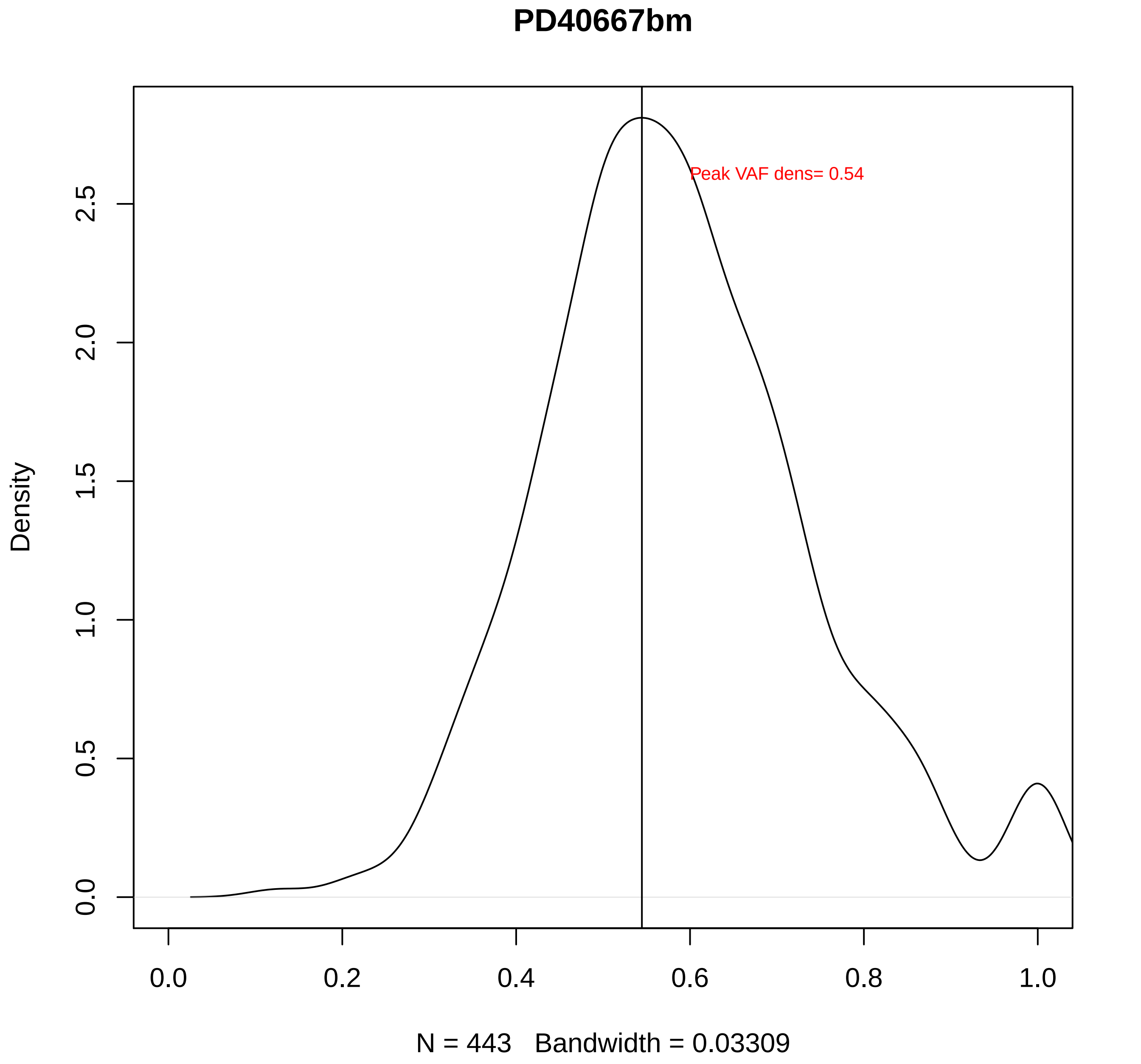
PD40667bm
Peak VAF dens= 0.54
Density
0.0
0.5
1.0
1.5
2.0
2.5
0.0
0.2
0.4
0.6
0.8
1.0
N = 443   Bandwidth = 0.03309

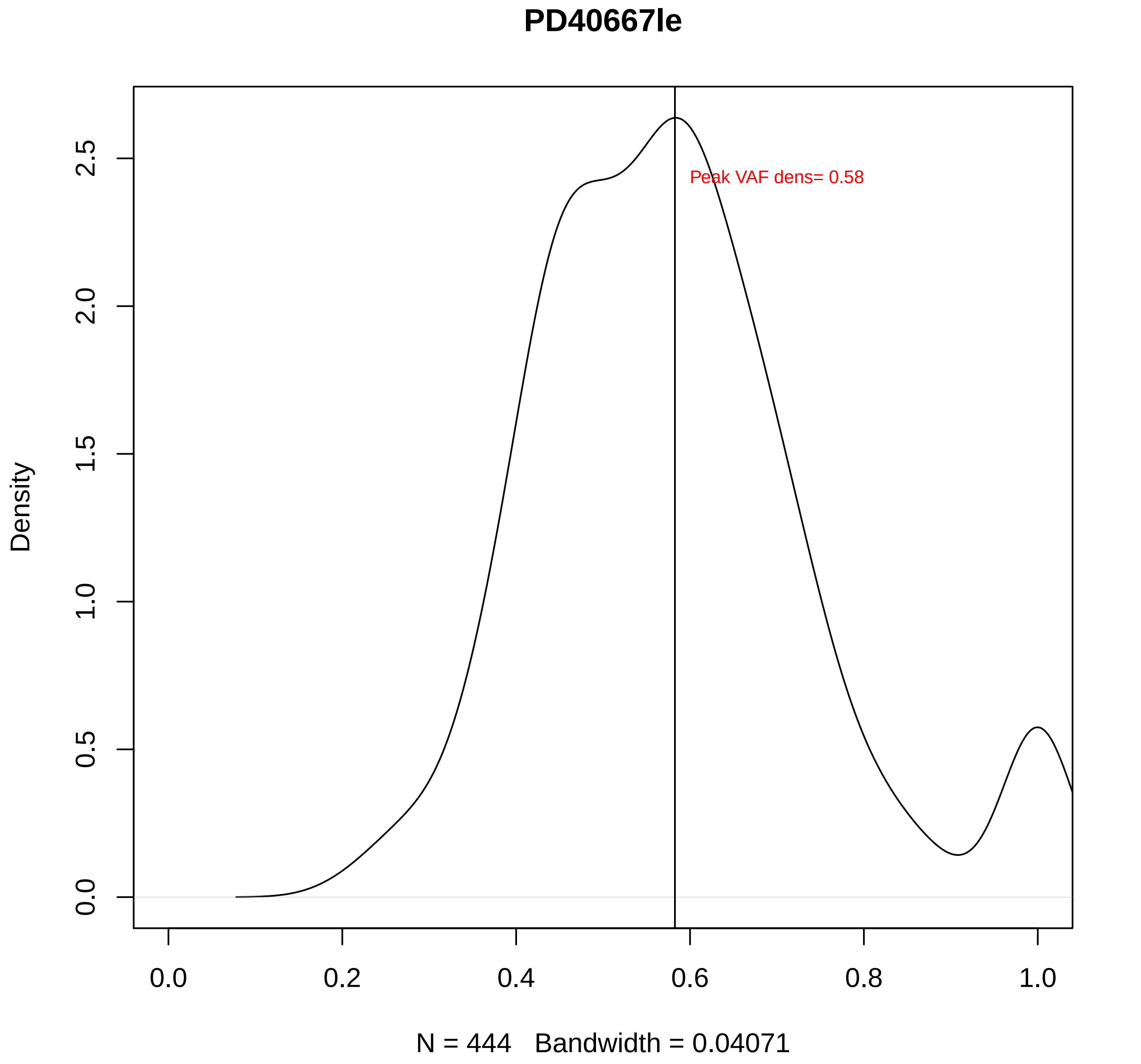

PD40667le
Peak VAF dens= 0.58
Density
0.0
0.5
1.0
1.5
2.0
2.5
0.0
0.2
0.4
0.6
0.8
1.0
N = 444   Bandwidth = 0.04071

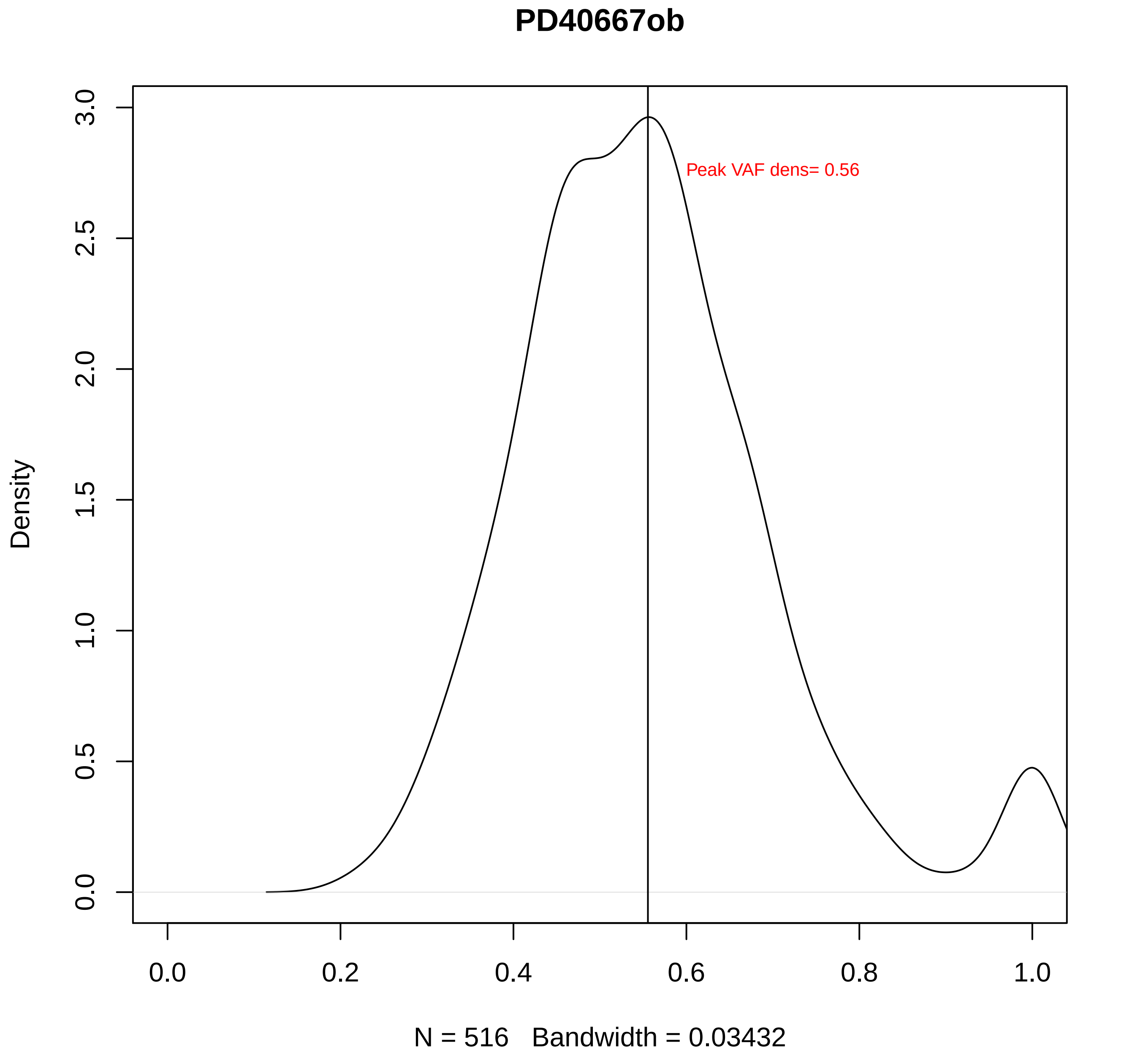
PD40667ob
Peak VAF dens= 0.56
Density
3.0
2.5
2.0
1.5
1.0
0.5
0.0
0.0
0.2
0.4
0.6
0.8
1.0
N = 516   Bandwidth = 0.03432

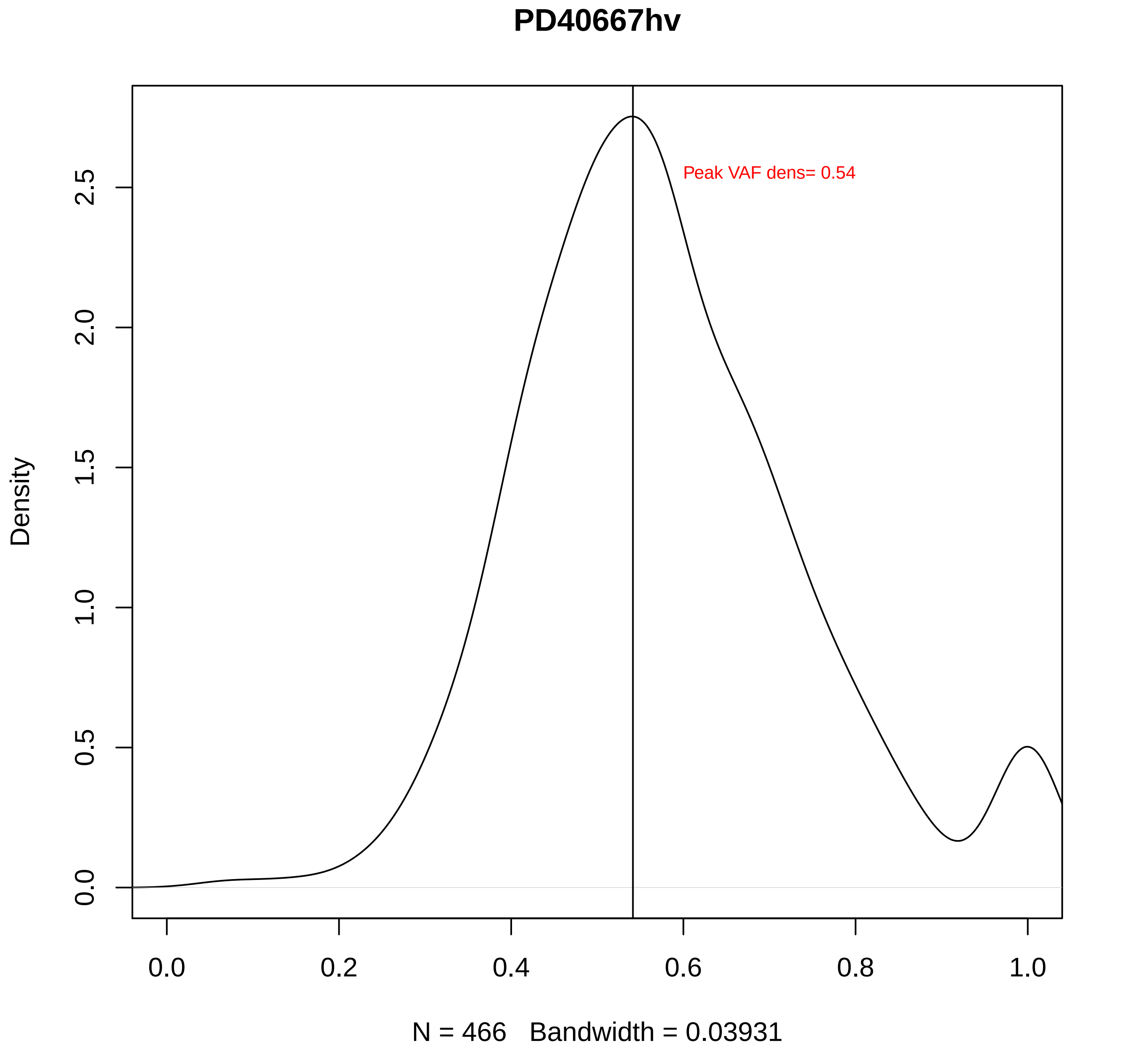
PD40667hv
Peak VAF dens= 0.54
Density
0.0
0.5
1.0
1.5
2.0
2.5
0.0
0.2
0.4
0.6
0.8
1.0
N = 466   Bandwidth = 0.03931

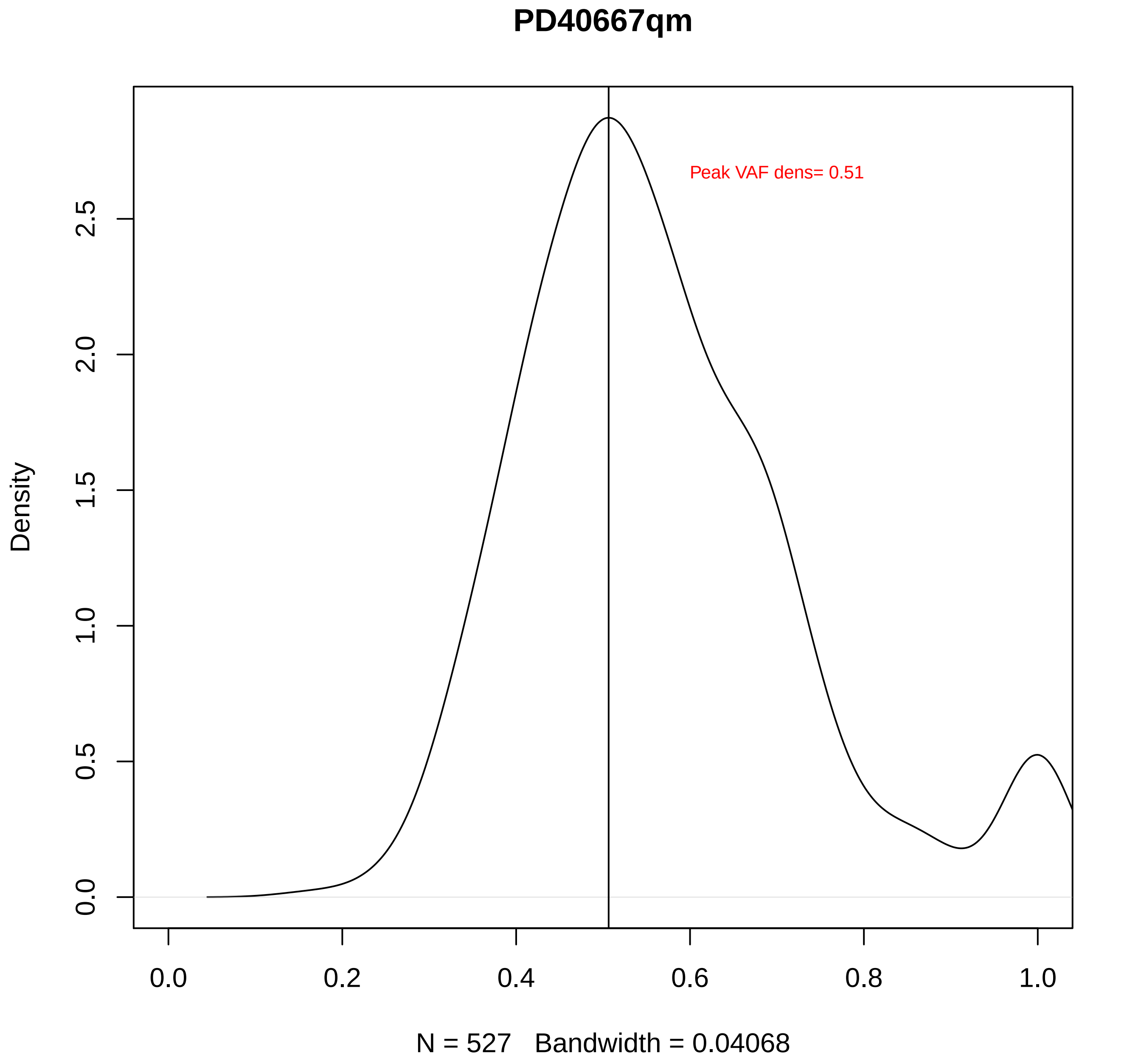

PD40667qm
Peak VAF dens= 0.51
Density
0.0
0.5
1.0
1.5
2.0
2.5
0.0
0.2
0.4
0.6
0.8
1.0
N = 527 Bandwidth = 0.04068

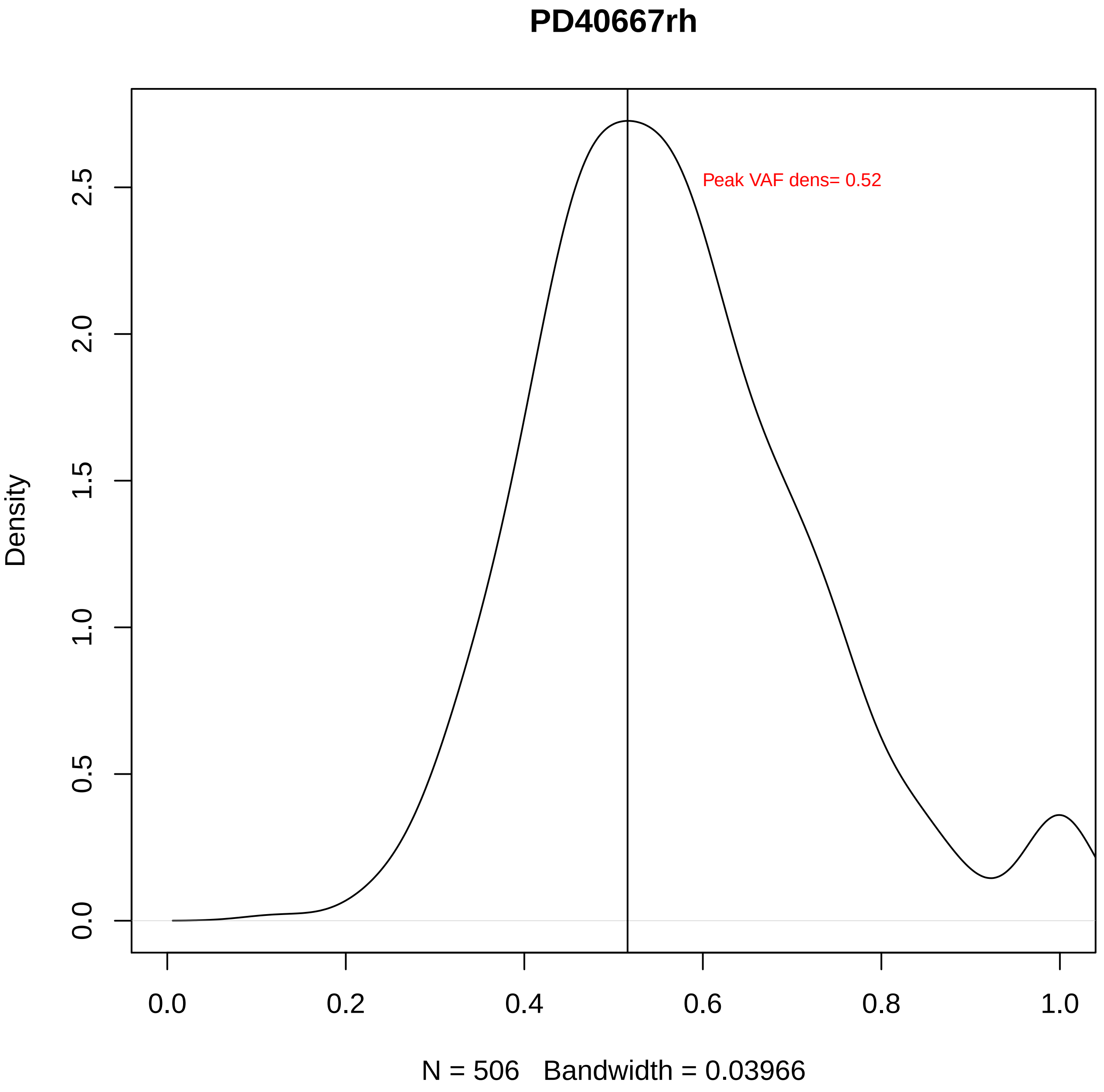

PD40667rh
Peak VAF dens= 0.52
Density
0.0
0.5
1.0
1.5
2.0
2.5
0.0
0.2
0.4
0.6
0.8
1.0
N = 506 Bandwidth = 0.03966

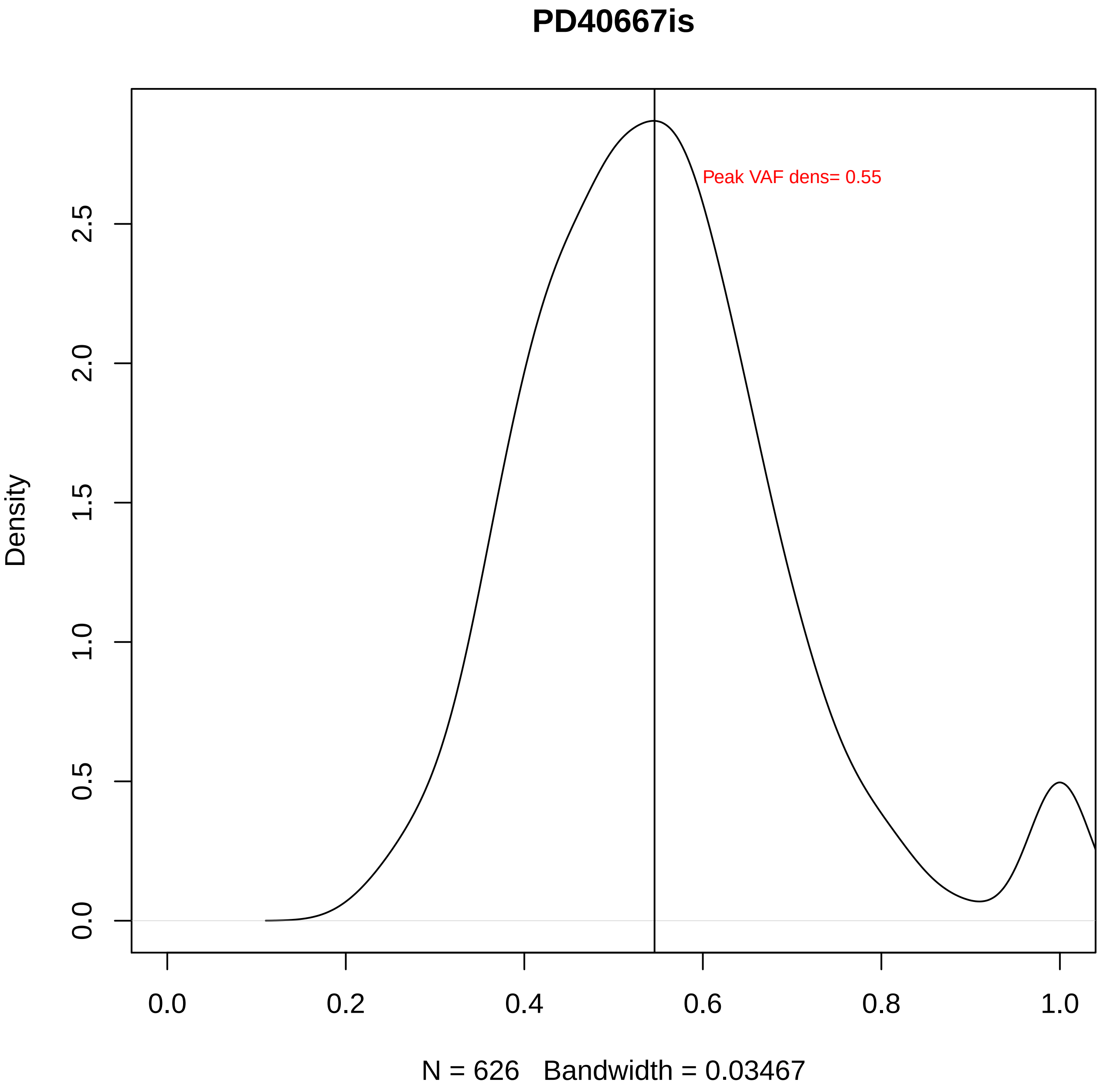
PD40667is
Peak VAF dens= 0.55
Density
0.0
0.5
1.0
1.5
2.0
2.5
0.0
0.2
0.4
0.6
0.8
1.0
N = 626   Bandwidth = 0.03467

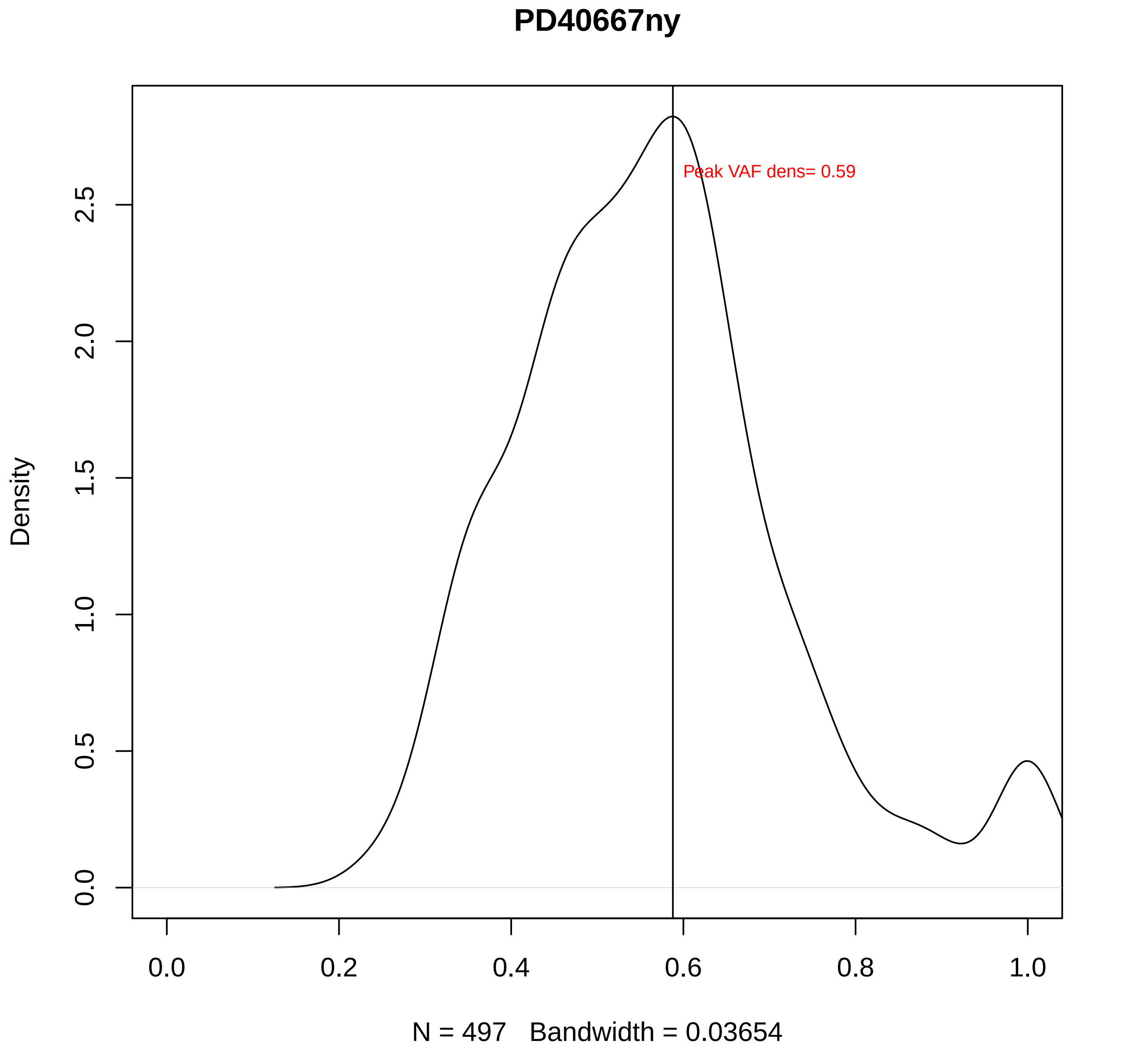
PD40667ny
Peak VAF dens= 0.59
Density
0.0
0.5
1.0
1.5
2.0
2.5
0.0
0.2
0.4
0.6
0.8
1.0
N = 497   Bandwidth = 0.03654

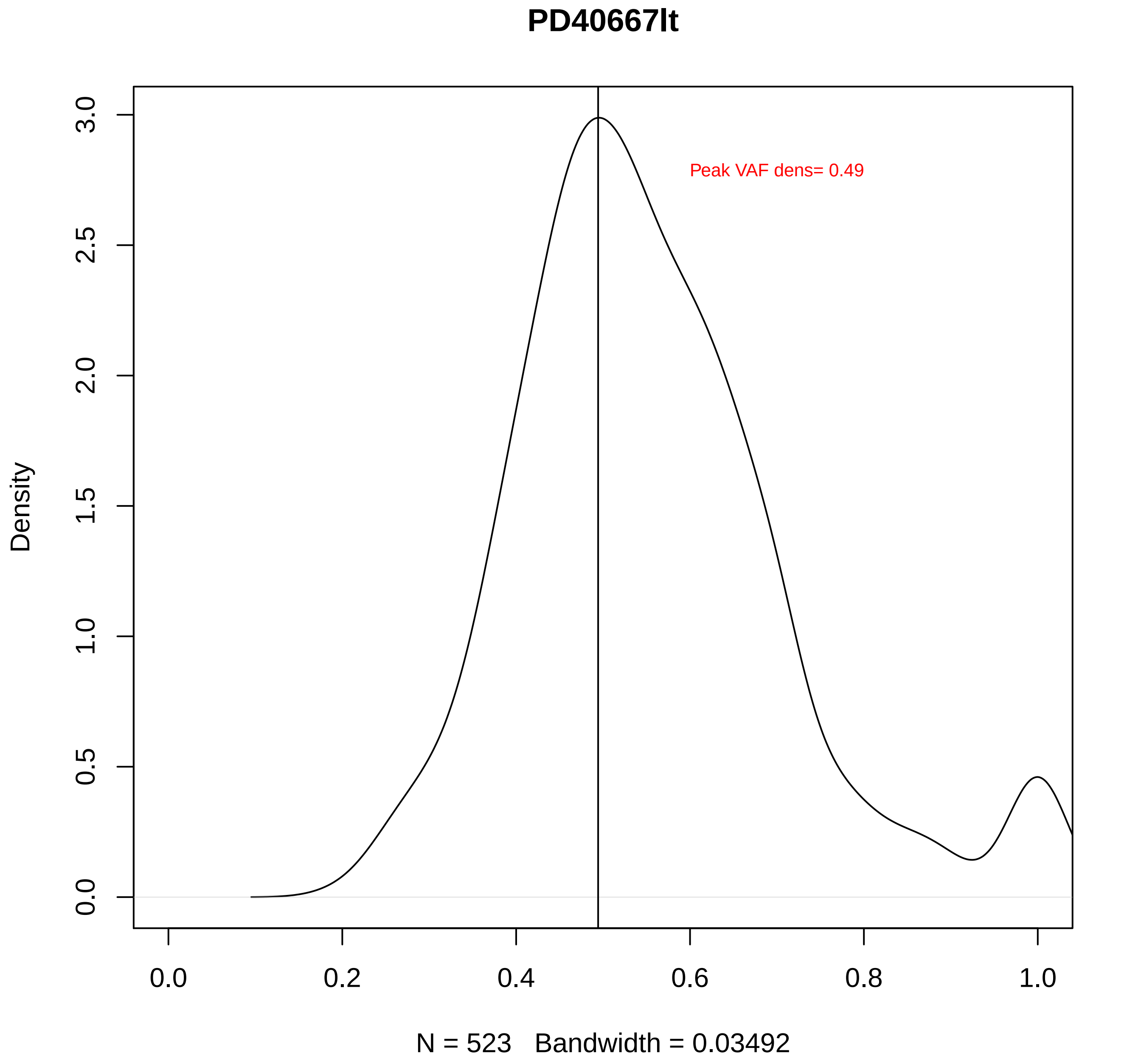
PD40667lt
Peak VAF dens= 0.49
Density
0.0
0.5
1.0
1.5
2.0
2.5
3.0
0.0
0.2
0.4
0.6
0.8
1.0
N = 523   Bandwidth = 0.03492

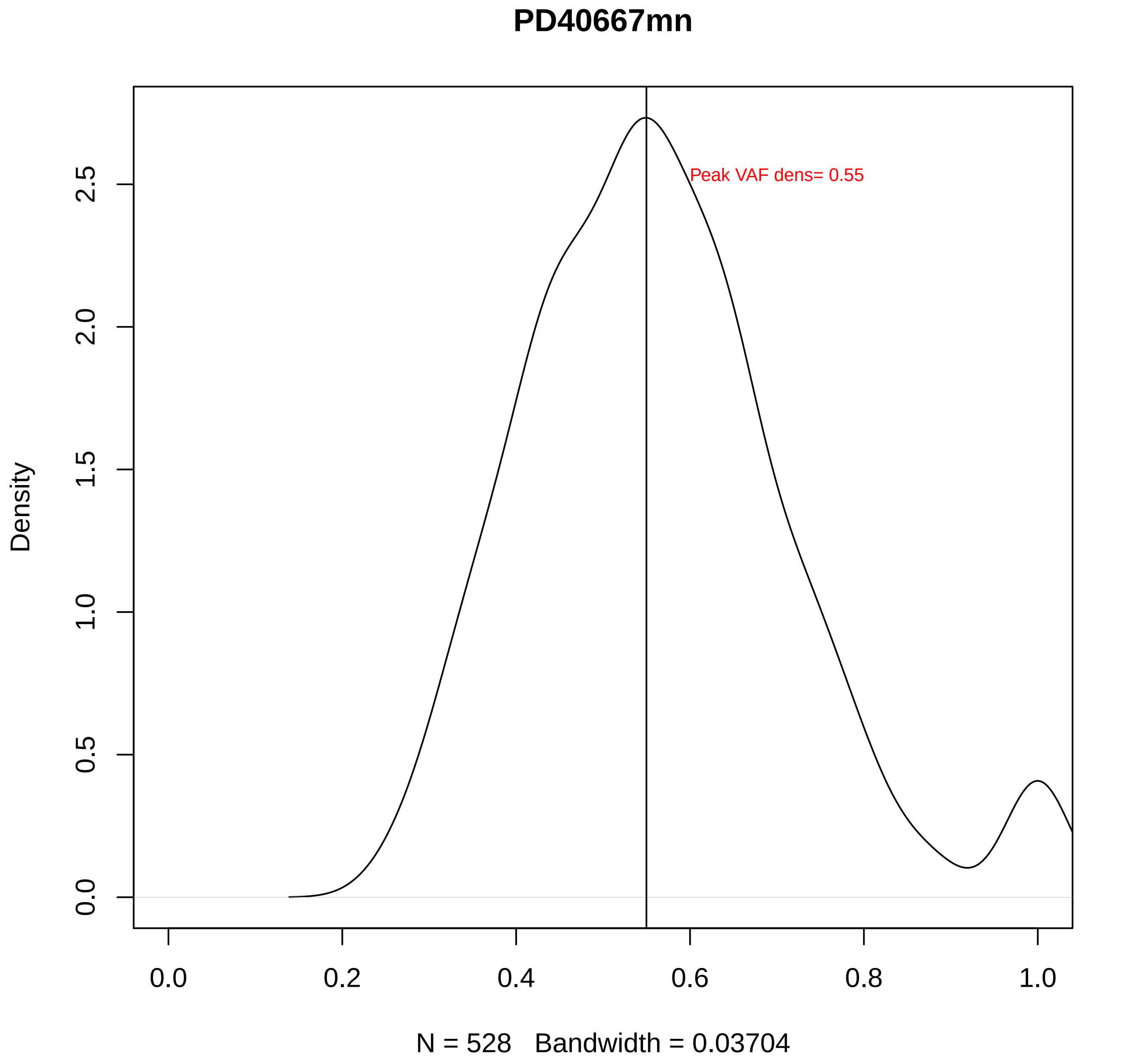

PD40667mn
Peak VAF dens= 0.55
Density
0.0
0.5
1.0
1.5
2.0
2.5
0.0
0.2
0.4
0.6
0.8
1.0
N = 528   Bandwidth = 0.03704

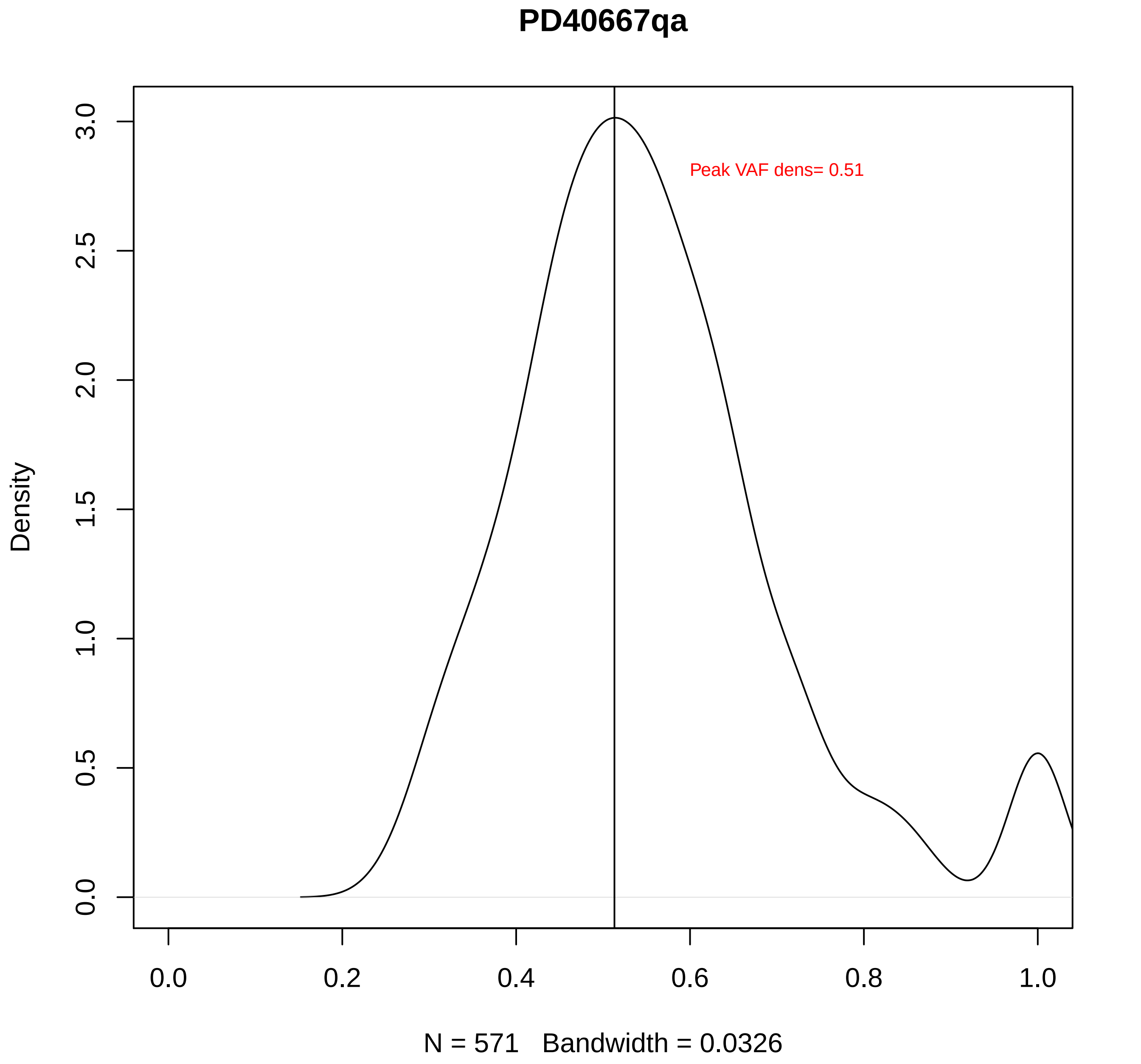

PD40667qa
Peak VAF dens= 0.51
Density
0.0
0.5
1.0
1.5
2.0
2.5
3.0
0.0
0.2
0.4
0.6
0.8
1.0
N = 571   Bandwidth = 0.0326

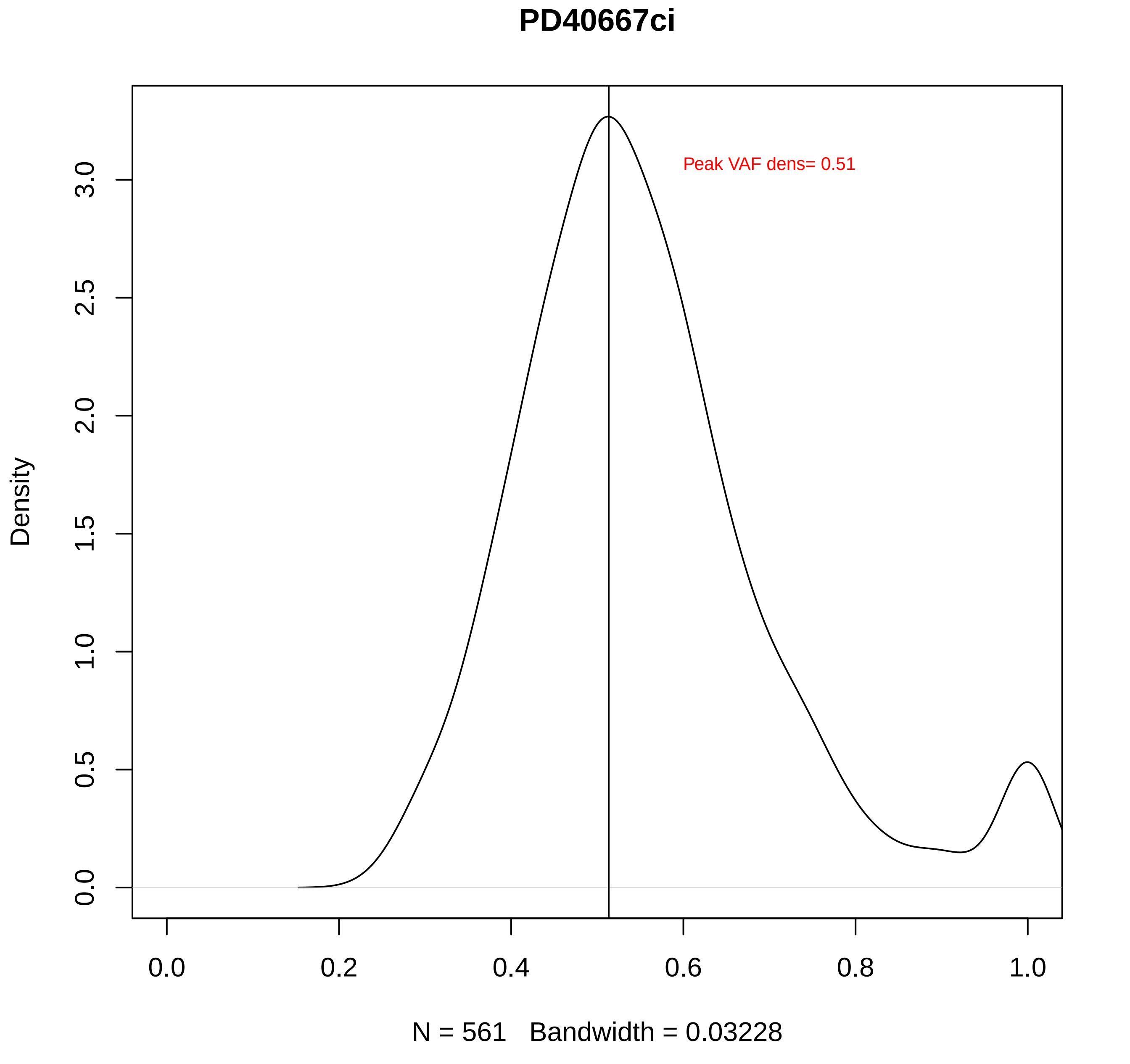
PD40667ci
Peak VAF dens= 0.51
Density
0.0
0.5
1.0
1.5
2.0
2.5
3.0
0.0
0.2
0.4
0.6
0.8
1.0
N = 561   Bandwidth = 0.03228

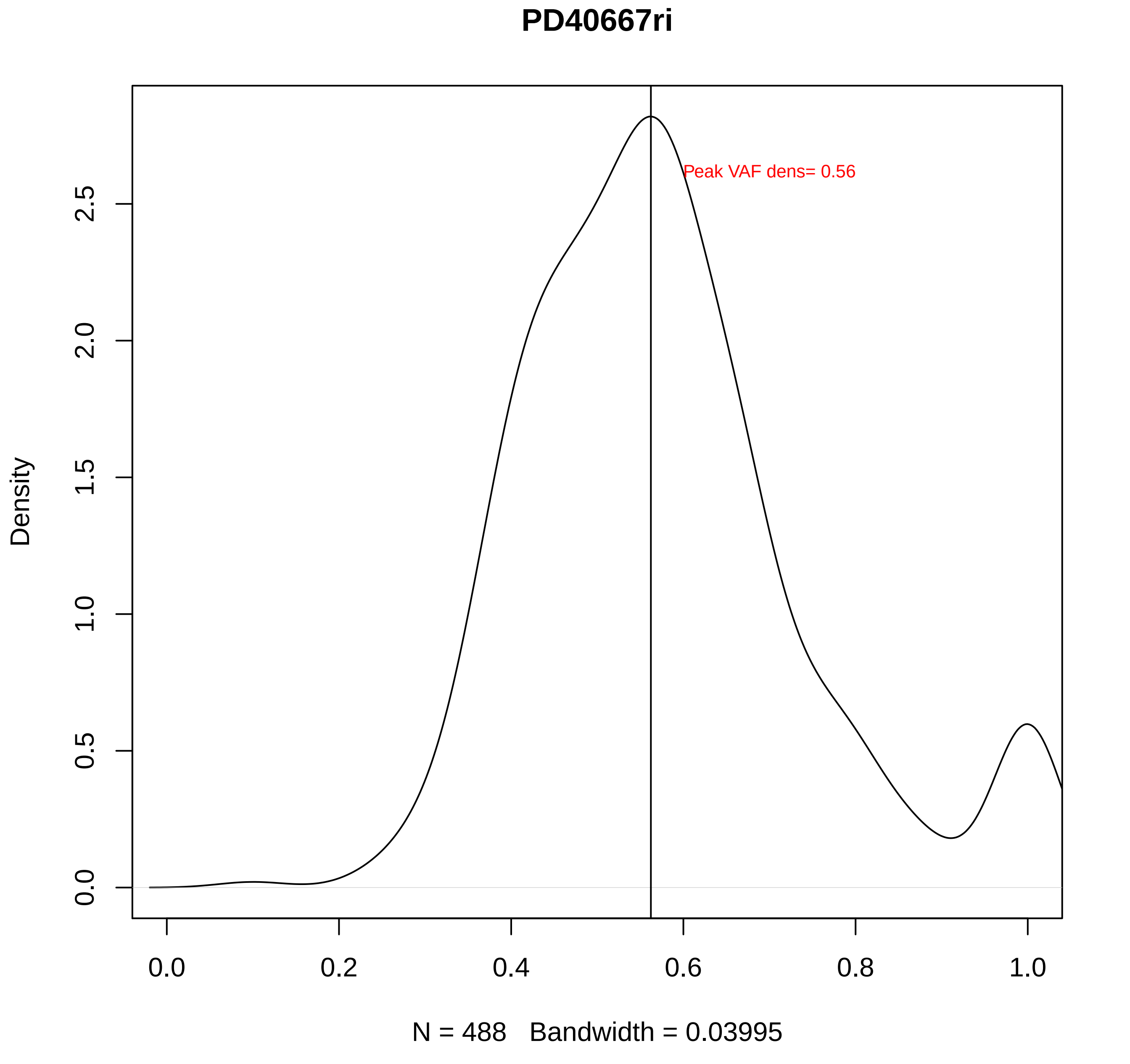
PD40667ri
Peak VAF dens= 0.56
Density
0.0
0.5
1.0
1.5
2.0
2.5
0.0
0.2
0.4
0.6
0.8
1.0
N = 488   Bandwidth = 0.03995

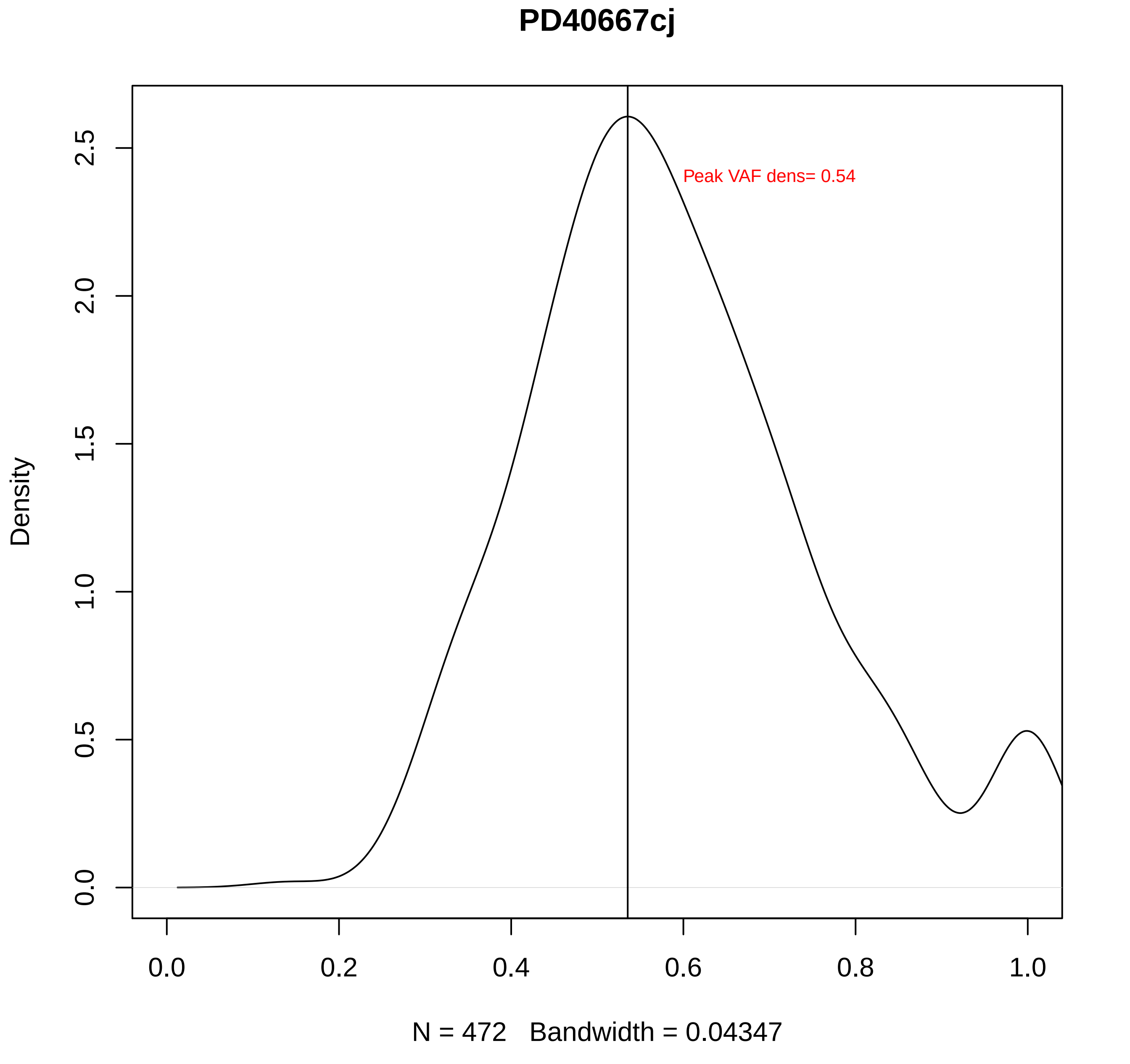
PD40667cj
Peak VAF dens= 0.54
Density
0.0
0.5
1.0
1.5
2.0
2.5
0.0
0.2
0.4
0.6
0.8
1.0
N = 472   Bandwidth = 0.04347

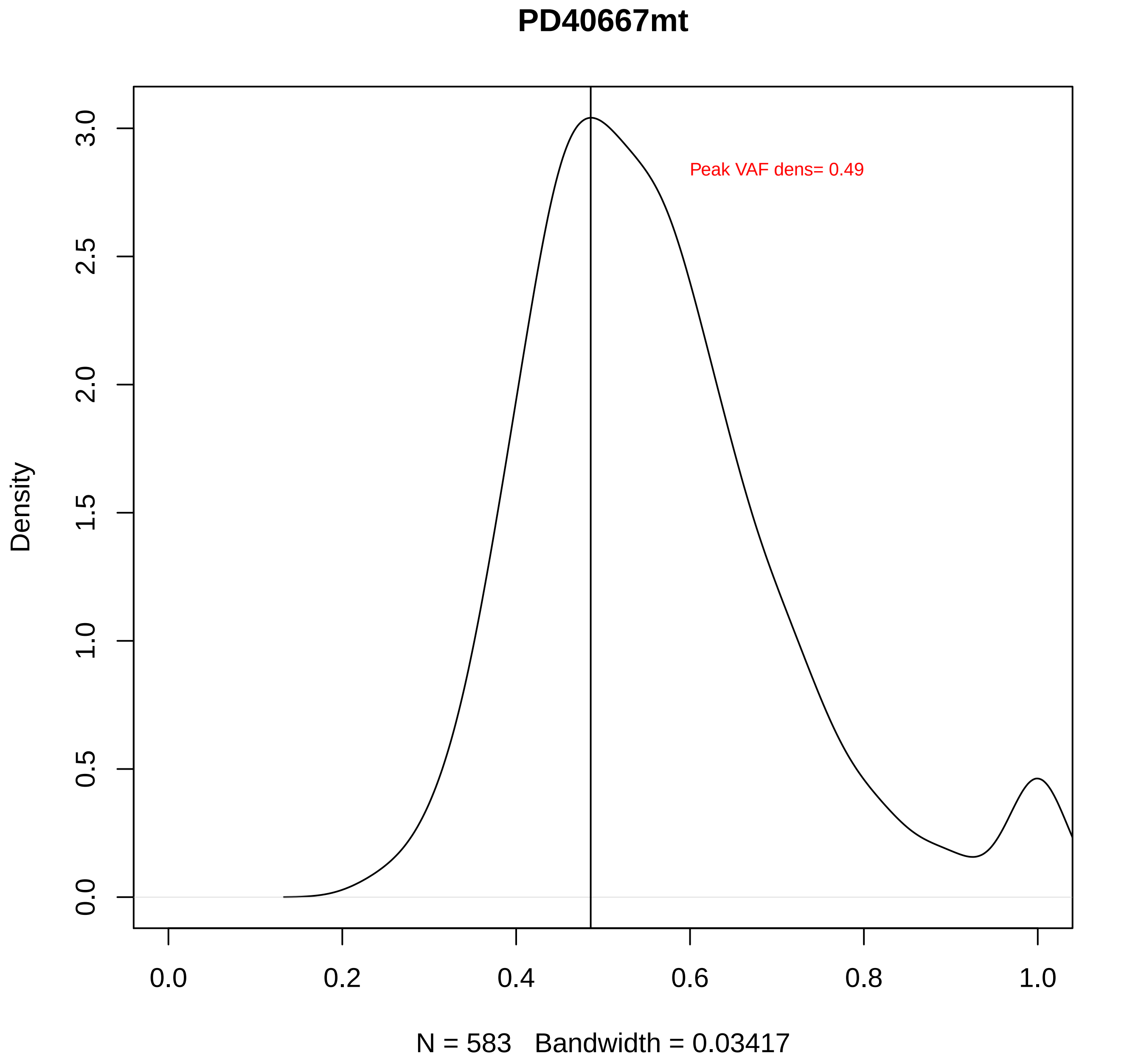

PD40667mt
Peak VAF dens= 0.49
Density
0.0
0.5
1.0
1.5
2.0
2.5
3.0
0.0
0.2
0.4
0.6
0.8
1.0
N = 583 Bandwidth = 0.03417

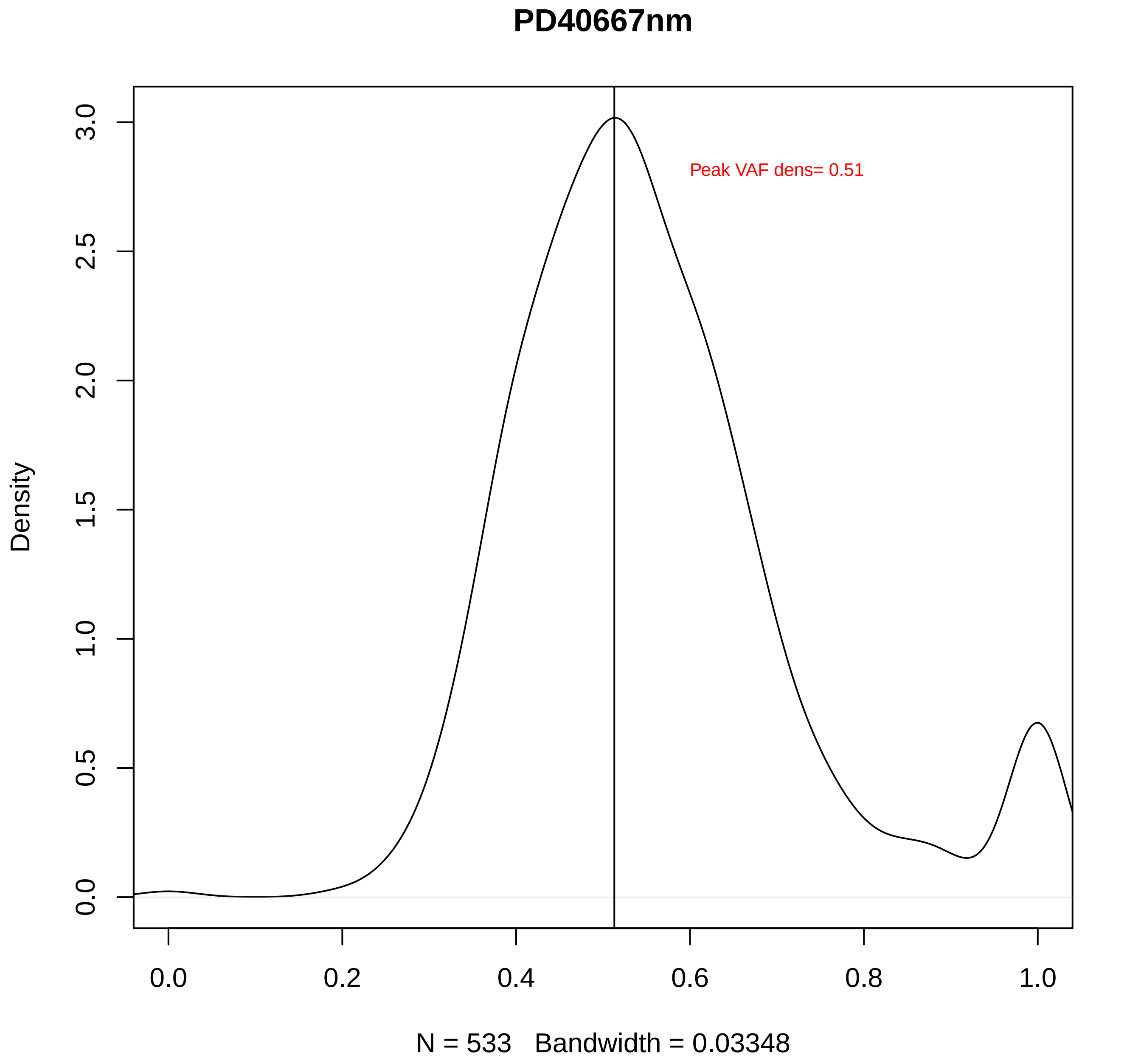

PD40667nm
Peak VAF dens= 0.51
Density
0.0
0.5
1.0
1.5
2.0
2.5
3.0
0.0
0.2
0.4
0.6
0.8
1.0
N = 533 Bandwidth = 0.03348

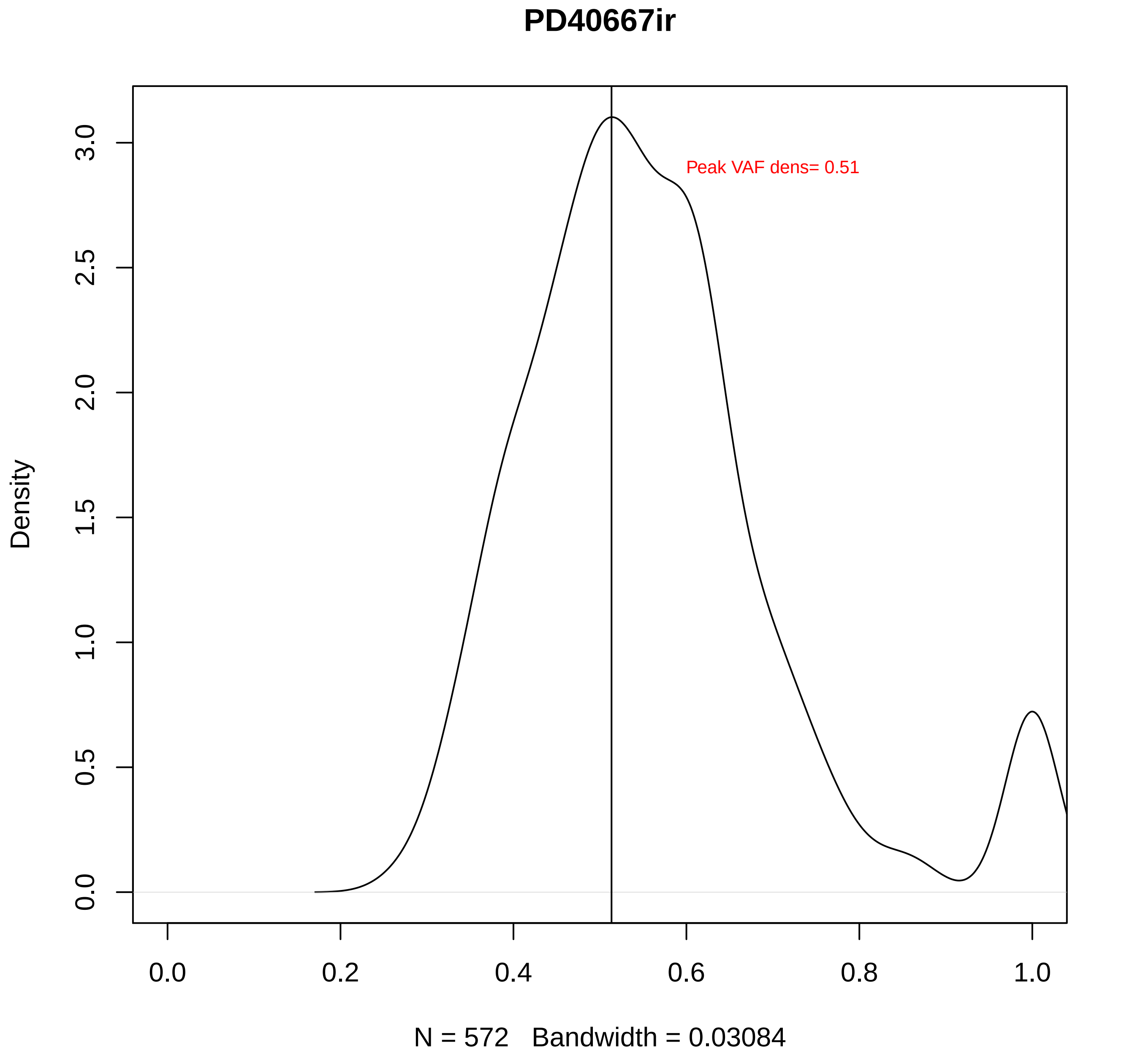

PD40667ir
Peak VAF dens= 0.51
Density
0.0
0.5
1.0
1.5
2.0
2.5
3.0
0.0
0.2
0.4
0.6
0.8
1.0
N = 572 Bandwidth = 0.03084

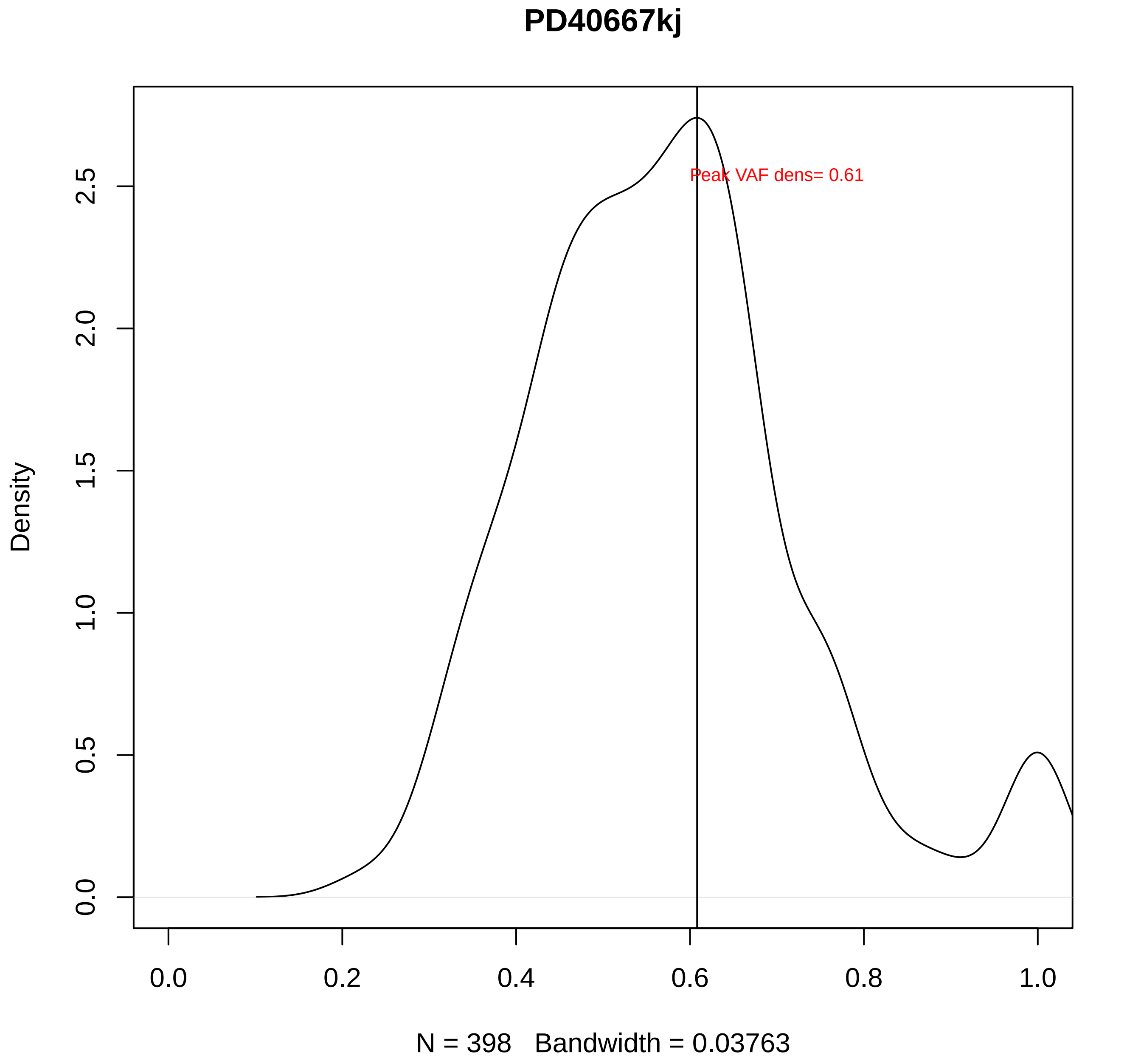
PD40667kj
Peak VAF dens= 0.61
Density
0.0
0.5
1.0
1.5
2.0
2.5
0.0
0.2
0.4
0.6
0.8
1.0
N = 398 Bandwidth = 0.03763

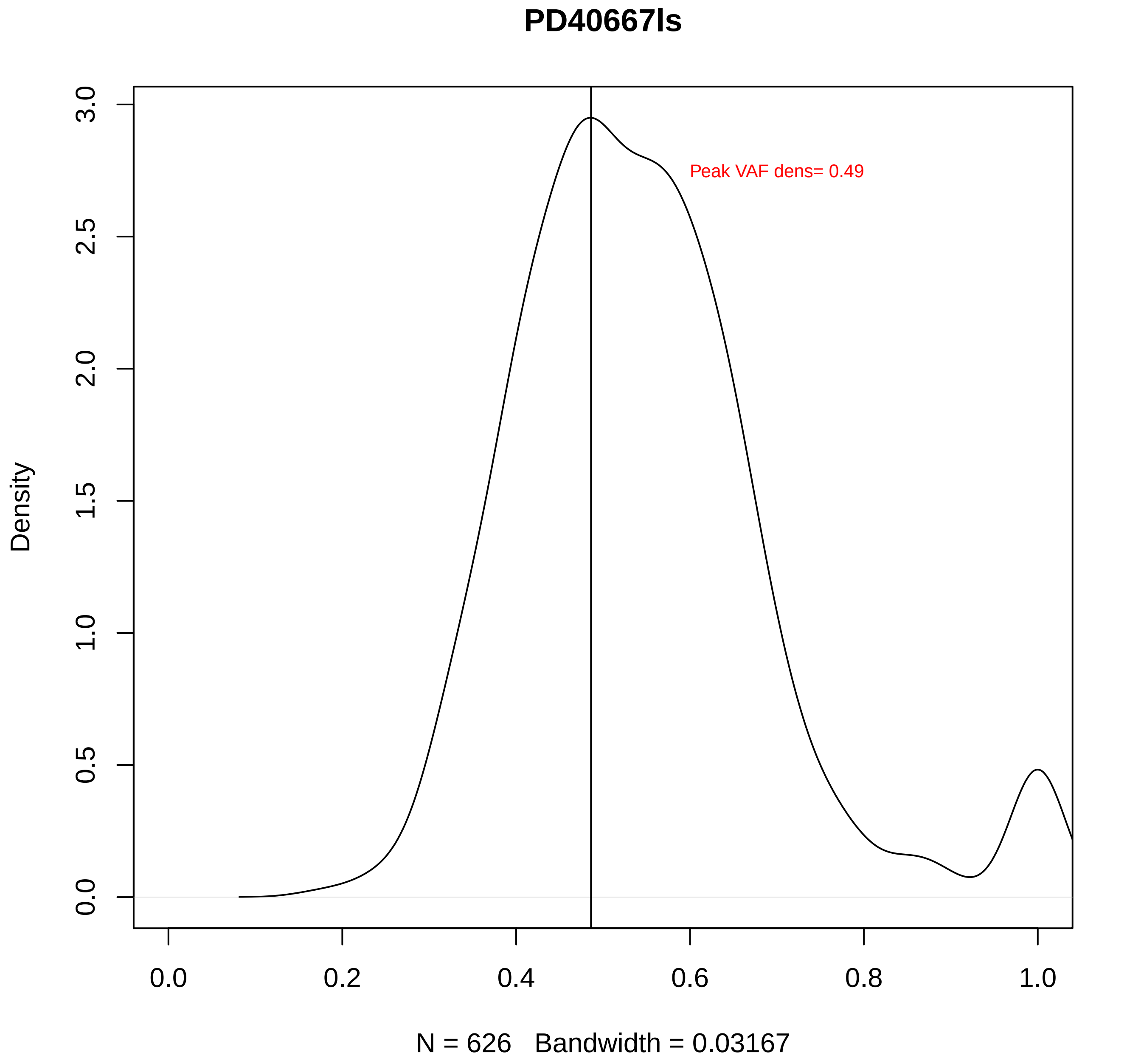
PD40667ls
Peak VAF dens= 0.49
Density
0.0
0.5
1.0
1.5
2.0
2.5
3.0
0.0
0.2
0.4
0.6
0.8
1.0
N = 626   Bandwidth = 0.03167

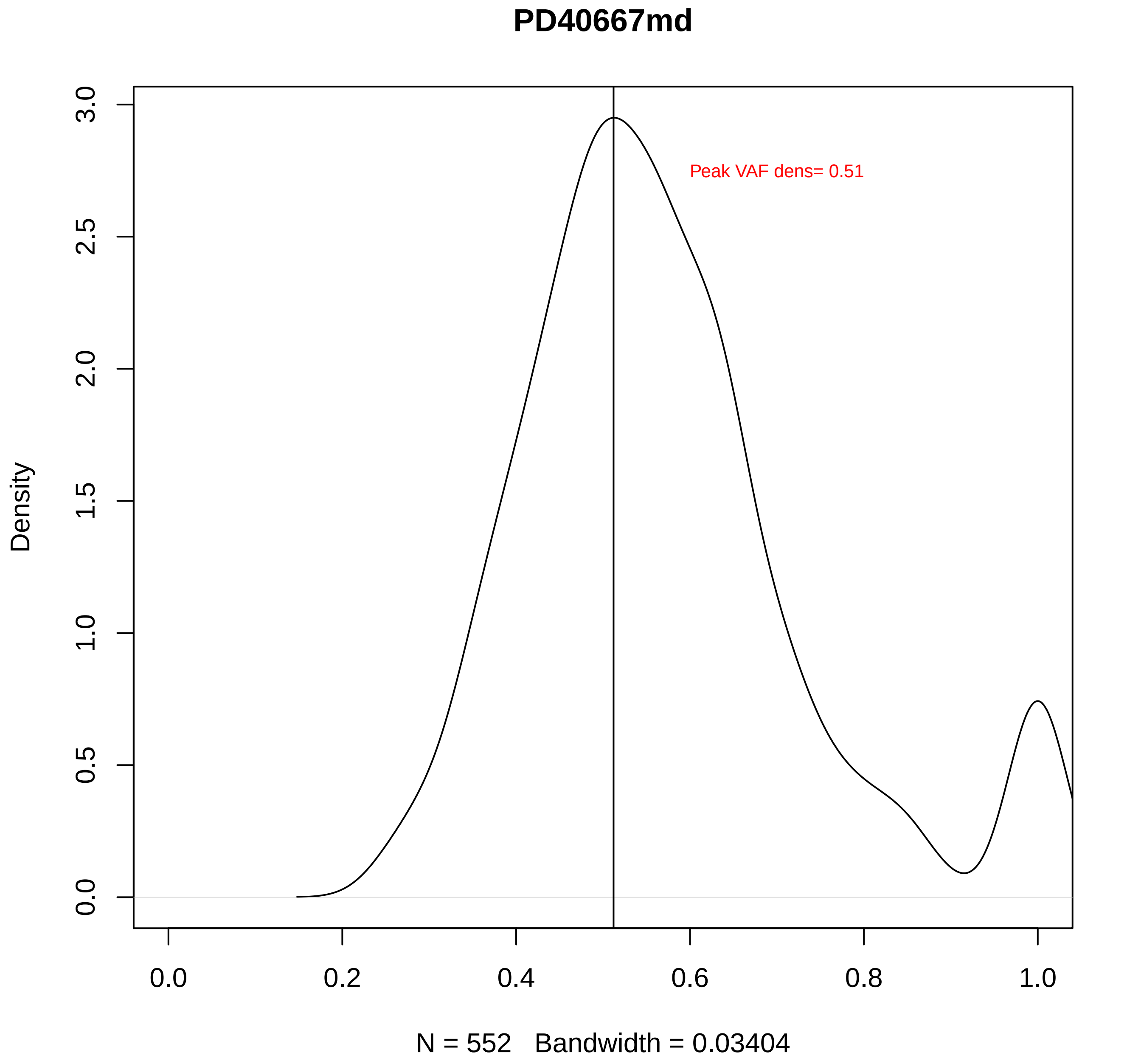

PD40667md
Peak VAF dens= 0.51
Density
3.0
2.5
2.0
1.5
1.0
0.5
0.0
0.0
0.2
0.4
0.6
0.8
1.0
N = 552   Bandwidth = 0.03404

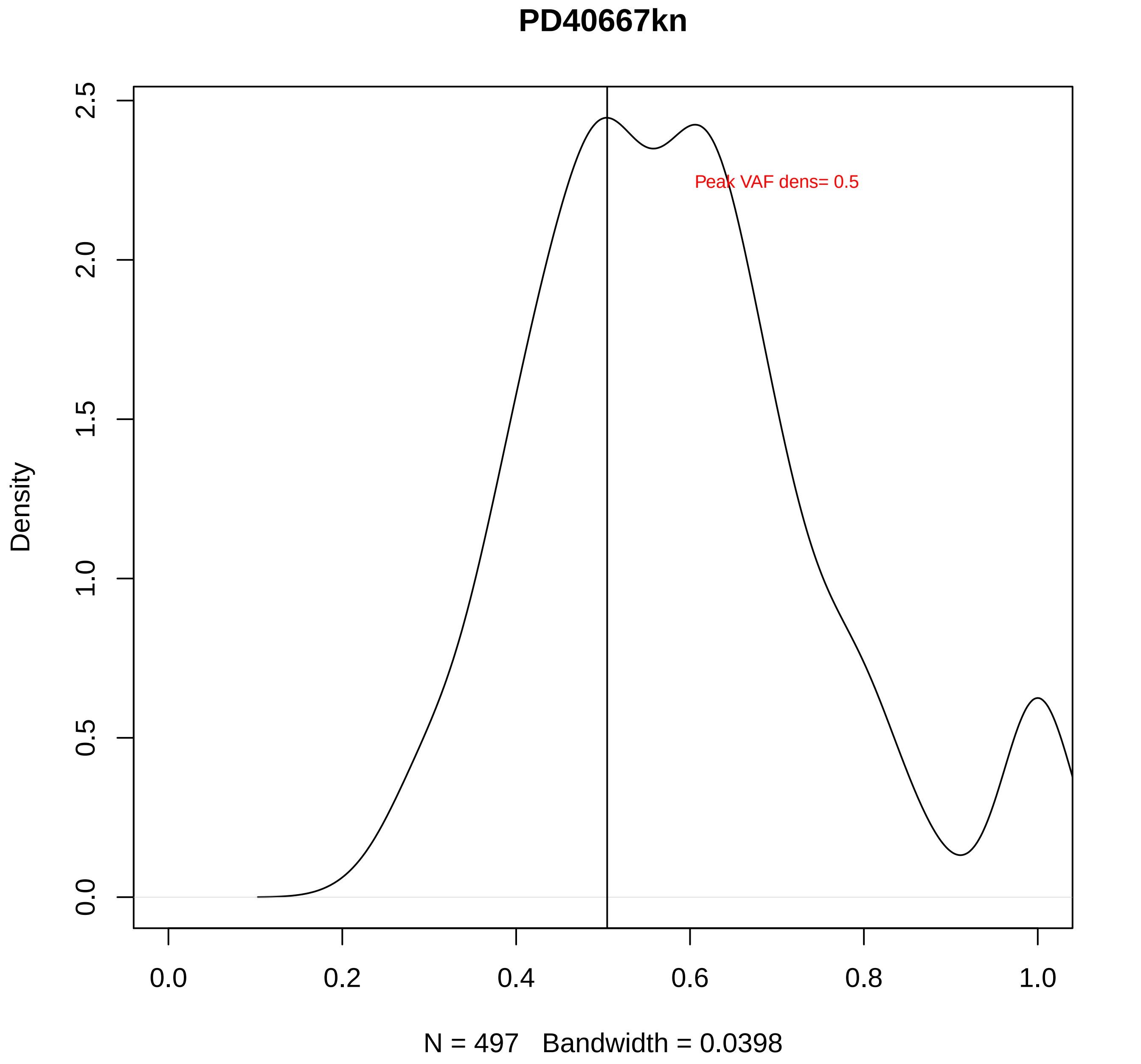
PD40667kn
Peak VAF dens= 0.5
Density
0.0
0.5
1.0
1.5
2.0
2.5
0.0
0.2
0.4
0.6
0.8
1.0
N = 497   Bandwidth = 0.0398

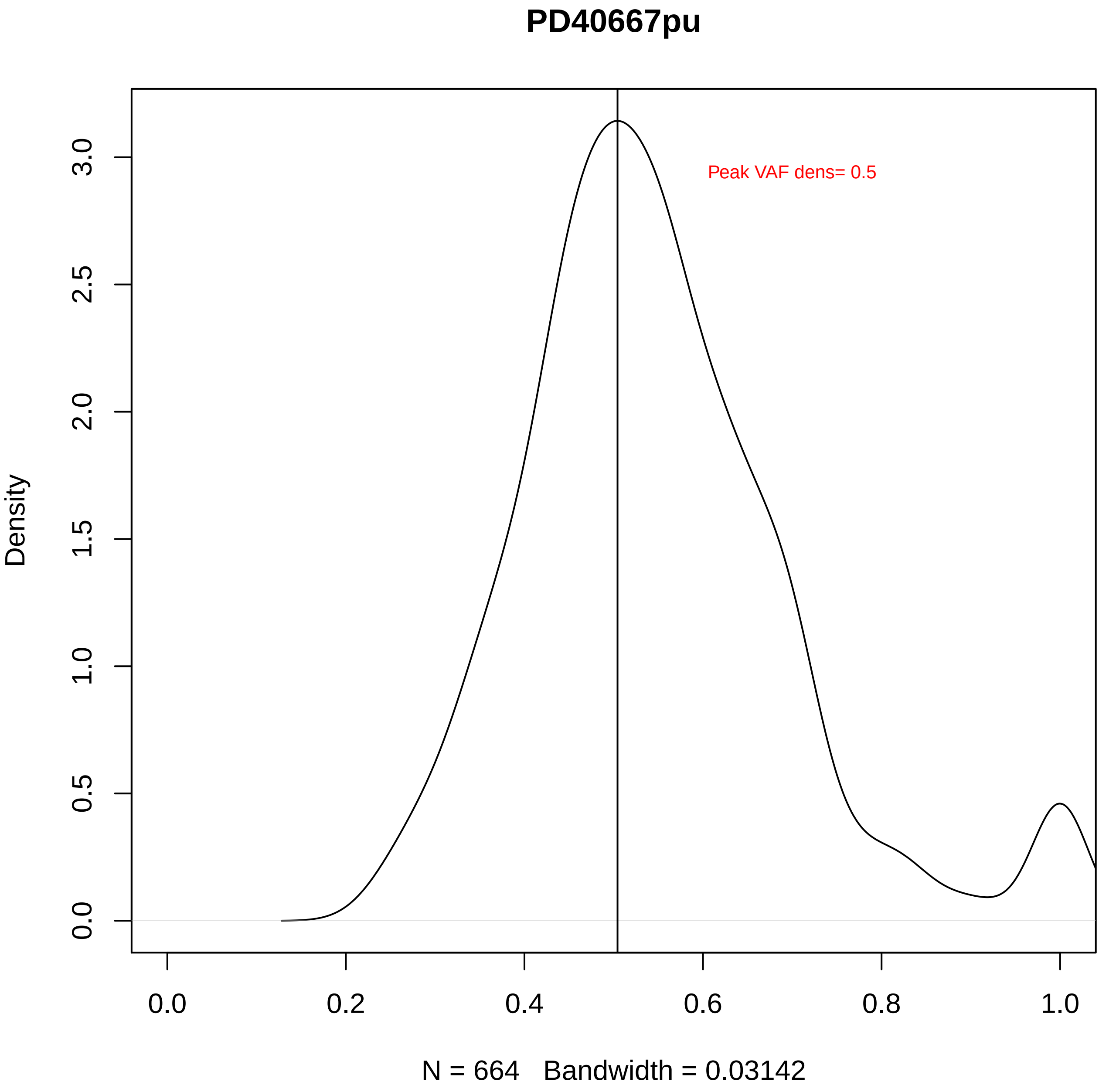
PD40667pu
Peak VAF dens= 0.5
Density
0.0
0.5
1.0
1.5
2.0
2.5
3.0
0.0
0.2
0.4
0.6
0.8
1.0
N = 664 Bandwidth = 0.03142

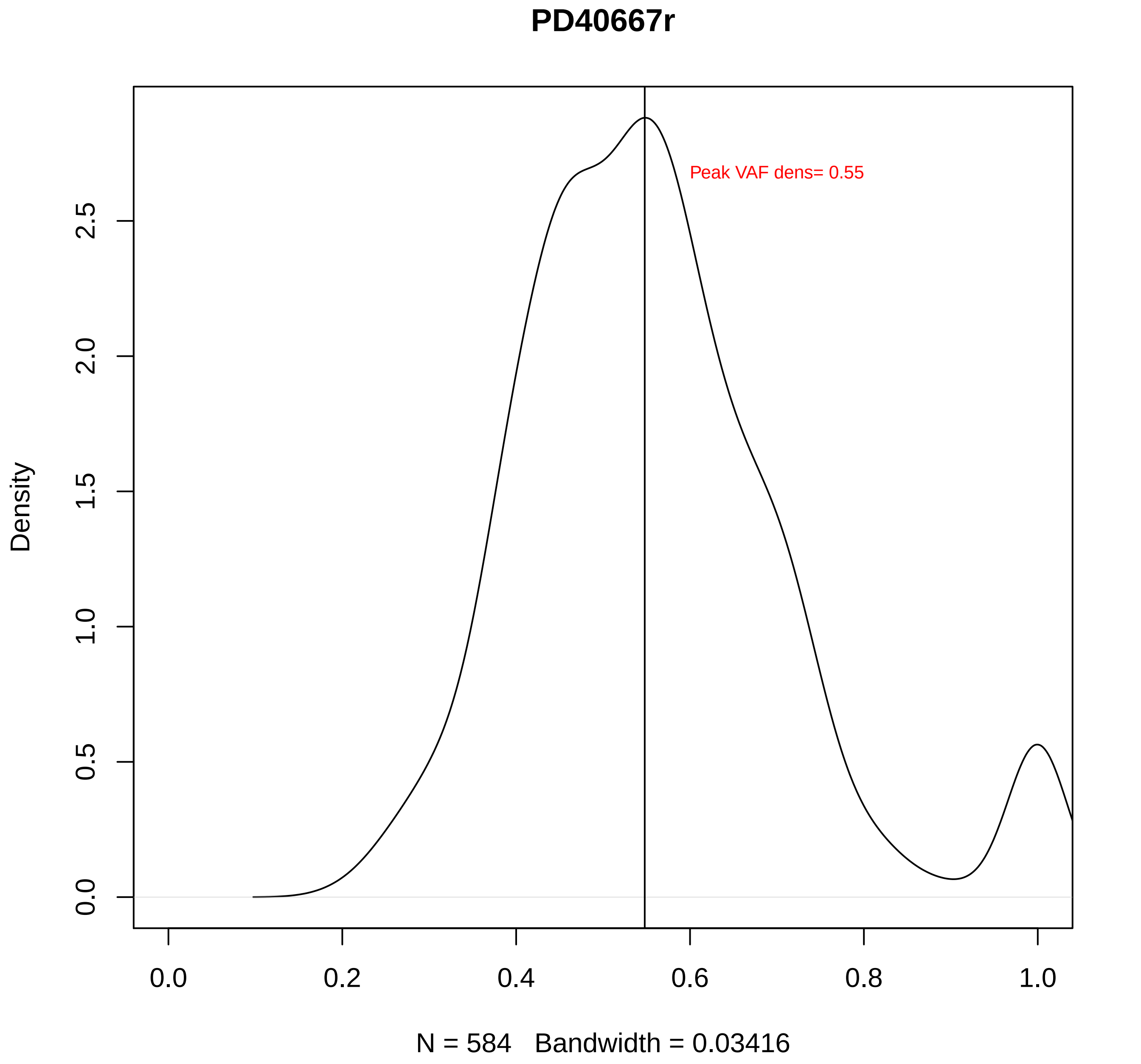

PD40667r
Peak VAF dens= 0.55
Density
0.0
0.5
1.0
1.5
2.0
2.5
0.0
0.2
0.4
0.6
0.8
1.0
N = 584 Bandwidth = 0.03416

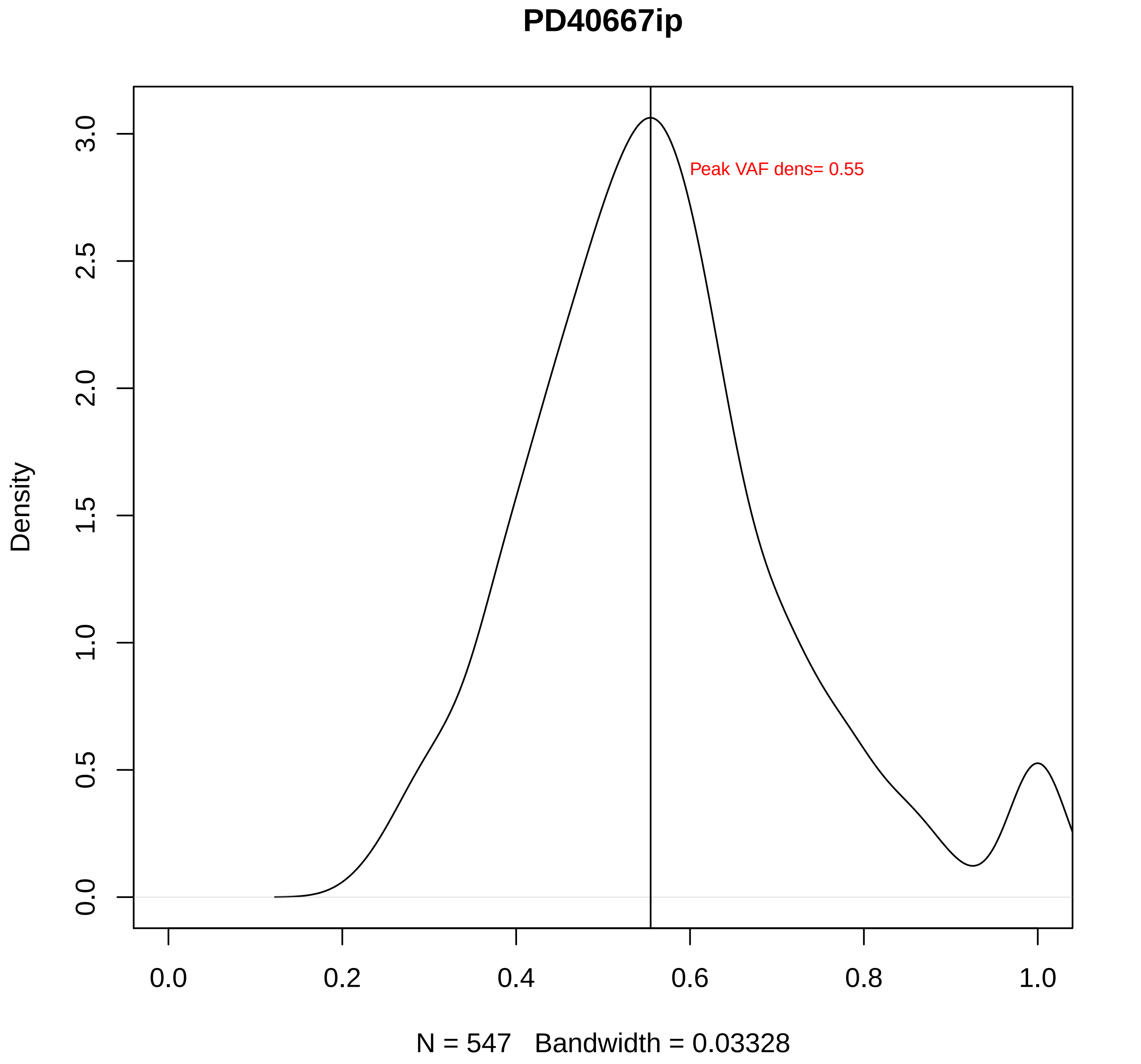
PD40667ip
Peak VAF dens= 0.55
Density
0.0
0.5
1.0
1.5
2.0
2.5
3.0
0.0
0.2
0.4
0.6
0.8
1.0
N = 547 Bandwidth = 0.03328

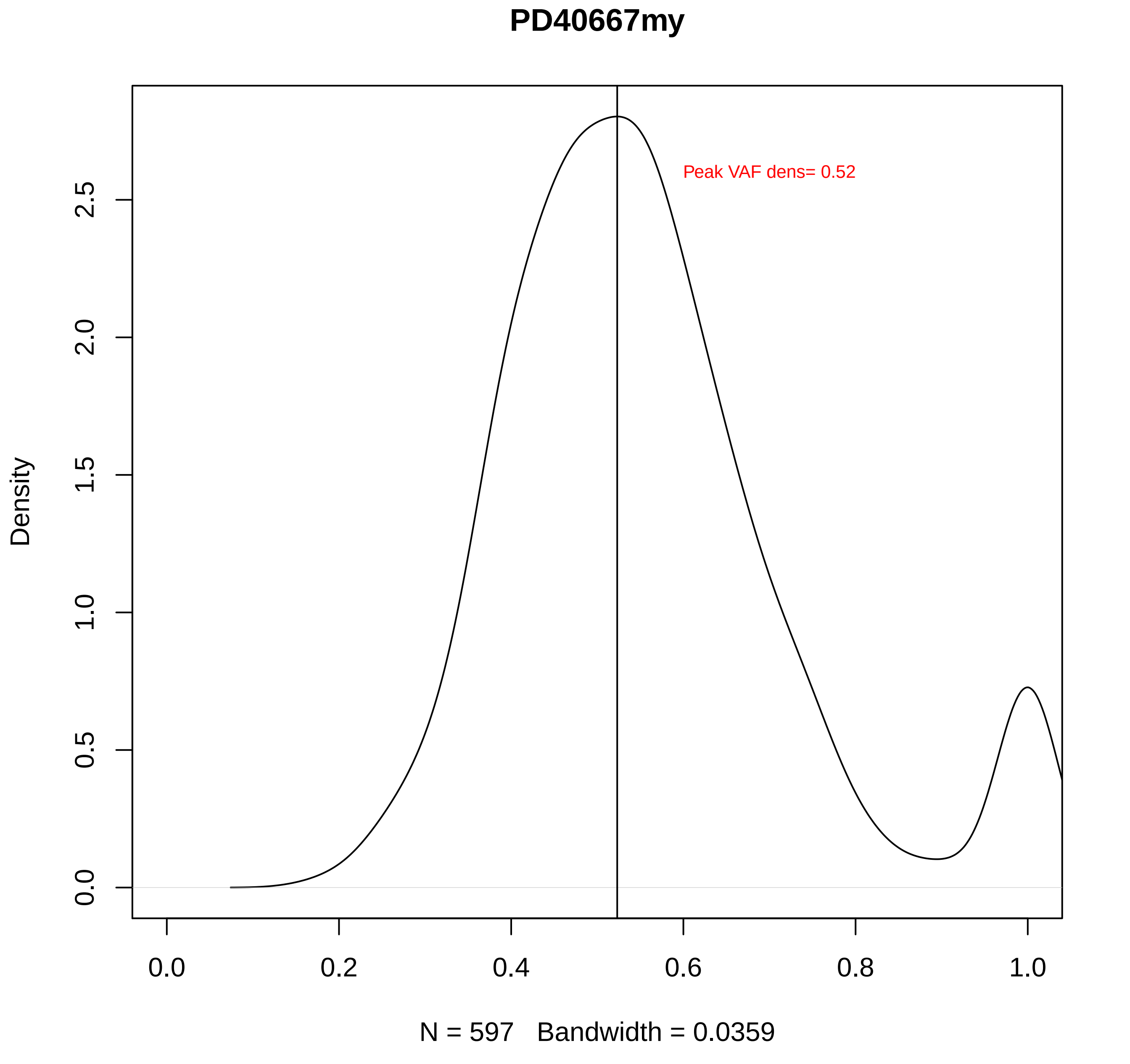
PD40667my
Peak VAF dens= 0.52
Density
0.0
0.5
1.0
1.5
2.0
2.5
0.0
0.2
0.4
0.6
0.8
1.0
N = 597   Bandwidth = 0.0359

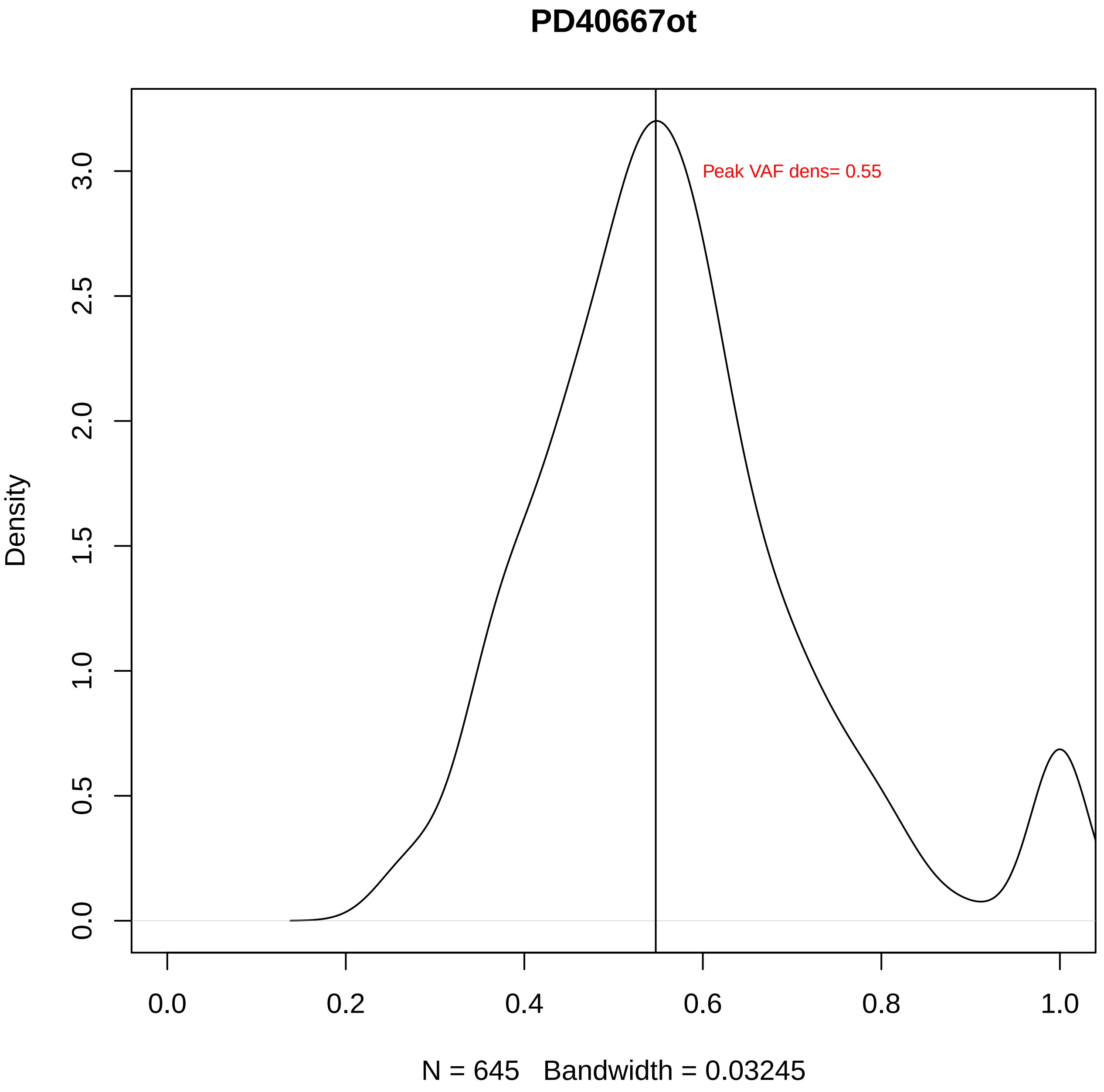

PD40667ot
Peak VAF dens= 0.55
Density
0.0
0.5
1.0
1.5
2.0
2.5
3.0
0.0
0.2
0.4
0.6
0.8
1.0
N = 645 Bandwidth = 0.03245

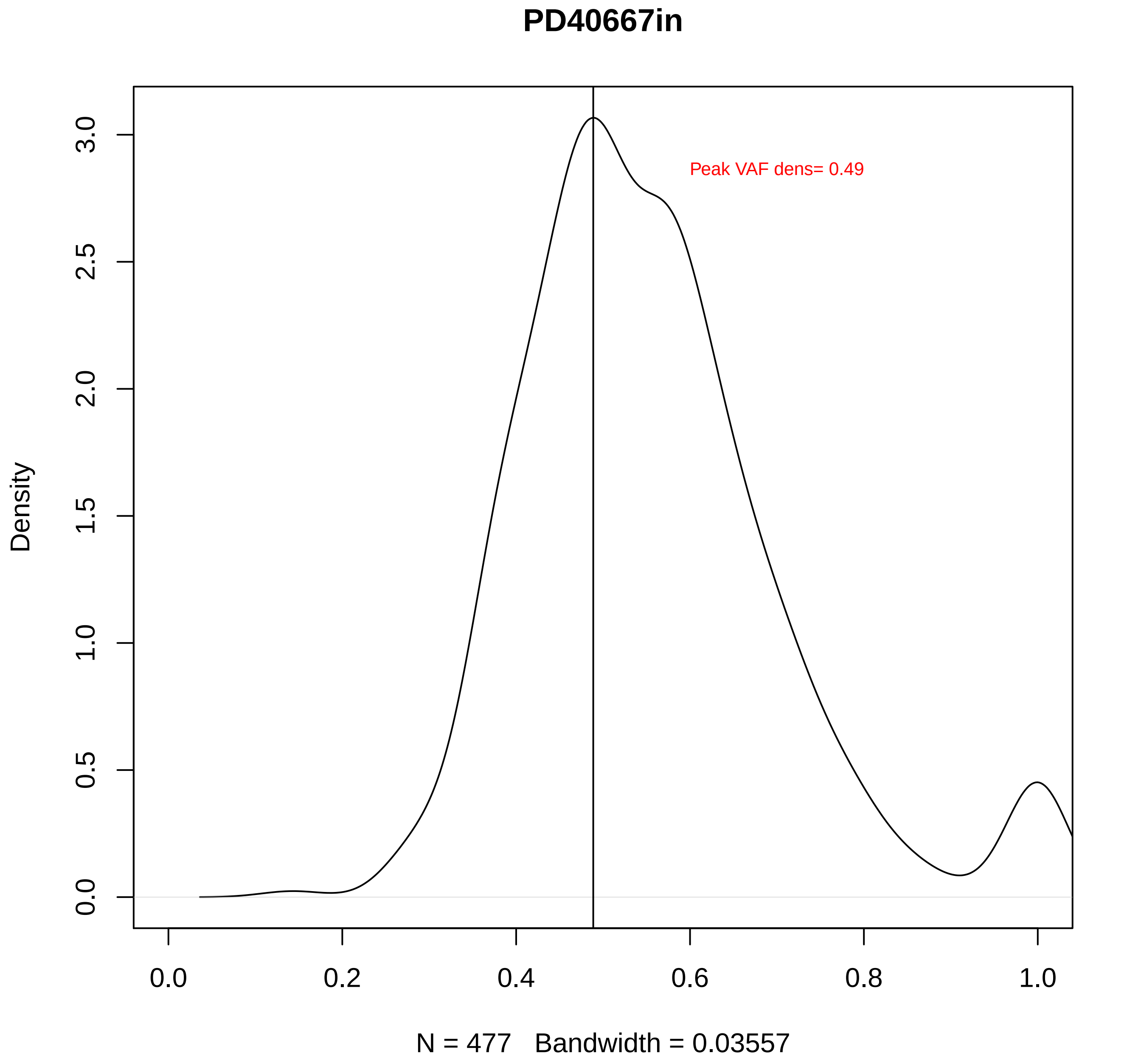

PD40667in
Peak VAF dens= 0.49
Density
3.0
2.5
2.0
1.5
1.0
0.5
0.0
0.0
0.2
0.4
0.6
0.8
1.0
N = 477   Bandwidth = 0.03557

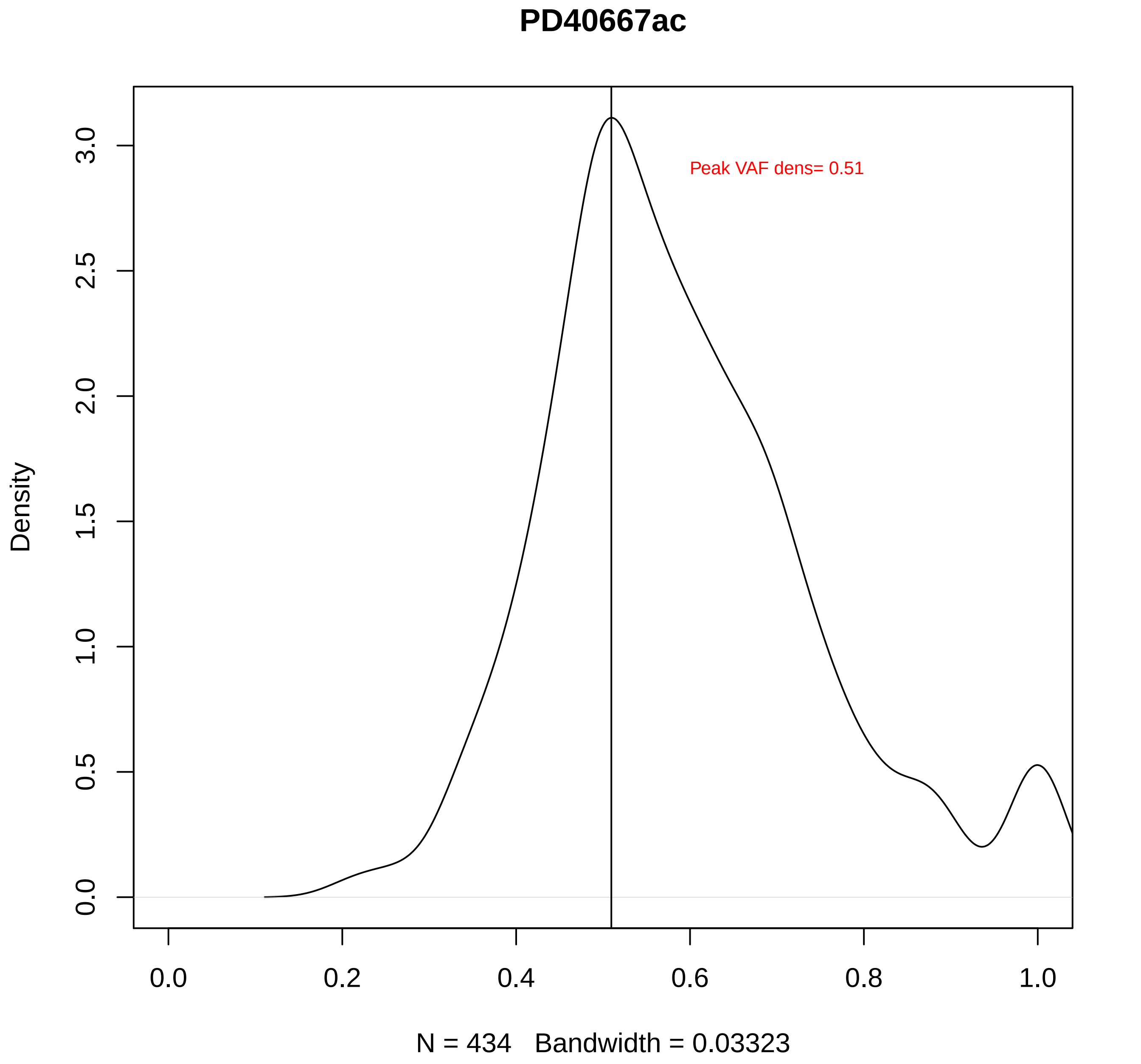

PD40667ac
Peak VAF dens= 0.51
Density
0.0
0.5
1.0
1.5
2.0
2.5
3.0
0.0
0.2
0.4
0.6
0.8
1.0
N = 434   Bandwidth = 0.03323

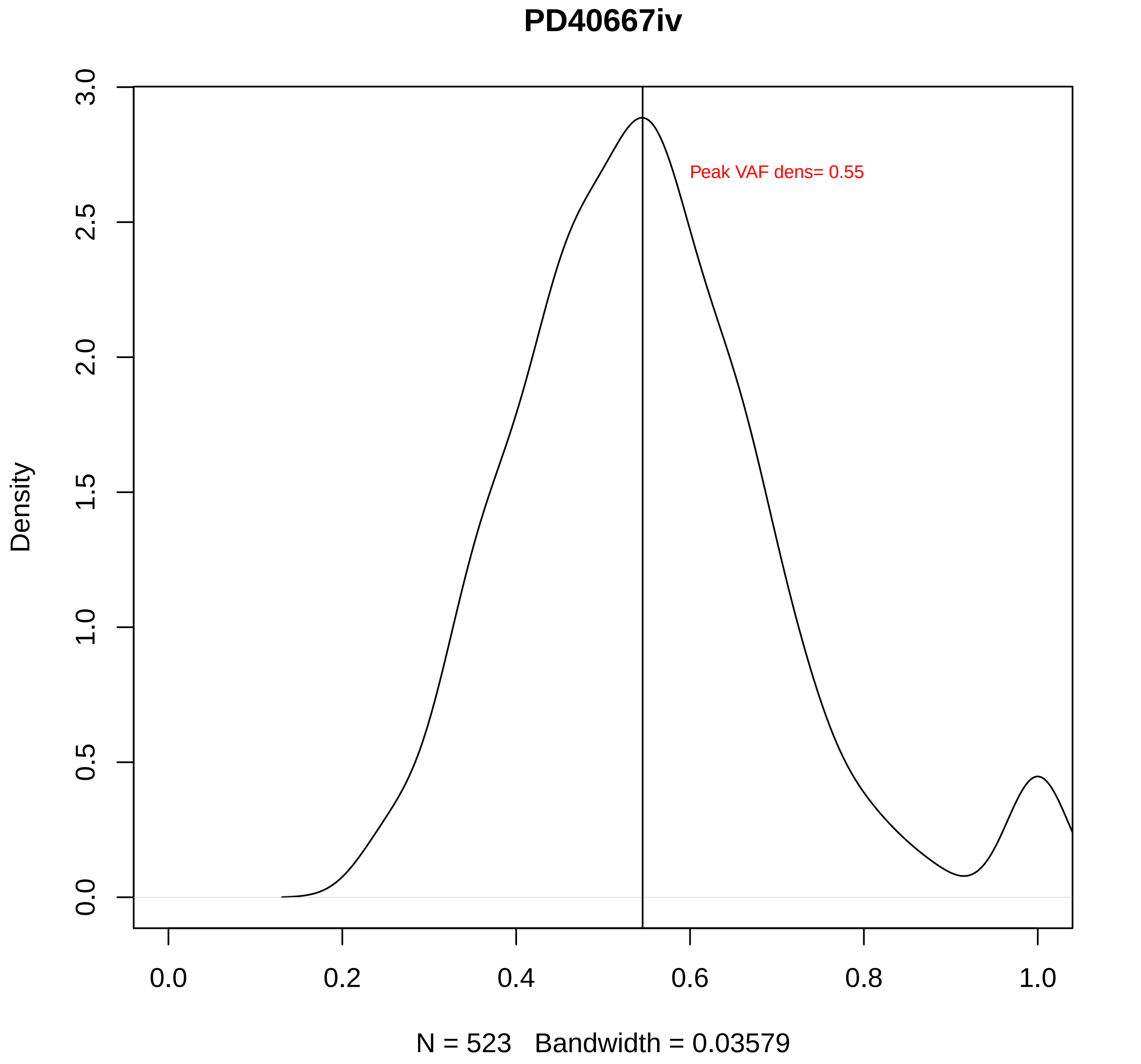

PD40667iv
Peak VAF dens= 0.55
Density
3.0
2.5
2.0
1.5
1.0
0.5
0.0
0.0
0.2
0.4
0.6
0.8
1.0
N = 523 Bandwidth = 0.03579

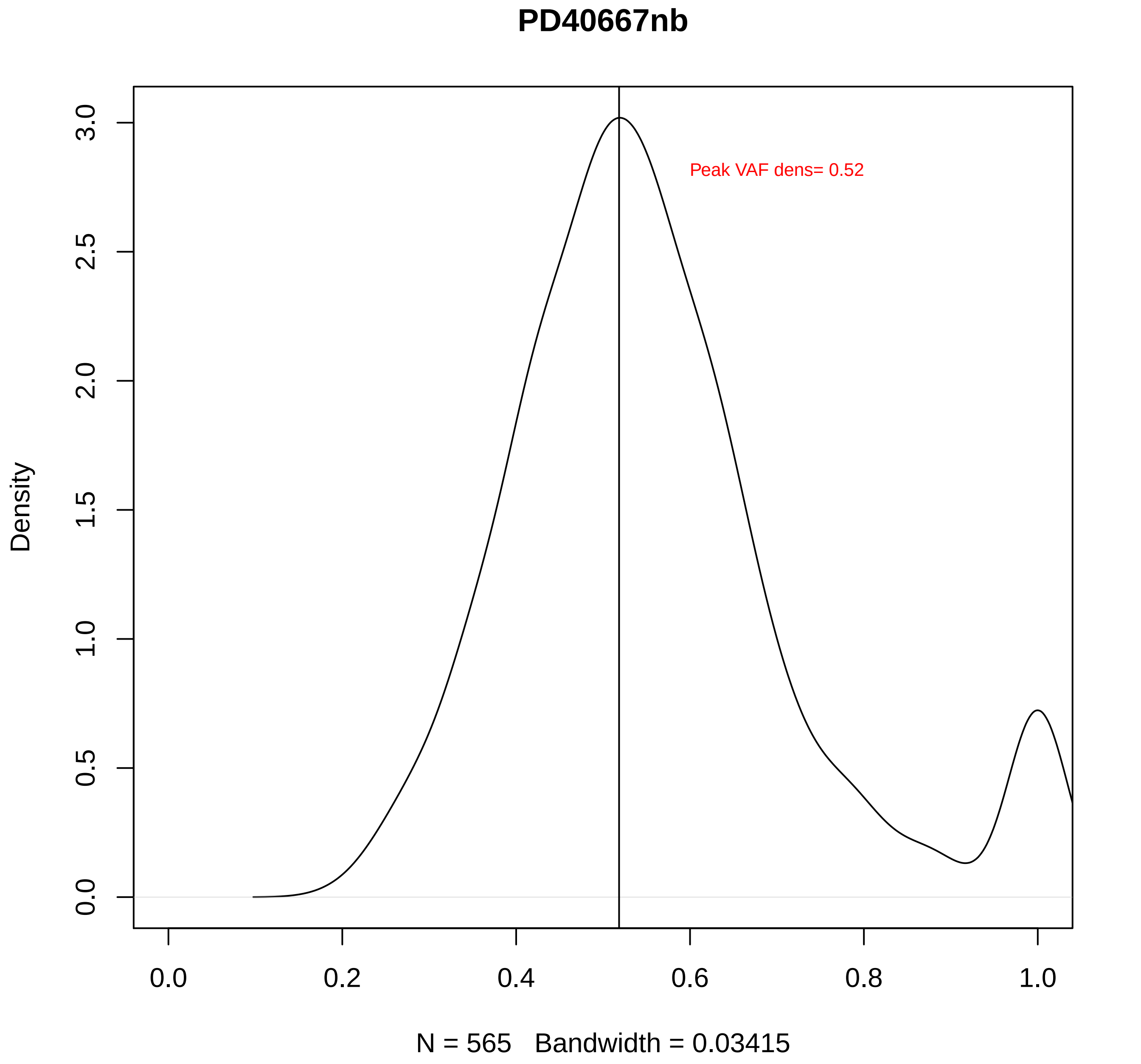

PD40667nb
Peak VAF dens= 0.52
Density
0.0
0.5
1.0
1.5
2.0
2.5
3.0
0.0
0.2
0.4
0.6
0.8
1.0
N = 565 Bandwidth = 0.03415

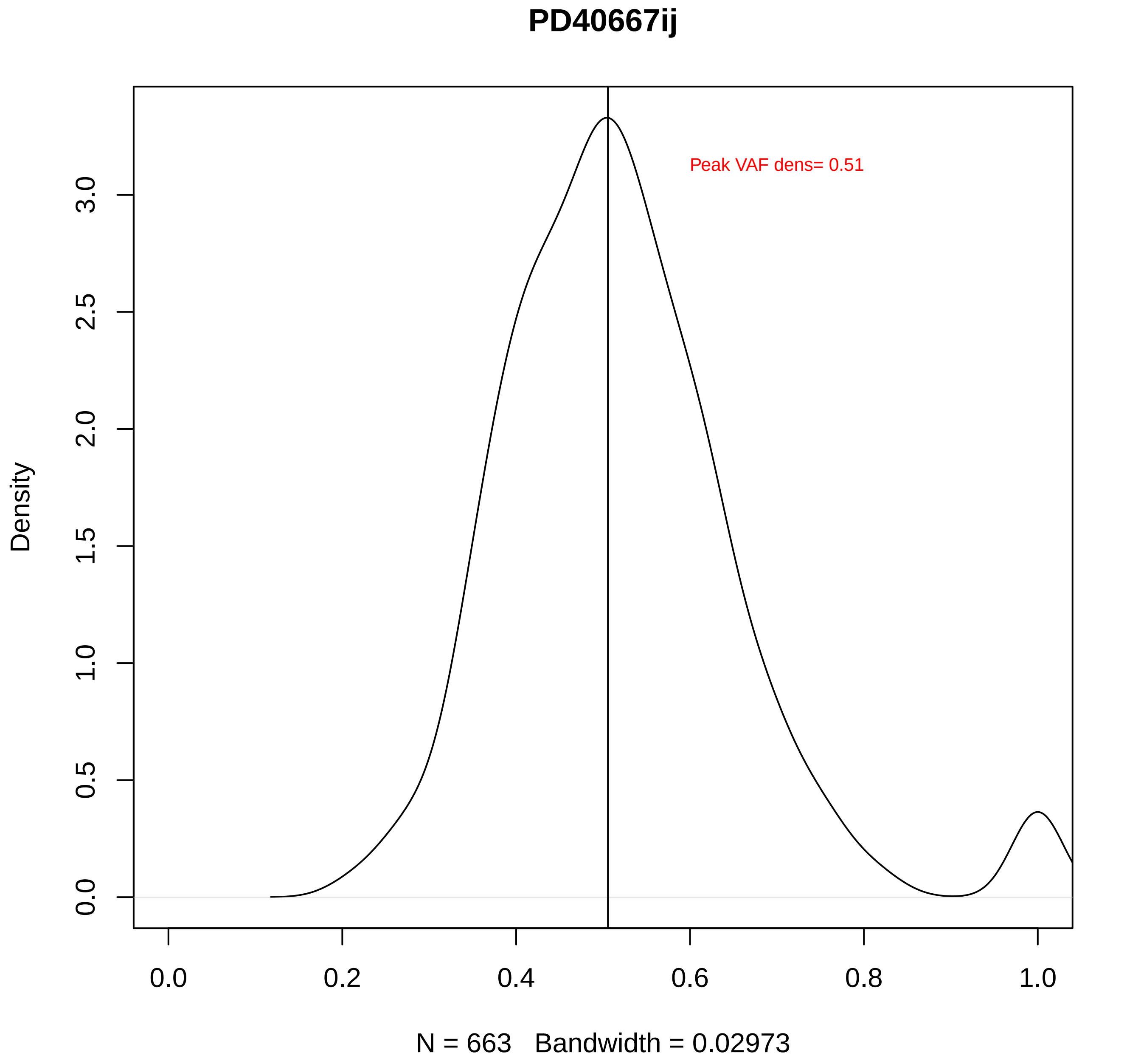
PD40667ij
Peak VAF dens= 0.51
Density
0.0
0.5
1.0
1.5
2.0
2.5
3.0
0.0
0.2
0.4
0.6
0.8
1.0
N = 663   Bandwidth = 0.02973

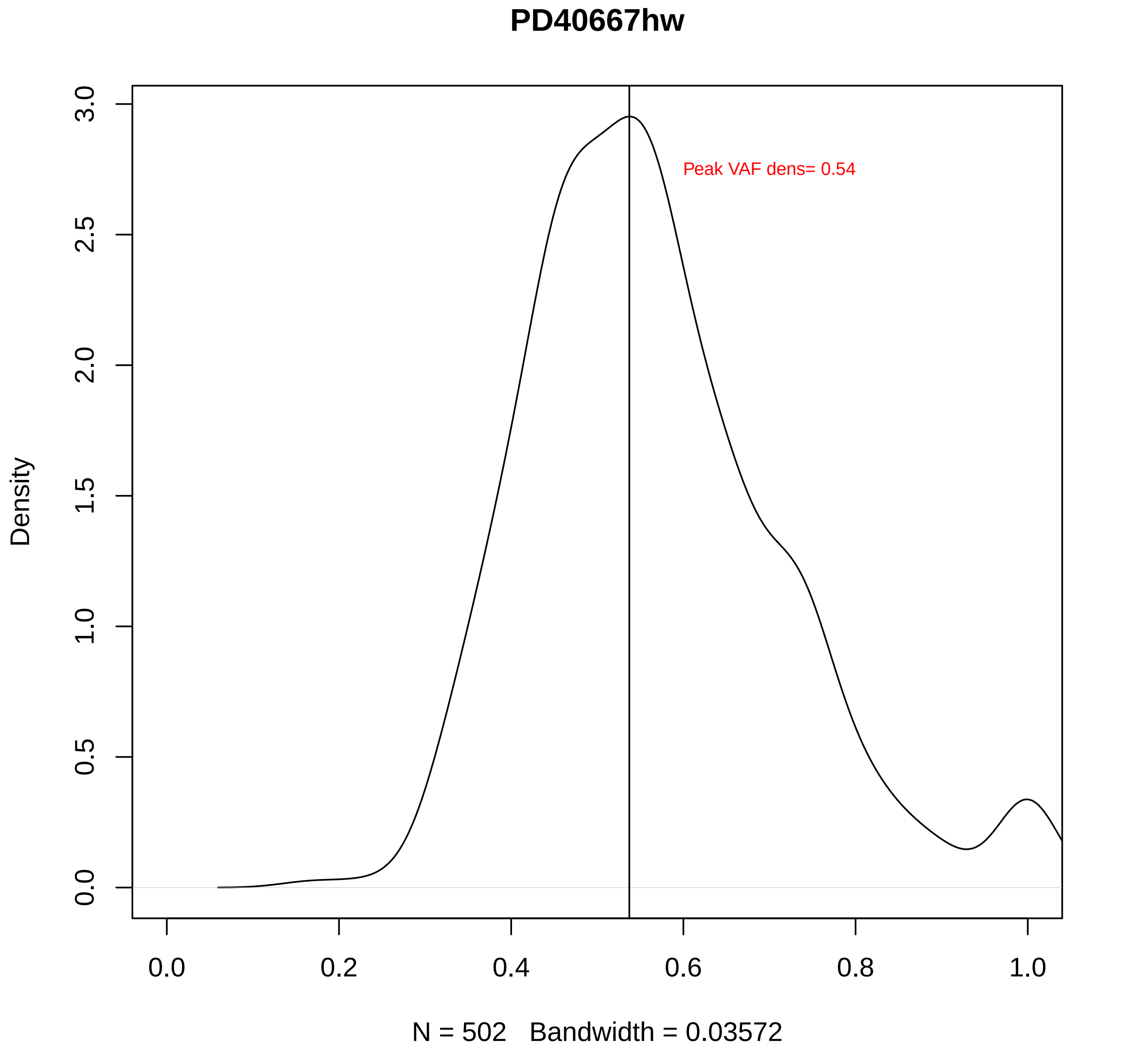
PD40667hw
Peak VAF dens= 0.54
Density
0.0
0.5
1.0
1.5
2.0
2.5
3.0
0.0
0.2
0.4
0.6
0.8
1.0
N = 502   Bandwidth = 0.03572

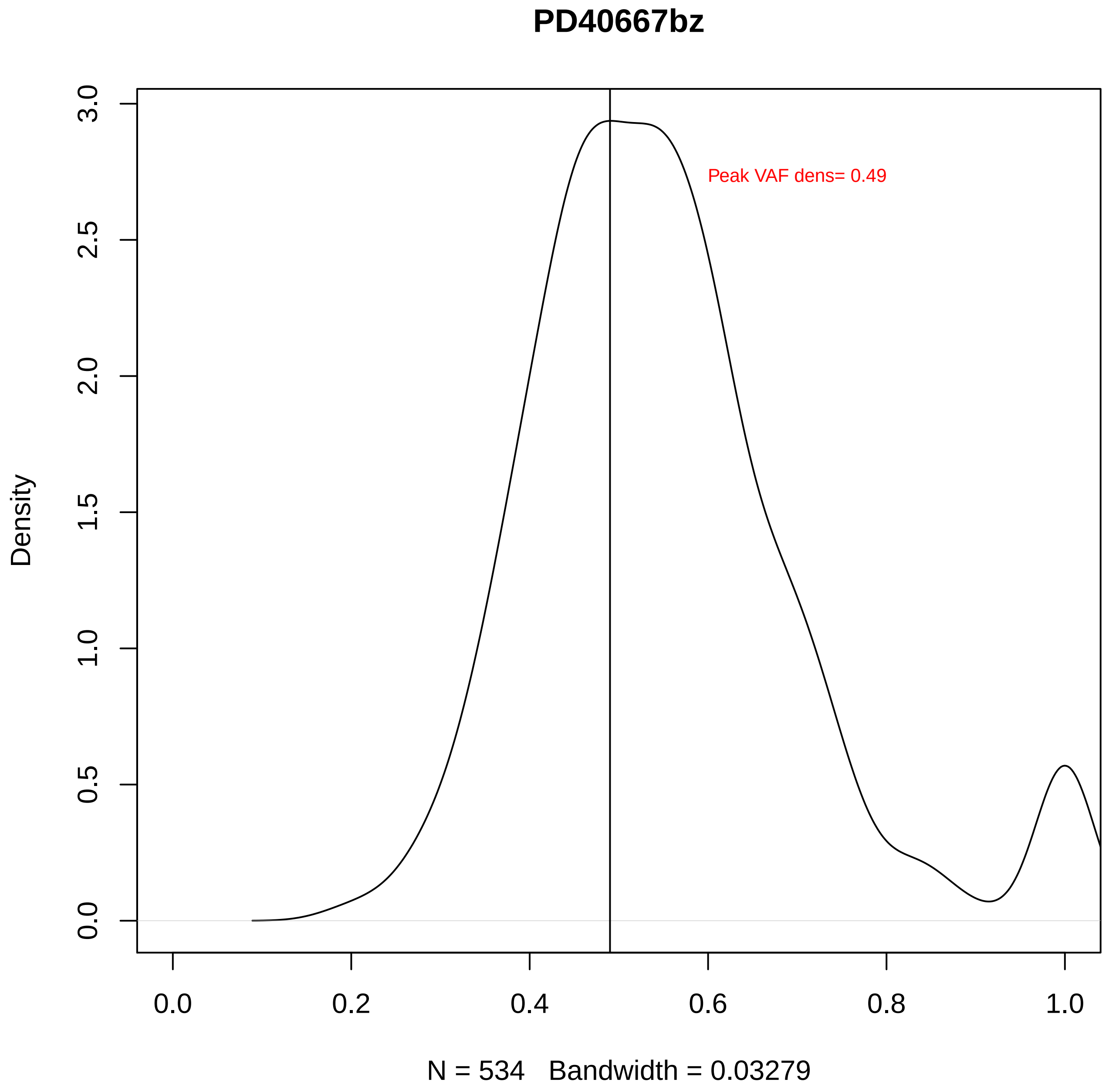

PD40667bz
Peak VAF dens= 0.49
Density
0.0
0.5
1.0
1.5
2.0
2.5
3.0
0.0
0.2
0.4
0.6
0.8
1.0
N = 534   Bandwidth = 0.03279

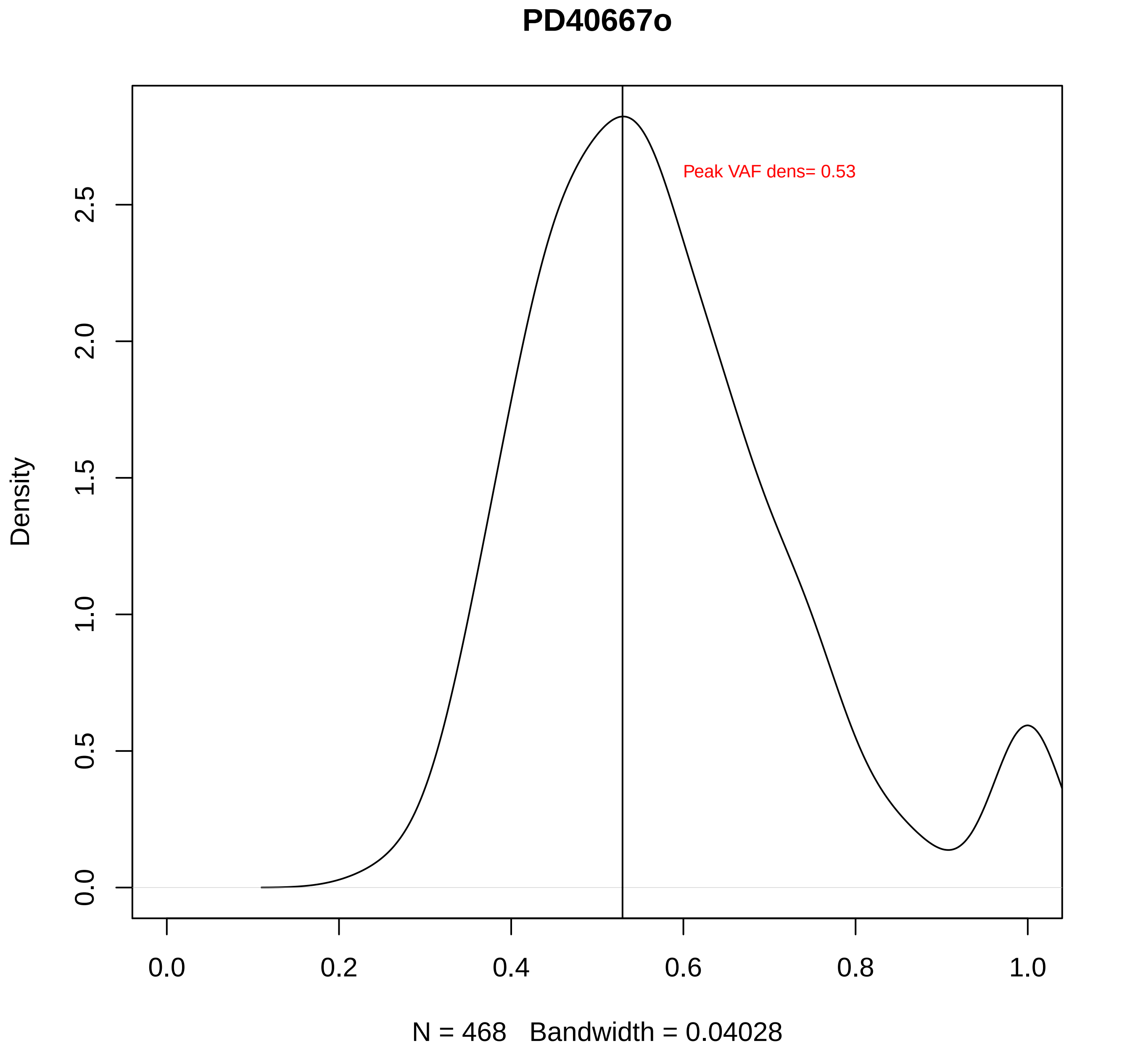
PD40667o
Peak VAF dens= 0.53
Density
0.0
0.5
1.0
1.5
2.0
2.5
0.0
0.2
0.4
0.6
0.8
1.0
N = 468 Bandwidth = 0.04028

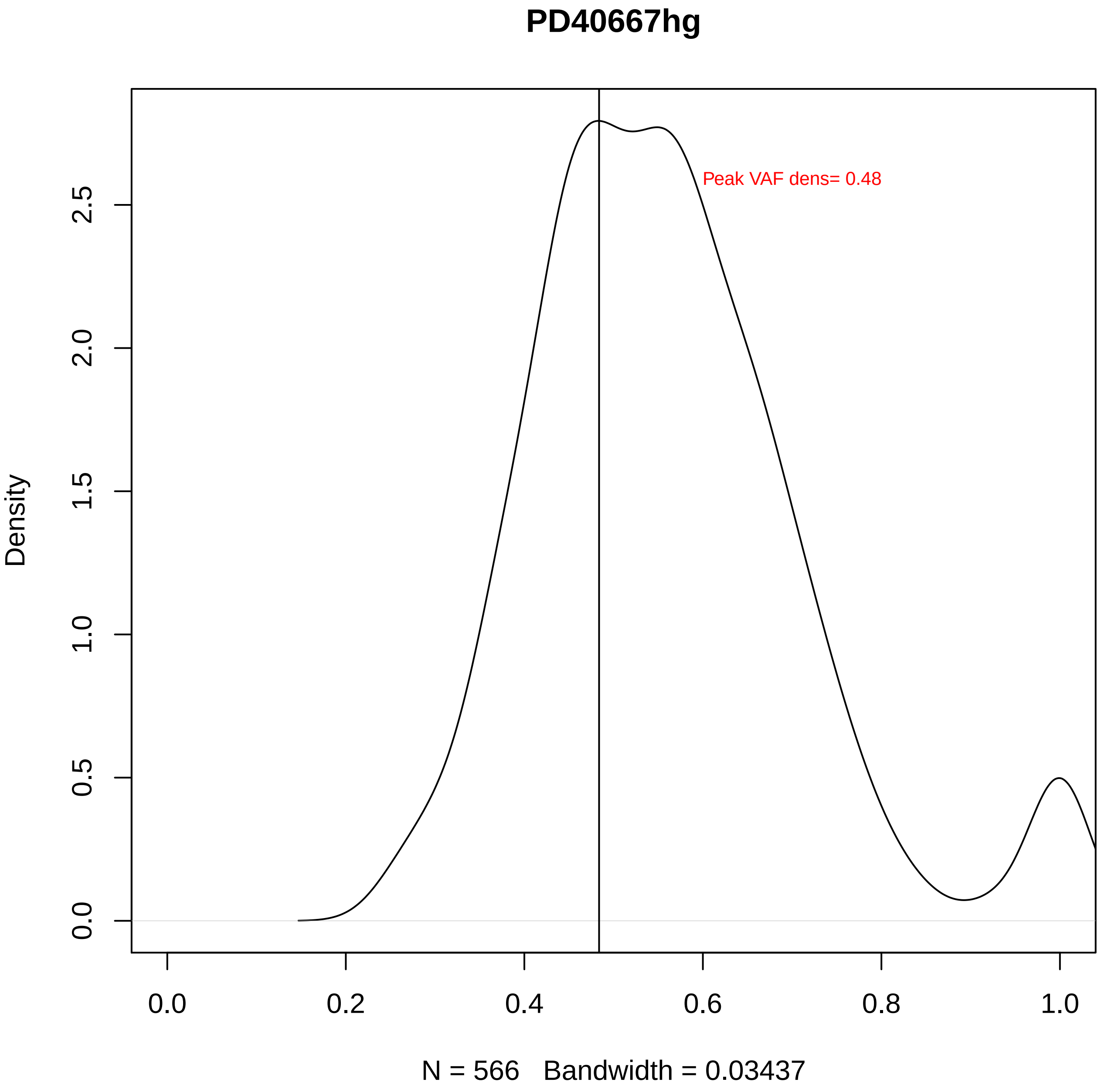
PD40667hg
Peak VAF dens= 0.48
Density
0.0
0.5
1.0
1.5
2.0
2.5
0.0
0.2
0.4
0.6
0.8
1.0
N = 566   Bandwidth = 0.03437

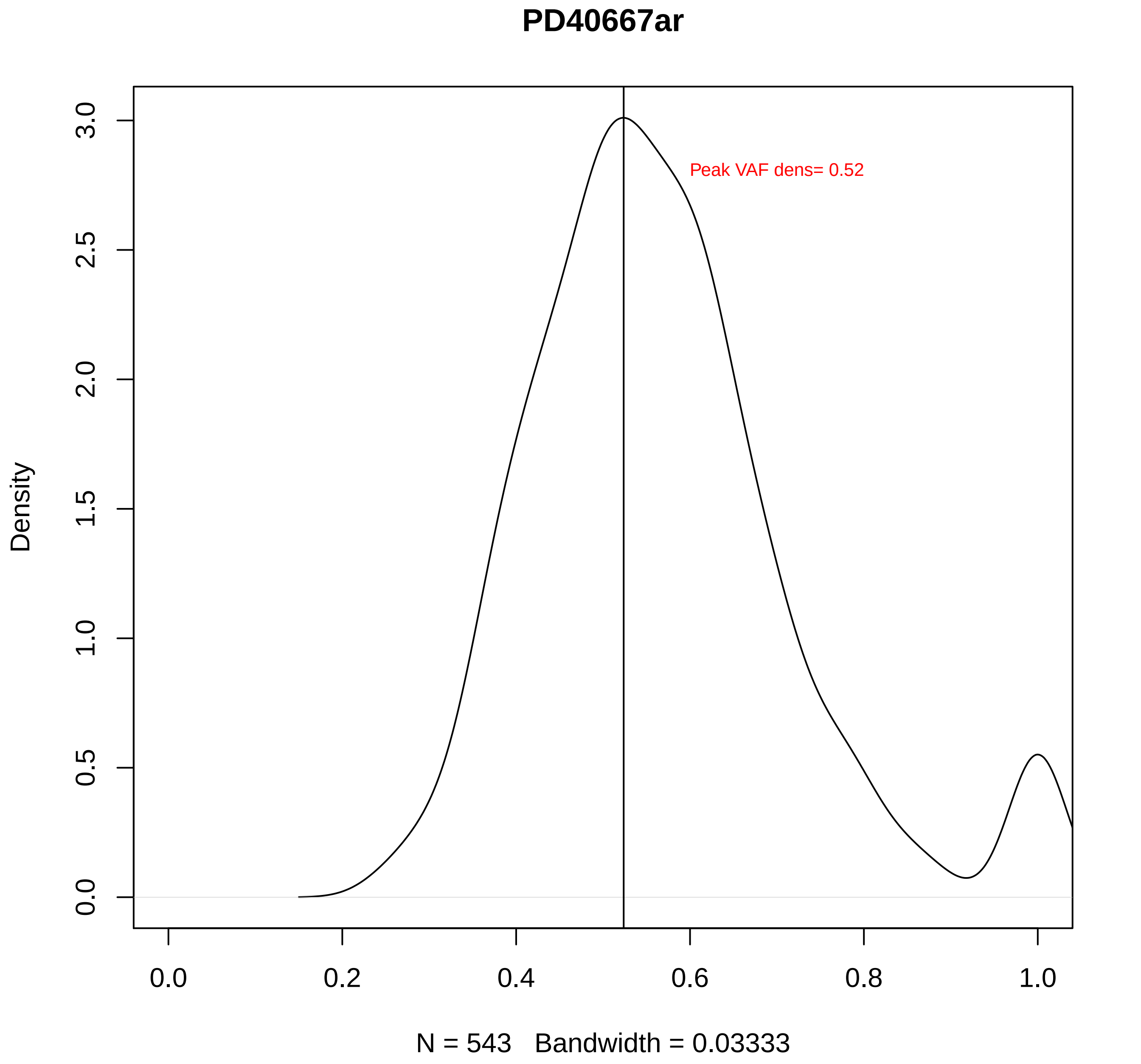

PD40667ar
Peak VAF dens= 0.52
Density
3.0
2.5
2.0
1.5
1.0
0.5
0.0
0.0
0.2
0.4
0.6
0.8
1.0
N = 543   Bandwidth = 0.03333

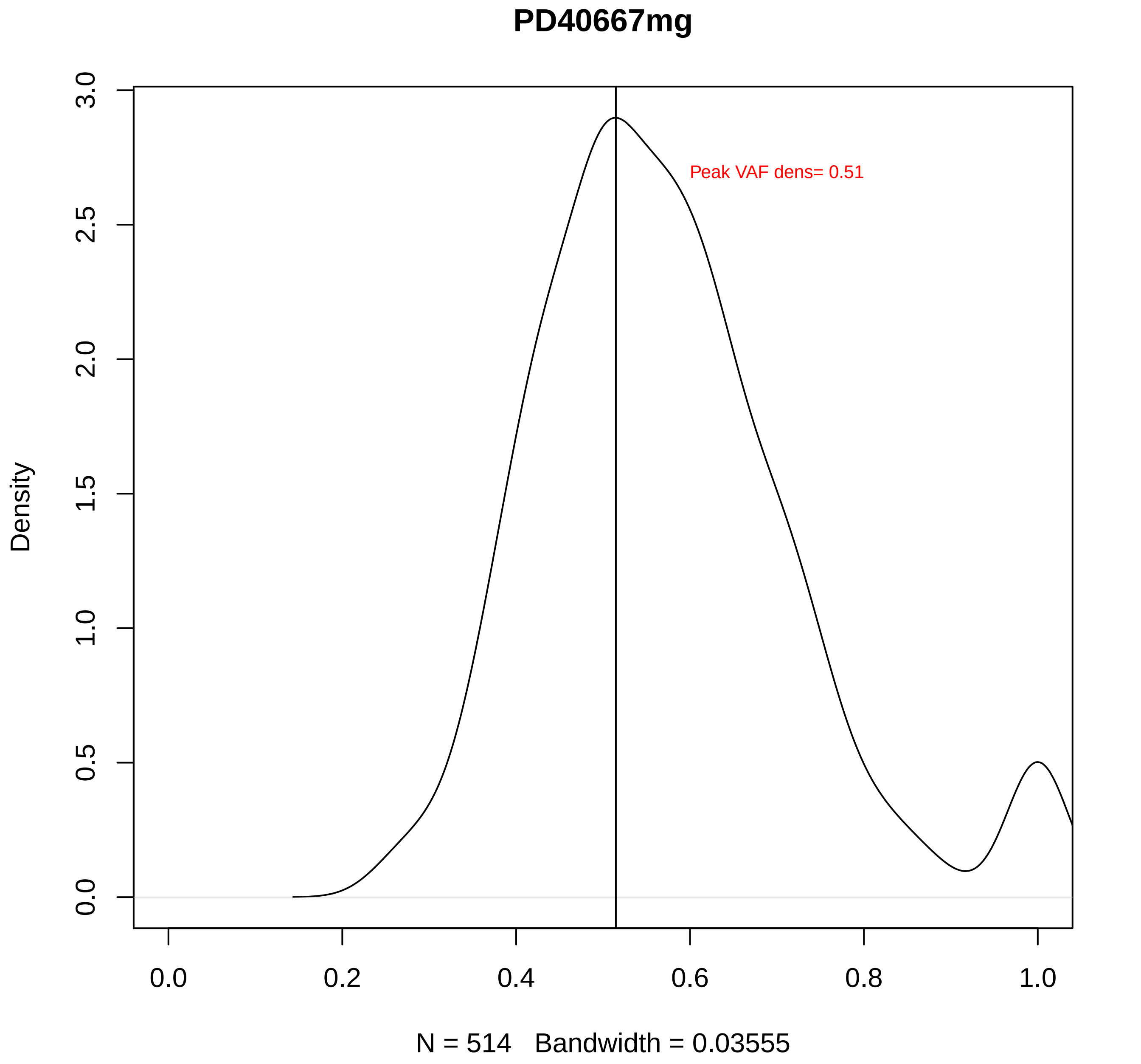
PD40667mg
Peak VAF dens= 0.51
Density
0.0
0.5
1.0
1.5
2.0
2.5
3.0
0.0
0.2
0.4
0.6
0.8
1.0
N = 514   Bandwidth = 0.03555

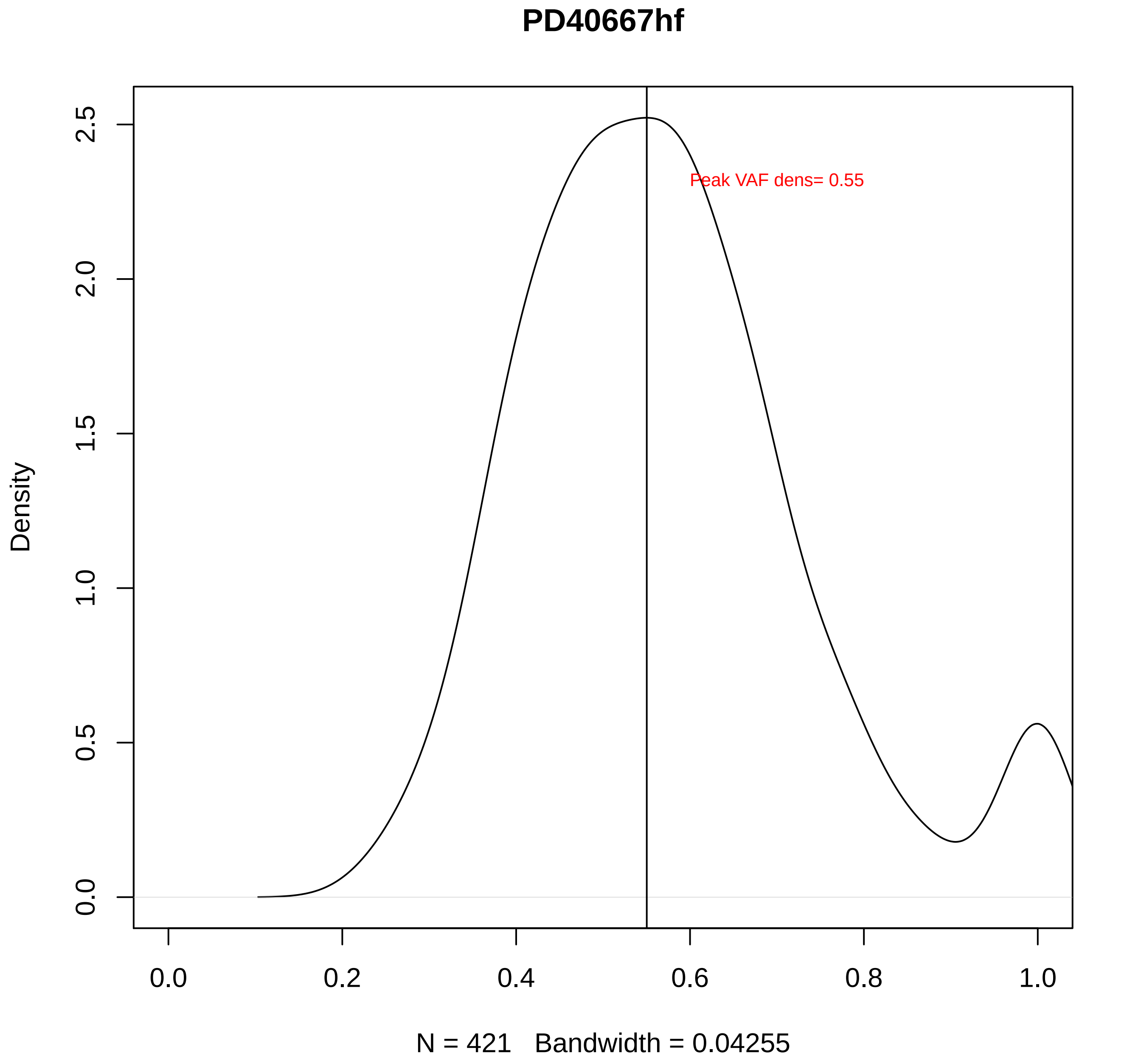
PD40667hf
Peak VAF dens= 0.55
Density
0.0
0.5
1.0
1.5
2.0
2.5
0.0
0.2
0.4
0.6
0.8
1.0
N = 421 Bandwidth = 0.04255

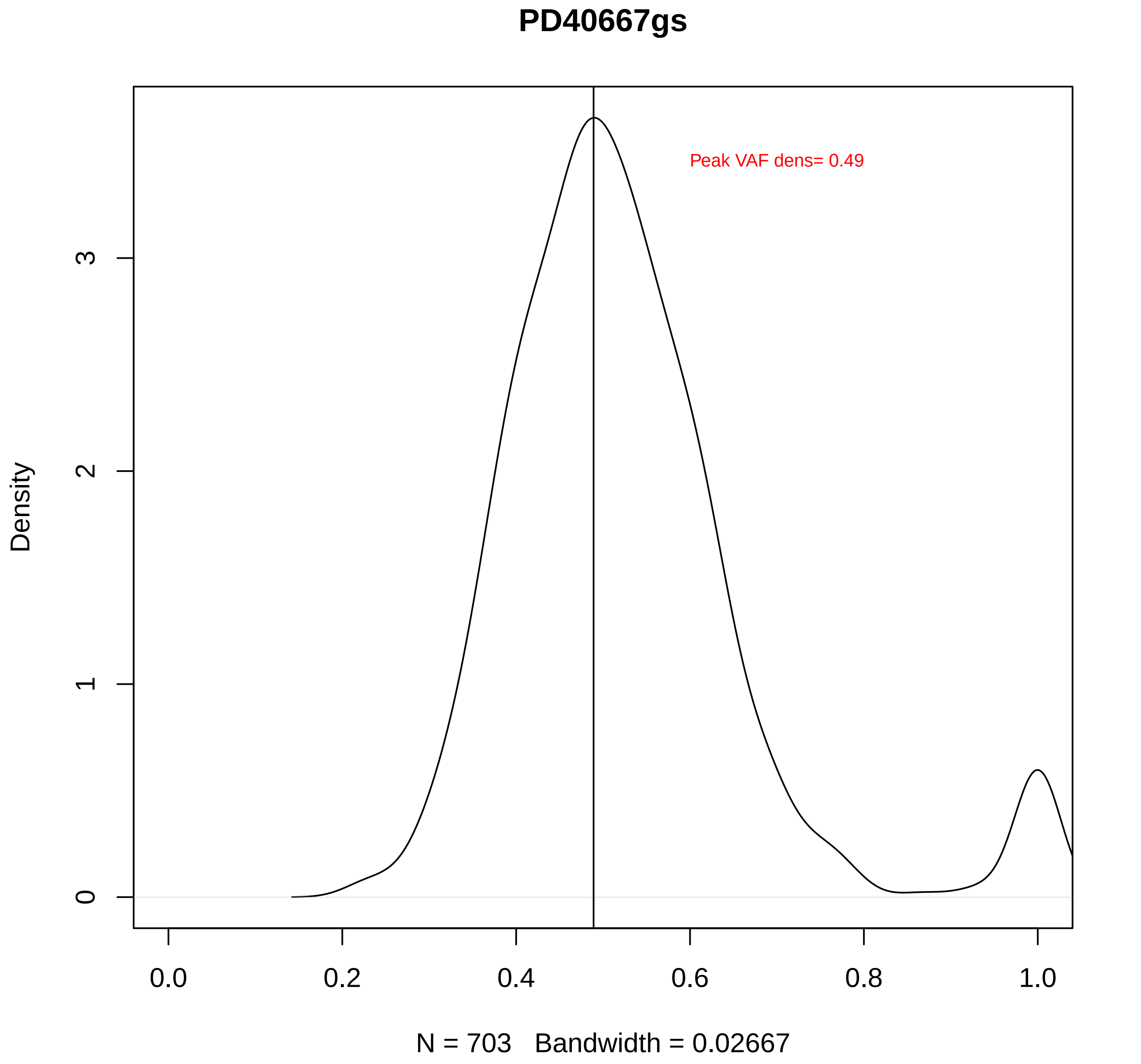

PD40667gs
Peak VAF dens= 0.49
Density
0
1
2
3
0.0
0.2
0.4
0.6
0.8
1.0
N = 703 Bandwidth = 0.02667

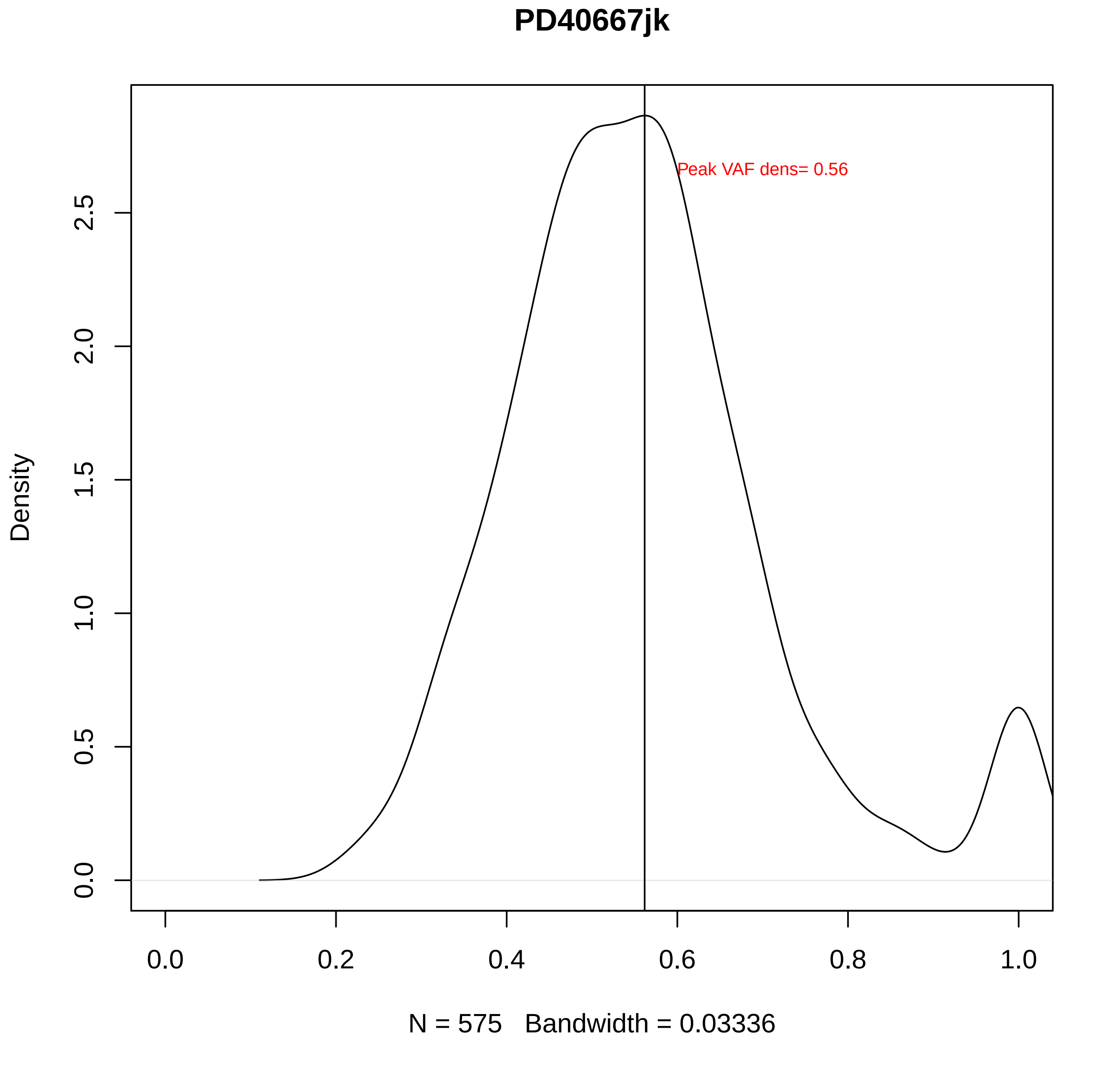
PD40667jk
Peak VAF dens= 0.56
Density
0.0
0.5
1.0
1.5
2.0
2.5
0.0
0.2
0.4
0.6
0.8
1.0
N = 575   Bandwidth = 0.03336

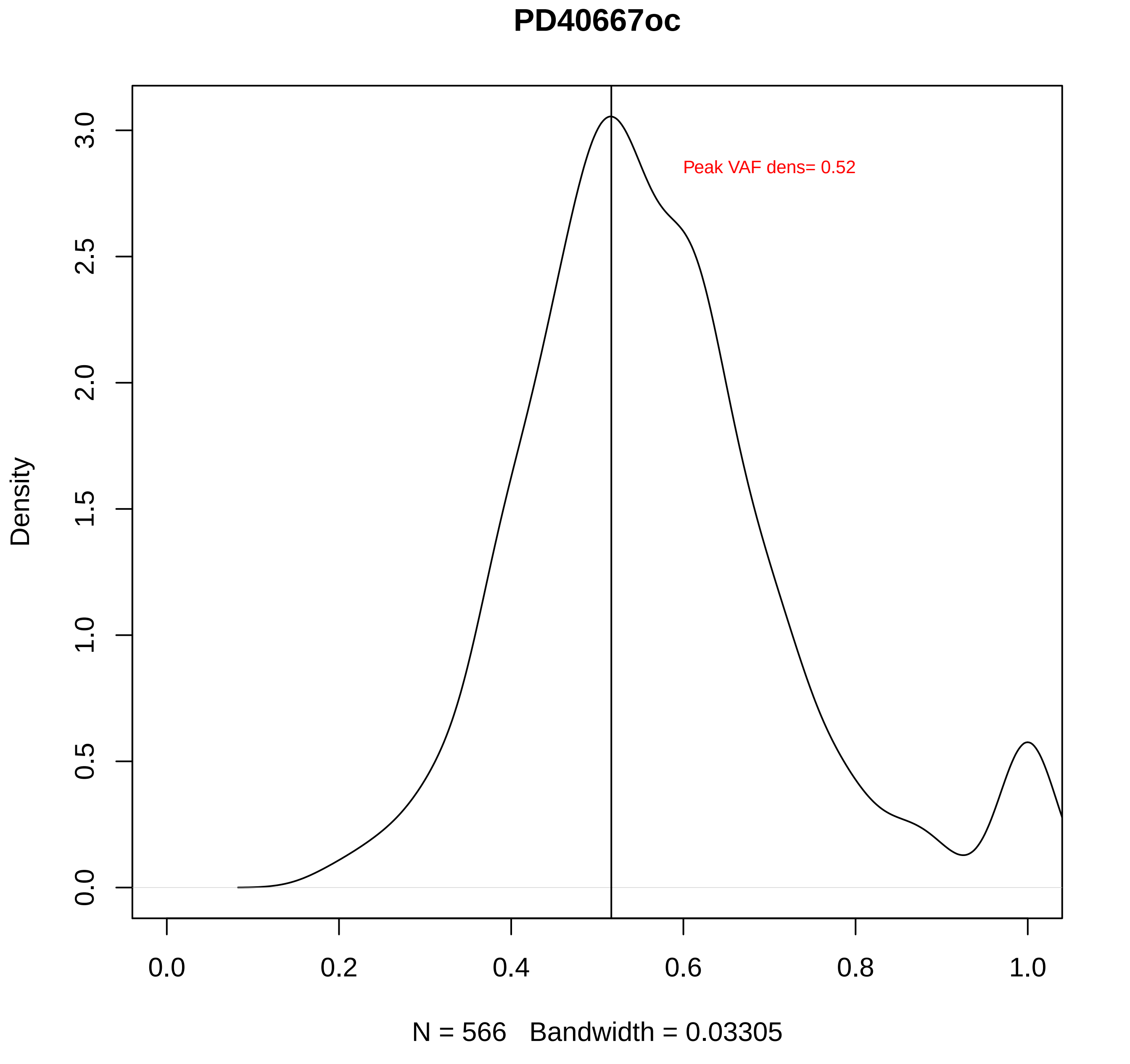
PD40667oc
Peak VAF dens= 0.52
Density
0.0
0.5
1.0
1.5
2.0
2.5
3.0
0.0
0.2
0.4
0.6
0.8
1.0
N = 566   Bandwidth = 0.03305

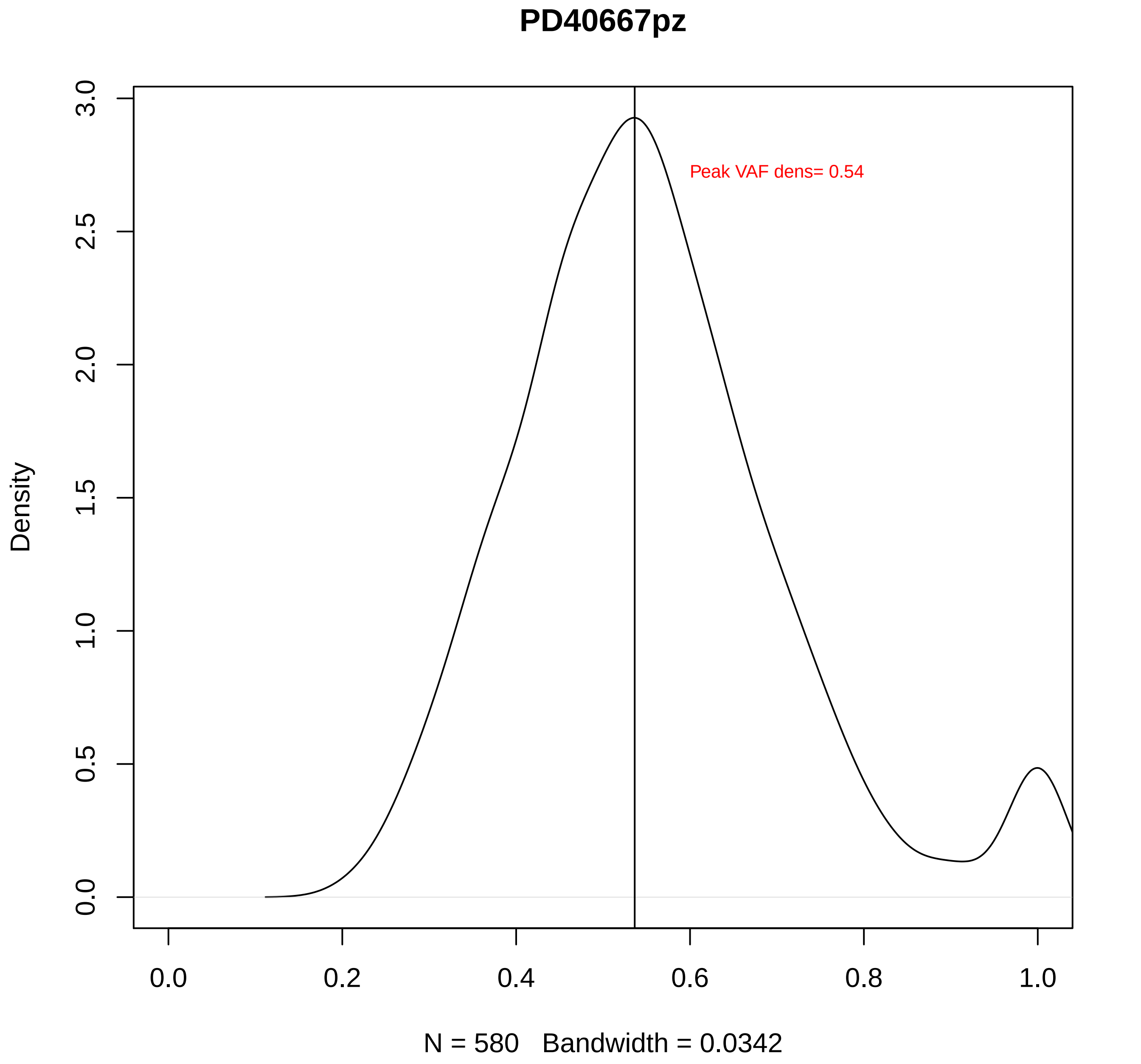
PD40667pz
Peak VAF dens= 0.54
Density
0.0
0.5
1.0
1.5
2.0
2.5
3.0
0.0
0.2
0.4
0.6
0.8
1.0
N = 580 Bandwidth = 0.0342

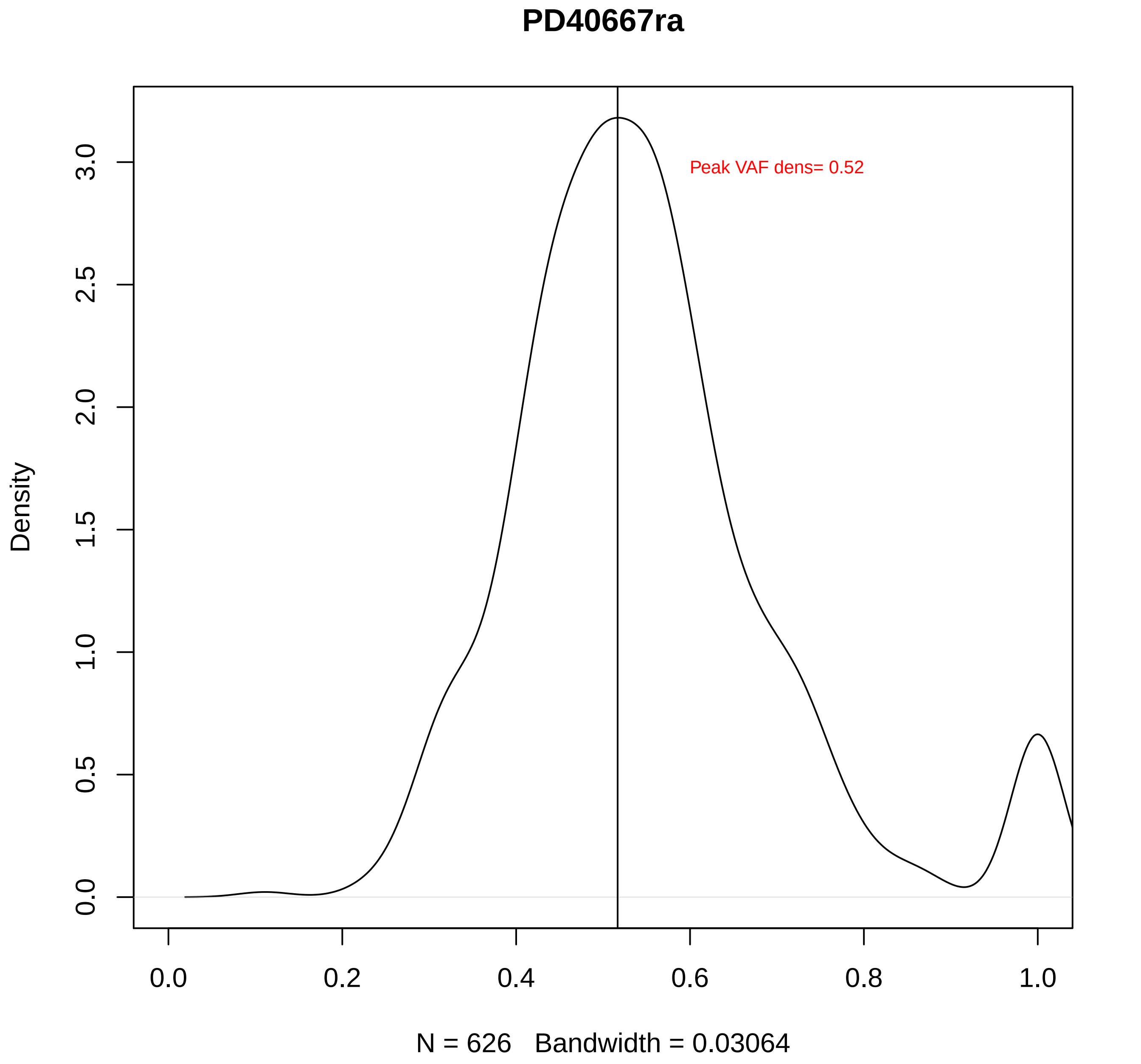

PD40667ra
Peak VAF dens= 0.52
Density
0.0
0.5
1.0
1.5
2.0
2.5
3.0
0.0
0.2
0.4
0.6
0.8
1.0
N = 626   Bandwidth = 0.03064

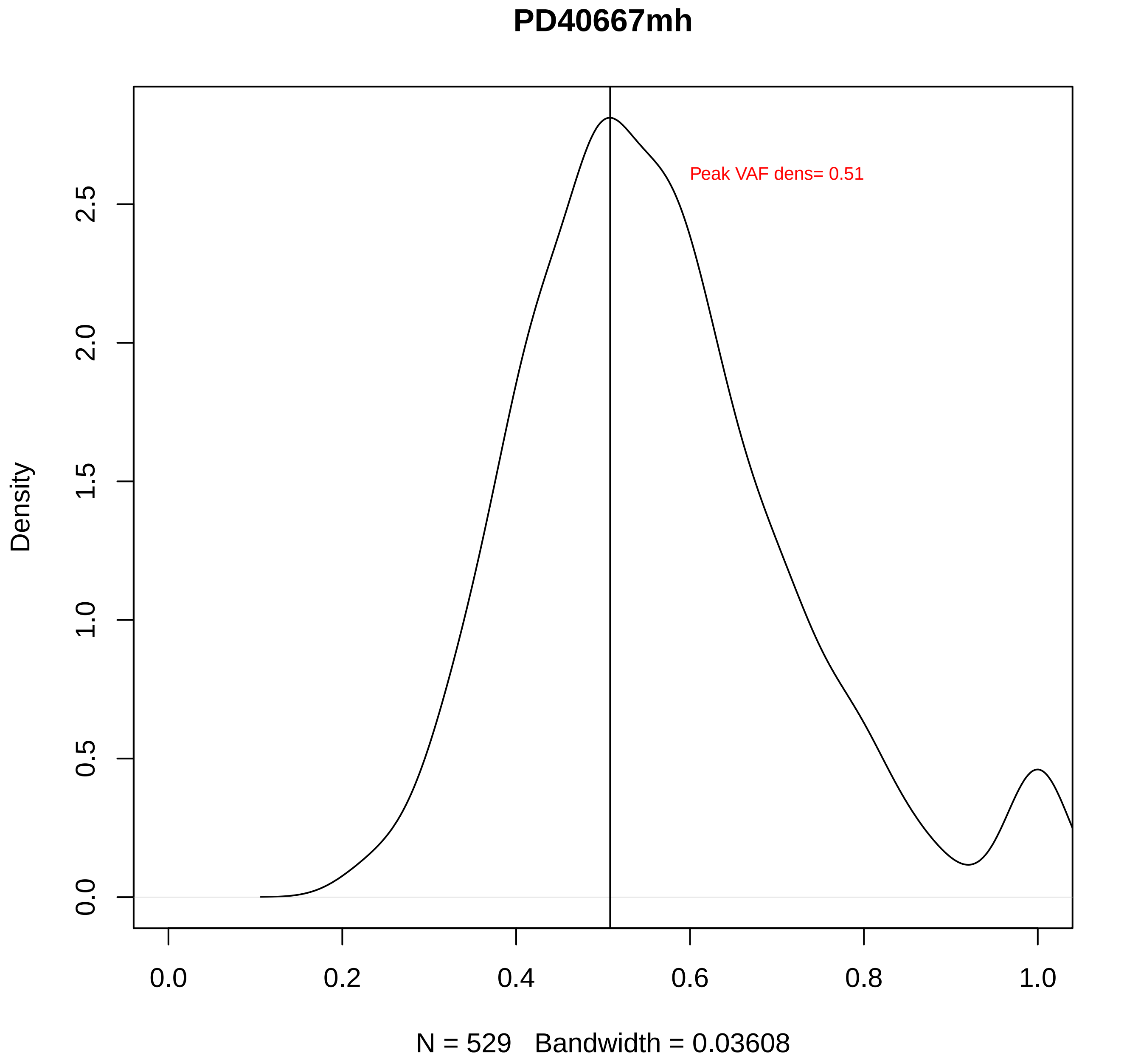
PD40667mh
Peak VAF dens= 0.51
Density
0.0
0.5
1.0
1.5
2.0
2.5
0.0
0.2
0.4
0.6
0.8
1.0
N = 529   Bandwidth = 0.03608

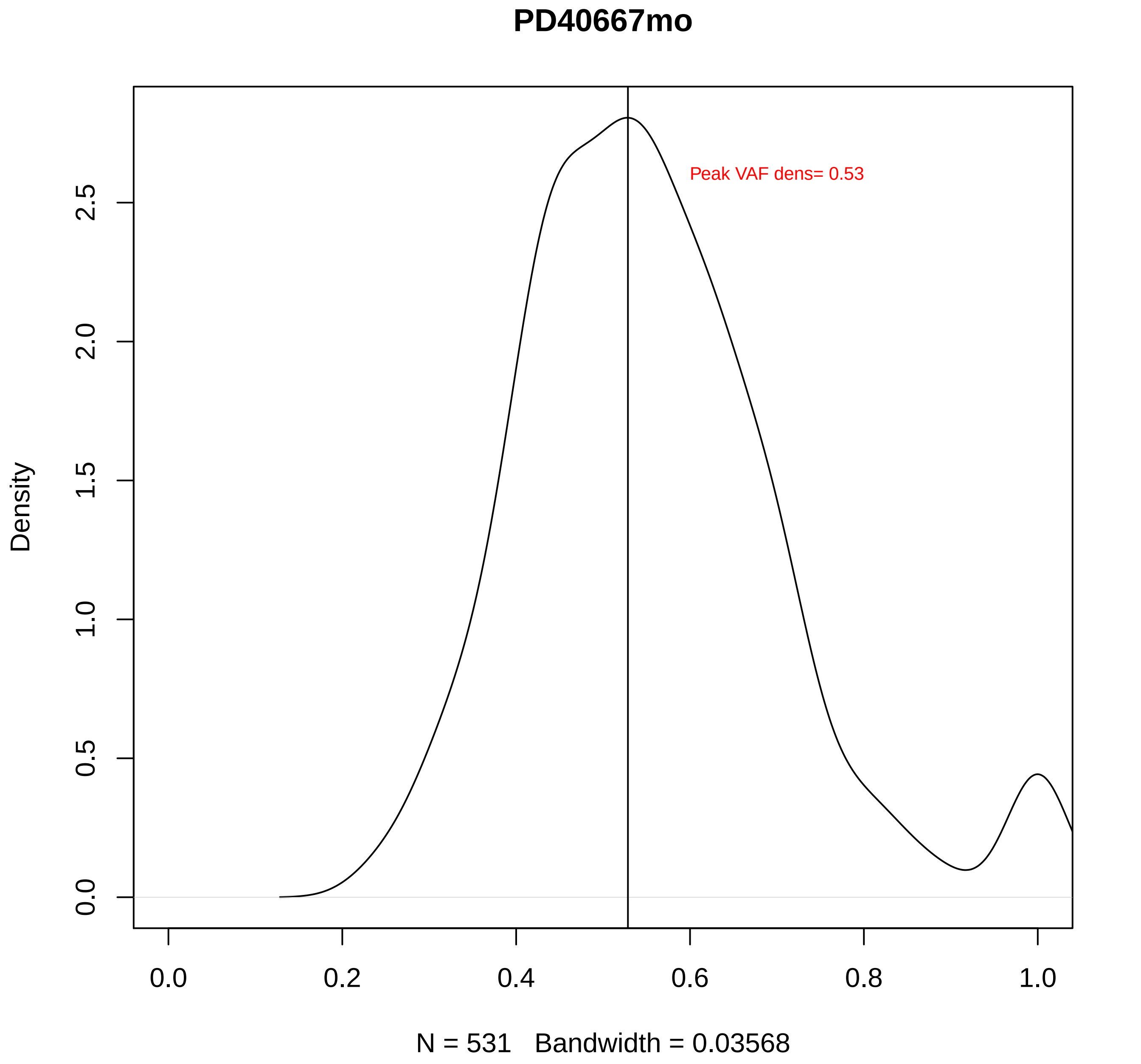

PD40667mo
Peak VAF dens= 0.53
Density
0.0
0.5
1.0
1.5
2.0
2.5
0.0
0.2
0.4
0.6
0.8
1.0
N = 531   Bandwidth = 0.03568

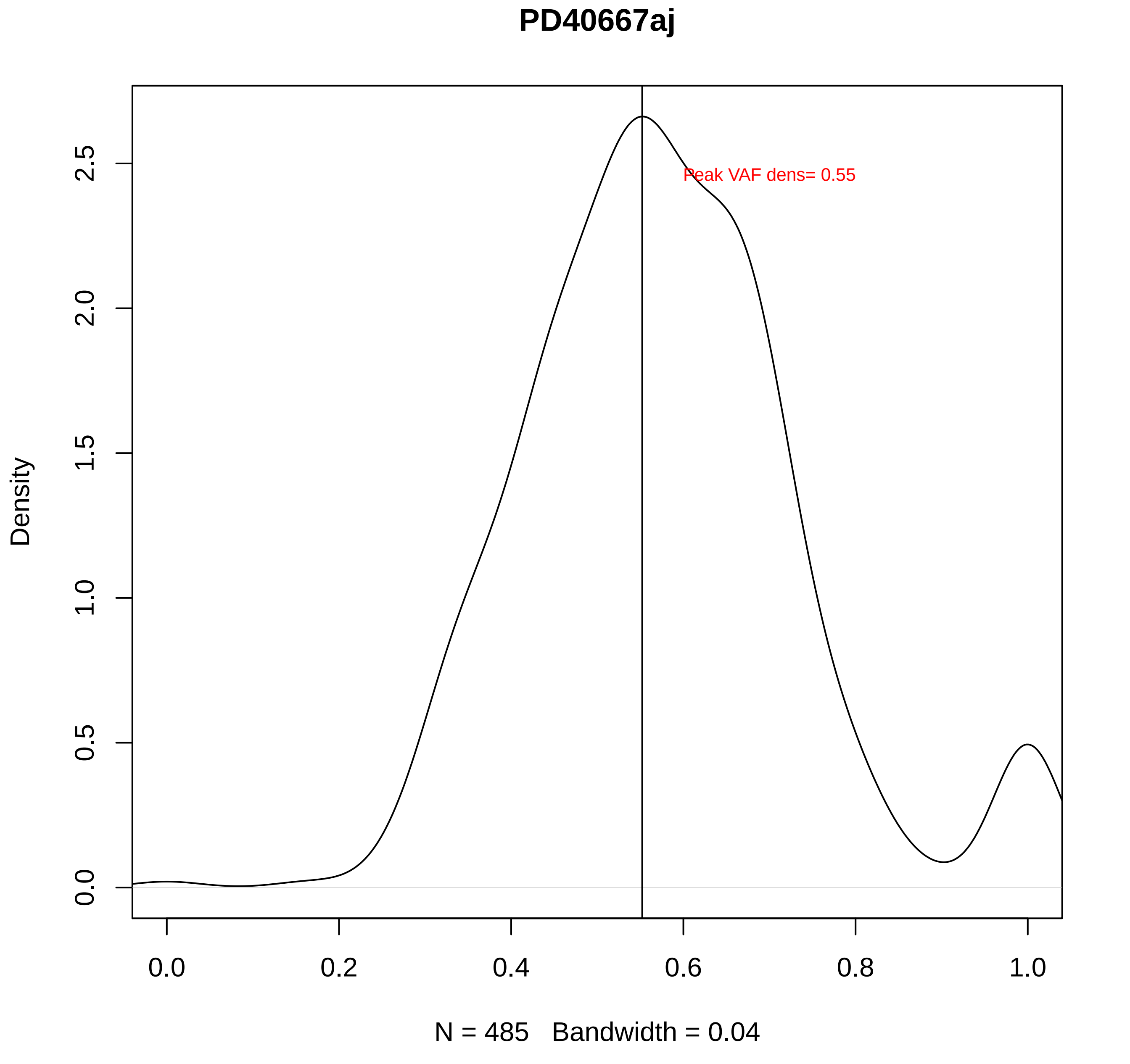

PD40667aj
Peak VAF dens= 0.55
Density
0.0
0.5
1.0
1.5
2.0
2.5
0.0
0.2
0.4
0.6
0.8
1.0
N = 485   Bandwidth = 0.04

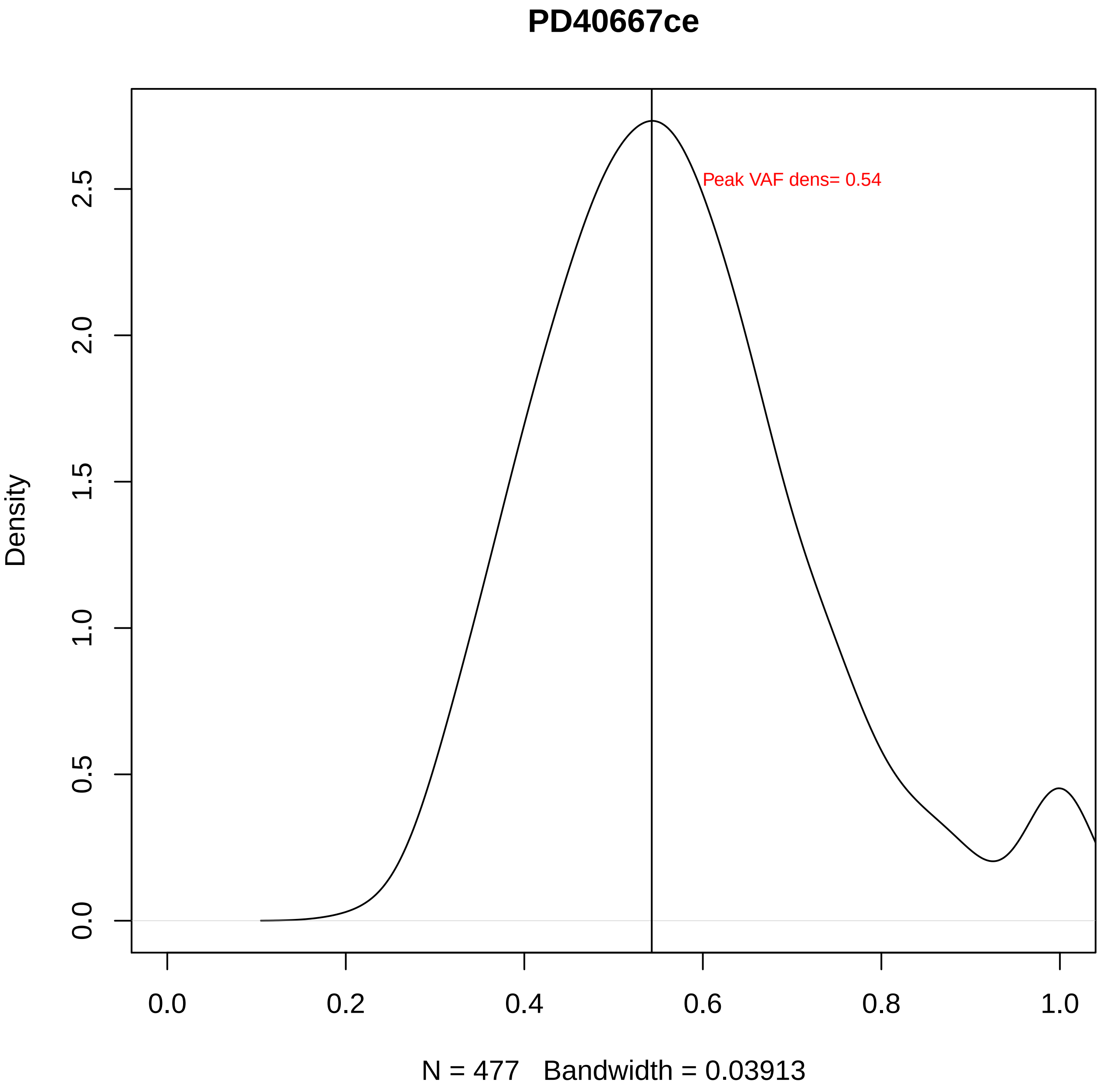
PD40667ce
Peak VAF dens= 0.54
Density
0.0
0.5
1.0
1.5
2.0
2.5
0.0
0.2
0.4
0.6
0.8
1.0
N = 477   Bandwidth = 0.03913

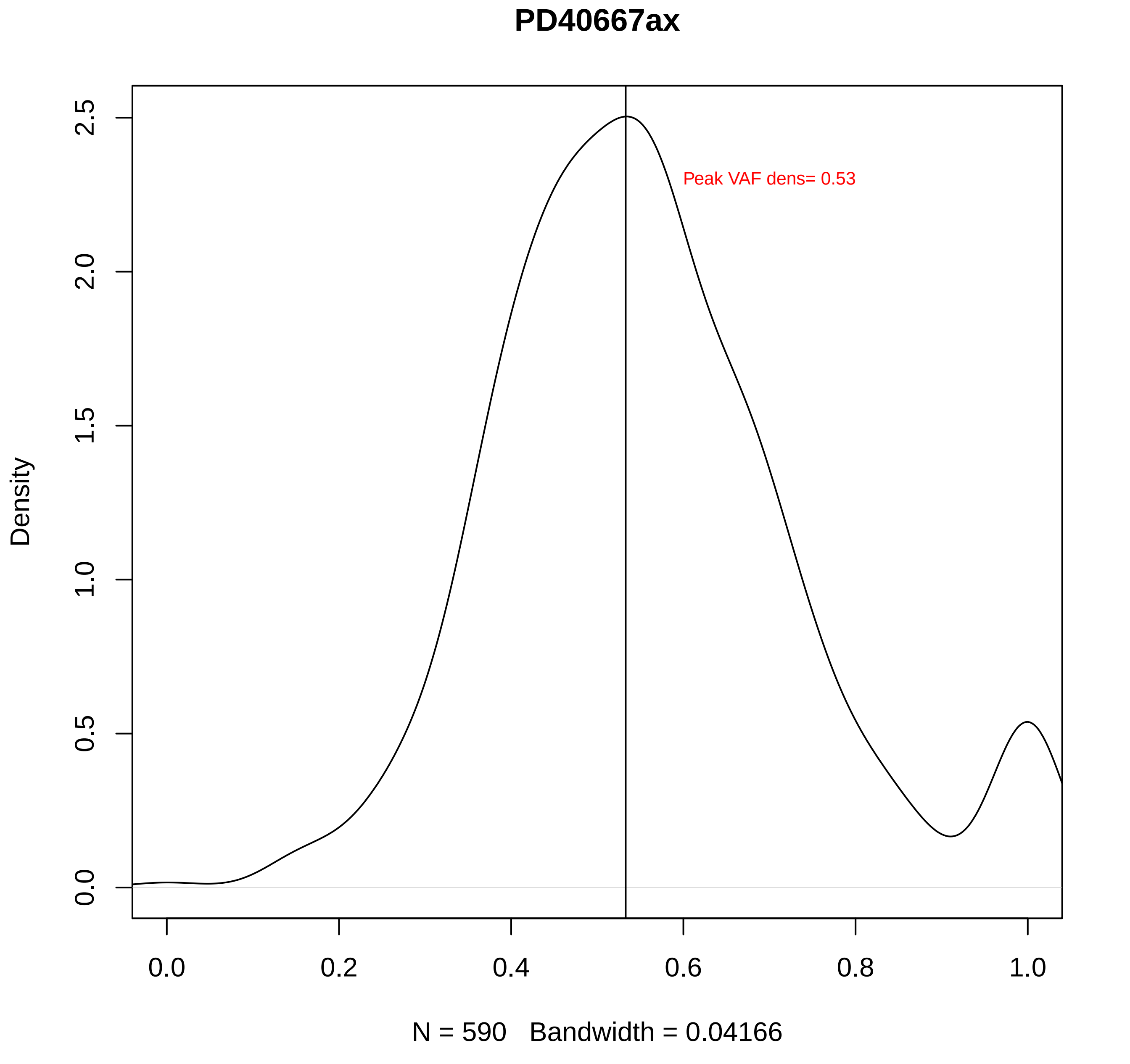
PD40667ax
Peak VAF dens= 0.53
Density
0.0
0.5
1.0
1.5
2.0
2.5
0.0
0.2
0.4
0.6
0.8
1.0
N = 590 Bandwidth = 0.04166

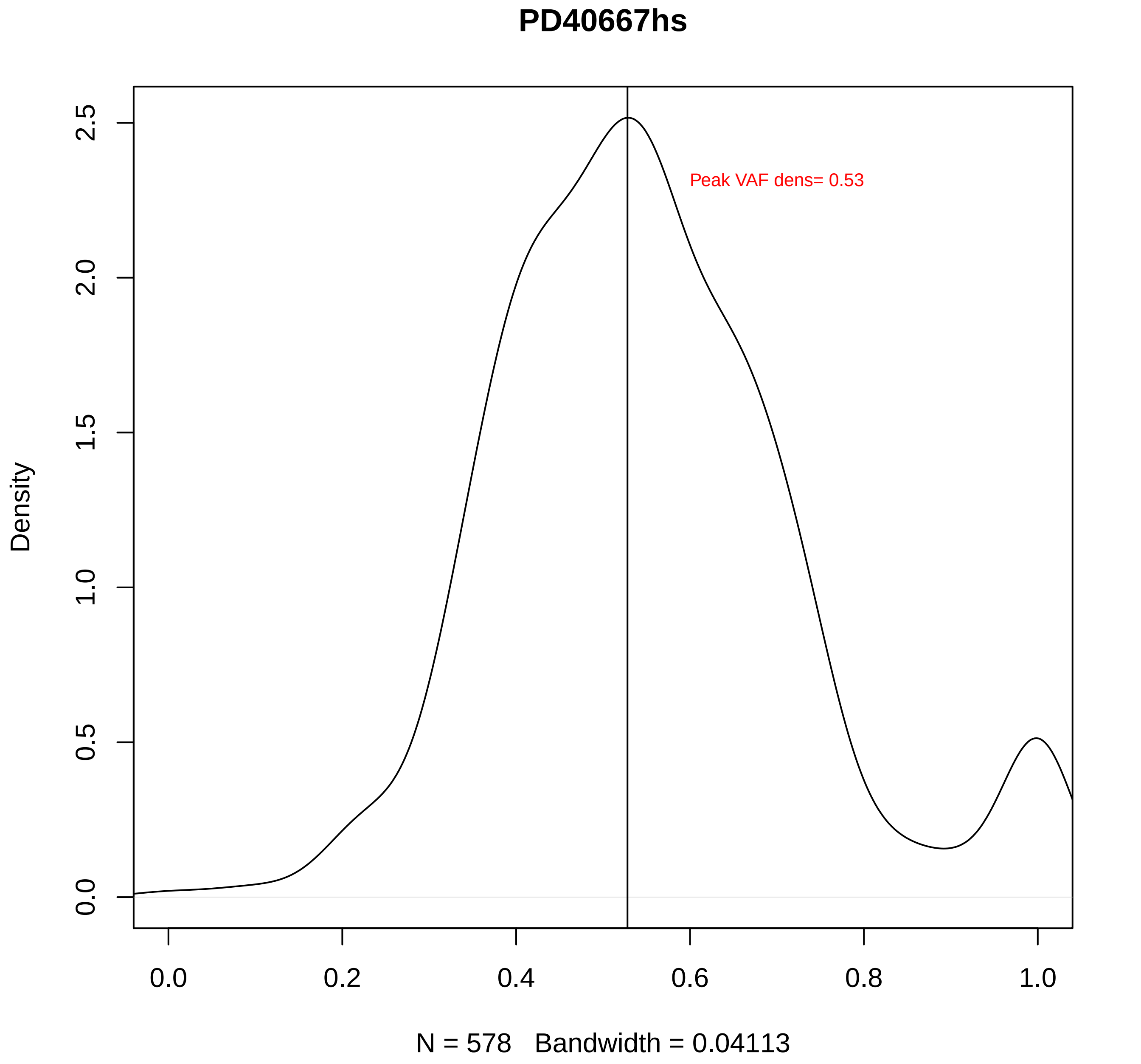
PD40667hs
Peak VAF dens= 0.53
Density
0.0
0.5
1.0
1.5
2.0
2.5
0.0
0.2
0.4
0.6
0.8
1.0
N = 578 Bandwidth = 0.04113

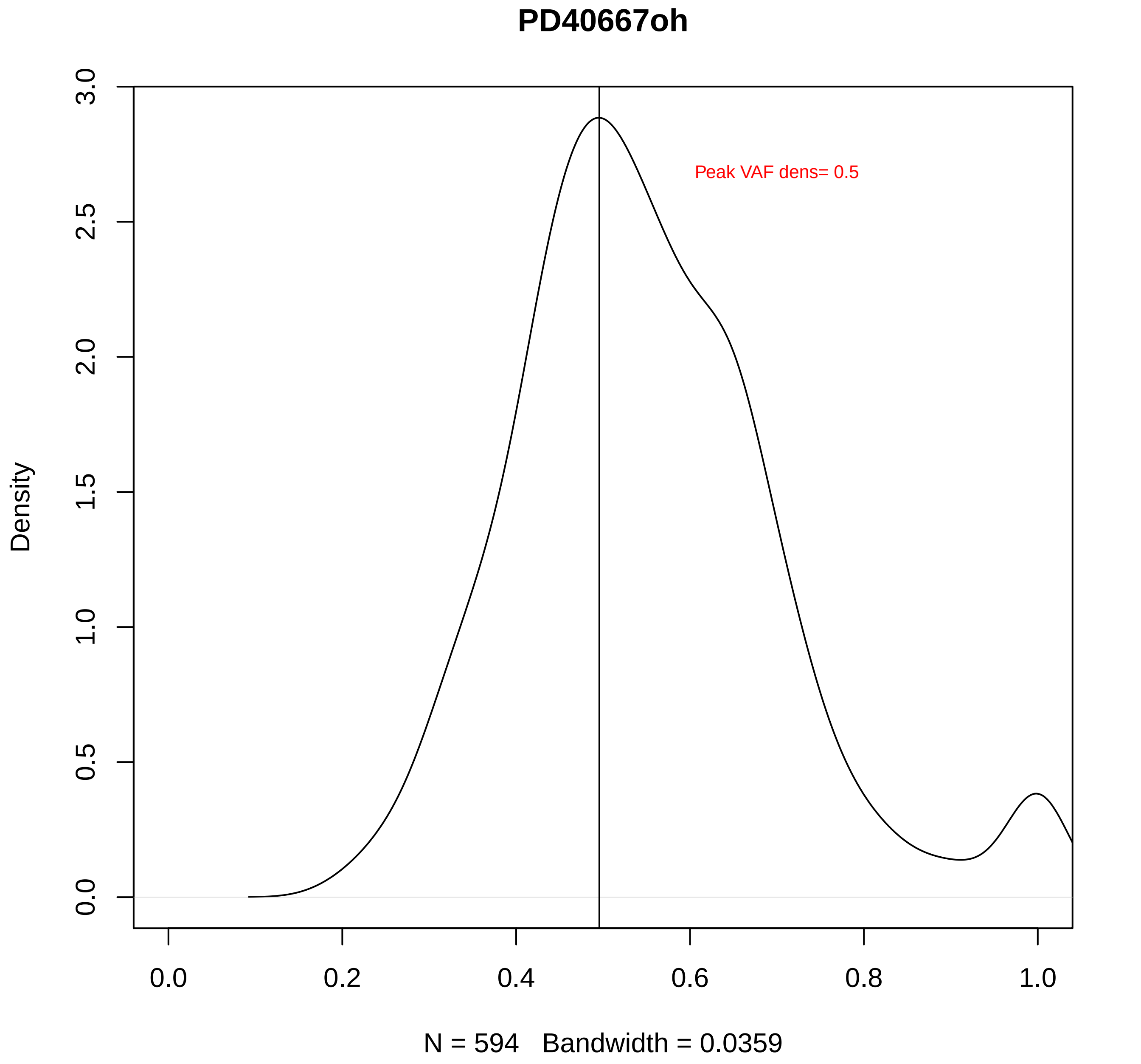
PD40667oh
Peak VAF dens= 0.5
Density
0.0
0.5
1.0
1.5
2.0
2.5
3.0
0.0
0.2
0.4
0.6
0.8
1.0
N = 594   Bandwidth = 0.0359

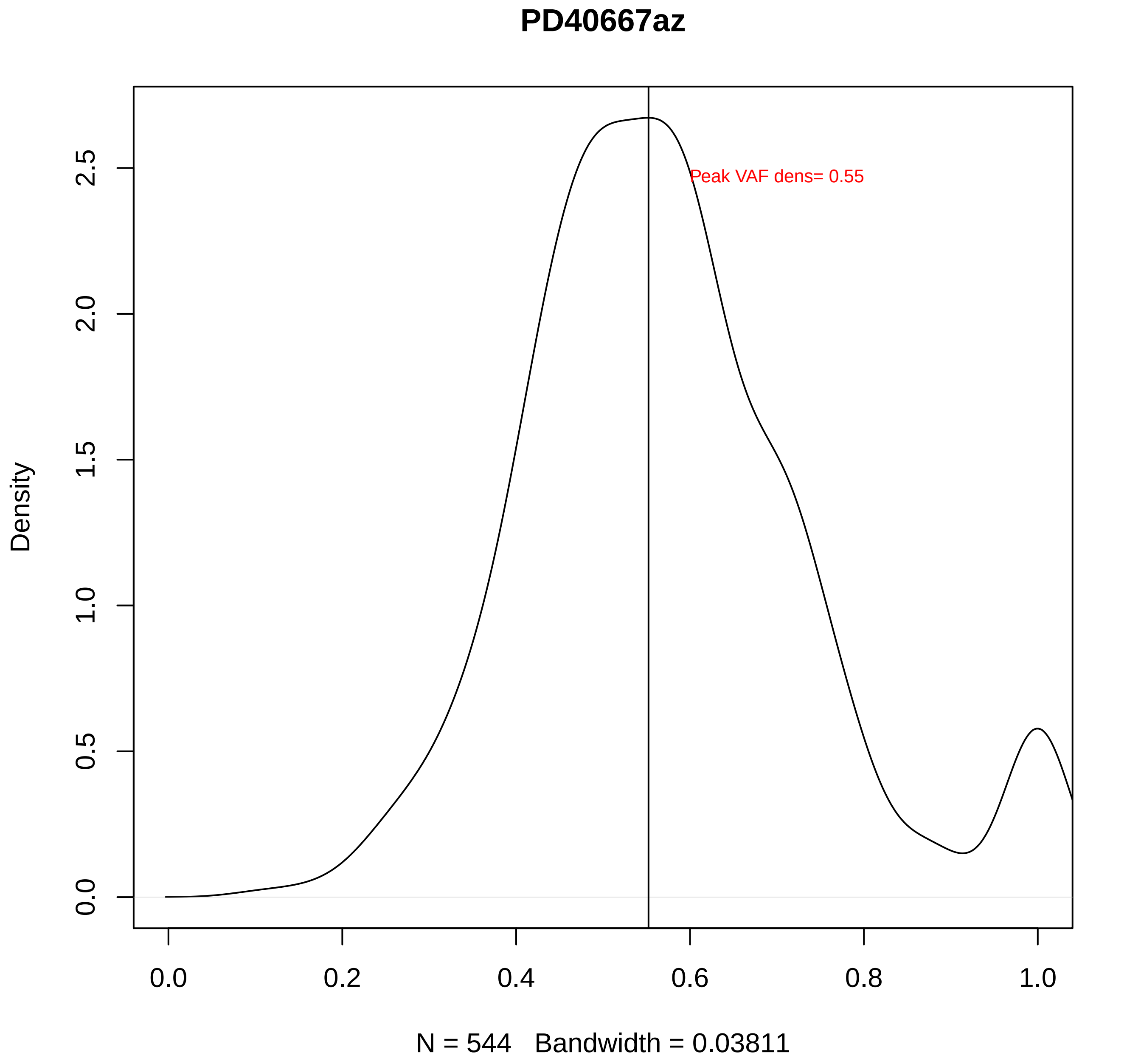

PD40667az
Peak VAF dens= 0.55
Density
0.0
0.5
1.0
1.5
2.0
2.5
0.0
0.2
0.4
0.6
0.8
1.0
N = 544   Bandwidth = 0.03811

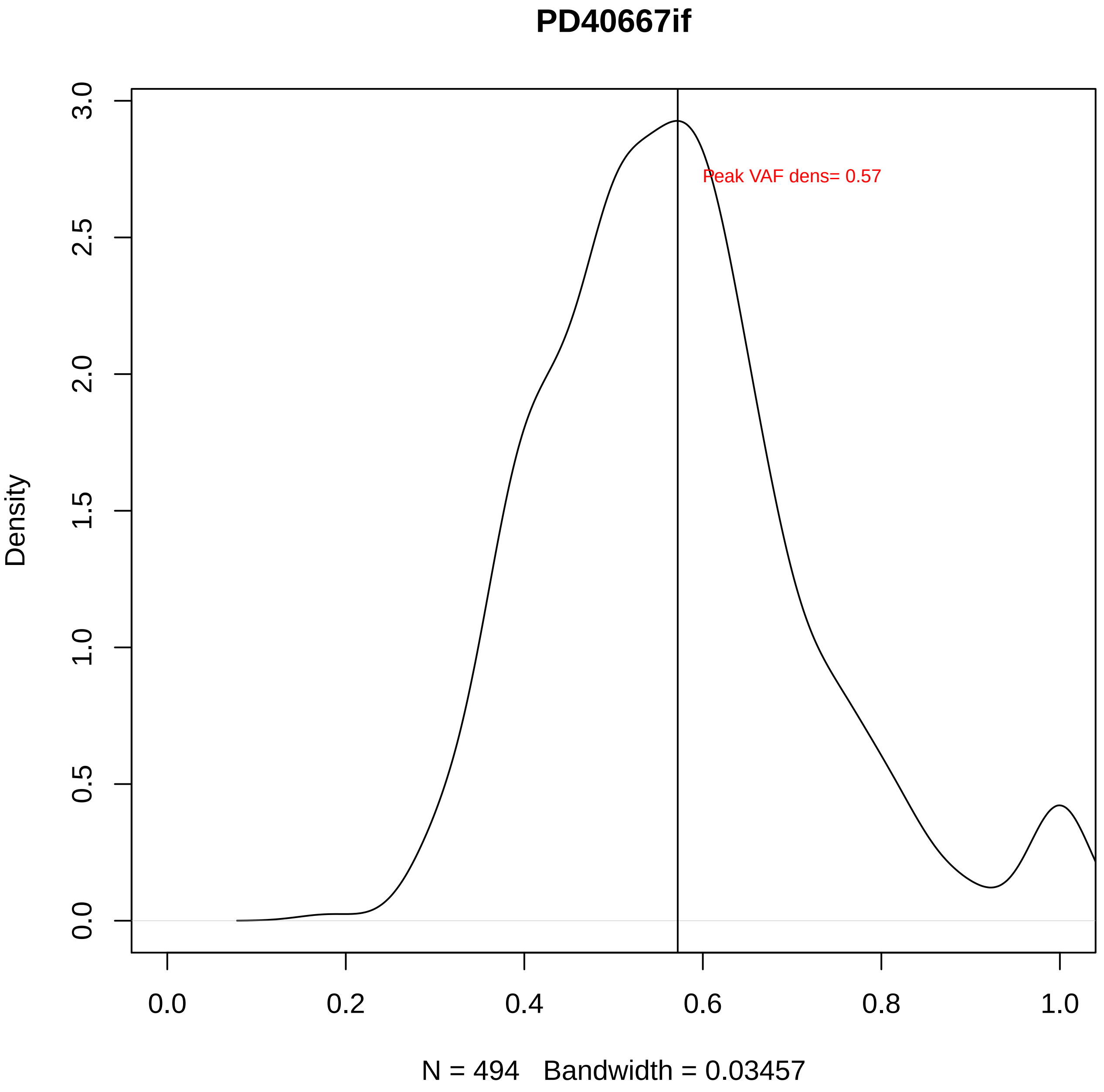
PD40667if
Density
3.0
2.5
2.0
1.5
1.0
0.5
0.0
0.0
0.2
0.4
0.6
0.8
1.0
Peak VAF dens= 0.57
N = 494   Bandwidth = 0.03457

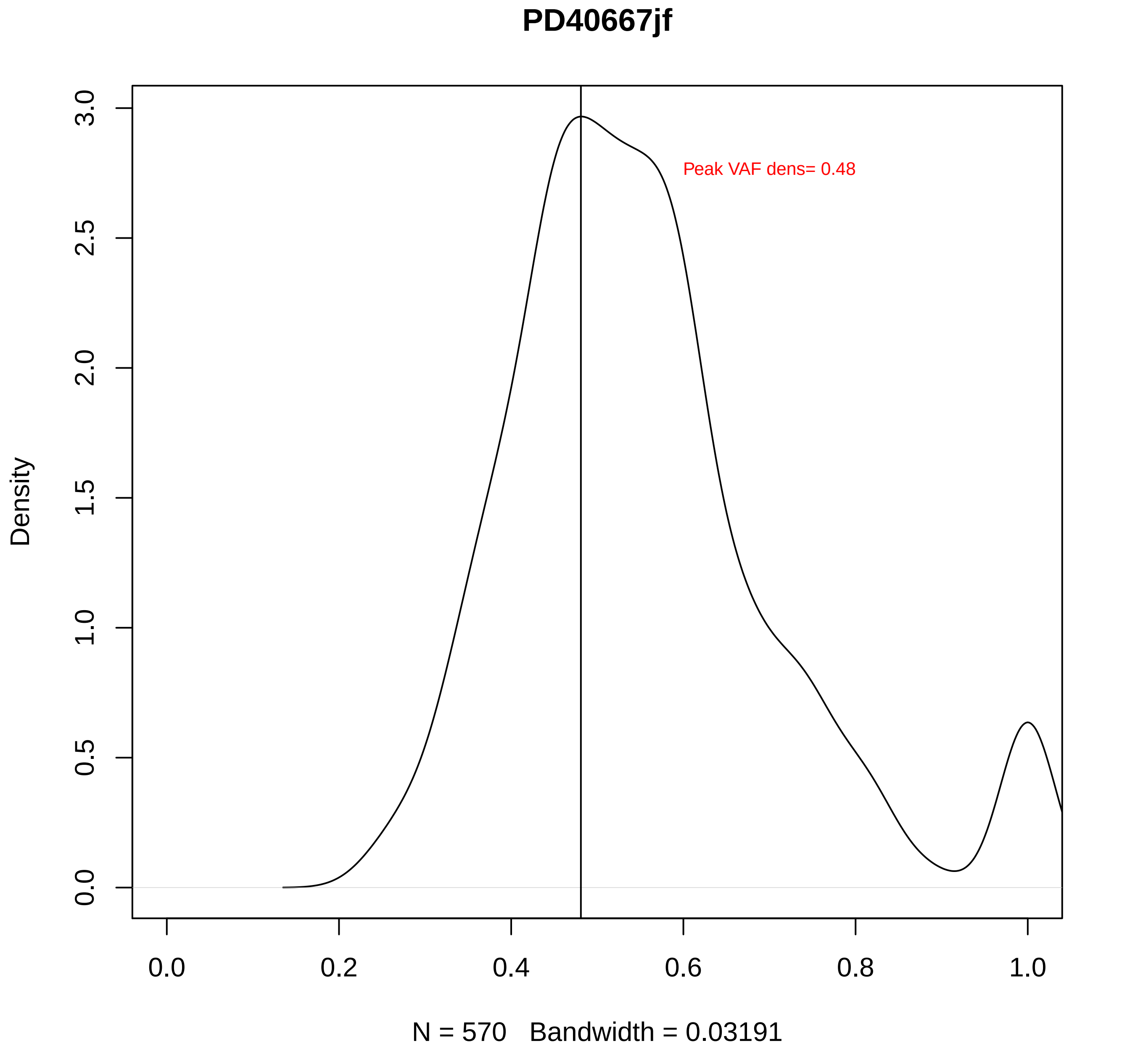
PD40667jf
Peak VAF dens= 0.48
Density
3.0
2.5
2.0
1.5
1.0
0.5
0.0
0.0
0.2
0.4
0.6
0.8
1.0
N = 570 Bandwidth = 0.03191

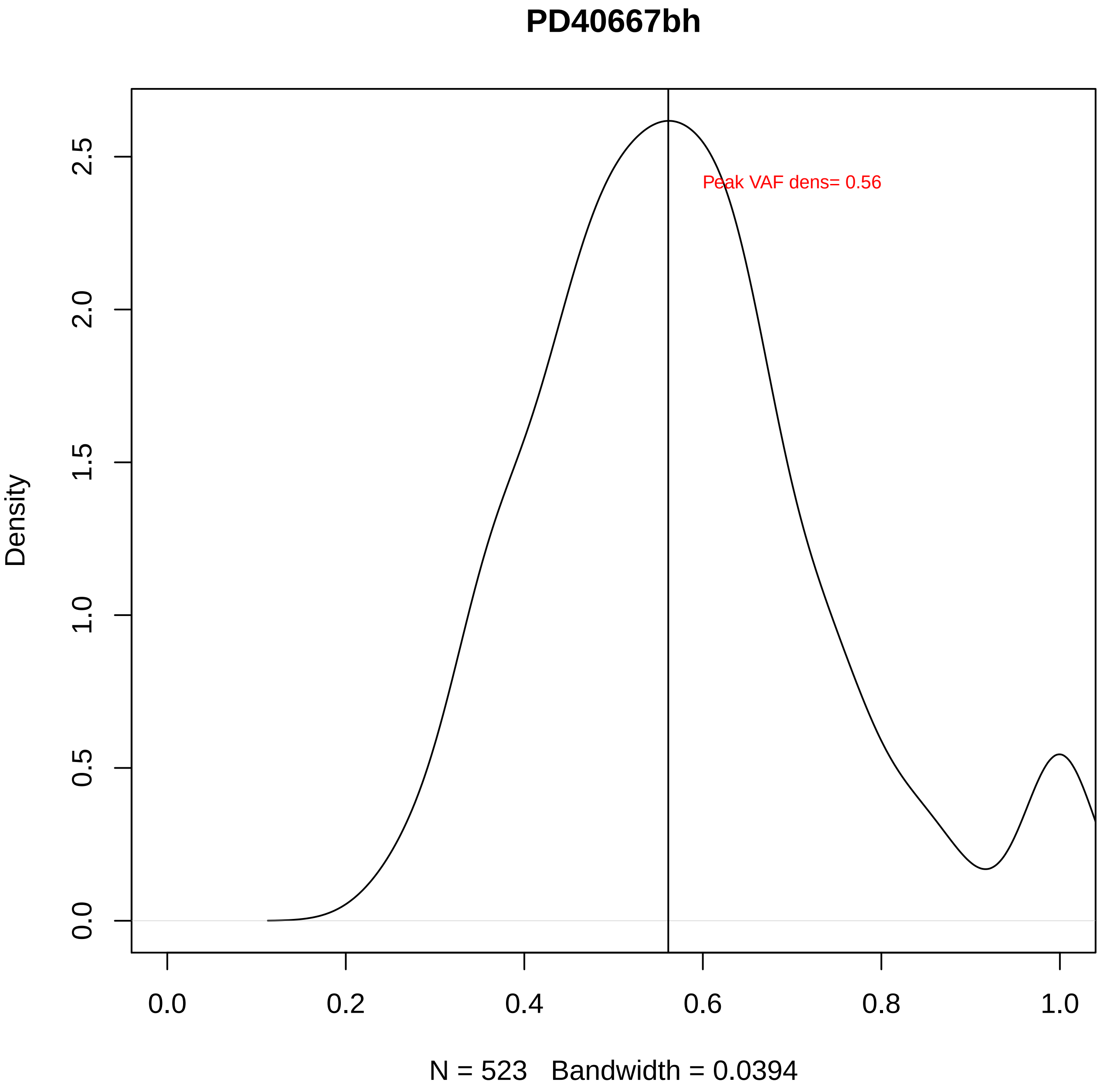

PD40667bh
Peak VAF dens= 0.56
Density
0.0
0.5
1.0
1.5
2.0
2.5
0.0
0.2
0.4
0.6
0.8
1.0
N = 523   Bandwidth = 0.0394

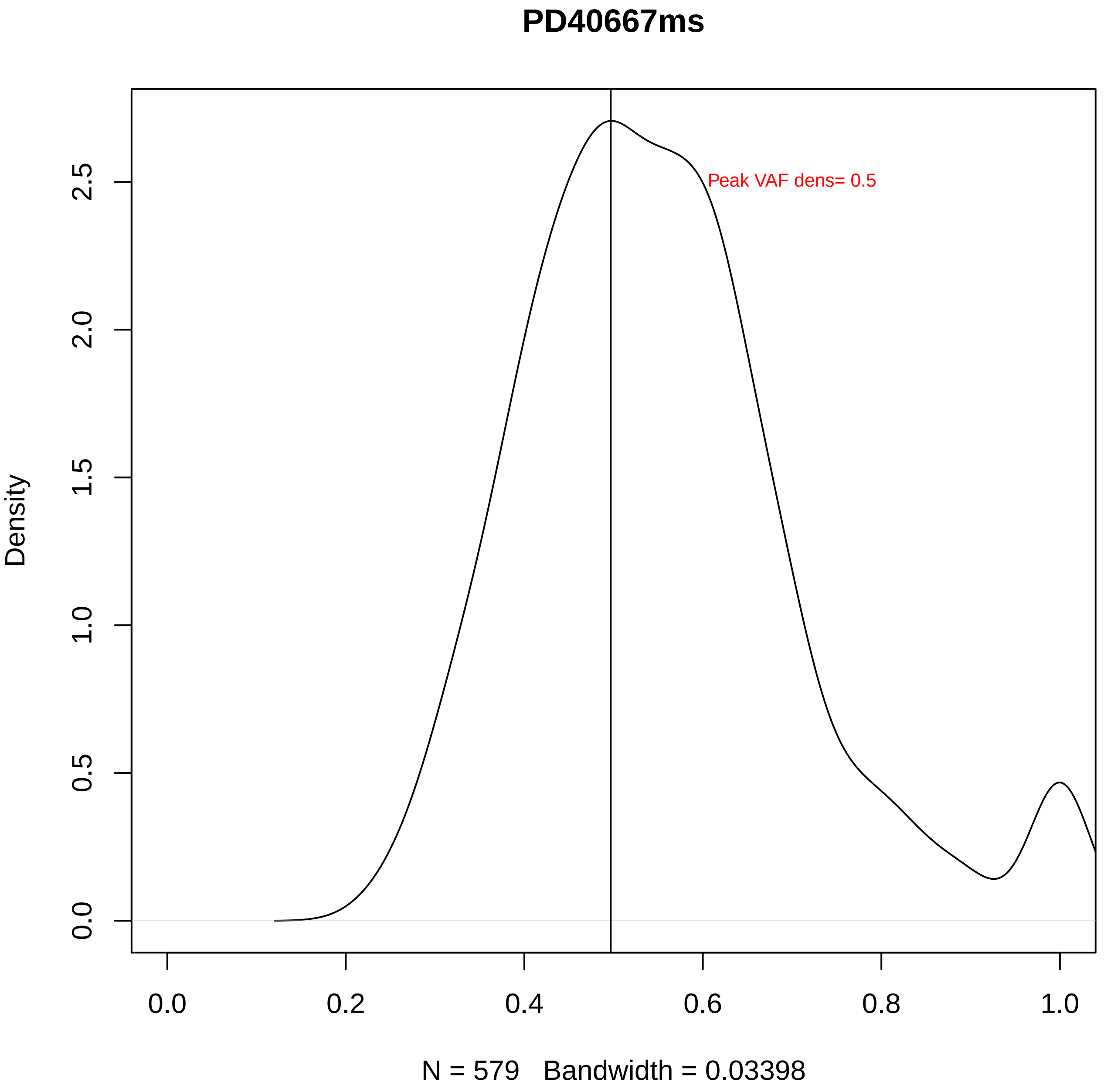
PD40667ms
Peak VAF dens= 0.5
Density
0.0
0.5
1.0
1.5
2.0
2.5
0.0
0.2
0.4
0.6
0.8
1.0
N = 579 Bandwidth = 0.03398

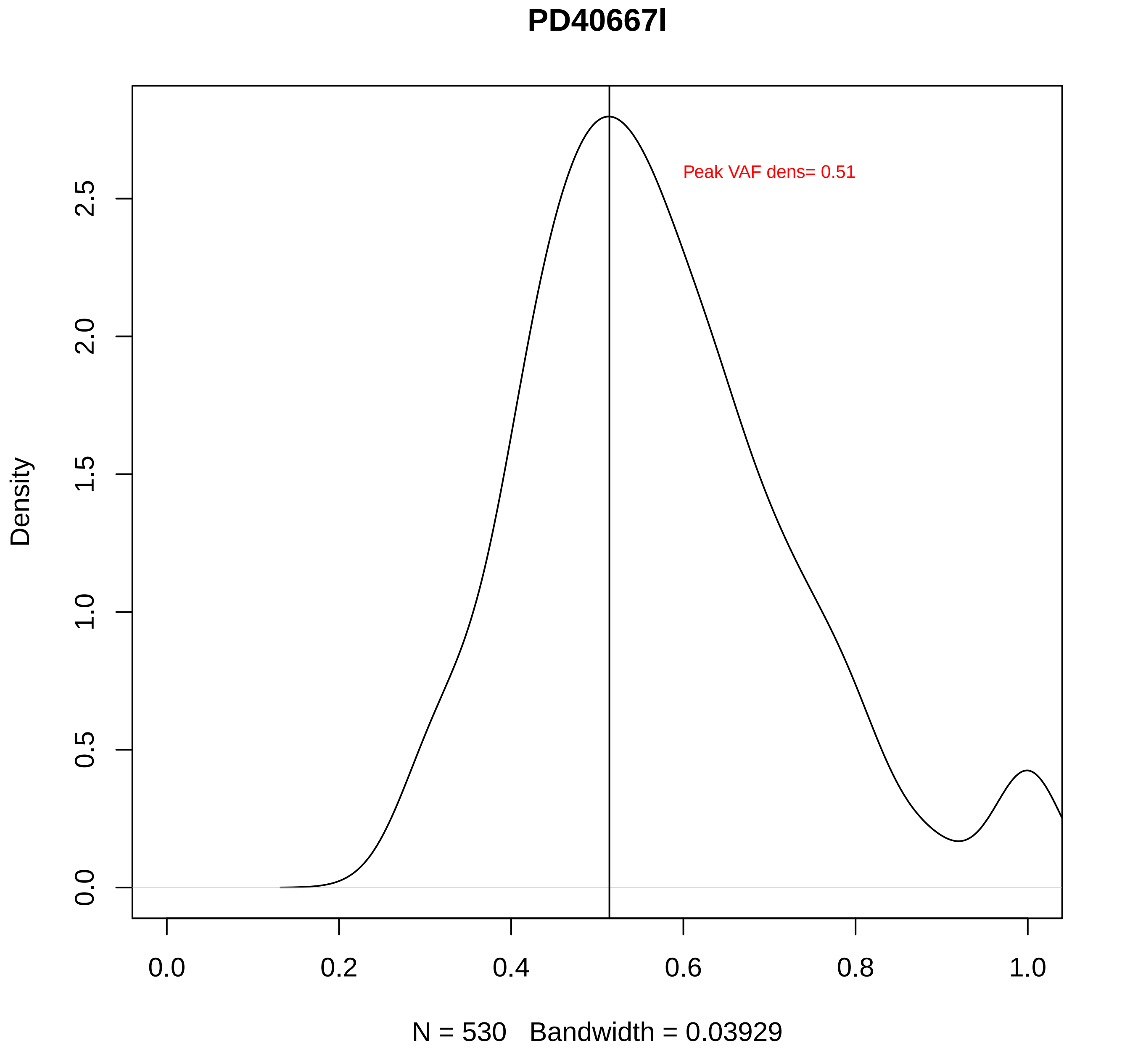
PD40667l
Peak VAF dens= 0.51
Density
0.0
0.5
1.0
1.5
2.0
2.5
0.0
0.2
0.4
0.6
0.8
1.0
N = 530   Bandwidth = 0.03929

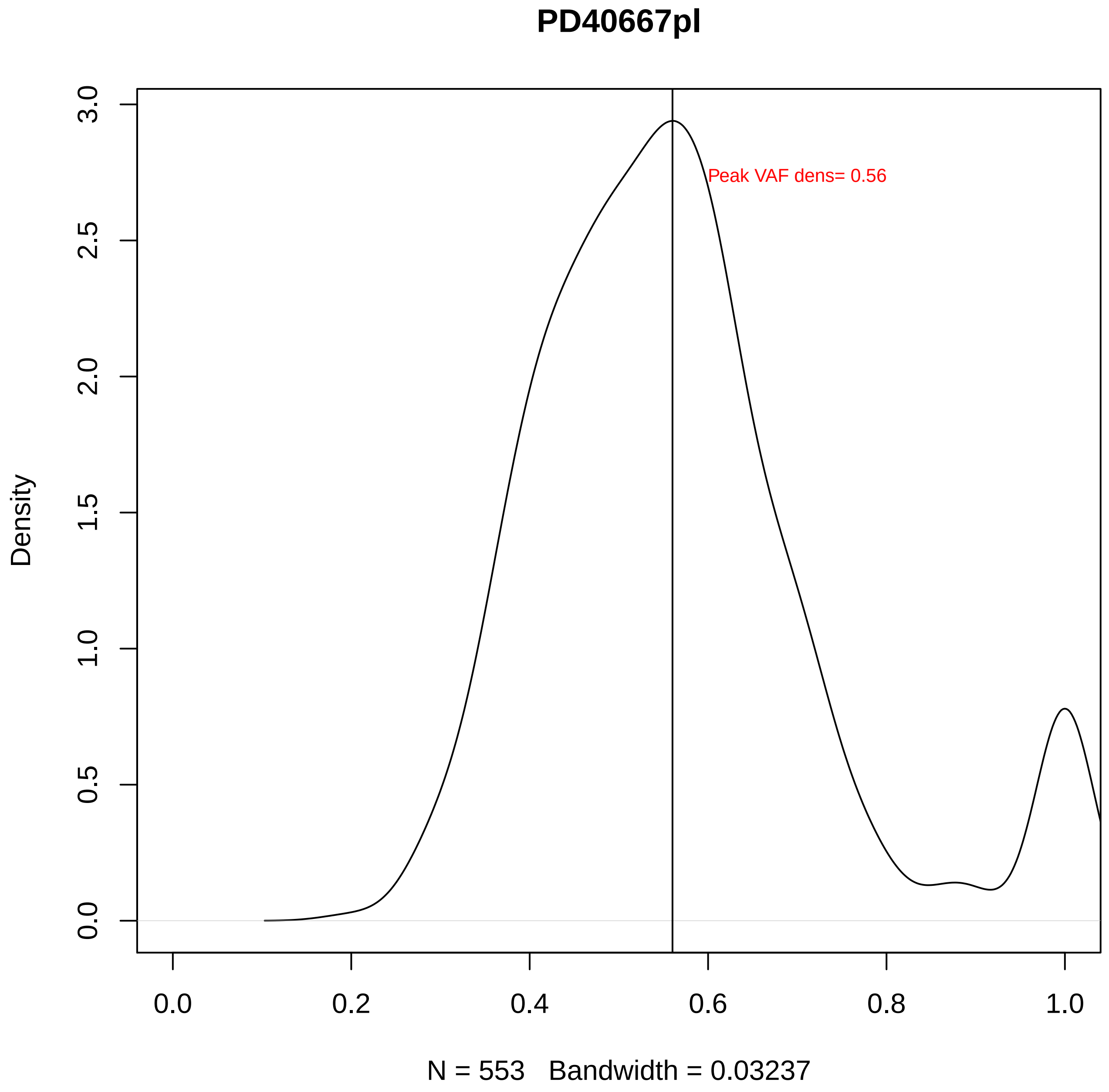
PD40667pl
Peak VAF dens= 0.56
Density
3.0
2.5
2.0
1.5
1.0
0.5
0.0
0.0
0.2
0.4
0.6
0.8
1.0
N = 553   Bandwidth = 0.03237

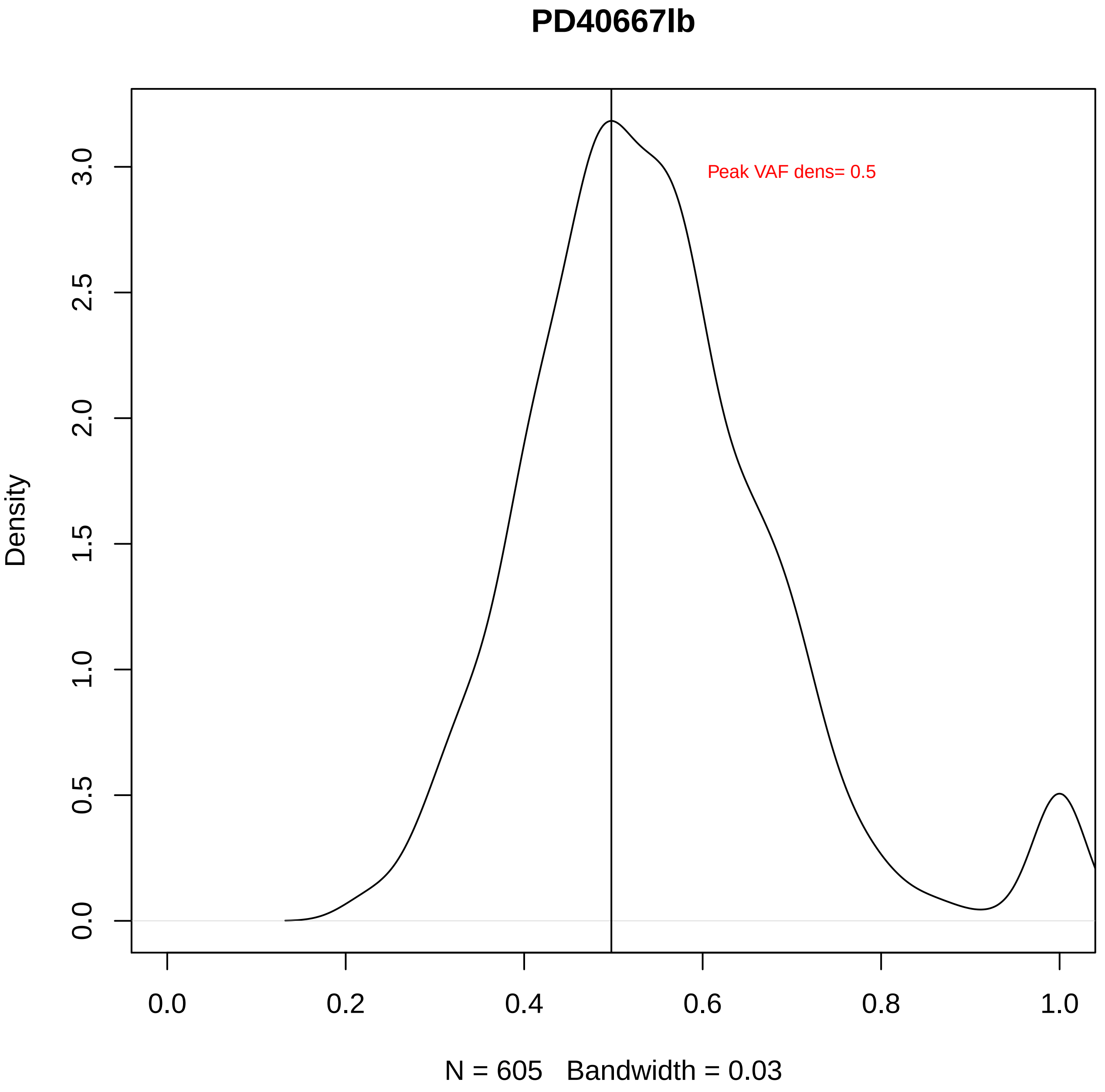

PD40667lb
Peak VAF dens= 0.5
Density
0.0
0.5
1.0
1.5
2.0
2.5
3.0
0.0
0.2
0.4
0.6
0.8
1.0
N = 605 Bandwidth = 0.03

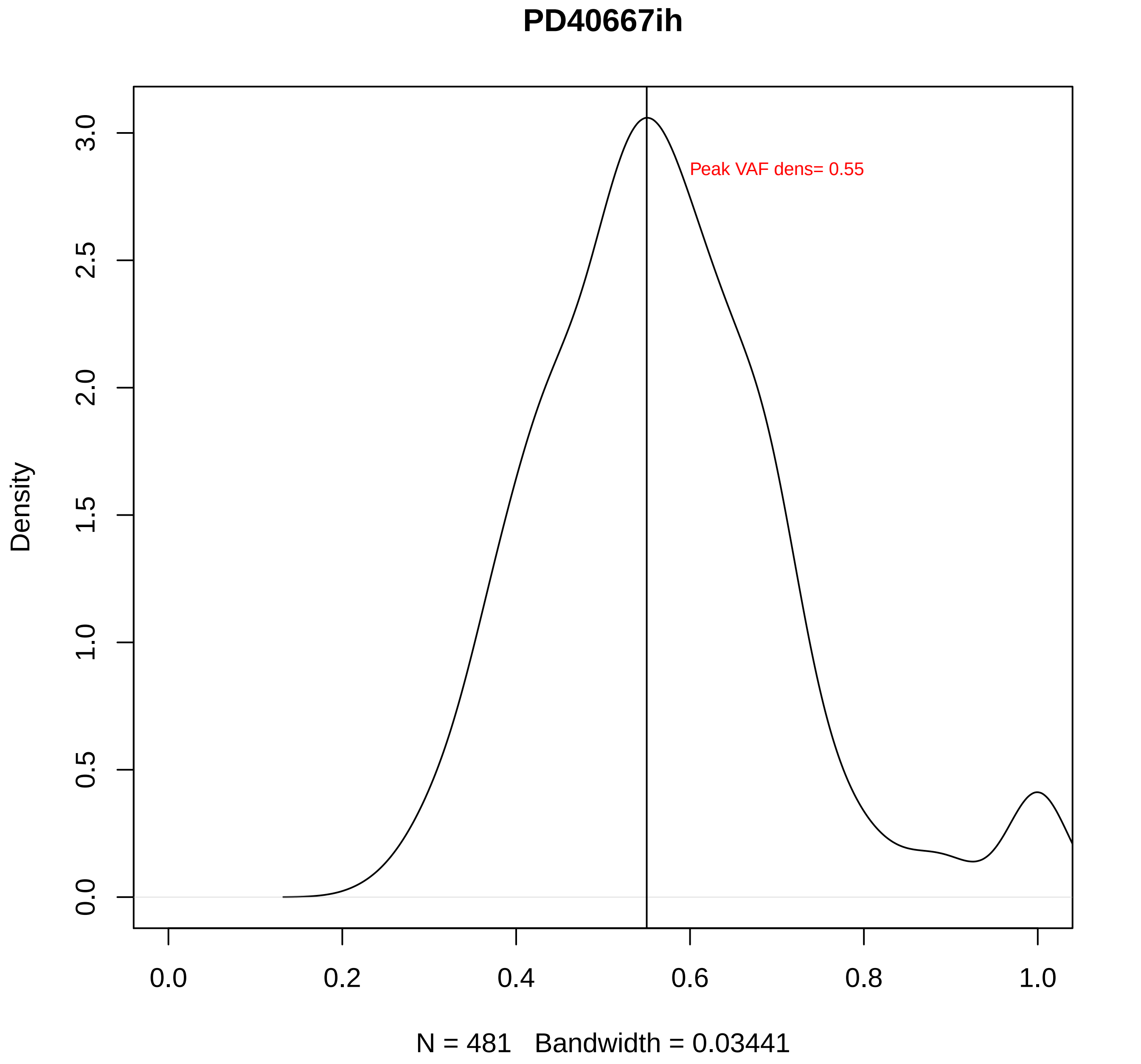

PD40667ih
Peak VAF dens= 0.55
Density
3.0
2.5
2.0
1.5
1.0
0.5
0.0
0.0
0.2
0.4
0.6
0.8
1.0
N = 481   Bandwidth = 0.03441

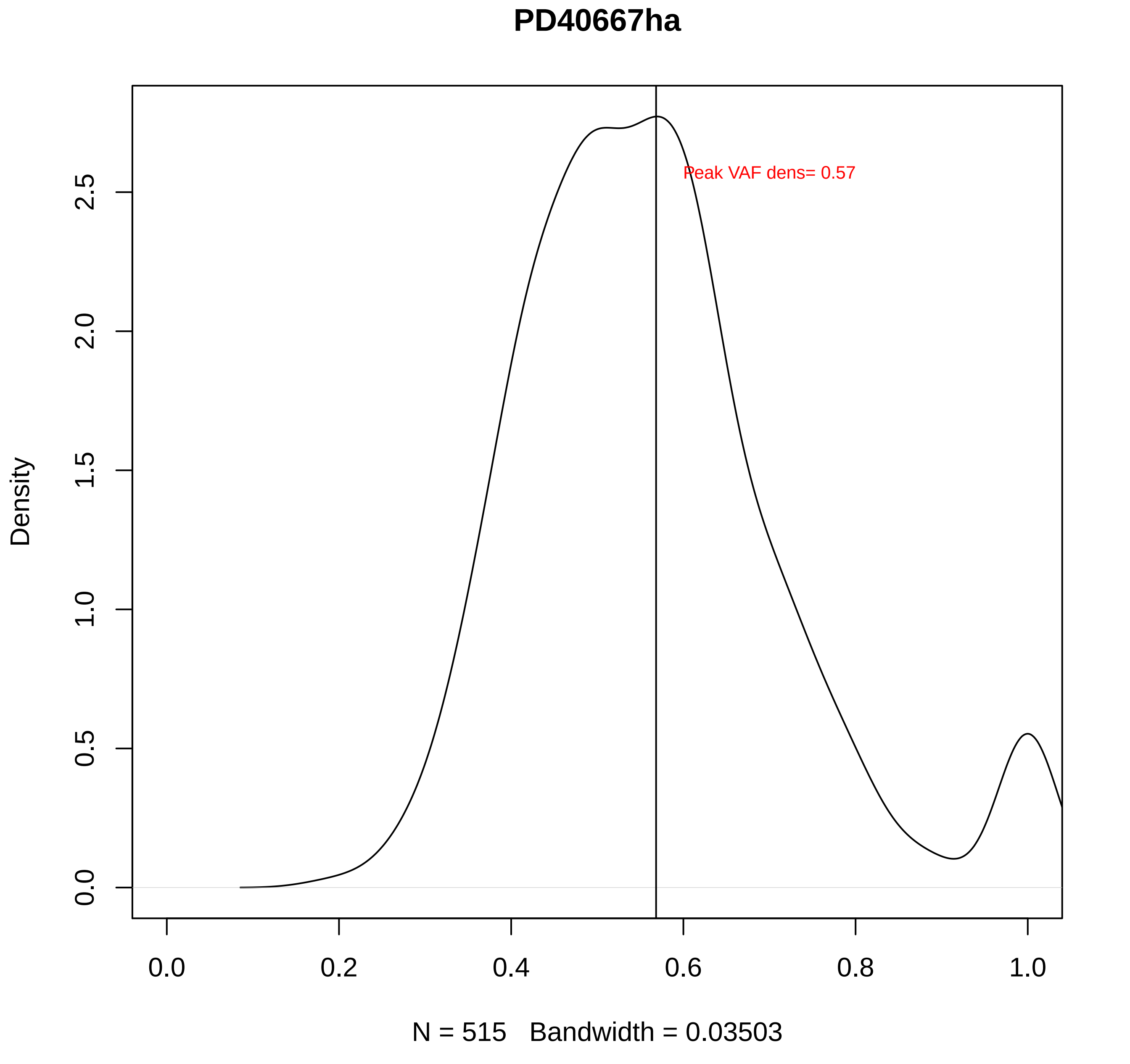

PD40667ha
Peak VAF dens= 0.57
Density
0.0
0.5
1.0
1.5
2.0
2.5
0.0
0.2
0.4
0.6
0.8
1.0
N = 515   Bandwidth = 0.03503

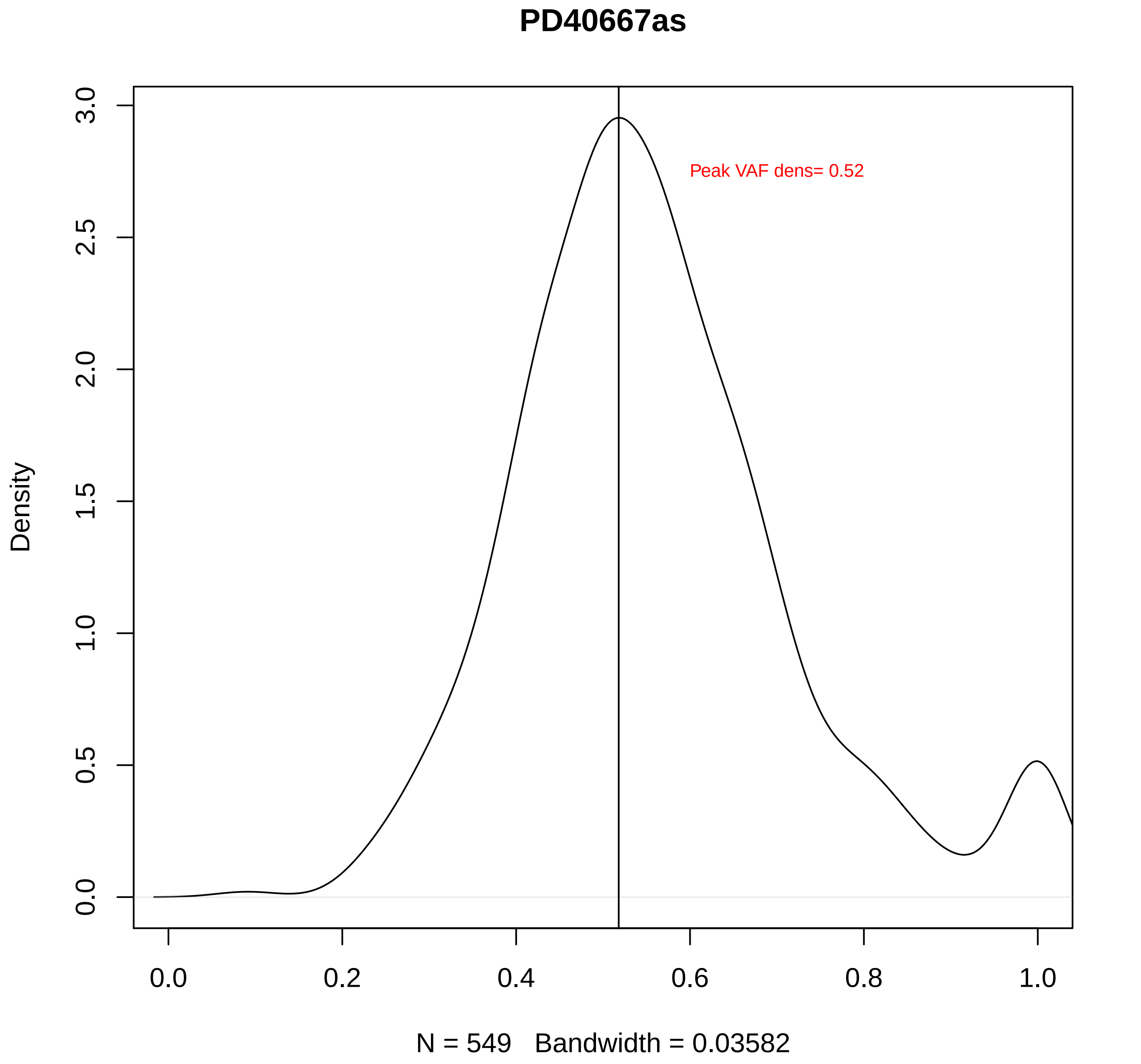
PD40667as
Peak VAF dens= 0.52
Density
3.0
2.5
2.0
1.5
1.0
0.5
0.0
0.0
0.2
0.4
0.6
0.8
1.0
N = 549   Bandwidth = 0.03582

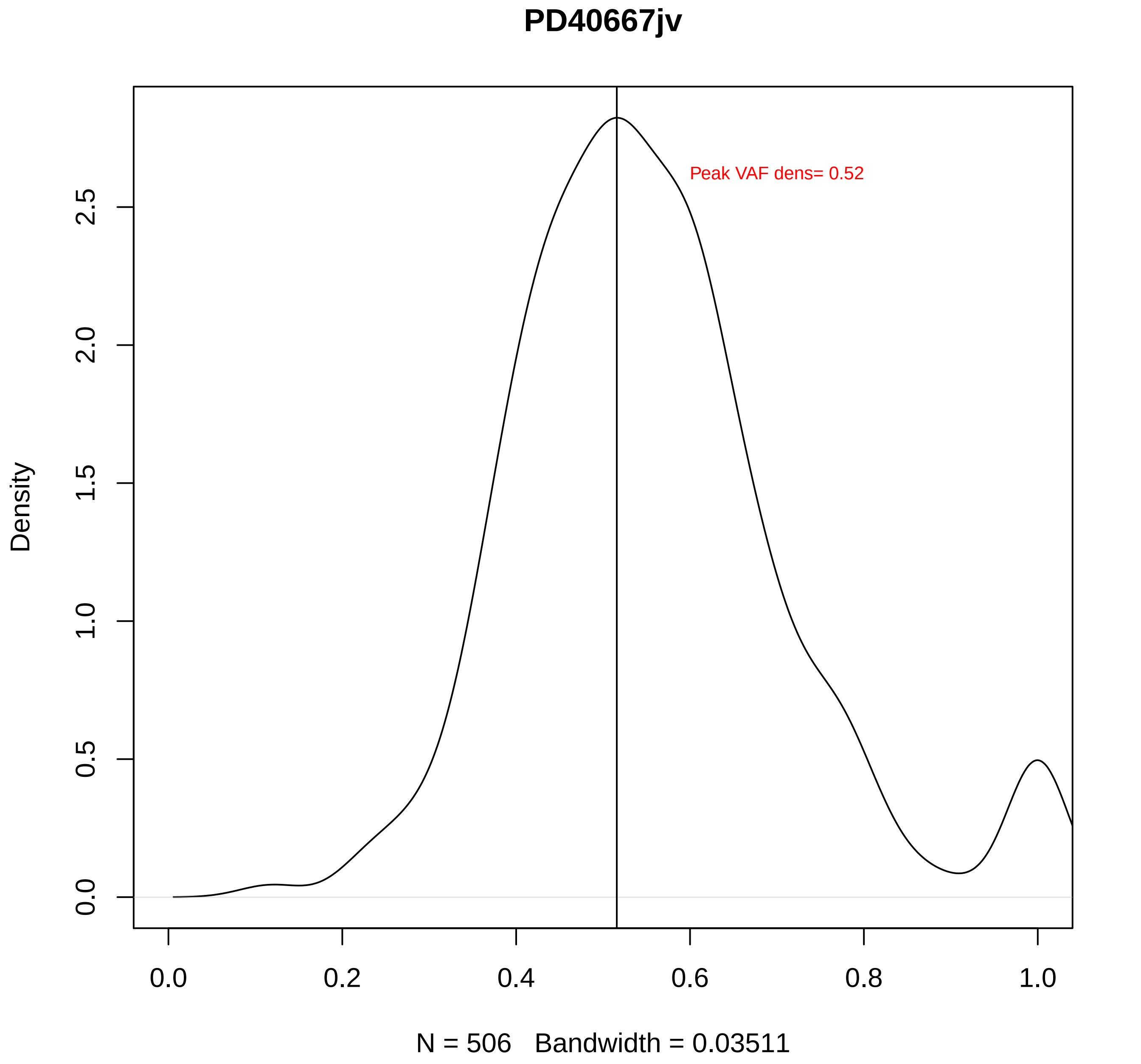

PD40667jv
Peak VAF dens= 0.52
Density
0.0
0.5
1.0
1.5
2.0
2.5
0.0
0.2
0.4
0.6
0.8
1.0
N = 506   Bandwidth = 0.03511

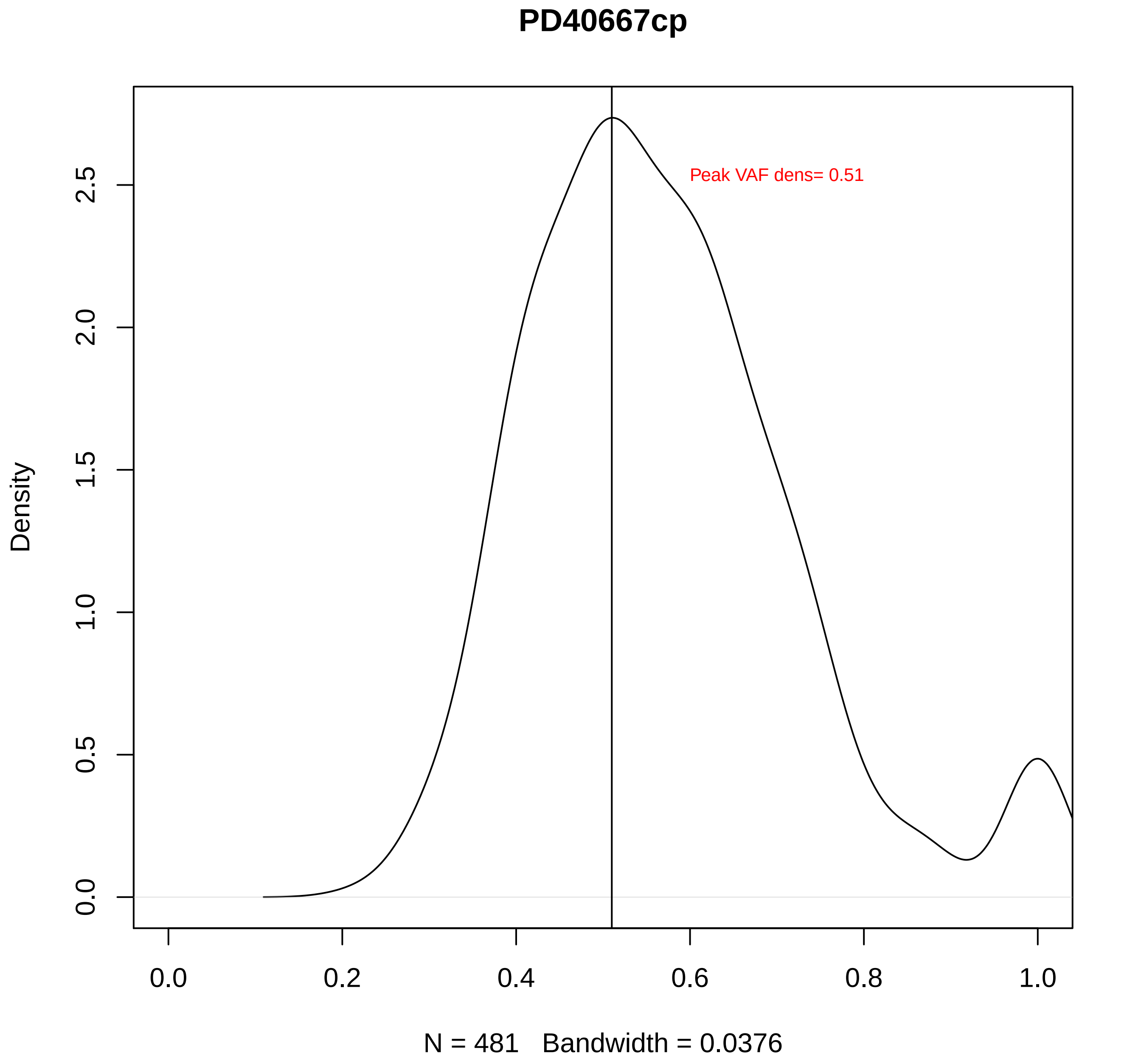

PD40667cp
Peak VAF dens= 0.51
Density
0.0
0.5
1.0
1.5
2.0
2.5
0.0
0.2
0.4
0.6
0.8
1.0
N = 481   Bandwidth = 0.0376

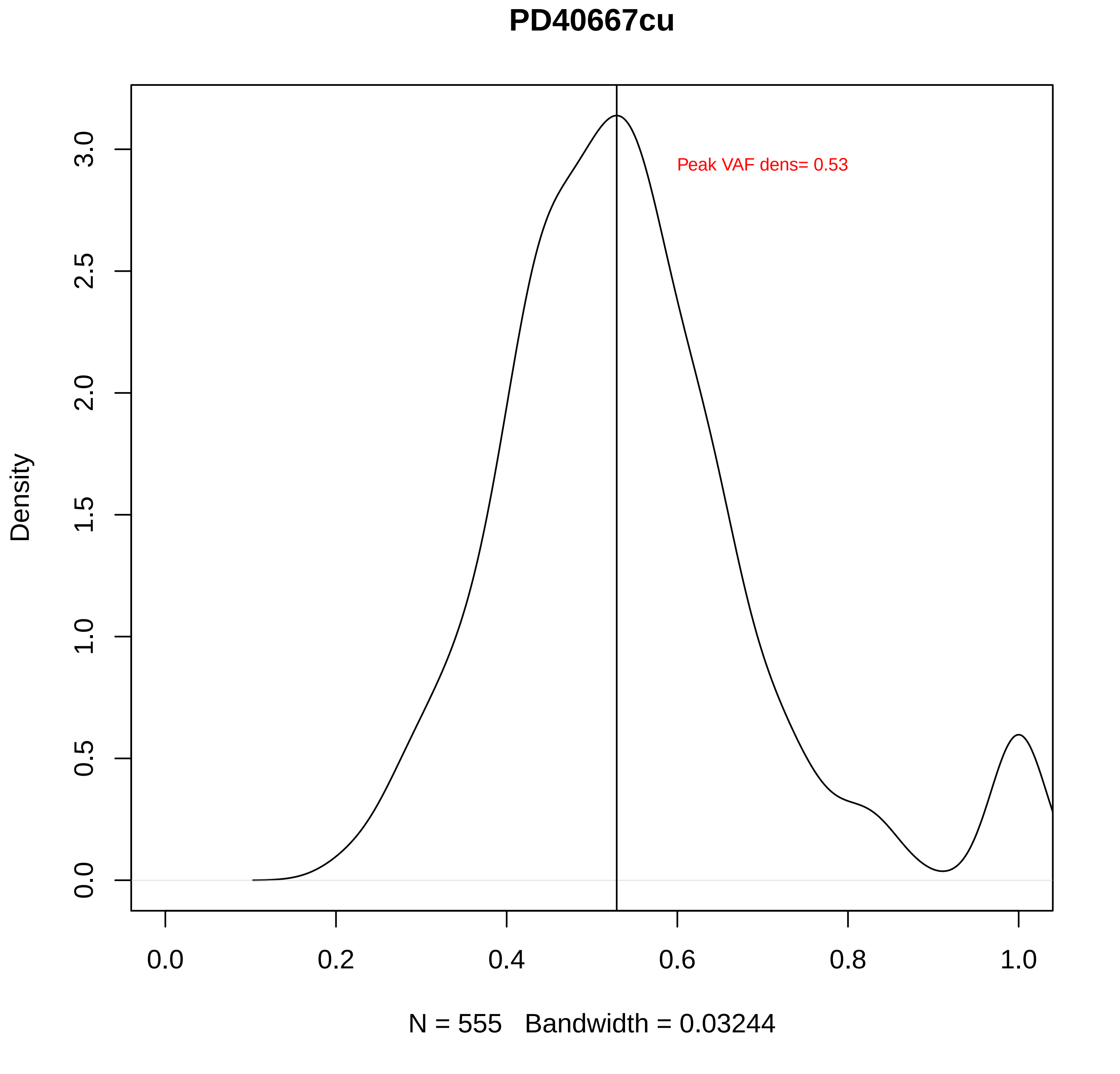
PD40667cu
Peak VAF dens= 0.53
Density
0.0
0.5
1.0
1.5
2.0
2.5
3.0
0.0
0.2
0.4
0.6
0.8
1.0
N = 555 Bandwidth = 0.03244

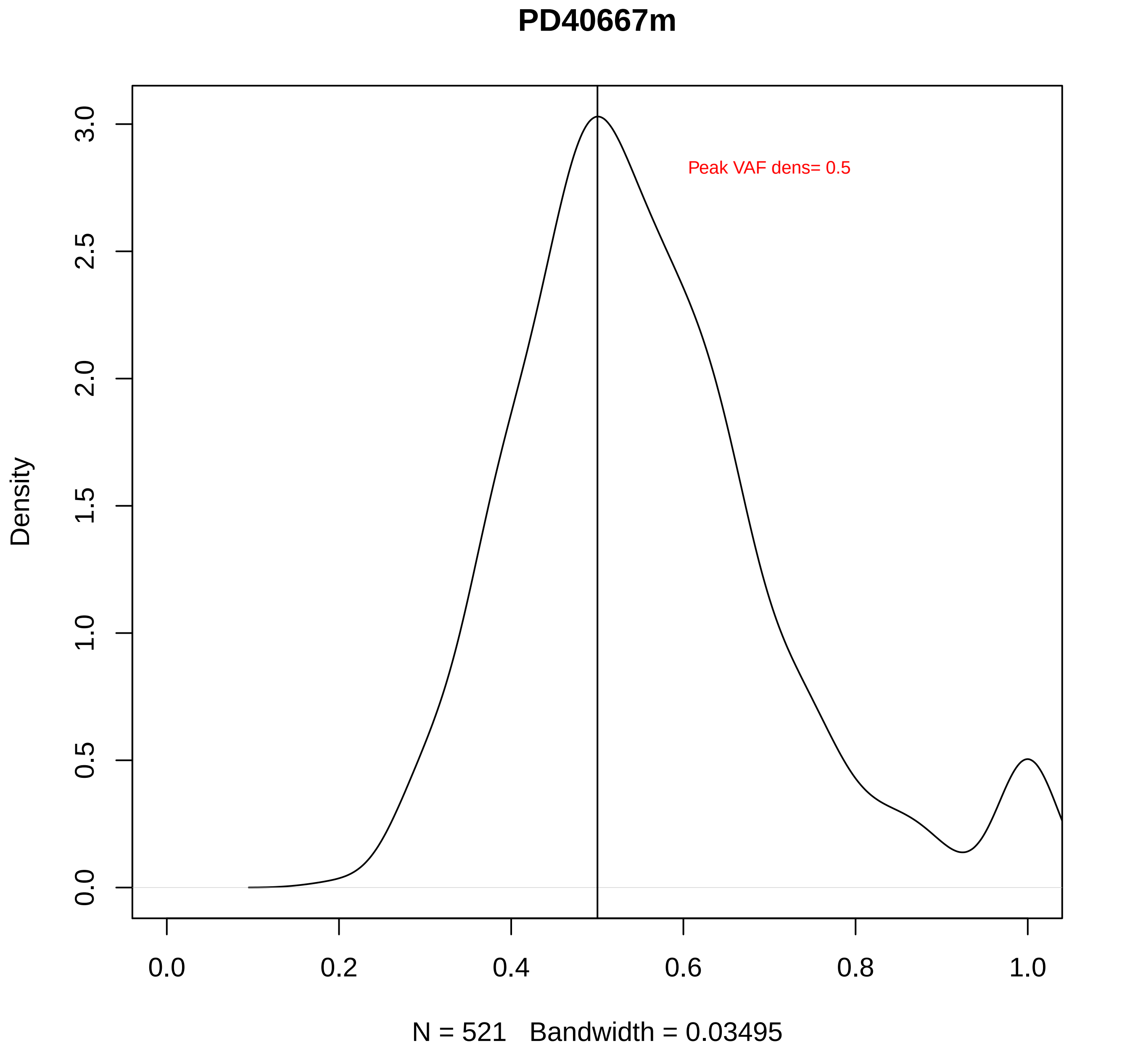

PD40667m
Peak VAF dens= 0.5
Density
0.0
0.5
1.0
1.5
2.0
2.5
3.0
0.0
0.2
0.4
0.6
0.8
1.0
N = 521 Bandwidth = 0.03495

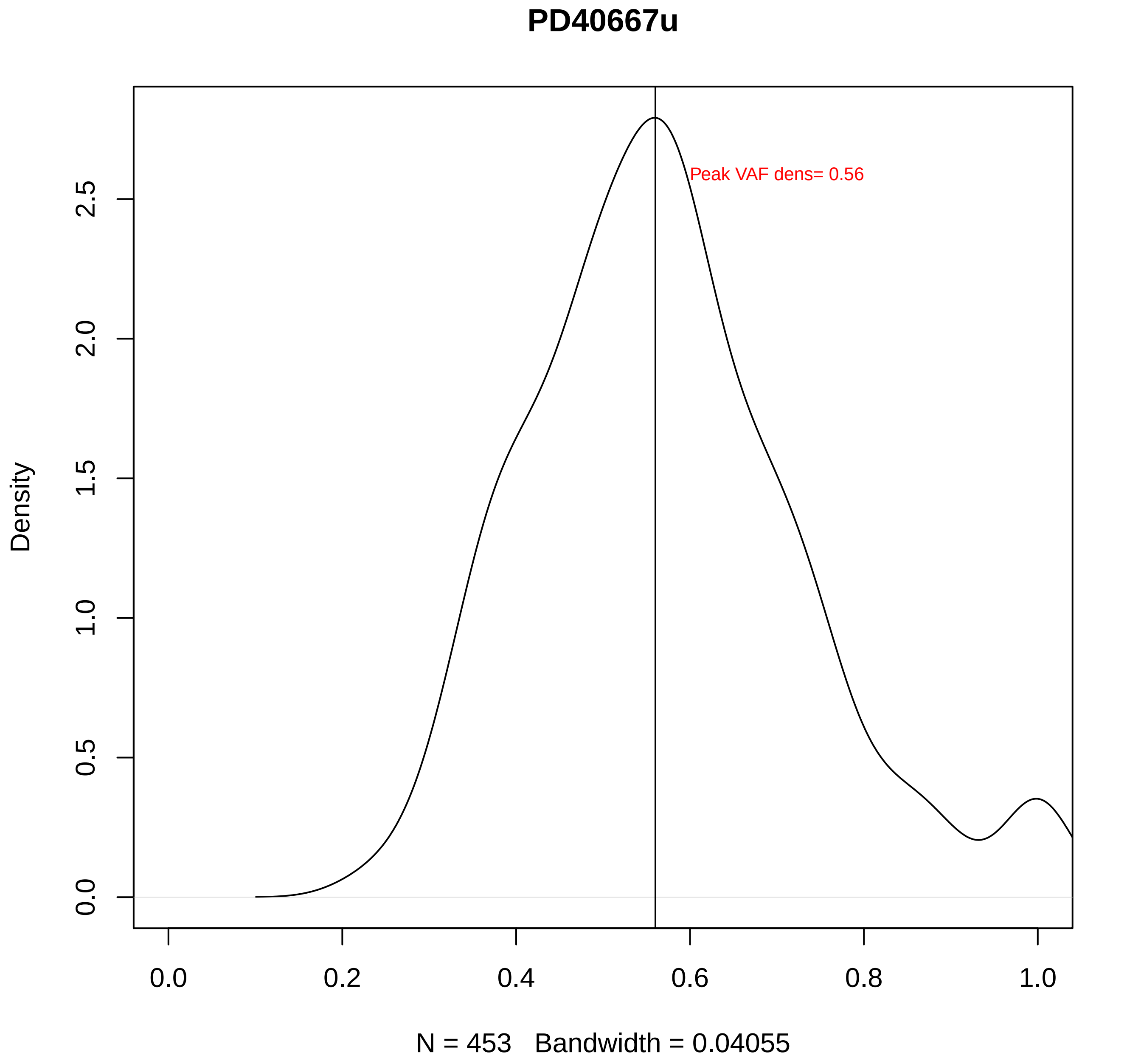

PD40667u
Peak VAF dens= 0.56
Density
0.0
0.5
1.0
1.5
2.0
2.5
0.0
0.2
0.4
0.6
0.8
1.0
N = 453 Bandwidth = 0.04055

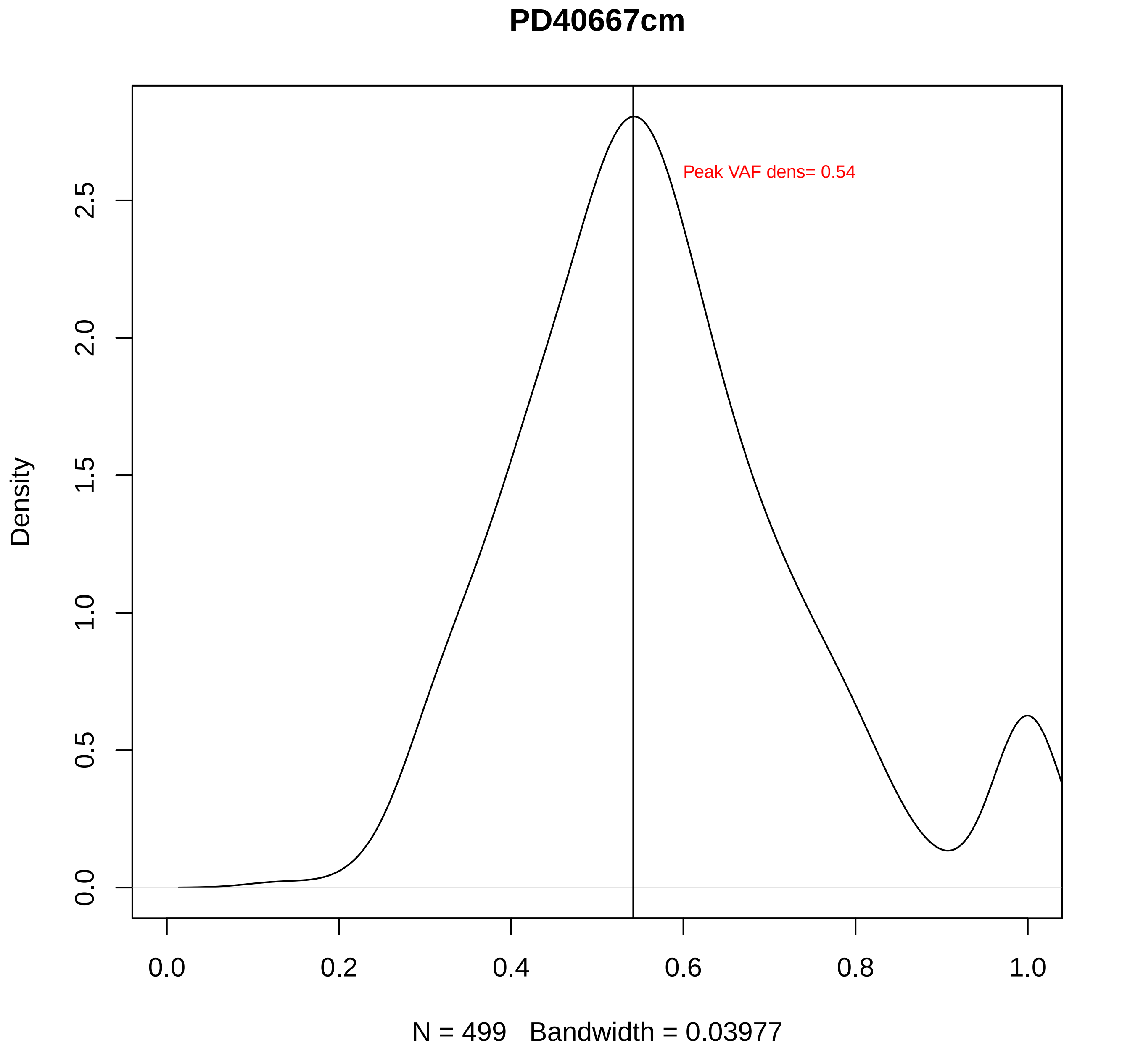

PD40667cm
Peak VAF dens= 0.54
Density
0.0
0.5
1.0
1.5
2.0
2.5
0.0
0.2
0.4
0.6
0.8
1.0
N = 499   Bandwidth = 0.03977

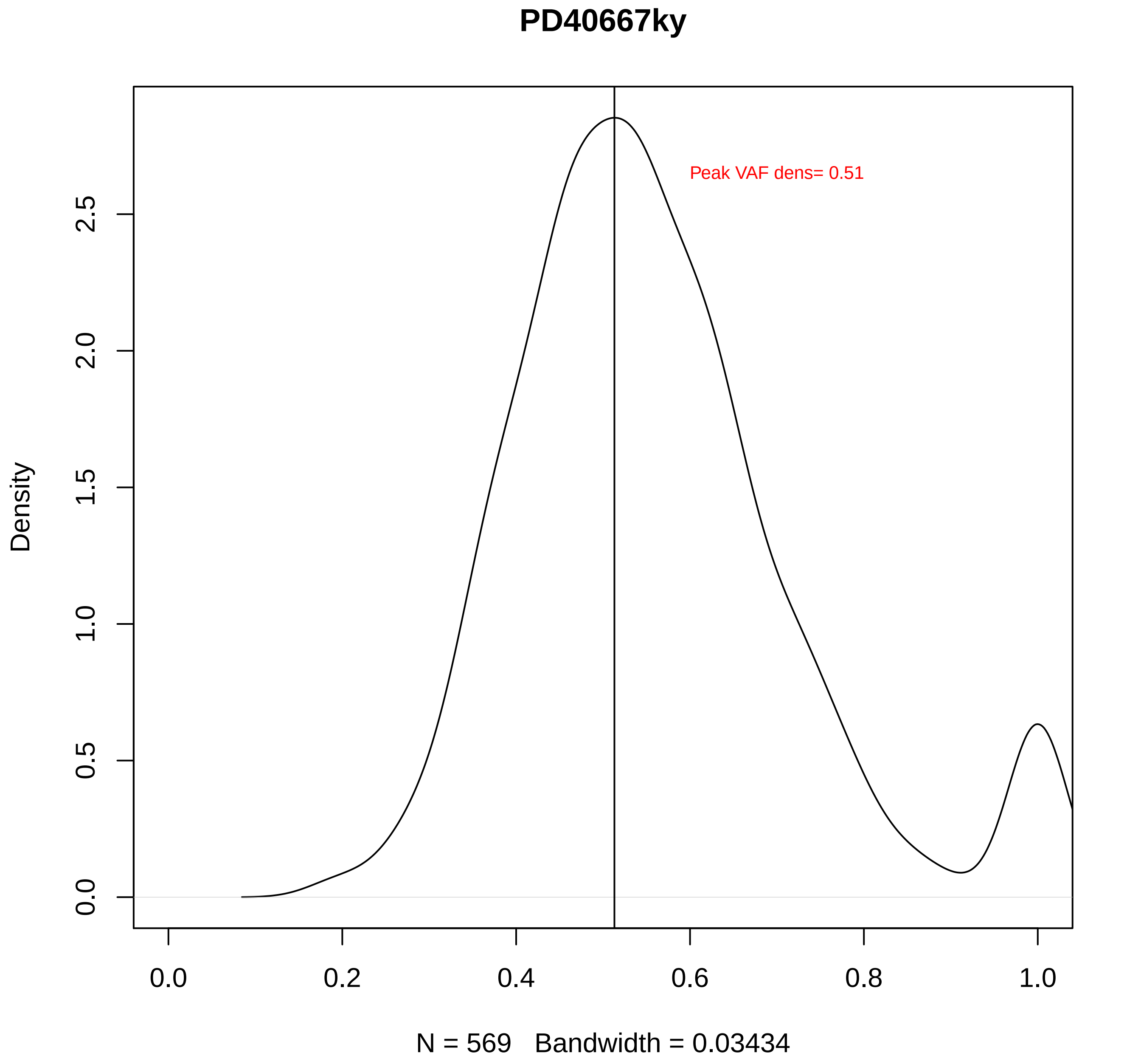
PD40667ky
Peak VAF dens= 0.51
Density
0.0
0.5
1.0
1.5
2.0
2.5
0.0
0.2
0.4
0.6
0.8
1.0
N = 569   Bandwidth = 0.03434

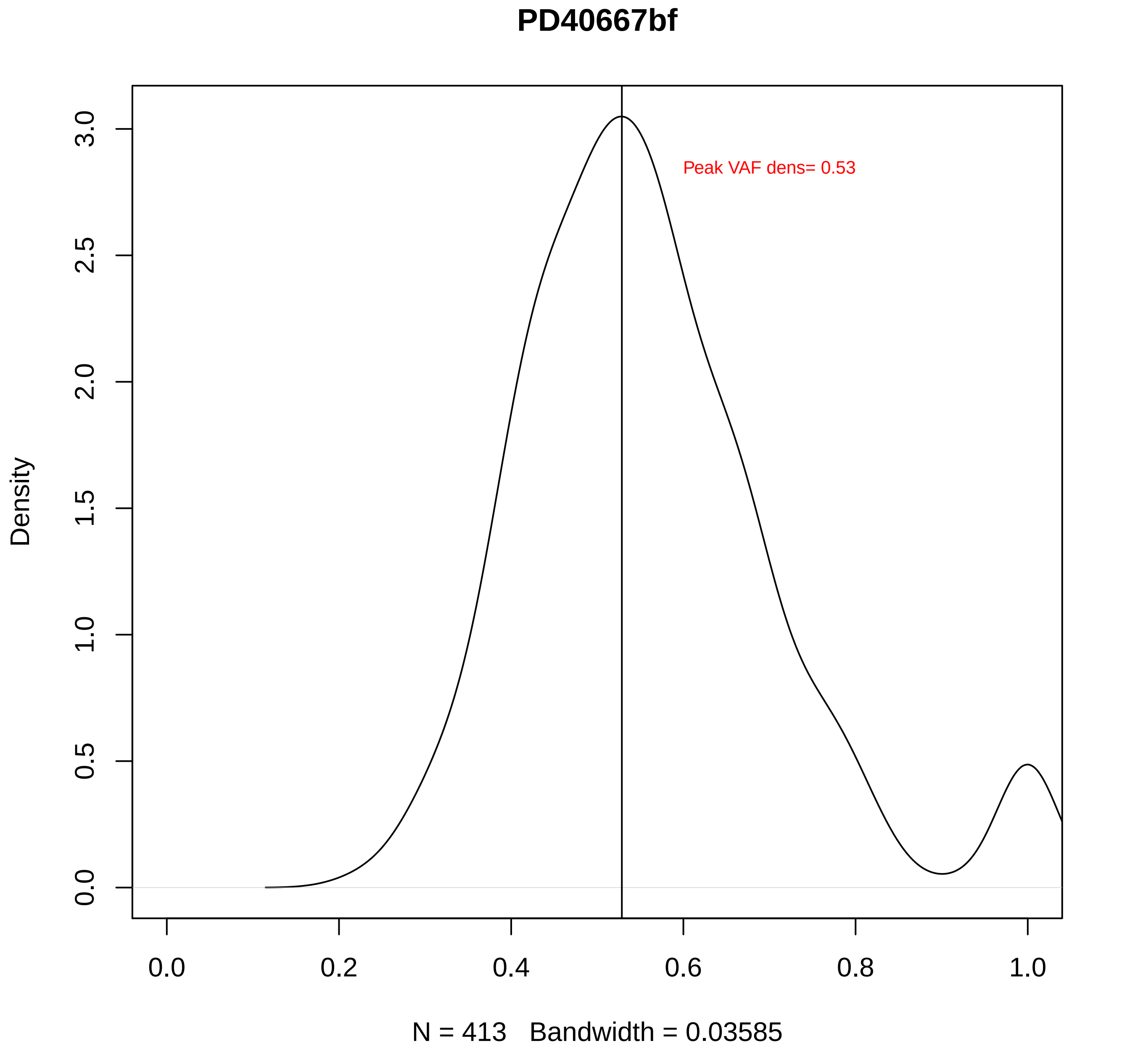

PD40667bf
Peak VAF dens= 0.53
Density
0.0
0.5
1.0
1.5
2.0
2.5
3.0
0.0
0.2
0.4
0.6
0.8
1.0
N = 413 Bandwidth = 0.03585

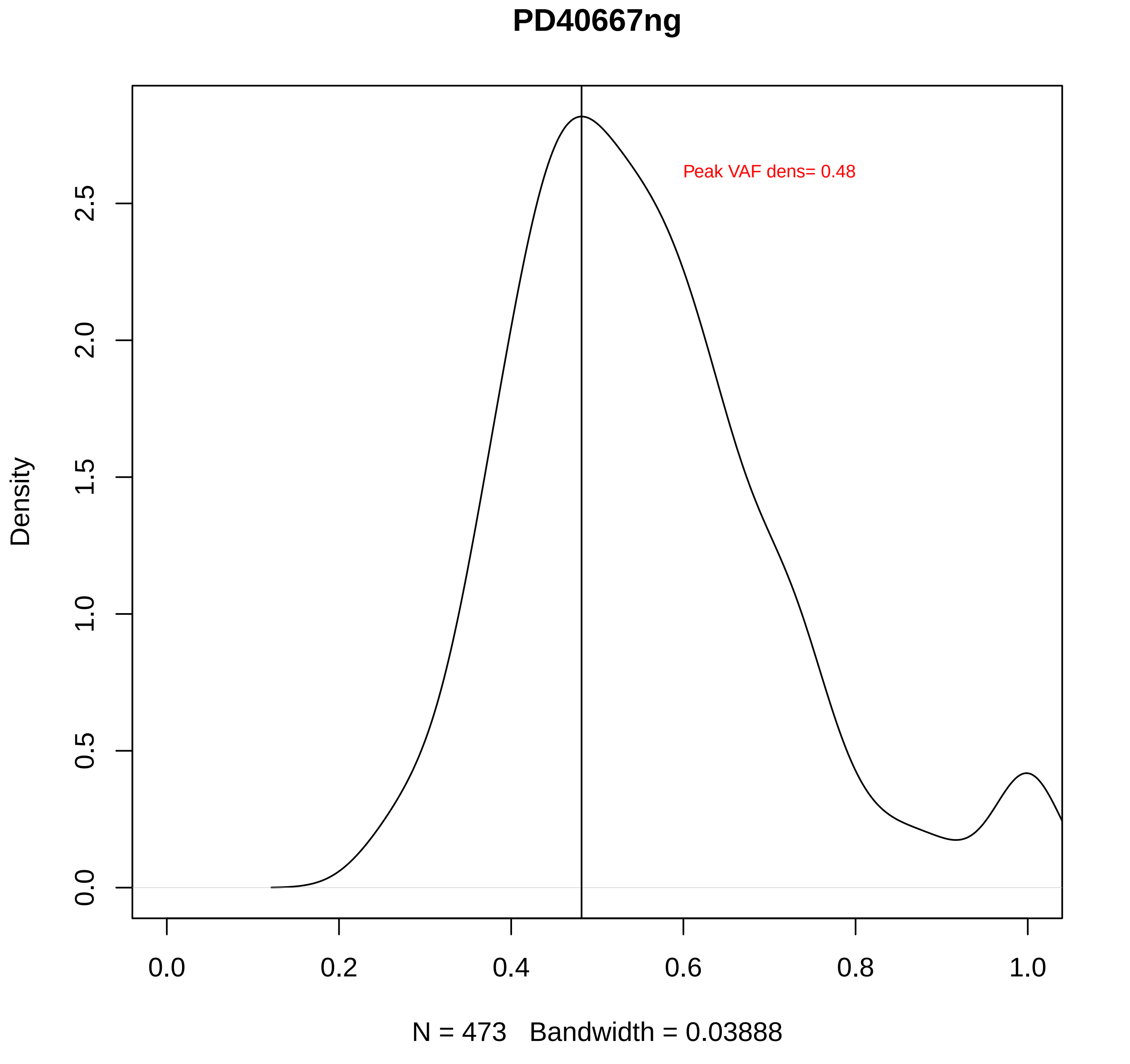

PD40667ng
Peak VAF dens= 0.48
Density
0.0
0.5
1.0
1.5
2.0
2.5
0.0
0.2
0.4
0.6
0.8
1.0
N = 473   Bandwidth = 0.03888

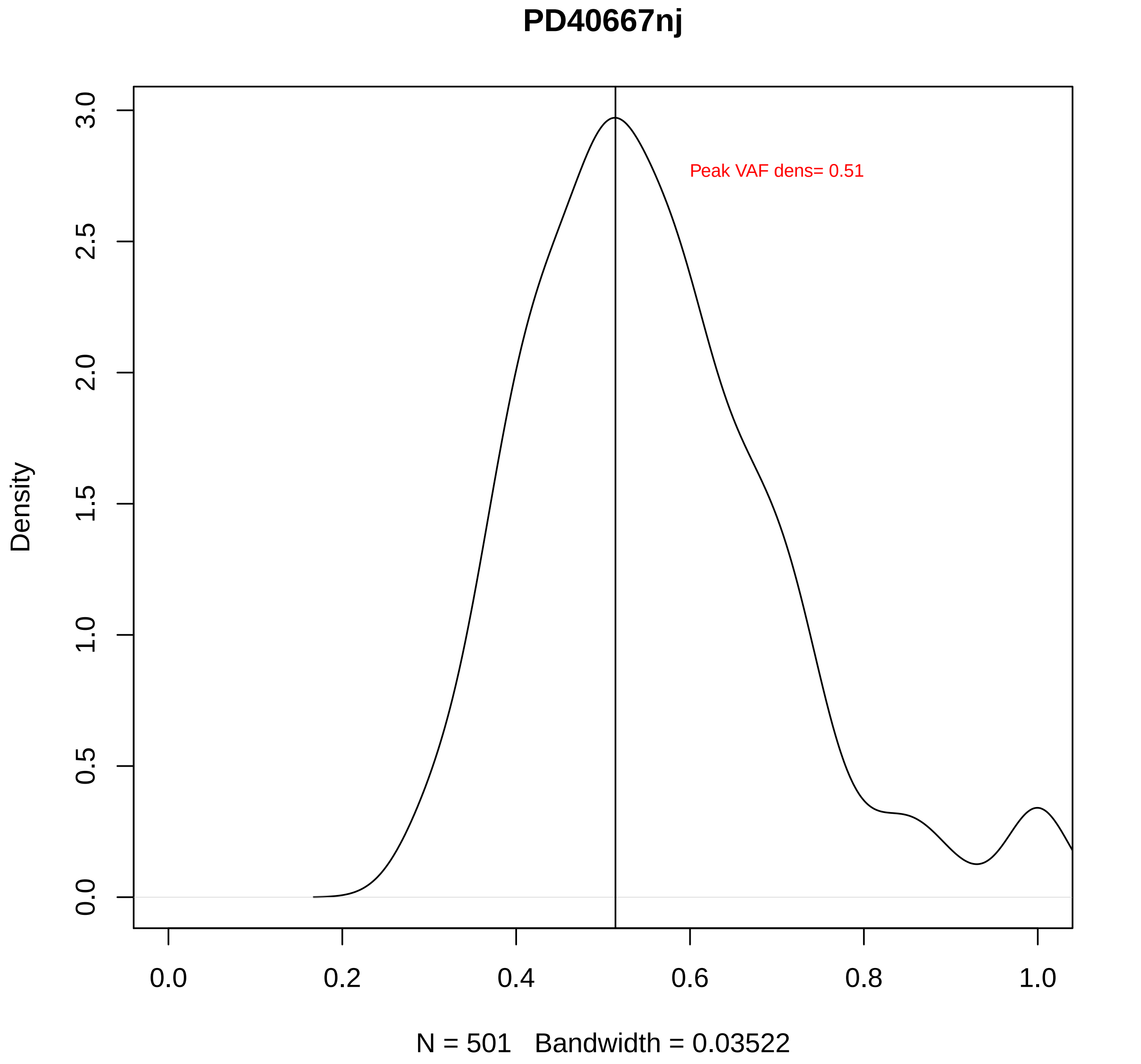

PD40667nj
Peak VAF dens= 0.51
Density
0.0
0.5
1.0
1.5
2.0
2.5
3.0
0.0
0.2
0.4
0.6
0.8
1.0
N = 501   Bandwidth = 0.03522

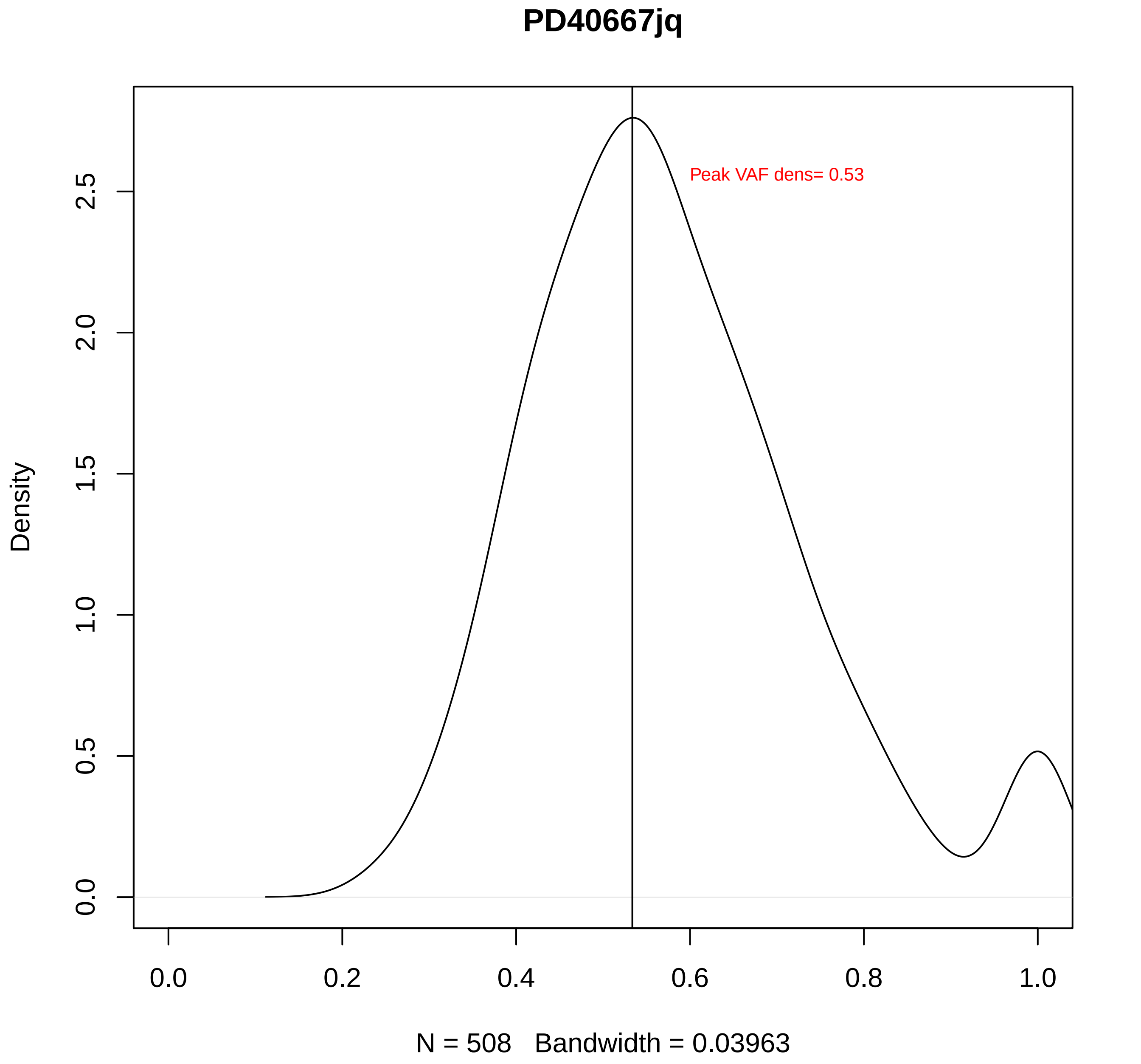
PD40667jq
Peak VAF dens= 0.53
Density
0.0
0.5
1.0
1.5
2.0
2.5
0.0
0.2
0.4
0.6
0.8
1.0
N = 508   Bandwidth = 0.03963

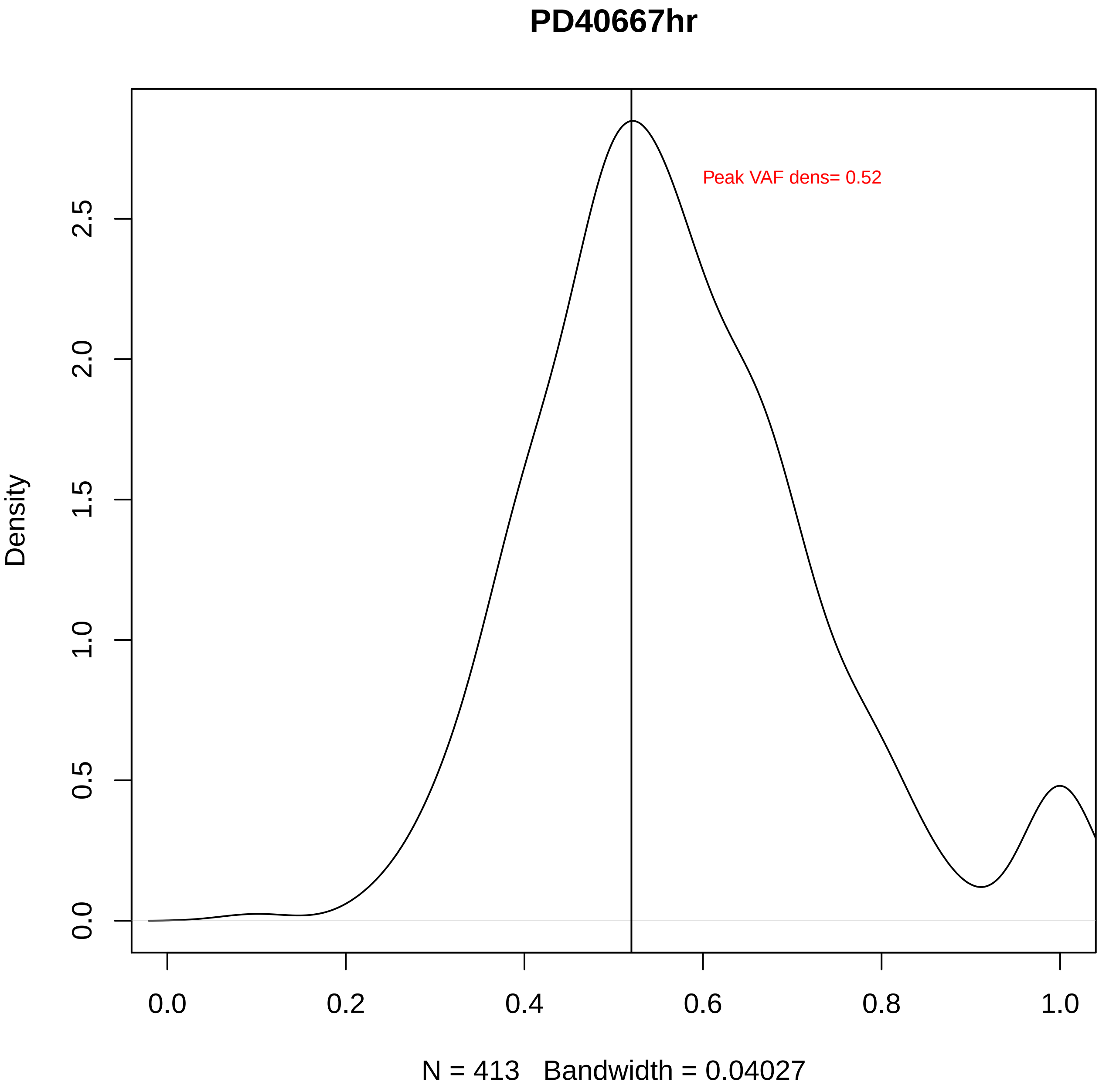
PD40667hr
Peak VAF dens= 0.52
Density
0.0
0.5
1.0
1.5
2.0
2.5
0.0
0.2
0.4
0.6
0.8
1.0
N = 413   Bandwidth = 0.04027

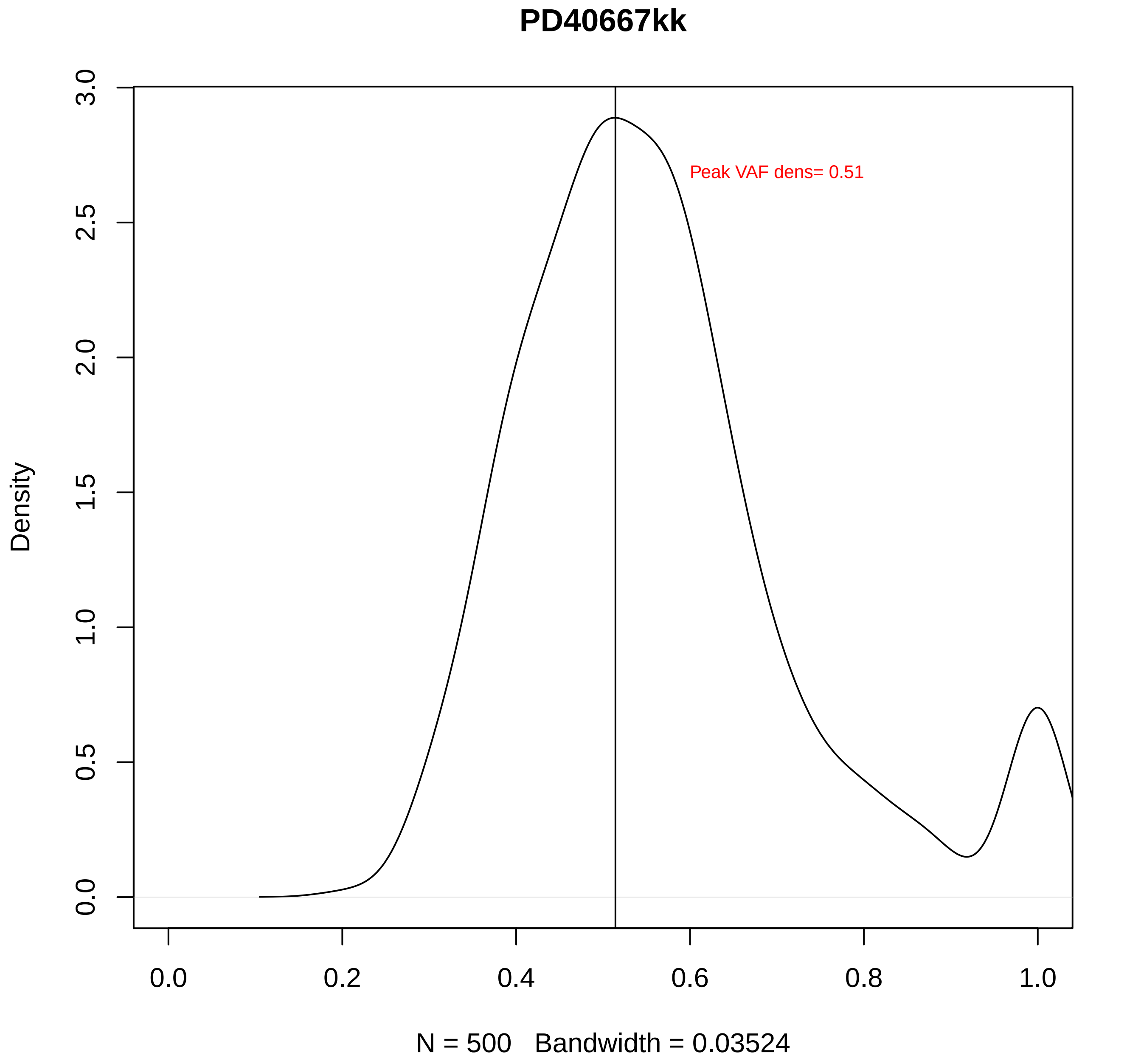

PD40667kk
Peak VAF dens= 0.51
Density
0.0
0.5
1.0
1.5
2.0
2.5
3.0
0.0
0.2
0.4
0.6
0.8
1.0
N = 500 Bandwidth = 0.03524

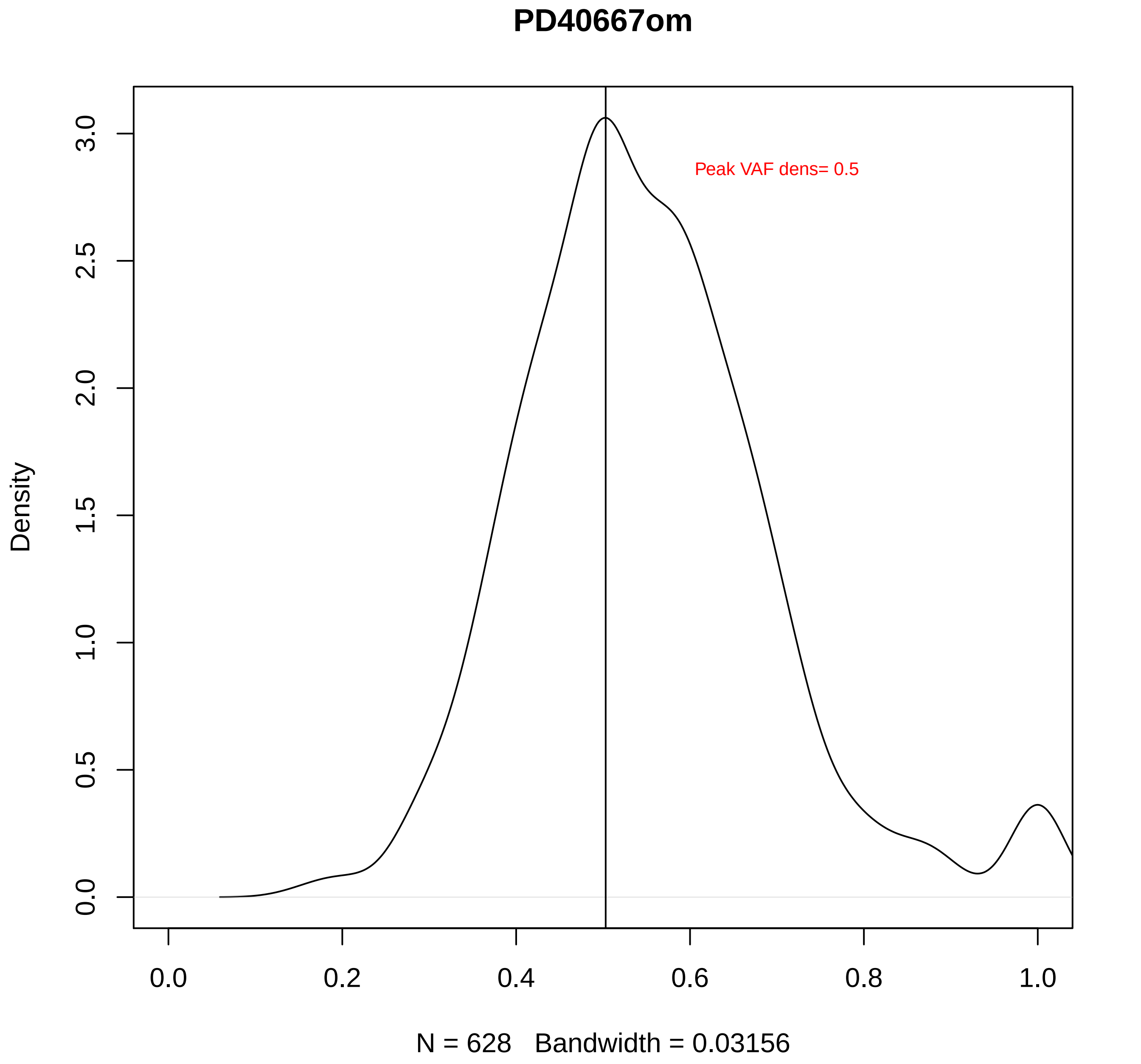
PD40667om
Peak VAF dens= 0.5
Density
3.0
2.5
2.0
1.5
1.0
0.5
0.0
0.0
0.2
0.4
0.6
0.8
1.0
N = 628   Bandwidth = 0.03156

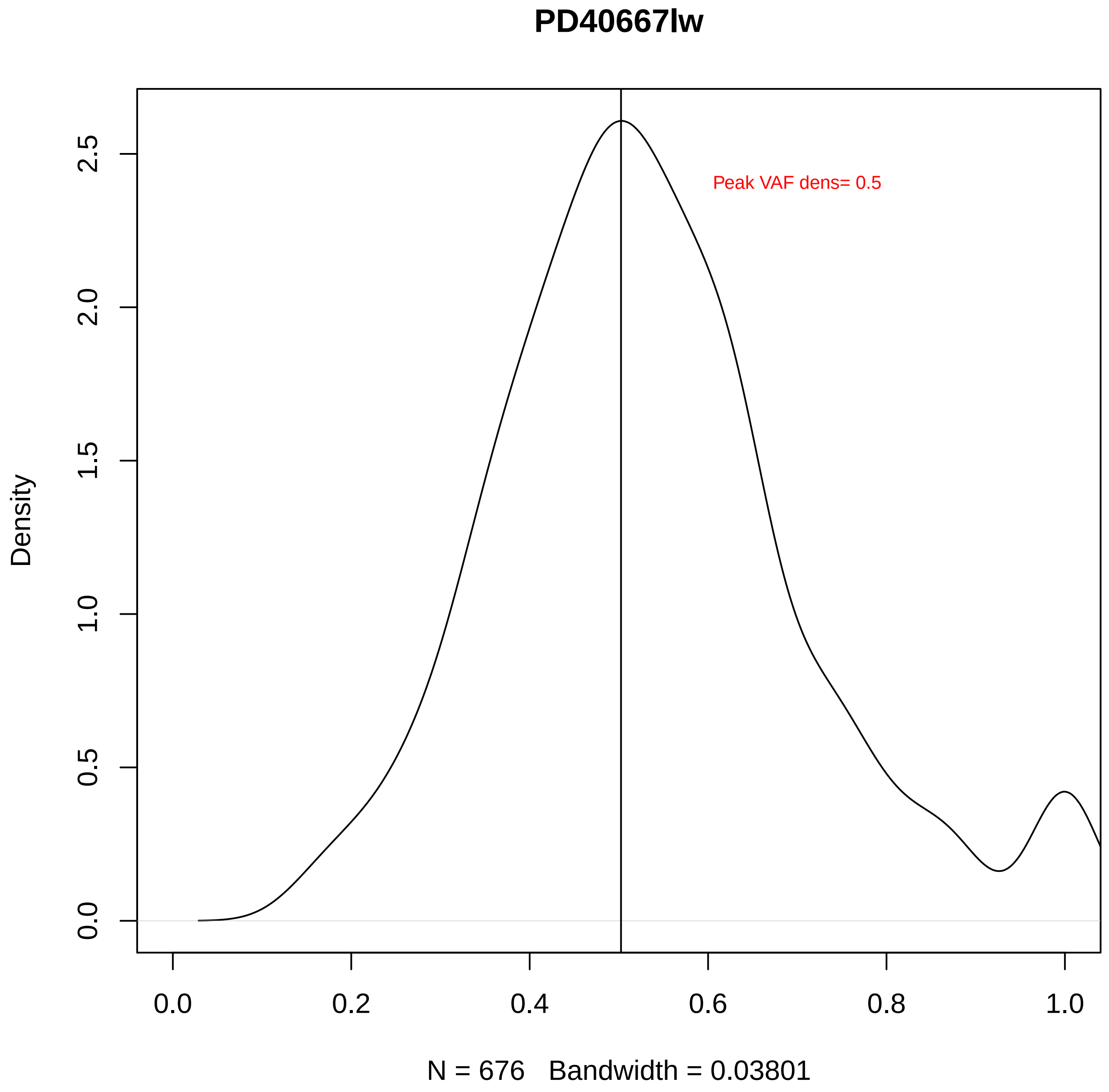
PD40667lw
Peak VAF dens= 0.5
Density
0.0
0.5
1.0
1.5
2.0
2.5
0.0
0.2
0.4
0.6
0.8
1.0
N = 676   Bandwidth = 0.03801

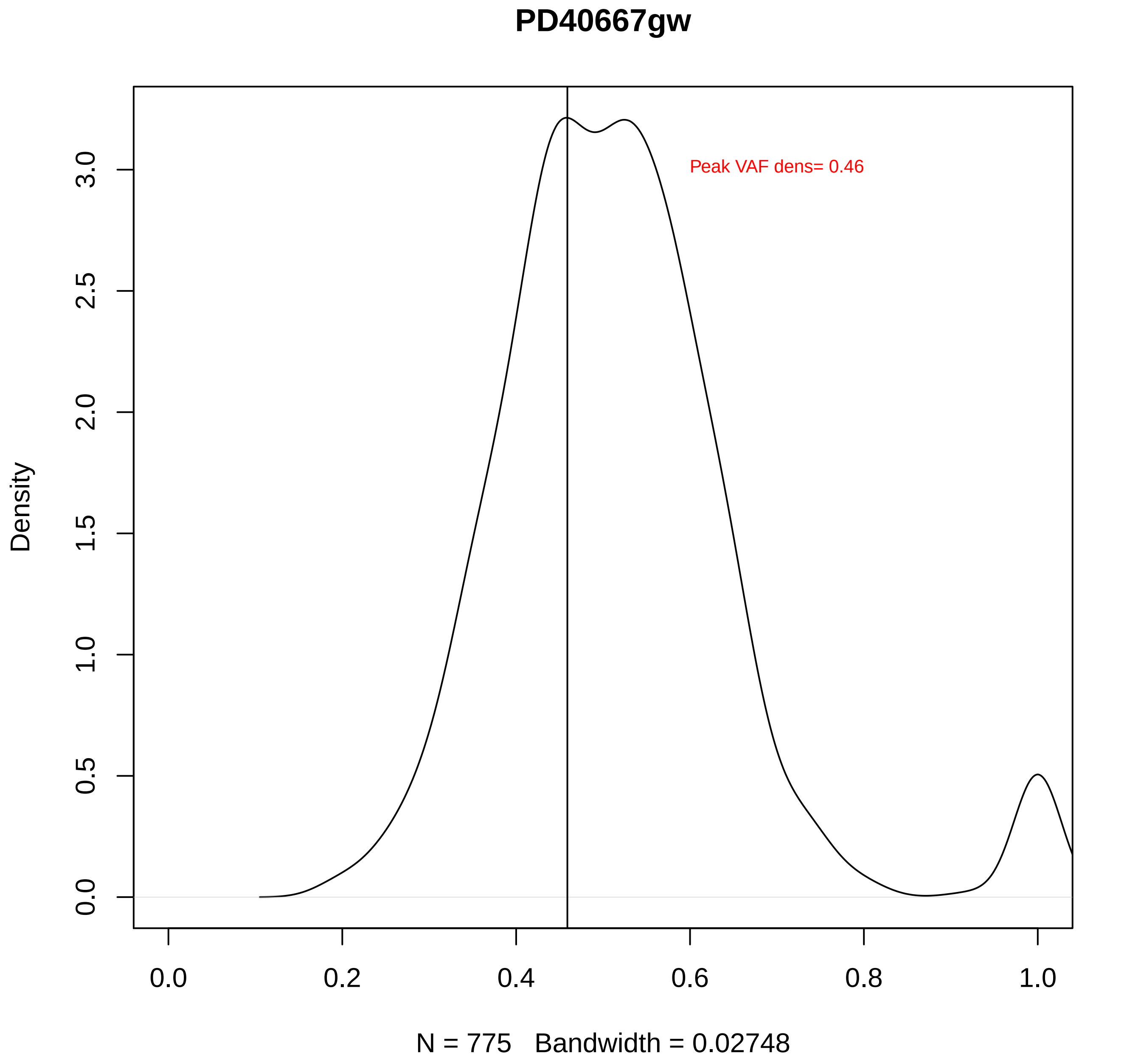
PD40667gw
Peak VAF dens= 0.46
Density
0.0
0.5
1.0
1.5
2.0
2.5
3.0
0.0
0.2
0.4
0.6
0.8
1.0
N = 775   Bandwidth = 0.02748

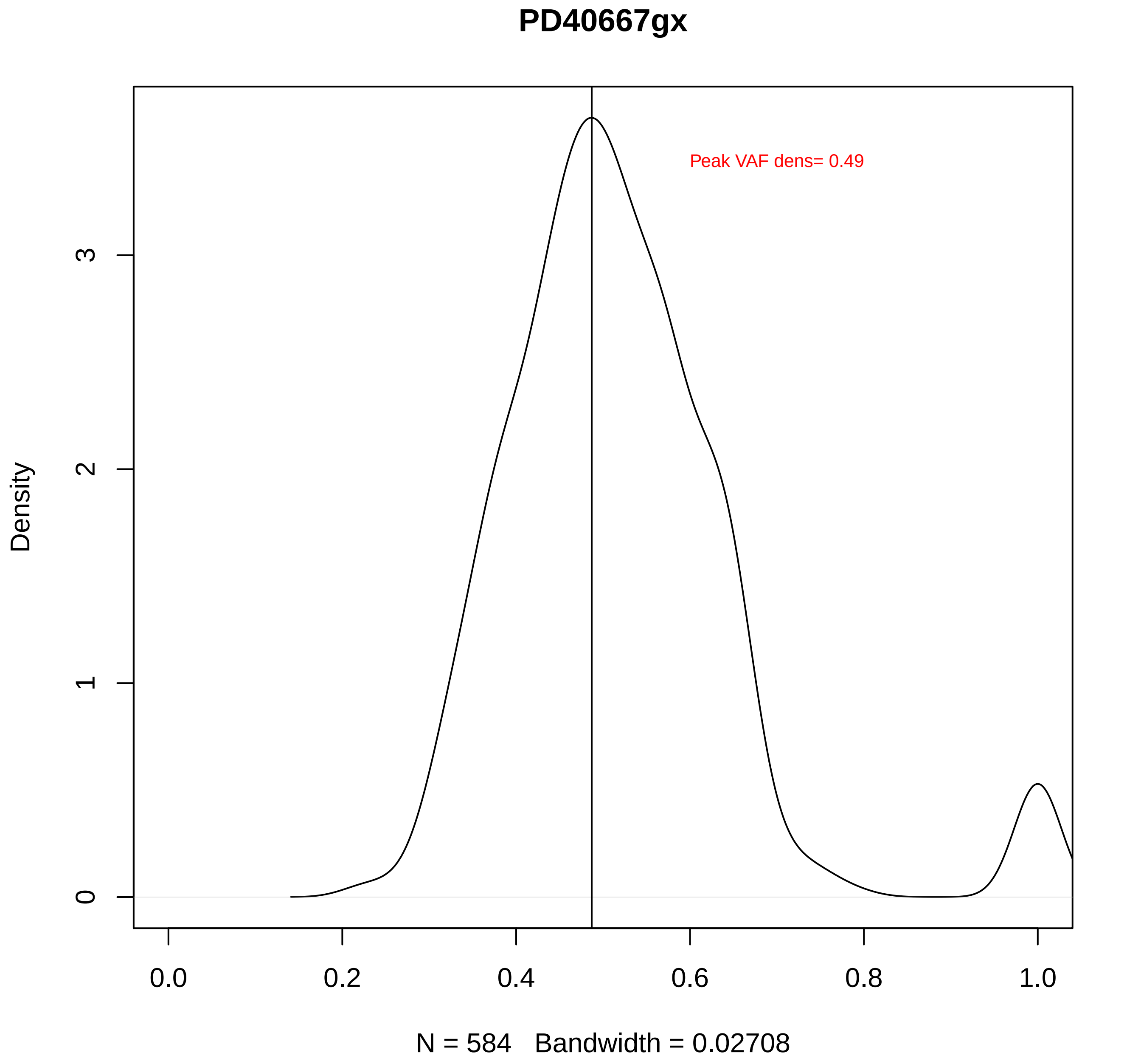

PD40667gx
Peak VAF dens= 0.49
Density
0
1
2
3
0.0
0.2
0.4
0.6
0.8
1.0
N = 584   Bandwidth = 0.02708

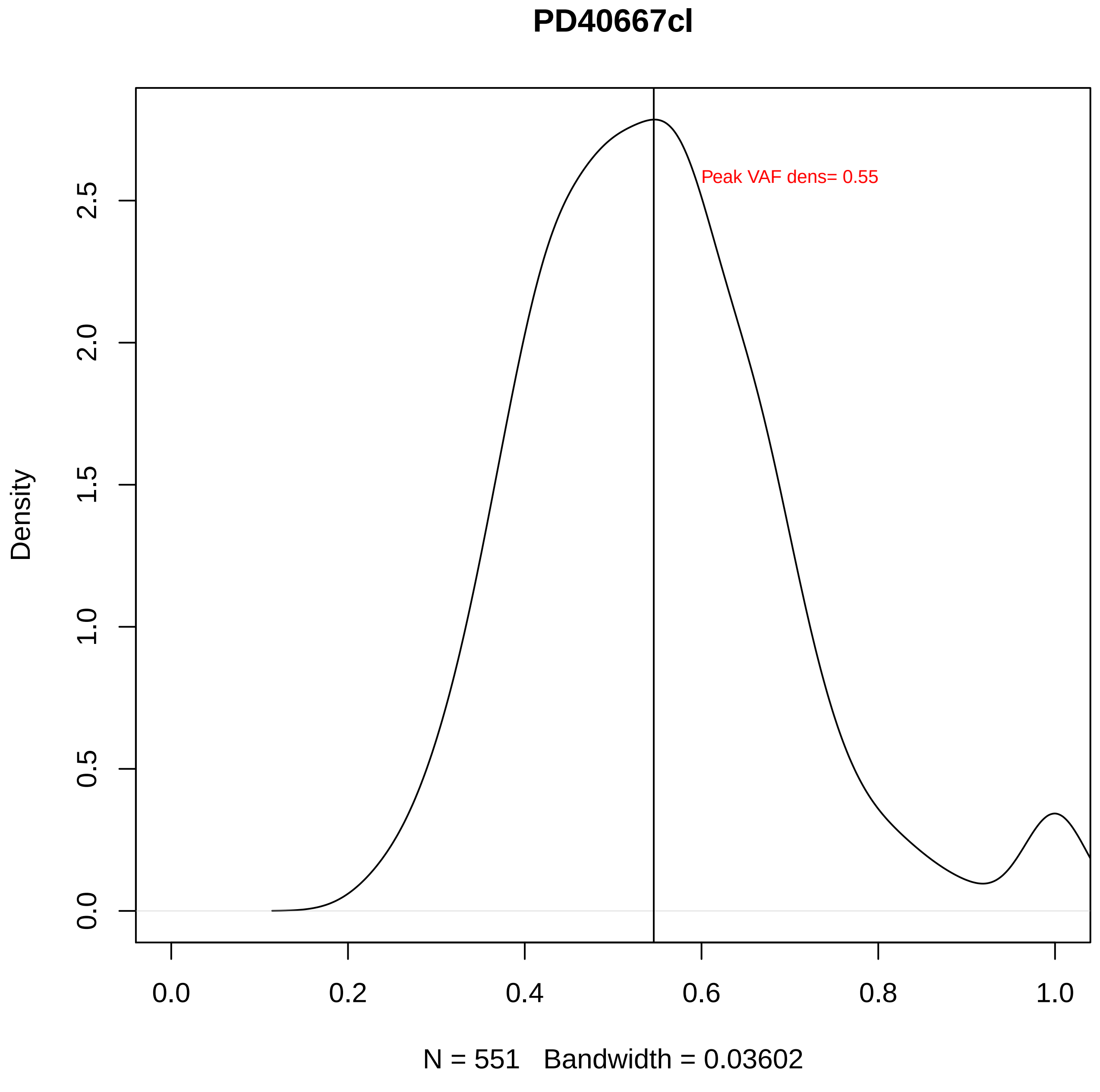

PD40667cl
Peak VAF dens= 0.55
Density
0.0
0.5
1.0
1.5
2.0
2.5
0.0
0.2
0.4
0.6
0.8
1.0
N = 551   Bandwidth = 0.03602

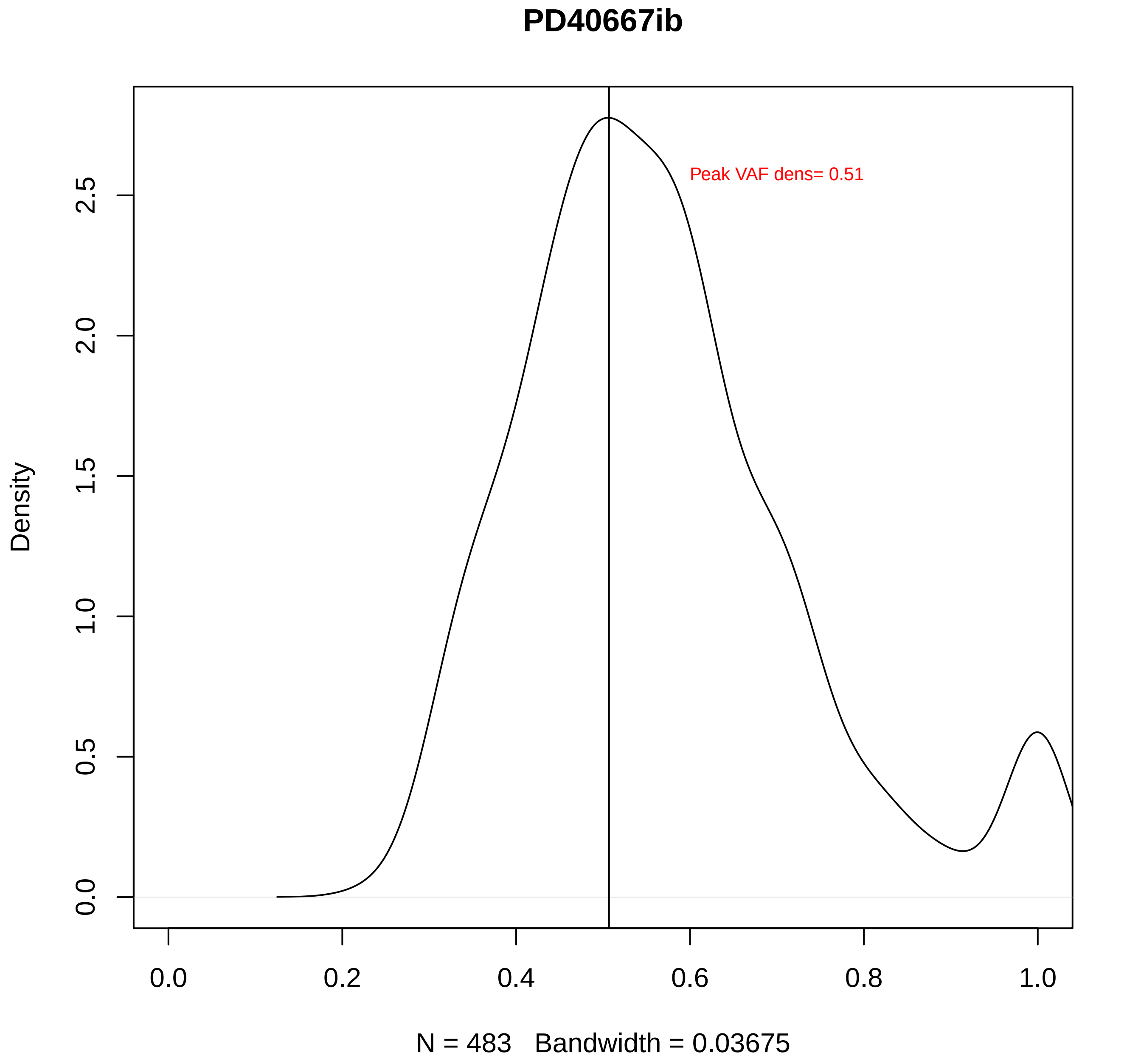

PD40667ib
Peak VAF dens= 0.51
Density
0.0
0.5
1.0
1.5
2.0
2.5
0.0
0.2
0.4
0.6
0.8
1.0
N = 483   Bandwidth = 0.03675

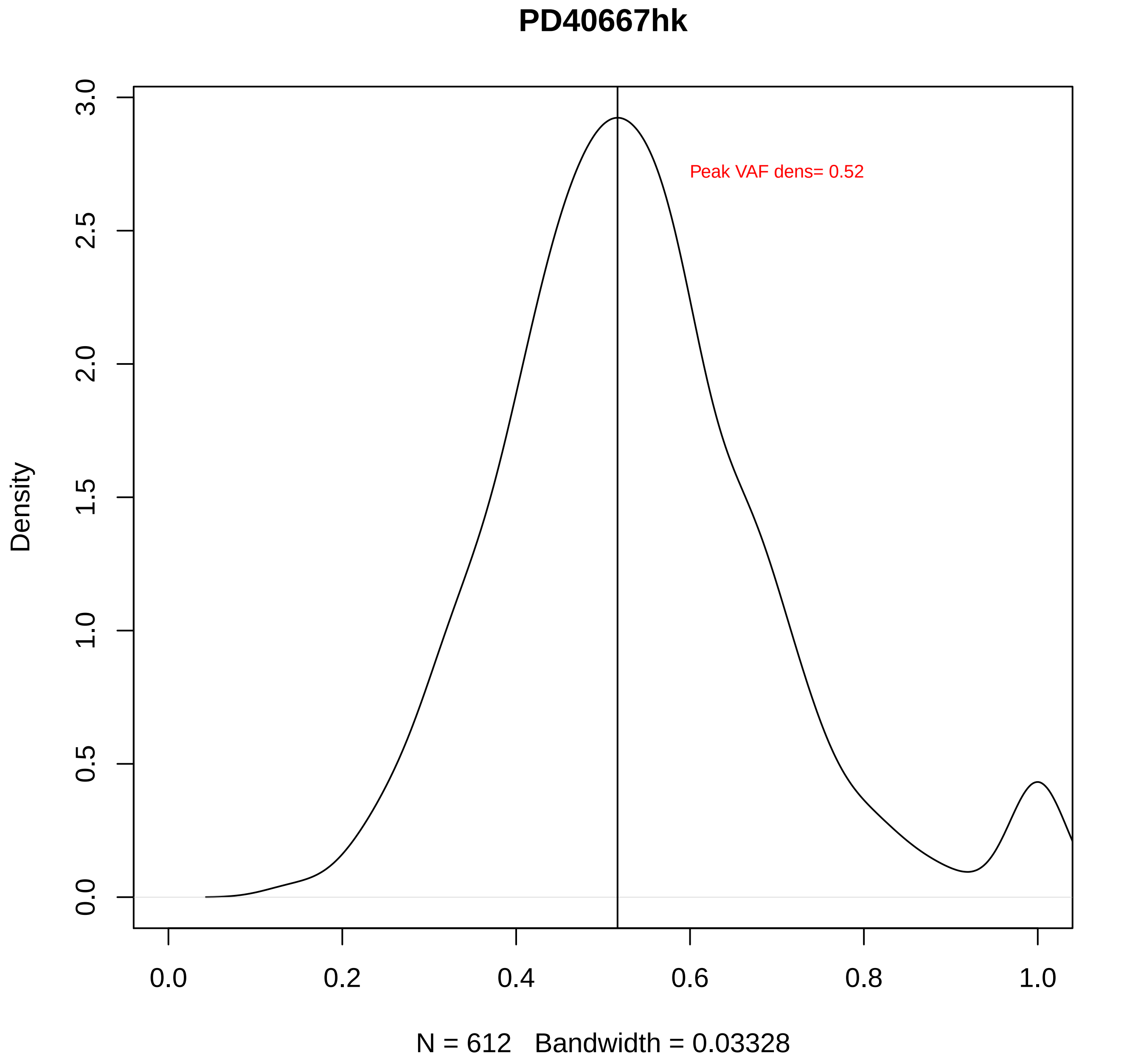
PD40667hk
Peak VAF dens= 0.52
Density
3.0
2.5
2.0
1.5
1.0
0.5
0.0
0.0
0.2
0.4
0.6
0.8
1.0
N = 612   Bandwidth = 0.03328

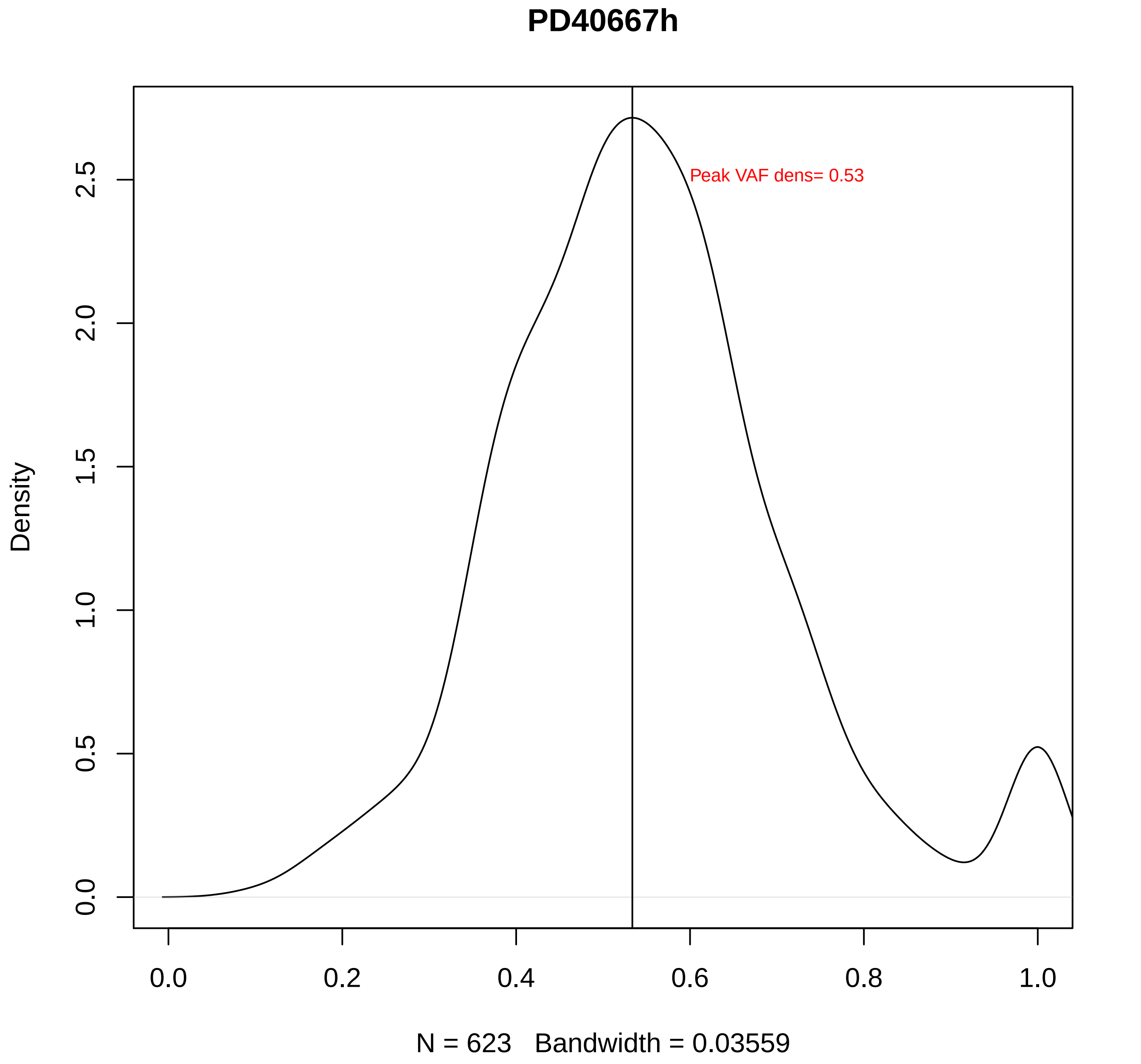
PD40667h
Density
Peak VAF dens= 0.53
0.0
0.5
1.0
1.5
2.0
2.5
0.0
0.2
0.4
0.6
0.8
1.0
N = 623   Bandwidth = 0.03559

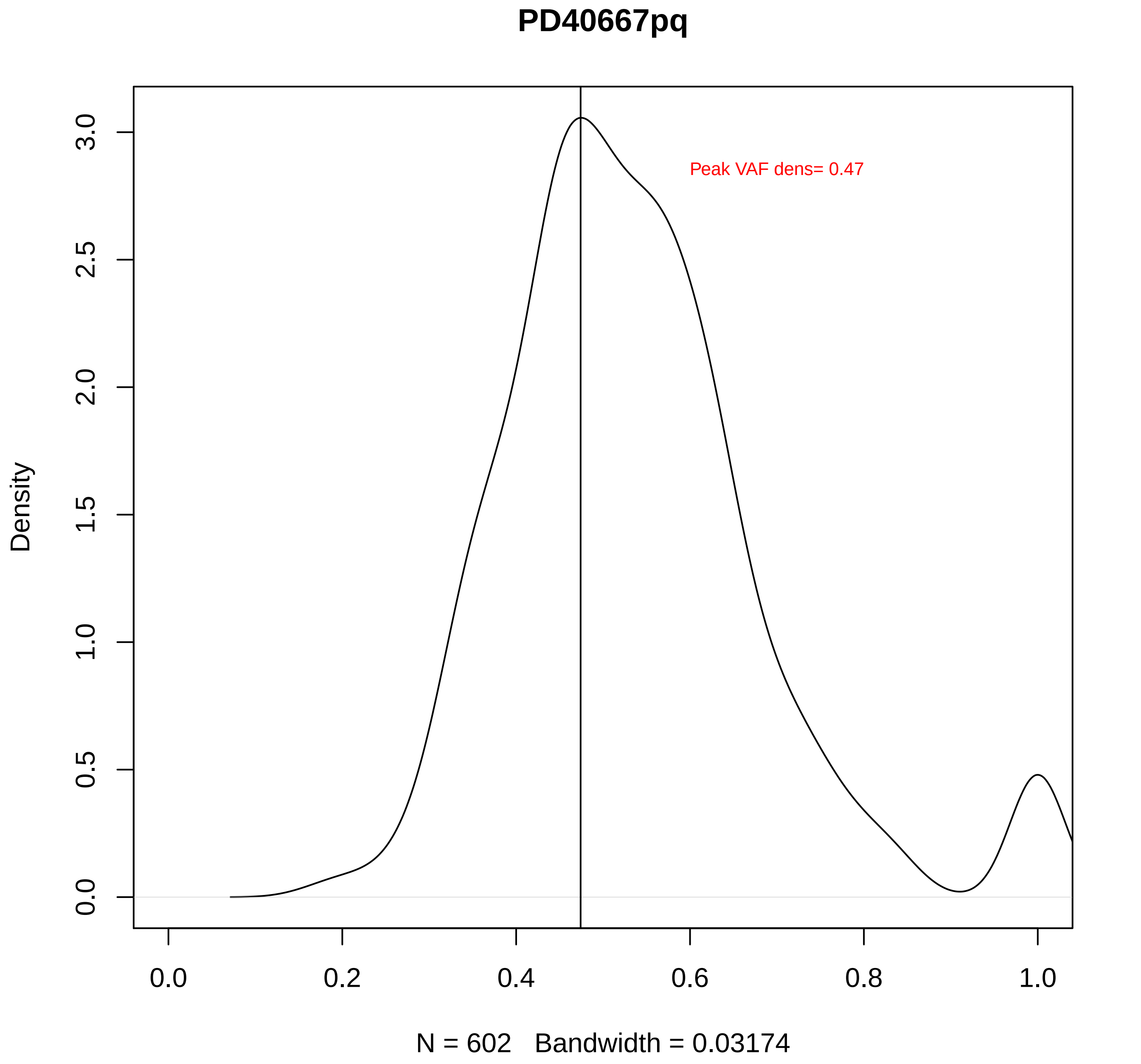
PD40667pq
Peak VAF dens= 0.47
Density
3.0
2.5
2.0
1.5
1.0
0.5
0.0
0.0
0.2
0.4
0.6
0.8
1.0
N = 602   Bandwidth = 0.03174

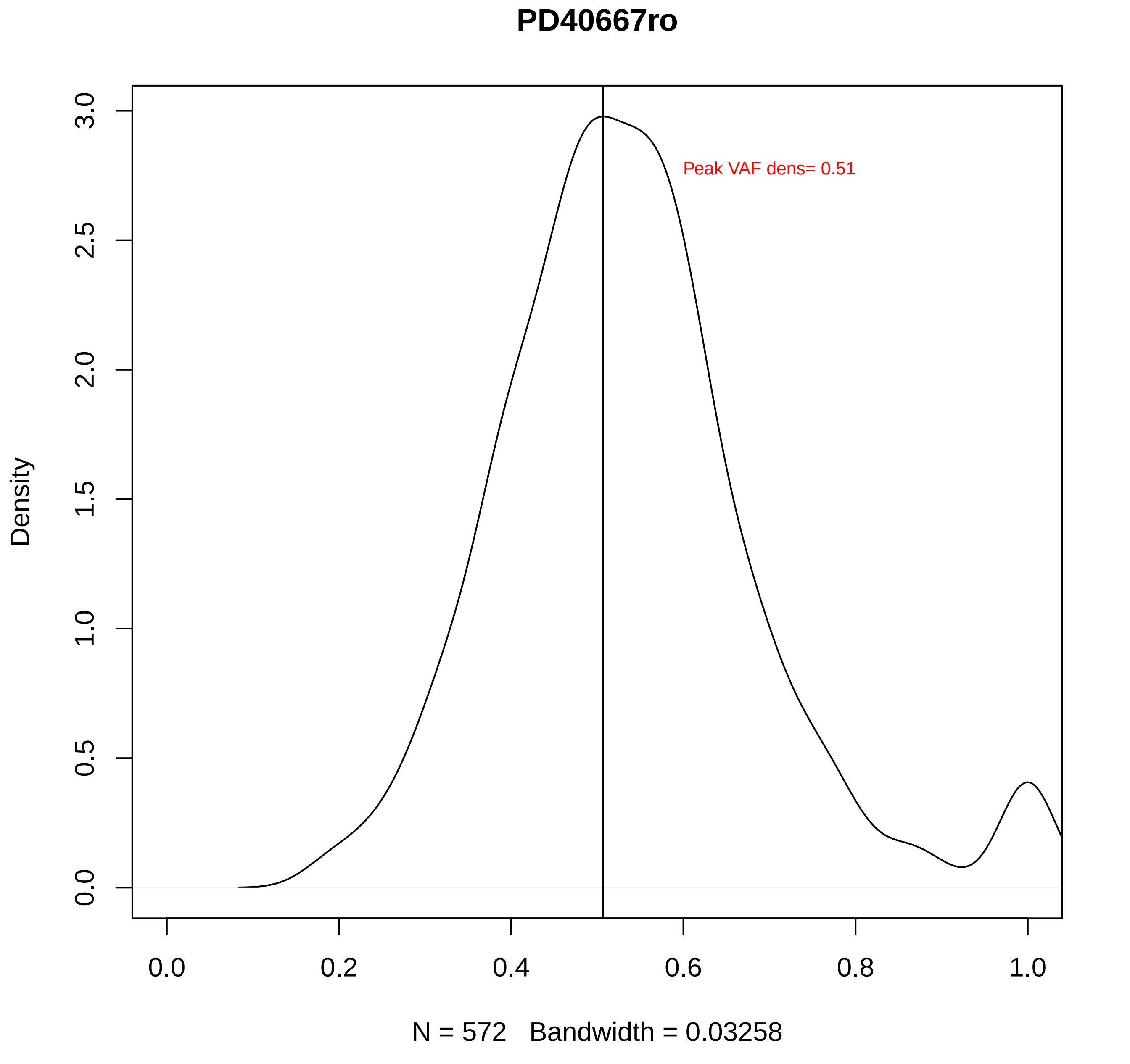
PD40667ro
Peak VAF dens= 0.51
Density
0.0
0.5
1.0
1.5
2.0
2.5
3.0
0.0
0.2
0.4
0.6
0.8
1.0
N = 572   Bandwidth = 0.03258

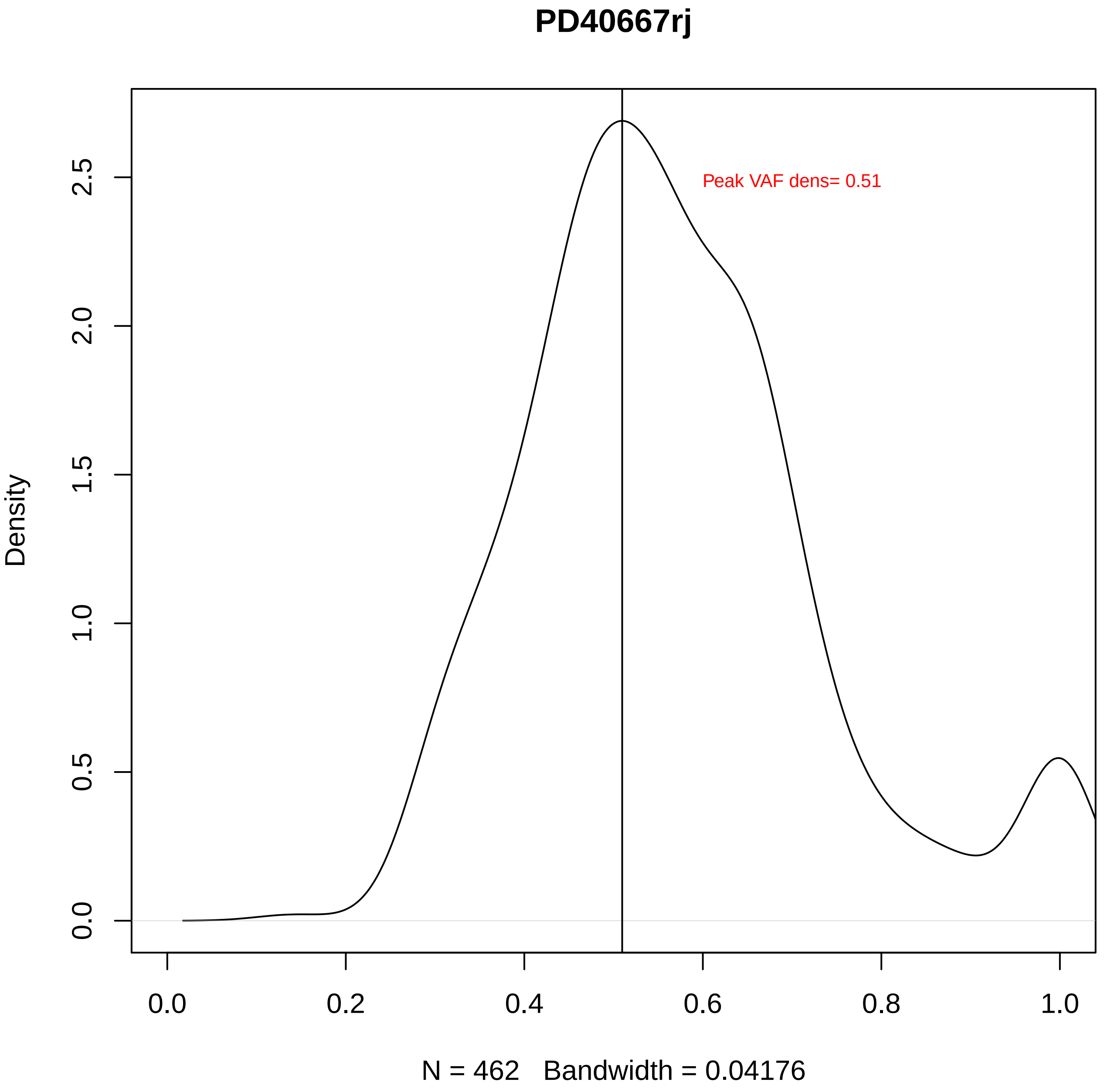
PD40667rj
Peak VAF dens= 0.51
Density
0.0
0.5
1.0
1.5
2.0
2.5
0.0
0.2
0.4
0.6
0.8
1.0
N = 462   Bandwidth = 0.04176

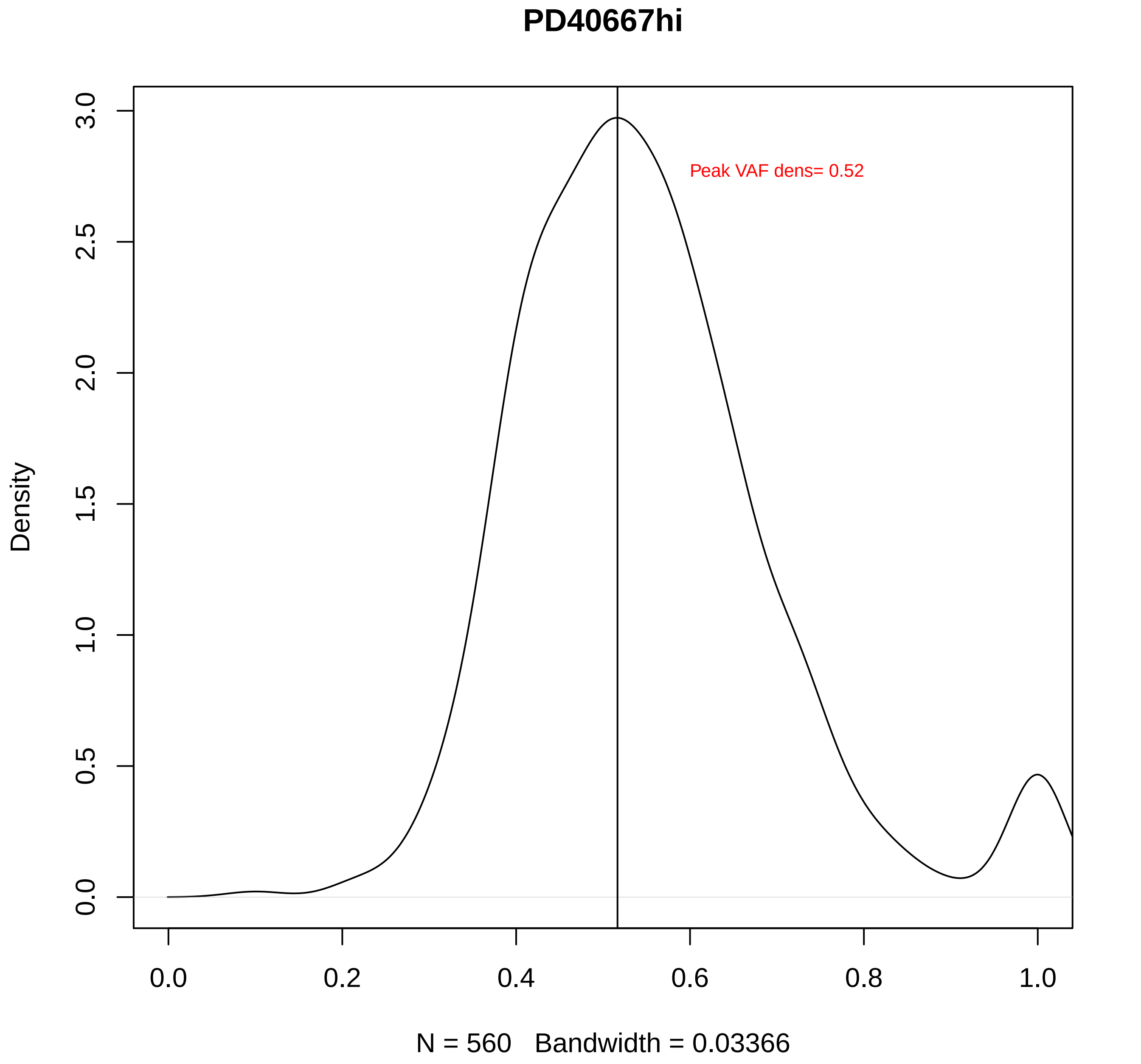
PD40667hi
Peak VAF dens= 0.52
Density
3.0
2.5
2.0
1.5
1.0
0.5
0.0
0.0
0.2
0.4
0.6
0.8
1.0
N = 560   Bandwidth = 0.03366

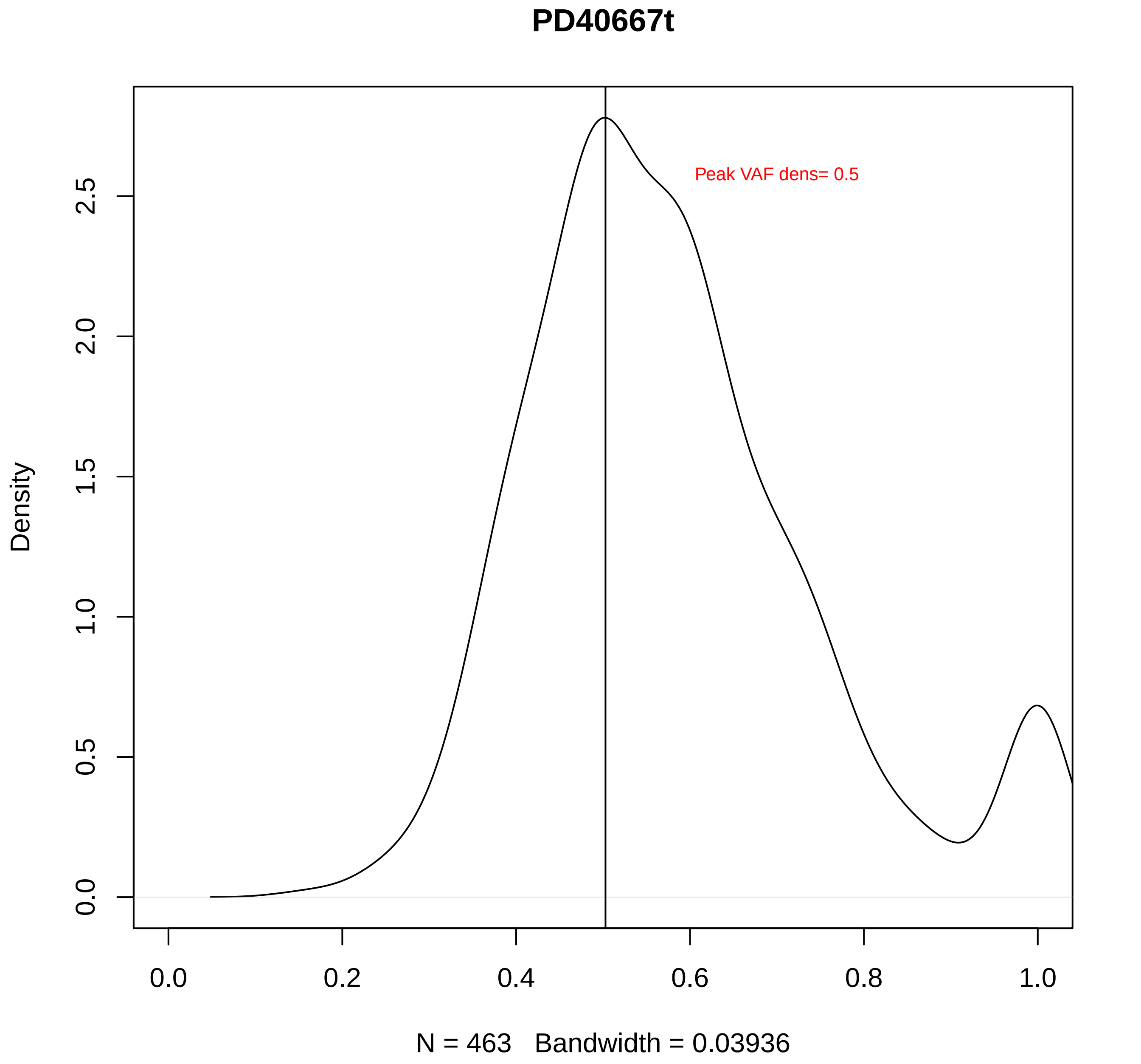
PD40667t
Peak VAF dens= 0.5
Density
0.0
0.5
1.0
1.5
2.0
2.5
0.0
0.2
0.4
0.6
0.8
1.0
N = 463   Bandwidth = 0.03936

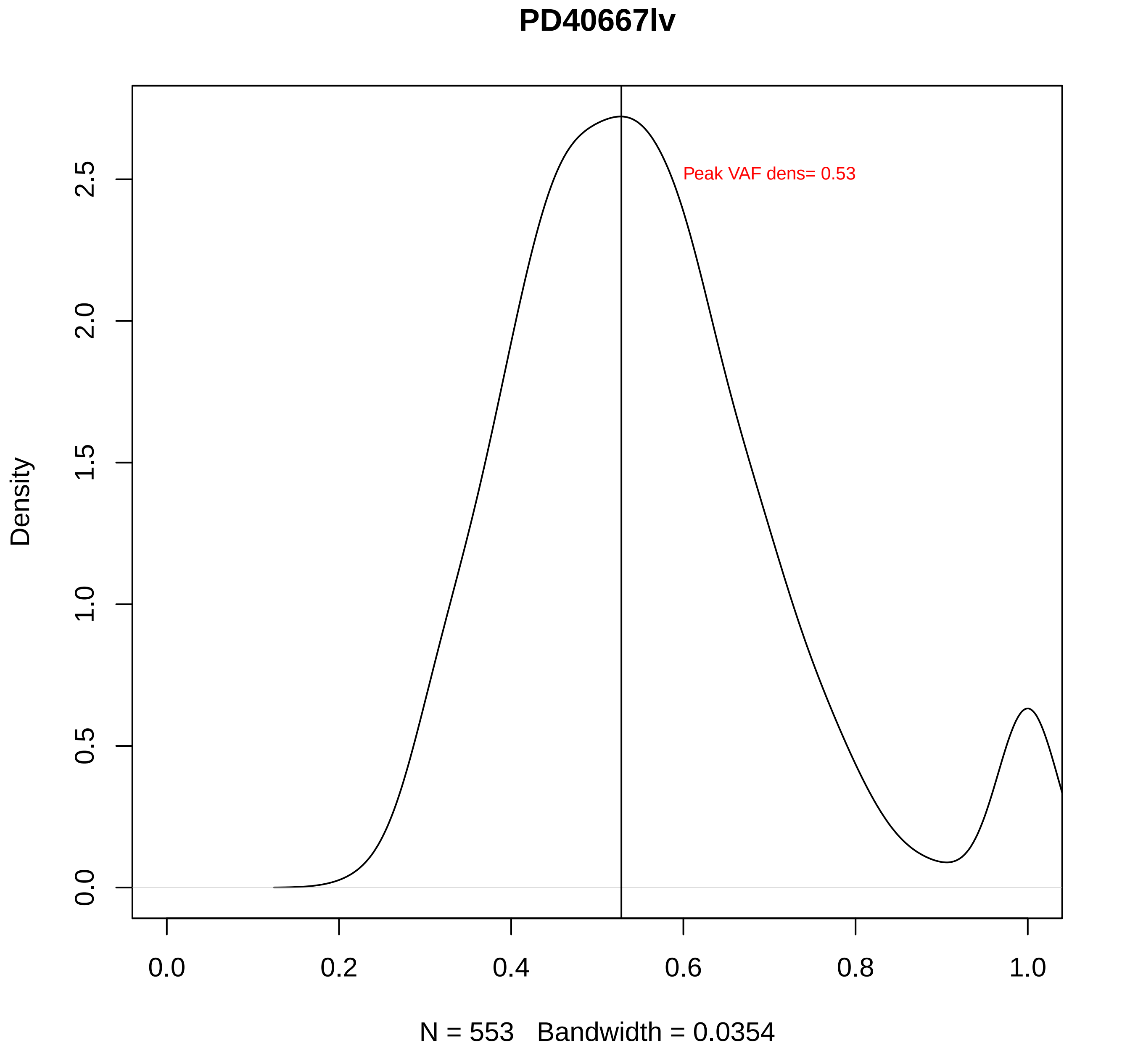
PD40667lv
Peak VAF dens= 0.53
Density
0.0
0.5
1.0
1.5
2.0
2.5
0.0
0.2
0.4
0.6
0.8
1.0
N = 553   Bandwidth = 0.0354

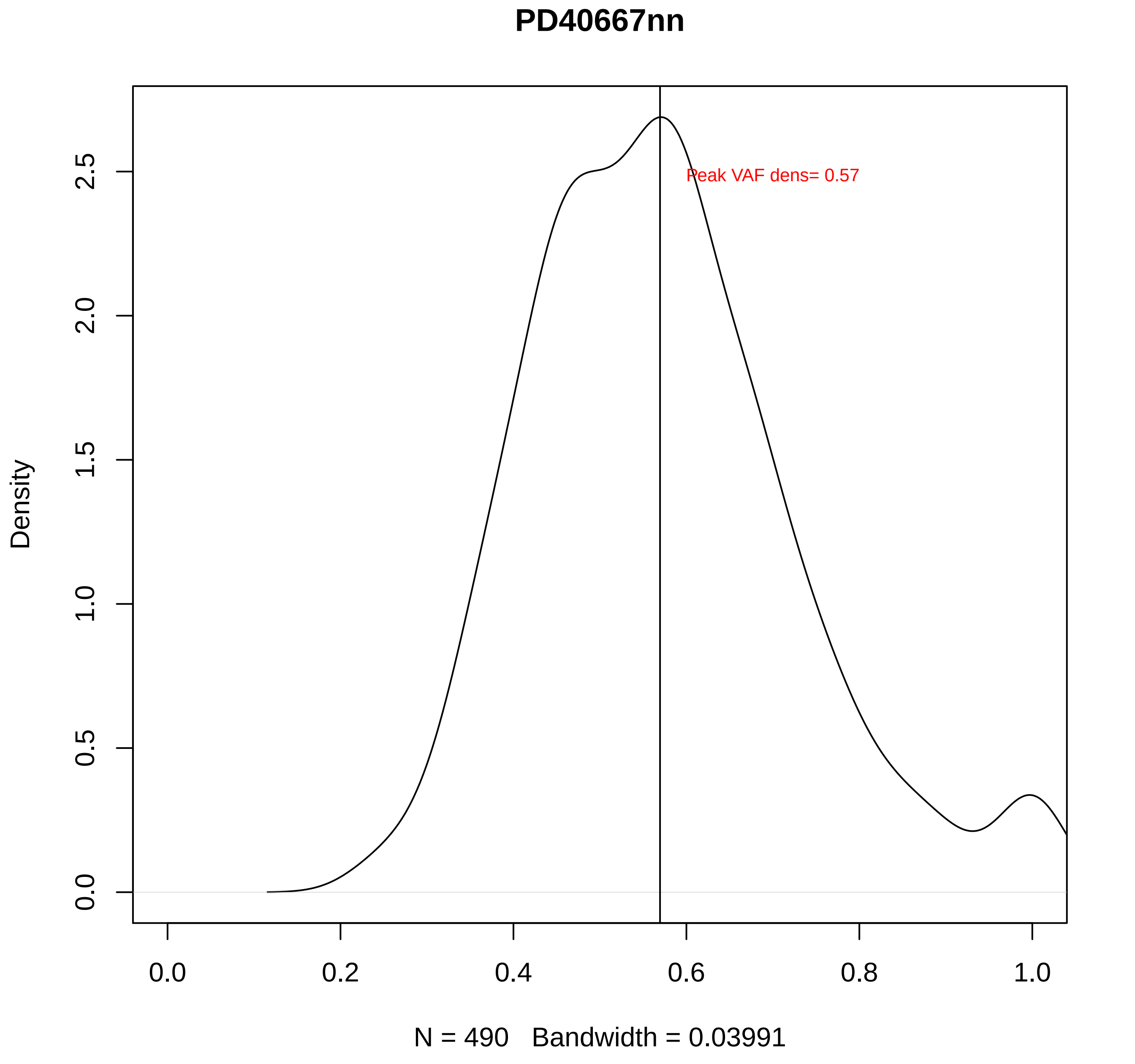

PD40667nn
Peak VAF dens= 0.57
Density
0.0
0.5
1.0
1.5
2.0
2.5
0.0
0.2
0.4
0.6
0.8
1.0
N = 490 Bandwidth = 0.03991

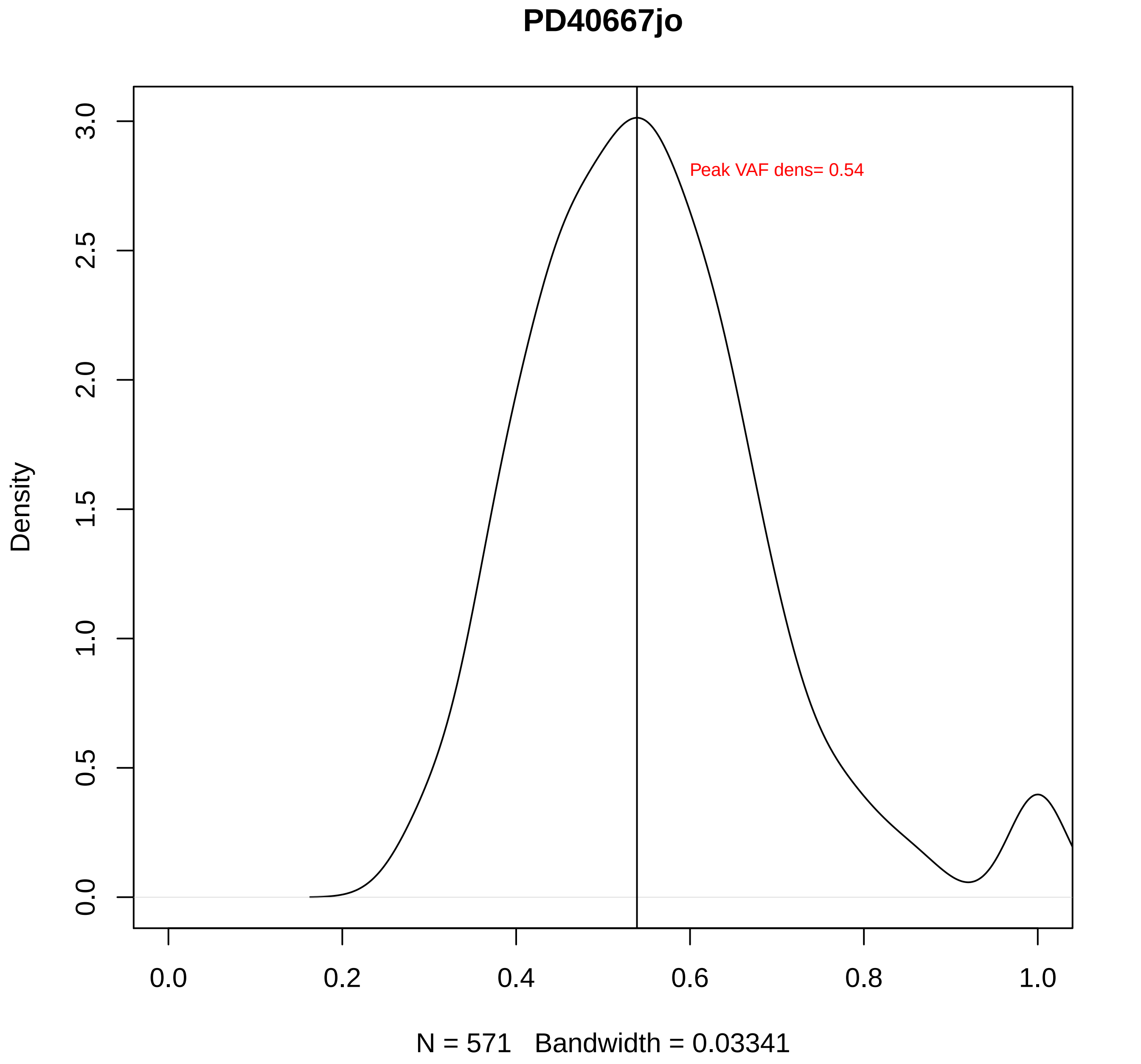

PD40667jo
Peak VAF dens= 0.54
Density
3.0
2.5
2.0
1.5
1.0
0.5
0.0
0.0
0.2
0.4
0.6
0.8
1.0
N = 571   Bandwidth = 0.03341

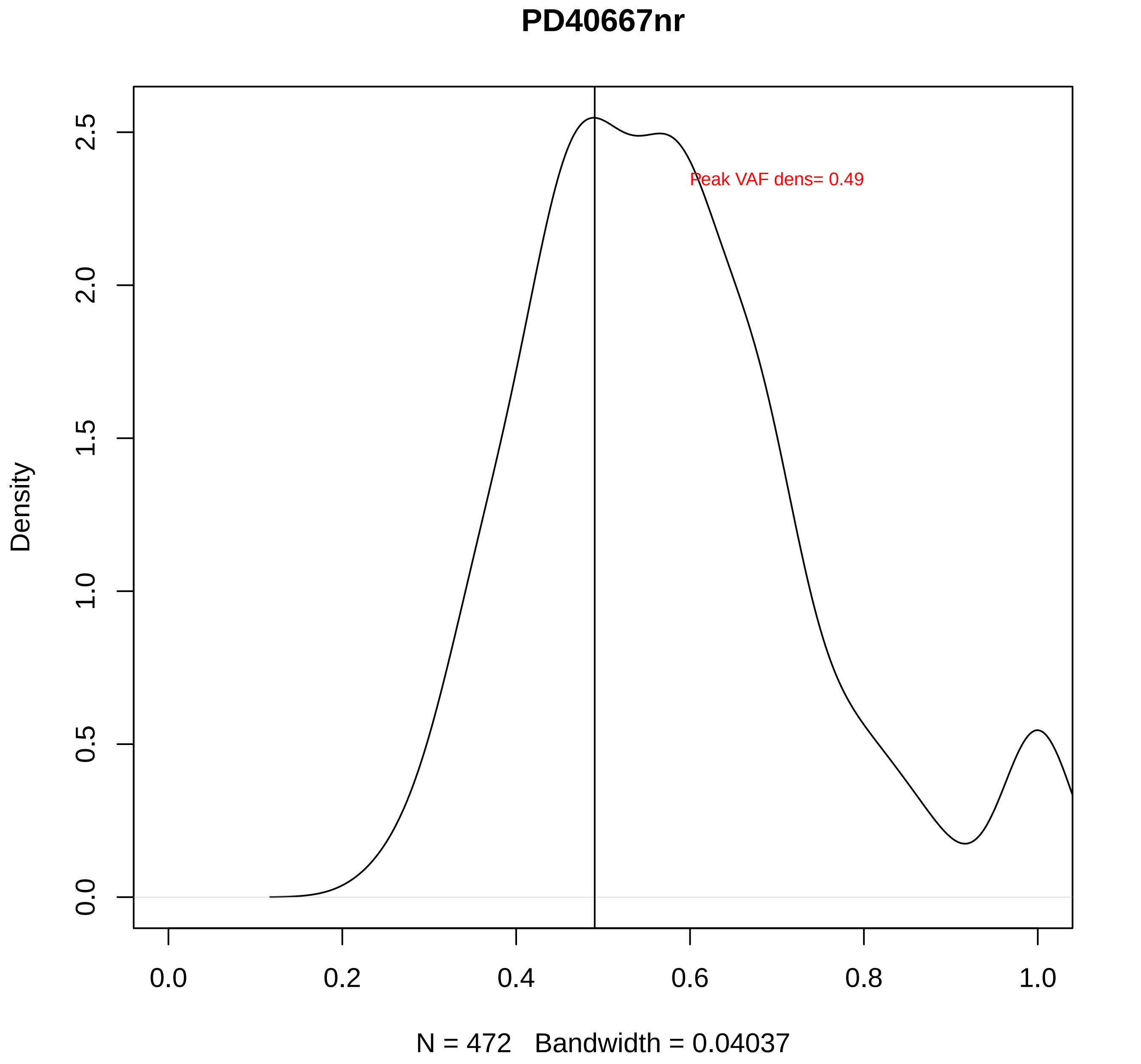

PD40667nr
Peak VAF dens= 0.49
Density
2.5
2.0
1.5
1.0
0.5
0.0
0.0
0.2
0.4
0.6
0.8
1.0
N = 472   Bandwidth = 0.04037

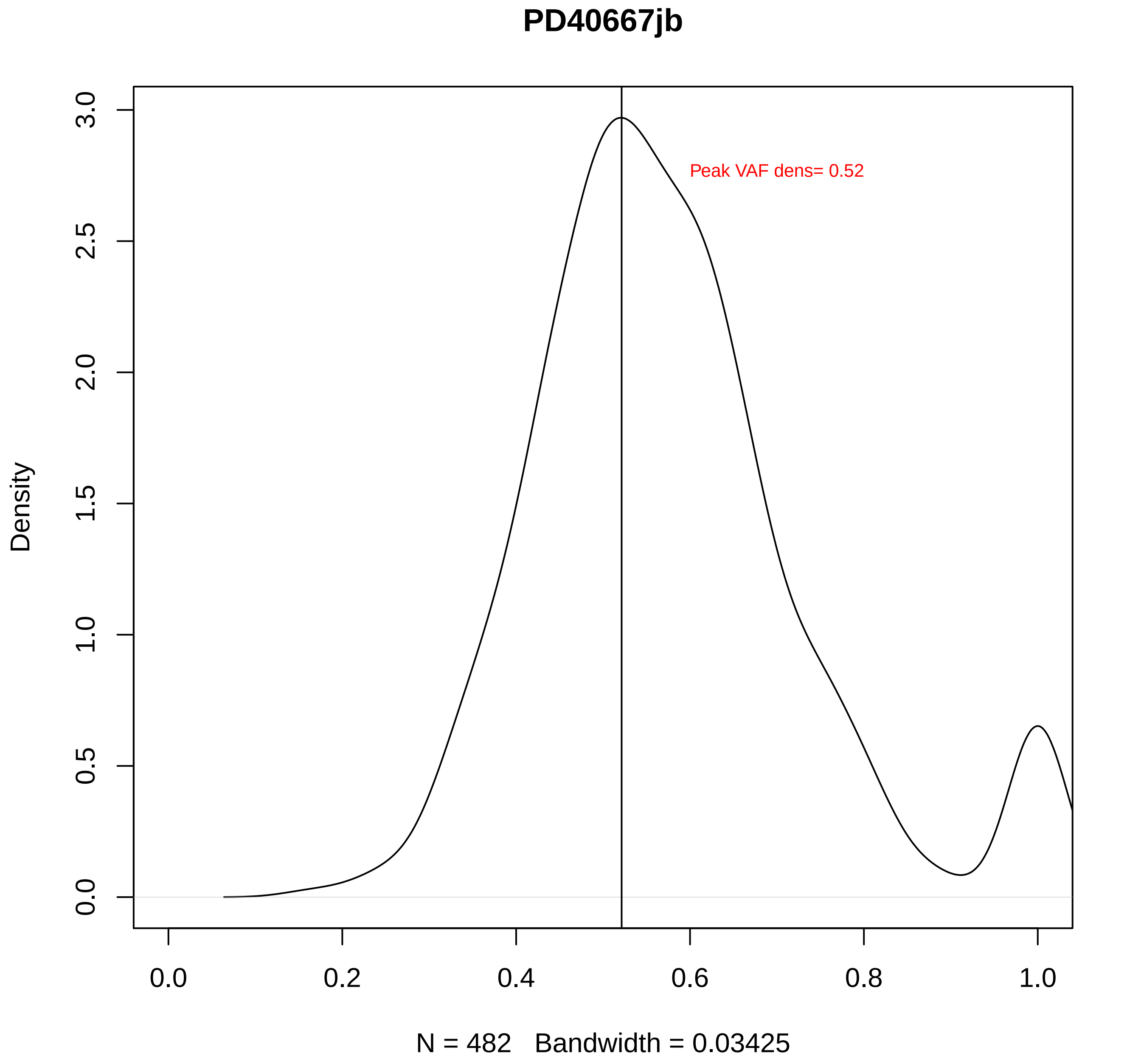

PD40667jb
Peak VAF dens= 0.52
Density
0.0
0.5
1.0
1.5
2.0
2.5
3.0
0.0
0.2
0.4
0.6
0.8
1.0
N = 482   Bandwidth = 0.03425

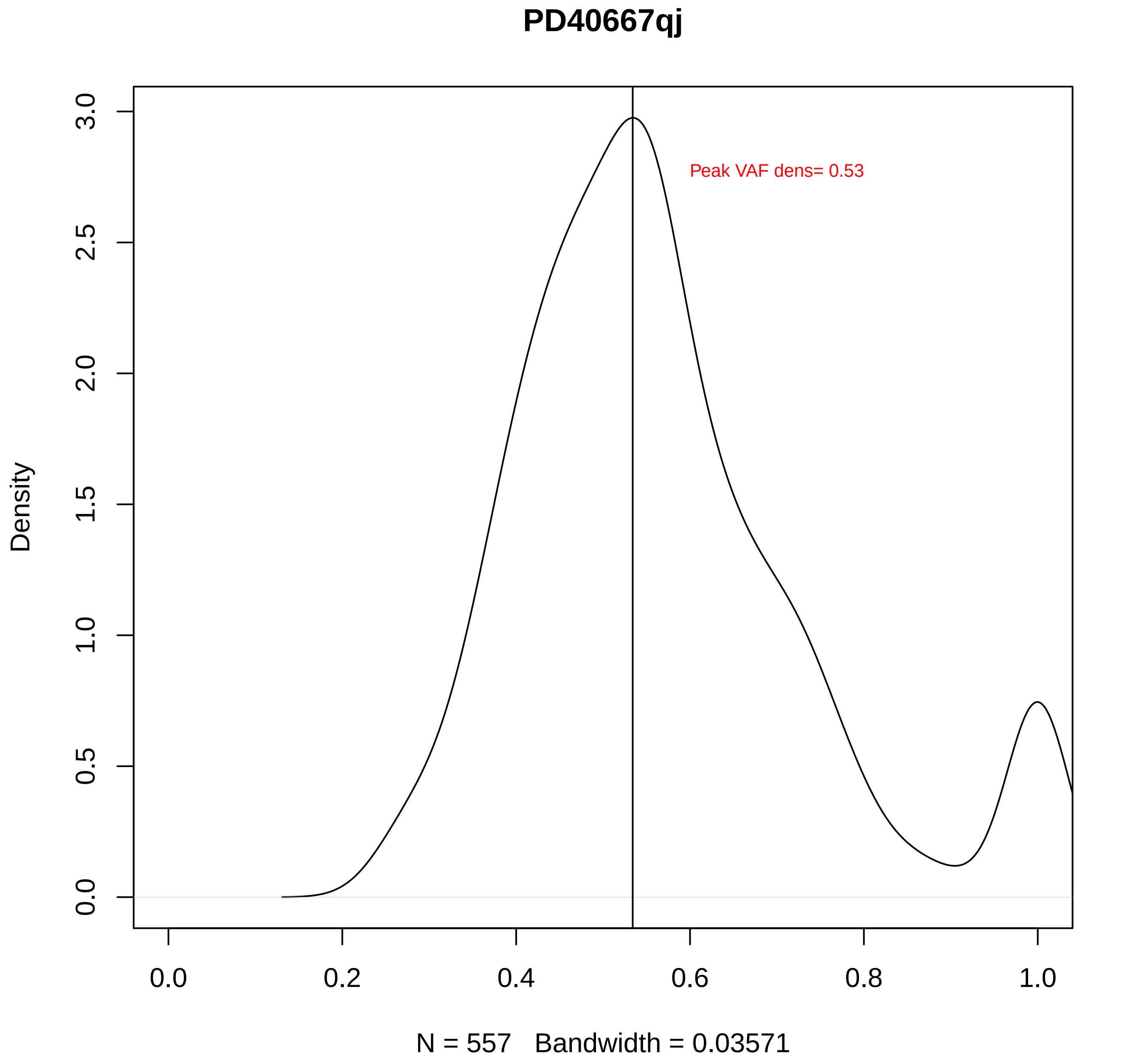

PD40667qj
Peak VAF dens= 0.53
Density
0.0
0.5
1.0
1.5
2.0
2.5
3.0
0.0
0.2
0.4
0.6
0.8
1.0
N = 557   Bandwidth = 0.03571

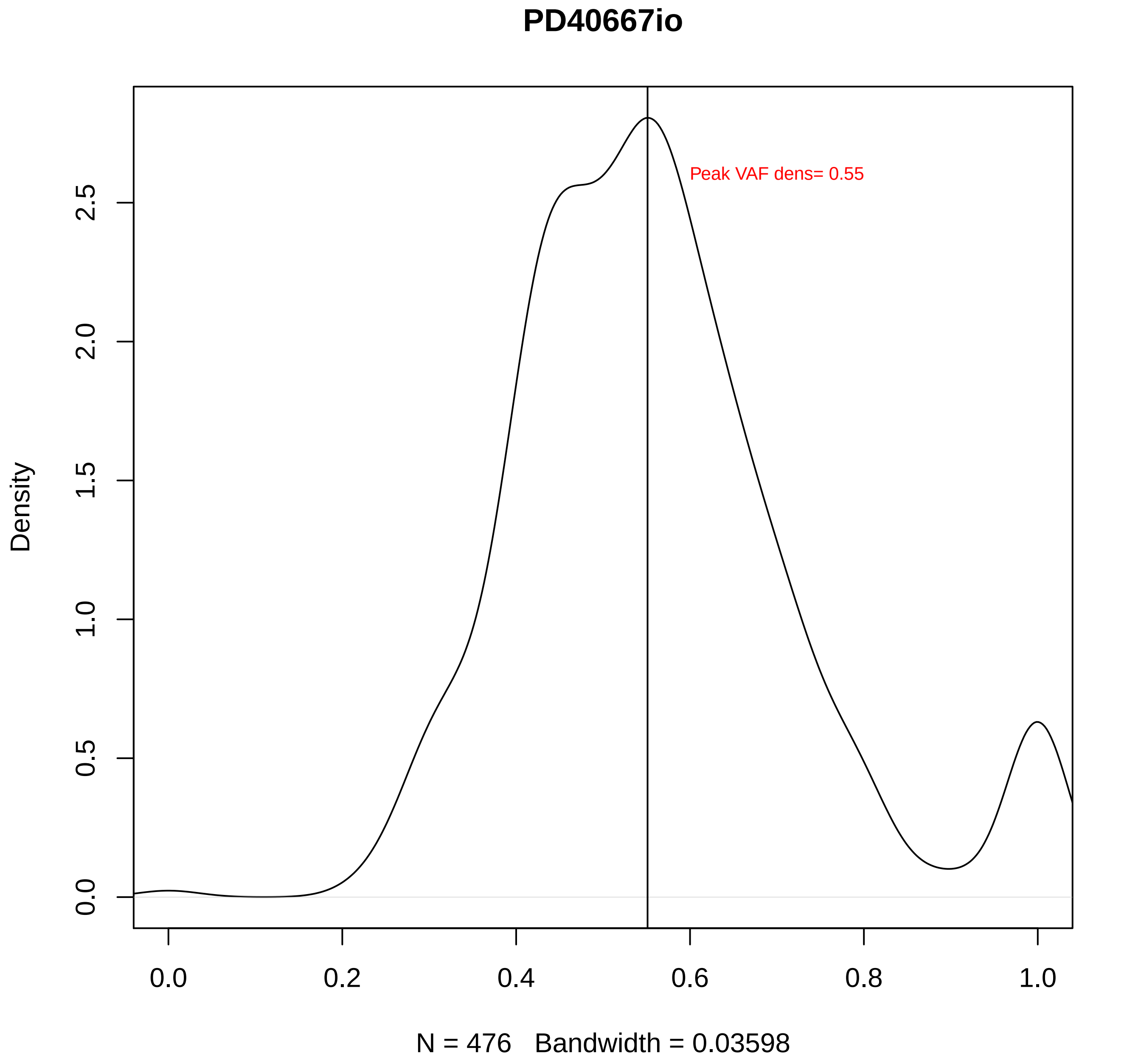

PD40667io
Peak VAF dens= 0.55
Density
0.0
0.5
1.0
1.5
2.0
2.5
0.0
0.2
0.4
0.6
0.8
1.0
N = 476   Bandwidth = 0.03598

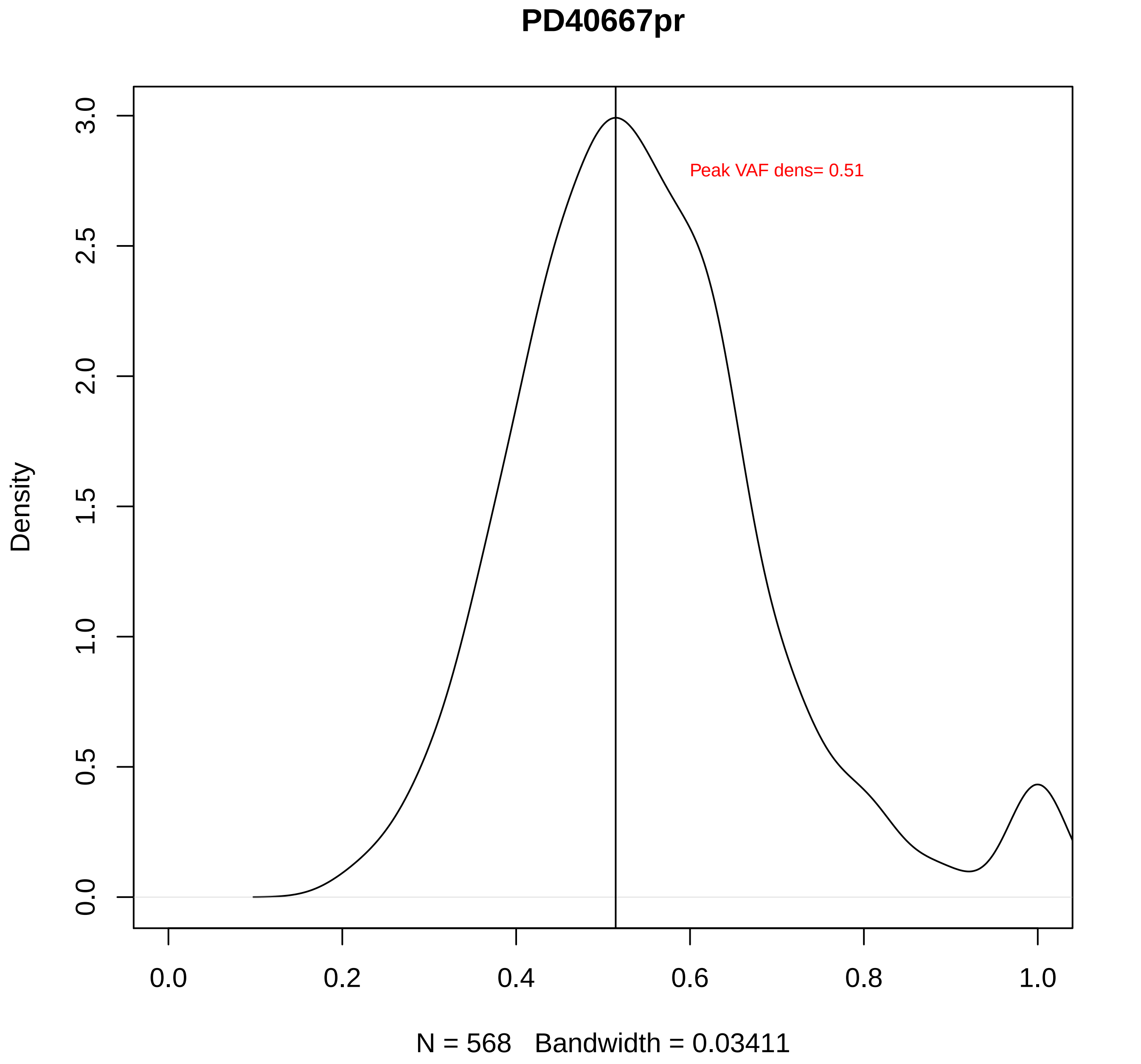
PD40667pr
Peak VAF dens= 0.51
Density
0.0
0.5
1.0
1.5
2.0
2.5
3.0
0.0
0.2
0.4
0.6
0.8
1.0
N = 568   Bandwidth = 0.03411

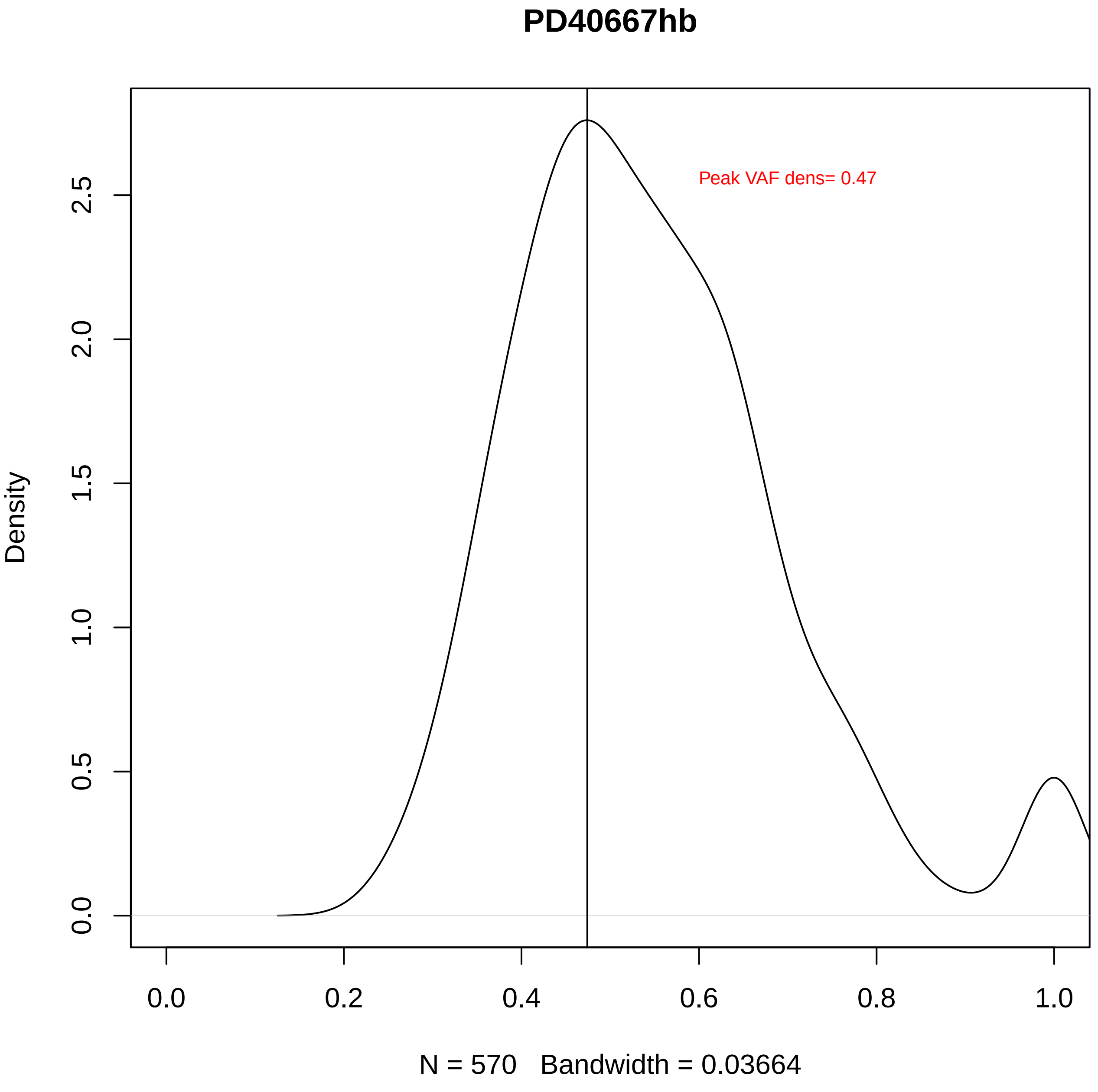
PD40667hb
Peak VAF dens= 0.47
Density
0.0
0.5
1.0
1.5
2.0
2.5
0.0
0.2
0.4
0.6
0.8
1.0
N = 570 Bandwidth = 0.03664

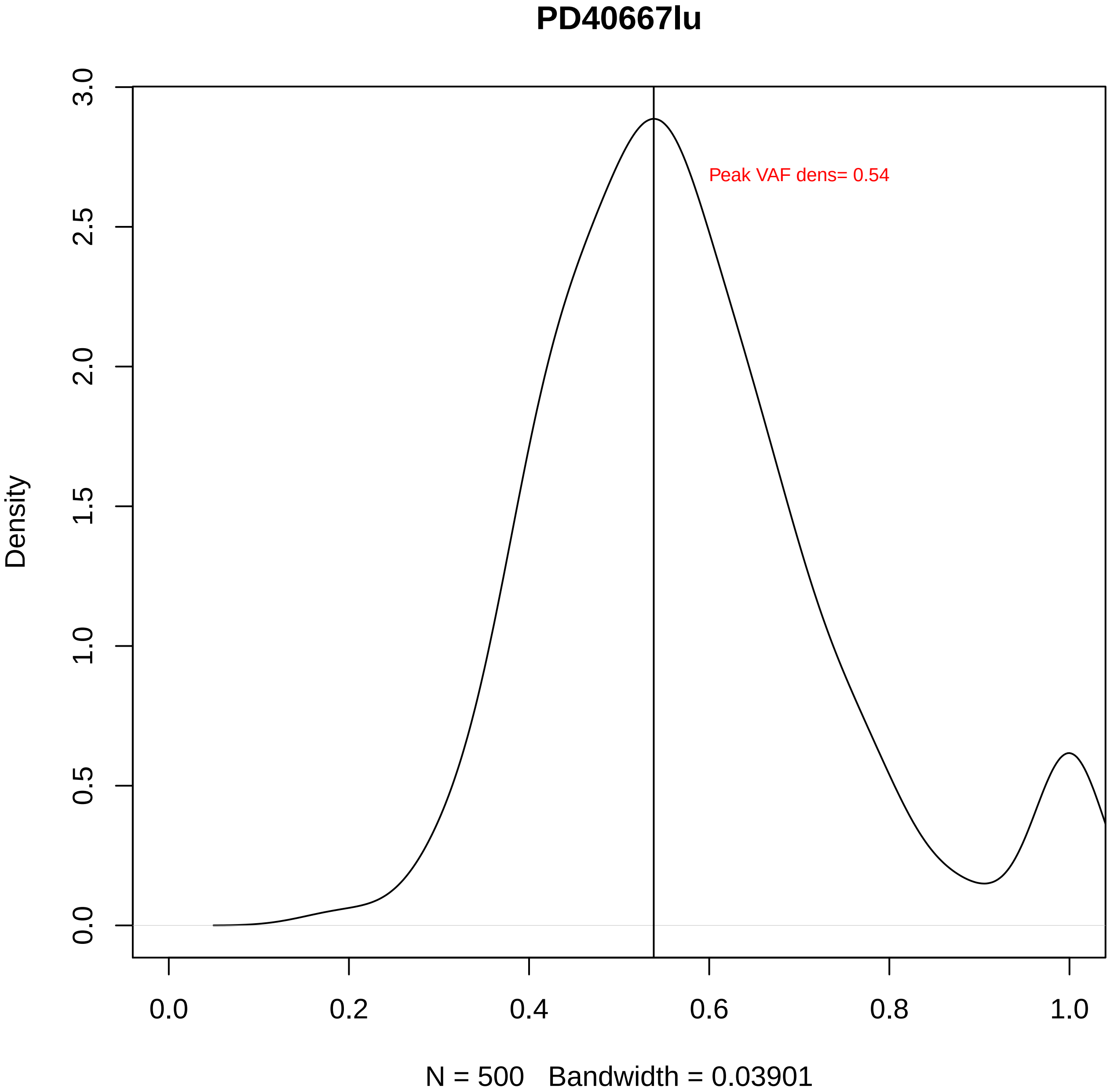
PD40667lu
Peak VAF dens= 0.54
Density
0.0
0.5
1.0
1.5
2.0
2.5
3.0
0.0
0.2
0.4
0.6
0.8
1.0
N = 500   Bandwidth = 0.03901

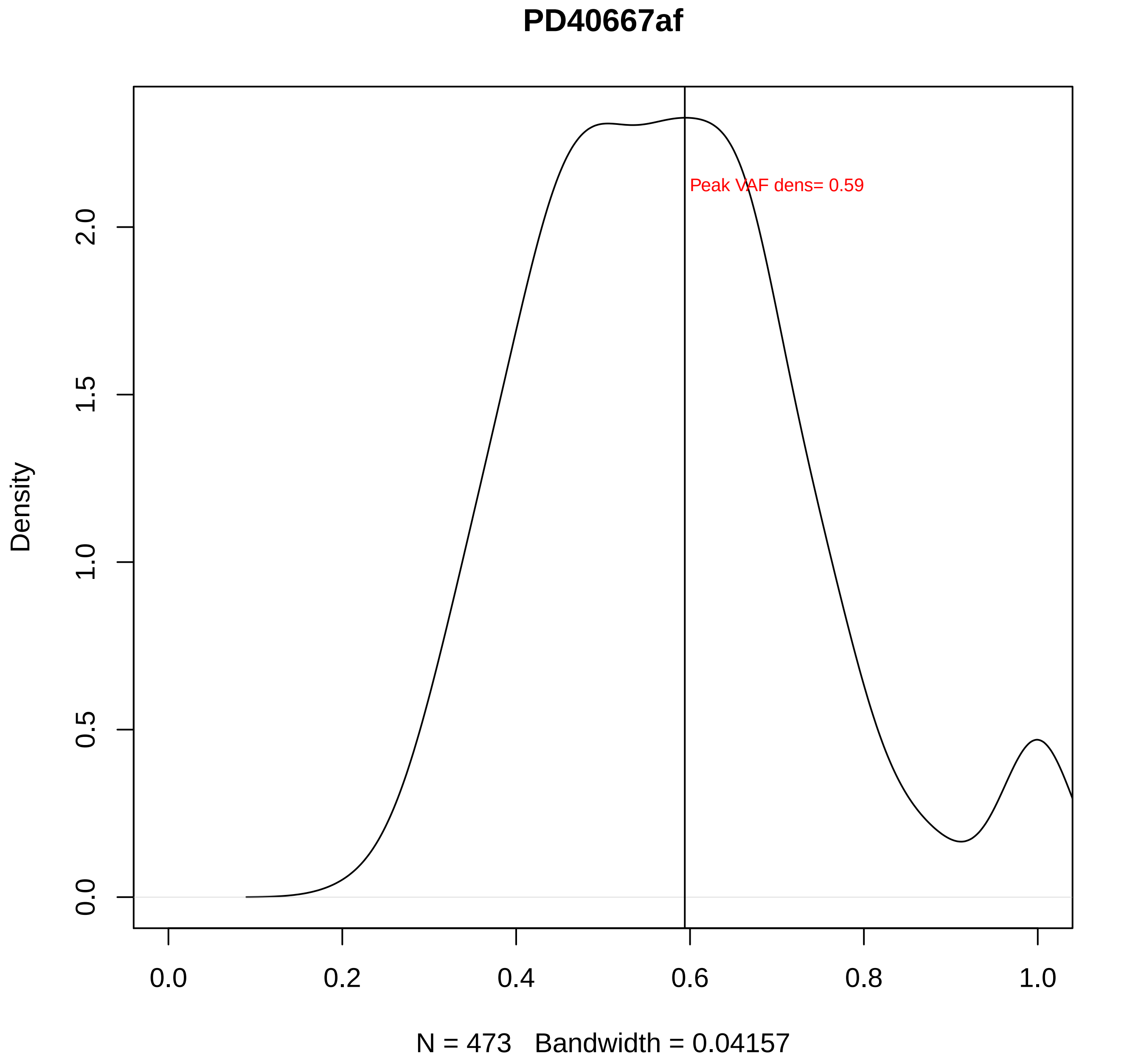

PD40667af
Peak VAF dens= 0.59
Density
0.0
0.5
1.0
1.5
2.0
0.0
0.2
0.4
0.6
0.8
1.0
N = 473   Bandwidth = 0.04157

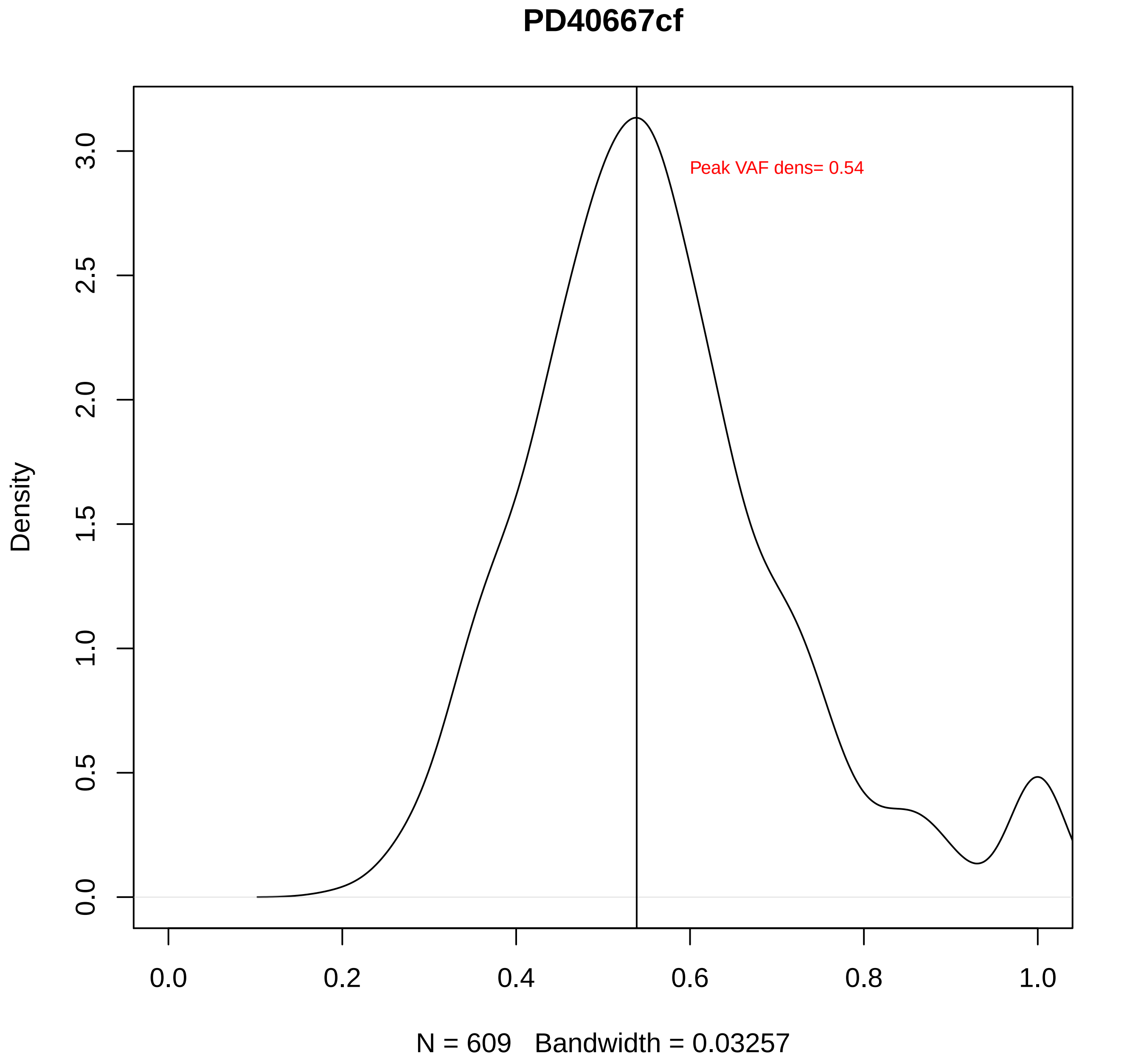
PD40667cf
Peak VAF dens= 0.54
Density
0.0
0.5
1.0
1.5
2.0
2.5
3.0
0.0
0.2
0.4
0.6
0.8
1.0
N = 609 Bandwidth = 0.03257

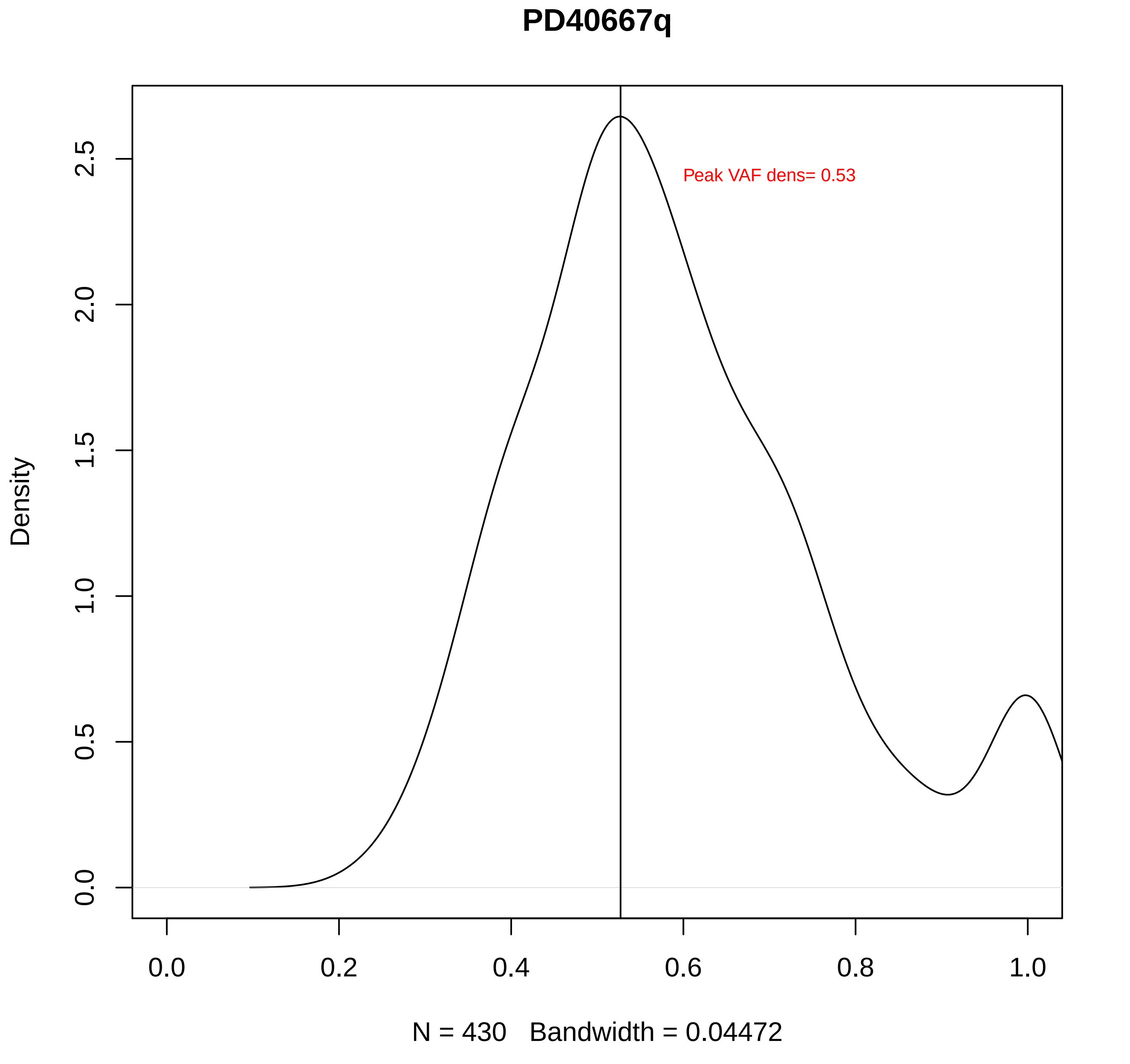
PD40667q
Peak VAF dens= 0.53
Density
0.0
0.5
1.0
1.5
2.0
2.5
0.0
0.2
0.4
0.6
0.8
1.0
N = 430   Bandwidth = 0.04472

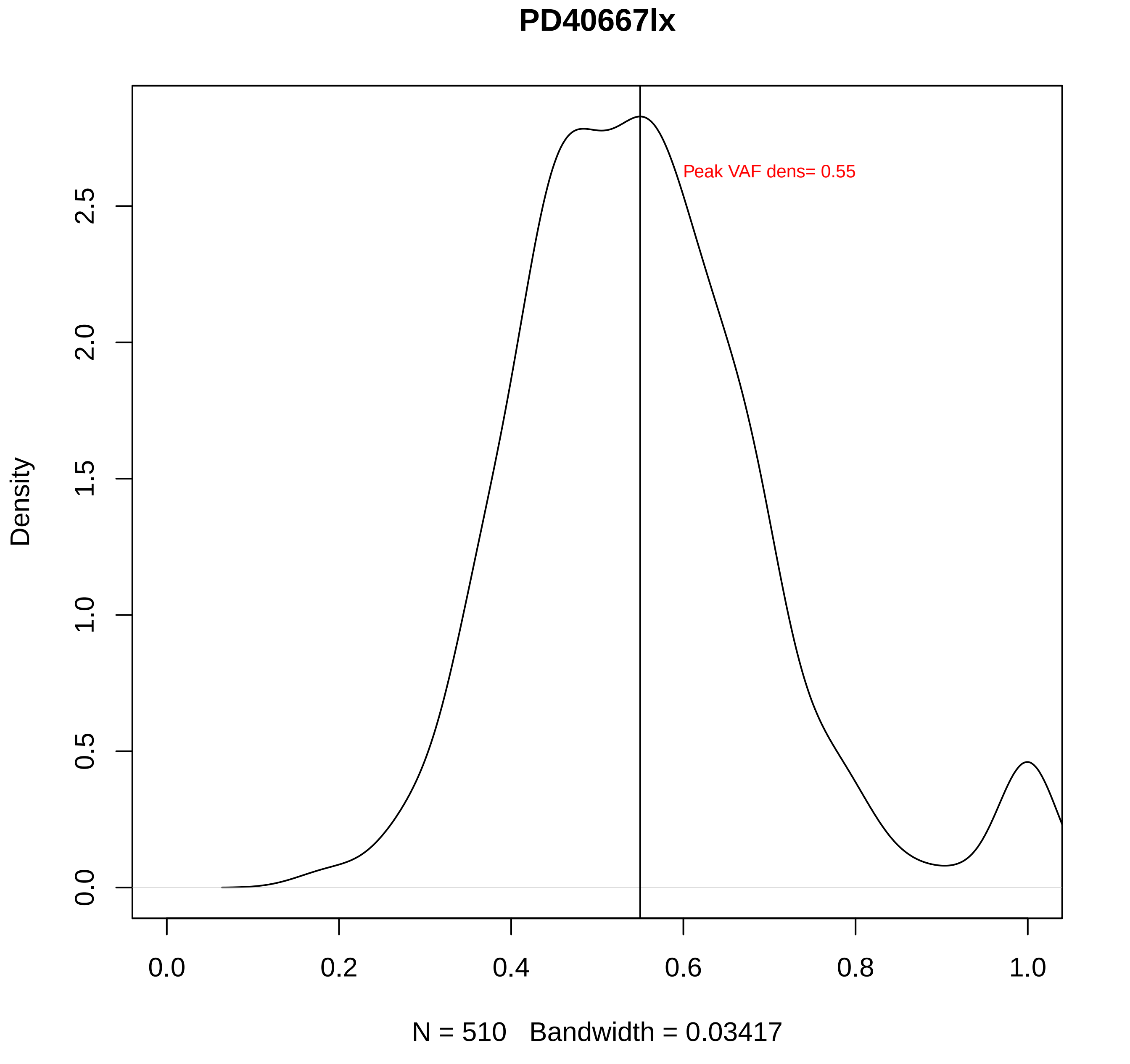
PD40667lx
Peak VAF dens= 0.55
Density
0.0
0.5
1.0
1.5
2.0
2.5
0.0
0.2
0.4
0.6
0.8
1.0
N = 510   Bandwidth = 0.03417

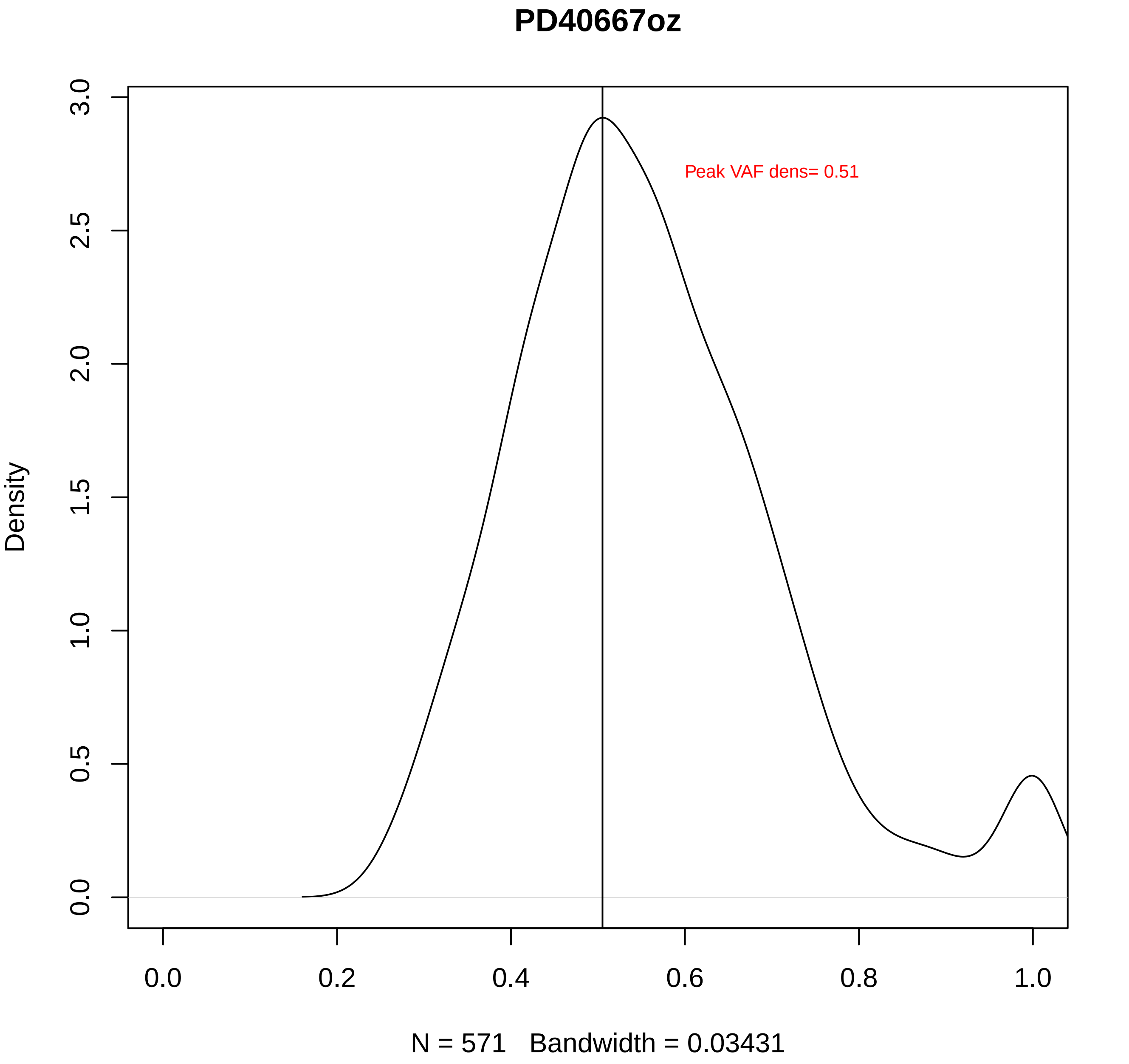

PD40667oz
Peak VAF dens= 0.51
Density
3.0
2.5
2.0
1.5
1.0
0.5
0.0
0.0
0.2
0.4
0.6
0.8
1.0
N = 571   Bandwidth = 0.03431

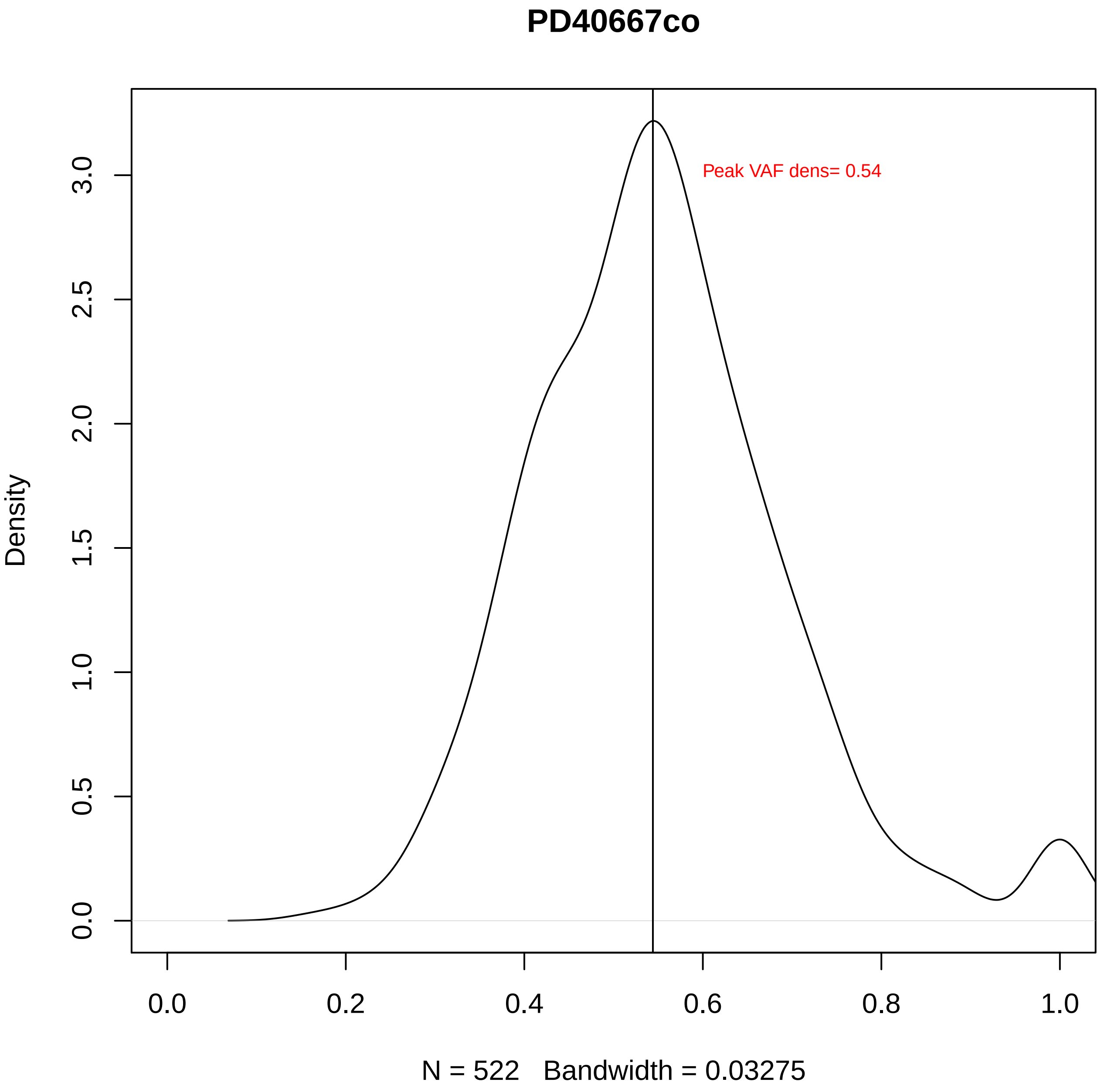

PD40667co
Peak VAF dens= 0.54
Density
0.0
0.5
1.0
1.5
2.0
2.5
3.0
0.0
0.2
0.4
0.6
0.8
1.0
N = 522   Bandwidth = 0.03275

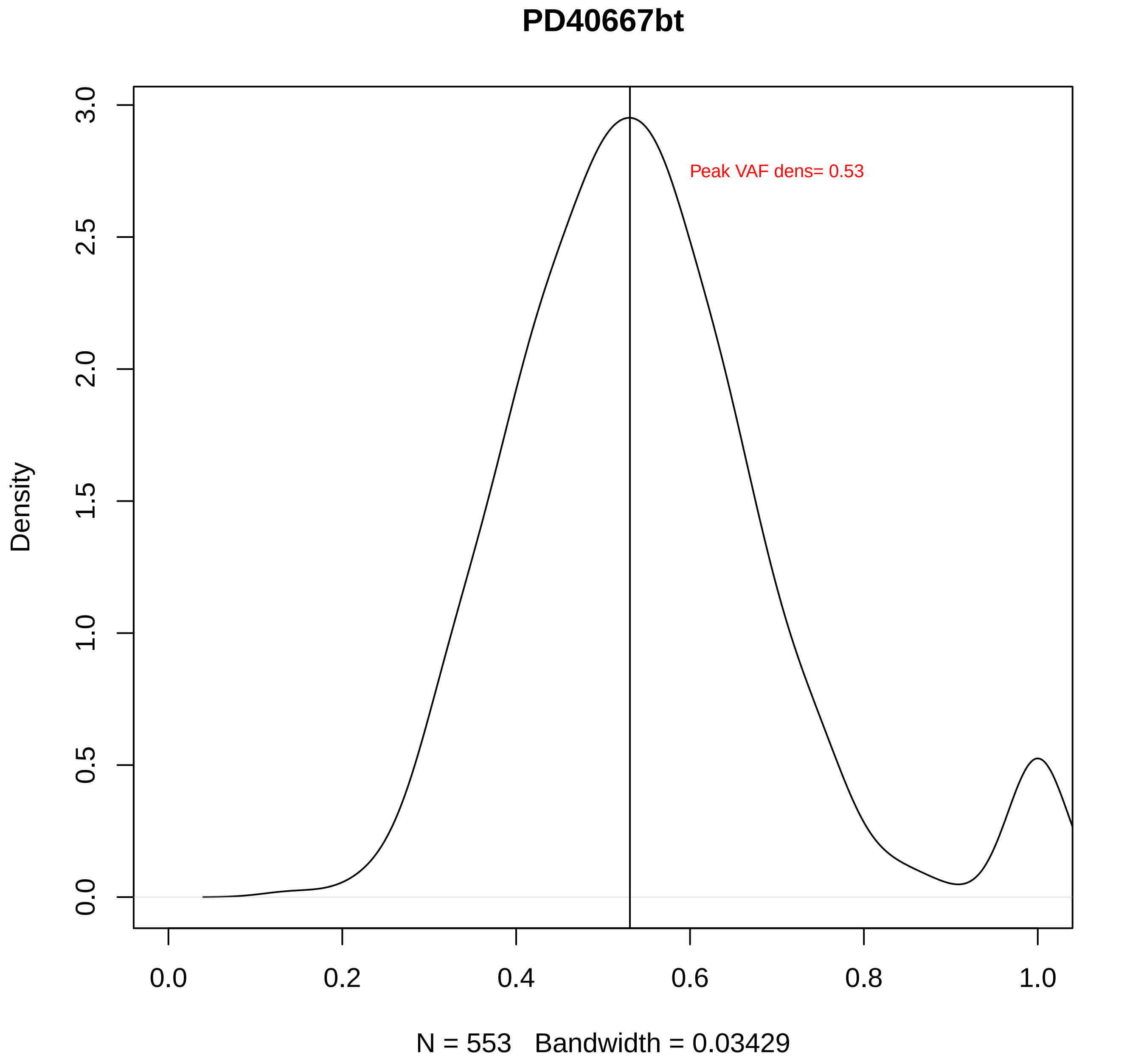

PD40667bt
Peak VAF dens= 0.53
Density
3.0
2.5
2.0
1.5
1.0
0.5
0.0
0.0
0.2
0.4
0.6
0.8
1.0
N = 553 Bandwidth = 0.03429

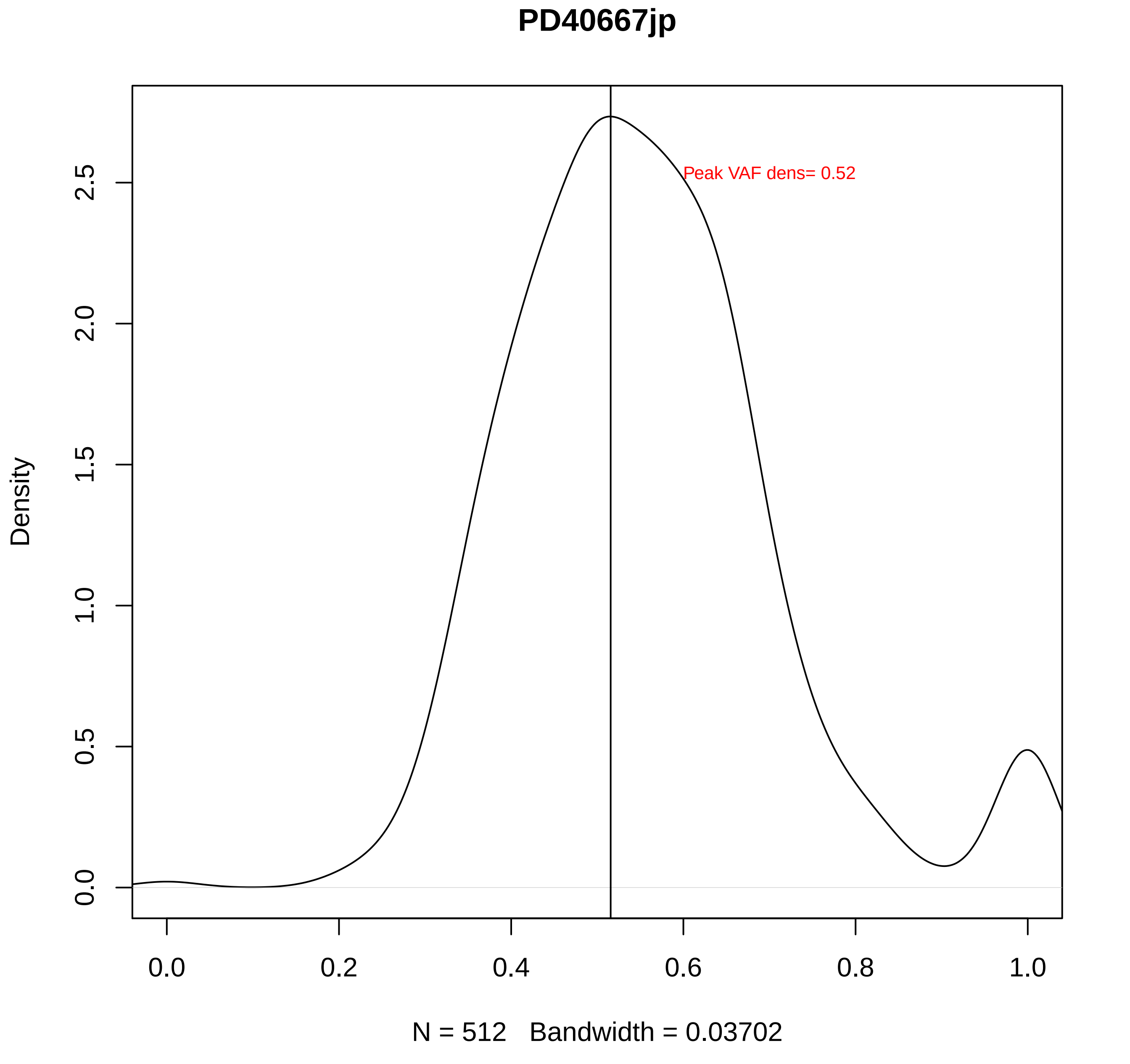
PD40667jp
Peak VAF dens= 0.52
Density
0.0
0.5
1.0
1.5
2.0
2.5
0.0
0.2
0.4
0.6
0.8
1.0
N = 512   Bandwidth = 0.03702

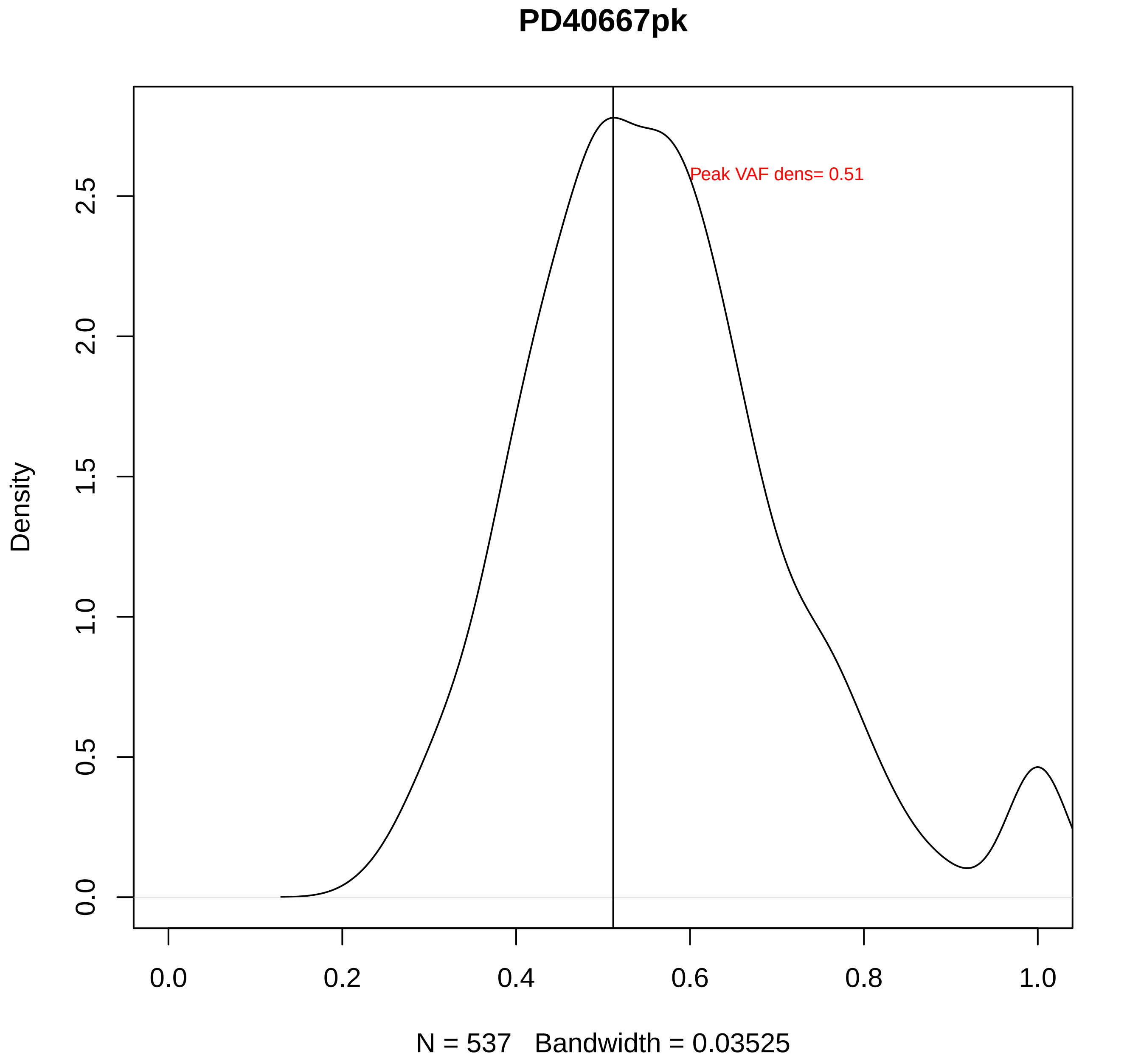
PD40667pk
Peak VAF dens= 0.51
Density
0.0
0.5
1.0
1.5
2.0
2.5
0.0
0.2
0.4
0.6
0.8
1.0
N = 537   Bandwidth = 0.03525

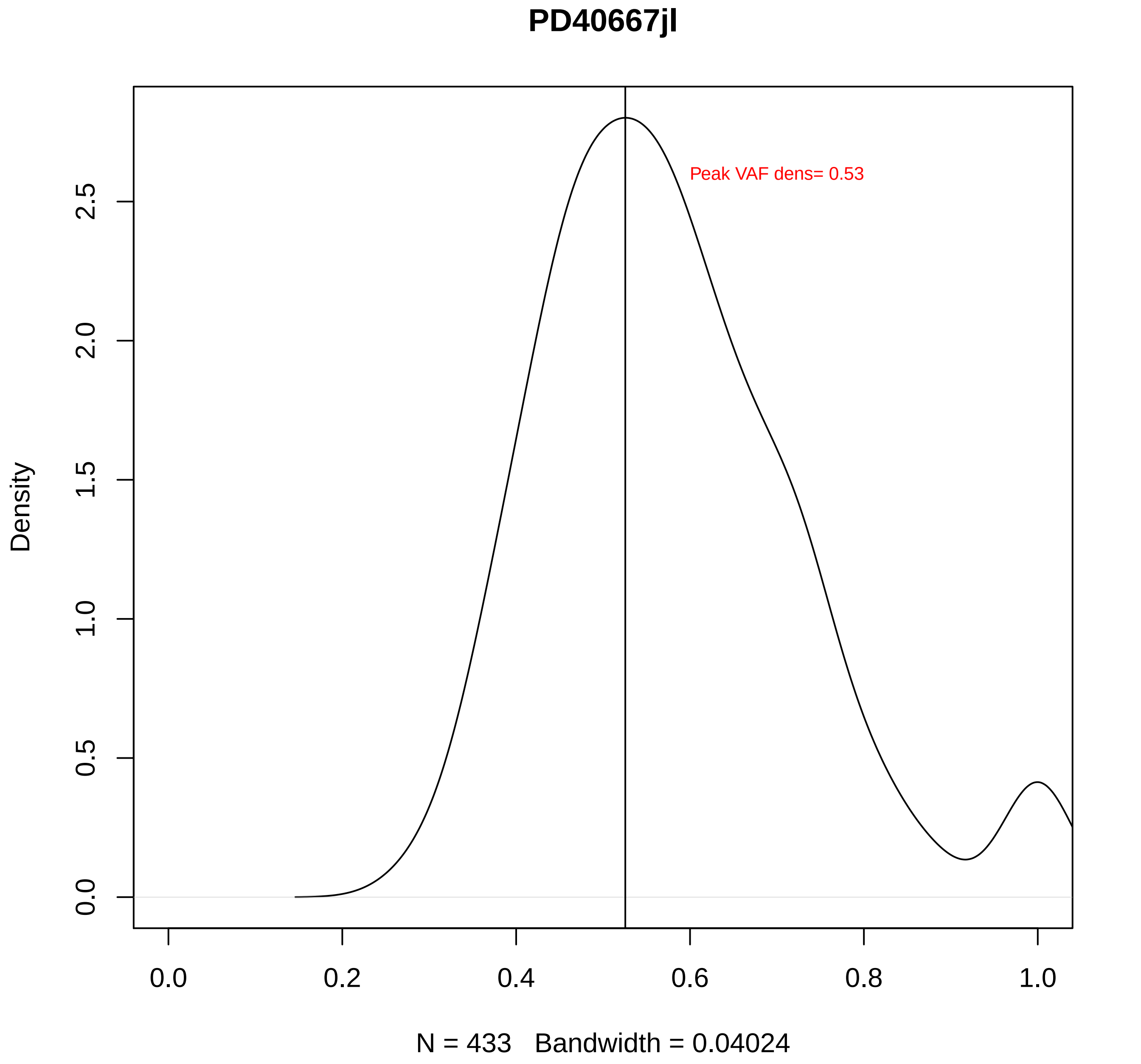
PD40667jl
Peak VAF dens= 0.53
Density
0.0
0.5
1.0
1.5
2.0
2.5
0.0
0.2
0.4
0.6
0.8
1.0
N = 433   Bandwidth = 0.04024

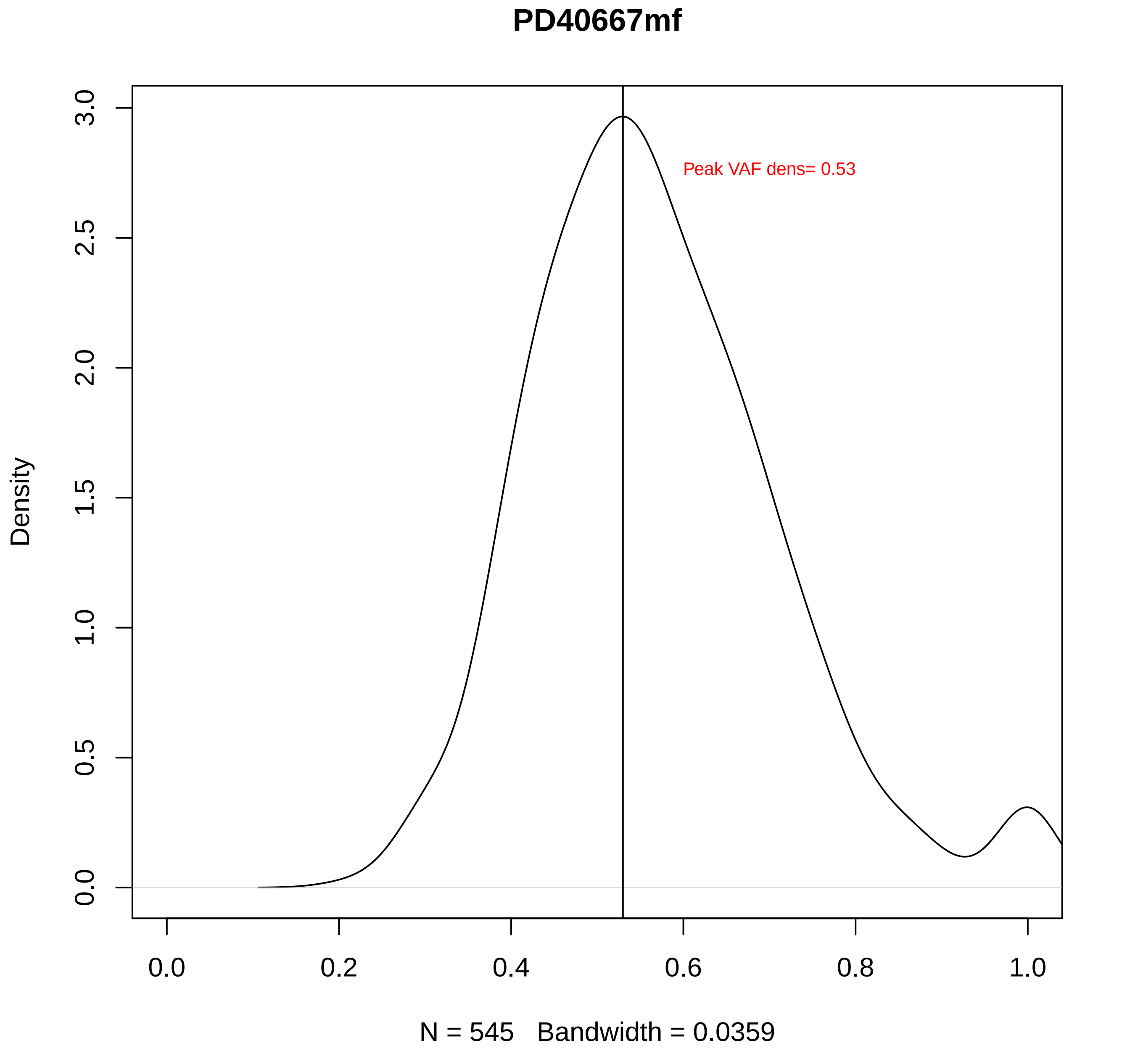
PD40667mf
Peak VAF dens= 0.53
Density
0.0
0.5
1.0
1.5
2.0
2.5
3.0
0.0
0.2
0.4
0.6
0.8
1.0
N = 545 Bandwidth = 0.0359

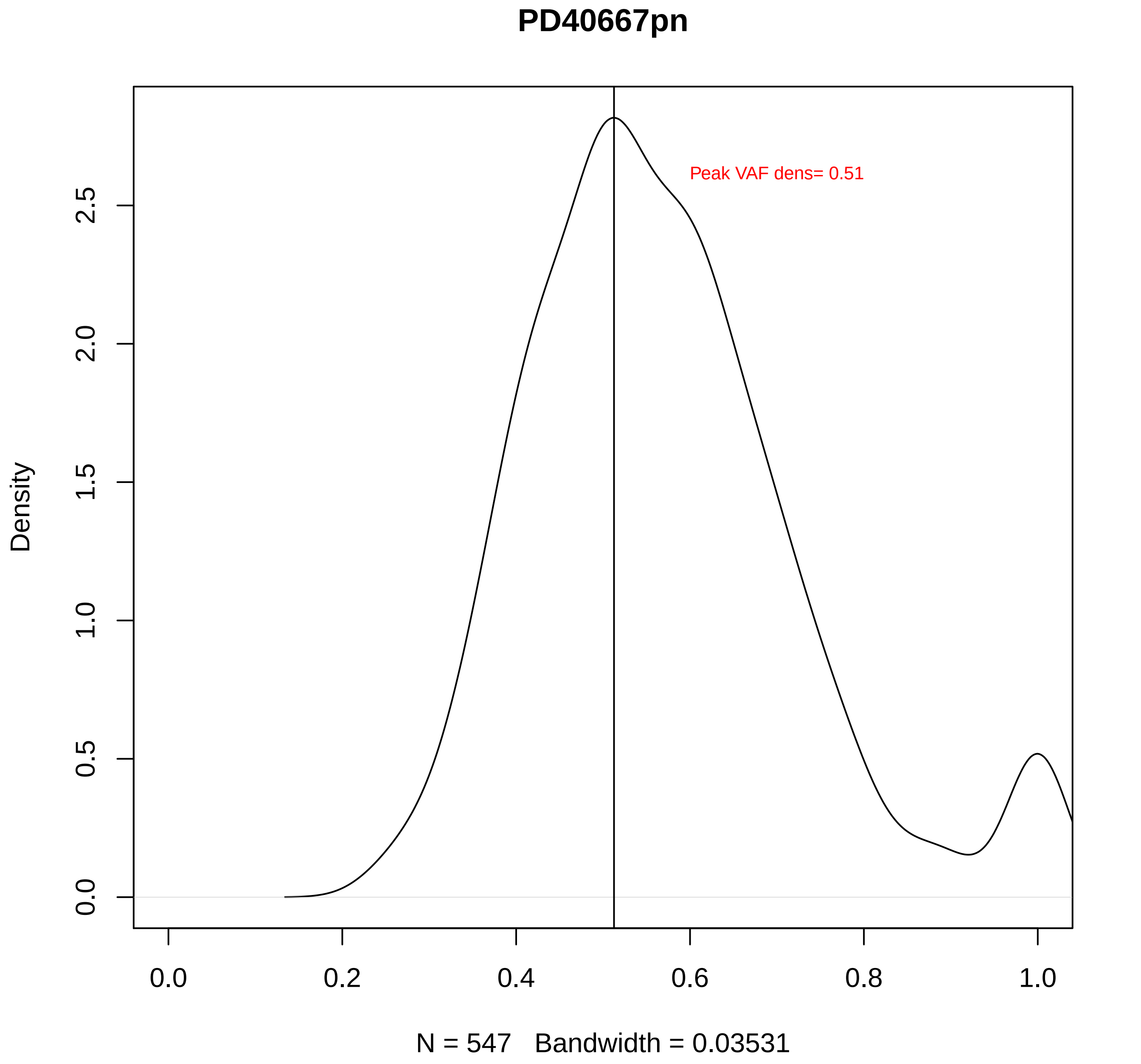

PD40667pn
Peak VAF dens= 0.51
Density
0.0
0.5
1.0
1.5
2.0
2.5
0.0
0.2
0.4
0.6
0.8
1.0
N = 547 Bandwidth = 0.03531

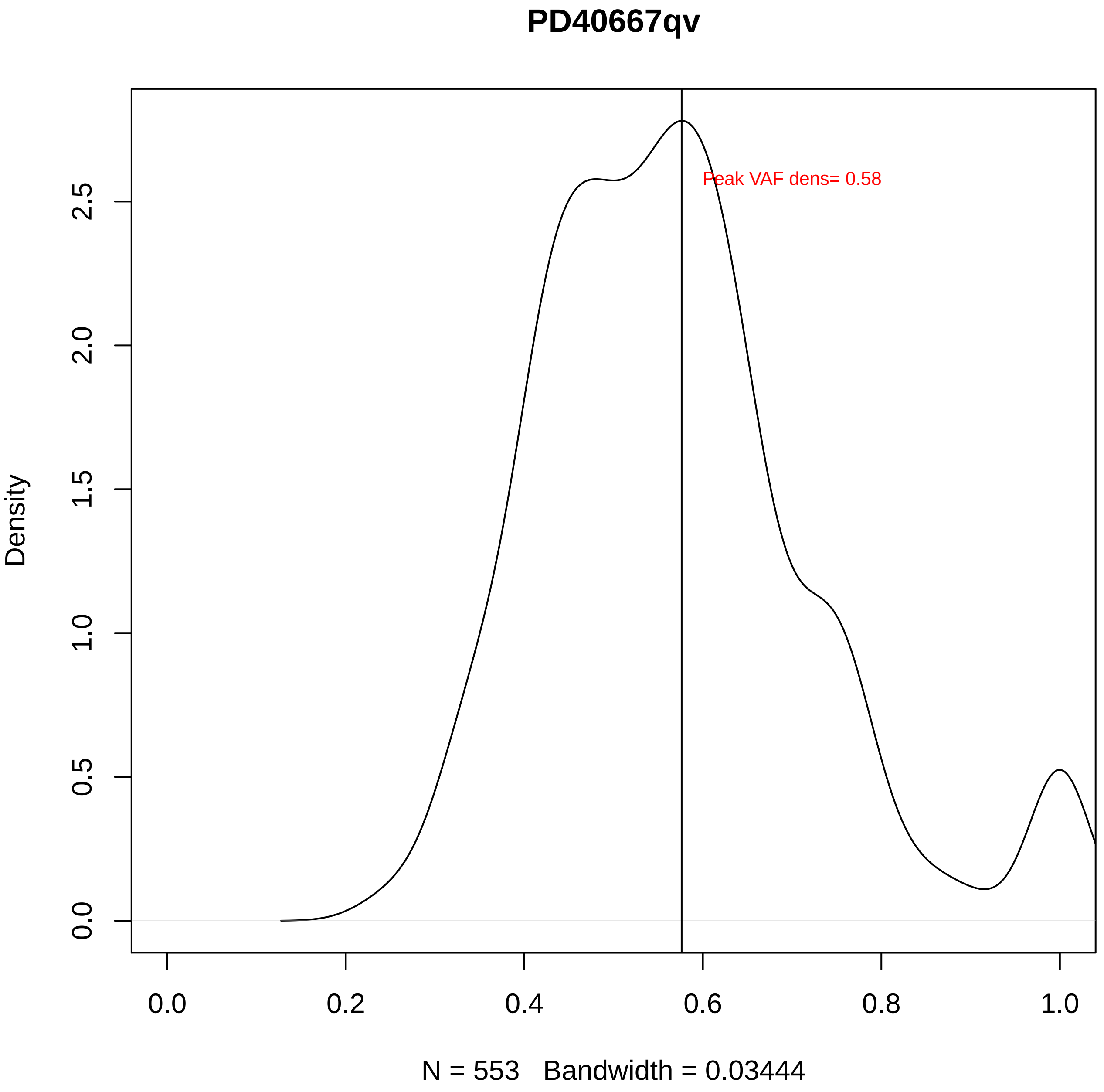
PD40667qv
Peak VAF dens= 0.58
Density
0.0
0.5
1.0
1.5
2.0
2.5
0.0
0.2
0.4
0.6
0.8
1.0
N = 553   Bandwidth = 0.03444

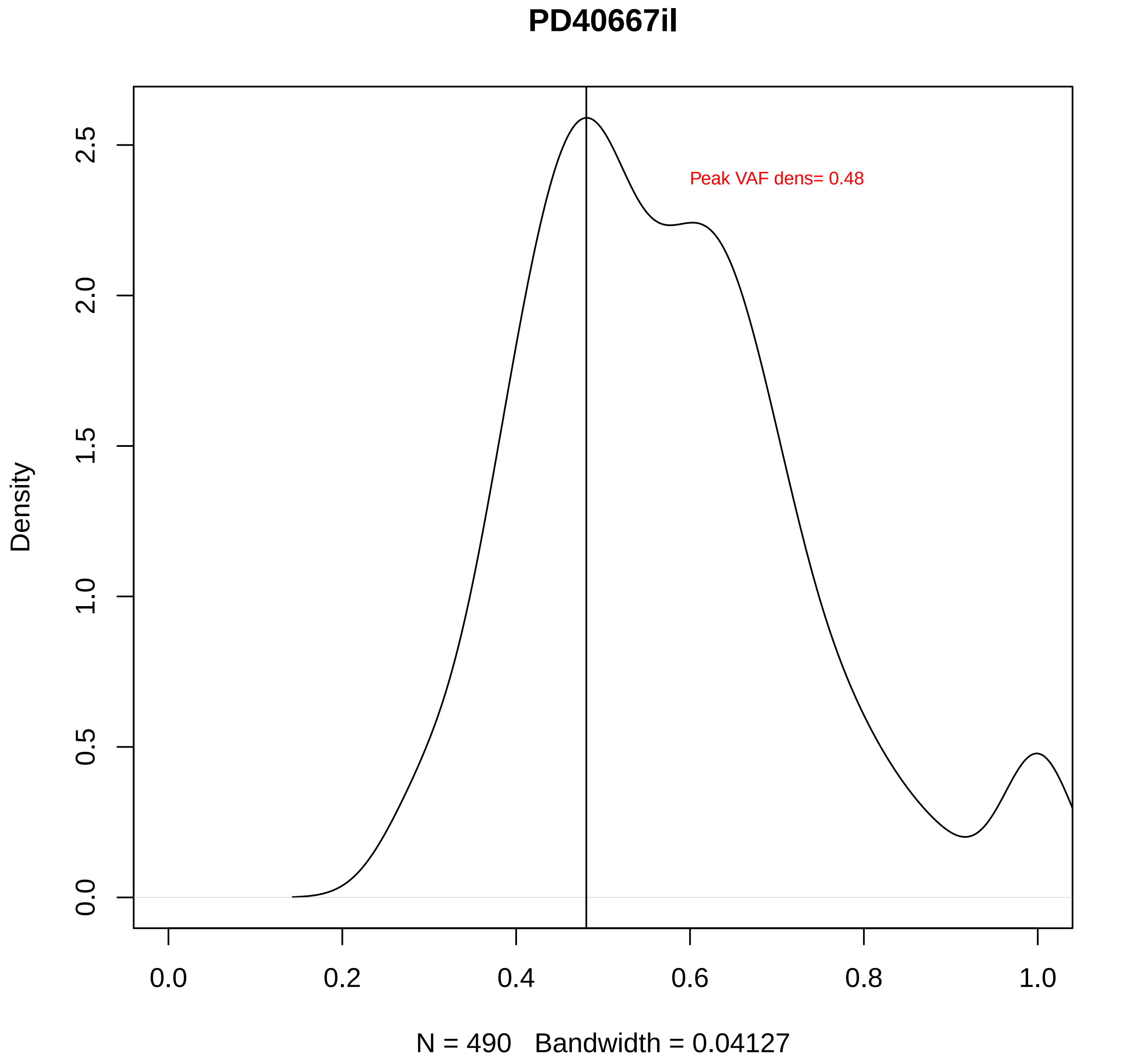
PD40667il
Peak VAF dens= 0.48
Density
0.0
0.5
1.0
1.5
2.0
2.5
0.0
0.2
0.4
0.6
0.8
1.0
N = 490 Bandwidth = 0.04127

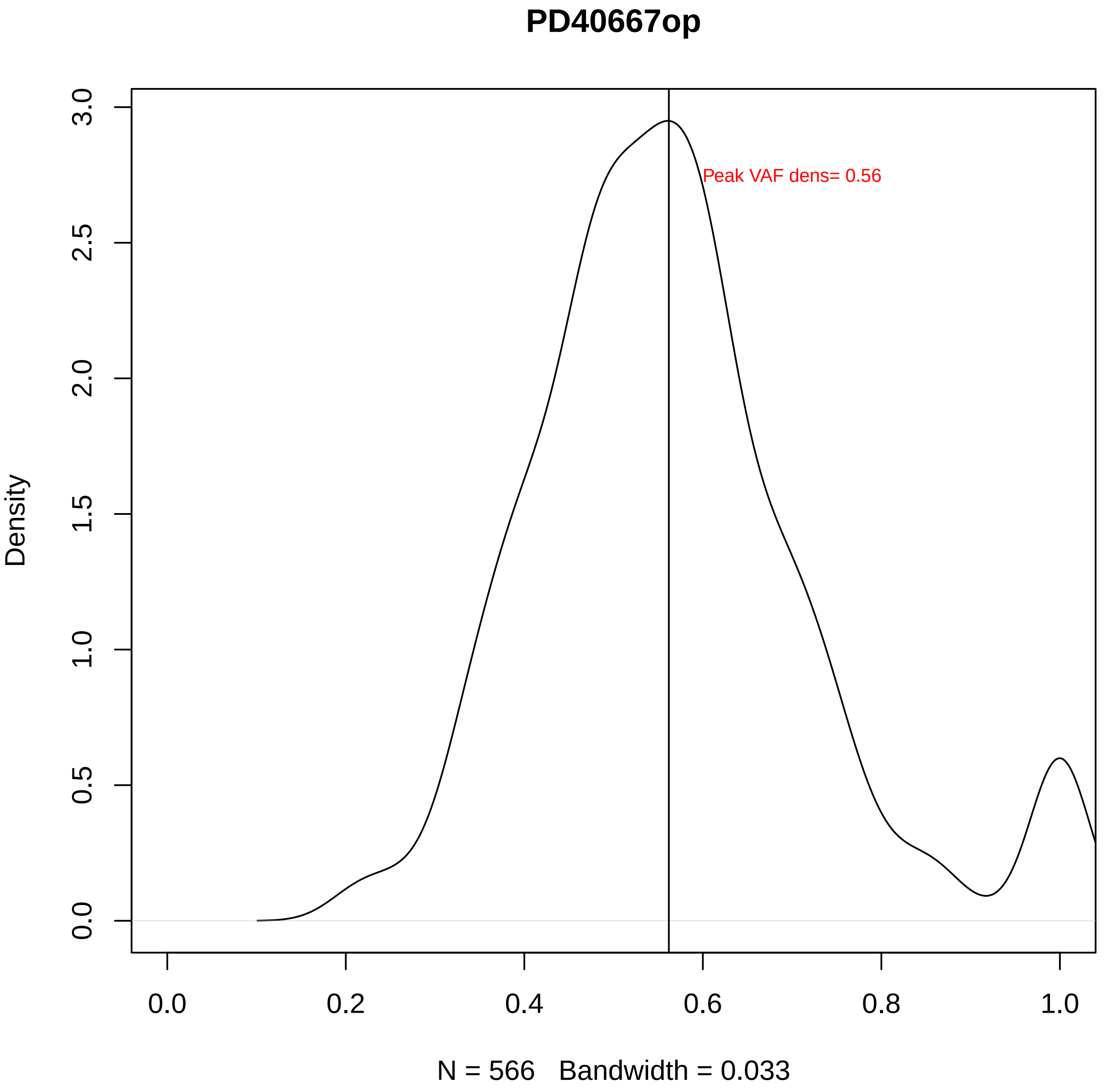

PD40667op
Peak VAF dens= 0.56
Density
0.0
0.5
1.0
1.5
2.0
2.5
3.0
0.0
0.2
0.4
0.6
0.8
1.0
N = 566 Bandwidth = 0.033

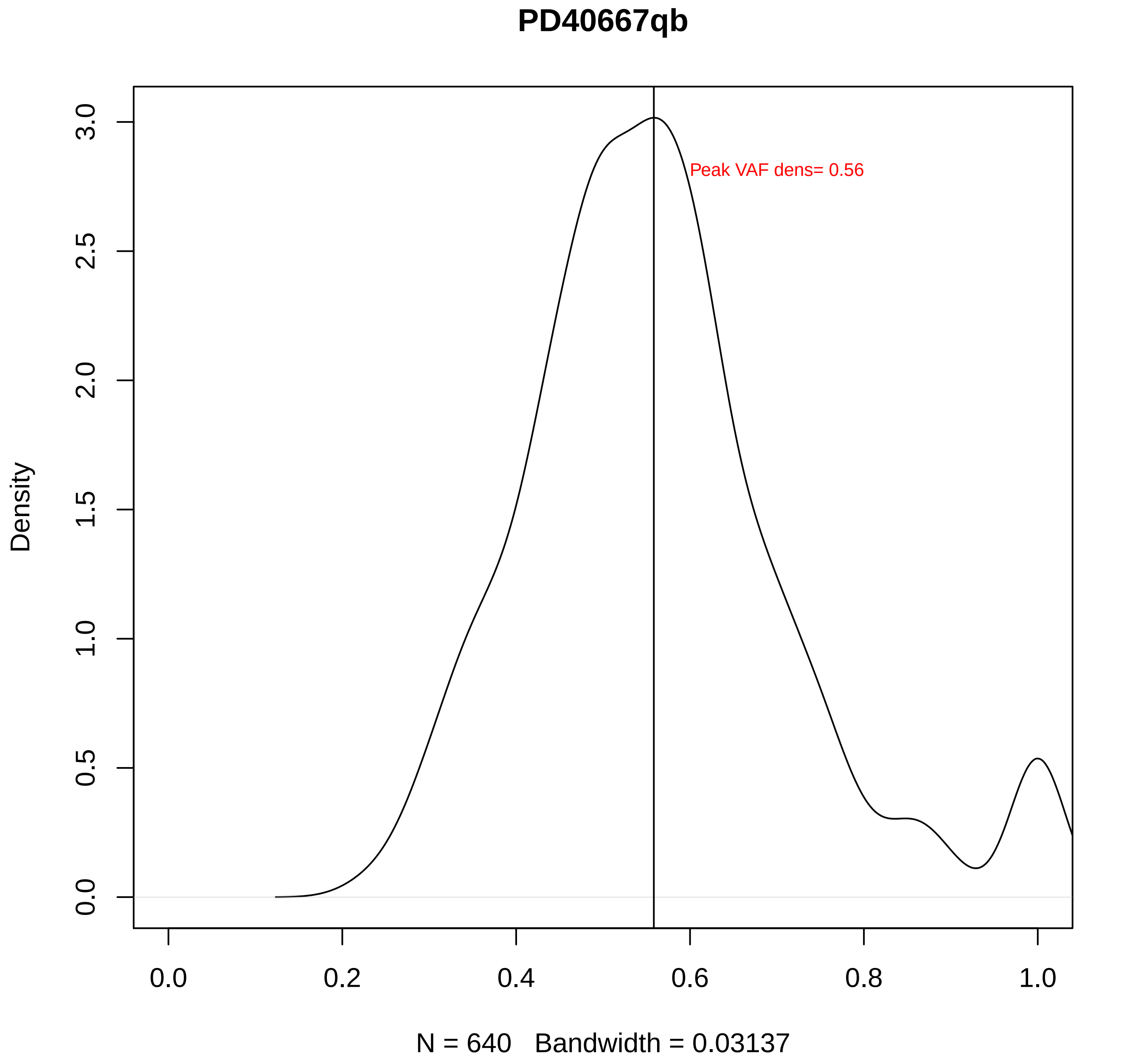
PD40667qb
Peak VAF dens= 0.56
Density
0.0
0.5
1.0
1.5
2.0
2.5
3.0
0.0
0.2
0.4
0.6
0.8
1.0
N = 640   Bandwidth = 0.03137

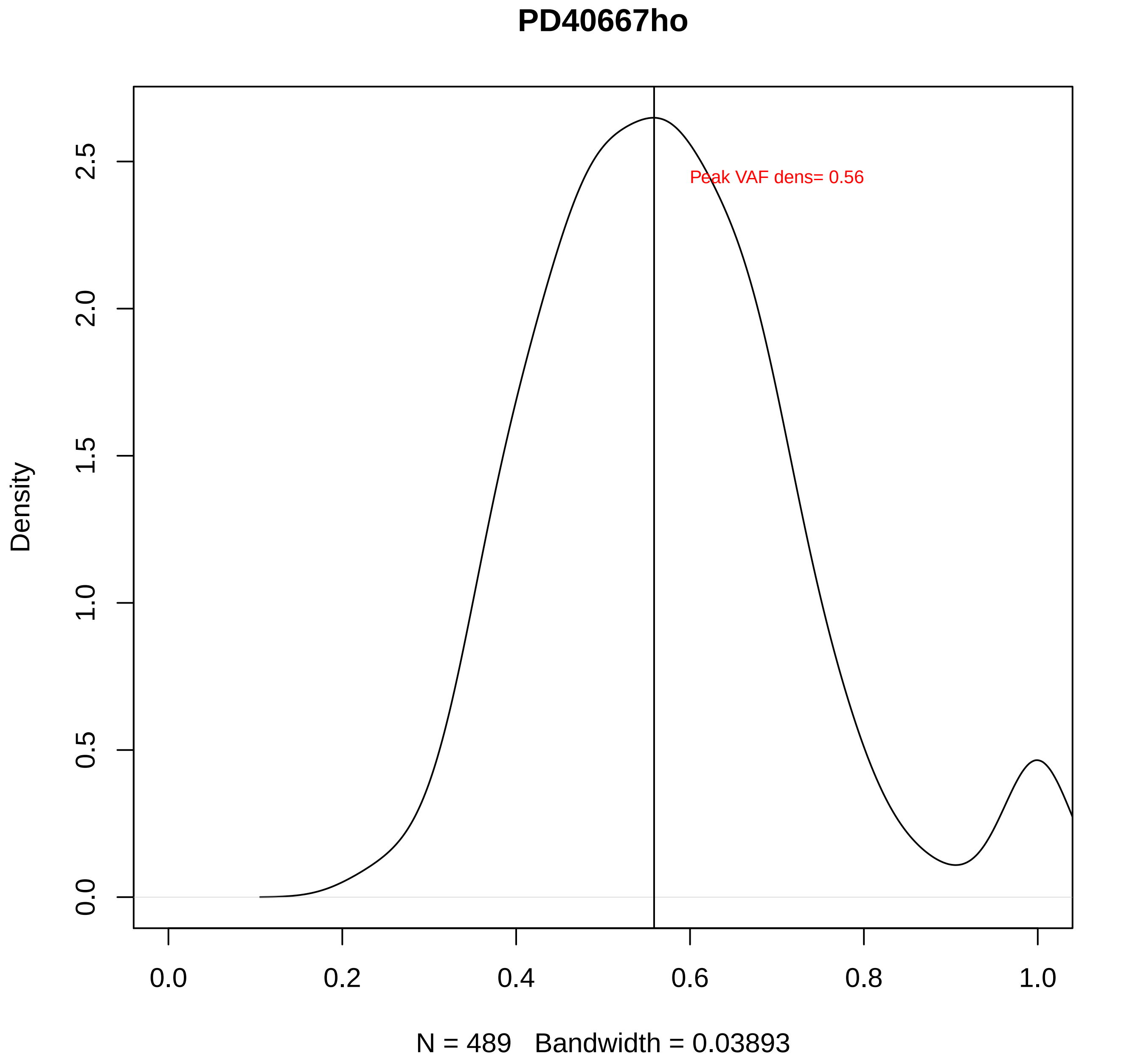
PD40667ho
Peak VAF dens= 0.56
Density
0.0
0.5
1.0
1.5
2.0
2.5
0.0
0.2
0.4
0.6
0.8
1.0
N = 489   Bandwidth = 0.03893

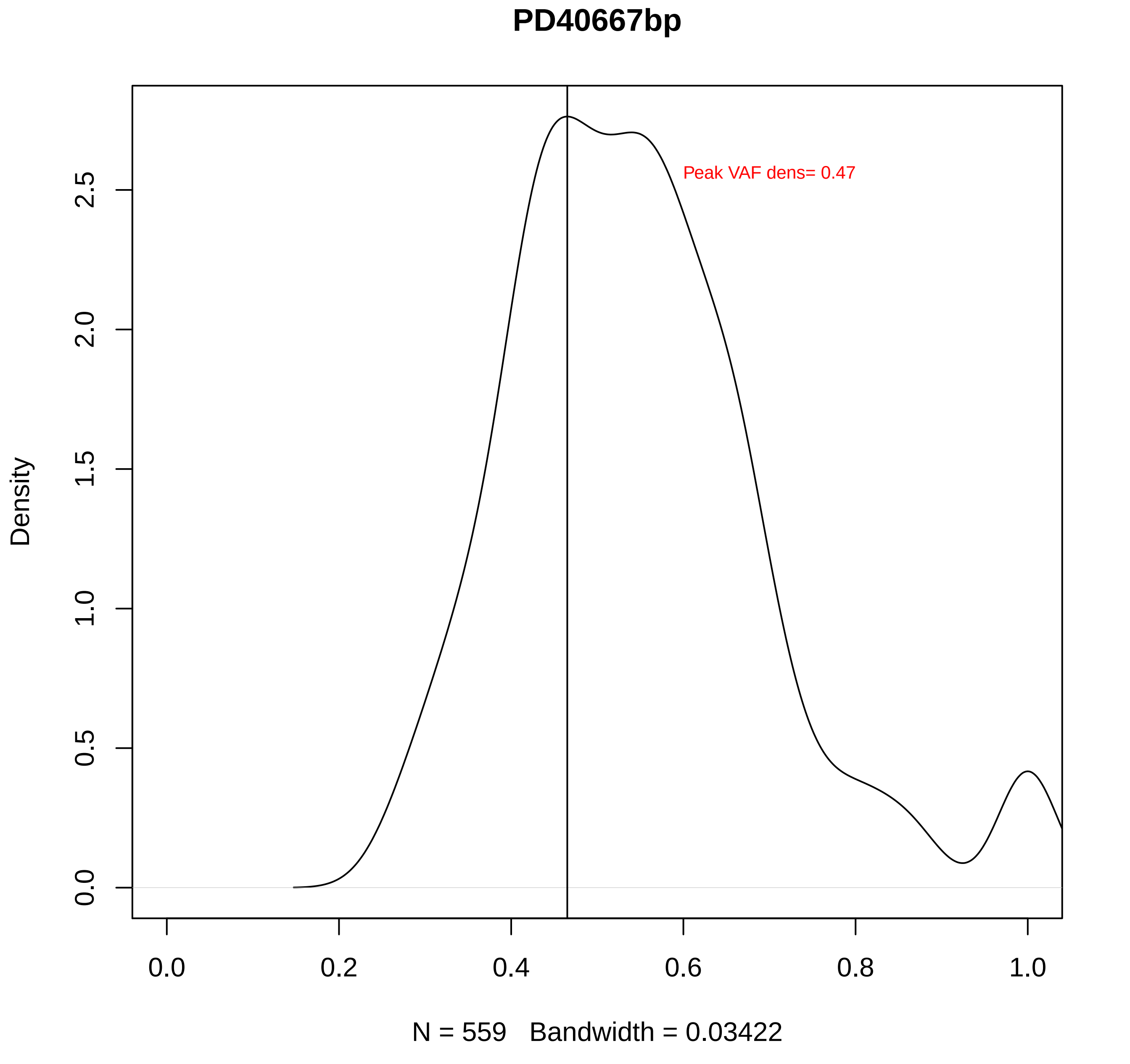

PD40667bp
Peak VAF dens= 0.47
Density
0.0
0.5
1.0
1.5
2.0
2.5
0.0
0.2
0.4
0.6
0.8
1.0
N = 559 Bandwidth = 0.03422

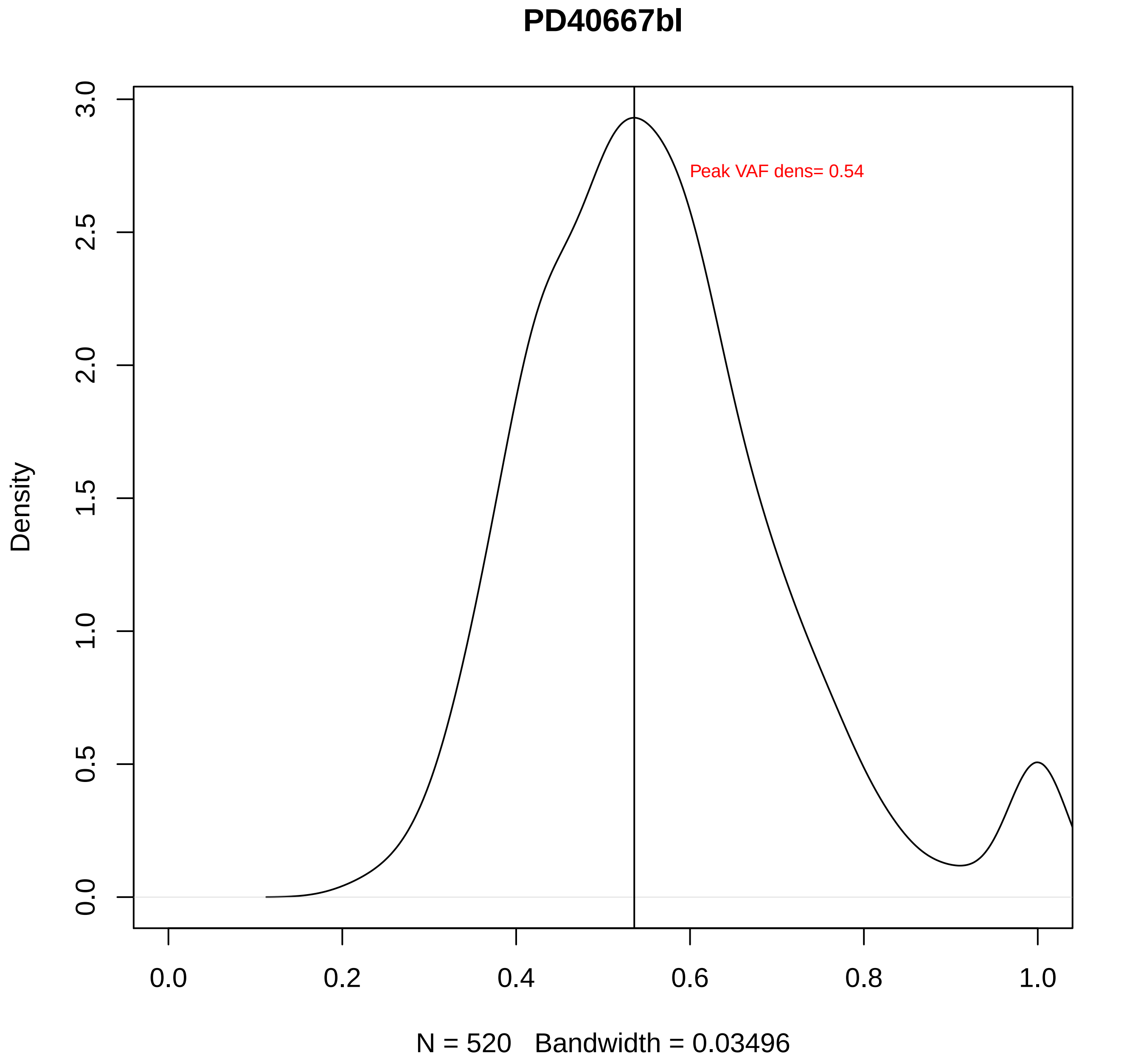
PD40667bl
Peak VAF dens= 0.54
Density
0.0
0.5
1.0
1.5
2.0
2.5
3.0
0.0
0.2
0.4
0.6
0.8
1.0
N = 520 Bandwidth = 0.03496

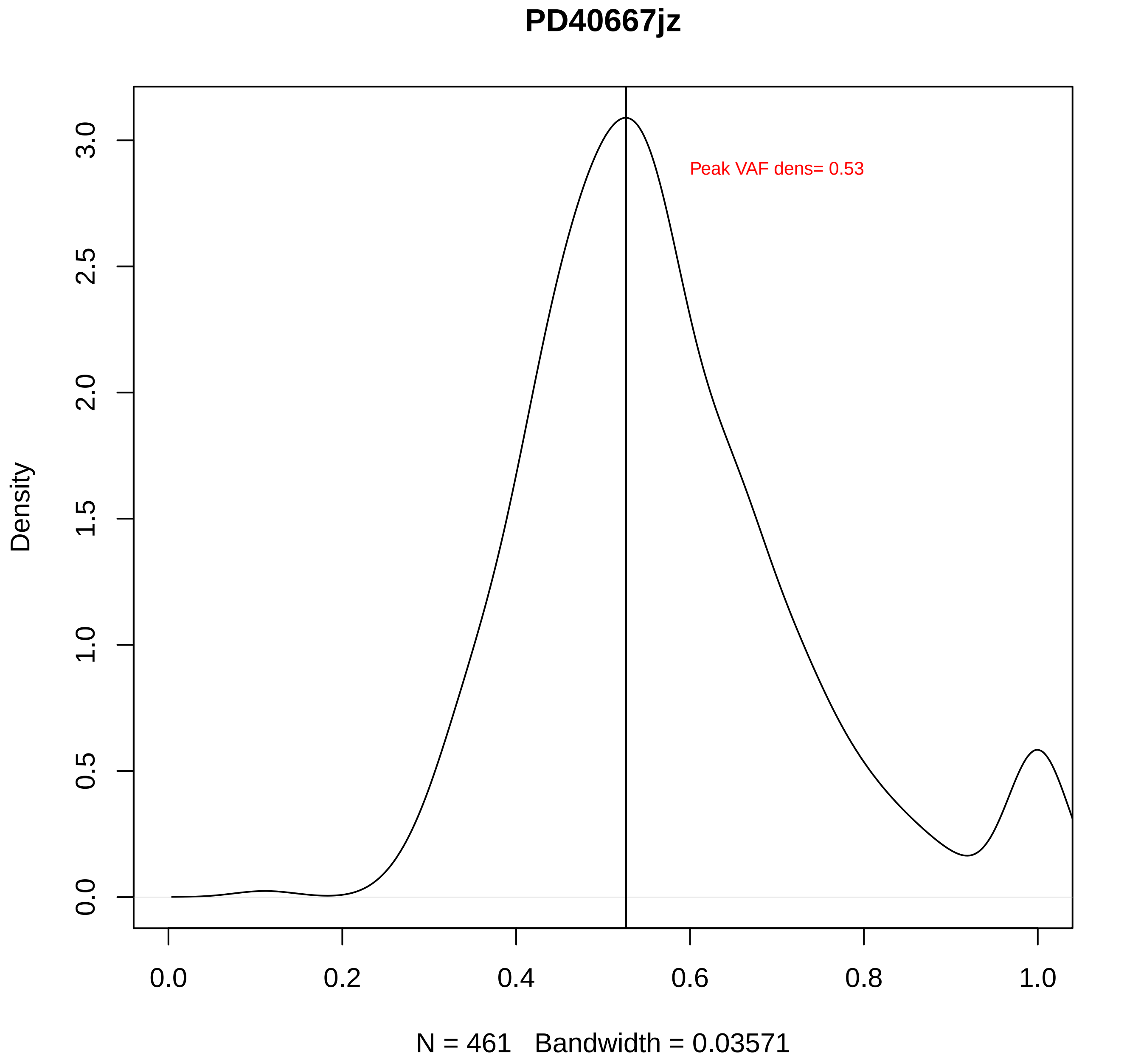

PD40667jz
Peak VAF dens= 0.53
Density
0.0
0.5
1.0
1.5
2.0
2.5
3.0
0.0
0.2
0.4
0.6
0.8
1.0
N = 461 Bandwidth = 0.03571

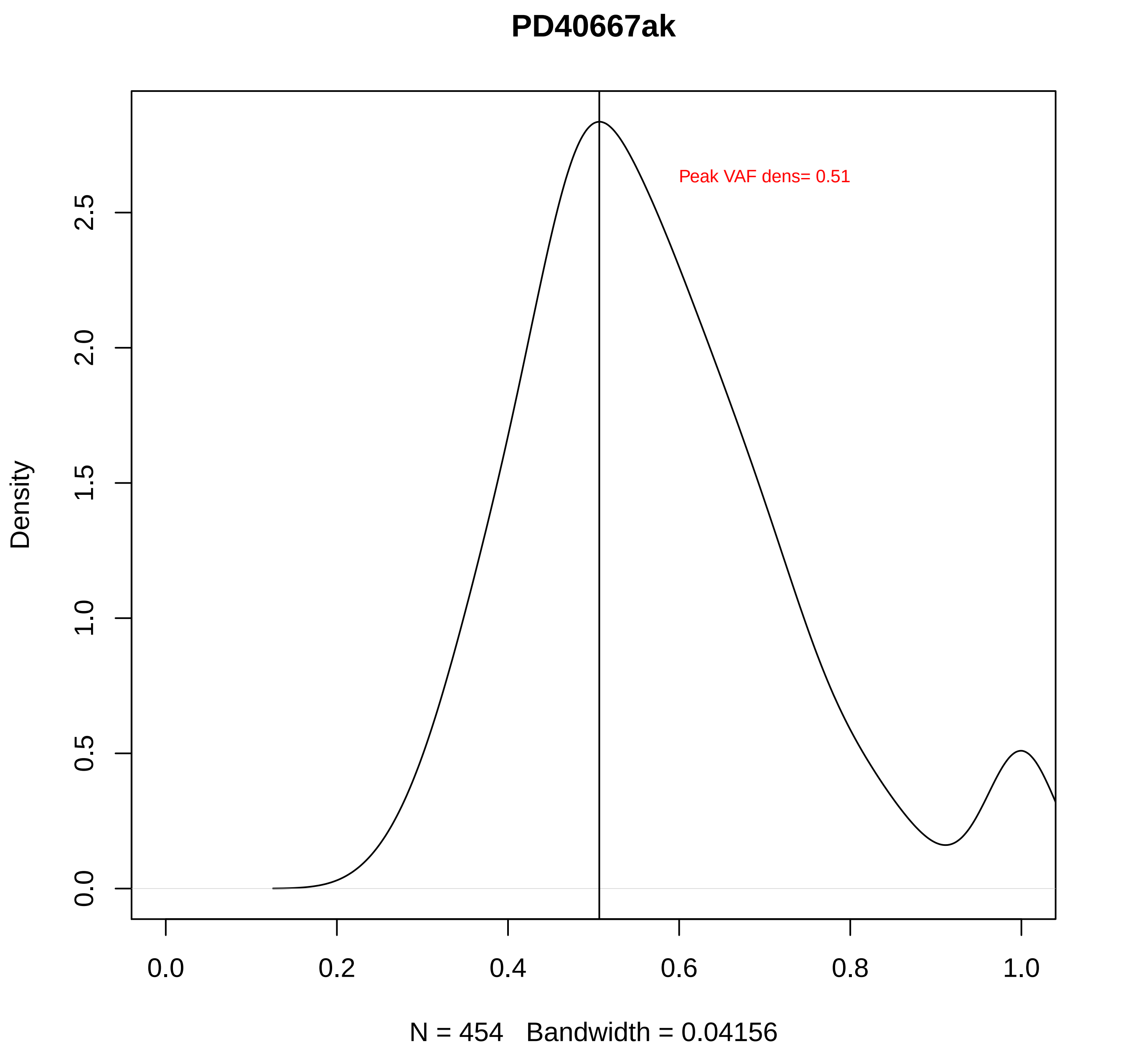

PD40667ak
Peak VAF dens= 0.51
Density
0.0
0.5
1.0
1.5
2.0
2.5
0.0
0.2
0.4
0.6
0.8
1.0
N = 454   Bandwidth = 0.04156

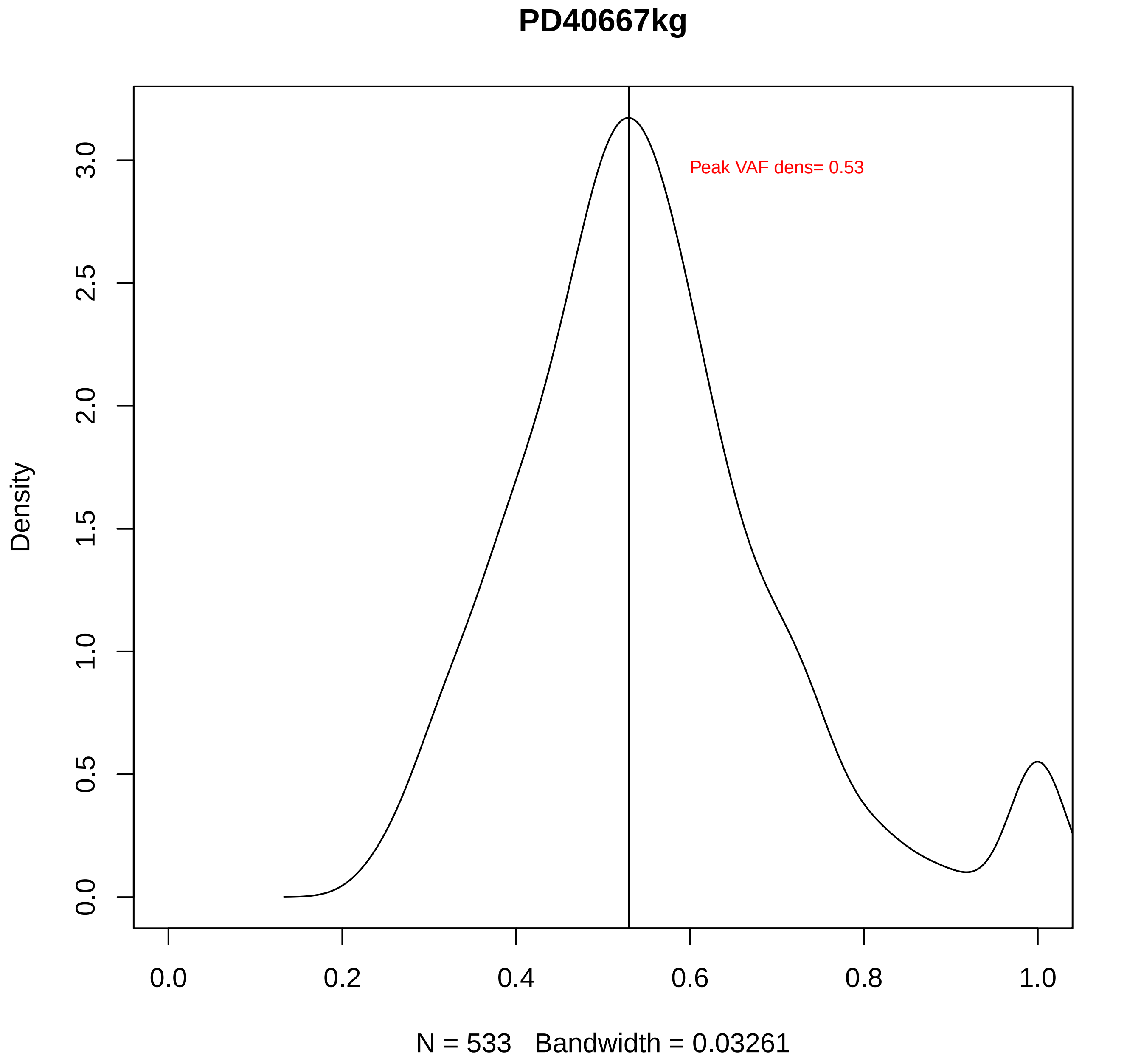
PD40667kg
Peak VAF dens= 0.53
Density
0.0
0.5
1.0
1.5
2.0
2.5
3.0
0.0
0.2
0.4
0.6
0.8
1.0
N = 533 Bandwidth = 0.03261

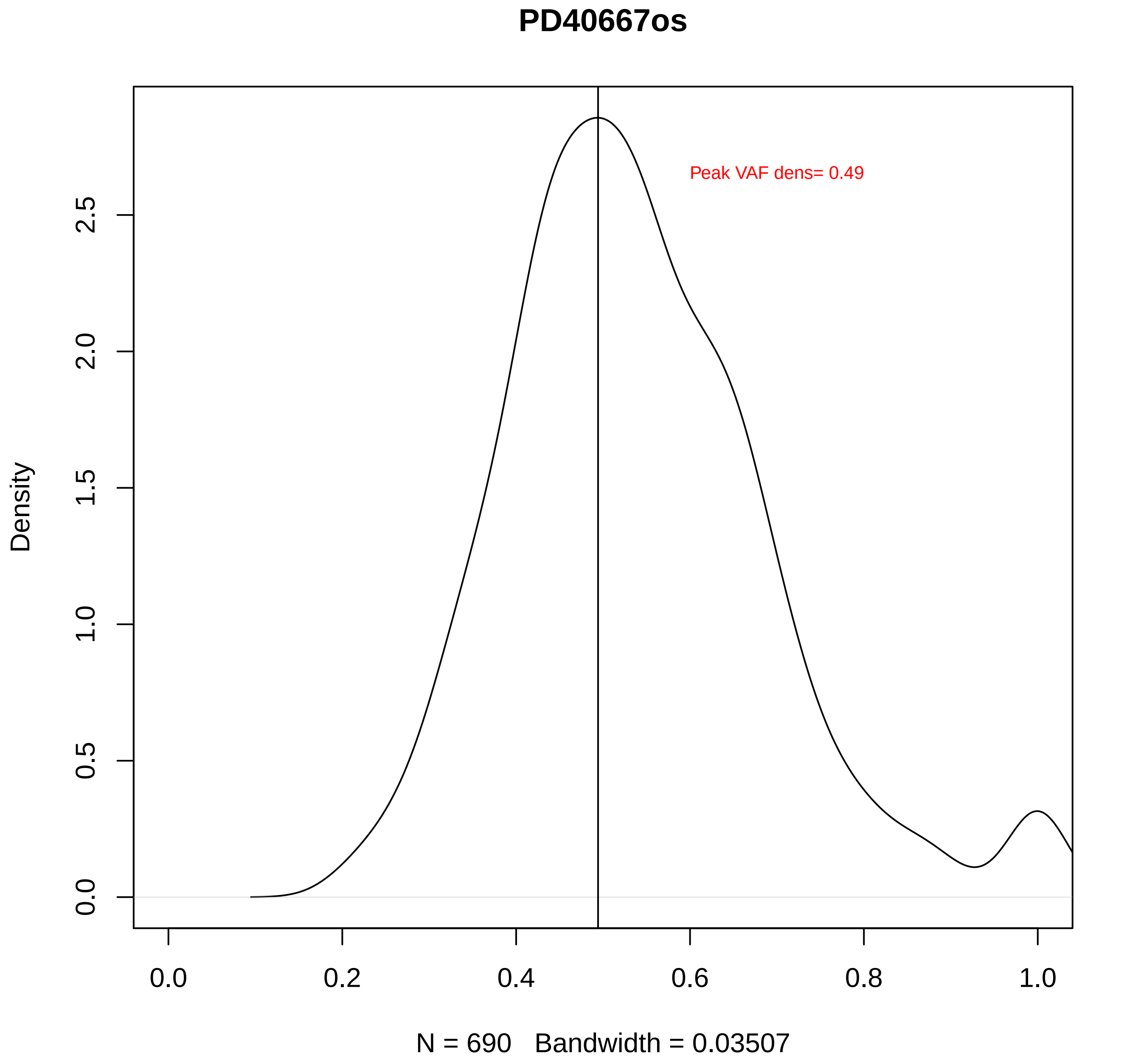

PD40667os
Peak VAF dens= 0.49
Density
0.0
0.5
1.0
1.5
2.0
2.5
0.0
0.2
0.4
0.6
0.8
1.0
N = 690   Bandwidth = 0.03507

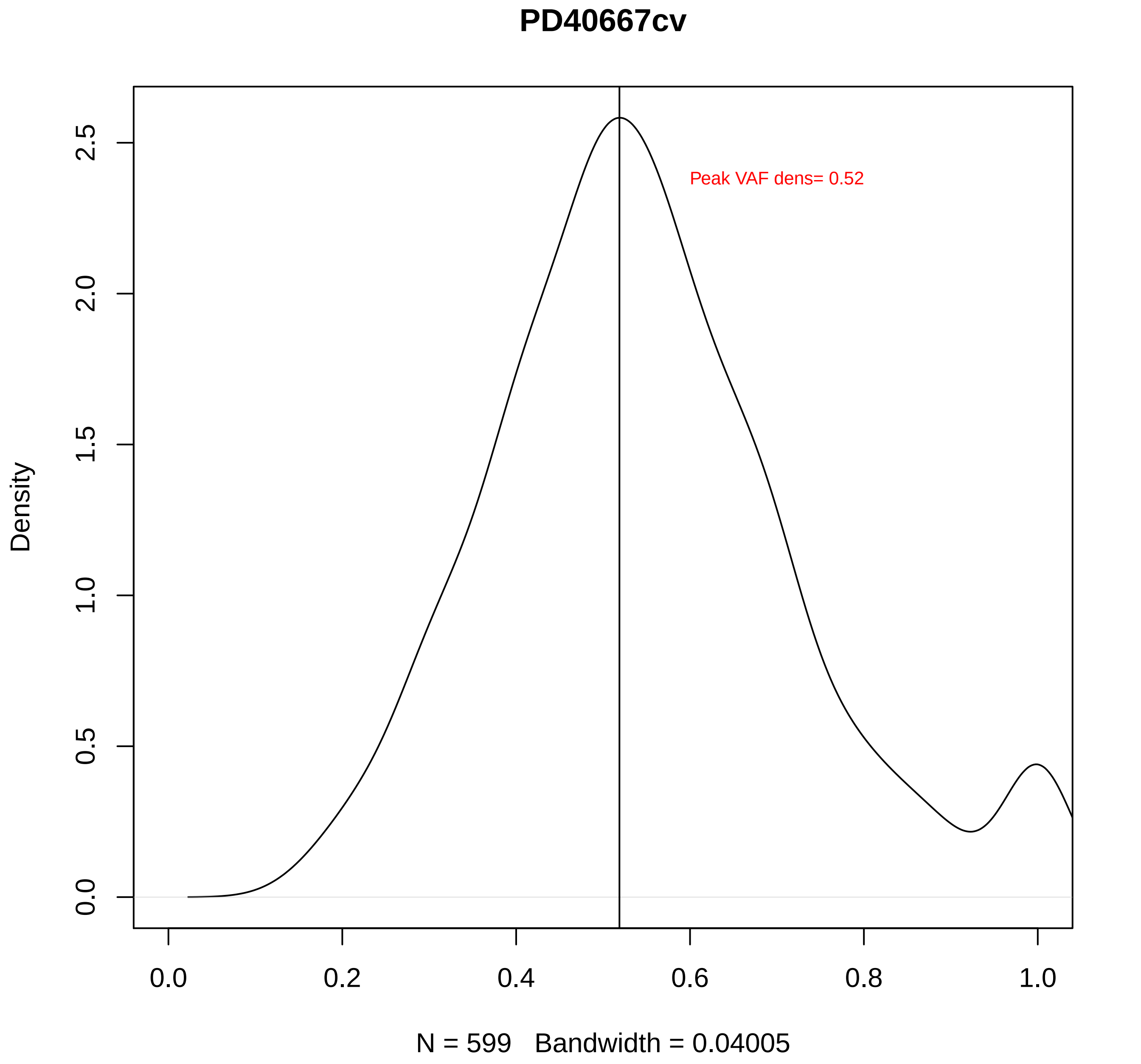
PD40667cv
Peak VAF dens= 0.52
Density
0.0
0.5
1.0
1.5
2.0
2.5
0.0
0.2
0.4
0.6
0.8
1.0
N = 599   Bandwidth = 0.04005

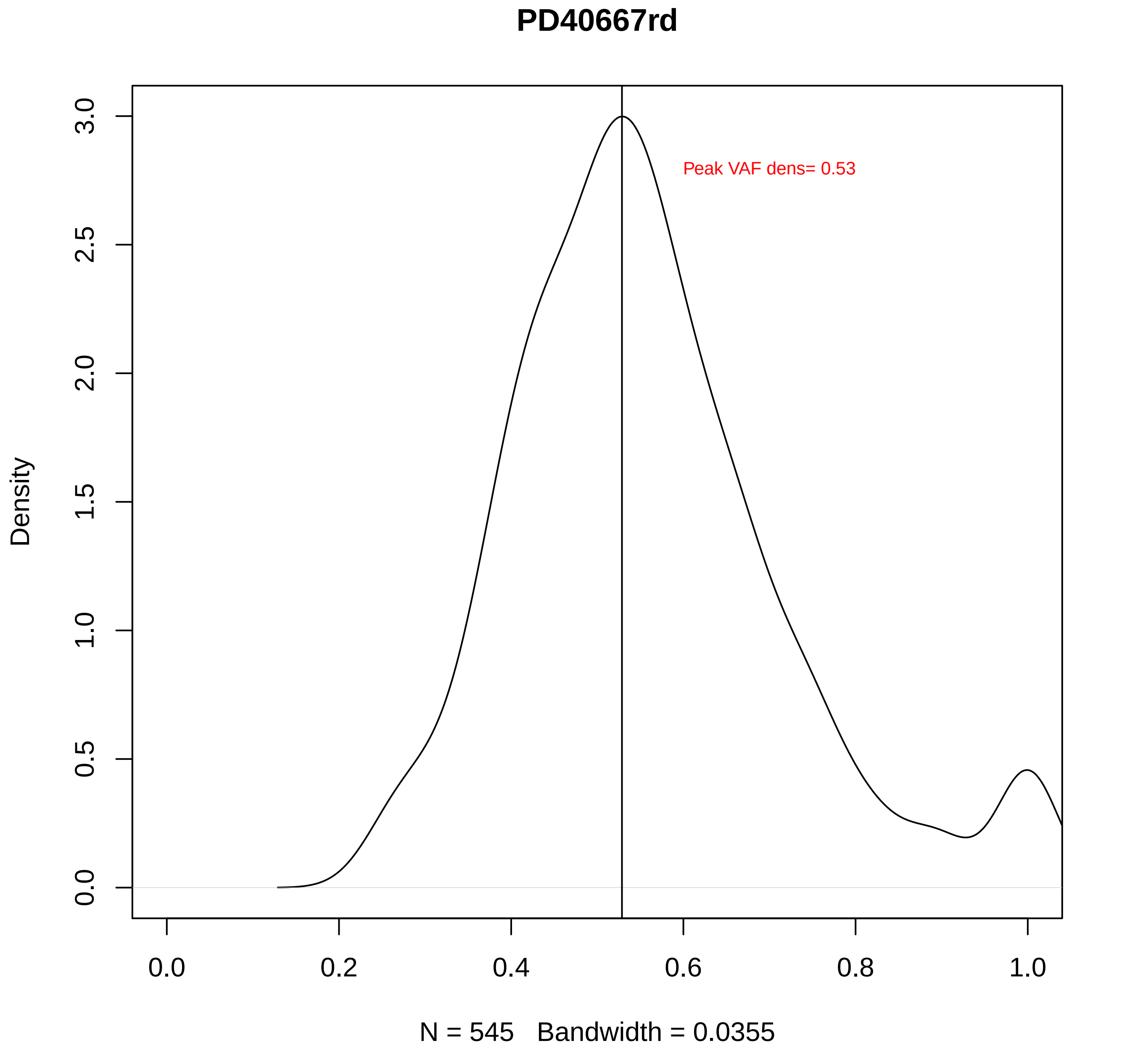

PD40667rd
Peak VAF dens= 0.53
Density
3.0
2.5
2.0
1.5
1.0
0.5
0.0
0.0
0.2
0.4
0.6
0.8
1.0
N = 545 Bandwidth = 0.0355

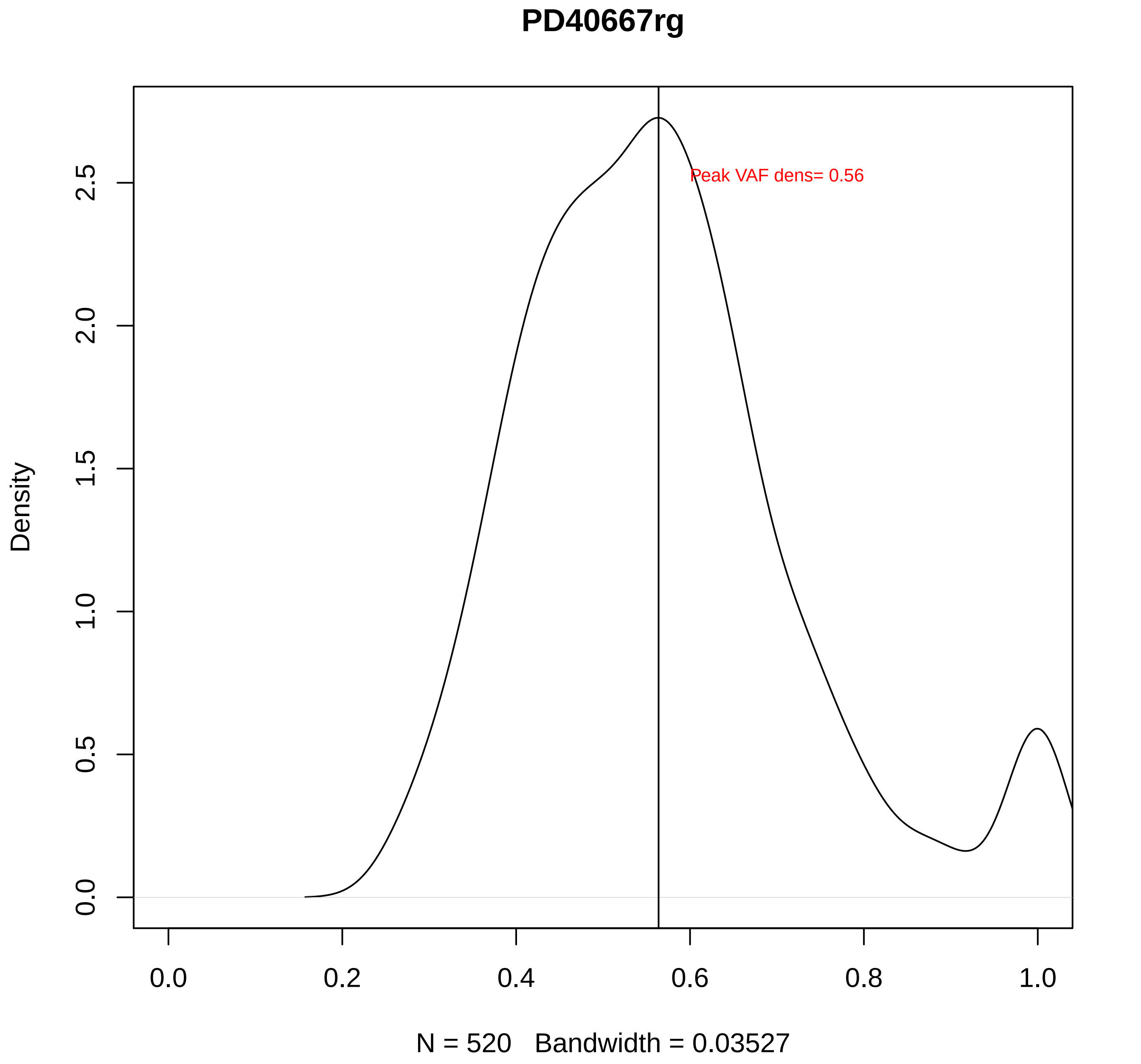
PD40667rg
Peak VAF dens= 0.56
Density
0.0
0.5
1.0
1.5
2.0
2.5
0.0
0.2
0.4
0.6
0.8
1.0
N = 520   Bandwidth = 0.03527

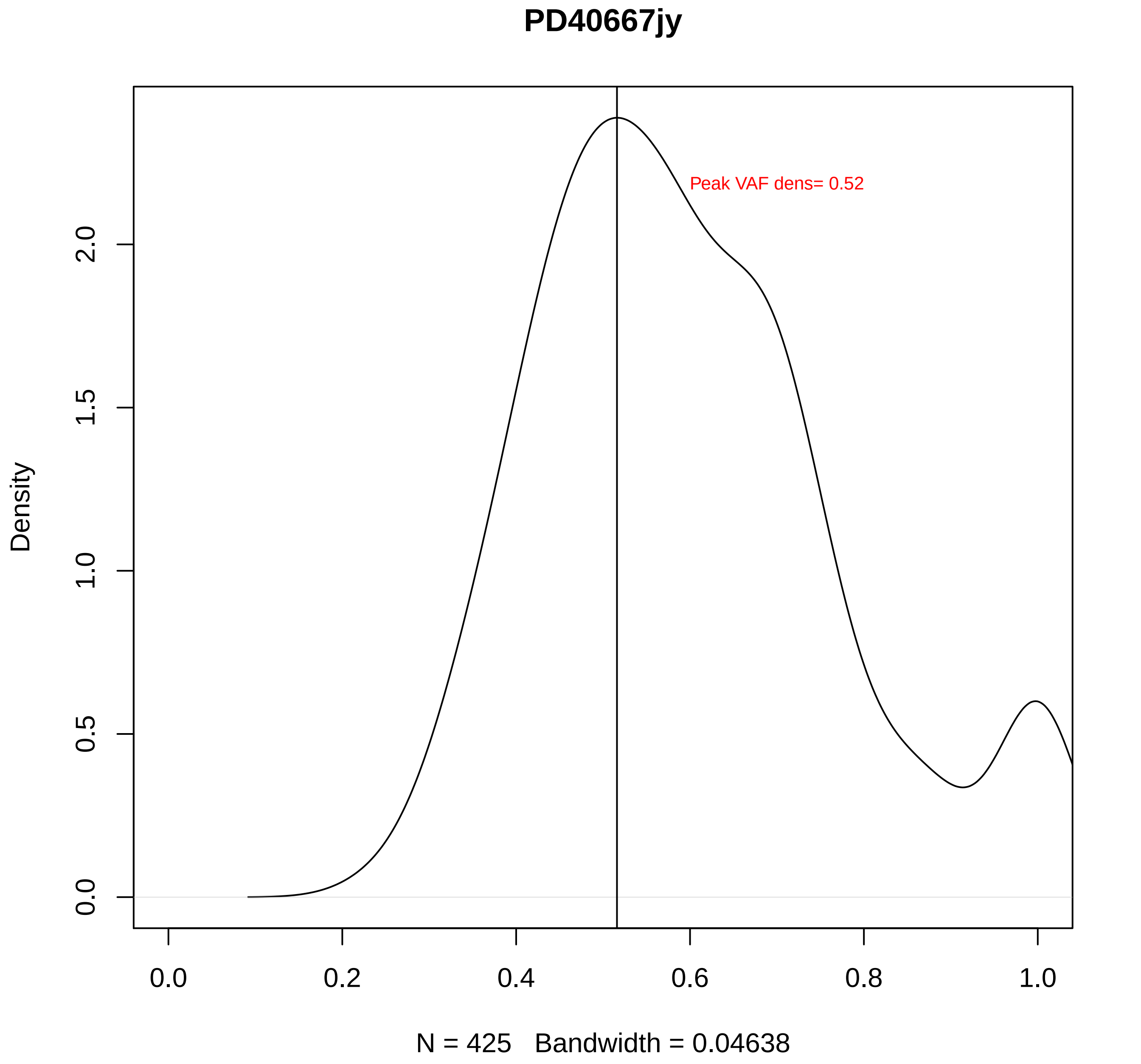
PD40667jy
Peak VAF dens= 0.52
Density
0.0
0.5
1.0
1.5
2.0
0.0
0.2
0.4
0.6
0.8
1.0
N = 425   Bandwidth = 0.04638

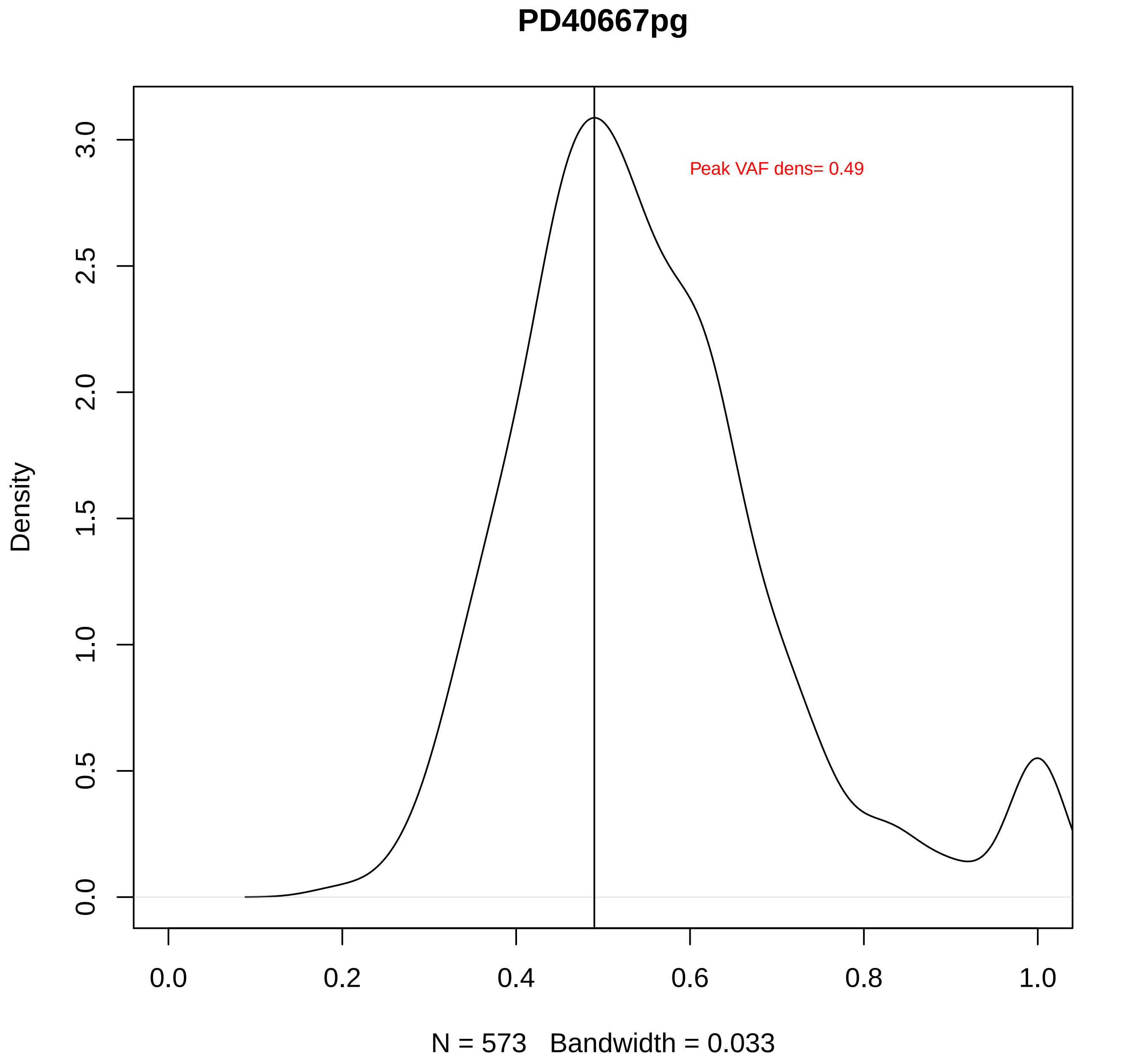

PD40667pg
Peak VAF dens= 0.49
Density
0.0
0.5
1.0
1.5
2.0
2.5
3.0
0.0
0.2
0.4
0.6
0.8
1.0
N = 573   Bandwidth = 0.033

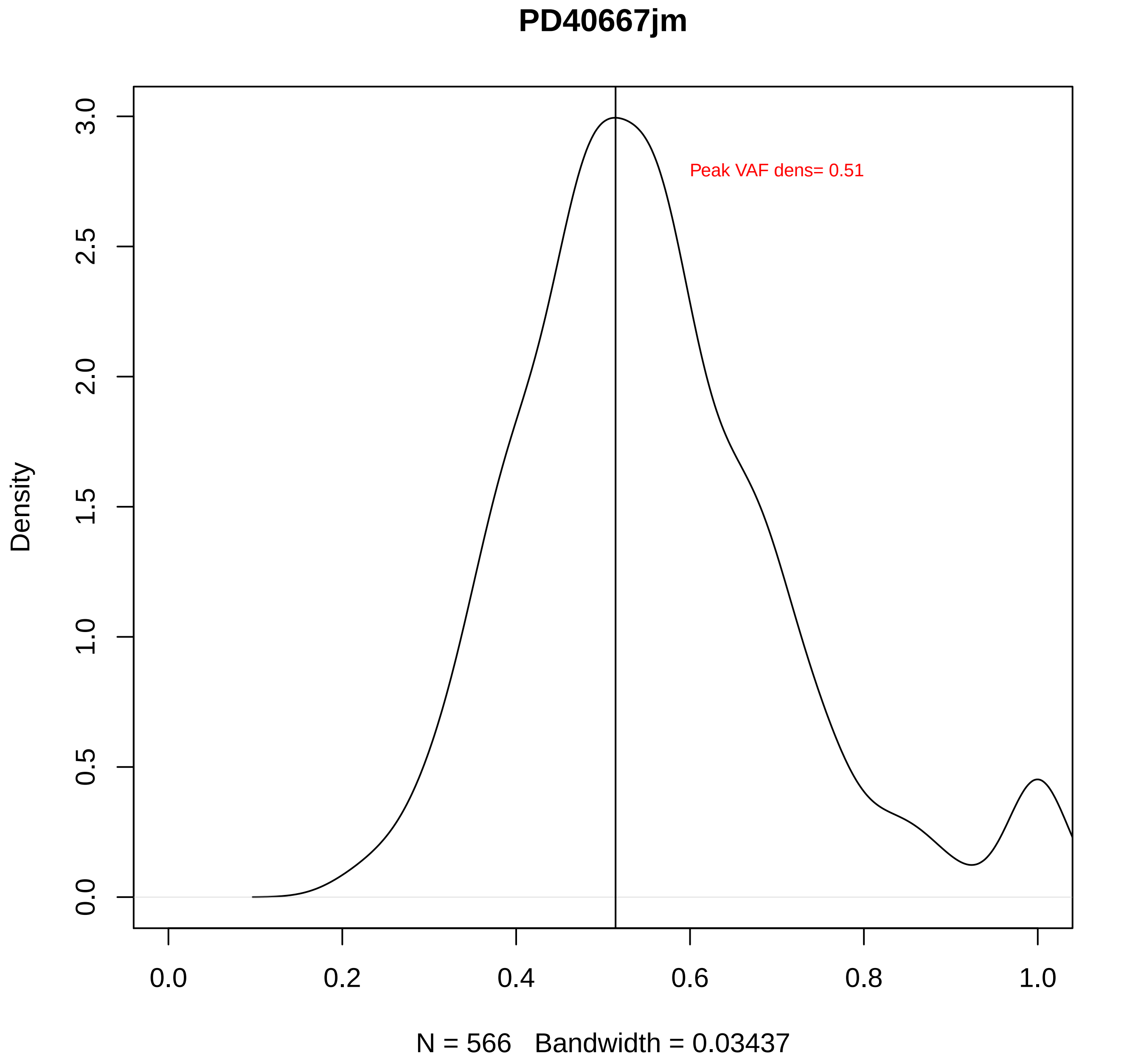

PD40667jm
Peak VAF dens= 0.51
Density
0.0
0.5
1.0
1.5
2.0
2.5
3.0
0.0
0.2
0.4
0.6
0.8
1.0
N = 566   Bandwidth = 0.03437

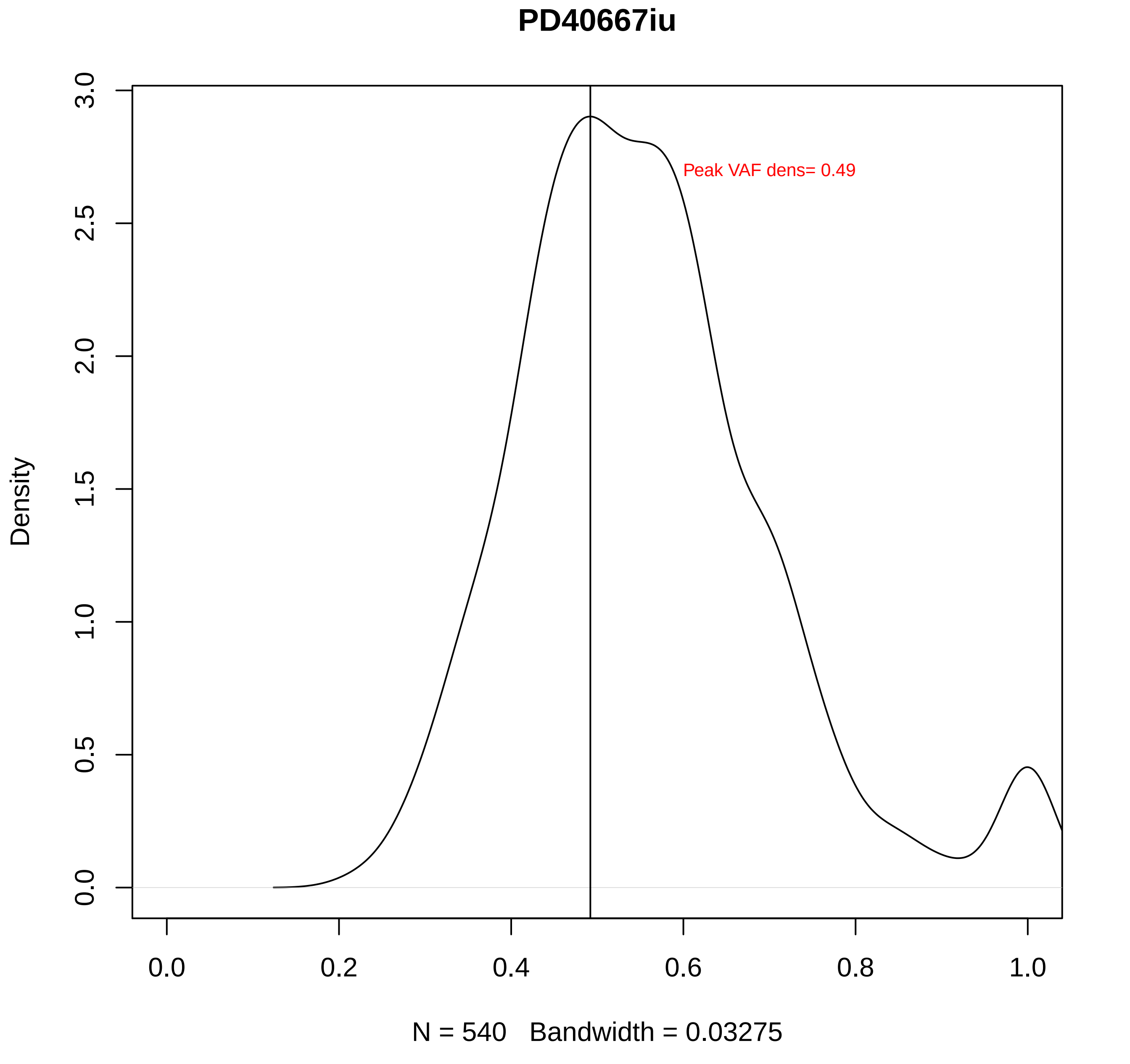

PD40667iu
Peak VAF dens= 0.49
Density
0.0
0.5
1.0
1.5
2.0
2.5
3.0
0.0
0.2
0.4
0.6
0.8
1.0
N = 540   Bandwidth = 0.03275

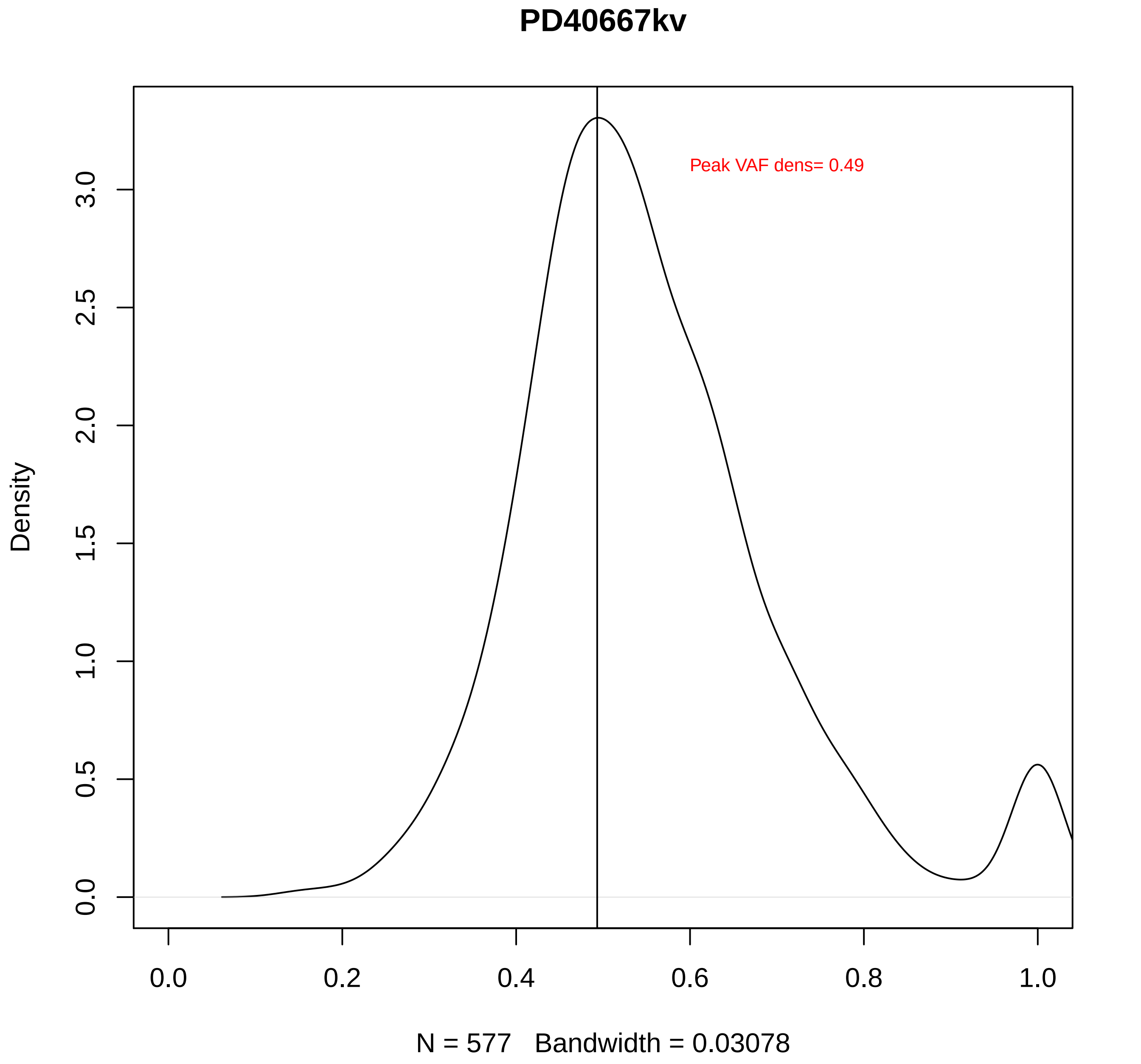
PD40667kv
Peak VAF dens= 0.49
Density
0.0
0.5
1.0
1.5
2.0
2.5
3.0
0.0
0.2
0.4
0.6
0.8
1.0
N = 577 Bandwidth = 0.03078

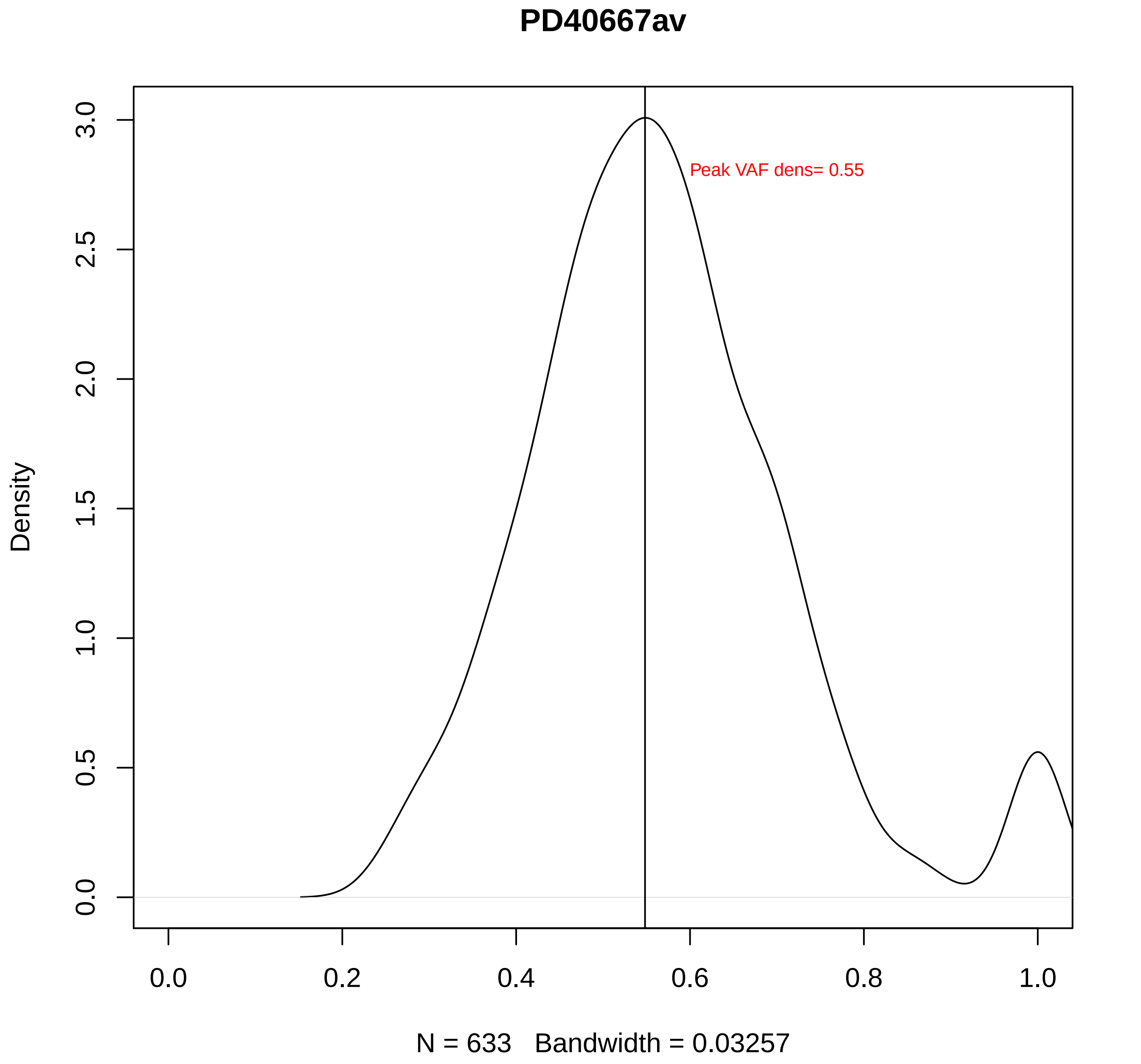

PD40667av
Peak VAF dens= 0.55
Density
0.0
0.5
1.0
1.5
2.0
2.5
3.0
0.0
0.2
0.4
0.6
0.8
1.0
N = 633 Bandwidth = 0.03257

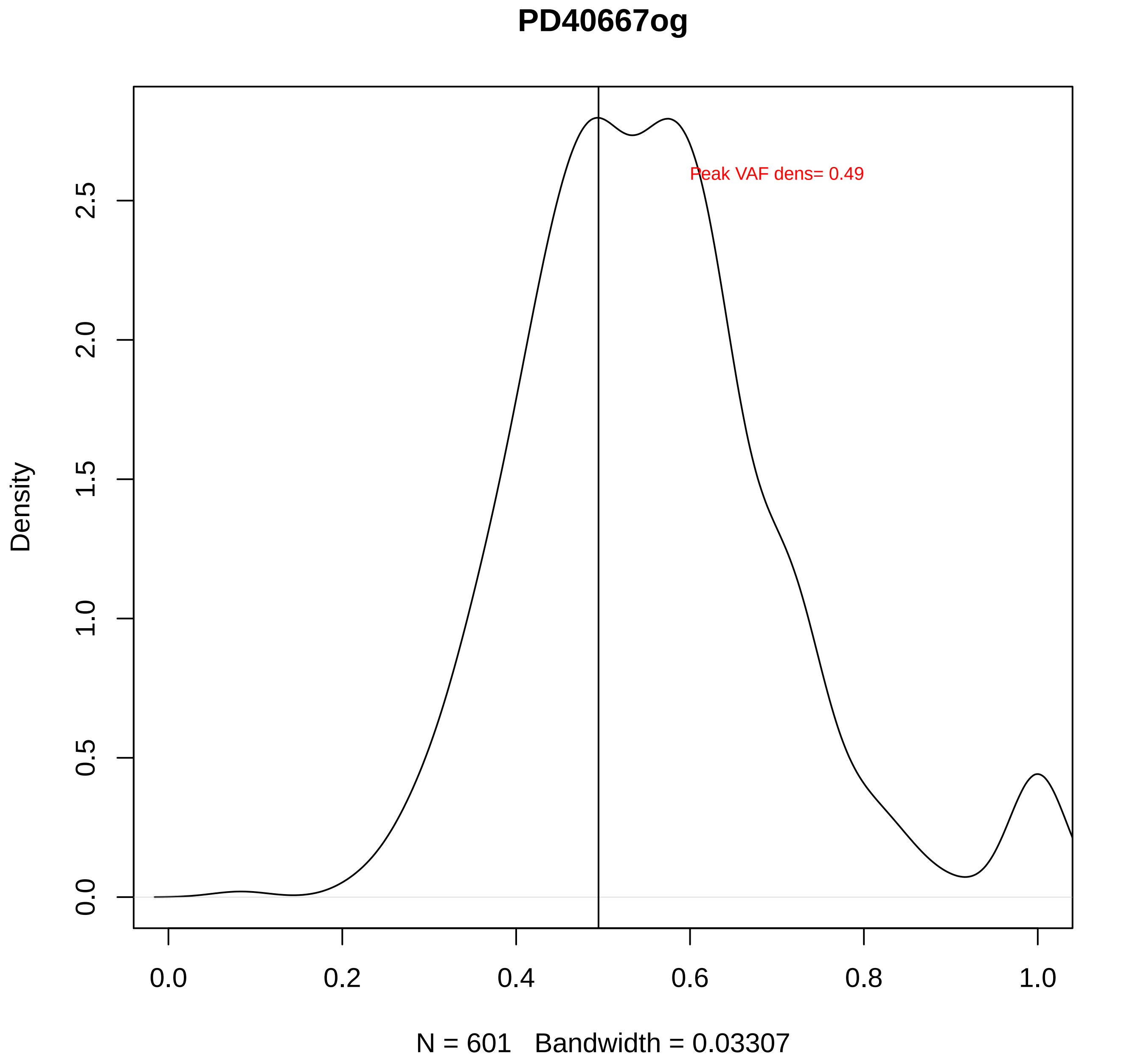
PD40667og
Peak VAF dens= 0.49
Density
0.0
0.5
1.0
1.5
2.0
2.5
0.0
0.2
0.4
0.6
0.8
1.0
N = 601 Bandwidth = 0.03307

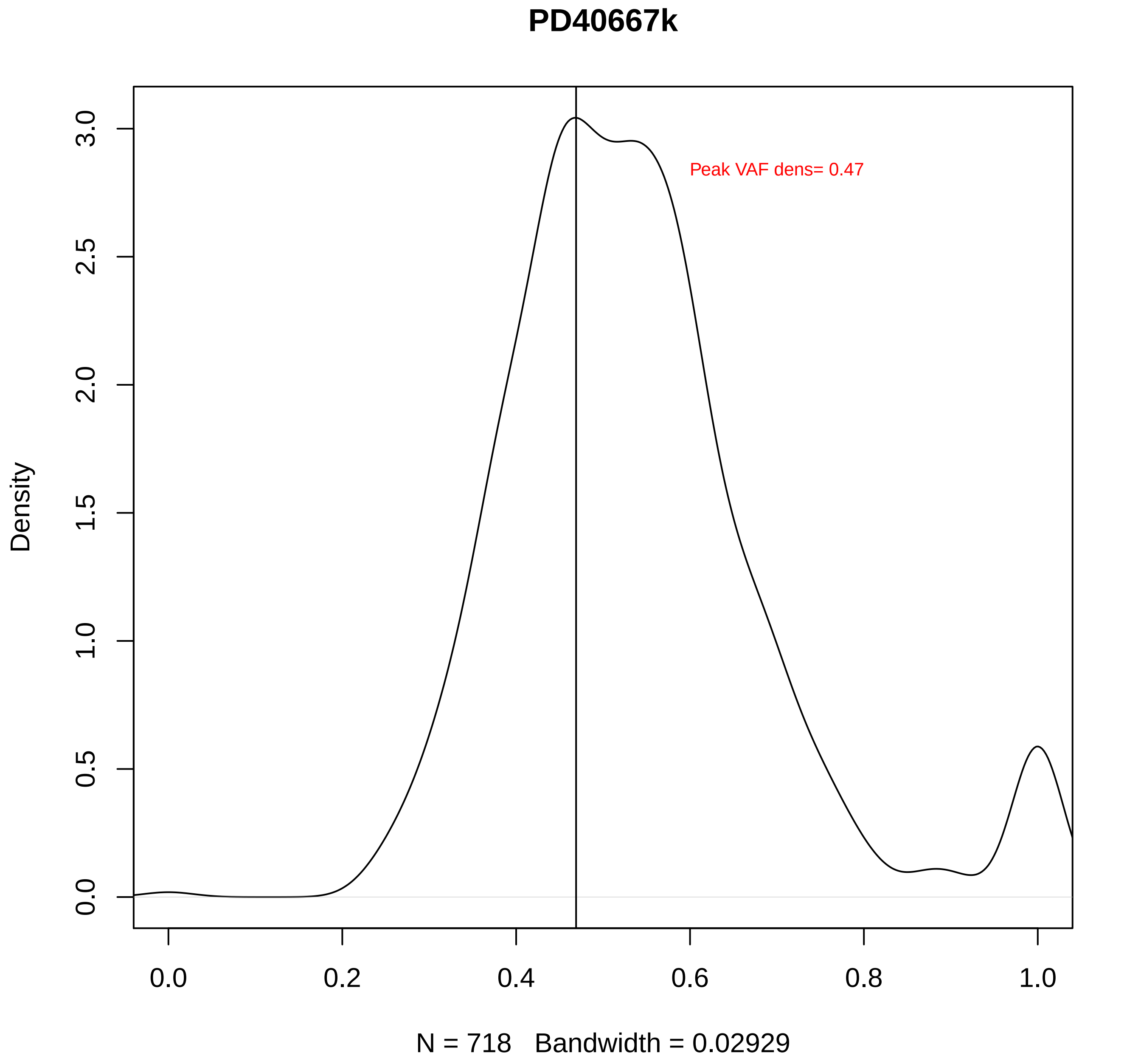
PD40667k
Peak VAF dens= 0.47
Density
0.0
0.5
1.0
1.5
2.0
2.5
3.0
0.0
0.2
0.4
0.6
0.8
1.0
N = 718   Bandwidth = 0.02929

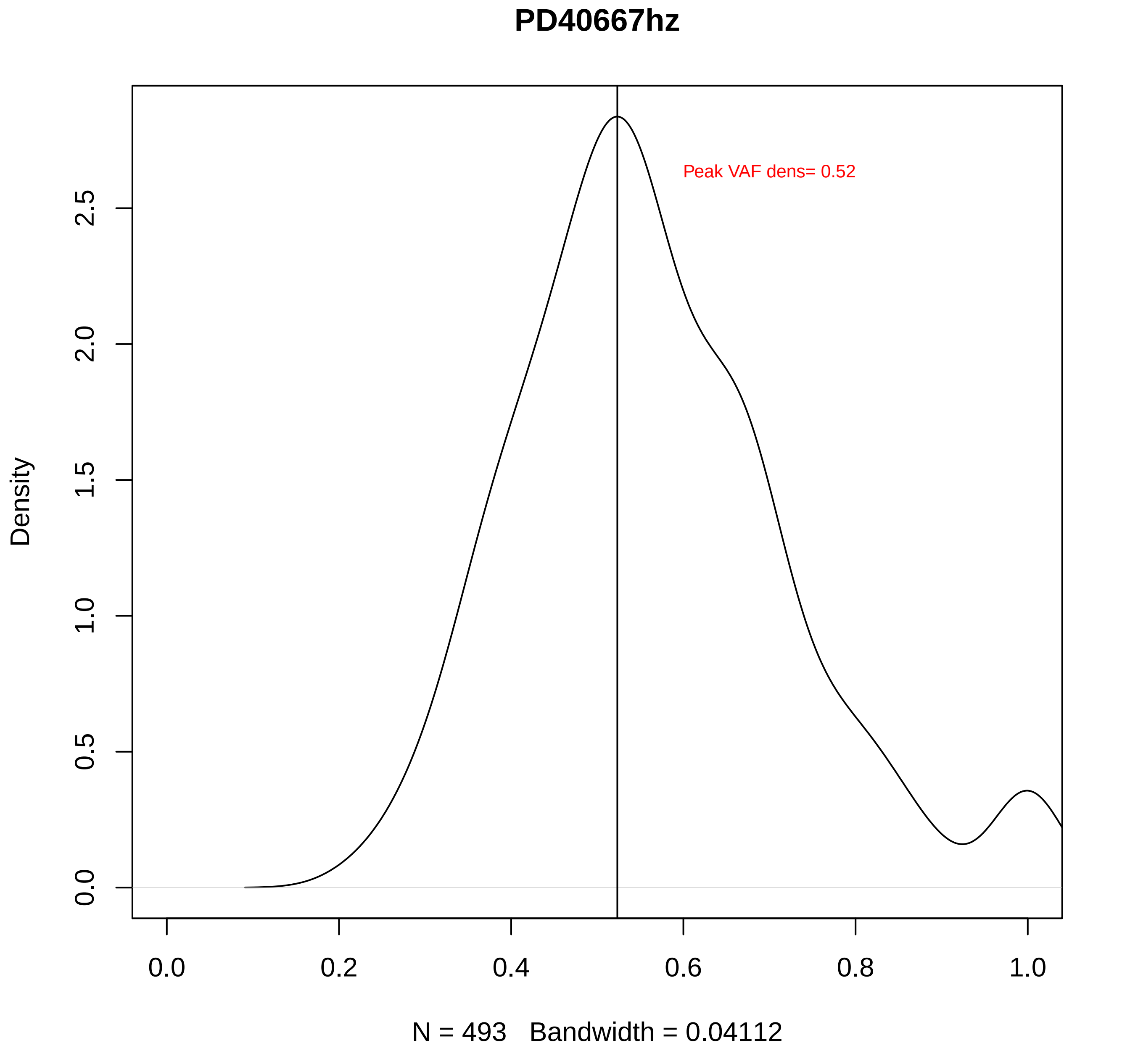
PD40667hz
Peak VAF dens= 0.52
Density
0.0
0.5
1.0
1.5
2.0
2.5
0.0
0.2
0.4
0.6
0.8
1.0
N = 493   Bandwidth = 0.04112

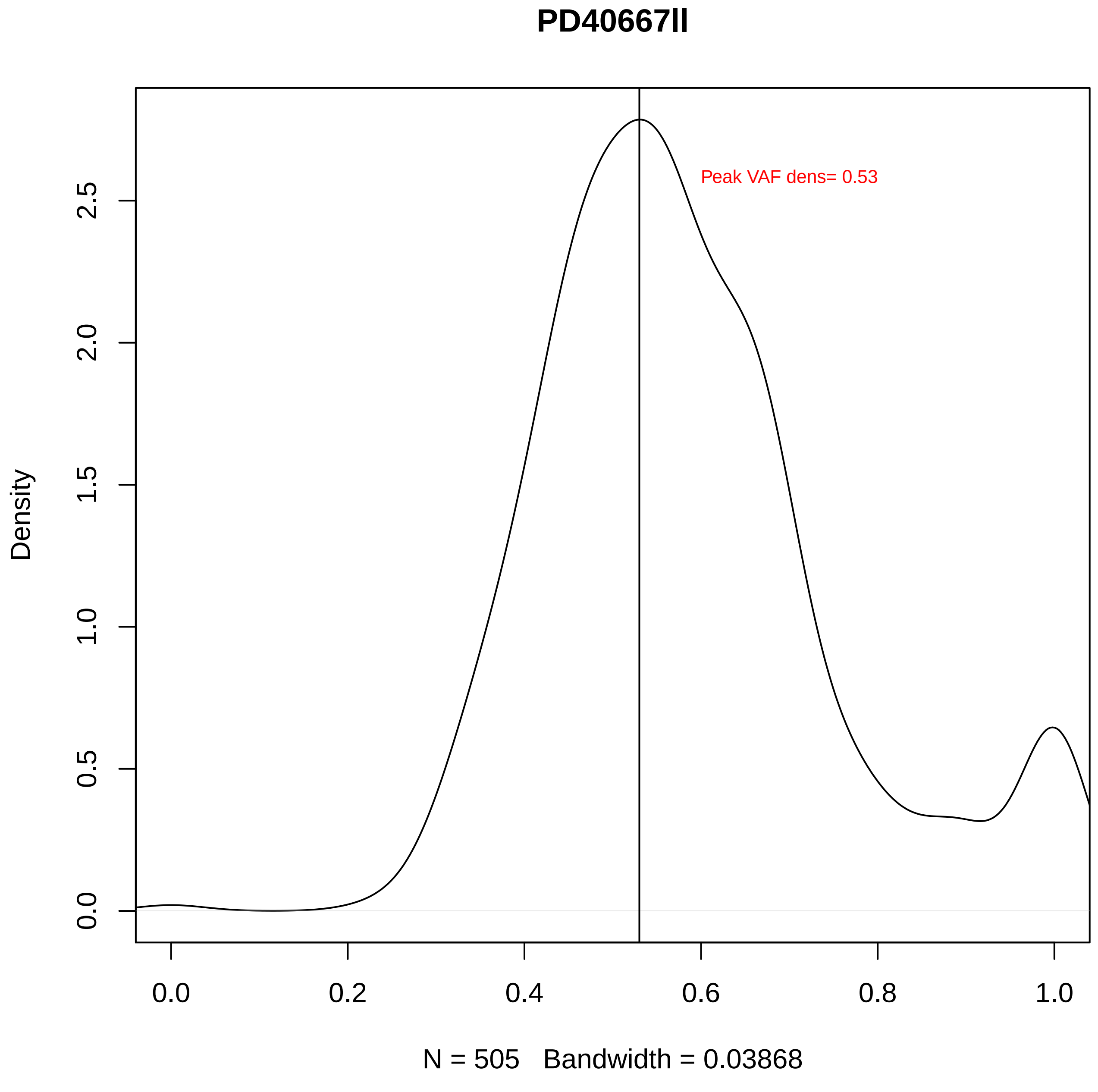

PD40667ll
Peak VAF dens= 0.53
Density
0.0
0.5
1.0
1.5
2.0
2.5
0.0
0.2
0.4
0.6
0.8
1.0
N = 505 Bandwidth = 0.03868

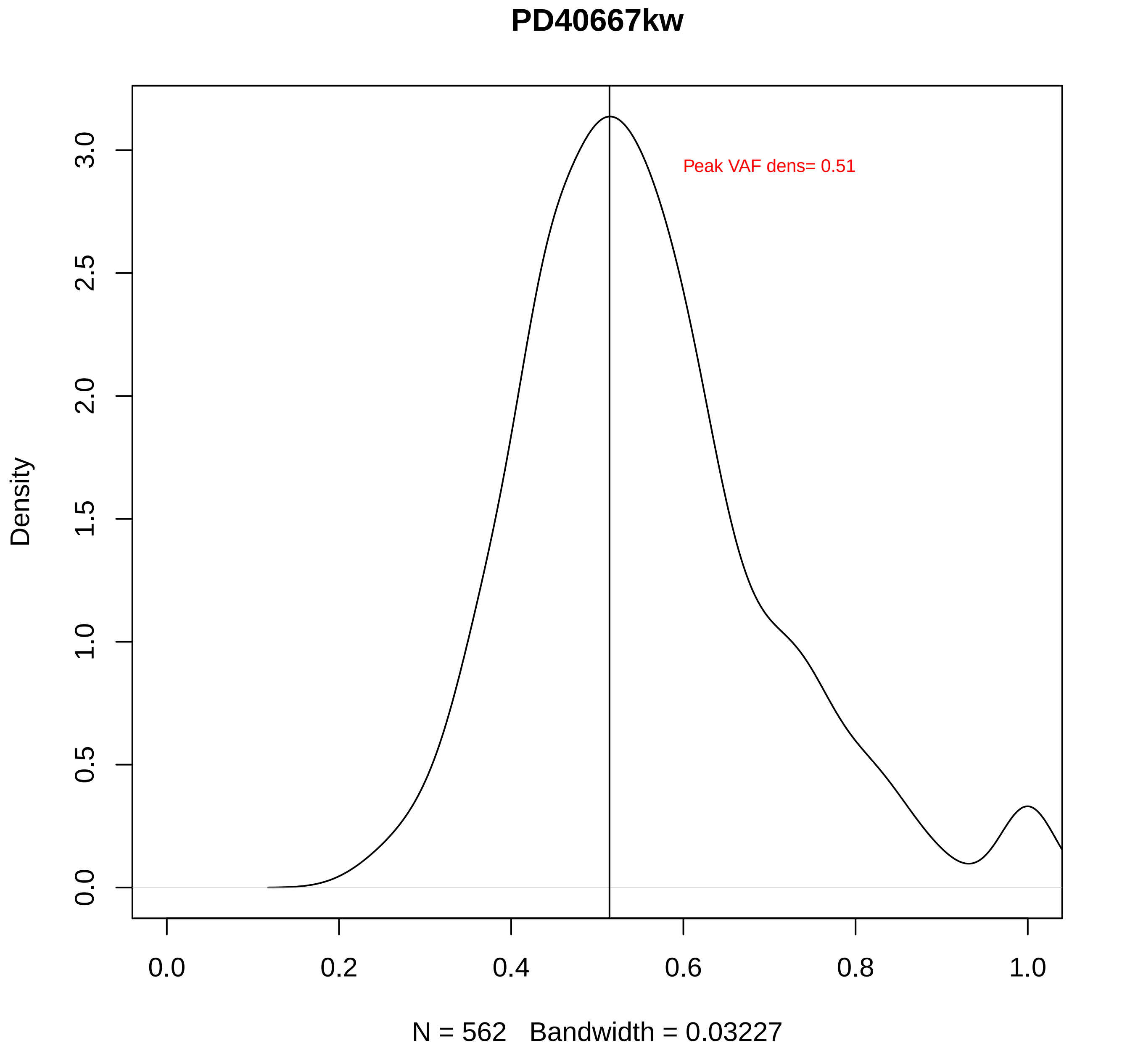

PD40667kw
Peak VAF dens= 0.51
Density
0.0
0.5
1.0
1.5
2.0
2.5
3.0
0.0
0.2
0.4
0.6
0.8
1.0
N = 562 Bandwidth = 0.03227

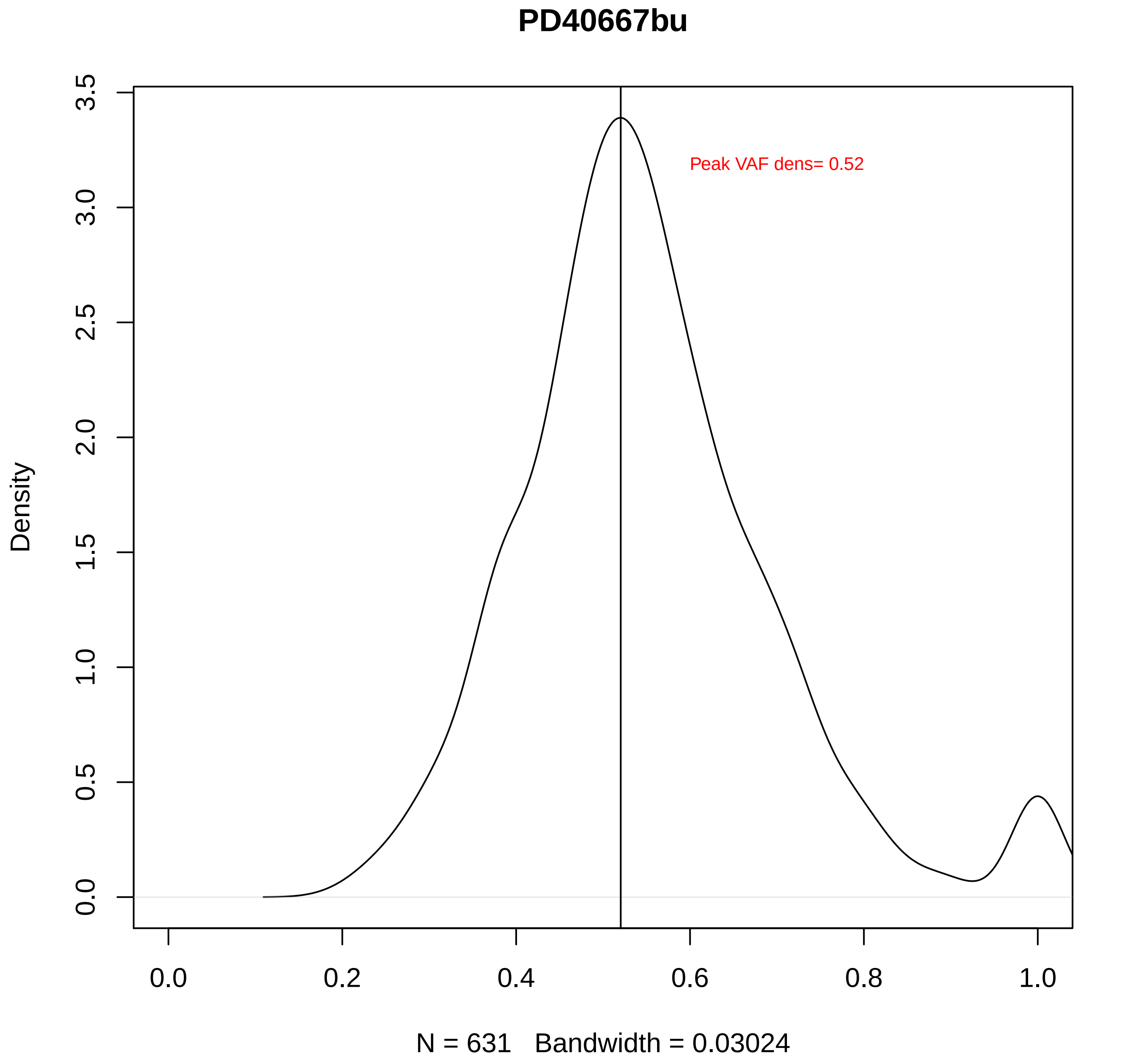
PD40667bu
Peak VAF dens= 0.52
Density
3.5
3.0
2.5
2.0
1.5
1.0
0.5
0.0
0.0
0.2
0.4
0.6
0.8
1.0
N = 631   Bandwidth = 0.03024

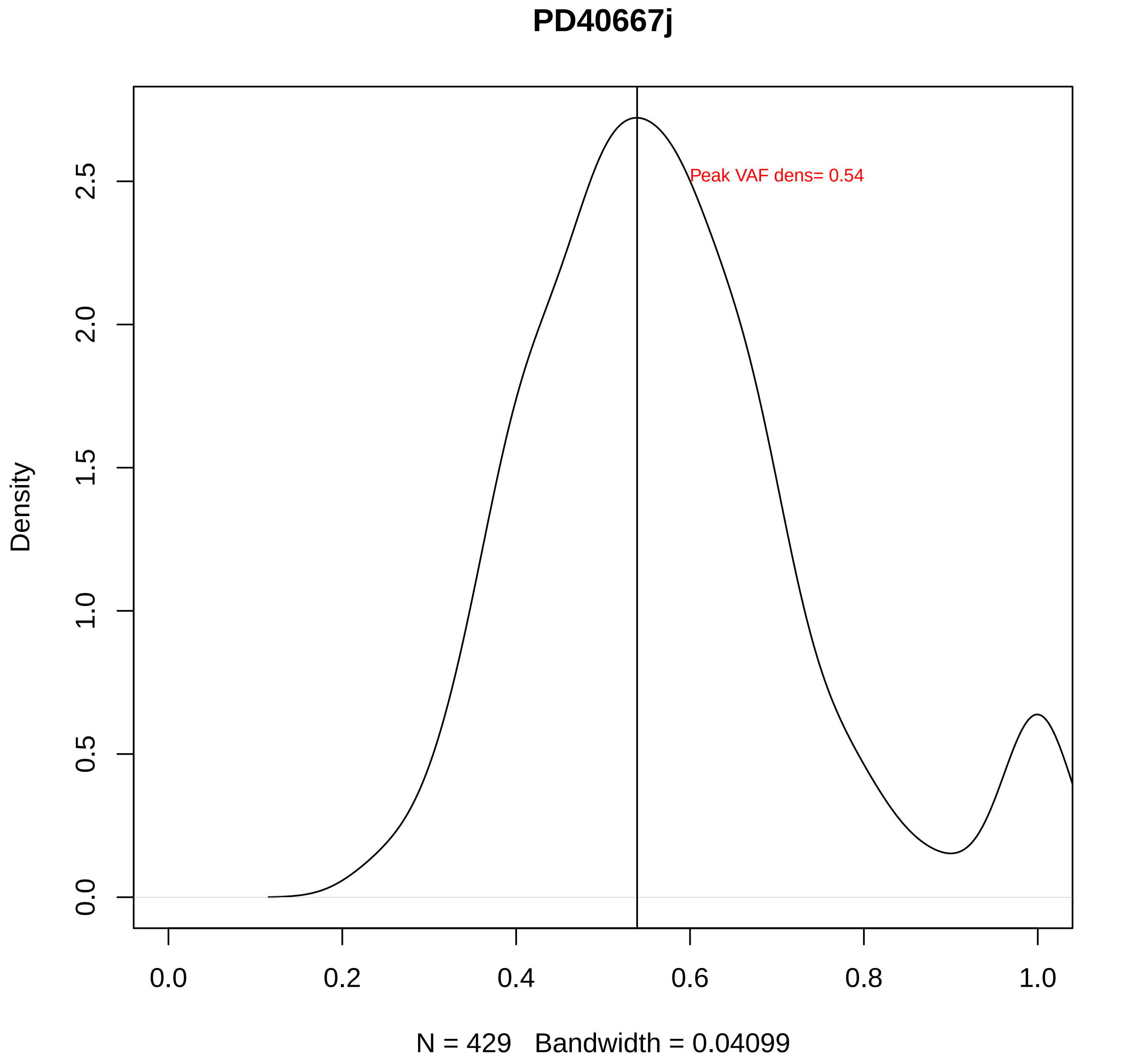
PD40667j
Peak VAF dens= 0.54
Density
0.0
0.5
1.0
1.5
2.0
2.5
0.0
0.2
0.4
0.6
0.8
1.0
N = 429   Bandwidth = 0.04099

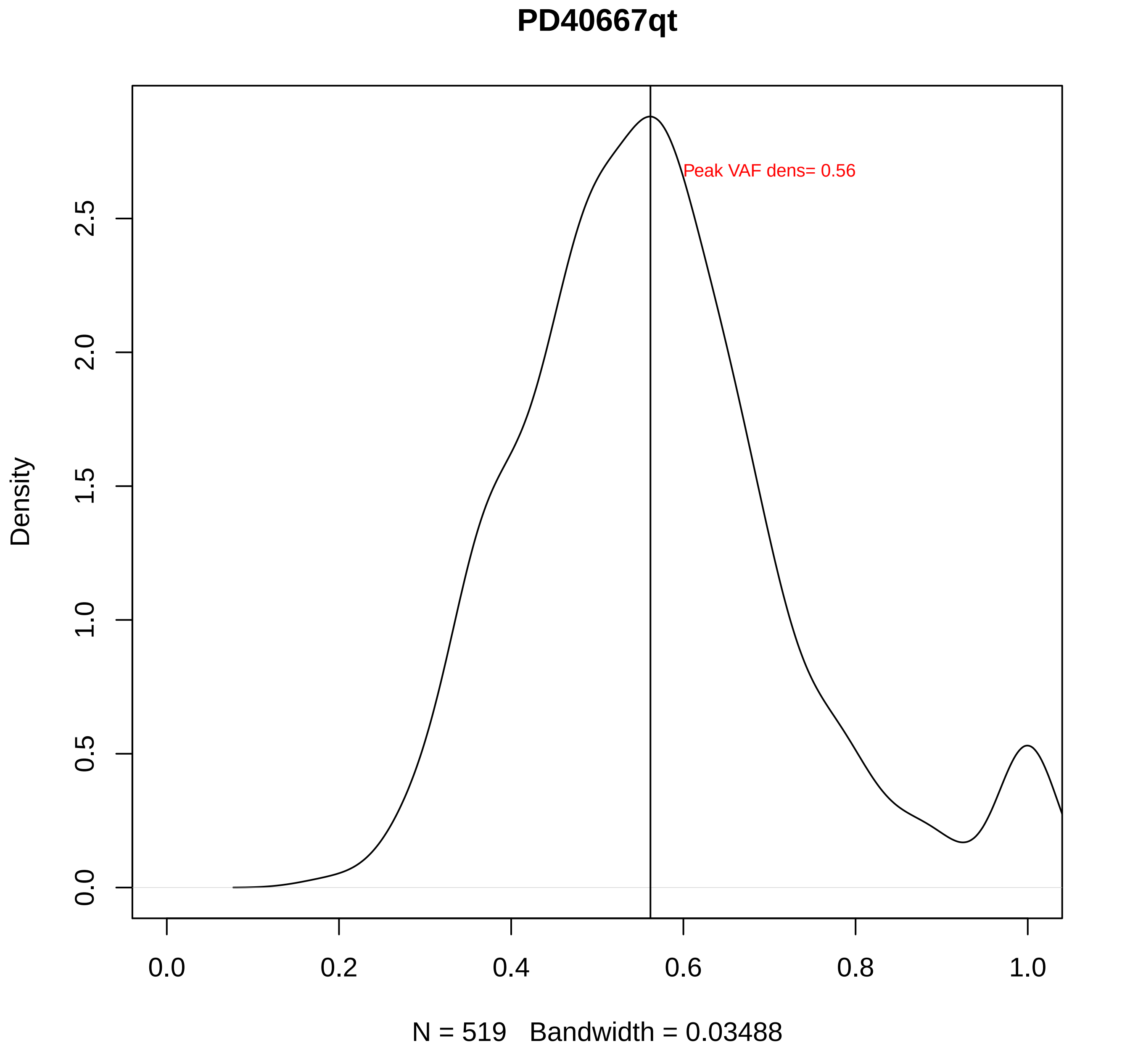
PD40667qt
Peak VAF dens= 0.56
Density
0.0
0.5
1.0
1.5
2.0
2.5
0.0
0.2
0.4
0.6
0.8
1.0
N = 519   Bandwidth = 0.03488

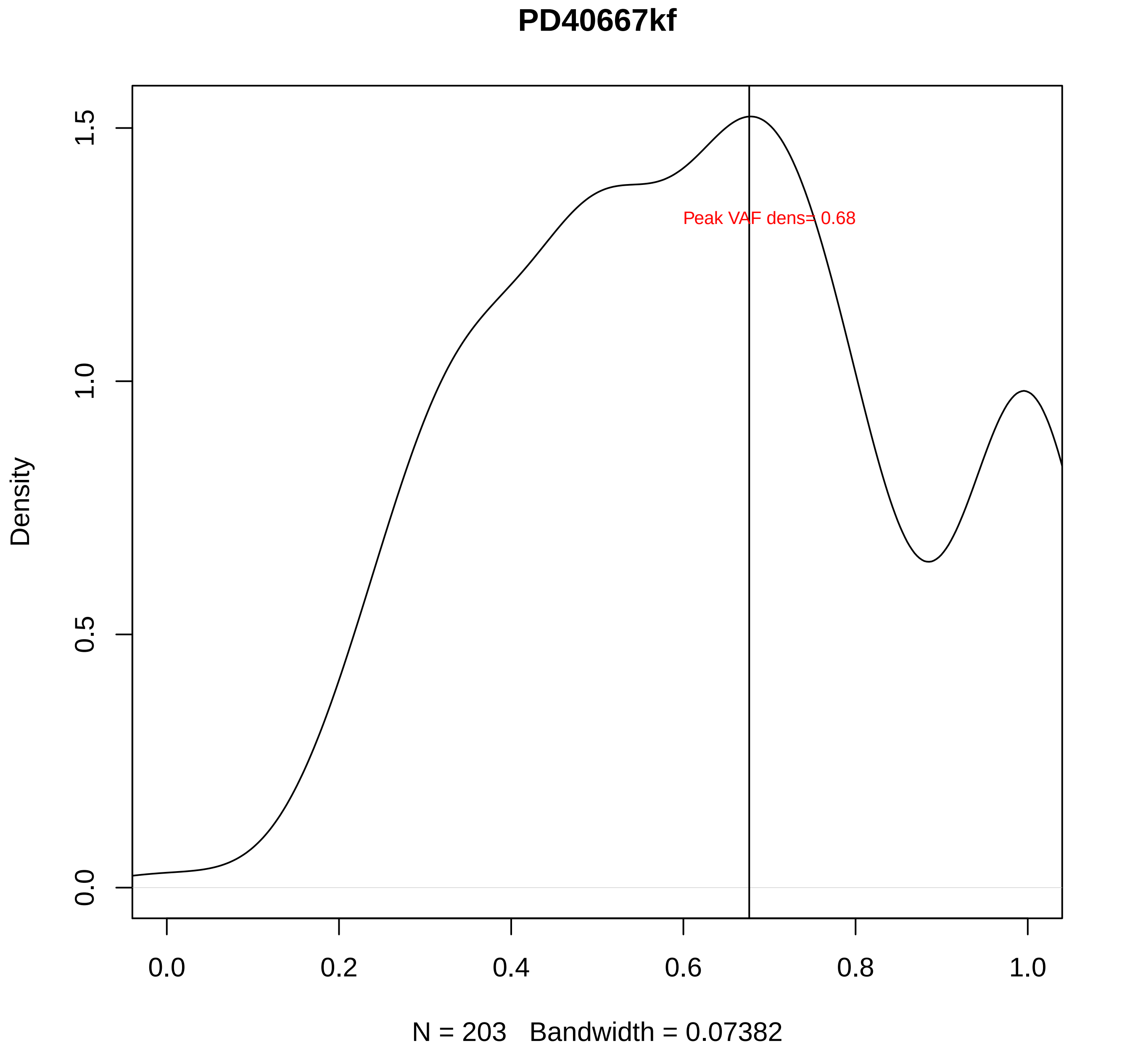

PD40667kf
Density
Peak VAF dens= 0.68
0.0
0.5
1.0
1.5
0.0
0.2
0.4
0.6
0.8
1.0
N = 203 Bandwidth = 0.07382

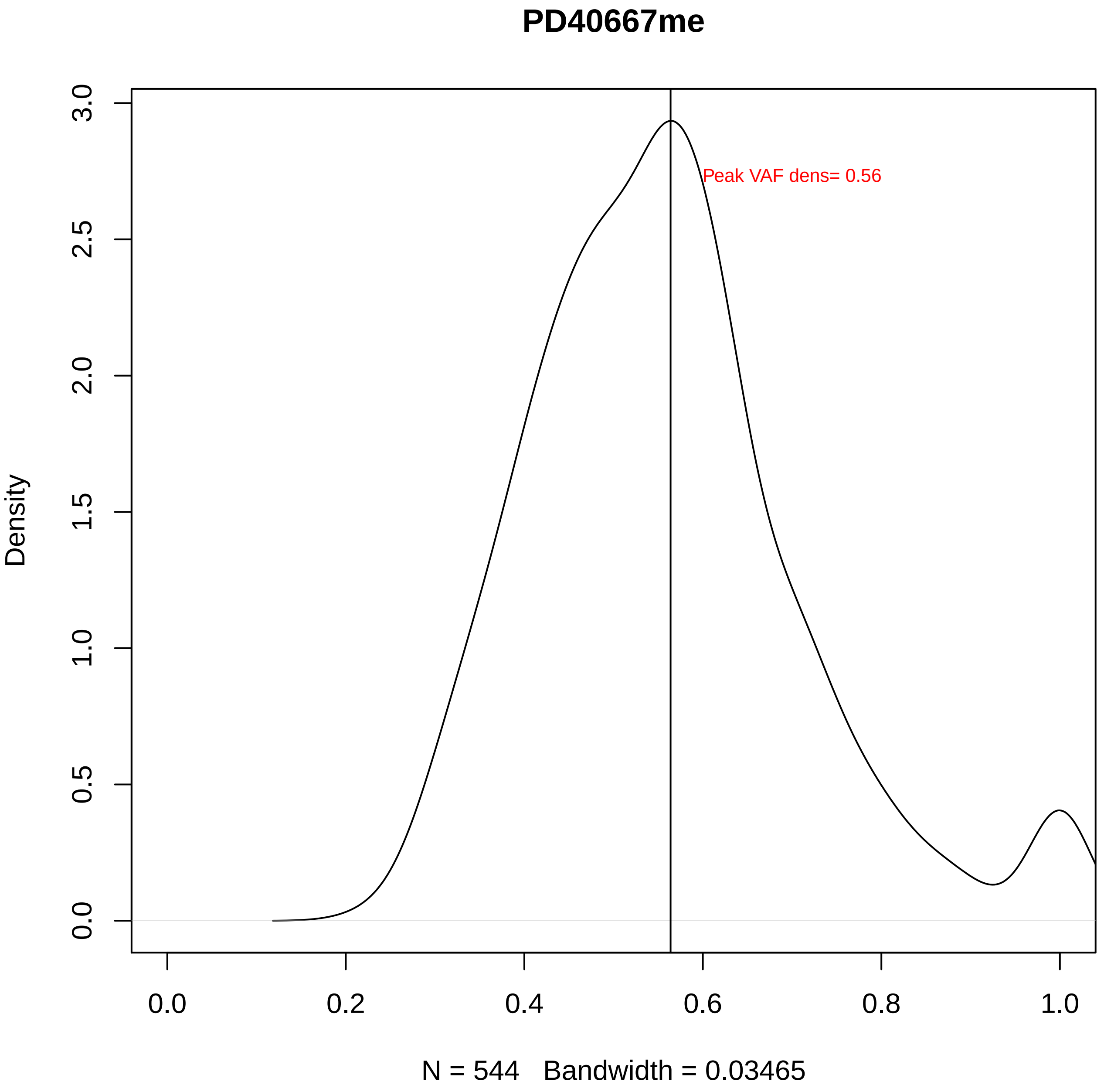
PD40667me
Peak VAF dens= 0.56
Density
0.0
0.5
1.0
1.5
2.0
2.5
3.0
0.0
0.2
0.4
0.6
0.8
1.0
N = 544 Bandwidth = 0.03465

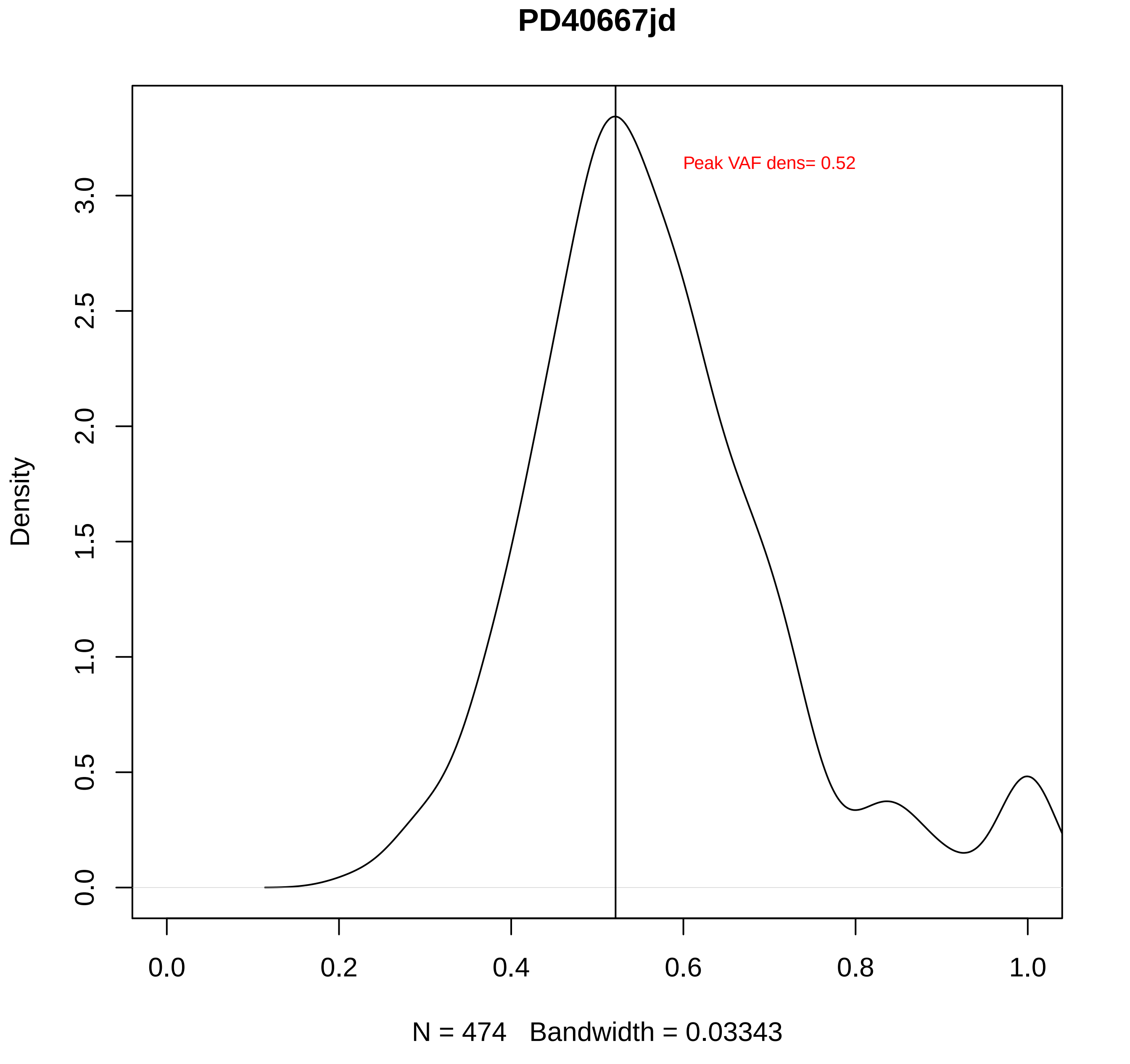

PD40667jd
Peak VAF dens= 0.52
Density
0.0
0.5
1.0
1.5
2.0
2.5
3.0
0.0
0.2
0.4
0.6
0.8
1.0
N = 474 Bandwidth = 0.03343

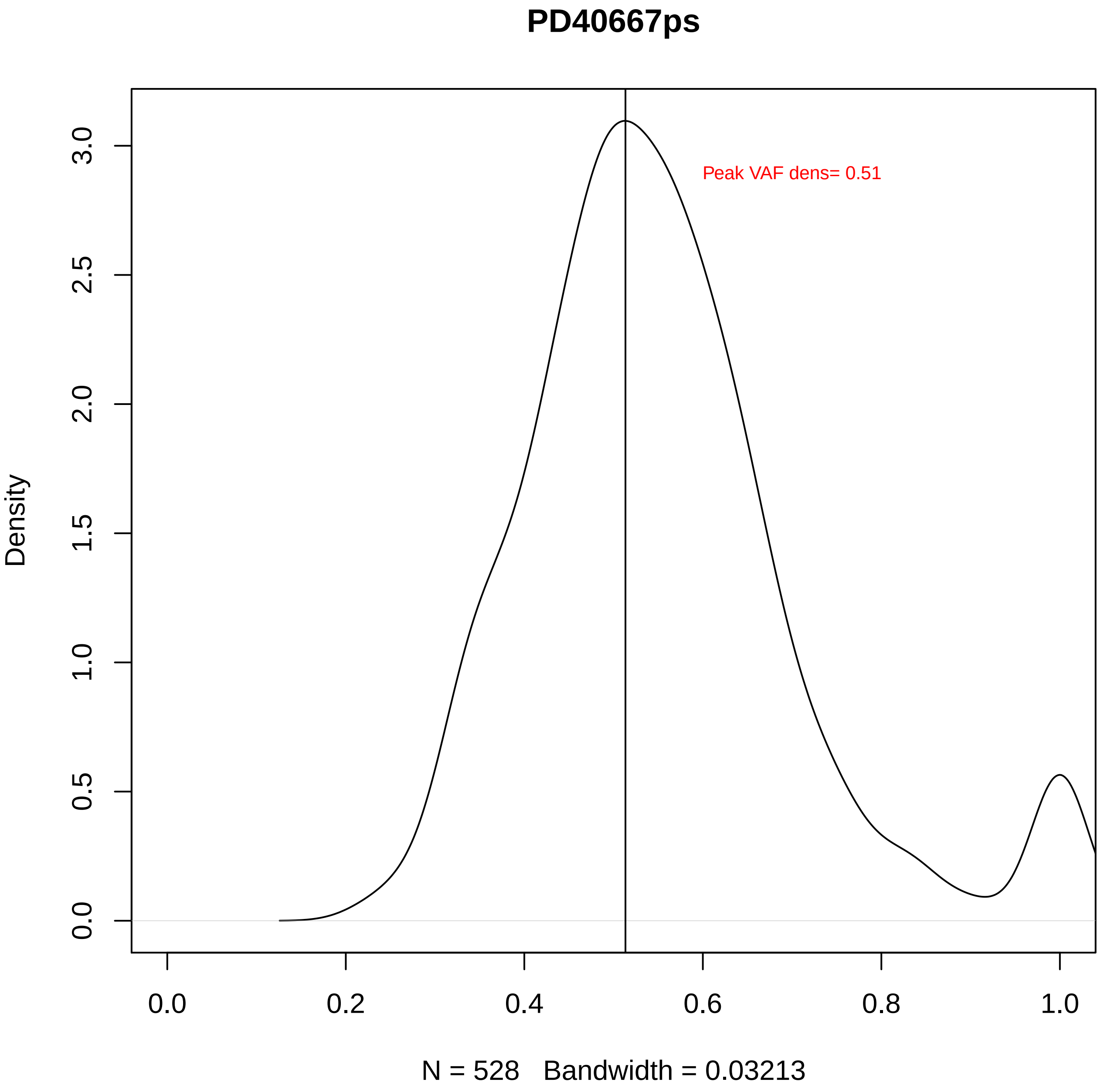

PD40667ps
Peak VAF dens= 0.51
Density
0.0
0.5
1.0
1.5
2.0
2.5
3.0
0.0
0.2
0.4
0.6
0.8
1.0
N = 528   Bandwidth = 0.03213

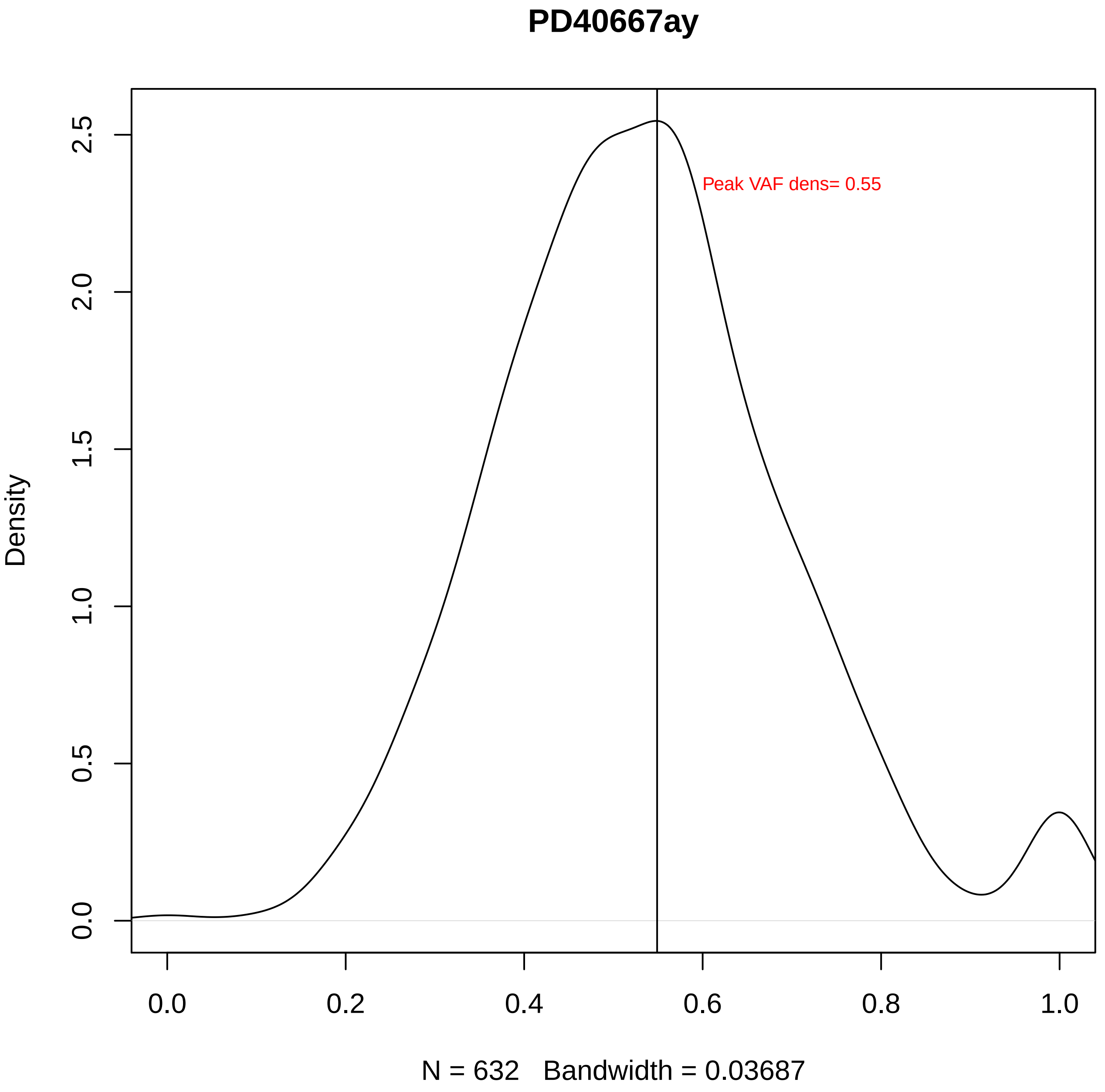

PD40667ay
Peak VAF dens= 0.55
Density
0.0
0.5
1.0
1.5
2.0
2.5
0.0
0.2
0.4
0.6
0.8
1.0
N = 632 Bandwidth = 0.03687

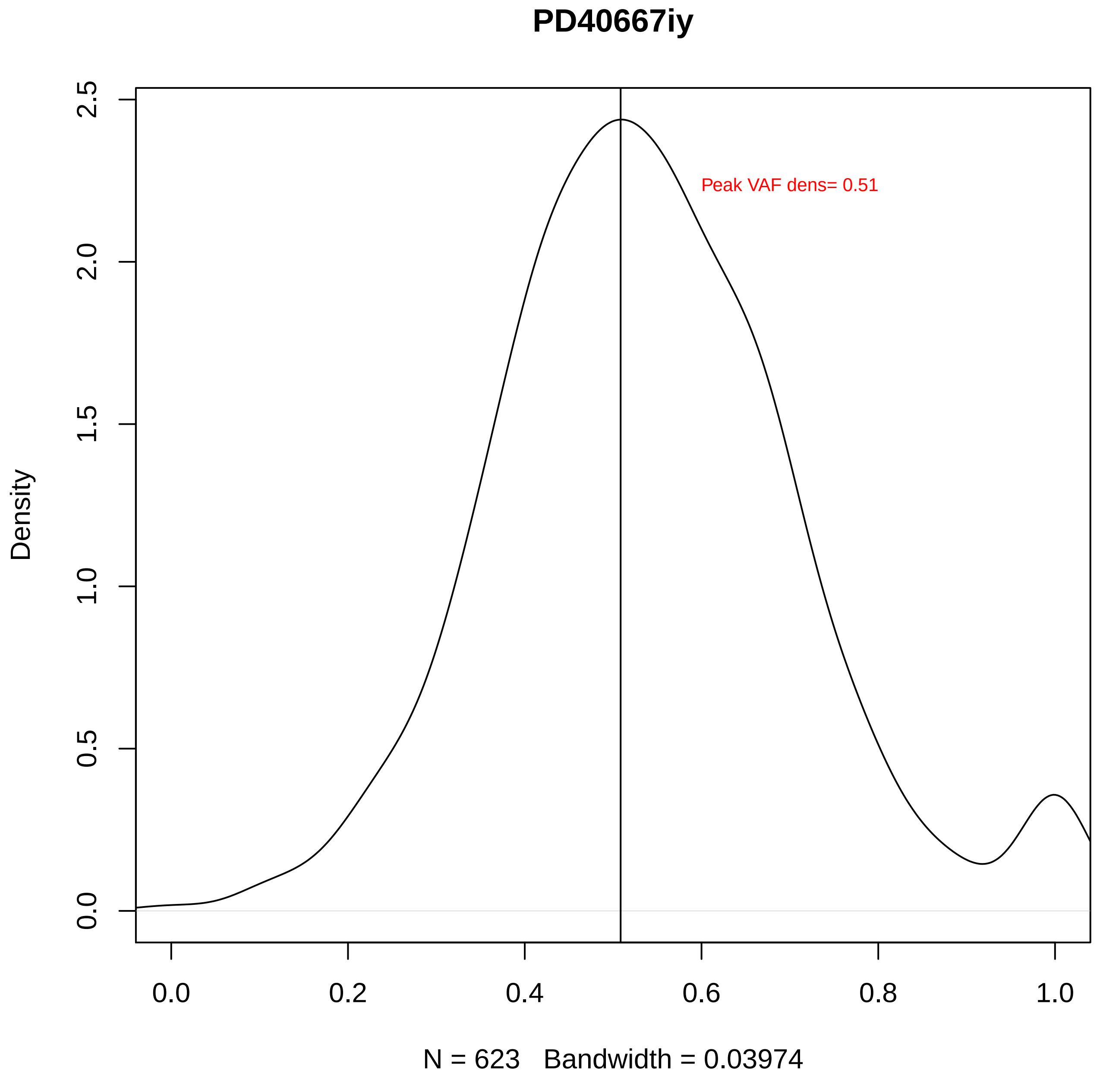

PD40667iy
Peak VAF dens= 0.51
Density
2.5
2.0
1.5
1.0
0.5
0.0
0.0
0.2
0.4
0.6
0.8
1.0
N = 623 Bandwidth = 0.03974

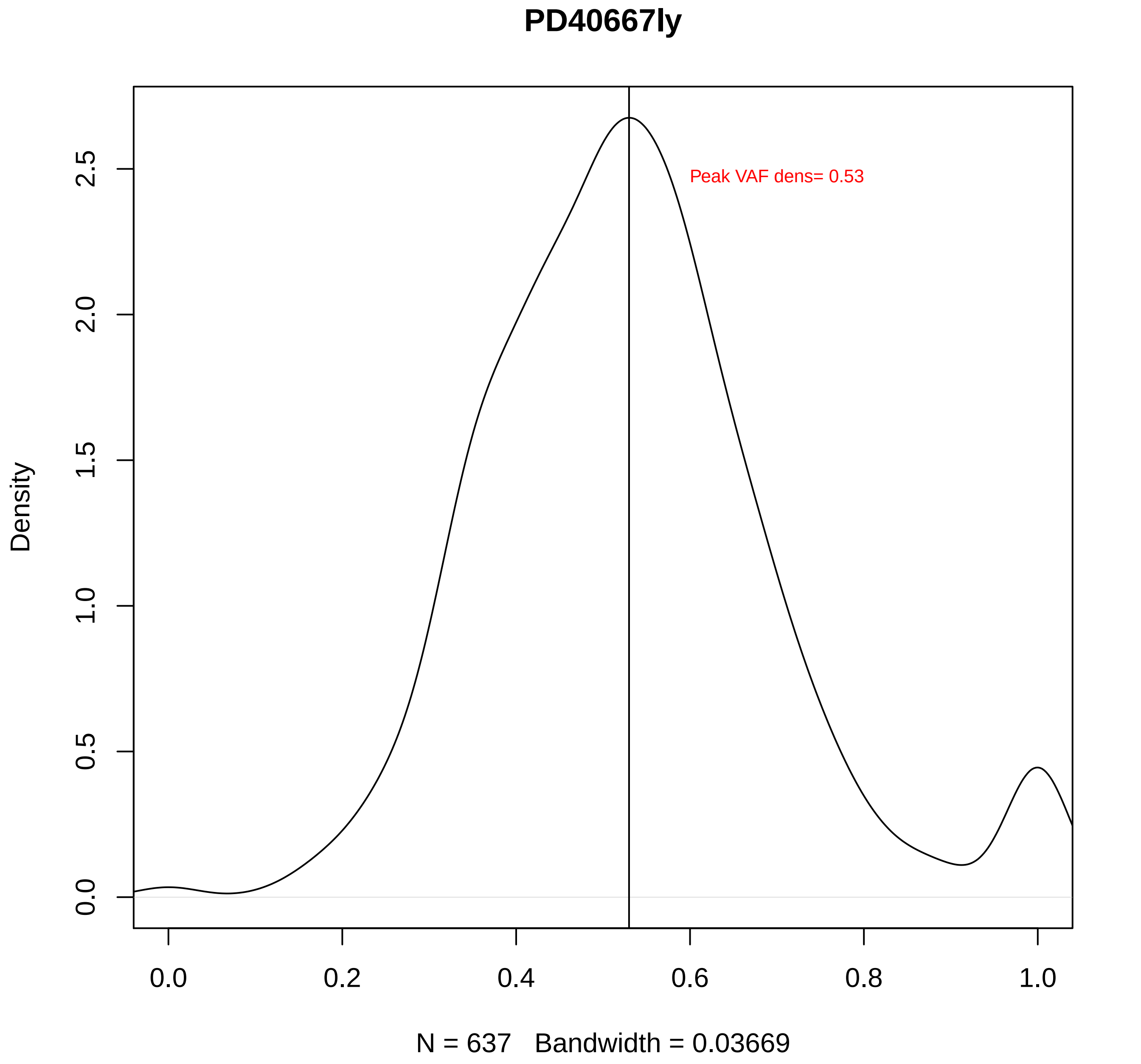
PD40667ly
Peak VAF dens= 0.53
Density
0.0
0.5
1.0
1.5
2.0
2.5
0.0
0.2
0.4
0.6
0.8
1.0
N = 637   Bandwidth = 0.03669

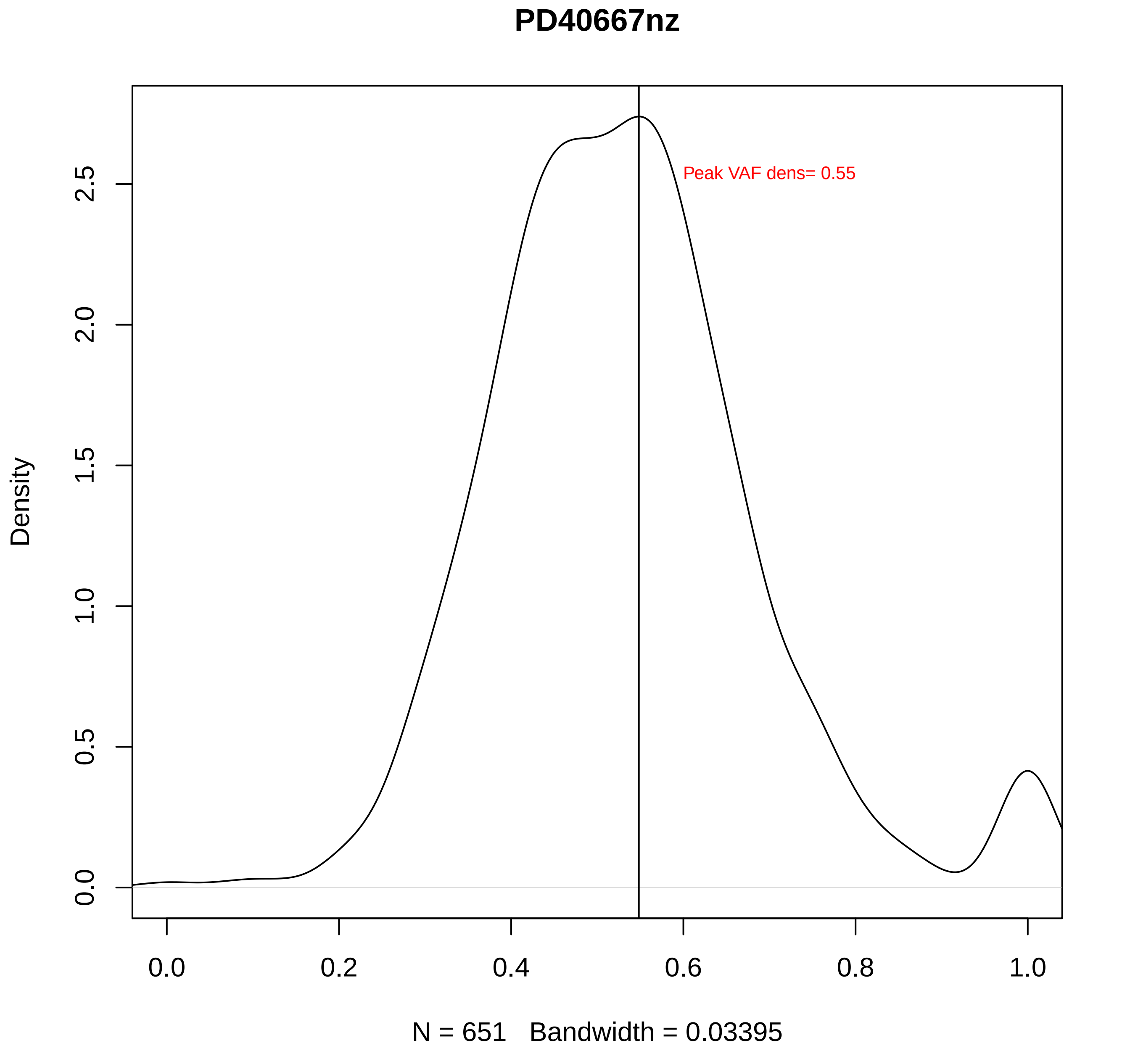
PD40667nz
Peak VAF dens= 0.55
Density
0.0
0.5
1.0
1.5
2.0
2.5
0.0
0.2
0.4
0.6
0.8
1.0
N = 651   Bandwidth = 0.03395

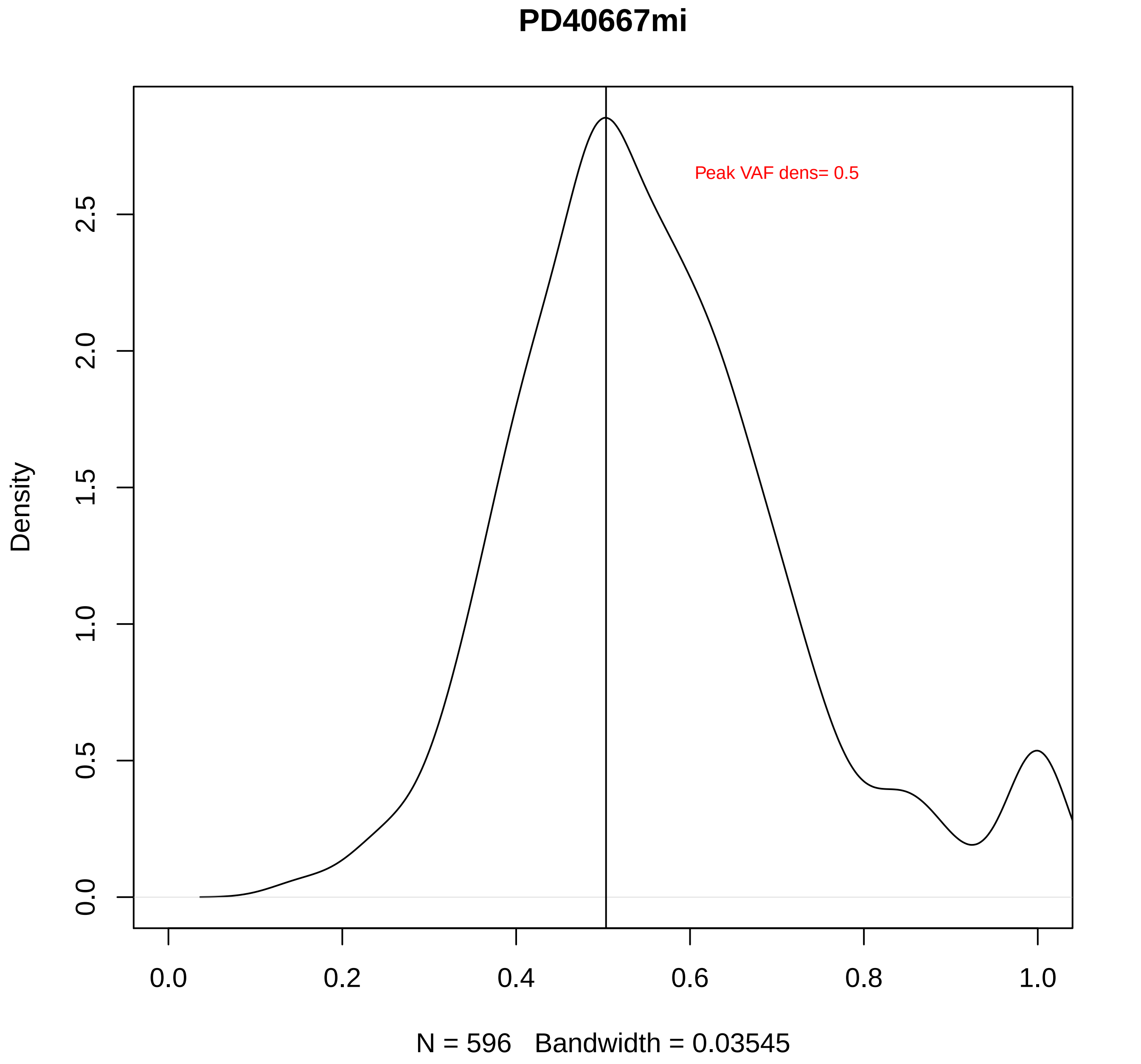

PD40667mi
Peak VAF dens= 0.5
Density
0.0
0.5
1.0
1.5
2.0
2.5
0.0
0.2
0.4
0.6
0.8
1.0
N = 596 Bandwidth = 0.03545

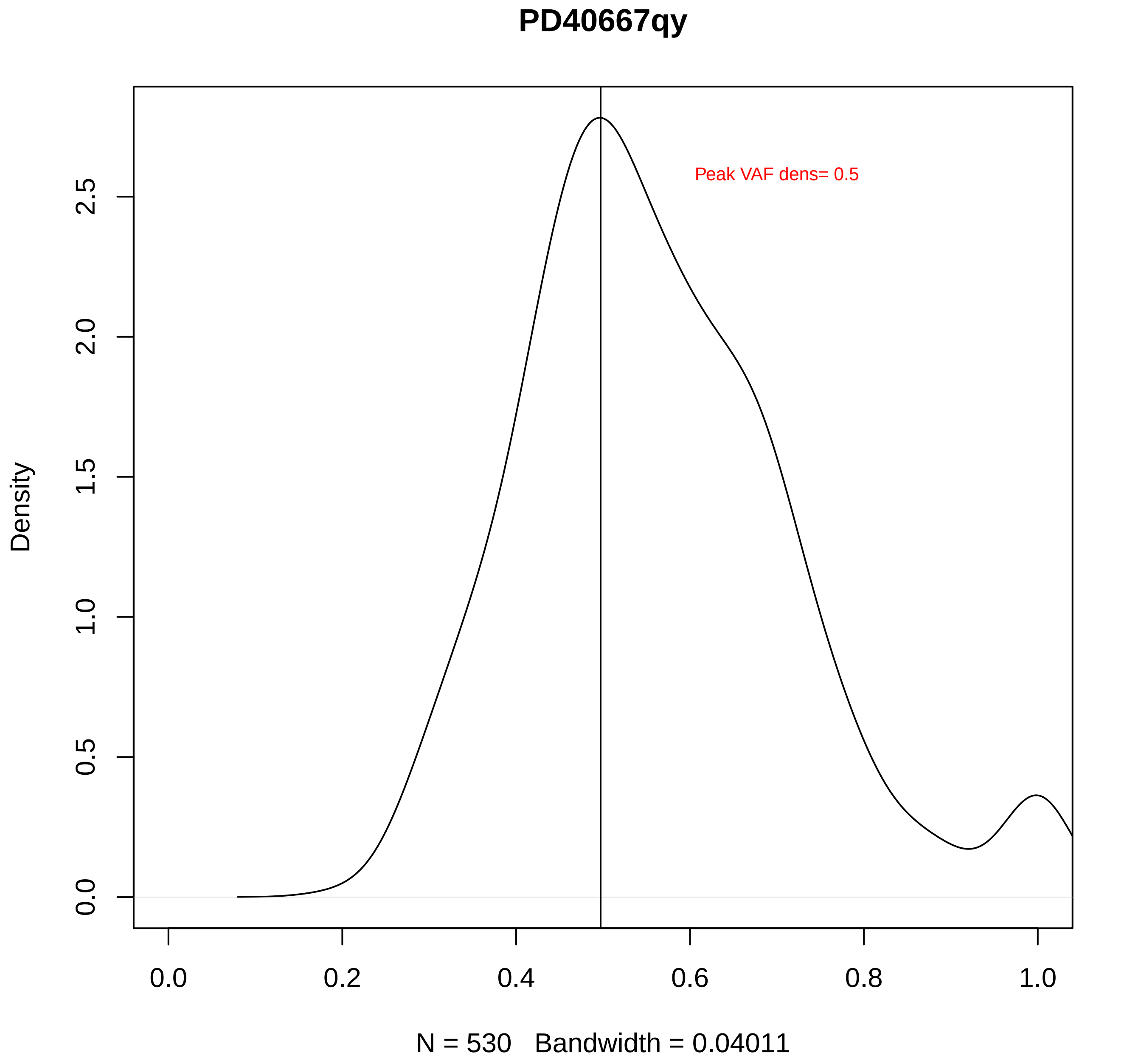
PD40667qy
Peak VAF dens= 0.5
Density
0.0
0.5
1.0
1.5
2.0
2.5
0.0
0.2
0.4
0.6
0.8
1.0
N = 530   Bandwidth = 0.04011

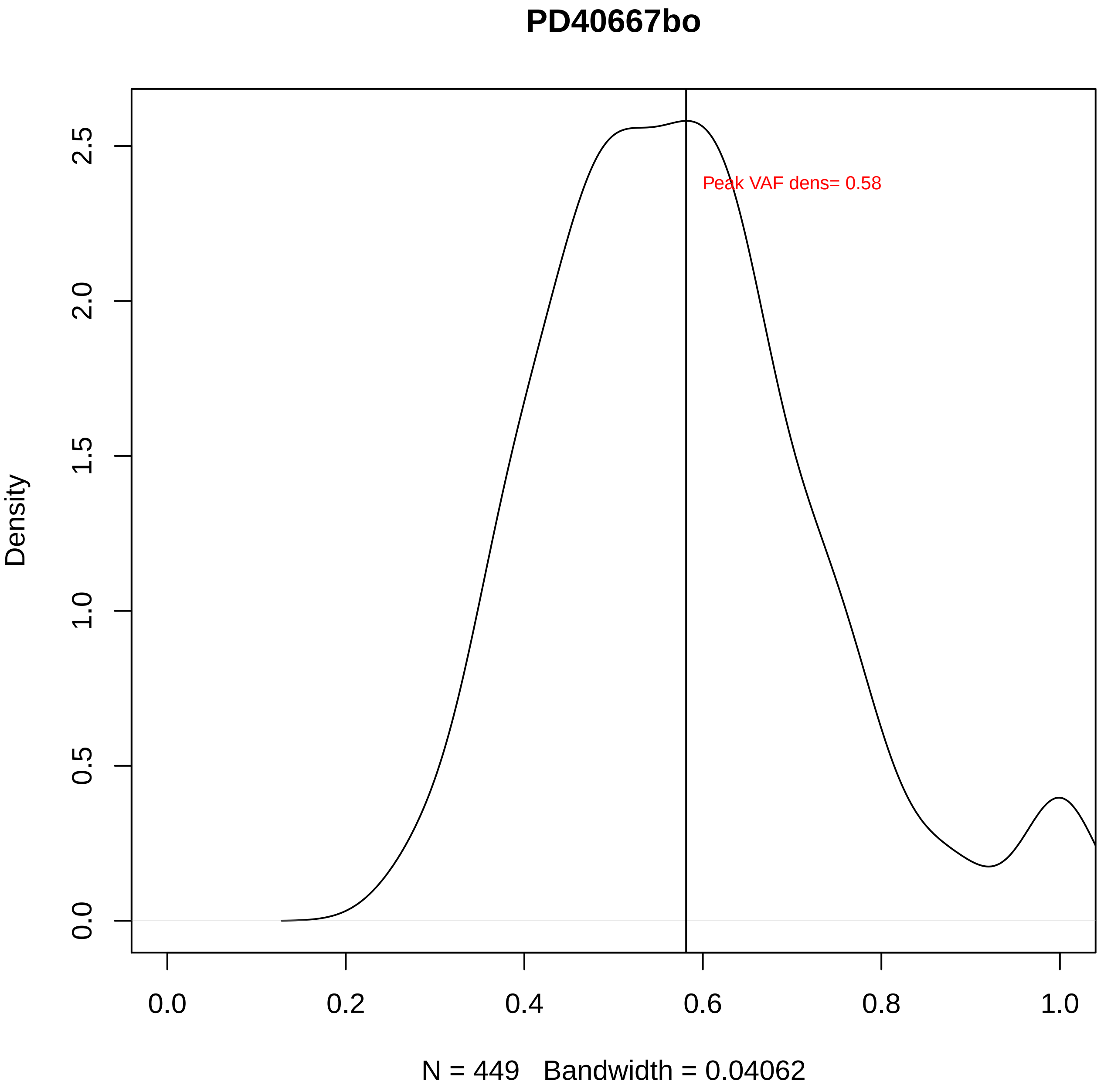

PD40667bo
Density
Peak VAF dens= 0.58
0.0
0.5
1.0
1.5
2.0
2.5
0.0
0.2
0.4
0.6
0.8
1.0
N = 449   Bandwidth = 0.04062

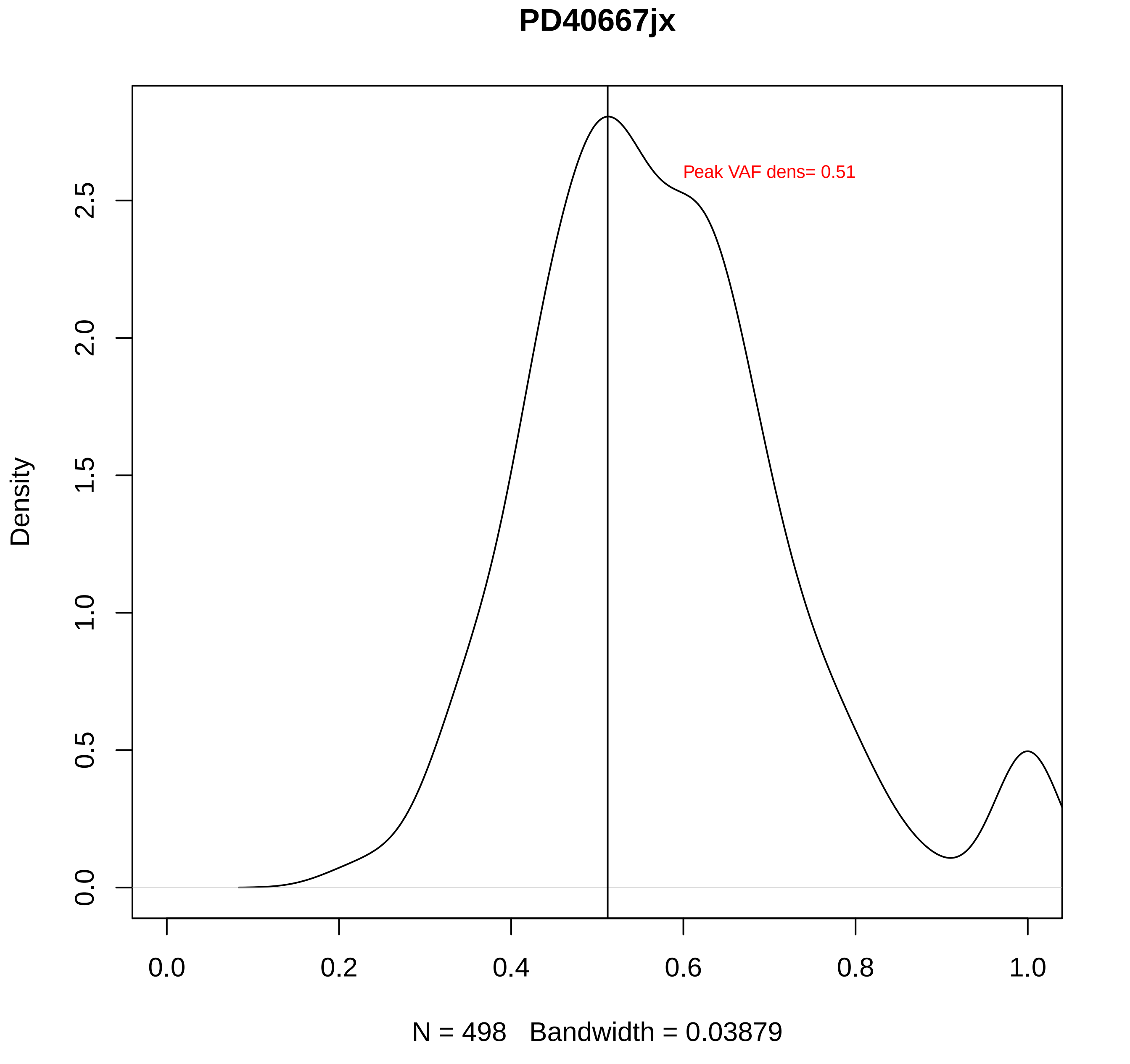
PD40667jx
Peak VAF dens= 0.51
Density
0.0
0.5
1.0
1.5
2.0
2.5
0.0
0.2
0.4
0.6
0.8
1.0
N = 498   Bandwidth = 0.03879

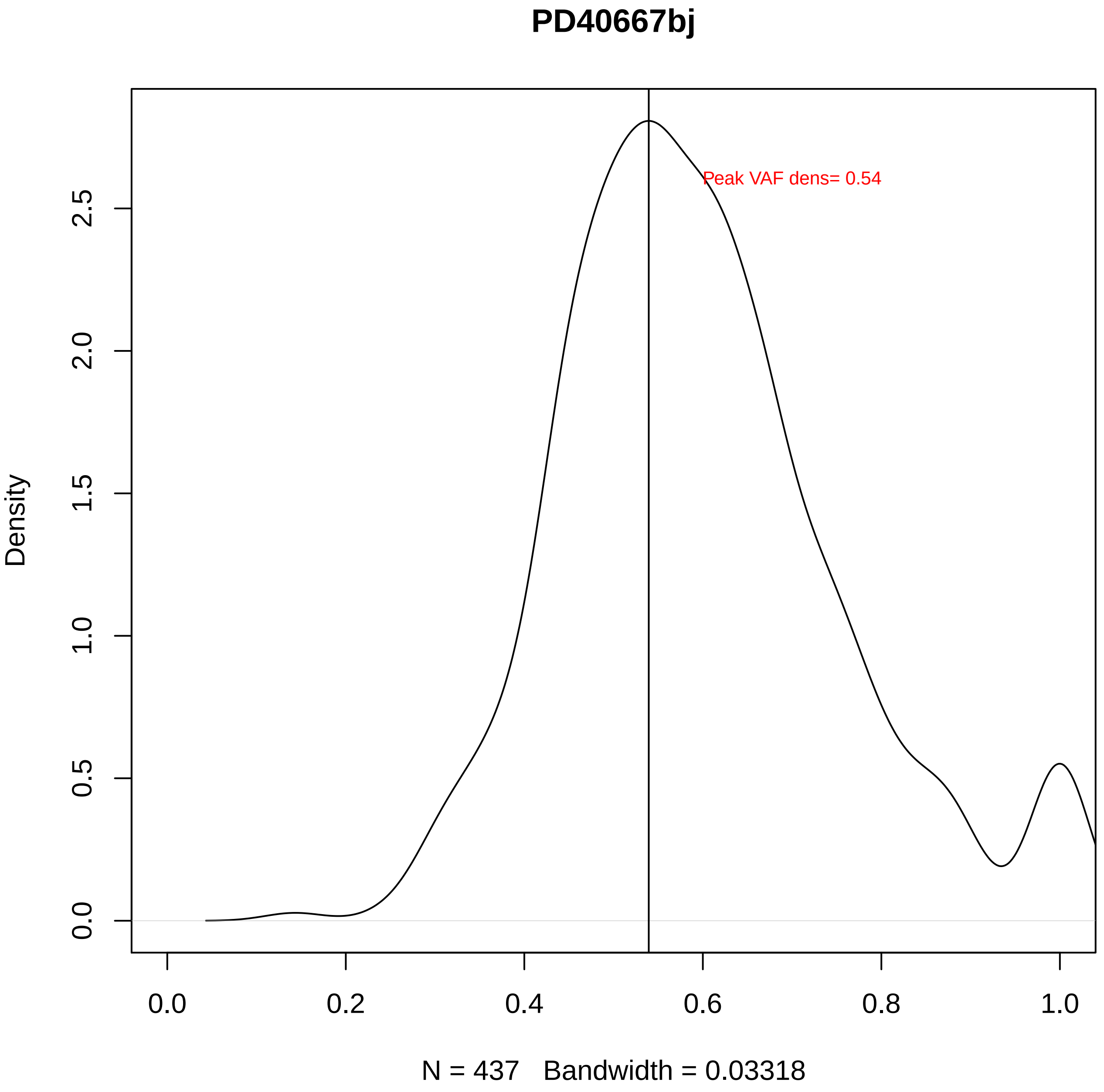
PD40667bj
Peak VAF dens= 0.54
Density
0.0
0.5
1.0
1.5
2.0
2.5
0.0
0.2
0.4
0.6
0.8
1.0
N = 437 Bandwidth = 0.03318

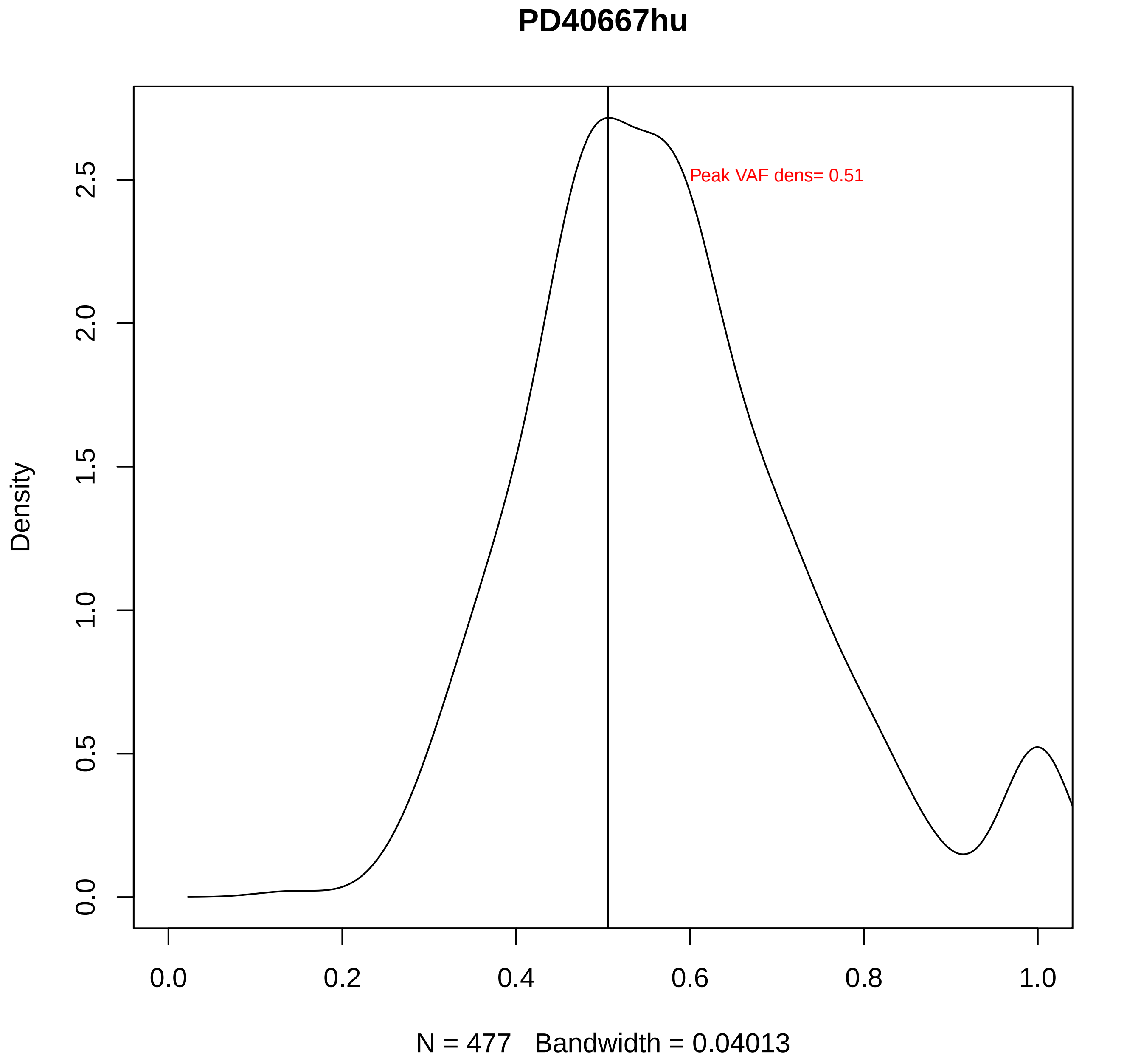
PD40667hu
Peak VAF dens= 0.51
Density
0.0
0.5
1.0
1.5
2.0
2.5
0.0
0.2
0.4
0.6
0.8
1.0
N = 477   Bandwidth = 0.04013

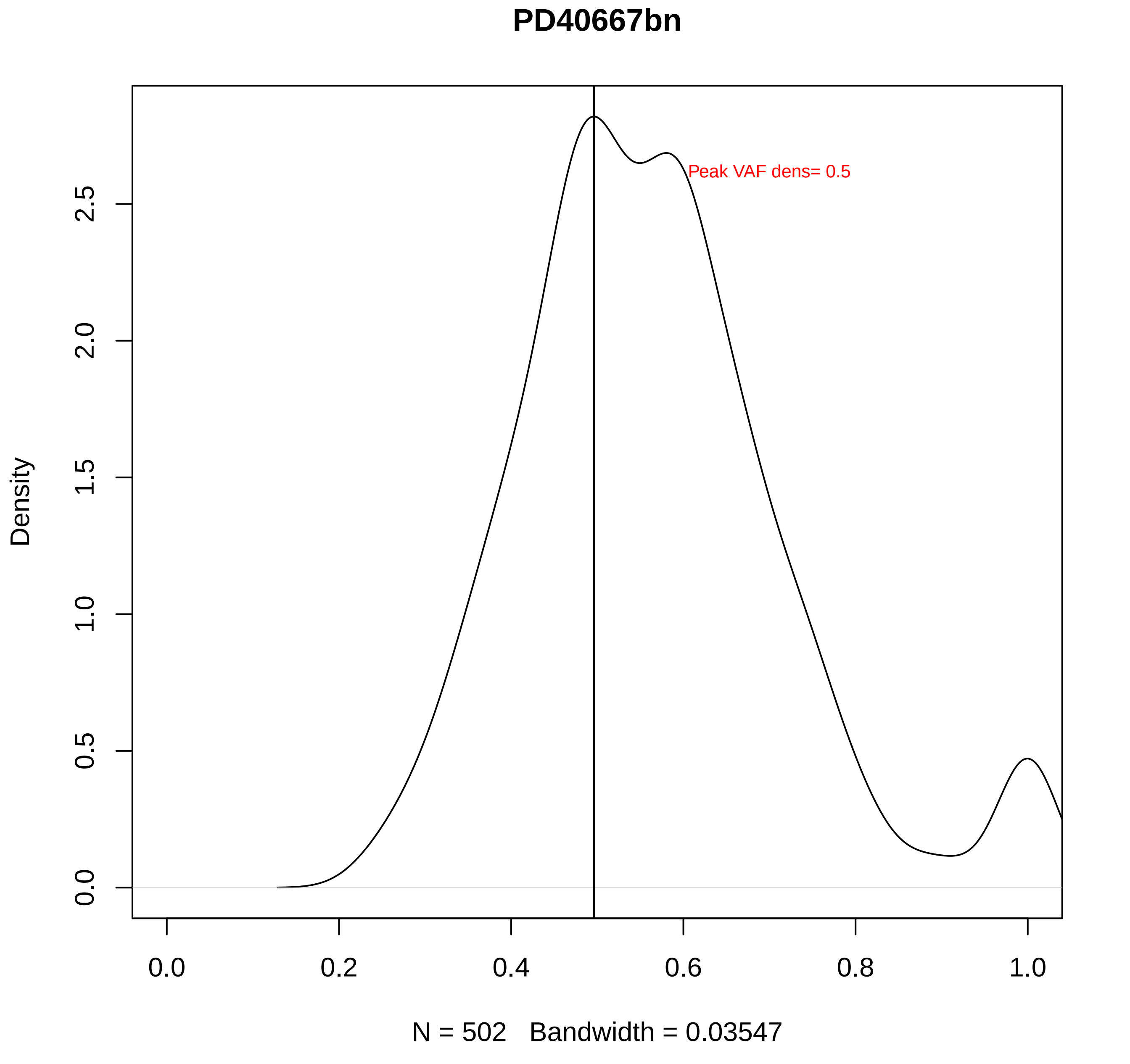

PD40667bn
Peak VAF dens= 0.5
Density
0.0
0.5
1.0
1.5
2.0
2.5
0.0
0.2
0.4
0.6
0.8
1.0
N = 502   Bandwidth = 0.03547

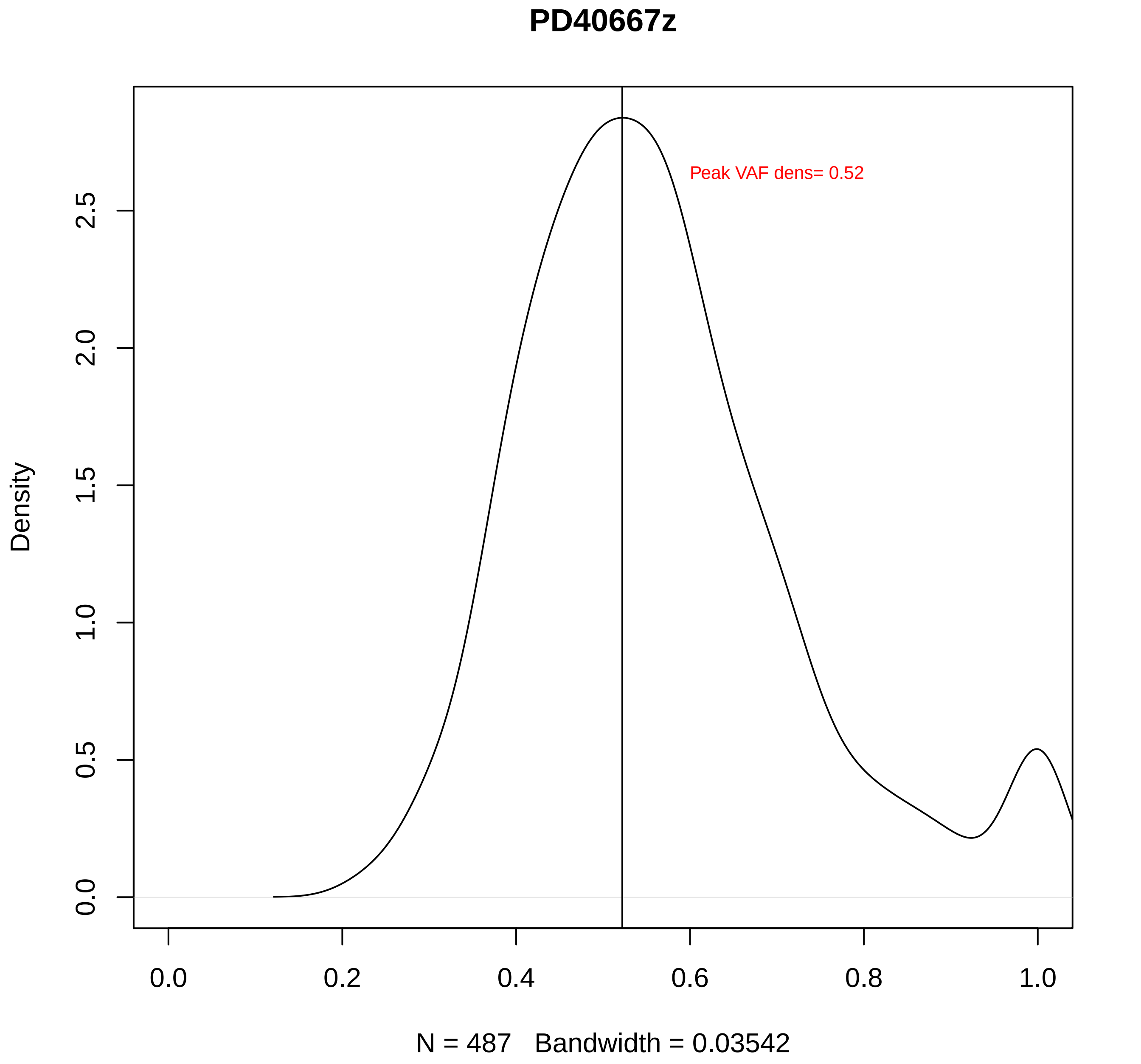
PD40667z
Peak VAF dens= 0.52
Density
0.0
0.5
1.0
1.5
2.0
2.5
0.0
0.2
0.4
0.6
0.8
1.0
N = 487   Bandwidth = 0.03542

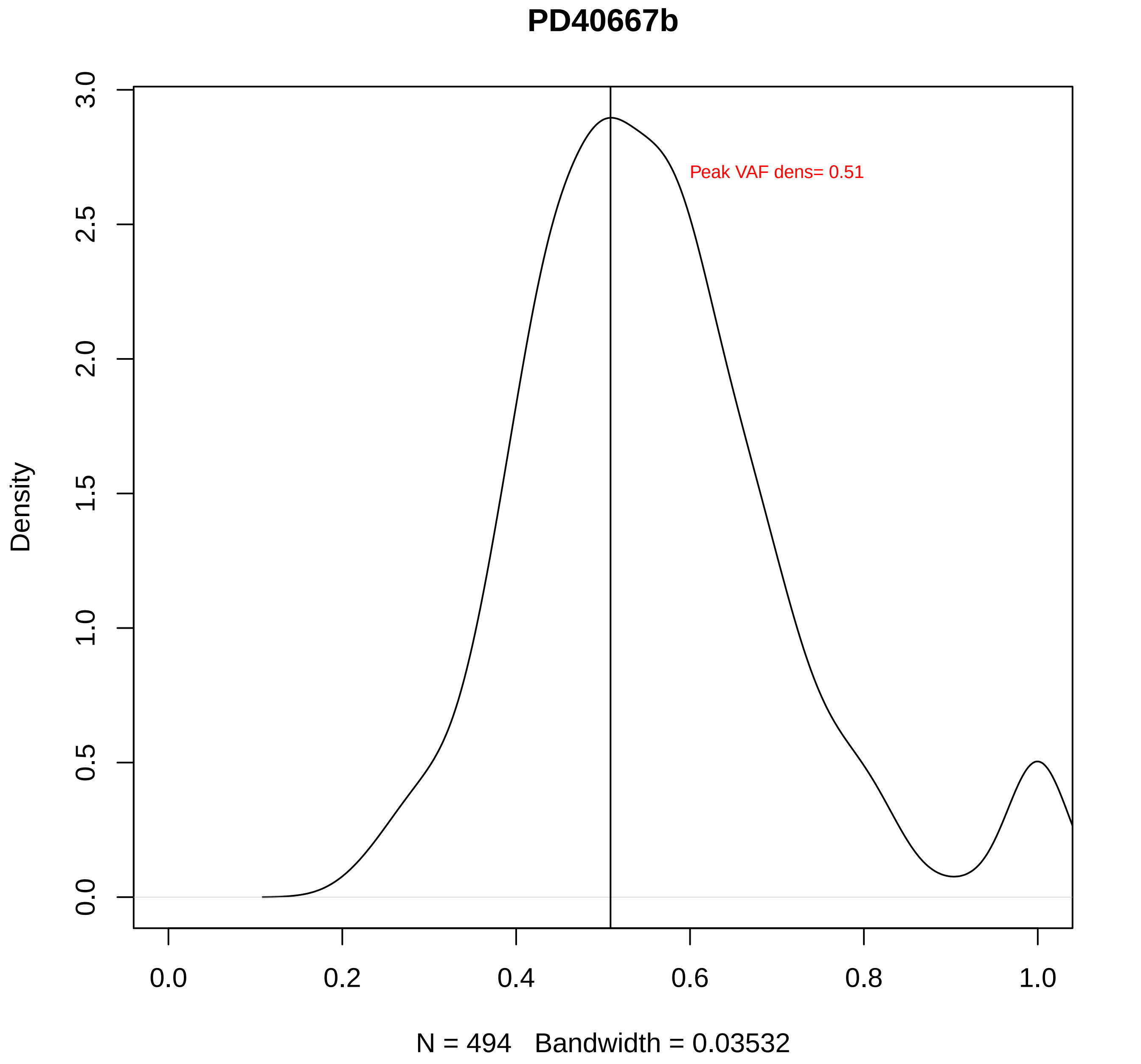
PD40667b
Peak VAF dens= 0.51
Density
3.0
2.5
2.0
1.5
1.0
0.5
0.0
0.0
0.2
0.4
0.6
0.8
1.0
N = 494   Bandwidth = 0.03532

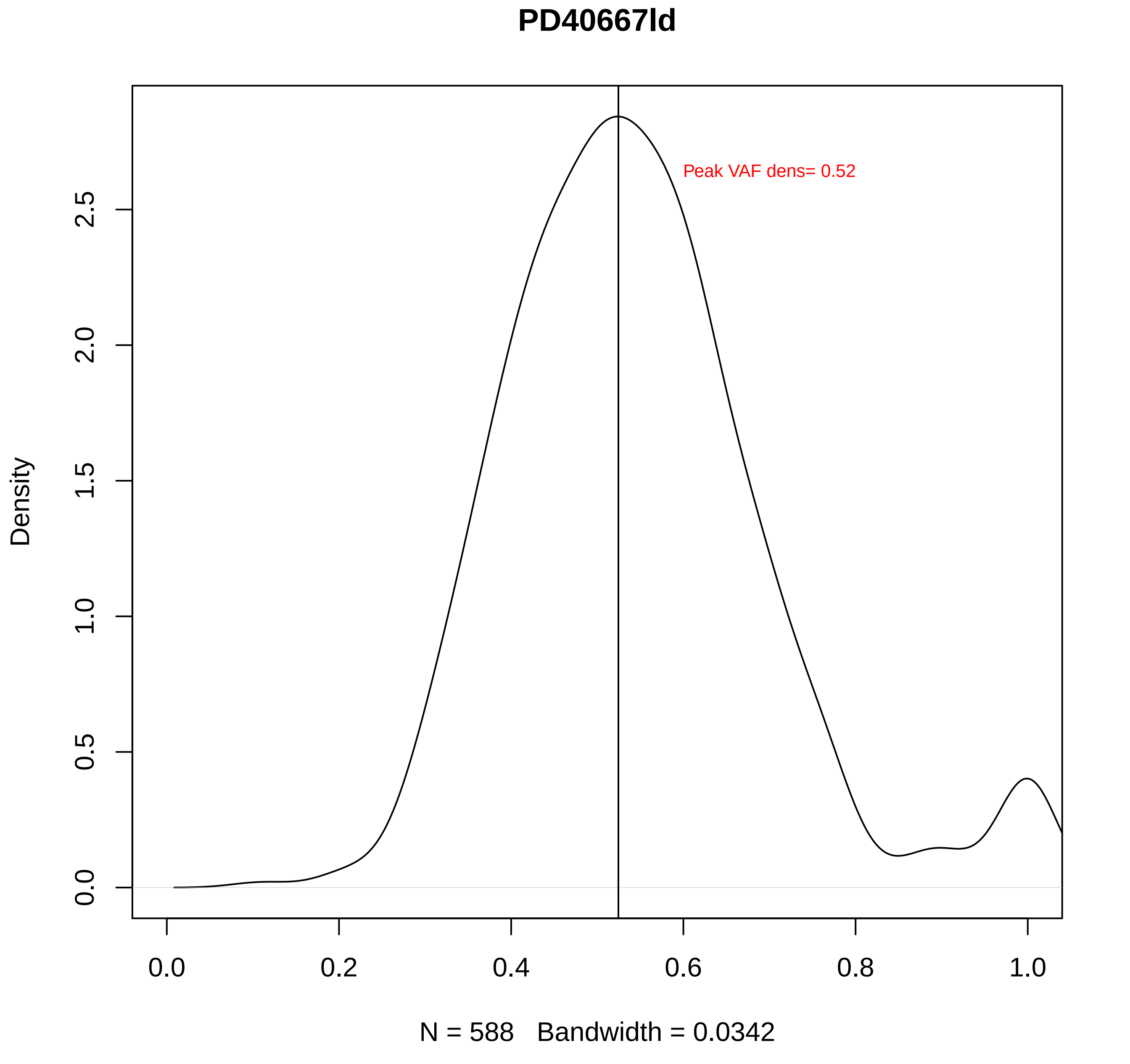

PD40667ld
Peak VAF dens= 0.52
Density
0.0
0.5
1.0
1.5
2.0
2.5
0.0
0.2
0.4
0.6
0.8
1.0
N = 588   Bandwidth = 0.0342

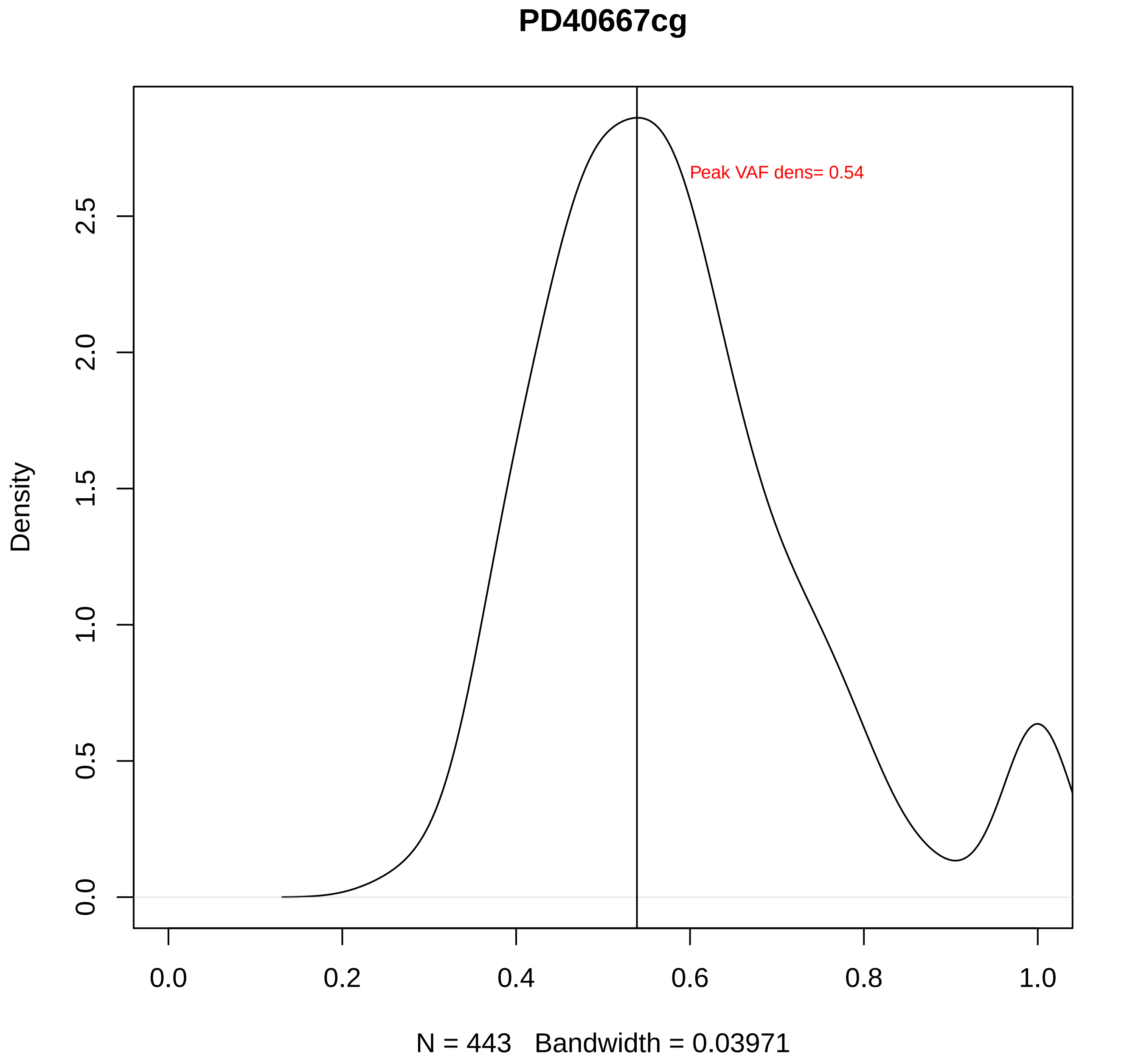

PD40667cg
Peak VAF dens= 0.54
Density
0.0
0.5
1.0
1.5
2.0
2.5
0.0
0.2
0.4
0.6
0.8
1.0
N = 443   Bandwidth = 0.03971

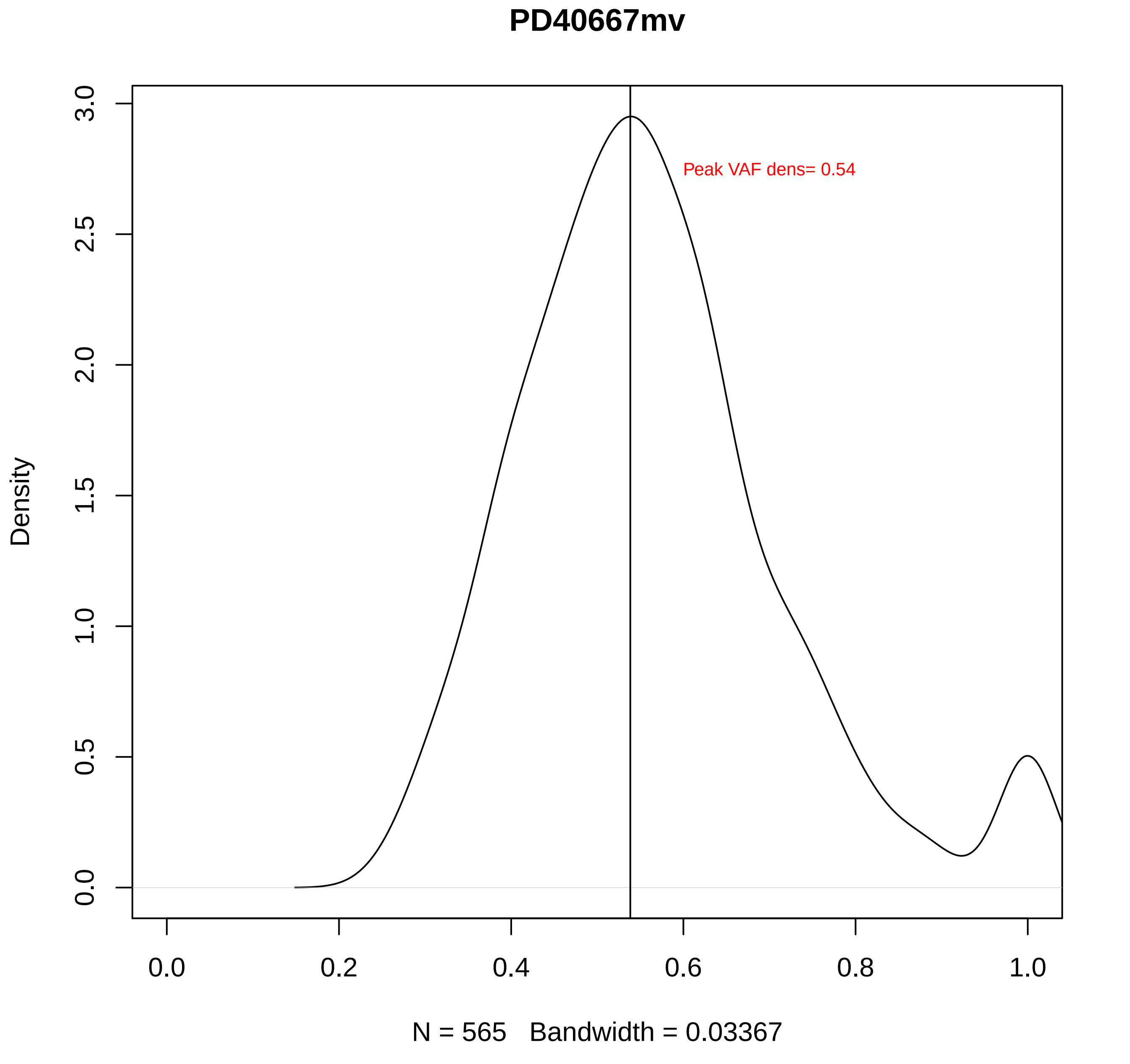

PD40667mv
Peak VAF dens= 0.54
Density
0.0
0.5
1.0
1.5
2.0
2.5
3.0
0.0
0.2
0.4
0.6
0.8
1.0
N = 565 Bandwidth = 0.03367

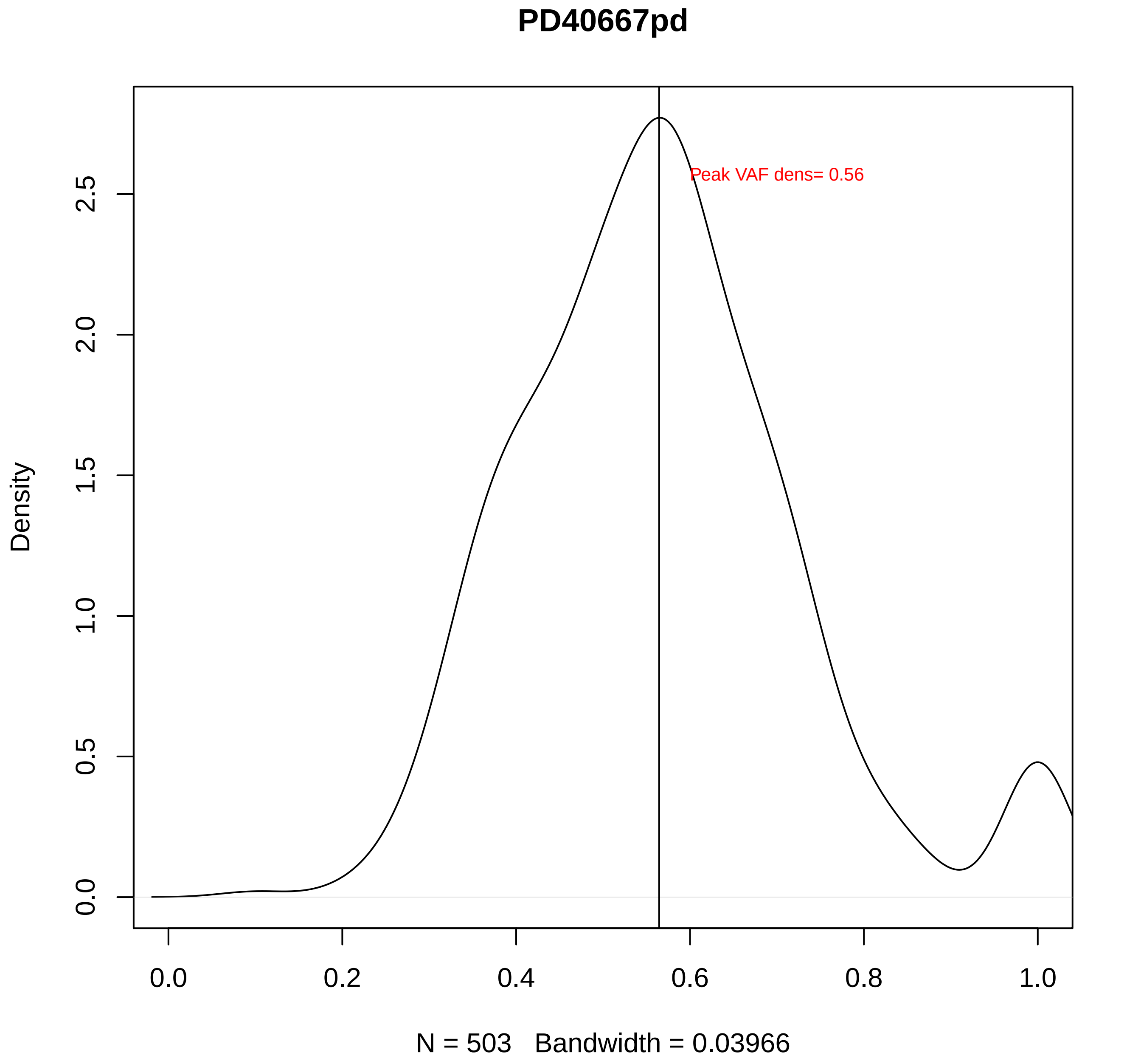
PD40667pd
Peak VAF dens= 0.56
Density
0.0
0.5
1.0
1.5
2.0
2.5
0.0
0.2
0.4
0.6
0.8
1.0
N = 503 Bandwidth = 0.03966

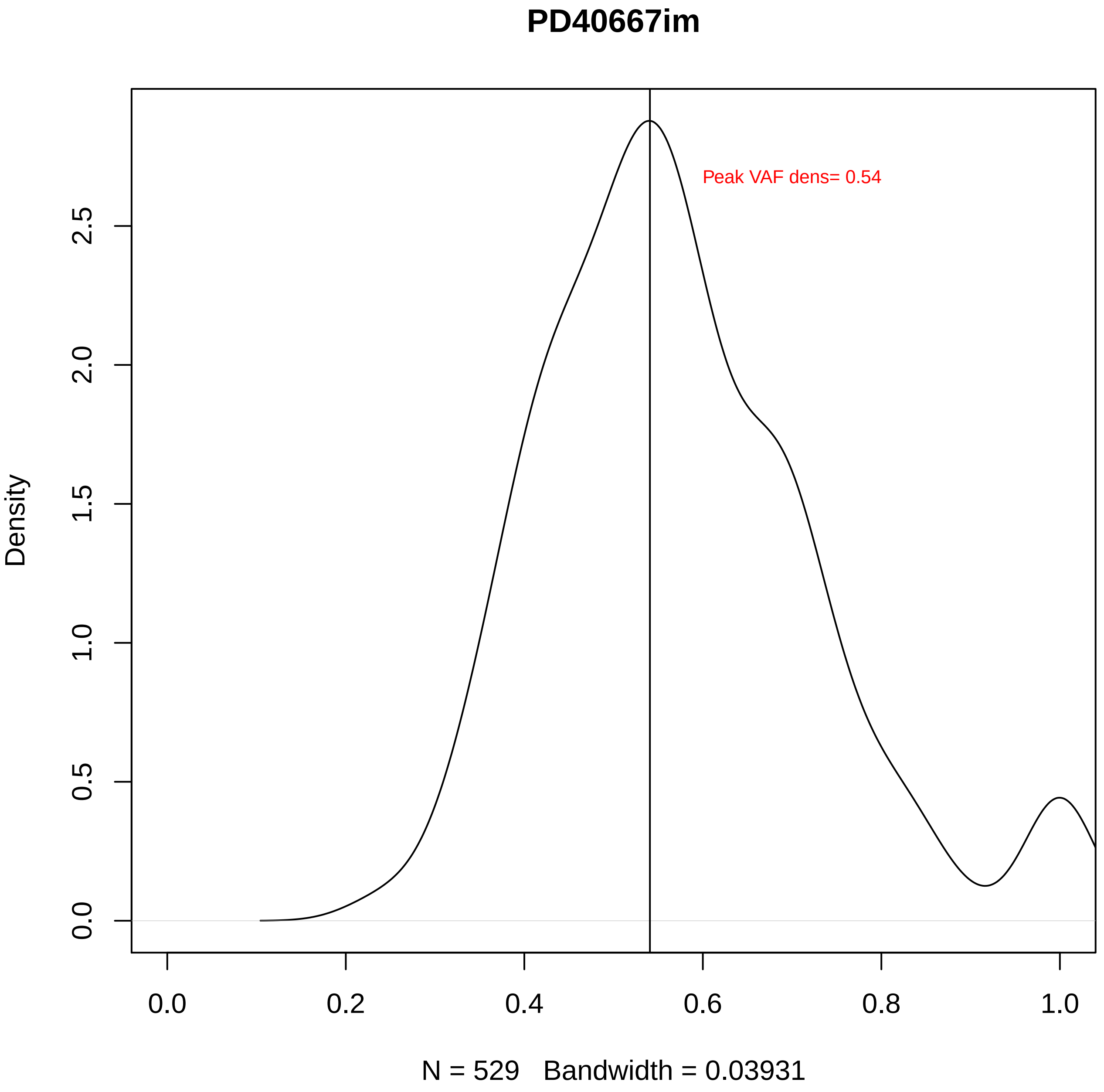

PD40667im
Peak VAF dens= 0.54
Density
0.0
0.5
1.0
1.5
2.0
2.5
0.0
0.2
0.4
0.6
0.8
1.0
N = 529 Bandwidth = 0.03931

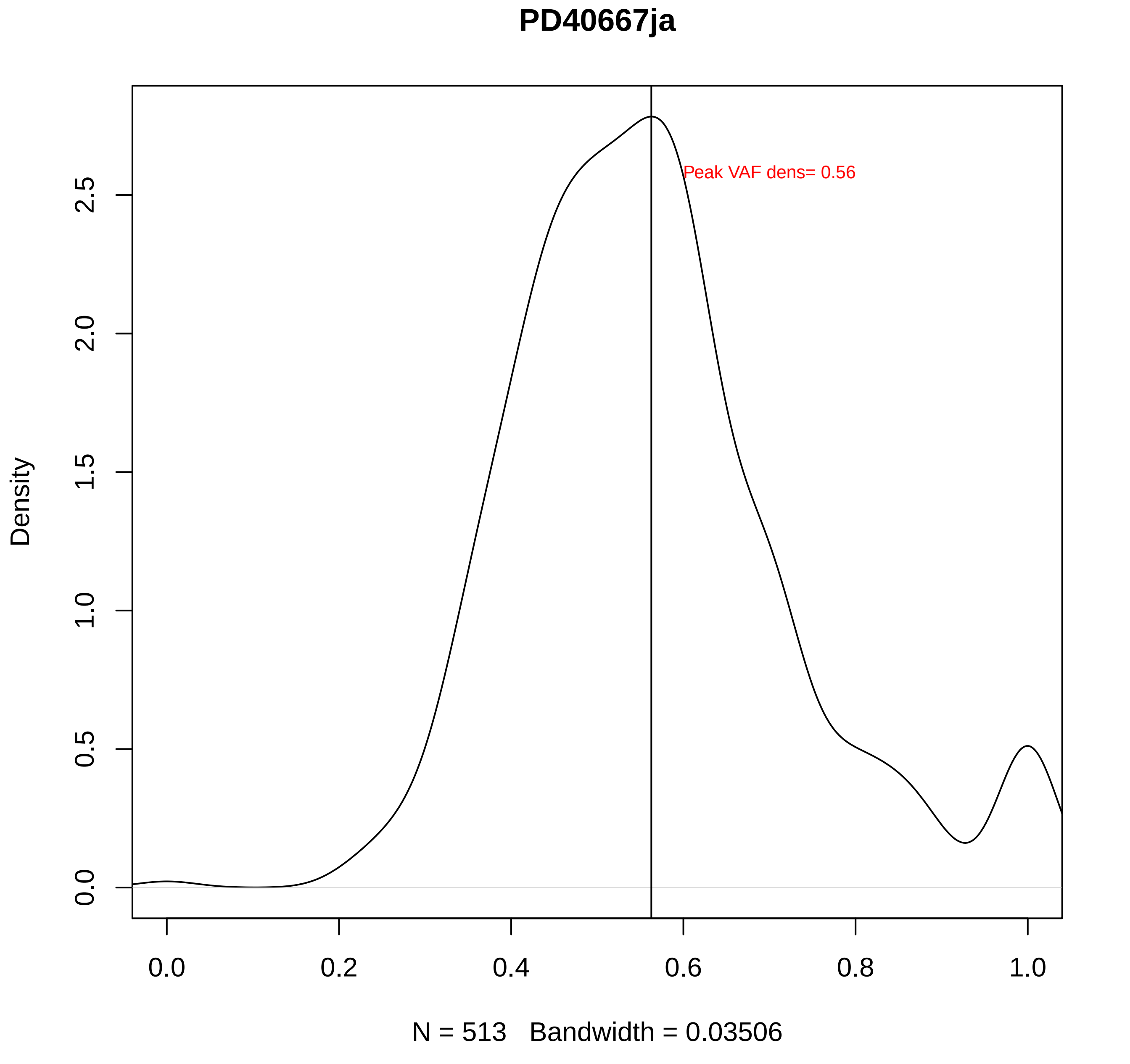
PD40667ja
Peak VAF dens= 0.56
Density
0.0
0.5
1.0
1.5
2.0
2.5
0.0
0.2
0.4
0.6
0.8
1.0
N = 513   Bandwidth = 0.03506

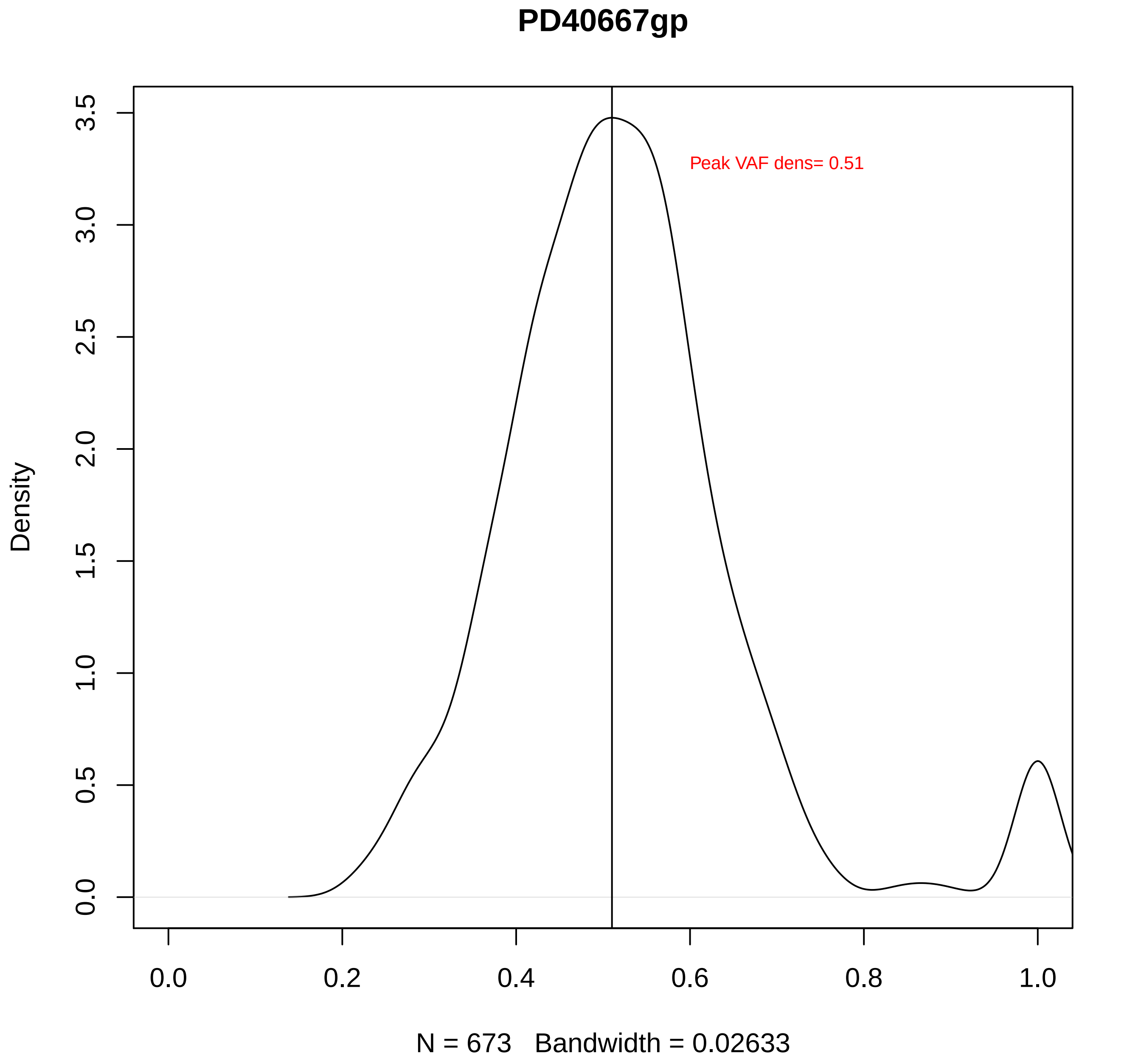

PD40667gp
Peak VAF dens= 0.51
Density
0.0
0.5
1.0
1.5
2.0
2.5
3.0
3.5
0.0
0.2
0.4
0.6
0.8
1.0
N = 673   Bandwidth = 0.02633

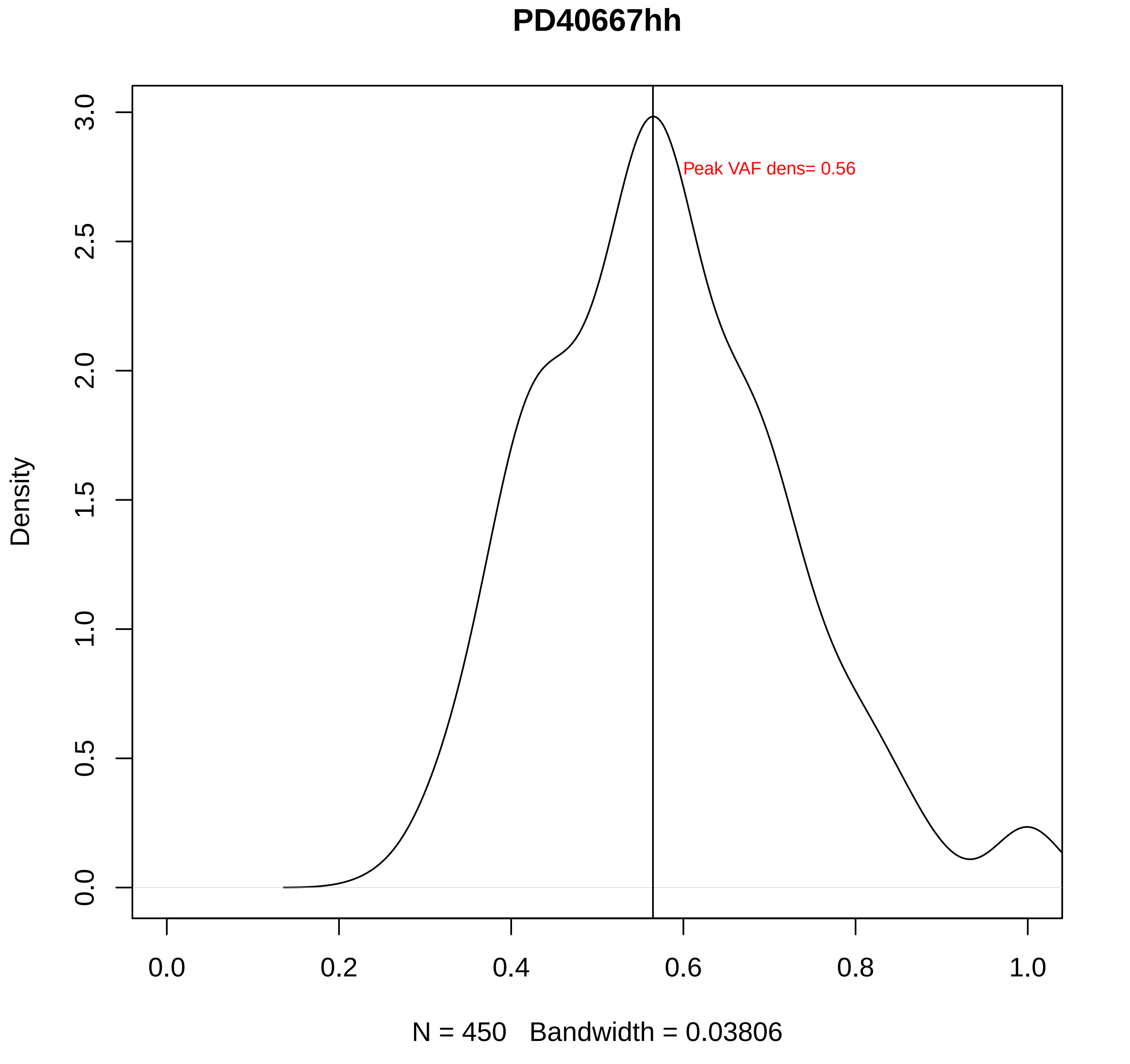
PD40667hh
Peak VAF dens= 0.56
Density
0.0
0.5
1.0
1.5
2.0
2.5
3.0
0.0
0.2
0.4
0.6
0.8
1.0
N = 450   Bandwidth = 0.03806

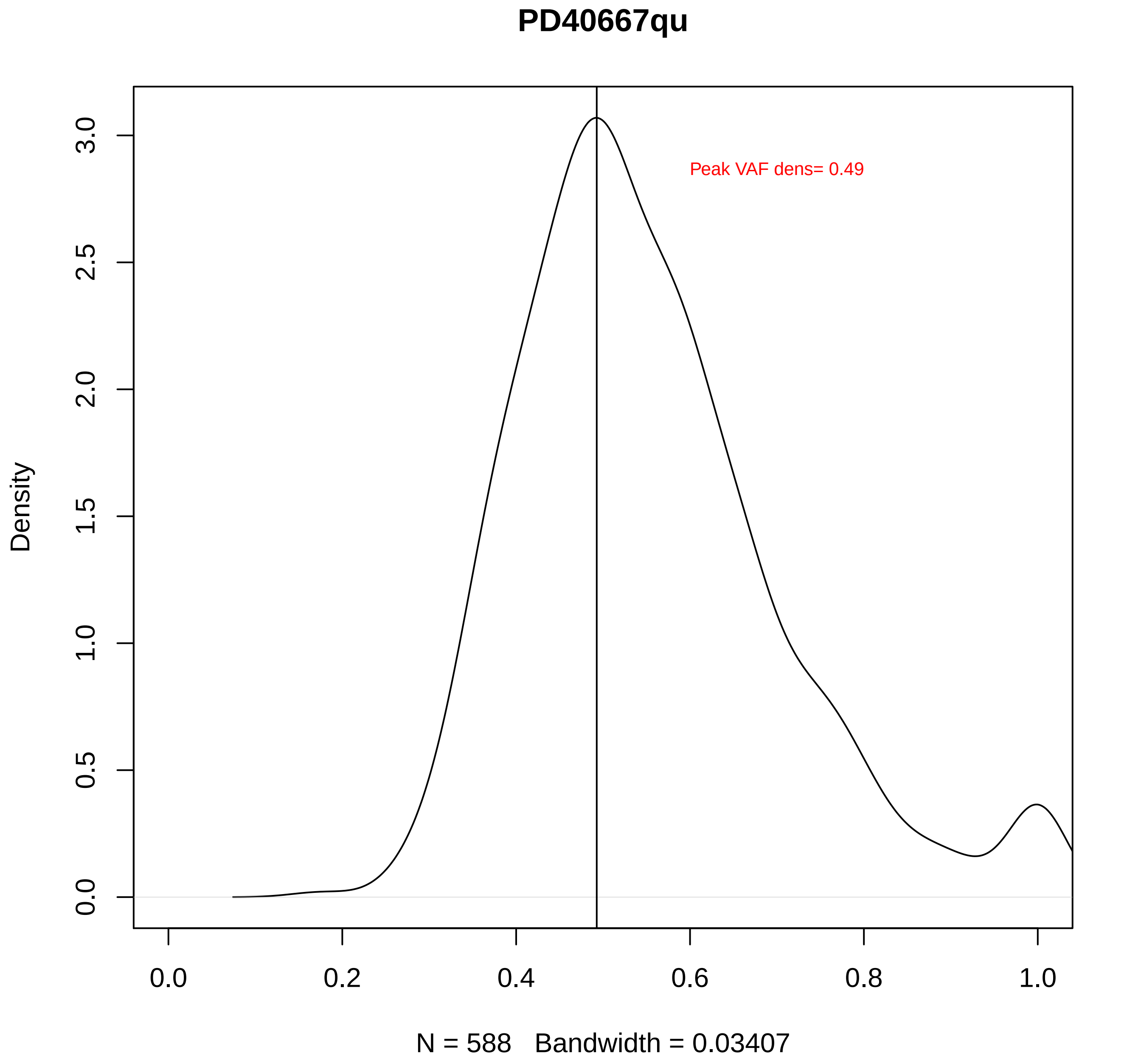
PD40667qu
Peak VAF dens= 0.49
Density
0.0
0.5
1.0
1.5
2.0
2.5
3.0
0.0
0.2
0.4
0.6
0.8
1.0
N = 588   Bandwidth = 0.03407

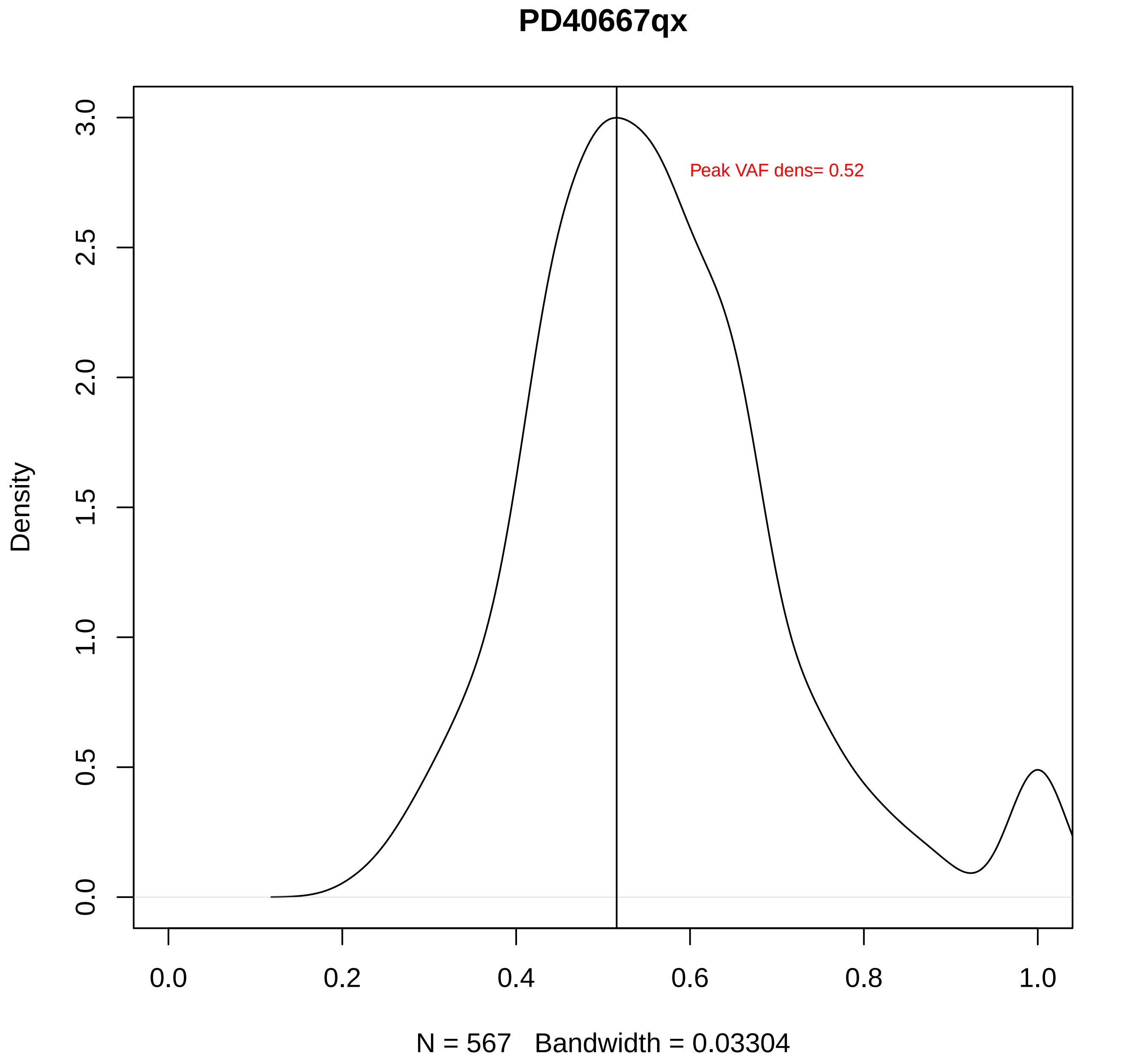
PD40667qx
Peak VAF dens= 0.52
Density
3.0
2.5
2.0
1.5
1.0
0.5
0.0
0.0
0.2
0.4
0.6
0.8
1.0
N = 567   Bandwidth = 0.03304

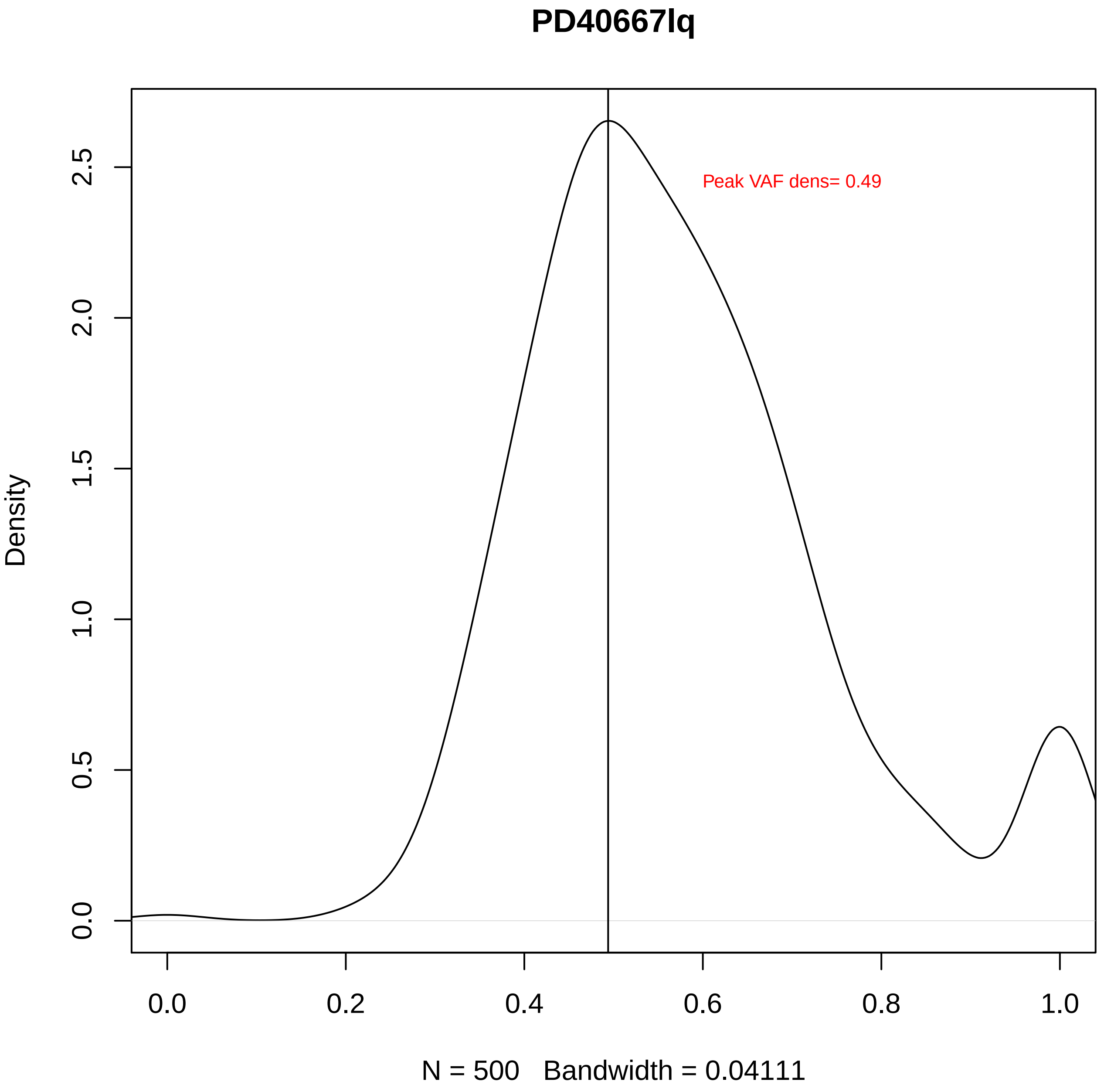
PD40667lq
Peak VAF dens= 0.49
Density
0.0
0.5
1.0
1.5
2.0
2.5
0.0
0.2
0.4
0.6
0.8
1.0
N = 500   Bandwidth = 0.04111

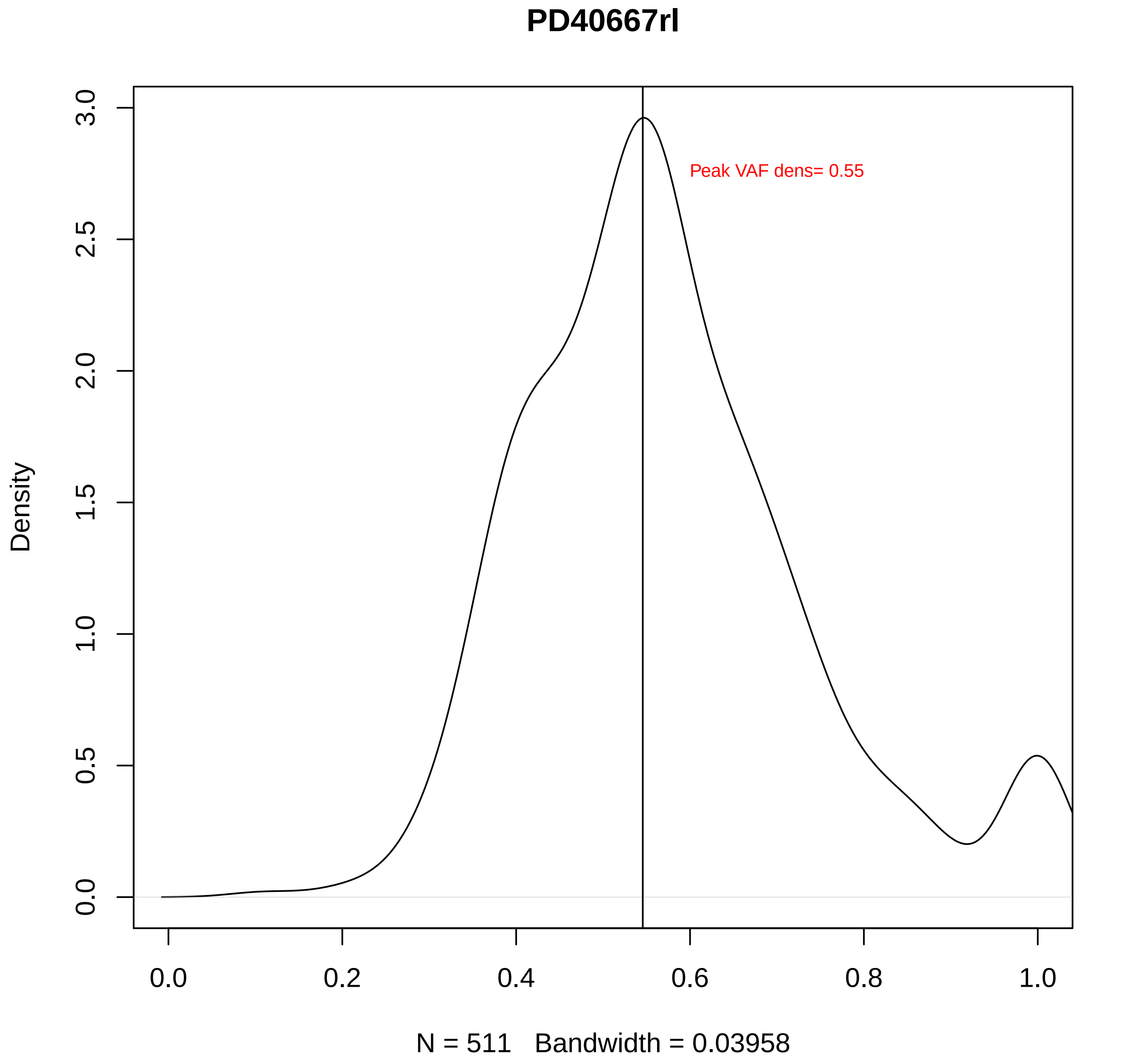
PD40667rl
Peak VAF dens= 0.55
Density
0.0
0.5
1.0
1.5
2.0
2.5
3.0
0.0
0.2
0.4
0.6
0.8
1.0
N = 511 Bandwidth = 0.03958

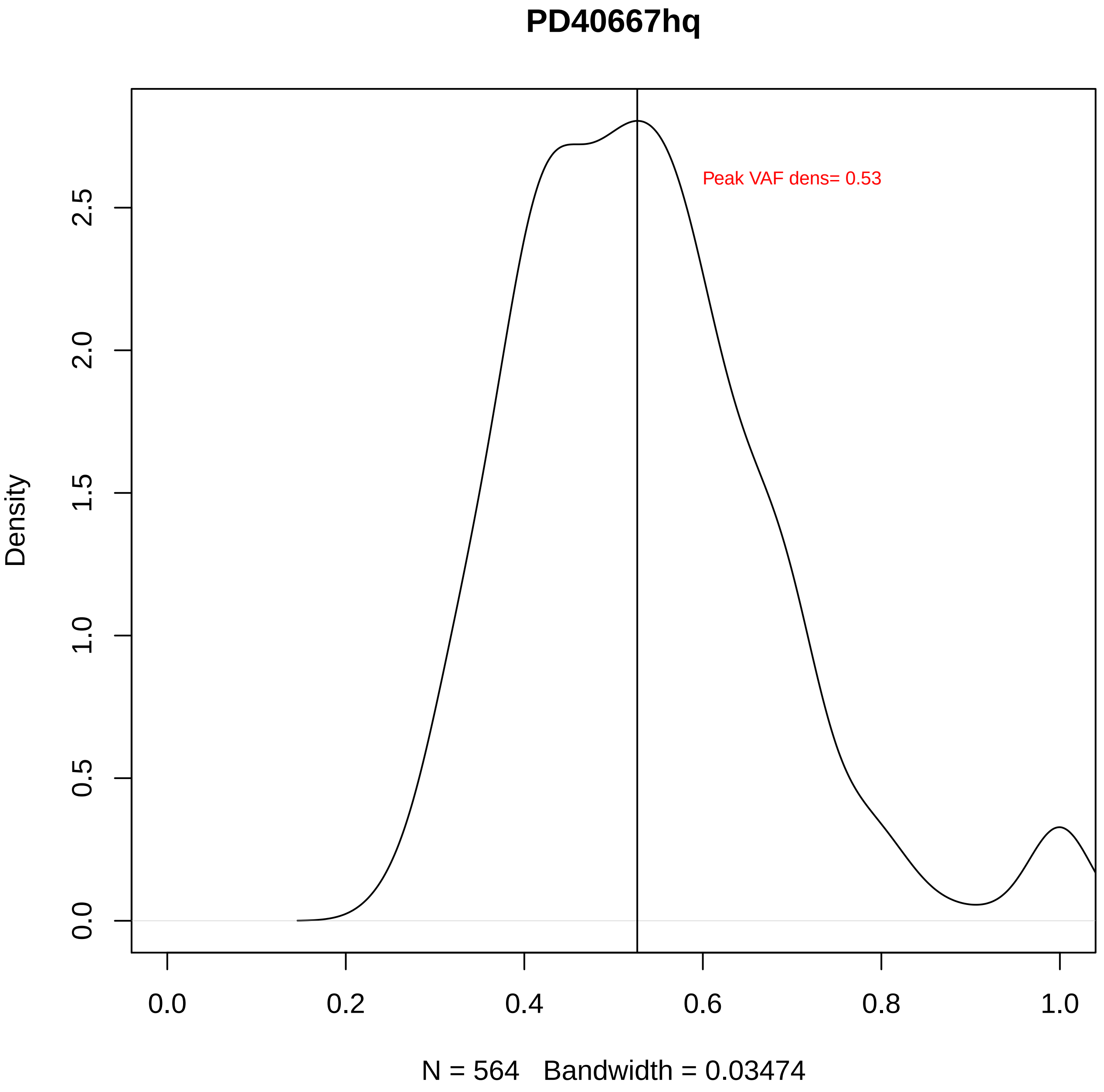
PD40667hq
Peak VAF dens= 0.53
Density
0.0
0.5
1.0
1.5
2.0
2.5
0.0
0.2
0.4
0.6
0.8
1.0
N = 564   Bandwidth = 0.03474

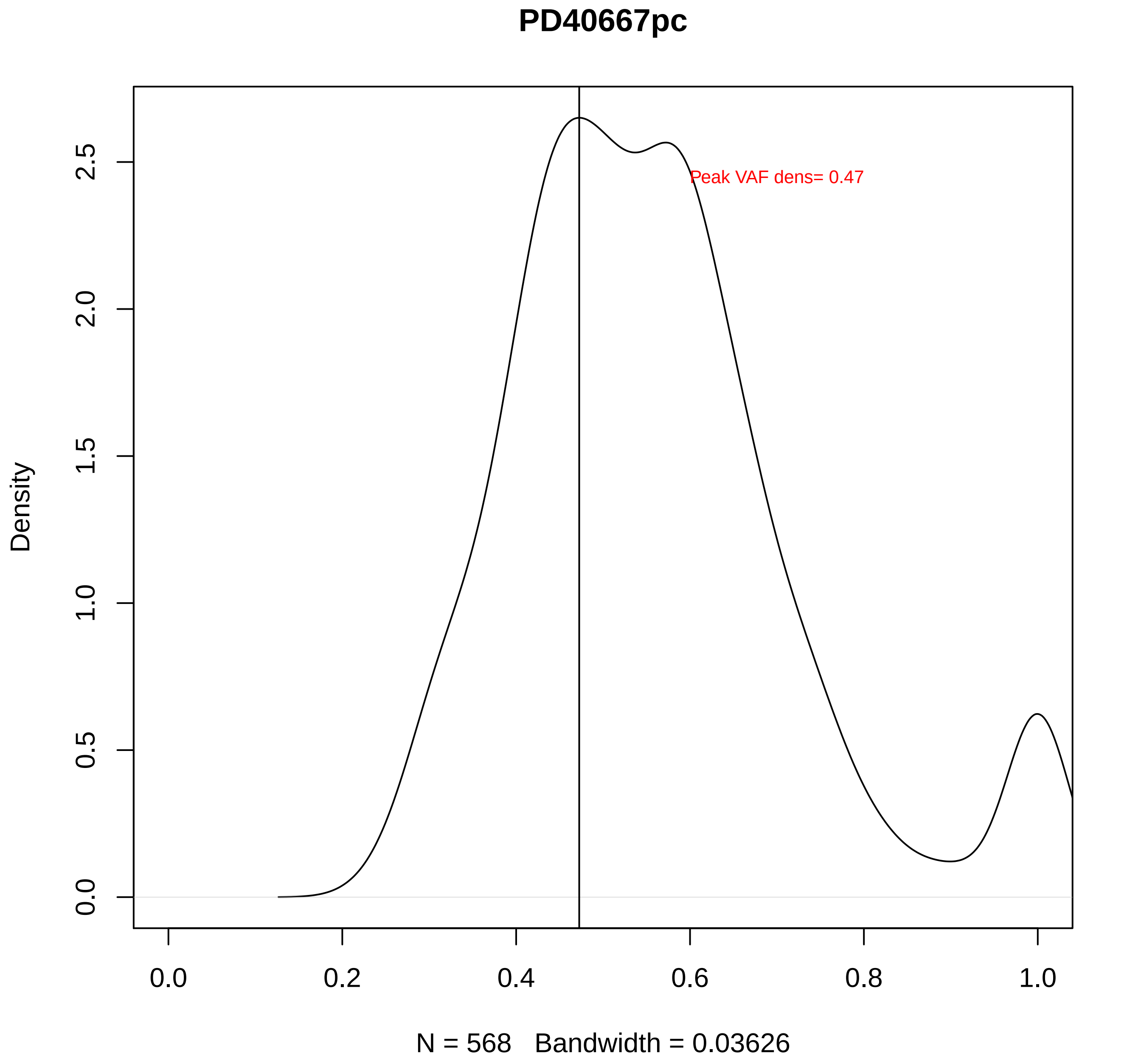
PD40667pc
Density
0.0
0.5
1.0
1.5
2.0
2.5
Peak VAF dens= 0.47
0.0
0.2
0.4
0.6
0.8
1.0
N = 568   Bandwidth = 0.03626

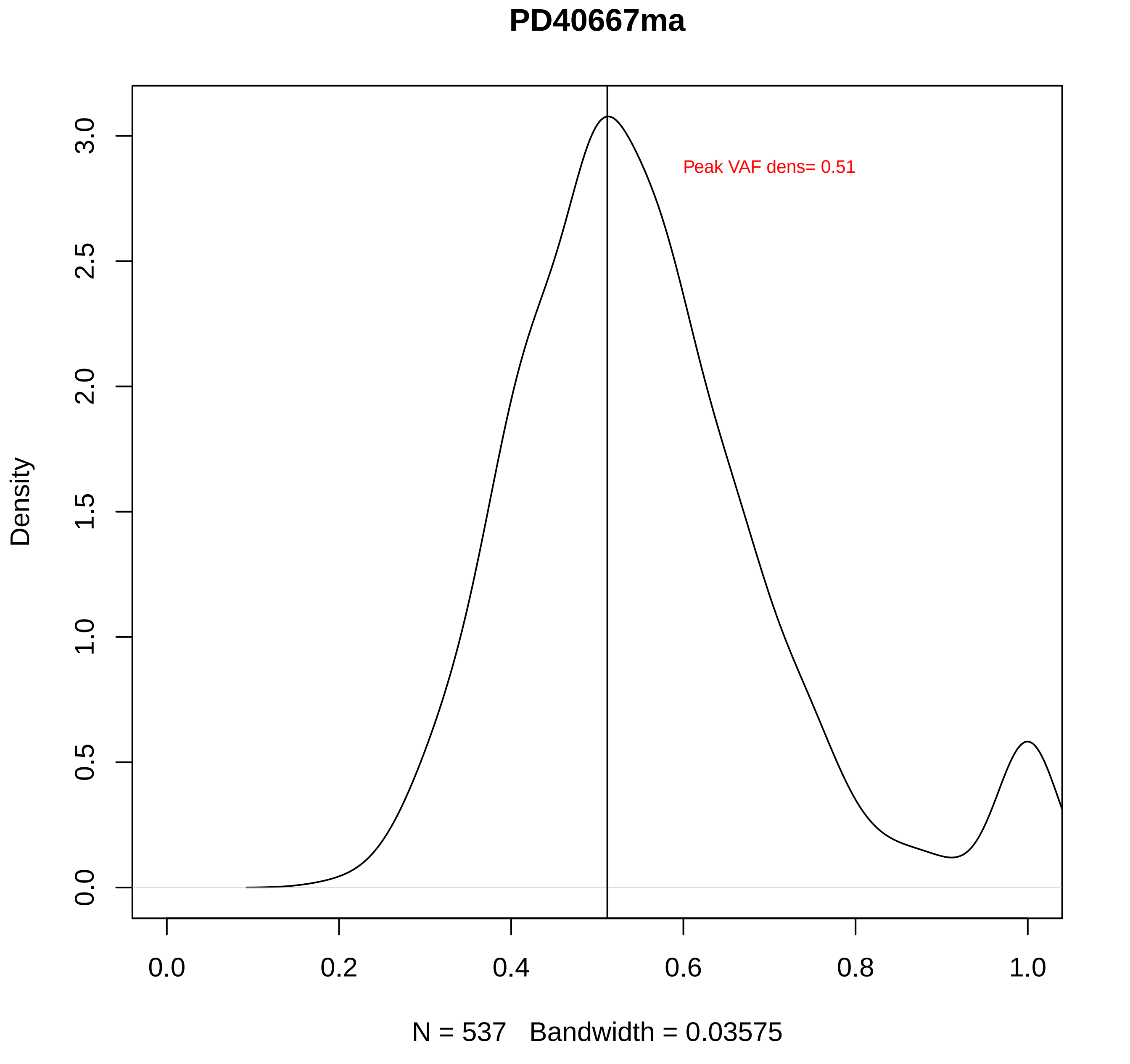
PD40667ma
Peak VAF dens= 0.51
Density
3.0
2.5
2.0
1.5
1.0
0.5
0.0
0.0
0.2
0.4
0.6
0.8
1.0
N = 537   Bandwidth = 0.03575

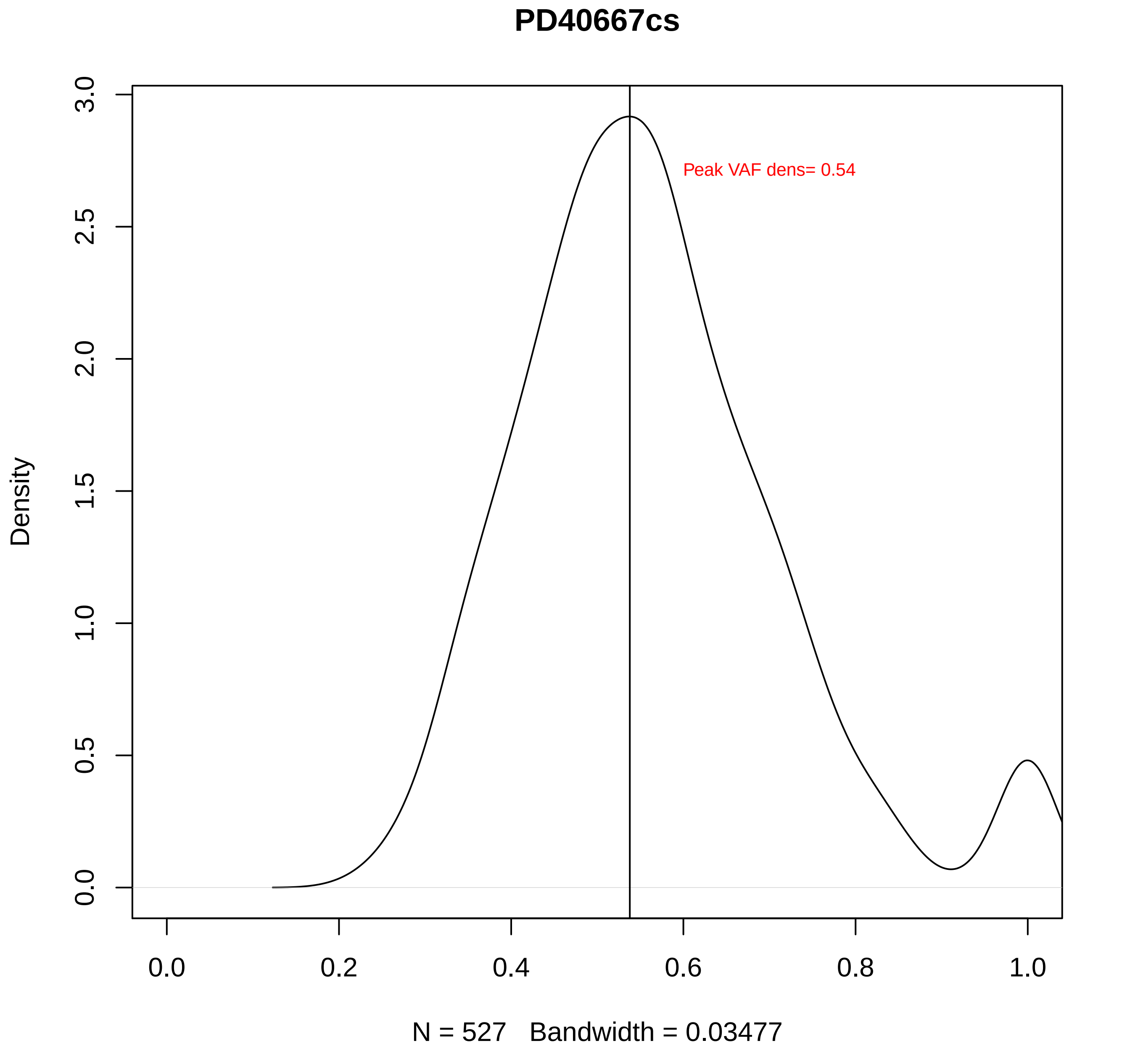

PD40667cs
Peak VAF dens= 0.54
Density
0.0
0.5
1.0
1.5
2.0
2.5
3.0
0.0
0.2
0.4
0.6
0.8
1.0
N = 527   Bandwidth = 0.03477

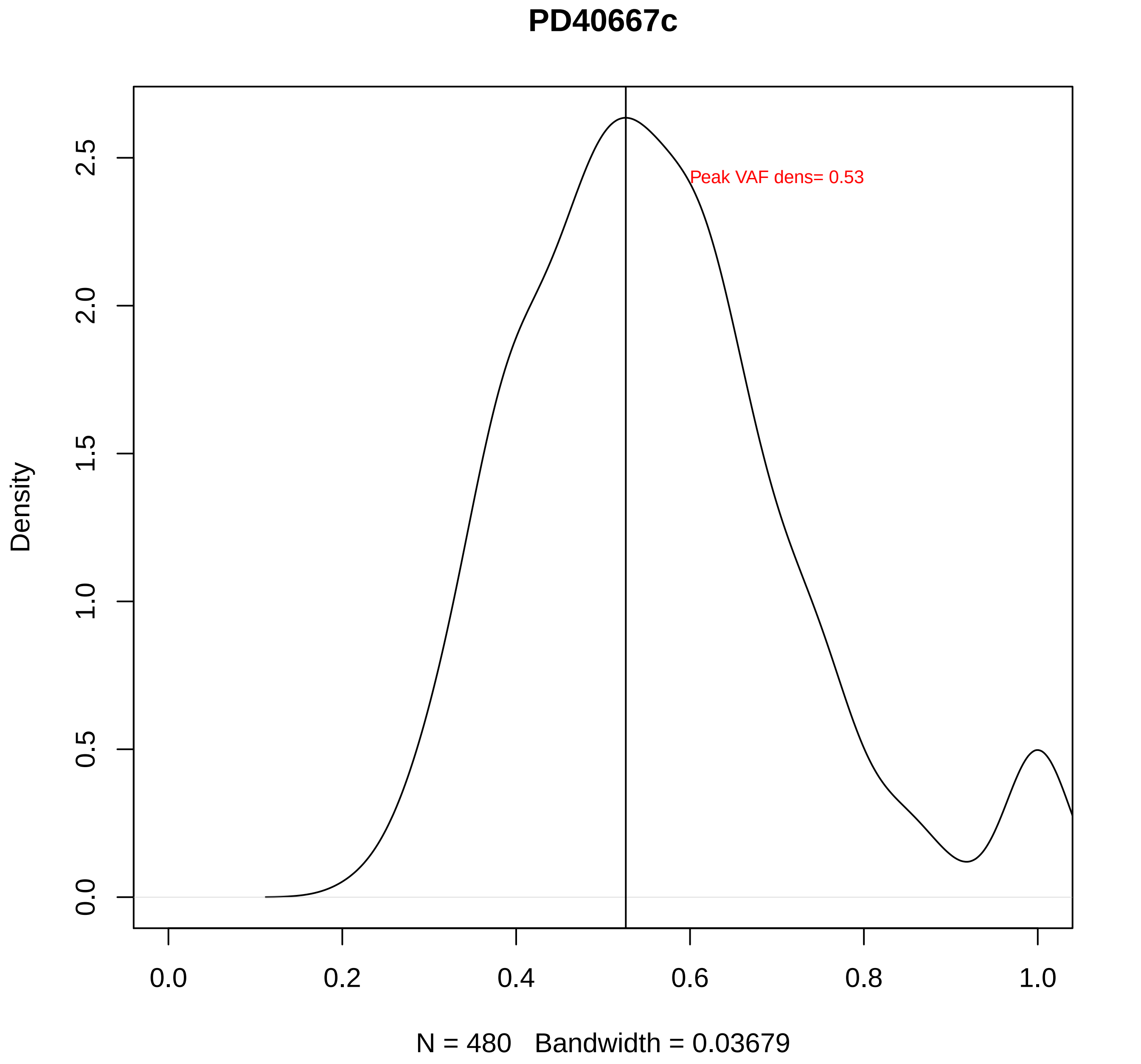
PD40667c
Peak VAF dens= 0.53
Density
0.0
0.5
1.0
1.5
2.0
2.5
0.0
0.2
0.4
0.6
0.8
1.0
N = 480   Bandwidth = 0.03679

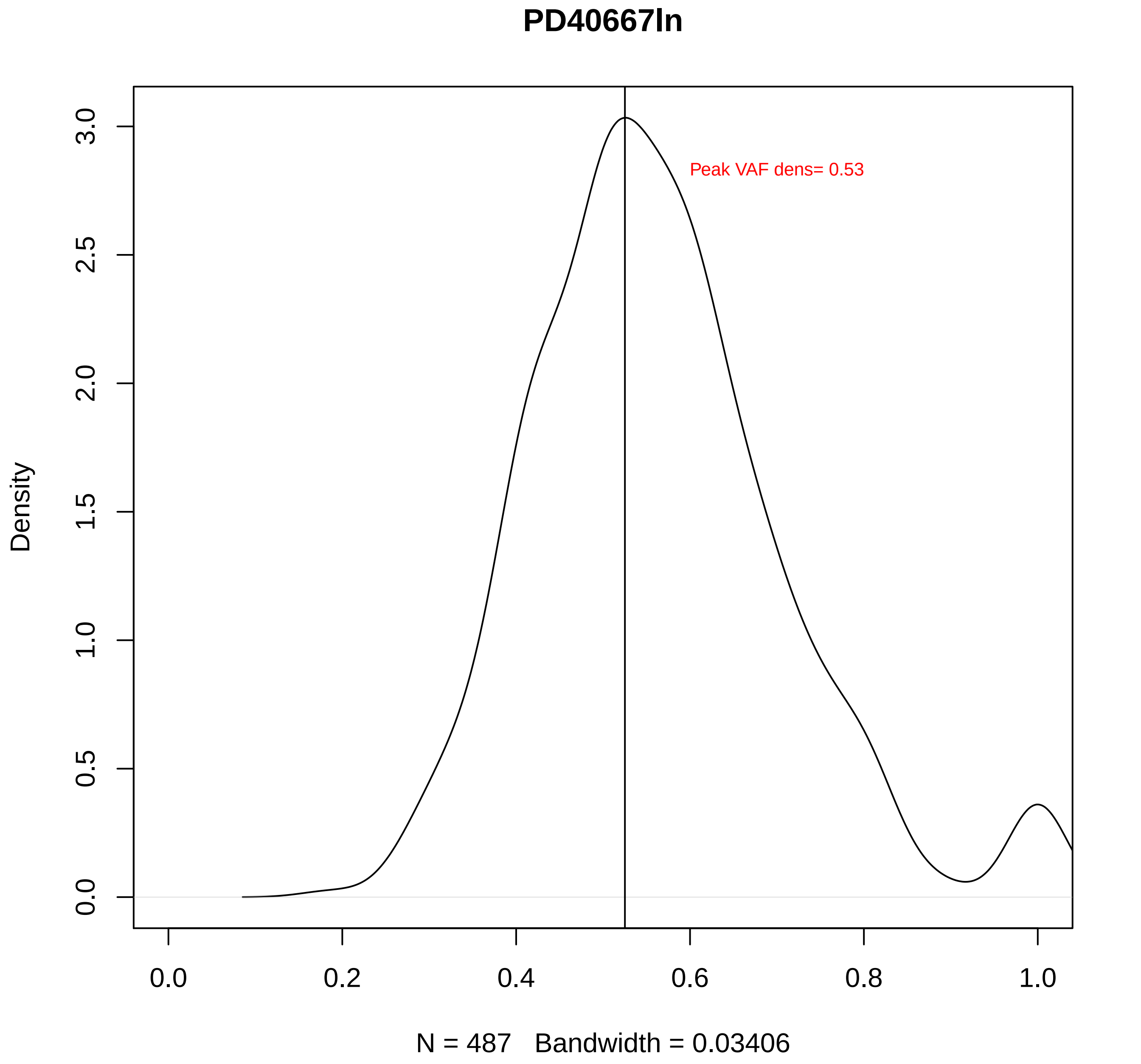
PD40667ln
Peak VAF dens= 0.53
Density
0.0
0.5
1.0
1.5
2.0
2.5
3.0
0.0
0.2
0.4
0.6
0.8
1.0
N = 487   Bandwidth = 0.03406

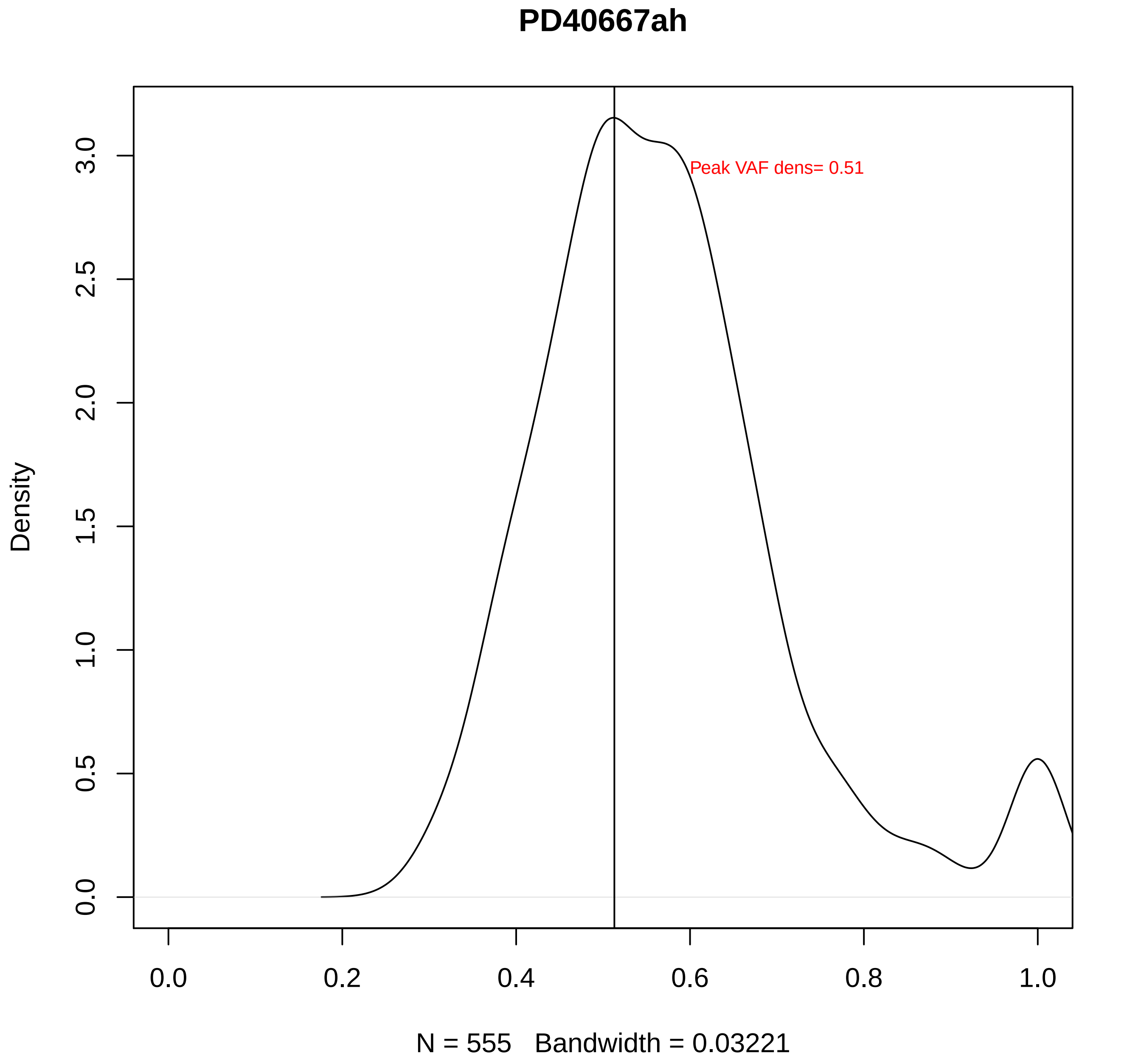

PD40667ah
Peak VAF dens= 0.51
Density
0.0
0.5
1.0
1.5
2.0
2.5
3.0
0.0
0.2
0.4
0.6
0.8
1.0
N = 555   Bandwidth = 0.03221

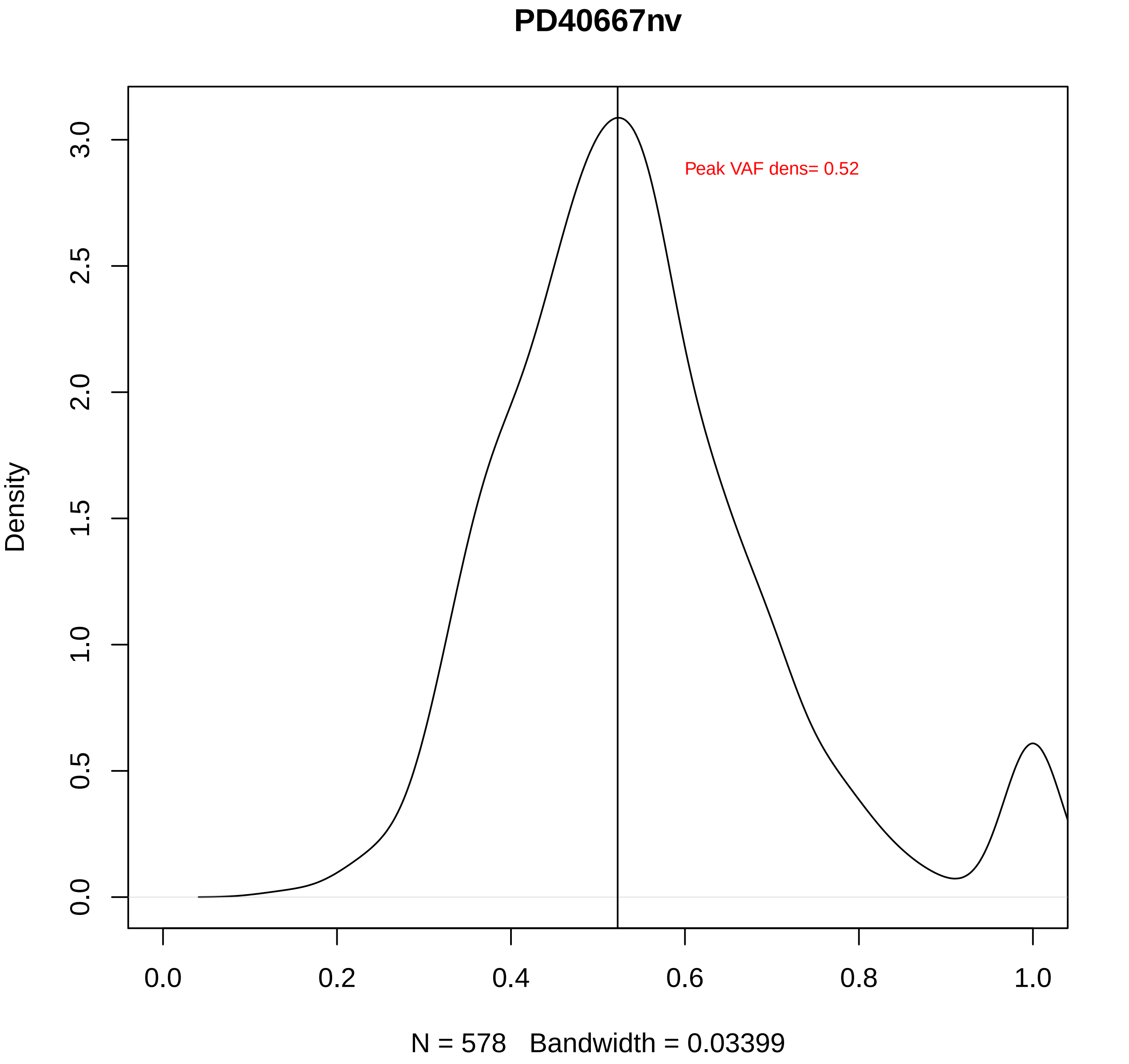
PD40667nv
Peak VAF dens= 0.52
Density
3.0
2.5
2.0
1.5
1.0
0.5
0.0
0.0
0.2
0.4
0.6
0.8
1.0
N = 578   Bandwidth = 0.03399

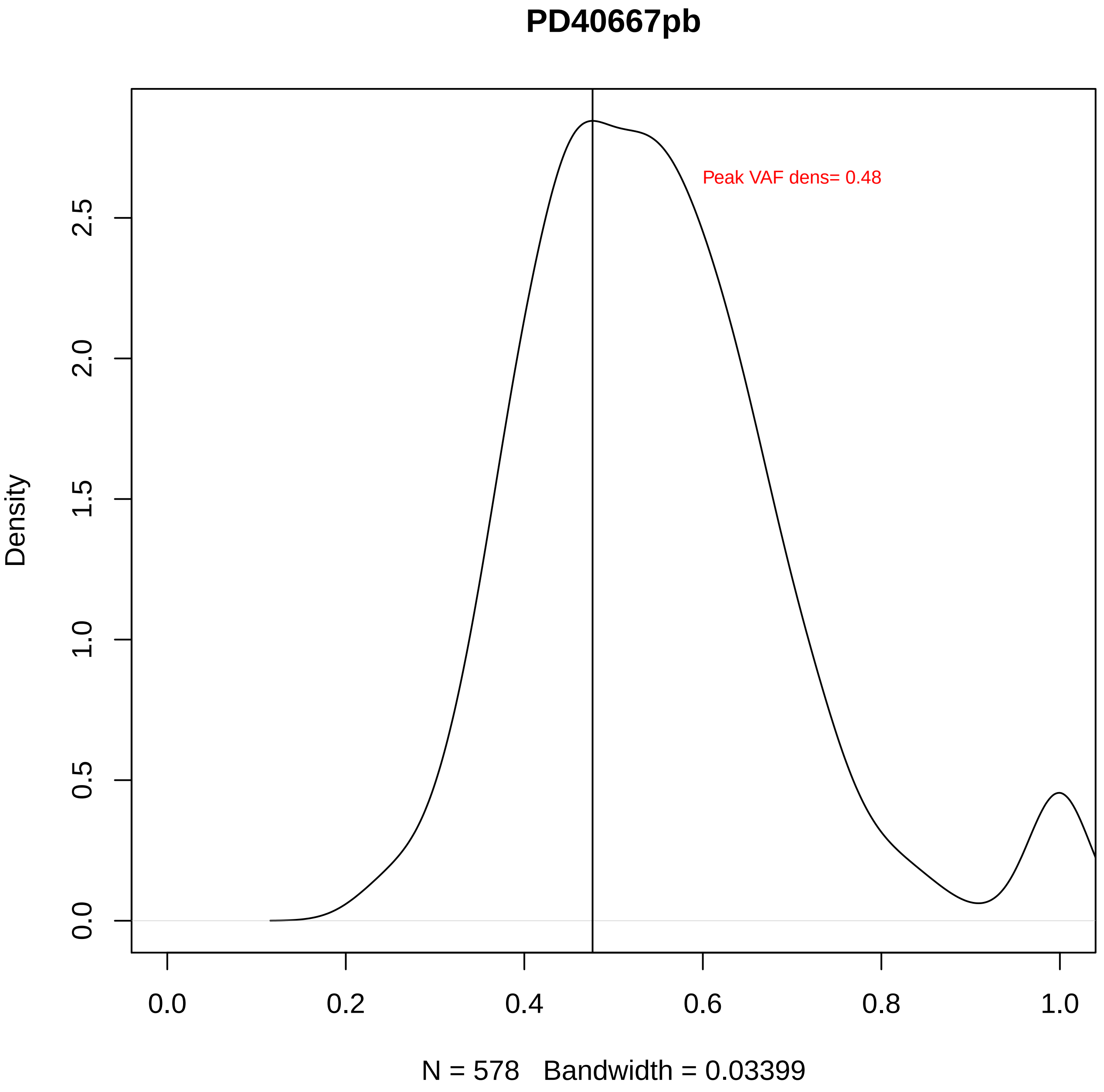
PD40667pb
Peak VAF dens= 0.48
Density
0.0
0.5
1.0
1.5
2.0
2.5
0.0
0.2
0.4
0.6
0.8
1.0
N = 578 Bandwidth = 0.03399

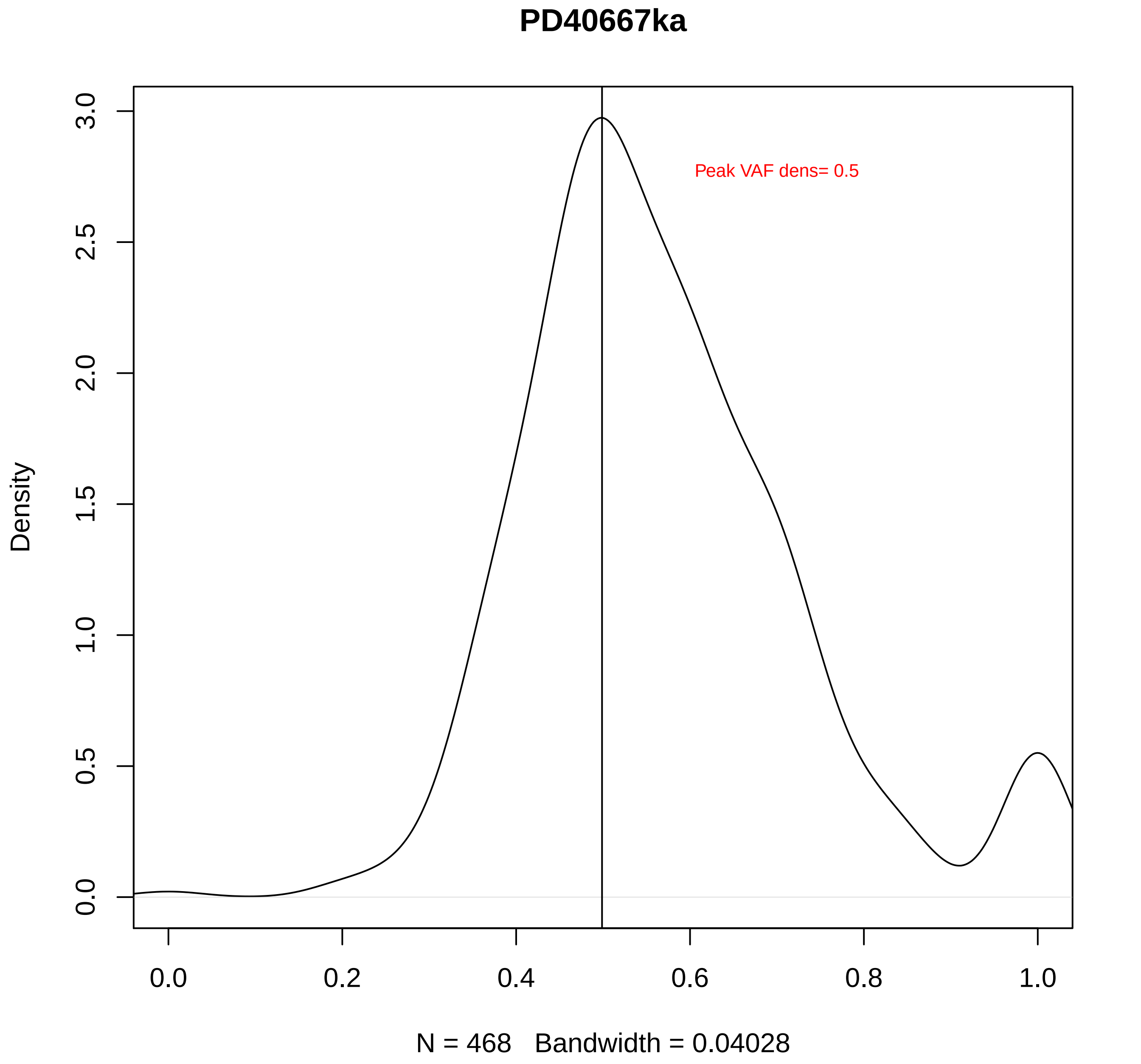
PD40667ka
Peak VAF dens= 0.5
Density
0.0
0.5
1.0
1.5
2.0
2.5
3.0
0.0
0.2
0.4
0.6
0.8
1.0
N = 468   Bandwidth = 0.04028

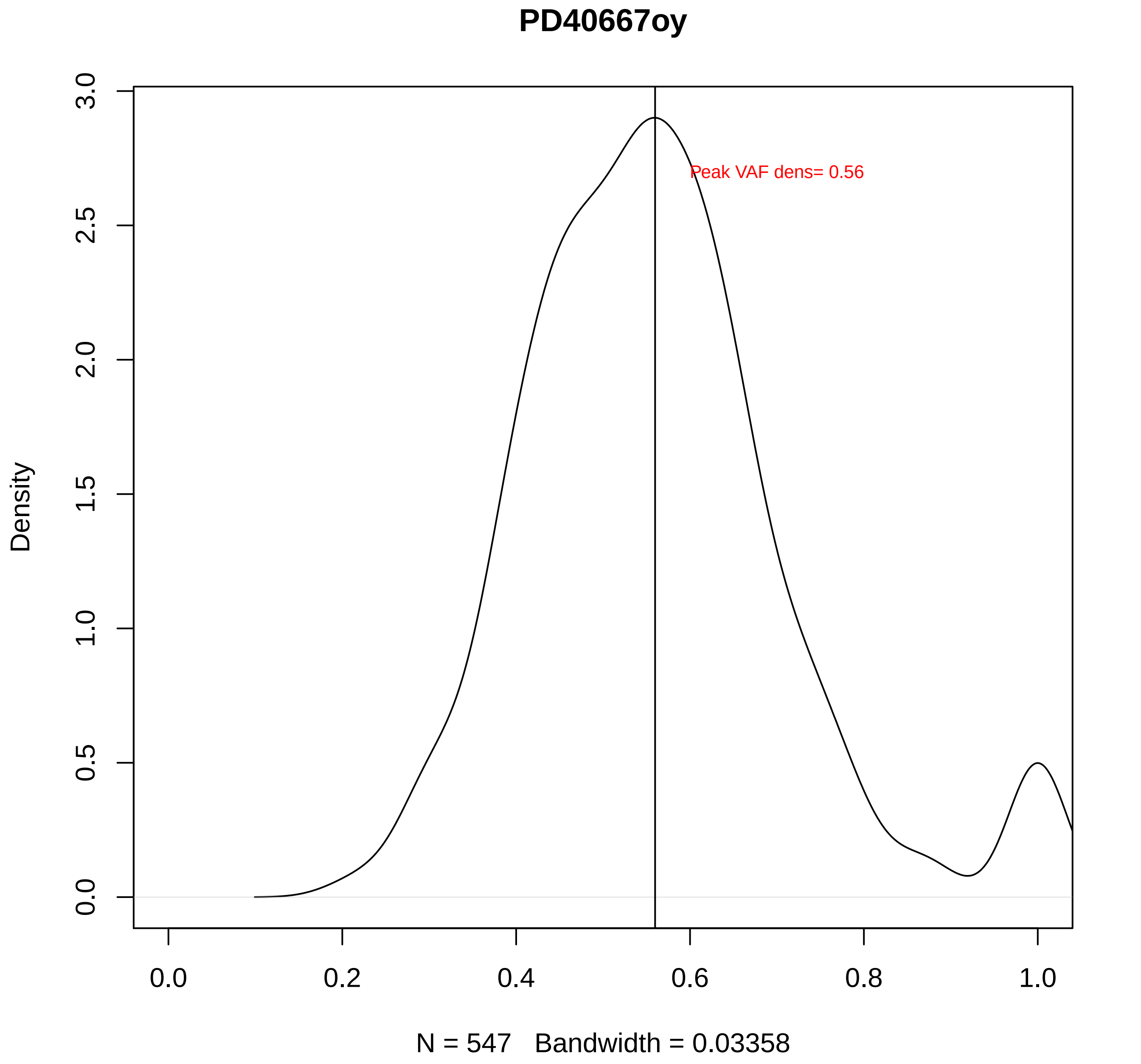

PD40667oy
Peak VAF dens= 0.56
Density
0.0
0.5
1.0
1.5
2.0
2.5
3.0
0.0
0.2
0.4
0.6
0.8
1.0
N = 547 Bandwidth = 0.03358

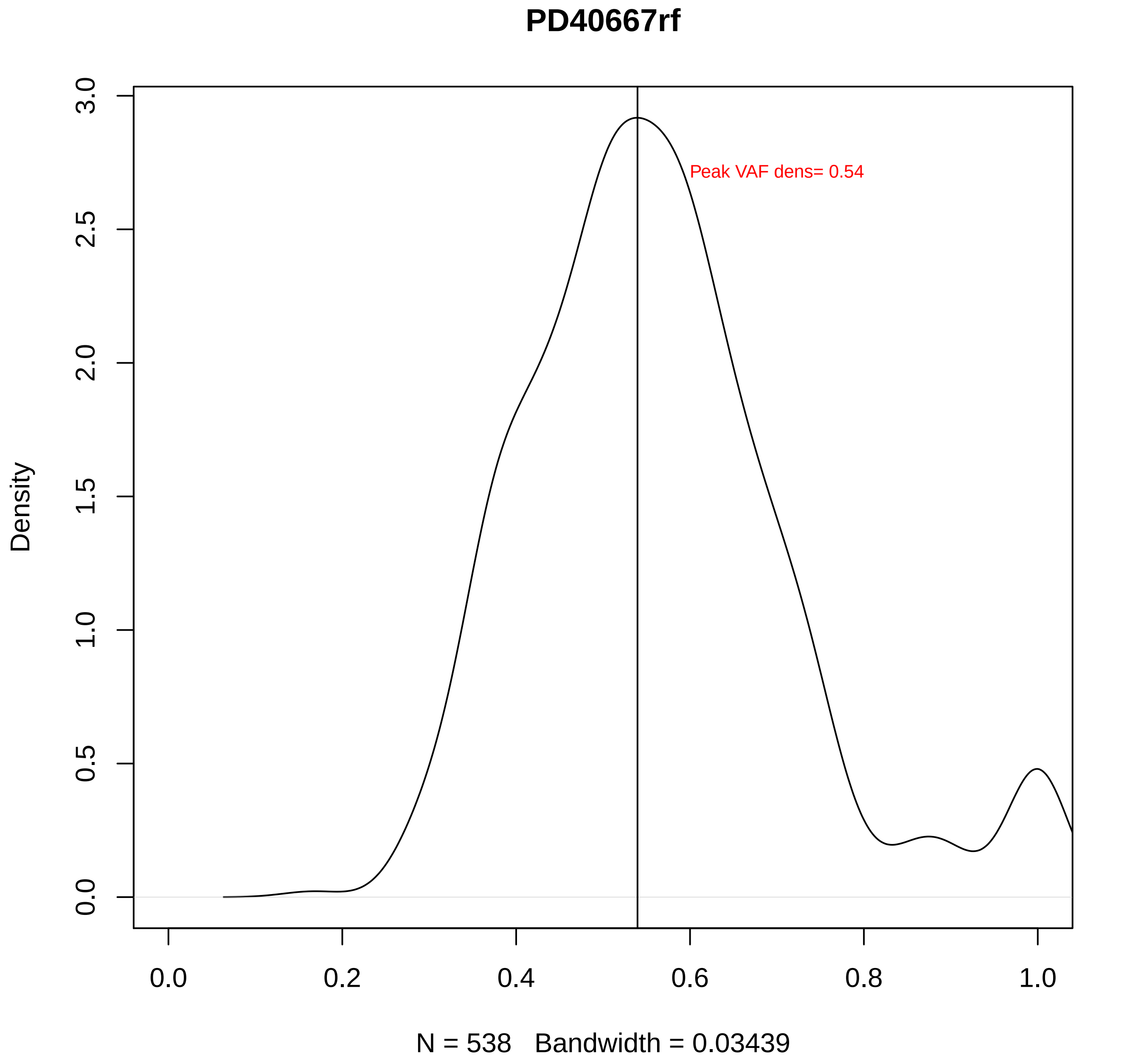

PD40667rf
Peak VAF dens= 0.54
Density
0.0
0.5
1.0
1.5
2.0
2.5
3.0
0.0
0.2
0.4
0.6
0.8
1.0
N = 538 Bandwidth = 0.03439

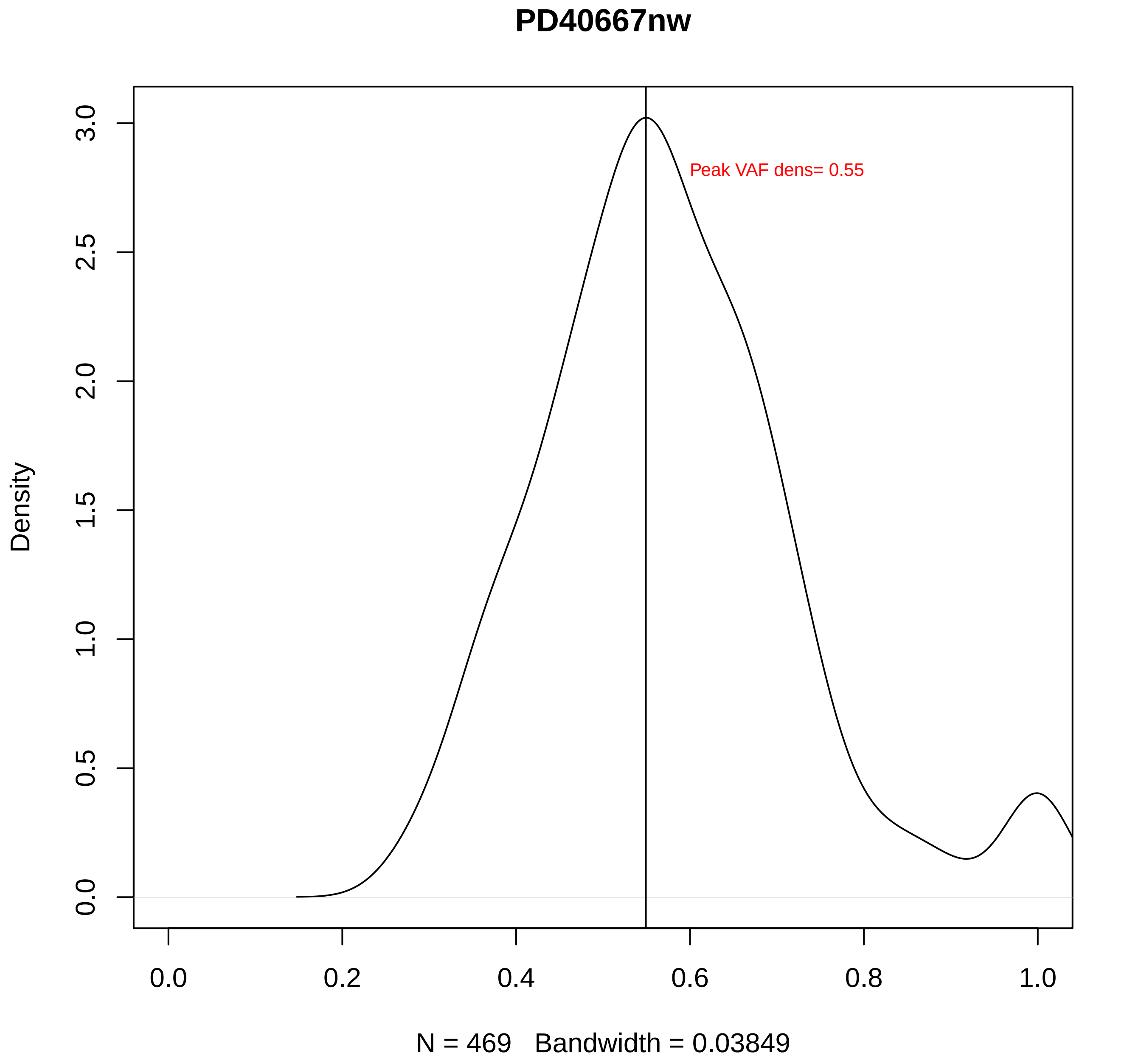
PD40667nw
Peak VAF dens= 0.55
Density
0.0
0.5
1.0
1.5
2.0
2.5
3.0
0.0
0.2
0.4
0.6
0.8
1.0
N = 469   Bandwidth = 0.03849

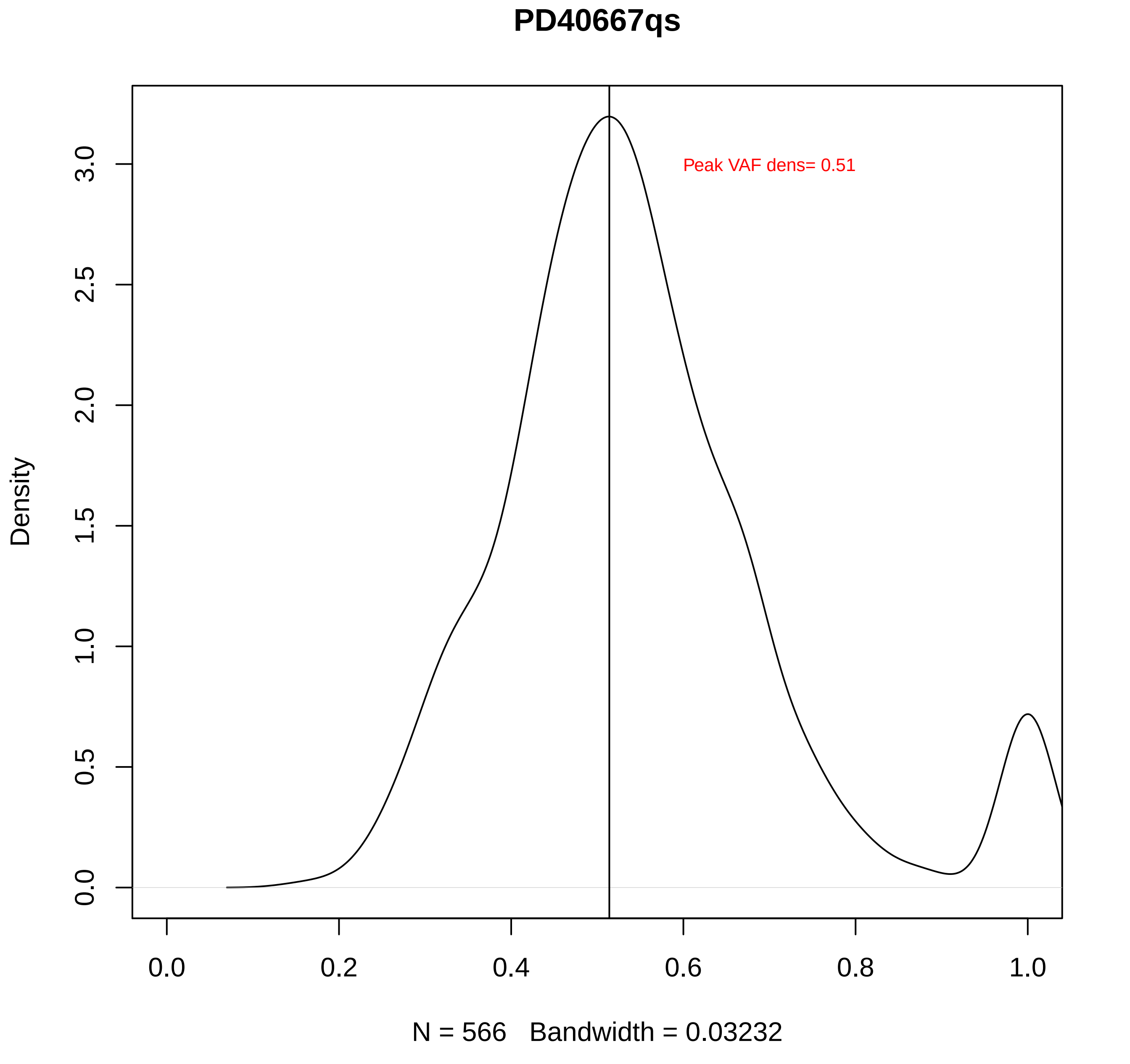
PD40667qs
Peak VAF dens= 0.51
Density
0.0
0.5
1.0
1.5
2.0
2.5
3.0
0.0
0.2
0.4
0.6
0.8
1.0
N = 566 Bandwidth = 0.03232

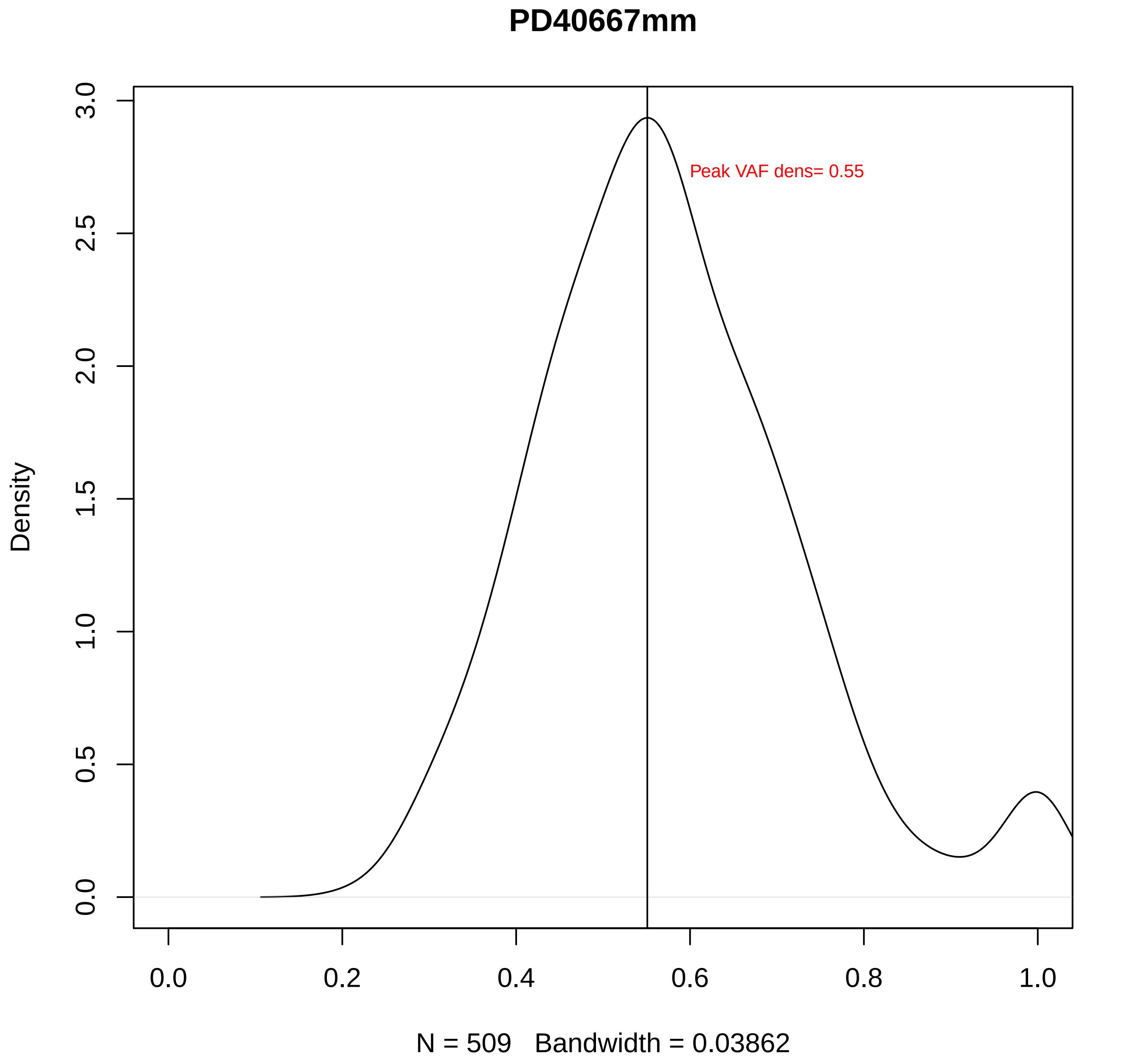

PD40667mm
Peak VAF dens= 0.55
Density
0.0
0.5
1.0
1.5
2.0
2.5
3.0
0.0
0.2
0.4
0.6
0.8
1.0
N = 509   Bandwidth = 0.03862

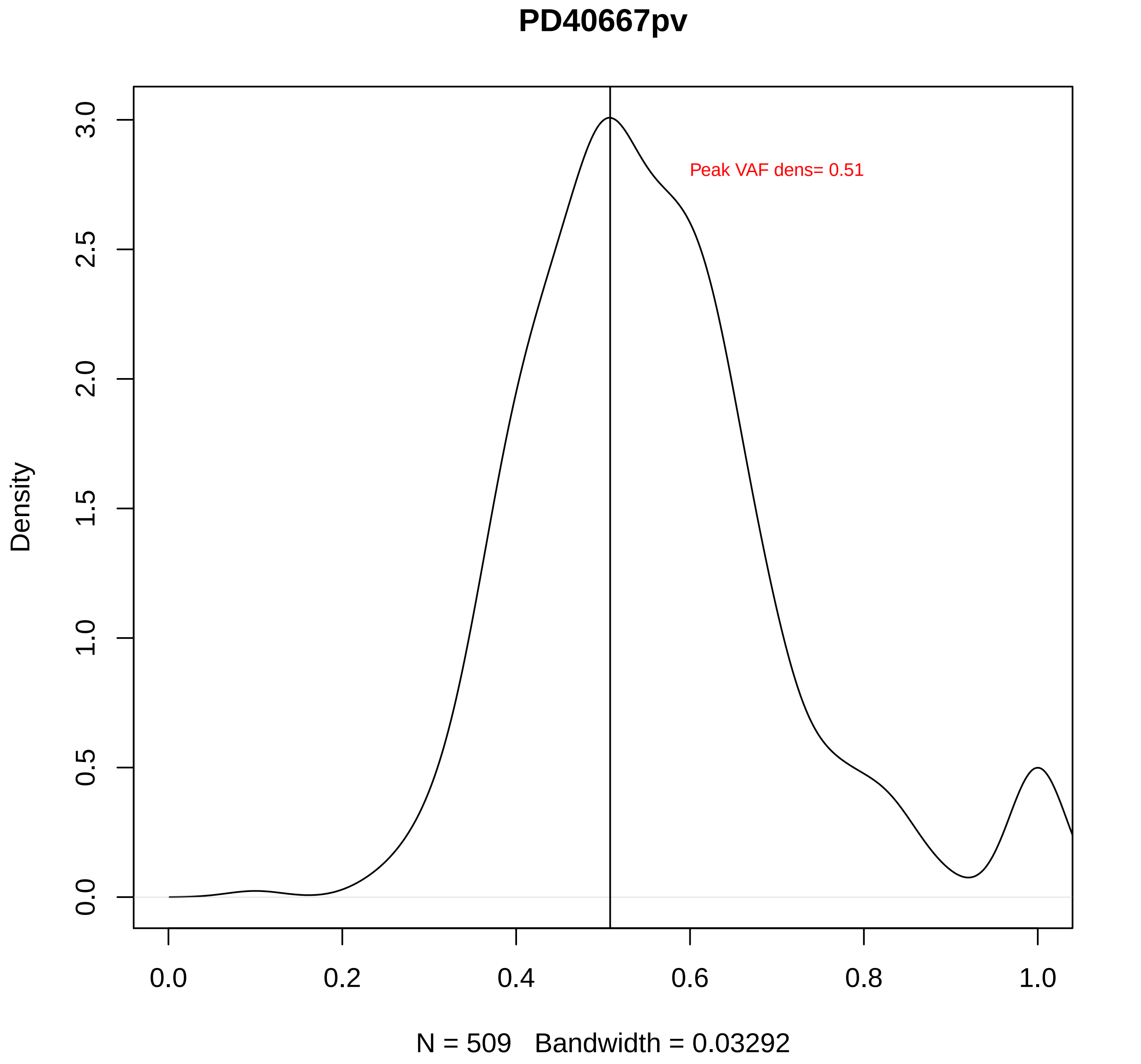

PD40667pv
Peak VAF dens= 0.51
Density
3.0
2.5
2.0
1.5
1.0
0.5
0.0
0.0
0.2
0.4
0.6
0.8
1.0
N = 509 Bandwidth = 0.03292

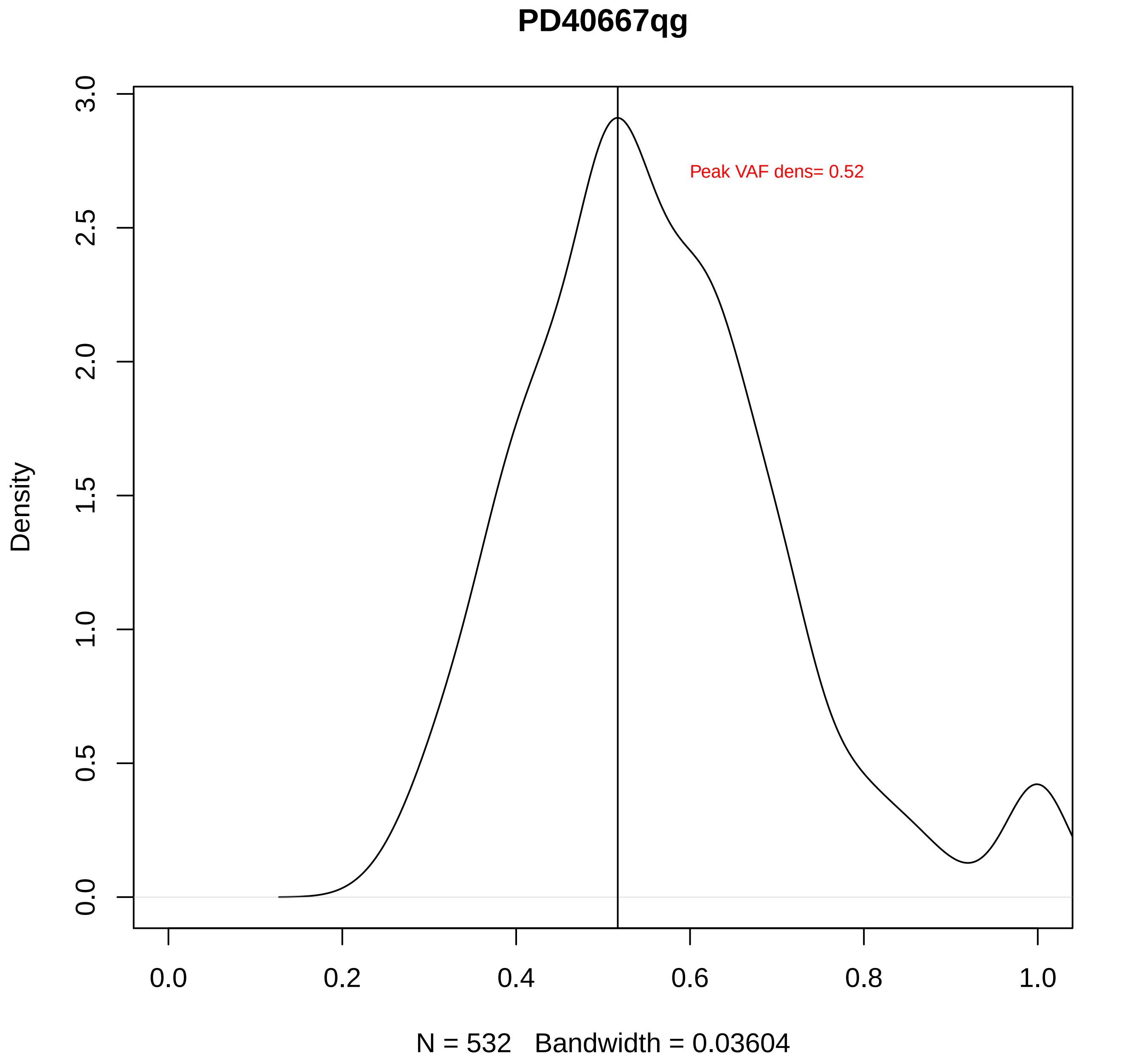
PD40667qg
Peak VAF dens= 0.52
Density
0.0
0.5
1.0
1.5
2.0
2.5
3.0
0.0
0.2
0.4
0.6
0.8
1.0
N = 532 Bandwidth = 0.03604

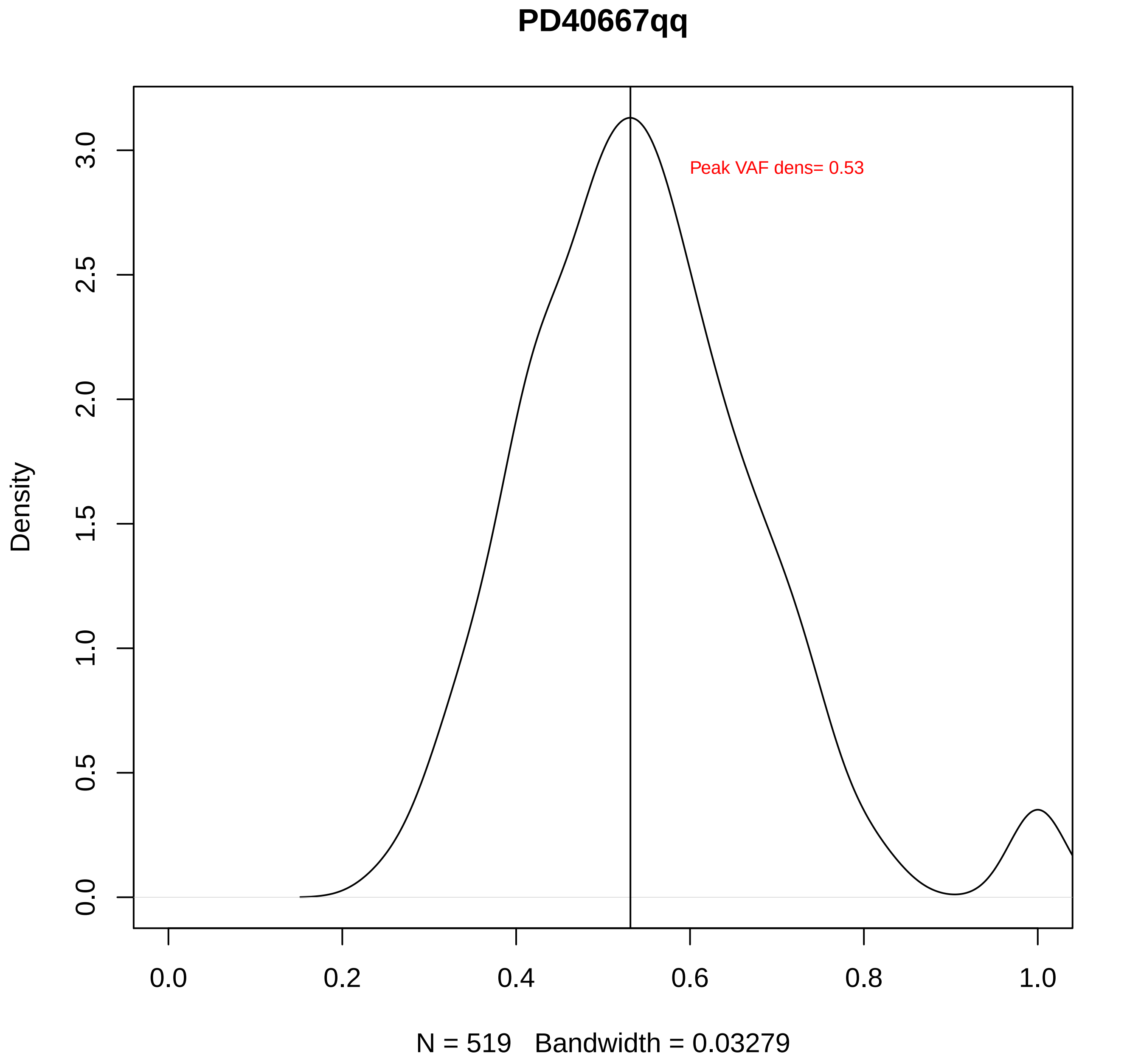

PD40667qq
Peak VAF dens= 0.53
Density
0.0
0.5
1.0
1.5
2.0
2.5
3.0
0.0
0.2
0.4
0.6
0.8
1.0
N = 519 Bandwidth = 0.03279

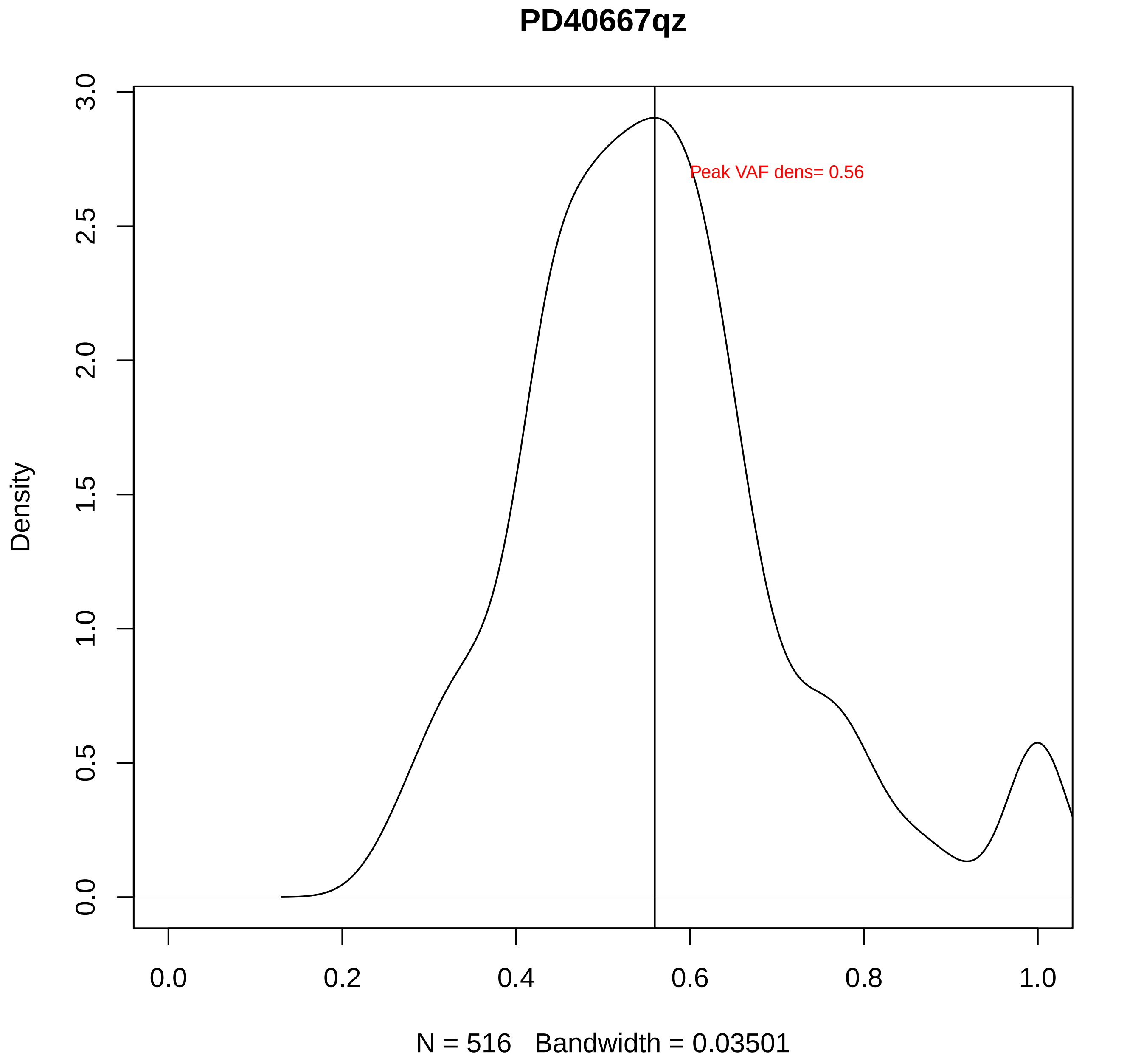
PD40667qz
Density
3.0
2.5
2.0
1.5
1.0
0.5
0.0
Peak VAF dens= 0.56
0.0
0.2
0.4
0.6
0.8
1.0
N = 516 Bandwidth = 0.03501

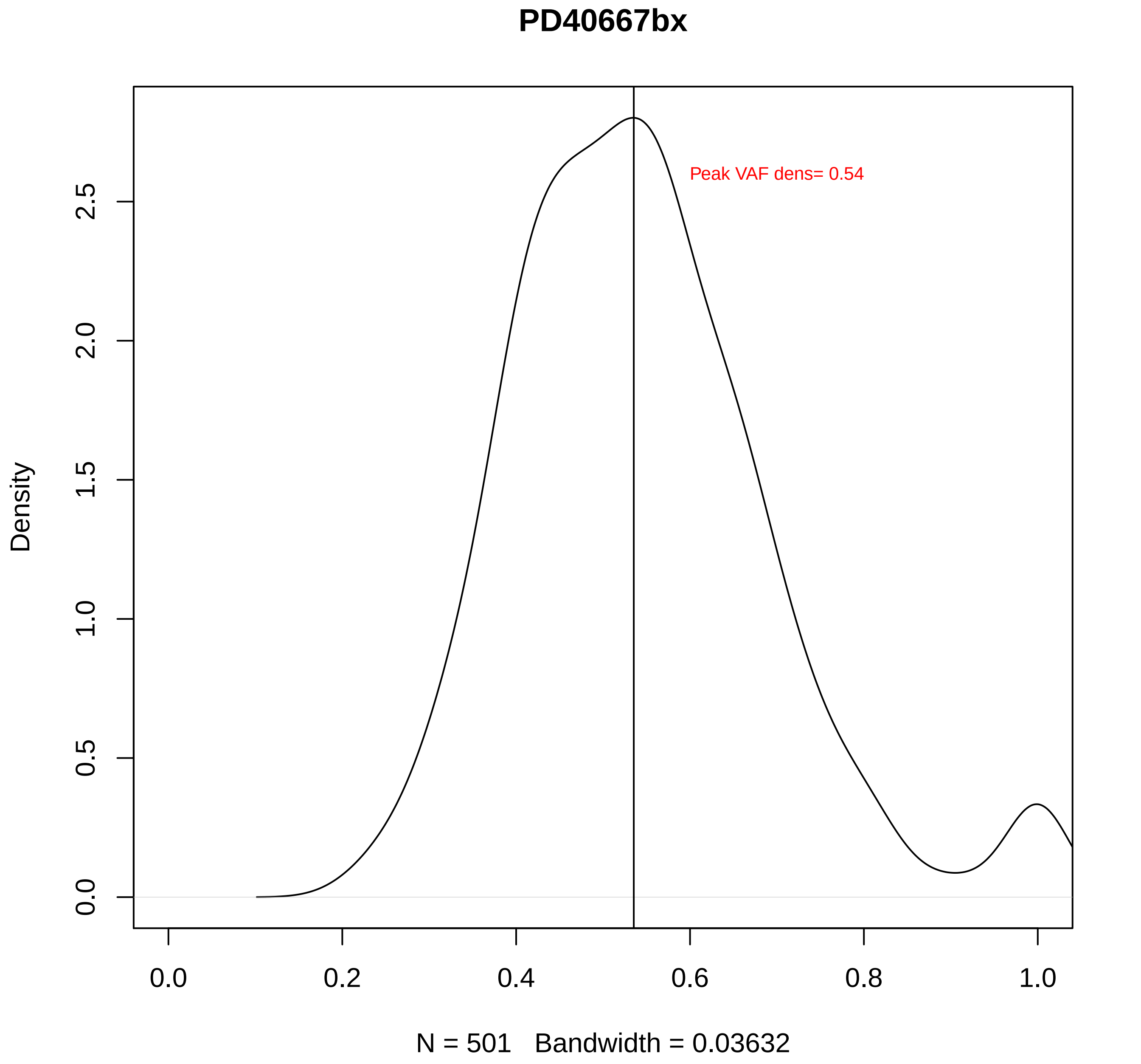

PD40667bx
Peak VAF dens= 0.54
Density
0.0
0.5
1.0
1.5
2.0
2.5
0.0
0.2
0.4
0.6
0.8
1.0
N = 501   Bandwidth = 0.03632

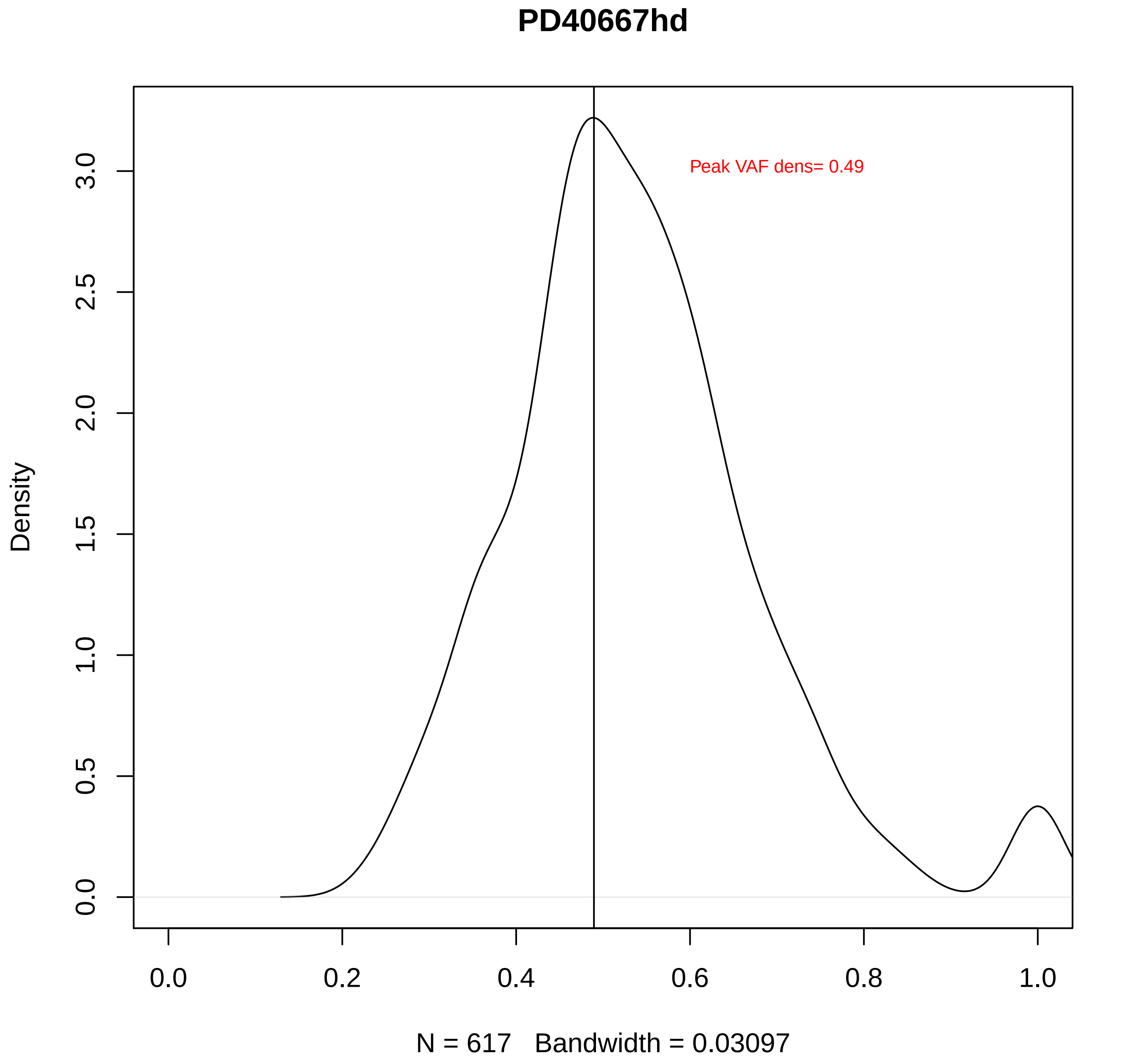
PD40667hd
Peak VAF dens= 0.49
Density
0.0
0.5
1.0
1.5
2.0
2.5
3.0
0.0
0.2
0.4
0.6
0.8
1.0
N = 617 Bandwidth = 0.03097

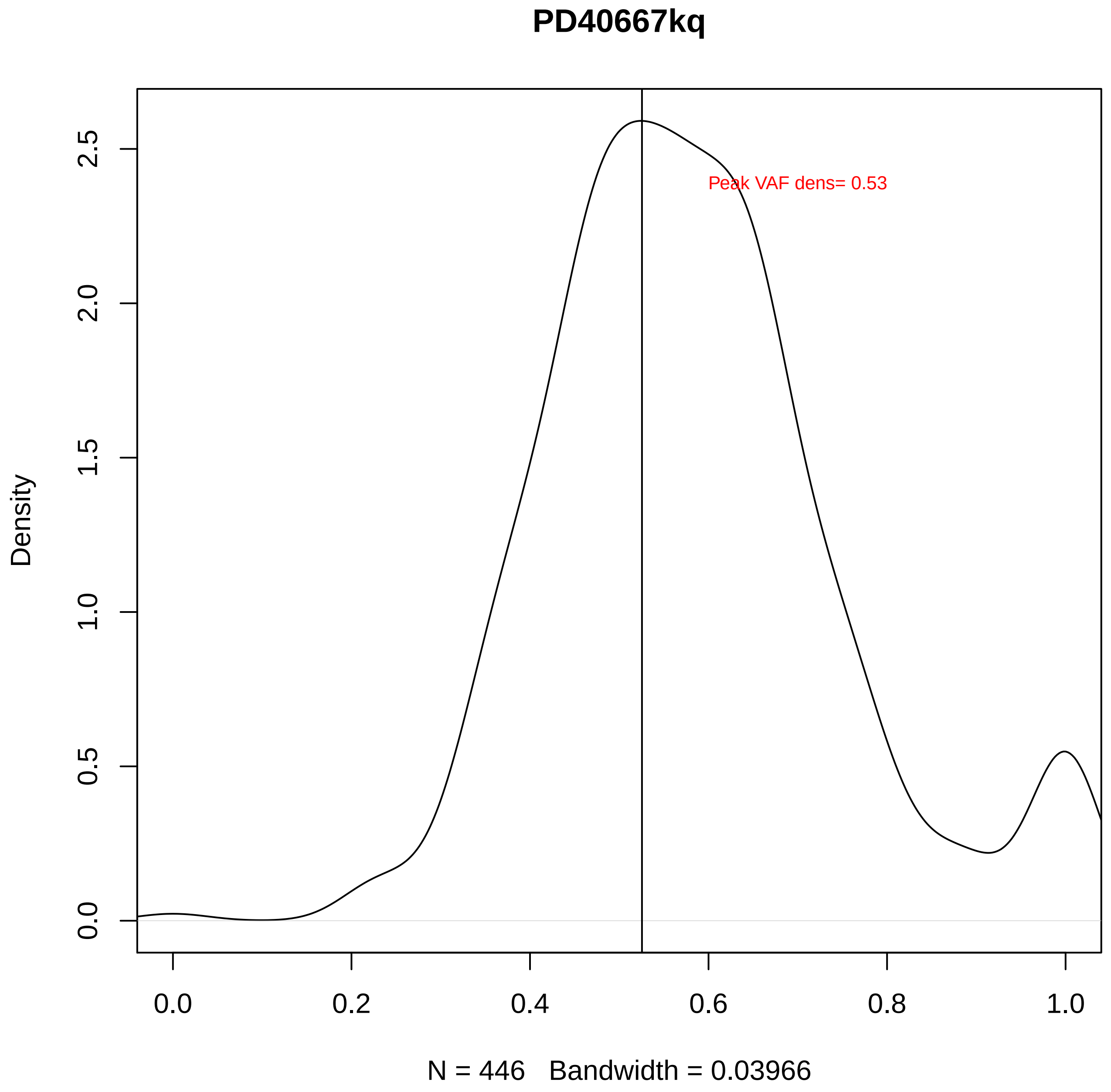

PD40667kq
Peak VAF dens= 0.53
Density
0.0
0.5
1.0
1.5
2.0
2.5
0.0
0.2
0.4
0.6
0.8
1.0
N = 446   Bandwidth = 0.03966

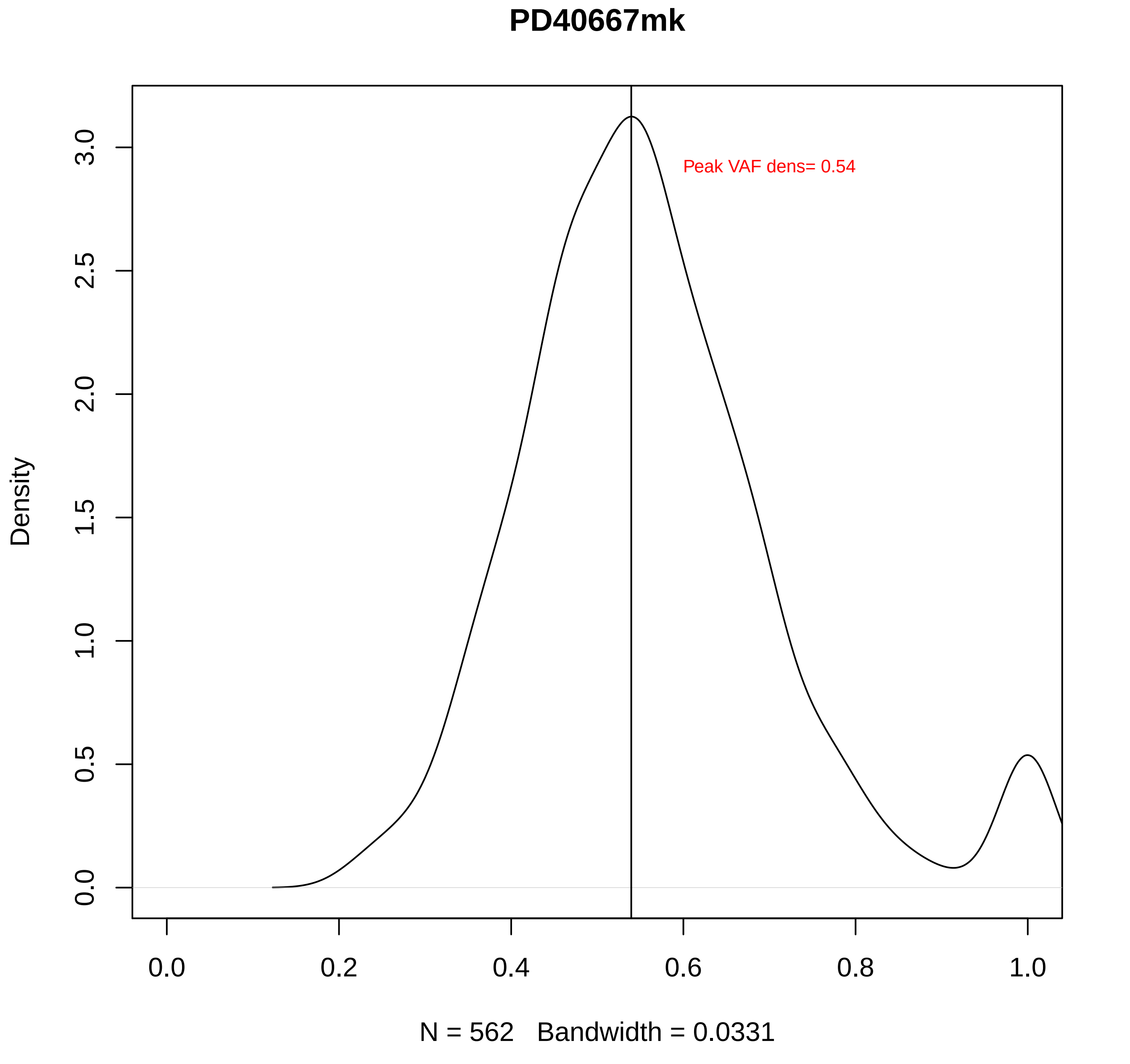

PD40667mk
Peak VAF dens= 0.54
Density
0.0
0.5
1.0
1.5
2.0
2.5
3.0
0.0
0.2
0.4
0.6
0.8
1.0
N = 562   Bandwidth = 0.0331

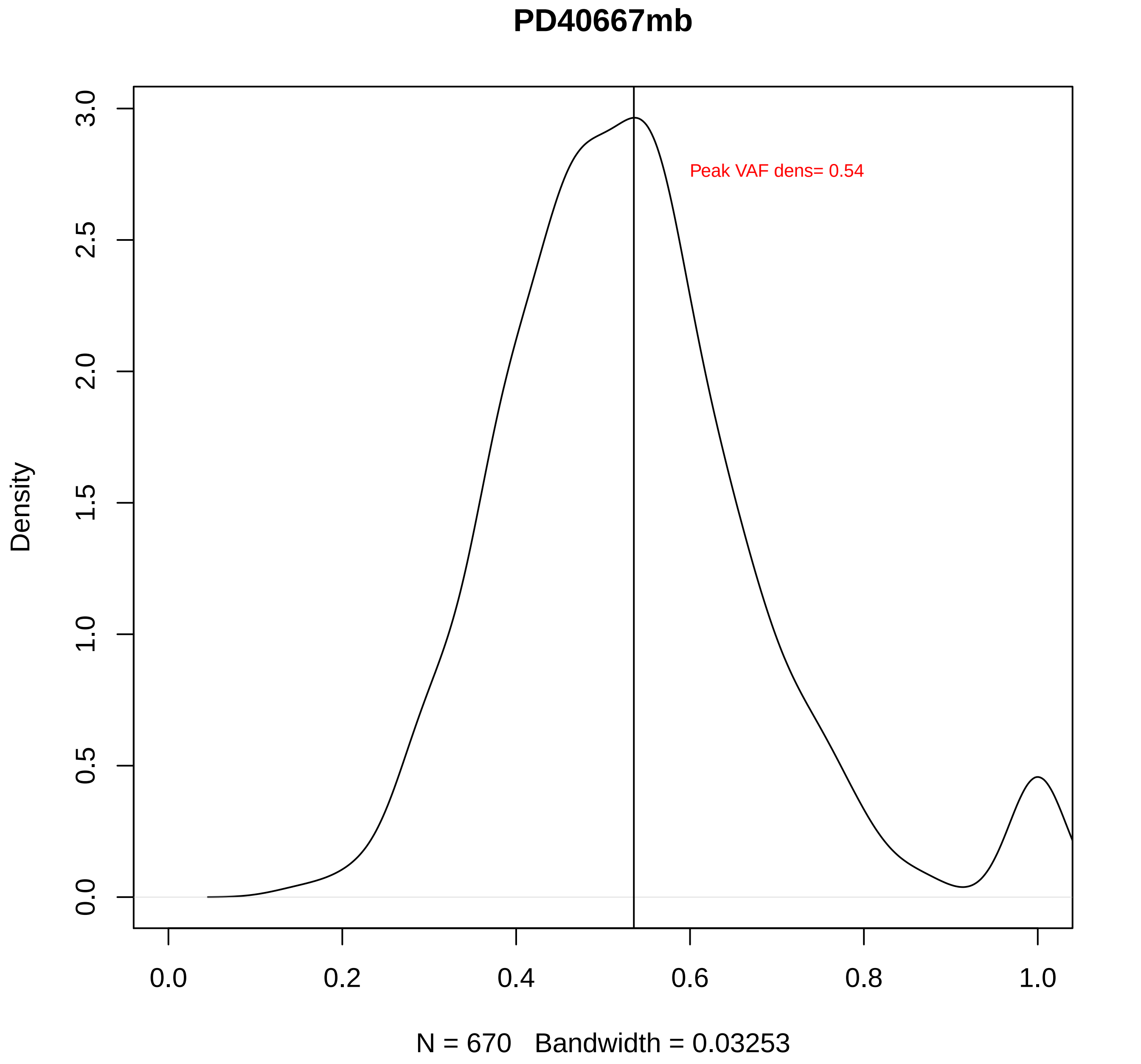

PD40667mb
Peak VAF dens= 0.54
Density
0.0
0.5
1.0
1.5
2.0
2.5
3.0
0.0
0.2
0.4
0.6
0.8
1.0
N = 670   Bandwidth = 0.03253

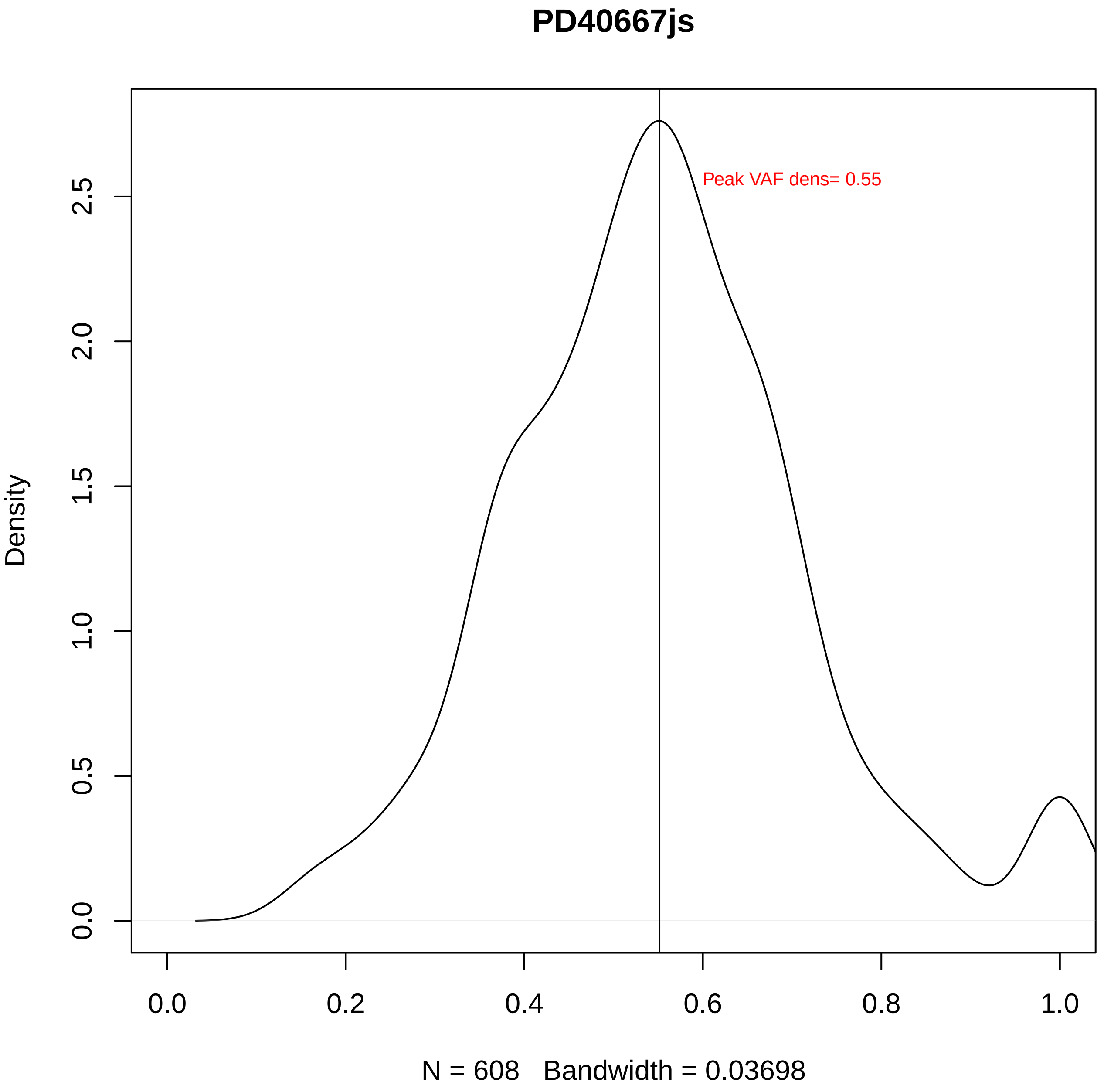
PD40667js
Peak VAF dens= 0.55
Density
0.0
0.5
1.0
1.5
2.0
2.5
0.0
0.2
0.4
0.6
0.8
1.0
N = 608   Bandwidth = 0.03698

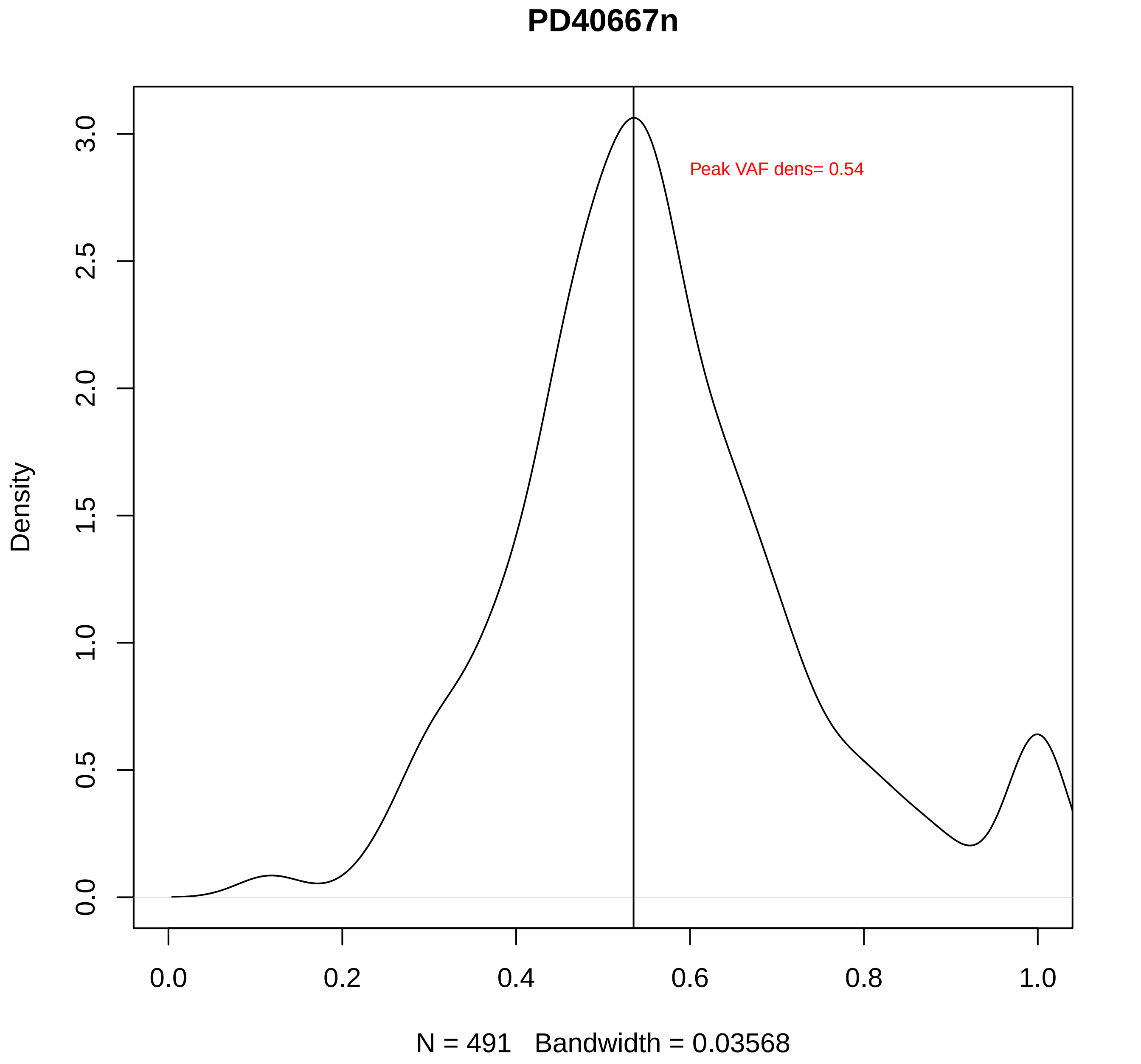
PD40667n
Peak VAF dens= 0.54
Density
0.0
0.5
1.0
1.5
2.0
2.5
3.0
0.0
0.2
0.4
0.6
0.8
1.0
N = 491   Bandwidth = 0.03568

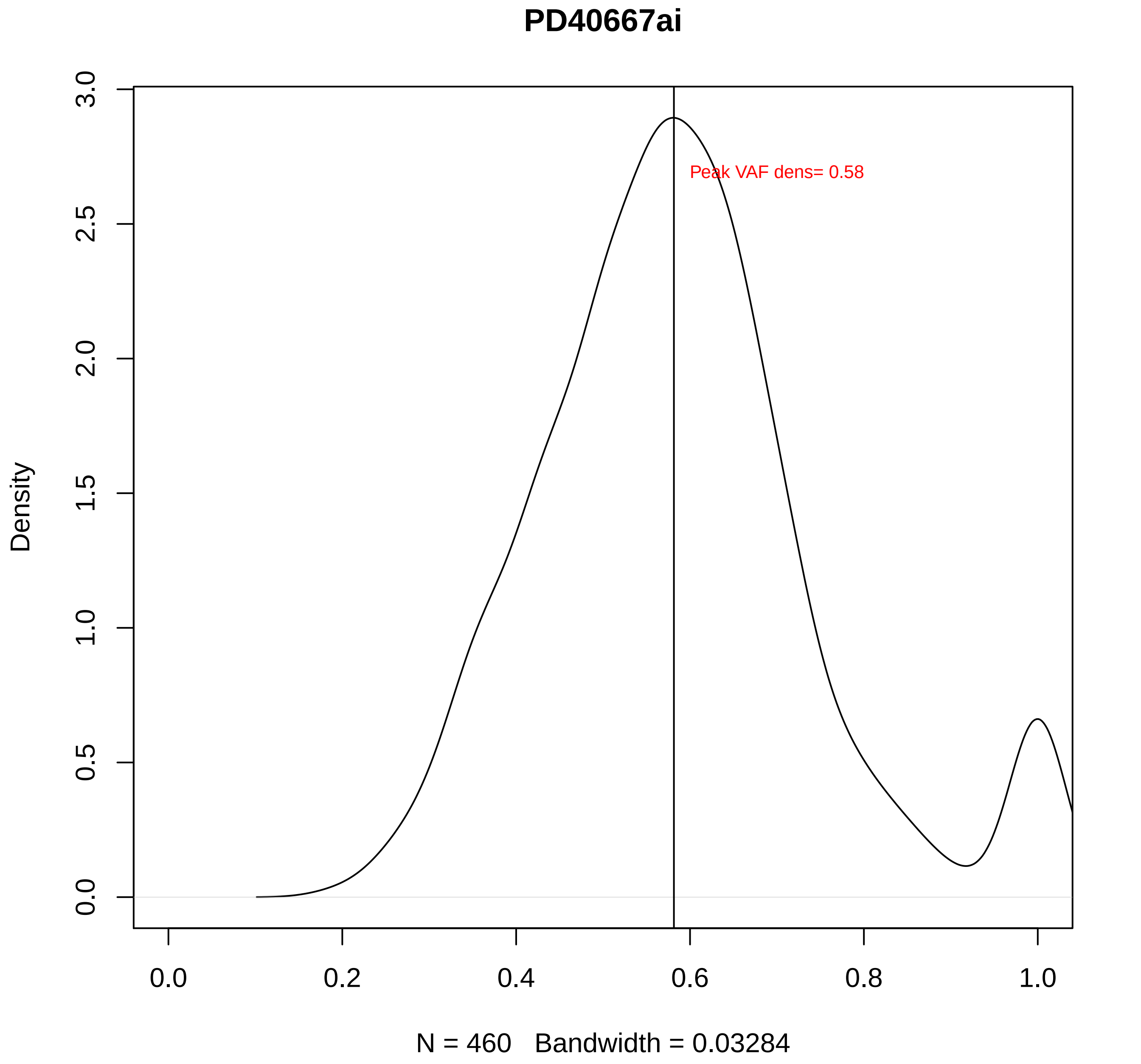
PD40667ai
Peak VAF dens= 0.58
Density
0.0
0.5
1.0
1.5
2.0
2.5
3.0
0.0
0.2
0.4
0.6
0.8
1.0
N = 460   Bandwidth = 0.03284

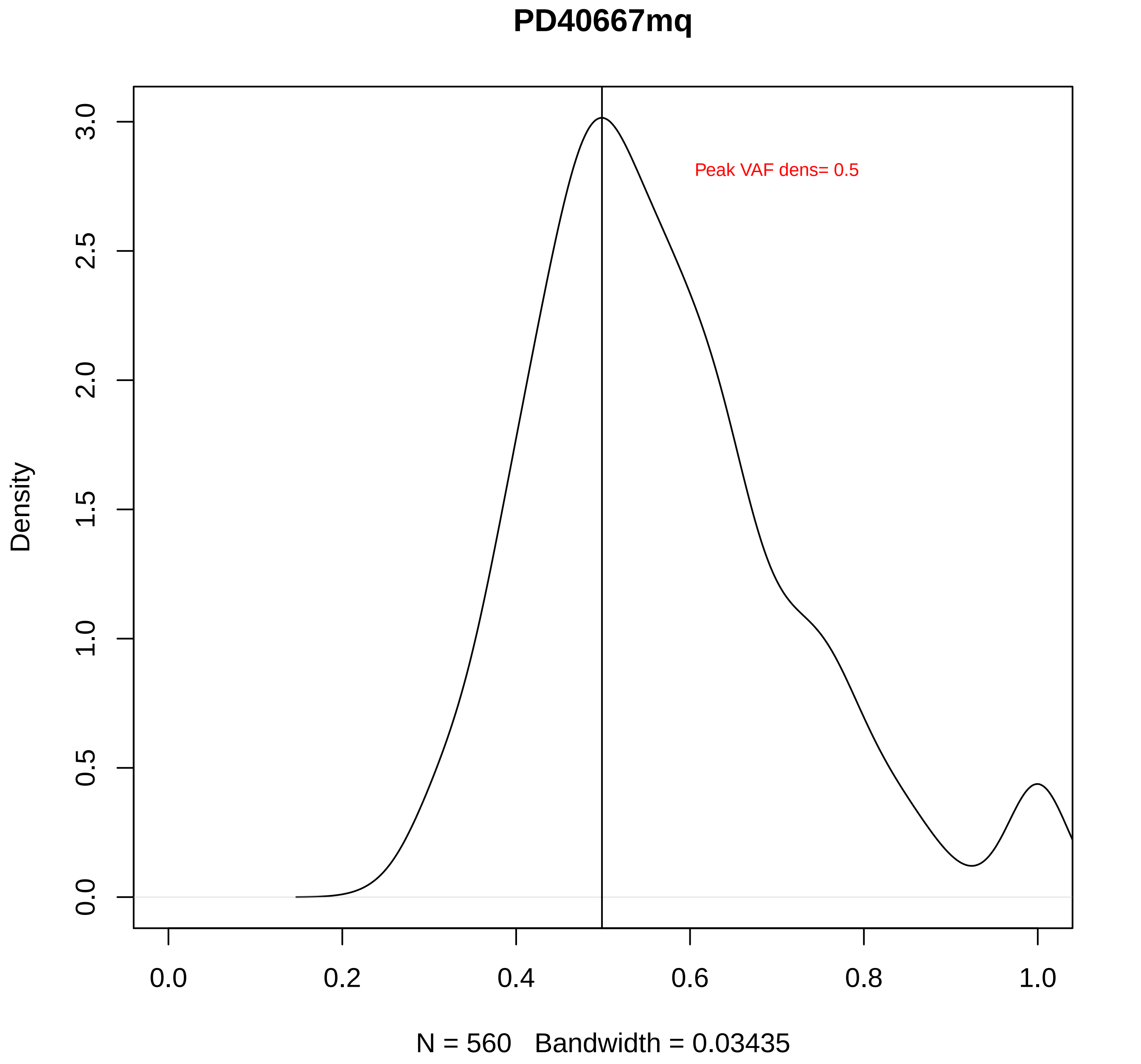
PD40667mq
Peak VAF dens= 0.5
Density
0.0
0.5
1.0
1.5
2.0
2.5
3.0
0.0
0.2
0.4
0.6
0.8
1.0
N = 560   Bandwidth = 0.03435

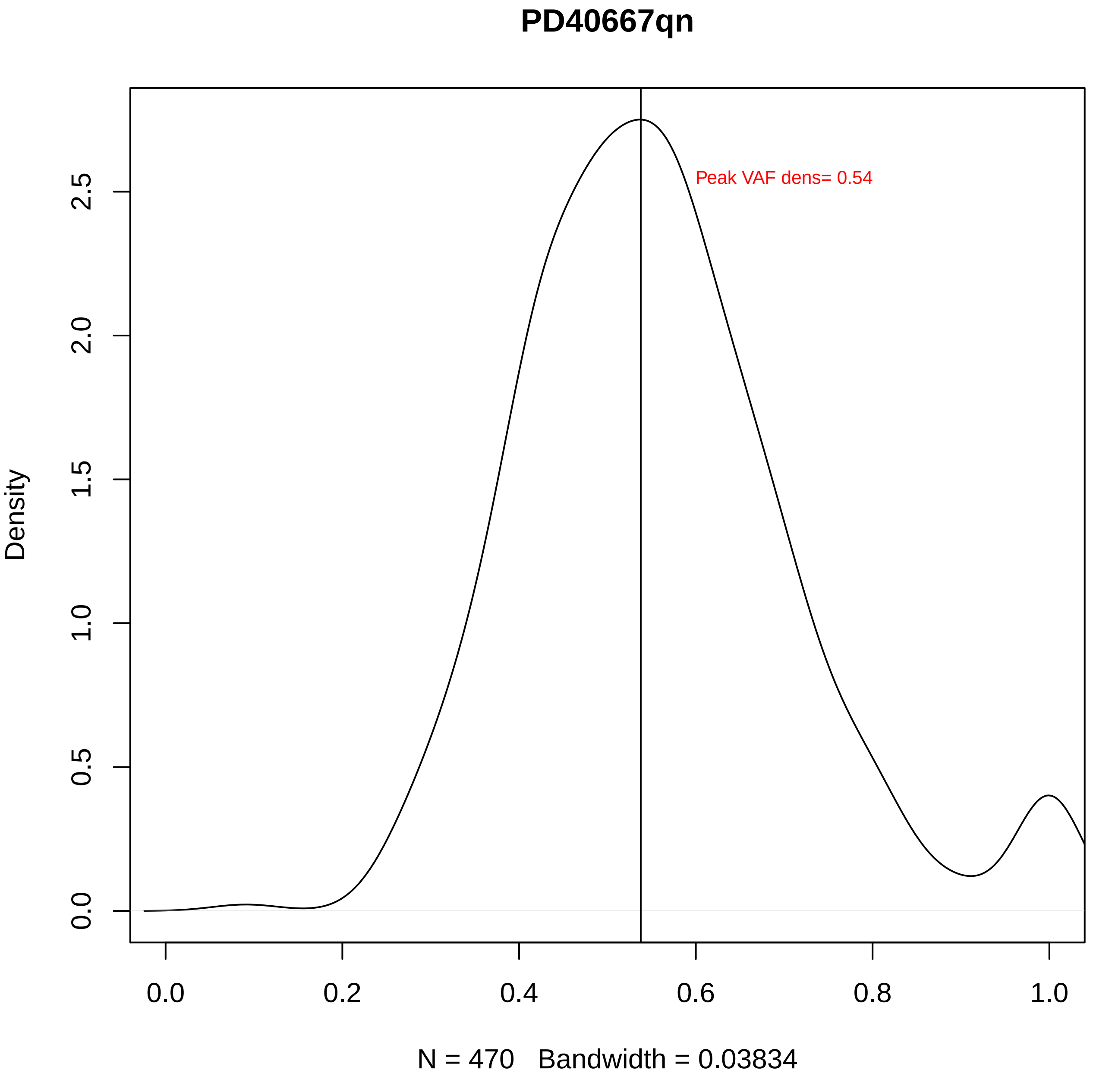

PD40667qn
Peak VAF dens= 0.54
Density
0.0
0.5
1.0
1.5
2.0
2.5
0.0
0.2
0.4
0.6
0.8
1.0
N = 470   Bandwidth = 0.03834

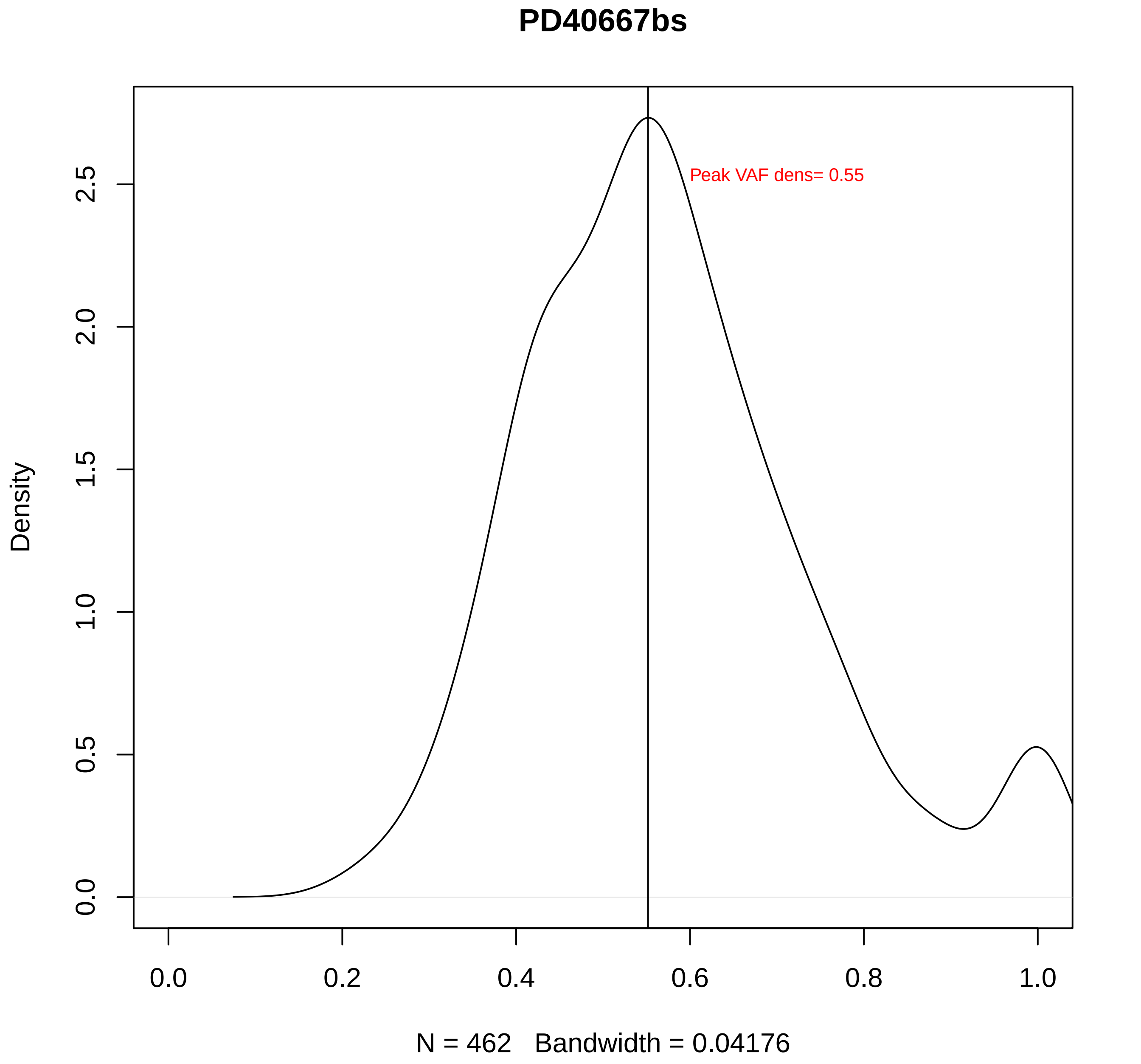
PD40667bs
Peak VAF dens= 0.55
Density
0.0
0.5
1.0
1.5
2.0
2.5
0.0
0.2
0.4
0.6
0.8
1.0
N = 462   Bandwidth = 0.04176

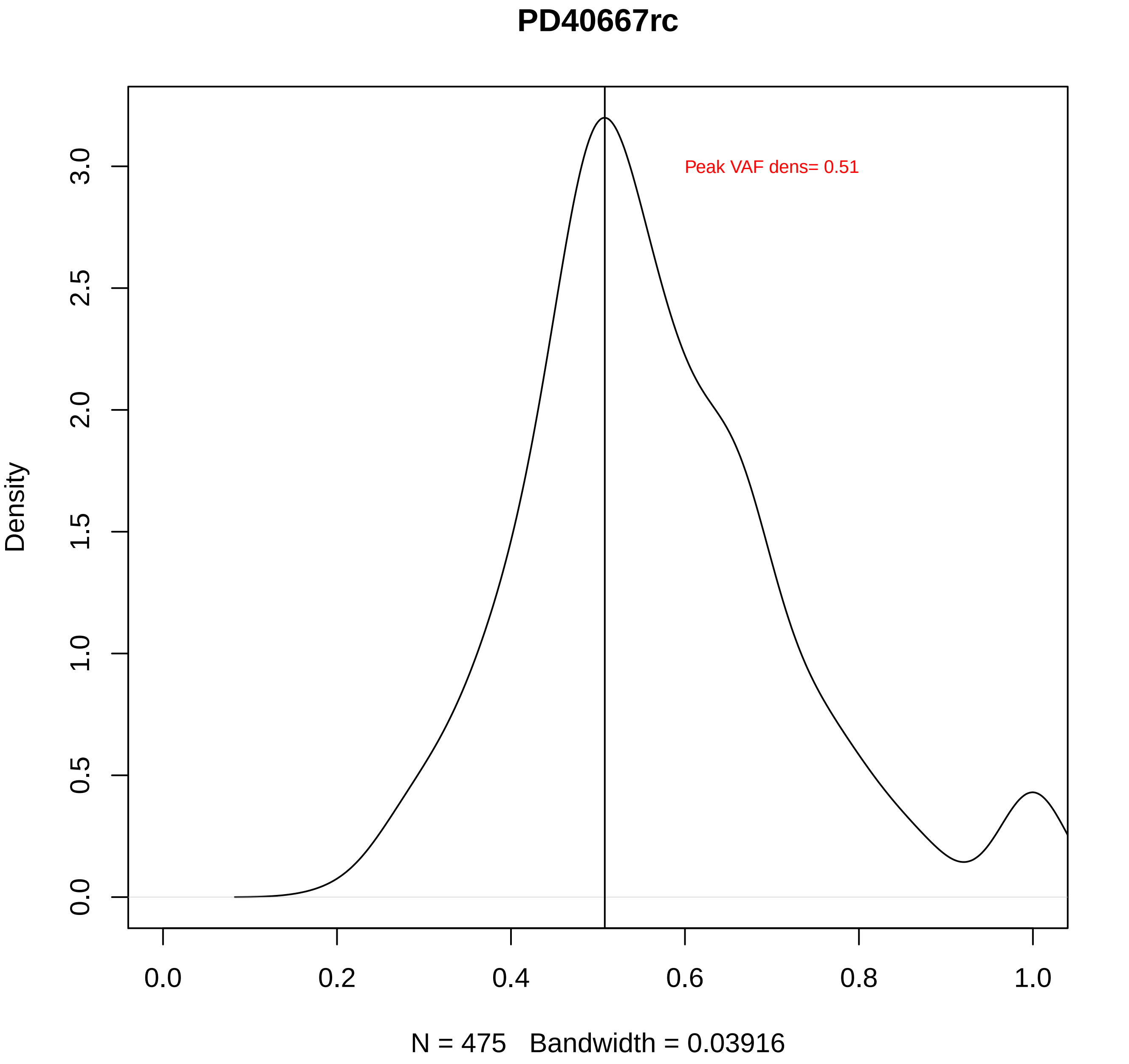
PD40667rc
Peak VAF dens= 0.51
Density
3.0
2.5
2.0
1.5
1.0
0.5
0.0
0.0
0.2
0.4
0.6
0.8
1.0
N = 475 Bandwidth = 0.03916

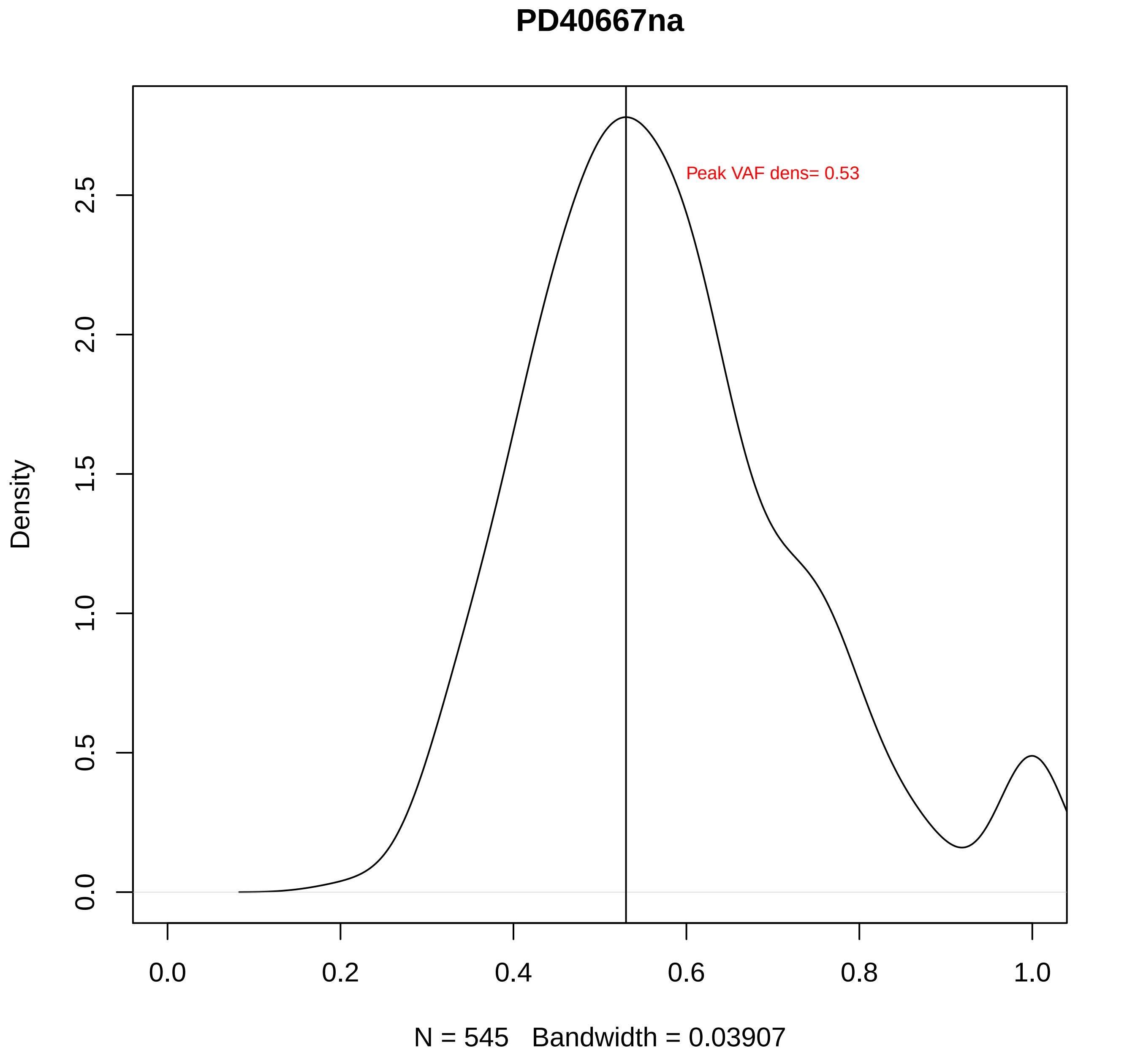

PD40667na
Peak VAF dens= 0.53
Density
0.0
0.5
1.0
1.5
2.0
2.5
0.0
0.2
0.4
0.6
0.8
1.0
N = 545   Bandwidth = 0.03907

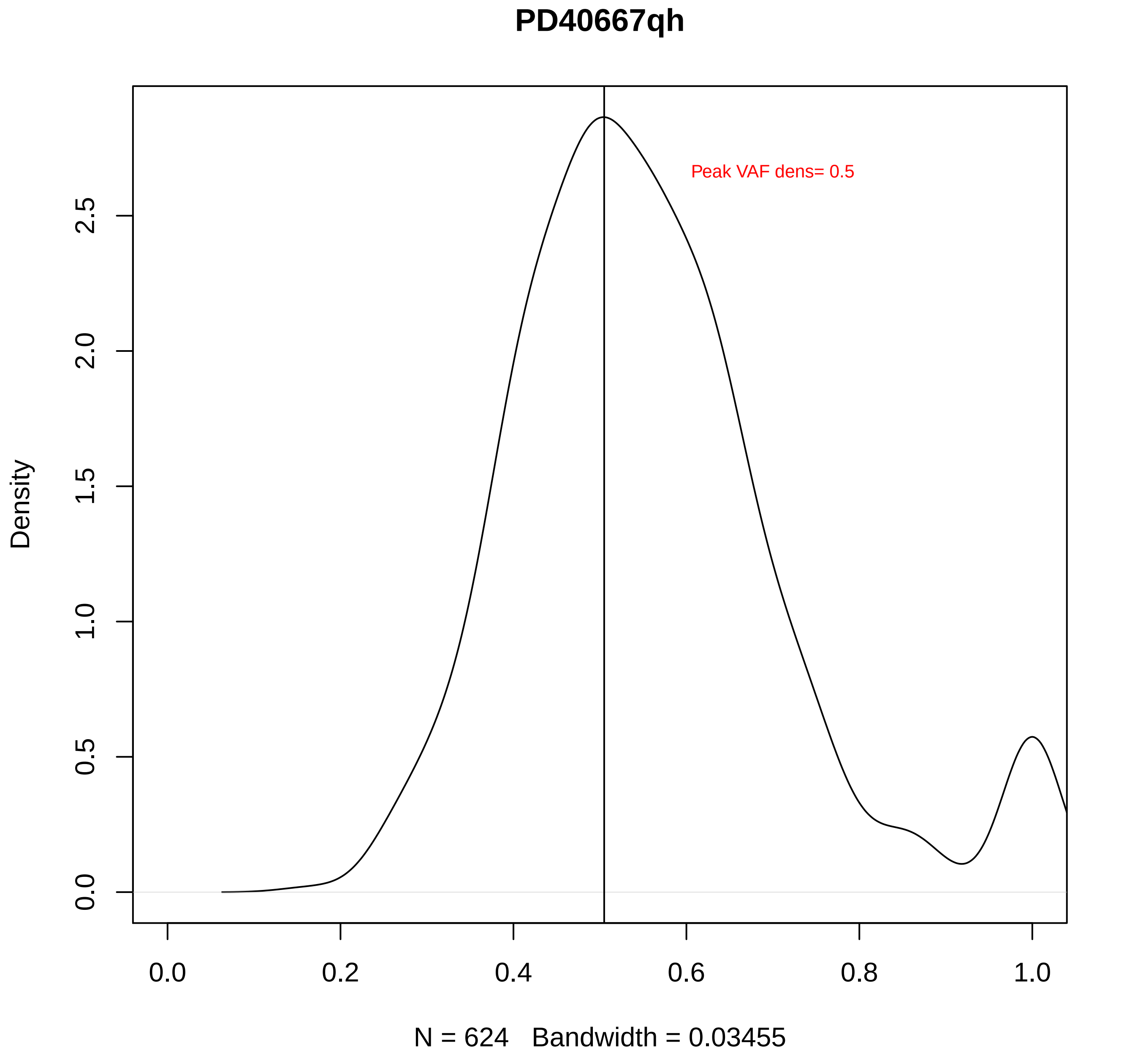

PD40667qh
Peak VAF dens= 0.5
Density
0.0
0.5
1.0
1.5
2.0
2.5
0.0
0.2
0.4
0.6
0.8
1.0
N = 624 Bandwidth = 0.03455

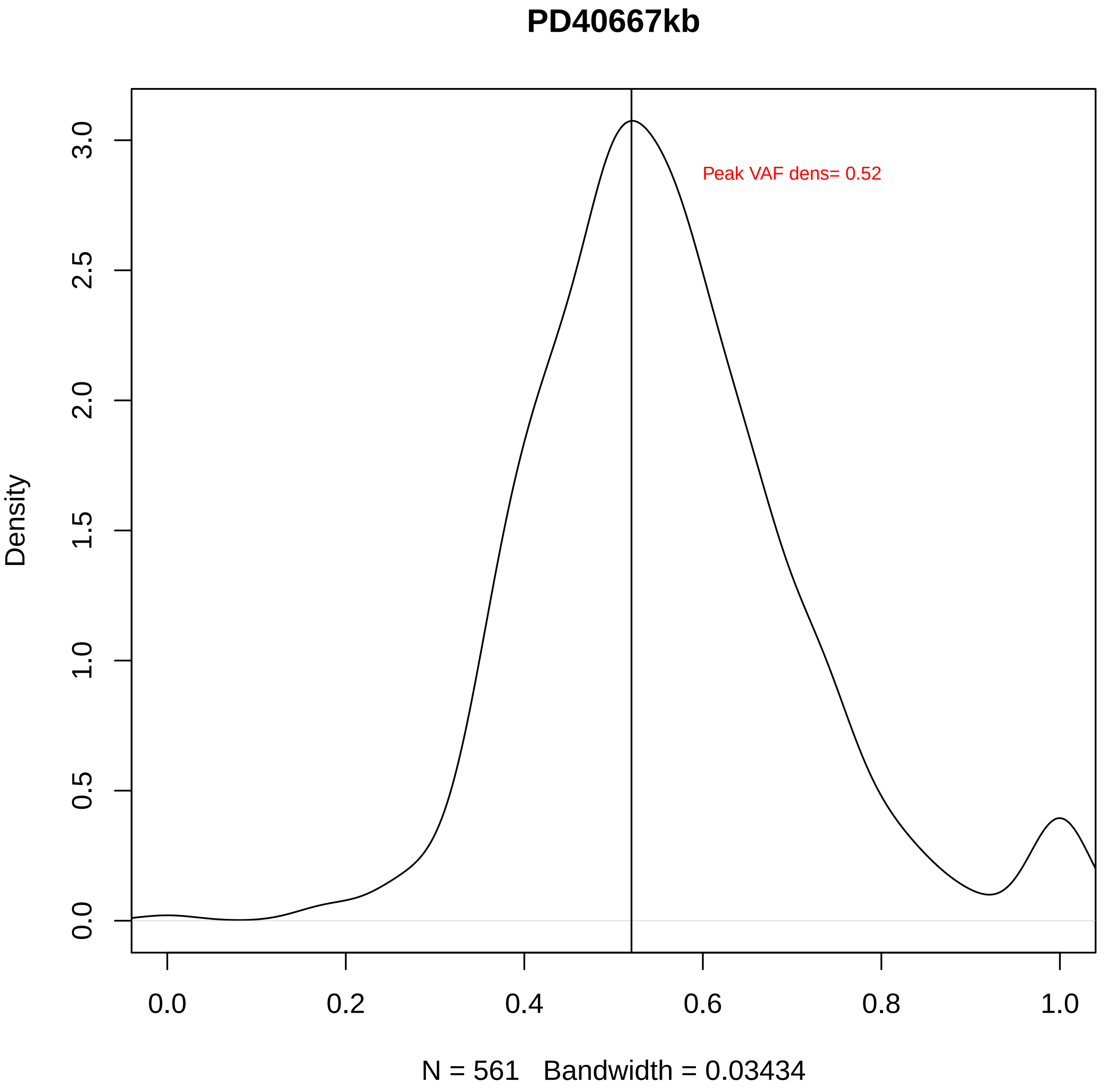

PD40667kb
Peak VAF dens= 0.52
Density
0.0
0.5
1.0
1.5
2.0
2.5
3.0
0.0
0.2
0.4
0.6
0.8
1.0
N = 561 Bandwidth = 0.03434

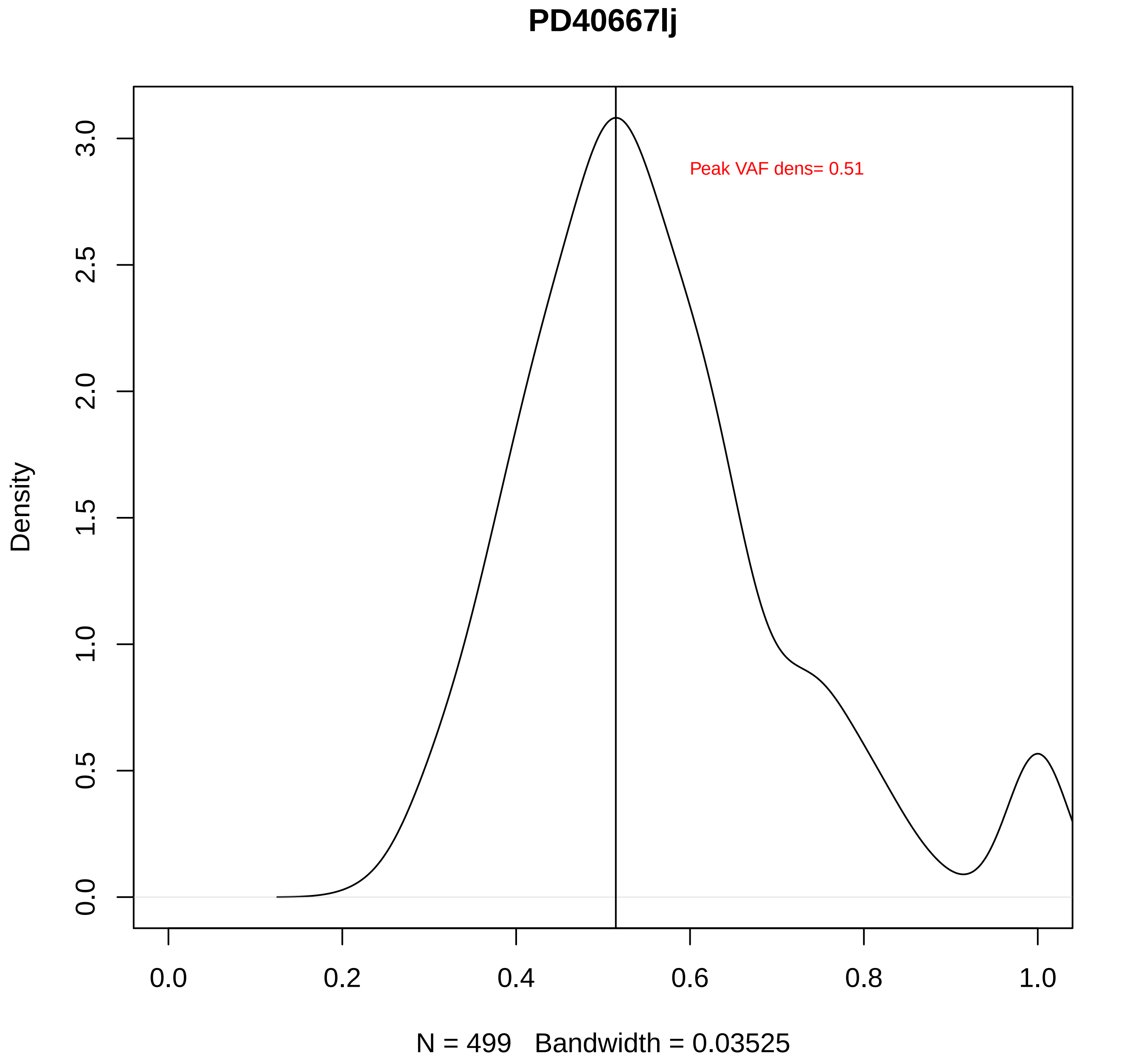

PD40667lj
Peak VAF dens= 0.51
Density
0.0
0.5
1.0
1.5
2.0
2.5
3.0
0.0
0.2
0.4
0.6
0.8
1.0
N = 499   Bandwidth = 0.03525

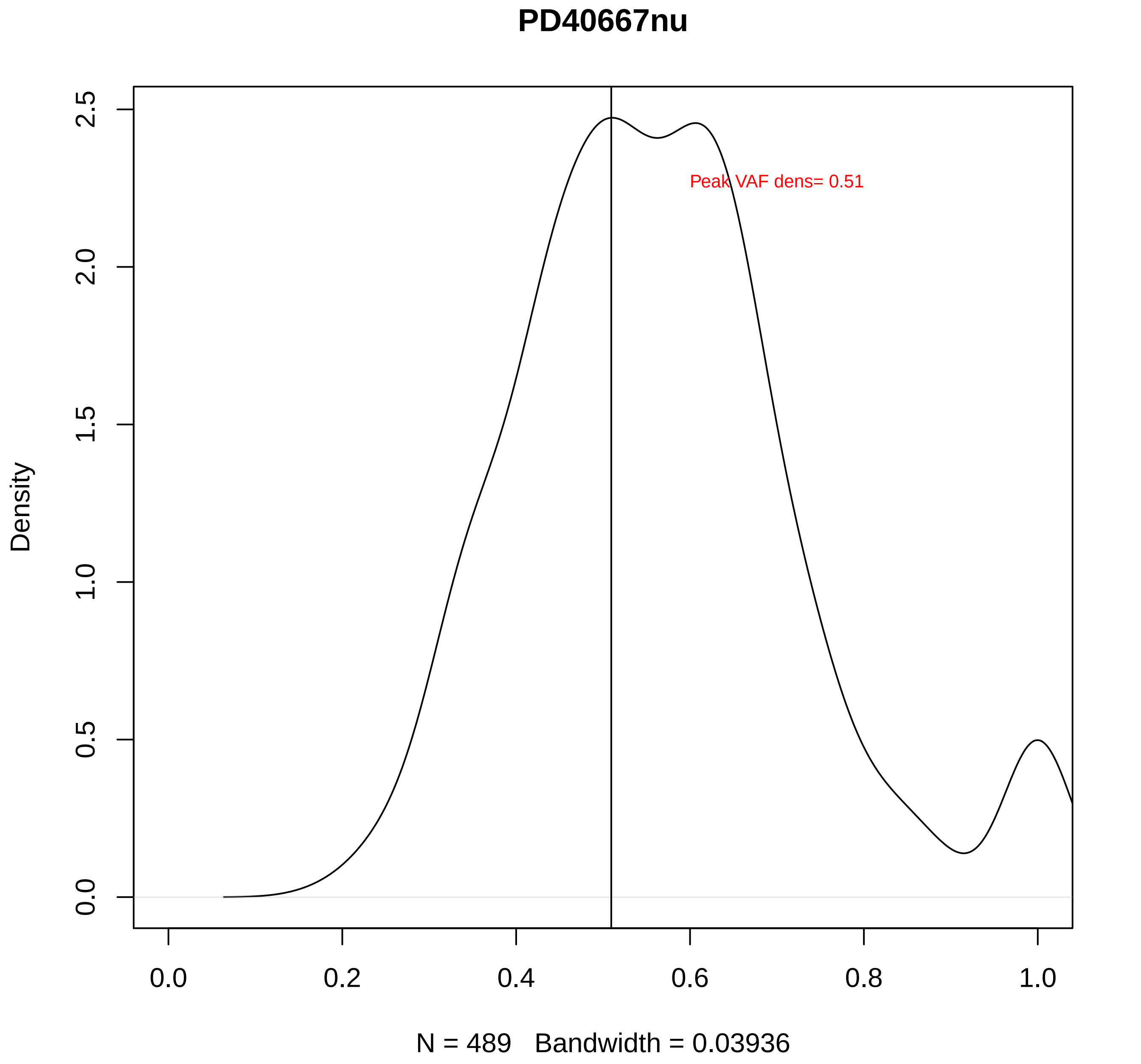
PD40667nu
Peak VAF dens= 0.51
Density
0.0
0.5
1.0
1.5
2.0
2.5
0.0
0.2
0.4
0.6
0.8
1.0
N = 489   Bandwidth = 0.03936

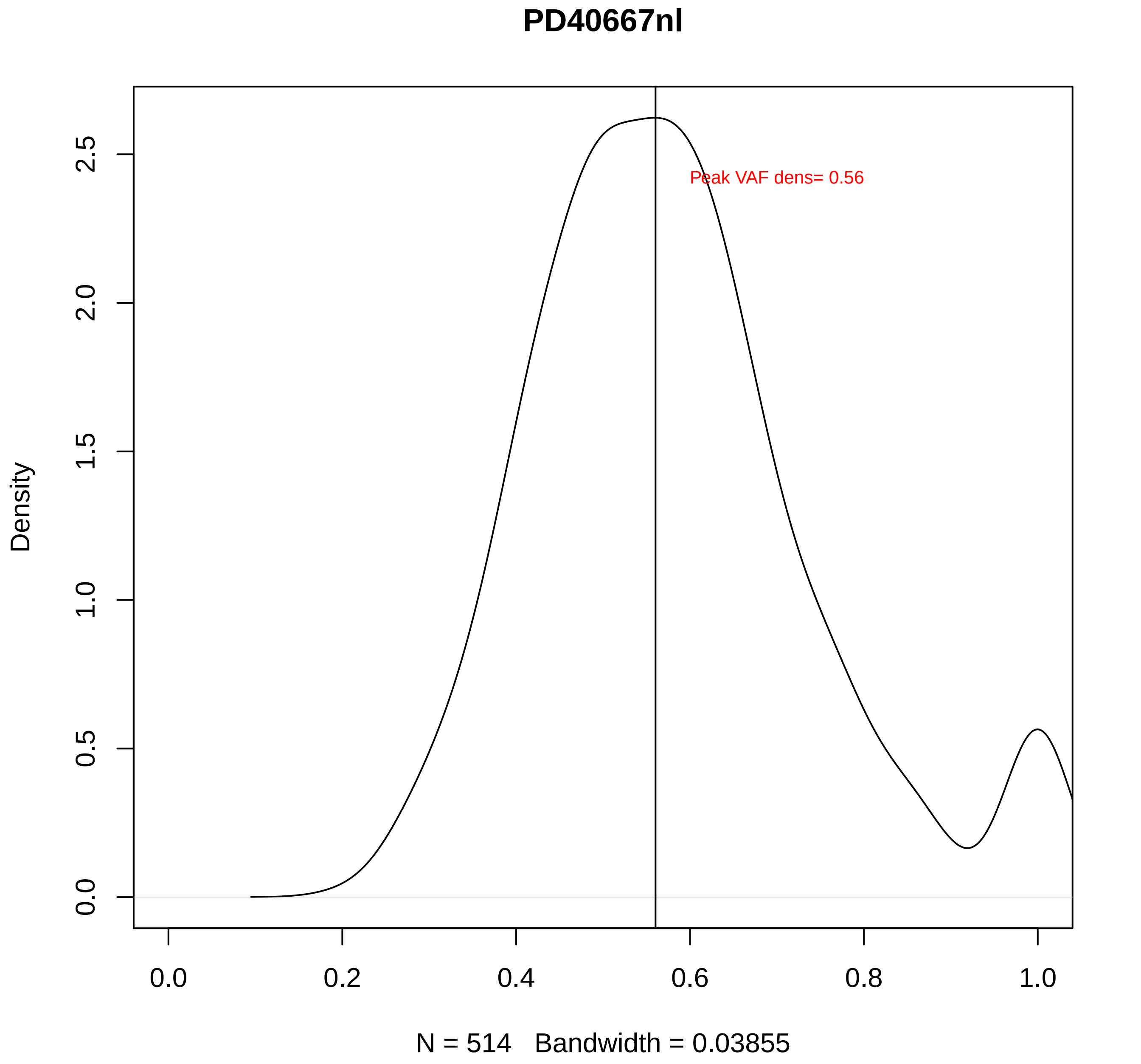

PD40667nl
Peak VAF dens= 0.56
Density
0.0
0.5
1.0
1.5
2.0
2.5
0.0
0.2
0.4
0.6
0.8
1.0
N = 514 Bandwidth = 0.03855

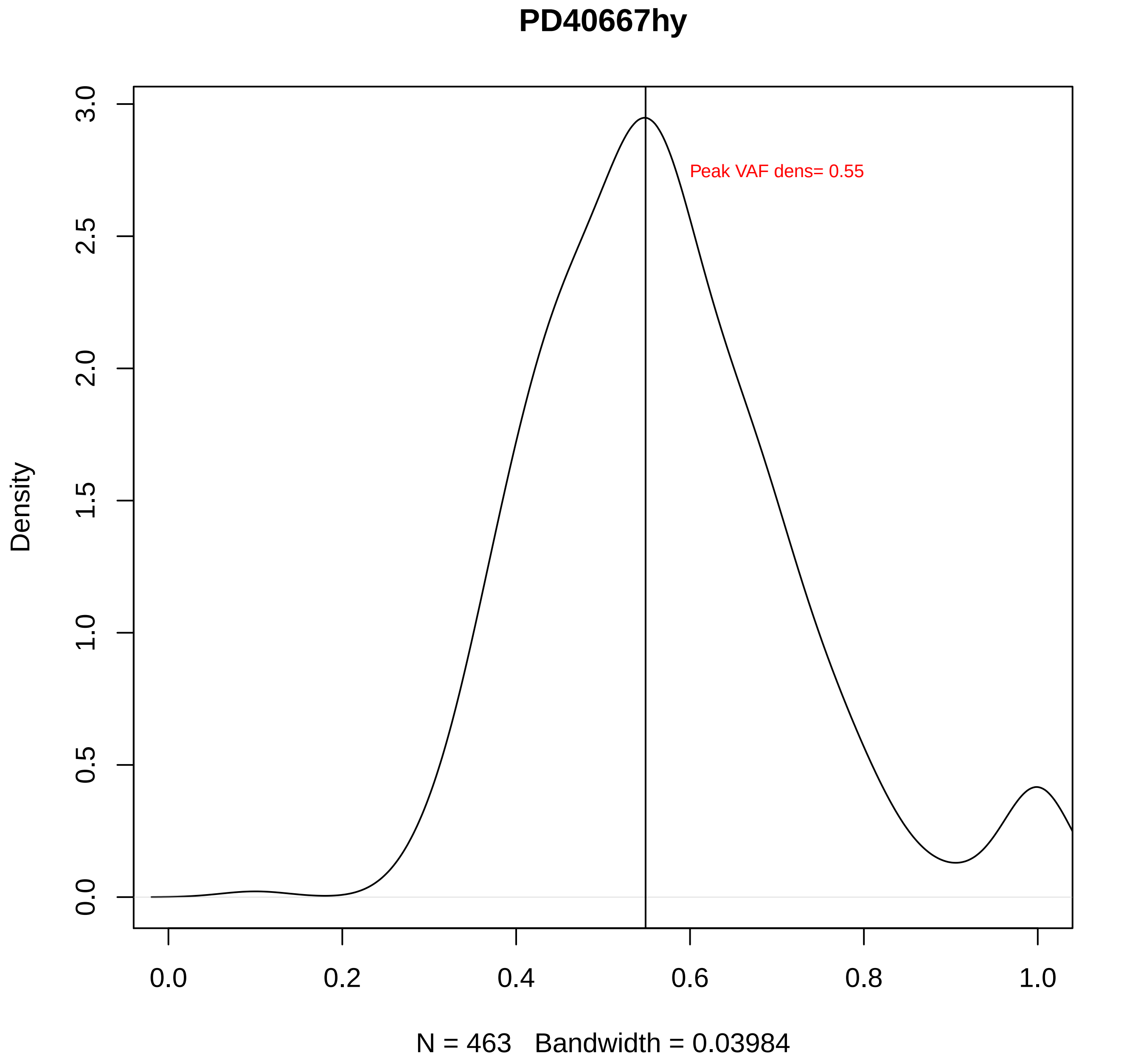

PD40667hy
Peak VAF dens= 0.55
Density
0.0
0.5
1.0
1.5
2.0
2.5
3.0
0.0
0.2
0.4
0.6
0.8
1.0
N = 463   Bandwidth = 0.03984

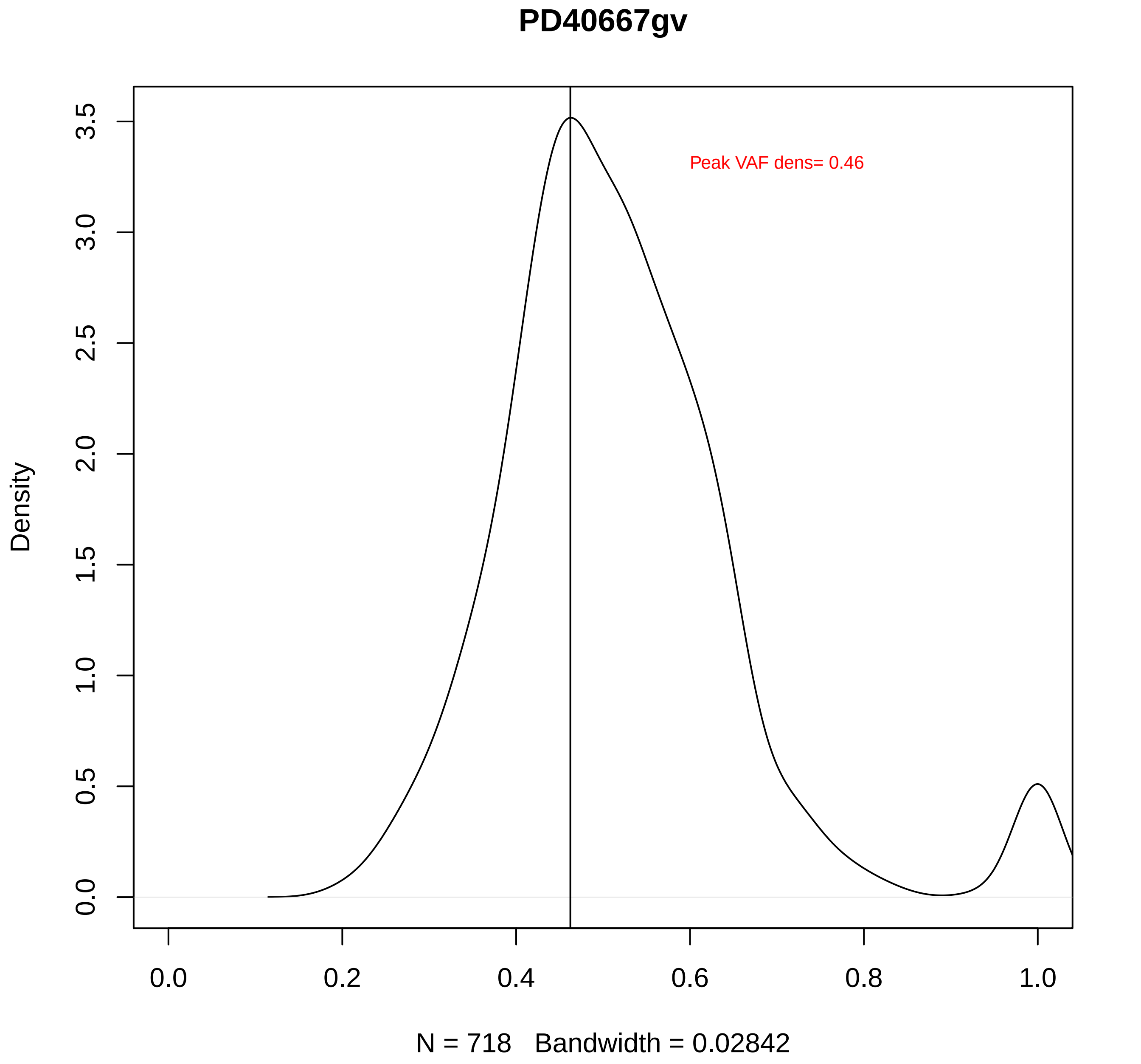
PD40667gv
Peak VAF dens= 0.46
Density
0.0
0.5
1.0
1.5
2.0
2.5
3.0
3.5
0.0
0.2
0.4
0.6
0.8
1.0
N = 718   Bandwidth = 0.02842

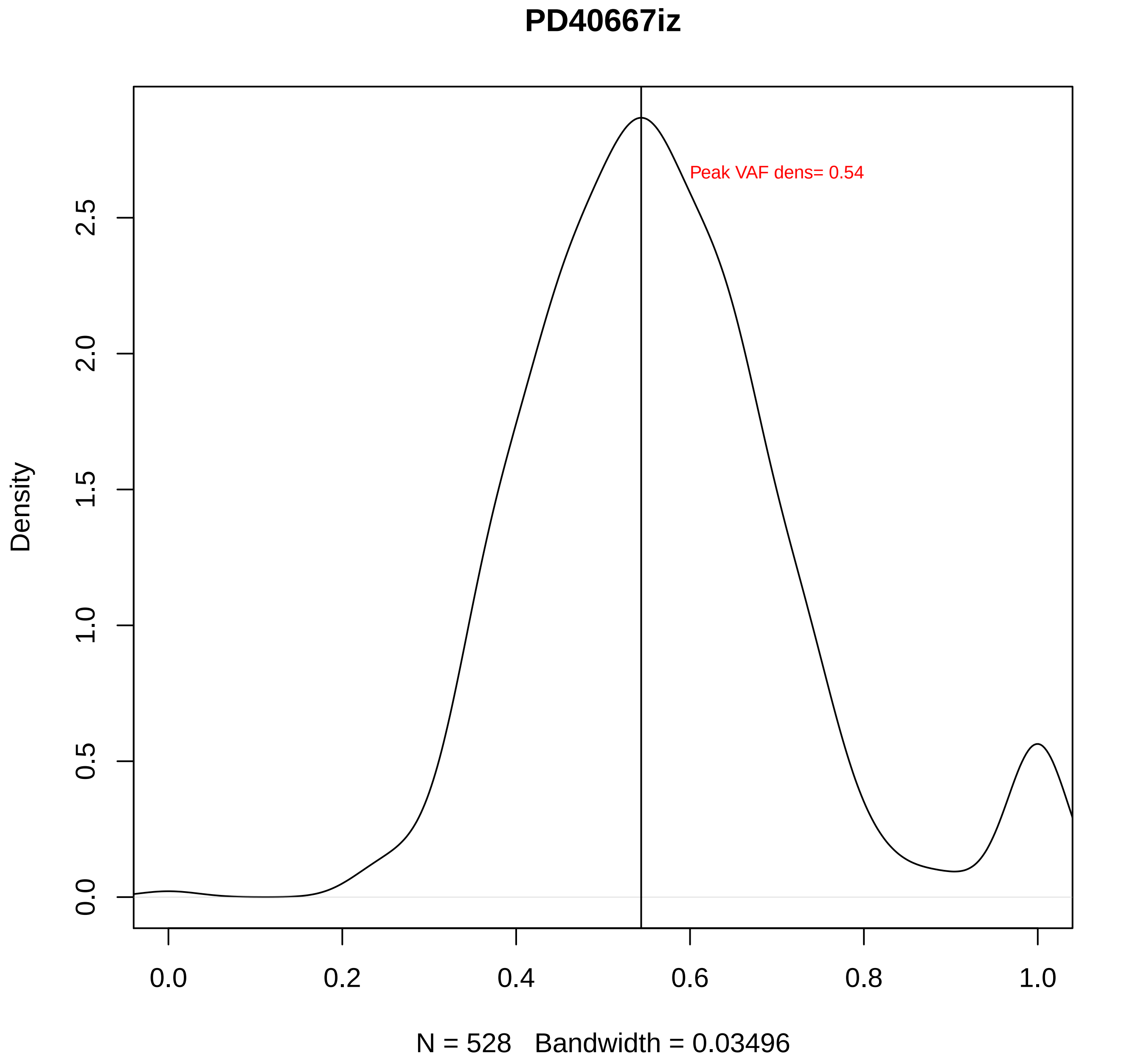
PD40667iz
Peak VAF dens= 0.54
Density
0.0
0.5
1.0
1.5
2.0
2.5
0.0
0.2
0.4
0.6
0.8
1.0
N = 528   Bandwidth = 0.03496

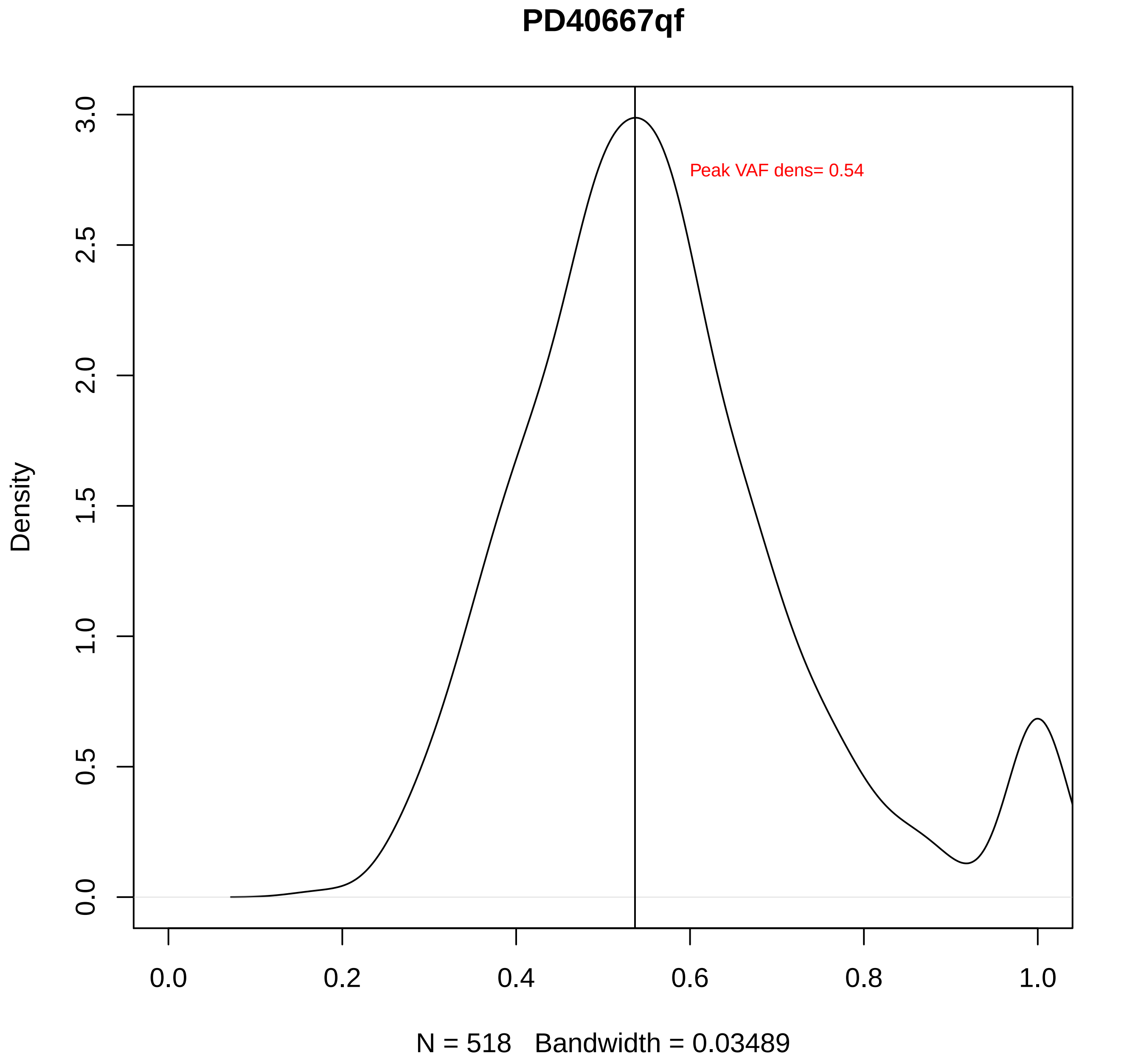
PD40667qf
Peak VAF dens= 0.54
Density
0.0
0.5
1.0
1.5
2.0
2.5
3.0
0.0
0.2
0.4
0.6
0.8
1.0
N = 518 Bandwidth = 0.03489

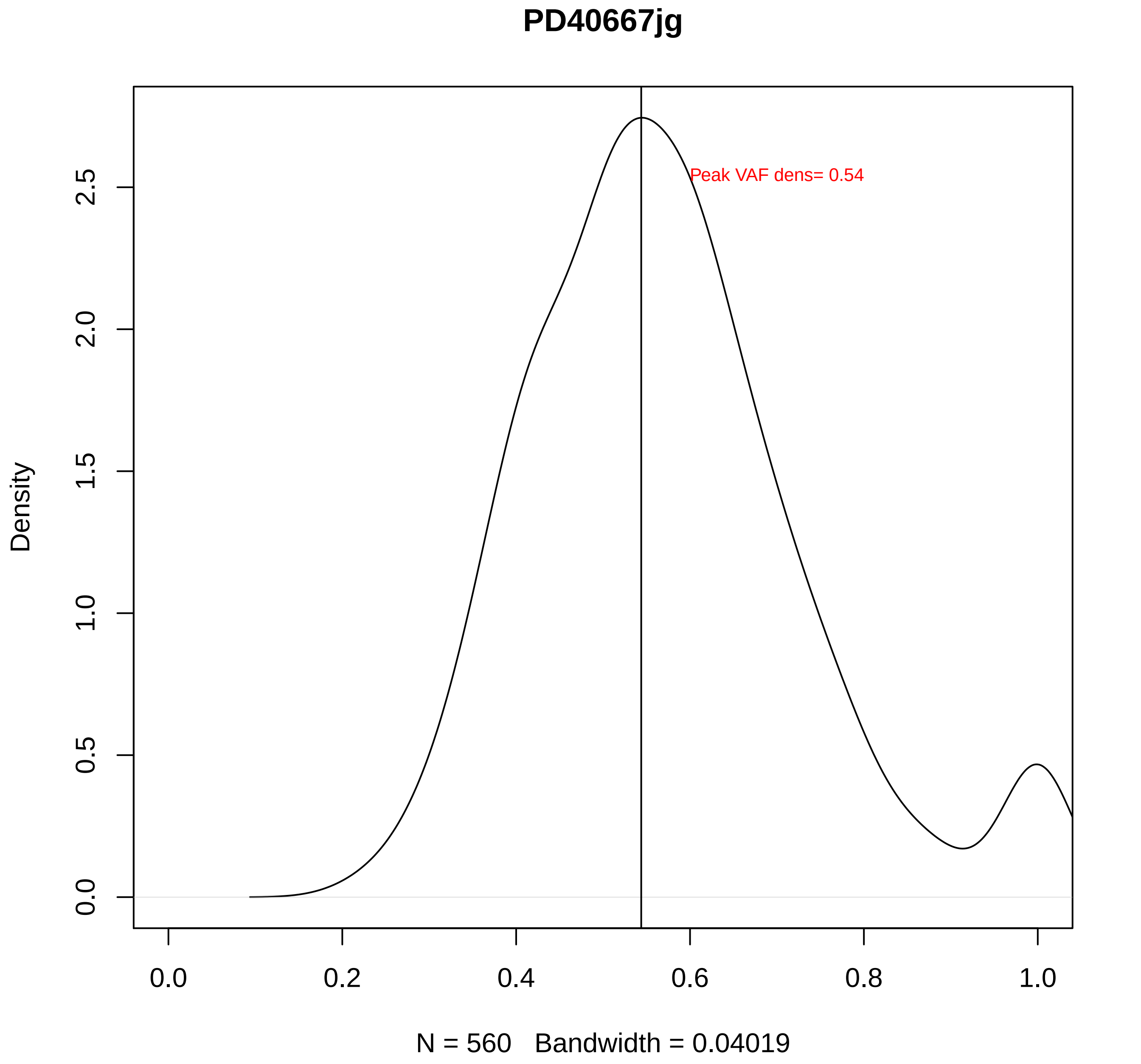
PD40667jg
Peak VAF dens= 0.54
Density
0.0
0.5
1.0
1.5
2.0
2.5
0.0
0.2
0.4
0.6
0.8
1.0
N = 560   Bandwidth = 0.04019

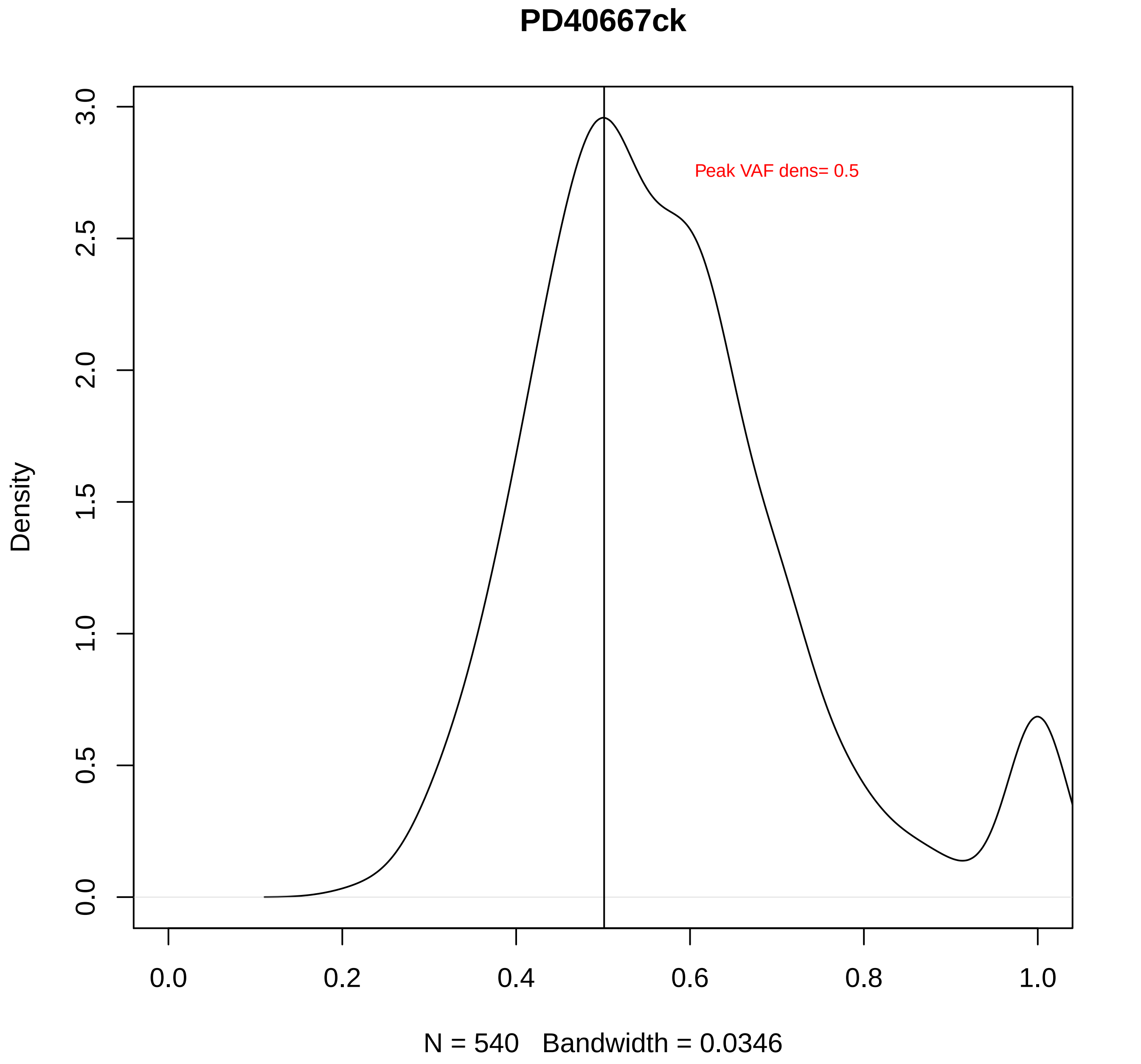
PD40667ck
Peak VAF dens= 0.5
Density
3.0
2.5
2.0
1.5
1.0
0.5
0.0
0.0
0.2
0.4
0.6
0.8
1.0
N = 540   Bandwidth = 0.0346

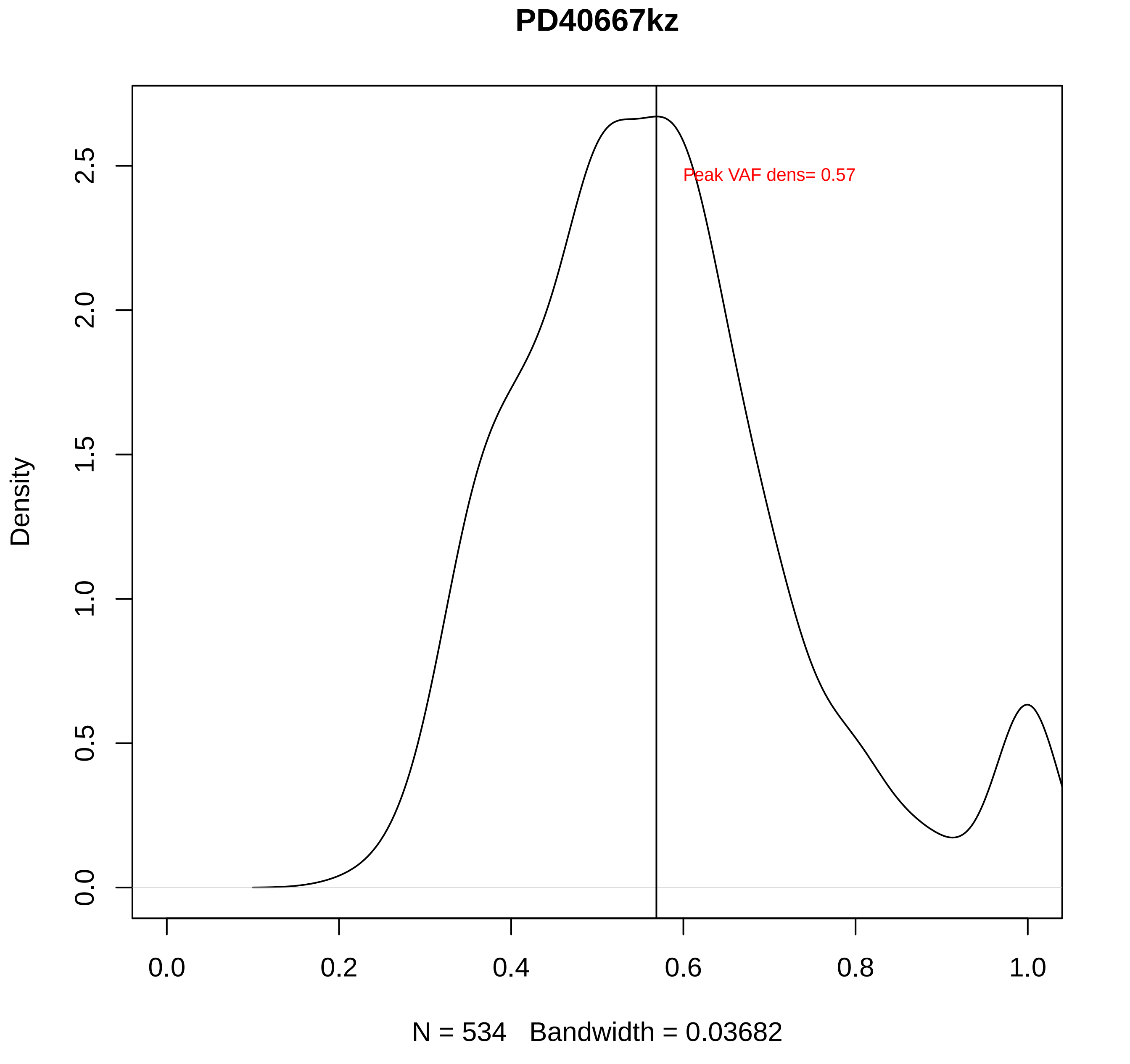
PD40667kz
Peak VAF dens= 0.57
Density
0.0
0.5
1.0
1.5
2.0
2.5
0.0
0.2
0.4
0.6
0.8
1.0
N = 534 Bandwidth = 0.03682

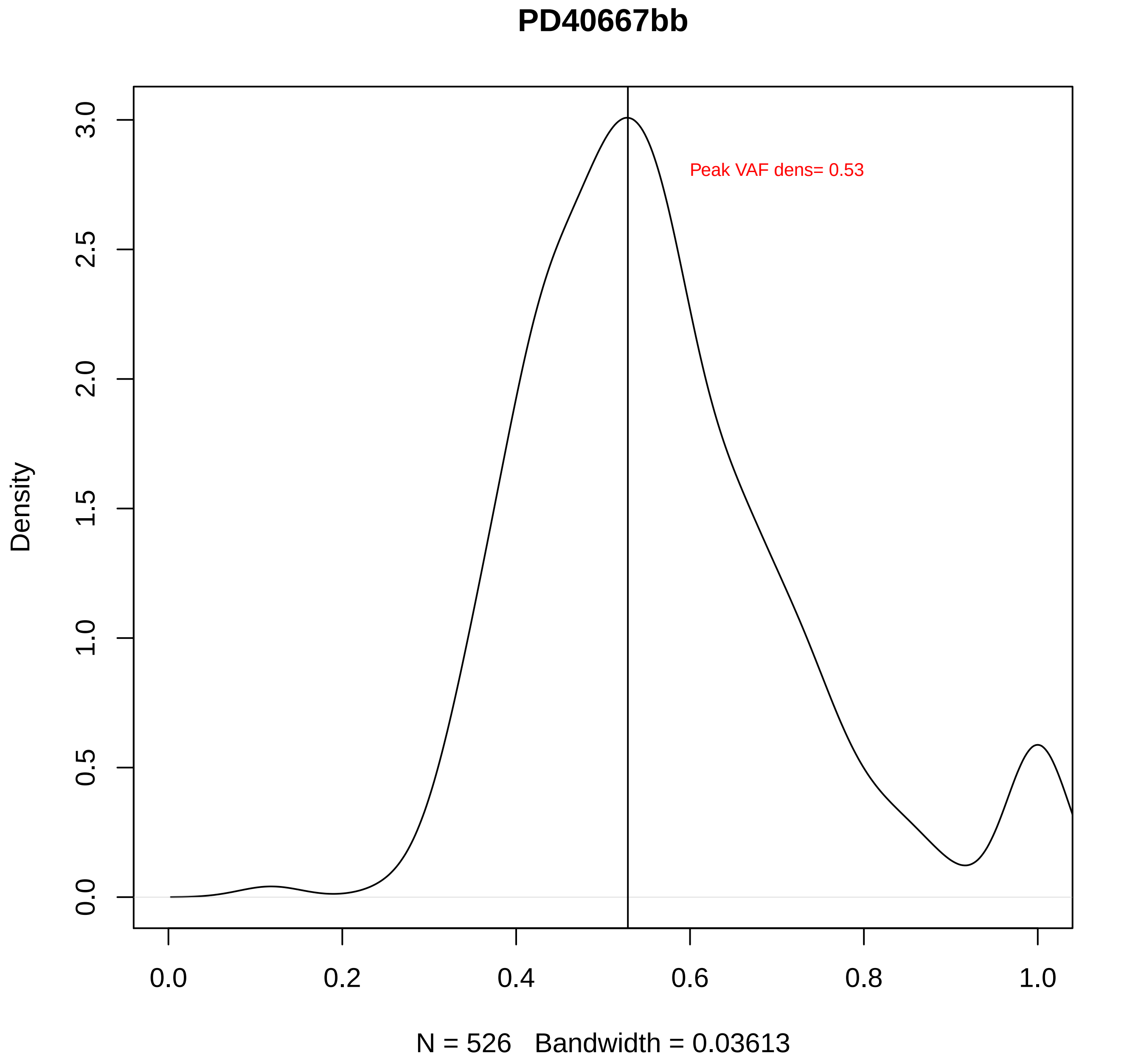
PD40667bb
Peak VAF dens= 0.53
Density
0.0
0.5
1.0
1.5
2.0
2.5
3.0
0.0
0.2
0.4
0.6
0.8
1.0
N = 526   Bandwidth = 0.03613

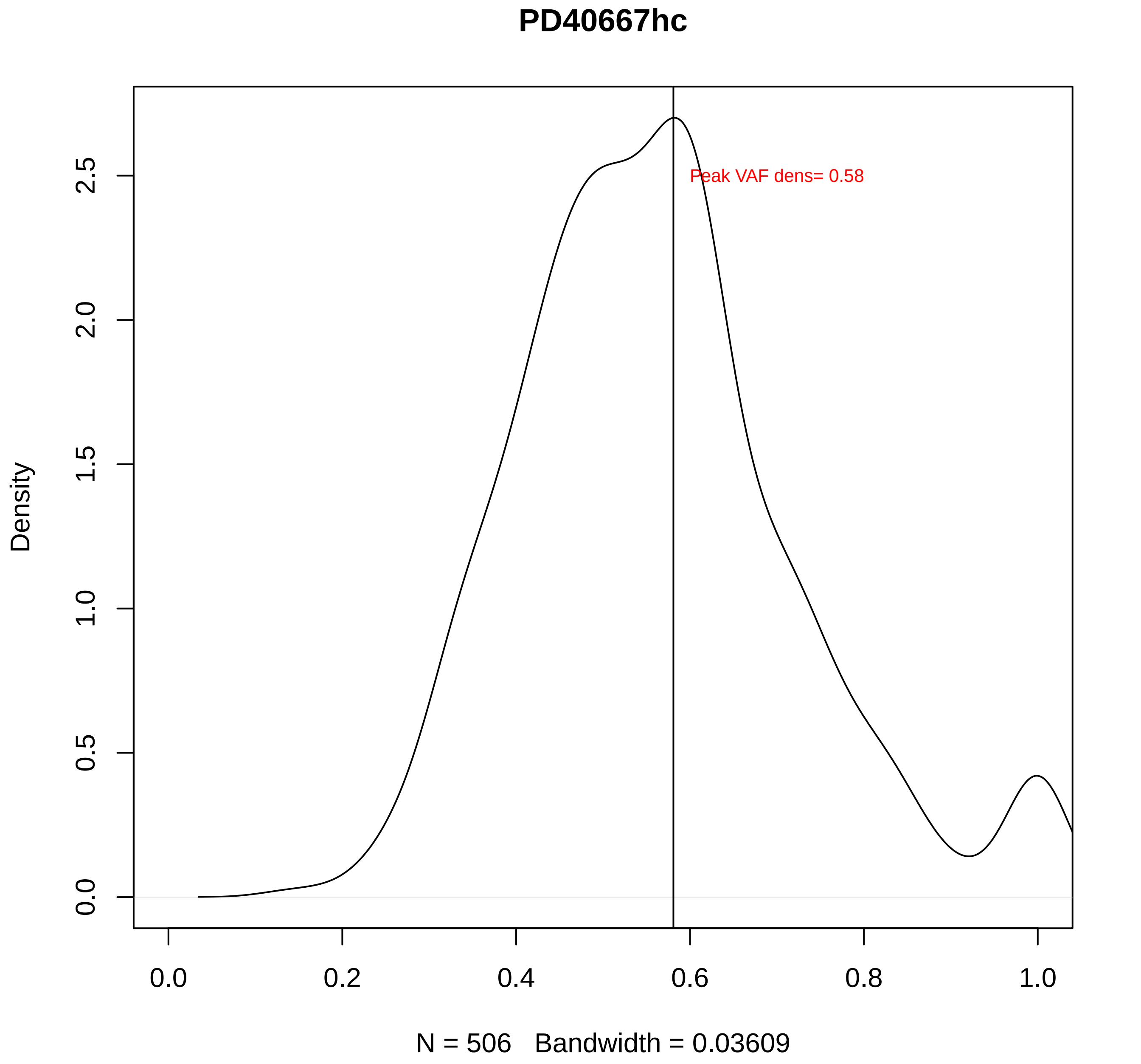
PD40667hc
Peak VAF dens= 0.58
Density
0.0
0.5
1.0
1.5
2.0
2.5
0.0
0.2
0.4
0.6
0.8
1.0
N = 506   Bandwidth = 0.03609

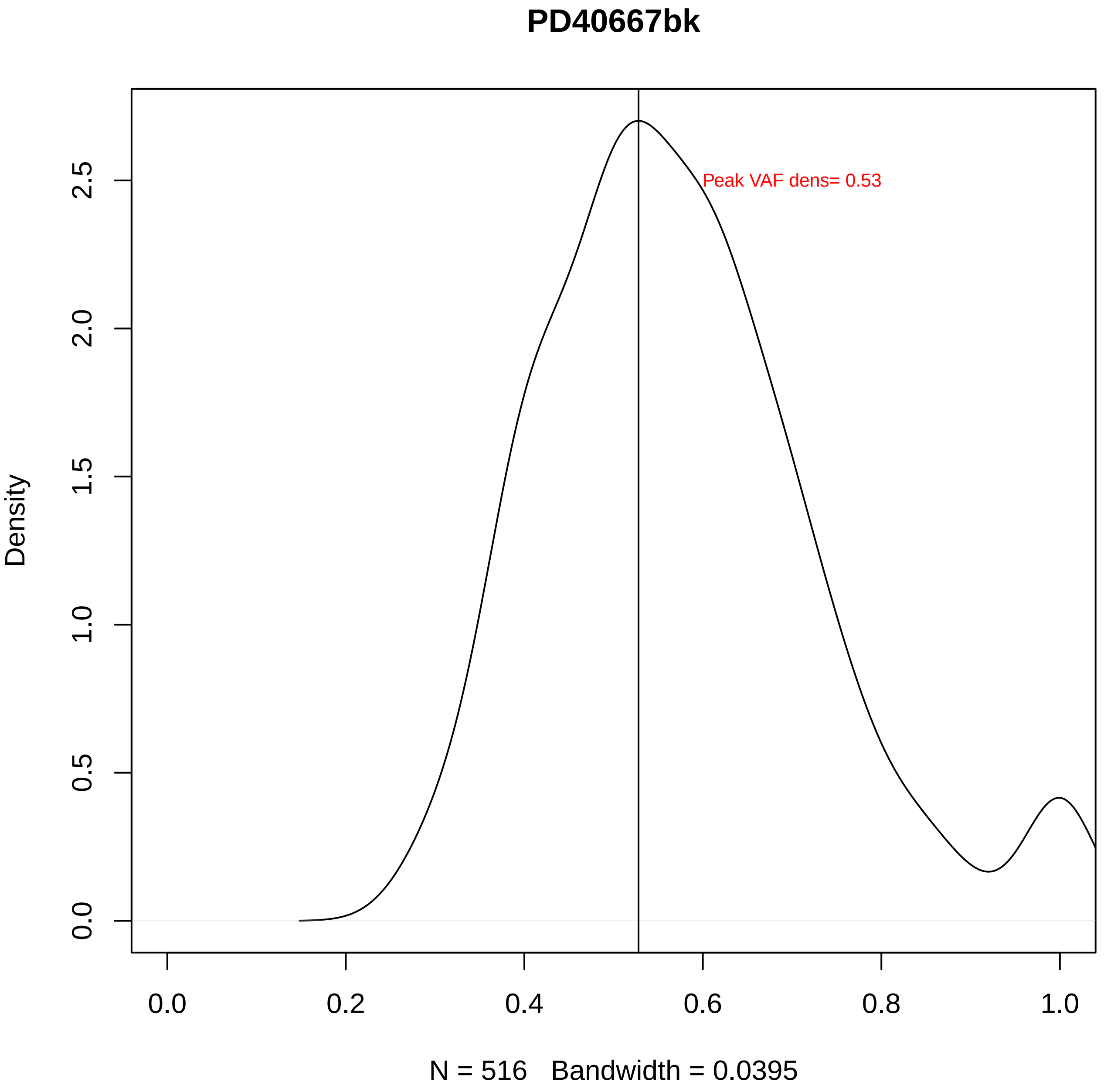
PD40667bk
Peak VAF dens= 0.53
Density
0.0
0.5
1.0
1.5
2.0
2.5
0.0
0.2
0.4
0.6
0.8
1.0
N = 516   Bandwidth = 0.0395

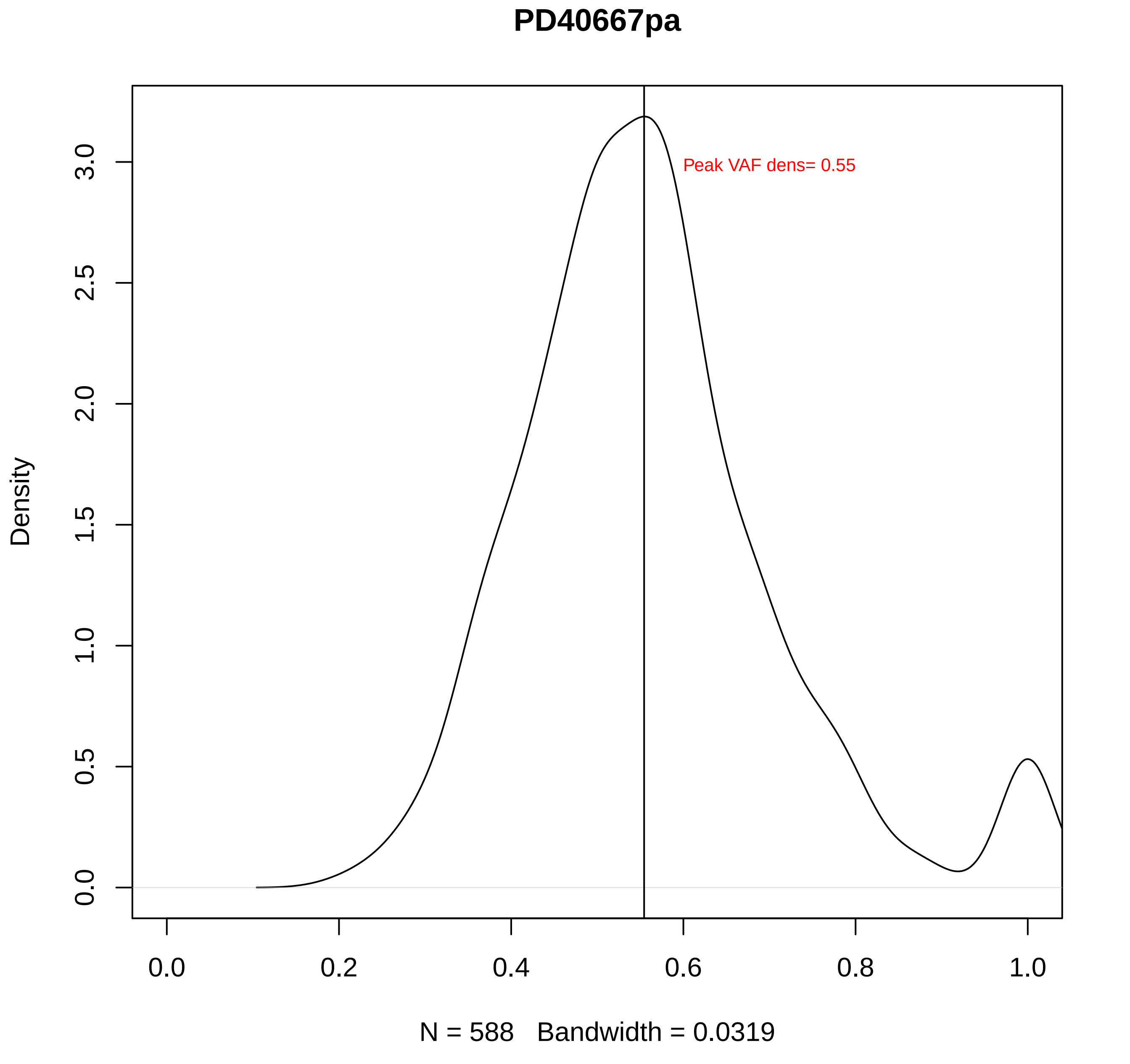
PD40667pa
Peak VAF dens= 0.55
Density
0.0
0.5
1.0
1.5
2.0
2.5
3.0
0.0
0.2
0.4
0.6
0.8
1.0
N = 588   Bandwidth = 0.0319

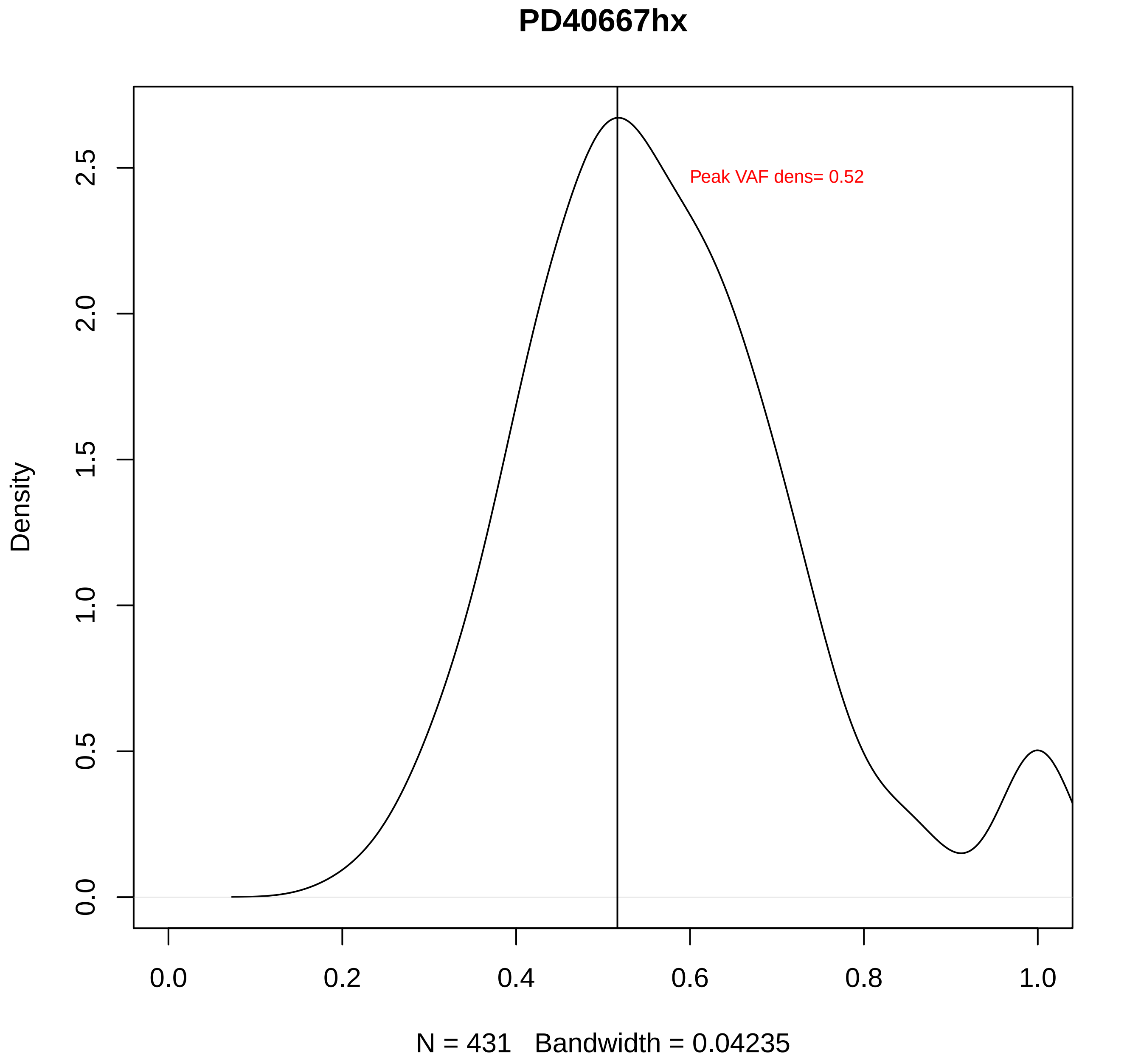
PD40667hx
Peak VAF dens= 0.52
Density
0.0
0.5
1.0
1.5
2.0
2.5
0.0
0.2
0.4
0.6
0.8
1.0
N = 431   Bandwidth = 0.04235

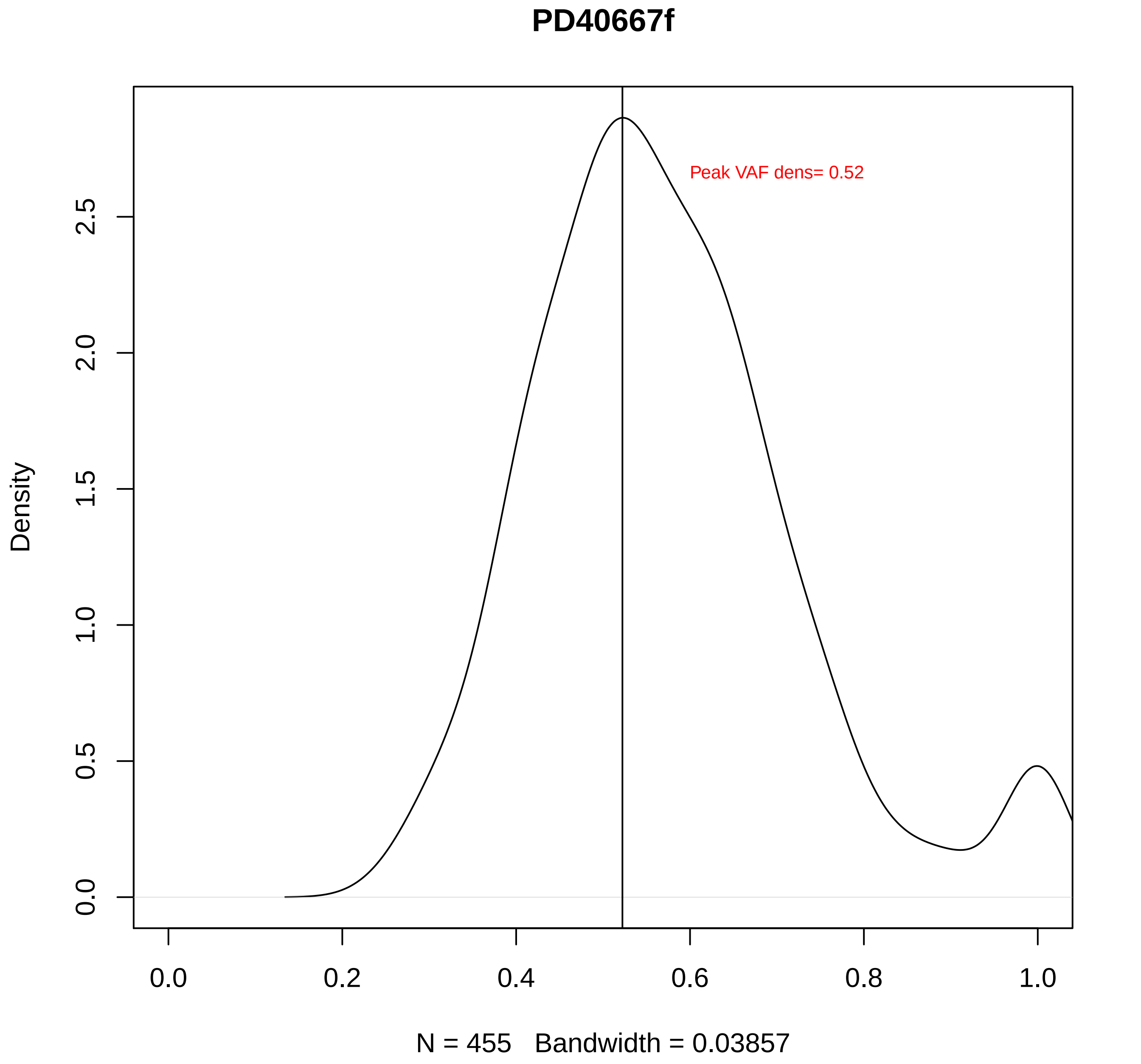
PD40667f
Peak VAF dens= 0.52
Density
0.0
0.5
1.0
1.5
2.0
2.5
0.0
0.2
0.4
0.6
0.8
1.0
N = 455   Bandwidth = 0.03857

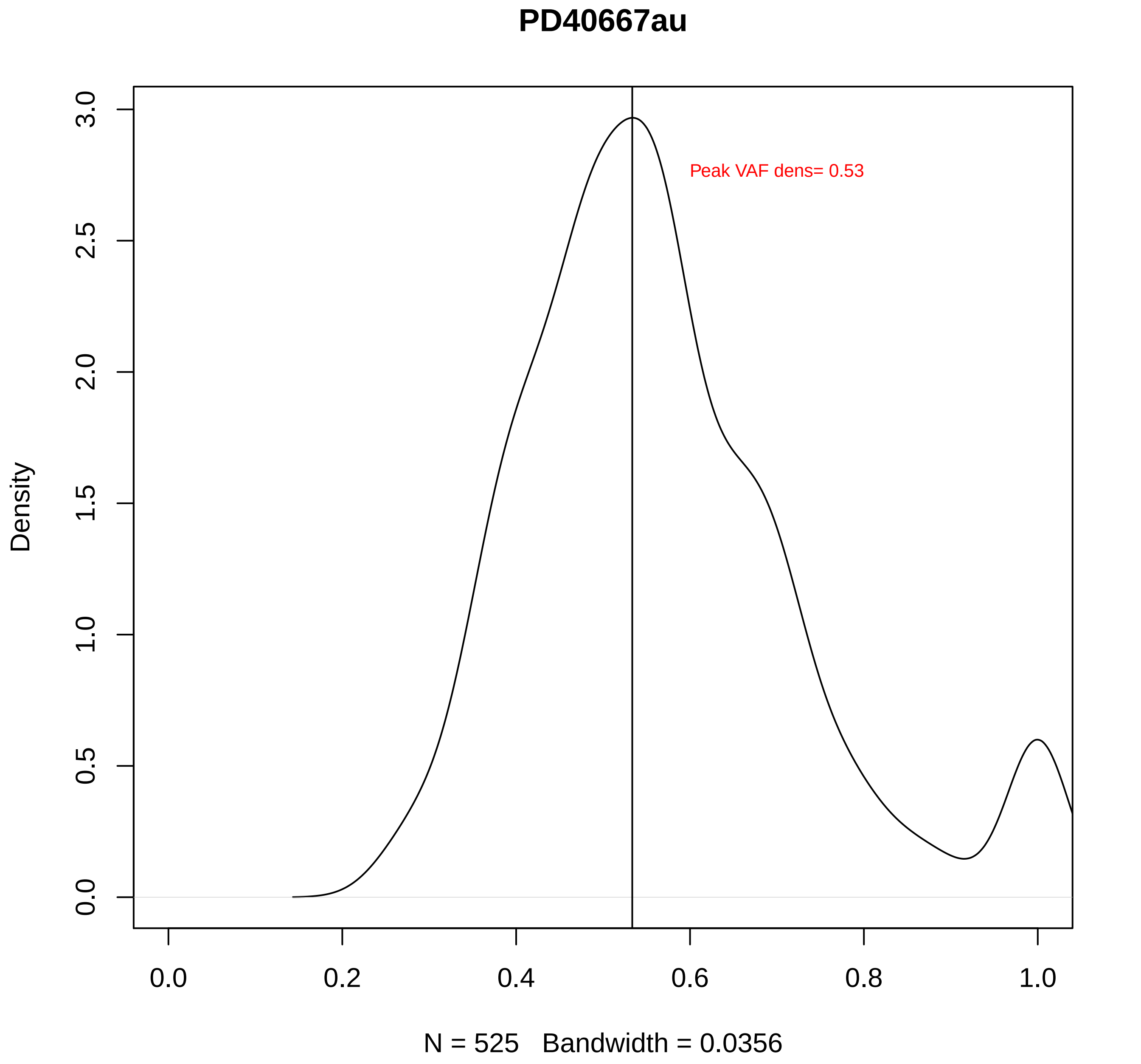
PD40667au
Peak VAF dens= 0.53
Density
0.0
0.5
1.0
1.5
2.0
2.5
3.0
0.0
0.2
0.4
0.6
0.8
1.0
N = 525 Bandwidth = 0.0356

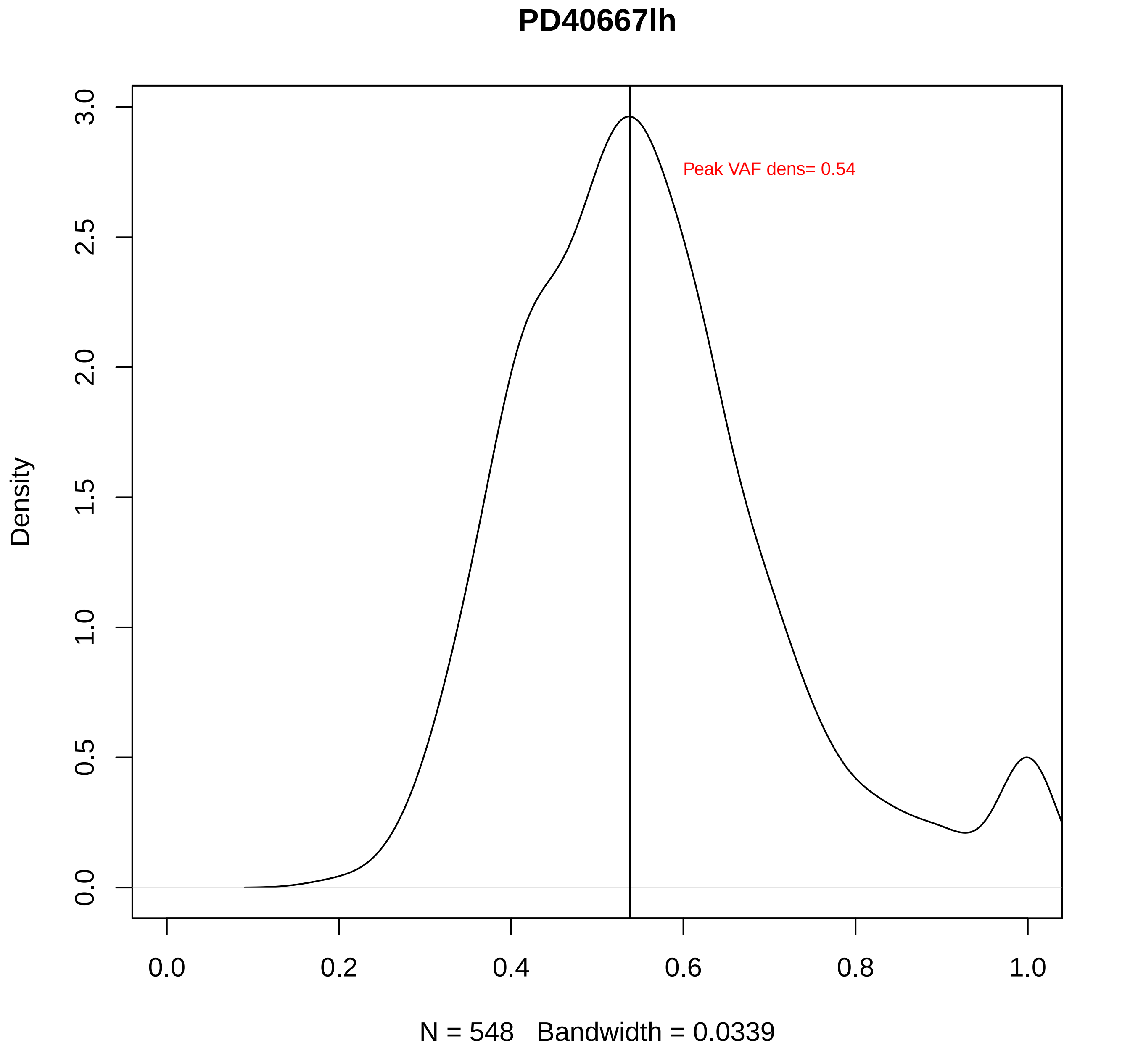
PD40667lh
Peak VAF dens= 0.54
Density
3.0
2.5
2.0
1.5
1.0
0.5
0.0
0.0
0.2
0.4
0.6
0.8
1.0
N = 548   Bandwidth = 0.0339

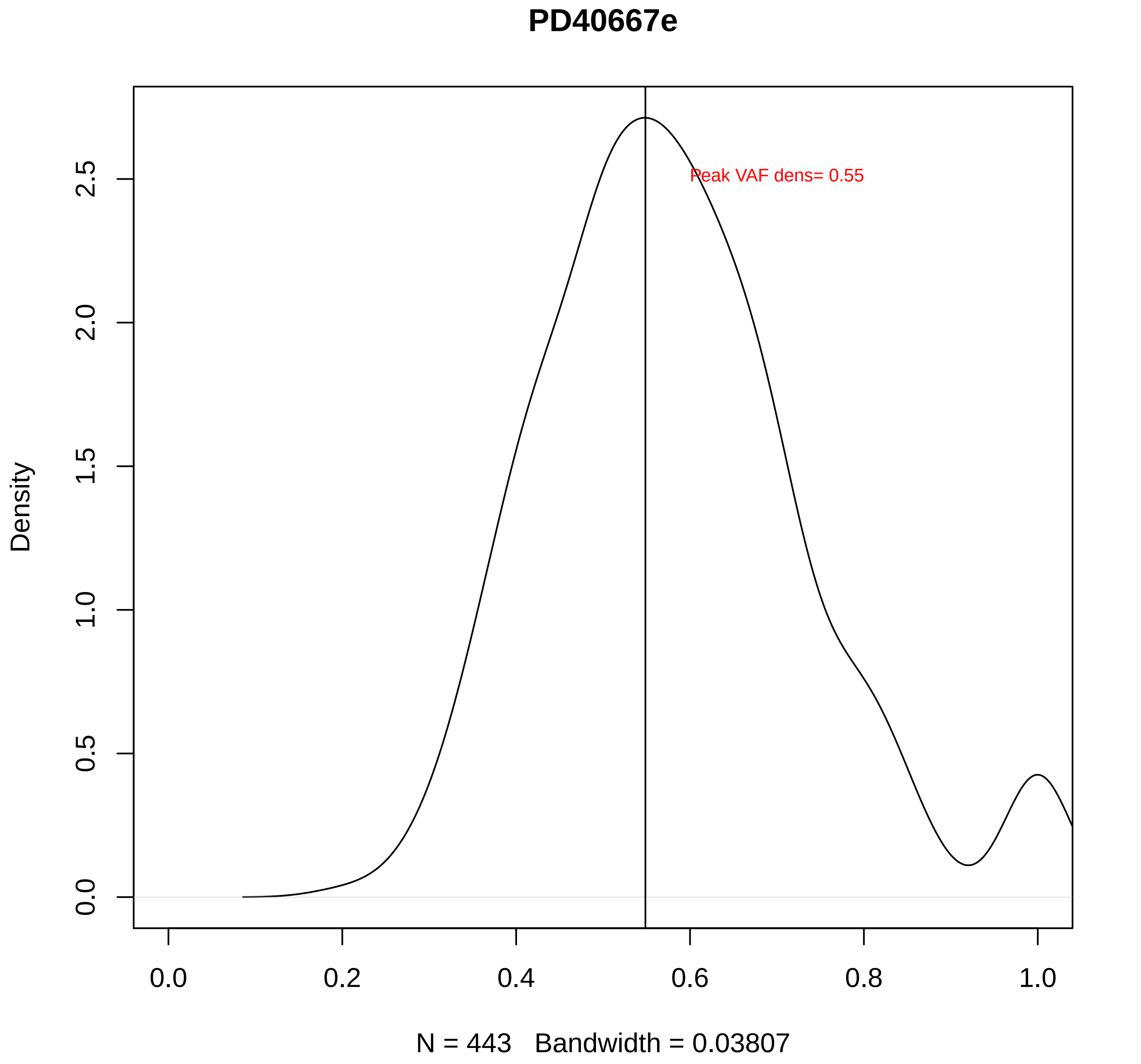
PD40667e
Peak VAF dens= 0.55
Density
0.0
0.5
1.0
1.5
2.0
2.5
0.0
0.2
0.4
0.6
0.8
1.0
N = 443 Bandwidth = 0.03807

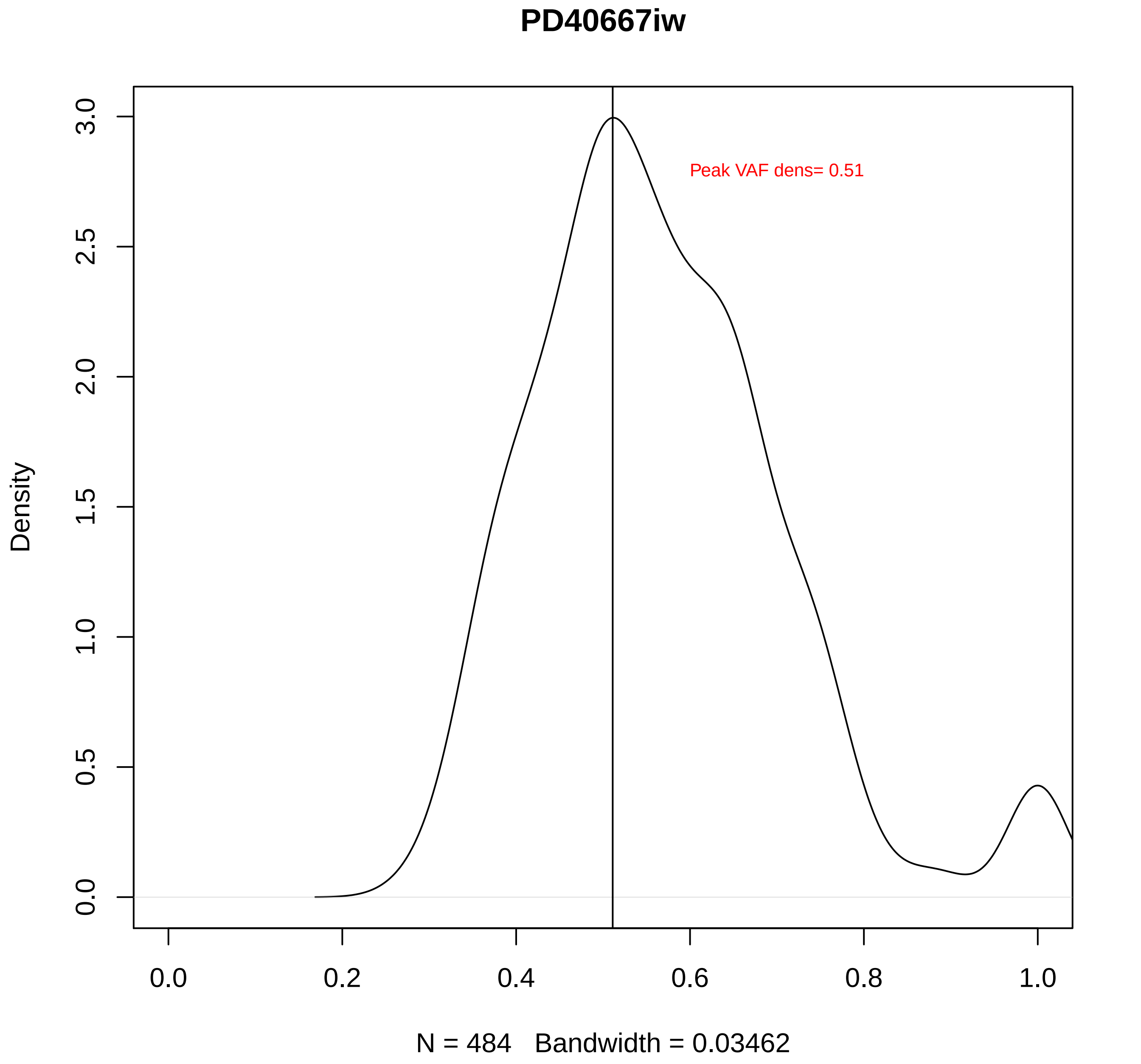

PD40667iw
Peak VAF dens= 0.51
Density
0.0
0.5
1.0
1.5
2.0
2.5
3.0
0.0
0.2
0.4
0.6
0.8
1.0
N = 484   Bandwidth = 0.03462

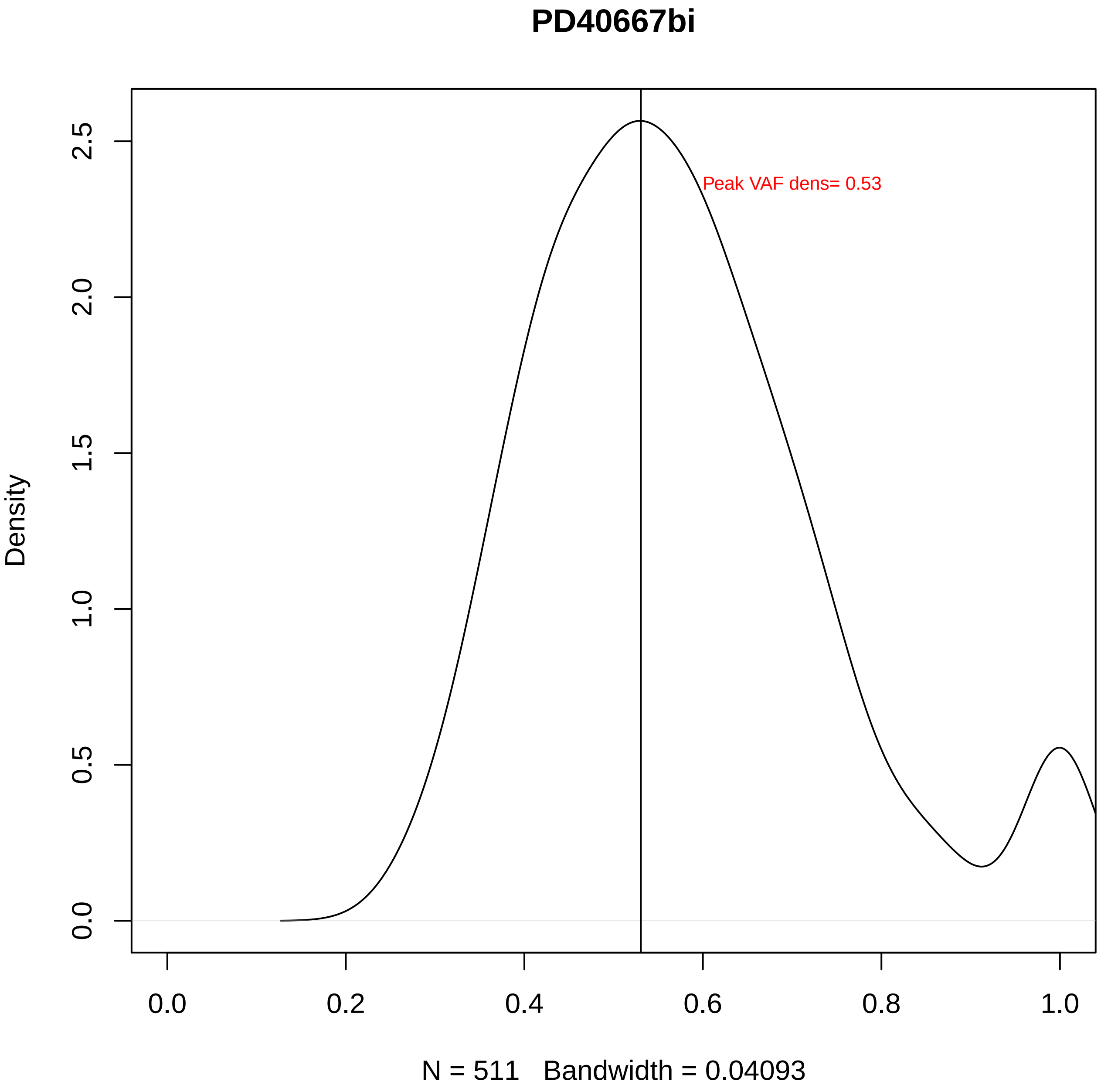
PD40667bi
Peak VAF dens= 0.53
Density
0.0
0.5
1.0
1.5
2.0
2.5
0.0
0.2
0.4
0.6
0.8
1.0
N = 511   Bandwidth = 0.04093

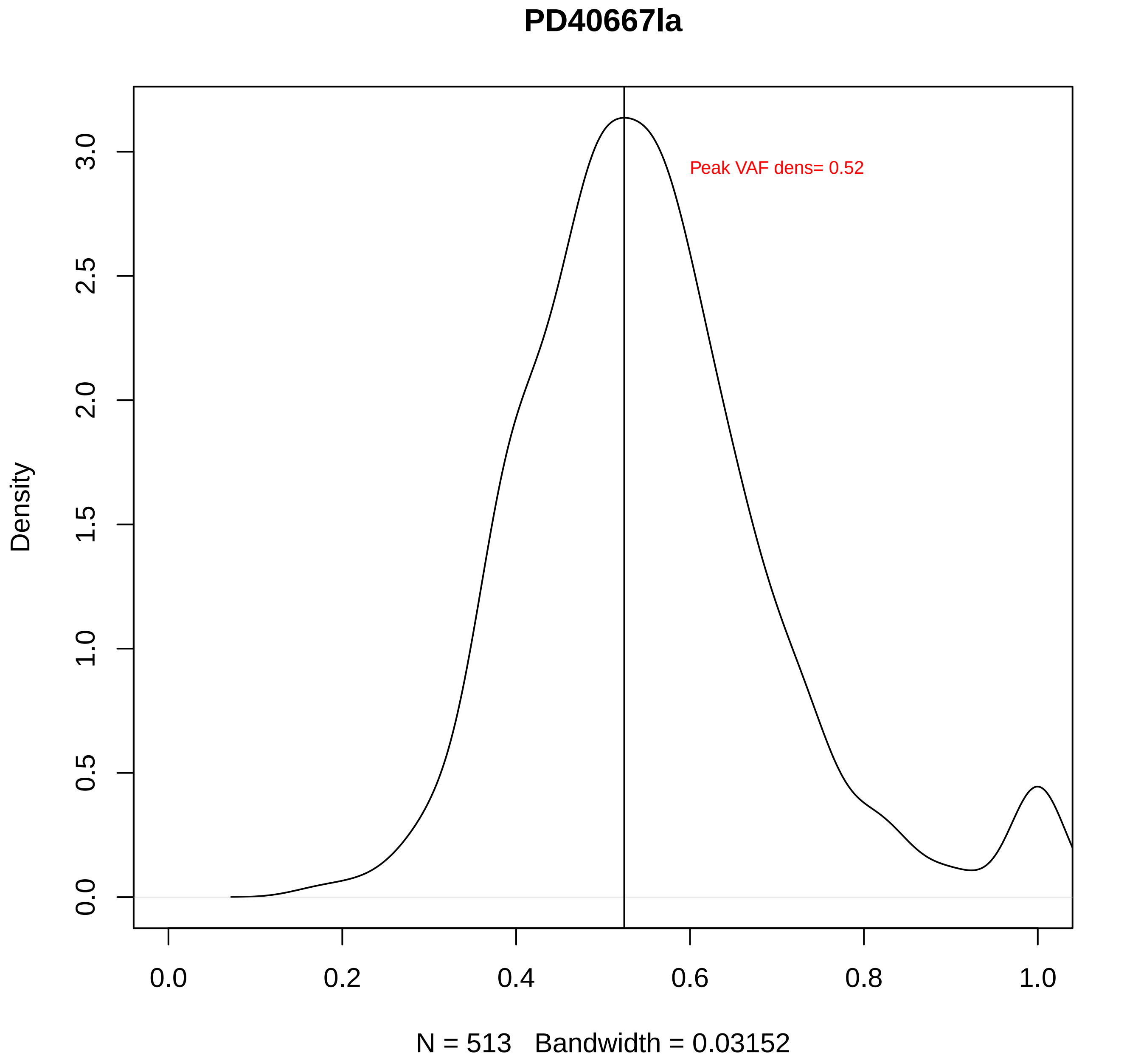

PD40667la
Peak VAF dens= 0.52
Density
0.0
0.5
1.0
1.5
2.0
2.5
3.0
0.0
0.2
0.4
0.6
0.8
1.0
N = 513   Bandwidth = 0.03152

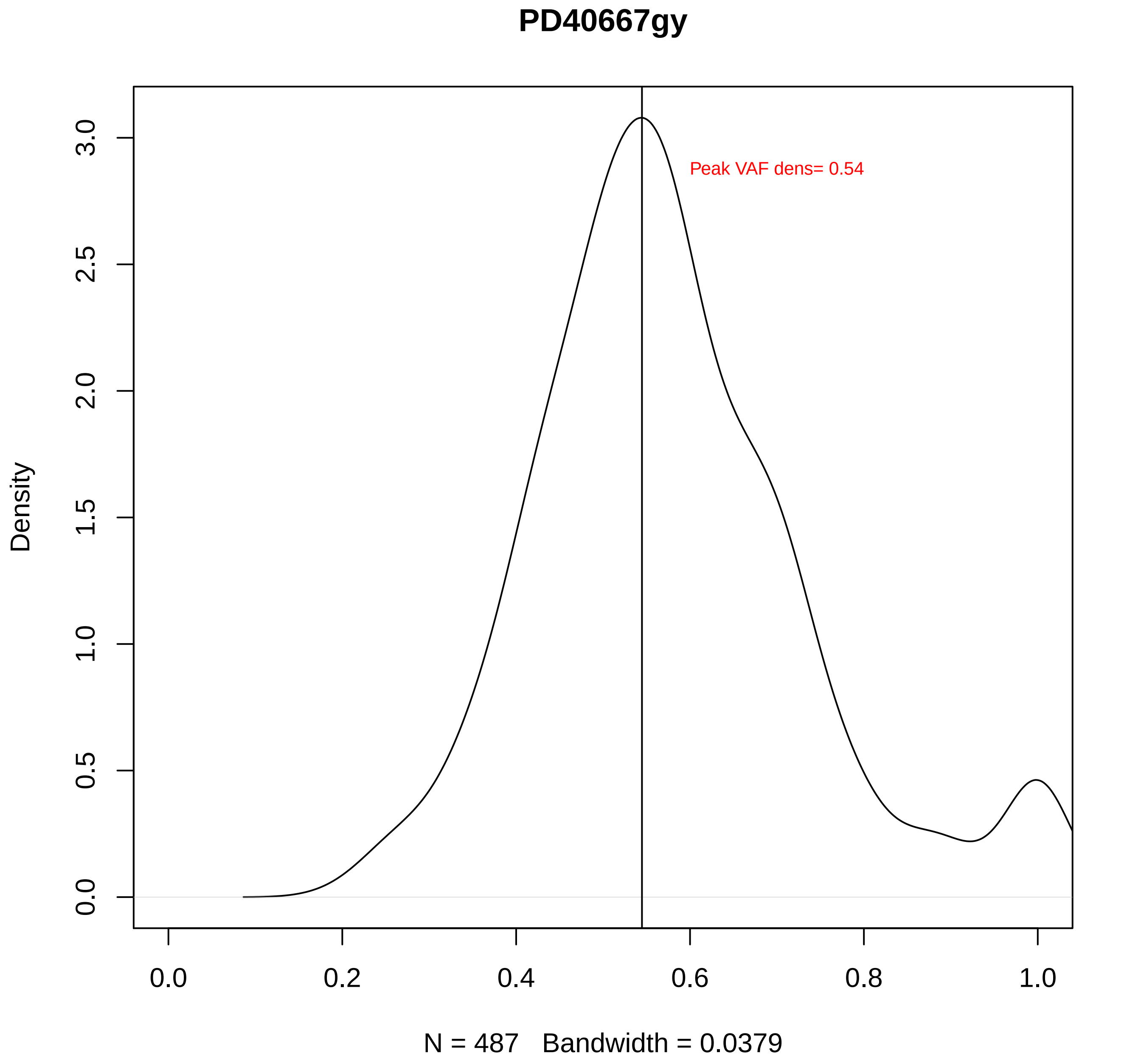
PD40667gy
Peak VAF dens= 0.54
Density
0.0
0.5
1.0
1.5
2.0
2.5
3.0
0.0
0.2
0.4
0.6
0.8
1.0
N = 487   Bandwidth = 0.0379

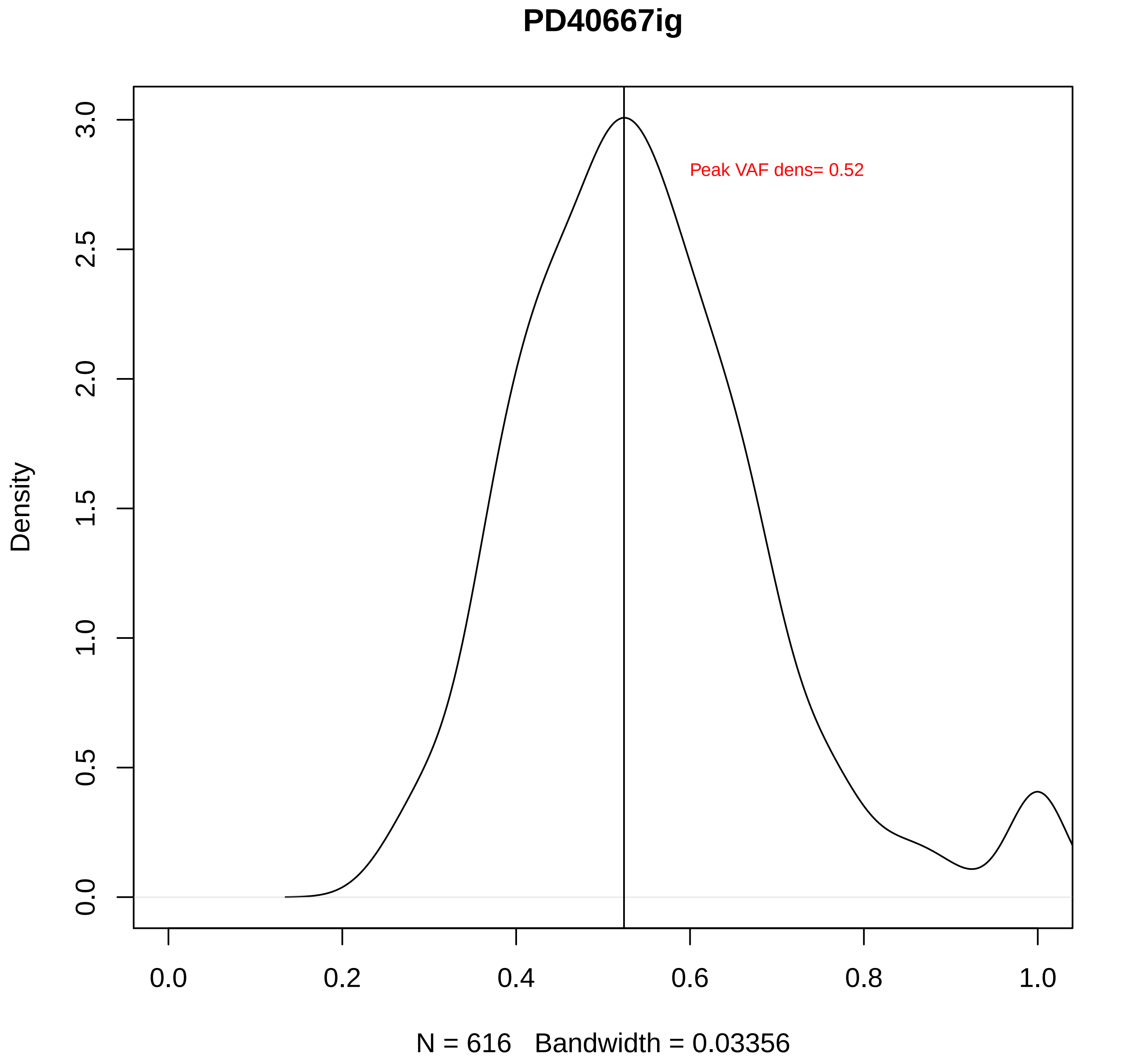

PD40667ig
Peak VAF dens= 0.52
Density
3.0
2.5
2.0
1.5
1.0
0.5
0.0
0.0
0.2
0.4
0.6
0.8
1.0
N = 616   Bandwidth = 0.03356

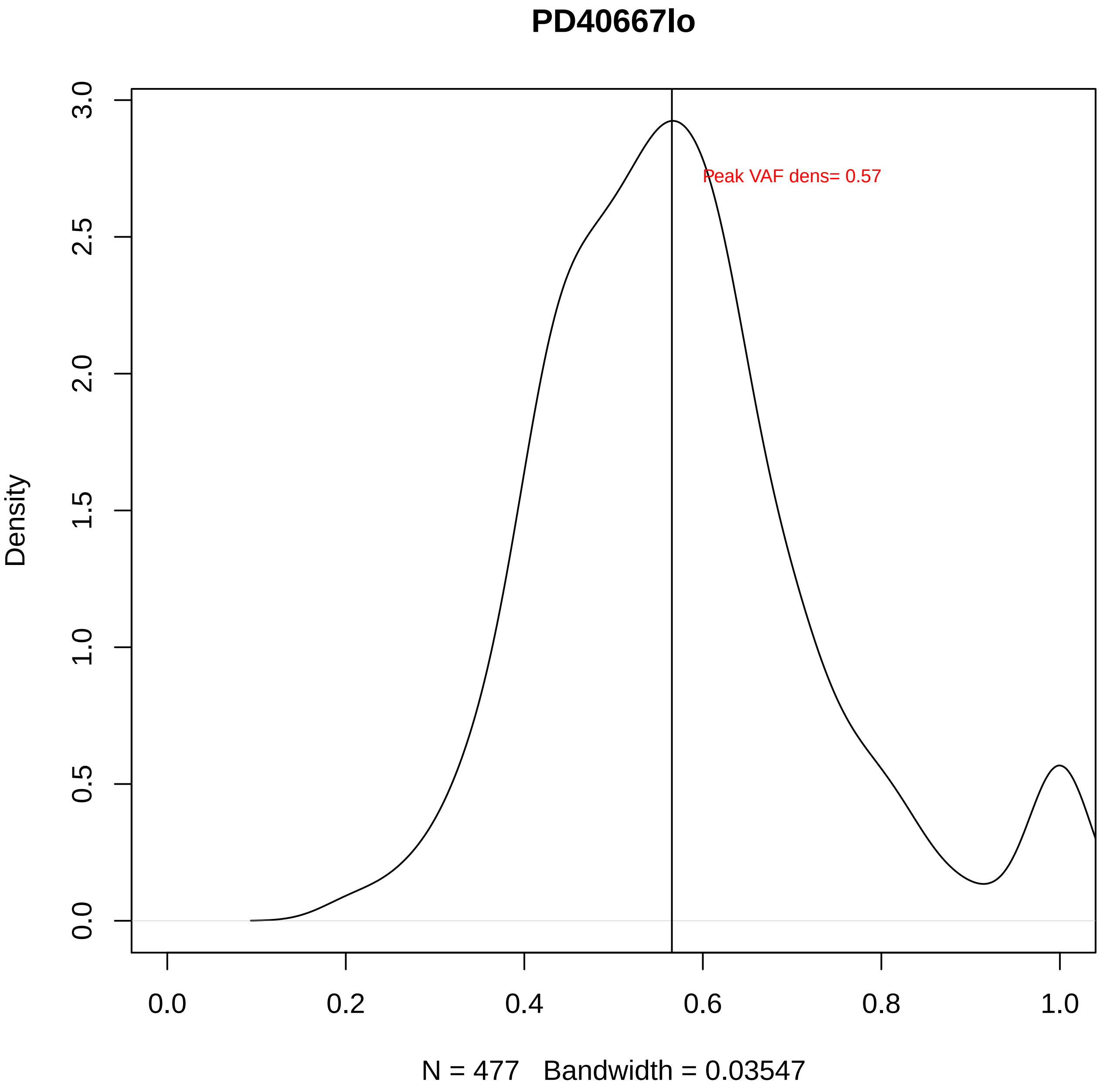
PD40667lo
Peak VAF dens= 0.57
Density
0.0
0.5
1.0
1.5
2.0
2.5
3.0
0.0
0.2
0.4
0.6
0.8
1.0
N = 477 Bandwidth = 0.03547

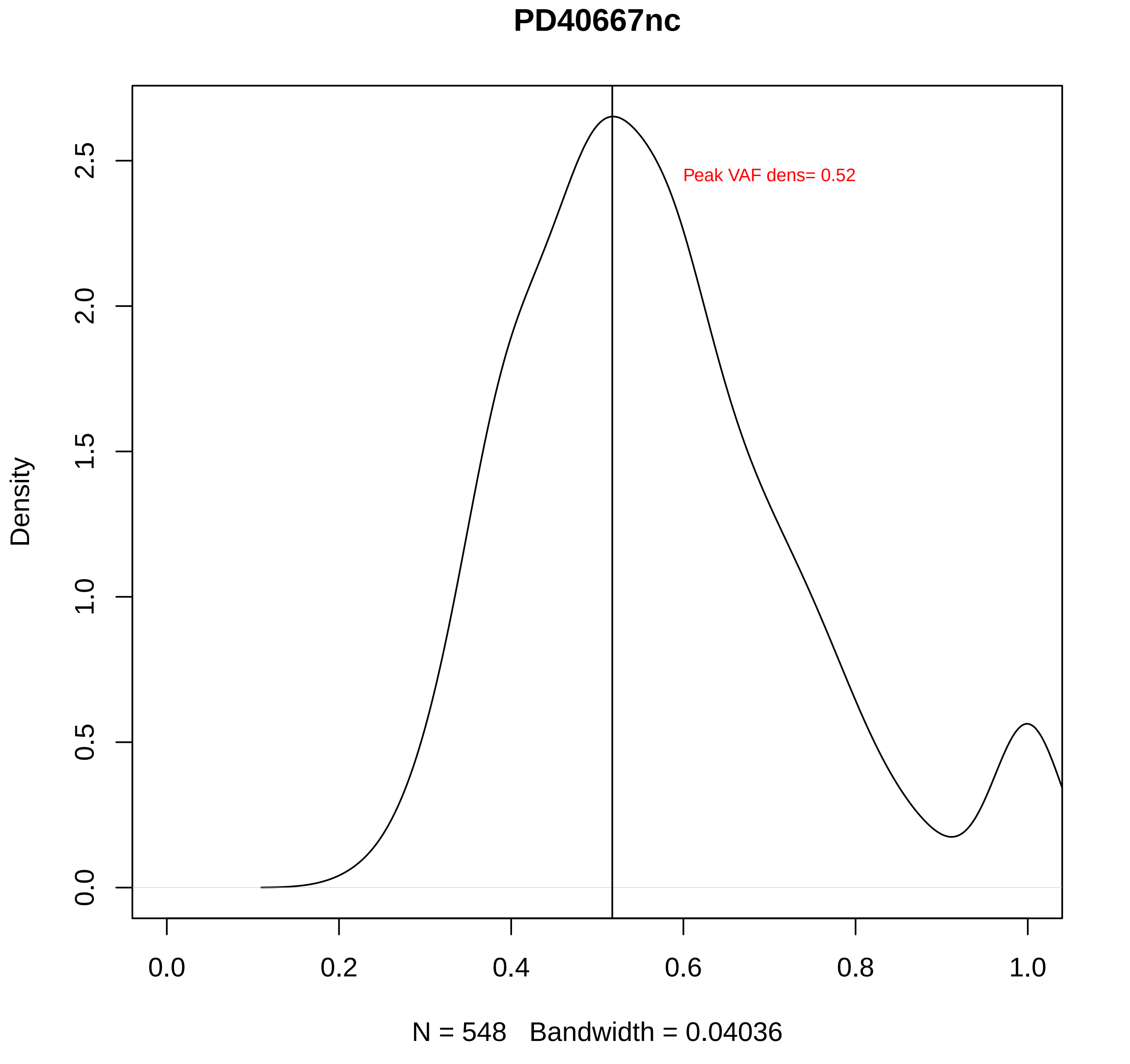
PD40667nc
Peak VAF dens= 0.52
Density
0.0
0.5
1.0
1.5
2.0
2.5
0.0
0.2
0.4
0.6
0.8
1.0
N = 548   Bandwidth = 0.04036

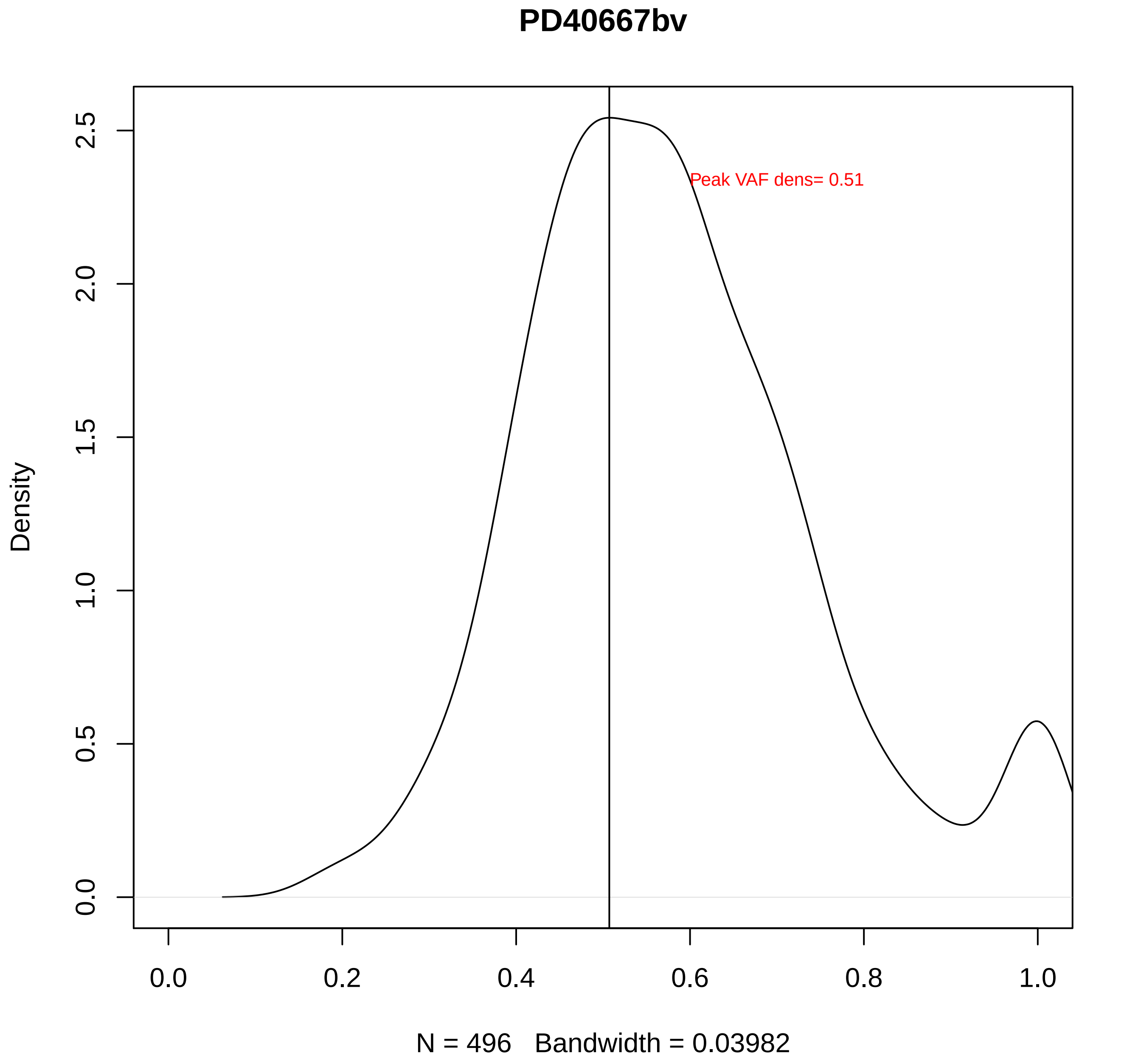
PD40667bv
Peak VAF dens= 0.51
Density
0.0
0.5
1.0
1.5
2.0
2.5
0.0
0.2
0.4
0.6
0.8
1.0
N = 496   Bandwidth = 0.03982

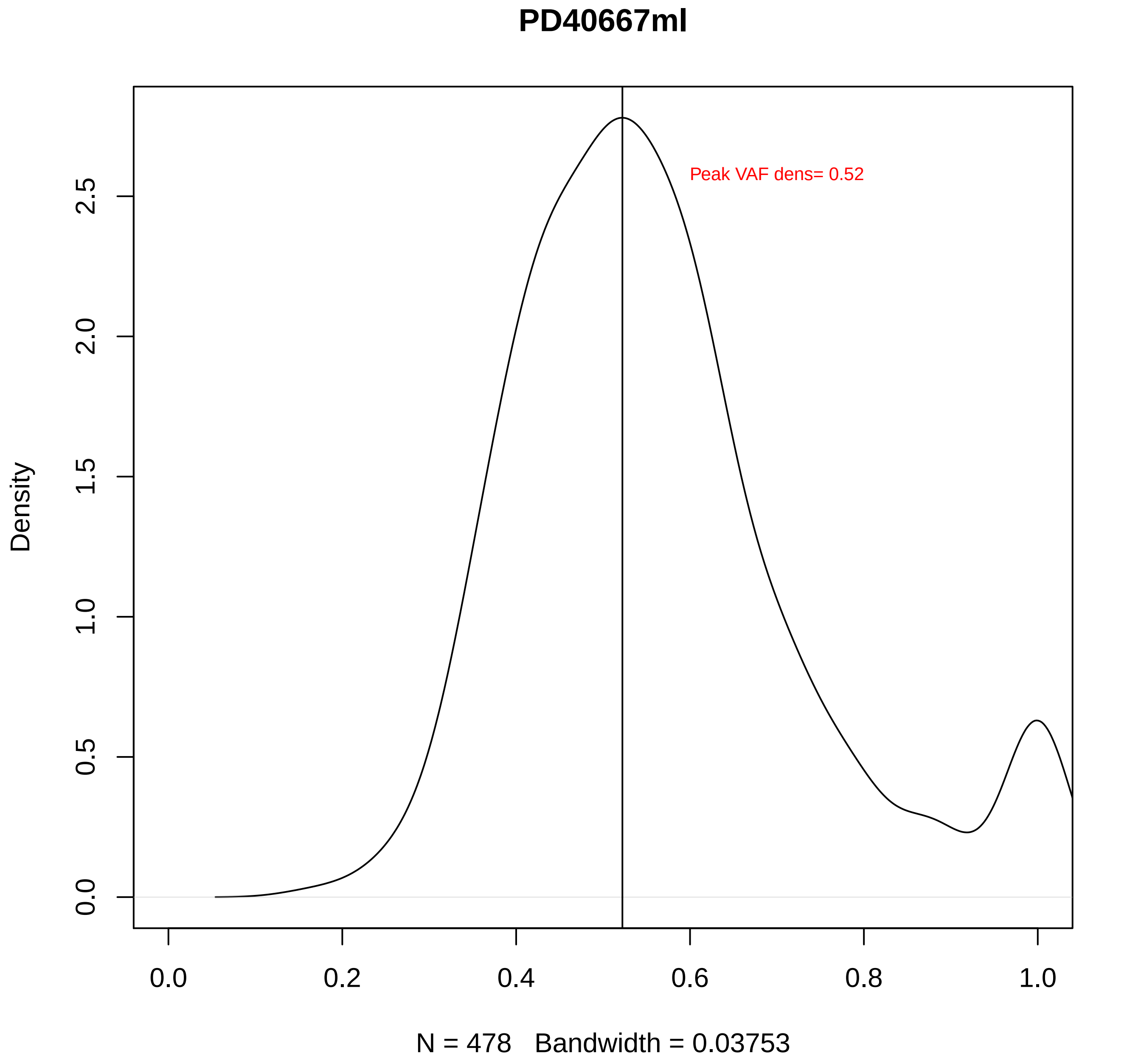
PD40667ml
Peak VAF dens= 0.52
Density
0.0
0.5
1.0
1.5
2.0
2.5
0.0
0.2
0.4
0.6
0.8
1.0
N = 478   Bandwidth = 0.03753

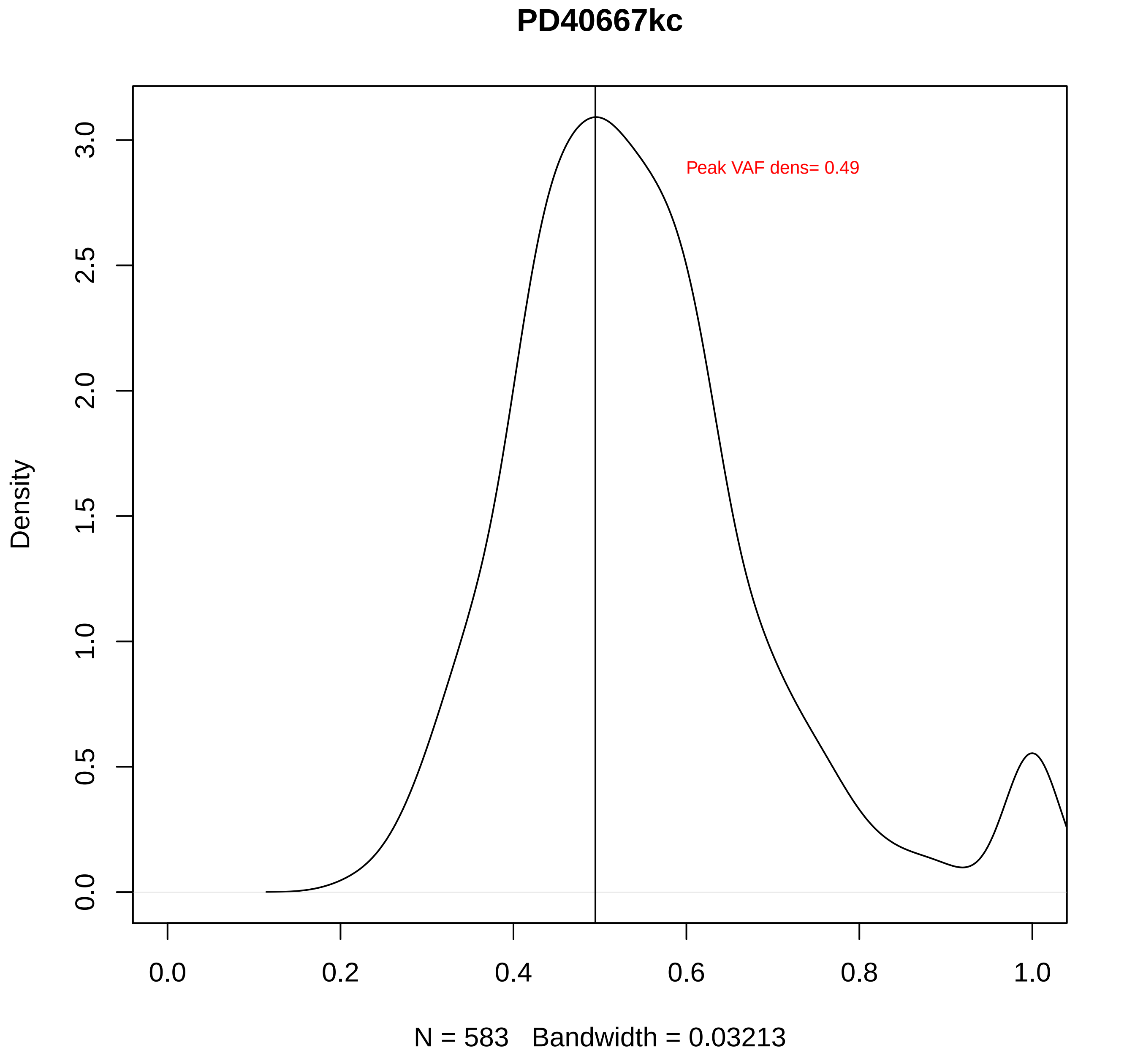
PD40667kc
Peak VAF dens= 0.49
Density
0.0
0.5
1.0
1.5
2.0
2.5
3.0
0.0
0.2
0.4
0.6
0.8
1.0
N = 583   Bandwidth = 0.03213

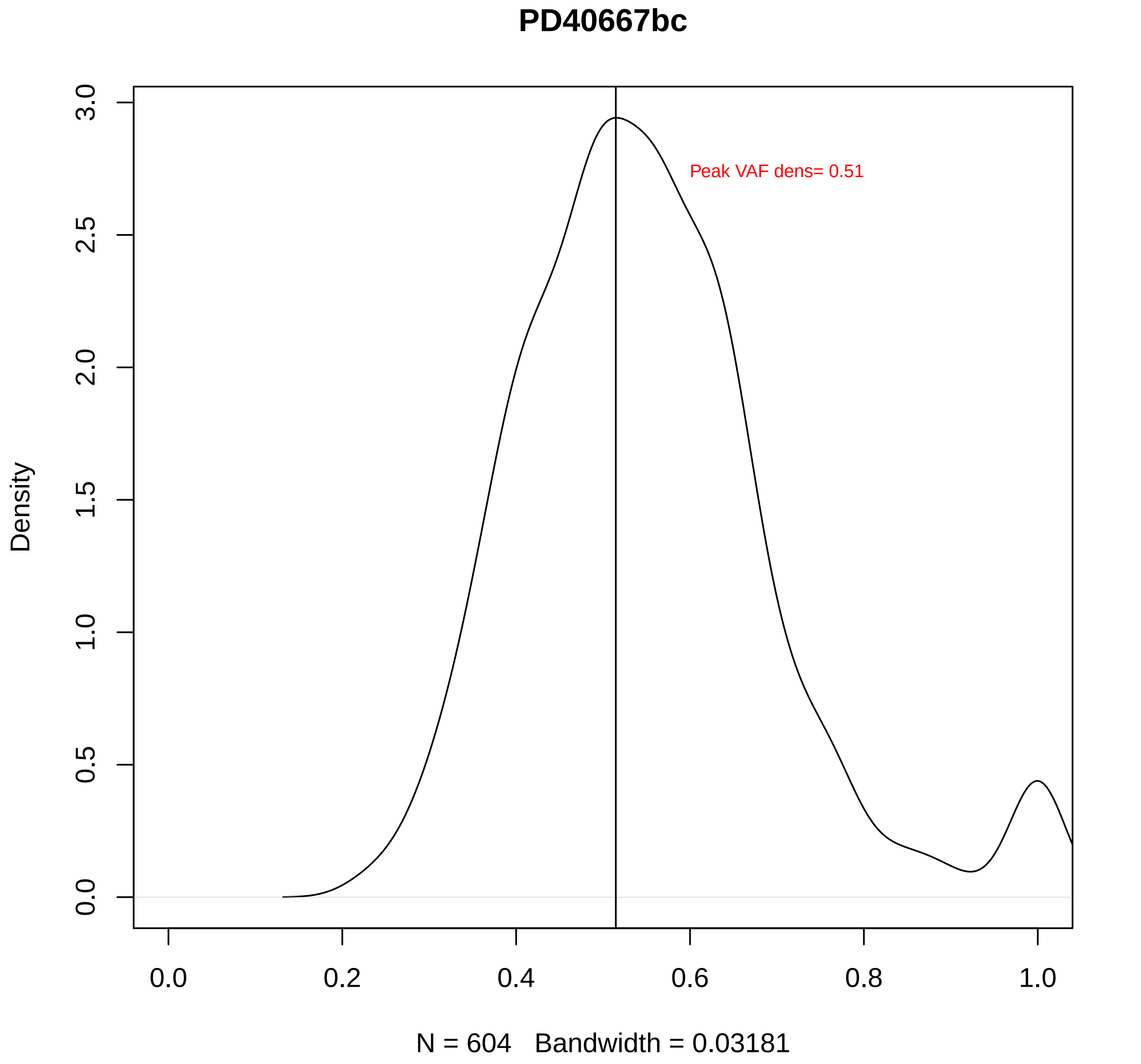
PD40667bc
Peak VAF dens= 0.51
Density
0.0
0.5
1.0
1.5
2.0
2.5
3.0
0.0
0.2
0.4
0.6
0.8
1.0
N = 604   Bandwidth = 0.03181

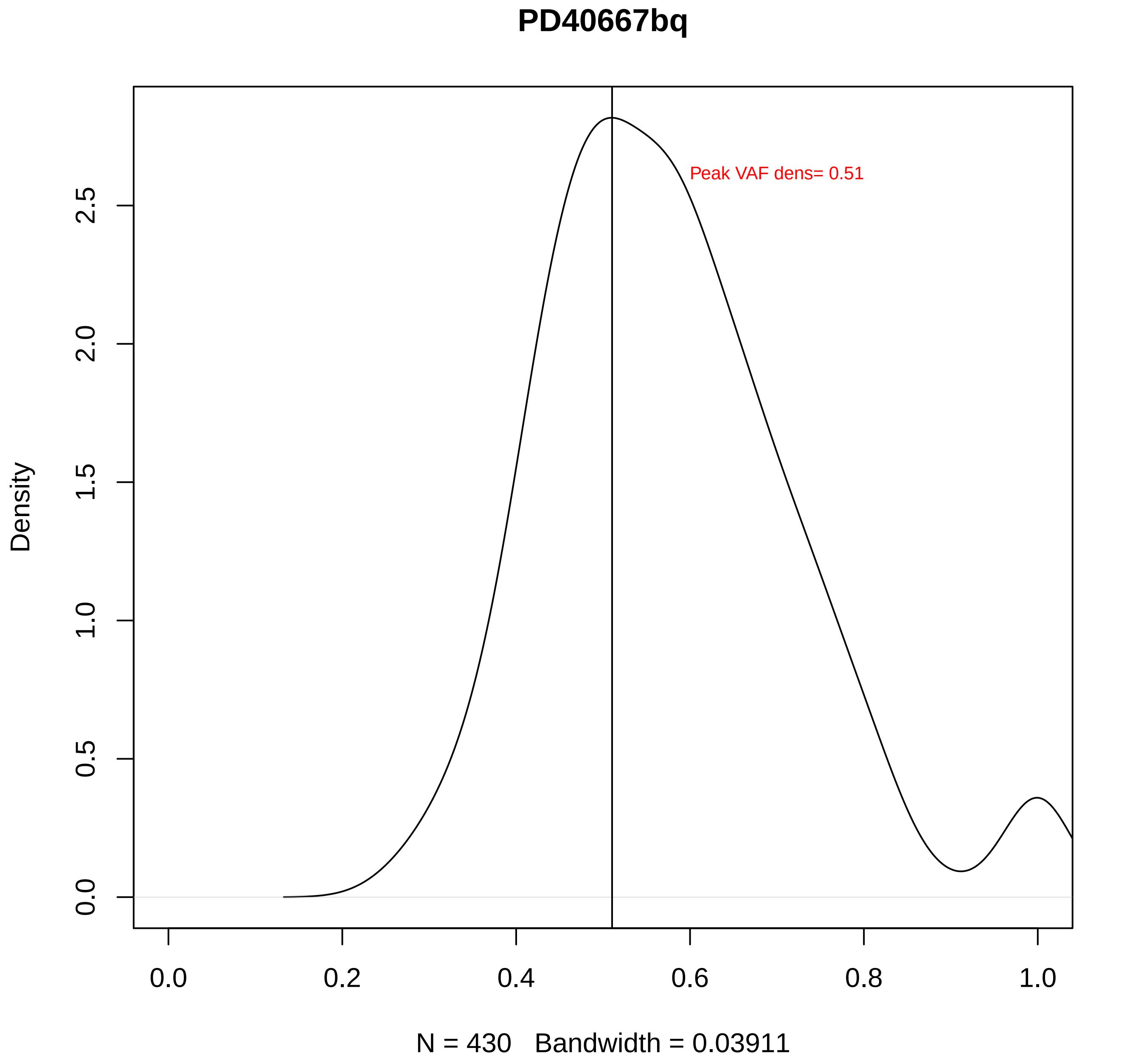
PD40667bq
Peak VAF dens= 0.51
Density
0.0
0.5
1.0
1.5
2.0
2.5
0.0
0.2
0.4
0.6
0.8
1.0
N = 430   Bandwidth = 0.03911

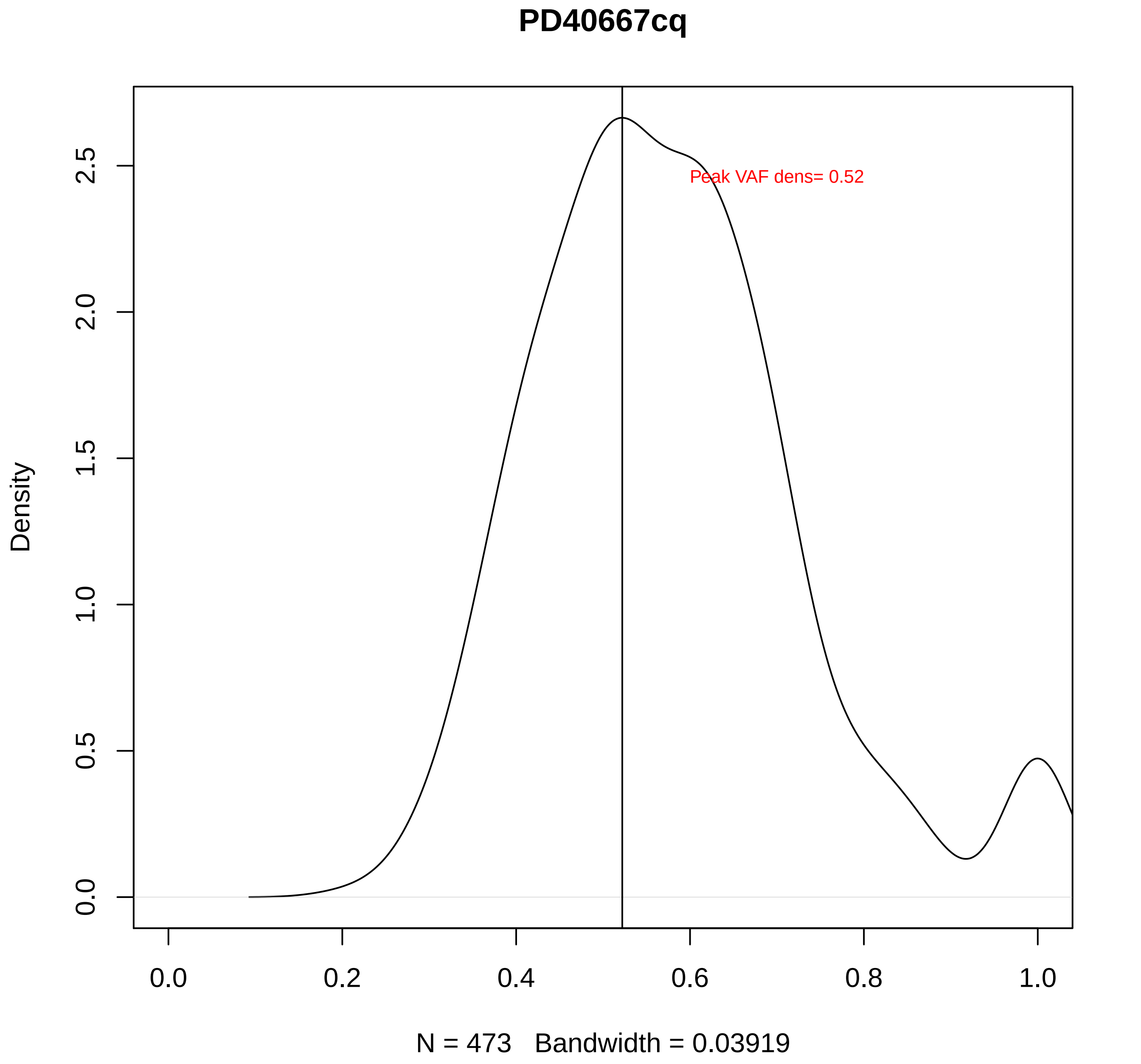

PD40667cq
Peak VAF dens= 0.52
Density
0.0
0.5
1.0
1.5
2.0
2.5
0.0
0.2
0.4
0.6
0.8
1.0
N = 473   Bandwidth = 0.03919

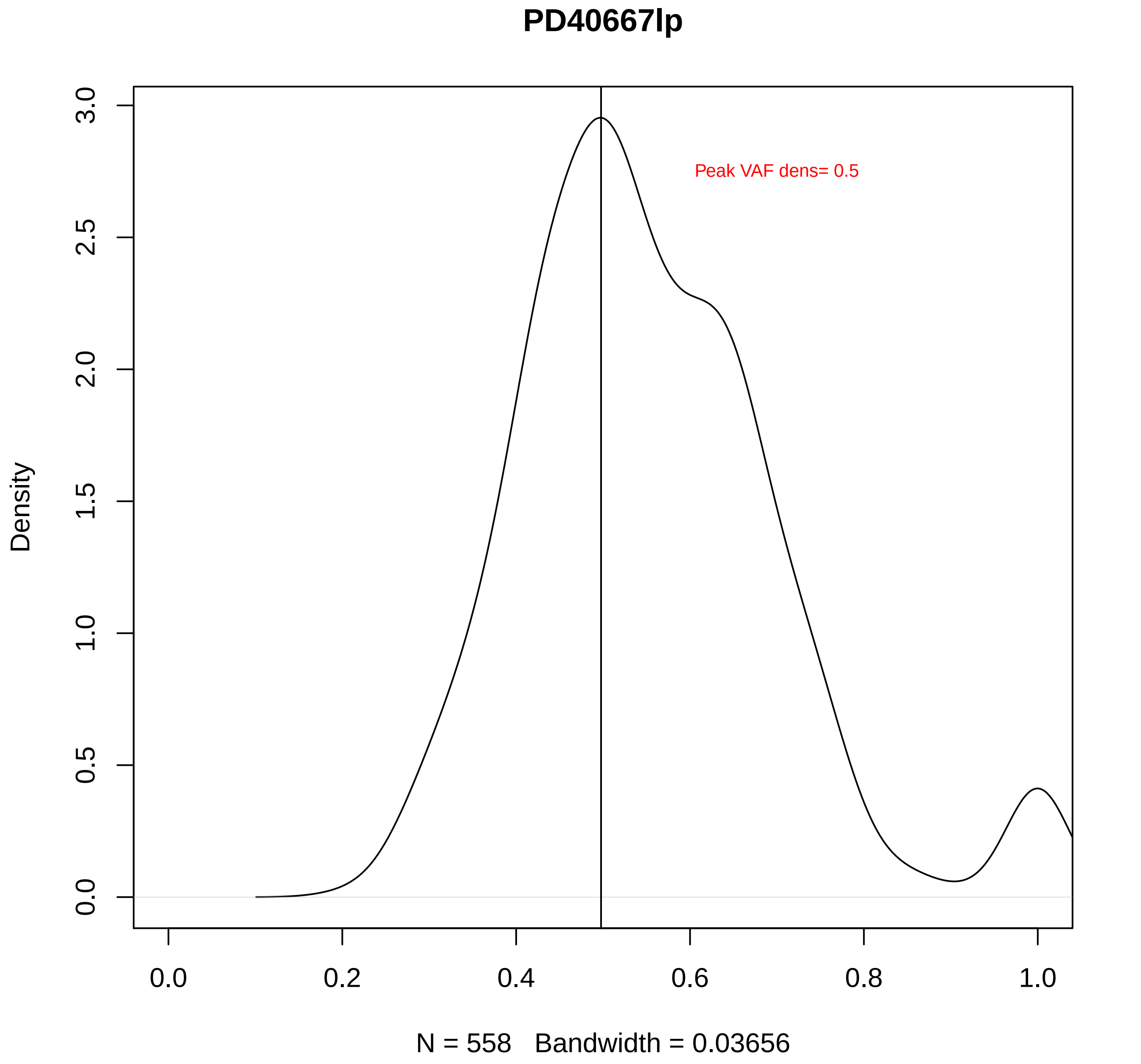
PD40667lp
Peak VAF dens= 0.5
Density
0.0
0.5
1.0
1.5
2.0
2.5
3.0
0.0
0.2
0.4
0.6
0.8
1.0
N = 558 Bandwidth = 0.03656

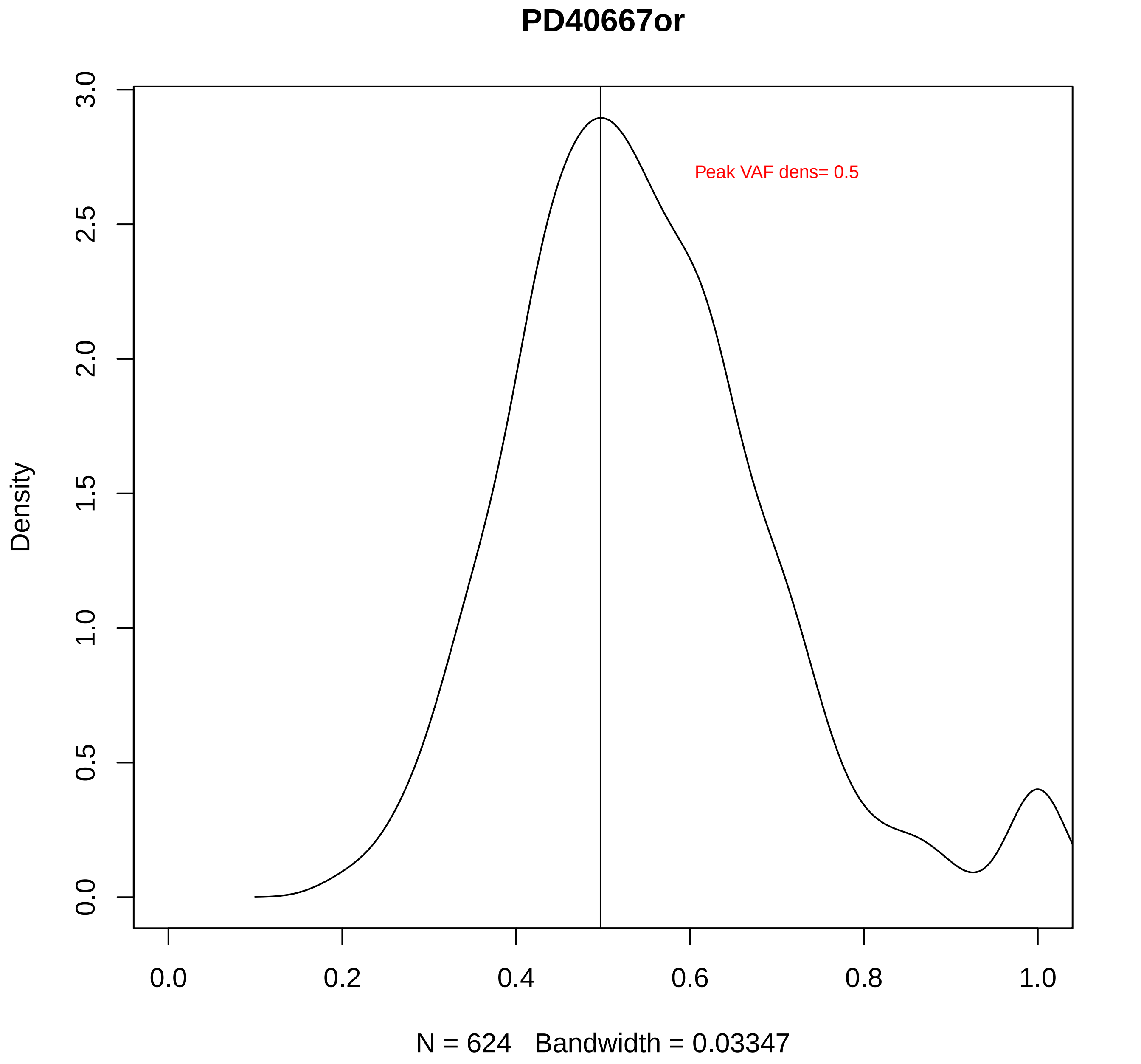

PD40667or
Peak VAF dens= 0.5
Density
0.0
0.5
1.0
1.5
2.0
2.5
3.0
0.0
0.2
0.4
0.6
0.8
1.0
N = 624 Bandwidth = 0.03347

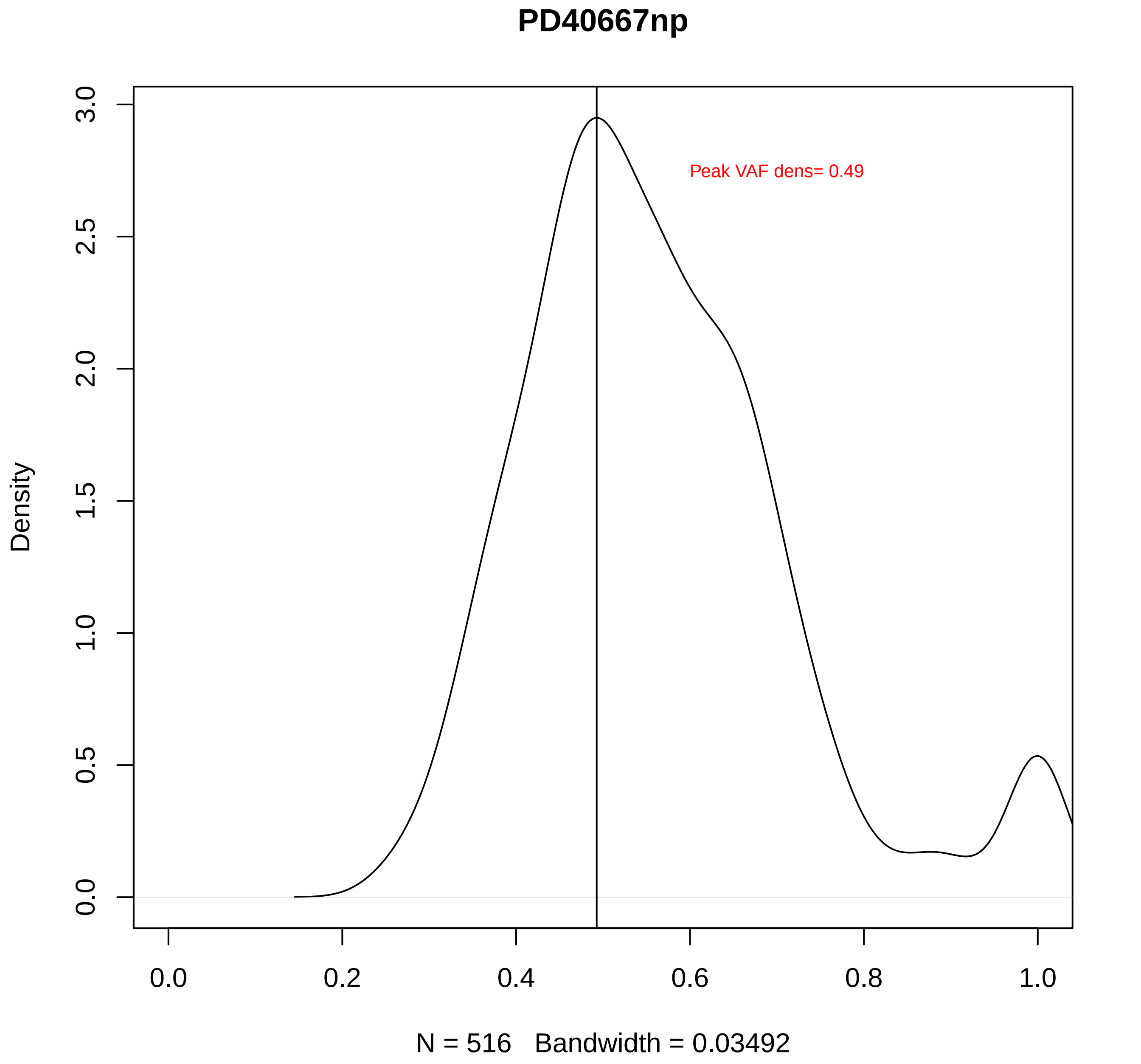
PD40667np
Peak VAF dens= 0.49
Density
3.0
2.5
2.0
1.5
1.0
0.5
0.0
0.0
0.2
0.4
0.6
0.8
1.0
N = 516   Bandwidth = 0.03492

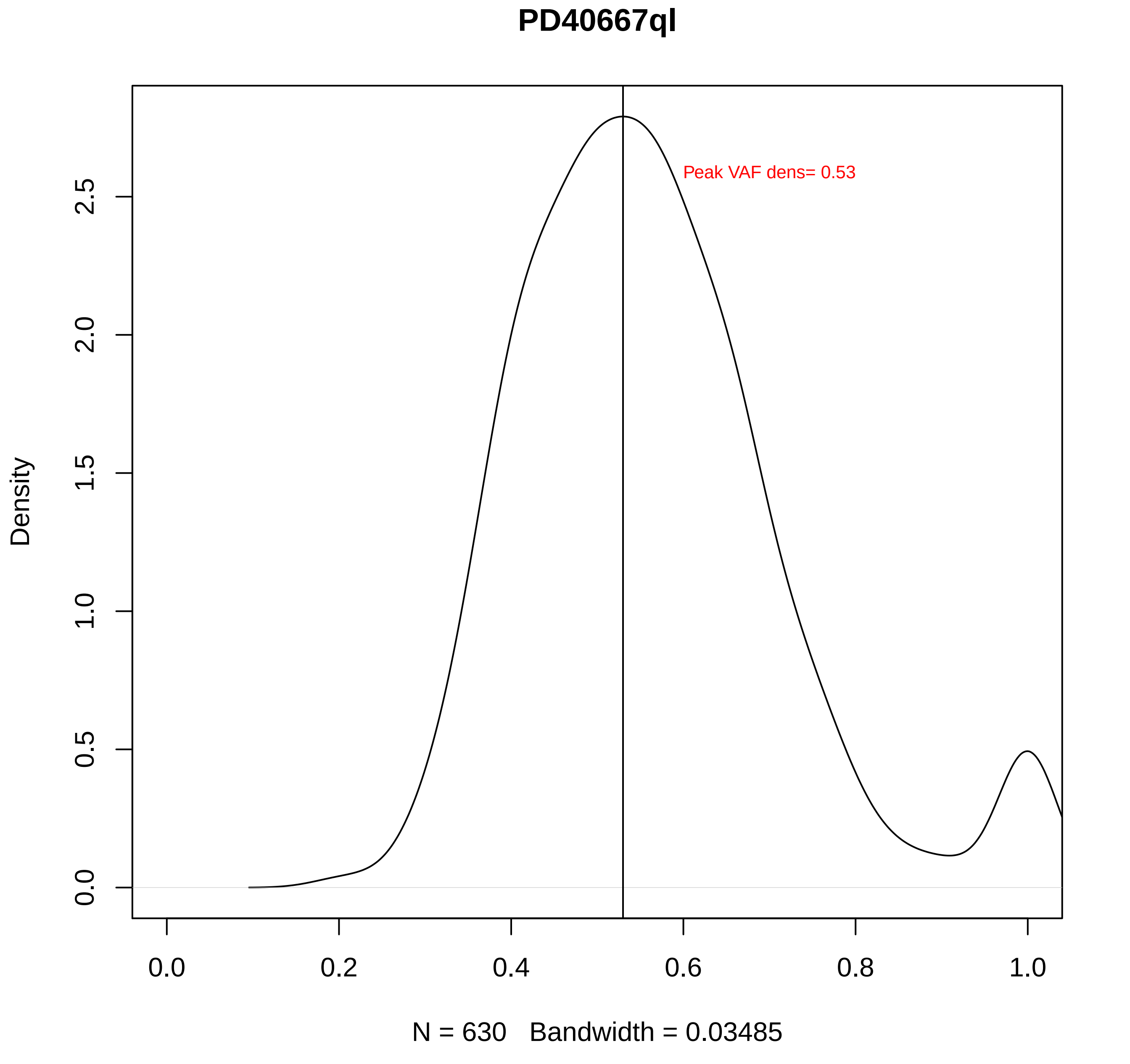

PD40667ql
Peak VAF dens= 0.53
Density
0.0
0.5
1.0
1.5
2.0
2.5
0.0
0.2
0.4
0.6
0.8
1.0
N = 630   Bandwidth = 0.03485

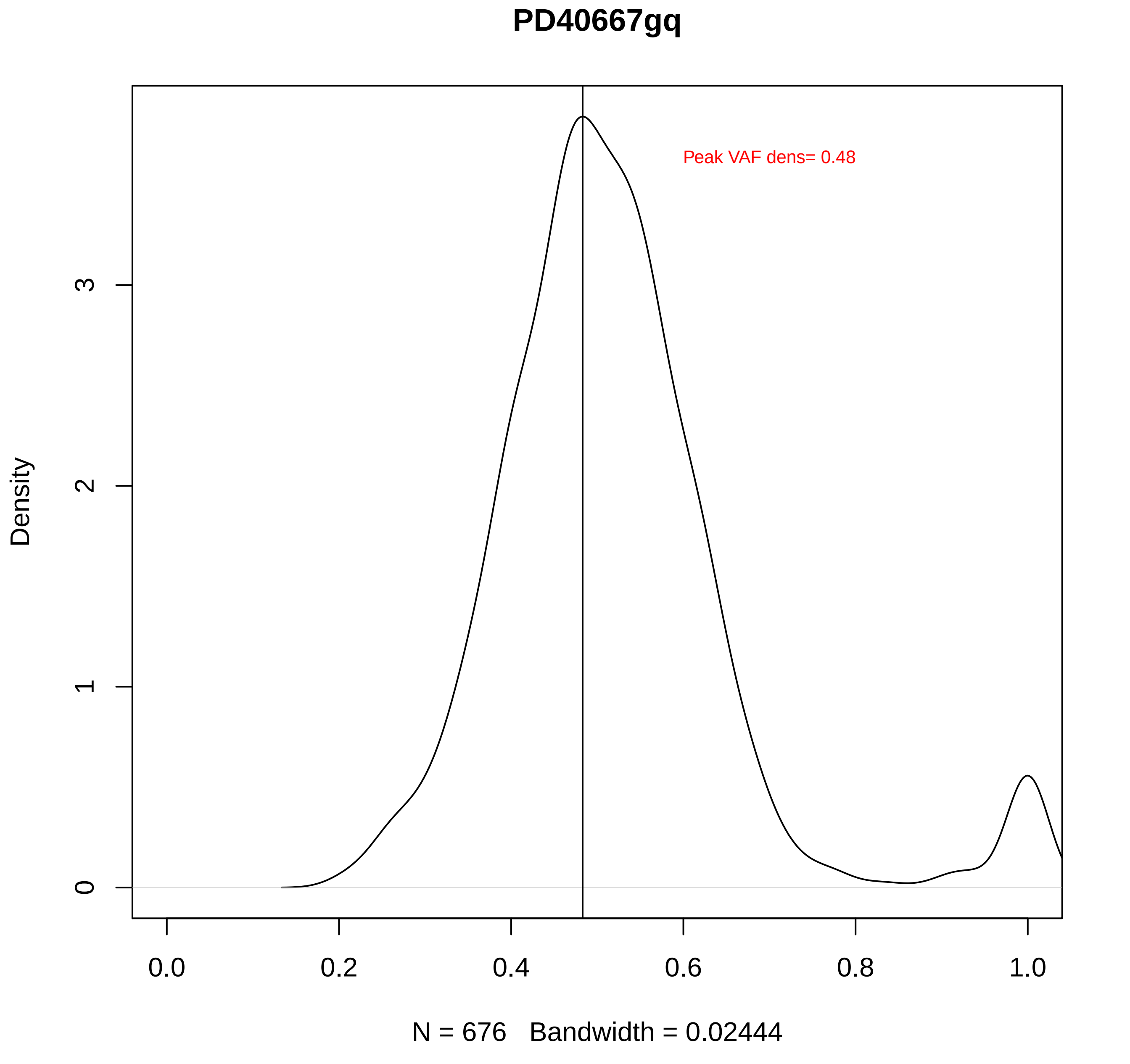
PD40667gq
Peak VAF dens= 0.48
Density
0
1
2
3
0.0
0.2
0.4
0.6
0.8
1.0
N = 676 Bandwidth = 0.02444

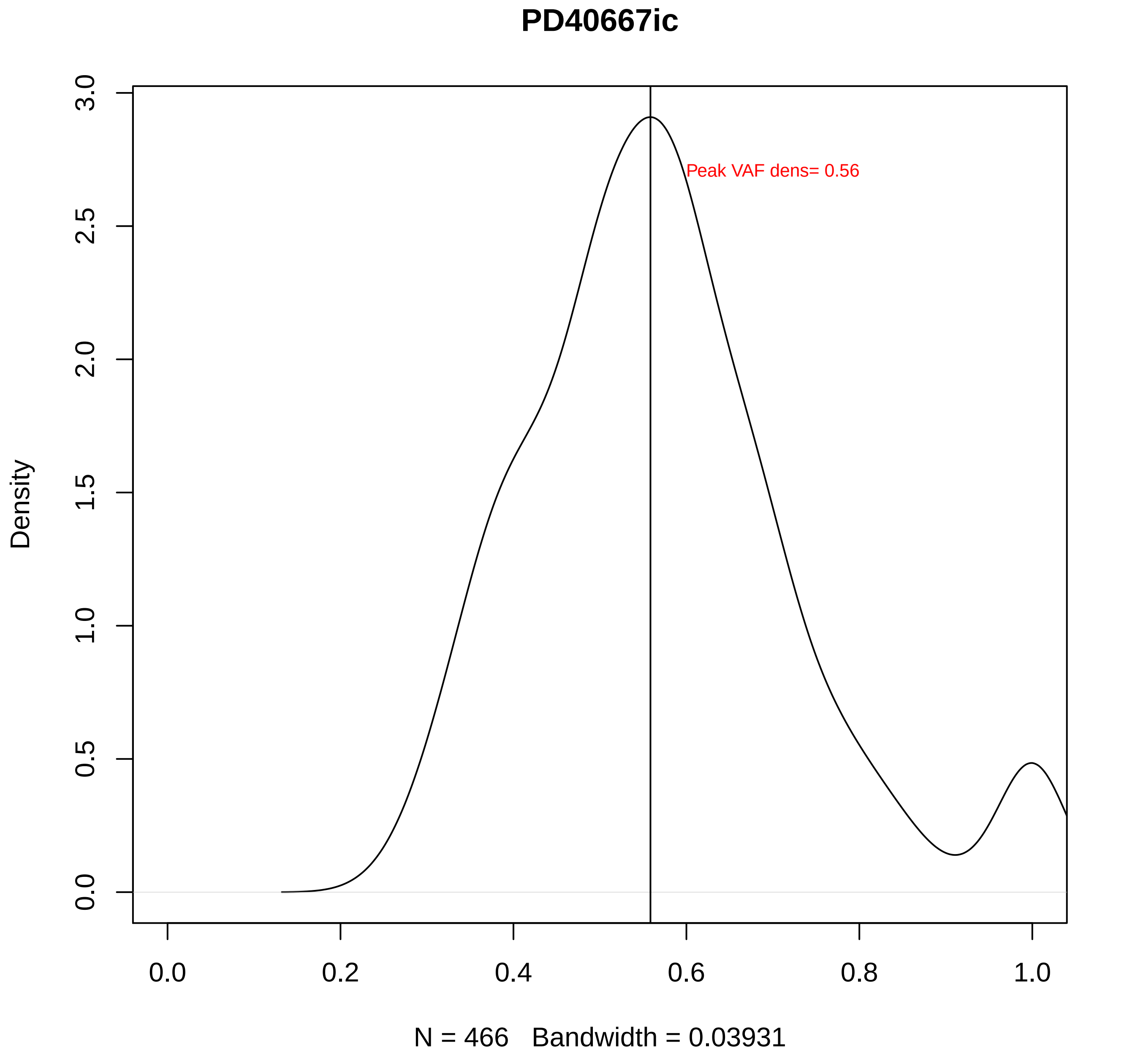
PD40667ic
Peak VAF dens= 0.56
Density
0.0
0.5
1.0
1.5
2.0
2.5
3.0
0.0
0.2
0.4
0.6
0.8
1.0
N = 466   Bandwidth = 0.03931

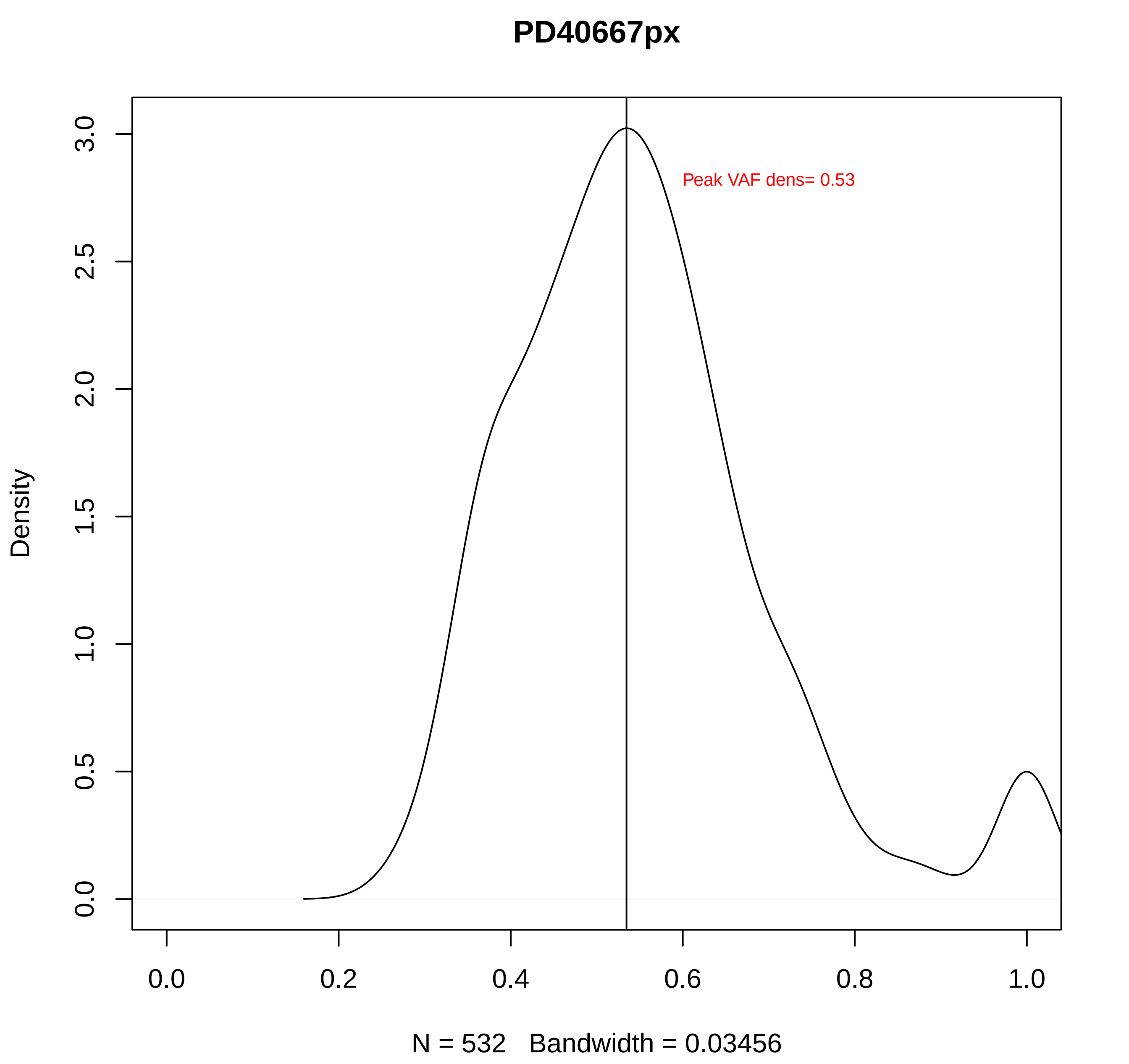

PD40667px
Peak VAF dens= 0.53
Density
0.0
0.5
1.0
1.5
2.0
2.5
3.0
0.0
0.2
0.4
0.6
0.8
1.0
N = 532 Bandwidth = 0.03456

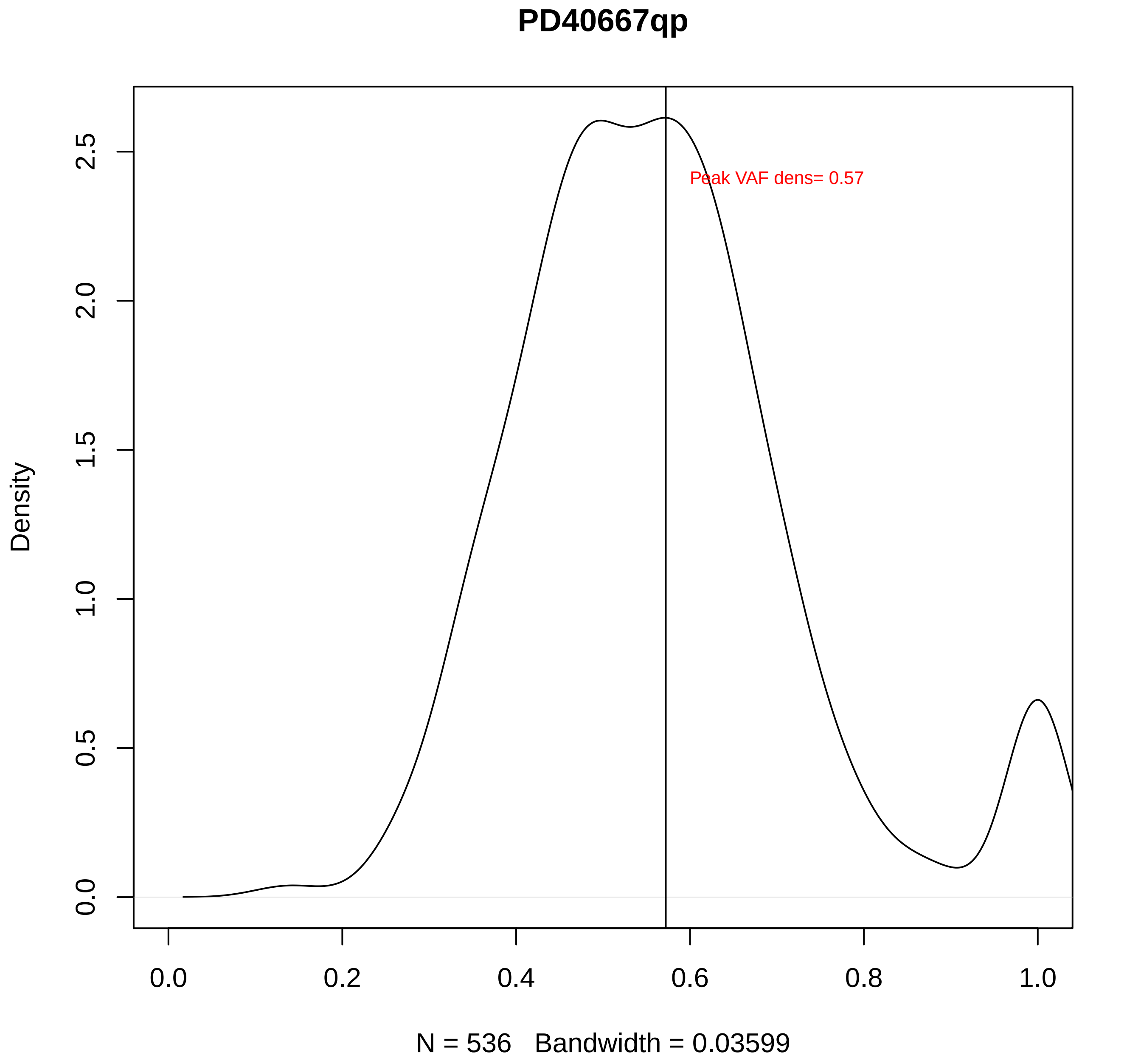

PD40667qp
Peak VAF dens= 0.57
Density
0.0
0.5
1.0
1.5
2.0
2.5
0.0
0.2
0.4
0.6
0.8
1.0
N = 536 Bandwidth = 0.03599

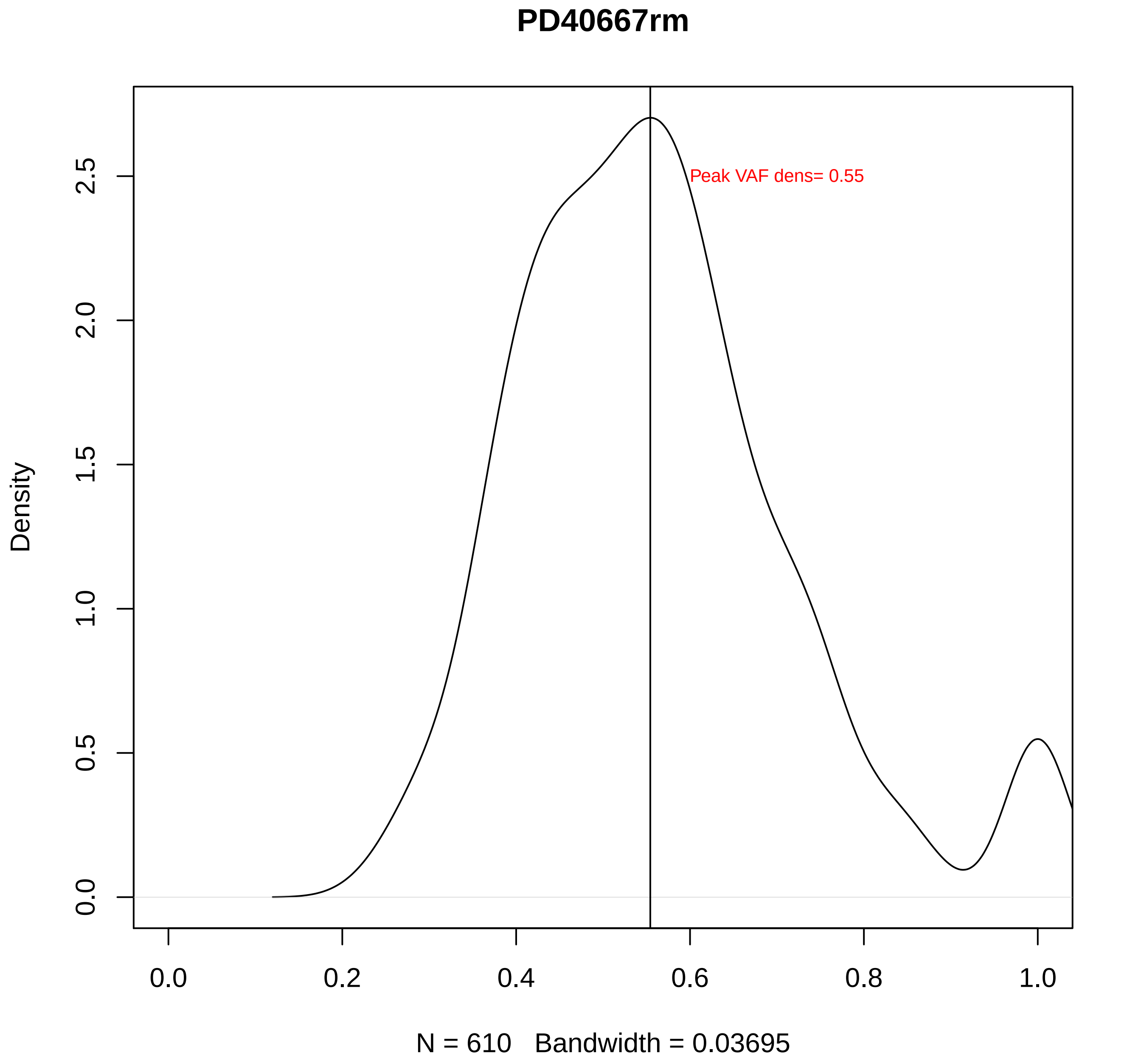
PD40667rm
Peak VAF dens= 0.55
Density
0.0
0.5
1.0
1.5
2.0
2.5
0.0
0.2
0.4
0.6
0.8
1.0
N = 610   Bandwidth = 0.03695

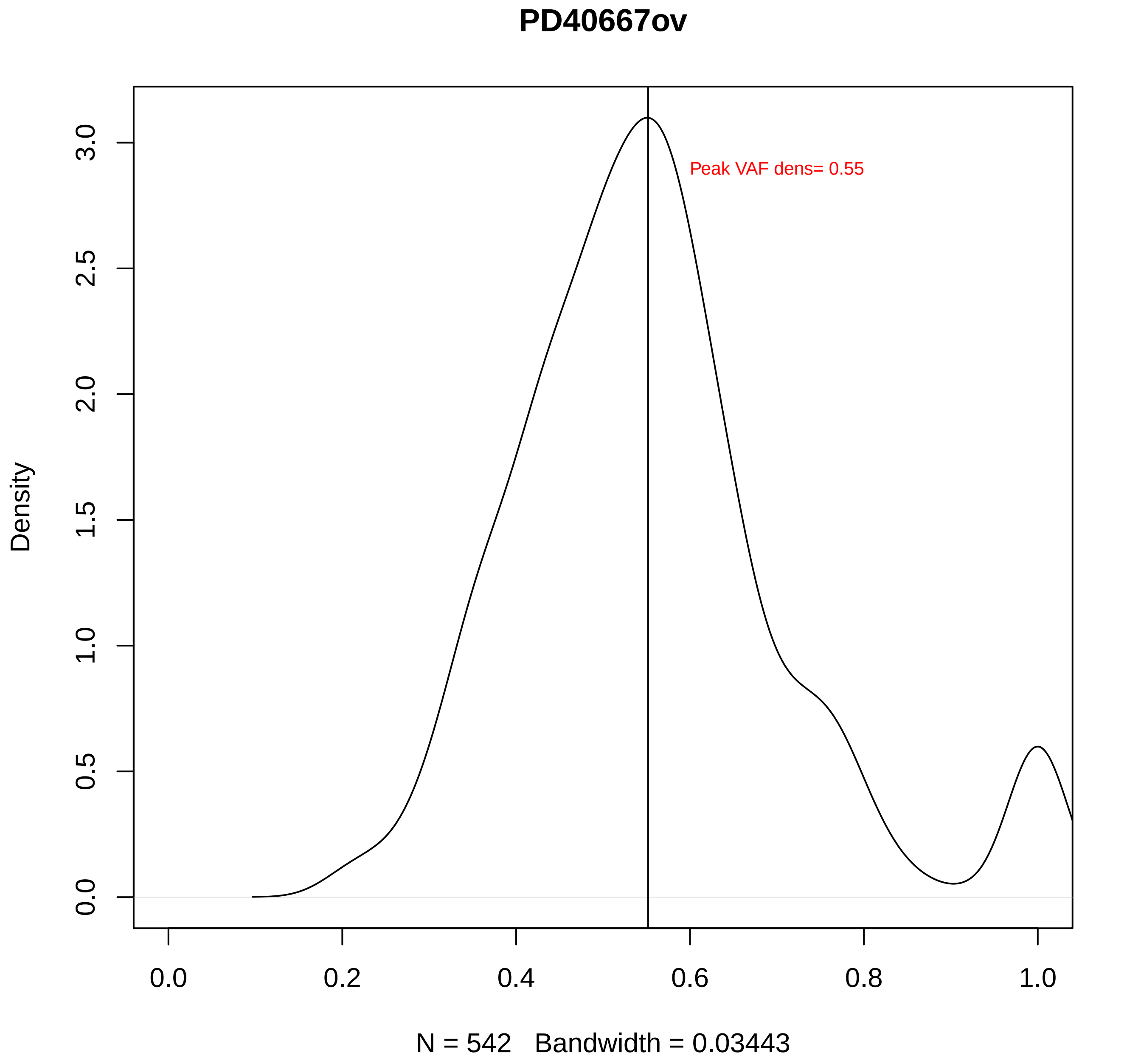
PD40667ov
Peak VAF dens= 0.55
Density
0.0
0.5
1.0
1.5
2.0
2.5
3.0
0.0
0.2
0.4
0.6
0.8
1.0
N = 542   Bandwidth = 0.03443

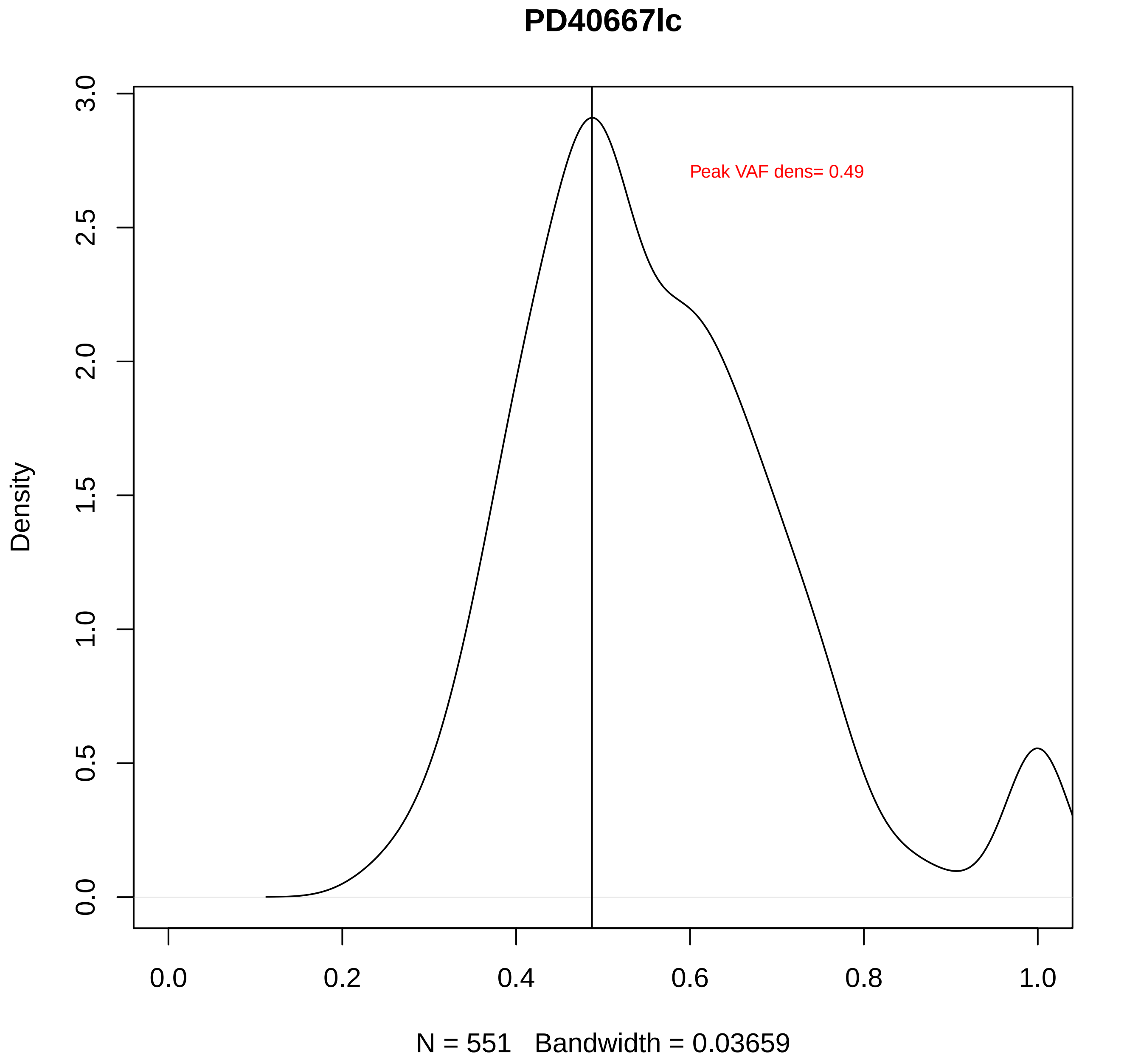

PD40667lc
Peak VAF dens= 0.49
Density
0.0
0.5
1.0
1.5
2.0
2.5
3.0
0.0
0.2
0.4
0.6
0.8
1.0
N = 551 Bandwidth = 0.03659

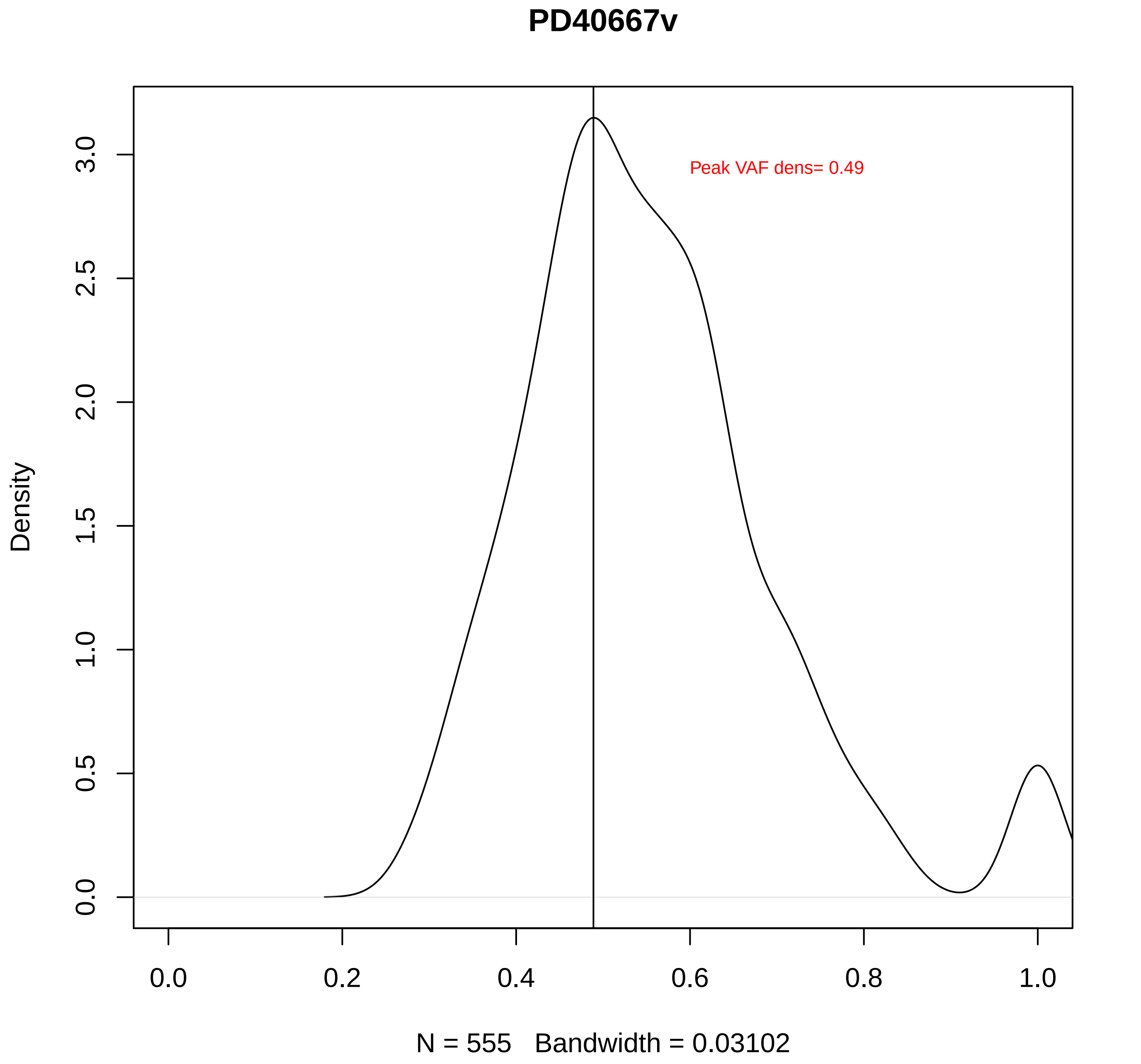
PD40667v
Peak VAF dens= 0.49
Density
0.0
0.5
1.0
1.5
2.0
2.5
3.0
0.0
0.2
0.4
0.6
0.8
1.0
N = 555 Bandwidth = 0.03102

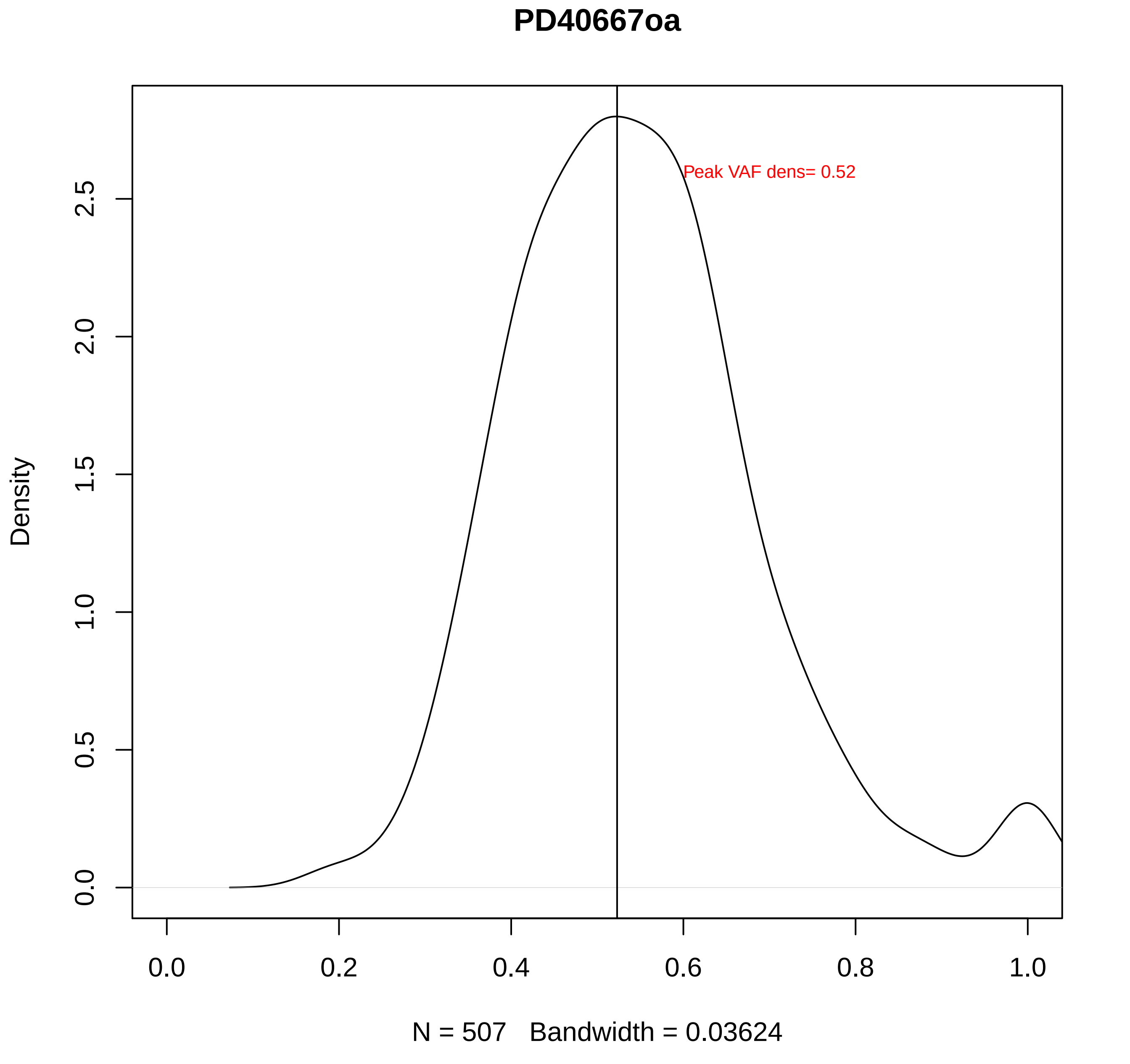

PD40667oa
Peak VAF dens= 0.52
Density
0.0
0.5
1.0
1.5
2.0
2.5
0.0
0.2
0.4
0.6
0.8
1.0
N = 507   Bandwidth = 0.03624

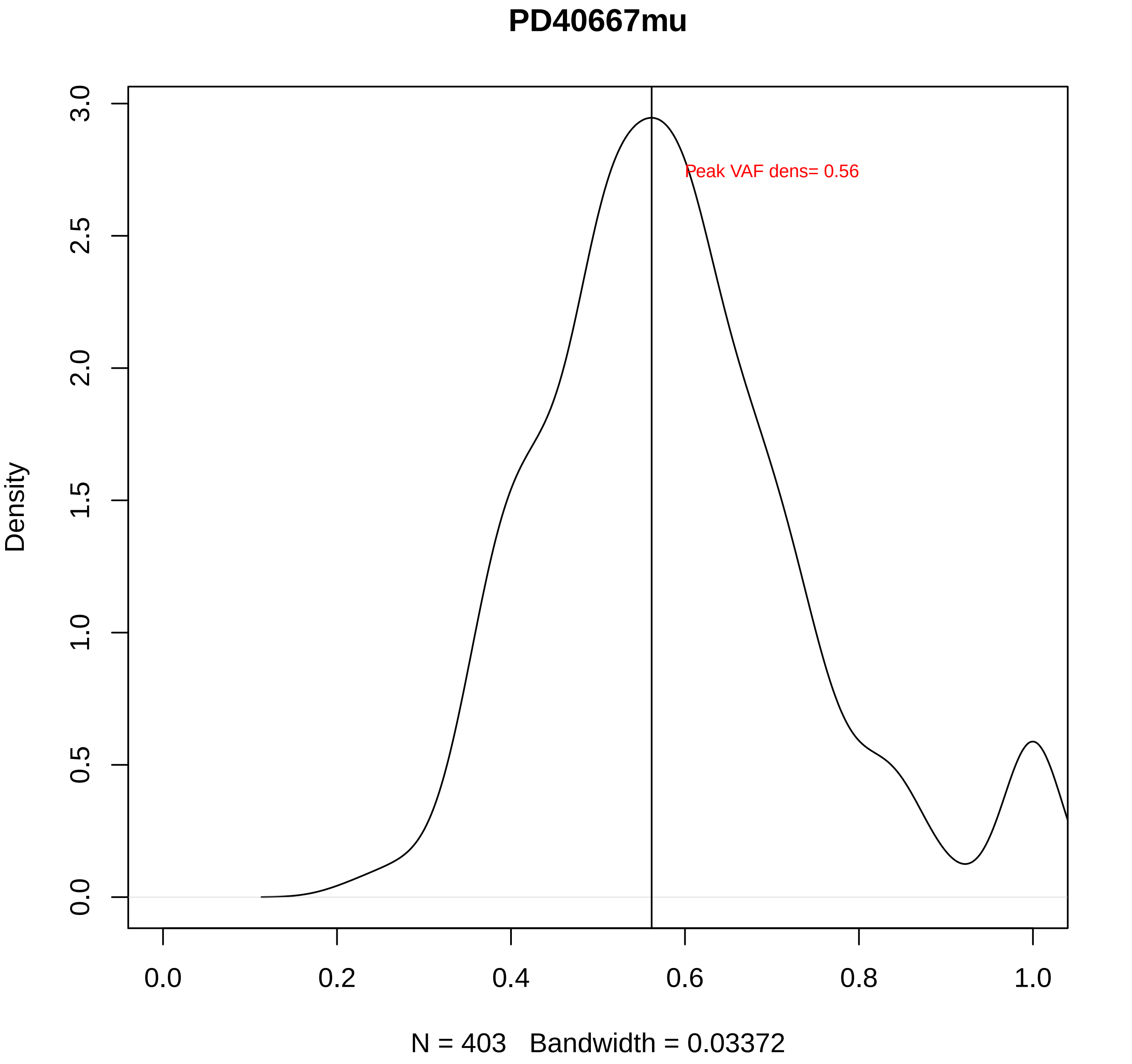
PD40667mu
Peak VAF dens= 0.56
Density
0.0
0.5
1.0
1.5
2.0
2.5
3.0
0.0
0.2
0.4
0.6
0.8
1.0
N = 403   Bandwidth = 0.03372

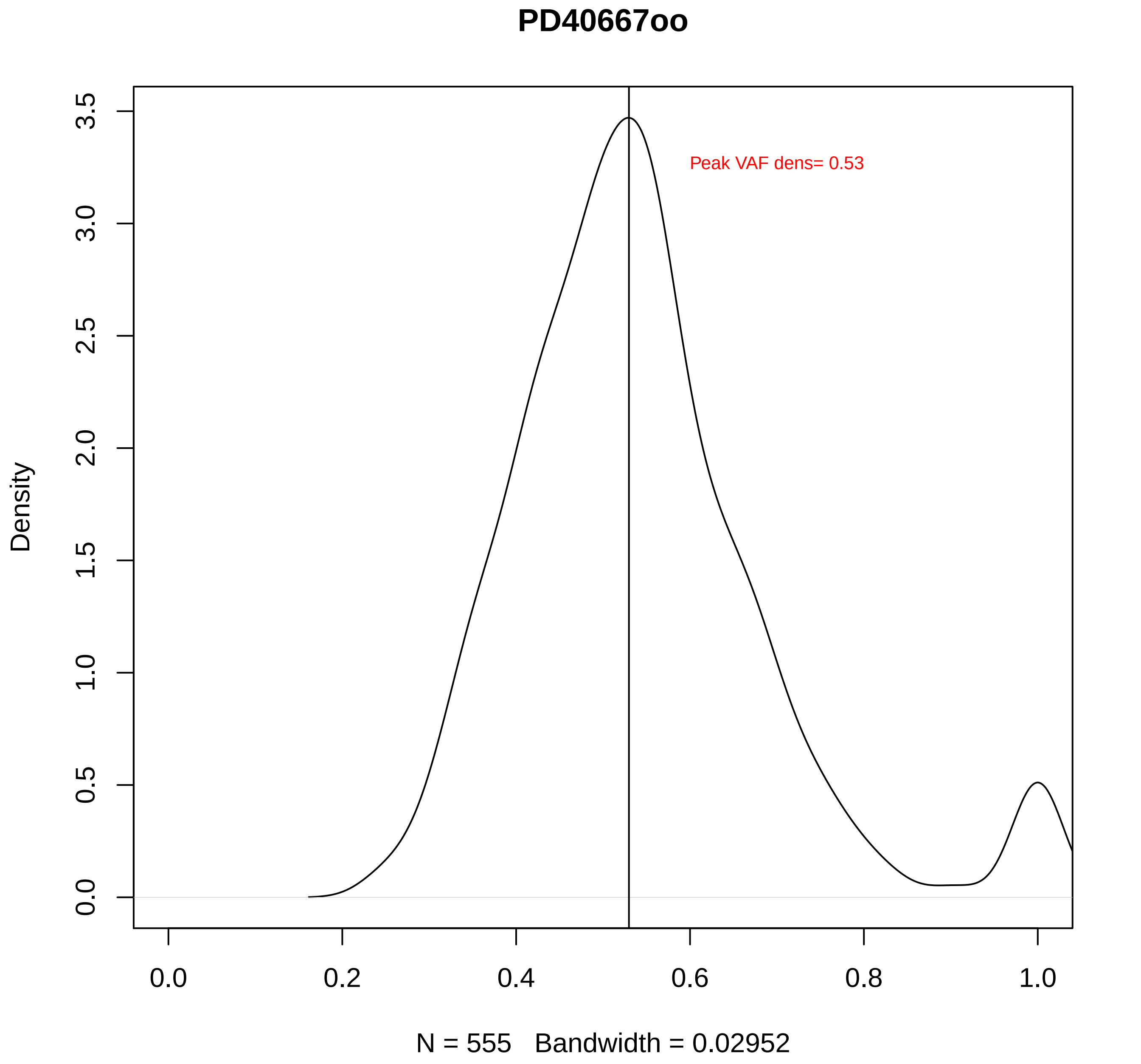

PD40667oo
Peak VAF dens= 0.53
Density
0.0
0.5
1.0
1.5
2.0
2.5
3.0
3.5
0.0
0.2
0.4
0.6
0.8
1.0
N = 555   Bandwidth = 0.02952

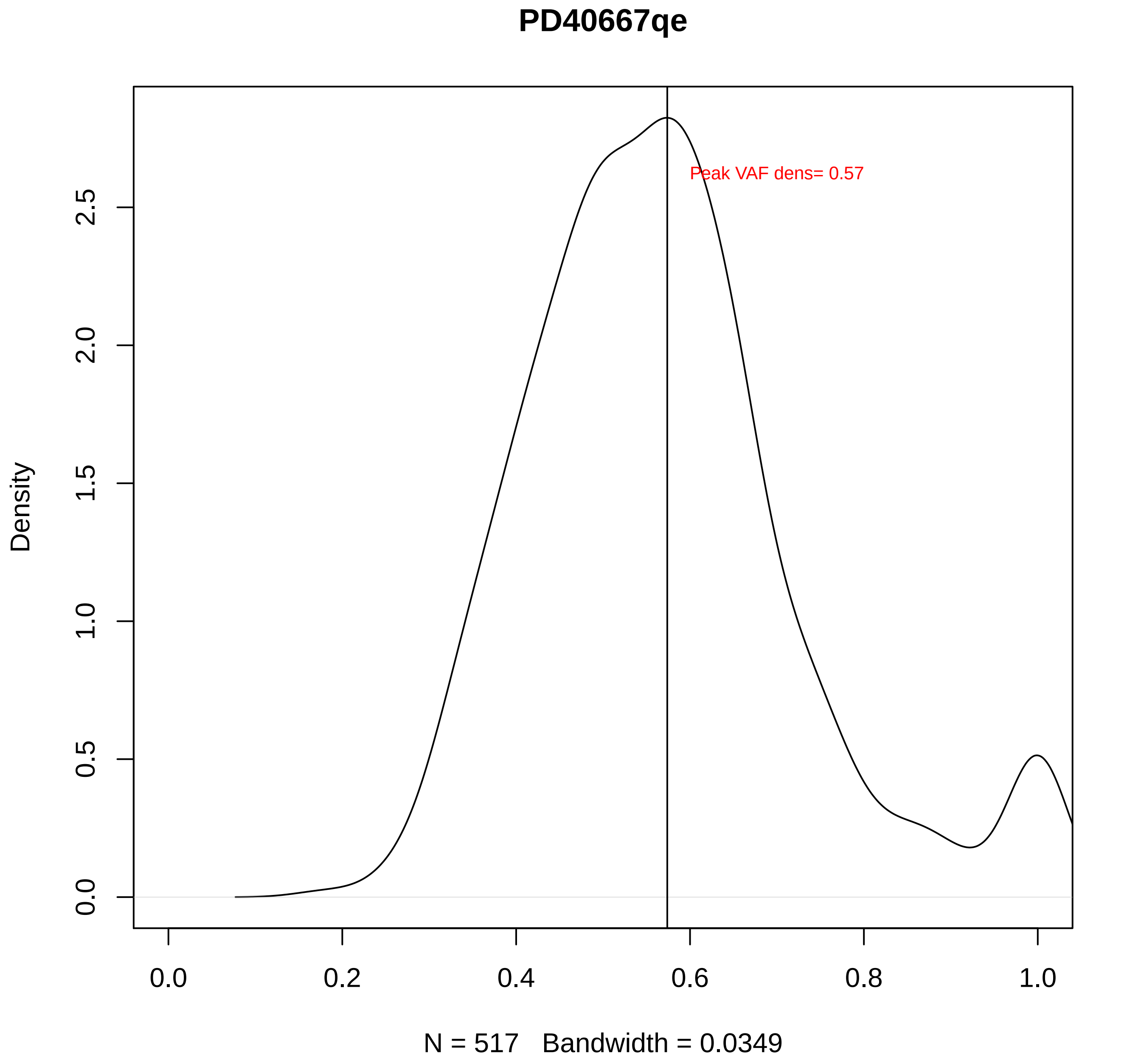
PD40667qe
Peak VAF dens= 0.57
Density
0.0
0.5
1.0
1.5
2.0
2.5
0.0
0.2
0.4
0.6
0.8
1.0
N = 517 Bandwidth = 0.0349

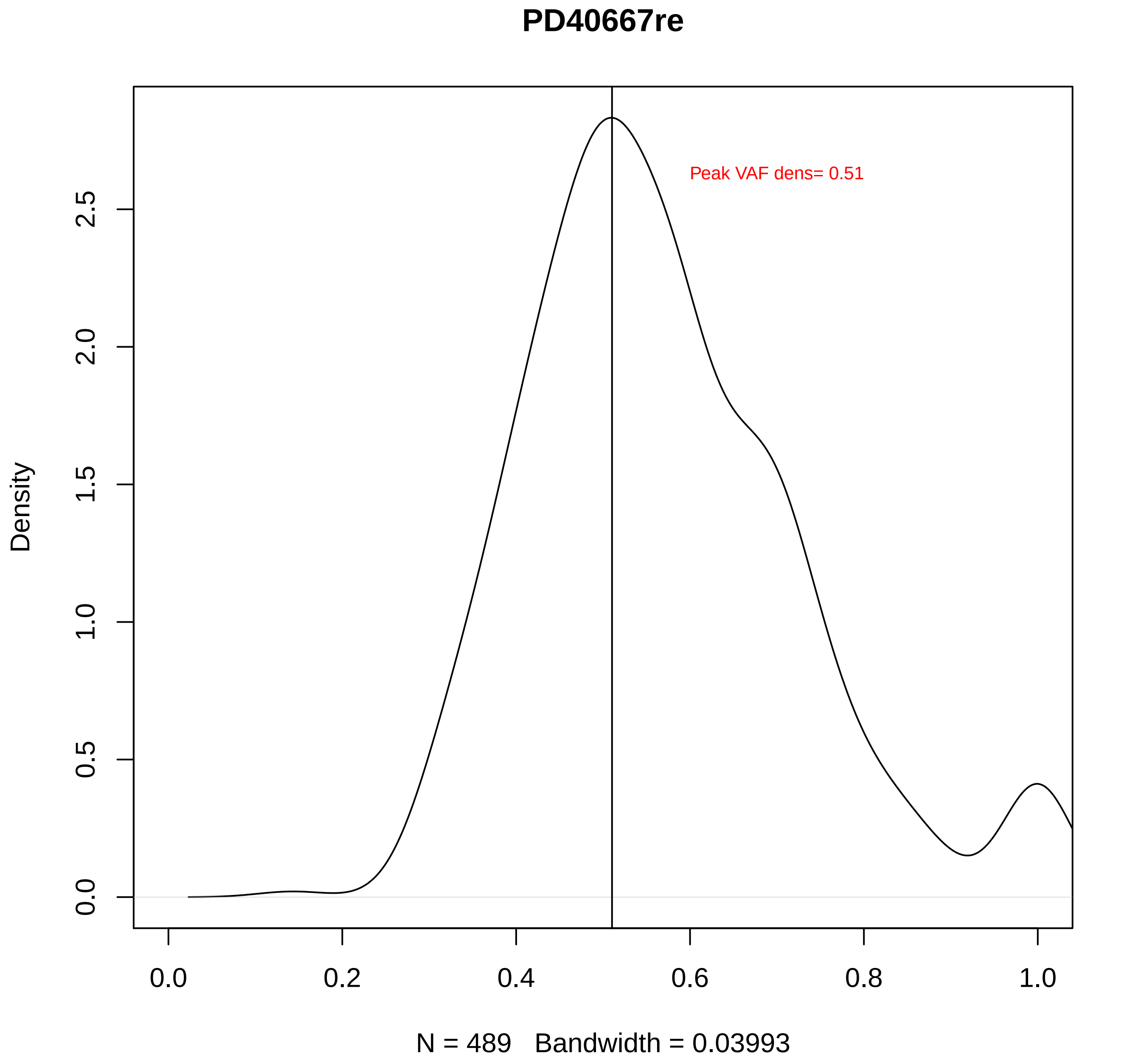
PD40667re
Peak VAF dens= 0.51
Density
0.0
0.5
1.0
1.5
2.0
2.5
0.0
0.2
0.4
0.6
0.8
1.0
N = 489   Bandwidth = 0.03993

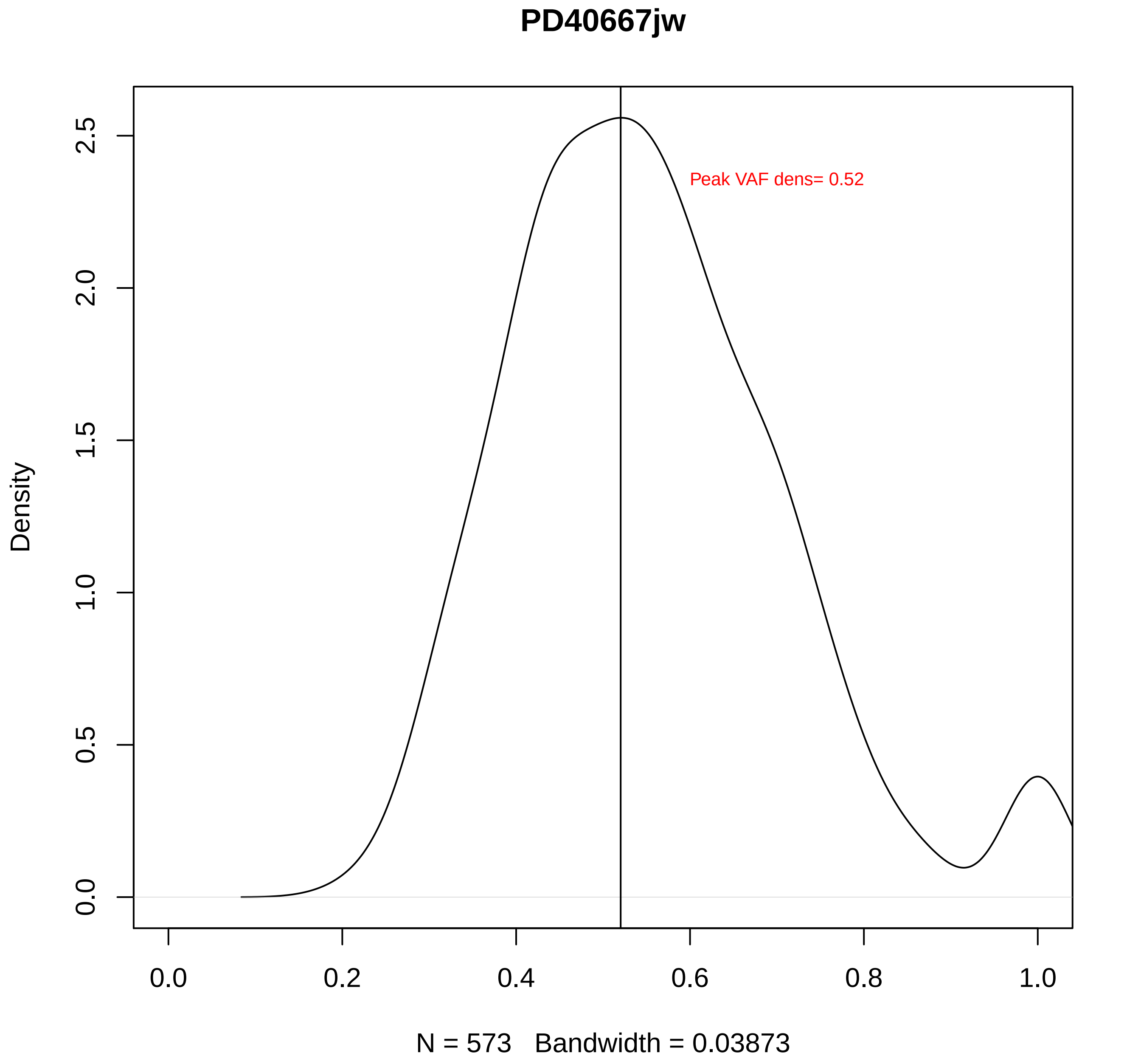
PD40667jw
Peak VAF dens= 0.52
Density
0.0
0.5
1.0
1.5
2.0
2.5
0.0
0.2
0.4
0.6
0.8
1.0
N = 573 Bandwidth = 0.03873

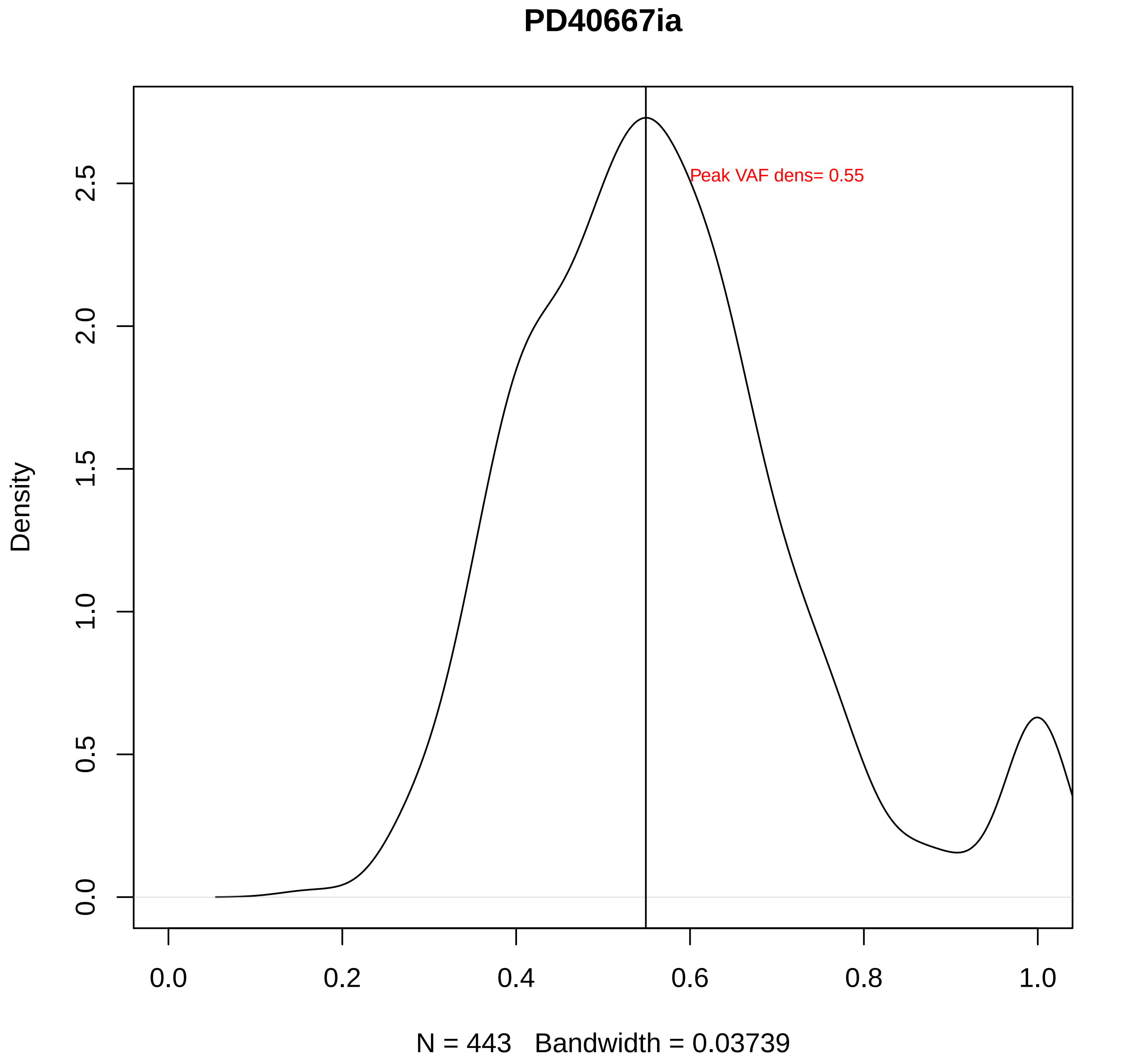
PD40667ia
Peak VAF dens= 0.55
Density
0.0
0.5
1.0
1.5
2.0
2.5
0.0
0.2
0.4
0.6
0.8
1.0
N = 443   Bandwidth = 0.03739

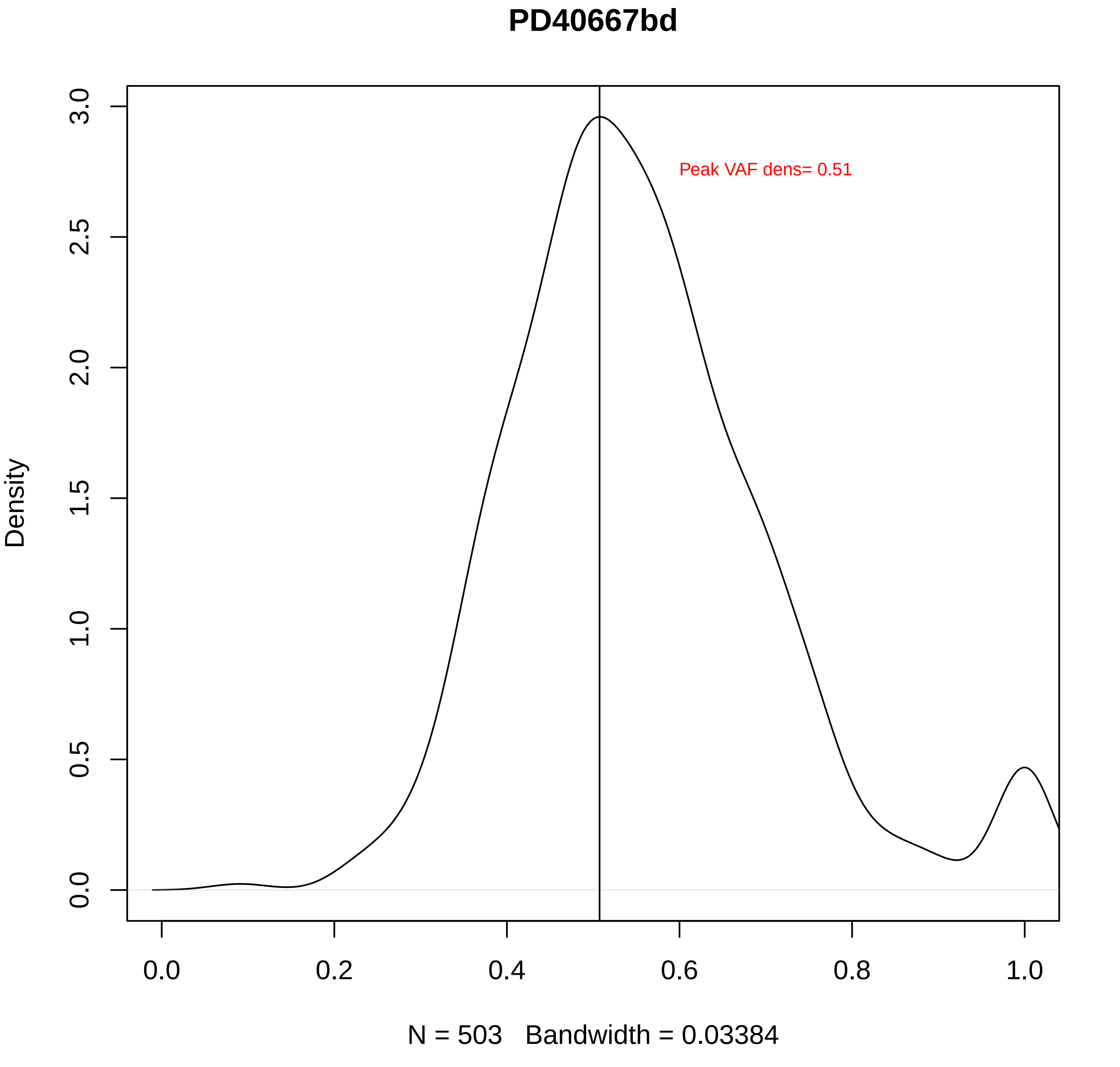

PD40667bd
Peak VAF dens= 0.51
Density
3.0
2.5
2.0
1.5
1.0
0.5
0.0
0.0
0.2
0.4
0.6
0.8
1.0
N = 503 Bandwidth = 0.03384

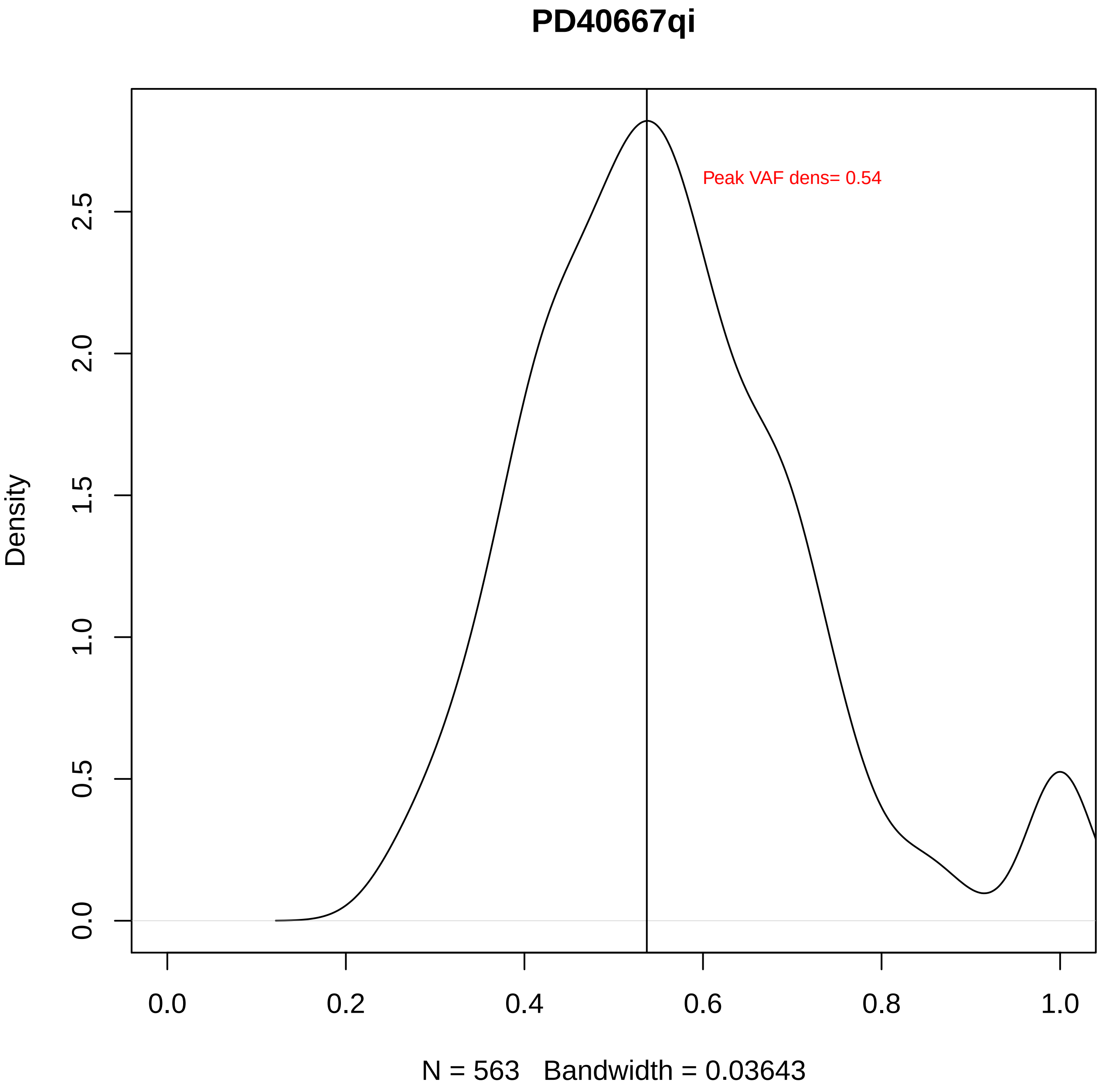

PD40667qi
Peak VAF dens= 0.54
Density
0.0
0.5
1.0
1.5
2.0
2.5
0.0
0.2
0.4
0.6
0.8
1.0
N = 563   Bandwidth = 0.03643

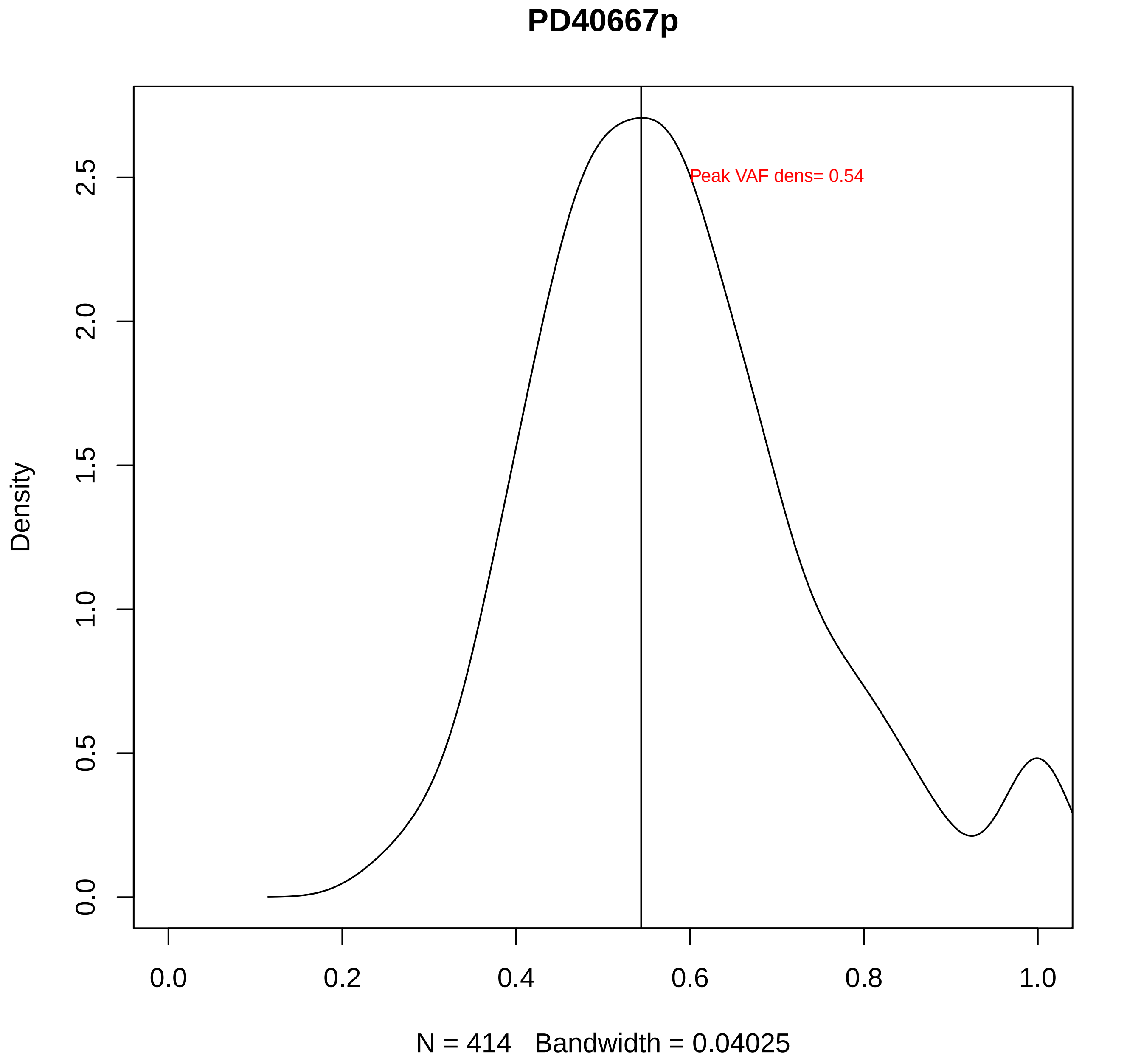

PD40667p
Peak VAF dens= 0.54
Density
0.0
0.5
1.0
1.5
2.0
2.5
0.0
0.2
0.4
0.6
0.8
1.0
N = 414   Bandwidth = 0.04025

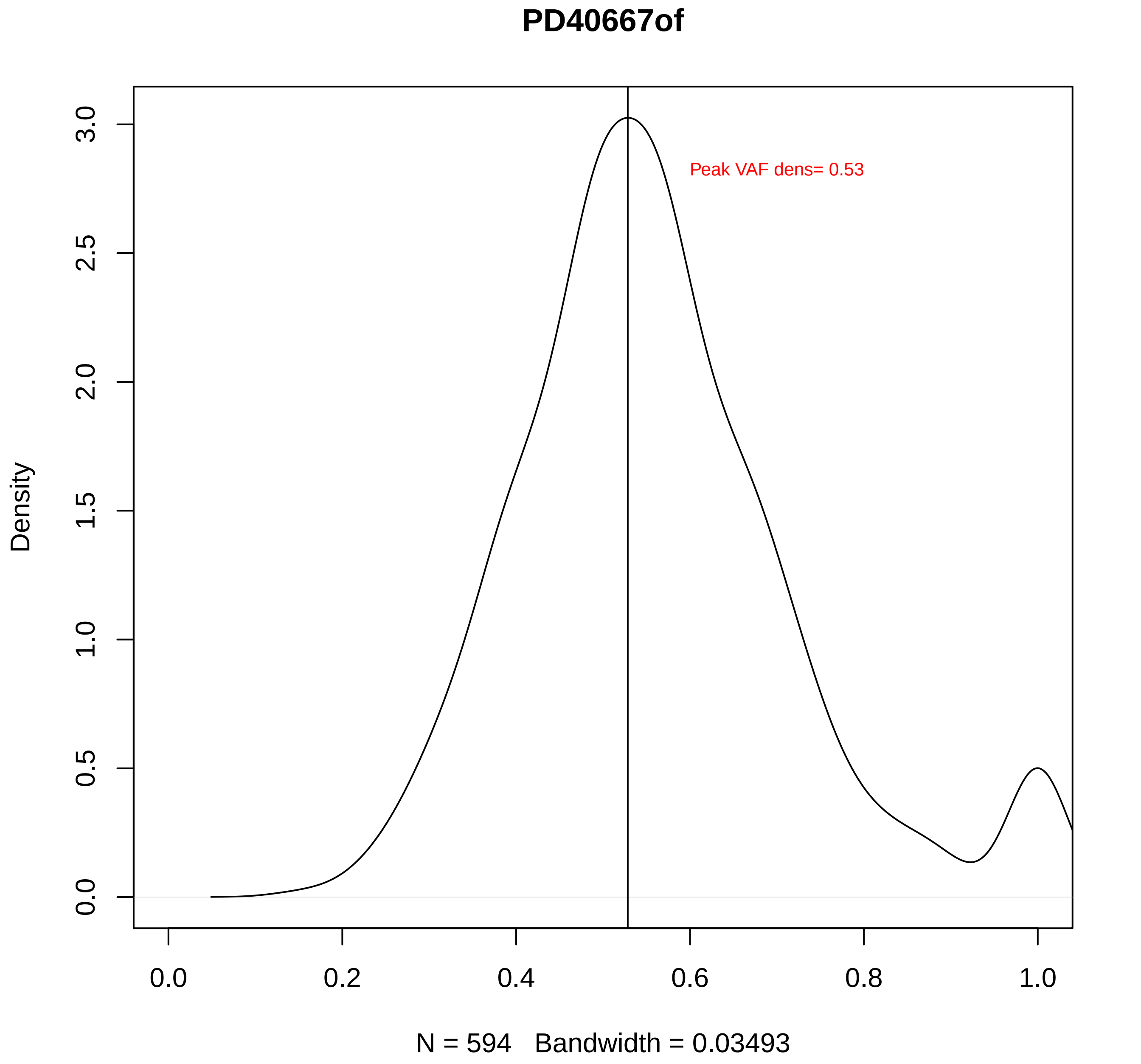

PD40667of
Peak VAF dens= 0.53
Density
0.0
0.5
1.0
1.5
2.0
2.5
3.0
0.0
0.2
0.4
0.6
0.8
1.0
N = 594   Bandwidth = 0.03493

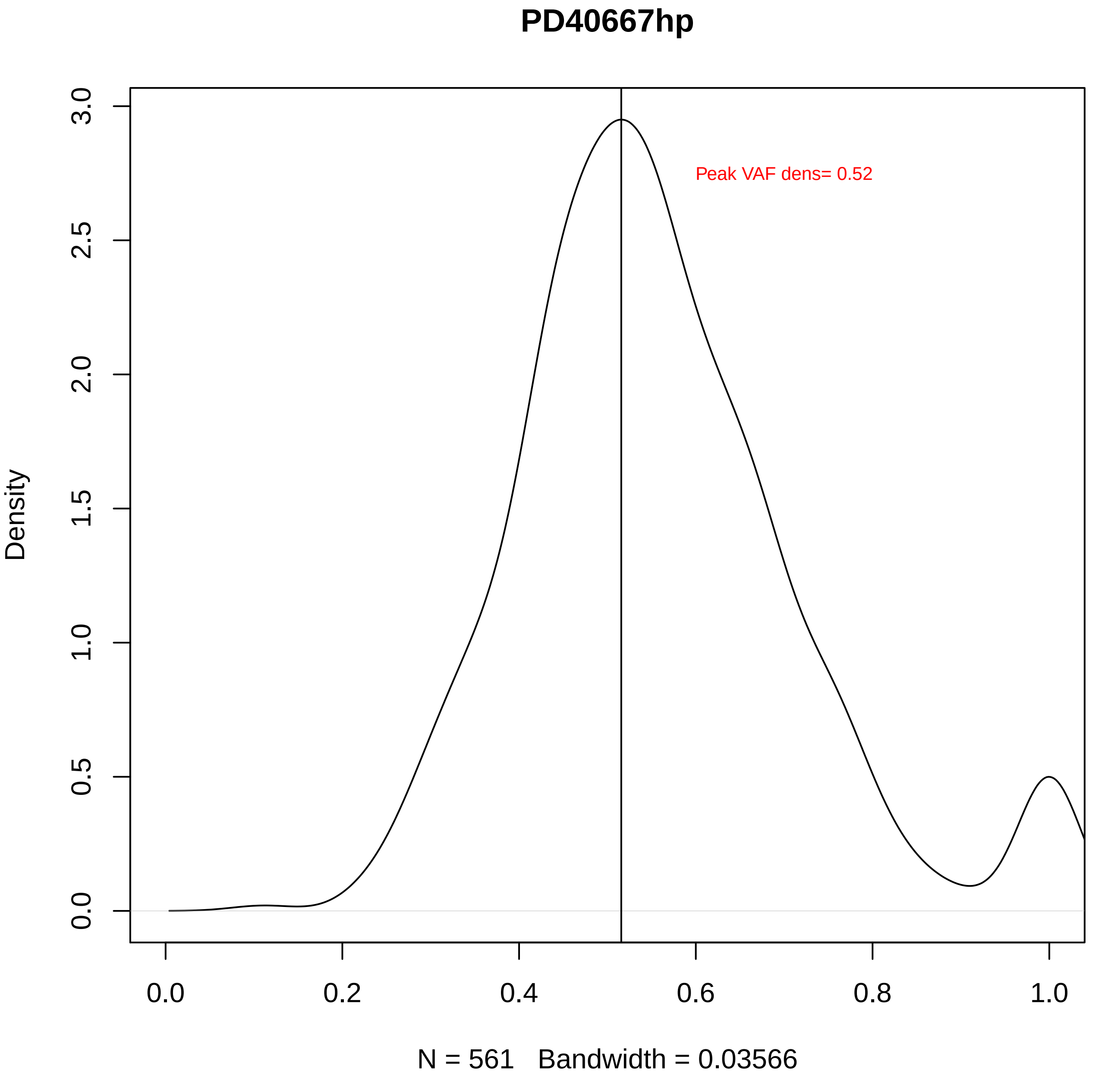

PD40667hp
Peak VAF dens= 0.52
Density
0.0
0.5
1.0
1.5
2.0
2.5
3.0
0.0
0.2
0.4
0.6
0.8
1.0
N = 561 Bandwidth = 0.03566

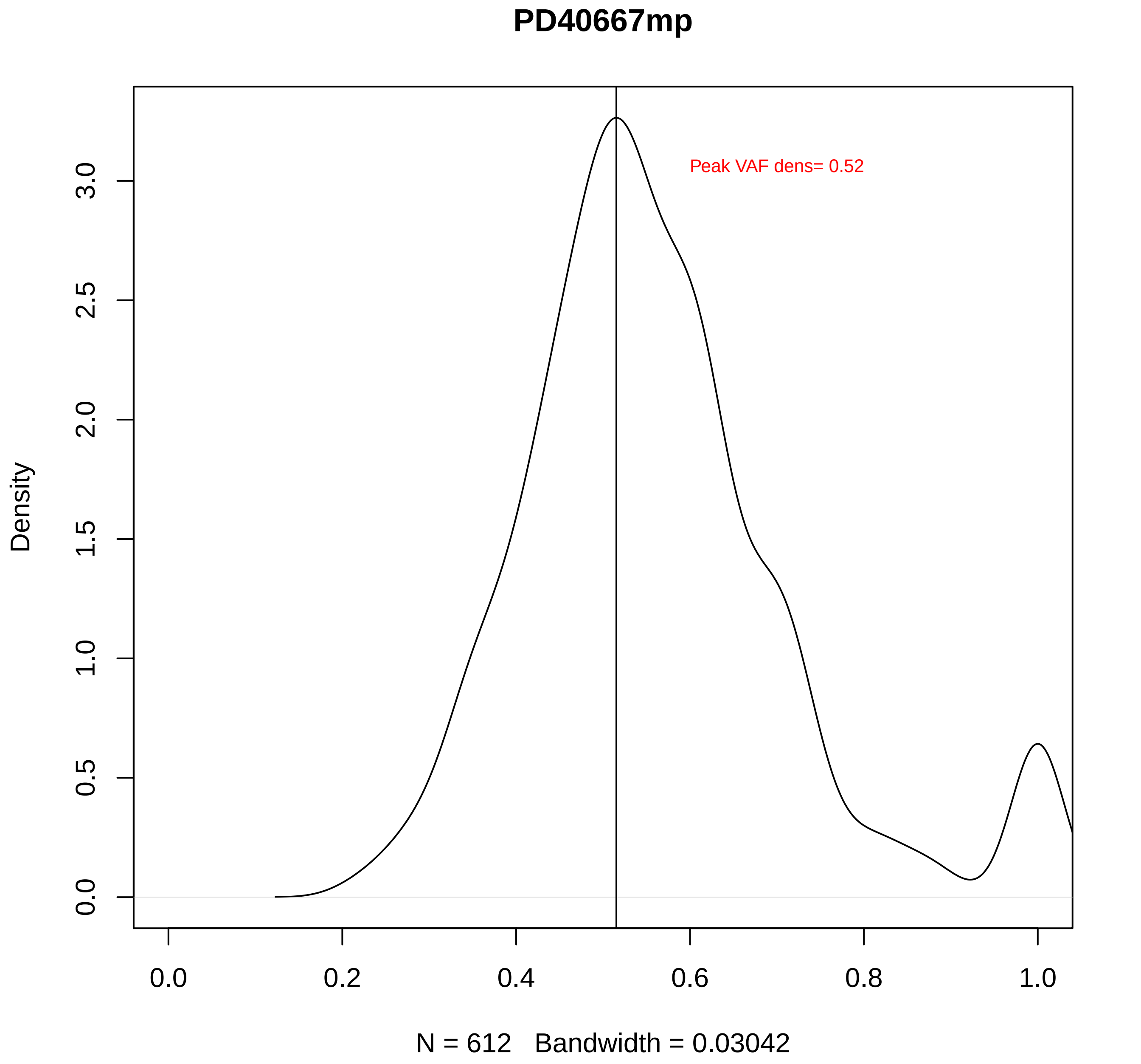
PD40667mp
Peak VAF dens= 0.52
Density
0.0
0.5
1.0
1.5
2.0
2.5
3.0
0.0
0.2
0.4
0.6
0.8
1.0
N = 612   Bandwidth = 0.03042

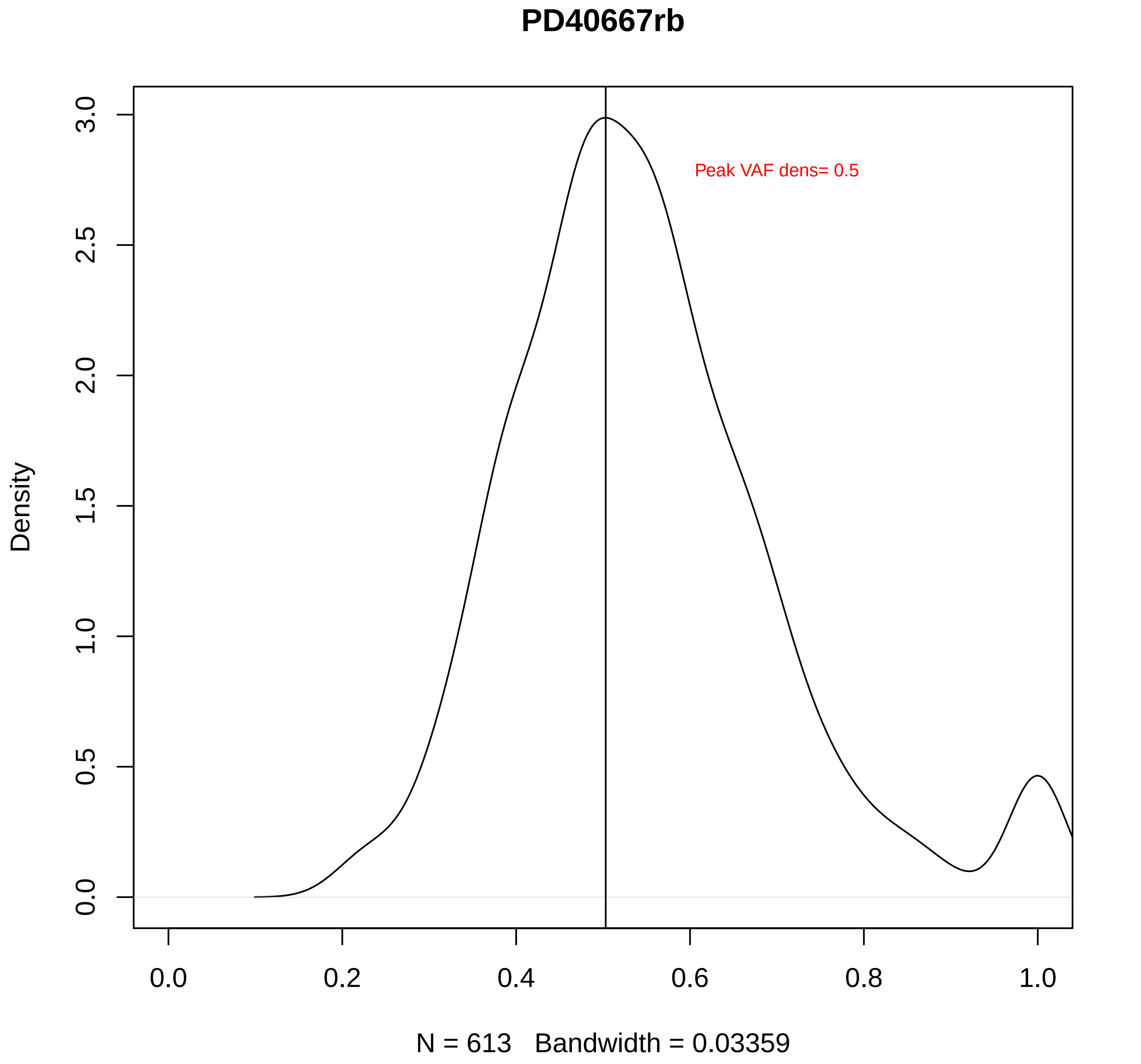
PD40667rb
Peak VAF dens= 0.5
Density
3.0
2.5
2.0
1.5
1.0
0.5
0.0
0.2
0.4
0.6
0.8
1.0
N = 613 Bandwidth = 0.03359

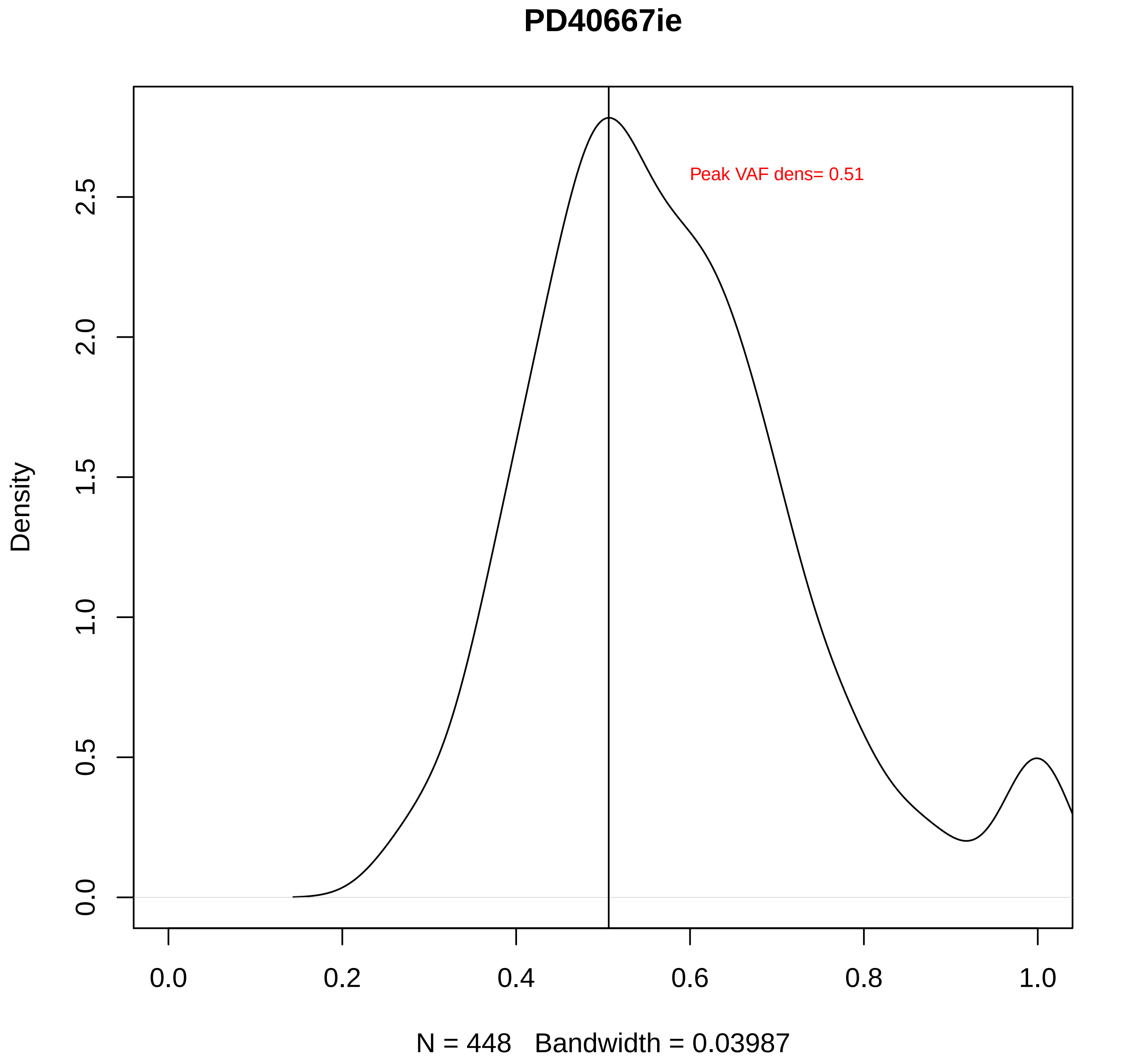
PD40667ie
Peak VAF dens= 0.51
Density
0.0
0.5
1.0
1.5
2.0
2.5
0.0
0.2
0.4
0.6
0.8
1.0
N = 448   Bandwidth = 0.03987

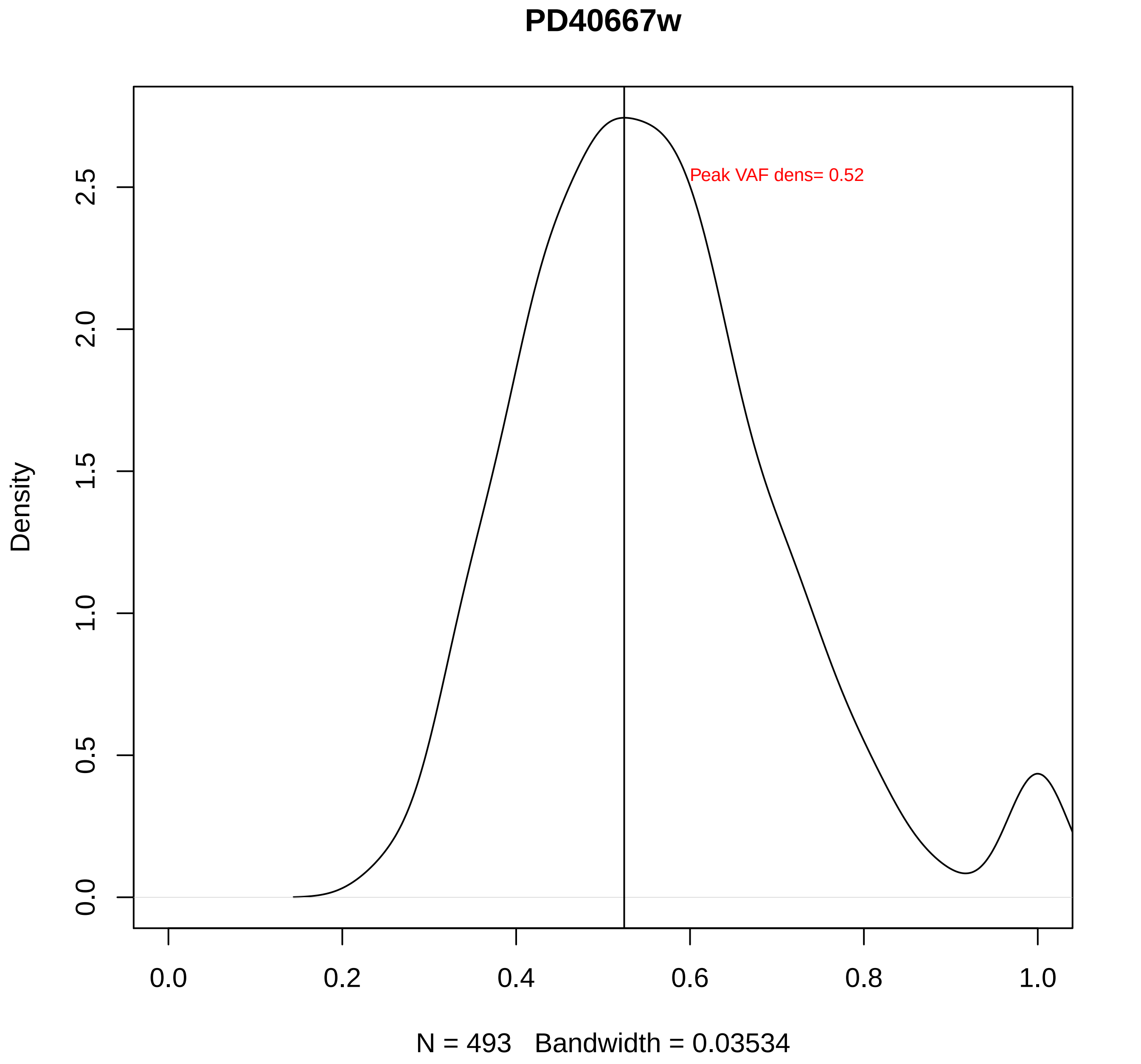
PD40667w
Peak VAF dens= 0.52
Density
0.0
0.5
1.0
1.5
2.0
2.5
0.0
0.2
0.4
0.6
0.8
1.0
N = 493   Bandwidth = 0.03534

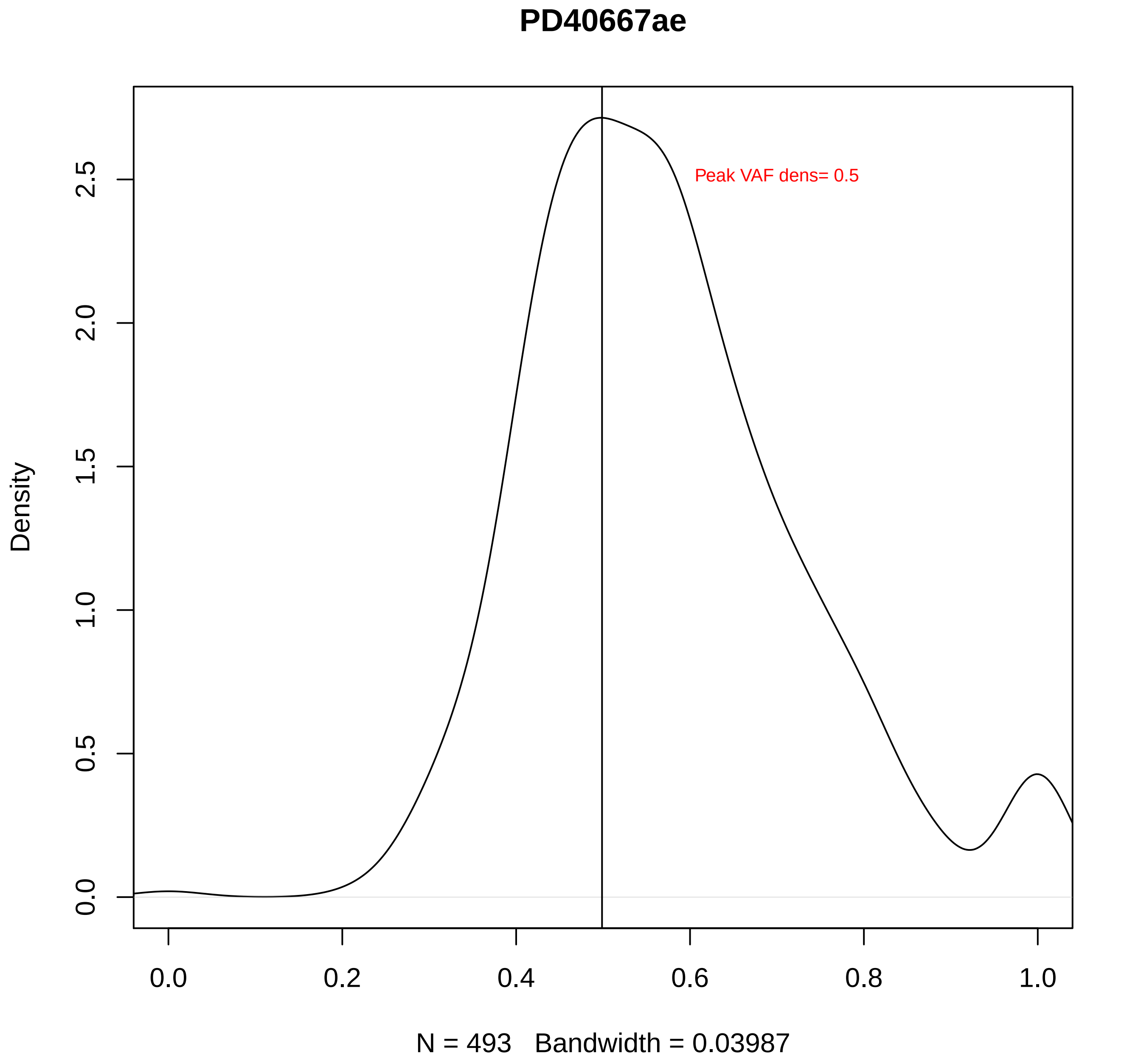

PD40667ae
Peak VAF dens= 0.5
Density
0.0
0.5
1.0
1.5
2.0
2.5
0.0
0.2
0.4
0.6
0.8
1.0
N = 493 Bandwidth = 0.03987

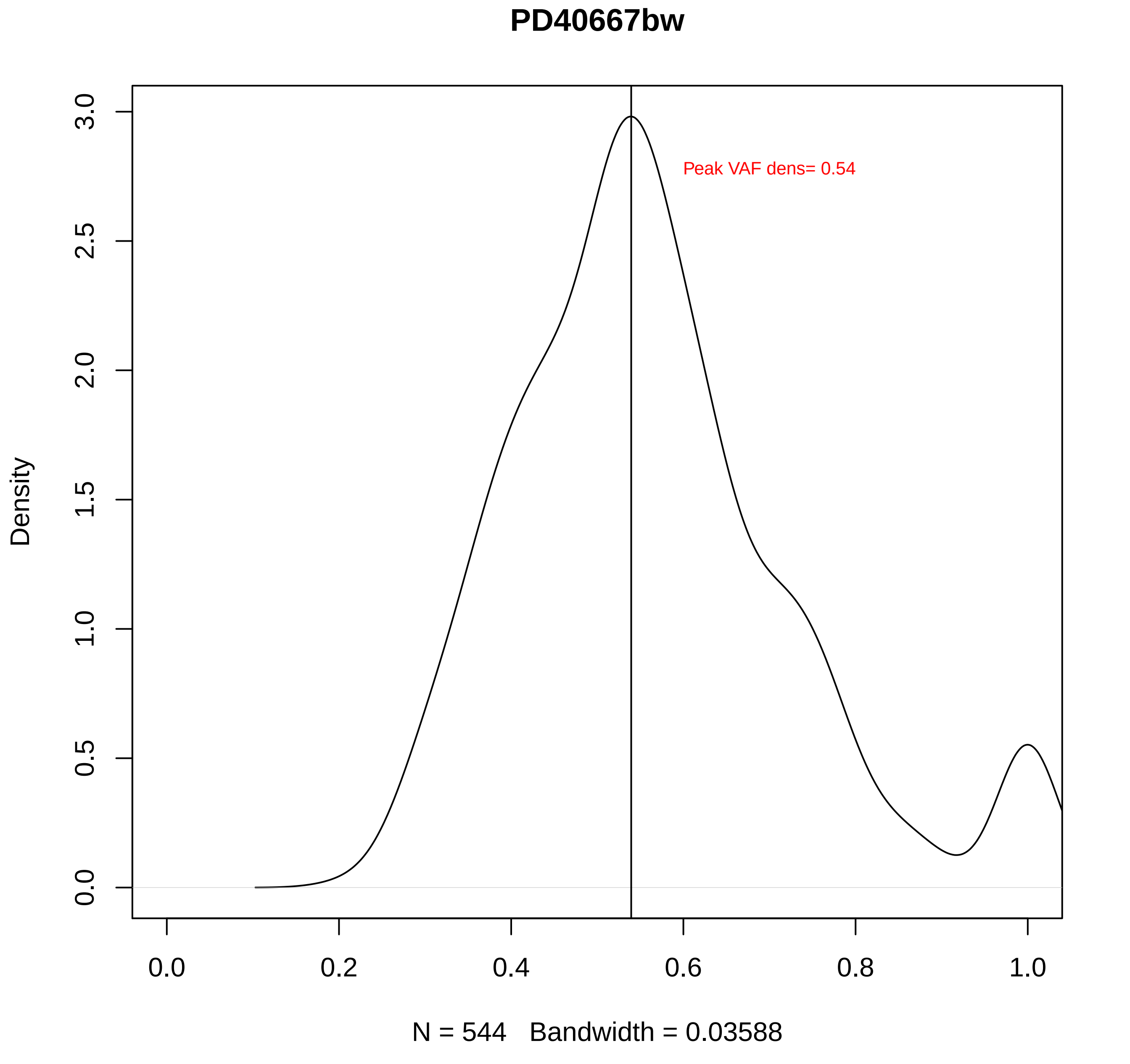
PD40667bw
Peak VAF dens= 0.54
Density
0.0
0.5
1.0
1.5
2.0
2.5
3.0
0.0
0.2
0.4
0.6
0.8
1.0
N = 544   Bandwidth = 0.03588

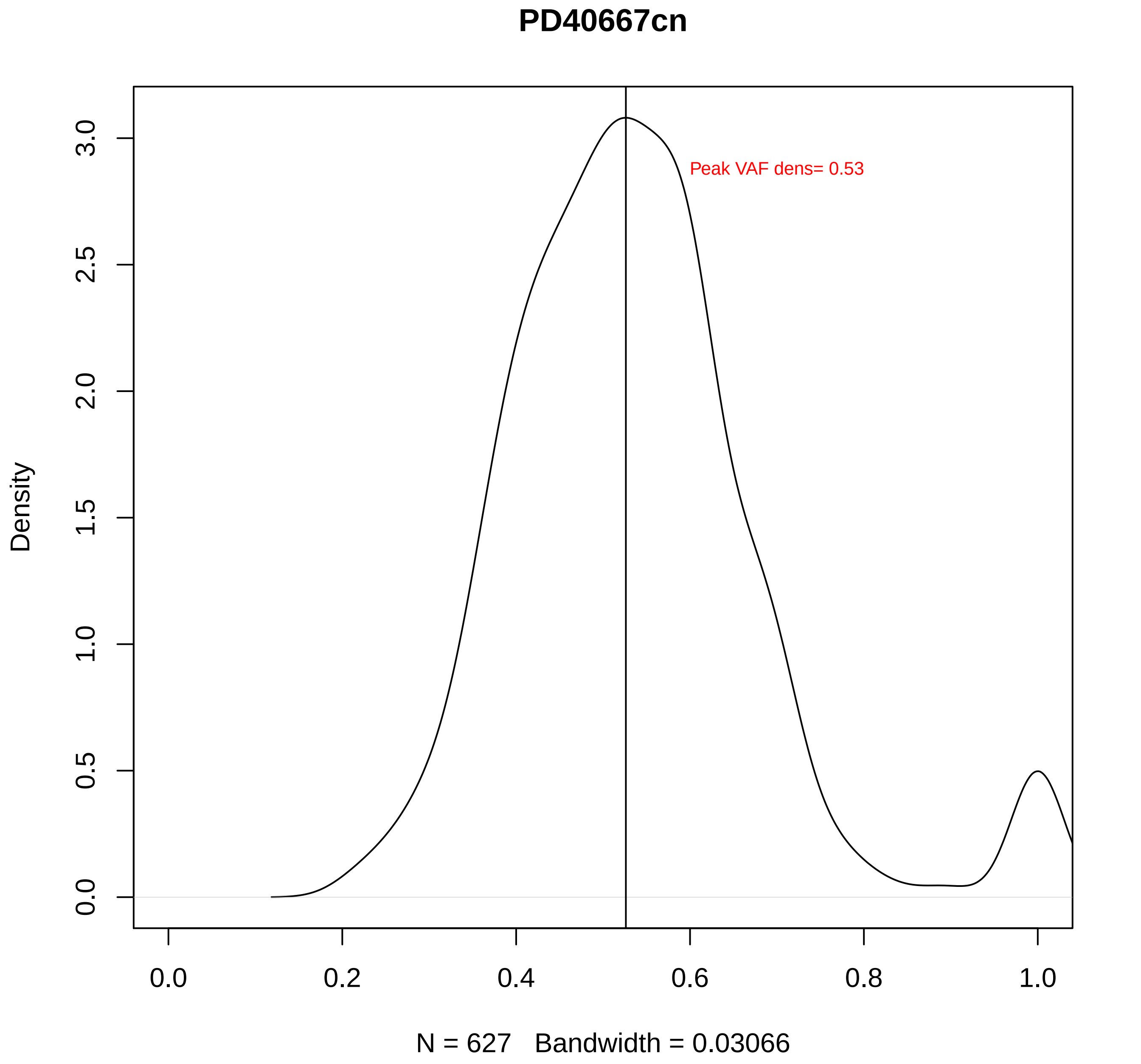
PD40667cn
Peak VAF dens= 0.53
Density
0.0
0.5
1.0
1.5
2.0
2.5
3.0
0.0
0.2
0.4
0.6
0.8
1.0
N = 627   Bandwidth = 0.03066

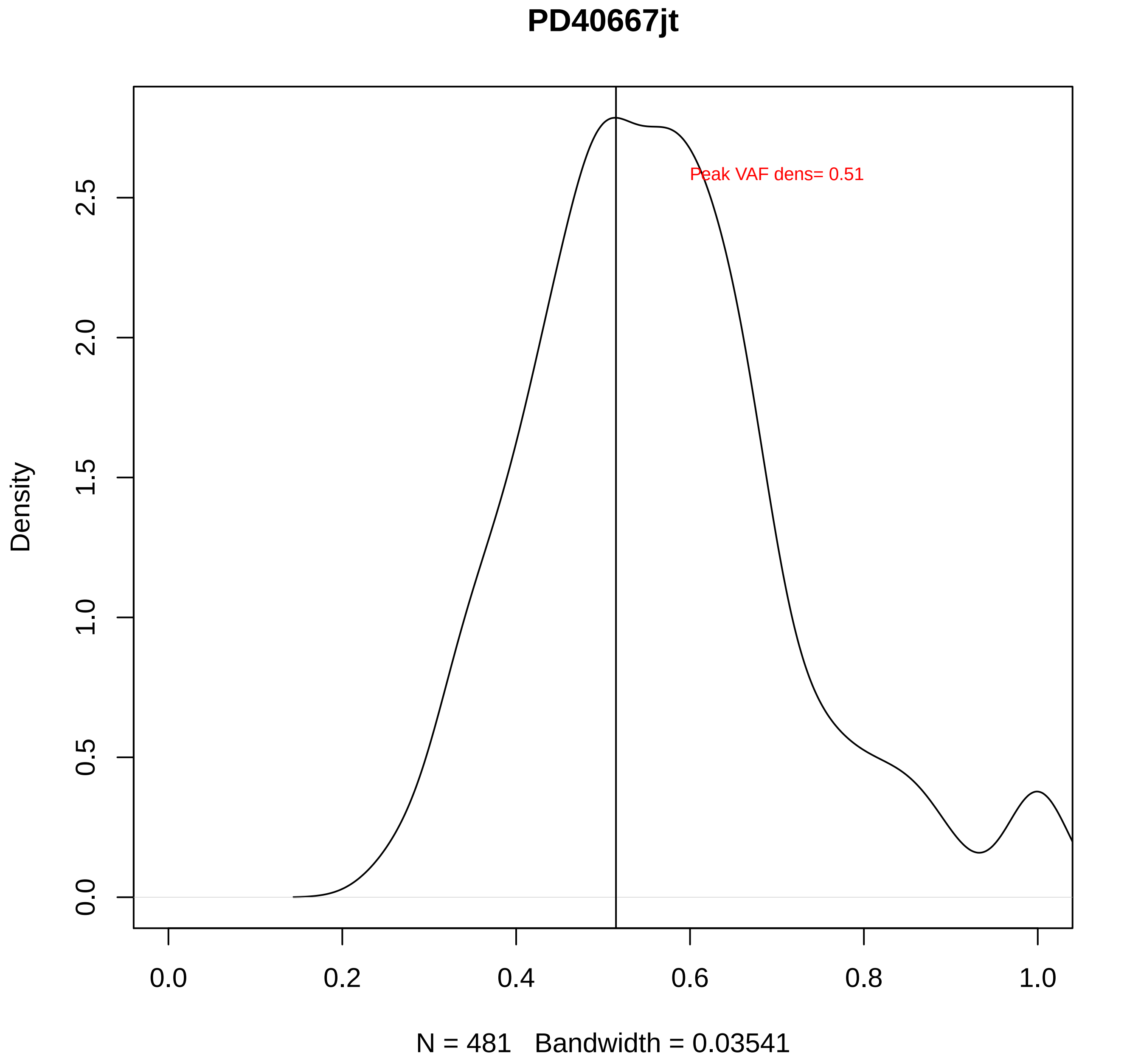

PD40667jt
Peak VAF dens= 0.51
Density
0.0
0.5
1.0
1.5
2.0
2.5
0.0
0.2
0.4
0.6
0.8
1.0
N = 481 Bandwidth = 0.03541

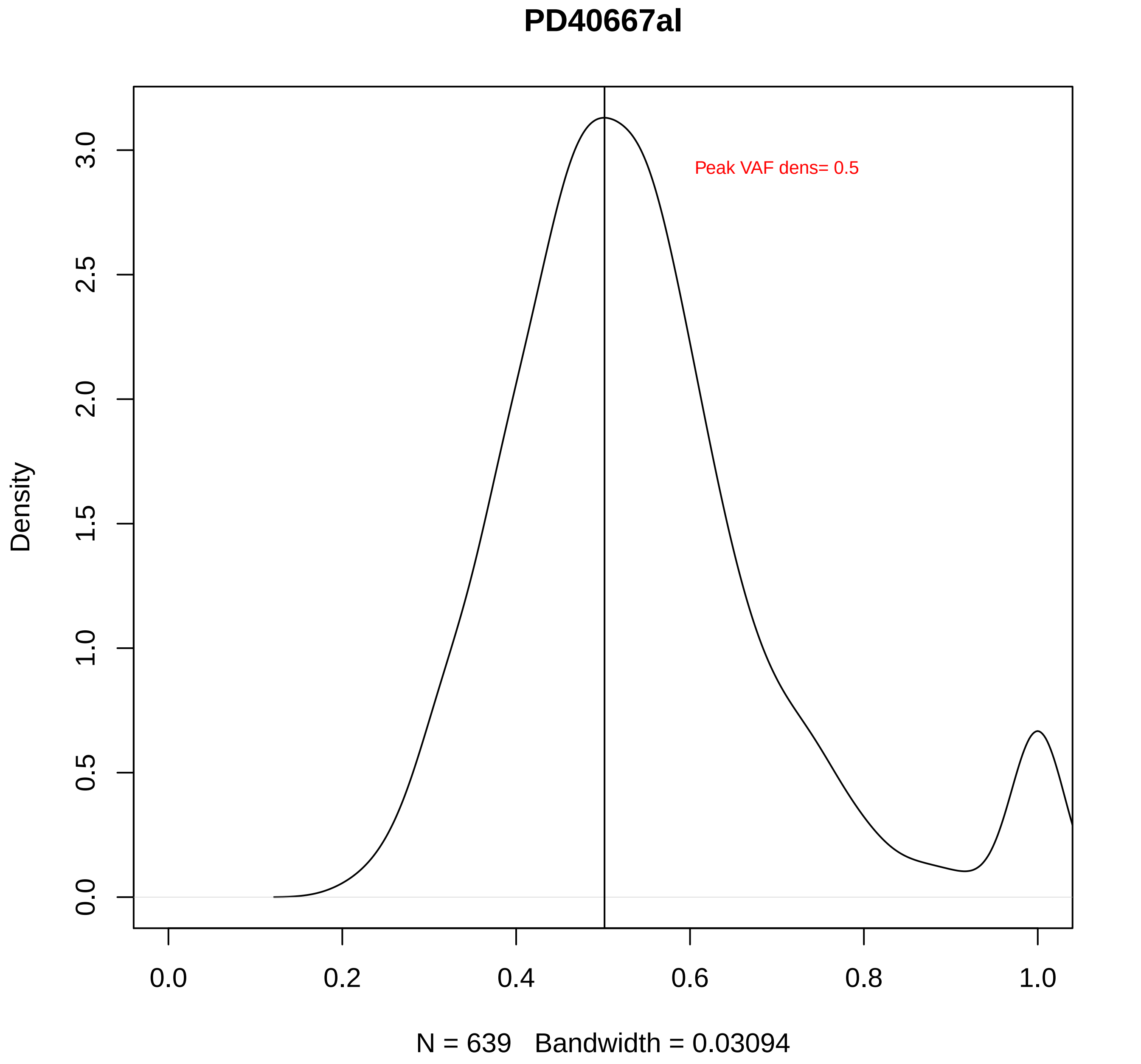

PD40667al
Peak VAF dens= 0.5
Density
0.0
0.5
1.0
1.5
2.0
2.5
3.0
0.0
0.2
0.4
0.6
0.8
1.0
N = 639   Bandwidth = 0.03094

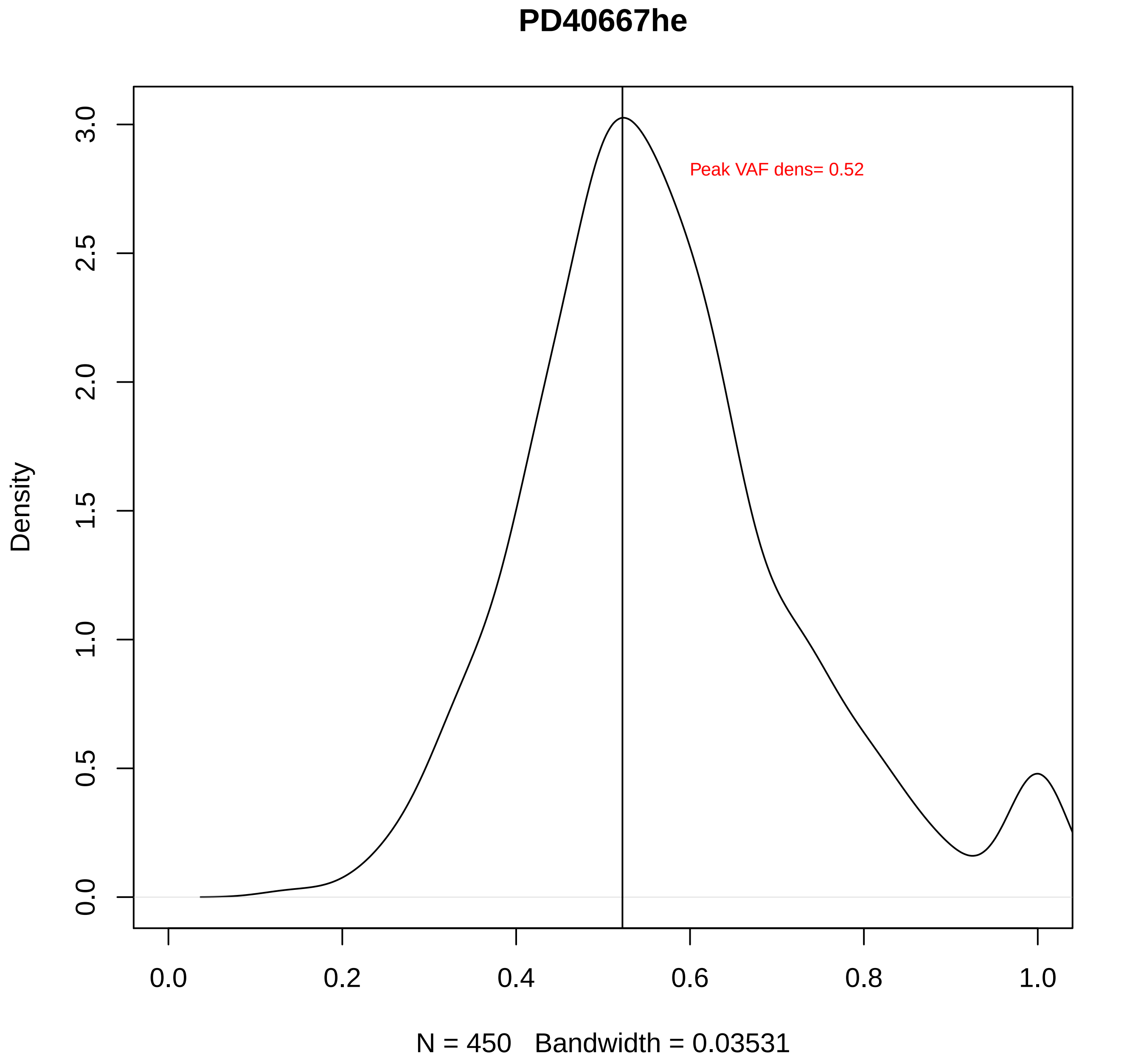

PD40667he
Peak VAF dens= 0.52
Density
3.0
2.5
2.0
1.5
1.0
0.5
0.0
0.0
0.2
0.4
0.6
0.8
1.0
N = 450 Bandwidth = 0.03531

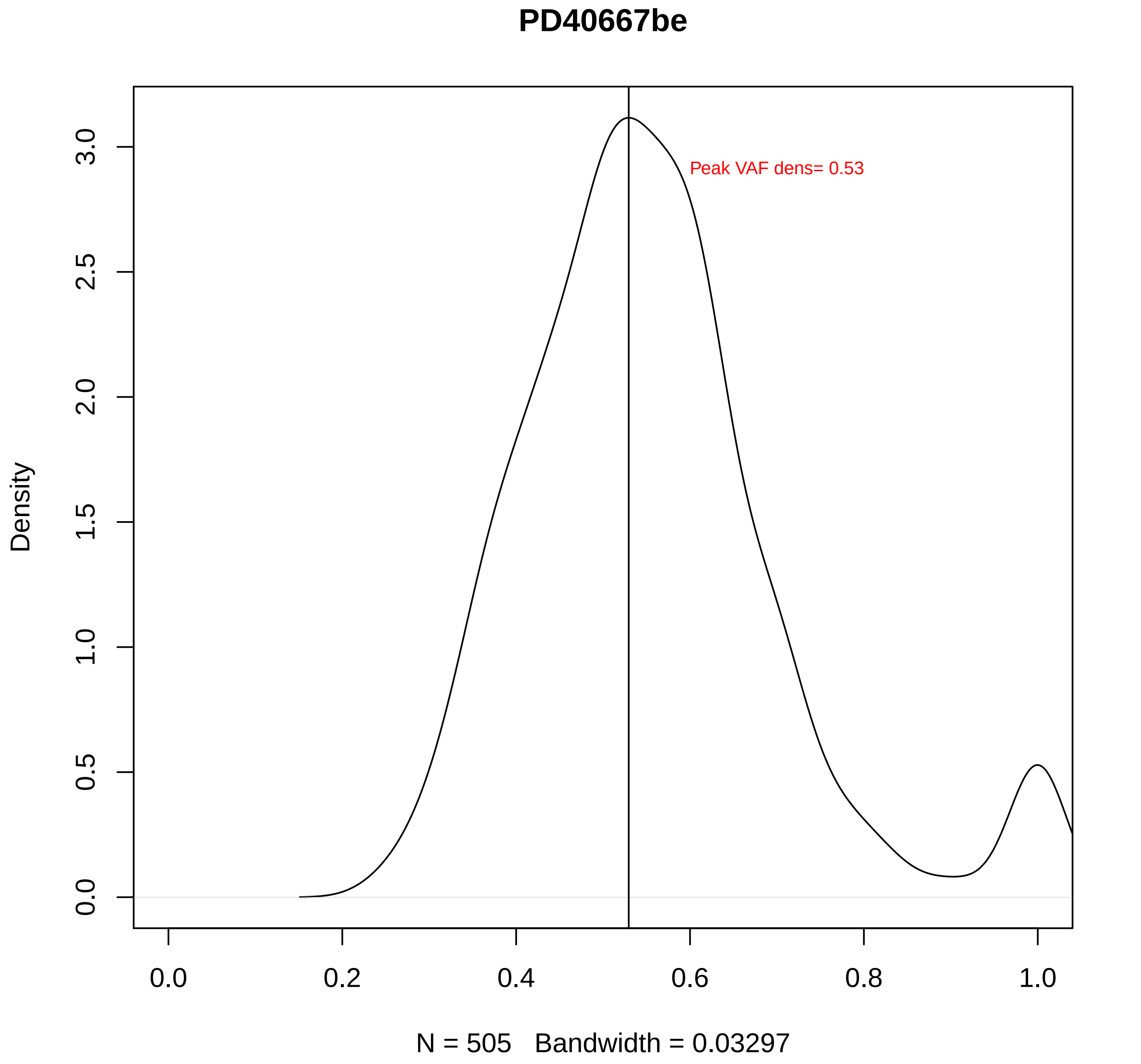

PD40667be
Peak VAF dens= 0.53
Density
0.0
0.5
1.0
1.5
2.0
2.5
3.0
0.0
0.2
0.4
0.6
0.8
1.0
N = 505   Bandwidth = 0.03297

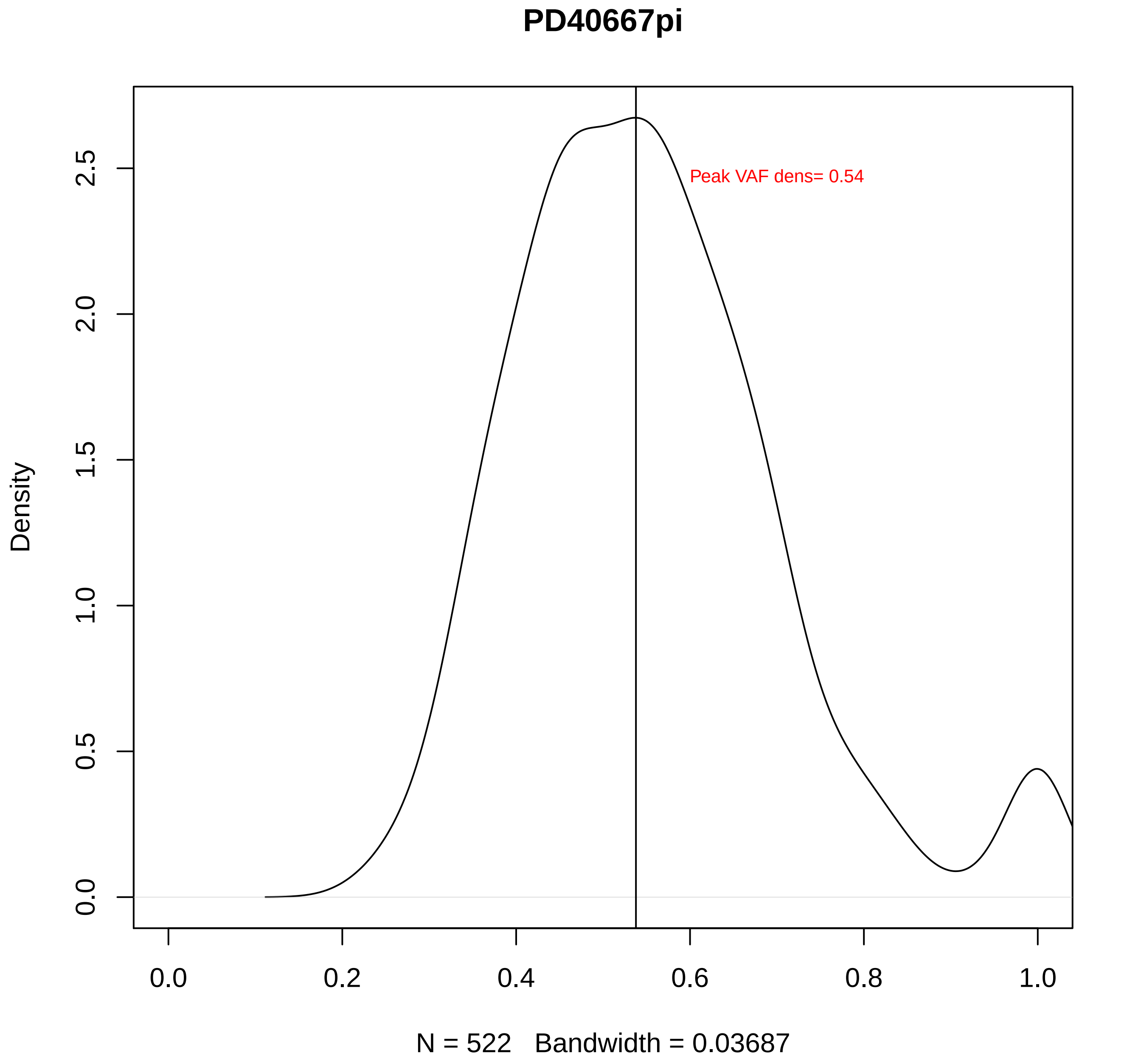
PD40667pi
Peak VAF dens= 0.54
Density
0.0
0.5
1.0
1.5
2.0
2.5
0.0
0.2
0.4
0.6
0.8
1.0
N = 522   Bandwidth = 0.03687

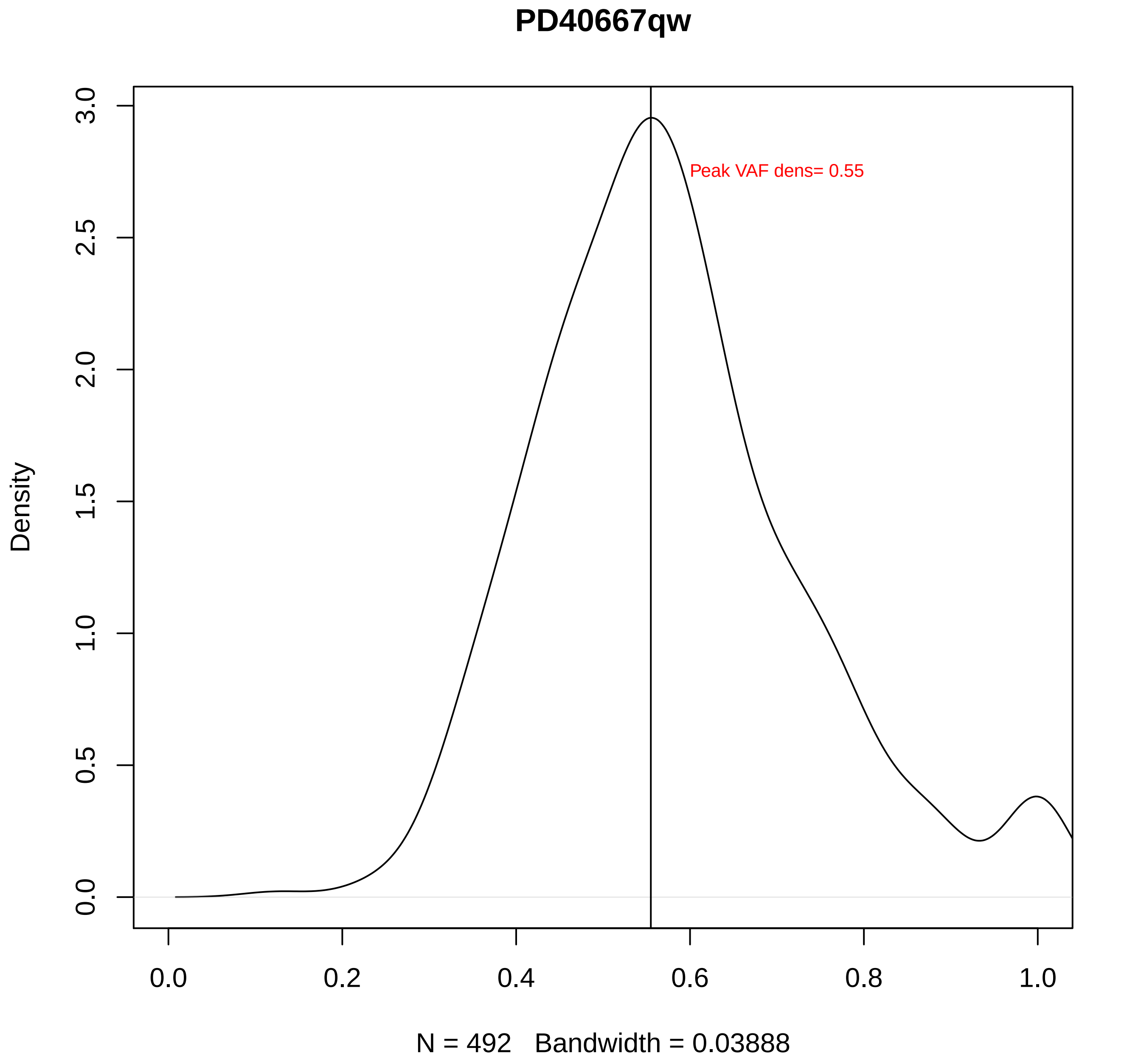

PD40667qw
Density
3.0
2.5
2.0
1.5
1.0
0.5
0.0
Peak VAF dens= 0.55
0.0
0.2
0.4
0.6
0.8
1.0
N = 492 Bandwidth = 0.03888

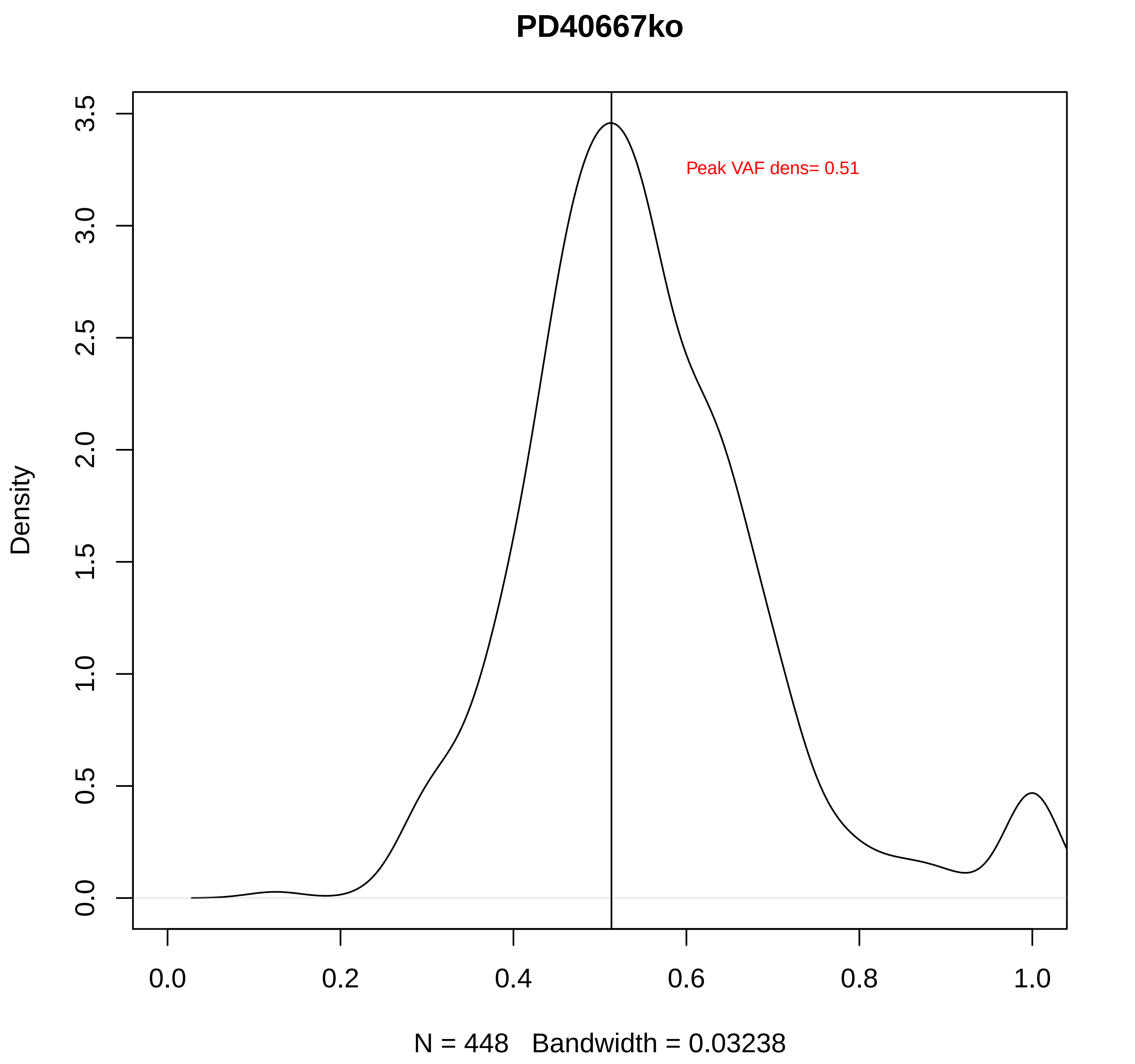
PD40667ko
Peak VAF dens= 0.51
Density
0.0
0.5
1.0
1.5
2.0
2.5
3.0
3.5
0.0
0.2
0.4
0.6
0.8
1.0
N = 448   Bandwidth = 0.03238

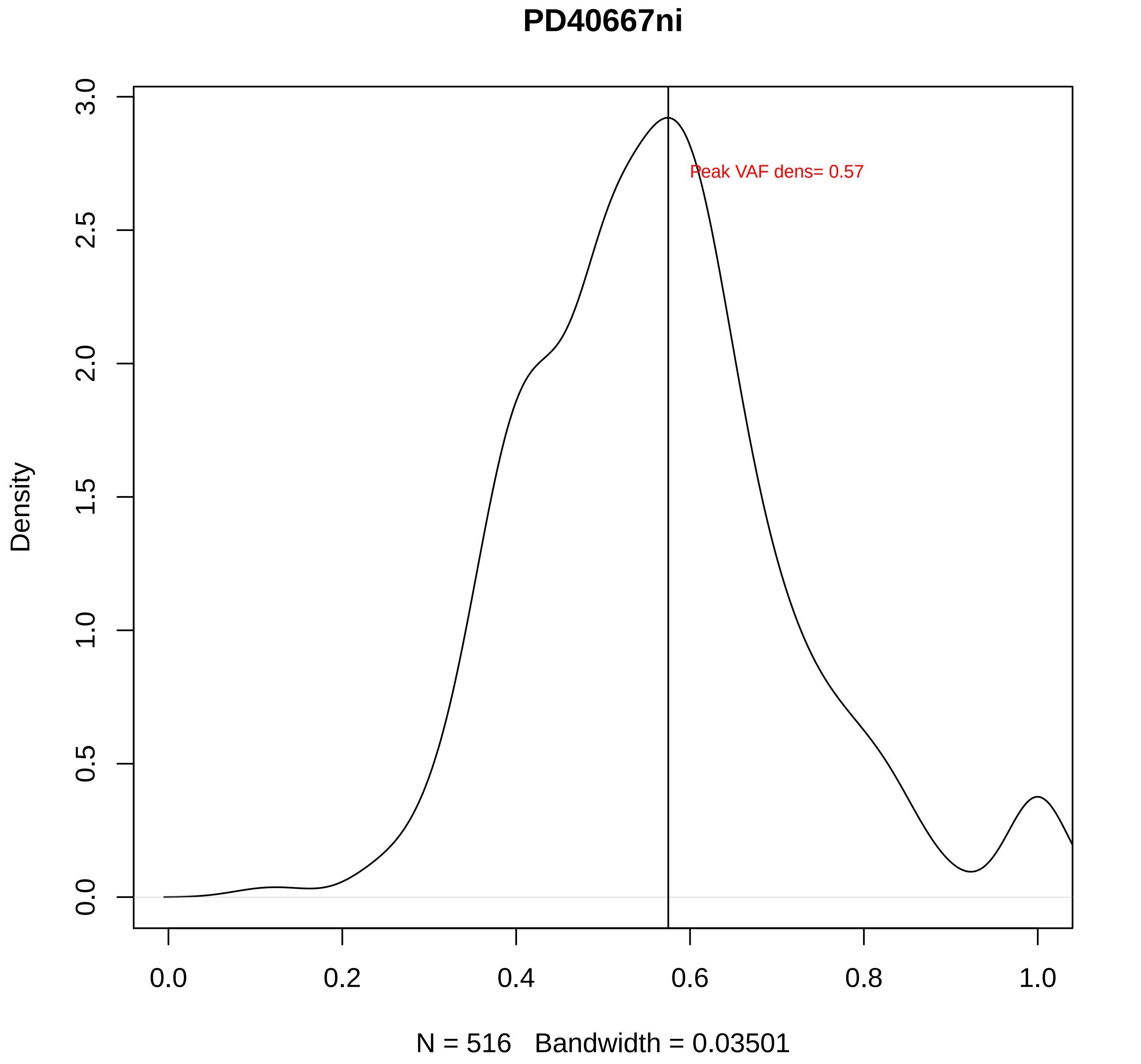
PD40667ni
Peak VAF dens= 0.57
Density
0.0
0.5
1.0
1.5
2.0
2.5
3.0
0.0
0.2
0.4
0.6
0.8
1.0
N = 516 Bandwidth = 0.03501

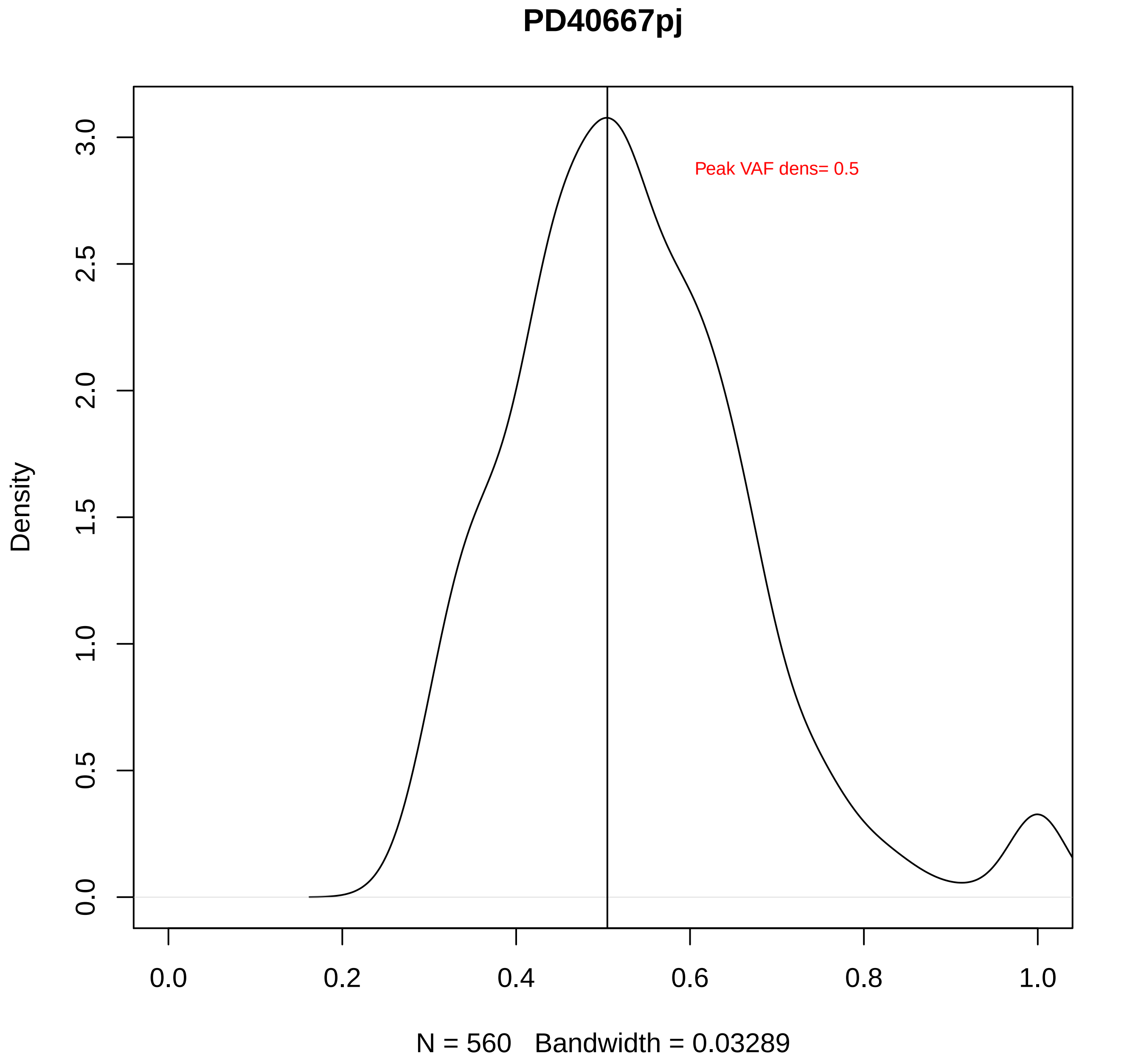

PD40667pj
Peak VAF dens= 0.5
Density
0.0
0.5
1.0
1.5
2.0
2.5
3.0
0.0
0.2
0.4
0.6
0.8
1.0
N = 560 Bandwidth = 0.03289

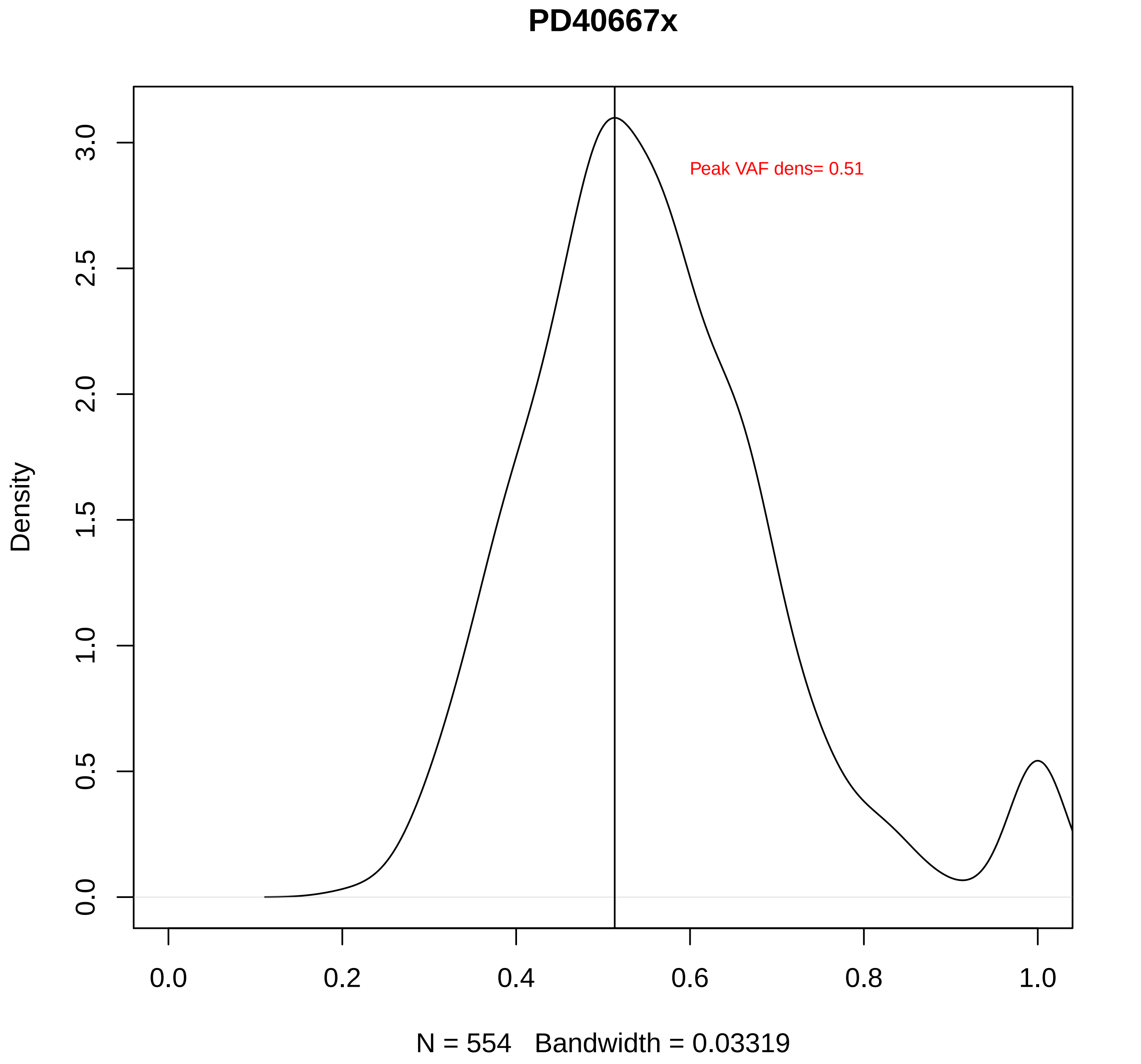

PD40667x
Peak VAF dens= 0.51
Density
3.0
2.5
2.0
1.5
1.0
0.5
0.0
0.0
0.2
0.4
0.6
0.8
1.0
N = 554   Bandwidth = 0.03319

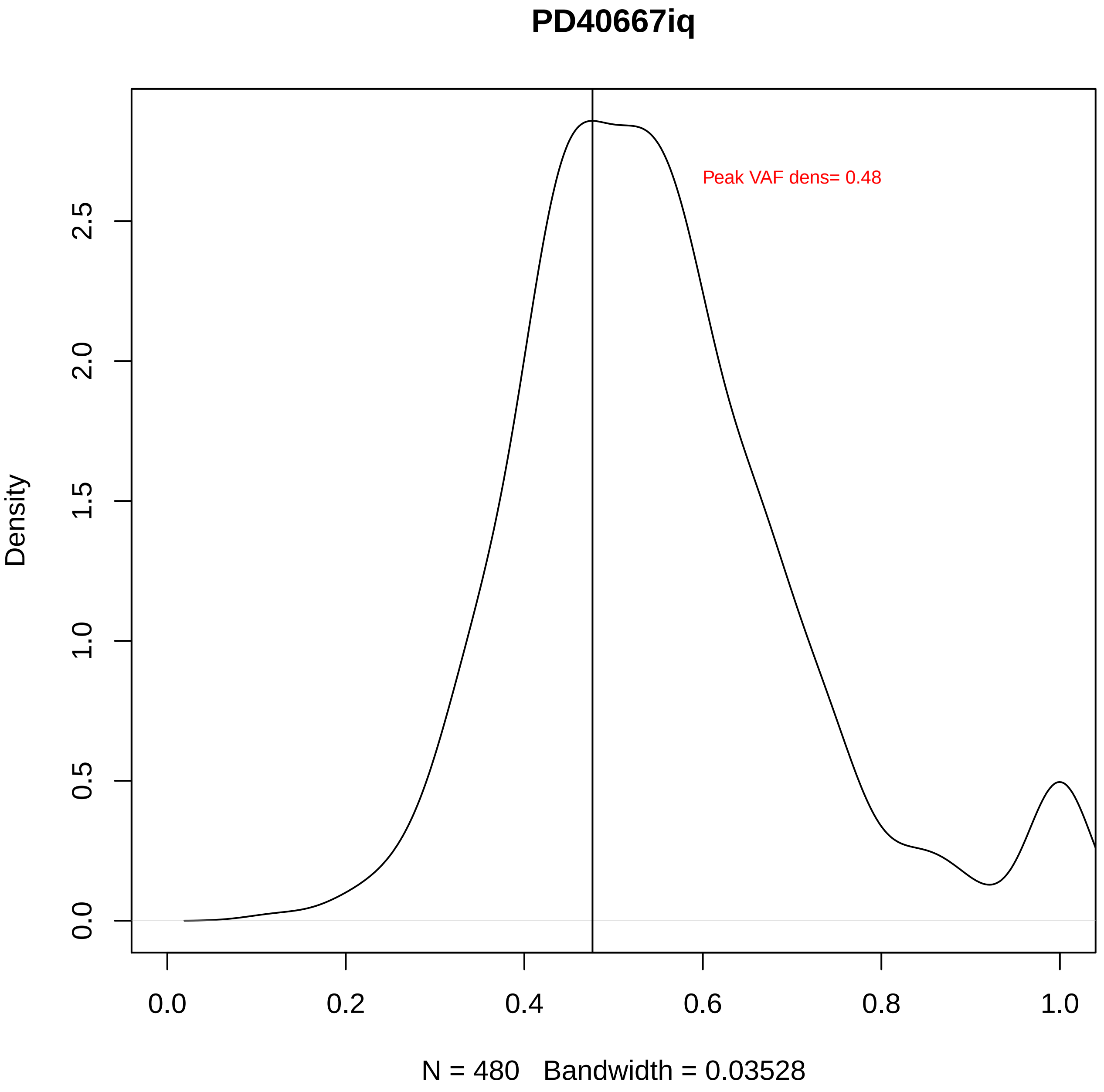

PD40667iq
Peak VAF dens= 0.48
Density
0.0
0.5
1.0
1.5
2.0
2.5
0.0
0.2
0.4
0.6
0.8
1.0
N = 480 Bandwidth = 0.03528

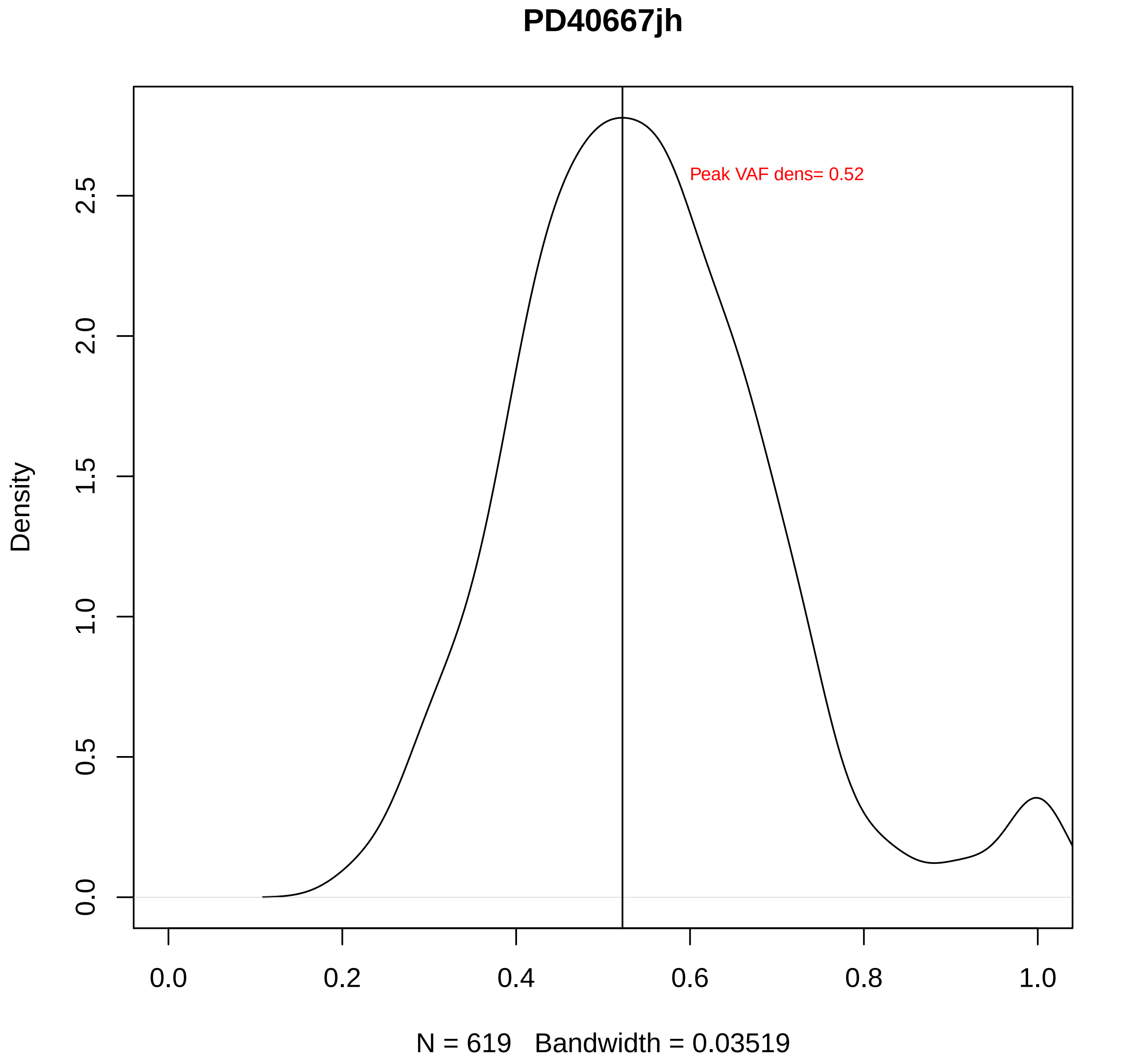

PD40667jh
Peak VAF dens= 0.52
Density
0.0
0.5
1.0
1.5
2.0
2.5
0.0
0.2
0.4
0.6
0.8
1.0
N = 619   Bandwidth = 0.03519

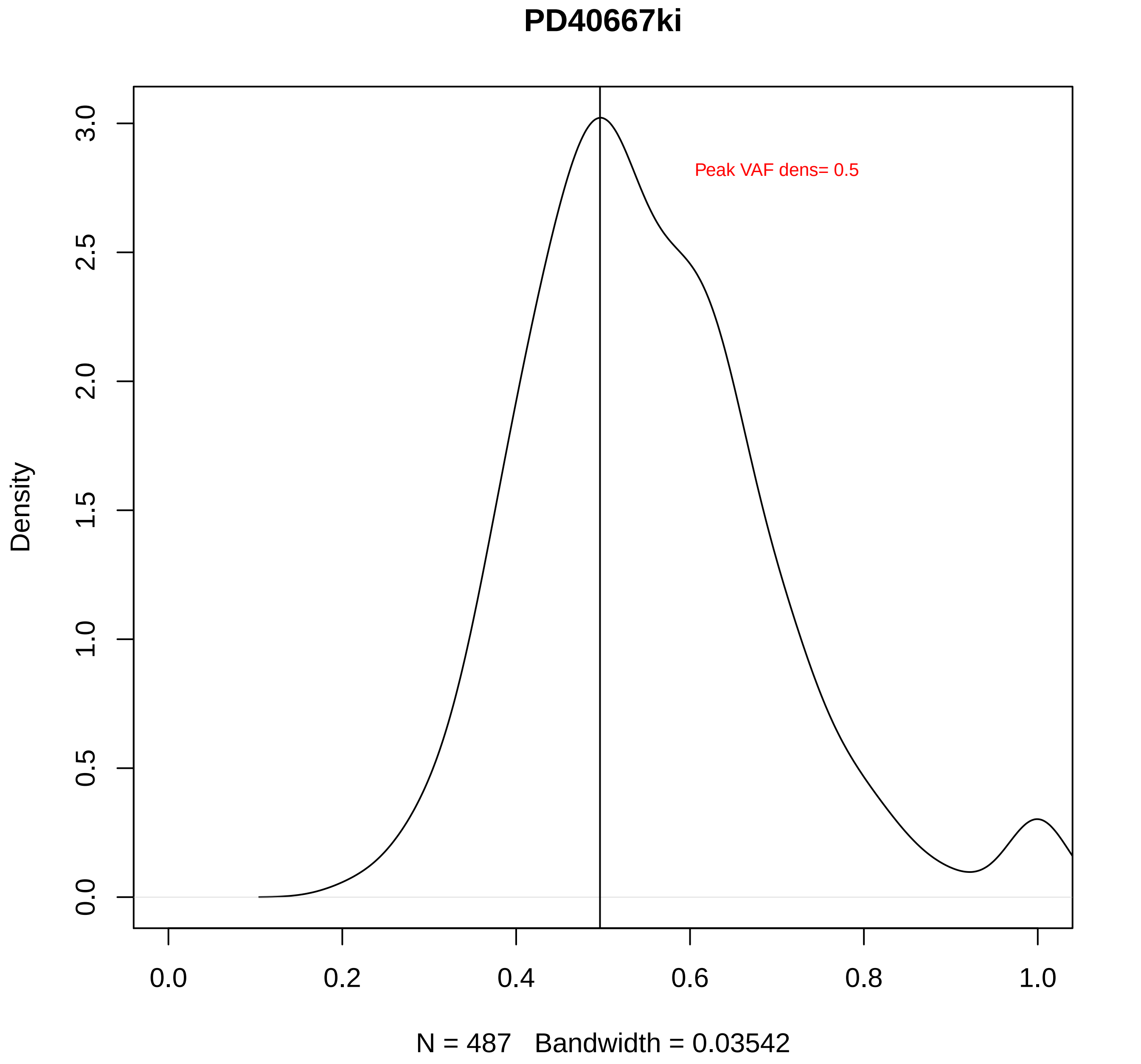

PD40667ki
Peak VAF dens= 0.5
Density
3.0
2.5
2.0
1.5
1.0
0.5
0.0
0.0
0.2
0.4
0.6
0.8
1.0
N = 487   Bandwidth = 0.03542

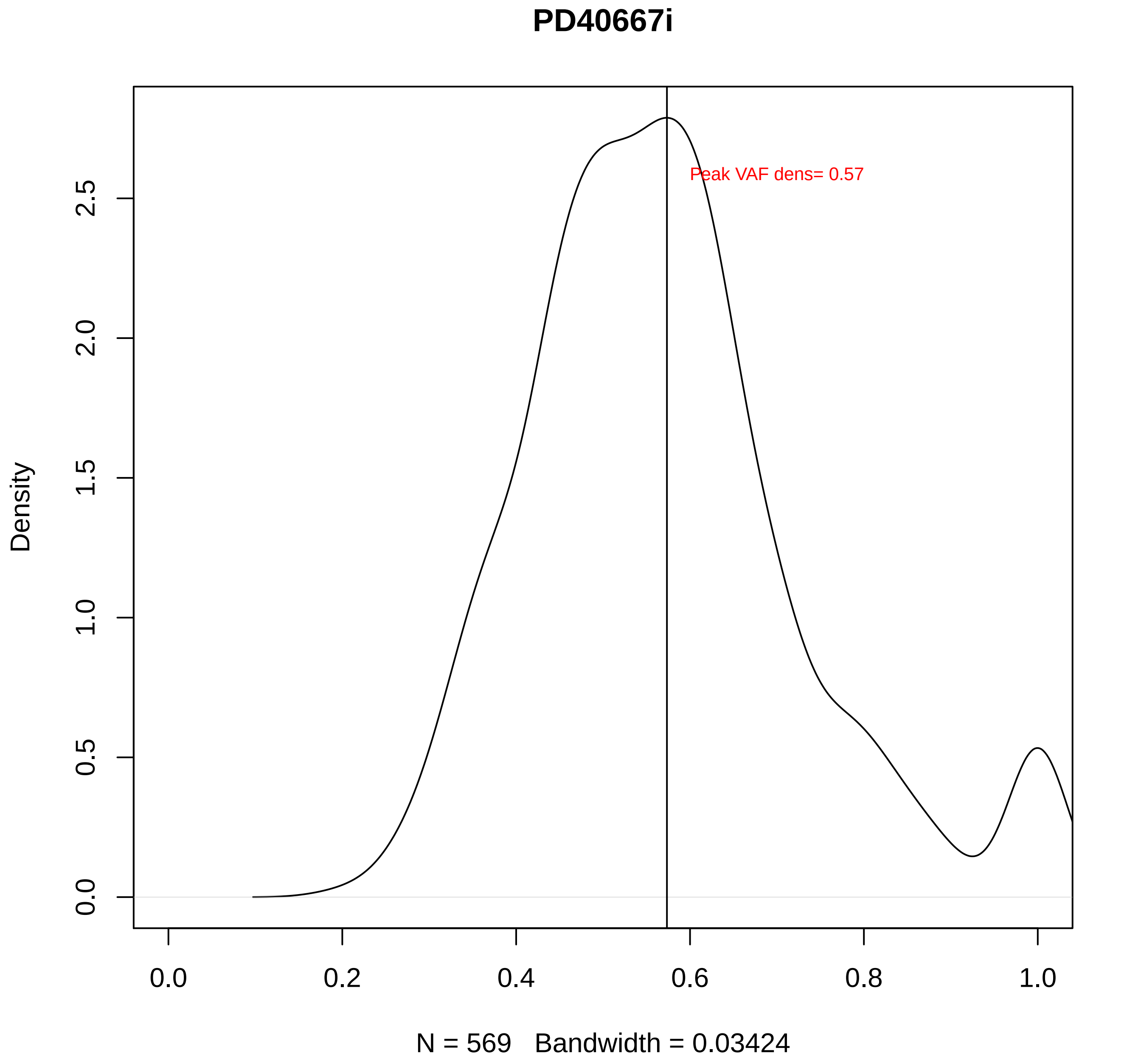

PD40667i
Peak VAF dens= 0.57
Density
0.0
0.5
1.0
1.5
2.0
2.5
0.0
0.2
0.4
0.6
0.8
1.0
N = 569   Bandwidth = 0.03424

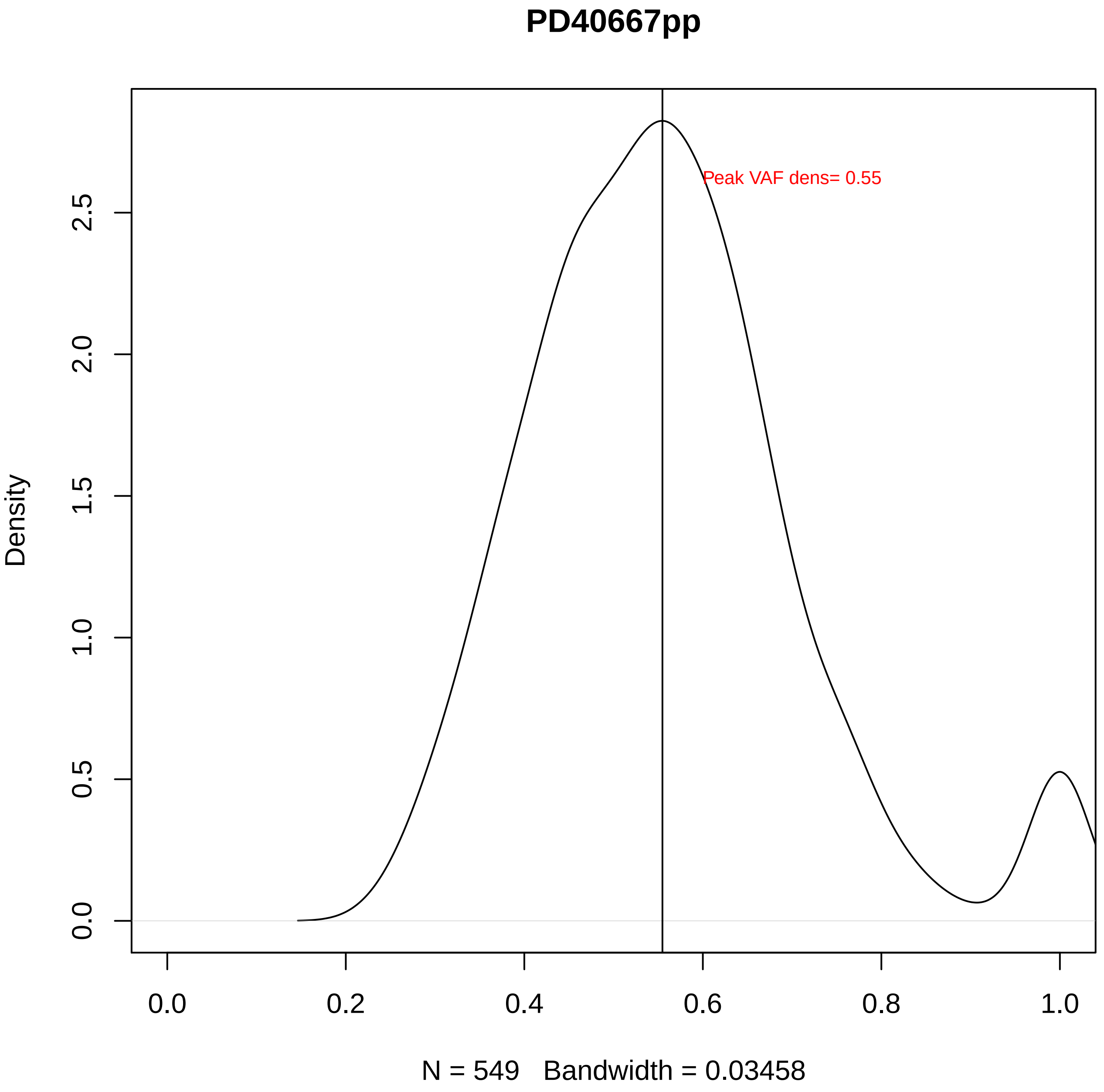
PD40667pp
Peak VAF dens= 0.55
Density
0.0
0.5
1.0
1.5
2.0
2.5
0.0
0.2
0.4
0.6
0.8
1.0
N = 549   Bandwidth = 0.03458

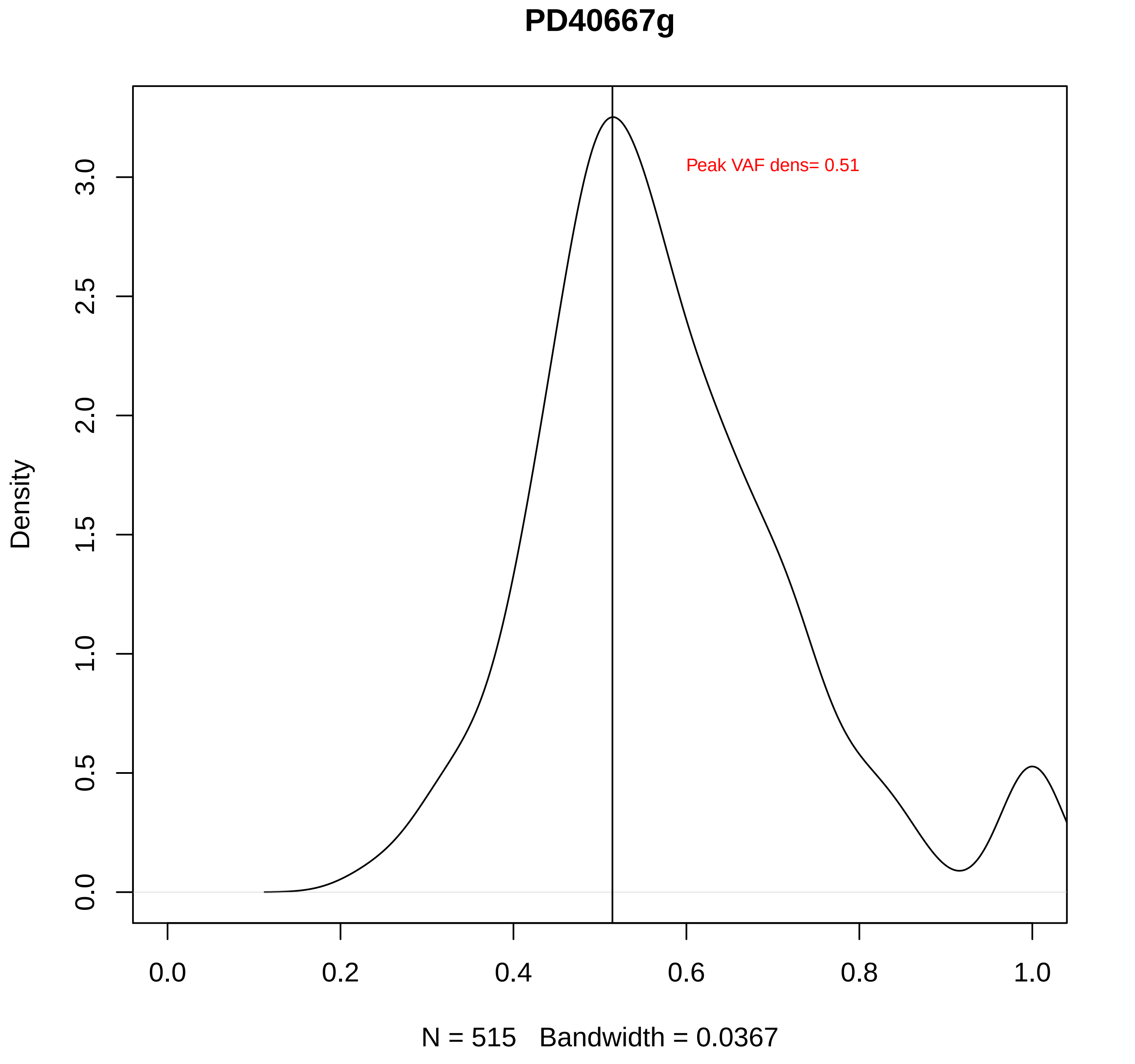
PD40667g
Peak VAF dens= 0.51
Density
0.0
0.5
1.0
1.5
2.0
2.5
3.0
0.0
0.2
0.4
0.6
0.8
1.0
N = 515   Bandwidth = 0.0367

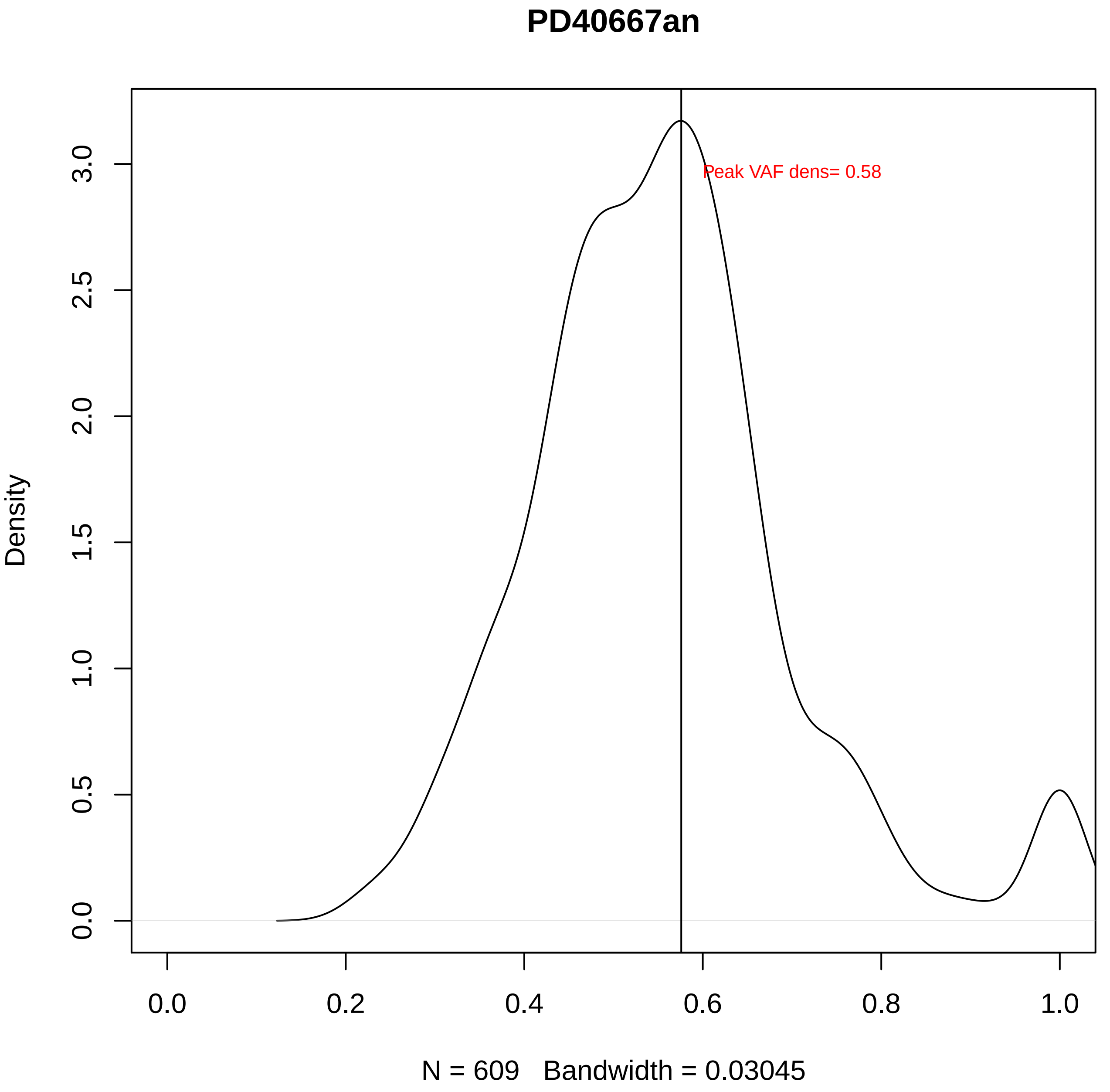
PD40667an
Peak VAF dens= 0.58
Density
3.0
2.5
2.0
1.5
1.0
0.5
0.0
0.0
0.2
0.4
0.6
0.8
1.0
N = 609   Bandwidth = 0.03045

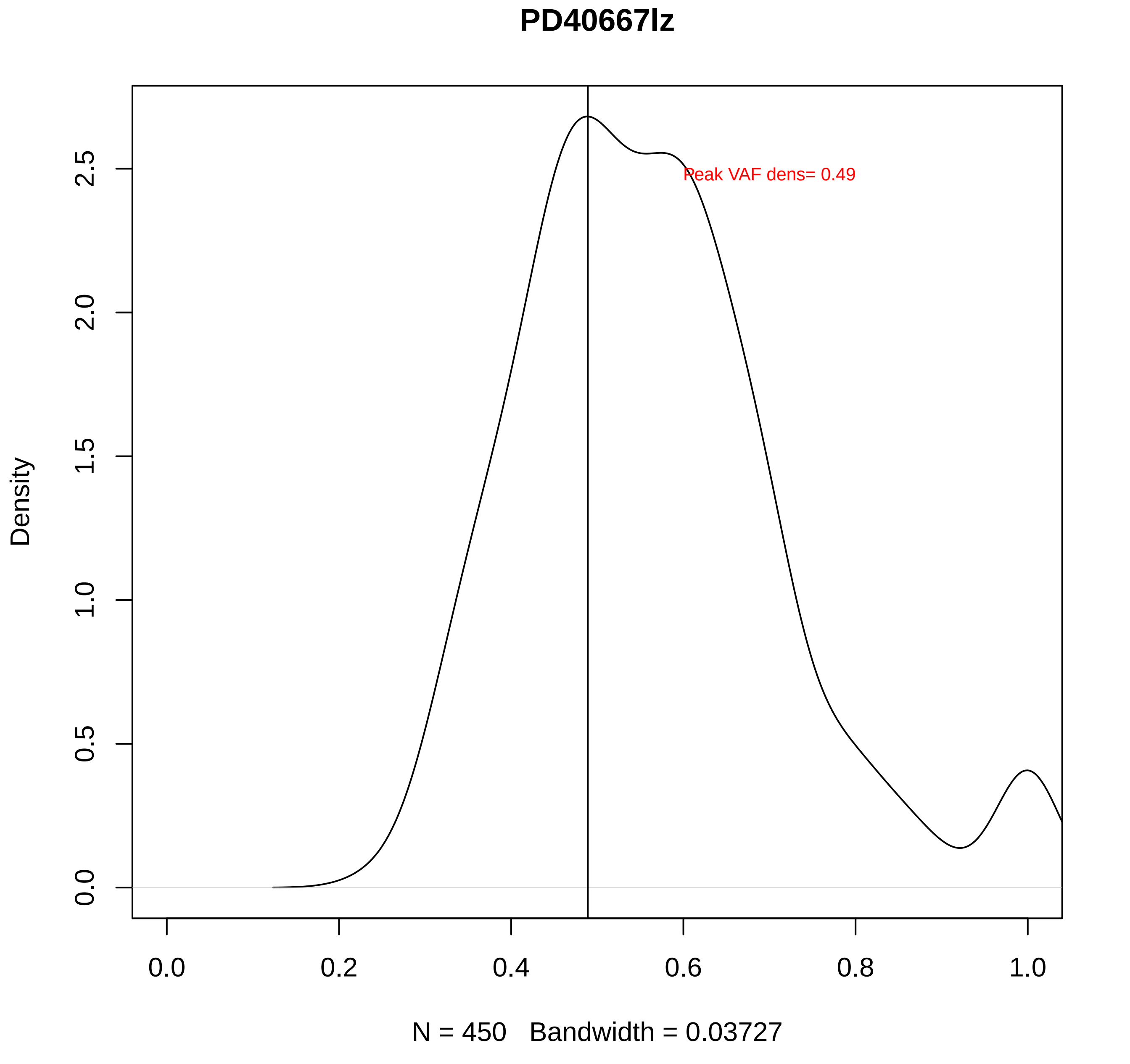

PD40667lz
Peak VAF dens= 0.49
Density
2.5
2.0
1.5
1.0
0.5
0.0
0.0
0.2
0.4
0.6
0.8
1.0
N = 450   Bandwidth = 0.03727

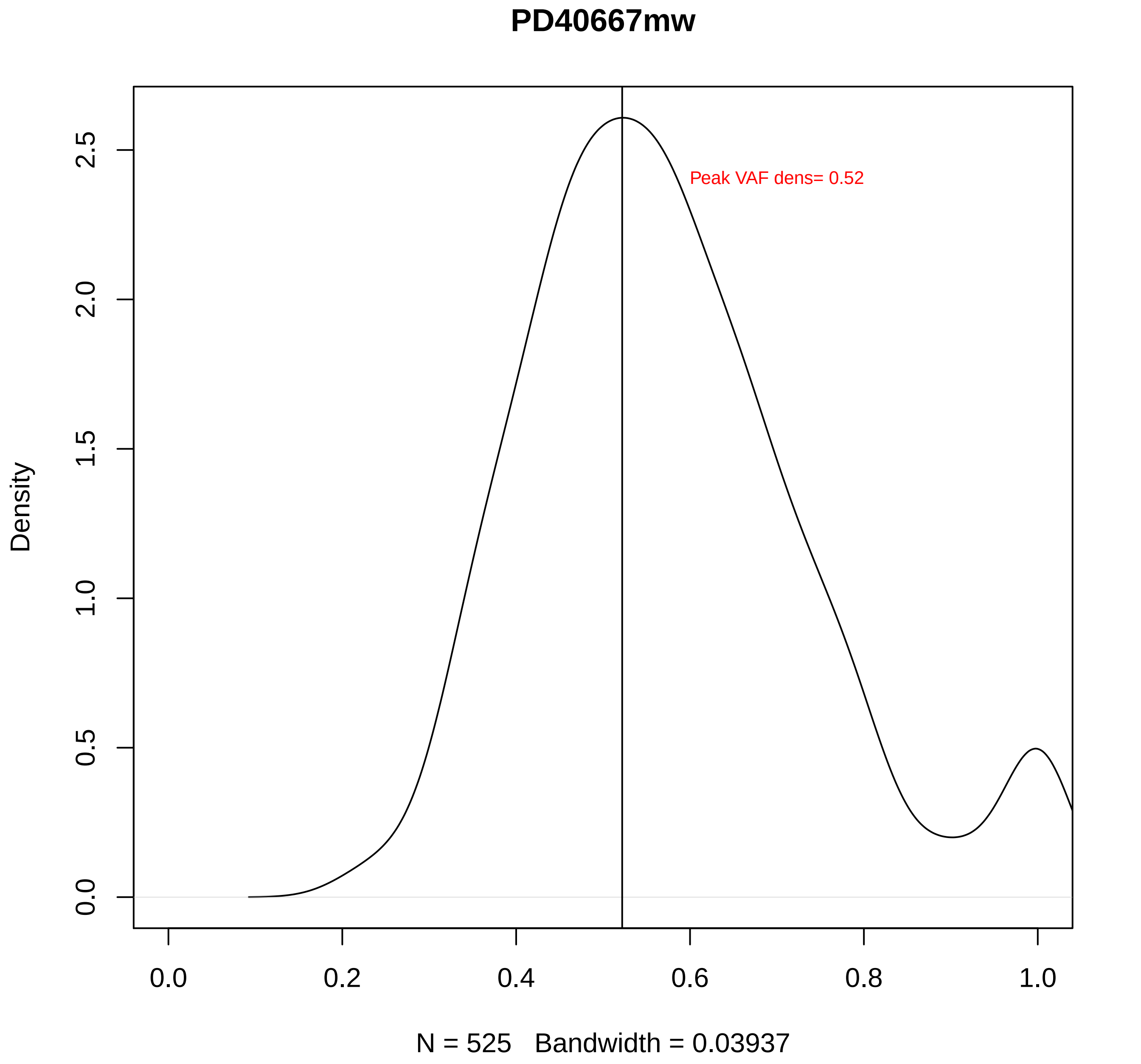

PD40667mw
Peak VAF dens= 0.52
Density
0.0
0.5
1.0
1.5
2.0
2.5
0.0
0.2
0.4
0.6
0.8
1.0
N = 525   Bandwidth = 0.03937

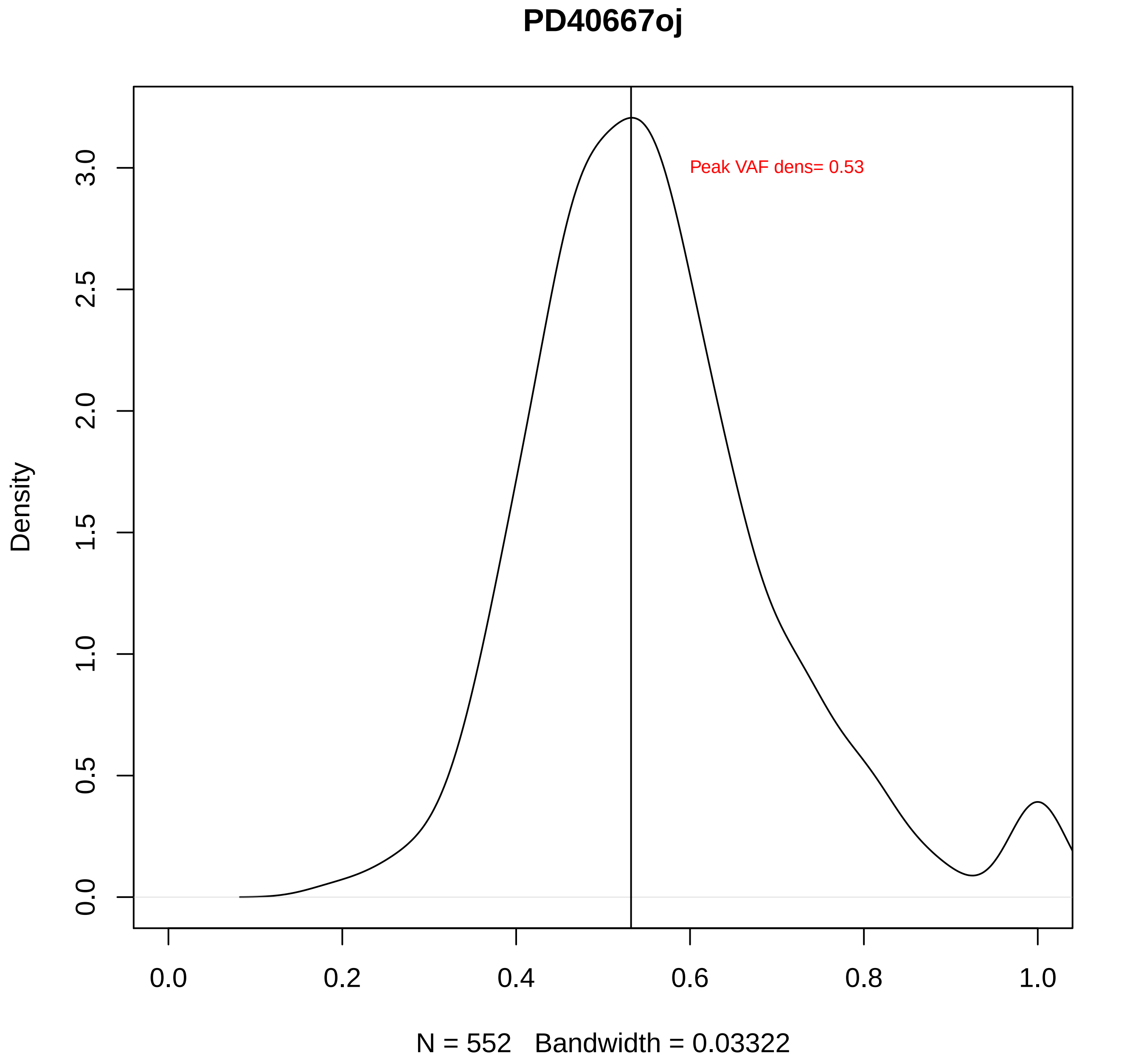
PD40667oj
Peak VAF dens= 0.53
Density
0.0
0.5
1.0
1.5
2.0
2.5
3.0
0.0
0.2
0.4
0.6
0.8
1.0
N = 552   Bandwidth = 0.03322

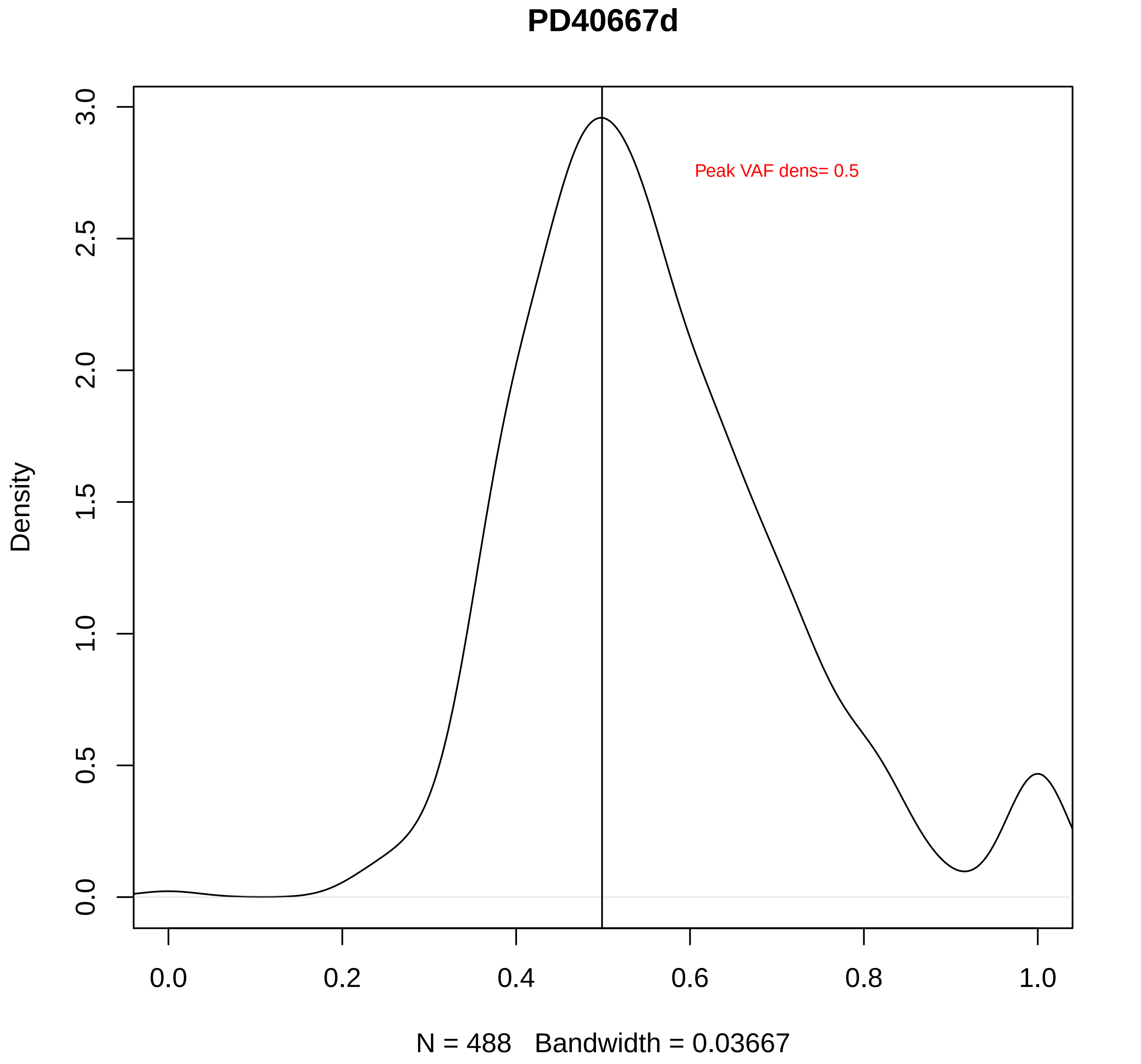

PD40667d
Peak VAF dens= 0.5
Density
0.0
0.5
1.0
1.5
2.0
2.5
3.0
0.0
0.2
0.4
0.6
0.8
1.0
N = 488   Bandwidth = 0.03667

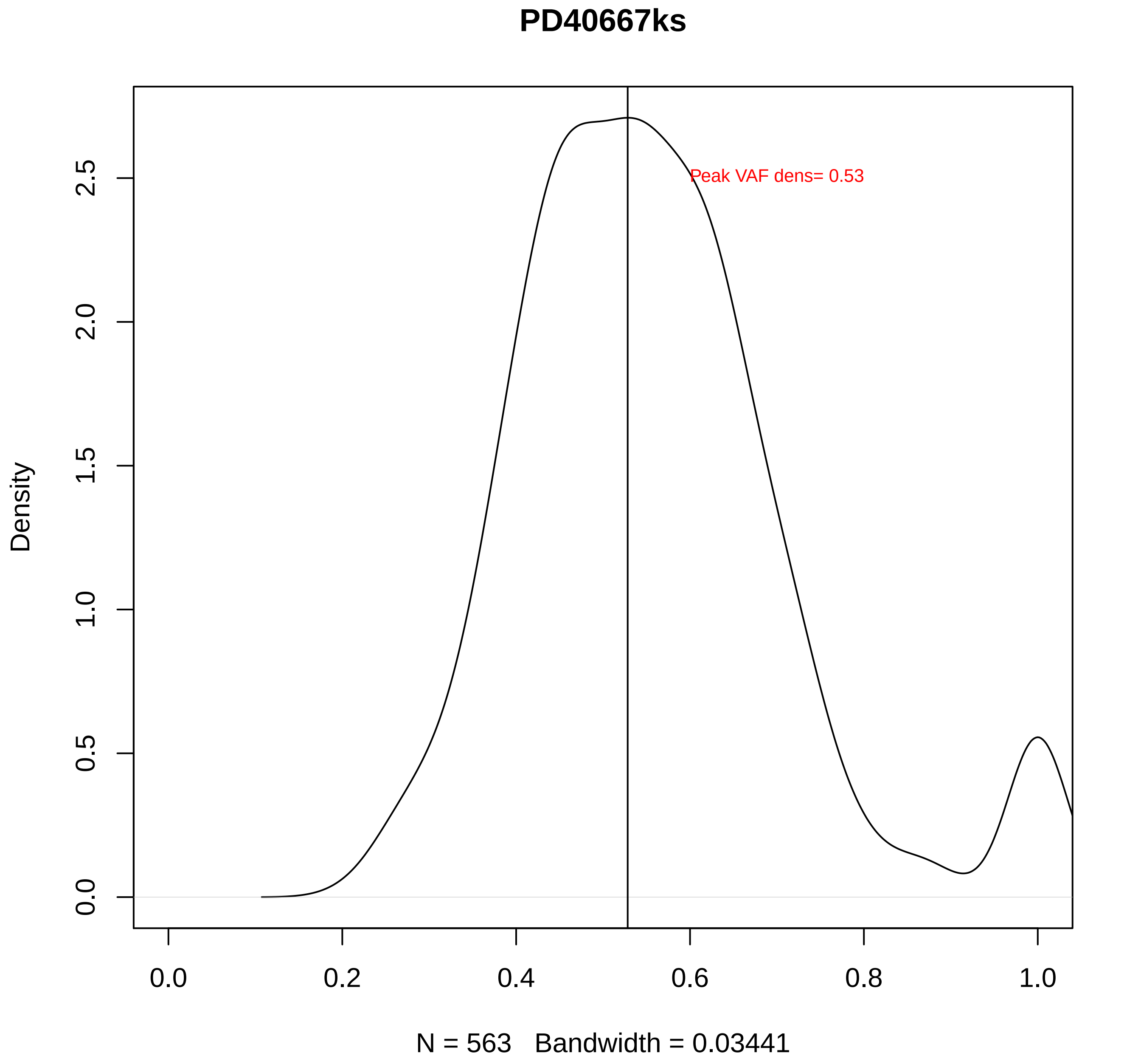
PD40667ks
Peak VAF dens= 0.53
Density
0.0
0.5
1.0
1.5
2.0
2.5
0.0
0.2
0.4
0.6
0.8
1.0
N = 563   Bandwidth = 0.03441

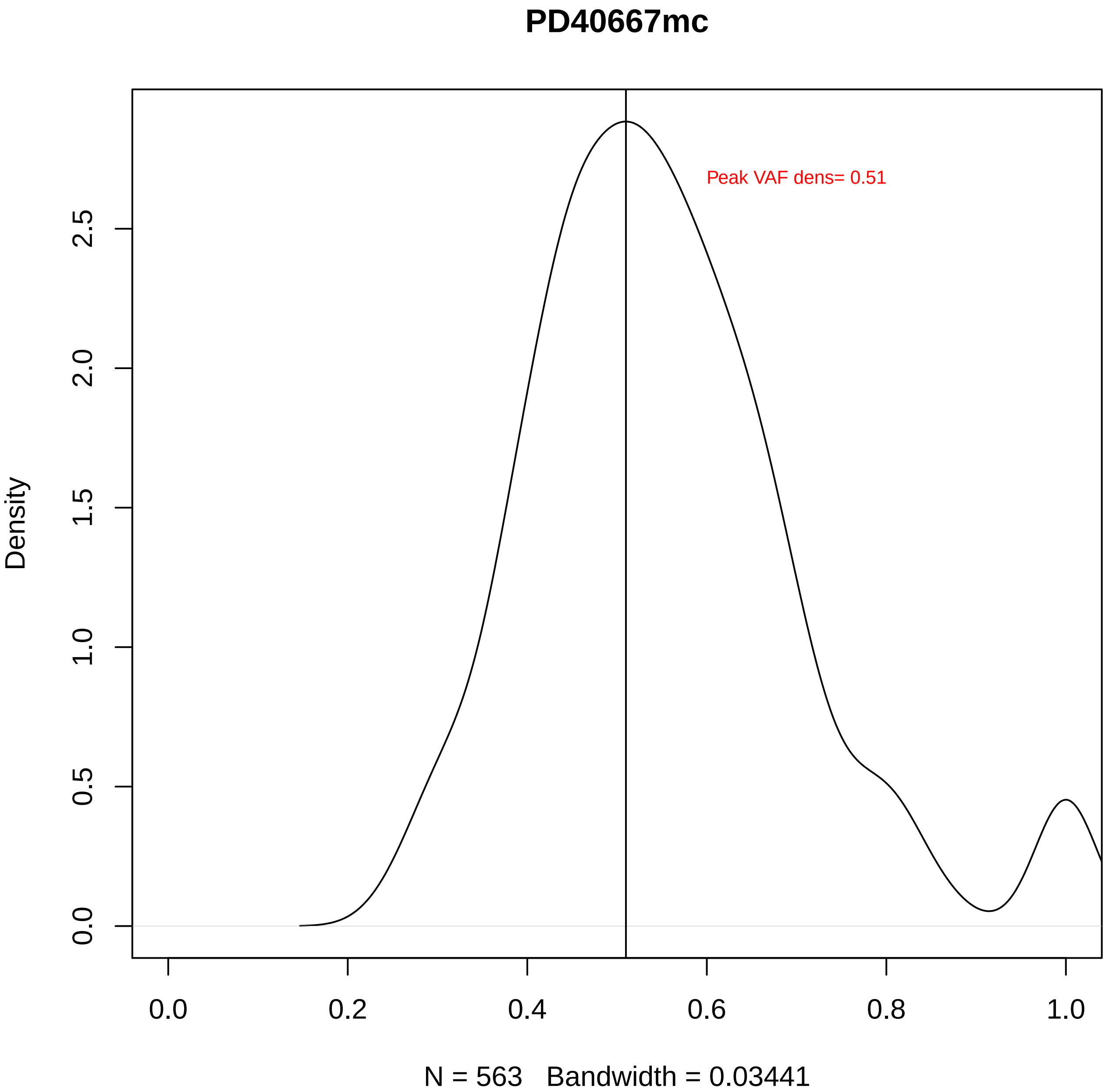
PD40667mc
Peak VAF dens= 0.51
Density
0.0
0.5
1.0
1.5
2.0
2.5
0.0
0.2
0.4
0.6
0.8
1.0
N = 563 Bandwidth = 0.03441

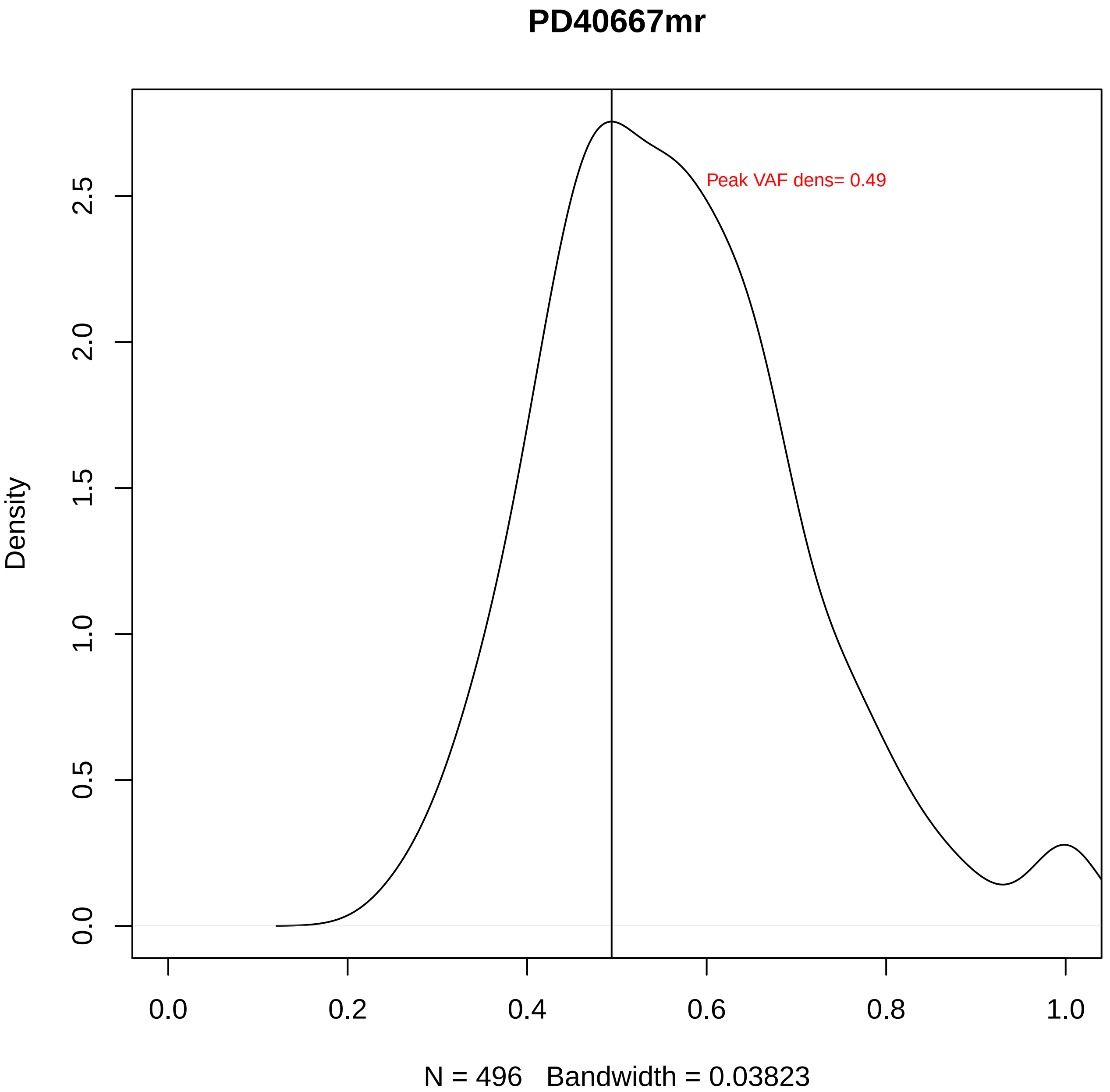
PD40667mr
Peak VAF dens= 0.49
Density
0.0
0.5
1.0
1.5
2.0
2.5
0.0
0.2
0.4
0.6
0.8
1.0
N = 496 Bandwidth = 0.03823

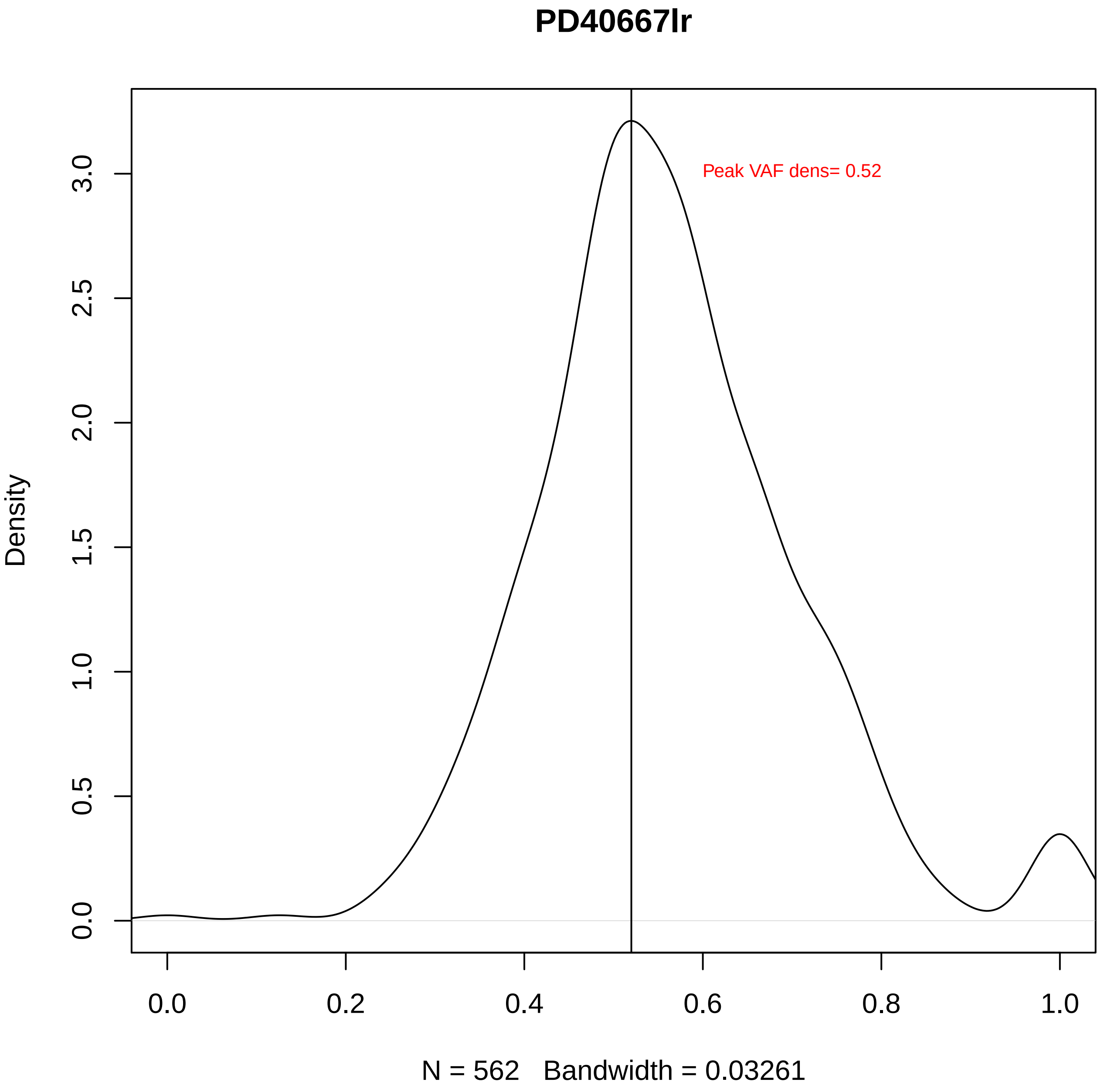
PD40667lr
Peak VAF dens= 0.52
Density
0.0
0.5
1.0
1.5
2.0
2.5
3.0
0.0
0.2
0.4
0.6
0.8
1.0
N = 562   Bandwidth = 0.03261

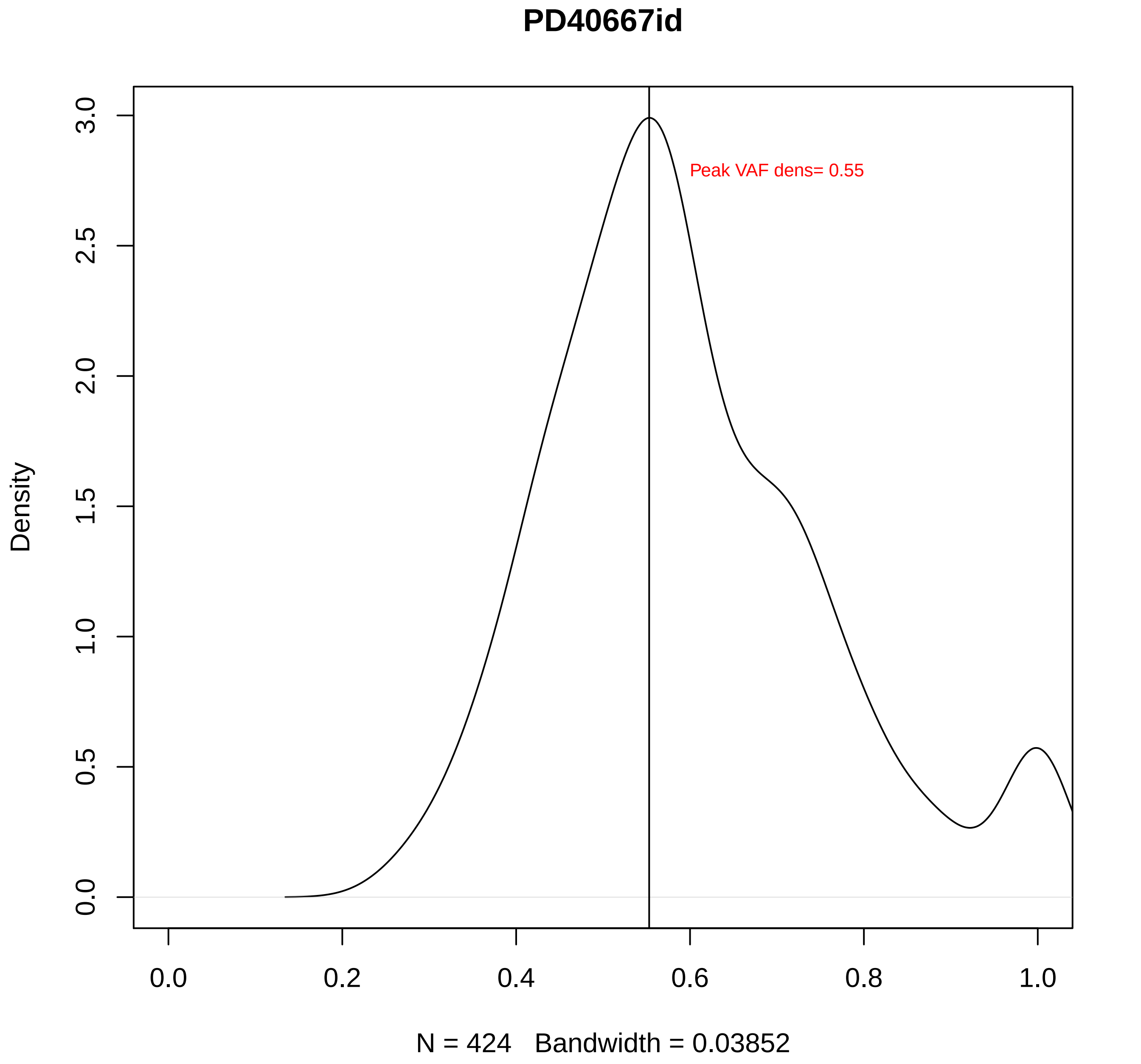

PD40667id
Peak VAF dens= 0.55
Density
0.0
0.5
1.0
1.5
2.0
2.5
3.0
0.0
0.2
0.4
0.6
0.8
1.0
N = 424   Bandwidth = 0.03852

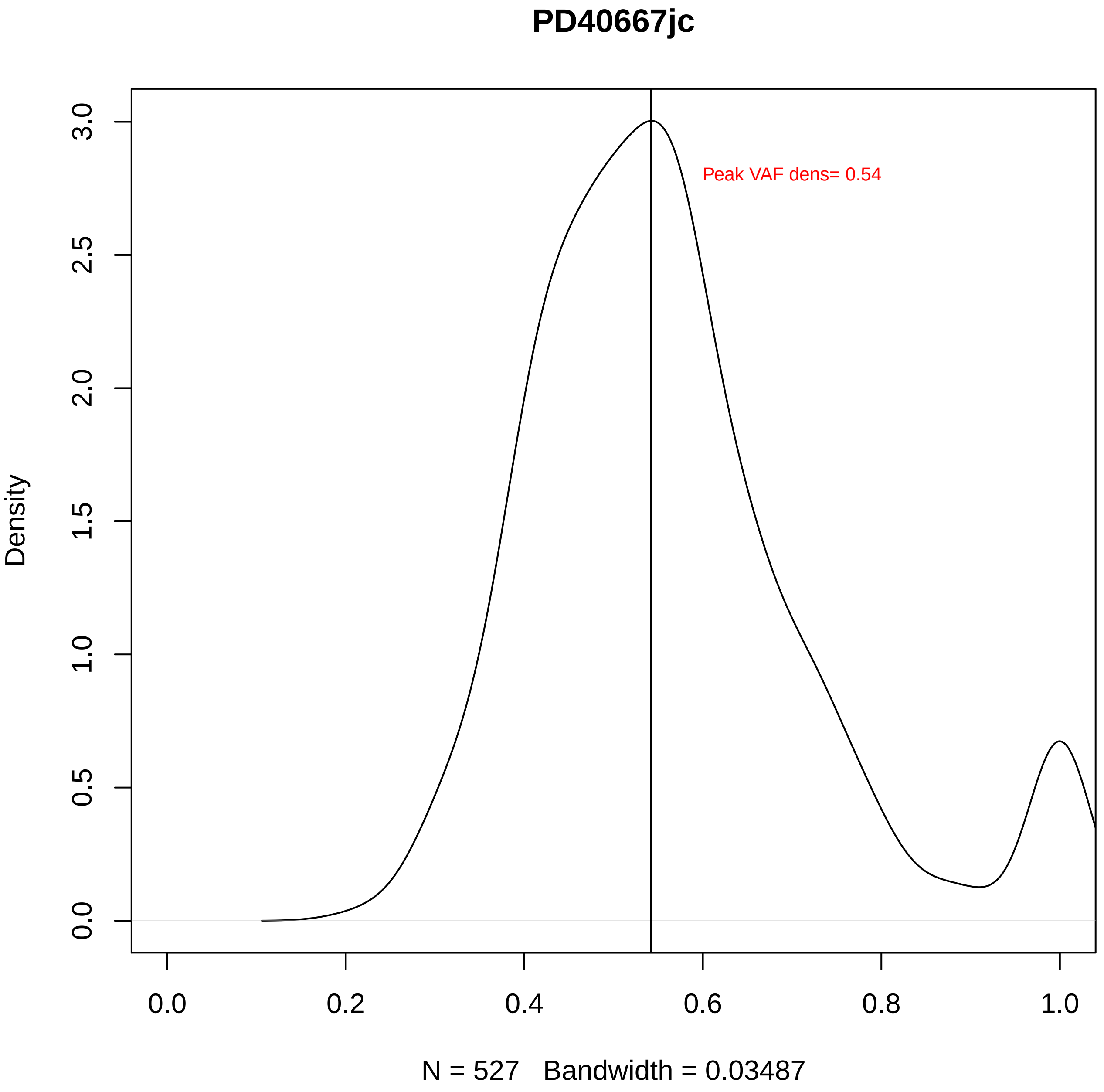

PD40667jc
Peak VAF dens= 0.54
Density
3.0
2.5
2.0
1.5
1.0
0.5
0.0
0.0
0.2
0.4
0.6
0.8
1.0
N = 527   Bandwidth = 0.03487

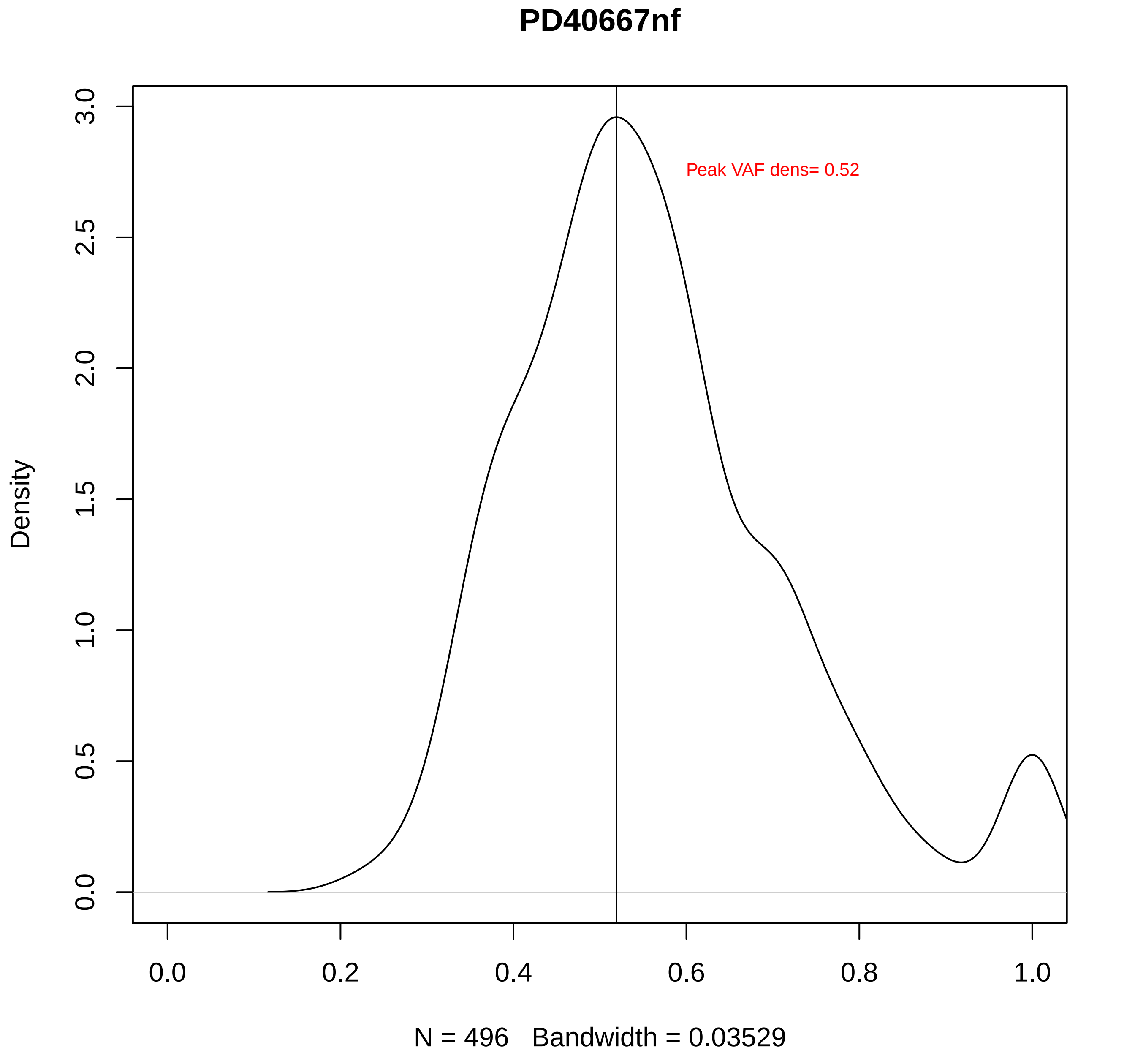

PD40667nf
Peak VAF dens= 0.52
Density
3.0
2.5
2.0
1.5
1.0
0.5
0.0
0.0
0.2
0.4
0.6
0.8
1.0
N = 496   Bandwidth = 0.03529

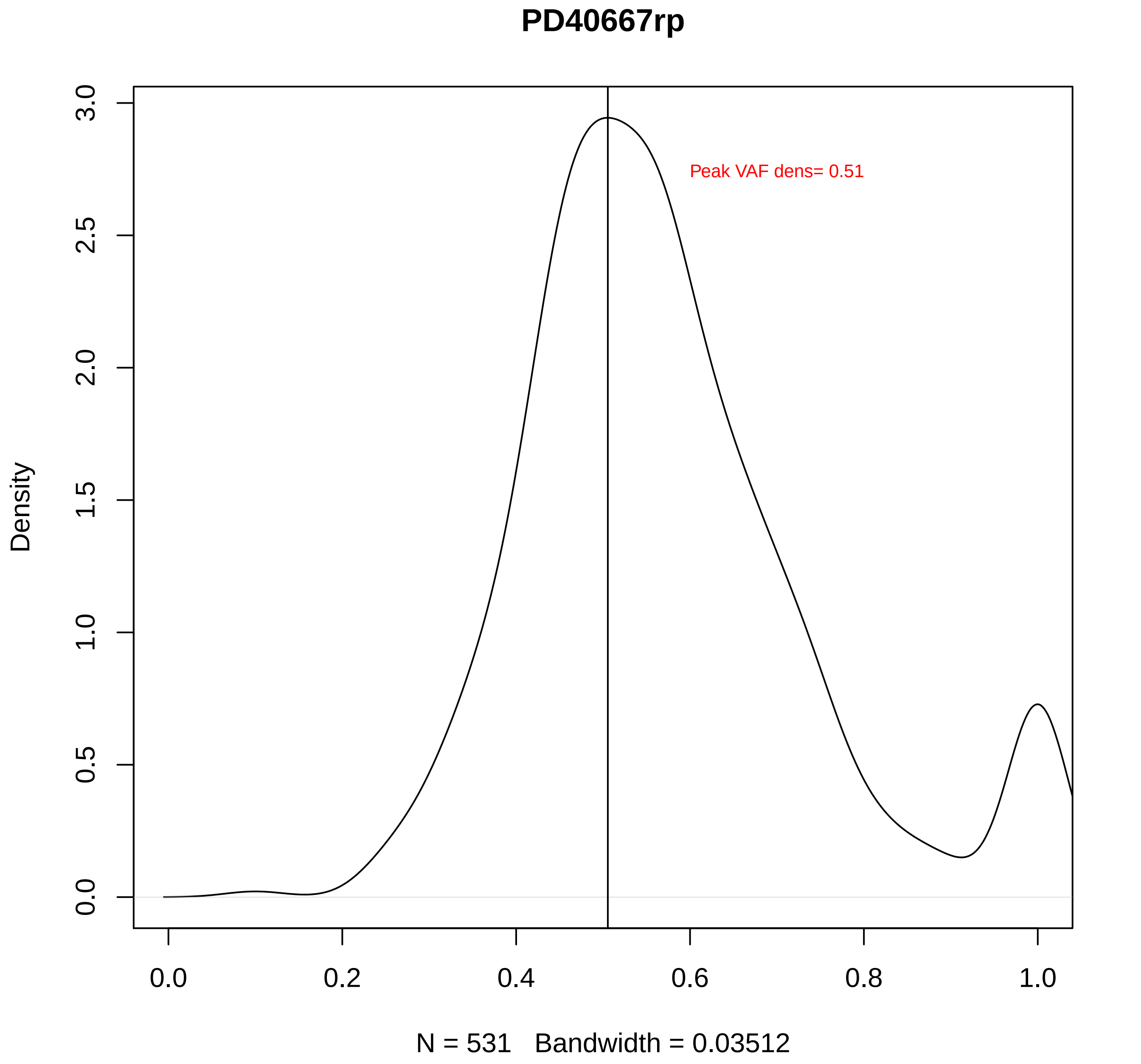
PD40667rp
Peak VAF dens= 0.51
Density
0.0
0.5
1.0
1.5
2.0
2.5
3.0
0.0
0.2
0.4
0.6
0.8
1.0
N = 531   Bandwidth = 0.03512

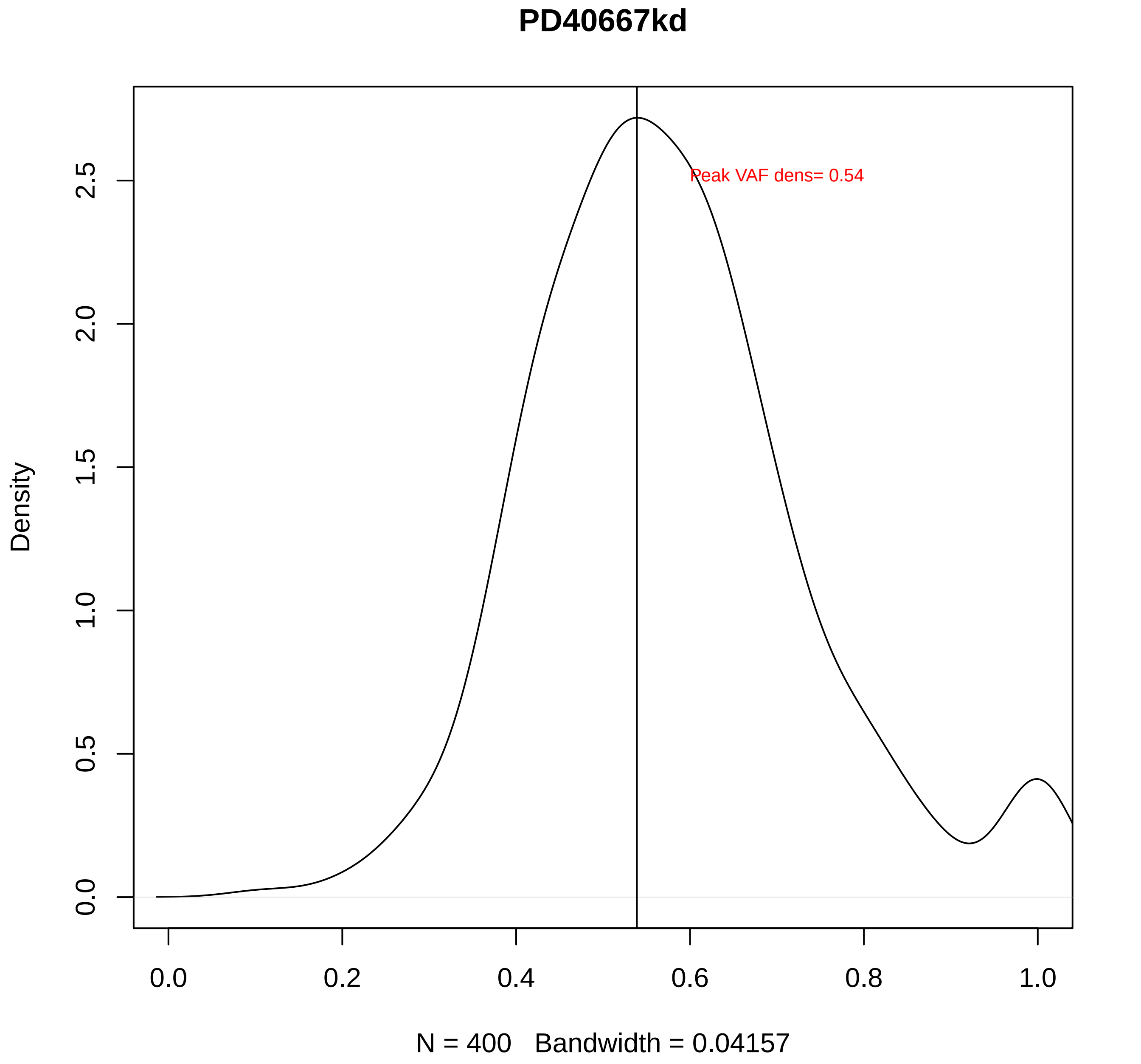
PD40667kd
Peak VAF dens= 0.54
Density
0.0
0.5
1.0
1.5
2.0
2.5
0.0
0.2
0.4
0.6
0.8
1.0
N = 400 Bandwidth = 0.04157

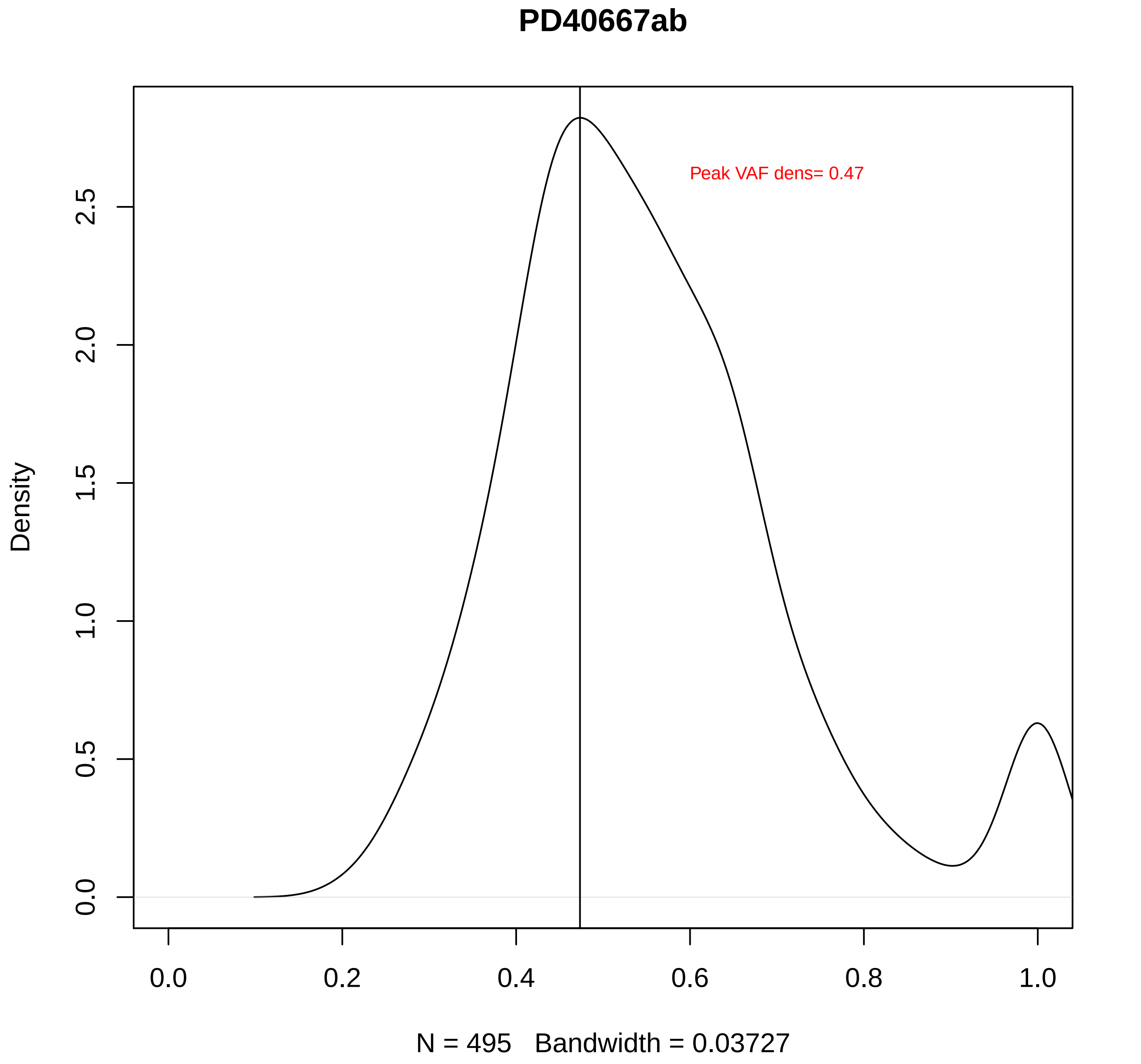

PD40667ab
Peak VAF dens= 0.47
Density
0.0
0.5
1.0
1.5
2.0
2.5
0.0
0.2
0.4
0.6
0.8
1.0
N = 495 Bandwidth = 0.03727

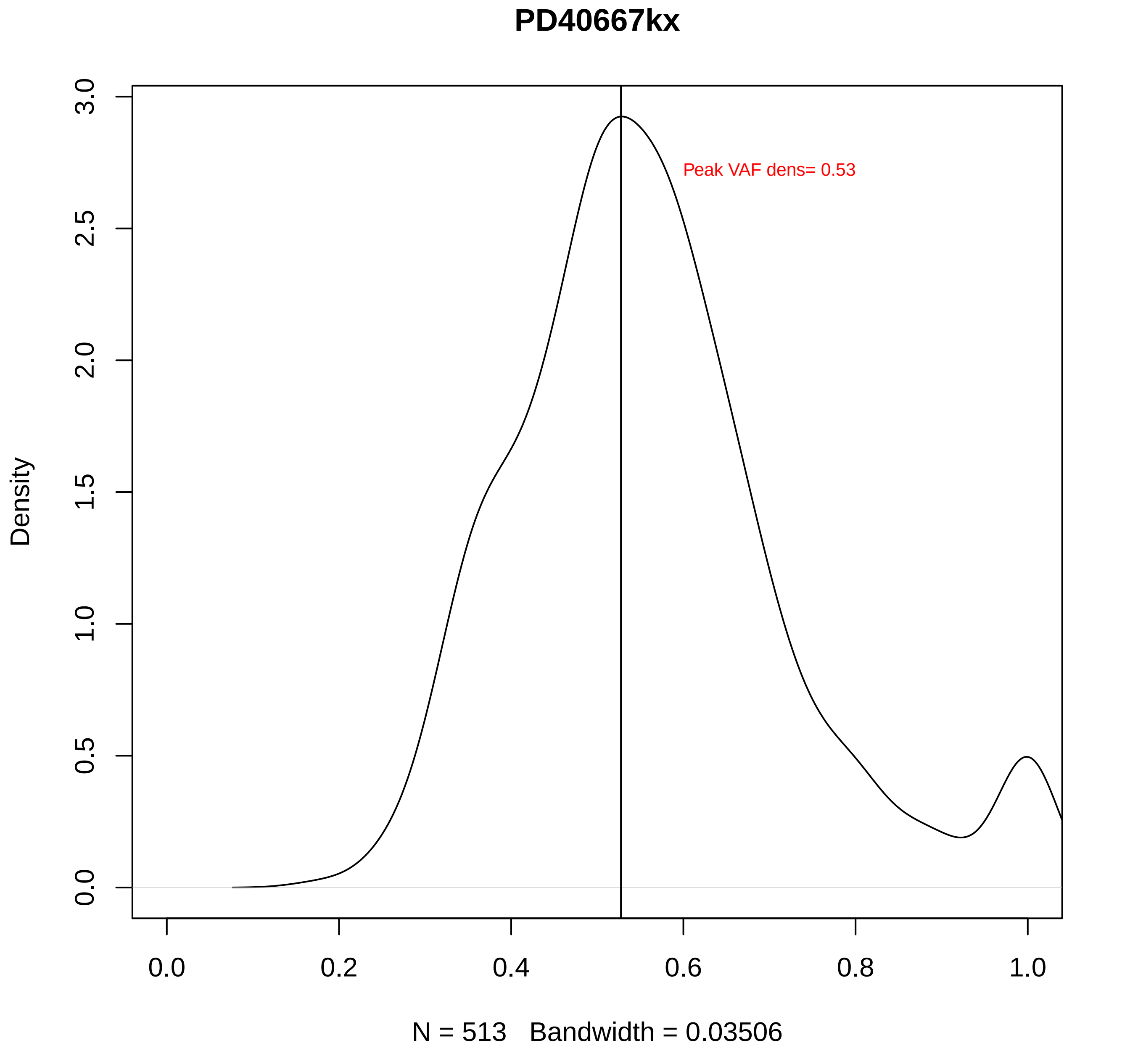

PD40667kx
Peak VAF dens= 0.53
Density
0.0
0.5
1.0
1.5
2.0
2.5
3.0
0.0
0.2
0.4
0.6
0.8
1.0
N = 513   Bandwidth = 0.03506

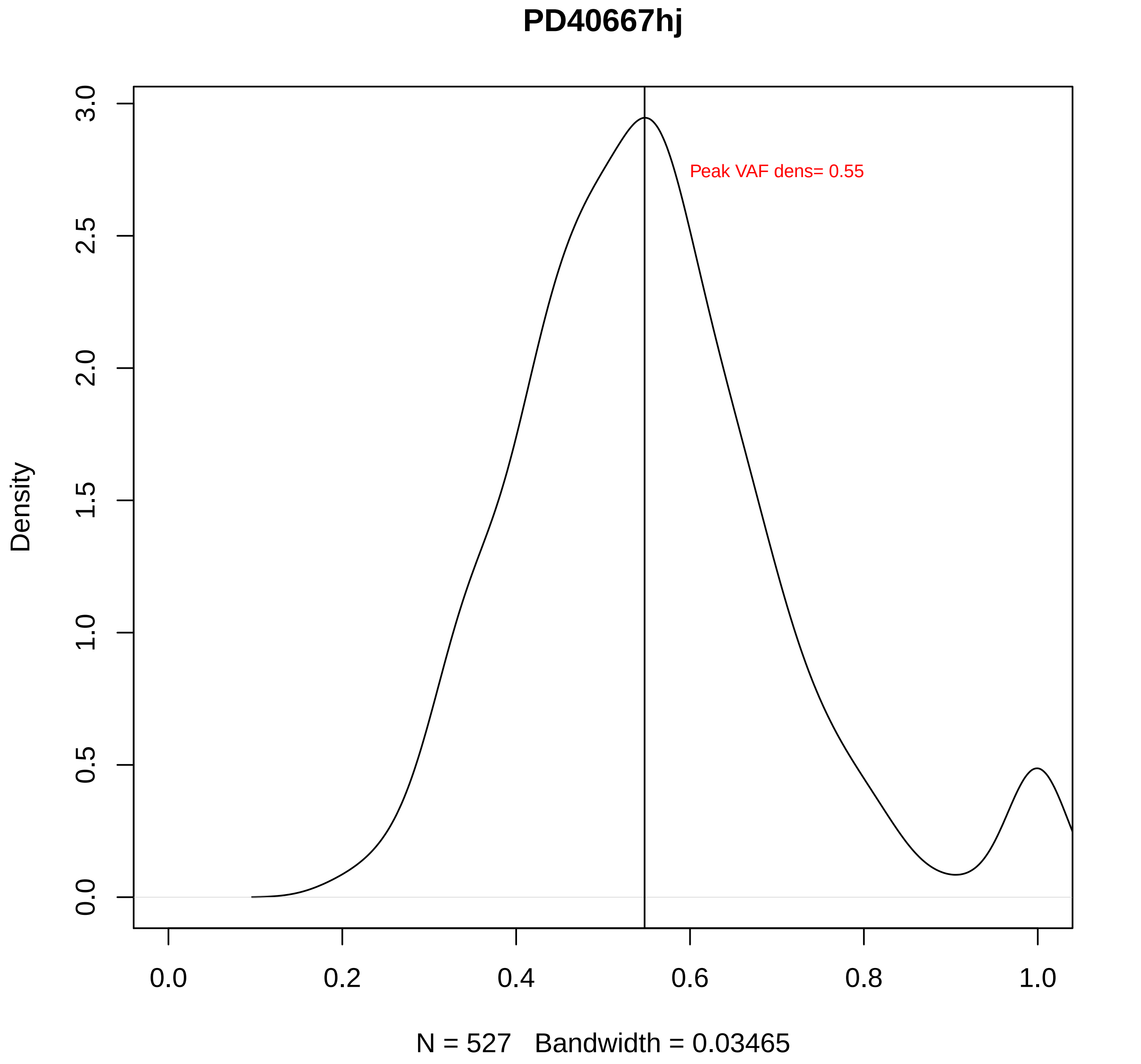
PD40667hj
Peak VAF dens= 0.55
Density
0.0
0.5
1.0
1.5
2.0
2.5
3.0
0.0
0.2
0.4
0.6
0.8
1.0
N = 527   Bandwidth = 0.03465

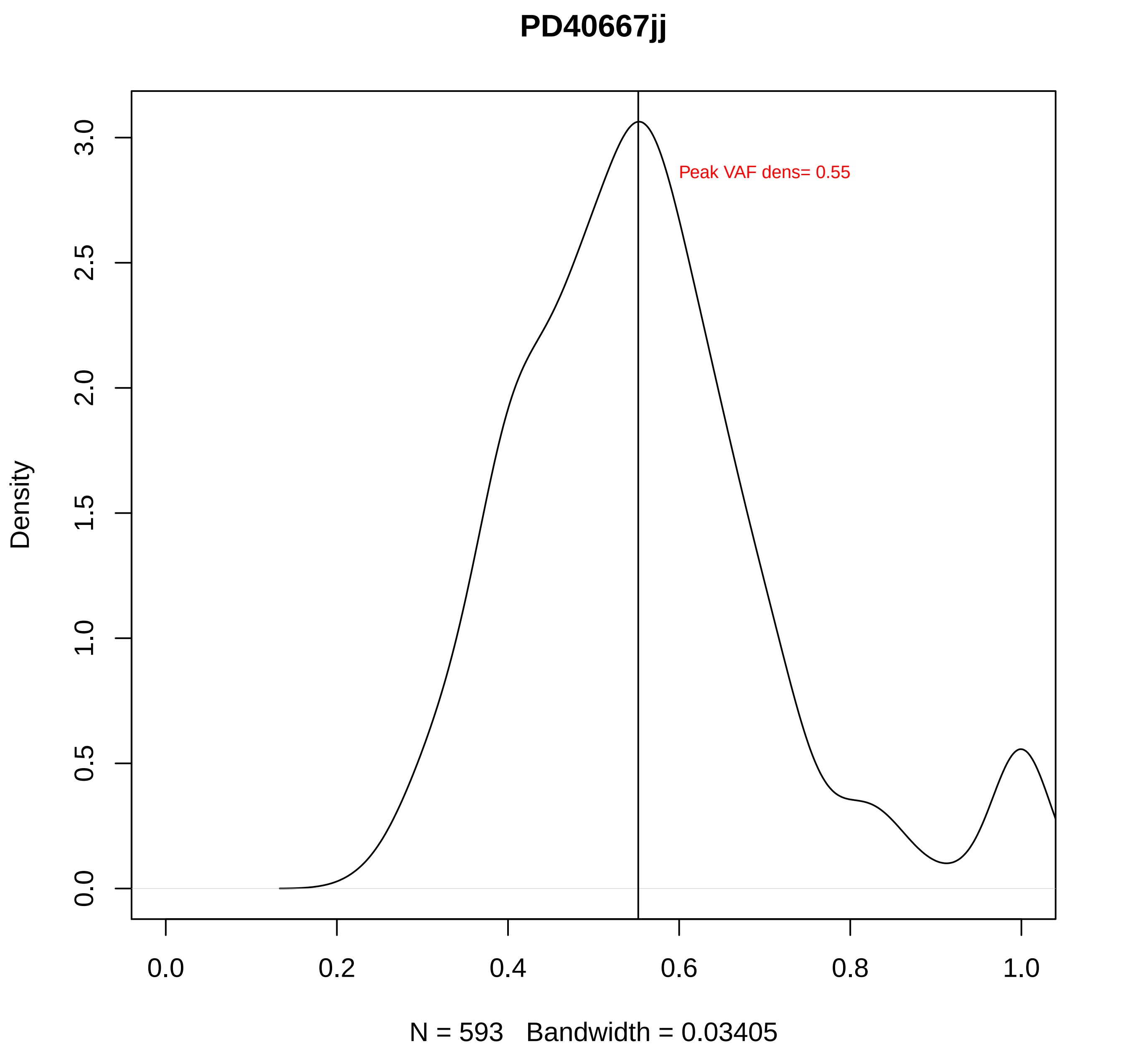

PD40667jj
Peak VAF dens= 0.55
Density
3.0
2.5
2.0
1.5
1.0
0.5
0.0
0.0
0.2
0.4
0.6
0.8
1.0
N = 593   Bandwidth = 0.03405

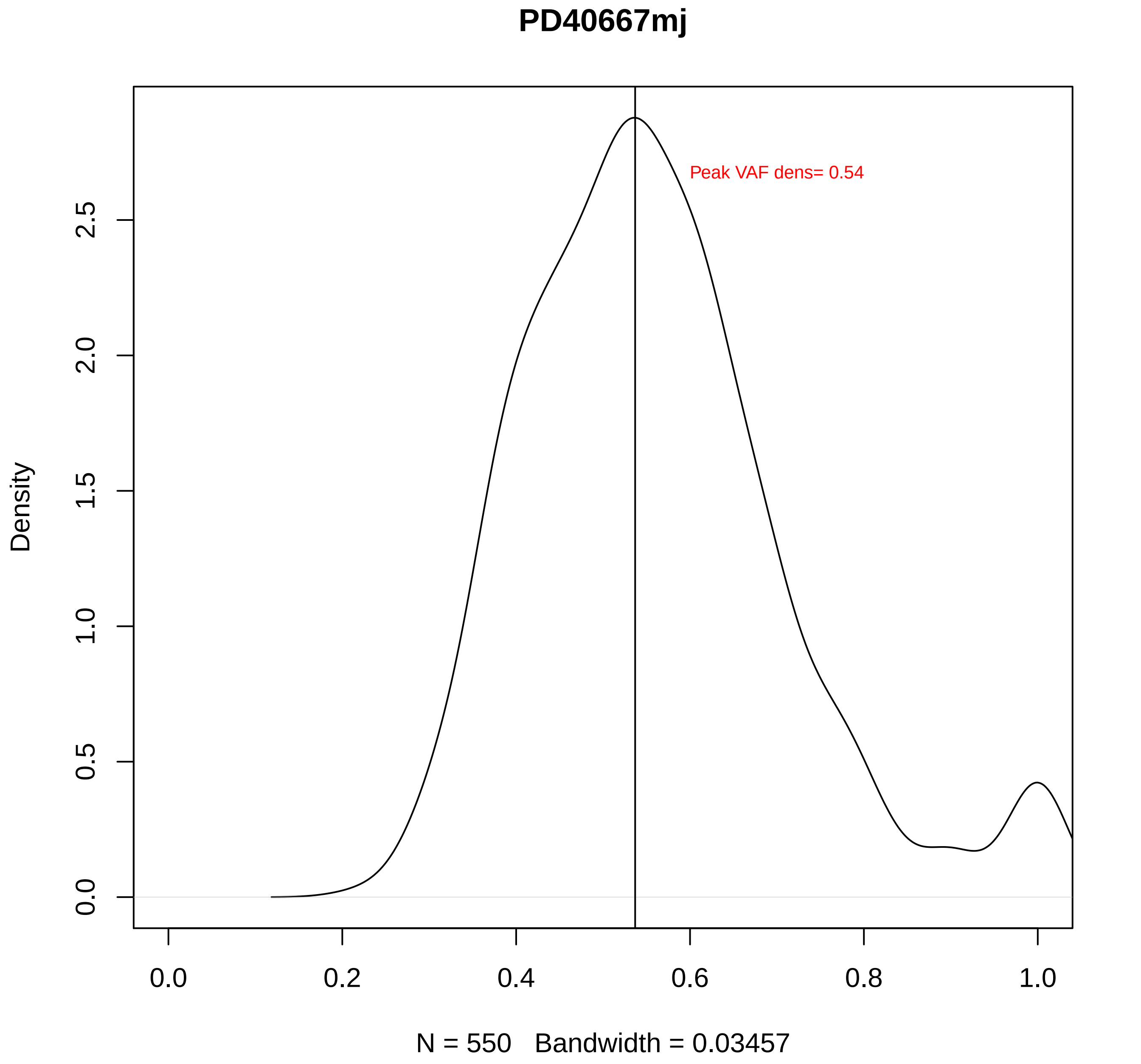

PD40667mj
Peak VAF dens= 0.54
Density
0.0
0.5
1.0
1.5
2.0
2.5
0.0
0.2
0.4
0.6
0.8
1.0
N = 550   Bandwidth = 0.03457

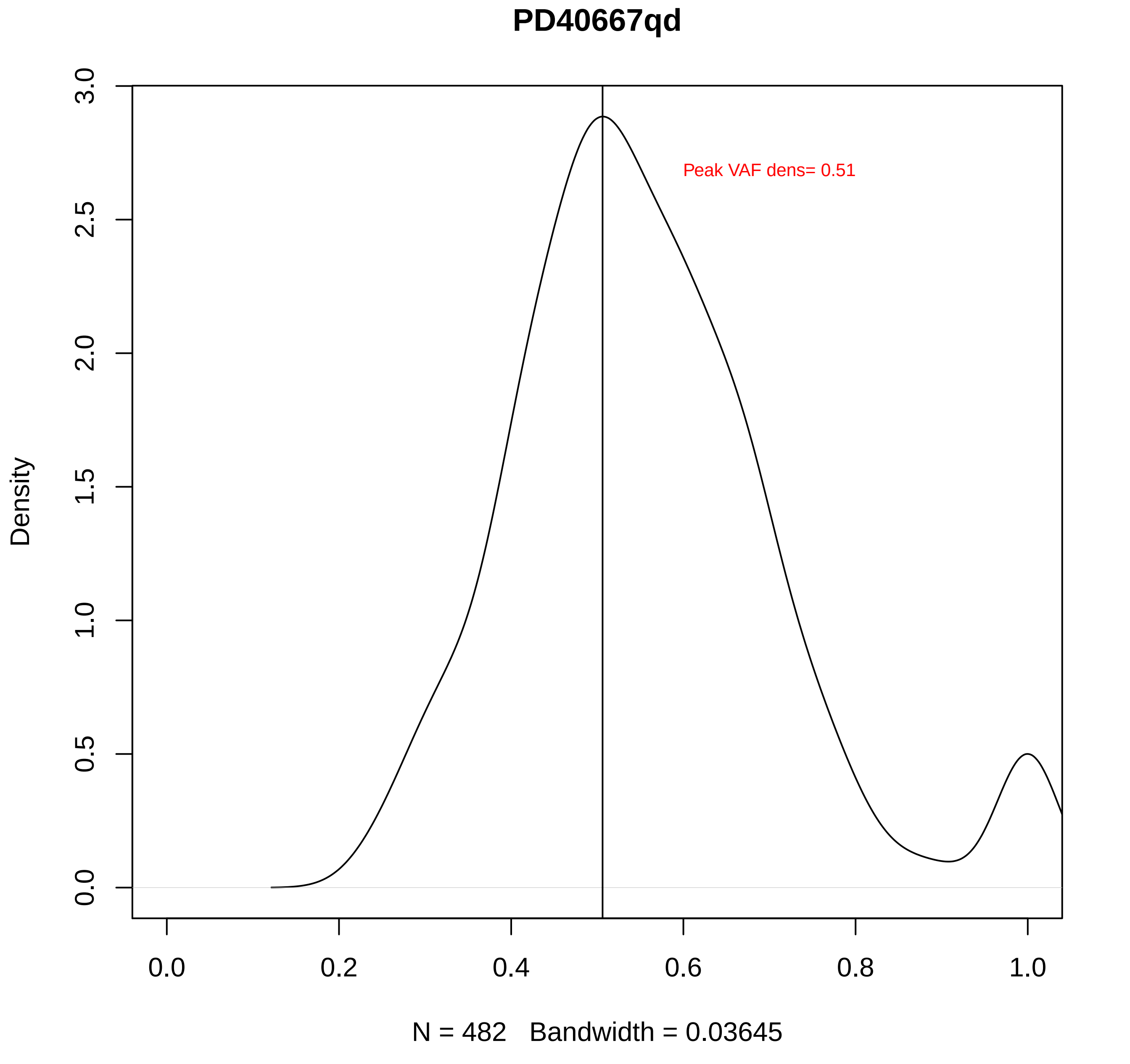
PD40667qd
Peak VAF dens= 0.51
Density
3.0
2.5
2.0
1.5
1.0
0.5
0.0
0.0
0.2
0.4
0.6
0.8
1.0
N = 482   Bandwidth = 0.03645

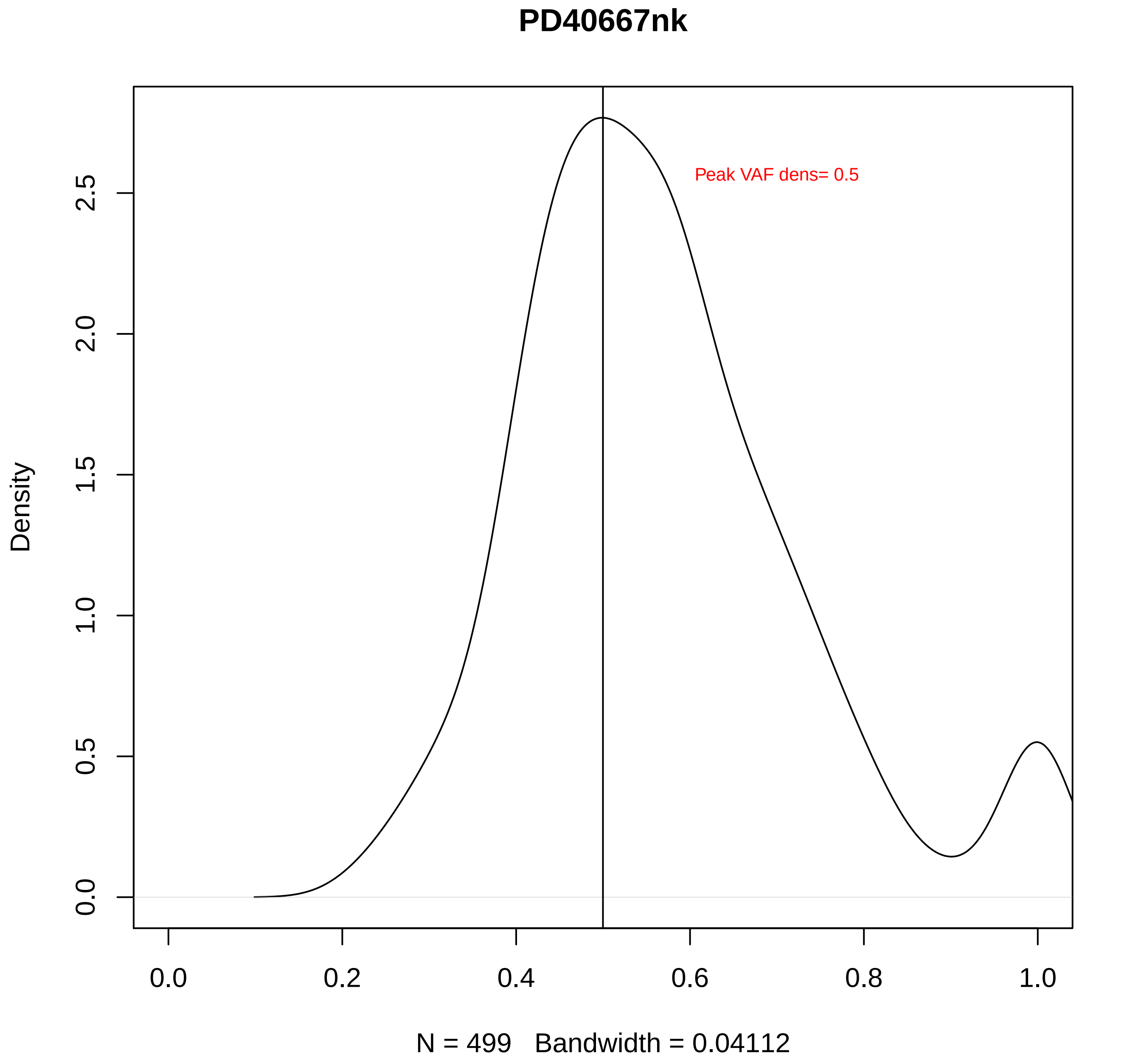

PD40667nk
Peak VAF dens= 0.5
Density
0.0
0.5
1.0
1.5
2.0
2.5
0.0
0.2
0.4
0.6
0.8
1.0
N = 499   Bandwidth = 0.04112

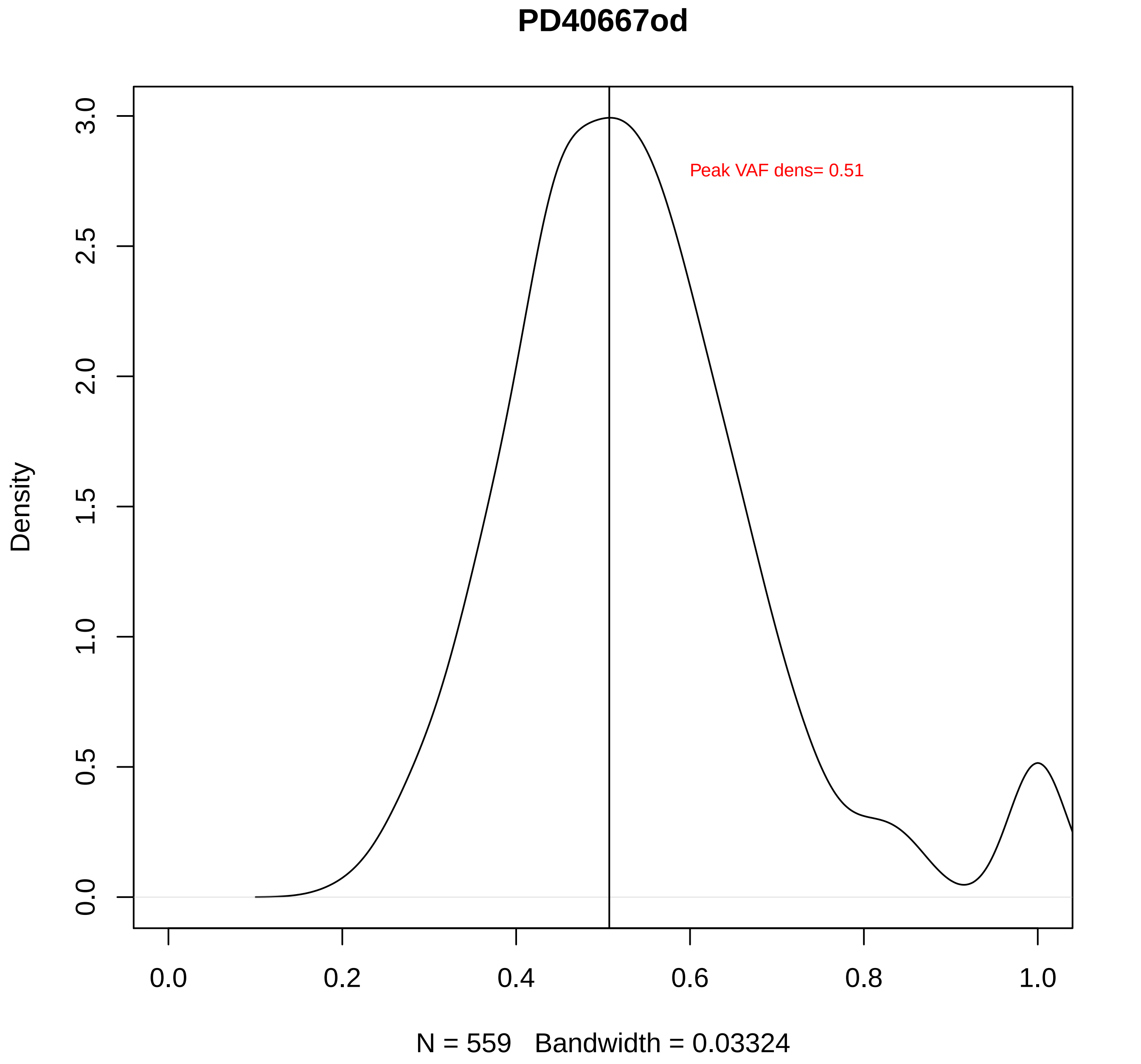
PD40667od
Peak VAF dens= 0.51
Density
0.0
0.5
1.0
1.5
2.0
2.5
3.0
0.0
0.2
0.4
0.6
0.8
1.0
N = 559 Bandwidth = 0.03324

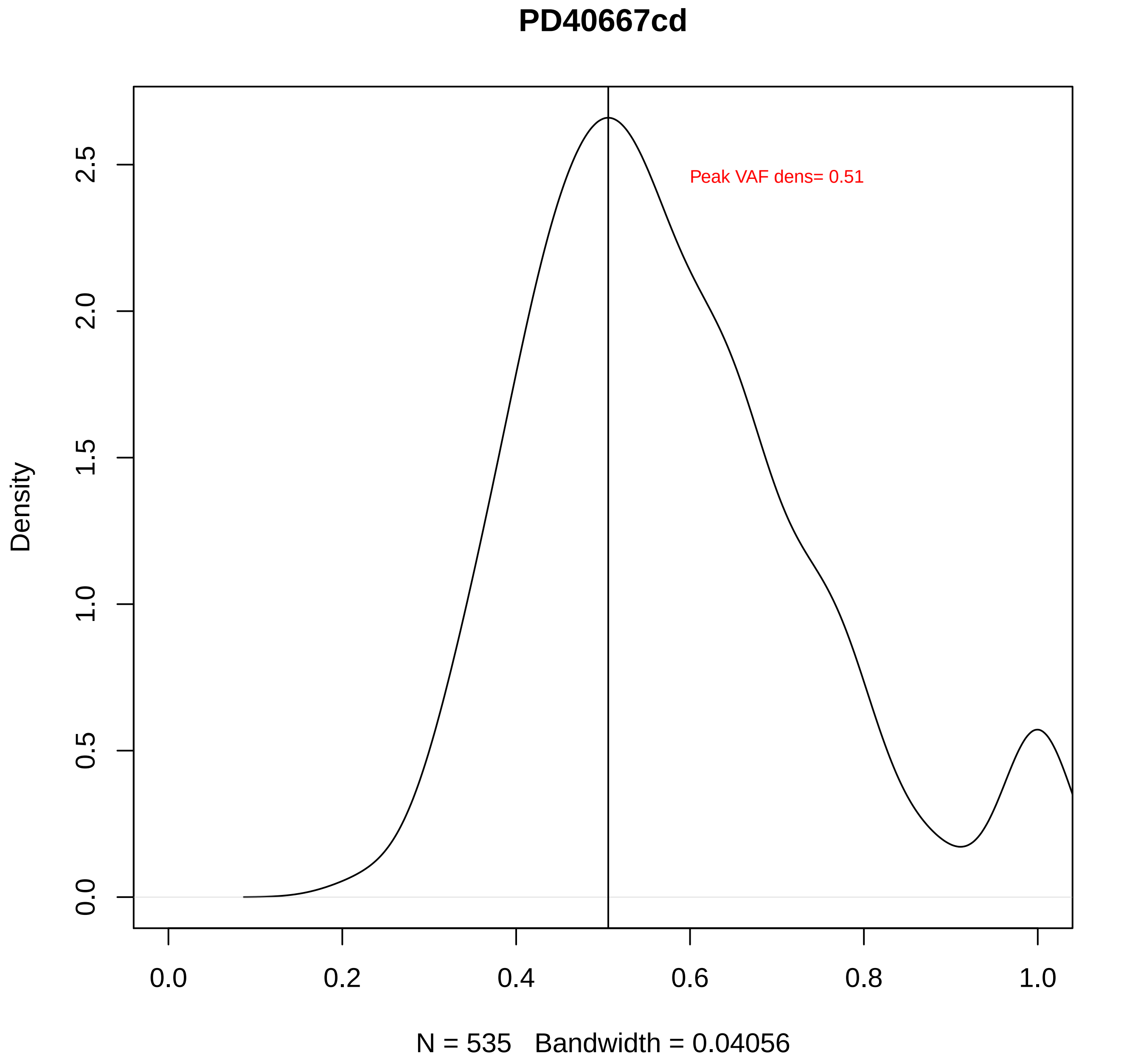
PD40667cd
Peak VAF dens= 0.51
Density
0.0
0.5
1.0
1.5
2.0
2.5
0.0
0.2
0.4
0.6
0.8
1.0
N = 535   Bandwidth = 0.04056

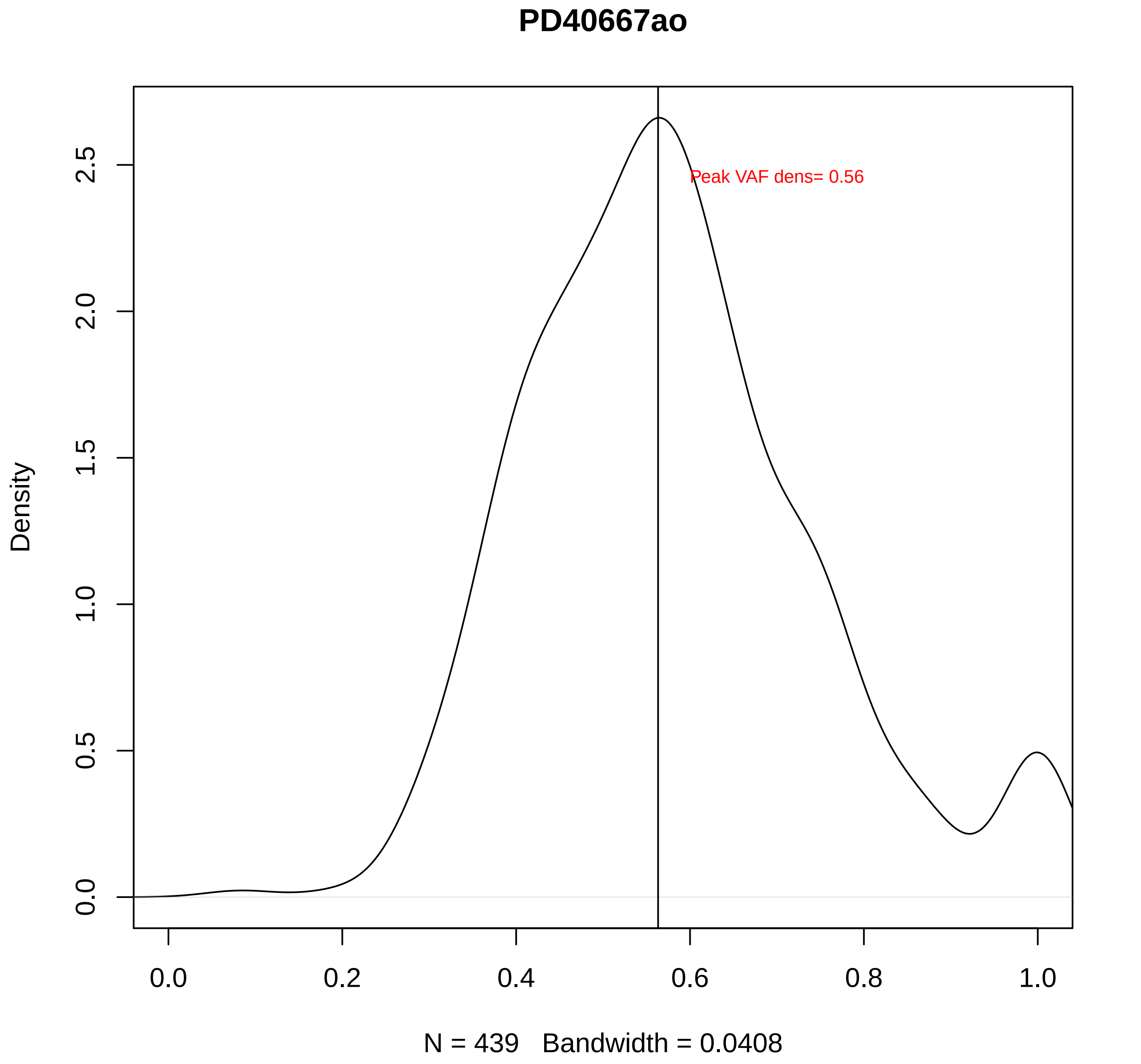
PD40667ao
Peak VAF dens= 0.56
Density
0.0
0.5
1.0
1.5
2.0
2.5
0.0
0.2
0.4
0.6
0.8
1.0
N = 439 Bandwidth = 0.0408

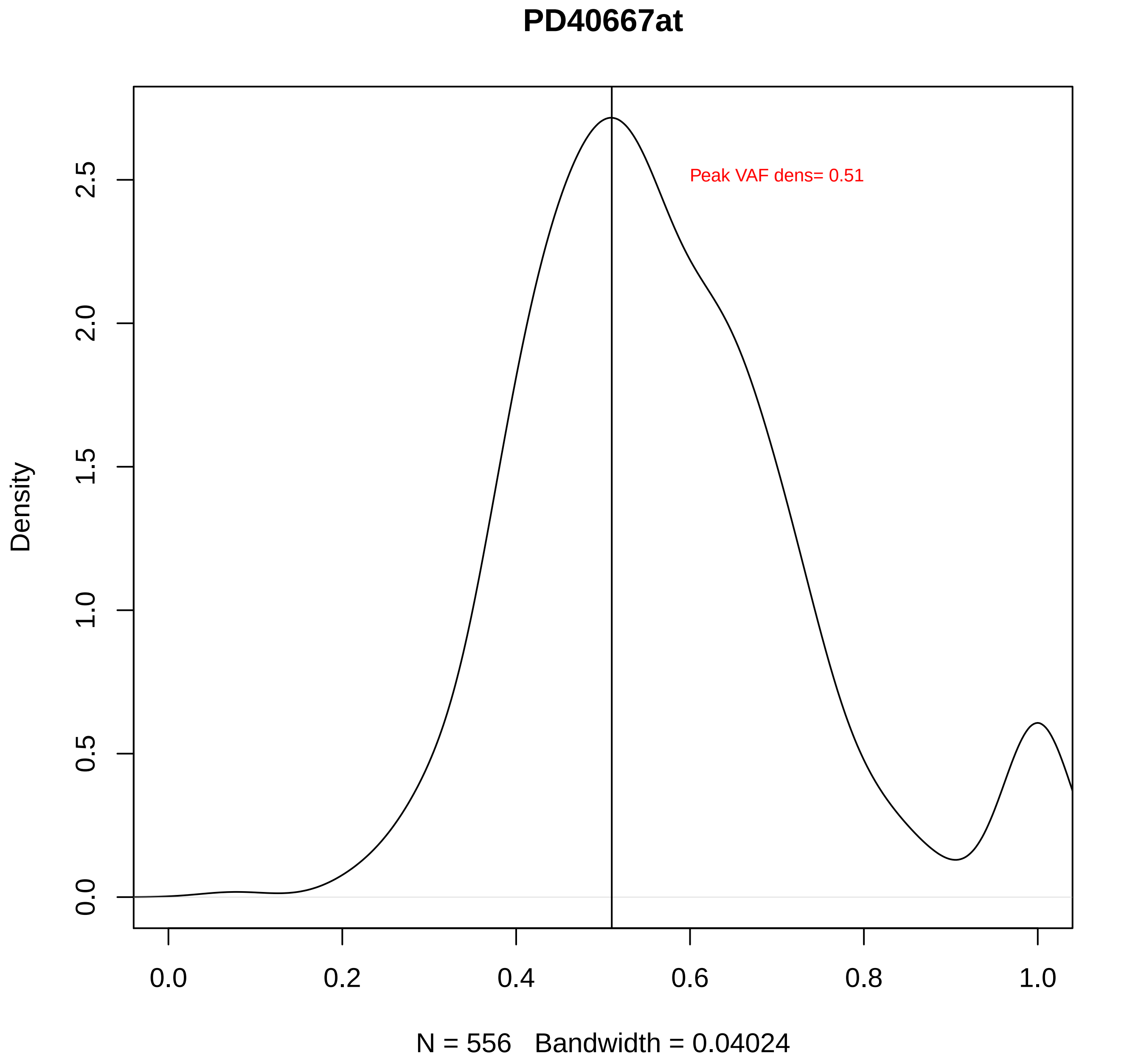
PD40667at
Peak VAF dens= 0.51
Density
0.0
0.5
1.0
1.5
2.0
2.5
0.0
0.2
0.4
0.6
0.8
1.0
N = 556   Bandwidth = 0.04024

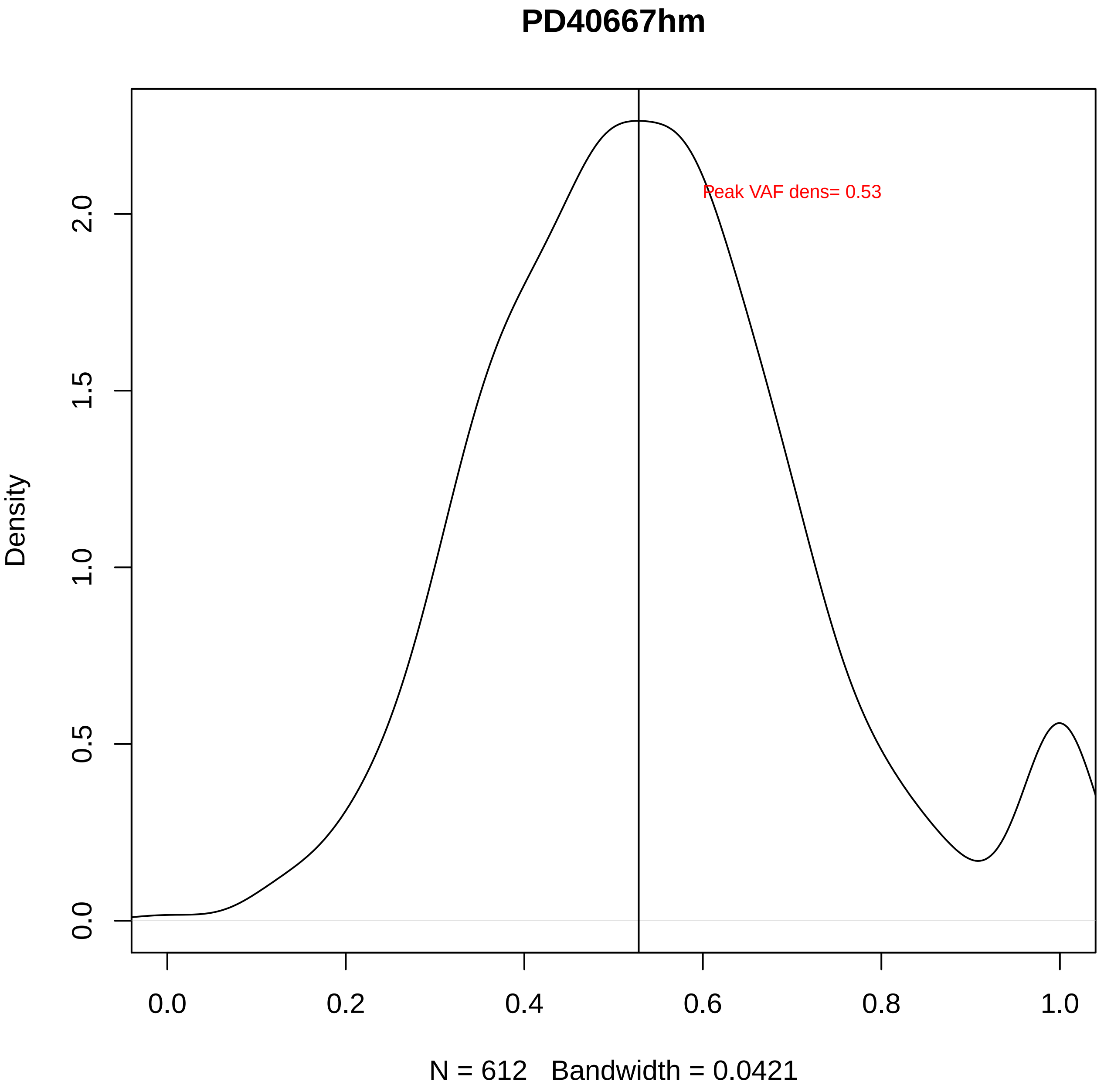
PD40667hm
Peak VAF dens= 0.53
Density
0.0
0.5
1.0
1.5
2.0
0.0
0.2
0.4
0.6
0.8
1.0
N = 612   Bandwidth = 0.0421

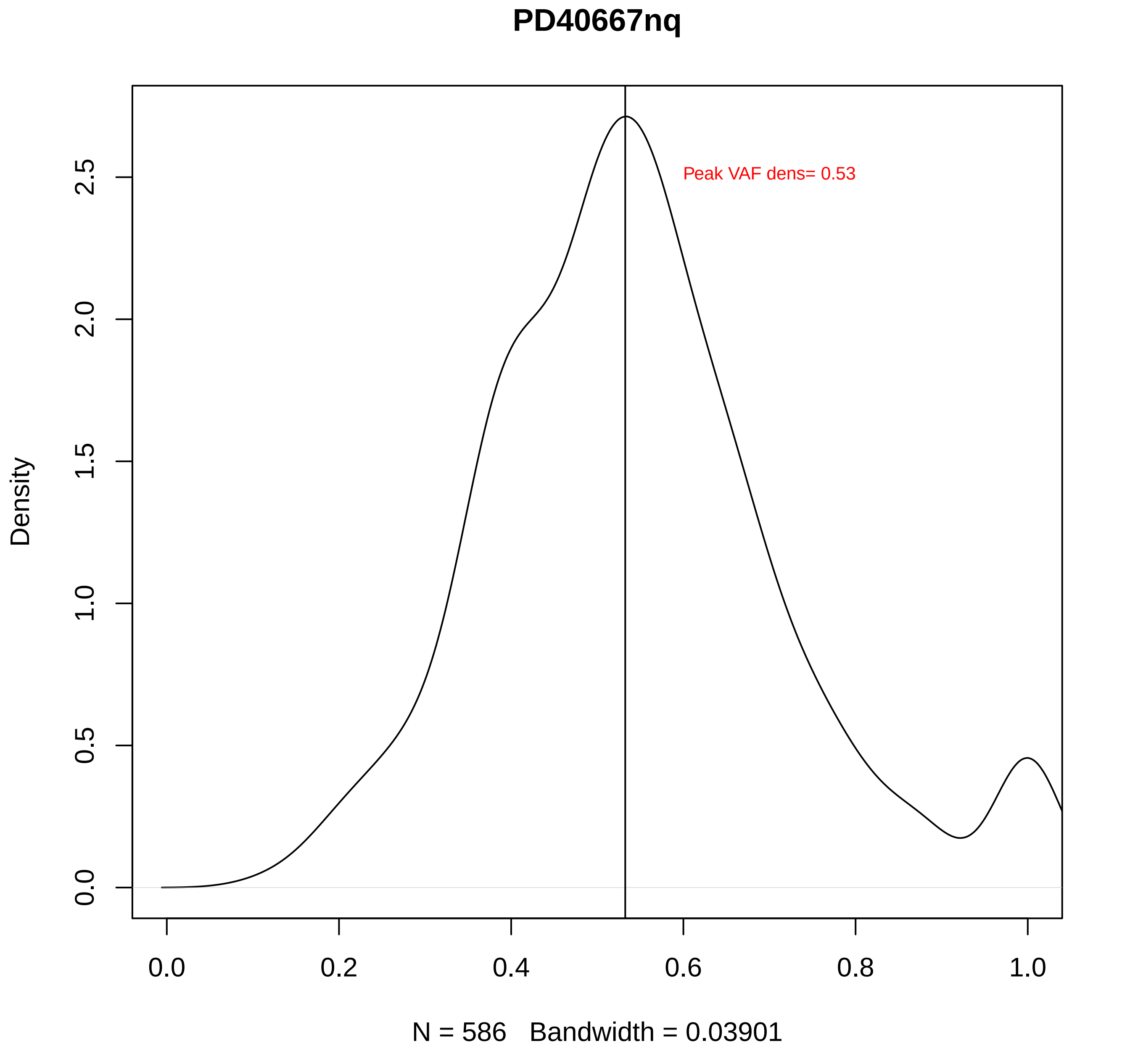
PD40667nq
Peak VAF dens= 0.53
Density
0.0
0.5
1.0
1.5
2.0
2.5
0.0
0.2
0.4
0.6
0.8
1.0
N = 586   Bandwidth = 0.03901

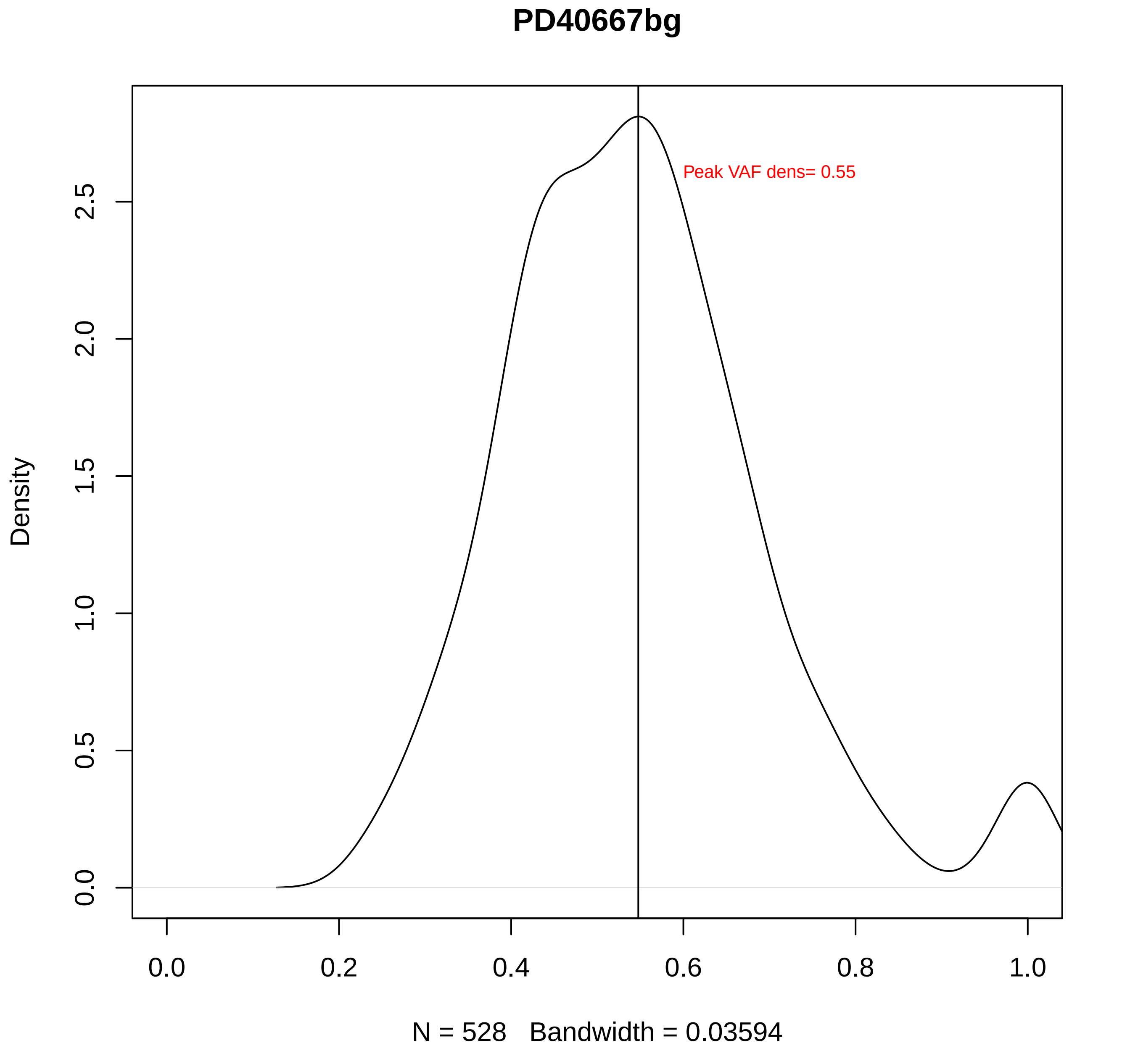

PD40667bg
Peak VAF dens= 0.55
Density
0.0
0.5
1.0
1.5
2.0
2.5
0.0
0.2
0.4
0.6
0.8
1.0
N = 528   Bandwidth = 0.03594

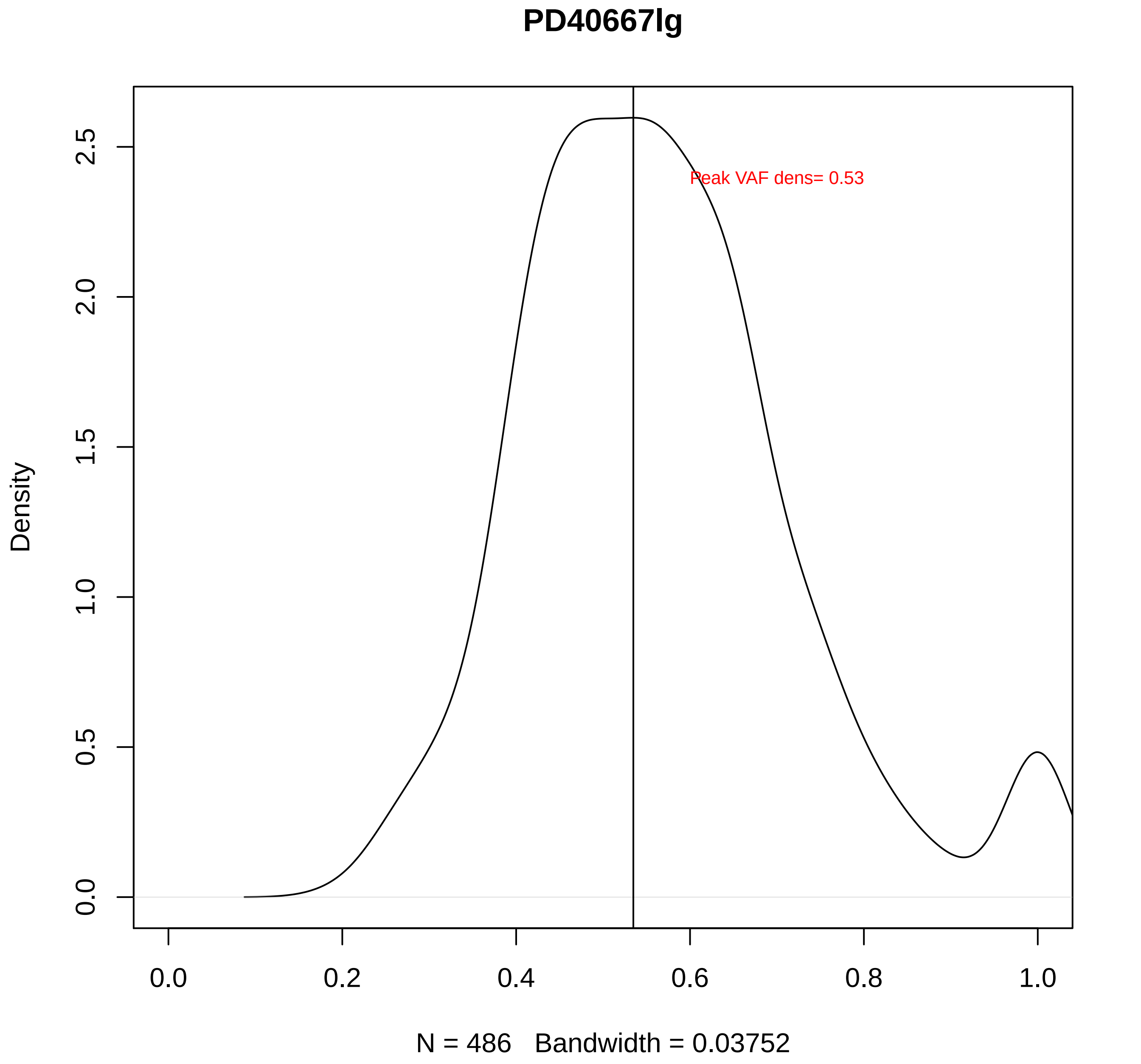

PD40667lg
Density
2.5
2.0
1.5
1.0
0.5
0.0
Peak VAF dens= 0.53
0.0
0.2
0.4
0.6
0.8
1.0
N = 486 Bandwidth = 0.03752

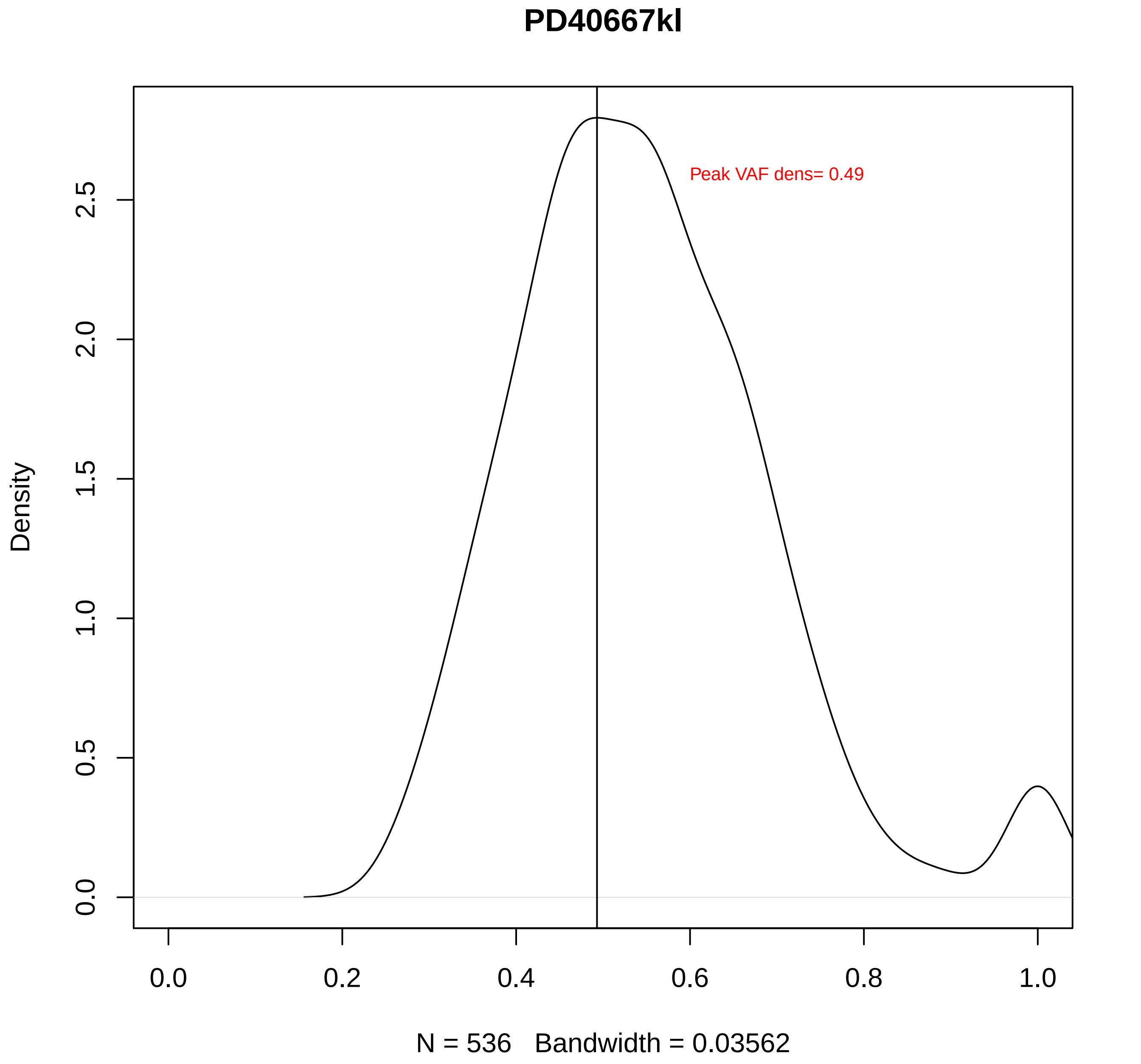
PD40667kl
Peak VAF dens= 0.49
Density
0.0
0.5
1.0
1.5
2.0
2.5
0.0
0.2
0.4
0.6
0.8
1.0
N = 536   Bandwidth = 0.03562

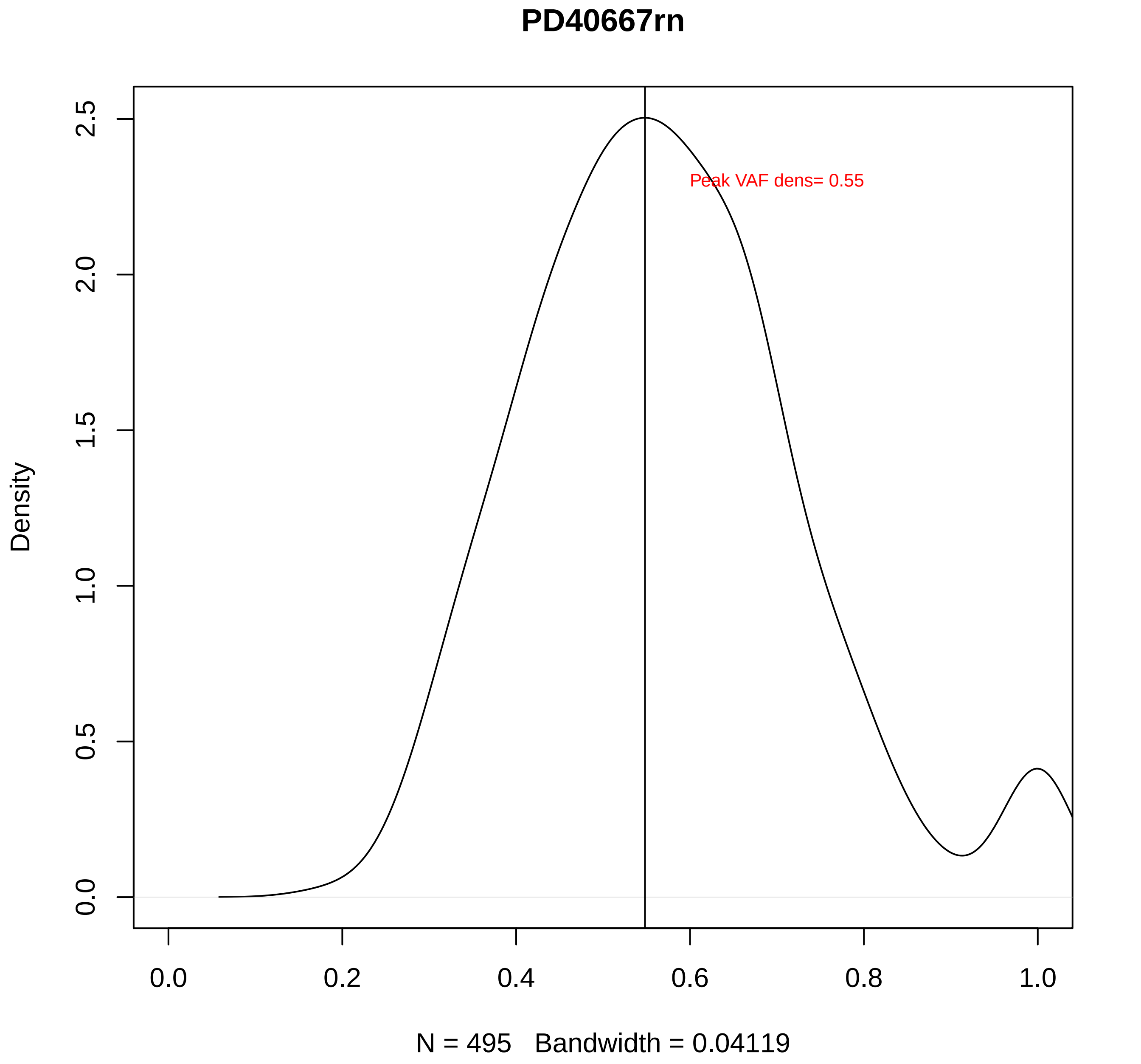
PD40667rn
Peak VAF dens= 0.55
Density
0.0
0.5
1.0
1.5
2.0
2.5
0.0
0.2
0.4
0.6
0.8
1.0
N = 495   Bandwidth = 0.04119

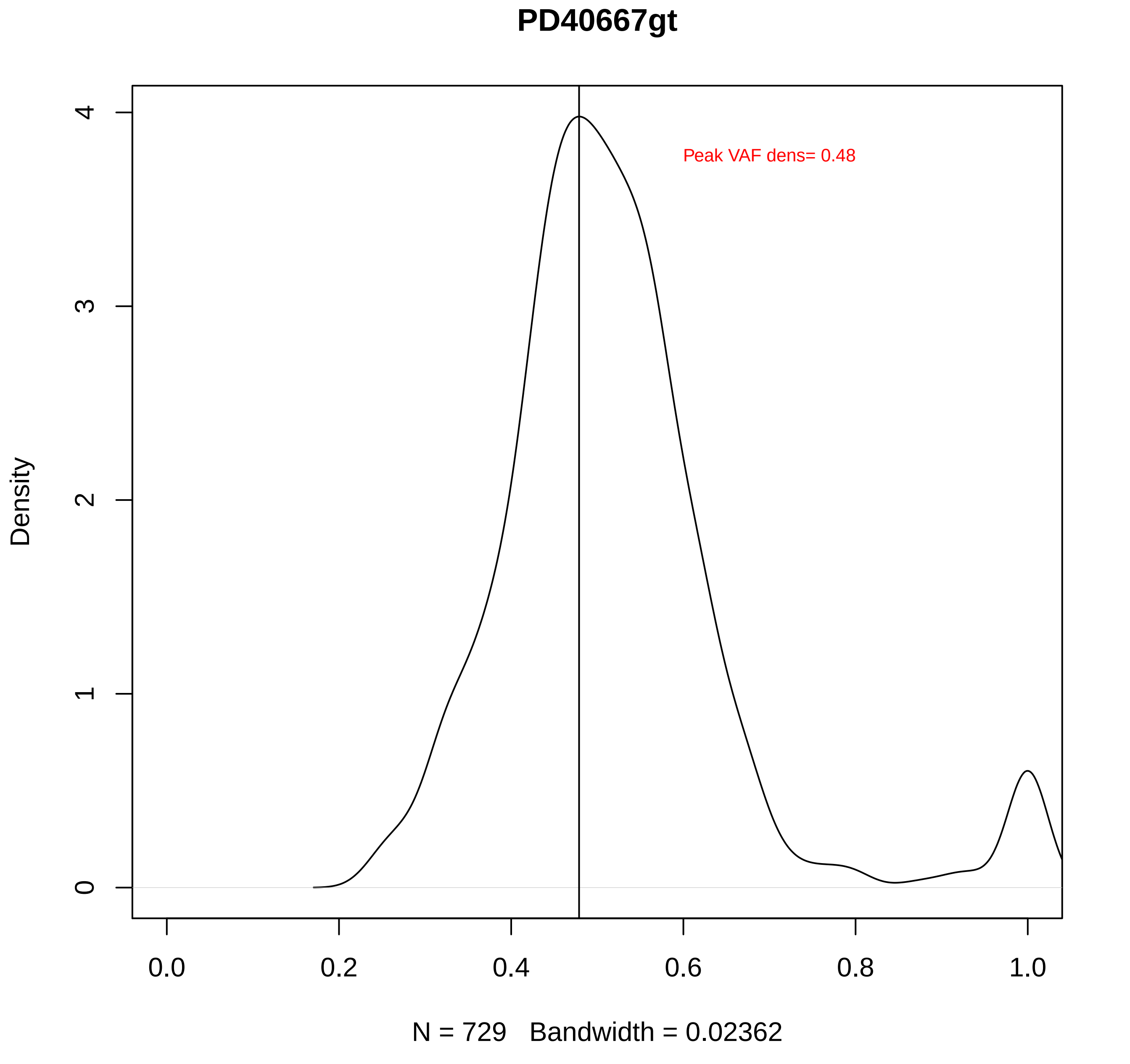
PD40667gt
Peak VAF dens= 0.48
Density
0
1
2
3
4
0.0
0.2
0.4
0.6
0.8
1.0
N = 729 Bandwidth = 0.02362

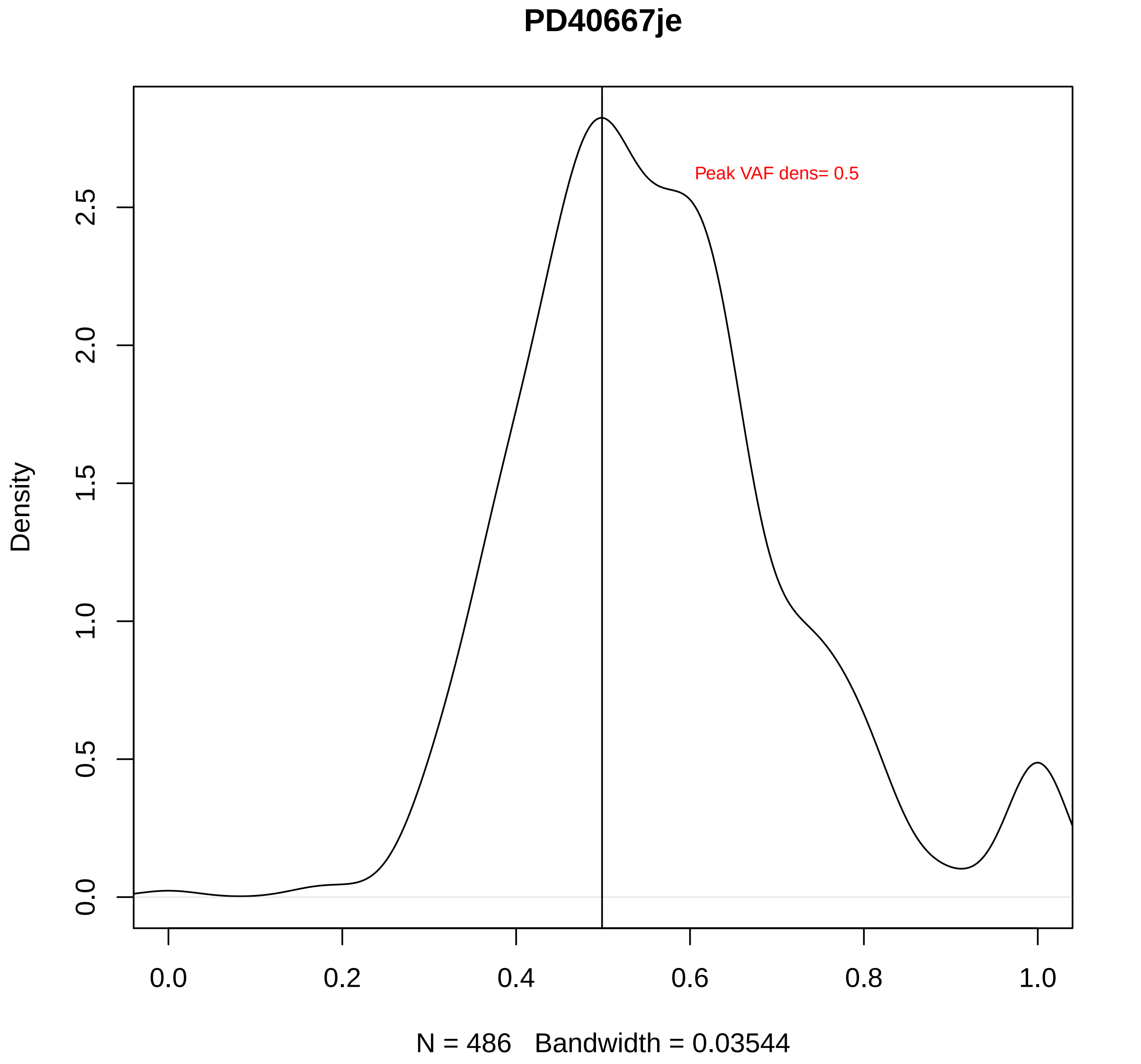

PD40667je
Peak VAF dens= 0.5
Density
0.0
0.5
1.0
1.5
2.0
2.5
0.0
0.2
0.4
0.6
0.8
1.0
N = 486 Bandwidth = 0.03544

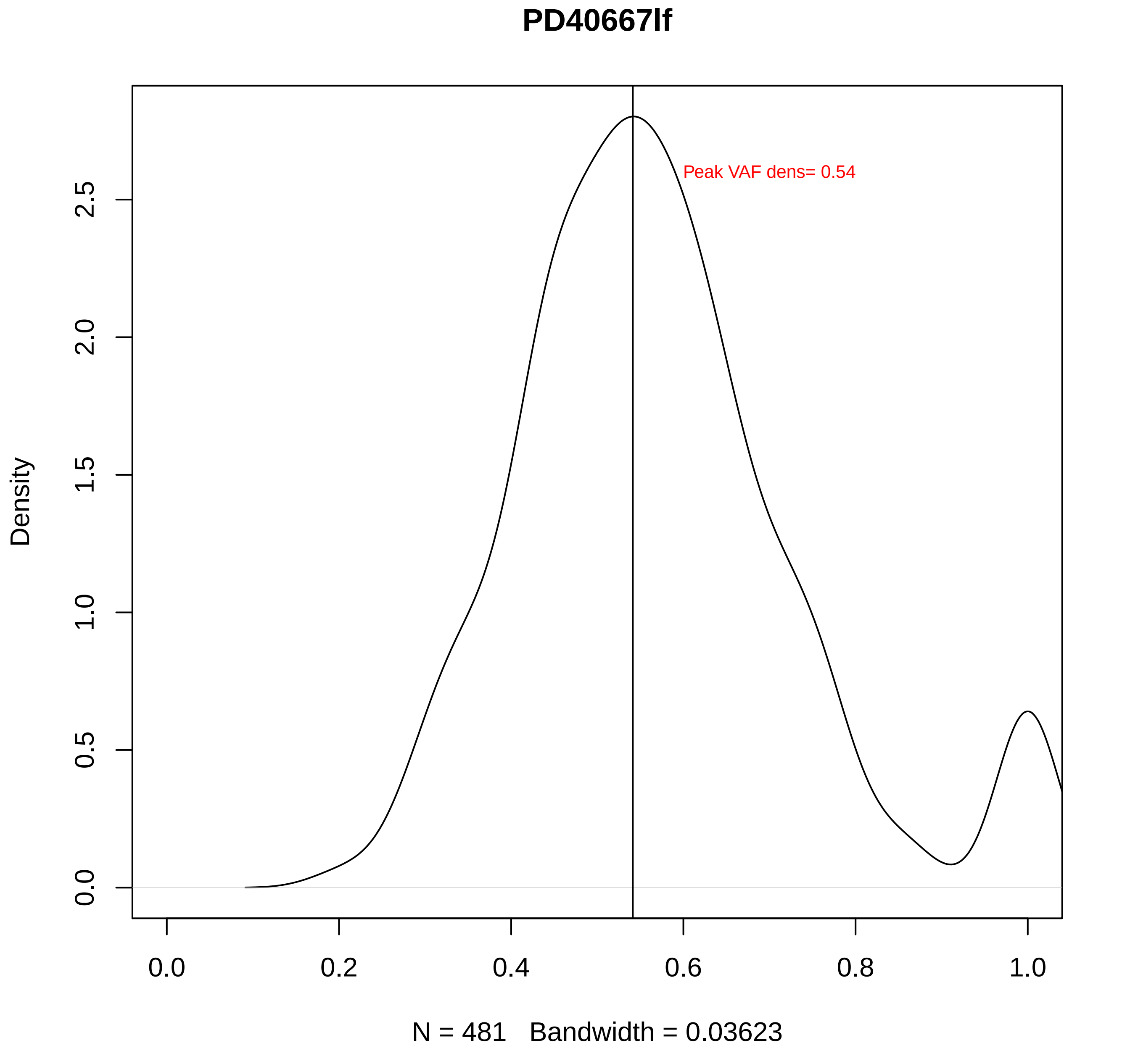

PD40667lf
Peak VAF dens= 0.54
Density
0.0
0.5
1.0
1.5
2.0
2.5
0.0
0.2
0.4
0.6
0.8
1.0
N = 481 Bandwidth = 0.03623

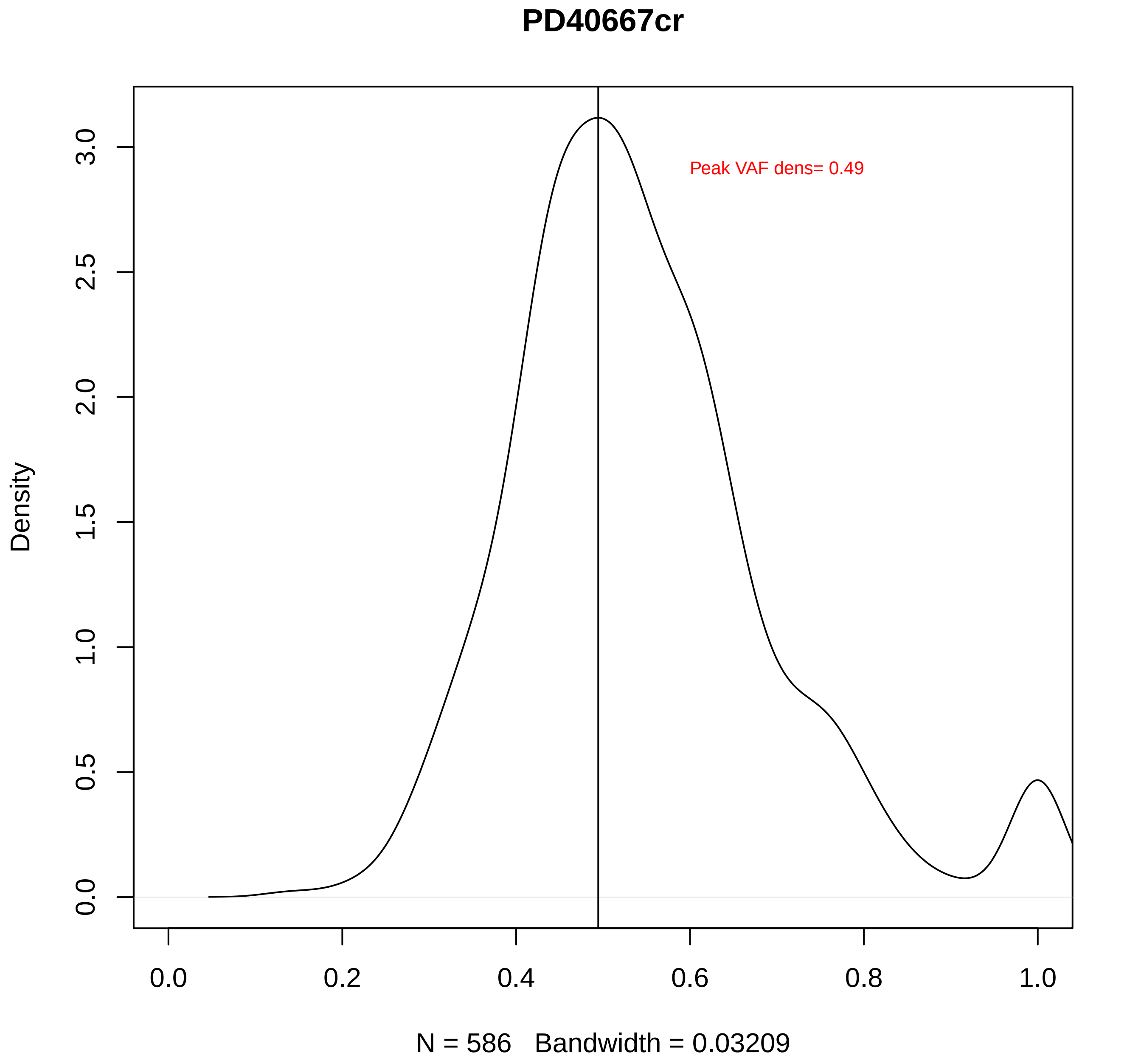
PD40667cr
Peak VAF dens= 0.49
Density
3.0
2.5
2.0
1.5
1.0
0.5
0.0
0.0
0.2
0.4
0.6
0.8
1.0
N = 586 Bandwidth = 0.03209

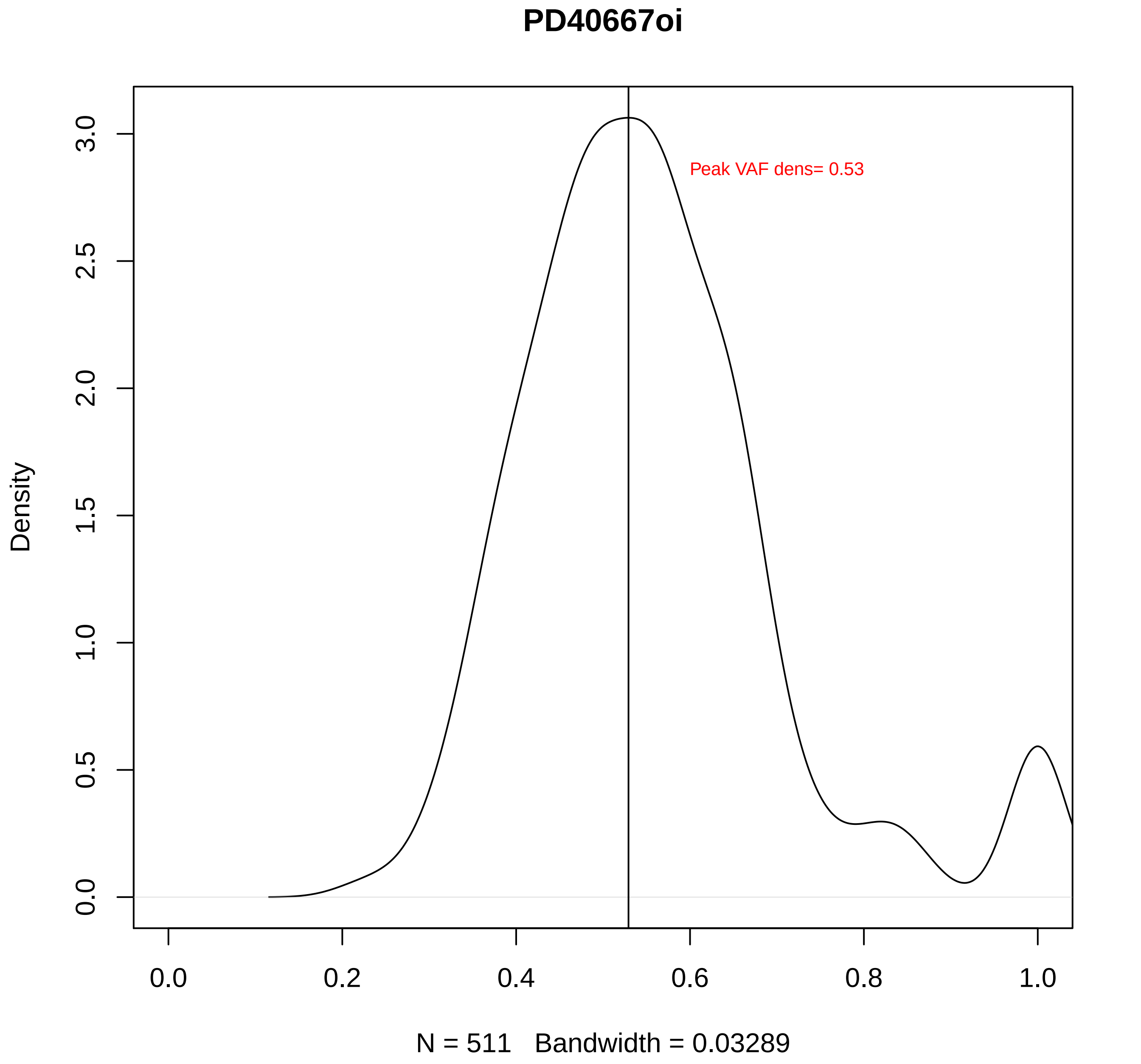

PD40667oi
Peak VAF dens= 0.53
Density
0.0
0.5
1.0
1.5
2.0
2.5
3.0
0.0
0.2
0.4
0.6
0.8
1.0
N = 511 Bandwidth = 0.03289

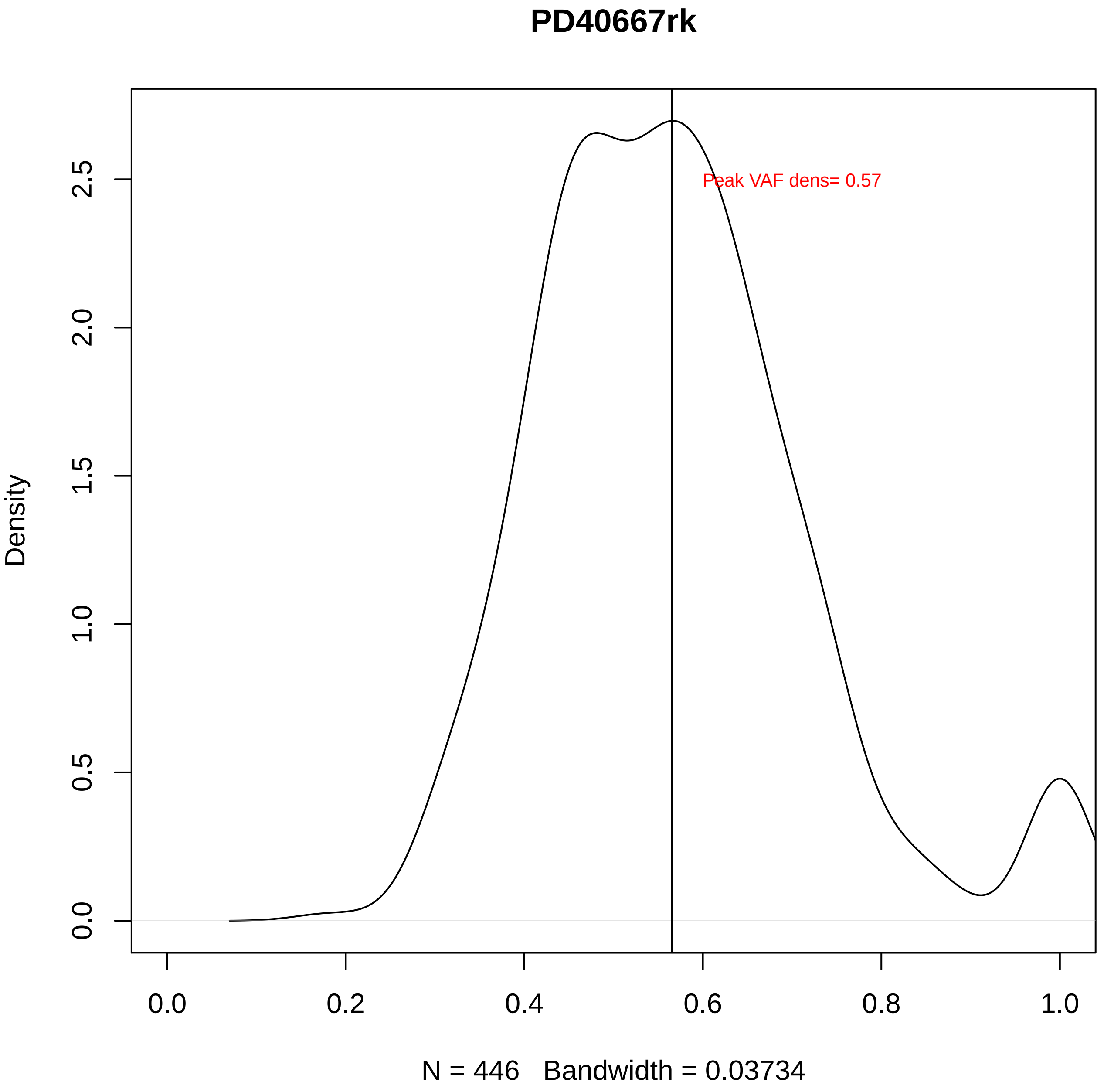

PD40667rk
Peak VAF dens= 0.57
Density
0.0
0.5
1.0
1.5
2.0
2.5
0.0
0.2
0.4
0.6
0.8
1.0
N = 446   Bandwidth = 0.03734

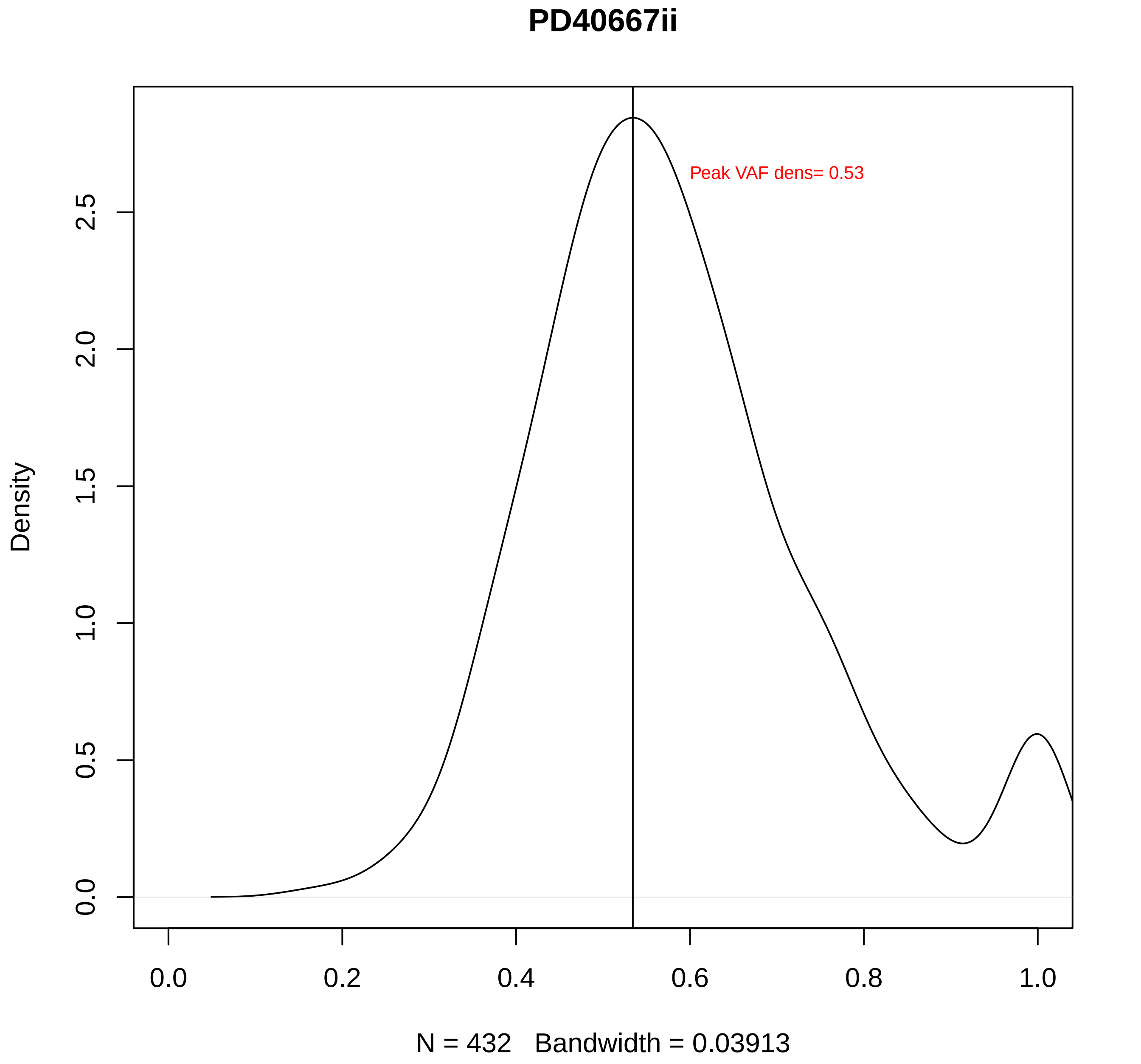
PD40667ii
Peak VAF dens= 0.53
Density
0.0
0.5
1.0
1.5
2.0
2.5
0.0
0.2
0.4
0.6
0.8
1.0
N = 432 Bandwidth = 0.03913

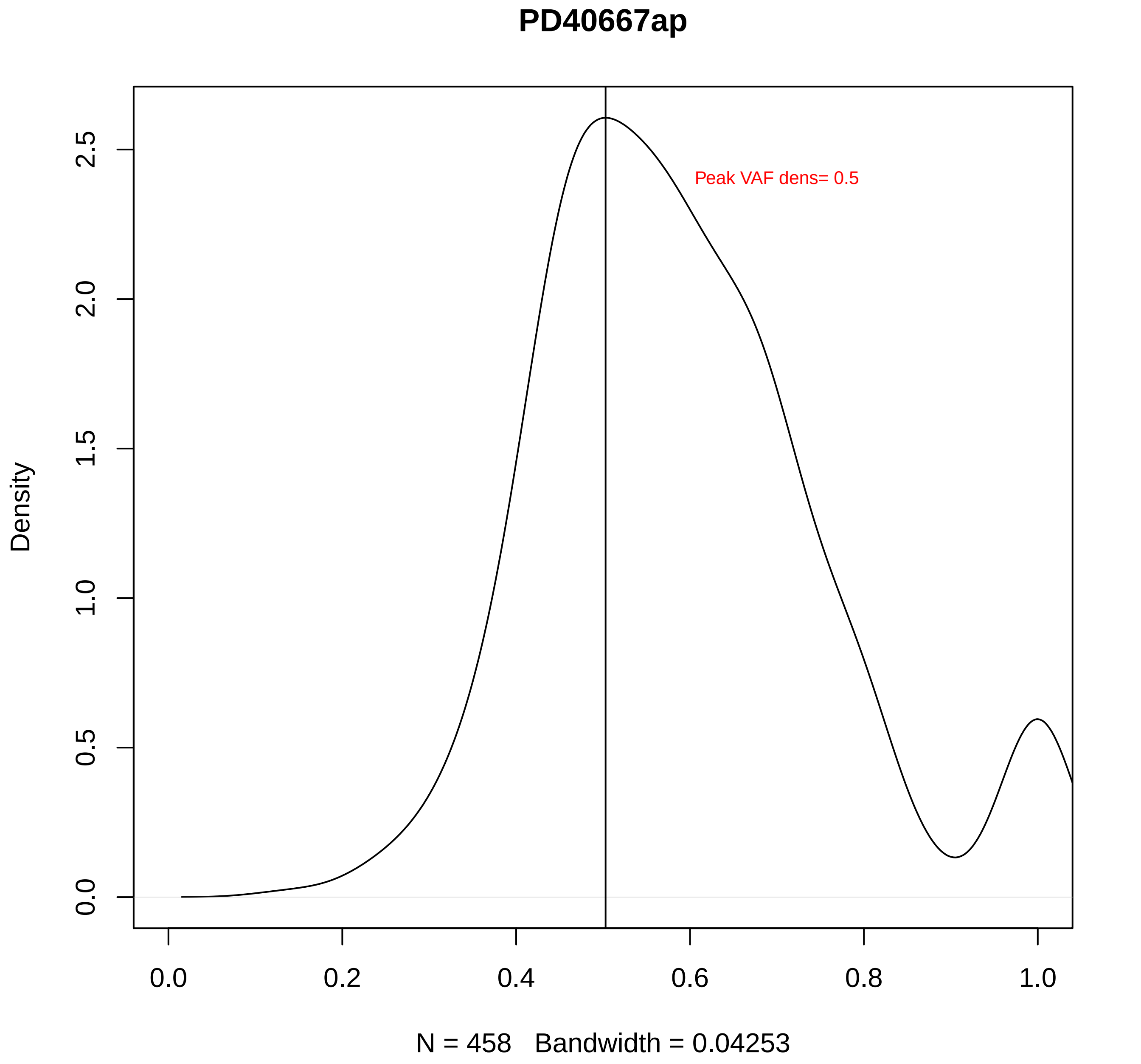
PD40667ap
Peak VAF dens= 0.5
Density
0.0
0.5
1.0
1.5
2.0
2.5
0.0
0.2
0.4
0.6
0.8
1.0
N = 458   Bandwidth = 0.04253

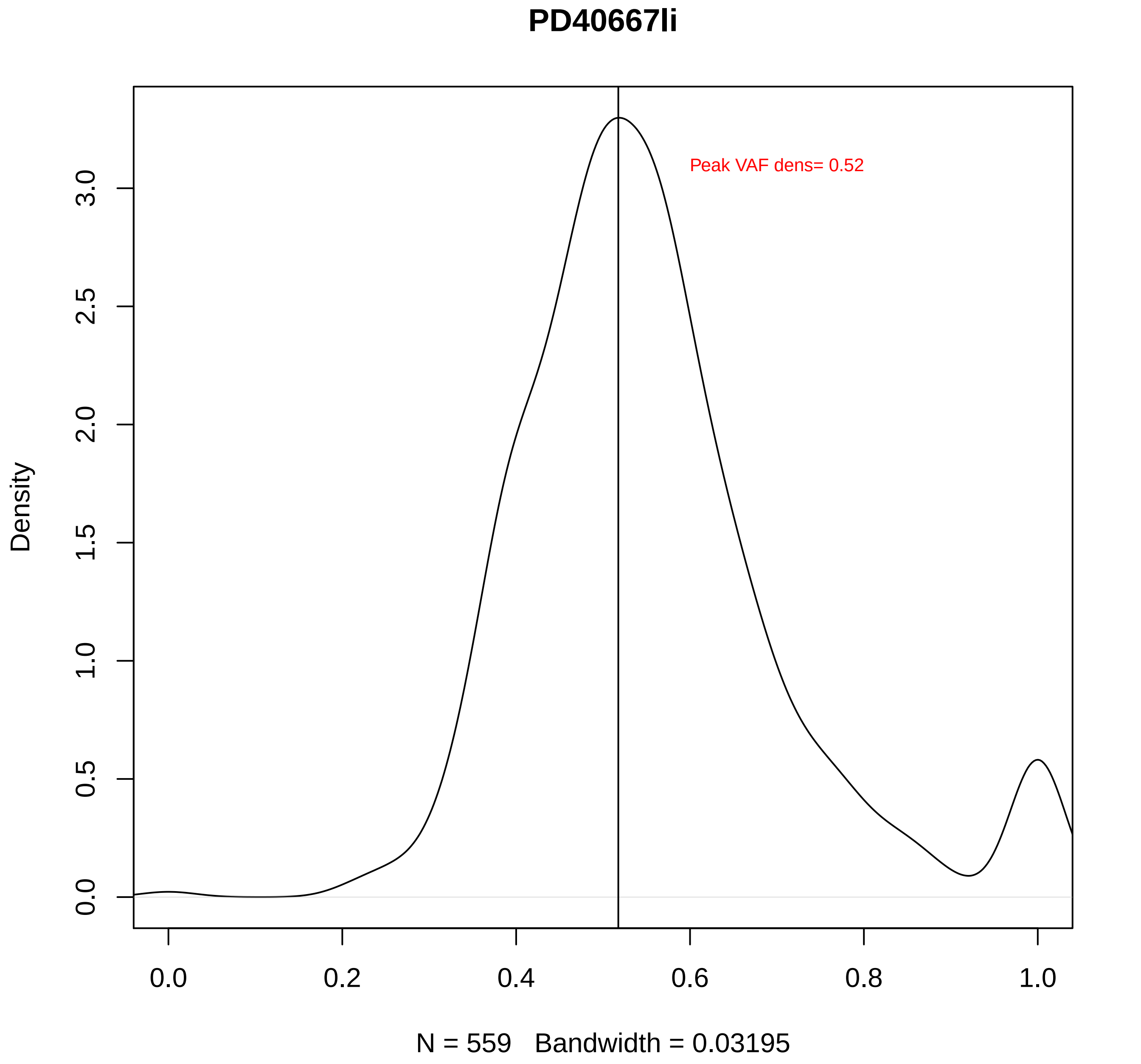

PD40667li
Peak VAF dens= 0.52
Density
0.0
0.5
1.0
1.5
2.0
2.5
3.0
0.0
0.2
0.4
0.6
0.8
1.0
N = 559   Bandwidth = 0.03195

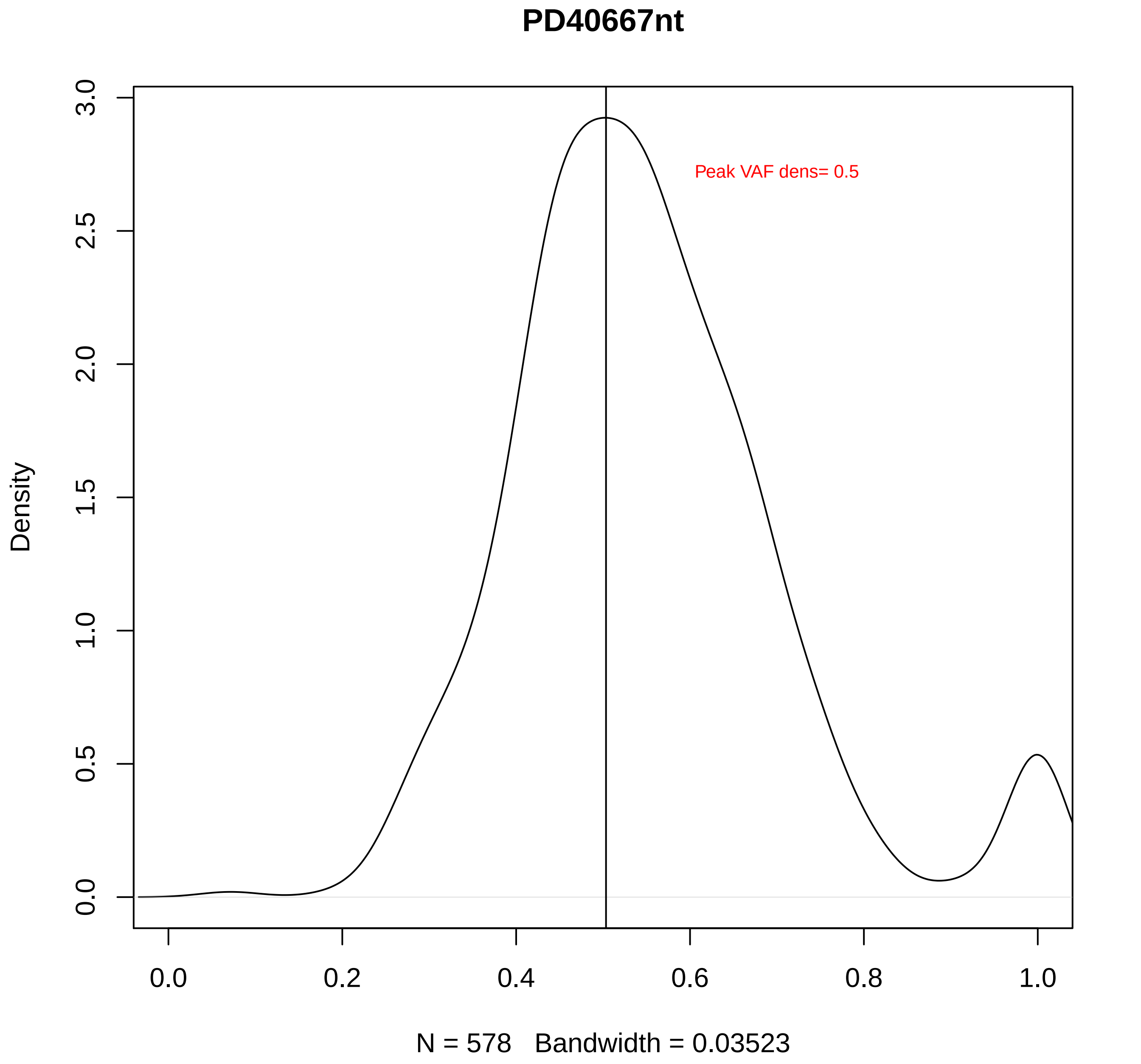

PD40667nt
Peak VAF dens= 0.5
Density
3.0
2.5
2.0
1.5
1.0
0.5
0.0
0.0
0.2
0.4
0.6
0.8
1.0
N = 578 Bandwidth = 0.03523

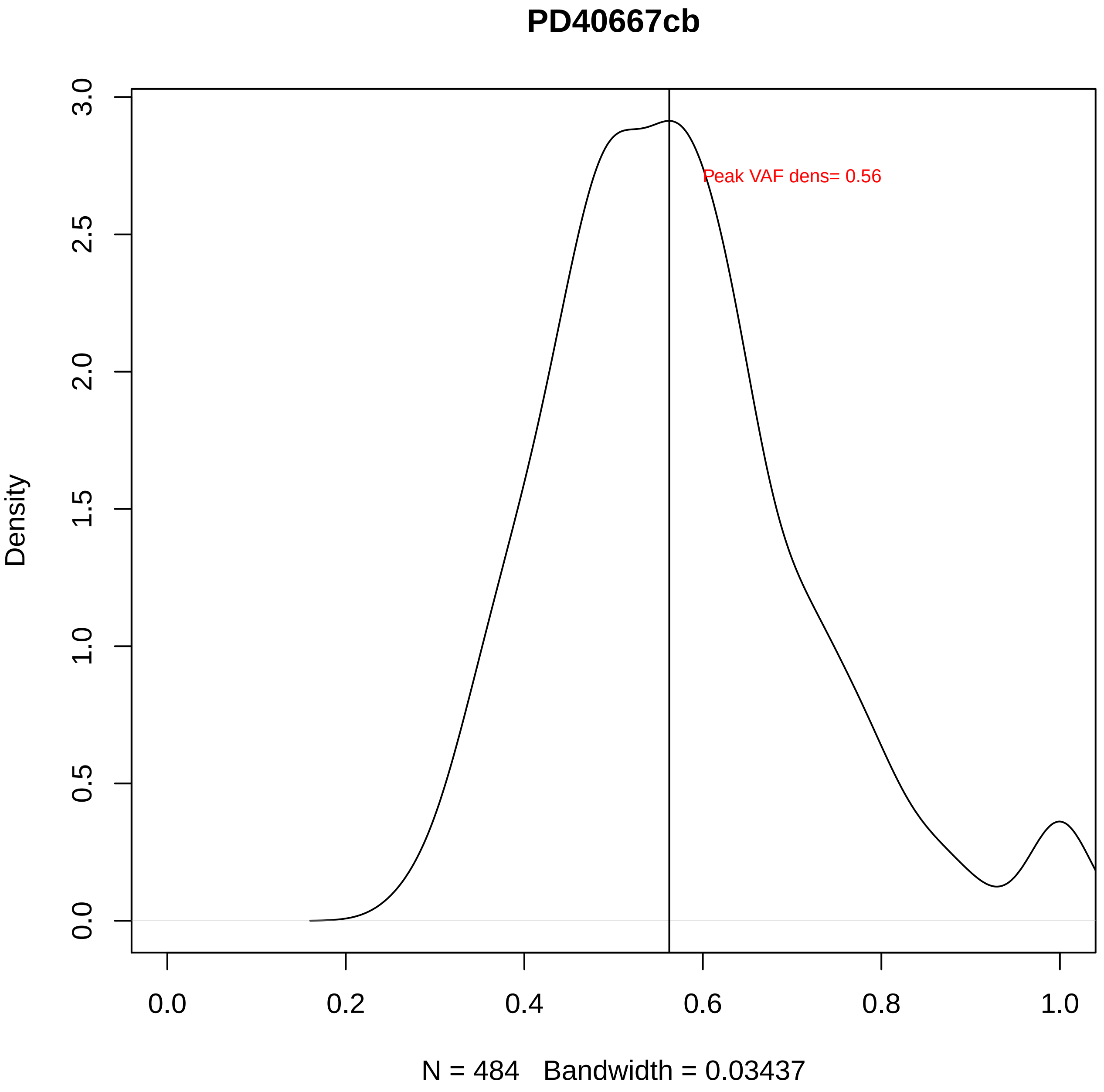
PD40667cb
Peak VAF dens= 0.56
Density
0.0
0.5
1.0
1.5
2.0
2.5
3.0
0.0
0.2
0.4
0.6
0.8
1.0
N = 484   Bandwidth = 0.03437

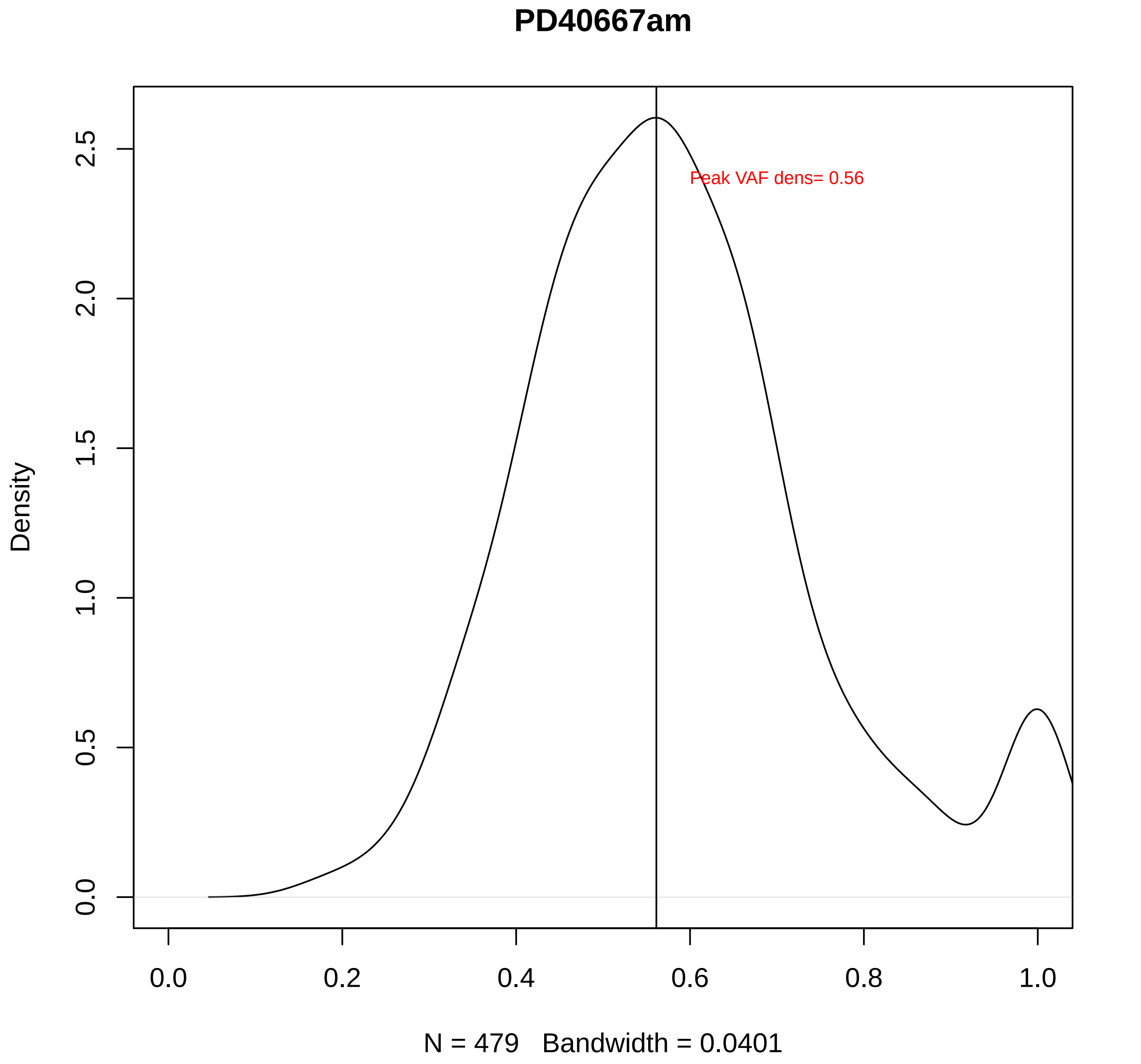

PD40667am
Peak VAF dens= 0.56
Density
0.0
0.5
1.0
1.5
2.0
2.5
0.0
0.2
0.4
0.6
0.8
1.0
N = 479 Bandwidth = 0.0401

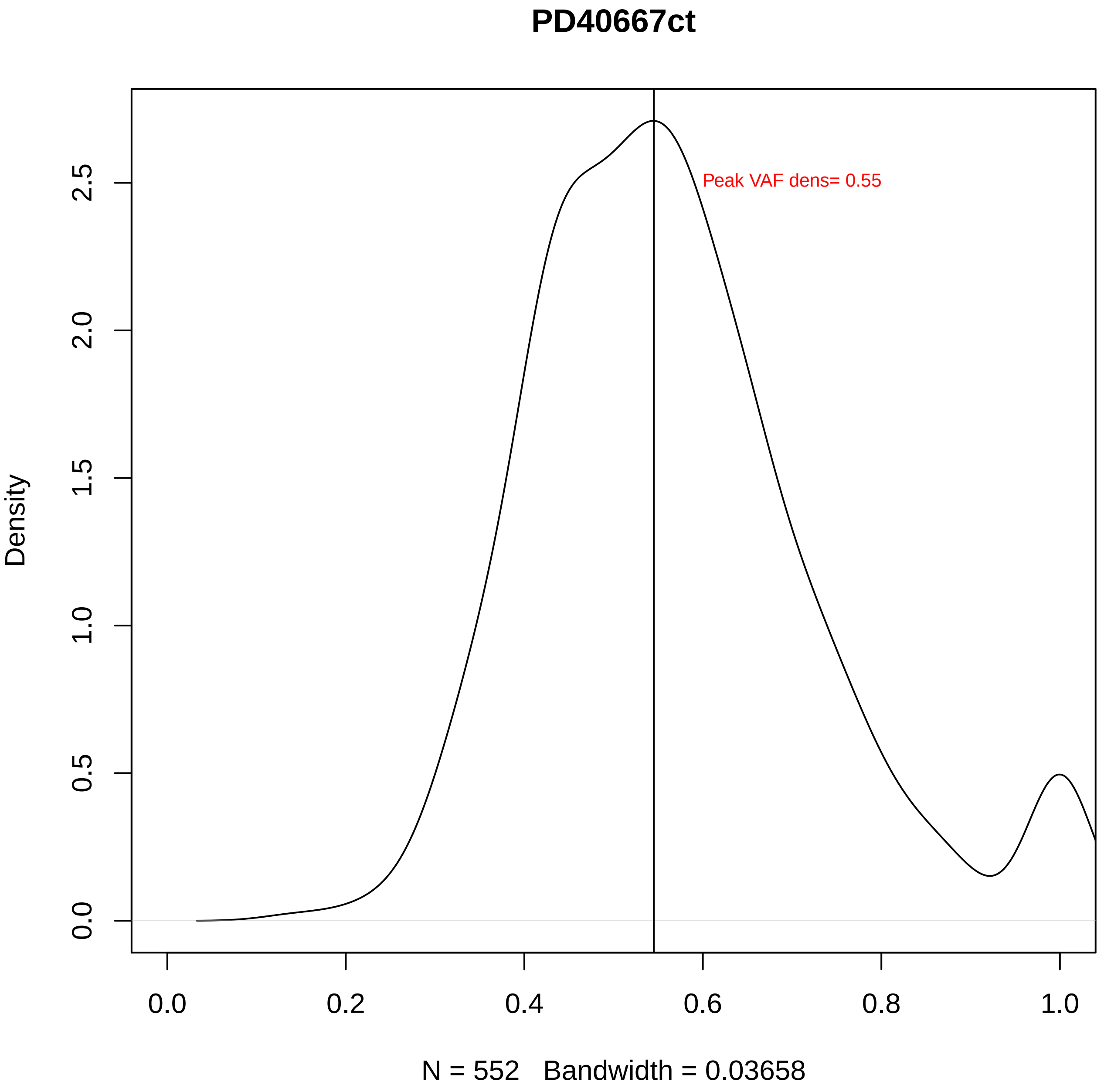
PD40667ct
Peak VAF dens= 0.55
Density
0.0
0.5
1.0
1.5
2.0
2.5
0.0
0.2
0.4
0.6
0.8
1.0
N = 552 Bandwidth = 0.03658

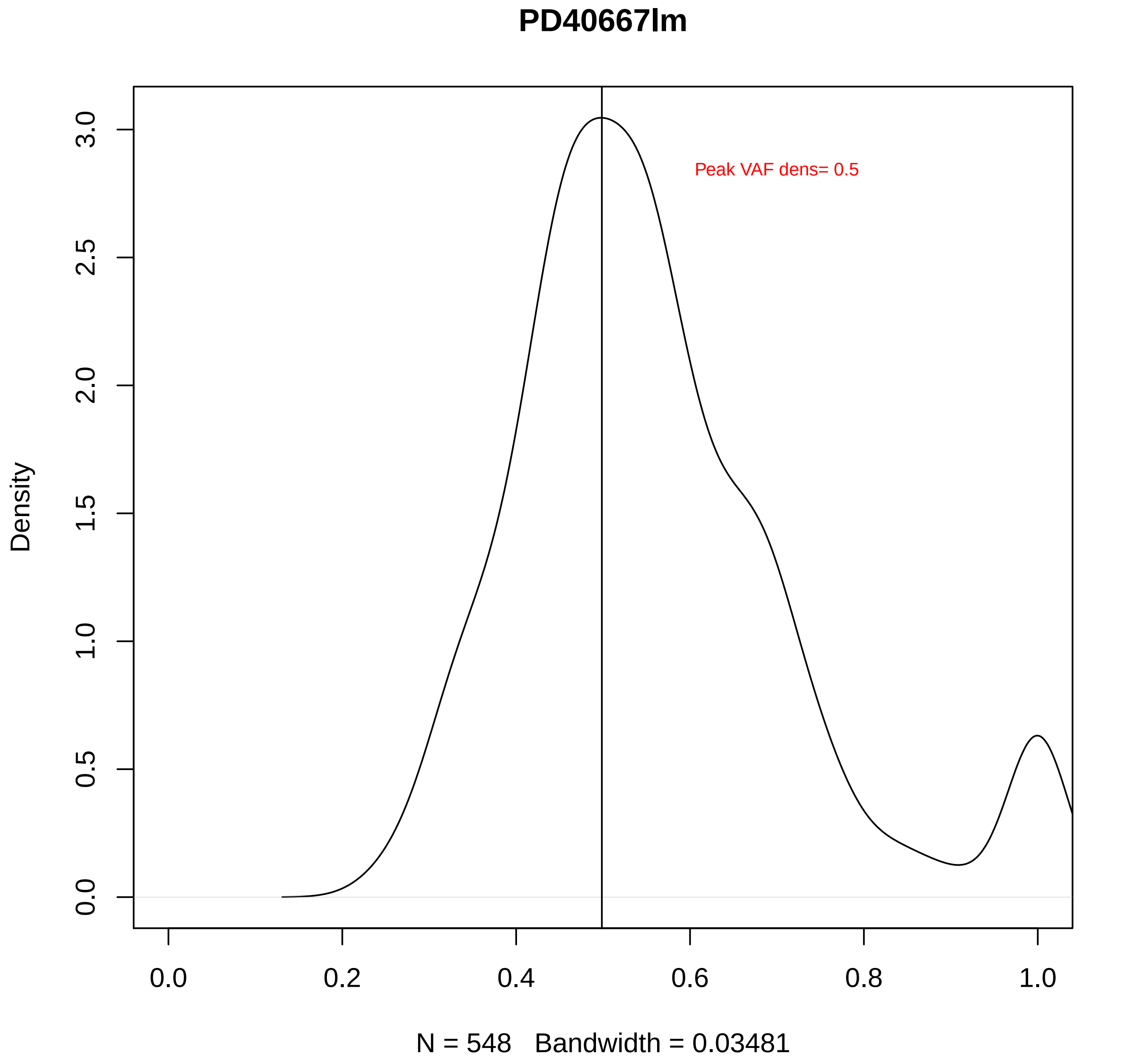
PD40667lm
Peak VAF dens= 0.5
Density
0.0
0.5
1.0
1.5
2.0
2.5
3.0
0.0
0.2
0.4
0.6
0.8
1.0
N = 548 Bandwidth = 0.03481

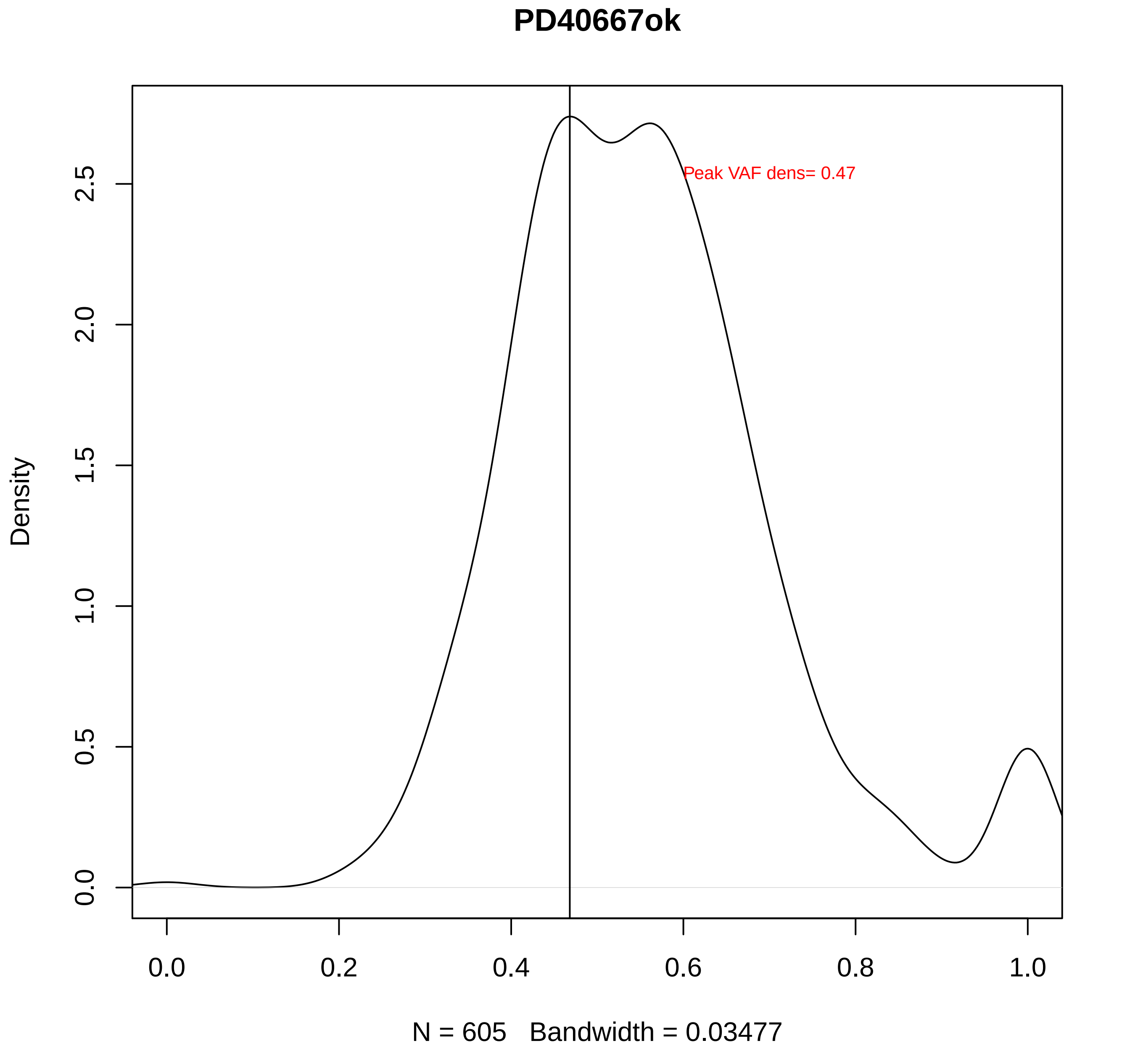
PD40667ok
Peak VAF dens= 0.47
Density
0.0
0.5
1.0
1.5
2.0
2.5
0.0
0.2
0.4
0.6
0.8
1.0
N = 605 Bandwidth = 0.03477

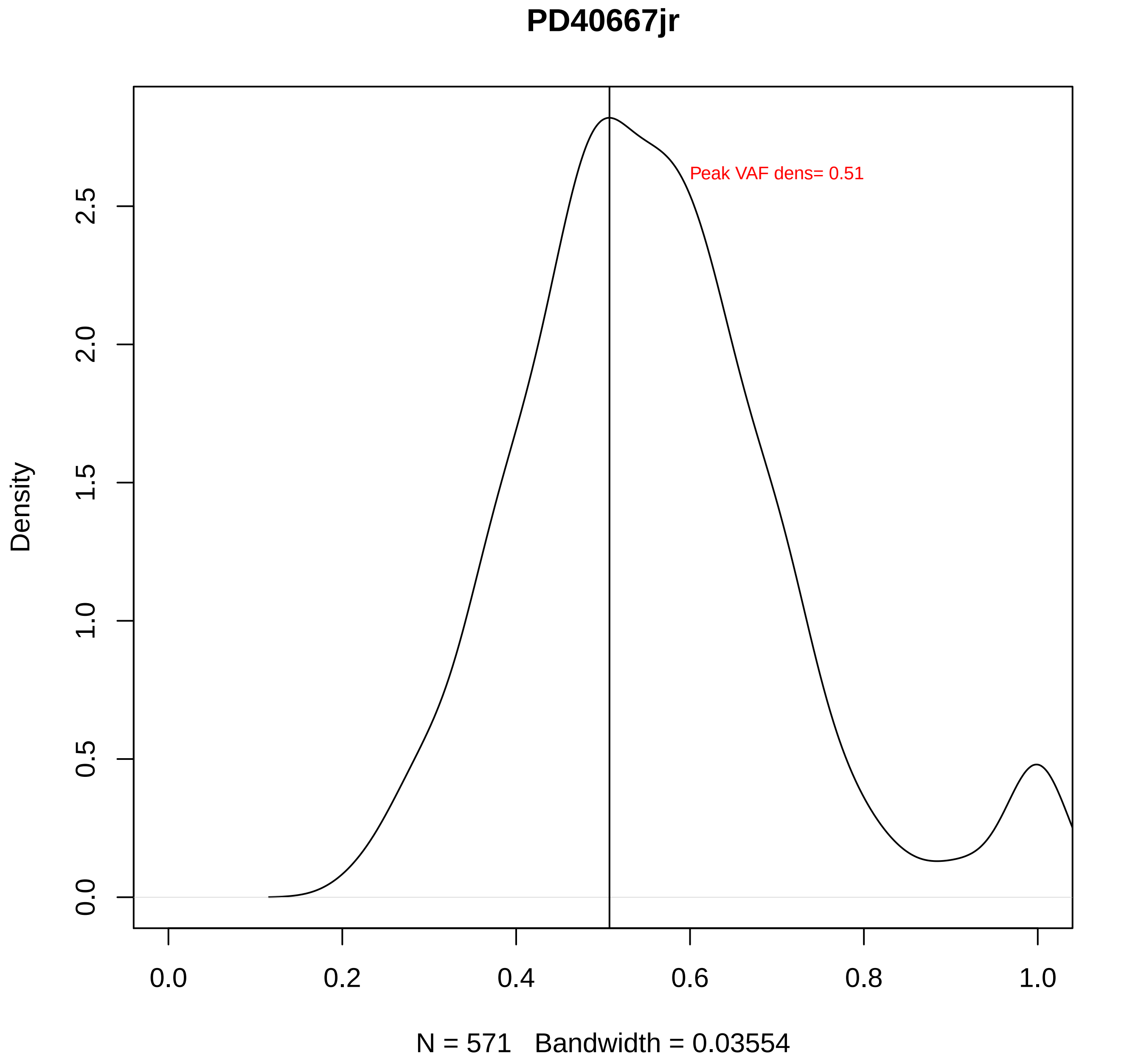
PD40667jr
Peak VAF dens= 0.51
Density
0.0
0.5
1.0
1.5
2.0
2.5
0.0
0.2
0.4
0.6
0.8
1.0
N = 571   Bandwidth = 0.03554

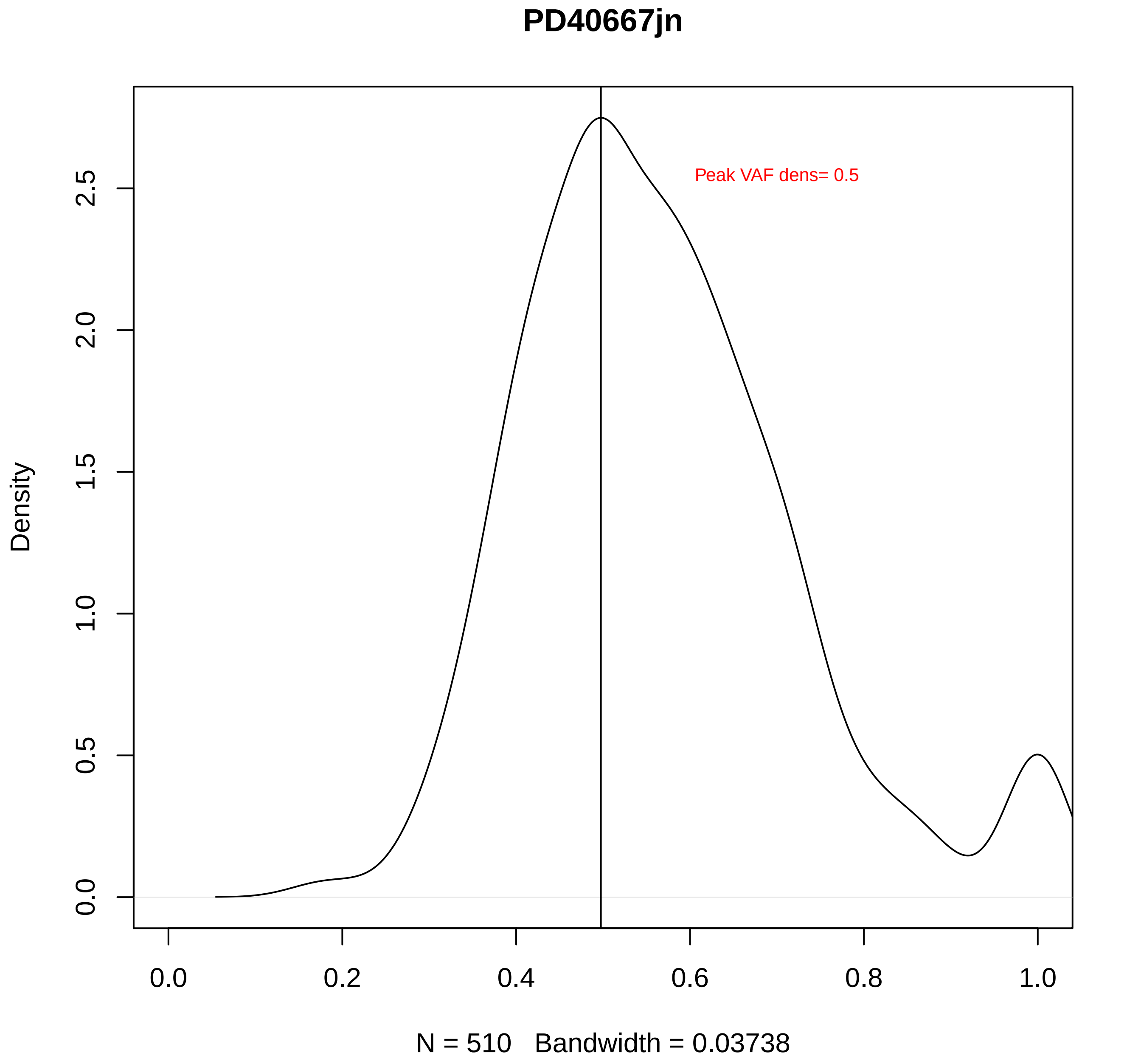

PD40667jn
Peak VAF dens= 0.5
Density
0.0
0.5
1.0
1.5
2.0
2.5
0.0
0.2
0.4
0.6
0.8
1.0
N = 510   Bandwidth = 0.03738

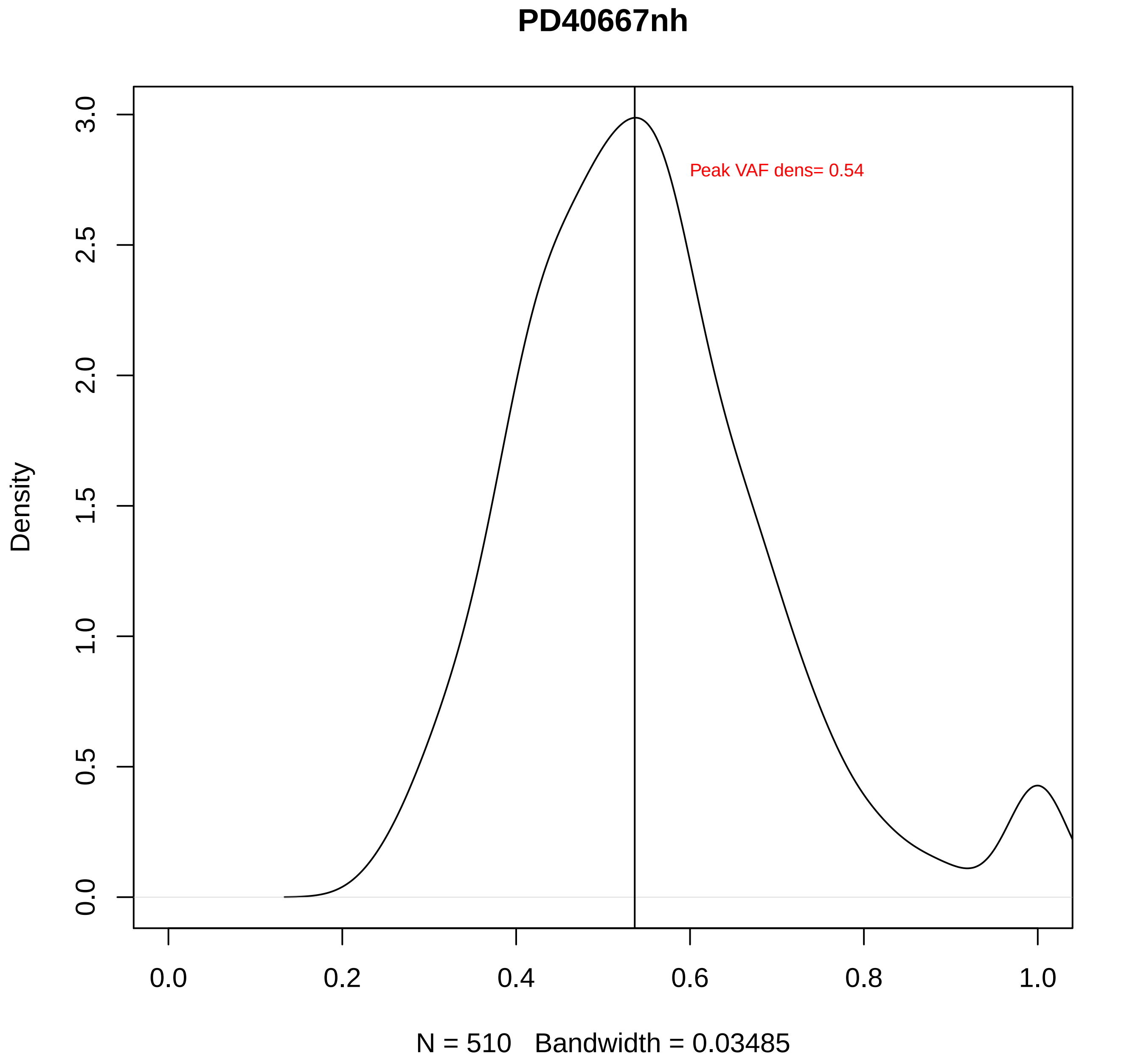
PD40667nh
Peak VAF dens= 0.54
Density
3.0
2.5
2.0
1.5
1.0
0.5
0.0
0.0
0.2
0.4
0.6
0.8
1.0
N = 510   Bandwidth = 0.03485

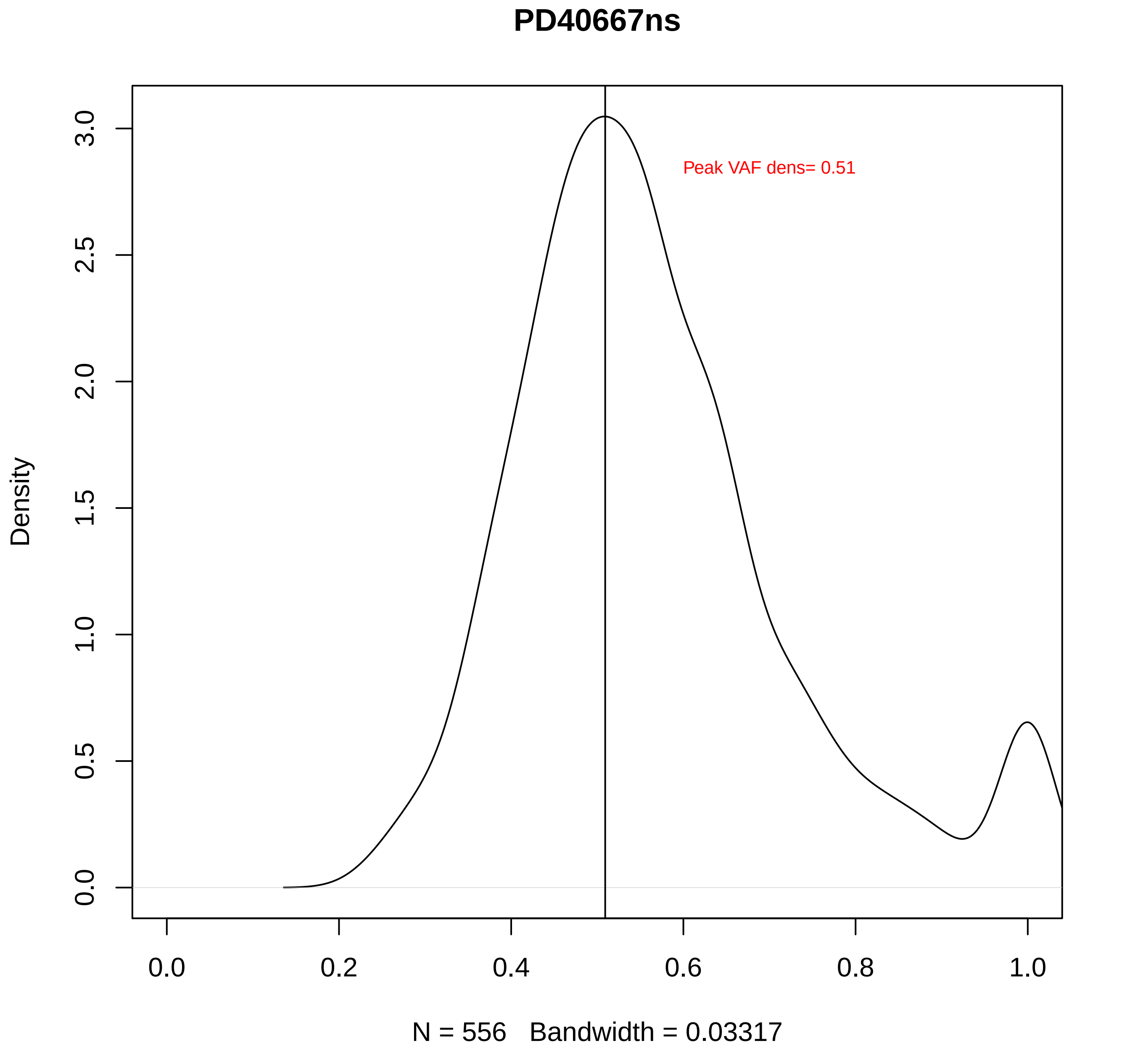

PD40667ns
Peak VAF dens= 0.51
Density
0.0
0.5
1.0
1.5
2.0
2.5
3.0
0.0
0.2
0.4
0.6
0.8
1.0
N = 556   Bandwidth = 0.03317

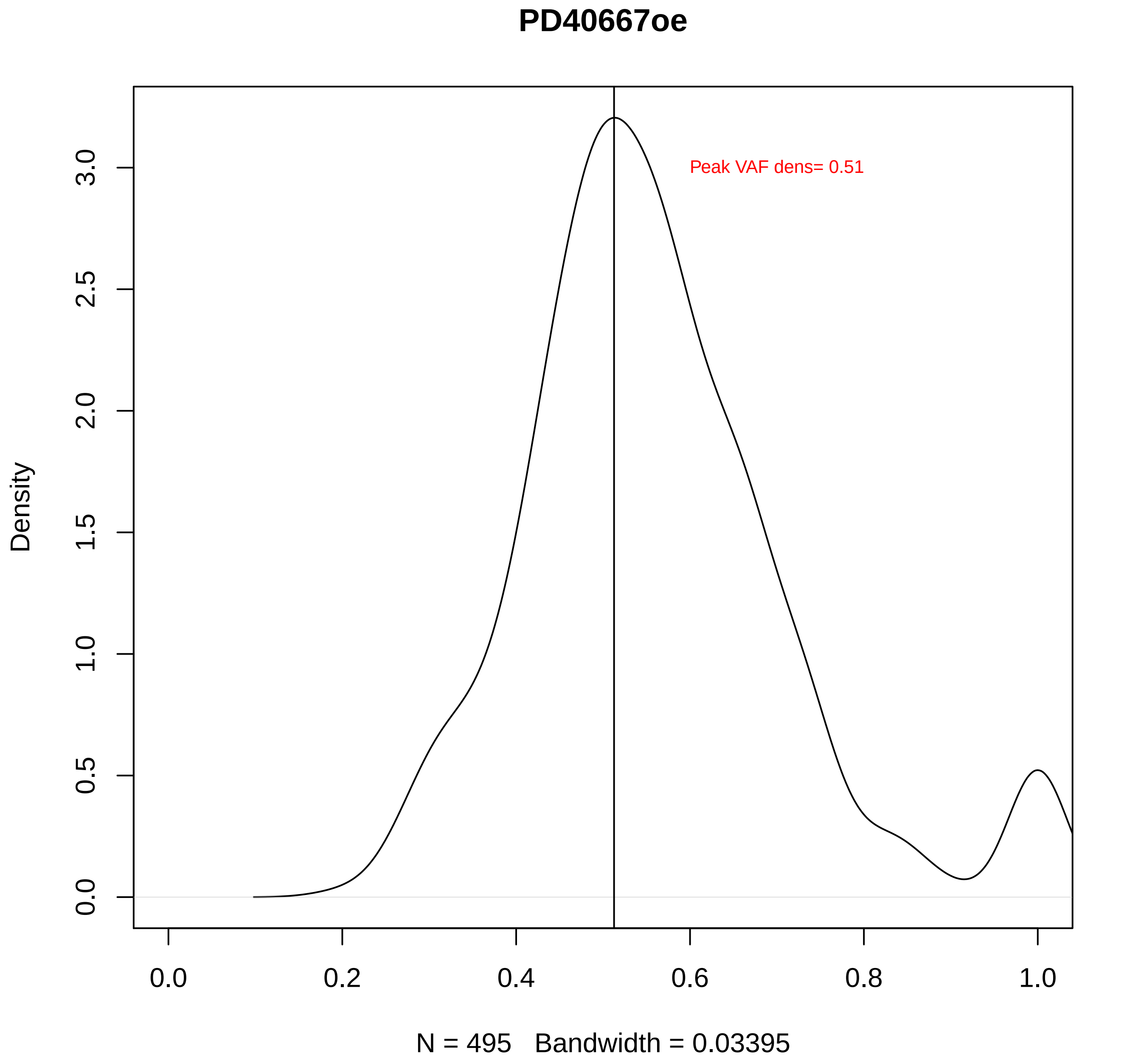
PD40667oe
Peak VAF dens= 0.51
Density
0.0
0.5
1.0
1.5
2.0
2.5
3.0
0.0
0.2
0.4
0.6
0.8
1.0
N = 495   Bandwidth = 0.03395

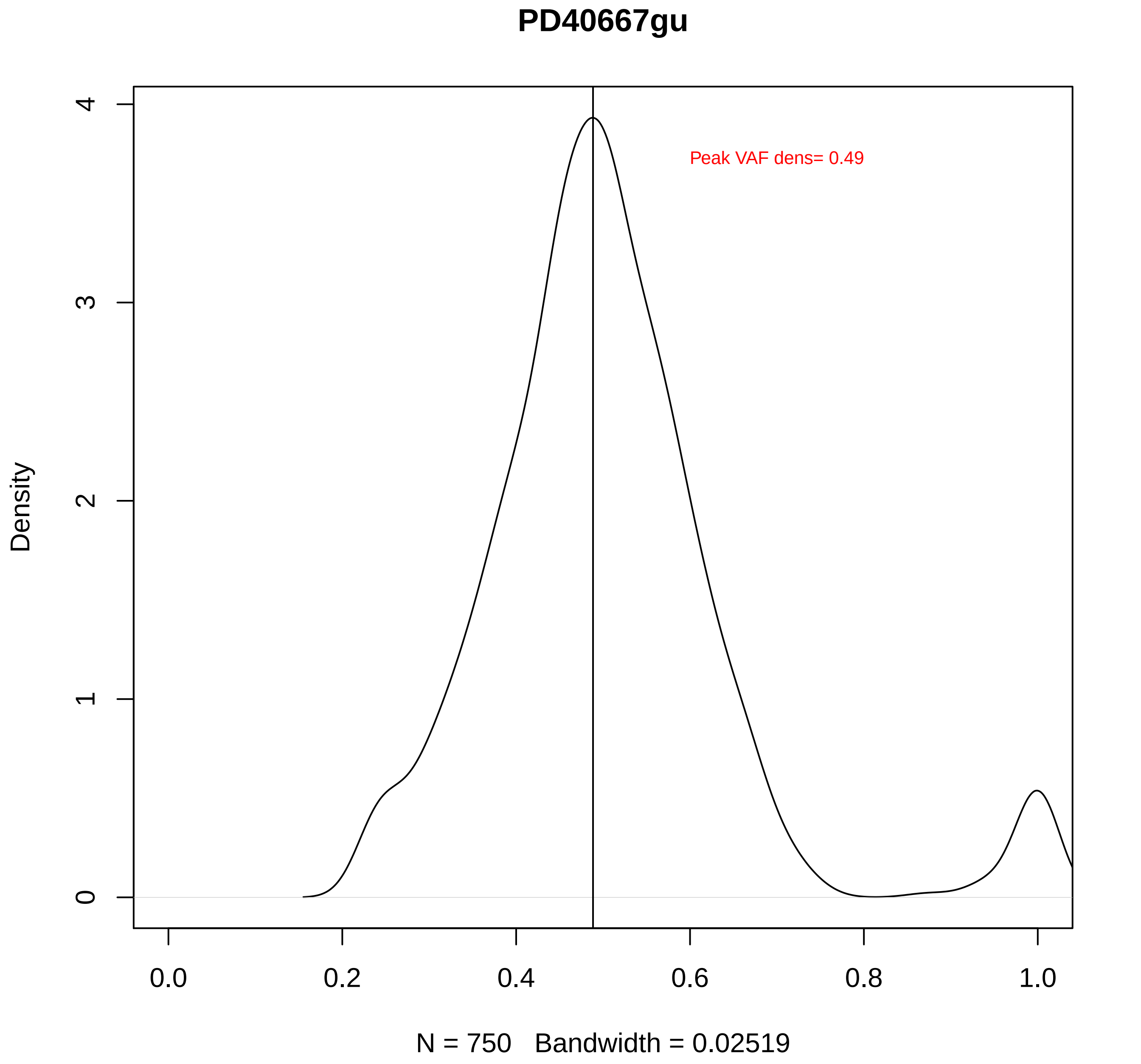

PD40667gu
Peak VAF dens= 0.49
Density
0
1
2
3
4
0.0
0.2
0.4
0.6
0.8
1.0
N = 750 Bandwidth = 0.02519

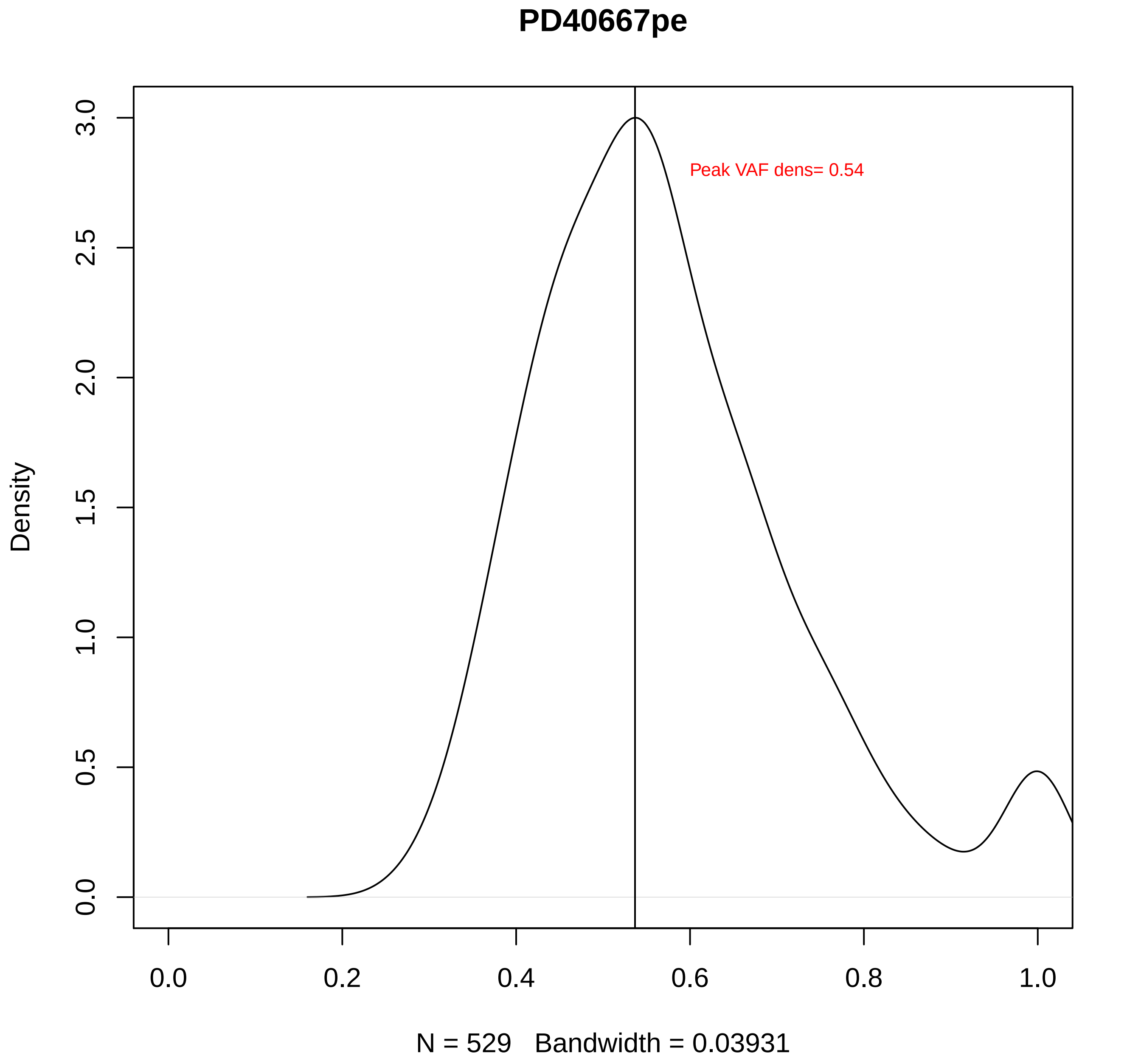

PD40667pe
Peak VAF dens= 0.54
Density
3.0
2.5
2.0
1.5
1.0
0.5
0.0
0.0
0.2
0.4
0.6
0.8
1.0
N = 529 Bandwidth = 0.03931

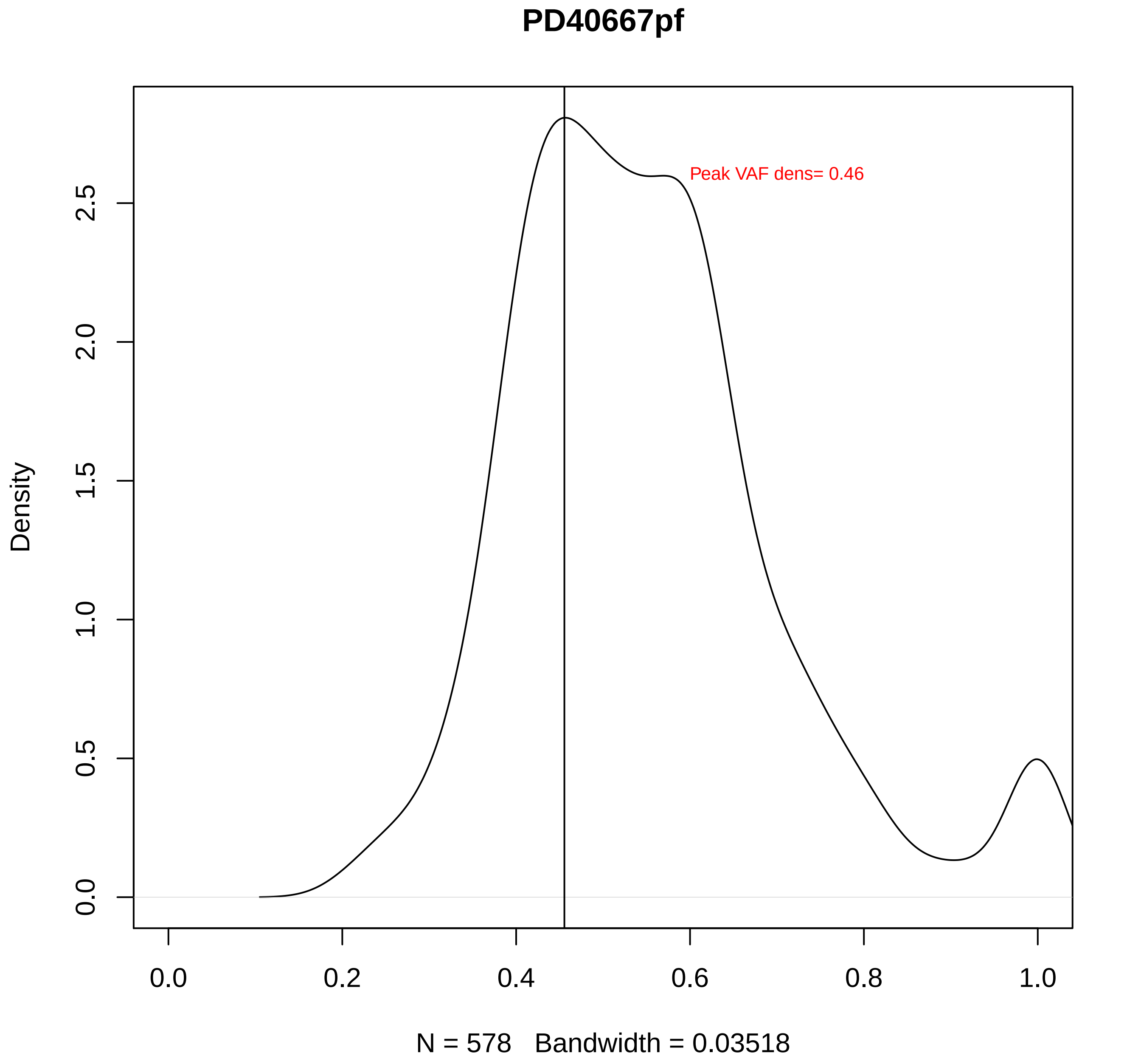

PD40667pf
Peak VAF dens= 0.46
Density
0.0
0.5
1.0
1.5
2.0
2.5
0.0
0.2
0.4
0.6
0.8
1.0
N = 578   Bandwidth = 0.03518

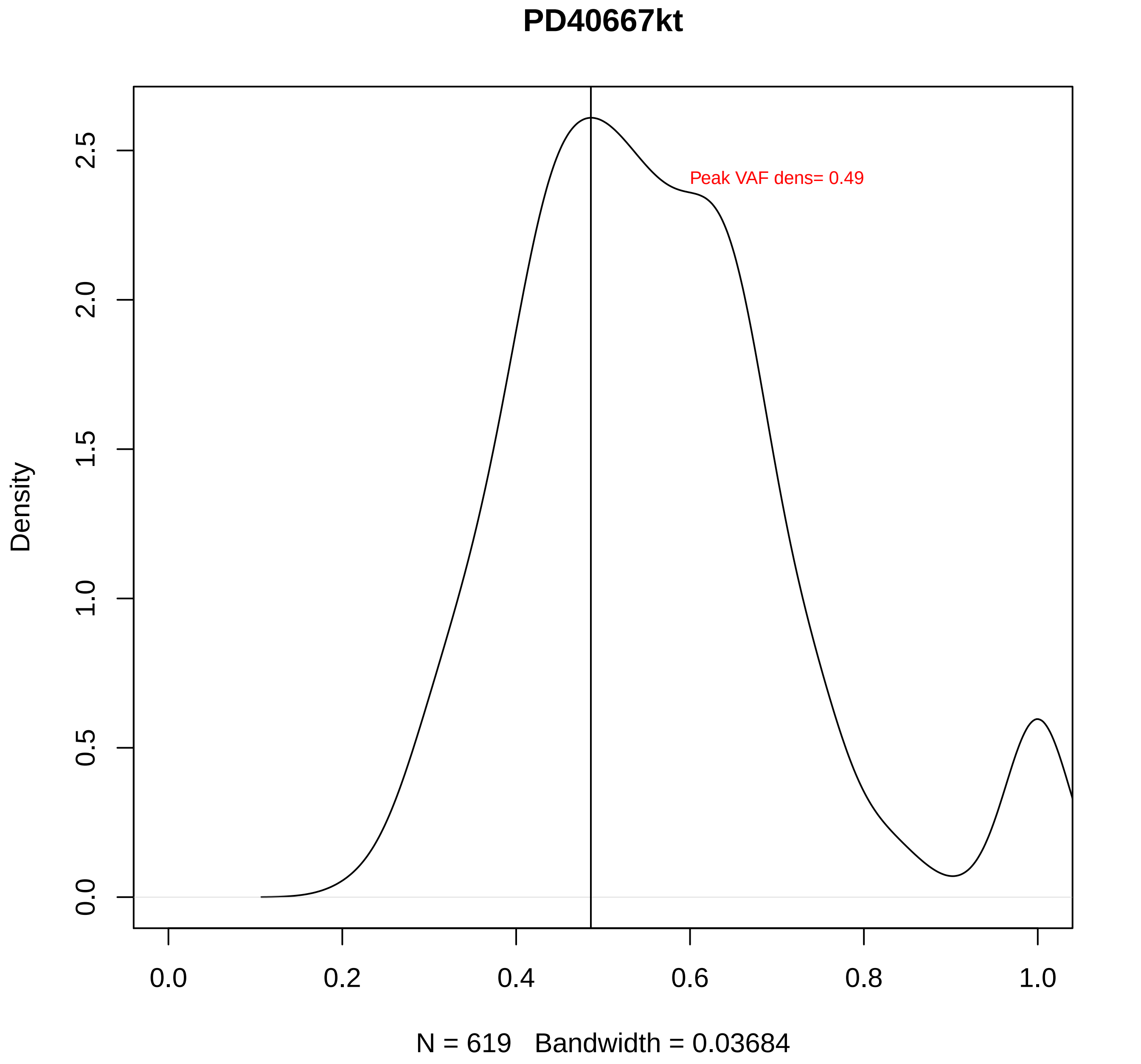
PD40667kt
Peak VAF dens= 0.49
Density
0.0
0.5
1.0
1.5
2.0
2.5
0.0
0.2
0.4
0.6
0.8
1.0
N = 619   Bandwidth = 0.03684

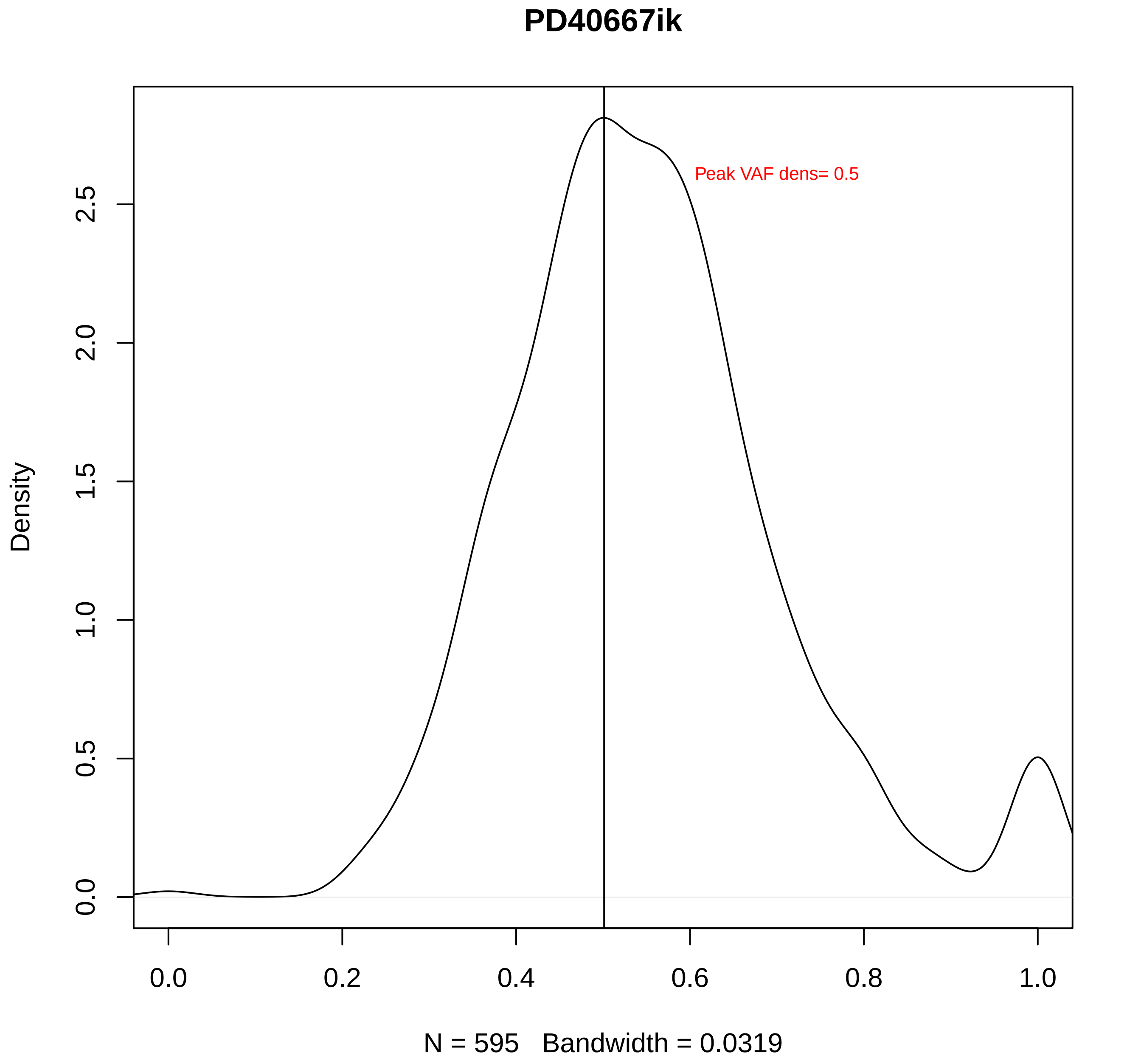
PD40667ik
Peak VAF dens= 0.5
Density
0.0
0.5
1.0
1.5
2.0
2.5
0.0
0.2
0.4
0.6
0.8
1.0
N = 595   Bandwidth = 0.0319

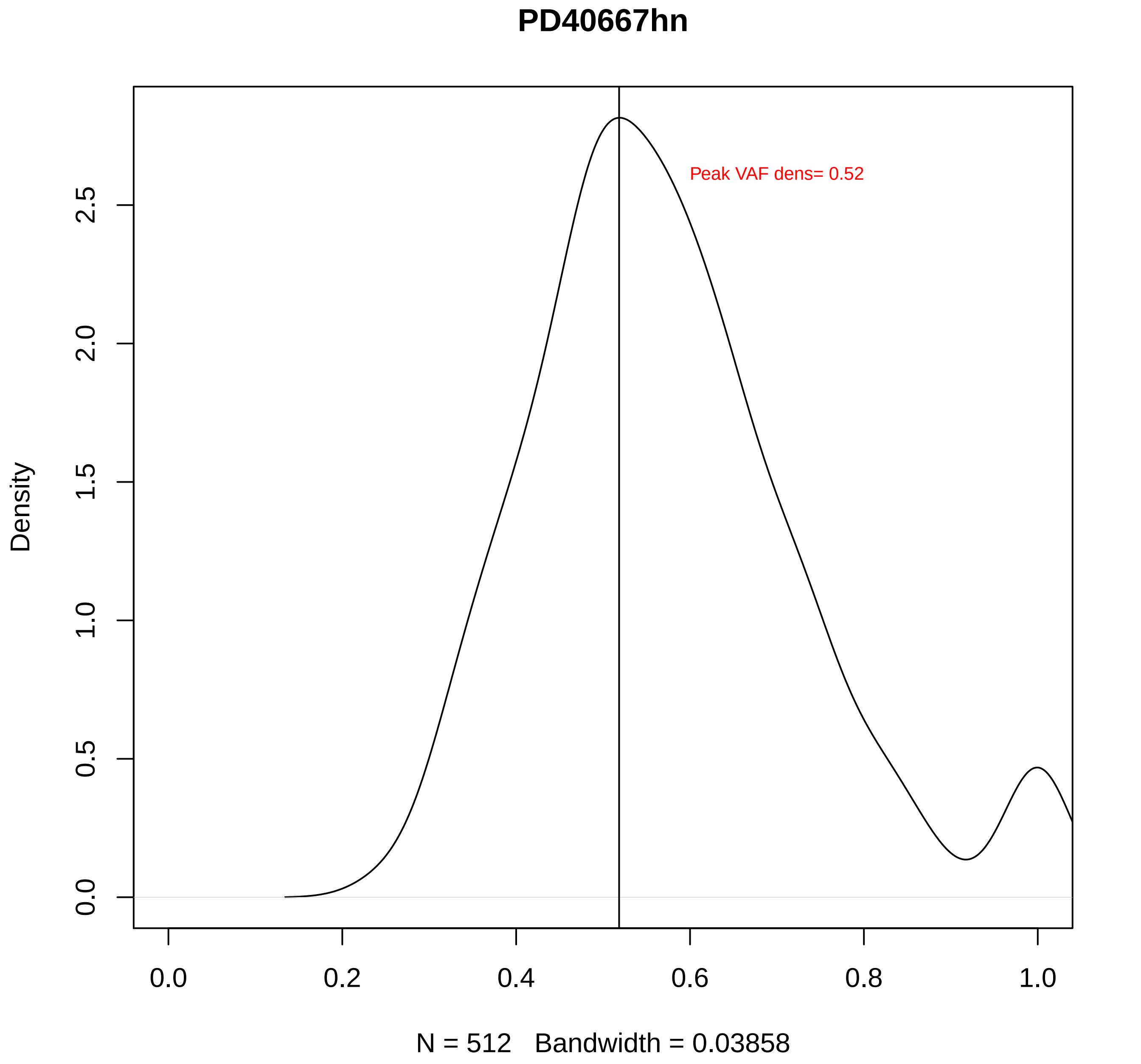
PD40667hn
Peak VAF dens= 0.52
Density
0.0
0.5
1.0
1.5
2.0
2.5
0.0
0.2
0.4
0.6
0.8
1.0
N = 512 Bandwidth = 0.03858

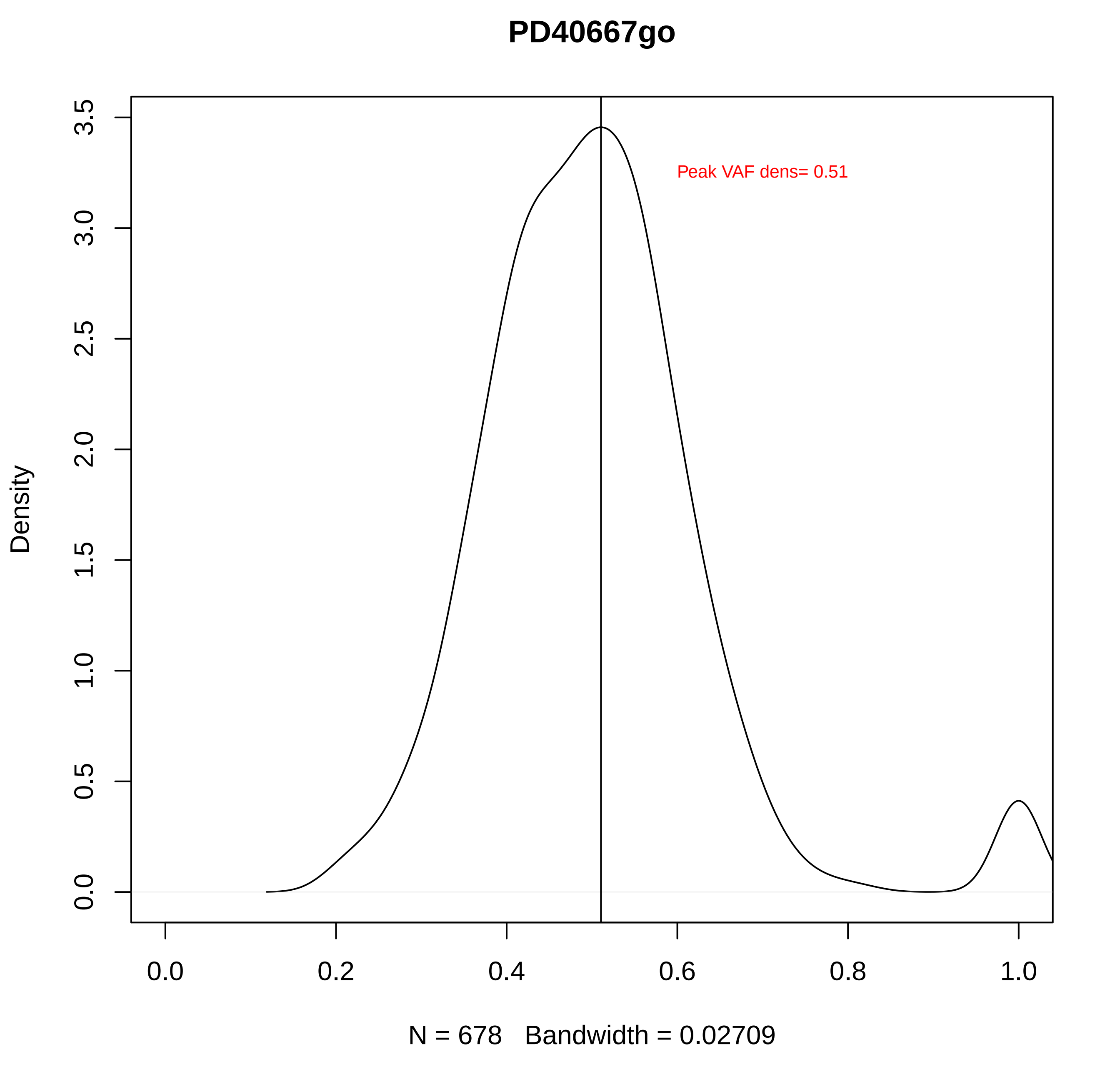

PD40667go
Peak VAF dens= 0.51
Density
0.0
0.5
1.0
1.5
2.0
2.5
3.0
3.5
0.0
0.2
0.4
0.6
0.8
1.0
N = 678   Bandwidth = 0.02709

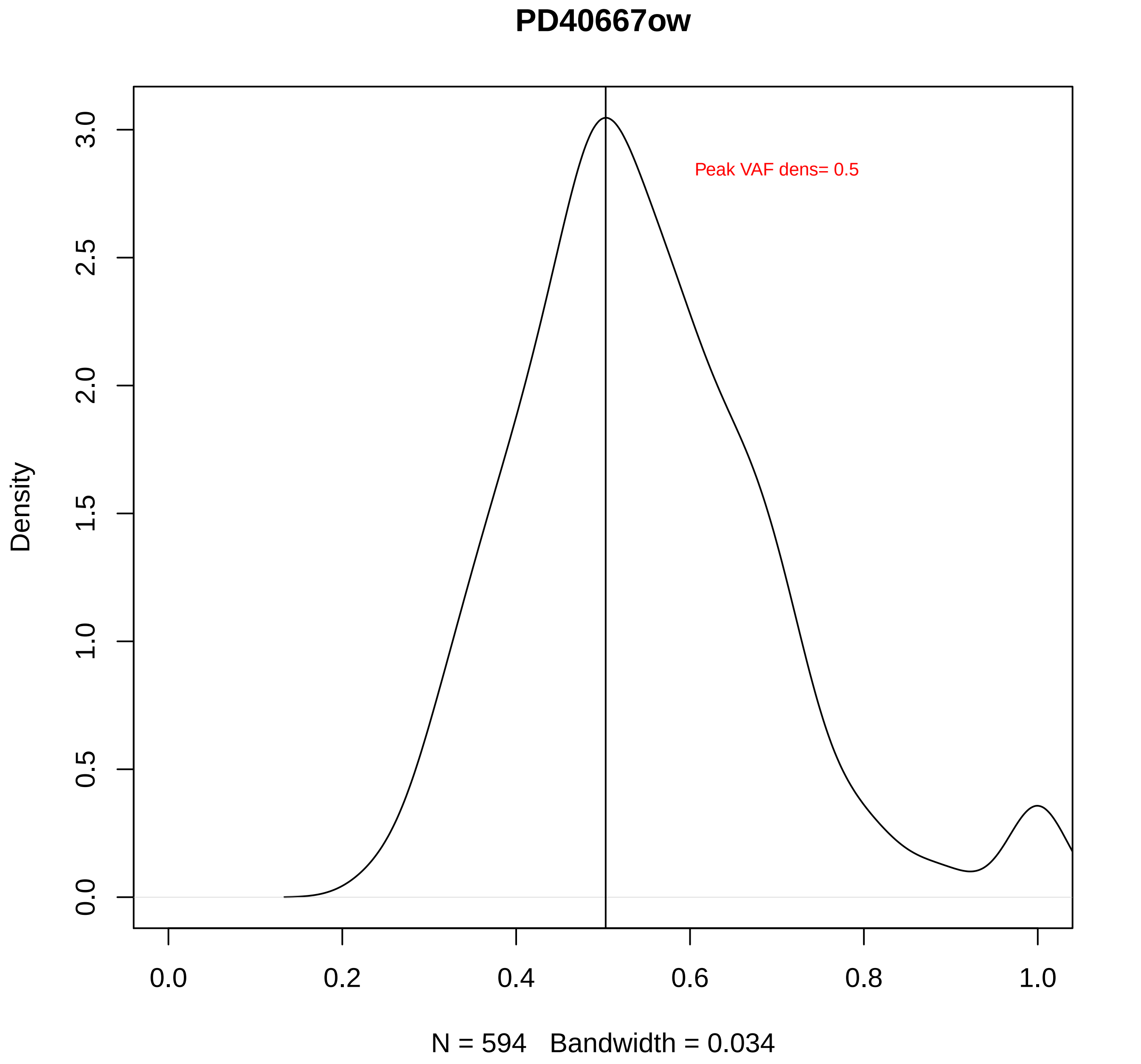

PD40667ow
Peak VAF dens= 0.5
Density
0.0
0.5
1.0
1.5
2.0
2.5
3.0
0.0
0.2
0.4
0.6
0.8
1.0
N = 594   Bandwidth = 0.034

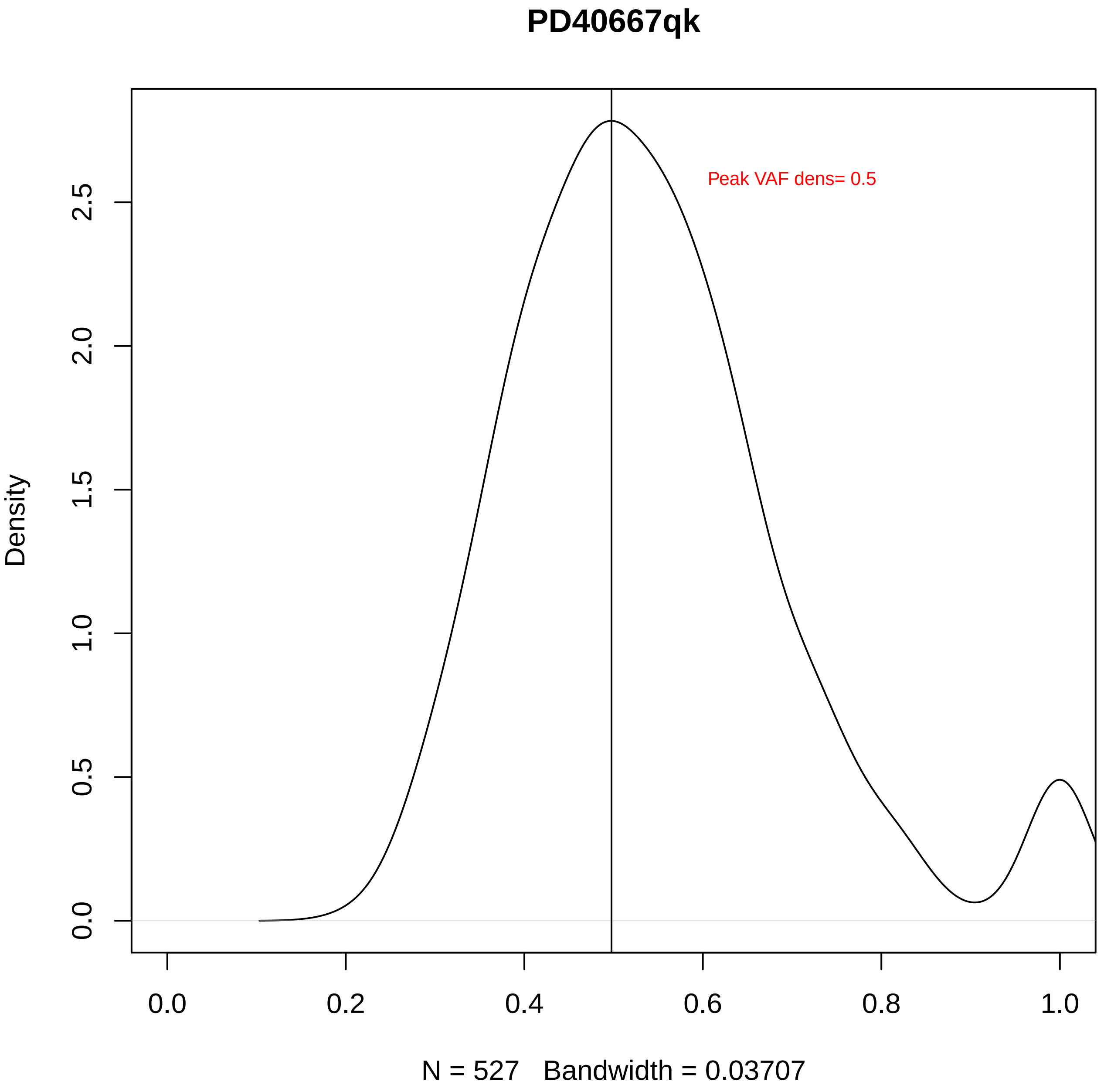
PD40667qk
Peak VAF dens= 0.5
Density
0.0
0.5
1.0
1.5
2.0
2.5
0.0
0.2
0.4
0.6
0.8
1.0
N = 527   Bandwidth = 0.03707

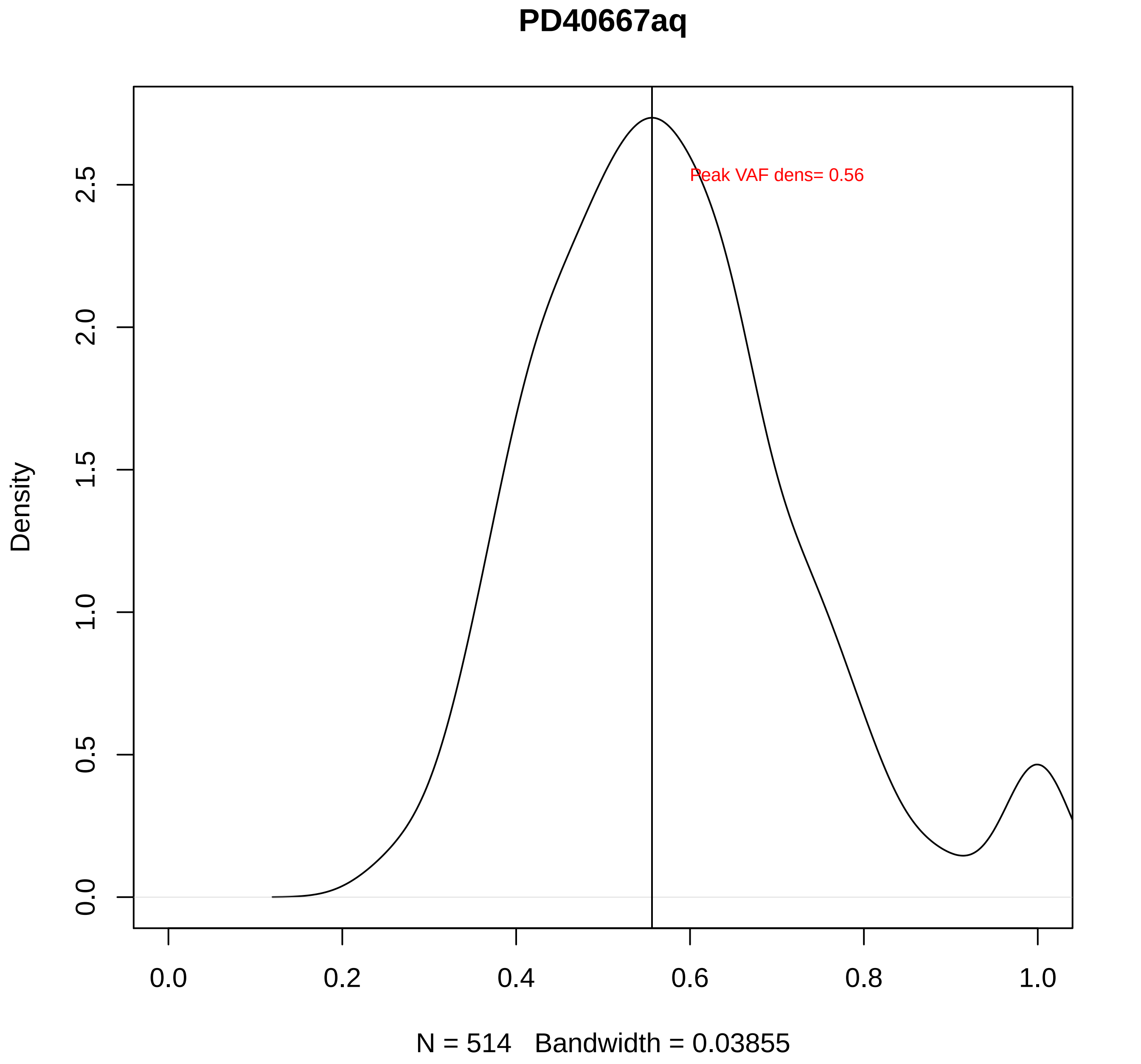
PD40667aq
Peak VAF dens= 0.56
Density
0.0
0.5
1.0
1.5
2.0
2.5
0.0
0.2
0.4
0.6
0.8
1.0
N = 514   Bandwidth = 0.03855

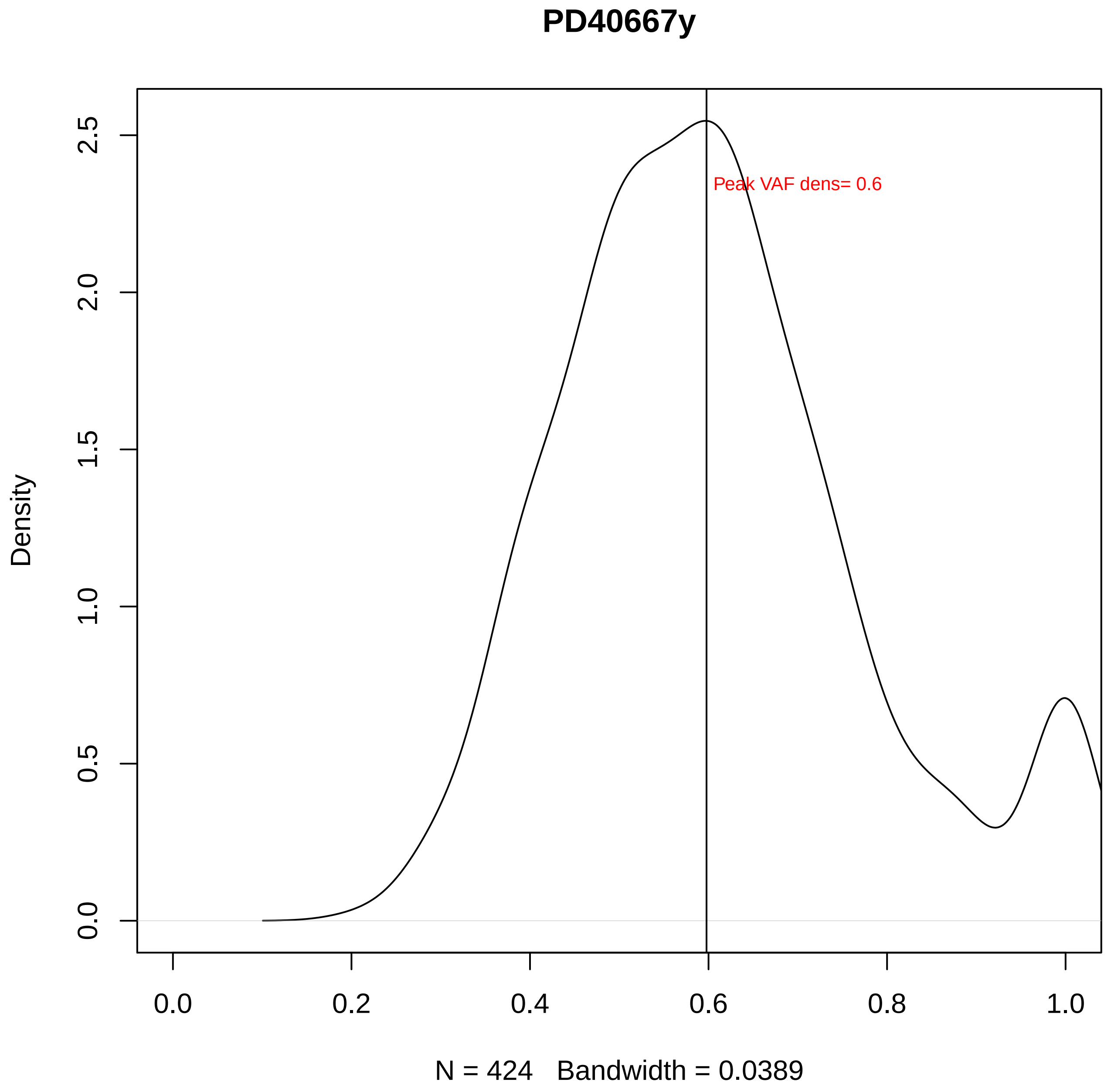
PD40667y
Peak VAF dens= 0.6
Density
0.0
0.5
1.0
1.5
2.0
2.5
0.0
0.2
0.4
0.6
0.8
1.0
N = 424   Bandwidth = 0.0389

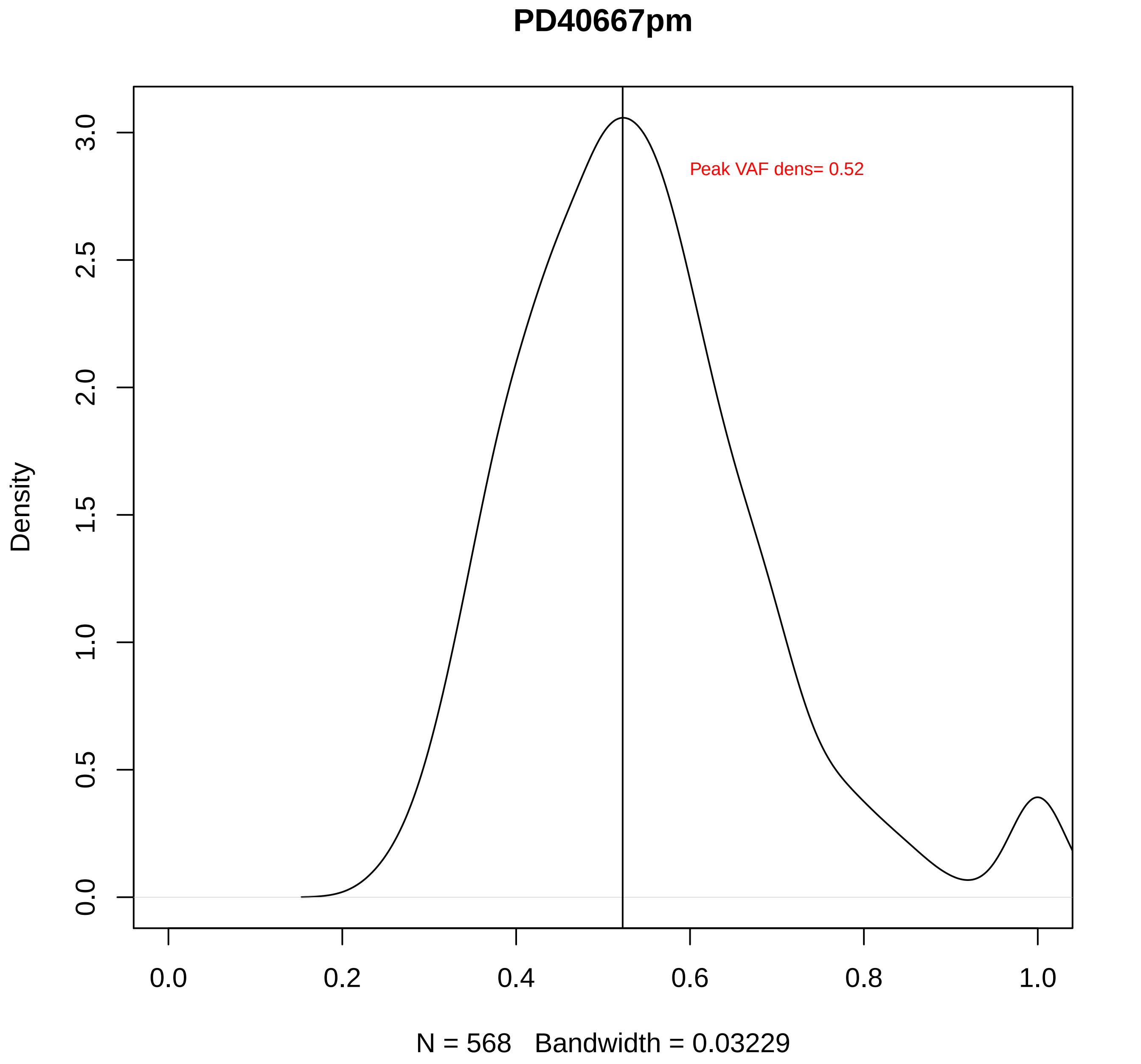
PD40667pm
Peak VAF dens= 0.52
Density
3.0
2.5
2.0
1.5
1.0
0.5
0.0
0.0
0.2
0.4
0.6
0.8
1.0
N = 568   Bandwidth = 0.03229

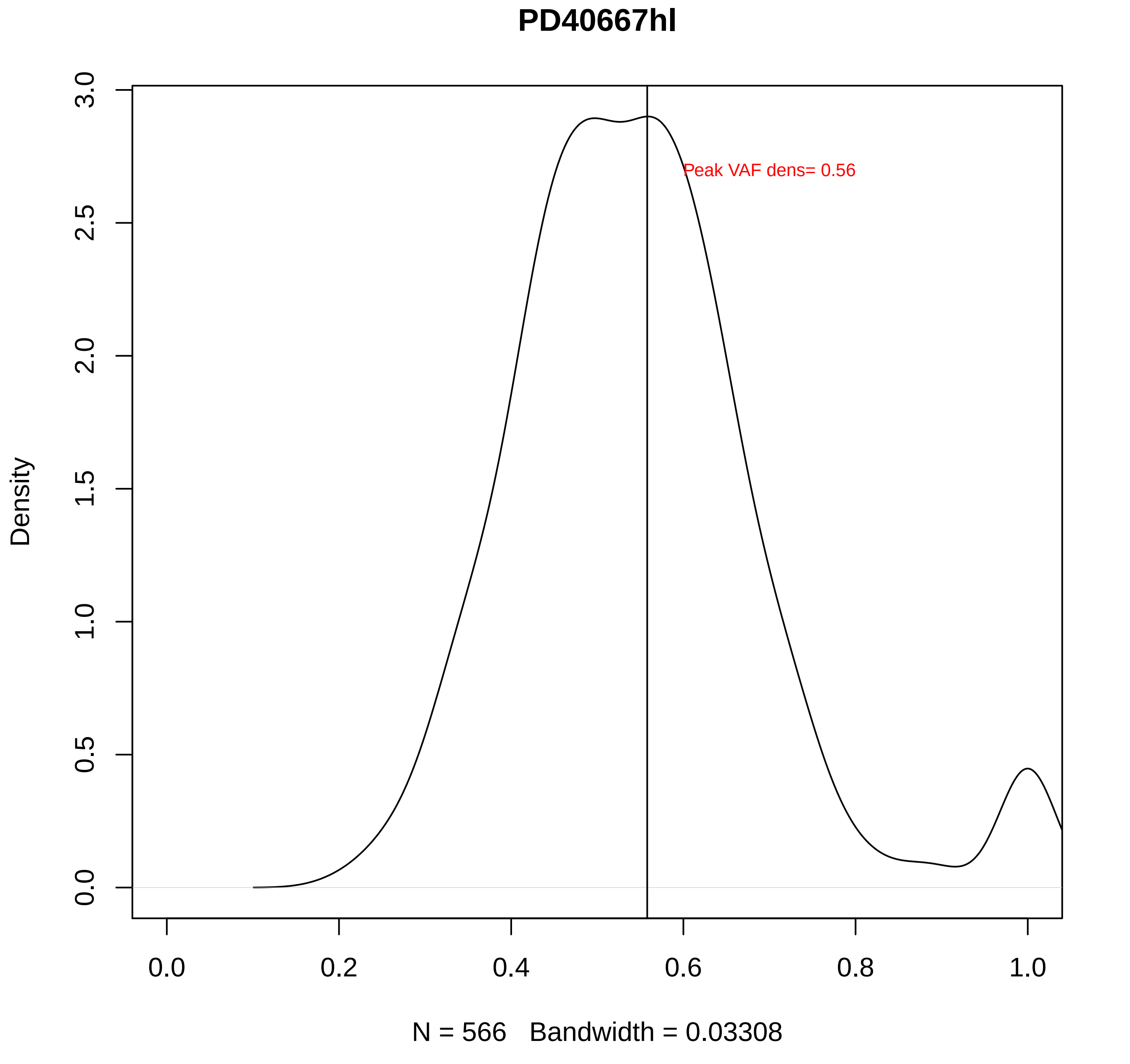

PD40667hl
Peak VAF dens= 0.56
Density
3.0
2.5
2.0
1.5
1.0
0.5
0.0
0.0
0.2
0.4
0.6
0.8
1.0
N = 566   Bandwidth = 0.03308

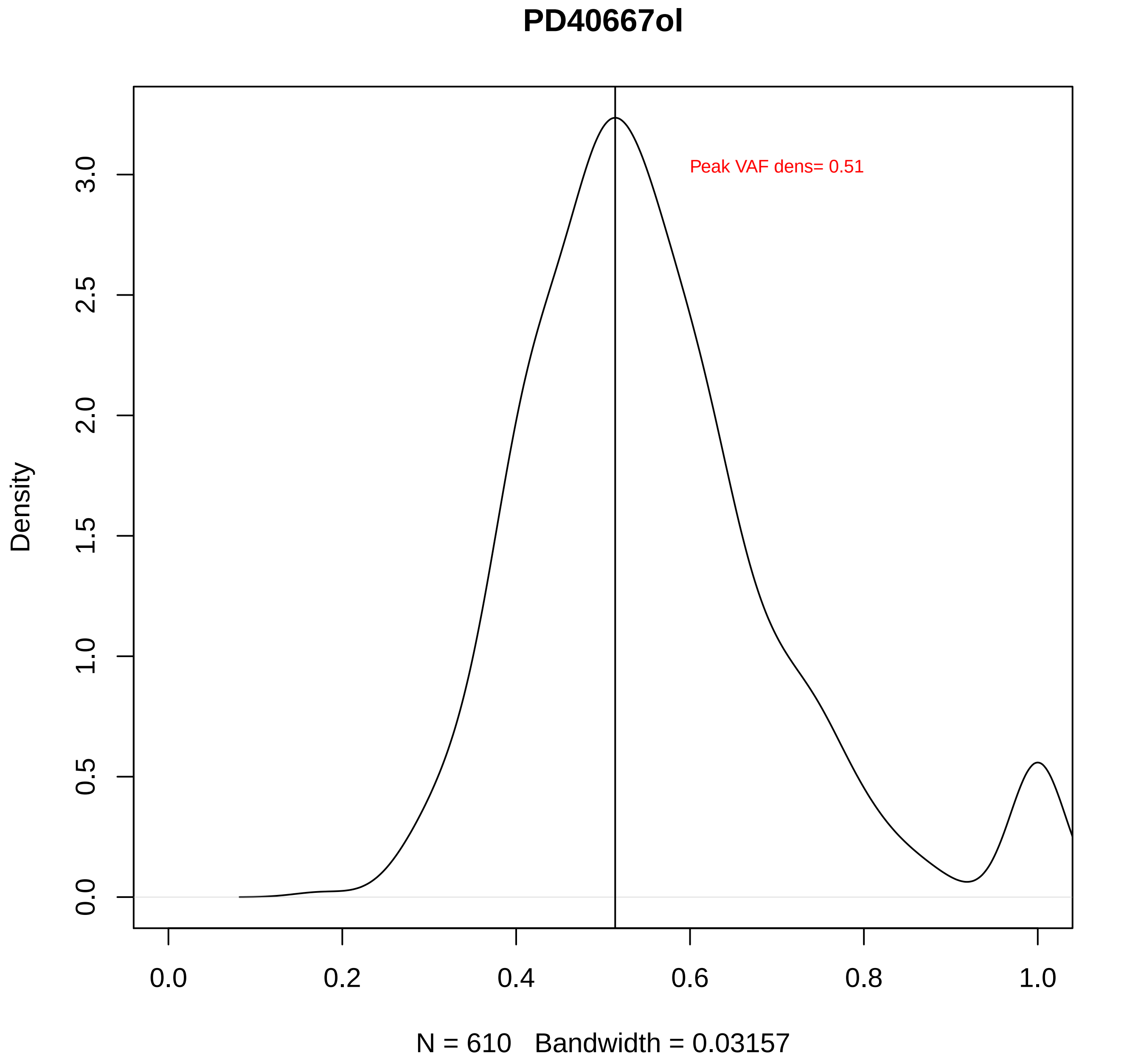

PD40667ol
Peak VAF dens= 0.51
Density
0.0
0.5
1.0
1.5
2.0
2.5
3.0
0.0
0.2
0.4
0.6
0.8
1.0
N = 610   Bandwidth = 0.03157

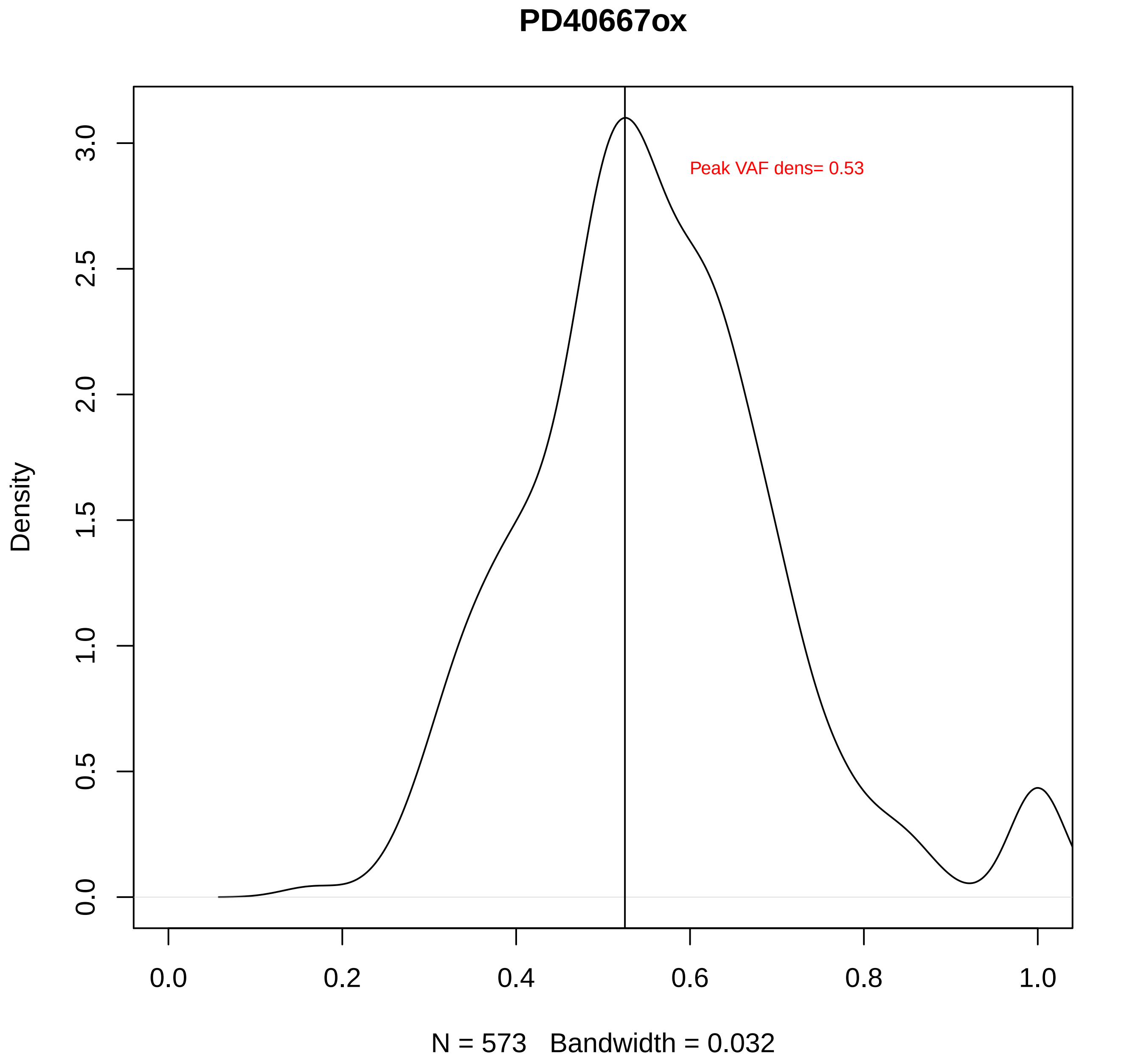
PD40667ox
Peak VAF dens= 0.53
Density
0.0
0.5
1.0
1.5
2.0
2.5
3.0
0.0
0.2
0.4
0.6
0.8
1.0
N = 573 Bandwidth = 0.032

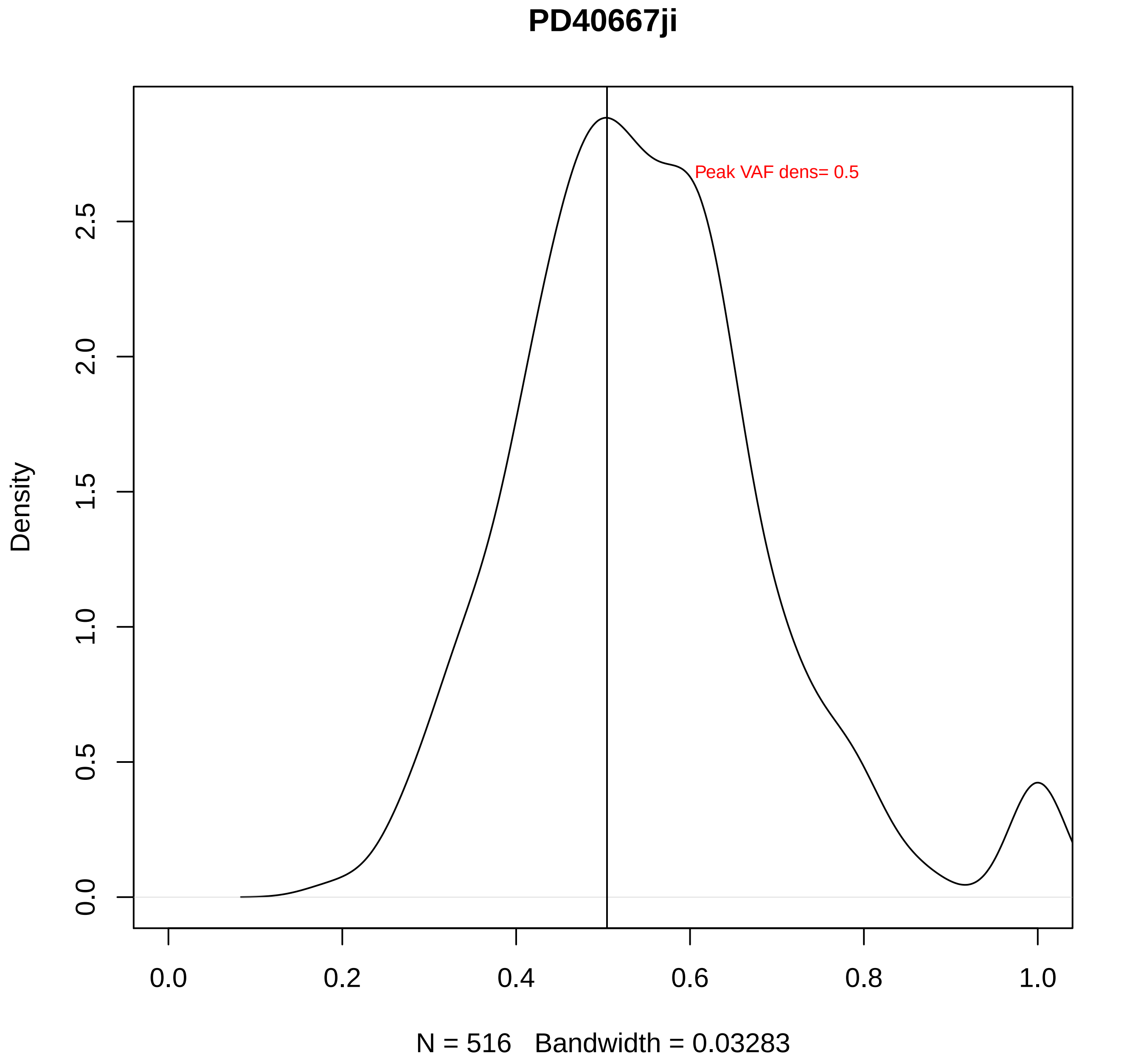

PD40667ji
Peak VAF dens= 0.5
Density
0.0
0.5
1.0
1.5
2.0
2.5
0.0
0.2
0.4
0.6
0.8
1.0
N = 516   Bandwidth = 0.03283

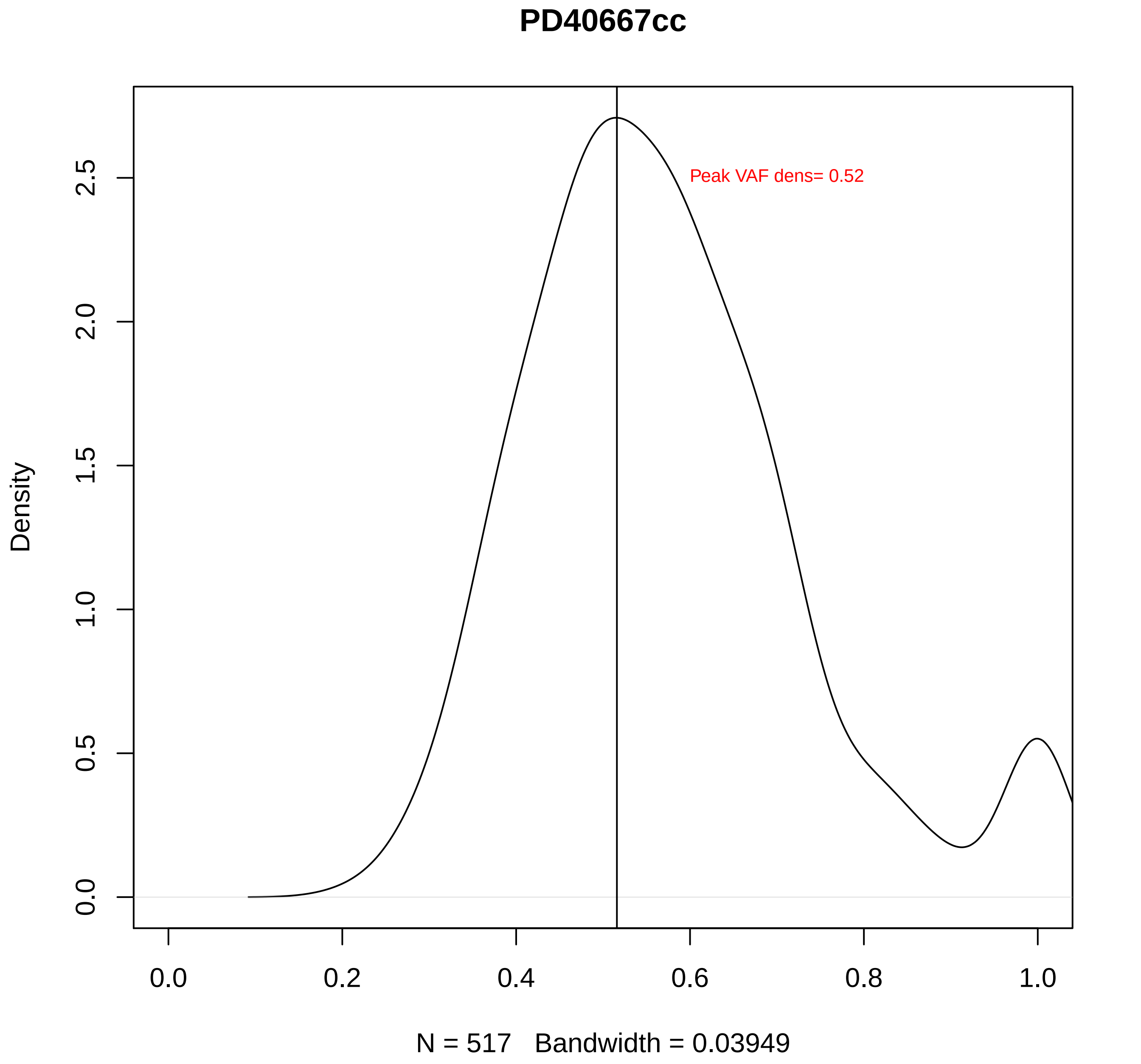

PD40667cc
Peak VAF dens= 0.52
Density
0.0
0.5
1.0
1.5
2.0
2.5
0.0
0.2
0.4
0.6
0.8
1.0
N = 517   Bandwidth = 0.03949

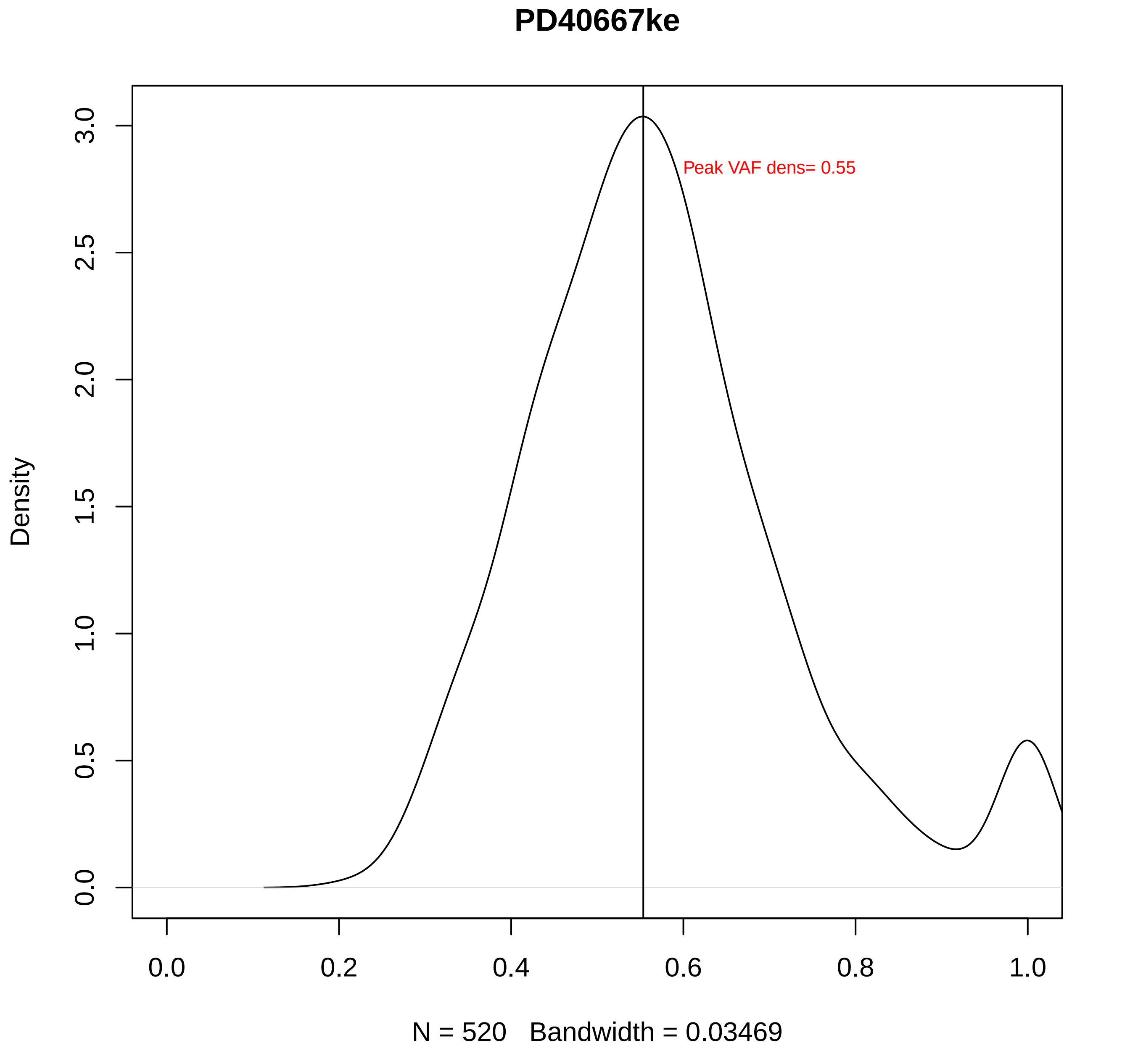
PD40667ke
Peak VAF dens= 0.55
Density
0.0
0.5
1.0
1.5
2.0
2.5
3.0
0.0
0.2
0.4
0.6
0.8
1.0
N = 520   Bandwidth = 0.03469

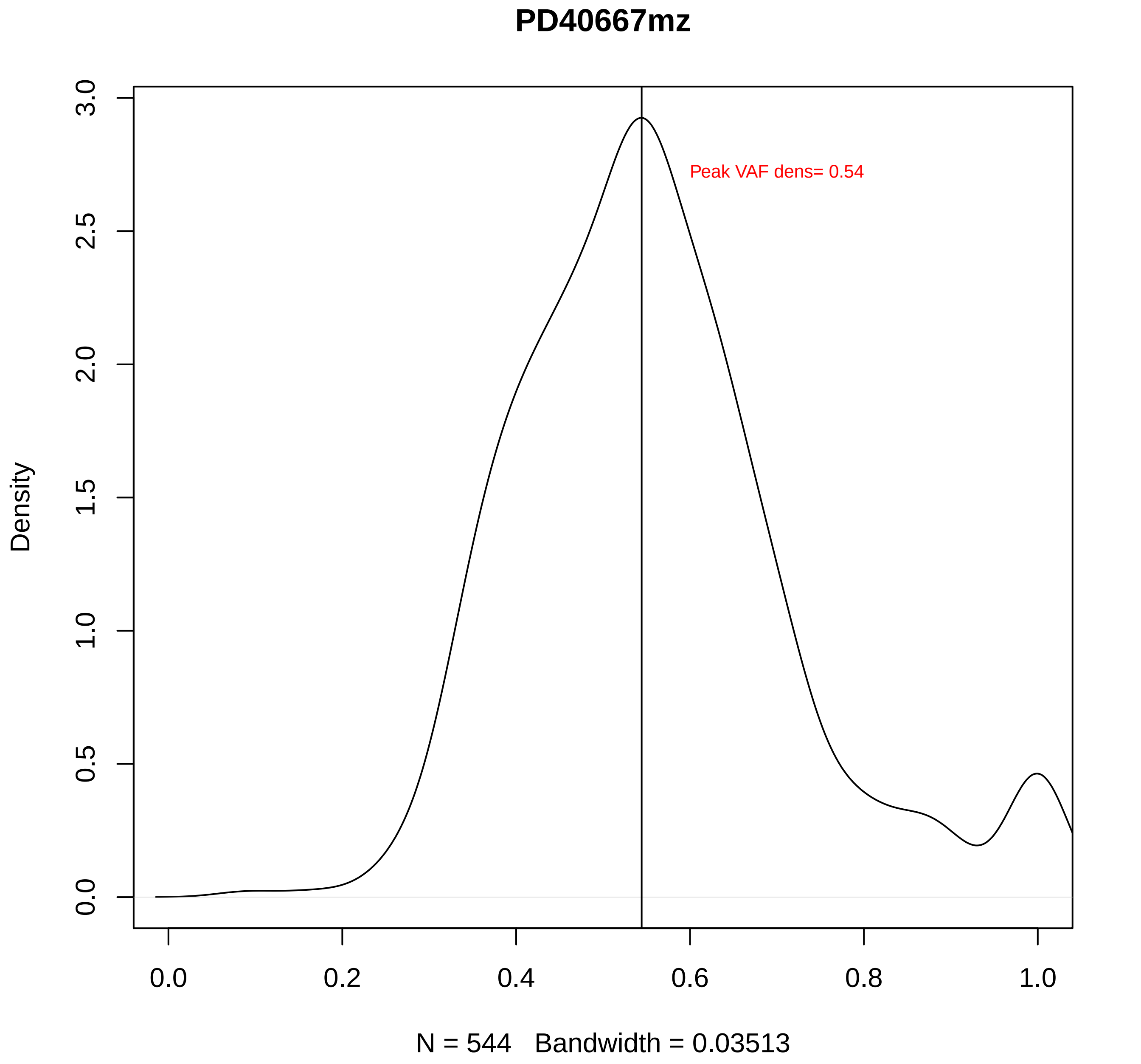

PD40667mz
Peak VAF dens= 0.54
Density
3.0
2.5
2.0
1.5
1.0
0.5
0.0
0.0
0.2
0.4
0.6
0.8
1.0
N = 544   Bandwidth = 0.03513

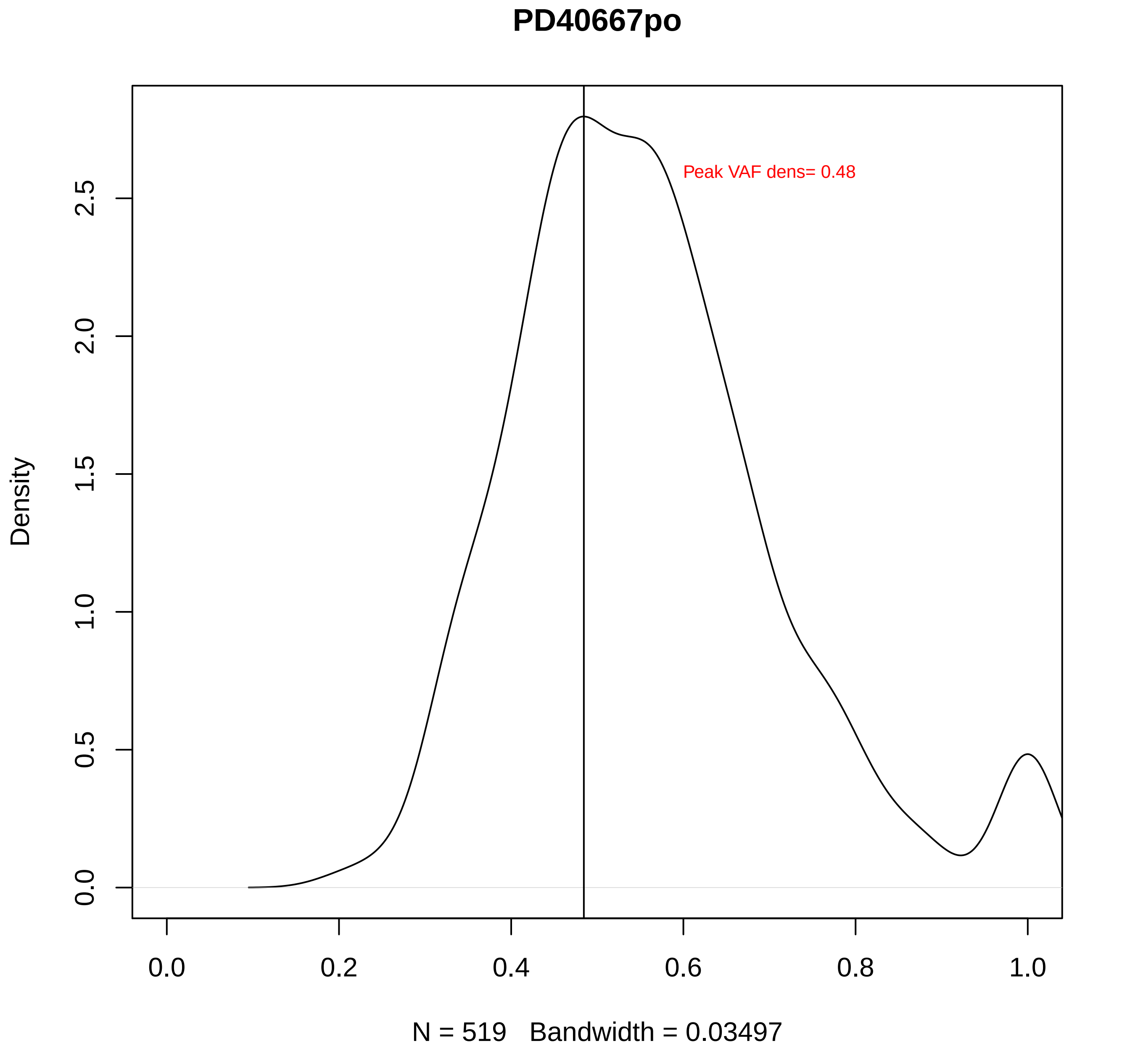

PD40667po
Peak VAF dens= 0.48
Density
0.0
0.5
1.0
1.5
2.0
2.5
0.0
0.2
0.4
0.6
0.8
1.0
N = 519   Bandwidth = 0.03497

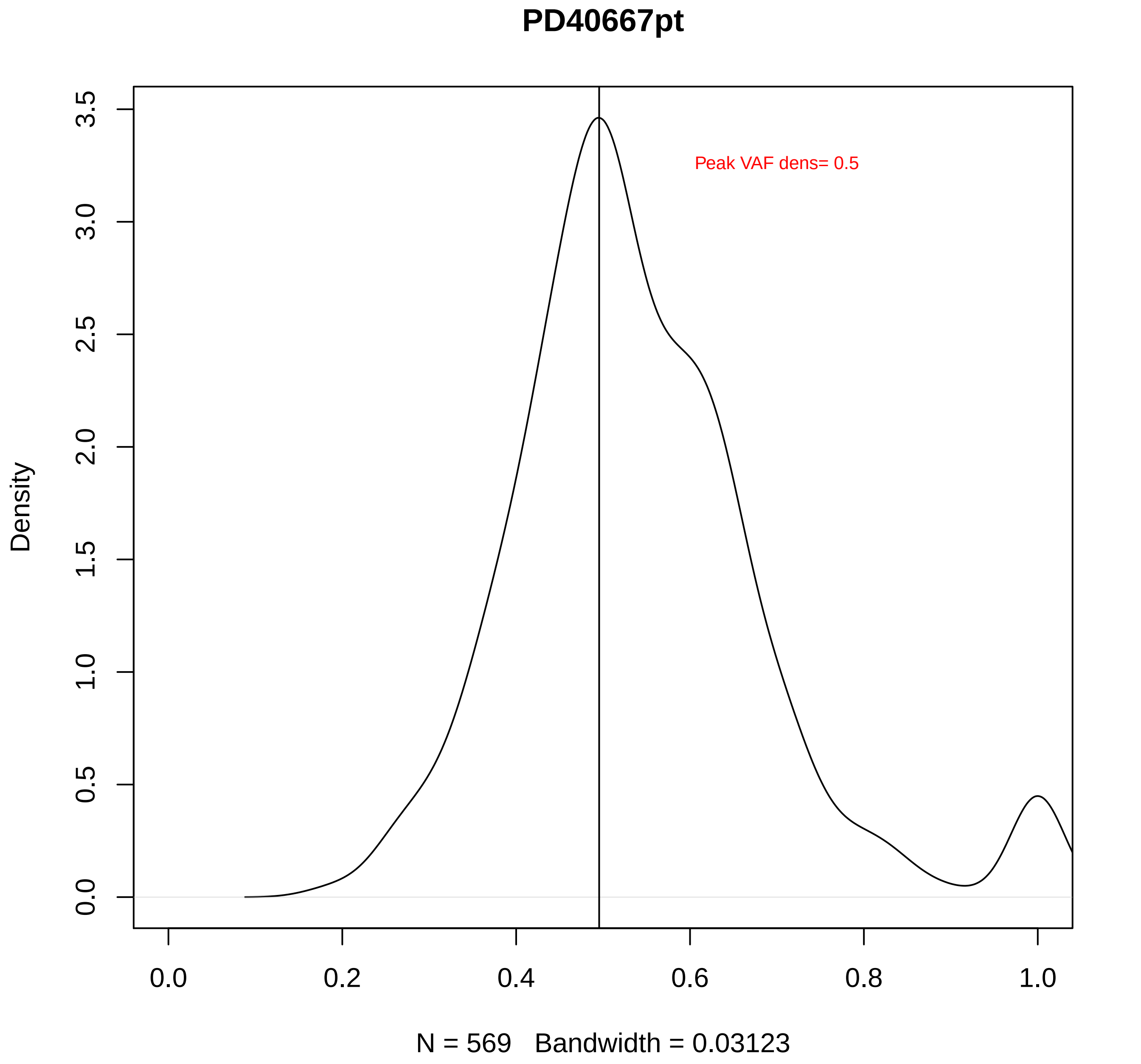

PD40667pt
Peak VAF dens= 0.5
Density
0.0
0.5
1.0
1.5
2.0
2.5
3.0
3.5
0.0
0.2
0.4
0.6
0.8
1.0
N = 569   Bandwidth = 0.03123

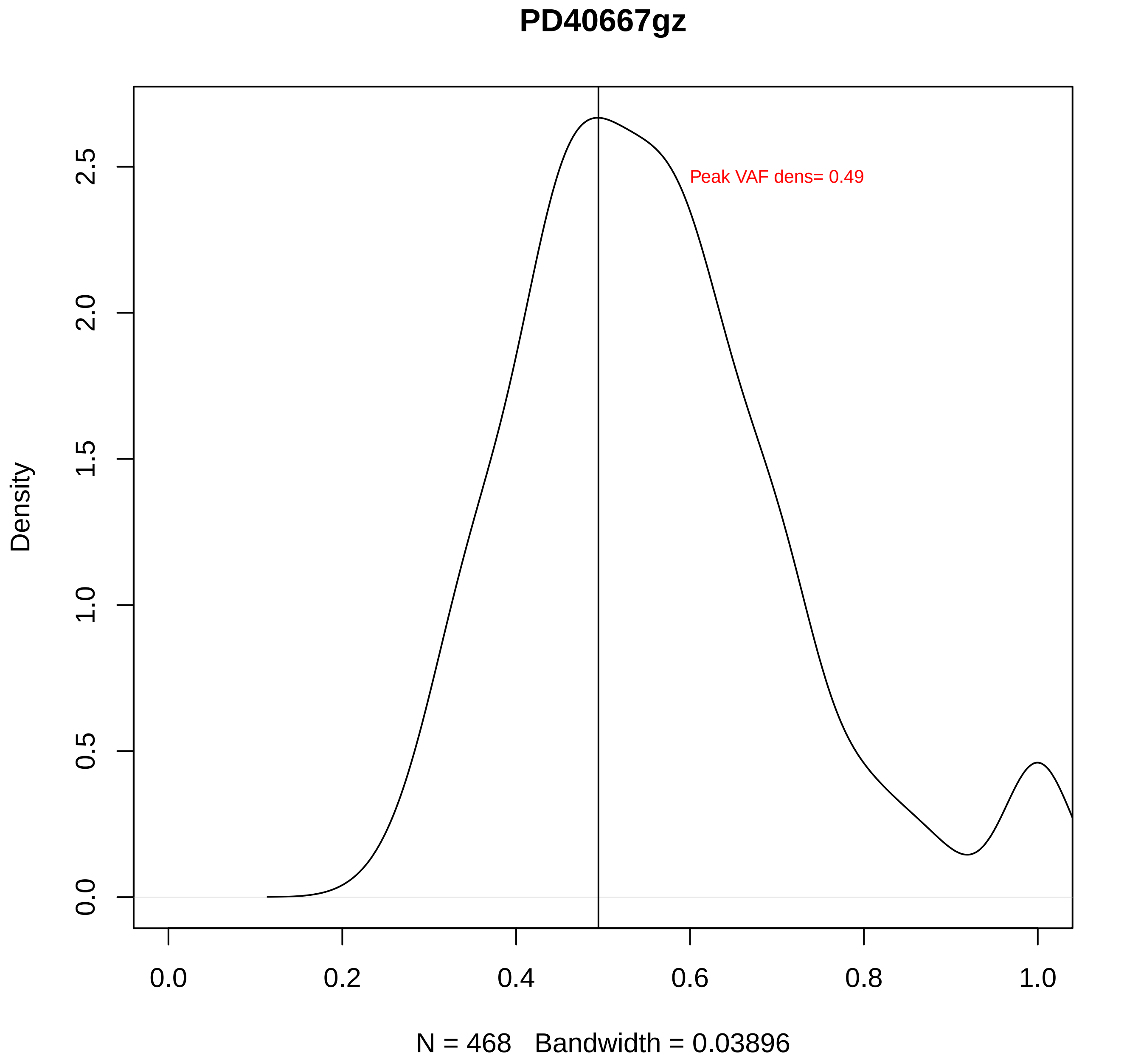

PD40667gz
Peak VAF dens= 0.49
Density
2.5
2.0
1.5
1.0
0.5
0.0
0.0
0.2
0.4
0.6
0.8
1.0
N = 468 Bandwidth = 0.03896

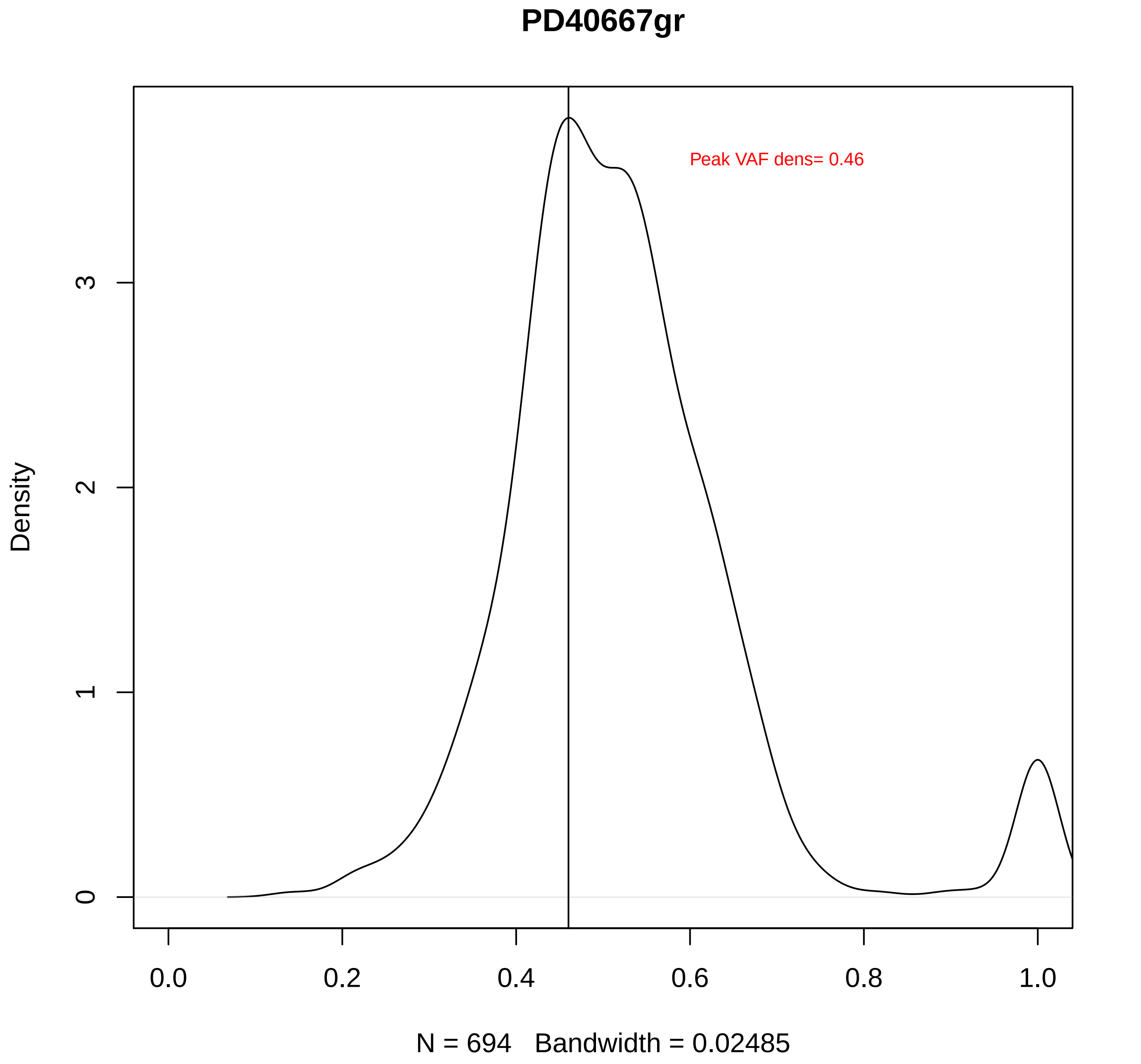
PD40667gr
Peak VAF dens= 0.46
Density
0
1
2
3
0.0
0.2
0.4
0.6
0.8
1.0
N = 694 Bandwidth = 0.02485

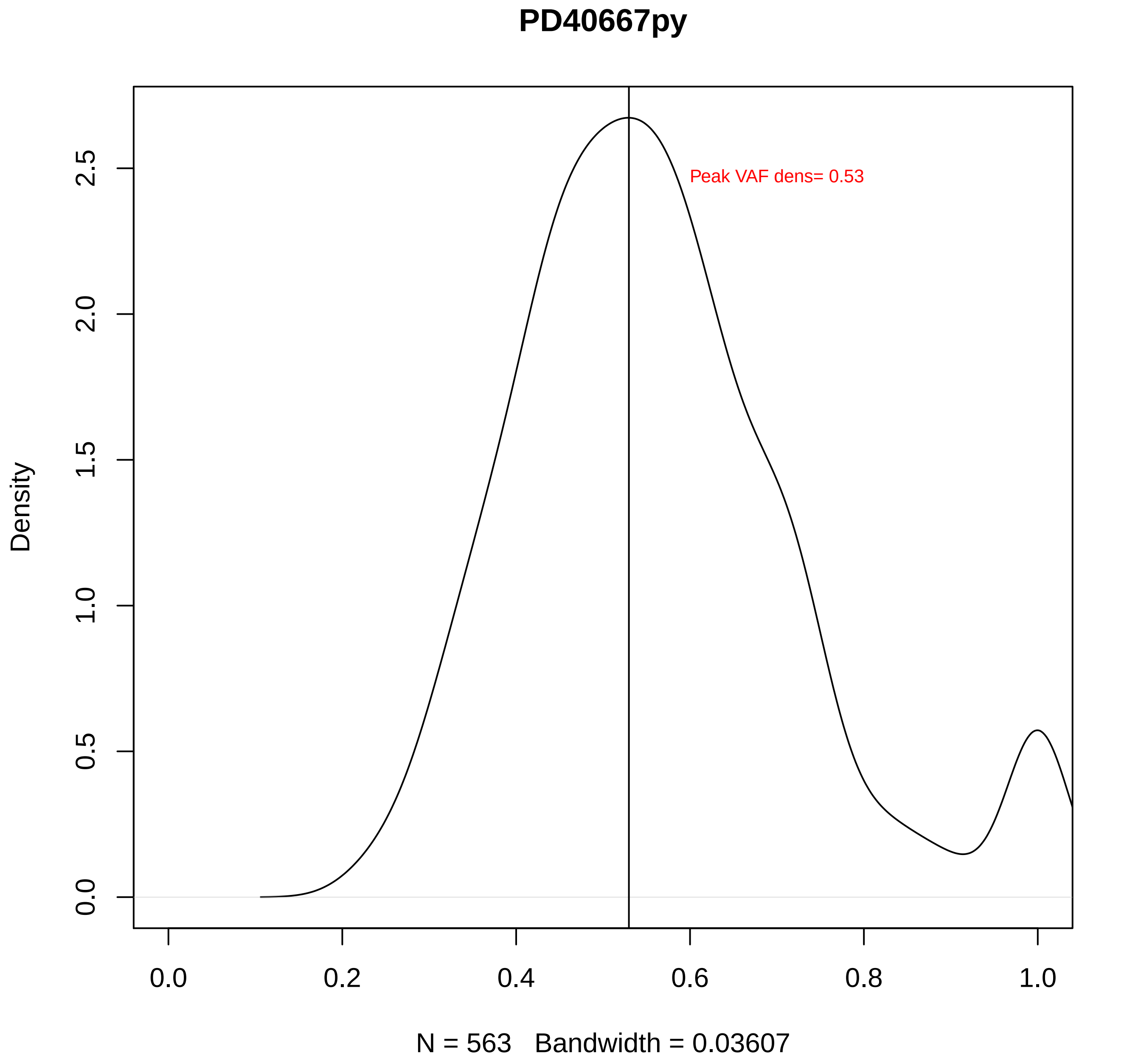
PD40667py
Peak VAF dens= 0.53
Density
0.0
0.5
1.0
1.5
2.0
2.5
0.0
0.2
0.4
0.6
0.8
1.0
N = 563 Bandwidth = 0.03607

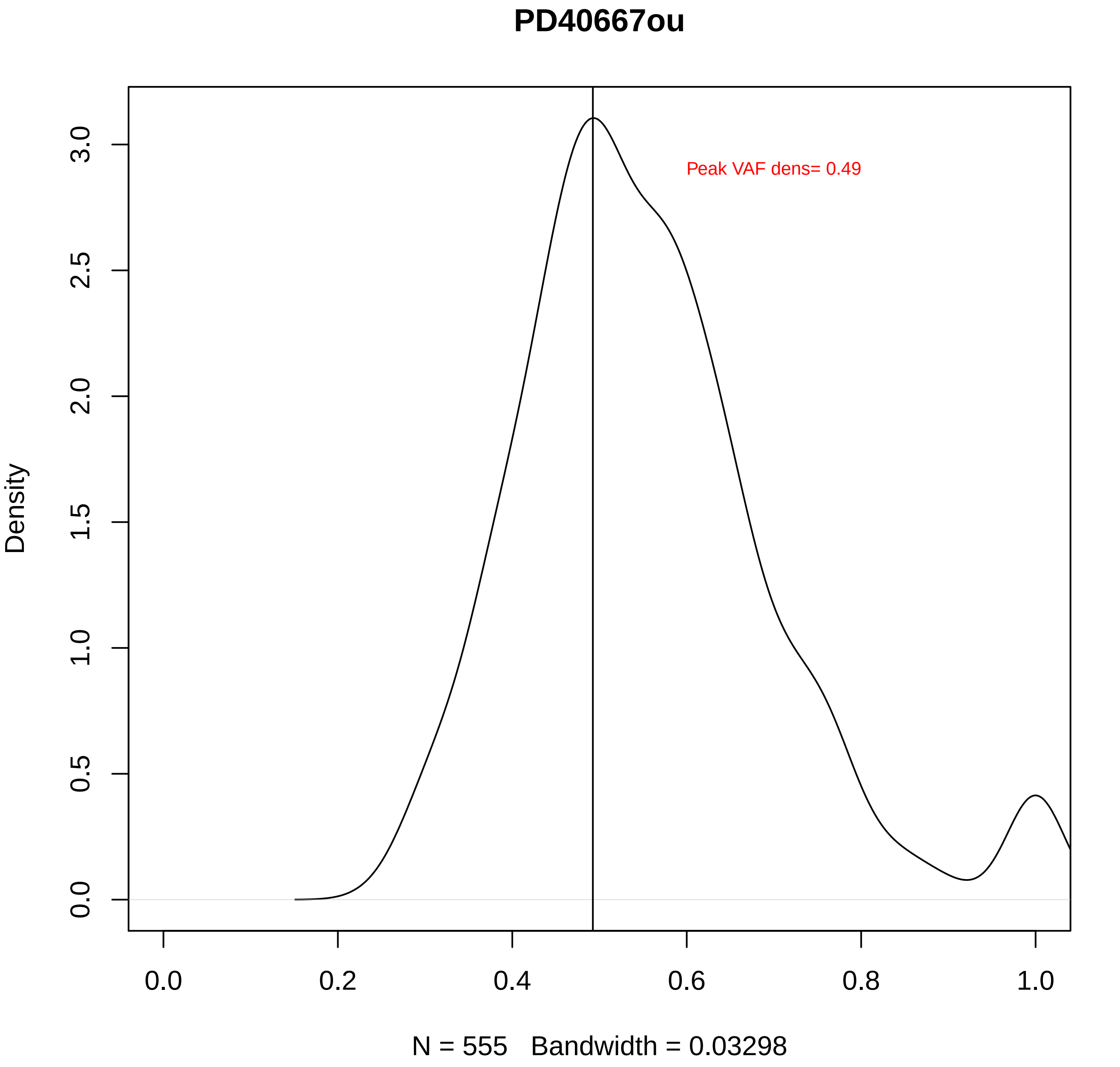
PD40667ou
Peak VAF dens= 0.49
Density
0.0
0.5
1.0
1.5
2.0
2.5
3.0
0.0
0.2
0.4
0.6
0.8
1.0
N = 555 Bandwidth = 0.03298

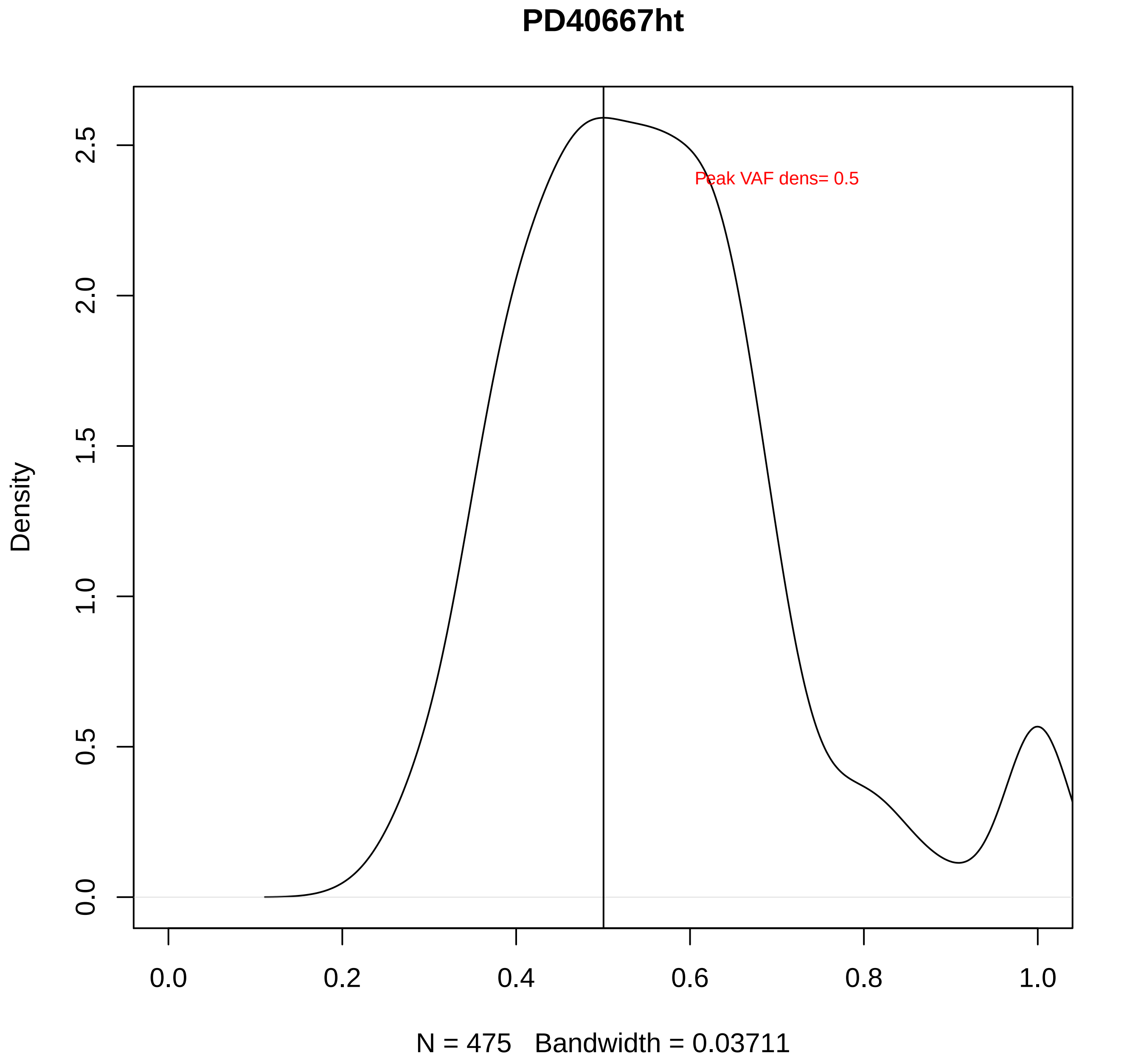

PD40667ht
Peak VAF dens= 0.5
Density
0.0
0.5
1.0
1.5
2.0
2.5
0.0
0.2
0.4
0.6
0.8
1.0
N = 475 Bandwidth = 0.03711

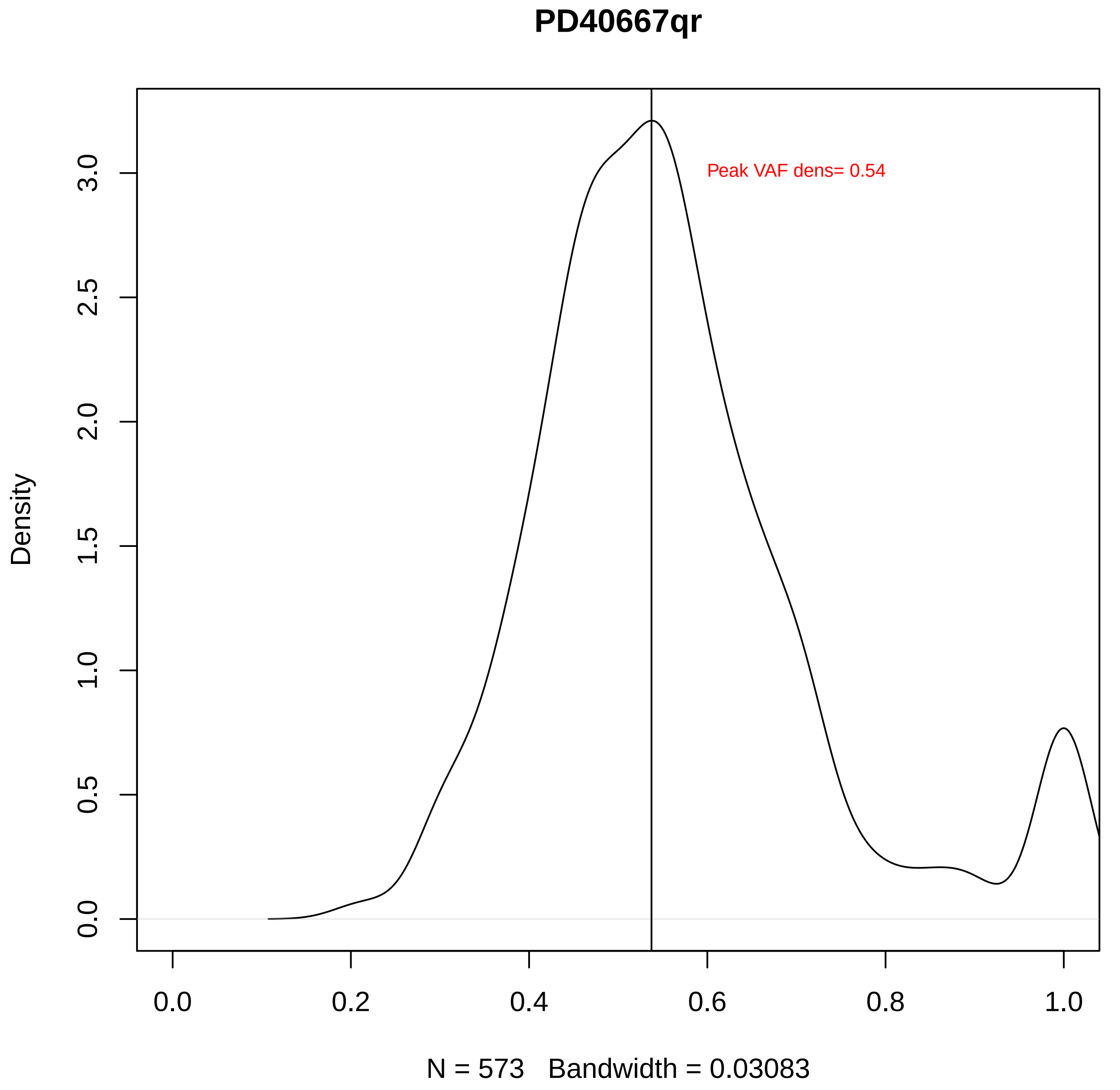
PD40667qr
Peak VAF dens= 0.54
Density
0.0
0.5
1.0
1.5
2.0
2.5
3.0
0.0
0.2
0.4
0.6
0.8
1.0
N = 573   Bandwidth = 0.03083

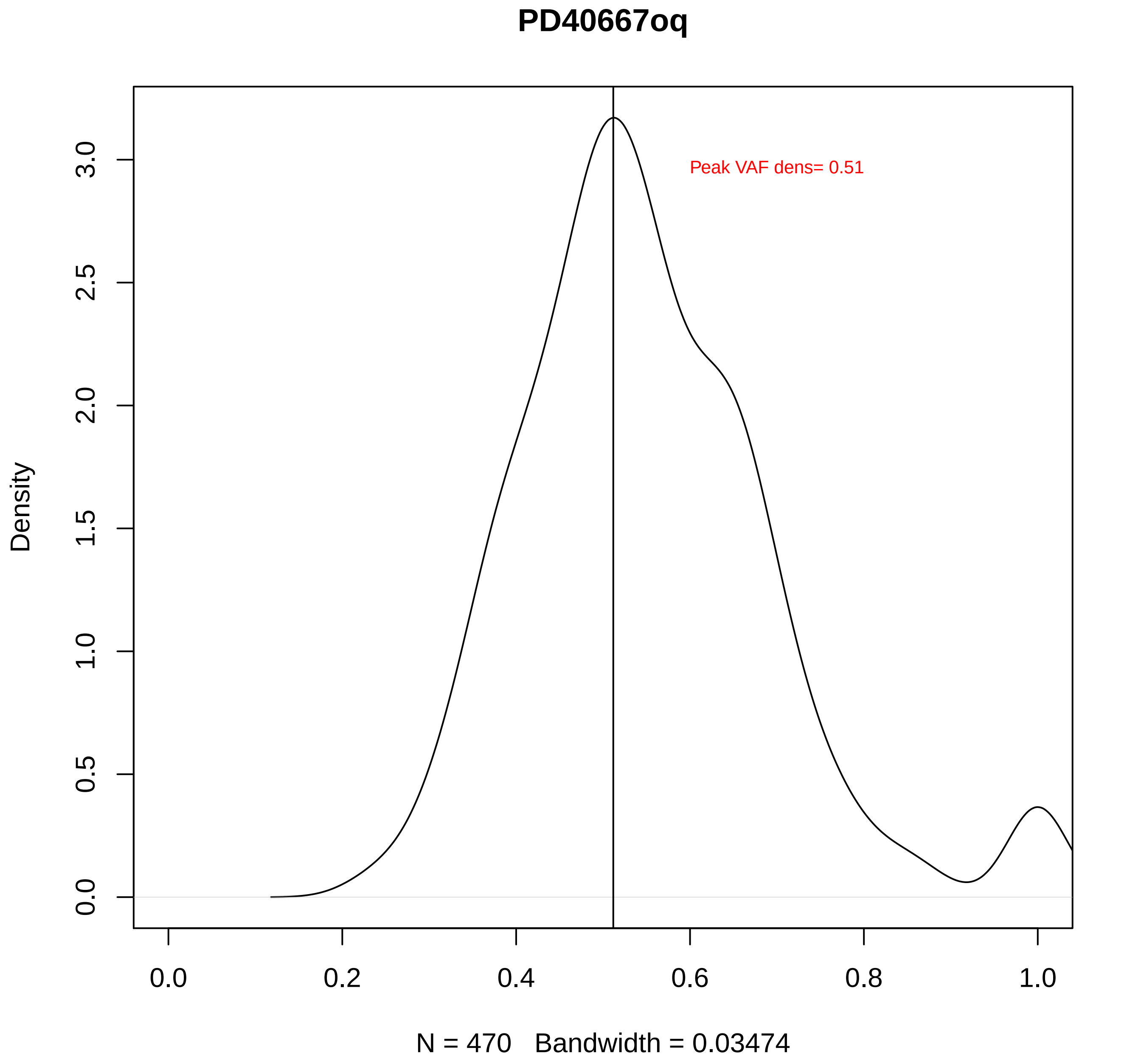

PD40667oq
Peak VAF dens= 0.51
Density
0.0
0.5
1.0
1.5
2.0
2.5
3.0
0.0
0.2
0.4
0.6
0.8
1.0
N = 470   Bandwidth = 0.03474

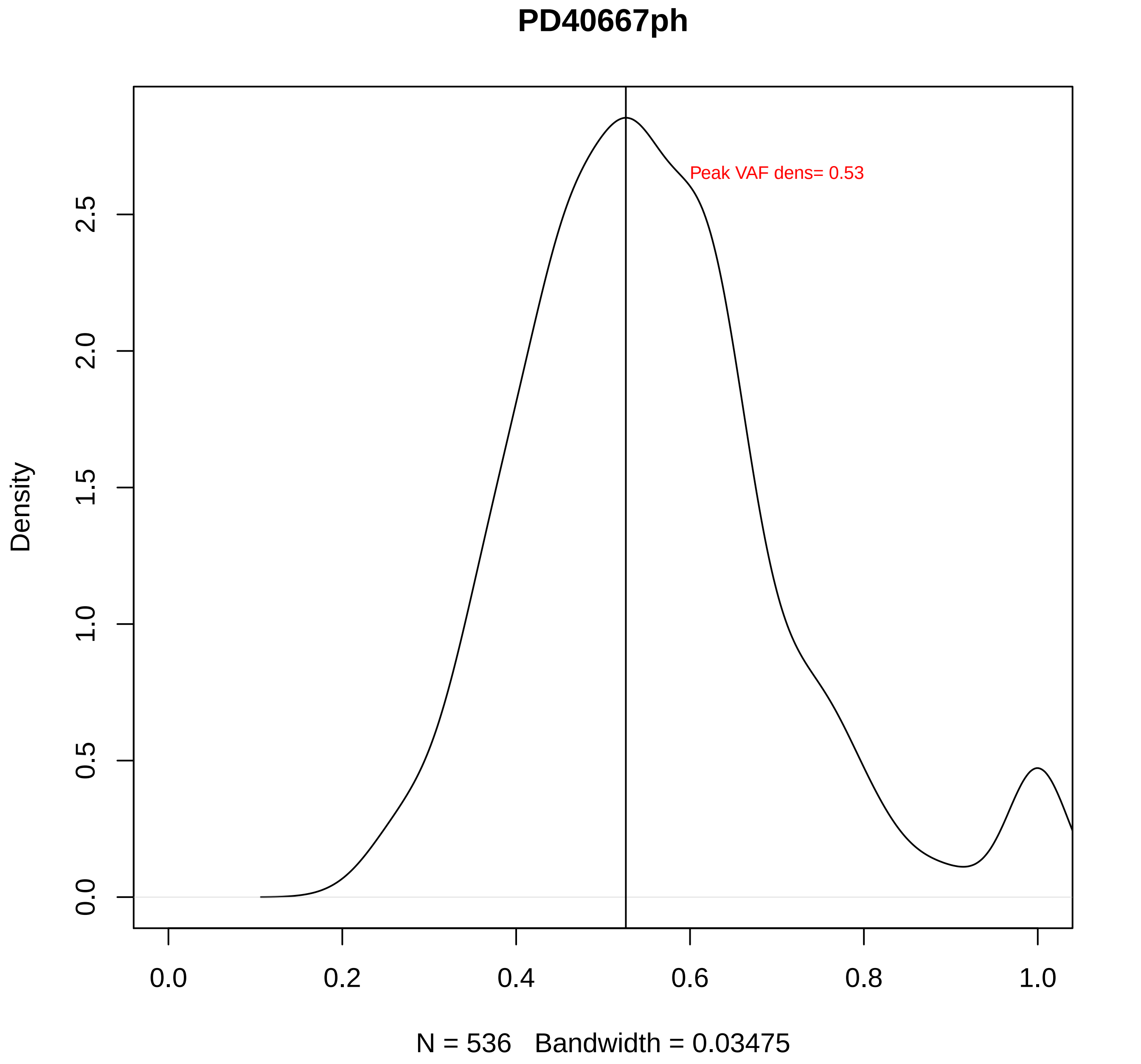

PD40667ph
Peak VAF dens= 0.53
Density
0.0
0.5
1.0
1.5
2.0
2.5
0.0
0.2
0.4
0.6
0.8
1.0
N = 536   Bandwidth = 0.03475

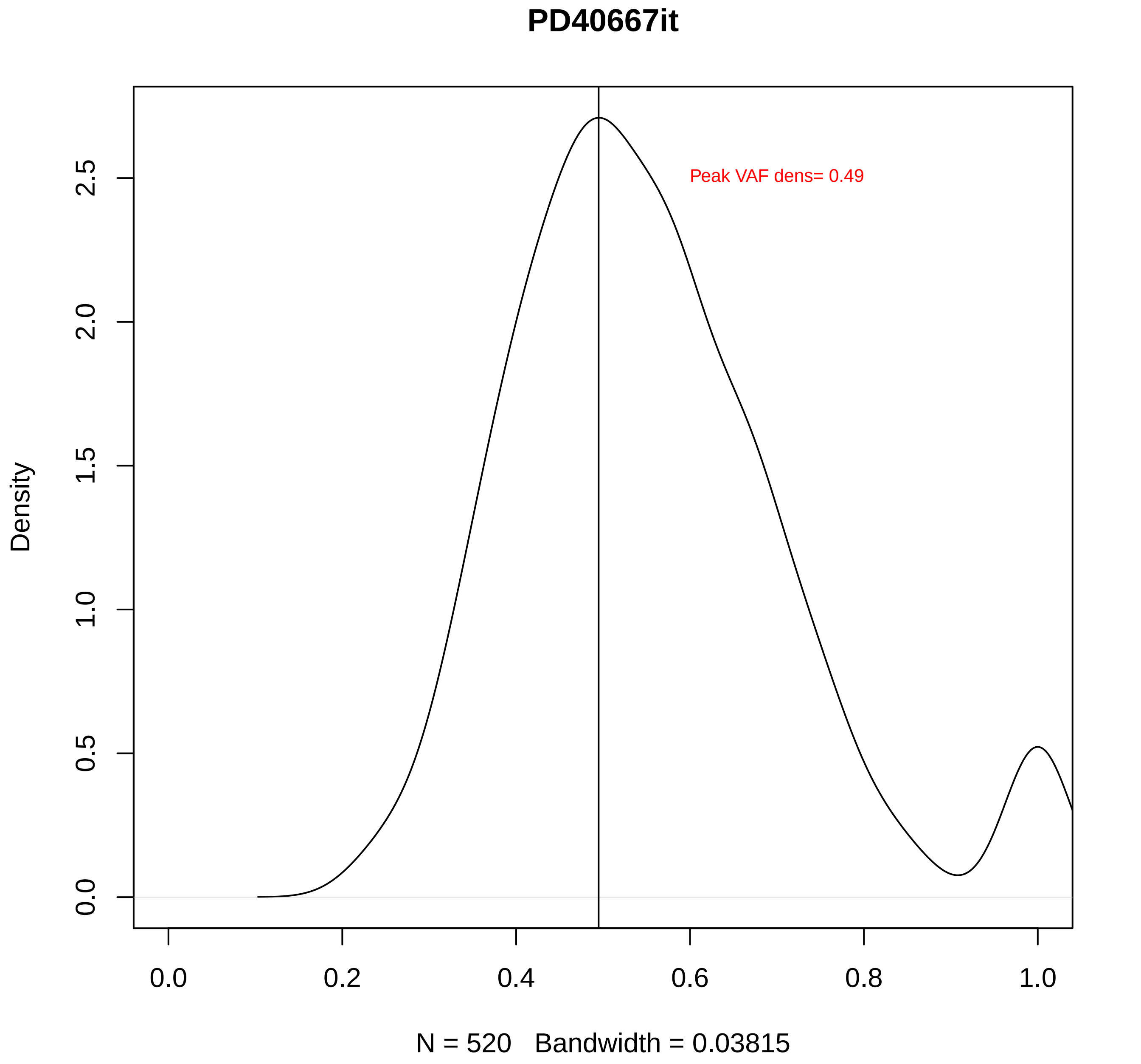
PD40667it
Peak VAF dens= 0.49
Density
0.0
0.5
1.0
1.5
2.0
2.5
0.0
0.2
0.4
0.6
0.8
1.0
N = 520   Bandwidth = 0.03815

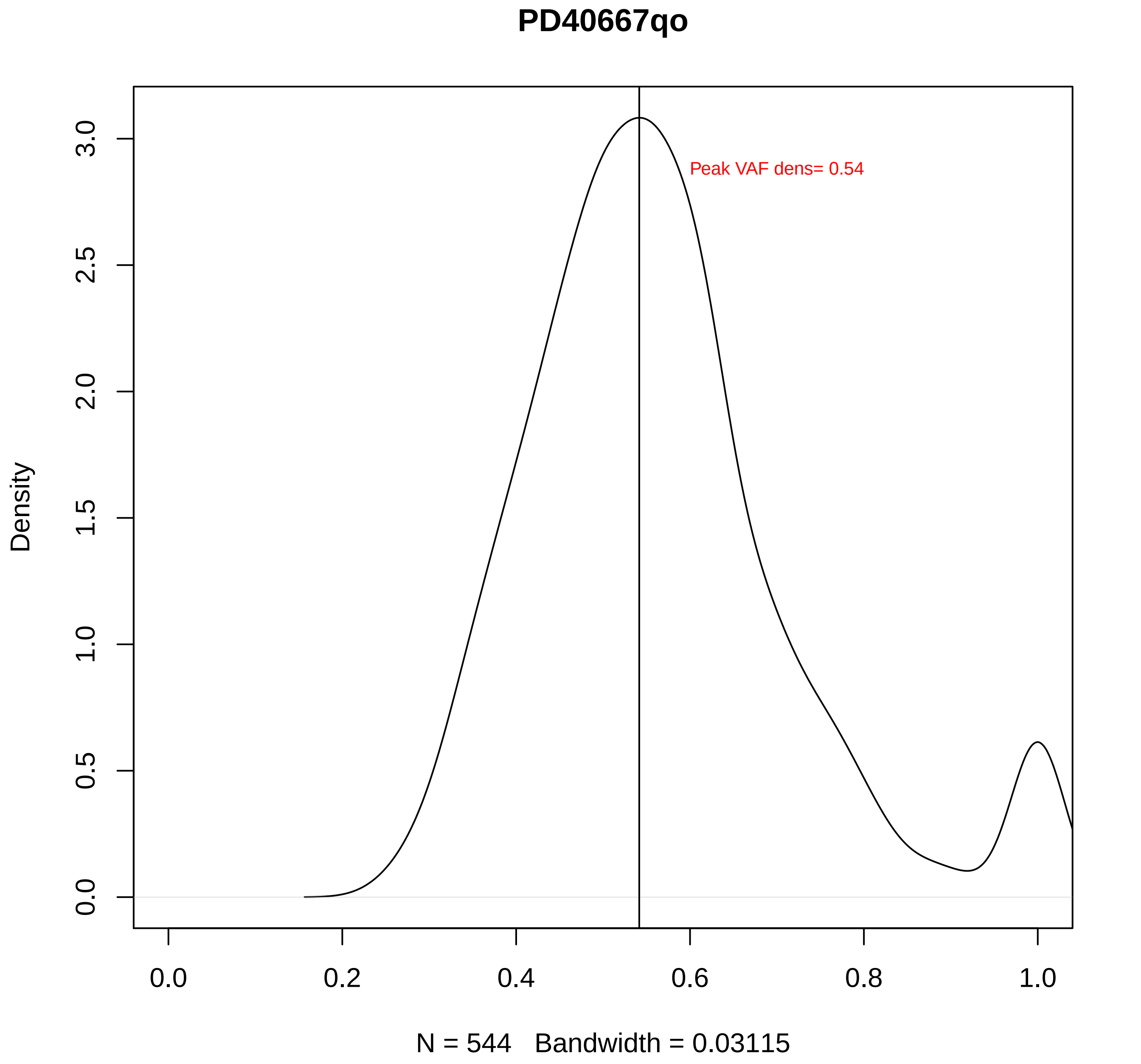
PD40667qo
Peak VAF dens= 0.54
Density
0.0
0.5
1.0
1.5
2.0
2.5
3.0
0.0
0.2
0.4
0.6
0.8
1.0
N = 544 Bandwidth = 0.03115
