# Supplementary material for: Clonal dynamics of haematopoiesis across the human lifespan
Source: Nature. 2022 Jun 1;606(7913):343–50. doi: 10.1038/s41586-022-04786-y (PMC9177428; doi:10.1038/s41586-022-04786-y)
Supplement: Supplementary file 4 — HTMLs of notebooks outlining key statistical analyses presented in the manuscript, including analysis of phylogenetic trees. [file 41586_2022_4786_MOESM4_ESM.zip › Supplementary_code/SNV_indel_analysis/KX008_subs_cut_analysis.html]

Analysis of SNVs and indels (non-clonal samples removed)


# Analysis of SNVs and indels (non-clonal samples removed)

#### Emily Mitchell

### Summary

This script performs analysis and visualisation of SNV and indels in the dataset of a single individual.  
1. Calculation of per colony SNV and indel burden per sample.  
2. Adjustment of SNV and indel burdens to account for sequencing depth using asymptotic regression.  
3. Generate input matrix for dN/dS analysis.  
4. Visualise mutation spectrum of the pooled dataset.  
5. Calculation number of non-synonymous mutations per sample.

##### Open libraries

```
suppressMessages(library(GenomicRanges))
```

```
## Warning: package 'GenomicRanges' was built under R version 4.0.3
```

```
## Warning: package 'BiocGenerics' was built under R version 4.0.3
```

```
## Warning: package 'S4Vectors' was built under R version 4.0.3
```

```
## Warning: package 'IRanges' was built under R version 4.0.3
```

```
## Warning: package 'GenomeInfoDb' was built under R version 4.0.3
```

```
suppressMessages(library(dplyr))
suppressMessages(library(tidyr))
suppressMessages(library(ggplot2))
suppressMessages(library(BiocGenerics))
suppressMessages(library(knitr))
suppressMessages(library(stringr))
suppressMessages(library(ggtree))
```

```
## Warning: package 'ggtree' was built under R version 4.0.3
```

```
suppressMessages(library(ape))
suppressMessages(library(seqinr))
suppressMessages(library(data.table))
suppressMessages(library(phytools))
suppressMessages(library(Rsamtools))
```

```
## Warning: package 'Rsamtools' was built under R version 4.0.3
```

```
## Warning: package 'Biostrings' was built under R version 4.0.3
```

```
## Warning: package 'XVector' was built under R version 4.0.3
```

```
suppressMessages(library(MASS))
```

### 1. Set working directory and define files and paths

```
ID = "KX008" #Edit
Iteration = "KX008_2" #Edit
Run_ID = "KX008_2_01" #Edit
filtering_ID = "standard_rho01" #Edit
lim_snv = 2500 #Edit- used for ylim_snv on graphs of mutation burden
br_snv = c(400,800,1200,1600,2000,2400)
lim_indel = 400 #Edit- used for ylim_snv on graphs of mutation burden
br_indel = c(40,80,120,160,200,240,280,320,360,400)
setwd = paste0("~/Documents/PhD/Sequencing_results/DNA_seq/",ID,"/",Iteration,"/subs/vaf_cut/")
mats_and_param_file = paste0("~/Documents/PhD/Sequencing_results/DNA_seq/",ID,"/",Iteration,"/trees/vaf_cut/output/mats_and_params_", Run_ID)
tree_file_path = paste0("~/Documents/PhD/Sequencing_results/DNA_seq/",ID,"/",Iteration,"/trees/vaf_cut/output/tree_", Run_ID,"_",filtering_ID, ".tree")
file_annot = paste0("~/Documents/PhD/Sequencing_results/DNA_seq/",ID,"/",Iteration,"/trees/vaf_cut/output/annotated_mut_set_", Run_ID,"_",filtering_ID)
sensitivity_df = paste0("~/Documents/PhD/Sequencing_results/DNA_seq/",ID,"/",Iteration,"/trees/vaf_cut/output/",Iteration,"_sensitivity")
```

### 2. Source functions

```
function_files=list.files('~/Documents/Bioinformatics/CGP/Functions/', full.names = TRUE, pattern = ".R")
suppressMessages(sapply(function_files, source))
```

```
##         /Users/em16/Documents/Bioinformatics/CGP/Functions//filters_parallel_functions.R
## value   ?                                                                               
## visible FALSE                                                                           
##         /Users/em16/Documents/Bioinformatics/CGP/Functions//targeted_analysis_functions.R
## value   ?                                                                                
## visible FALSE                                                                            
##         /Users/em16/Documents/Bioinformatics/CGP/Functions//tree_functions.R
## value   ?                                                                   
## visible FALSE
```

### 3. Load relevant files

```
load(file_annot)
load(mats_and_param_file)
tree <- read.tree(tree_file_path)
sensitivity <- read.table(sensitivity_df, stringsAsFactors = F, header = T)
```

### 4. Assessing SNV burden per colony

```
SNVs_per_sample = colSums(filtered_muts$COMB_mats.tree.build$Genotype_bin[filtered_muts$COMB_mats.tree.build$mat$Mut_type == "SNV",] == 1)
mean(SNVs_per_sample); sd(SNVs_per_sample); range(SNVs_per_sample)
```

```
## [1] 1207.161
```

```
## [1] 106.293
```

```
## [1]  807 1455
```

```
Mutation_burden <- as.data.frame(colSums(filtered_muts$COMB_mats.tree.build$Genotype_bin[filtered_muts$COMB_mats.tree.build$mat$Mut_type == "SNV",] == 1))
Mutation_burden$Mean_depth <- (colSums(filtered_muts$COMB_mats.tree.build$NR)/nrow(filtered_muts$COMB_mats.tree.build$NR))
colnames(Mutation_burden)[1] <- "Number_mutations"
Mutation_burden$Sample <- rownames(Mutation_burden)
Summary_snvs <- as.data.frame(cbind(Mutation_burden[,c(3,1,2)]))
```

```
ggplot(Summary_snvs, aes(x = Mean_depth, y = Number_mutations))+
  scale_x_continuous(limits = c(0,50))+
   scale_y_continuous(limits = c(0,lim_snv), breaks = br_snv)+
  theme_bw() +
  labs(x="Mean depth", y="Mutation number")+
  geom_point()
```

### 5. Assessing Indel burden per colony

```
INDELs_per_sample = colSums(filtered_muts$COMB_mats.tree.build$Genotype_bin[filtered_muts$COMB_mats.tree.build$mat$Mut_type == "INDEL",] == 1)
mean(INDELs_per_sample); sd(INDELs_per_sample); range(INDELs_per_sample)
```

```
## [1] 41.14986
```

```
## [1] 10.76104
```

```
## [1] 12 68
```

```
Indel_burden <- as.data.frame(colSums(filtered_muts$COMB_mats.tree.build$Genotype_bin[filtered_muts$COMB_mats.tree.build$mat$Mut_type == "INDEL",] == 1))
Indel_burden$Mean_depth <- (colSums(filtered_muts$COMB_mats.tree.build$NR)/nrow(filtered_muts$COMB_mats.tree.build$NR))
colnames(Indel_burden)[1] <- "Number_indels"
Indel_burden$Sample <- rownames(Indel_burden)
Summary_indels <- as.data.frame(cbind(Indel_burden[,c(3,1,2)]))
```

```
ggplot(Summary_indels, aes(x = Mean_depth, y = Number_indels))+
  scale_x_continuous(limits = c(0,50))+
   scale_y_continuous(limits = c(0,lim_indel), breaks = br_indel)+
  theme_bw() +
  labs(x="Mean depth", y="Mutation number")+
  geom_point()
```

### 6. Asymptotic model to correct SNV burden for sequencing depth of coverage

```
# INPUT
# Summary_snvs: data.frame with one row per sample and the following columns (additional columns are fine)
#   Mean_depth: the average coverage per sample
#   Number_mutations: the number of mutations per sample


### Asymptotic model
# Sort data
sorted = sortedXyData(Summary_snvs$Mean_depth, Summary_snvs$Number_mutations)

# Run model
model.as = NLSstAsymptotic(sorted)
# Note: you will be alerted if the model does not converge (typically if data are not asymptotic or there is too little data)
# If data are more linear then a linear regression is reasonable.

# Look at results
model.as
```

```
##          b0          b1         lrc 
##   54.268773 1289.515252   -1.836608
```

##### Create model function

```
mymodel = function(x){
  b0 = model.as[1]
  b1 = model.as[2]
  lrc = model.as[3]
  b0 + b1*(1-exp(-exp(lrc) * x))  
}
```

##### Plot model

```
myrange=1:round(max(Summary_snvs$Mean_depth))
asp = data.frame(x=myrange, y=unlist(lapply(myrange, FUN=mymodel)))
ggplot(Summary_snvs) +
  ylim(1,lim_snv)+
  xlim(0,50)+
  geom_point(aes(x=Mean_depth,y=Number_mutations), data=Summary_snvs, pch=21) + 
  theme_bw() +
  scale_fill_brewer(type="qual", palette=3) + 
  ylab("# Mutations") +
  xlab("Coverage per colony")+
  geom_line(aes(x=x, y=y), data=asp)
```

##### Normalize mutation burden to a specific sequencing depth (30x)

```
new_Number_mutations = function(rd, Number_mutations){
  Number_mutations + ( mymodel(30) - mymodel(rd)) ## can change depth here
}
Summary_snvs$Number_mutations_adj_as = apply(Summary_snvs, MARGIN=1, FUN=function(X)  new_Number_mutations(rd=as.numeric(X[which(colnames(Summary_snvs)=="Mean_depth")]), Number_mutations=as.numeric(X[which(colnames(Summary_snvs)=="Number_mutations")]) ) )
ggplot(Summary_snvs) +
  ylim(0,lim_snv)+
  xlim(0,50)+
  theme_bw() +
  geom_point(aes(Mean_depth,Number_mutations_adj_as), pch=21) + 
  ylab("# Mutations") +
  xlab("Coverage per colony")
```

```
ggplot(Summary_snvs) +
  ylim(0,lim_snv)+
  xlim(0,50)+
  theme_bw() +
  geom_smooth(aes(Mean_depth,Number_mutations_adj_as), pch=21) + 
  ylab("# Mutations") +
  xlab("Coverage per colony")
```

```
## Warning: Ignoring unknown parameters: shape
```

```
## `geom_smooth()` using method = 'loess' and formula 'y ~ x'
```

```
write.table(Summary_snvs, paste0("~/Documents/PhD/Sequencing_results/DNA_seq/",ID,"/",Iteration,"/subs/vaf_cut/mutation_burden/",Iteration,"_sub_dep_adj.txt"), sep="\t", col.names = T, row.names = F, quote=F)
```

### 7. Asymptotic model to correct indel burden for sequencing depth of coverage

```
# INPUT
# Summary_snvs: data.frame with one row per sample and the following columns (additional columns are fine)
#   Mean_depth: the average coverage per sample
#   Number_mutations: the number of mutations per sample


### Asymptotic model
# Sort data
sorted = sortedXyData(Summary_indels$Mean_depth, Summary_indels$Number_indels)

# Run model
model.as = NLSstAsymptotic(sorted)
# Note: you will be alerted if the model does not converge (typically if data are not asymptotic or there is too little data)
# If data are more linear then a linear regression is reasonable.

# Look at results
model.as
```

```
##        b0        b1       lrc 
## -34.87494  98.16920  -2.25838
```

##### Create model function

```
mymodel = function(x){
  b0 = model.as[1]
  b1 = model.as[2]
  lrc = model.as[3]
  b0 + b1*(1-exp(-exp(lrc) * x))  
}
```

##### Plot model

```
myrange=1:round(max(Summary_indels$Mean_depth))
asp = data.frame(x=myrange, y=unlist(lapply(myrange, FUN=mymodel)))
ggplot(Summary_indels) +
  ylim(1,lim_indel)+
  xlim(0,50)+
  geom_point(aes(x=Mean_depth,y=Number_indels), data=Summary_indels, pch=21) + 
  theme_bw() +
  scale_fill_brewer(type="qual", palette=3) + 
  ylab("# Indels") +
  xlab("Coverage per colony")+
  geom_line(aes(x=x, y=y), data=asp)
```

```
## Warning: Removed 4 row(s) containing missing values (geom_path).
```

##### Normalize mutation burden to a specific sequencing depth (30x)

```
new_Number_indels = function(rd, Number_indels){
  Number_indels + ( mymodel(30) - mymodel(rd)) ## can change depth here
}
Summary_indels$Number_indels_adj_as = apply(Summary_indels, MARGIN=1, FUN=function(X)  new_Number_indels(rd=as.numeric(X[which(colnames(Summary_indels)=="Mean_depth")]), Number_indels=as.numeric(X[which(colnames(Summary_indels)=="Number_indels")]) ) )
ggplot(Summary_indels) +
  ylim(0,lim_indel)+
  xlim(0,50)+
  theme_bw() +
  geom_point(aes(Mean_depth,Number_indels_adj_as), pch=21) + 
  ylab("# Indels") +
  xlab("Coverage per colony")
```

```
ggplot(Summary_indels) +
  ylim(0,lim_indel)+
  xlim(0,50)+
  theme_bw() +
  geom_smooth(aes(Mean_depth,Number_indels_adj_as), pch=21) + 
  ylab("# Mutations") +
  xlab("Coverage per colony")
```

```
## Warning: Ignoring unknown parameters: shape
```

```
## `geom_smooth()` using method = 'loess' and formula 'y ~ x'
```

```
write.table(Summary_indels, paste0("~/Documents/PhD/Sequencing_results/DNA_seq/",ID,"/",Iteration,"/subs/vaf_cut/mutation_burden/",Iteration,"_indel_dep_adj.txt"), sep="\t", col.names = T, row.names = F, quote=F)
```

### 8. Generate input for dnds - all variants

```
dnds_input <- filtered_muts$COMB_mats.tree.build$mat[,2:5]
dnds_input$SampleID <- ID
colnames(dnds_input) <- c("Chr","Pos","Ref","Alt","SampleID")
dnds_input <- dnds_input[,c("SampleID","Chr","Pos","Ref","Alt")]
write.table(dnds_input, paste0("~/Documents/PhD/Sequencing_results/DNA_seq/",ID,"/",Iteration,"/subs/vaf_cut/dnds/",Iteration, "_dnds_all.txt"), sep="\t", col.names = T, row.names = F, quote=F)

create_dnds_files = function(mat, select_vector = NULL) {
  if(is.null(select_vector)) {dnds_file = mat[,2:5]} else {dnds_file = mat[select_vector,2:5]}
  dnds_file$SampleID <- ID
  colnames(dnds_file) <- c("Chr","Pos","Ref","Alt","SampleID")
  dnds_file <- dnds_file[,c("SampleID","Chr","Pos","Ref","Alt")]
  return(dnds_file)
}

shared_dnds_file = create_dnds_files(filtered_muts$COMB_mats.tree.build$mat, select_vector = filtered_muts$COMB_mats.tree.build$mat$mut_ref %in% rownames(filtered_muts$Genotype_shared_bin))

private_dnds_file = create_dnds_files(filtered_muts$COMB_mats.tree.build$mat, select_vector = !filtered_muts$COMB_mats.tree.build$mat$mut_ref %in% rownames(filtered_muts$Genotype_shared_bin))

write.table(shared_dnds_file, paste0("~/Documents/PhD/Sequencing_results/DNA_seq/",ID,"/",Iteration,"/subs/vaf_cut/dnds/",Iteration, "_dnds_shared.txt"), sep="\t", col.names = T, row.names = F, quote=F)

write.table(private_dnds_file, paste0("~/Documents/PhD/Sequencing_results/DNA_seq/",ID,"/",Iteration,"/subs/vaf_cut/dnds/",Iteration, "_dnds_private.txt"), sep="\t", col.names = T, row.names = F, quote=F)
```

### 9. Generate input for signature analysis

```
all_muts <- filtered_muts$COMB_mats.tree.build$mat[filtered_muts$COMB_mats.tree.build$mat$Mut_type == "SNV",]
all_muts <- all_muts[,c(2:5,7)]
all_muts$SampleID <- paste0(ID, "_", all_muts$node)
all_muts <- all_muts[,c(1:4,6)]
colnames(all_muts) <- c("Chr","Pos","Ref","Alt","SampleID")
write.table(all_muts, paste0("~/Documents/PhD/Sequencing_results/DNA_seq/",ID,"/",Iteration,"/subs/vaf_cut/hdp/",Iteration, "_hdp_input_branches.txt"), sep="\t", col.names = T, row.names = F, quote=F)
```

### 10. Mutation spectrum - samples pooled

```
genomeFile = "/Users/em16/Documents/Bioinformatics/CGP/Reference_genomes/hs37d5.fa"

subs_only = all_muts
nrow(subs_only)
```

```
## [1] 319444
```

```
  colnames(subs_only) = c("chr", "pos", "ref", "mut")
  subs_only = subs_only[(subs_only$ref %in% c("A","C","G","T")) & (subs_only$mut %in% c("A","C","G","T")) & subs_only$chr %in% c(1:22,"X","Y"),]
  subs_only$trinuc_ref = as.vector(scanFa(genomeFile, GRanges(subs_only$chr, IRanges(subs_only$pos-1, subs_only$pos+1))))
  
  # 2. Annotating the mutation from the pyrimidine base
  ntcomp = c(T="A",G="C",C="G",A="T")
  subs_only$sub = paste(subs_only$ref,subs_only$mut,sep=">")
  subs_only$trinuc_ref_py = subs_only$trinuc_ref
  for (j in 1:nrow(subs_only)) {
    if (subs_only$ref[j] %in% c("A","G")) { # Purine base
      subs_only$sub[j] = paste(ntcomp[subs_only$ref[j]],ntcomp[subs_only$mut[j]],sep=">")
      subs_only$trinuc_ref_py[j] = paste(ntcomp[rev(strsplit(subs_only$trinuc_ref[j],split="")[[1]])],collapse="")
    }
  }
  
 # 3. Counting subs
  freqs = table(paste(subs_only$sub,paste(substr(subs_only$trinuc_ref_py,1,1),substr(subs_only$trinuc_ref_py,3,3),sep="-"),sep=","))
  sub_vec = c("C>A","C>G","C>T","T>A","T>C","T>G")
  ctx_vec = paste(rep(c("A","C","G","T"),each=4),rep(c("A","C","G","T"),times=4),sep="-")
  full_vec = paste(rep(sub_vec,each=16),rep(ctx_vec,times=6),sep=",")
  freqs_full = freqs[full_vec]
  freqs_full[is.na(freqs_full)] = 0
  names(freqs_full) = full_vec
  
  xstr = paste(substr(full_vec,5,5), substr(full_vec,1,1), substr(full_vec,7,7), sep="")
  
  colvec = rep(c("dodgerblue","black","red","grey70","olivedrab3","plum2"),each=16)
  y = freqs_full
  maxy = max(y)
  
  h=barplot(y, las=2, col=colvec, border=NA, ylim_snv=c(0,maxy*1.5), space=1, cex.names=0.6, names.arg=xstr, ylab="# SNVs")
```

```
## Warning in plot.window(xlim, ylim, log = log, ...): "ylim_snv" is not a
## graphical parameter
```

```
## Warning in axis(if (horiz) 2 else 1, at = at.l, labels = names.arg, lty =
## axis.lty, : "ylim_snv" is not a graphical parameter
```

```
## Warning in title(main = main, sub = sub, xlab = xlab, ylab = ylab, ...):
## "ylim_snv" is not a graphical parameter
```

```
## Warning in axis(if (horiz) 1 else 2, cex.axis = cex.axis, ...): "ylim_snv" is
## not a graphical parameter
```

```
  mtext(side=4, text= ID)
  for (j in 1:length(sub_vec)) {
    xpos = h[c((j-1)*16+1,j*16)]
    rect(xpos[1]-0.5, maxy*1.2, xpos[2]+0.5, maxy*1.3, border=NA, col=colvec[j*16])
    text(x=mean(xpos), y=maxy*1.3, pos=3, label=sub_vec[j])
  }
```

### 11. Calculating number of non-synonymous mutations per sample

```
details <- filtered_muts$COMB_mats.tree.build$mat
details$non_syn_mutation <- ifelse(details$Type %in% c("protein_coding:exon:CDS:substitution:codon_variant:non_synonymous_codon"),
                              "Non synonymous mutation", "no")

Non_syn_burden <- as.data.frame(colSums(filtered_muts$COMB_mats.tree.build$Genotype_bin[details$non_syn_mutation == "Non synonymous mutation",] == 1))


colnames(Non_syn_burden)[1] <- "Number_non-syn"
Non_syn_burden$Sample <- rownames(Non_syn_burden)
Summary_non_syn <- as.data.frame(cbind(Non_syn_burden[,c(2,1)]))

write.table(Summary_non_syn, paste0("~/Documents/PhD/Sequencing_results/DNA_seq/",ID,"/",Iteration,"/subs/vaf_cut/mutation_burden/",Iteration,"_non_syn.txt"), sep="\t", col.names = T, row.names = F, quote=F)
```
