# Supplementary material for: Clonal dynamics of haematopoiesis across the human lifespan
Source: Nature. 2022 Jun 1;606(7913):343–50. doi: 10.1038/s41586-022-04786-y (PMC9177428; doi:10.1038/s41586-022-04786-y)
Supplement: Supplementary file 4 — HTMLs of notebooks outlining key statistical analyses presented in the manuscript, including analysis of phylogenetic trees. [file 41586_2022_4786_MOESM4_ESM.zip › Supplementary_code/SNV_indel_analysis/KX001_vaf_plots.pdf]

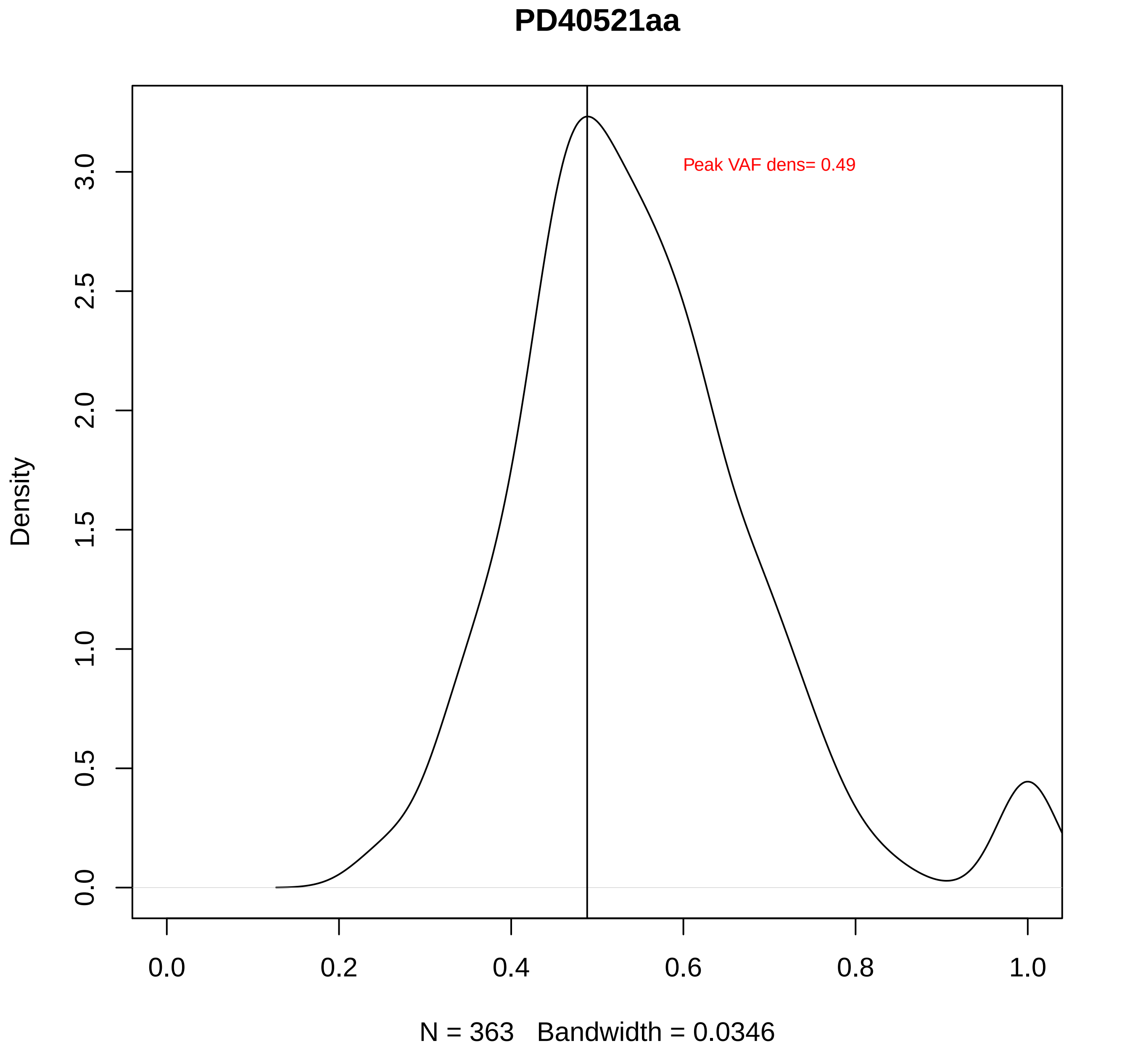
PD40521aa
Peak VAF dens= 0.49
Density
0.0
0.5
1.0
1.5
2.0
2.5
3.0
0.0
0.2
0.4
0.6
0.8
1.0
N = 363 Bandwidth = 0.0346

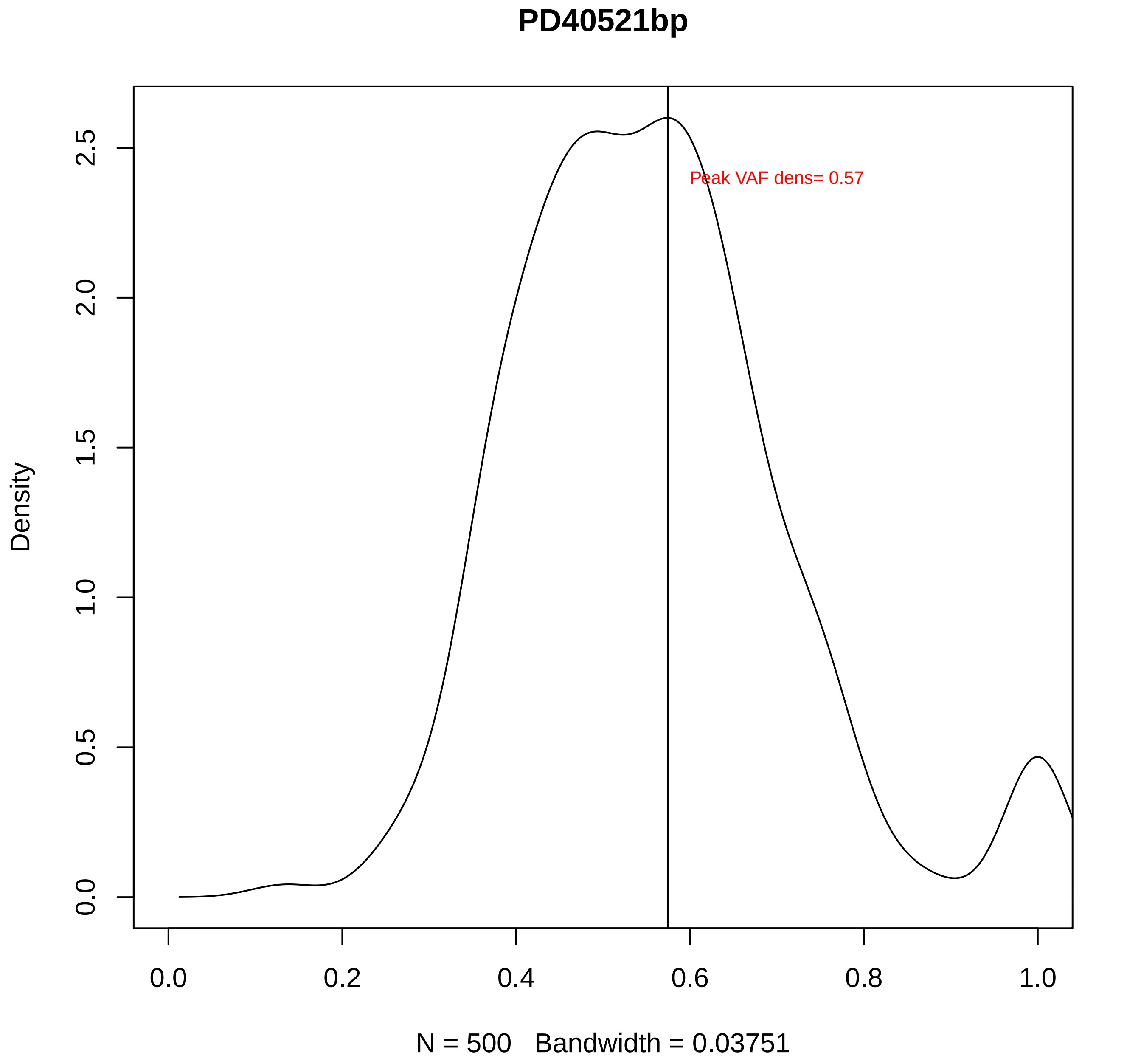
PD40521bp
Density
2.5
2.0
1.5
1.0
0.5
0.0
Peak VAF dens= 0.57
0.0
0.2
0.4
0.6
0.8
1.0
N = 500   Bandwidth = 0.03751

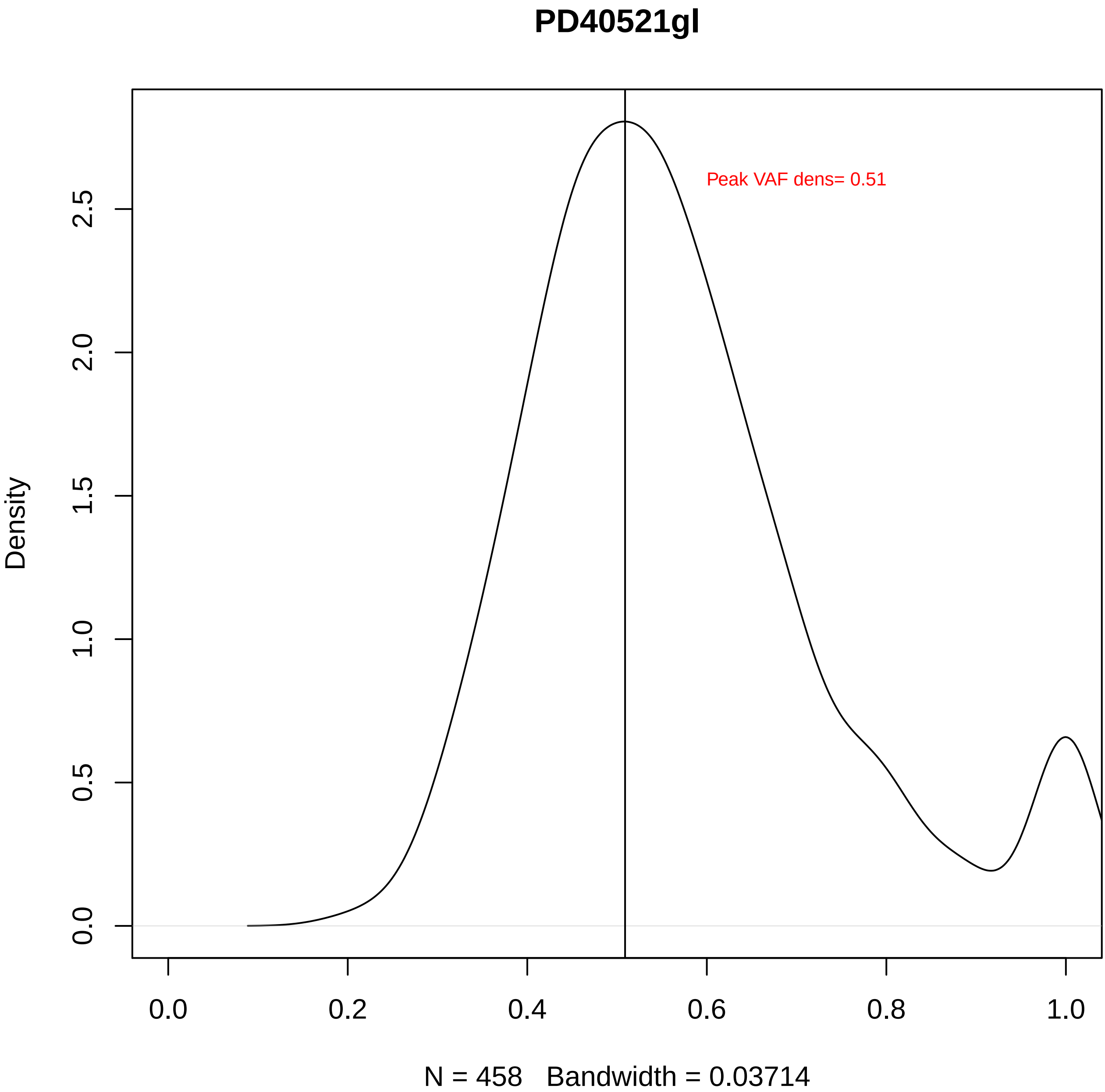
PD40521gl
Peak VAF dens= 0.51
Density
0.0
0.5
1.0
1.5
2.0
2.5
0.0
0.2
0.4
0.6
0.8
1.0
N = 458   Bandwidth = 0.03714

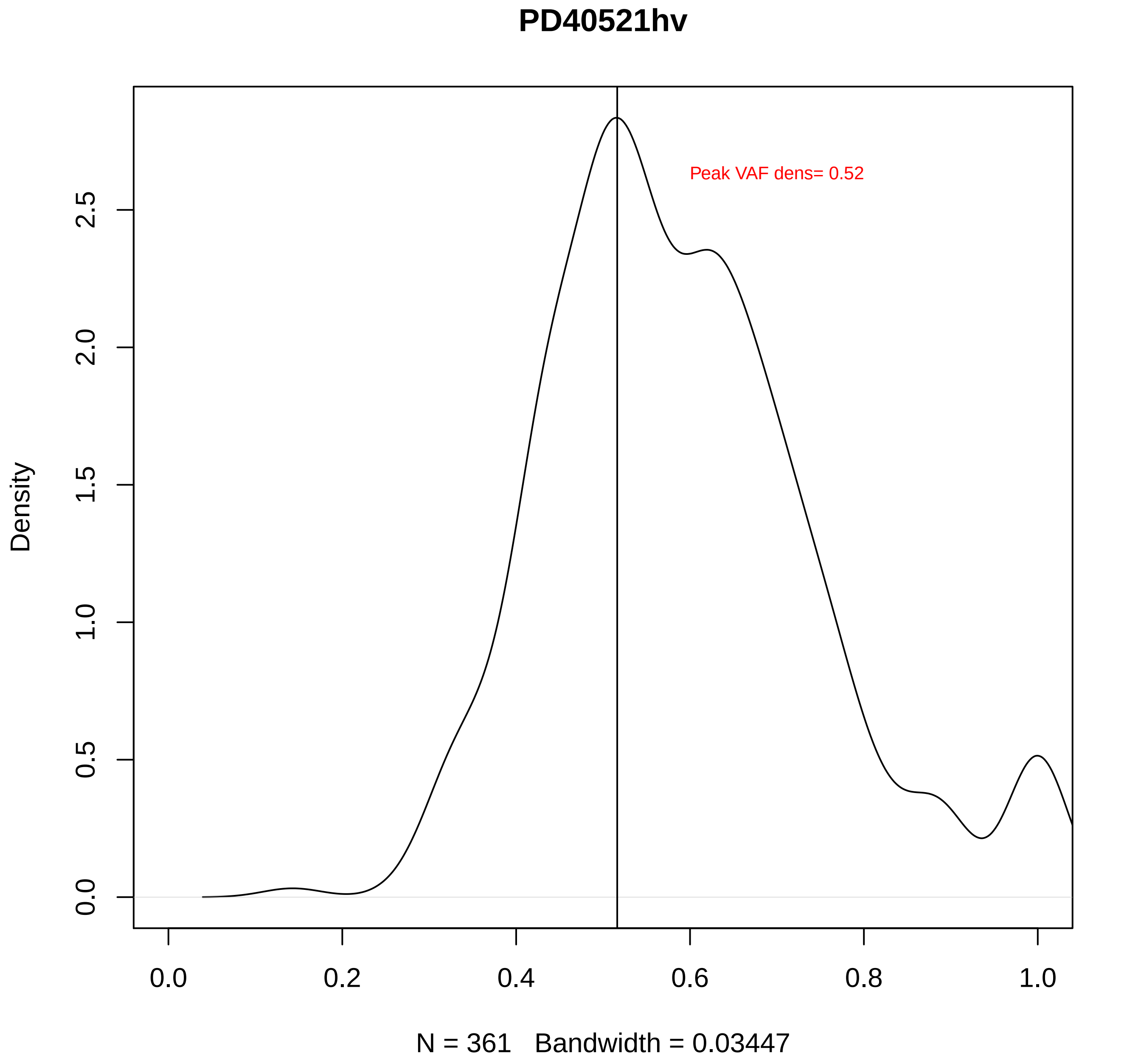

PD40521hv
Peak VAF dens= 0.52
Density
2.5
2.0
1.5
1.0
0.5
0.0
0.0
0.2
0.4
0.6
0.8
1.0
N = 361   Bandwidth = 0.03447

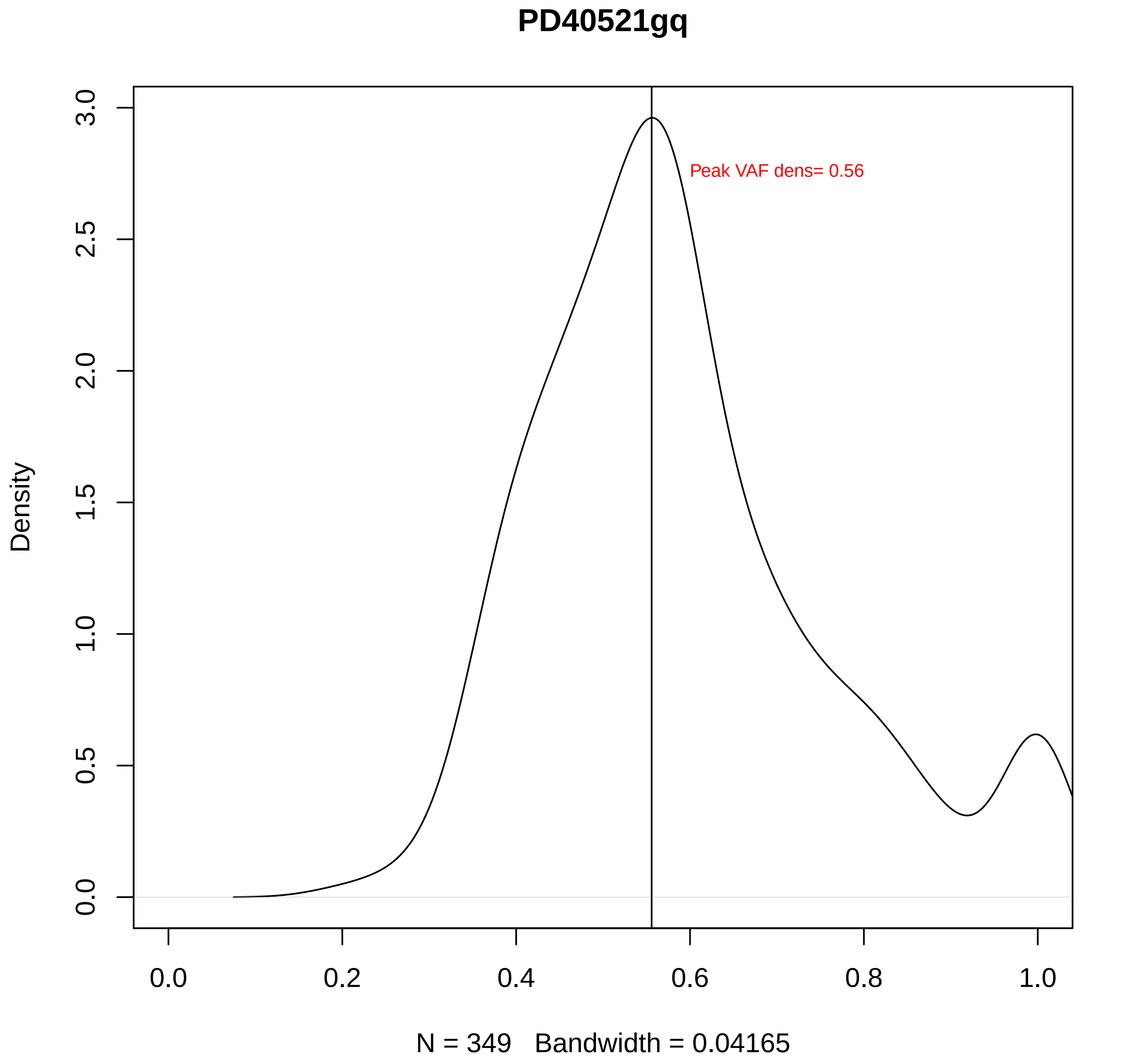
PD40521gq
Peak VAF dens= 0.56
Density
0.0
0.5
1.0
1.5
2.0
2.5
3.0
0.0
0.2
0.4
0.6
0.8
1.0
N = 349   Bandwidth = 0.04165

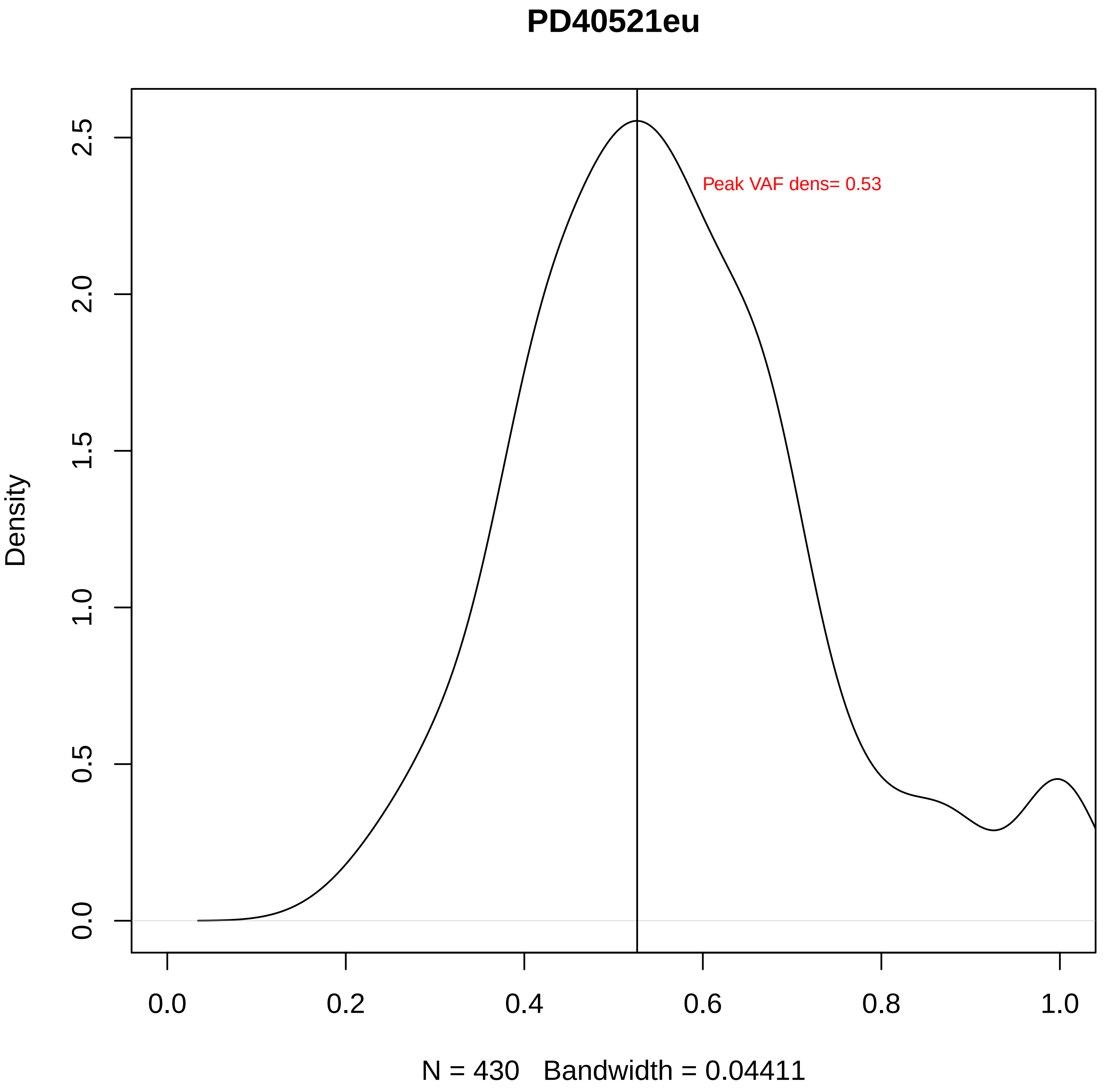
PD40521eu
Peak VAF dens= 0.53
Density
2.5
2.0
1.5
1.0
0.5
0.0
0.0
0.2
0.4
0.6
0.8
1.0
N = 430   Bandwidth = 0.04411

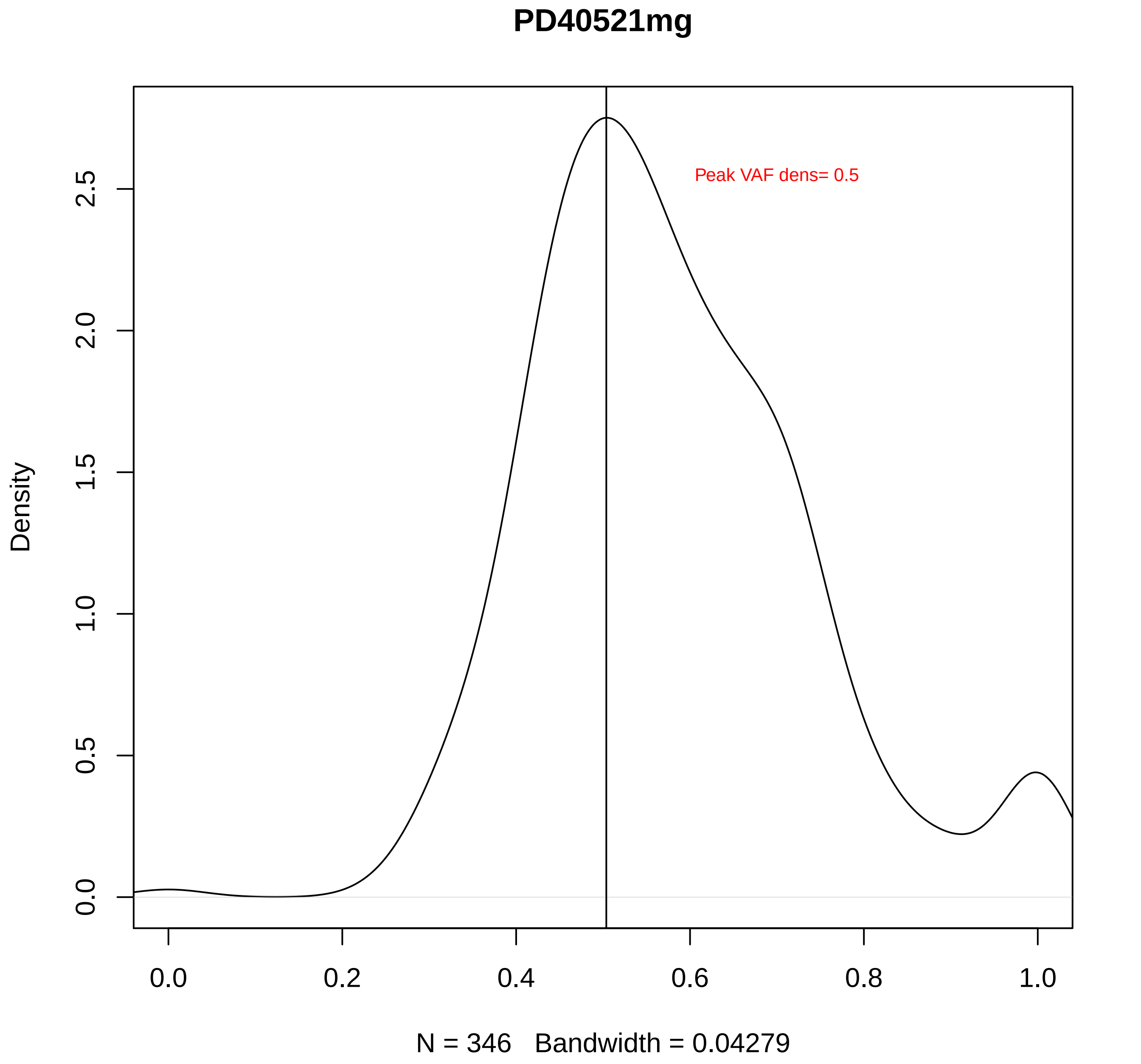
PD40521mg
Peak VAF dens= 0.5
Density
0.0
0.5
1.0
1.5
2.0
2.5
0.0
0.2
0.4
0.6
0.8
1.0
N = 346   Bandwidth = 0.04279

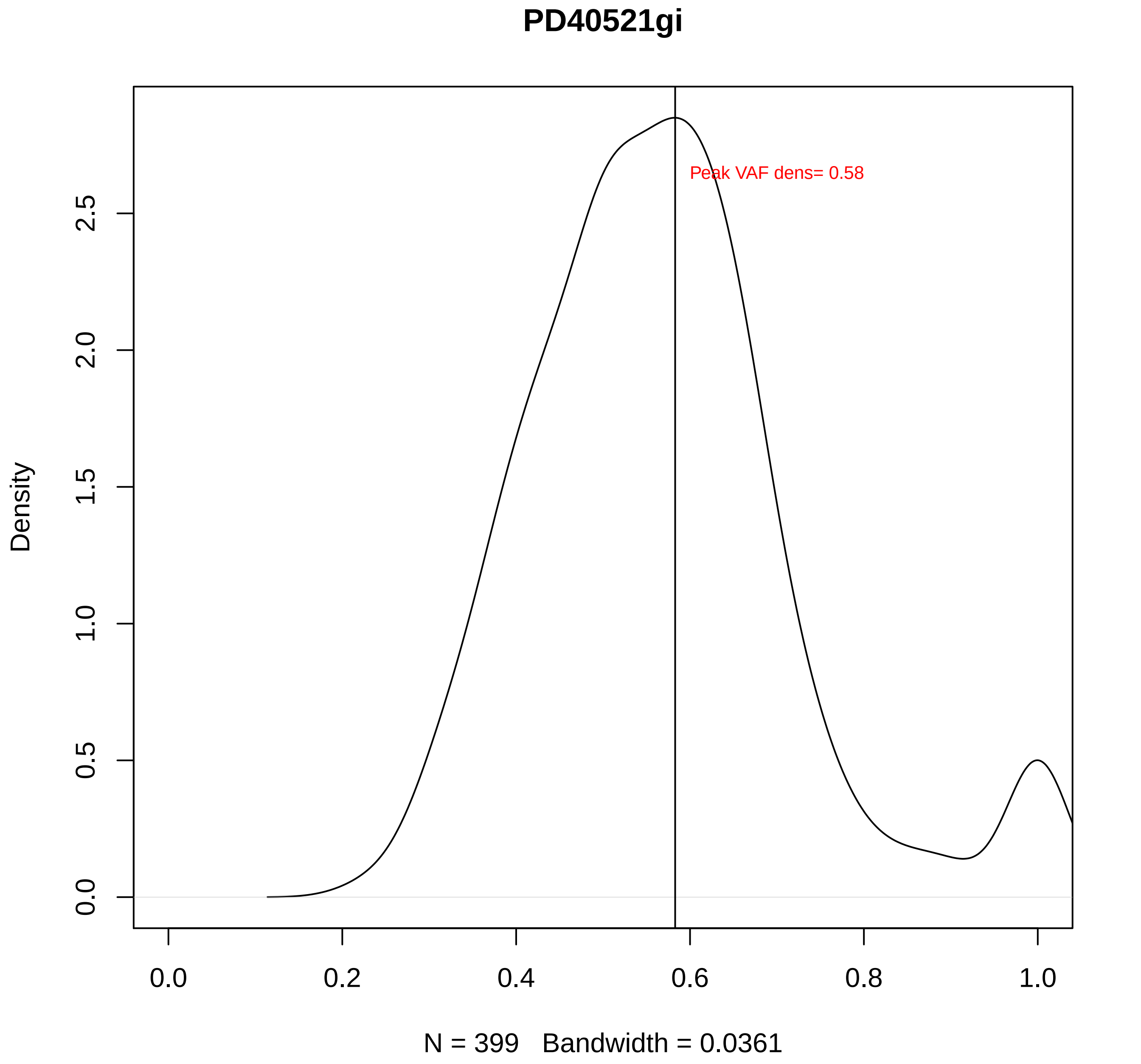

PD40521gi
Peak VAF dens= 0.58
Density
0.0
0.5
1.0
1.5
2.0
2.5
0.0
0.2
0.4
0.6
0.8
1.0
N = 399   Bandwidth = 0.0361

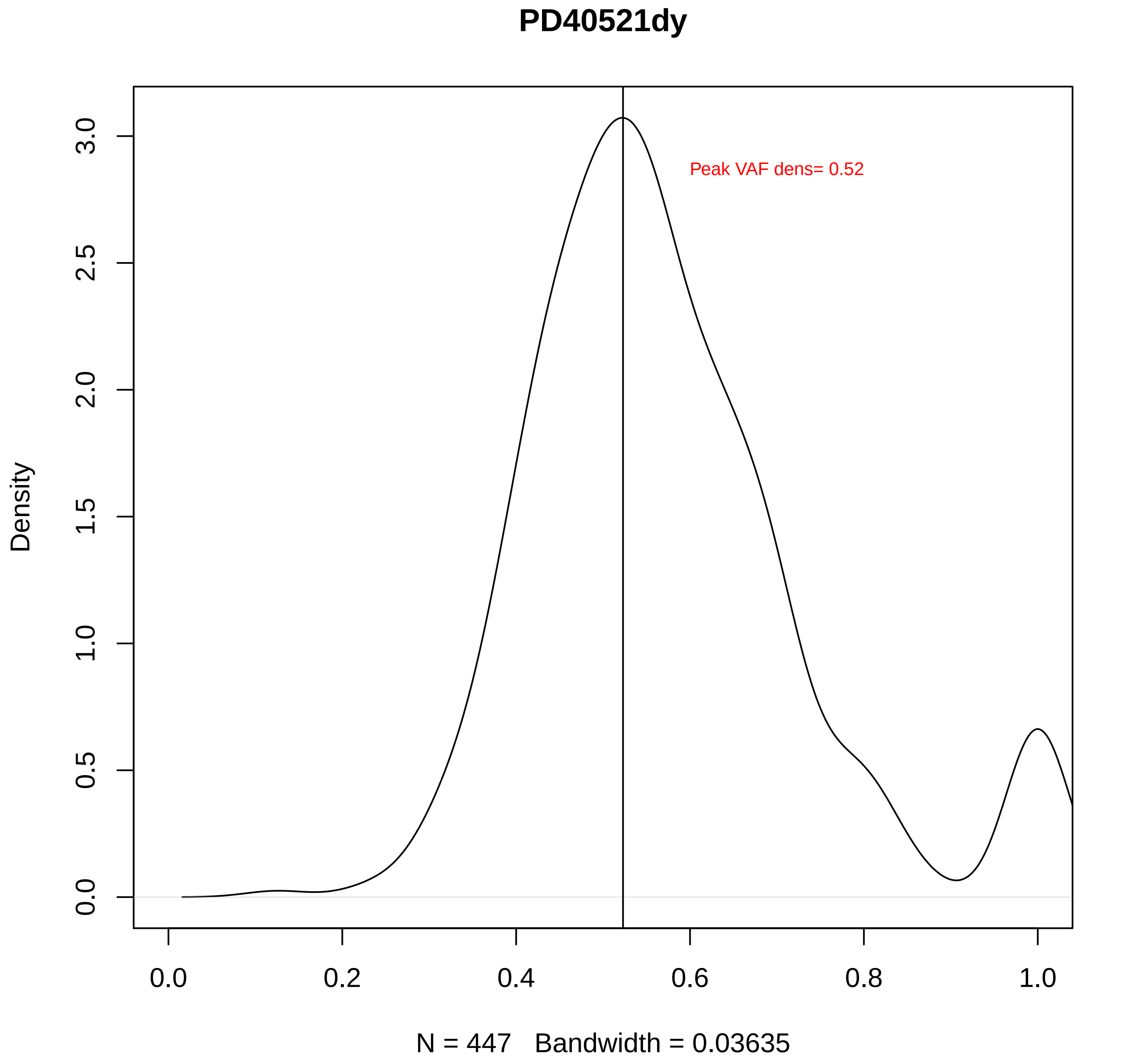
PD40521dy
Peak VAF dens= 0.52
Density
0.0
0.5
1.0
1.5
2.0
2.5
3.0
0.0
0.2
0.4
0.6
0.8
1.0
N = 447   Bandwidth = 0.03635

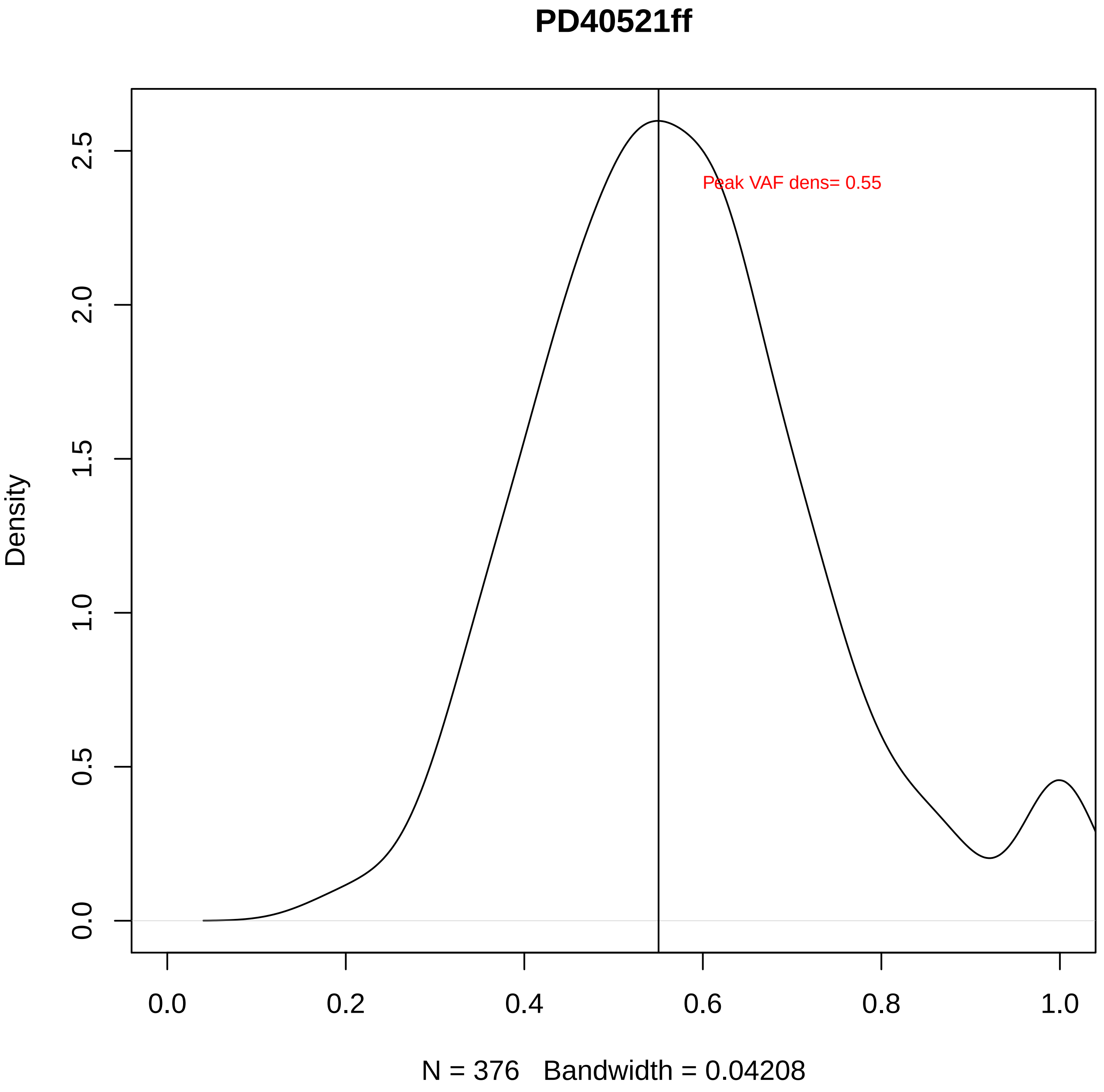
PD40521ff
Density
0.0
0.5
1.0
1.5
2.0
2.5
Peak VAF dens= 0.55
0.0
0.2
0.4
0.6
0.8
1.0
N = 376   Bandwidth = 0.04208

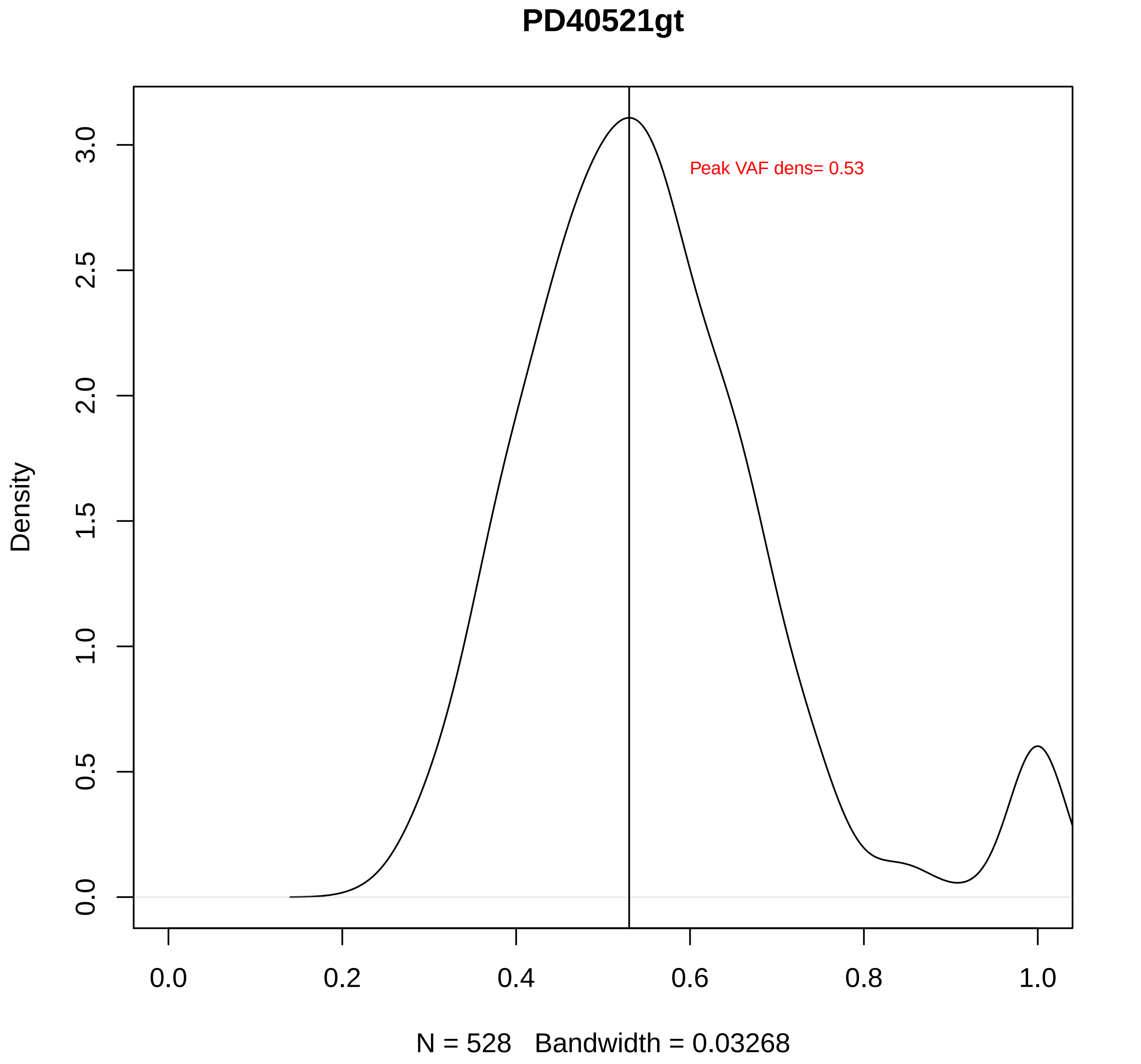

PD40521gt
Peak VAF dens= 0.53
Density
0.0
0.5
1.0
1.5
2.0
2.5
3.0
0.0
0.2
0.4
0.6
0.8
1.0
N = 528 Bandwidth = 0.03268

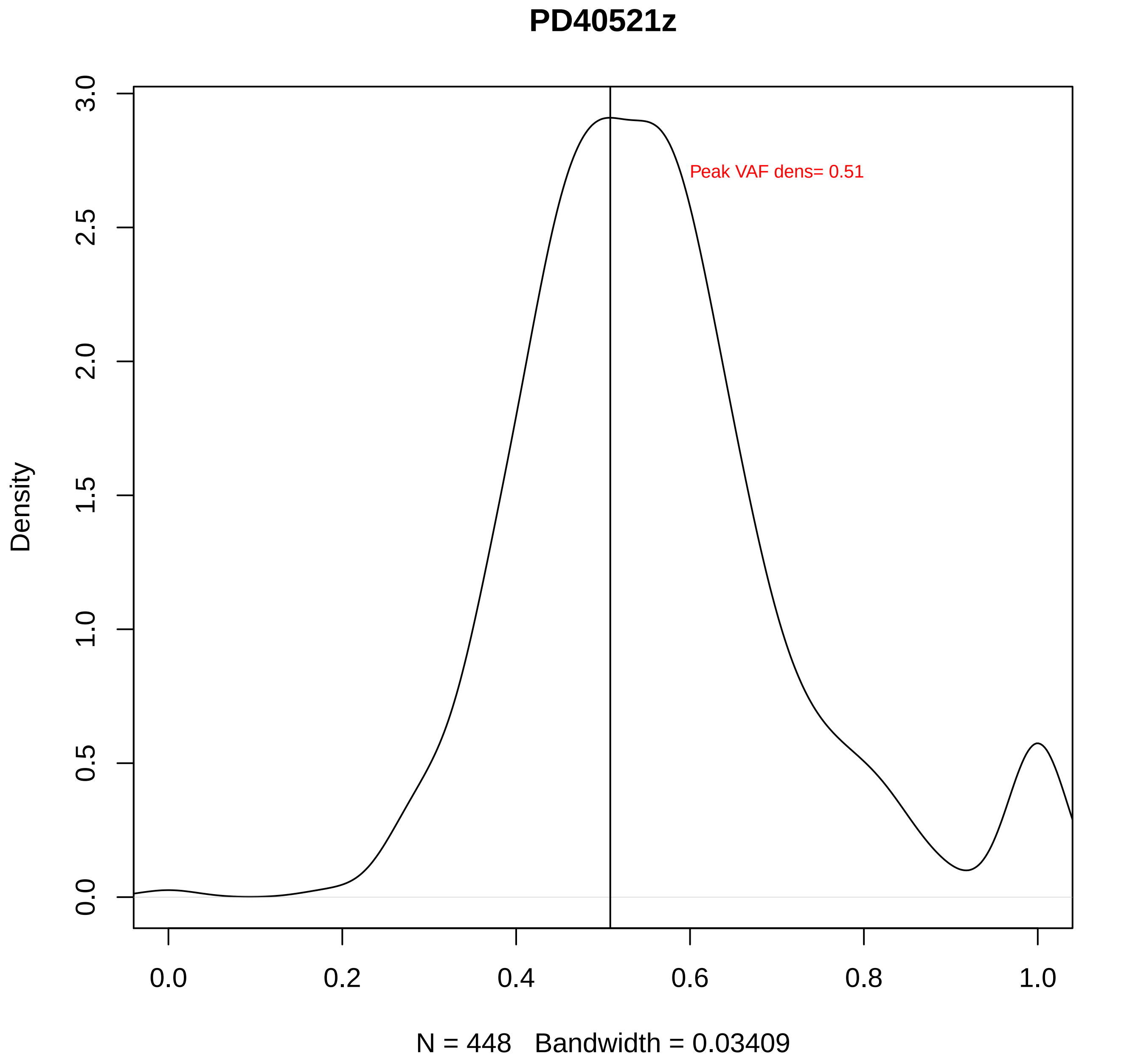

PD40521z
Peak VAF dens= 0.51
Density
0.0
0.5
1.0
1.5
2.0
2.5
3.0
0.0
0.2
0.4
0.6
0.8
1.0
N = 448   Bandwidth = 0.03409

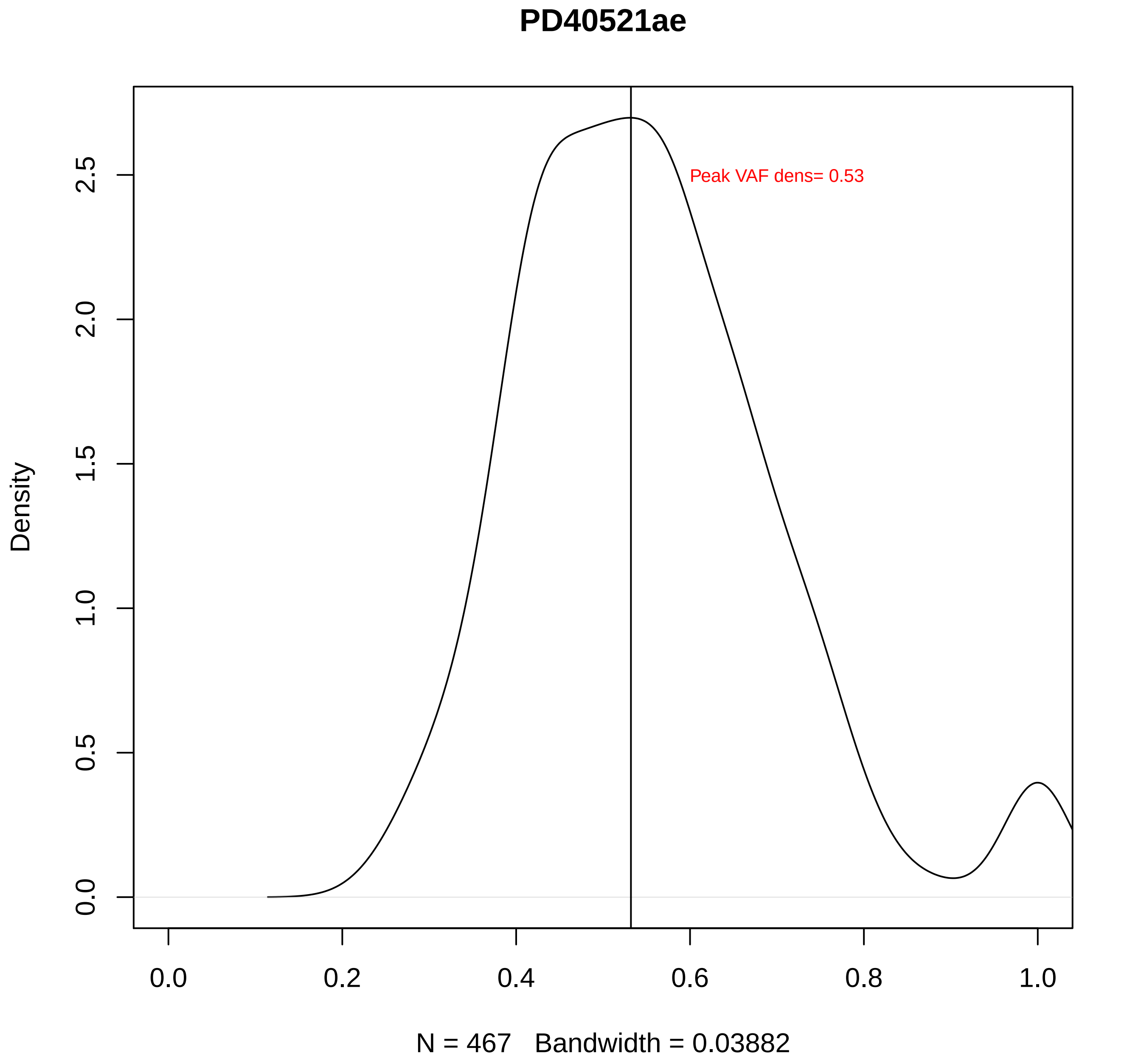
PD40521ae
Peak VAF dens= 0.53
Density
0.0
0.5
1.0
1.5
2.0
2.5
0.0
0.2
0.4
0.6
0.8
1.0
N = 467   Bandwidth = 0.03882

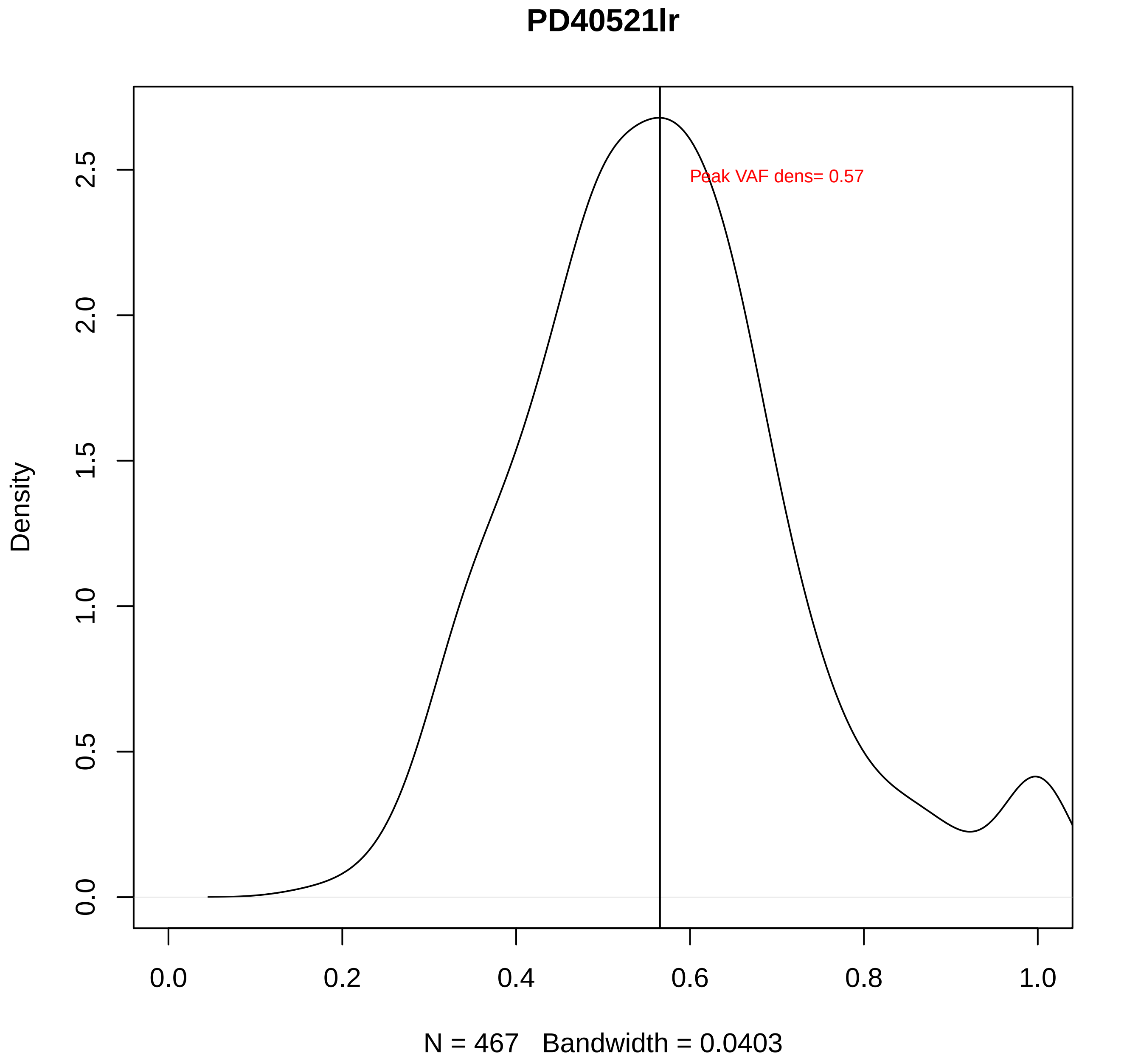

PD40521lr
Peak VAF dens= 0.57
Density
0.0
0.5
1.0
1.5
2.0
2.5
0.0
0.2
0.4
0.6
0.8
1.0
N = 467   Bandwidth = 0.0403

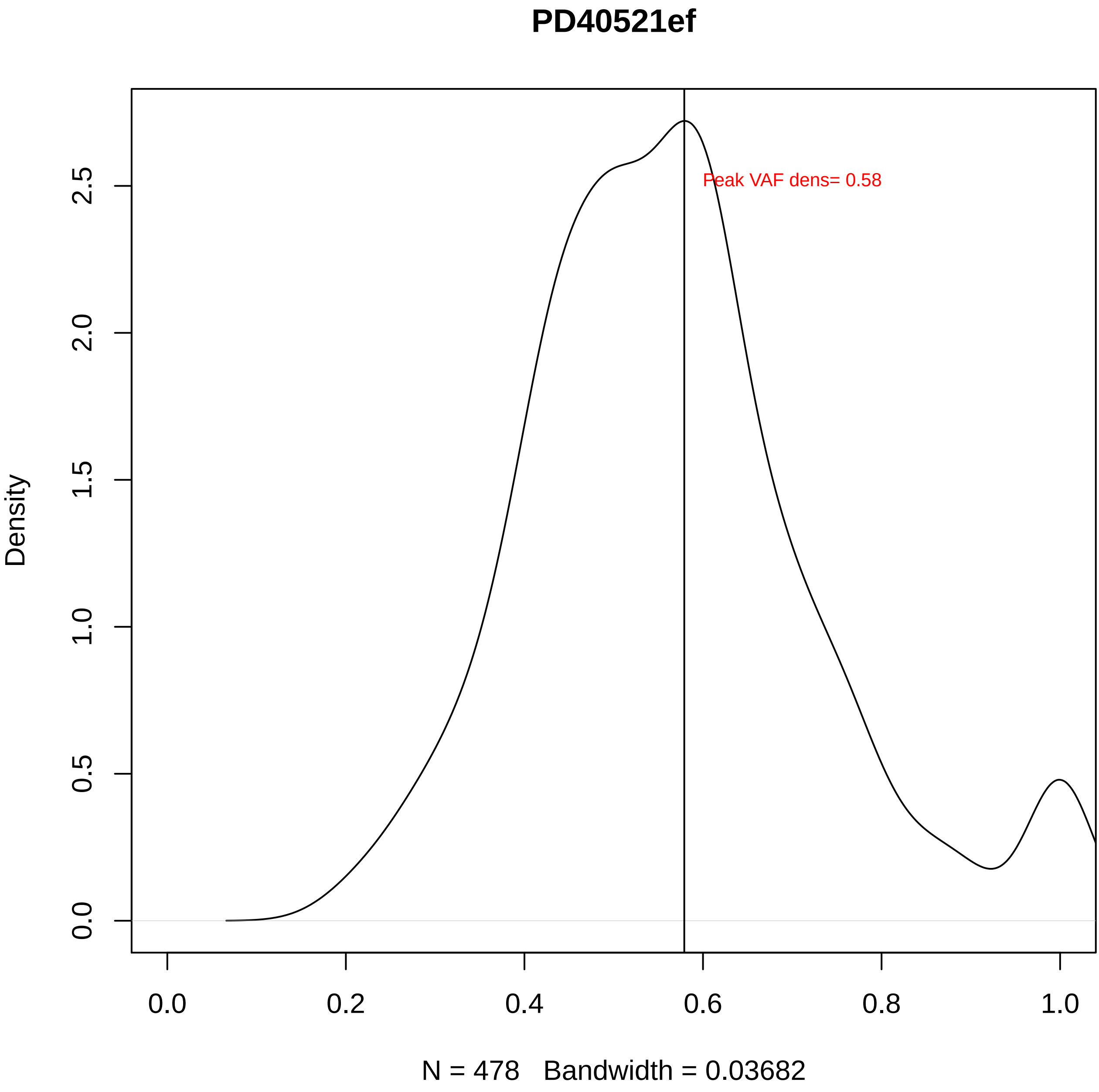
PD40521ef
Peak VAF dens= 0.58
Density
0.0
0.5
1.0
1.5
2.0
2.5
0.0
0.2
0.4
0.6
0.8
1.0
N = 478   Bandwidth = 0.03682

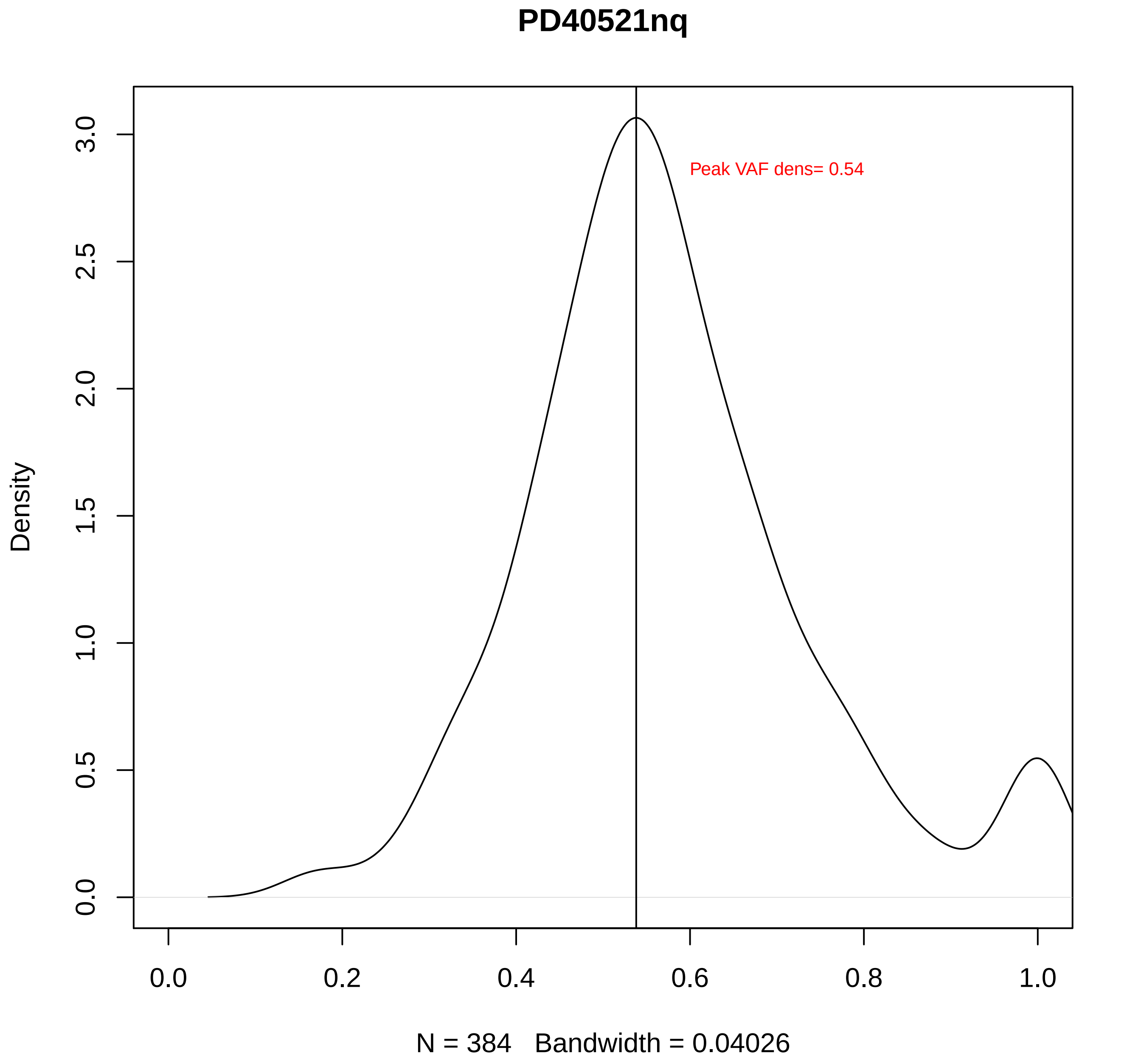
PD40521nq
Peak VAF dens= 0.54
Density
0.0
0.5
1.0
1.5
2.0
2.5
3.0
0.0
0.2
0.4
0.6
0.8
1.0
N = 384   Bandwidth = 0.04026

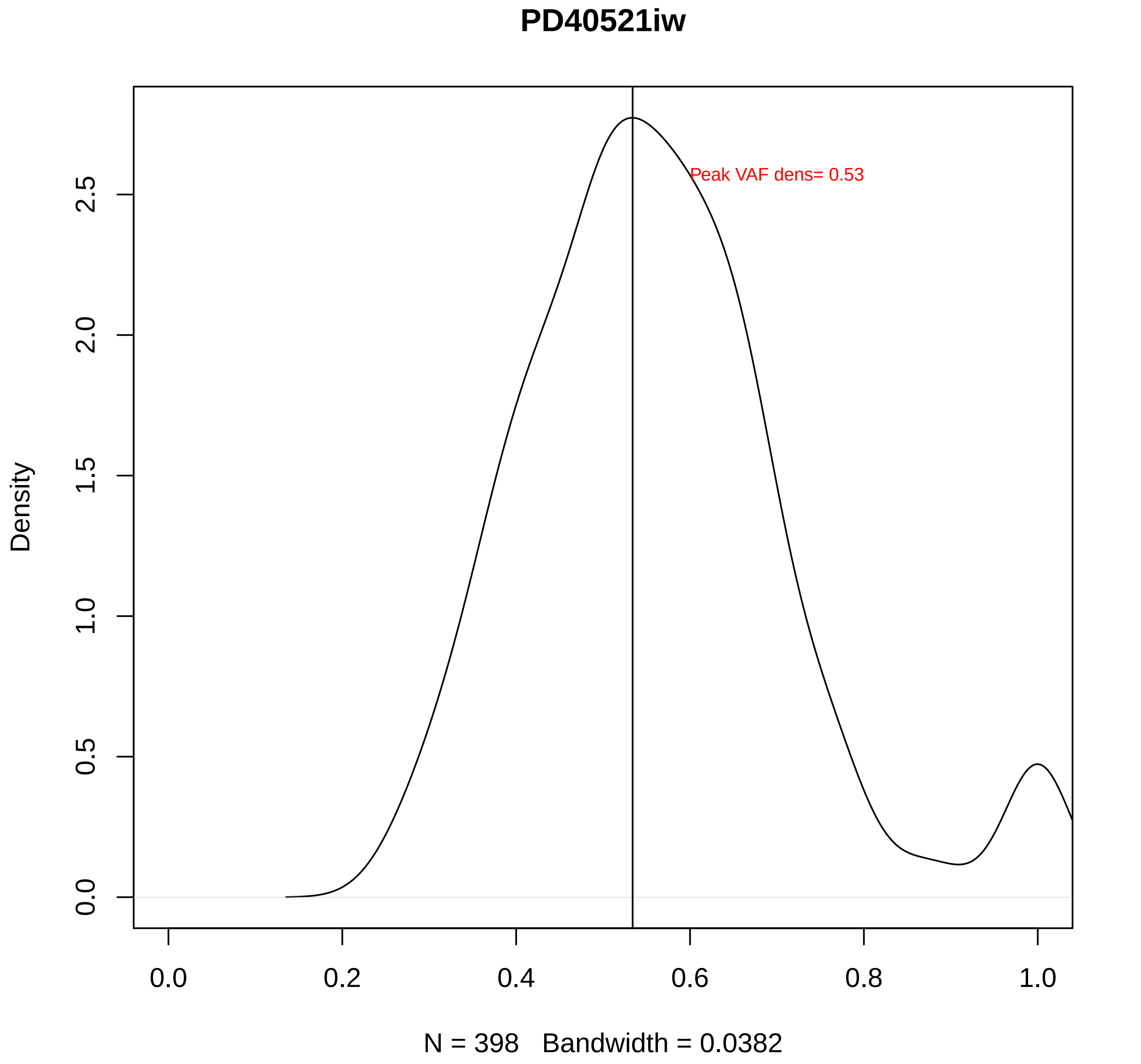
PD40521iw
Peak VAF dens= 0.53
Density
0.0
0.5
1.0
1.5
2.0
2.5
0.0
0.2
0.4
0.6
0.8
1.0
N = 398   Bandwidth = 0.0382

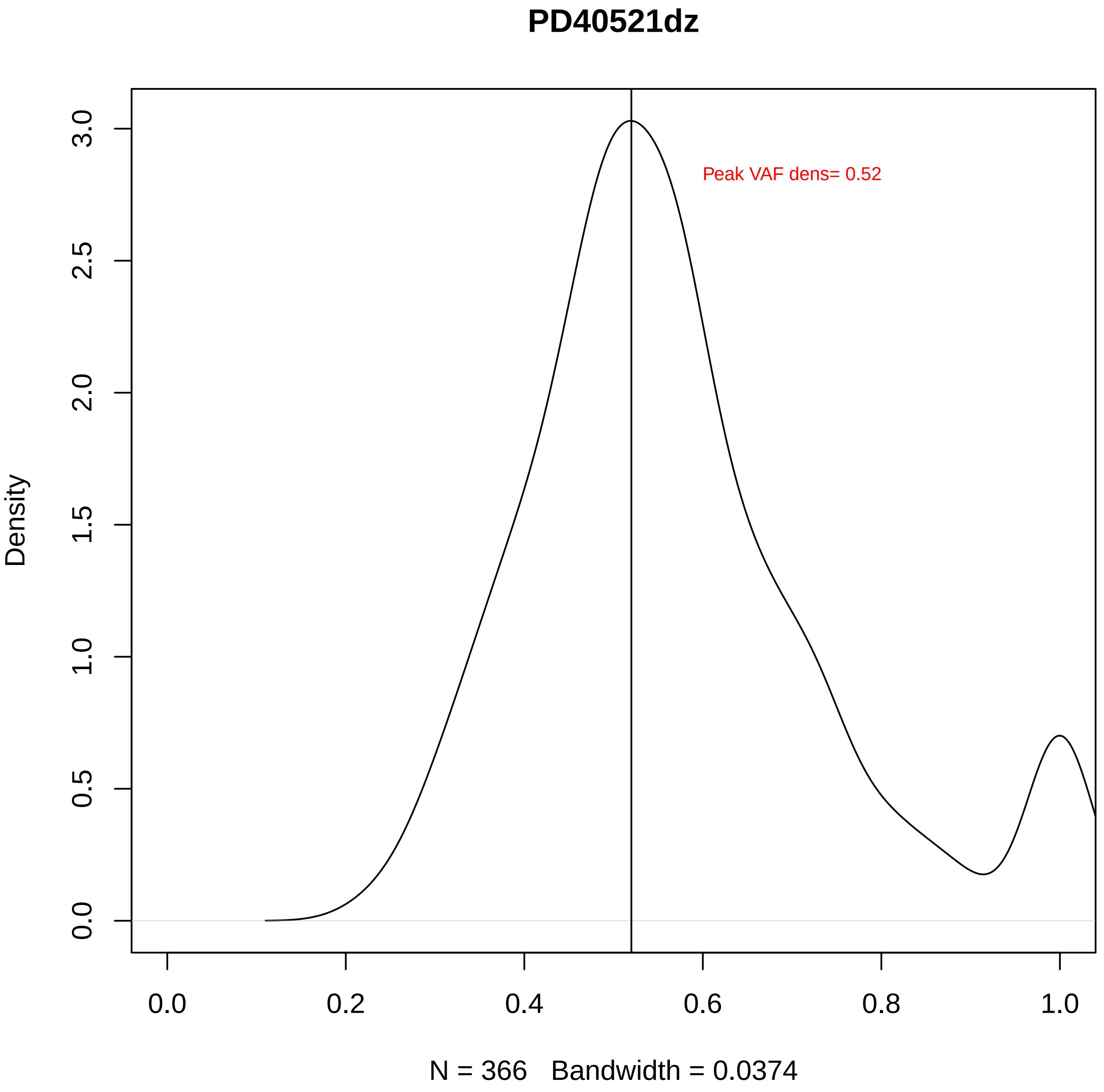

PD40521dz
Peak VAF dens= 0.52
Density
0.0
0.5
1.0
1.5
2.0
2.5
3.0
0.0
0.2
0.4
0.6
0.8
1.0
N = 366 Bandwidth = 0.0374

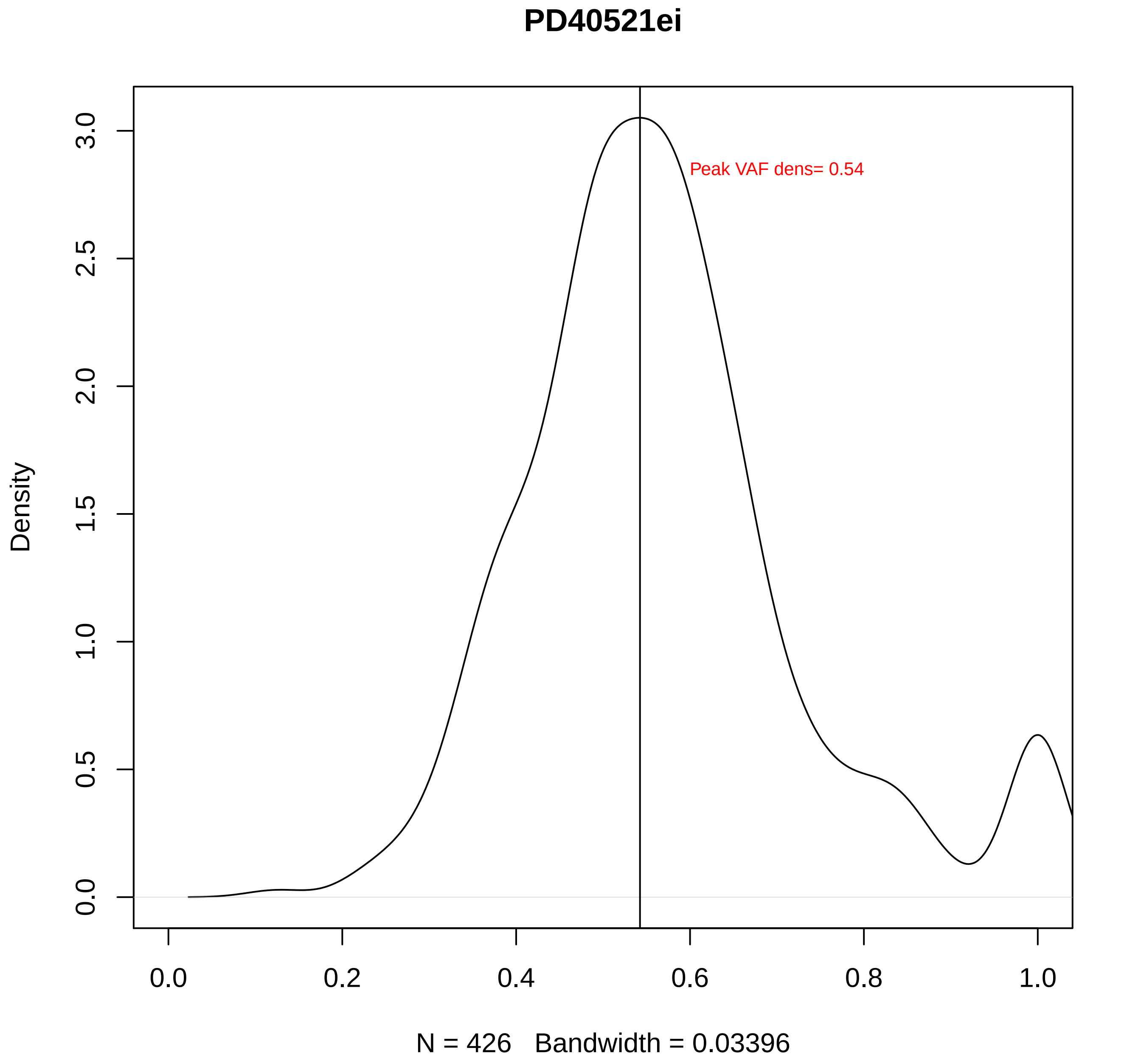
PD40521ei
Peak VAF dens= 0.54
Density
0.0
0.5
1.0
1.5
2.0
2.5
3.0
0.0
0.2
0.4
0.6
0.8
1.0
N = 426 Bandwidth = 0.03396

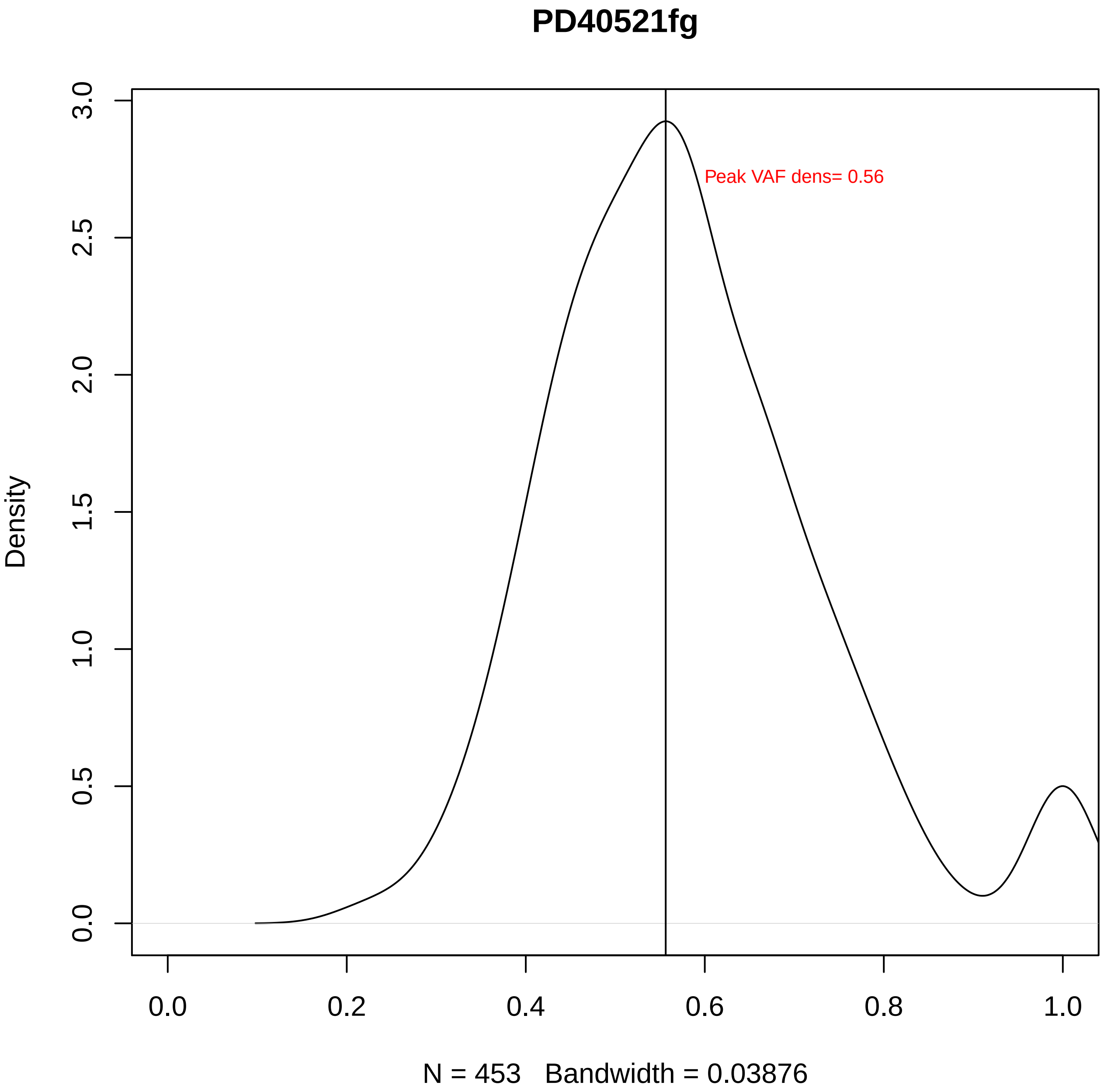

PD40521fg
Peak VAF dens= 0.56
Density
0.0
0.5
1.0
1.5
2.0
2.5
3.0
0.0
0.2
0.4
0.6
0.8
1.0
N = 453   Bandwidth = 0.03876

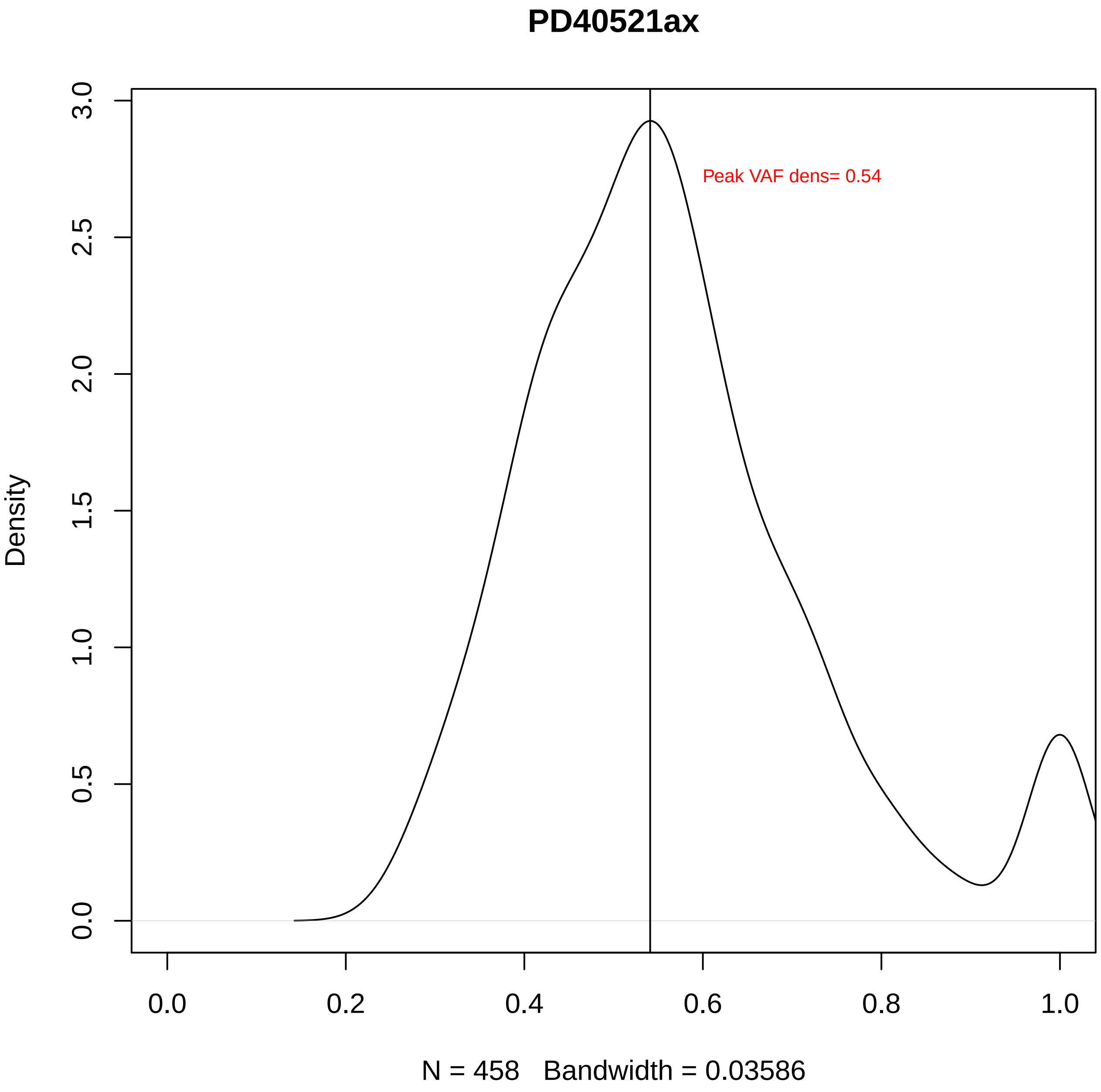
PD40521ax
Peak VAF dens= 0.54
Density
0.0
0.5
1.0
1.5
2.0
2.5
3.0
0.0
0.2
0.4
0.6
0.8
1.0
N = 458   Bandwidth = 0.03586

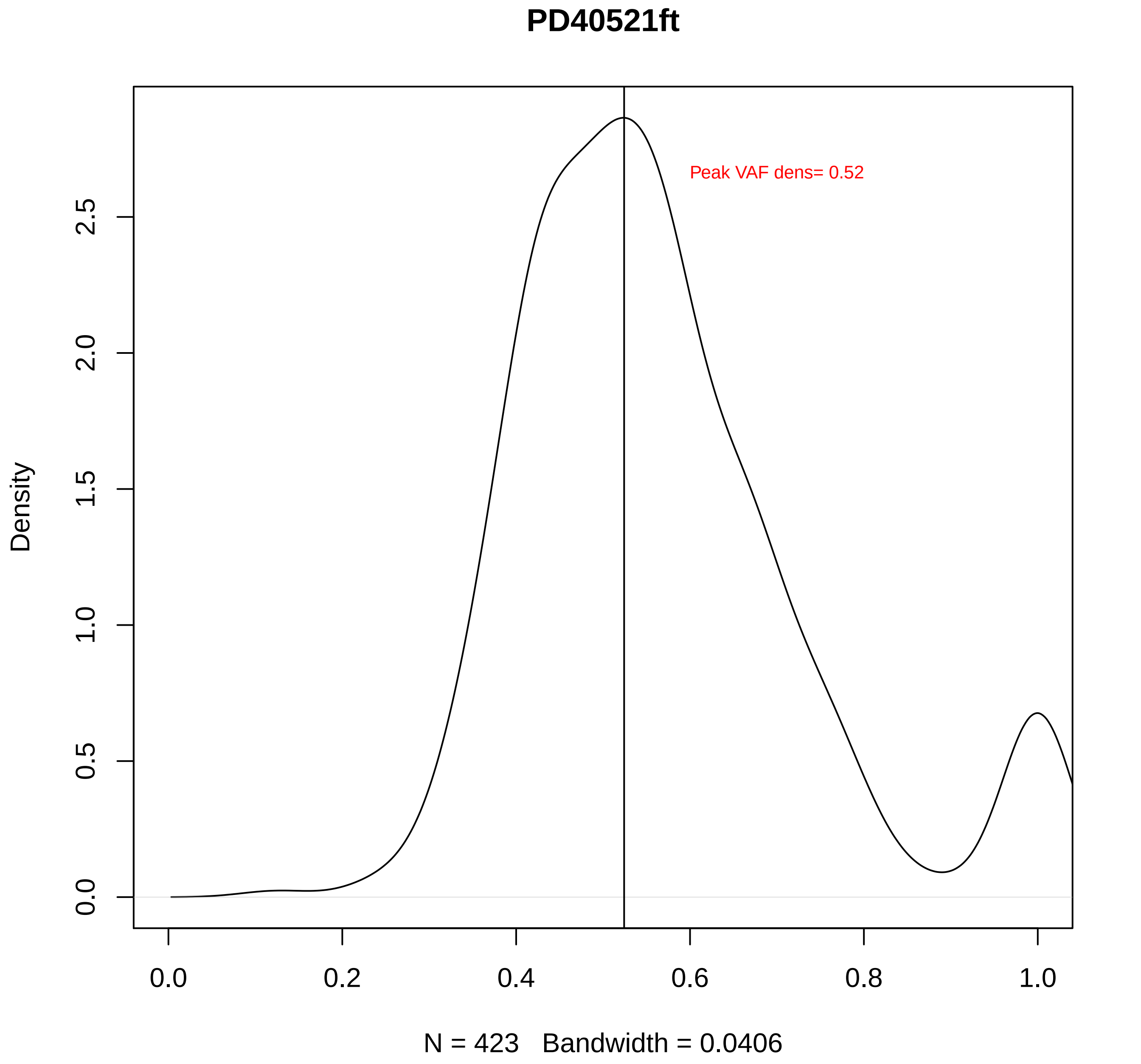
PD40521ft
Peak VAF dens= 0.52
Density
0.0
0.5
1.0
1.5
2.0
2.5
0.0
0.2
0.4
0.6
0.8
1.0
N = 423   Bandwidth = 0.0406

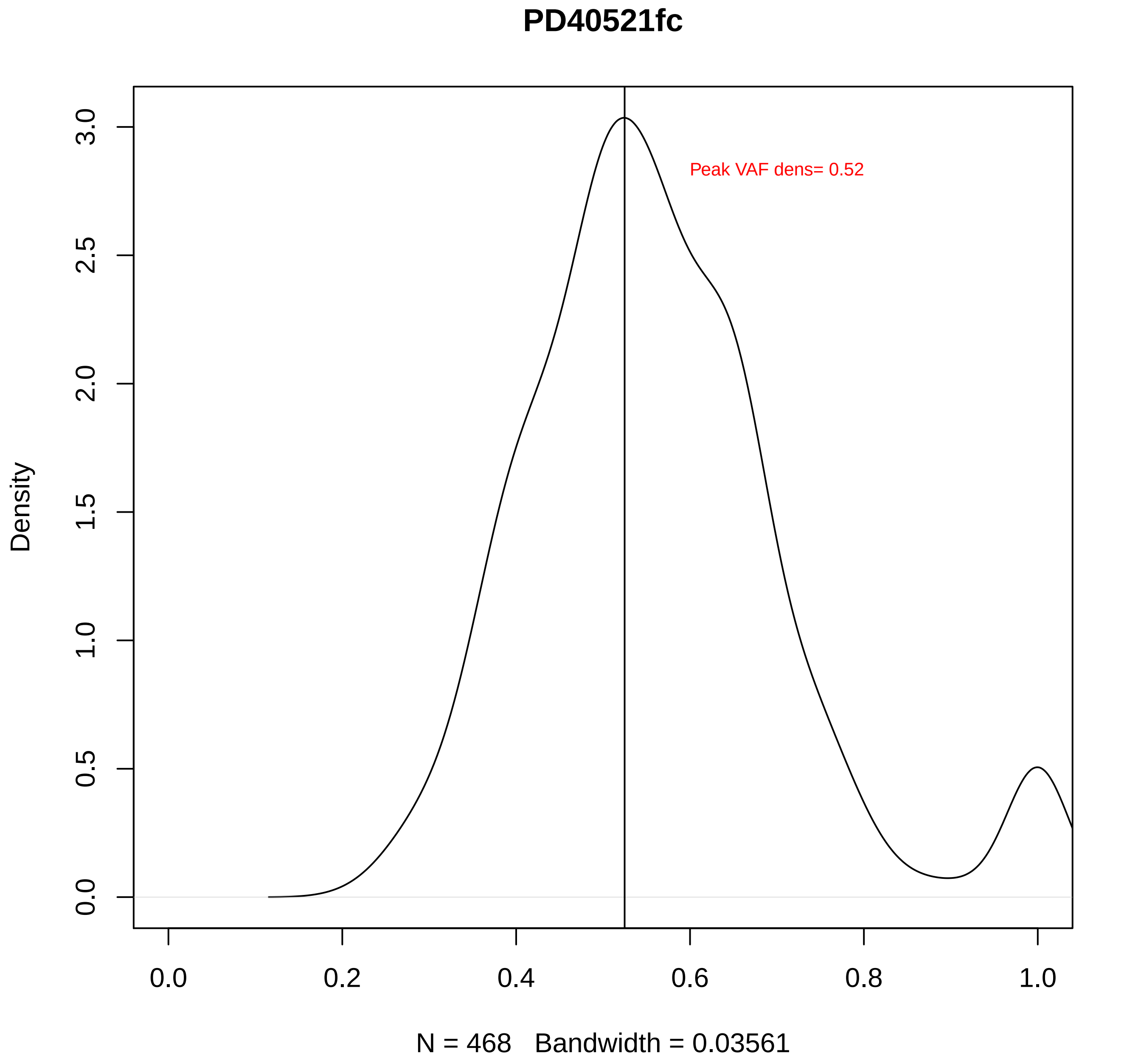
PD40521fc
Peak VAF dens= 0.52
Density
3.0
2.5
2.0
1.5
1.0
0.5
0.0
0.0
0.2
0.4
0.6
0.8
1.0
N = 468   Bandwidth = 0.03561

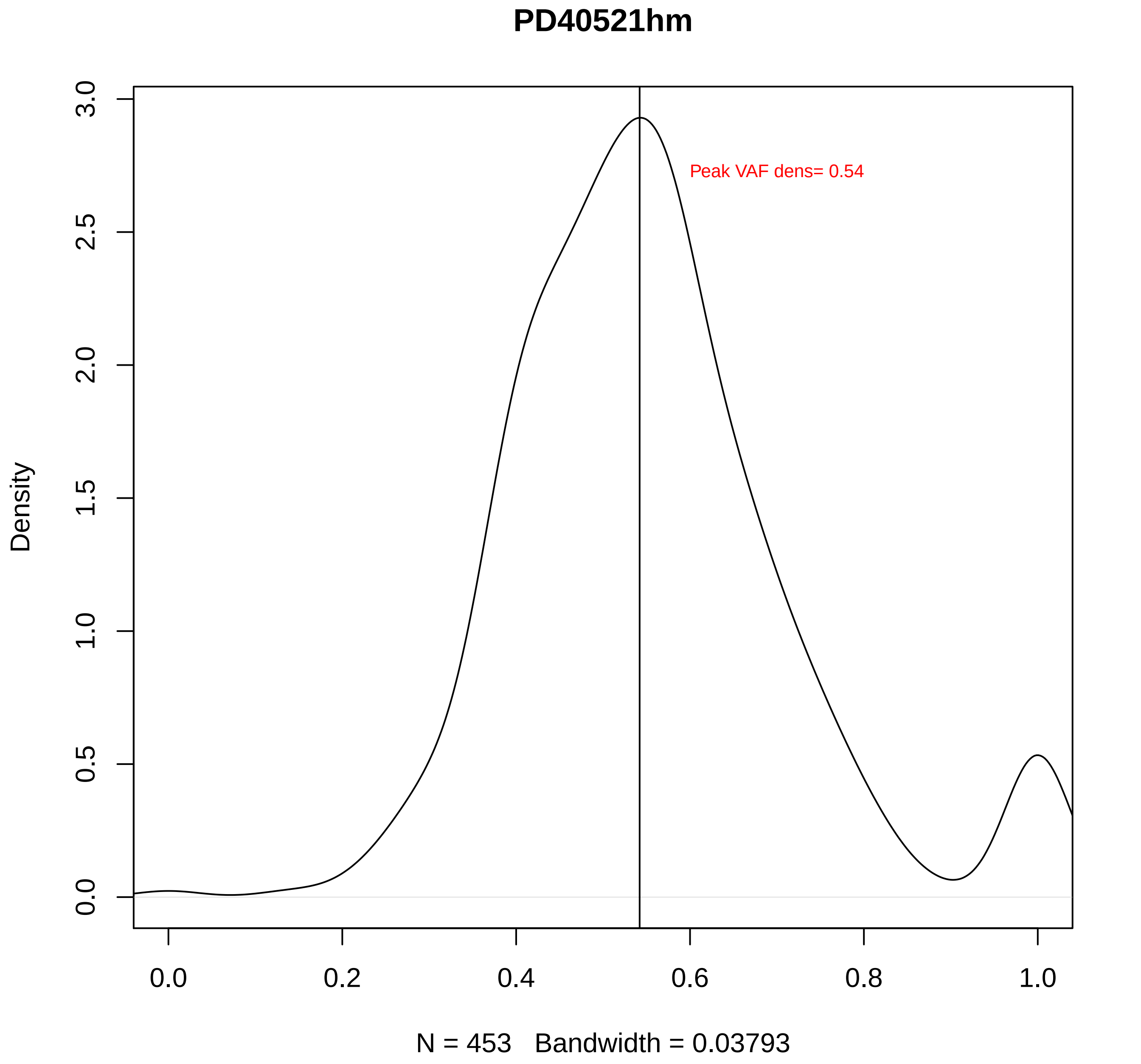

PD40521hm
Peak VAF dens= 0.54
Density
0.0
0.5
1.0
1.5
2.0
2.5
3.0
0.0
0.2
0.4
0.6
0.8
1.0
N = 453   Bandwidth = 0.03793

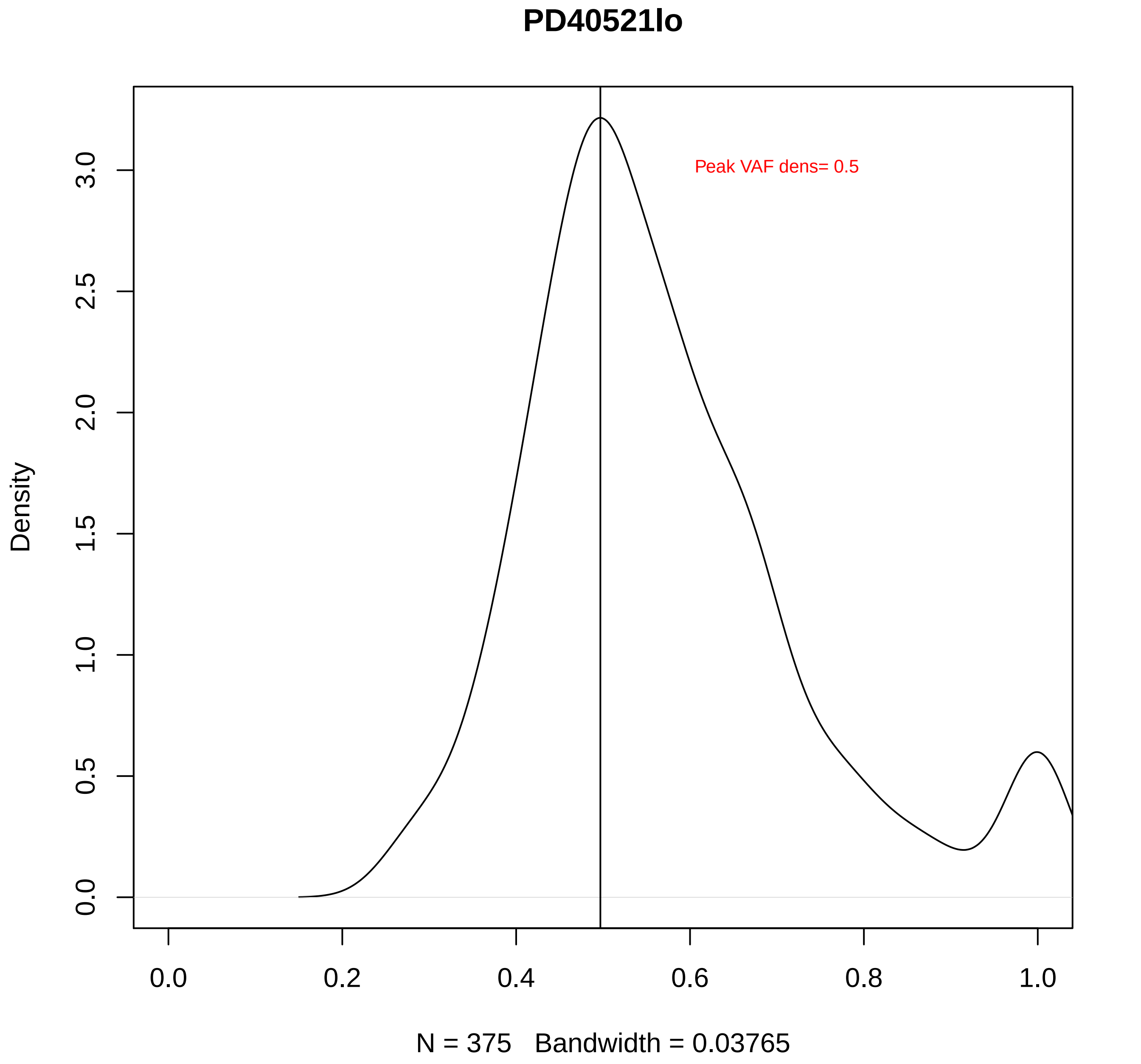
PD40521lo
Peak VAF dens= 0.5
Density
0.0
0.5
1.0
1.5
2.0
2.5
3.0
0.0
0.2
0.4
0.6
0.8
1.0
N = 375   Bandwidth = 0.03765

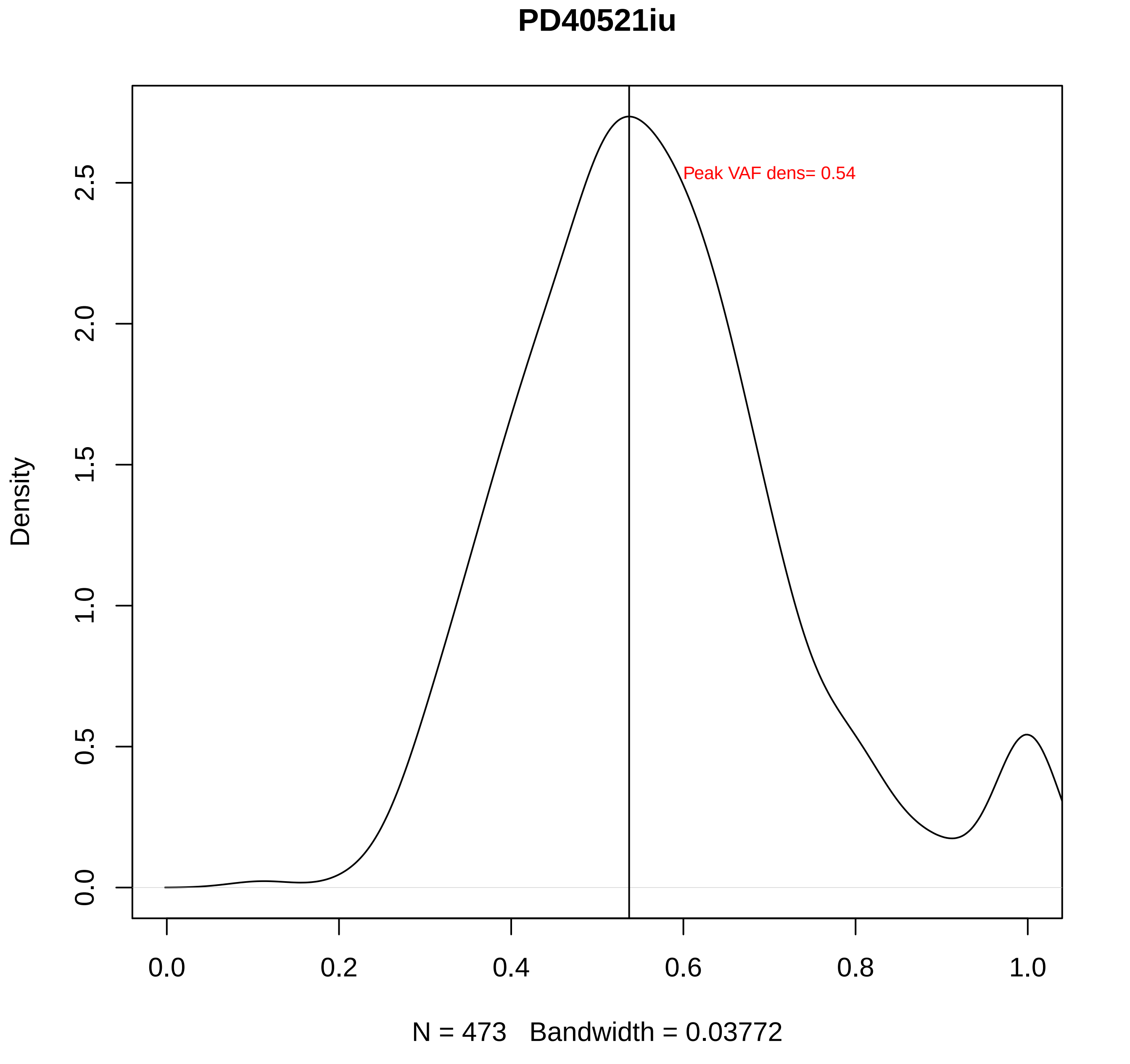

PD40521iu
Peak VAF dens= 0.54
Density
0.0
0.5
1.0
1.5
2.0
2.5
0.0
0.2
0.4
0.6
0.8
1.0
N = 473 Bandwidth = 0.03772

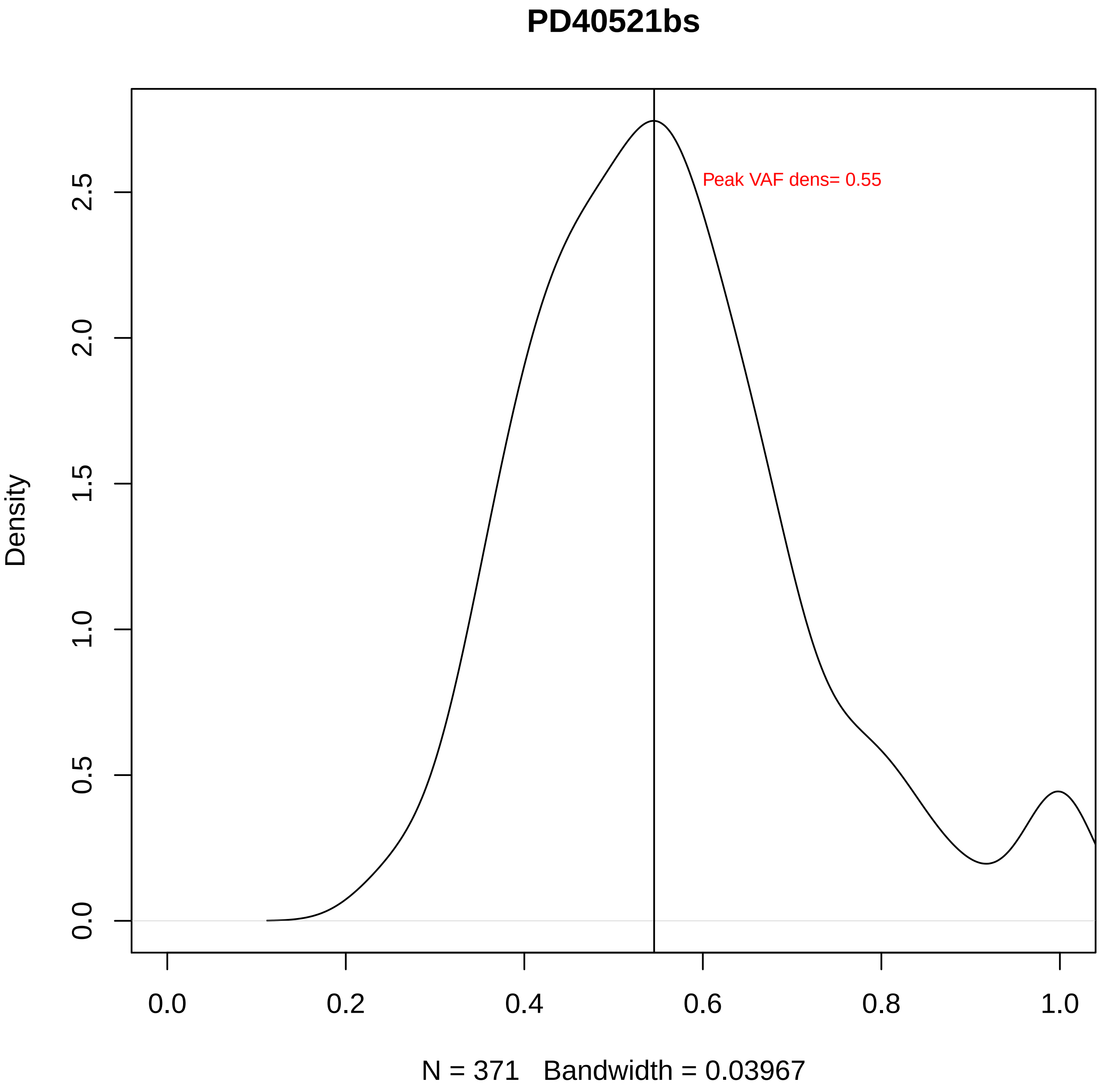
PD40521bs
Peak VAF dens= 0.55
Density
0.0
0.5
1.0
1.5
2.0
2.5
0.0
0.2
0.4
0.6
0.8
1.0
N = 371   Bandwidth = 0.03967

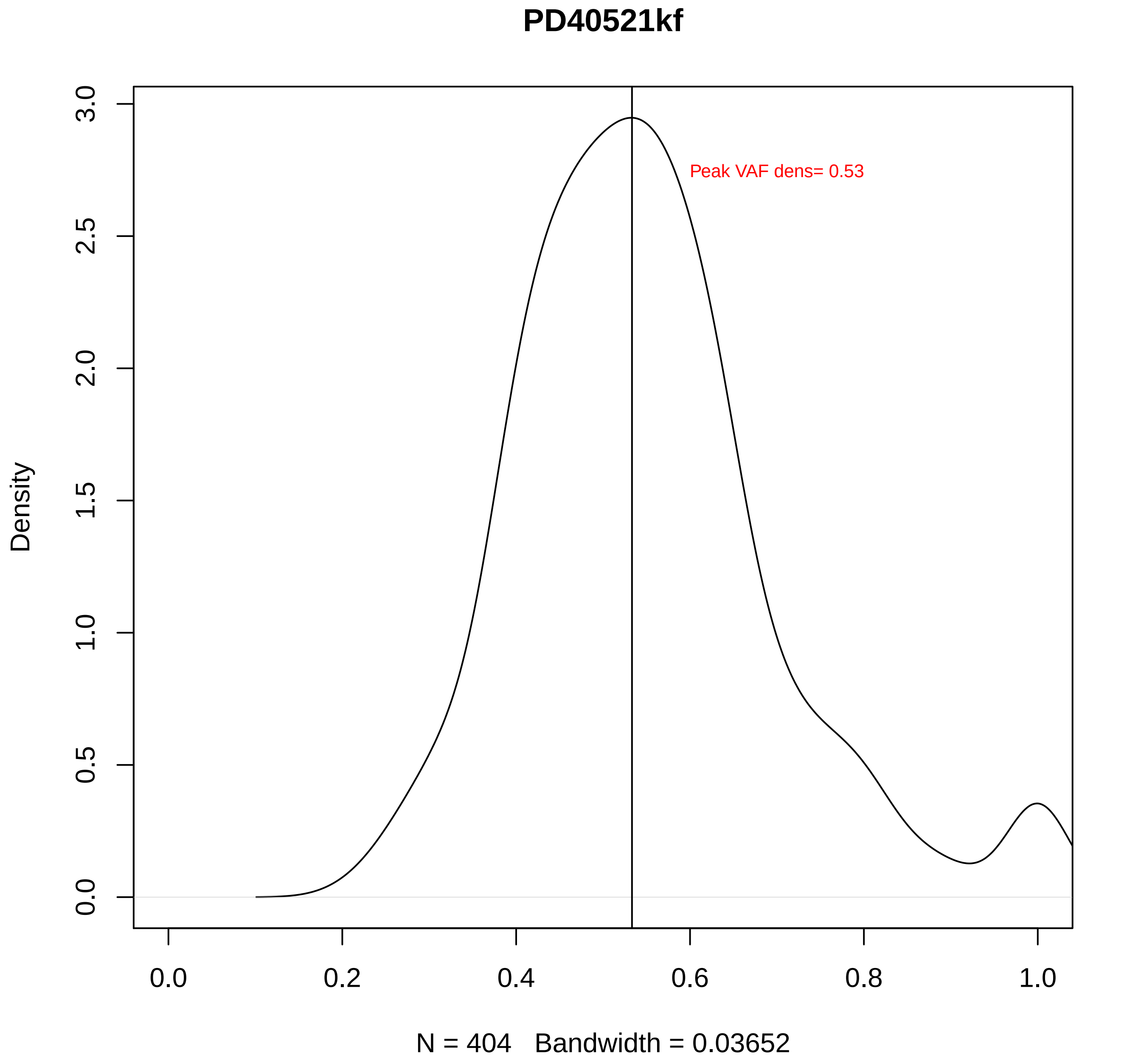
PD40521kf
Peak VAF dens= 0.53
Density
0.0
0.5
1.0
1.5
2.0
2.5
3.0
0.0
0.2
0.4
0.6
0.8
1.0
N = 404   Bandwidth = 0.03652

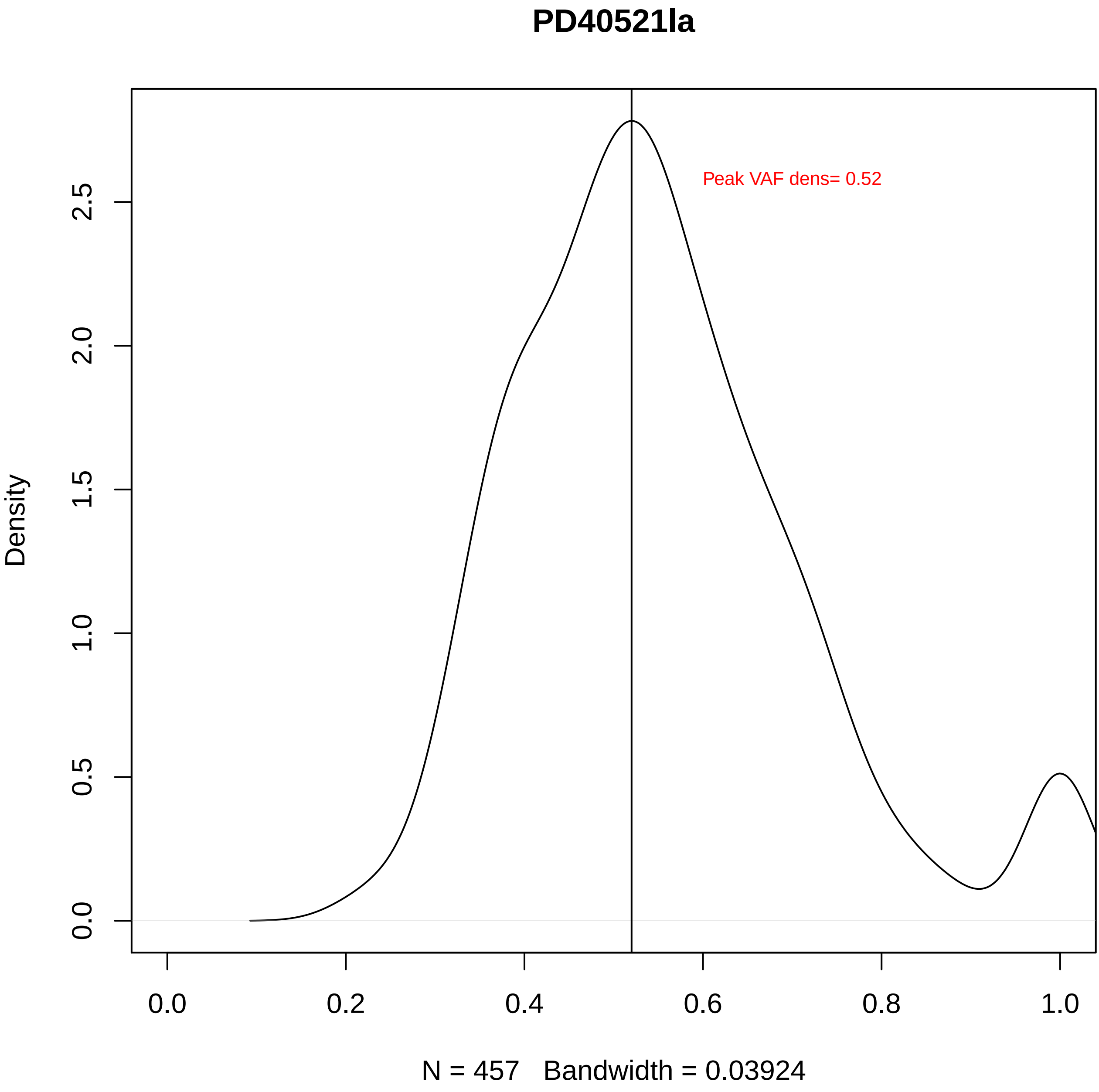

PD40521la
Peak VAF dens= 0.52
Density
0.0
0.5
1.0
1.5
2.0
2.5
0.0
0.2
0.4
0.6
0.8
1.0
N = 457   Bandwidth = 0.03924

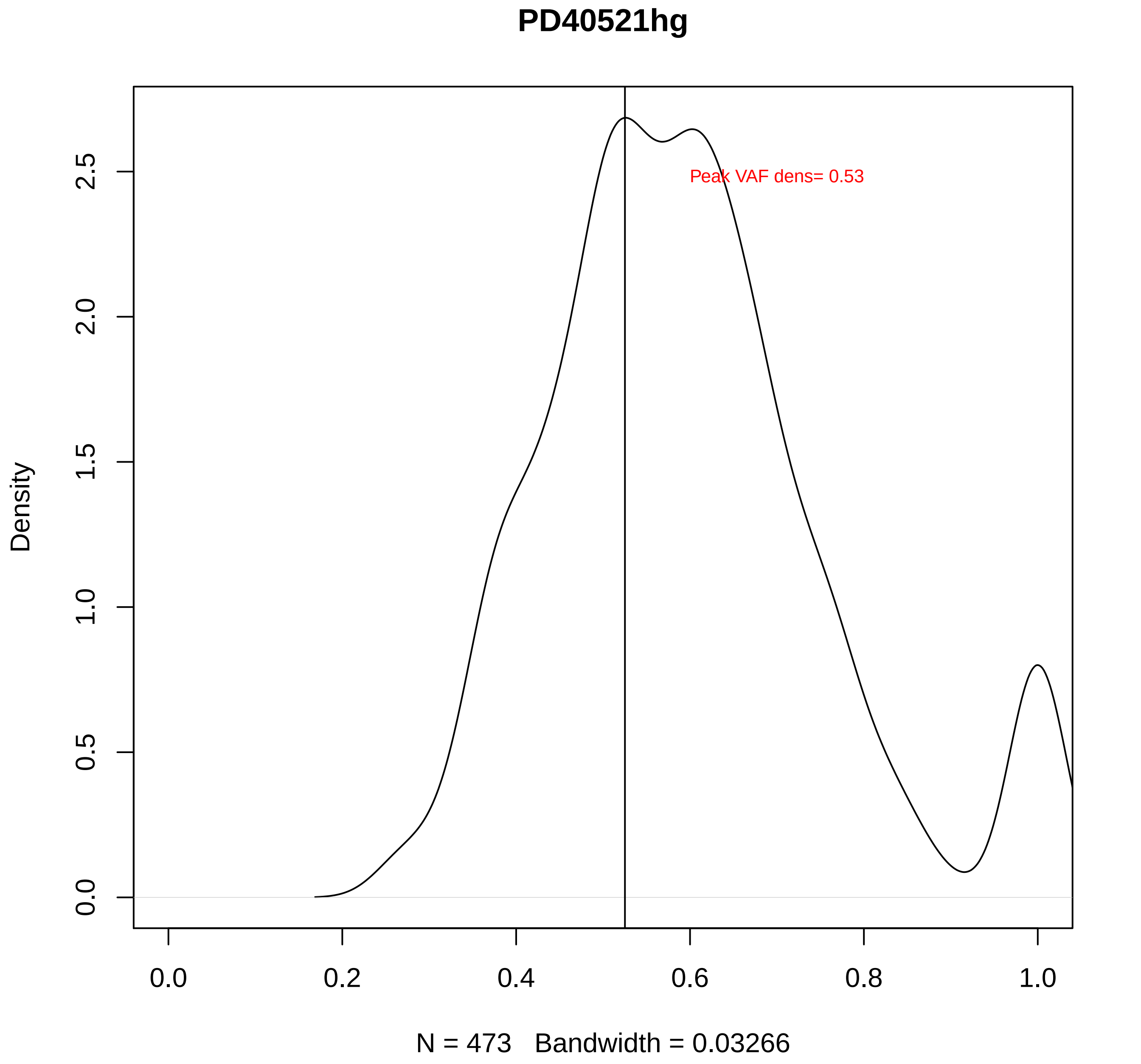
PD40521hg
Peak VAF dens= 0.53
Density
0.0
0.5
1.0
1.5
2.0
2.5
0.0
0.2
0.4
0.6
0.8
1.0
N = 473   Bandwidth = 0.03266

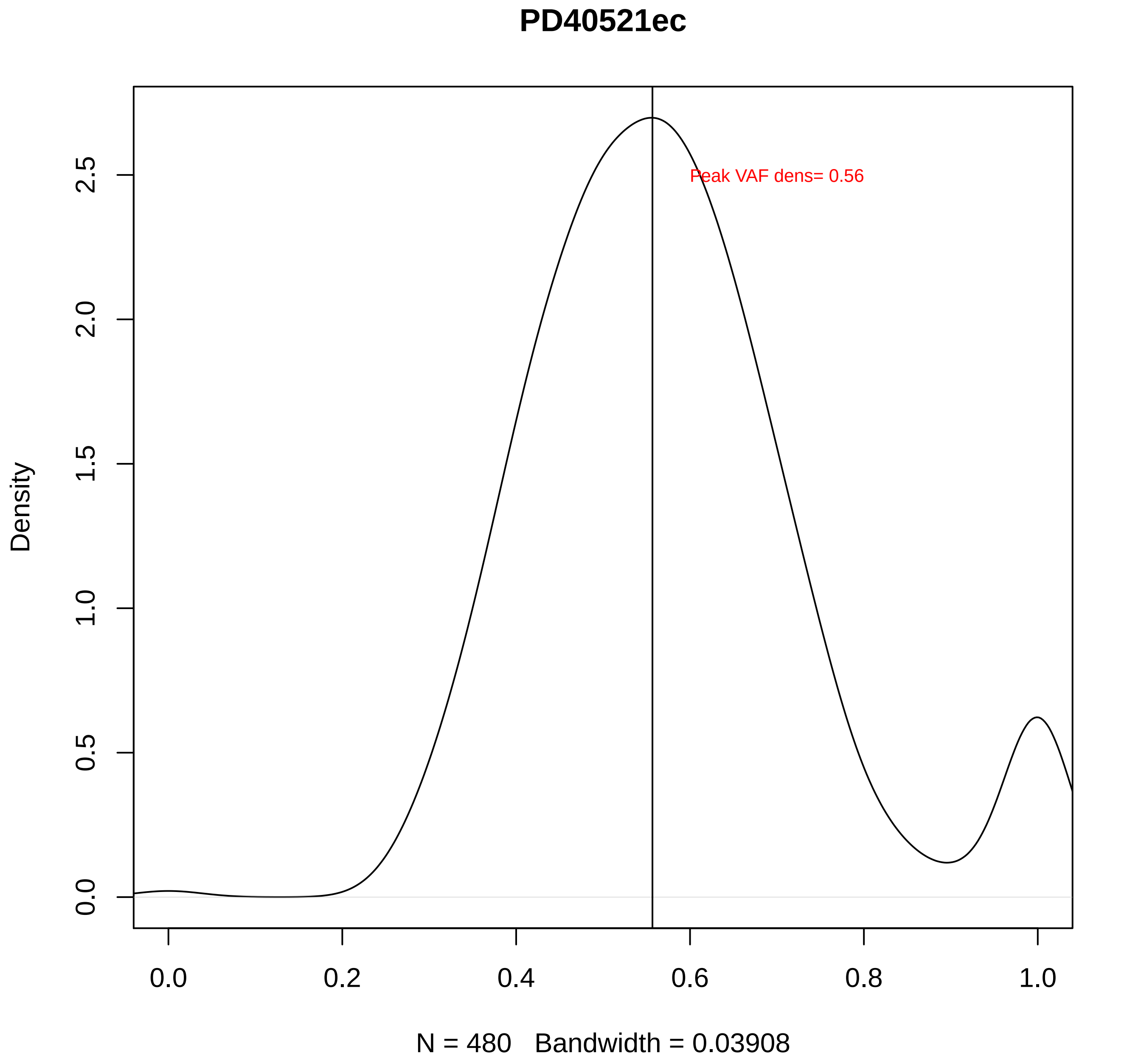
PD40521ec
Peak VAF dens= 0.56
Density
0.0
0.5
1.0
1.5
2.0
2.5
0.0
0.2
0.4
0.6
0.8
1.0
N = 480   Bandwidth = 0.03908

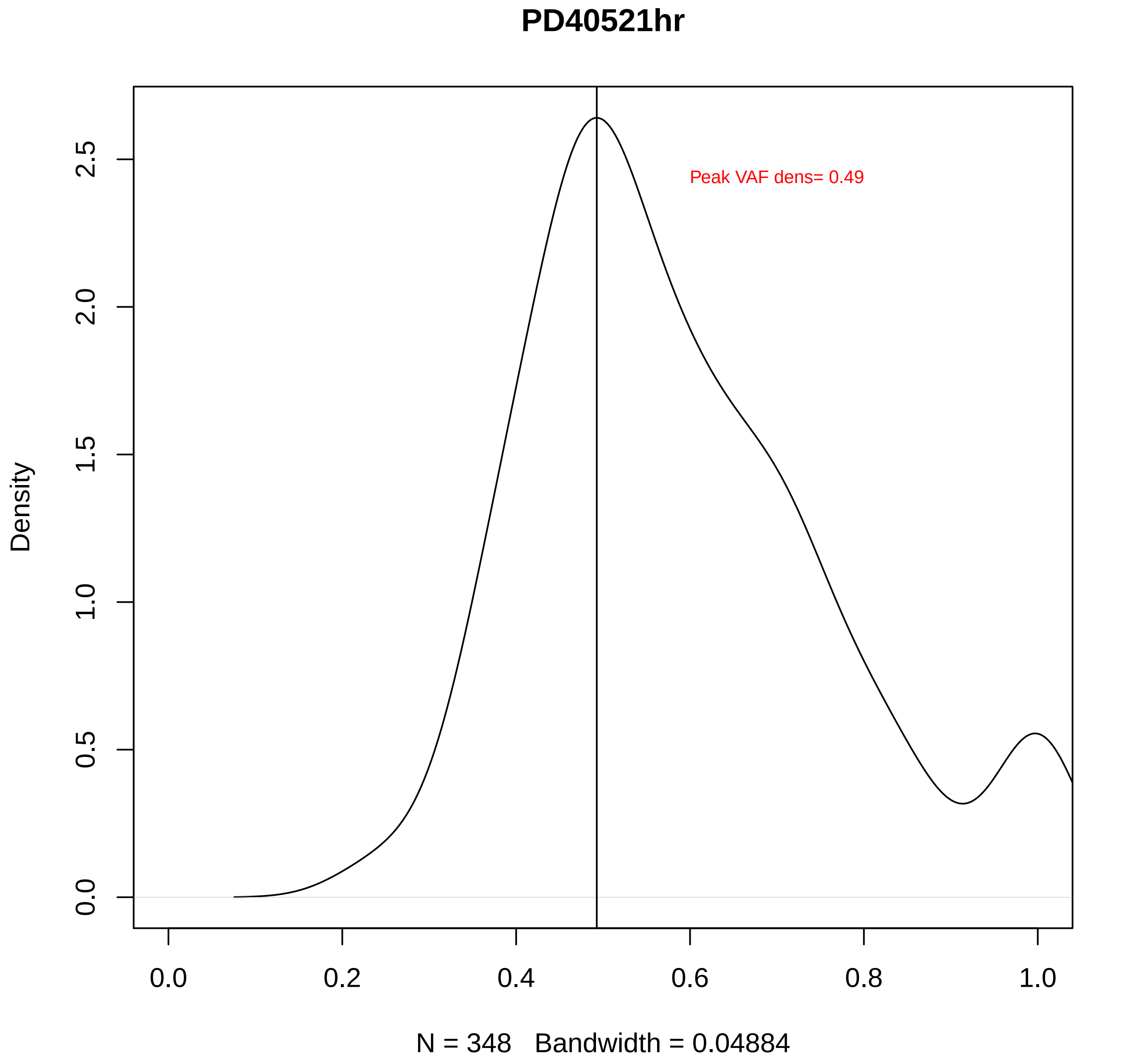
PD40521hr
Peak VAF dens= 0.49
Density
0.0
0.5
1.0
1.5
2.0
2.5
0.0
0.2
0.4
0.6
0.8
1.0
N = 348   Bandwidth = 0.04884

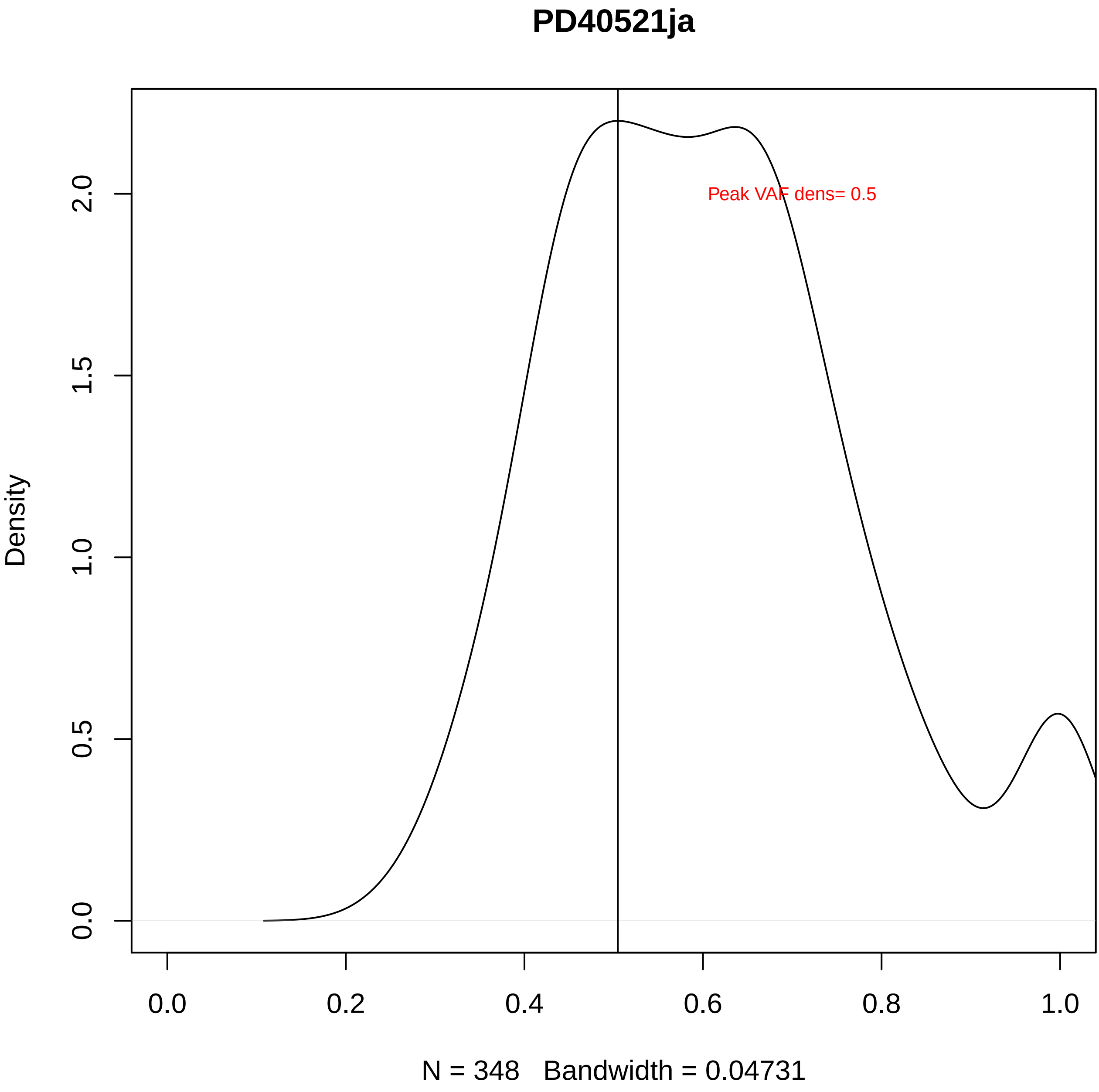
PD40521ja
Peak VAF dens= 0.5
Density
0.0
0.5
1.0
1.5
2.0
0.0
0.2
0.4
0.6
0.8
1.0
N = 348   Bandwidth = 0.04731

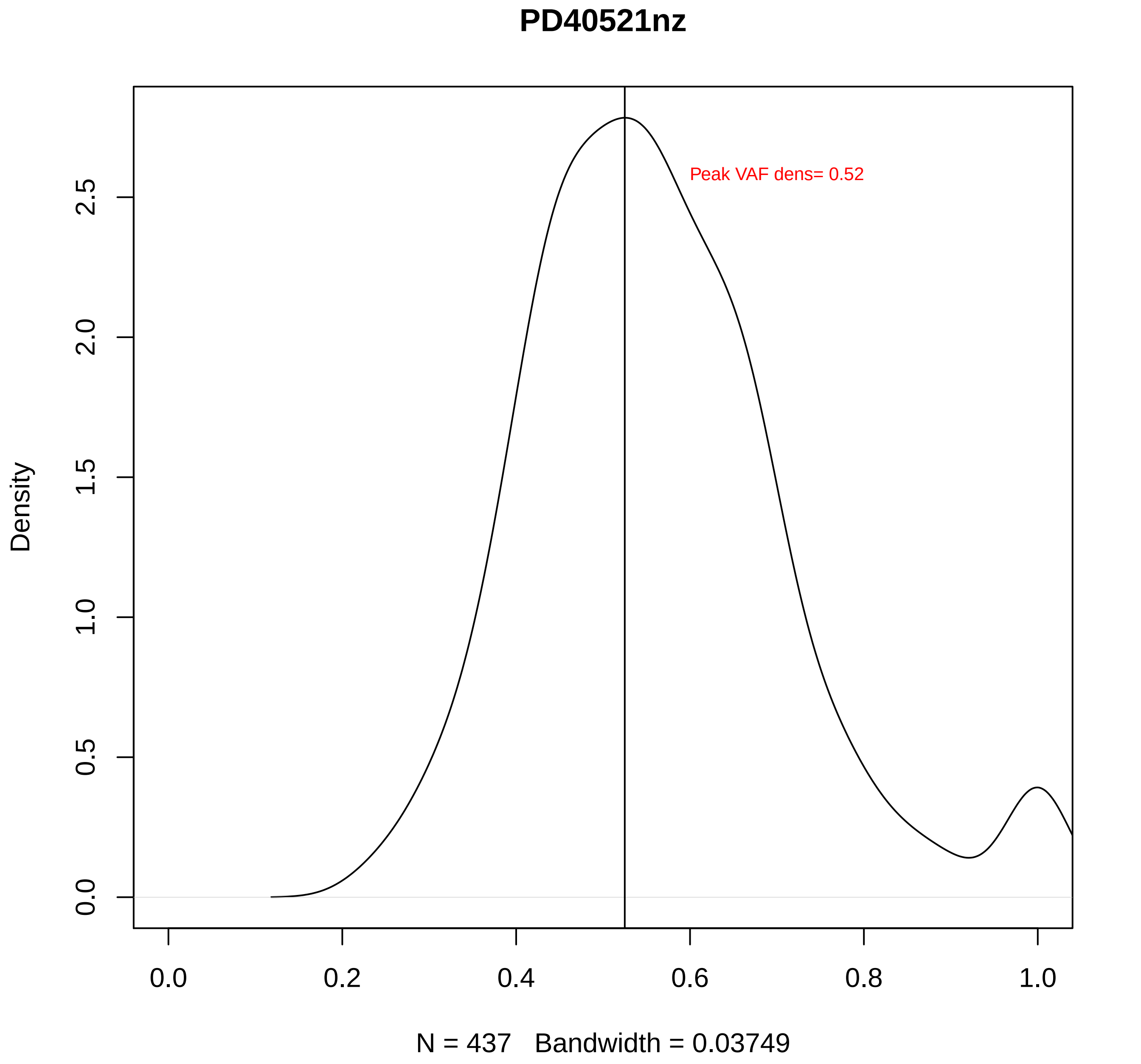
PD40521nz
Peak VAF dens= 0.52
Density
0.0
0.5
1.0
1.5
2.0
2.5
0.0
0.2
0.4
0.6
0.8
1.0
N = 437   Bandwidth = 0.03749

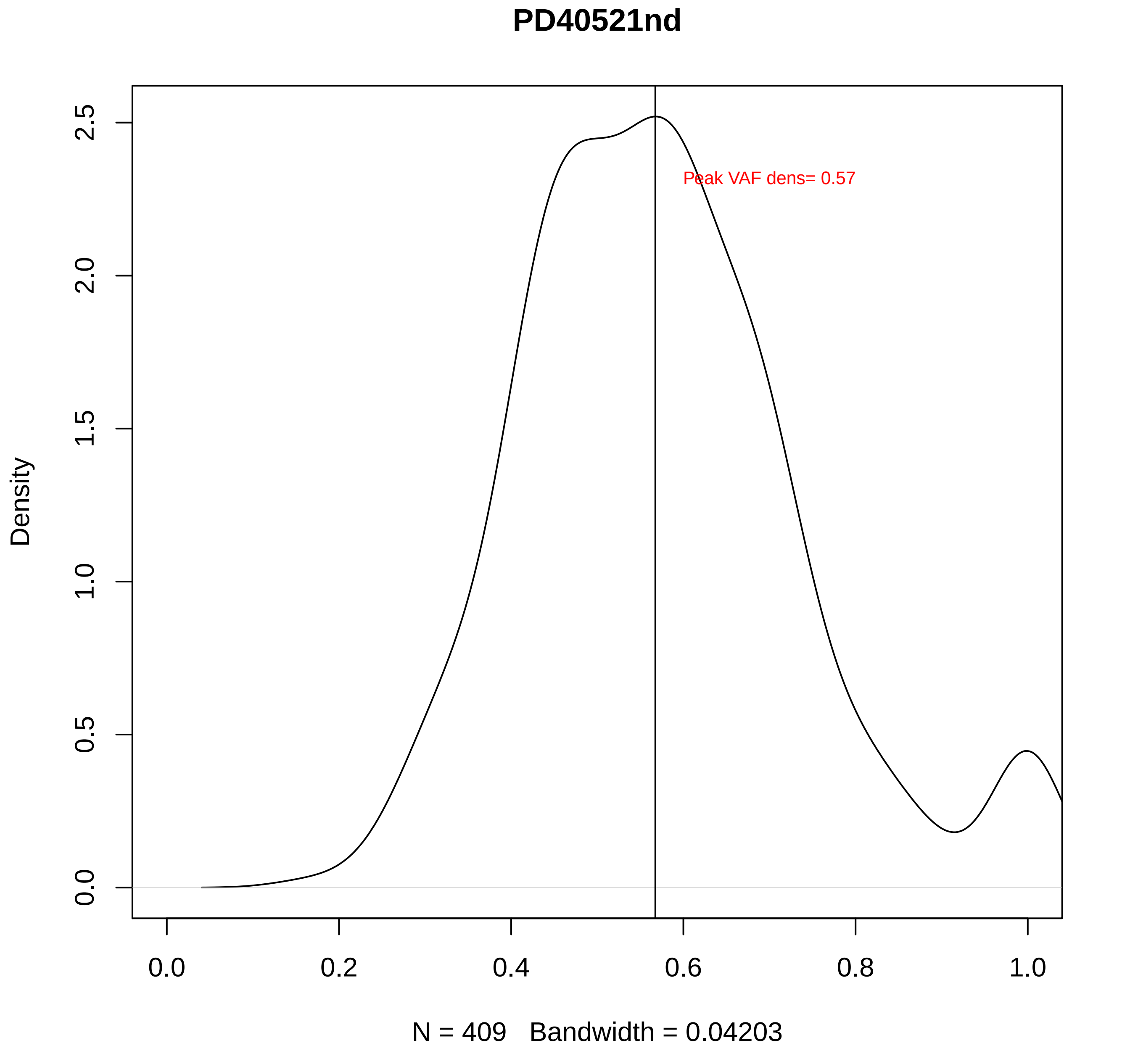

PD40521nd
Peak VAF dens= 0.57
Density
0.0
0.5
1.0
1.5
2.0
2.5
0.0
0.2
0.4
0.6
0.8
1.0
N = 409 Bandwidth = 0.04203

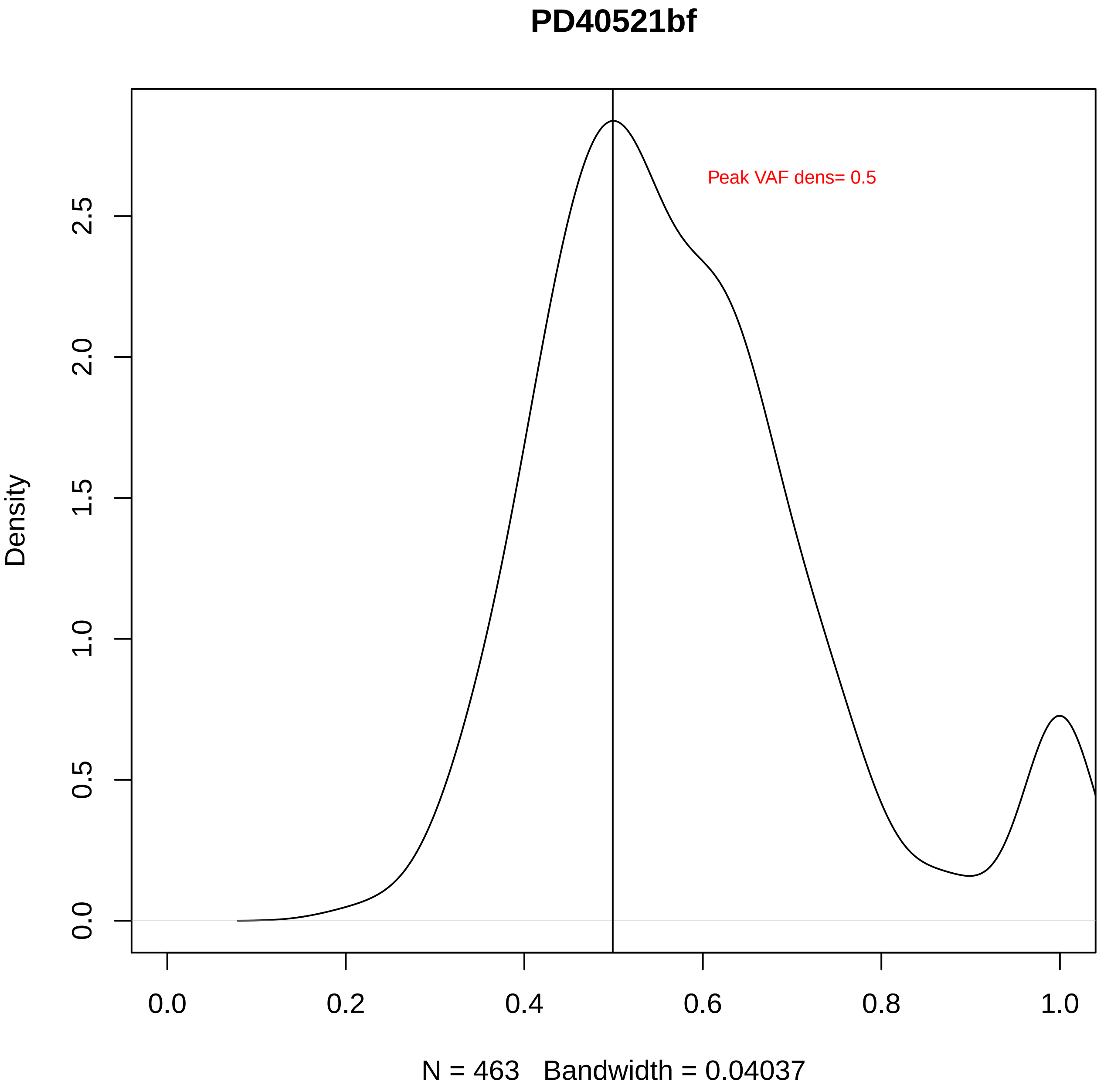

PD40521bf
Peak VAF dens= 0.5
Density
0.0
0.5
1.0
1.5
2.0
2.5
0.0
0.2
0.4
0.6
0.8
1.0
N = 463   Bandwidth = 0.04037

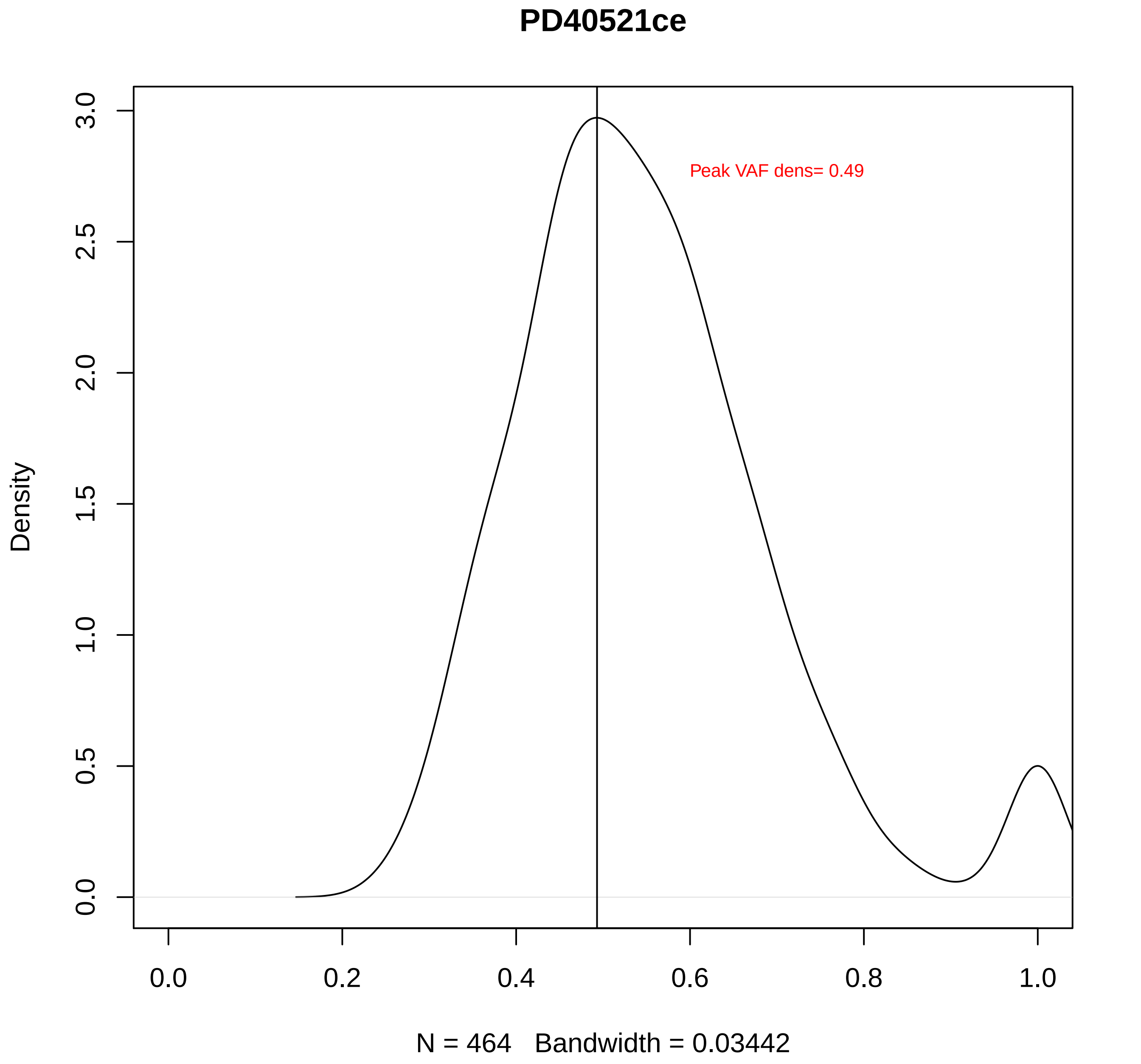

PD40521ce
Peak VAF dens= 0.49
Density
0.0
0.5
1.0
1.5
2.0
2.5
3.0
0.0
0.2
0.4
0.6
0.8
1.0
N = 464   Bandwidth = 0.03442

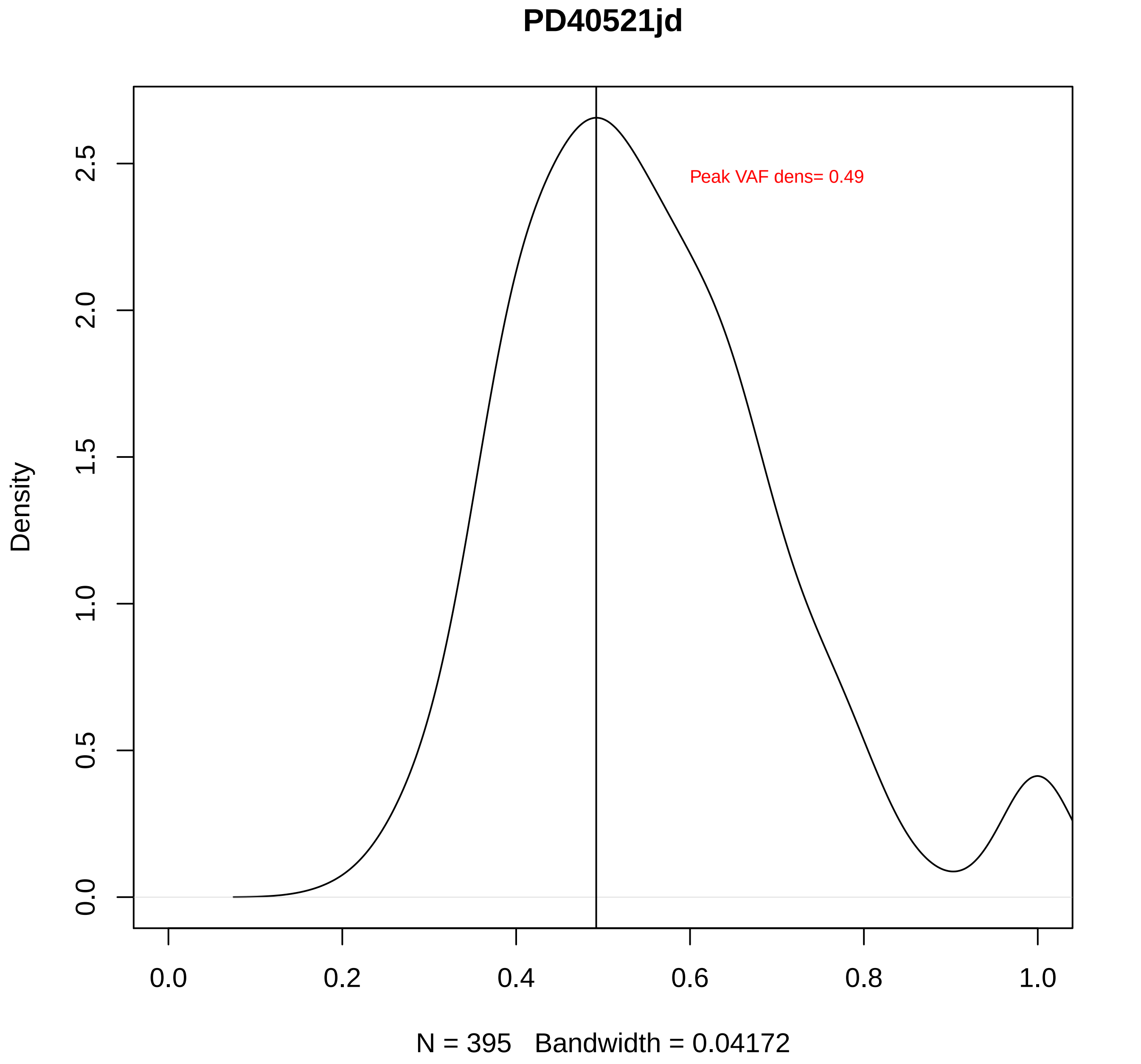

PD40521jd
Peak VAF dens= 0.49
Density
0.0
0.5
1.0
1.5
2.0
2.5
0.0
0.2
0.4
0.6
0.8
1.0
N = 395 Bandwidth = 0.04172

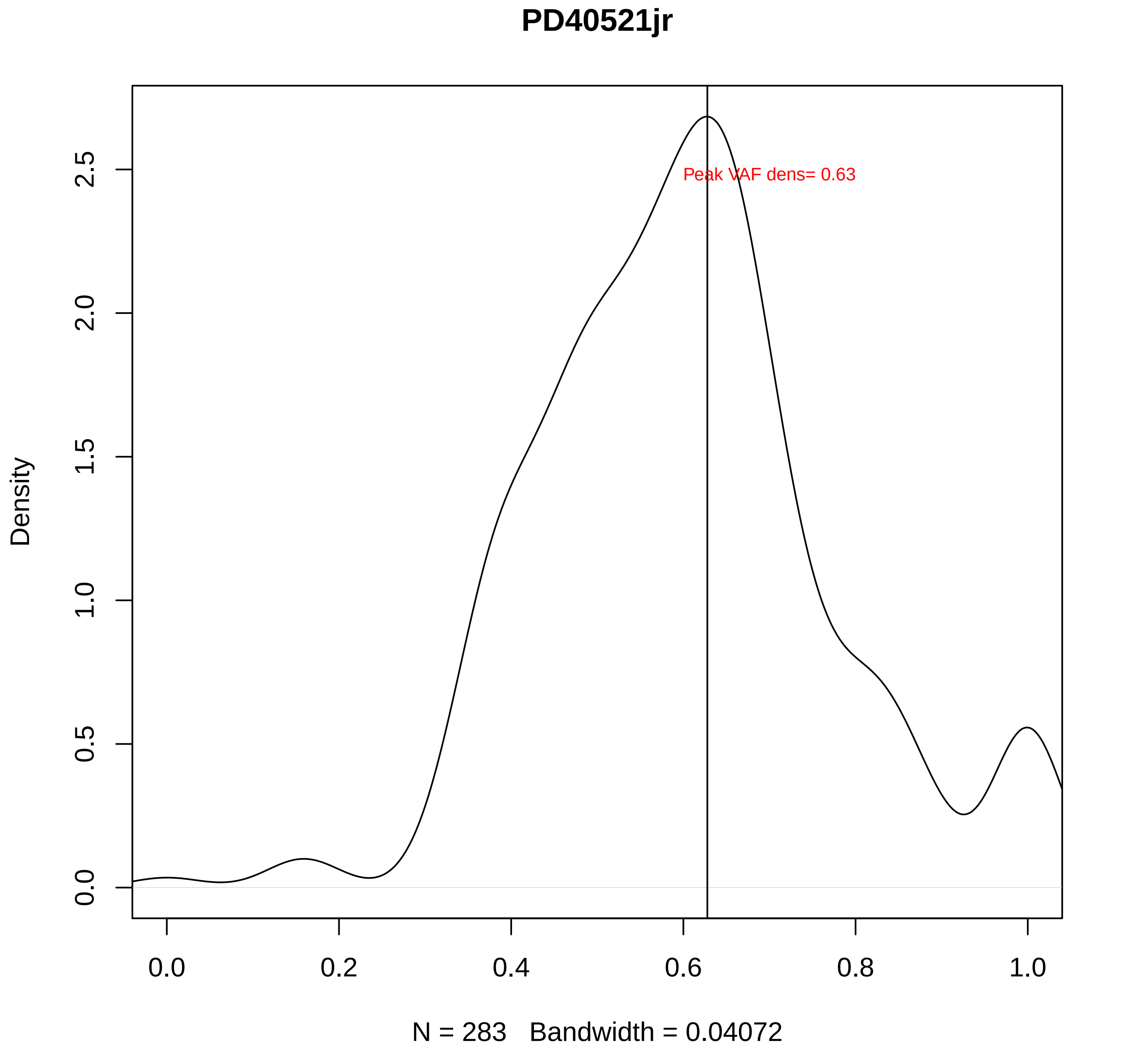

PD40521jr
Peak VAF dens= 0.63
Density
0.0
0.5
1.0
1.5
2.0
2.5
0.0
0.2
0.4
0.6
0.8
1.0
N = 283   Bandwidth = 0.04072

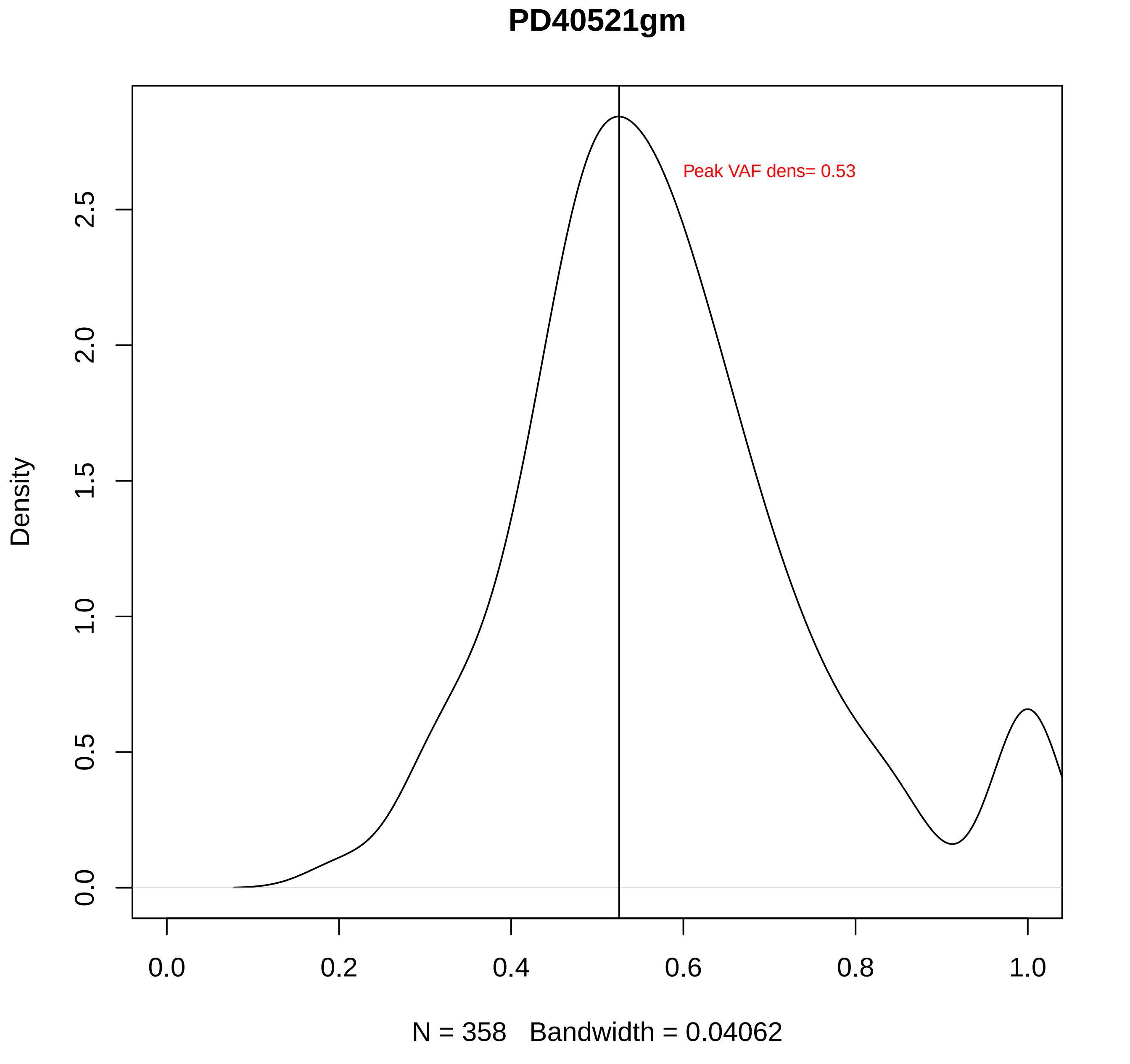
PD40521gm
Peak VAF dens= 0.53
Density
0.0
0.5
1.0
1.5
2.0
2.5
0.0
0.2
0.4
0.6
0.8
1.0
N = 358   Bandwidth = 0.04062

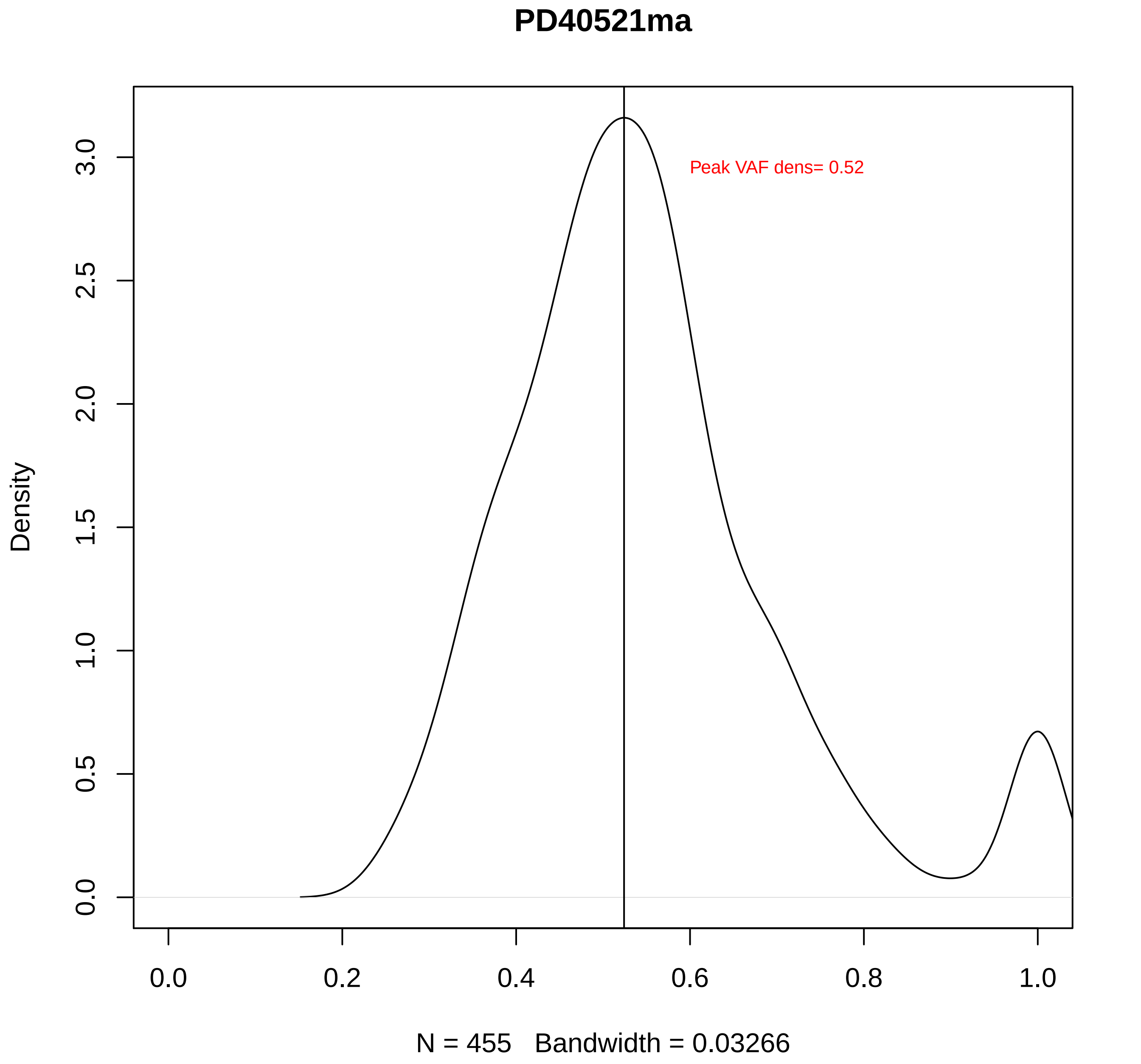
PD40521ma
Peak VAF dens= 0.52
Density
0.0
0.5
1.0
1.5
2.0
2.5
3.0
0.0
0.2
0.4
0.6
0.8
1.0
N = 455   Bandwidth = 0.03266

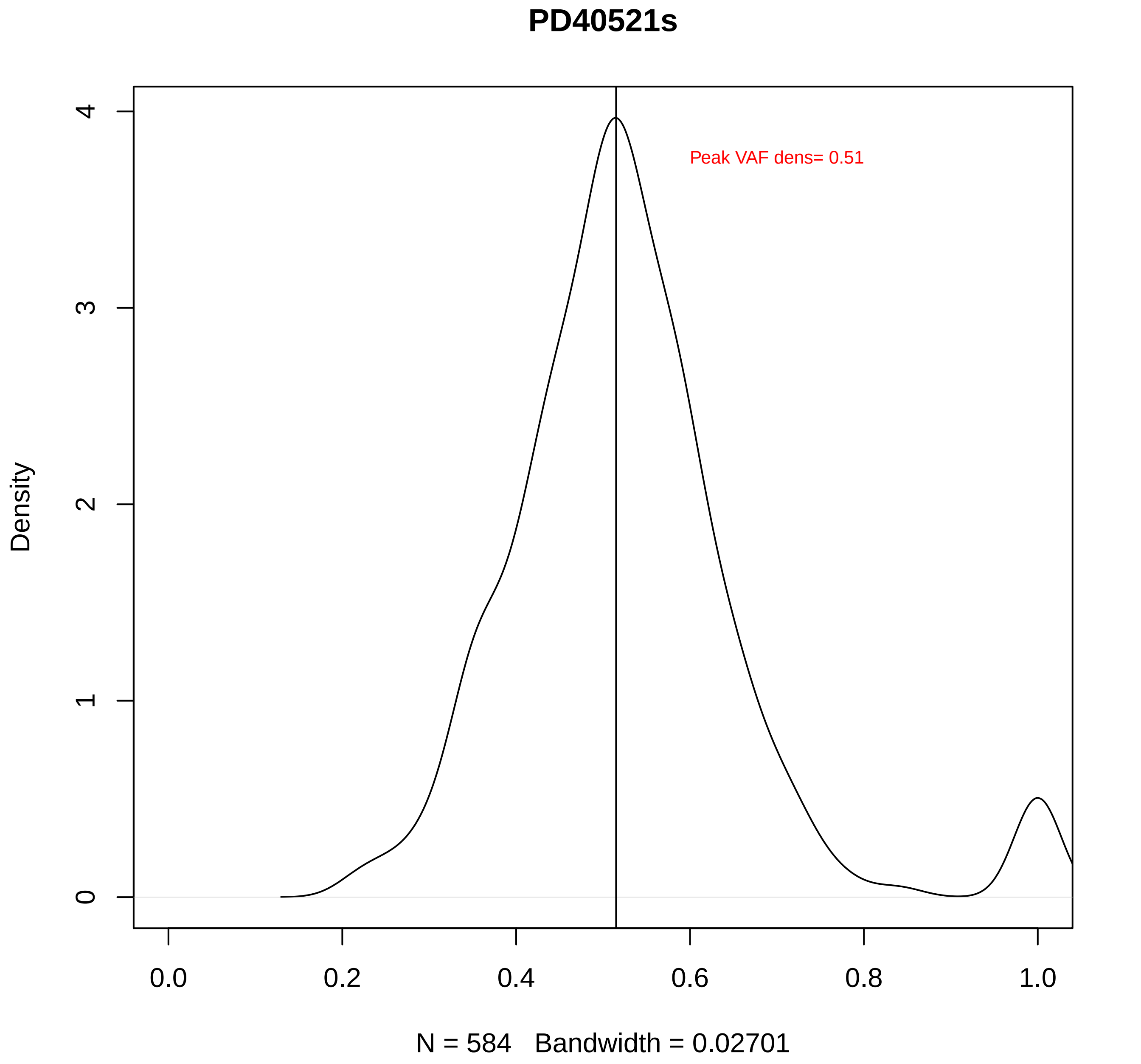
PD40521s
Peak VAF dens= 0.51
Density
0
1
2
3
4
0.0
0.2
0.4
0.6
0.8
1.0
N = 584   Bandwidth = 0.02701

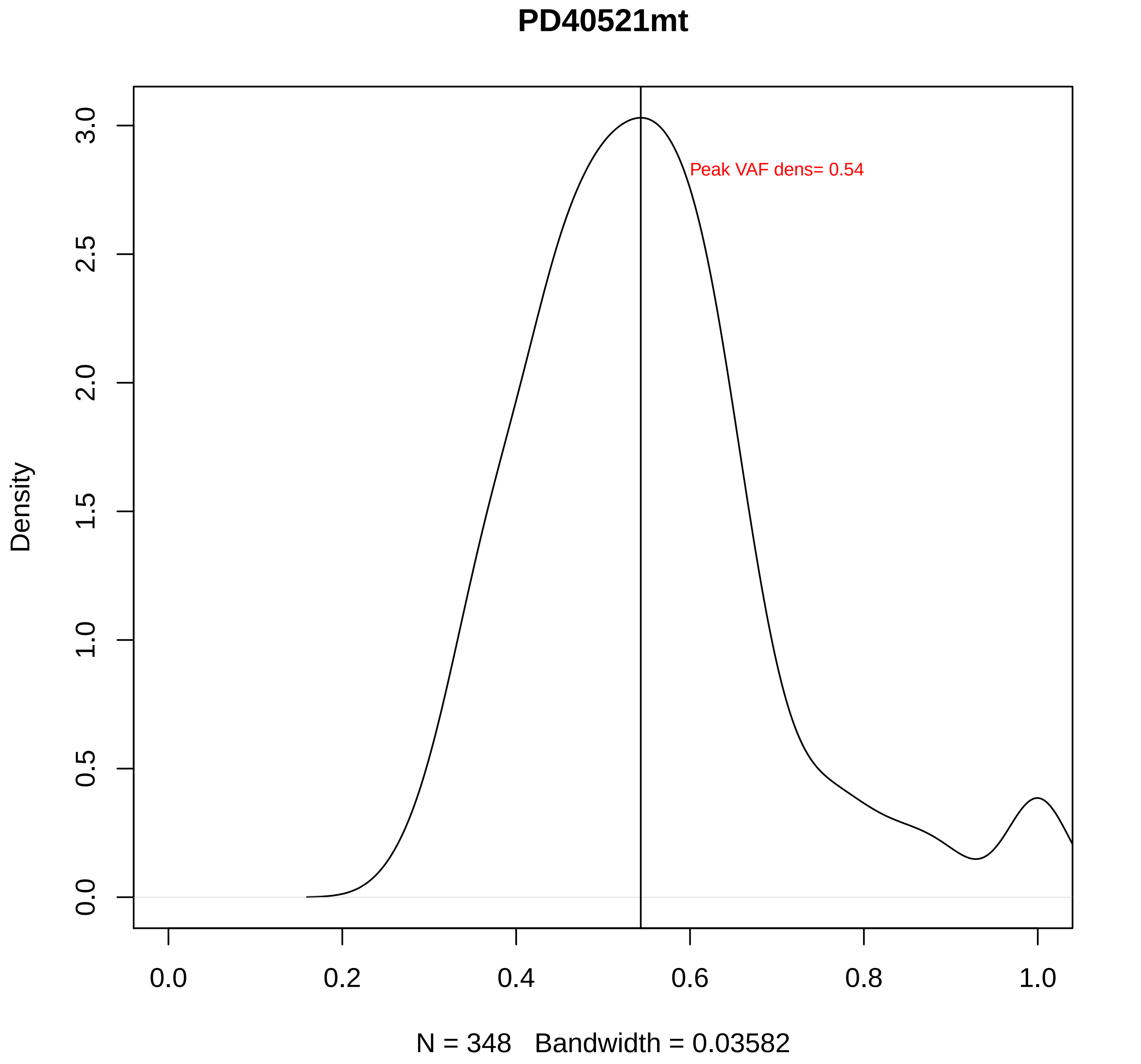
PD40521mt
Peak VAF dens= 0.54
Density
0.0
0.5
1.0
1.5
2.0
2.5
3.0
0.0
0.2
0.4
0.6
0.8
1.0
N = 348   Bandwidth = 0.03582

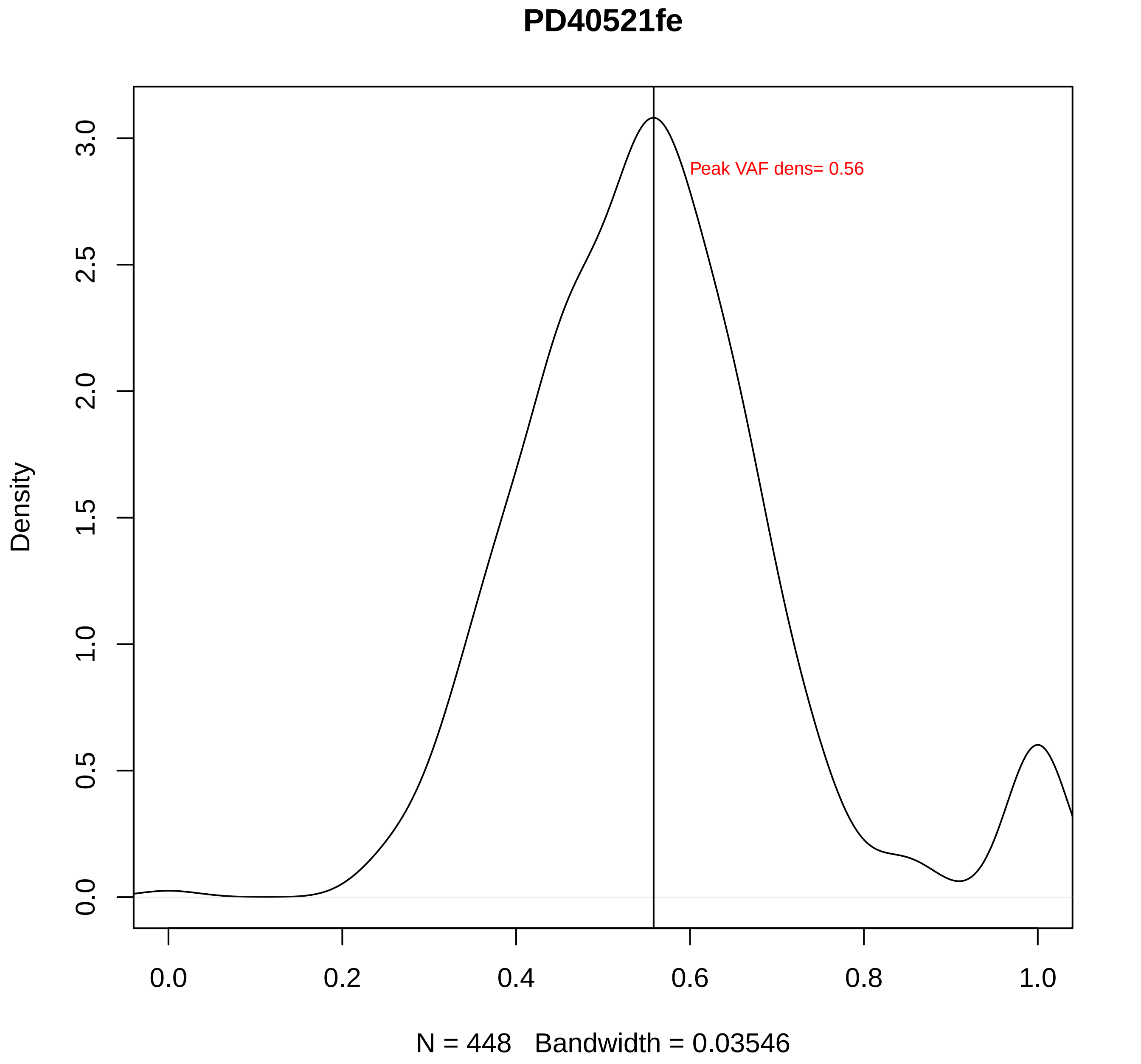
PD40521fe
Peak VAF dens= 0.56
Density
0.0
0.5
1.0
1.5
2.0
2.5
3.0
0.0
0.2
0.4
0.6
0.8
1.0
N = 448   Bandwidth = 0.03546

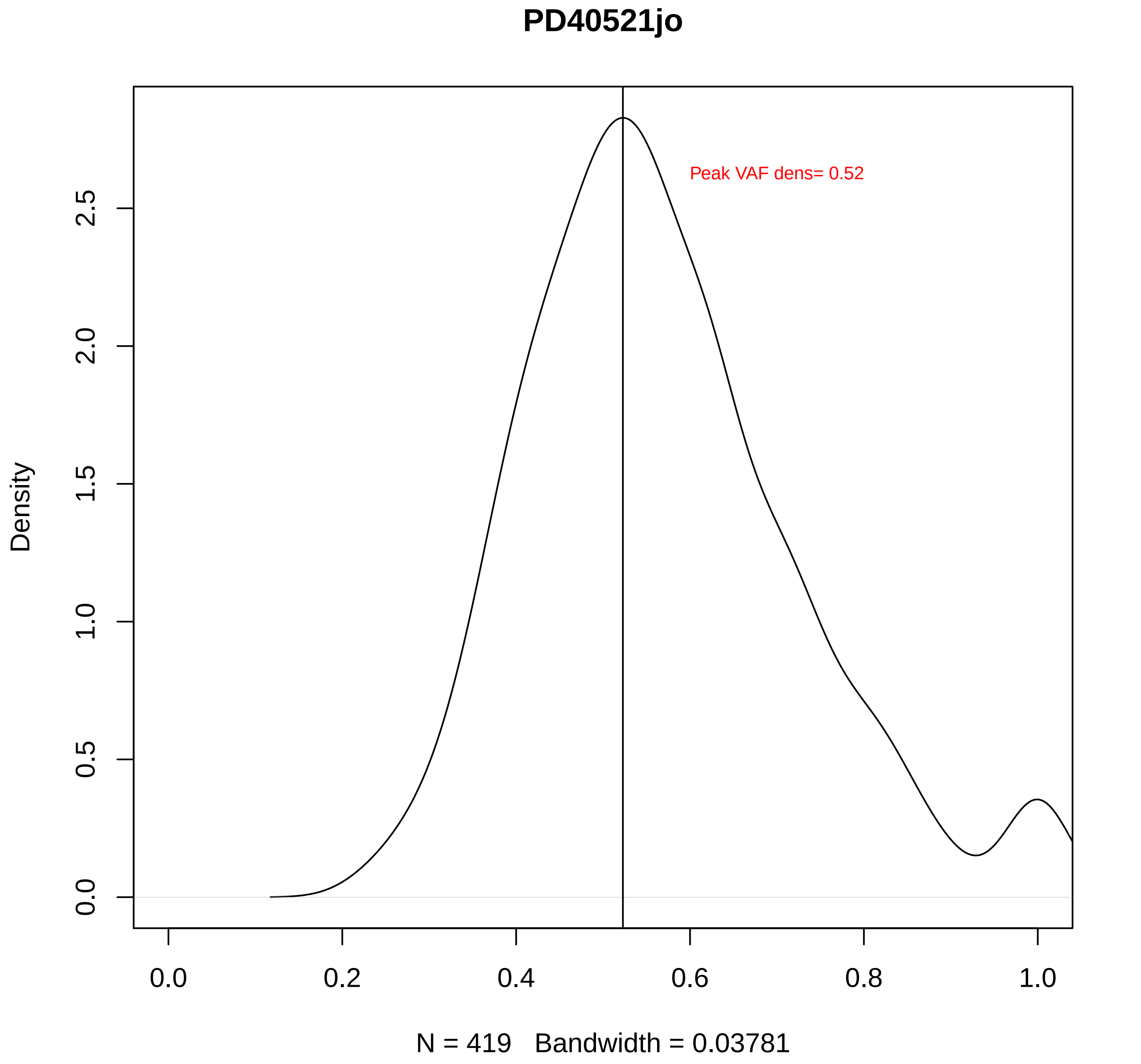
PD40521jo
Peak VAF dens= 0.52
Density
0.0
0.5
1.0
1.5
2.0
2.5
0.0
0.2
0.4
0.6
0.8
1.0
N = 419   Bandwidth = 0.03781

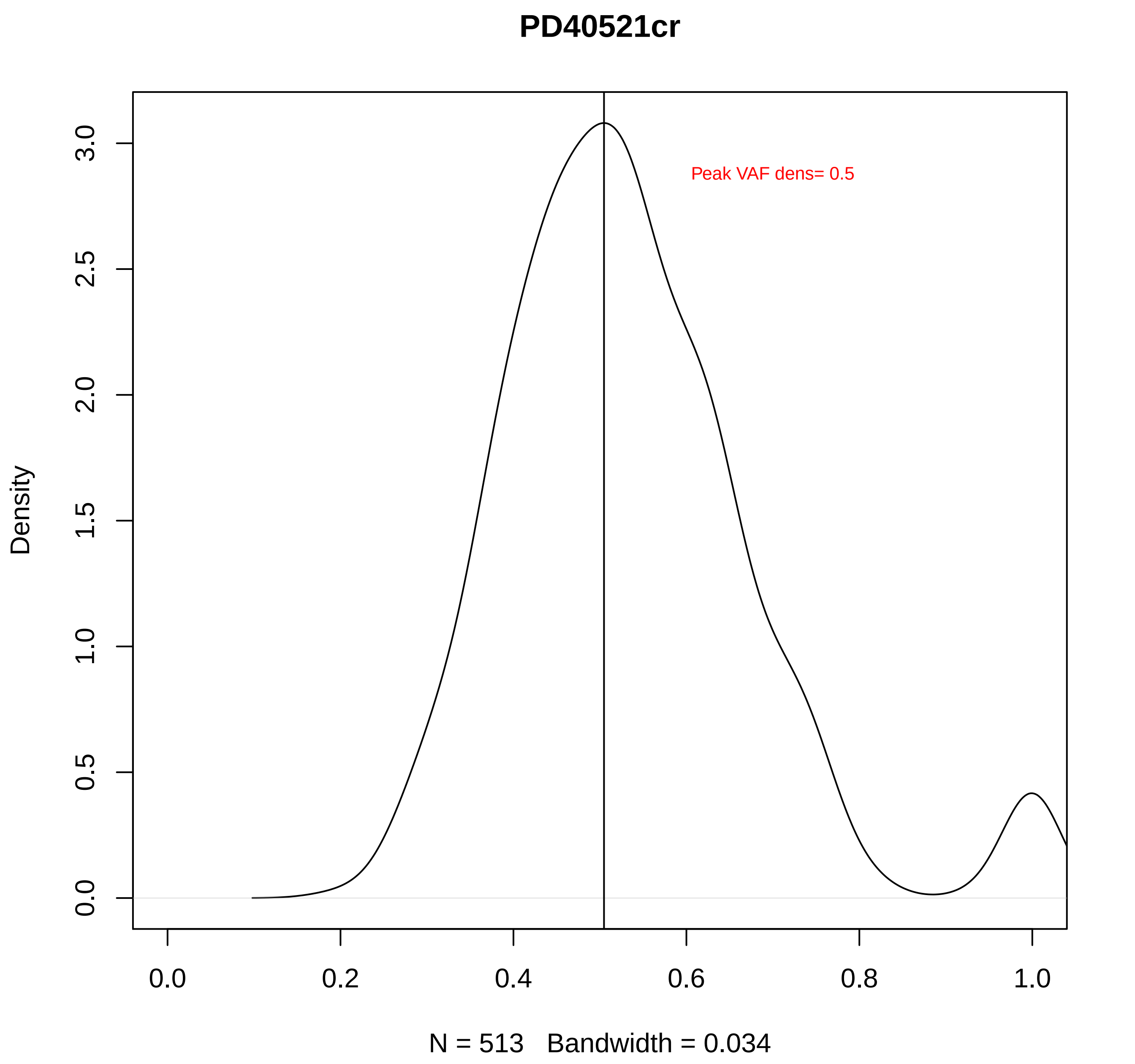
PD40521cr
Peak VAF dens= 0.5
Density
0.0
0.5
1.0
1.5
2.0
2.5
3.0
0.0
0.2
0.4
0.6
0.8
1.0
N = 513   Bandwidth = 0.034

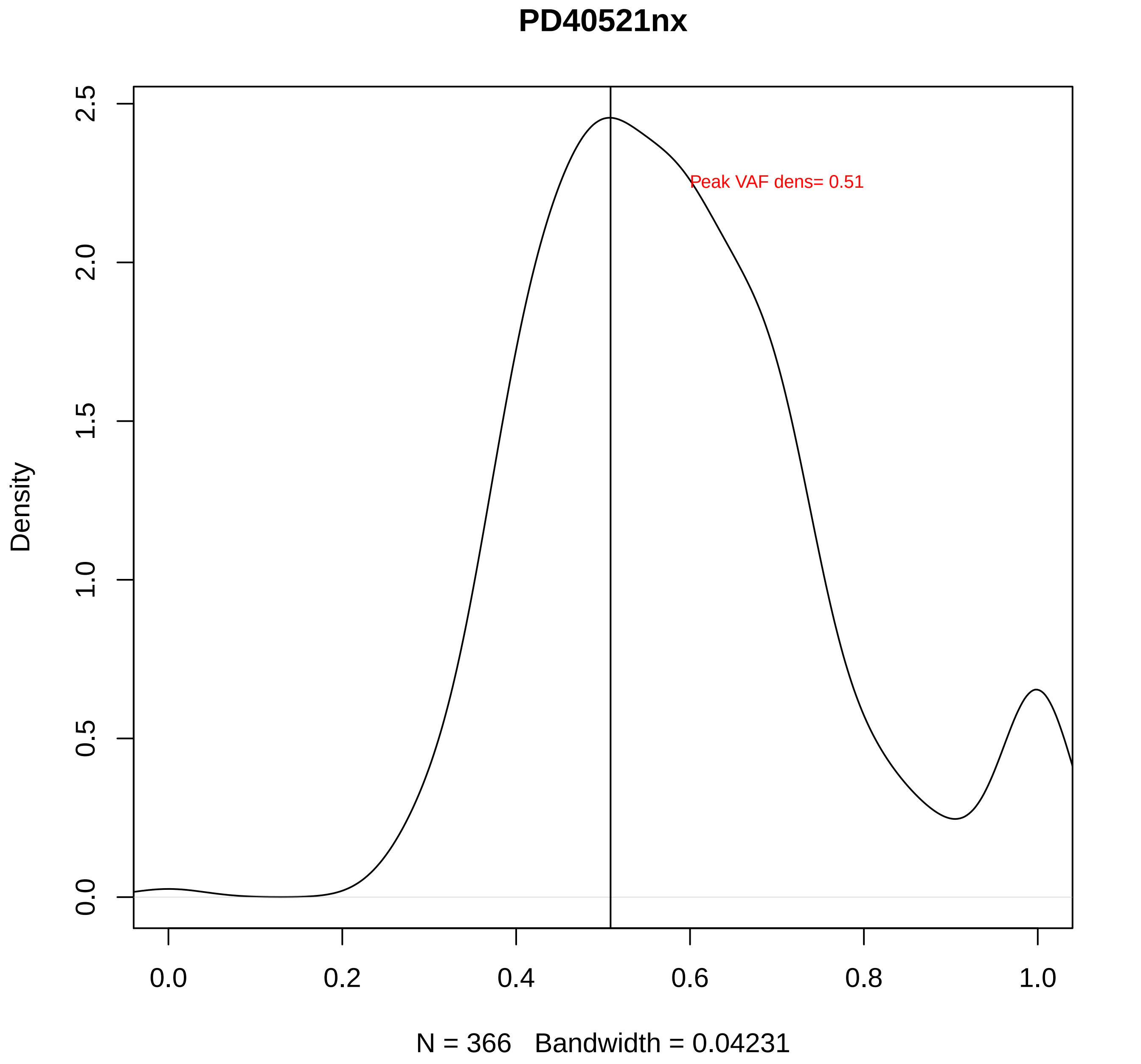
PD40521nx
Peak VAF dens= 0.51
Density
0.0
0.5
1.0
1.5
2.0
2.5
0.0
0.2
0.4
0.6
0.8
1.0
N = 366   Bandwidth = 0.04231

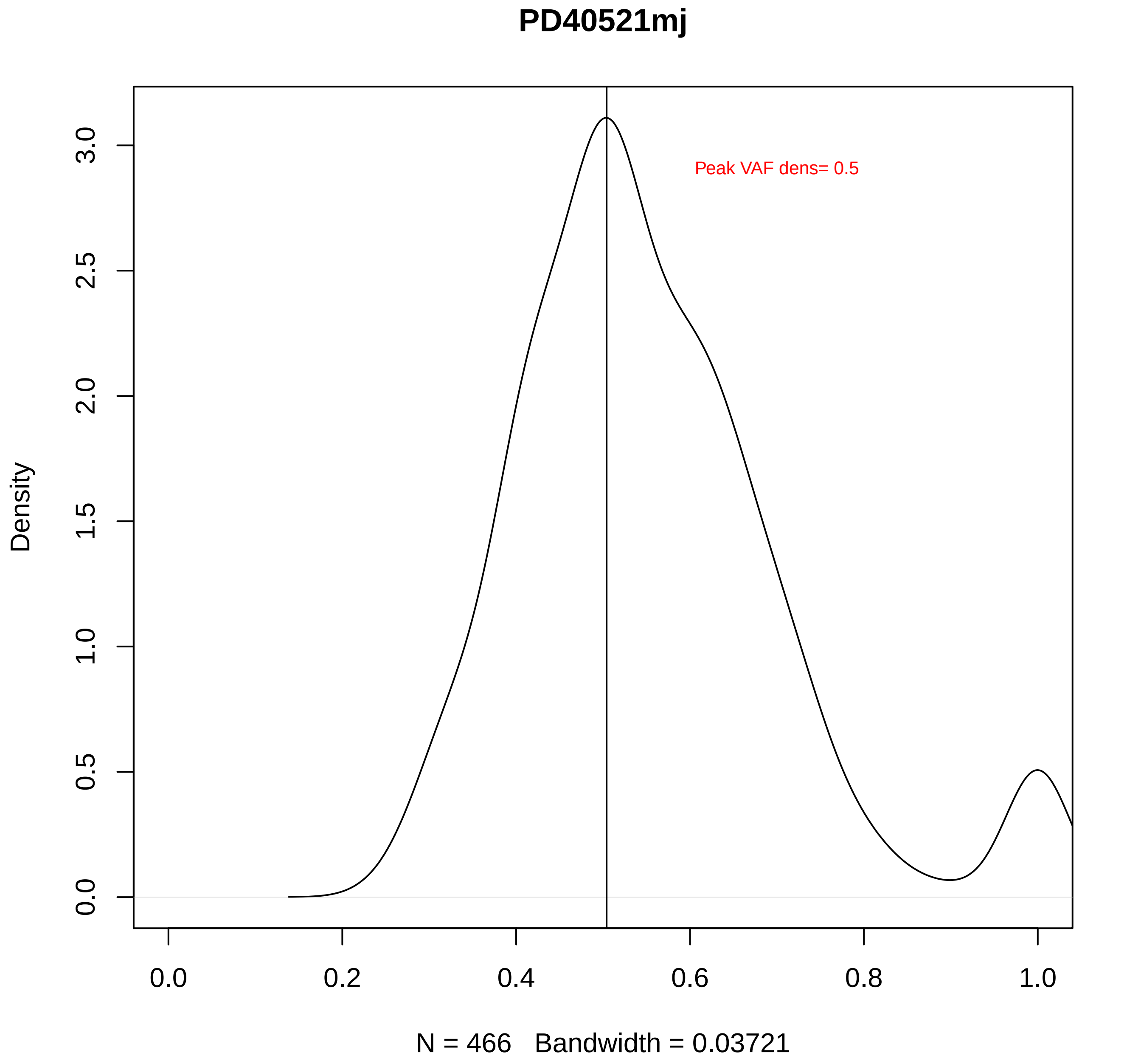
PD40521mj
Peak VAF dens= 0.5
Density
0.0
0.5
1.0
1.5
2.0
2.5
3.0
0.0
0.2
0.4
0.6
0.8
1.0
N = 466   Bandwidth = 0.03721

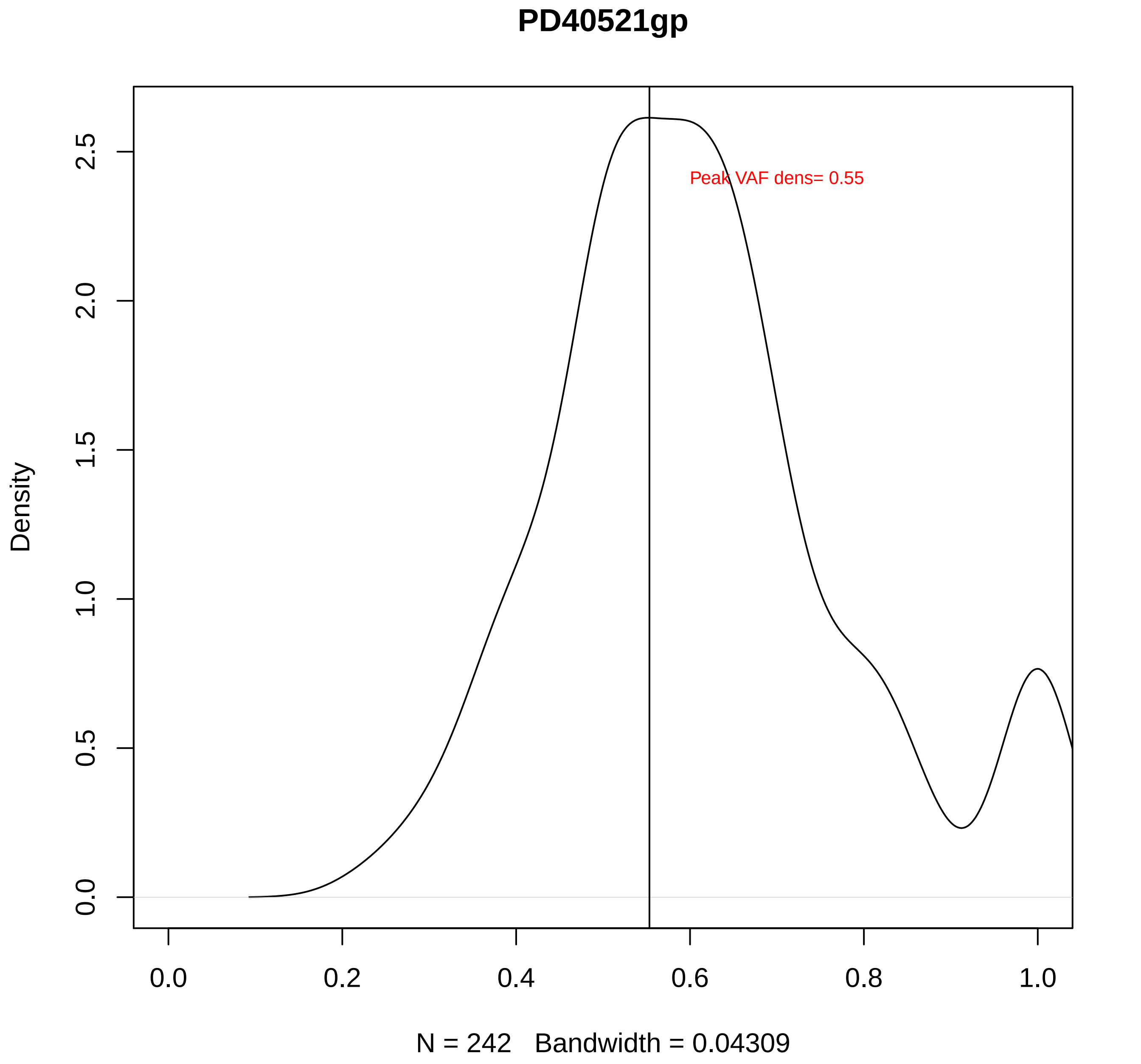
PD40521gp
Peak VAF dens= 0.55
Density
0.0
0.5
1.0
1.5
2.0
2.5
0.0
0.2
0.4
0.6
0.8
1.0
N = 242   Bandwidth = 0.04309

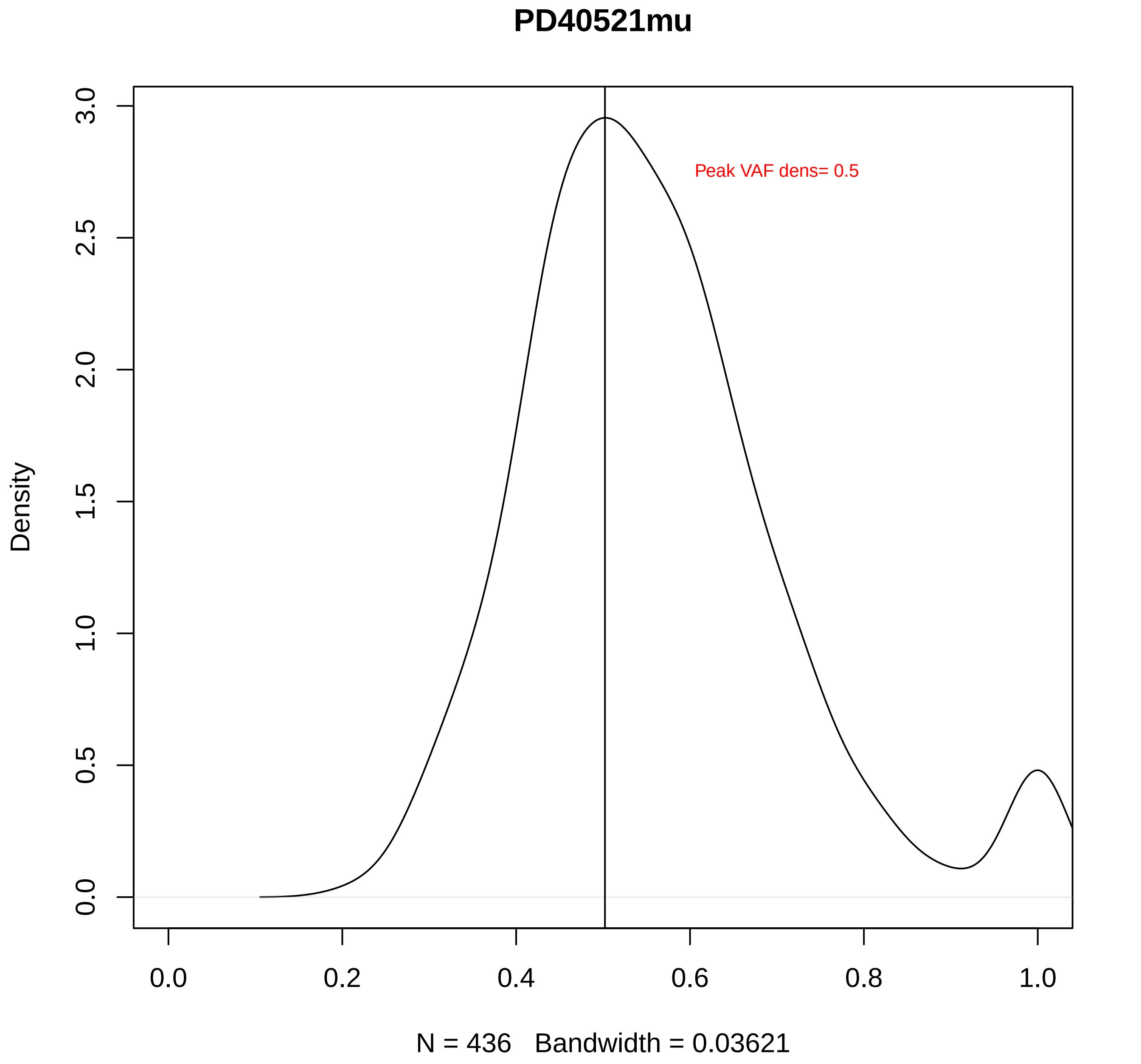
PD40521mu
Peak VAF dens= 0.5
Density
0.0
0.5
1.0
1.5
2.0
2.5
3.0
0.0
0.2
0.4
0.6
0.8
1.0
N = 436 Bandwidth = 0.03621

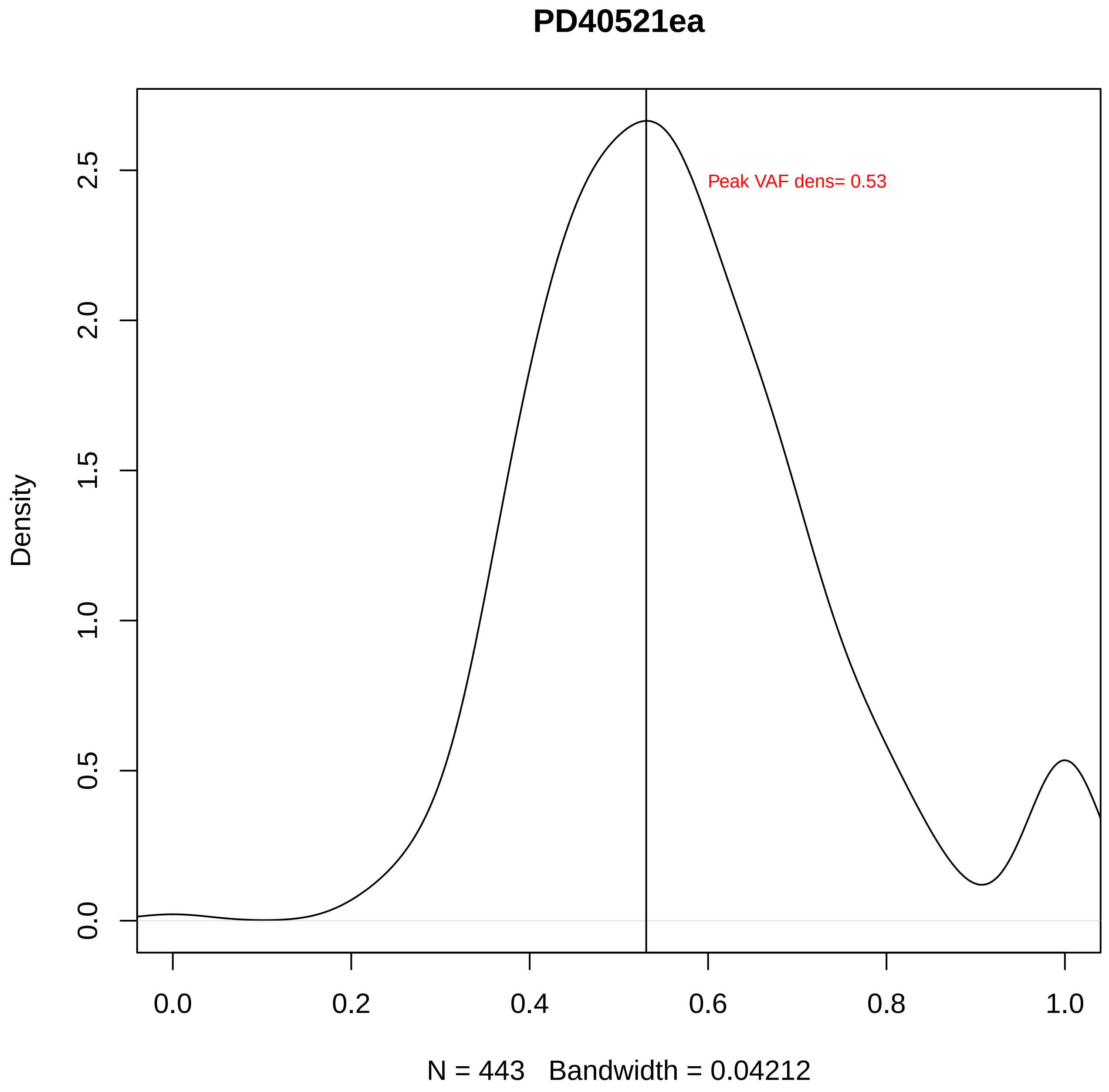

PD40521ea
Peak VAF dens= 0.53
Density
0.0
0.5
1.0
1.5
2.0
2.5
0.0
0.2
0.4
0.6
0.8
1.0
N = 443 Bandwidth = 0.04212

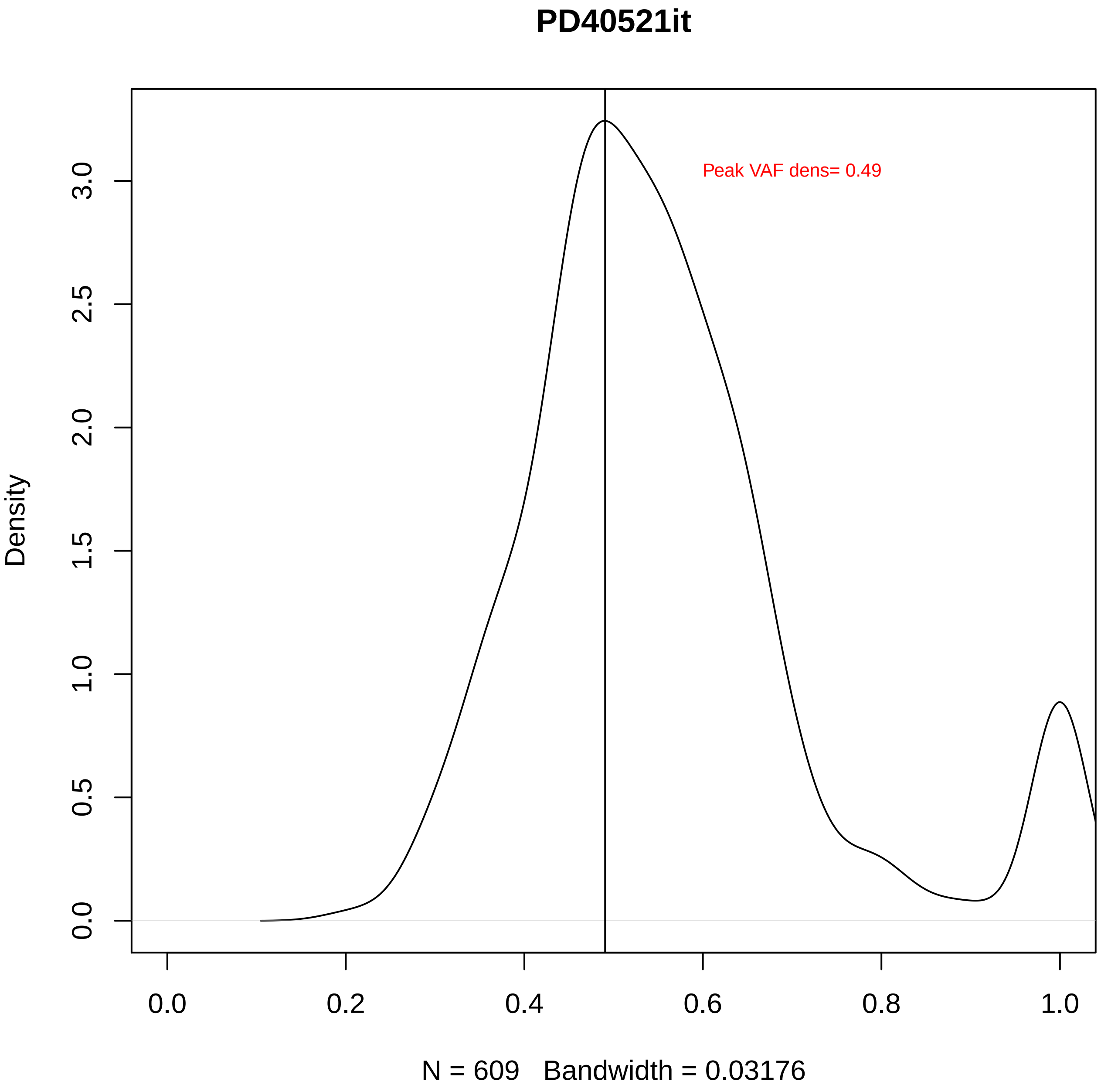

PD40521it
Peak VAF dens= 0.49
Density
0.0
0.5
1.0
1.5
2.0
2.5
3.0
0.0
0.2
0.4
0.6
0.8
1.0
N = 609   Bandwidth = 0.03176

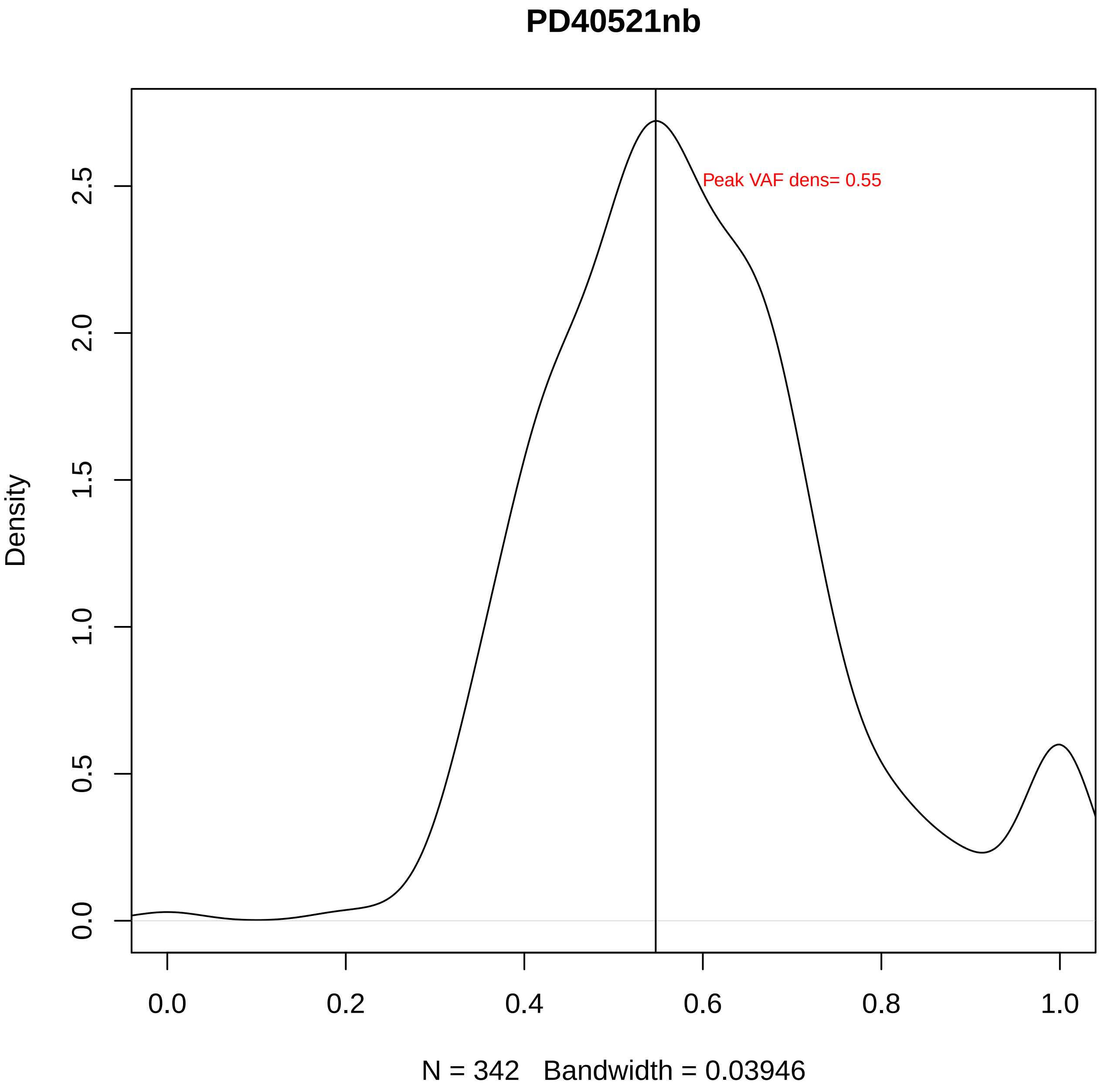
PD40521nb
Peak VAF dens= 0.55
Density
0.0
0.5
1.0
1.5
2.0
2.5
0.0
0.2
0.4
0.6
0.8
1.0
N = 342   Bandwidth = 0.03946

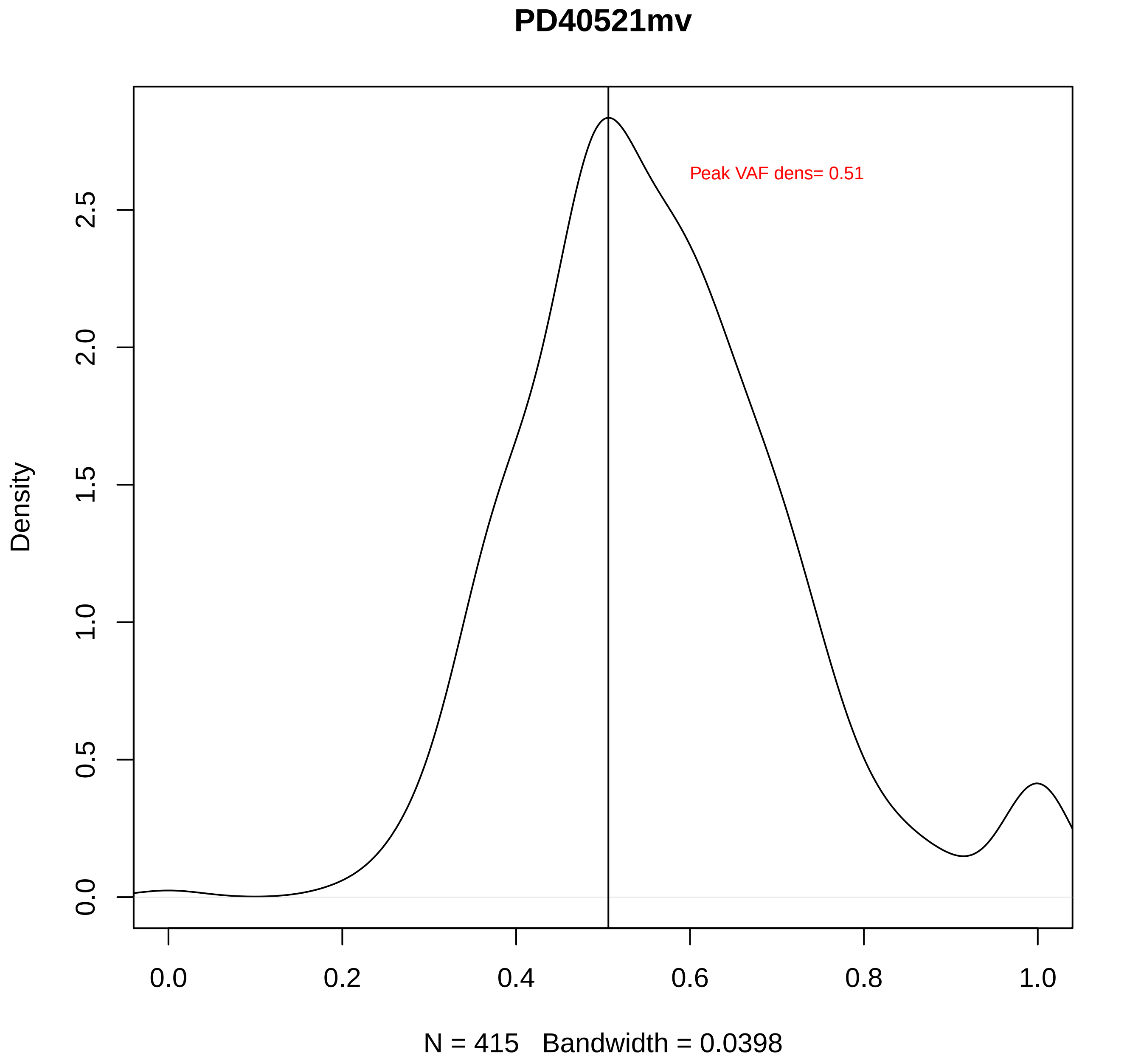
PD40521mv
Peak VAF dens= 0.51
Density
0.0
0.5
1.0
1.5
2.0
2.5
0.0
0.2
0.4
0.6
0.8
1.0
N = 415   Bandwidth = 0.0398

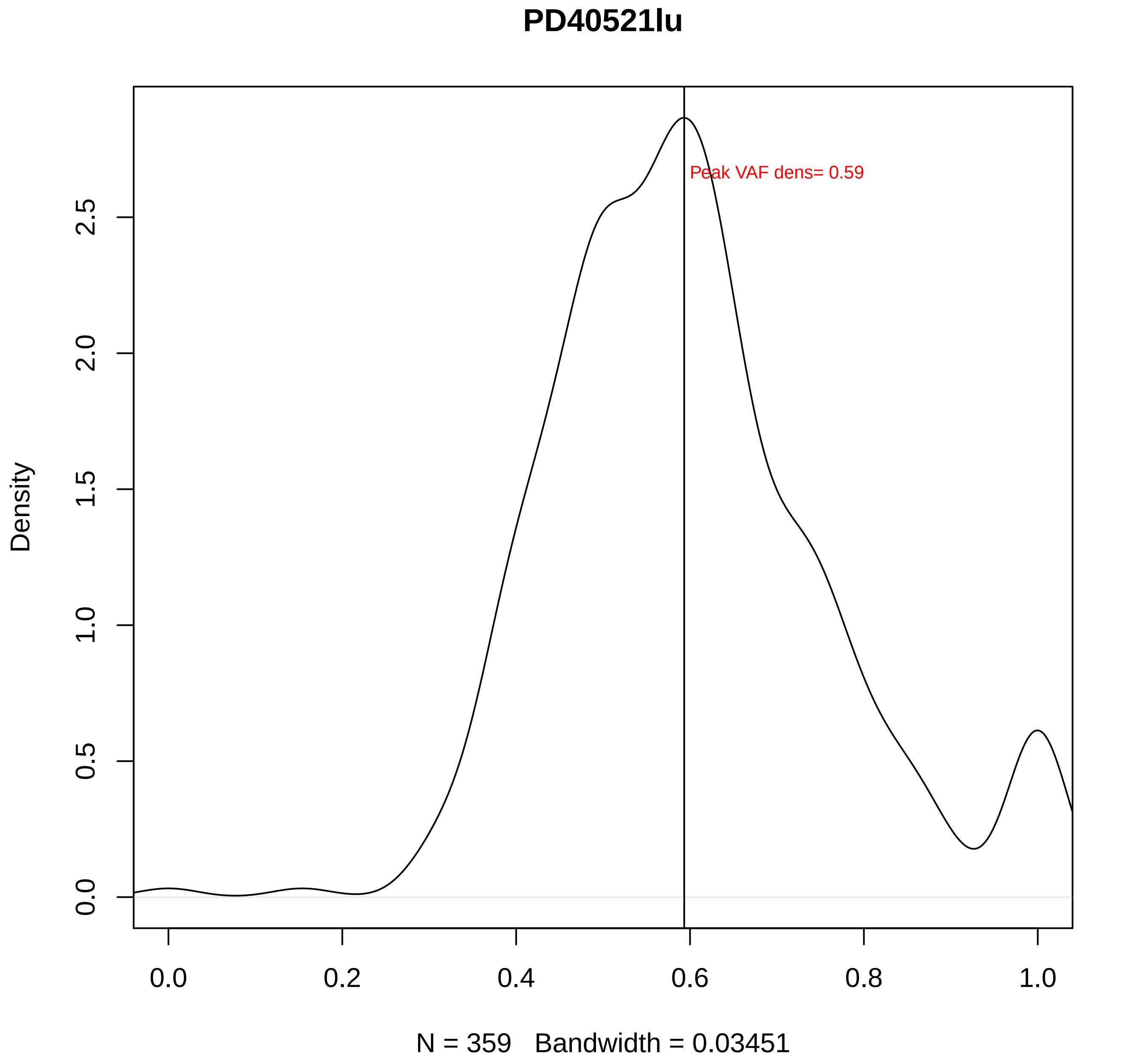

PD40521lu
Peak VAF dens= 0.59
Density
0.0
0.5
1.0
1.5
2.0
2.5
0.0
0.2
0.4
0.6
0.8
1.0
N = 359   Bandwidth = 0.03451

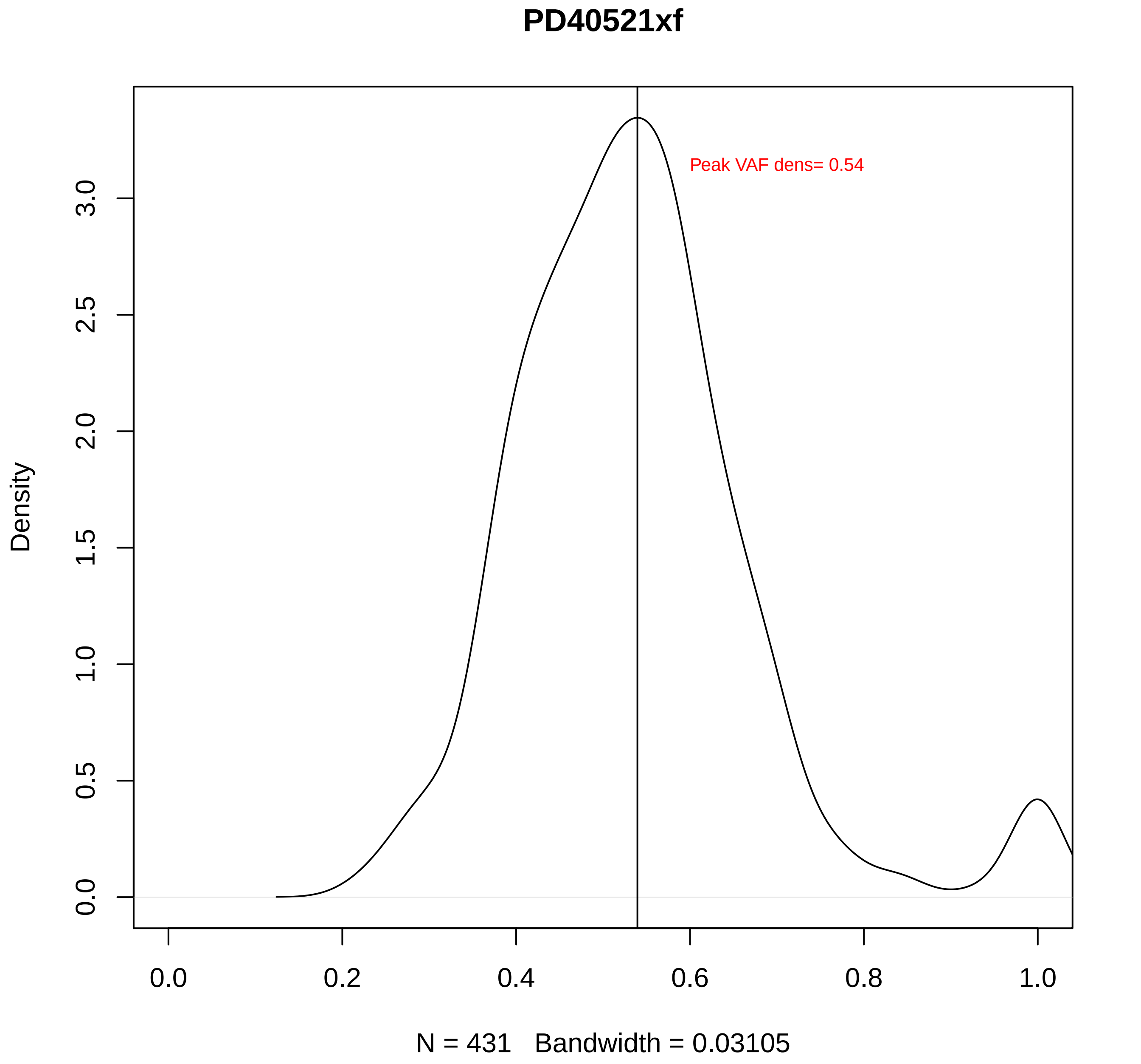

PD40521xf
Peak VAF dens= 0.54
Density
0.0
0.5
1.0
1.5
2.0
2.5
3.0
0.0
0.2
0.4
0.6
0.8
1.0
N = 431   Bandwidth = 0.03105

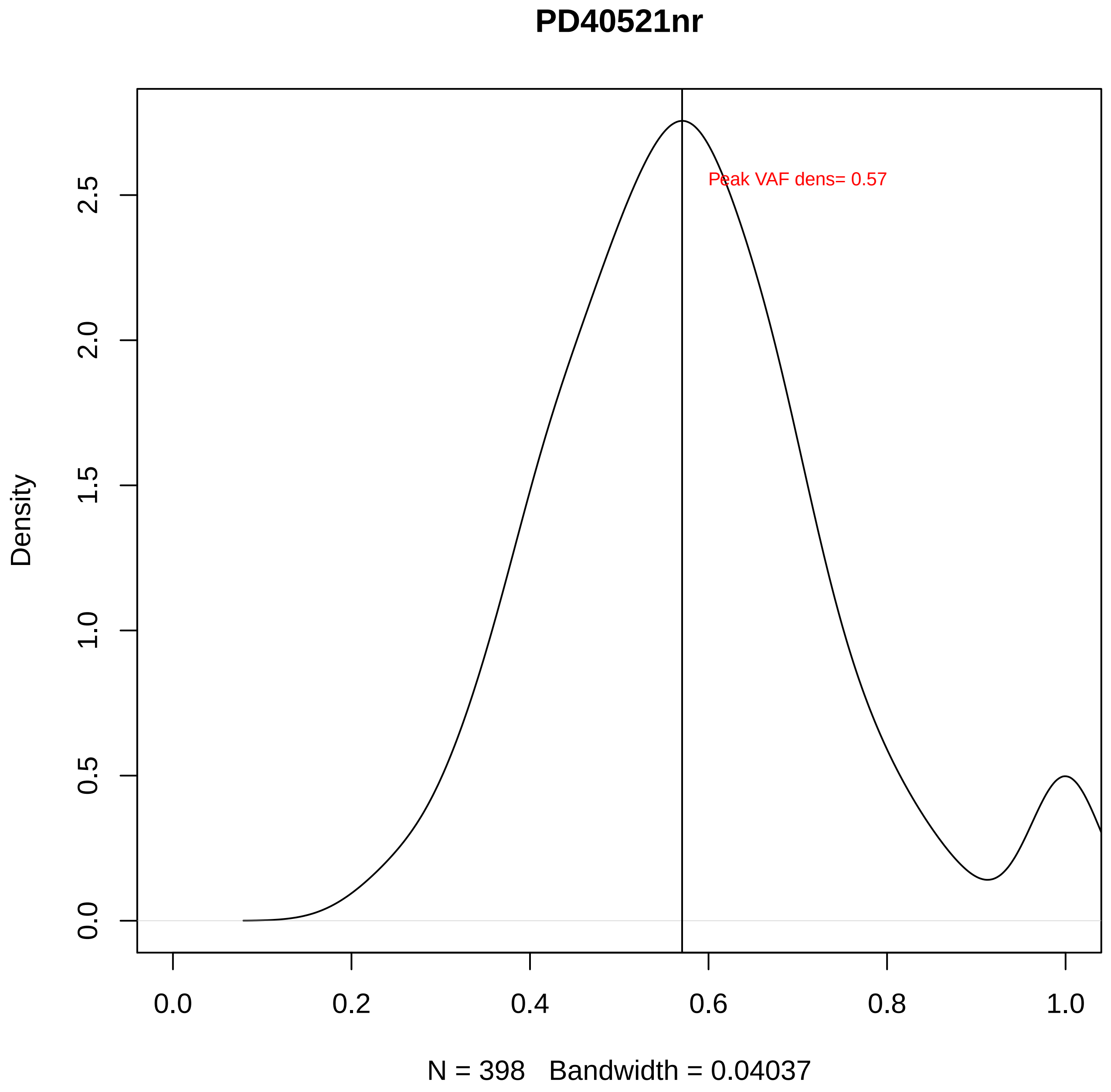
PD40521nr
Peak VAF dens= 0.57
Density
0.0
0.5
1.0
1.5
2.0
2.5
0.0
0.2
0.4
0.6
0.8
1.0
N = 398   Bandwidth = 0.04037

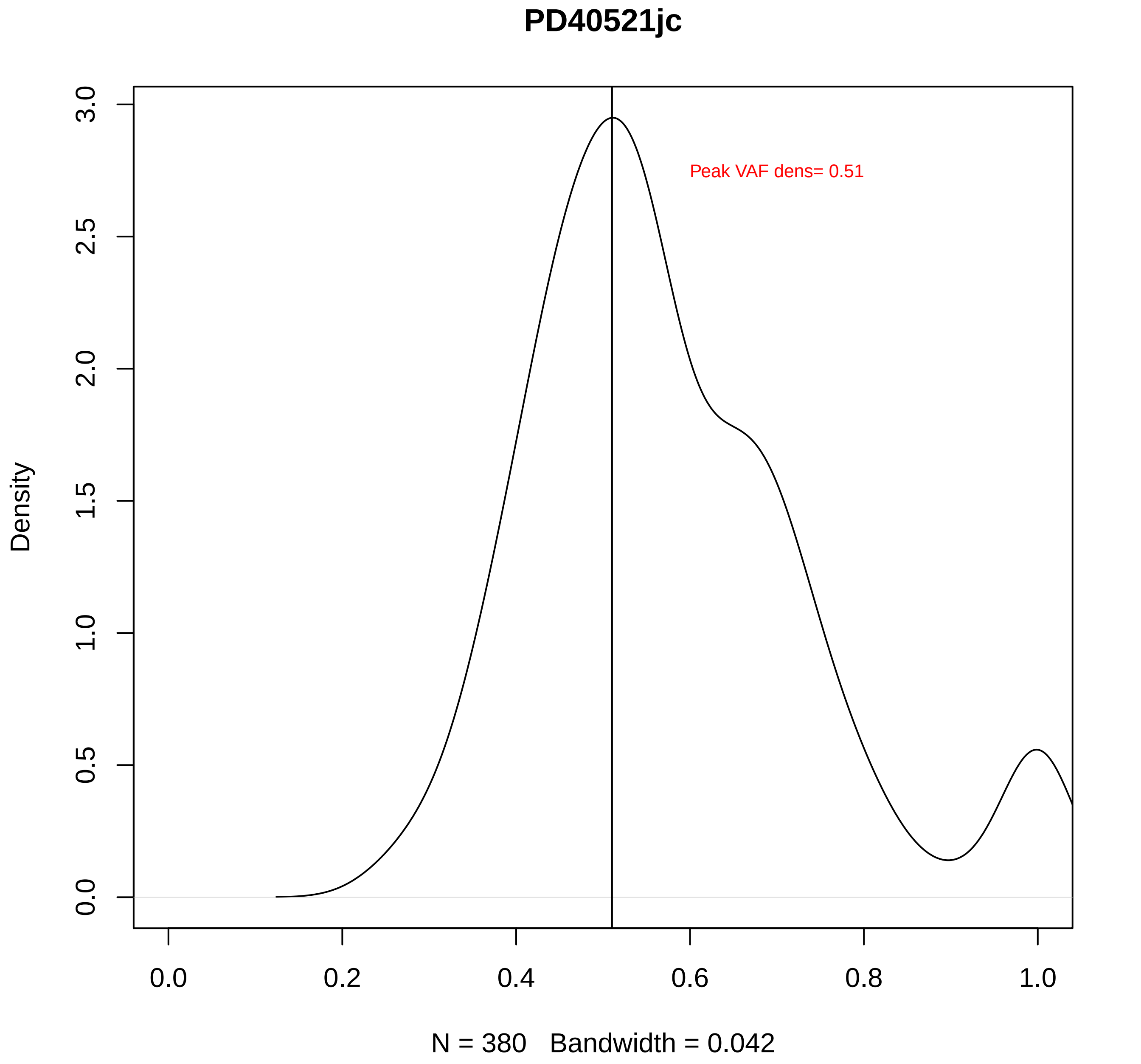
PD40521jc
Peak VAF dens= 0.51
Density
0.0
0.5
1.0
1.5
2.0
2.5
3.0
0.0
0.2
0.4
0.6
0.8
1.0
N = 380   Bandwidth = 0.042

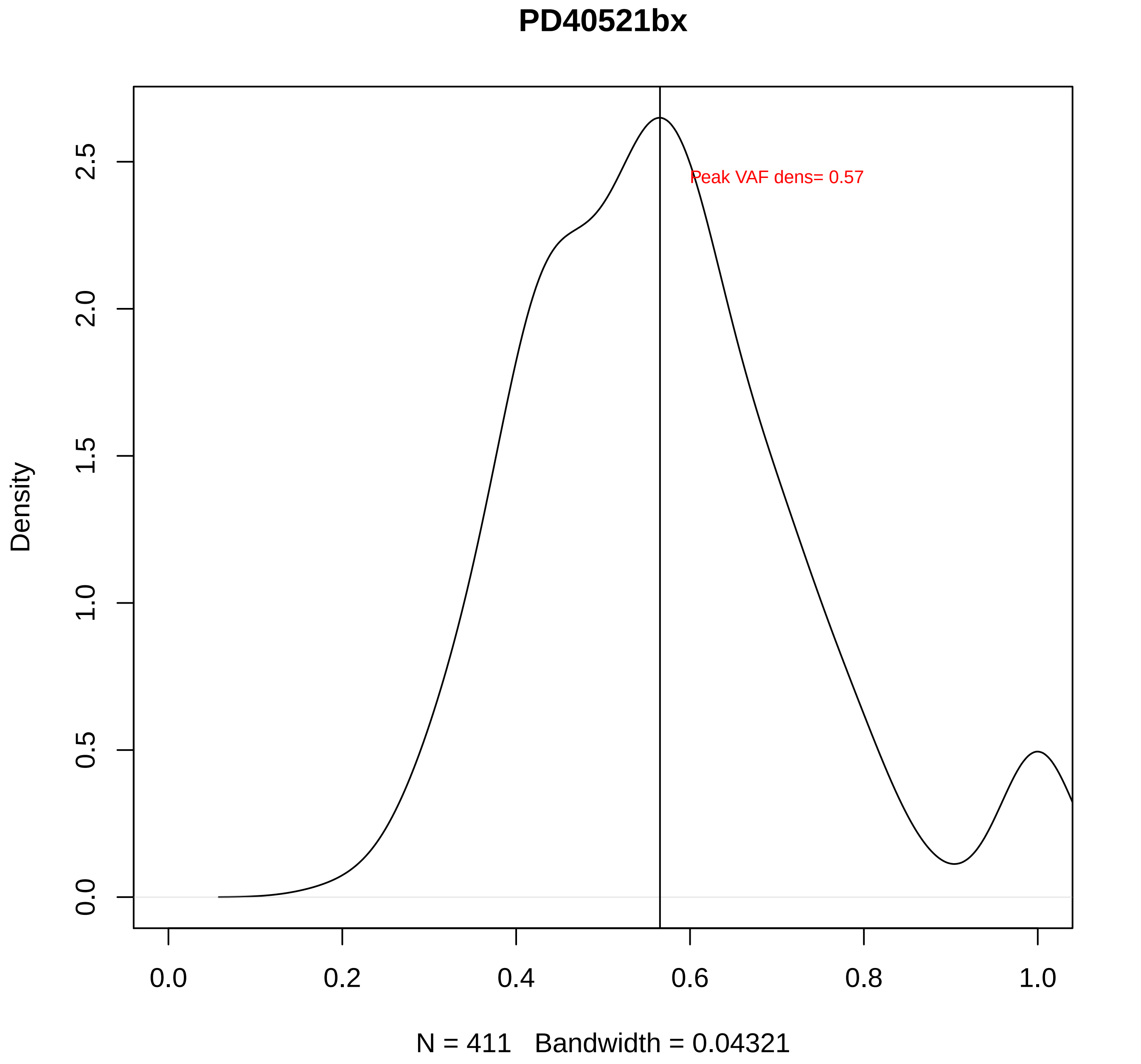
PD40521bx
Peak VAF dens= 0.57
Density
0.0
0.5
1.0
1.5
2.0
2.5
0.0
0.2
0.4
0.6
0.8
1.0
N = 411   Bandwidth = 0.04321

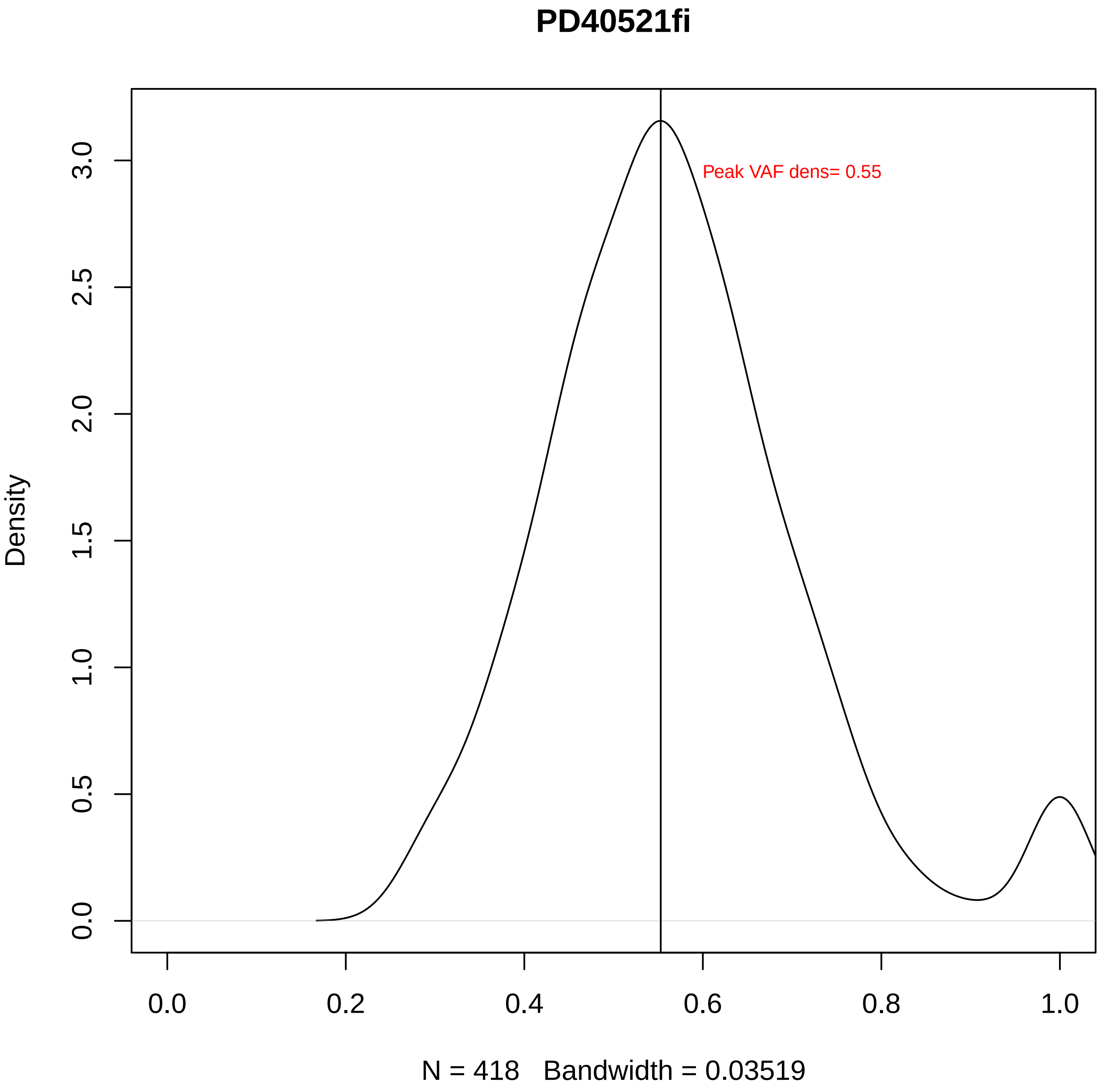
PD40521fi
Peak VAF dens= 0.55
Density
0.0
0.5
1.0
1.5
2.0
2.5
3.0
0.0
0.2
0.4
0.6
0.8
1.0
N = 418   Bandwidth = 0.03519

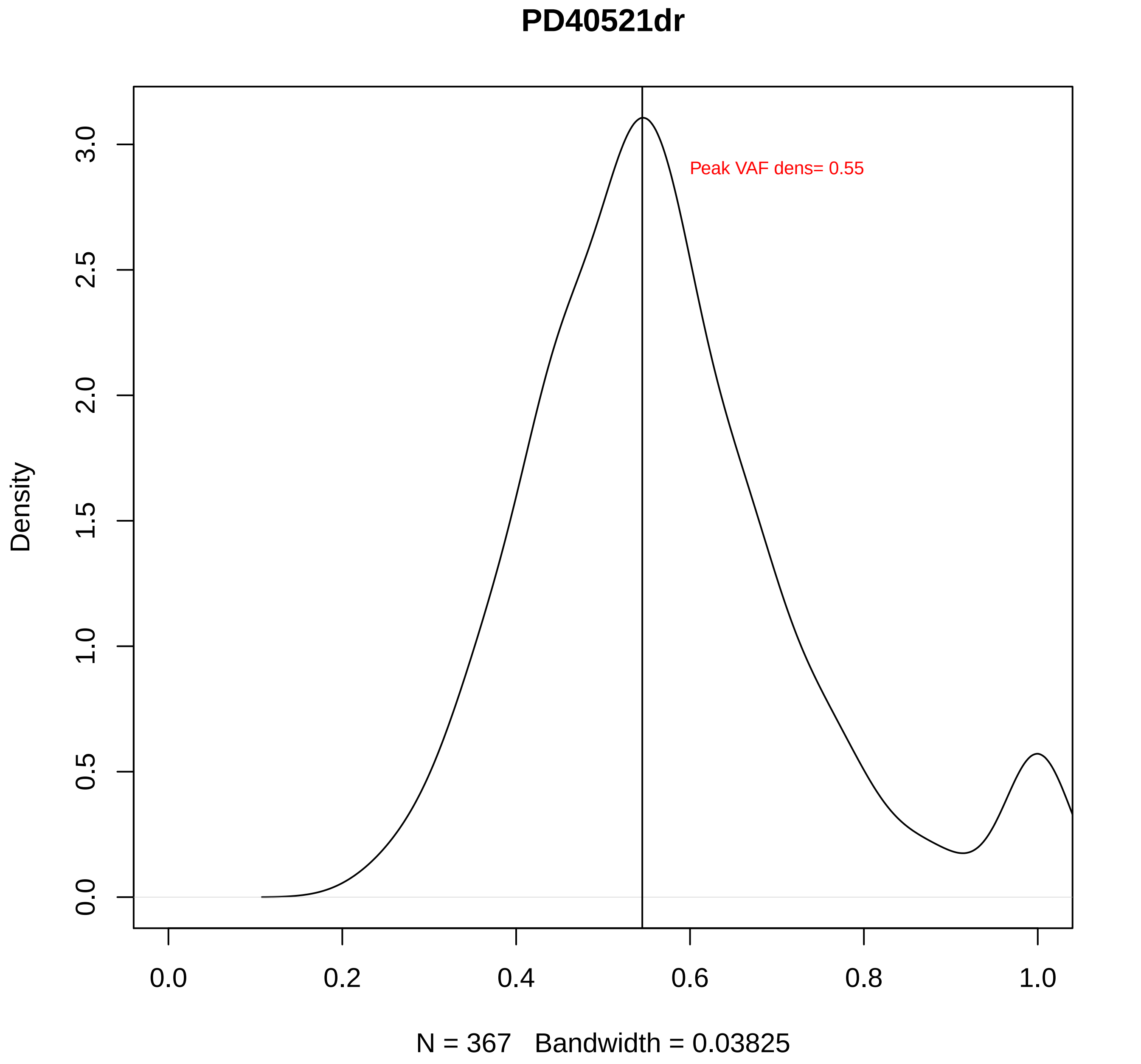
PD40521dr
Peak VAF dens= 0.55
Density
0.0
0.5
1.0
1.5
2.0
2.5
3.0
0.0
0.2
0.4
0.6
0.8
1.0
N = 367 Bandwidth = 0.03825

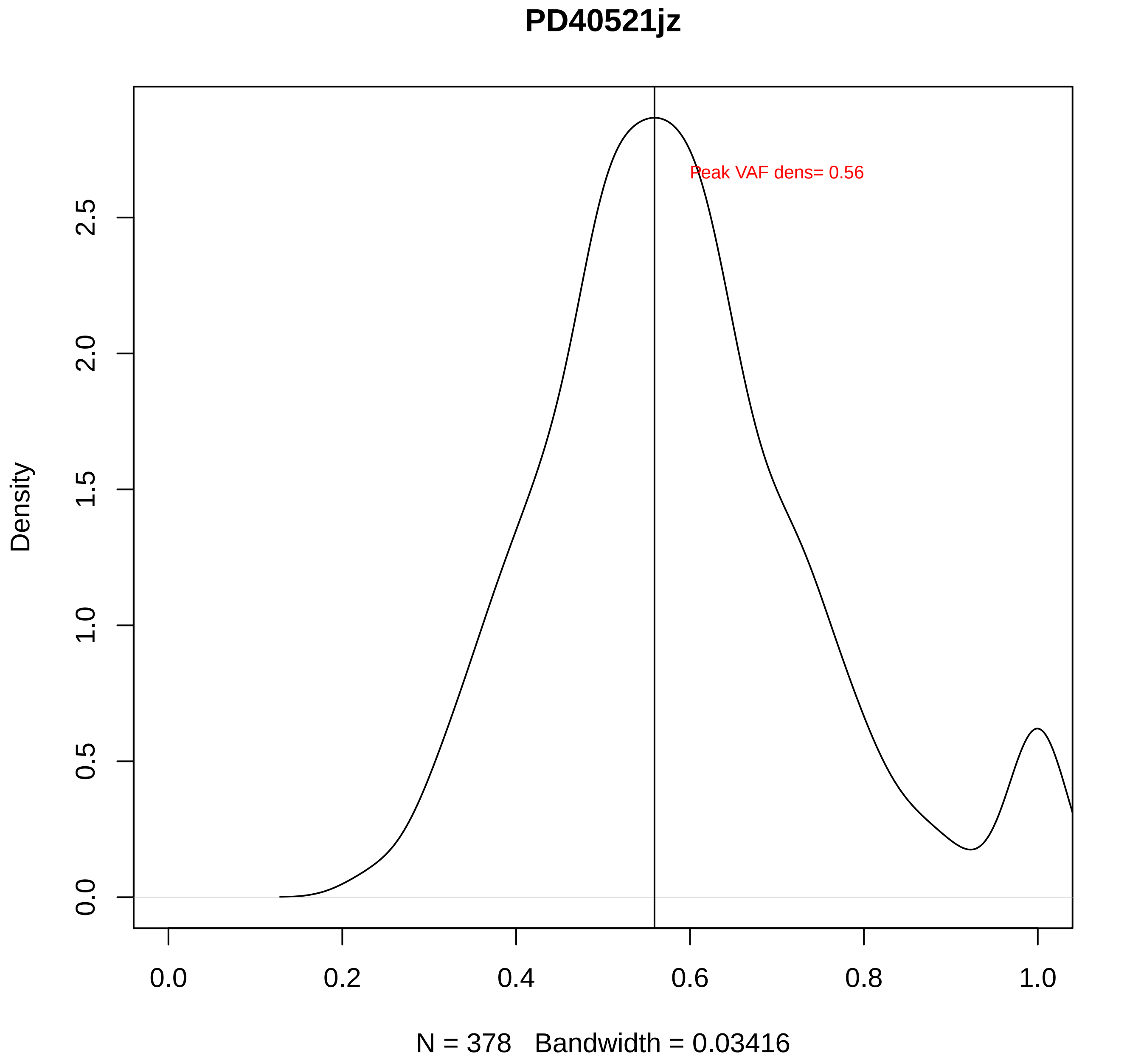
PD40521jz
Peak VAF dens= 0.56
Density
0.0
0.5
1.0
1.5
2.0
2.5
0.0
0.2
0.4
0.6
0.8
1.0
N = 378   Bandwidth = 0.03416

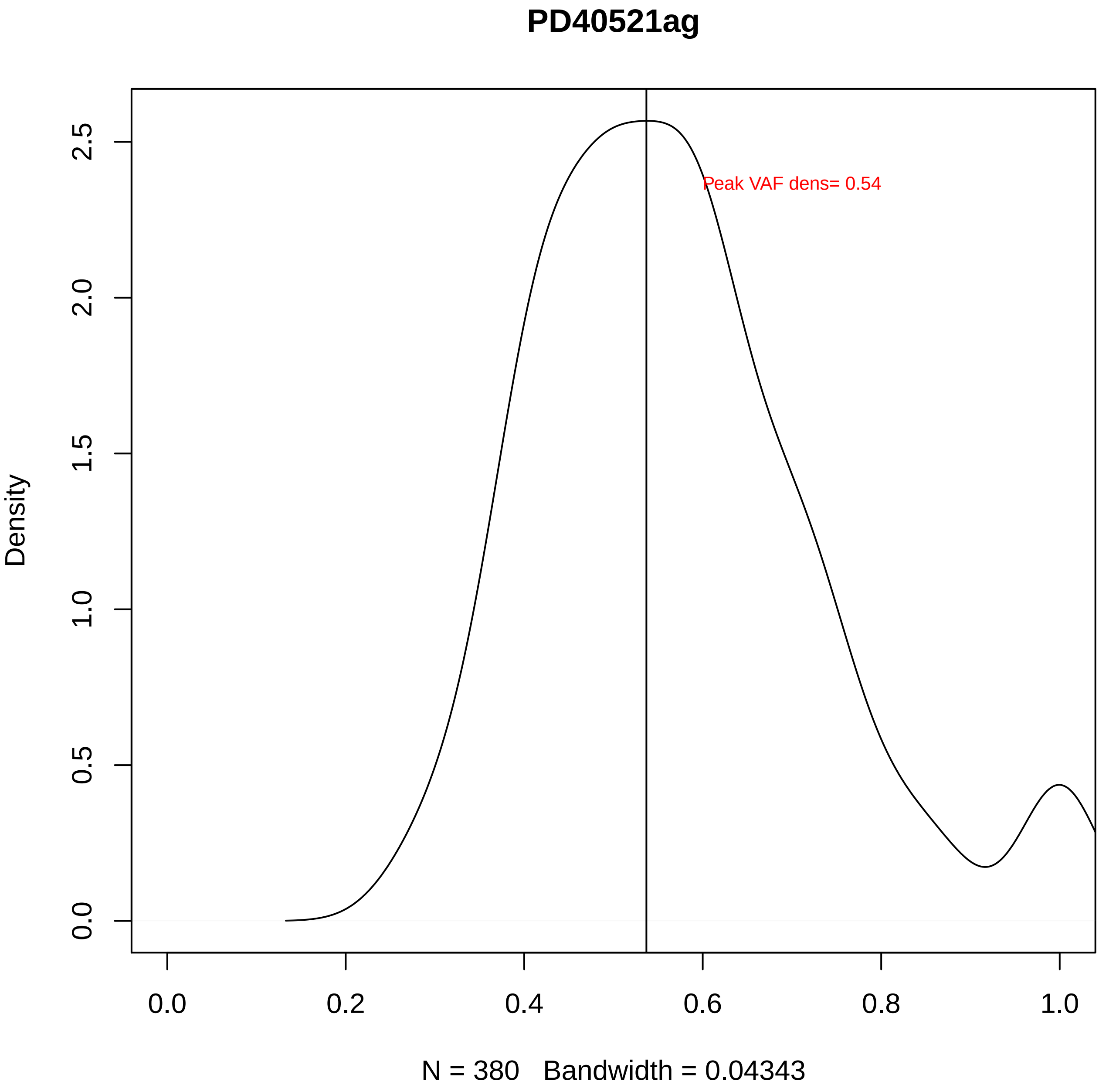
PD40521ag
Peak VAF dens= 0.54
Density
0.0
0.5
1.0
1.5
2.0
2.5
0.0
0.2
0.4
0.6
0.8
1.0
N = 380   Bandwidth = 0.04343

PD40521fn

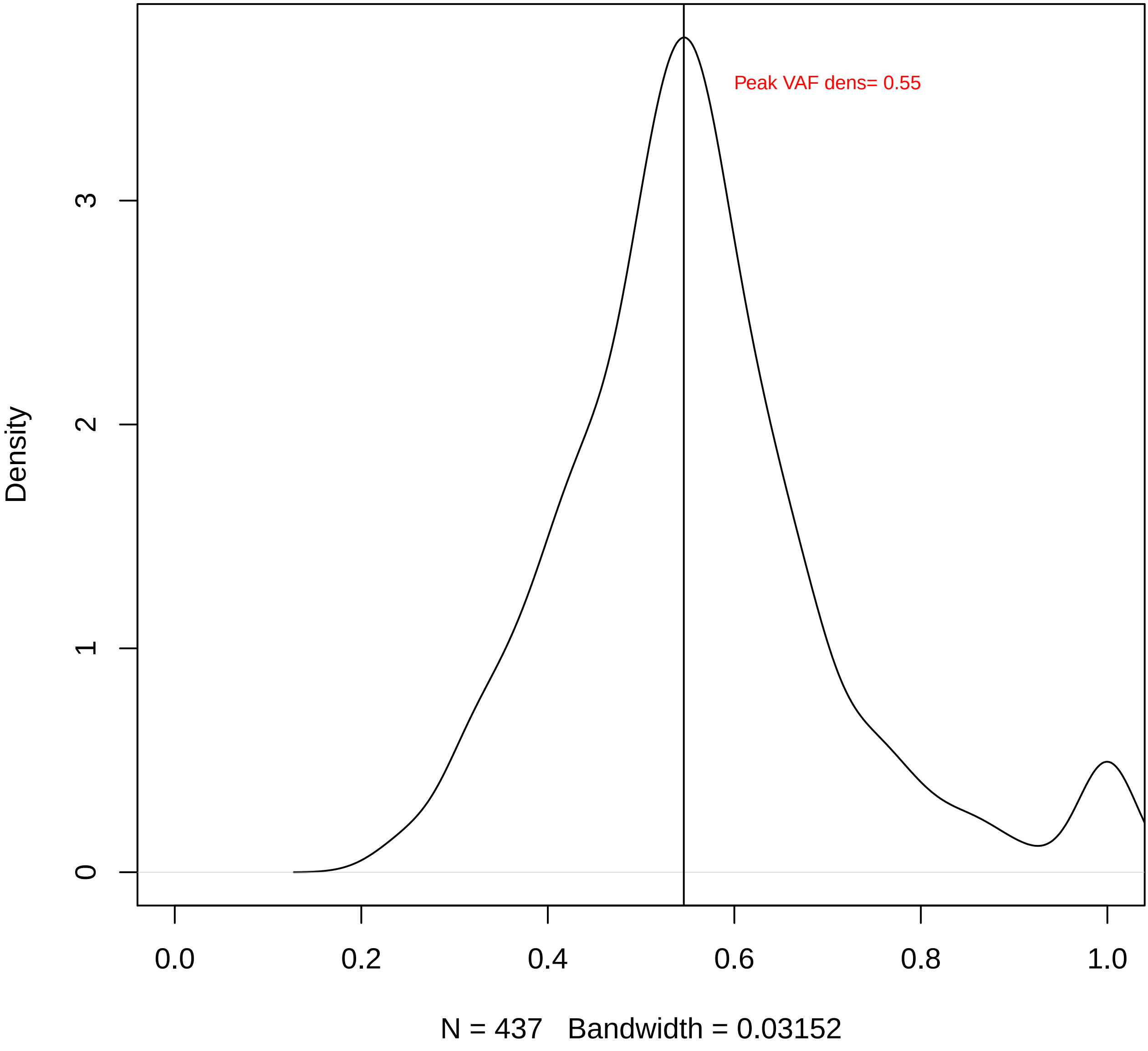
Peak VAF dens= 0.55
Density
0
1
2
3
0.0
0.2
0.4
0.6
0.8
1.0
N = 437 Bandwidth = 0.03152

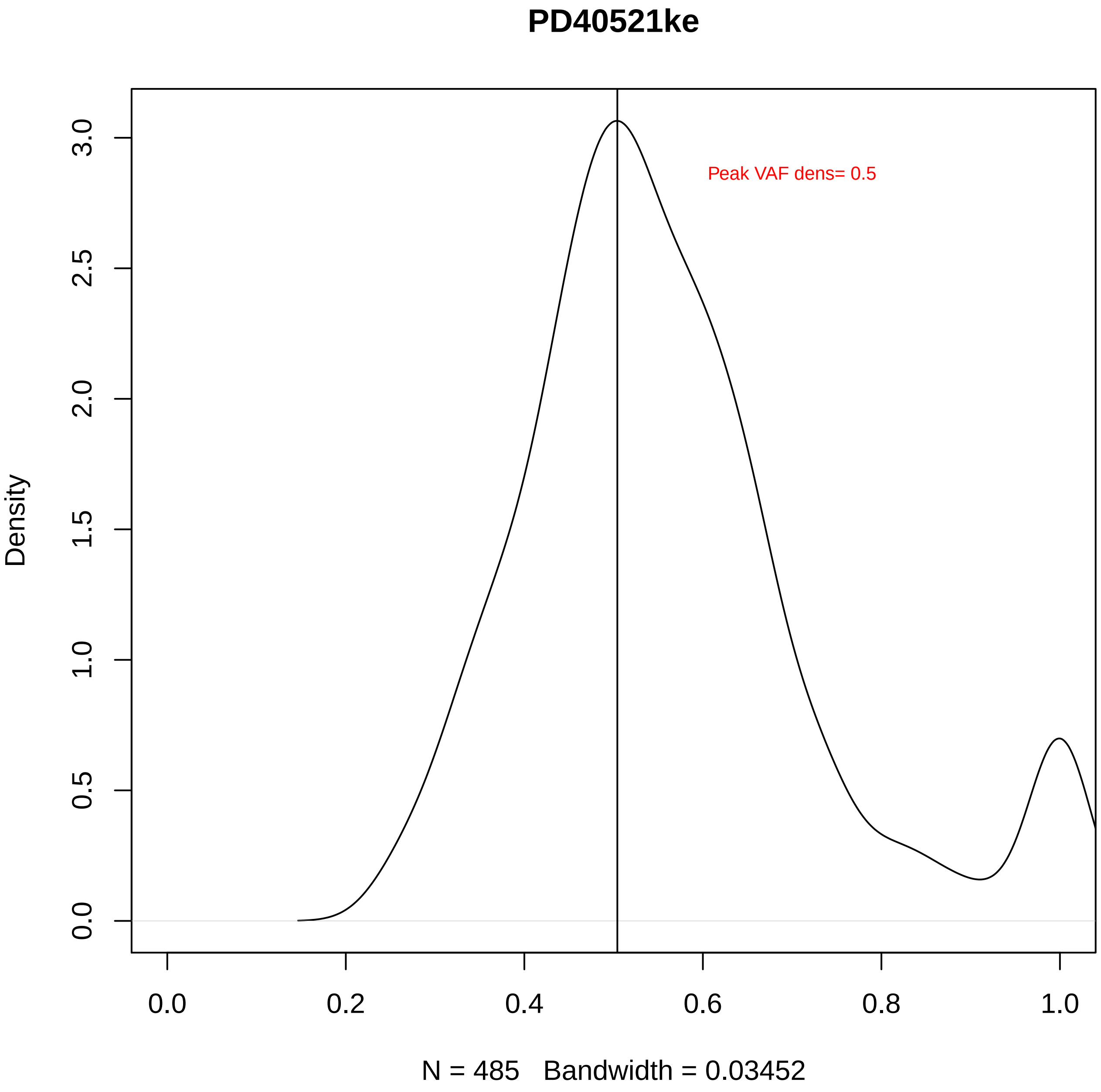

PD40521ke
Peak VAF dens= 0.5
Density
0.0
0.5
1.0
1.5
2.0
2.5
3.0
0.0
0.2
0.4
0.6
0.8
1.0
N = 485   Bandwidth = 0.03452

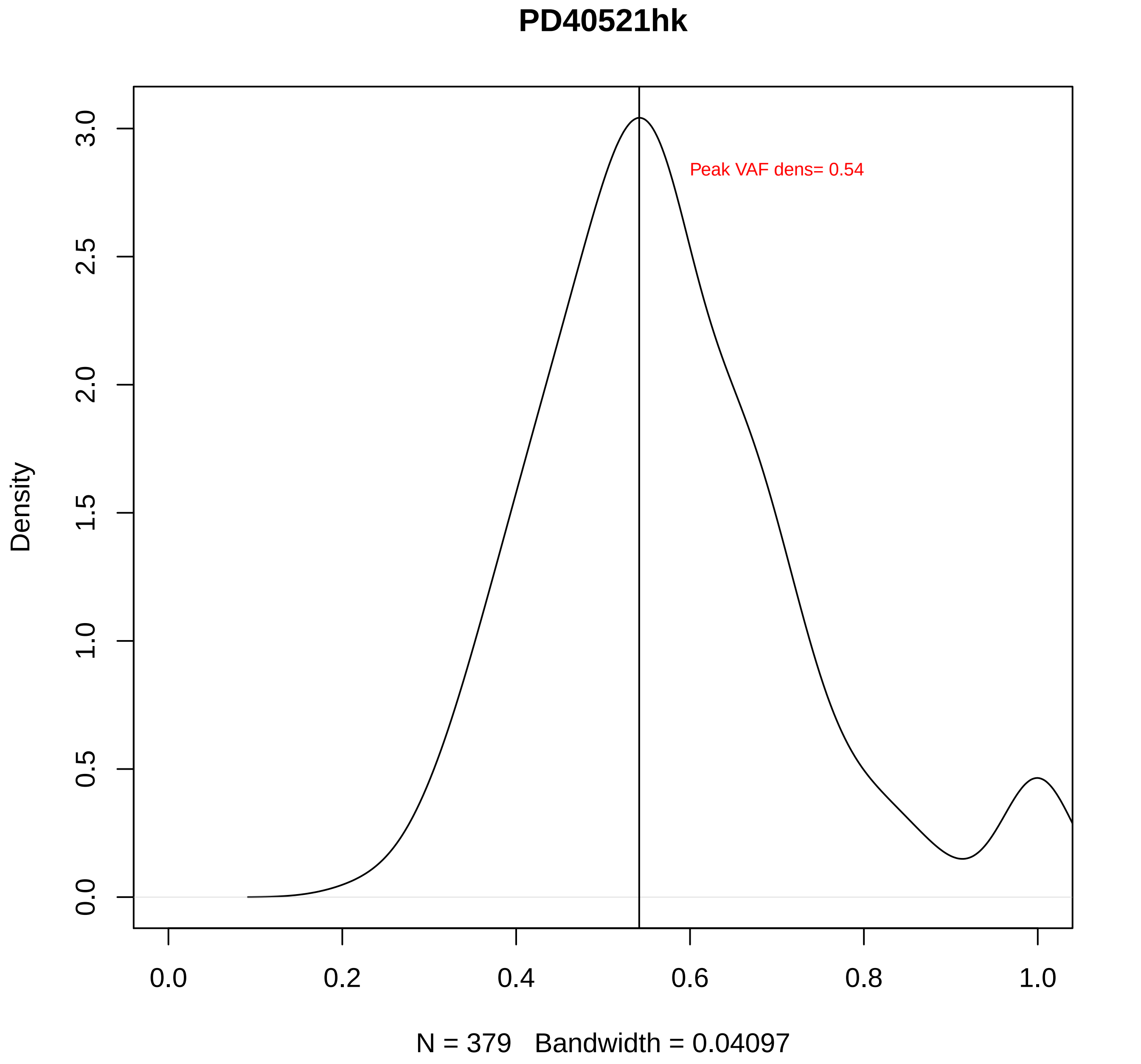

PD40521hk
Peak VAF dens= 0.54
Density
0.0
0.5
1.0
1.5
2.0
2.5
3.0
0.0
0.2
0.4
0.6
0.8
1.0
N = 379   Bandwidth = 0.04097

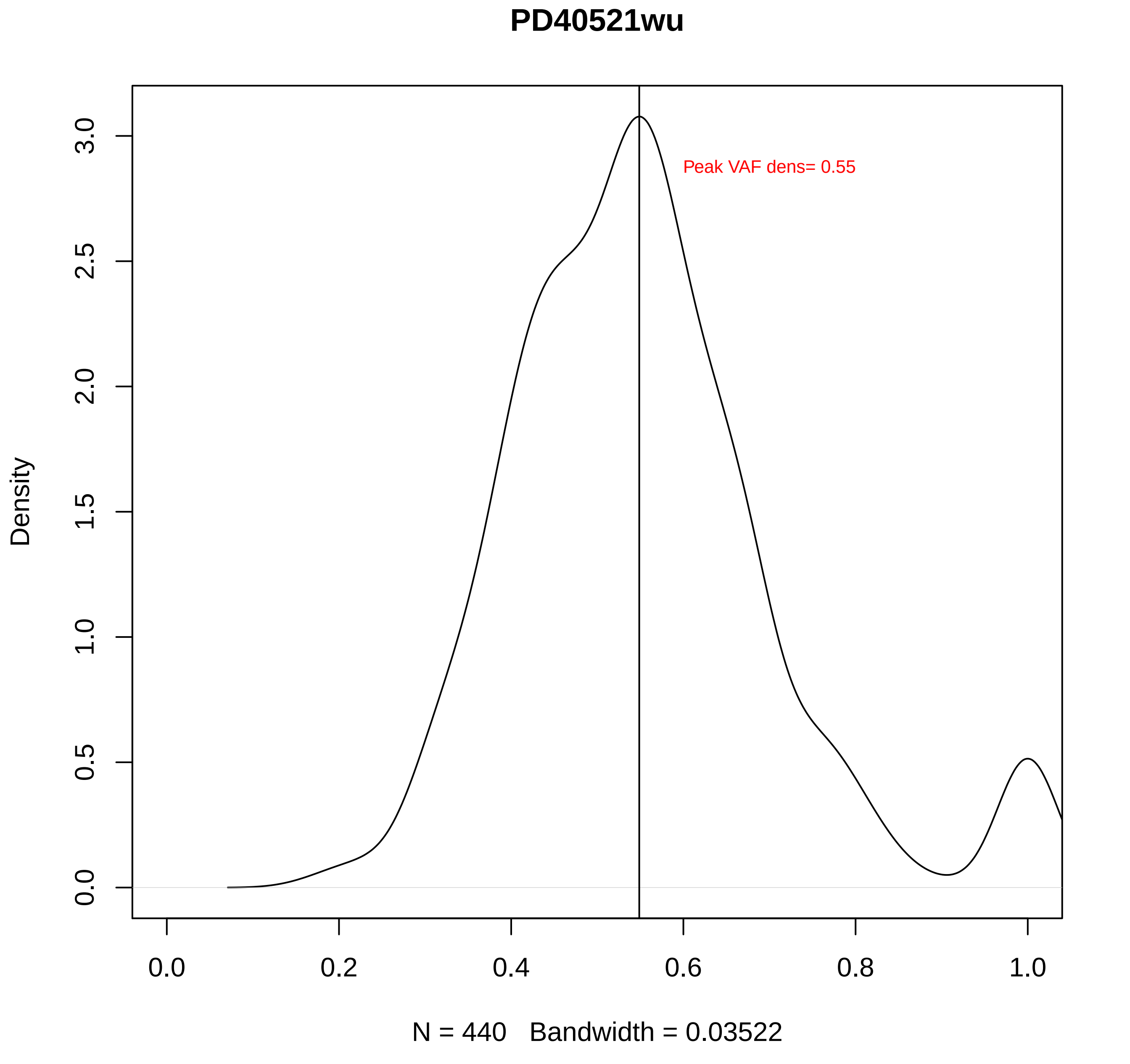
PD40521wu
Peak VAF dens= 0.55
Density
0.0
0.5
1.0
1.5
2.0
2.5
3.0
0.0
0.2
0.4
0.6
0.8
1.0
N = 440 Bandwidth = 0.03522

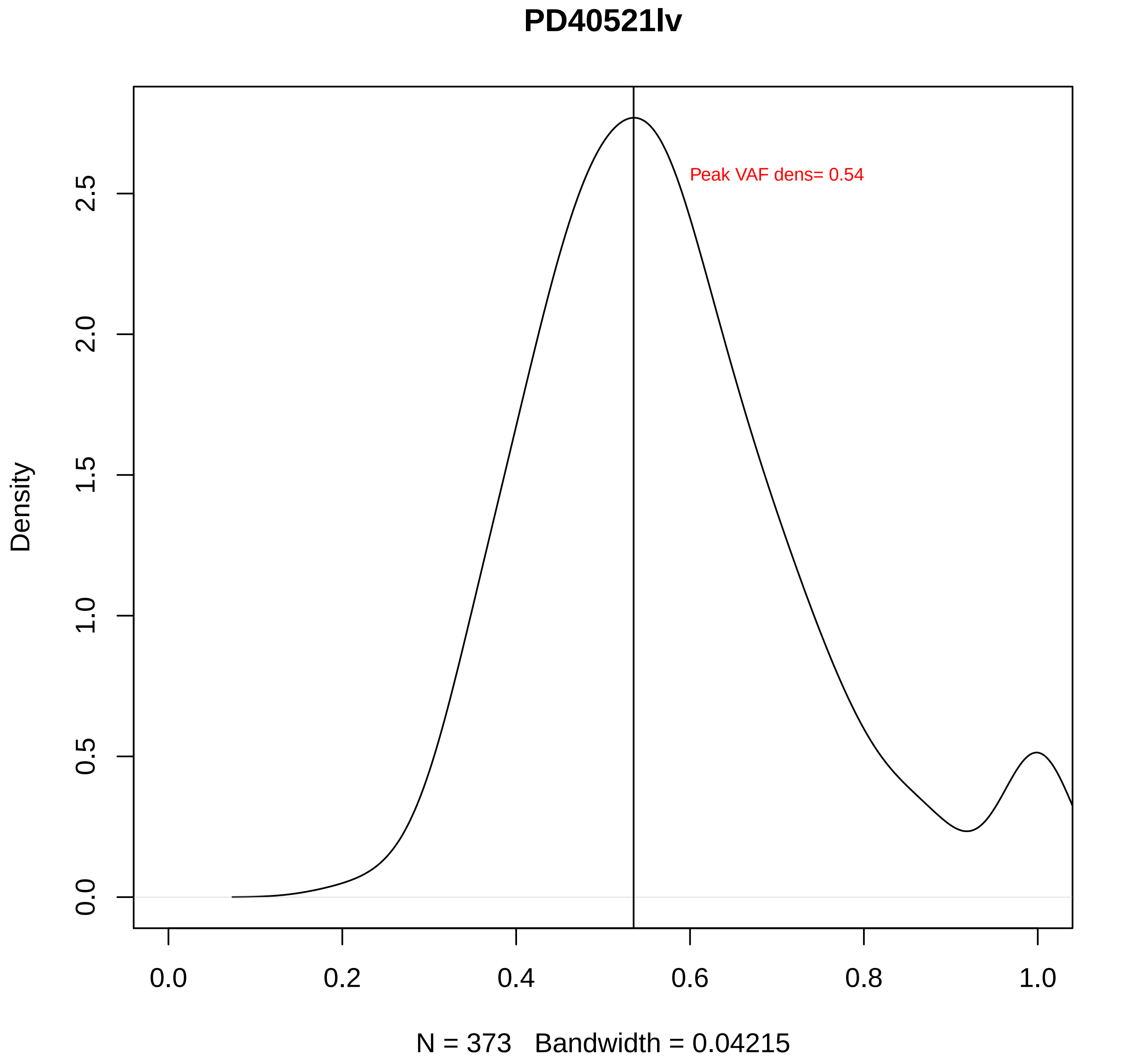
PD40521lv
Peak VAF dens= 0.54
Density
0.0
0.5
1.0
1.5
2.0
2.5
0.0
0.2
0.4
0.6
0.8
1.0
N = 373   Bandwidth = 0.04215

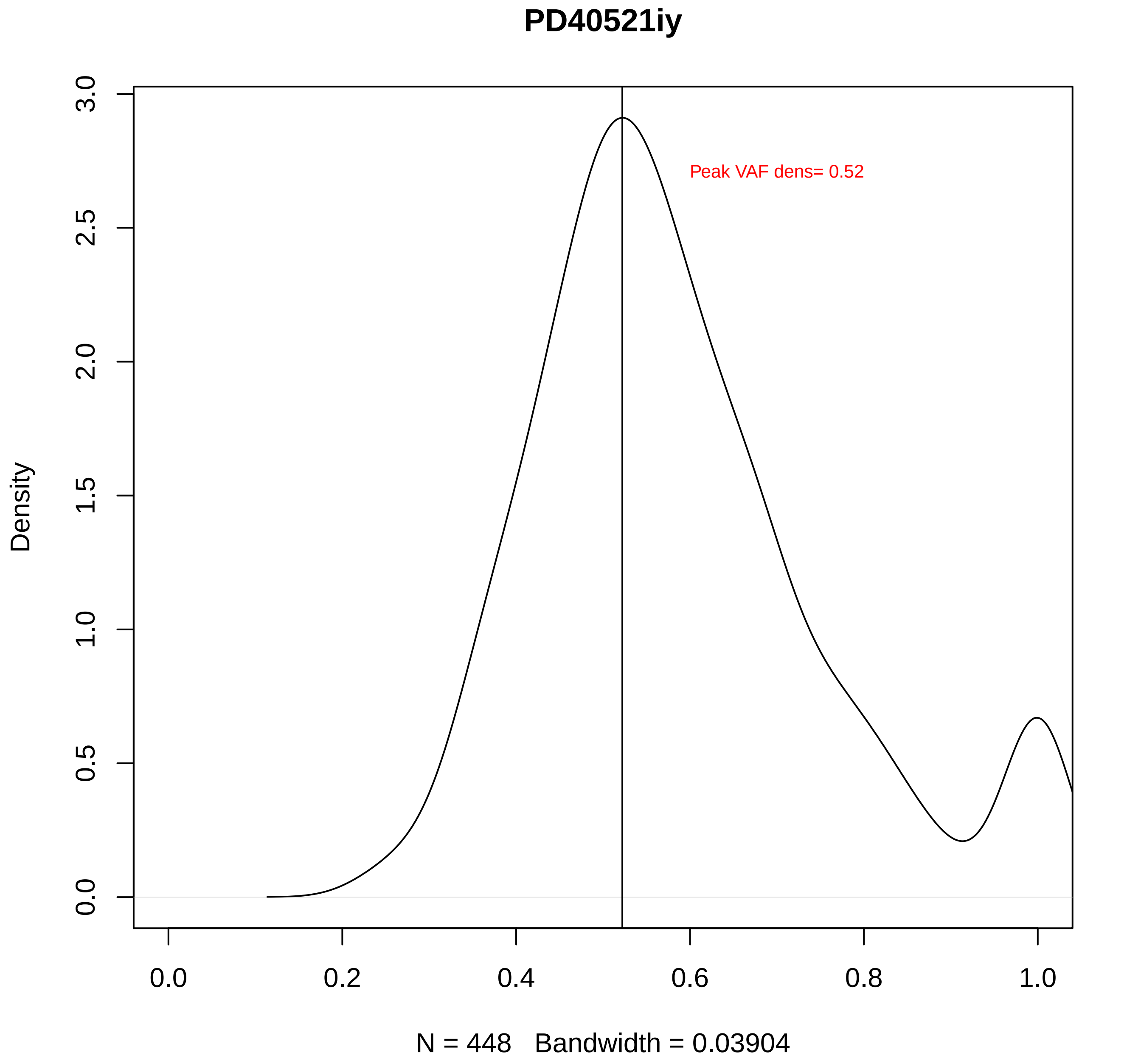
PD40521iy
Peak VAF dens= 0.52
Density
3.0
2.5
2.0
1.5
1.0
0.5
0.0
0.0
0.2
0.4
0.6
0.8
1.0
N = 448   Bandwidth = 0.03904

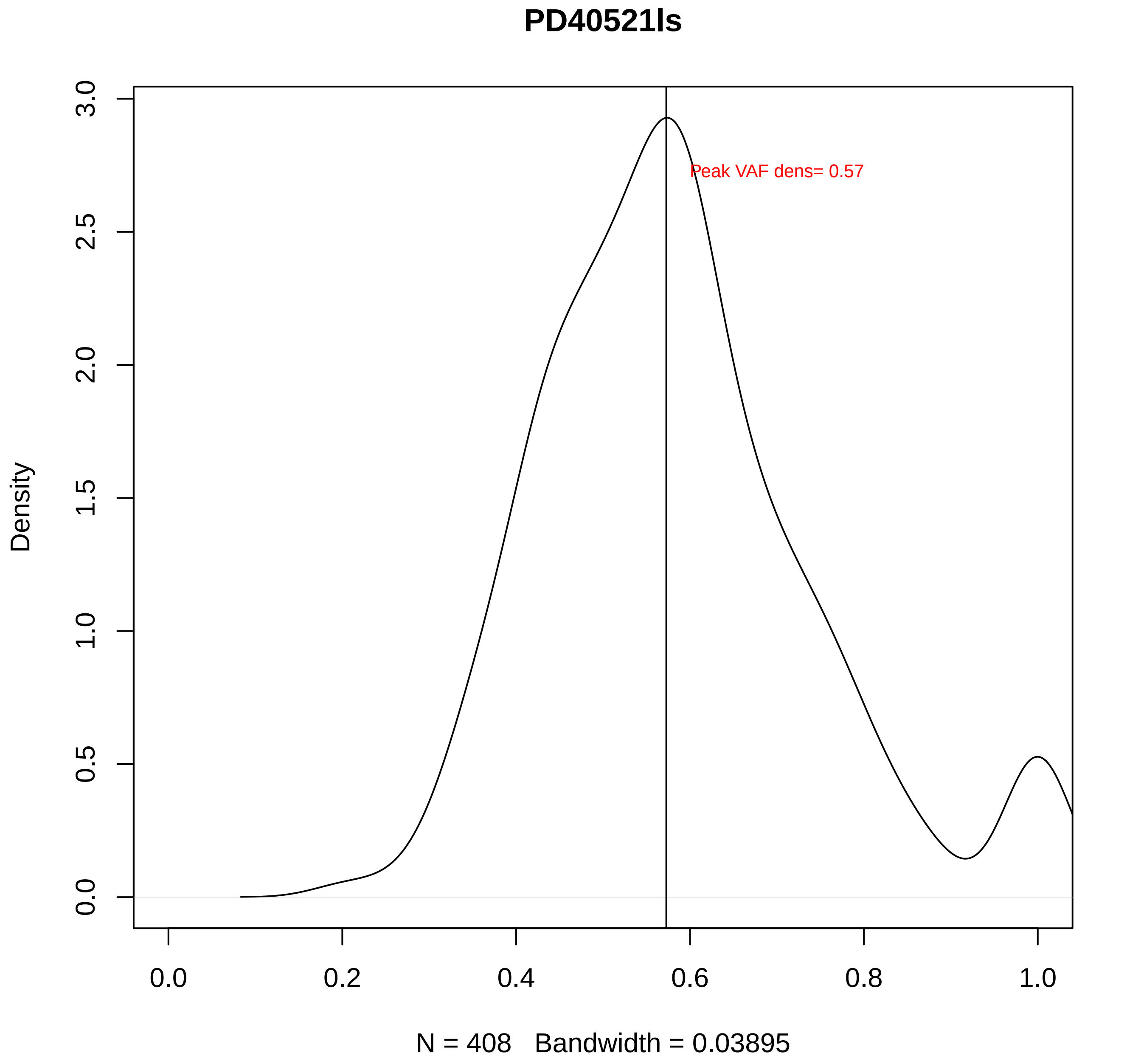
PD40521ls
Peak VAF dens= 0.57
Density
0.0
0.5
1.0
1.5
2.0
2.5
3.0
0.0
0.2
0.4
0.6
0.8
1.0
N = 408   Bandwidth = 0.03895

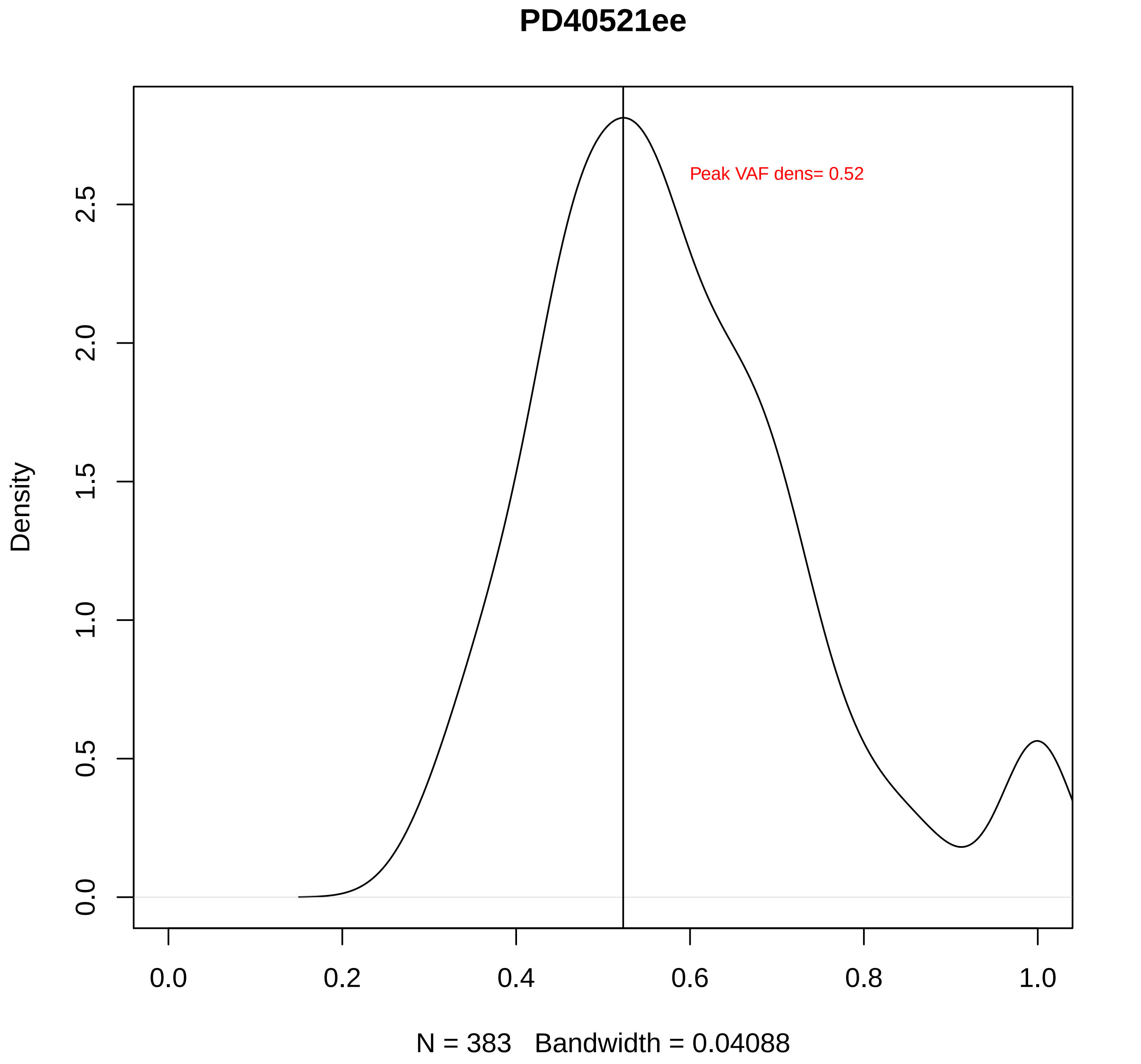
PD40521ee
Peak VAF dens= 0.52
Density
0.0
0.5
1.0
1.5
2.0
2.5
0.0
0.2
0.4
0.6
0.8
1.0
N = 383   Bandwidth = 0.04088

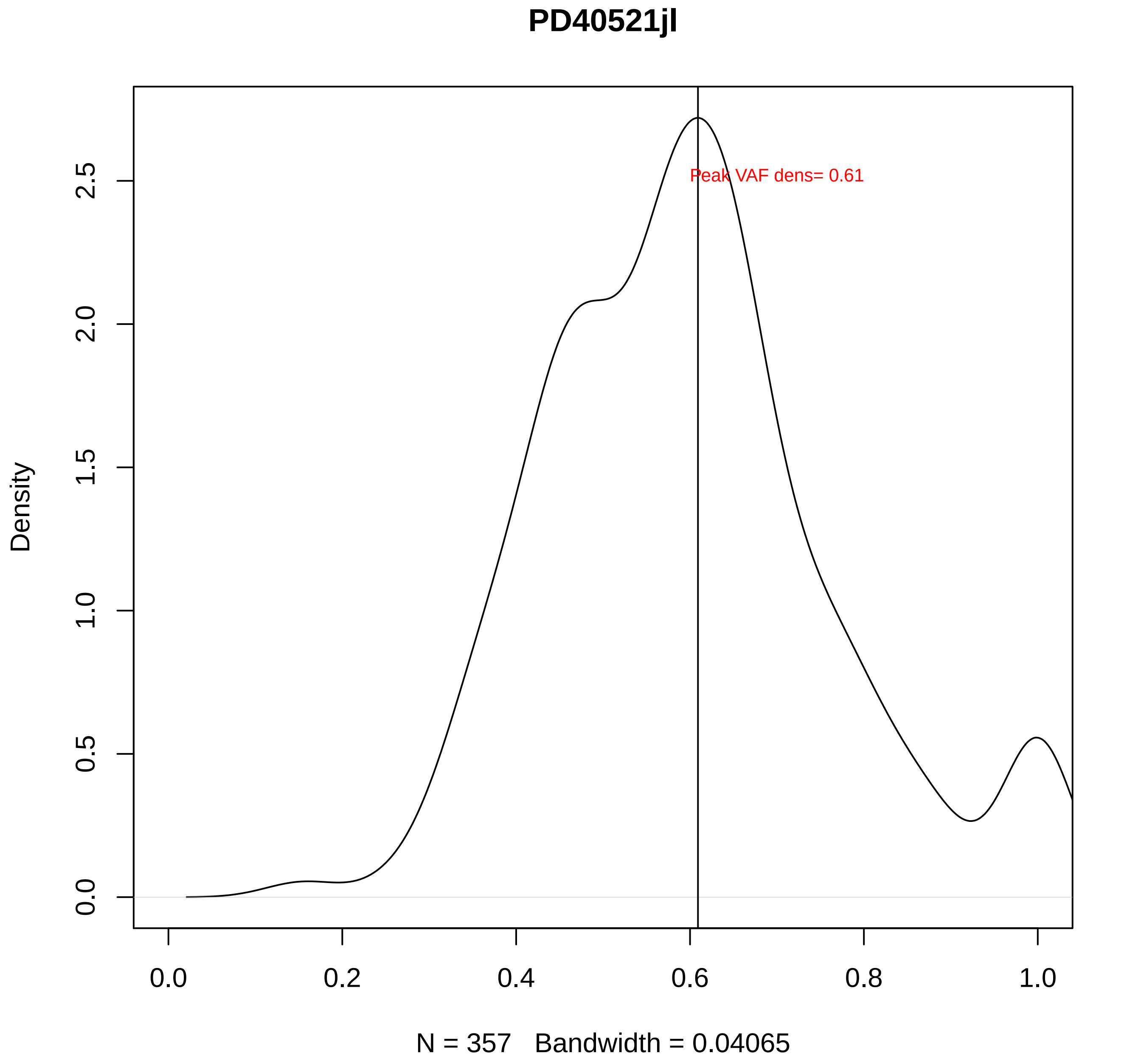
PD40521jl
Peak VAF dens= 0.61
Density
0.0
0.5
1.0
1.5
2.0
2.5
0.0
0.2
0.4
0.6
0.8
1.0
N = 357   Bandwidth = 0.04065

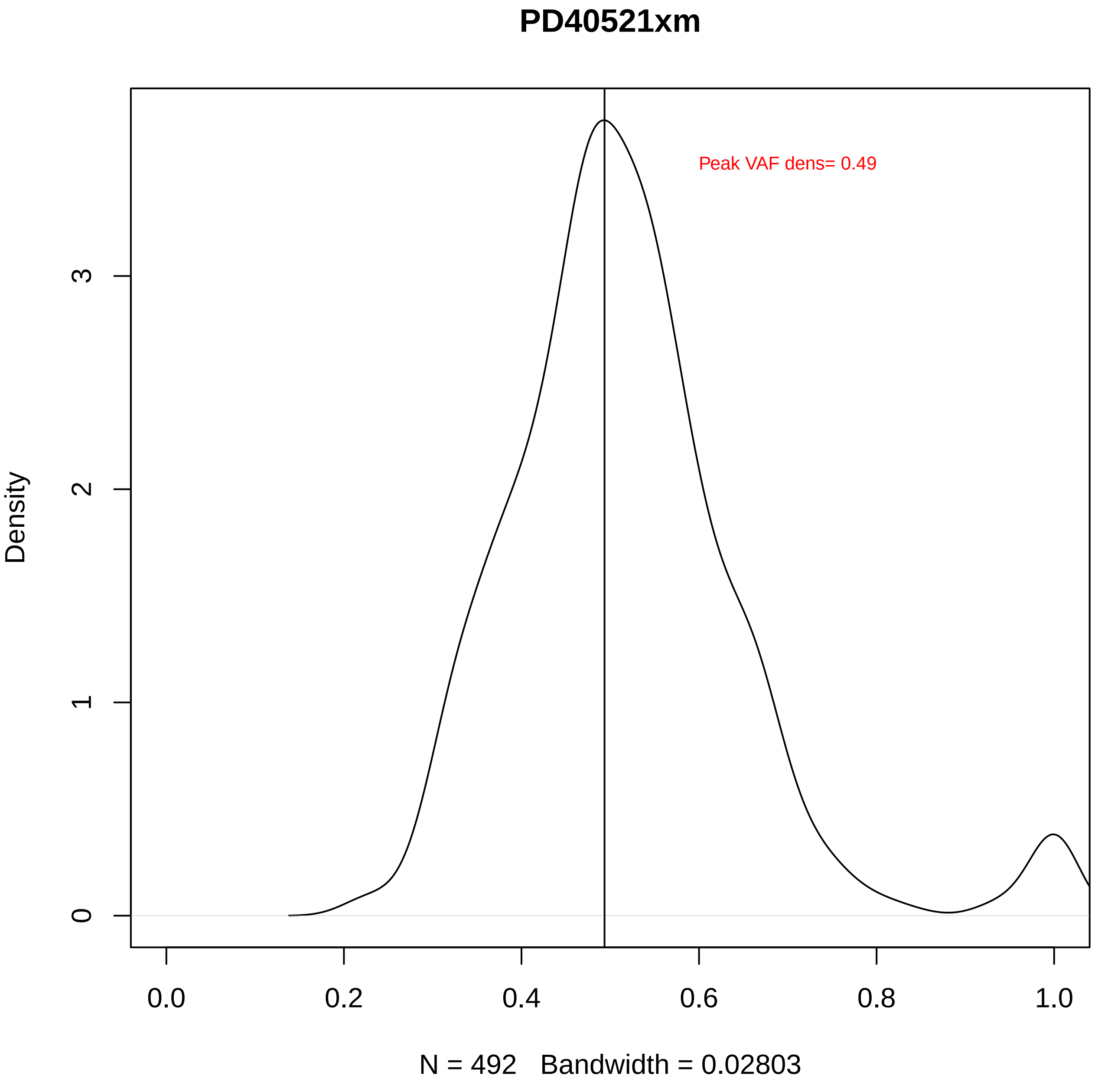
PD40521xm
Peak VAF dens= 0.49
Density
0
1
2
3
0.0
0.2
0.4
0.6
0.8
1.0
N = 492 Bandwidth = 0.02803

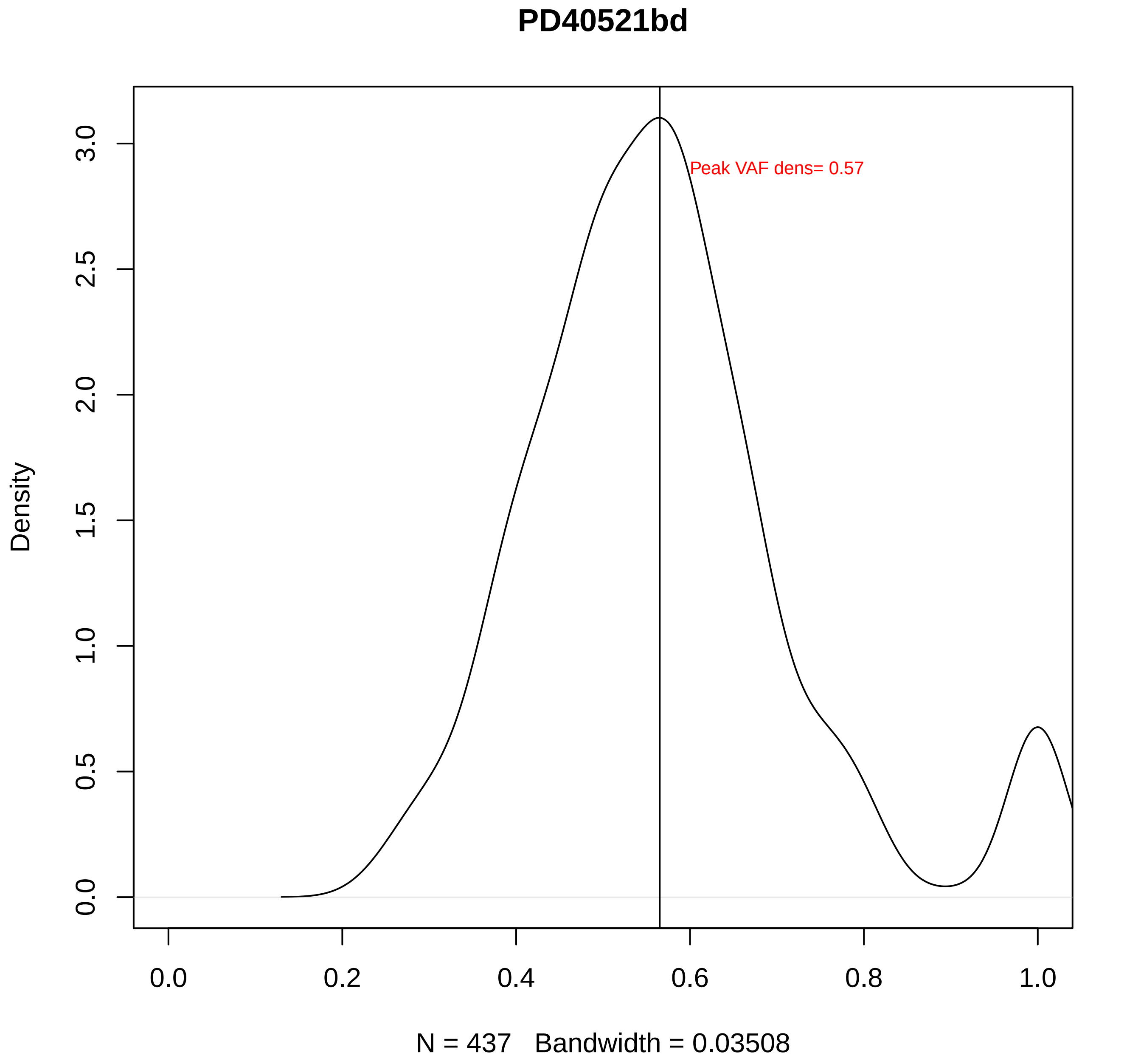

PD40521bd
Peak VAF dens= 0.57
Density
0.0
0.5
1.0
1.5
2.0
2.5
3.0
0.0
0.2
0.4
0.6
0.8
1.0
N = 437   Bandwidth = 0.03508

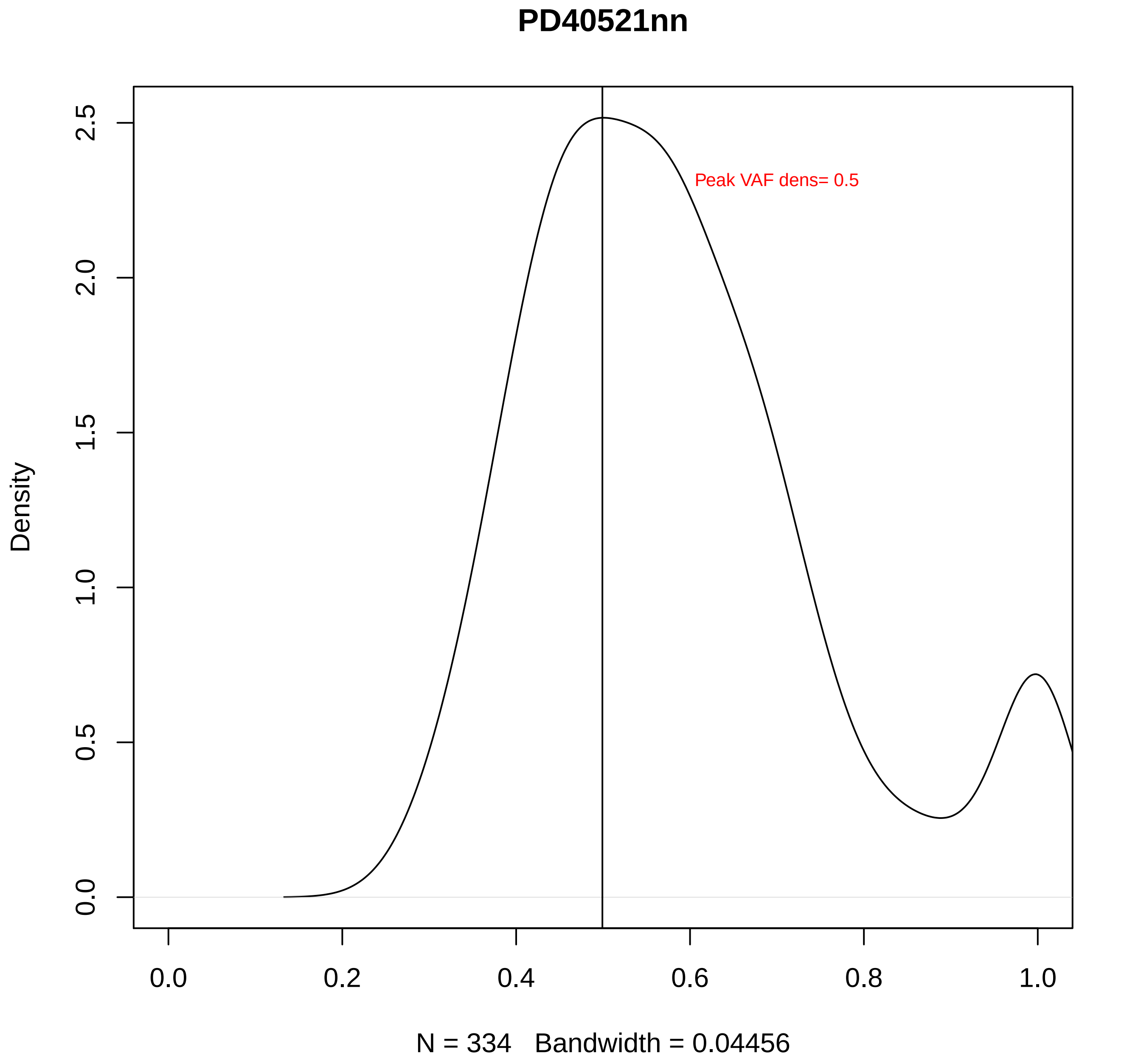
PD40521nn
Peak VAF dens= 0.5
Density
2.5
2.0
1.5
1.0
0.5
0.0
0.0
0.2
0.4
0.6
0.8
1.0
N = 334 Bandwidth = 0.04456

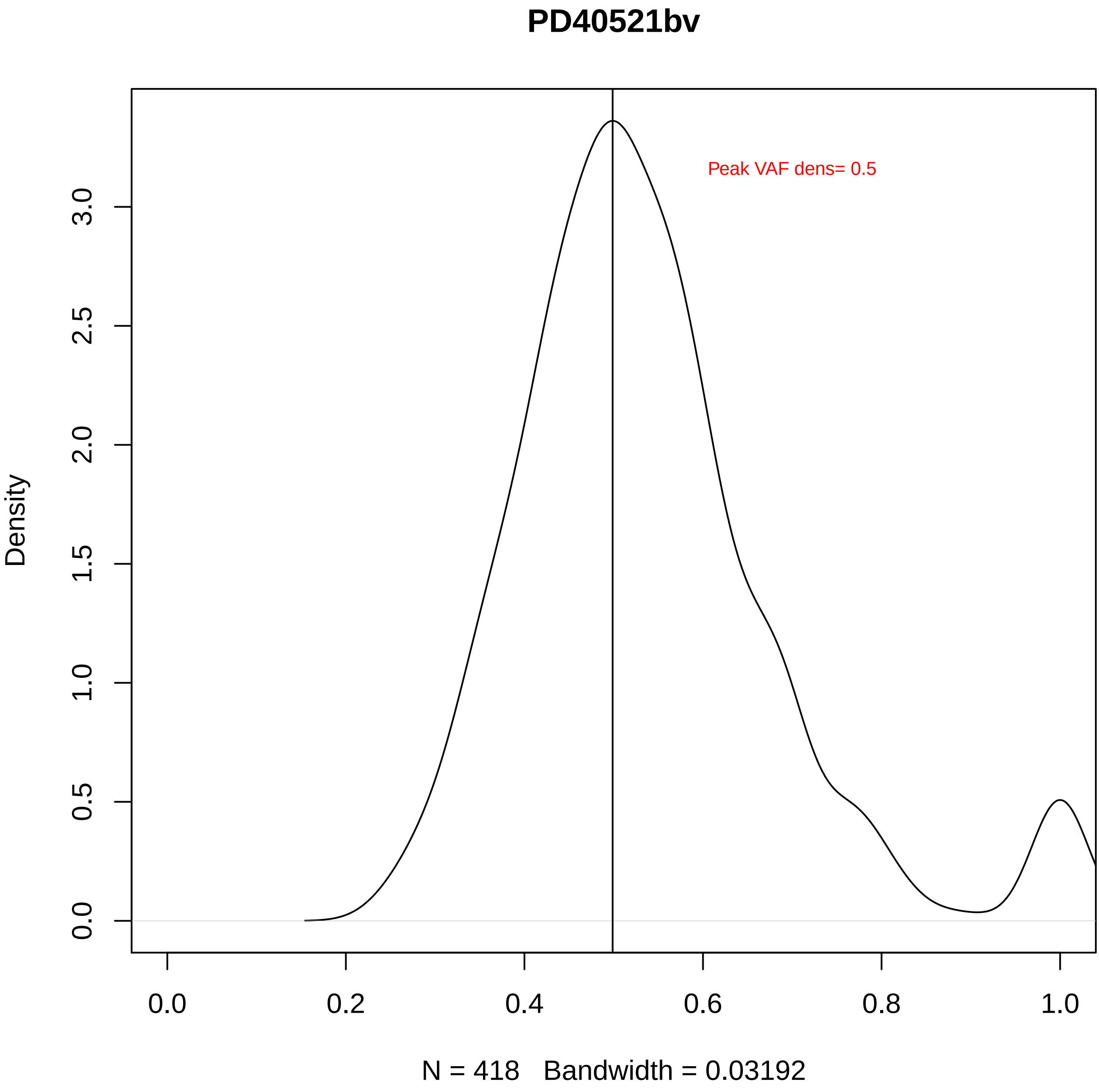
PD40521bv
Peak VAF dens= 0.5
Density
0.0
0.5
1.0
1.5
2.0
2.5
3.0
0.0
0.2
0.4
0.6
0.8
1.0
N = 418 Bandwidth = 0.03192

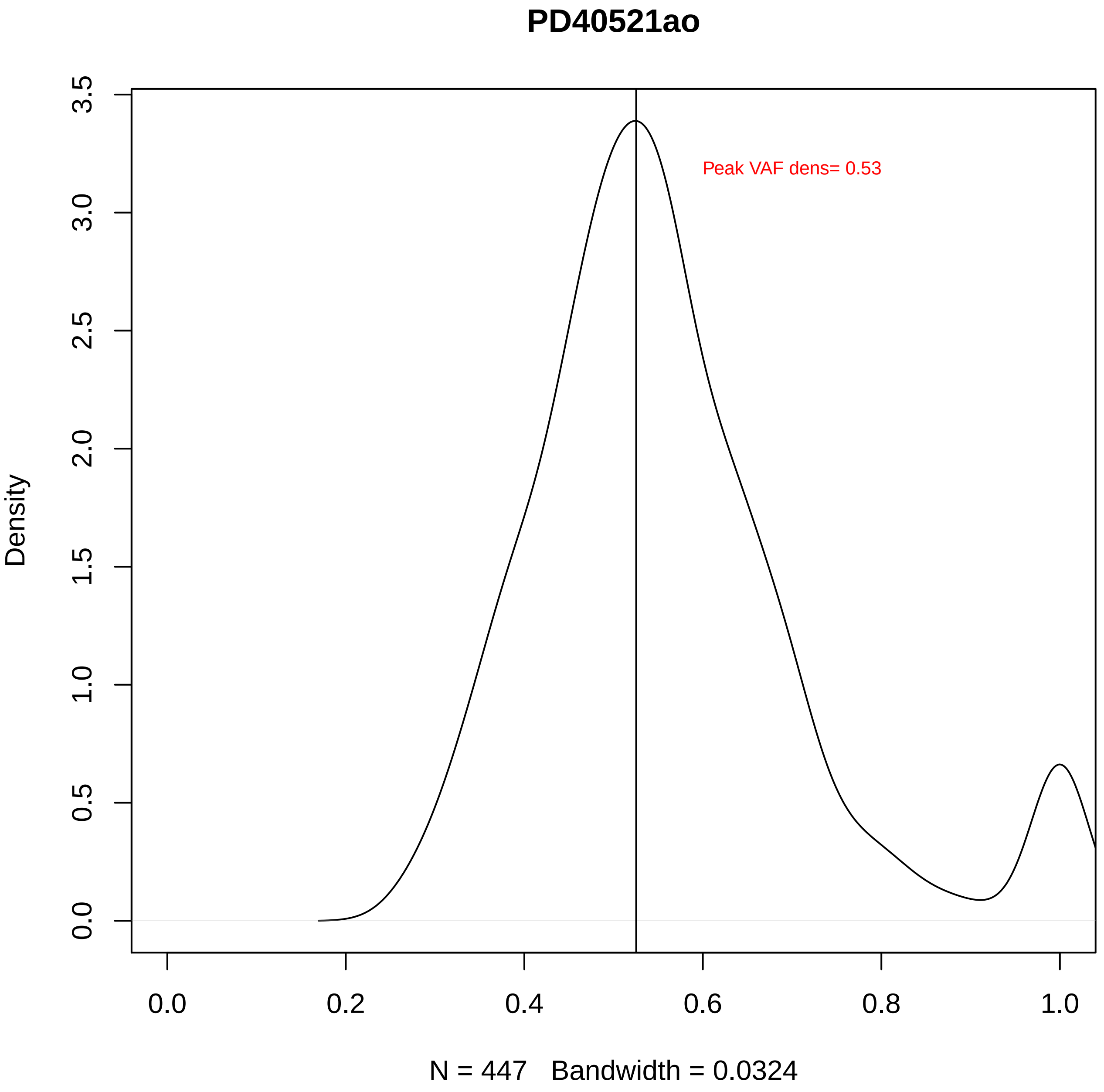
PD40521ao
Peak VAF dens= 0.53
Density
0.0
0.5
1.0
1.5
2.0
2.5
3.0
3.5
0.0
0.2
0.4
0.6
0.8
1.0
N = 447   Bandwidth = 0.0324

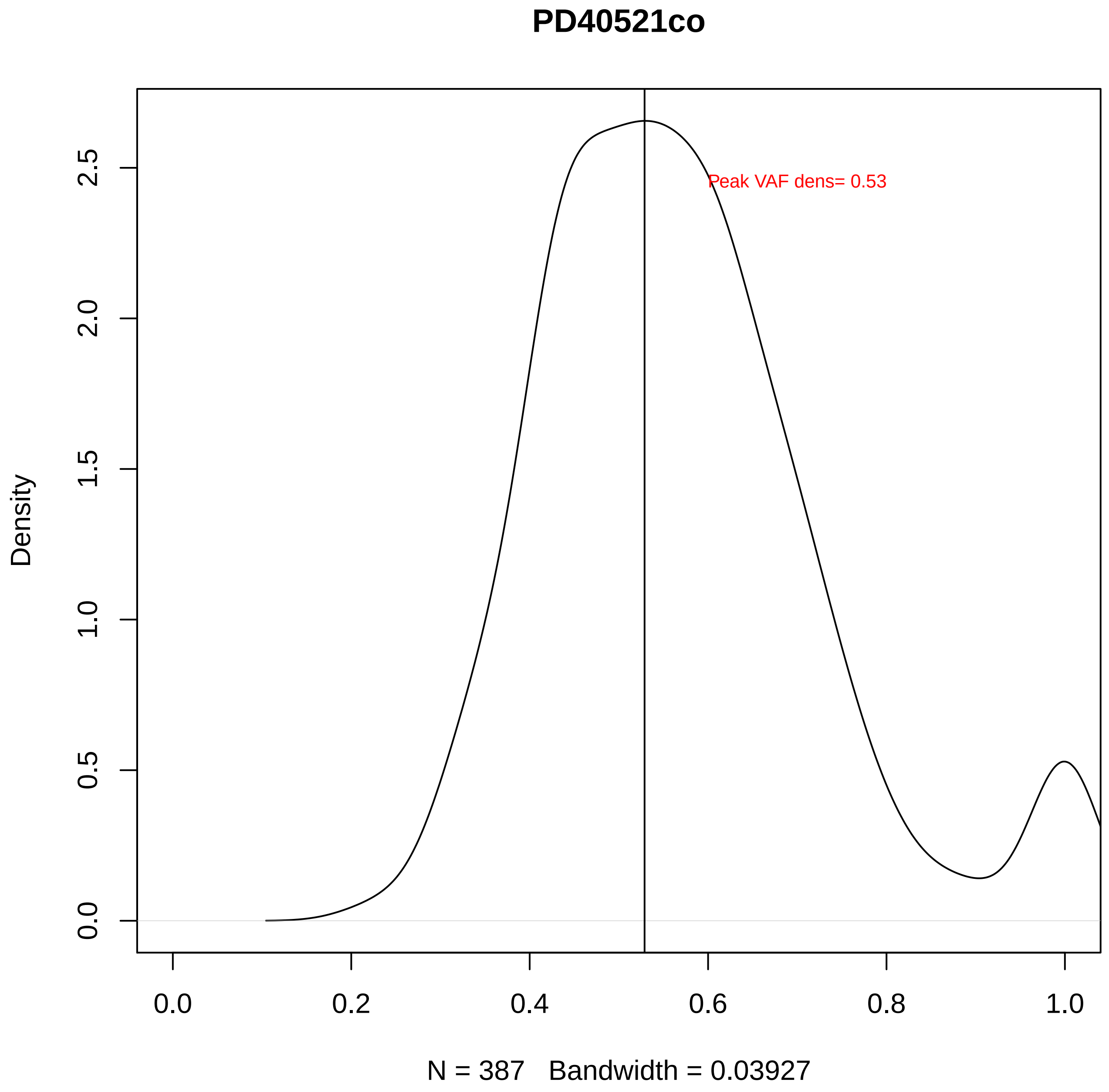

PD40521co
Density
Peak VAF dens= 0.53
N = 387 Bandwidth = 0.03927
0.0
0.5
1.0
1.5
2.0
2.5
0.0
0.2
0.4
0.6
0.8
1.0

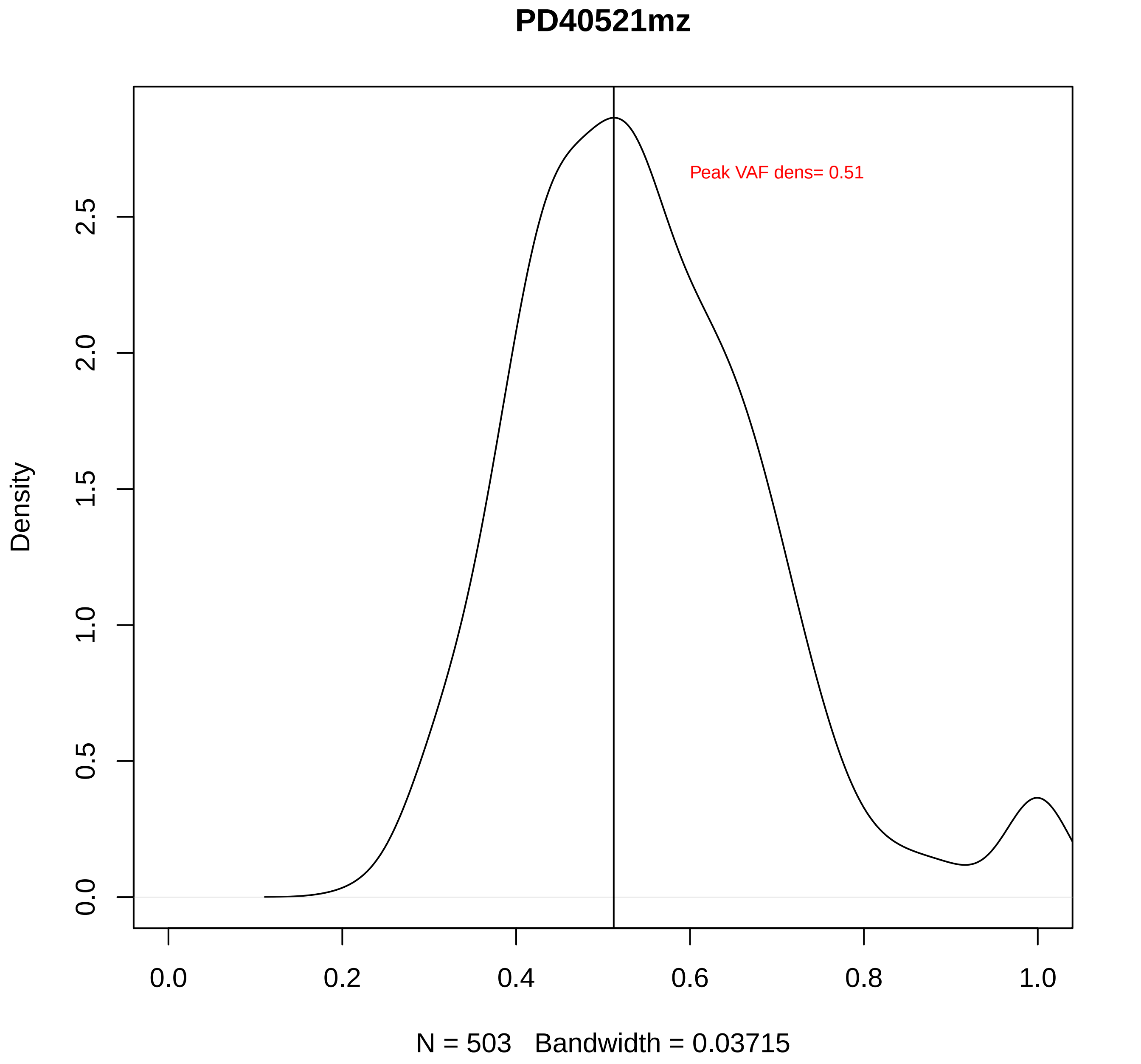
PD40521mz
Peak VAF dens= 0.51
Density
0.0
0.5
1.0
1.5
2.0
2.5
0.0
0.2
0.4
0.6
0.8
1.0
N = 503   Bandwidth = 0.03715

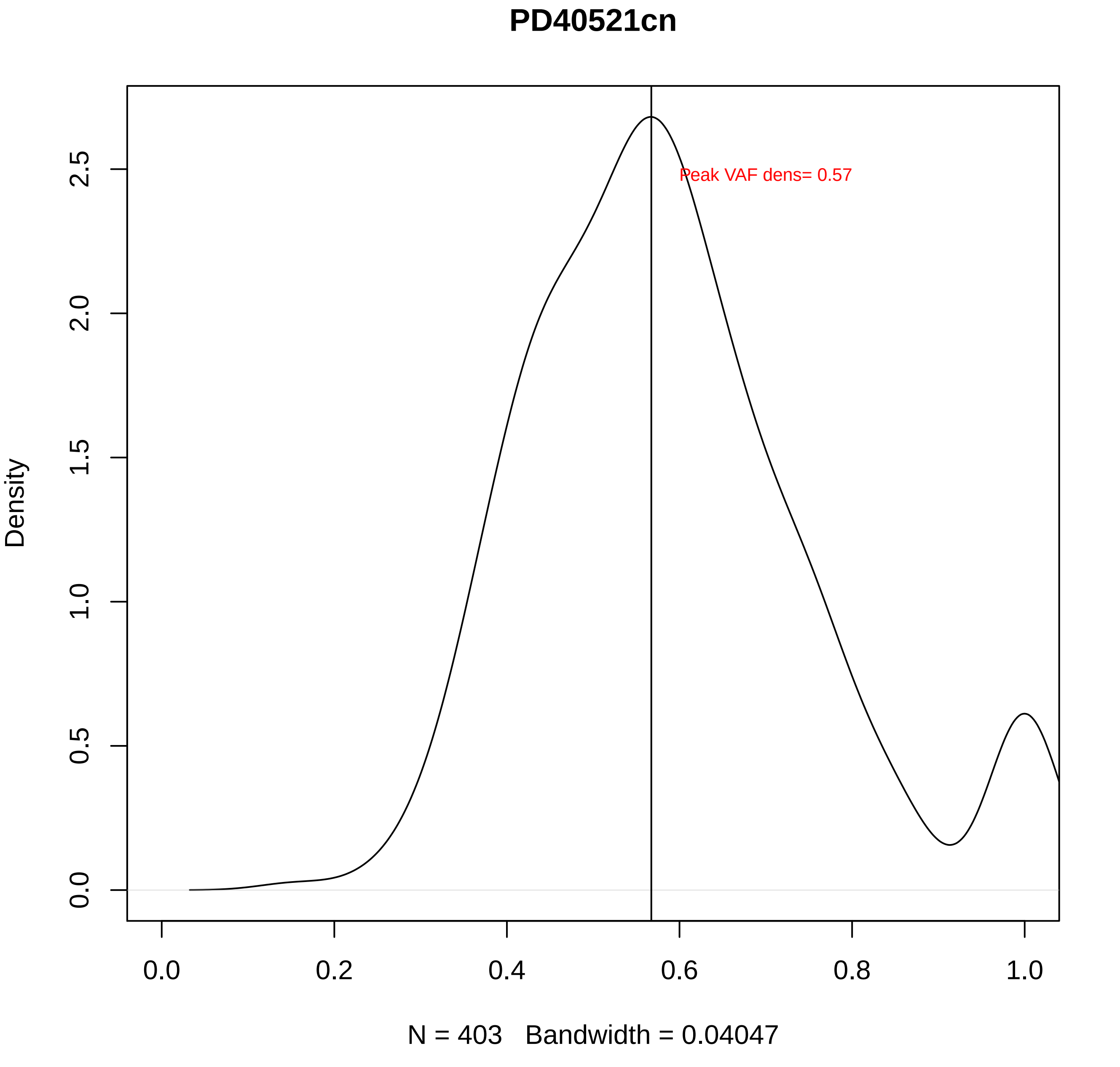
PD40521cn
Density
0.0
0.5
1.0
1.5
2.0
2.5
0.0
0.2
0.4
0.6
0.8
1.0
Peak VAF dens= 0.57
N = 403   Bandwidth = 0.04047

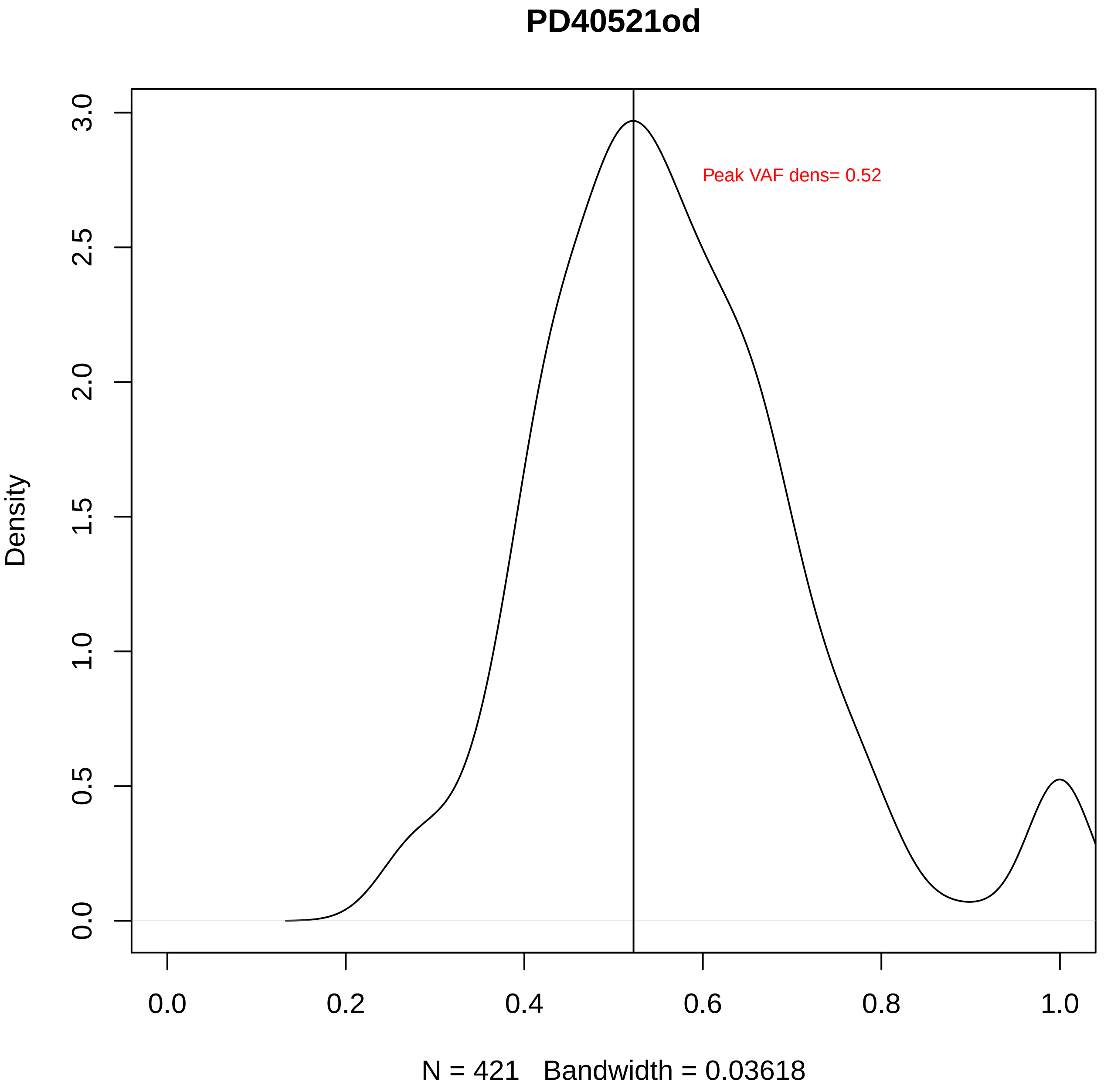

PD40521od
Peak VAF dens= 0.52
Density
0.0
0.5
1.0
1.5
2.0
2.5
3.0
0.0
0.2
0.4
0.6
0.8
1.0
N = 421   Bandwidth = 0.03618

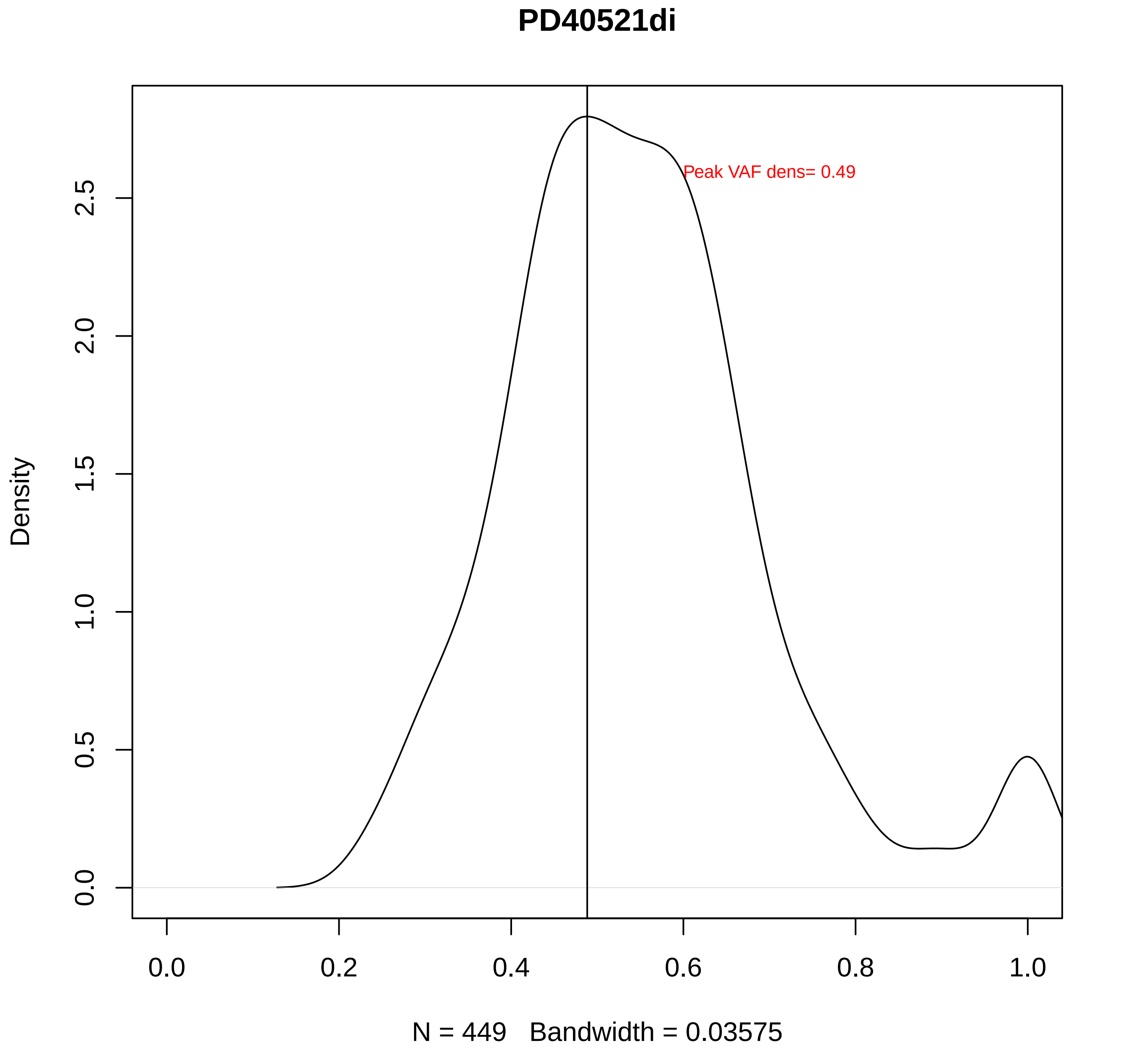

PD40521di
Peak VAF dens= 0.49
Density
0.0
0.5
1.0
1.5
2.0
2.5
0.0
0.2
0.4
0.6
0.8
1.0
N = 449   Bandwidth = 0.03575

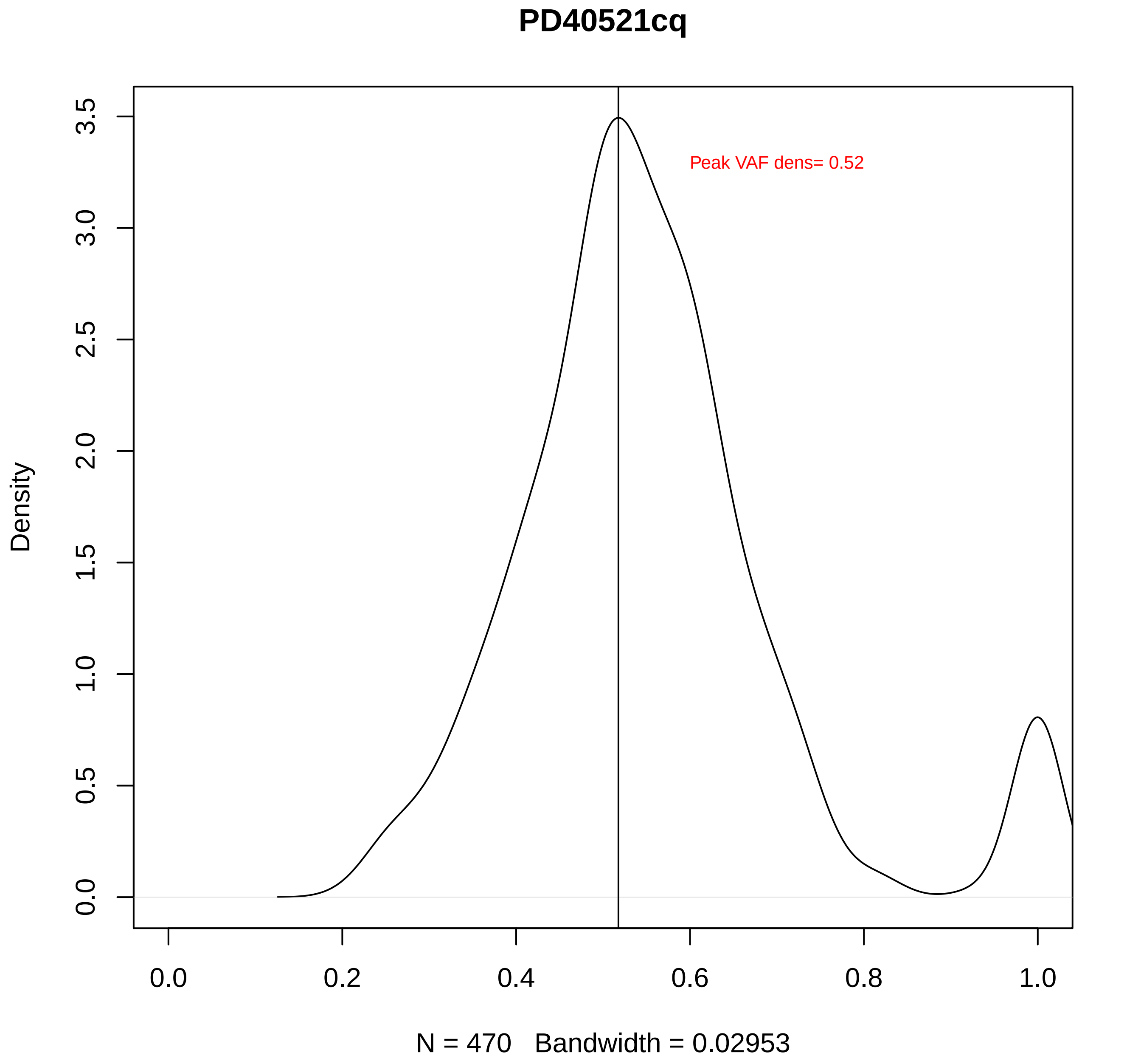
PD40521cq
Peak VAF dens= 0.52
Density
0.0
0.5
1.0
1.5
2.0
2.5
3.0
3.5
0.0
0.2
0.4
0.6
0.8
1.0
N = 470   Bandwidth = 0.02953

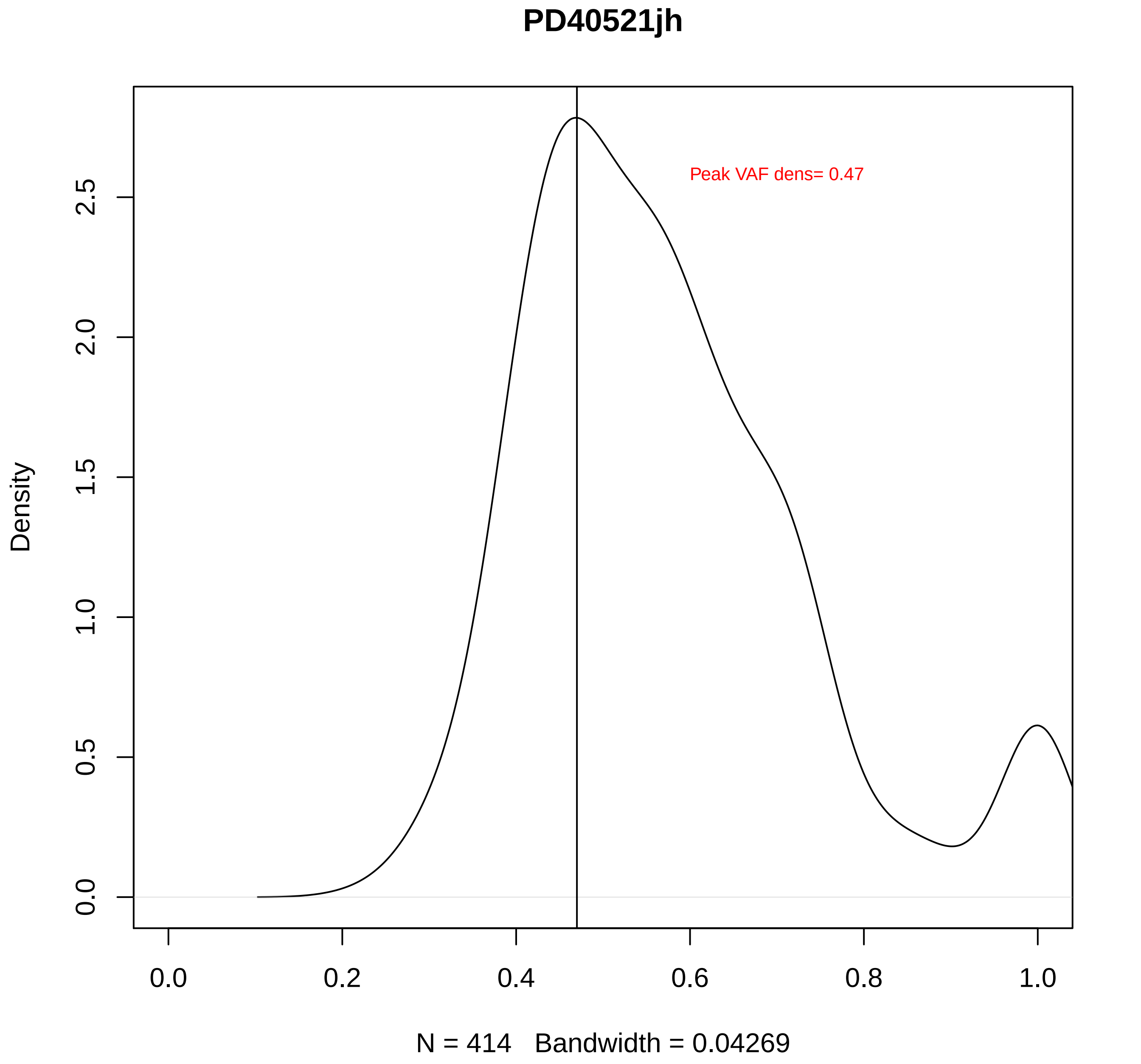

PD40521jh
Peak VAF dens= 0.47
Density
0.0
0.5
1.0
1.5
2.0
2.5
0.0
0.2
0.4
0.6
0.8
1.0
N = 414   Bandwidth = 0.04269

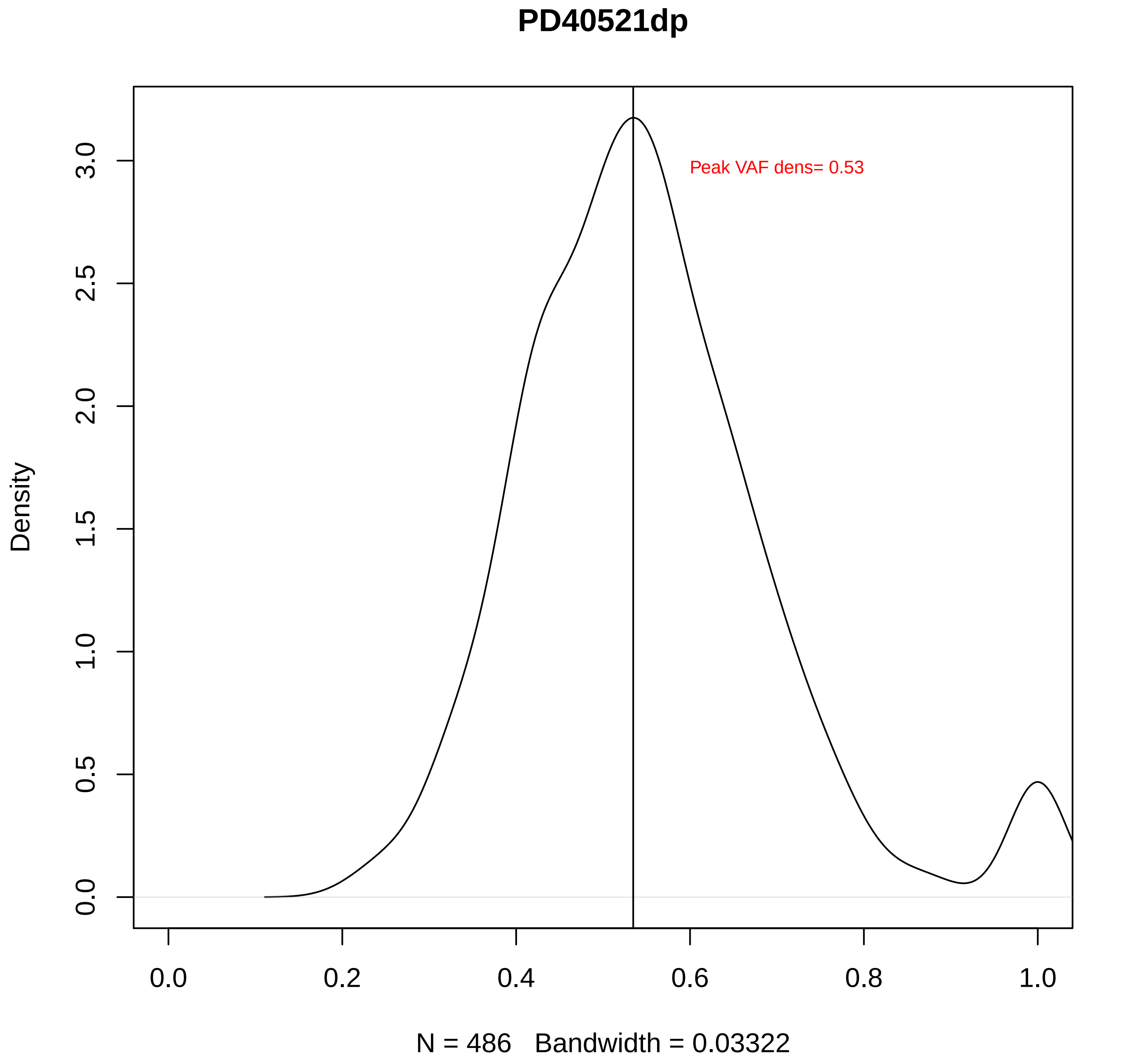
PD40521dp
Peak VAF dens= 0.53
Density
0.0
0.5
1.0
1.5
2.0
2.5
3.0
0.0
0.2
0.4
0.6
0.8
1.0
N = 486 Bandwidth = 0.03322

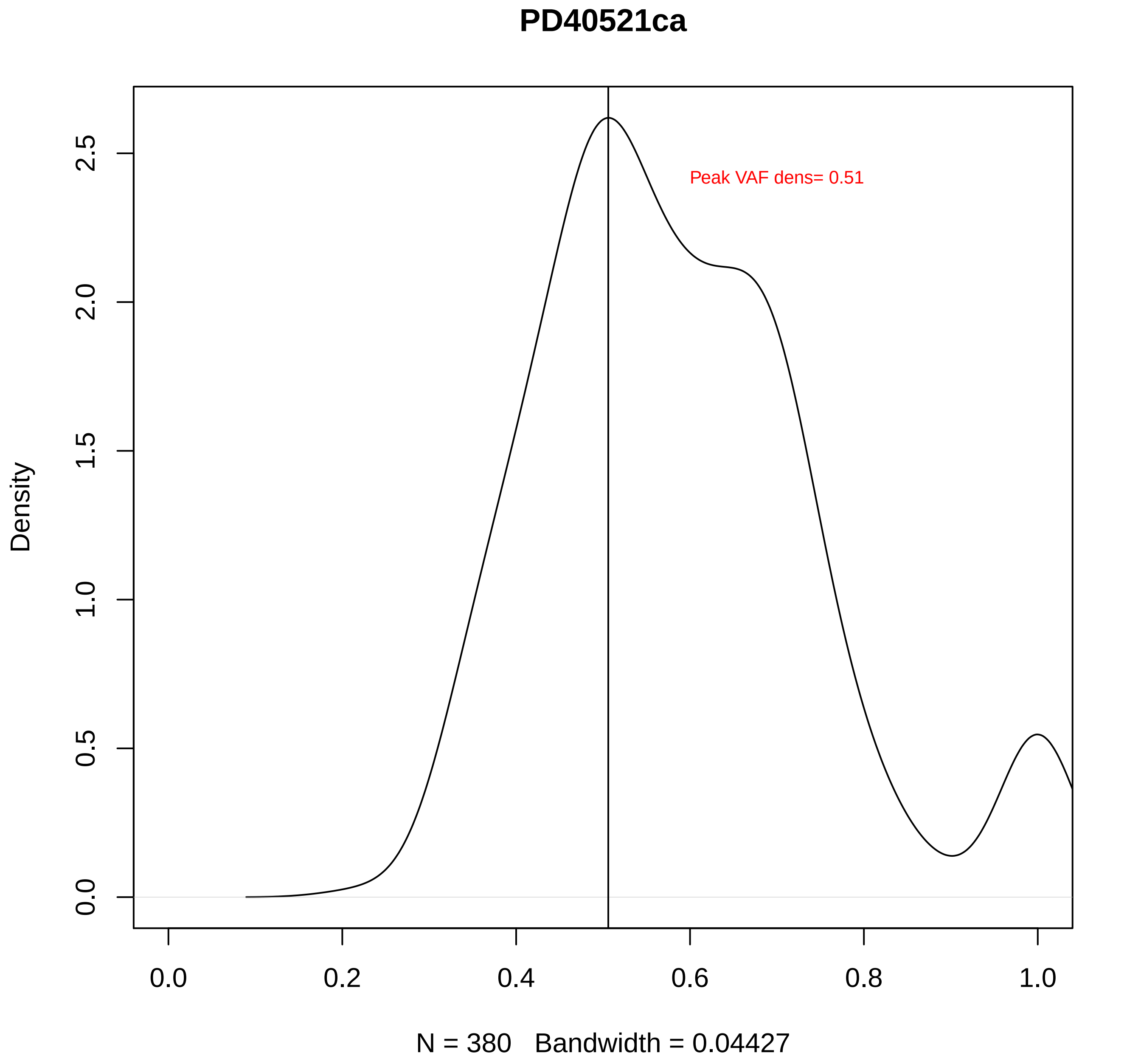

PD40521ca
Peak VAF dens= 0.51
Density
0.0
0.5
1.0
1.5
2.0
2.5
0.0
0.2
0.4
0.6
0.8
1.0
N = 380   Bandwidth = 0.04427

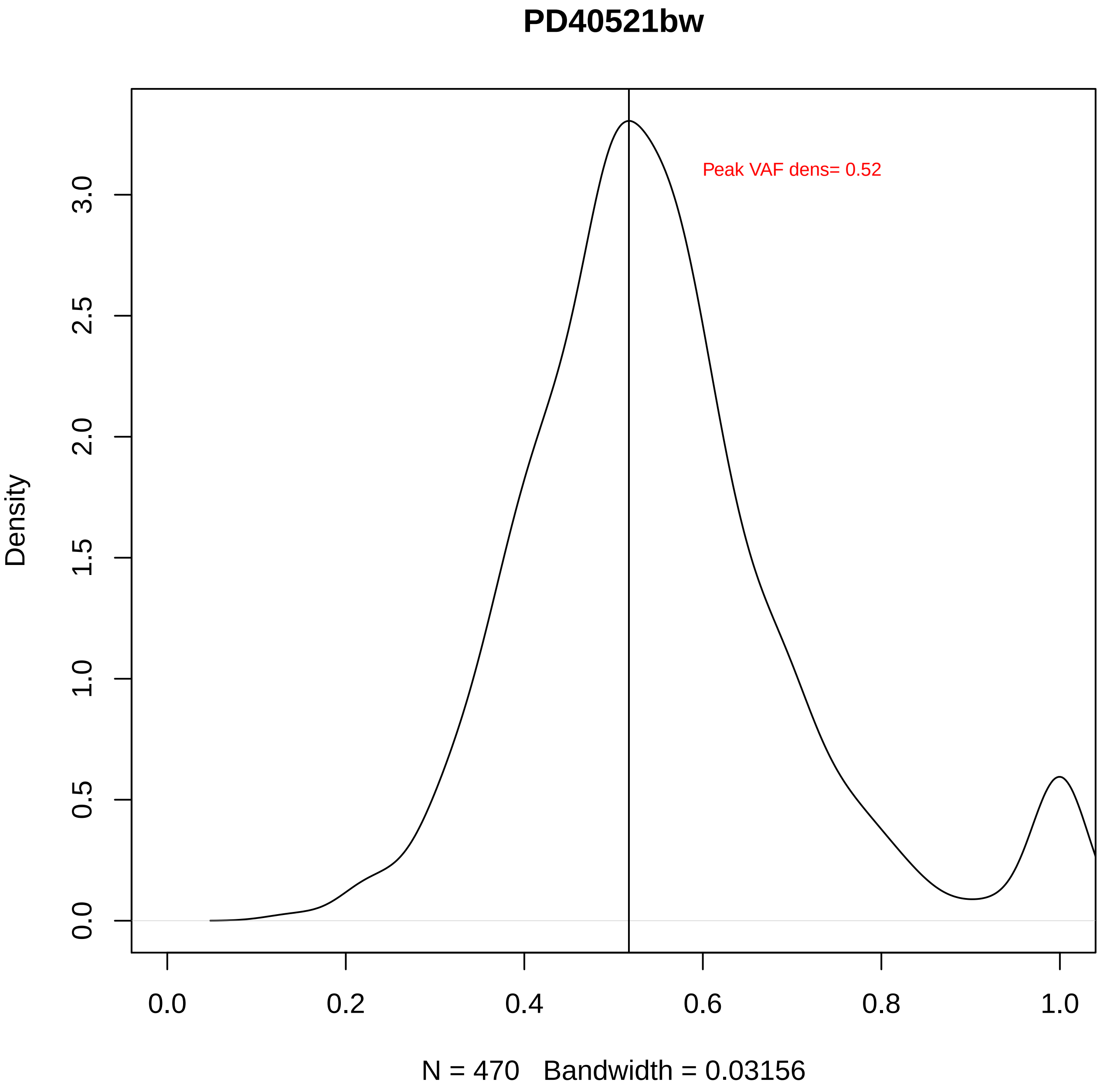
PD40521bw
Peak VAF dens= 0.52
Density
0.0
0.5
1.0
1.5
2.0
2.5
3.0
0.0
0.2
0.4
0.6
0.8
1.0
N = 470   Bandwidth = 0.03156

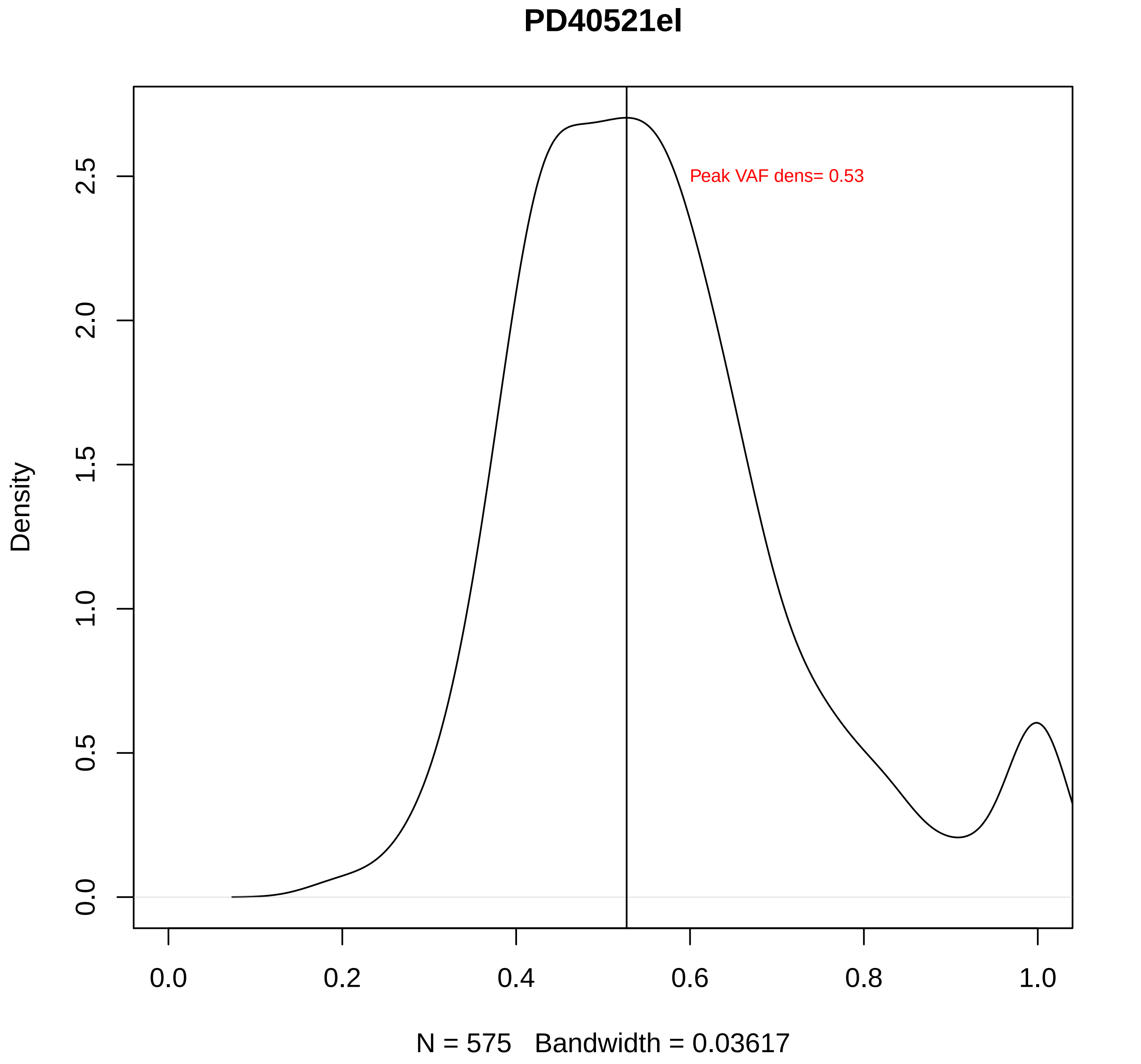
PD40521el
Peak VAF dens= 0.53
Density
0.0
0.5
1.0
1.5
2.0
2.5
0.0
0.2
0.4
0.6
0.8
1.0
N = 575 Bandwidth = 0.03617

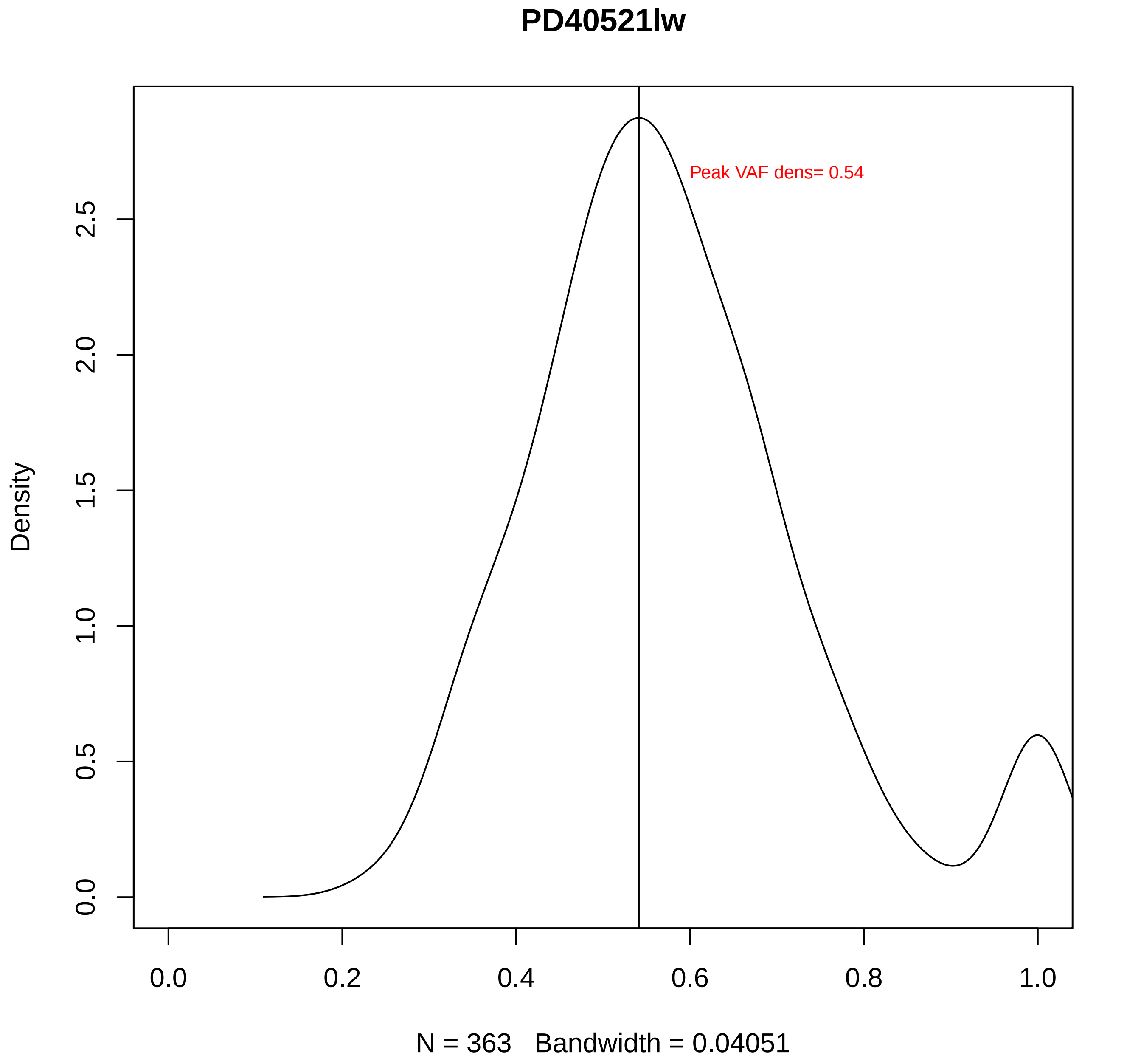
PD40521lw
Peak VAF dens= 0.54
Density
0.0
0.5
1.0
1.5
2.0
2.5
0.0
0.2
0.4
0.6
0.8
1.0
N = 363   Bandwidth = 0.04051

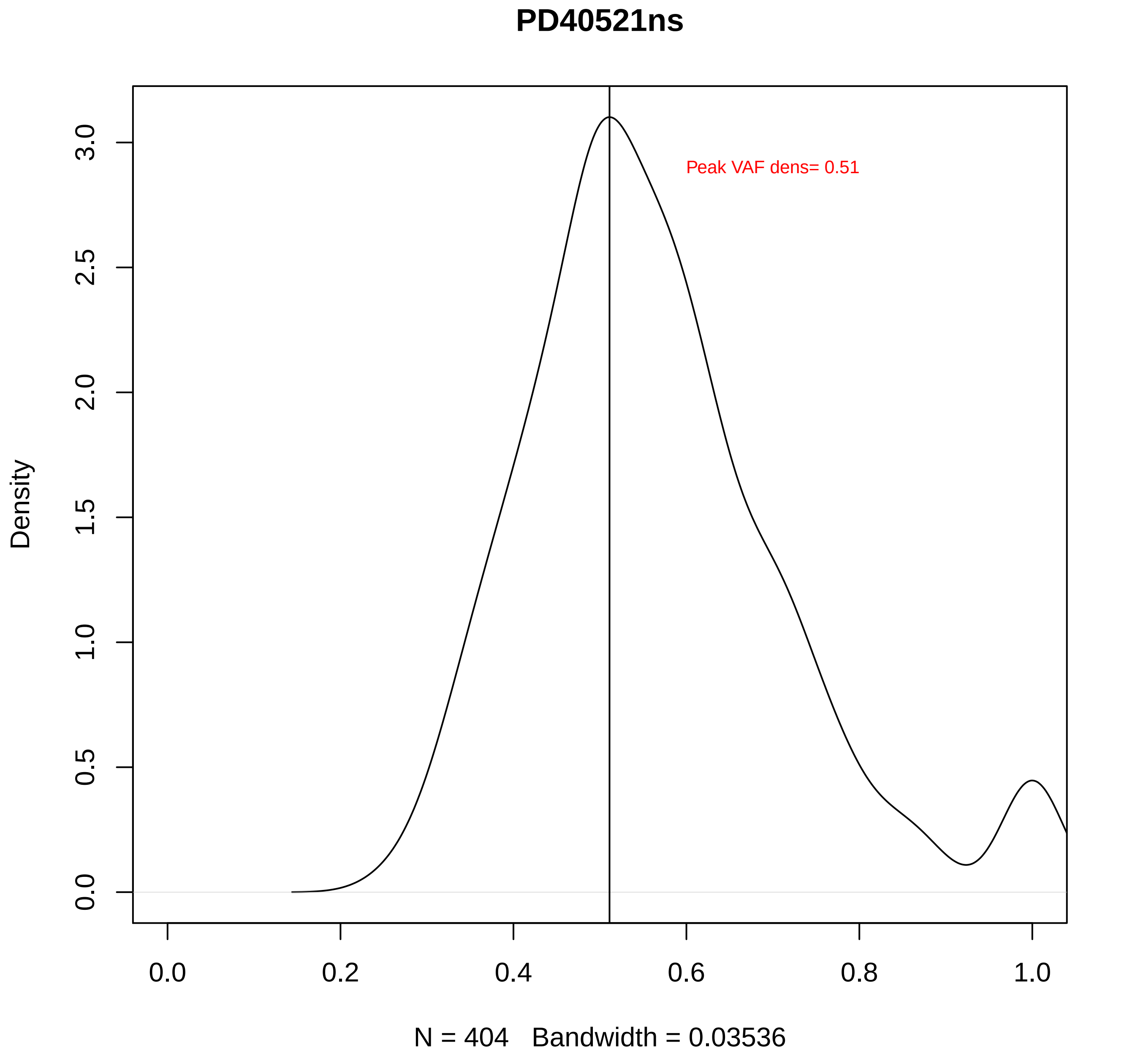

PD40521ns
Peak VAF dens= 0.51
Density
0.0
0.5
1.0
1.5
2.0
2.5
3.0
0.0
0.2
0.4
0.6
0.8
1.0
N = 404   Bandwidth = 0.03536

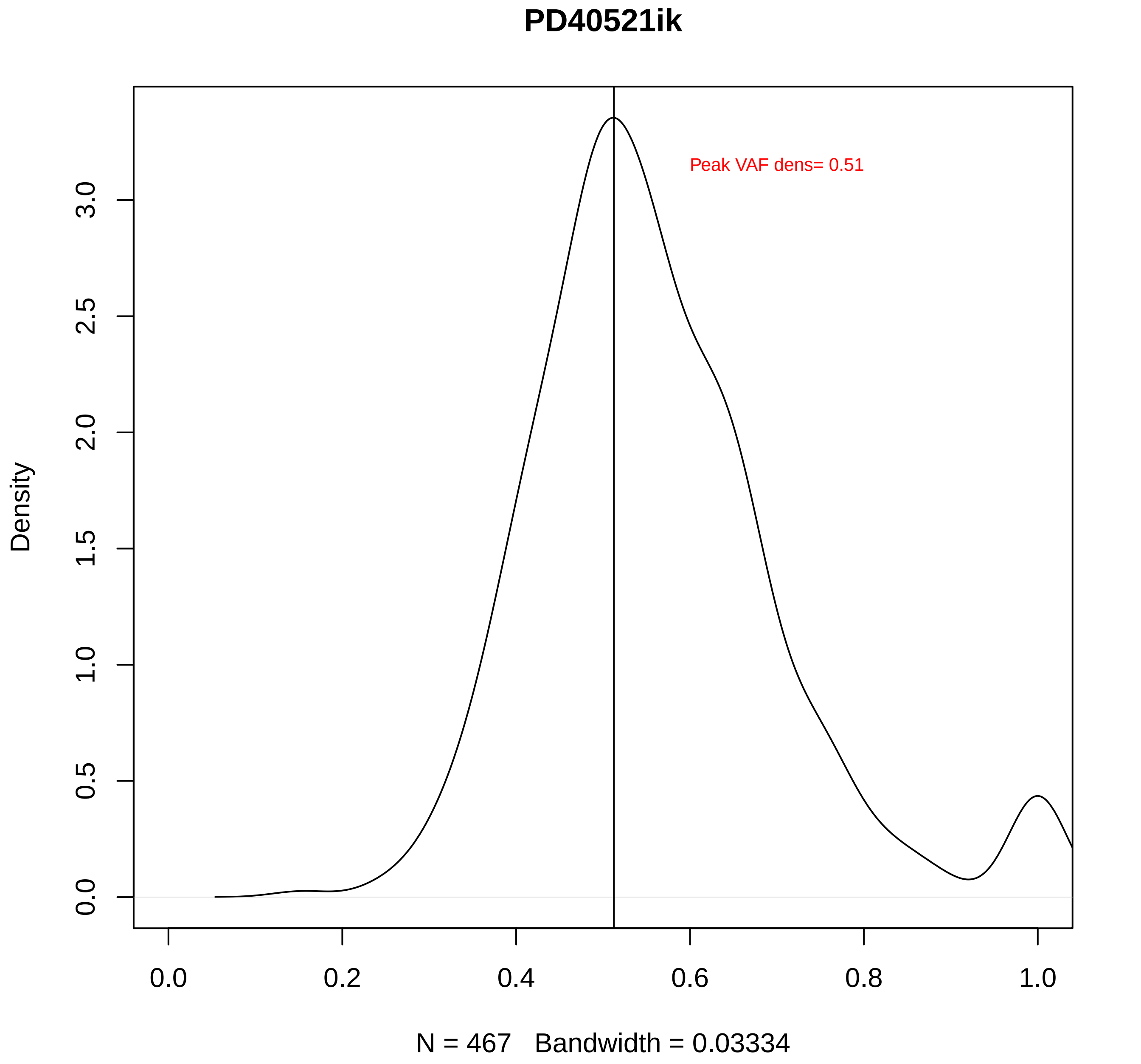
PD40521ik
Peak VAF dens= 0.51
Density
0.0
0.5
1.0
1.5
2.0
2.5
3.0
0.0
0.2
0.4
0.6
0.8
1.0
N = 467   Bandwidth = 0.03334

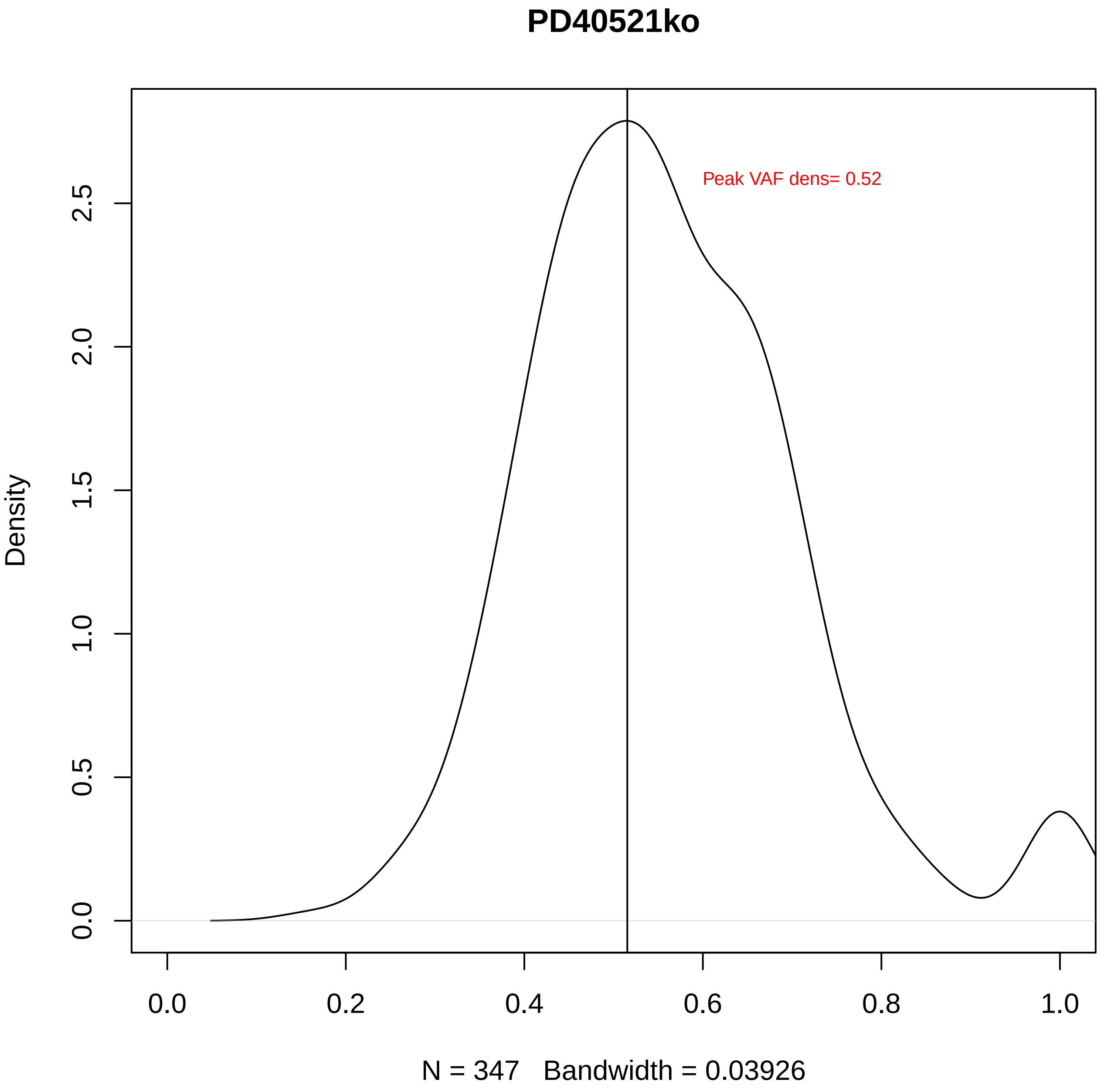
PD40521ko
Peak VAF dens= 0.52
Density
0.0
0.5
1.0
1.5
2.0
2.5
0.0
0.2
0.4
0.6
0.8
1.0
N = 347   Bandwidth = 0.03926

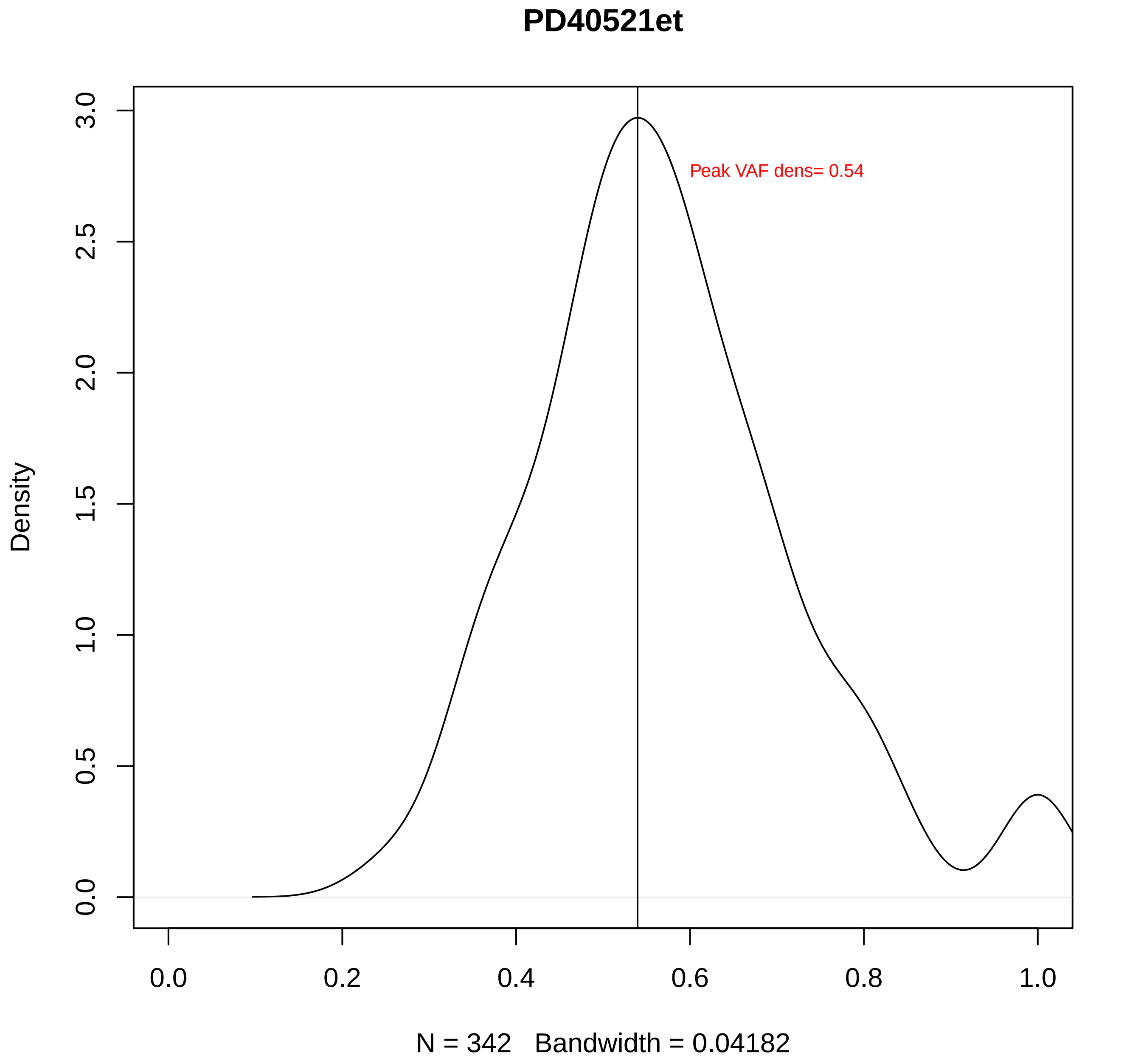

PD40521et
Peak VAF dens= 0.54
Density
3.0
2.5
2.0
1.5
1.0
0.5
0.0
0.0
0.2
0.4
0.6
0.8
1.0
N = 342   Bandwidth = 0.04182

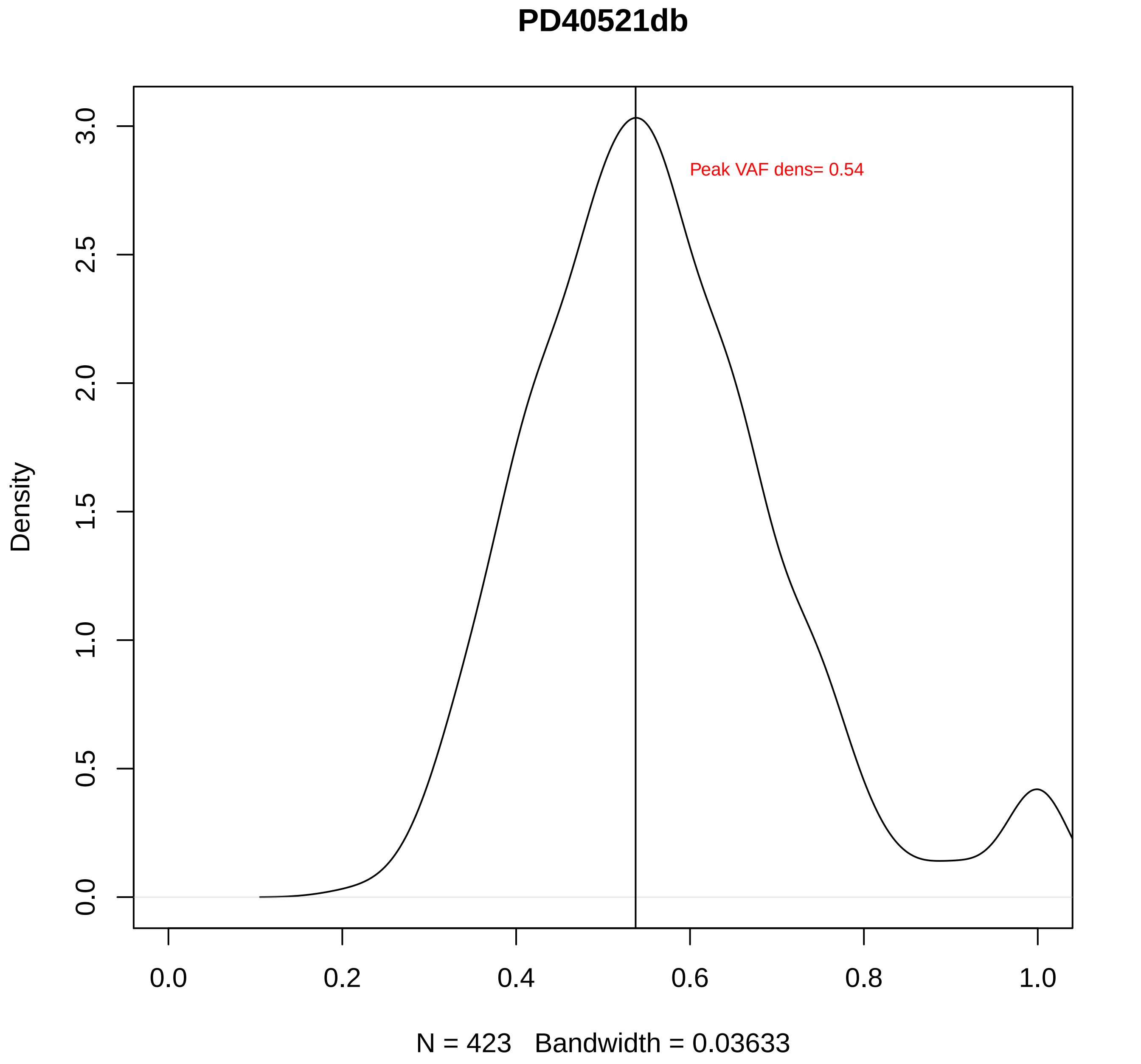
PD40521db
Peak VAF dens= 0.54
Density
0.0
0.5
1.0
1.5
2.0
2.5
3.0
0.0
0.2
0.4
0.6
0.8
1.0
N = 423   Bandwidth = 0.03633

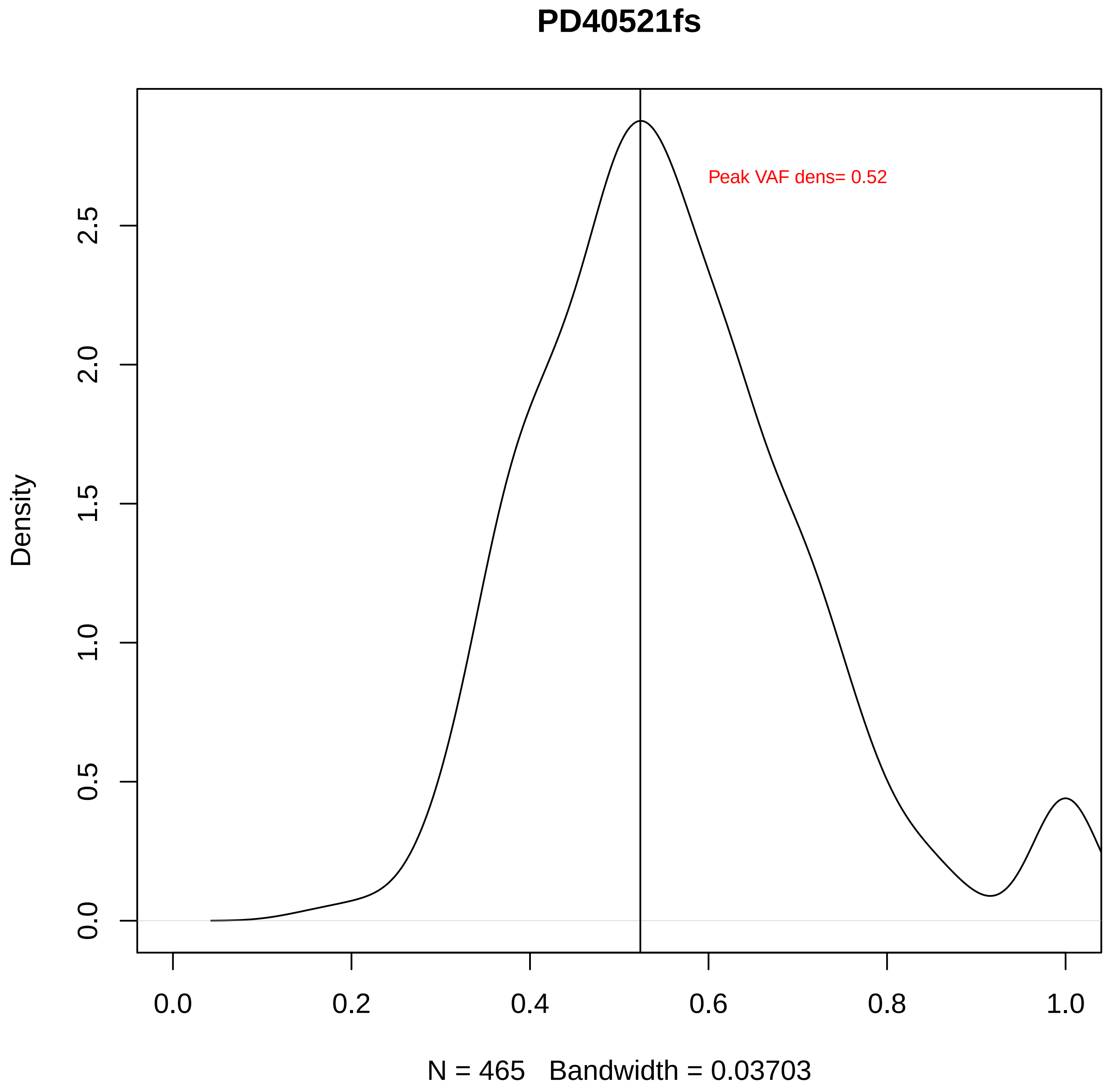
PD40521fs
Peak VAF dens= 0.52
Density
0.0
0.5
1.0
1.5
2.0
2.5
0.0
0.2
0.4
0.6
0.8
1.0
N = 465   Bandwidth = 0.03703

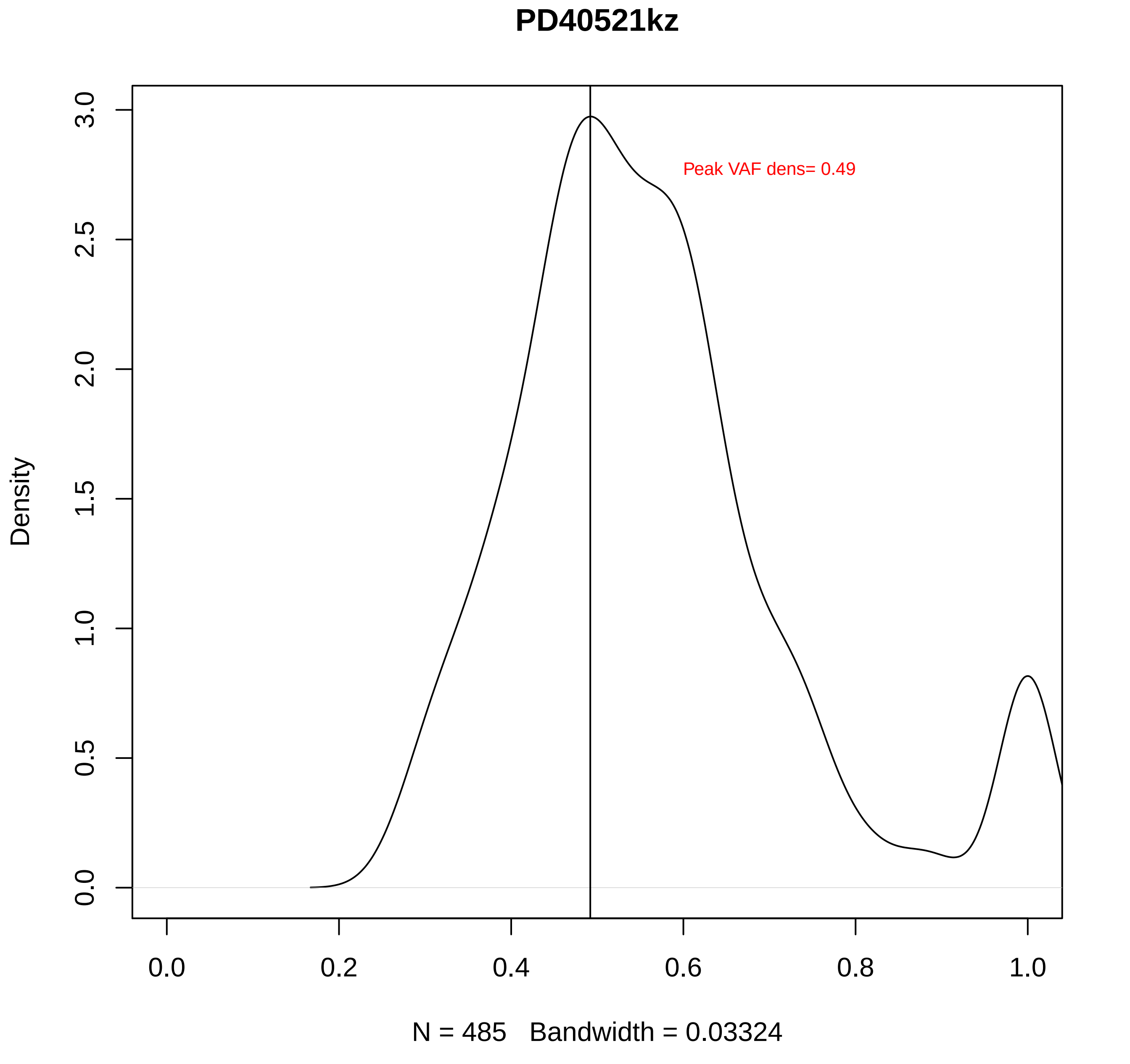
PD40521kz
Peak VAF dens= 0.49
Density
3.0
2.5
2.0
1.5
1.0
0.5
0.0
0.0
0.2
0.4
0.6
0.8
1.0
N = 485 Bandwidth = 0.03324

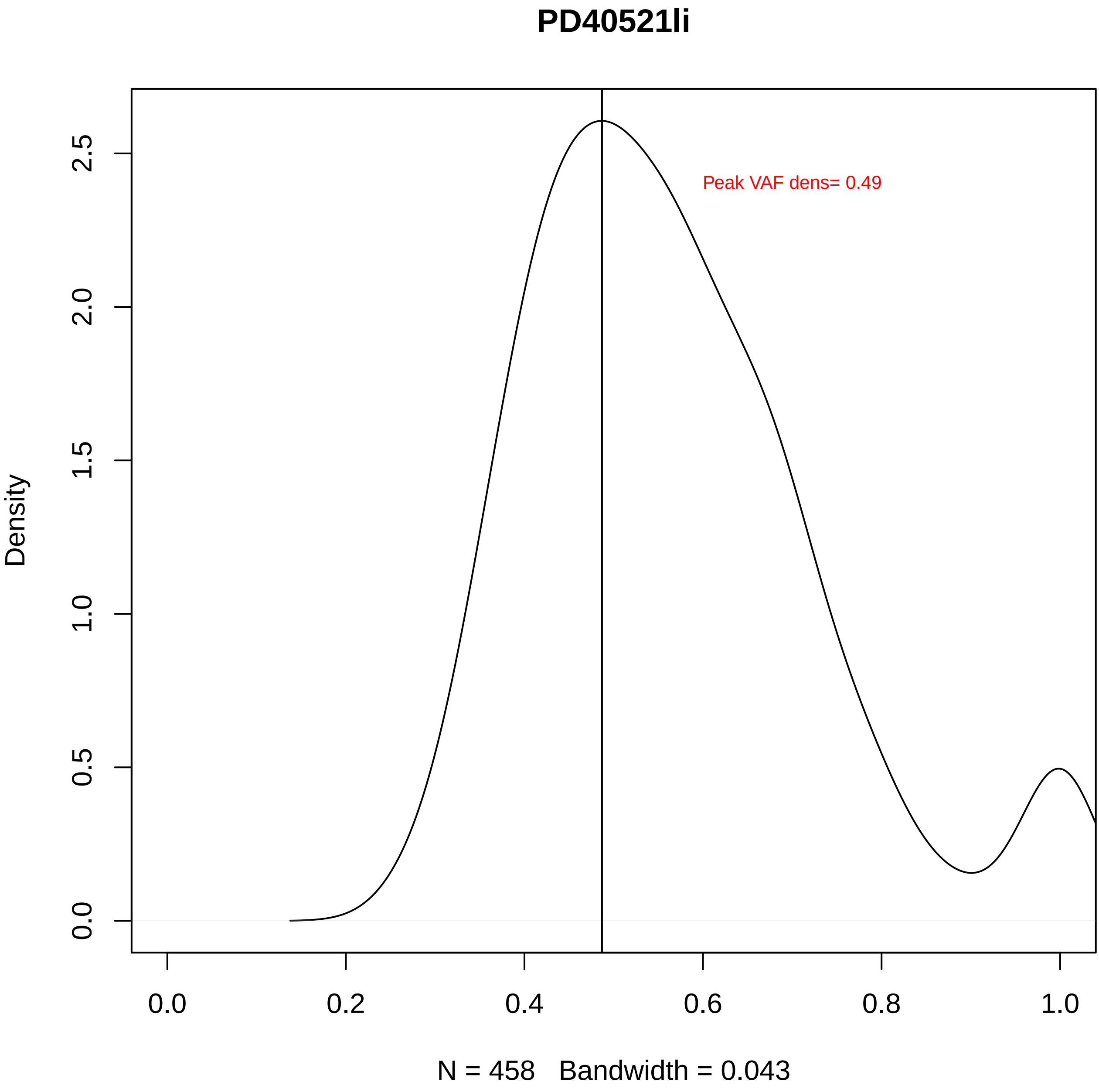
PD40521li
Peak VAF dens= 0.49
Density
0.0
0.5
1.0
1.5
2.0
2.5
0.0
0.2
0.4
0.6
0.8
1.0
N = 458   Bandwidth = 0.043

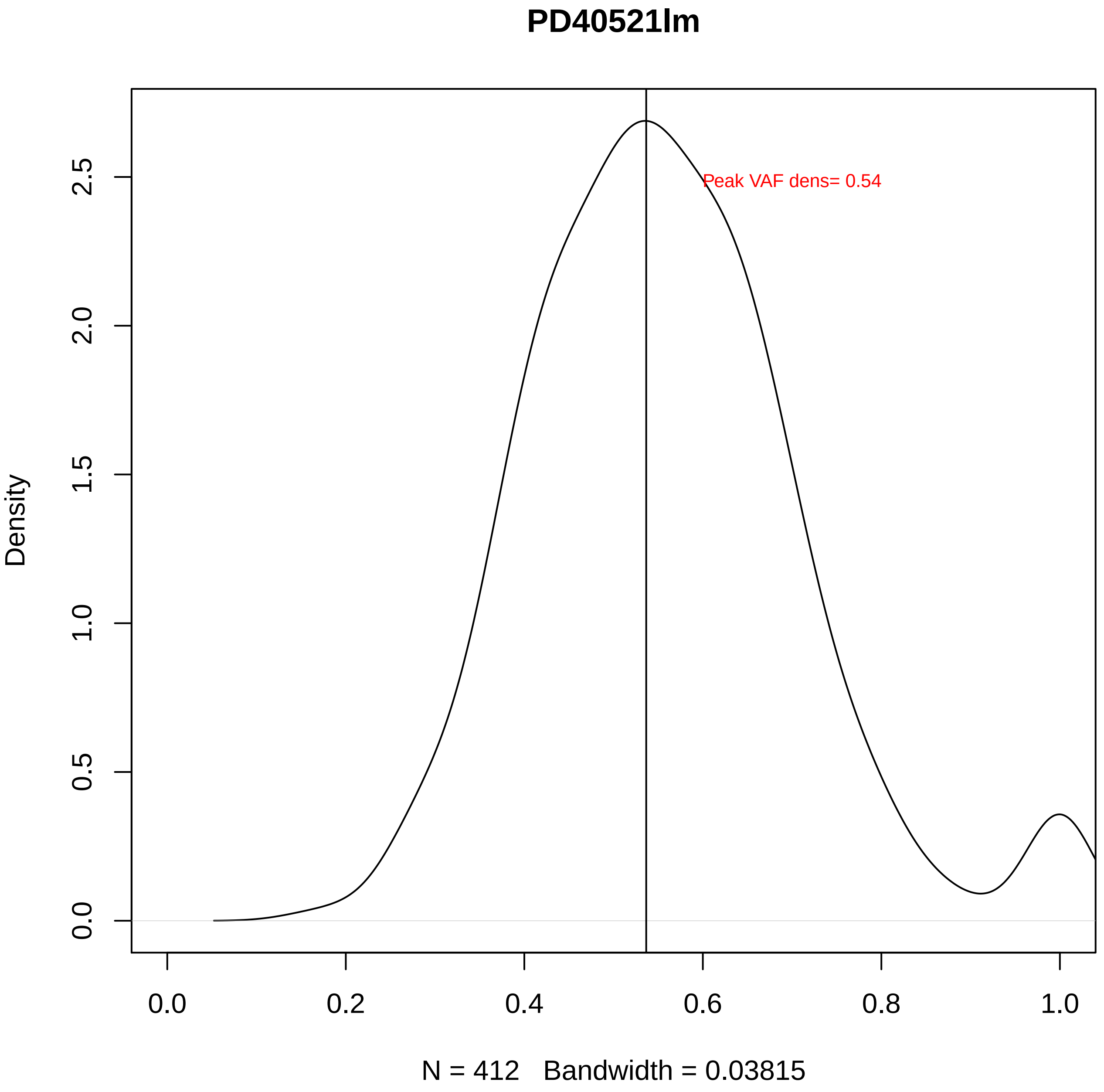
PD40521lm
Peak VAF dens= 0.54
Density
0.0
0.5
1.0
1.5
2.0
2.5
0.0
0.2
0.4
0.6
0.8
1.0
N = 412 Bandwidth = 0.03815

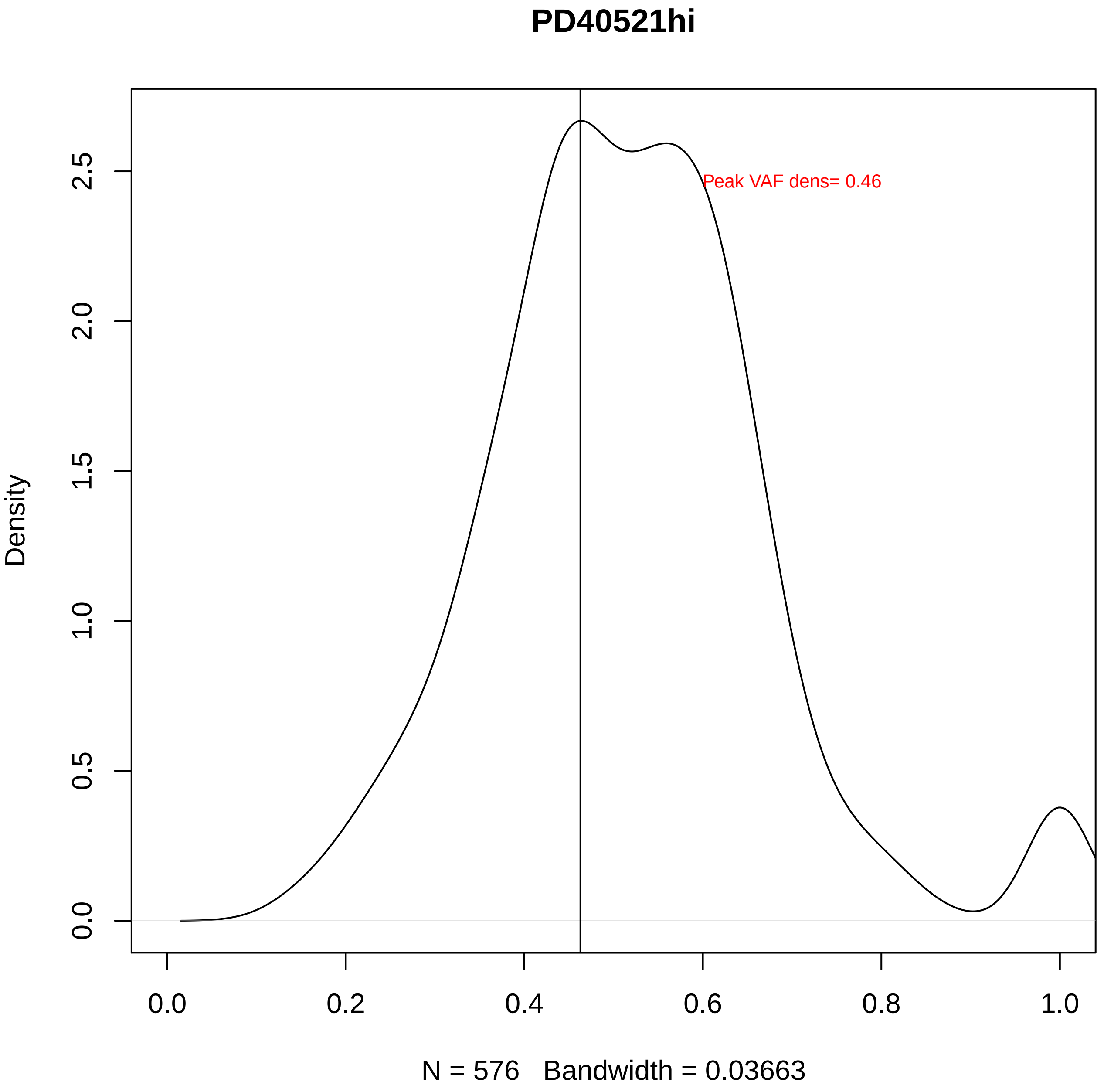

PD40521hi
Peak VAF dens= 0.46
Density
0.0
0.5
1.0
1.5
2.0
2.5
0.0
0.2
0.4
0.6
0.8
1.0
N = 576 Bandwidth = 0.03663

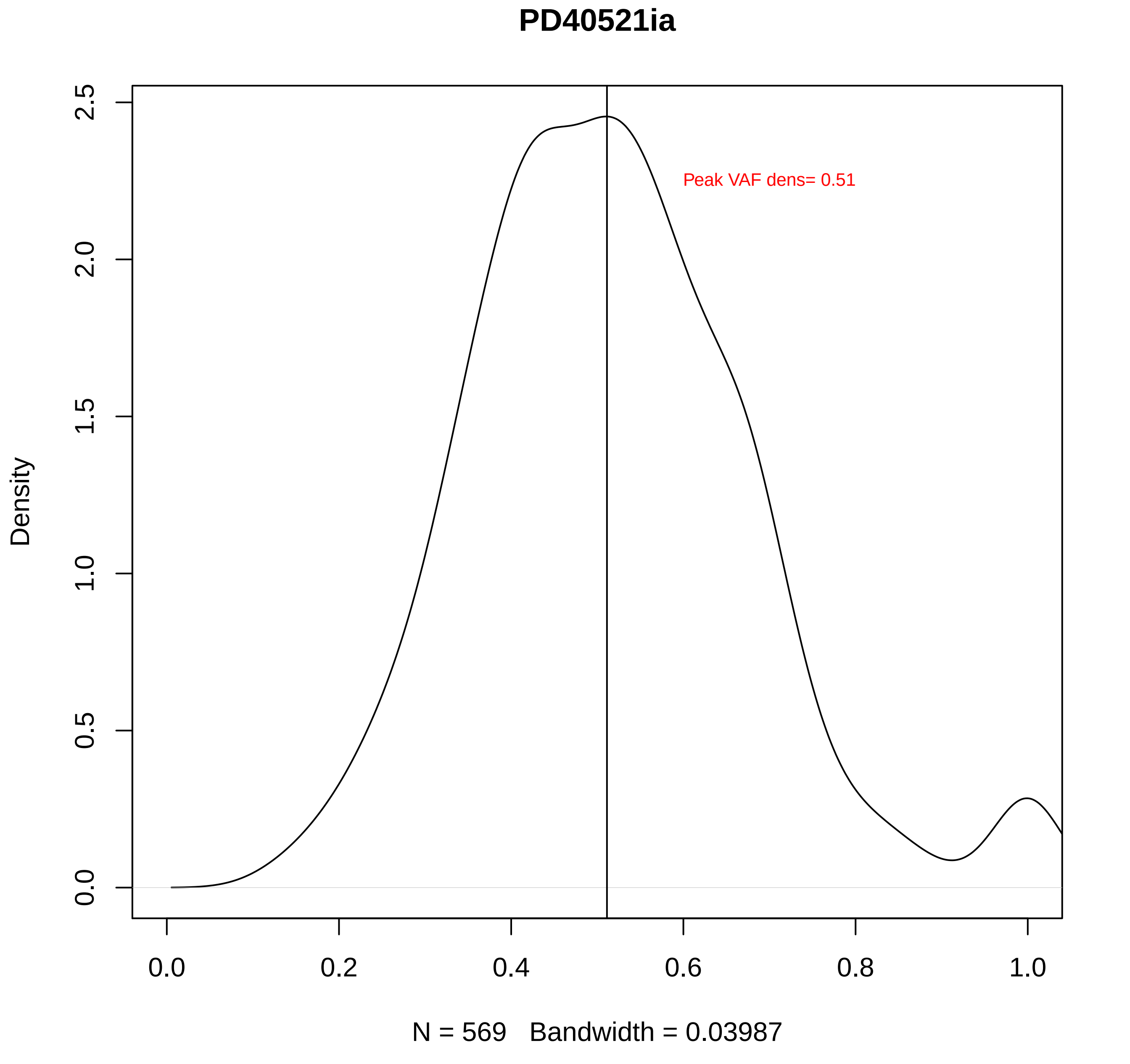
PD40521ia
Peak VAF dens= 0.51
Density
0.0
0.5
1.0
1.5
2.0
2.5
0.0
0.2
0.4
0.6
0.8
1.0
N = 569   Bandwidth = 0.03987

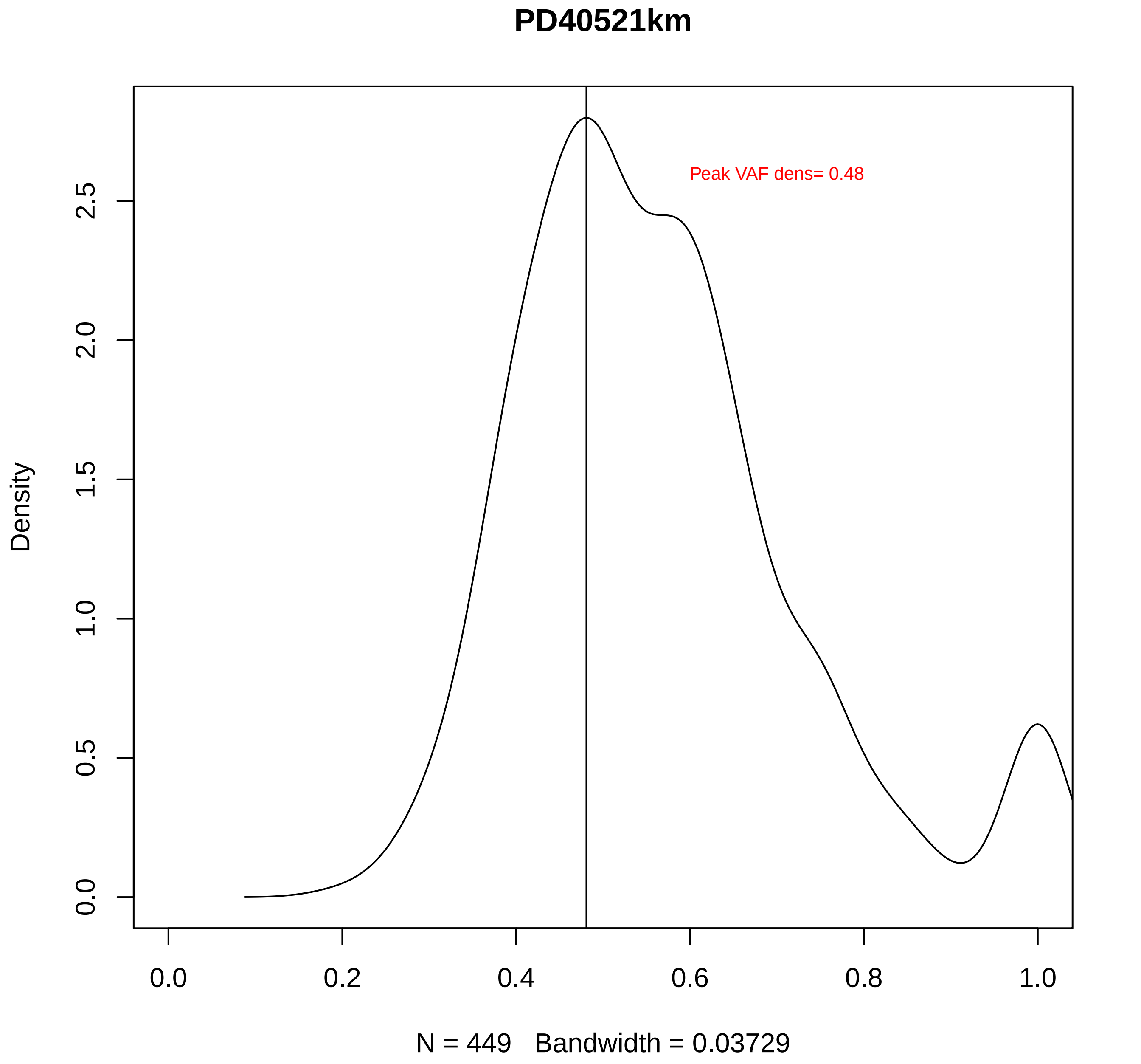
PD40521km
Peak VAF dens= 0.48
Density
0.0
0.5
1.0
1.5
2.0
2.5
0.0
0.2
0.4
0.6
0.8
1.0
N = 449   Bandwidth = 0.03729

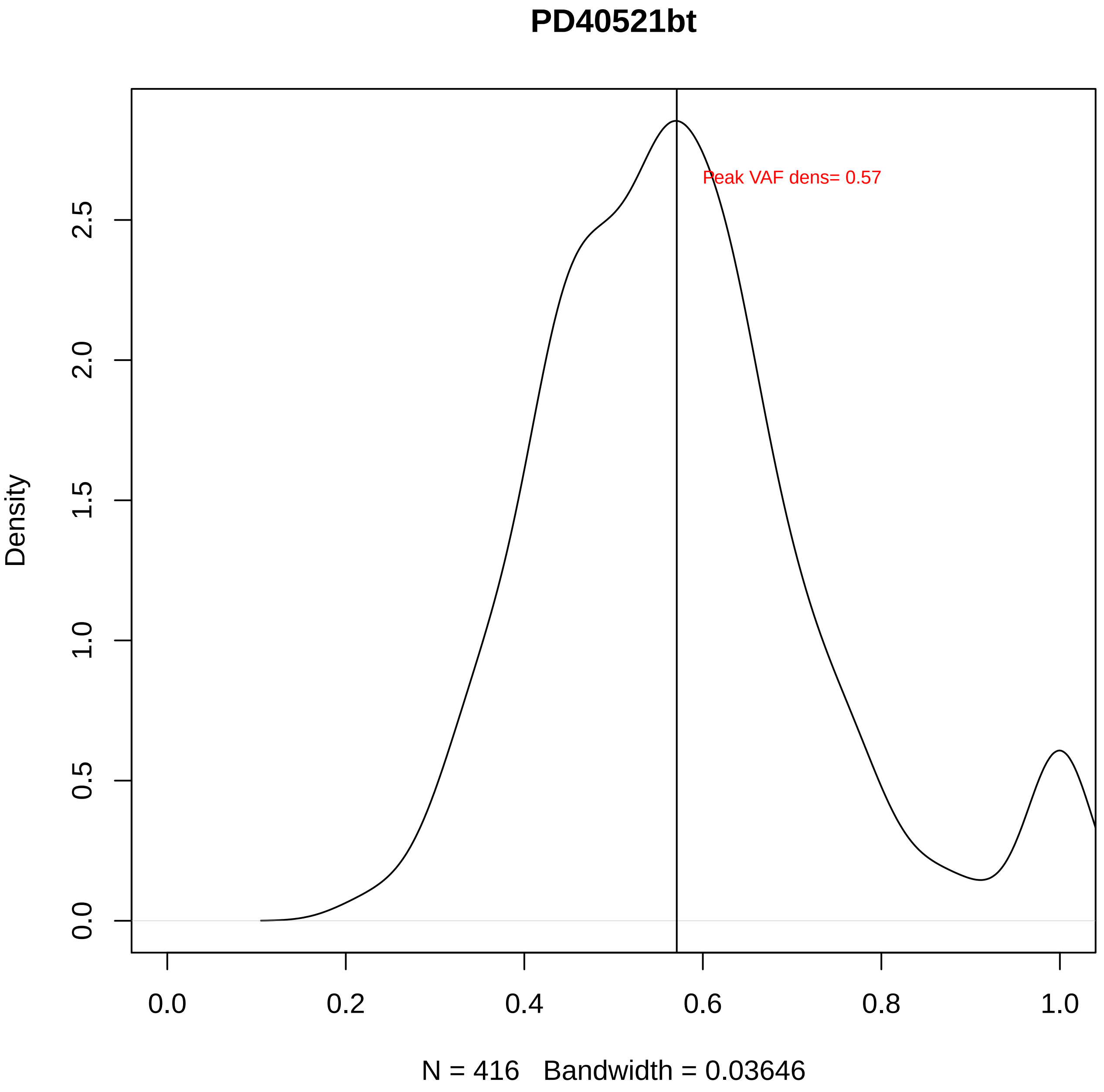
PD40521bt
Peak VAF dens= 0.57
Density
0.0
0.5
1.0
1.5
2.0
2.5
0.0
0.2
0.4
0.6
0.8
1.0
N = 416 Bandwidth = 0.03646

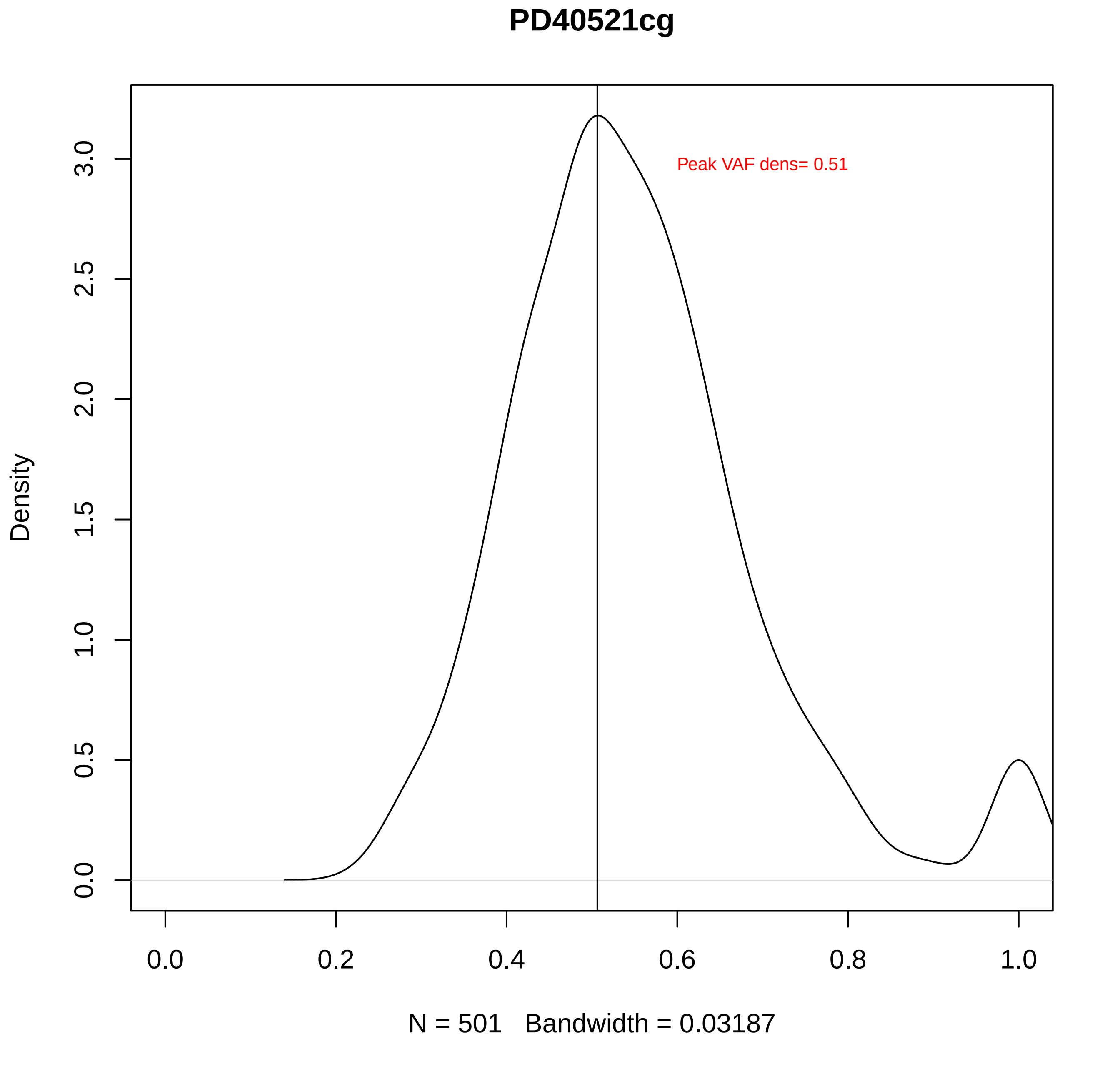
PD40521cg
Peak VAF dens= 0.51
Density
0.0
0.5
1.0
1.5
2.0
2.5
3.0
0.0
0.2
0.4
0.6
0.8
1.0
N = 501   Bandwidth = 0.03187

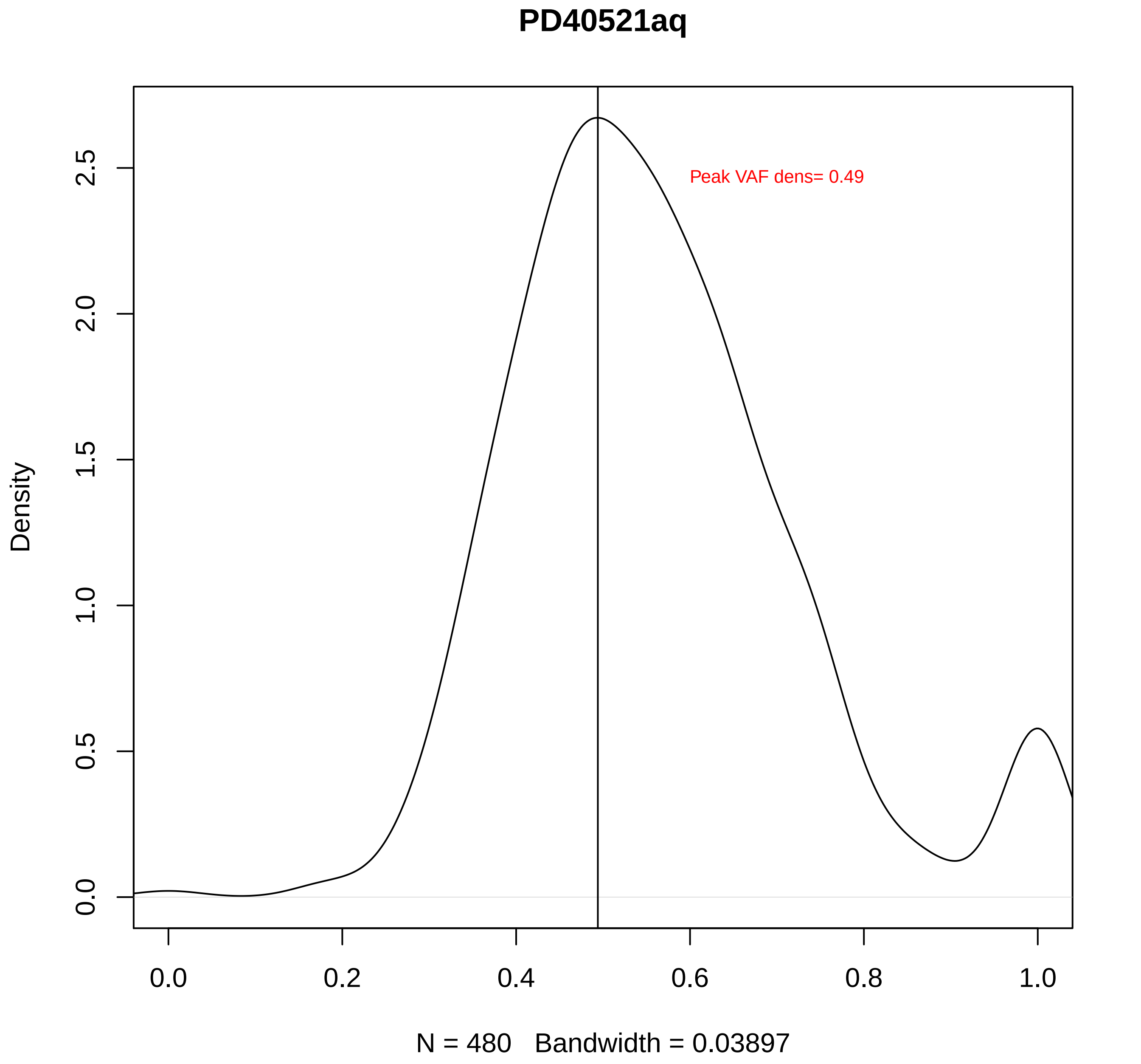

PD40521aq
Peak VAF dens= 0.49
Density
0.0
0.5
1.0
1.5
2.0
2.5
0.0
0.2
0.4
0.6
0.8
1.0
N = 480   Bandwidth = 0.03897

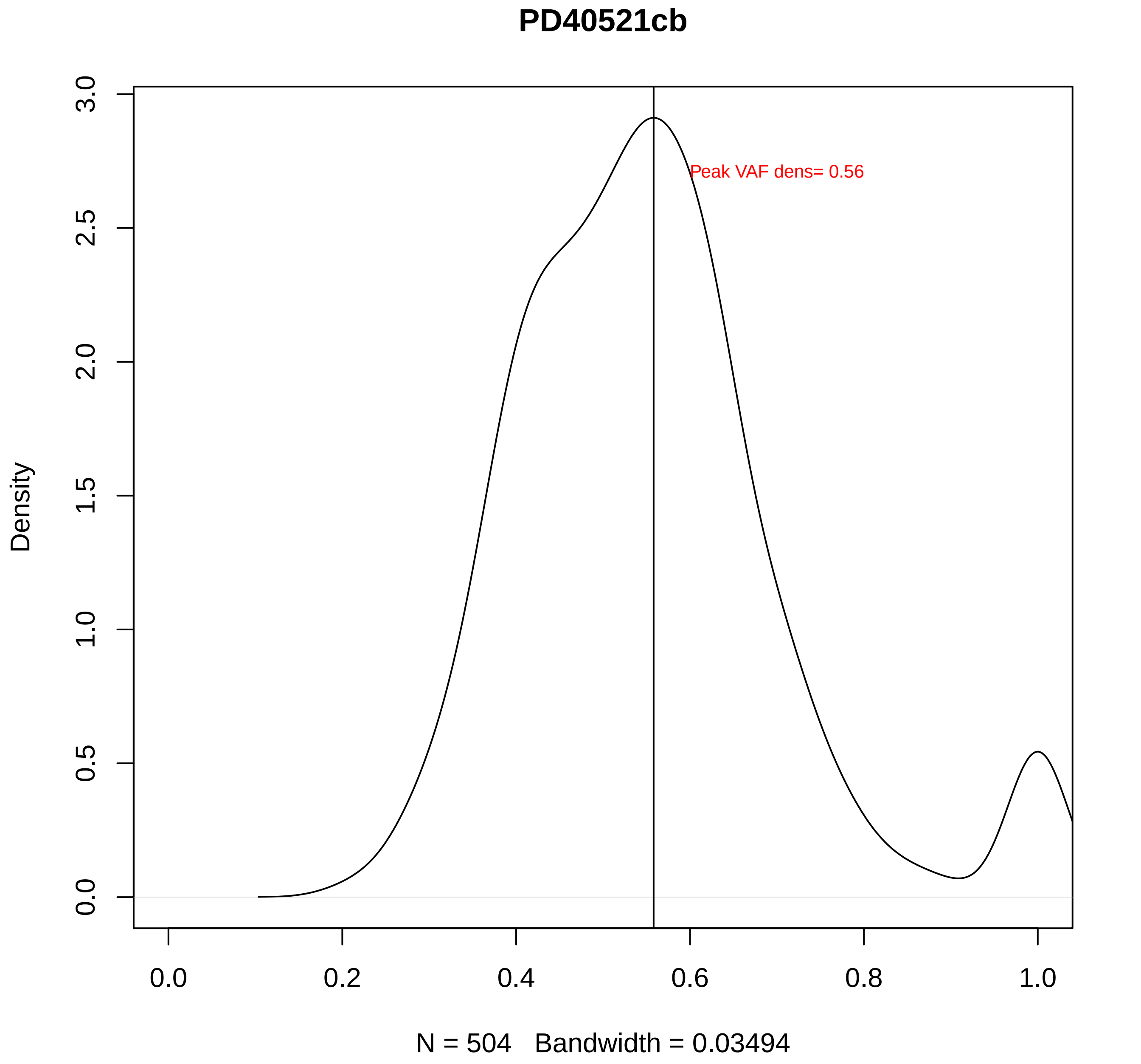

PD40521cb
Peak VAF dens= 0.56
Density
0.0
0.5
1.0
1.5
2.0
2.5
3.0
0.0
0.2
0.4
0.6
0.8
1.0
N = 504   Bandwidth = 0.03494

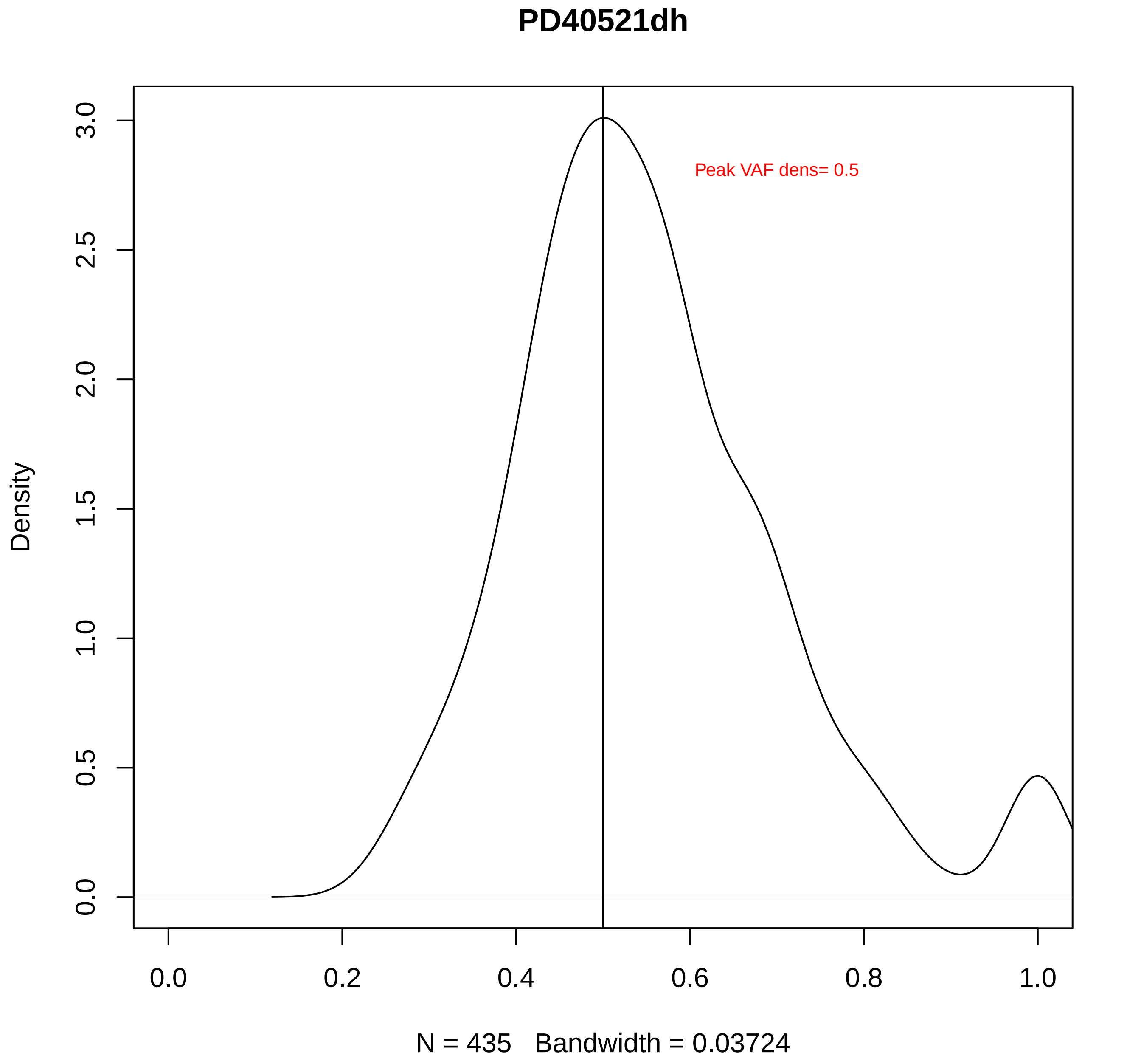

PD40521dh
Peak VAF dens= 0.5
Density
3.0
2.5
2.0
1.5
1.0
0.5
0.0
0.0
0.2
0.4
0.6
0.8
1.0
N = 435   Bandwidth = 0.03724

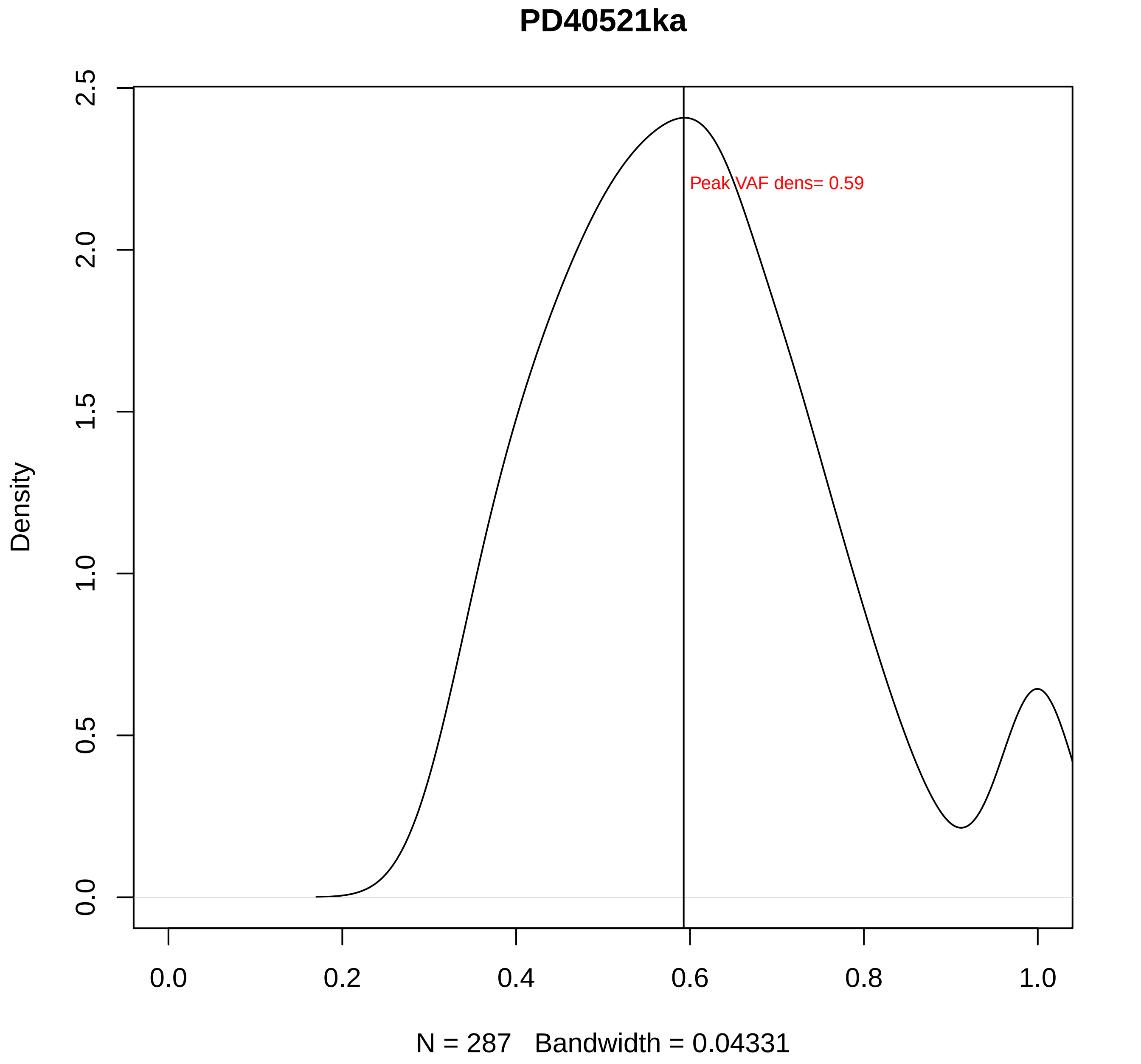
PD40521ka
Density
Peak VAF dens= 0.59
0.0
0.5
1.0
1.5
2.0
2.5
0.0
0.2
0.4
0.6
0.8
1.0
N = 287   Bandwidth = 0.04331

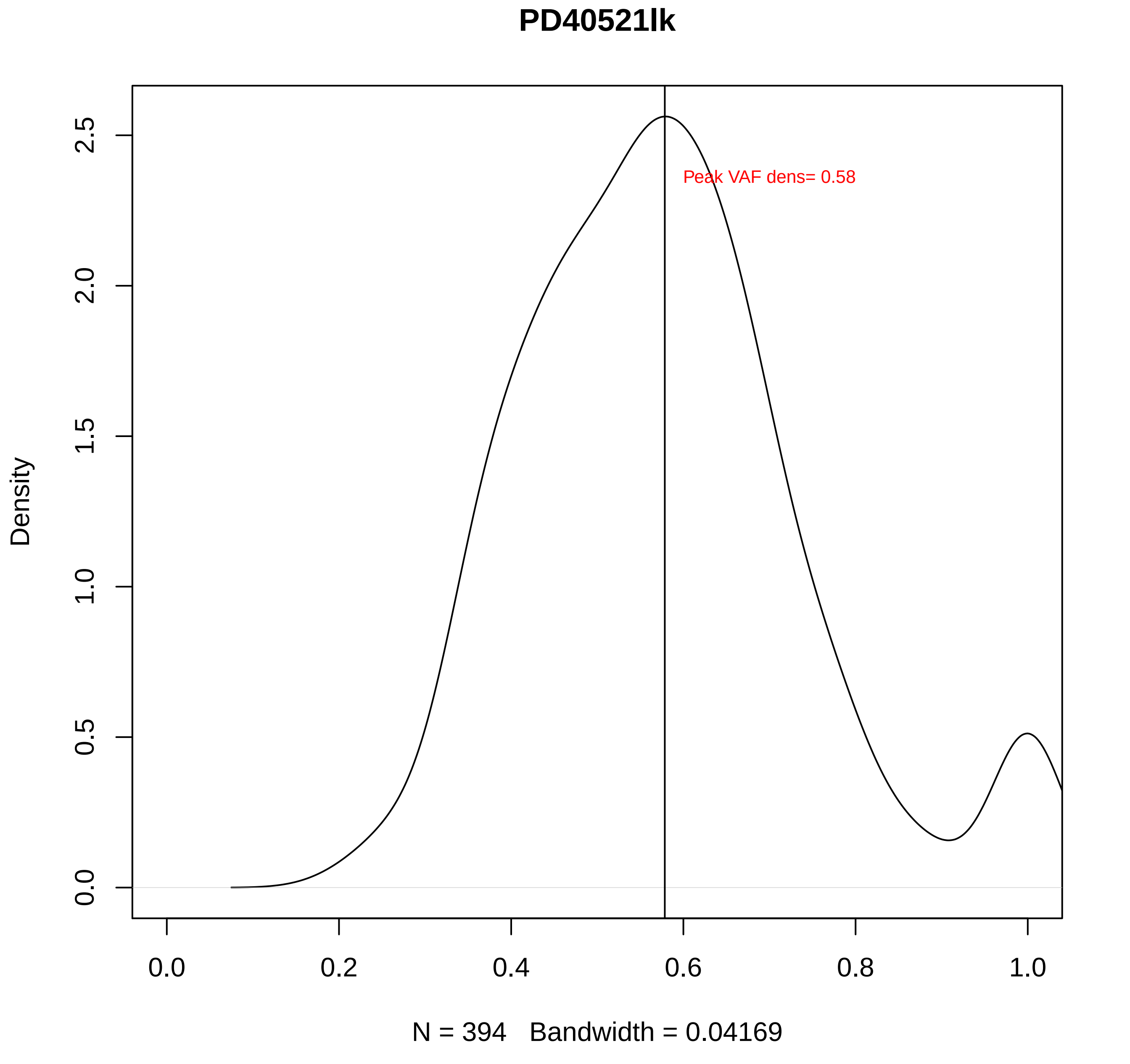

PD40521lk
Peak VAF dens= 0.58
Density
0.0
0.5
1.0
1.5
2.0
2.5
0.0
0.2
0.4
0.6
0.8
1.0
N = 394 Bandwidth = 0.04169

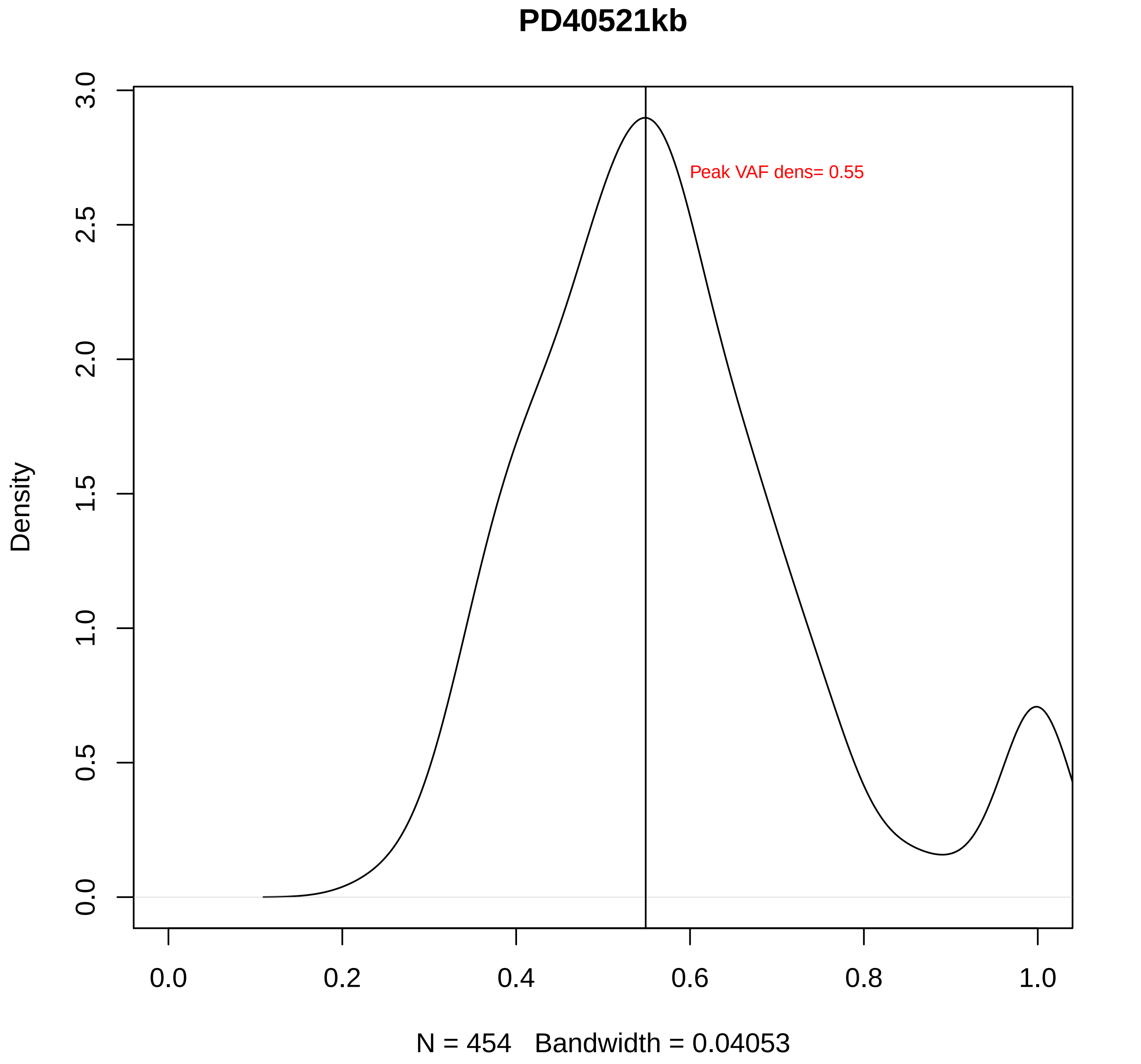

PD40521kb
Peak VAF dens= 0.55
Density
3.0
2.5
2.0
1.5
1.0
0.5
0.0
0.0
0.2
0.4
0.6
0.8
1.0
N = 454   Bandwidth = 0.04053

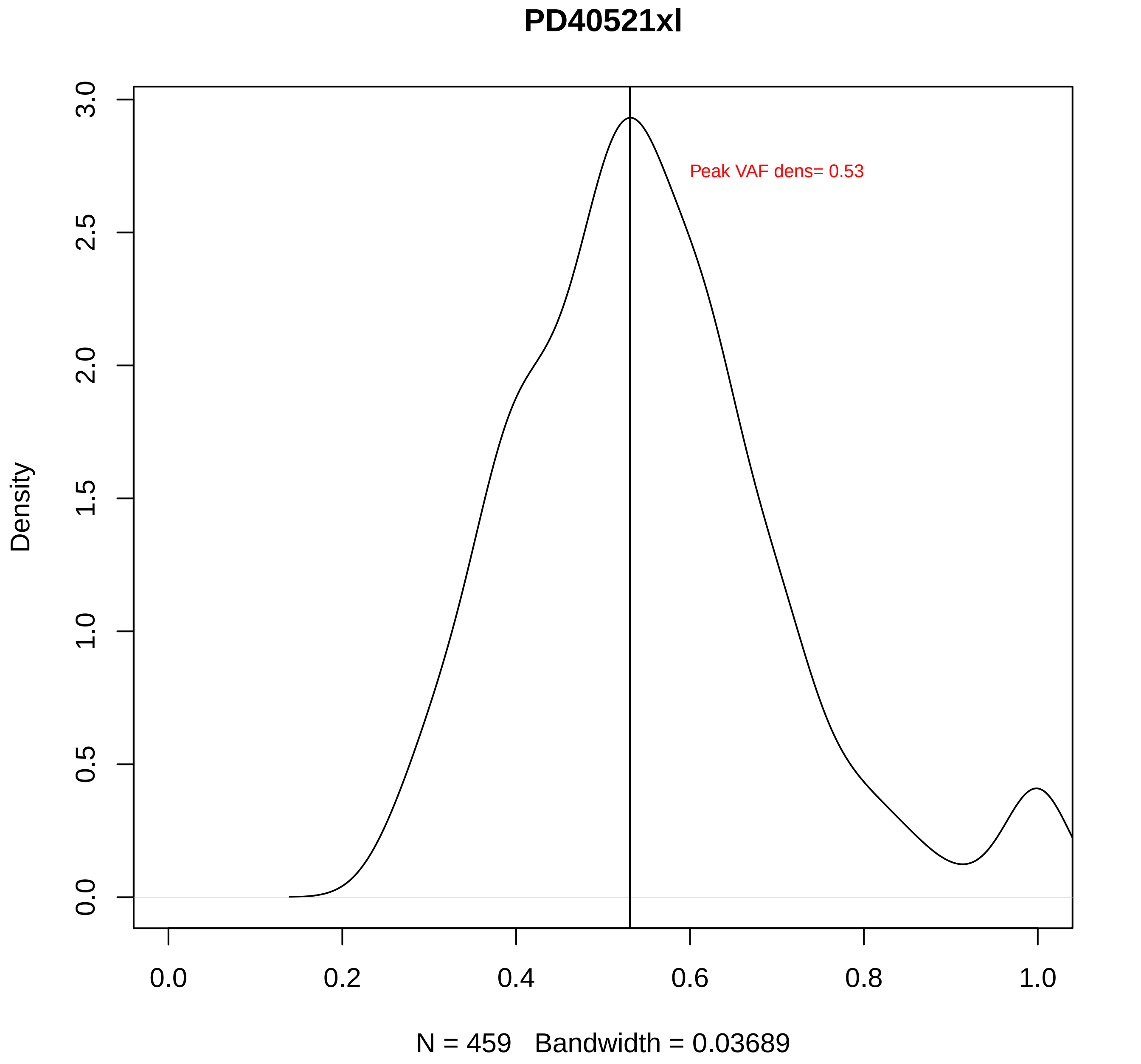

PD40521xl
Peak VAF dens= 0.53
Density
0.0
0.5
1.0
1.5
2.0
2.5
3.0
0.0
0.2
0.4
0.6
0.8
1.0
N = 459   Bandwidth = 0.03689

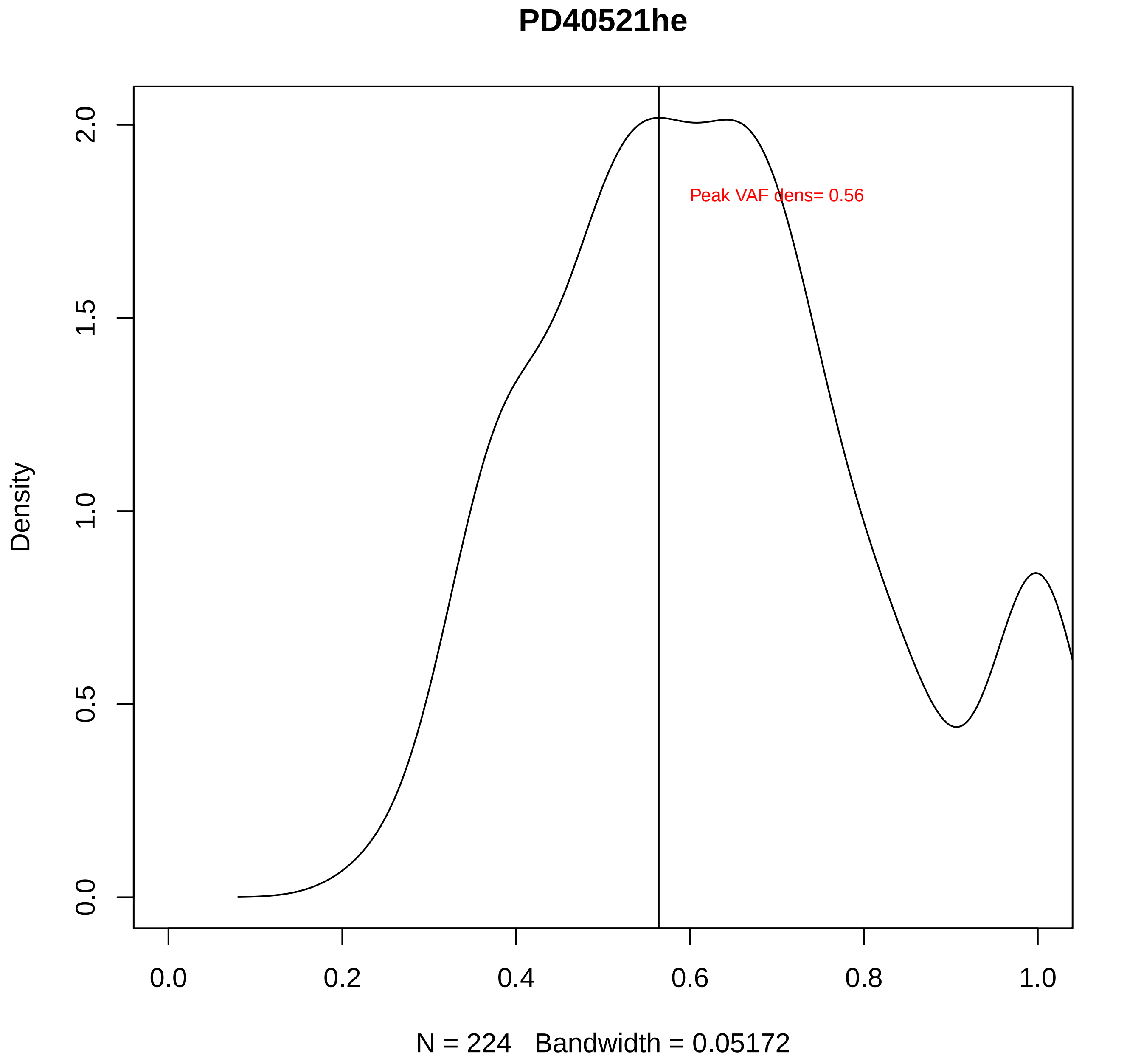
PD40521he
Peak VAF dens= 0.56
Density
0.0
0.5
1.0
1.5
2.0
0.0
0.2
0.4
0.6
0.8
1.0
N = 224   Bandwidth = 0.05172

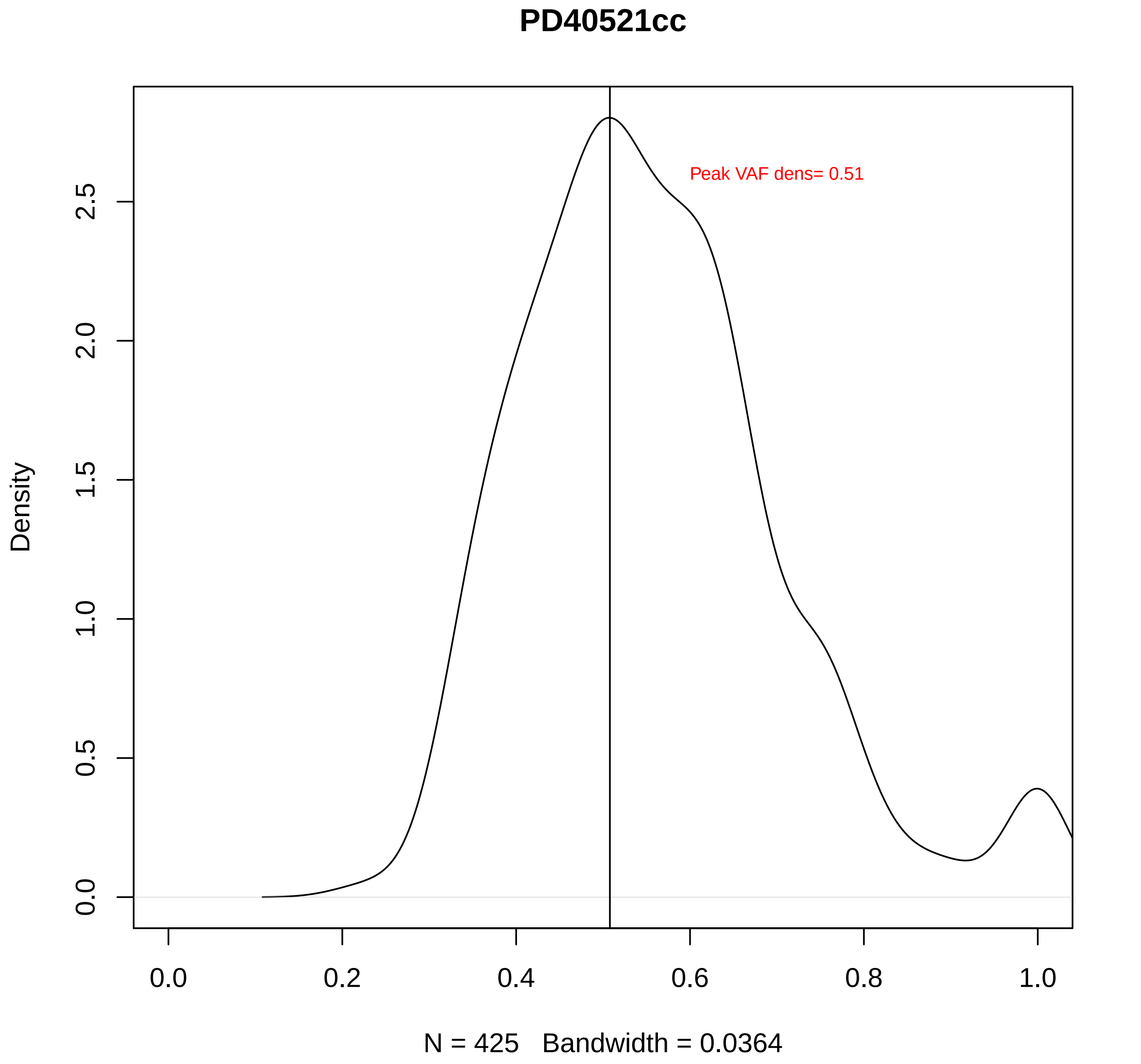
PD40521cc
Peak VAF dens= 0.51
Density
0.0
0.5
1.0
1.5
2.0
2.5
0.0
0.2
0.4
0.6
0.8
1.0
N = 425   Bandwidth = 0.0364

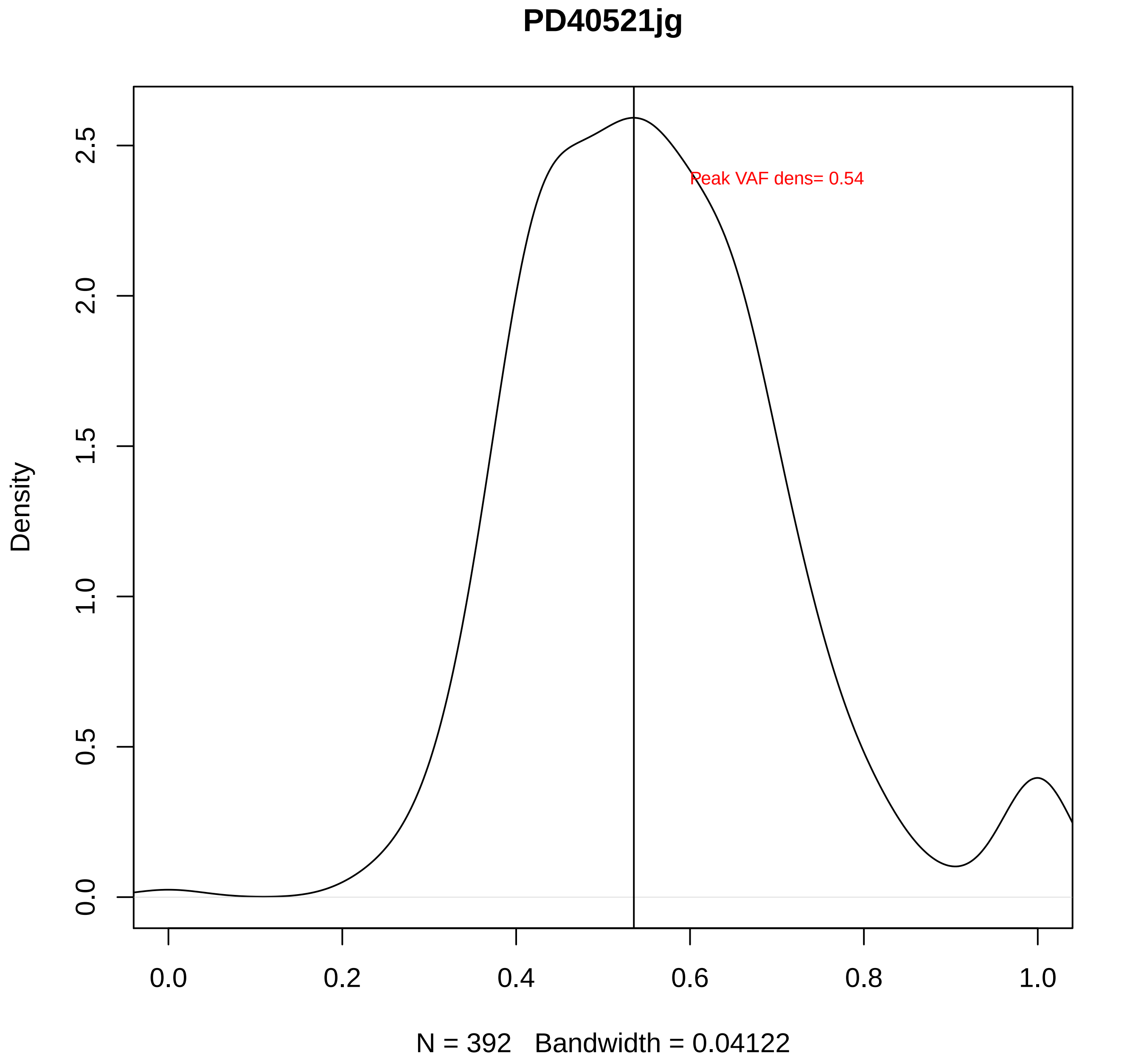
PD40521jg
Peak VAF dens= 0.54
Density
0.0
0.5
1.0
1.5
2.0
2.5
0.0
0.2
0.4
0.6
0.8
1.0
N = 392   Bandwidth = 0.04122

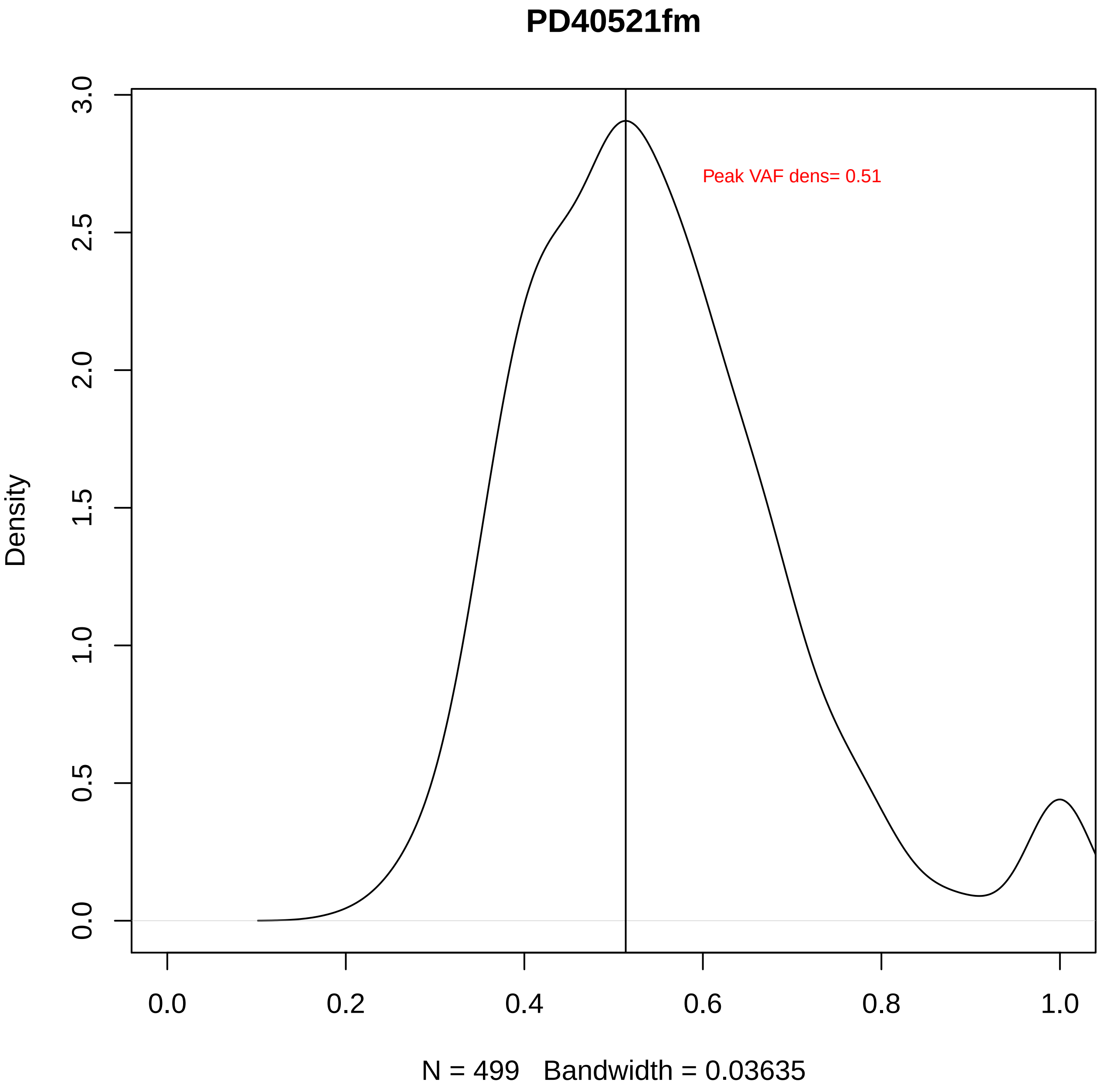

PD40521fm
Peak VAF dens= 0.51
Density
0.0
0.5
1.0
1.5
2.0
2.5
3.0
0.0
0.2
0.4
0.6
0.8
1.0
N = 499 Bandwidth = 0.03635

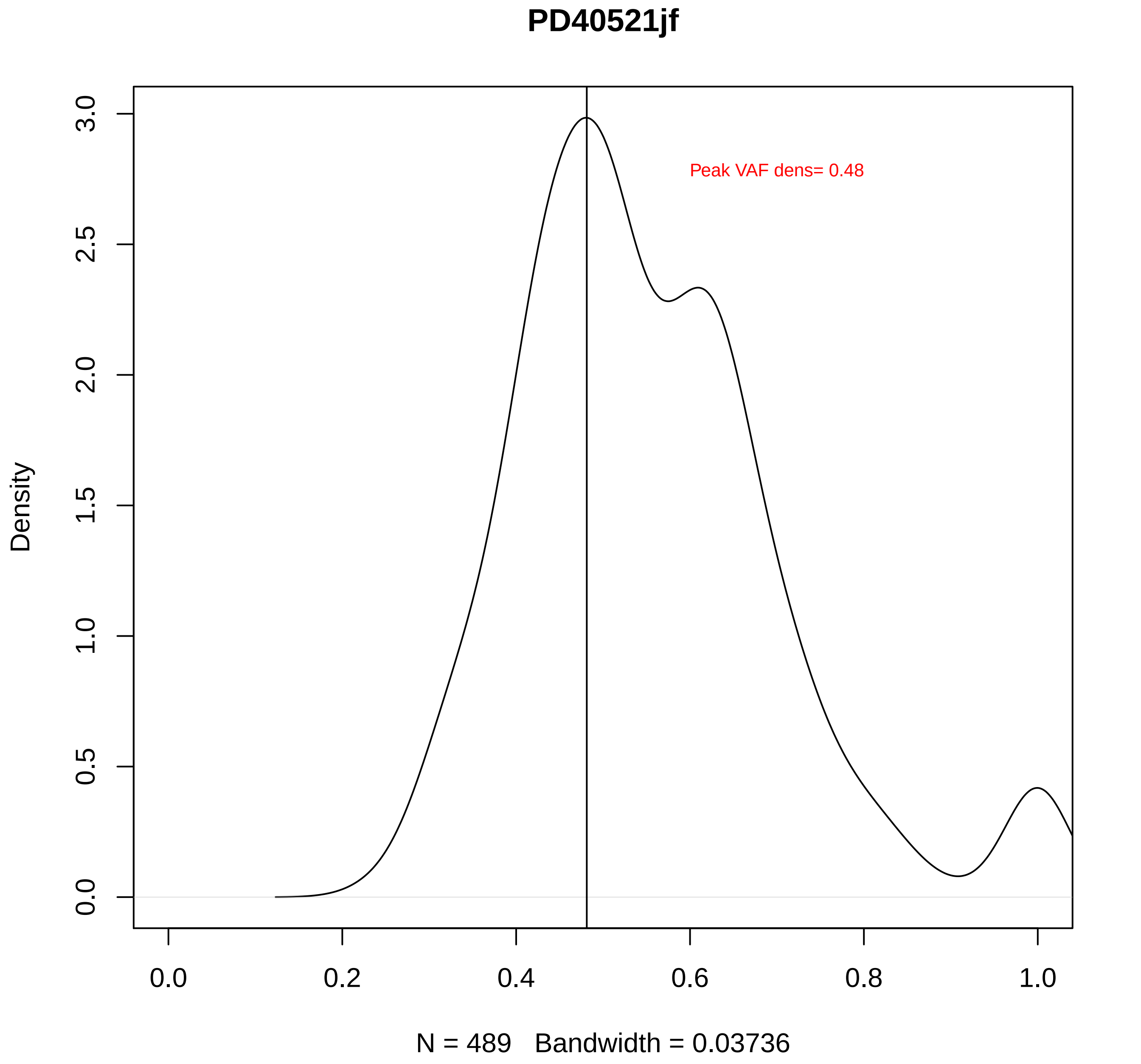
PD40521jf
Peak VAF dens= 0.48
Density
3.0
2.5
2.0
1.5
1.0
0.5
0.0
0.0
0.2
0.4
0.6
0.8
1.0
N = 489   Bandwidth = 0.03736

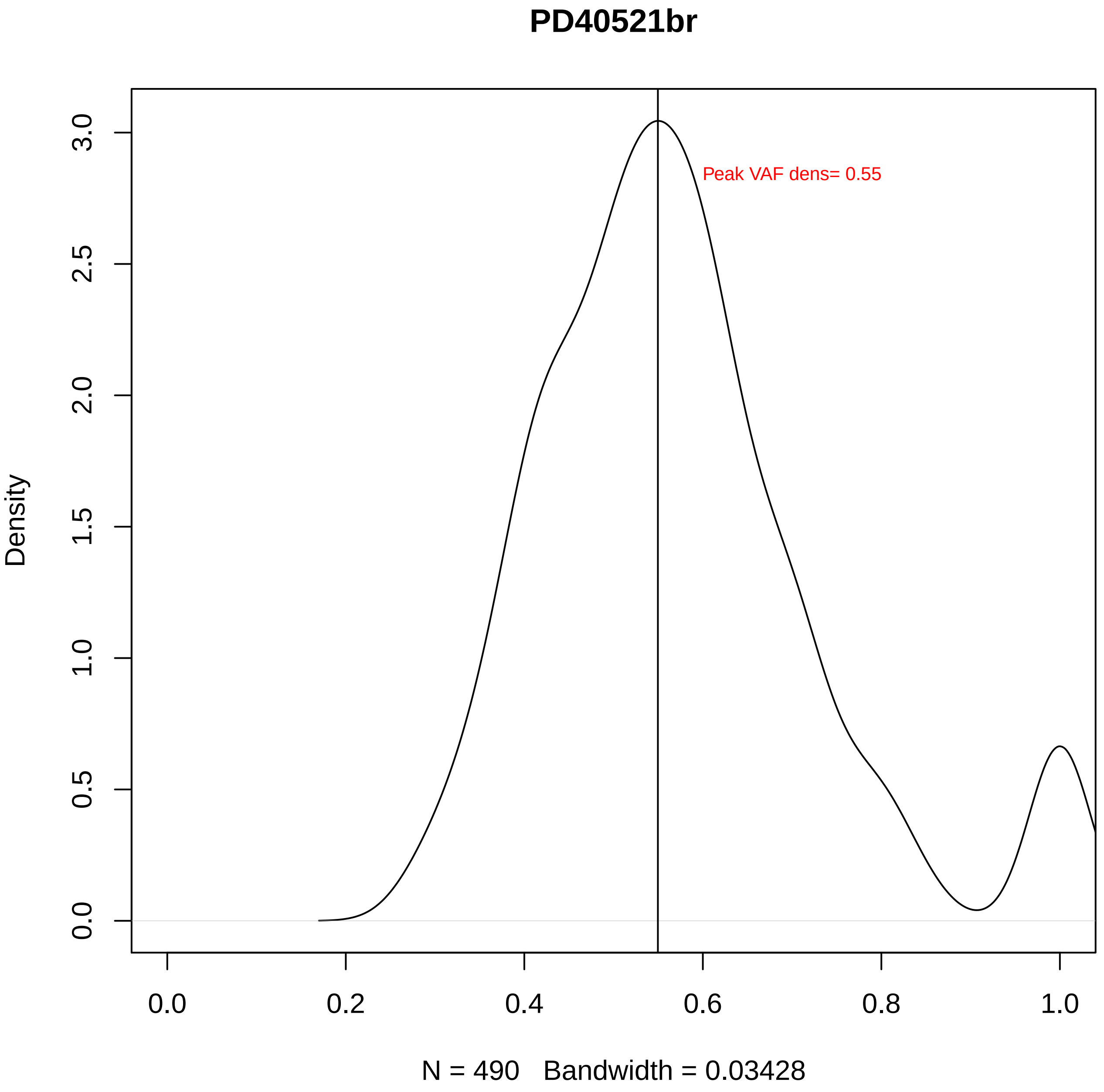
PD40521br
Peak VAF dens= 0.55
Density
0.0
0.5
1.0
1.5
2.0
2.5
3.0
0.0
0.2
0.4
0.6
0.8
1.0
N = 490   Bandwidth = 0.03428

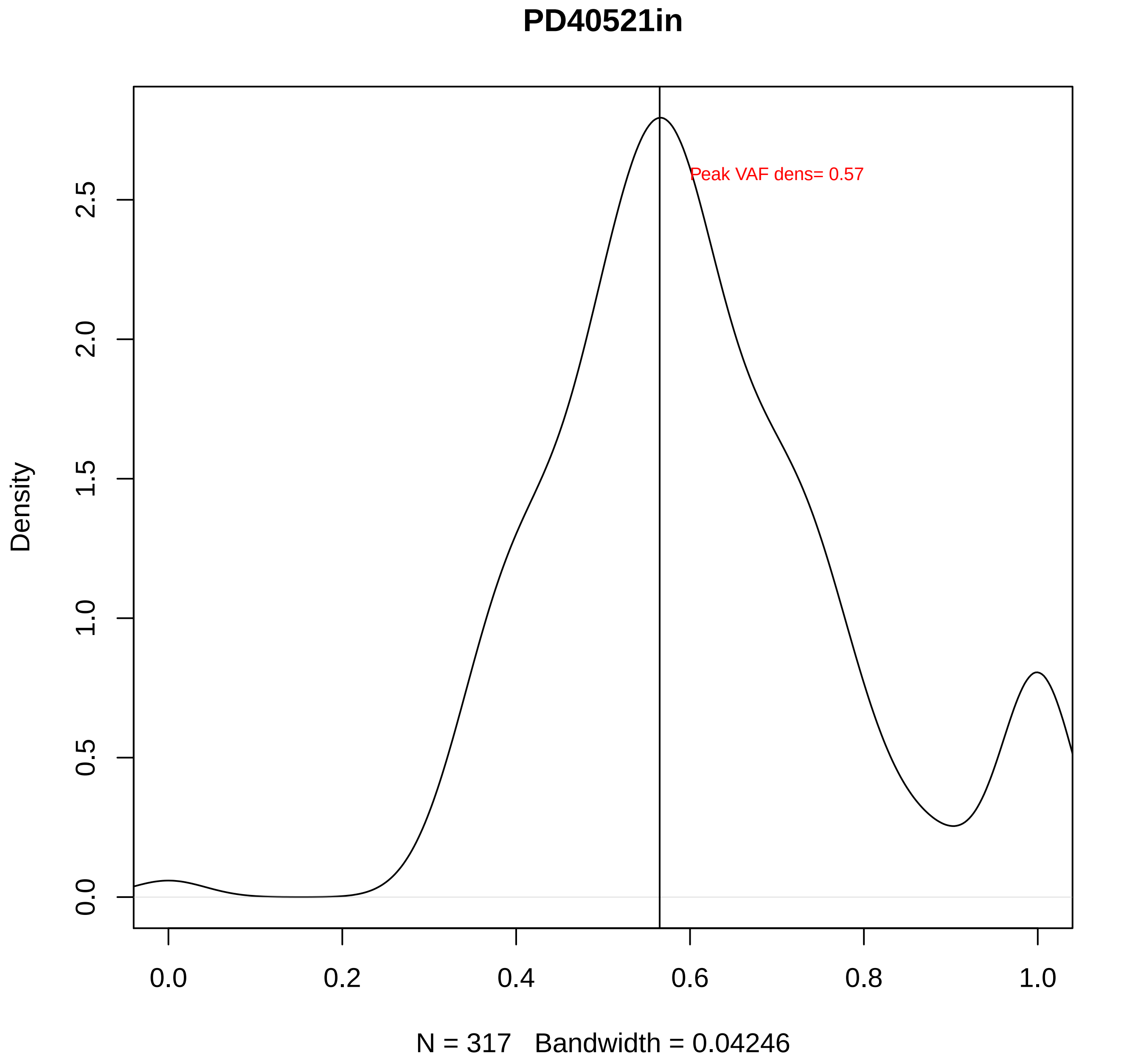
PD40521in
Peak VAF dens= 0.57
Density
0.0
0.5
1.0
1.5
2.0
2.5
0.0
0.2
0.4
0.6
0.8
1.0
N = 317 Bandwidth = 0.04246

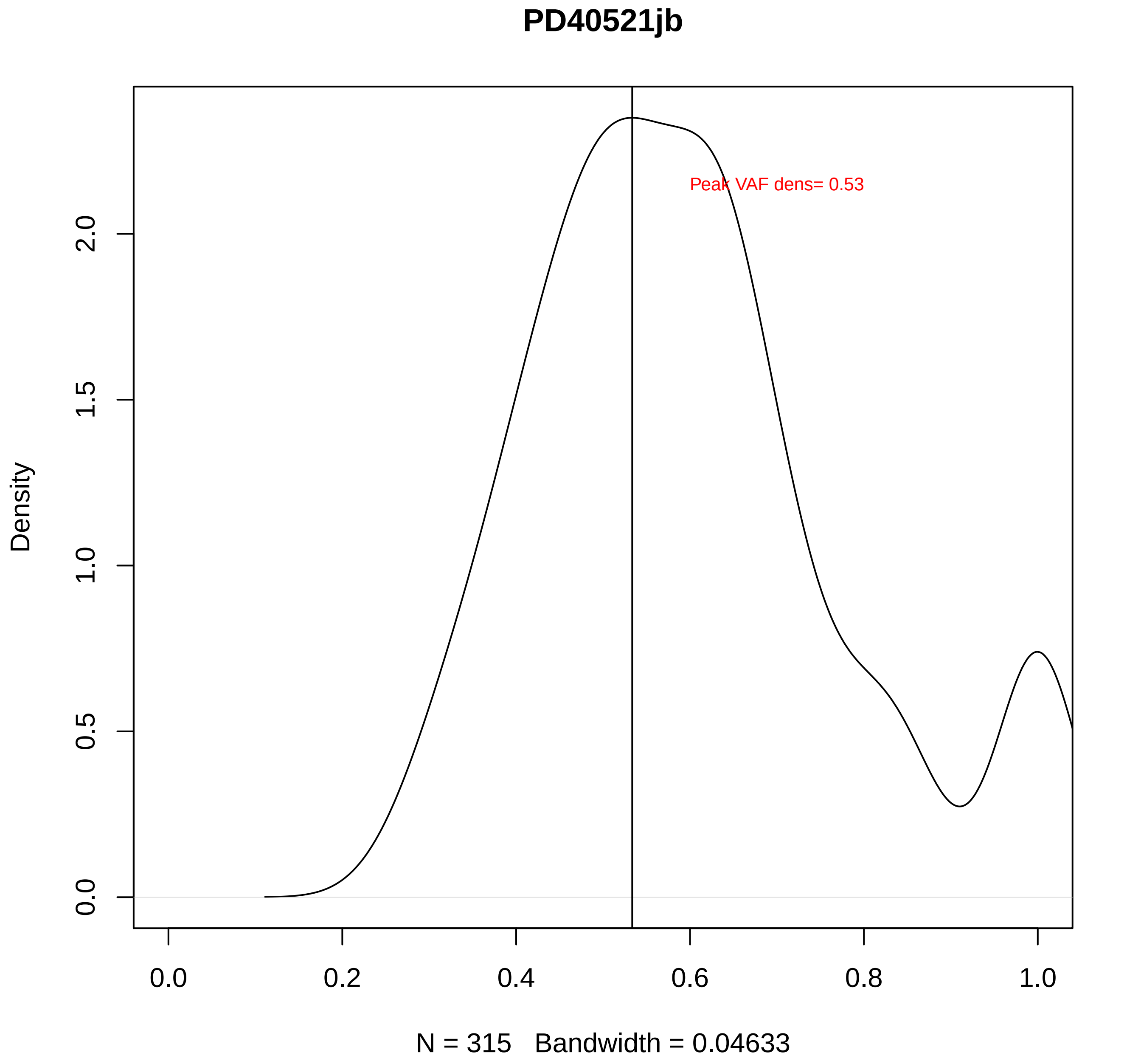
PD40521jb
Peak VAF dens= 0.53
Density
0.0
0.5
1.0
1.5
2.0
0.0
0.2
0.4
0.6
0.8
1.0
N = 315   Bandwidth = 0.04633

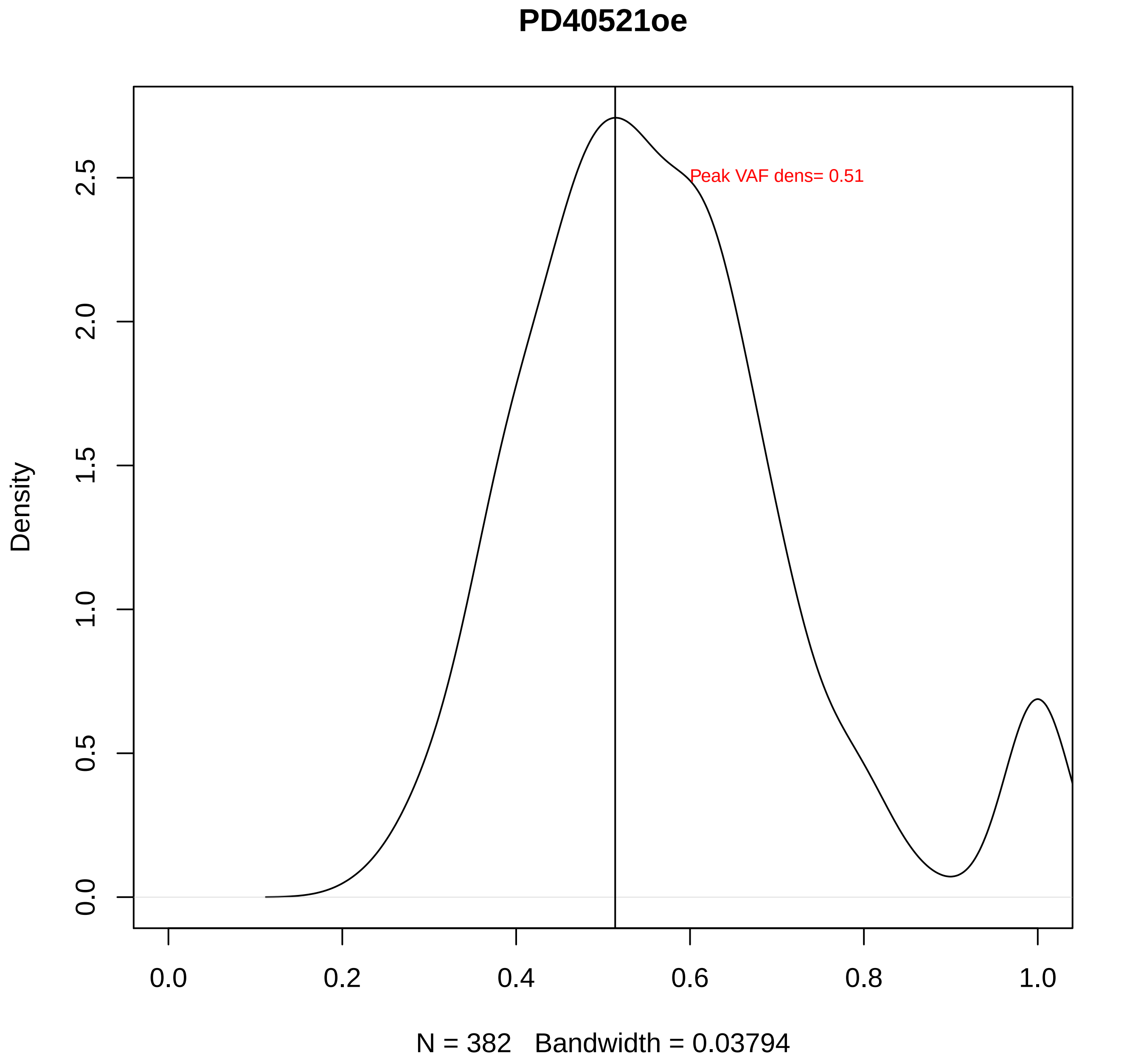

PD40521oe
Peak VAF dens= 0.51
Density
0.0
0.5
1.0
1.5
2.0
2.5
0.0
0.2
0.4
0.6
0.8
1.0
N = 382   Bandwidth = 0.03794

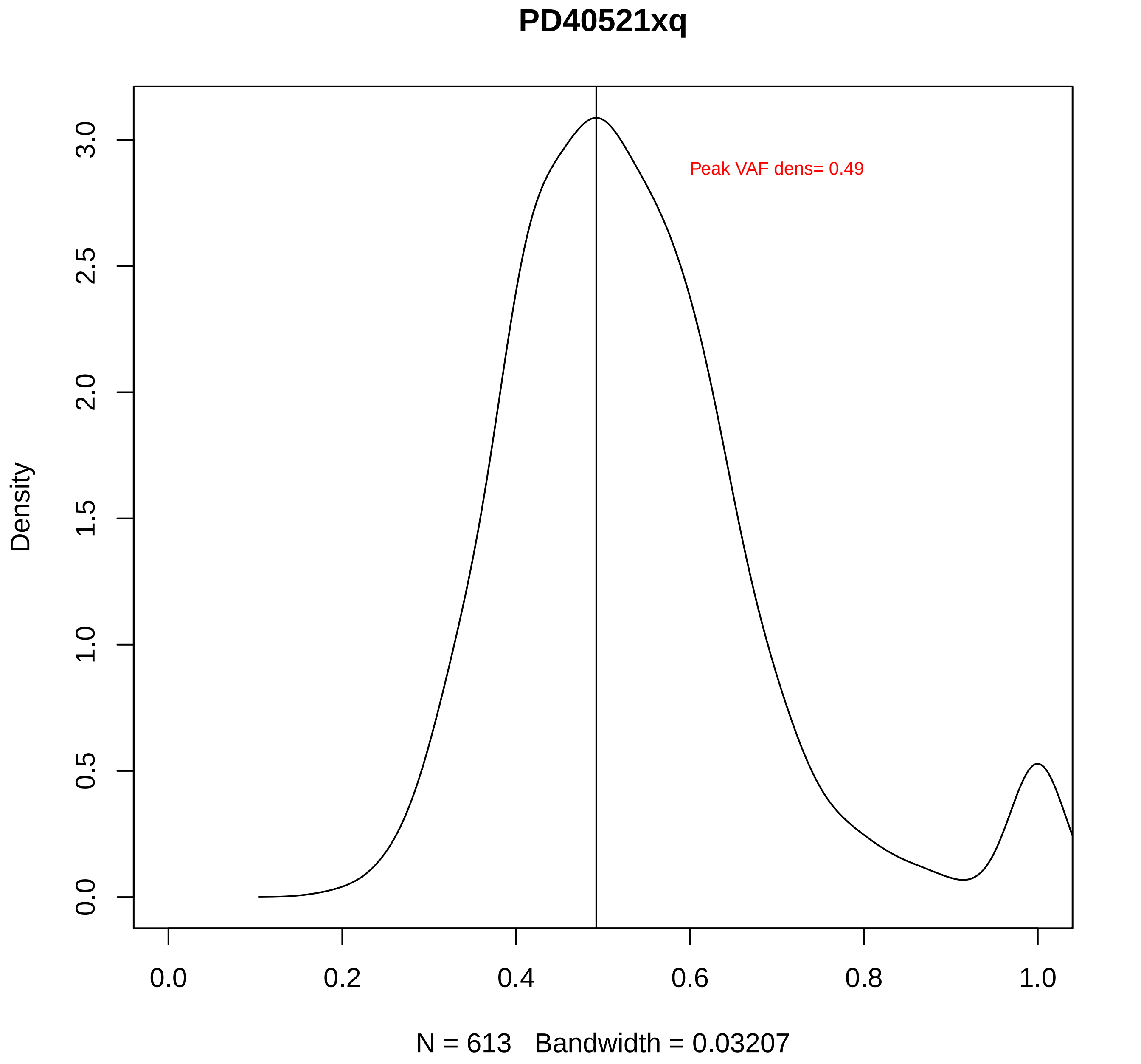
PD40521xq
Peak VAF dens= 0.49
Density
0.0
0.5
1.0
1.5
2.0
2.5
3.0
0.0
0.2
0.4
0.6
0.8
1.0
N = 613   Bandwidth = 0.03207

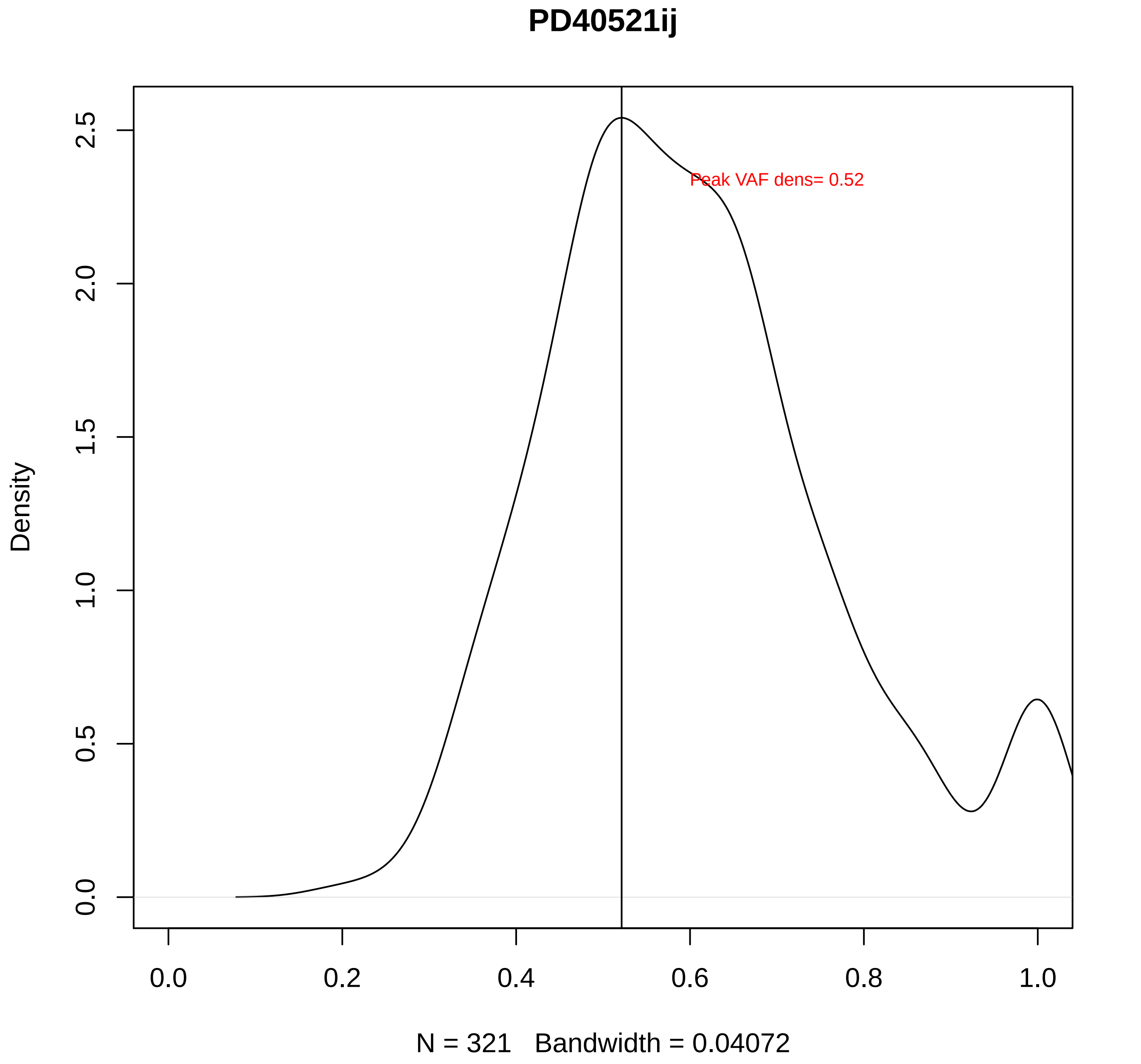

PD40521ij
Peak VAF dens= 0.52
Density
0.0
0.5
1.0
1.5
2.0
2.5
0.0
0.2
0.4
0.6
0.8
1.0
N = 321 Bandwidth = 0.04072

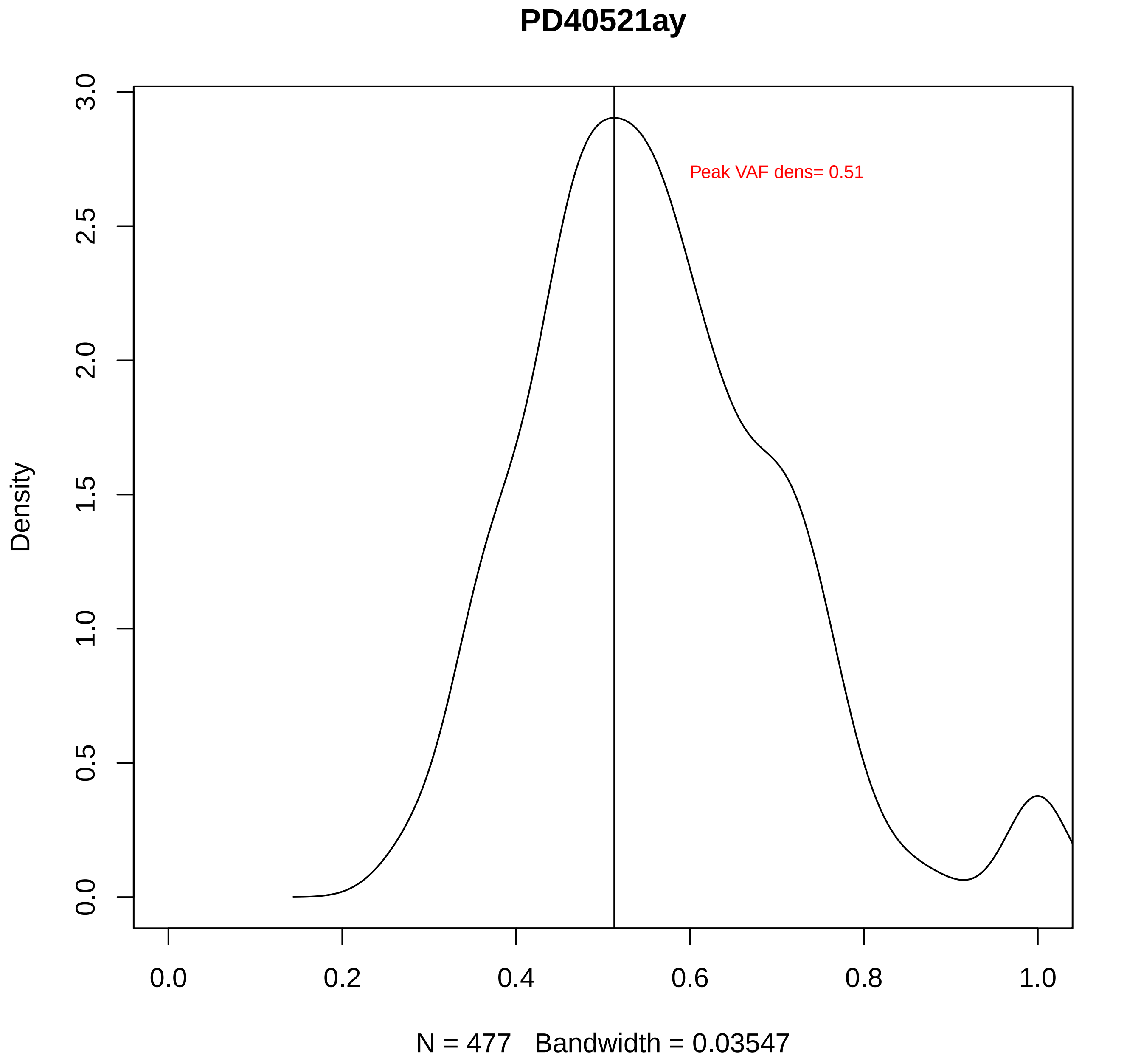
PD40521ay
Peak VAF dens= 0.51
Density
0.0
0.5
1.0
1.5
2.0
2.5
3.0
0.0
0.2
0.4
0.6
0.8
1.0
N = 477   Bandwidth = 0.03547

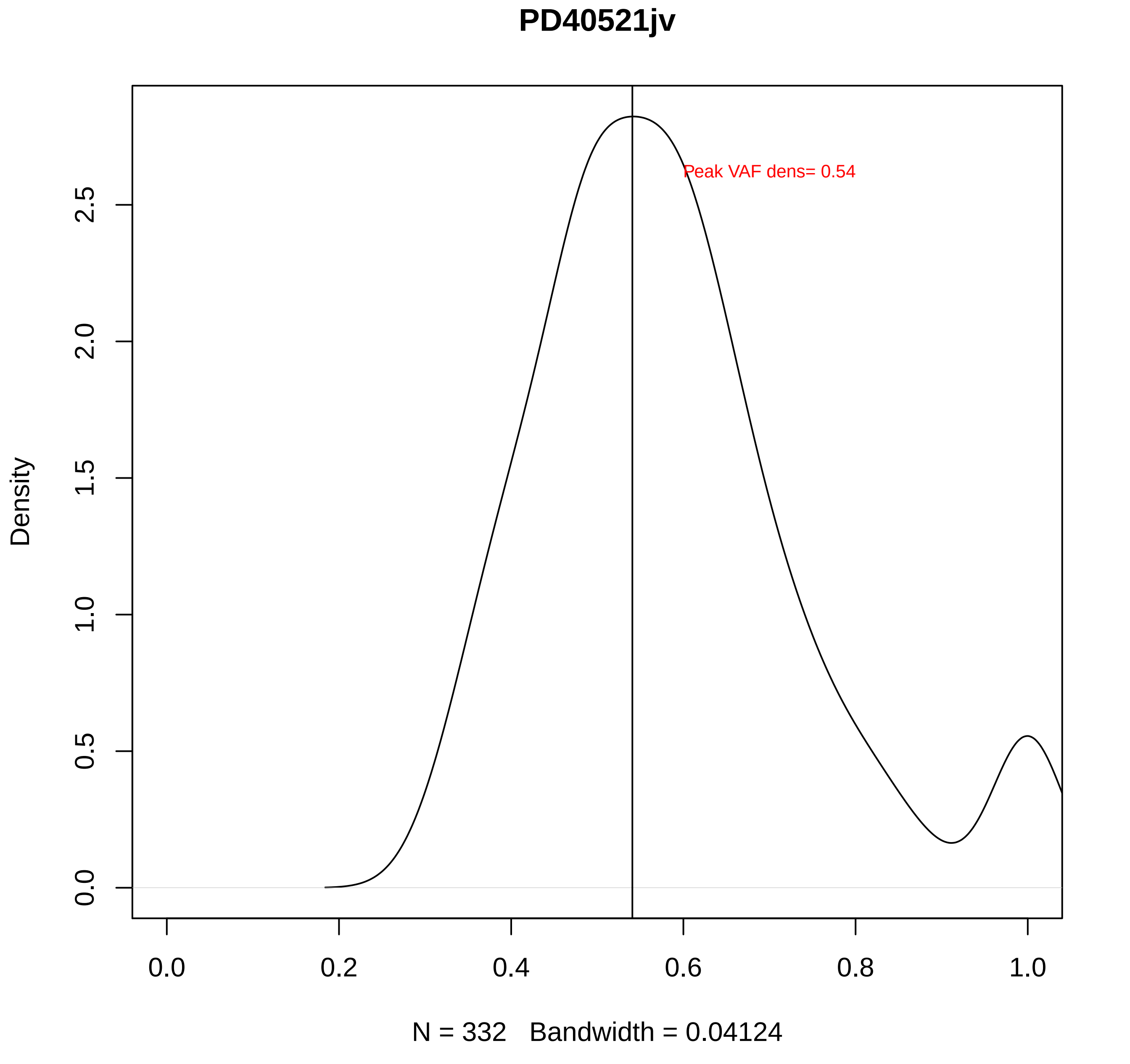
PD40521jv
Peak VAF dens= 0.54
Density
0.0
0.5
1.0
1.5
2.0
2.5
0.0
0.2
0.4
0.6
0.8
1.0
N = 332 Bandwidth = 0.04124

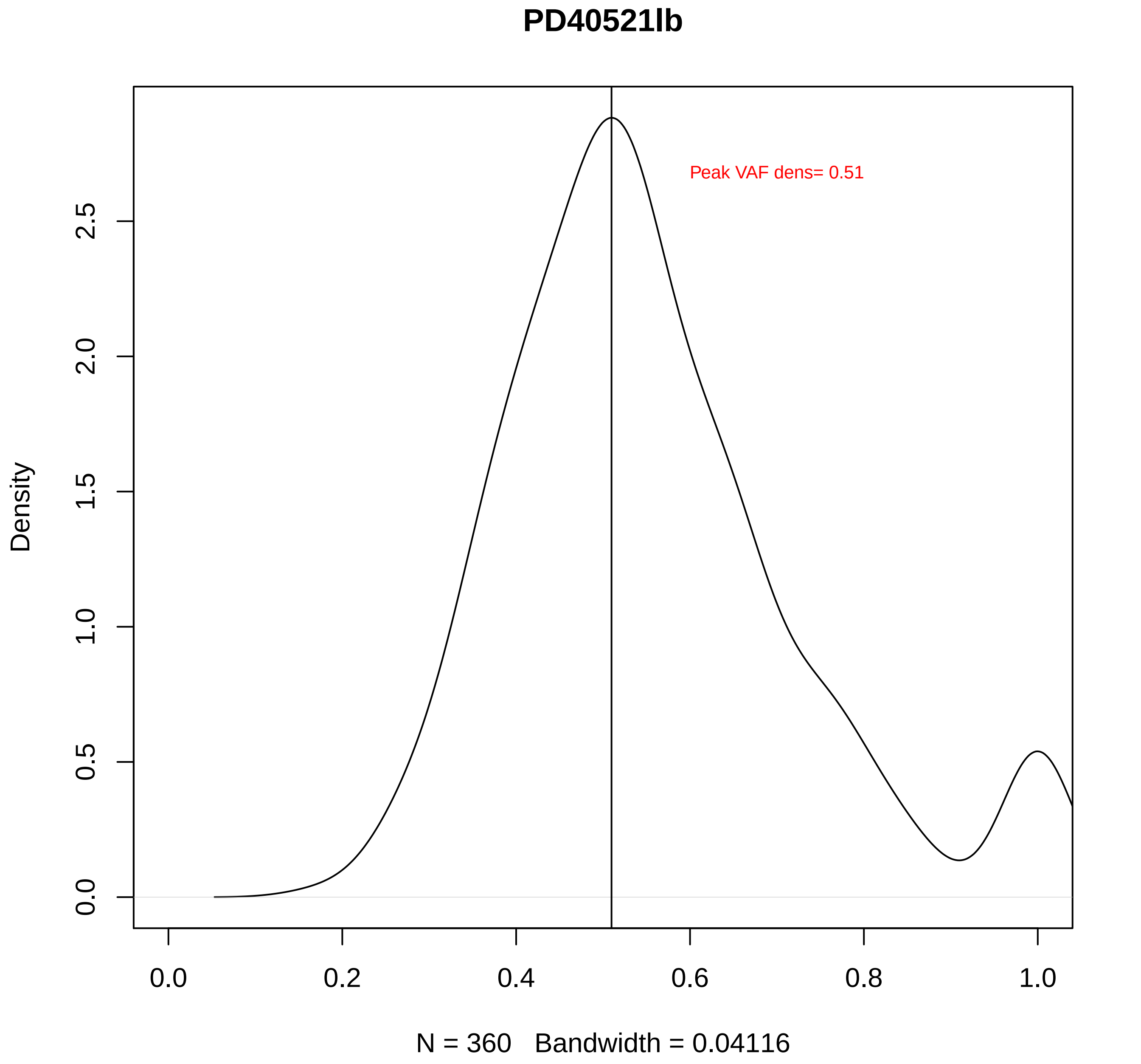

PD40521lb
Peak VAF dens= 0.51
Density
0.0
0.5
1.0
1.5
2.0
2.5
0.0
0.2
0.4
0.6
0.8
1.0
N = 360   Bandwidth = 0.04116

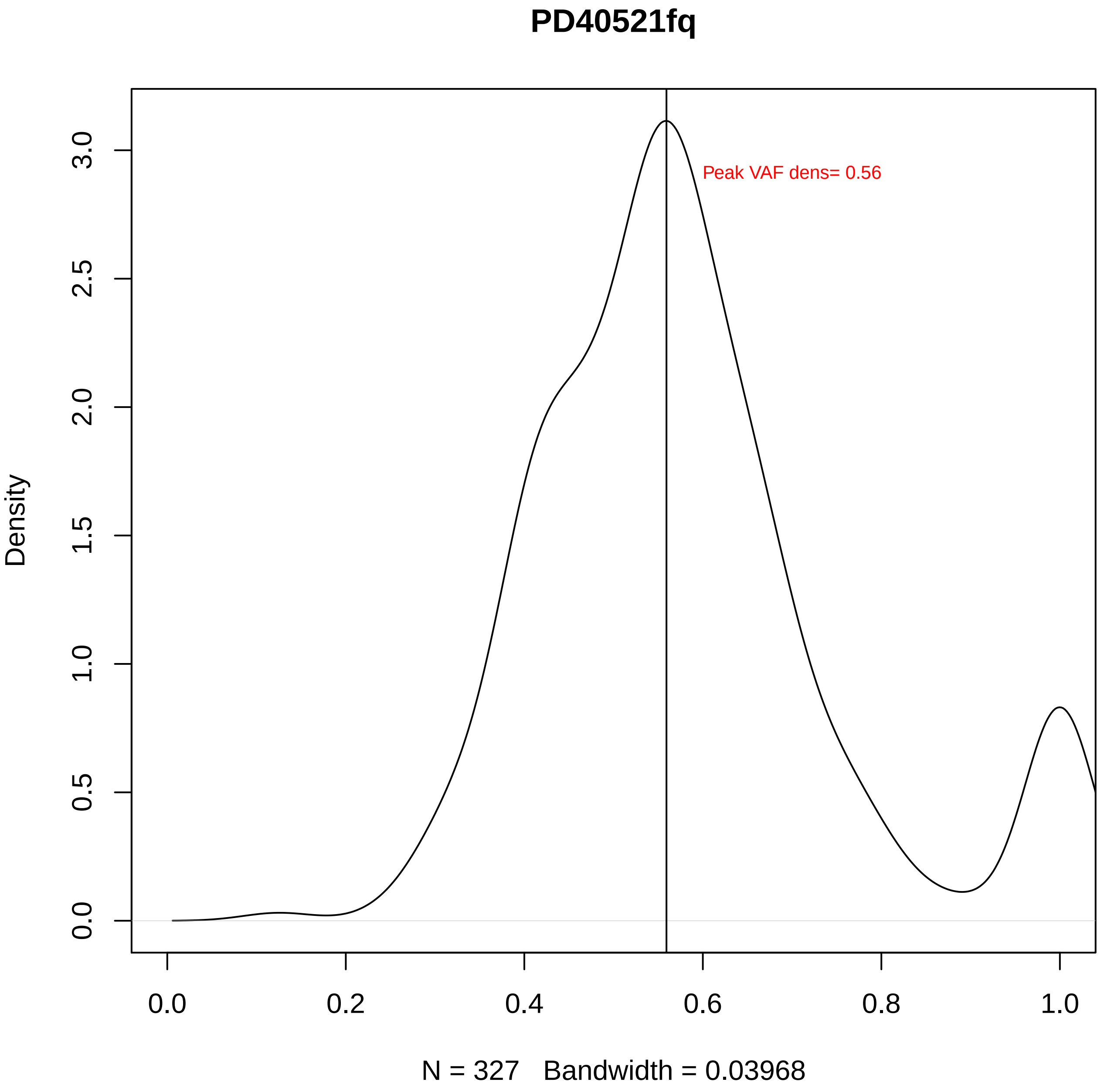

PD40521fq
Peak VAF dens= 0.56
Density
0.0
0.5
1.0
1.5
2.0
2.5
3.0
0.0
0.2
0.4
0.6
0.8
1.0
N = 327 Bandwidth = 0.03968

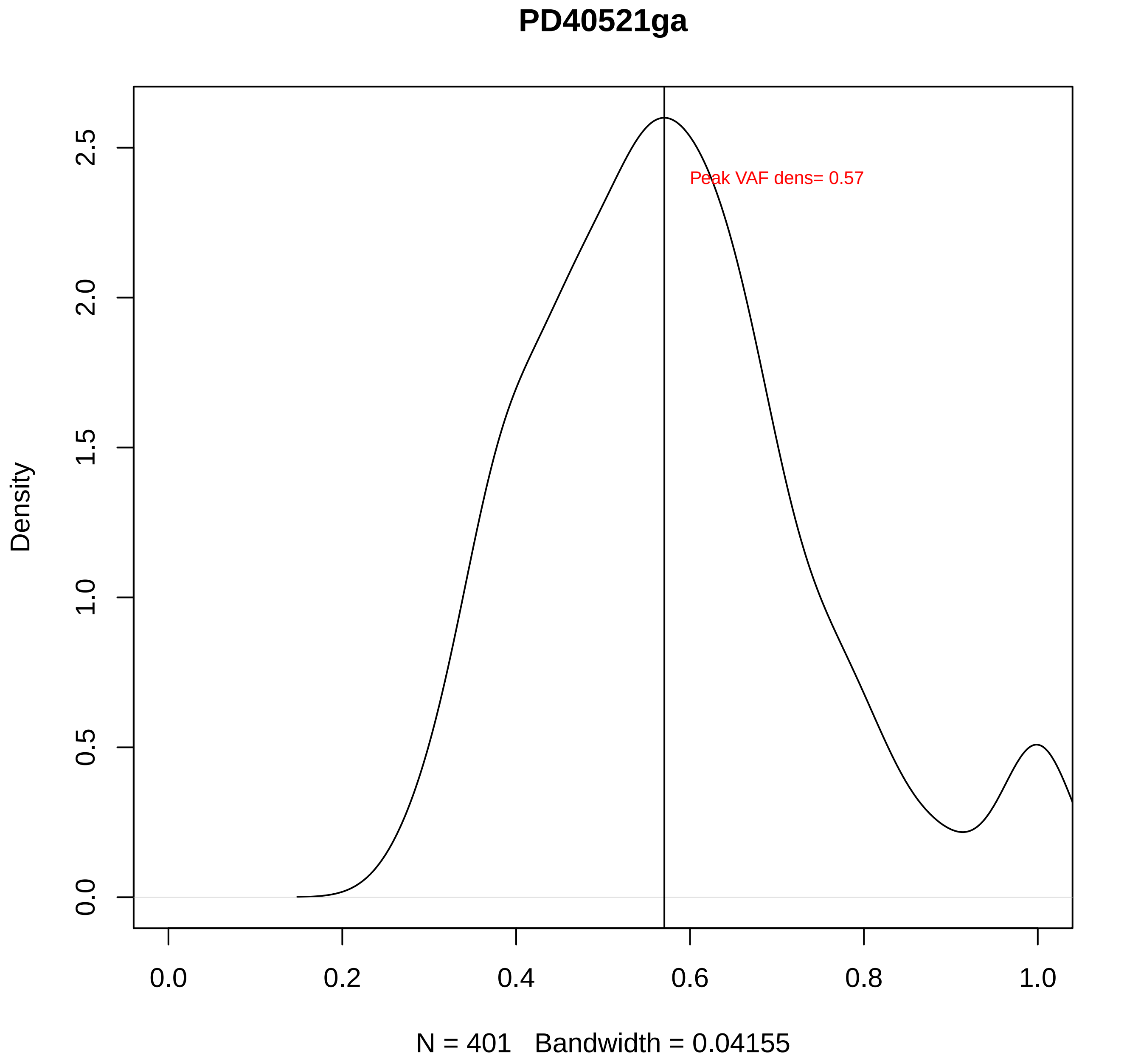
PD40521ga
Peak VAF dens= 0.57
Density
0.0
0.5
1.0
1.5
2.0
2.5
0.0
0.2
0.4
0.6
0.8
1.0
N = 401   Bandwidth = 0.04155

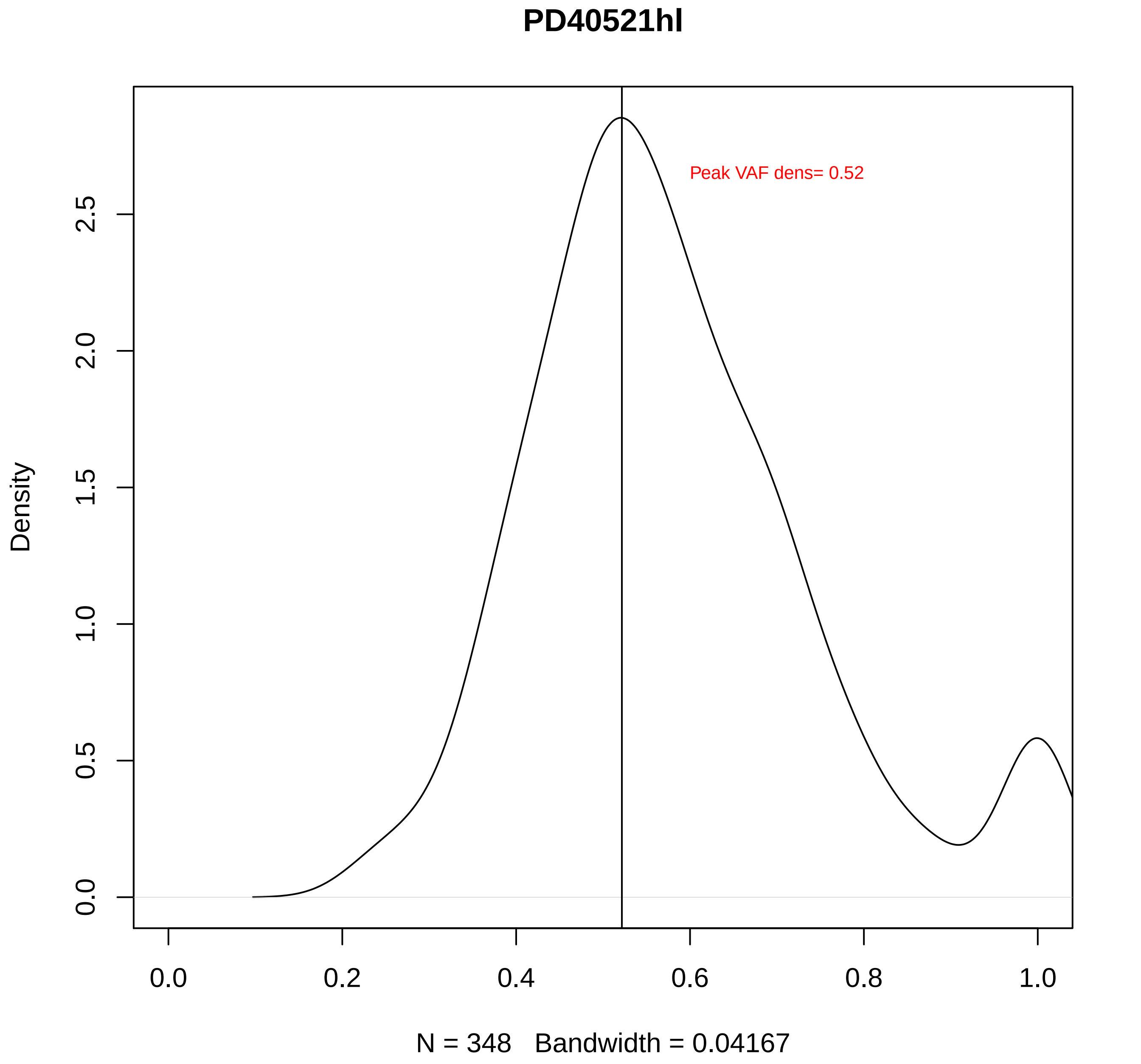
PD40521hl
Peak VAF dens= 0.52
Density
0.0
0.5
1.0
1.5
2.0
2.5
0.0
0.2
0.4
0.6
0.8
1.0
N = 348   Bandwidth = 0.04167

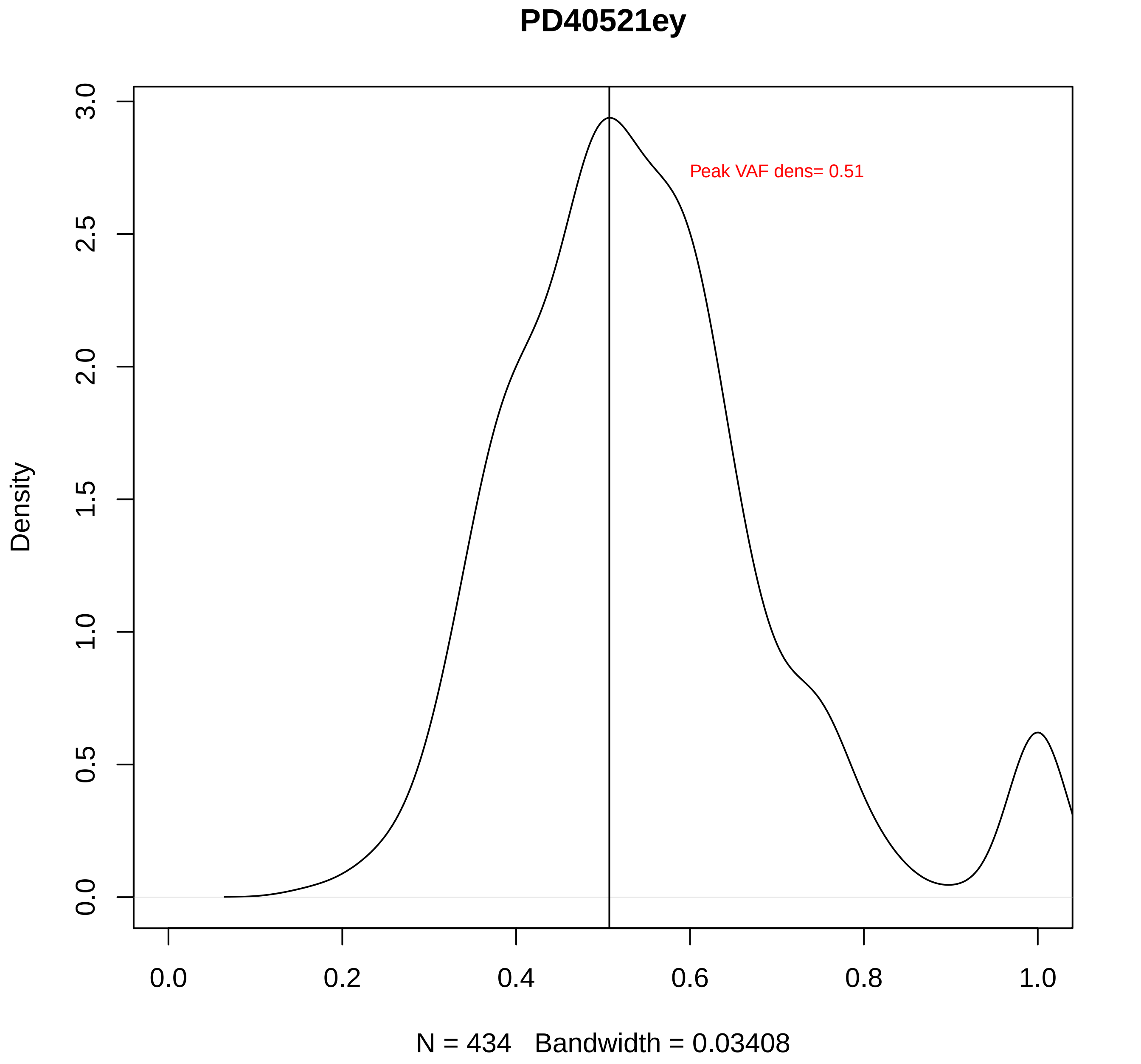

PD40521ey
Peak VAF dens= 0.51
Density
3.0
2.5
2.0
1.5
1.0
0.5
0.0
0.0
0.2
0.4
0.6
0.8
1.0
N = 434   Bandwidth = 0.03408

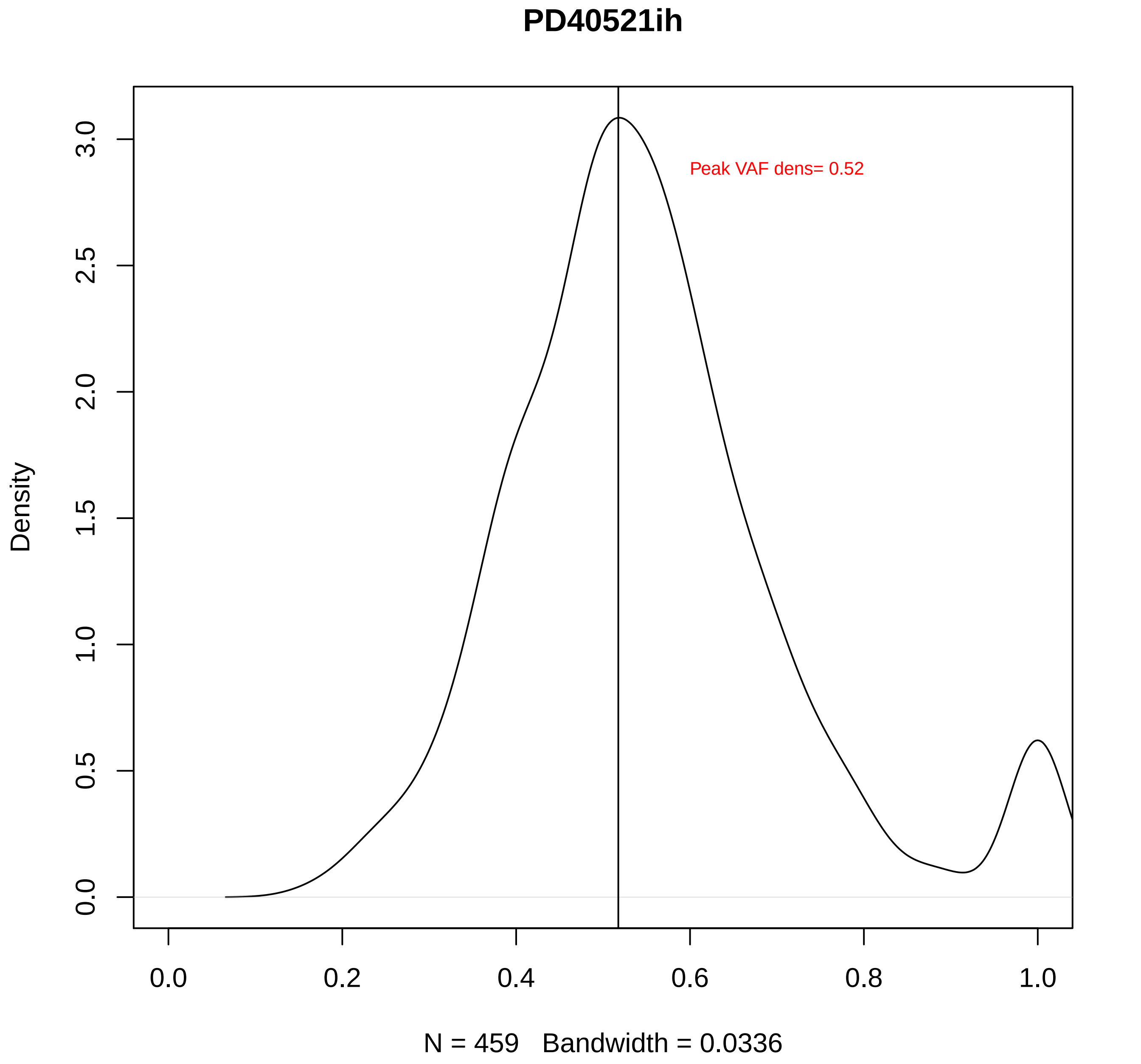

PD40521ih
Peak VAF dens= 0.52
Density
0.0
0.5
1.0
1.5
2.0
2.5
3.0
0.0
0.2
0.4
0.6
0.8
1.0
N = 459   Bandwidth = 0.0336

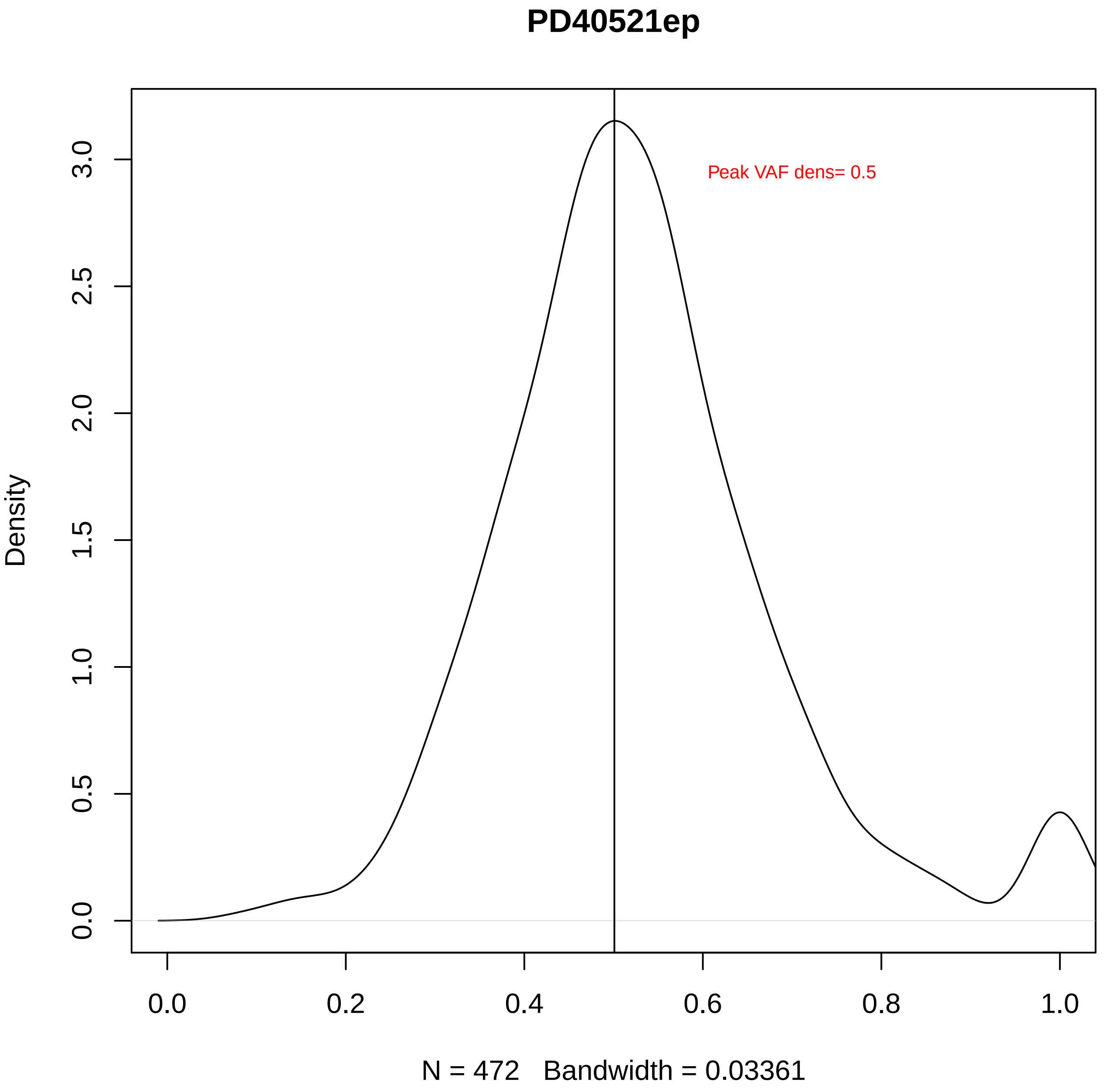
PD40521ep
Peak VAF dens= 0.5
Density
0.0
0.5
1.0
1.5
2.0
2.5
3.0
0.0
0.2
0.4
0.6
0.8
1.0
N = 472   Bandwidth = 0.03361

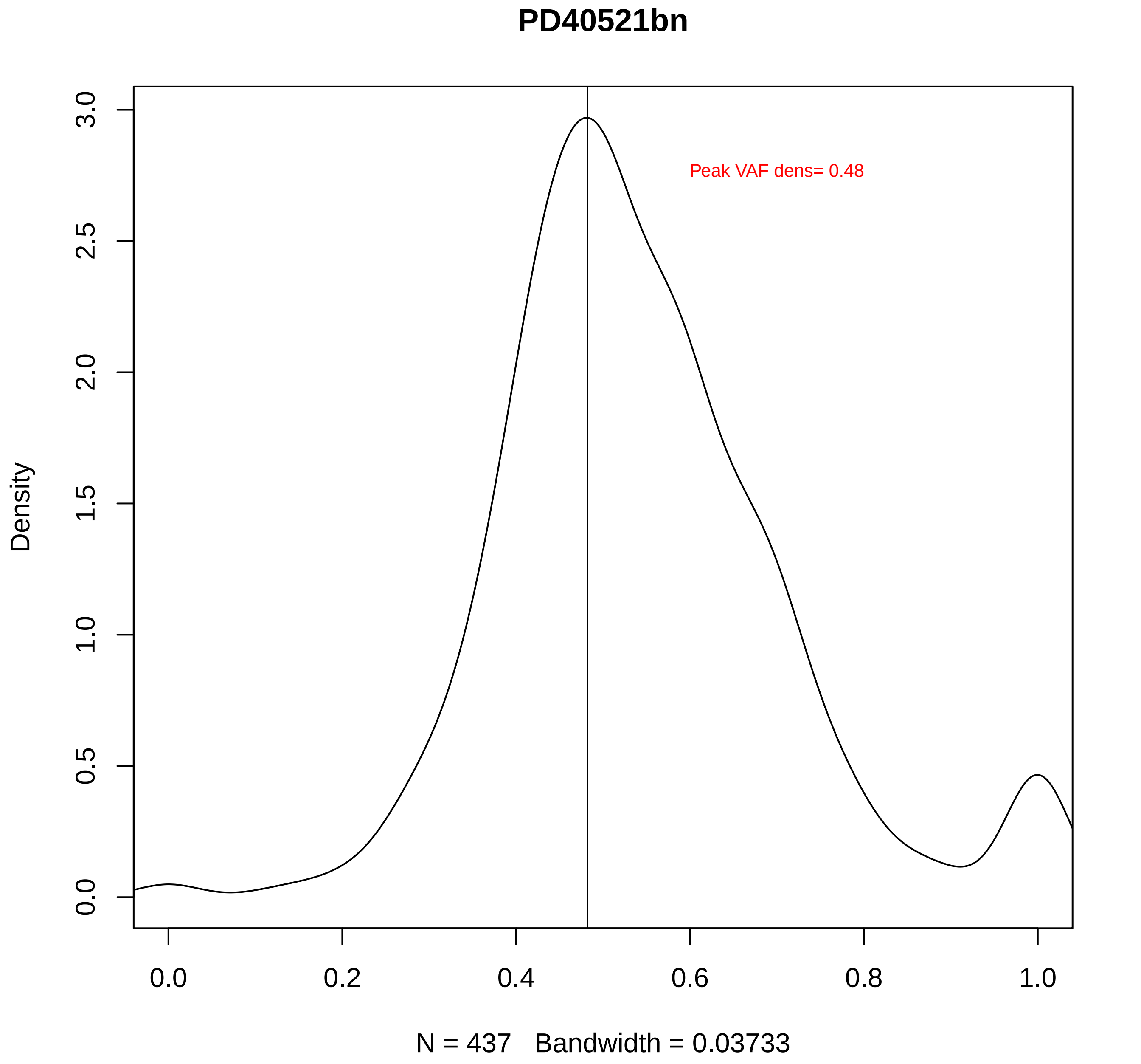
PD40521bn
Peak VAF dens= 0.48
Density
0.0
0.5
1.0
1.5
2.0
2.5
3.0
0.0
0.2
0.4
0.6
0.8
1.0
N = 437   Bandwidth = 0.03733

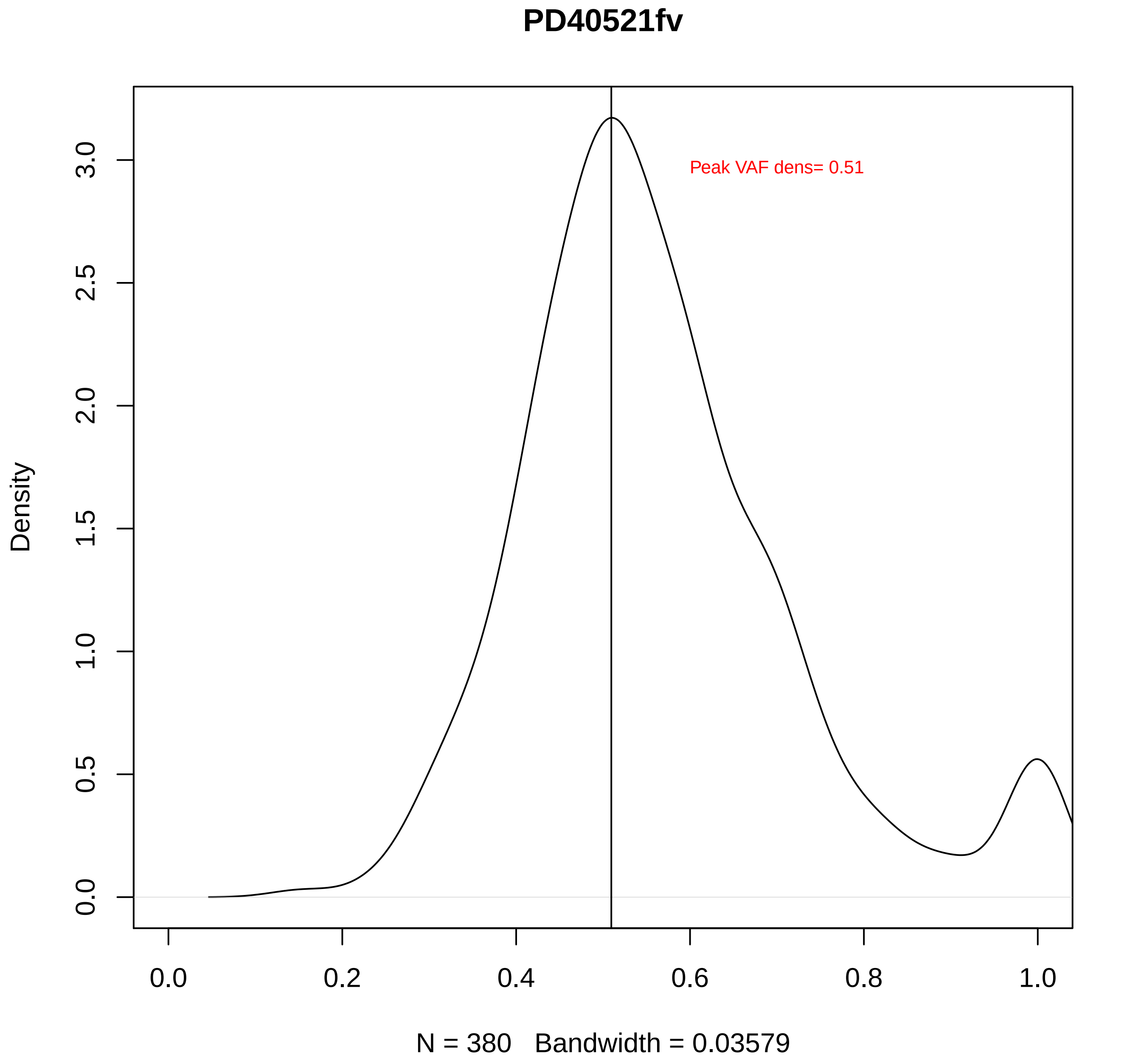
PD40521fv
Peak VAF dens= 0.51
Density
0.0
0.5
1.0
1.5
2.0
2.5
3.0
0.0
0.2
0.4
0.6
0.8
1.0
N = 380   Bandwidth = 0.03579

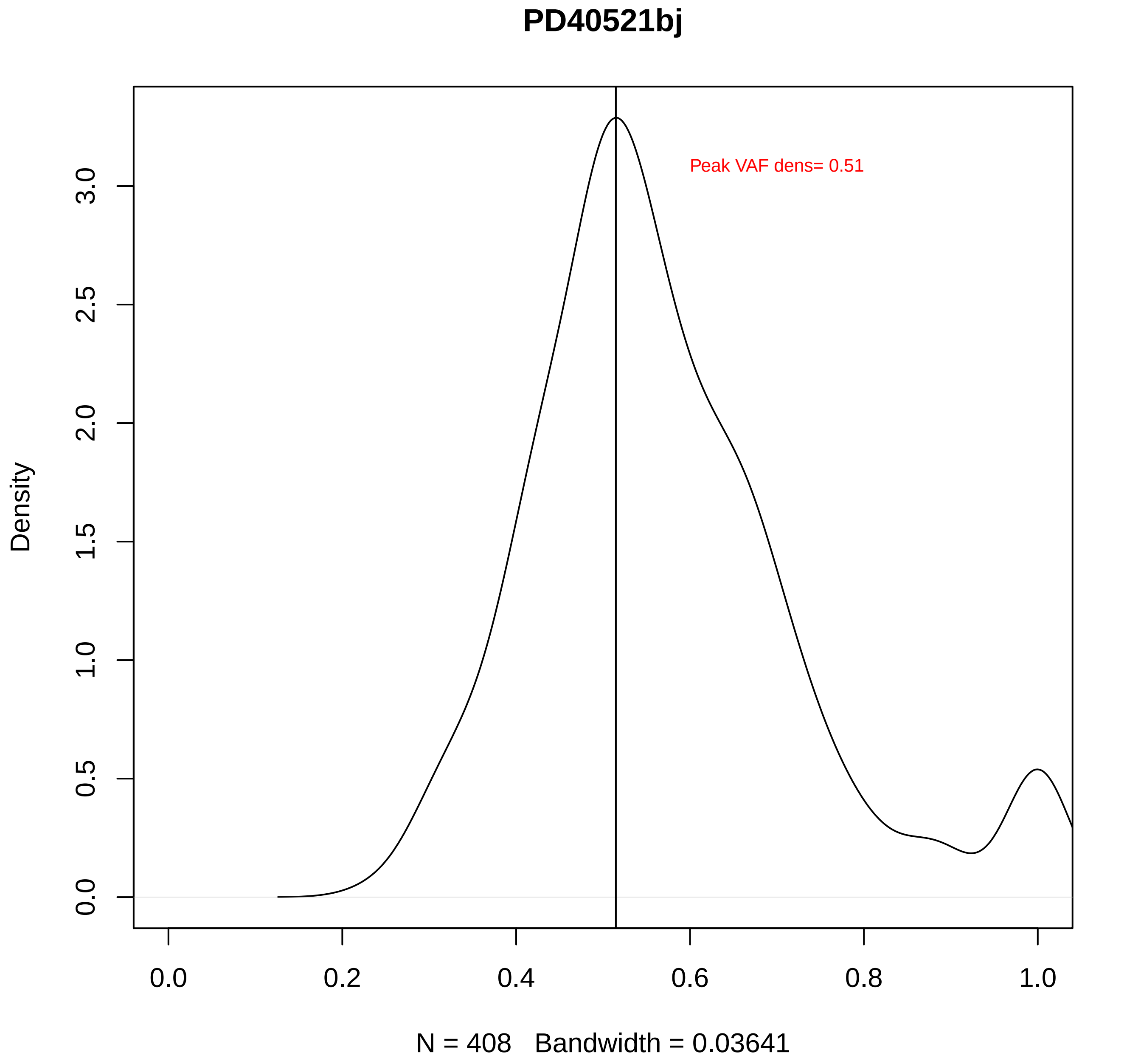

PD40521bj
Peak VAF dens= 0.51
Density
0.0
0.5
1.0
1.5
2.0
2.5
3.0
0.0
0.2
0.4
0.6
0.8
1.0
N = 408 Bandwidth = 0.03641

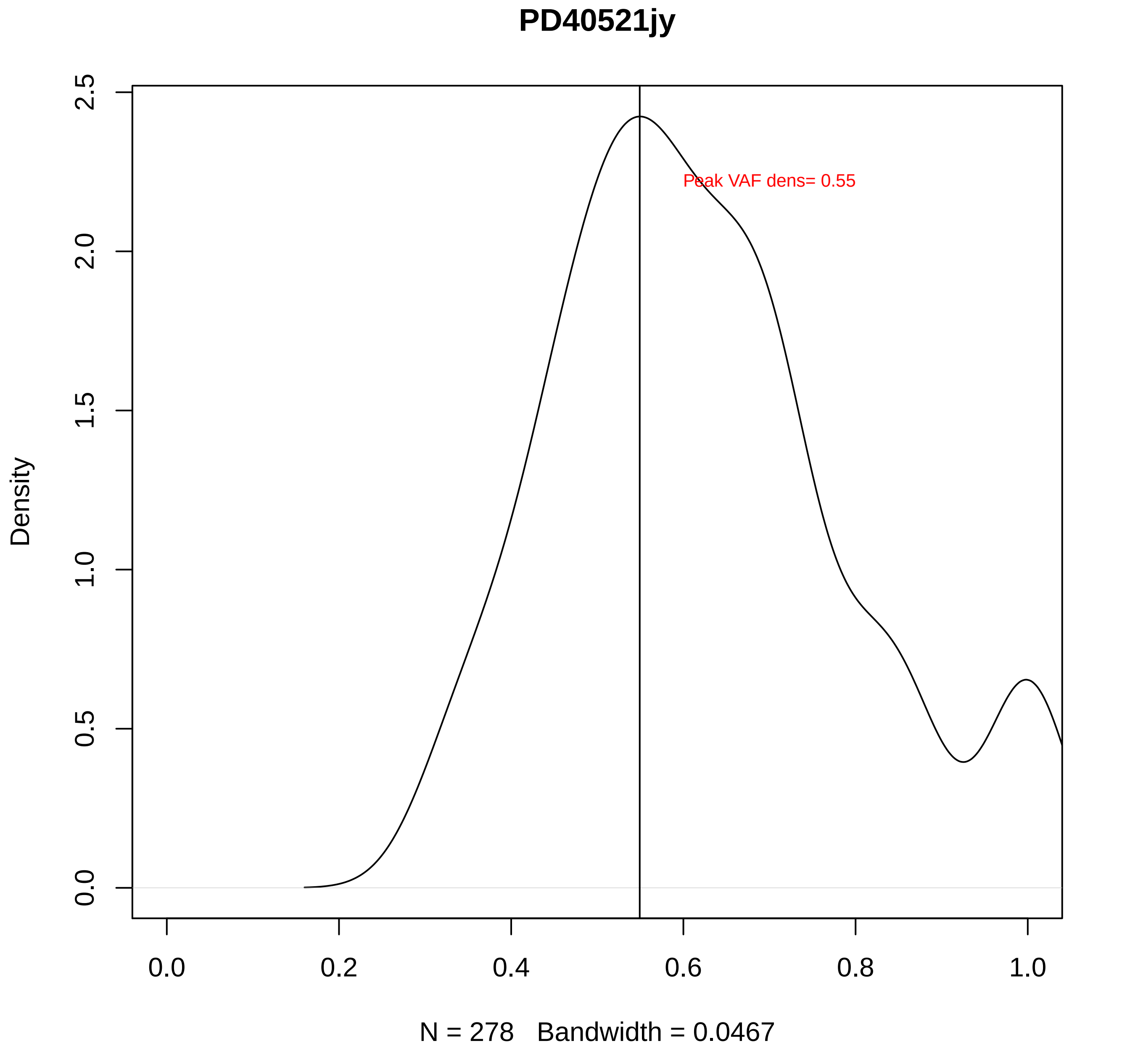

PD40521jy
Density
2.5
2.0
1.5
1.0
0.5
0.0
Peak VAF dens= 0.55
0.0
0.2
0.4
0.6
0.8
1.0
N = 278   Bandwidth = 0.0467

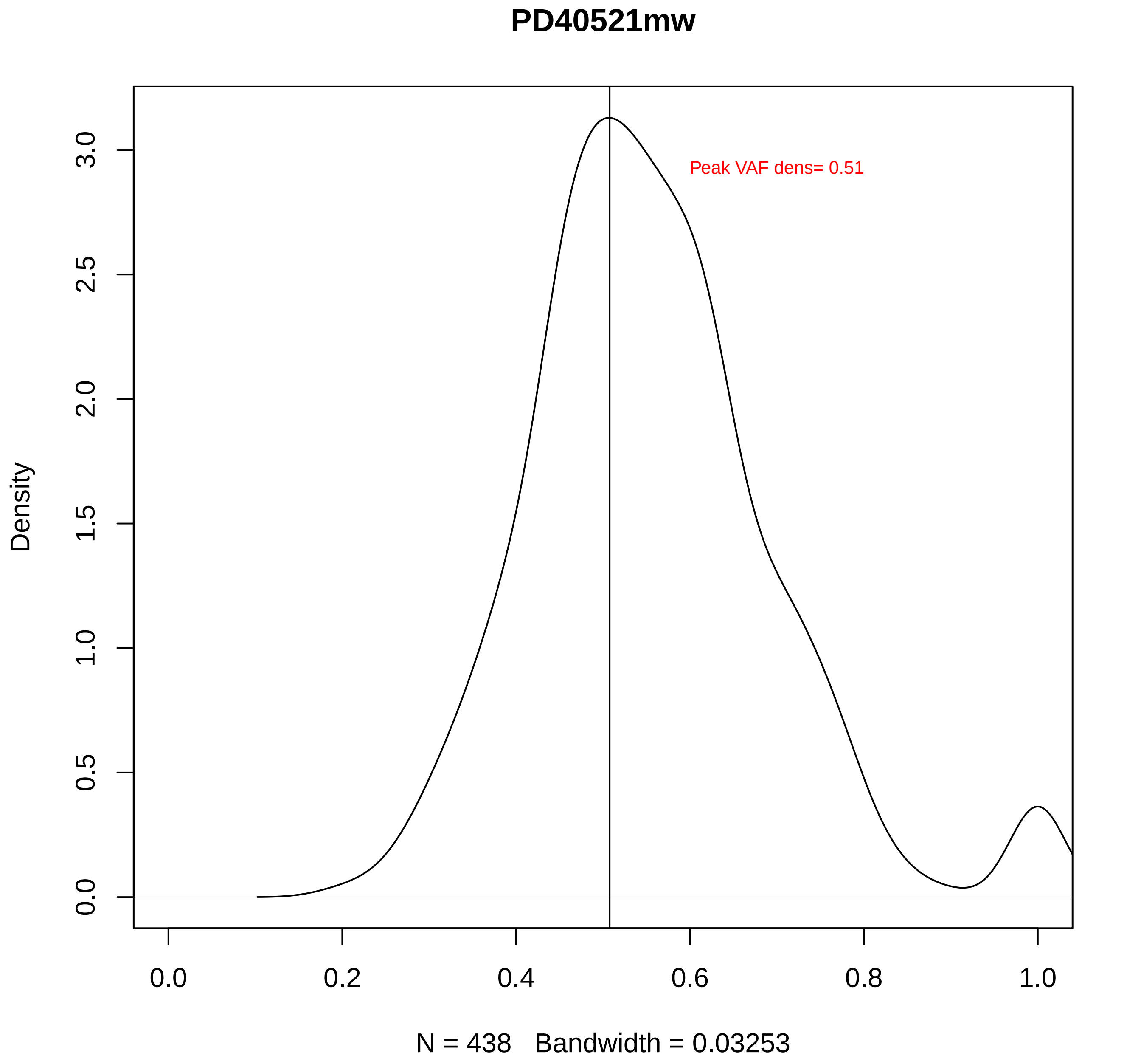
PD40521mw
Peak VAF dens= 0.51
Density
0.0
0.5
1.0
1.5
2.0
2.5
3.0
0.0
0.2
0.4
0.6
0.8
1.0
N = 438   Bandwidth = 0.03253

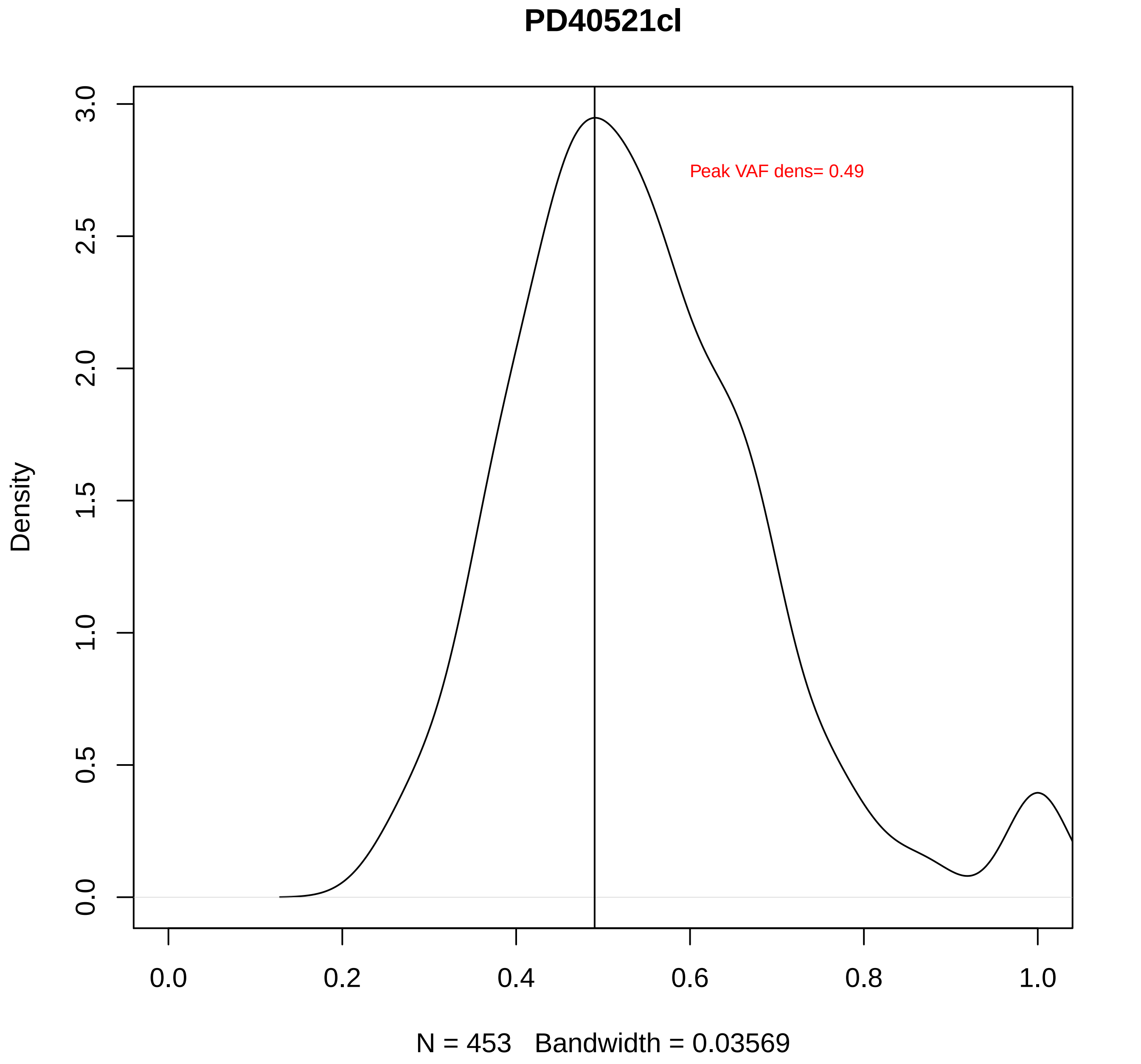
PD40521cl
Peak VAF dens= 0.49
Density
0.0
0.5
1.0
1.5
2.0
2.5
3.0
0.0
0.2
0.4
0.6
0.8
1.0
N = 453   Bandwidth = 0.03569

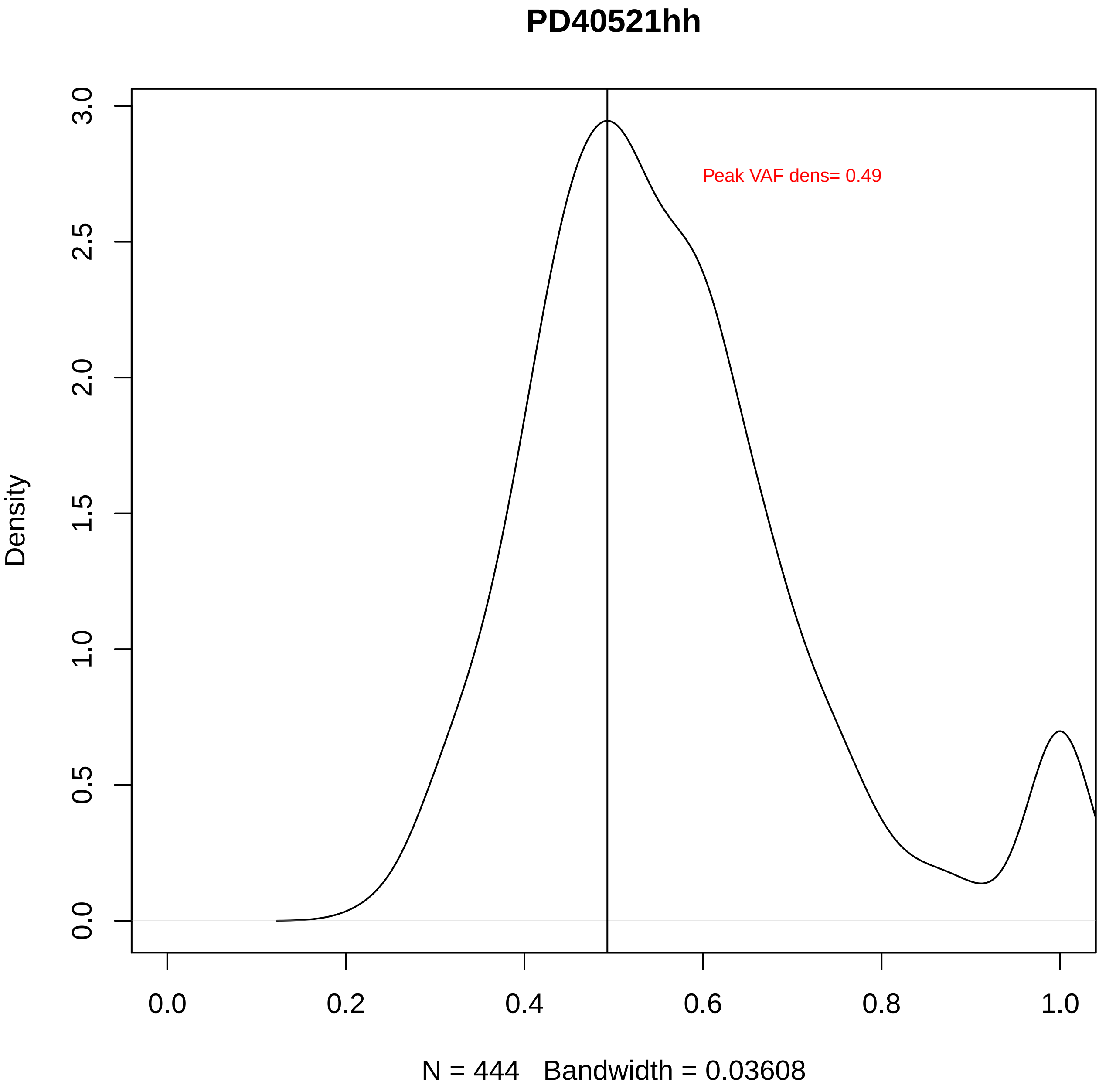
PD40521hh
Peak VAF dens= 0.49
Density
0.0
0.5
1.0
1.5
2.0
2.5
3.0
0.0
0.2
0.4
0.6
0.8
1.0
N = 444   Bandwidth = 0.03608

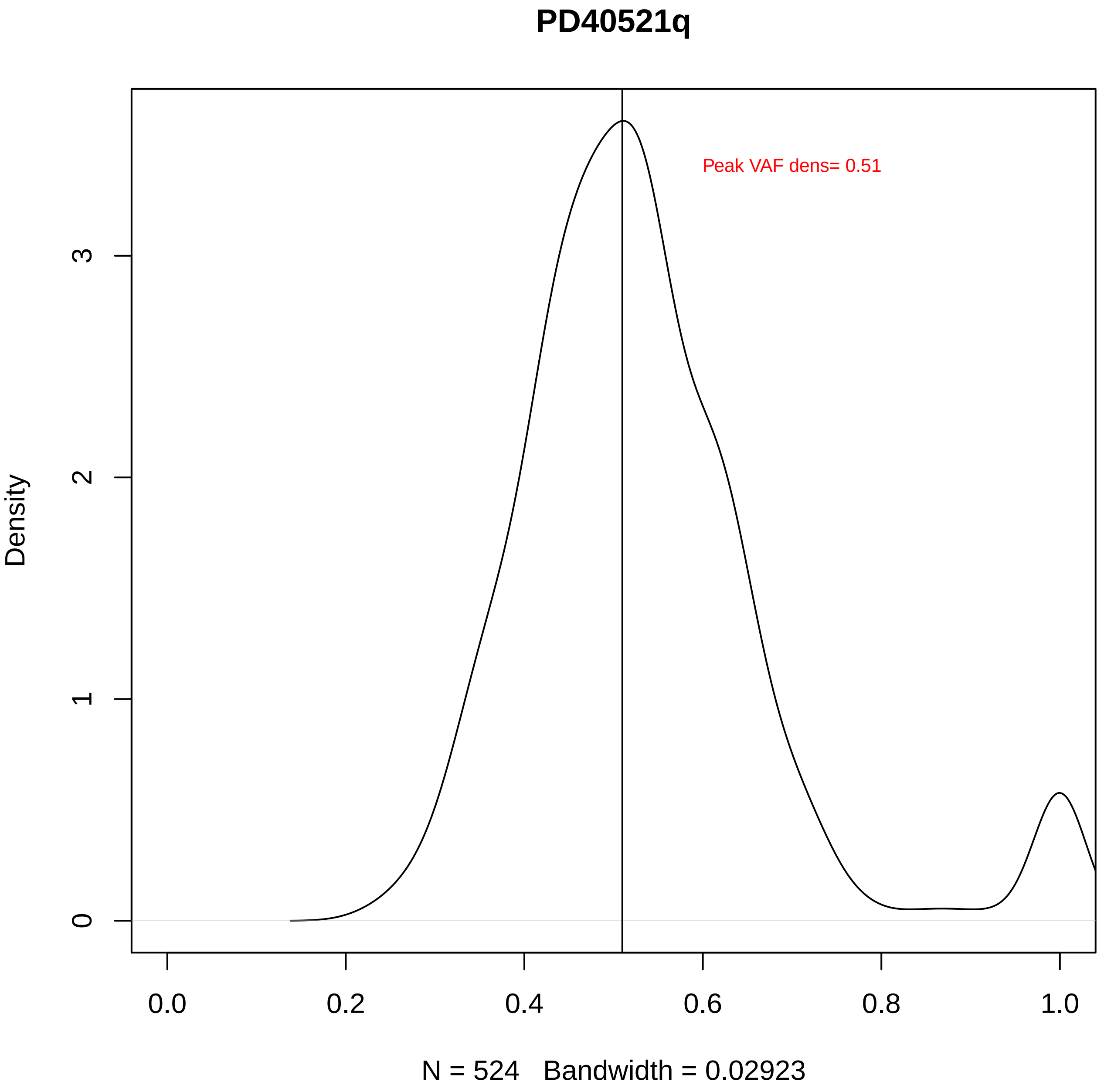
PD40521q
Peak VAF dens= 0.51
Density
0
1
2
3
0.0
0.2
0.4
0.6
0.8
1.0
N = 524   Bandwidth = 0.02923

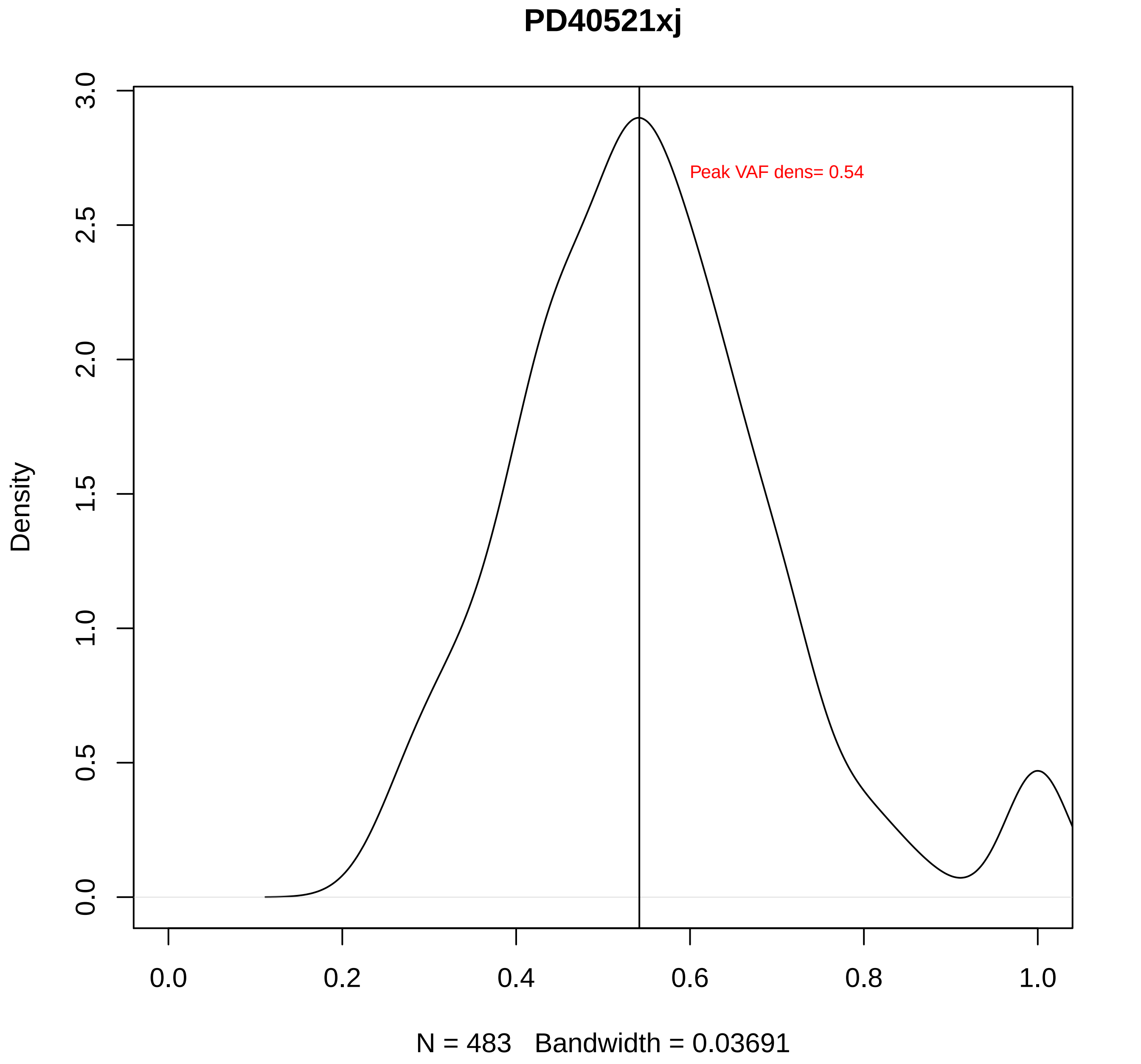
PD40521xj
Peak VAF dens= 0.54
Density
0.0
0.5
1.0
1.5
2.0
2.5
3.0
0.0
0.2
0.4
0.6
0.8
1.0
N = 483   Bandwidth = 0.03691

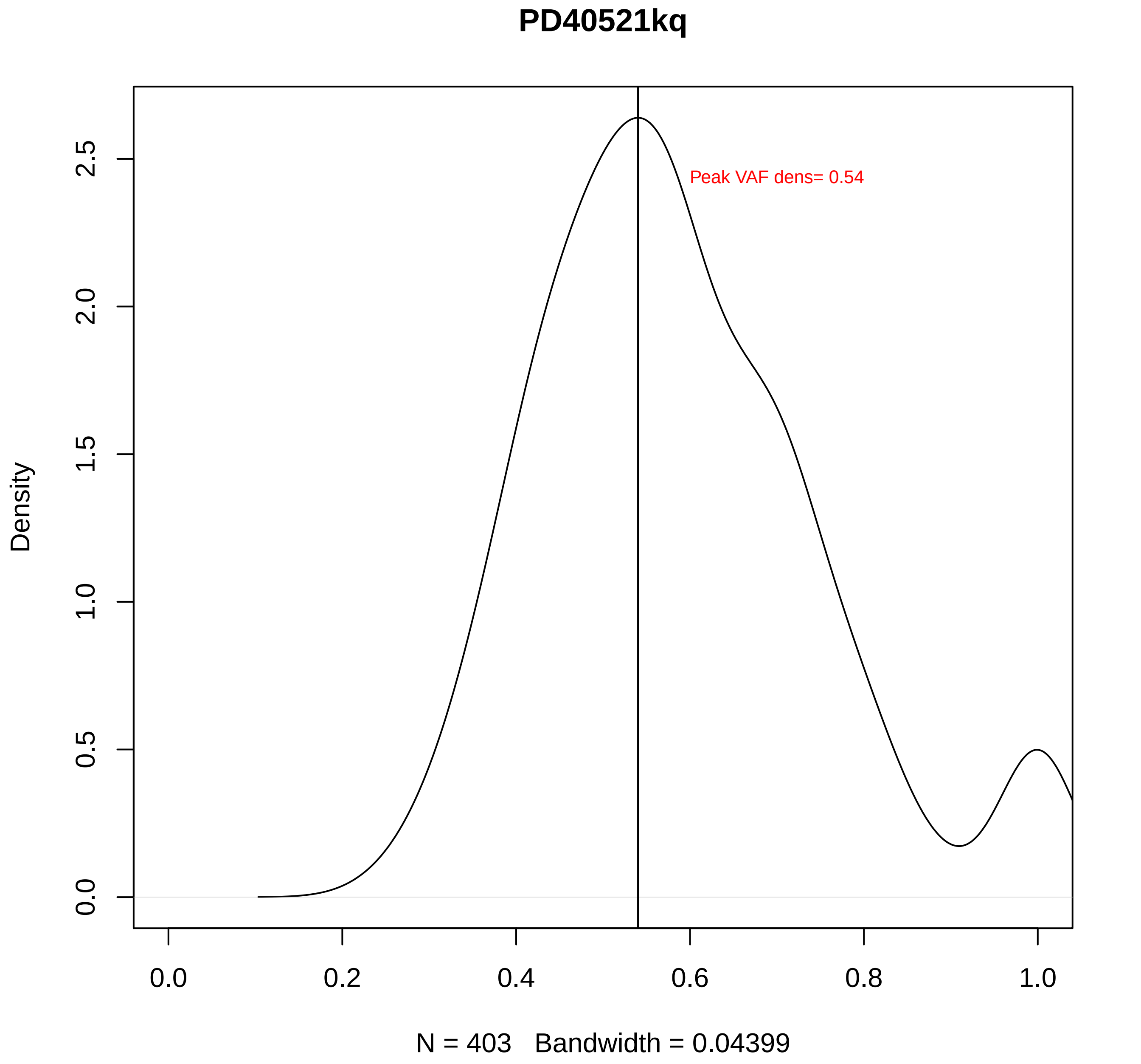
PD40521kq
Peak VAF dens= 0.54
Density
0.0
0.5
1.0
1.5
2.0
2.5
0.0
0.2
0.4
0.6
0.8
1.0
N = 403   Bandwidth = 0.04399

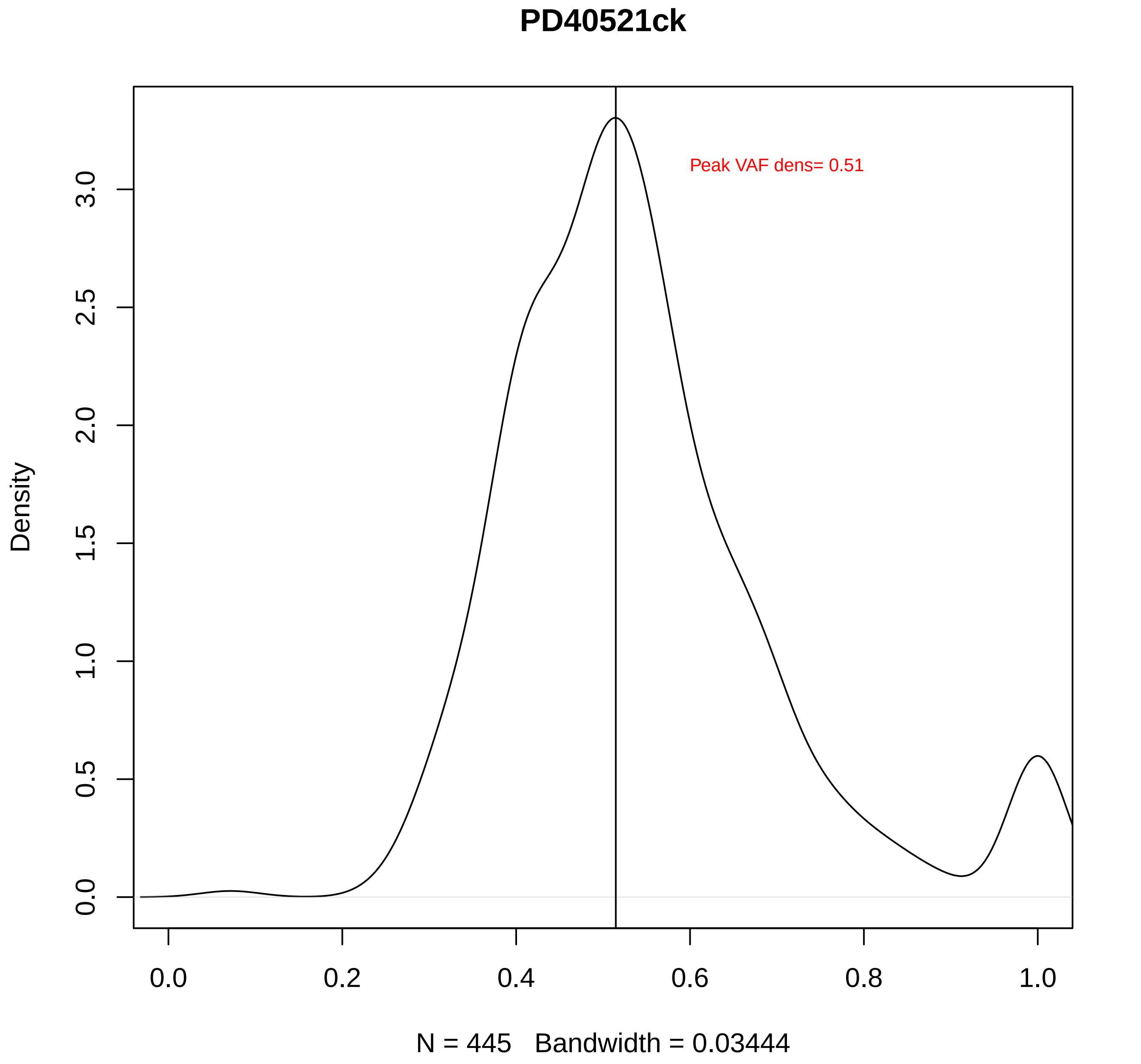

PD40521ck
Peak VAF dens= 0.51
Density
0.0
0.5
1.0
1.5
2.0
2.5
3.0
0.0
0.2
0.4
0.6
0.8
1.0
N = 445   Bandwidth = 0.03444

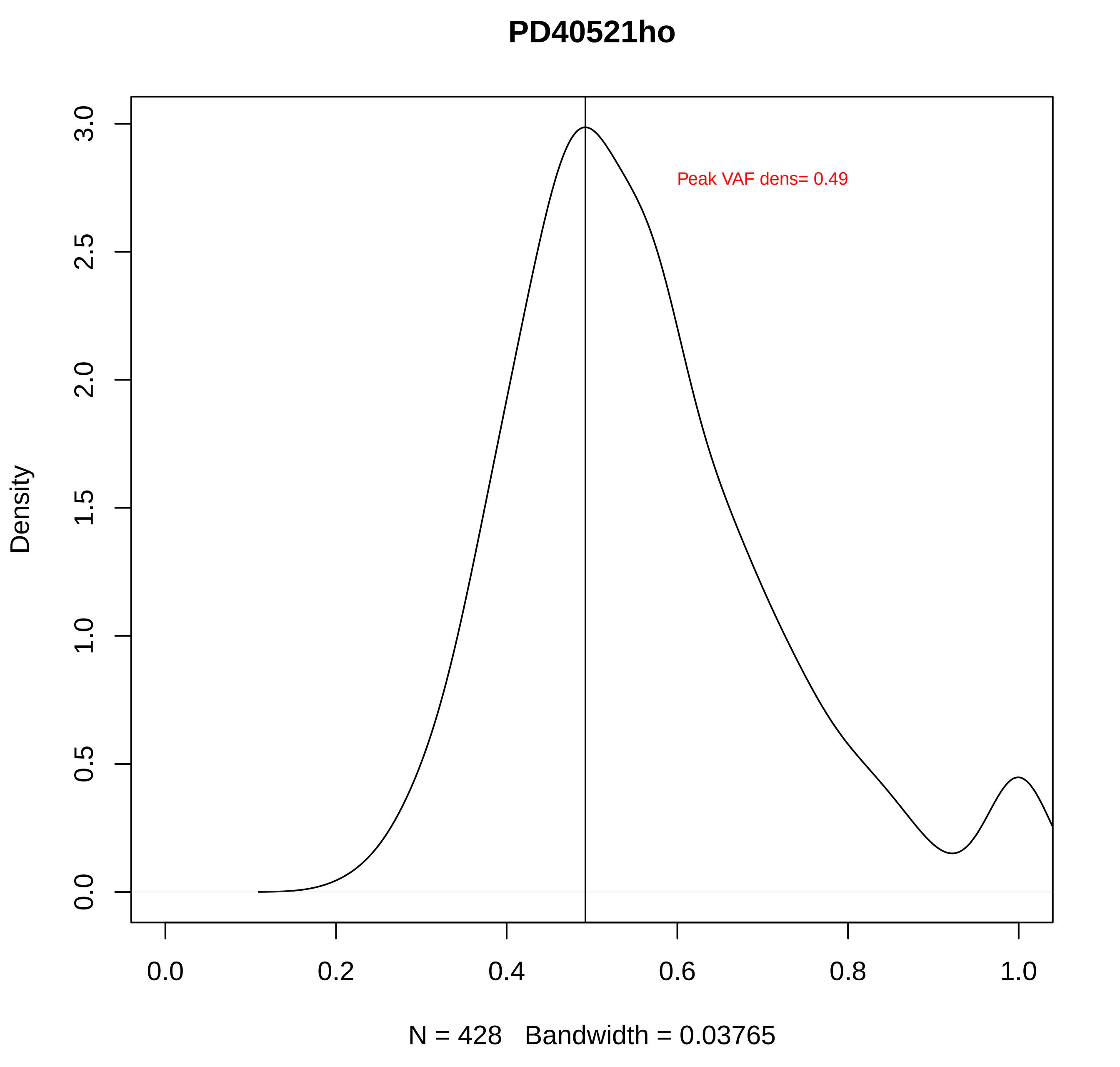
PD40521ho
Peak VAF dens= 0.49
Density
0.0
0.5
1.0
1.5
2.0
2.5
3.0
0.0
0.2
0.4
0.6
0.8
1.0
N = 428 Bandwidth = 0.03765

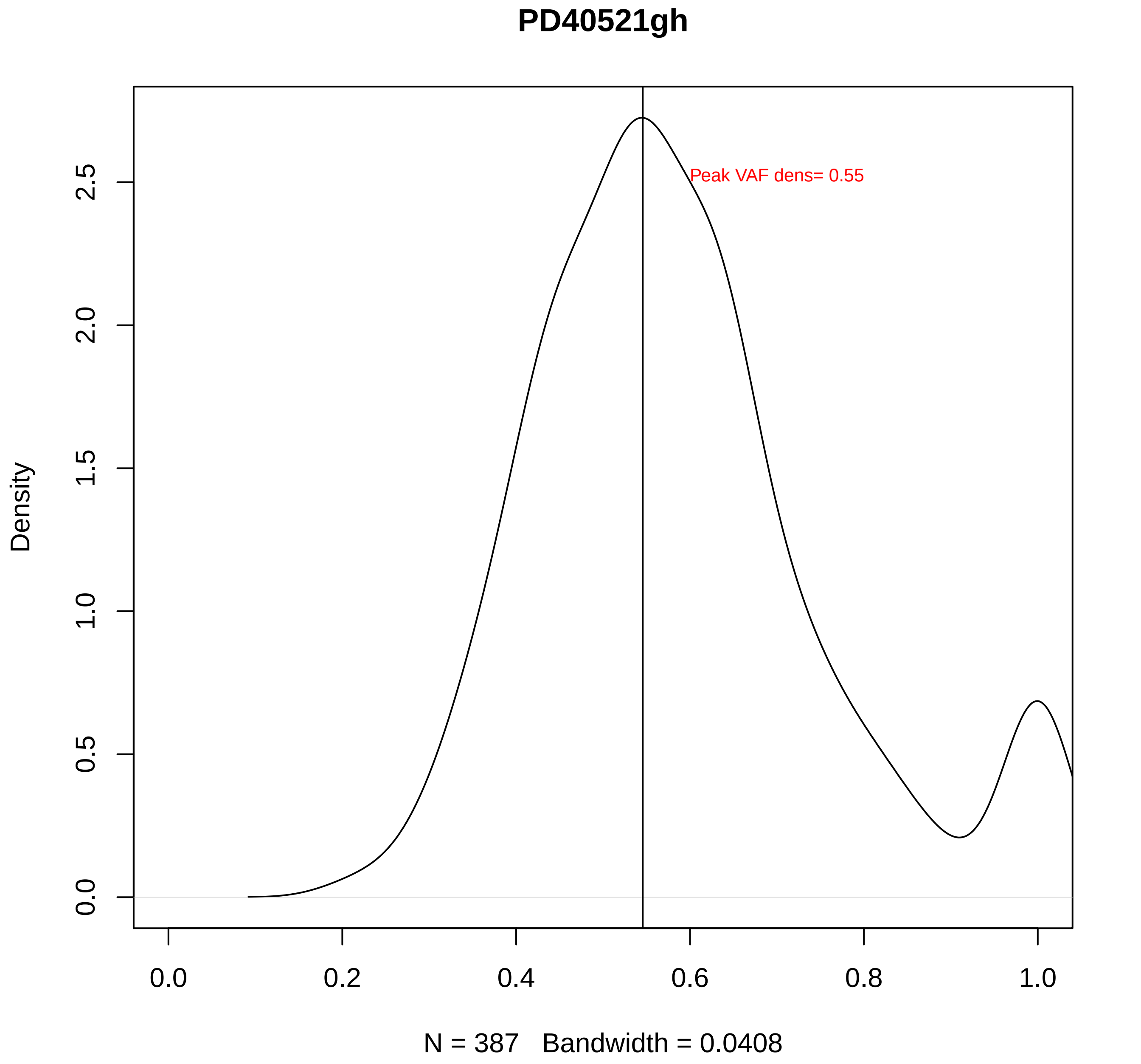
PD40521gh
Peak VAF dens= 0.55
Density
0.0
0.5
1.0
1.5
2.0
2.5
0.0
0.2
0.4
0.6
0.8
1.0
N = 387   Bandwidth = 0.0408

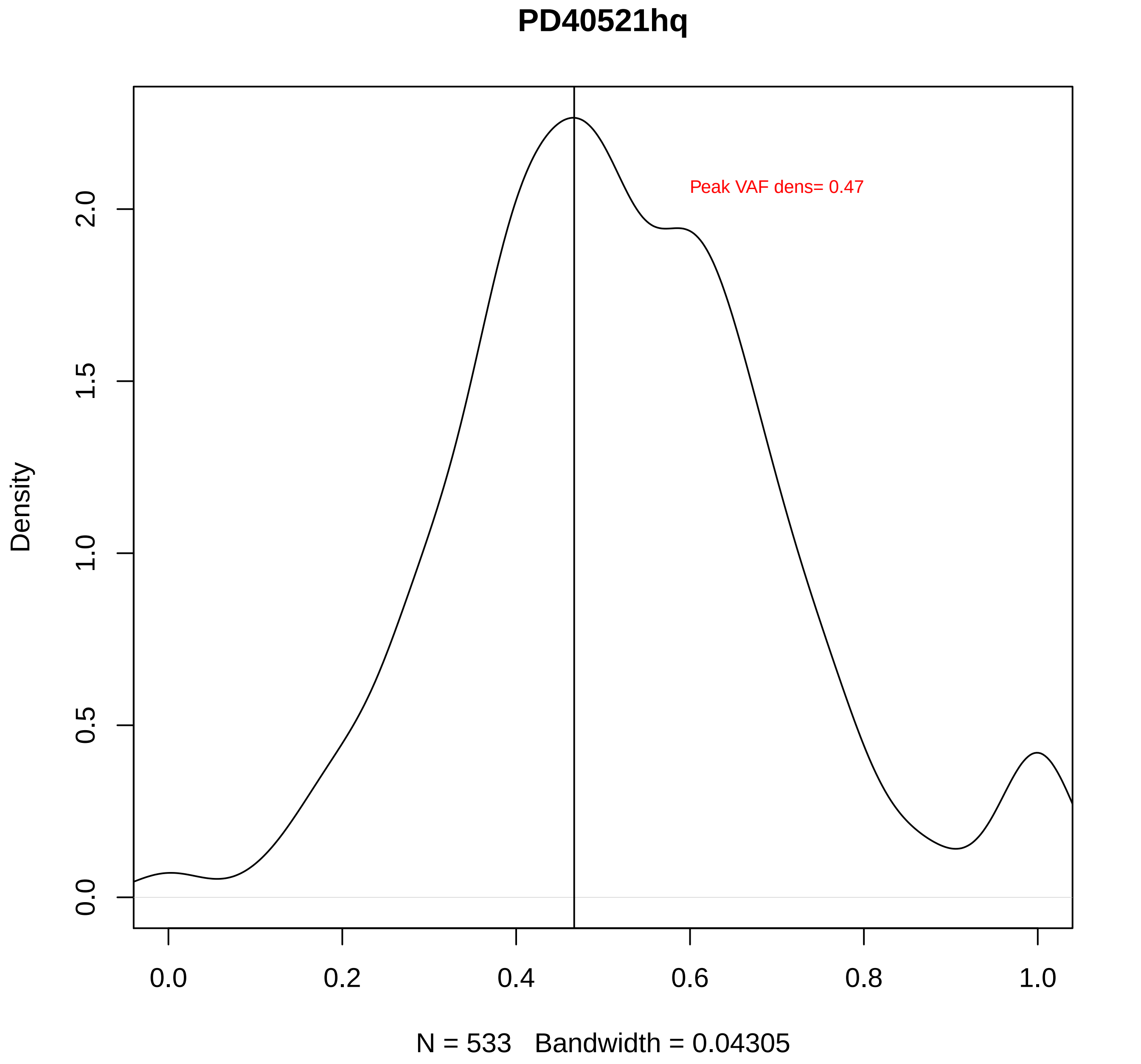

PD40521hq
Peak VAF dens= 0.47
Density
0.0
0.5
1.0
1.5
2.0
0.0
0.2
0.4
0.6
0.8
1.0
N = 533 Bandwidth = 0.04305

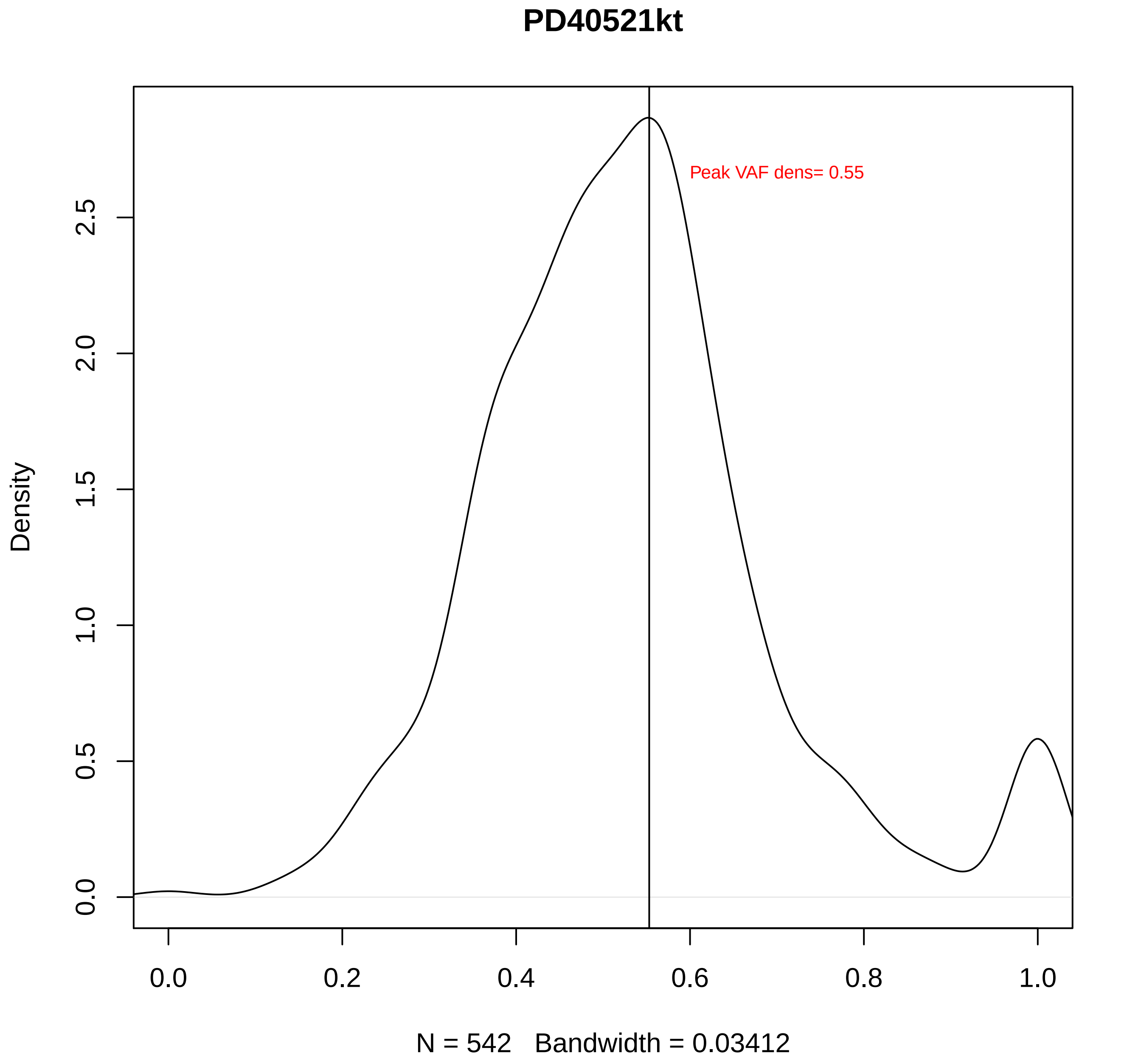

PD40521kt
Peak VAF dens= 0.55
Density
0.0
0.5
1.0
1.5
2.0
2.5
0.0
0.2
0.4
0.6
0.8
1.0
N = 542 Bandwidth = 0.03412

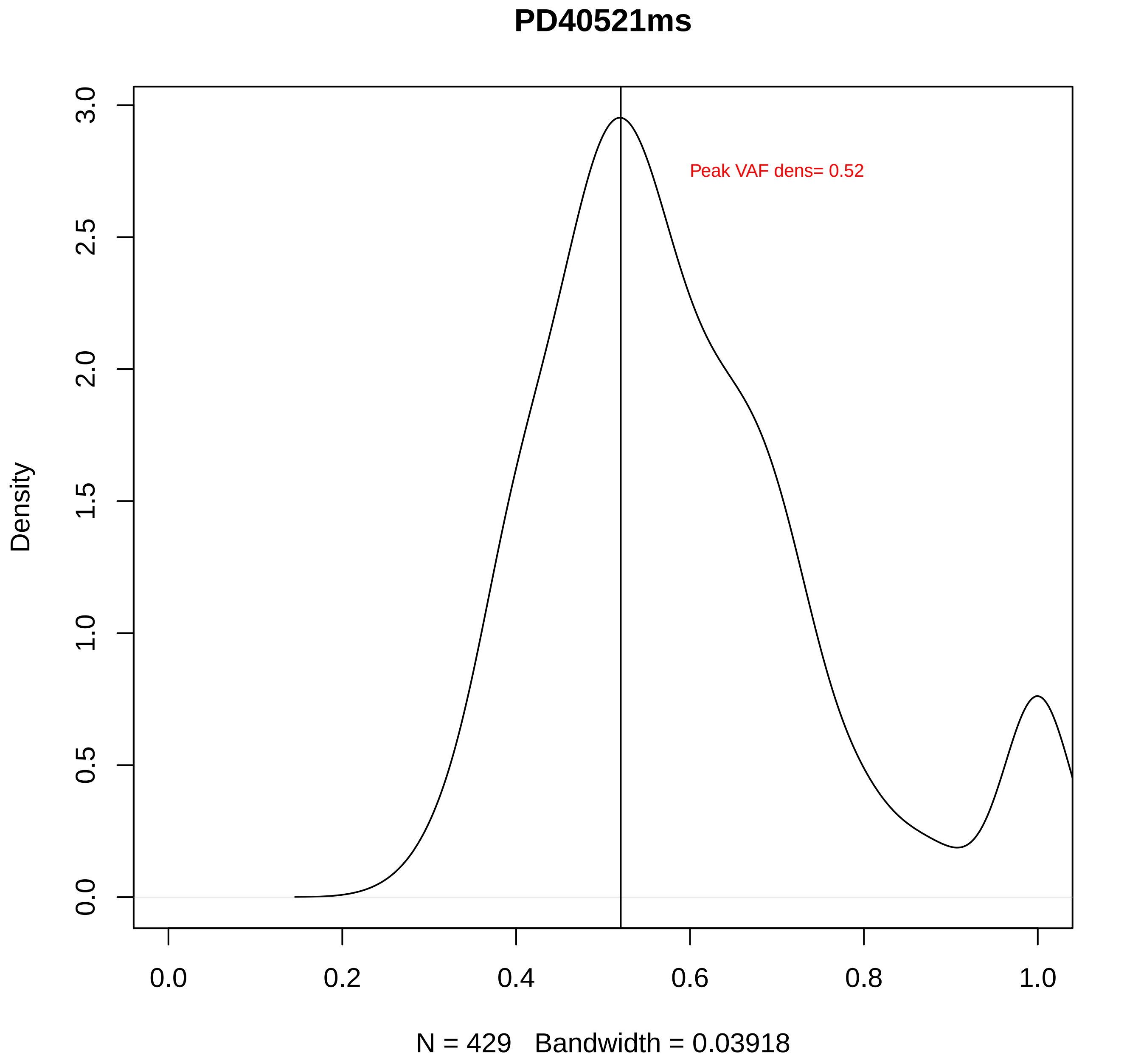
PD40521ms
Peak VAF dens= 0.52
Density
0.0
0.5
1.0
1.5
2.0
2.5
3.0
0.0
0.2
0.4
0.6
0.8
1.0
N = 429 Bandwidth = 0.03918

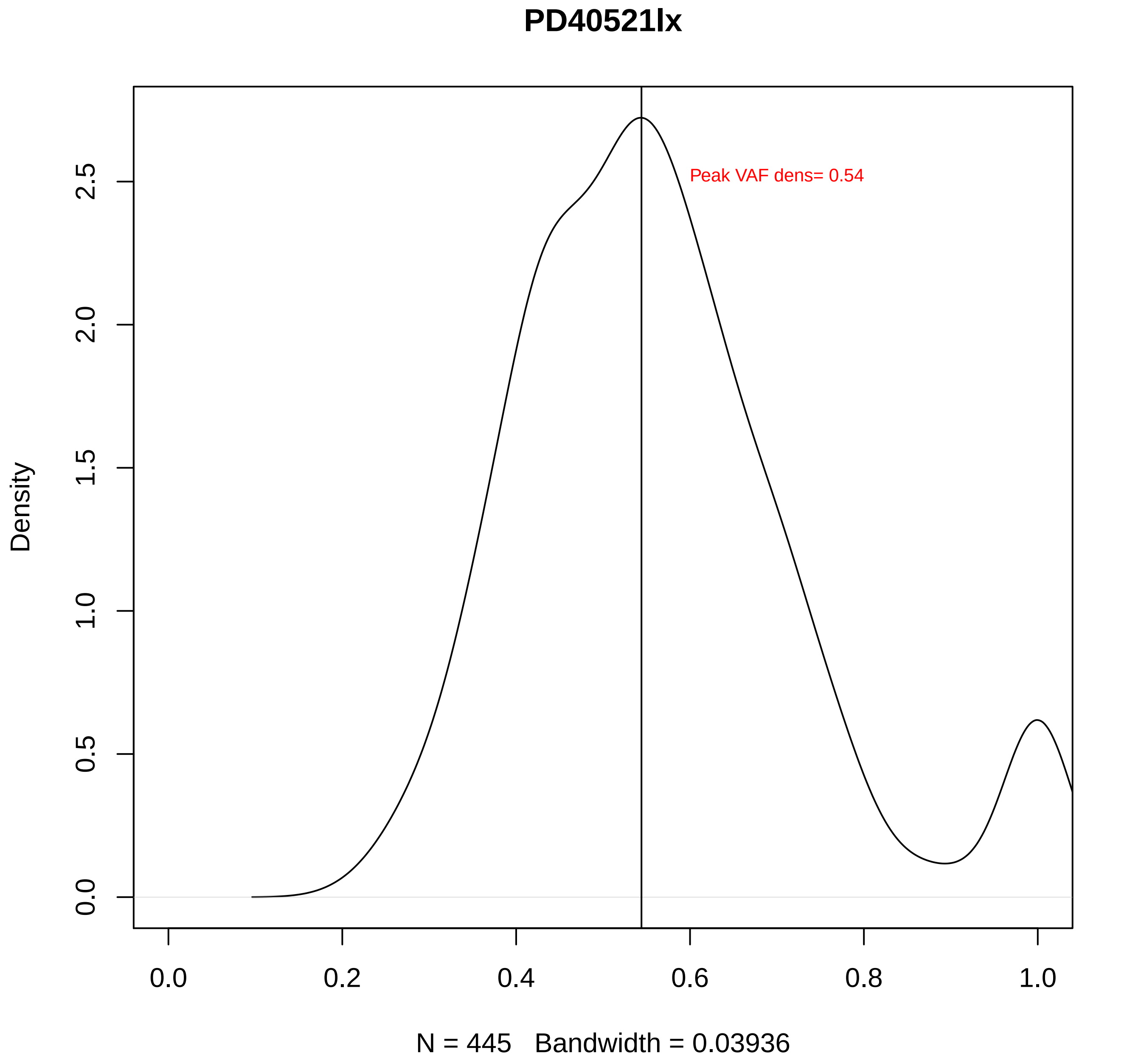
PD40521lx
Peak VAF dens= 0.54
Density
0.0
0.5
1.0
1.5
2.0
2.5
0.0
0.2
0.4
0.6
0.8
1.0
N = 445   Bandwidth = 0.03936

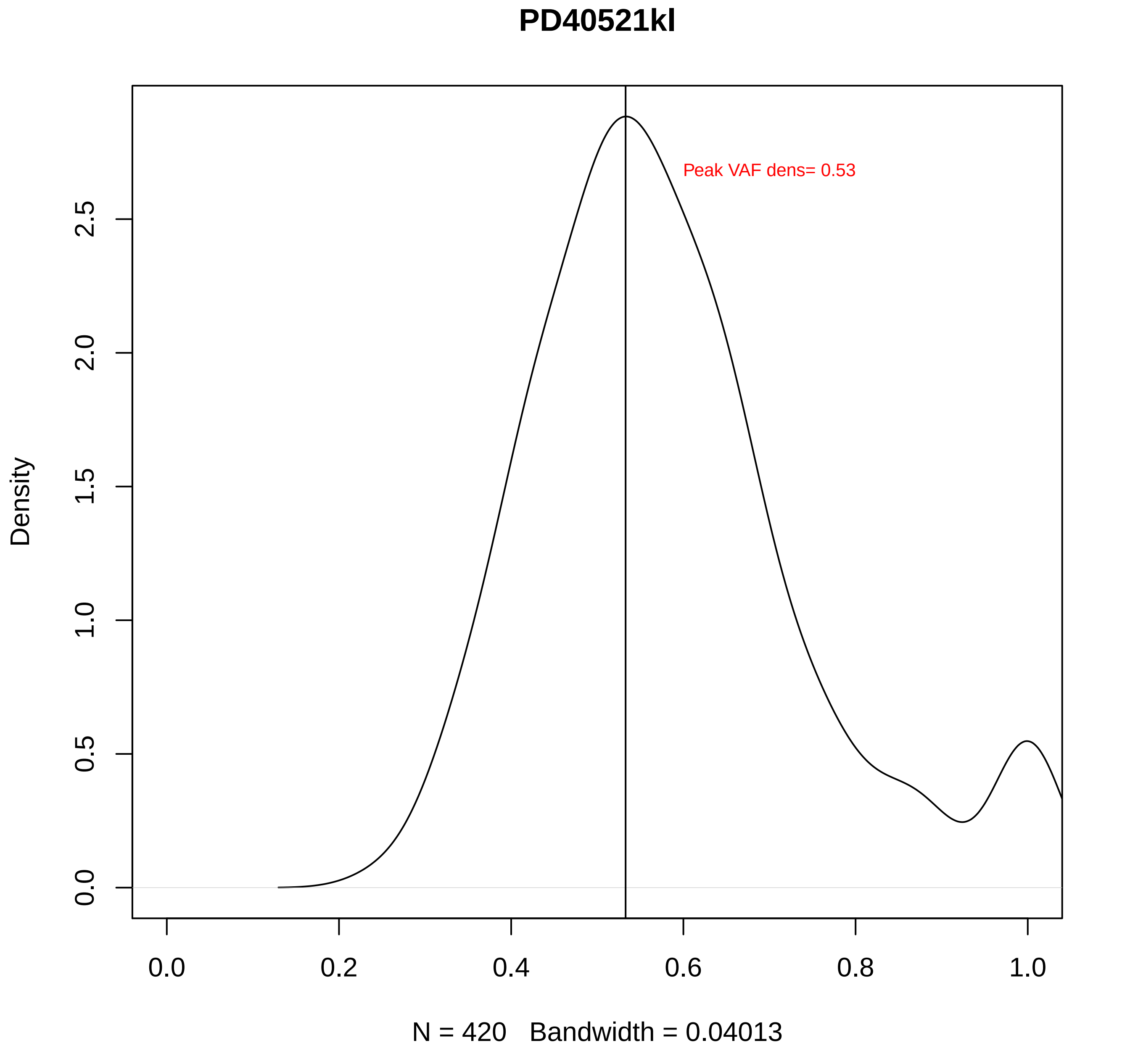
PD40521kl
Peak VAF dens= 0.53
Density
0.0
0.5
1.0
1.5
2.0
2.5
0.0
0.2
0.4
0.6
0.8
1.0
N = 420 Bandwidth = 0.04013

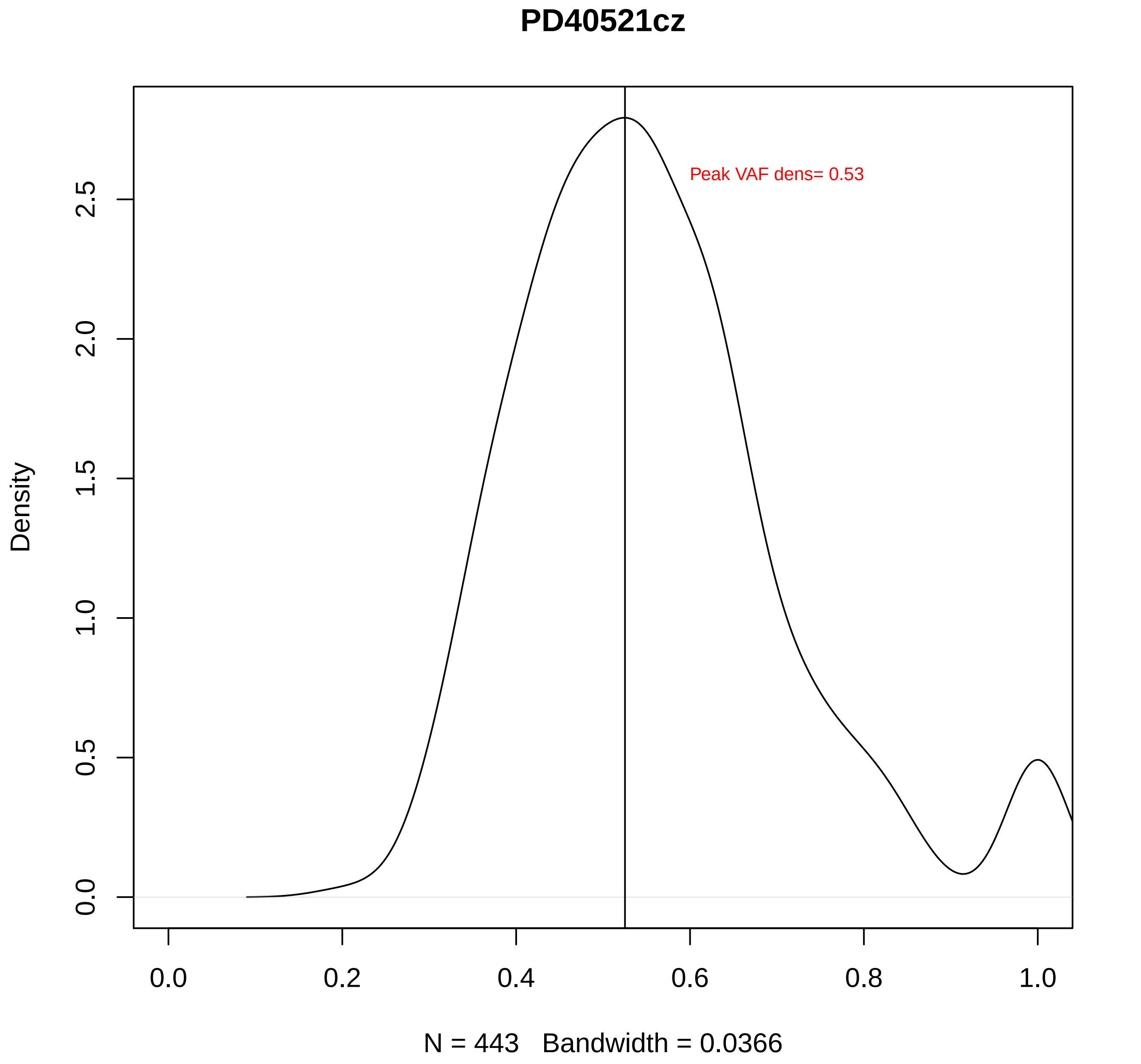

PD40521cz
Peak VAF dens= 0.53
Density
0.0
0.5
1.0
1.5
2.0
2.5
0.0
0.2
0.4
0.6
0.8
1.0
N = 443   Bandwidth = 0.0366

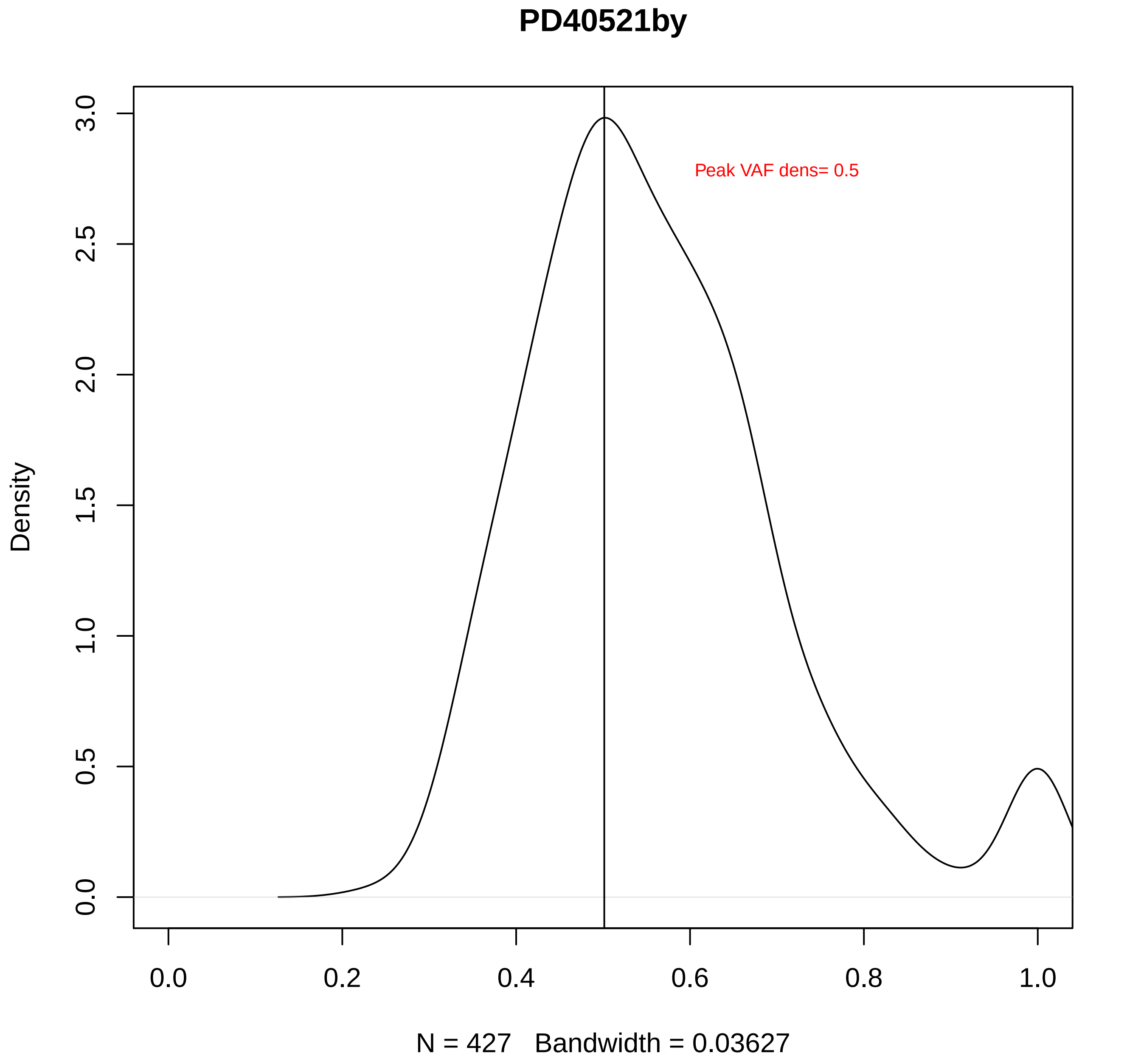
PD40521by
Peak VAF dens= 0.5
Density
0.0
0.5
1.0
1.5
2.0
2.5
3.0
0.0
0.2
0.4
0.6
0.8
1.0
N = 427   Bandwidth = 0.03627

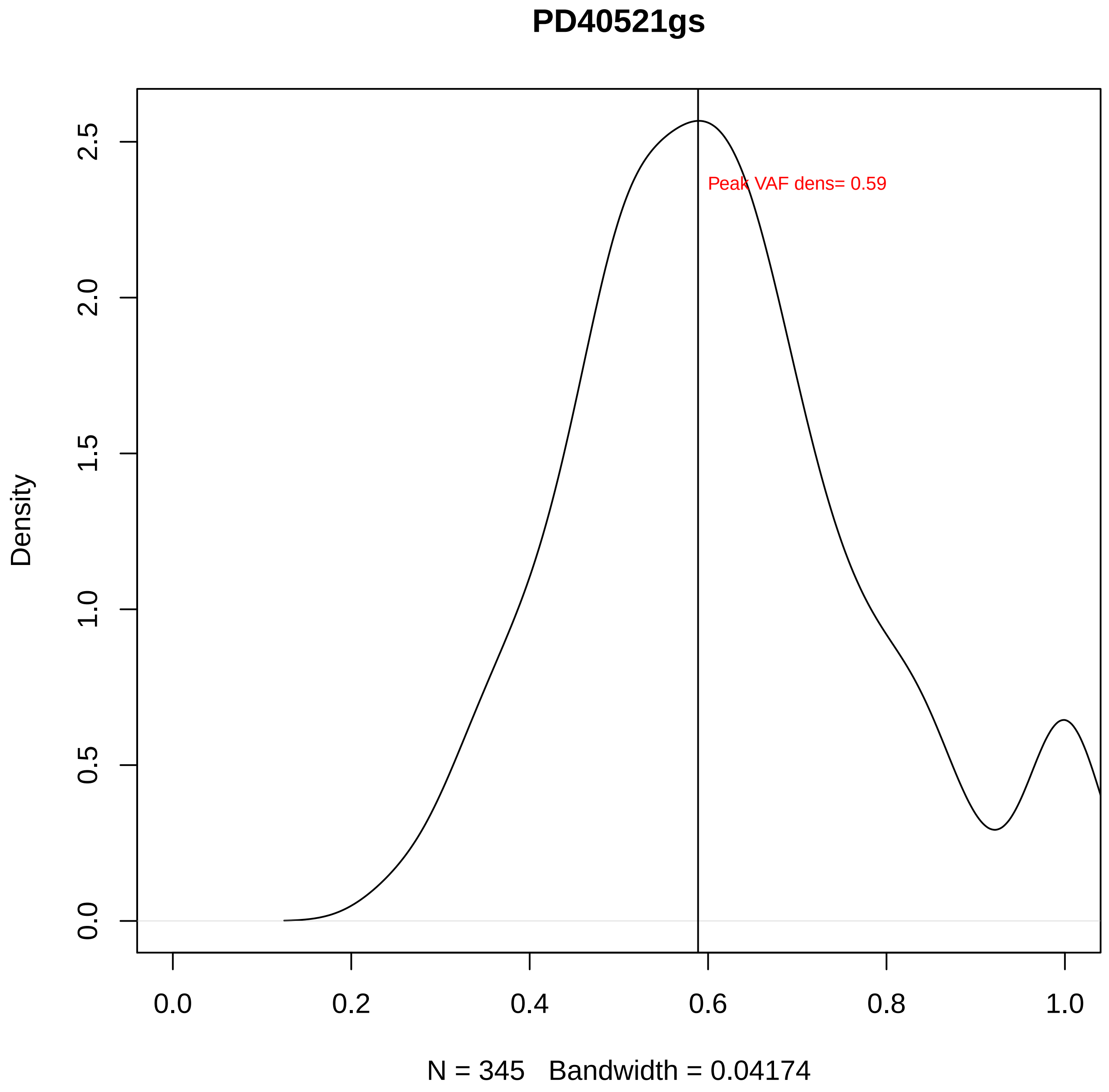
PD40521gs
Peak VAF dens= 0.59
Density
0.0
0.5
1.0
1.5
2.0
2.5
0.0
0.2
0.4
0.6
0.8
1.0
N = 345 Bandwidth = 0.04174

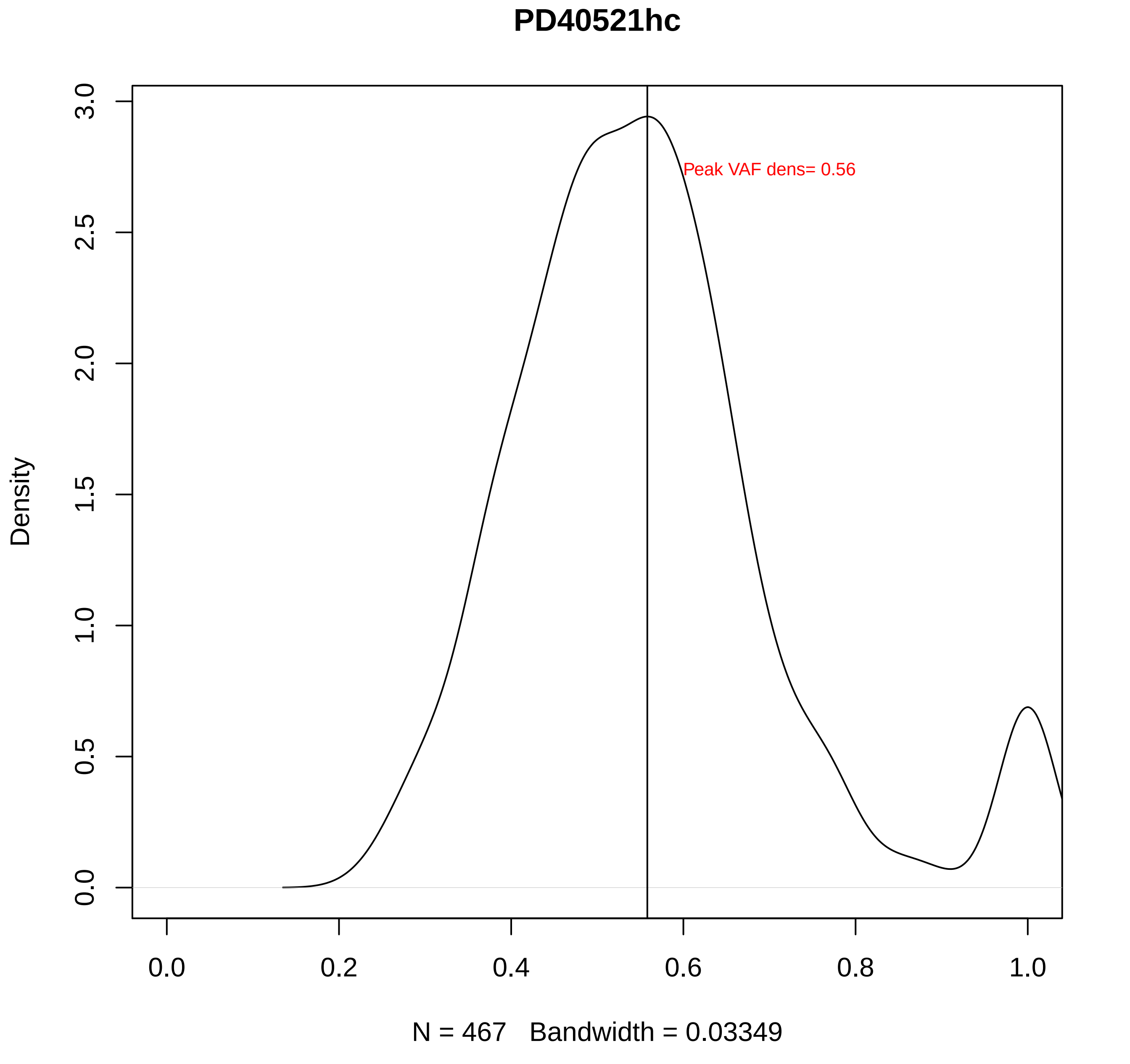

PD40521hc
Peak VAF dens= 0.56
Density
0.0
0.5
1.0
1.5
2.0
2.5
3.0
0.0
0.2
0.4
0.6
0.8
1.0
N = 467 Bandwidth = 0.03349

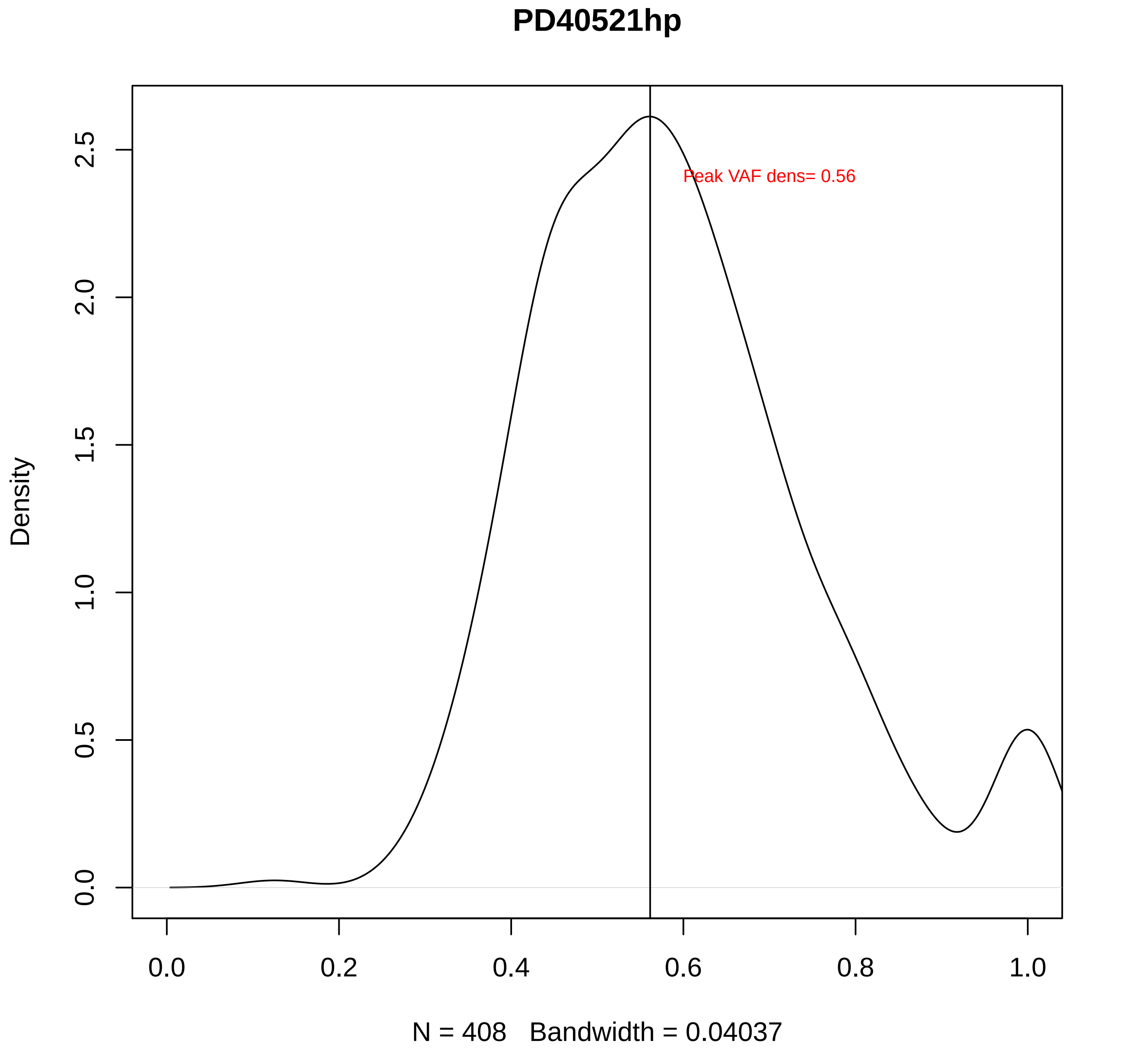
PD40521hp
Peak VAF dens= 0.56
Density
0.0
0.5
1.0
1.5
2.0
2.5
0.0
0.2
0.4
0.6
0.8
1.0
N = 408   Bandwidth = 0.04037

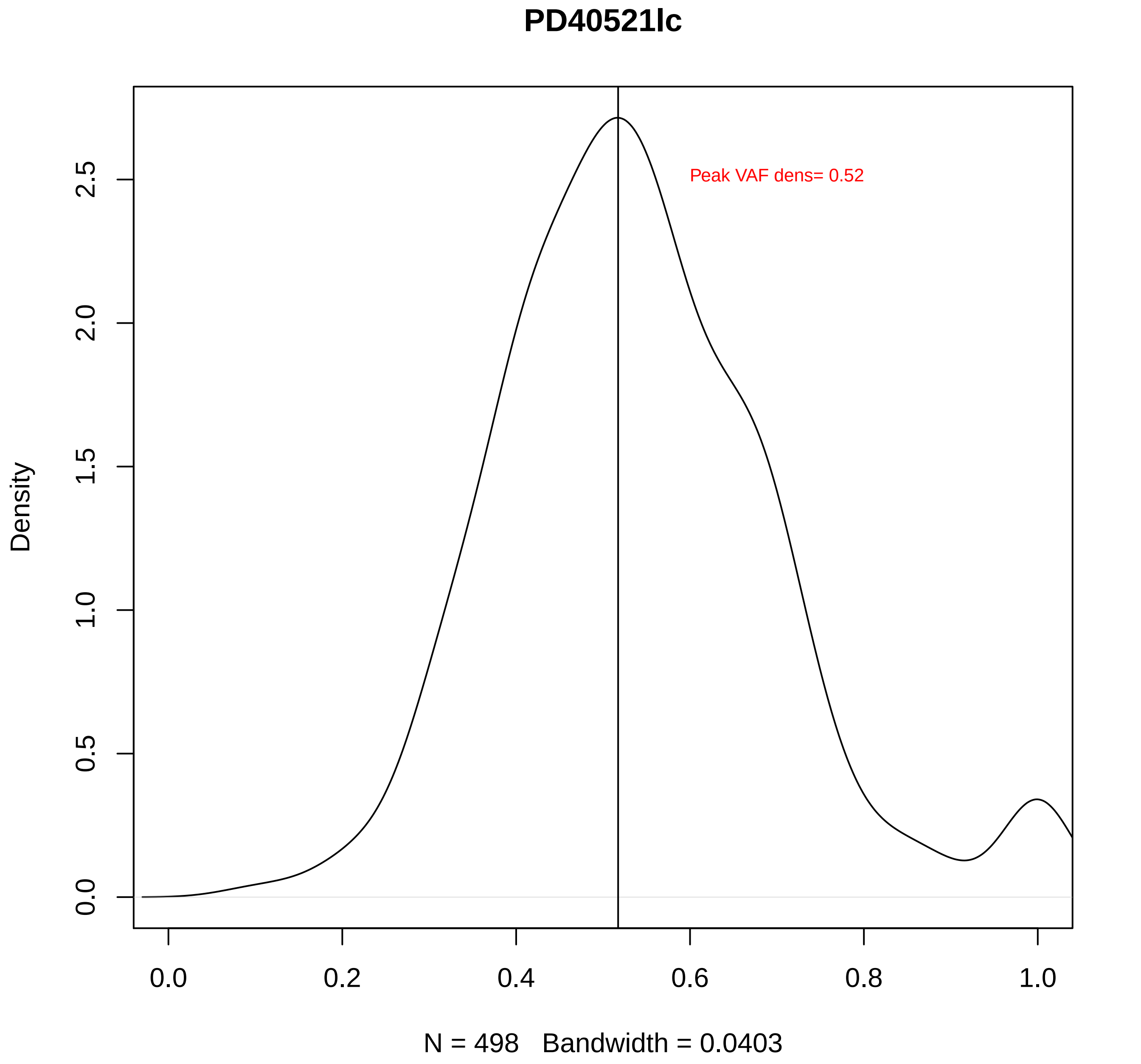
PD40521lc
Peak VAF dens= 0.52
Density
0.0
0.5
1.0
1.5
2.0
2.5
0.0
0.2
0.4
0.6
0.8
1.0
N = 498   Bandwidth = 0.0403

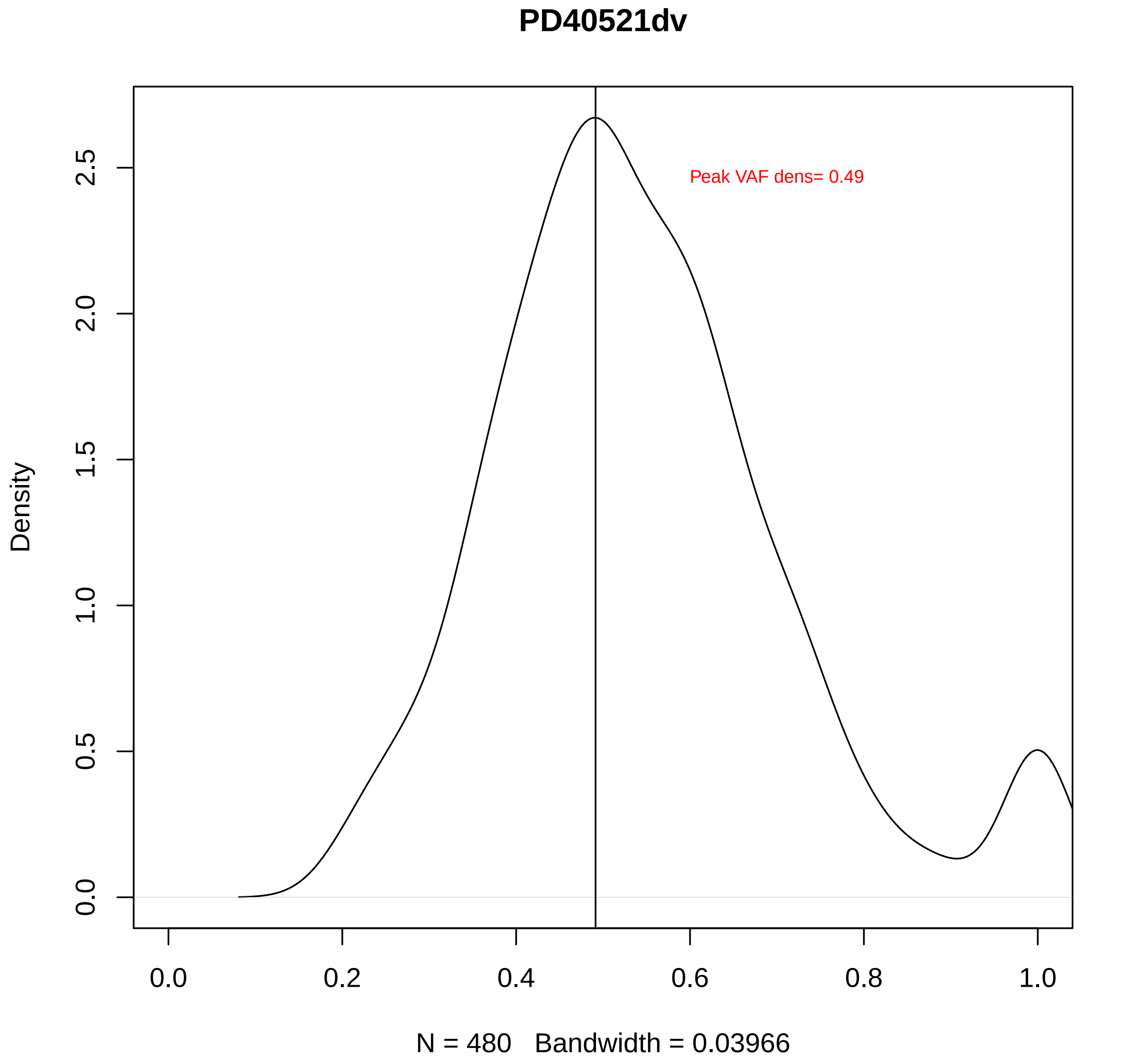

PD40521dv
Peak VAF dens= 0.49
Density
0.0
0.5
1.0
1.5
2.0
2.5
0.0
0.2
0.4
0.6
0.8
1.0
N = 480   Bandwidth = 0.03966

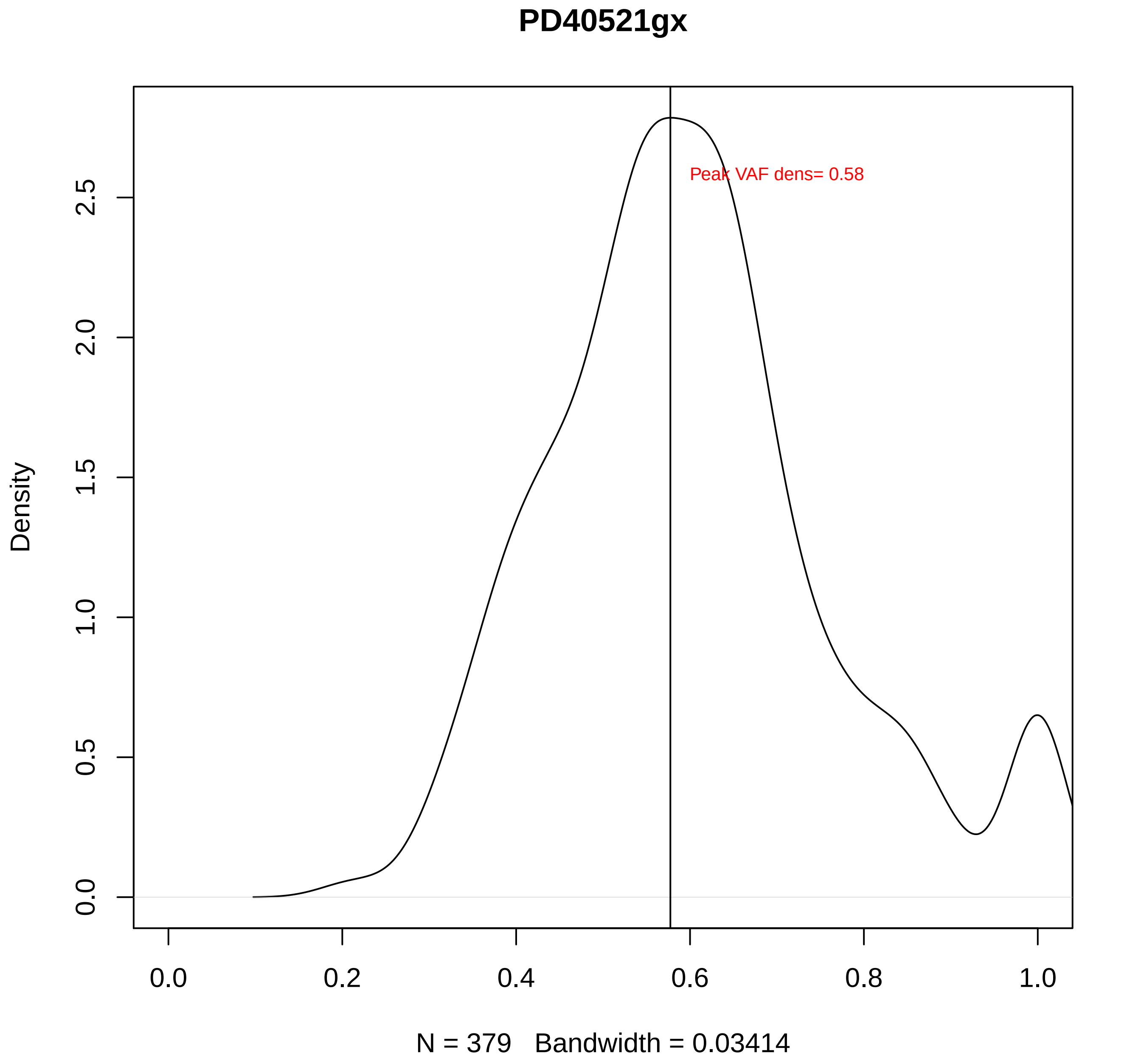
PD40521gx
Peak VAF dens= 0.58
Density
0.0
0.5
1.0
1.5
2.0
2.5
0.0
0.2
0.4
0.6
0.8
1.0
N = 379   Bandwidth = 0.03414

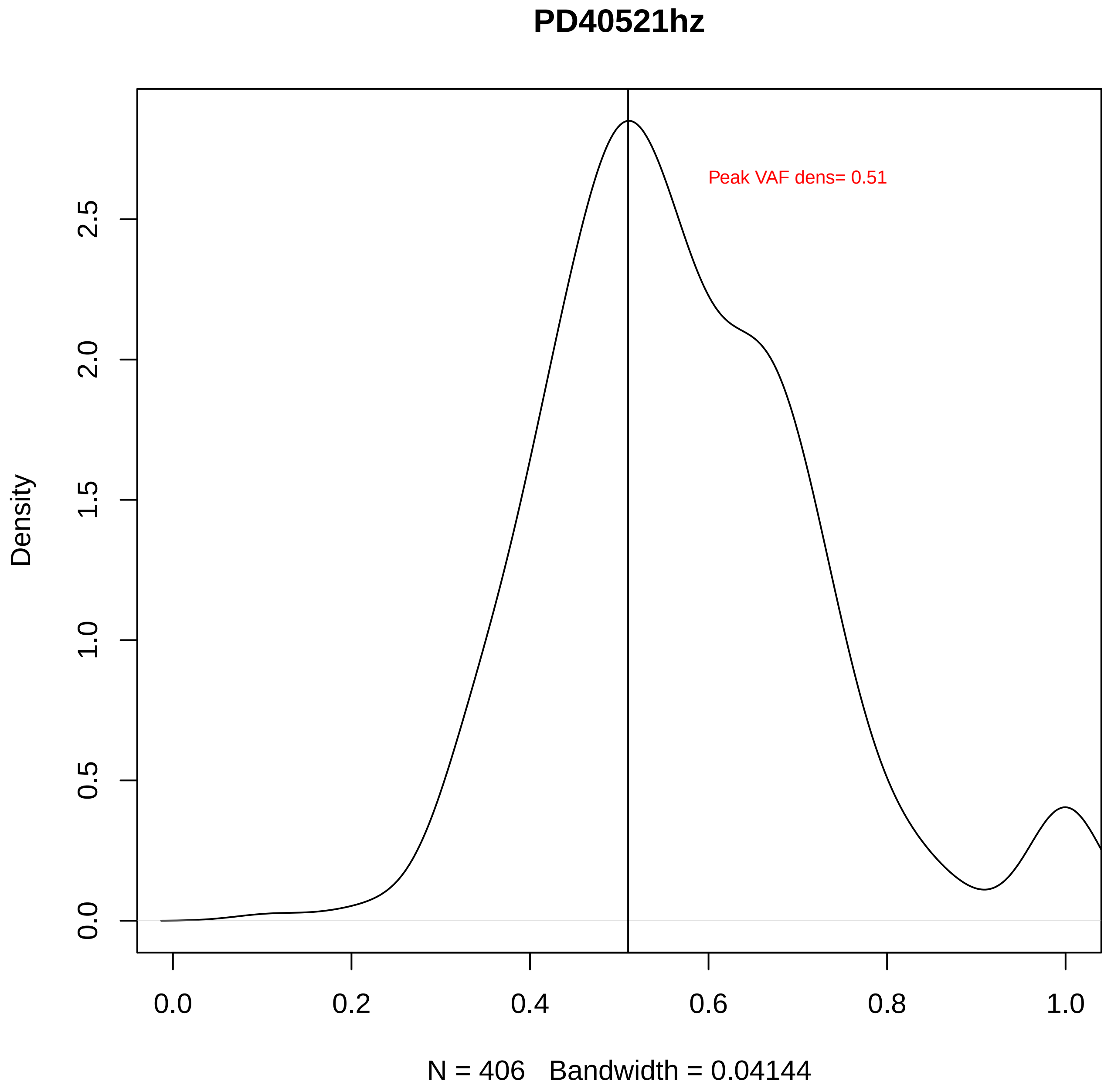

PD40521hz
Peak VAF dens= 0.51
Density
0.0
0.5
1.0
1.5
2.0
2.5
0.0
0.2
0.4
0.6
0.8
1.0
N = 406   Bandwidth = 0.04144

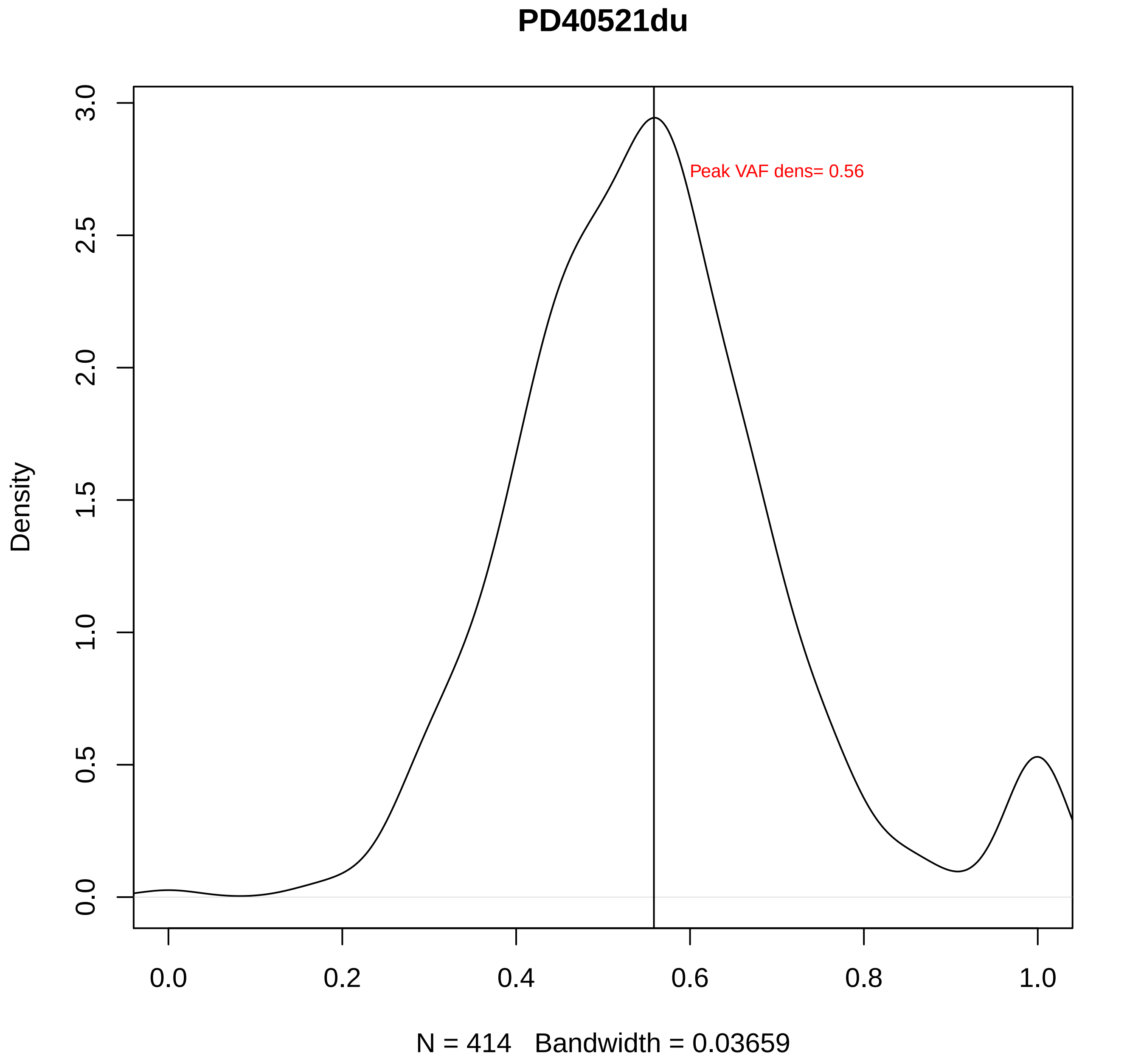

PD40521du
Peak VAF dens= 0.56
Density
0.0
0.5
1.0
1.5
2.0
2.5
3.0
0.0
0.2
0.4
0.6
0.8
1.0
N = 414   Bandwidth = 0.03659

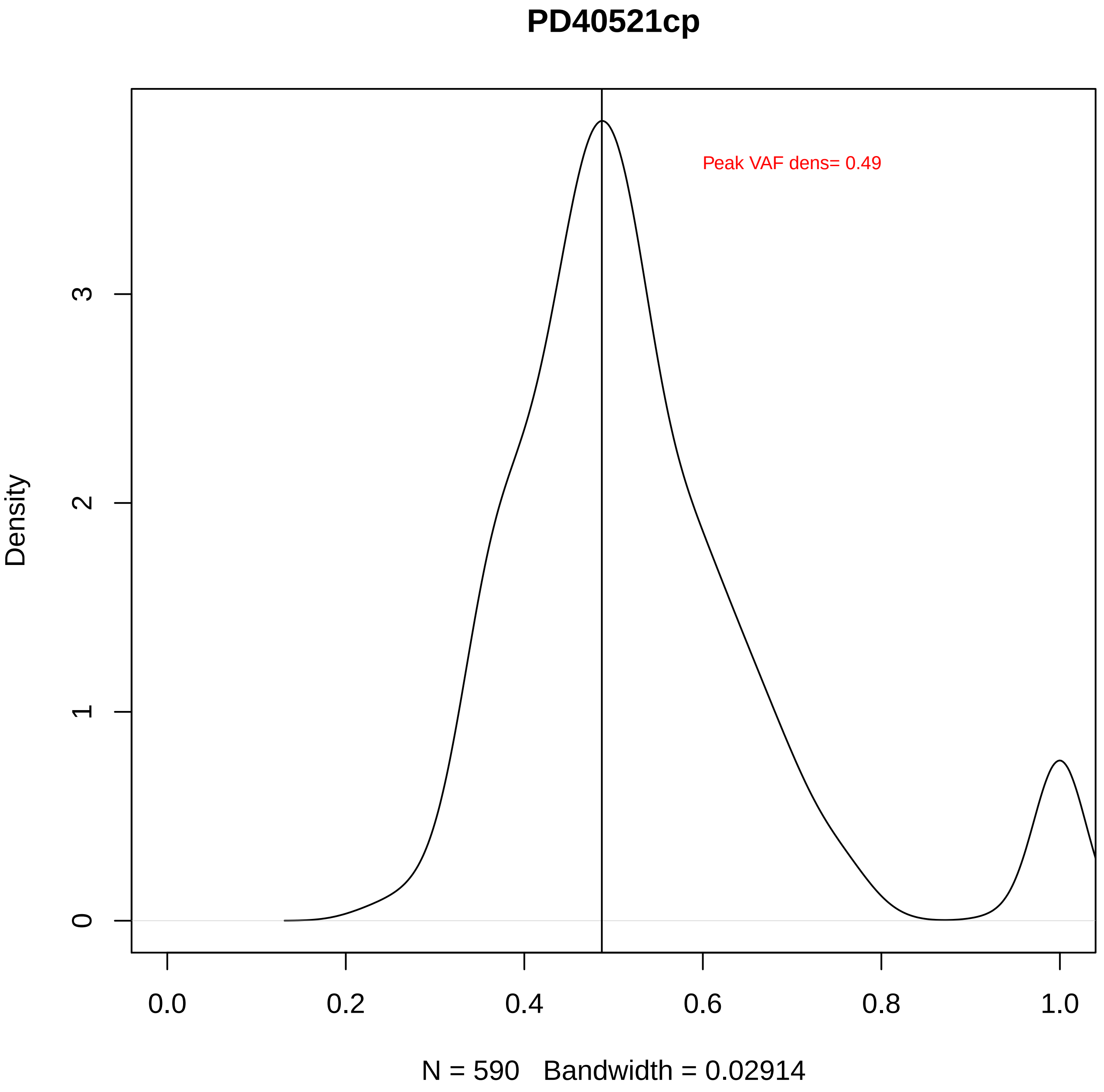

PD40521cp
Peak VAF dens= 0.49
Density
0
1
2
3
0.0
0.2
0.4
0.6
0.8
1.0
N = 590   Bandwidth = 0.02914

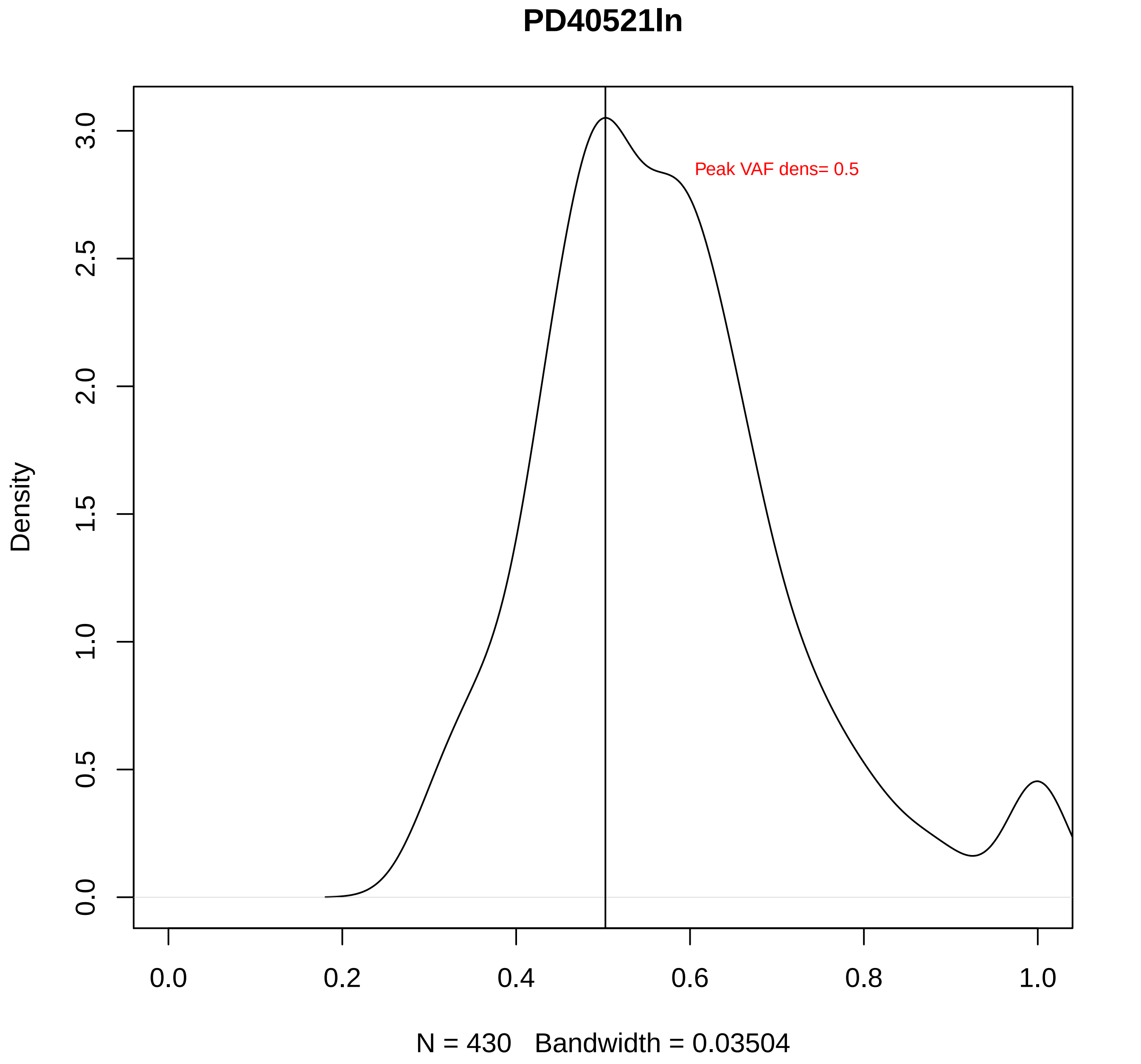
PD40521ln
Peak VAF dens= 0.5
Density
0.0
0.5
1.0
1.5
2.0
2.5
3.0
0.0
0.2
0.4
0.6
0.8
1.0
N = 430   Bandwidth = 0.03504

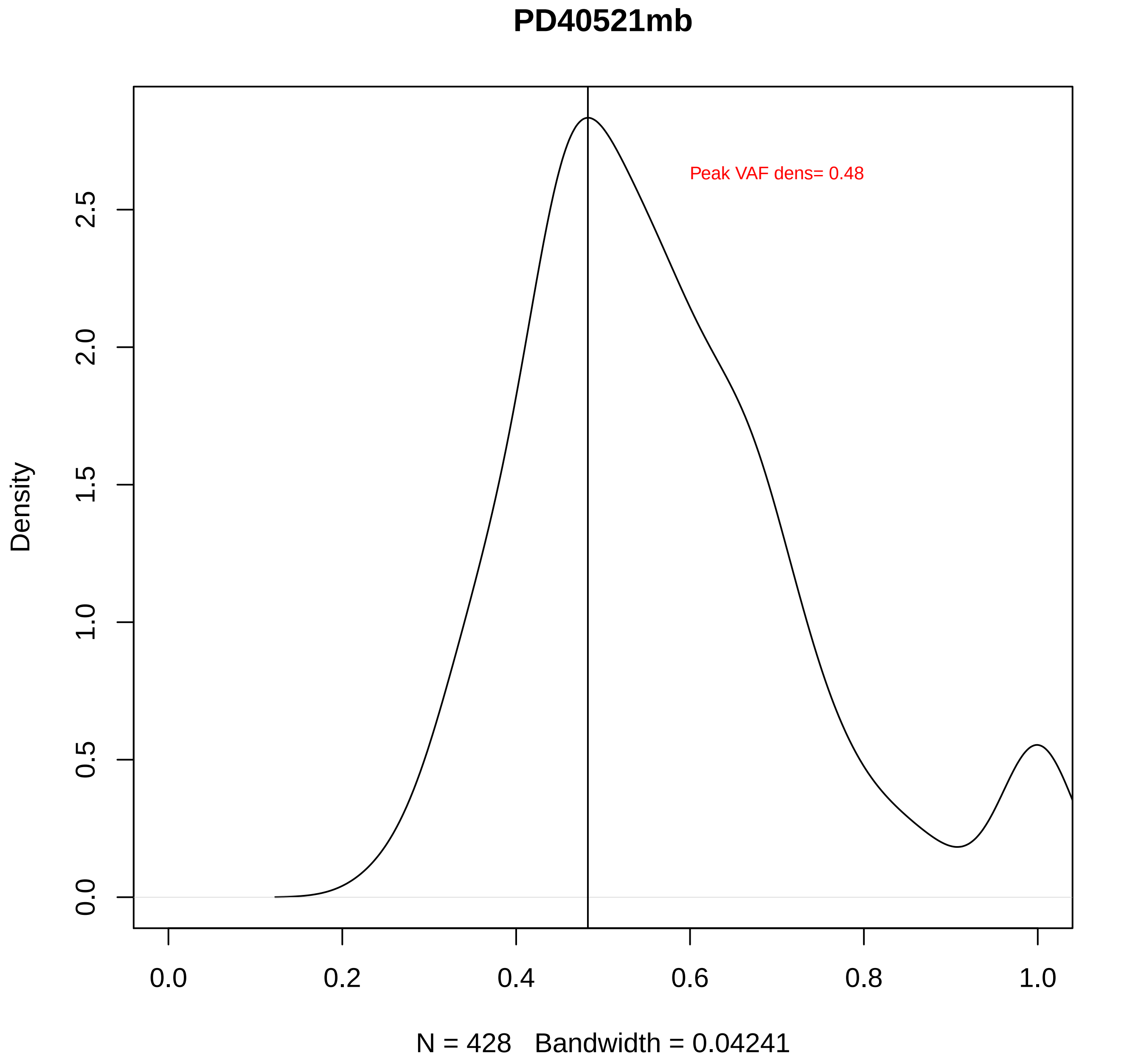
PD40521mb
Peak VAF dens= 0.48
Density
0.0
0.5
1.0
1.5
2.0
2.5
0.0
0.2
0.4
0.6
0.8
1.0
N = 428   Bandwidth = 0.04241

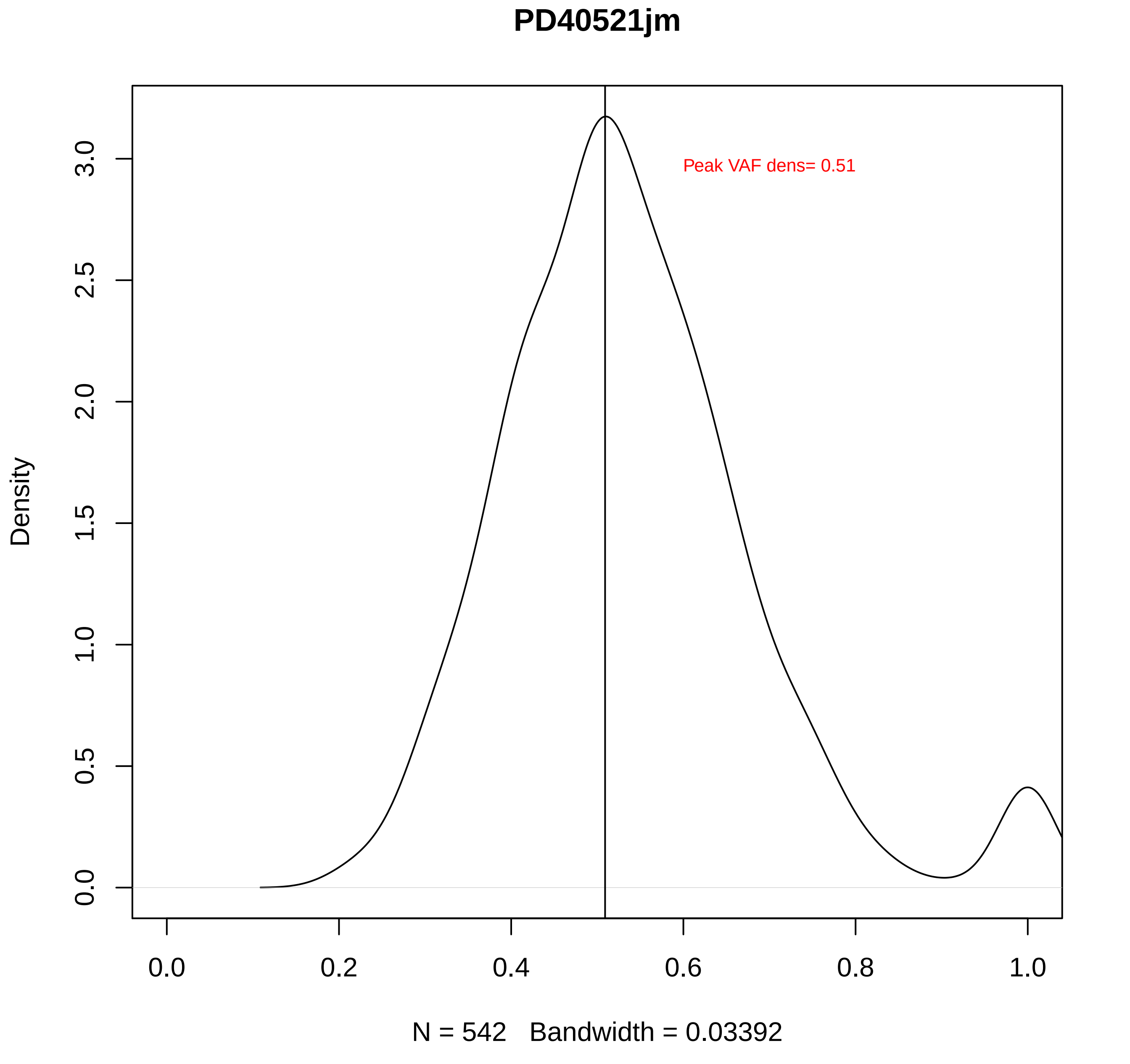
PD40521jm
Peak VAF dens= 0.51
Density
0.0
0.5
1.0
1.5
2.0
2.5
3.0
0.0
0.2
0.4
0.6
0.8
1.0
N = 542 Bandwidth = 0.03392

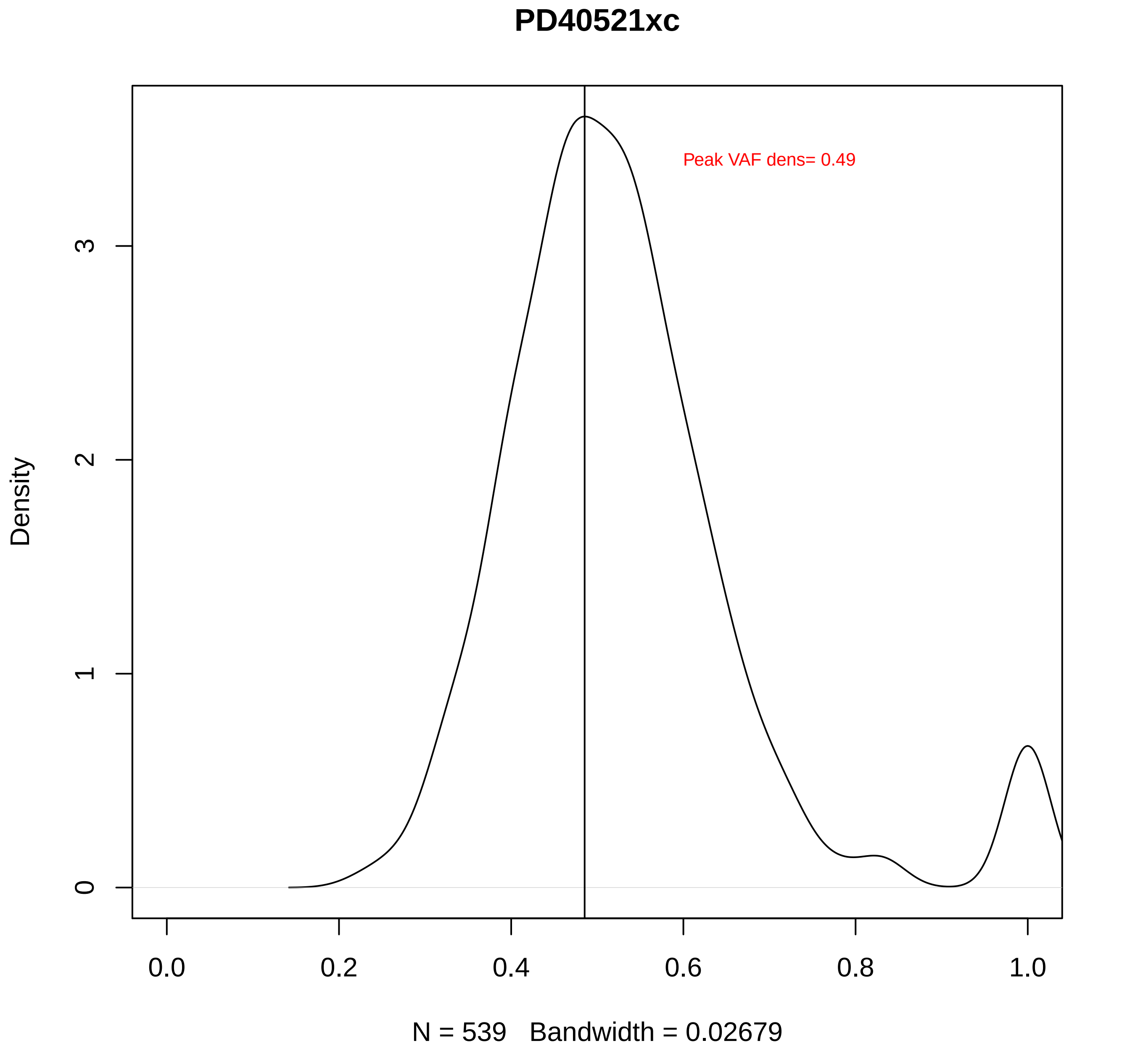

PD40521xc
Peak VAF dens= 0.49
Density
0
1
2
3
0.0
0.2
0.4
0.6
0.8
1.0
N = 539   Bandwidth = 0.02679

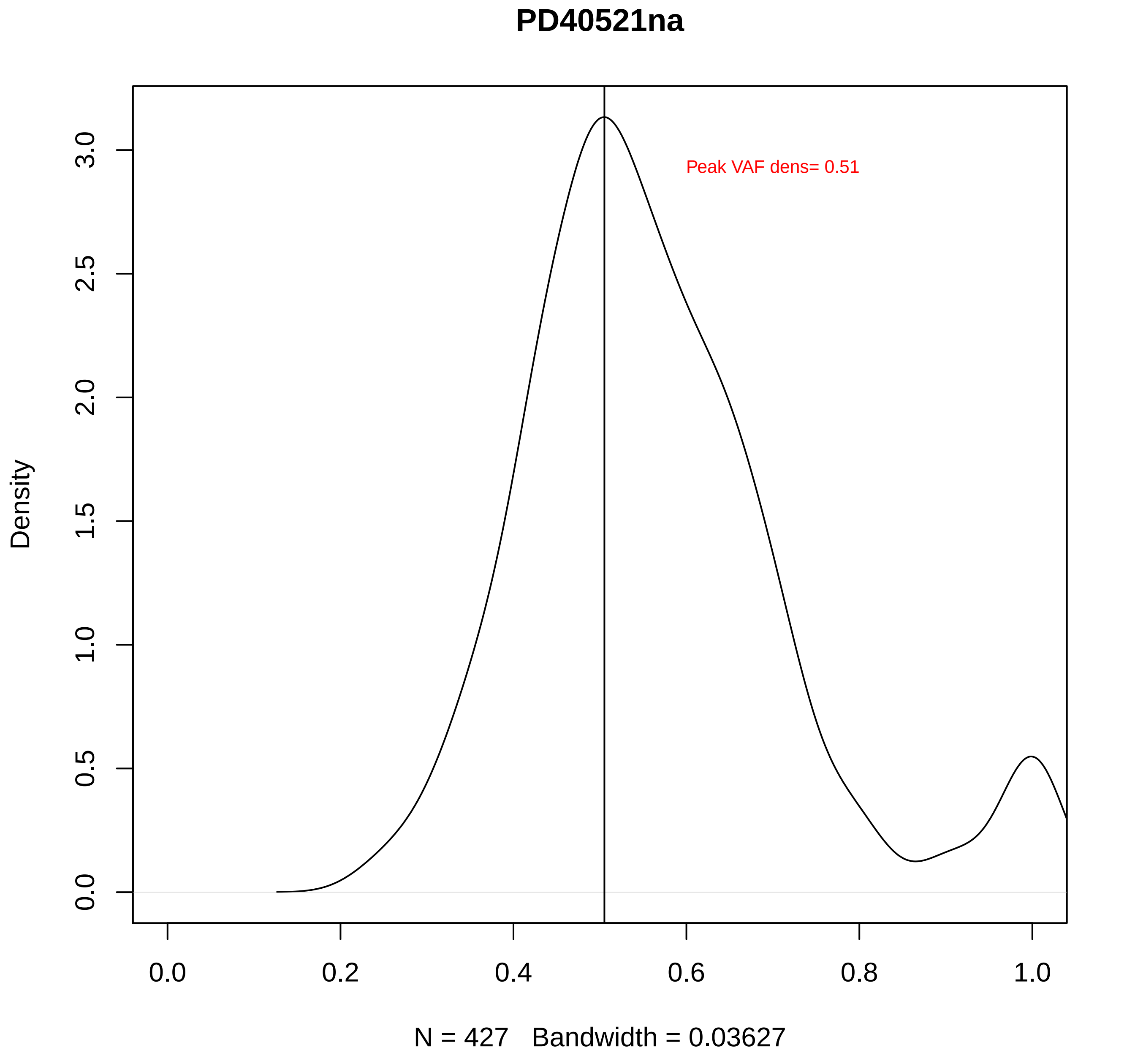

PD40521na
Peak VAF dens= 0.51
Density
0.0
0.5
1.0
1.5
2.0
2.5
3.0
0.0
0.2
0.4
0.6
0.8
1.0
N = 427 Bandwidth = 0.03627

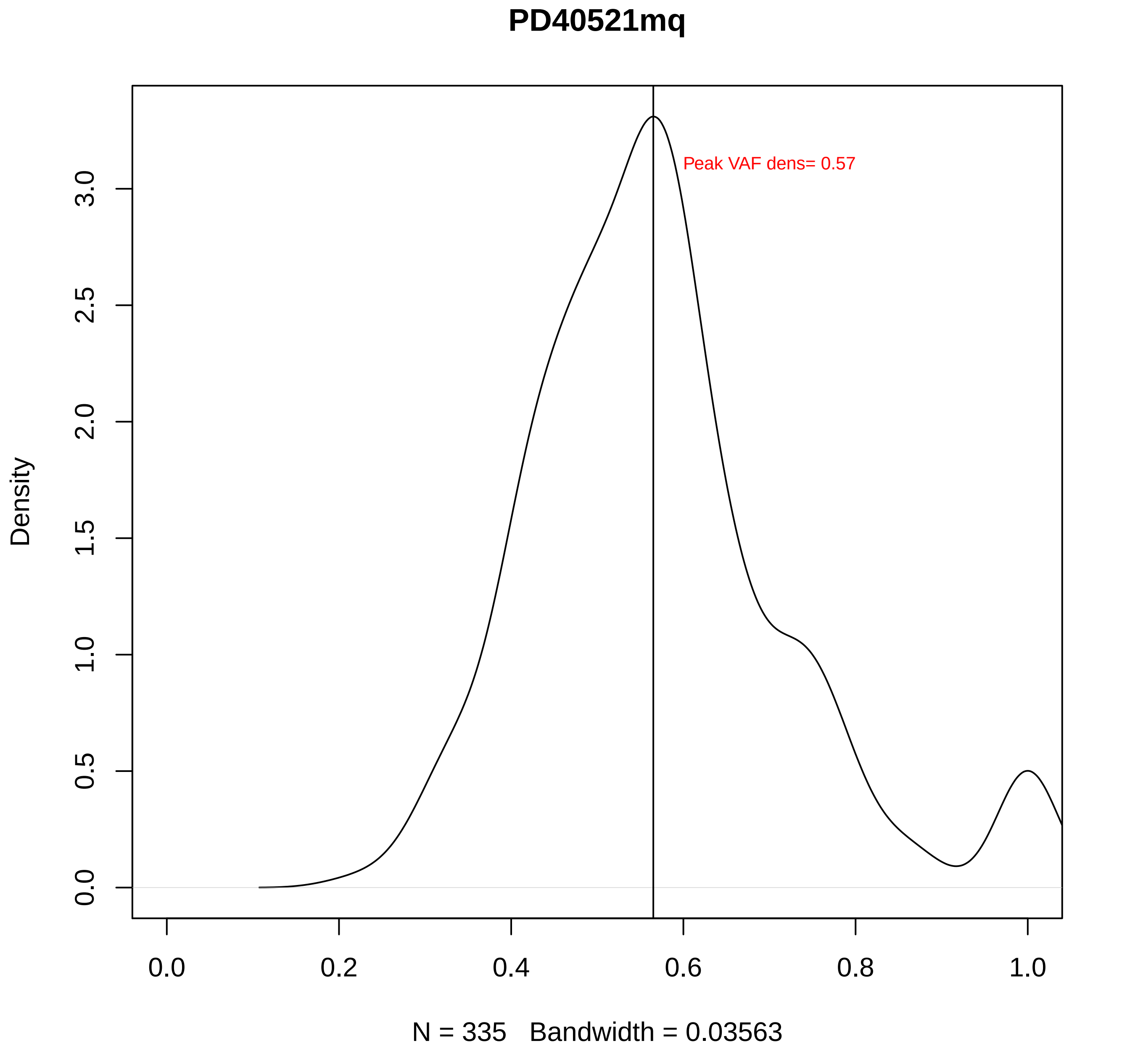
PD40521mq
Peak VAF dens= 0.57
Density
0.0
0.5
1.0
1.5
2.0
2.5
3.0
0.0
0.2
0.4
0.6
0.8
1.0
N = 335   Bandwidth = 0.03563

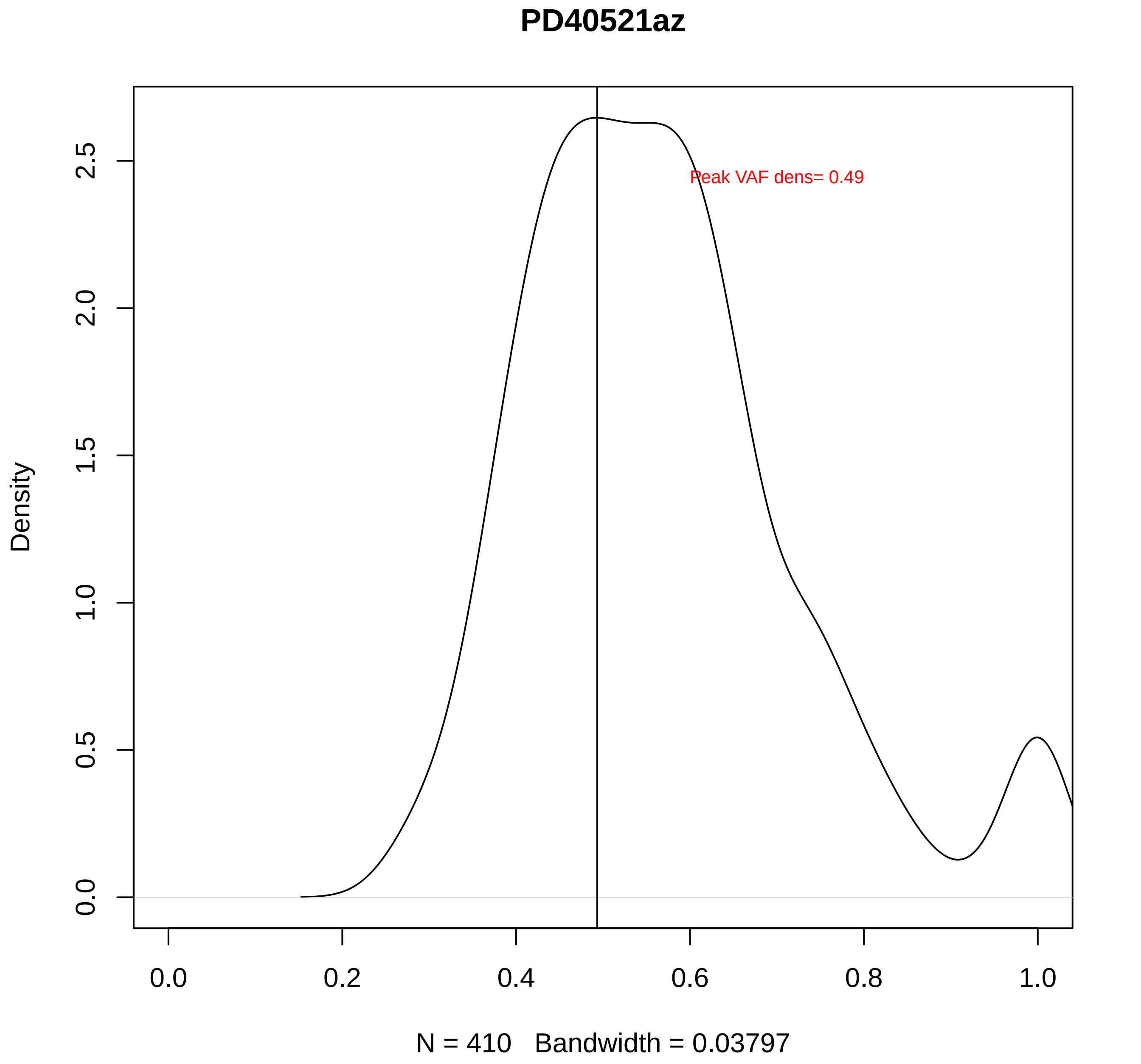
PD40521az
Peak VAF dens= 0.49
Density
0.0
0.5
1.0
1.5
2.0
2.5
0.0
0.2
0.4
0.6
0.8
1.0
N = 410   Bandwidth = 0.03797

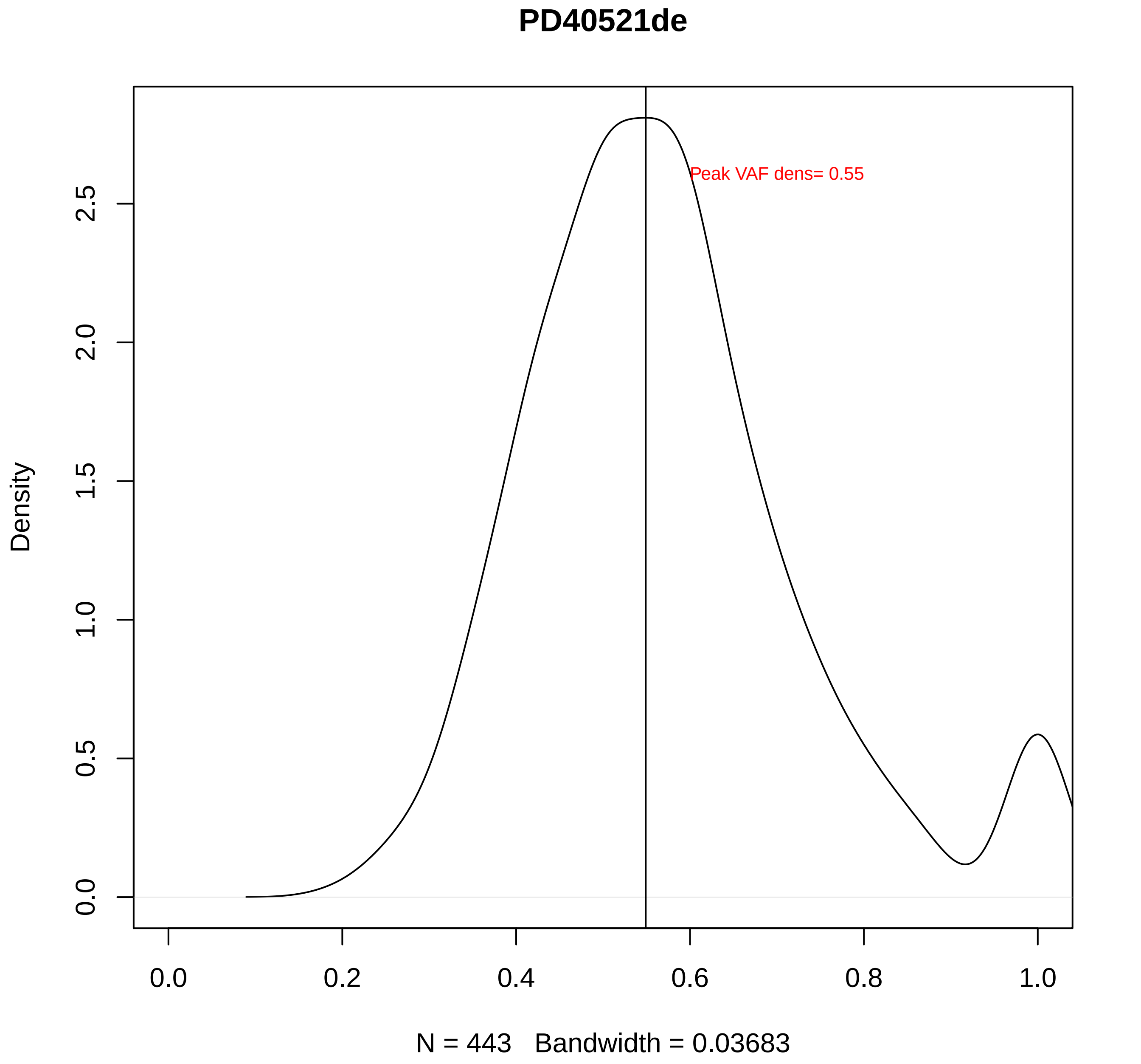

PD40521de
Peak VAF dens= 0.55
Density
0.0
0.5
1.0
1.5
2.0
2.5
0.0
0.2
0.4
0.6
0.8
1.0
N = 443   Bandwidth = 0.03683

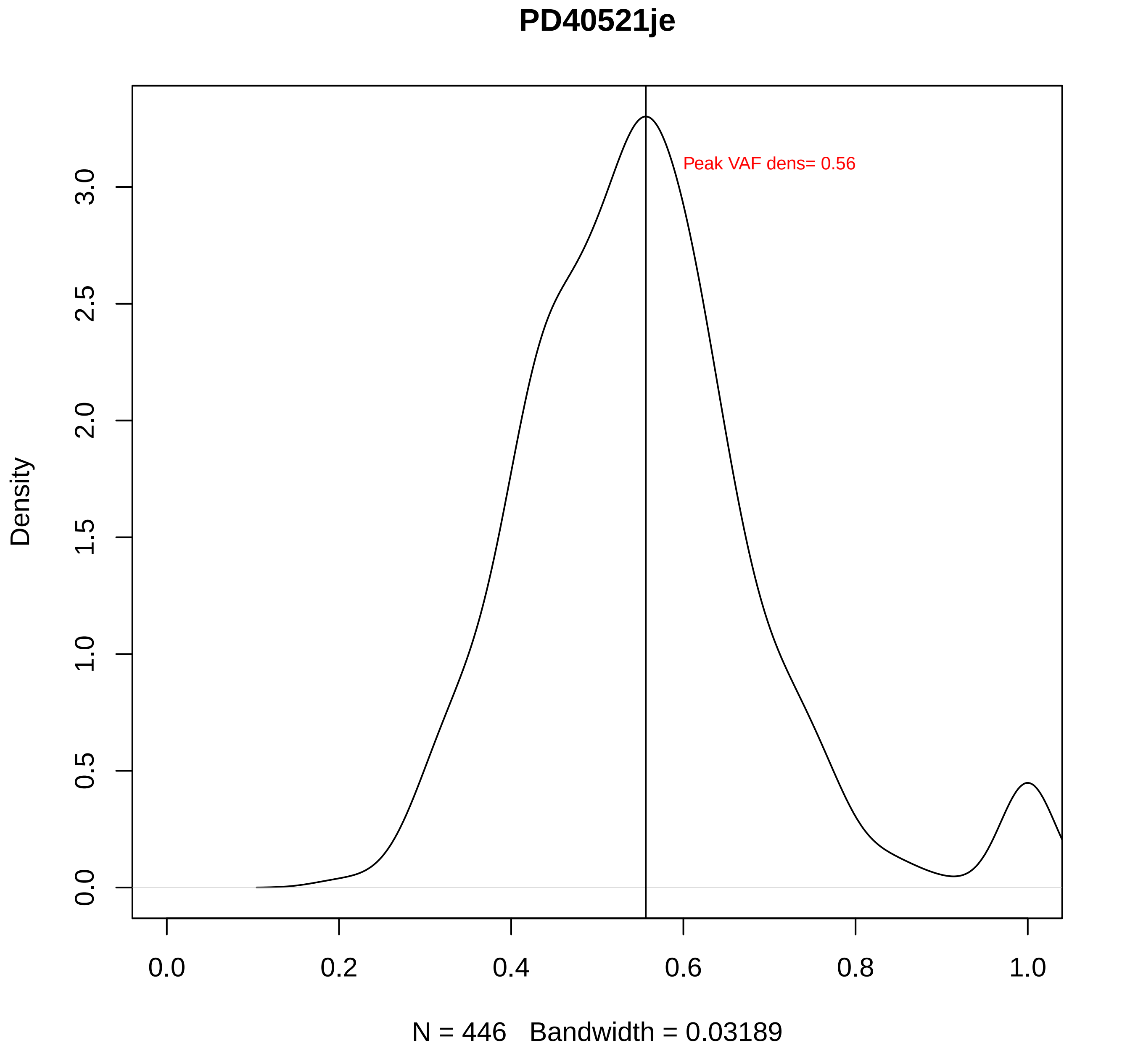
PD40521je
Peak VAF dens= 0.56
Density
0.0
0.5
1.0
1.5
2.0
2.5
3.0
0.0
0.2
0.4
0.6
0.8
1.0
N = 446   Bandwidth = 0.03189

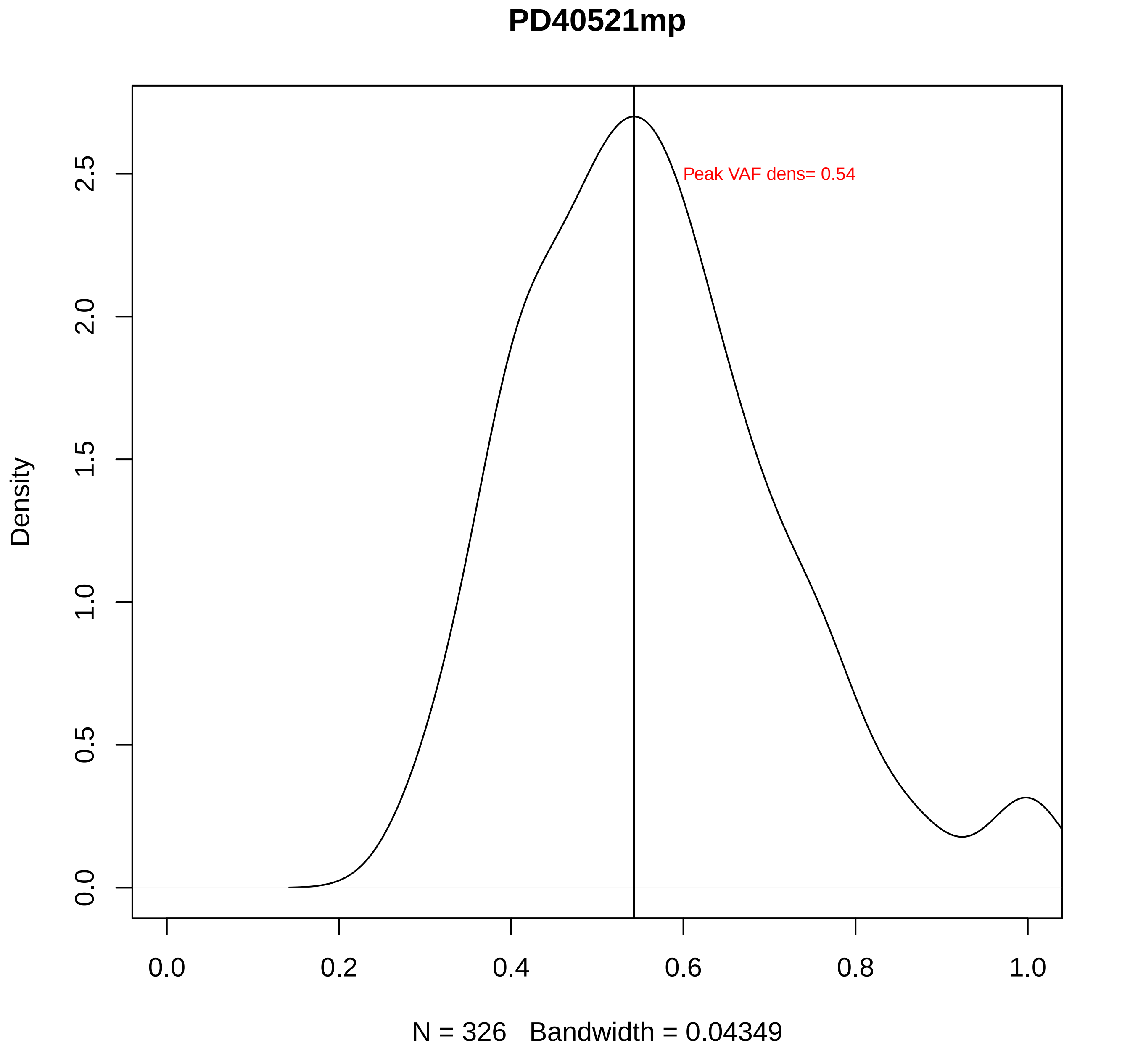
PD40521mp
Peak VAF dens= 0.54
Density
0.0
0.5
1.0
1.5
2.0
2.5
0.0
0.2
0.4
0.6
0.8
1.0
N = 326   Bandwidth = 0.04349

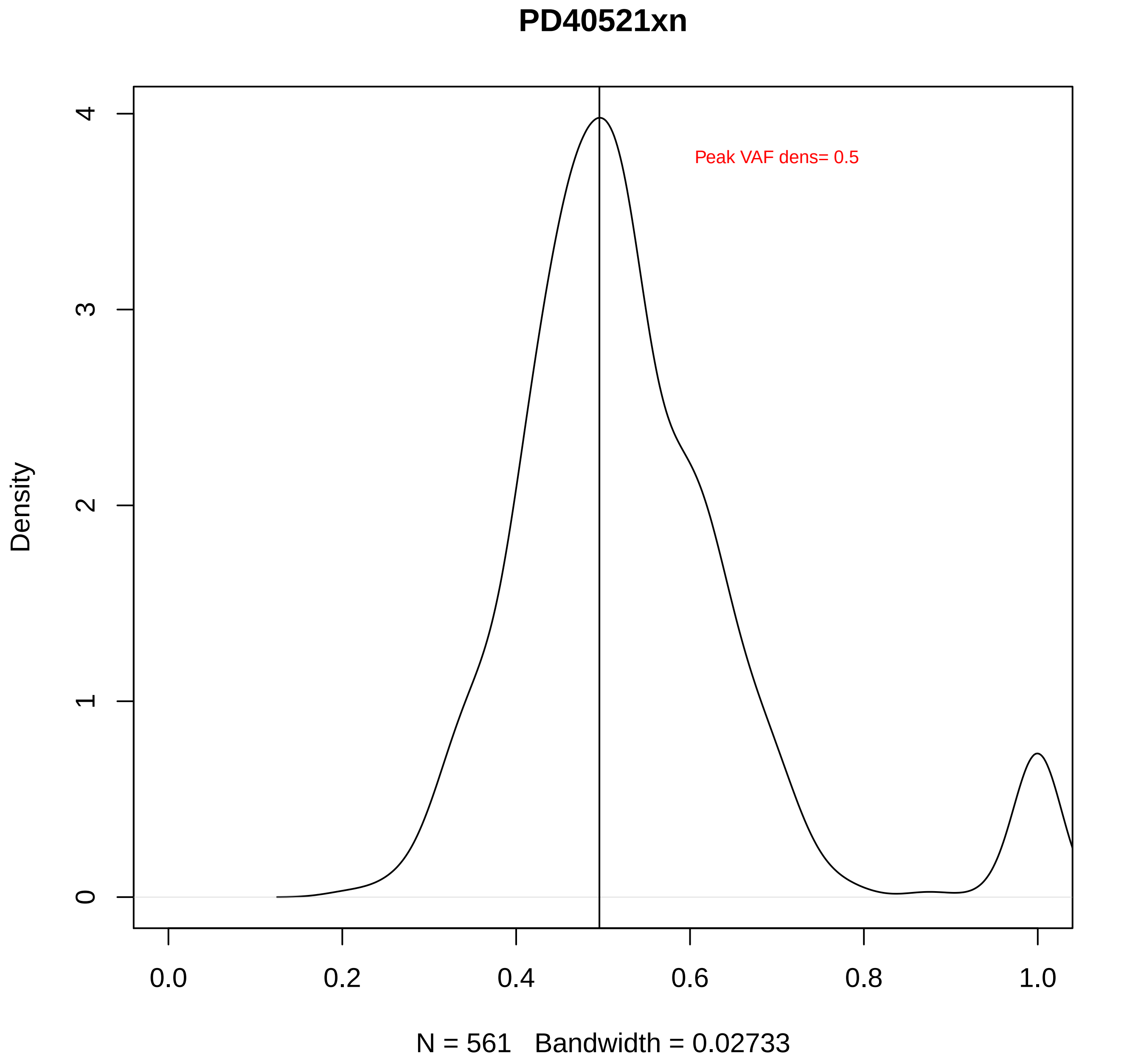
PD40521xn
Peak VAF dens= 0.5
Density
0
1
2
3
4
0.0
0.2
0.4
0.6
0.8
1.0
N = 561   Bandwidth = 0.02733

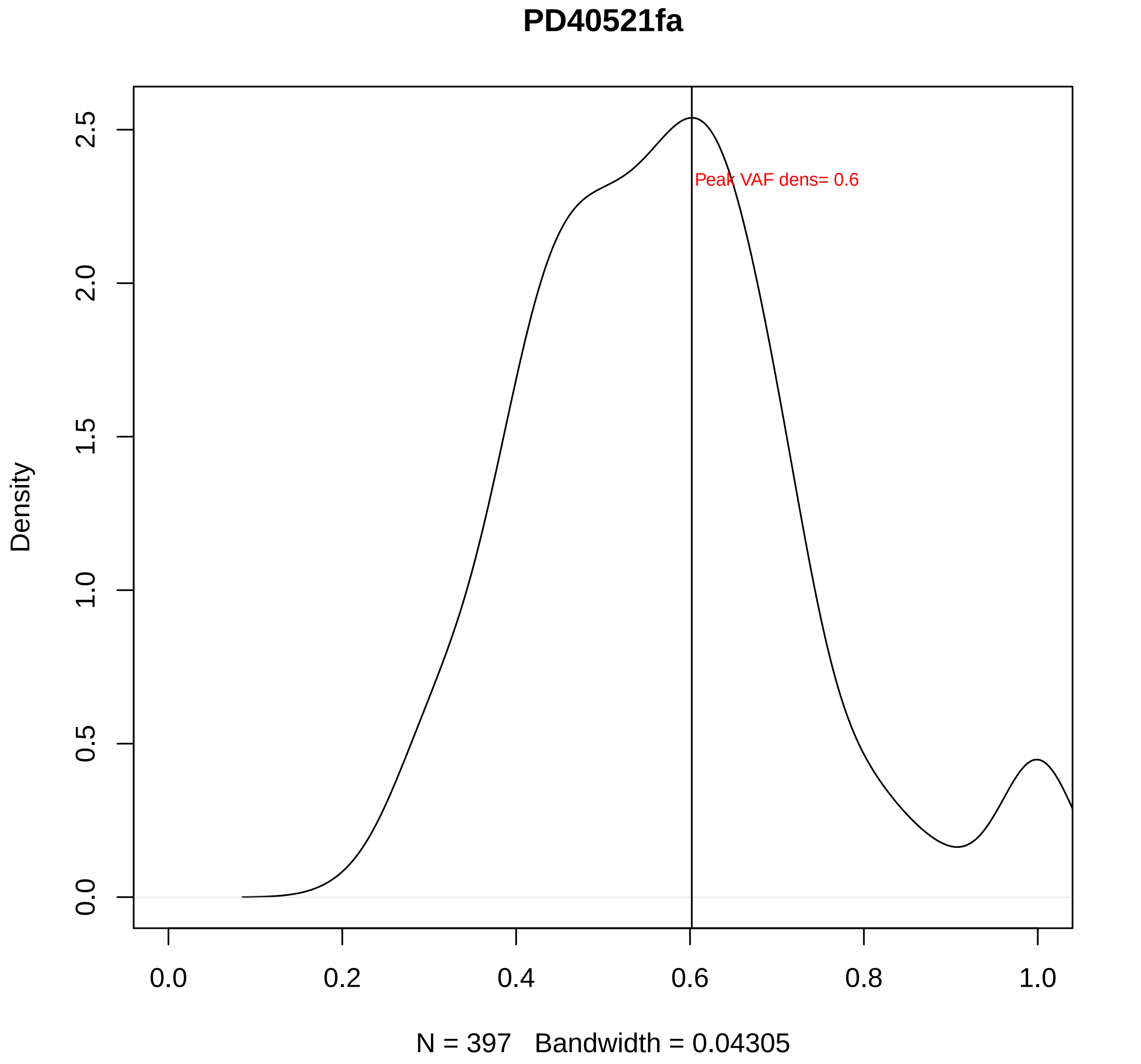
PD40521fa
Peak VAF dens= 0.6
Density
0.0
0.5
1.0
1.5
2.0
2.5
0.0
0.2
0.4
0.6
0.8
1.0
N = 397   Bandwidth = 0.04305

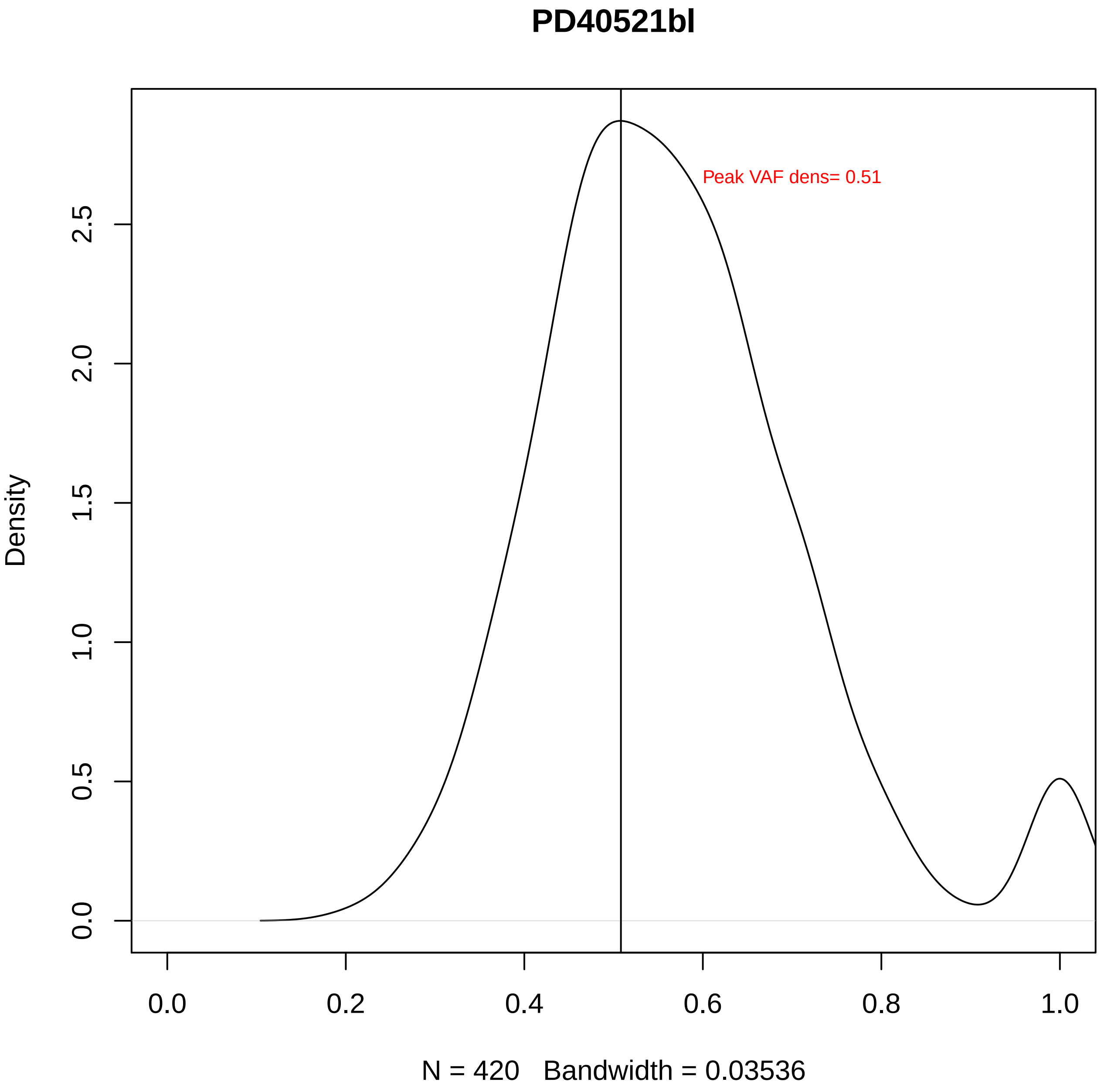

PD40521bl
Peak VAF dens= 0.51
Density
0.0
0.5
1.0
1.5
2.0
2.5
0.0
0.2
0.4
0.6
0.8
1.0
N = 420   Bandwidth = 0.03536

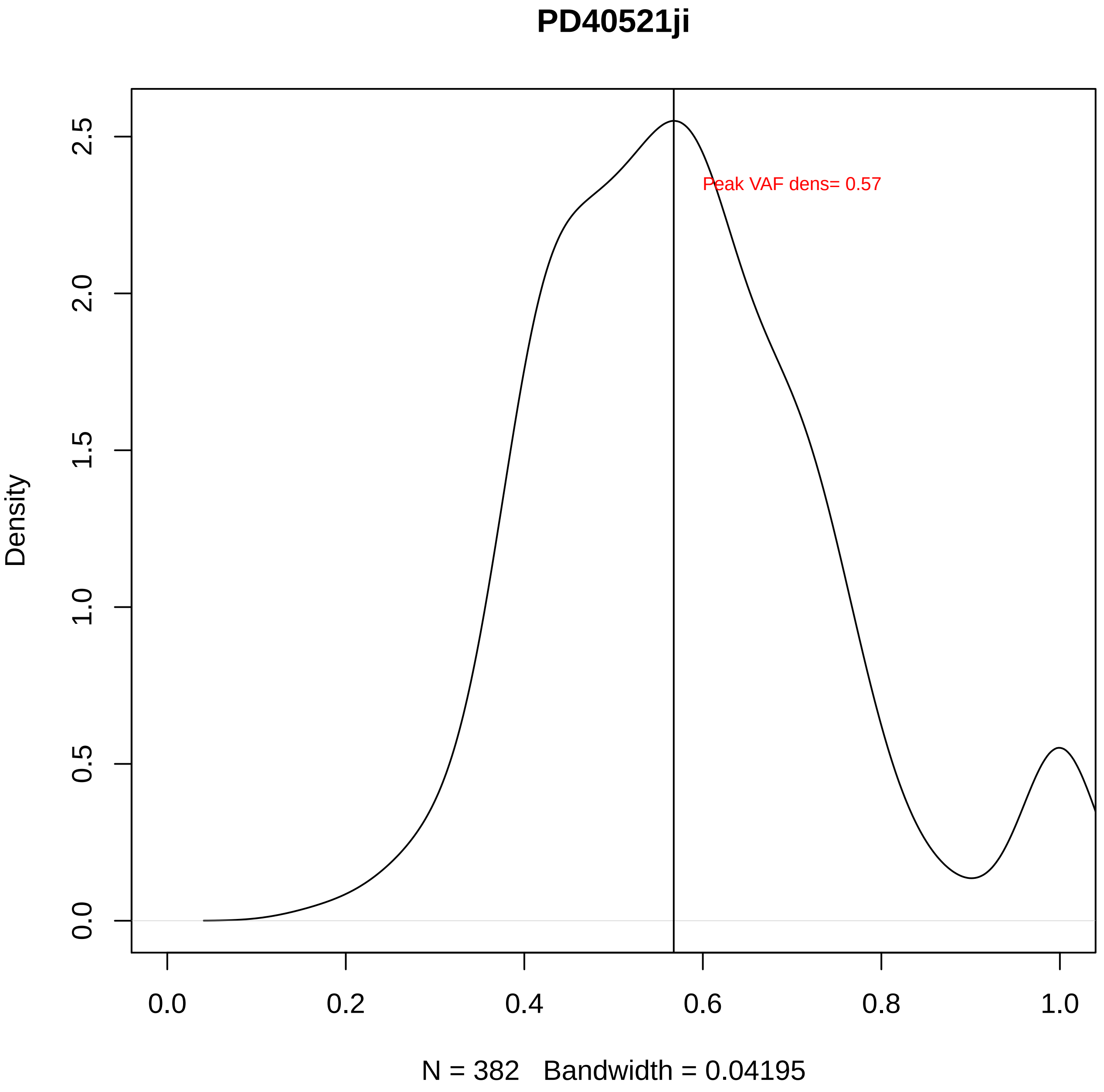
PD40521ji
Peak VAF dens= 0.57
Density
0.0
0.5
1.0
1.5
2.0
2.5
0.0
0.2
0.4
0.6
0.8
1.0
N = 382   Bandwidth = 0.04195

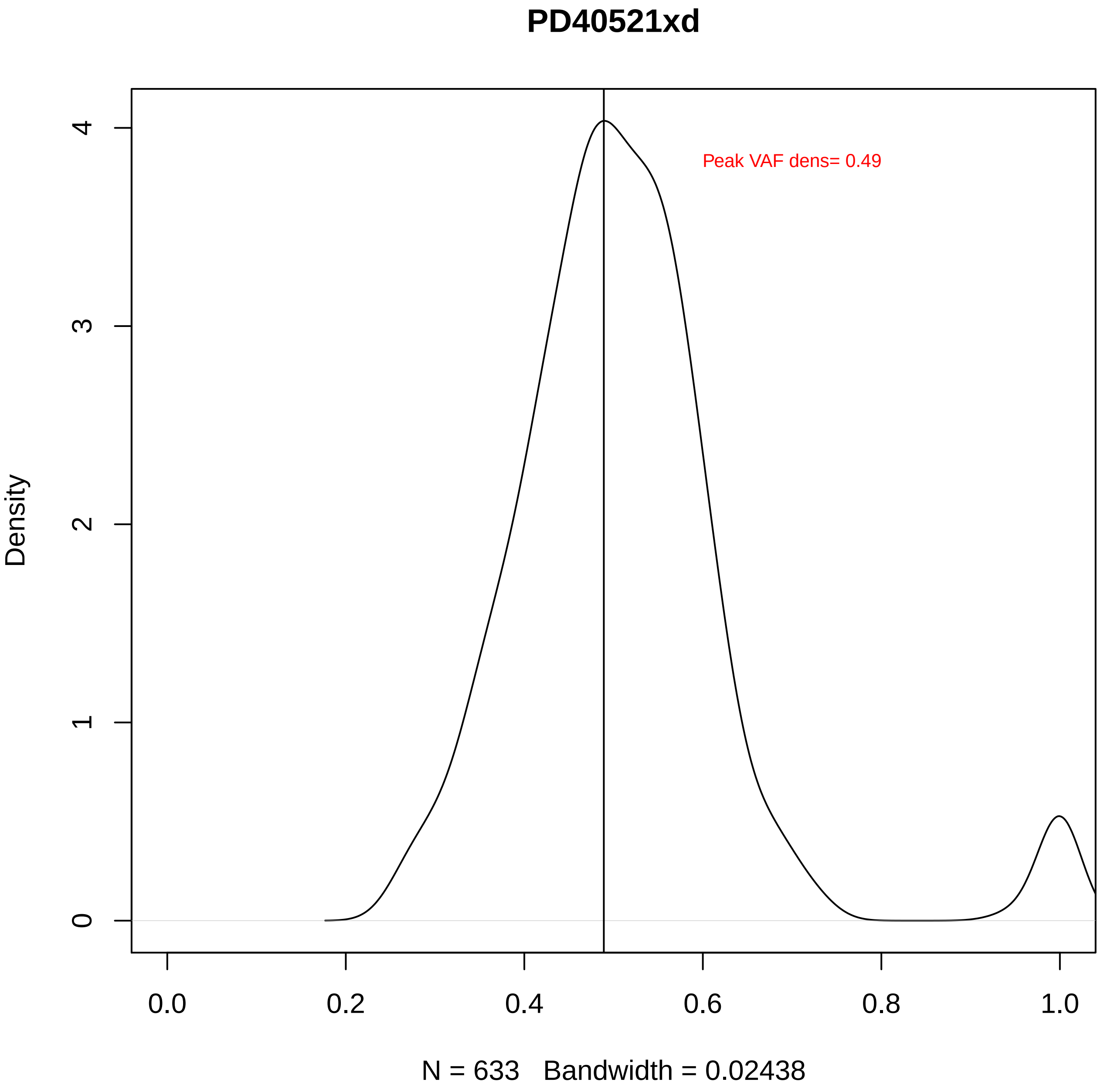

PD40521xd
Peak VAF dens= 0.49
Density
0
1
2
3
4
0.0
0.2
0.4
0.6
0.8
1.0
N = 633 Bandwidth = 0.02438

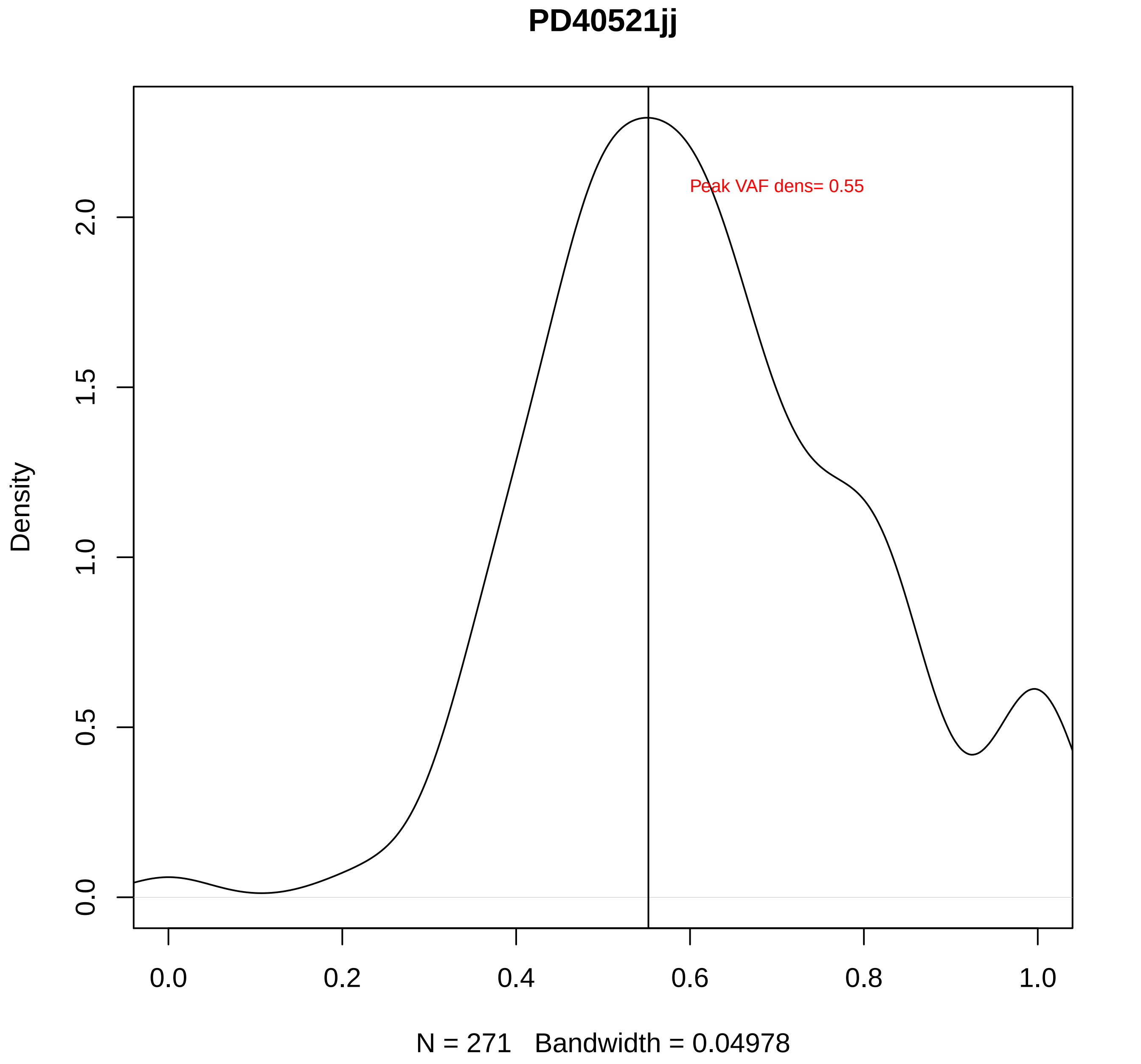
PD40521jj
Peak VAF dens= 0.55
Density
0.0
0.5
1.0
1.5
2.0
0.0
0.2
0.4
0.6
0.8
1.0
N = 271 Bandwidth = 0.04978

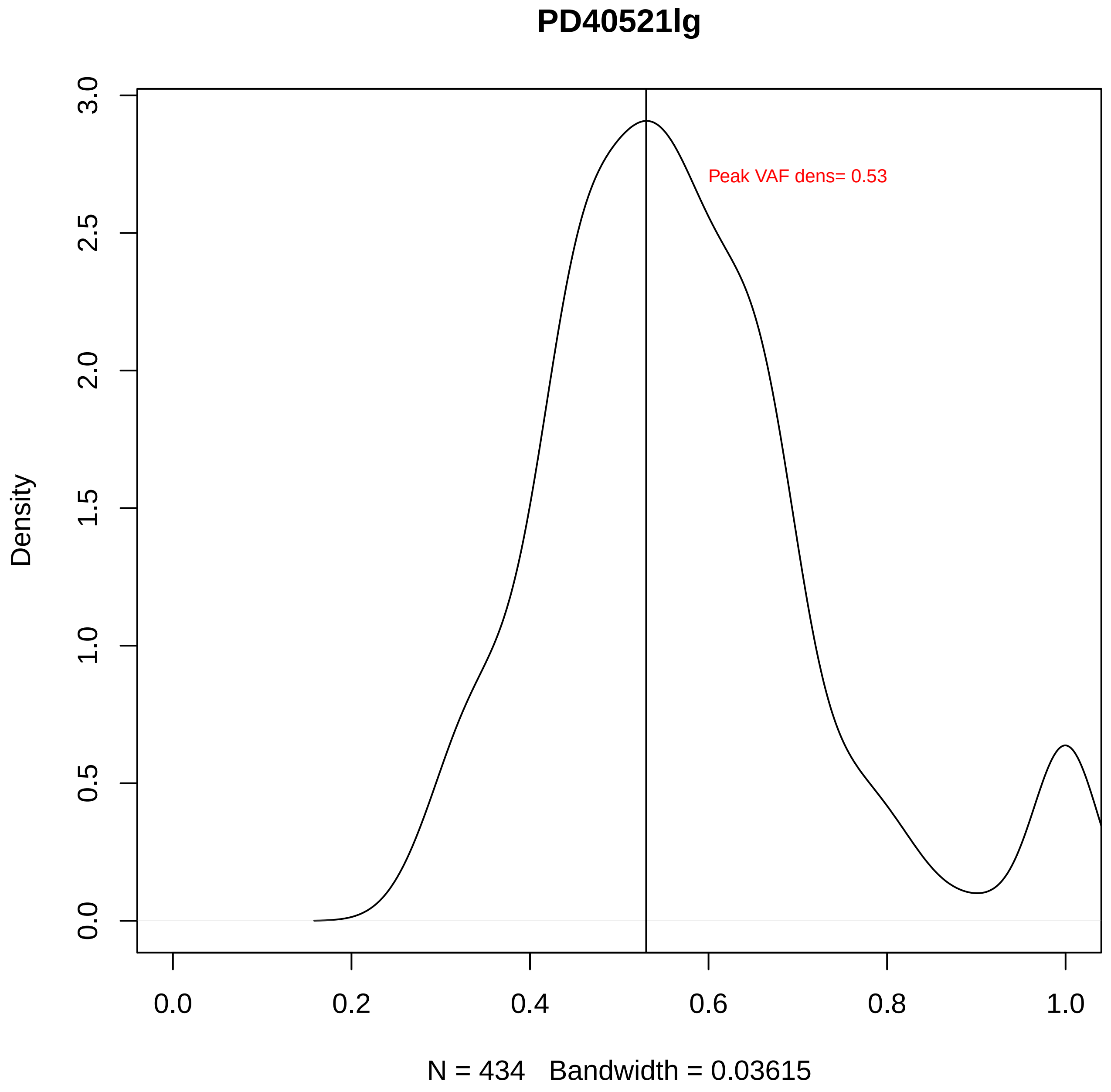
PD40521lg
Peak VAF dens= 0.53
Density
0.0
0.5
1.0
1.5
2.0
2.5
3.0
0.0
0.2
0.4
0.6
0.8
1.0
N = 434 Bandwidth = 0.03615

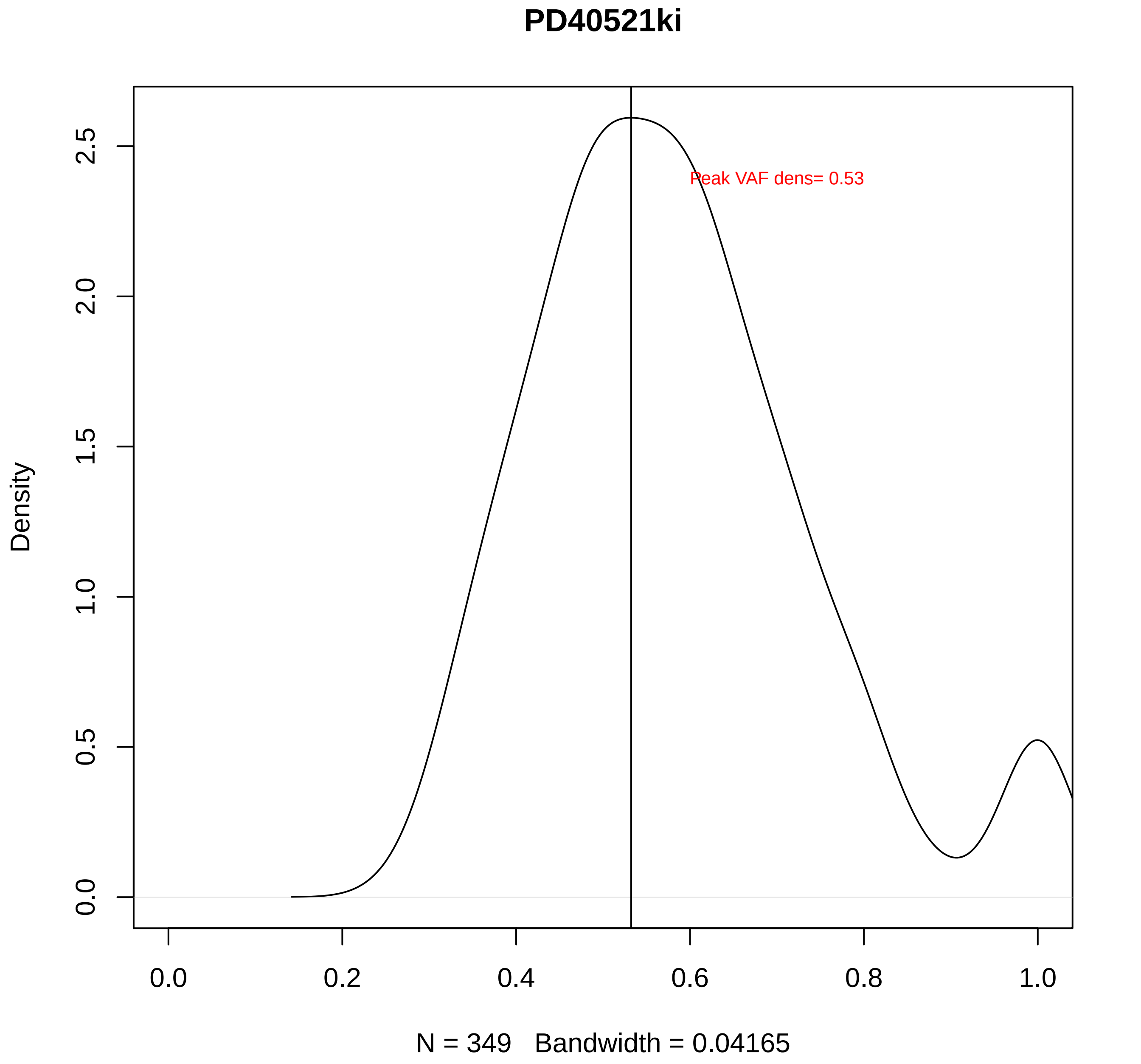
PD40521ki
Peak VAF dens= 0.53
Density
0.0
0.5
1.0
1.5
2.0
2.5
0.0
0.2
0.4
0.6
0.8
1.0
N = 349   Bandwidth = 0.04165

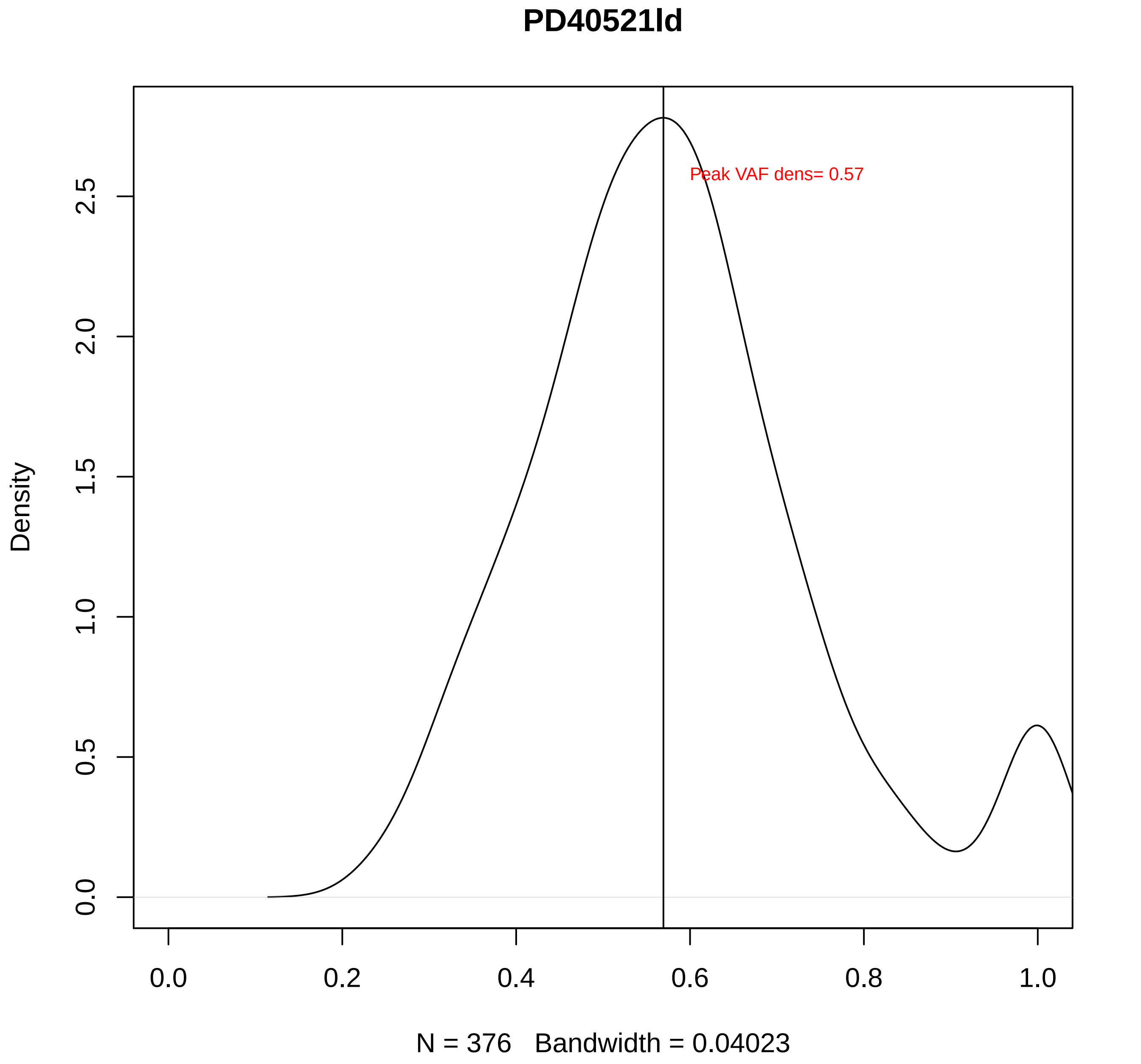
PD40521ld
Peak VAF dens= 0.57
Density
0.0
0.5
1.0
1.5
2.0
2.5
0.0
0.2
0.4
0.6
0.8
1.0
N = 376   Bandwidth = 0.04023

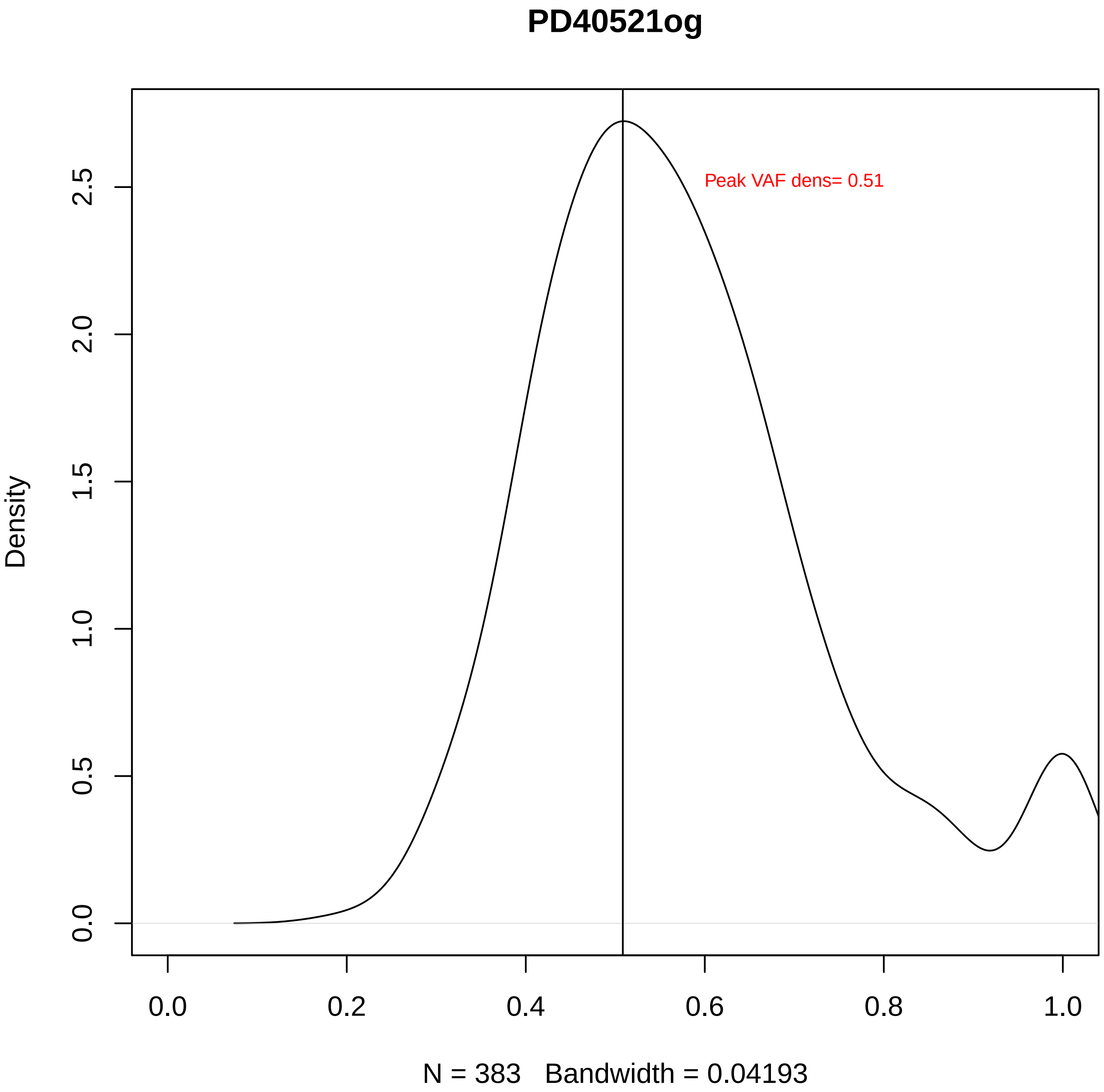
PD40521og
Peak VAF dens= 0.51
Density
0.0
0.5
1.0
1.5
2.0
2.5
0.0
0.2
0.4
0.6
0.8
1.0
N = 383 Bandwidth = 0.04193

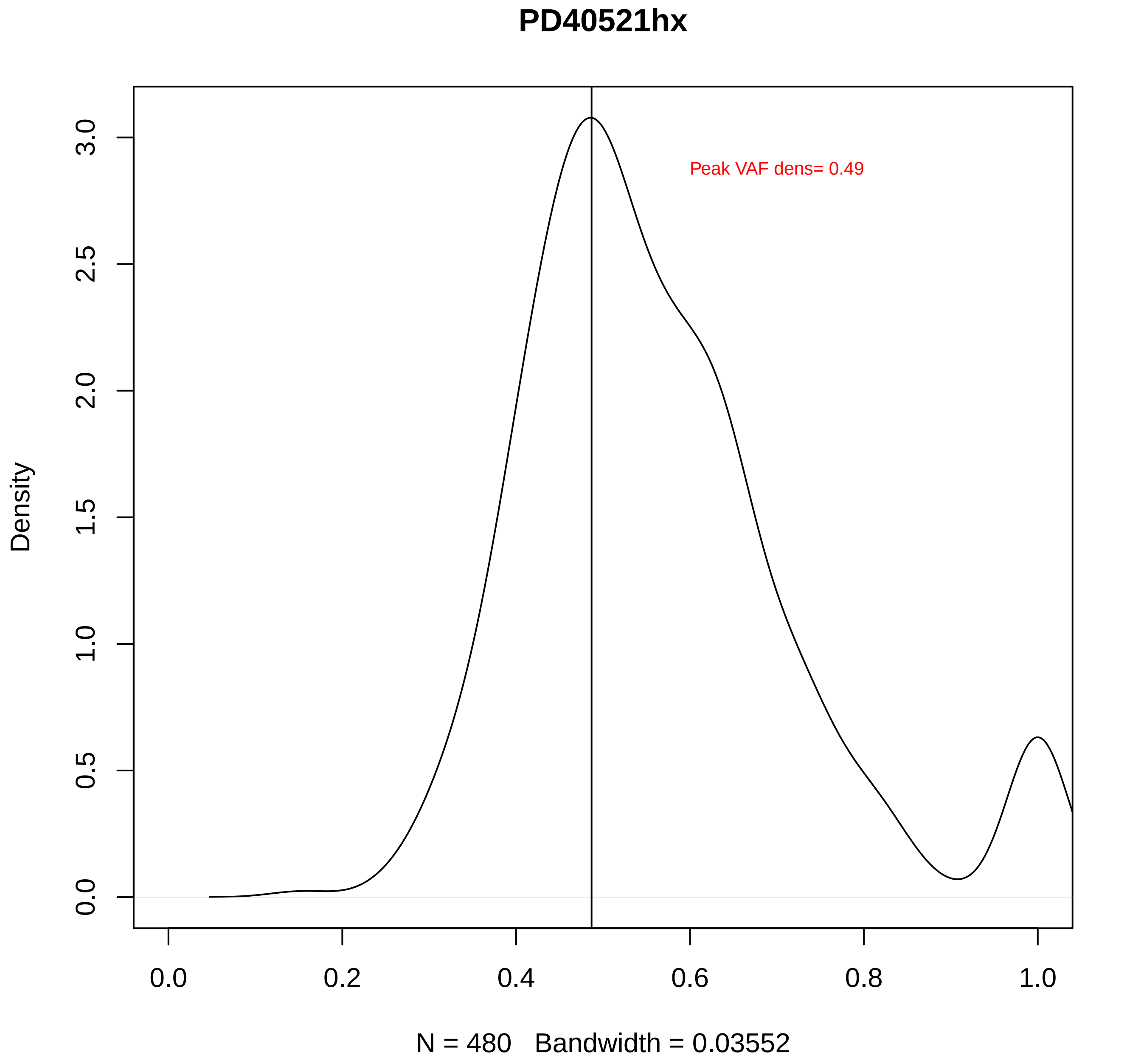

PD40521hx
Peak VAF dens= 0.49
Density
0.0
0.5
1.0
1.5
2.0
2.5
3.0
0.0
0.2
0.4
0.6
0.8
1.0
N = 480   Bandwidth = 0.03552

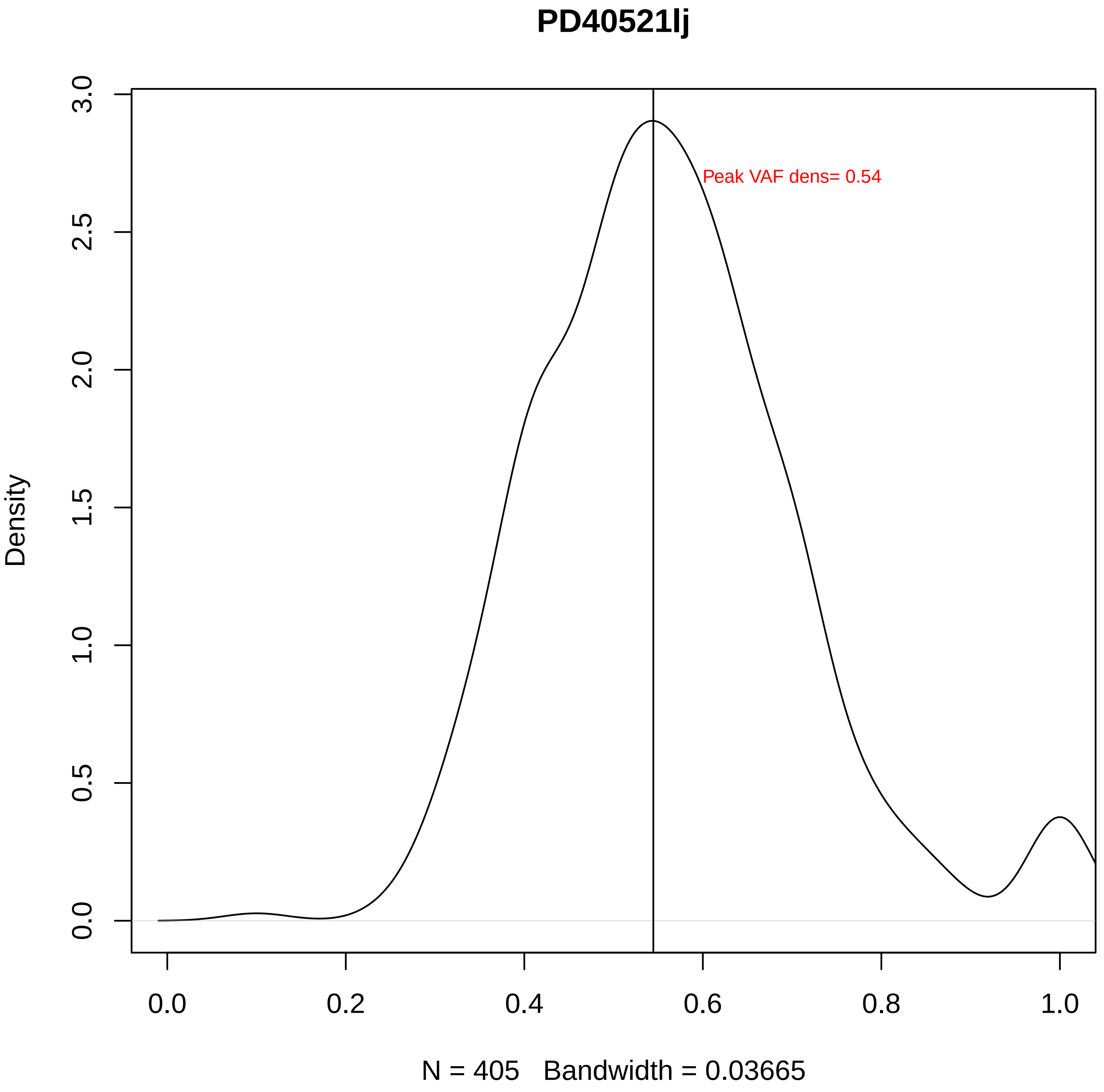

PD40521lj
Peak VAF dens= 0.54
Density
0.0
0.5
1.0
1.5
2.0
2.5
3.0
0.0
0.2
0.4
0.6
0.8
1.0
N = 405 Bandwidth = 0.03665

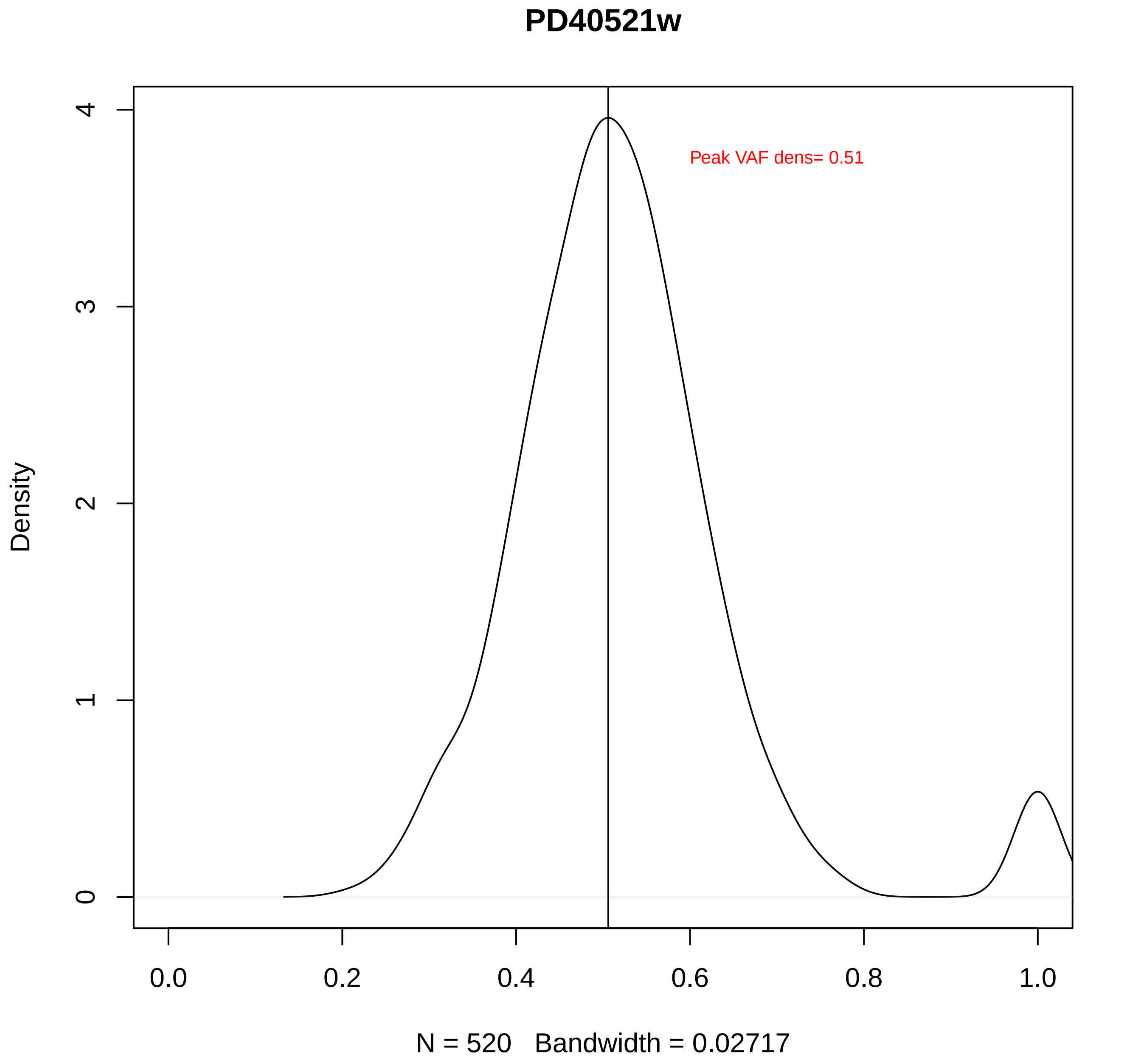
PD40521w
Peak VAF dens= 0.51
Density
0
1
2
3
4
0.0
0.2
0.4
0.6
0.8
1.0
N = 520   Bandwidth = 0.02717

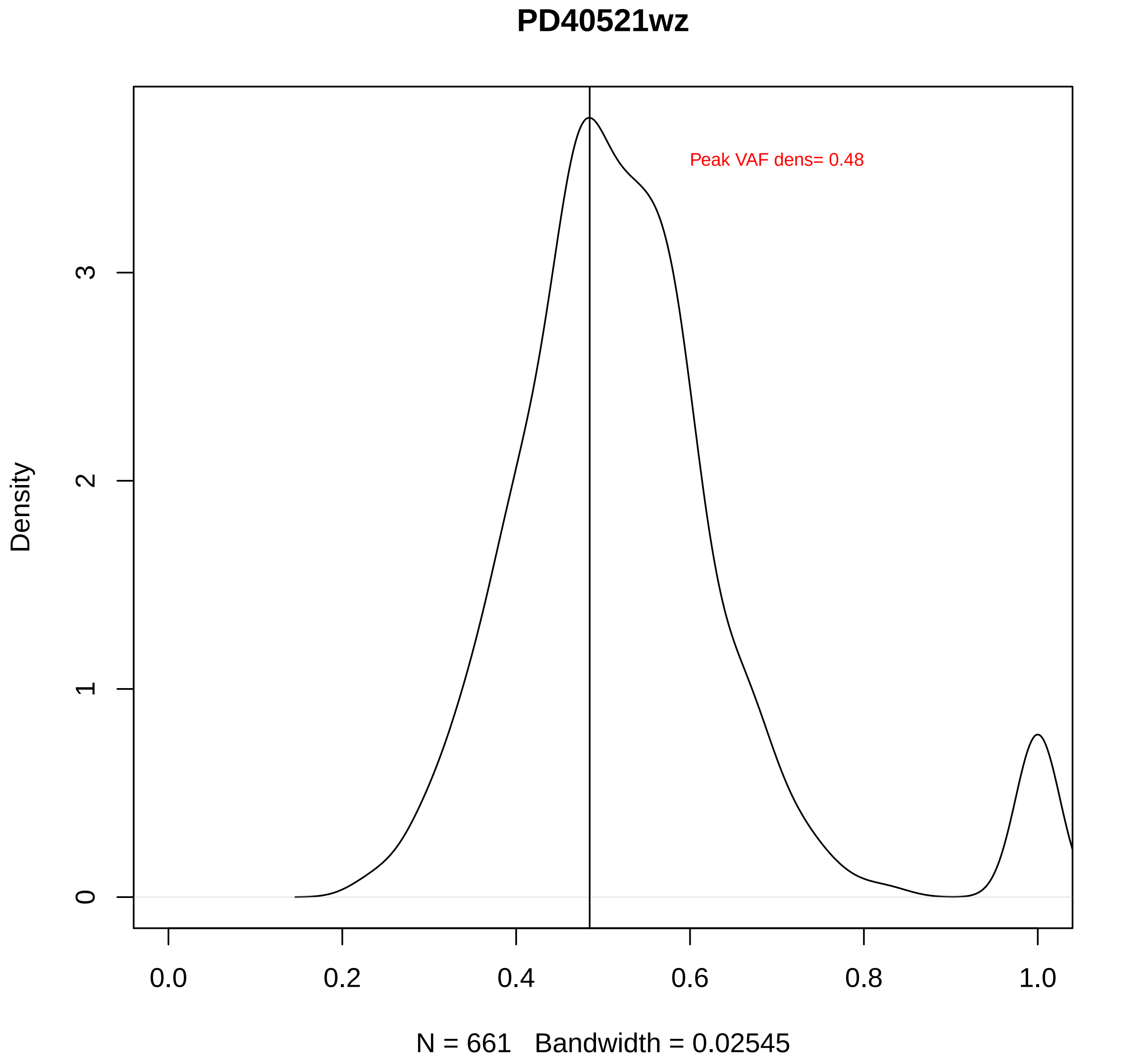
PD40521wz
Peak VAF dens= 0.48
Density
0
1
2
3
0.0
0.2
0.4
0.6
0.8
1.0
N = 661   Bandwidth = 0.02545

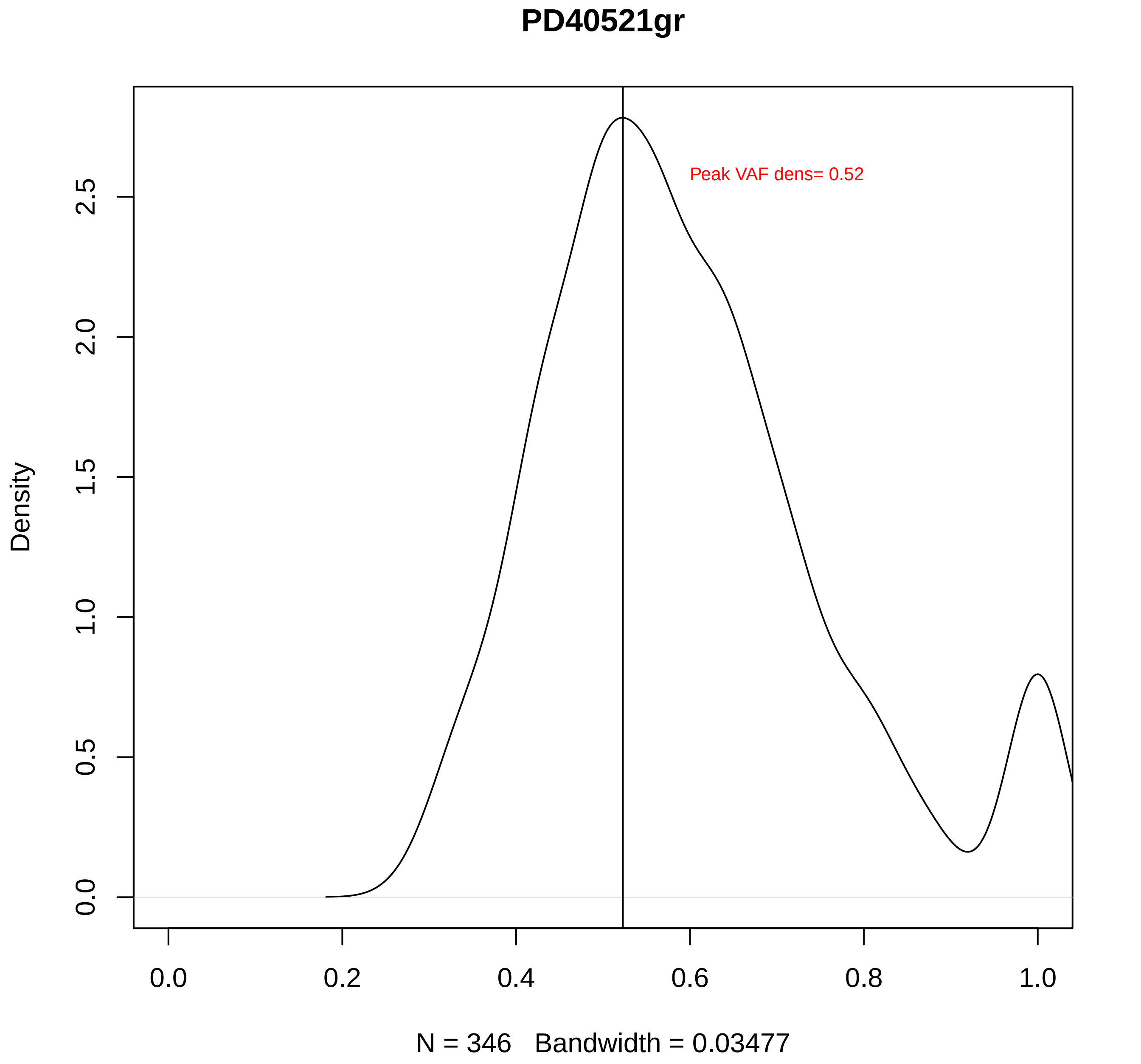

PD40521gr
Peak VAF dens= 0.52
Density
0.0
0.5
1.0
1.5
2.0
2.5
0.0
0.2
0.4
0.6
0.8
1.0
N = 346   Bandwidth = 0.03477

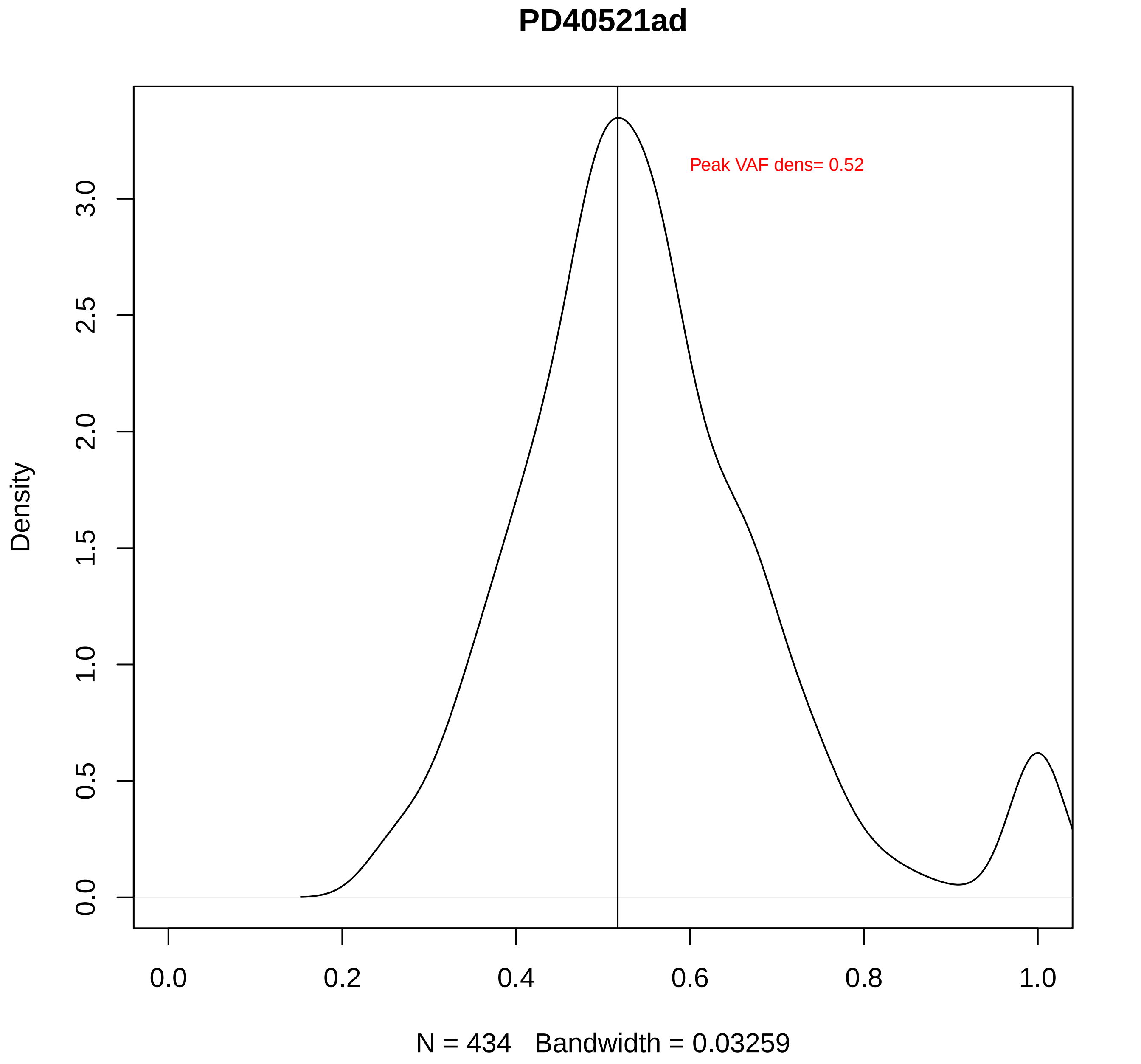

PD40521ad
Peak VAF dens= 0.52
Density
0.0
0.5
1.0
1.5
2.0
2.5
3.0
0.0
0.2
0.4
0.6
0.8
1.0
N = 434 Bandwidth = 0.03259

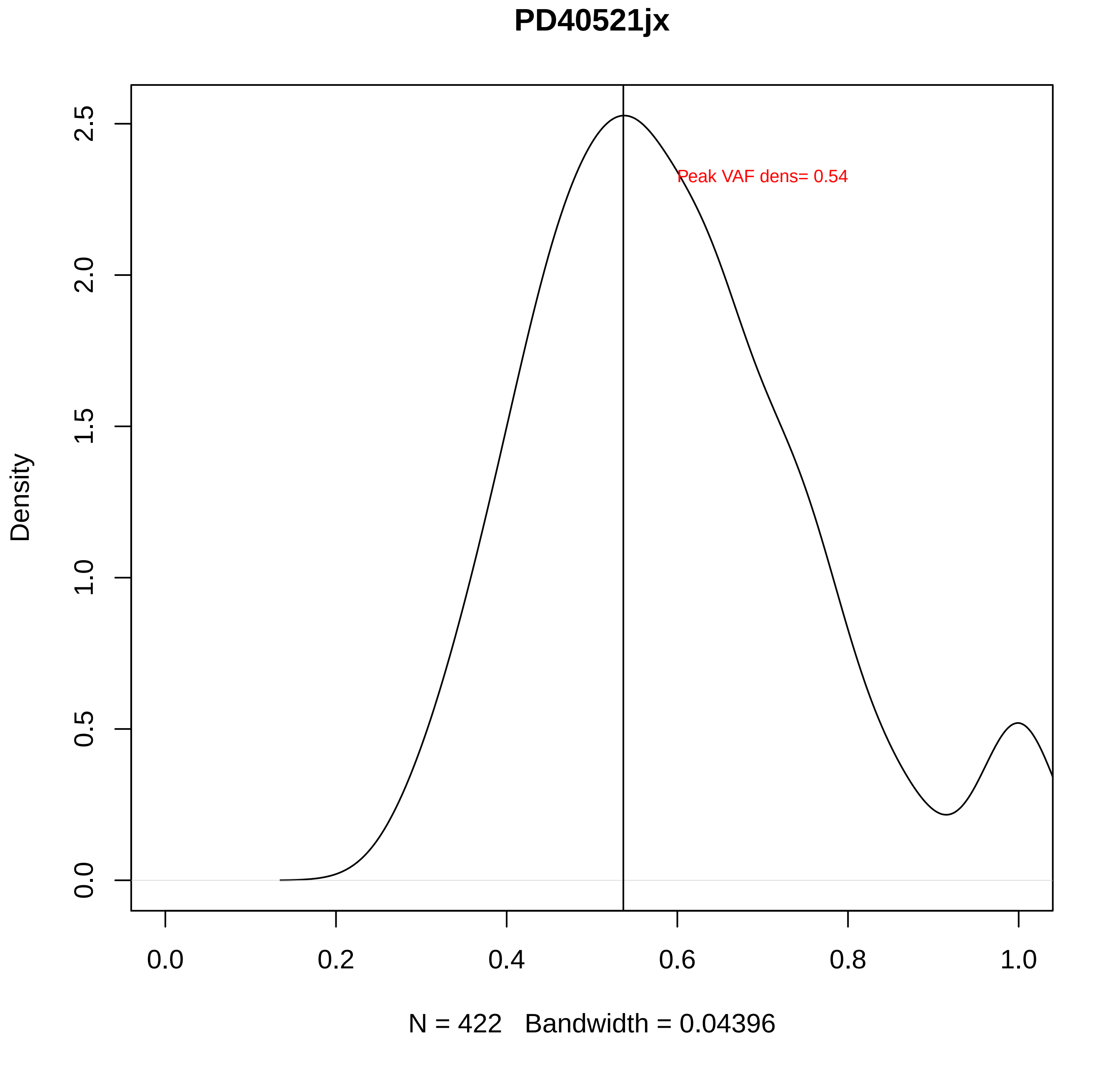

PD40521jx
Peak VAF dens= 0.54
Density
0.0
0.5
1.0
1.5
2.0
2.5
0.0
0.2
0.4
0.6
0.8
1.0
N = 422   Bandwidth = 0.04396

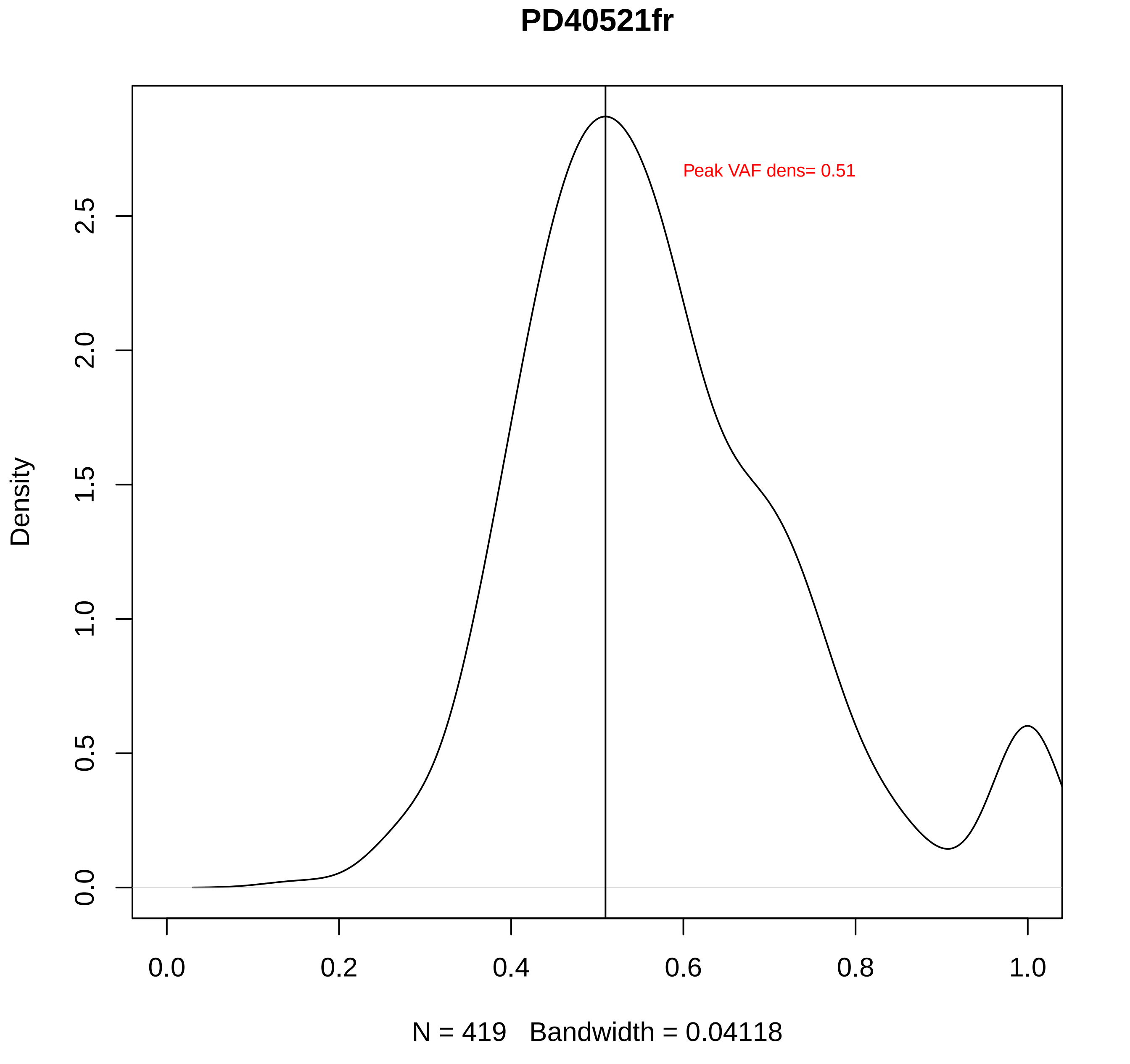
PD40521fr
Peak VAF dens= 0.51
Density
0.0
0.5
1.0
1.5
2.0
2.5
0.0
0.2
0.4
0.6
0.8
1.0
N = 419   Bandwidth = 0.04118

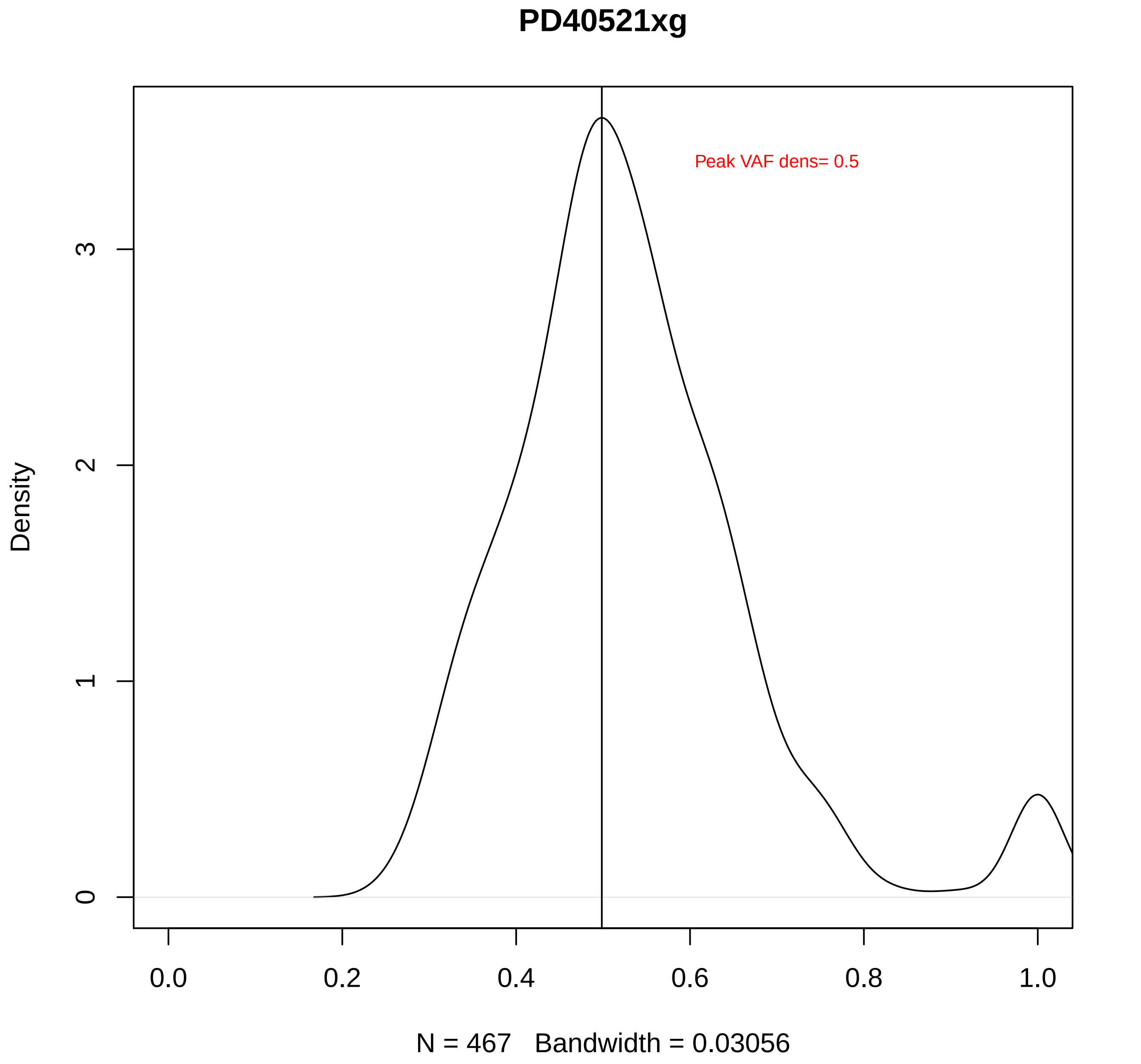
PD40521xg
Peak VAF dens= 0.5
Density
0
1
2
3
0.0
0.2
0.4
0.6
0.8
1.0
N = 467   Bandwidth = 0.03056

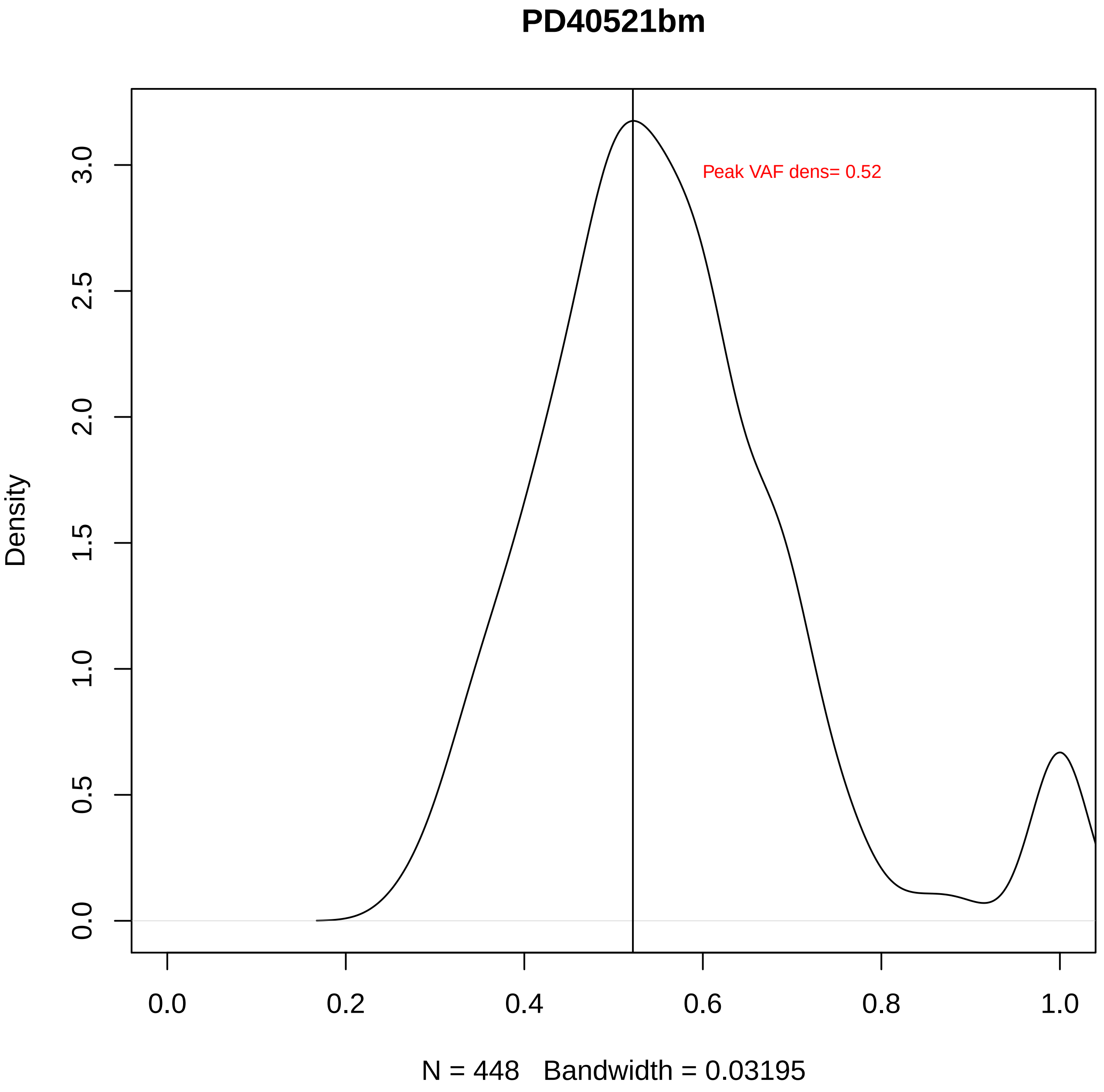
PD40521bm
Peak VAF dens= 0.52
Density
0.0
0.5
1.0
1.5
2.0
2.5
3.0
0.0
0.2
0.4
0.6
0.8
1.0
N = 448 Bandwidth = 0.03195

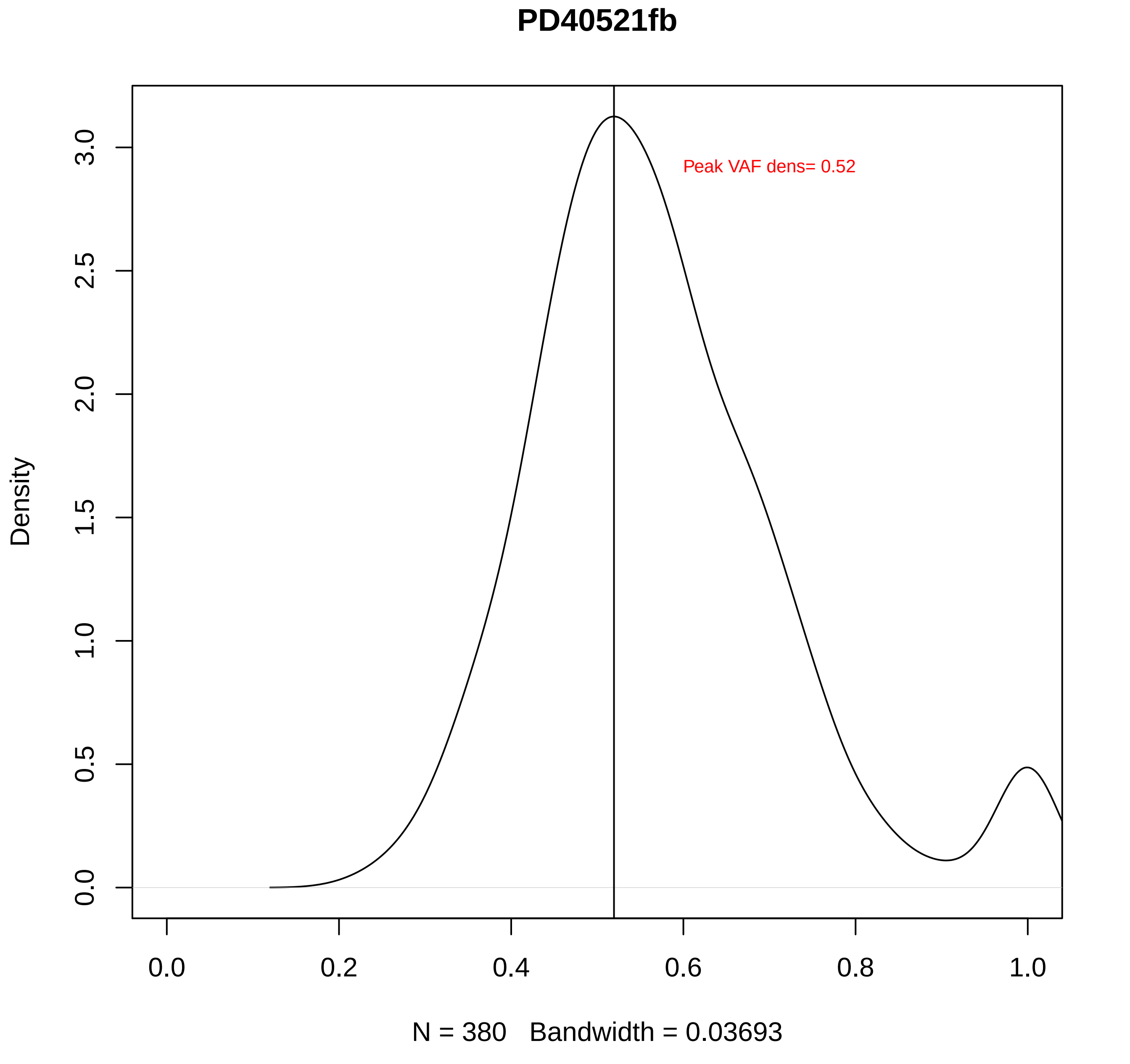

PD40521fb
Peak VAF dens= 0.52
Density
0.0
0.5
1.0
1.5
2.0
2.5
3.0
0.0
0.2
0.4
0.6
0.8
1.0
N = 380   Bandwidth = 0.03693

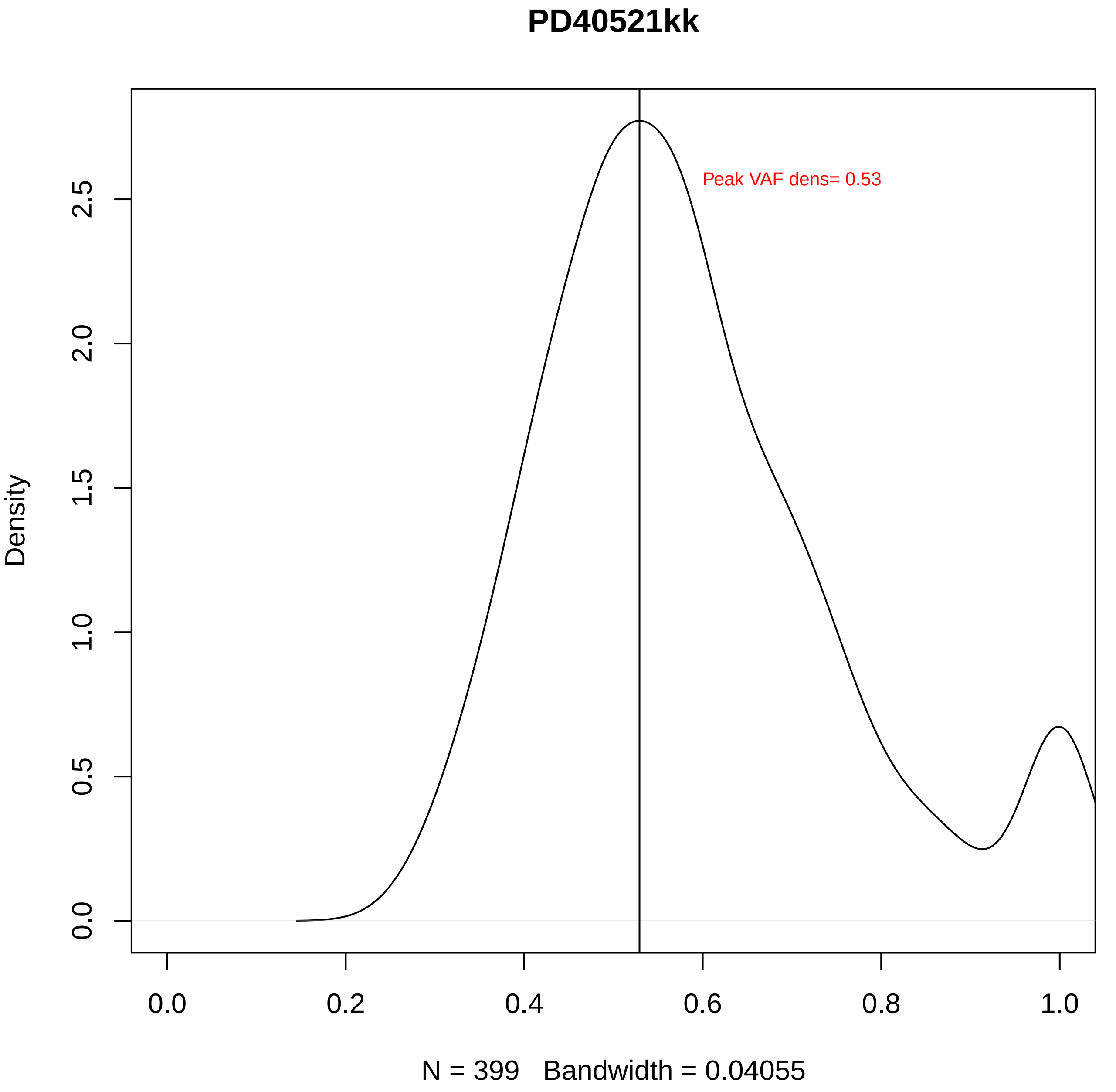
PD40521kk
Peak VAF dens= 0.53
Density
0.0
0.5
1.0
1.5
2.0
2.5
0.0
0.2
0.4
0.6
0.8
1.0
N = 399   Bandwidth = 0.04055

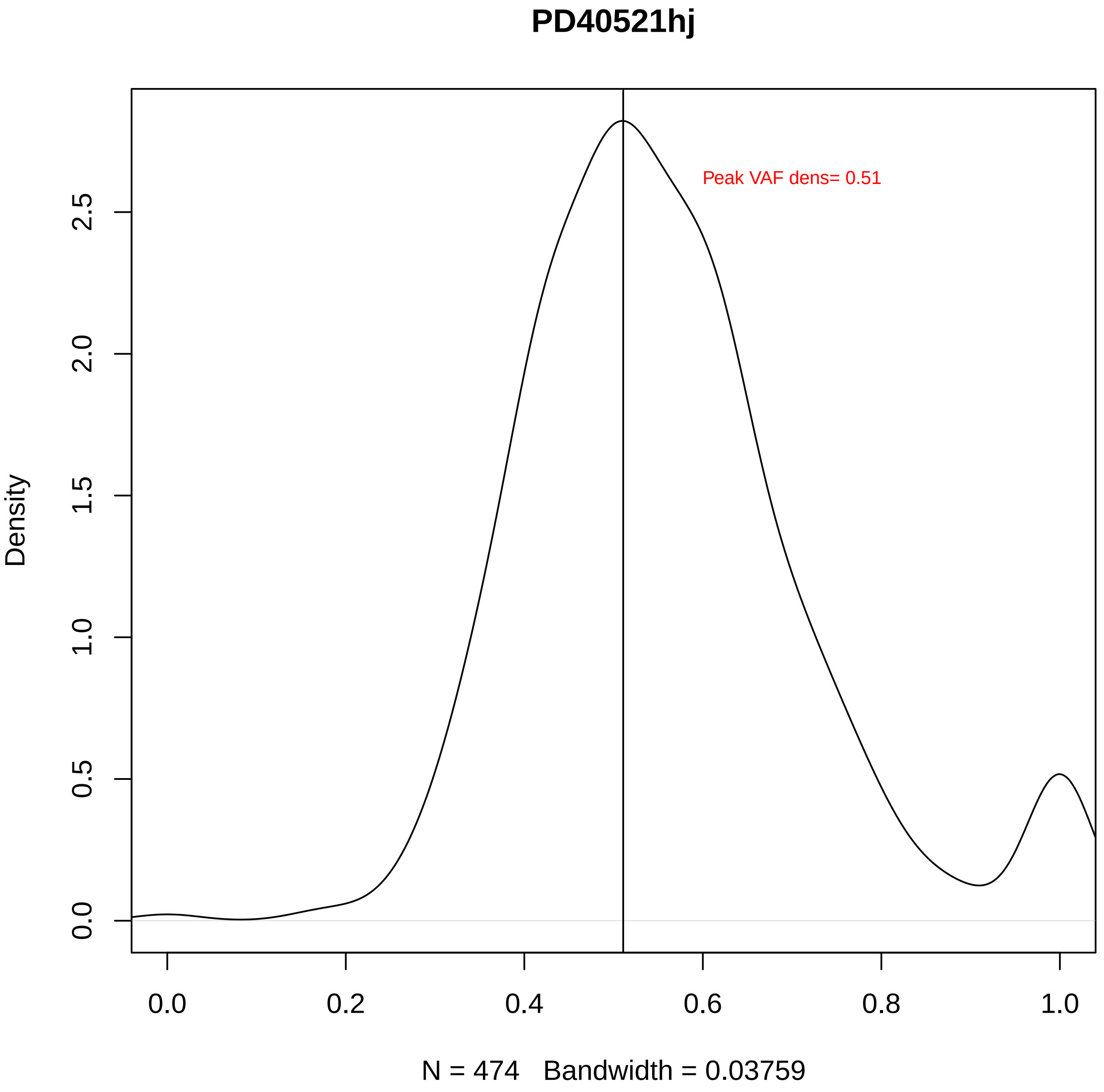
PD40521hj
Peak VAF dens= 0.51
Density
0.0
0.5
1.0
1.5
2.0
2.5
0.0
0.2
0.4
0.6
0.8
1.0
N = 474   Bandwidth = 0.03759

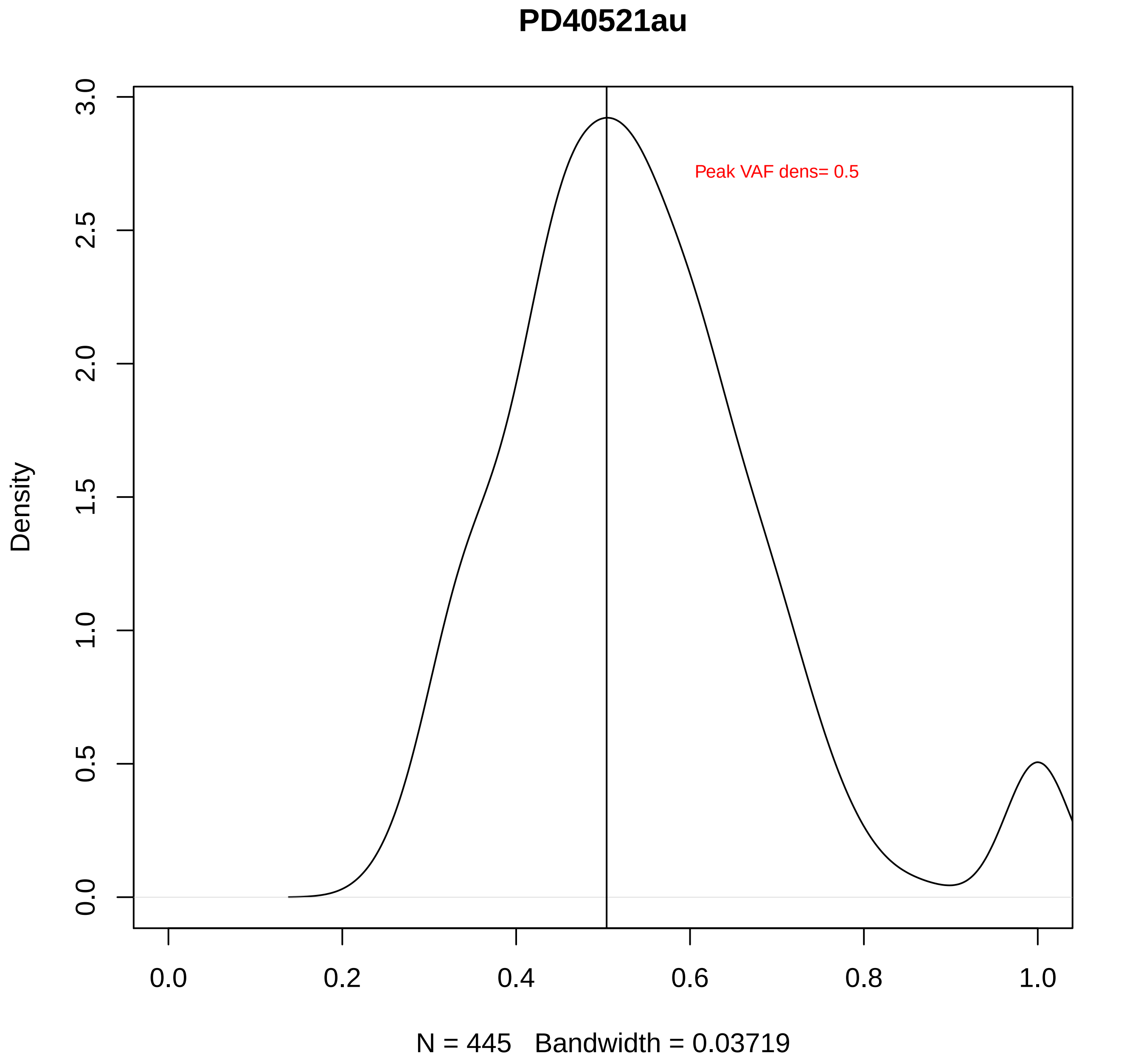

PD40521au
Peak VAF dens= 0.5
Density
0.0
0.5
1.0
1.5
2.0
2.5
3.0
0.0
0.2
0.4
0.6
0.8
1.0
N = 445 Bandwidth = 0.03719

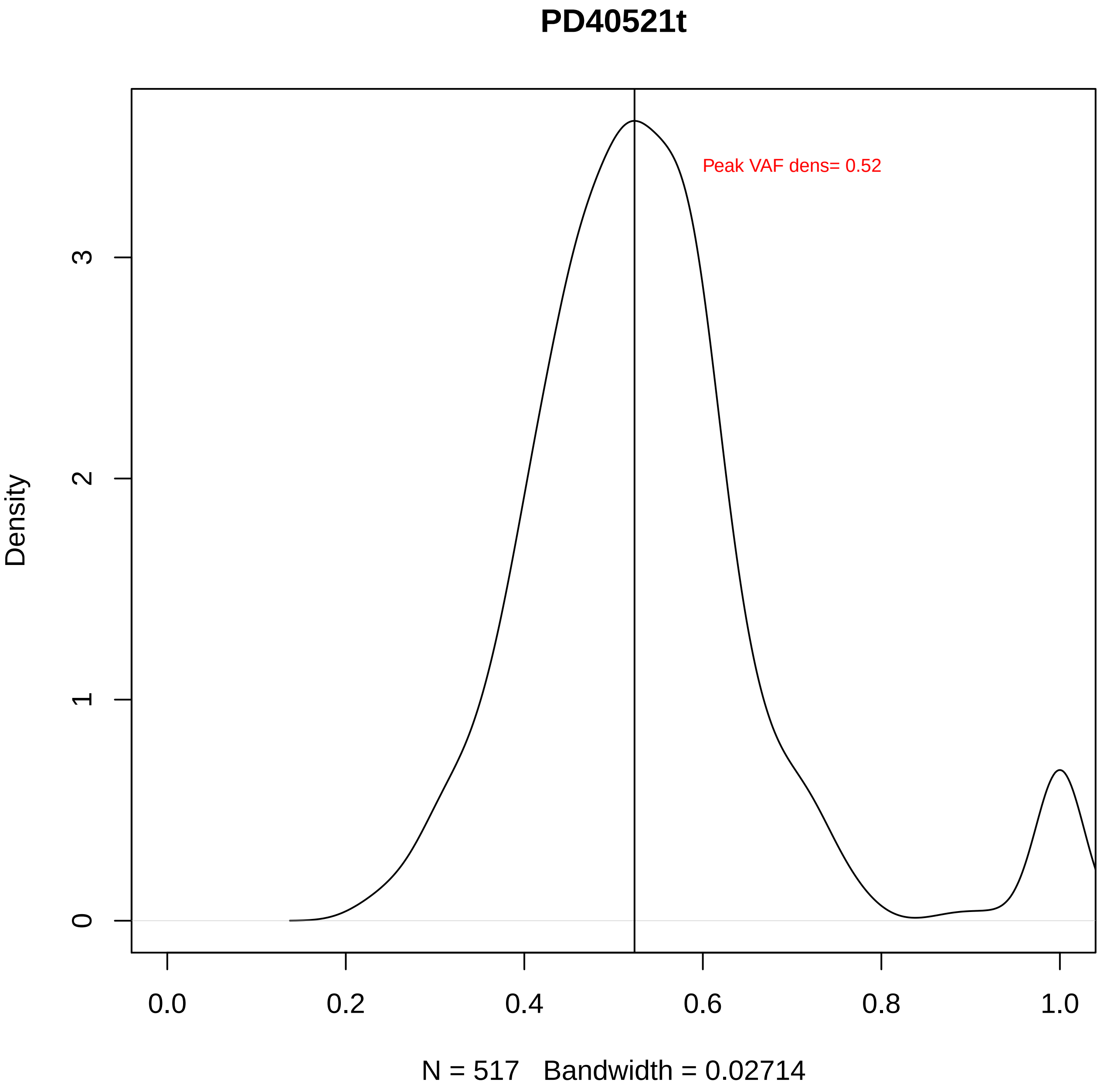
PD40521t
Peak VAF dens= 0.52
Density
0
1
2
3
0.0
0.2
0.4
0.6
0.8
1.0
N = 517   Bandwidth = 0.02714

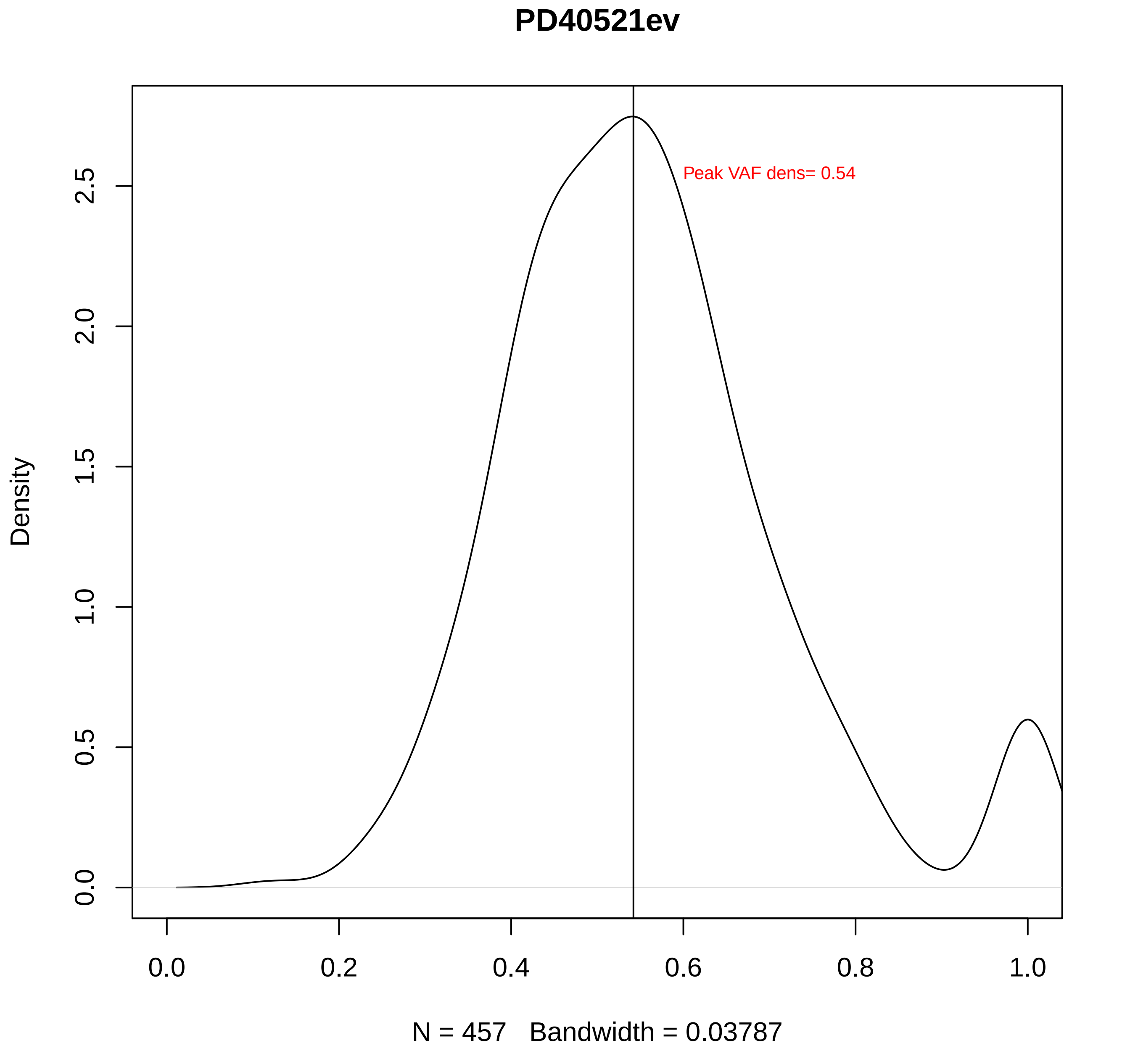
PD40521ev
Peak VAF dens= 0.54
Density
0.0
0.5
1.0
1.5
2.0
2.5
0.0
0.2
0.4
0.6
0.8
1.0
N = 457   Bandwidth = 0.03787

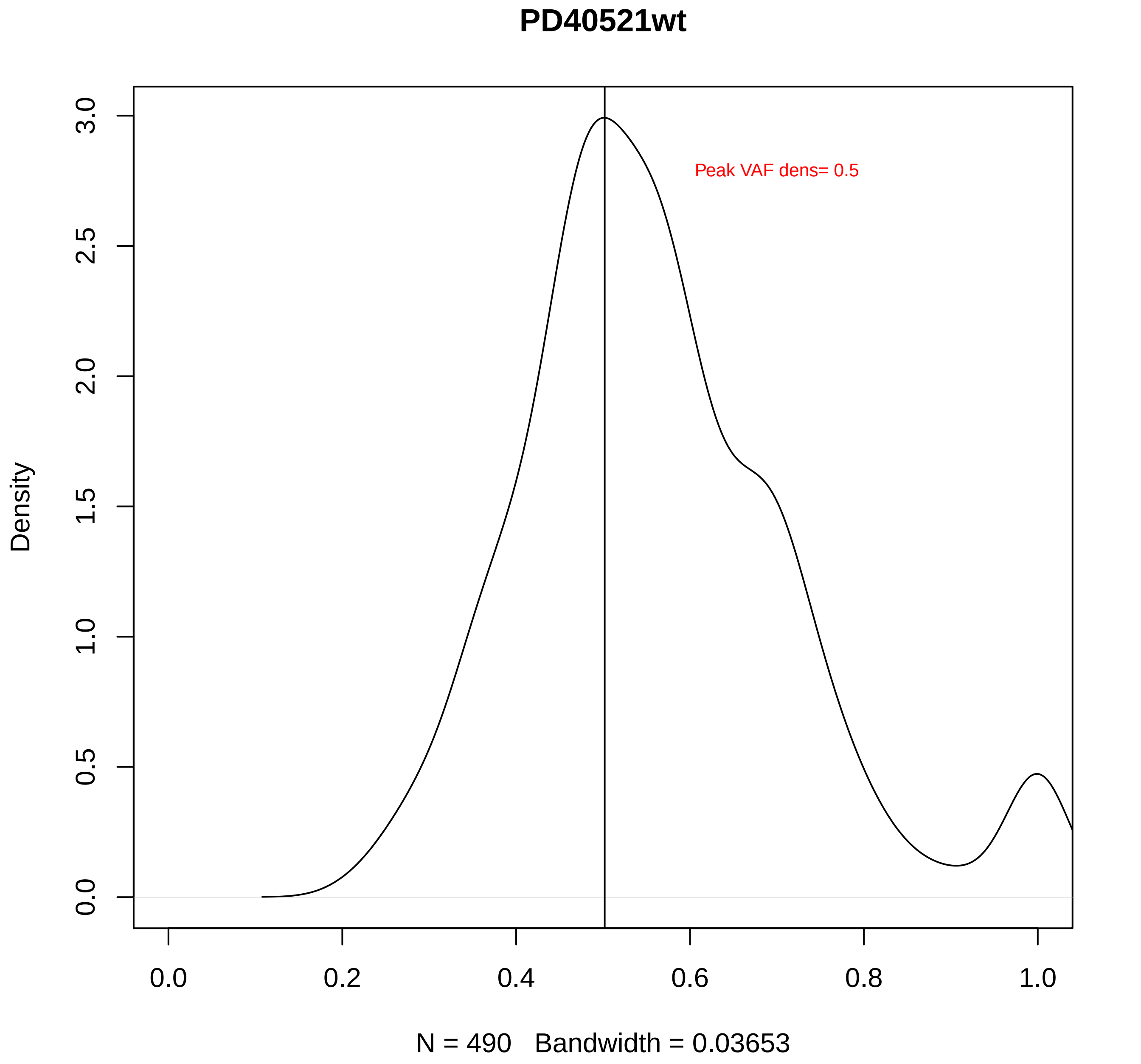

PD40521wt
Peak VAF dens= 0.5
Density
3.0
2.5
2.0
1.5
1.0
0.5
0.0
0.0
0.2
0.4
0.6
0.8
1.0
N = 490   Bandwidth = 0.03653

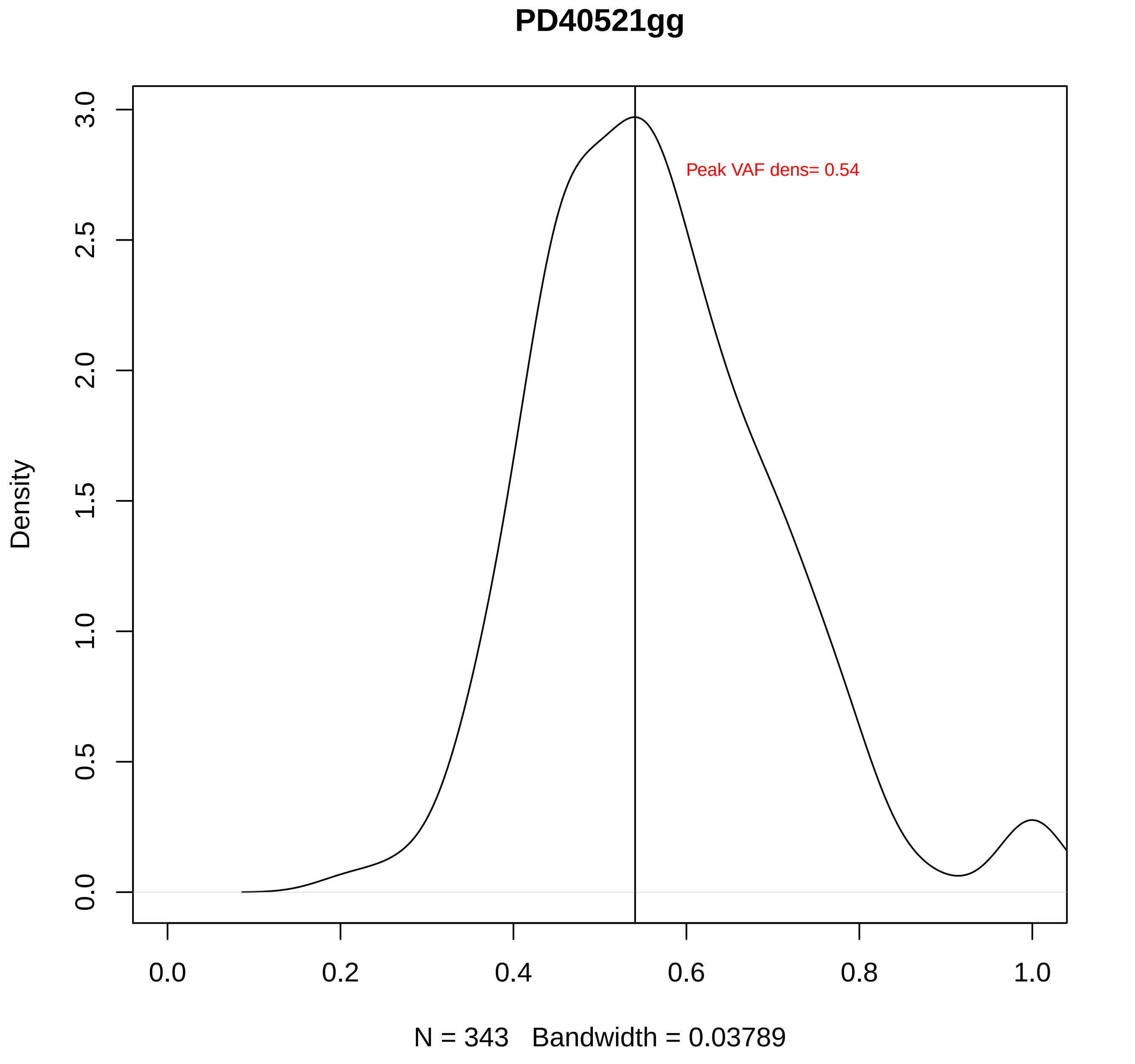
PD40521gg
Peak VAF dens= 0.54
Density
3.0
2.5
2.0
1.5
1.0
0.5
0.0
0.0
0.2
0.4
0.6
0.8
1.0
N = 343 Bandwidth = 0.03789

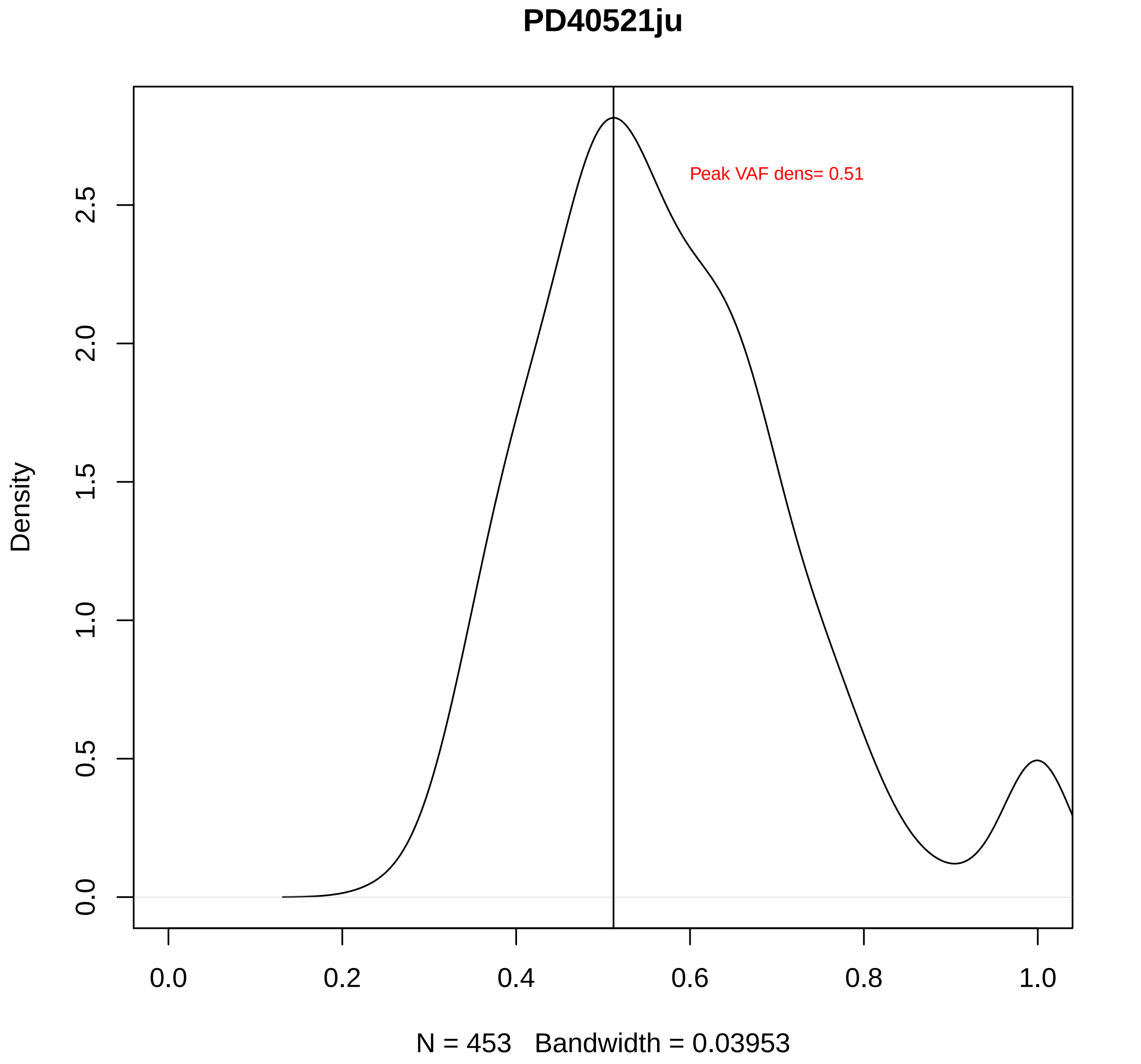

PD40521ju
Peak VAF dens= 0.51
Density
0.0
0.5
1.0
1.5
2.0
2.5
0.0
0.2
0.4
0.6
0.8
1.0
N = 453   Bandwidth = 0.03953

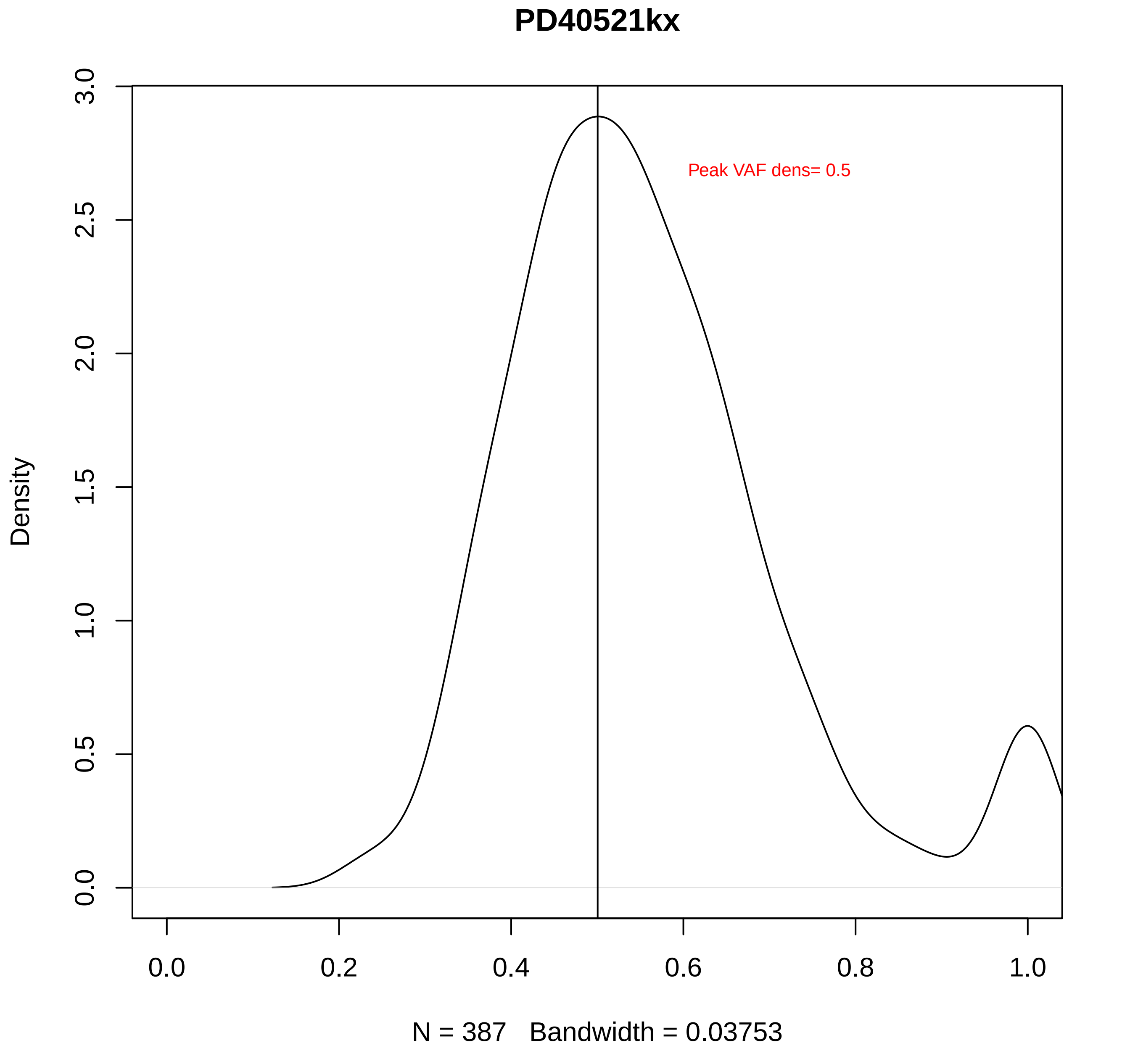
PD40521kx
Peak VAF dens= 0.5
Density
0.0
0.5
1.0
1.5
2.0
2.5
3.0
0.0
0.2
0.4
0.6
0.8
1.0
N = 387   Bandwidth = 0.03753

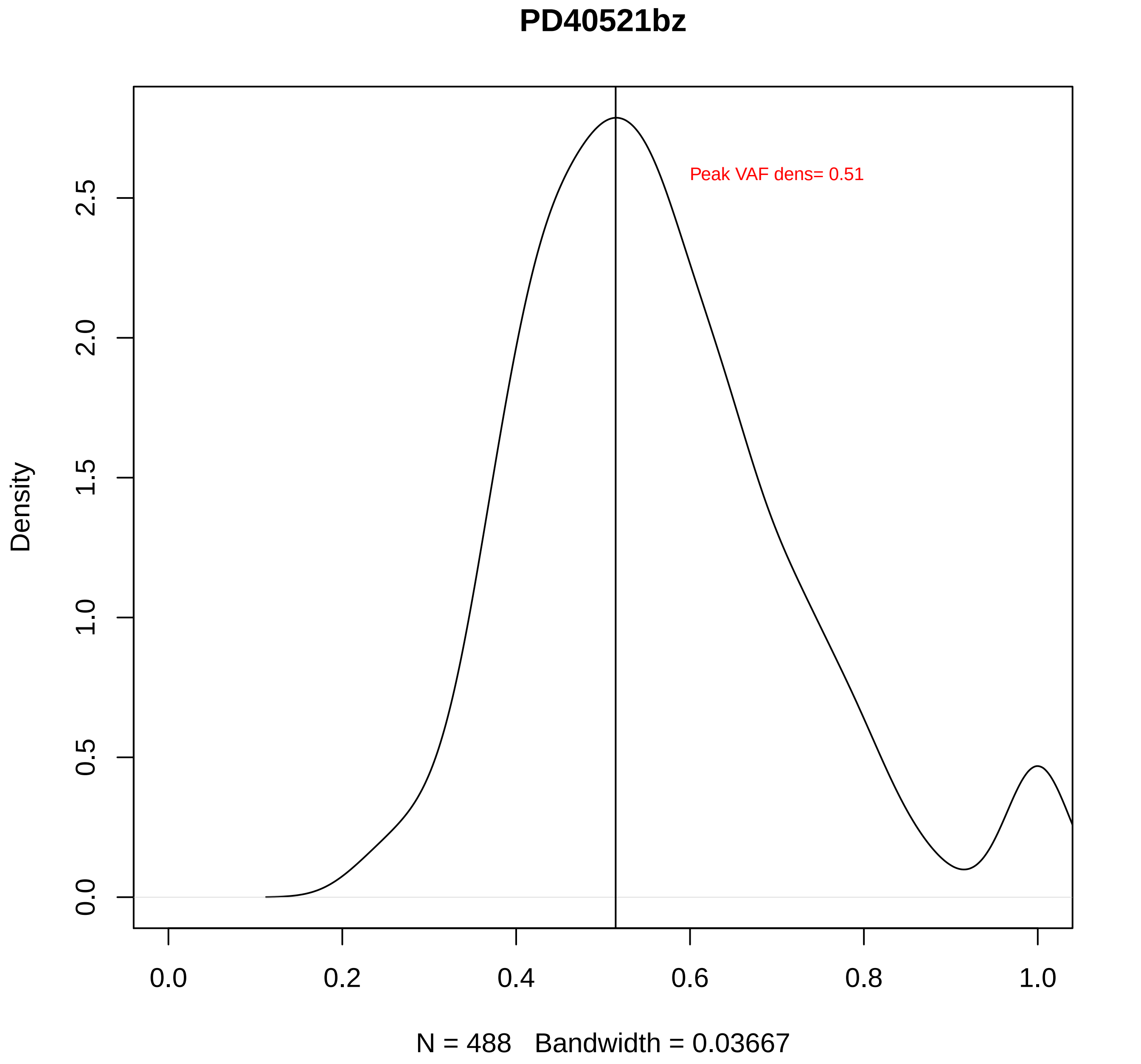
PD40521bz
Peak VAF dens= 0.51
Density
0.0
0.5
1.0
1.5
2.0
2.5
0.0
0.2
0.4
0.6
0.8
1.0
N = 488   Bandwidth = 0.03667

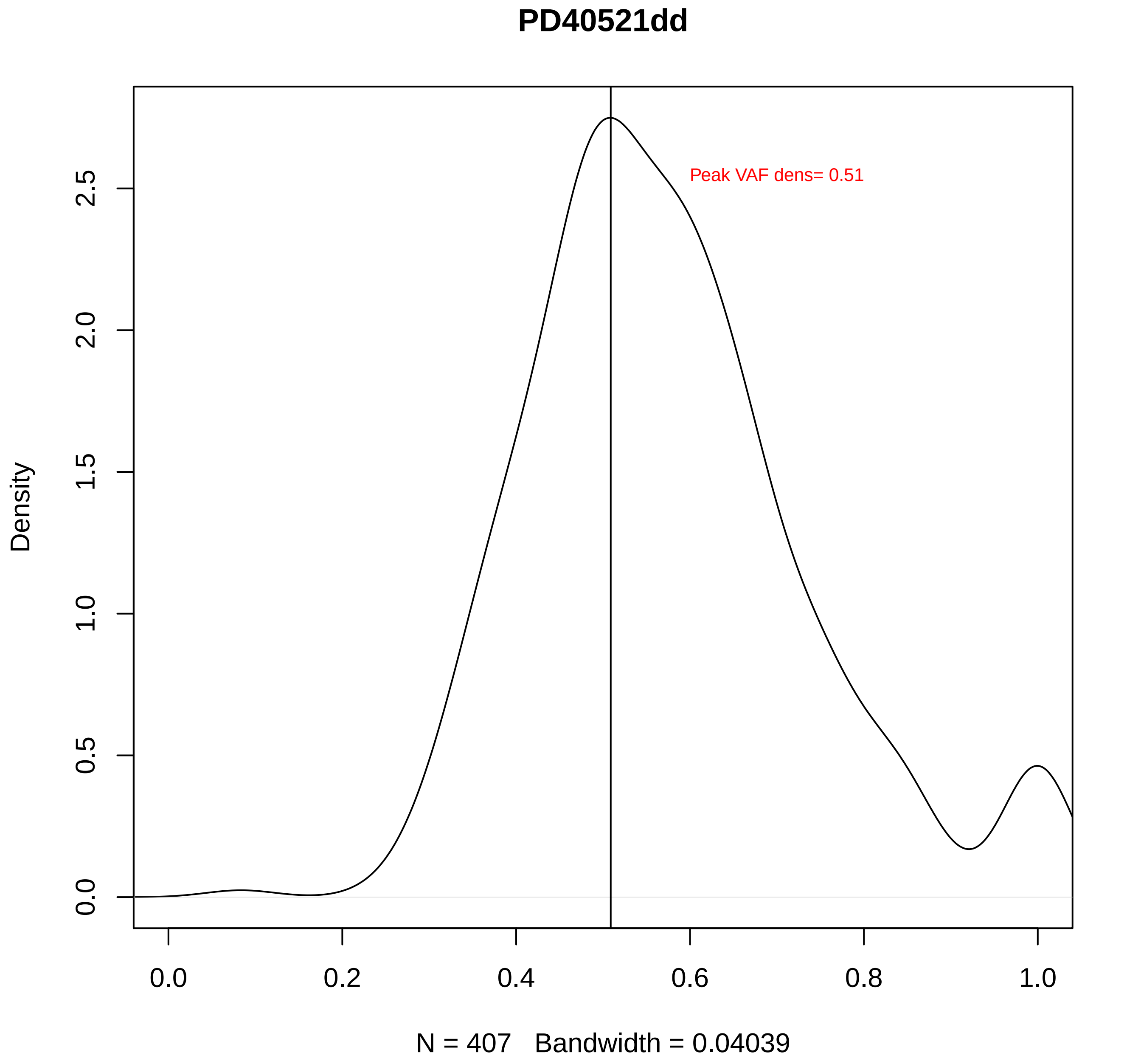

PD40521dd
Peak VAF dens= 0.51
Density
0.0
0.5
1.0
1.5
2.0
2.5
0.0
0.2
0.4
0.6
0.8
1.0
N = 407   Bandwidth = 0.04039

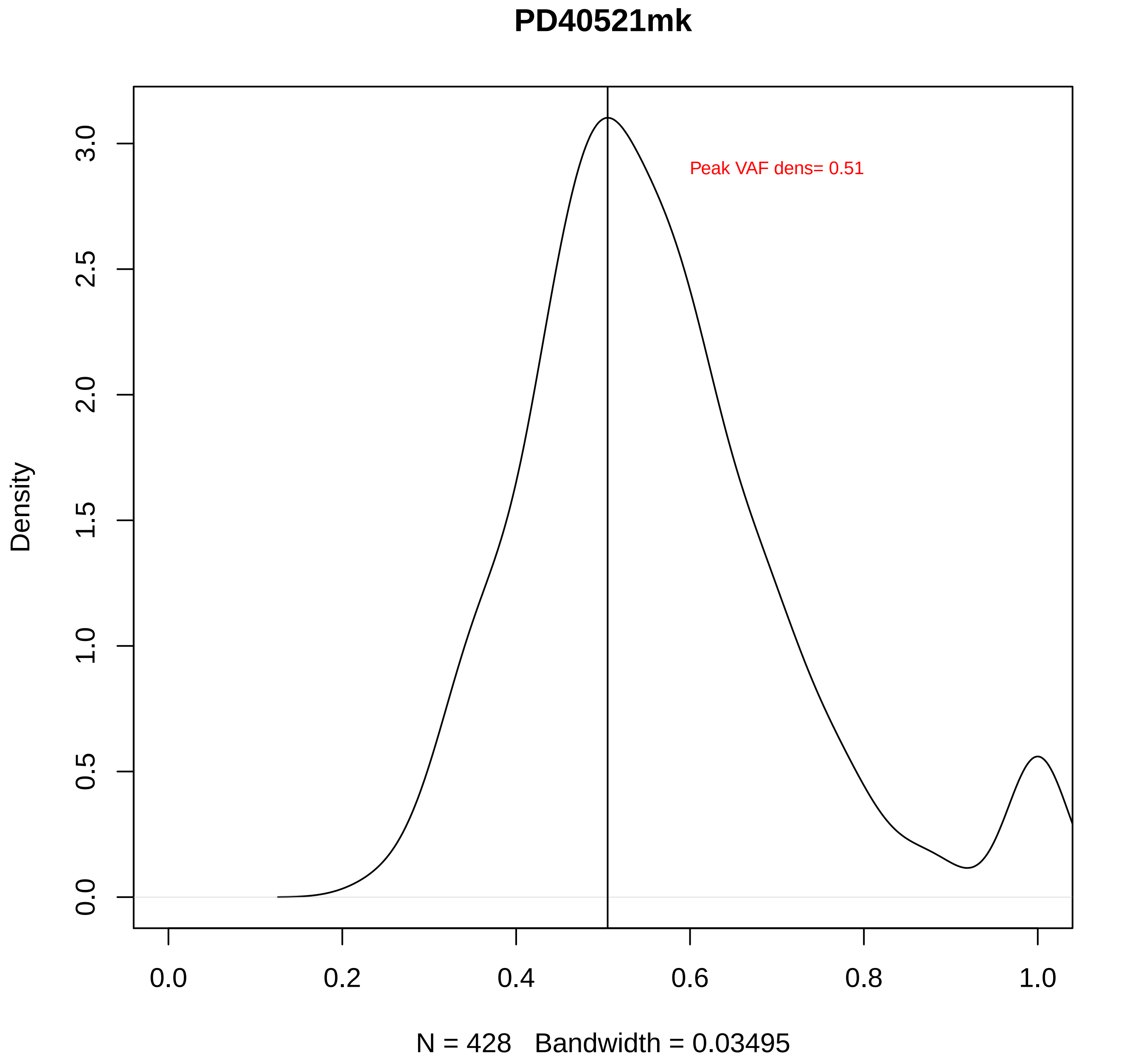

PD40521mk
Peak VAF dens= 0.51
Density
0.0
0.5
1.0
1.5
2.0
2.5
3.0
0.0
0.2
0.4
0.6
0.8
1.0
N = 428   Bandwidth = 0.03495

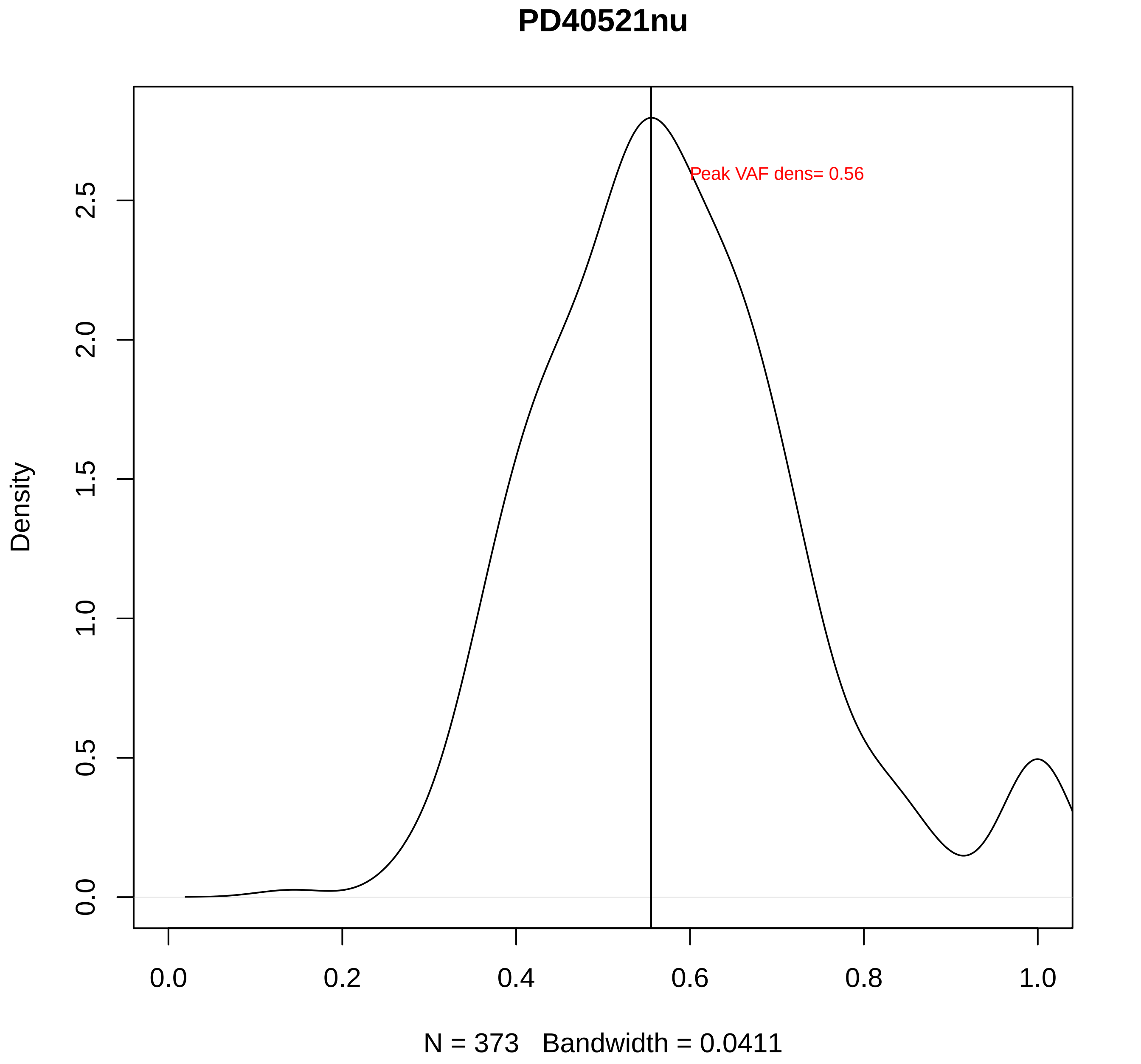

PD40521nu
Peak VAF dens= 0.56
Density
0.0
0.5
1.0
1.5
2.0
2.5
0.0
0.2
0.4
0.6
0.8
1.0
N = 373   Bandwidth = 0.0411

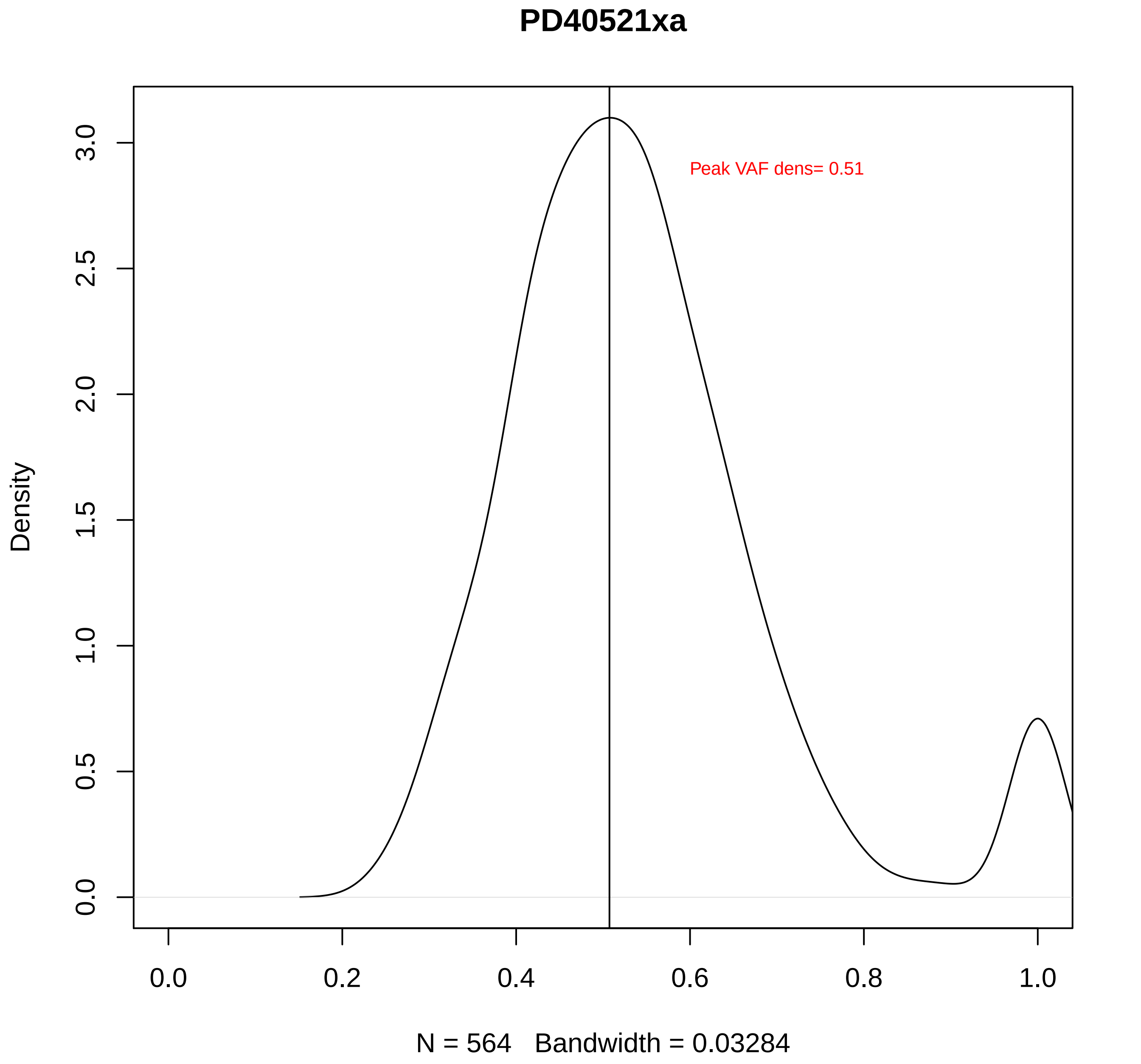
PD40521xa
Peak VAF dens= 0.51
Density
0.0
0.5
1.0
1.5
2.0
2.5
3.0
0.0
0.2
0.4
0.6
0.8
1.0
N = 564   Bandwidth = 0.03284

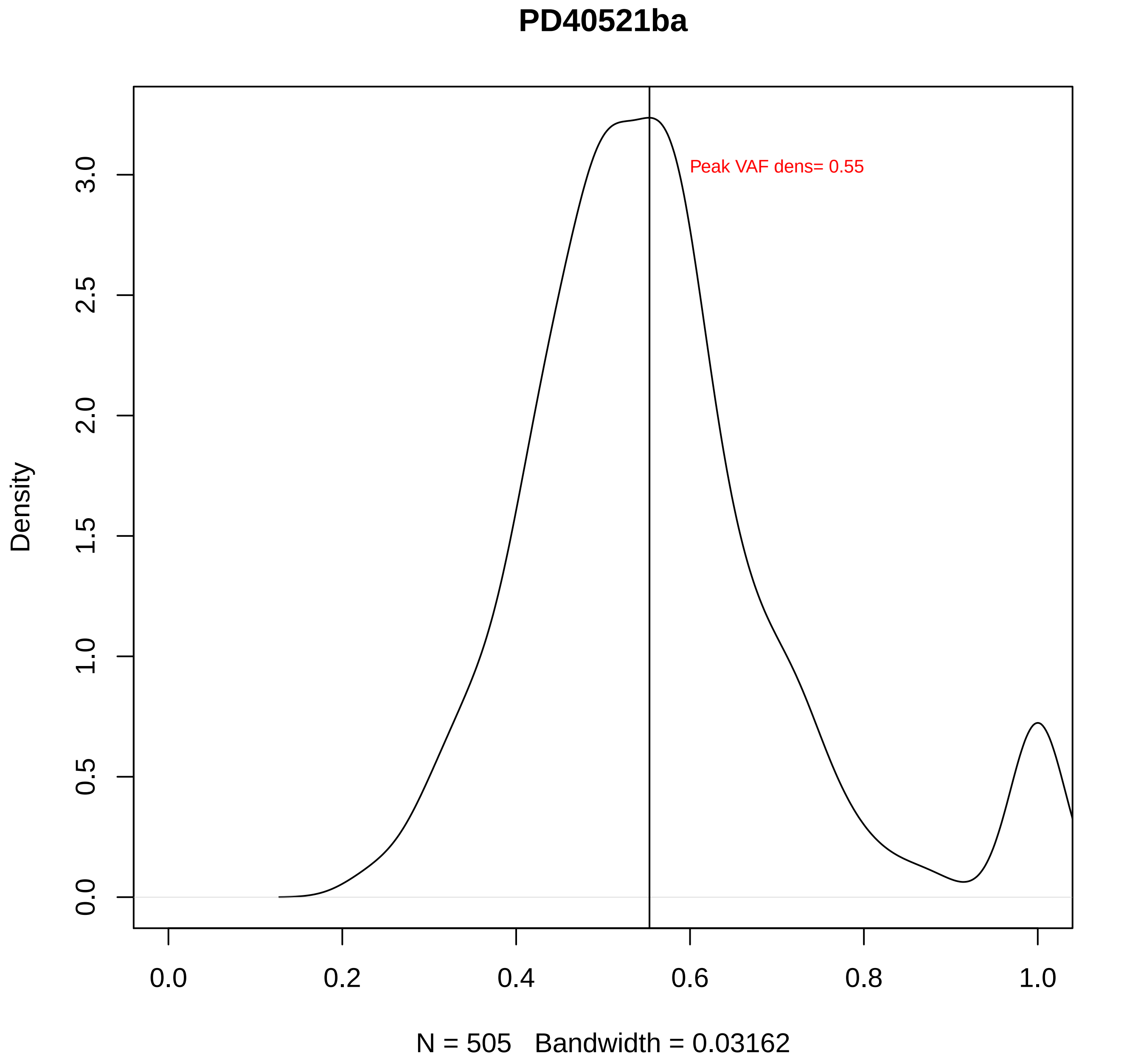

PD40521ba
Peak VAF dens= 0.55
Density
0.0
0.5
1.0
1.5
2.0
2.5
3.0
0.0
0.2
0.4
0.6
0.8
1.0
N = 505 Bandwidth = 0.03162

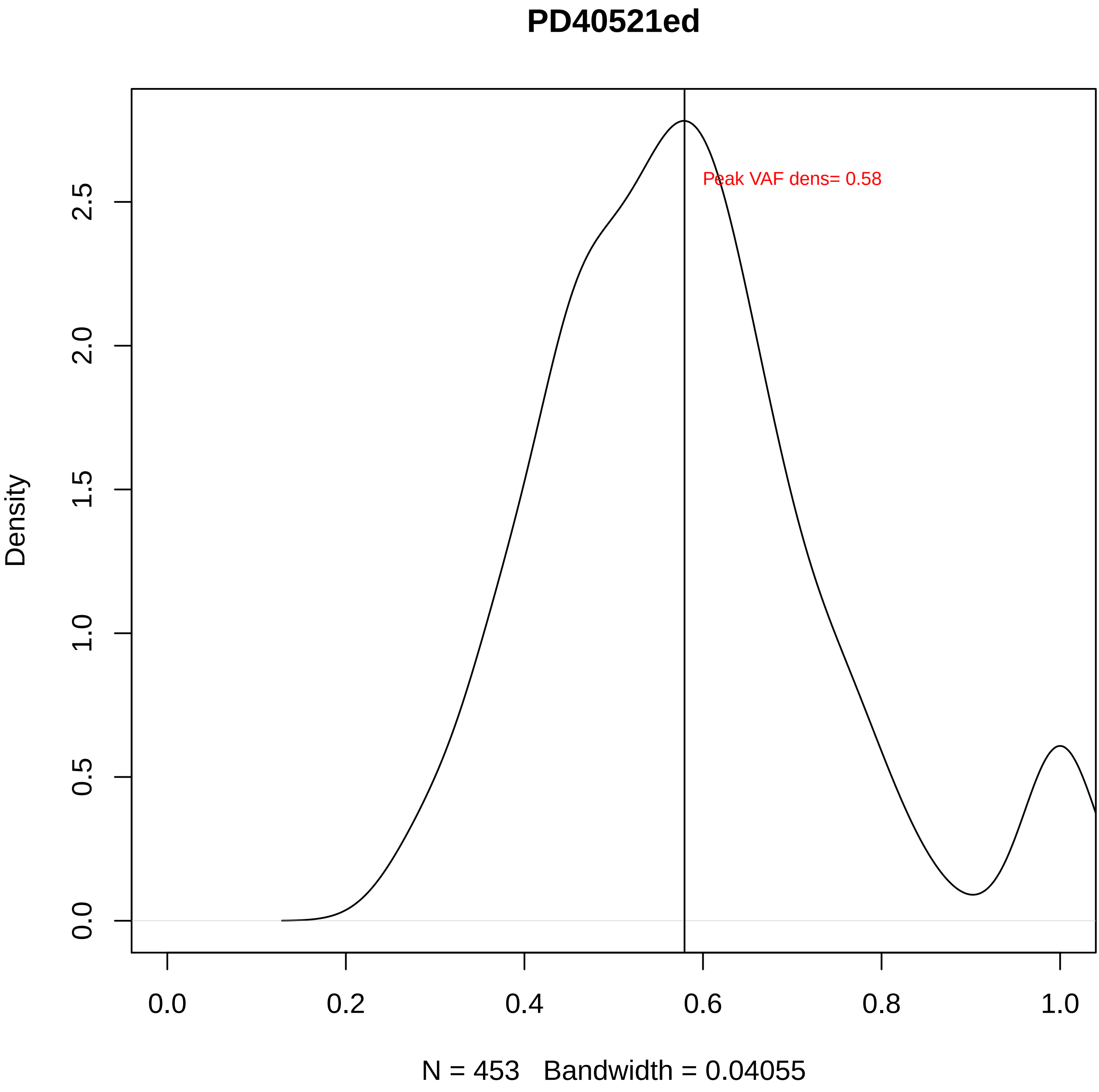
PD40521ed
Peak VAF dens= 0.58
Density
0.0
0.5
1.0
1.5
2.0
2.5
0.0
0.2
0.4
0.6
0.8
1.0
N = 453   Bandwidth = 0.04055

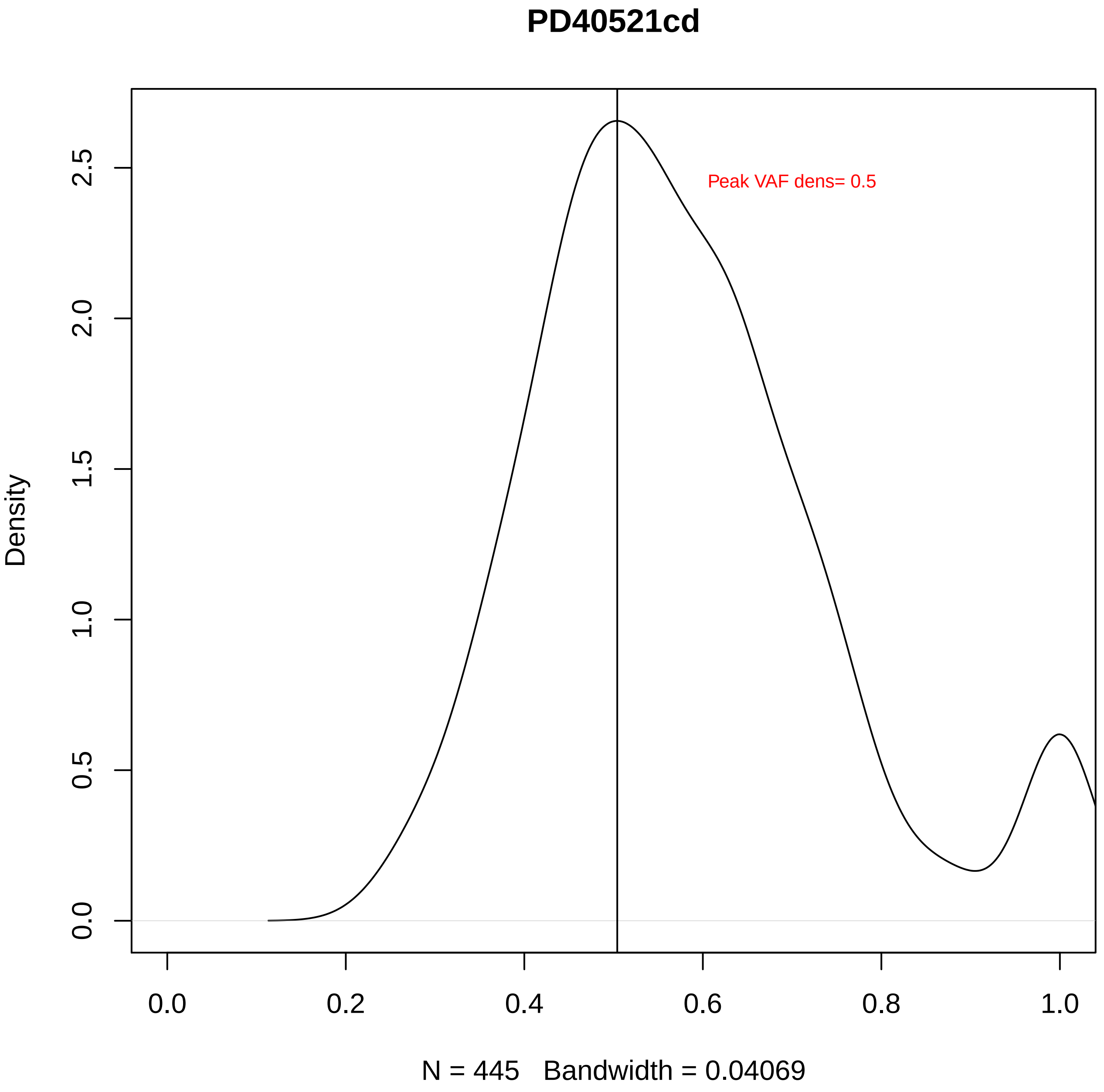
PD40521cd
Peak VAF dens= 0.5
Density
0.0
0.5
1.0
1.5
2.0
2.5
0.0
0.2
0.4
0.6
0.8
1.0
N = 445   Bandwidth = 0.04069

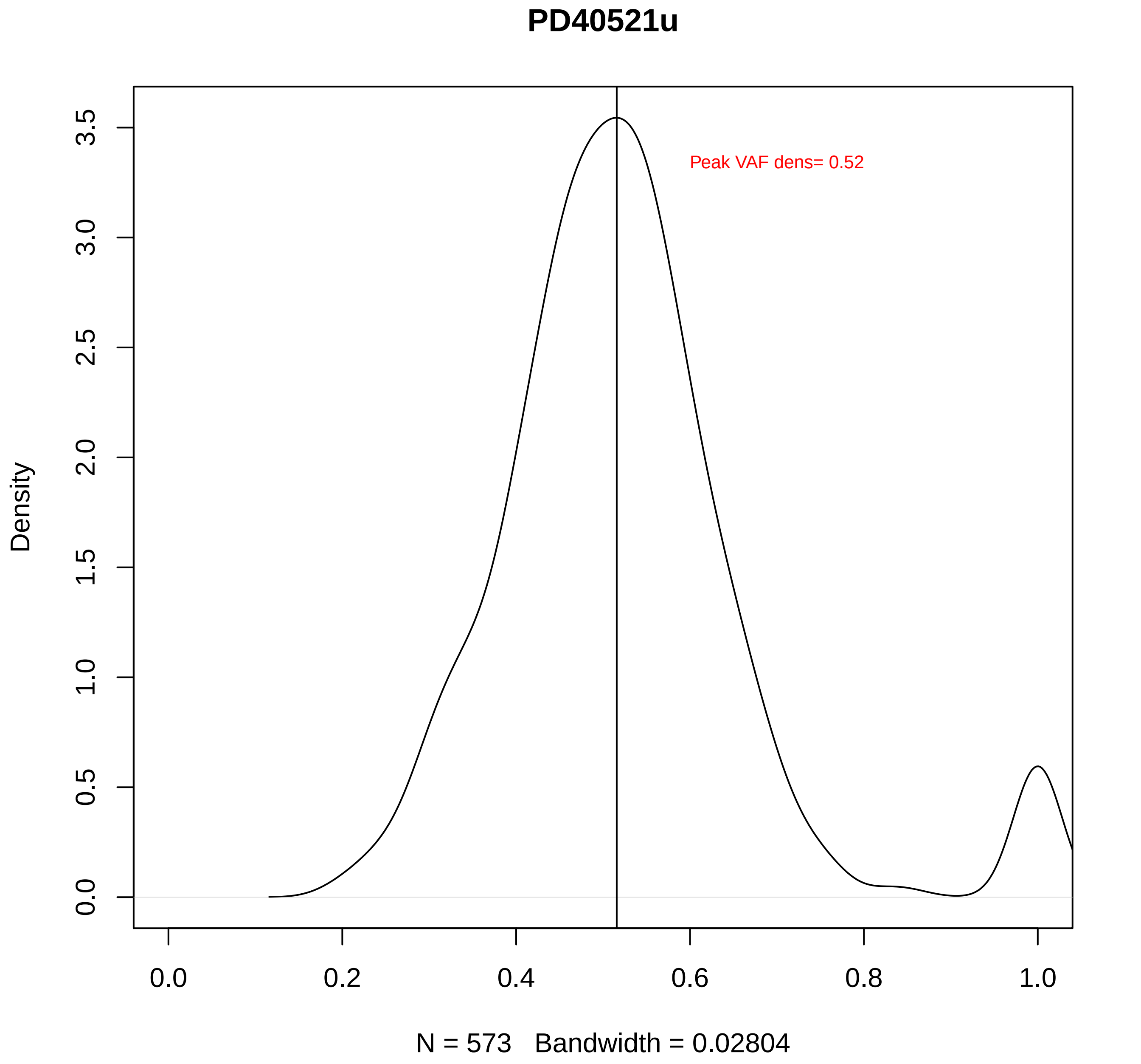
PD40521u
Peak VAF dens= 0.52
Density
0.0
0.5
1.0
1.5
2.0
2.5
3.0
3.5
0.0
0.2
0.4
0.6
0.8
1.0
N = 573   Bandwidth = 0.02804

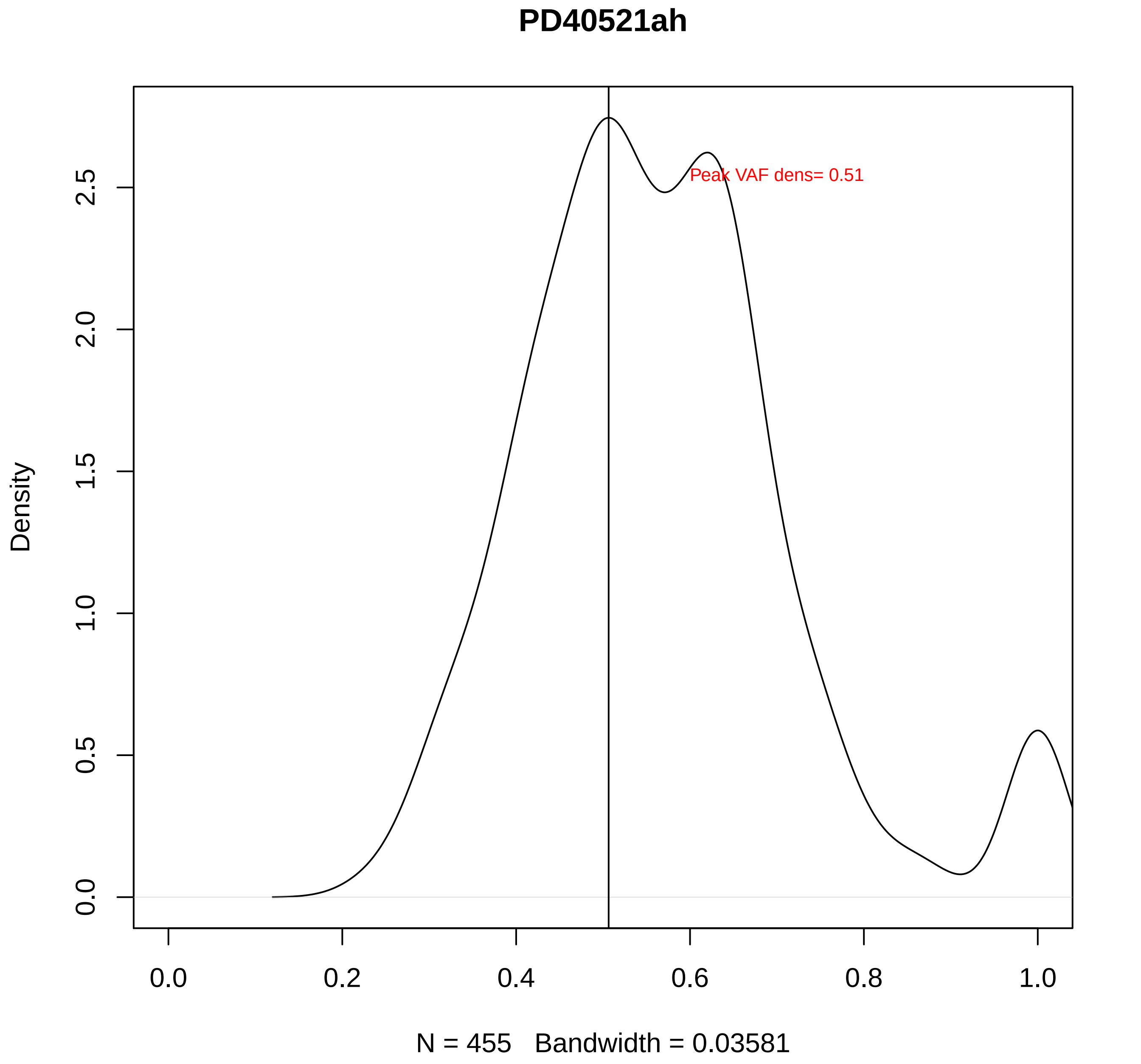
PD40521ah
Density
0.0
0.5
1.0
1.5
2.0
2.5
Peak VAF dens= 0.51
0.0
0.2
0.4
0.6
0.8
1.0
N = 455   Bandwidth = 0.03581

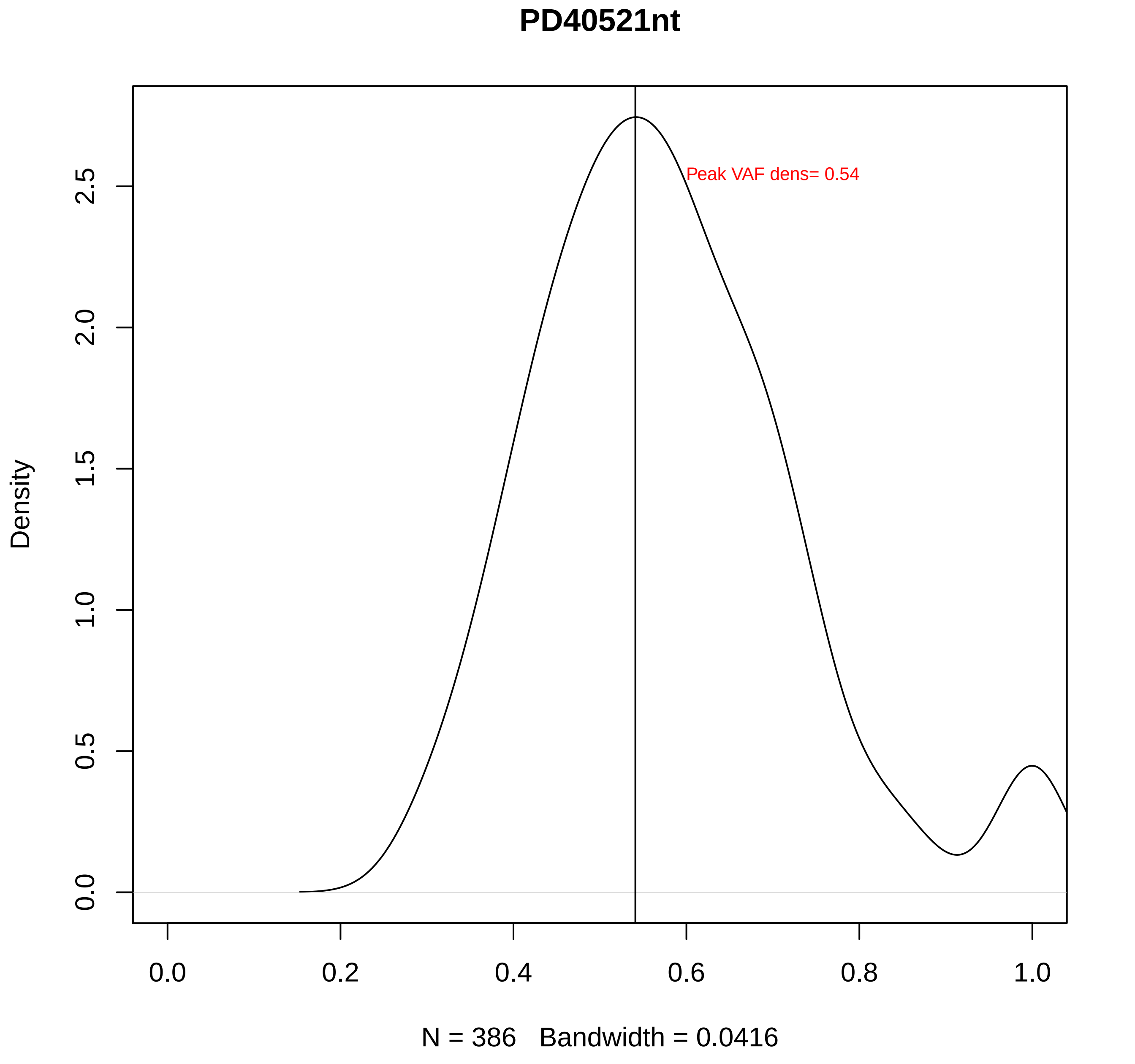
PD40521nt
Peak VAF dens= 0.54
Density
0.0
0.5
1.0
1.5
2.0
2.5
0.0
0.2
0.4
0.6
0.8
1.0
N = 386   Bandwidth = 0.0416

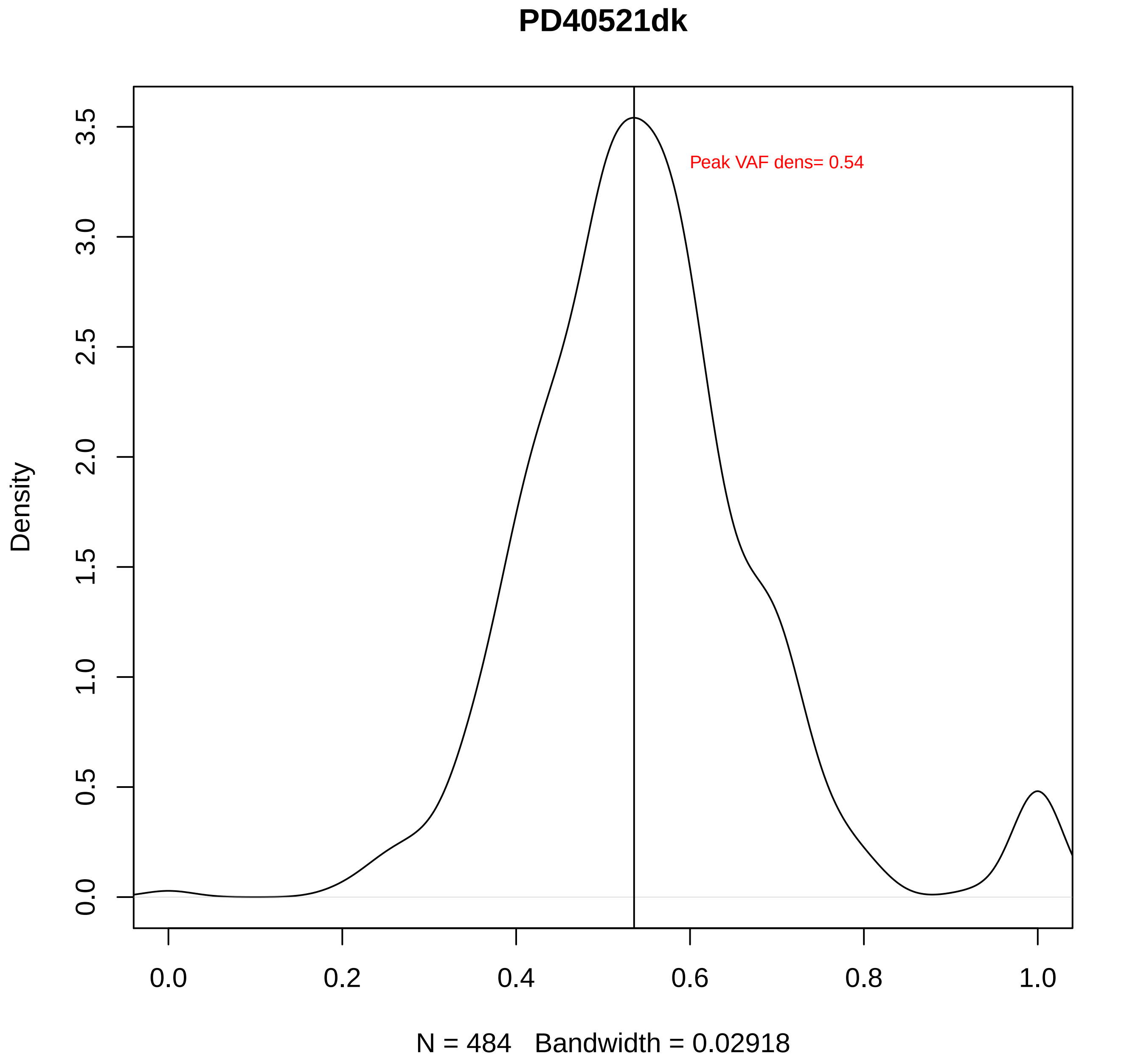
PD40521dk
Peak VAF dens= 0.54
Density
0.0
0.5
1.0
1.5
2.0
2.5
3.0
3.5
0.0
0.2
0.4
0.6
0.8
1.0
N = 484 Bandwidth = 0.02918

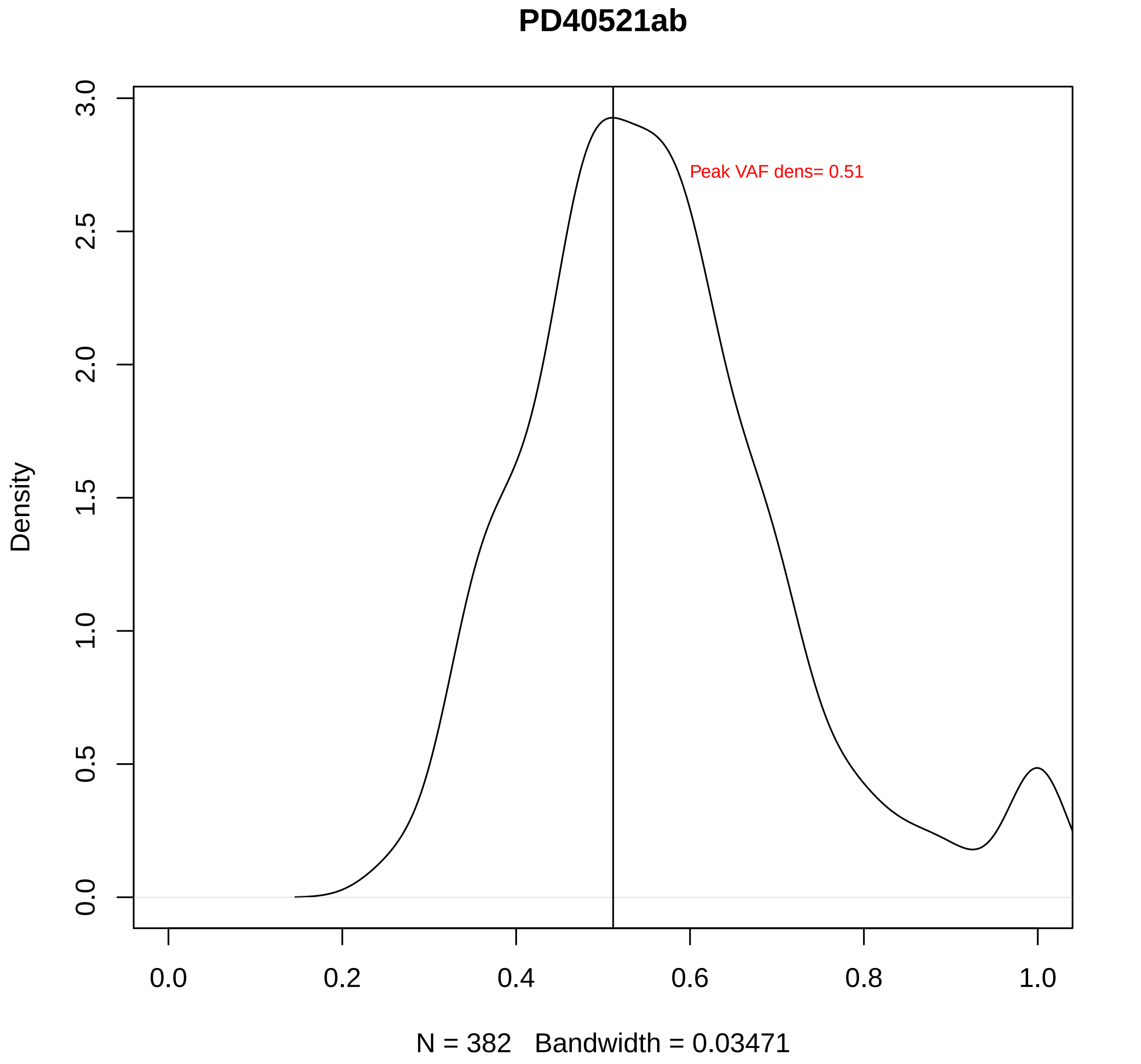
PD40521ab
Peak VAF dens= 0.51
Density
0.0
0.5
1.0
1.5
2.0
2.5
3.0
0.0
0.2
0.4
0.6
0.8
1.0
N = 382   Bandwidth = 0.03471

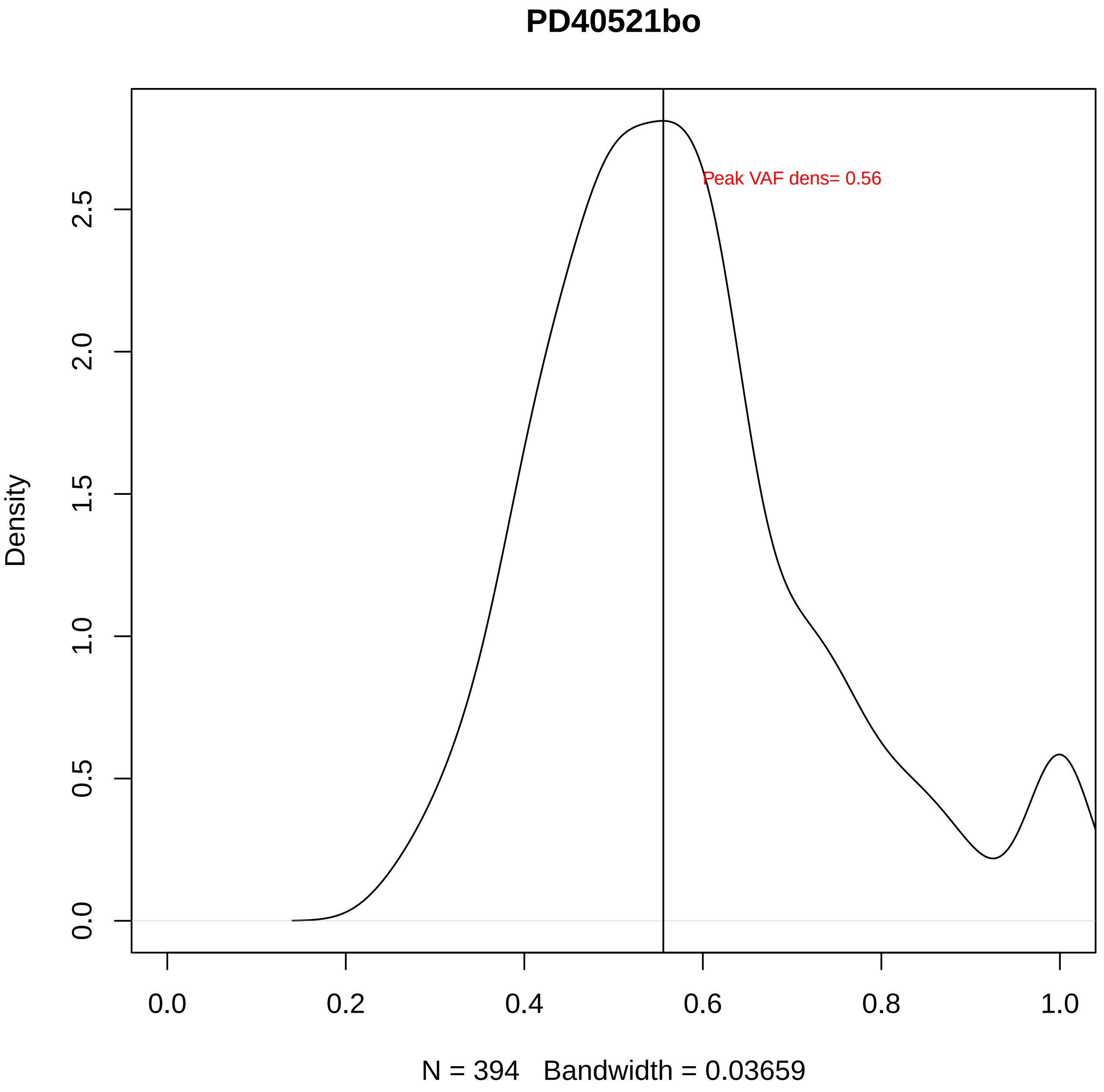

PD40521bo
Peak VAF dens= 0.56
Density
0.0
0.5
1.0
1.5
2.0
2.5
0.0
0.2
0.4
0.6
0.8
1.0
N = 394 Bandwidth = 0.03659

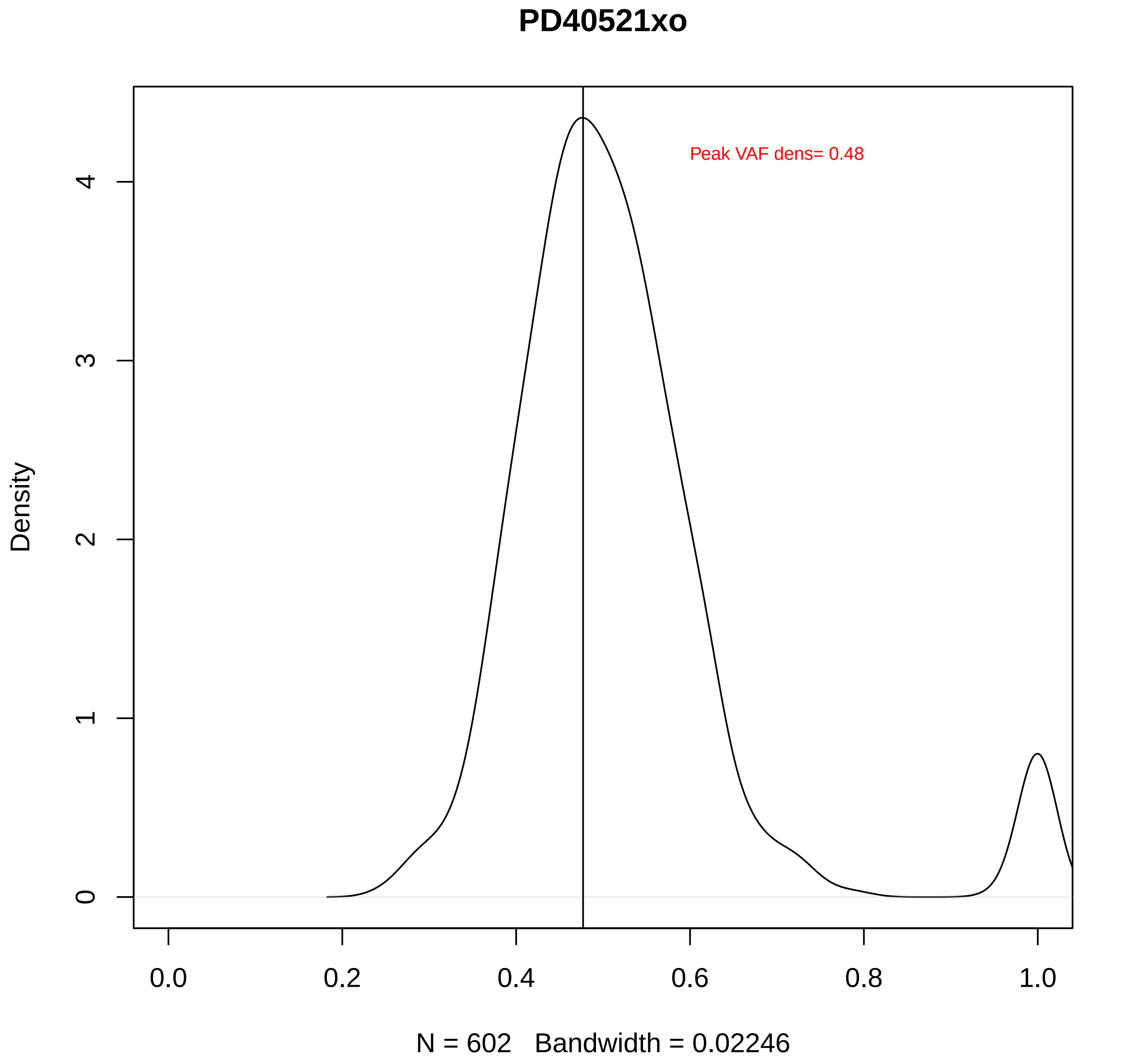
PD40521xo
Peak VAF dens= 0.48
Density
0
1
2
3
4
0.0
0.2
0.4
0.6
0.8
1.0
N = 602   Bandwidth = 0.02246

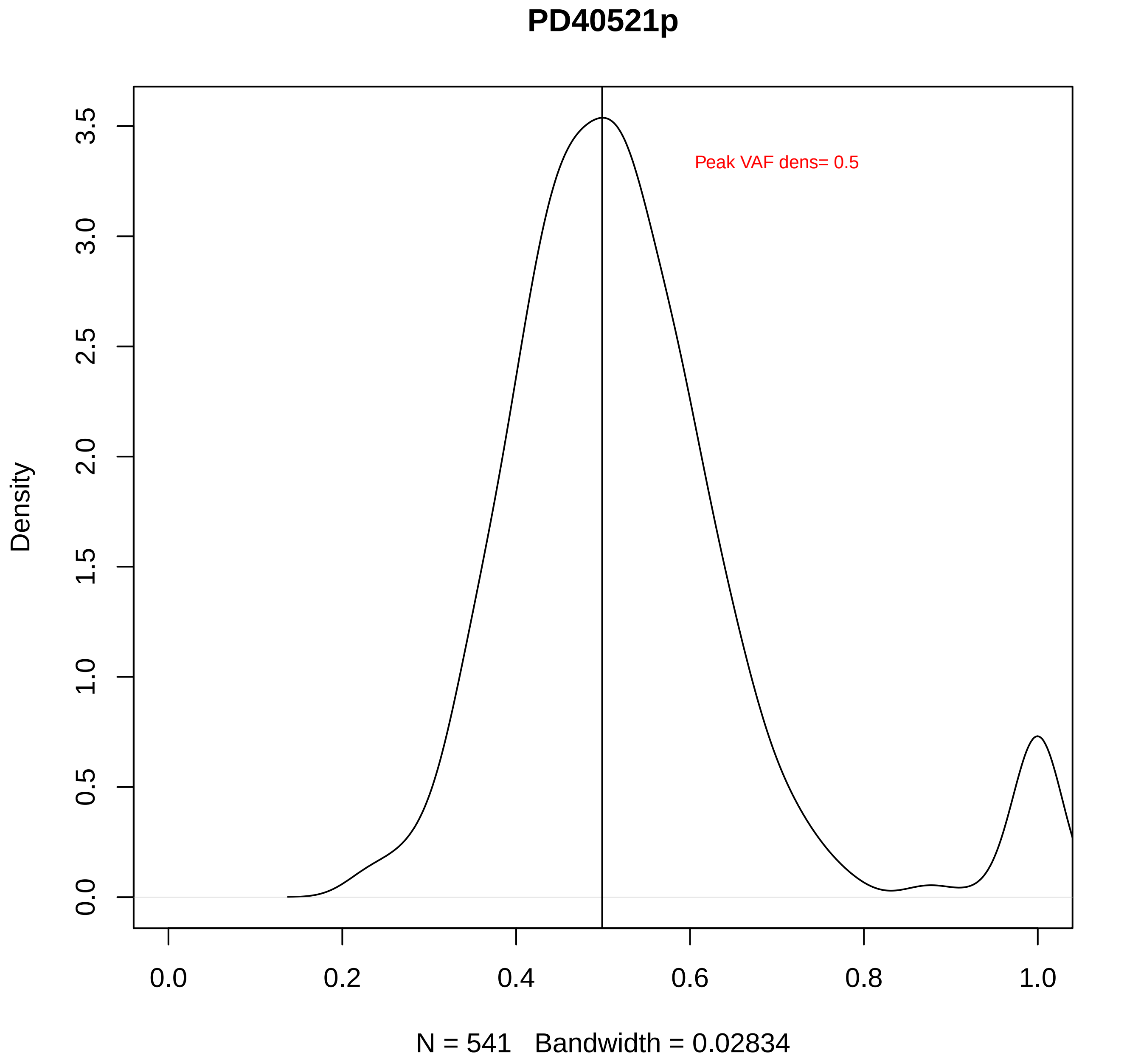
PD40521p
Peak VAF dens= 0.5
Density
0.0
0.5
1.0
1.5
2.0
2.5
3.0
3.5
0.0
0.2
0.4
0.6
0.8
1.0
N = 541 Bandwidth = 0.02834

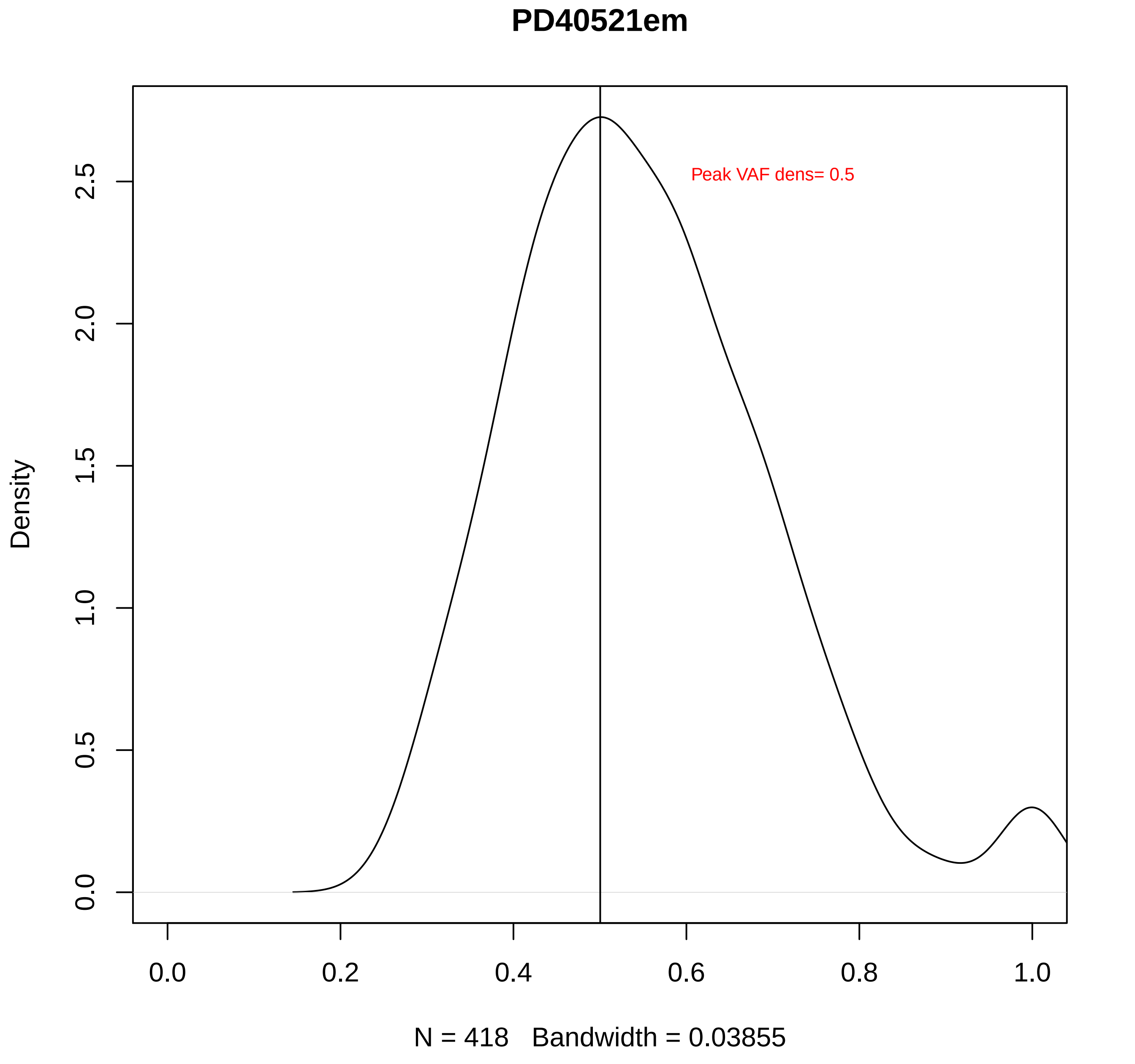

PD40521em
Peak VAF dens= 0.5
Density
0.0
0.5
1.0
1.5
2.0
2.5
0.0
0.2
0.4
0.6
0.8
1.0
N = 418   Bandwidth = 0.03855

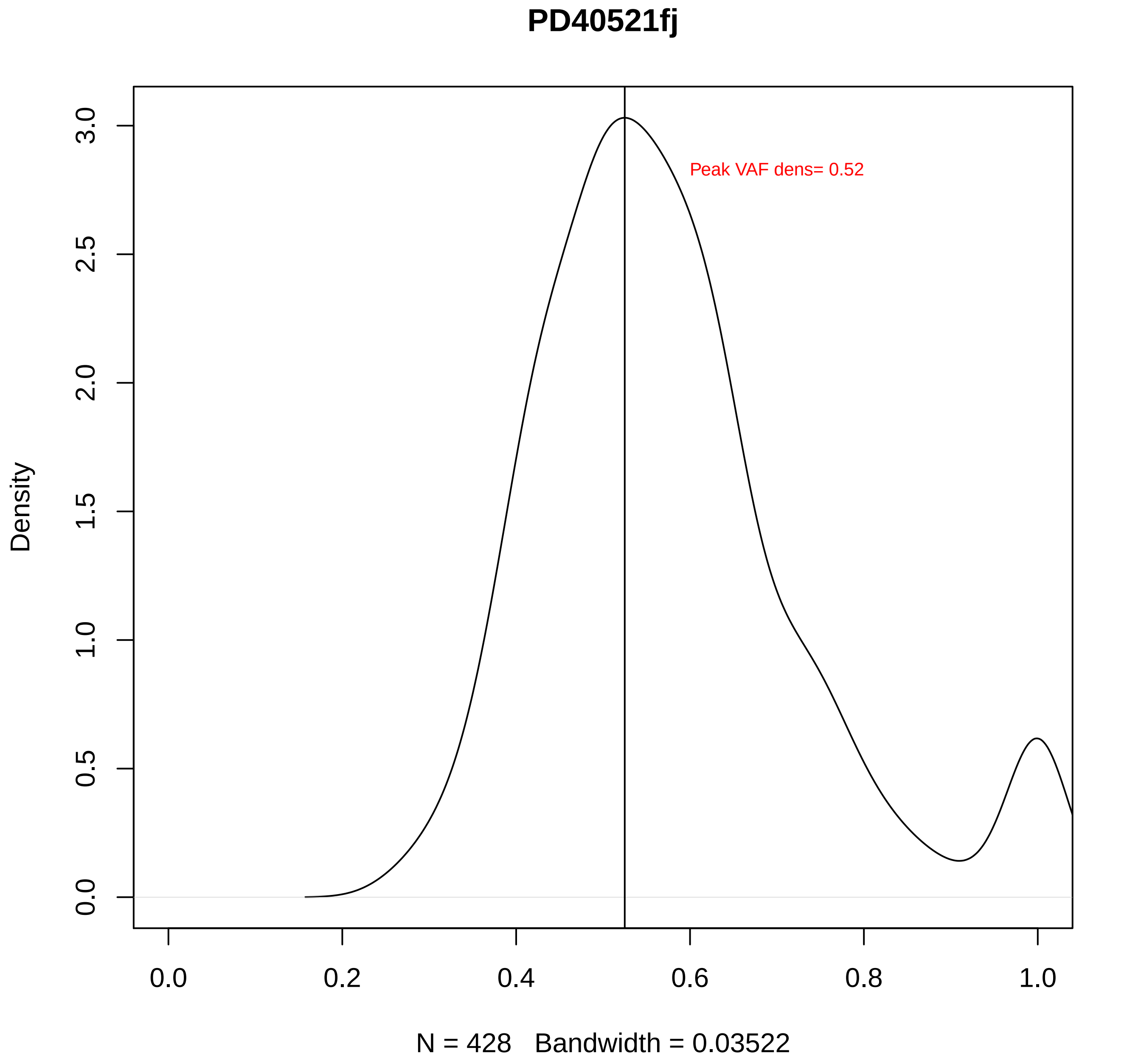
PD40521fj
Peak VAF dens= 0.52
Density
3.0
2.5
2.0
1.5
1.0
0.5
0.0
0.0
0.2
0.4
0.6
0.8
1.0
N = 428   Bandwidth = 0.03522

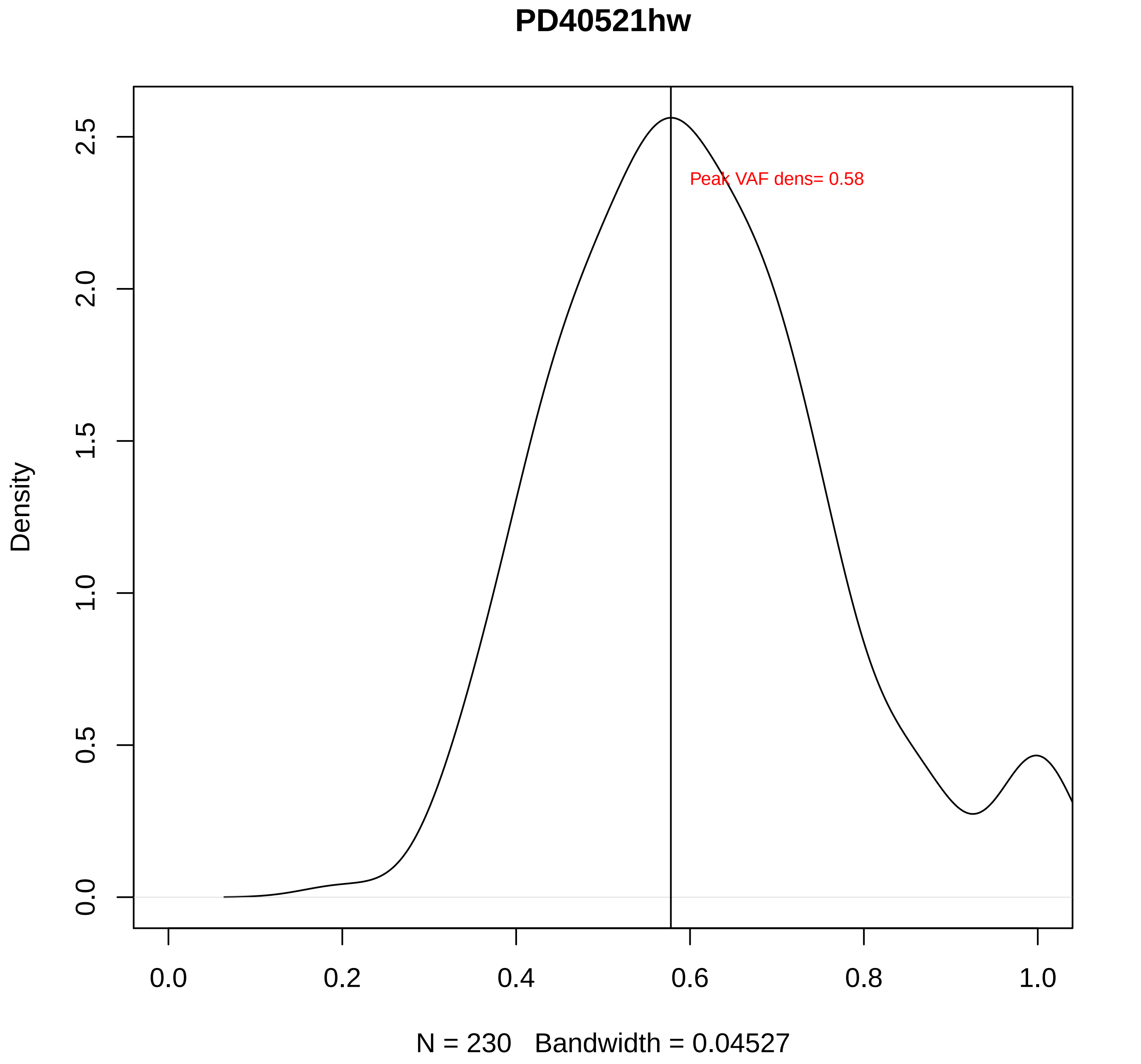
PD40521hw
Peak VAF dens= 0.58
Density
0.0
0.5
1.0
1.5
2.0
2.5
0.0
0.2
0.4
0.6
0.8
1.0
N = 230   Bandwidth = 0.04527

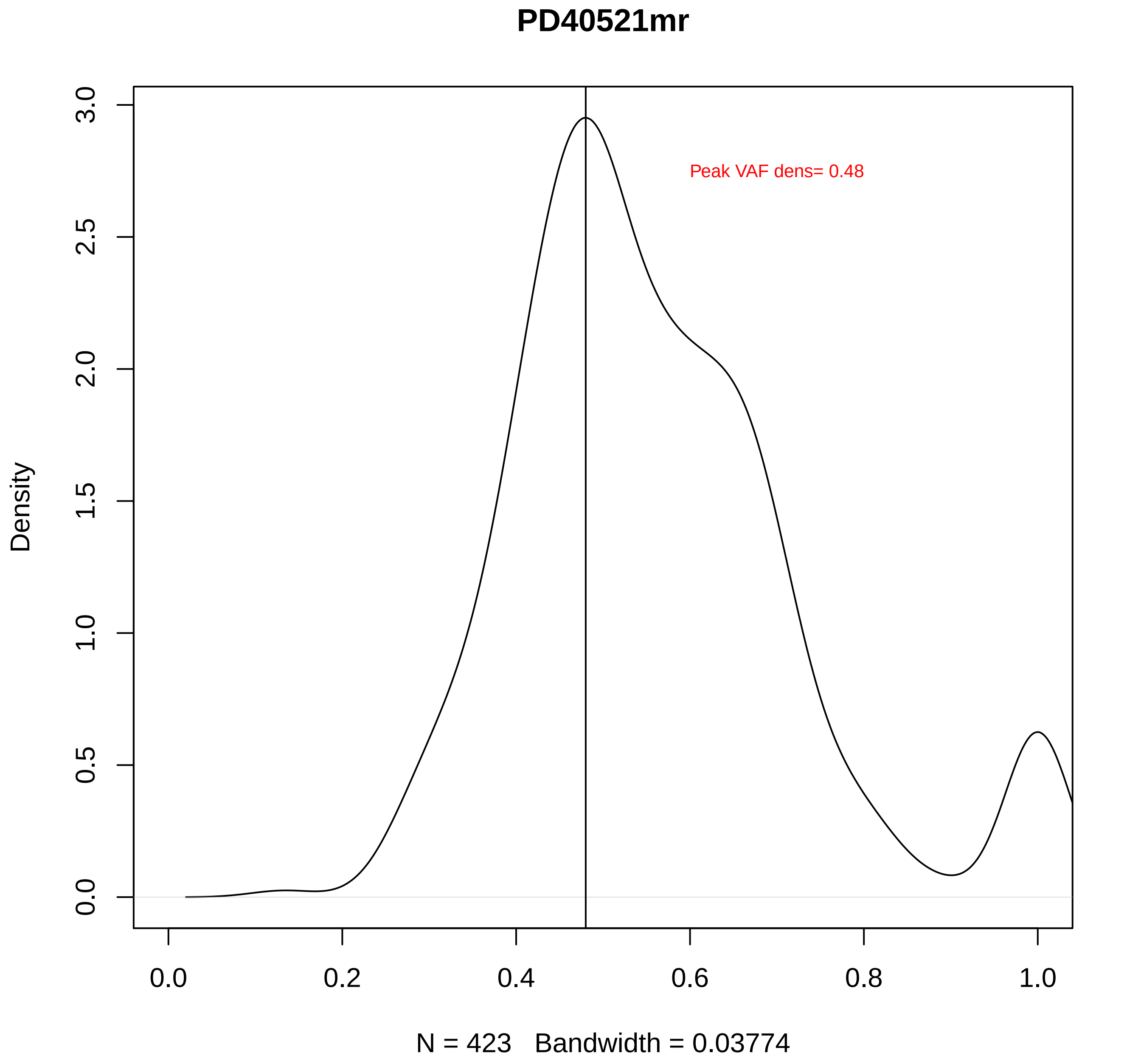
PD40521mr
Peak VAF dens= 0.48
Density
3.0
2.5
2.0
1.5
1.0
0.5
0.0
0.0
0.2
0.4
0.6
0.8
1.0
N = 423   Bandwidth = 0.03774

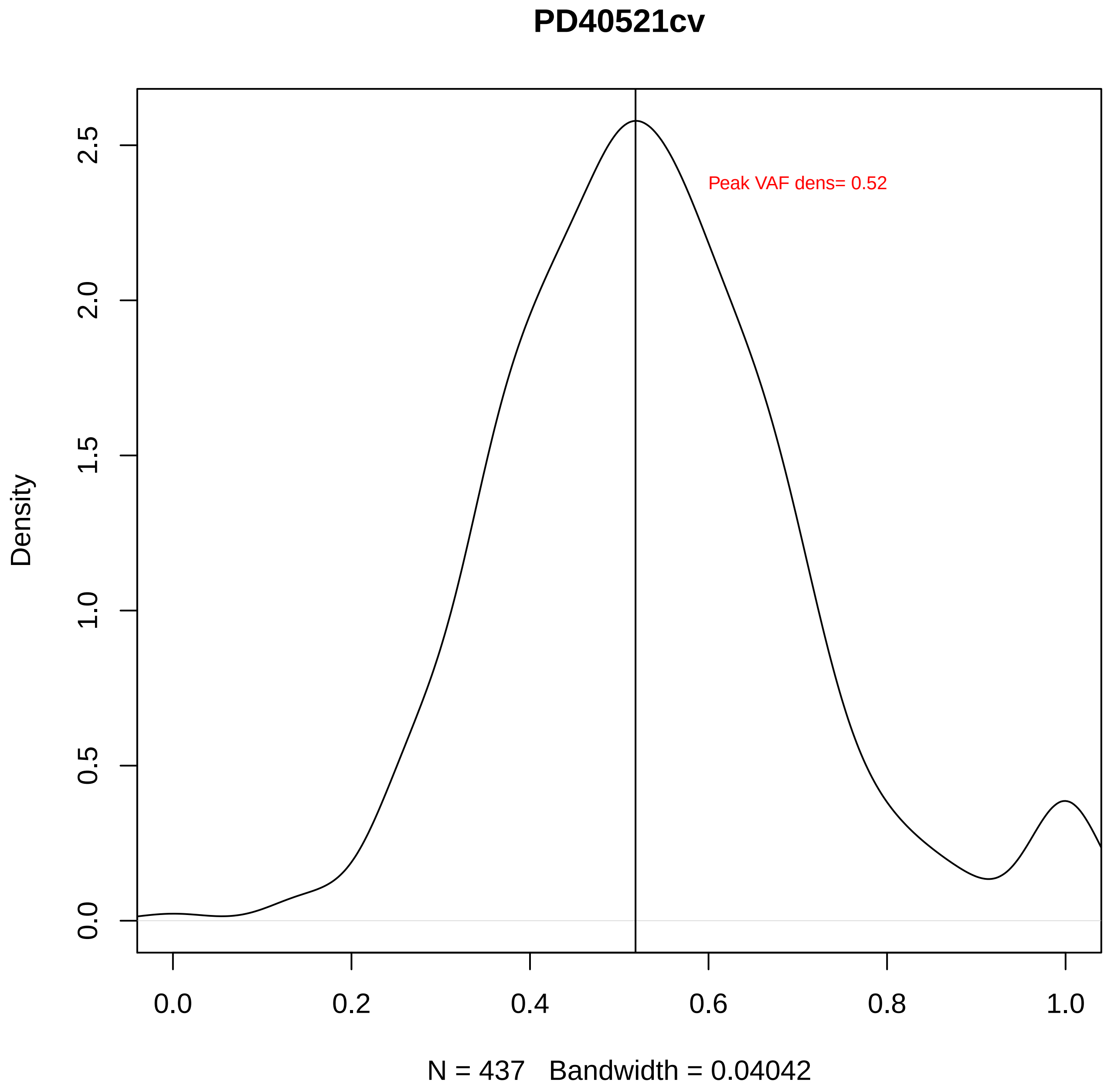
PD40521cv
Peak VAF dens= 0.52
Density
0.0
0.5
1.0
1.5
2.0
2.5
0.0
0.2
0.4
0.6
0.8
1.0
N = 437   Bandwidth = 0.04042

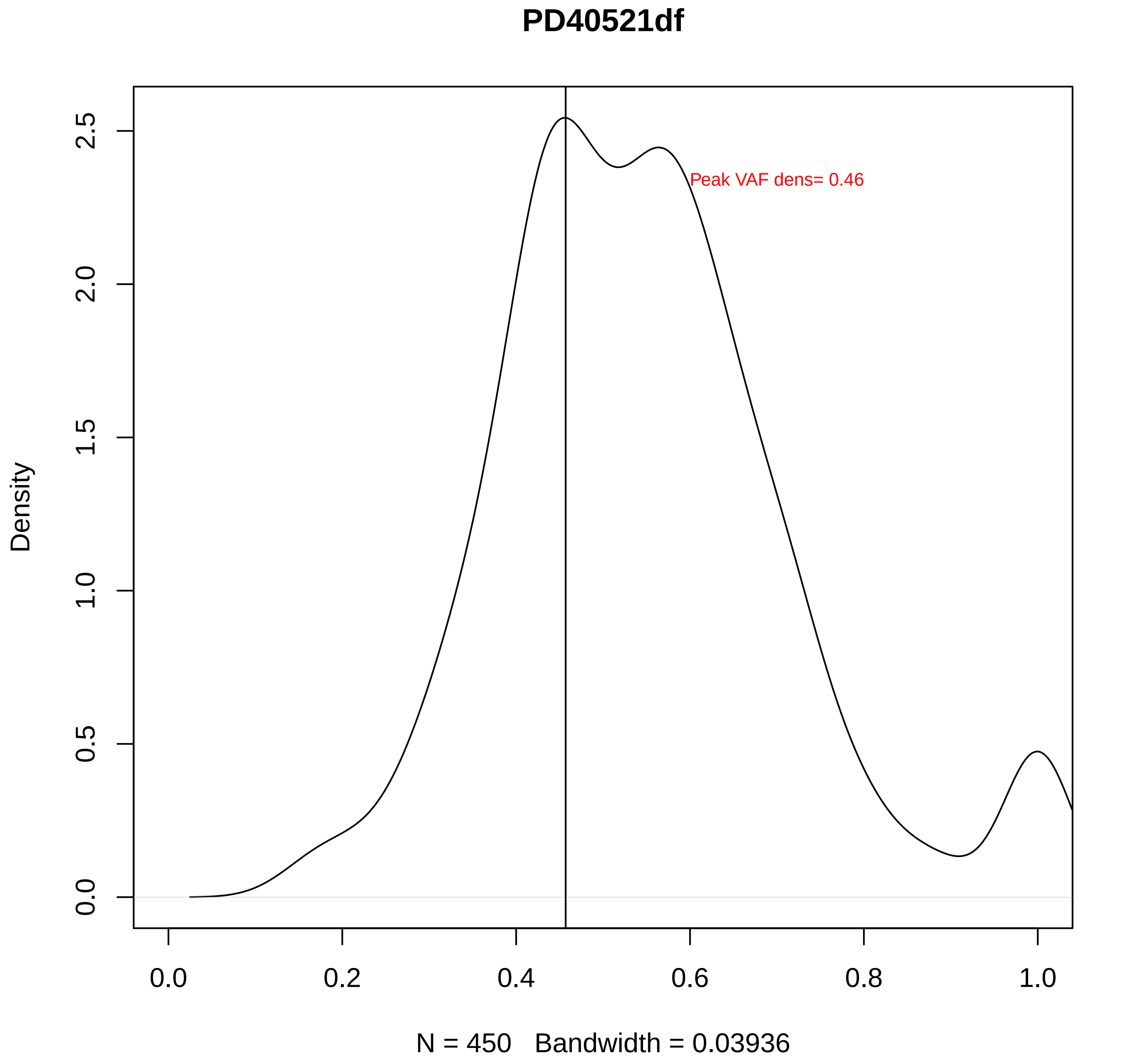
PD40521df
Peak VAF dens= 0.46
Density
0.0
0.5
1.0
1.5
2.0
2.5
0.0
0.2
0.4
0.6
0.8
1.0
N = 450   Bandwidth = 0.03936

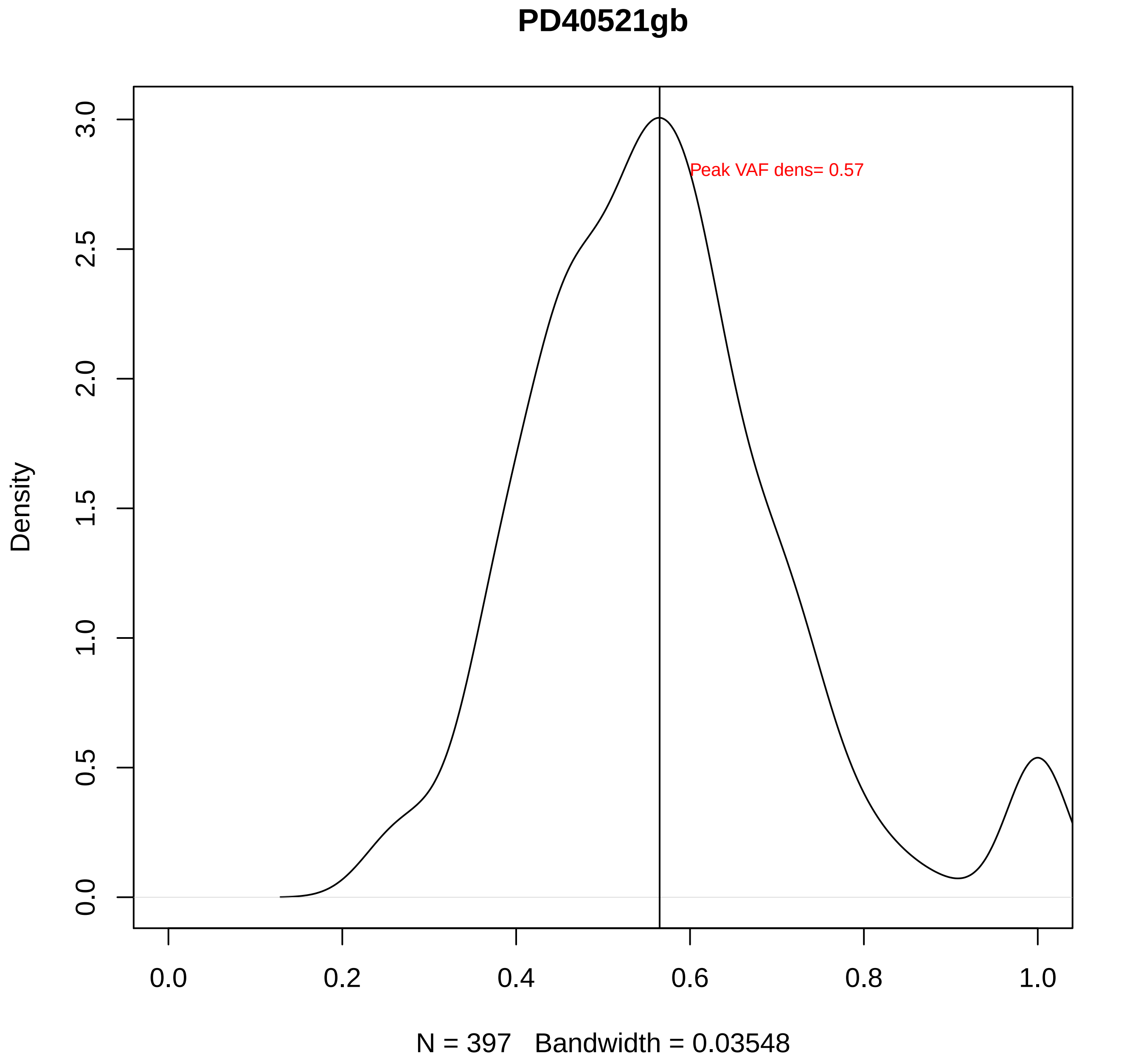
PD40521gb
Peak VAF dens= 0.57
Density
3.0
2.5
2.0
1.5
1.0
0.5
0.0
0.0
0.2
0.4
0.6
0.8
1.0
N = 397   Bandwidth = 0.03548

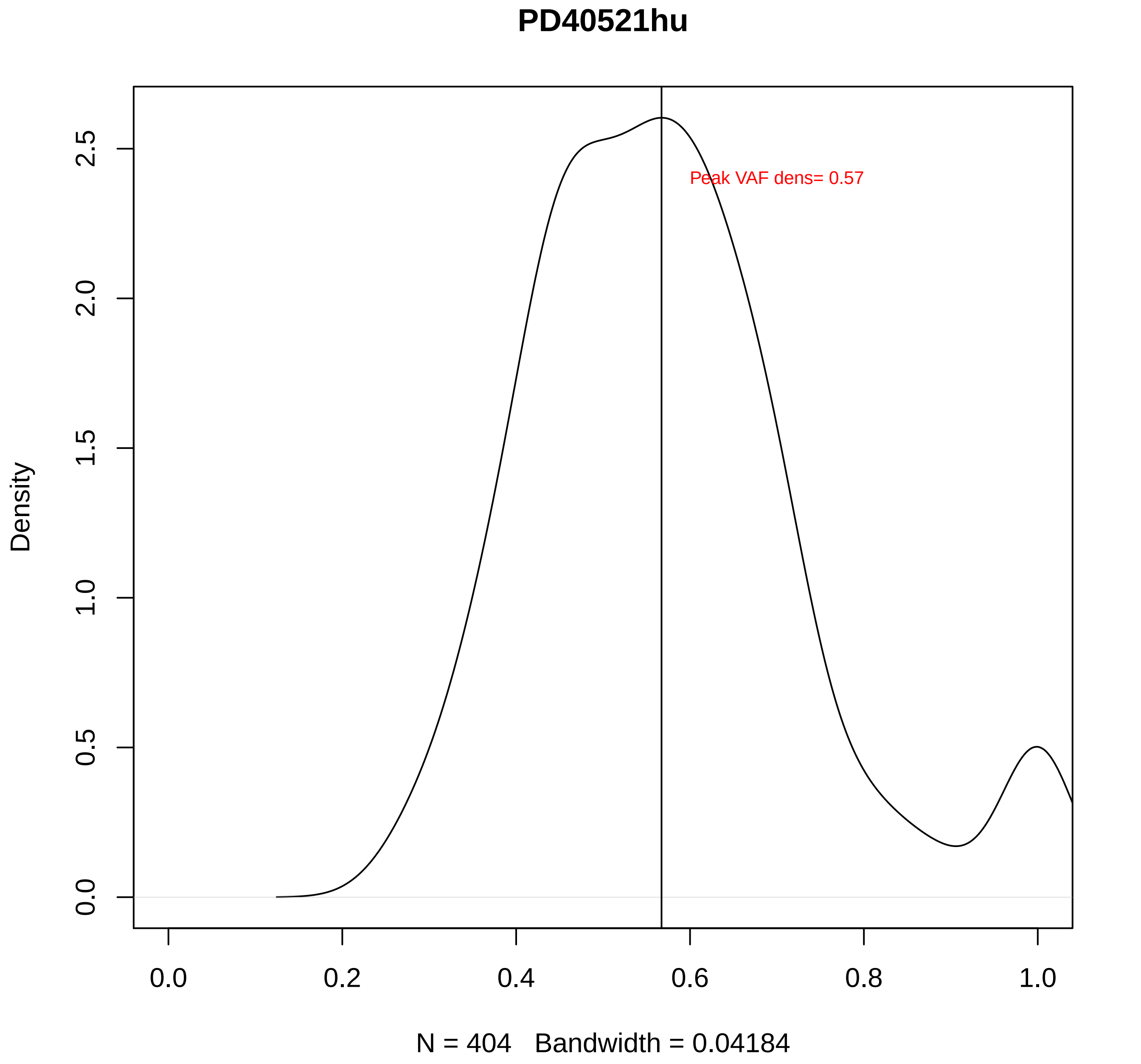
PD40521hu
Peak VAF dens= 0.57
Density
0.0
0.5
1.0
1.5
2.0
2.5
0.0
0.2
0.4
0.6
0.8
1.0
N = 404   Bandwidth = 0.04184

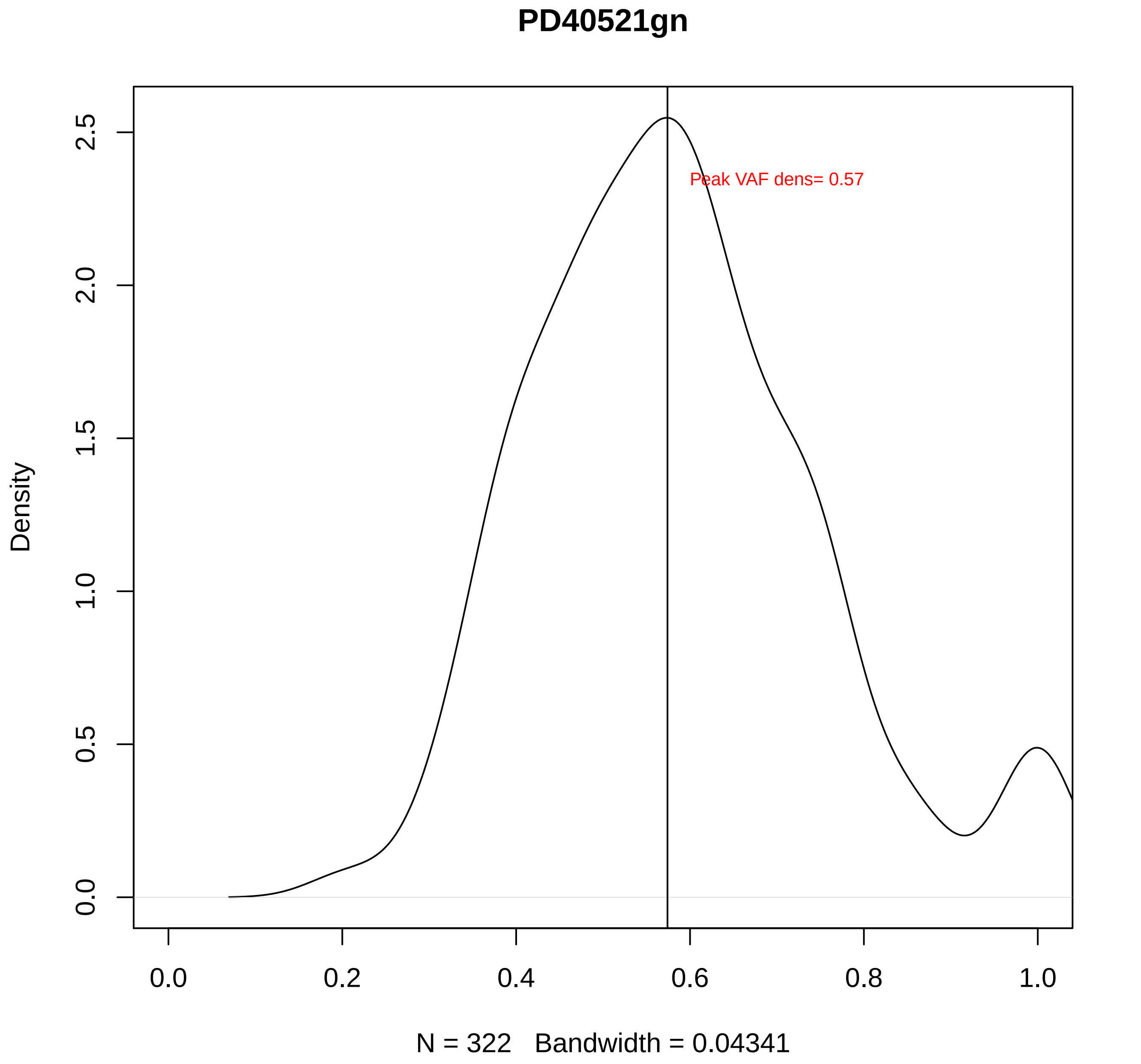
PD40521gn
Peak VAF dens= 0.57
Density
0.0
0.5
1.0
1.5
2.0
2.5
0.0
0.2
0.4
0.6
0.8
1.0
N = 322   Bandwidth = 0.04341

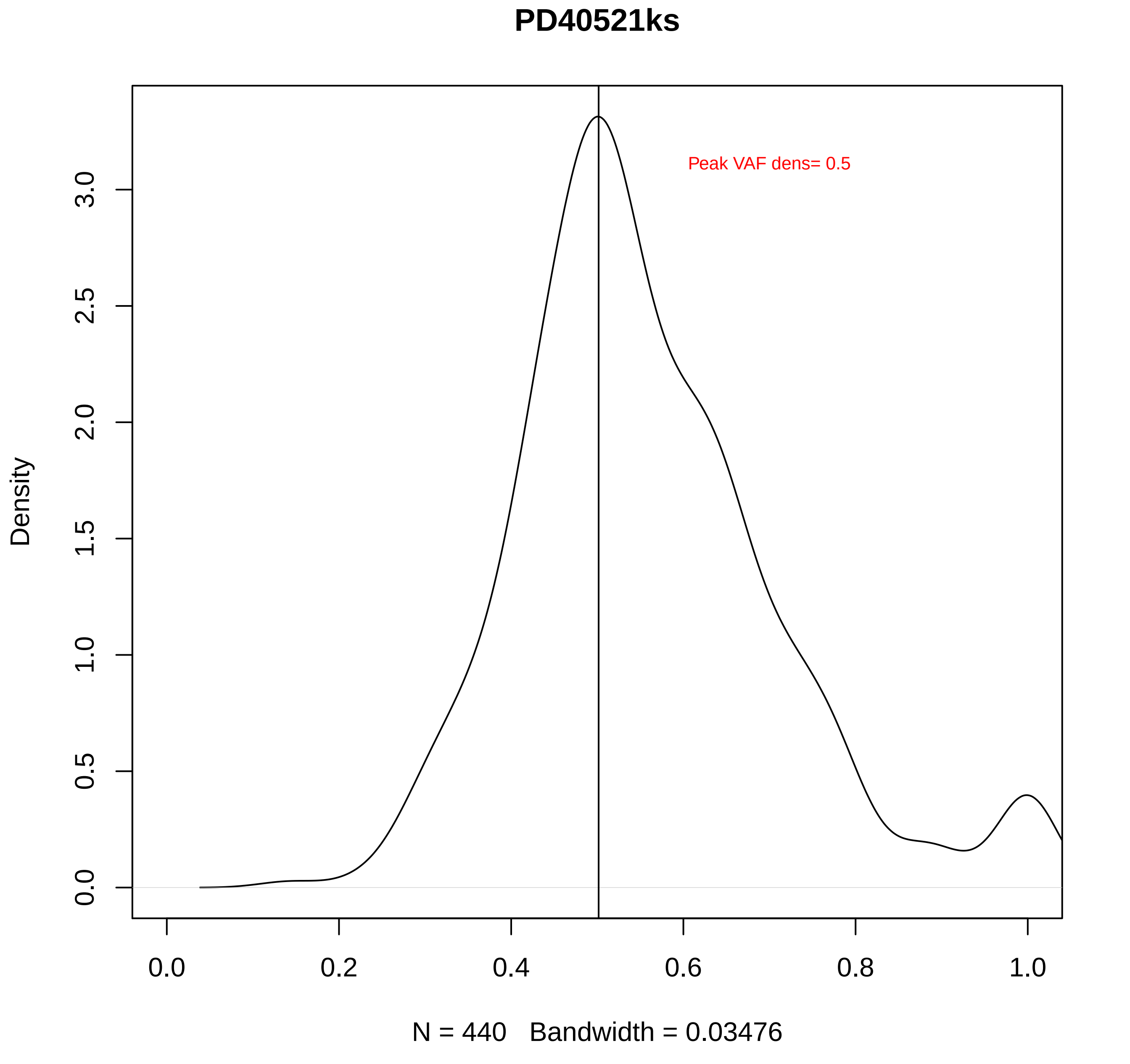

PD40521ks
Peak VAF dens= 0.5
Density
0.0
0.5
1.0
1.5
2.0
2.5
3.0
0.0
0.2
0.4
0.6
0.8
1.0
N = 440   Bandwidth = 0.03476

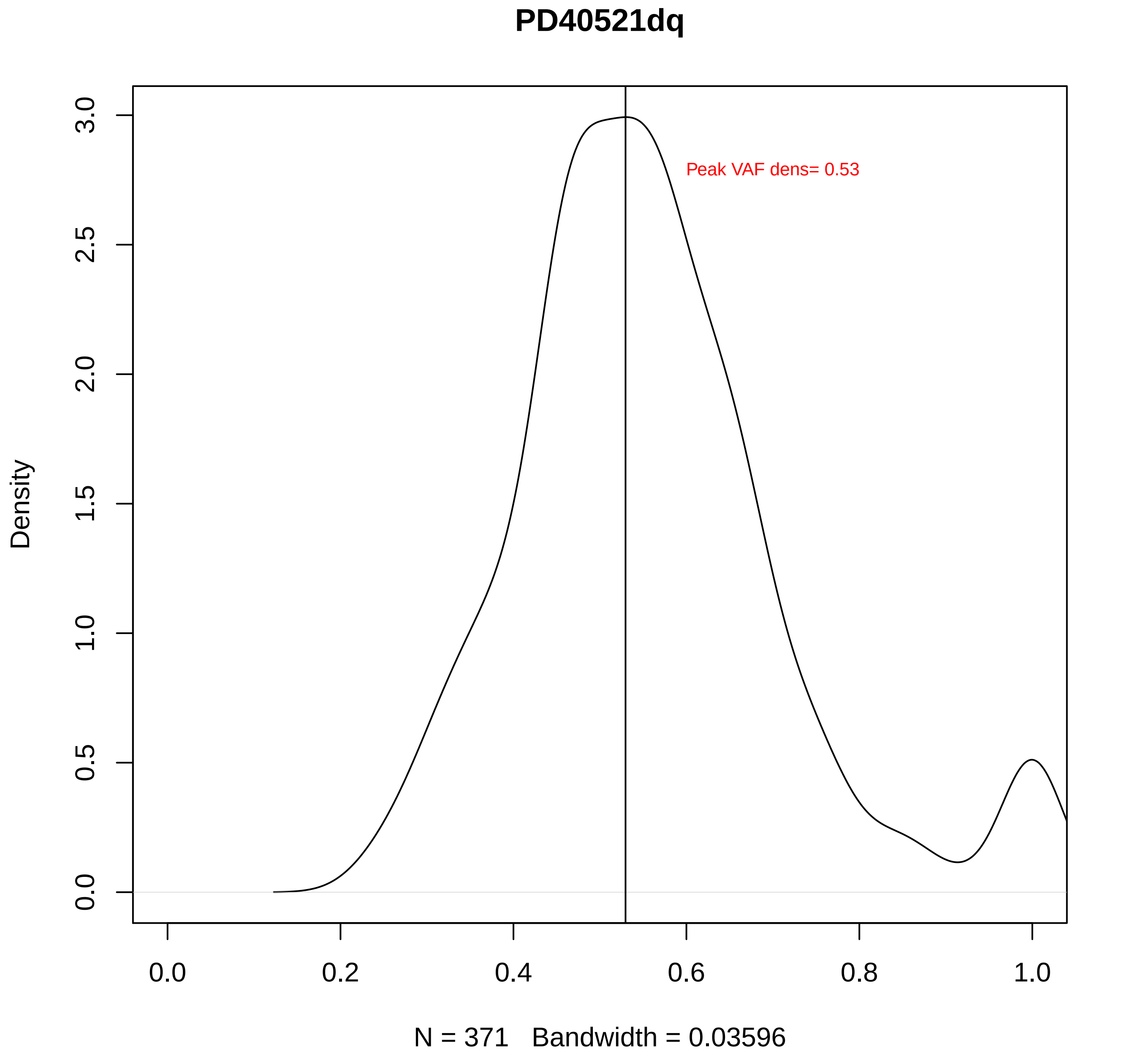
PD40521dq
Peak VAF dens= 0.53
Density
3.0
2.5
2.0
1.5
1.0
0.5
0.0
0.0
0.2
0.4
0.6
0.8
1.0
N = 371   Bandwidth = 0.03596

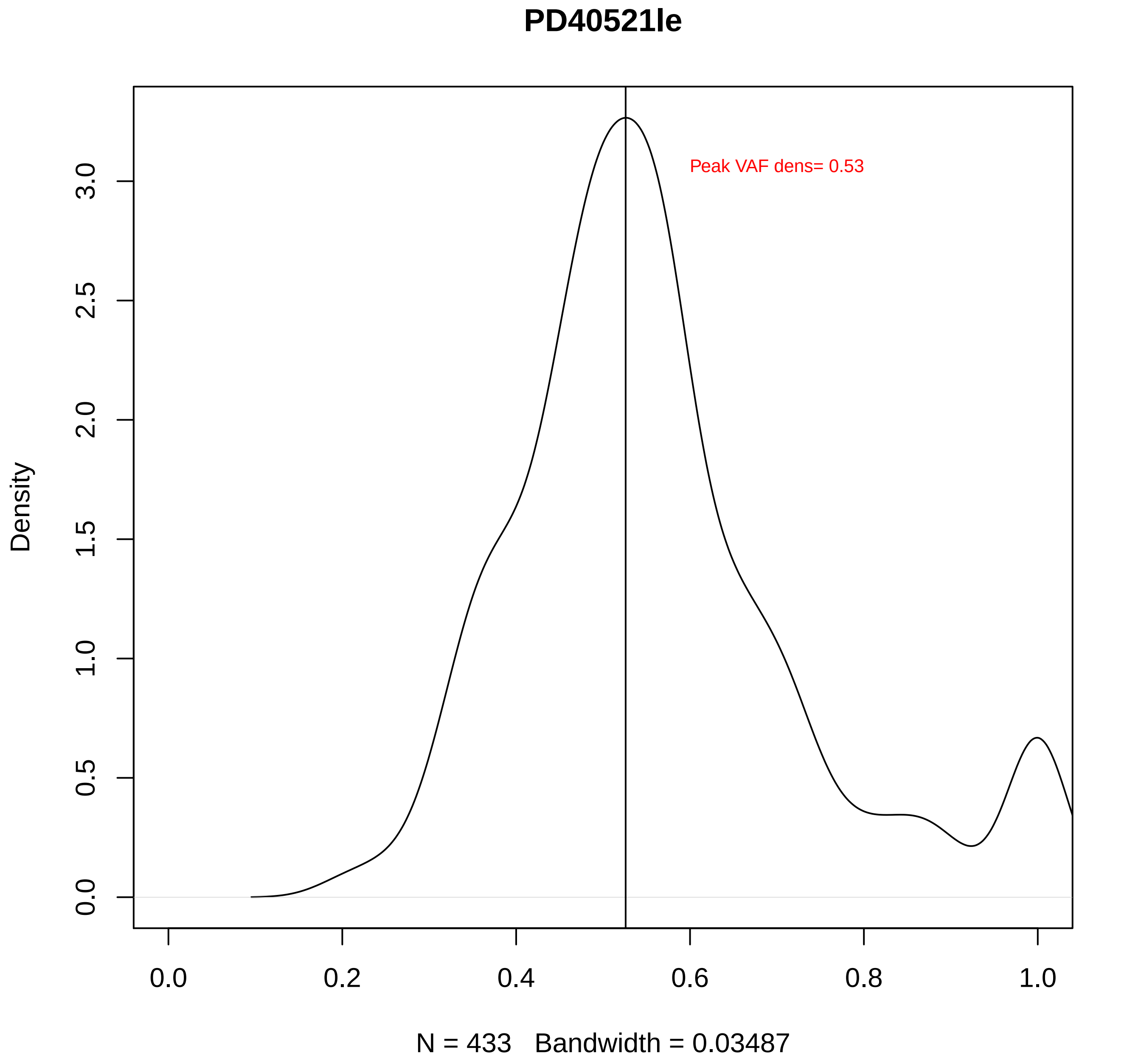
PD40521le
Peak VAF dens= 0.53
Density
0.0
0.5
1.0
1.5
2.0
2.5
3.0
0.0
0.2
0.4
0.6
0.8
1.0
N = 433   Bandwidth = 0.03487

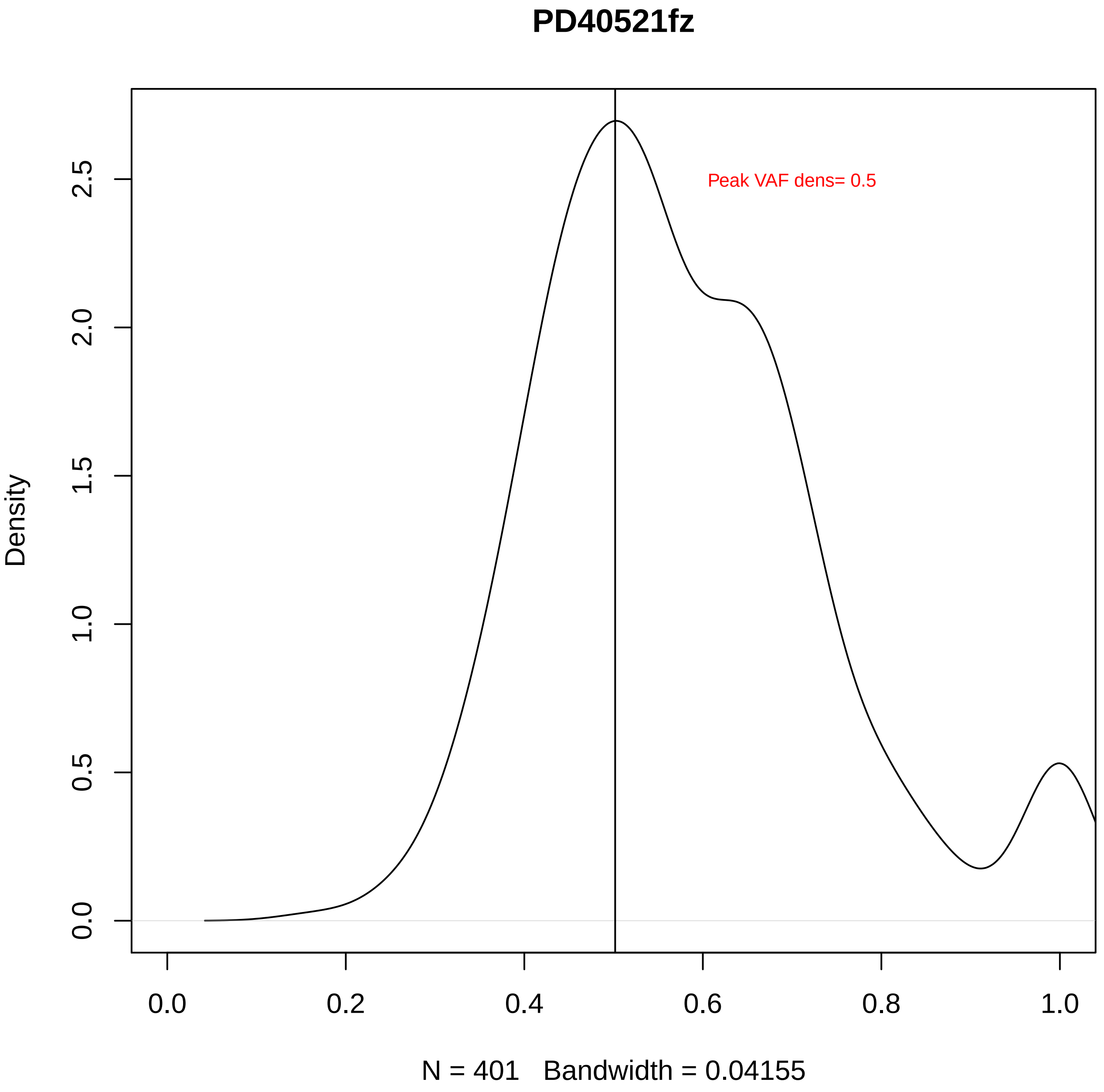
PD40521fz
Peak VAF dens= 0.5
Density
0.0
0.5
1.0
1.5
2.0
2.5
0.0
0.2
0.4
0.6
0.8
1.0
N = 401   Bandwidth = 0.04155

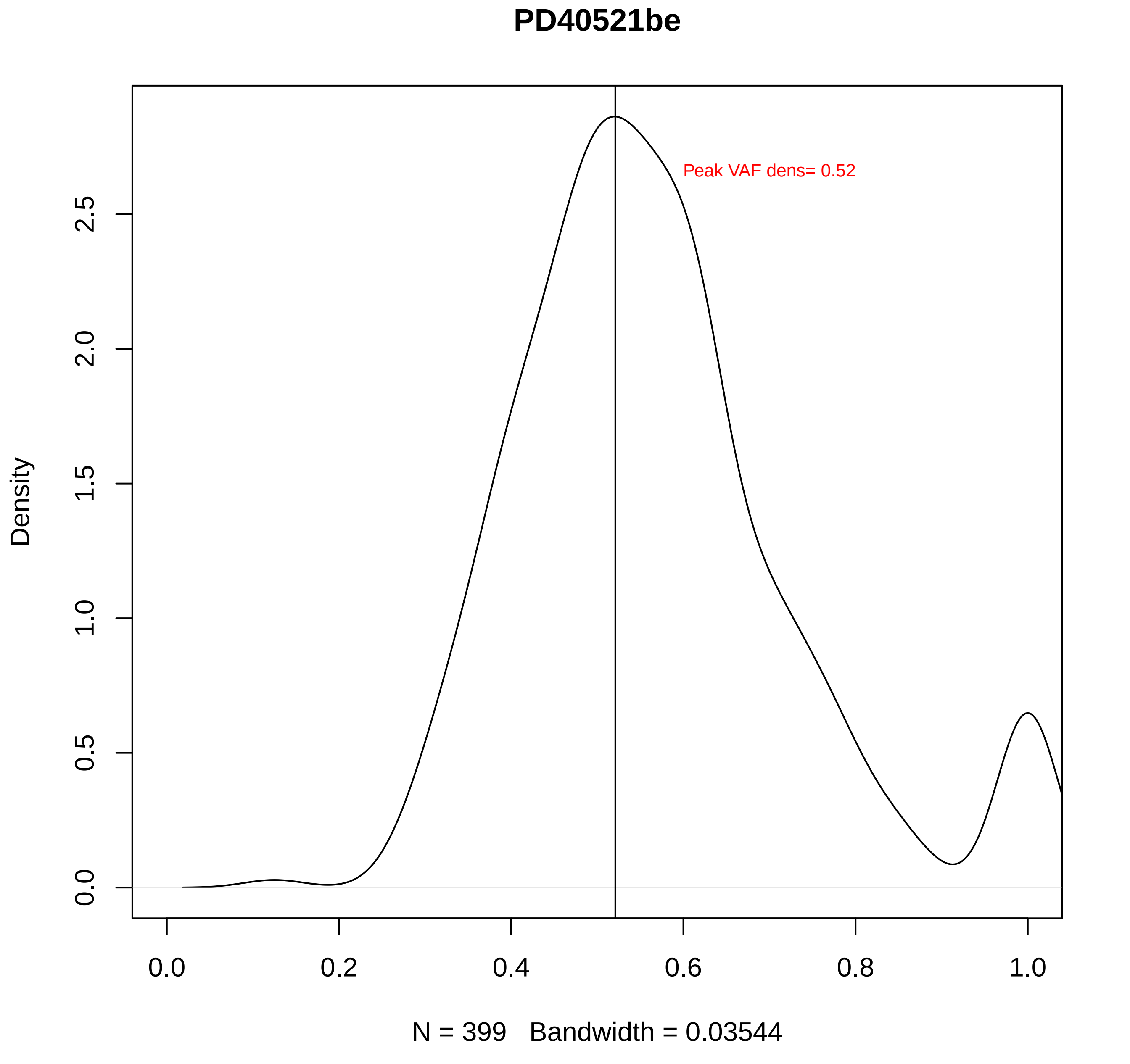

PD40521be
Peak VAF dens= 0.52
Density
0.0
0.5
1.0
1.5
2.0
2.5
0.0
0.2
0.4
0.6
0.8
1.0
N = 399   Bandwidth = 0.03544

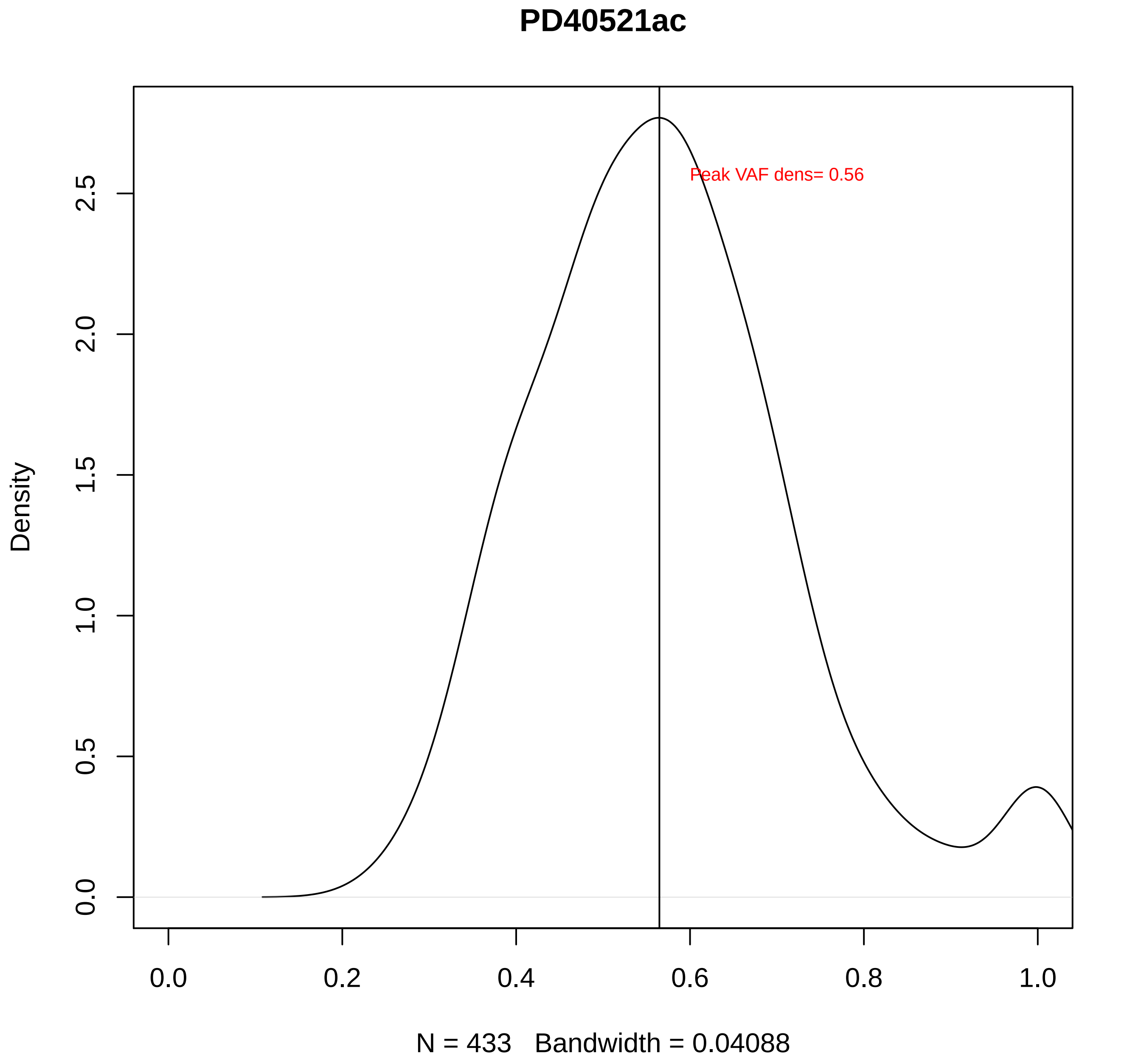
PD40521ac
Peak VAF dens= 0.56
Density
0.0
0.5
1.0
1.5
2.0
2.5
0.0
0.2
0.4
0.6
0.8
1.0
N = 433   Bandwidth = 0.04088

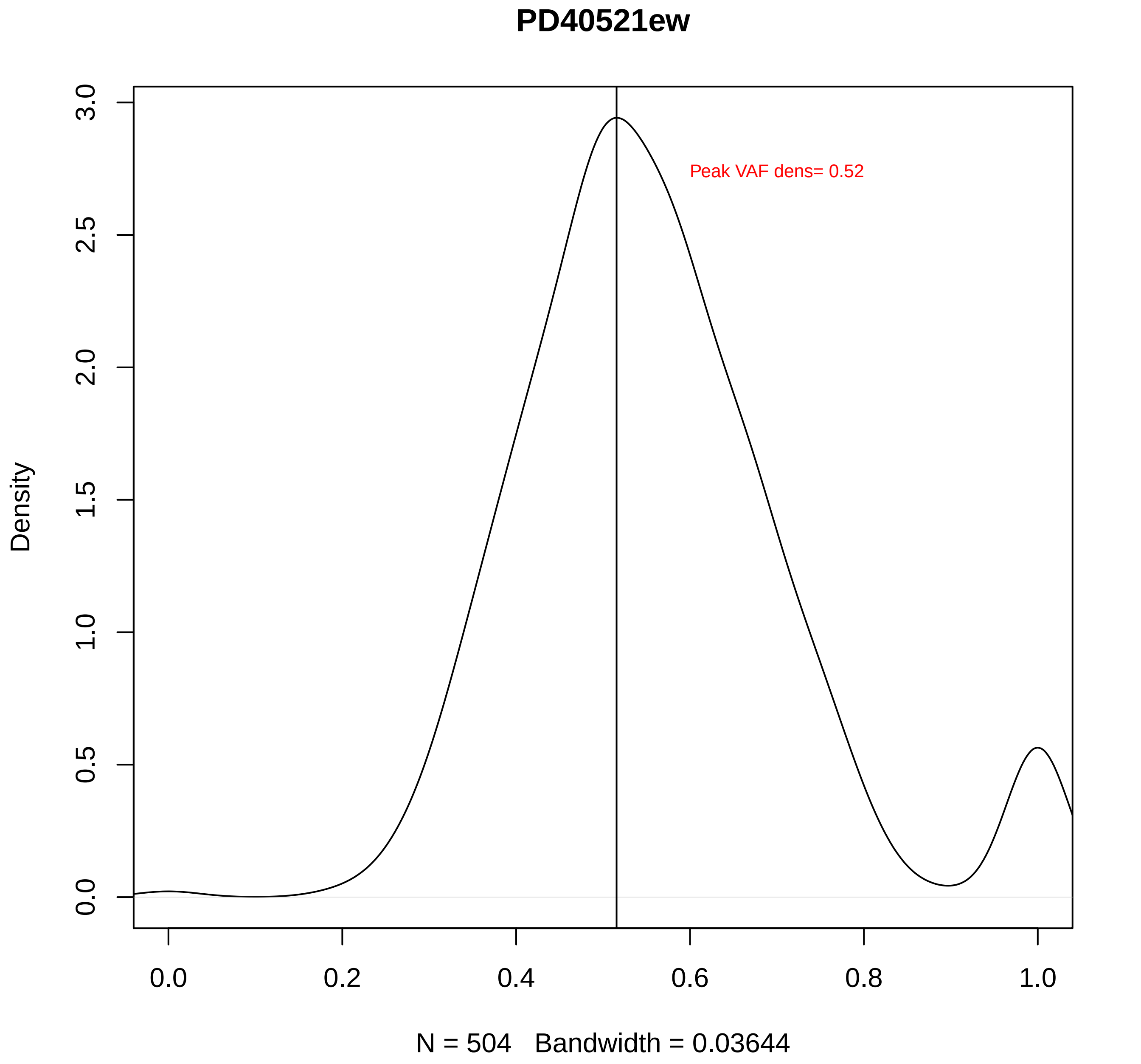
PD40521ew
Peak VAF dens= 0.52
Density
0.0
0.5
1.0
1.5
2.0
2.5
3.0
0.0
0.2
0.4
0.6
0.8
1.0
N = 504   Bandwidth = 0.03644

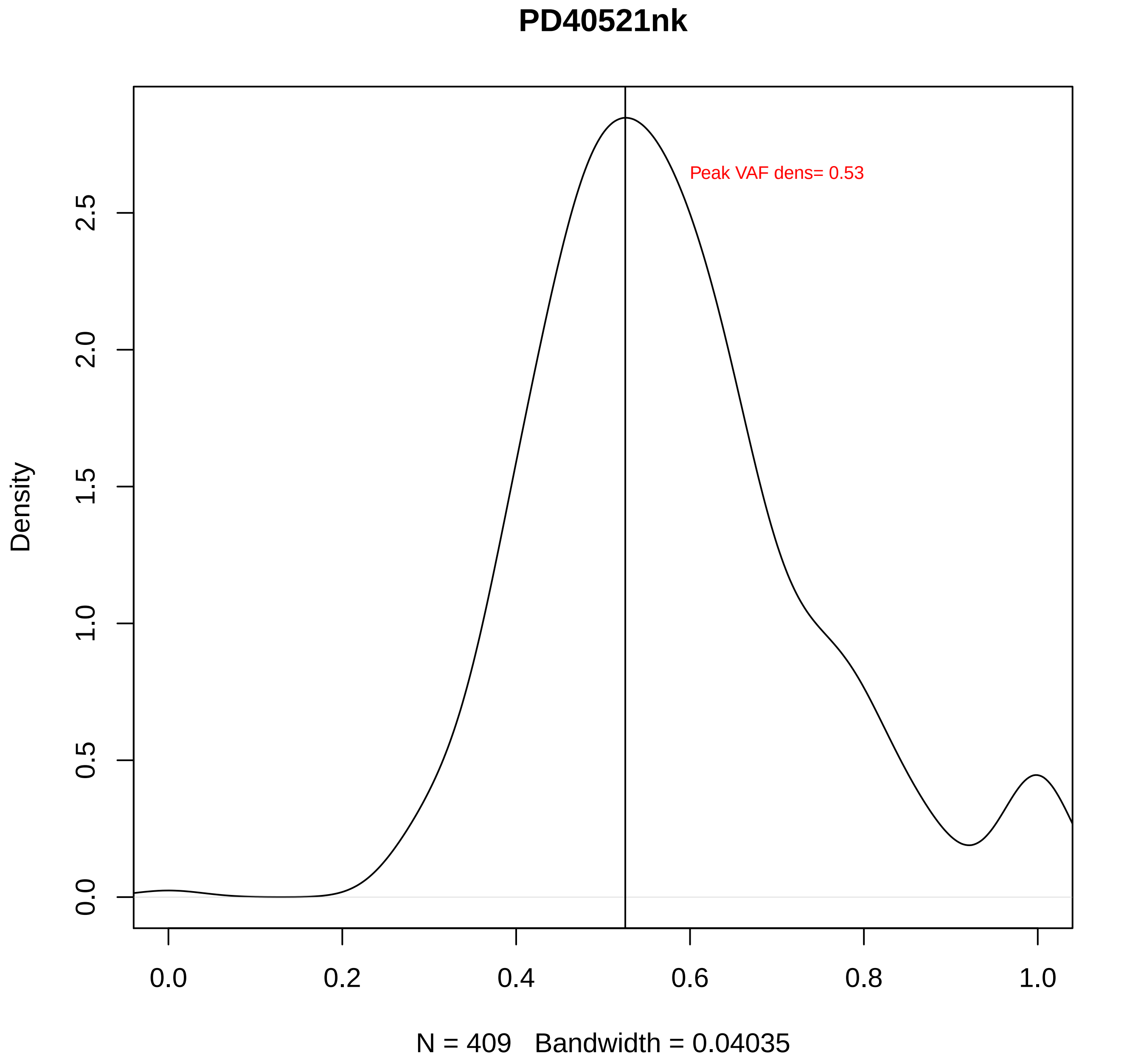
PD40521nk
Peak VAF dens= 0.53
Density
0.0
0.5
1.0
1.5
2.0
2.5
0.0
0.2
0.4
0.6
0.8
1.0
N = 409   Bandwidth = 0.04035

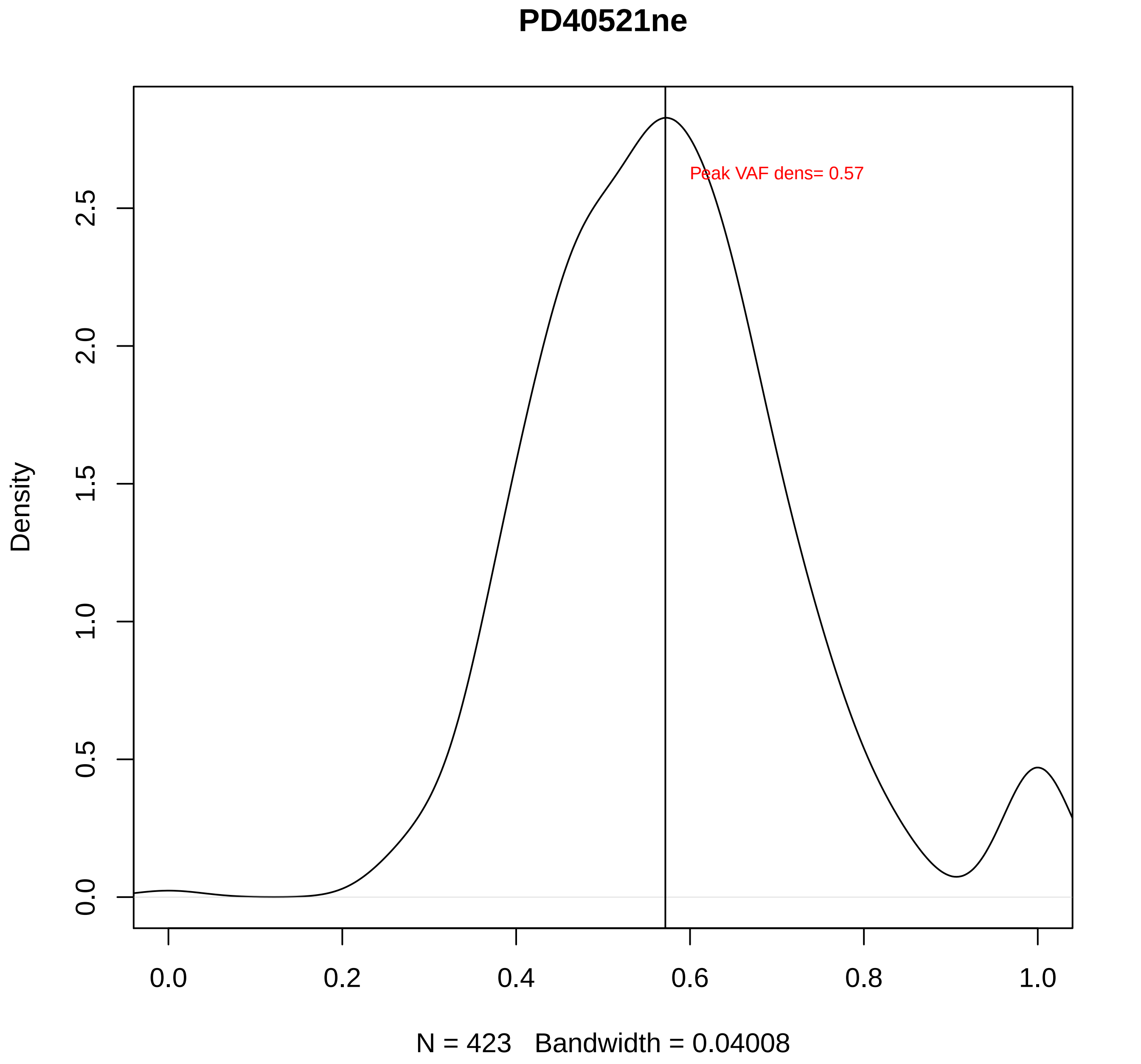
PD40521ne
Peak VAF dens= 0.57
Density
0.0
0.5
1.0
1.5
2.0
2.5
0.0
0.2
0.4
0.6
0.8
1.0
N = 423   Bandwidth = 0.04008

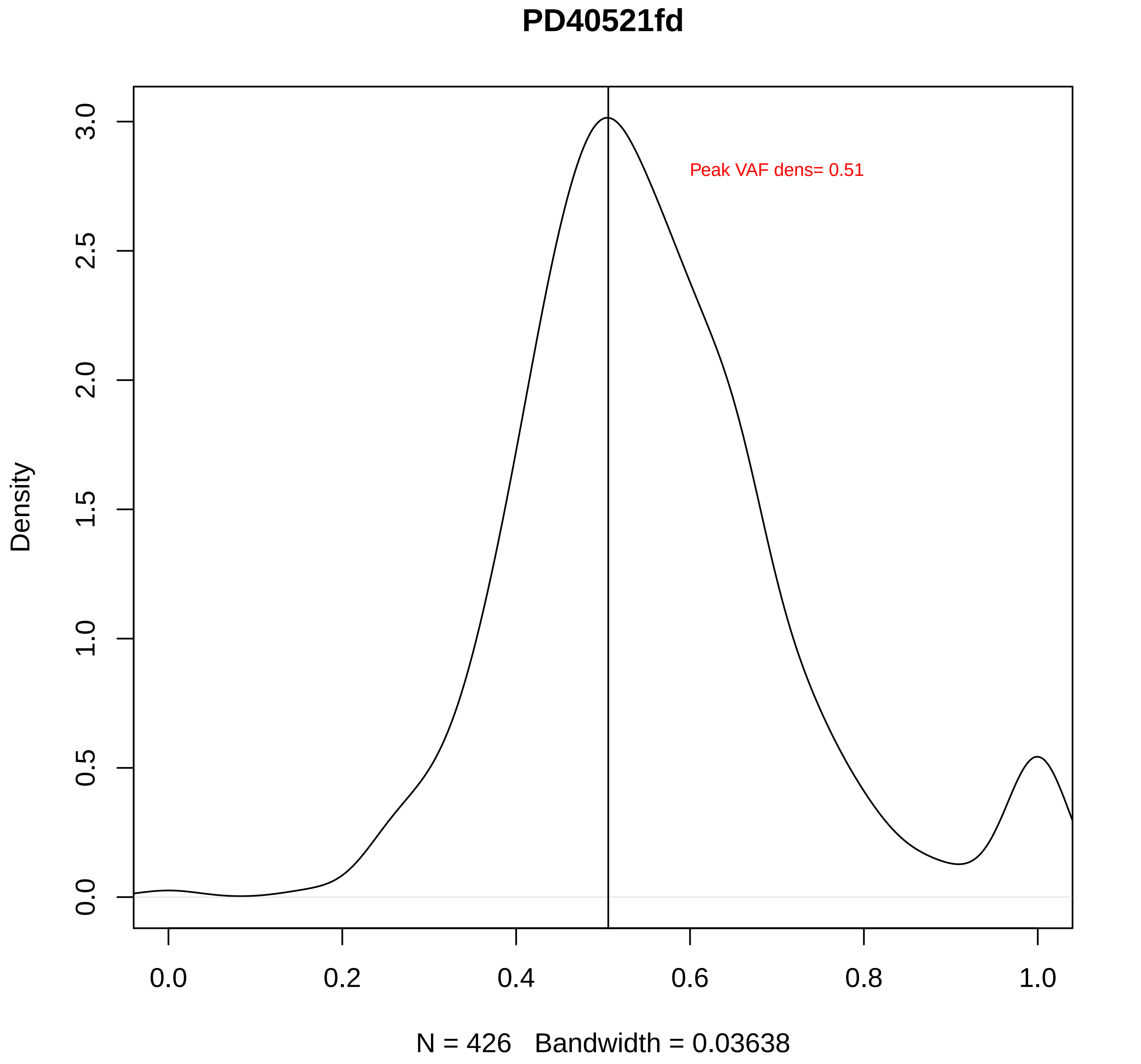
PD40521fd
Peak VAF dens= 0.51
Density
0.0
0.5
1.0
1.5
2.0
2.5
3.0
0.0
0.2
0.4
0.6
0.8
1.0
N = 426   Bandwidth = 0.03638

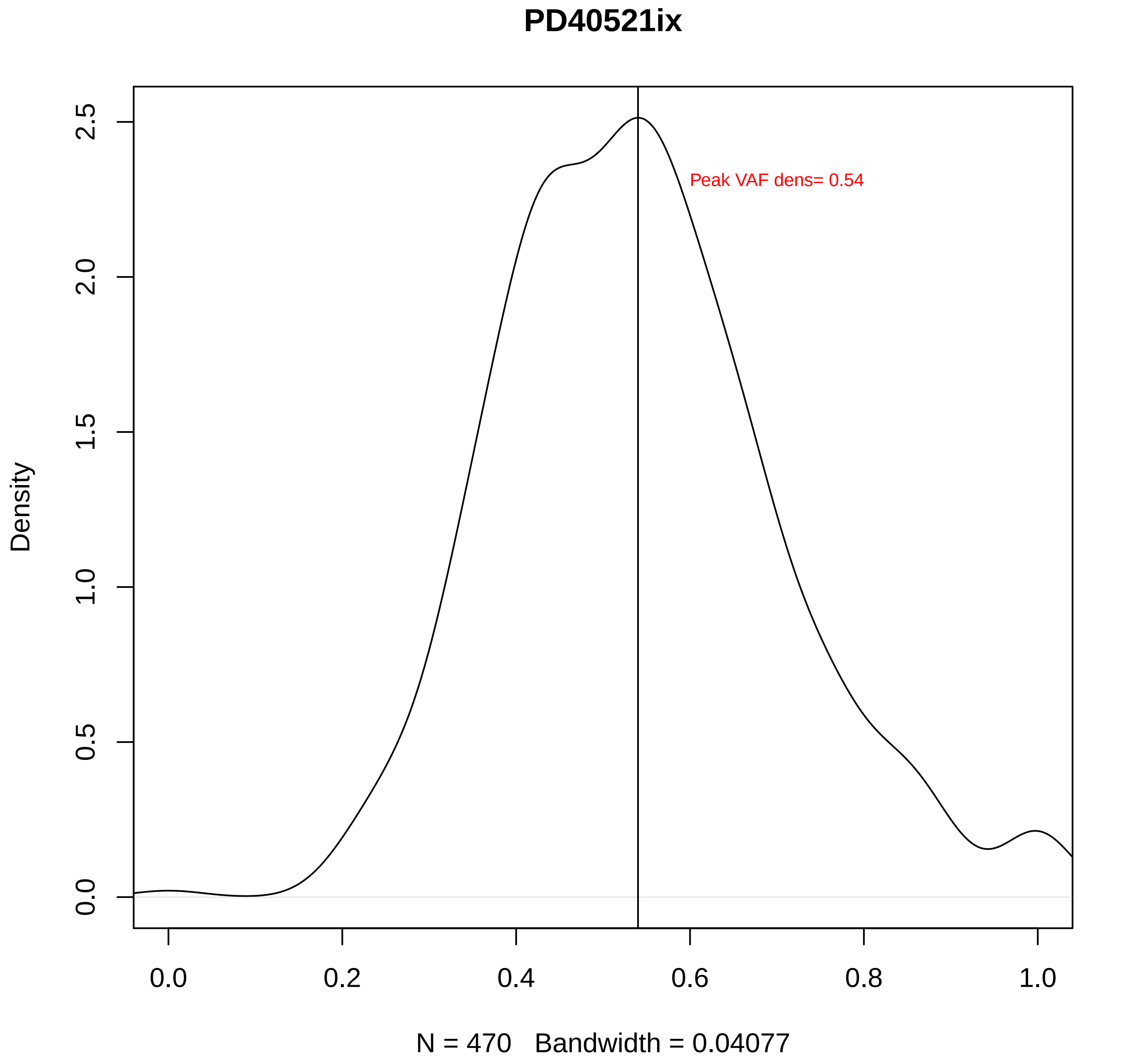
PD40521ix
Peak VAF dens= 0.54
Density
0.0
0.5
1.0
1.5
2.0
2.5
0.0
0.2
0.4
0.6
0.8
1.0
N = 470   Bandwidth = 0.04077

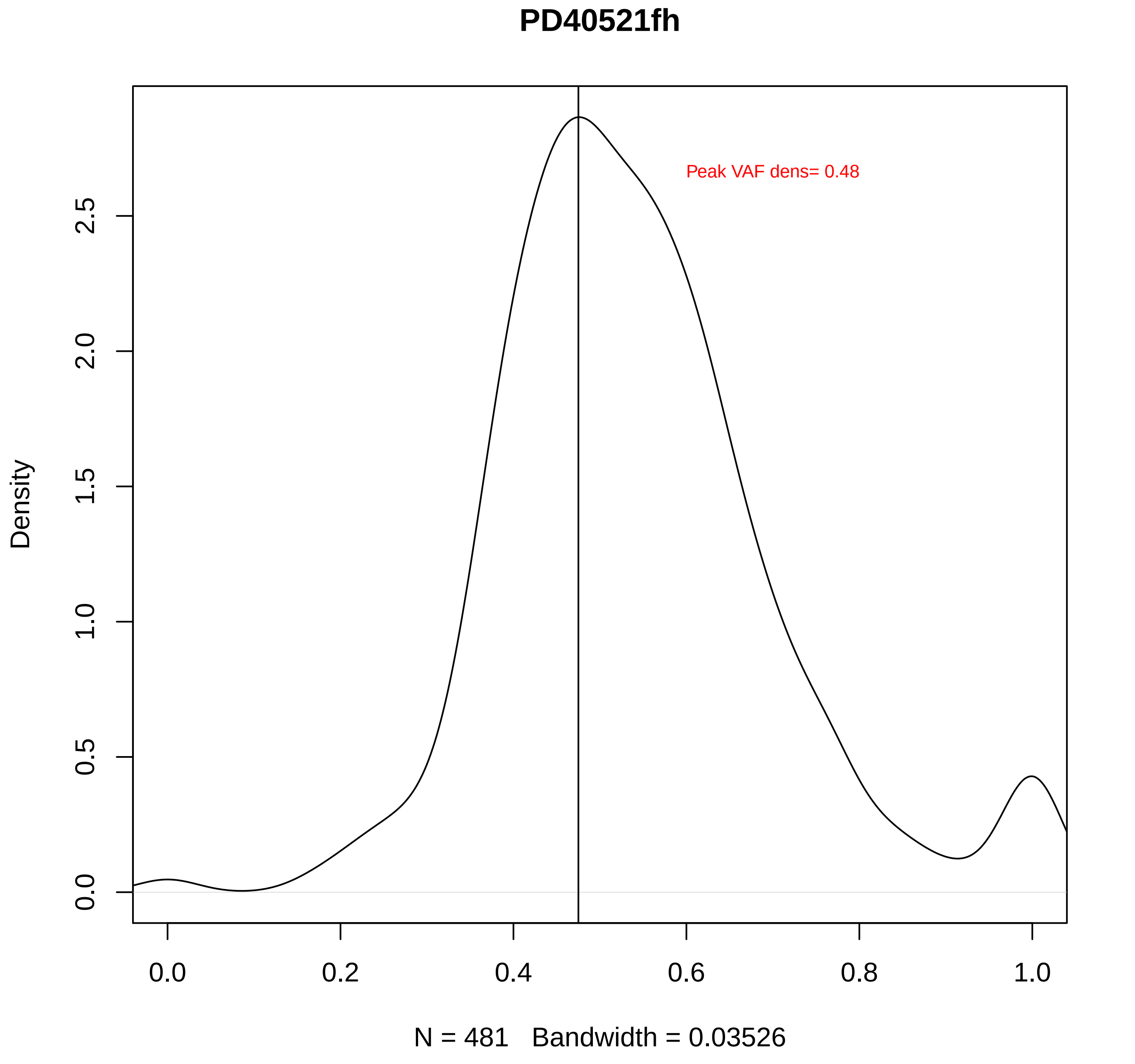

PD40521fh
Peak VAF dens= 0.48
Density
0.0
0.5
1.0
1.5
2.0
2.5
0.0
0.2
0.4
0.6
0.8
1.0
N = 481   Bandwidth = 0.03526

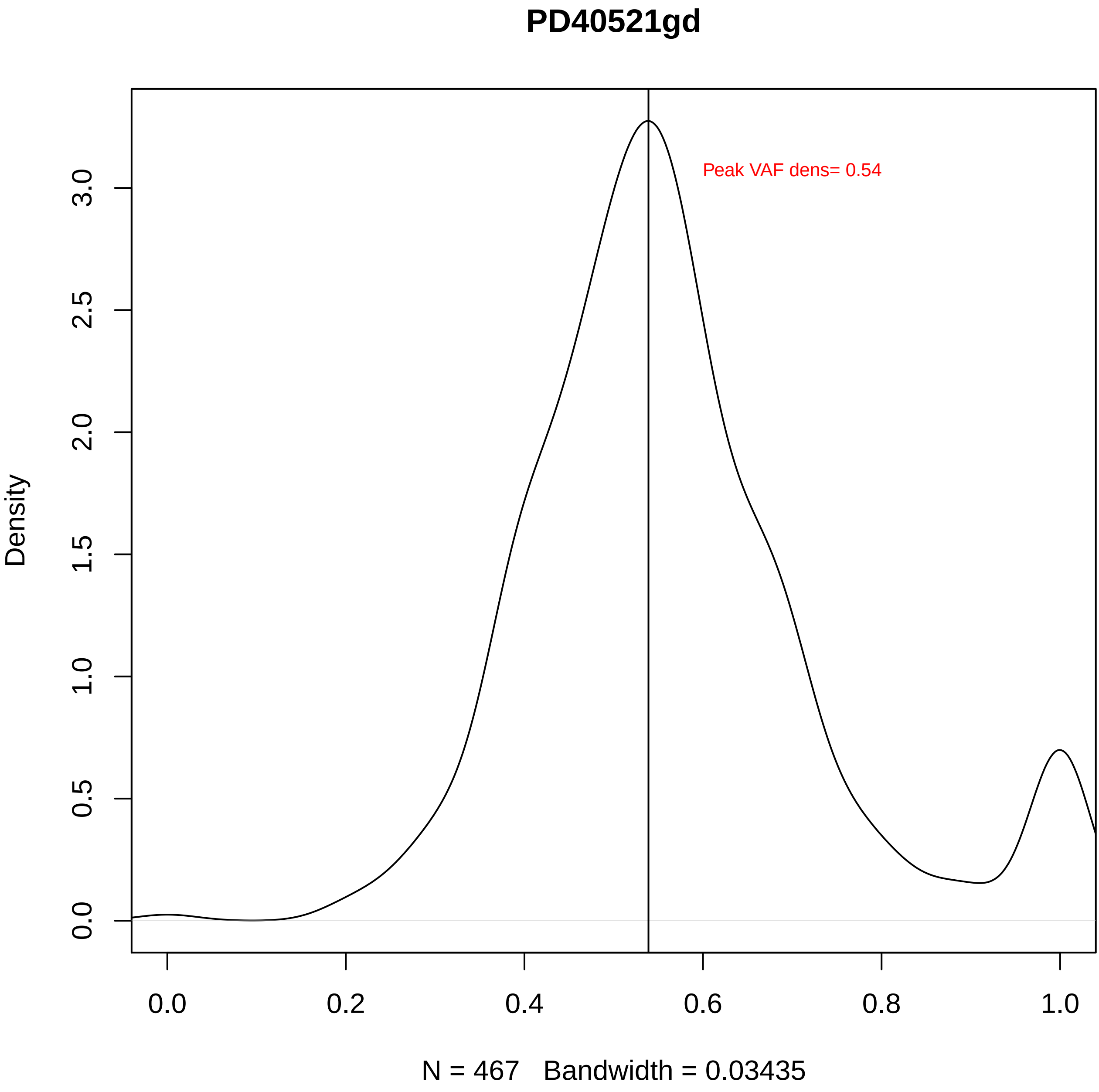

PD40521gd
Peak VAF dens= 0.54
Density
0.0
0.5
1.0
1.5
2.0
2.5
3.0
0.0
0.2
0.4
0.6
0.8
1.0
N = 467   Bandwidth = 0.03435

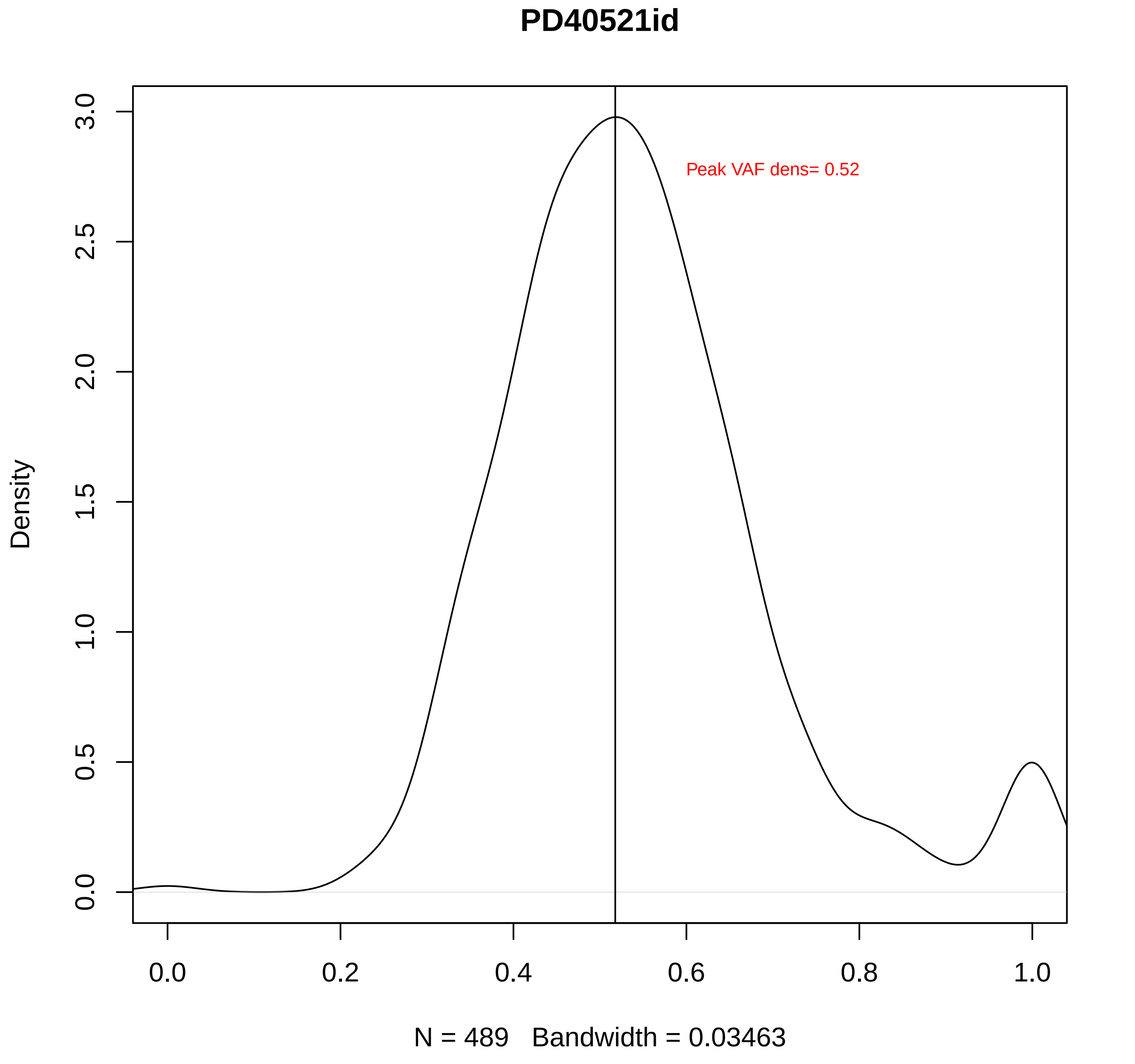

PD40521id
Peak VAF dens= 0.52
Density
3.0
2.5
2.0
1.5
1.0
0.5
0.0
0.0
0.2
0.4
0.6
0.8
1.0
N = 489   Bandwidth = 0.03463

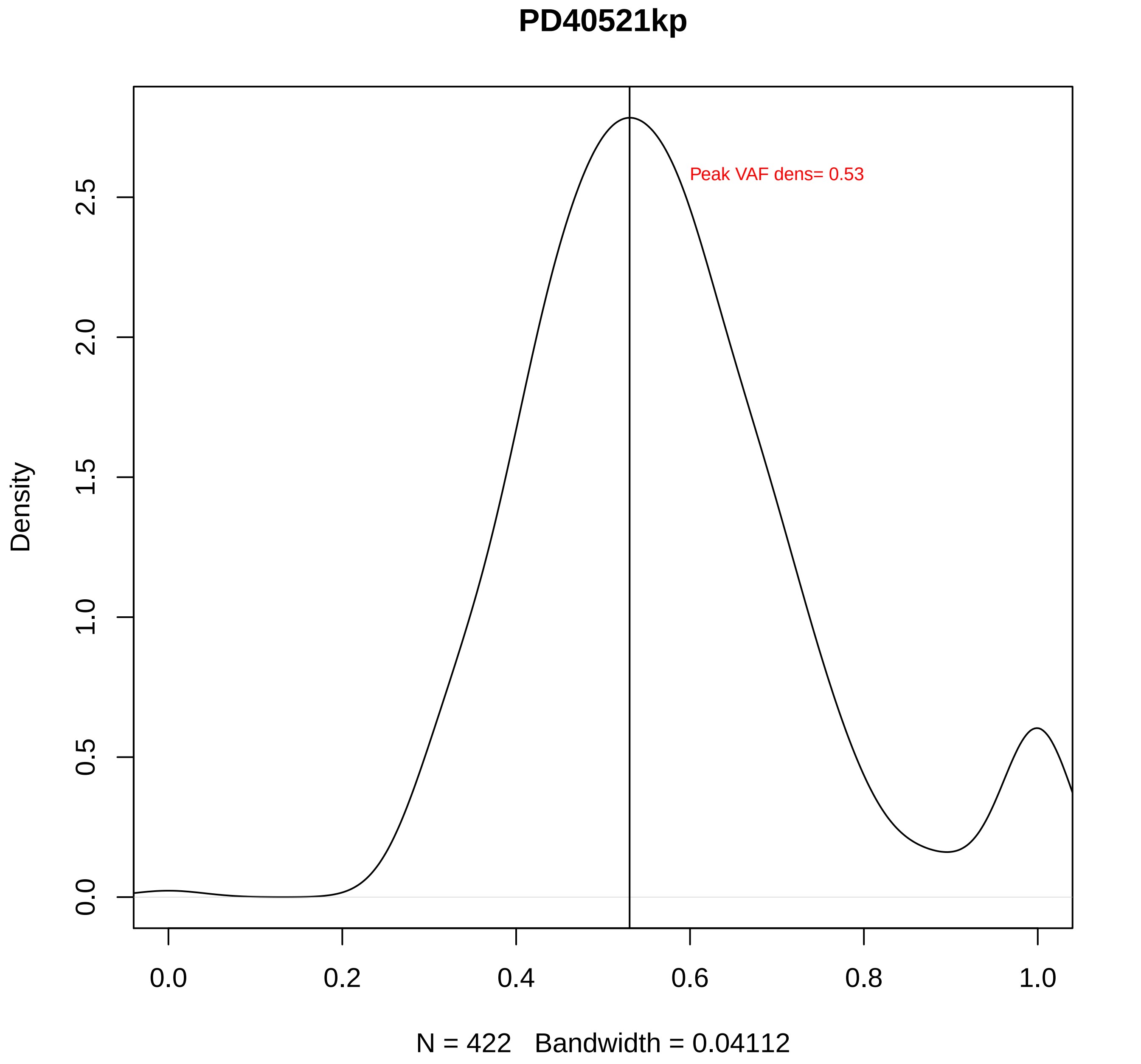
PD40521kp
Peak VAF dens= 0.53
Density
0.0
0.5
1.0
1.5
2.0
2.5
0.0
0.2
0.4
0.6
0.8
1.0
N = 422   Bandwidth = 0.04112

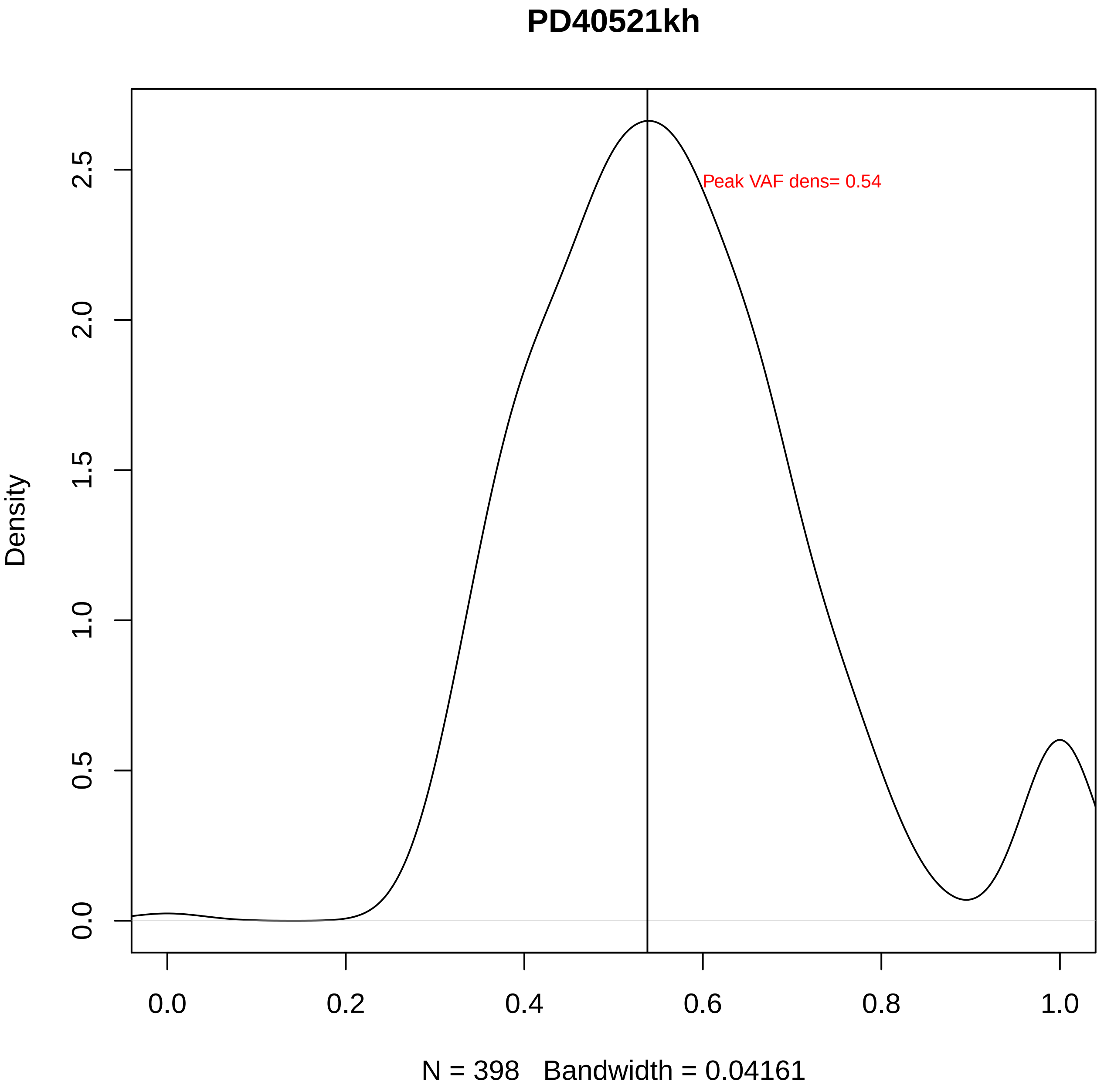
PD40521kh
Peak VAF dens= 0.54
Density
0.0
0.5
1.0
1.5
2.0
2.5
0.0
0.2
0.4
0.6
0.8
1.0
N = 398   Bandwidth = 0.04161

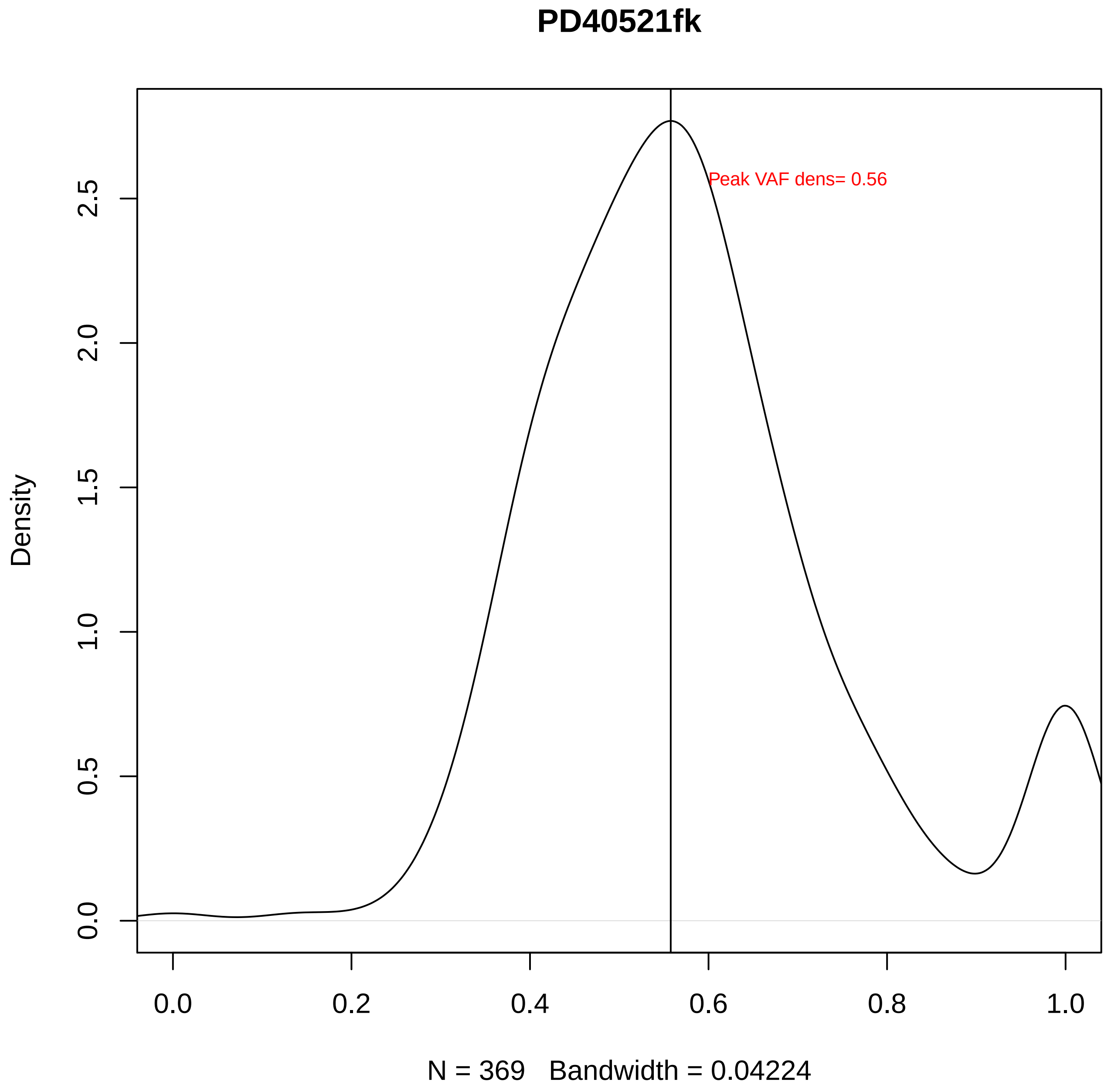
PD40521fk
Peak VAF dens= 0.56
Density
0.0
0.5
1.0
1.5
2.0
2.5
0.0
0.2
0.4
0.6
0.8
1.0
N = 369 Bandwidth = 0.04224

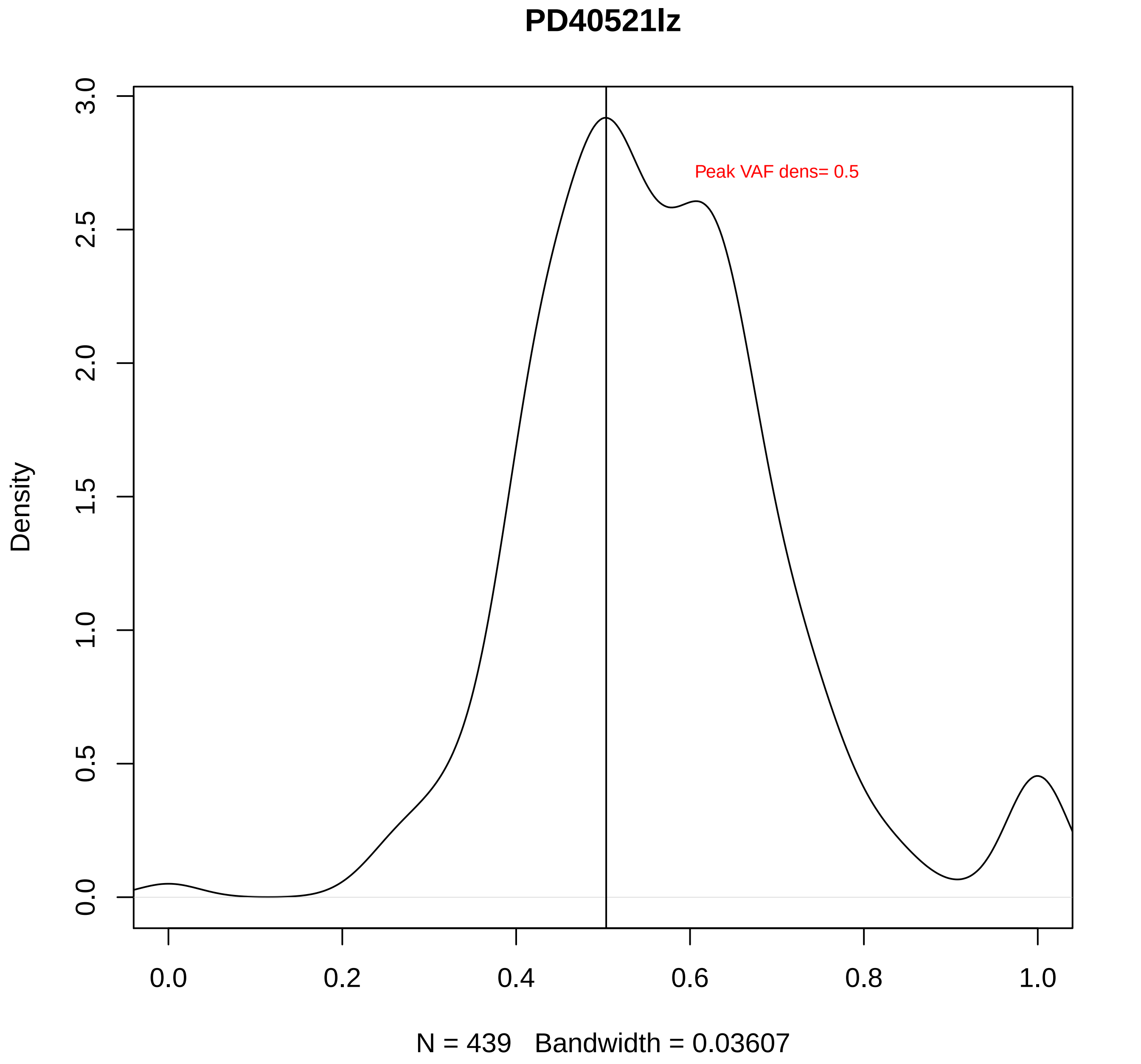

PD40521lz
Peak VAF dens= 0.5
Density
0.0
0.5
1.0
1.5
2.0
2.5
3.0
0.0
0.2
0.4
0.6
0.8
1.0
N = 439 Bandwidth = 0.03607

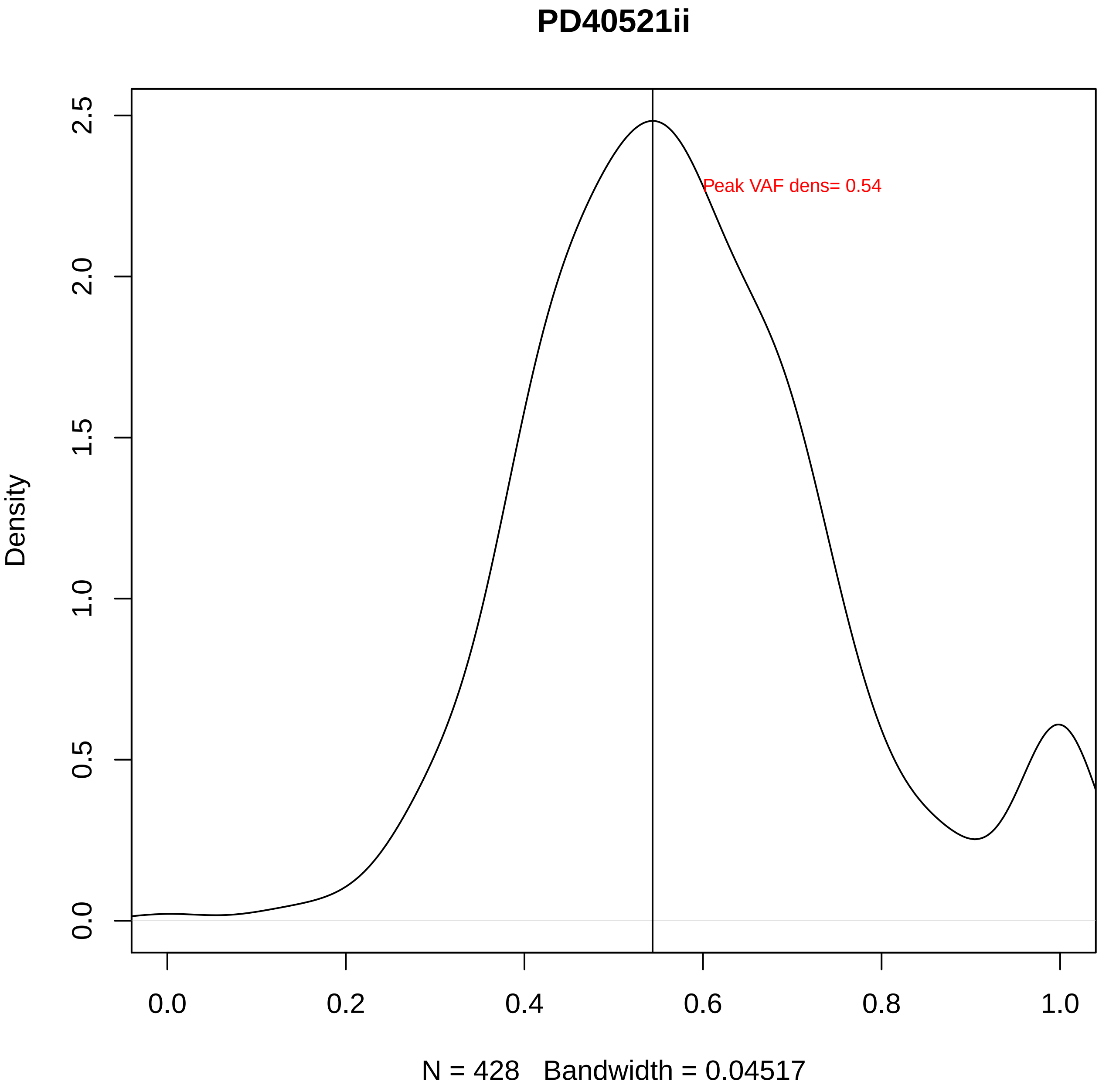

PD40521ii
Peak VAF dens= 0.54
Density
0.0
0.5
1.0
1.5
2.0
2.5
0.0
0.2
0.4
0.6
0.8
1.0
N = 428   Bandwidth = 0.04517

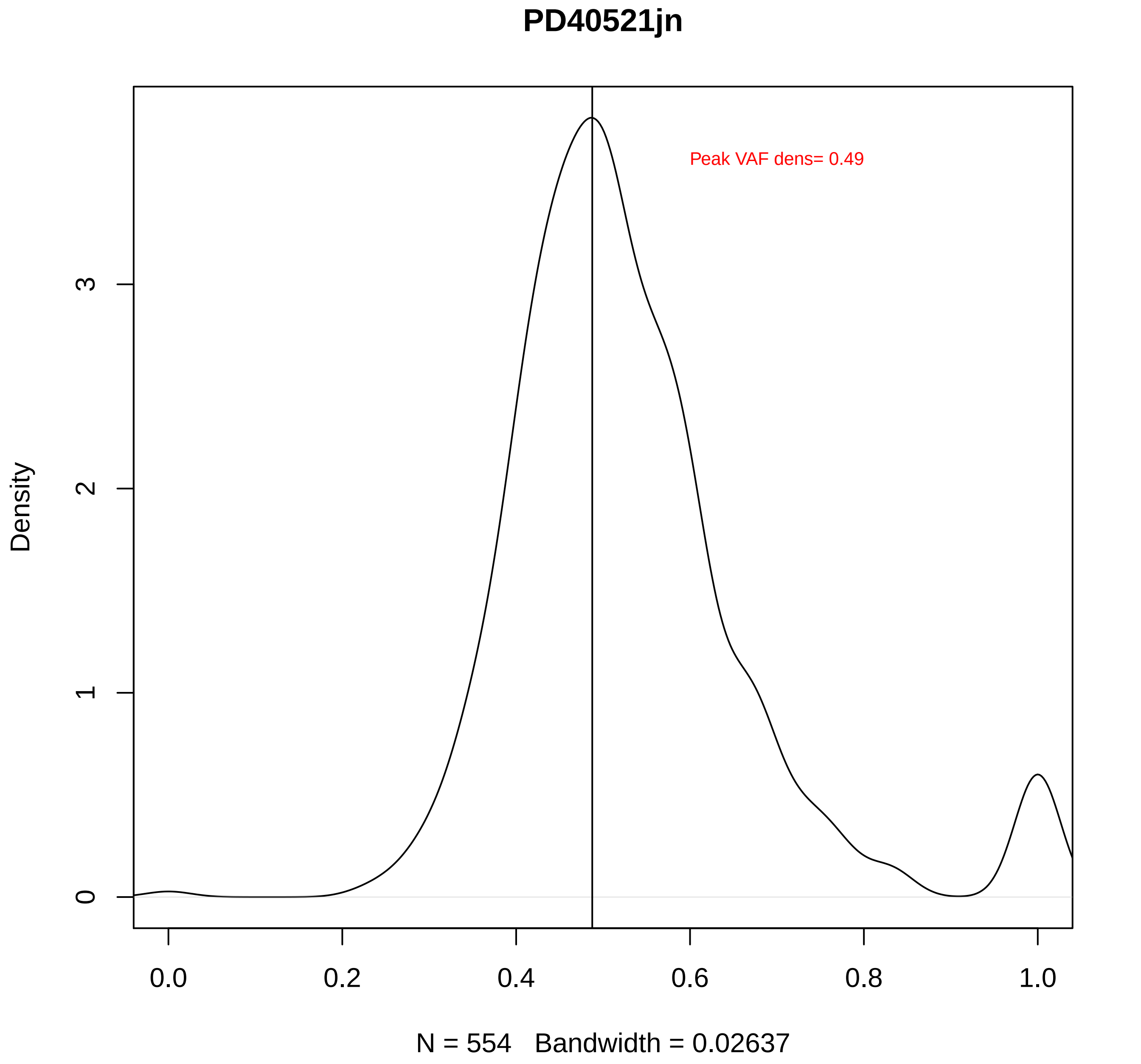
PD40521jn
Peak VAF dens= 0.49
Density
0
1
2
3
0.0
0.2
0.4
0.6
0.8
1.0
N = 554 Bandwidth = 0.02637

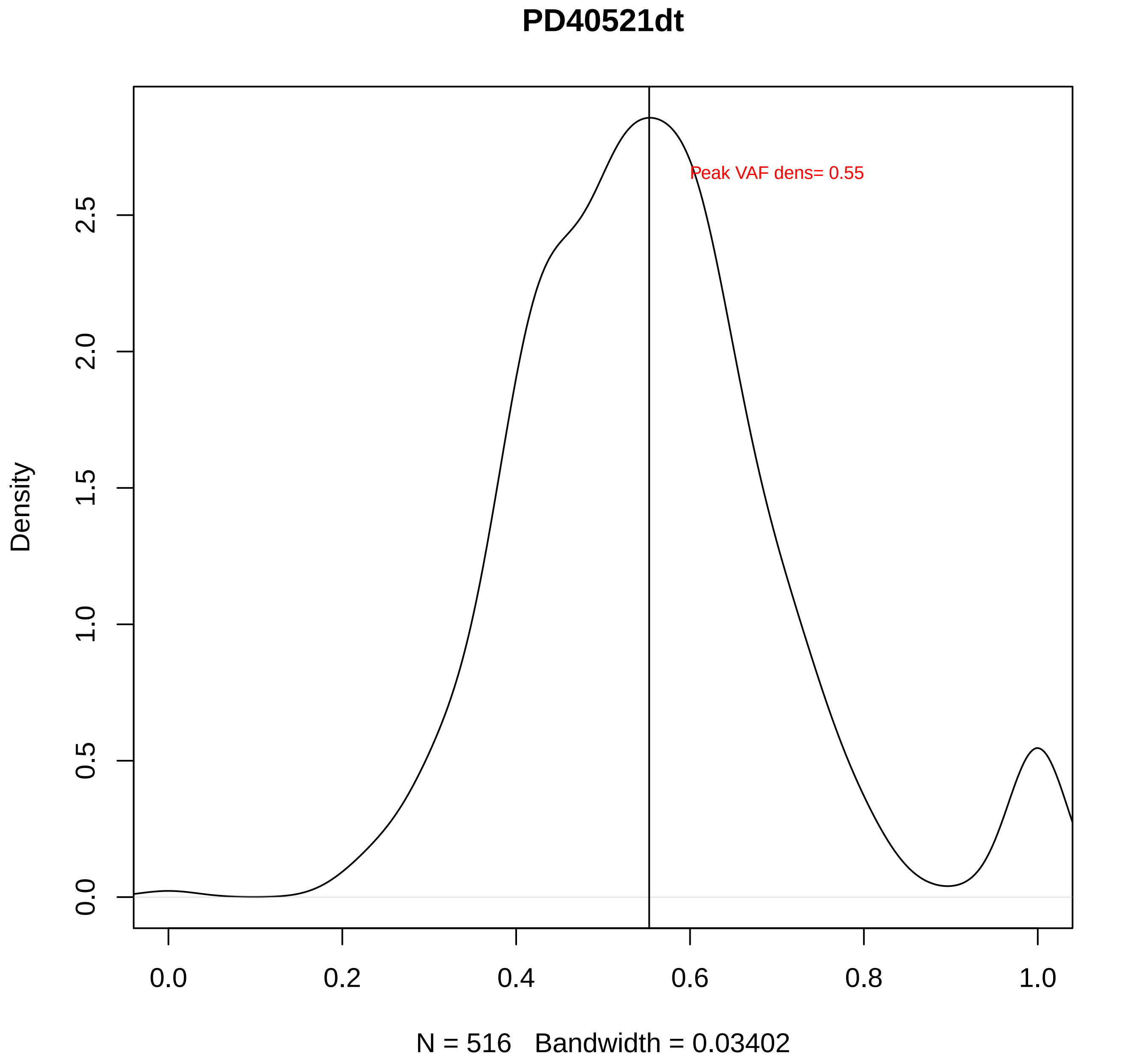

PD40521dt
Peak VAF dens= 0.55
Density
0.0
0.5
1.0
1.5
2.0
2.5
0.0
0.2
0.4
0.6
0.8
1.0
N = 516   Bandwidth = 0.03402

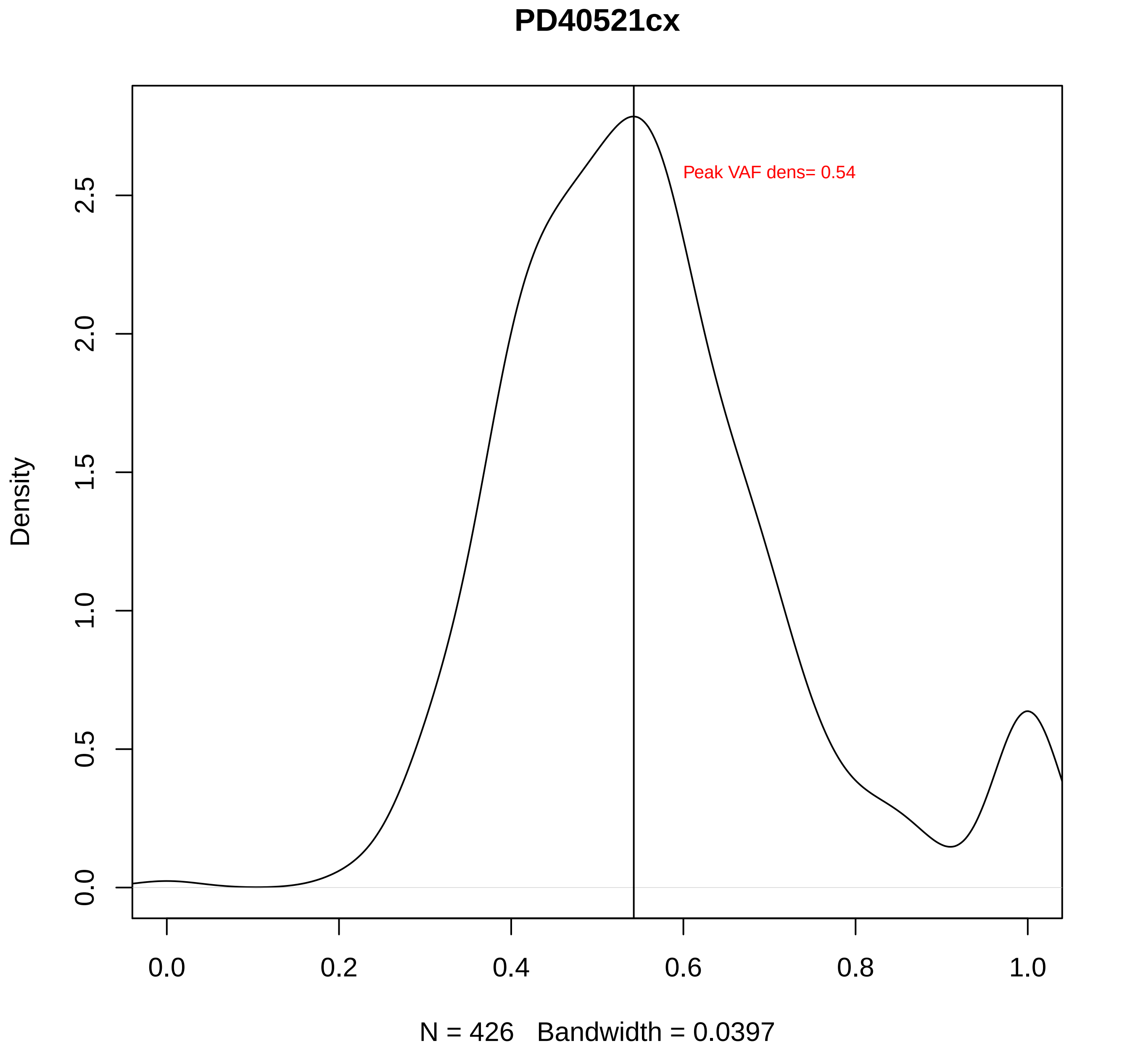
PD40521cx
Peak VAF dens= 0.54
Density
0.0
0.5
1.0
1.5
2.0
2.5
0.0
0.2
0.4
0.6
0.8
1.0
N = 426   Bandwidth = 0.0397

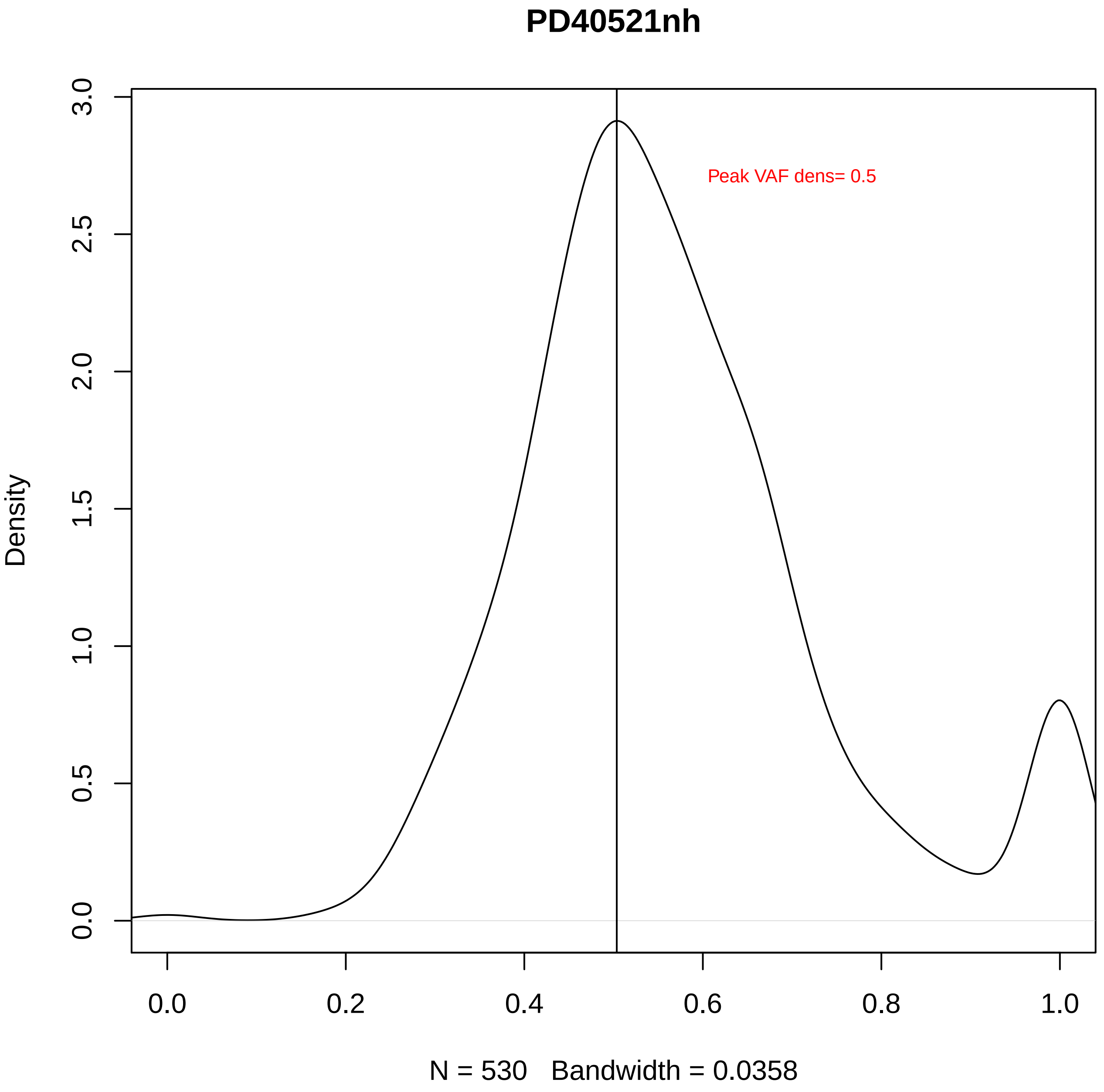
PD40521nh
Peak VAF dens= 0.5
Density
0.0
0.5
1.0
1.5
2.0
2.5
3.0
0.0
0.2
0.4
0.6
0.8
1.0
N = 530   Bandwidth = 0.0358

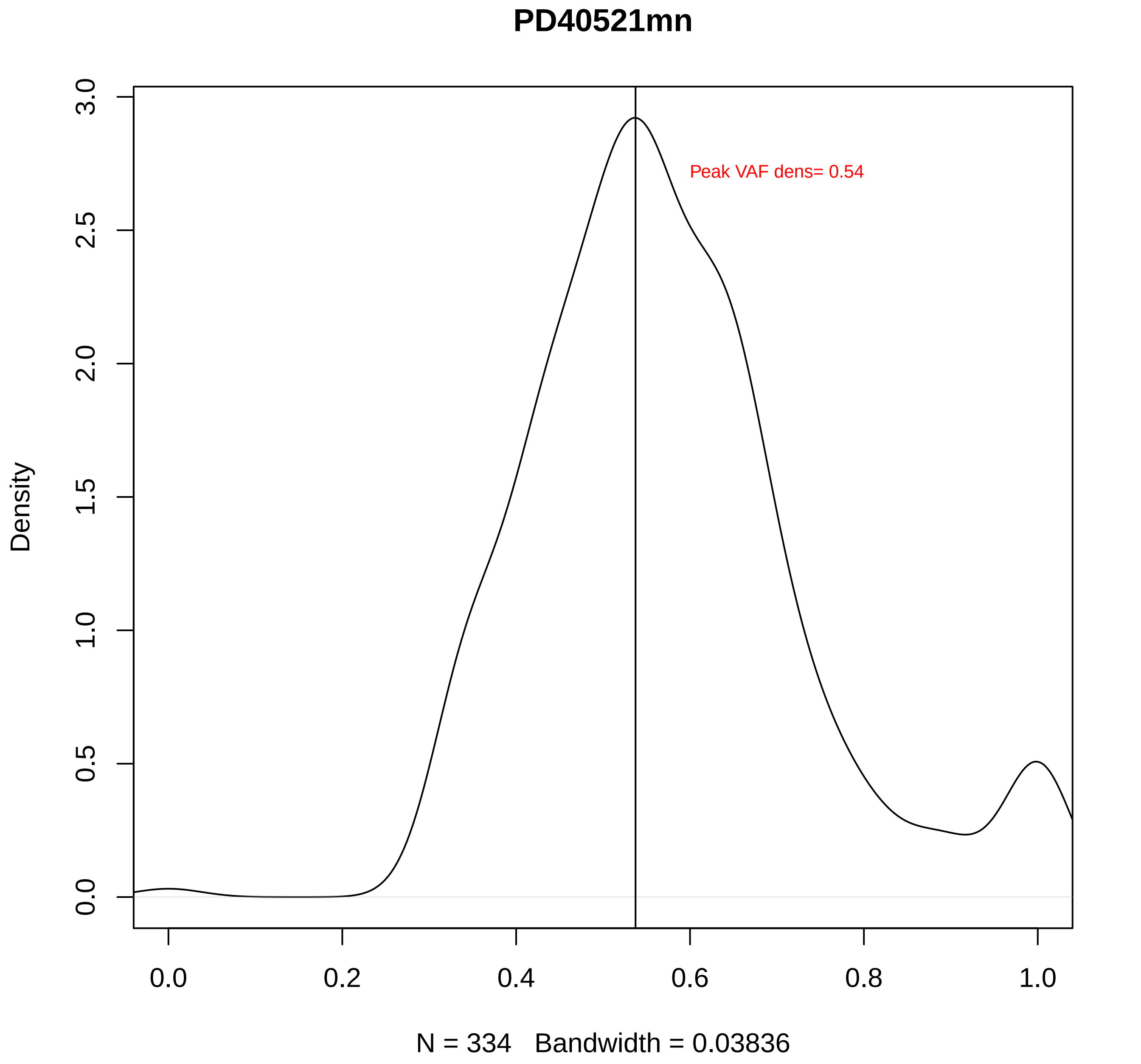
PD40521mn
Peak VAF dens= 0.54
Density
0.0
0.5
1.0
1.5
2.0
2.5
3.0
0.0
0.2
0.4
0.6
0.8
1.0
N = 334 Bandwidth = 0.03836

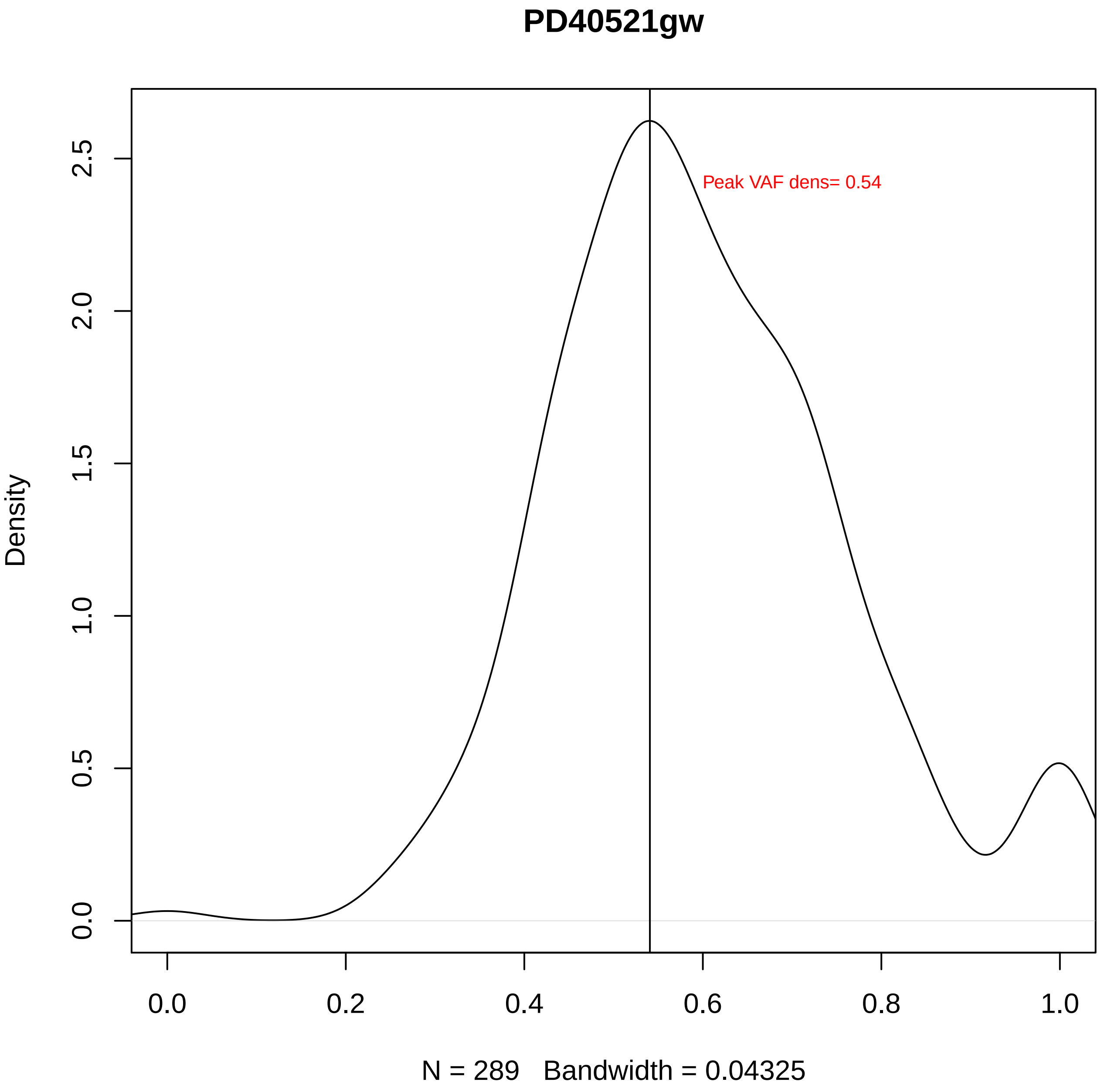
PD40521gw
Peak VAF dens= 0.54
Density
0.0
0.5
1.0
1.5
2.0
2.5
0.0
0.2
0.4
0.6
0.8
1.0
N = 289   Bandwidth = 0.04325

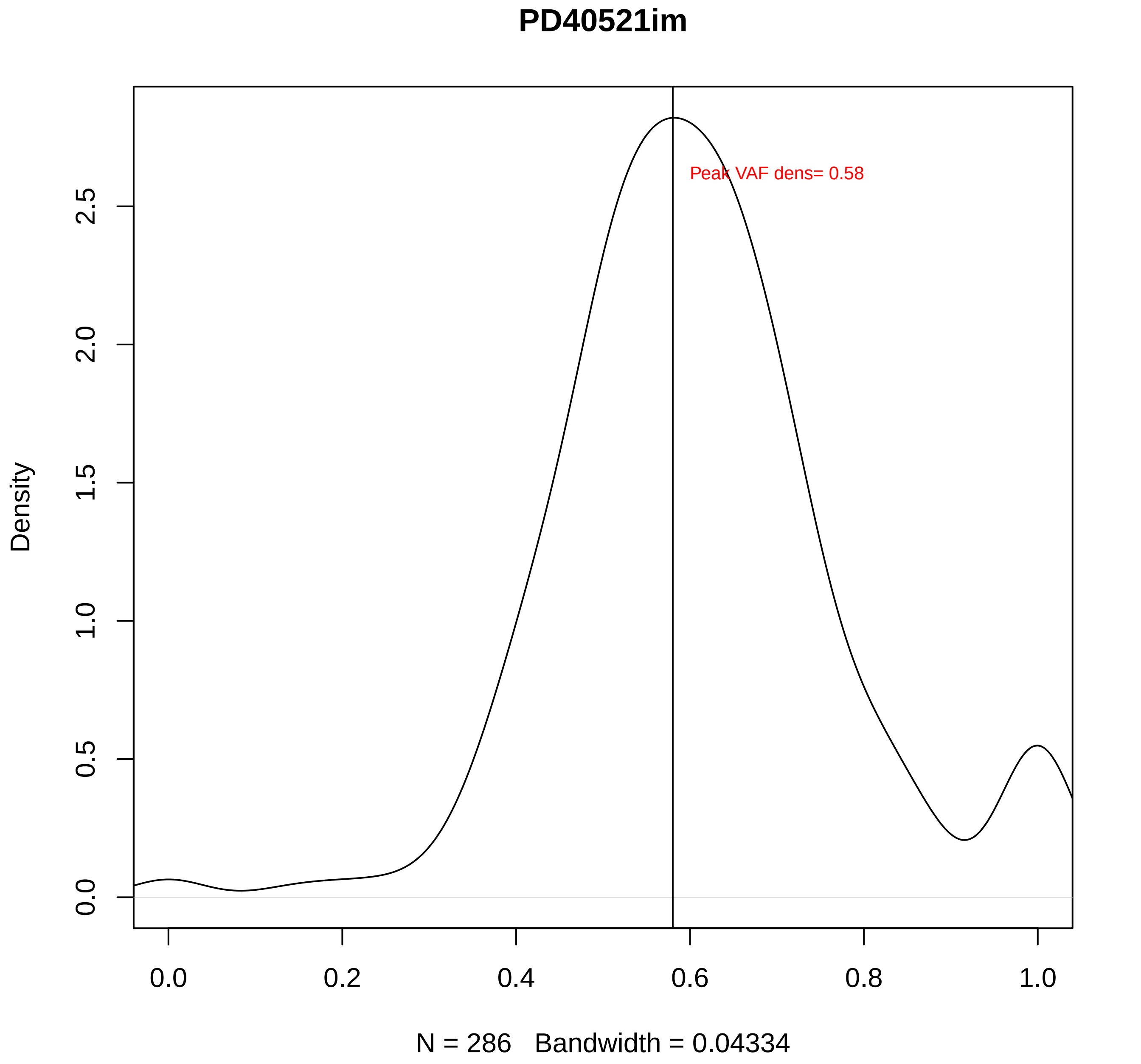

PD40521im
Peak VAF dens= 0.58
Density
0.0
0.5
1.0
1.5
2.0
2.5
0.0
0.2
0.4
0.6
0.8
1.0
N = 286   Bandwidth = 0.04334

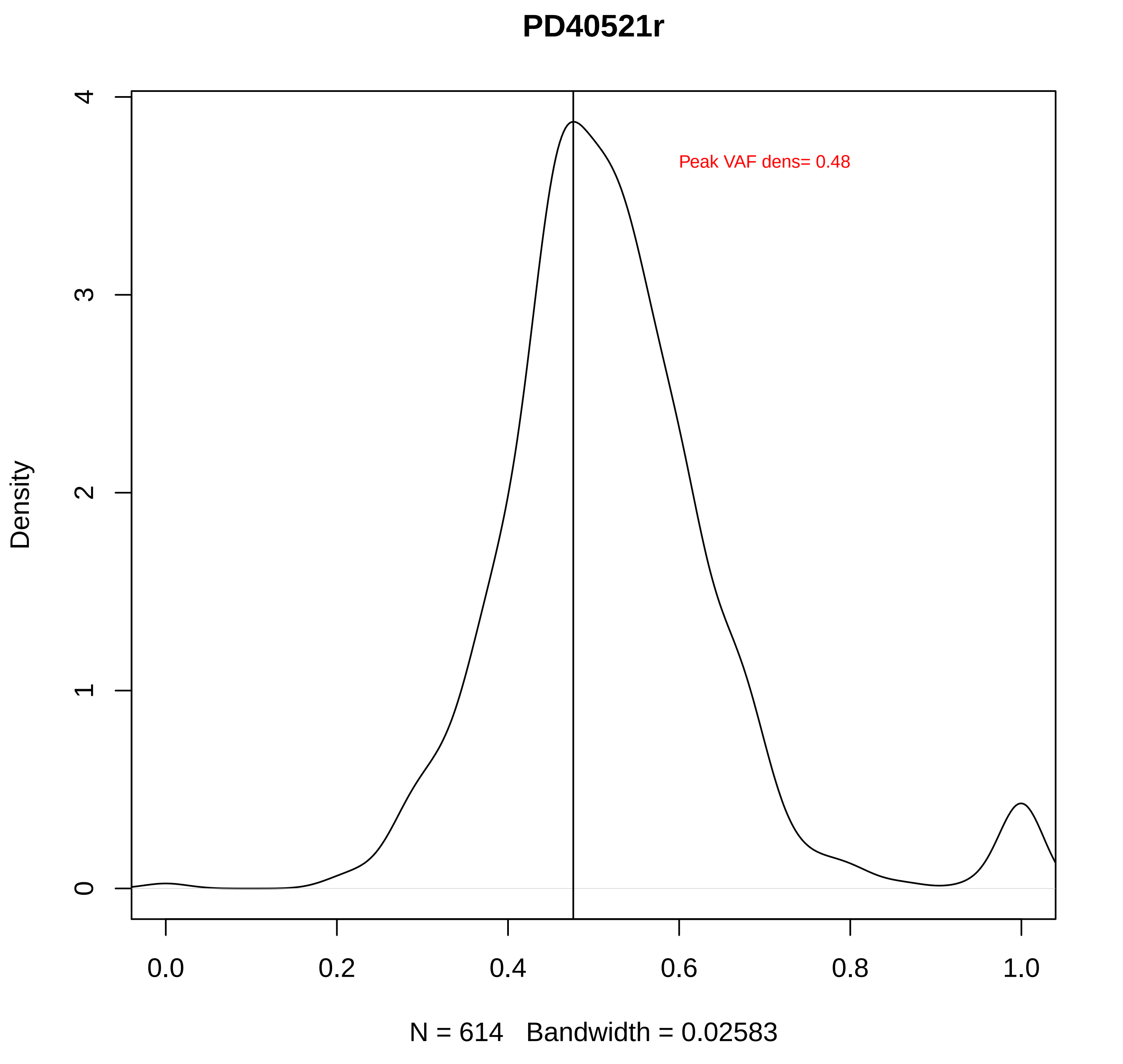
PD40521r
Peak VAF dens= 0.48
Density
0
1
2
3
4
0.0
0.2
0.4
0.6
0.8
1.0
N = 614   Bandwidth = 0.02583

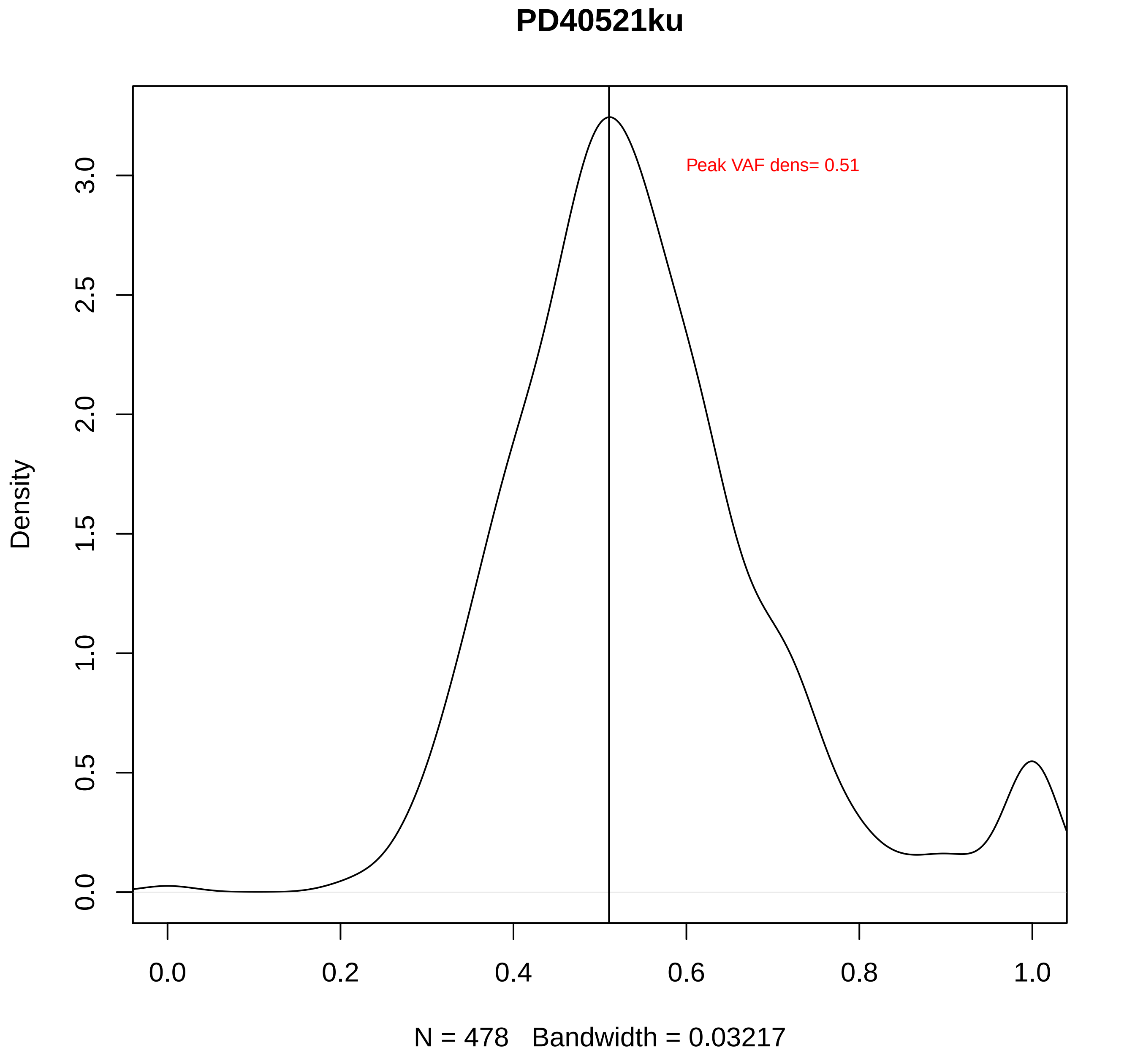
PD40521ku
Peak VAF dens= 0.51
Density
0.0
0.5
1.0
1.5
2.0
2.5
3.0
0.0
0.2
0.4
0.6
0.8
1.0
N = 478   Bandwidth = 0.03217

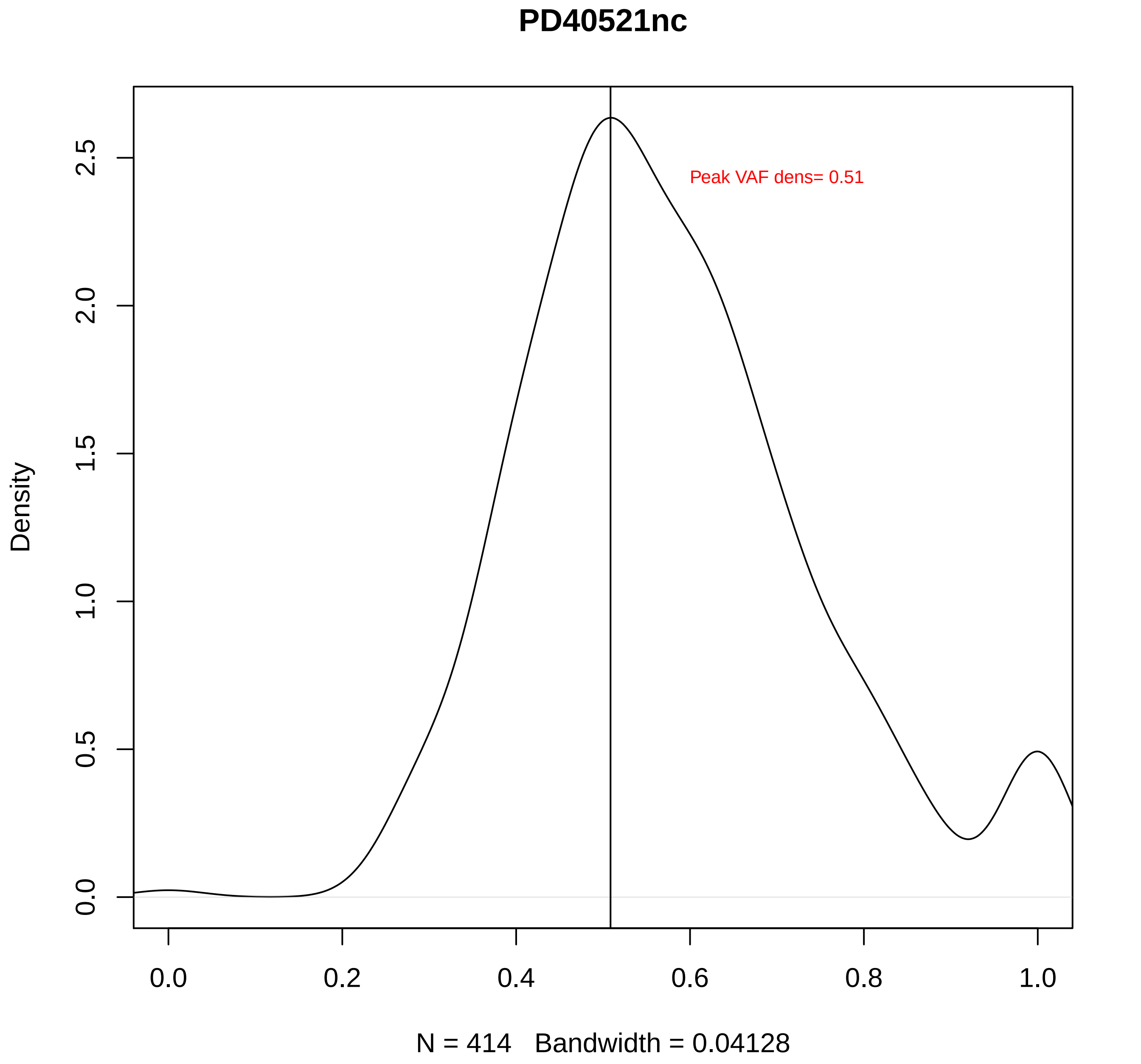

PD40521nc
Peak VAF dens= 0.51
Density
2.5
2.0
1.5
1.0
0.5
0.0
0.0
0.2
0.4
0.6
0.8
1.0
N = 414   Bandwidth = 0.04128

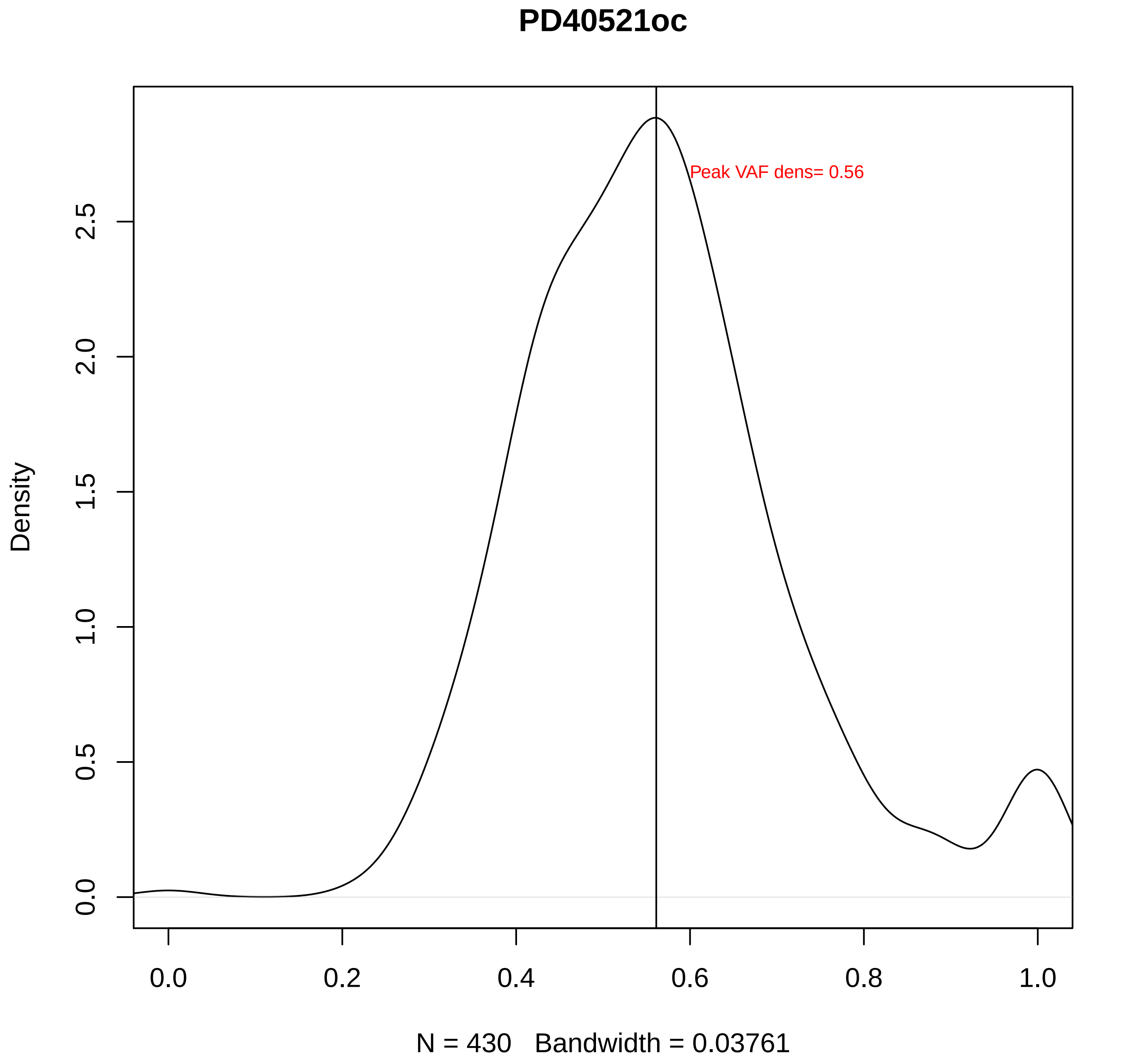
PD40521oc
Peak VAF dens= 0.56
Density
0.0
0.5
1.0
1.5
2.0
2.5
0.0
0.2
0.4
0.6
0.8
1.0
N = 430   Bandwidth = 0.03761

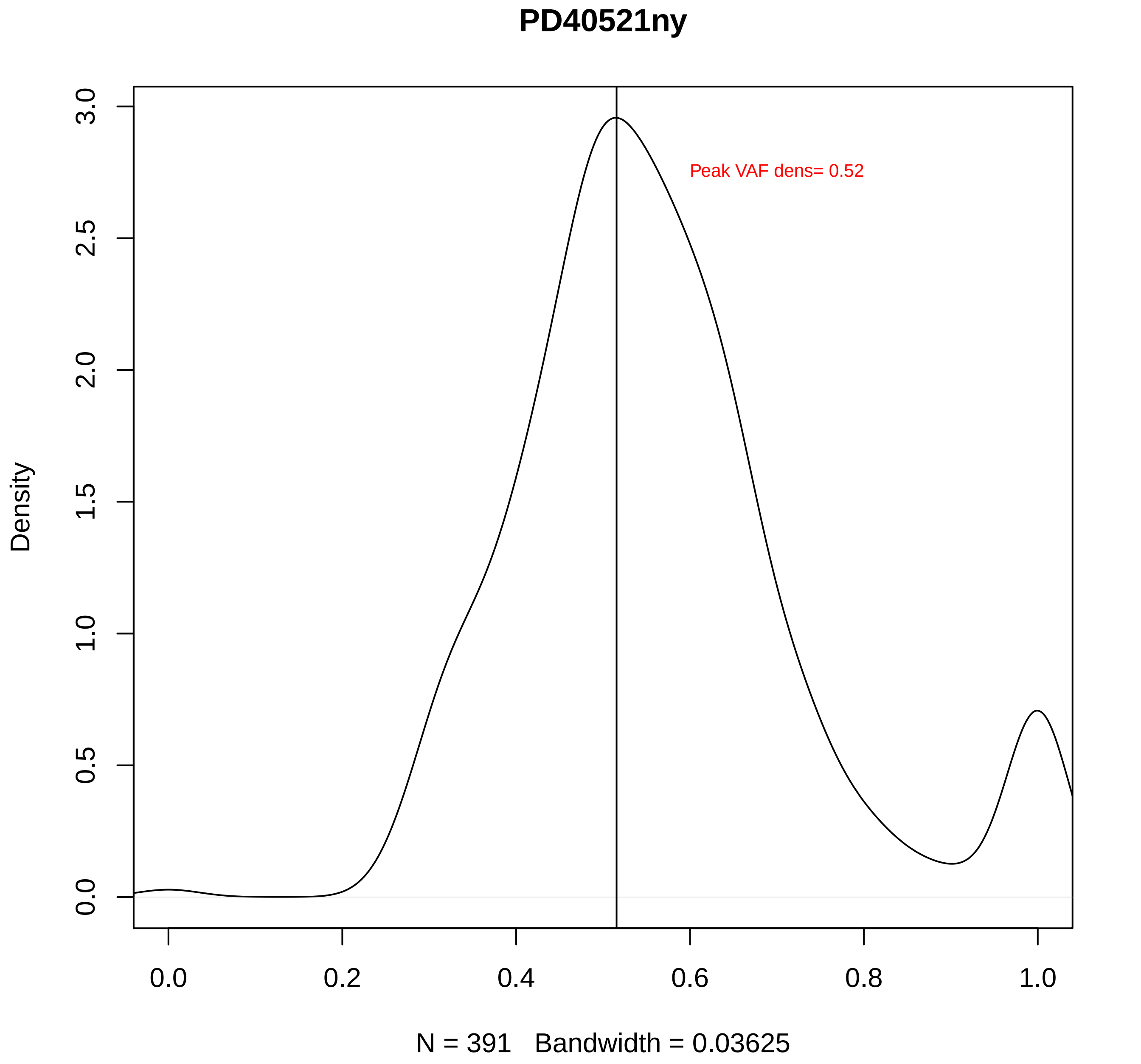
PD40521ny
Peak VAF dens= 0.52
Density
0.0
0.5
1.0
1.5
2.0
2.5
3.0
0.0
0.2
0.4
0.6
0.8
1.0
N = 391   Bandwidth = 0.03625

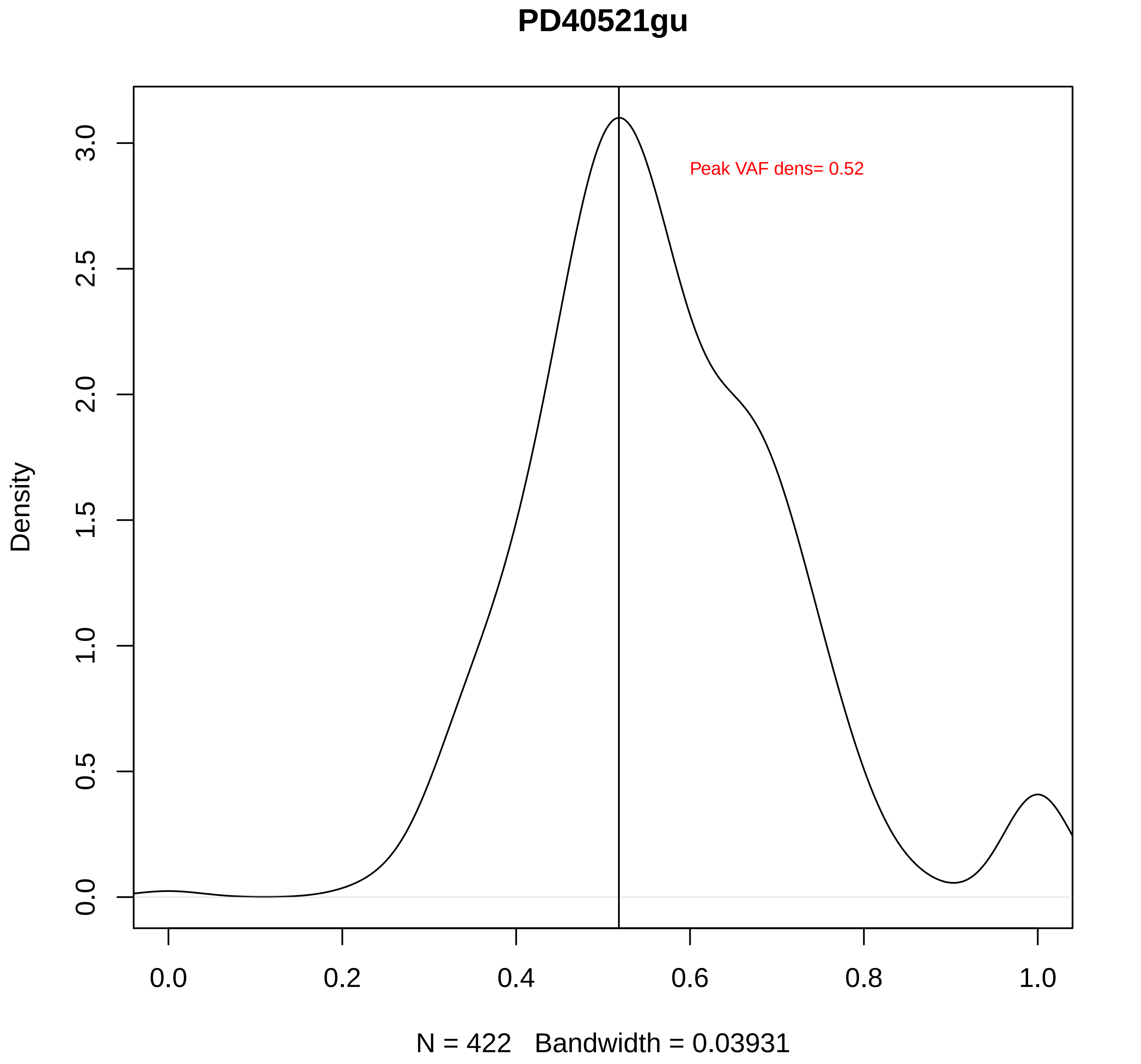
PD40521gu
Peak VAF dens= 0.52
Density
0.0
0.5
1.0
1.5
2.0
2.5
3.0
0.0
0.2
0.4
0.6
0.8
1.0
N = 422 Bandwidth = 0.03931

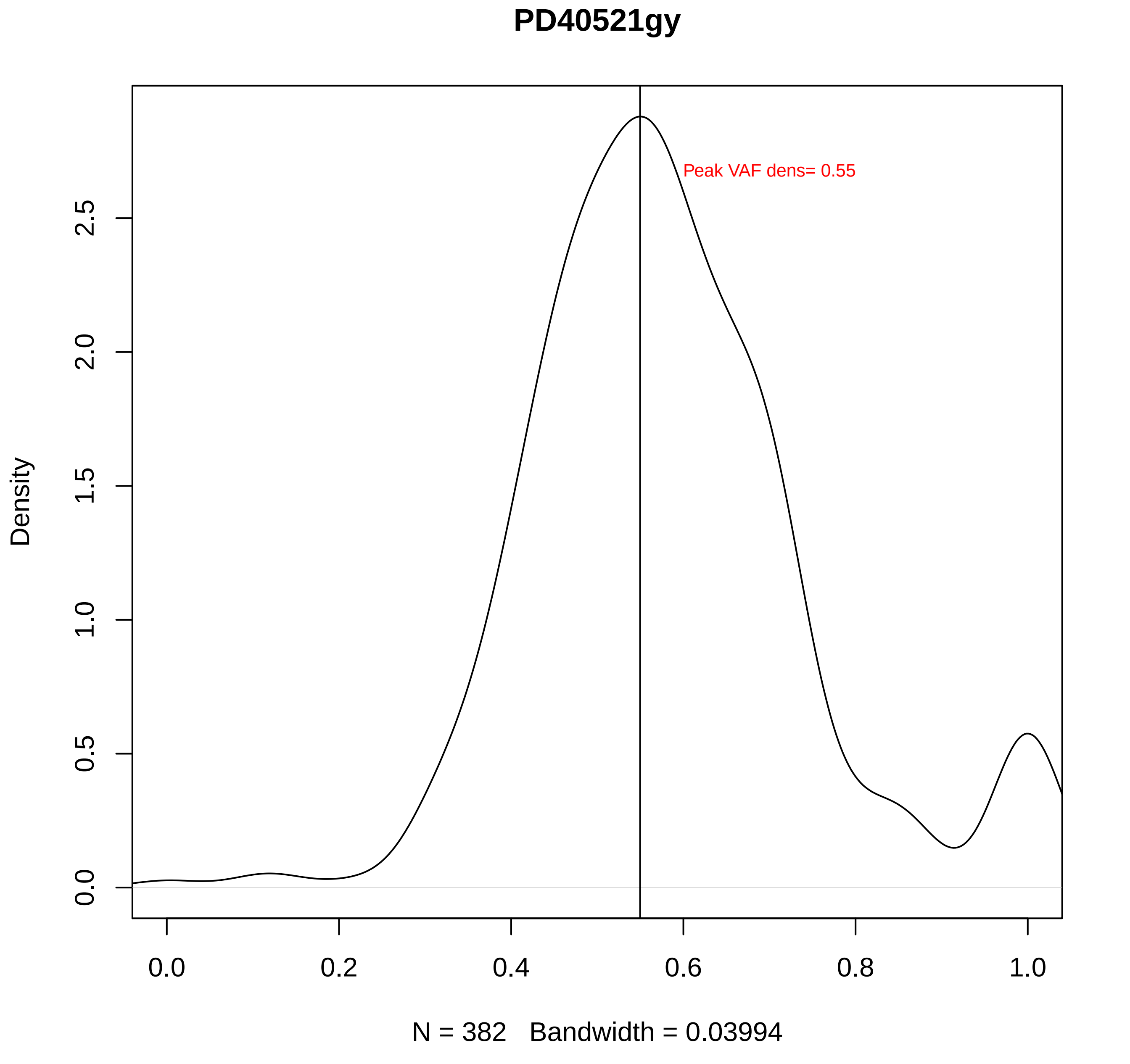
PD40521gy
Peak VAF dens= 0.55
Density
0.0
0.5
1.0
1.5
2.0
2.5
0.0
0.2
0.4
0.6
0.8
1.0
N = 382   Bandwidth = 0.03994

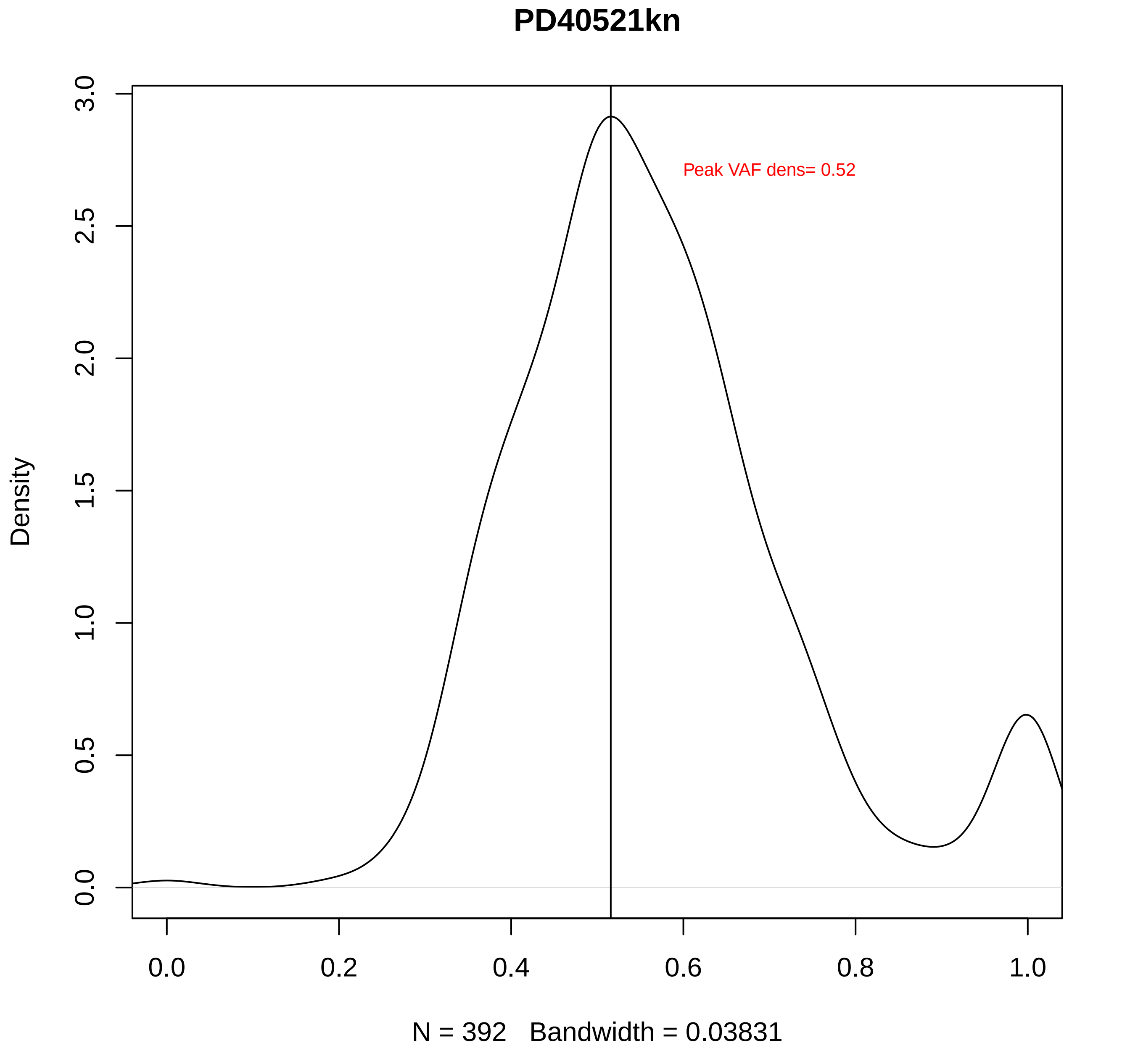
PD40521kn
Peak VAF dens= 0.52
Density
0.0
0.5
1.0
1.5
2.0
2.5
3.0
0.0
0.2
0.4
0.6
0.8
1.0
N = 392   Bandwidth = 0.03831

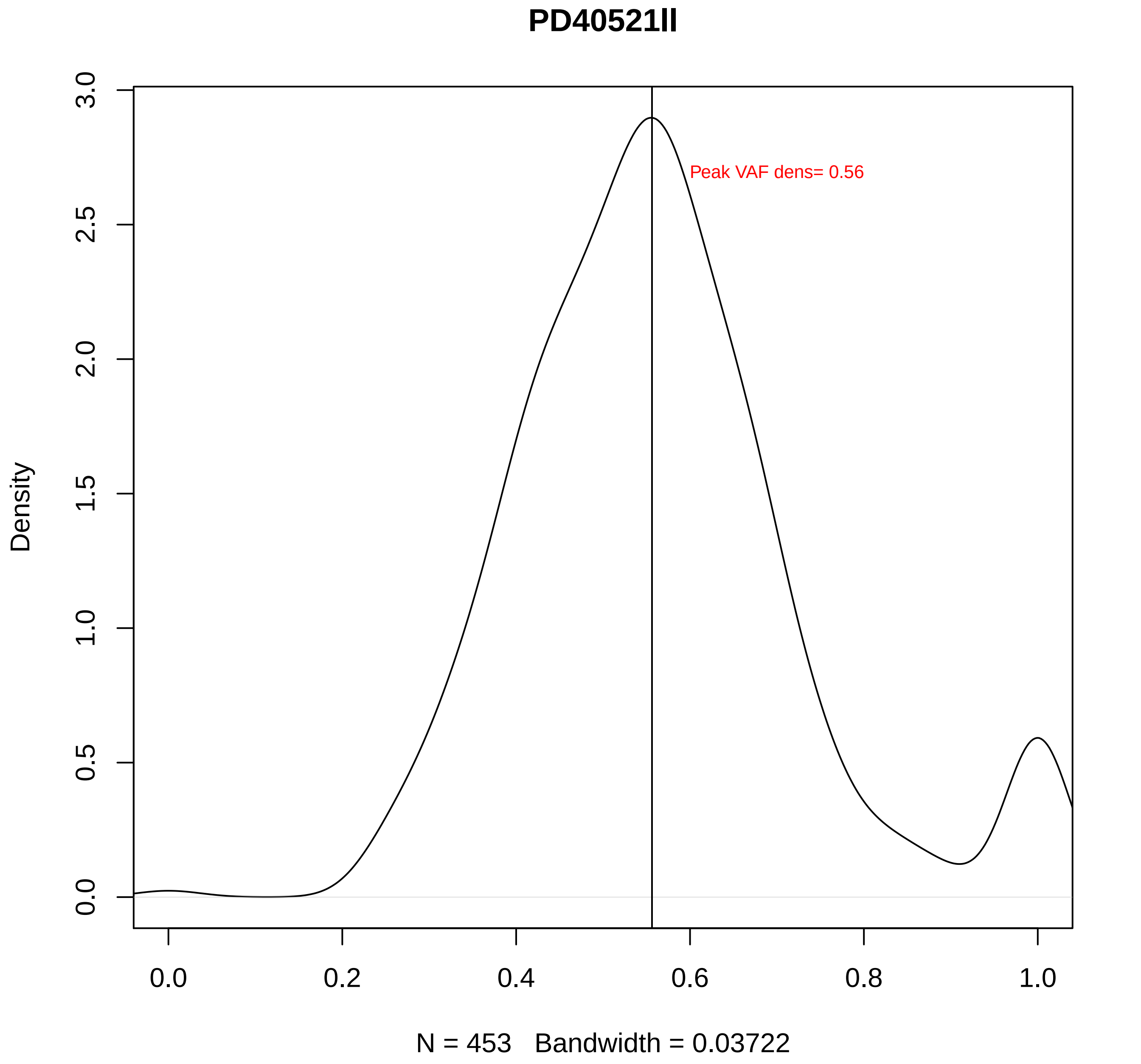
PD40521ll
Peak VAF dens= 0.56
Density
0.0
0.5
1.0
1.5
2.0
2.5
3.0
0.0
0.2
0.4
0.6
0.8
1.0
N = 453   Bandwidth = 0.03722

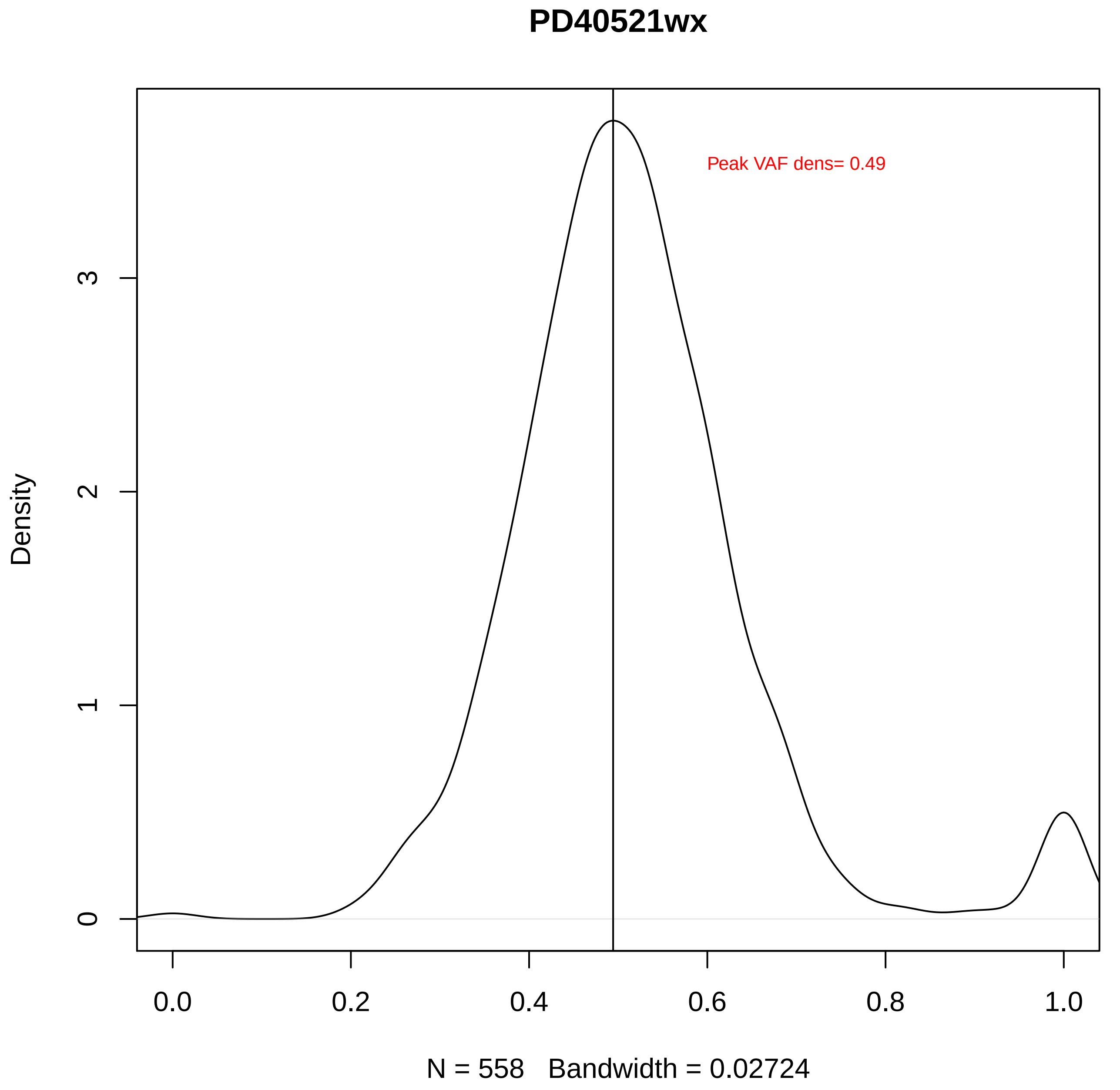
PD40521wx
Peak VAF dens= 0.49
Density
0
1
2
3
0.0
0.2
0.4
0.6
0.8
1.0
N = 558   Bandwidth = 0.02724

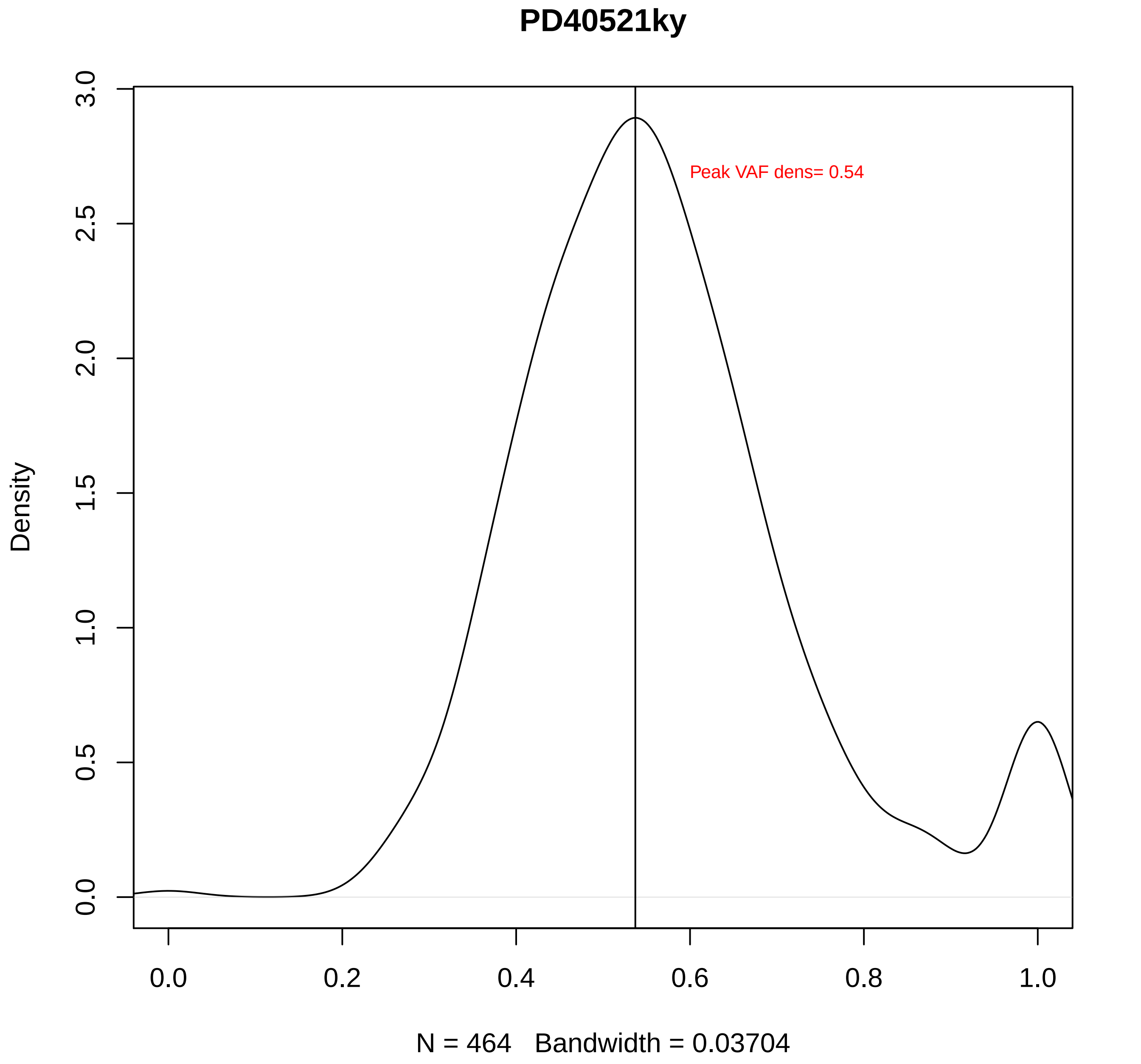
PD40521ky
Peak VAF dens= 0.54
Density
0.0
0.5
1.0
1.5
2.0
2.5
3.0
0.0
0.2
0.4
0.6
0.8
1.0
N = 464   Bandwidth = 0.03704

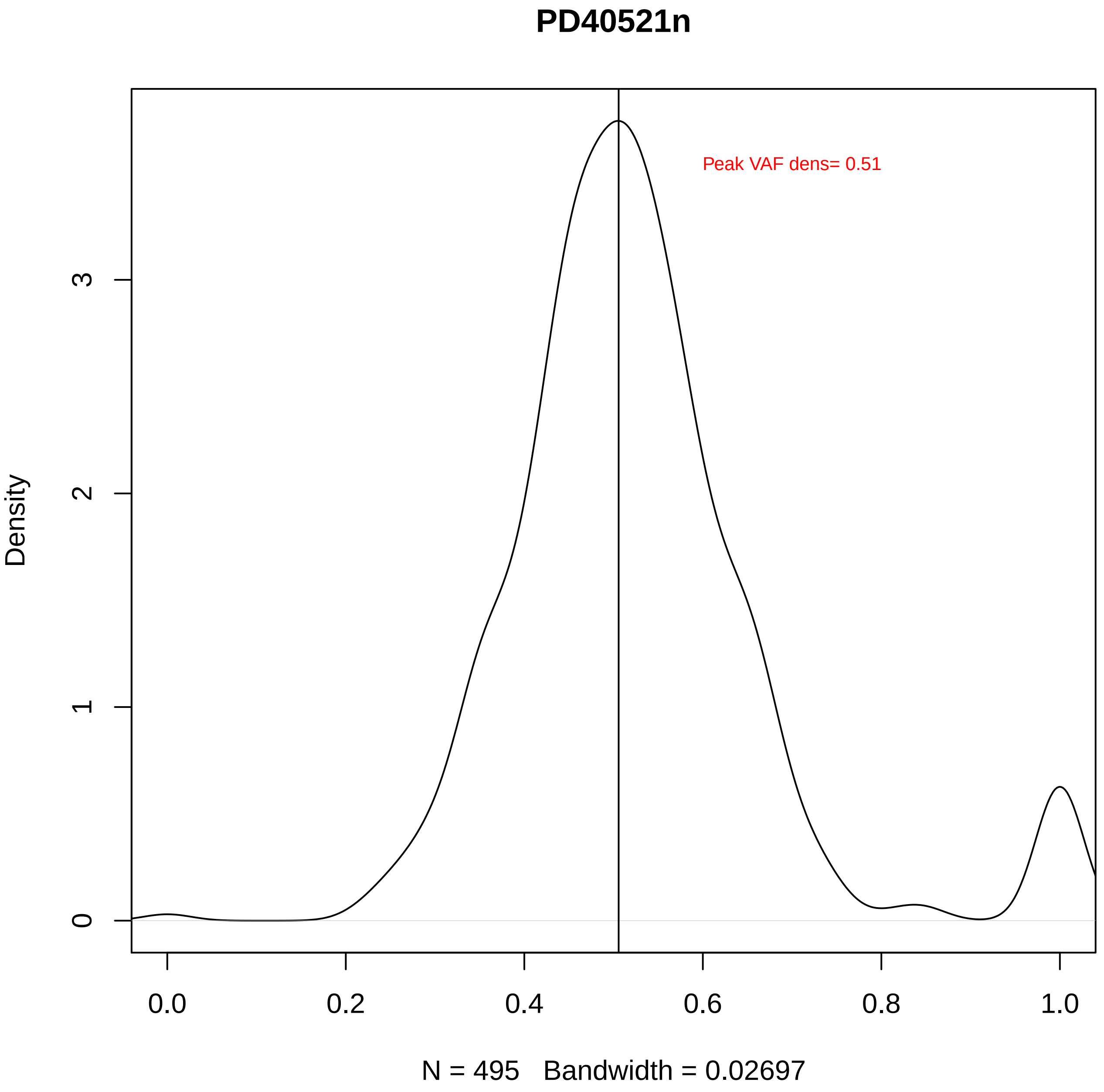

PD40521n
Peak VAF dens= 0.51
Density
0
1
2
3
0.0
0.2
0.4
0.6
0.8
1.0
N = 495 Bandwidth = 0.02697

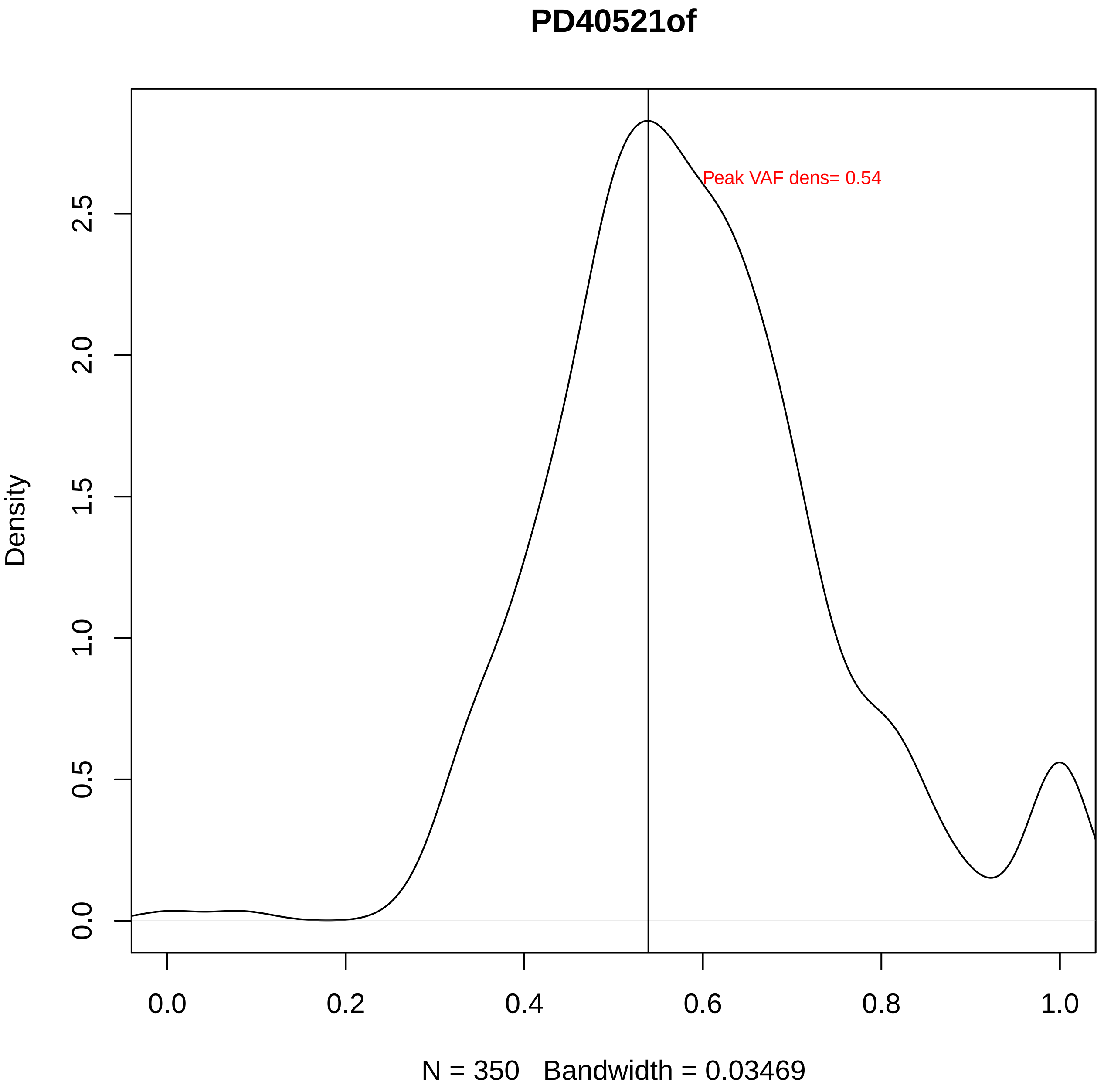
PD40521of
Peak VAF dens= 0.54
Density
0.0
0.5
1.0
1.5
2.0
2.5
0.0
0.2
0.4
0.6
0.8
1.0
N = 350 Bandwidth = 0.03469

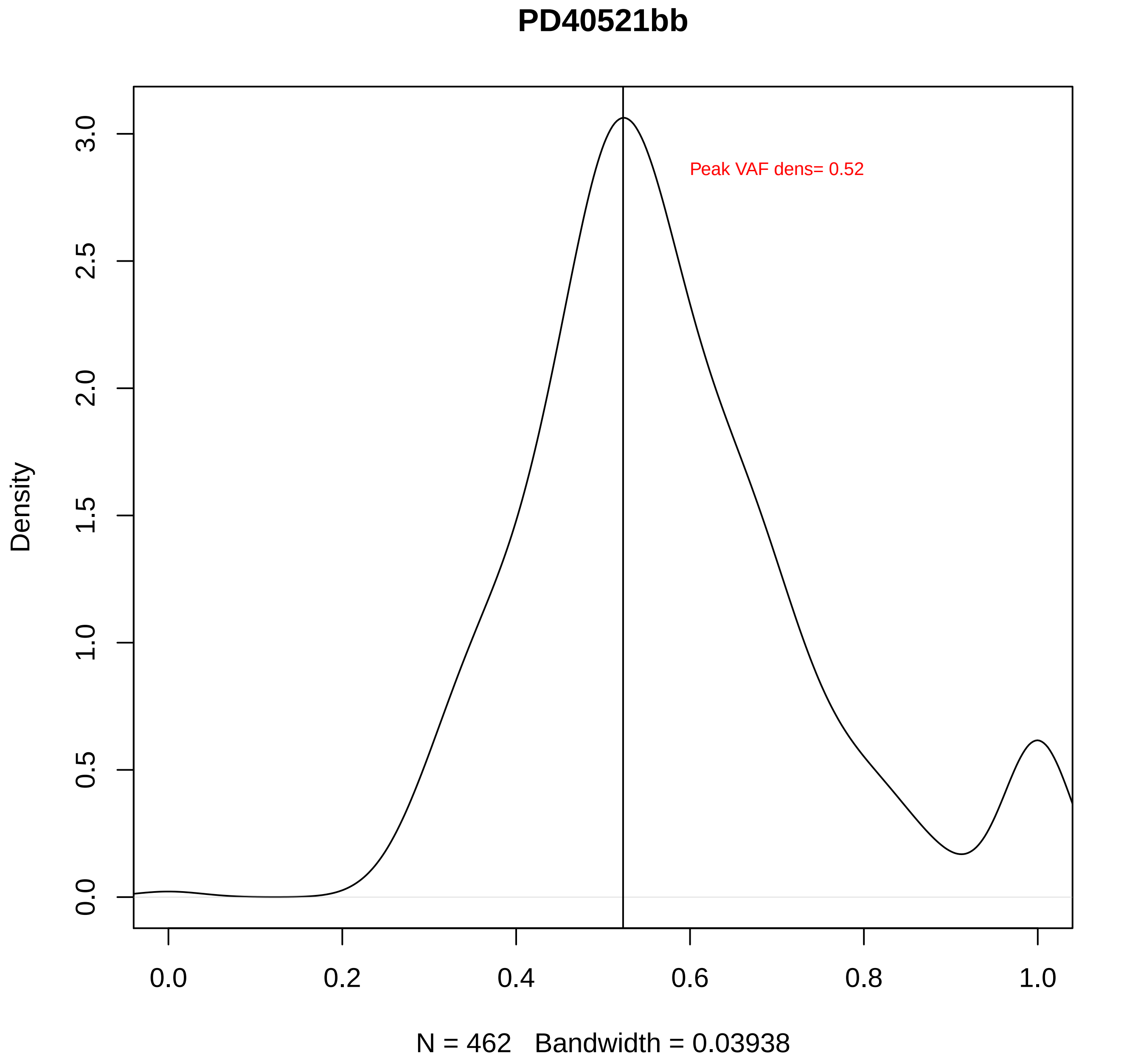
PD40521bb
Peak VAF dens= 0.52
Density
0.0
0.5
1.0
1.5
2.0
2.5
3.0
0.0
0.2
0.4
0.6
0.8
1.0
N = 462   Bandwidth = 0.03938

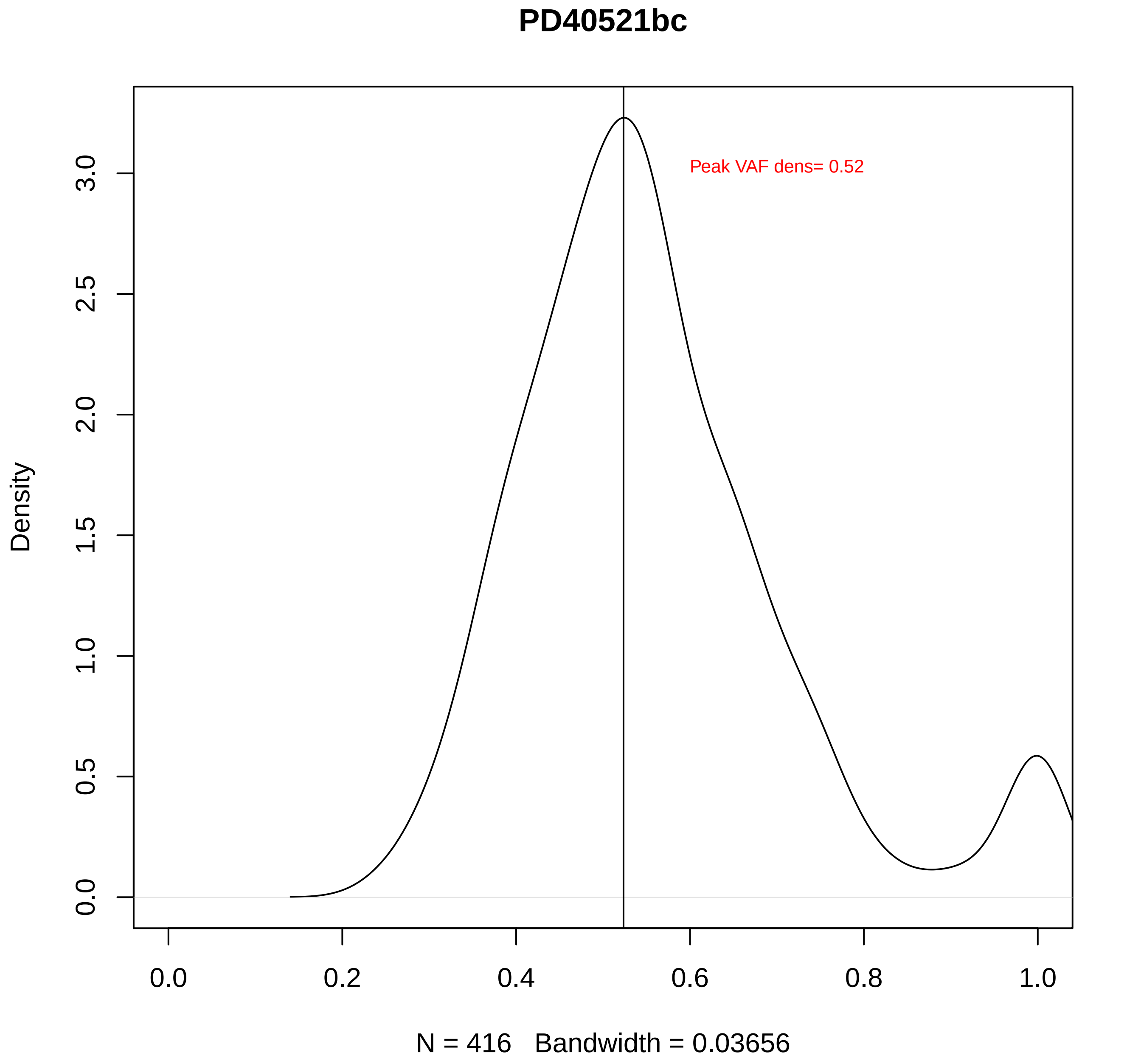
PD40521bc
Peak VAF dens= 0.52
Density
0.0
0.5
1.0
1.5
2.0
2.5
3.0
0.0
0.2
0.4
0.6
0.8
1.0
N = 416   Bandwidth = 0.03656

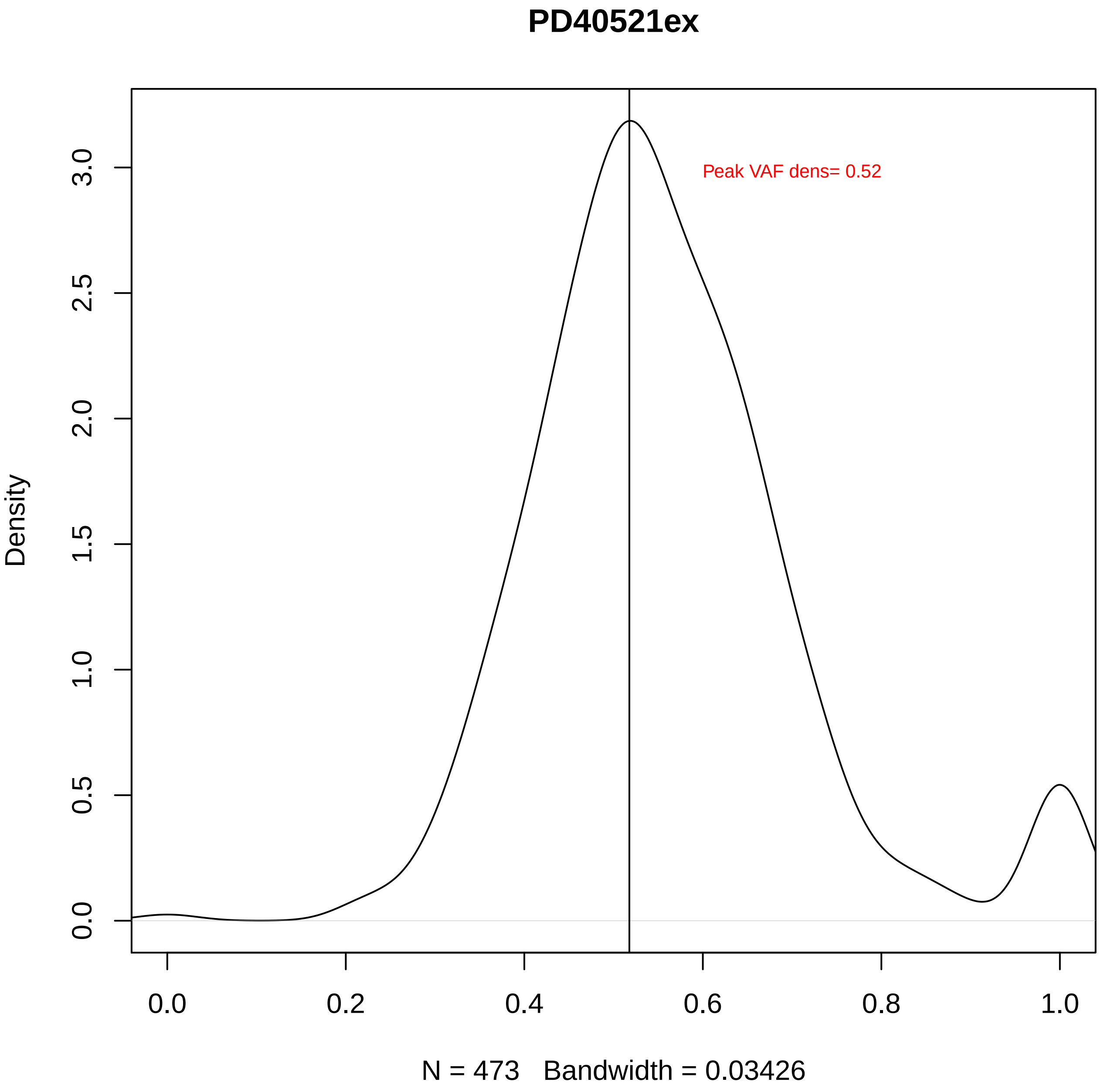
PD40521ex
Peak VAF dens= 0.52
Density
0.0
0.5
1.0
1.5
2.0
2.5
3.0
0.0
0.2
0.4
0.6
0.8
1.0
N = 473   Bandwidth = 0.03426

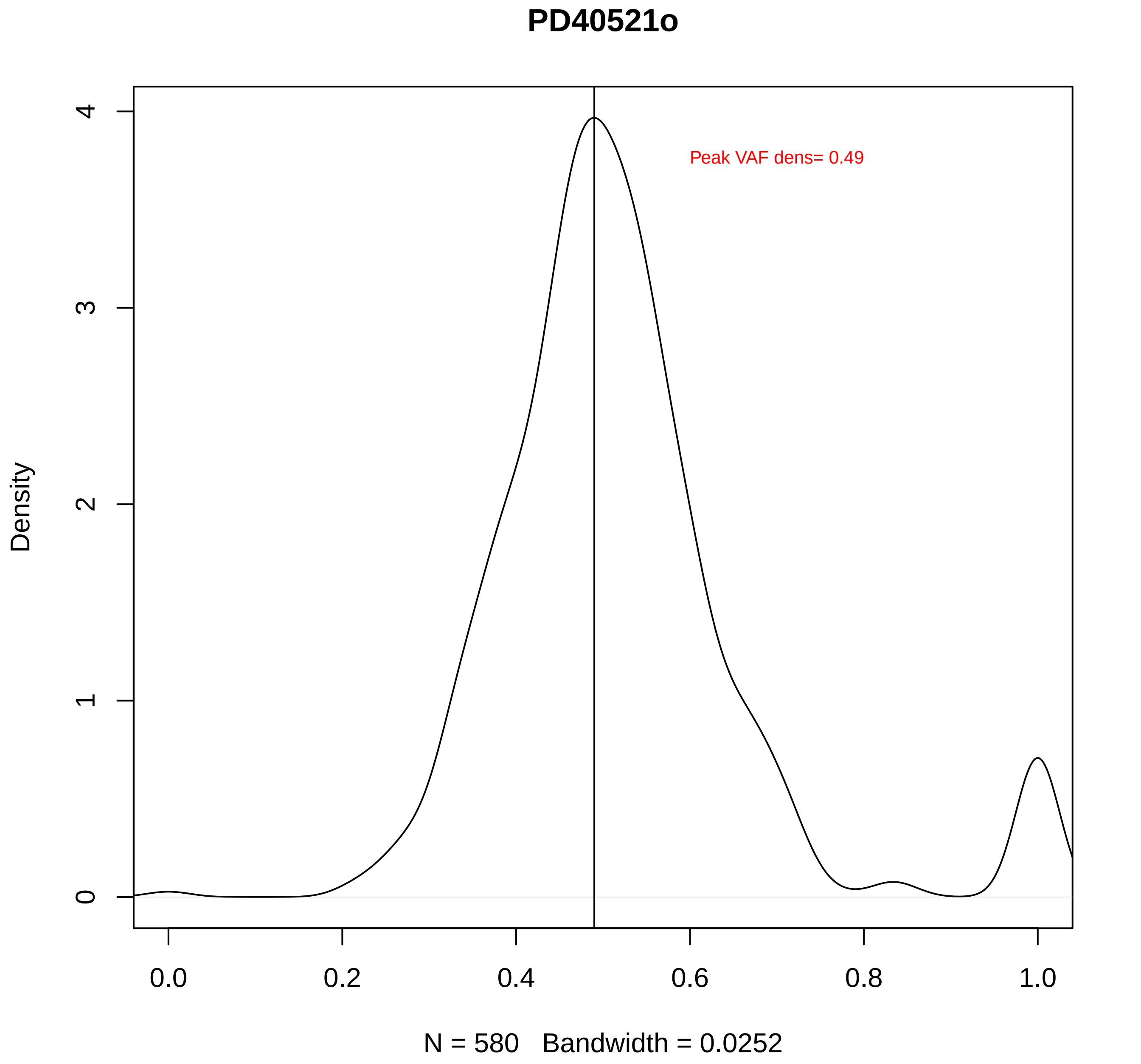
PD40521o
Peak VAF dens= 0.49
Density
0
1
2
3
4
0.0
0.2
0.4
0.6
0.8
1.0
N = 580   Bandwidth = 0.0252

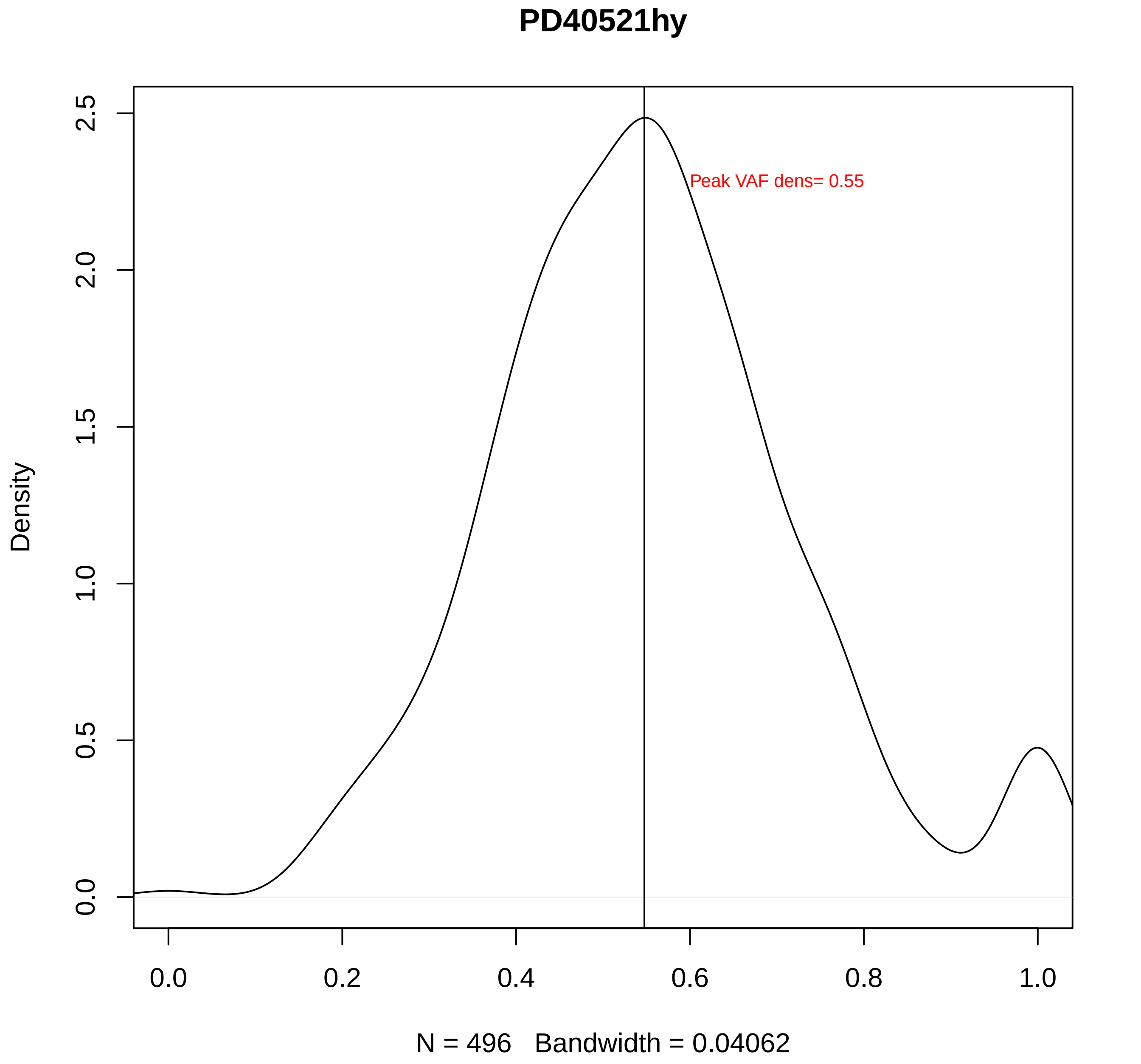
PD40521hy
Peak VAF dens= 0.55
Density
0.0
0.5
1.0
1.5
2.0
2.5
0.0
0.2
0.4
0.6
0.8
1.0
N = 496   Bandwidth = 0.04062

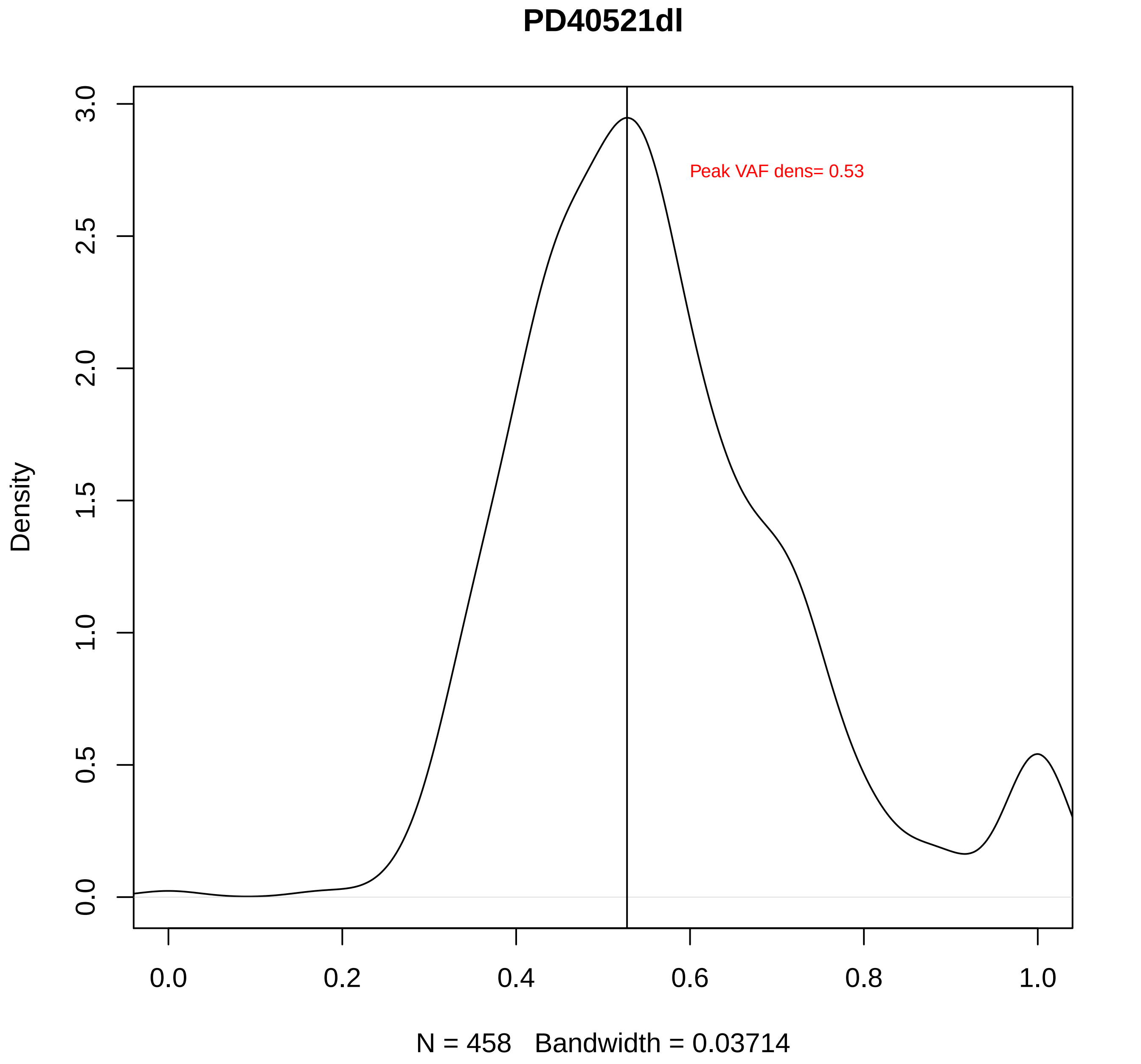
PD40521dl
Peak VAF dens= 0.53
Density
0.0
0.5
1.0
1.5
2.0
2.5
3.0
0.0
0.2
0.4
0.6
0.8
1.0
N = 458   Bandwidth = 0.03714

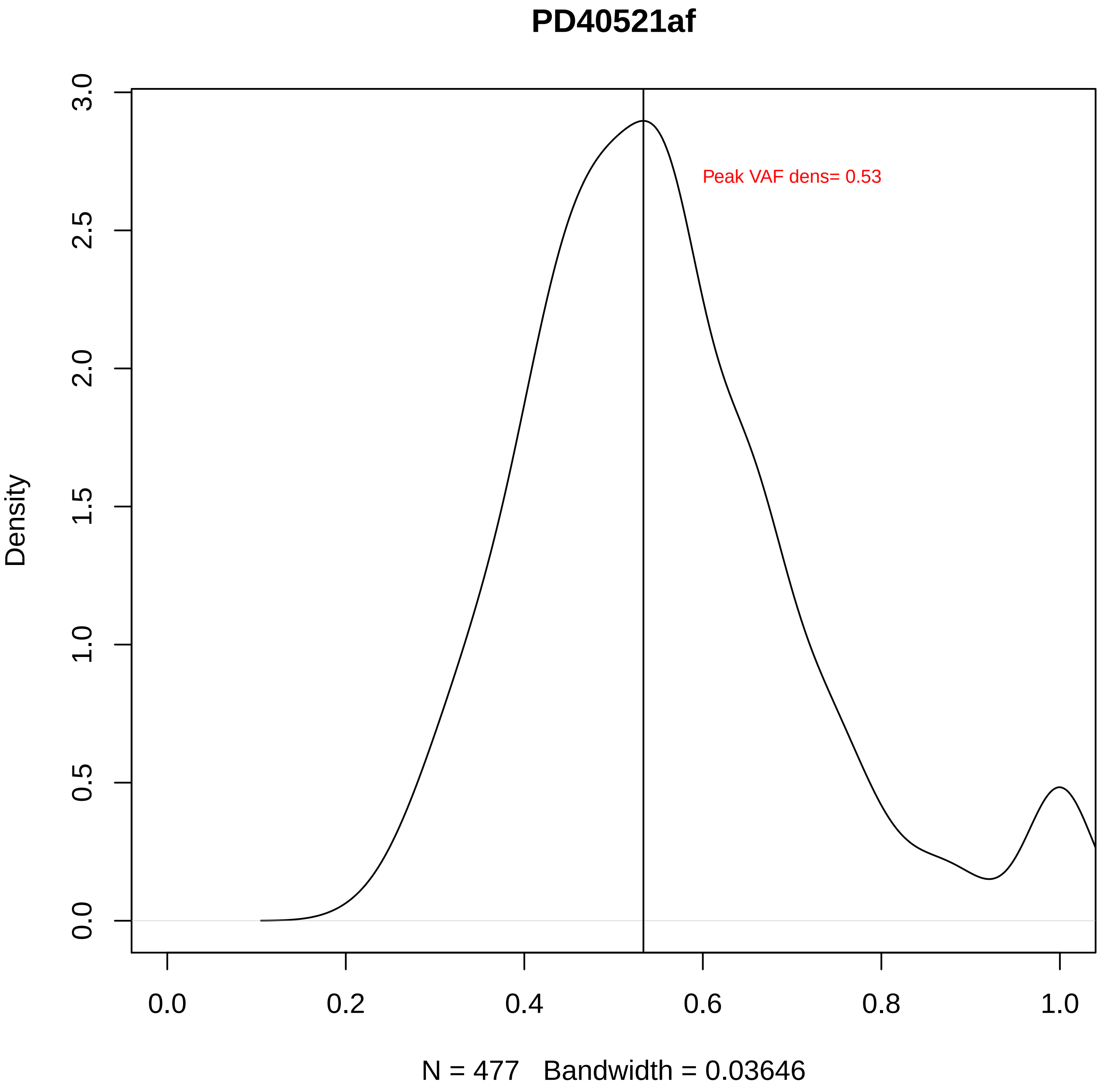

PD40521af
Peak VAF dens= 0.53
Density
0.0
0.5
1.0
1.5
2.0
2.5
3.0
0.0
0.2
0.4
0.6
0.8
1.0
N = 477 Bandwidth = 0.03646

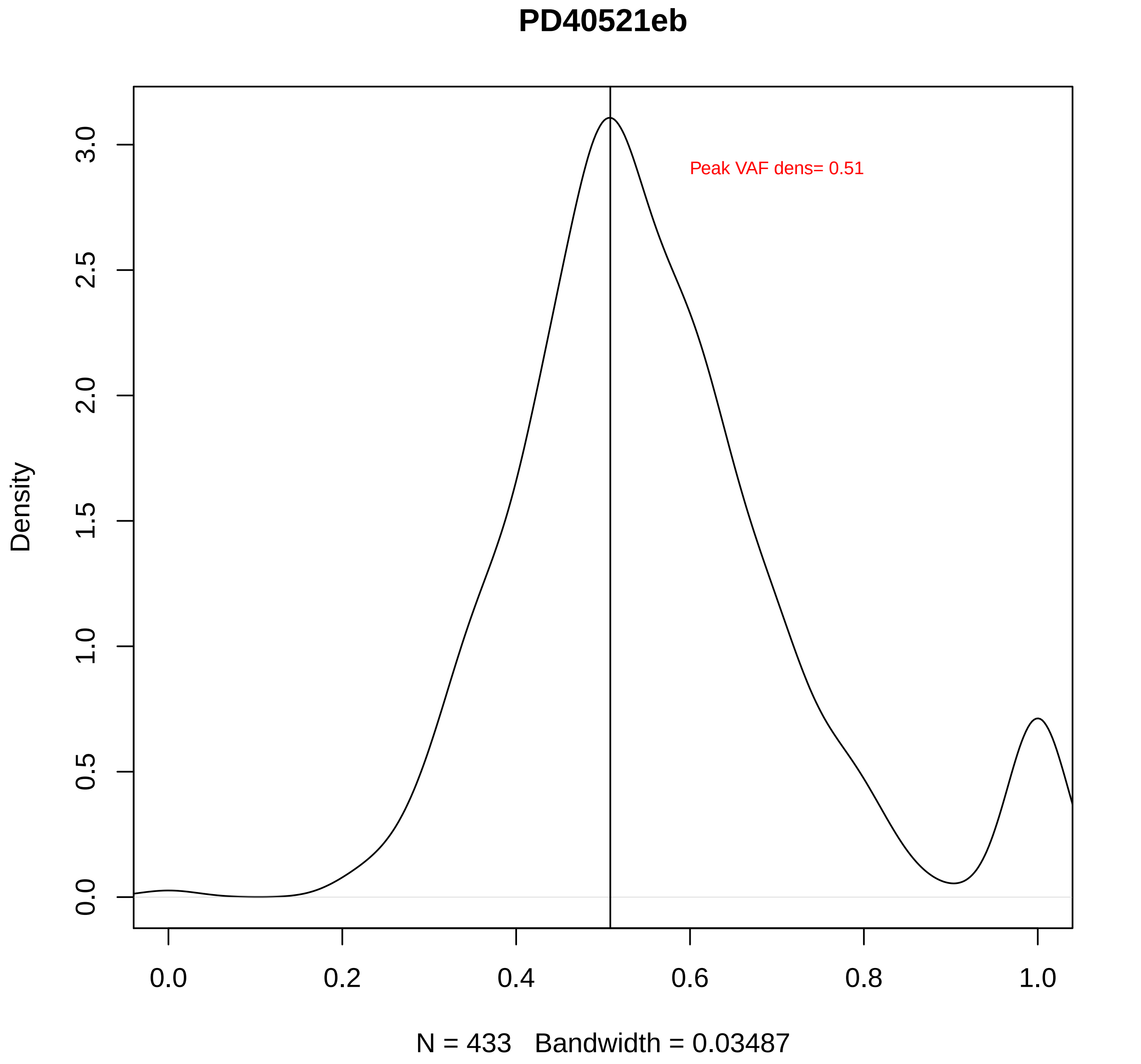
PD40521eb
Peak VAF dens= 0.51
Density
0.0
0.5
1.0
1.5
2.0
2.5
3.0
0.0
0.2
0.4
0.6
0.8
1.0
N = 433   Bandwidth = 0.03487

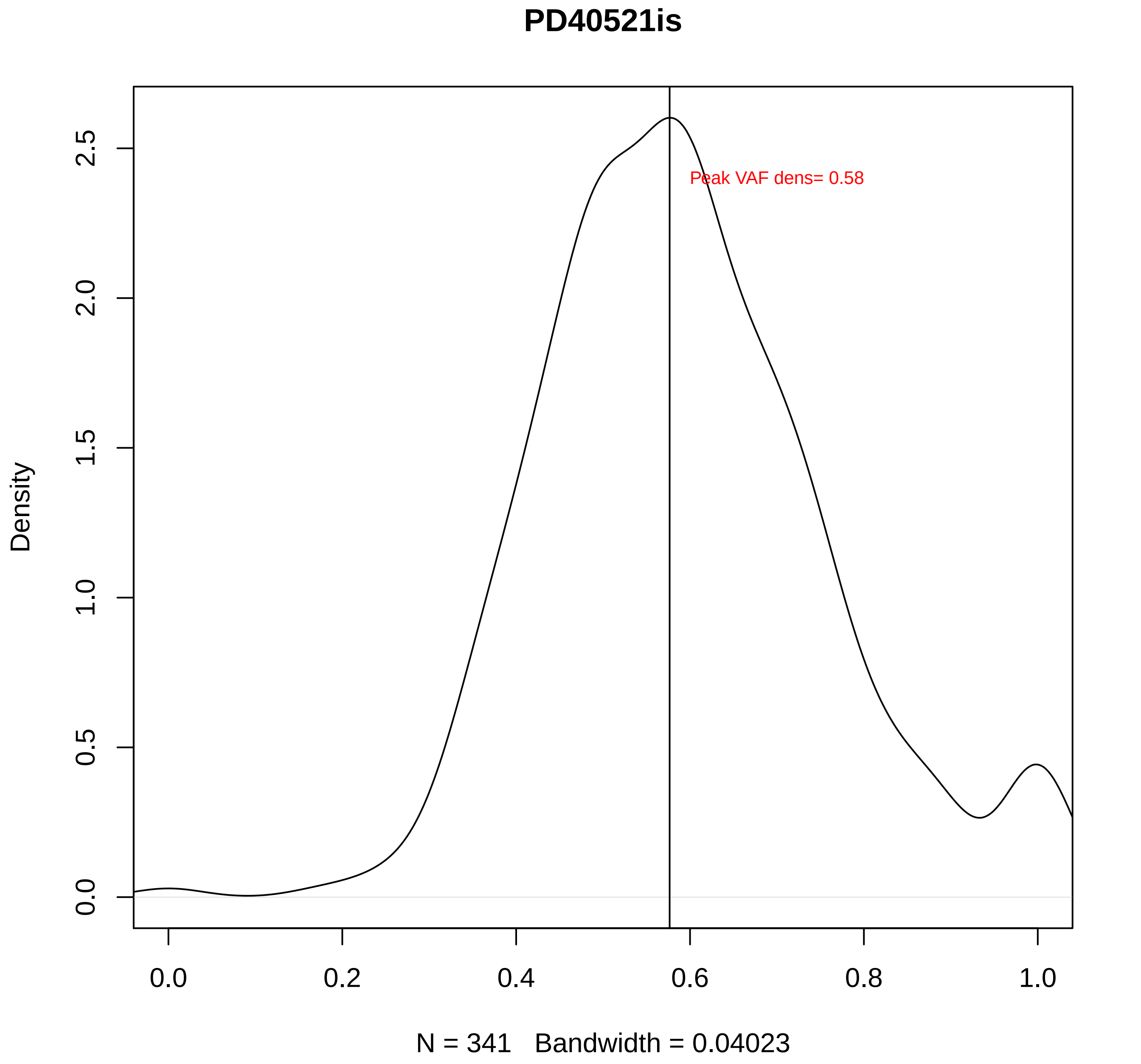
PD40521is
Peak VAF dens= 0.58
Density
0.0
0.5
1.0
1.5
2.0
2.5
0.0
0.2
0.4
0.6
0.8
1.0
N = 341   Bandwidth = 0.04023

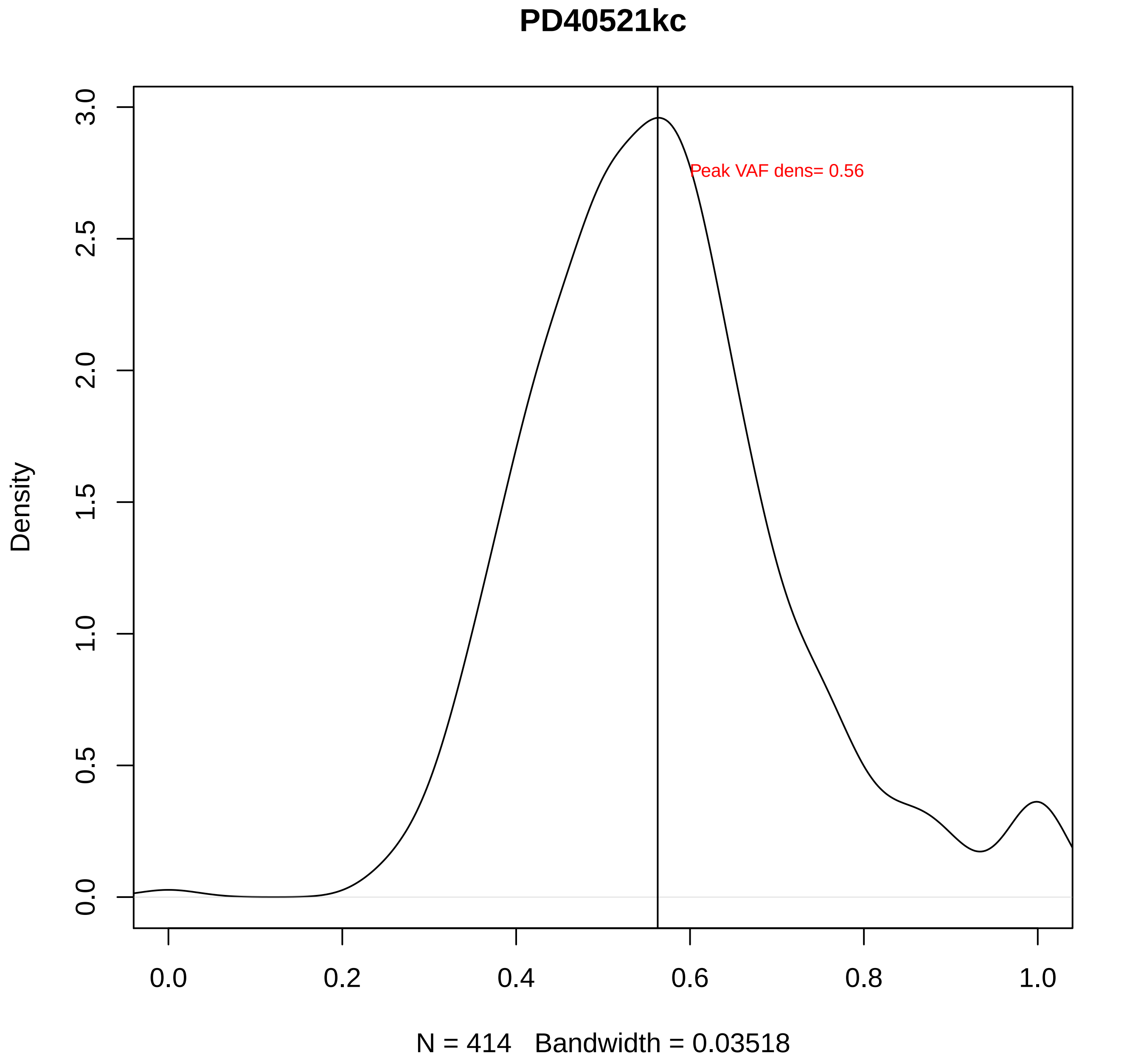

PD40521kc
Peak VAF dens= 0.56
Density
0.0
0.5
1.0
1.5
2.0
2.5
3.0
0.0
0.2
0.4
0.6
0.8
1.0
N = 414 Bandwidth = 0.03518

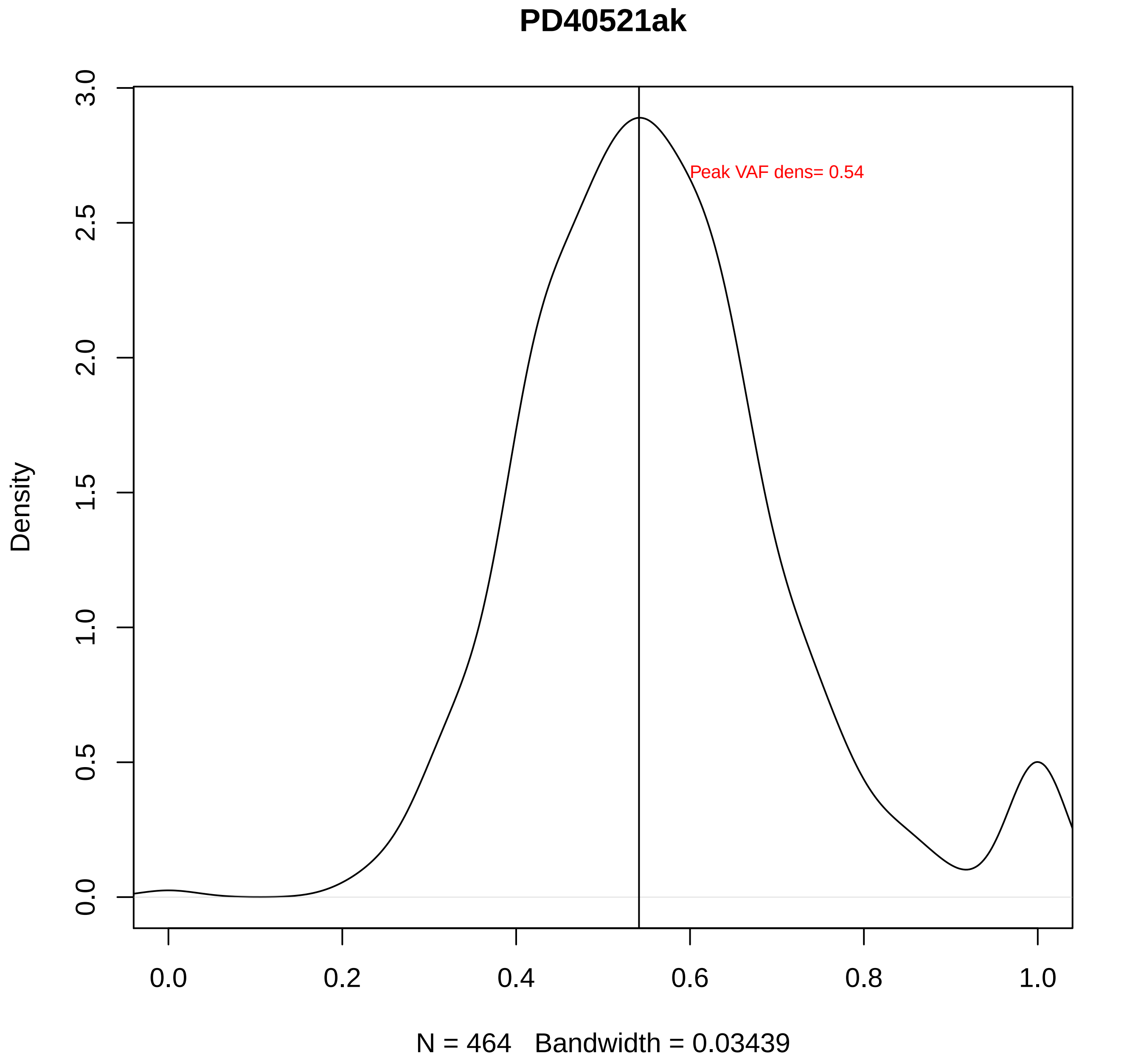

PD40521ak
Peak VAF dens= 0.54
Density
0.0
0.5
1.0
1.5
2.0
2.5
3.0
0.0
0.2
0.4
0.6
0.8
1.0
N = 464   Bandwidth = 0.03439

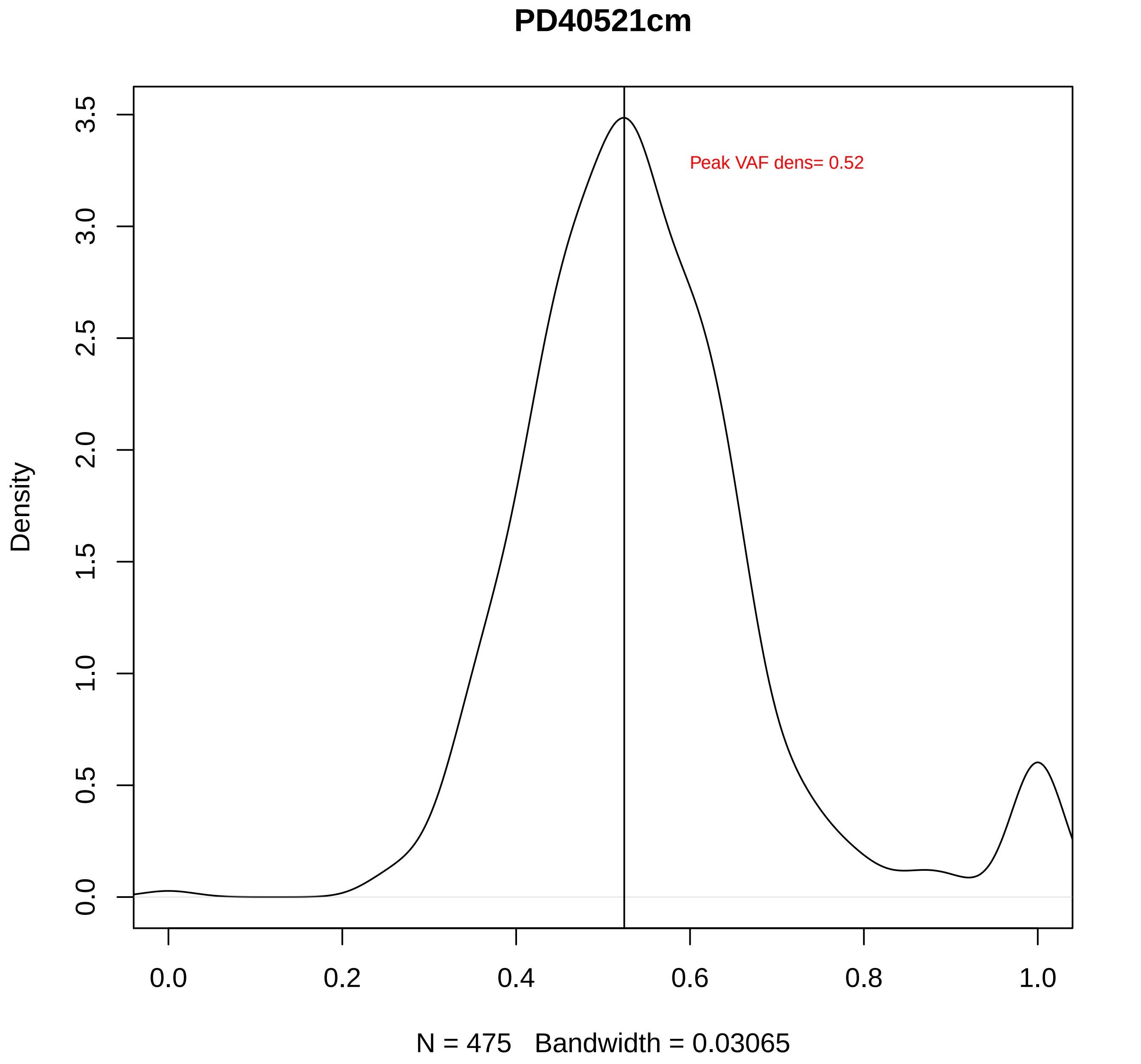

PD40521cm
Peak VAF dens= 0.52
Density
0.0
0.5
1.0
1.5
2.0
2.5
3.0
3.5
0.0
0.2
0.4
0.6
0.8
1.0
N = 475   Bandwidth = 0.03065

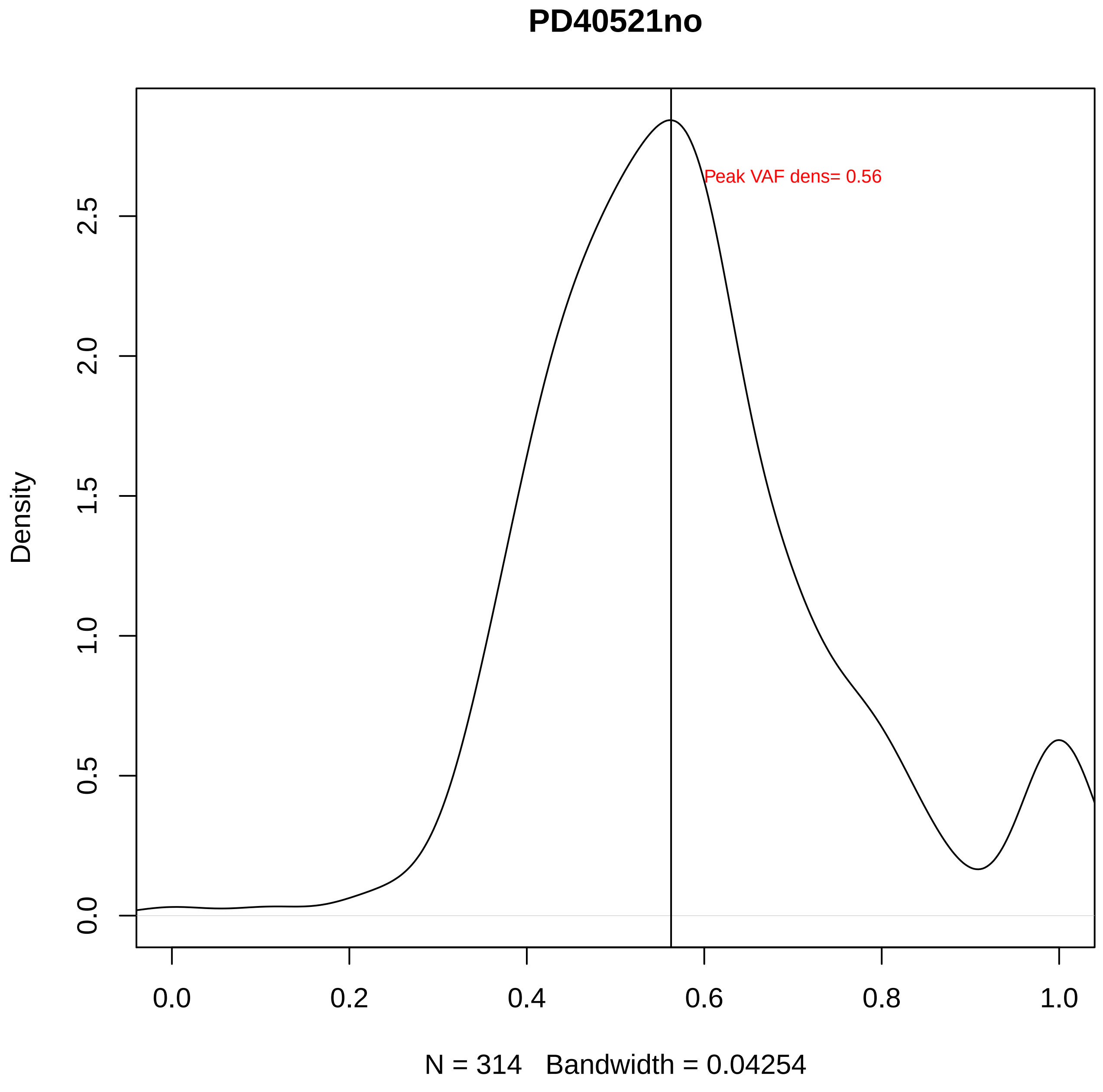

PD40521no
Peak VAF dens= 0.56
Density
0.0
0.5
1.0
1.5
2.0
2.5
0.0
0.2
0.4
0.6
0.8
1.0
N = 314 Bandwidth = 0.04254

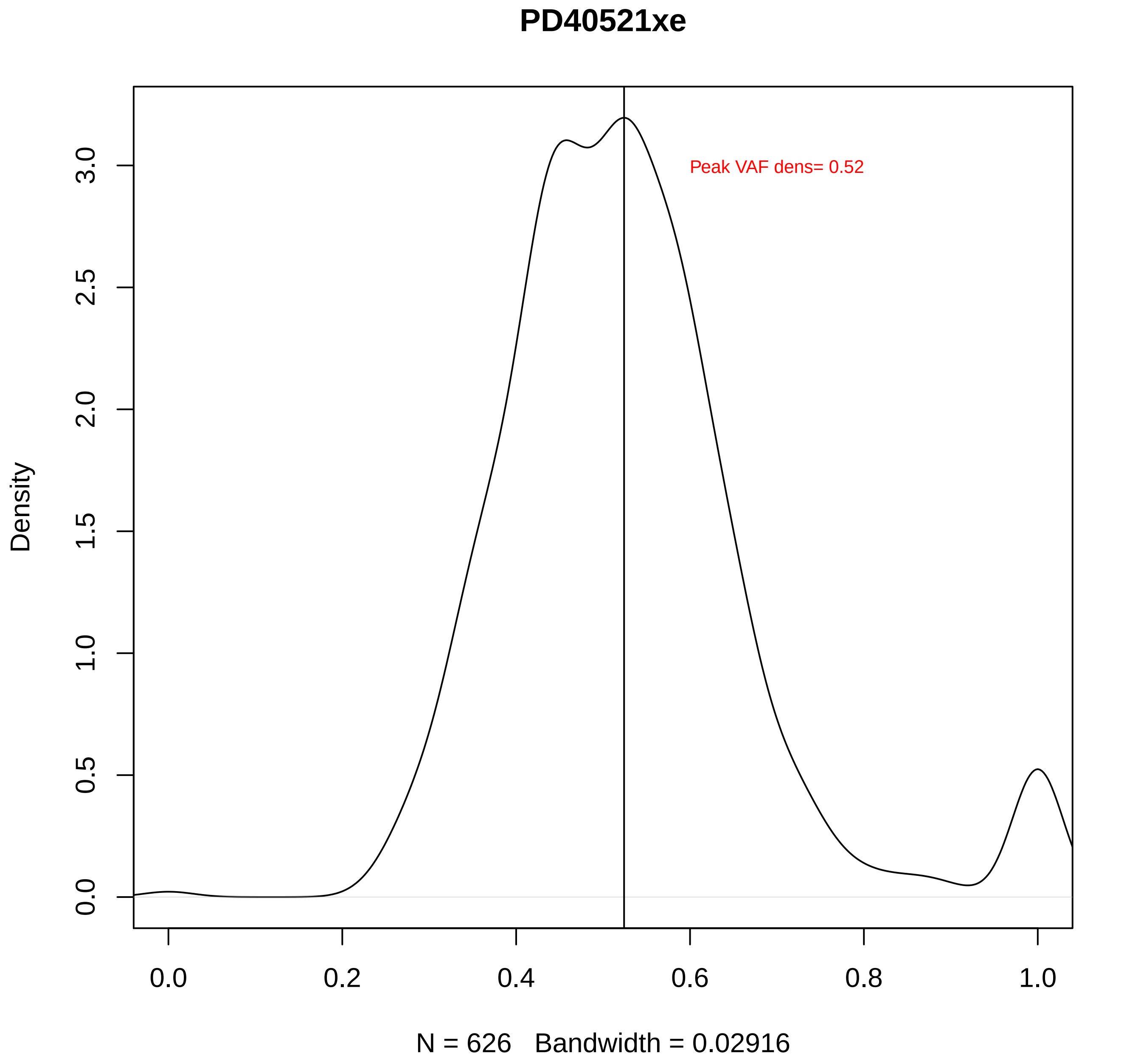
PD40521xe
Peak VAF dens= 0.52
Density
0.0
0.5
1.0
1.5
2.0
2.5
3.0
0.0
0.2
0.4
0.6
0.8
1.0
N = 626 Bandwidth = 0.02916

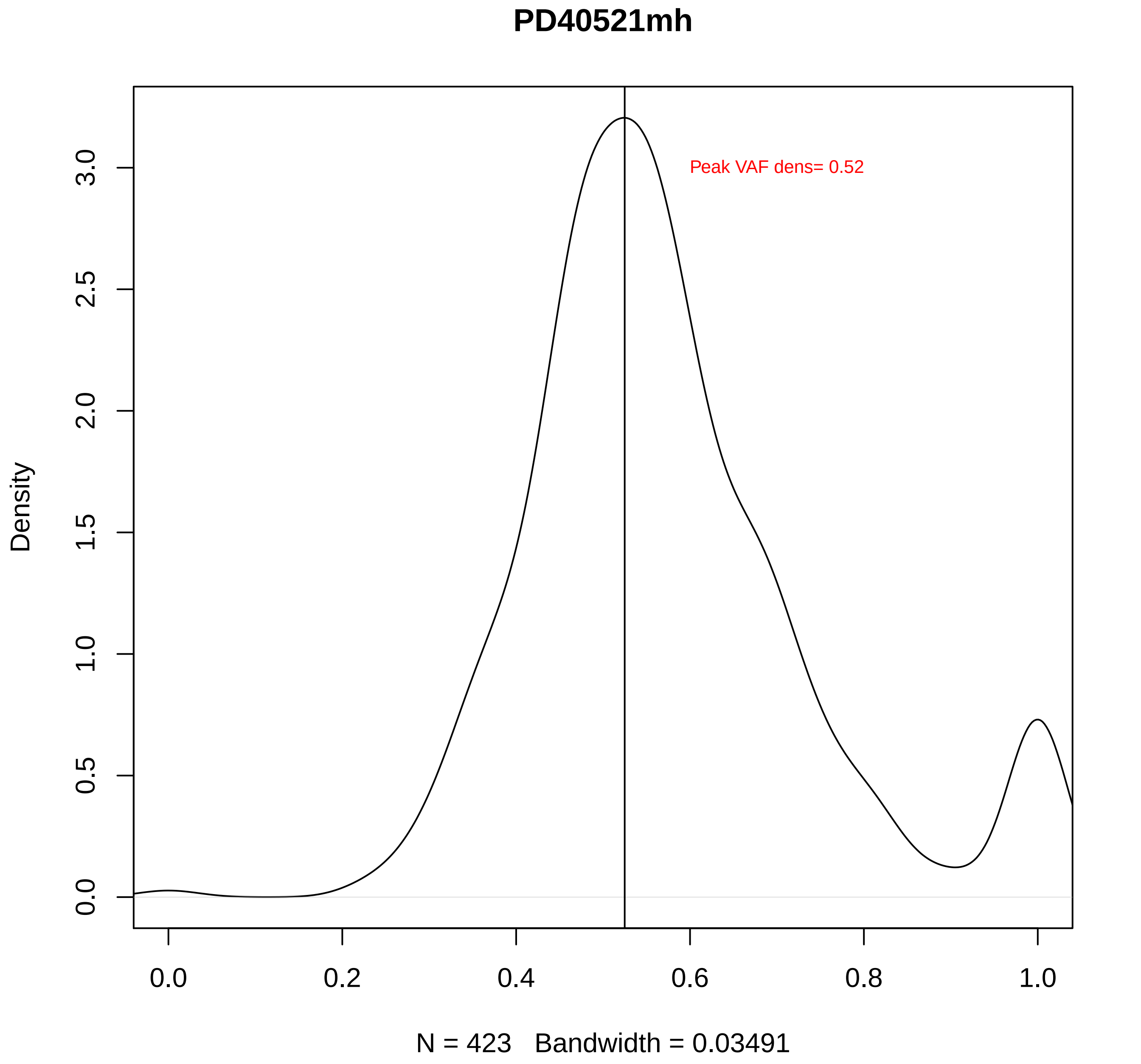
PD40521mh
Peak VAF dens= 0.52
Density
0.0
0.5
1.0
1.5
2.0
2.5
3.0
0.0
0.2
0.4
0.6
0.8
1.0
N = 423   Bandwidth = 0.03491

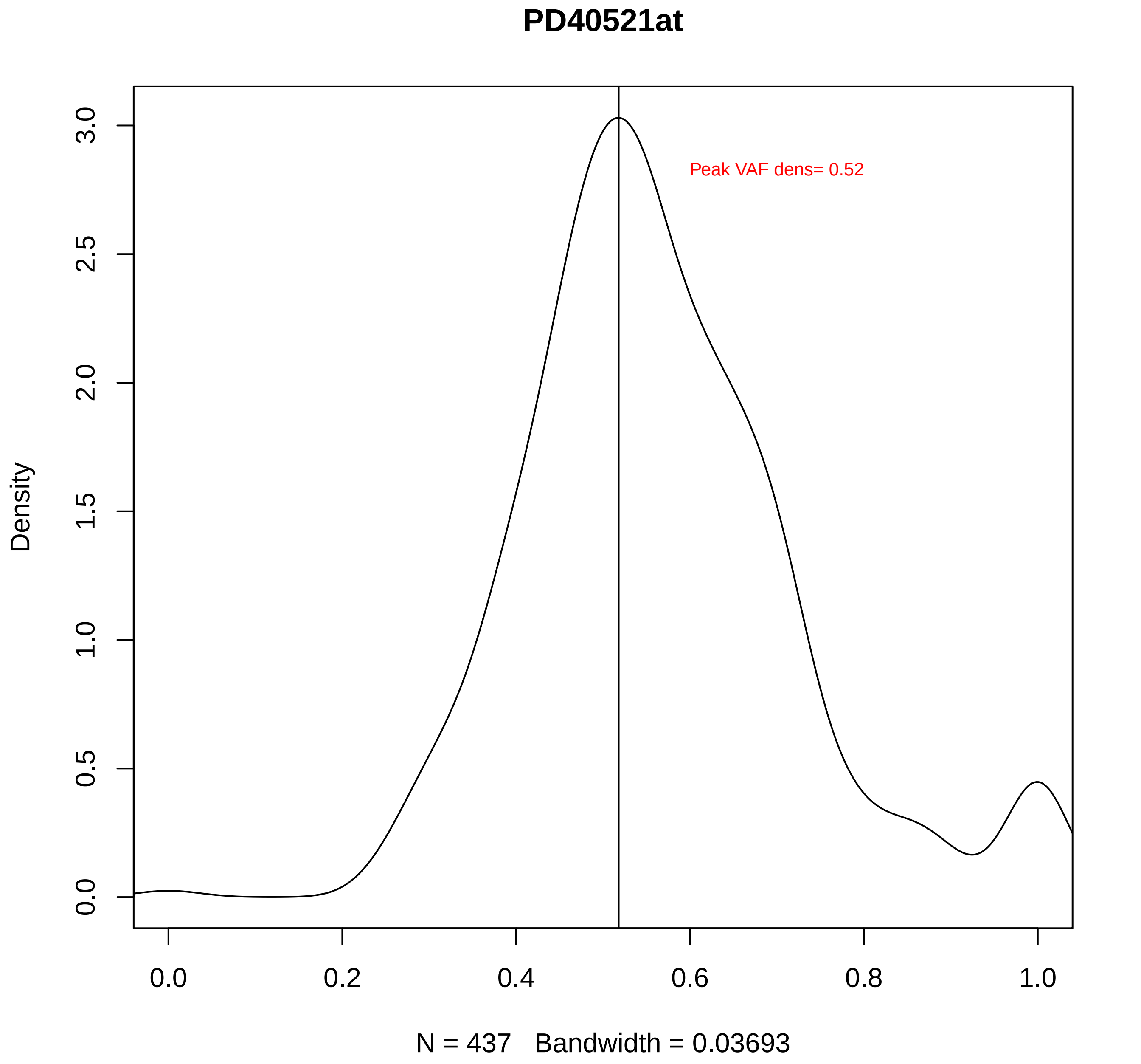
PD40521at
Peak VAF dens= 0.52
Density
3.0
2.5
2.0
1.5
1.0
0.5
0.0
0.0
0.2
0.4
0.6
0.8
1.0
N = 437   Bandwidth = 0.03693

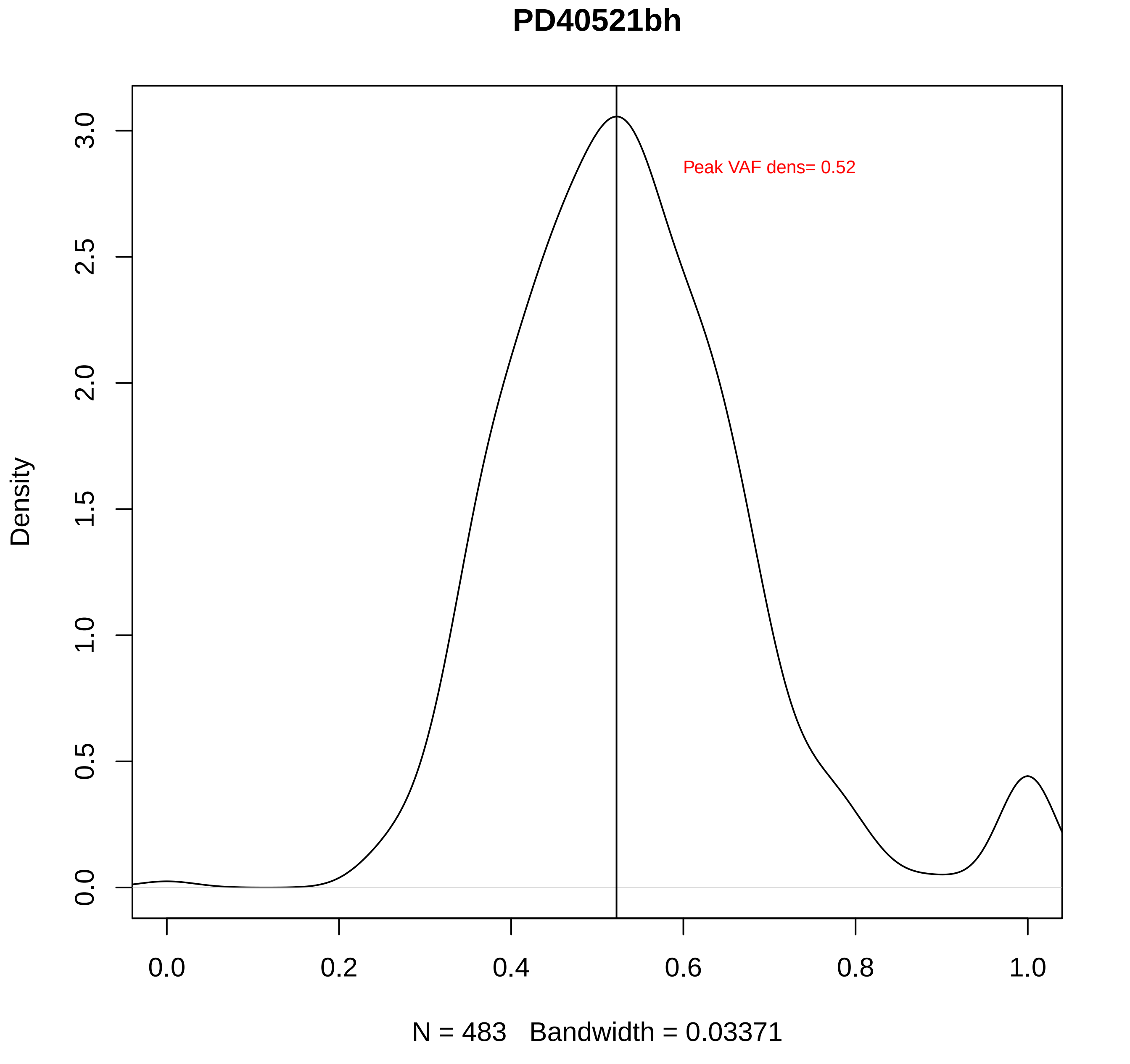
PD40521bh
Peak VAF dens= 0.52
Density
3.0
2.5
2.0
1.5
1.0
0.5
0.0
0.0
0.2
0.4
0.6
0.8
1.0
N = 483   Bandwidth = 0.03371

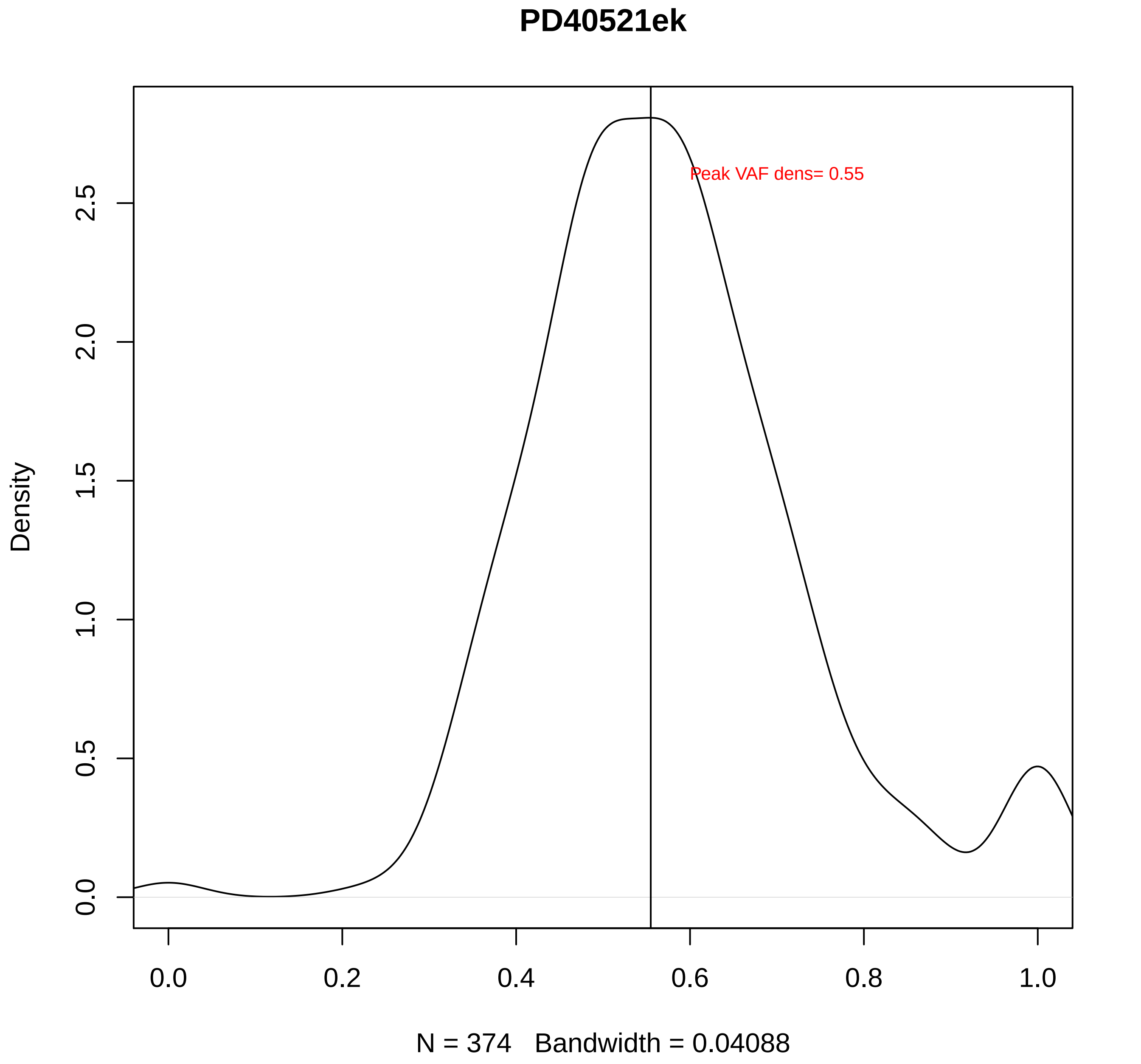
PD40521ek
Peak VAF dens= 0.55
Density
0.0
0.5
1.0
1.5
2.0
2.5
0.0
0.2
0.4
0.6
0.8
1.0
N = 374   Bandwidth = 0.04088

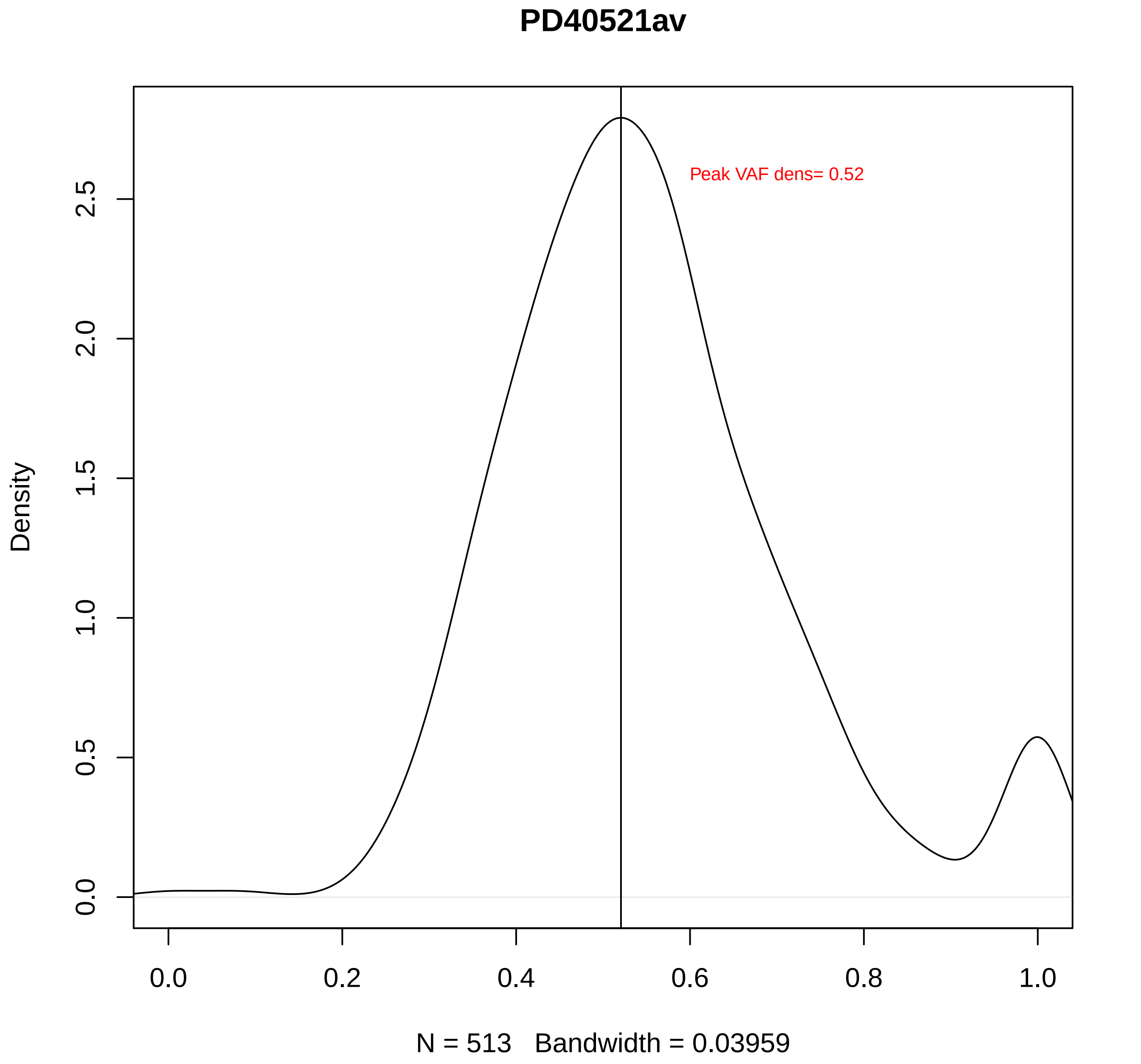
PD40521av
Peak VAF dens= 0.52
Density
0.0
0.5
1.0
1.5
2.0
2.5
0.0
0.2
0.4
0.6
0.8
1.0
N = 513 Bandwidth = 0.03959

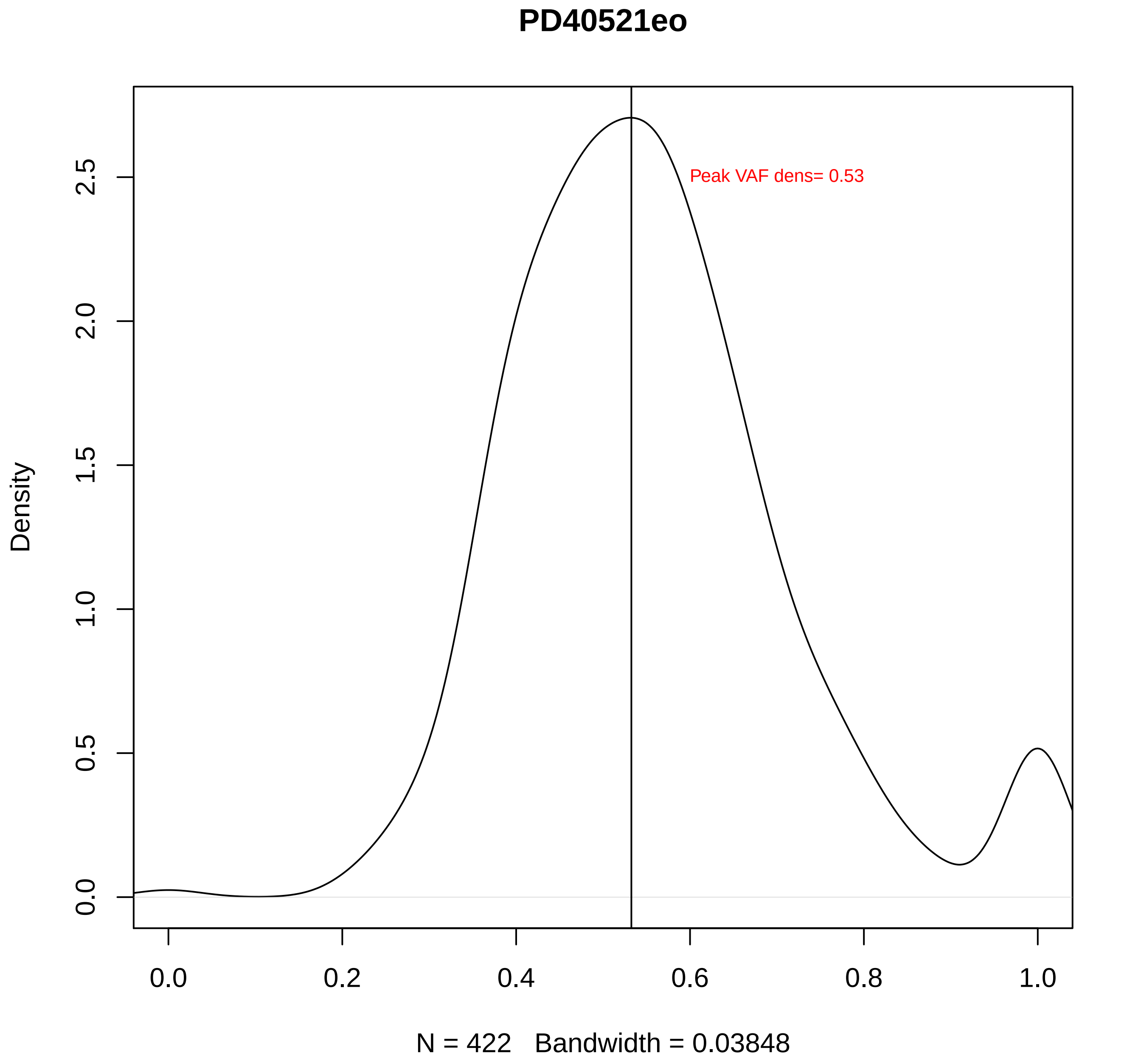

PD40521eo
Peak VAF dens= 0.53
Density
0.0
0.5
1.0
1.5
2.0
2.5
0.0
0.2
0.4
0.6
0.8
1.0
N = 422   Bandwidth = 0.03848

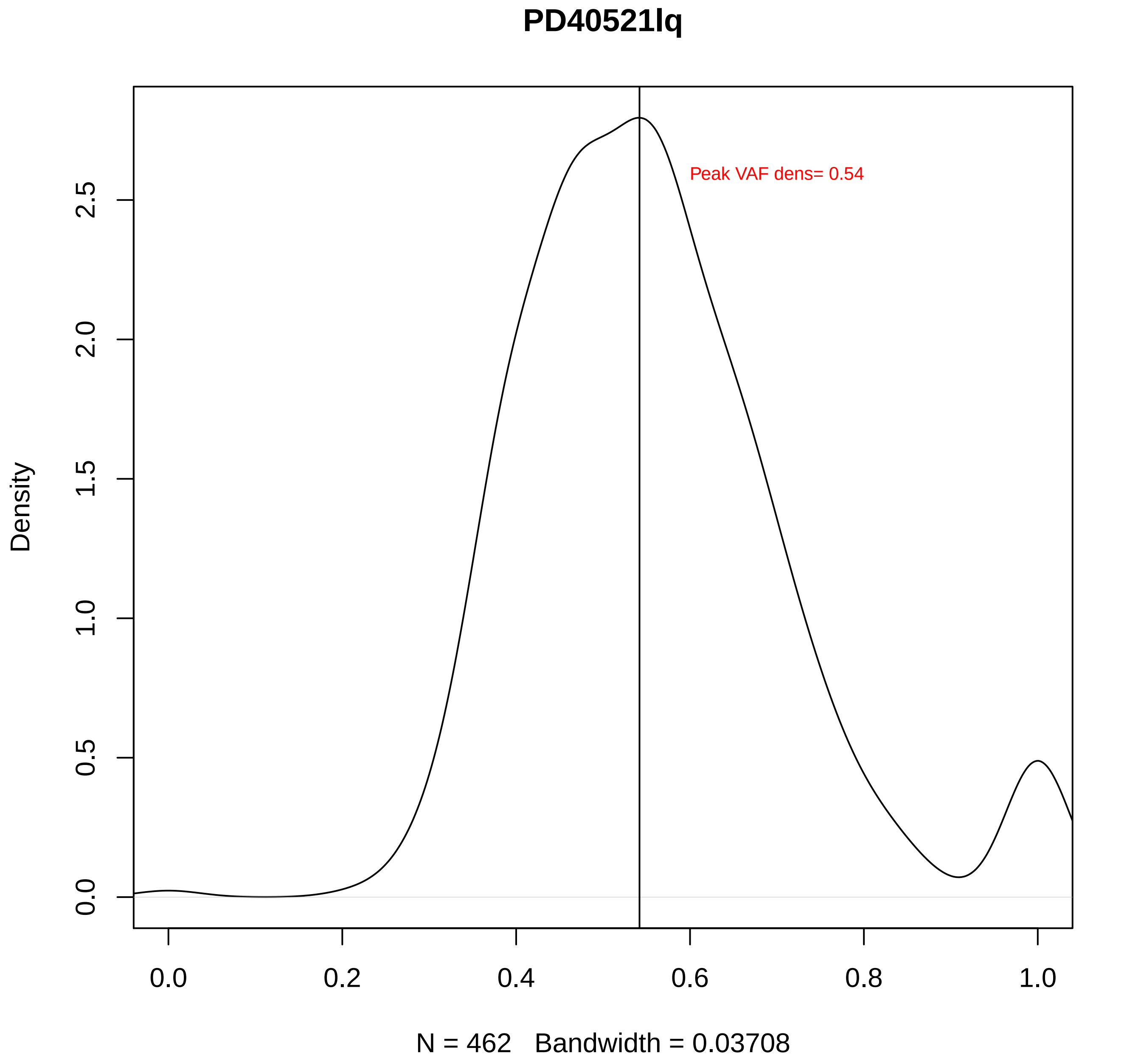
PD40521lq
Peak VAF dens= 0.54
Density
0.0
0.5
1.0
1.5
2.0
2.5
0.0
0.2
0.4
0.6
0.8
1.0
N = 462 Bandwidth = 0.03708

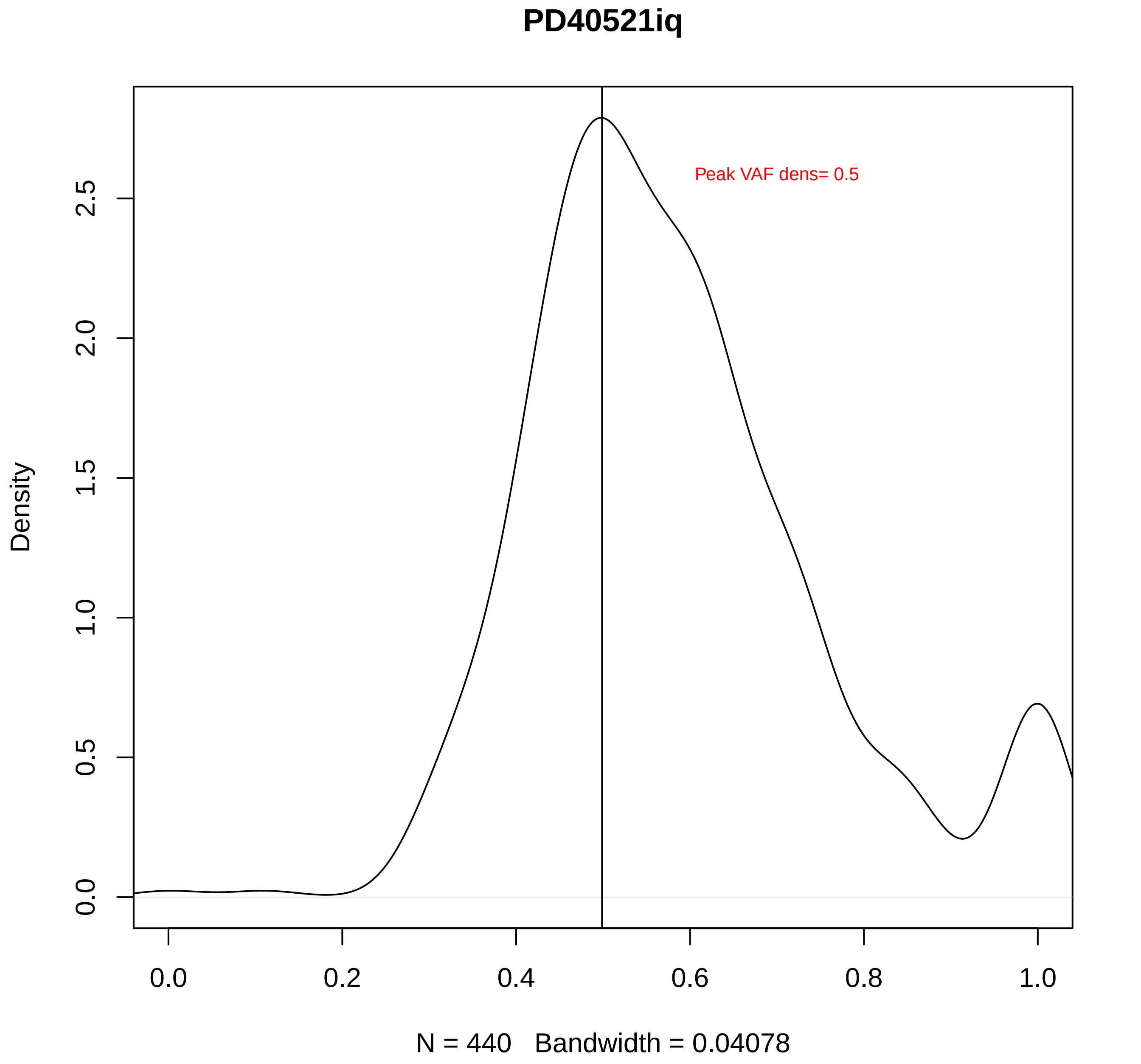
PD40521iq
Peak VAF dens= 0.5
Density
0.0
0.5
1.0
1.5
2.0
2.5
0.0
0.2
0.4
0.6
0.8
1.0
N = 440   Bandwidth = 0.04078

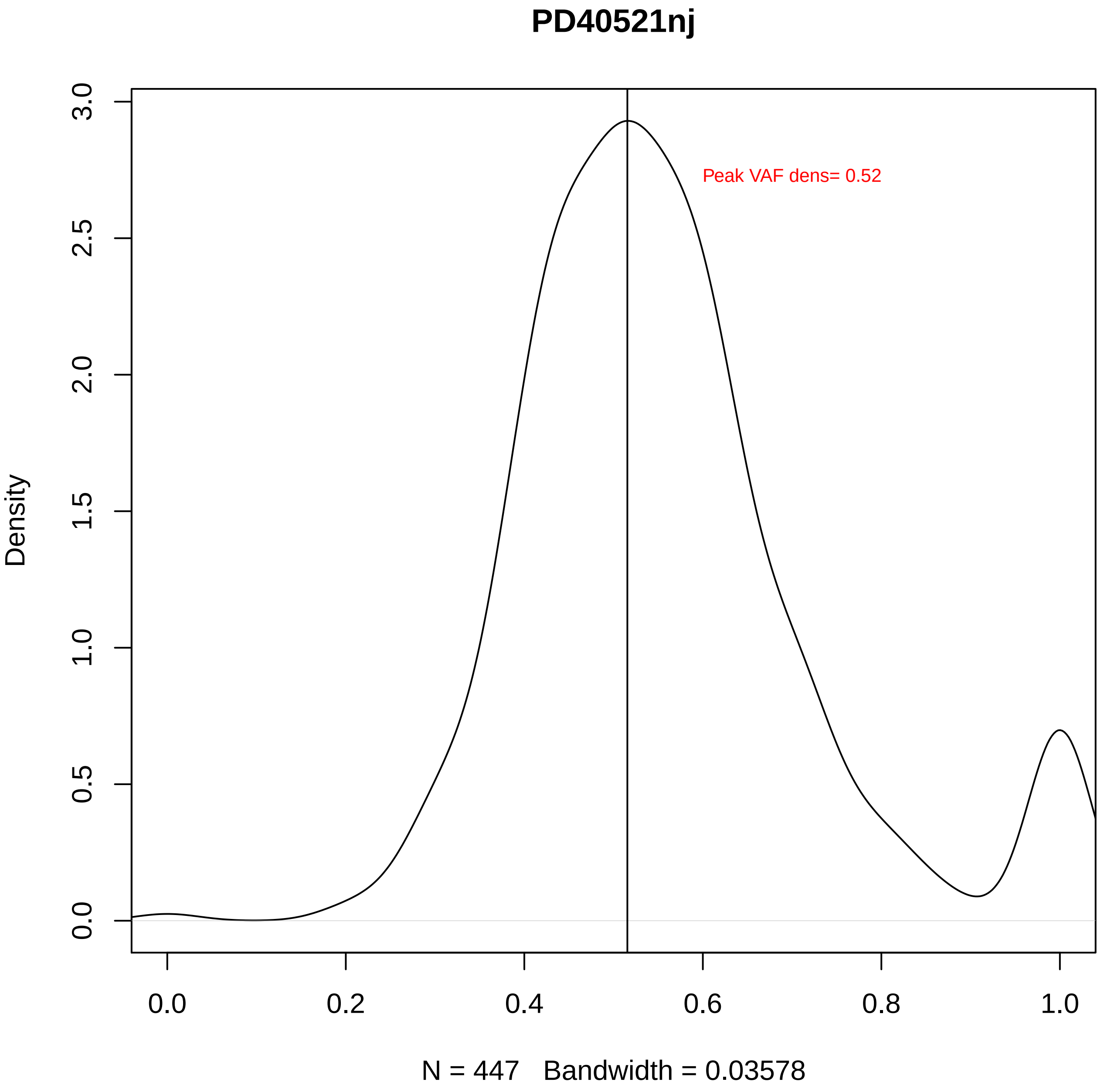

PD40521nj
Peak VAF dens= 0.52
Density
0.0
0.5
1.0
1.5
2.0
2.5
3.0
0.0
0.2
0.4
0.6
0.8
1.0
N = 447 Bandwidth = 0.03578

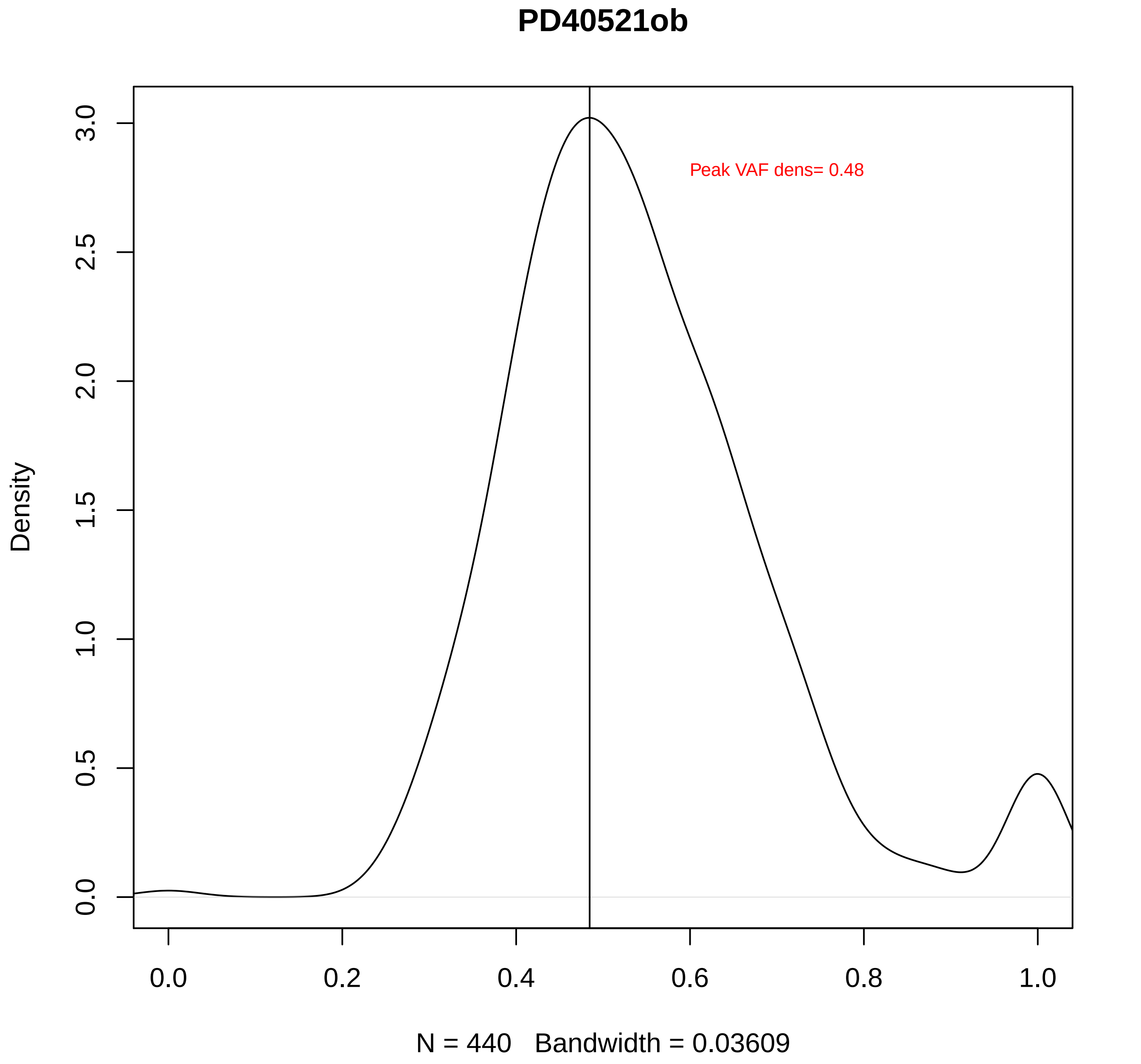
PD40521ob
Peak VAF dens= 0.48
Density
0.0
0.5
1.0
1.5
2.0
2.5
3.0
0.0
0.2
0.4
0.6
0.8
1.0
N = 440   Bandwidth = 0.03609

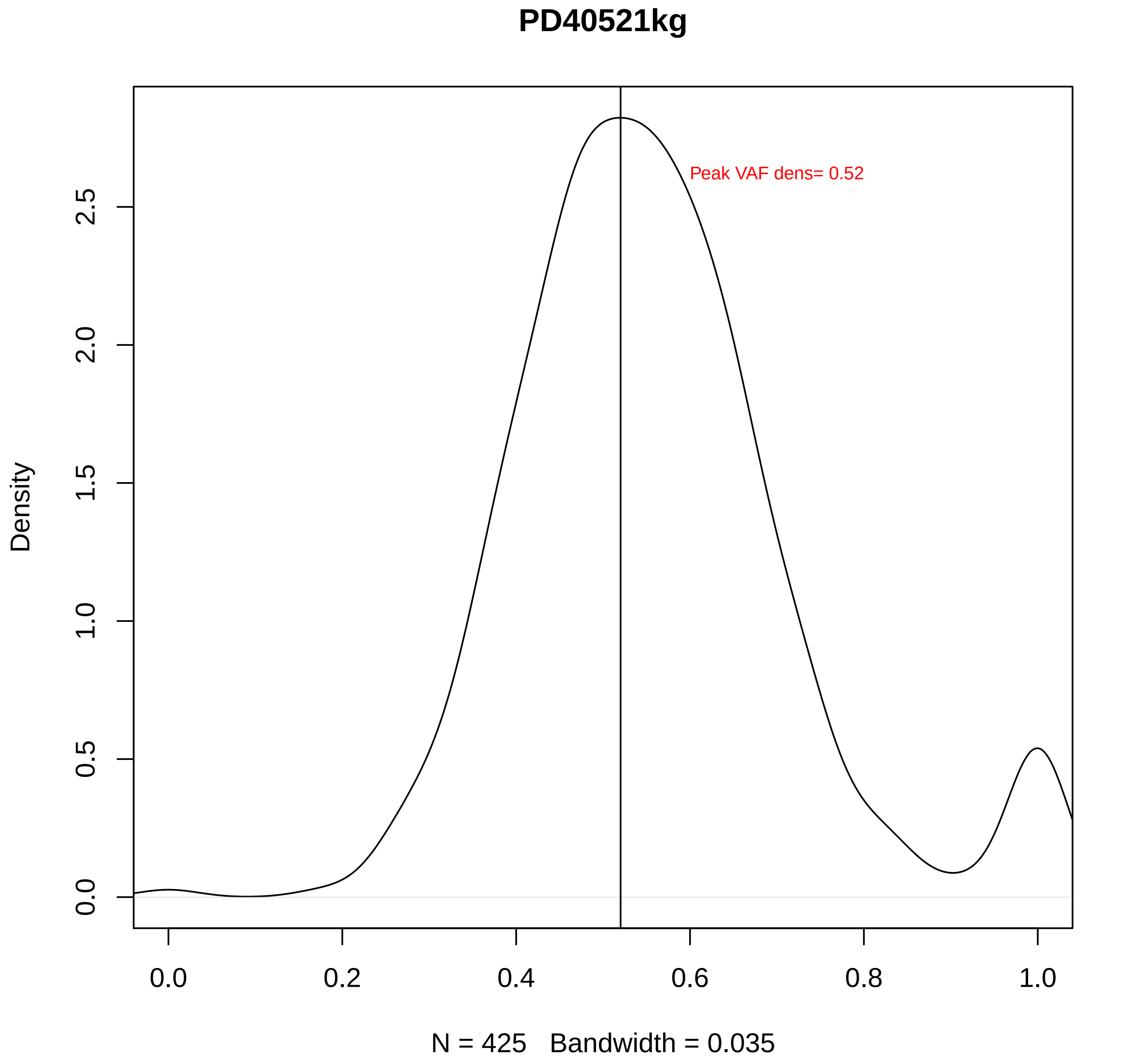
PD40521kg
Peak VAF dens= 0.52
Density
0.0
0.5
1.0
1.5
2.0
2.5
0.0
0.2
0.4
0.6
0.8
1.0
N = 425   Bandwidth = 0.035

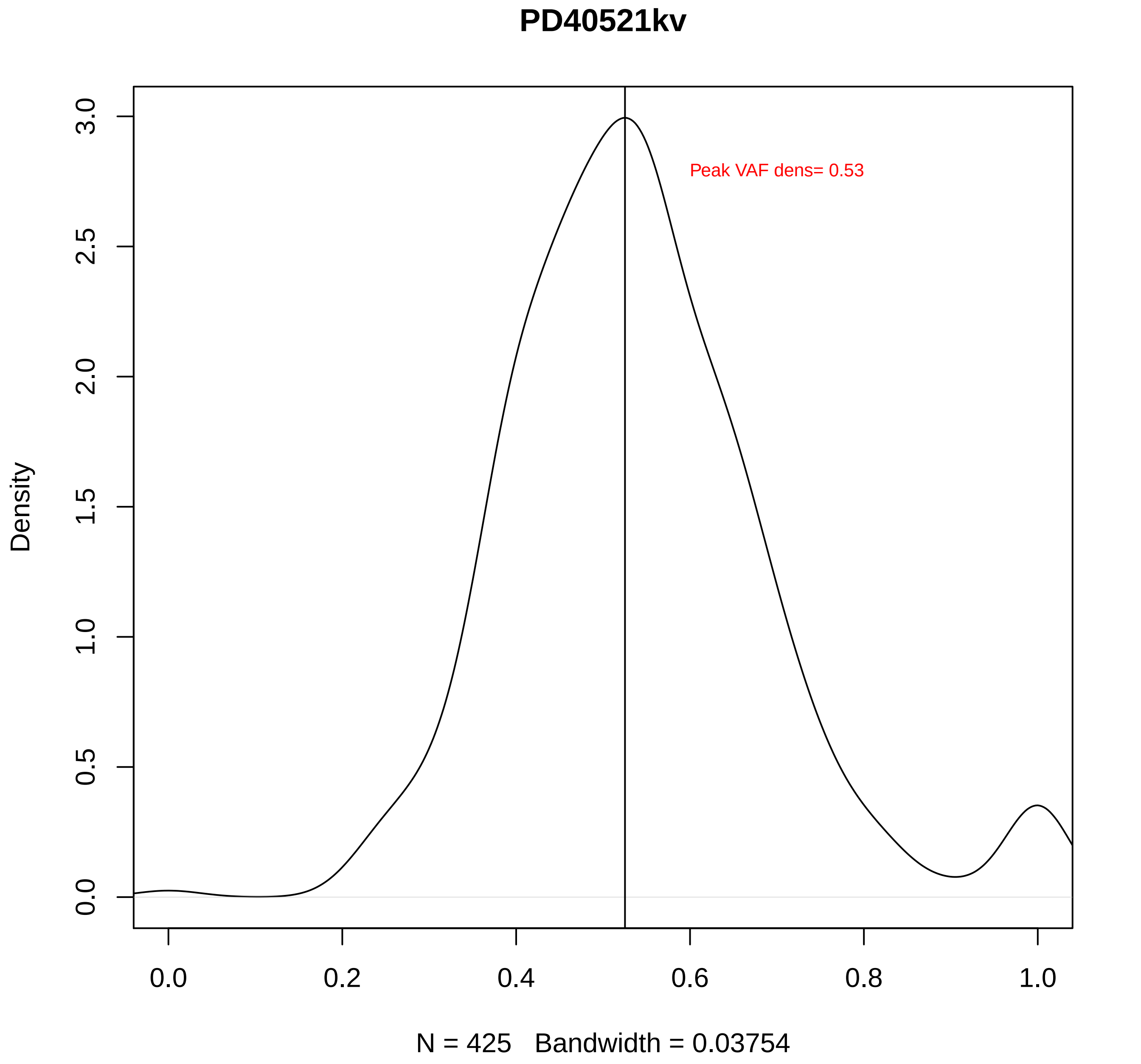
PD40521kv
Peak VAF dens= 0.53
Density
0.0
0.5
1.0
1.5
2.0
2.5
3.0
0.0
0.2
0.4
0.6
0.8
1.0
N = 425   Bandwidth = 0.03754

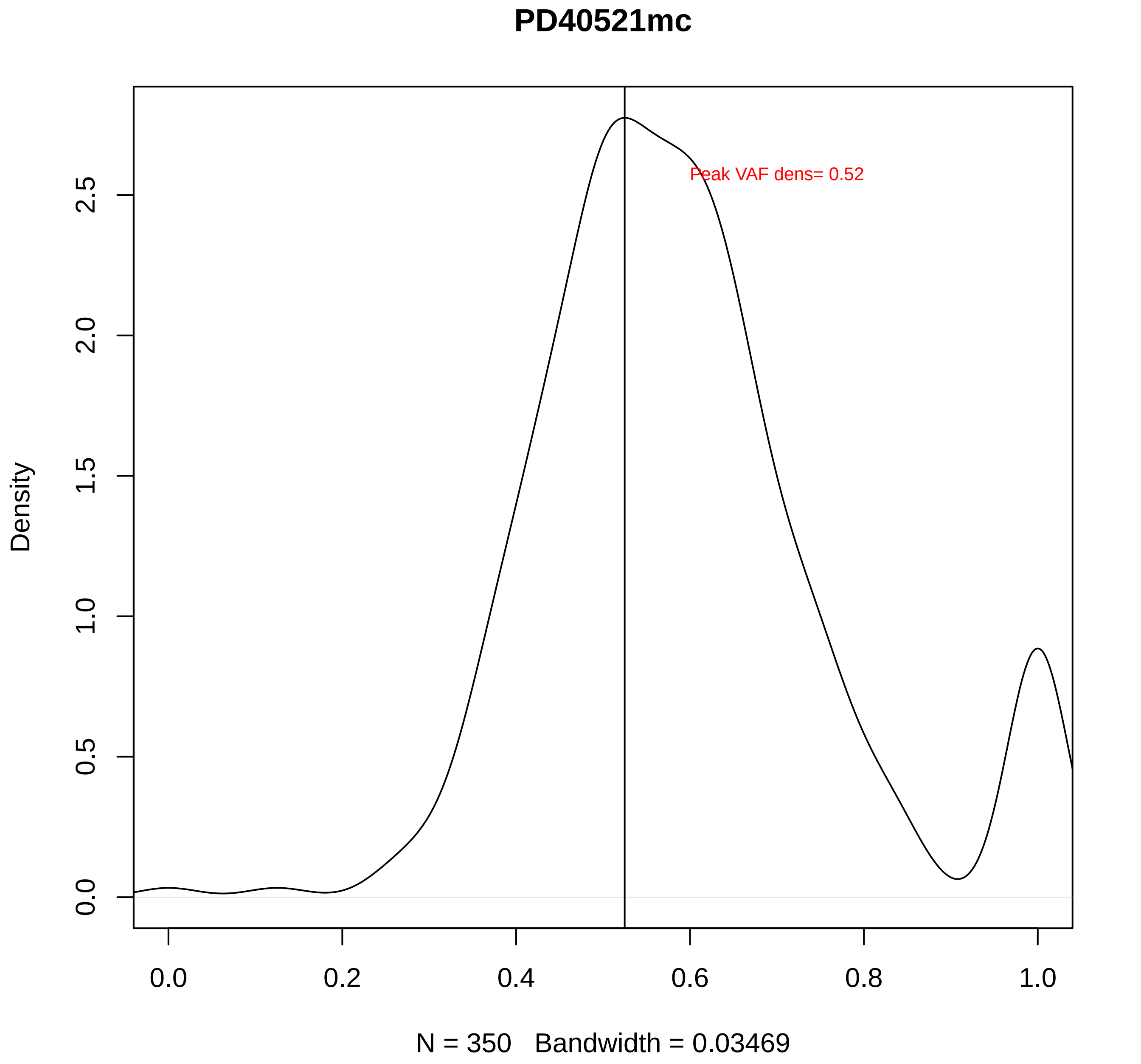

PD40521mc
Peak VAF dens= 0.52
Density
0.0
0.5
1.0
1.5
2.0
2.5
0.0
0.2
0.4
0.6
0.8
1.0
N = 350   Bandwidth = 0.03469

PD40521ni

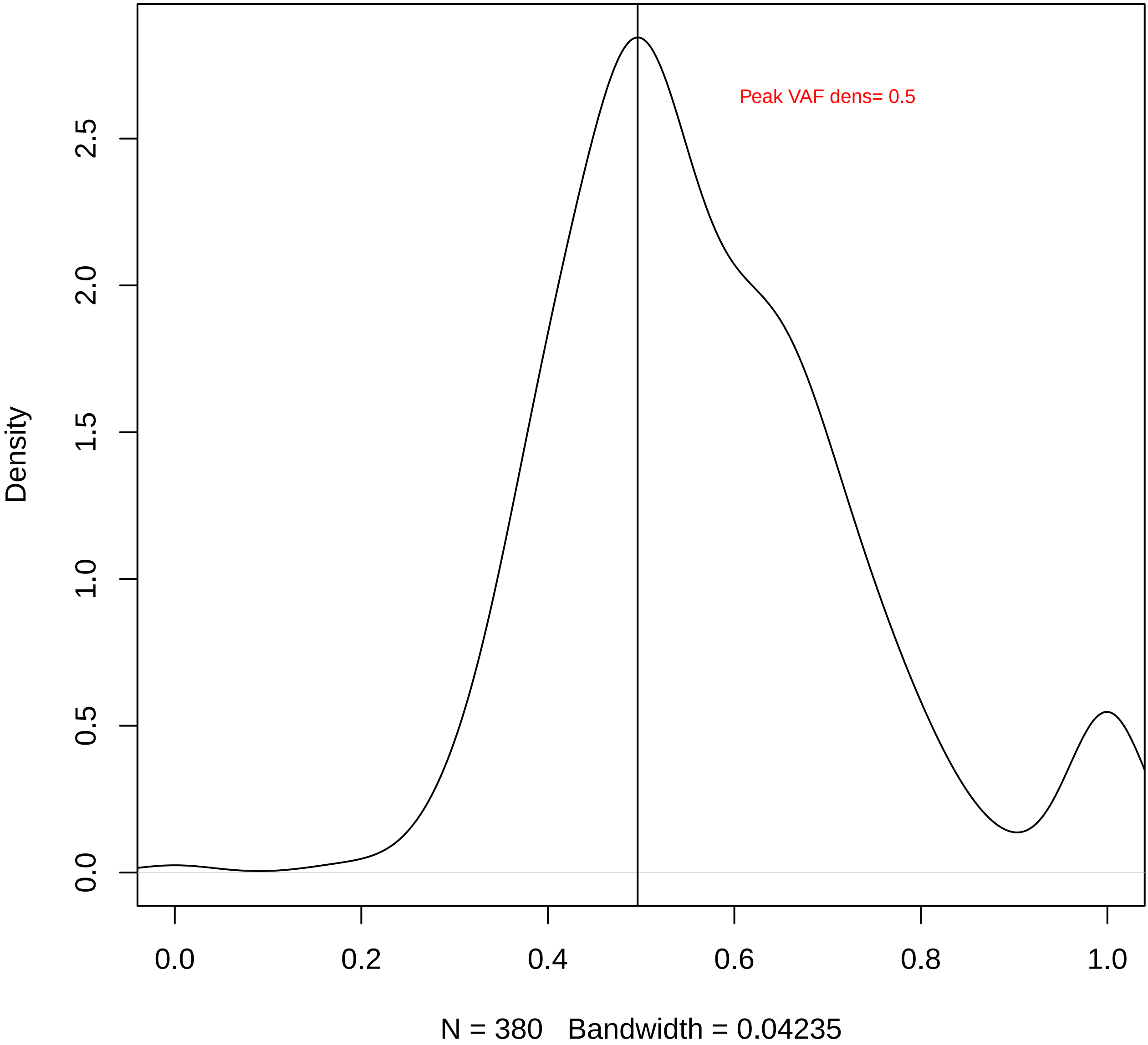

Peak VAF dens= 0.5
Density
0.0
0.5
1.0
1.5
2.0
2.5
0.0
0.2
0.4
0.6
0.8
1.0
N = 380 Bandwidth = 0.04235

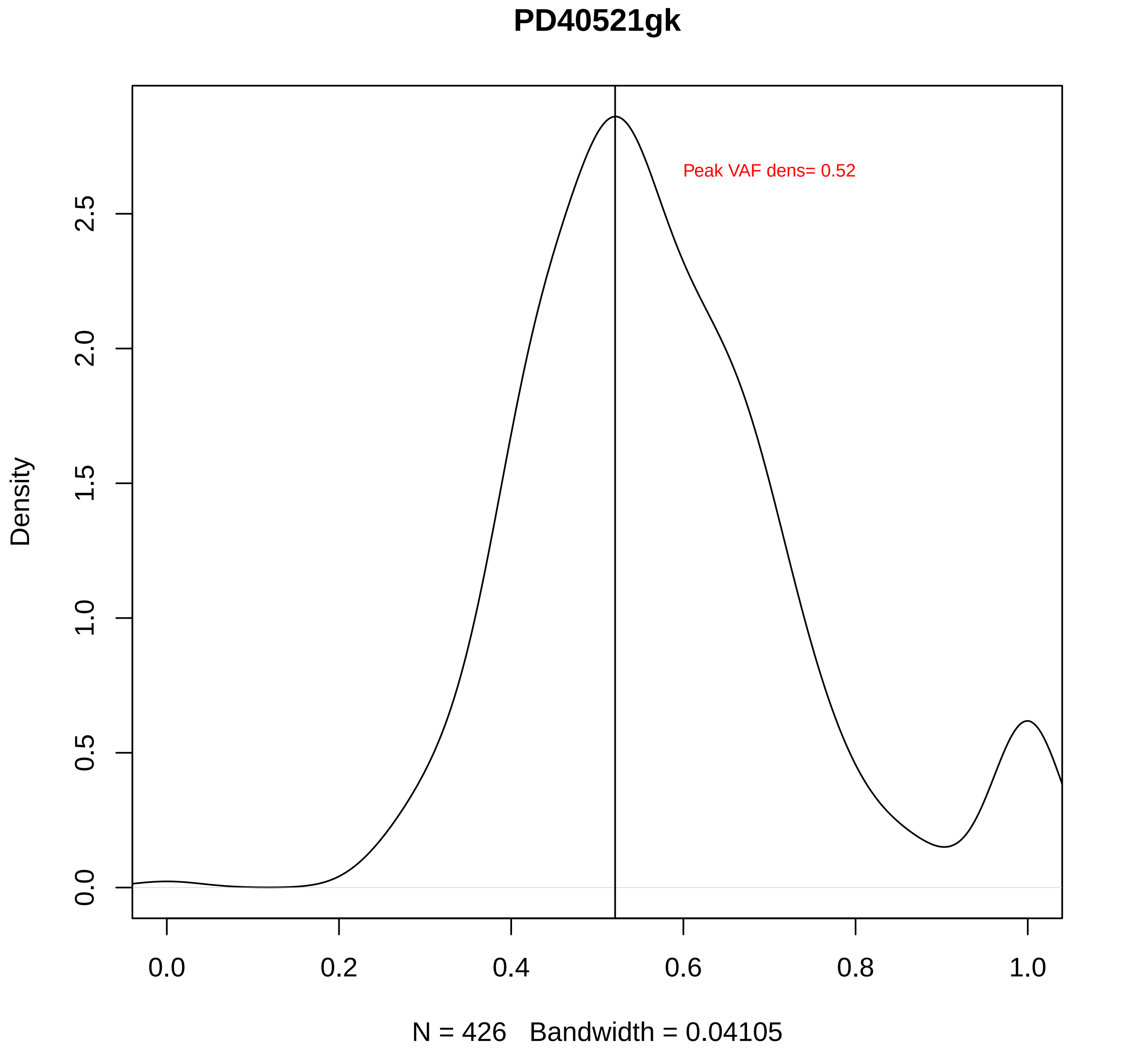
PD40521gk
Peak VAF dens= 0.52
Density
0.0
0.5
1.0
1.5
2.0
2.5
0.0
0.2
0.4
0.6
0.8
1.0
N = 426   Bandwidth = 0.04105

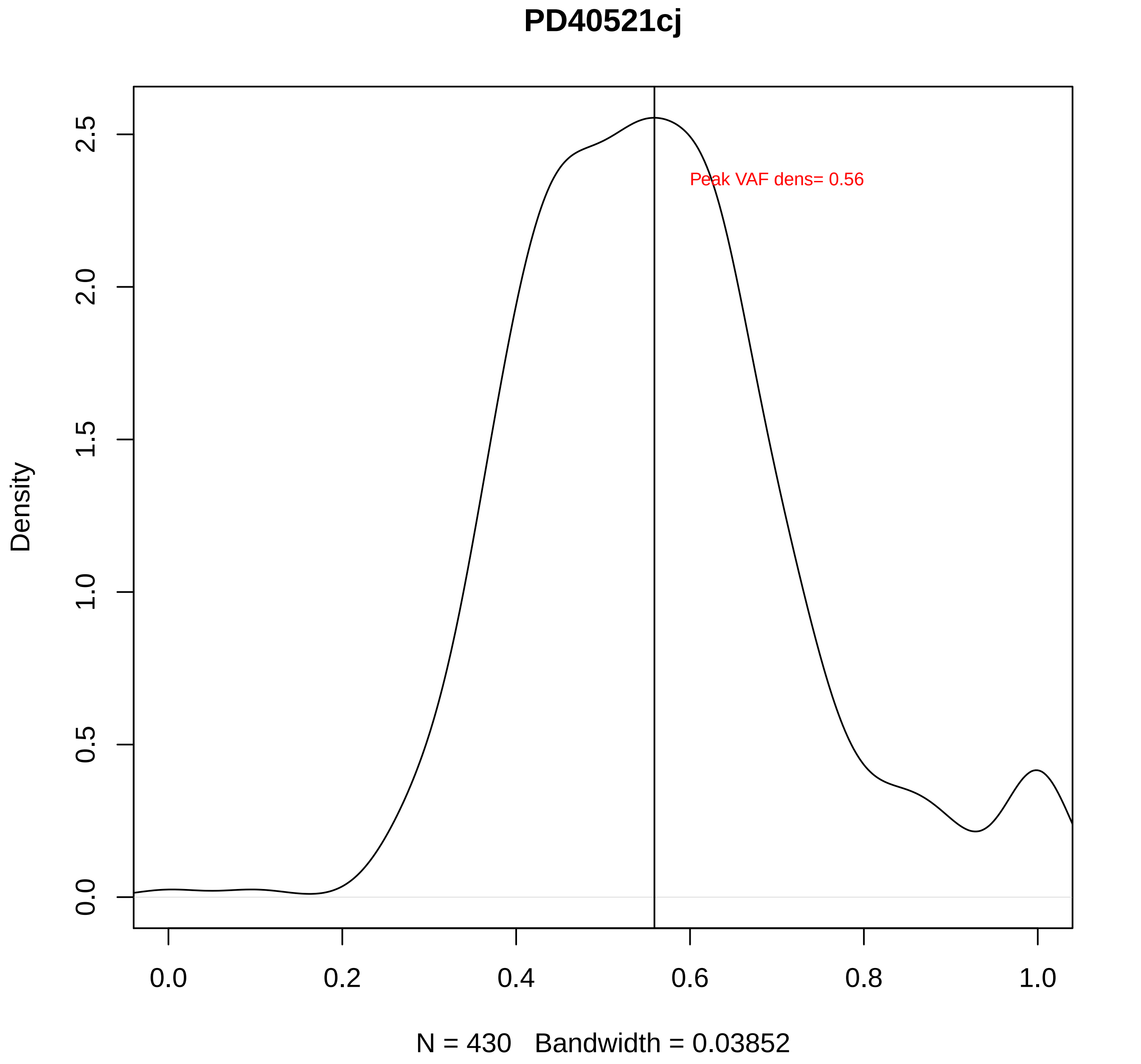

PD40521cj
Peak VAF dens= 0.56
Density
0.0
0.5
1.0
1.5
2.0
2.5
0.0
0.2
0.4
0.6
0.8
1.0
N = 430   Bandwidth = 0.03852

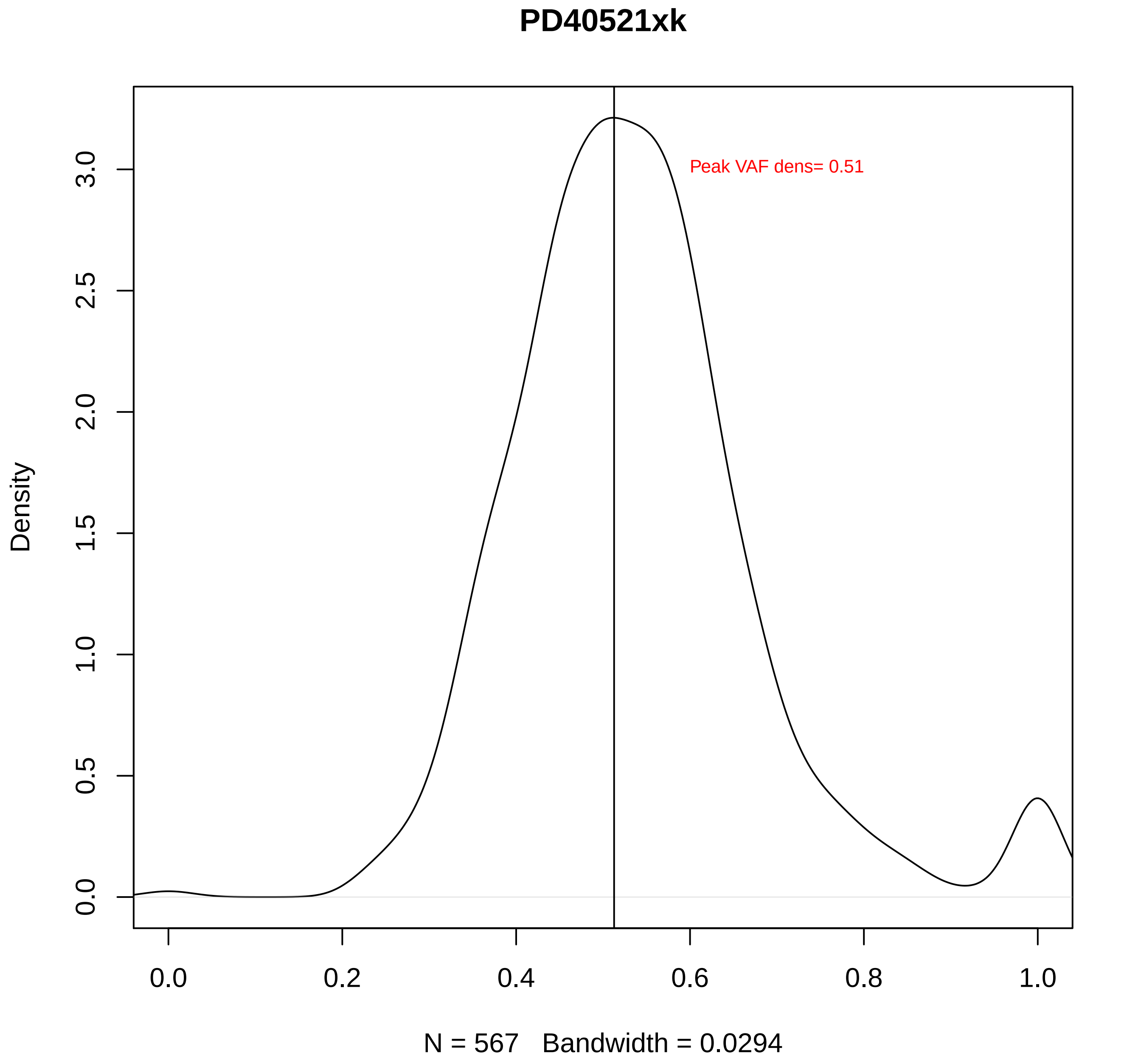
PD40521xk
Peak VAF dens= 0.51
Density
0.0
0.5
1.0
1.5
2.0
2.5
3.0
0.0
0.2
0.4
0.6
0.8
1.0
N = 567   Bandwidth = 0.0294

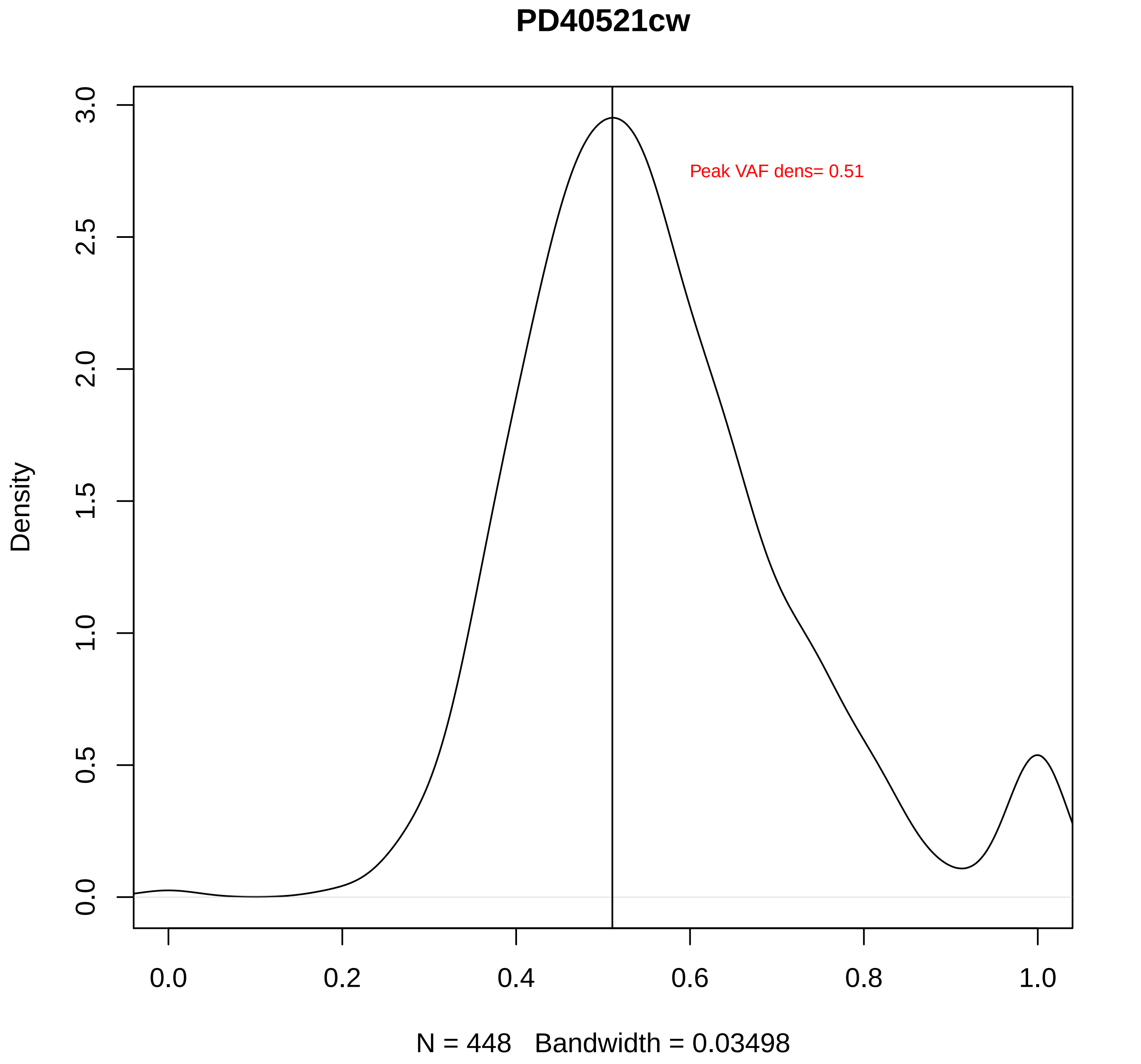
PD40521cw
Peak VAF dens= 0.51
Density
0.0
0.5
1.0
1.5
2.0
2.5
3.0
0.0
0.2
0.4
0.6
0.8
1.0
N = 448 Bandwidth = 0.03498

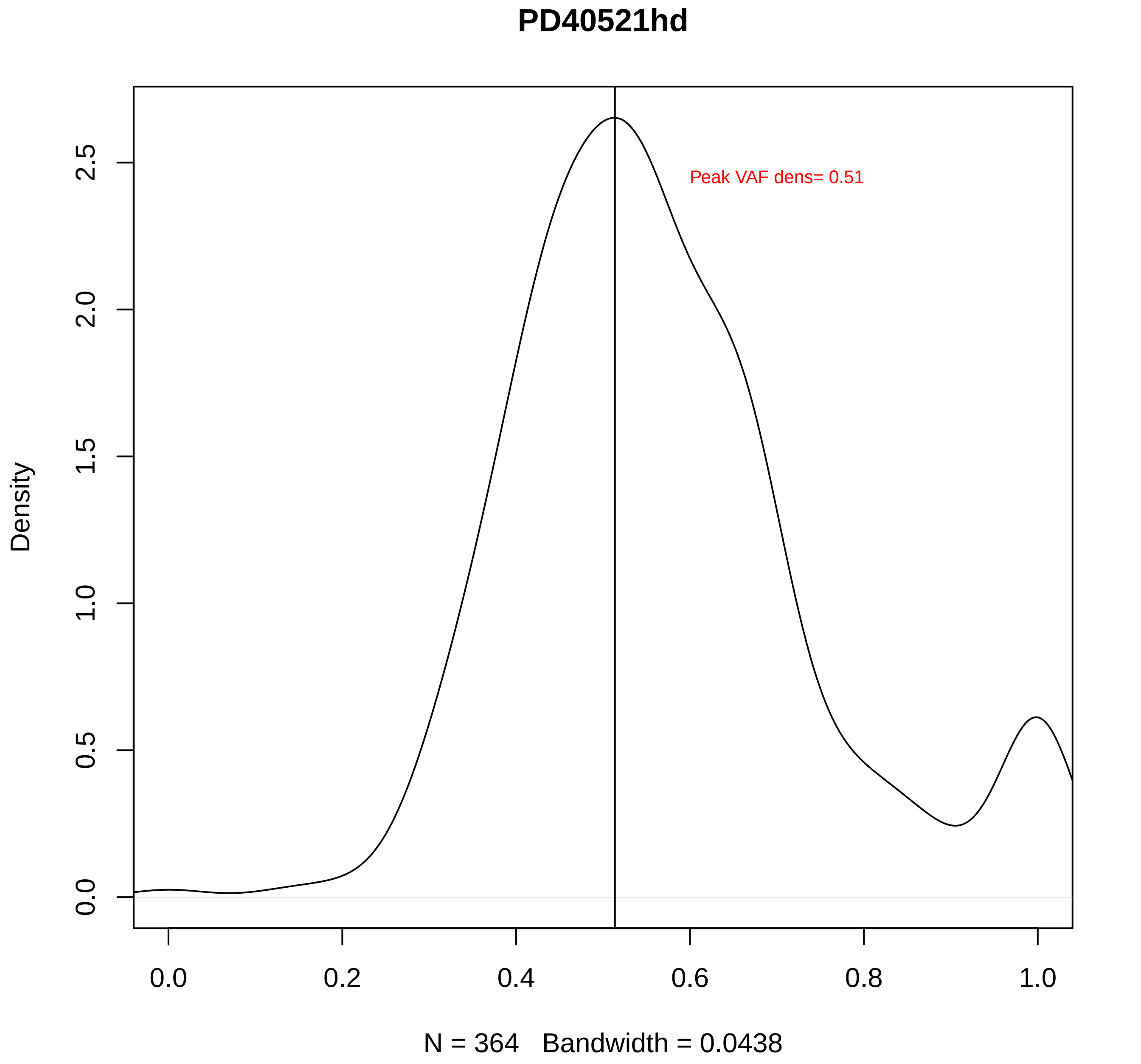
PD40521hd
Peak VAF dens= 0.51
Density
0.0
0.5
1.0
1.5
2.0
2.5
0.0
0.2
0.4
0.6
0.8
1.0
N = 364   Bandwidth = 0.0438

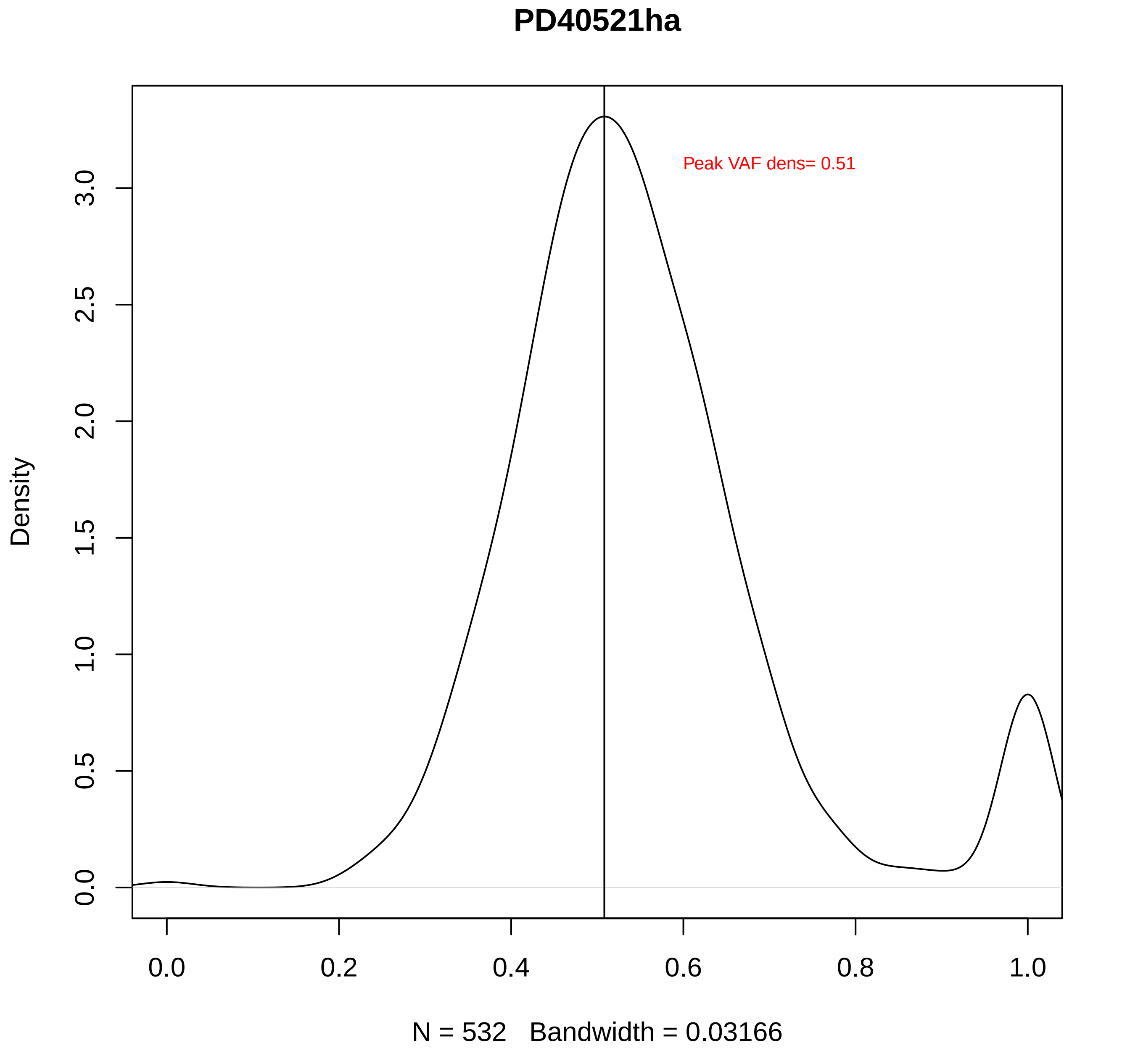
PD40521ha
Peak VAF dens= 0.51
Density
0.0
0.5
1.0
1.5
2.0
2.5
3.0
0.0
0.2
0.4
0.6
0.8
1.0
N = 532   Bandwidth = 0.03166

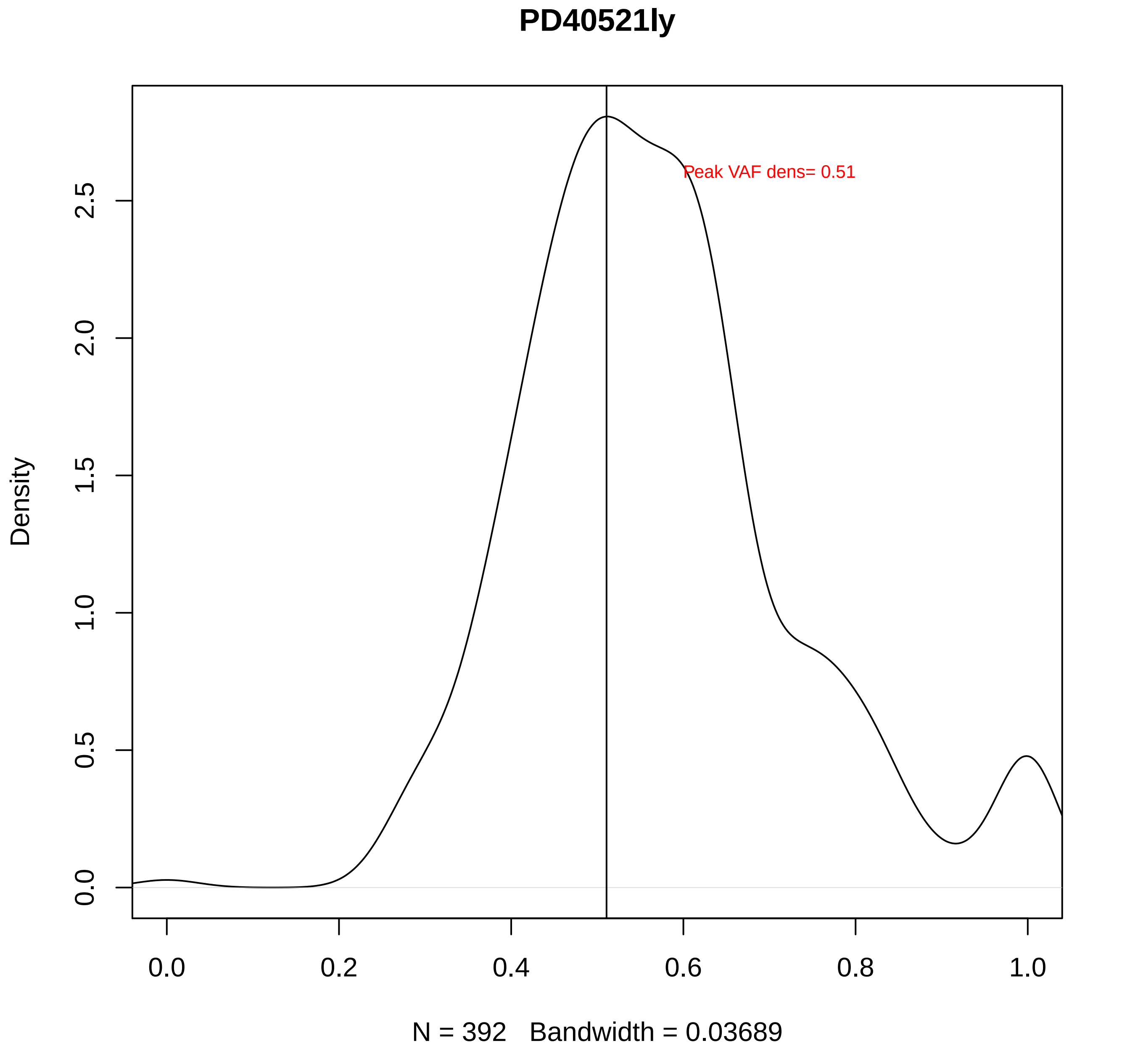
PD40521ly
Peak VAF dens= 0.51
Density
0.0
0.5
1.0
1.5
2.0
2.5
0.0
0.2
0.4
0.6
0.8
1.0
N = 392 Bandwidth = 0.03689

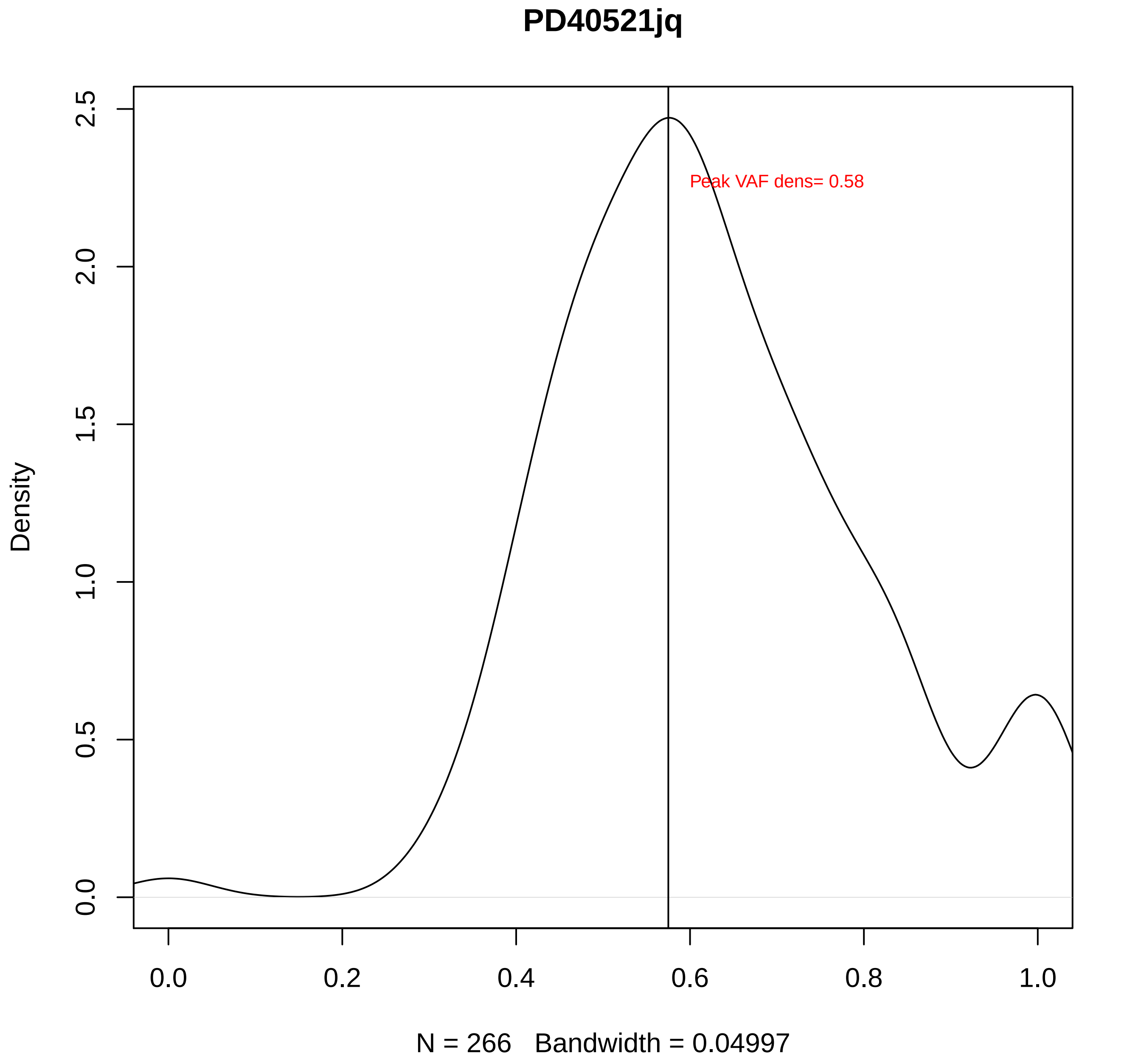

PD40521jq
Peak VAF dens= 0.58
Density
0.0
0.5
1.0
1.5
2.0
2.5
0.0
0.2
0.4
0.6
0.8
1.0
N = 266   Bandwidth = 0.04997

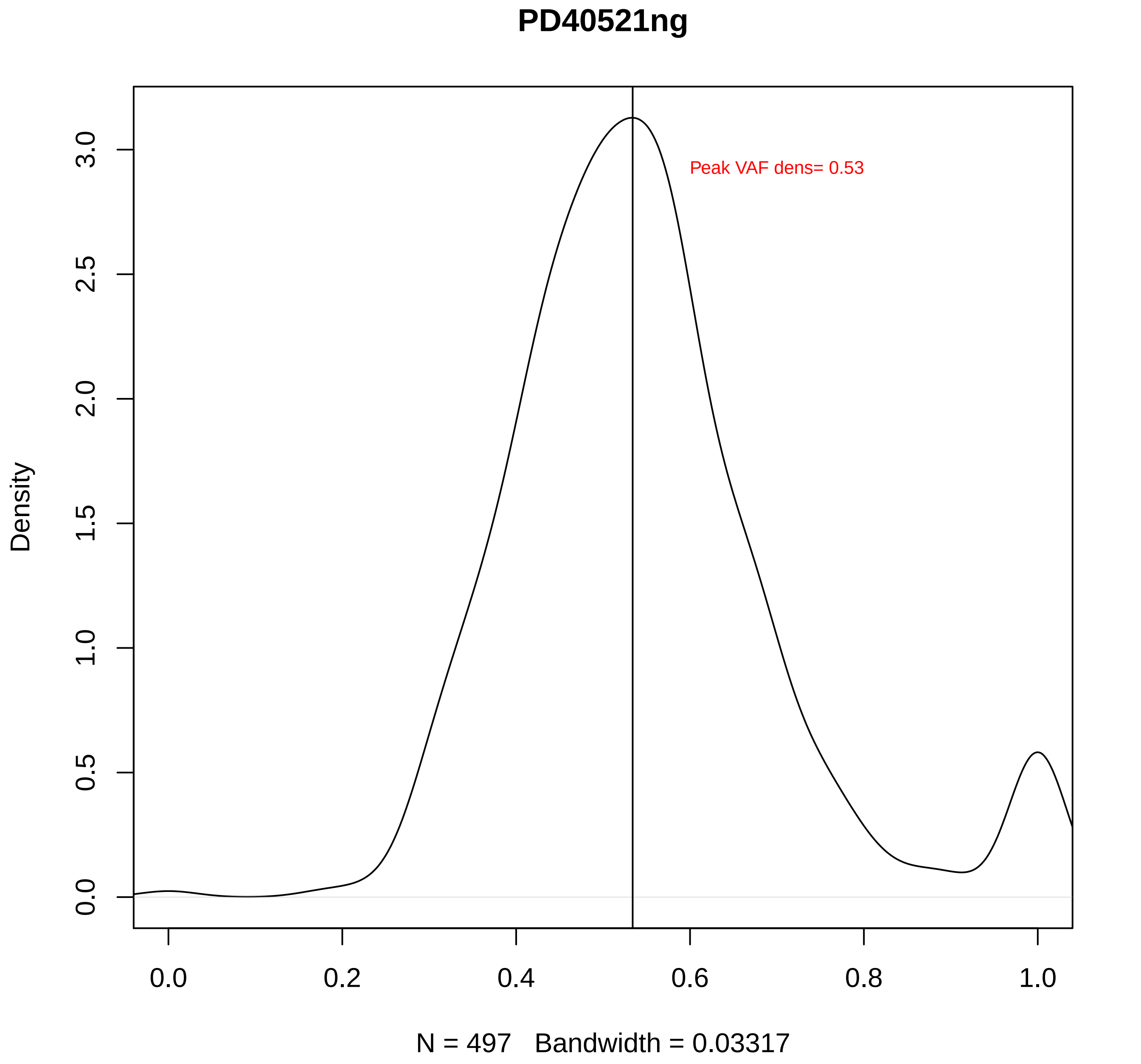

PD40521ng
Peak VAF dens= 0.53
Density
0.0
0.5
1.0
1.5
2.0
2.5
3.0
0.0
0.2
0.4
0.6
0.8
1.0
N = 497 Bandwidth = 0.03317

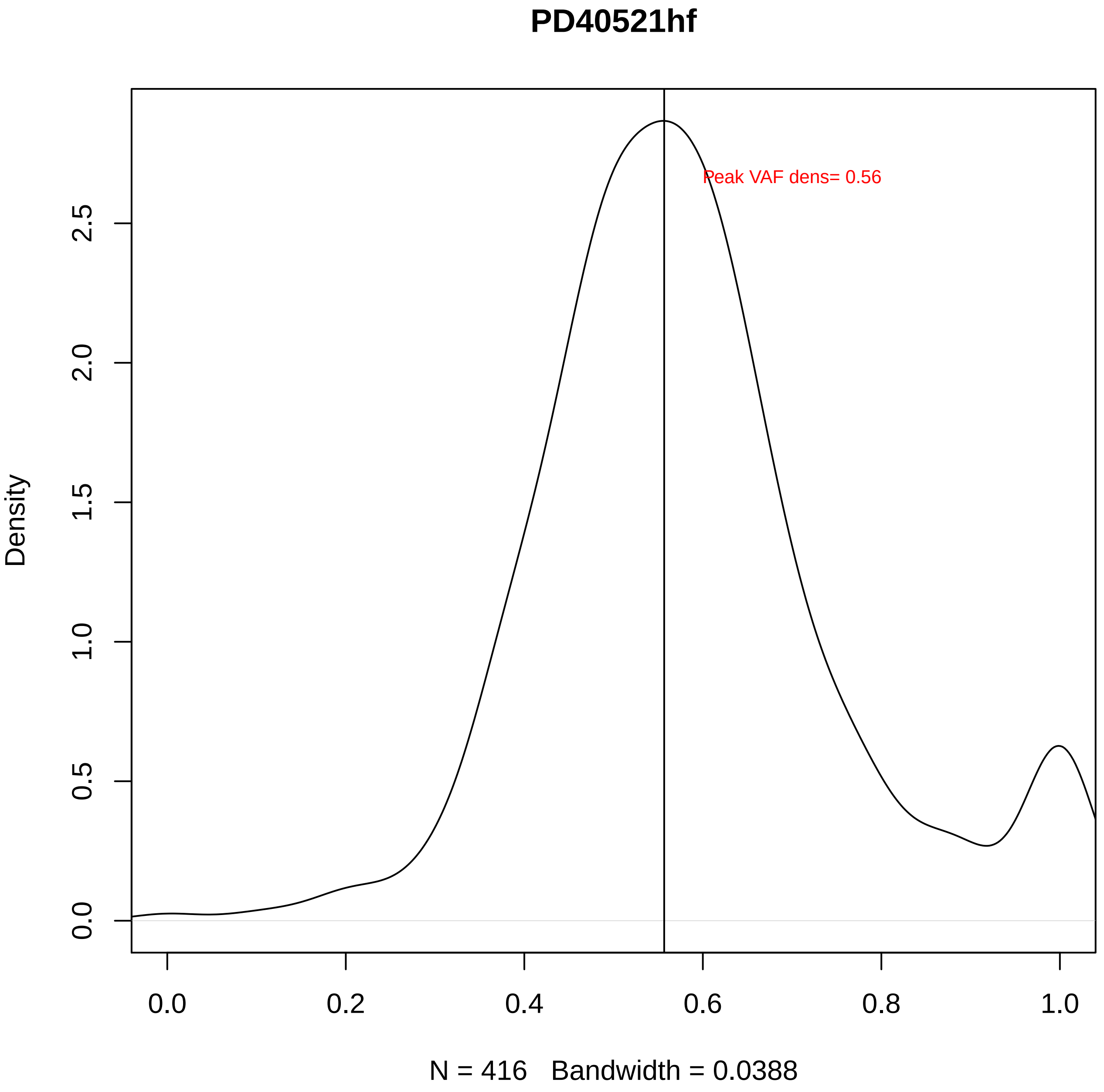
PD40521hf
Peak VAF dens= 0.56
Density
0.0
0.5
1.0
1.5
2.0
2.5
0.0
0.2
0.4
0.6
0.8
1.0
N = 416 Bandwidth = 0.0388

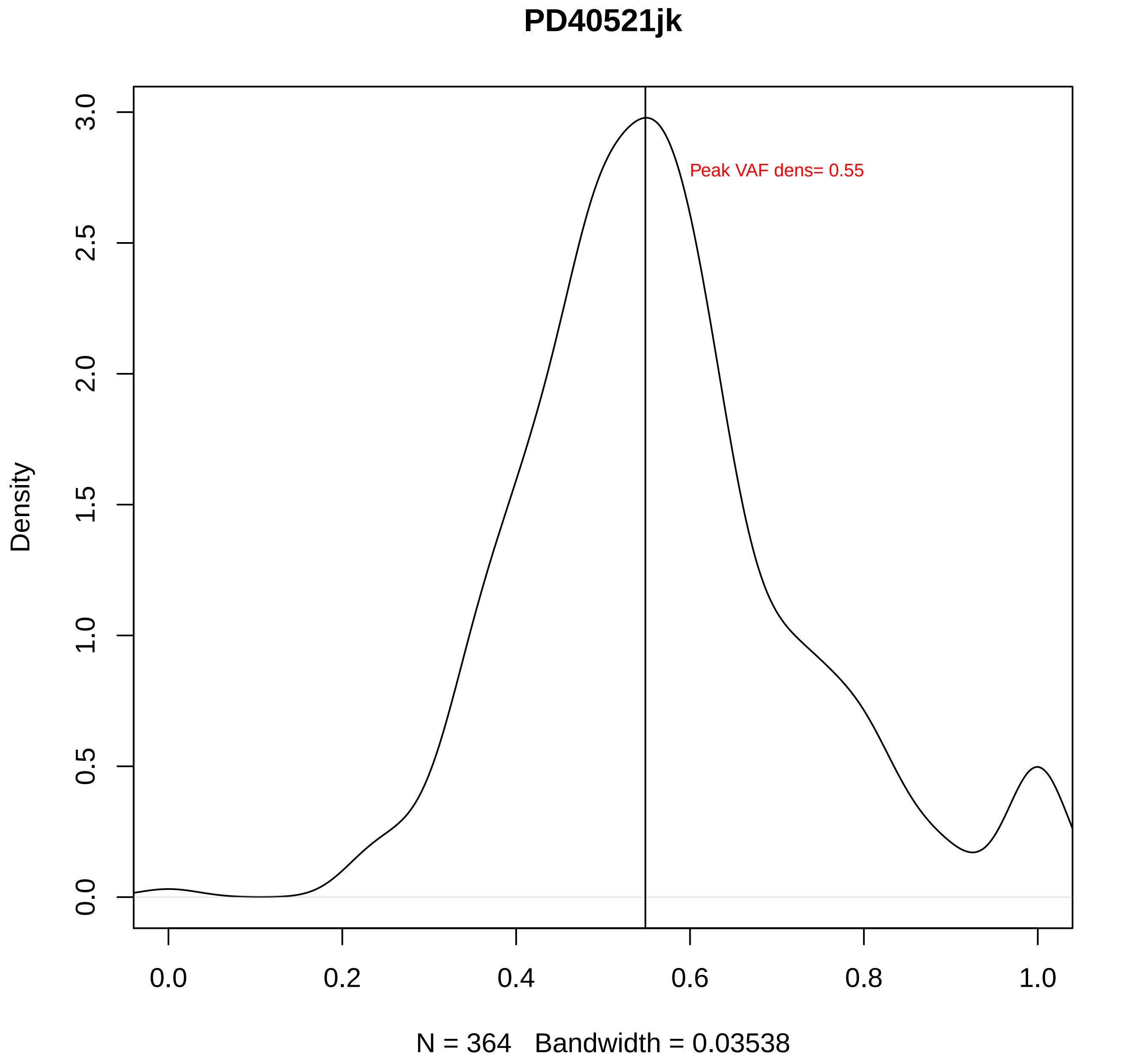
PD40521jk
Peak VAF dens= 0.55
Density
0.0
0.5
1.0
1.5
2.0
2.5
3.0
0.0
0.2
0.4
0.6
0.8
1.0
N = 364 Bandwidth = 0.03538

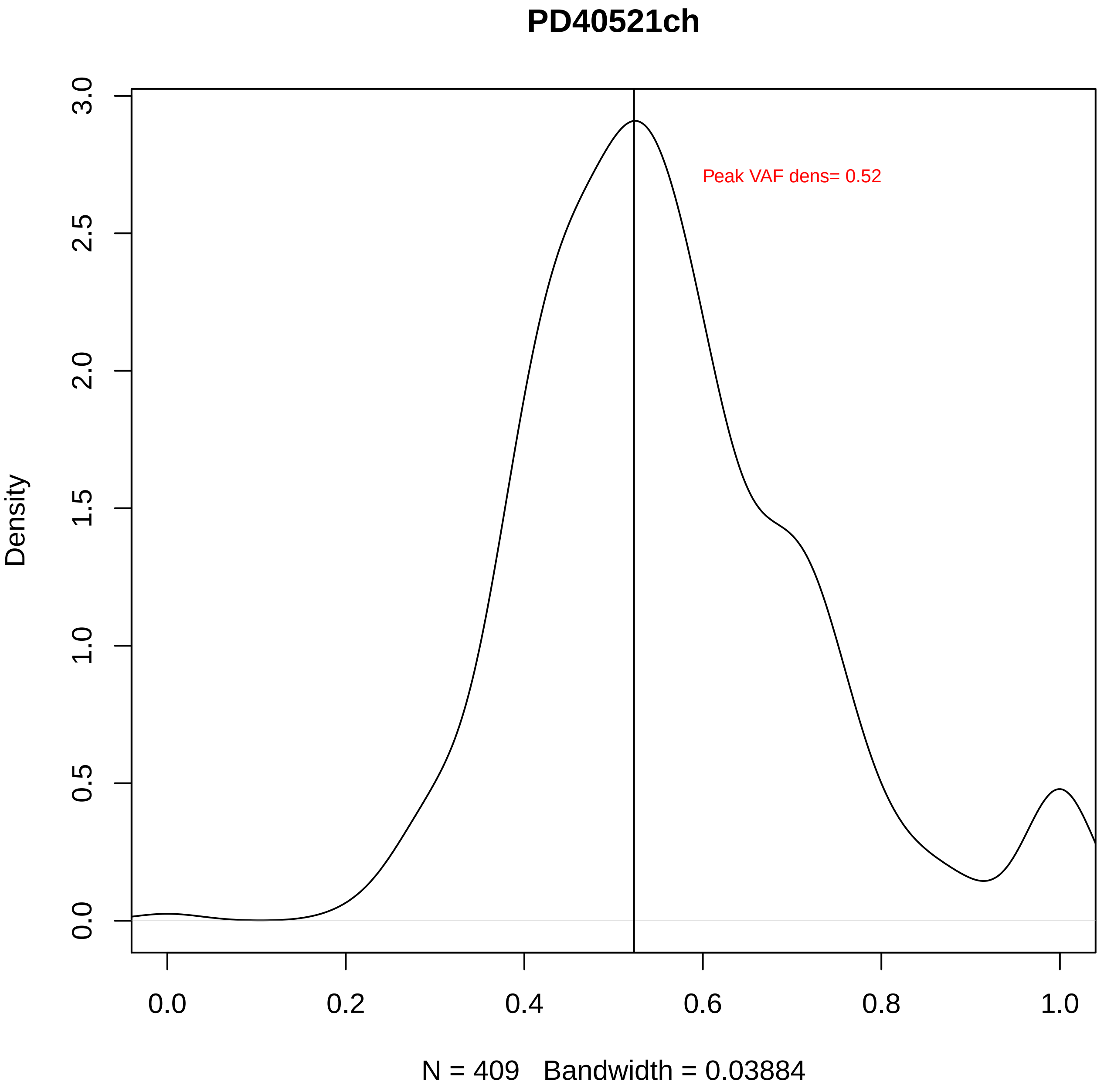
PD40521ch
Peak VAF dens= 0.52
Density
0.0
0.5
1.0
1.5
2.0
2.5
3.0
0.0
0.2
0.4
0.6
0.8
1.0
N = 409 Bandwidth = 0.03884

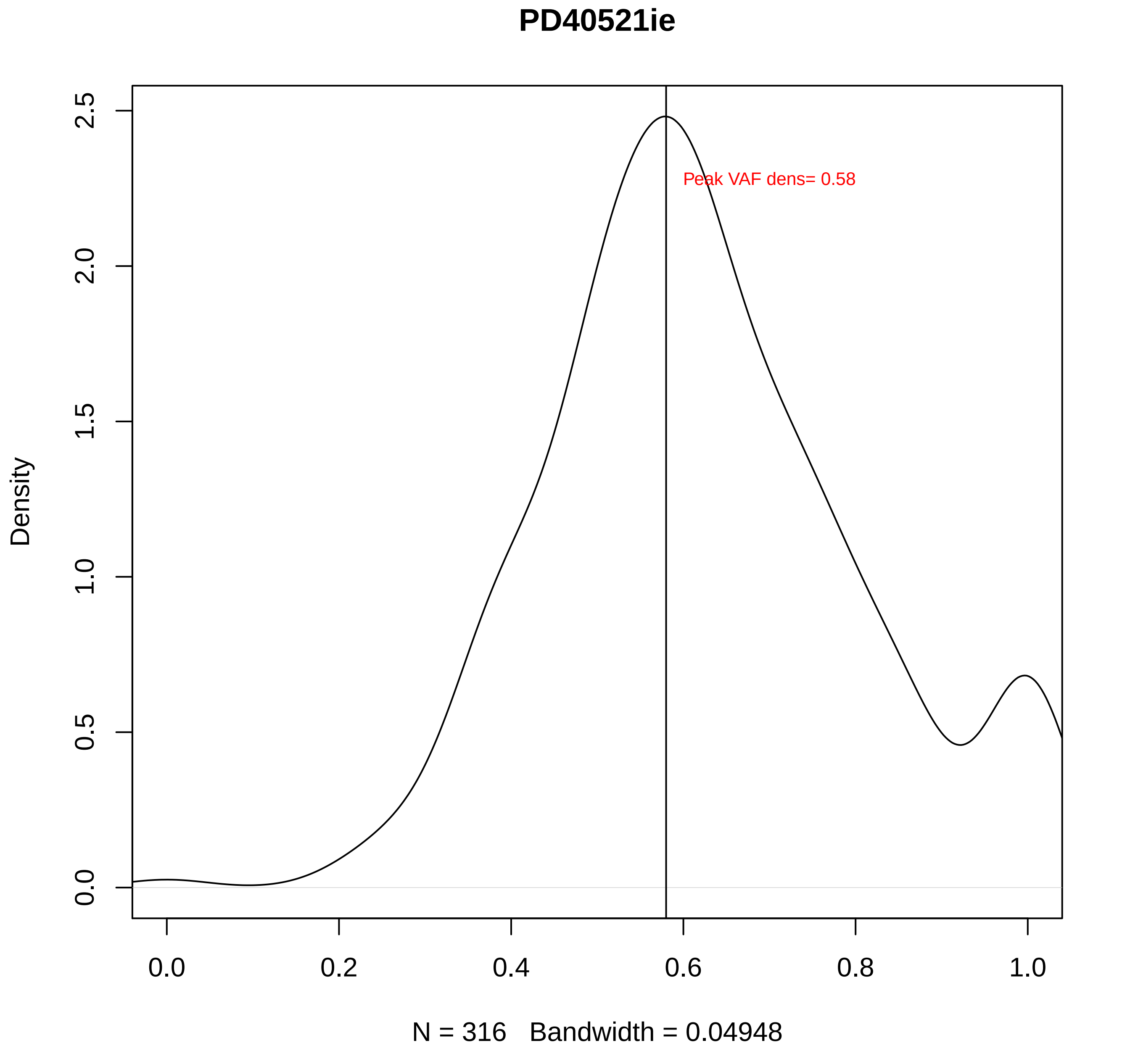
PD40521ie
Peak VAF dens= 0.58
Density
0.0
0.5
1.0
1.5
2.0
2.5
0.0
0.2
0.4
0.6
0.8
1.0
N = 316   Bandwidth = 0.04948

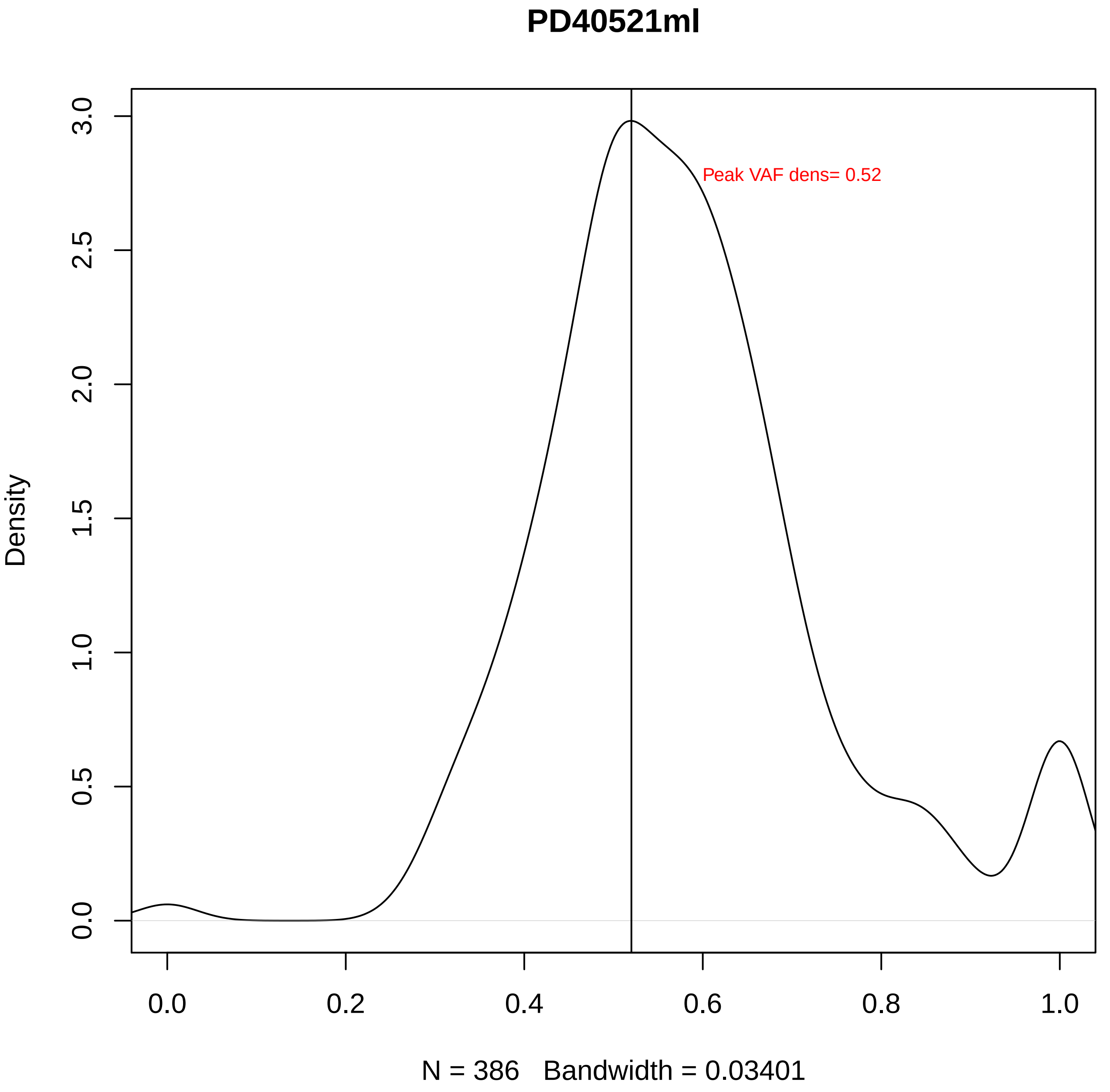
PD40521ml
Peak VAF dens= 0.52
Density
3.0
2.5
2.0
1.5
1.0
0.5
0.0
0.0
0.2
0.4
0.6
0.8
1.0
N = 386   Bandwidth = 0.03401

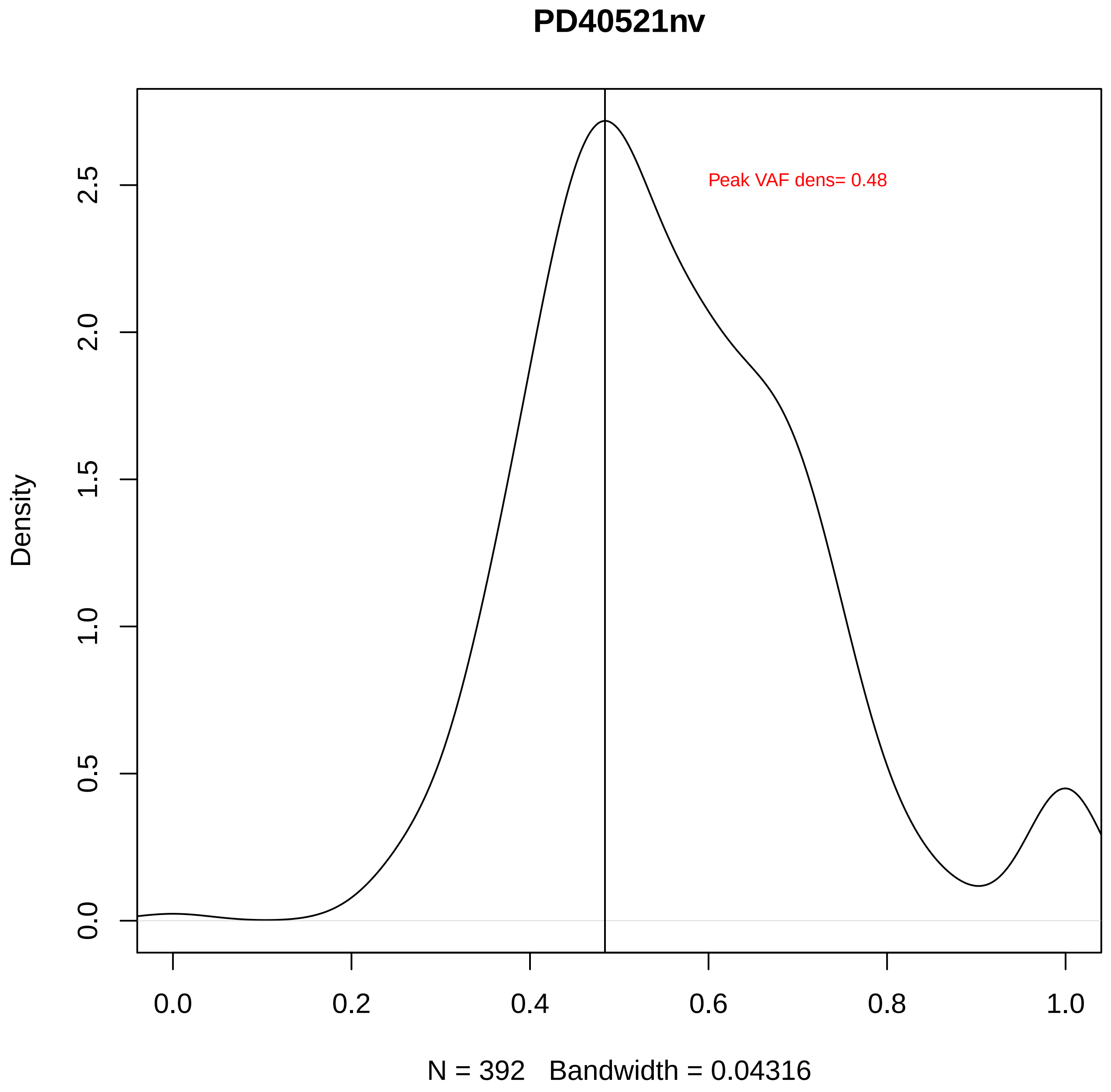

PD40521nv
Peak VAF dens= 0.48
Density
0.0
0.5
1.0
1.5
2.0
2.5
0.0
0.2
0.4
0.6
0.8
1.0
N = 392   Bandwidth = 0.04316

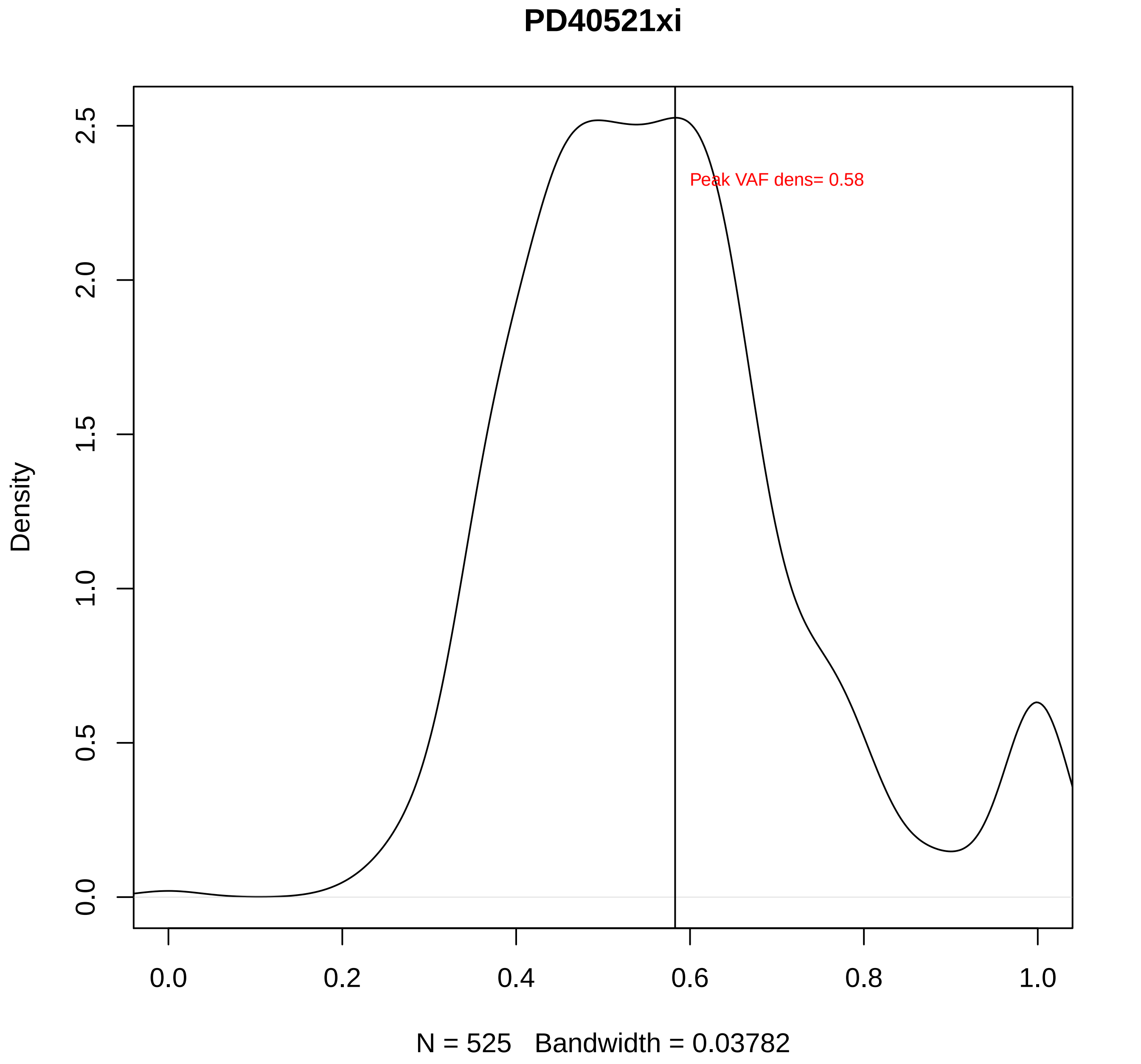
PD40521xi
Density
N = 525 Bandwidth = 0.03782
Peak VAF dens= 0.58
0.0
0.5
1.0
1.5
2.0
2.5
0.0
0.2
0.4
0.6
0.8
1.0

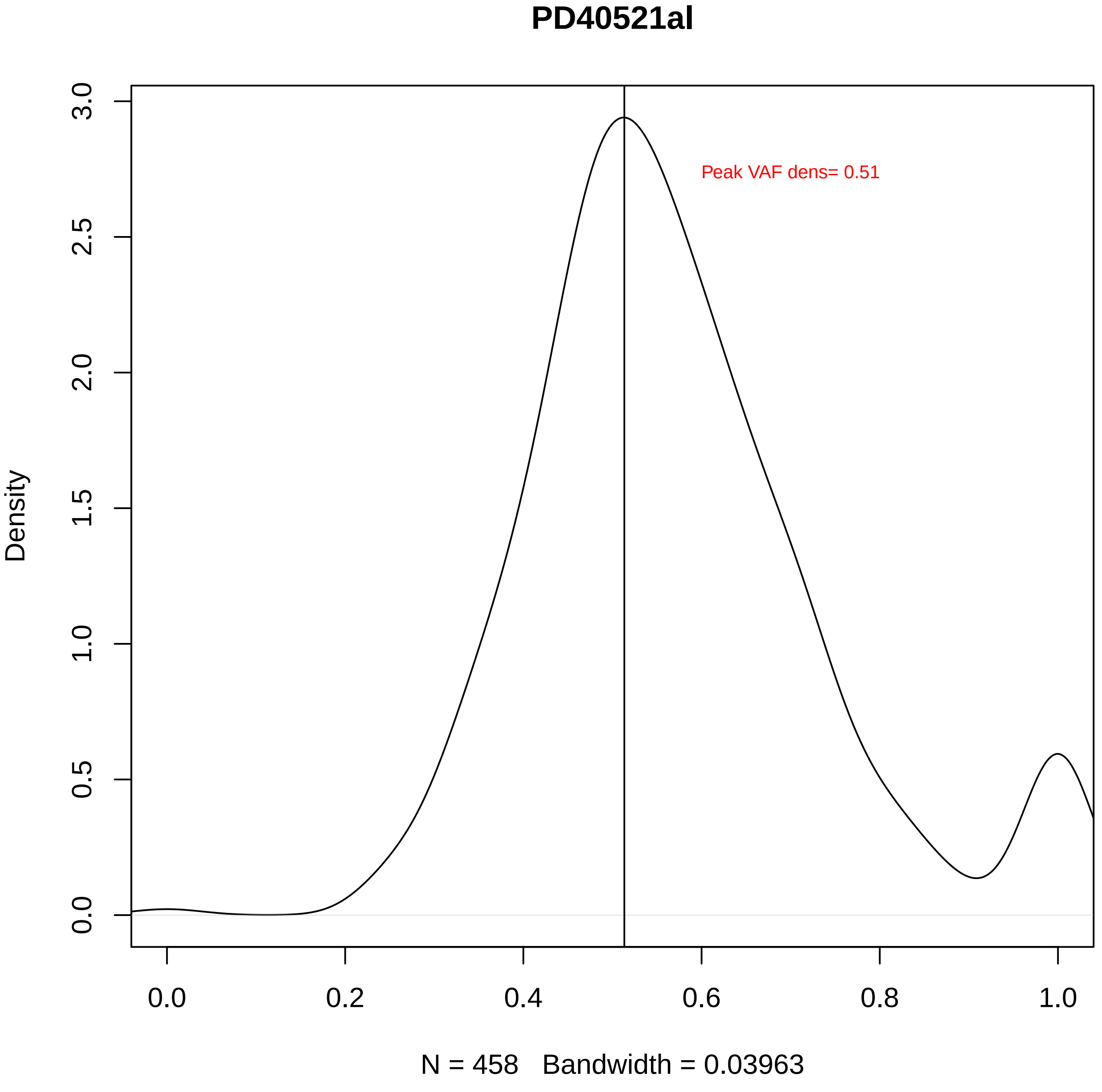
PD40521al
Peak VAF dens= 0.51
Density
0.0
0.5
1.0
1.5
2.0
2.5
3.0
0.0
0.2
0.4
0.6
0.8
1.0
N = 458 Bandwidth = 0.03963

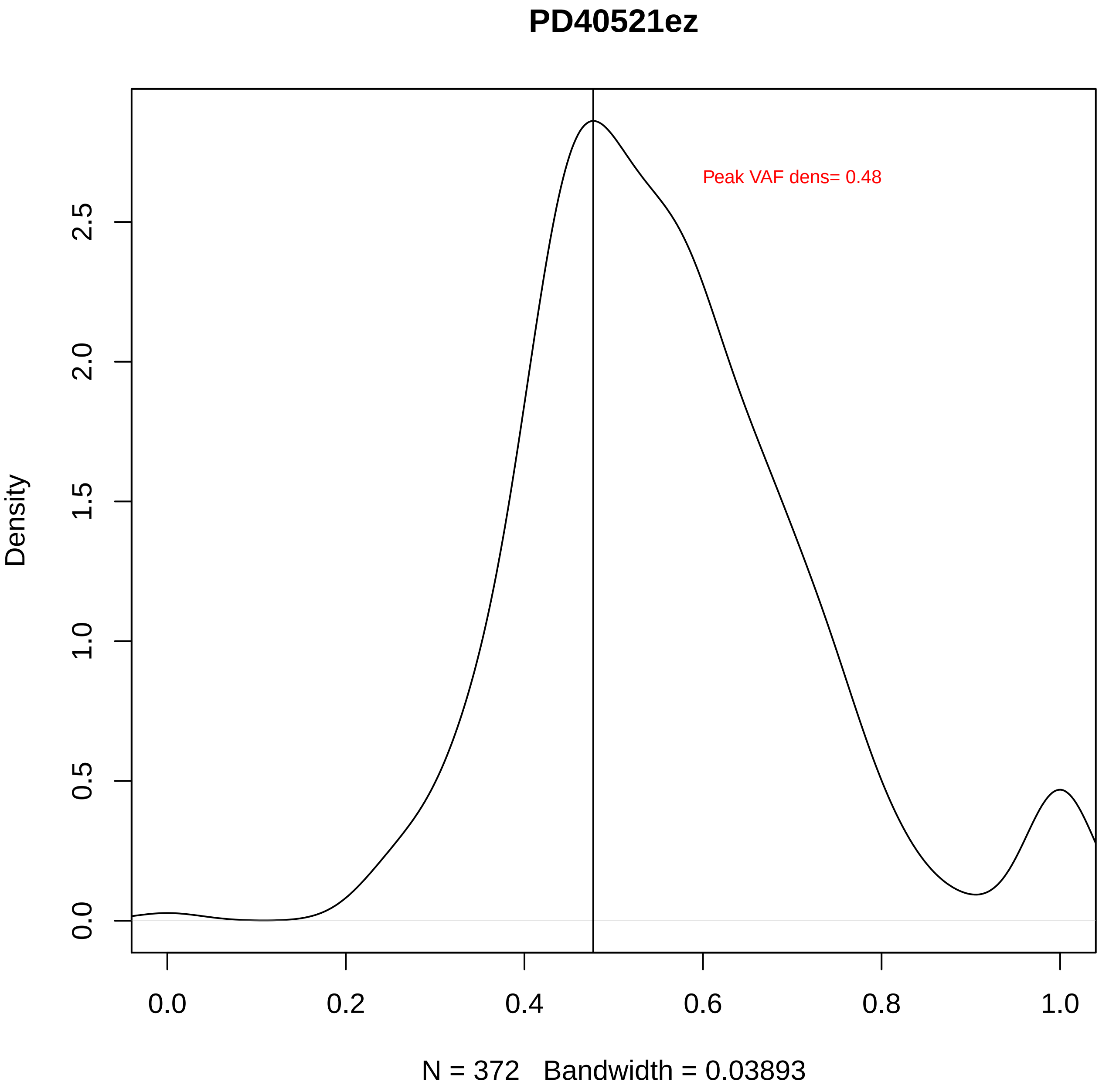
PD40521ez
Peak VAF dens= 0.48
Density
0.0
0.5
1.0
1.5
2.0
2.5
0.0
0.2
0.4
0.6
0.8
1.0
N = 372 Bandwidth = 0.03893

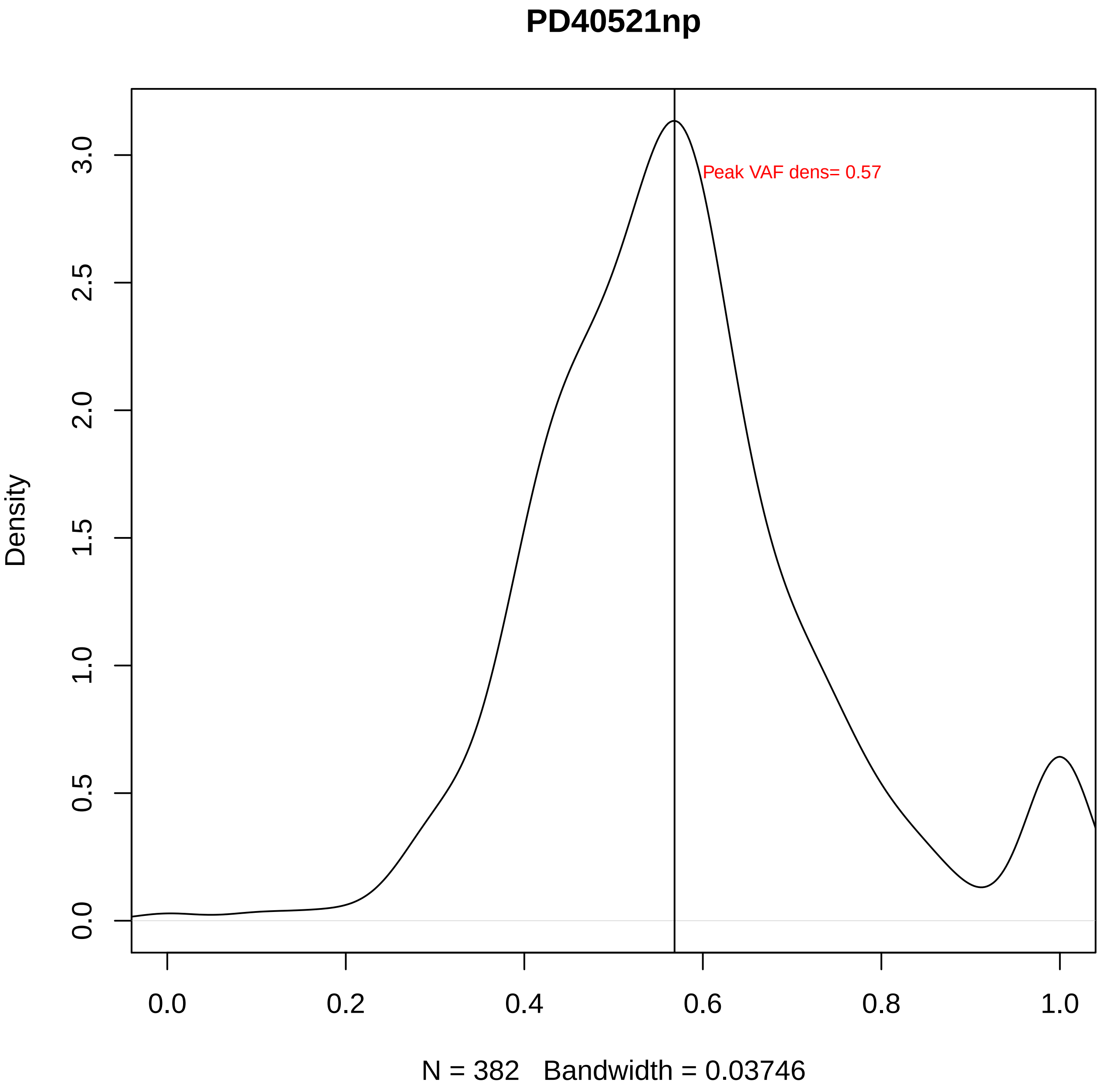

PD40521np
Peak VAF dens= 0.57
Density
0.0
0.5
1.0
1.5
2.0
2.5
3.0
0.0
0.2
0.4
0.6
0.8
1.0
N = 382   Bandwidth = 0.03746

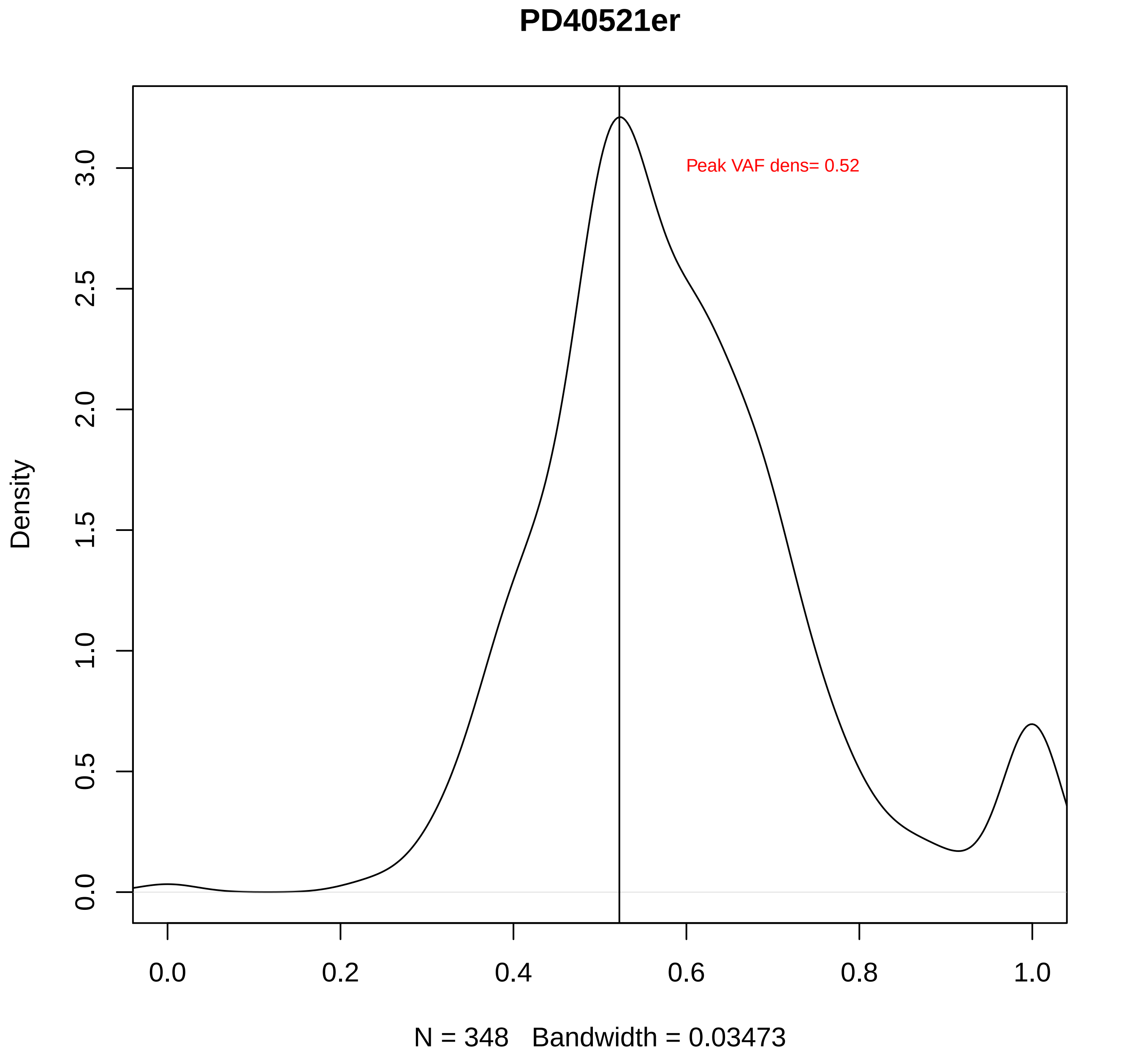
PD40521er
Peak VAF dens= 0.52
Density
0.0
0.5
1.0
1.5
2.0
2.5
3.0
0.0
0.2
0.4
0.6
0.8
1.0
N = 348   Bandwidth = 0.03473

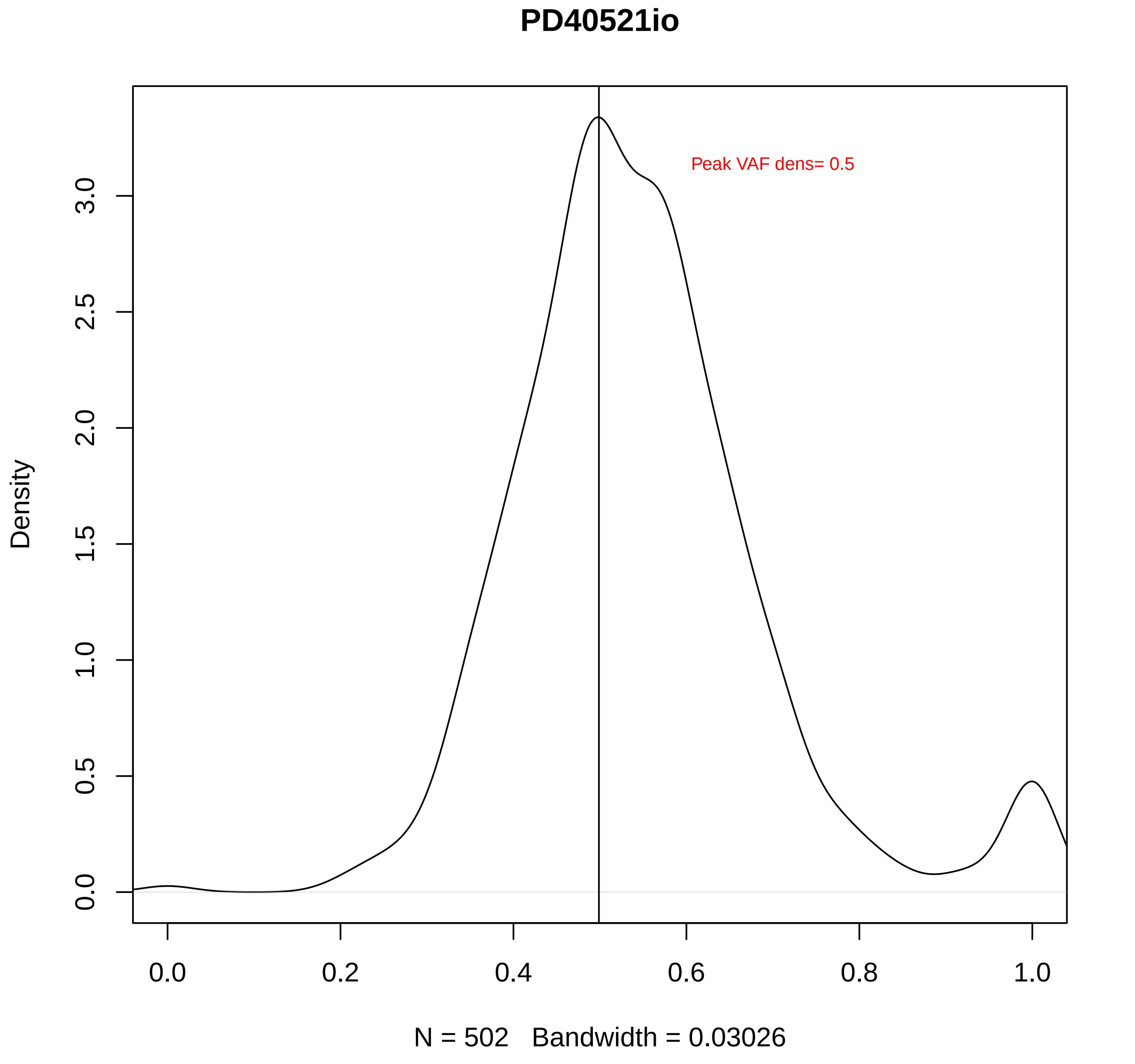
PD40521io
Peak VAF dens= 0.5
Density
0.0
0.5
1.0
1.5
2.0
2.5
3.0
0.0
0.2
0.4
0.6
0.8
1.0
N = 502   Bandwidth = 0.03026

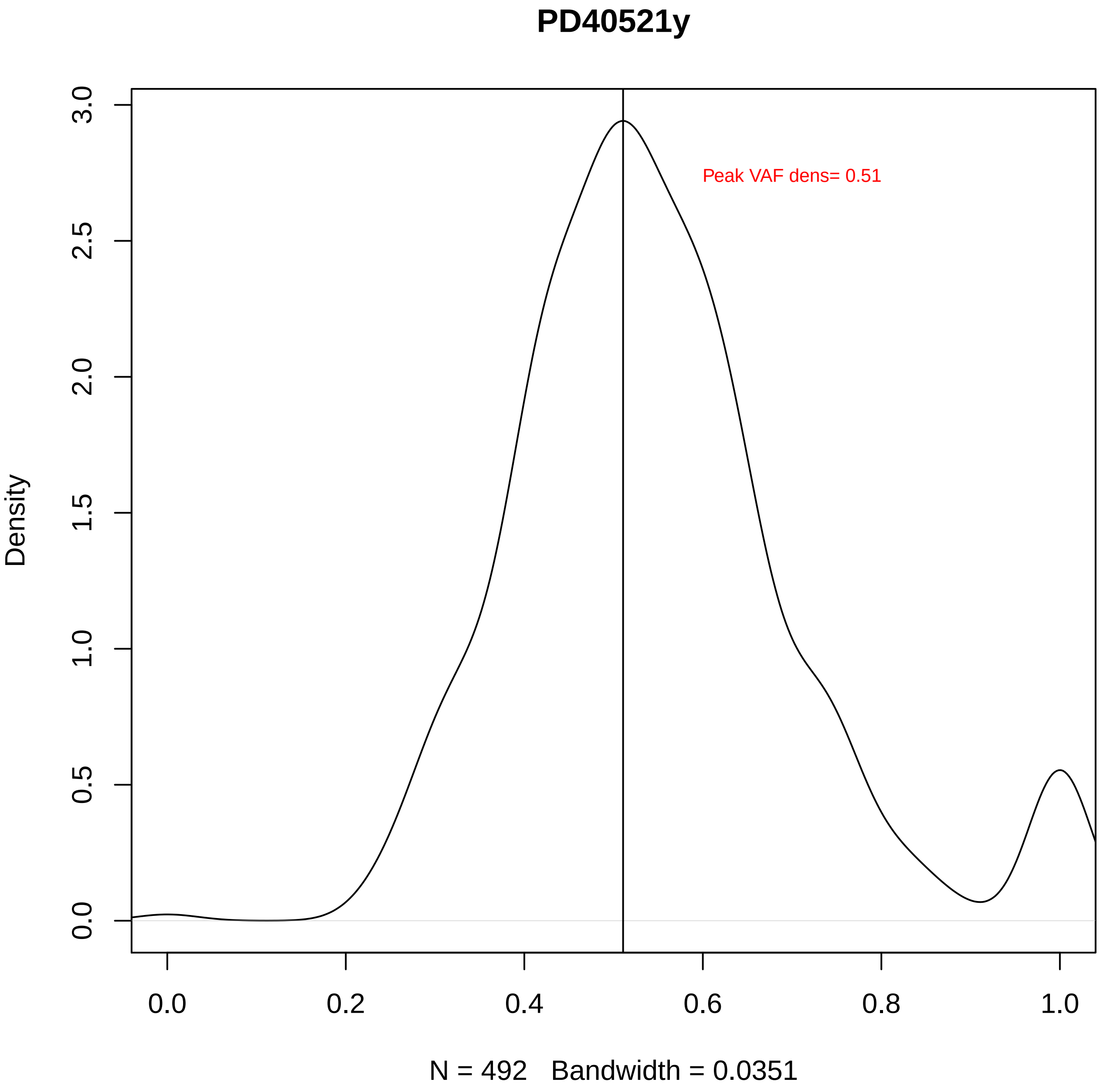
PD40521y
Peak VAF dens= 0.51
Density
0.0
0.5
1.0
1.5
2.0
2.5
3.0
0.0
0.2
0.4
0.6
0.8
1.0
N = 492   Bandwidth = 0.0351

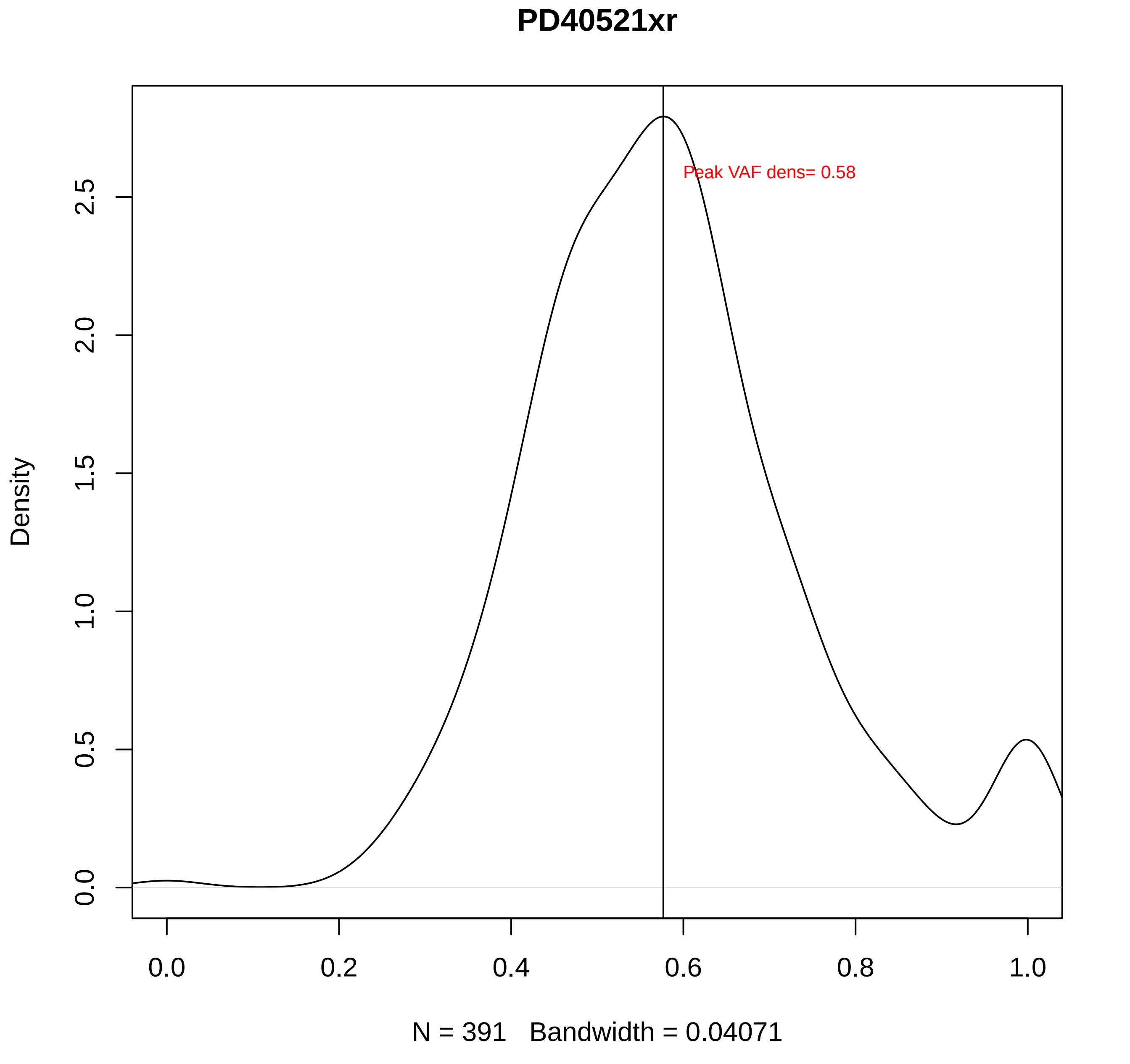
PD40521xr
Peak VAF dens= 0.58
Density
0.0
0.5
1.0
1.5
2.0
2.5
0.0
0.2
0.4
0.6
0.8
1.0
N = 391   Bandwidth = 0.04071

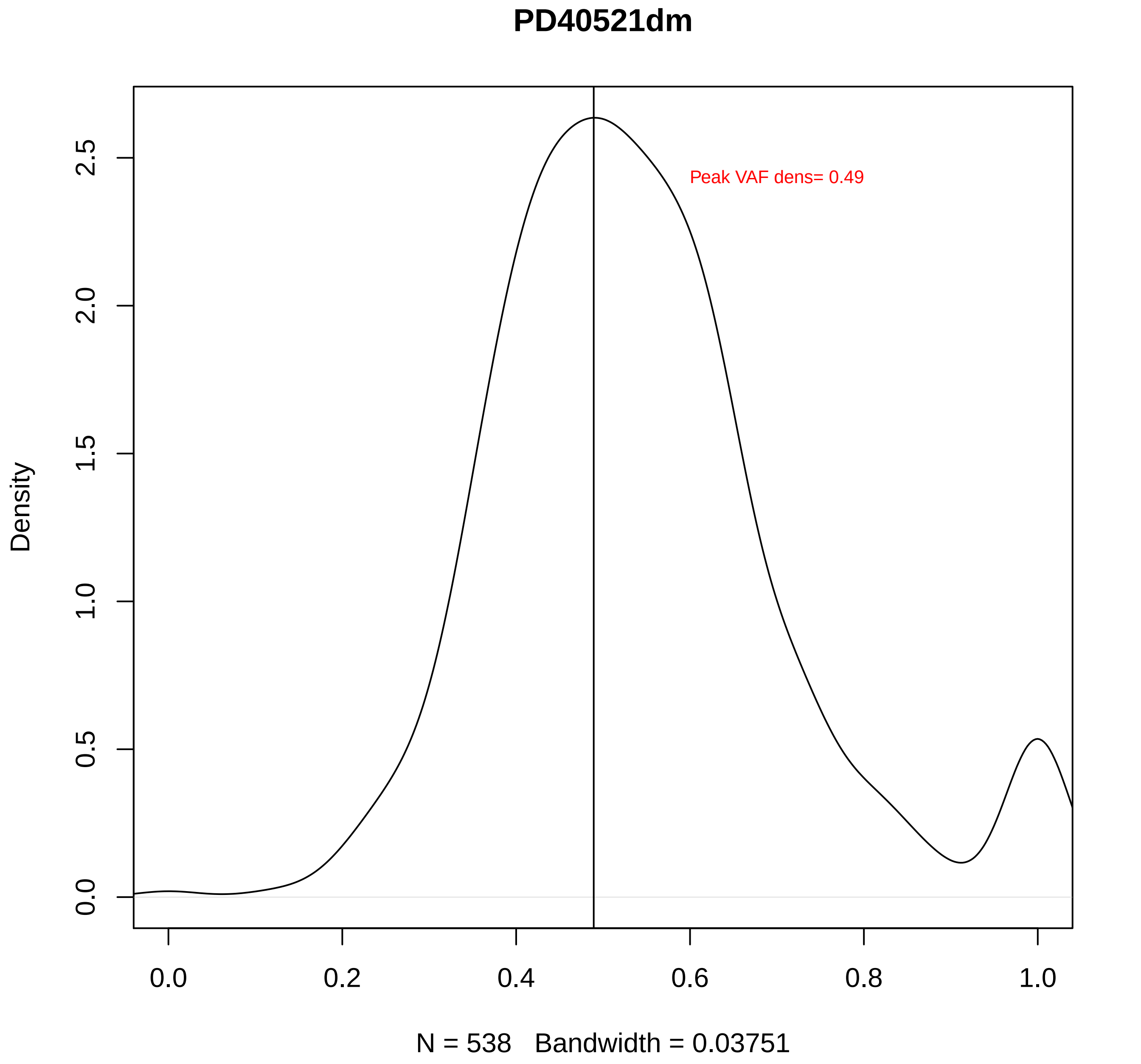
PD40521dm
Peak VAF dens= 0.49
Density
0.0
0.5
1.0
1.5
2.0
2.5
0.0
0.2
0.4
0.6
0.8
1.0
N = 538   Bandwidth = 0.03751

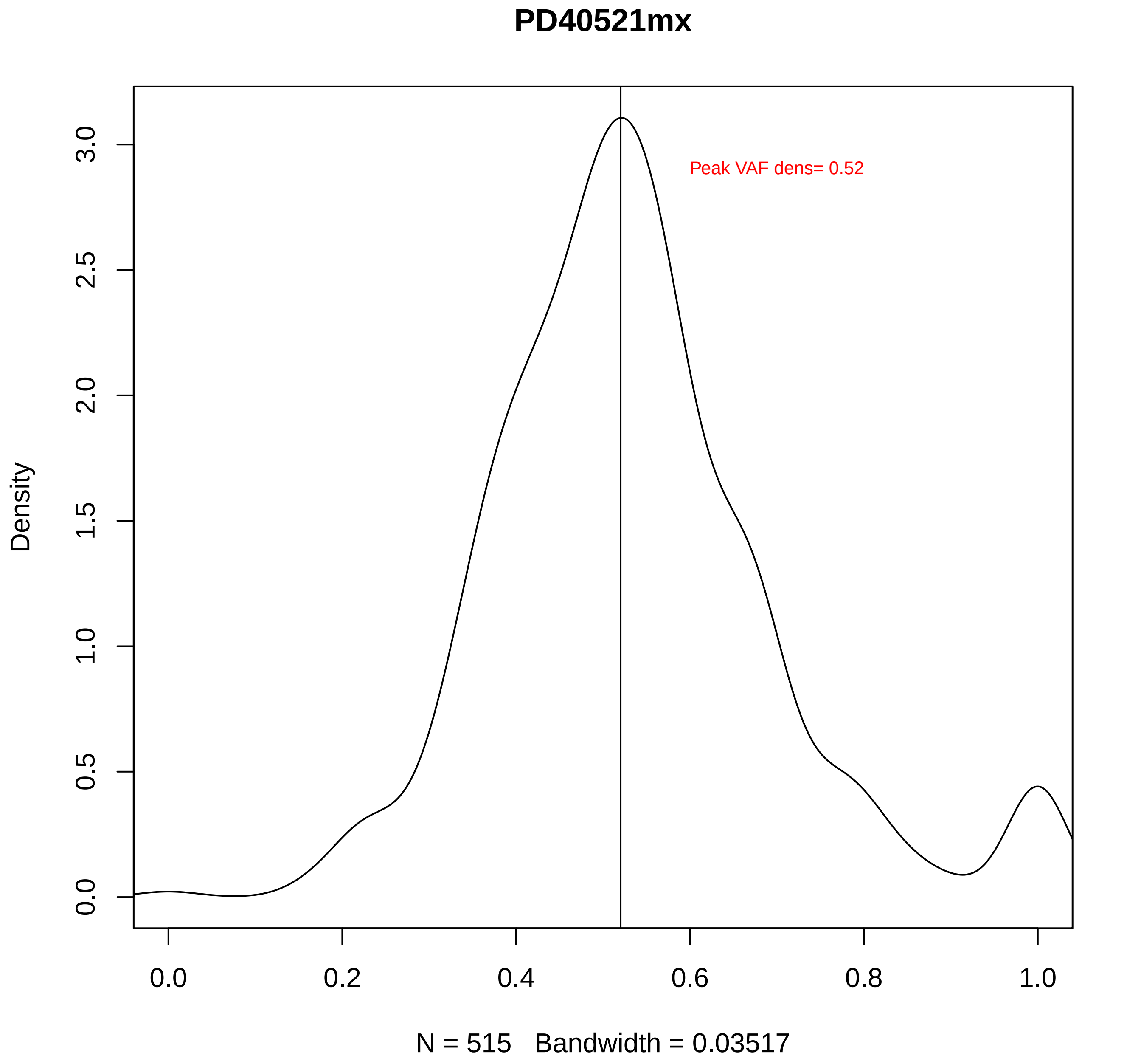

PD40521mx
Peak VAF dens= 0.52
Density
0.0
0.5
1.0
1.5
2.0
2.5
3.0
0.0
0.2
0.4
0.6
0.8
1.0
N = 515 Bandwidth = 0.03517

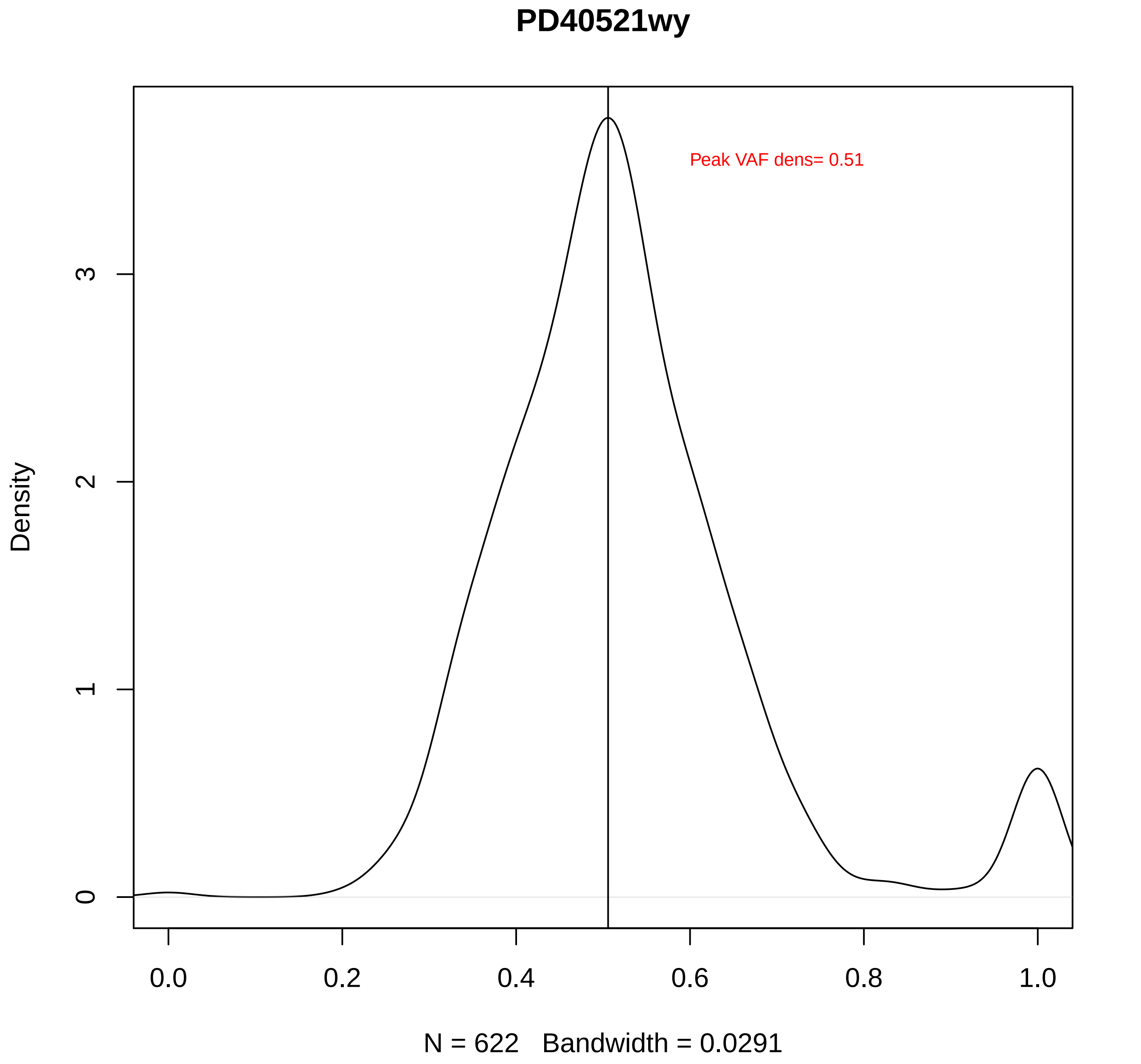
PD40521wy
Peak VAF dens= 0.51
Density
0
1
2
3
0.0
0.2
0.4
0.6
0.8
1.0
N = 622   Bandwidth = 0.0291

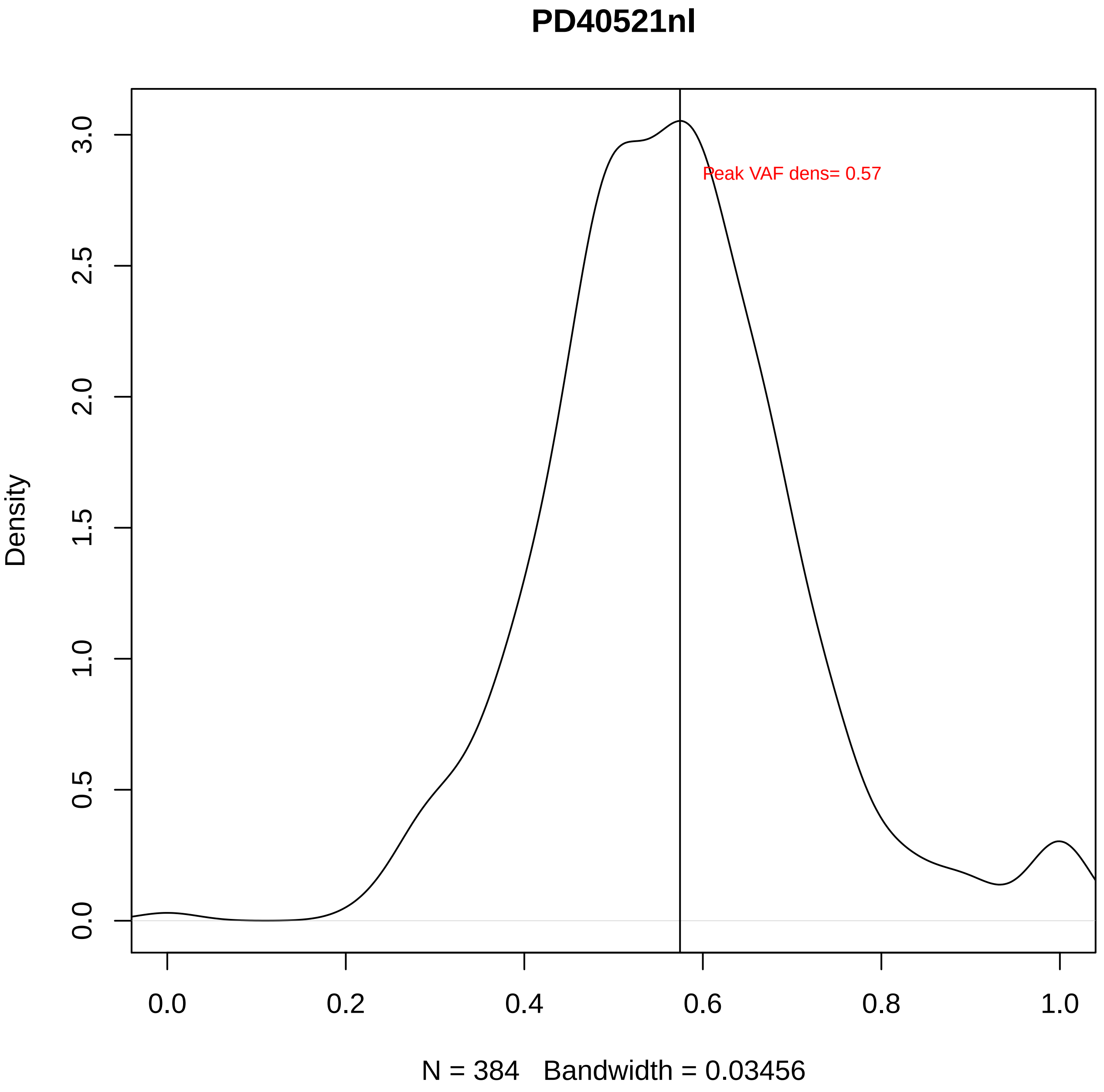
PD40521nl
Peak VAF dens= 0.57
Density
3.0
2.5
2.0
1.5
1.0
0.5
0.0
0.0
0.2
0.4
0.6
0.8
1.0
N = 384   Bandwidth = 0.03456

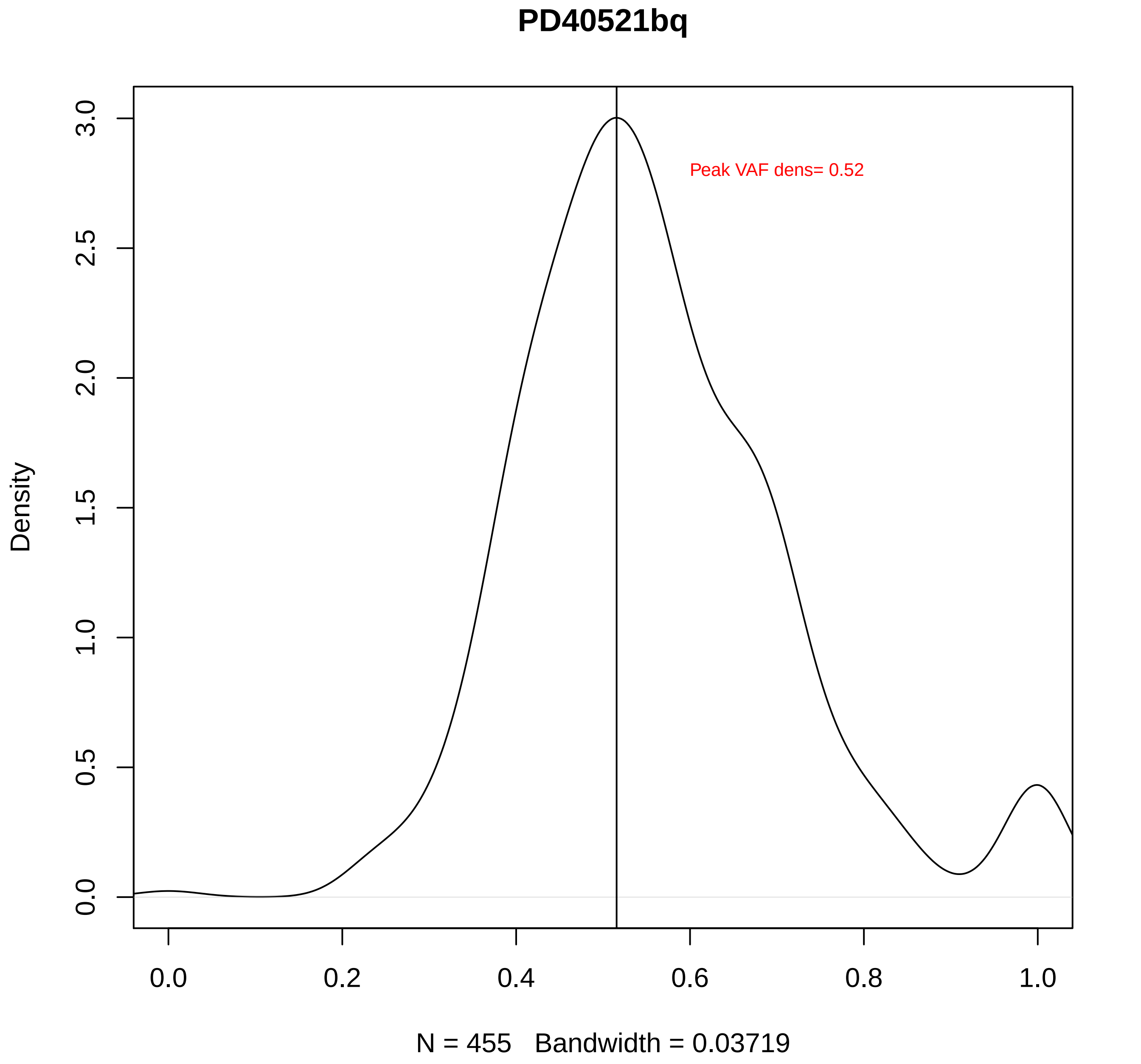

PD40521bq
Peak VAF dens= 0.52
Density
0.0
0.5
1.0
1.5
2.0
2.5
3.0
0.0
0.2
0.4
0.6
0.8
1.0
N = 455   Bandwidth = 0.03719

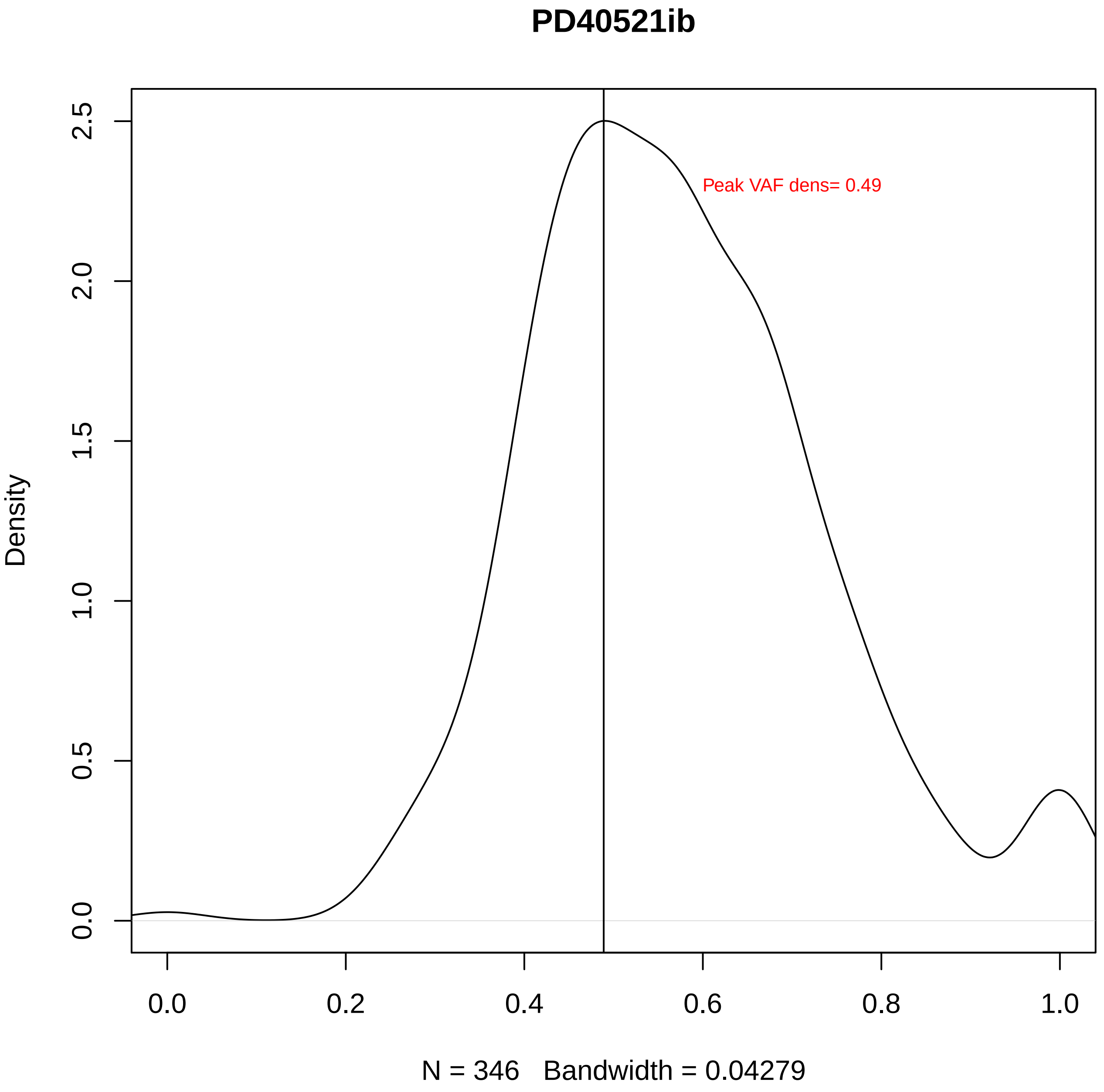

PD40521ib
Peak VAF dens= 0.49
Density
2.5
2.0
1.5
1.0
0.5
0.0
0.0
0.2
0.4
0.6
0.8
1.0
N = 346   Bandwidth = 0.04279

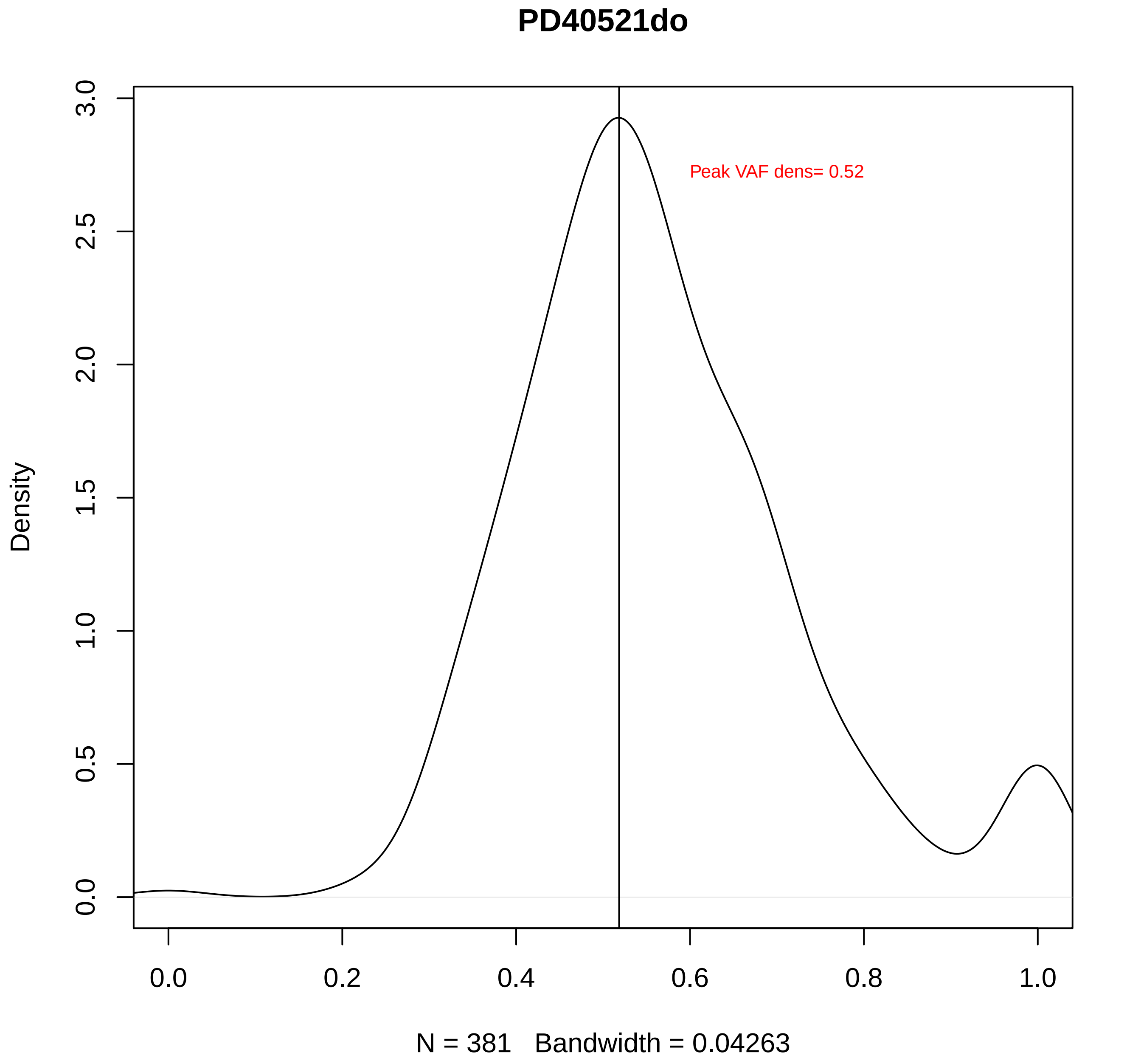

PD40521do
Peak VAF dens= 0.52
Density
3.0
2.5
2.0
1.5
1.0
0.5
0.0
0.0
0.2
0.4
0.6
0.8
1.0
N = 381 Bandwidth = 0.04263

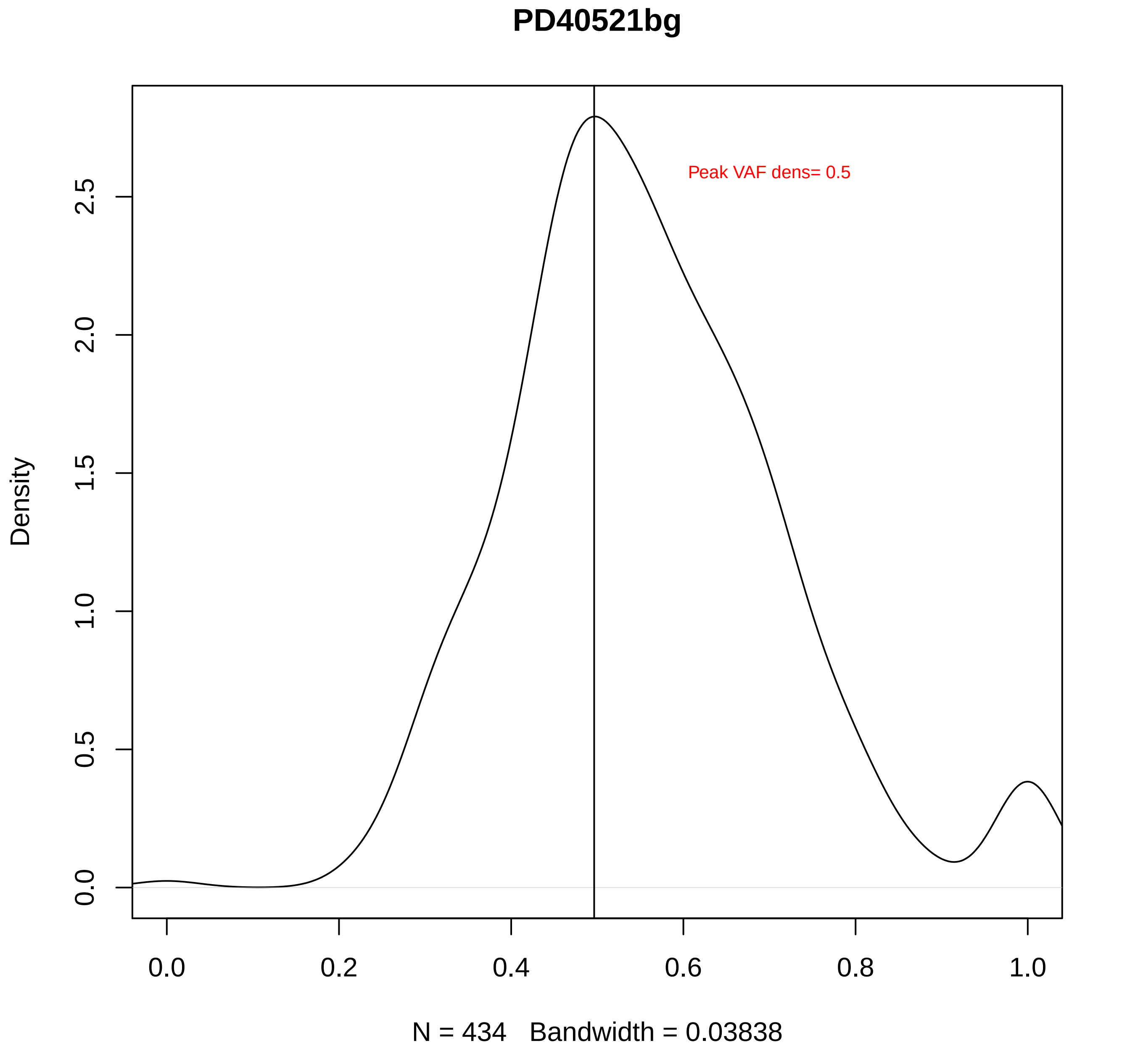

PD40521bg
Peak VAF dens= 0.5
Density
0.0
0.5
1.0
1.5
2.0
2.5
0.0
0.2
0.4
0.6
0.8
1.0
N = 434   Bandwidth = 0.03838

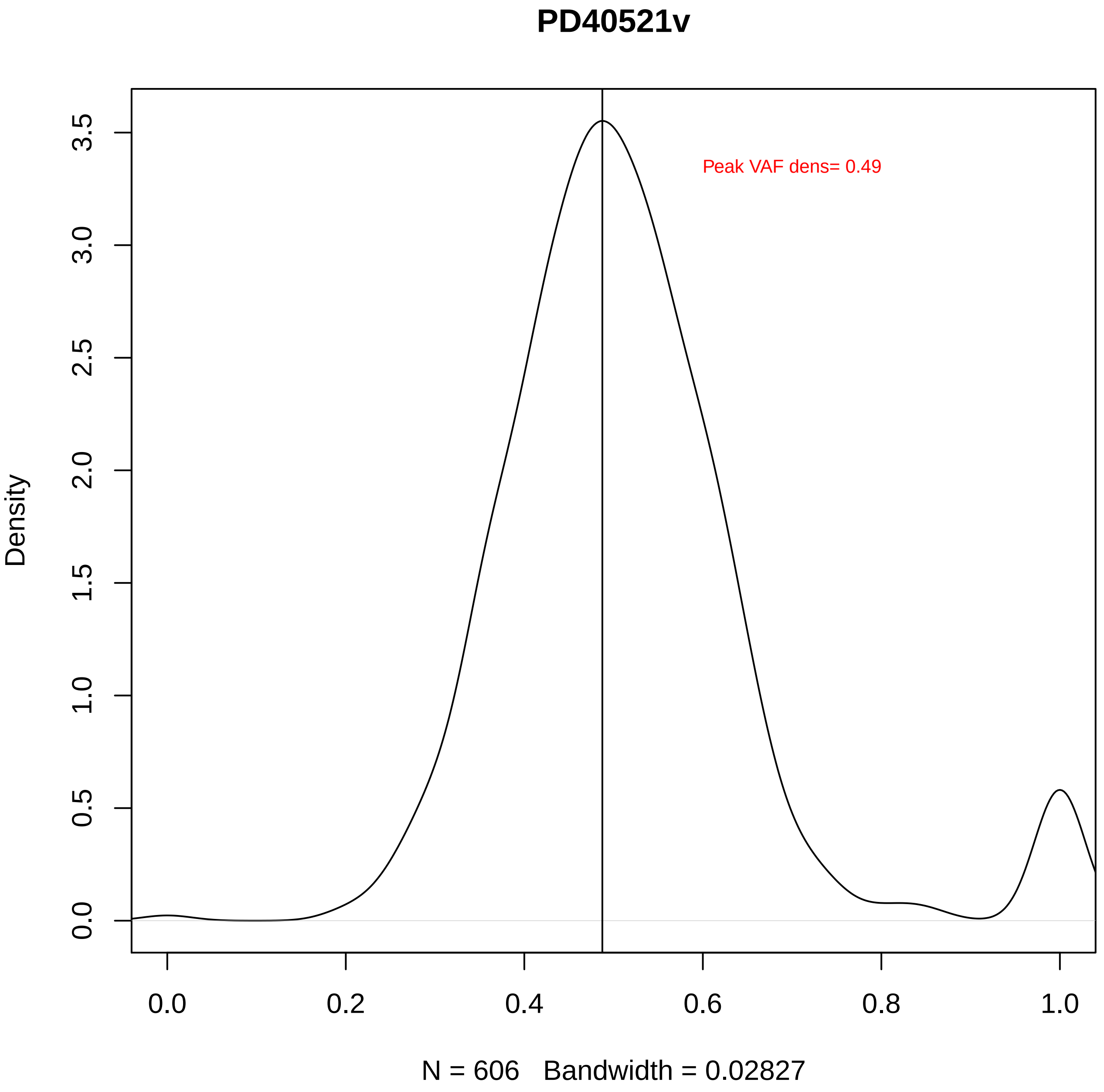

PD40521v
Peak VAF dens= 0.49
Density
3.5
3.0
2.5
2.0
1.5
1.0
0.5
0.0
0.0
0.2
0.4
0.6
0.8
1.0
N = 606   Bandwidth = 0.02827

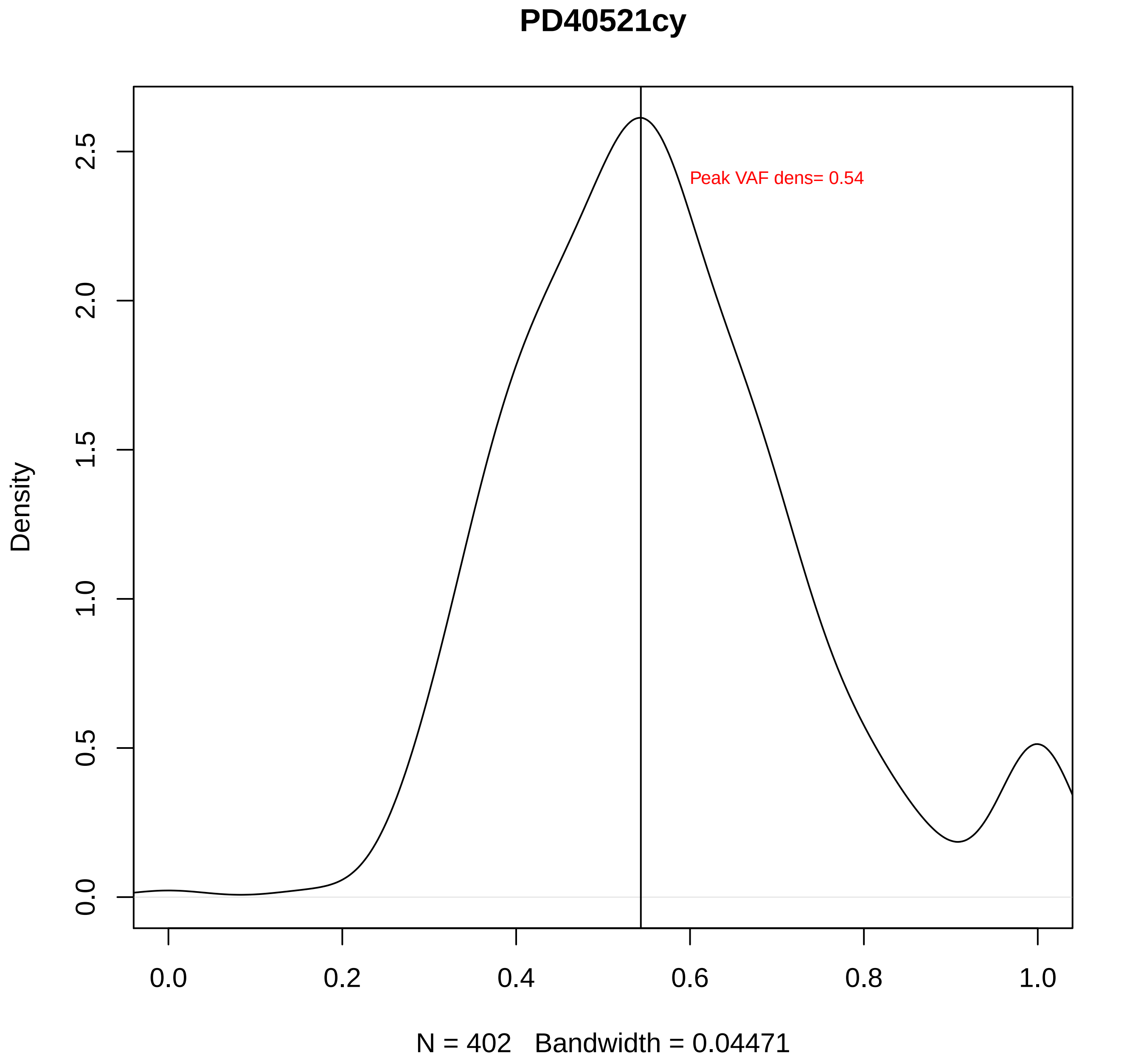
PD40521cy
Peak VAF dens= 0.54
Density
0.0
0.5
1.0
1.5
2.0
2.5
0.0
0.2
0.4
0.6
0.8
1.0
N = 402   Bandwidth = 0.04471

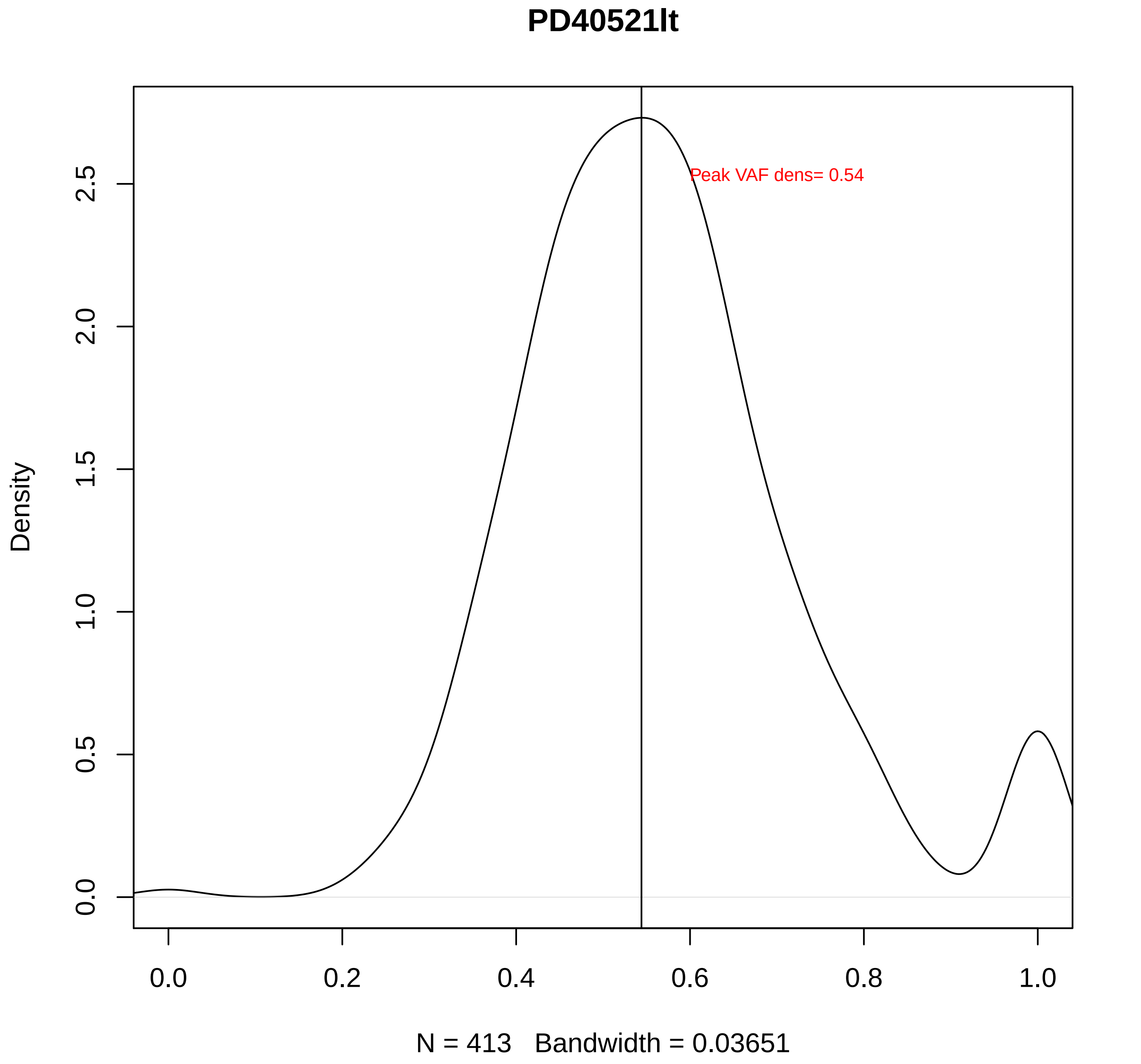
PD40521lt
Peak VAF dens= 0.54
Density
0.0
0.5
1.0
1.5
2.0
2.5
0.0
0.2
0.4
0.6
0.8
1.0
N = 413 Bandwidth = 0.03651

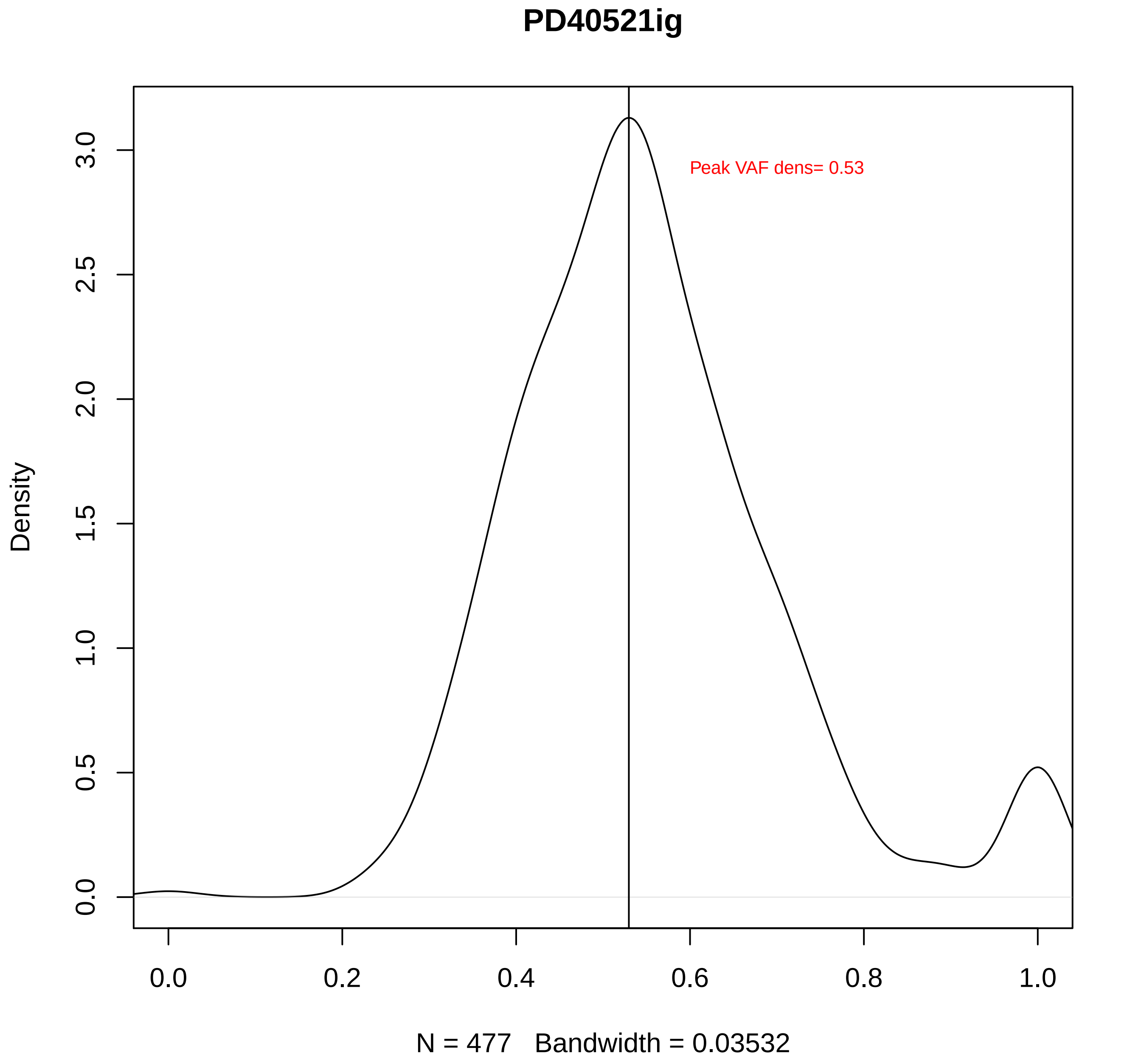

PD40521ig
Peak VAF dens= 0.53
Density
0.0
0.5
1.0
1.5
2.0
2.5
3.0
0.0
0.2
0.4
0.6
0.8
1.0
N = 477   Bandwidth = 0.03532

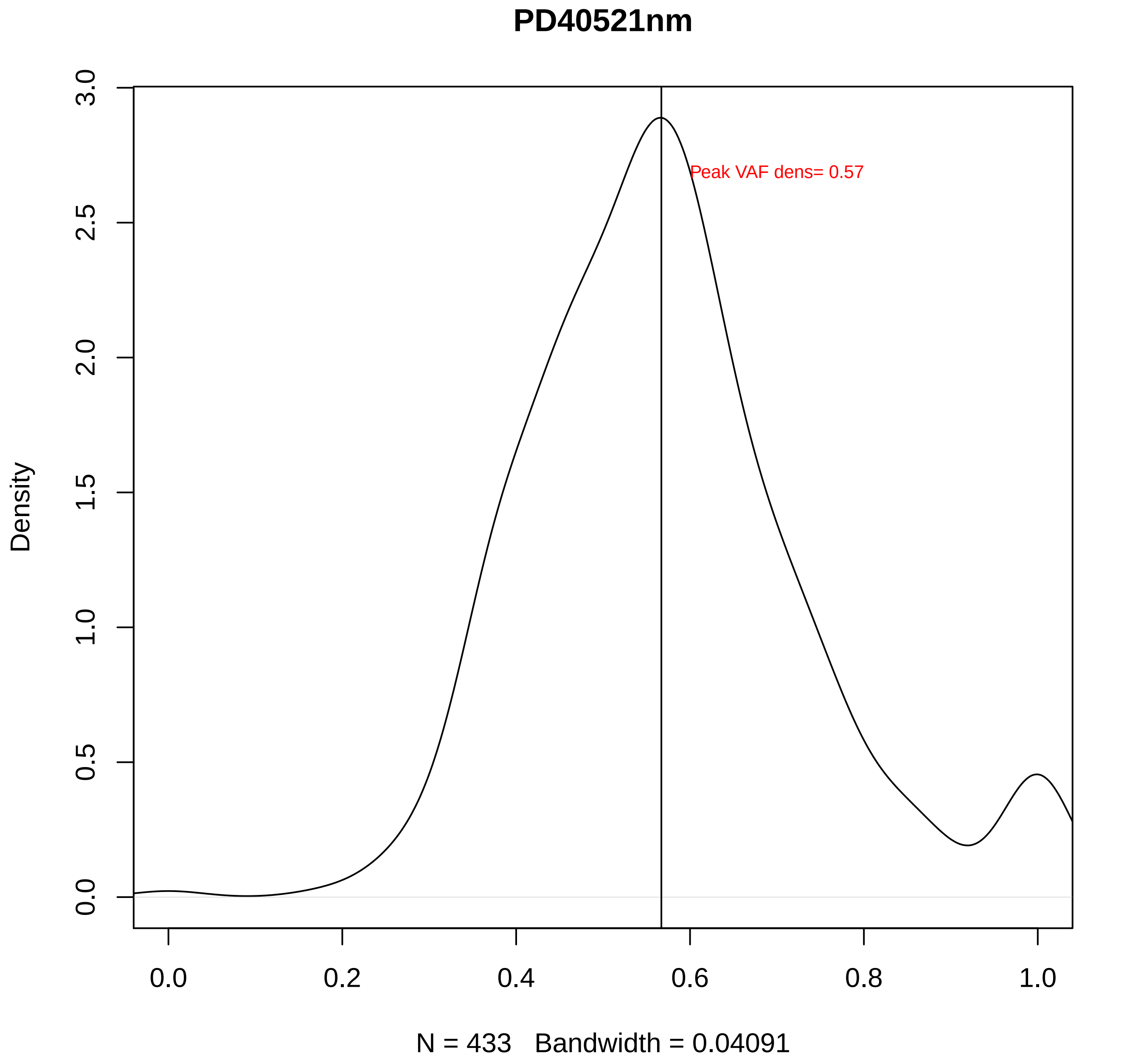

PD40521nm
Peak VAF dens= 0.57
Density
3.0
2.5
2.0
1.5
1.0
0.5
0.0
0.0
0.2
0.4
0.6
0.8
1.0
N = 433   Bandwidth = 0.04091

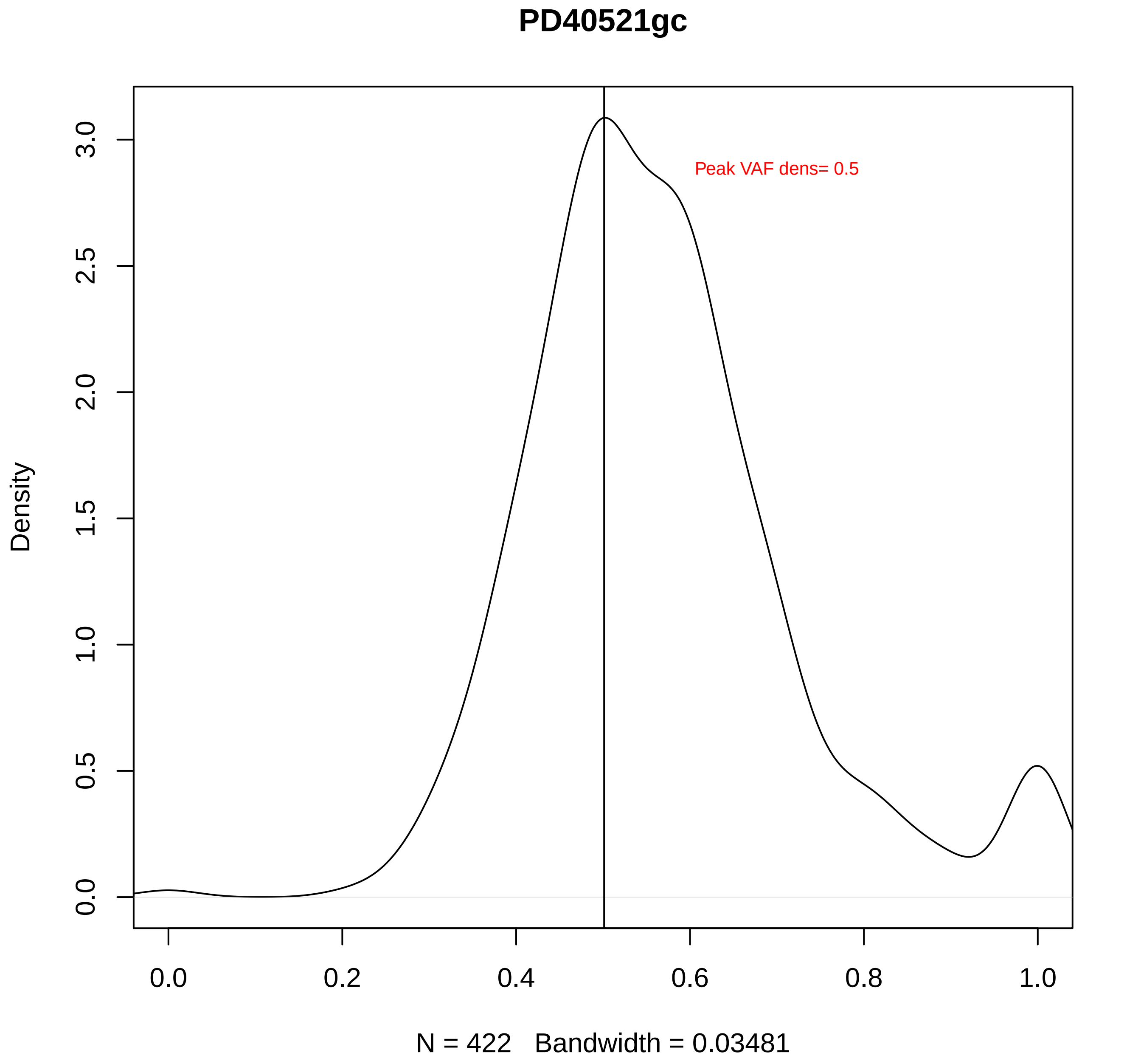
PD40521gc
Peak VAF dens= 0.5
Density
0.0
0.5
1.0
1.5
2.0
2.5
3.0
0.0
0.2
0.4
0.6
0.8
1.0
N = 422 Bandwidth = 0.03481

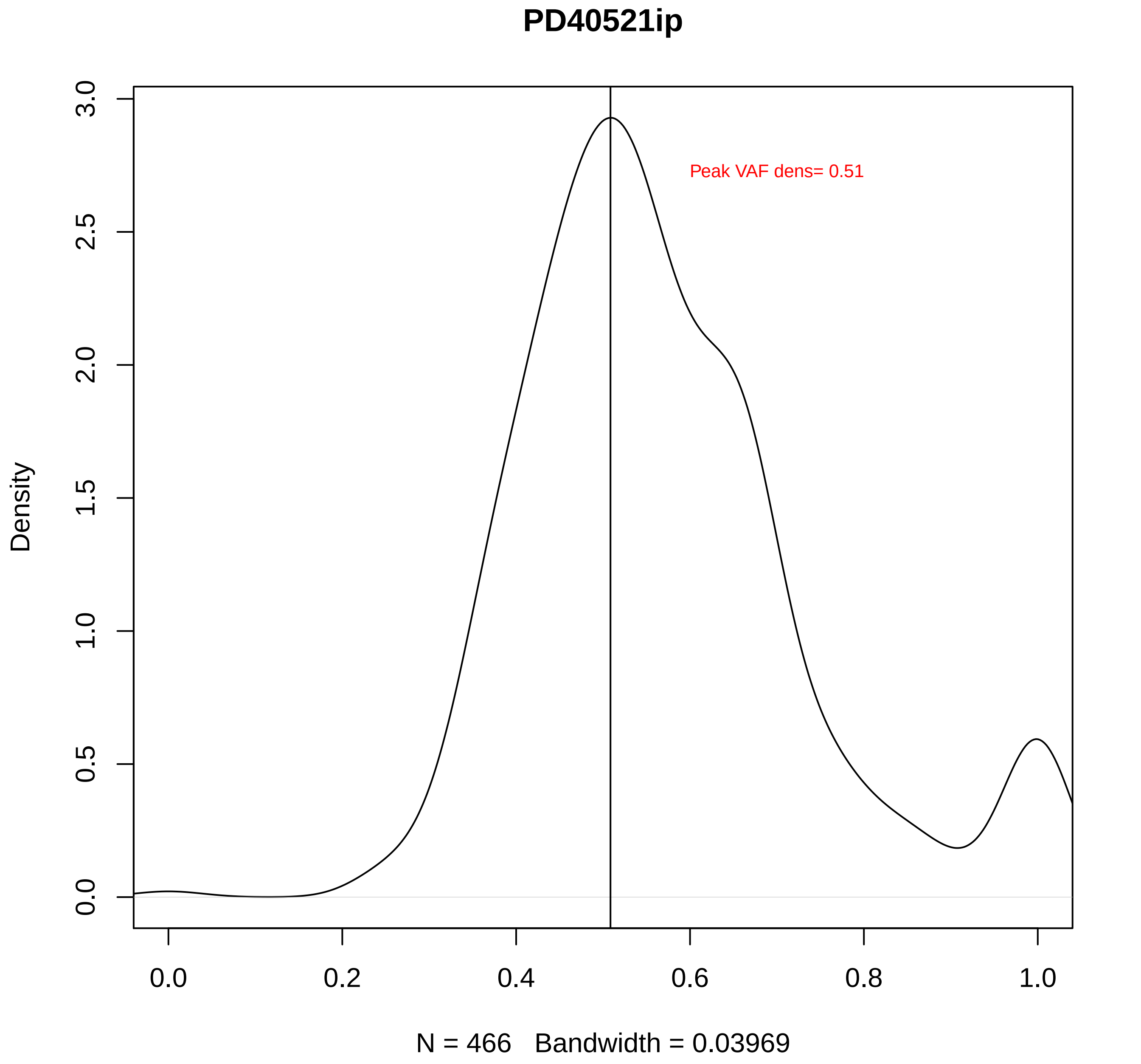
PD40521ip
Peak VAF dens= 0.51
Density
0.0
0.5
1.0
1.5
2.0
2.5
3.0
0.0
0.2
0.4
0.6
0.8
1.0
N = 466   Bandwidth = 0.03969

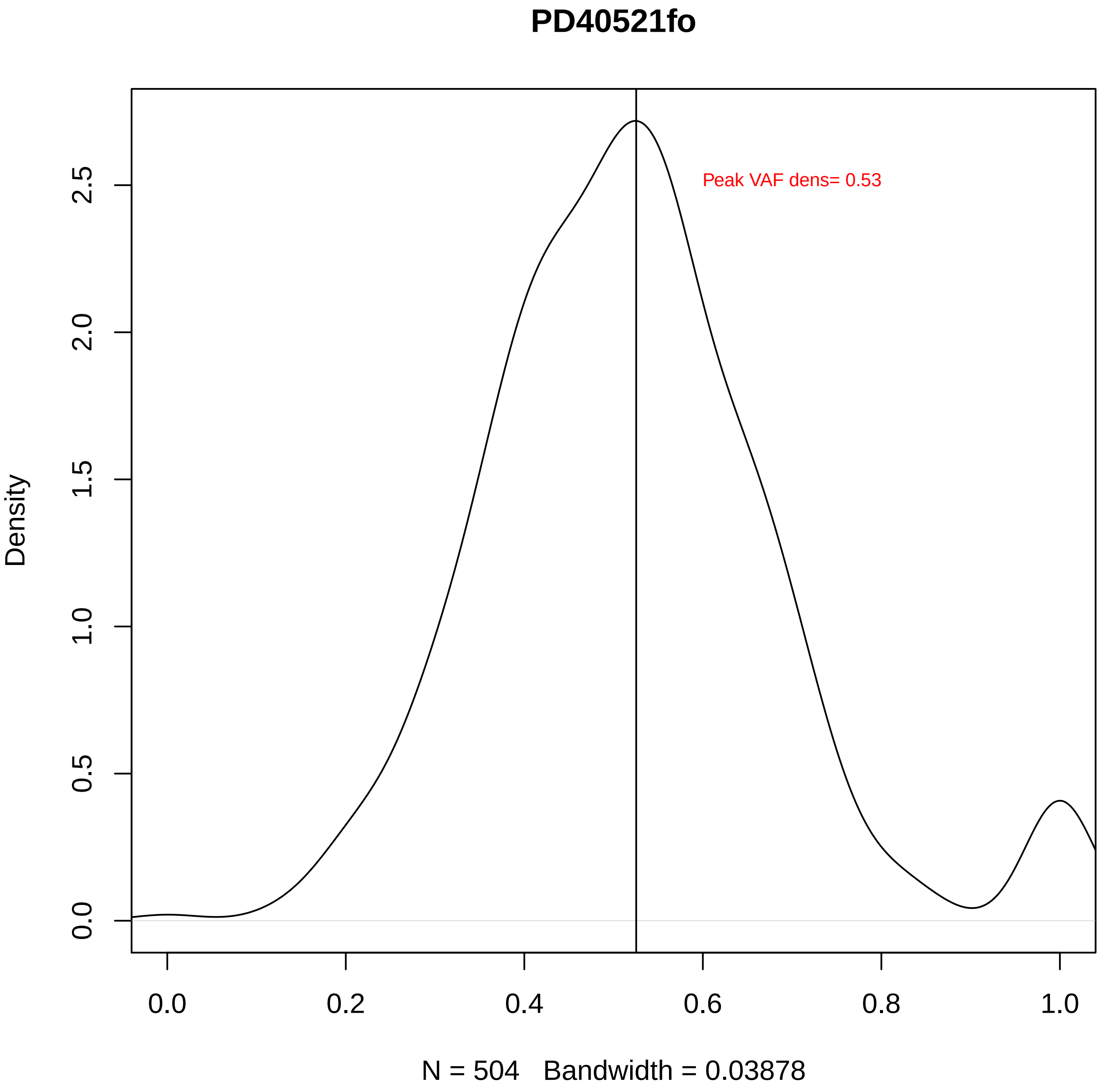
PD40521fo
Peak VAF dens= 0.53
Density
0.0
0.5
1.0
1.5
2.0
2.5
0.0
0.2
0.4
0.6
0.8
1.0
N = 504   Bandwidth = 0.03878

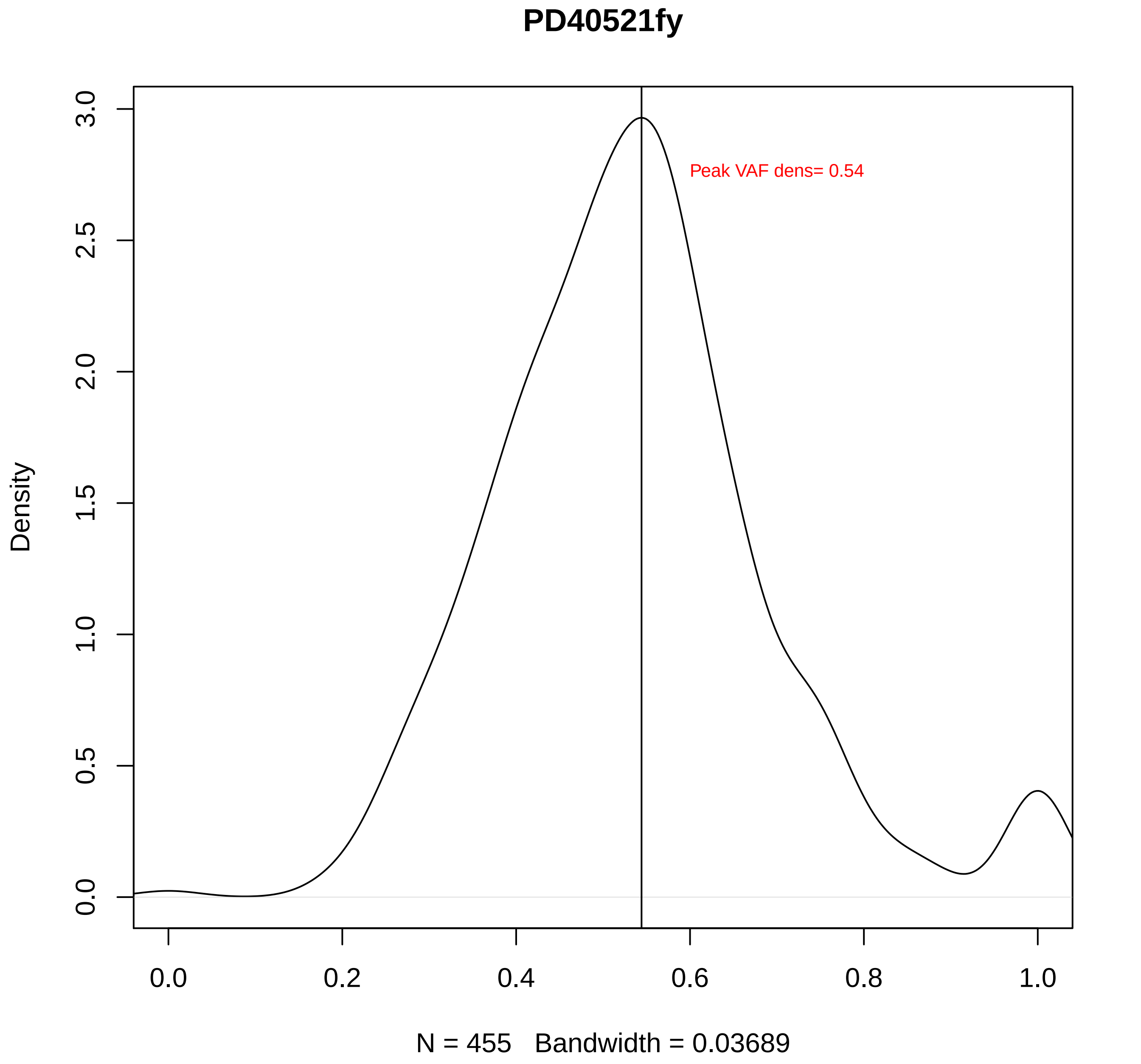

PD40521fy
Peak VAF dens= 0.54
Density
0.0
0.5
1.0
1.5
2.0
2.5
3.0
0.0
0.2
0.4
0.6
0.8
1.0
N = 455   Bandwidth = 0.03689

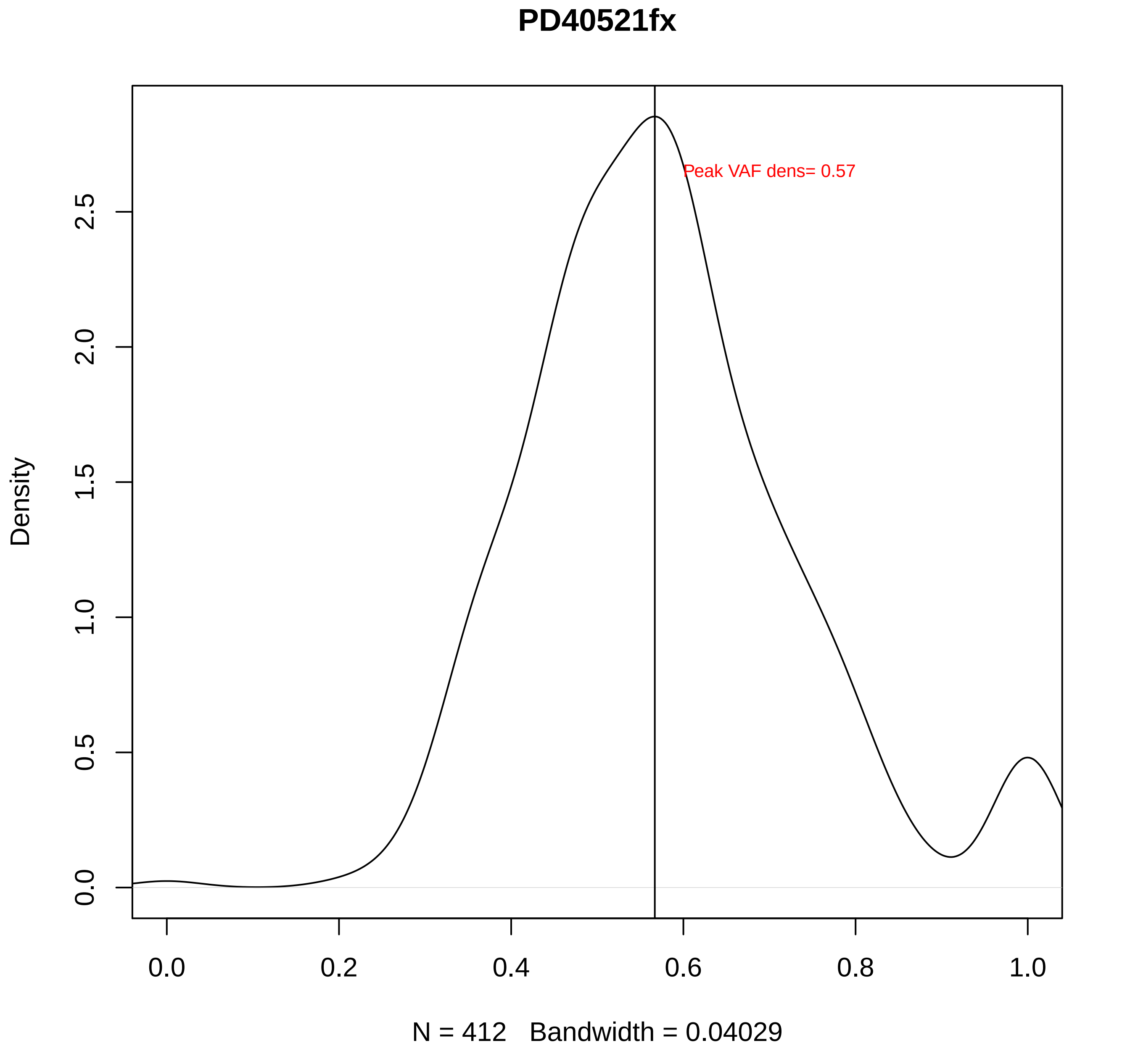

PD40521fx
Peak VAF dens= 0.57
Density
0.0
0.5
1.0
1.5
2.0
2.5
0.0
0.2
0.4
0.6
0.8
1.0
N = 412   Bandwidth = 0.04029

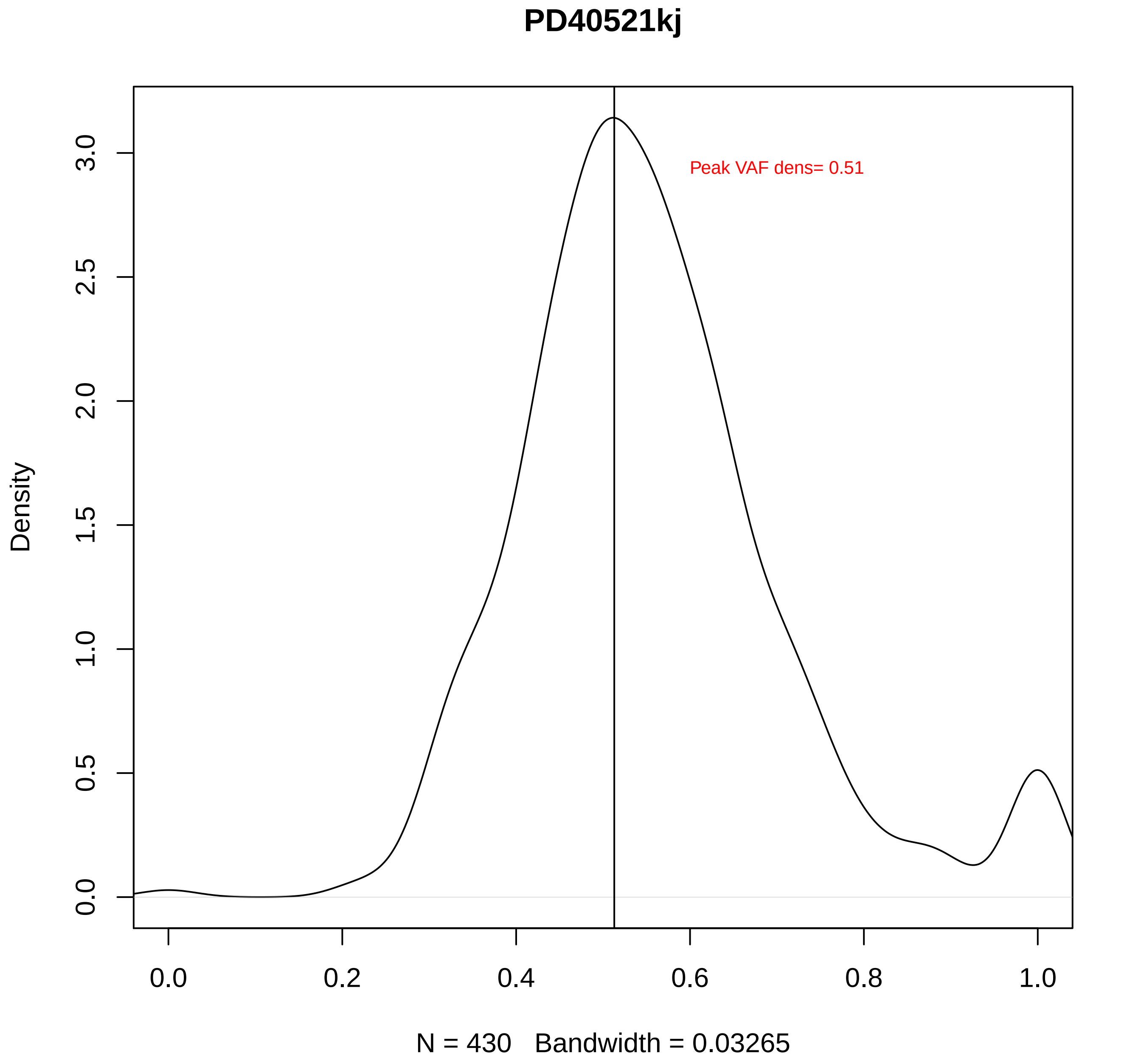

PD40521kj
Peak VAF dens= 0.51
Density
0.0
0.5
1.0
1.5
2.0
2.5
3.0
0.0
0.2
0.4
0.6
0.8
1.0
N = 430   Bandwidth = 0.03265

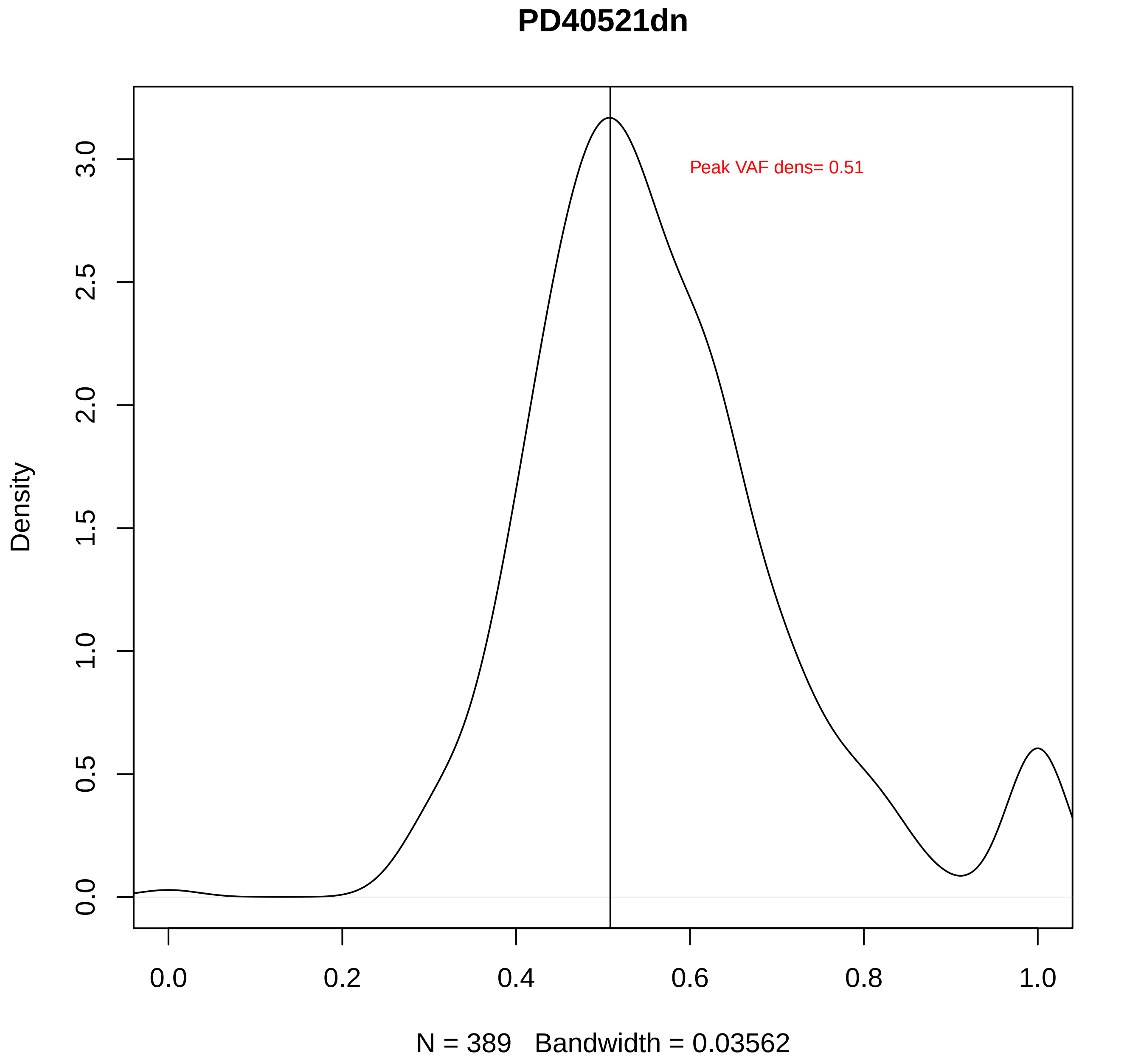
PD40521dn
Peak VAF dens= 0.51
Density
0.0
0.5
1.0
1.5
2.0
2.5
3.0
0.0
0.2
0.4
0.6
0.8
1.0
N = 389   Bandwidth = 0.03562

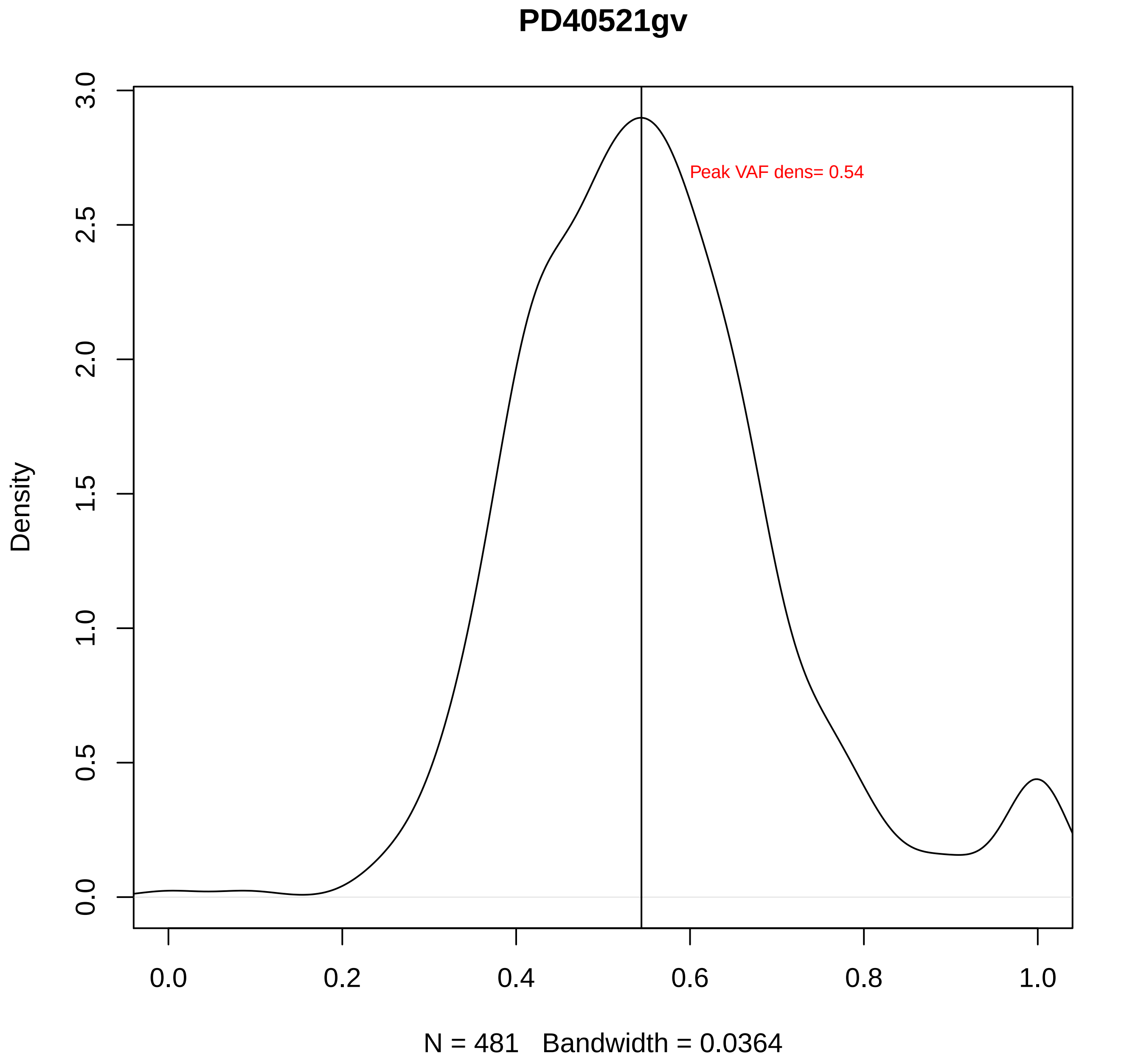

PD40521gv
Peak VAF dens= 0.54
Density
3.0
2.5
2.0
1.5
1.0
0.5
0.0
0.0
0.2
0.4
0.6
0.8
1.0
N = 481   Bandwidth = 0.0364

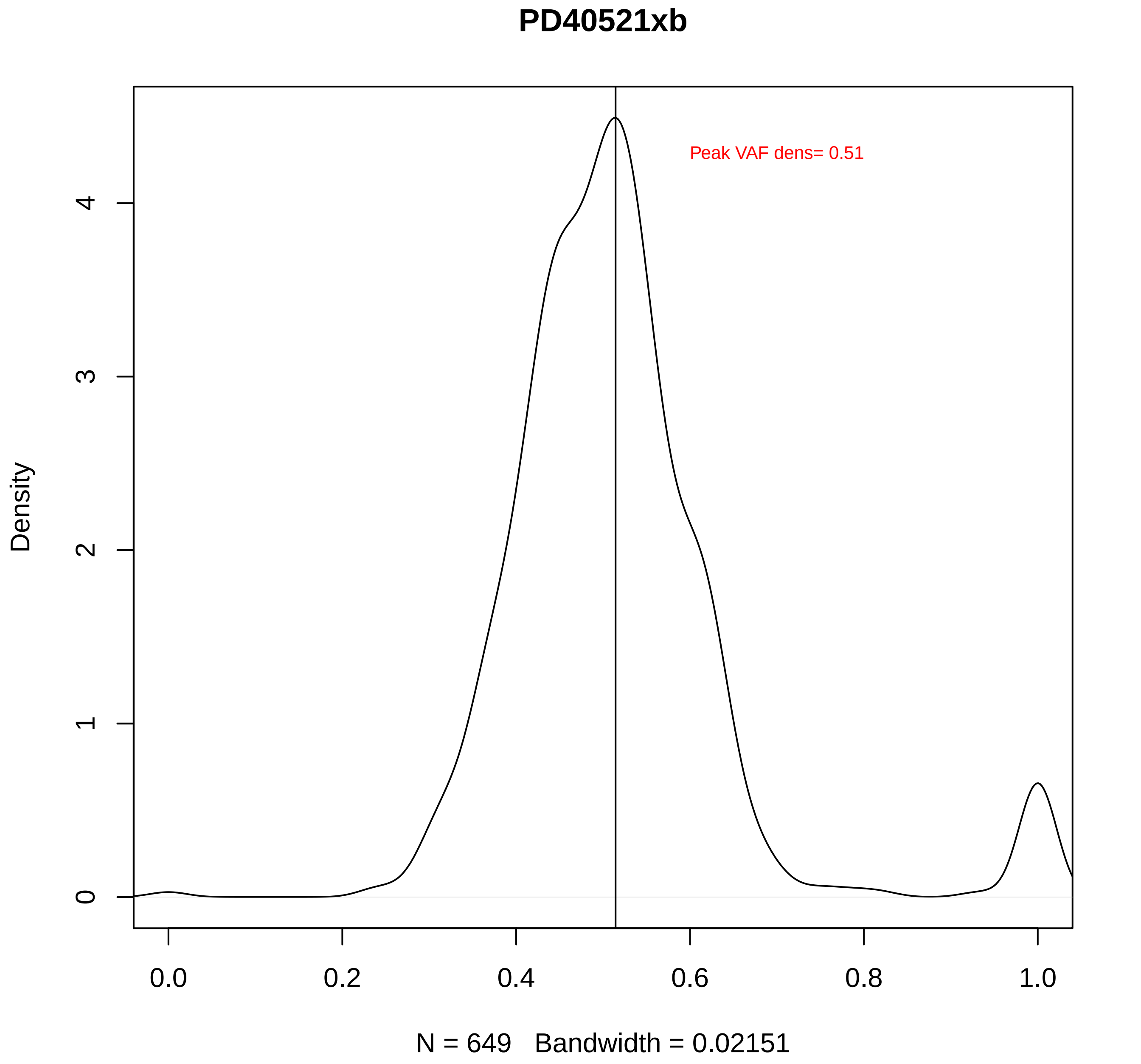

PD40521xb
Peak VAF dens= 0.51
Density
0
1
2
3
4
0.0
0.2
0.4
0.6
0.8
1.0
N = 649 Bandwidth = 0.02151

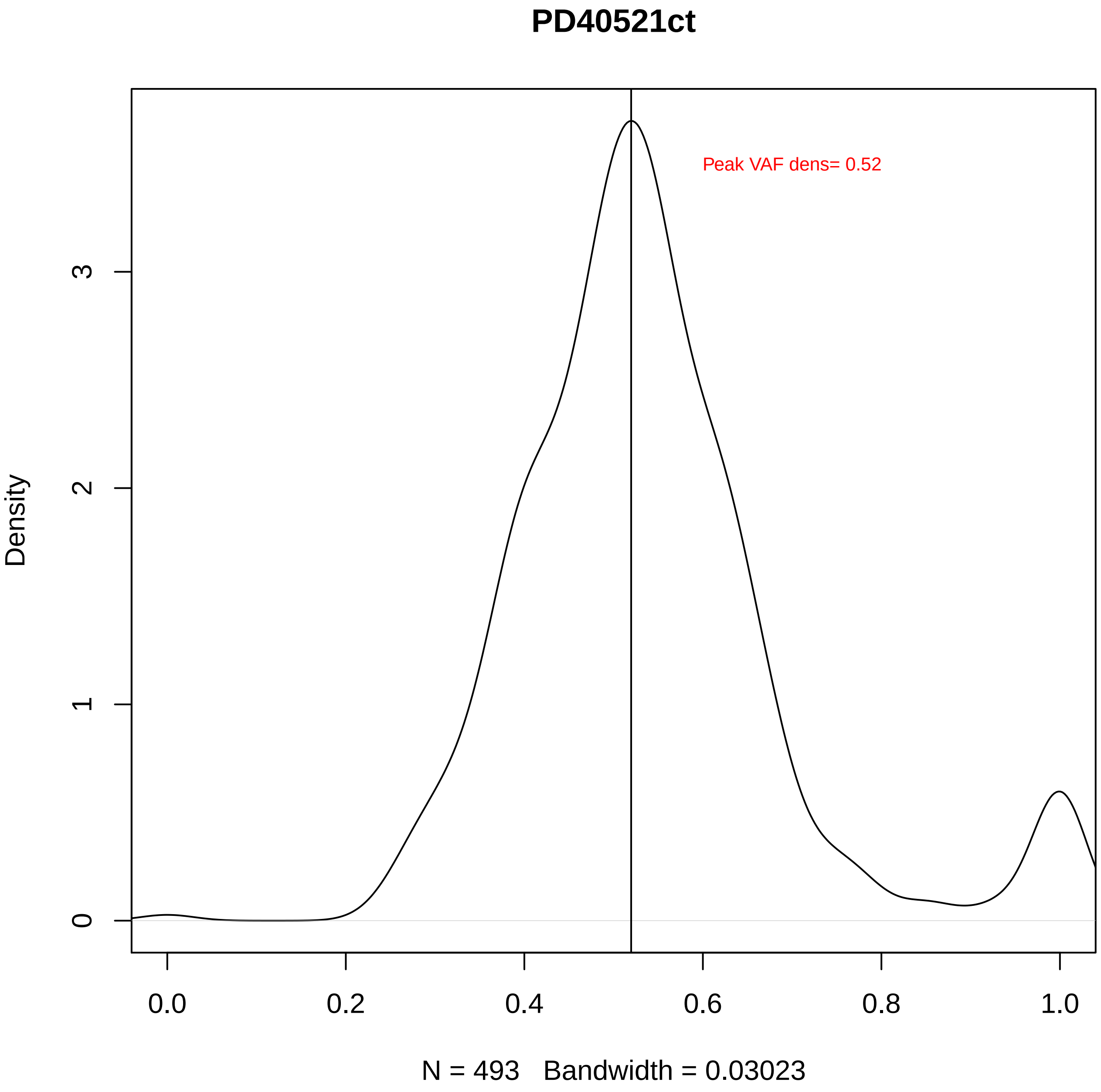

PD40521ct
Peak VAF dens= 0.52
Density
0
1
2
3
0.0
0.2
0.4
0.6
0.8
1.0
N = 493 Bandwidth = 0.03023

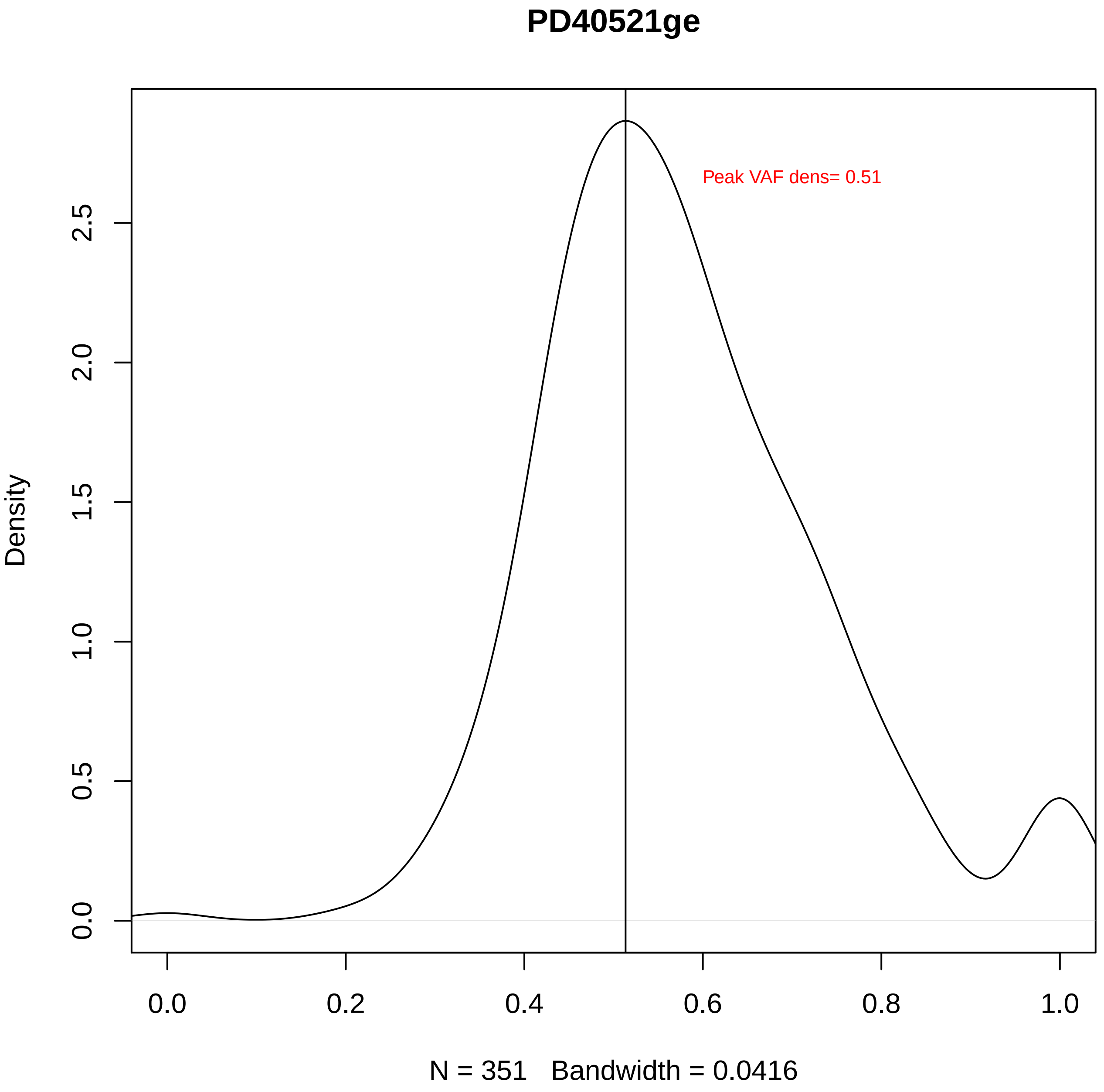
PD40521ge
Peak VAF dens= 0.51
Density
0.0
0.5
1.0
1.5
2.0
2.5
0.0
0.2
0.4
0.6
0.8
1.0
N = 351   Bandwidth = 0.0416

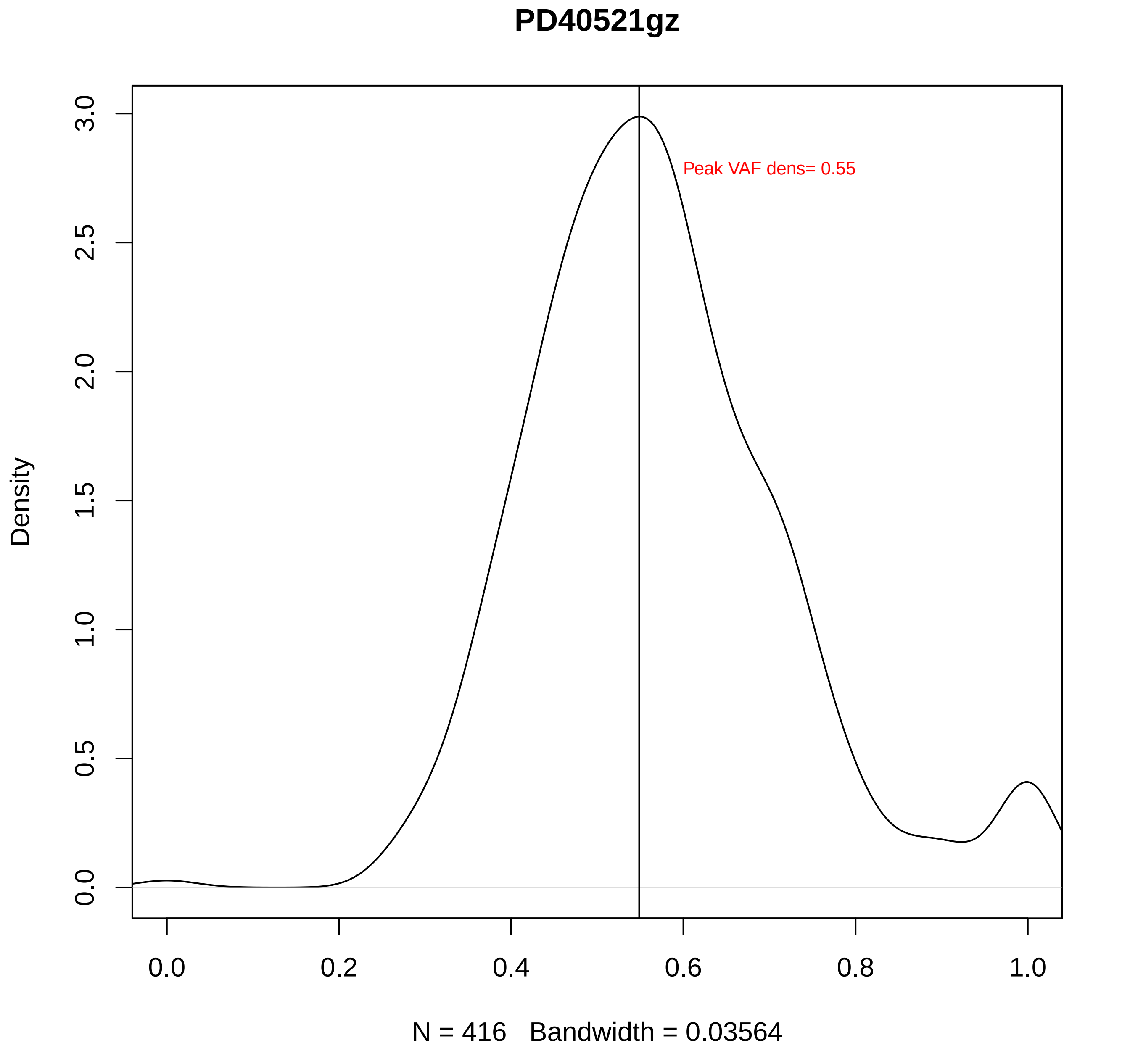

PD40521gz
Peak VAF dens= 0.55
Density
3.0
2.5
2.0
1.5
1.0
0.5
0.0
0.0
0.2
0.4
0.6
0.8
1.0
N = 416 Bandwidth = 0.03564

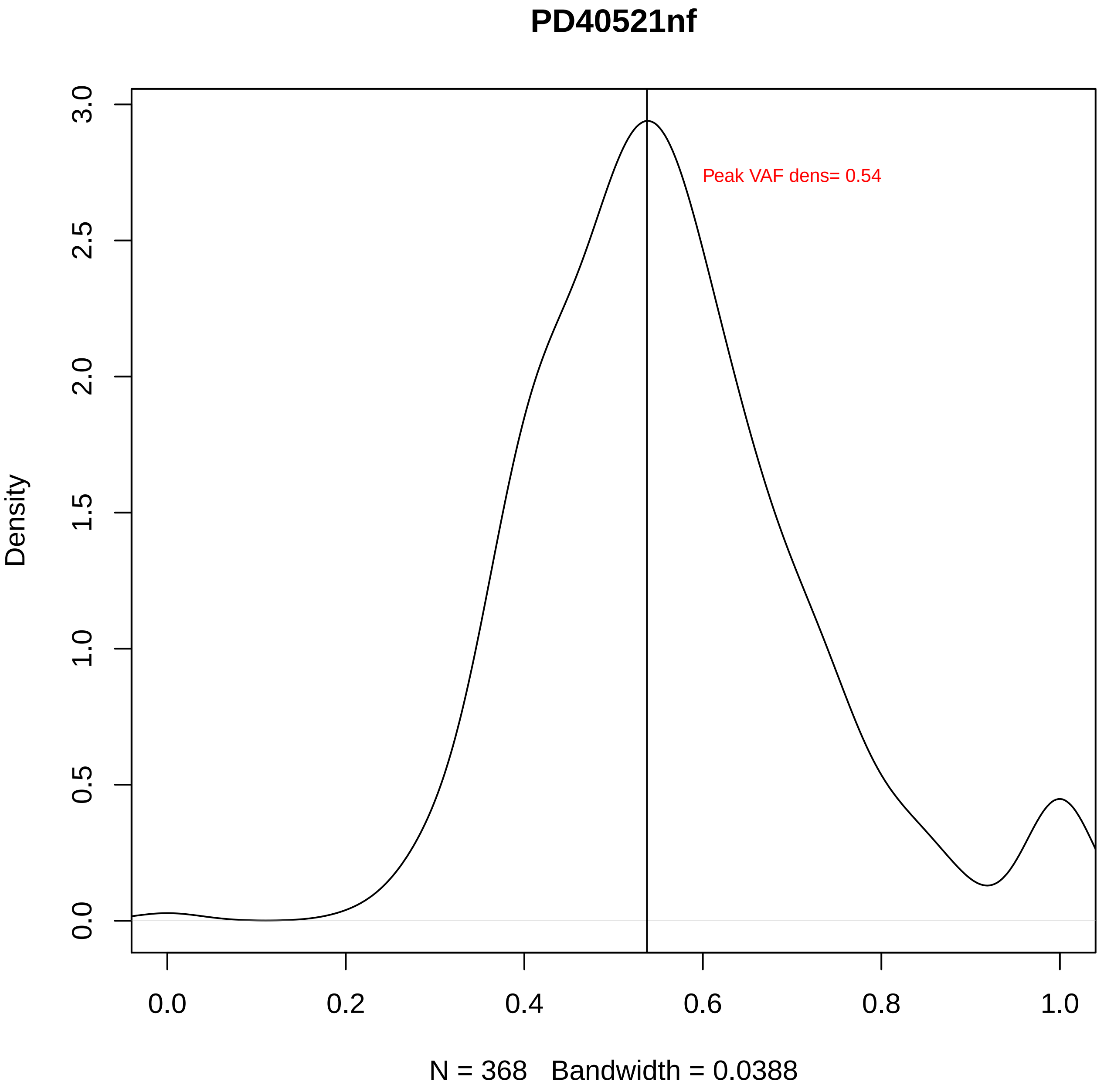
PD40521nf
Peak VAF dens= 0.54
Density
0.0
0.5
1.0
1.5
2.0
2.5
3.0
0.0
0.2
0.4
0.6
0.8
1.0
N = 368   Bandwidth = 0.0388

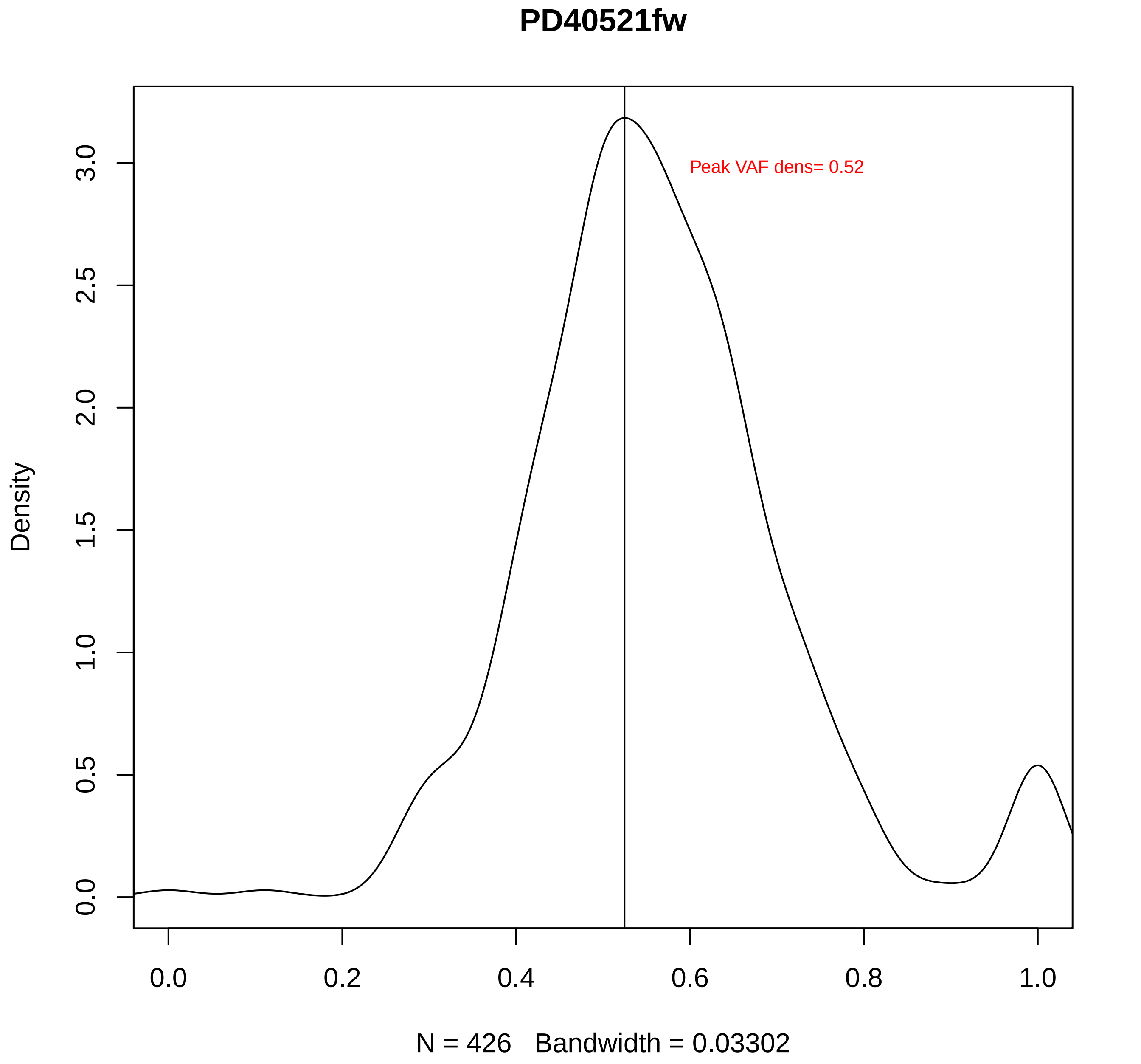

PD40521fw
Peak VAF dens= 0.52
Density
0.0
0.5
1.0
1.5
2.0
2.5
3.0
0.0
0.2
0.4
0.6
0.8
1.0
N = 426   Bandwidth = 0.03302

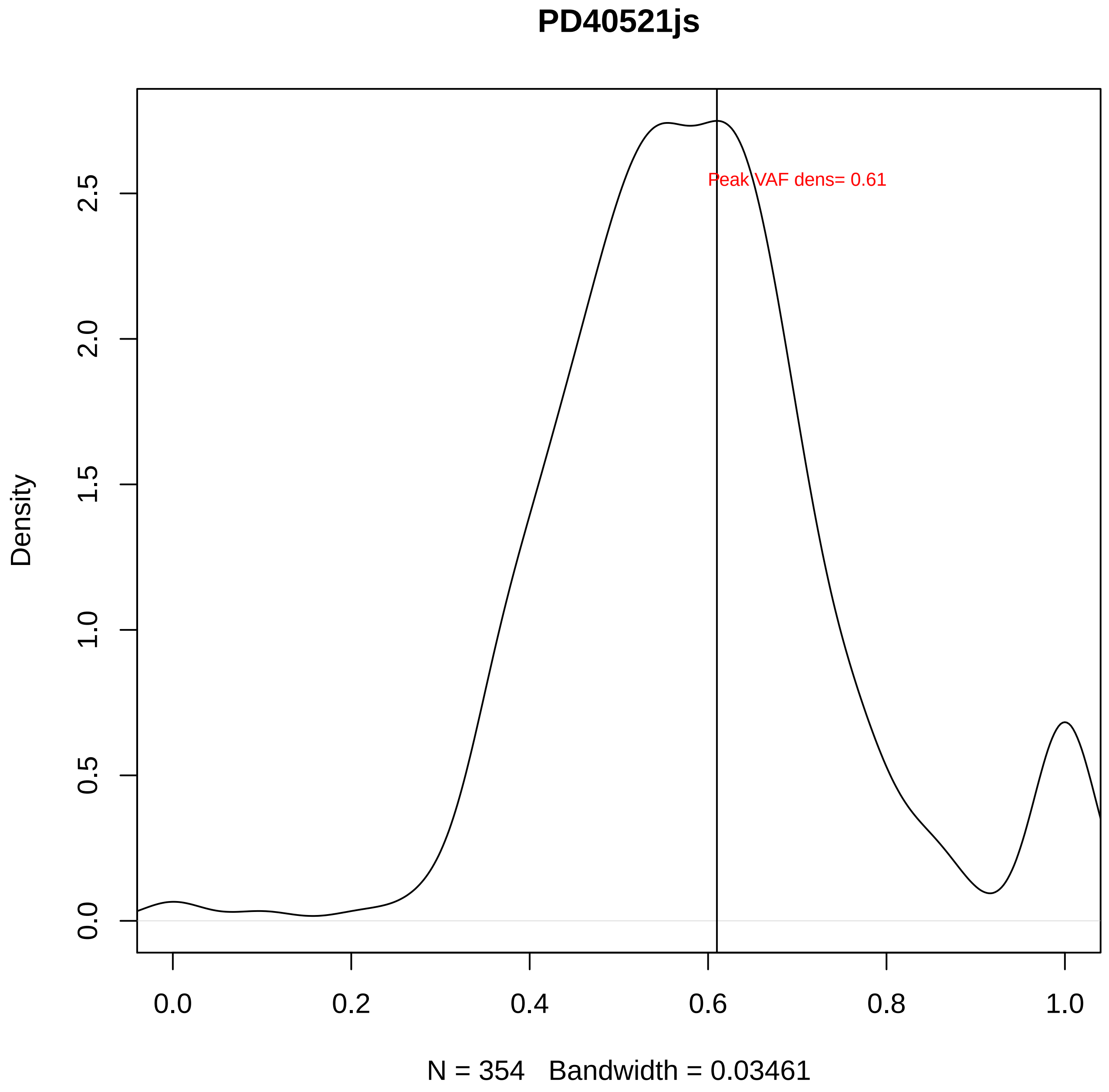
PD40521js
Peak VAF dens= 0.61
Density
0.0
0.5
1.0
1.5
2.0
2.5
0.0
0.2
0.4
0.6
0.8
1.0
N = 354   Bandwidth = 0.03461

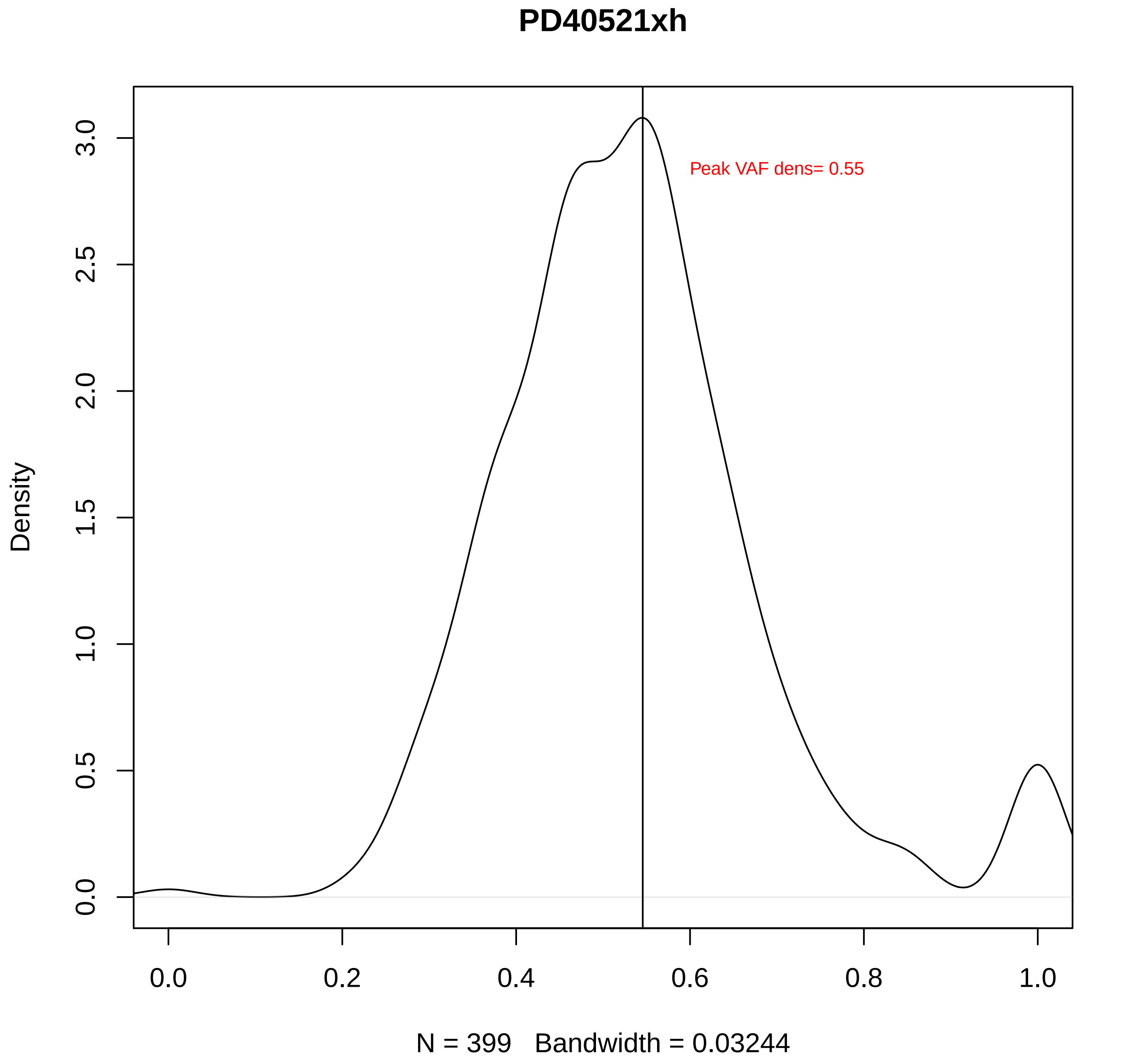
PD40521xh
Peak VAF dens= 0.55
Density
0.0
0.5
1.0
1.5
2.0
2.5
3.0
0.0
0.2
0.4
0.6
0.8
1.0
N = 399 Bandwidth = 0.03244

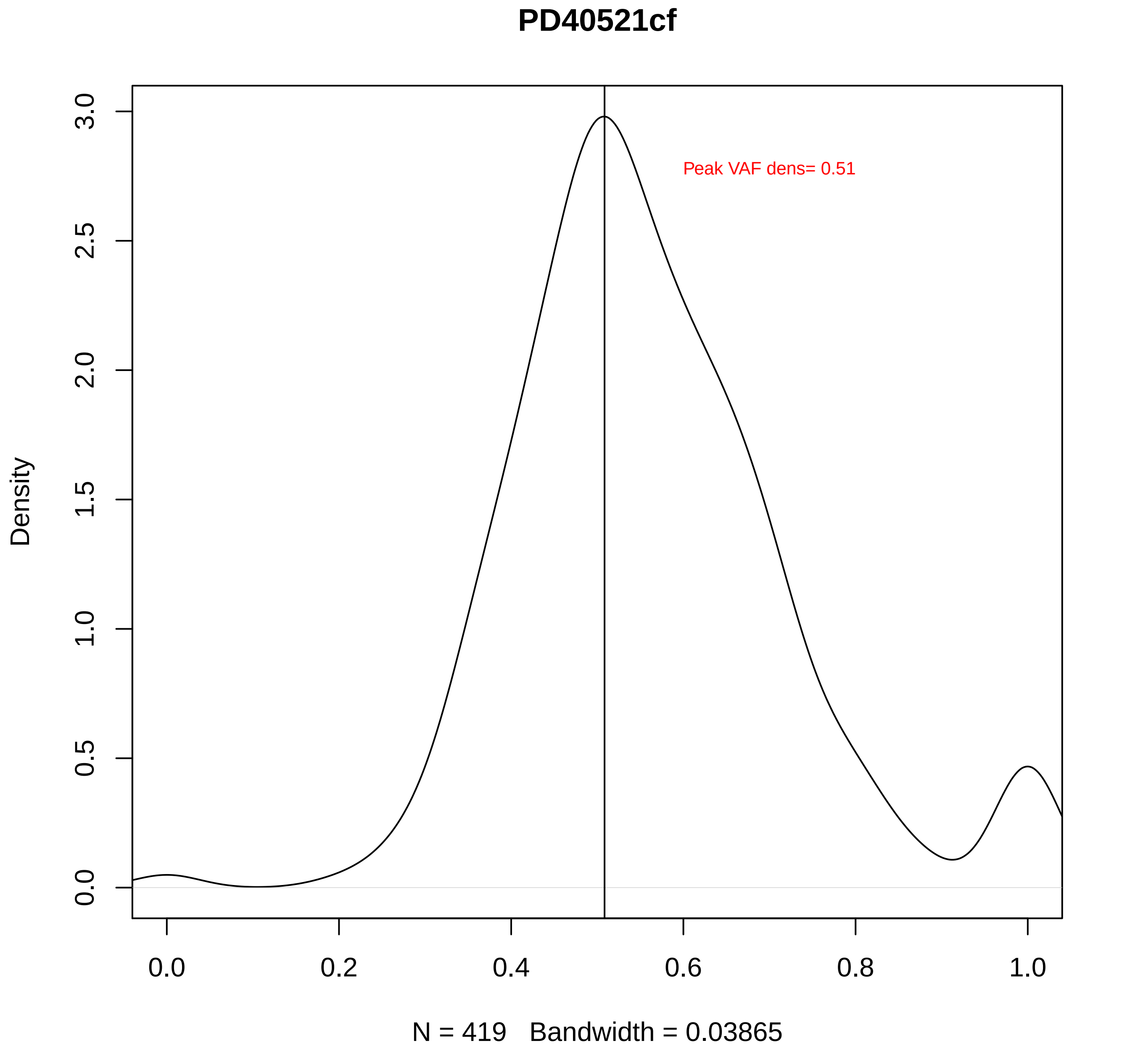
PD40521cf
Peak VAF dens= 0.51
Density
0.0
0.5
1.0
1.5
2.0
2.5
3.0
0.0
0.2
0.4
0.6
0.8
1.0
N = 419 Bandwidth = 0.03865

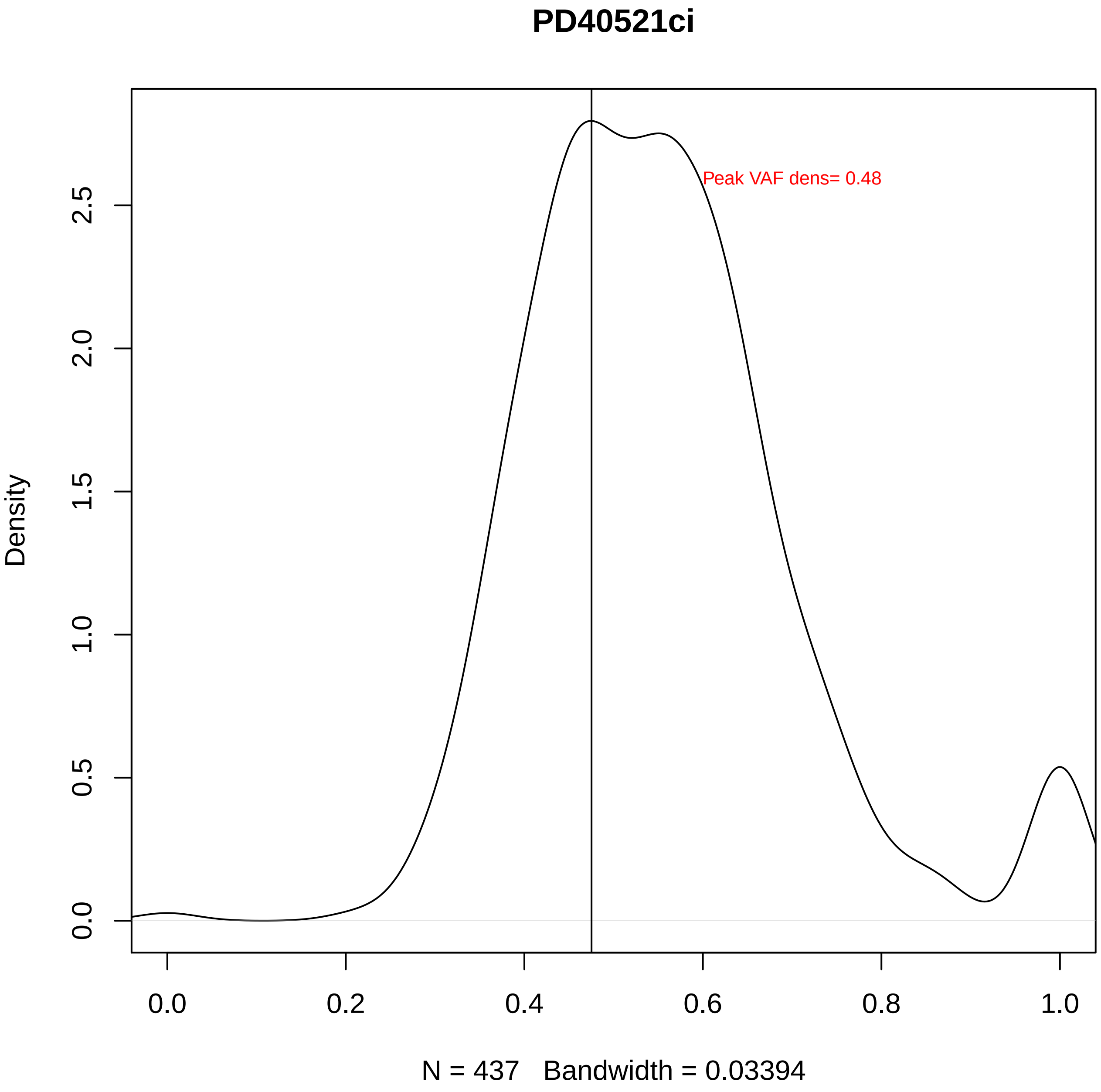
PD40521ci
Peak VAF dens= 0.48
Density
0.0
0.5
1.0
1.5
2.0
2.5
0.0
0.2
0.4
0.6
0.8
1.0
N = 437   Bandwidth = 0.03394

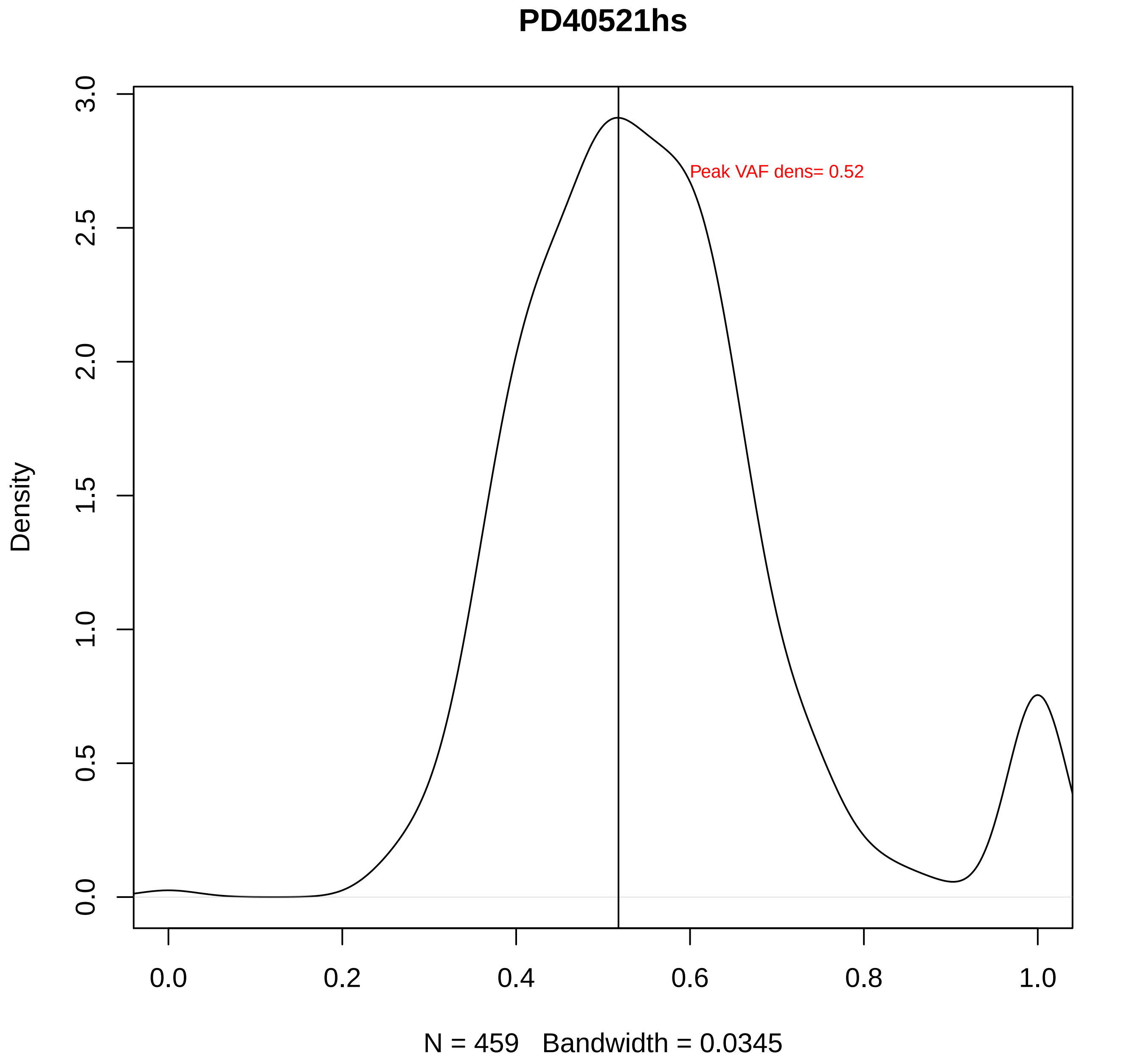
PD40521hs
Peak VAF dens= 0.52
Density
0.0
0.5
1.0
1.5
2.0
2.5
3.0
0.0
0.2
0.4
0.6
0.8
1.0
N = 459   Bandwidth = 0.0345

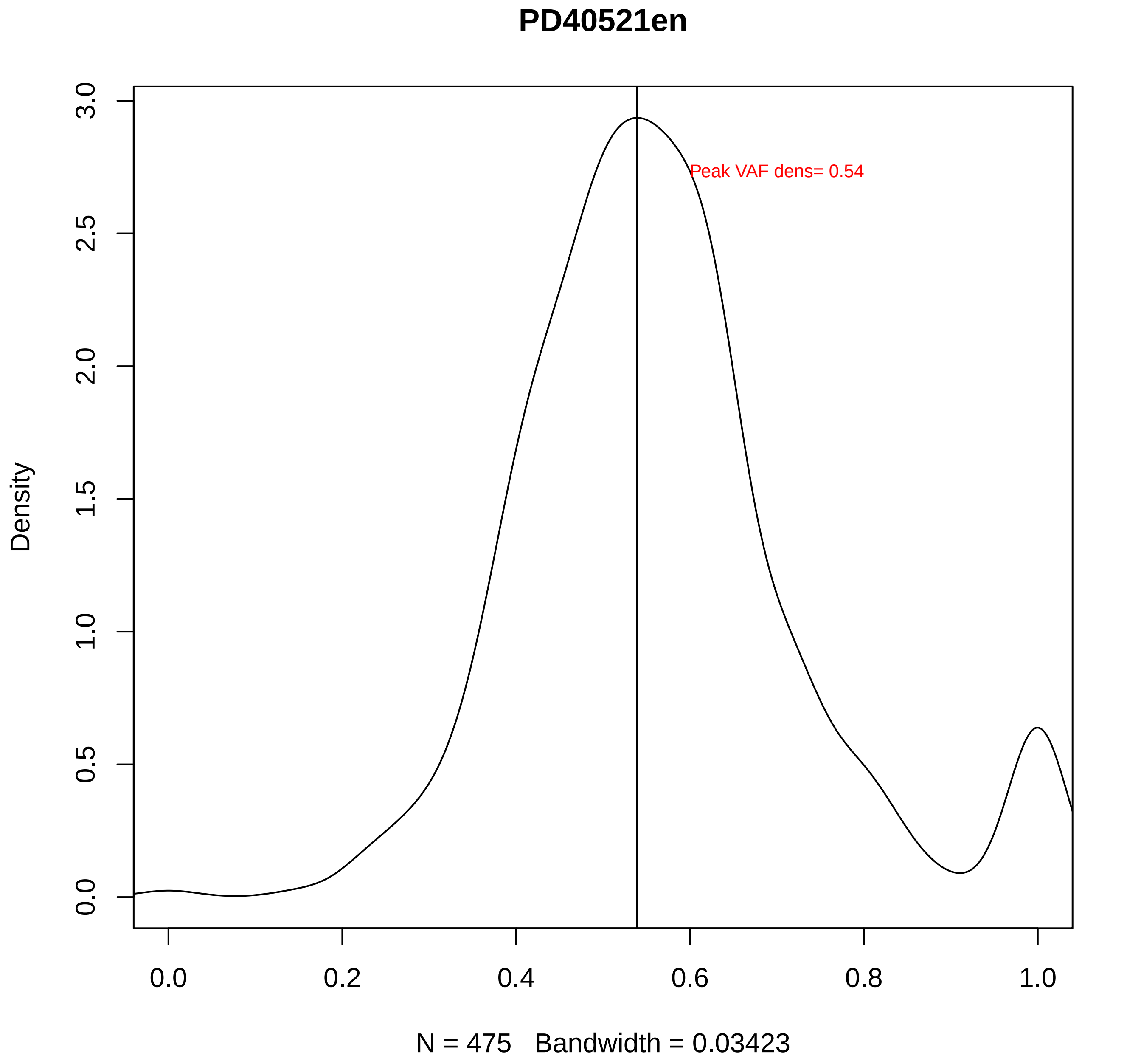
PD40521en
Peak VAF dens= 0.54
Density
0.0
0.5
1.0
1.5
2.0
2.5
3.0
0.0
0.2
0.4
0.6
0.8
1.0
N = 475   Bandwidth = 0.03423

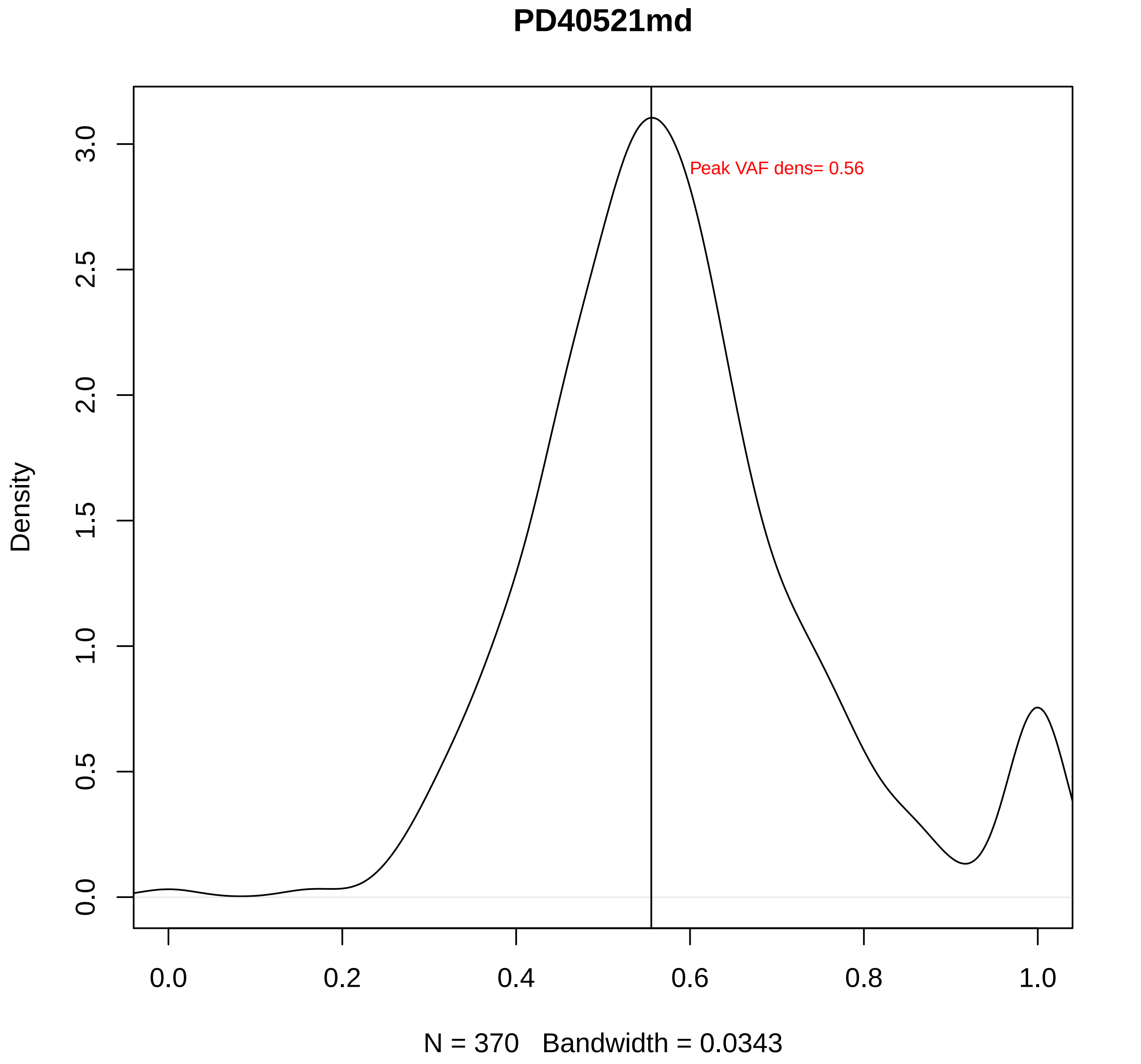

PD40521md
Peak VAF dens= 0.56
Density
0.0
0.5
1.0
1.5
2.0
2.5
3.0
0.0
0.2
0.4
0.6
0.8
1.0
N = 370 Bandwidth = 0.0343

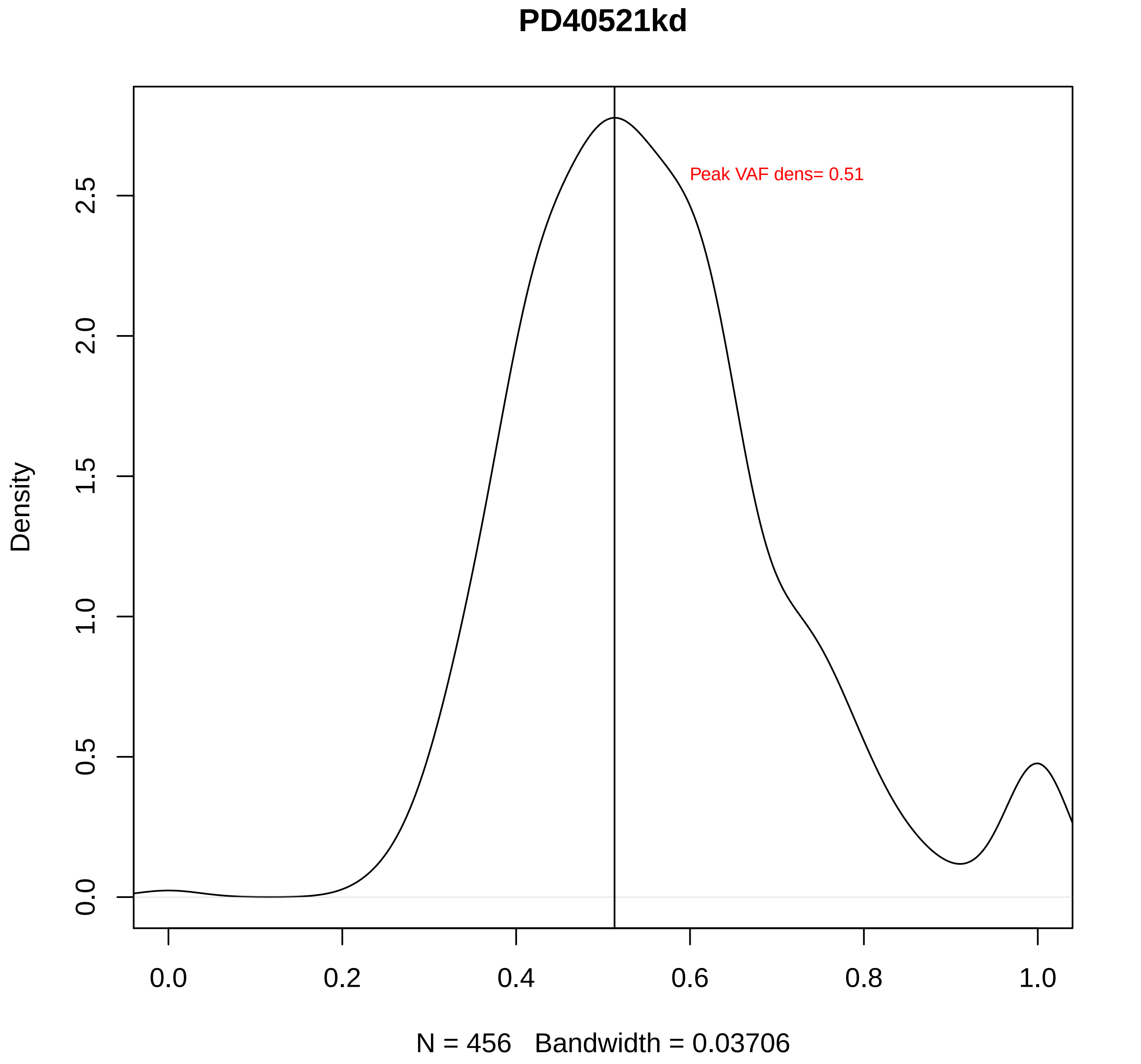
PD40521kd
Peak VAF dens= 0.51
Density
0.0
0.5
1.0
1.5
2.0
2.5
0.0
0.2
0.4
0.6
0.8
1.0
N = 456   Bandwidth = 0.03706

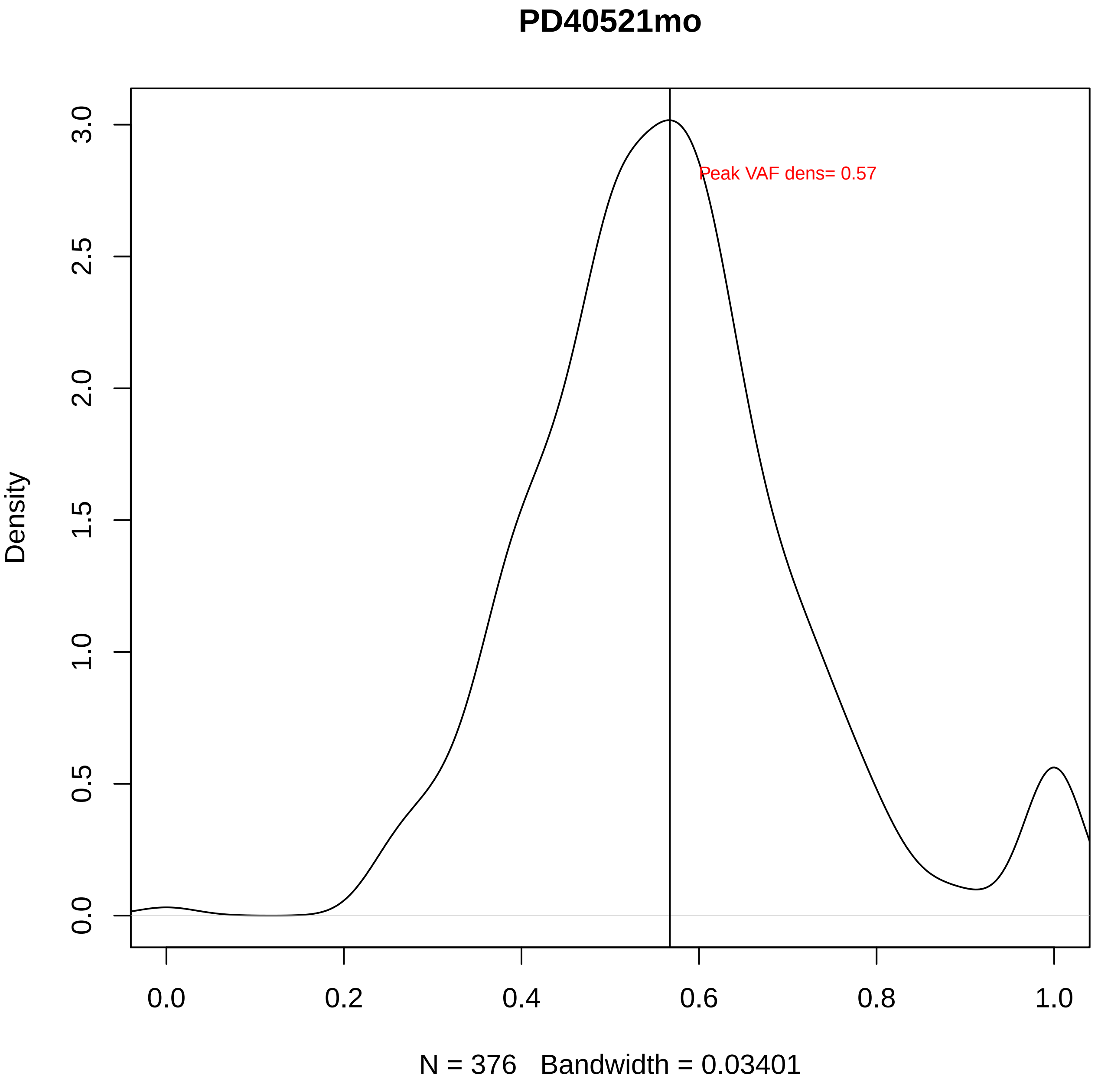

PD40521mo
Density
3.0
2.5
2.0
1.5
1.0
0.5
0.0
Peak VAF dens= 0.57
0.0
0.2
0.4
0.6
0.8
1.0
N = 376 Bandwidth = 0.03401

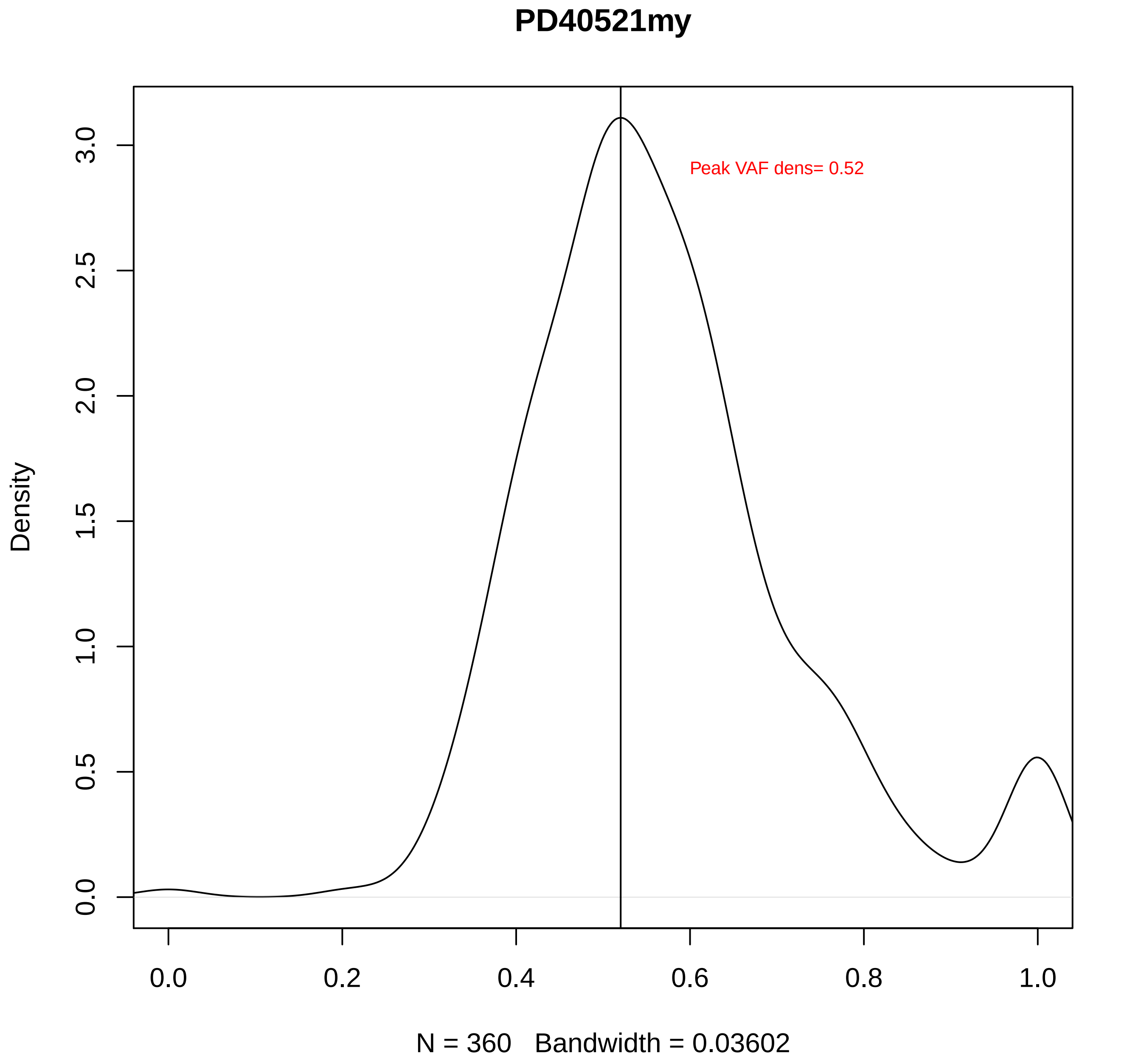

PD40521my
Peak VAF dens= 0.52
Density
0.0
0.5
1.0
1.5
2.0
2.5
3.0
0.0
0.2
0.4
0.6
0.8
1.0
N = 360   Bandwidth = 0.03602

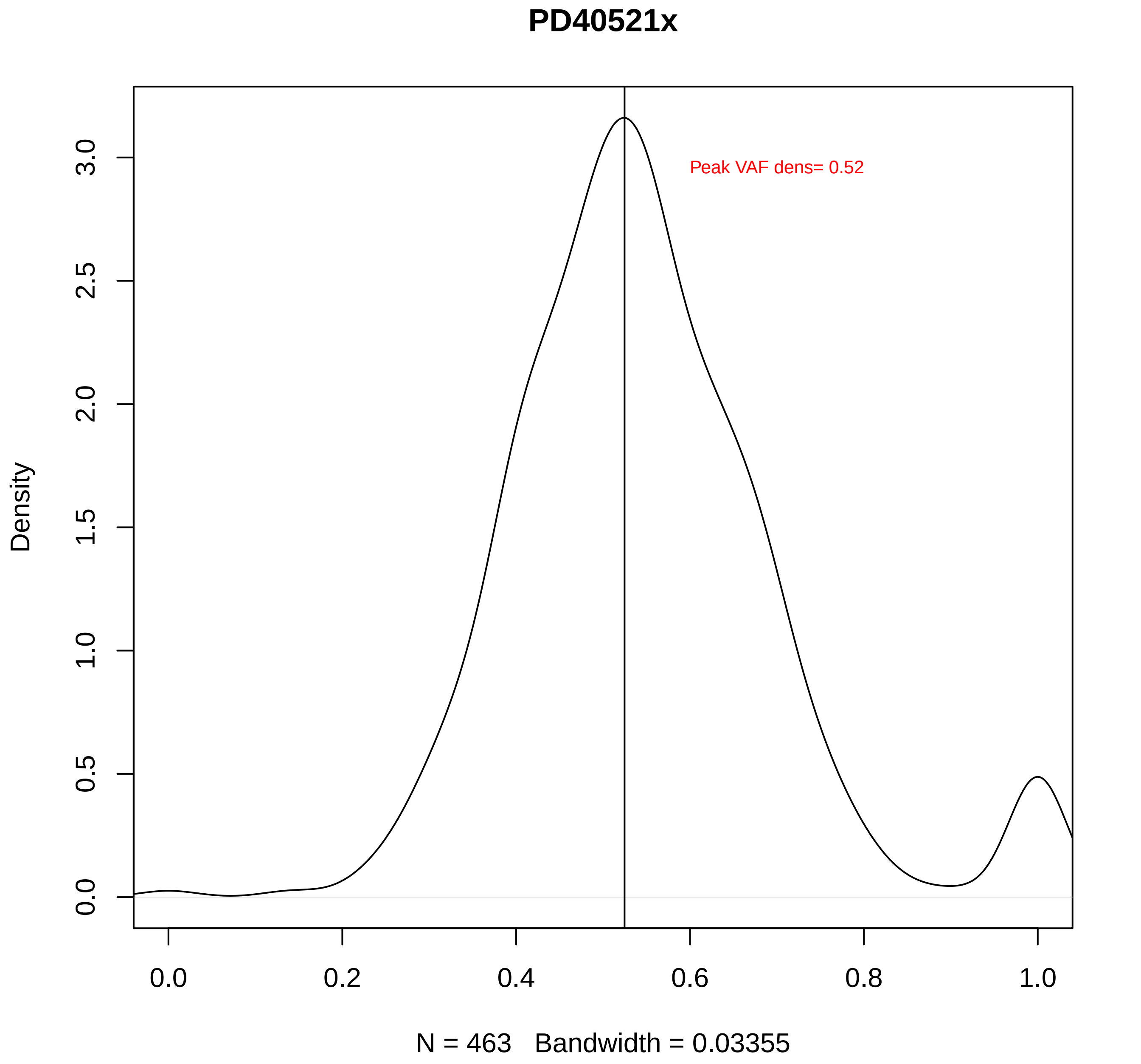
PD40521x
Peak VAF dens= 0.52
Density
0.0
0.5
1.0
1.5
2.0
2.5
3.0
0.0
0.2
0.4
0.6
0.8
1.0
N = 463 Bandwidth = 0.03355

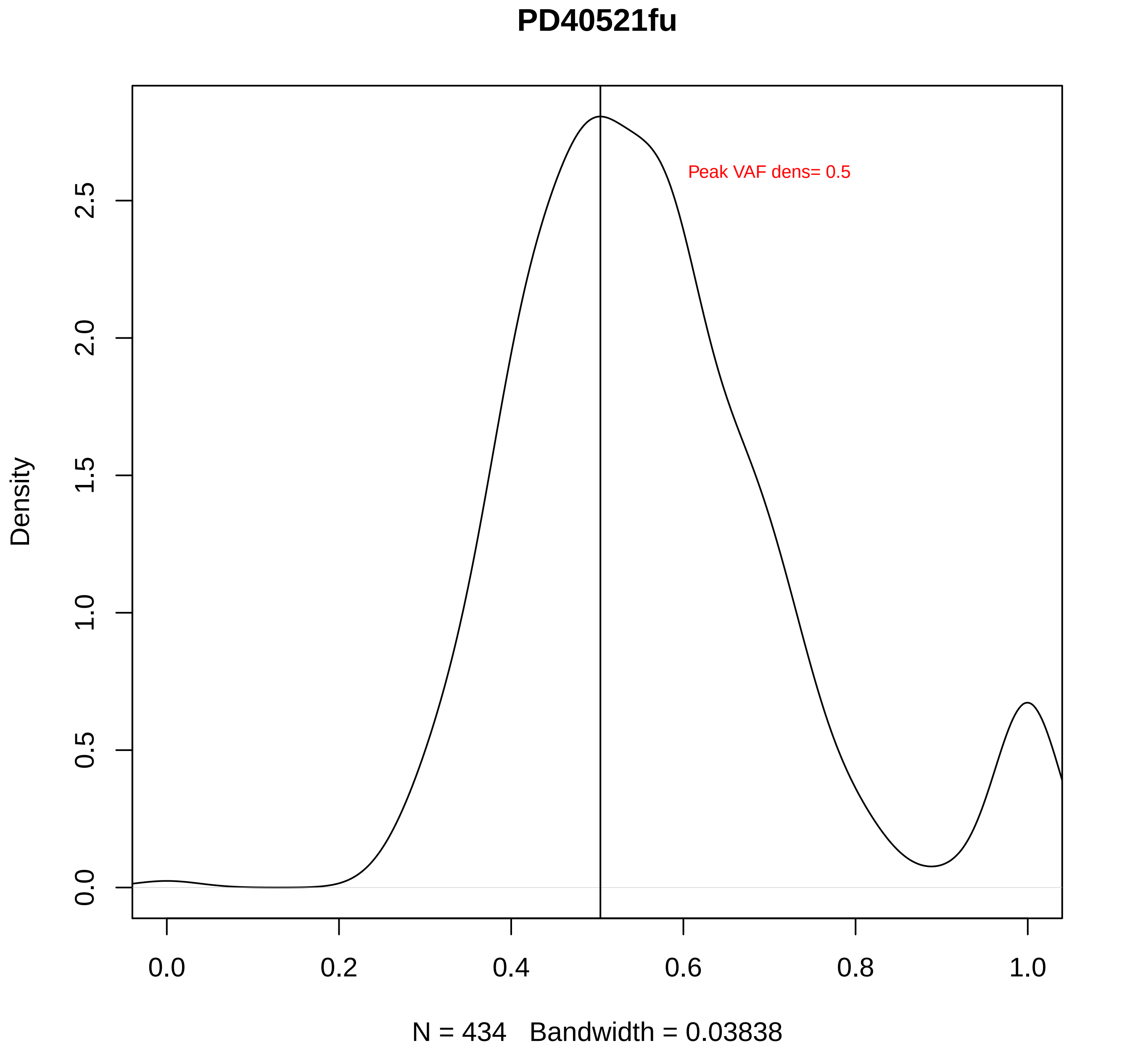
PD40521fu
Peak VAF dens= 0.5
Density
0.0
0.5
1.0
1.5
2.0
2.5
0.0
0.2
0.4
0.6
0.8
1.0
N = 434   Bandwidth = 0.03838

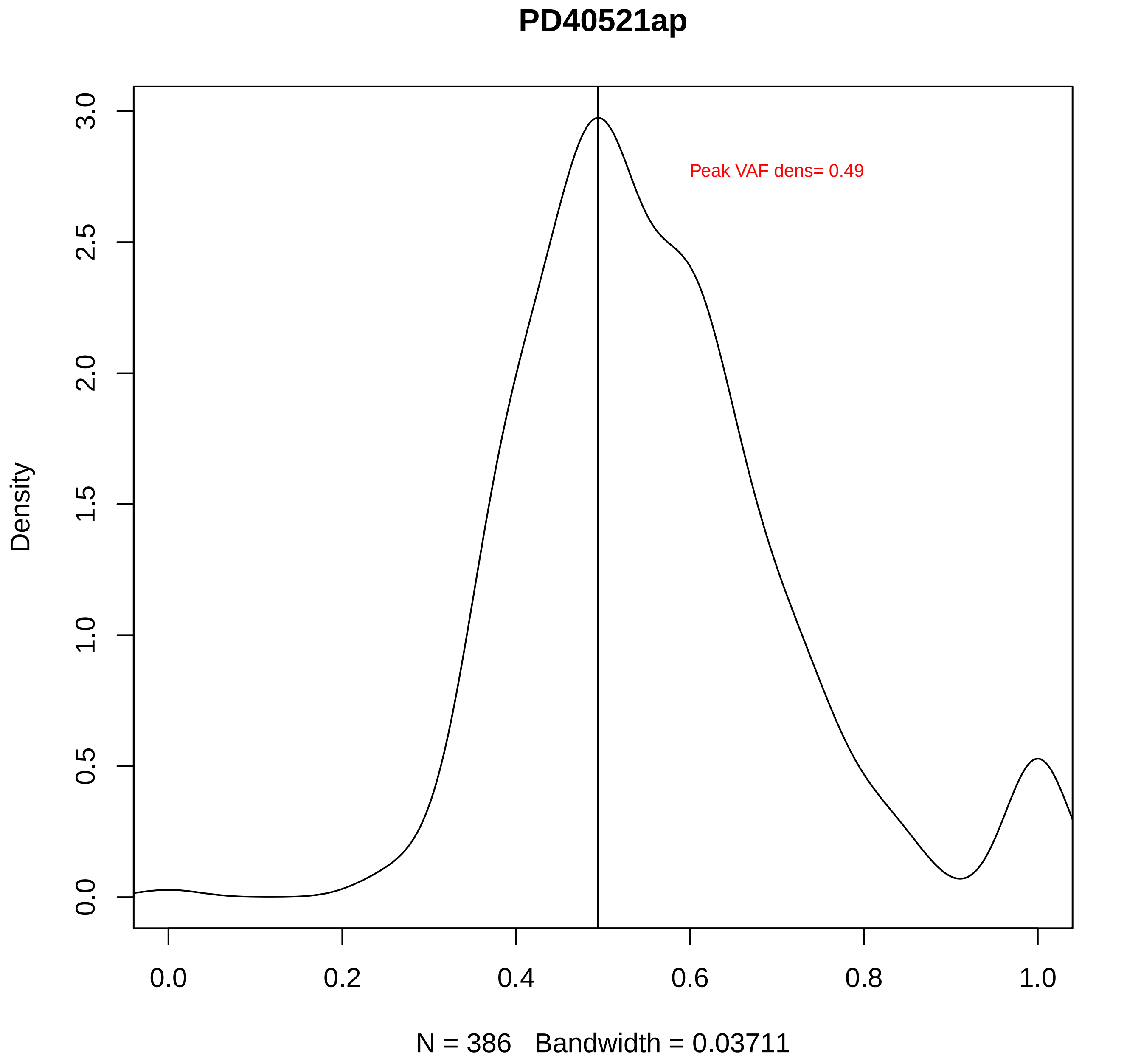
PD40521ap
Peak VAF dens= 0.49
Density
0.0
0.5
1.0
1.5
2.0
2.5
3.0
0.0
0.2
0.4
0.6
0.8
1.0
N = 386   Bandwidth = 0.03711

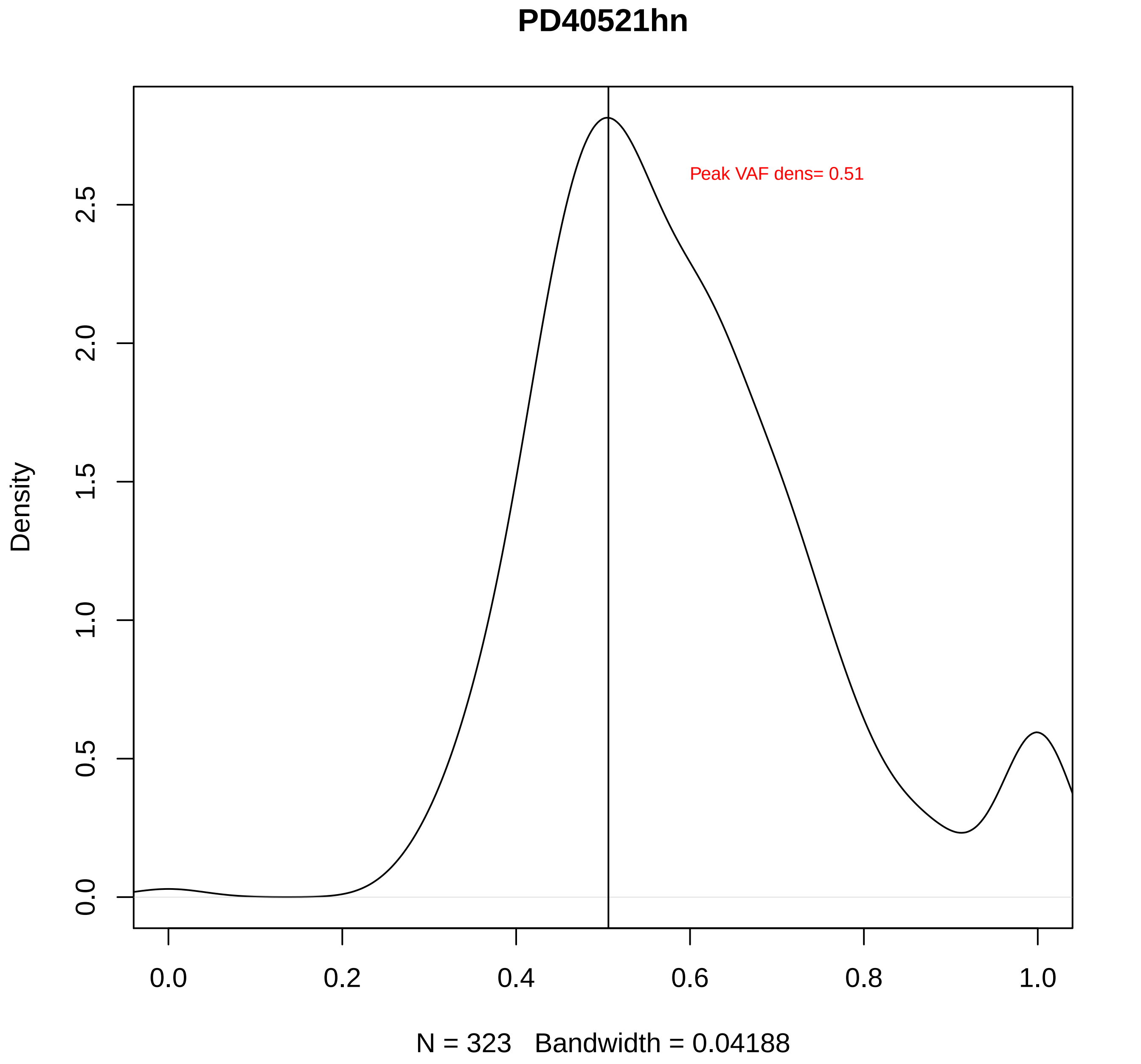
PD40521hn
Peak VAF dens= 0.51
Density
0.0
0.5
1.0
1.5
2.0
2.5
0.0
0.2
0.4
0.6
0.8
1.0
N = 323   Bandwidth = 0.04188

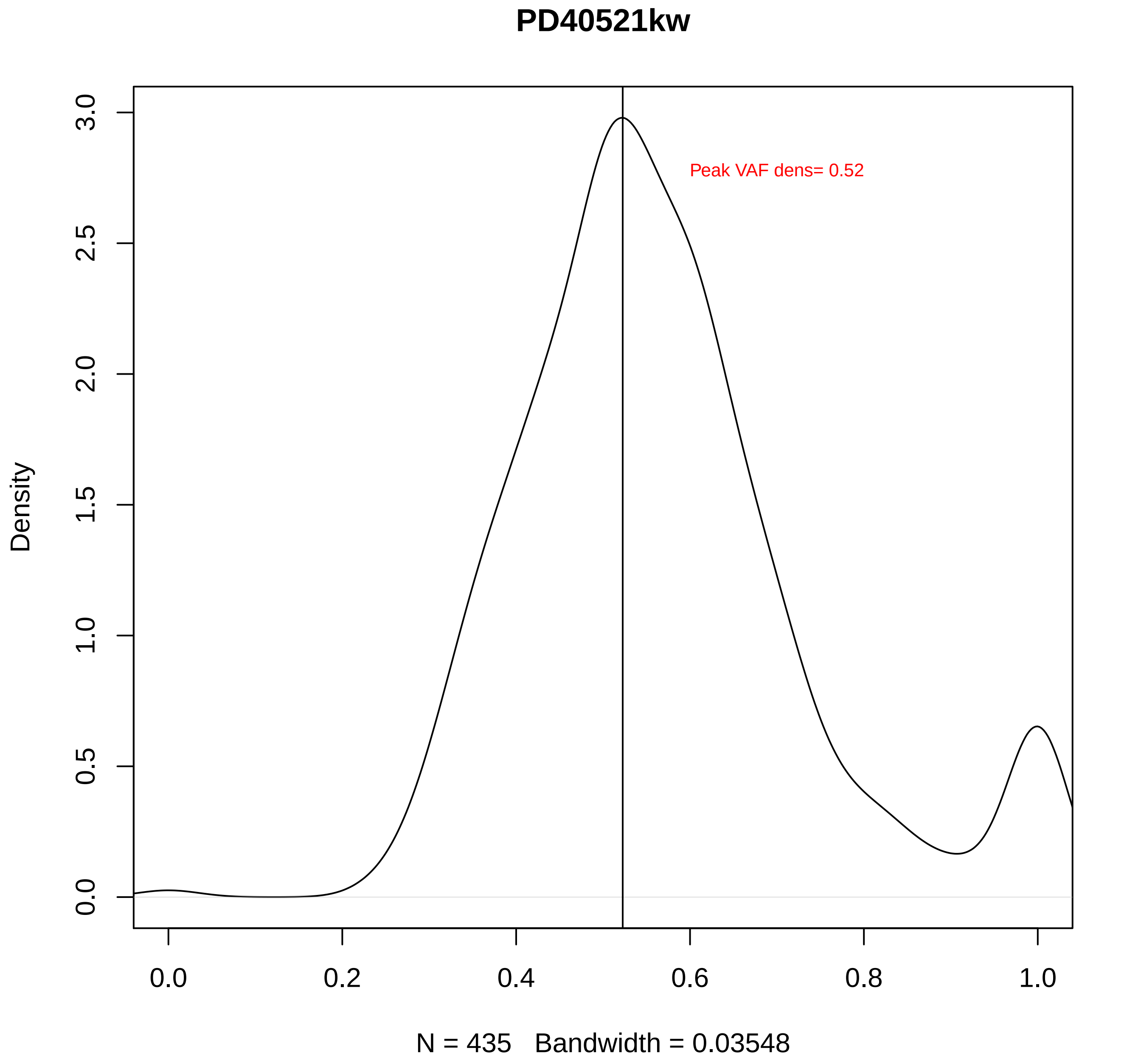
PD40521kw
Peak VAF dens= 0.52
Density
0.0
0.5
1.0
1.5
2.0
2.5
3.0
0.0
0.2
0.4
0.6
0.8
1.0
N = 435   Bandwidth = 0.03548

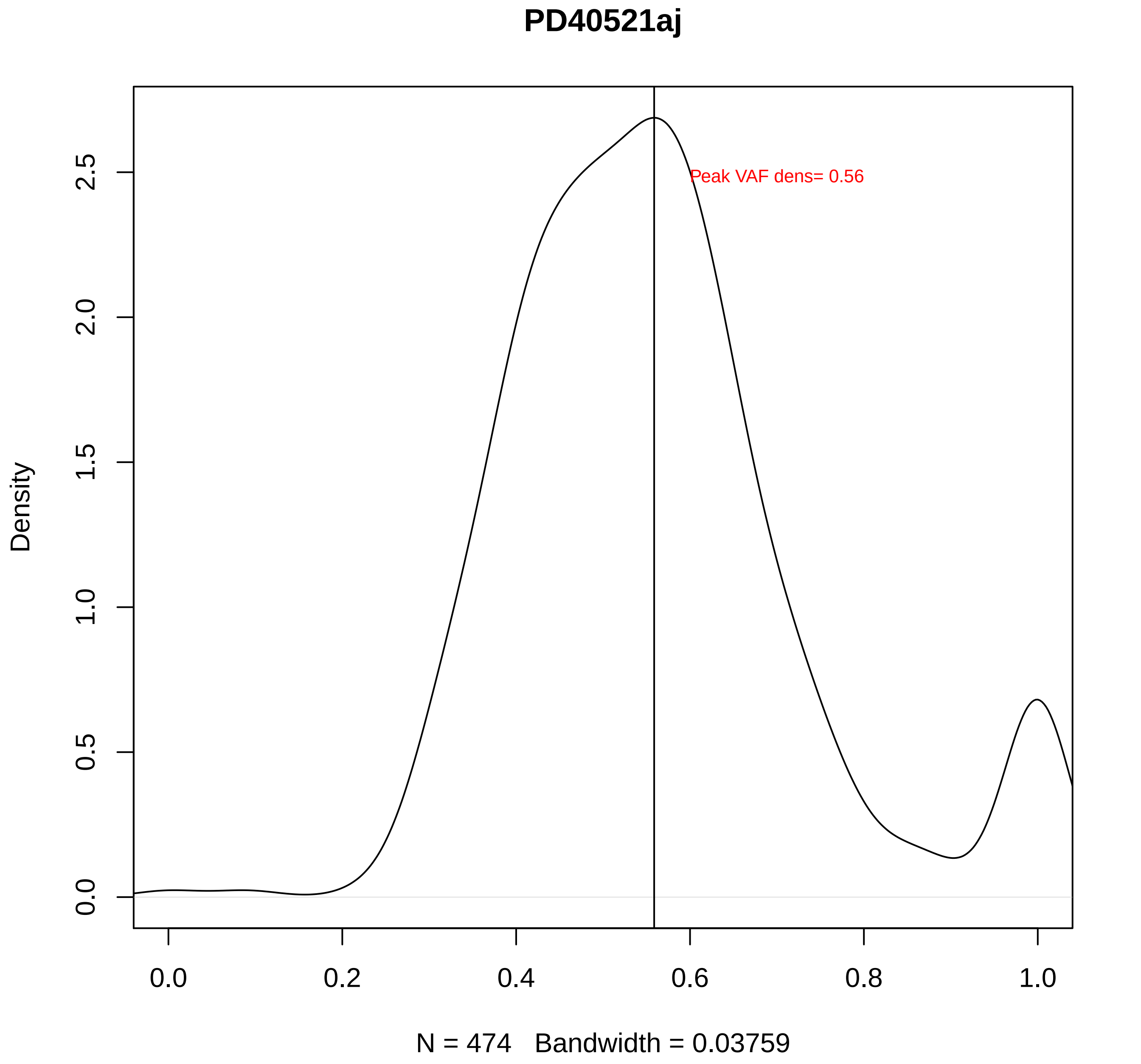

PD40521aj
Peak VAF dens= 0.56
Density
0.0
0.5
1.0
1.5
2.0
2.5
0.0
0.2
0.4
0.6
0.8
1.0
N = 474   Bandwidth = 0.03759

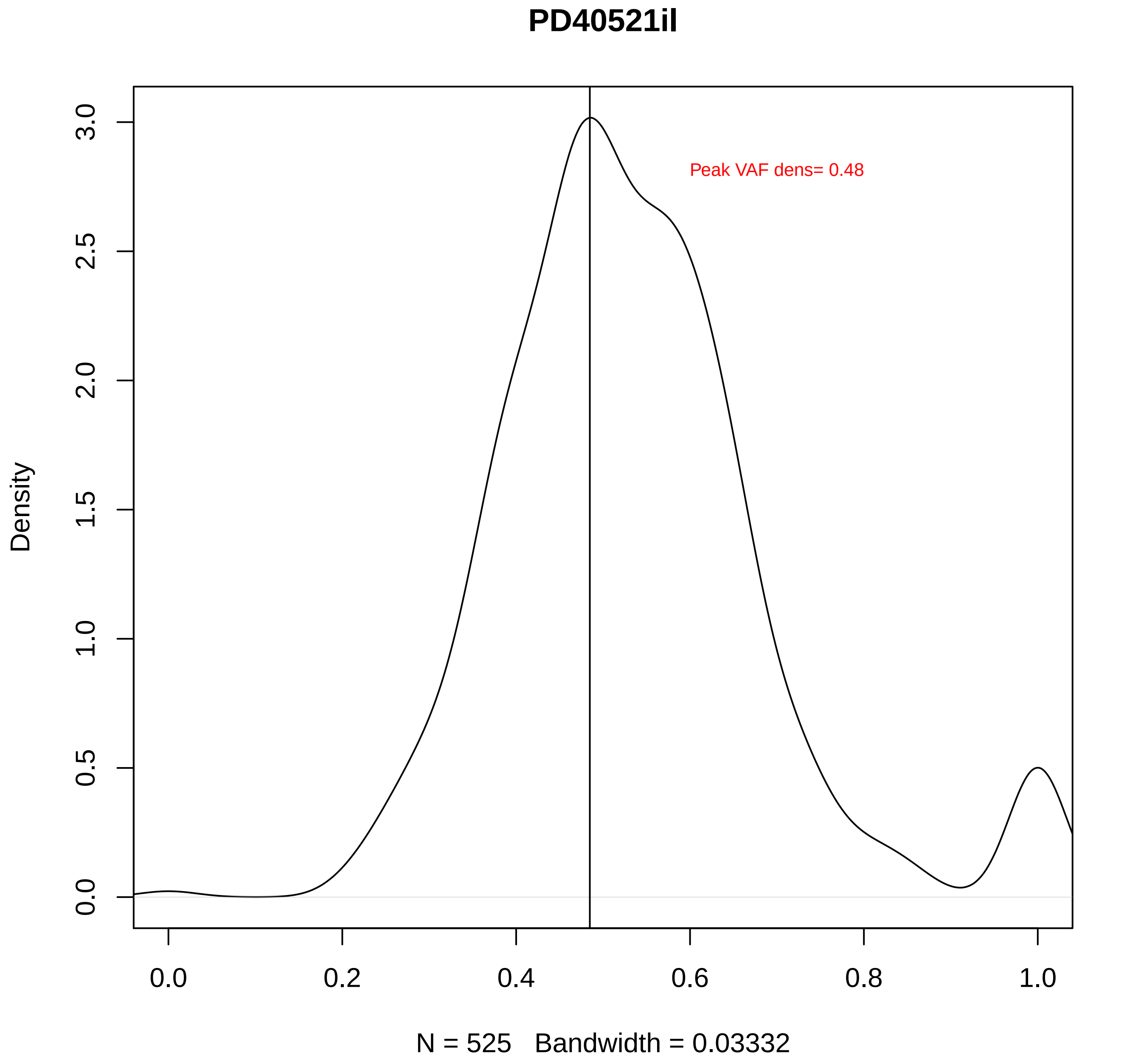
PD40521il
Peak VAF dens= 0.48
Density
0.0
0.5
1.0
1.5
2.0
2.5
3.0
0.0
0.2
0.4
0.6
0.8
1.0
N = 525   Bandwidth = 0.03332

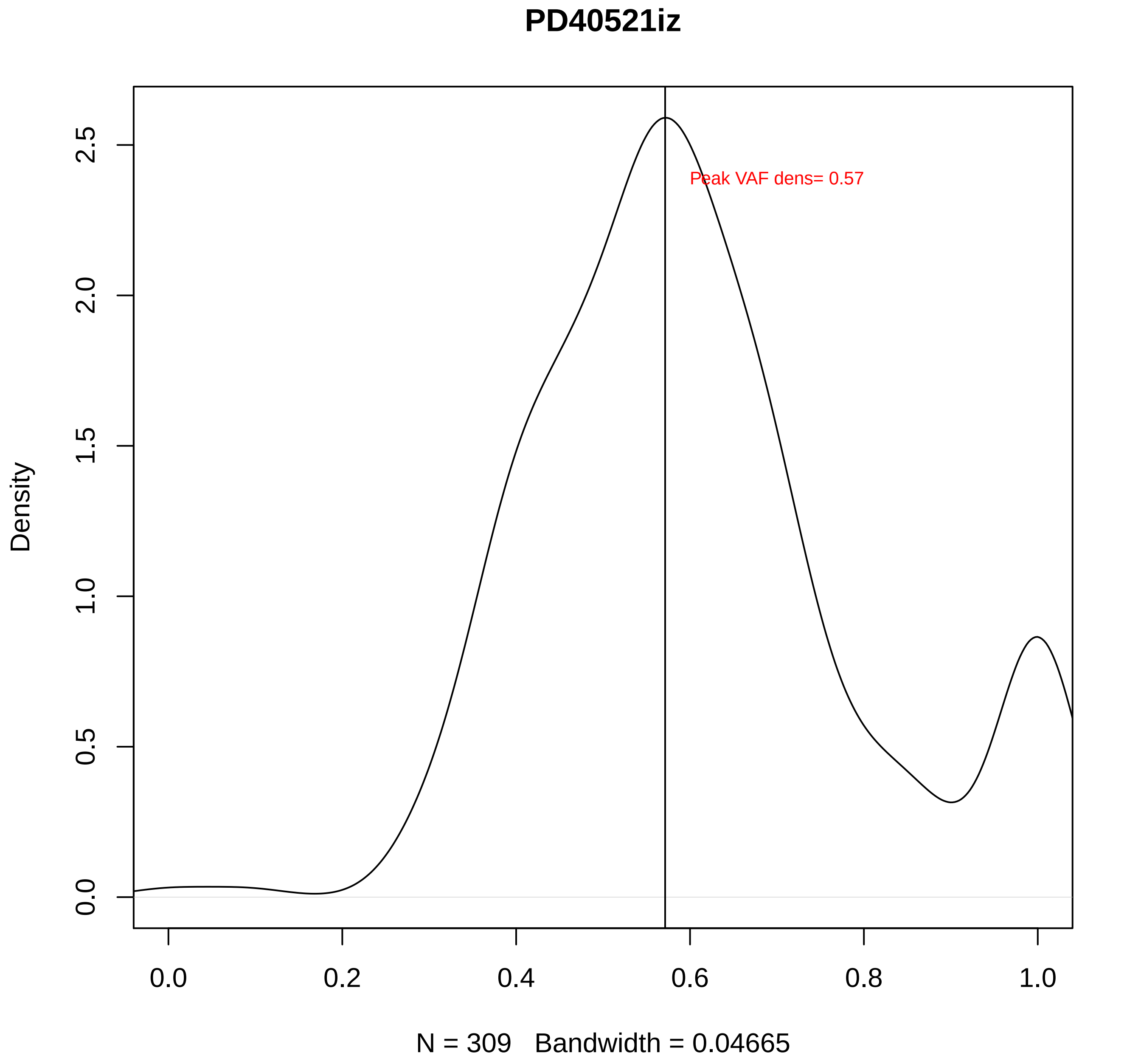

PD40521iz
Peak VAF dens= 0.57
Density
0.0
0.5
1.0
1.5
2.0
2.5
0.0
0.2
0.4
0.6
0.8
1.0
N = 309   Bandwidth = 0.04665

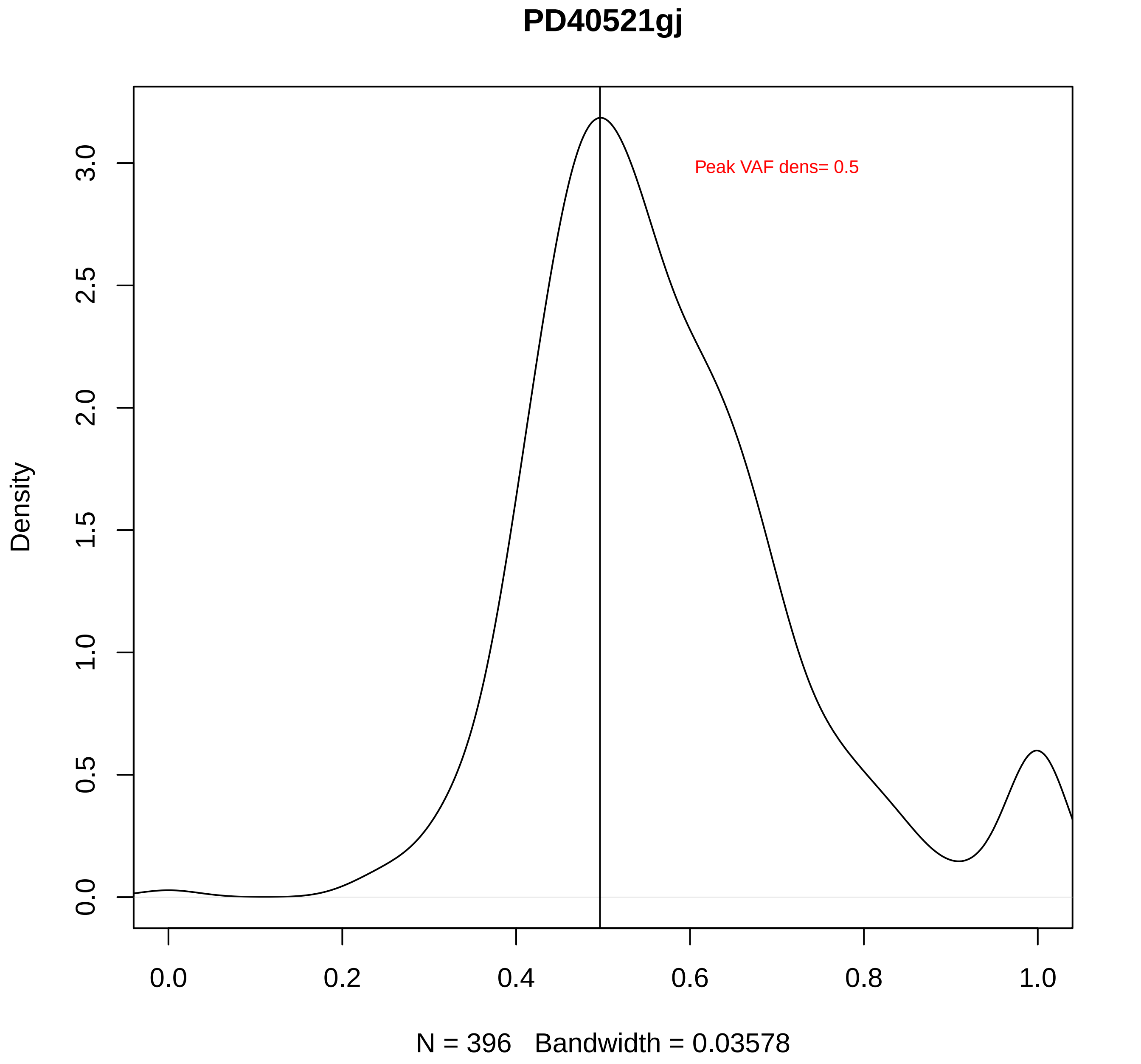

PD40521gj
Peak VAF dens= 0.5
Density
0.0
0.5
1.0
1.5
2.0
2.5
3.0
0.0
0.2
0.4
0.6
0.8
1.0
N = 396   Bandwidth = 0.03578

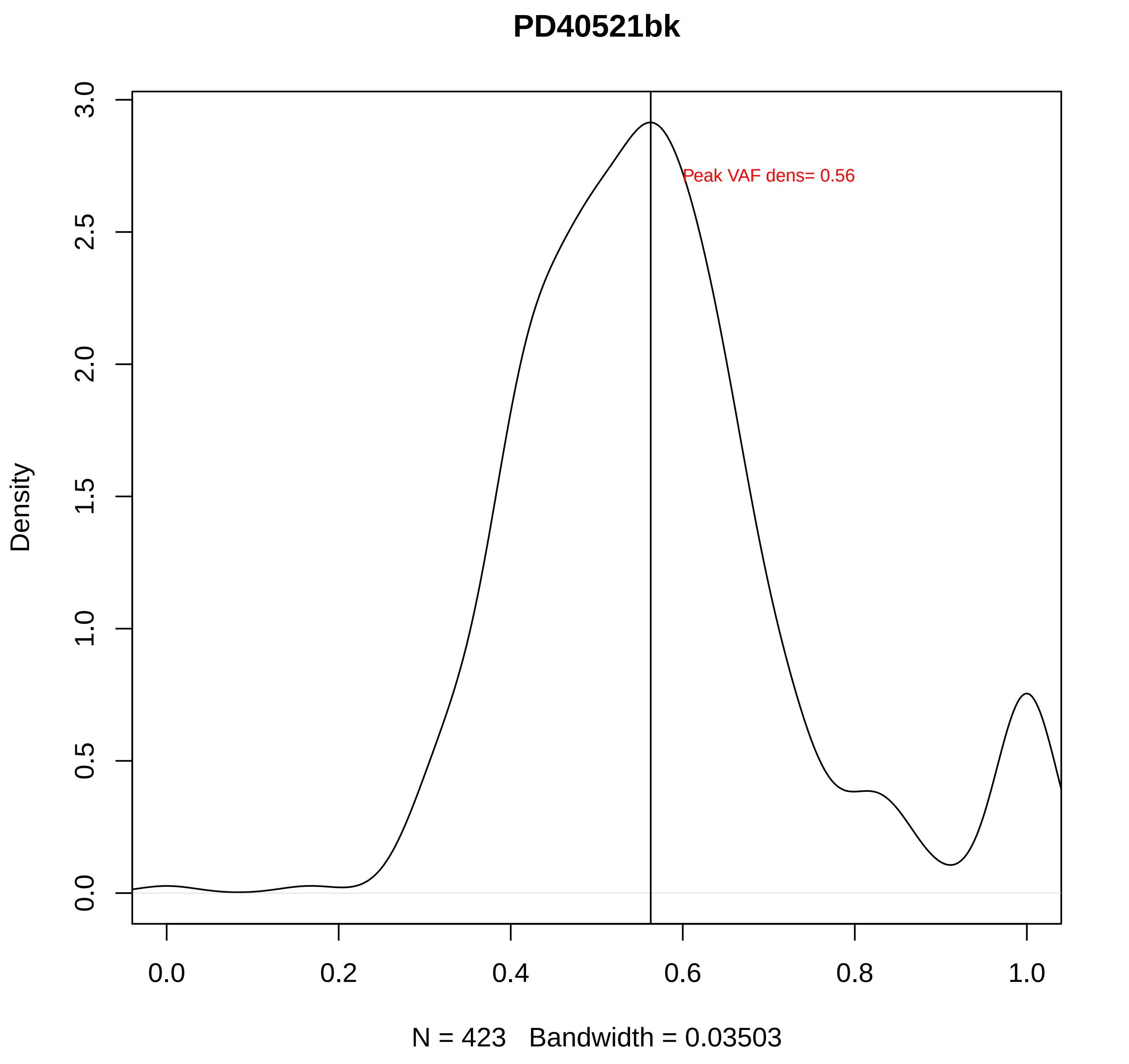
PD40521bk
Peak VAF dens= 0.56
Density
0.0
0.5
1.0
1.5
2.0
2.5
3.0
0.0
0.2
0.4
0.6
0.8
1.0
N = 423   Bandwidth = 0.03503

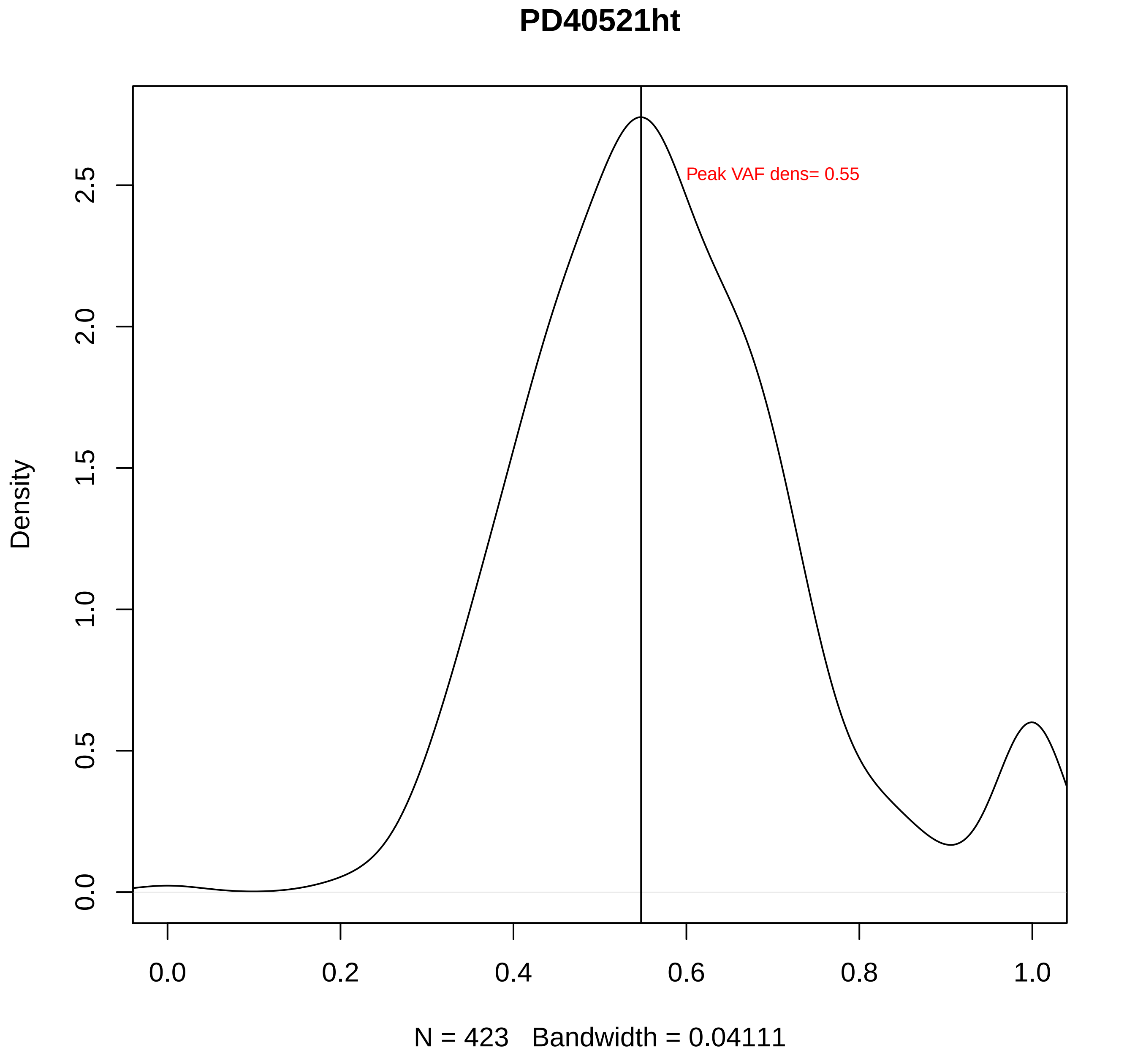
PD40521ht
Peak VAF dens= 0.55
Density
0.0
0.5
1.0
1.5
2.0
2.5
0.0
0.2
0.4
0.6
0.8
1.0
N = 423   Bandwidth = 0.04111

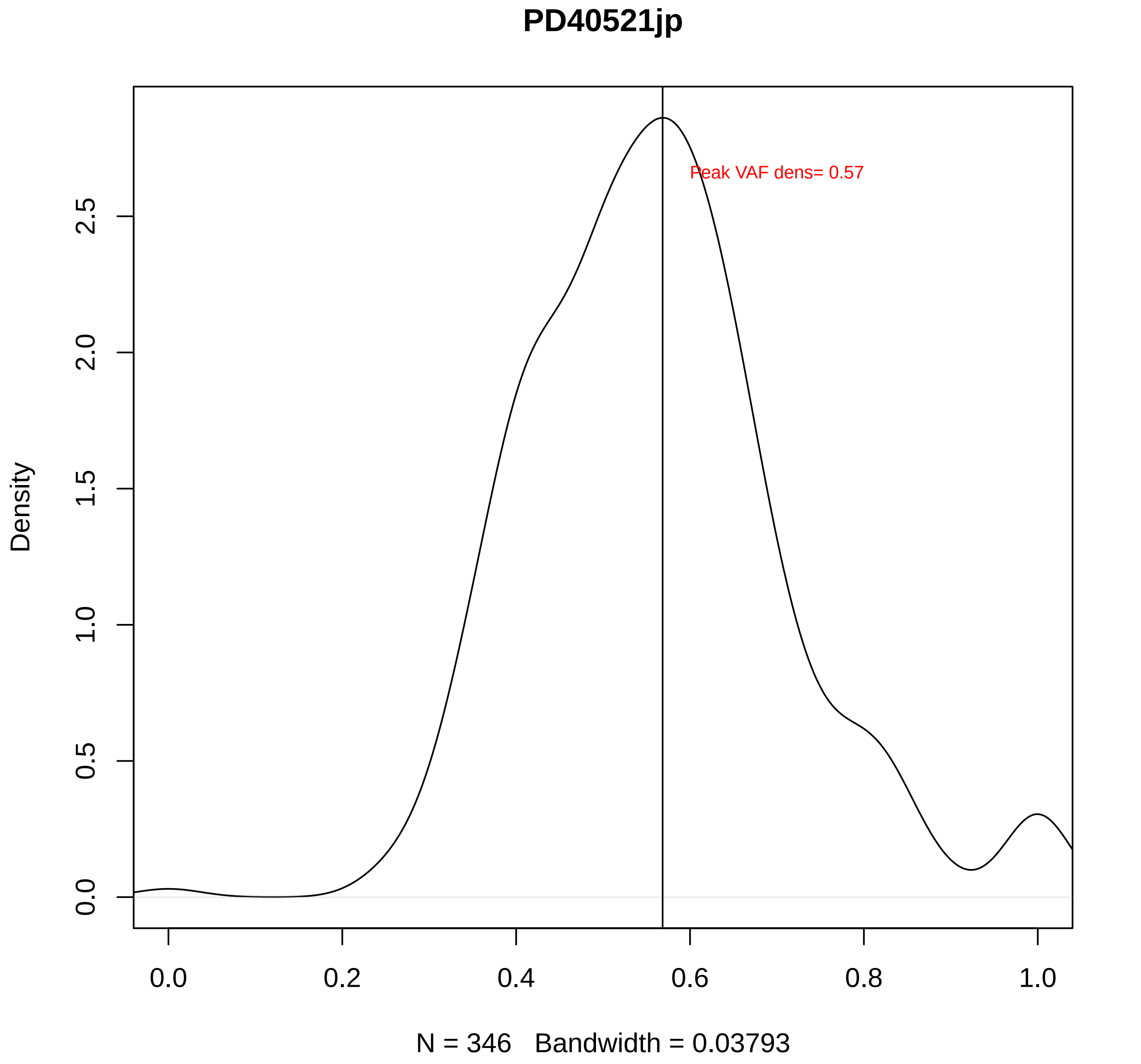

PD40521jp
Peak VAF dens= 0.57
Density
0.0
0.5
1.0
1.5
2.0
2.5
0.0
0.2
0.4
0.6
0.8
1.0
N = 346   Bandwidth = 0.03793

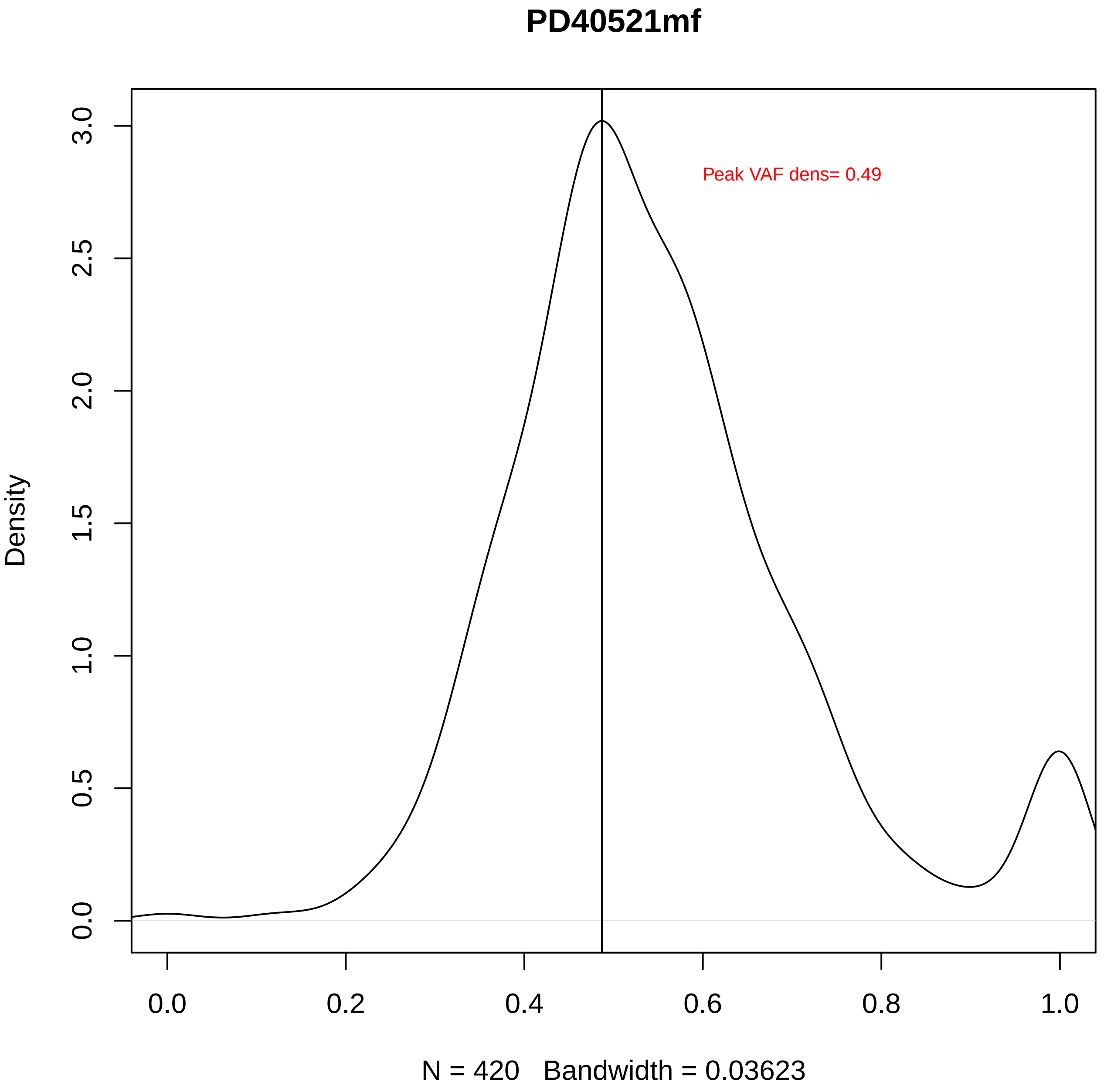

PD40521mf
Peak VAF dens= 0.49
Density
0.0
0.5
1.0
1.5
2.0
2.5
3.0
0.0
0.2
0.4
0.6
0.8
1.0
N = 420 Bandwidth = 0.03623

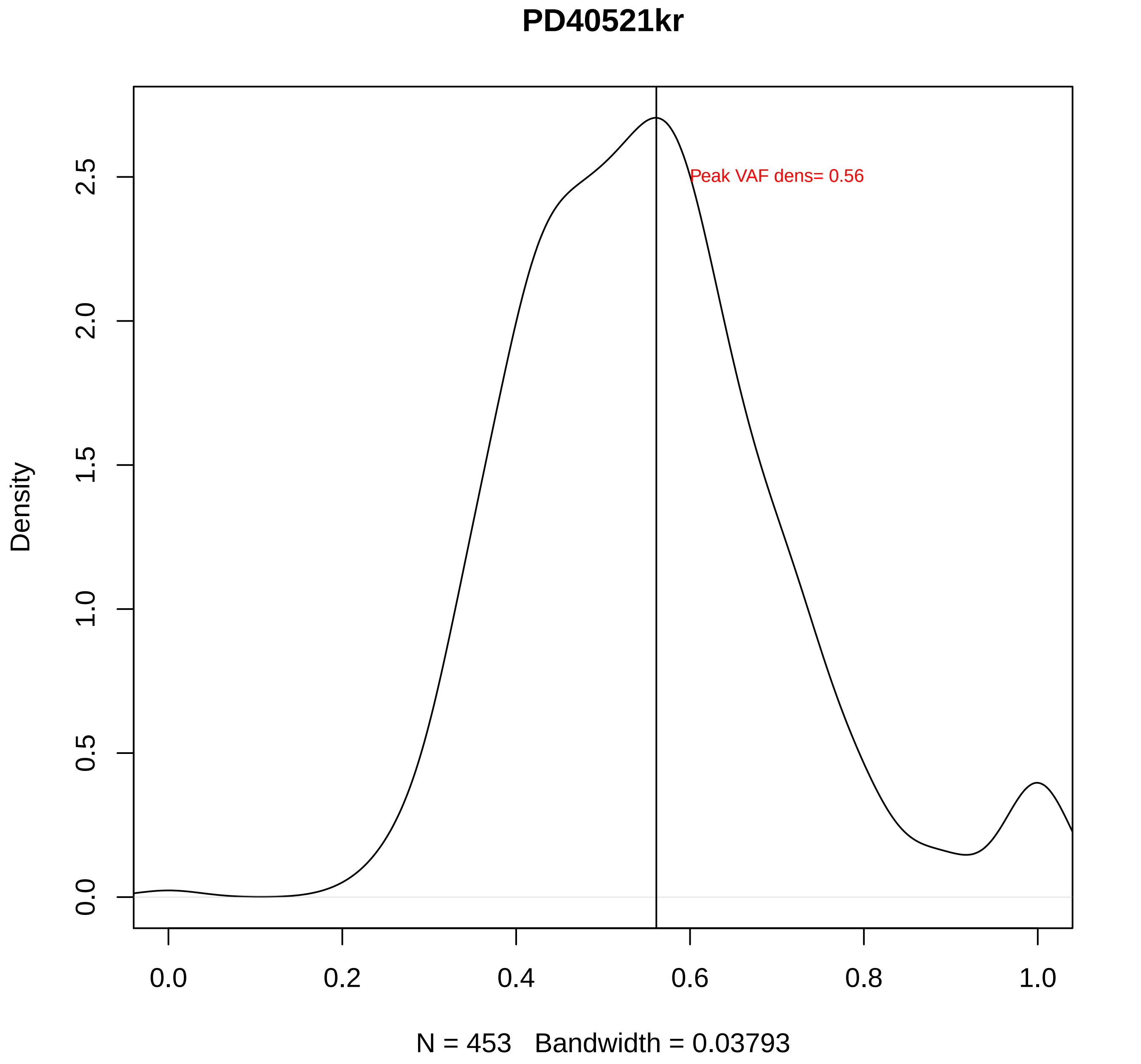

PD40521kr
Peak VAF dens= 0.56
Density
0.0
0.5
1.0
1.5
2.0
2.5
0.0
0.2
0.4
0.6
0.8
1.0
N = 453 Bandwidth = 0.03793

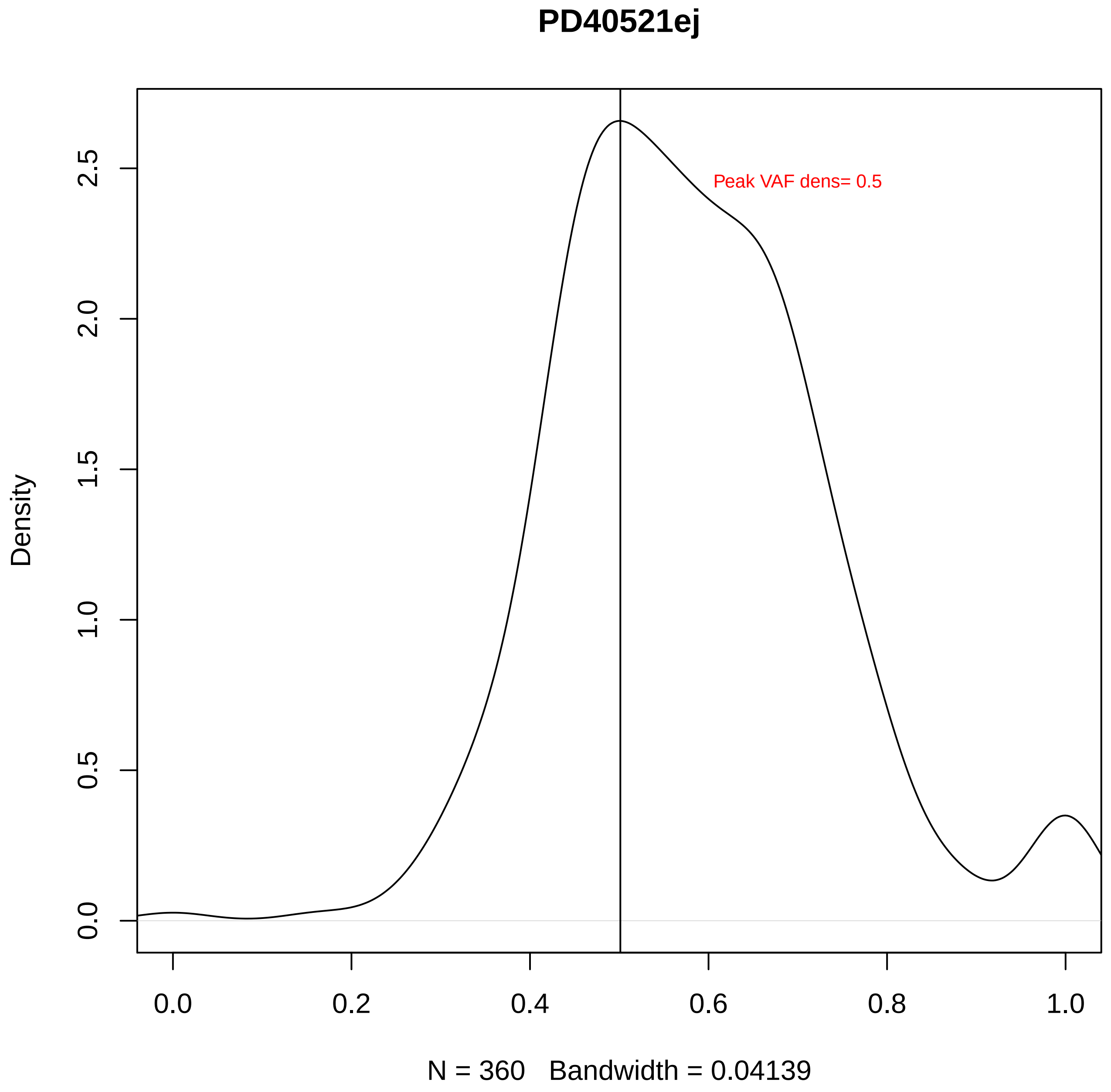

PD40521ej
Peak VAF dens= 0.5
Density
0.0
0.5
1.0
1.5
2.0
2.5
0.0
0.2
0.4
0.6
0.8
1.0
N = 360   Bandwidth = 0.04139

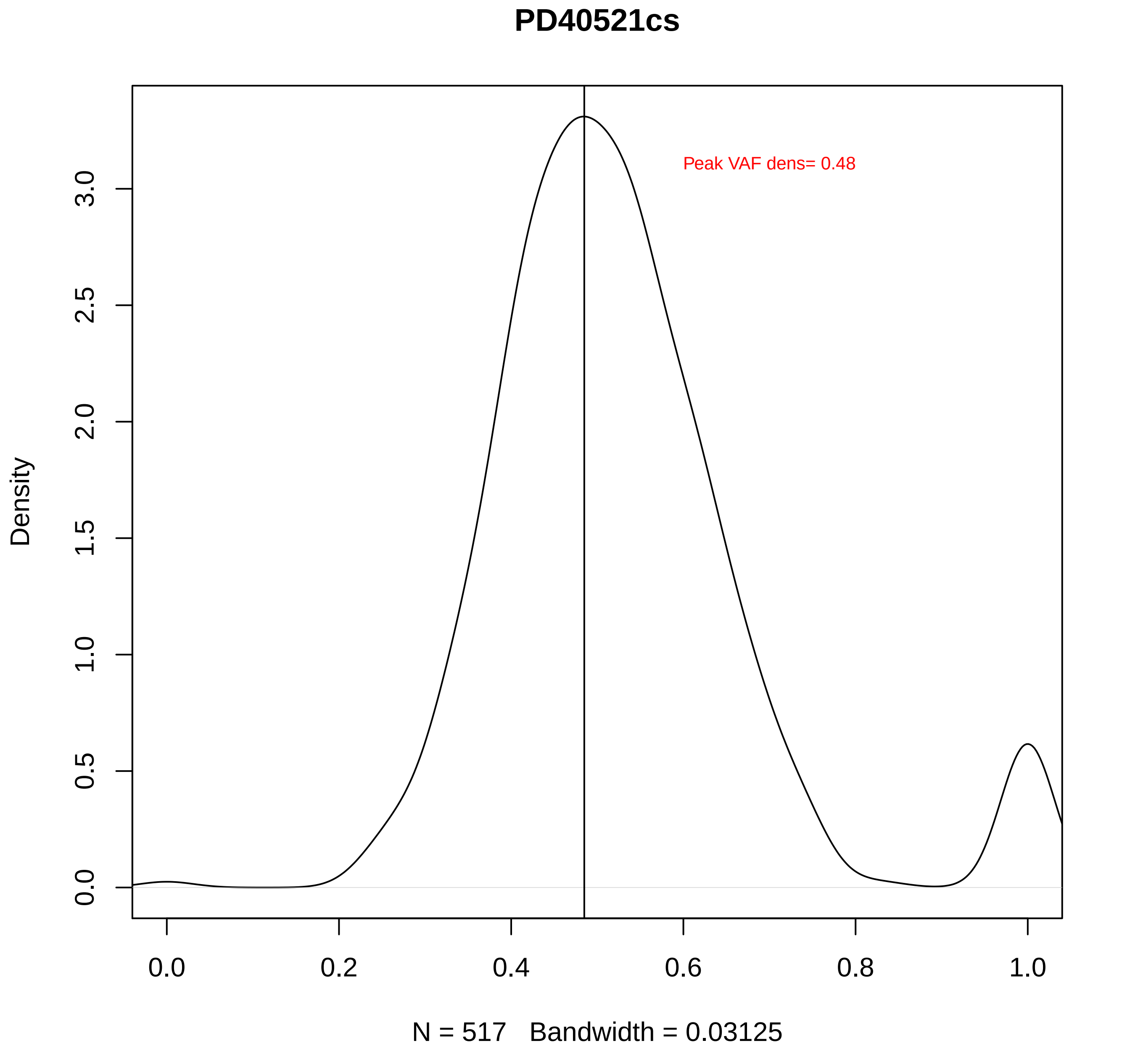
PD40521cs
Peak VAF dens= 0.48
Density
0.0
0.5
1.0
1.5
2.0
2.5
3.0
0.0
0.2
0.4
0.6
0.8
1.0
N = 517   Bandwidth = 0.03125

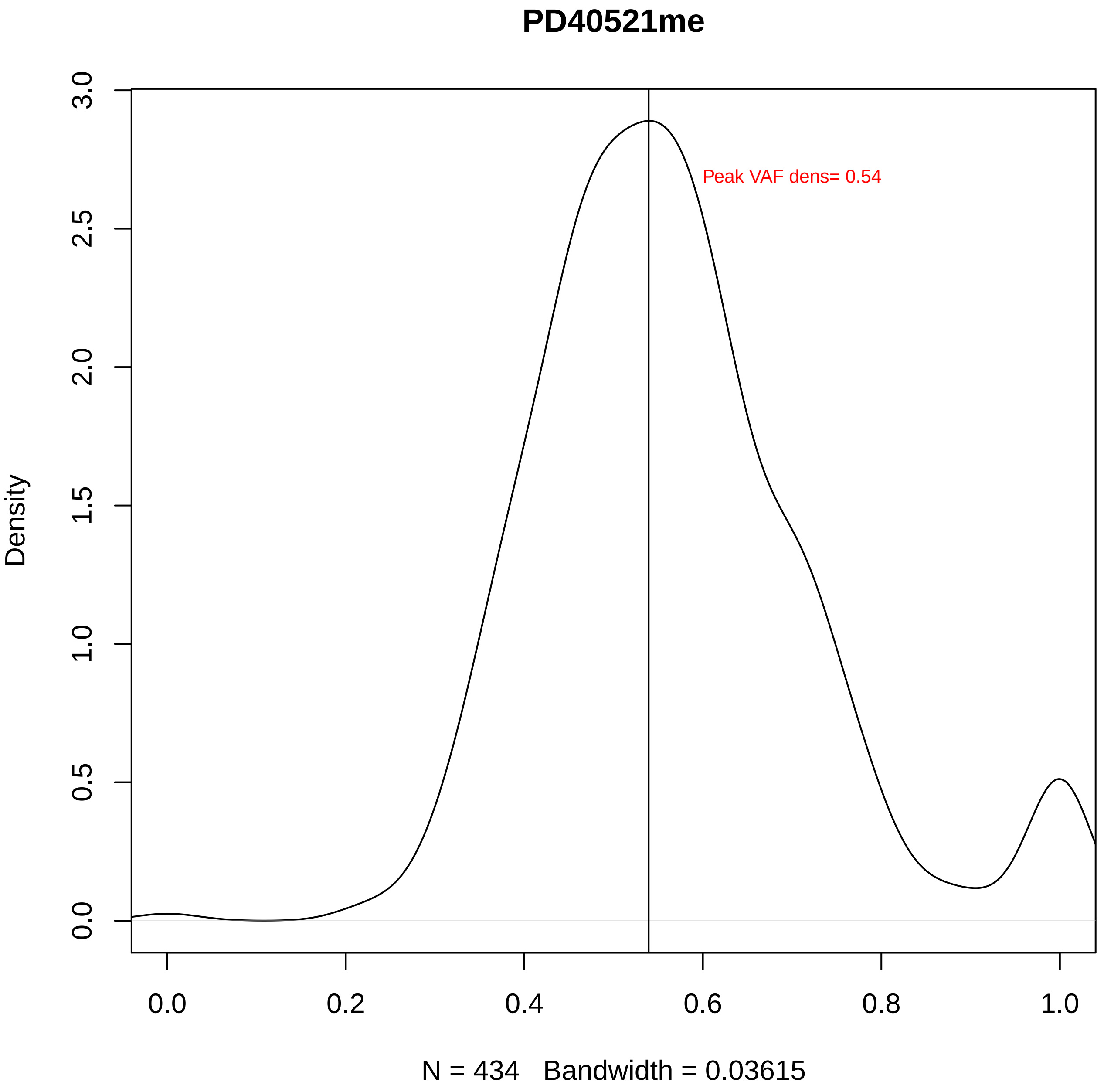
PD40521me
Peak VAF dens= 0.54
Density
0.0
0.5
1.0
1.5
2.0
2.5
3.0
0.0
0.2
0.4
0.6
0.8
1.0
N = 434   Bandwidth = 0.03615

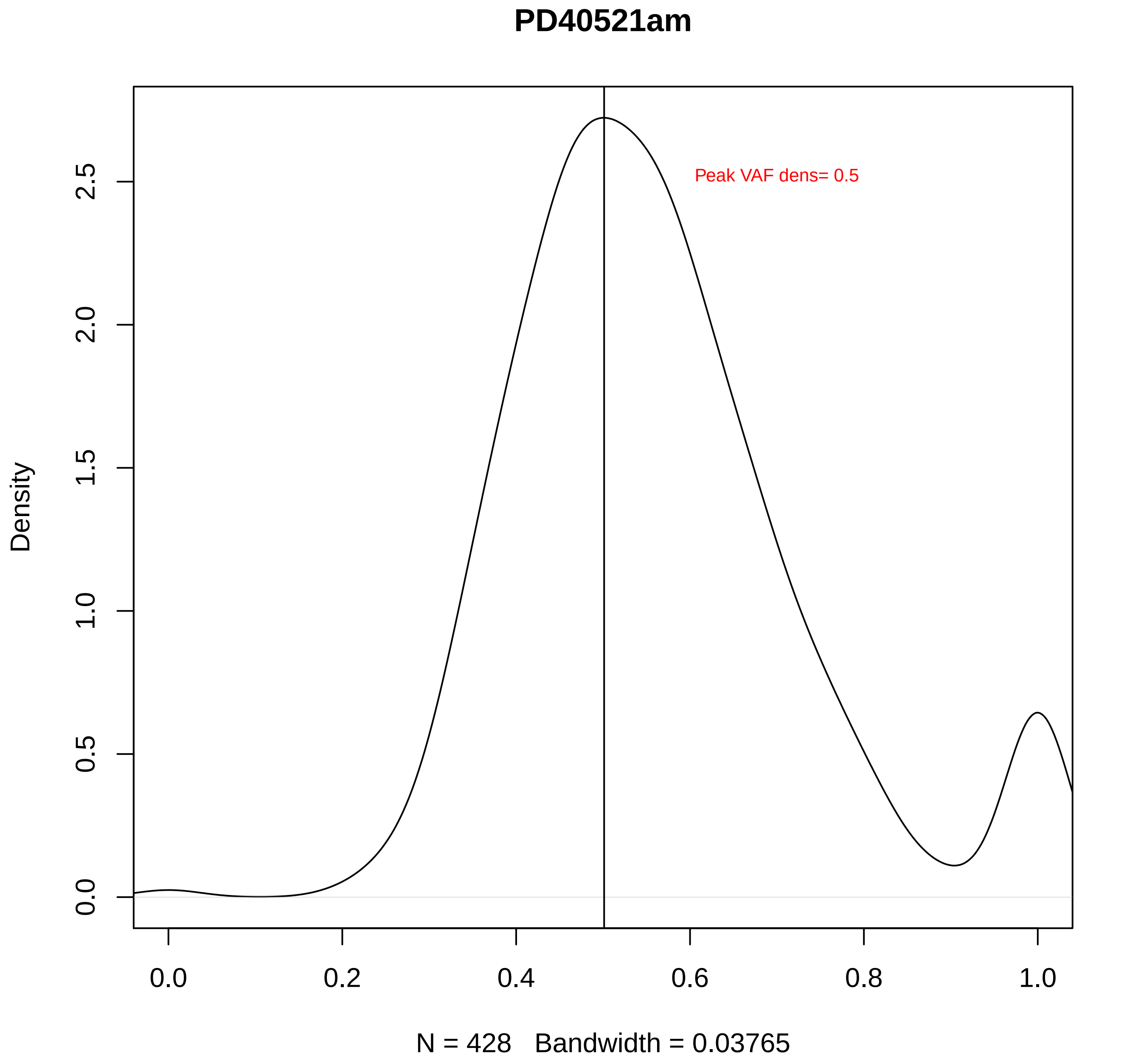
PD40521am
Peak VAF dens= 0.5
Density
0.0
0.5
1.0
1.5
2.0
2.5
0.0
0.2
0.4
0.6
0.8
1.0
N = 428   Bandwidth = 0.03765

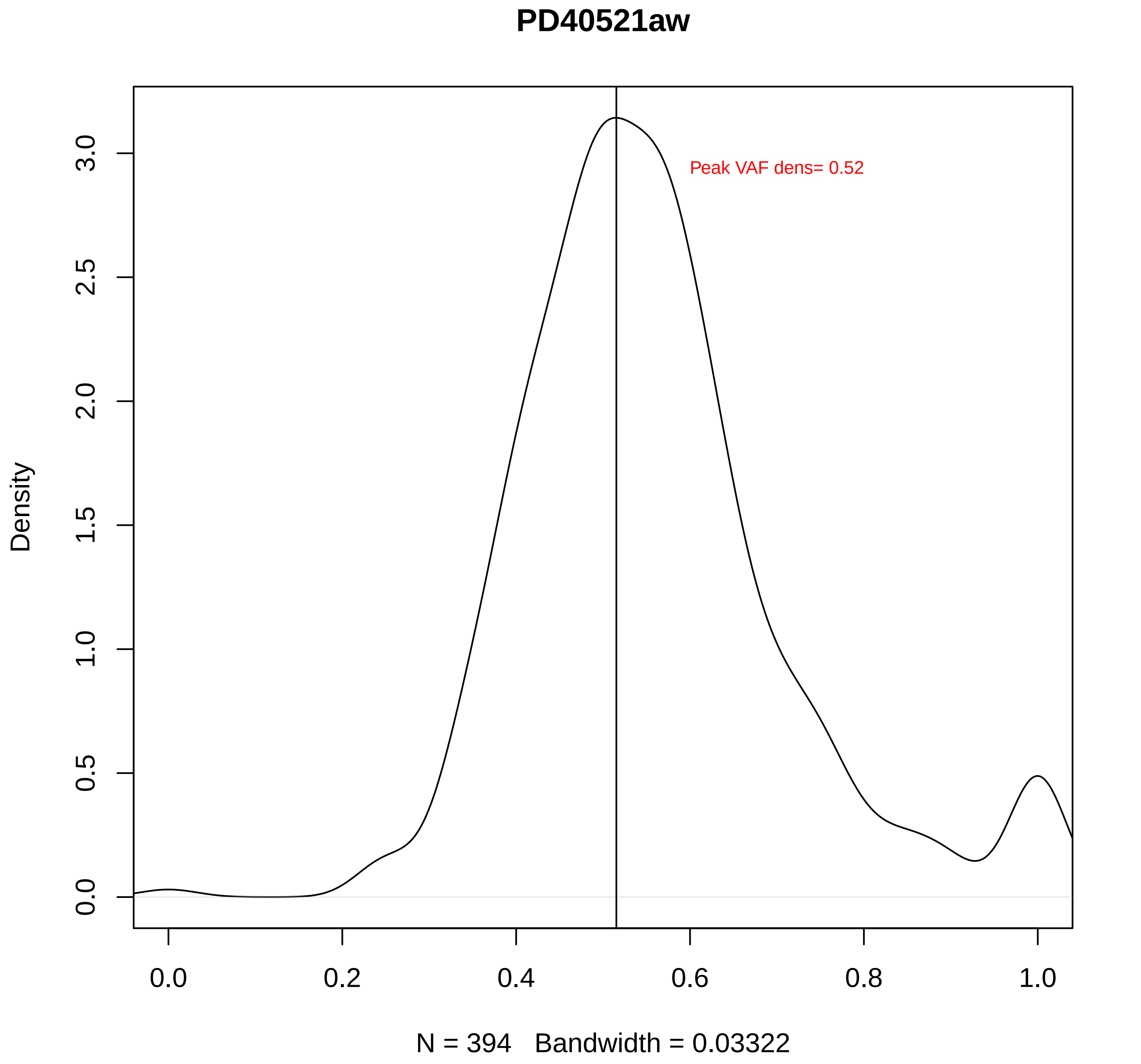
PD40521aw
Peak VAF dens= 0.52
Density
0.0
0.5
1.0
1.5
2.0
2.5
3.0
0.0
0.2
0.4
0.6
0.8
1.0
N = 394 Bandwidth = 0.03322

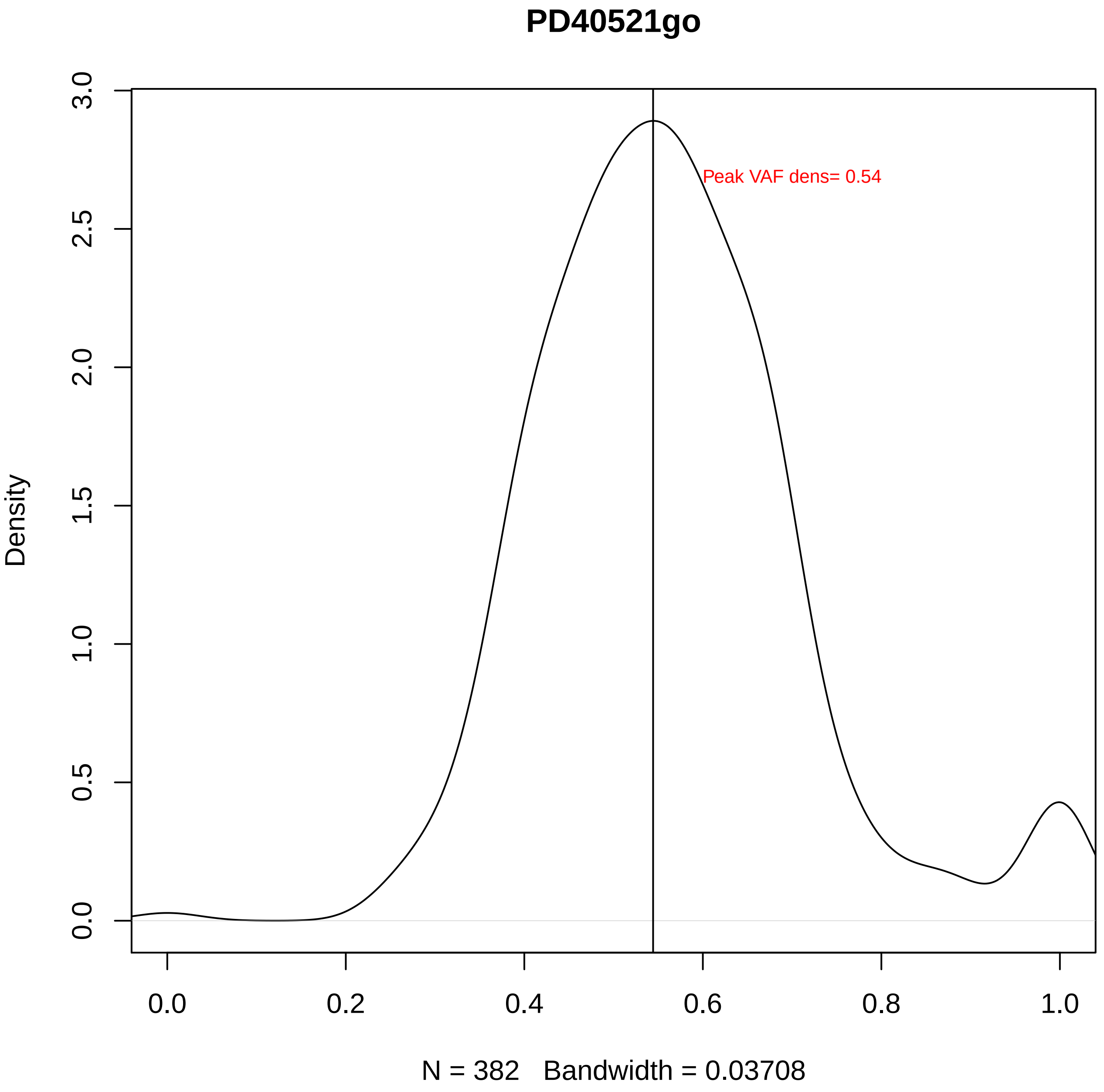
PD40521go
Peak VAF dens= 0.54
Density
3.0
2.5
2.0
1.5
1.0
0.5
0.0
0.0
0.2
0.4
0.6
0.8
1.0
N = 382   Bandwidth = 0.03708

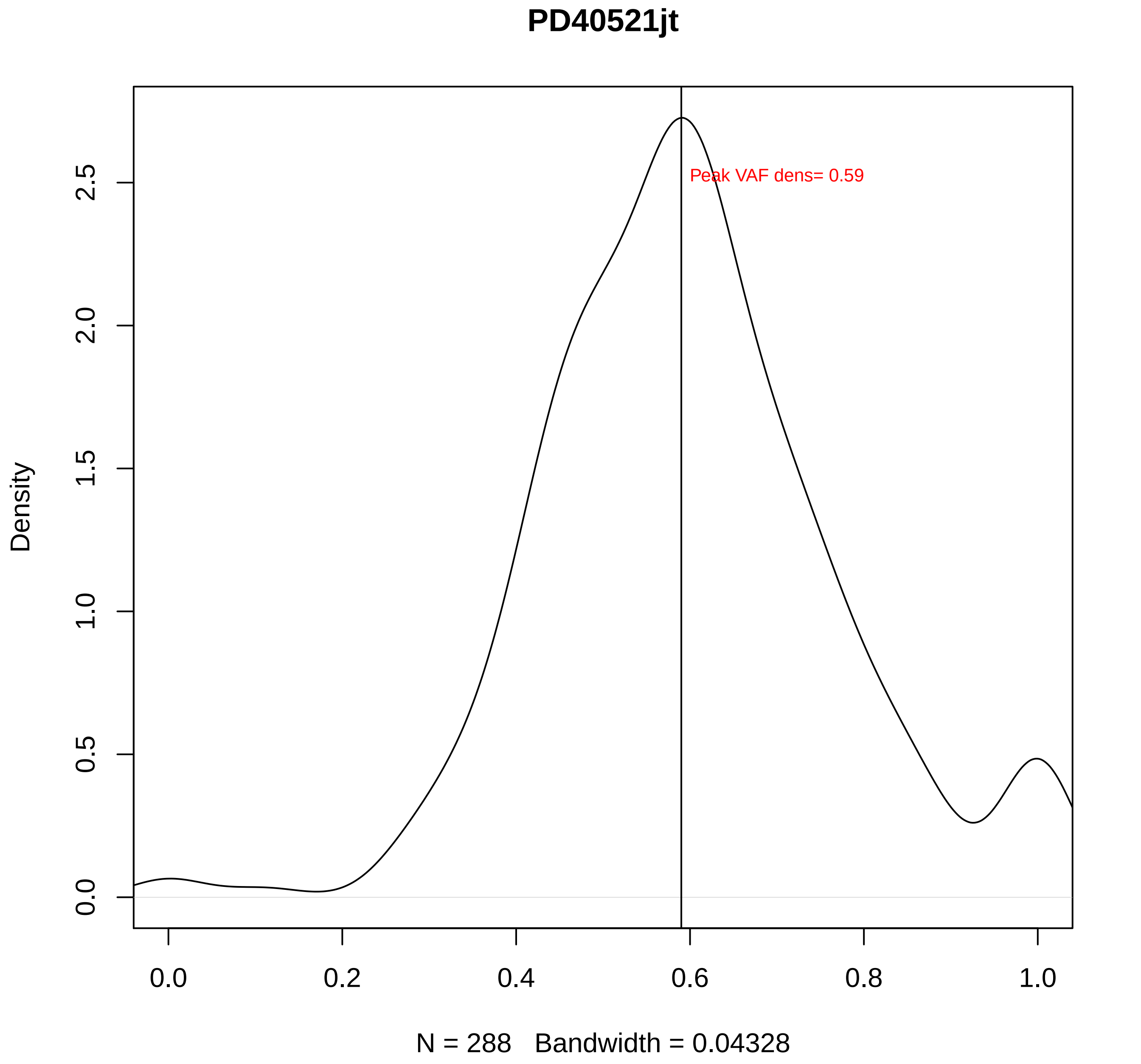
PD40521jt
Peak VAF dens= 0.59
Density
0.0
0.5
1.0
1.5
2.0
2.5
0.0
0.2
0.4
0.6
0.8
1.0
N = 288 Bandwidth = 0.04328

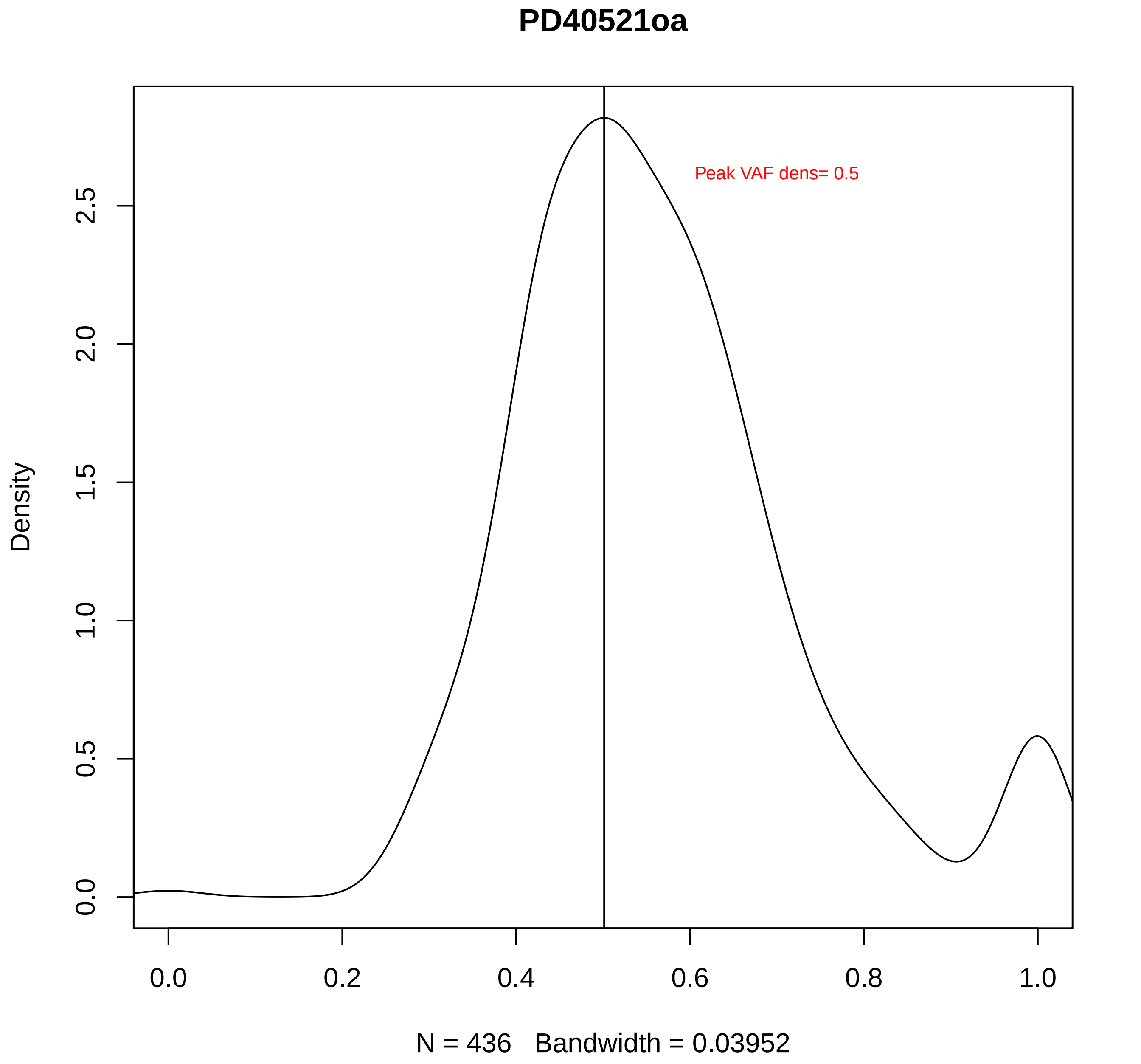
PD40521oa
Peak VAF dens= 0.5
Density
0.0
0.5
1.0
1.5
2.0
2.5
0.0
0.2
0.4
0.6
0.8
1.0
N = 436   Bandwidth = 0.03952

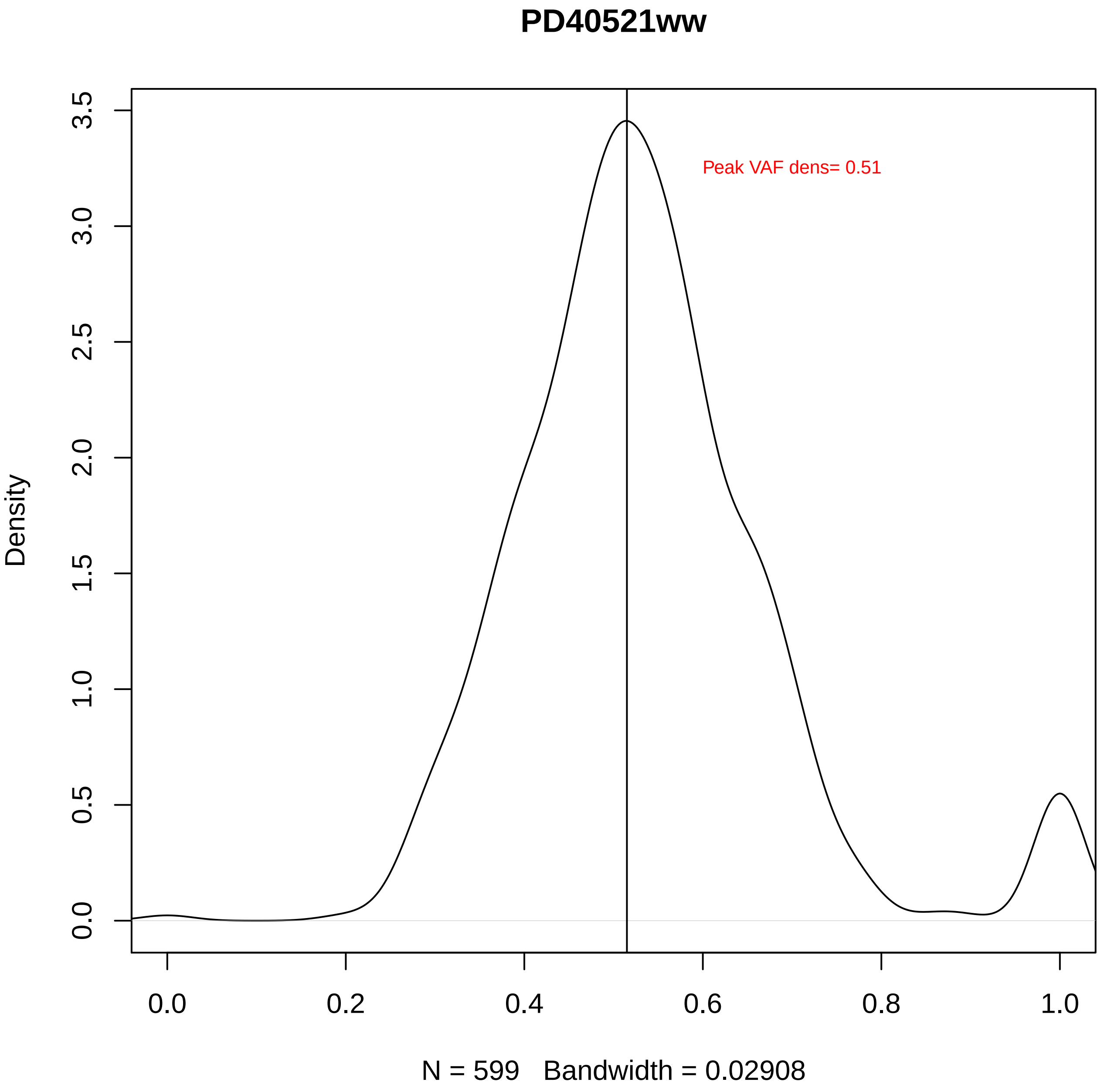
PD40521ww
Peak VAF dens= 0.51
Density
0.0
0.5
1.0
1.5
2.0
2.5
3.0
3.5
0.0
0.2
0.4
0.6
0.8
1.0
N = 599   Bandwidth = 0.02908

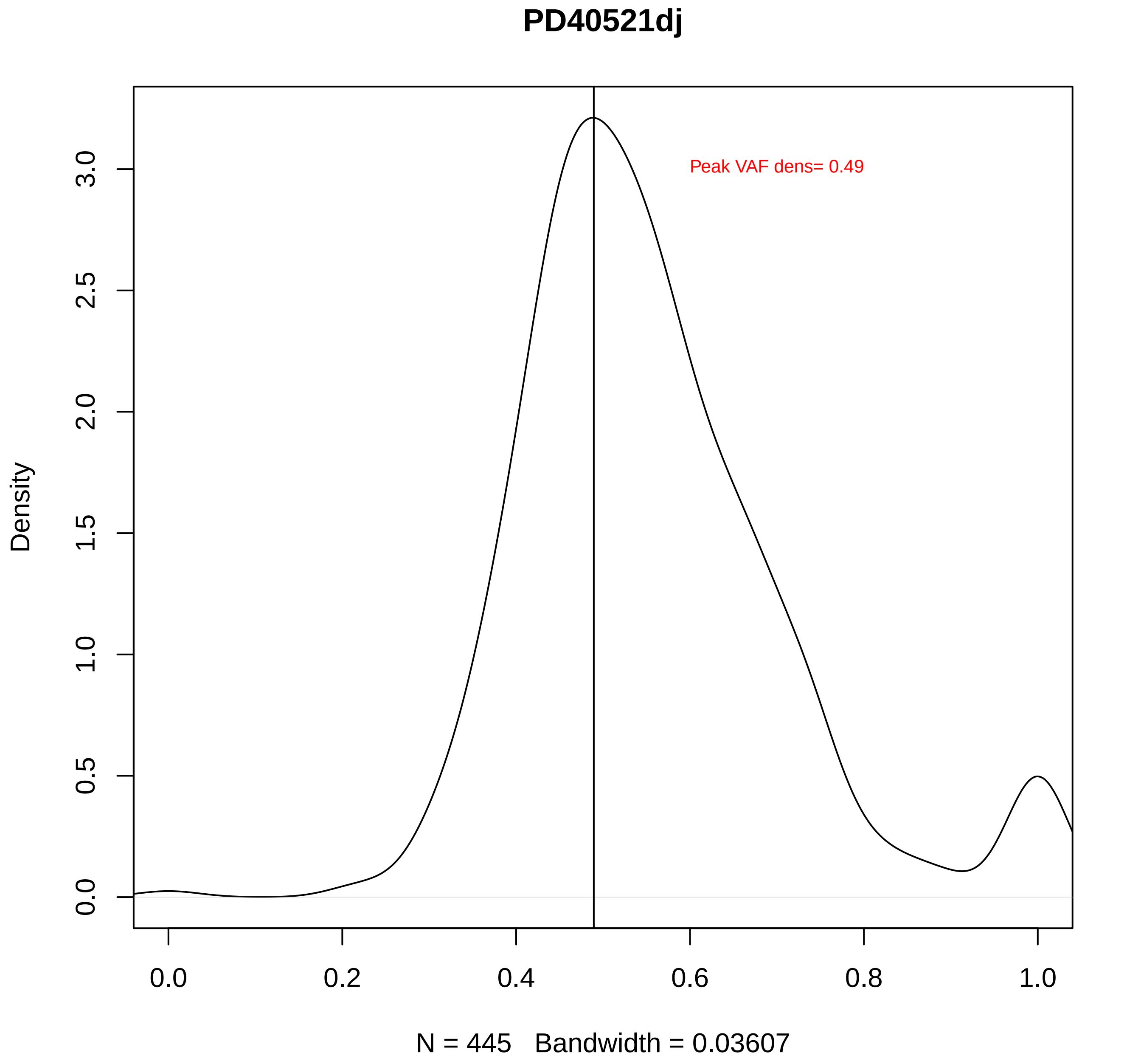
PD40521dj
Peak VAF dens= 0.49
Density
0.0
0.5
1.0
1.5
2.0
2.5
3.0
0.0
0.2
0.4
0.6
0.8
1.0
N = 445   Bandwidth = 0.03607

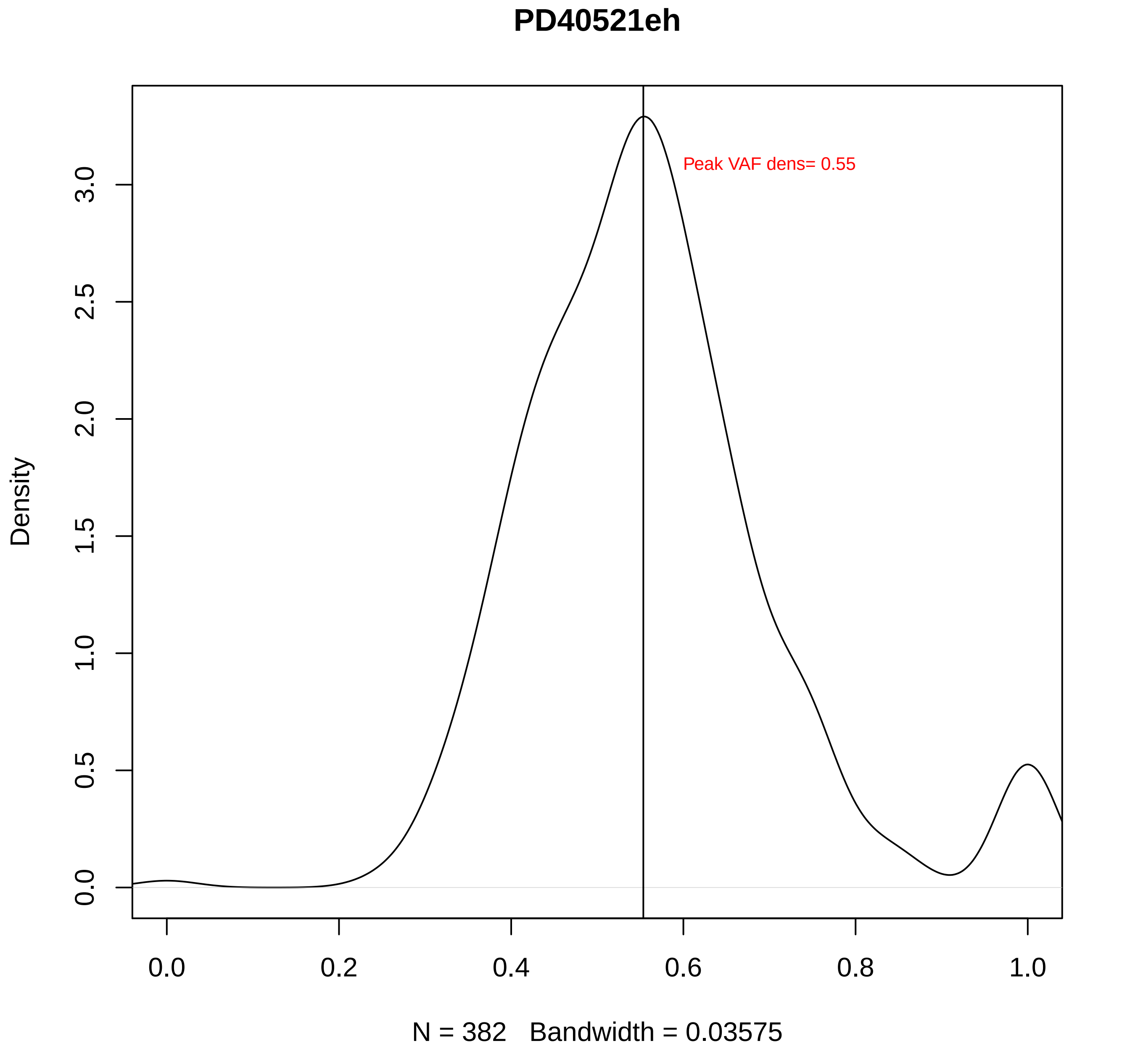
PD40521eh
Peak VAF dens= 0.55
Density
0.0
0.5
1.0
1.5
2.0
2.5
3.0
0.0
0.2
0.4
0.6
0.8
1.0
N = 382 Bandwidth = 0.03575

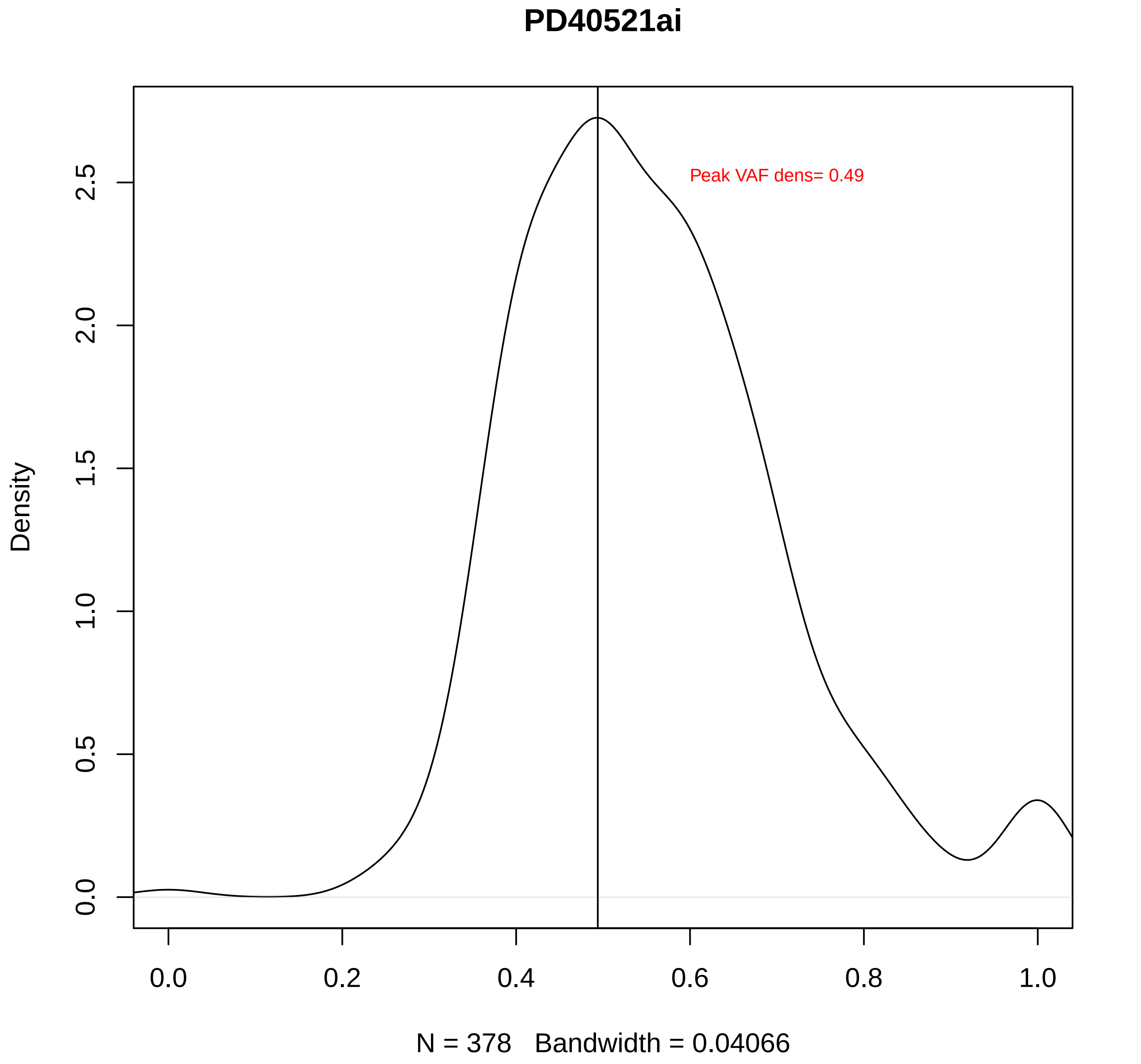

PD40521ai
Peak VAF dens= 0.49
Density
0.0
0.5
1.0
1.5
2.0
2.5
0.0
0.2
0.4
0.6
0.8
1.0
N = 378 Bandwidth = 0.04066

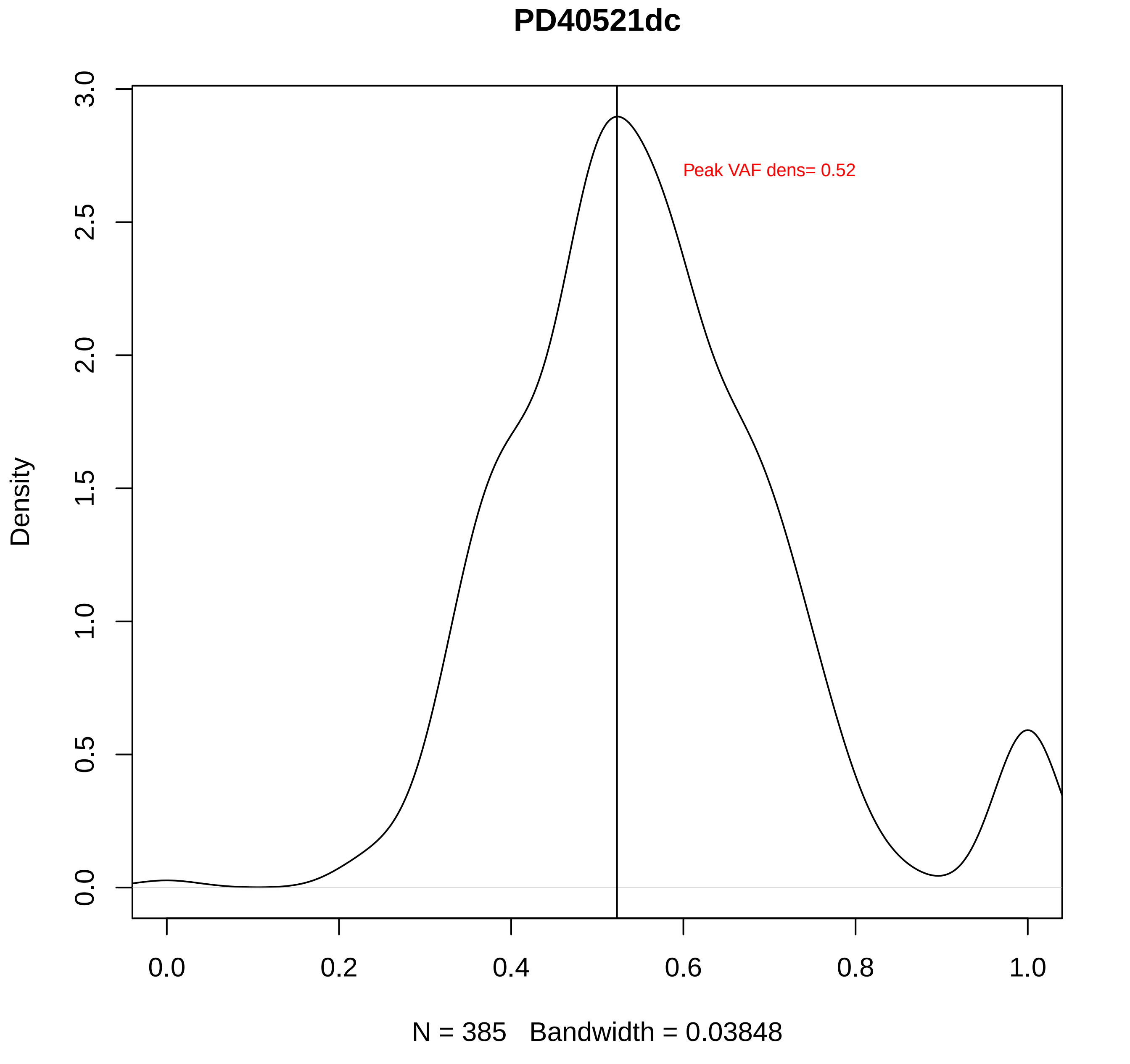
PD40521dc
Peak VAF dens= 0.52
Density
0.0
0.5
1.0
1.5
2.0
2.5
3.0
0.0
0.2
0.4
0.6
0.8
1.0
N = 385   Bandwidth = 0.03848

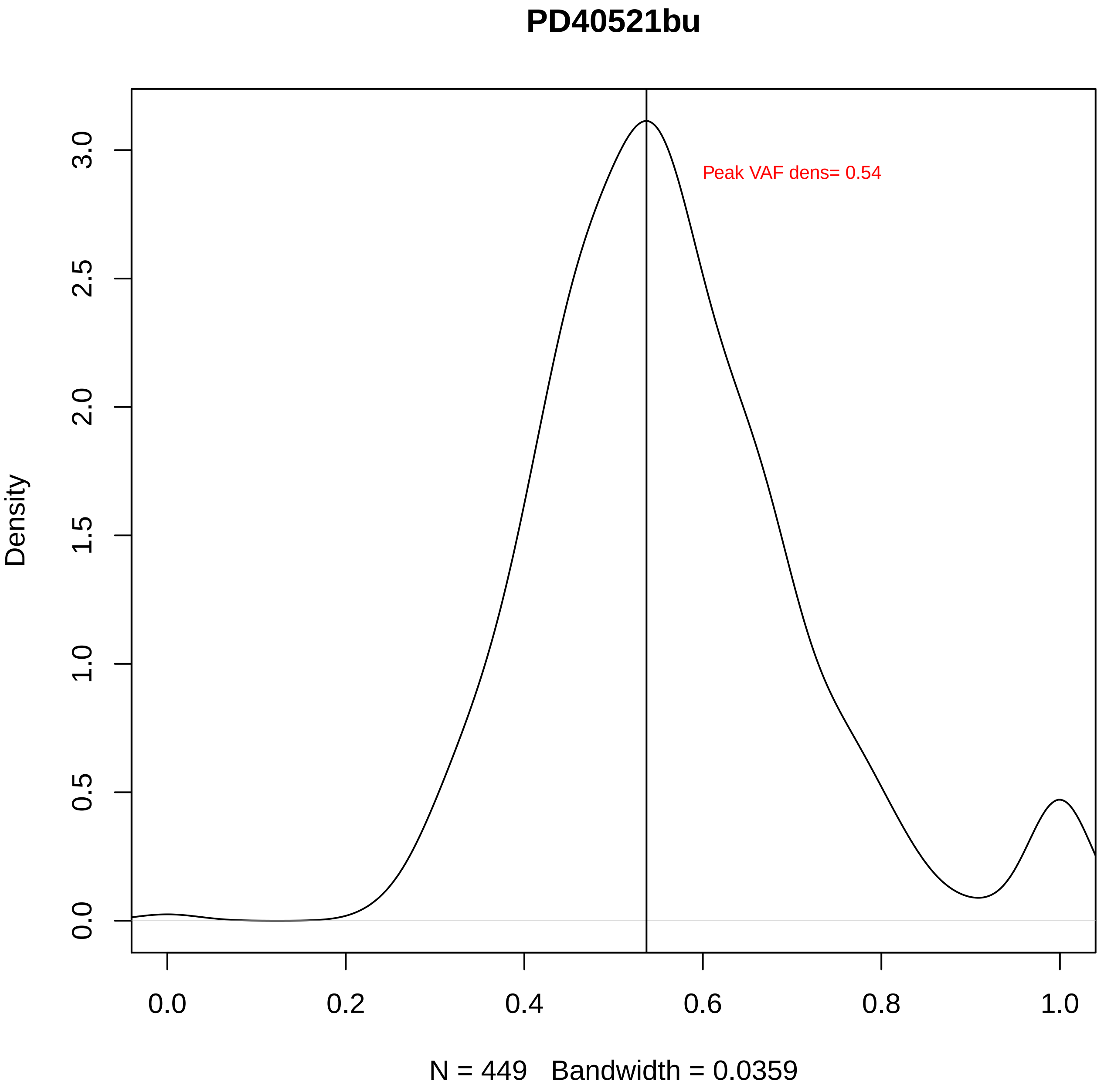
PD40521bu
Peak VAF dens= 0.54
Density
0.0
0.5
1.0
1.5
2.0
2.5
3.0
0.0
0.2
0.4
0.6
0.8
1.0
N = 449   Bandwidth = 0.0359

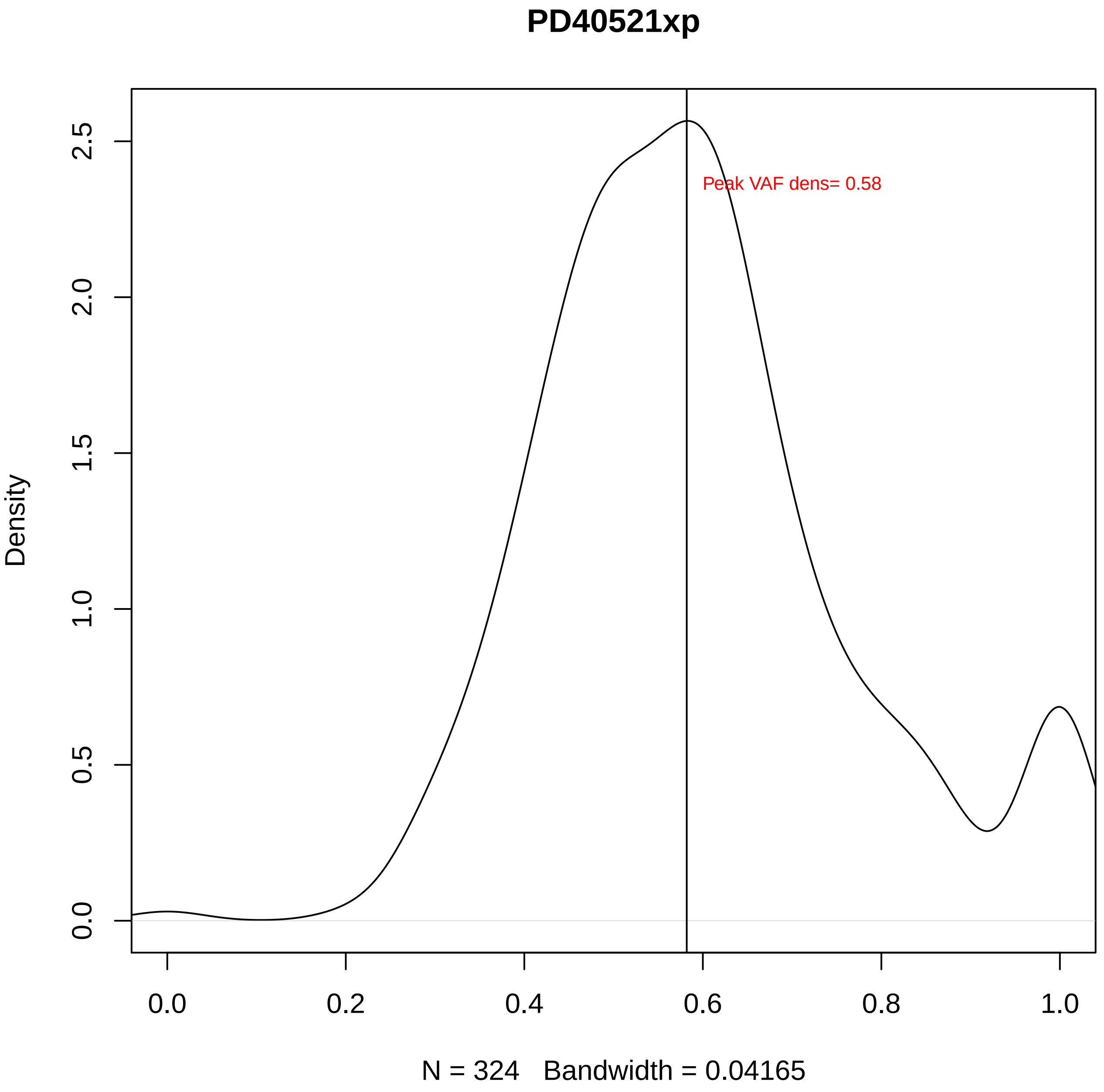

PD40521xp
Peak VAF dens= 0.58
Density
0.0
0.5
1.0
1.5
2.0
2.5
0.0
0.2
0.4
0.6
0.8
1.0
N = 324 Bandwidth = 0.04165

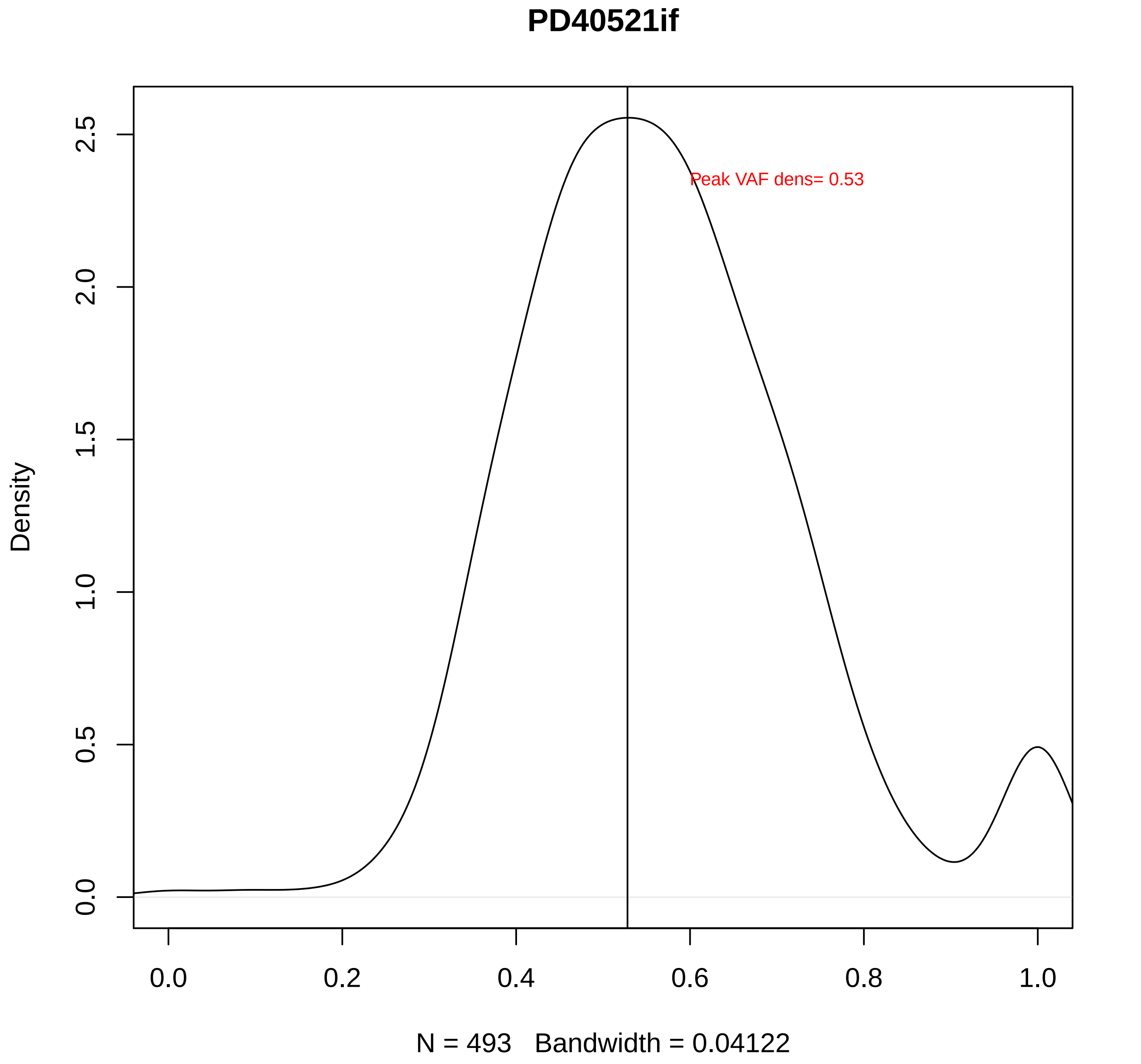
PD40521if
Peak VAF dens= 0.53
Density
0.0
0.5
1.0
1.5
2.0
2.5
0.0
0.2
0.4
0.6
0.8
1.0
N = 493 Bandwidth = 0.04122

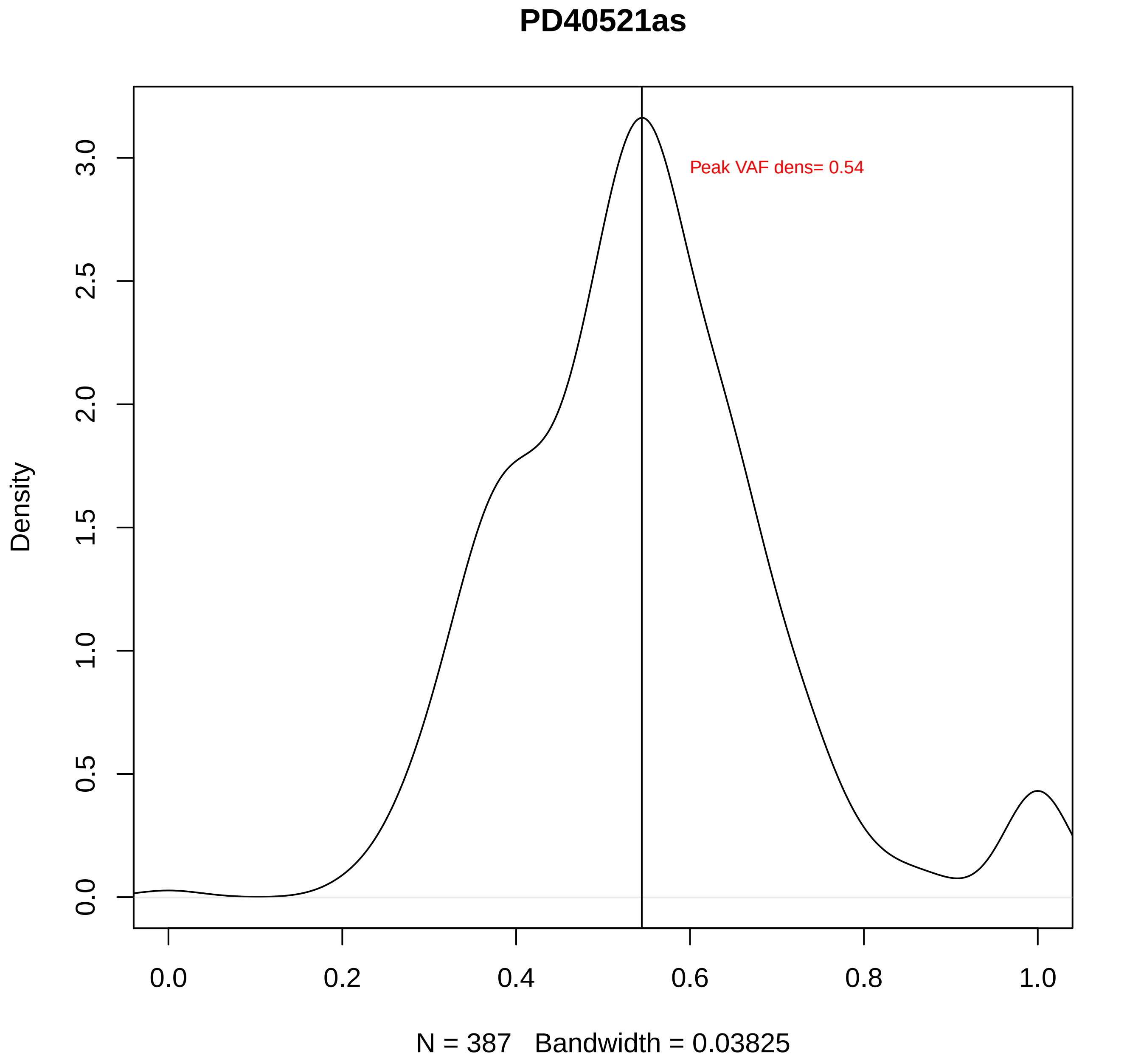
PD40521as
Peak VAF dens= 0.54
Density
0.0
0.5
1.0
1.5
2.0
2.5
3.0
0.0
0.2
0.4
0.6
0.8
1.0
N = 387   Bandwidth = 0.03825

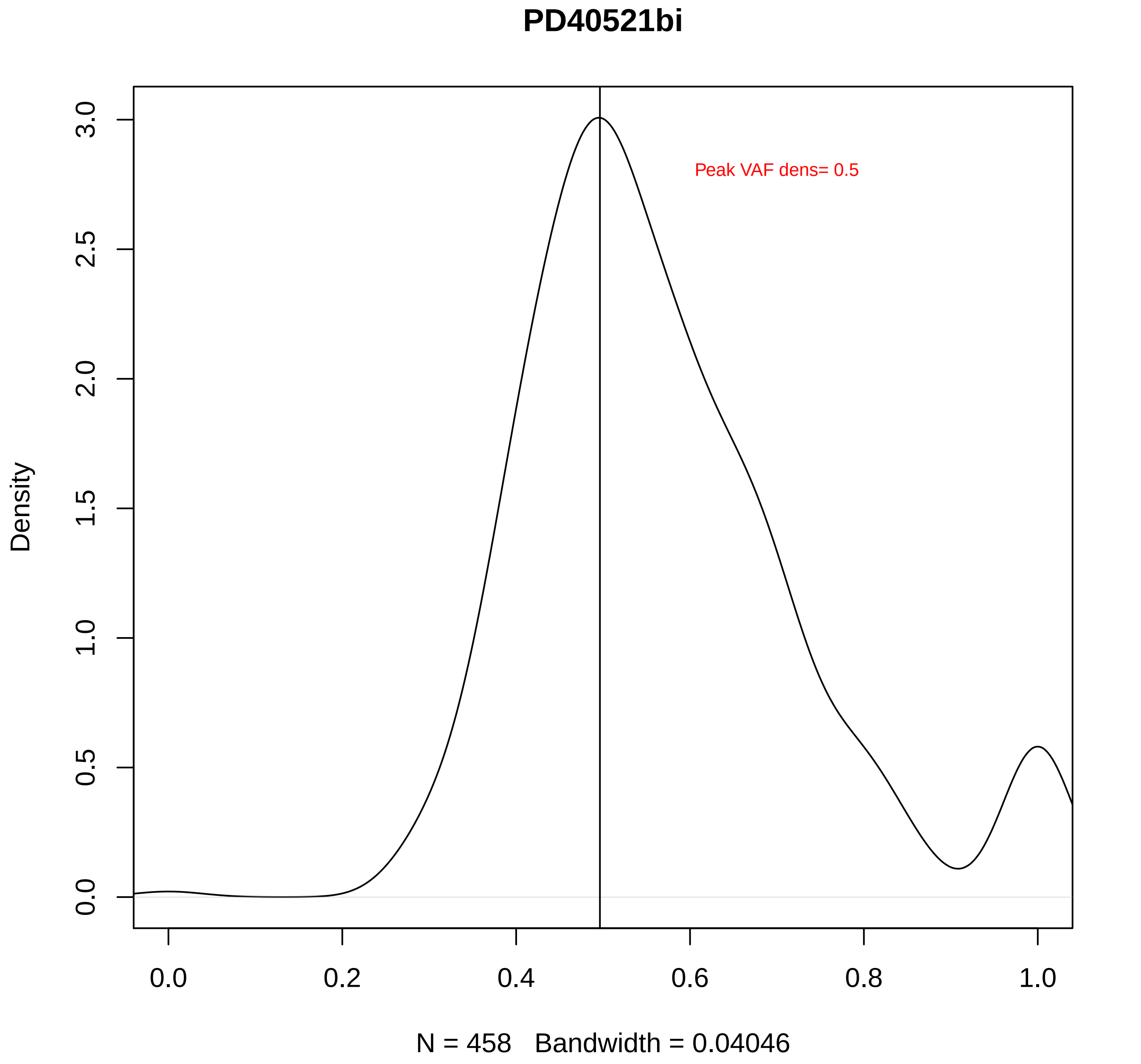
PD40521bi
Peak VAF dens= 0.5
Density
0.0
0.5
1.0
1.5
2.0
2.5
3.0
0.0
0.2
0.4
0.6
0.8
1.0
N = 458   Bandwidth = 0.04046

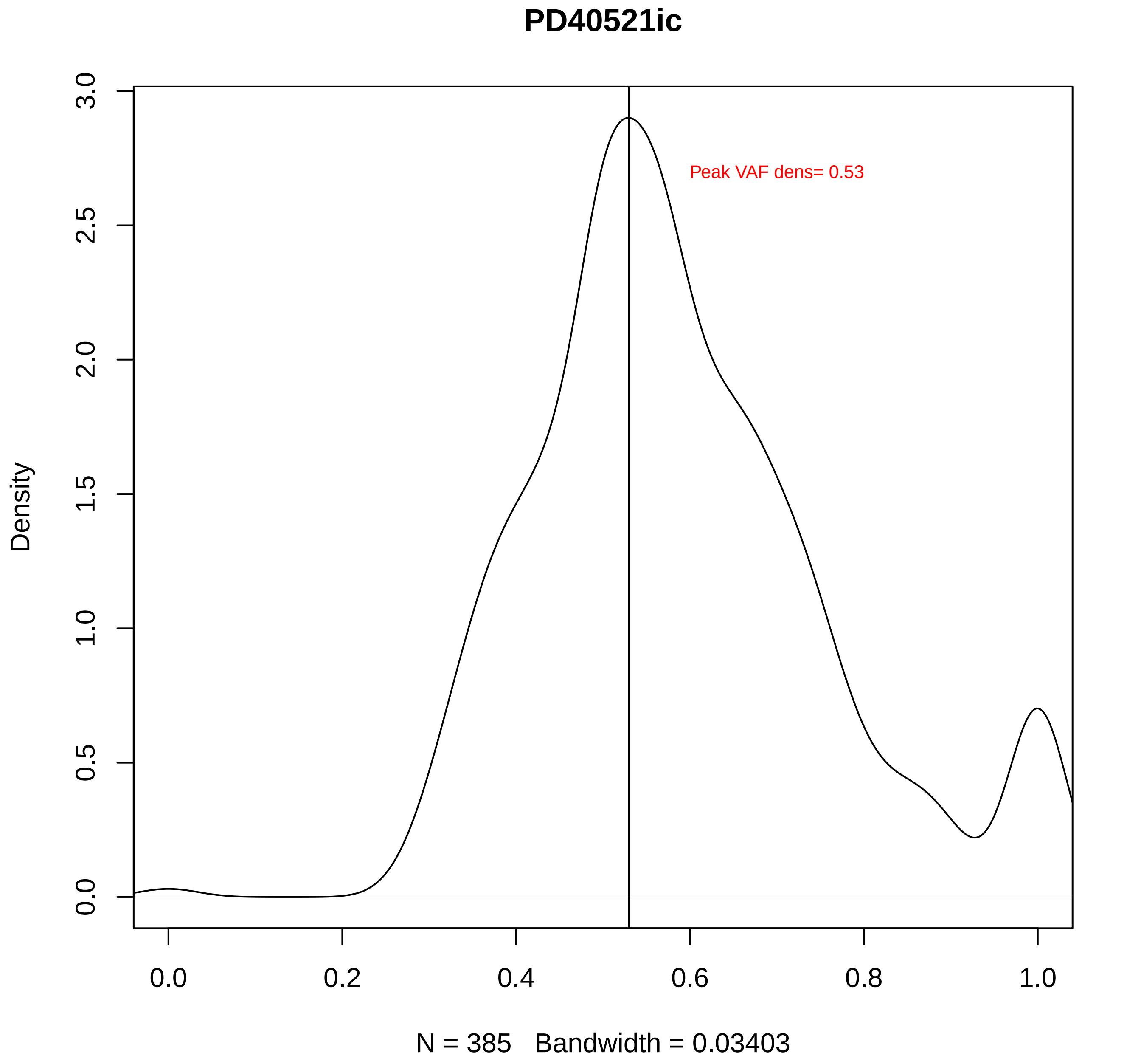
PD40521ic
Peak VAF dens= 0.53
Density
0.0
0.5
1.0
1.5
2.0
2.5
3.0
0.0
0.2
0.4
0.6
0.8
1.0
N = 385   Bandwidth = 0.03403

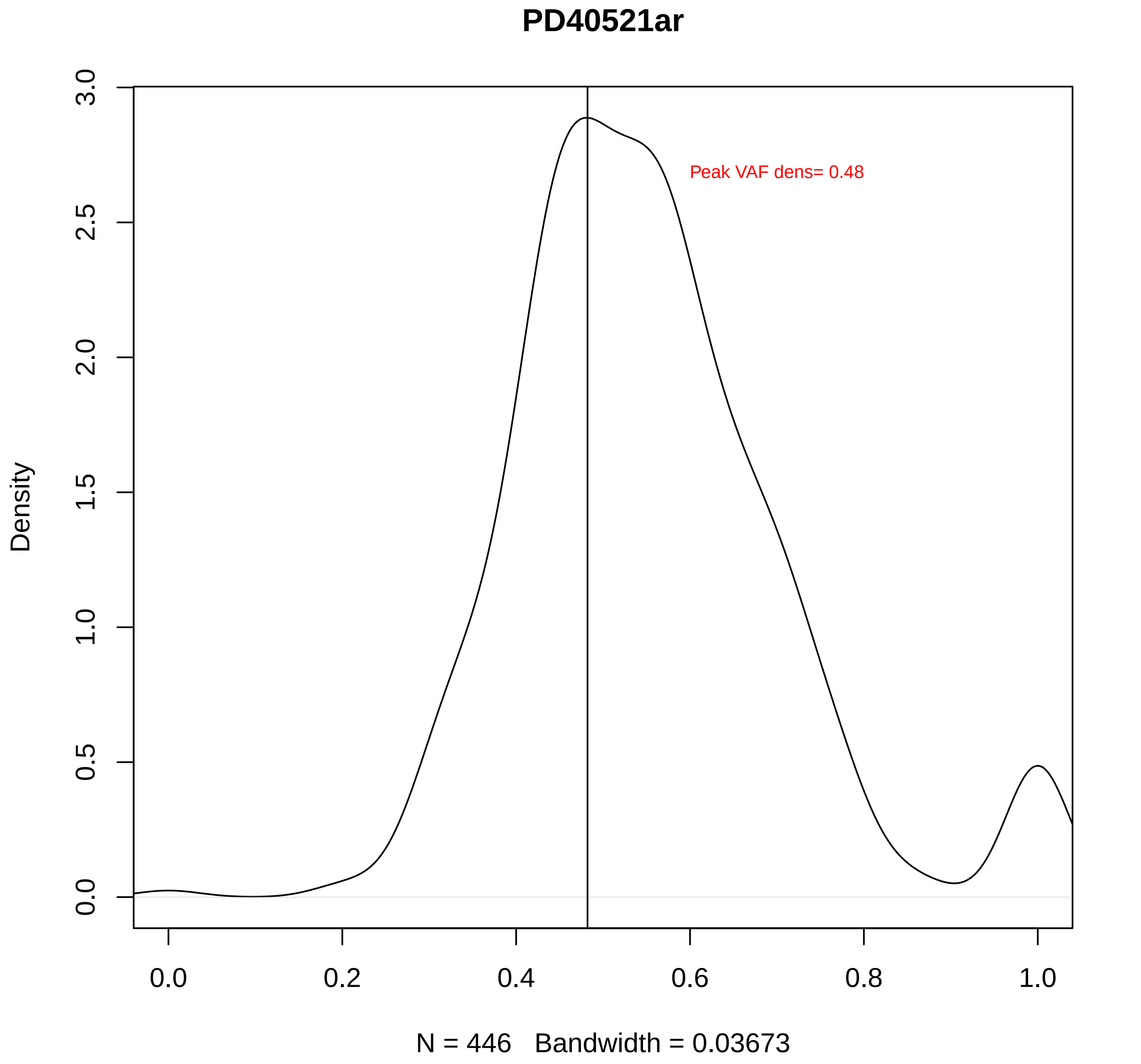
PD40521ar
Peak VAF dens= 0.48
Density
N = 446   Bandwidth = 0.03673
0.0
0.5
1.0
1.5
2.0
2.5
3.0
0.0
0.2
0.4
0.6
0.8
1.0

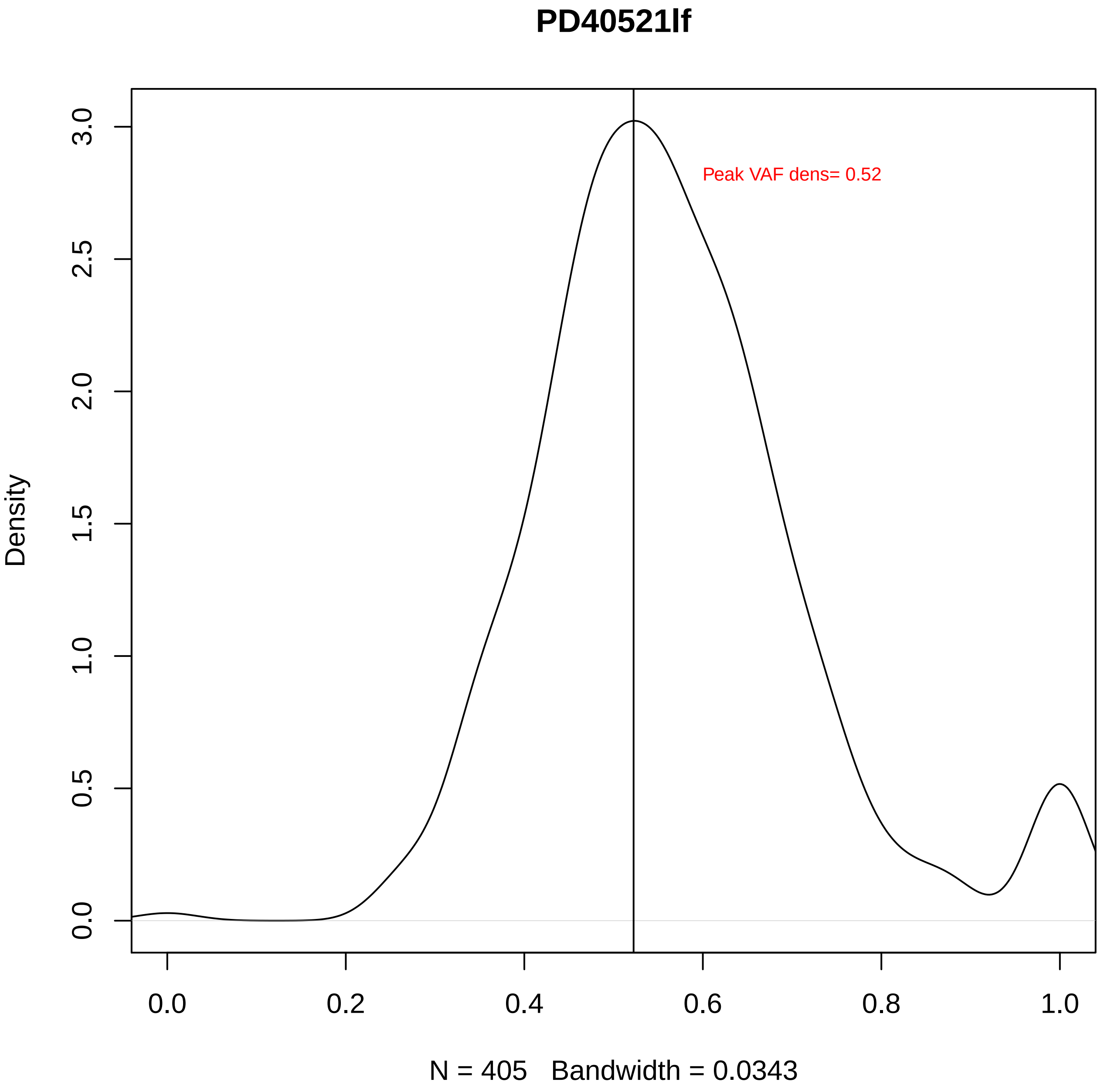

PD40521lf
Peak VAF dens= 0.52
Density
3.0
2.5
2.0
1.5
1.0
0.5
0.0
0.0
0.2
0.4
0.6
0.8
1.0
N = 405 Bandwidth = 0.0343

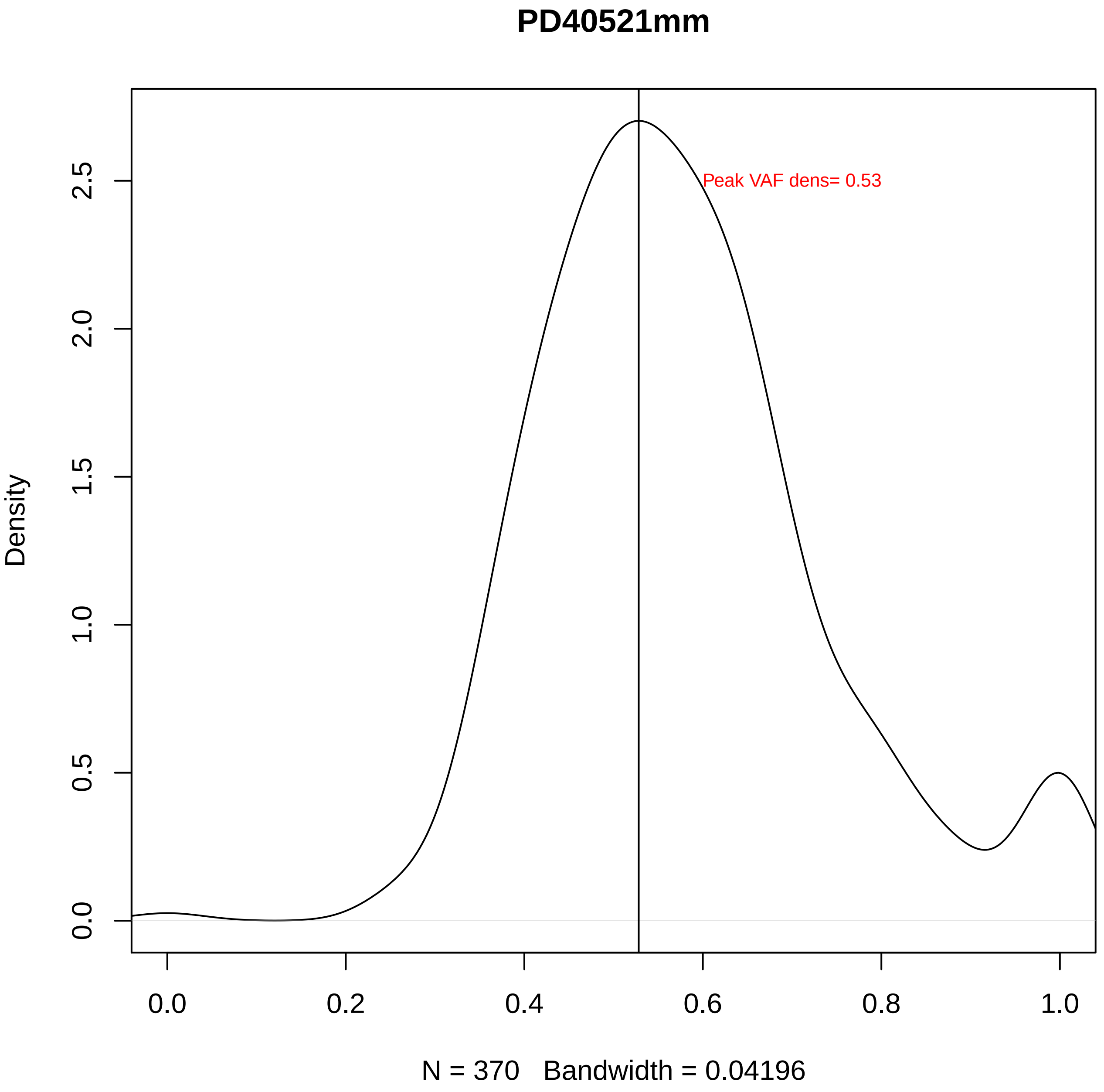

PD40521mm
Peak VAF dens= 0.53
Density
2.5
2.0
1.5
1.0
0.5
0.0
0.0
0.2
0.4
0.6
0.8
1.0
N = 370   Bandwidth = 0.04196

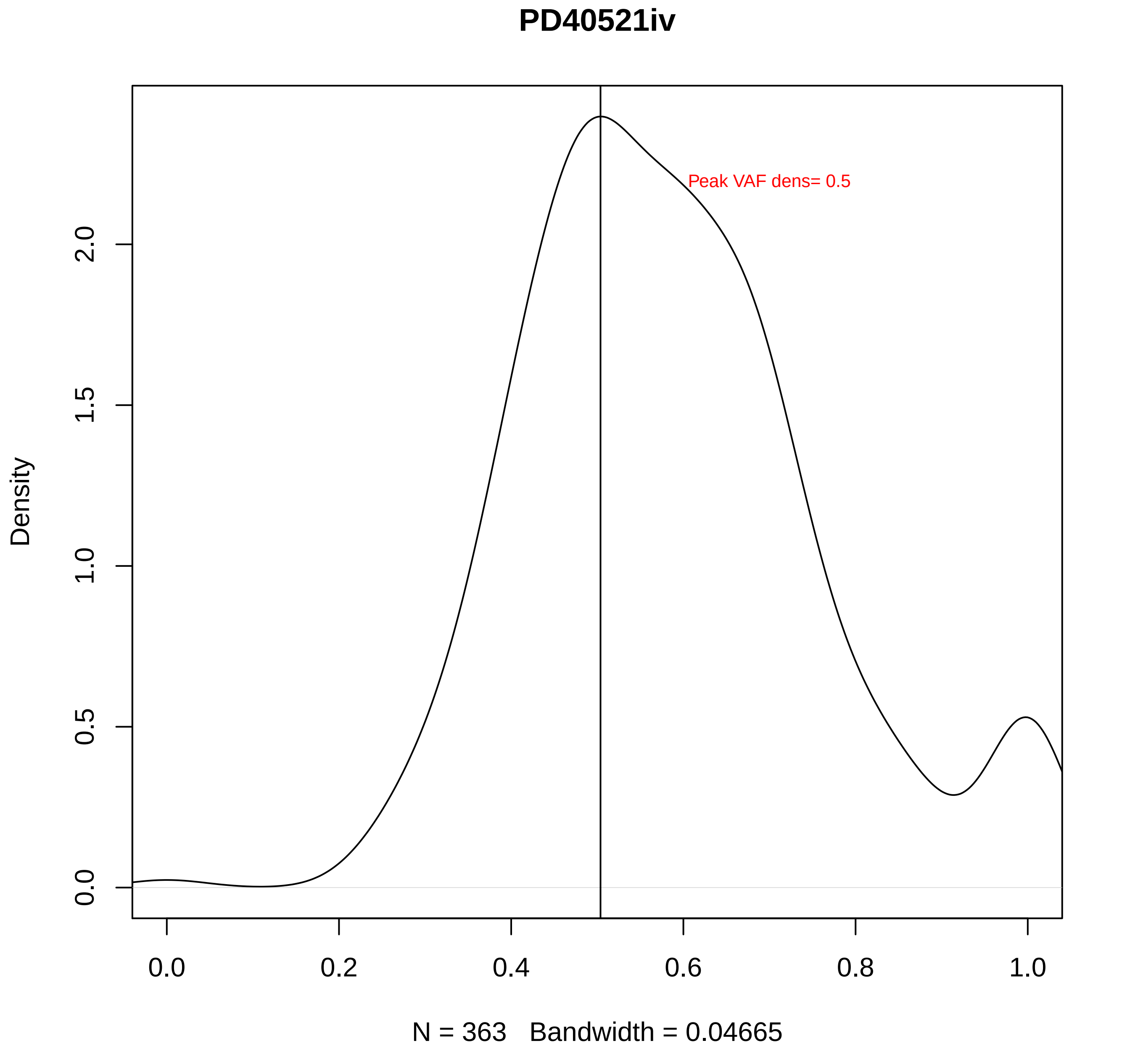
PD40521iv
Peak VAF dens= 0.5
Density
0.0
0.5
1.0
1.5
2.0
0.0
0.2
0.4
0.6
0.8
1.0
N = 363   Bandwidth = 0.04665

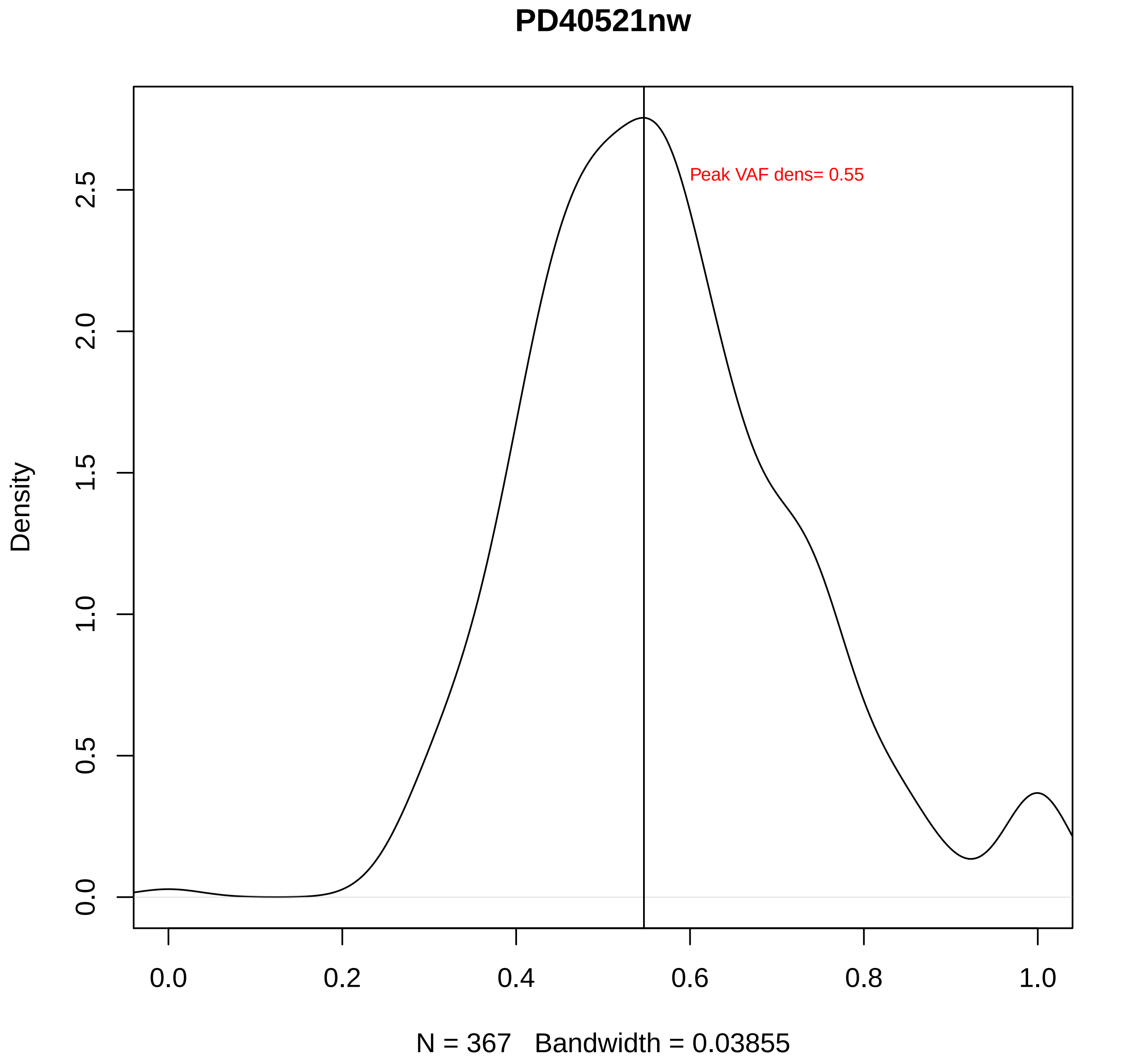
PD40521nw
Peak VAF dens= 0.55
Density
0.0
0.5
1.0
1.5
2.0
2.5
0.0
0.2
0.4
0.6
0.8
1.0
N = 367   Bandwidth = 0.03855

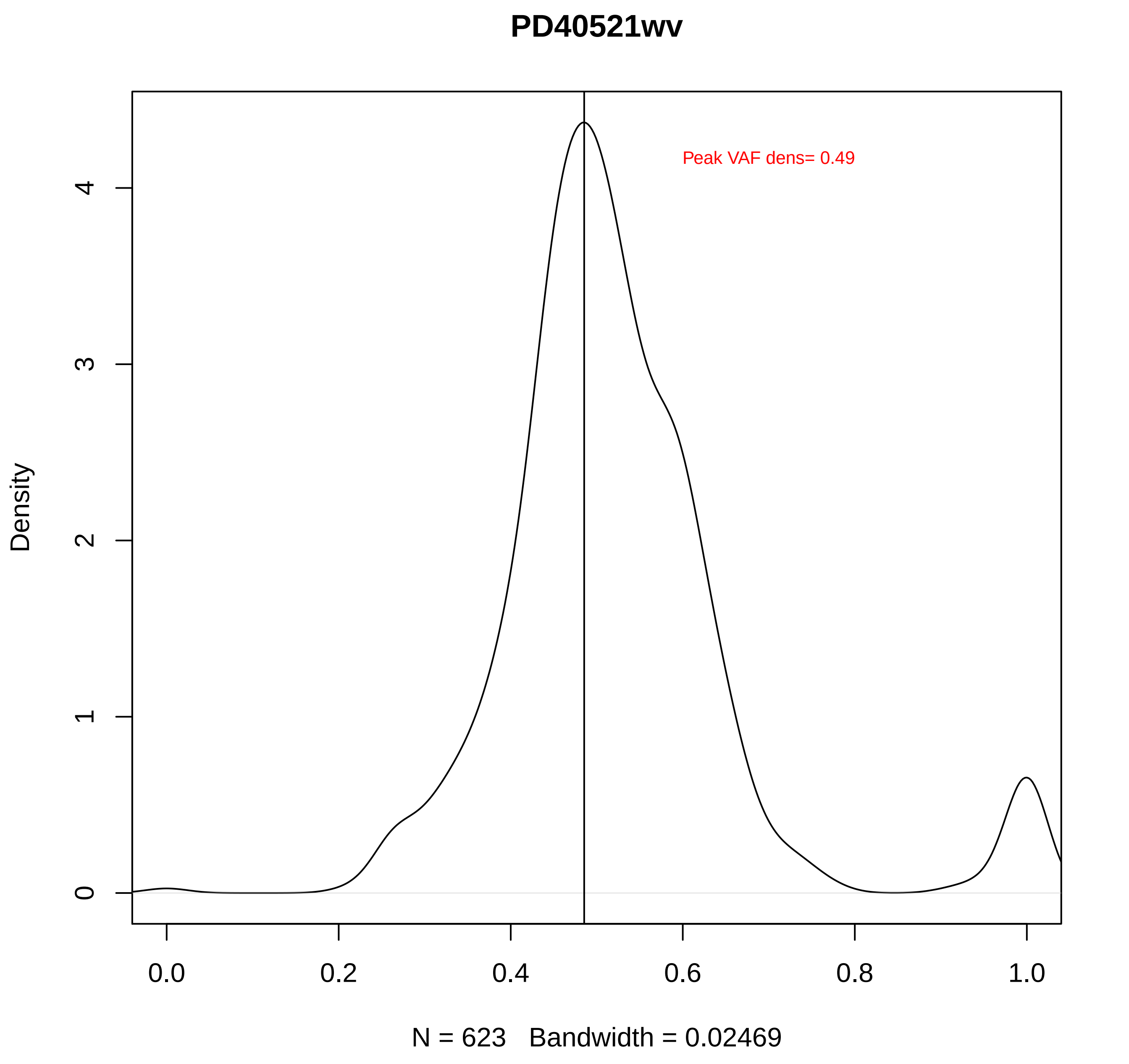
PD40521wv
Peak VAF dens= 0.49
Density
0
1
2
3
4
0.0
0.2
0.4
0.6
0.8
1.0
N = 623 Bandwidth = 0.02469

PD40521fl

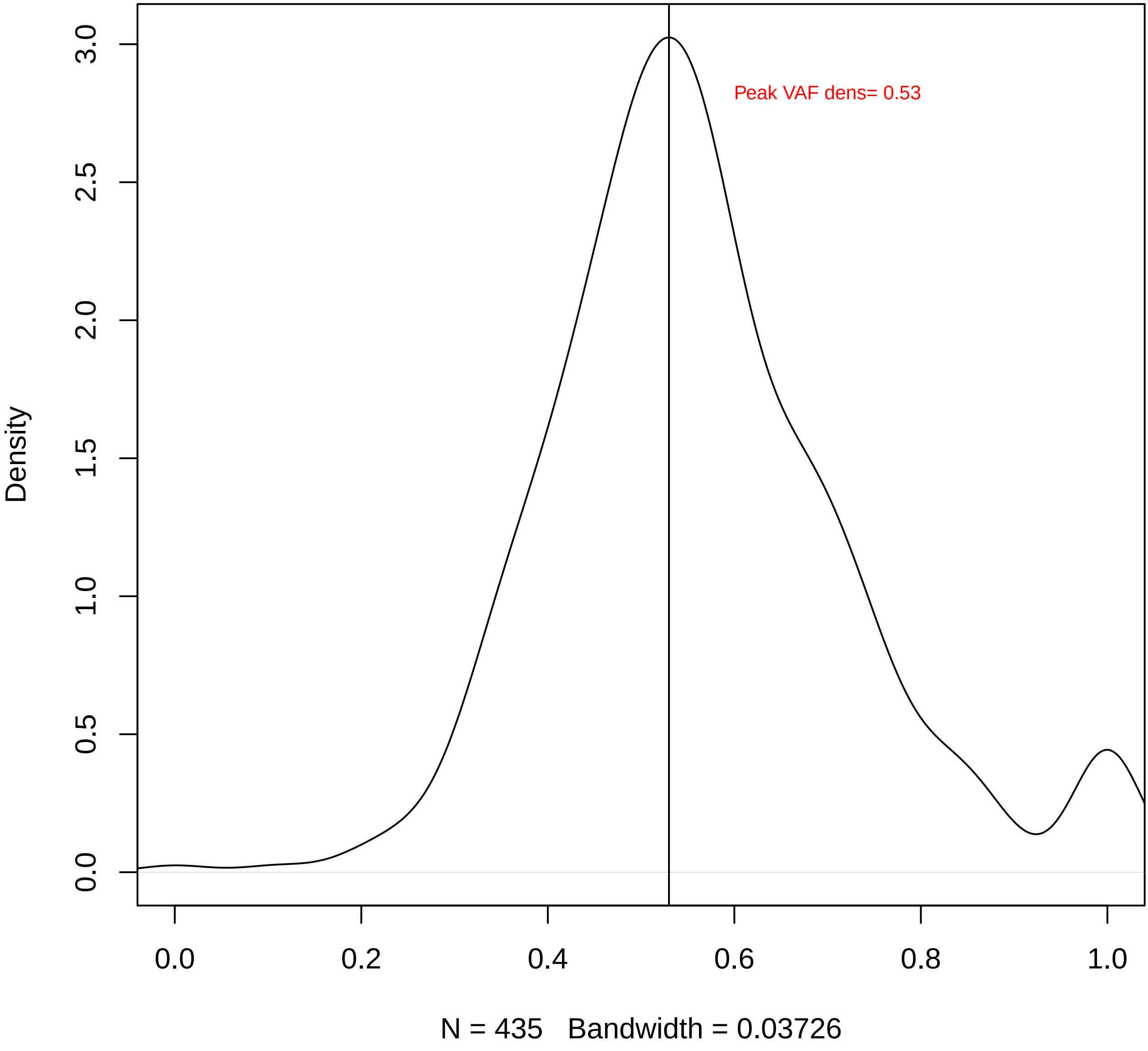

Peak VAF dens= 0.53
Density
3.0
2.5
2.0
1.5
1.0
0.5
0.0
0.0
0.2
0.4
0.6
0.8
1.0
N = 435   Bandwidth = 0.03726

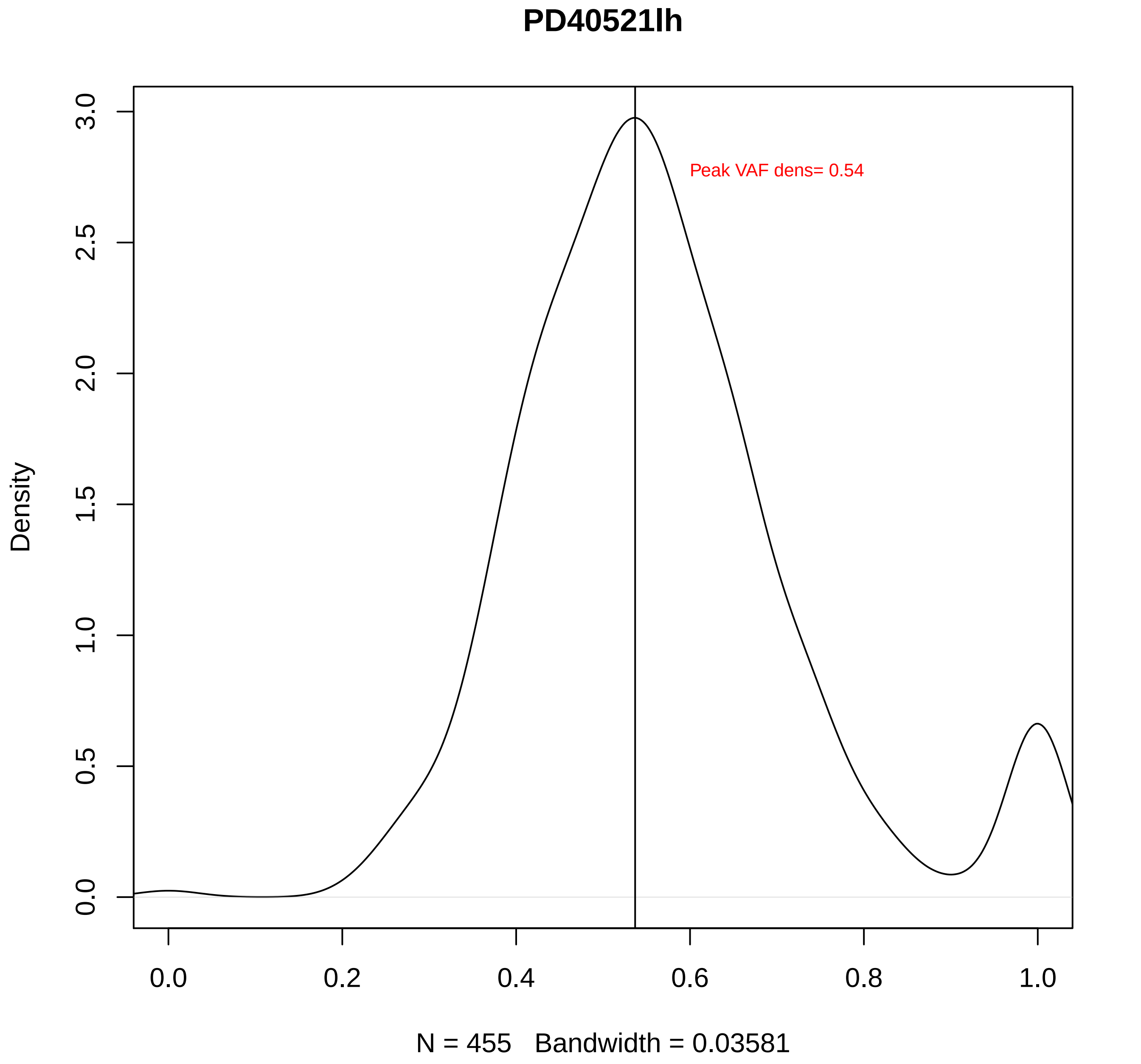
PD40521lh
Peak VAF dens= 0.54
Density
0.0
0.5
1.0
1.5
2.0
2.5
3.0
0.0
0.2
0.4
0.6
0.8
1.0
N = 455   Bandwidth = 0.03581

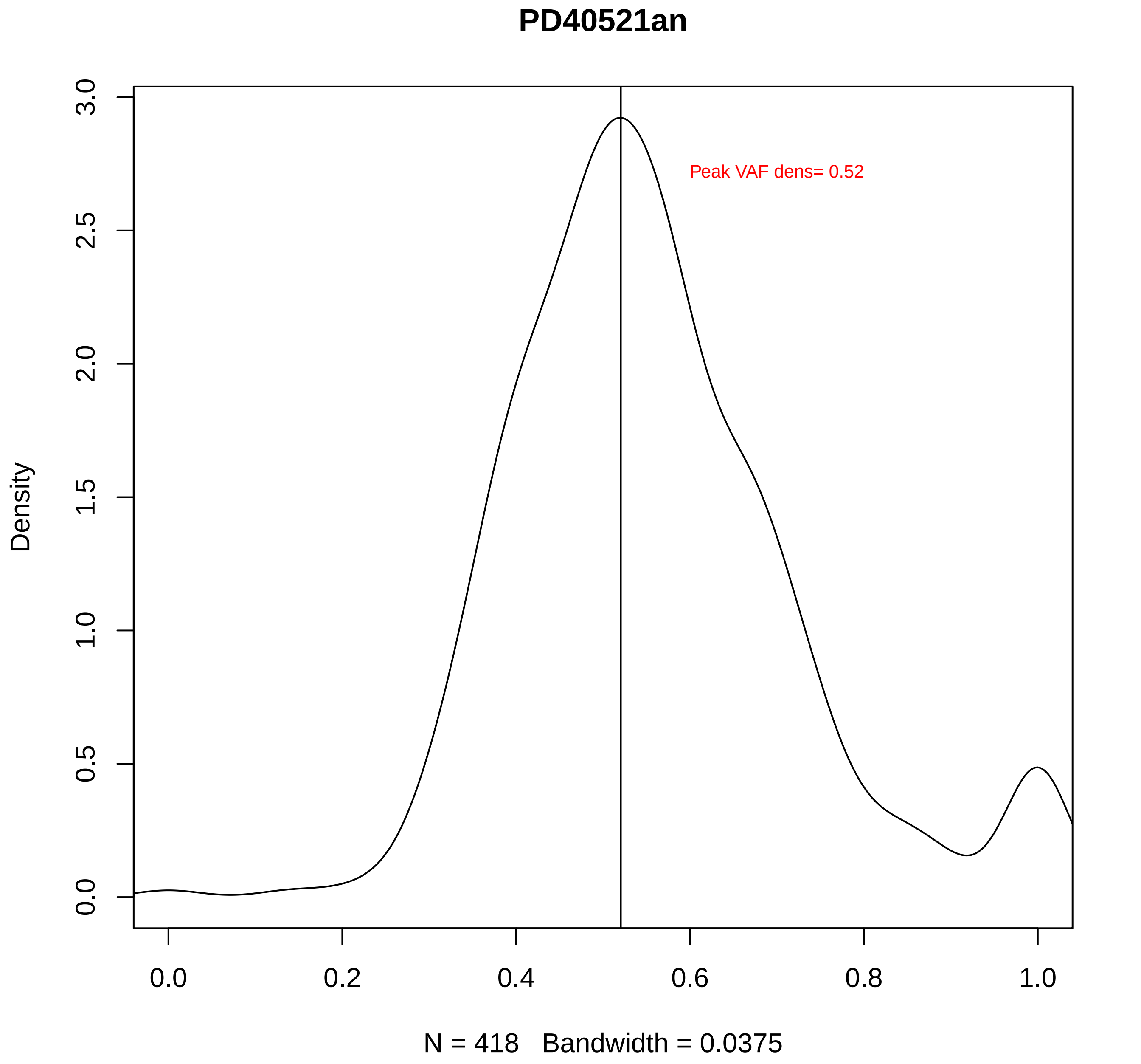
PD40521an
Peak VAF dens= 0.52
Density
0.0
0.5
1.0
1.5
2.0
2.5
3.0
0.0
0.2
0.4
0.6
0.8
1.0
N = 418   Bandwidth = 0.0375

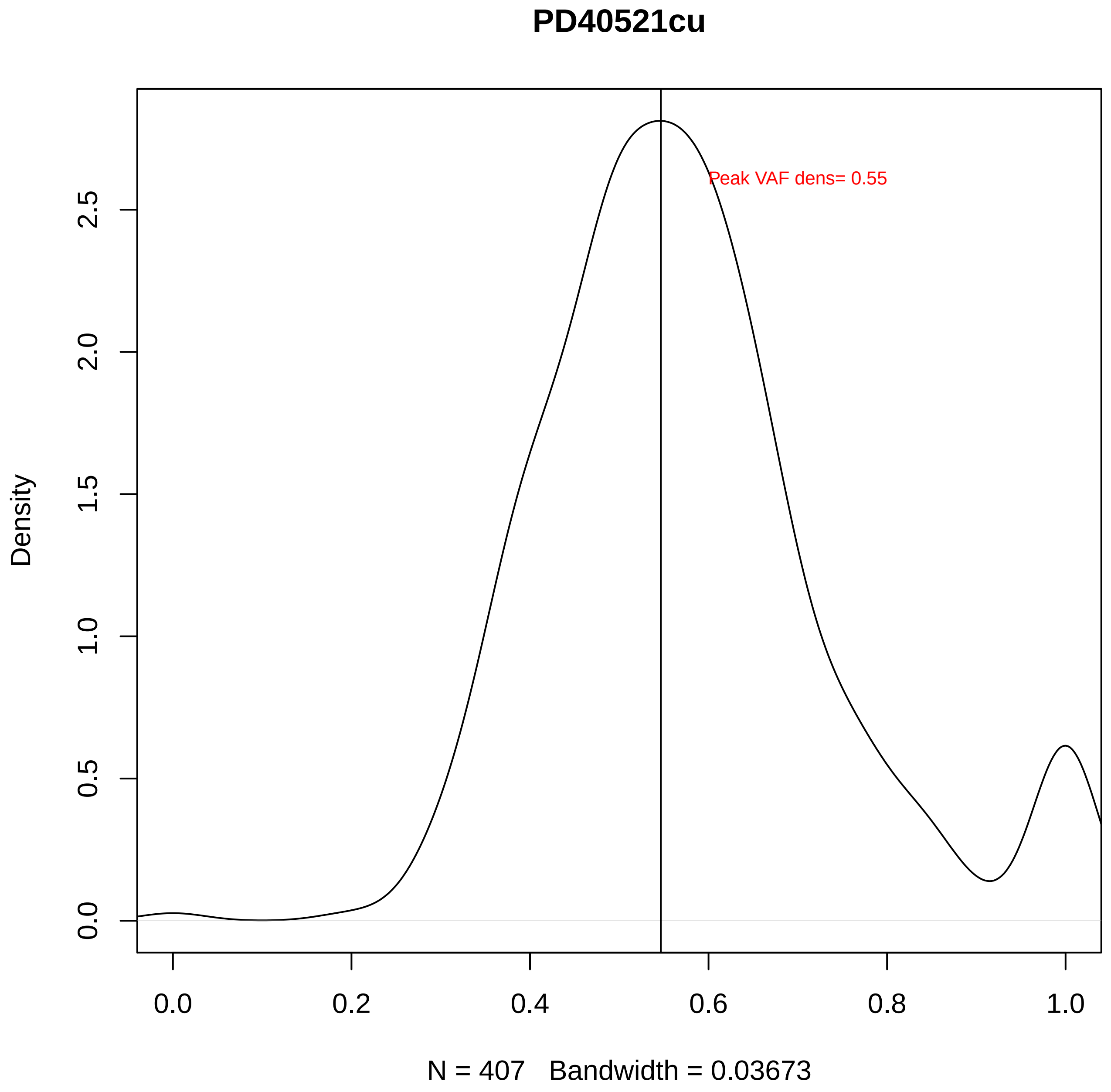

PD40521cu
Peak VAF dens= 0.55
Density
0.0
0.5
1.0
1.5
2.0
2.5
0.0
0.2
0.4
0.6
0.8
1.0
N = 407   Bandwidth = 0.03673

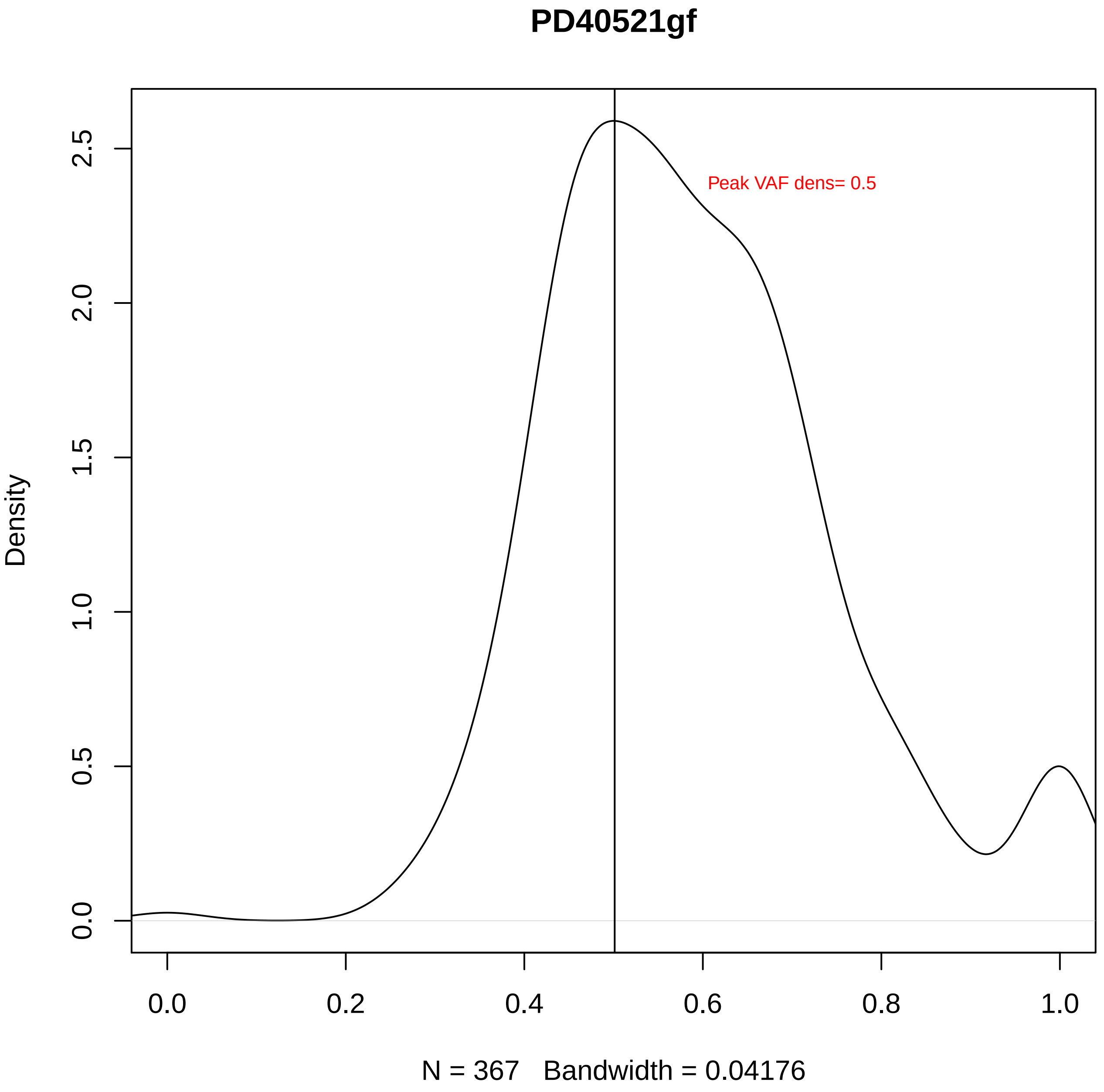
PD40521gf
Peak VAF dens= 0.5
Density
0.0
0.5
1.0
1.5
2.0
2.5
0.0
0.2
0.4
0.6
0.8
1.0
N = 367   Bandwidth = 0.04176

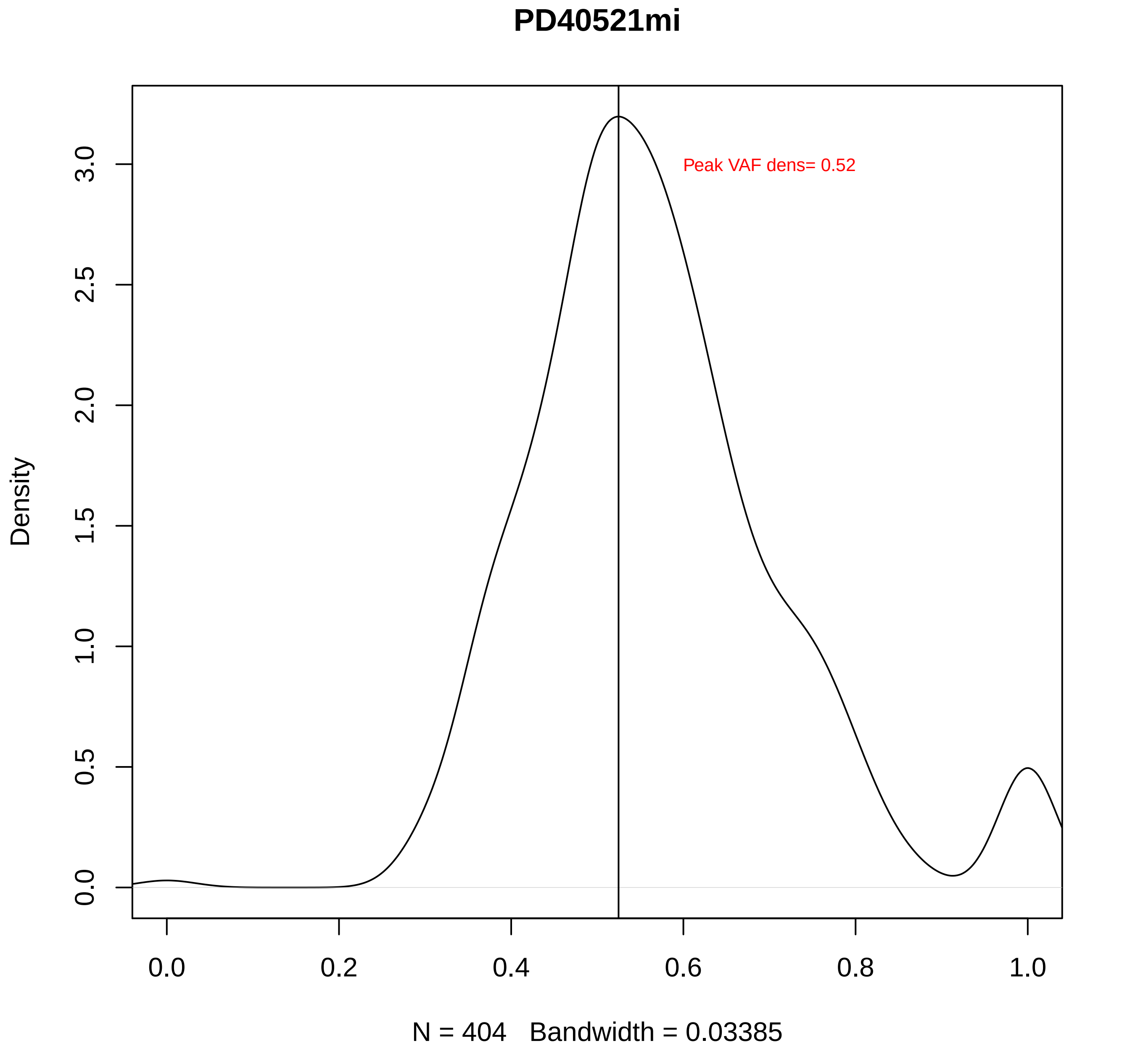
PD40521mi
Peak VAF dens= 0.52
Density
0.0
0.5
1.0
1.5
2.0
2.5
3.0
0.0
0.2
0.4
0.6
0.8
1.0
N = 404   Bandwidth = 0.03385

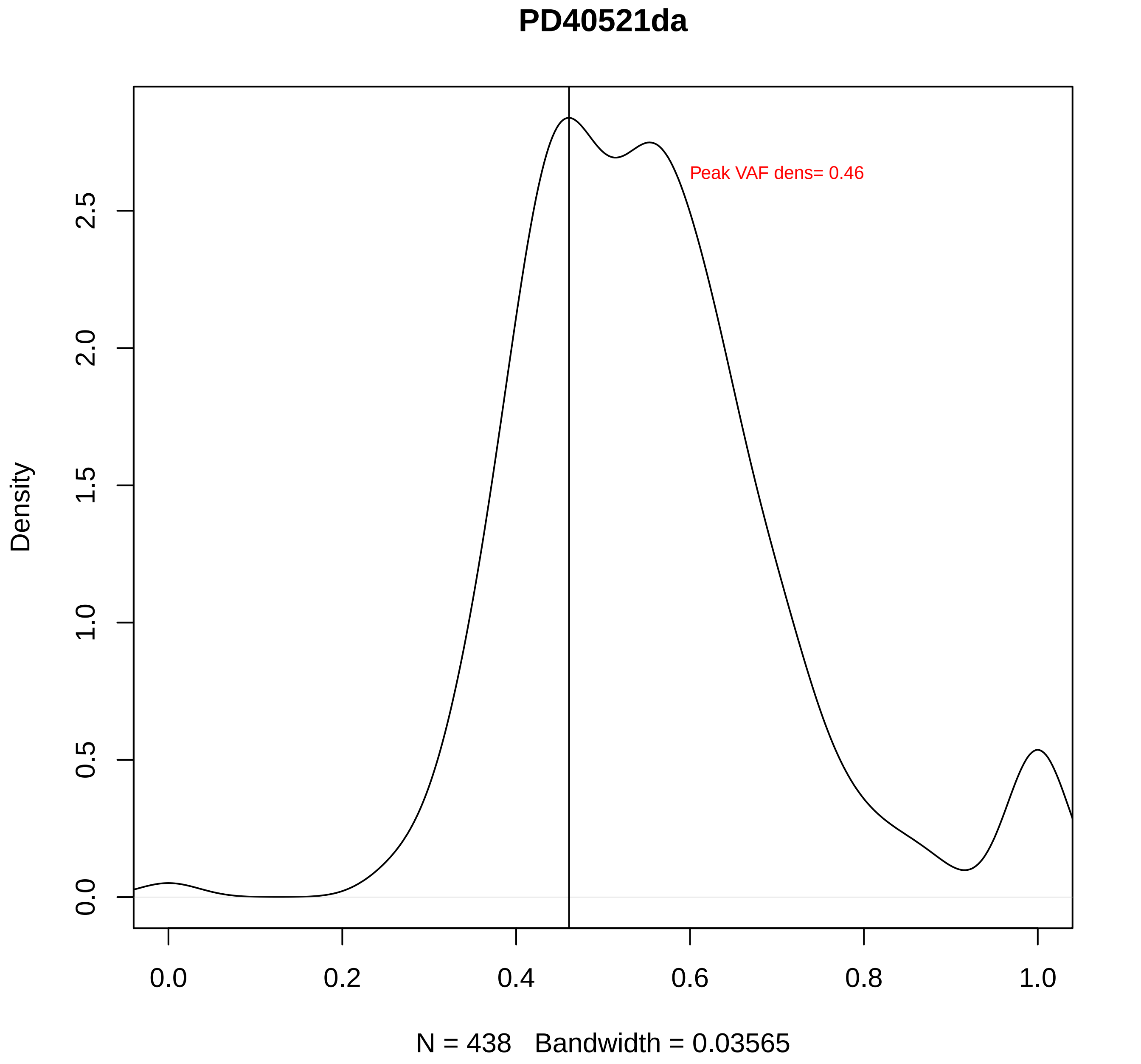

PD40521da
Peak VAF dens= 0.46
Density
0.0
0.5
1.0
1.5
2.0
2.5
0.0
0.2
0.4
0.6
0.8
1.0
N = 438   Bandwidth = 0.03565

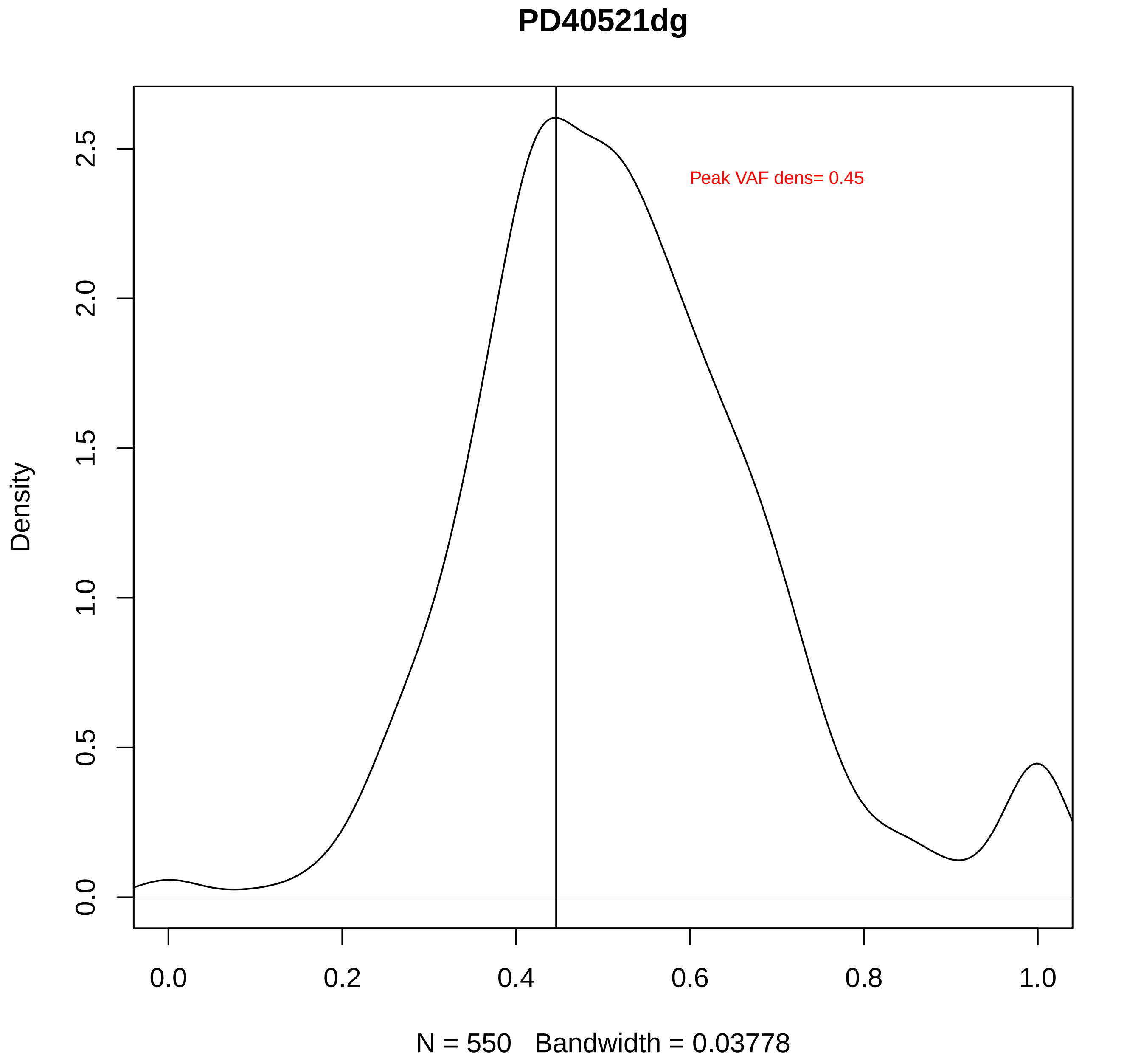
PD40521dg
Peak VAF dens= 0.45
Density
0.0
0.5
1.0
1.5
2.0
2.5
0.0
0.2
0.4
0.6
0.8
1.0
N = 550   Bandwidth = 0.03778

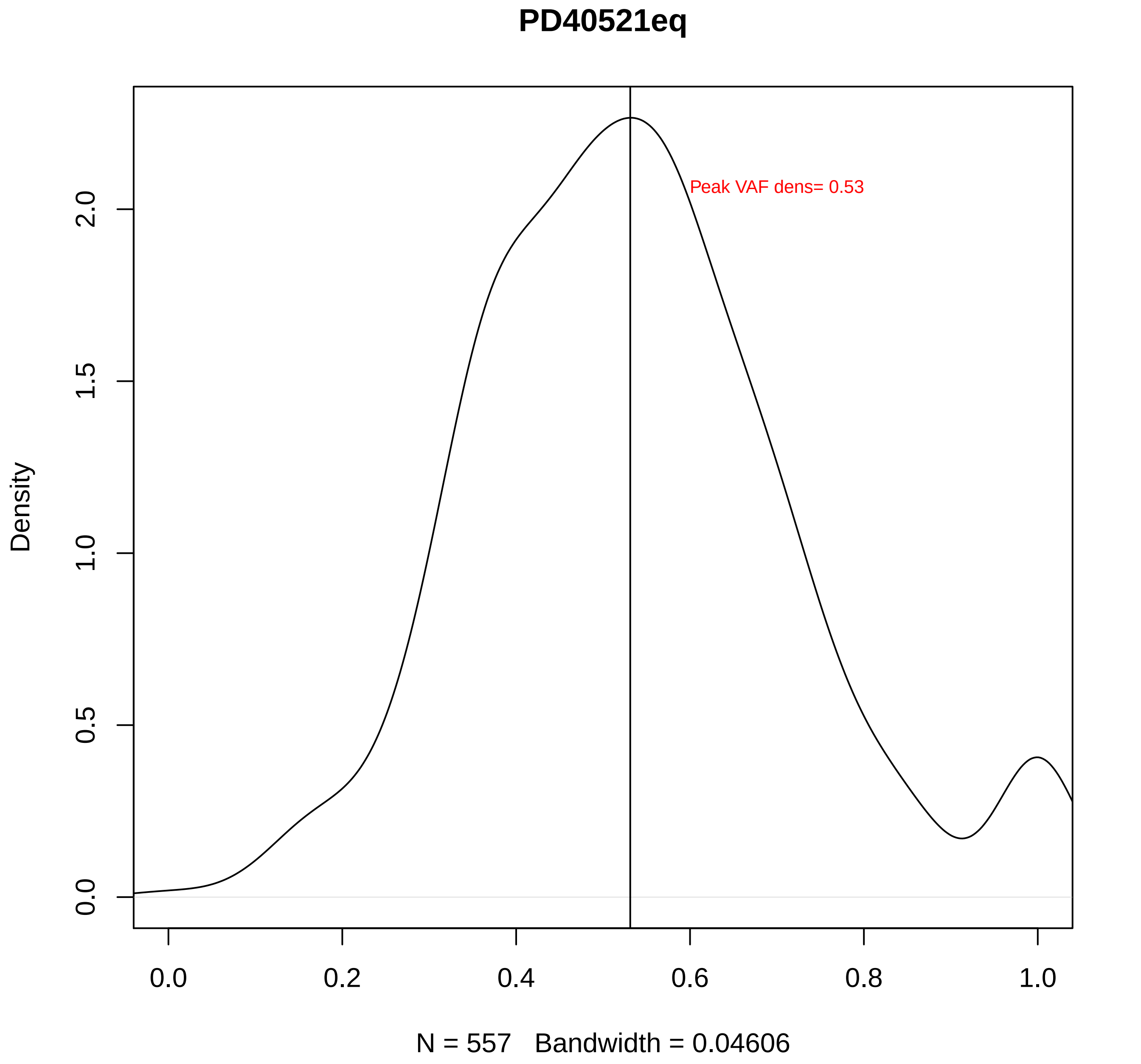
PD40521eq
Peak VAF dens= 0.53
Density
0.0
0.5
1.0
1.5
2.0
0.0
0.2
0.4
0.6
0.8
1.0
N = 557 Bandwidth = 0.04606

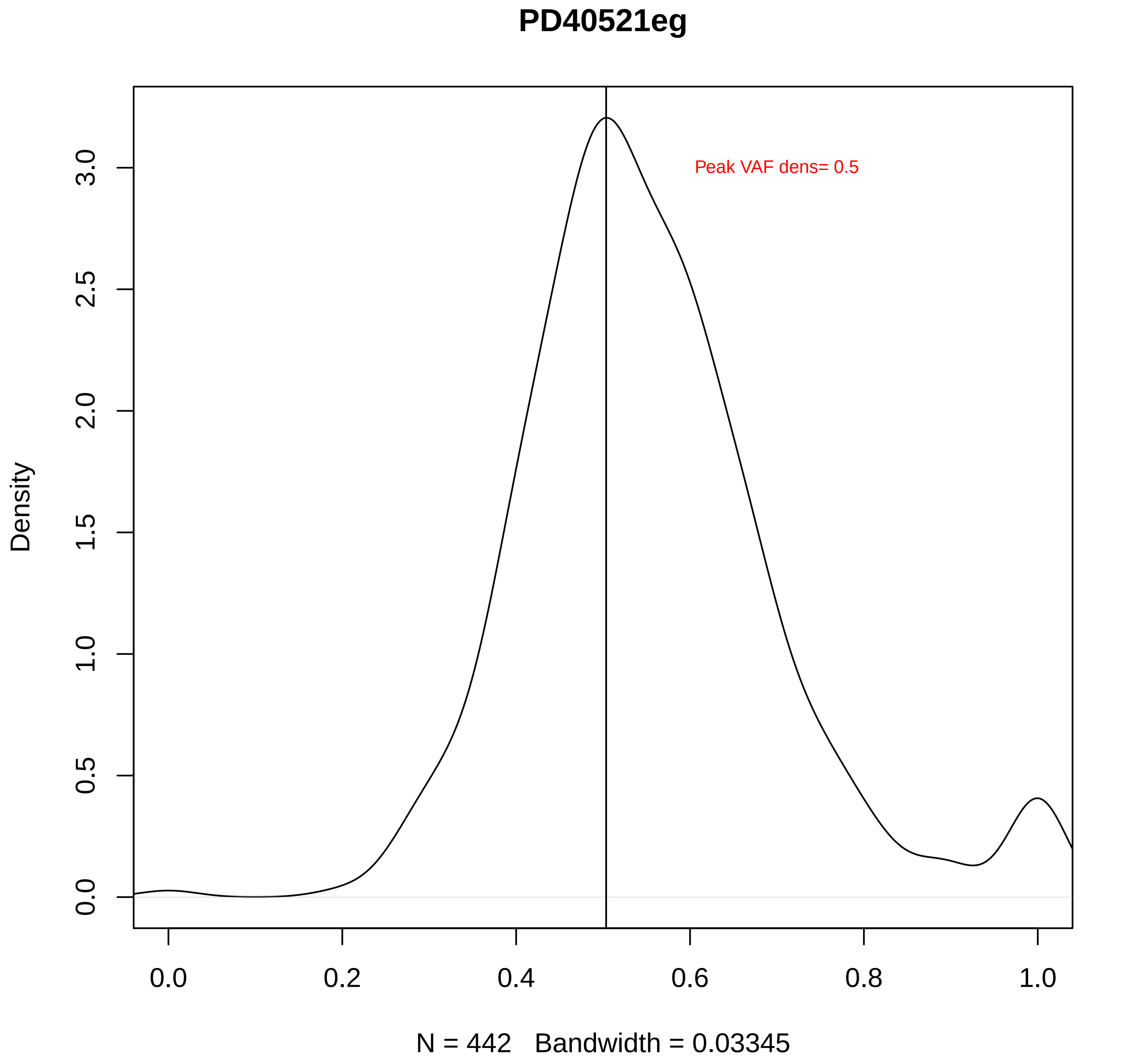
PD40521eg
Peak VAF dens= 0.5
Density
0.0
0.5
1.0
1.5
2.0
2.5
3.0
0.0
0.2
0.4
0.6
0.8
1.0
N = 442   Bandwidth = 0.03345

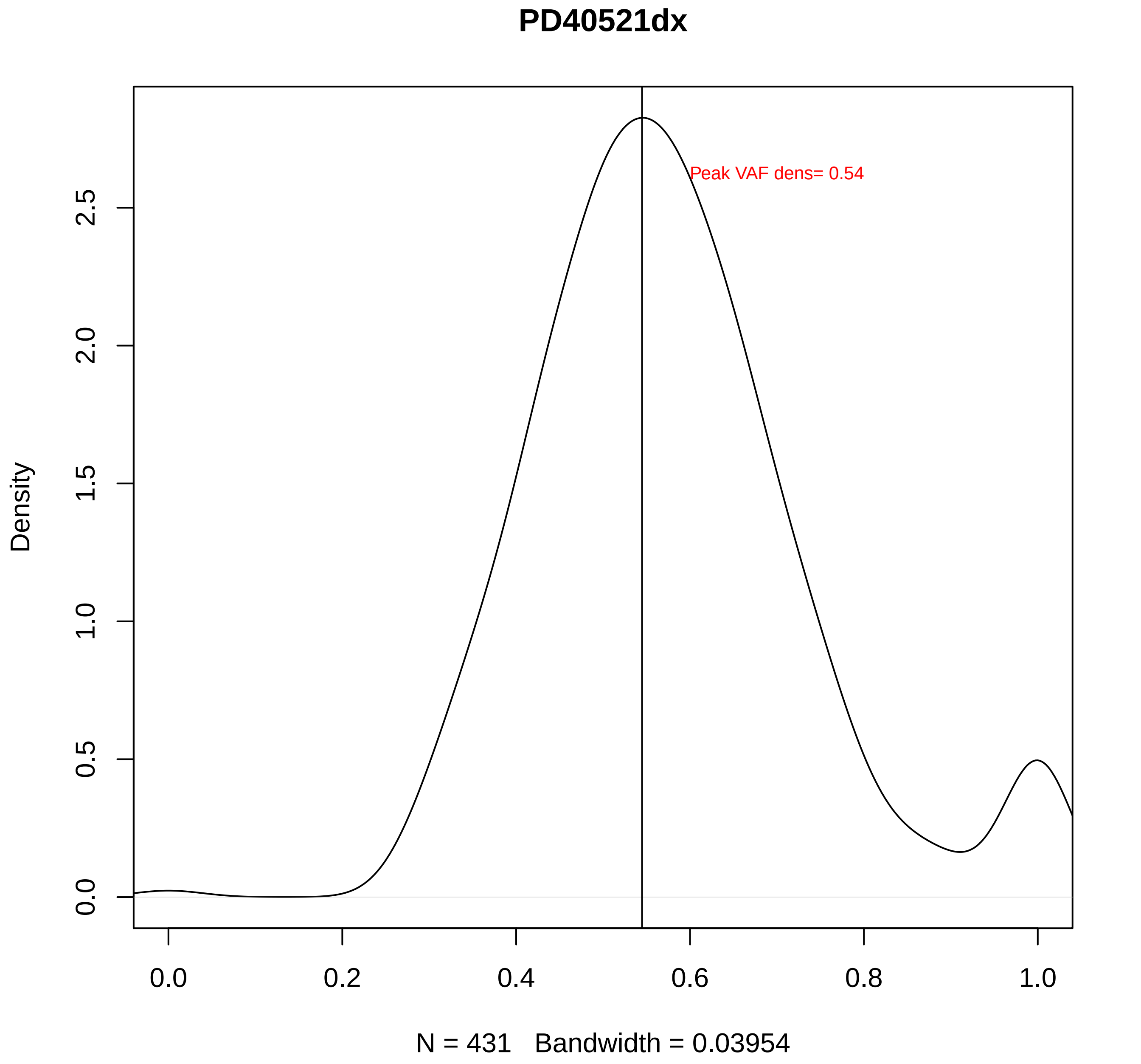
PD40521dx
Peak VAF dens= 0.54
Density
0.0
0.5
1.0
1.5
2.0
2.5
0.0
0.2
0.4
0.6
0.8
1.0
N = 431   Bandwidth = 0.03954

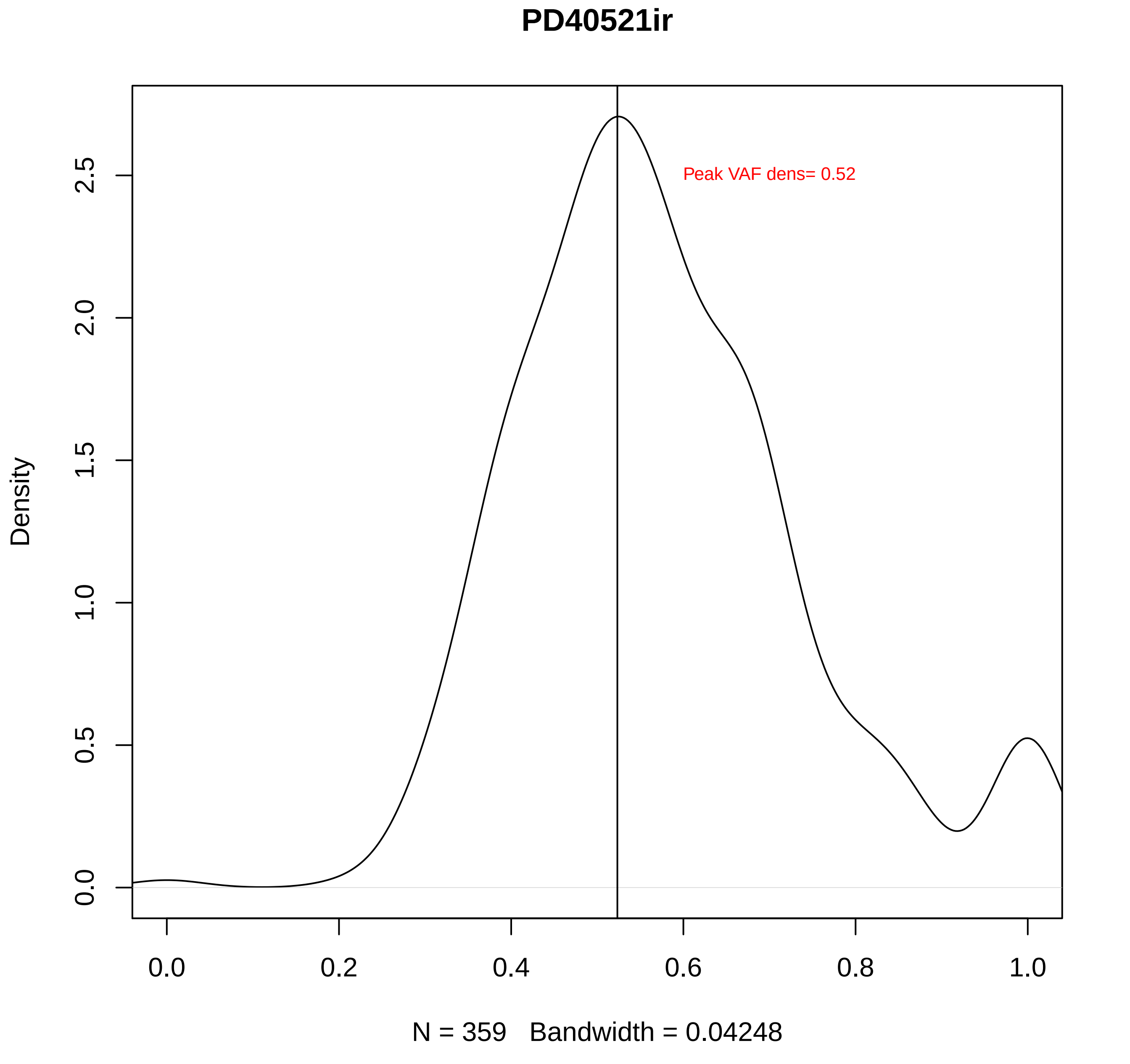
PD40521ir
Peak VAF dens= 0.52
Density
0.0
0.5
1.0
1.5
2.0
2.5
0.0
0.2
0.4
0.6
0.8
1.0
N = 359   Bandwidth = 0.04248

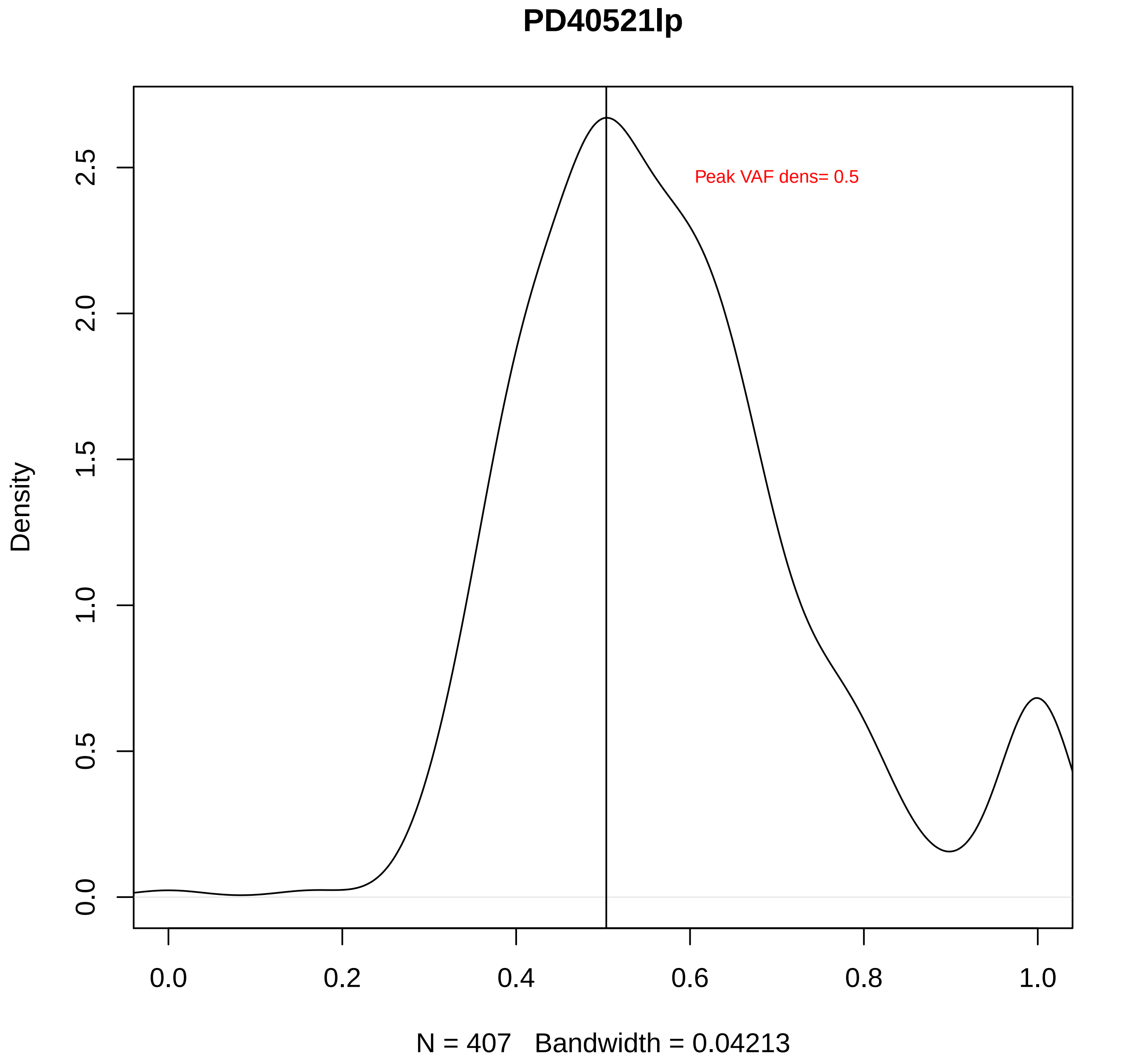

PD40521lp
Peak VAF dens= 0.5
Density
0.0
0.5
1.0
1.5
2.0
2.5
0.0
0.2
0.4
0.6
0.8
1.0
N = 407   Bandwidth = 0.04213

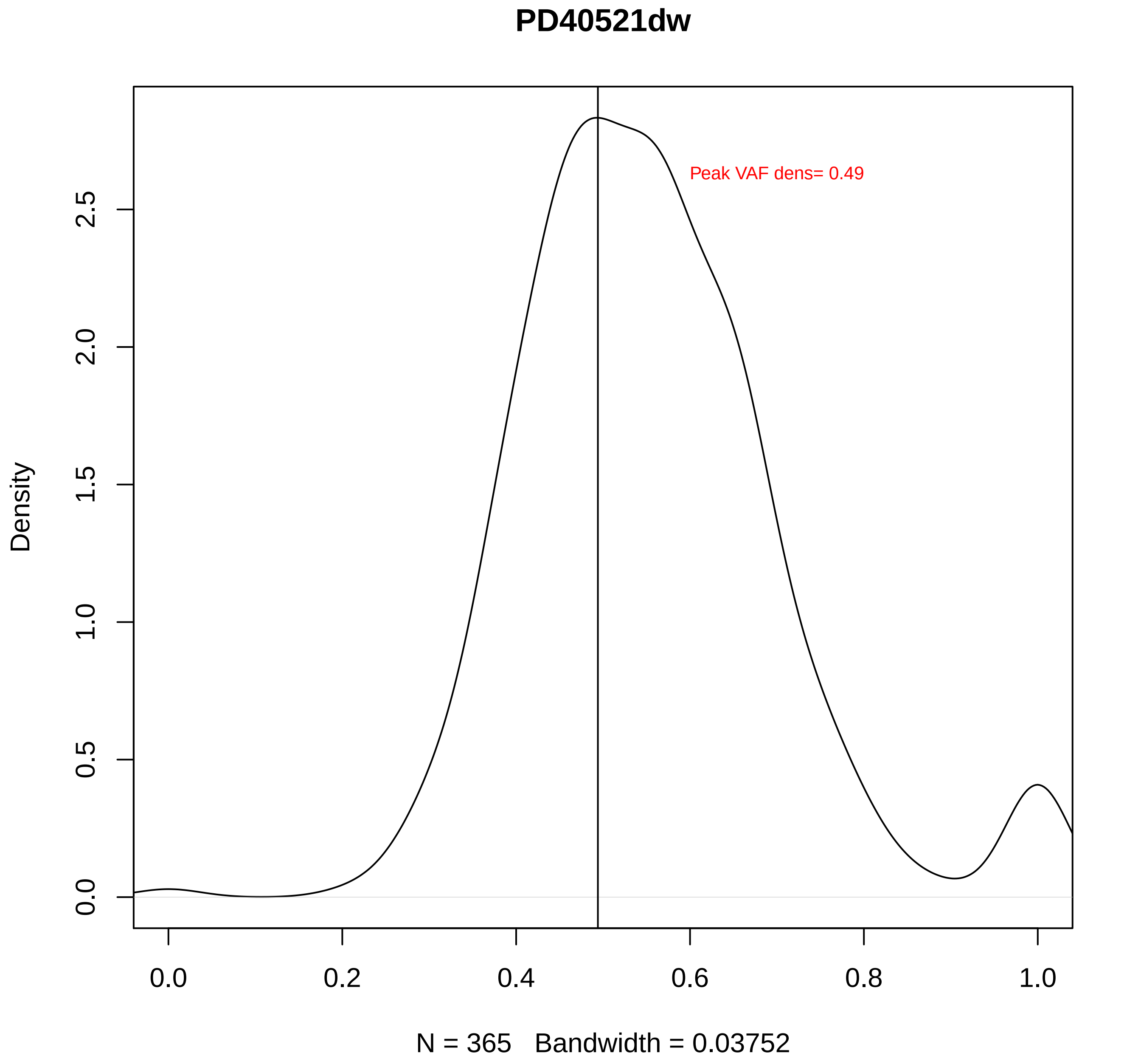
PD40521dw
Peak VAF dens= 0.49
Density
0.0
0.5
1.0
1.5
2.0
2.5
0.0
0.2
0.4
0.6
0.8
1.0
N = 365 Bandwidth = 0.03752

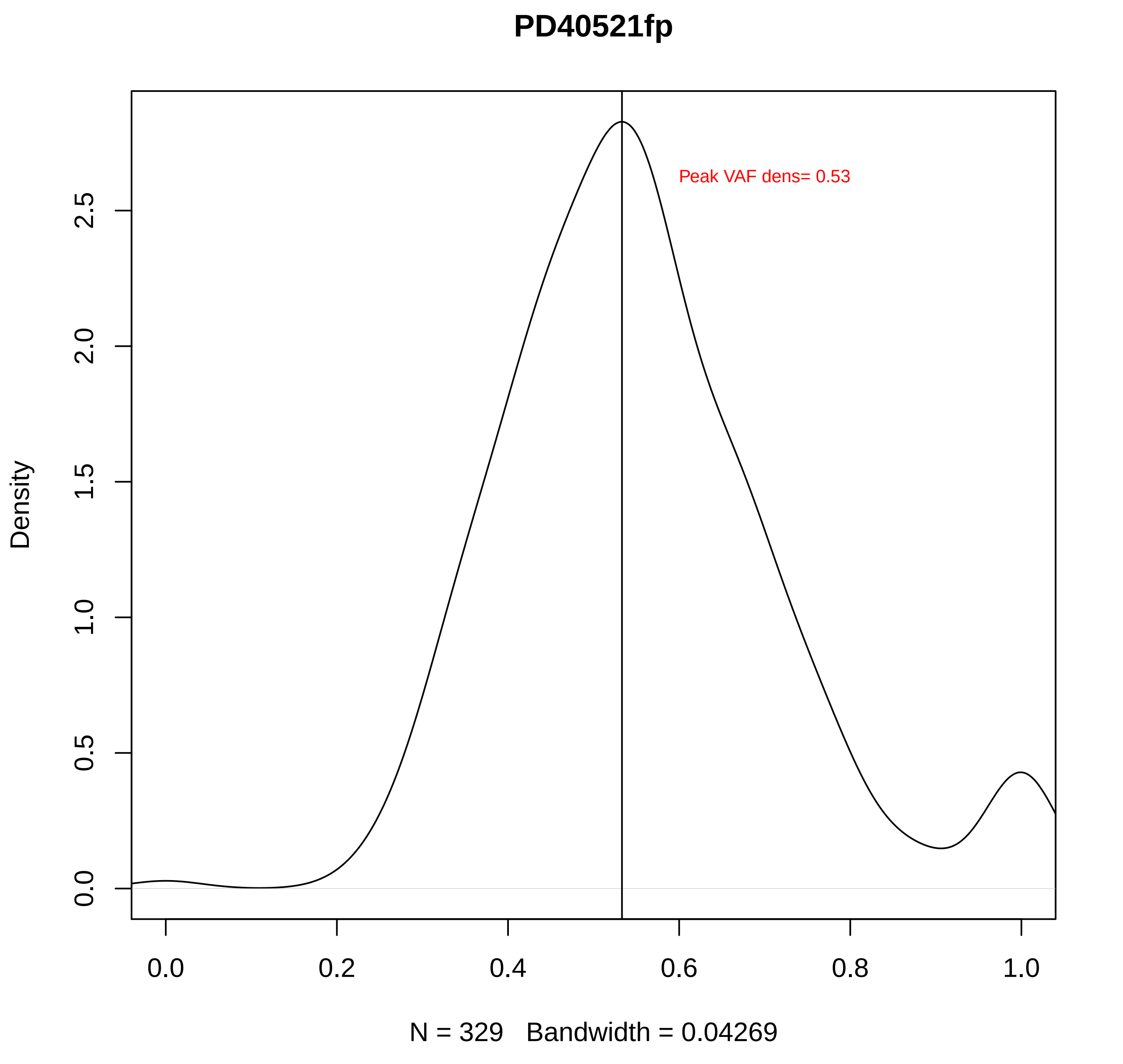
PD40521fp
Peak VAF dens= 0.53
Density
0.0
0.5
1.0
1.5
2.0
2.5
0.0
0.2
0.4
0.6
0.8
1.0
N = 329 Bandwidth = 0.04269

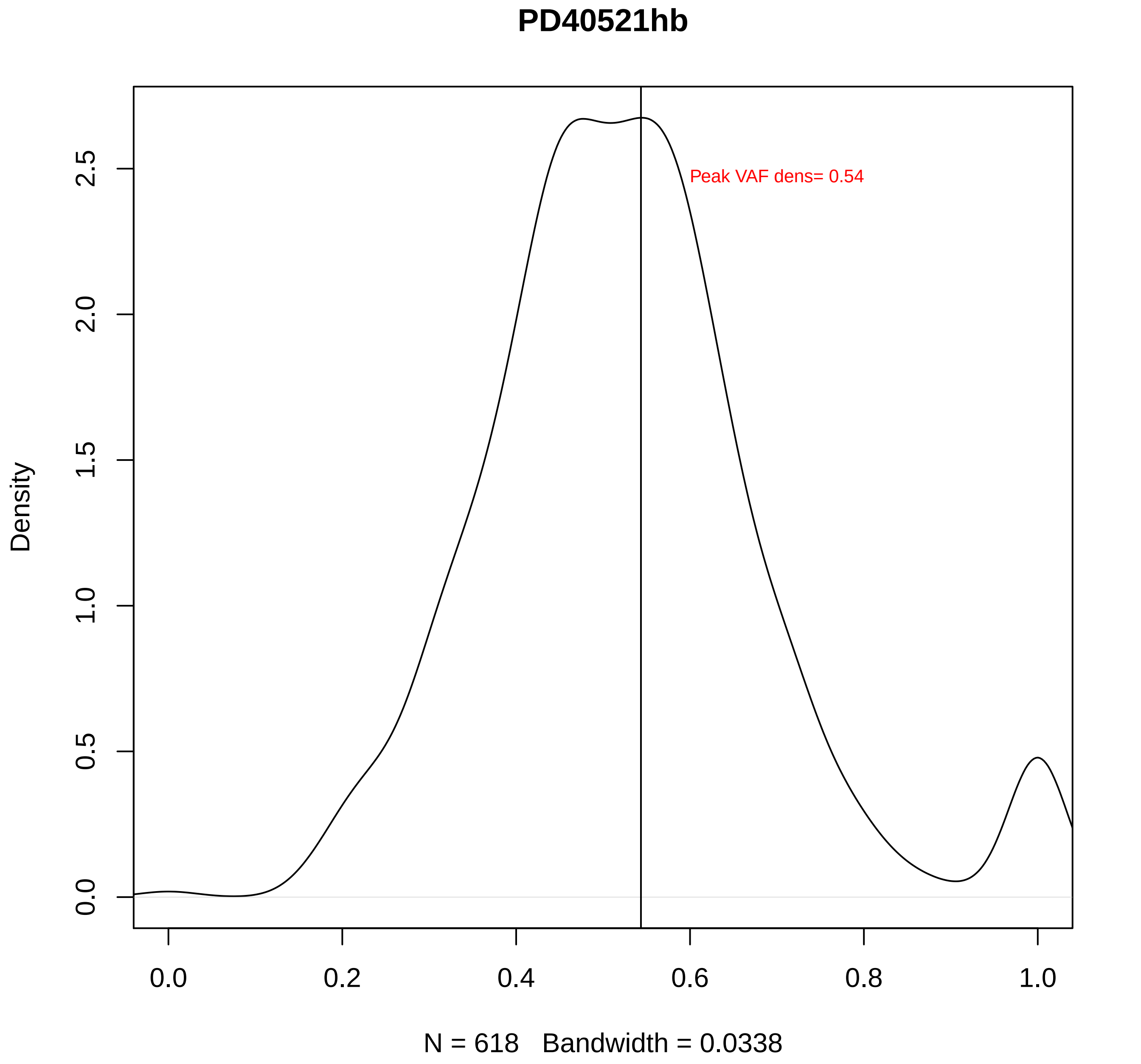

PD40521hb
Peak VAF dens= 0.54
Density
0.0
0.5
1.0
1.5
2.0
2.5
0.0
0.2
0.4
0.6
0.8
1.0
N = 618   Bandwidth = 0.0338

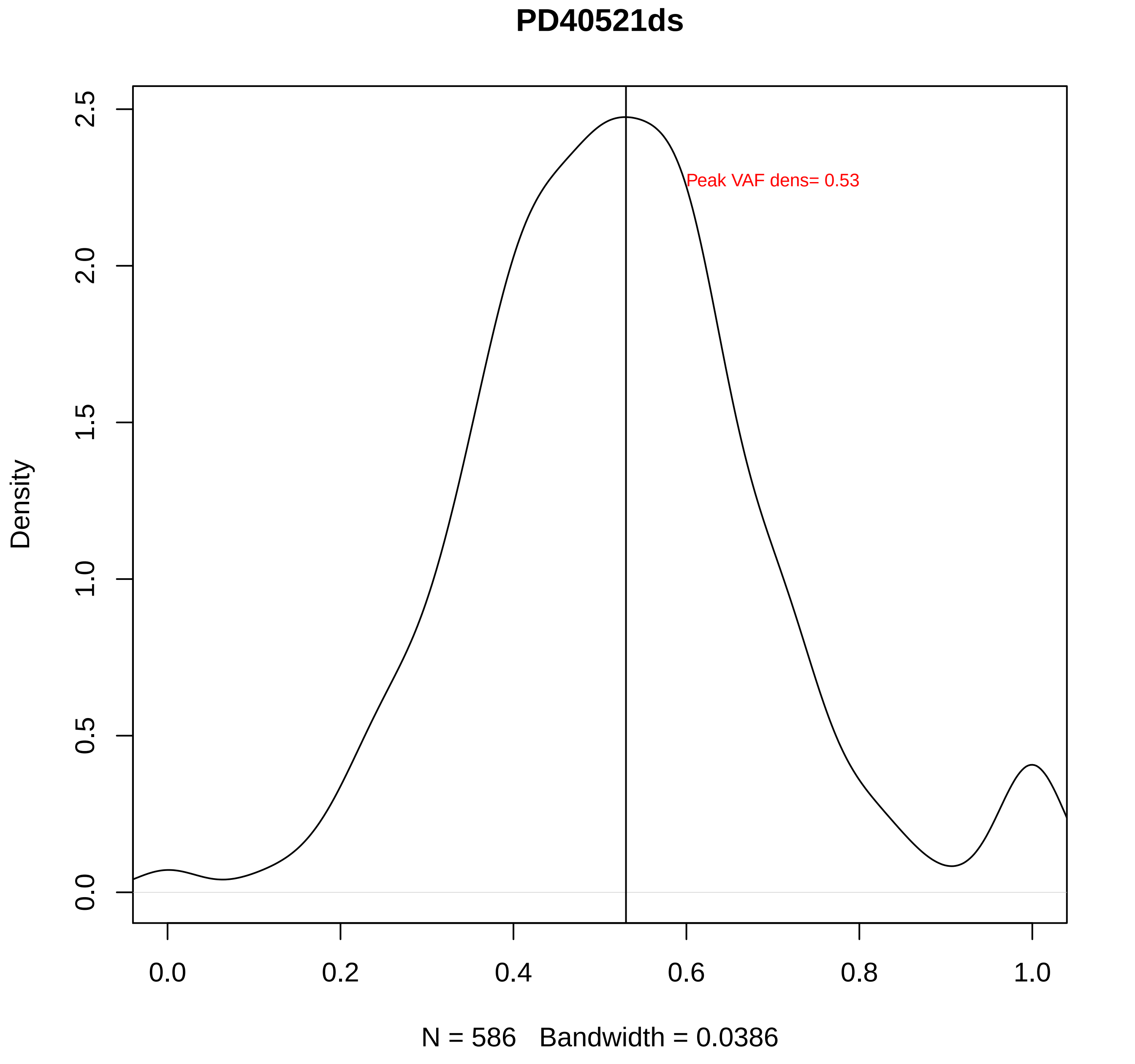
PD40521ds
Density
2.5
2.0
1.5
1.0
0.5
0.0
0.0
0.2
0.4
0.6
0.8
1.0
Peak VAF dens= 0.53
N = 586   Bandwidth = 0.0386

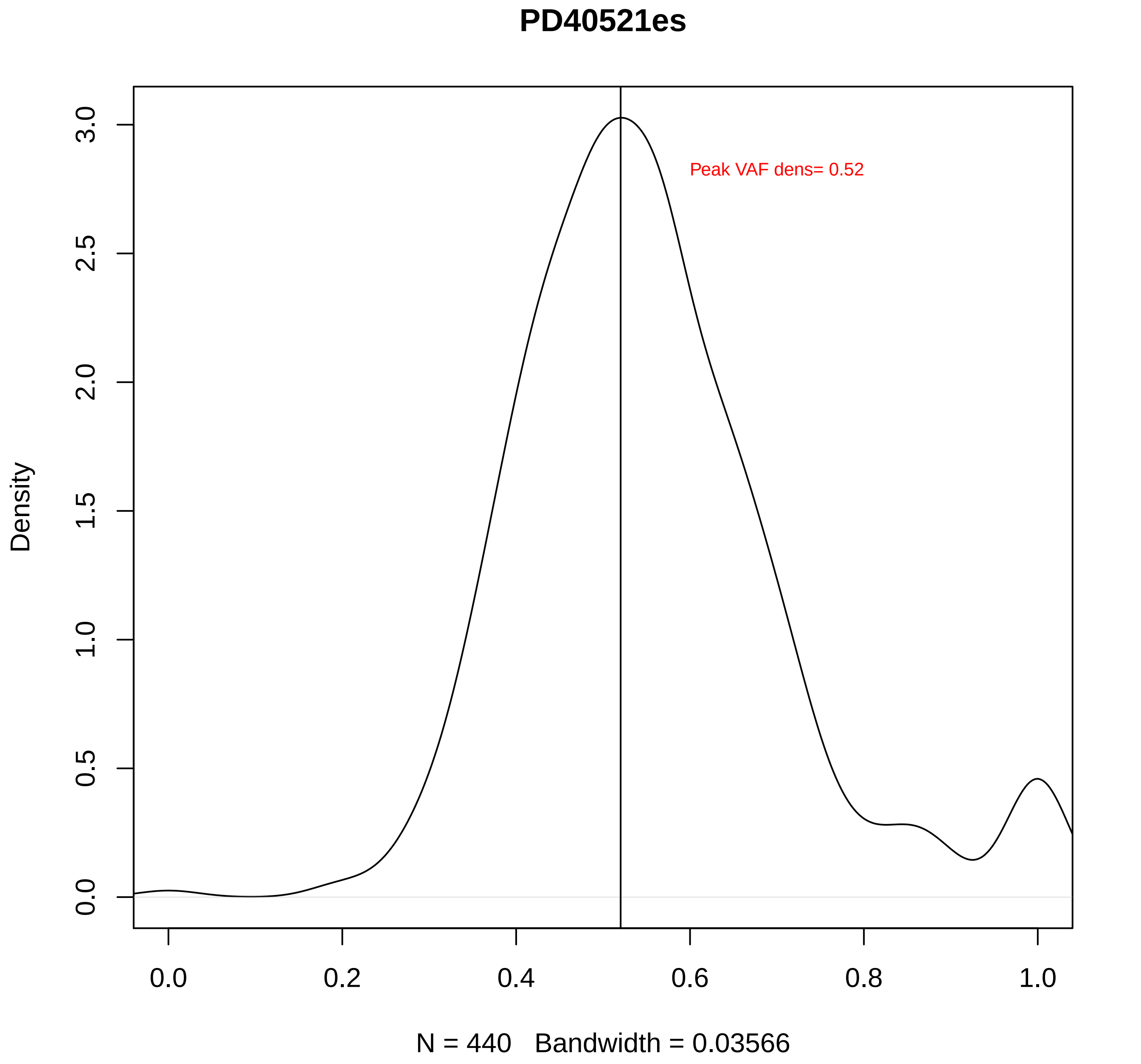
PD40521es
Peak VAF dens= 0.52
Density
3.0
2.5
2.0
1.5
1.0
0.5
0.0
0.0
0.2
0.4
0.6
0.8
1.0
N = 440   Bandwidth = 0.03566
