# Supplementary material for: Clonal dynamics of haematopoiesis across the human lifespan
Source: Nature. 2022 Jun 1;606(7913):343–50. doi: 10.1038/s41586-022-04786-y (PMC9177428; doi:10.1038/s41586-022-04786-y)
Supplement: Supplementary file 4 — HTMLs of notebooks outlining key statistical analyses presented in the manuscript, including analysis of phylogenetic trees. [file 41586_2022_4786_MOESM4_ESM.zip › Supplementary_code/SNV_indel_analysis/XX_subs_analysis.html]

Analysis of SNVs and indels (non-clonal samples included)


# Analysis of SNVs and indels (non-clonal samples included)

#### Emily Mitchell

### Summary

This script performs analysis and visualisation of SNV and indels in the dataset of a single individual. Plots of variant VAF distribution pre and post filtering are created per sample as a QC to enable identification of contaminated and non-clonal samples (as described in methods and illustrated in Extended Fig 2a,b).  
1. Calculation of per colony SNV and indel burden per sample.  
2. Graph of VAF distribution of unfiltered variants per sample (output not shown)  
3. Histogram of VAFs for filtered variants per sample (output not shown here but available for all individuals in pdf form separately “ID\_sample\_vaf\_plots.pdf”)

##### Open libraries

```
suppressMessages(library(GenomicRanges))
```

```
## Warning: package 'GenomicRanges' was built under R version 4.0.3
```

```
## Warning: package 'BiocGenerics' was built under R version 4.0.3
```

```
## Warning: package 'S4Vectors' was built under R version 4.0.3
```

```
## Warning: package 'IRanges' was built under R version 4.0.3
```

```
## Warning: package 'GenomeInfoDb' was built under R version 4.0.3
```

```
suppressMessages(library(dplyr))
suppressMessages(library(tidyr))
suppressMessages(library(ggplot2))
suppressMessages(library(BiocGenerics))
suppressMessages(library(knitr))
suppressMessages(library(stringr))
suppressMessages(library(ggtree))
```

```
## Warning: package 'ggtree' was built under R version 4.0.3
```

```
suppressMessages(library(ape))
suppressMessages(library(seqinr))
suppressMessages(library(data.table))
suppressMessages(library(phytools))
```

### 1. Set working directory and define files and paths

```
ID = "KX001" #Edit
Iteration = "KX001_4" #Edit
Run_ID = "KX001_4_01" #Edit
filtering_ID = "standard_rho01" #Edit
setwd = paste0("~/Documents/PhD/Sequencing_results/DNA_seq/",ID,"/",Iteration,"/subs/vaf/")
mats_and_param_file = paste0("~/Documents/PhD/Sequencing_results/DNA_seq/",ID,"/",Iteration,"/trees/vaf_cut/output/mats_and_params_", Run_ID)
file_annot = paste0("~/Documents/PhD/Sequencing_results/DNA_seq/",ID,"/",Iteration,"/trees/vaf/output/annotated_mut_set_",Run_ID,"_",filtering_ID)
tree_file_path = paste0("~/Documents/PhD/Sequencing_results/DNA_seq/",ID,"/",Iteration,"/trees/vaf/output/tree_", Run_ID,"_",filtering_ID, ".tree")
```

### 2. Load files

```
load(file_annot)
load(mats_and_param_file)
tree <- read.tree(tree_file_path)
```

### 3. Source functions

```
function_files=list.files('~/Documents/Bioinformatics/CGP/Functions/', full.names = TRUE, pattern = ".R")
suppressMessages(sapply(function_files, source))
```

```
##         /Users/em16/Documents/Bioinformatics/CGP/Functions//filters_parallel_functions.R
## value   ?                                                                               
## visible FALSE                                                                           
##         /Users/em16/Documents/Bioinformatics/CGP/Functions//targeted_analysis_functions.R
## value   ?                                                                                
## visible FALSE                                                                            
##         /Users/em16/Documents/Bioinformatics/CGP/Functions//tree_functions.R
## value   ?                                                                   
## visible FALSE
```

### 4. Assessing SNV burden per colony

```
SNVs_per_sample = colSums(filtered_muts$COMB_mats.tree.build$Genotype_bin[filtered_muts$COMB_mats.tree.build$mat$Mut_type == "SNV",] == 1)
mean(SNVs_per_sample); sd(SNVs_per_sample); range(SNVs_per_sample)
```

```
## [1] 427.1005
```

```
## [1] 65.7512
```

```
## [1] 228 632
```

```
Mutation_burden <- as.data.frame(colSums(filtered_muts$COMB_mats.tree.build$Genotype_bin[filtered_muts$COMB_mats.tree.build$mat$Mut_type == "SNV",] == 1))
Mutation_burden$Mean_depth <- (colSums(filtered_muts$COMB_mats.tree.build$NR)/nrow(filtered_muts$COMB_mats.tree.build$NR))
colnames(Mutation_burden)[1] <- "Number_mutations"
Mutation_burden$Sample <- rownames(Mutation_burden)
Summary_snvs <- as.data.frame(cbind(Mutation_burden[,c(3,1,2)]))

write.table(Summary_snvs, paste0("~/Documents/PhD/Sequencing_results/DNA_seq/",ID,"/",Iteration,"/subs/vaf/mutation_burden/",Iteration,"_sub_dep.txt"), sep="\t", col.names = T, row.names = F, quote=F)
```

### 5. Assessing Indel burden per colony

```
INDELs_per_sample = colSums(filtered_muts$COMB_mats.tree.build$Genotype_bin[filtered_muts$COMB_mats.tree.build$mat$Mut_type == "INDEL",] == 1)
mean(INDELs_per_sample); sd(INDELs_per_sample); range(INDELs_per_sample)
```

```
## [1] 10.52941
```

```
## [1] 4.872741
```

```
## [1]  1 29
```

```
Indel_burden <- as.data.frame(colSums(filtered_muts$COMB_mats.tree.build$Genotype_bin[filtered_muts$COMB_mats.tree.build$mat$Mut_type == "INDEL",] == 1))
Indel_burden$Mean_depth <- (colSums(filtered_muts$COMB_mats.tree.build$NR)/nrow(filtered_muts$COMB_mats.tree.build$NR))
colnames(Indel_burden)[1] <- "Number_indels"
Indel_burden$Sample <- rownames(Indel_burden)
Summary_indels <- as.data.frame(cbind(Indel_burden[,c(3,1,2)]))

write.table(Summary_indels, paste0("~/Documents/PhD/Sequencing_results/DNA_seq/",ID,"/",Iteration,"/subs/vaf/mutation_burden/",Iteration, "_indel_dep.txt"), sep="\t", col.names = T, row.names = F, quote=F)
```

### 6. Graph of VAF distribution of unfiltered variants per sample (output not shown)

```
xmtrs <- filtered_muts$COMB_mats.tree.build$NV
xdeps <- filtered_muts$COMB_mats.tree.build$NR
xvafs <- xmtrs/xdeps

#Create list of samples to loop through

samples <- colnames(xvafs)

for (sample in samples[1:ncol(xvafs)]) {
  raw_vafs <- xvafs[,sample]
  pos_vafs <- raw_vafs[raw_vafs>0 & !is.na(raw_vafs)]
  
  pdf(paste0("~/Documents/PhD/Sequencing_results/DNA_seq/",ID,"/",Iteration,"/subs/vaf/vaf_plots/",sample, "_VAF_dist_somatic.pdf"))
  hist(pos_vafs, main = paste0(sample, " histogram of somatic variants"), breaks =20)  
 dev.off()
 
}
```

### 7. Histogram of VAFs for filtered variants per sample (output not shown)

```
vaf_density_plot_final=function(sample,tree,COMB_mats){
  node <- which(tree$tip.label==sample)
  sample_muts <- COMB_mats$mat$mut_ref[COMB_mats$mat$node %in% get_ancestral_nodes(node,tree$edge)]
  COMB_mats$NR[COMB_mats$NR == 0] <- 1
  dens <- density((COMB_mats$NV/COMB_mats$NR)[sample_muts,sample])
  plot(dens,xlim = c(0,1),main=sample)
  abline(v = dens$x[which.max(dens$y)])
  text(0.7, max(dens$y) - 0.2, paste("Peak VAF dens=",round(dens$x[which.max(dens$y)], digits = 2)),col="red",cex = 0.7)
} 
 
pdf(paste0("~/Documents/PhD/Sequencing_results/DNA_seq/",ID,"/",Iteration,"/subs/vaf/vaf_plots/sample_vaf_plots.pdf"))
sapply(tree$tip.label, #a list of all the sample names – I get this from the tree tip labels
vaf_density_plot_final, #the function above
tree=tree, #the tree object
COMB_mats = filtered_muts$COMB_mats.tree.build)  #This is from the file_annot output which includes node numbers for each mutation
dev.off()
```
