# Supplementary material for: Clonal dynamics of haematopoiesis across the human lifespan
Source: Nature. 2022 Jun 1;606(7913):343–50. doi: 10.1038/s41586-022-04786-y (PMC9177428; doi:10.1038/s41586-022-04786-y)
Supplement: Supplementary file 4 — HTMLs of notebooks outlining key statistical analyses presented in the manuscript, including analysis of phylogenetic trees. [file 41586_2022_4786_MOESM4_ESM.zip › Supplementary_code/SNV_indel_analysis/KX004_vaf_plots.pdf]

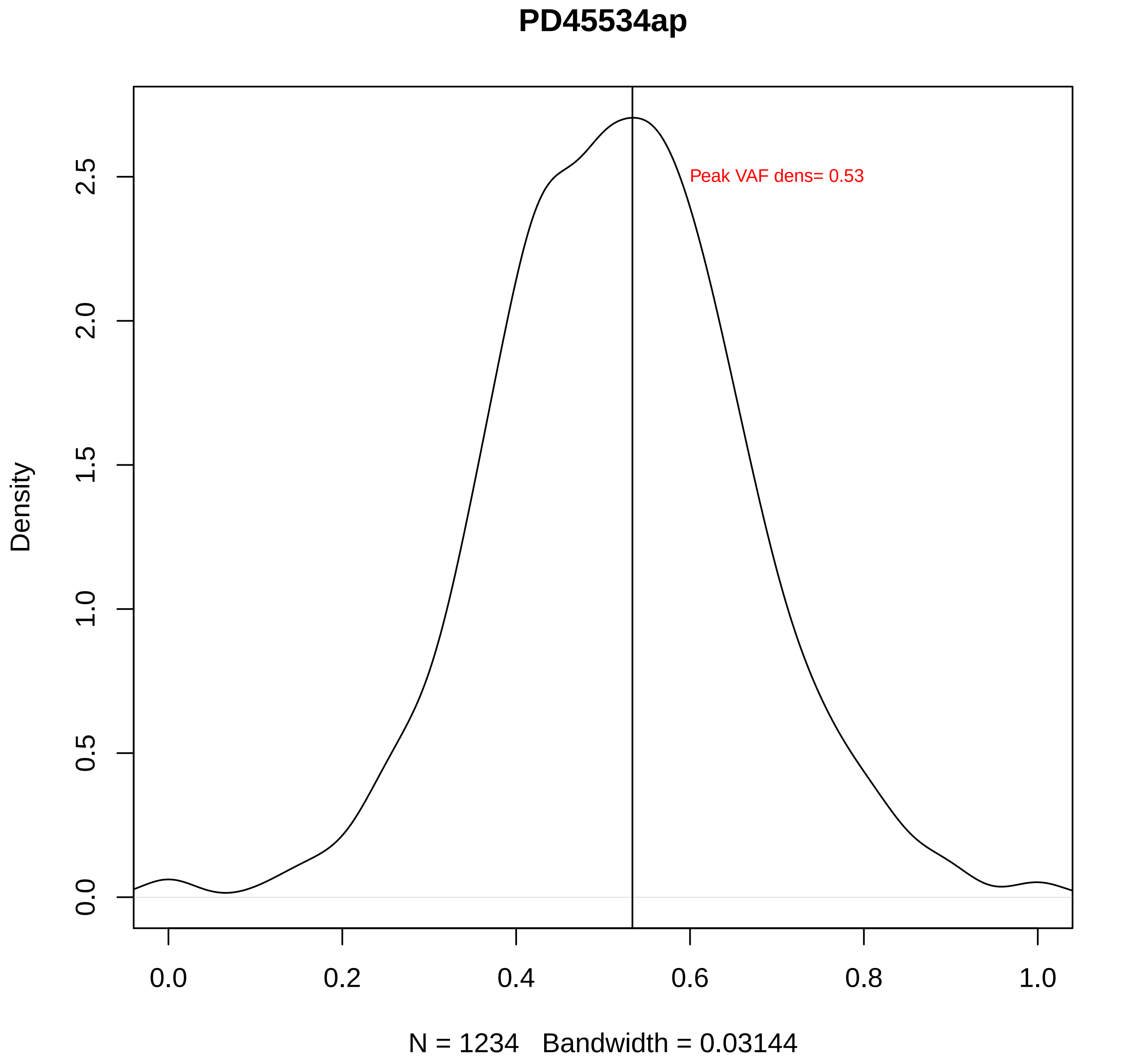
PD45534ap
Peak VAF dens= 0.53
Density
0.0
0.5
1.0
1.5
2.0
2.5
0.0
0.2
0.4
0.6
0.8
1.0
N = 1234   Bandwidth = 0.03144

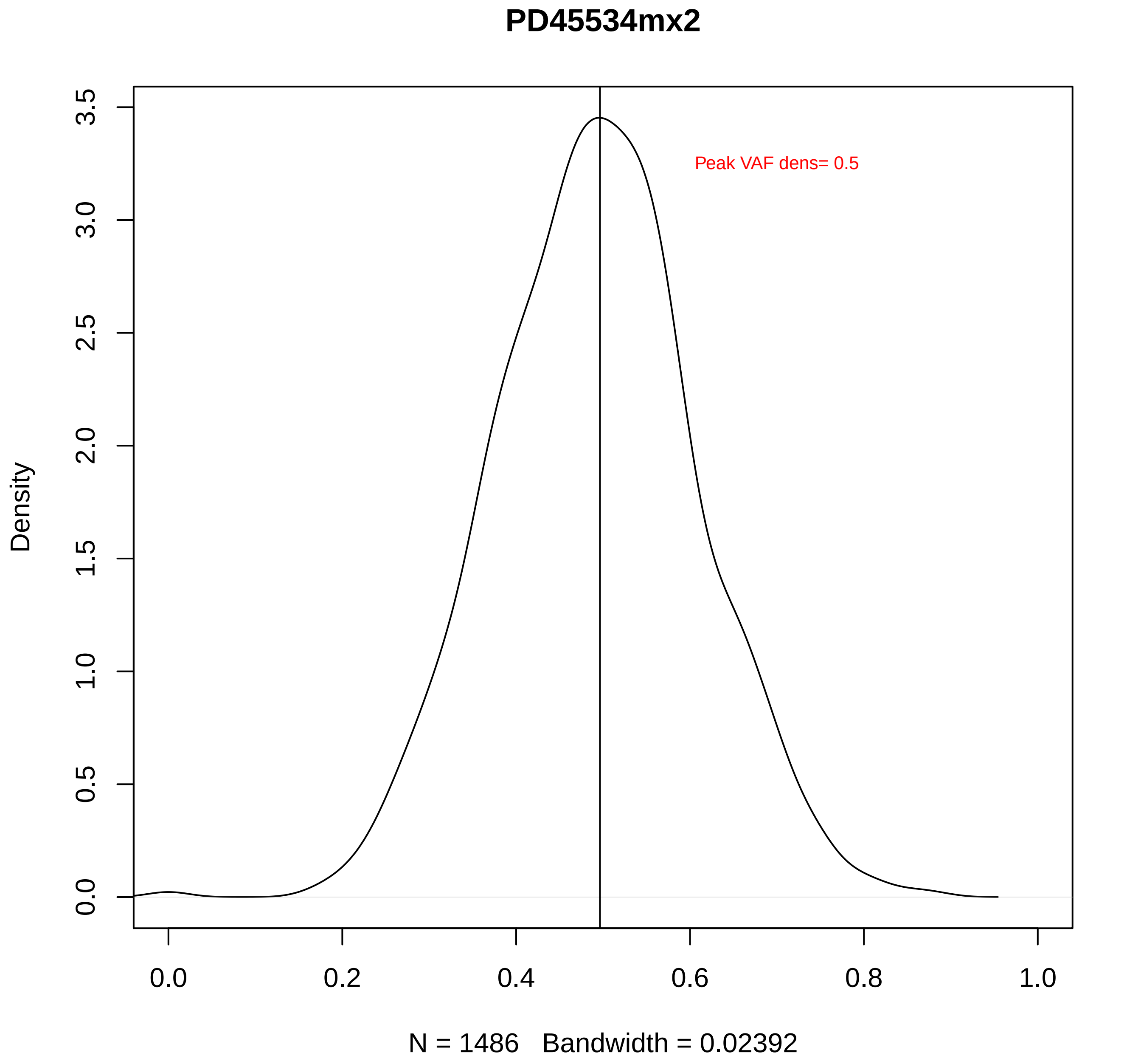

PD45534mx2
Peak VAF dens= 0.5
Density
0.0
0.5
1.0
1.5
2.0
2.5
3.0
3.5
0.0
0.2
0.4
0.6
0.8
1.0
N = 1486   Bandwidth = 0.02392

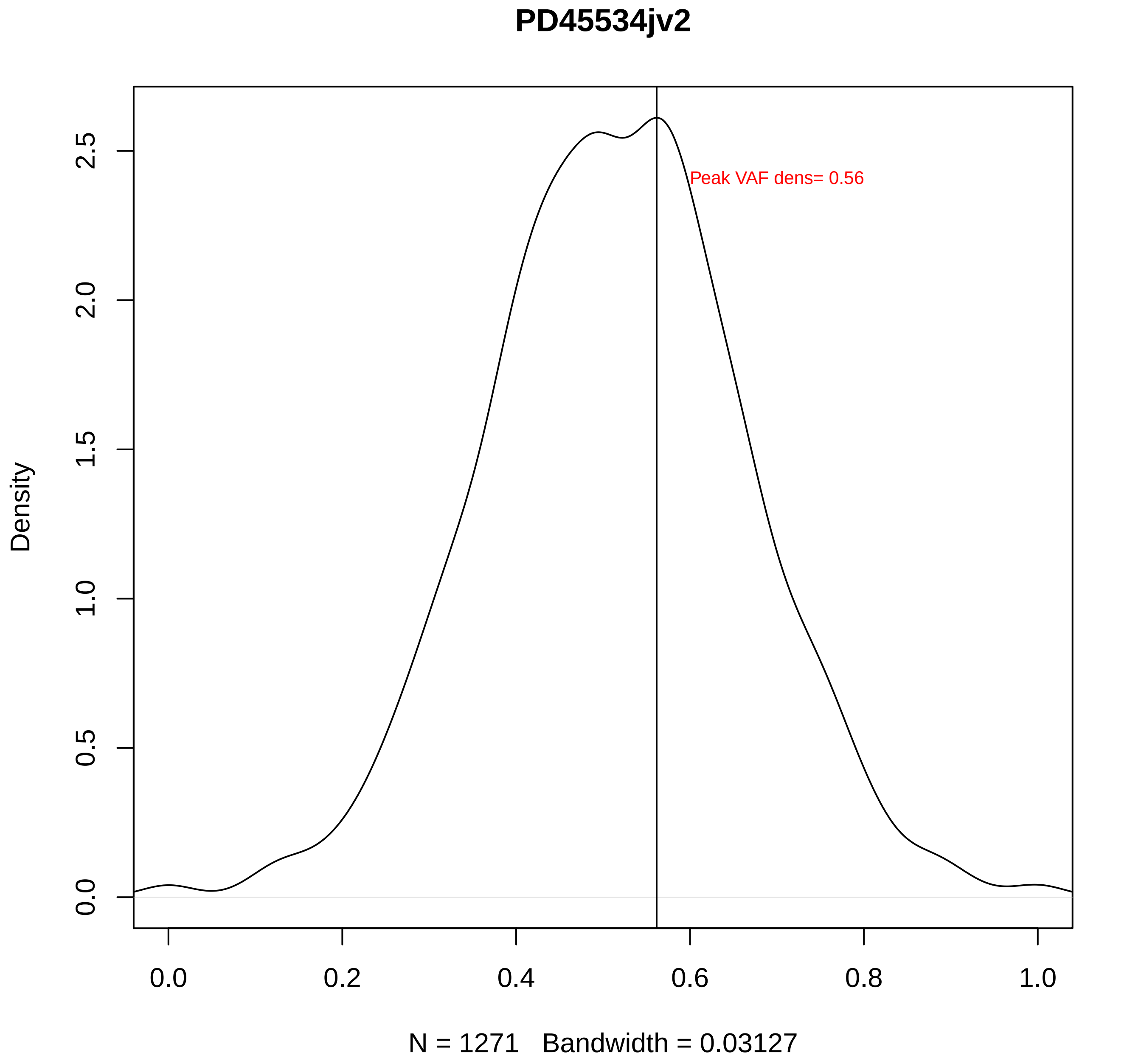

PD45534jv2
Peak VAF dens= 0.56
Density
0.0
0.5
1.0
1.5
2.0
2.5
0.0
0.2
0.4
0.6
0.8
1.0
N = 1271 Bandwidth = 0.03127

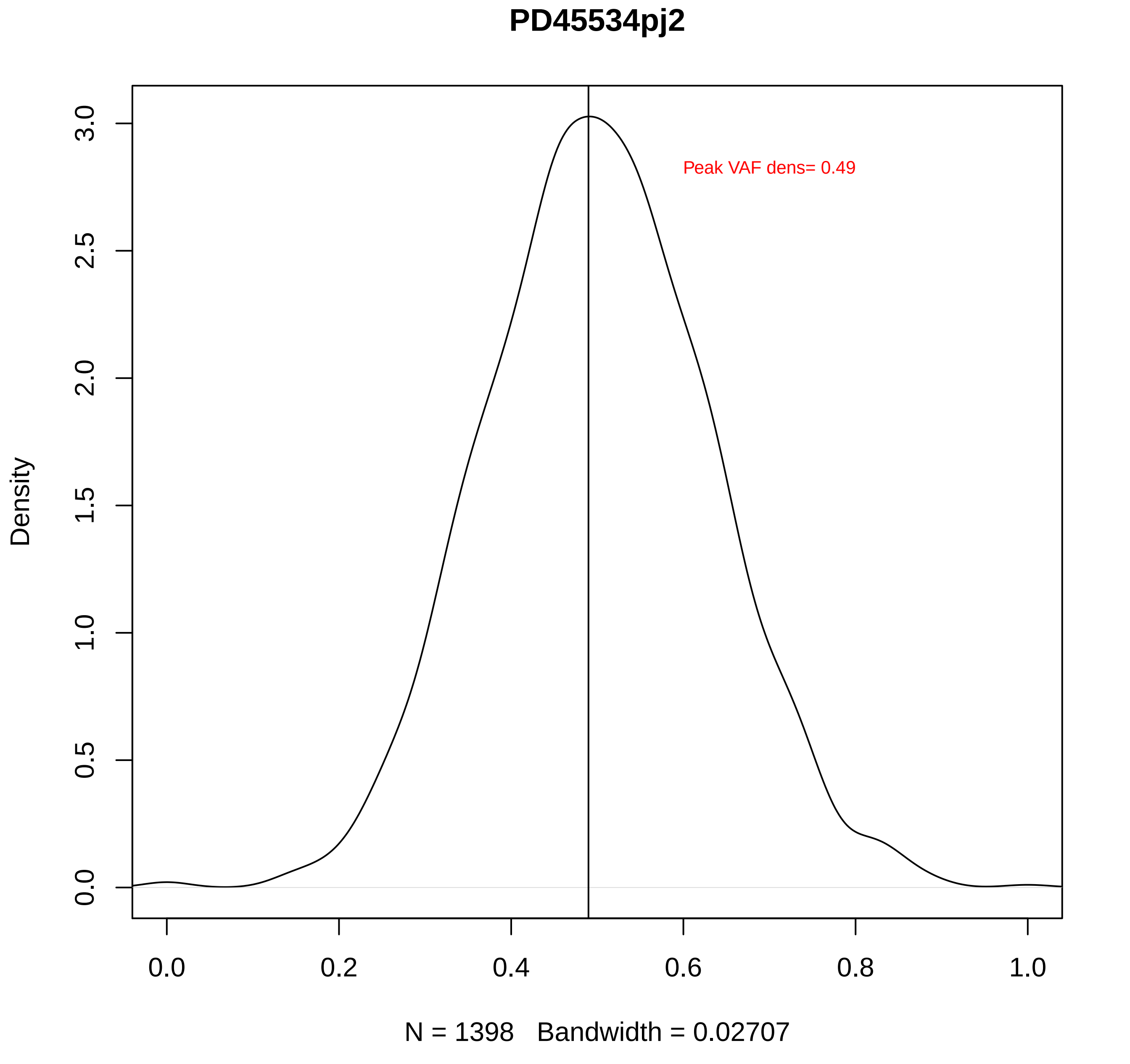
PD45534pj2
Peak VAF dens= 0.49
Density
0.0
0.5
1.0
1.5
2.0
2.5
3.0
0.0
0.2
0.4
0.6
0.8
1.0
N = 1398   Bandwidth = 0.02707

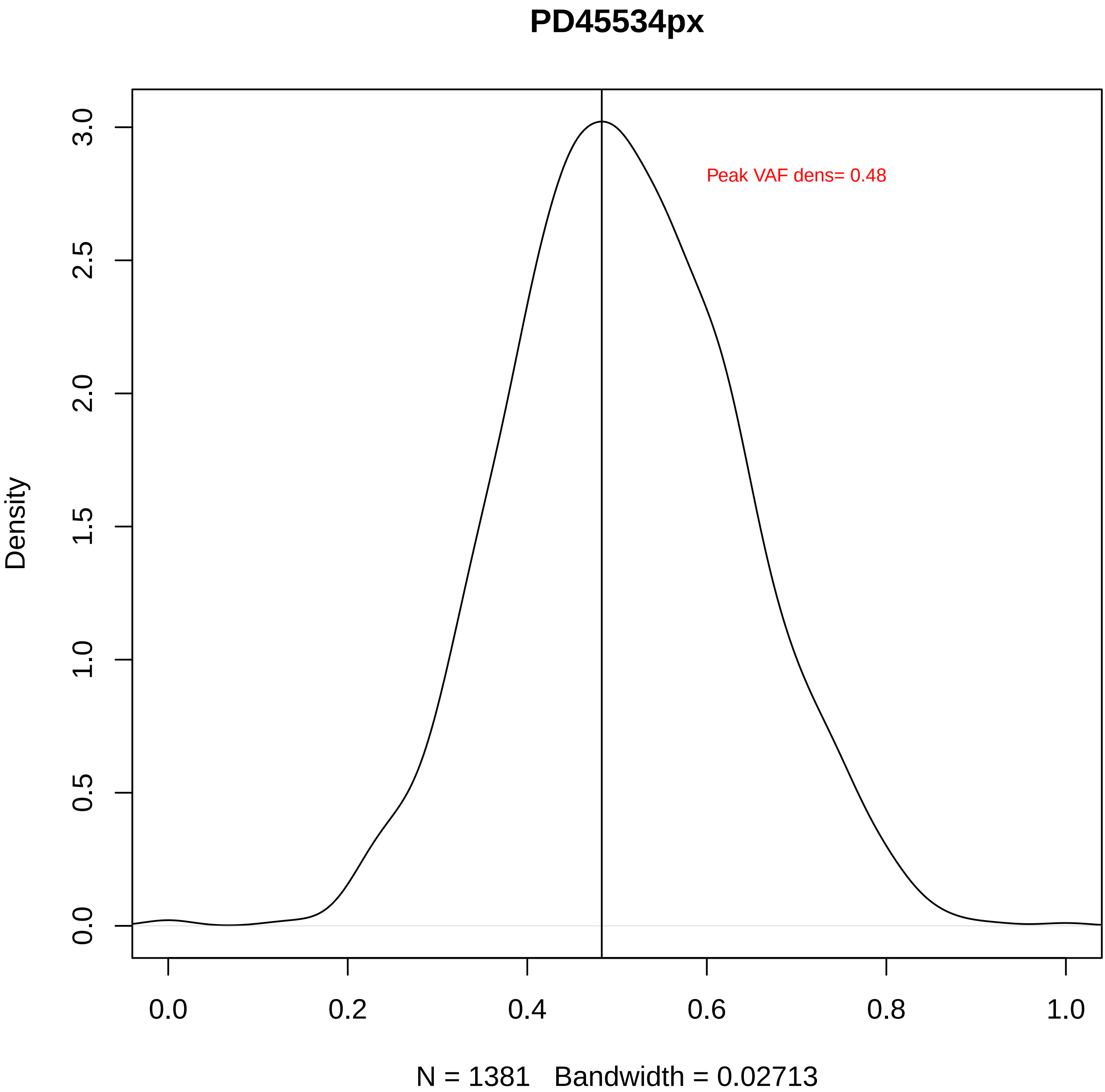
PD45534px
Peak VAF dens= 0.48
Density
0.0
0.5
1.0
1.5
2.0
2.5
3.0
0.0
0.2
0.4
0.6
0.8
1.0
N = 1381   Bandwidth = 0.02713

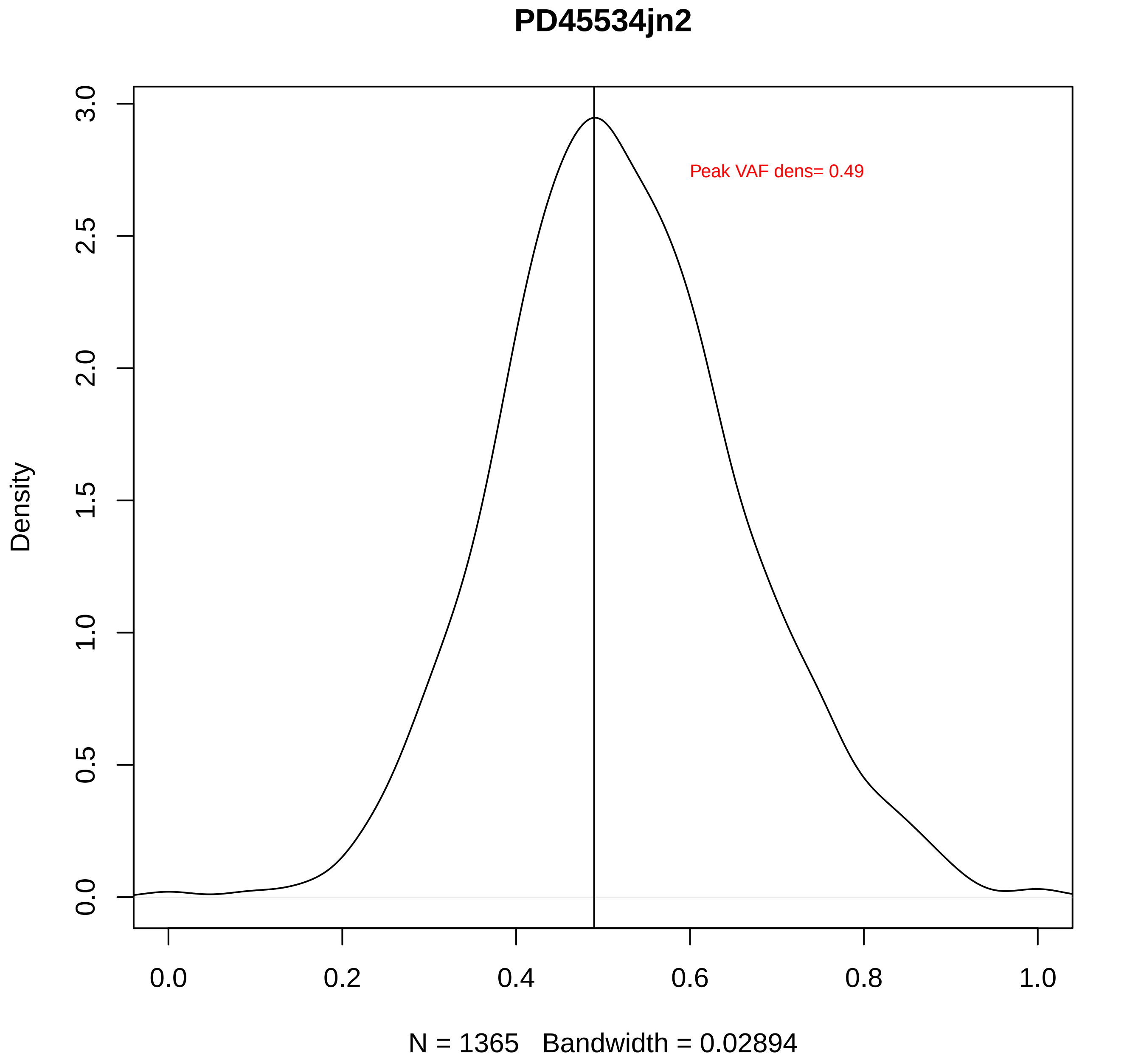
PD45534jn2
Peak VAF dens= 0.49
Density
0.0
0.5
1.0
1.5
2.0
2.5
3.0
0.0
0.2
0.4
0.6
0.8
1.0
N = 1365   Bandwidth = 0.02894

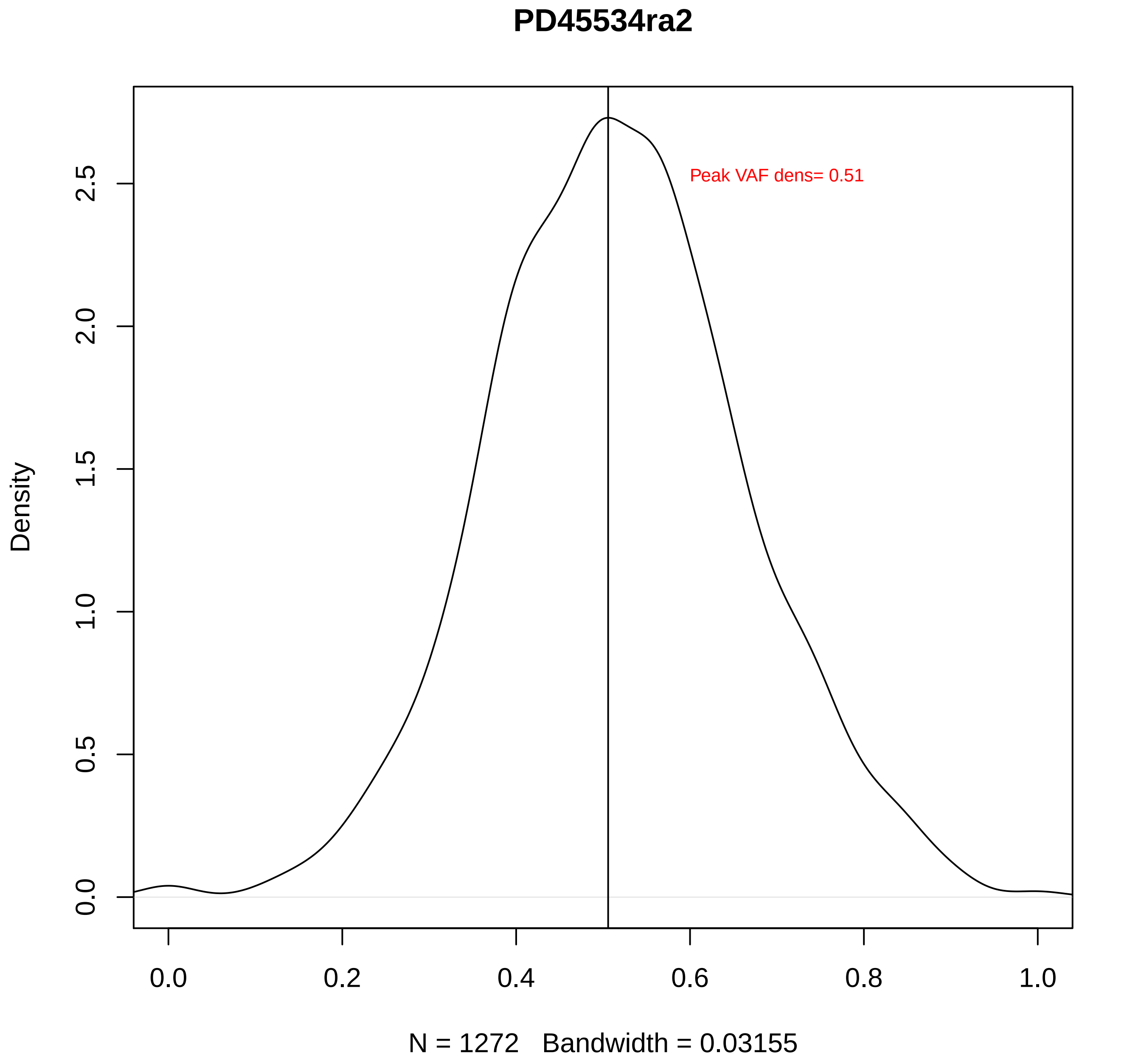

PD45534ra2
Peak VAF dens= 0.51
Density
0.0
0.5
1.0
1.5
2.0
2.5
0.0
0.2
0.4
0.6
0.8
1.0
N = 1272 Bandwidth = 0.03155

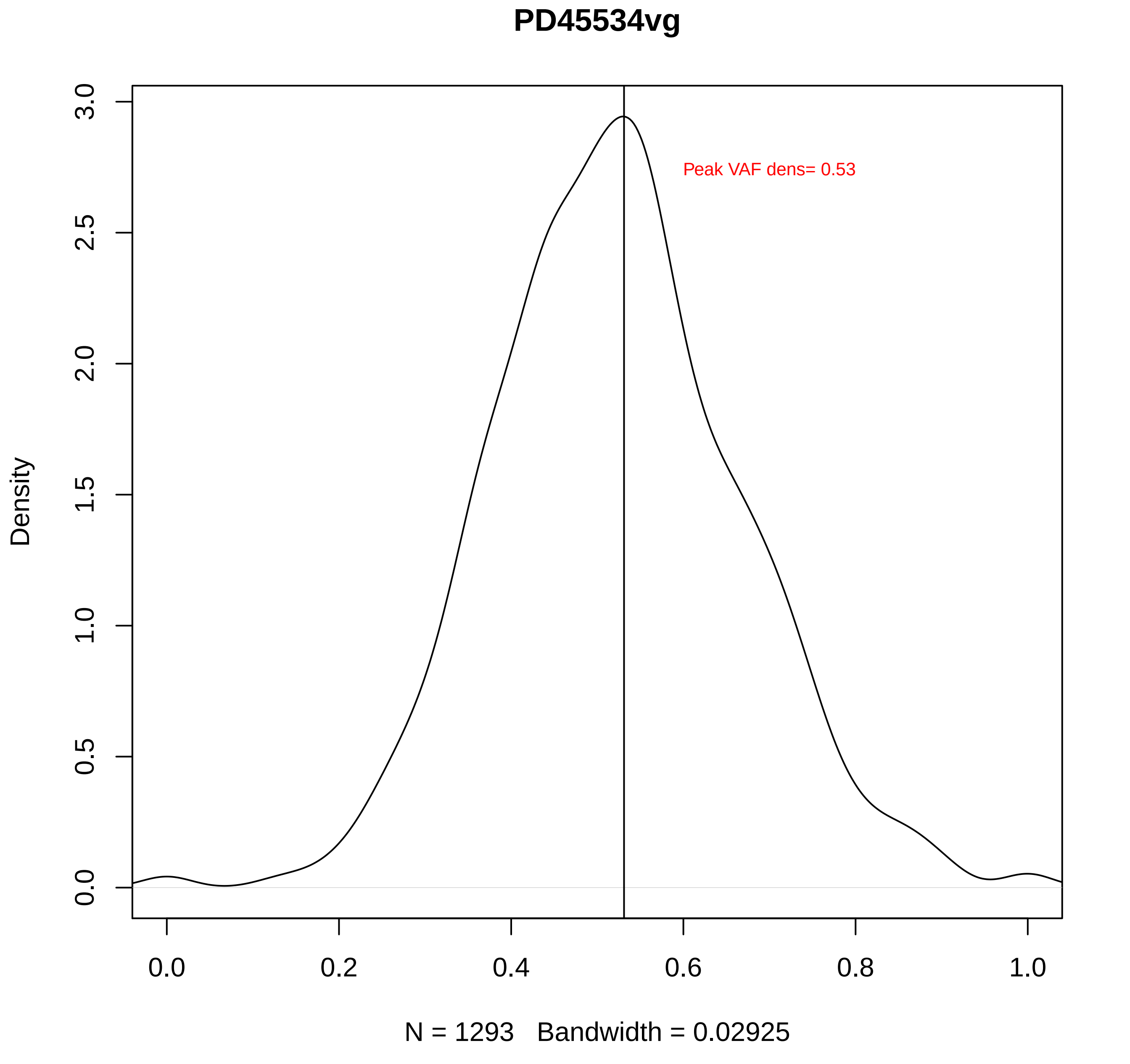
PD45534vg
Peak VAF dens= 0.53
Density
0.0
0.5
1.0
1.5
2.0
2.5
3.0
0.0
0.2
0.4
0.6
0.8
1.0
N = 1293   Bandwidth = 0.02925

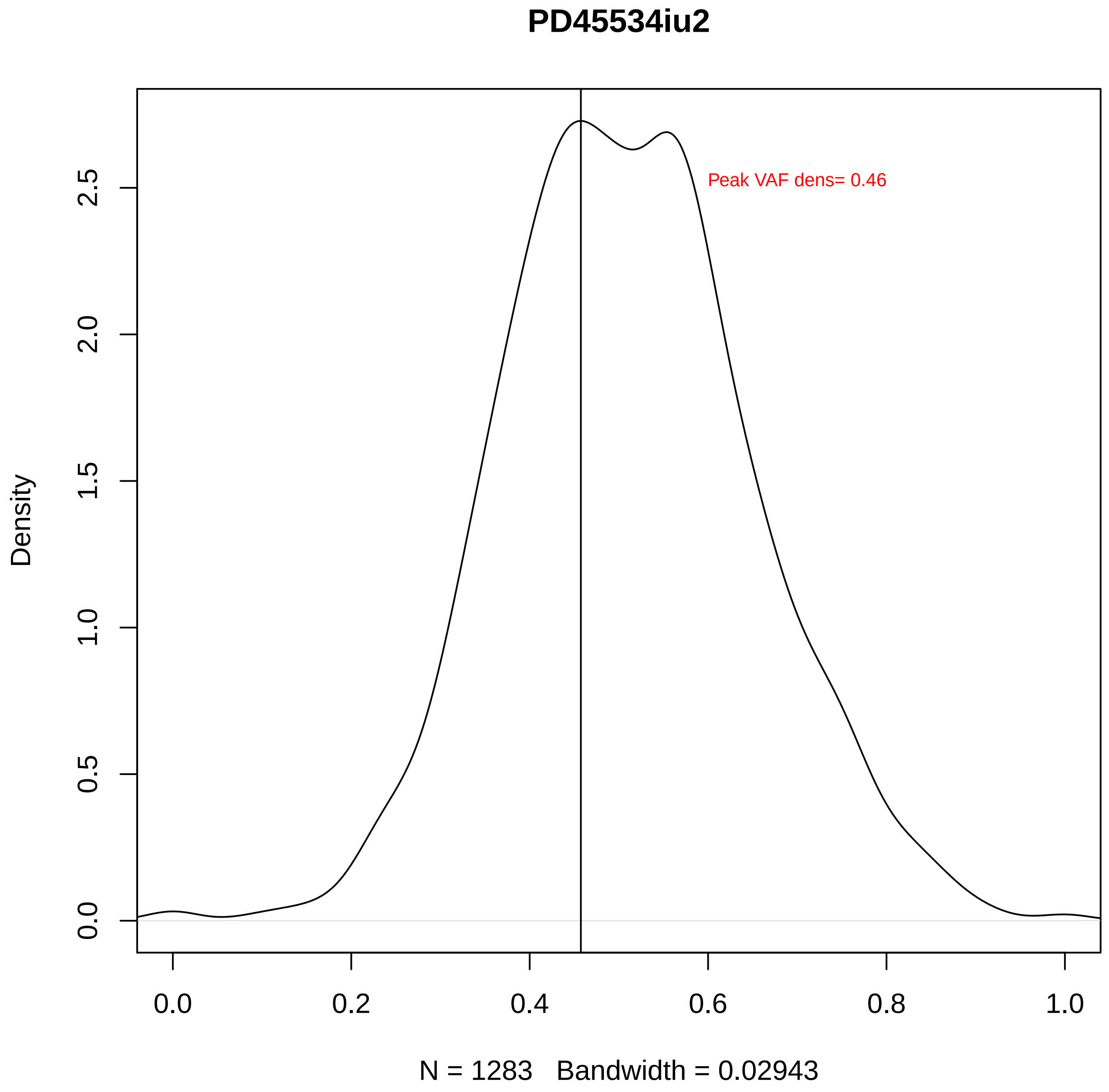

PD45534iu2
Peak VAF dens= 0.46
Density
0.0
0.5
1.0
1.5
2.0
2.5
0.0
0.2
0.4
0.6
0.8
1.0
N = 1283 Bandwidth = 0.02943

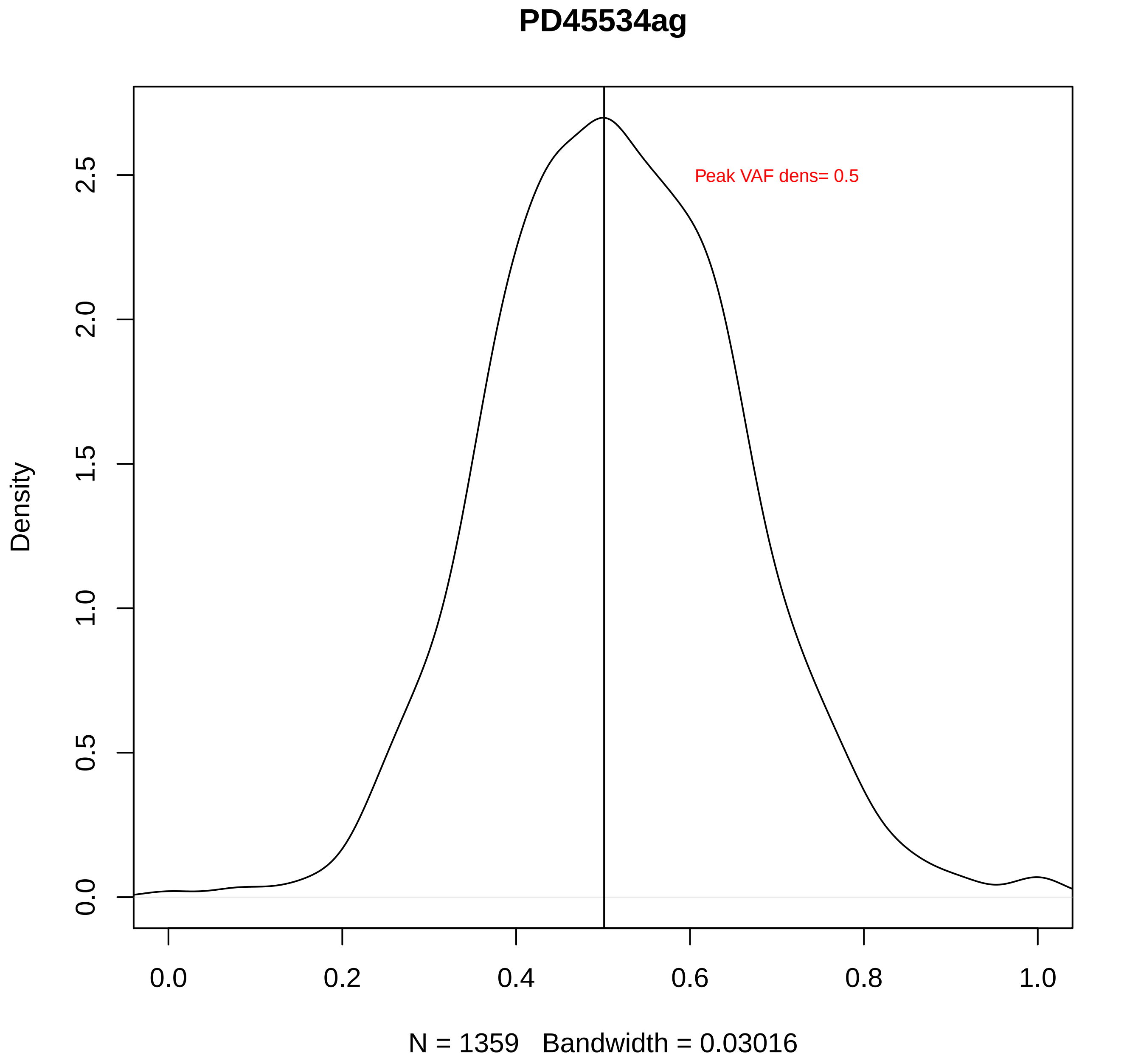

PD45534ag
Peak VAF dens= 0.5
Density
0.0
0.5
1.0
1.5
2.0
2.5
0.0
0.2
0.4
0.6
0.8
1.0
N = 1359 Bandwidth = 0.03016

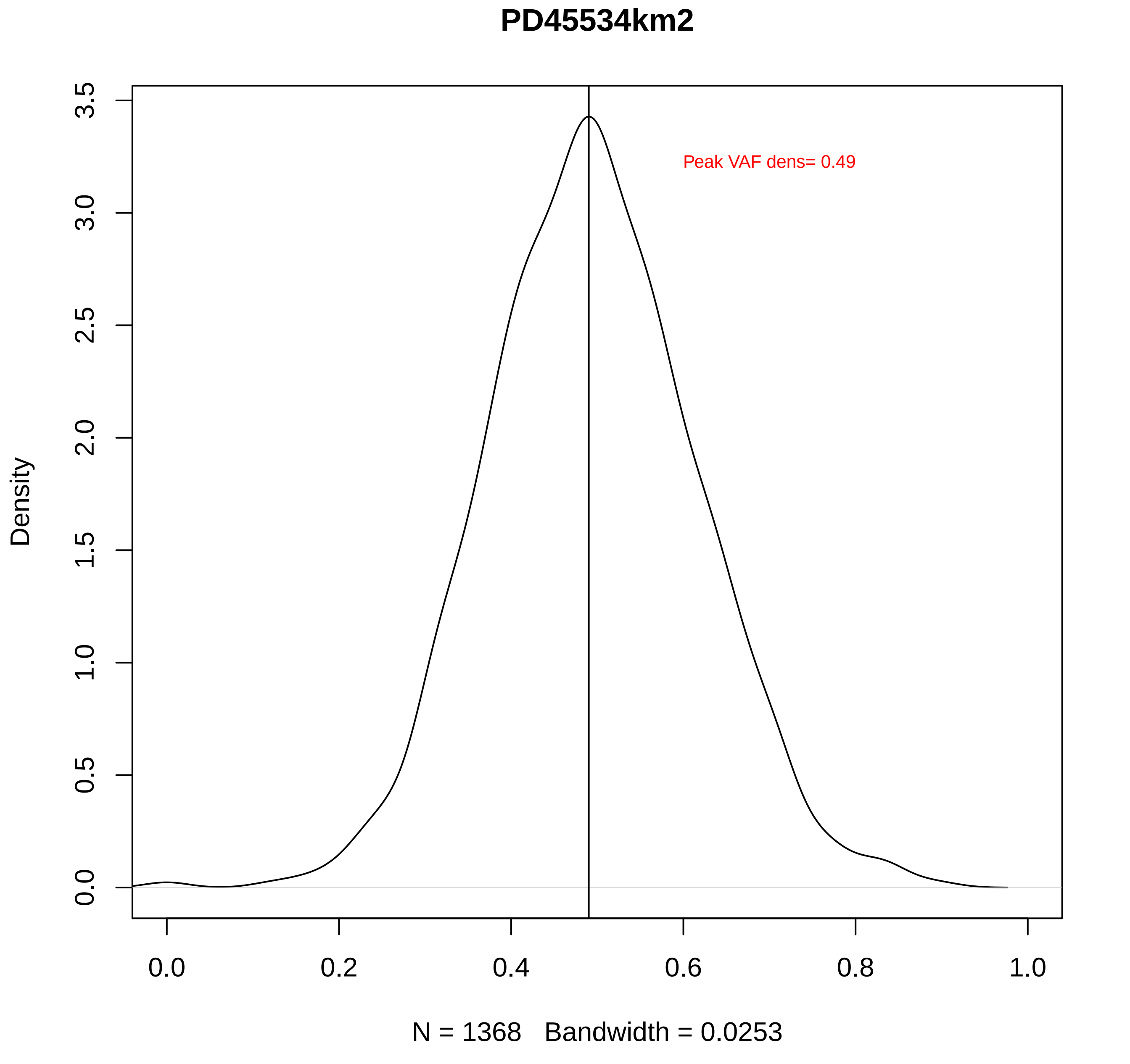
PD45534km2
Peak VAF dens= 0.49
Density
0.0
0.5
1.0
1.5
2.0
2.5
3.0
3.5
0.0
0.2
0.4
0.6
0.8
1.0
N = 1368   Bandwidth = 0.0253

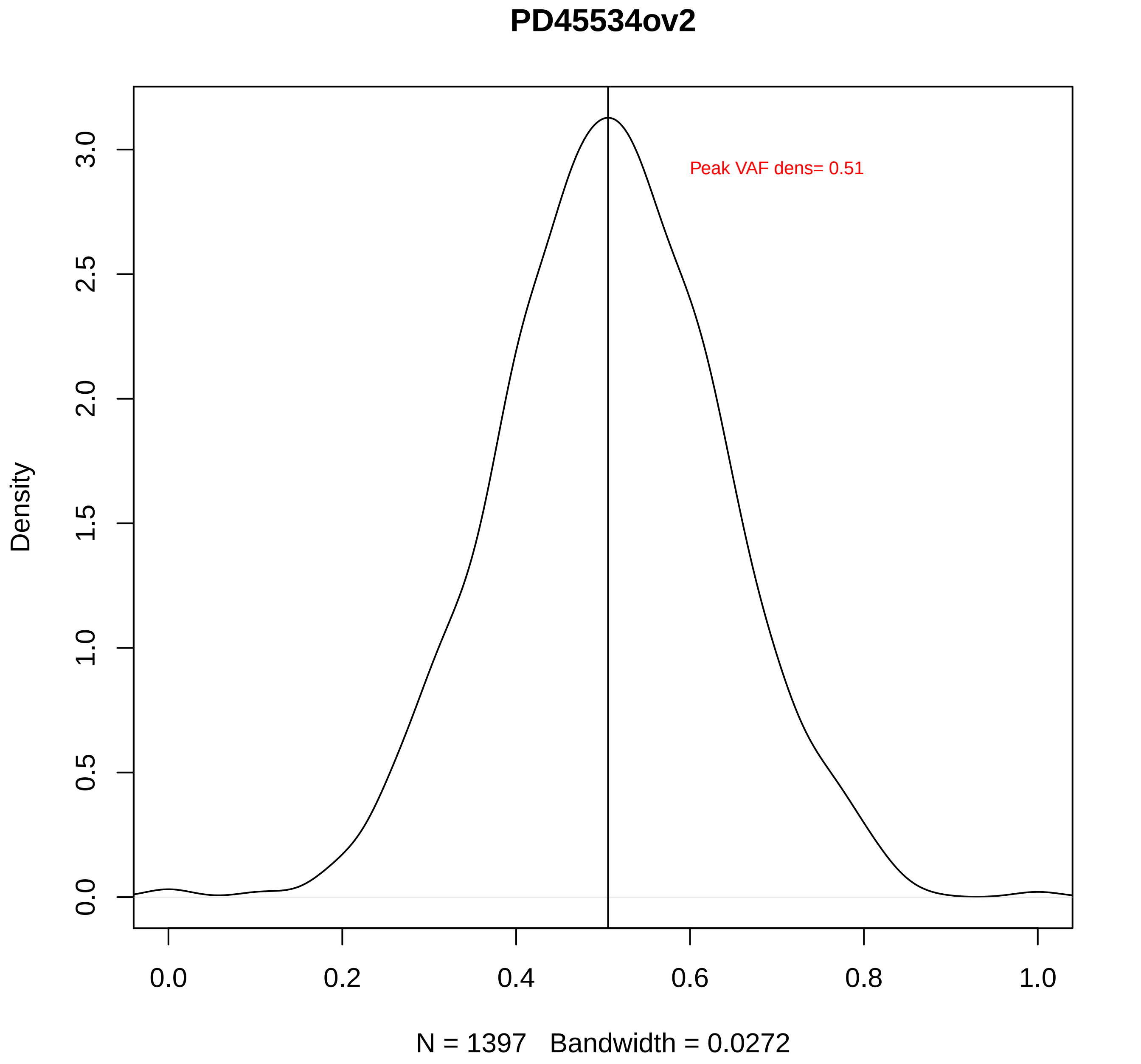
PD45534ov2
Peak VAF dens= 0.51
Density
0.0
0.5
1.0
1.5
2.0
2.5
3.0
0.0
0.2
0.4
0.6
0.8
1.0
N = 1397   Bandwidth = 0.0272

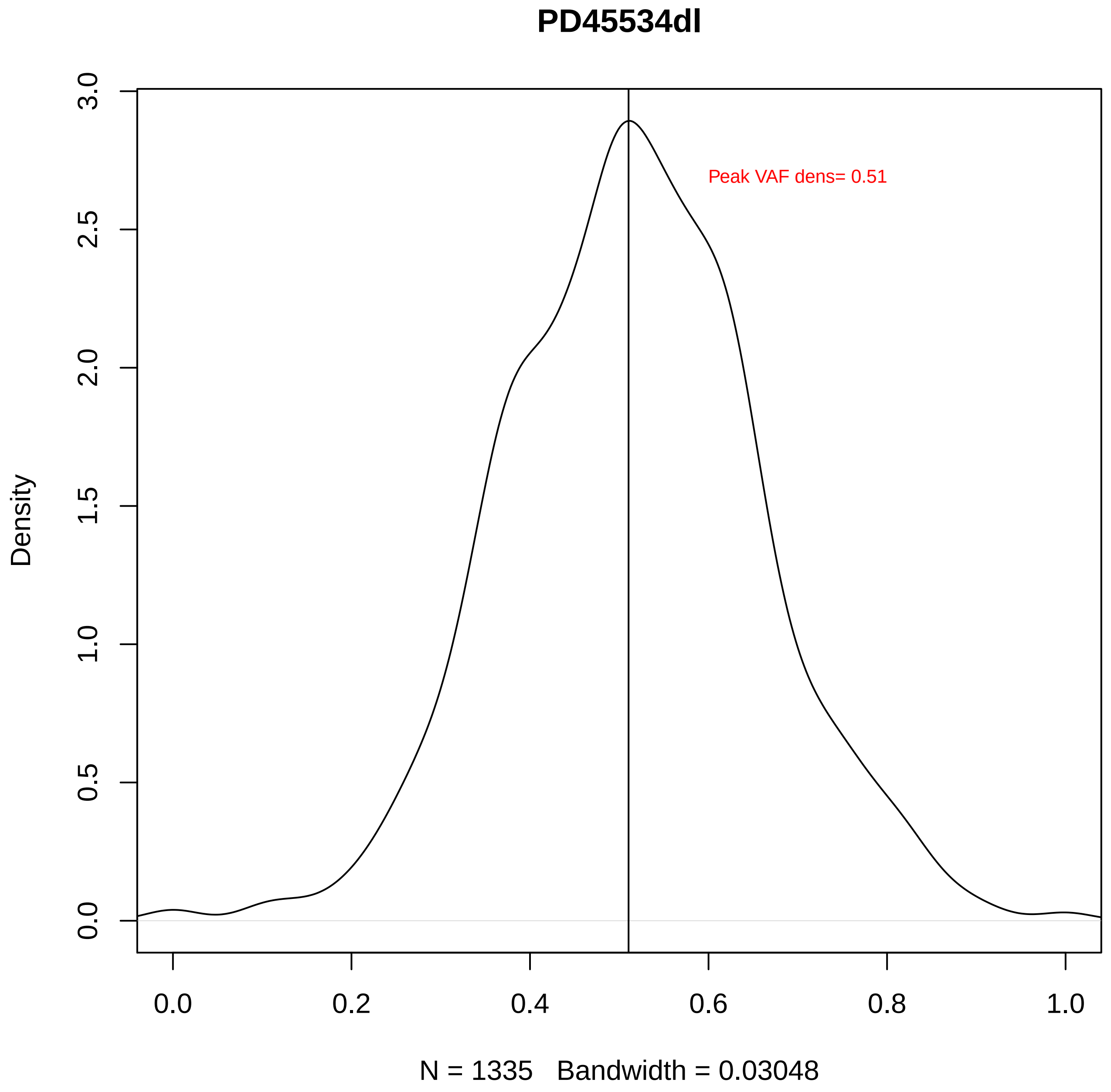
PD45534dl
Peak VAF dens= 0.51
Density
0.0
0.5
1.0
1.5
2.0
2.5
3.0
0.0
0.2
0.4
0.6
0.8
1.0
N = 1335   Bandwidth = 0.03048

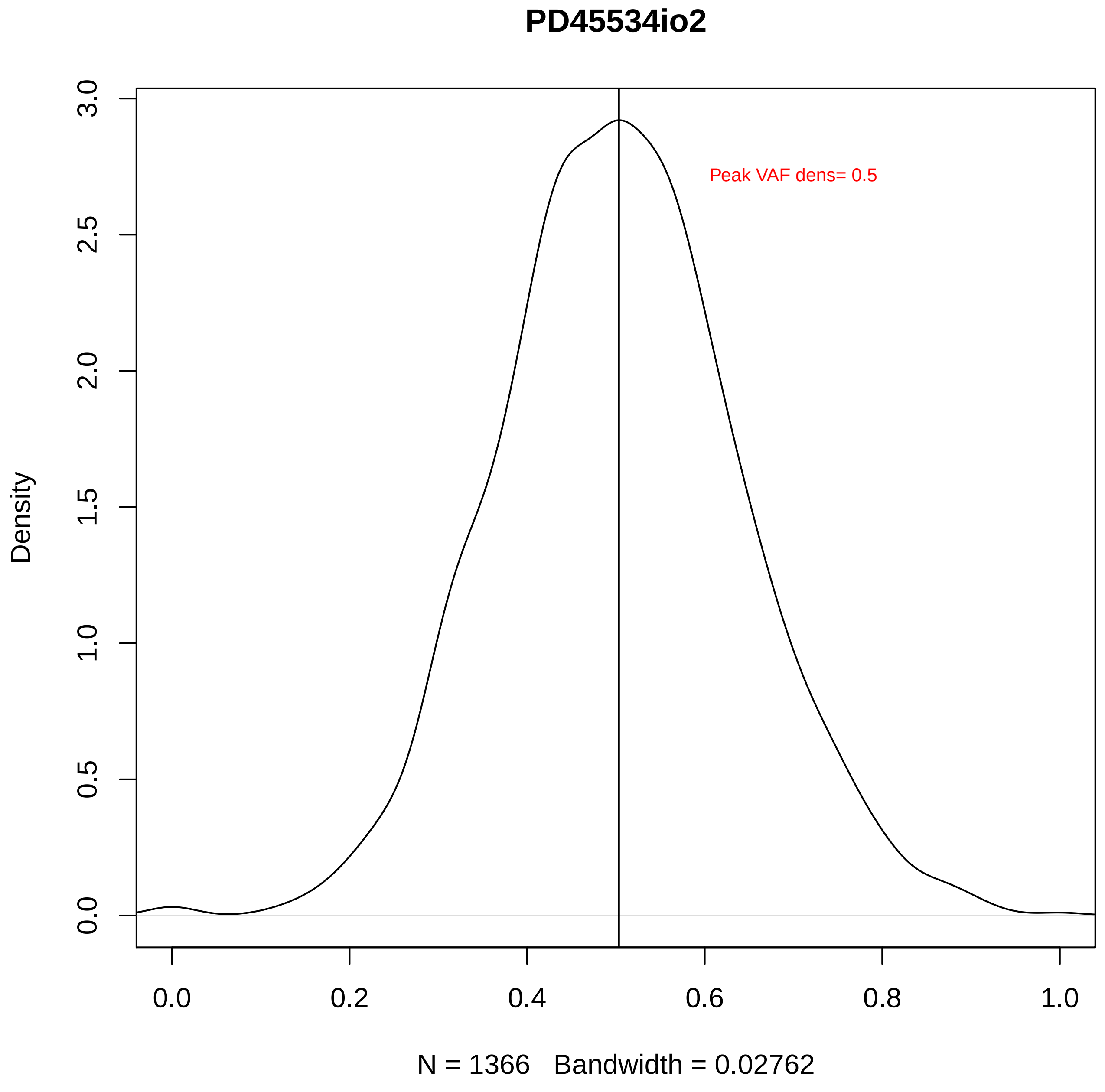

PD45534io2
Peak VAF dens= 0.5
Density
3.0
2.5
2.0
1.5
1.0
0.5
0.0
0.0
0.2
0.4
0.6
0.8
1.0
N = 1366 Bandwidth = 0.02762

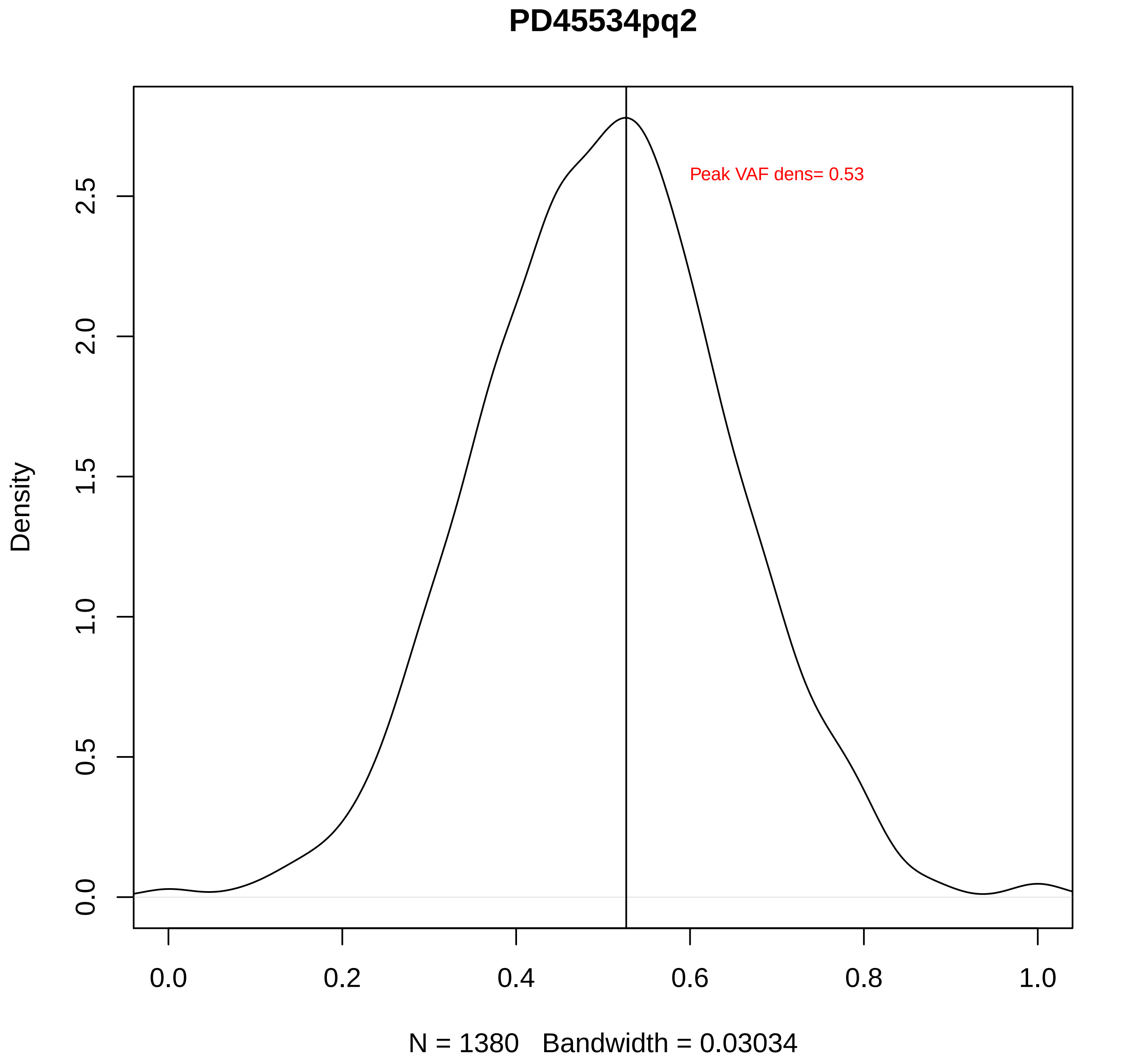
PD45534pq2
Peak VAF dens= 0.53
Density
0.0
0.5
1.0
1.5
2.0
2.5
0.0
0.2
0.4
0.6
0.8
1.0
N = 1380   Bandwidth = 0.03034

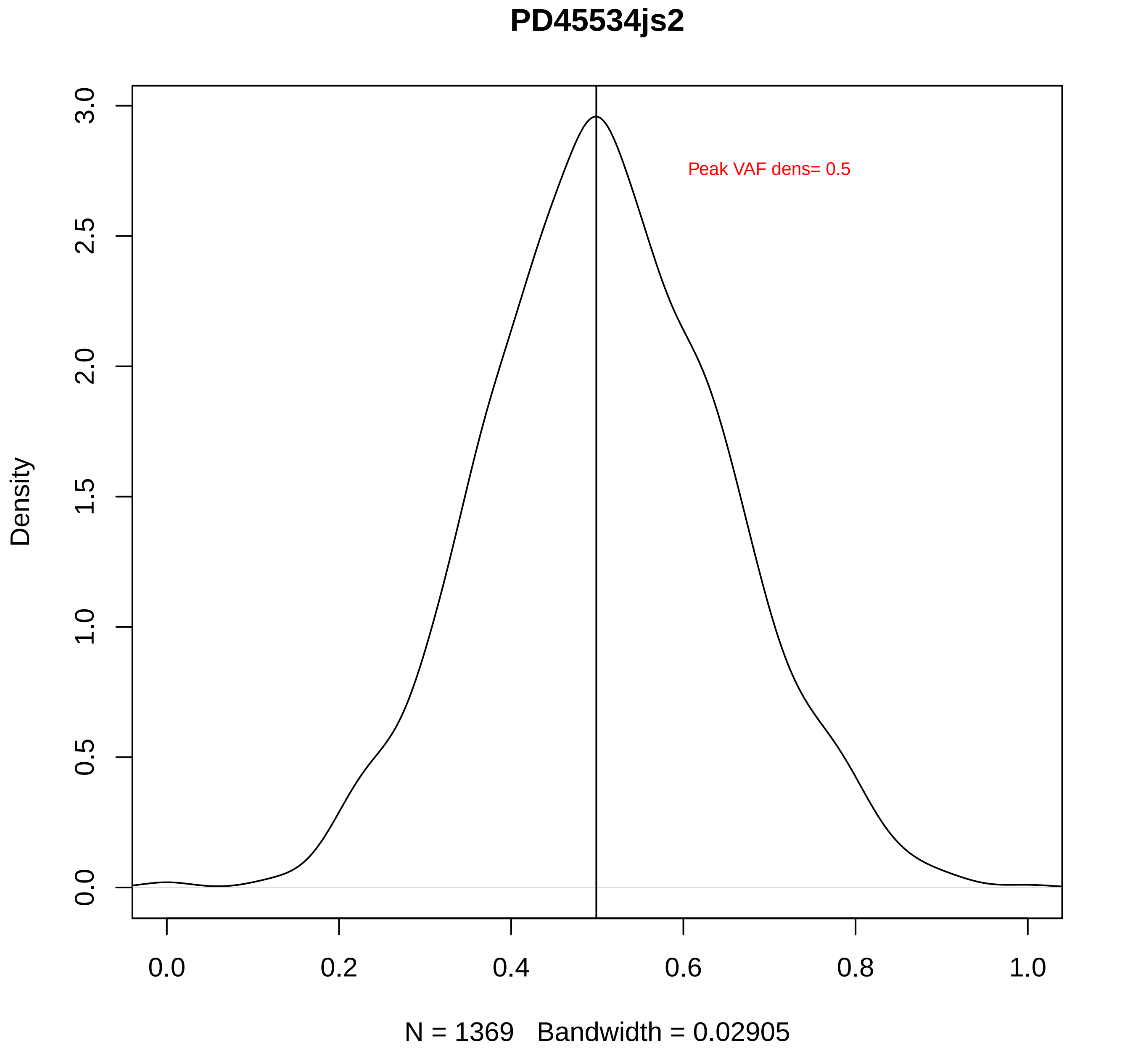

PD45534js2
Peak VAF dens= 0.5
Density
0.0
0.5
1.0
1.5
2.0
2.5
3.0
0.0
0.2
0.4
0.6
0.8
1.0
N = 1369   Bandwidth = 0.02905

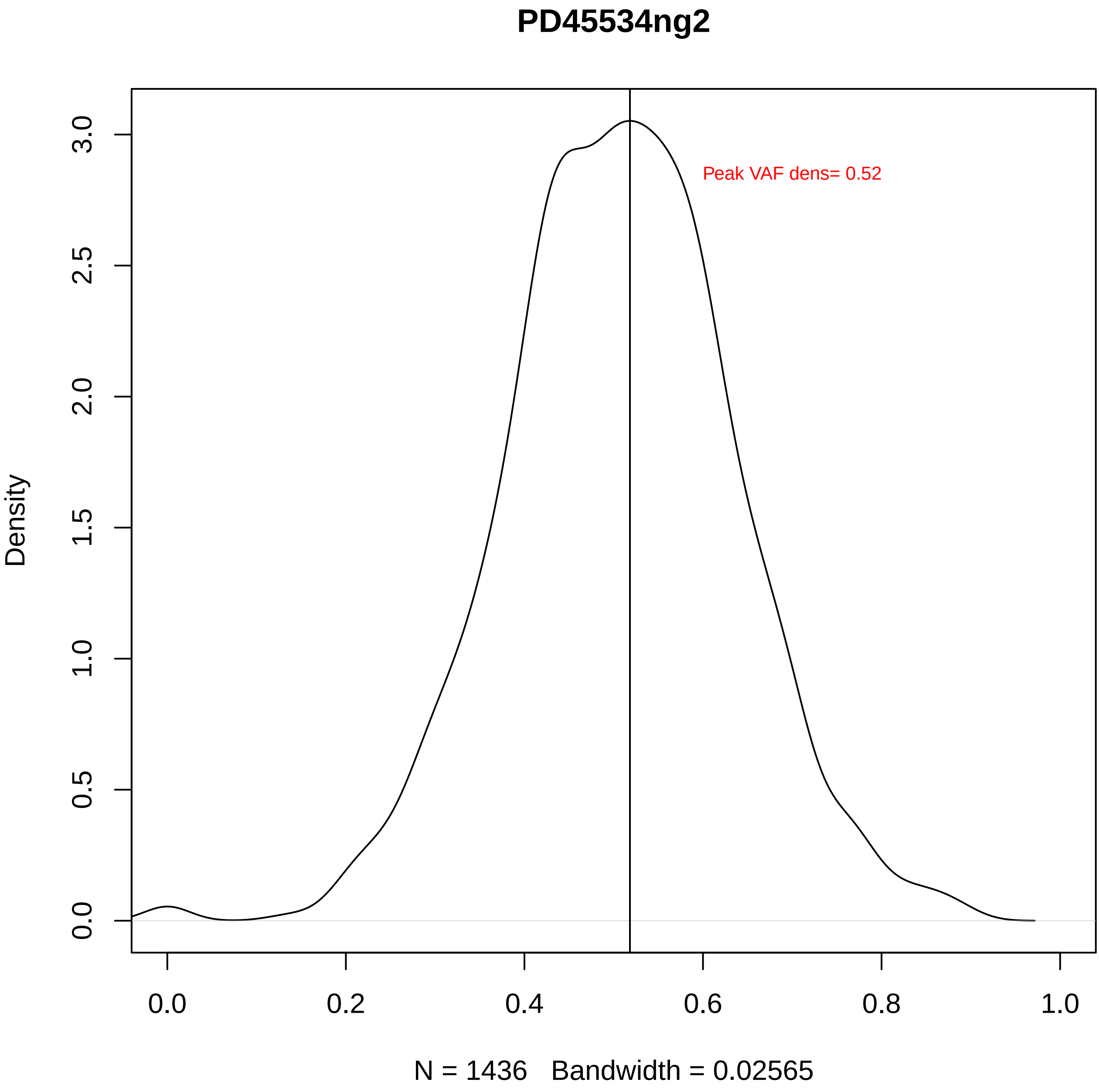
PD45534ng2
Density
3.0
2.5
2.0
1.5
1.0
0.5
0.0
0.0
0.2
0.4
0.6
0.8
1.0
Peak VAF dens= 0.52
N = 1436 Bandwidth = 0.02565

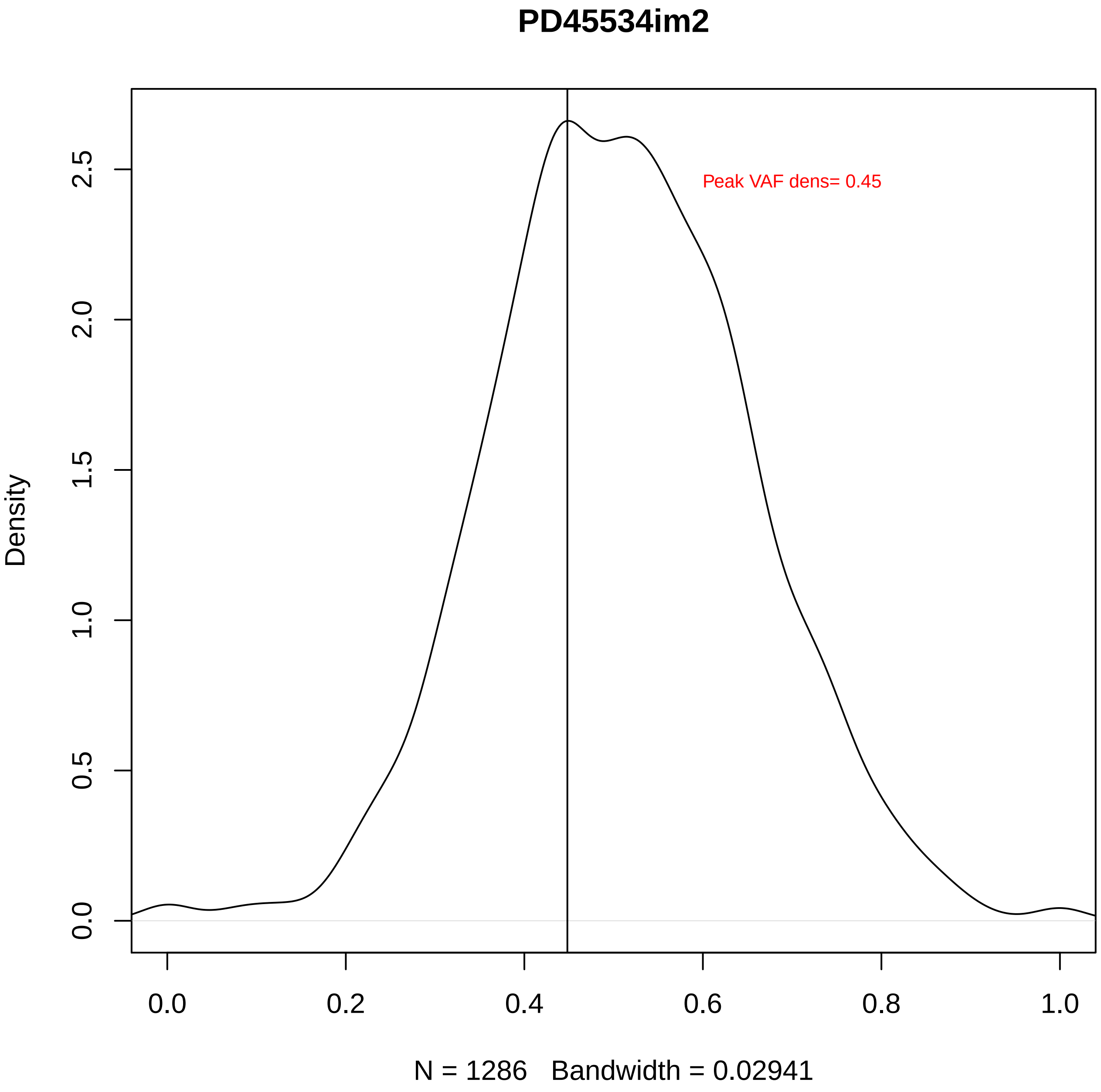
PD45534im2
Peak VAF dens= 0.45
Density
0.0
0.5
1.0
1.5
2.0
2.5
0.0
0.2
0.4
0.6
0.8
1.0
N = 1286   Bandwidth = 0.02941

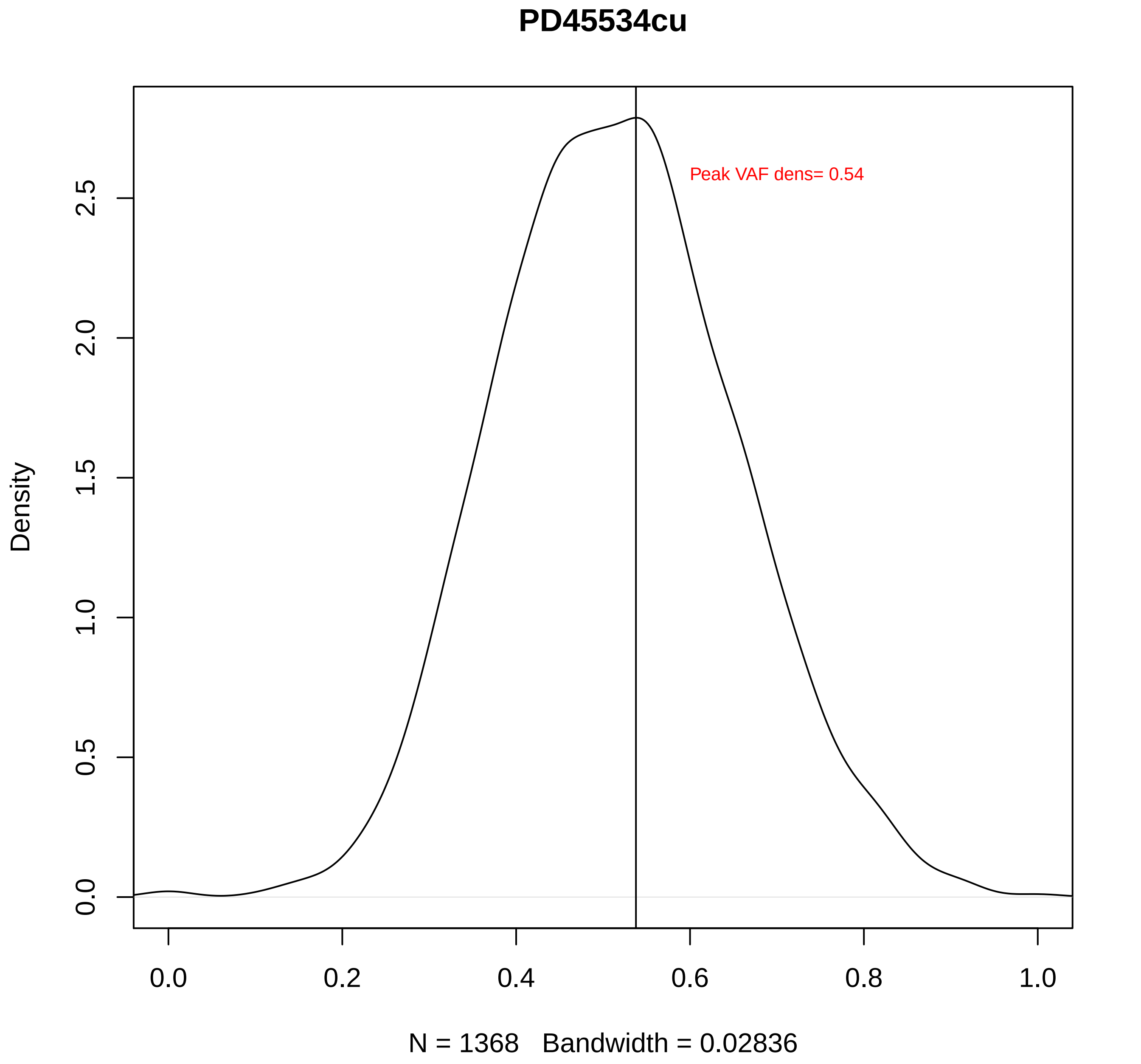
PD45534cu
Peak VAF dens= 0.54
Density
0.0
0.5
1.0
1.5
2.0
2.5
0.0
0.2
0.4
0.6
0.8
1.0
N = 1368   Bandwidth = 0.02836

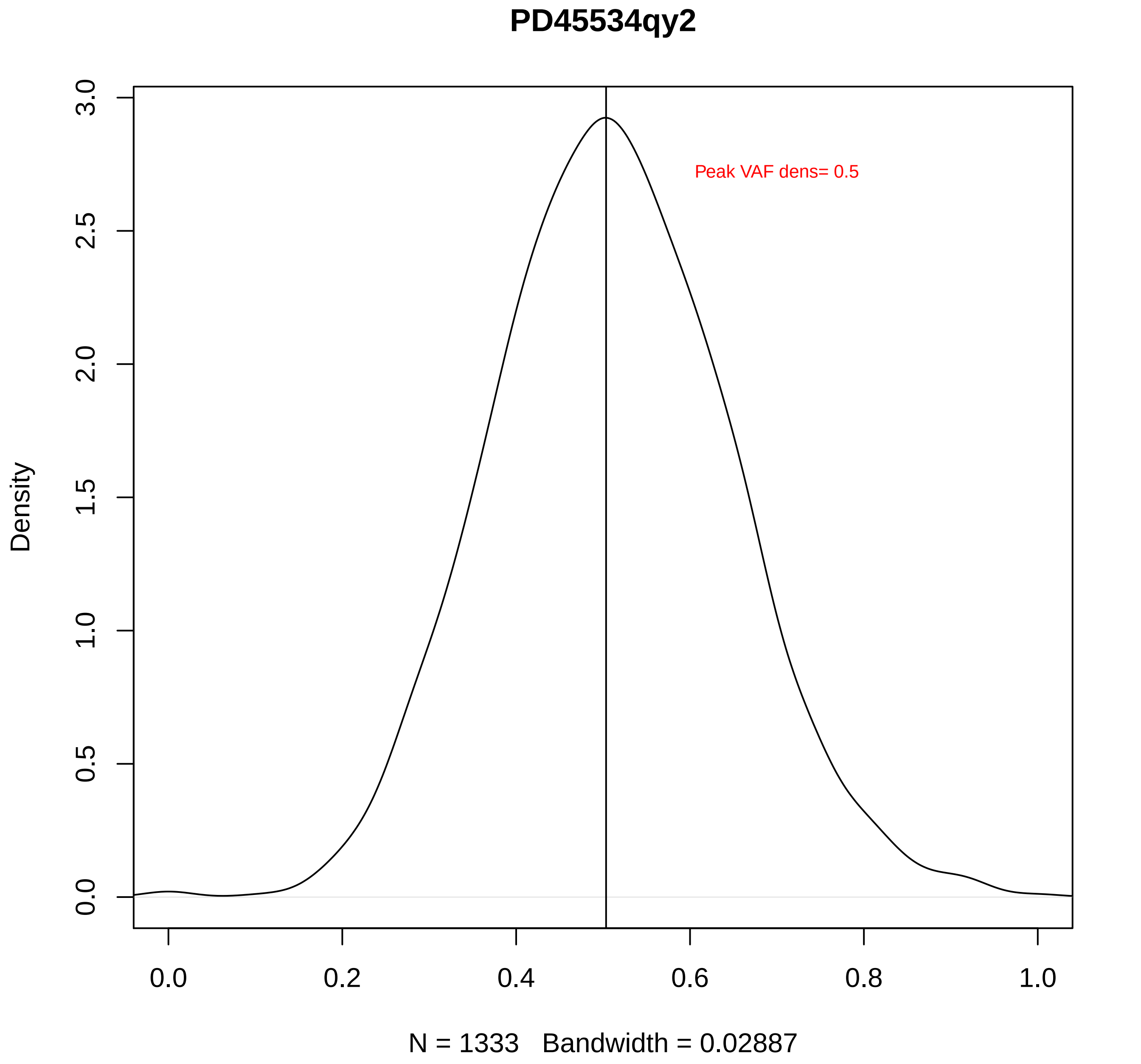
PD45534qy2
Peak VAF dens= 0.5
Density
0.0
0.5
1.0
1.5
2.0
2.5
3.0
0.0
0.2
0.4
0.6
0.8
1.0
N = 1333 Bandwidth = 0.02887

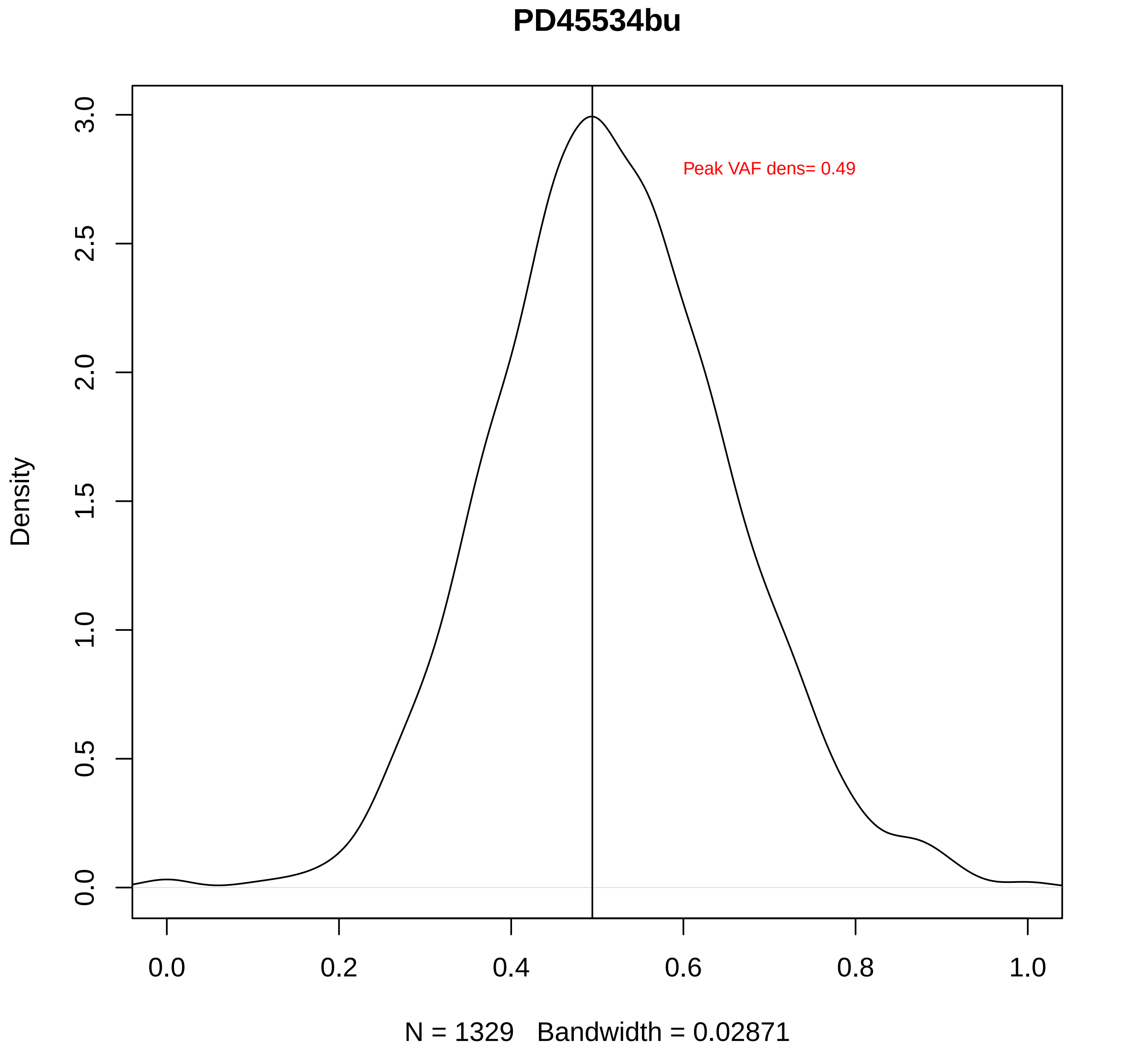

PD45534bu
Peak VAF dens= 0.49
Density
3.0
2.5
2.0
1.5
1.0
0.5
0.0
0.0
0.2
0.4
0.6
0.8
1.0
N = 1329   Bandwidth = 0.02871

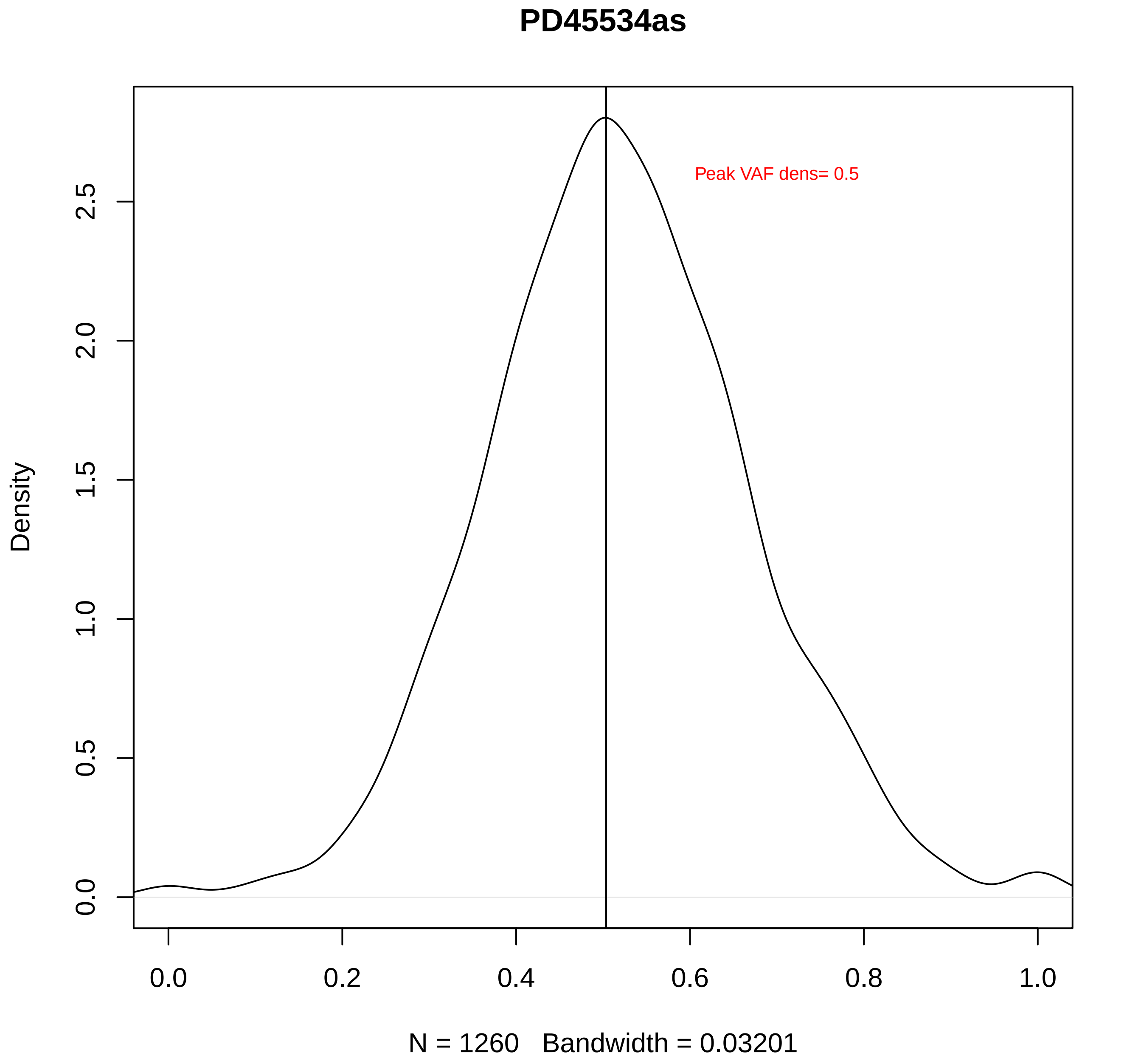

PD45534as
Peak VAF dens= 0.5
Density
0.0
0.5
1.0
1.5
2.0
2.5
0.0
0.2
0.4
0.6
0.8
1.0
N = 1260   Bandwidth = 0.03201

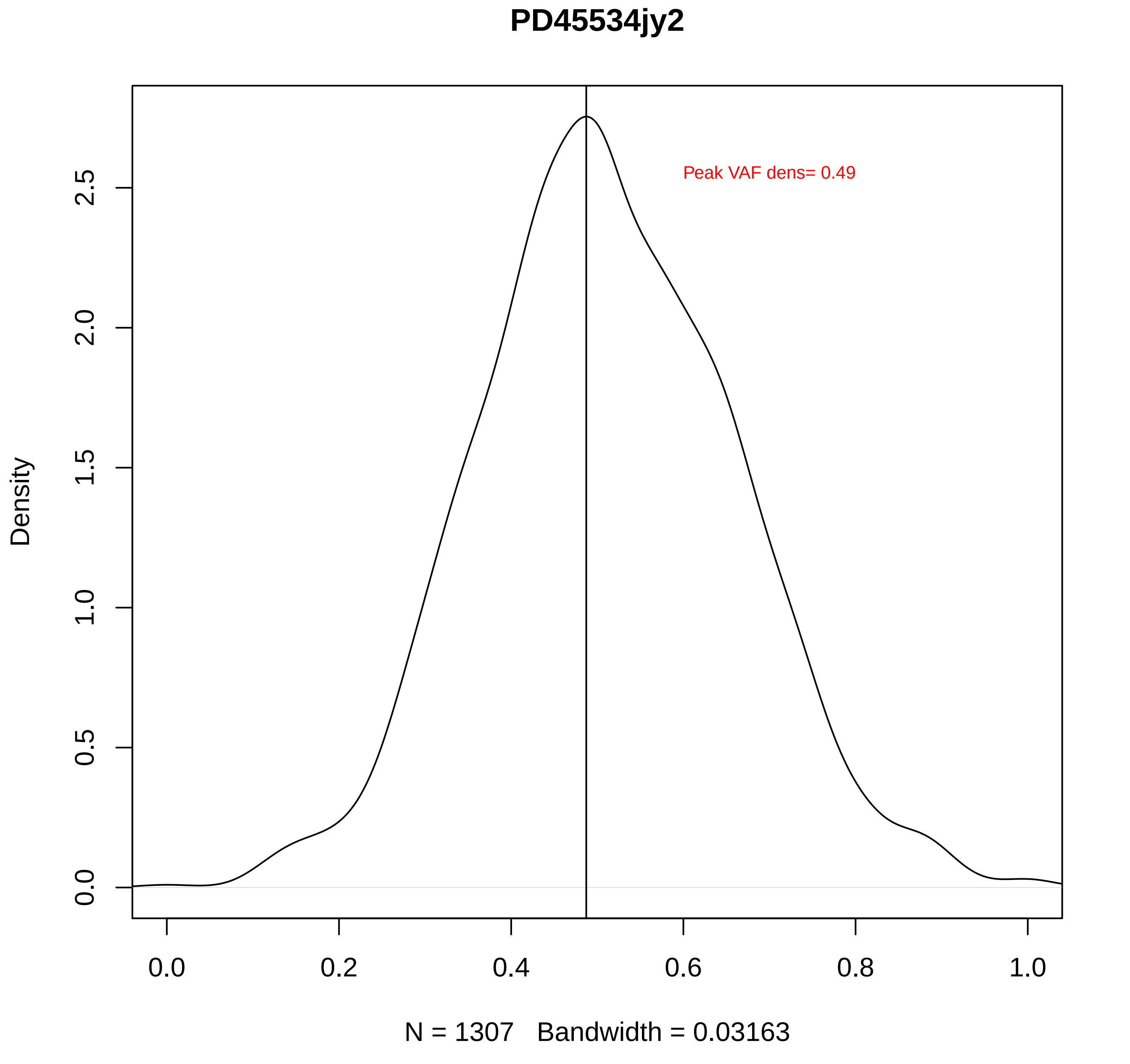
PD45534jy2
Peak VAF dens= 0.49
Density
0.0
0.5
1.0
1.5
2.0
2.5
0.0
0.2
0.4
0.6
0.8
1.0
N = 1307   Bandwidth = 0.03163

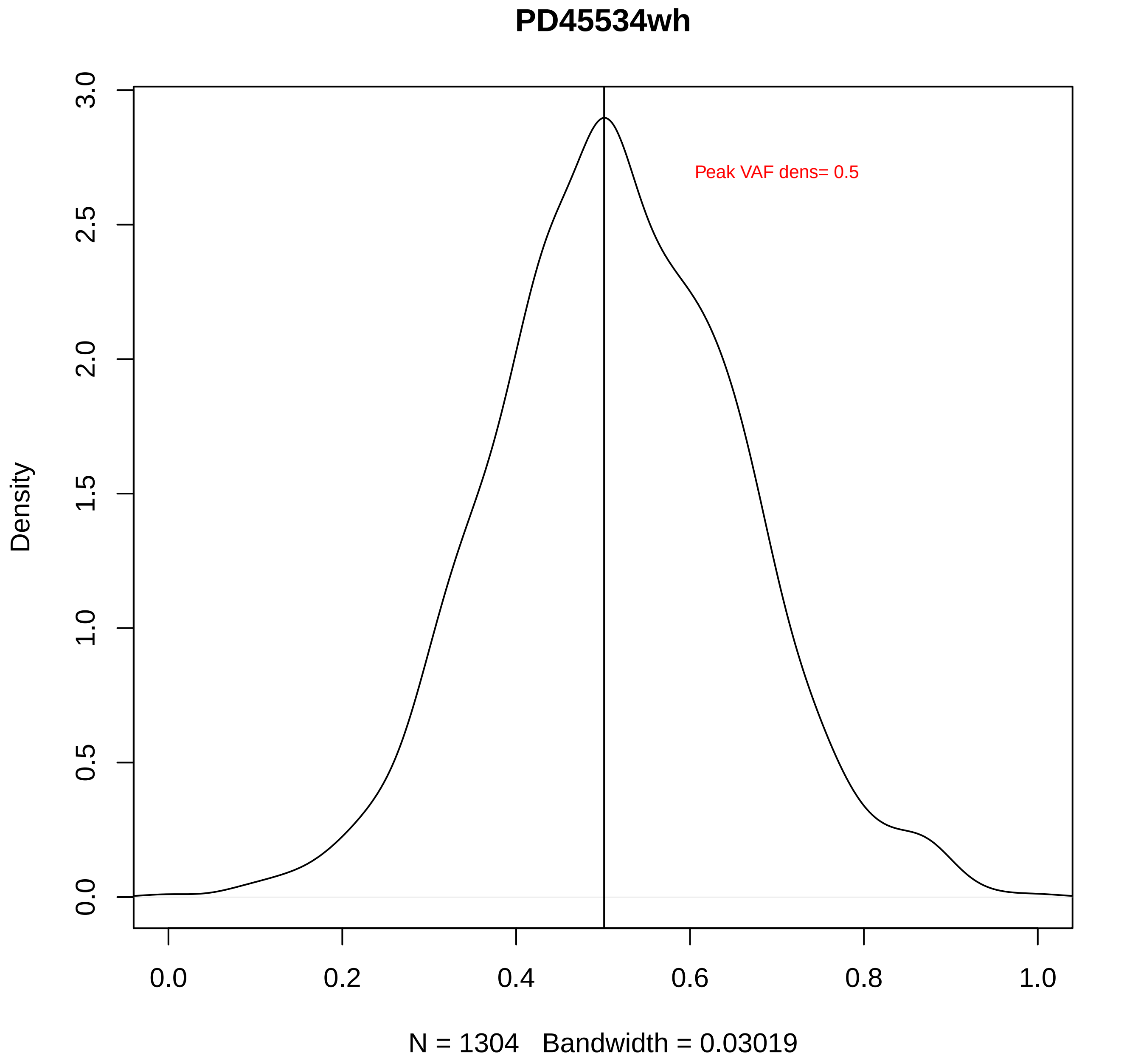
PD45534wh
Peak VAF dens= 0.5
Density
0.0
0.5
1.0
1.5
2.0
2.5
3.0
0.0
0.2
0.4
0.6
0.8
1.0
N = 1304 Bandwidth = 0.03019

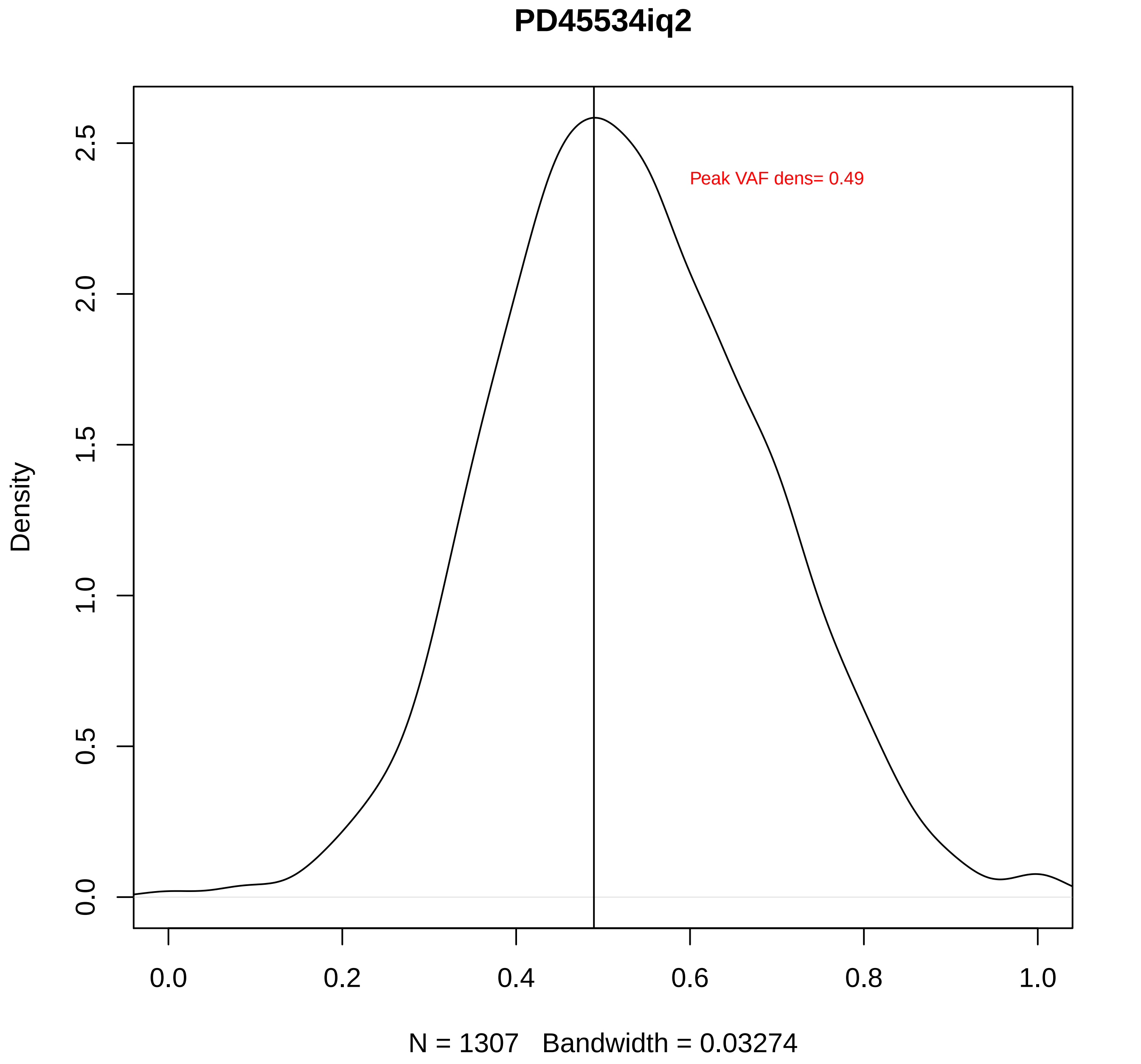
PD45534iq2
Peak VAF dens= 0.49
Density
0.0
0.5
1.0
1.5
2.0
2.5
0.0
0.2
0.4
0.6
0.8
1.0
N = 1307   Bandwidth = 0.03274

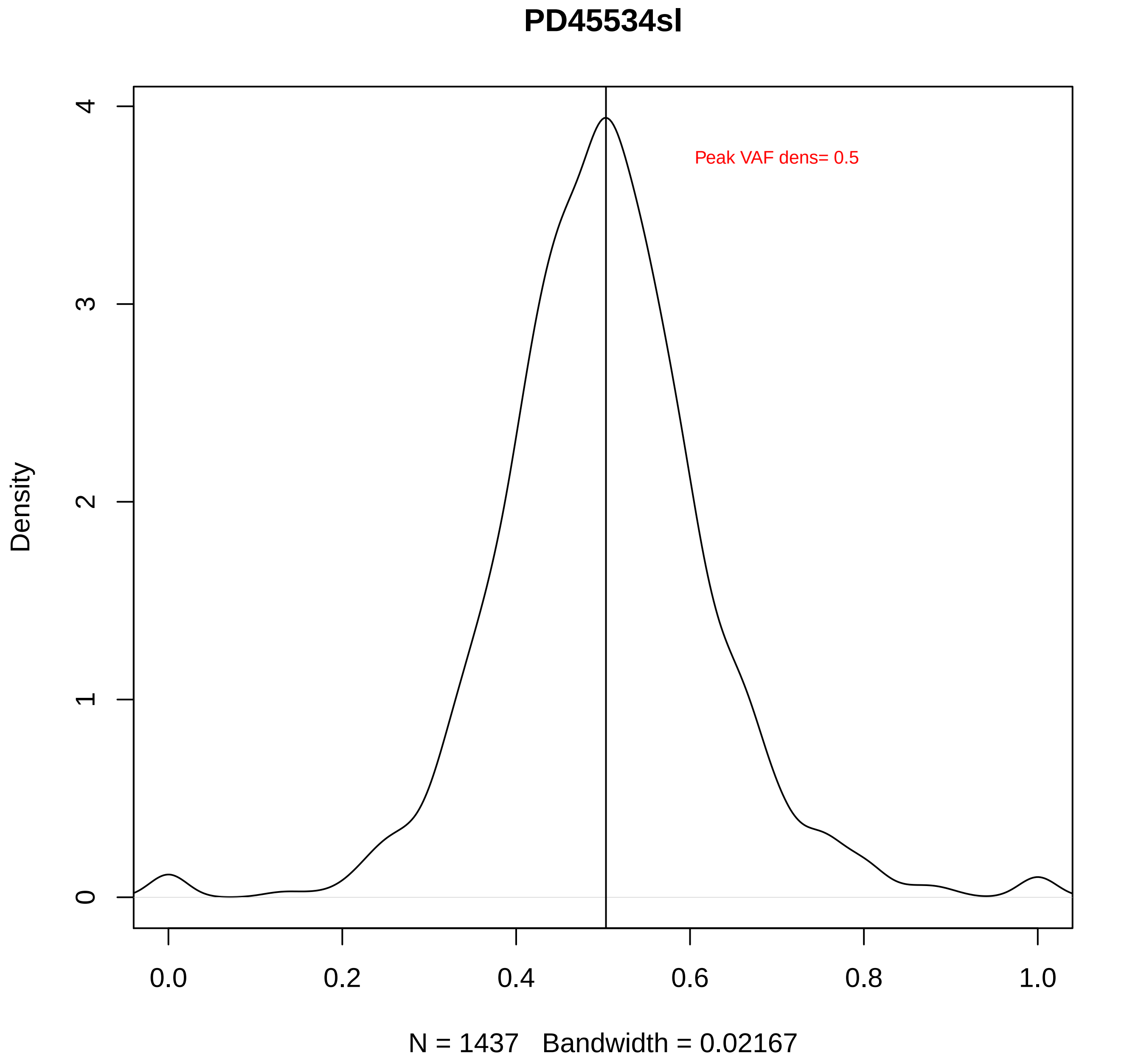
PD45534sl
Peak VAF dens= 0.5
Density
0
1
2
3
4
0.0
0.2
0.4
0.6
0.8
1.0
N = 1437   Bandwidth = 0.02167

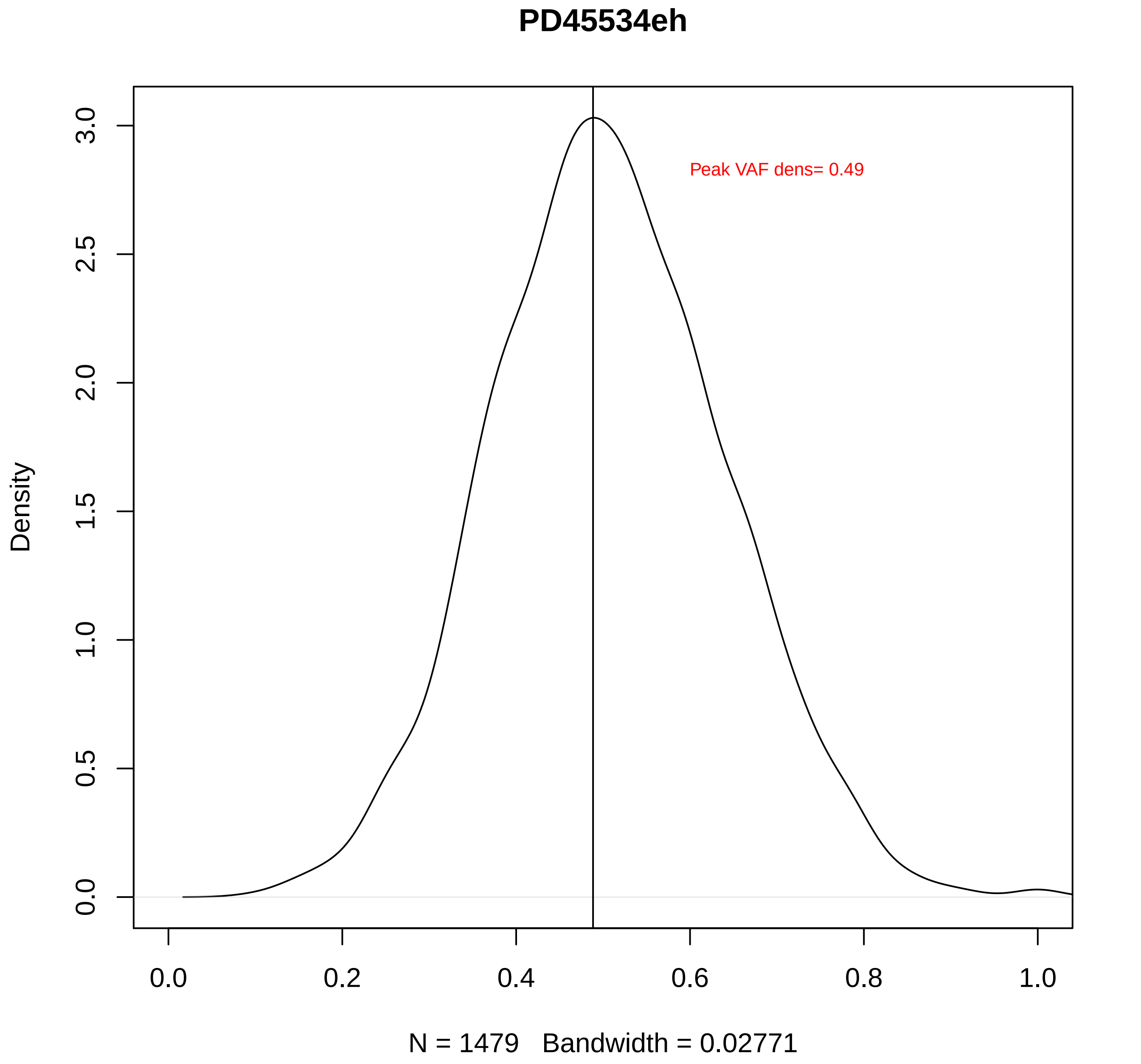
PD45534eh
Peak VAF dens= 0.49
Density
0.0
0.5
1.0
1.5
2.0
2.5
3.0
0.0
0.2
0.4
0.6
0.8
1.0
N = 1479   Bandwidth = 0.02771

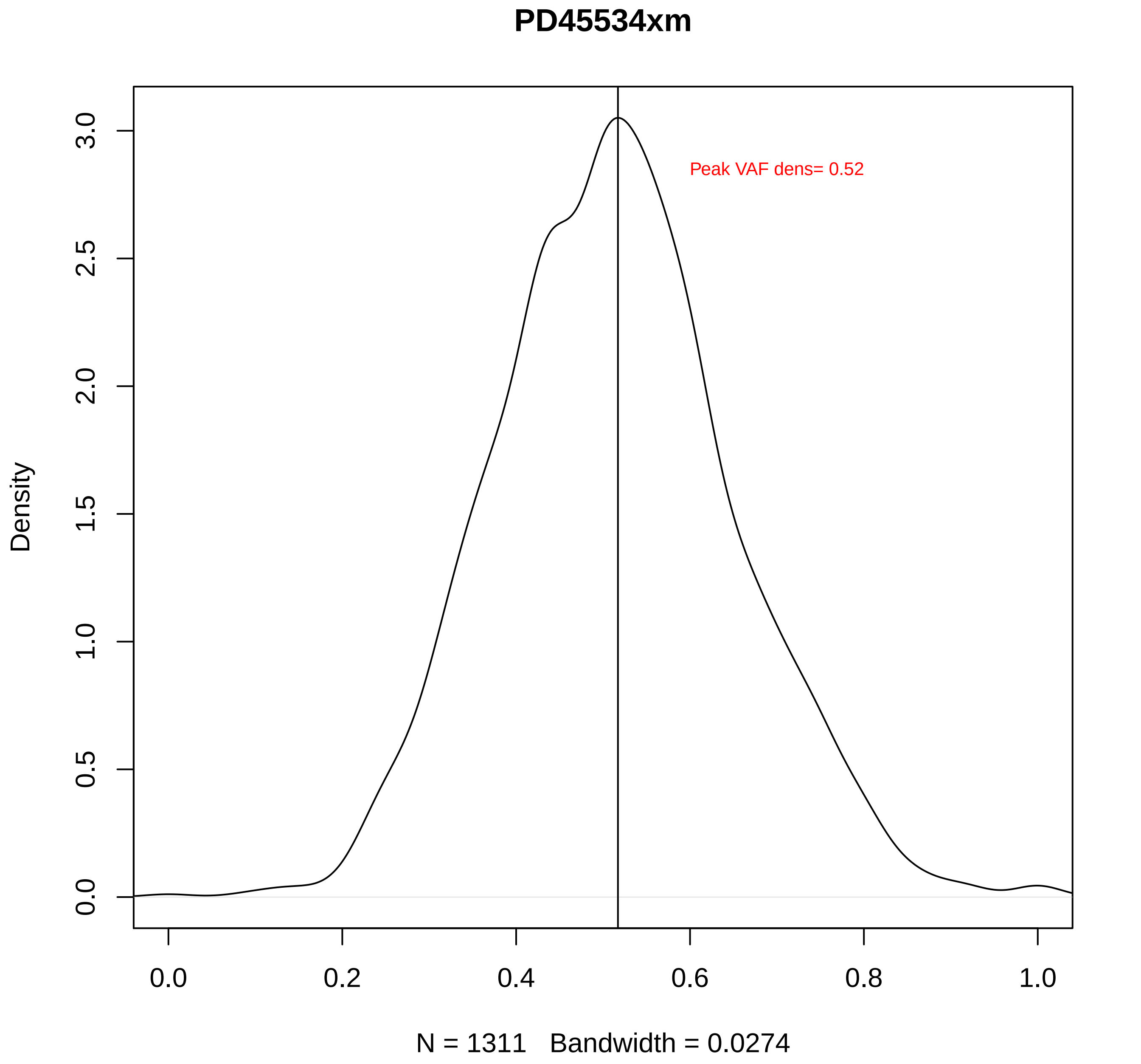
PD45534xm
Peak VAF dens= 0.52
Density
0.0
0.5
1.0
1.5
2.0
2.5
3.0
0.0
0.2
0.4
0.6
0.8
1.0
N = 1311   Bandwidth = 0.0274

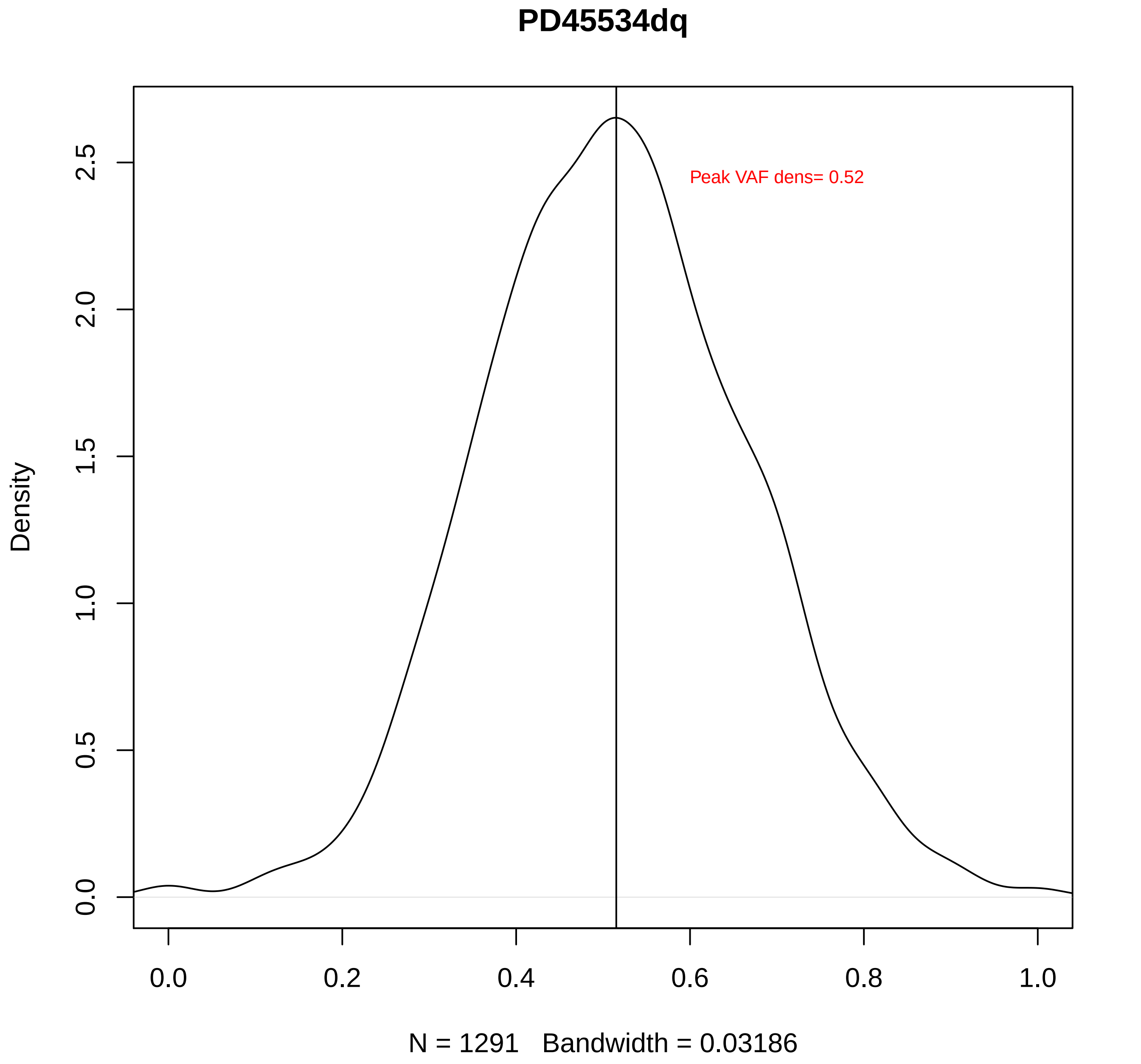

PD45534dq
Peak VAF dens= 0.52
Density
0.0
0.5
1.0
1.5
2.0
2.5
0.0
0.2
0.4
0.6
0.8
1.0
N = 1291   Bandwidth = 0.03186

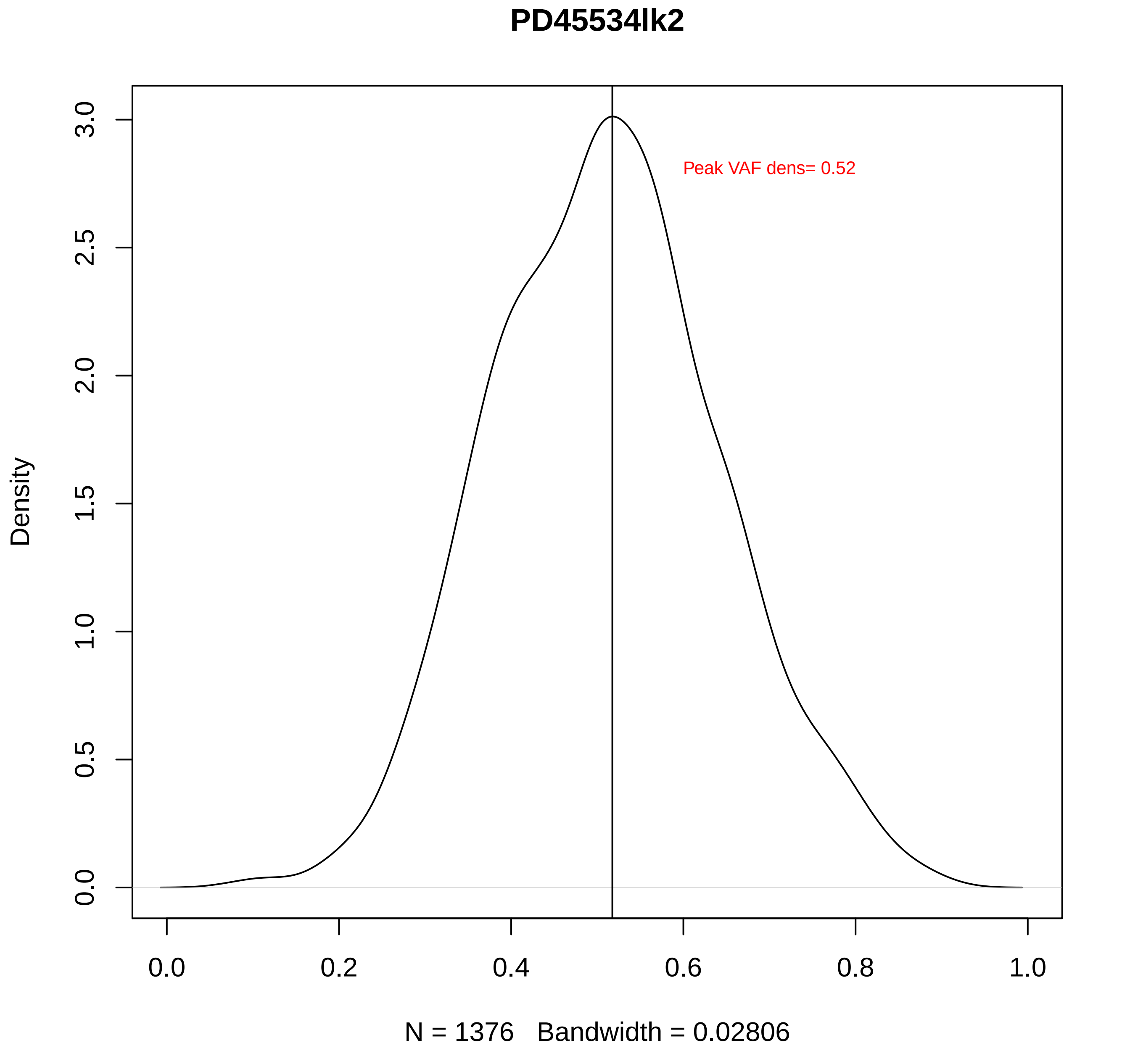
PD45534lk2
Peak VAF dens= 0.52
Density
0.0
0.5
1.0
1.5
2.0
2.5
3.0
0.0
0.2
0.4
0.6
0.8
1.0
N = 1376   Bandwidth = 0.02806

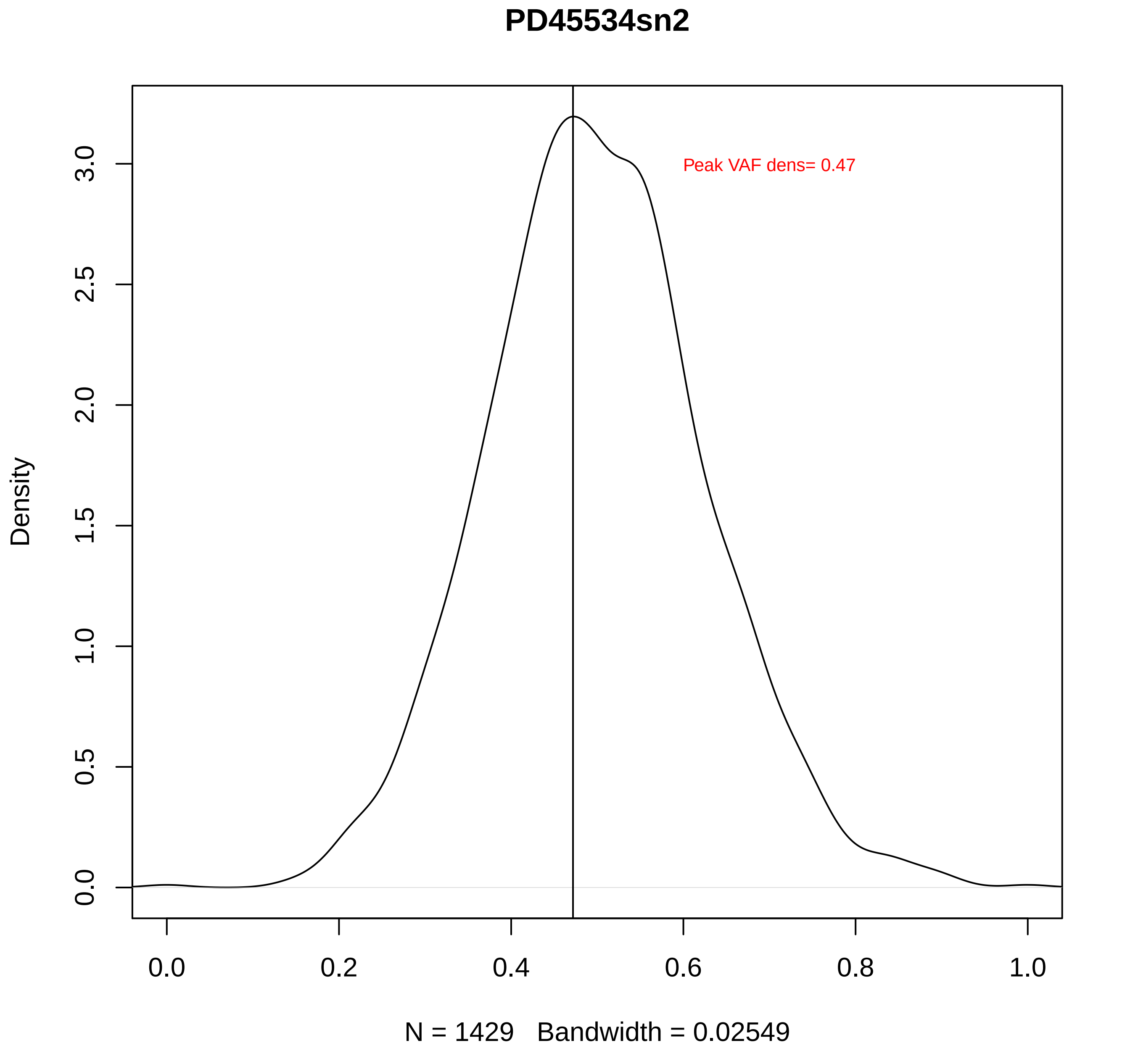
PD45534sn2
Peak VAF dens= 0.47
Density
0.0
0.5
1.0
1.5
2.0
2.5
3.0
0.0
0.2
0.4
0.6
0.8
1.0
N = 1429   Bandwidth = 0.02549

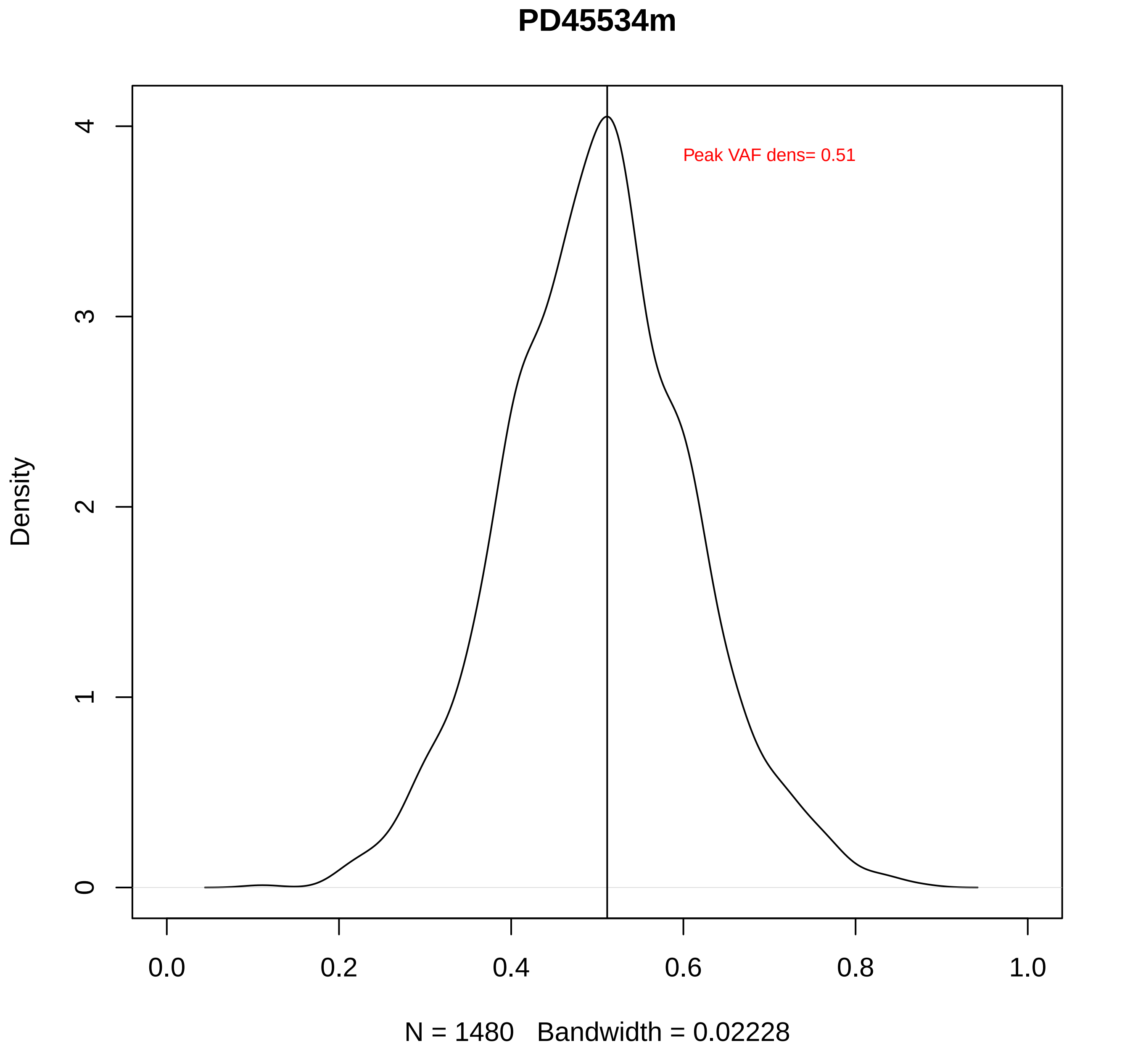

PD45534m
Peak VAF dens= 0.51
Density
0
1
2
3
4
0.0
0.2
0.4
0.6
0.8
1.0
N = 1480   Bandwidth = 0.02228

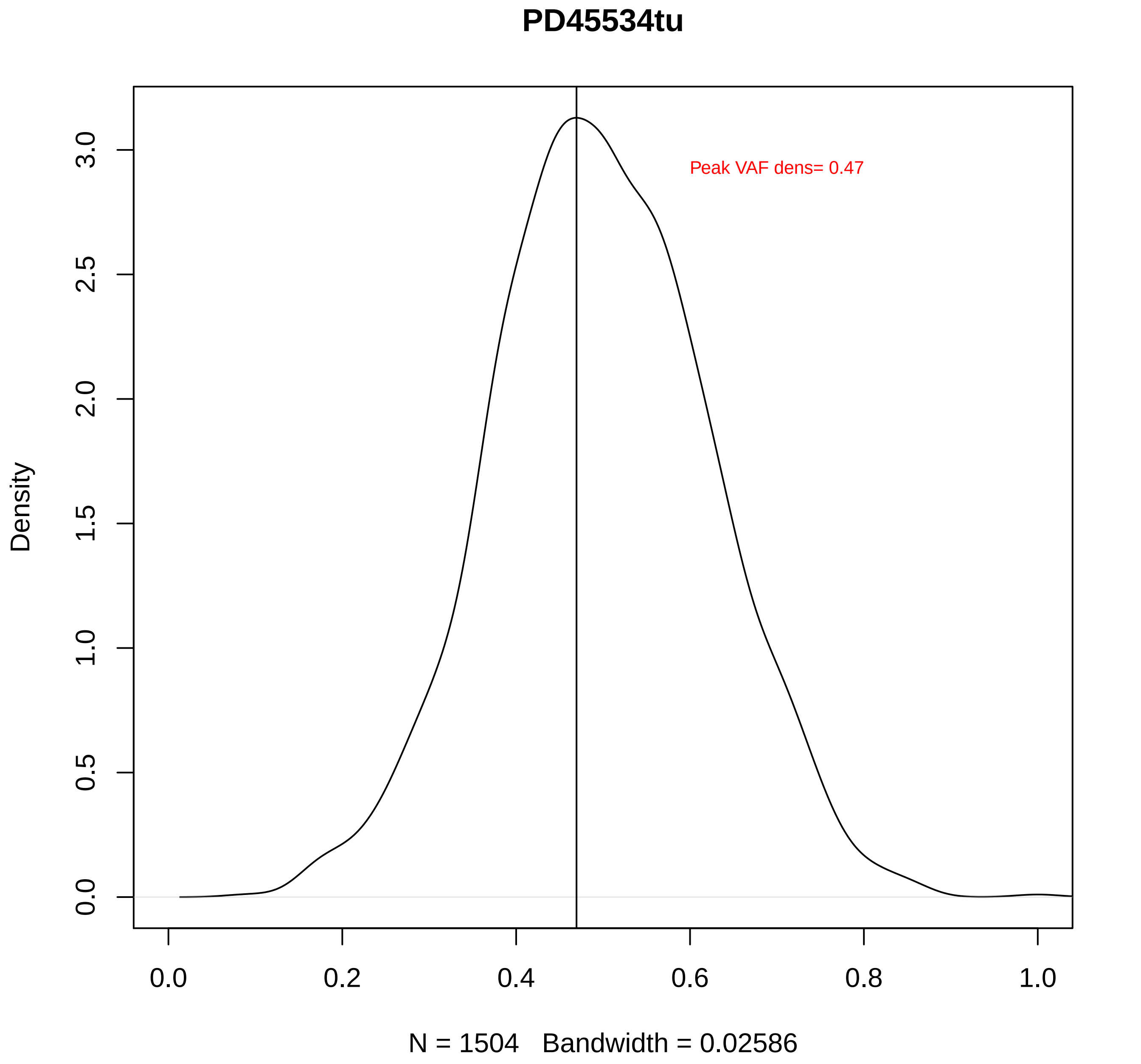
PD45534tu
Peak VAF dens= 0.47
Density
0.0
0.5
1.0
1.5
2.0
2.5
3.0
0.0
0.2
0.4
0.6
0.8
1.0
N = 1504   Bandwidth = 0.02586

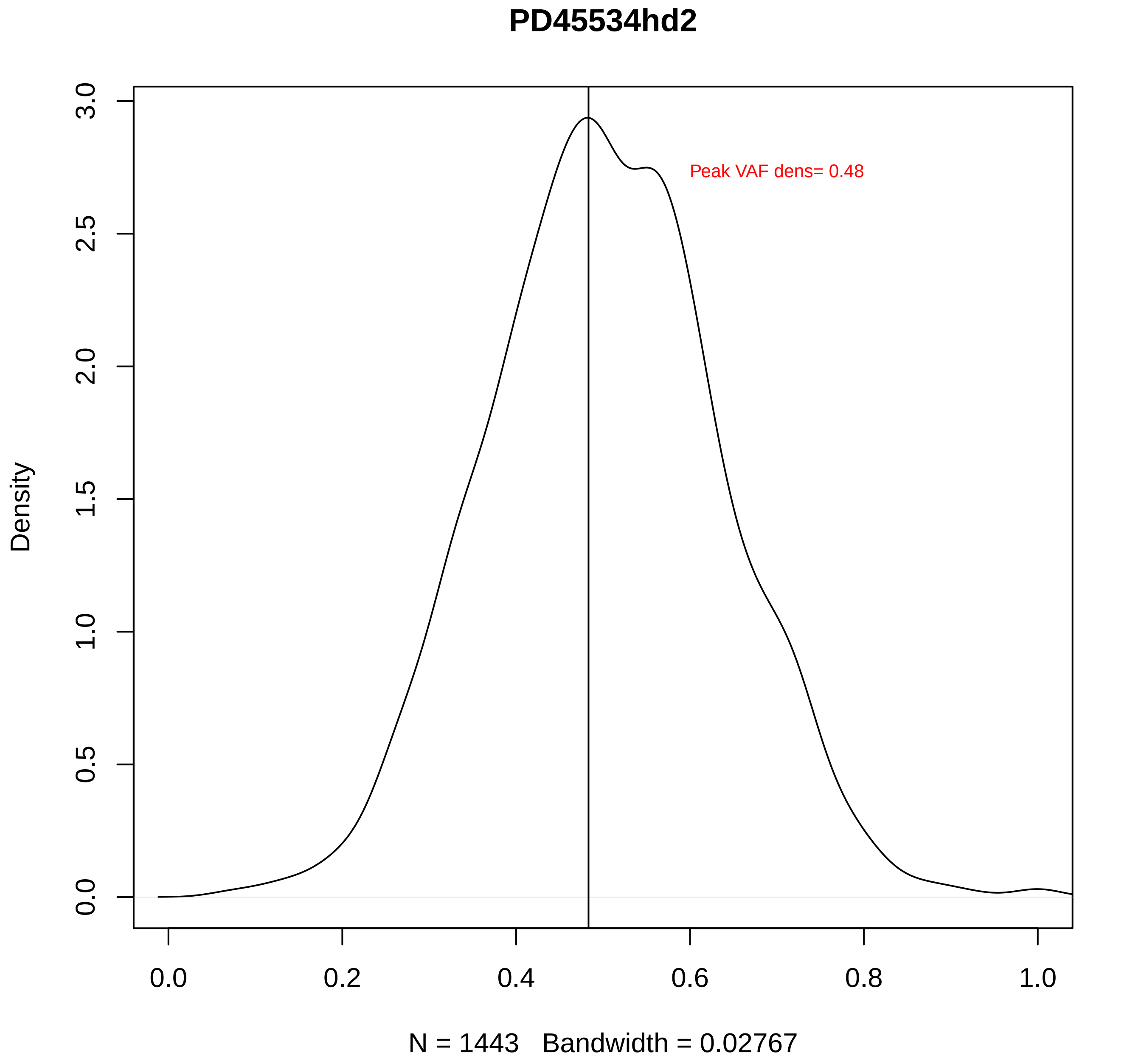

PD45534hd2
Peak VAF dens= 0.48
Density
0.0
0.5
1.0
1.5
2.0
2.5
3.0
0.0
0.2
0.4
0.6
0.8
1.0
N = 1443   Bandwidth = 0.02767

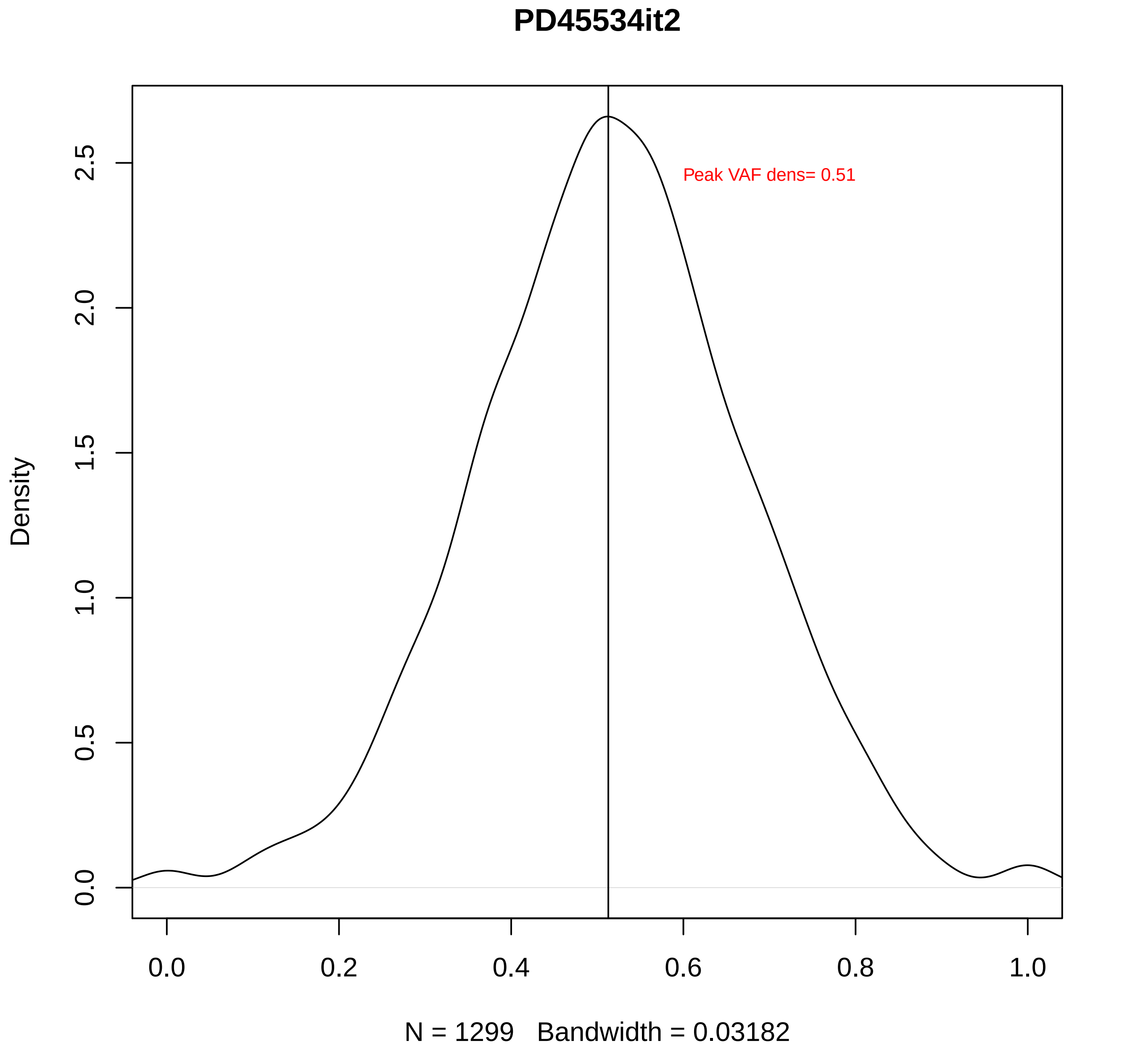
PD45534it2
Peak VAF dens= 0.51
Density
0.0
0.5
1.0
1.5
2.0
2.5
0.0
0.2
0.4
0.6
0.8
1.0
N = 1299   Bandwidth = 0.03182

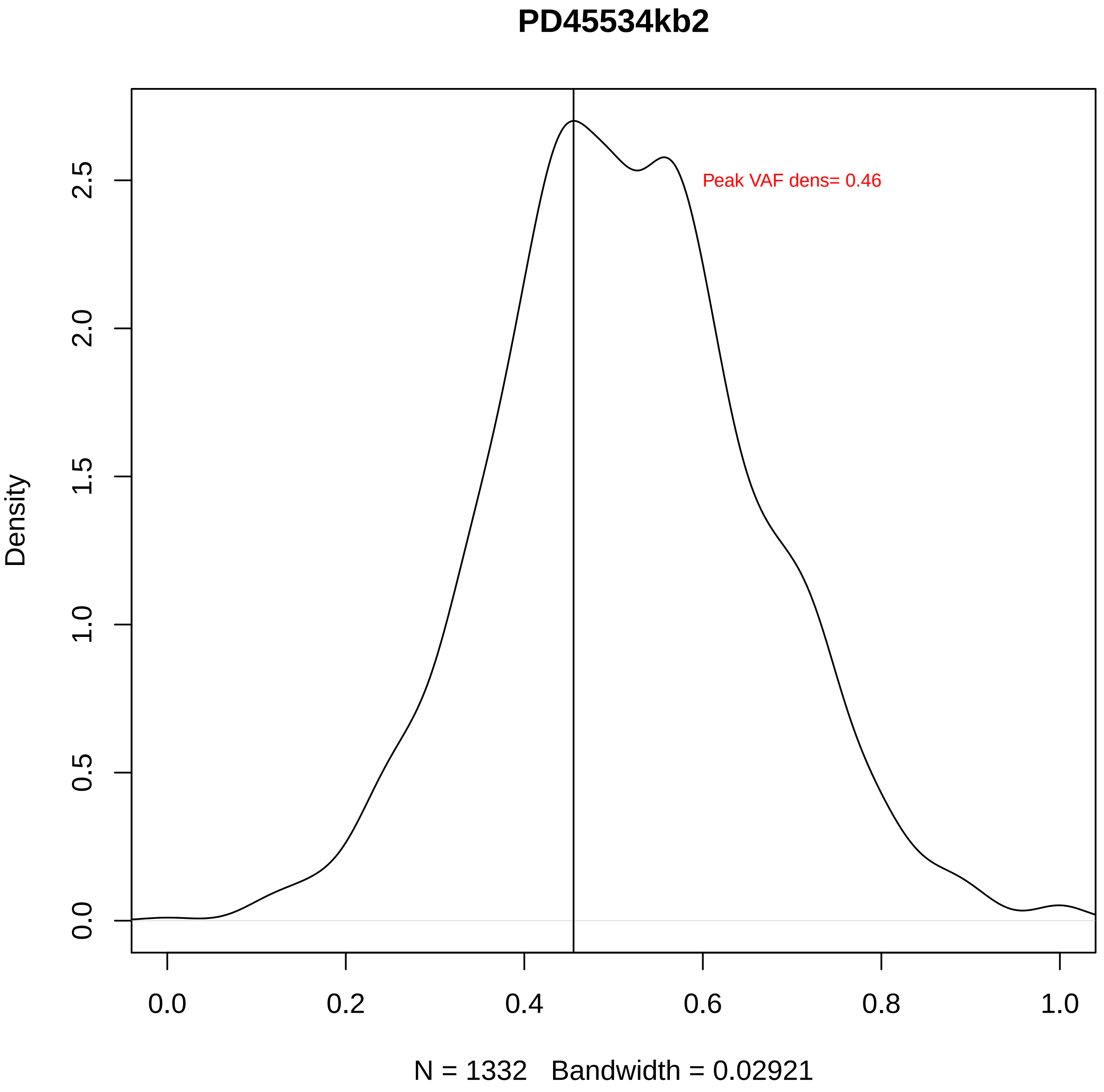

PD45534kb2
Peak VAF dens= 0.46
Density
0.0
0.5
1.0
1.5
2.0
2.5
0.0
0.2
0.4
0.6
0.8
1.0
N = 1332   Bandwidth = 0.02921

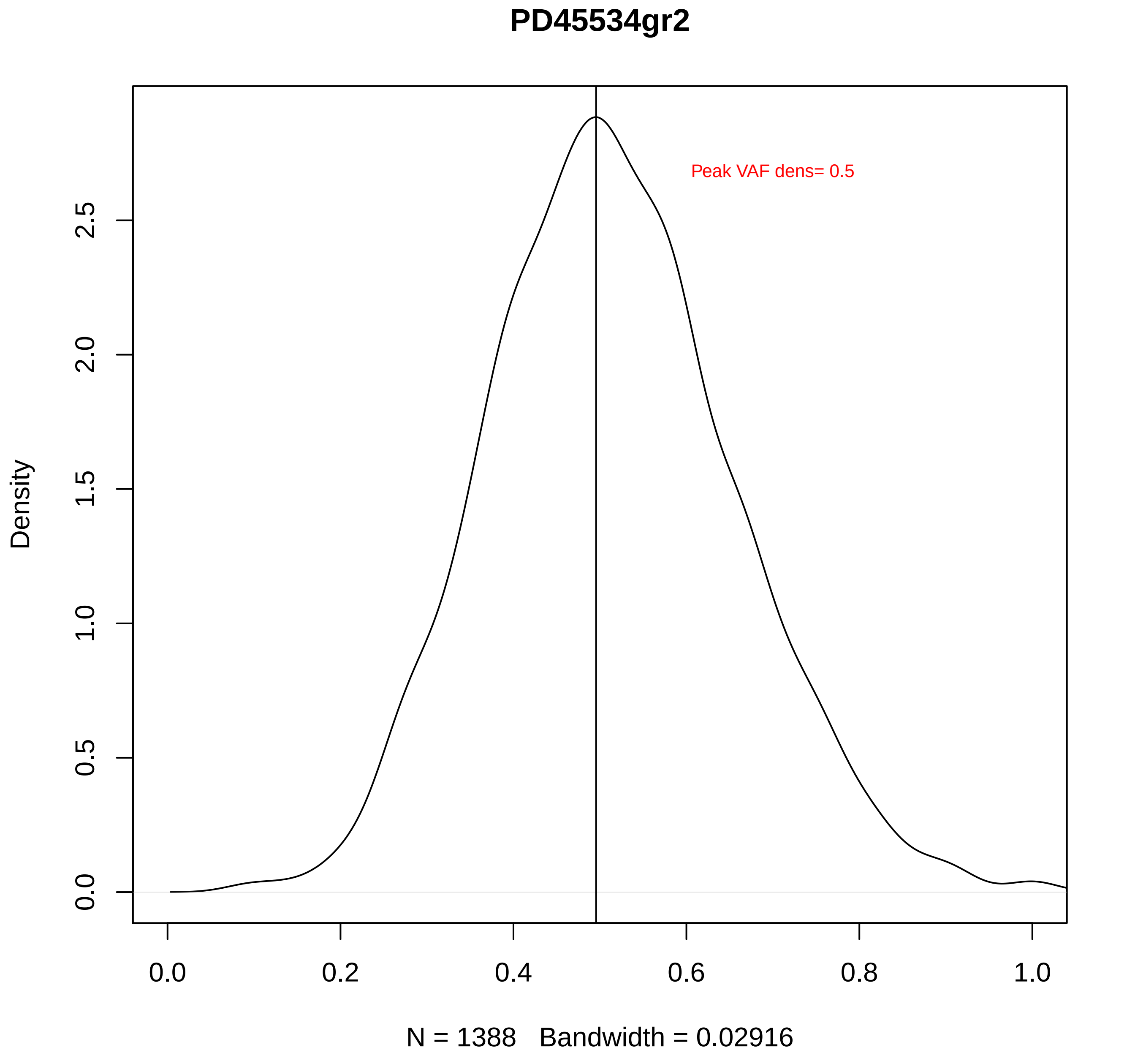
PD45534gr2
Peak VAF dens= 0.5
Density
0.0
0.5
1.0
1.5
2.0
2.5
0.0
0.2
0.4
0.6
0.8
1.0
N = 1388   Bandwidth = 0.02916

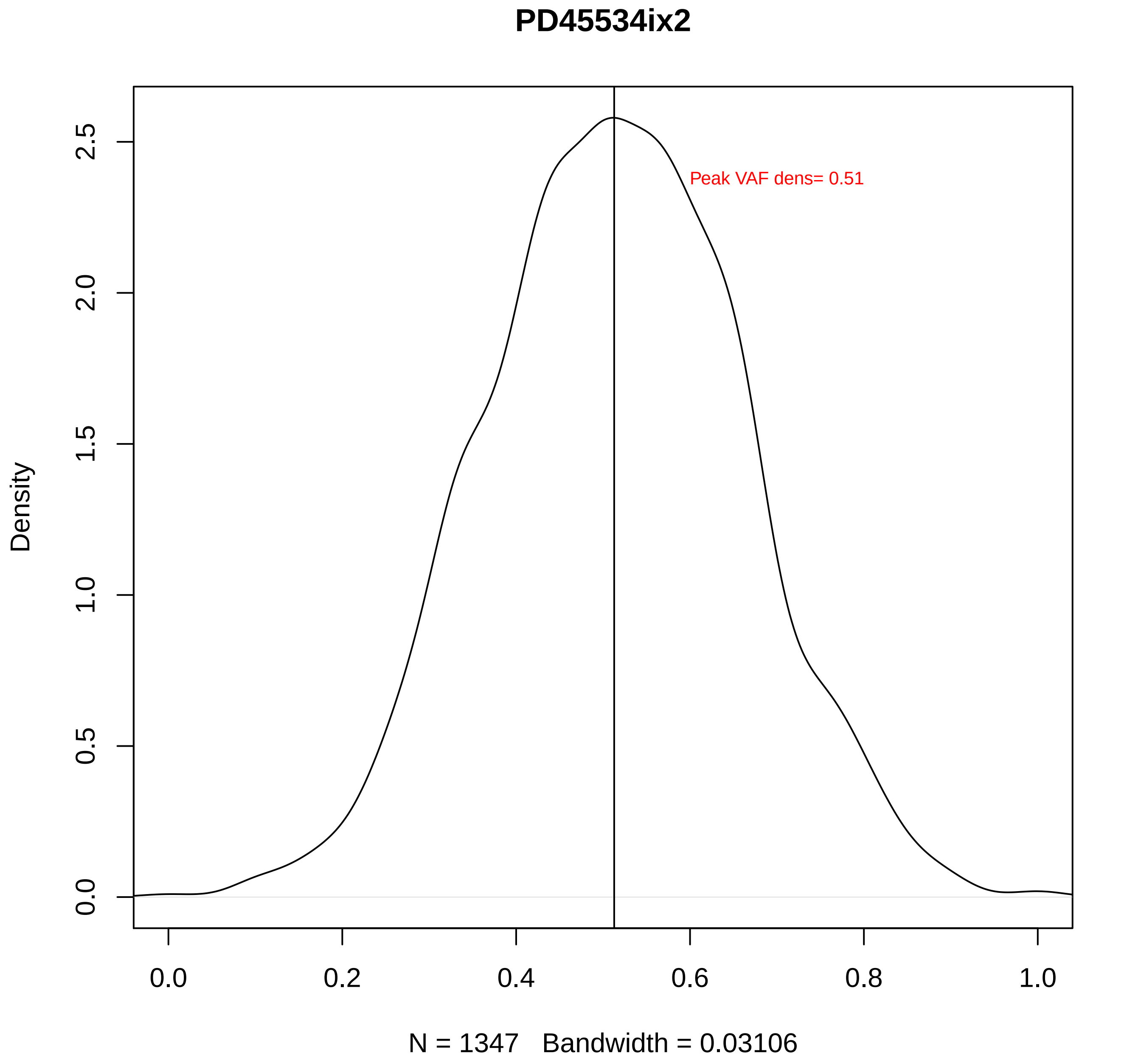
PD45534ix2
Peak VAF dens= 0.51
Density
0.0
0.5
1.0
1.5
2.0
2.5
0.0
0.2
0.4
0.6
0.8
1.0
N = 1347   Bandwidth = 0.03106

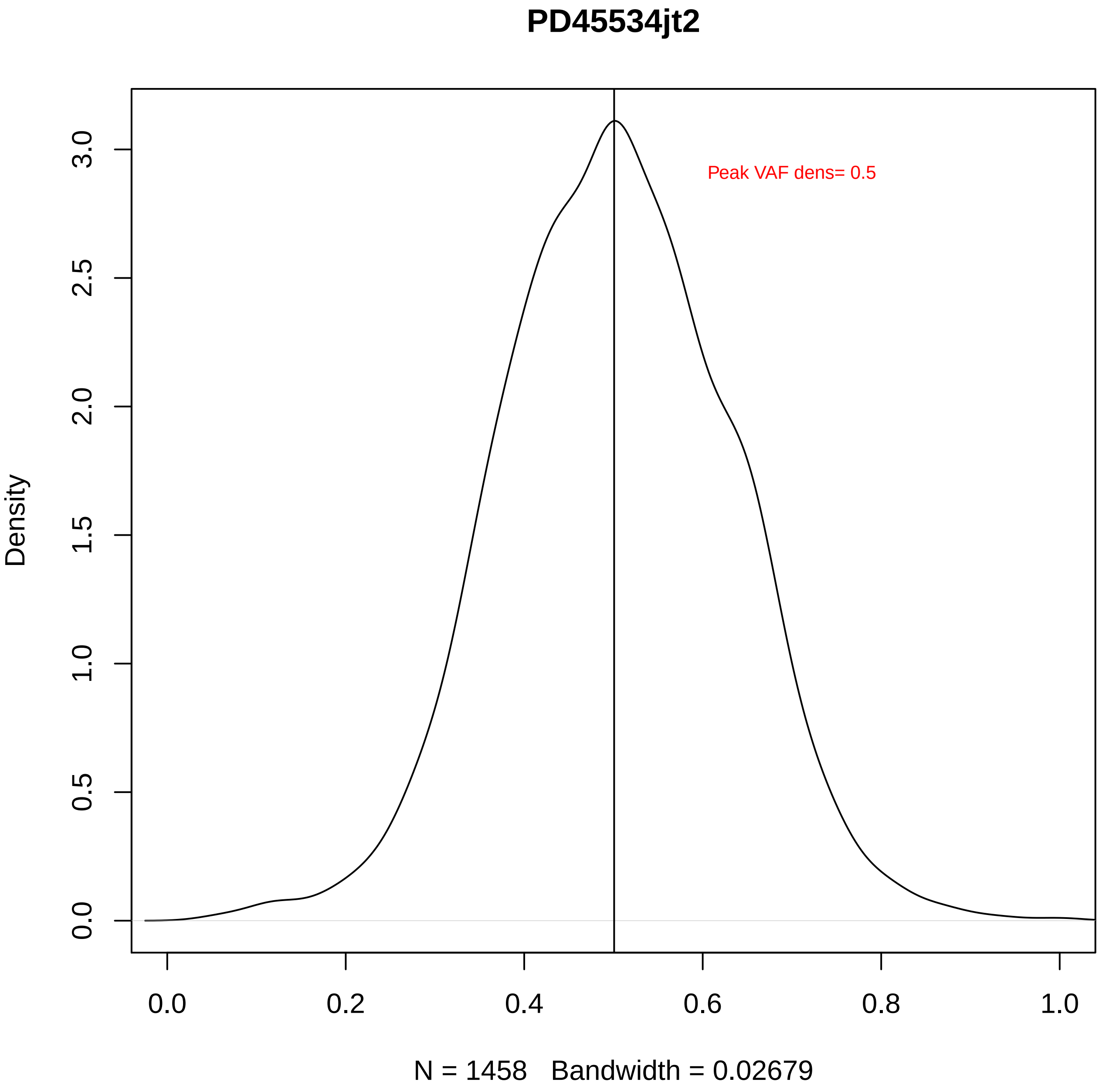
PD45534jt2
Peak VAF dens= 0.5
Density
0.0
0.5
1.0
1.5
2.0
2.5
3.0
0.0
0.2
0.4
0.6
0.8
1.0
N = 1458   Bandwidth = 0.02679

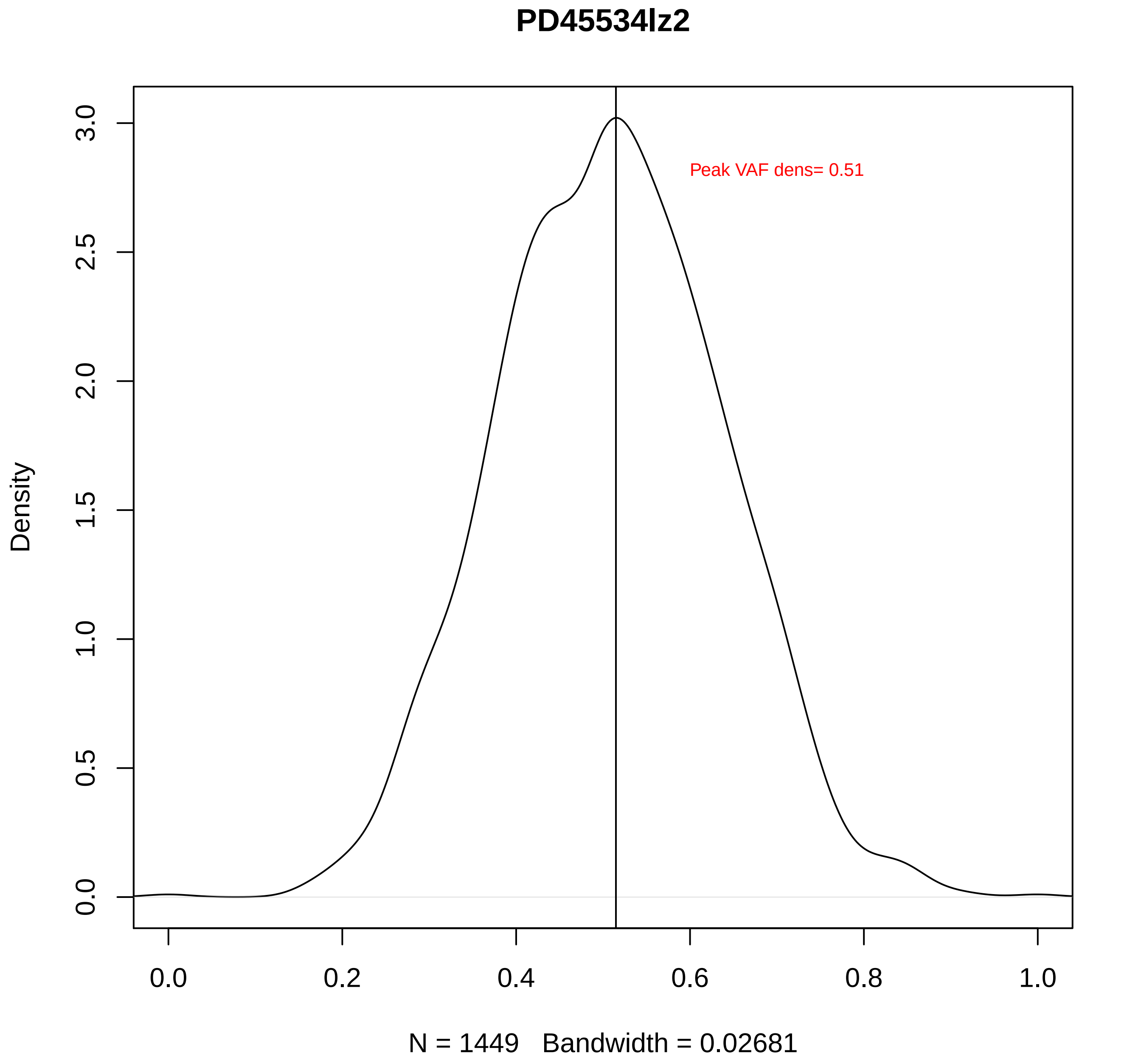

PD45534lz2
Peak VAF dens= 0.51
Density
0.0
0.5
1.0
1.5
2.0
2.5
3.0
0.0
0.2
0.4
0.6
0.8
1.0
N = 1449   Bandwidth = 0.02681

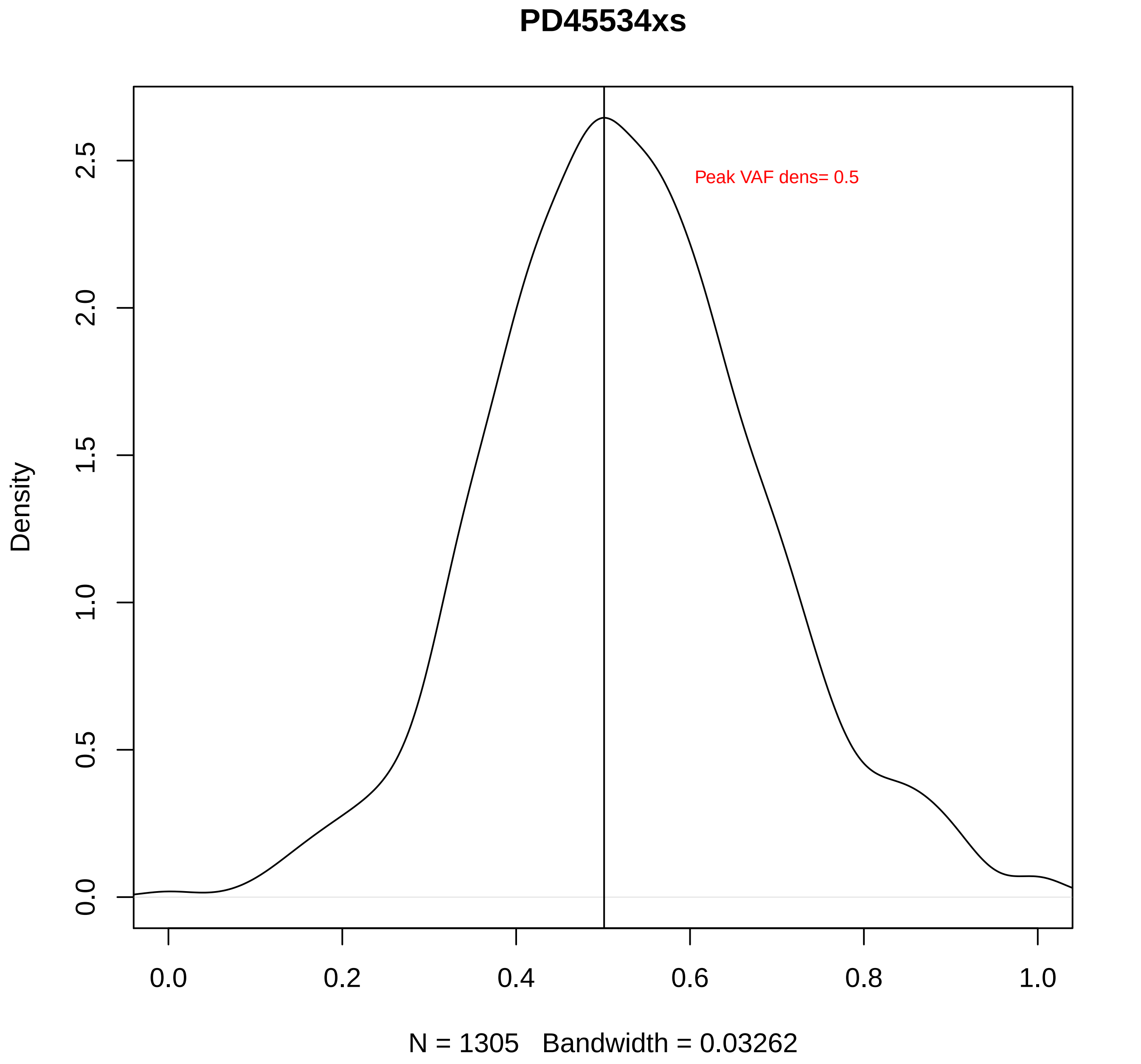

PD45534xs
Peak VAF dens= 0.5
Density
0.0
0.5
1.0
1.5
2.0
2.5
0.0
0.2
0.4
0.6
0.8
1.0
N = 1305 Bandwidth = 0.03262

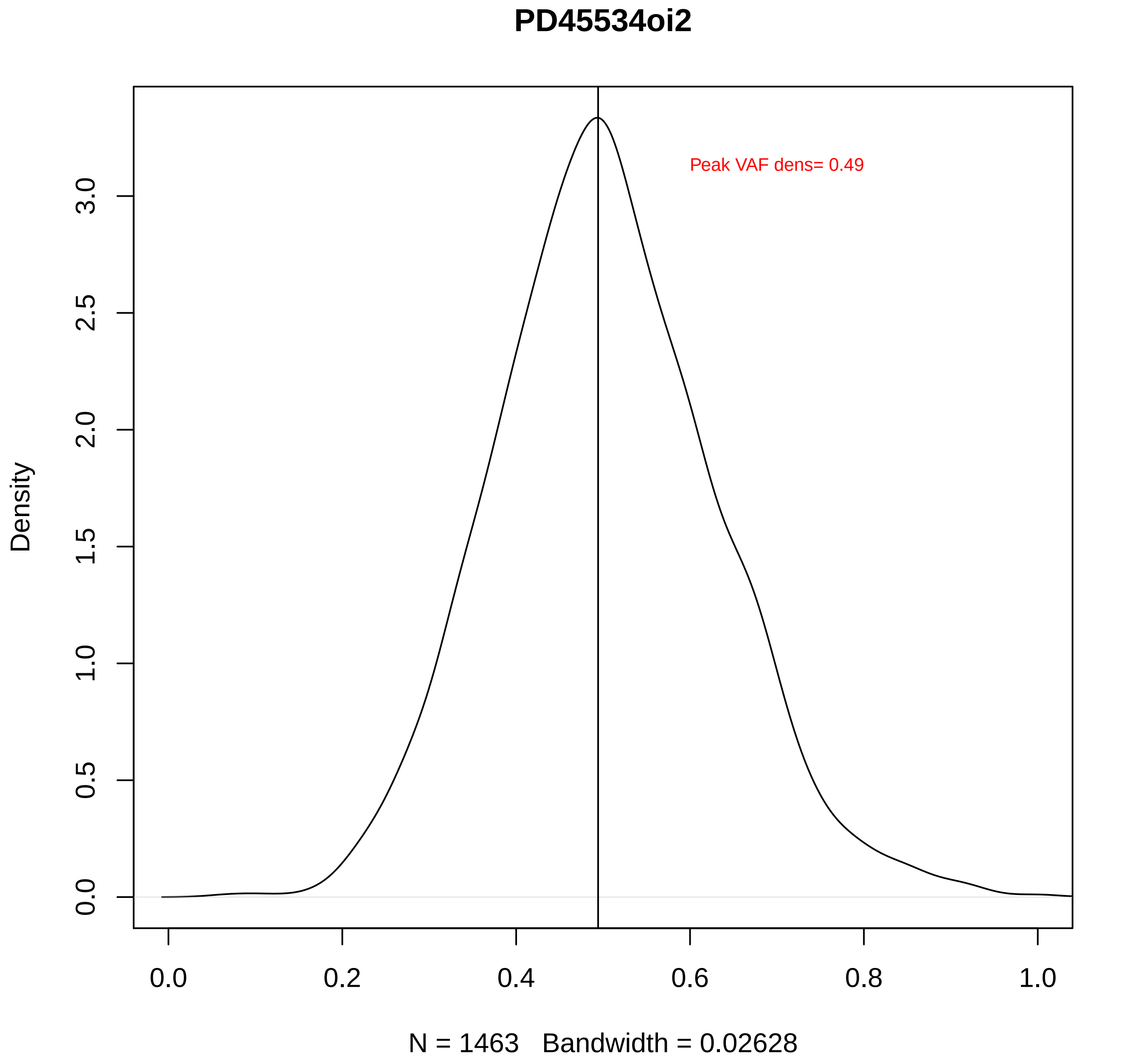

PD45534oi2
Peak VAF dens= 0.49
Density
0.0
0.5
1.0
1.5
2.0
2.5
3.0
0.0
0.2
0.4
0.6
0.8
1.0
N = 1463   Bandwidth = 0.02628

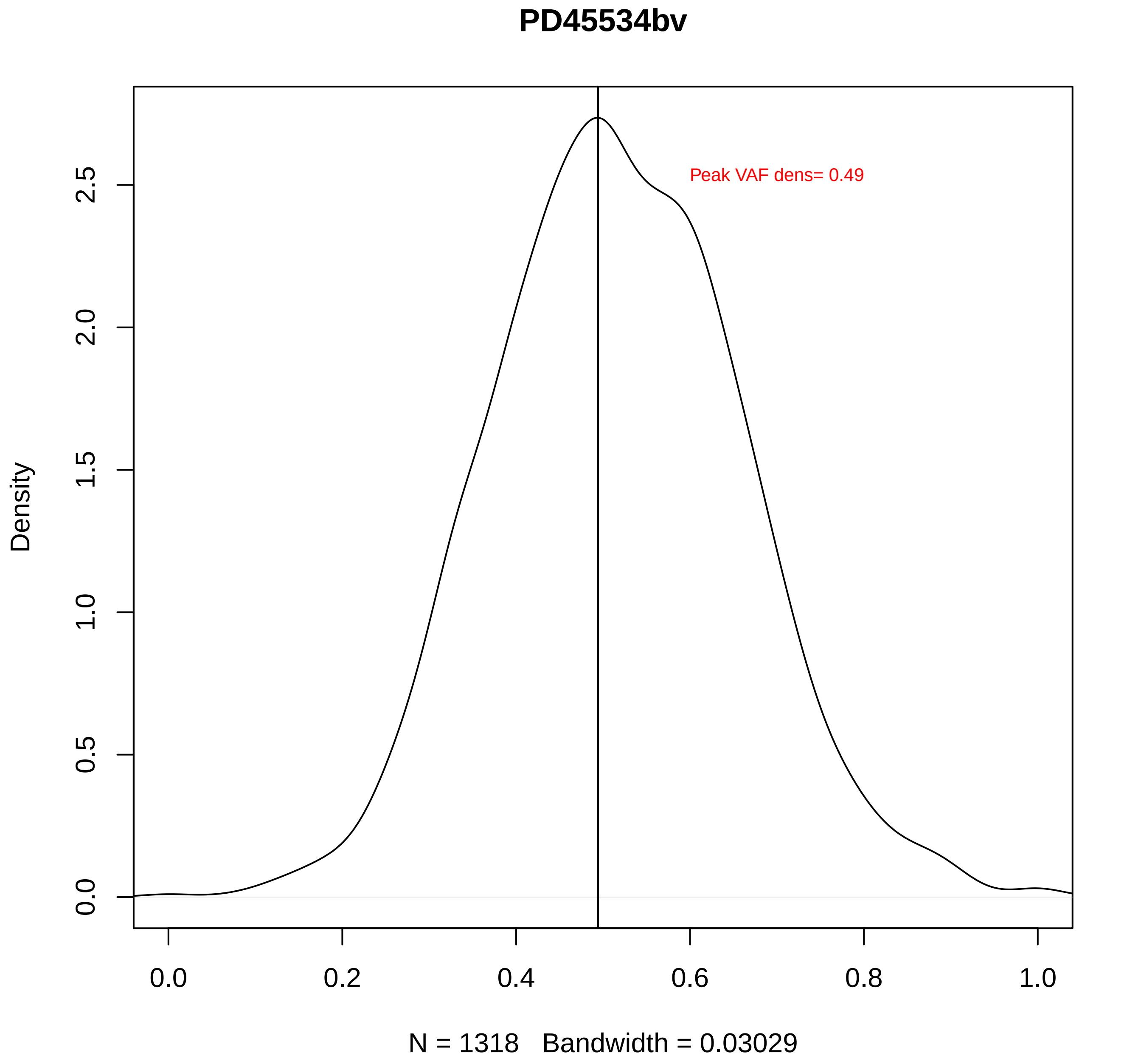

PD45534bv
Peak VAF dens= 0.49
Density
0.0
0.5
1.0
1.5
2.0
2.5
0.0
0.2
0.4
0.6
0.8
1.0
N = 1318   Bandwidth = 0.03029

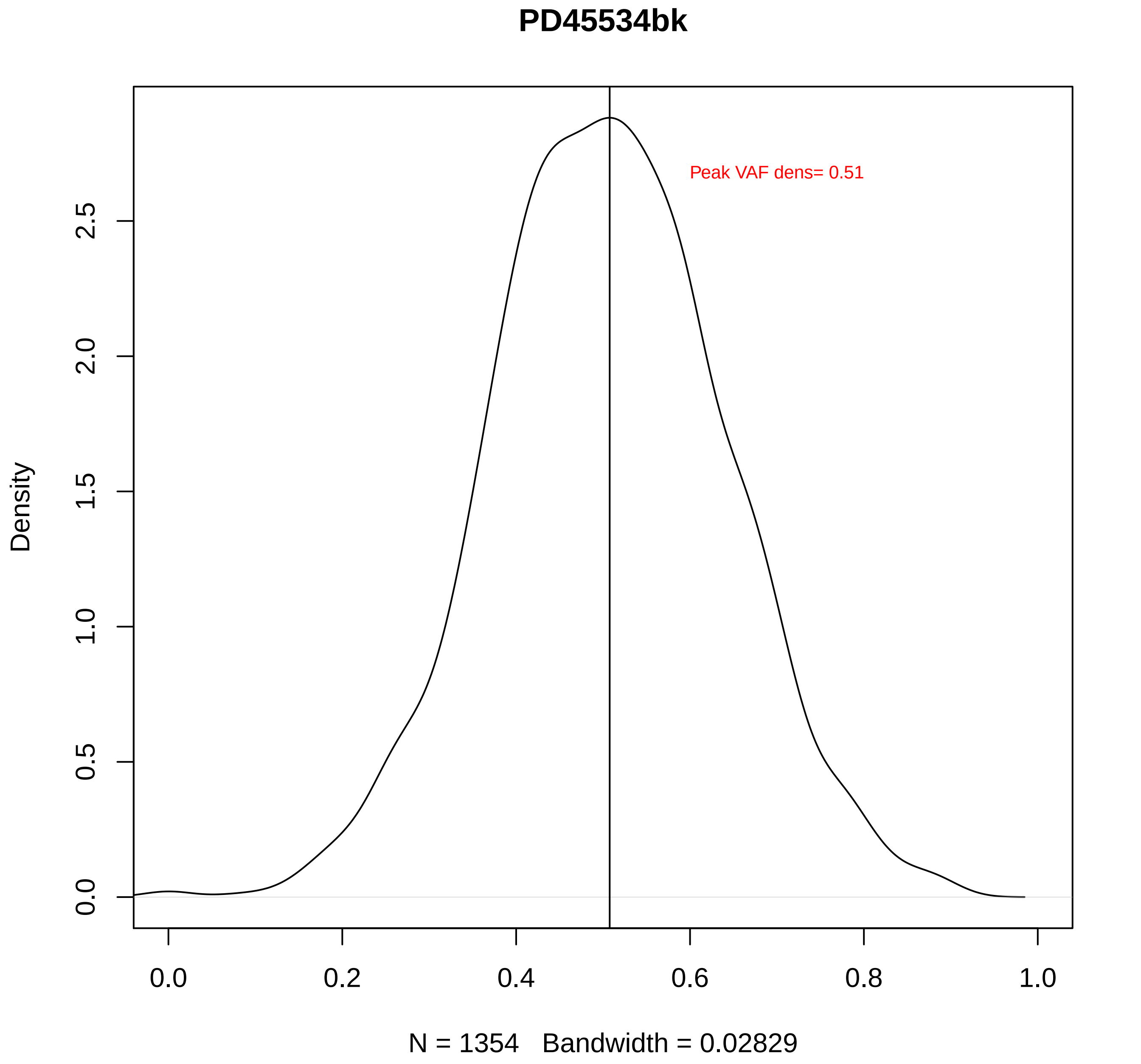
PD45534bk
Peak VAF dens= 0.51
Density
0.0
0.5
1.0
1.5
2.0
2.5
0.0
0.2
0.4
0.6
0.8
1.0
N = 1354 Bandwidth = 0.02829

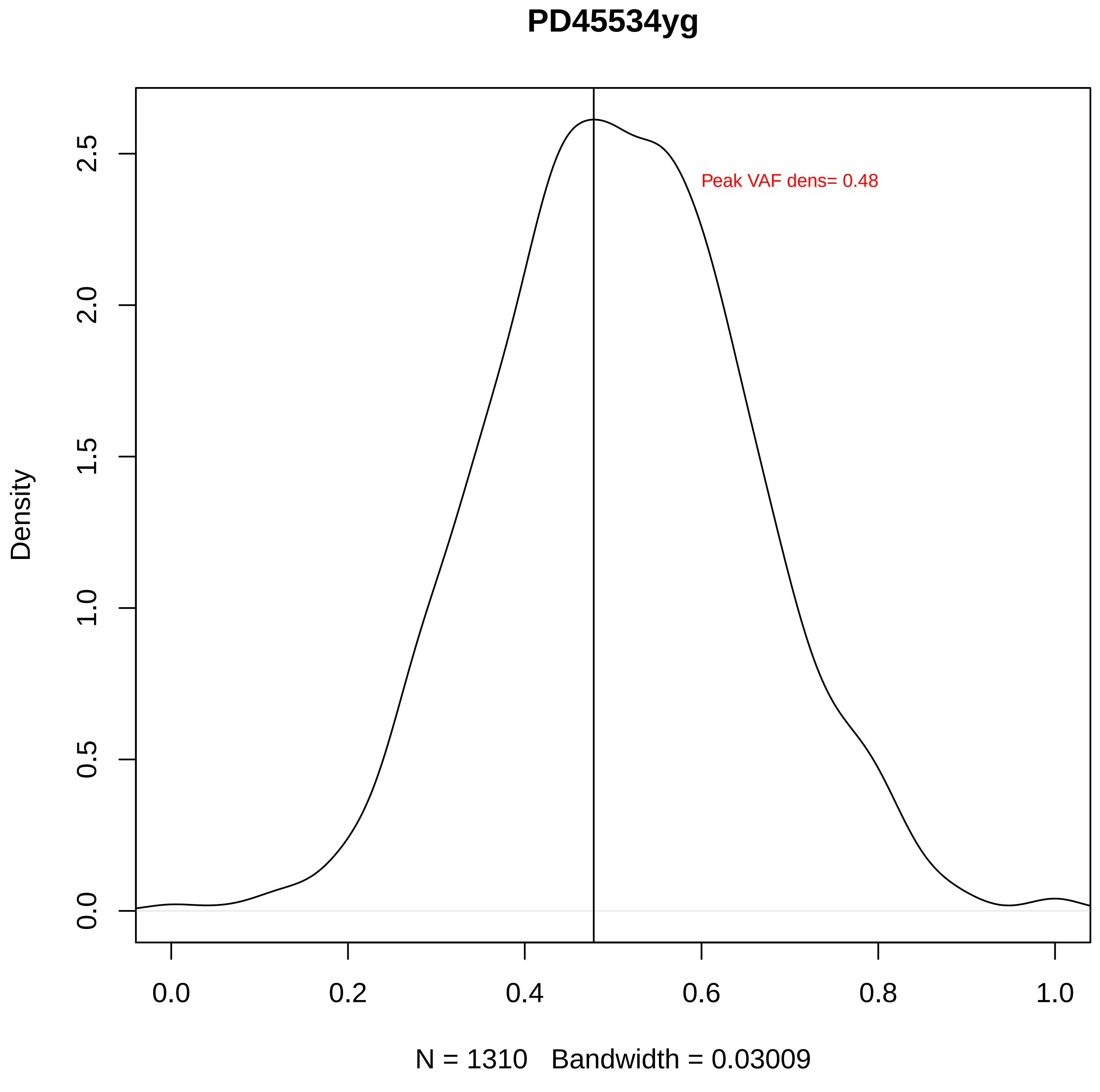
PD45534yg
Peak VAF dens= 0.48
Density
0.0
0.5
1.0
1.5
2.0
2.5
0.0
0.2
0.4
0.6
0.8
1.0
N = 1310   Bandwidth = 0.03009

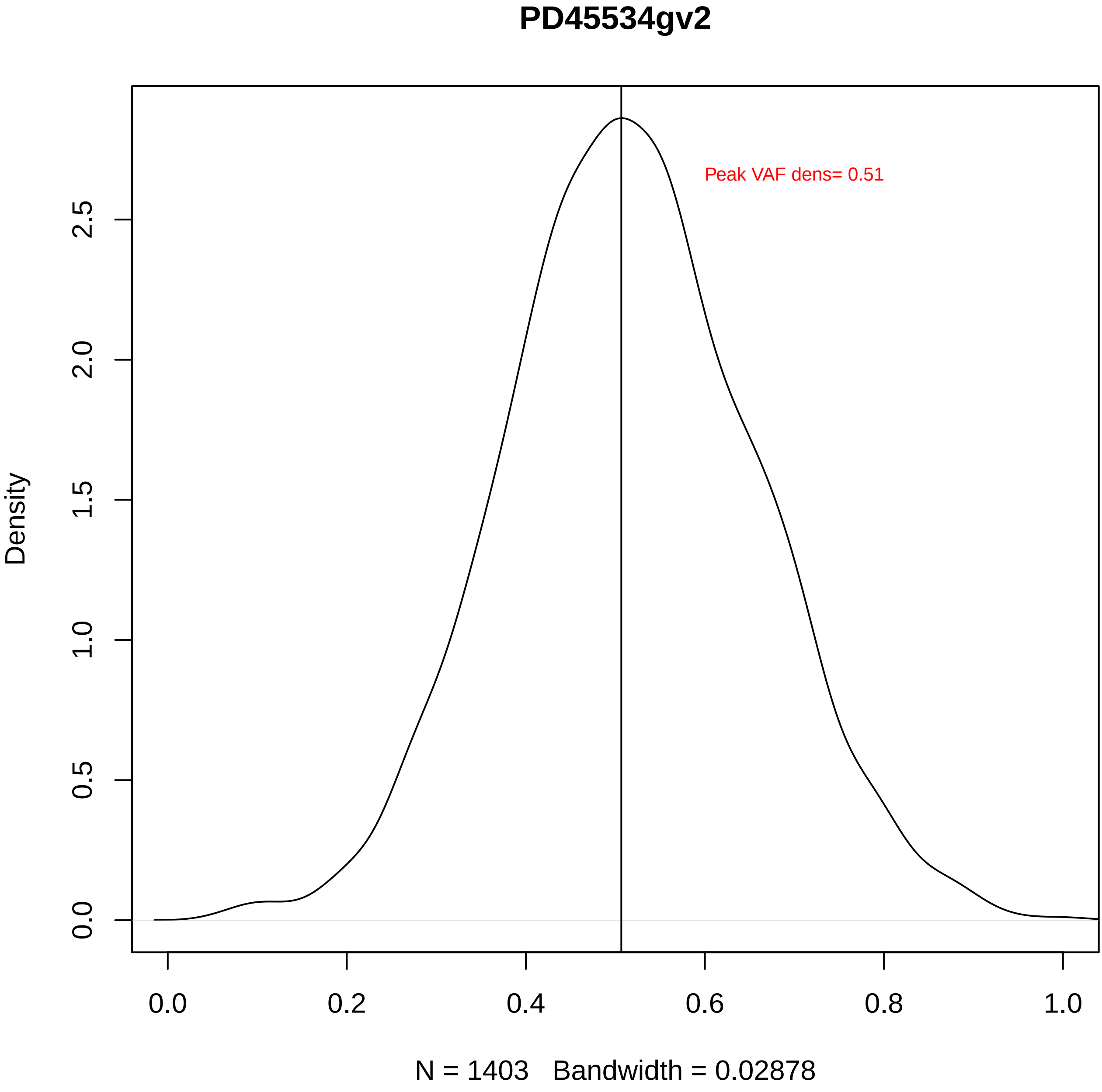
PD45534gv2
Peak VAF dens= 0.51
Density
0.0
0.5
1.0
1.5
2.0
2.5
0.0
0.2
0.4
0.6
0.8
1.0
N = 1403   Bandwidth = 0.02878

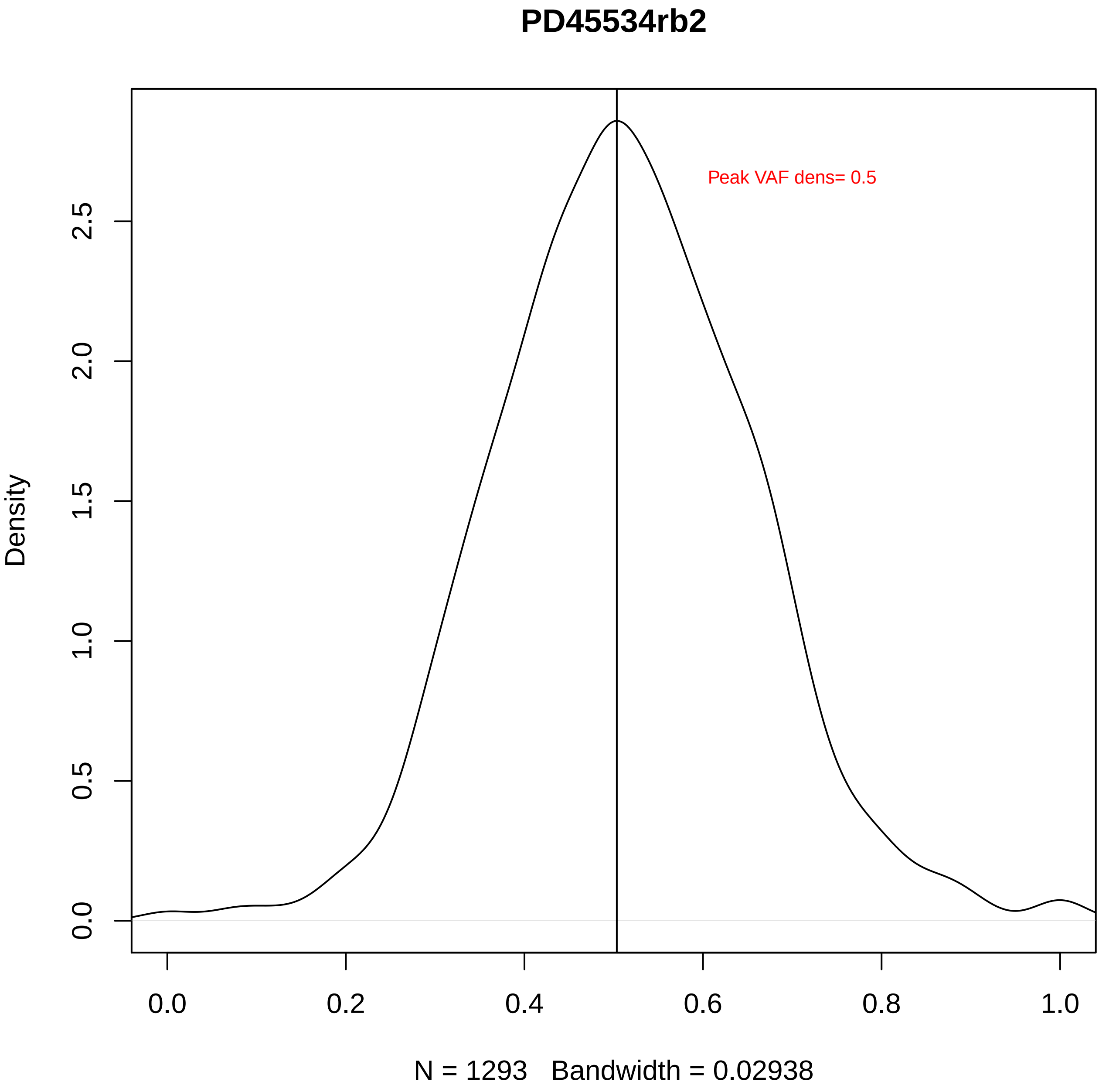
PD45534rb2
Peak VAF dens= 0.5
Density
0.0
0.5
1.0
1.5
2.0
2.5
0.0
0.2
0.4
0.6
0.8
1.0
N = 1293   Bandwidth = 0.02938

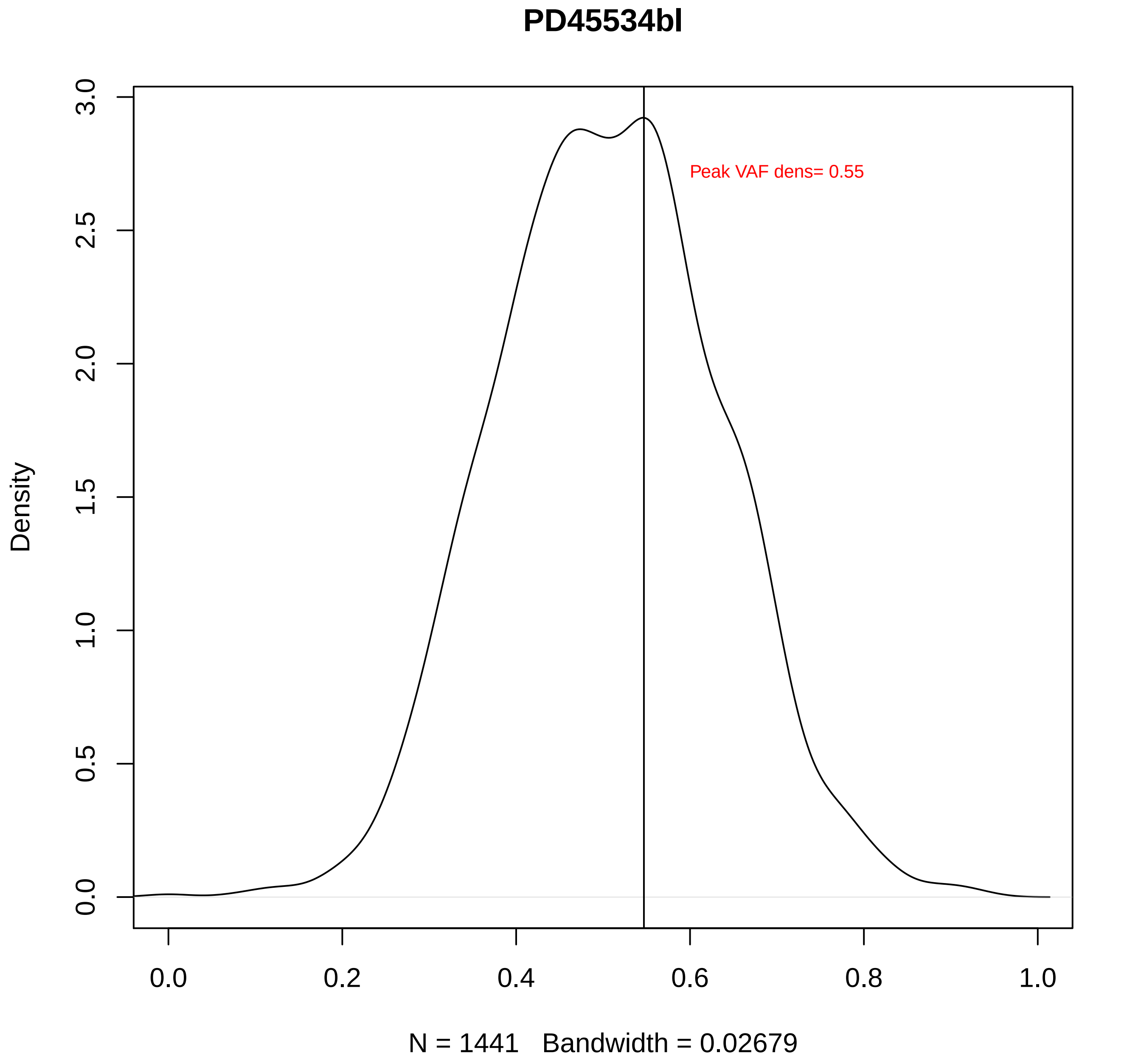

PD45534bl
Peak VAF dens= 0.55
Density
0.0
0.5
1.0
1.5
2.0
2.5
3.0
0.0
0.2
0.4
0.6
0.8
1.0
N = 1441   Bandwidth = 0.02679

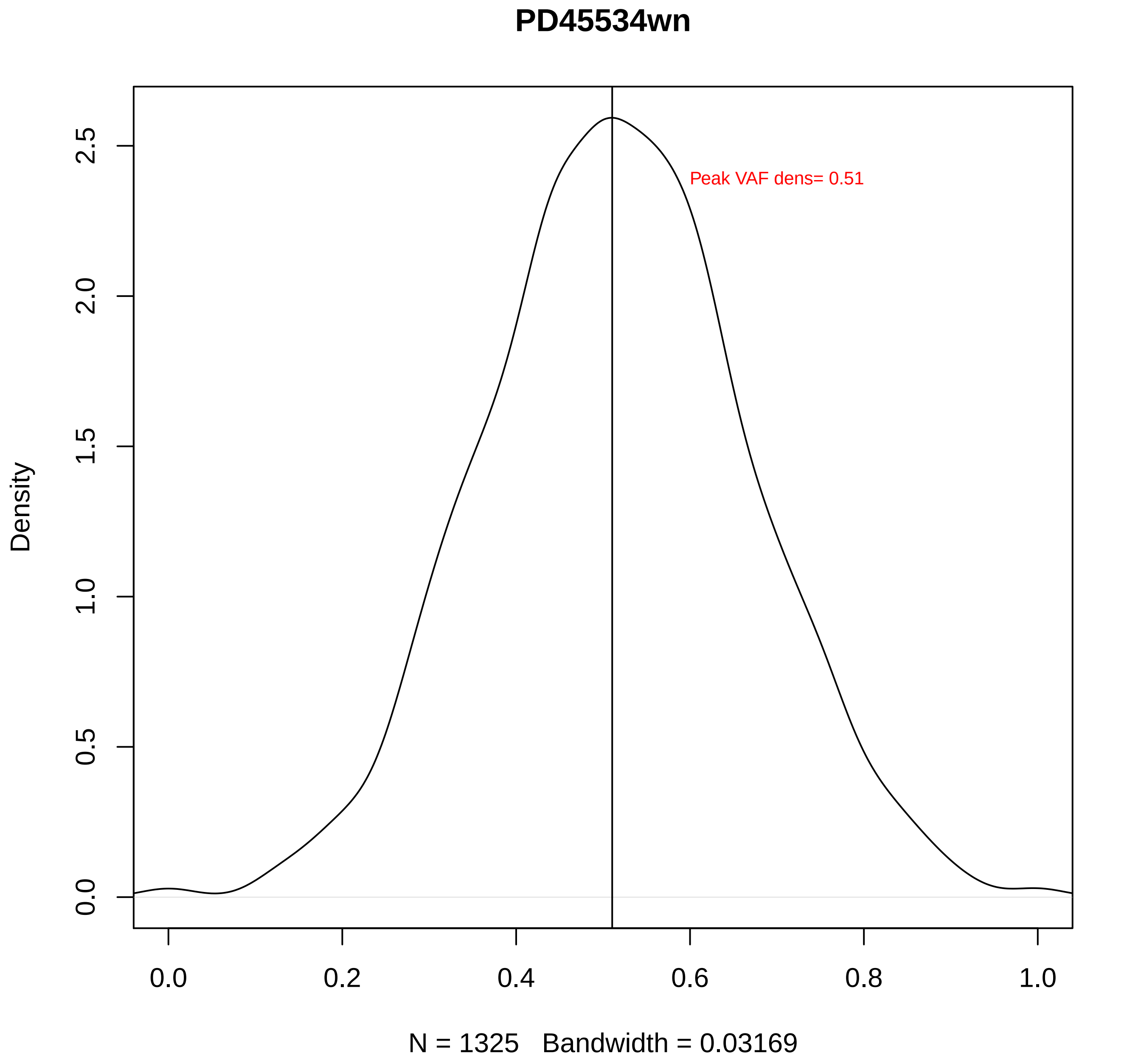
PD45534wn
Peak VAF dens= 0.51
Density
0.0
0.5
1.0
1.5
2.0
2.5
0.0
0.2
0.4
0.6
0.8
1.0
N = 1325   Bandwidth = 0.03169

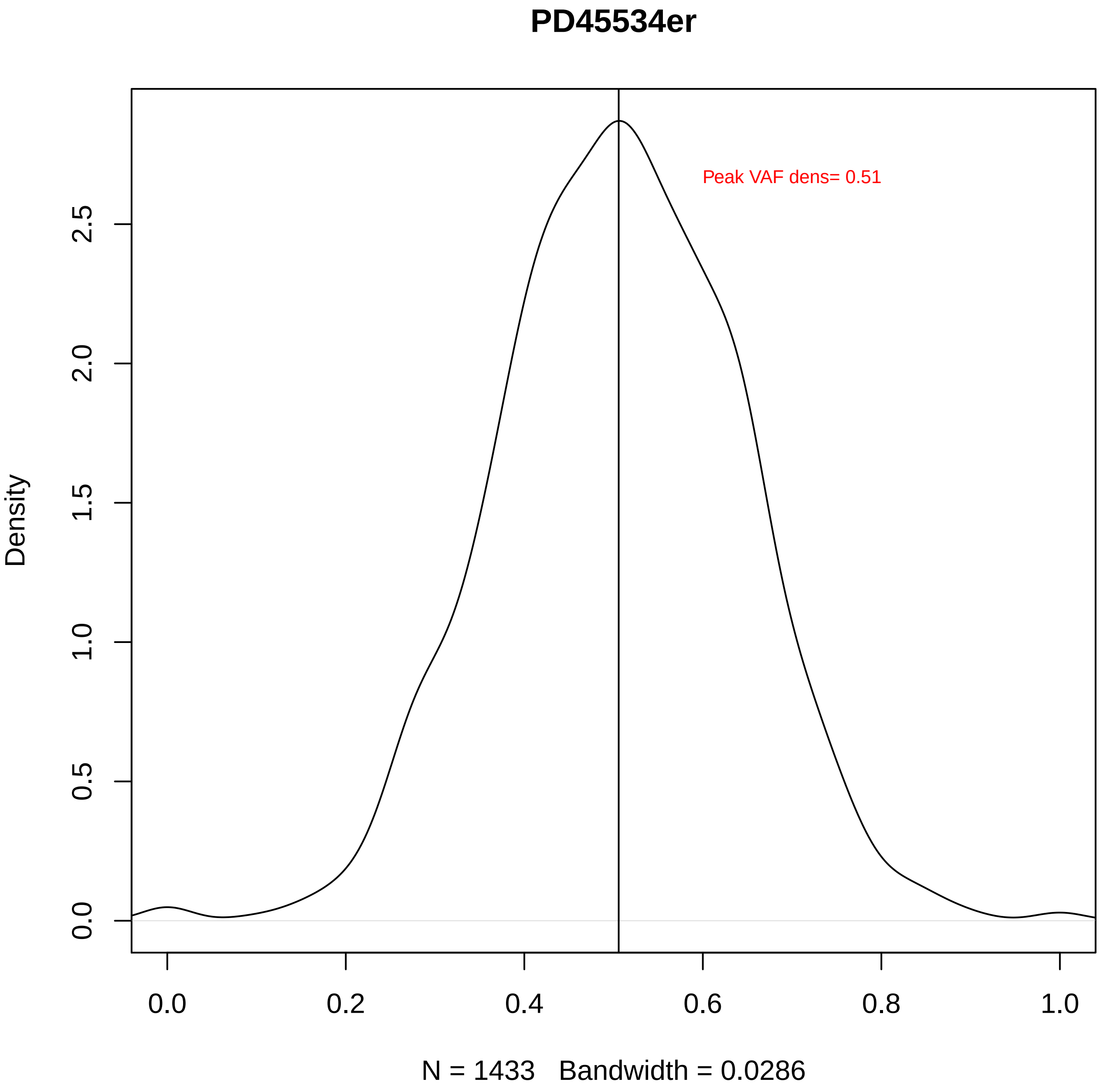
PD45534er
Peak VAF dens= 0.51
Density
0.0
0.5
1.0
1.5
2.0
2.5
0.0
0.2
0.4
0.6
0.8
1.0
N = 1433   Bandwidth = 0.0286

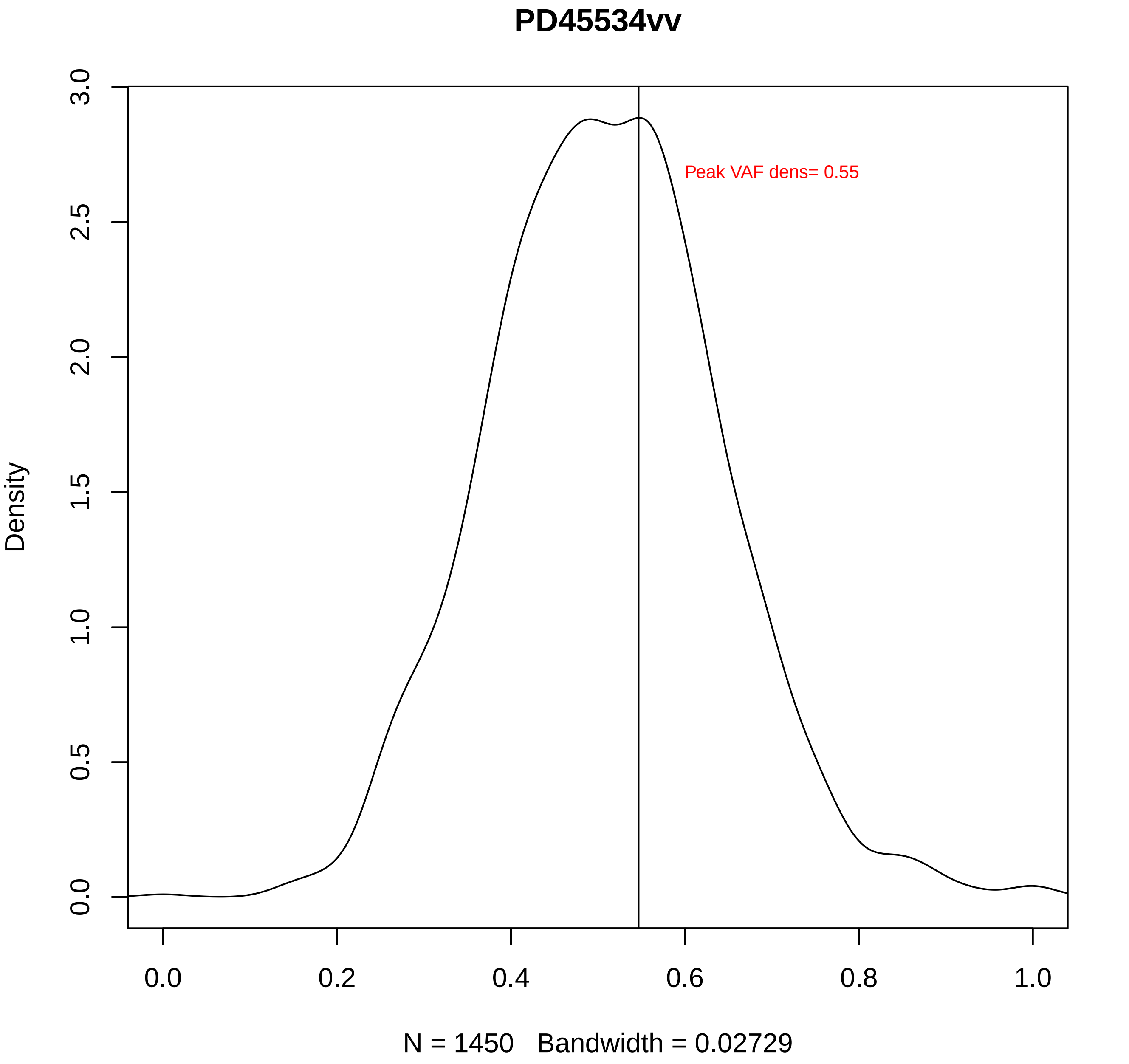
PD45534vv
Peak VAF dens= 0.55
Density
0.0
0.5
1.0
1.5
2.0
2.5
3.0
0.0
0.2
0.4
0.6
0.8
1.0
N = 1450   Bandwidth = 0.02729

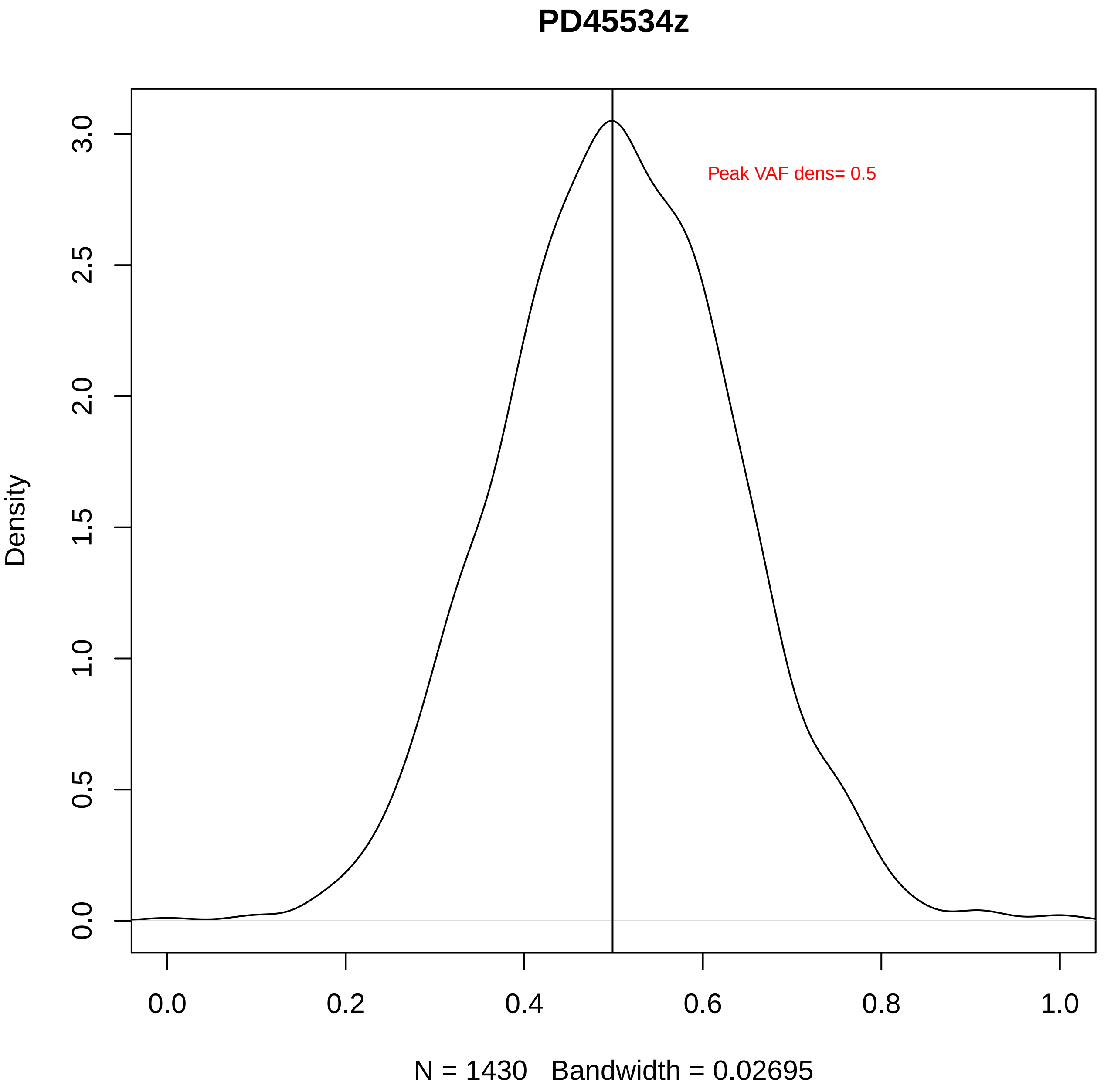
PD45534z
Peak VAF dens= 0.5
Density
0.0
0.5
1.0
1.5
2.0
2.5
3.0
0.0
0.2
0.4
0.6
0.8
1.0
N = 1430   Bandwidth = 0.02695

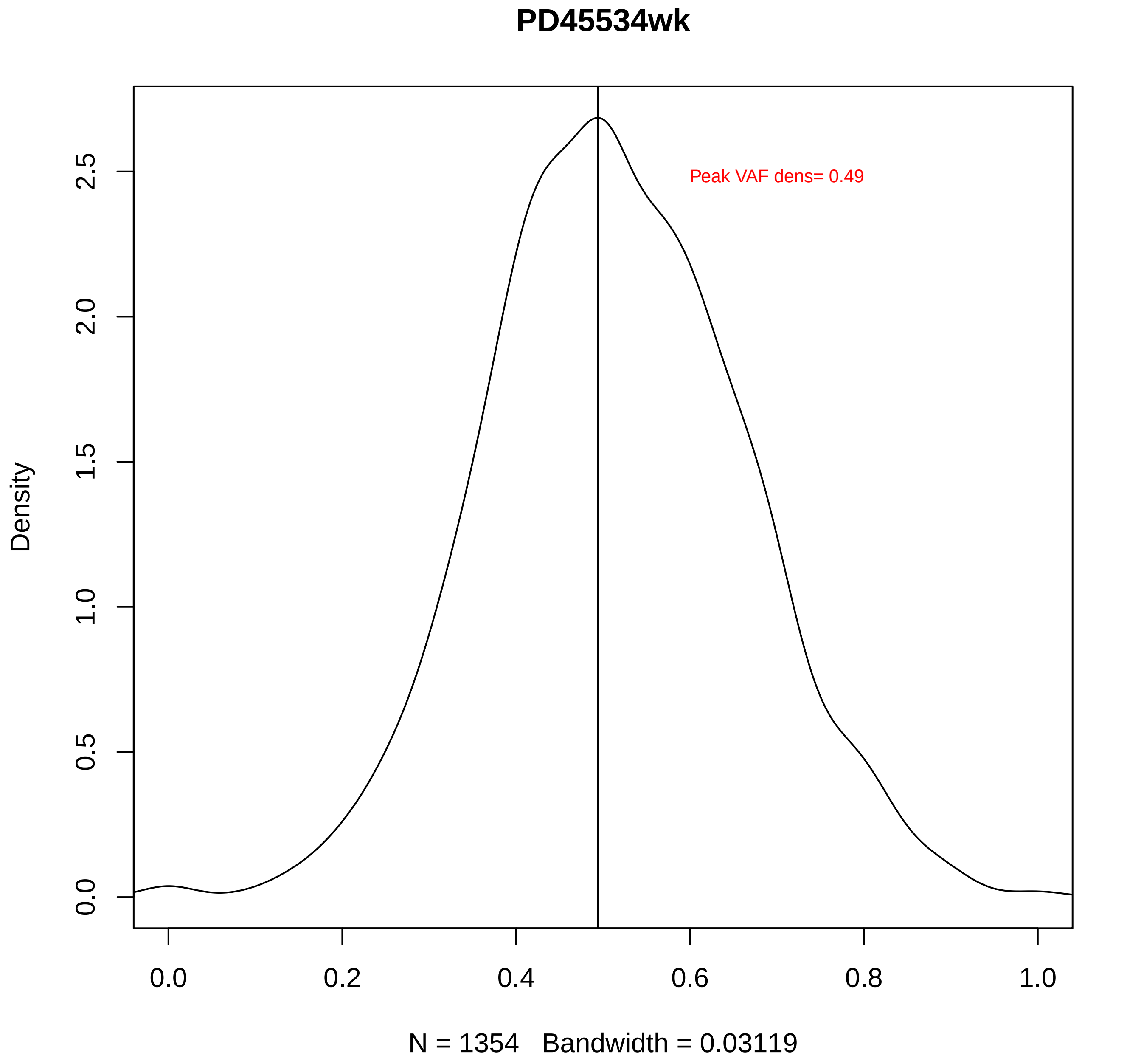
PD45534wk
Peak VAF dens= 0.49
Density
0.0
0.5
1.0
1.5
2.0
2.5
0.0
0.2
0.4
0.6
0.8
1.0
N = 1354   Bandwidth = 0.03119

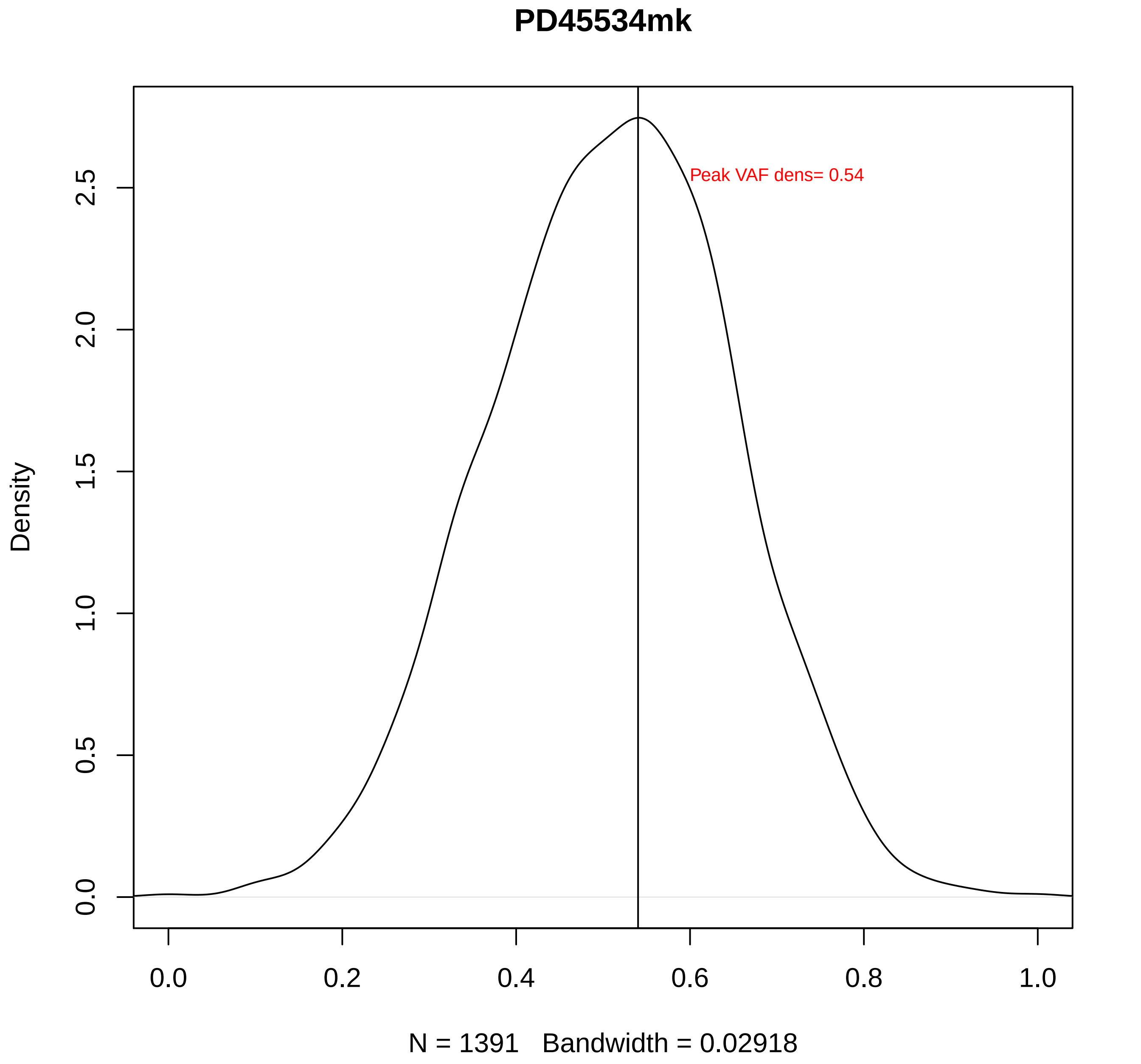

PD45534mk
Peak VAF dens= 0.54
Density
0.0
0.5
1.0
1.5
2.0
2.5
0.0
0.2
0.4
0.6
0.8
1.0
N = 1391 Bandwidth = 0.02918

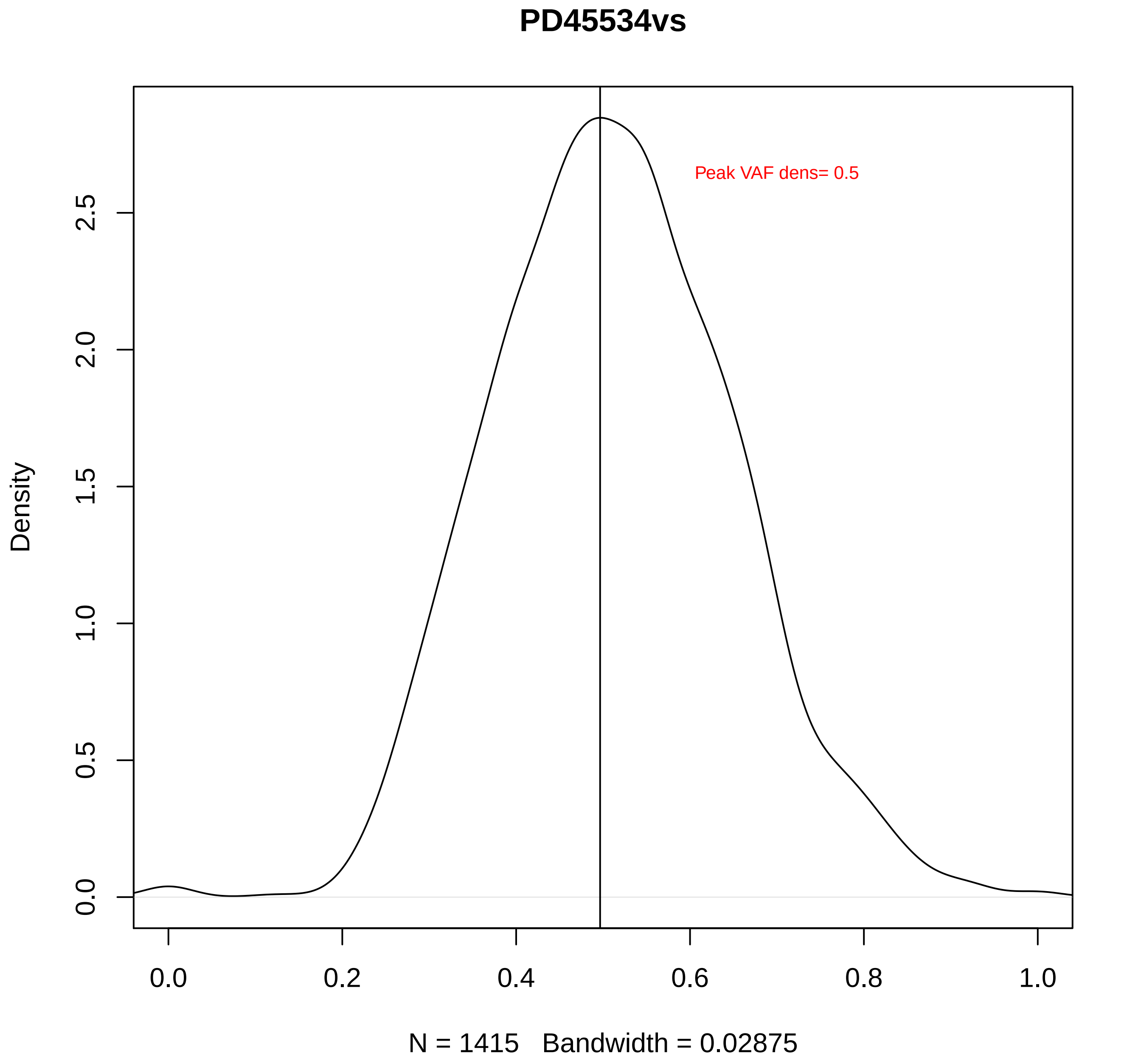

PD45534vs
Peak VAF dens= 0.5
Density
0.0
0.5
1.0
1.5
2.0
2.5
0.0
0.2
0.4
0.6
0.8
1.0
N = 1415   Bandwidth = 0.02875

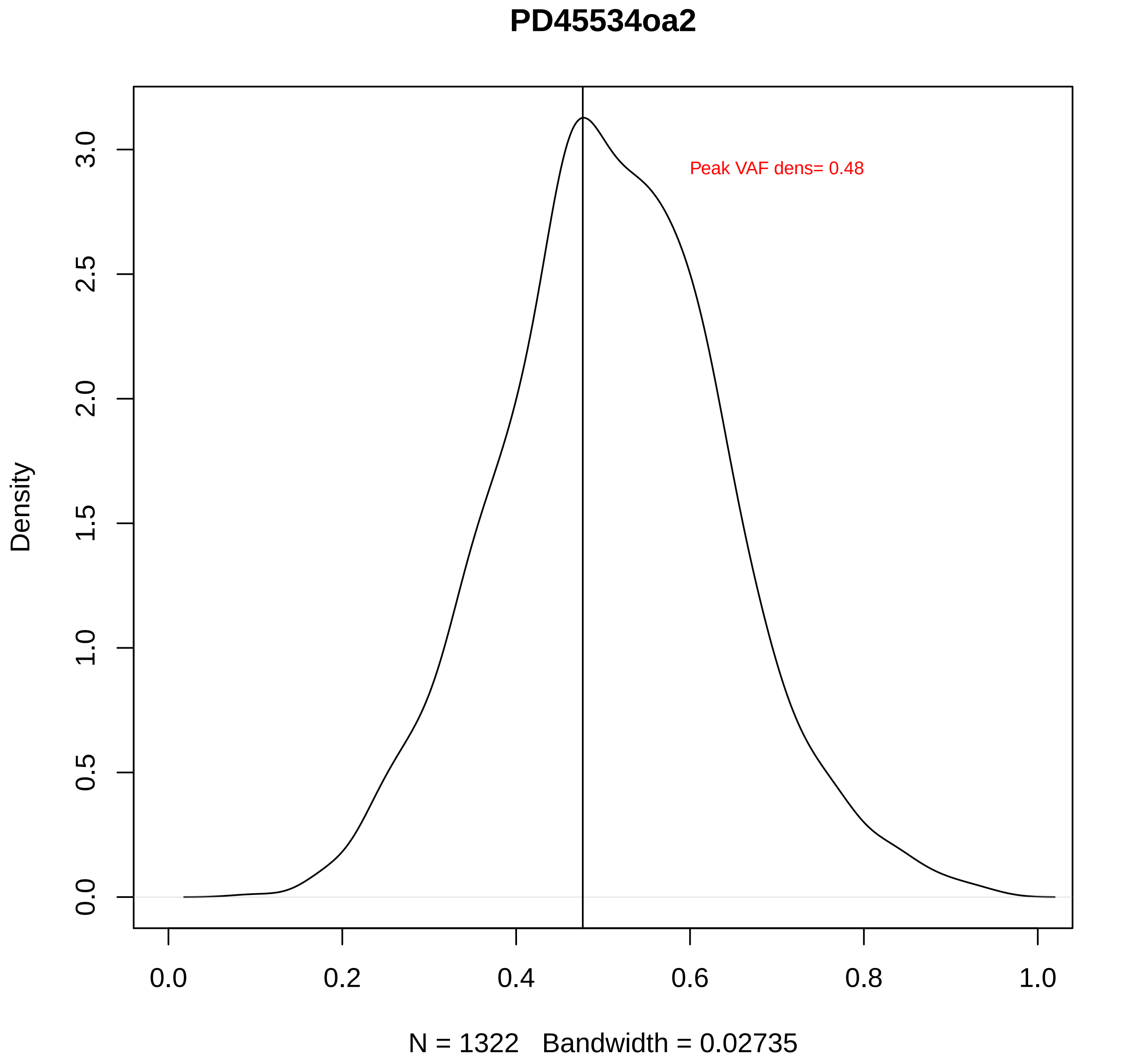
PD45534oa2
Peak VAF dens= 0.48
Density
0.0
0.5
1.0
1.5
2.0
2.5
3.0
0.0
0.2
0.4
0.6
0.8
1.0
N = 1322   Bandwidth = 0.02735

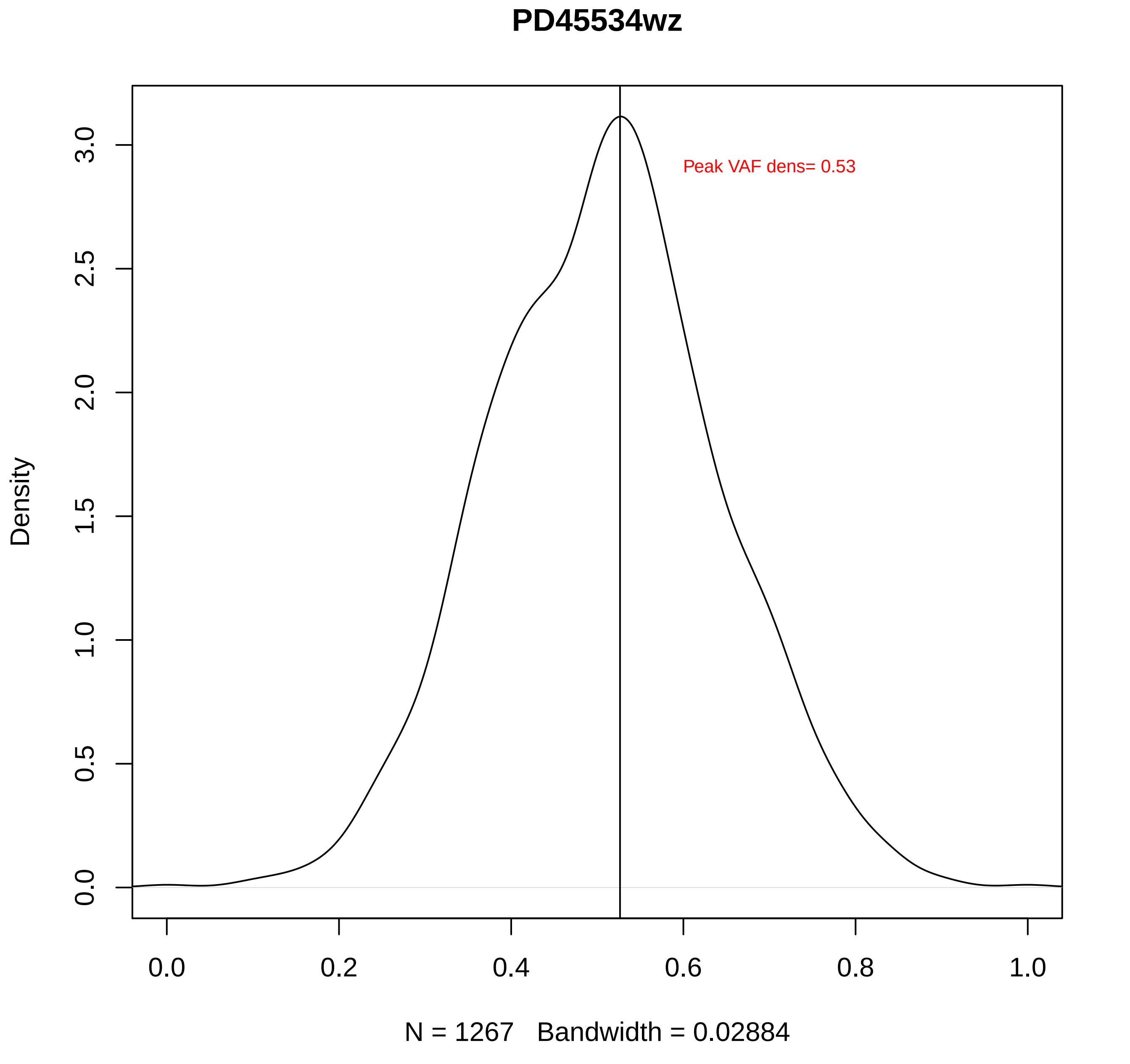
PD45534wz
Peak VAF dens= 0.53
Density
N = 1267 Bandwidth = 0.02884
0.0
0.5
1.0
1.5
2.0
2.5
3.0
0.0
0.2
0.4
0.6
0.8
1.0

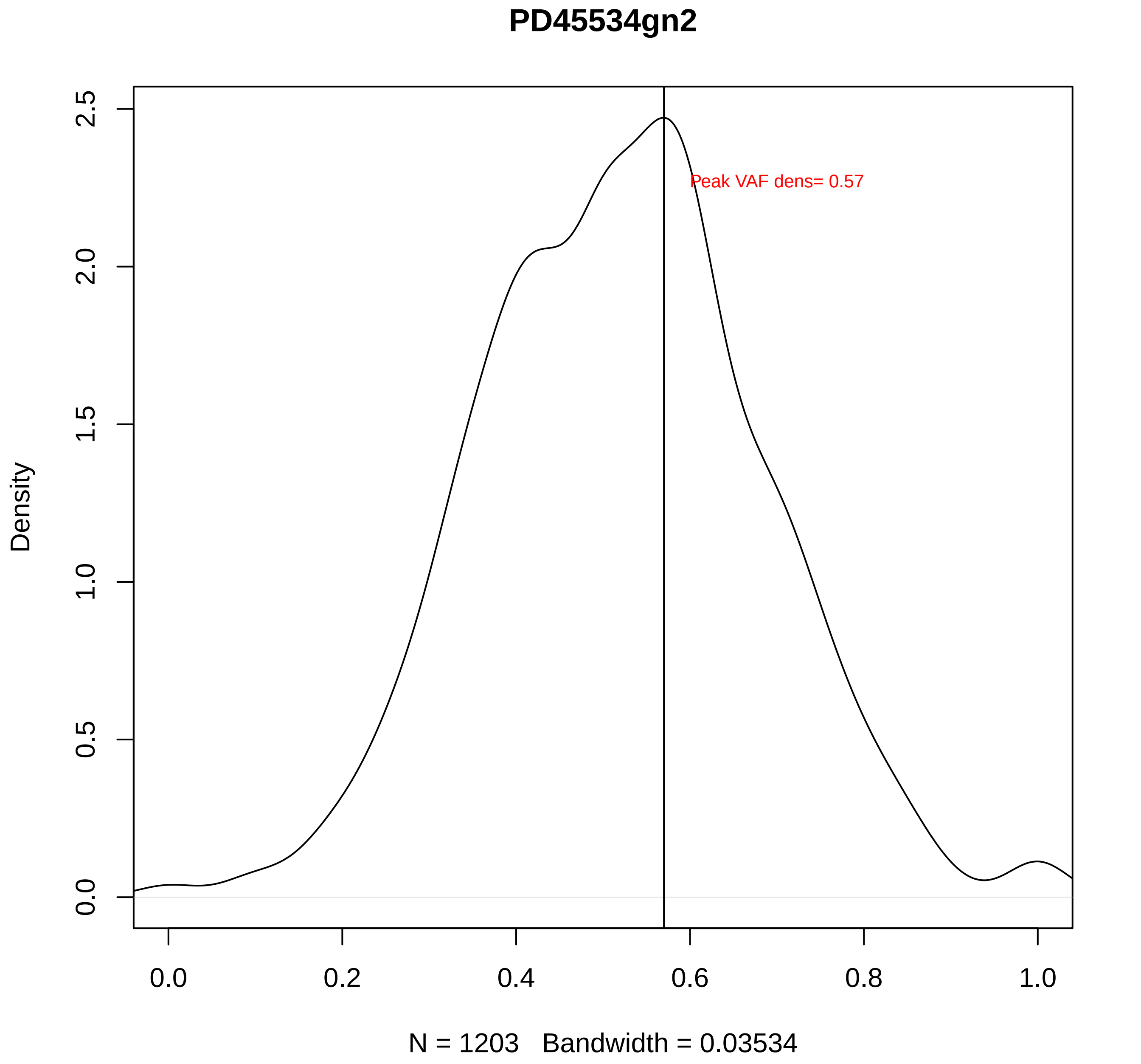
PD45534gn2
Peak VAF dens= 0.57
Density
0.0
0.5
1.0
1.5
2.0
2.5
0.0
0.2
0.4
0.6
0.8
1.0
N = 1203   Bandwidth = 0.03534

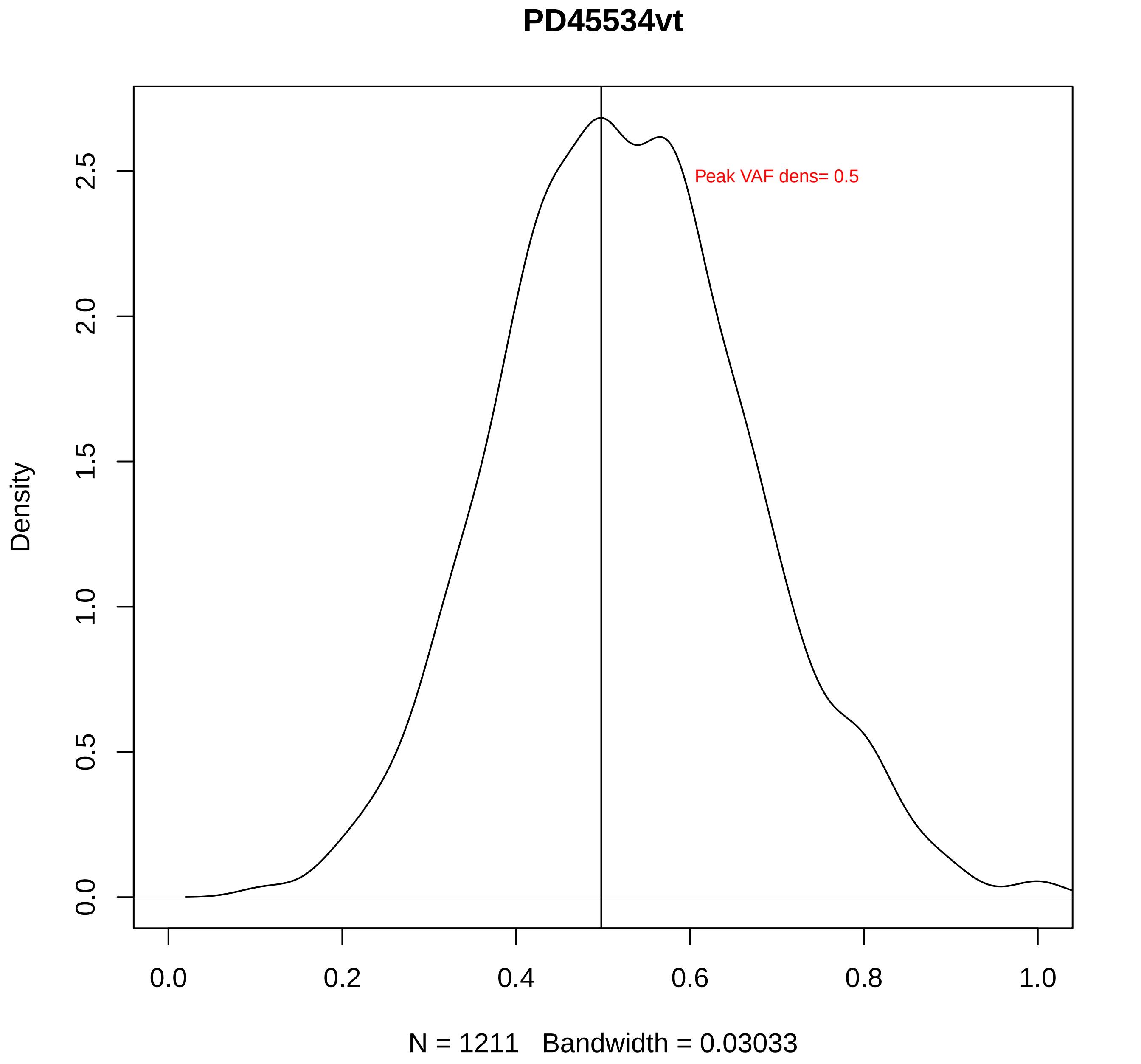

PD45534vt
Peak VAF dens= 0.5
Density
0.0
0.5
1.0
1.5
2.0
2.5
0.0
0.2
0.4
0.6
0.8
1.0
N = 1211   Bandwidth = 0.03033

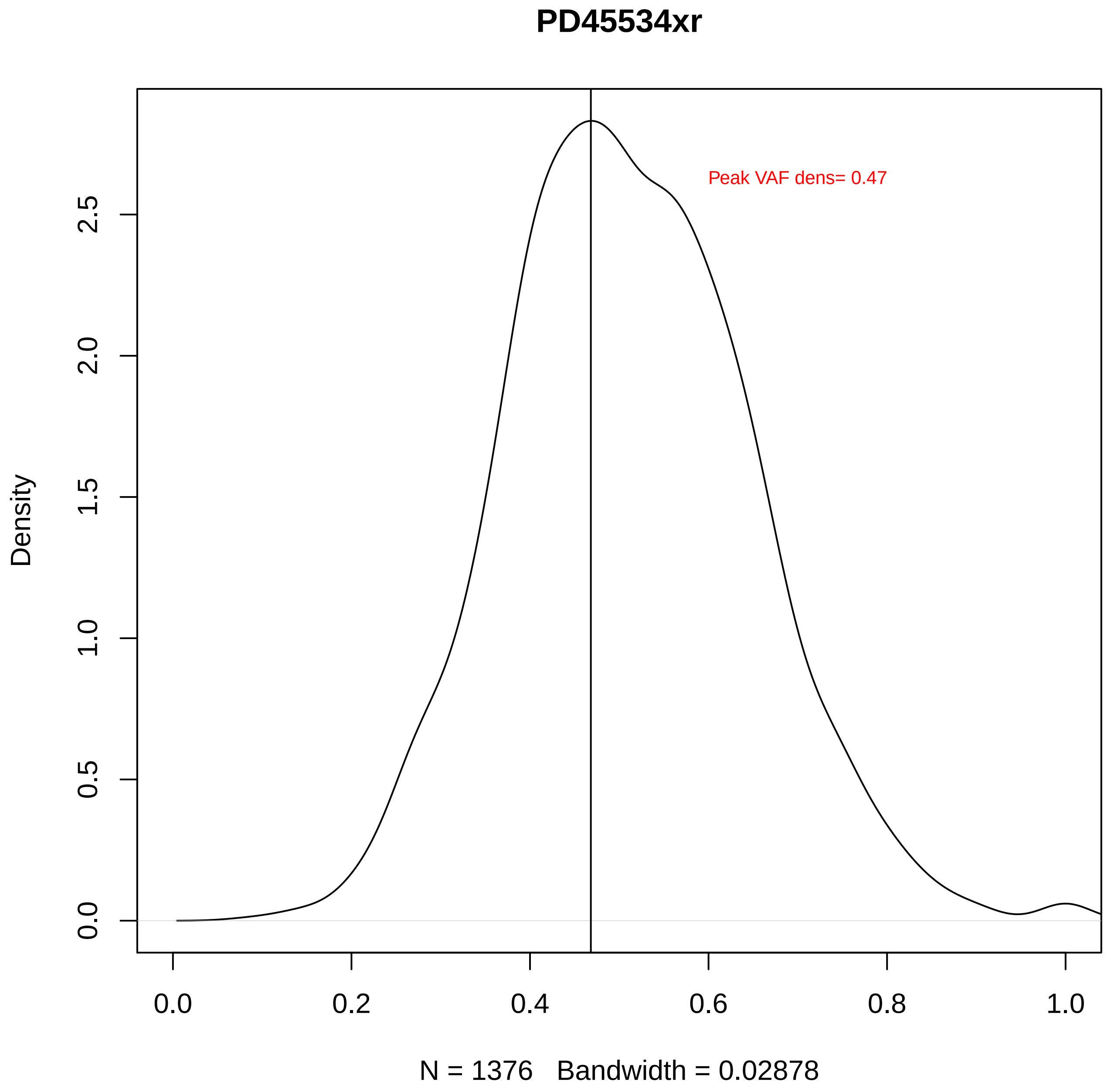

PD45534xr
Peak VAF dens= 0.47
Density
0.0
0.5
1.0
1.5
2.0
2.5
0.0
0.2
0.4
0.6
0.8
1.0
N = 1376   Bandwidth = 0.02878

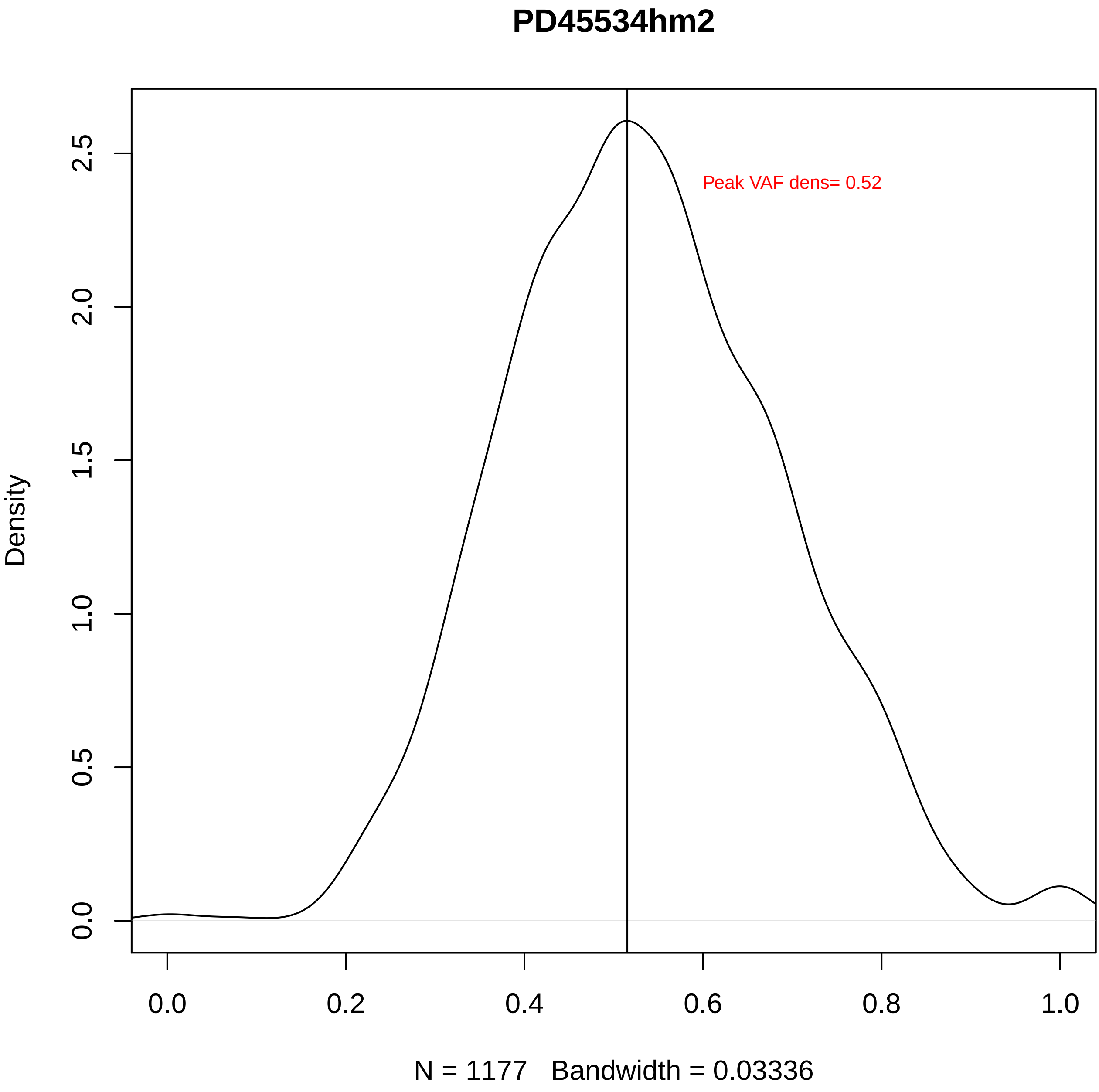
PD45534hm2
Peak VAF dens= 0.52
Density
0.0
0.5
1.0
1.5
2.0
2.5
0.0
0.2
0.4
0.6
0.8
1.0
N = 1177   Bandwidth = 0.03336

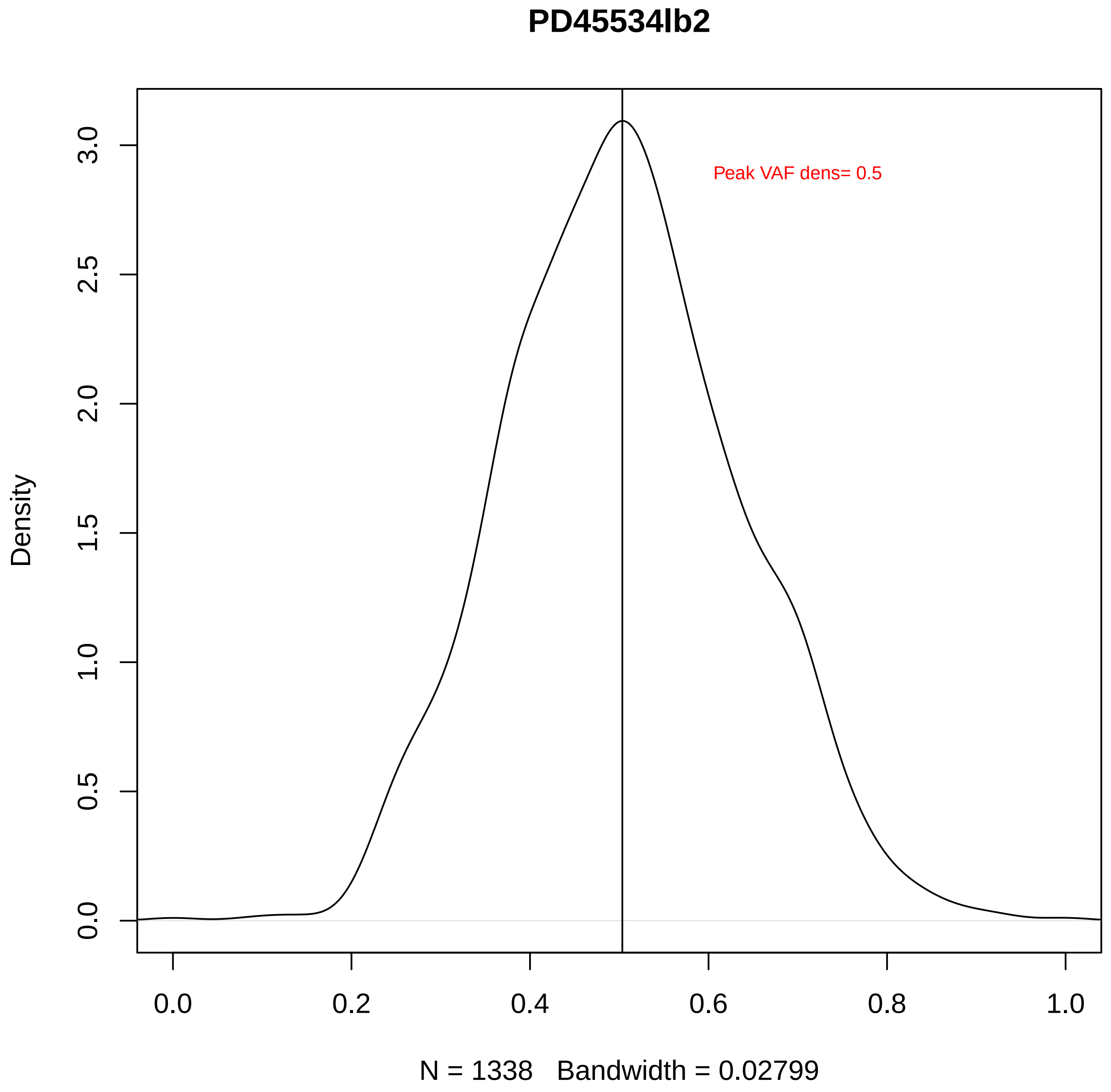
PD45534lb2
Peak VAF dens= 0.5
Density
0.0
0.5
1.0
1.5
2.0
2.5
3.0
0.0
0.2
0.4
0.6
0.8
1.0
N = 1338   Bandwidth = 0.02799

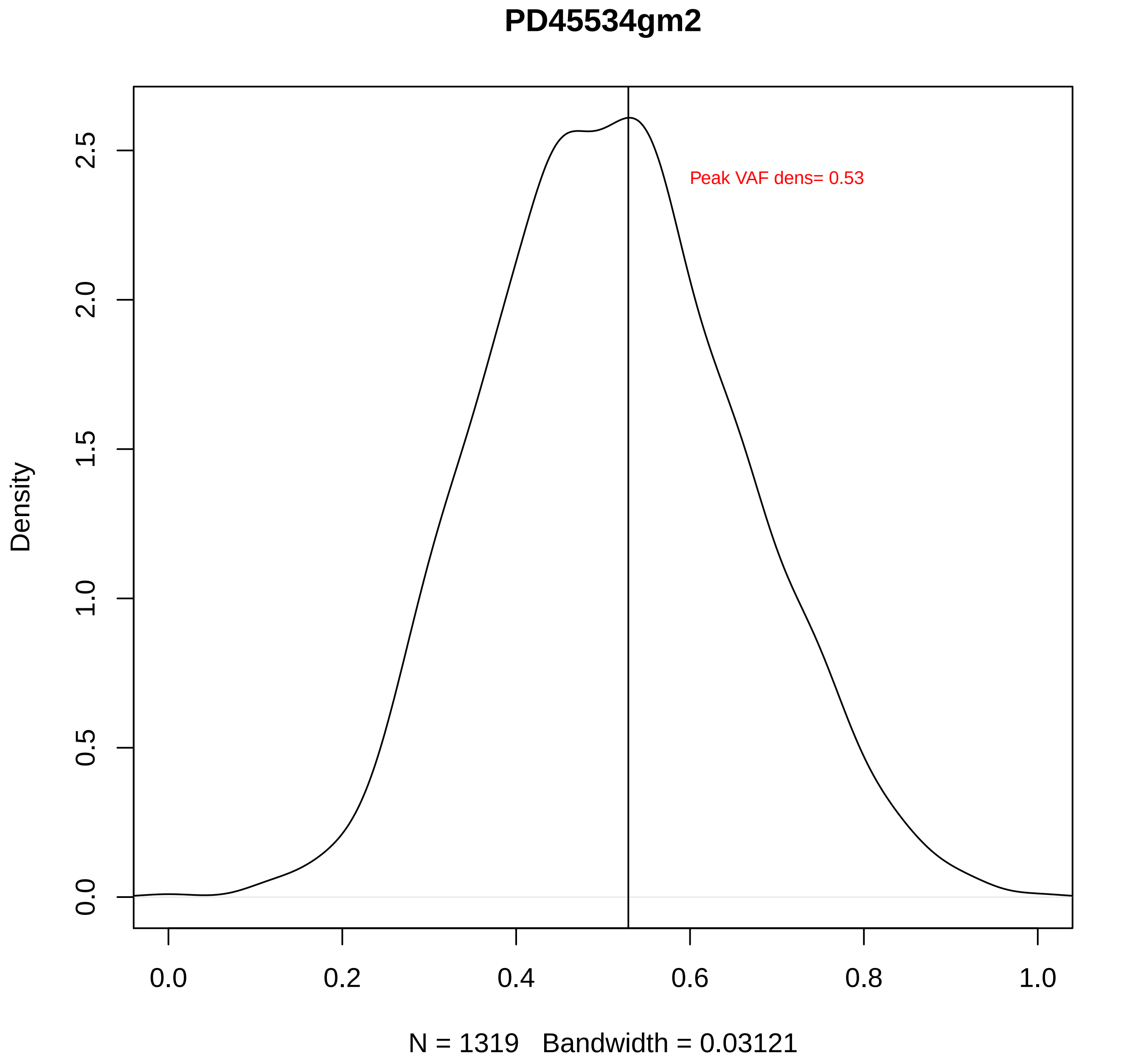
PD45534gm2
Peak VAF dens= 0.53
Density
0.0
0.5
1.0
1.5
2.0
2.5
0.0
0.2
0.4
0.6
0.8
1.0
N = 1319 Bandwidth = 0.03121

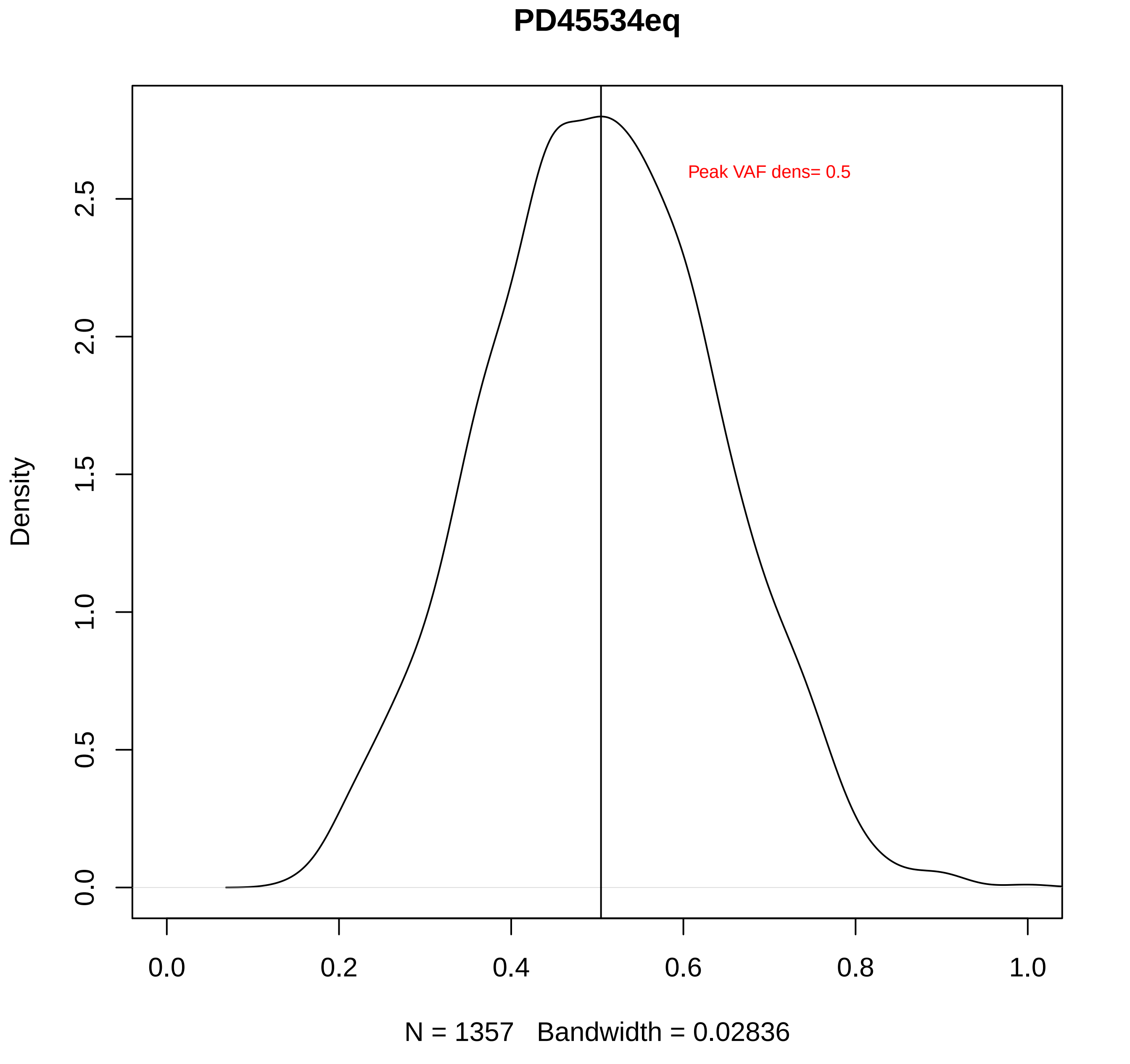
PD45534eq
Peak VAF dens= 0.5
Density
0.0
0.5
1.0
1.5
2.0
2.5
0.0
0.2
0.4
0.6
0.8
1.0
N = 1357   Bandwidth = 0.02836

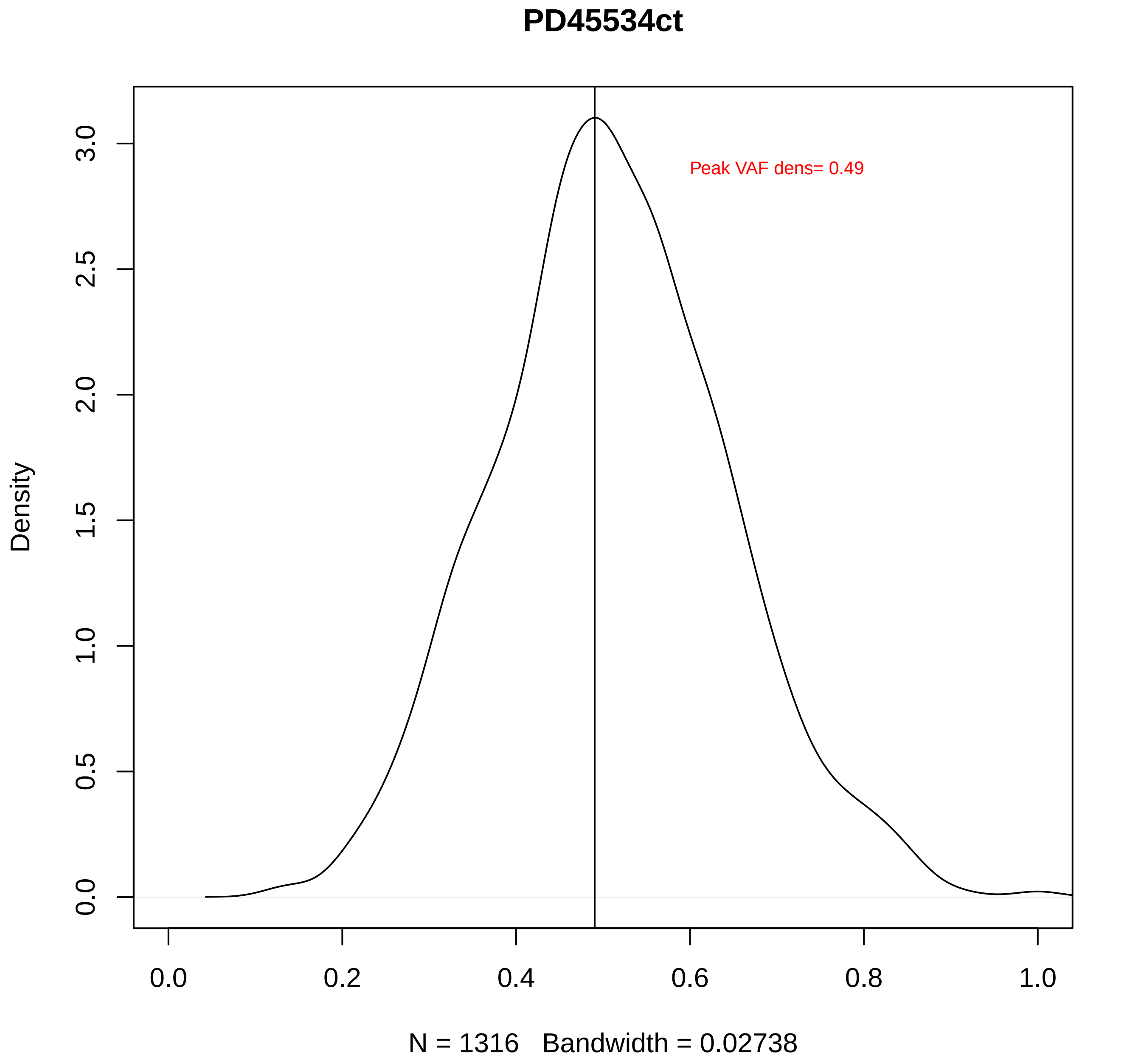
PD45534ct
Peak VAF dens= 0.49
Density
0.0
0.5
1.0
1.5
2.0
2.5
3.0
0.0
0.2
0.4
0.6
0.8
1.0
N = 1316   Bandwidth = 0.02738

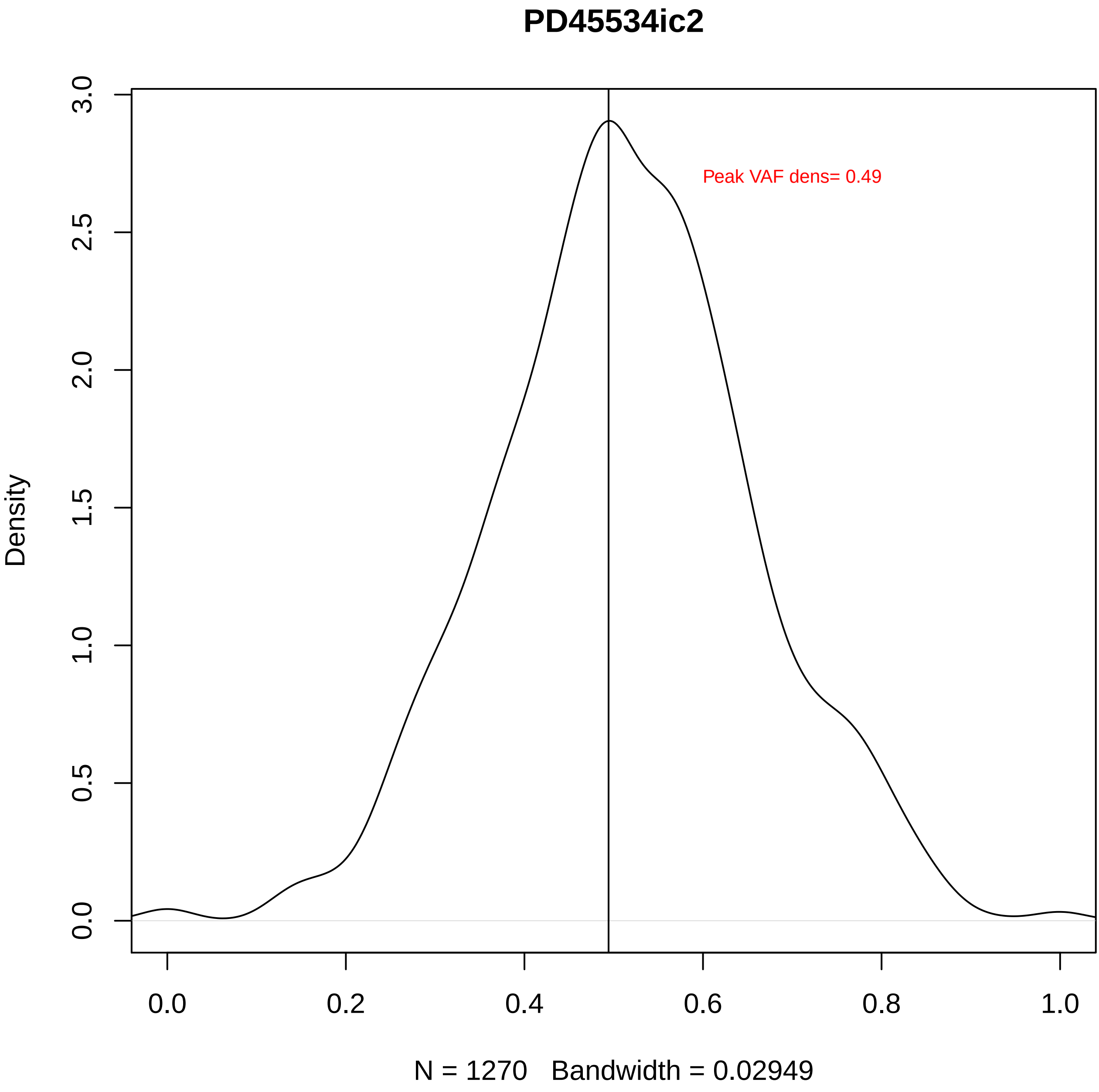
PD45534ic2
Peak VAF dens= 0.49
Density
0.0
0.5
1.0
1.5
2.0
2.5
3.0
0.0
0.2
0.4
0.6
0.8
1.0
N = 1270   Bandwidth = 0.02949

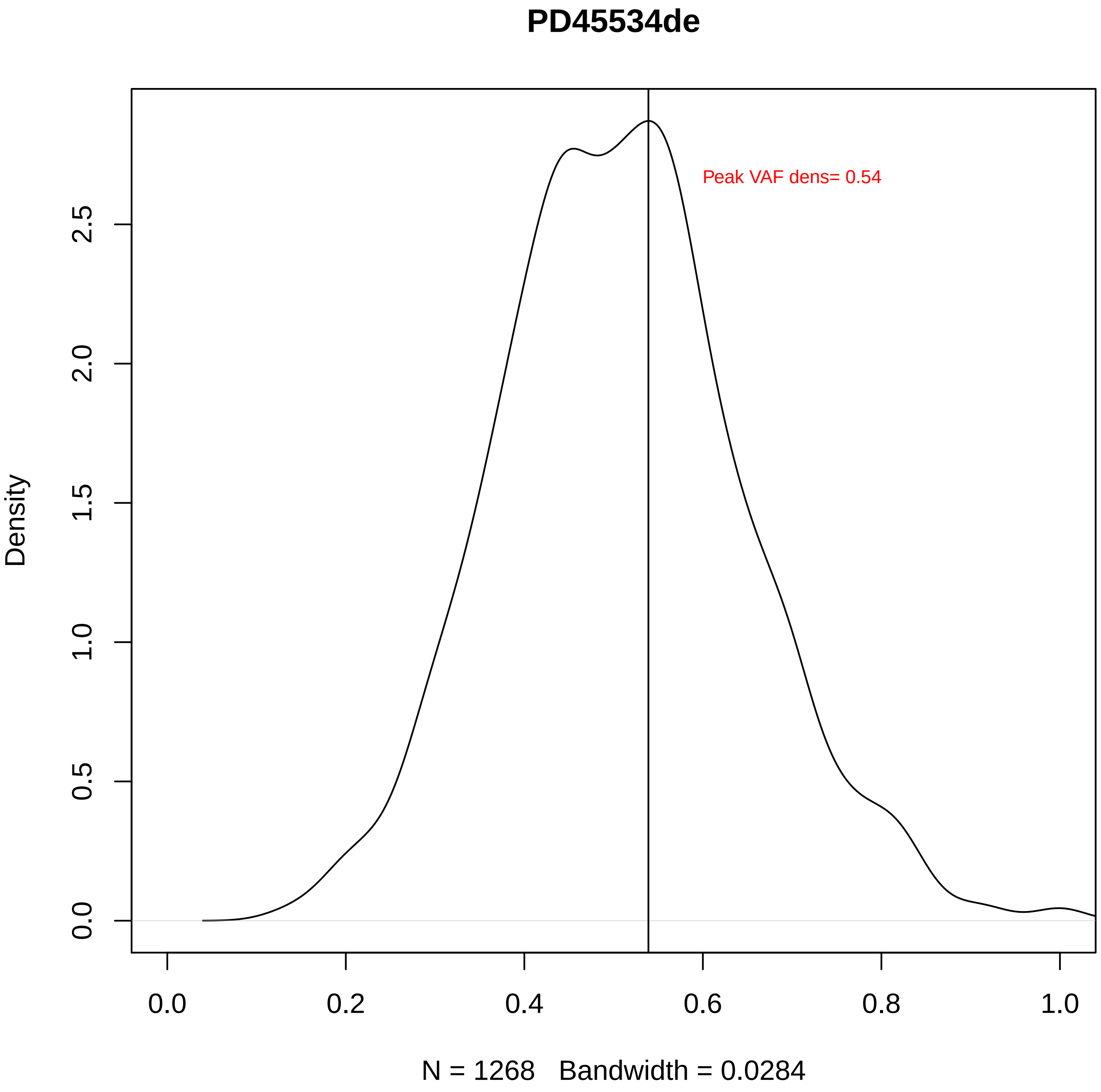

PD45534de
Peak VAF dens= 0.54
Density
0.0
0.5
1.0
1.5
2.0
2.5
0.0
0.2
0.4
0.6
0.8
1.0
N = 1268   Bandwidth = 0.0284

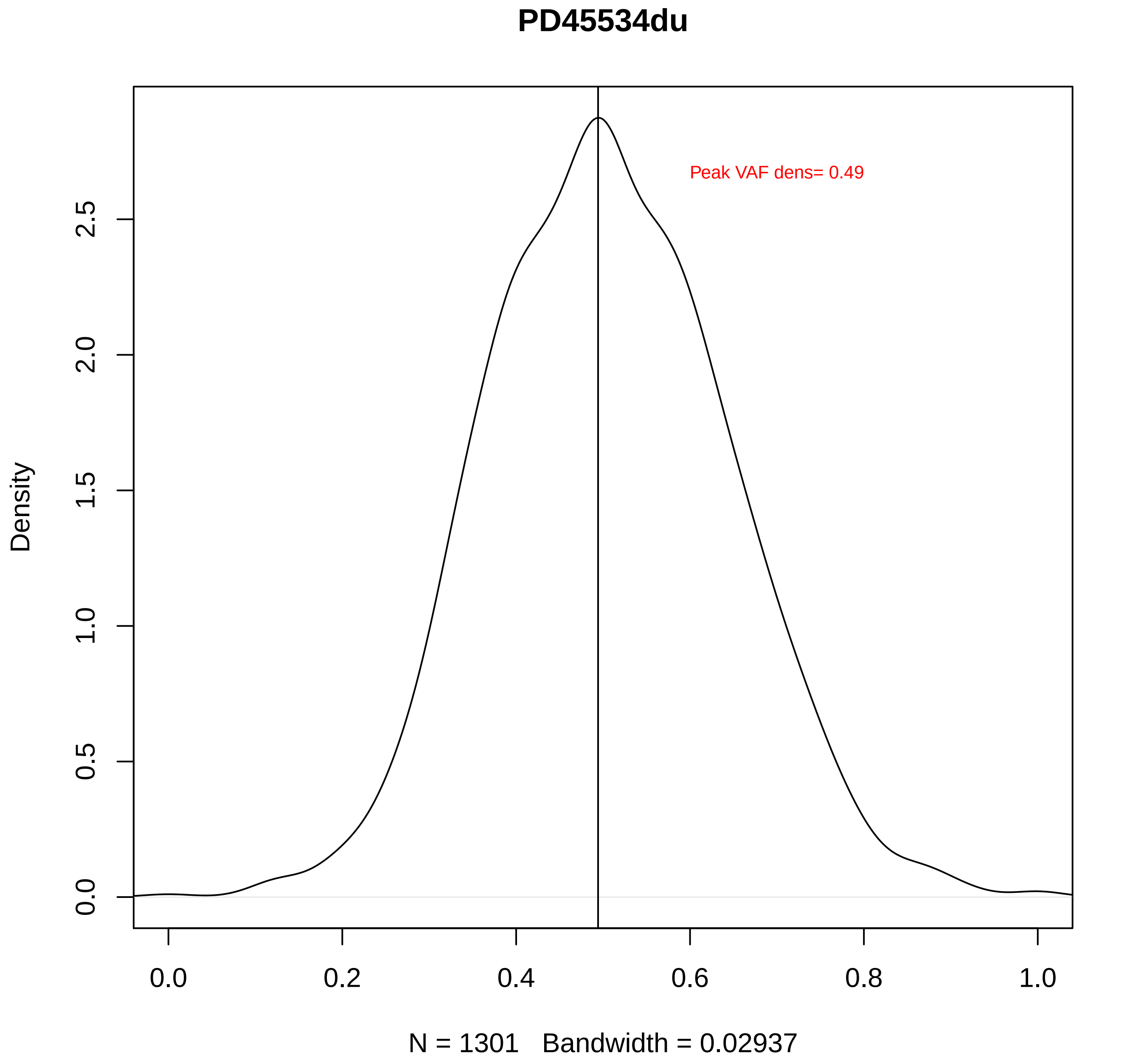
PD45534du
Peak VAF dens= 0.49
Density
0.0
0.5
1.0
1.5
2.0
2.5
0.0
0.2
0.4
0.6
0.8
1.0
N = 1301   Bandwidth = 0.02937

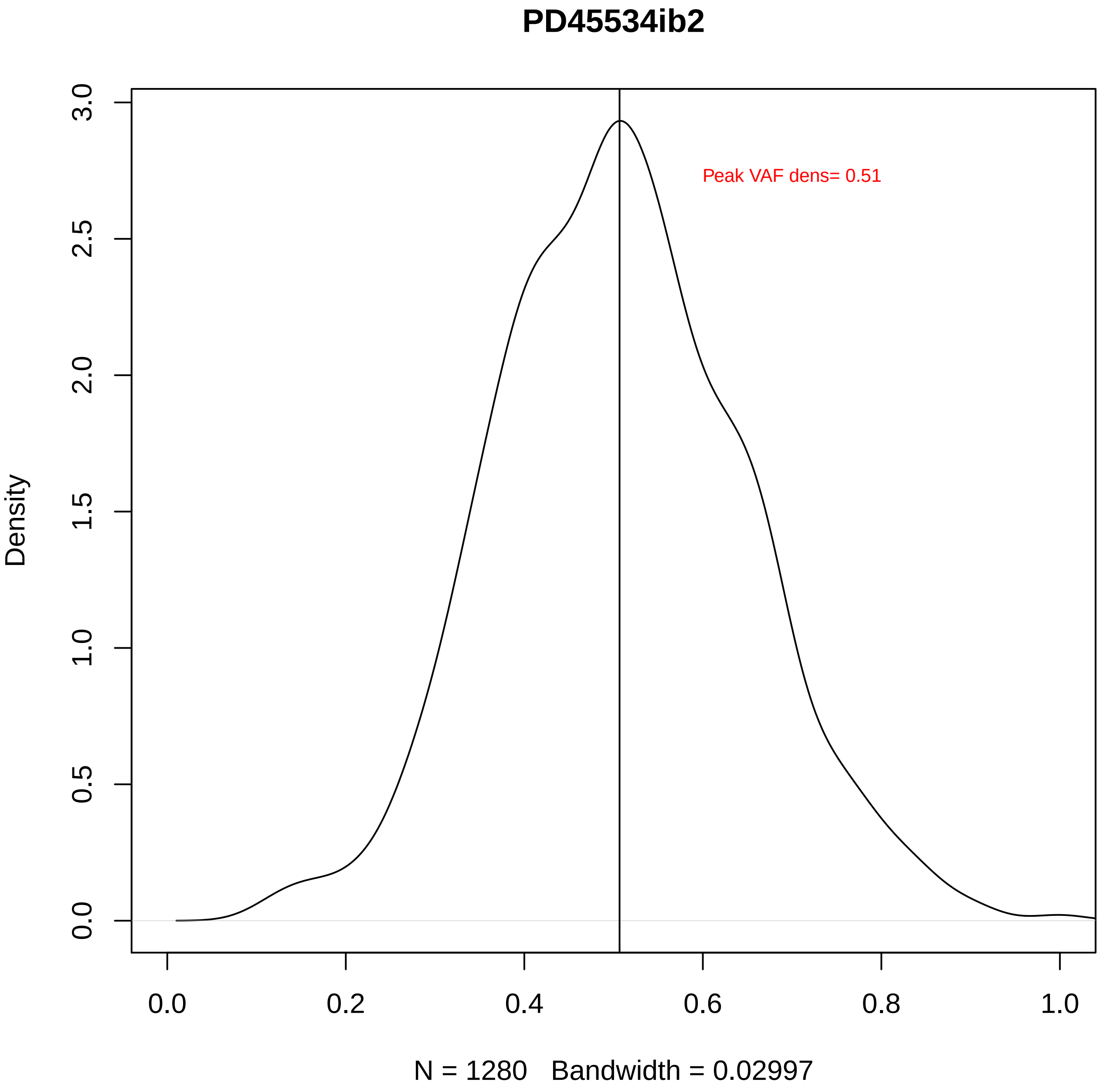
PD45534ib2
Peak VAF dens= 0.51
Density
3.0
2.5
2.0
1.5
1.0
0.5
0.0
0.0
0.2
0.4
0.6
0.8
1.0
N = 1280   Bandwidth = 0.02997

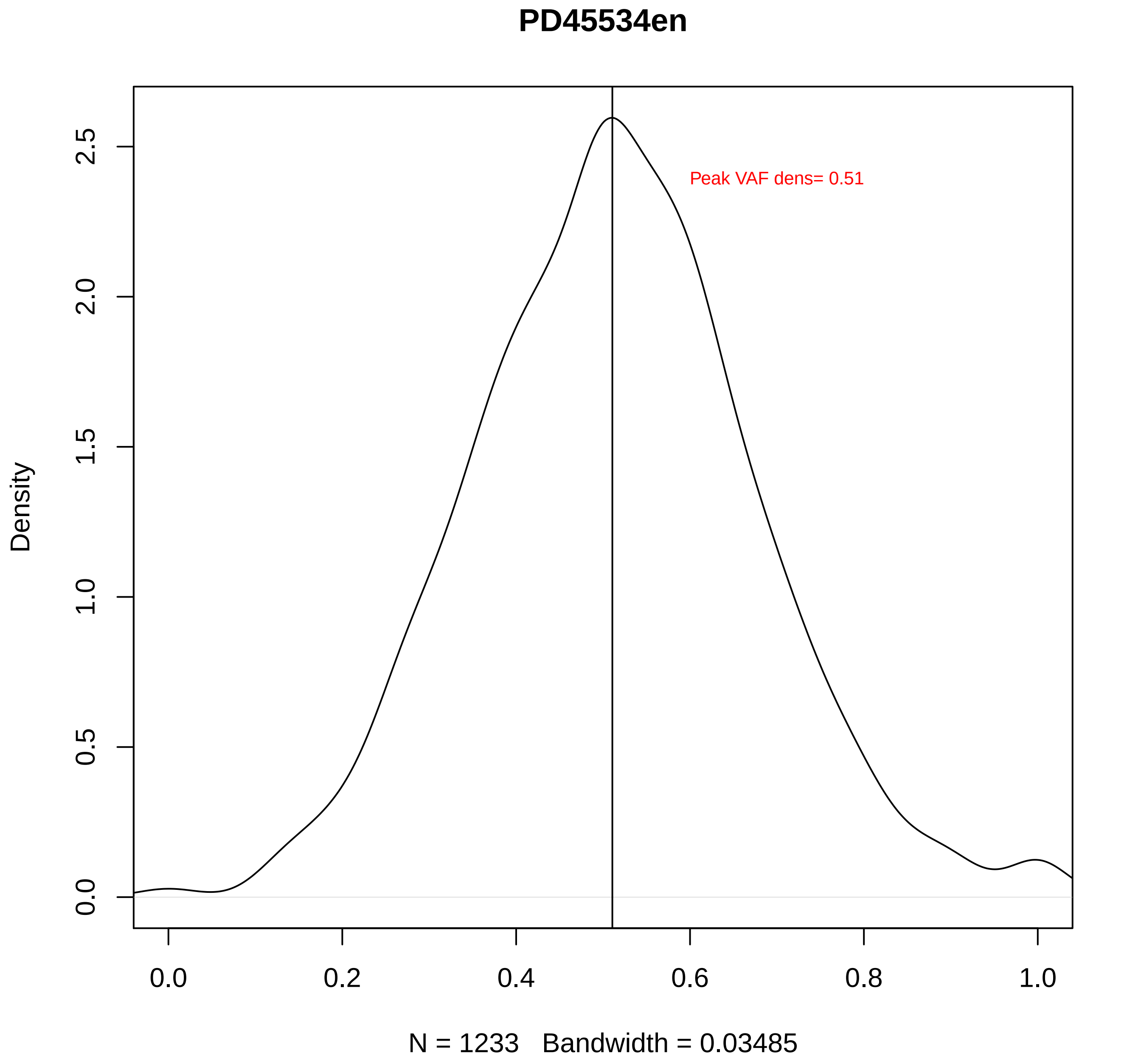
PD45534en
Peak VAF dens= 0.51
Density
0.0
0.5
1.0
1.5
2.0
2.5
0.0
0.2
0.4
0.6
0.8
1.0
N = 1233   Bandwidth = 0.03485

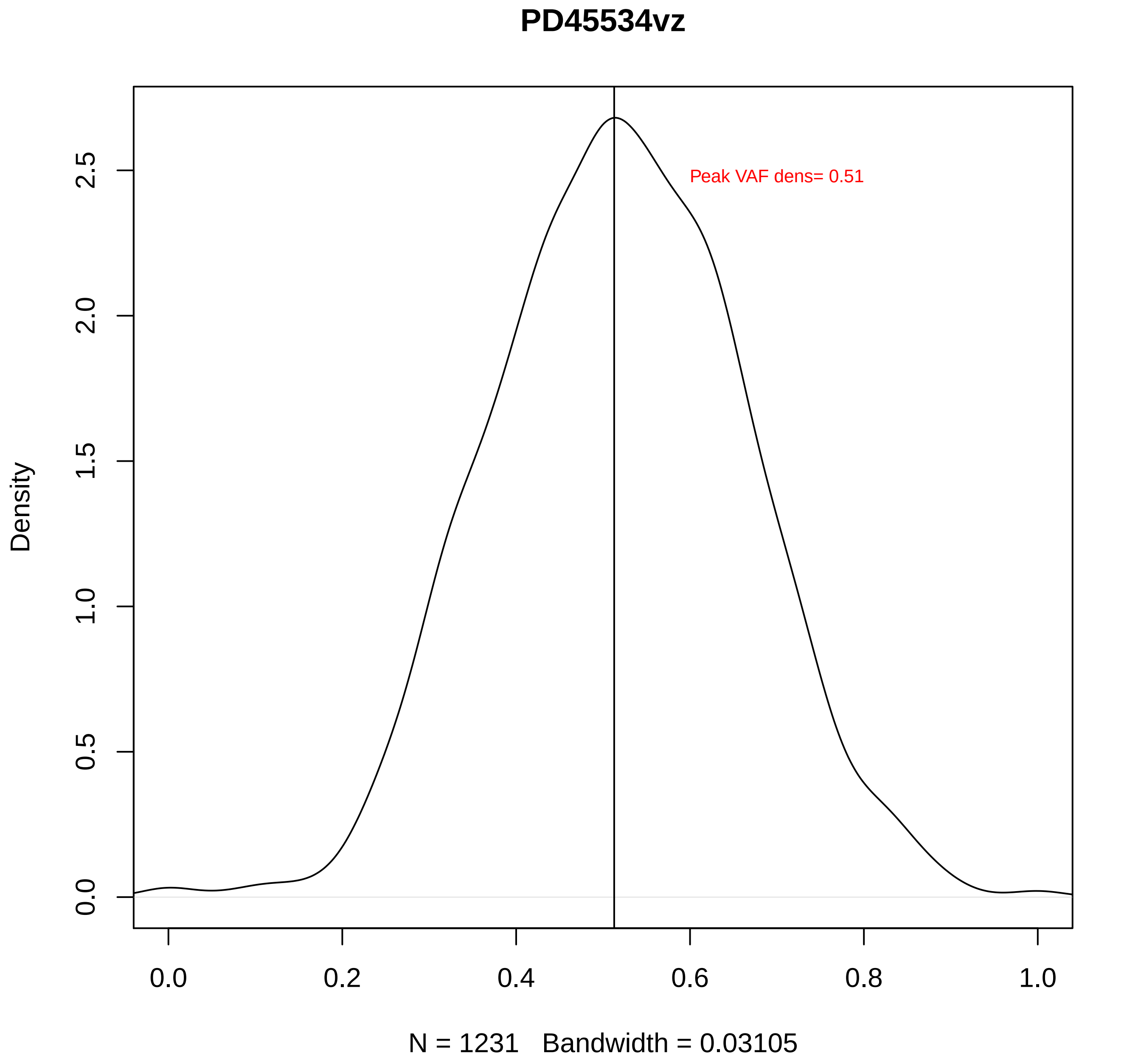

PD45534vz
Peak VAF dens= 0.51
Density
0.0
0.5
1.0
1.5
2.0
2.5
0.0
0.2
0.4
0.6
0.8
1.0
N = 1231 Bandwidth = 0.03105

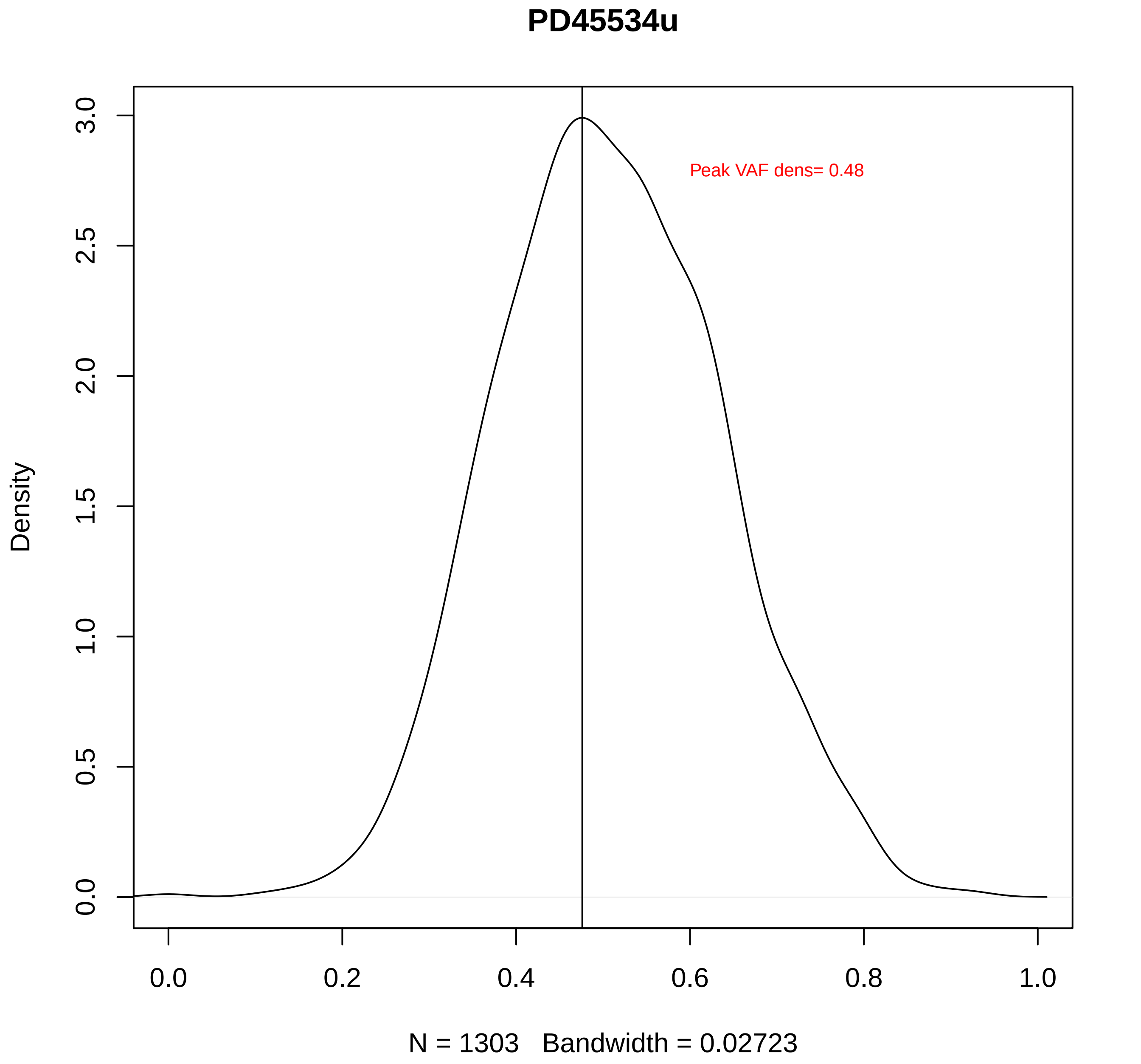

PD45534u
Peak VAF dens= 0.48
Density
0.0
0.5
1.0
1.5
2.0
2.5
3.0
0.0
0.2
0.4
0.6
0.8
1.0
N = 1303 Bandwidth = 0.02723

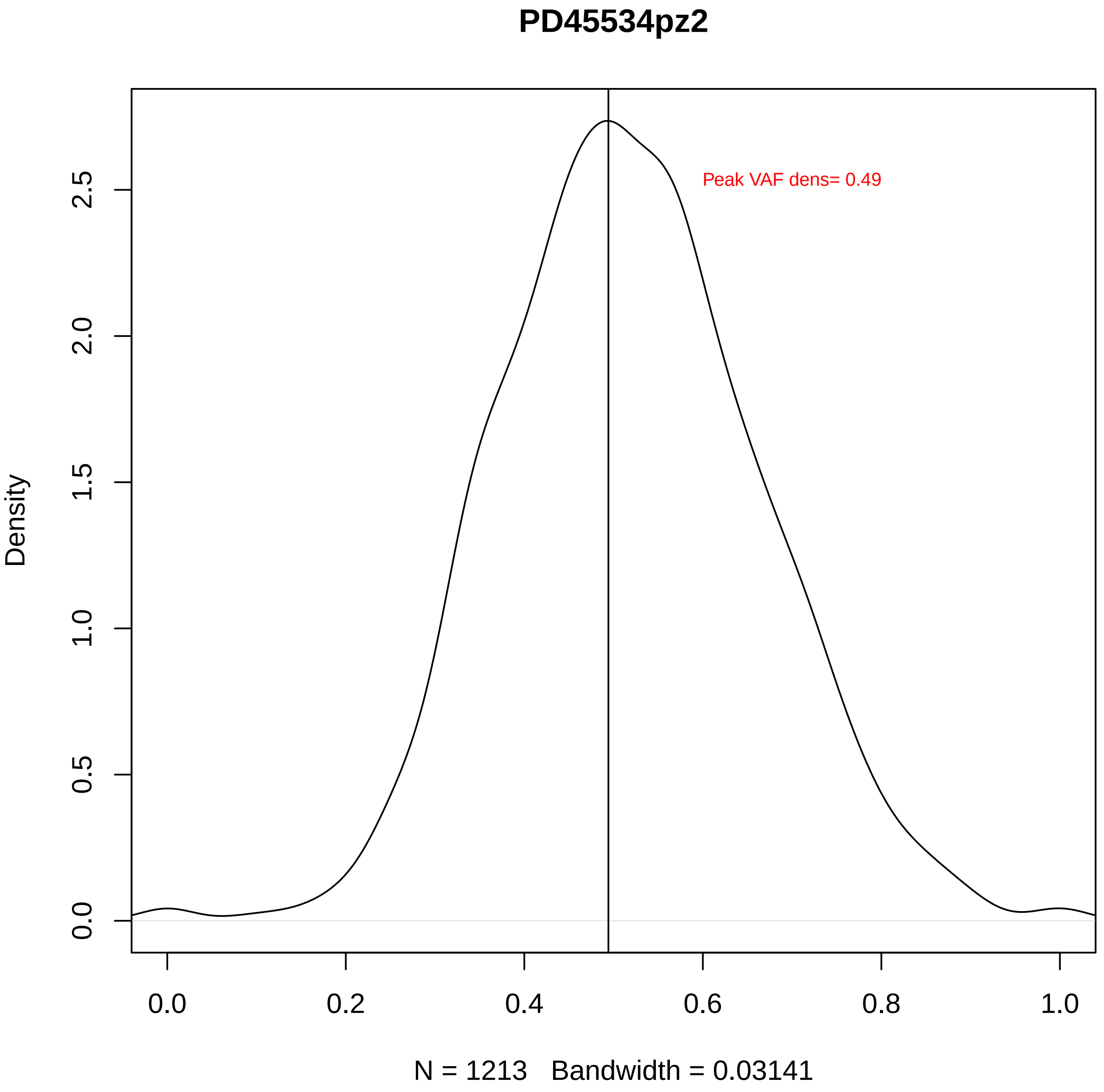
PD45534pz2
Peak VAF dens= 0.49
Density
0.0
0.5
1.0
1.5
2.0
2.5
0.0
0.2
0.4
0.6
0.8
1.0
N = 1213 Bandwidth = 0.03141

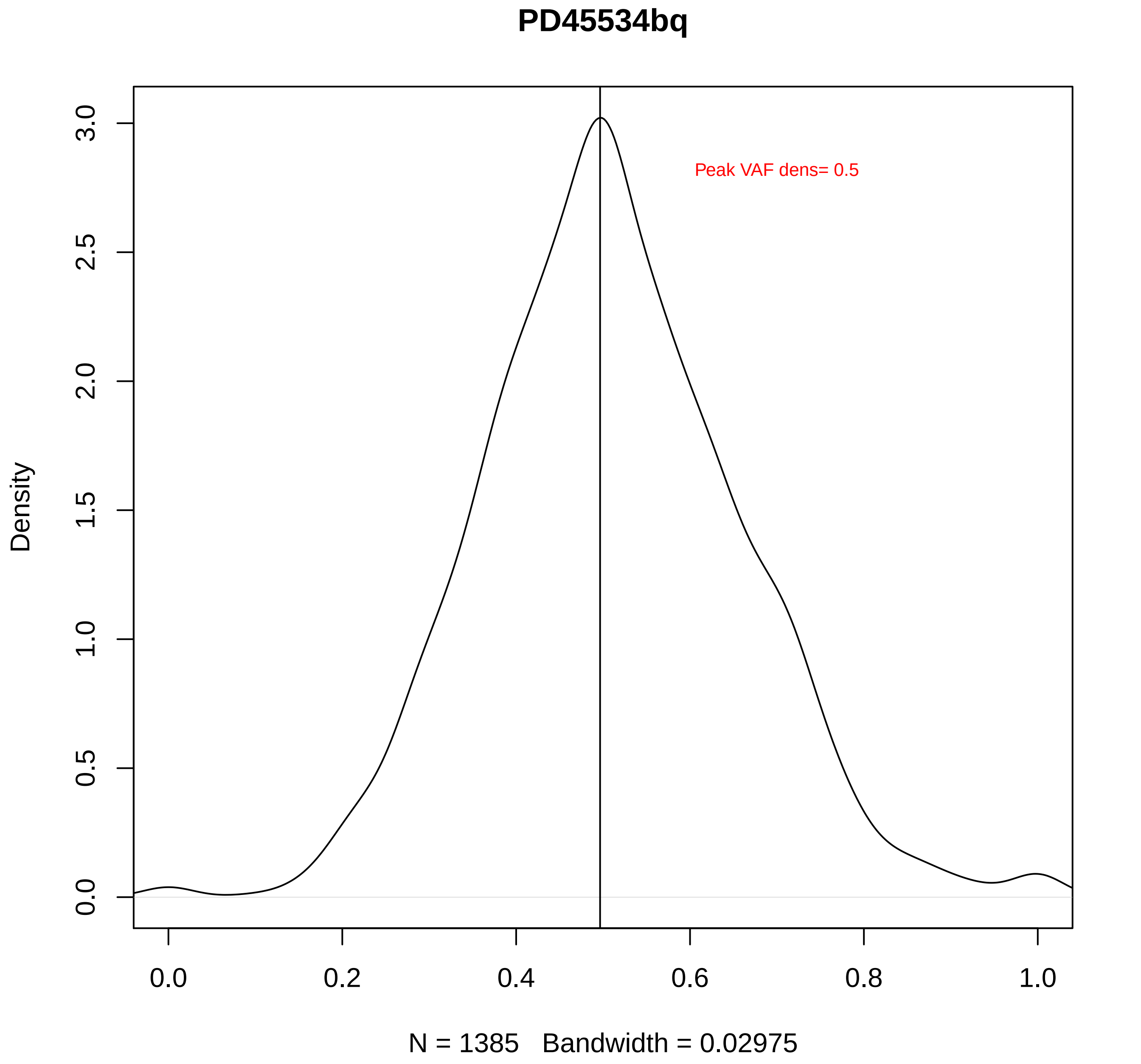
PD45534bq
Peak VAF dens= 0.5
Density
0.0
0.5
1.0
1.5
2.0
2.5
3.0
0.0
0.2
0.4
0.6
0.8
1.0
N = 1385   Bandwidth = 0.02975

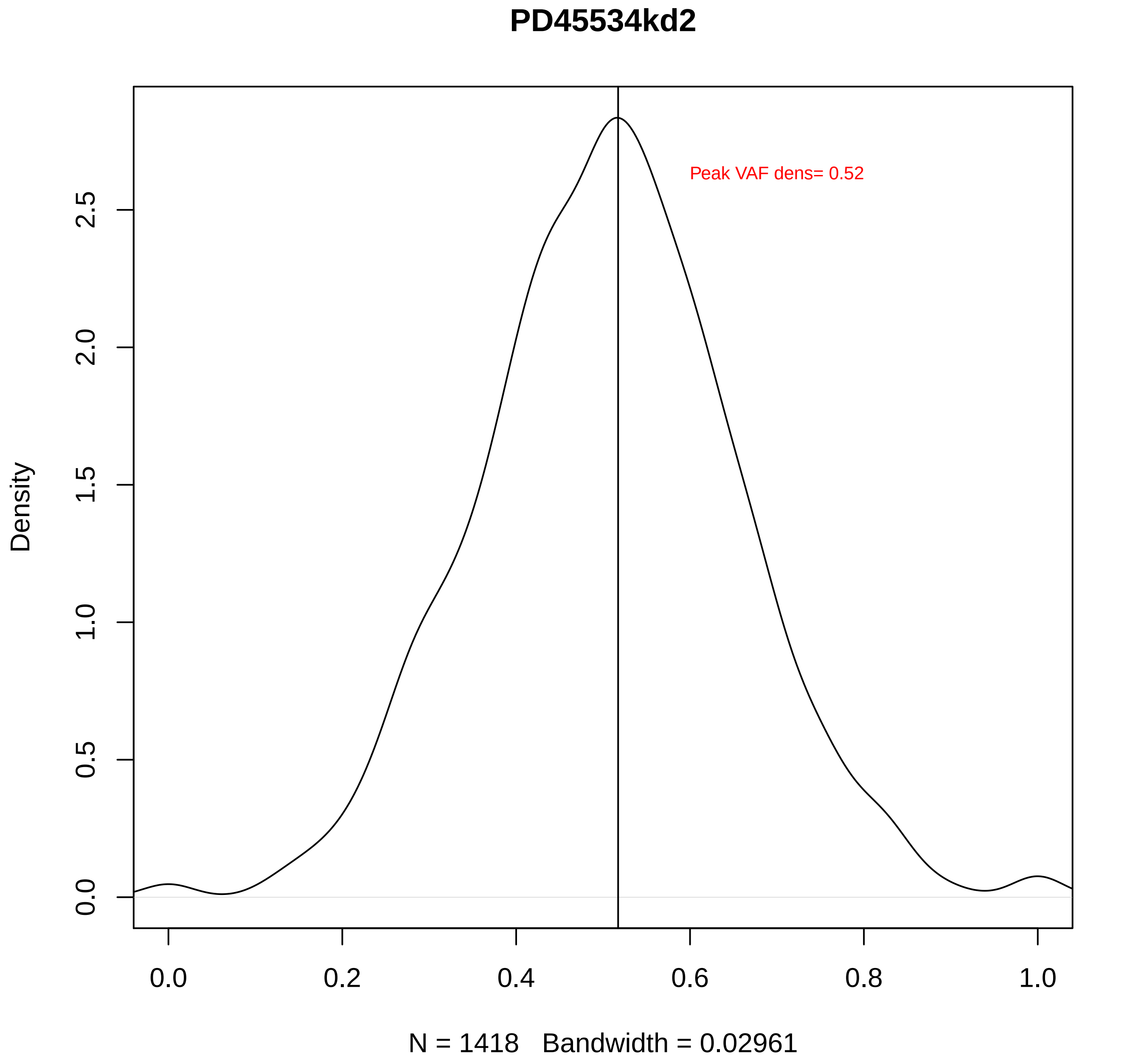

PD45534kd2
Peak VAF dens= 0.52
Density
0.0
0.5
1.0
1.5
2.0
2.5
0.0
0.2
0.4
0.6
0.8
1.0
N = 1418   Bandwidth = 0.02961

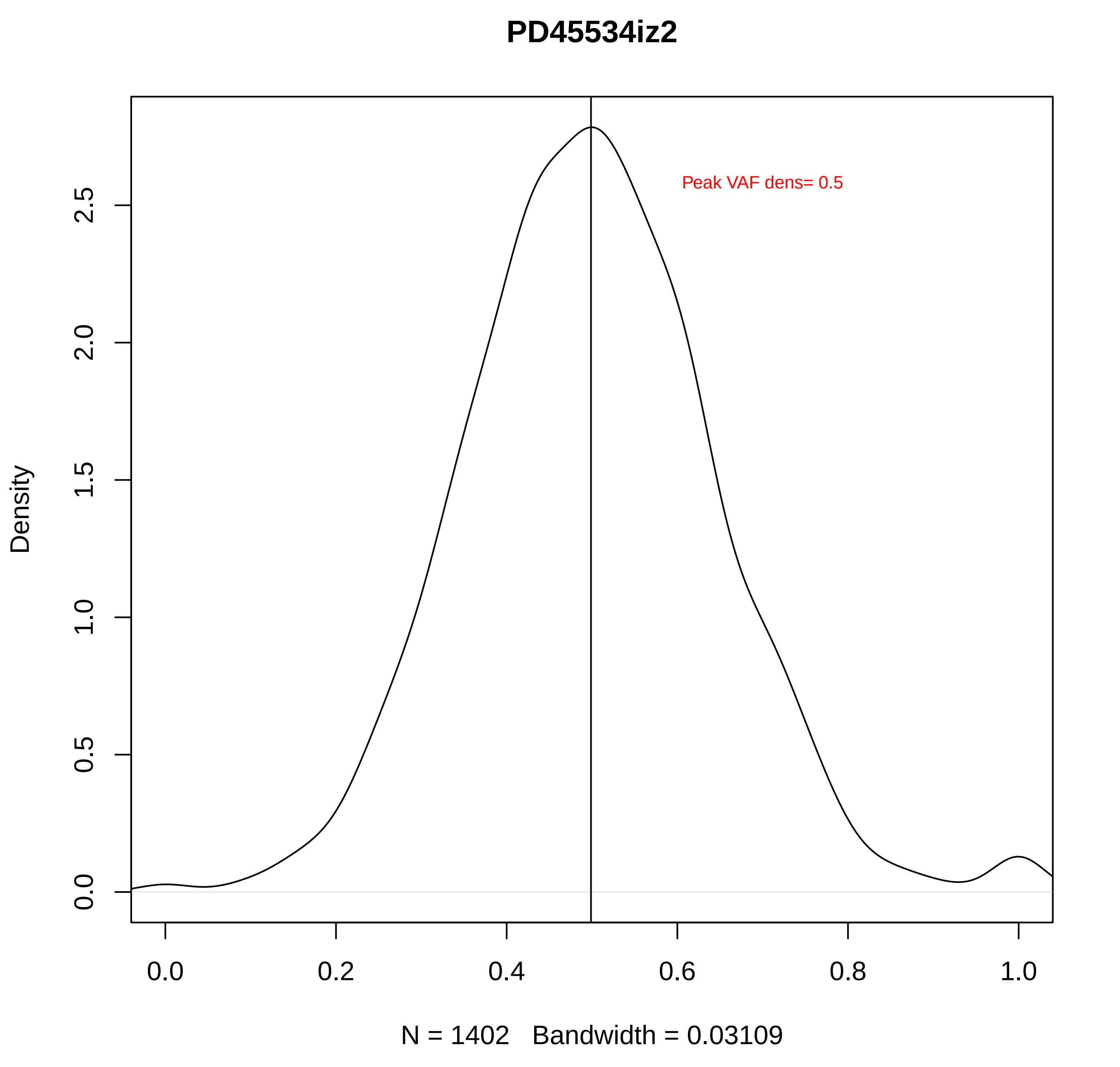
PD45534iz2
Peak VAF dens= 0.5
Density
0.0
0.5
1.0
1.5
2.0
2.5
0.0
0.2
0.4
0.6
0.8
1.0
N = 1402   Bandwidth = 0.03109

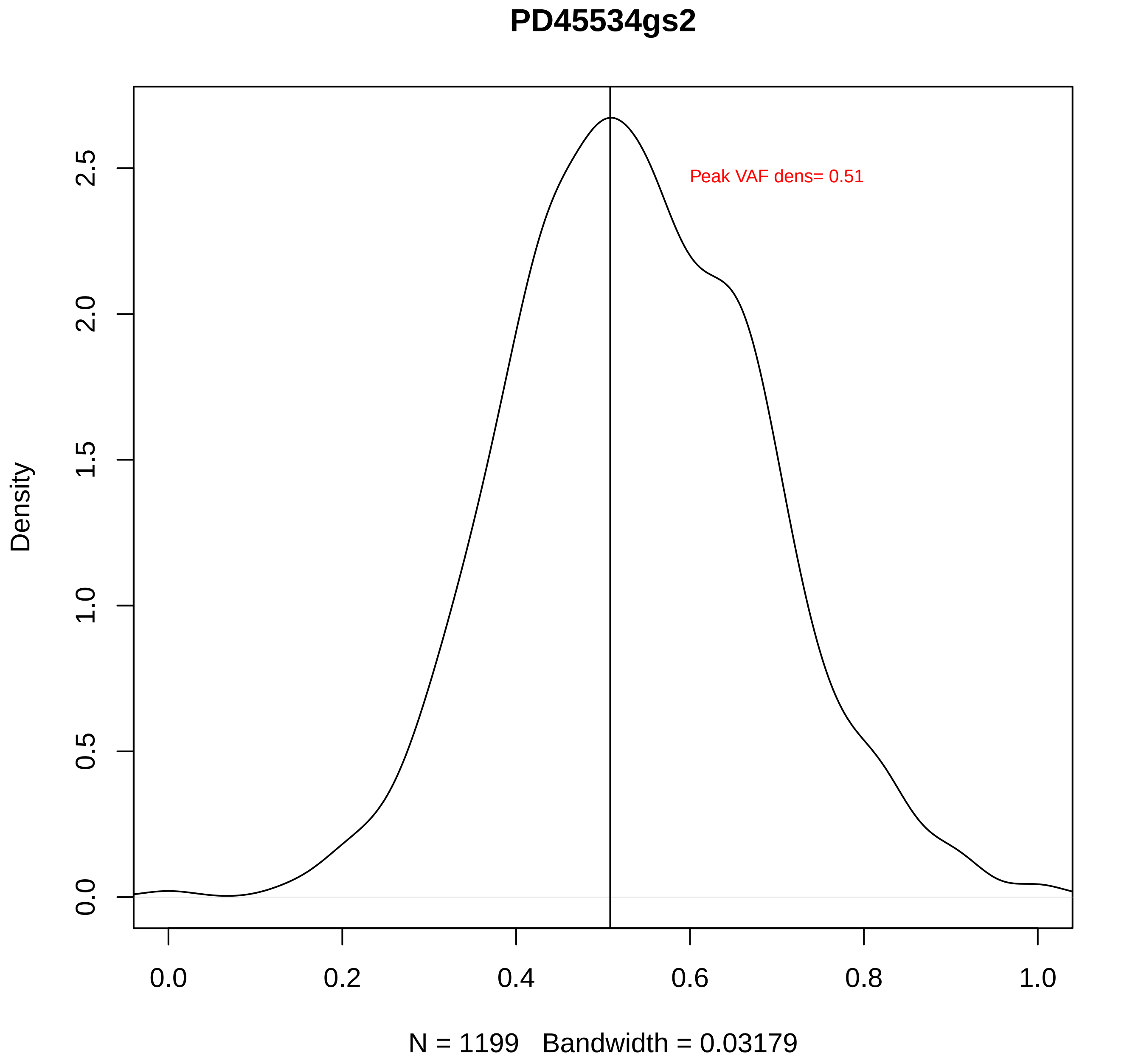

PD45534gs2
Peak VAF dens= 0.51
Density
0.0
0.5
1.0
1.5
2.0
2.5
0.0
0.2
0.4
0.6
0.8
1.0
N = 1199   Bandwidth = 0.03179

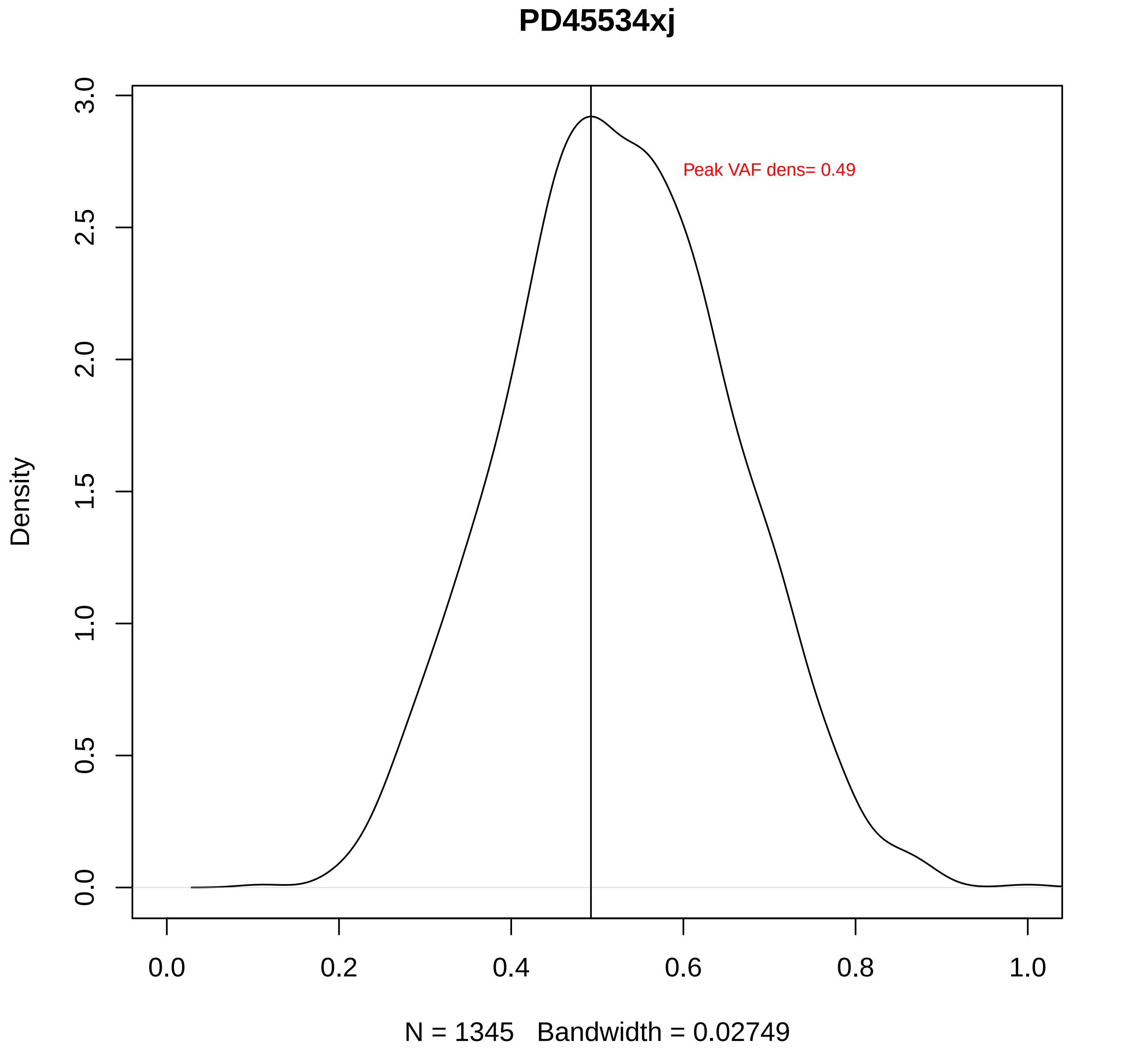
PD45534xj
Peak VAF dens= 0.49
Density
0.0
0.5
1.0
1.5
2.0
2.5
3.0
0.0
0.2
0.4
0.6
0.8
1.0
N = 1345   Bandwidth = 0.02749

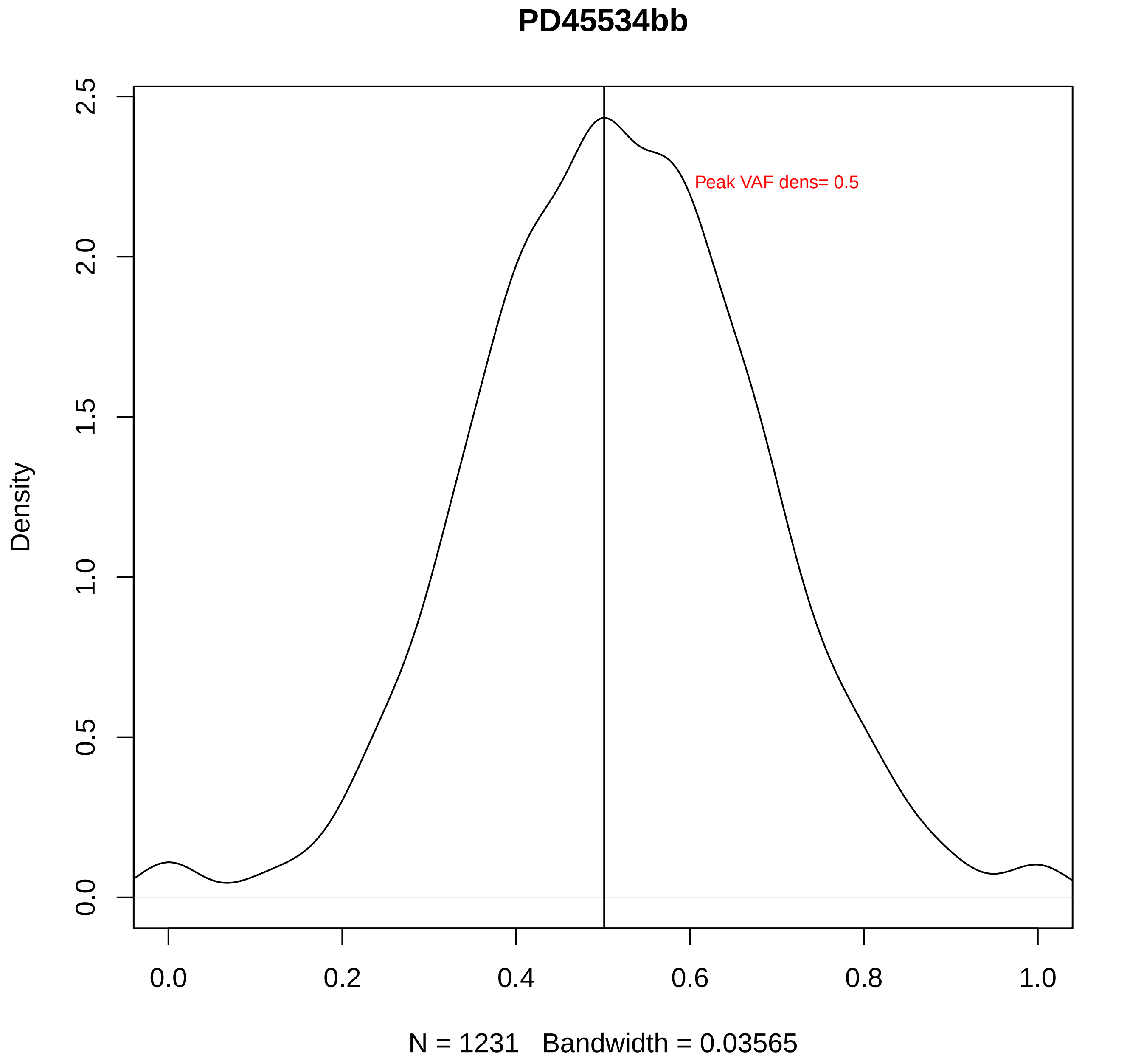
PD45534bb
Peak VAF dens= 0.5
Density
0.0
0.5
1.0
1.5
2.0
2.5
0.0
0.2
0.4
0.6
0.8
1.0
N = 1231   Bandwidth = 0.03565

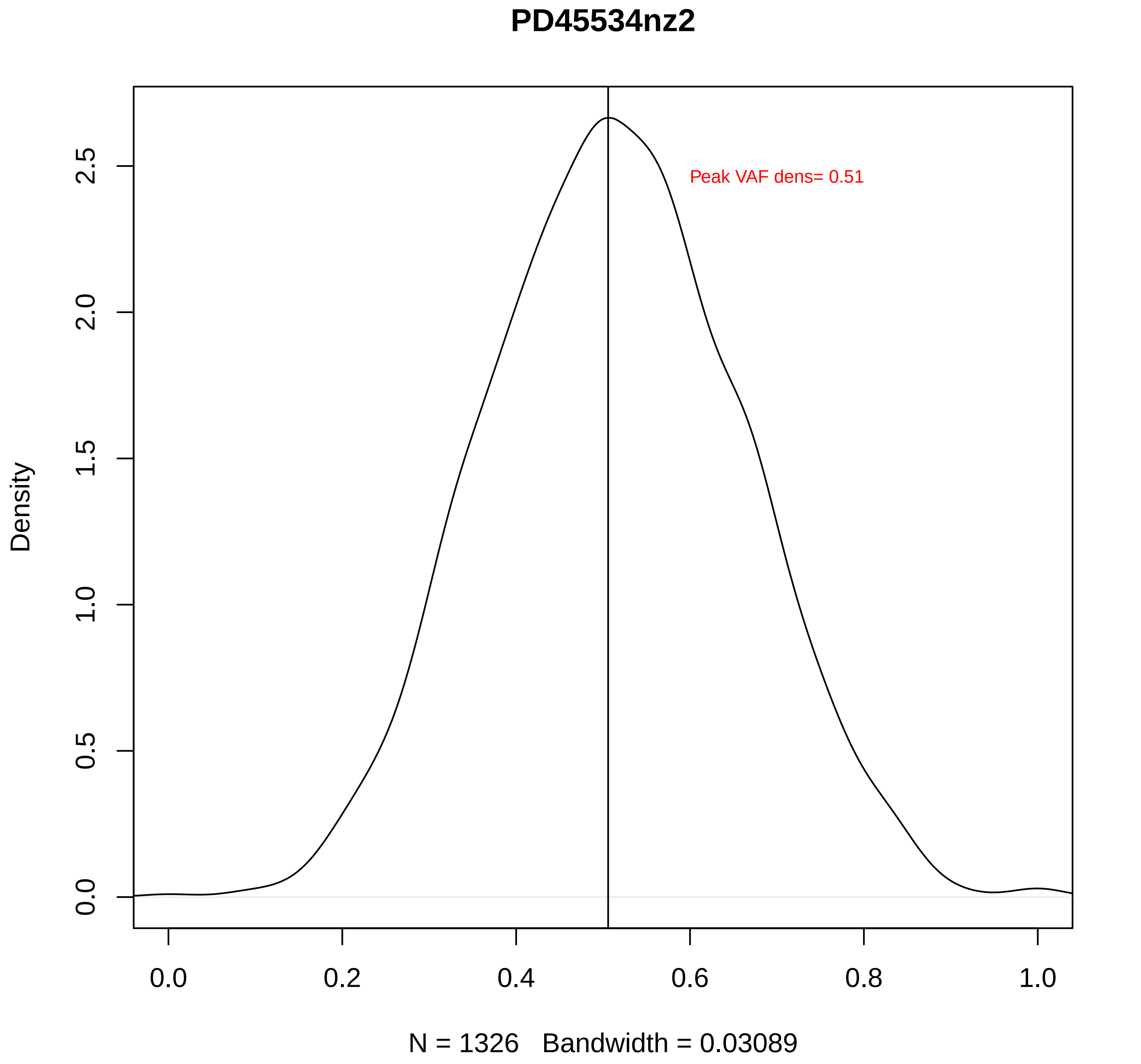
PD45534nz2
Peak VAF dens= 0.51
Density
0.0
0.5
1.0
1.5
2.0
2.5
0.0
0.2
0.4
0.6
0.8
1.0
N = 1326   Bandwidth = 0.03089

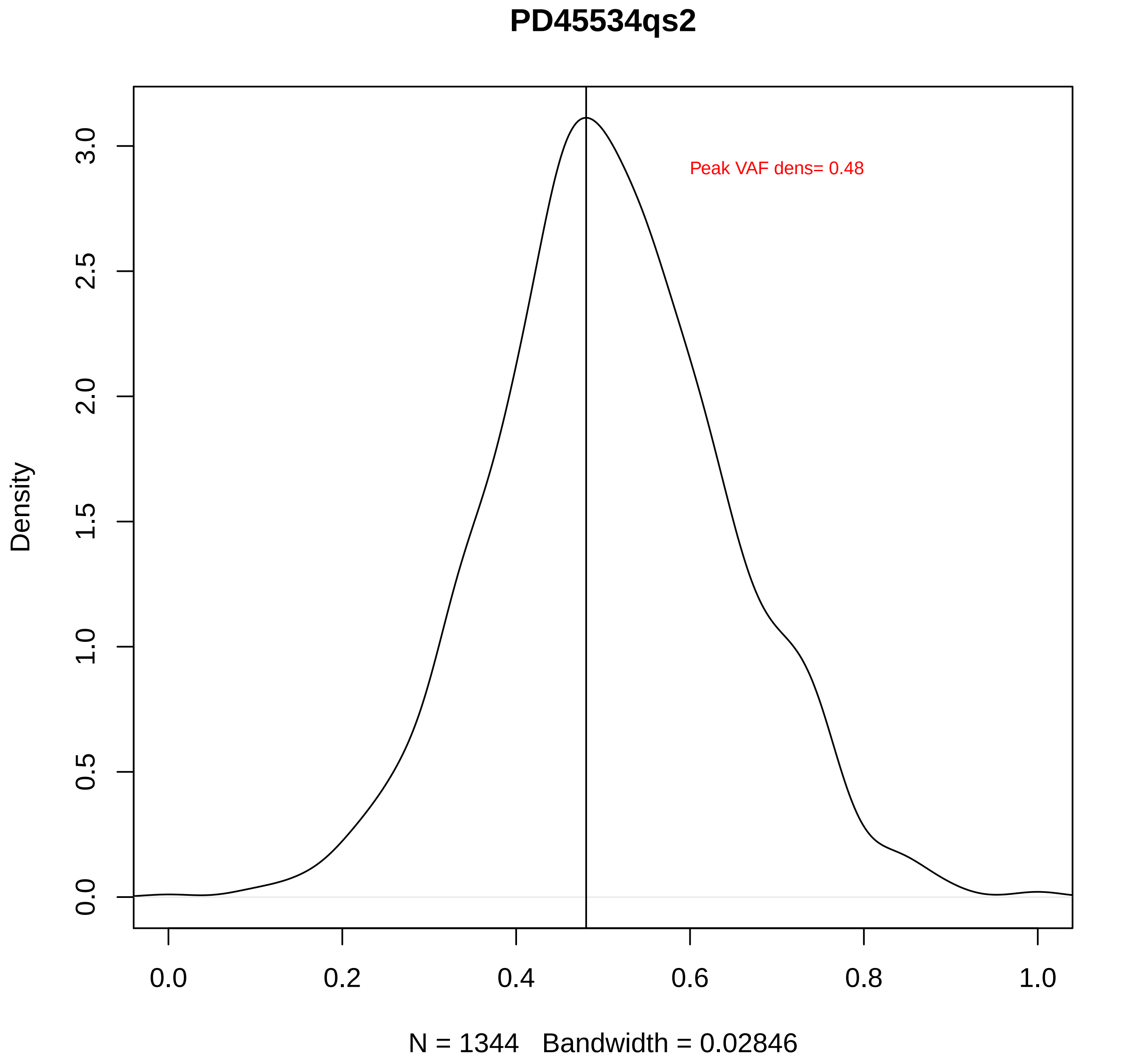
PD45534qs2
Peak VAF dens= 0.48
Density
0.0
0.5
1.0
1.5
2.0
2.5
3.0
0.0
0.2
0.4
0.6
0.8
1.0
N = 1344   Bandwidth = 0.02846

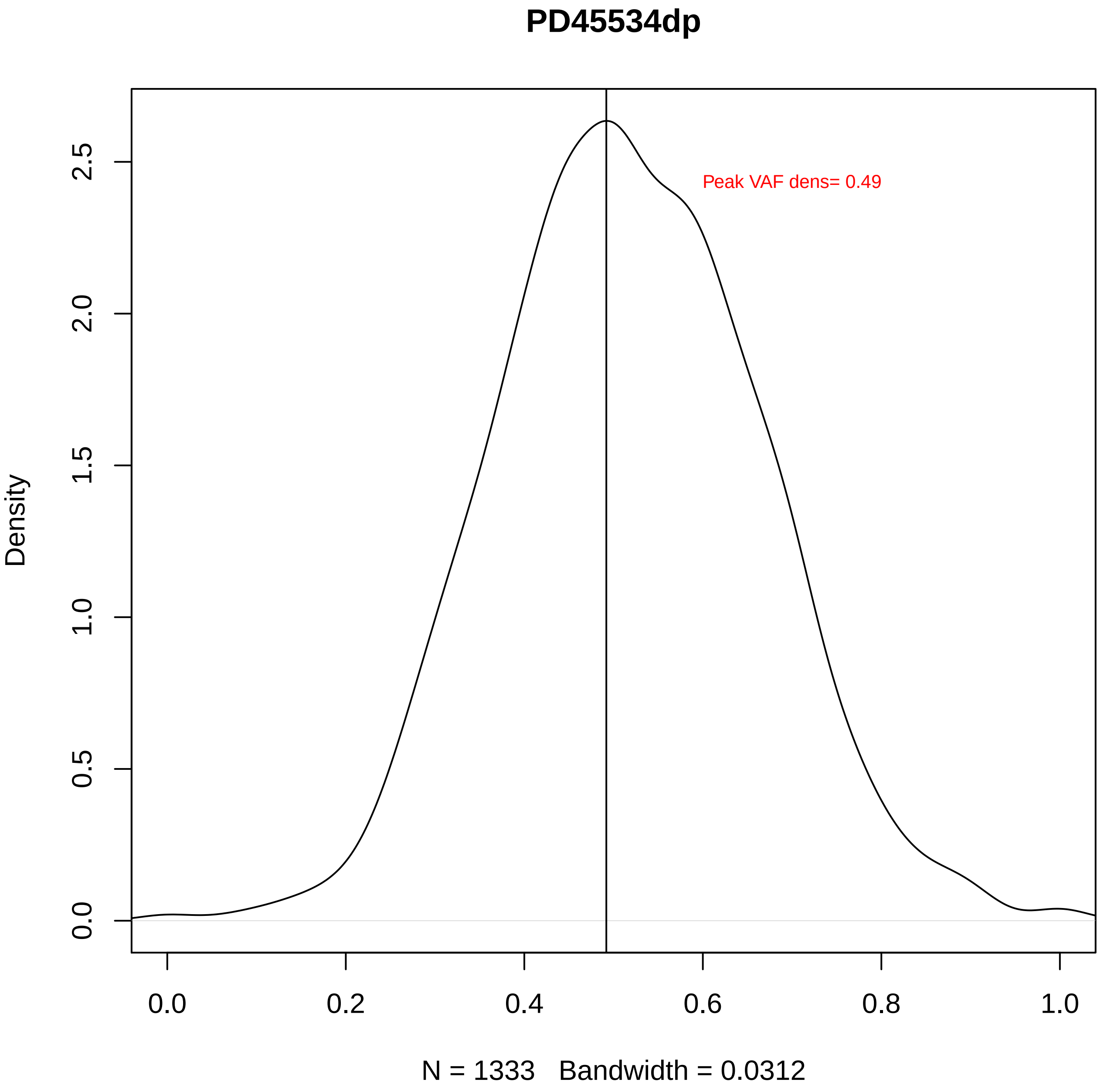

PD45534dp
Peak VAF dens= 0.49
Density
0.0
0.5
1.0
1.5
2.0
2.5
0.0
0.2
0.4
0.6
0.8
1.0
N = 1333   Bandwidth = 0.0312

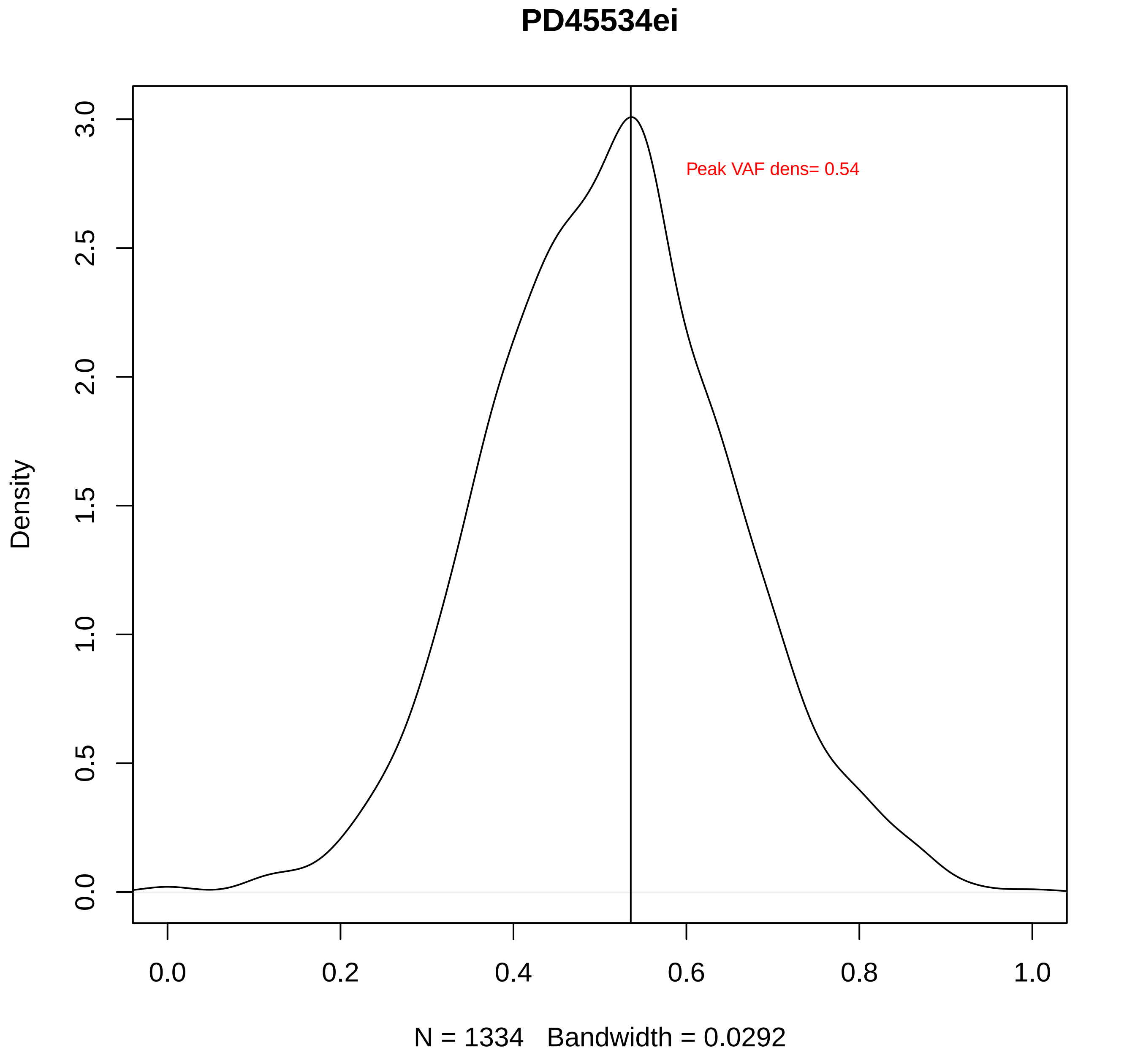

PD45534ei
Peak VAF dens= 0.54
Density
0.0
0.5
1.0
1.5
2.0
2.5
3.0
0.0
0.2
0.4
0.6
0.8
1.0
N = 1334 Bandwidth = 0.0292

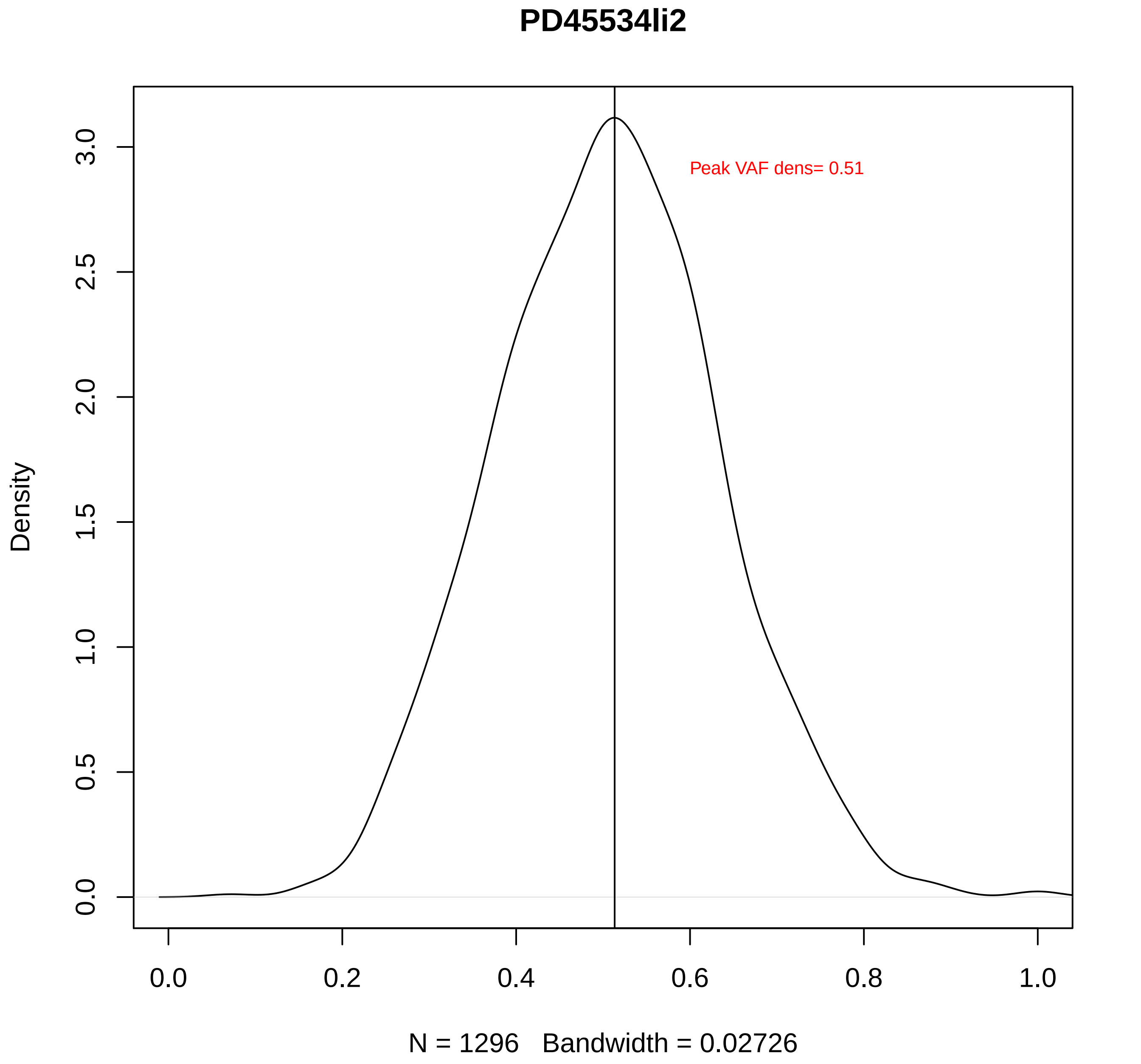
PD45534li2
Peak VAF dens= 0.51
Density
0.0
0.5
1.0
1.5
2.0
2.5
3.0
0.0
0.2
0.4
0.6
0.8
1.0
N = 1296   Bandwidth = 0.02726

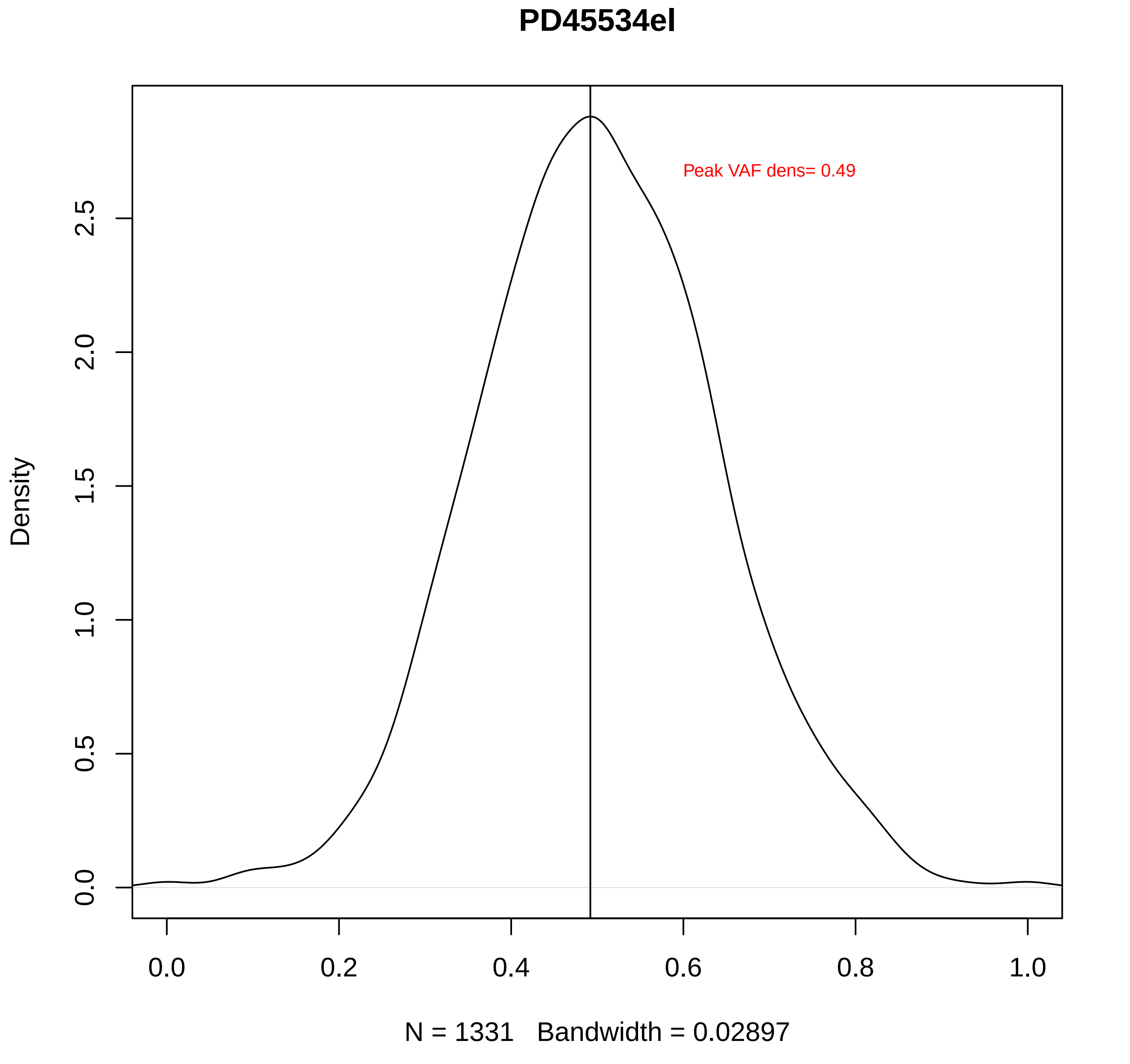
PD45534el
Peak VAF dens= 0.49
Density
0.0
0.5
1.0
1.5
2.0
2.5
0.0
0.2
0.4
0.6
0.8
1.0
N = 1331   Bandwidth = 0.02897

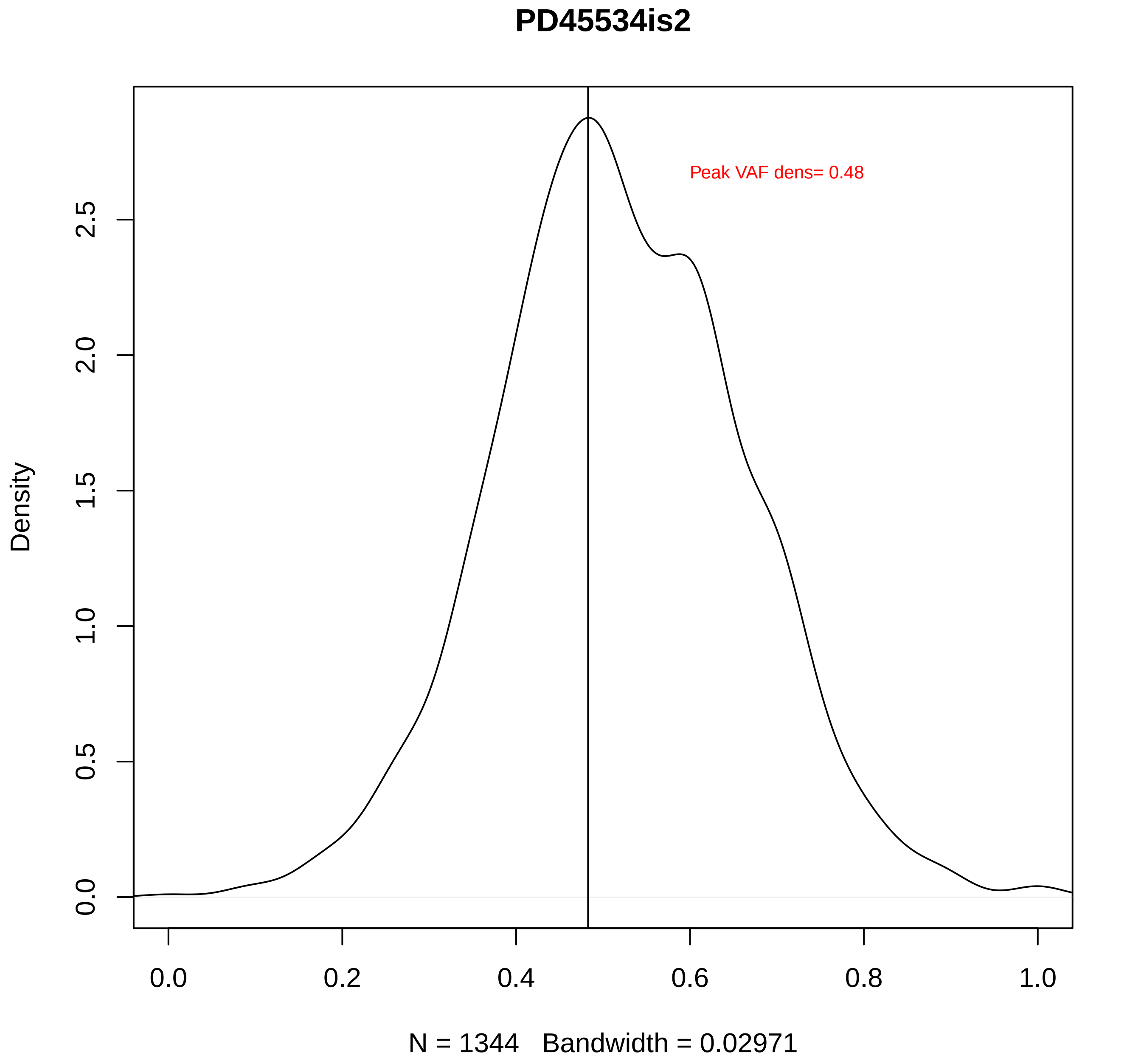
PD45534is2
Peak VAF dens= 0.48
Density
0.0
0.5
1.0
1.5
2.0
2.5
0.0
0.2
0.4
0.6
0.8
1.0
N = 1344   Bandwidth = 0.02971

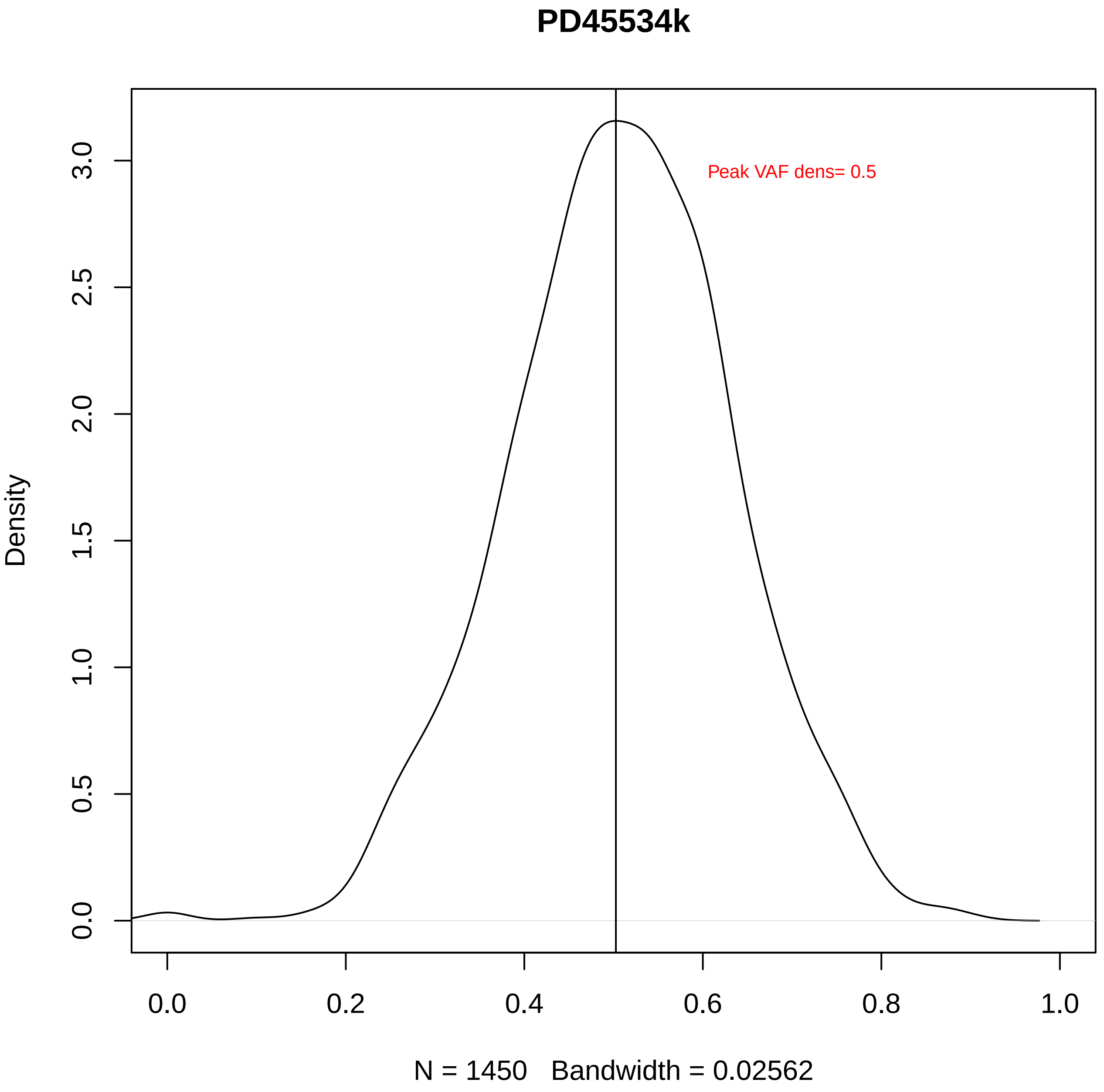
PD45534k
Peak VAF dens= 0.5
Density
0.0
0.5
1.0
1.5
2.0
2.5
3.0
0.0
0.2
0.4
0.6
0.8
1.0
N = 1450   Bandwidth = 0.02562

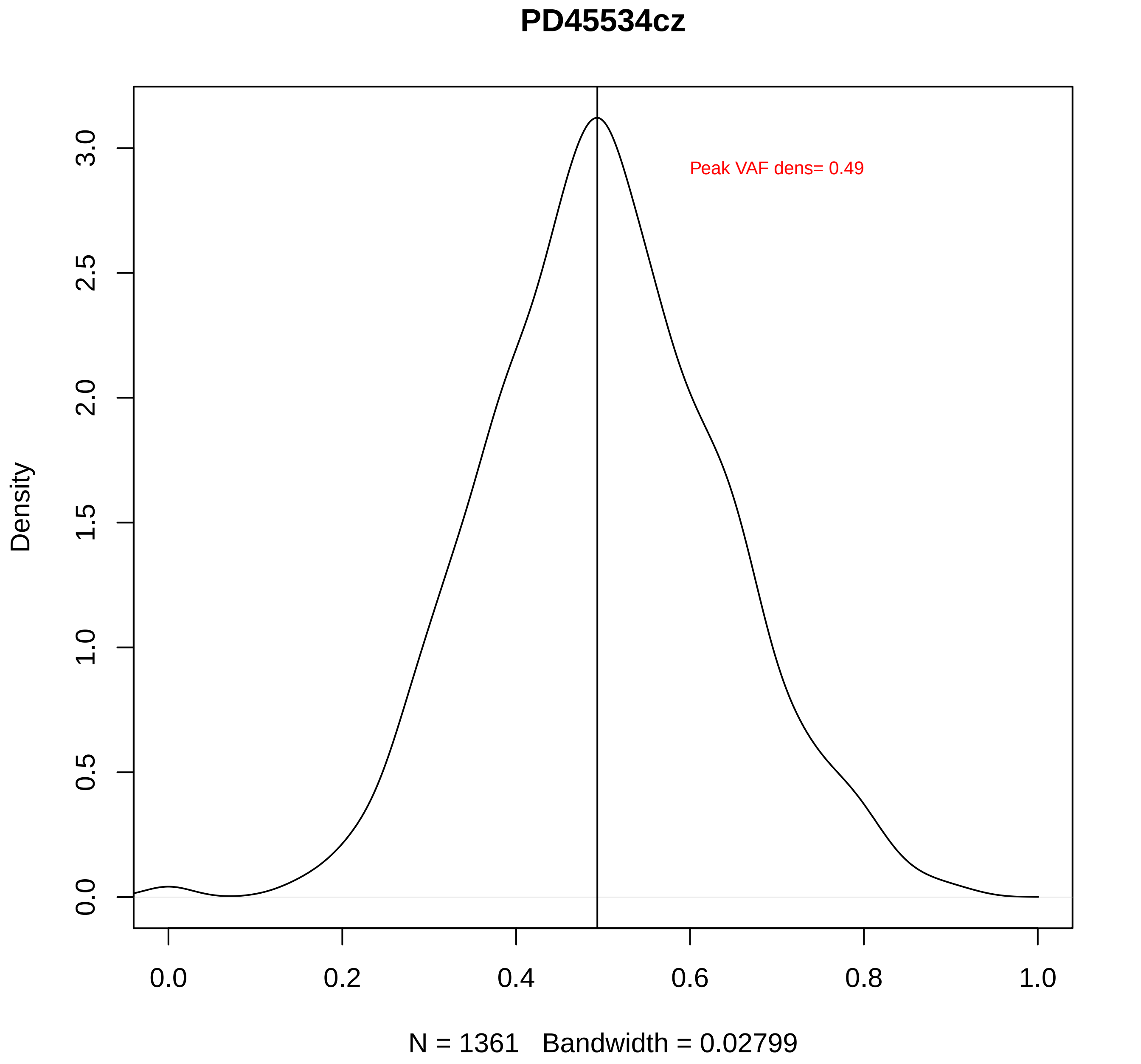

PD45534cz
Peak VAF dens= 0.49
Density
0.0
0.5
1.0
1.5
2.0
2.5
3.0
0.0
0.2
0.4
0.6
0.8
1.0
N = 1361 Bandwidth = 0.02799

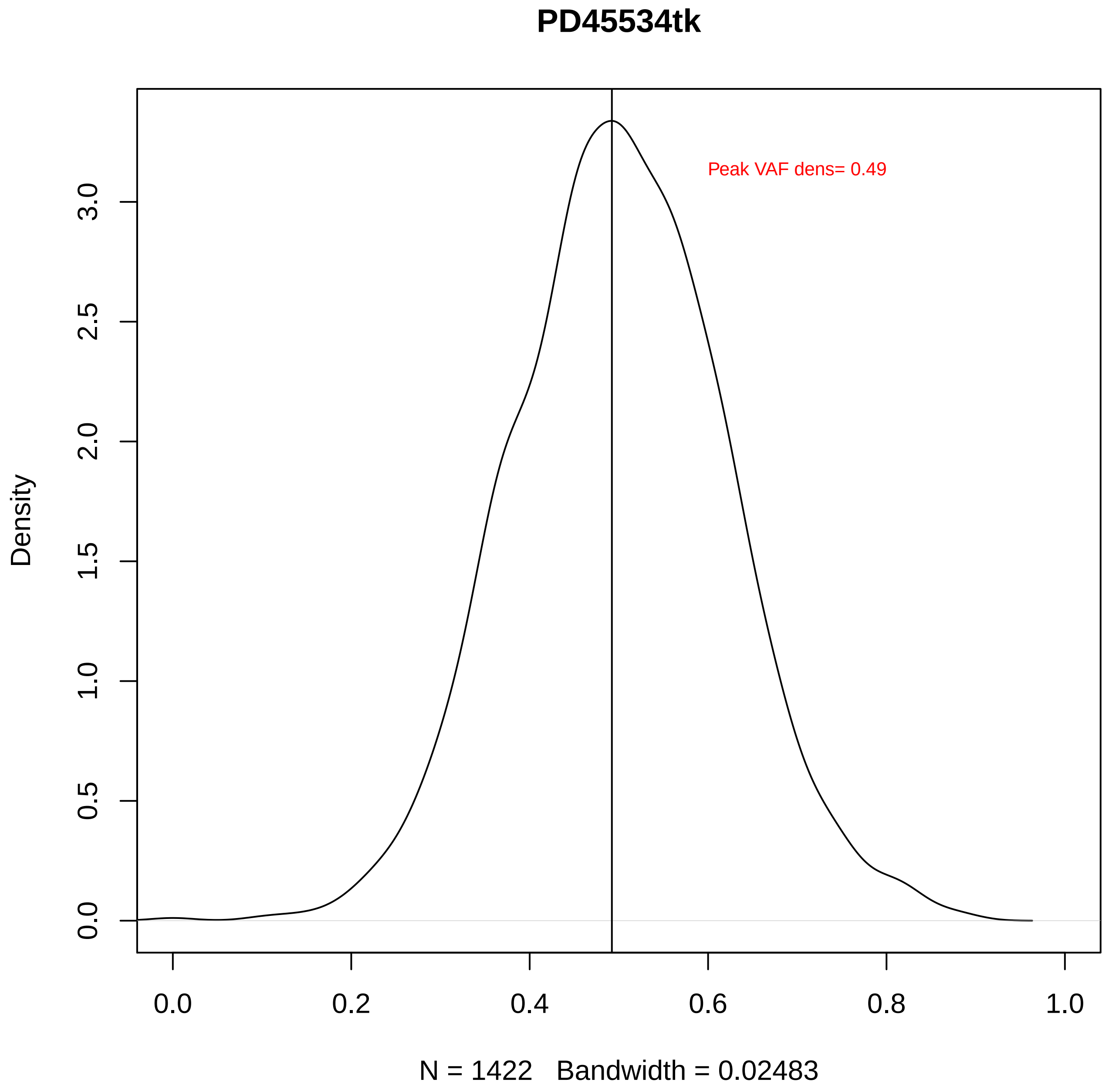
PD45534tk
Peak VAF dens= 0.49
Density
0.0
0.5
1.0
1.5
2.0
2.5
3.0
0.0
0.2
0.4
0.6
0.8
1.0
N = 1422   Bandwidth = 0.02483

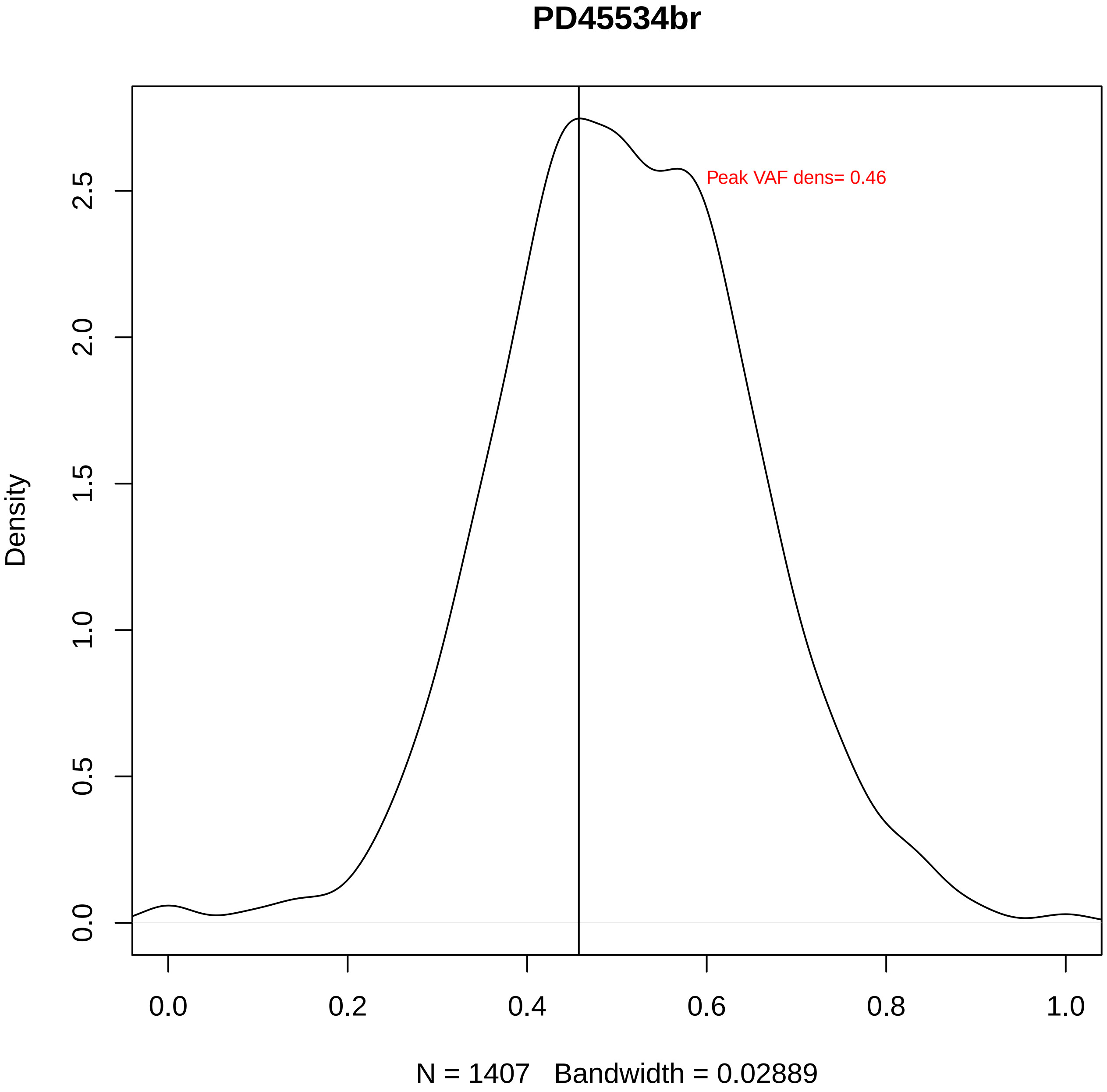
PD45534br
Peak VAF dens= 0.46
Density
0.0
0.5
1.0
1.5
2.0
2.5
0.0
0.2
0.4
0.6
0.8
1.0
N = 1407   Bandwidth = 0.02889

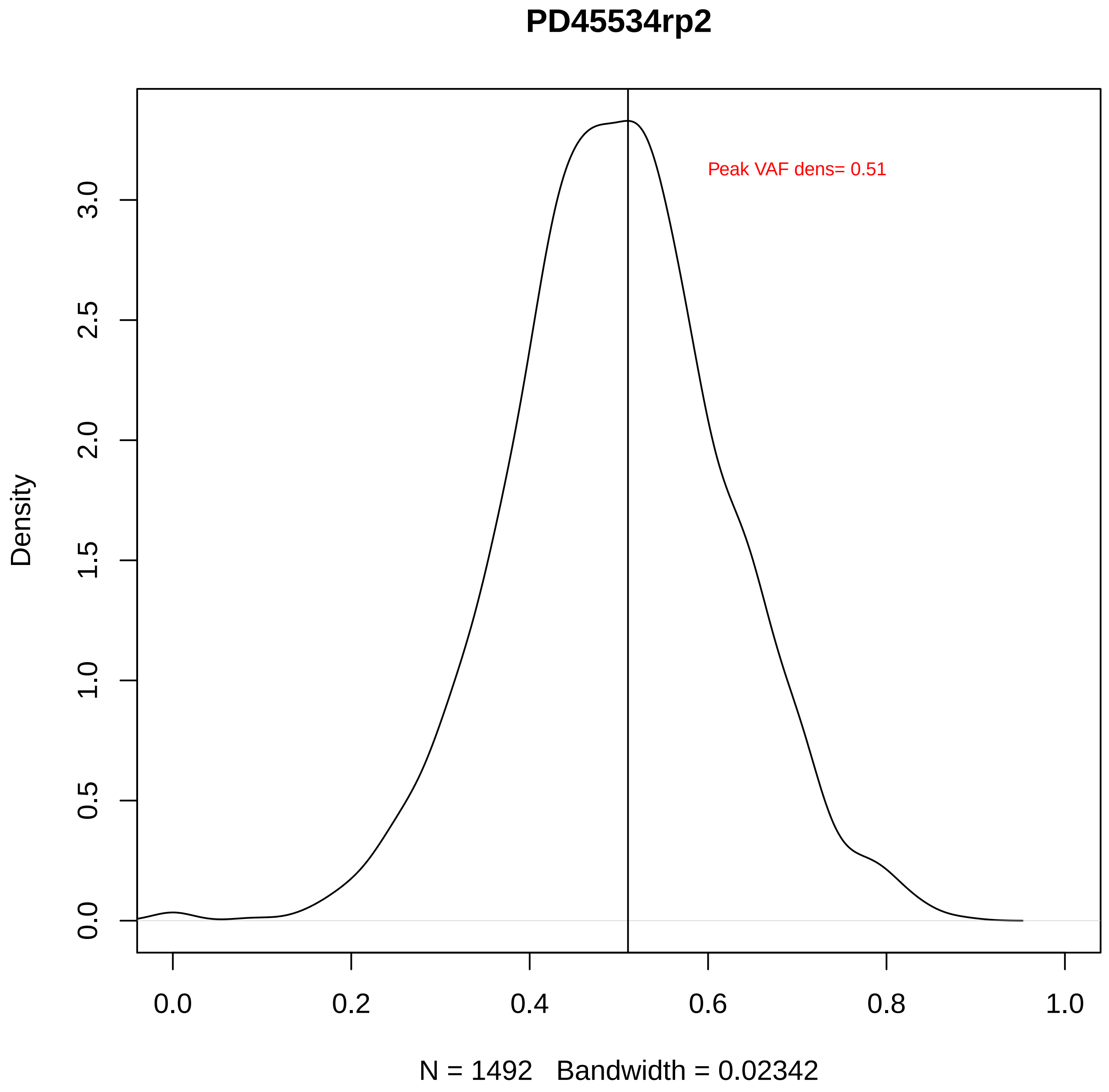
PD45534rp2
Peak VAF dens= 0.51
Density
0.0
0.5
1.0
1.5
2.0
2.5
3.0
0.0
0.2
0.4
0.6
0.8
1.0
N = 1492 Bandwidth = 0.02342

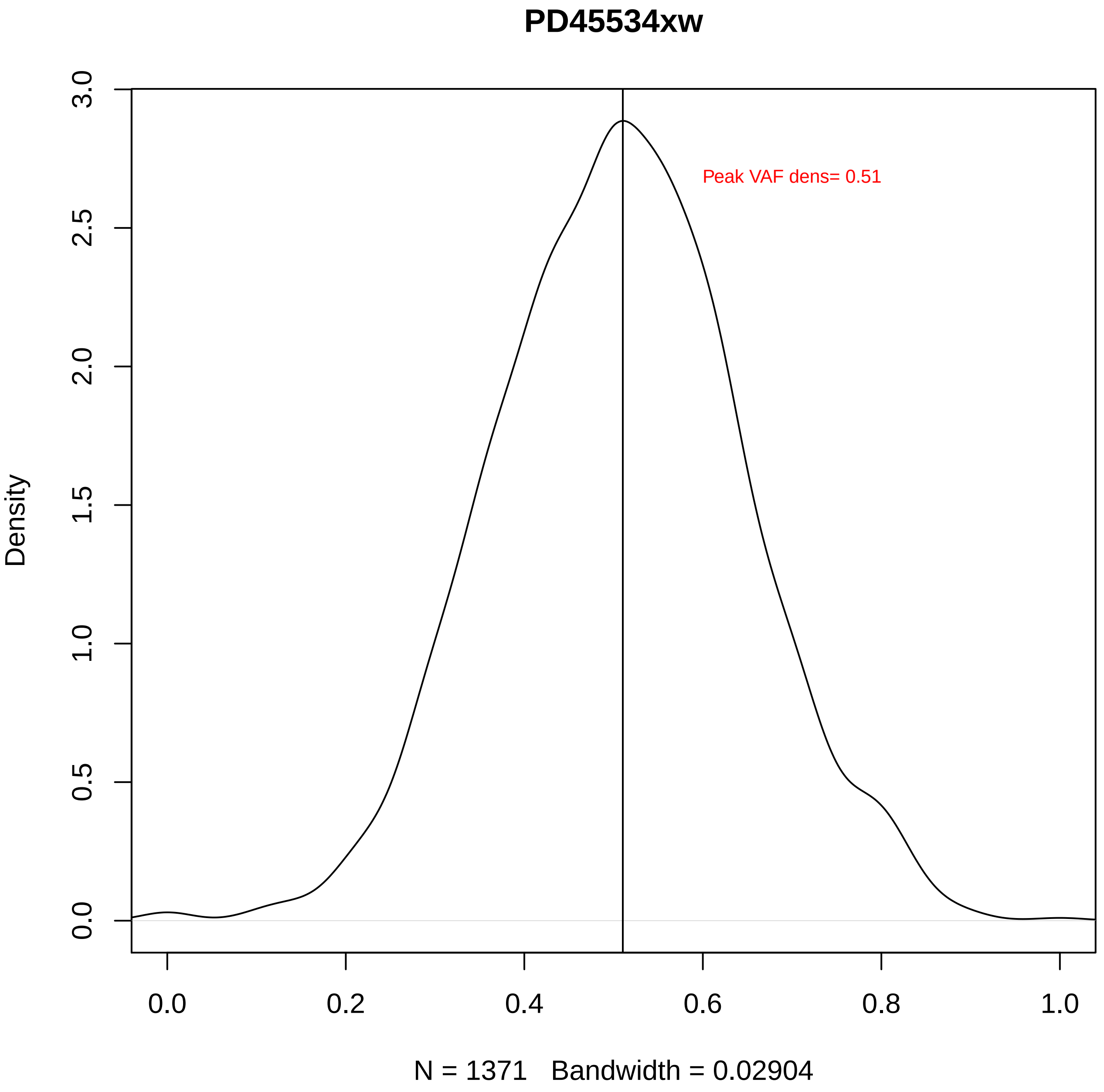

PD45534xw
Peak VAF dens= 0.51
Density
0.0
0.5
1.0
1.5
2.0
2.5
3.0
0.0
0.2
0.4
0.6
0.8
1.0
N = 1371 Bandwidth = 0.02904

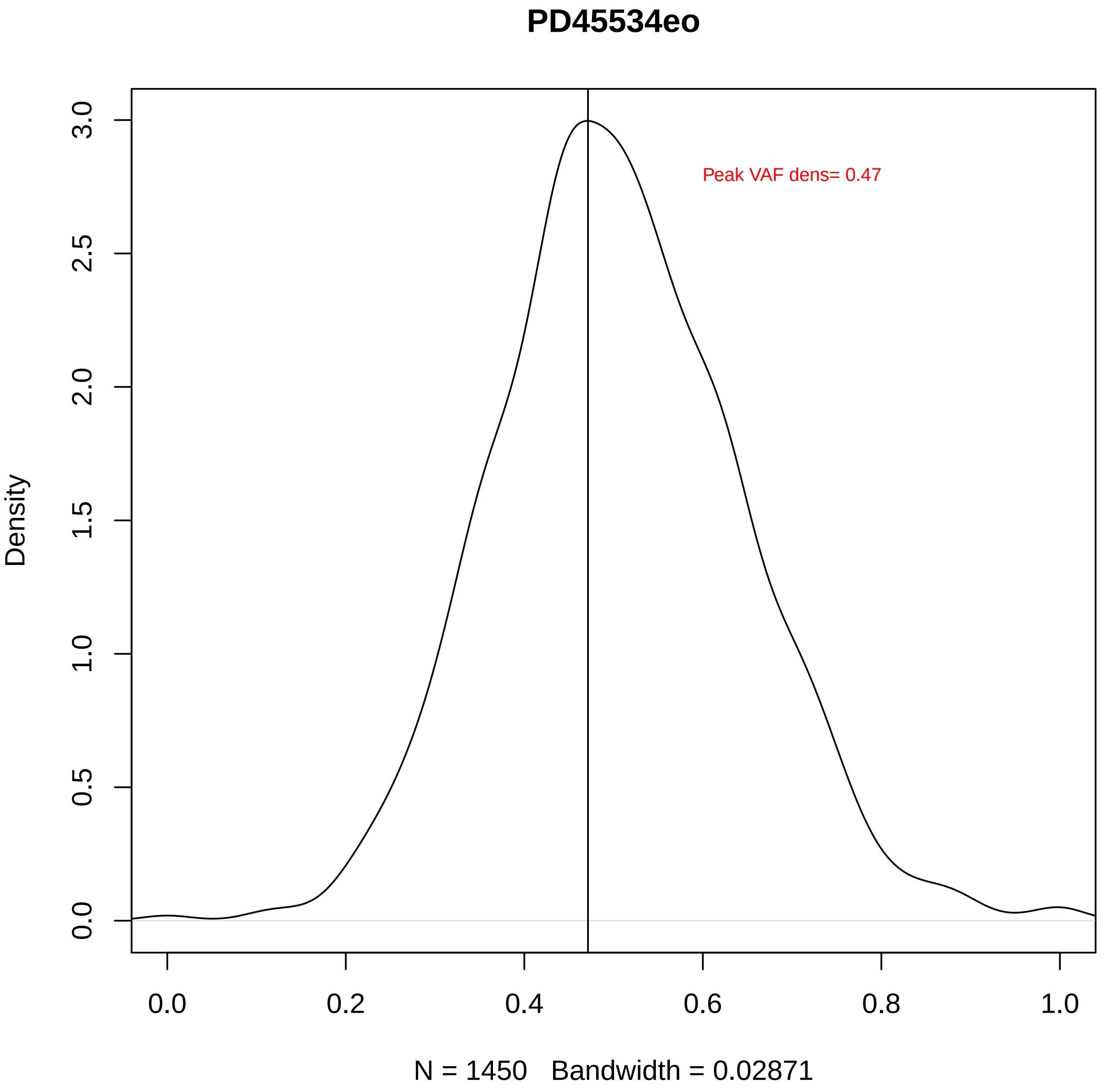

PD45534eo
Peak VAF dens= 0.47
Density
3.0
2.5
2.0
1.5
1.0
0.5
0.0
0.0
0.2
0.4
0.6
0.8
1.0
N = 1450 Bandwidth = 0.02871

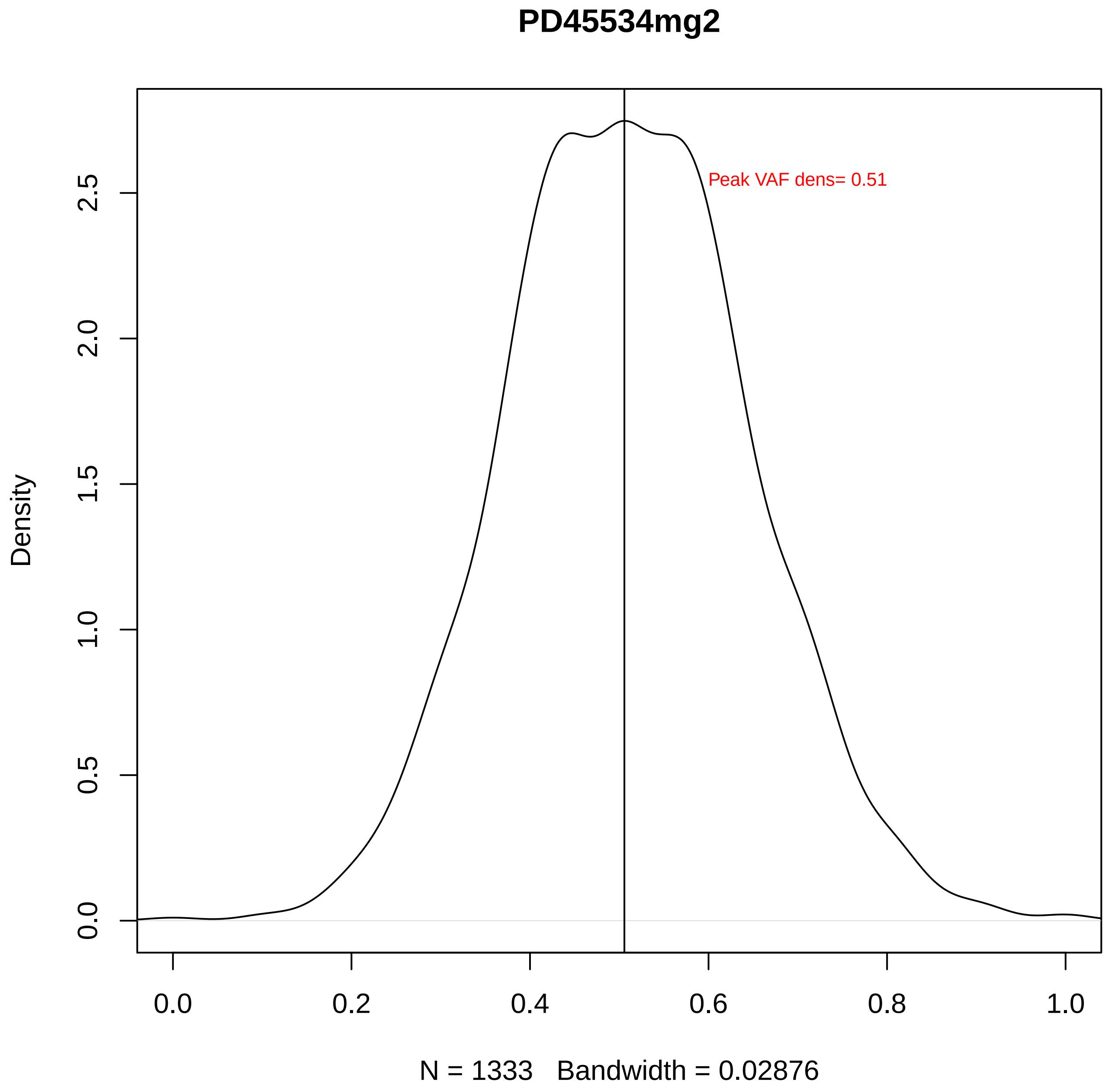
PD45534mg2
Peak VAF dens= 0.51
Density
0.0
0.5
1.0
1.5
2.0
2.5
0.0
0.2
0.4
0.6
0.8
1.0
N = 1333   Bandwidth = 0.02876

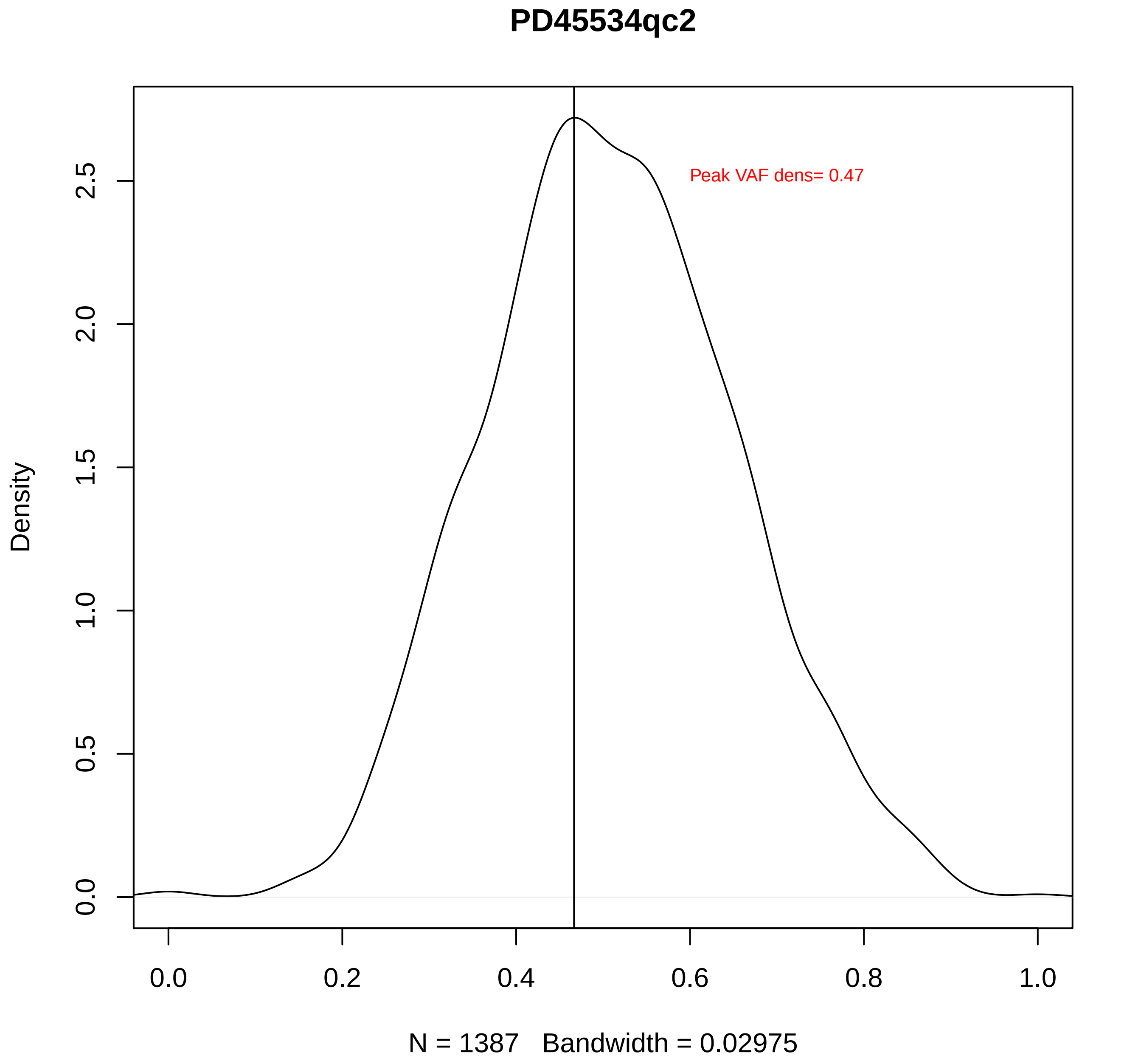
PD45534qc2
Peak VAF dens= 0.47
Density
0.0
0.5
1.0
1.5
2.0
2.5
0.0
0.2
0.4
0.6
0.8
1.0
N = 1387   Bandwidth = 0.02975

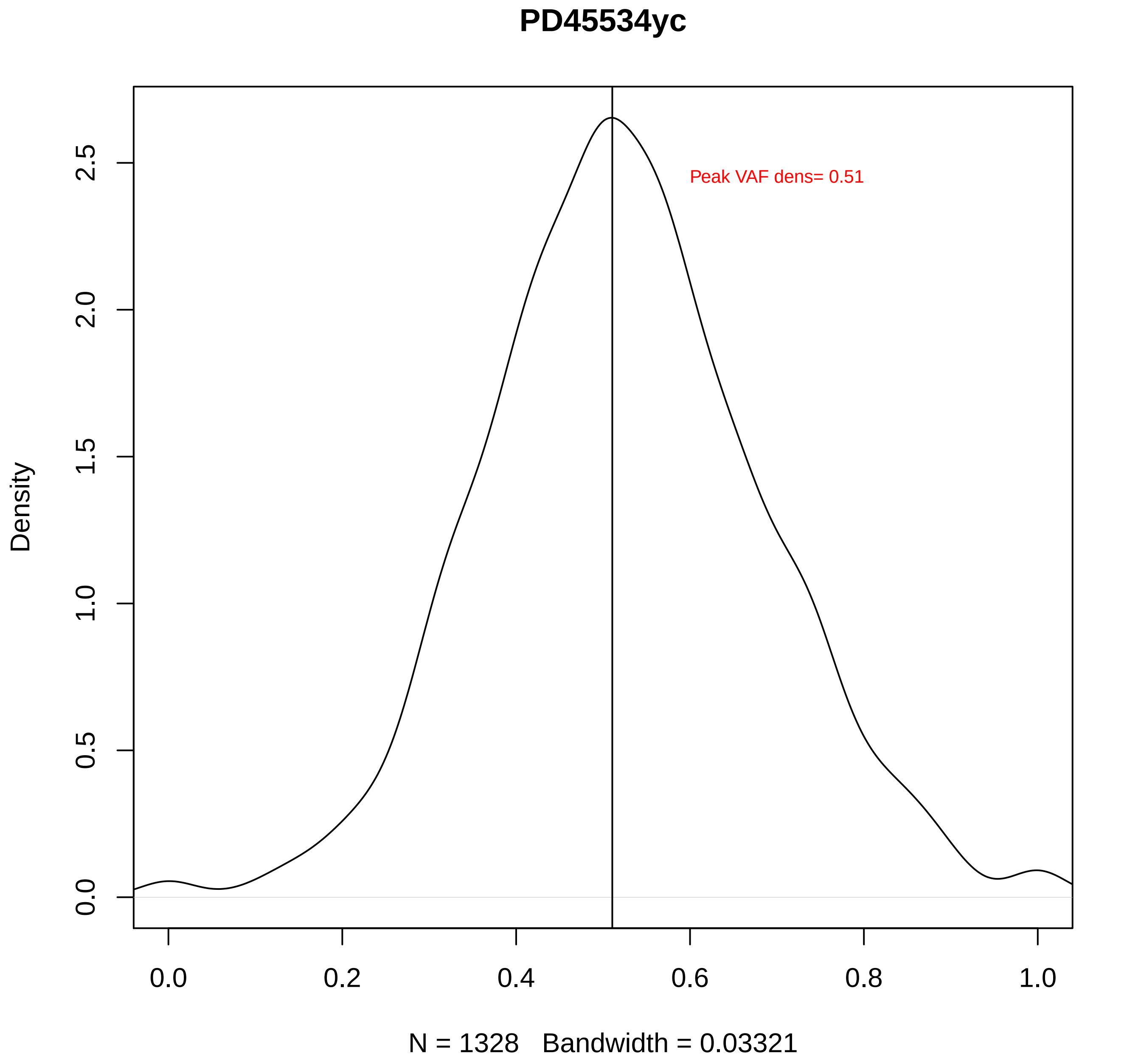

PD45534yc
Peak VAF dens= 0.51
Density
0.0
0.5
1.0
1.5
2.0
2.5
0.0
0.2
0.4
0.6
0.8
1.0
N = 1328   Bandwidth = 0.03321

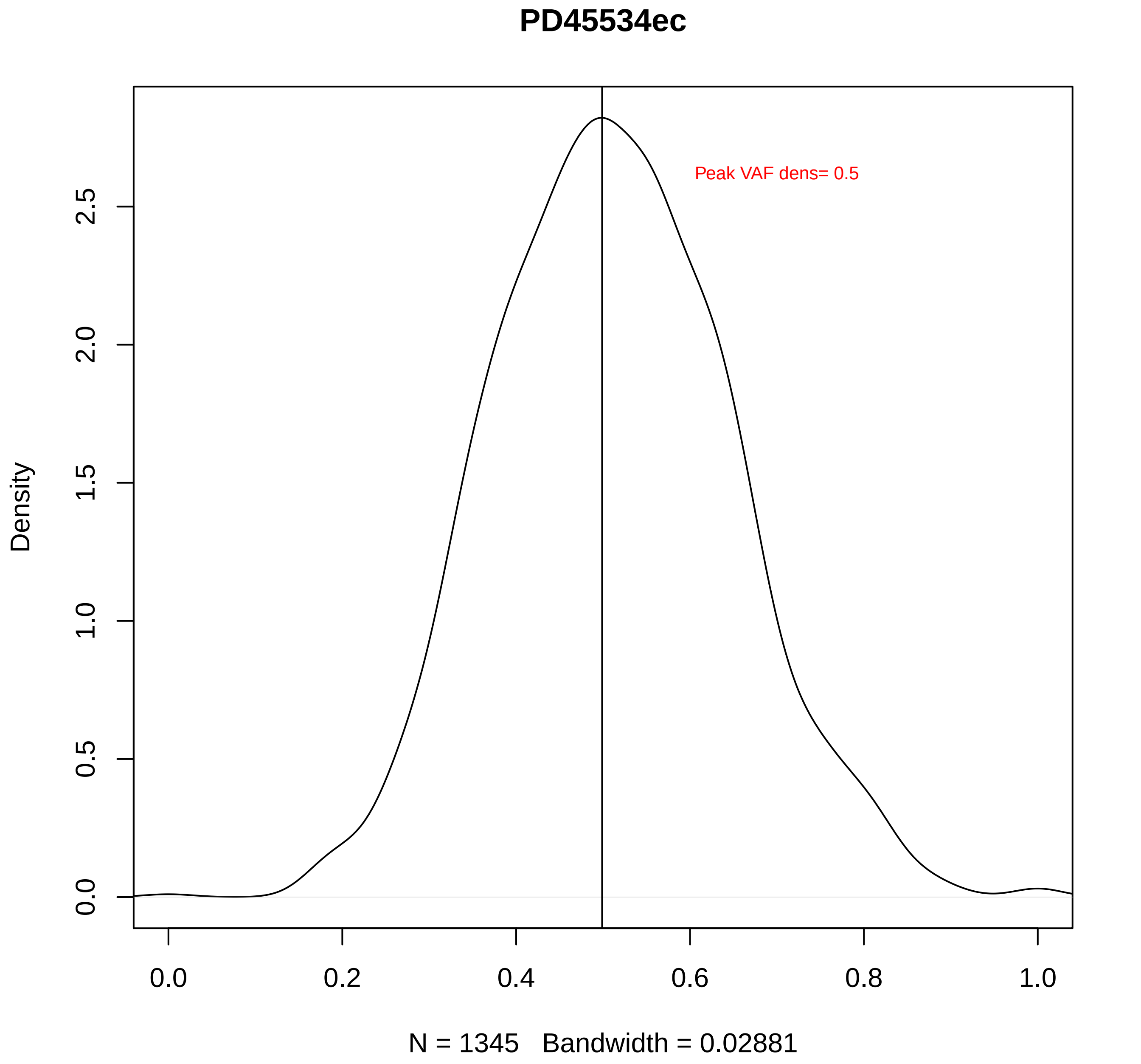

PD45534ec
Peak VAF dens= 0.5
Density
0.0
0.5
1.0
1.5
2.0
2.5
0.0
0.2
0.4
0.6
0.8
1.0
N = 1345   Bandwidth = 0.02881

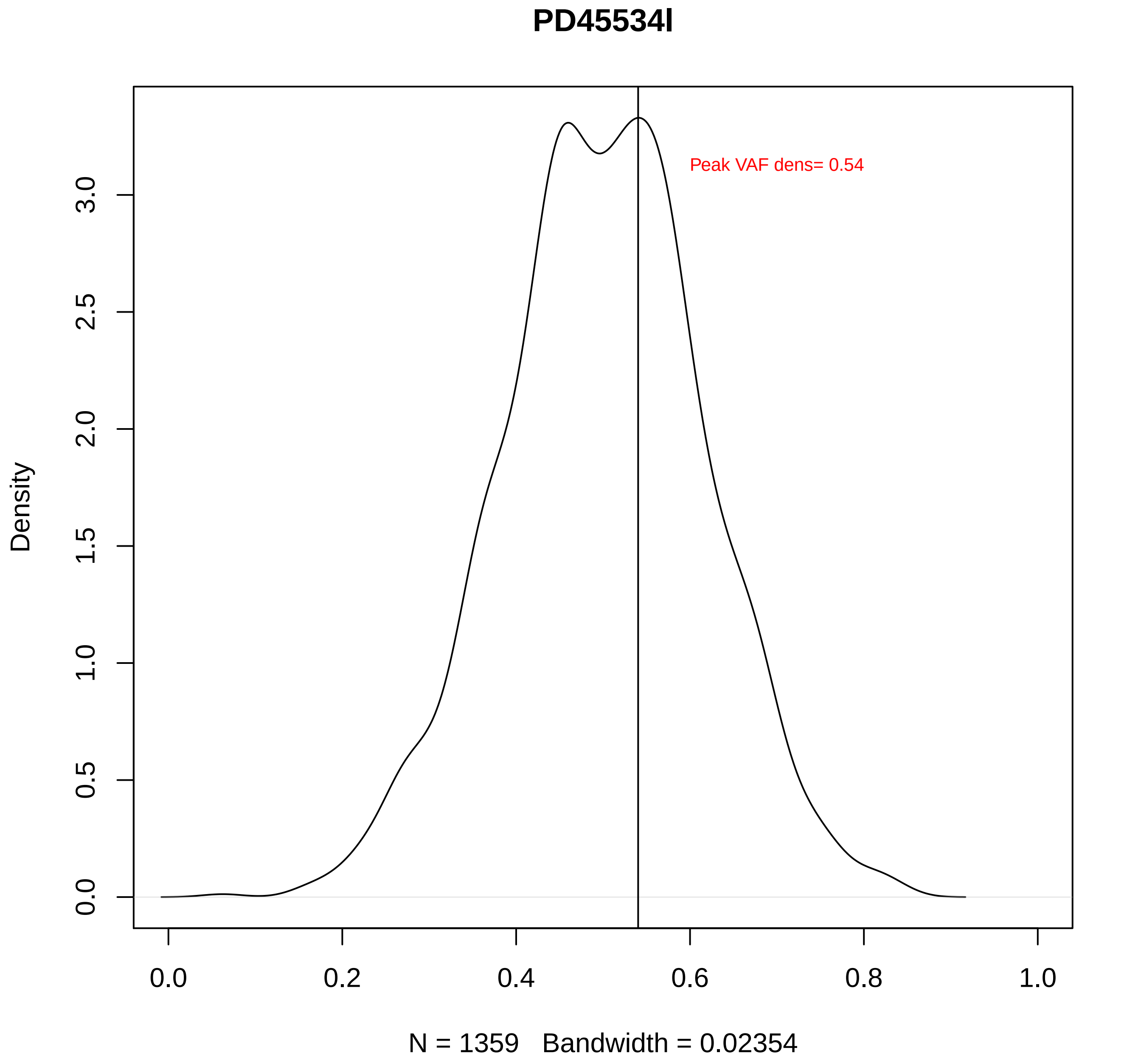

PD45534l
Peak VAF dens= 0.54
Density
0.0
0.5
1.0
1.5
2.0
2.5
3.0
0.0
0.2
0.4
0.6
0.8
1.0
N = 1359   Bandwidth = 0.02354

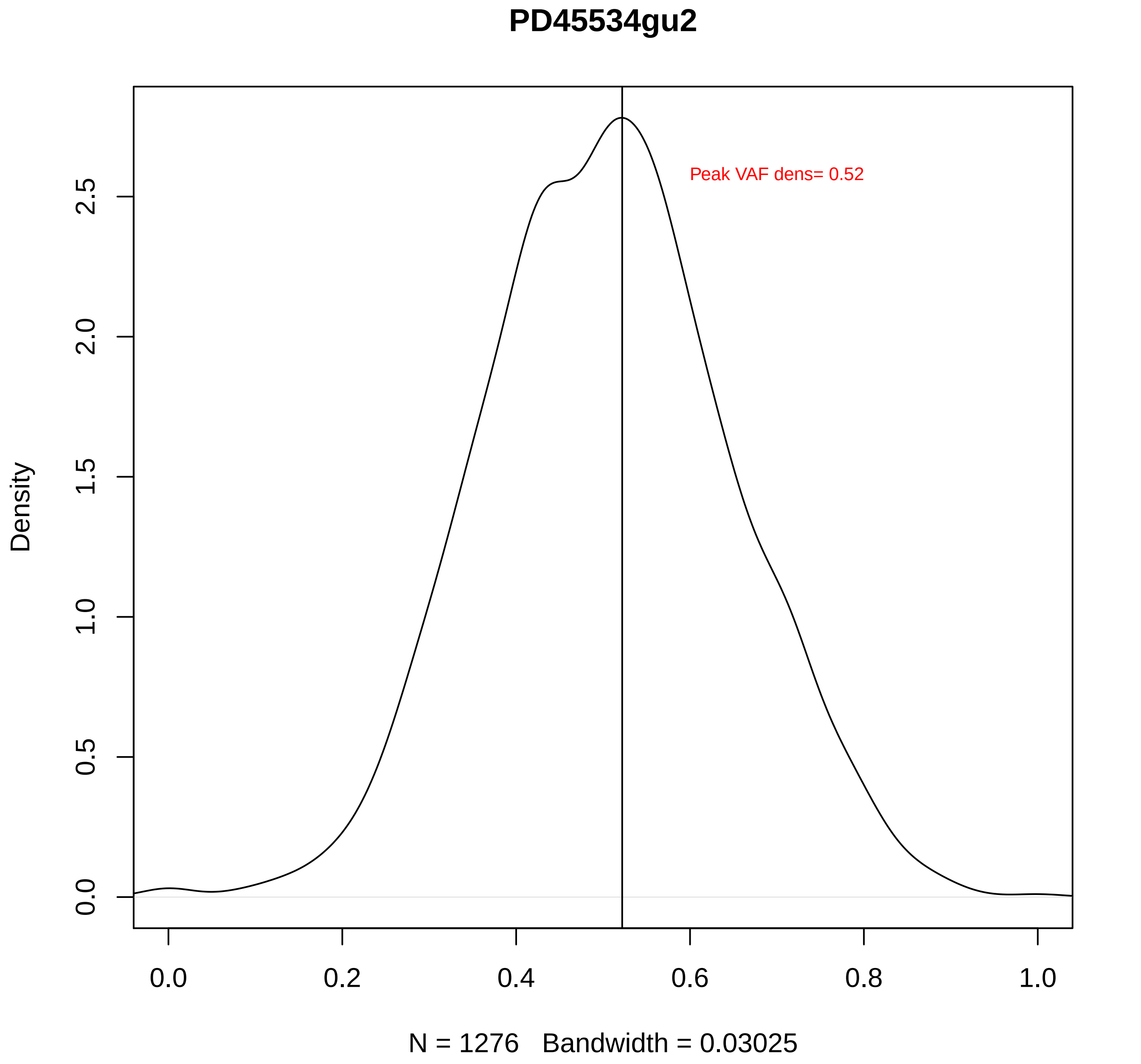
PD45534gu2
Peak VAF dens= 0.52
Density
0.0
0.5
1.0
1.5
2.0
2.5
0.0
0.2
0.4
0.6
0.8
1.0
N = 1276   Bandwidth = 0.03025

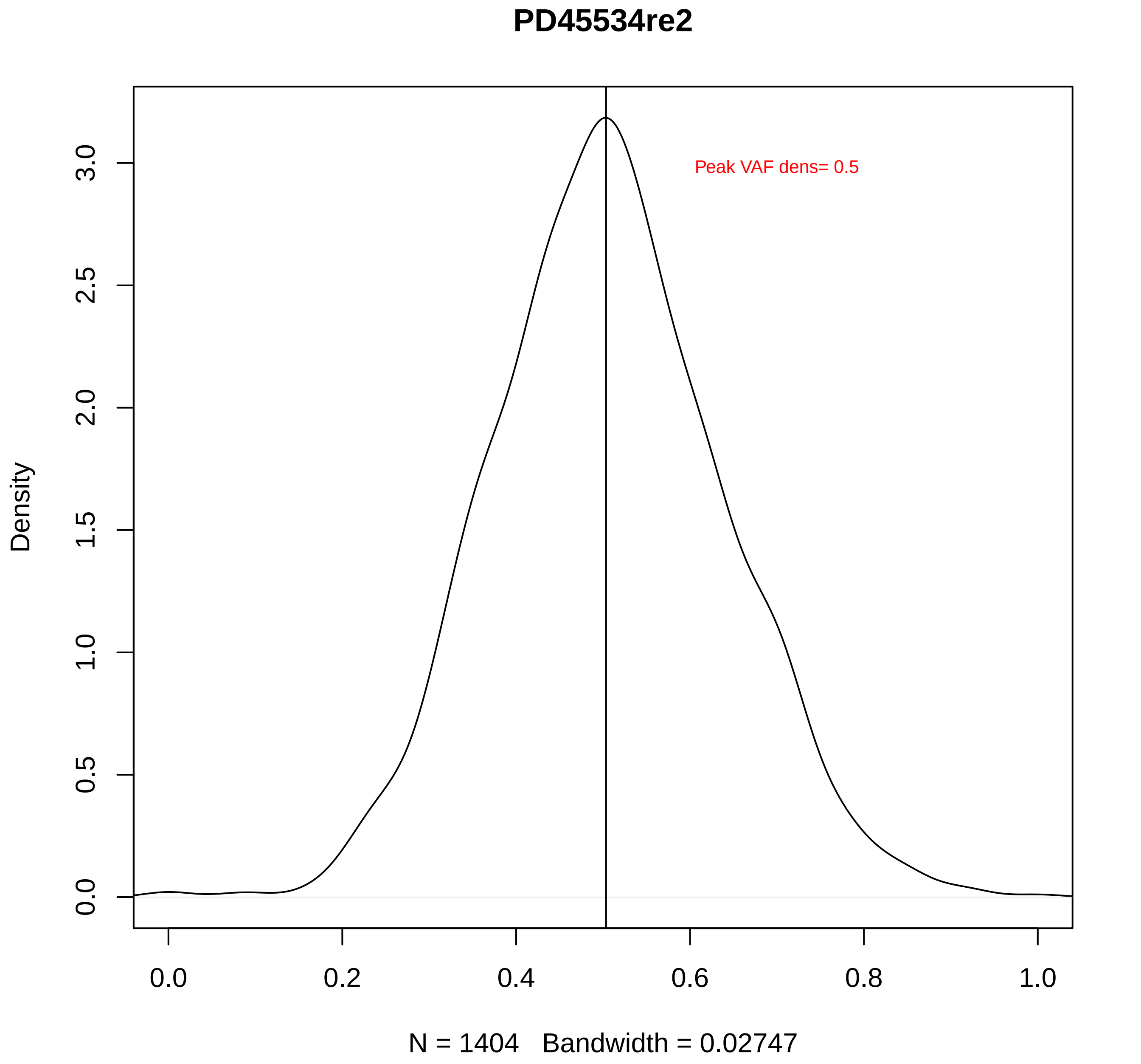

PD45534re2
Peak VAF dens= 0.5
Density
0.0
0.5
1.0
1.5
2.0
2.5
3.0
0.0
0.2
0.4
0.6
0.8
1.0
N = 1404 Bandwidth = 0.02747

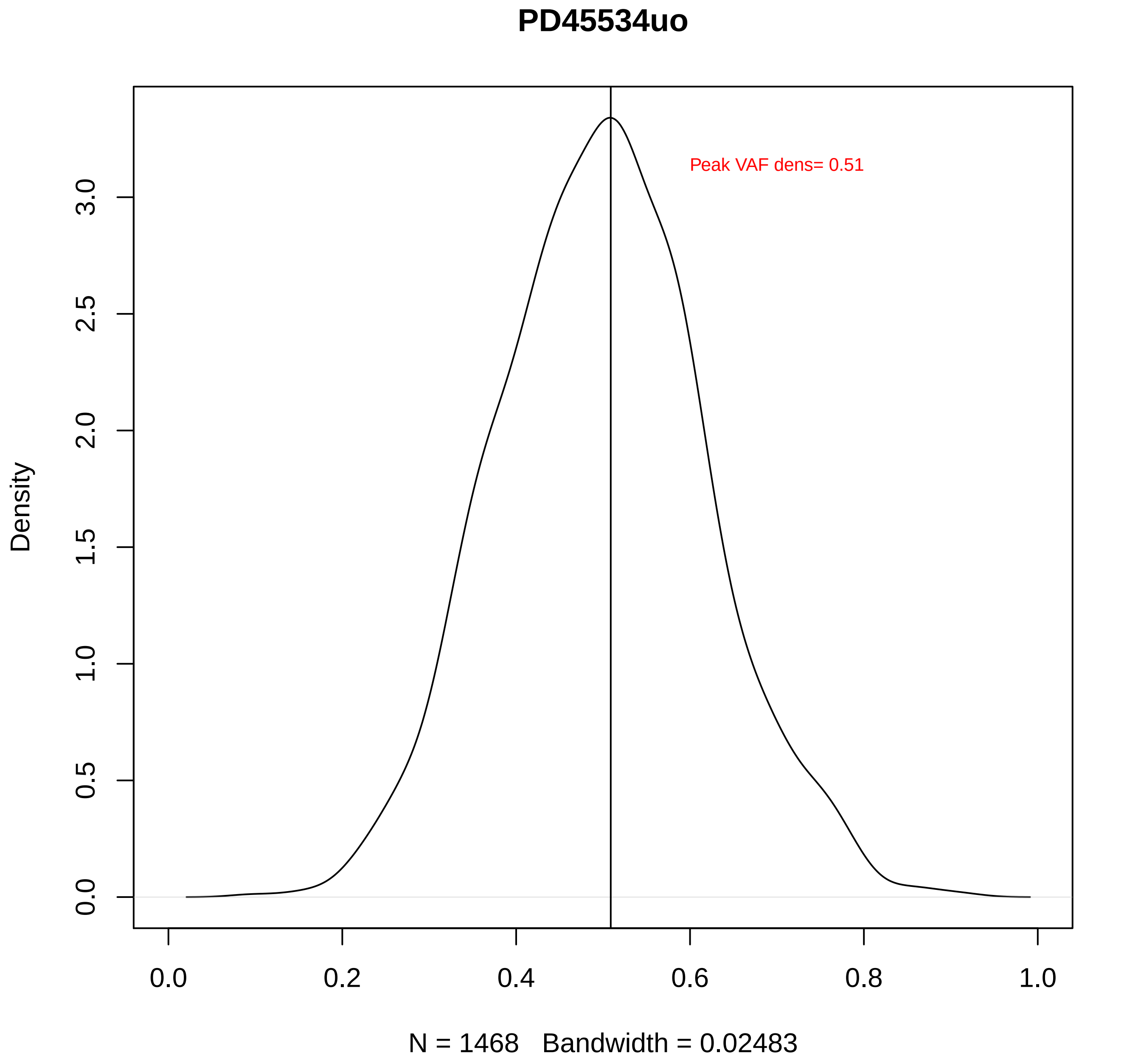
PD45534uo
Peak VAF dens= 0.51
Density
0.0
0.5
1.0
1.5
2.0
2.5
3.0
0.0
0.2
0.4
0.6
0.8
1.0
N = 1468   Bandwidth = 0.02483

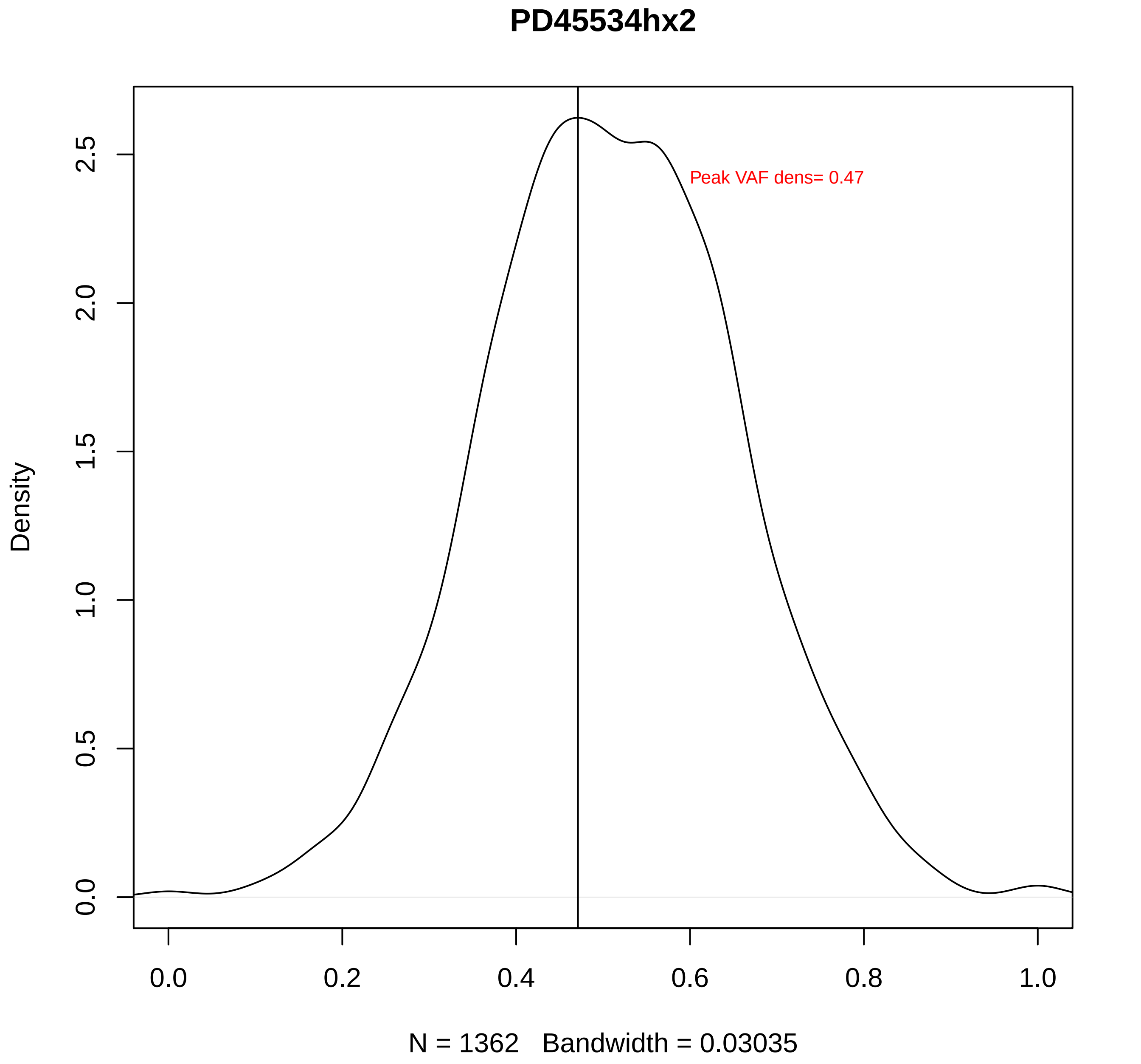
PD45534hx2
Peak VAF dens= 0.47
Density
0.0
0.5
1.0
1.5
2.0
2.5
0.0
0.2
0.4
0.6
0.8
1.0
N = 1362   Bandwidth = 0.03035

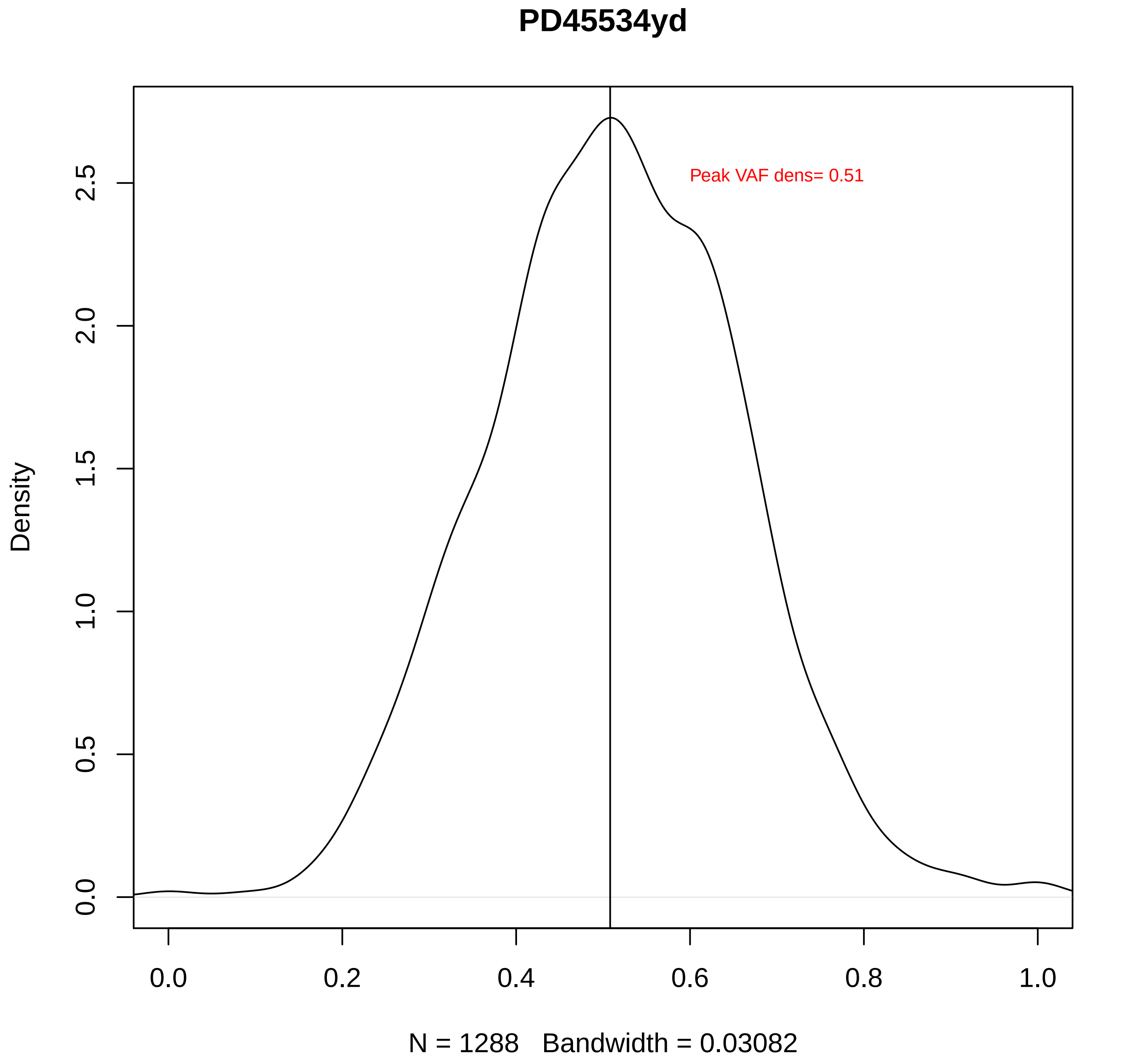
PD45534yd
Peak VAF dens= 0.51
Density
0.0
0.5
1.0
1.5
2.0
2.5
0.0
0.2
0.4
0.6
0.8
1.0
N = 1288   Bandwidth = 0.03082

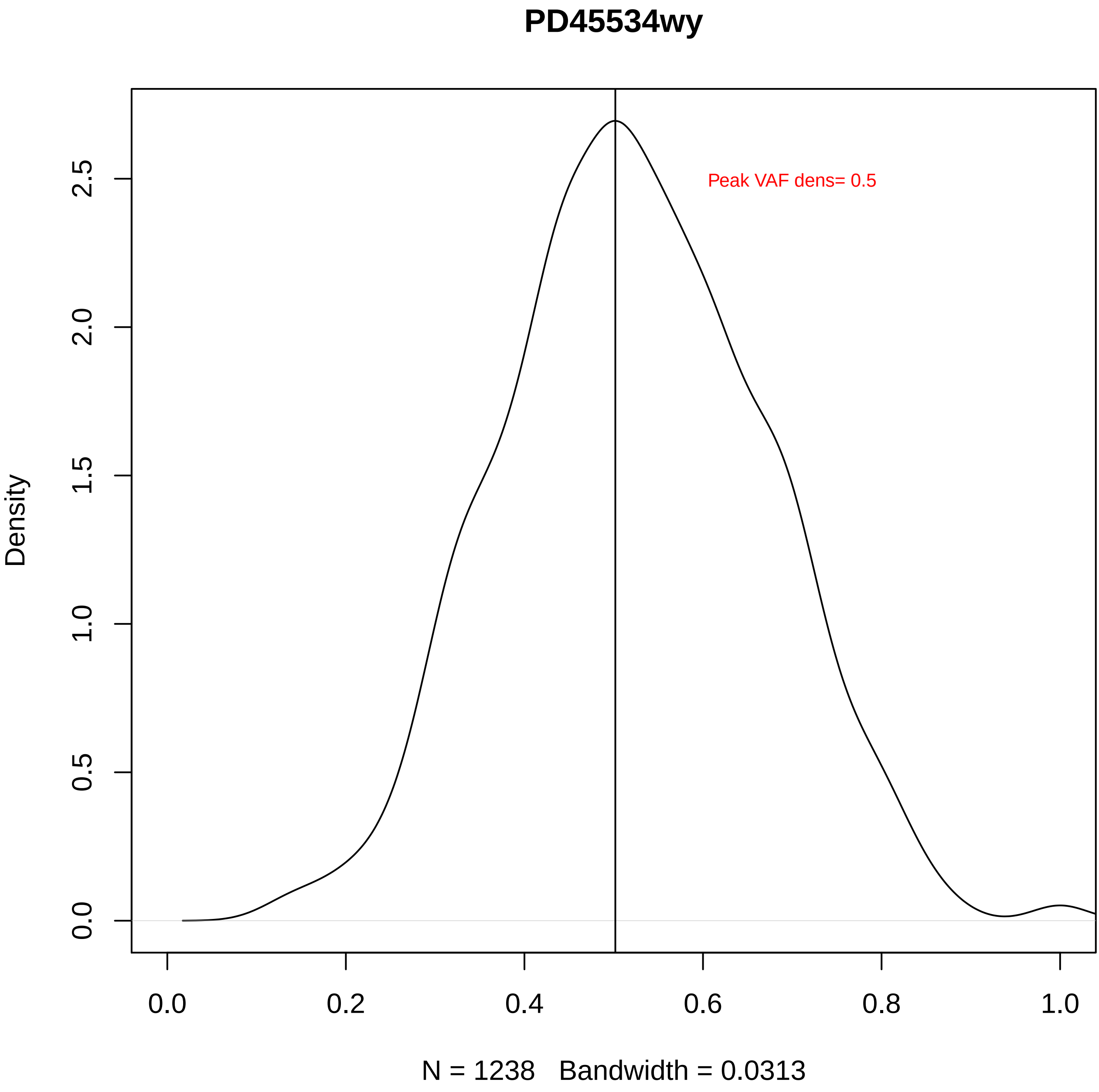

PD45534wy
Peak VAF dens= 0.5
Density
0.0
0.5
1.0
1.5
2.0
2.5
0.0
0.2
0.4
0.6
0.8
1.0
N = 1238   Bandwidth = 0.0313

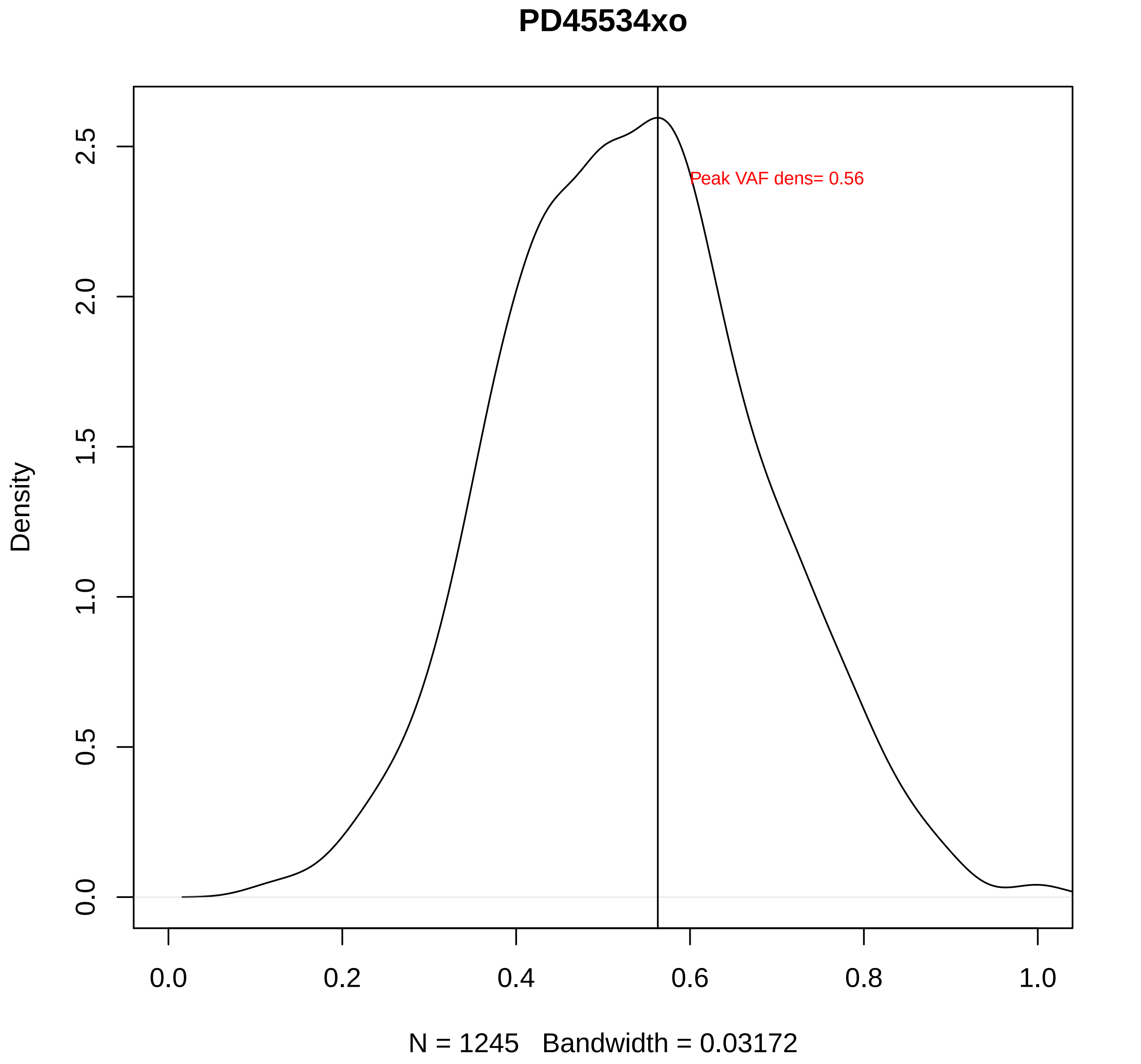
PD45534xo
Peak VAF dens= 0.56
Density
0.0
0.5
1.0
1.5
2.0
2.5
0.0
0.2
0.4
0.6
0.8
1.0
N = 1245   Bandwidth = 0.03172

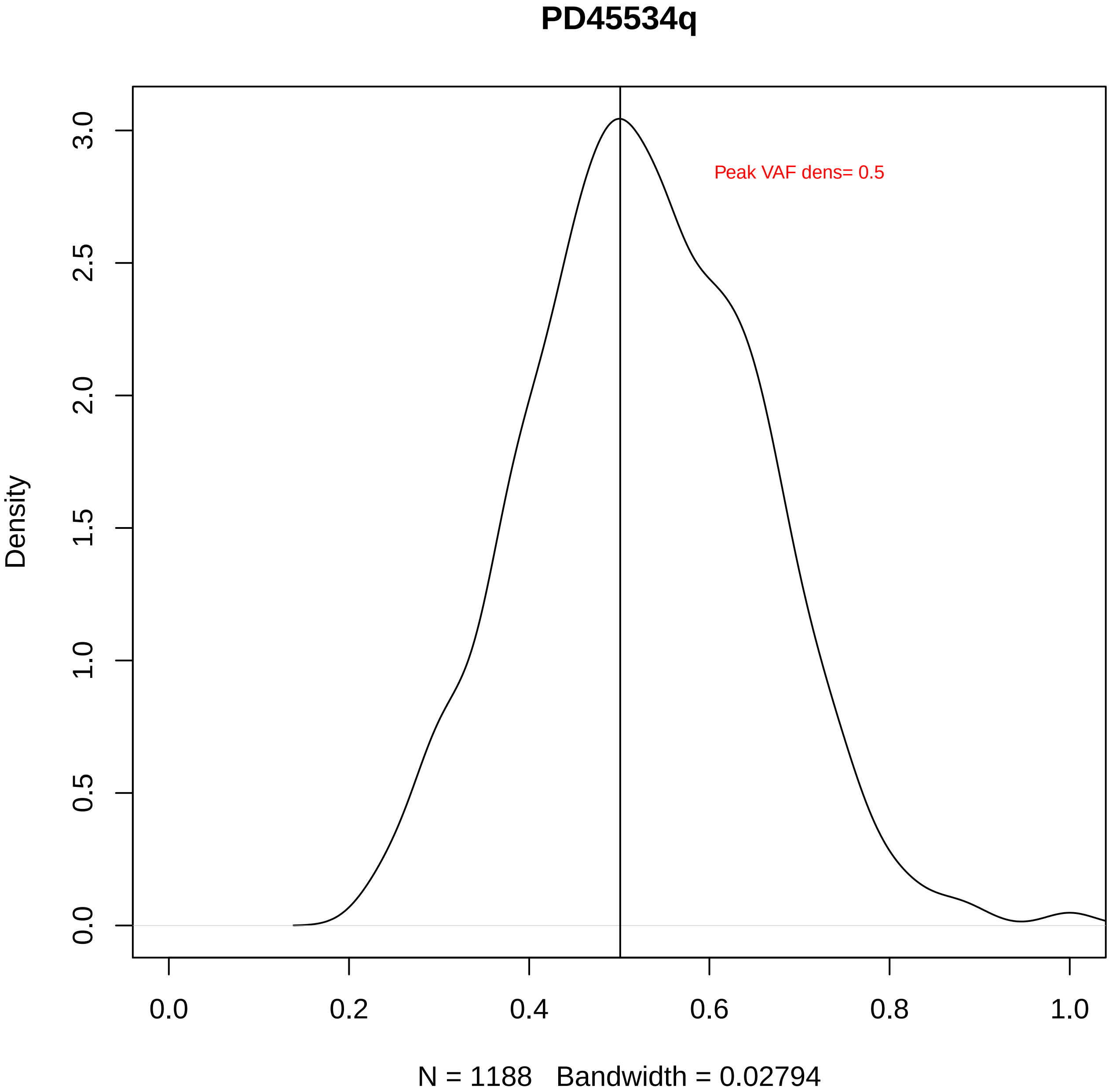
PD45534q
Peak VAF dens= 0.5
Density
0.0
0.5
1.0
1.5
2.0
2.5
3.0
0.0
0.2
0.4
0.6
0.8
1.0
N = 1188   Bandwidth = 0.02794

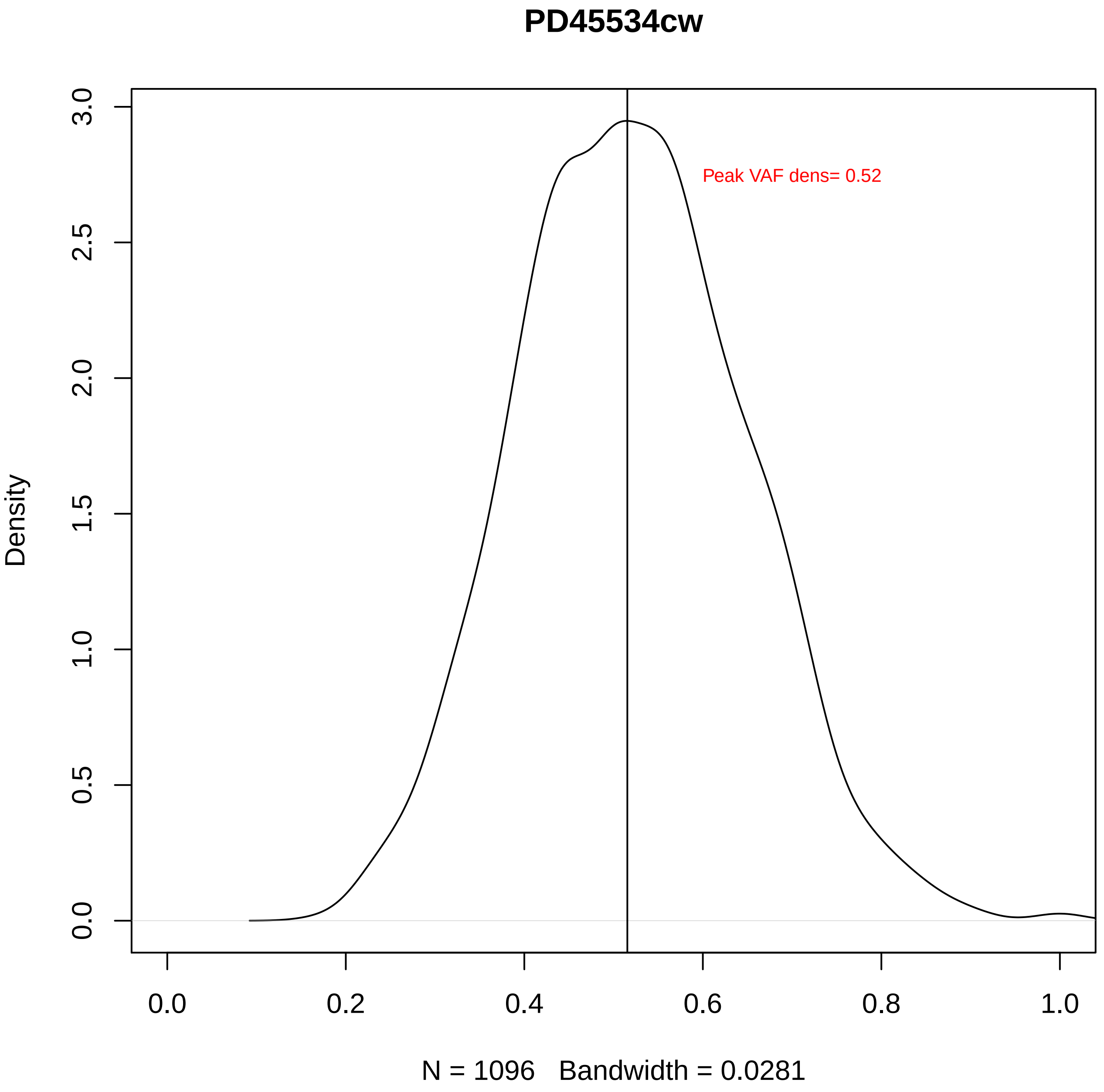
PD45534cw
Peak VAF dens= 0.52
Density
0.0
0.5
1.0
1.5
2.0
2.5
3.0
0.0
0.2
0.4
0.6
0.8
1.0
N = 1096   Bandwidth = 0.0281

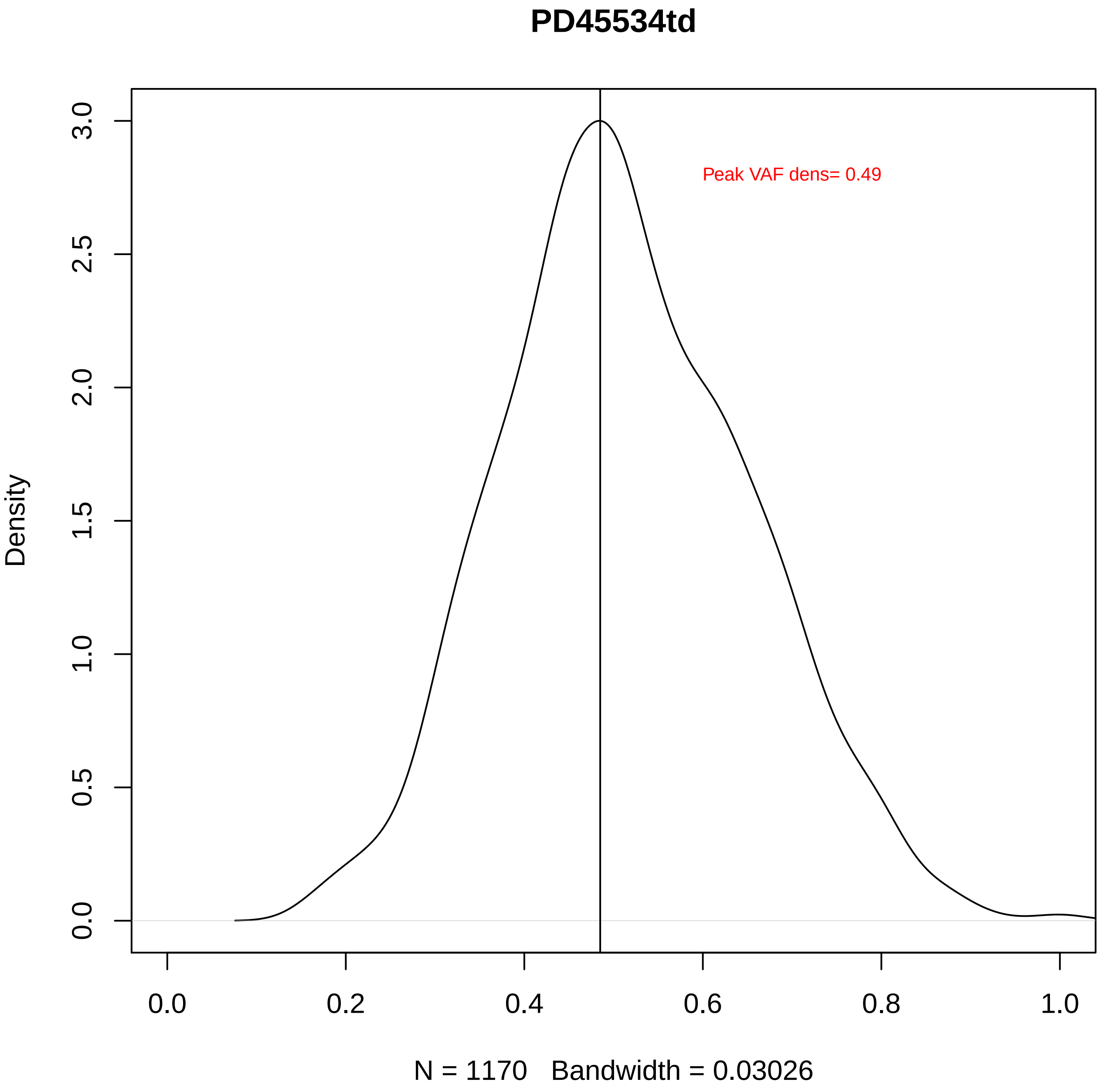
PD45534td
Peak VAF dens= 0.49
Density
0.0
0.5
1.0
1.5
2.0
2.5
3.0
0.0
0.2
0.4
0.6
0.8
1.0
N = 1170   Bandwidth = 0.03026

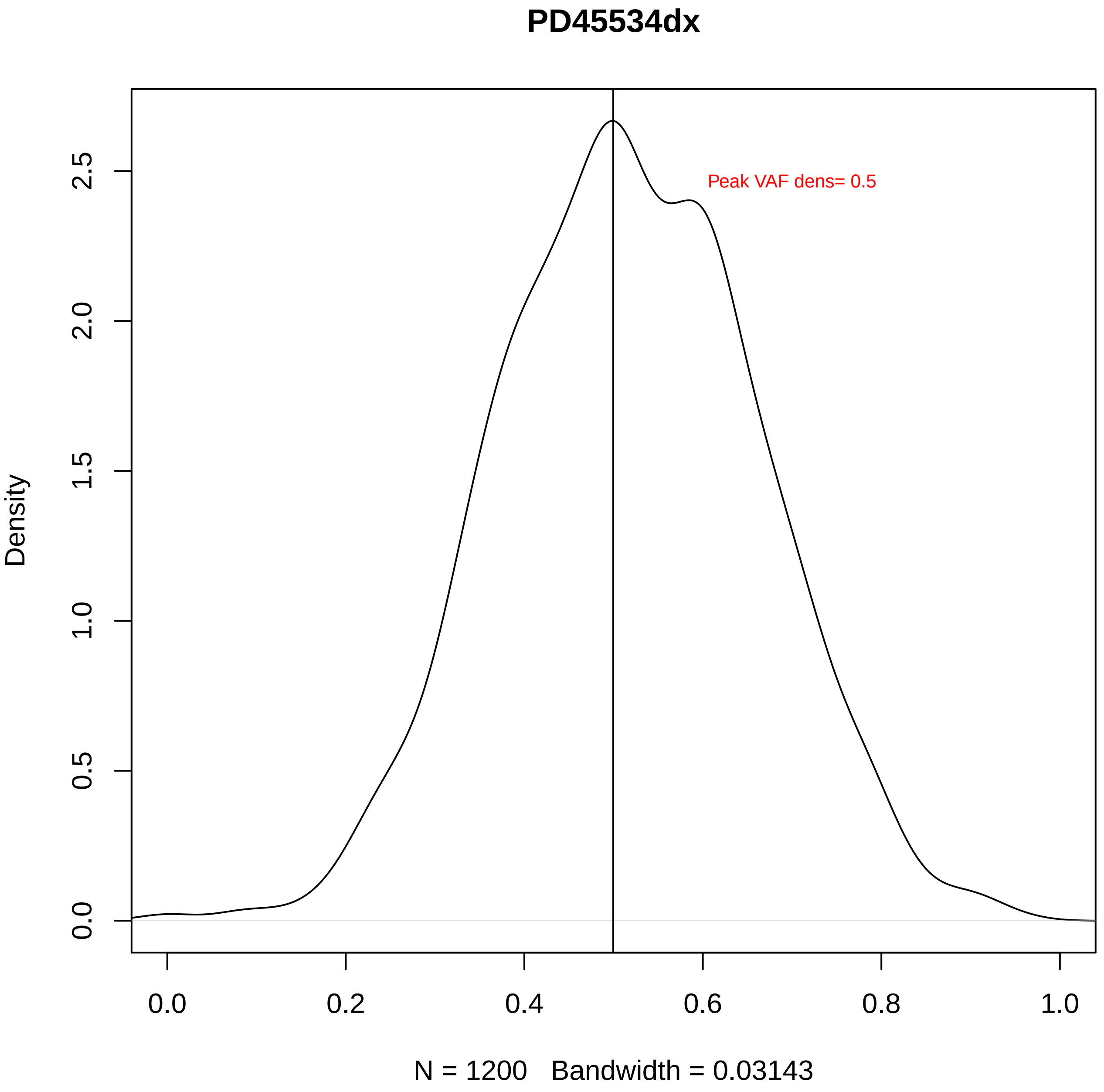
PD45534dx
Peak VAF dens= 0.5
Density
0.0
0.5
1.0
1.5
2.0
2.5
0.0
0.2
0.4
0.6
0.8
1.0
N = 1200   Bandwidth = 0.03143

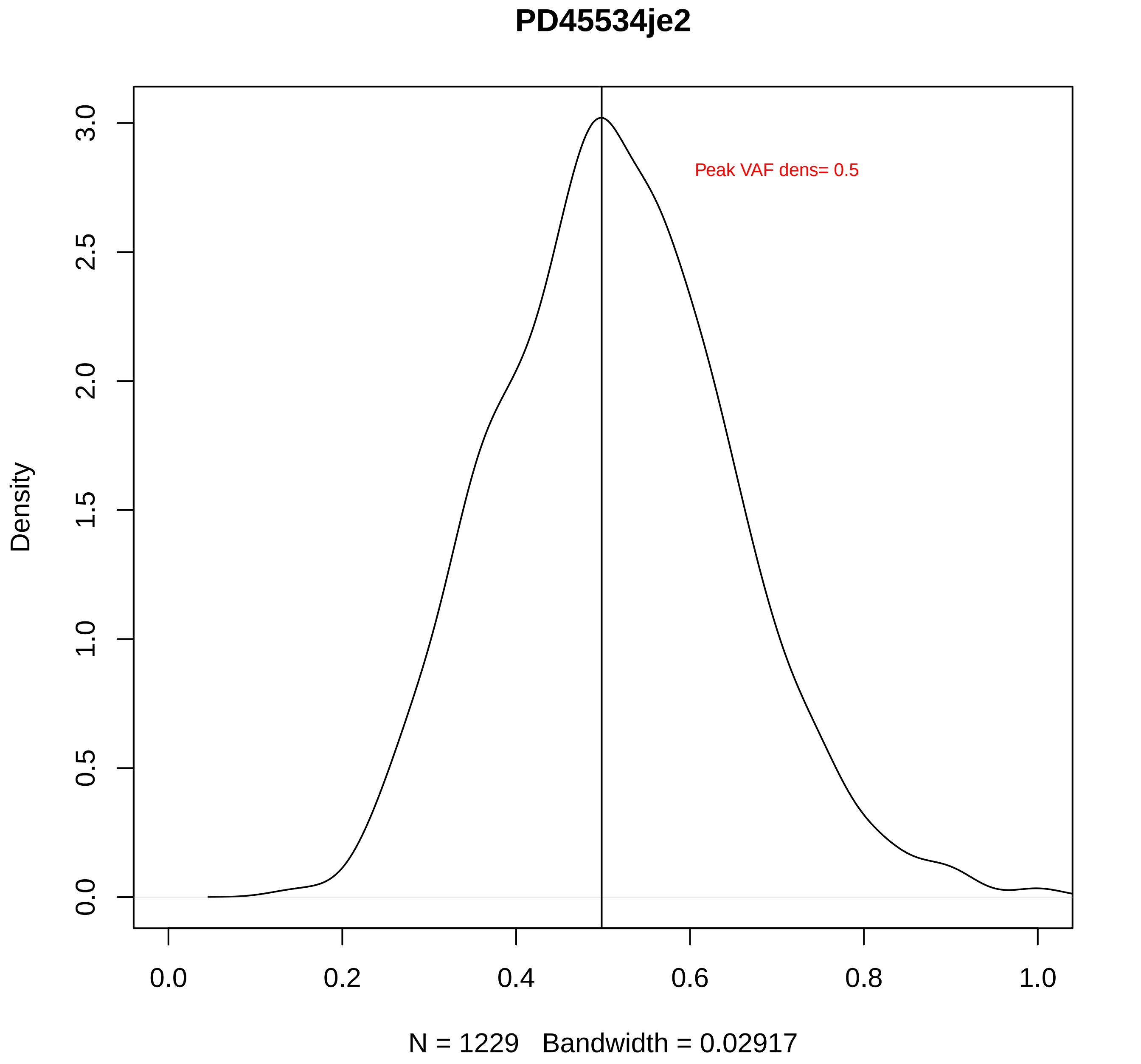

PD45534je2
Peak VAF dens= 0.5
Density
3.0
2.5
2.0
1.5
1.0
0.5
0.0
0.0
0.2
0.4
0.6
0.8
1.0
N = 1229   Bandwidth = 0.02917

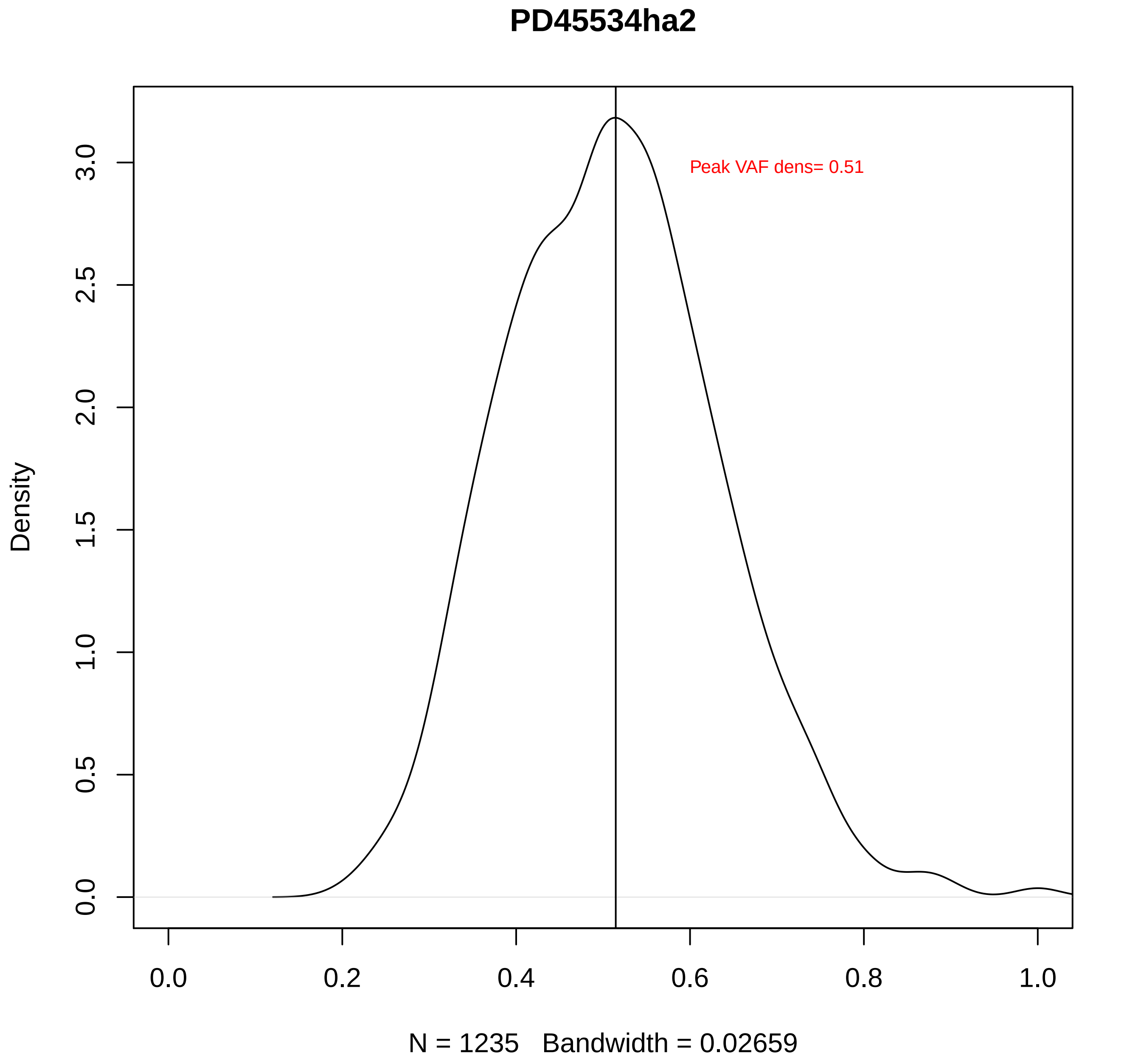

PD45534ha2
Peak VAF dens= 0.51
Density
0.0
0.5
1.0
1.5
2.0
2.5
3.0
0.0
0.2
0.4
0.6
0.8
1.0
N = 1235   Bandwidth = 0.02659

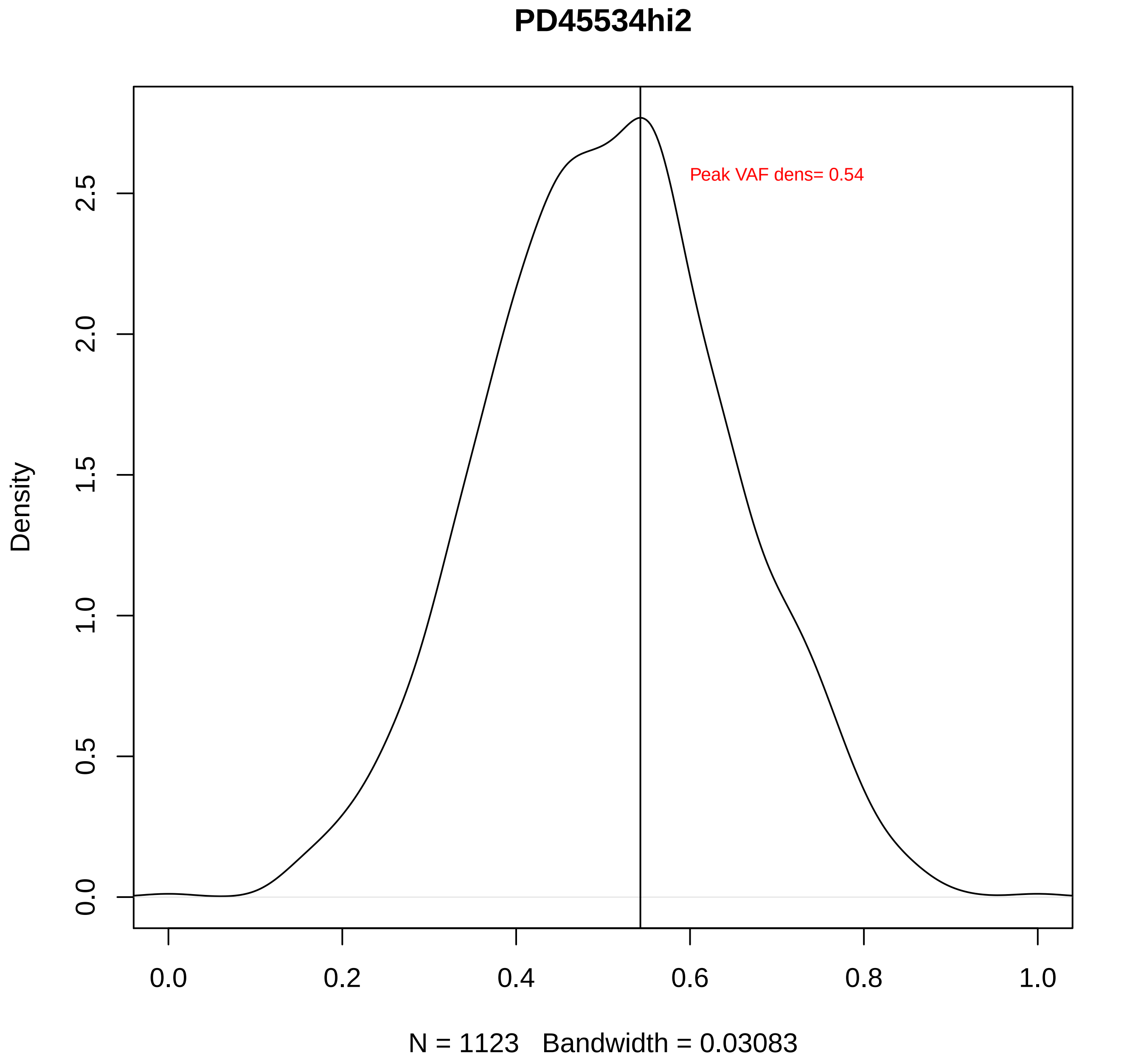

PD45534hi2
Peak VAF dens= 0.54
Density
0.0
0.5
1.0
1.5
2.0
2.5
0.0
0.2
0.4
0.6
0.8
1.0
N = 1123   Bandwidth = 0.03083

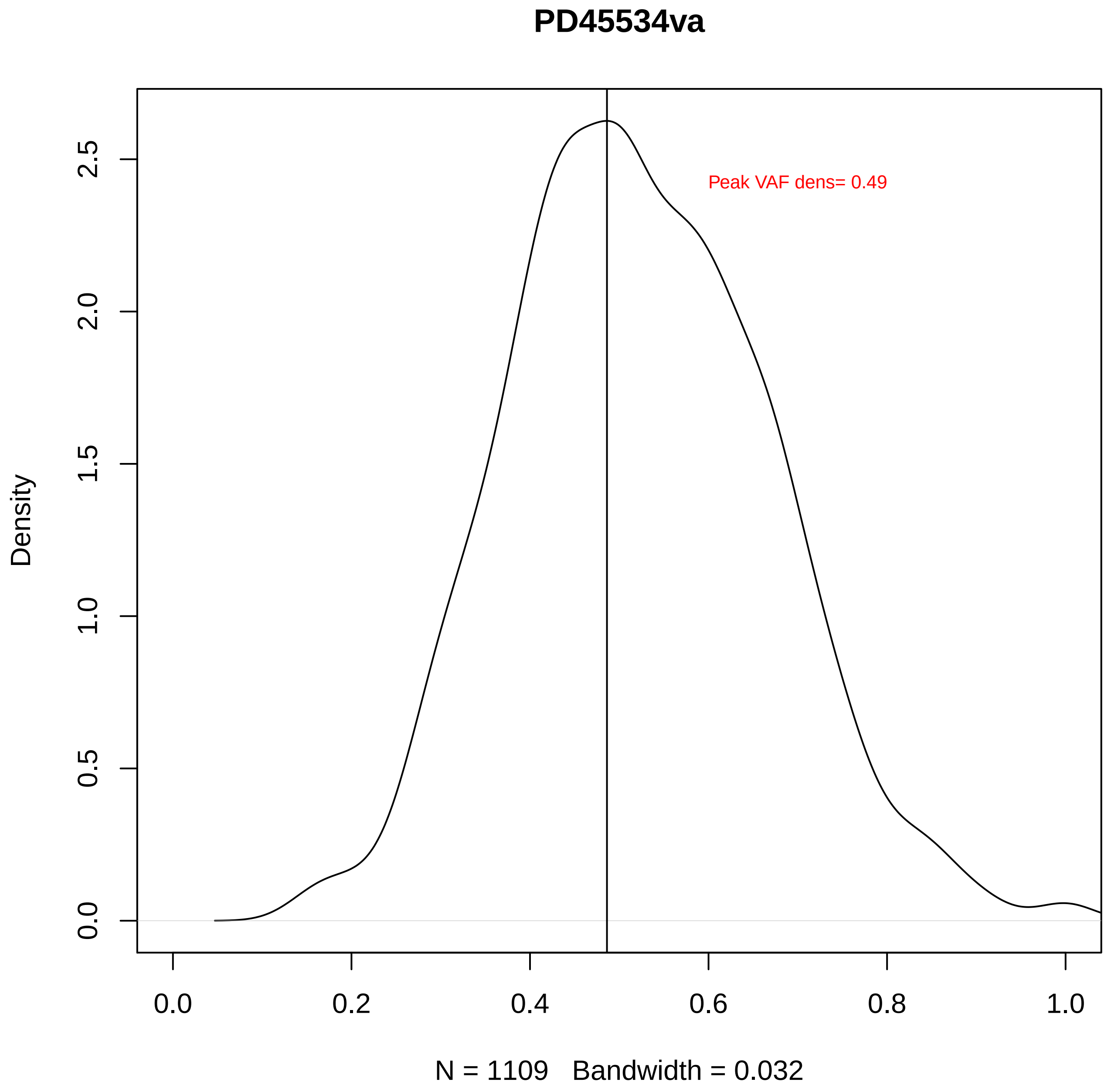

PD45534va
Peak VAF dens= 0.49
Density
0.0
0.5
1.0
1.5
2.0
2.5
0.0
0.2
0.4
0.6
0.8
1.0
N = 1109   Bandwidth = 0.032

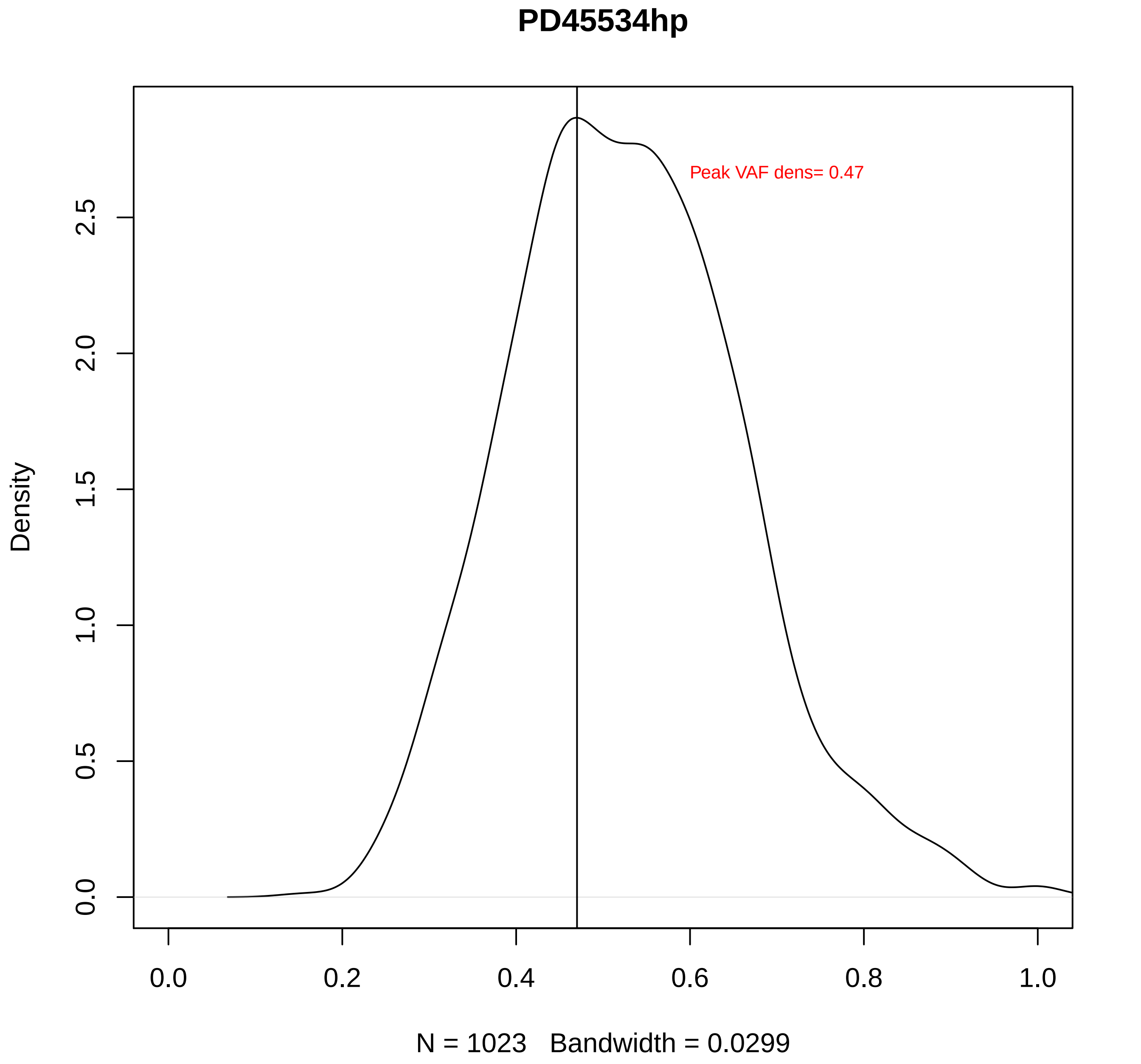
PD45534hp
Peak VAF dens= 0.47
Density
0.0
0.5
1.0
1.5
2.0
2.5
0.0
0.2
0.4
0.6
0.8
1.0
N = 1023   Bandwidth = 0.0299

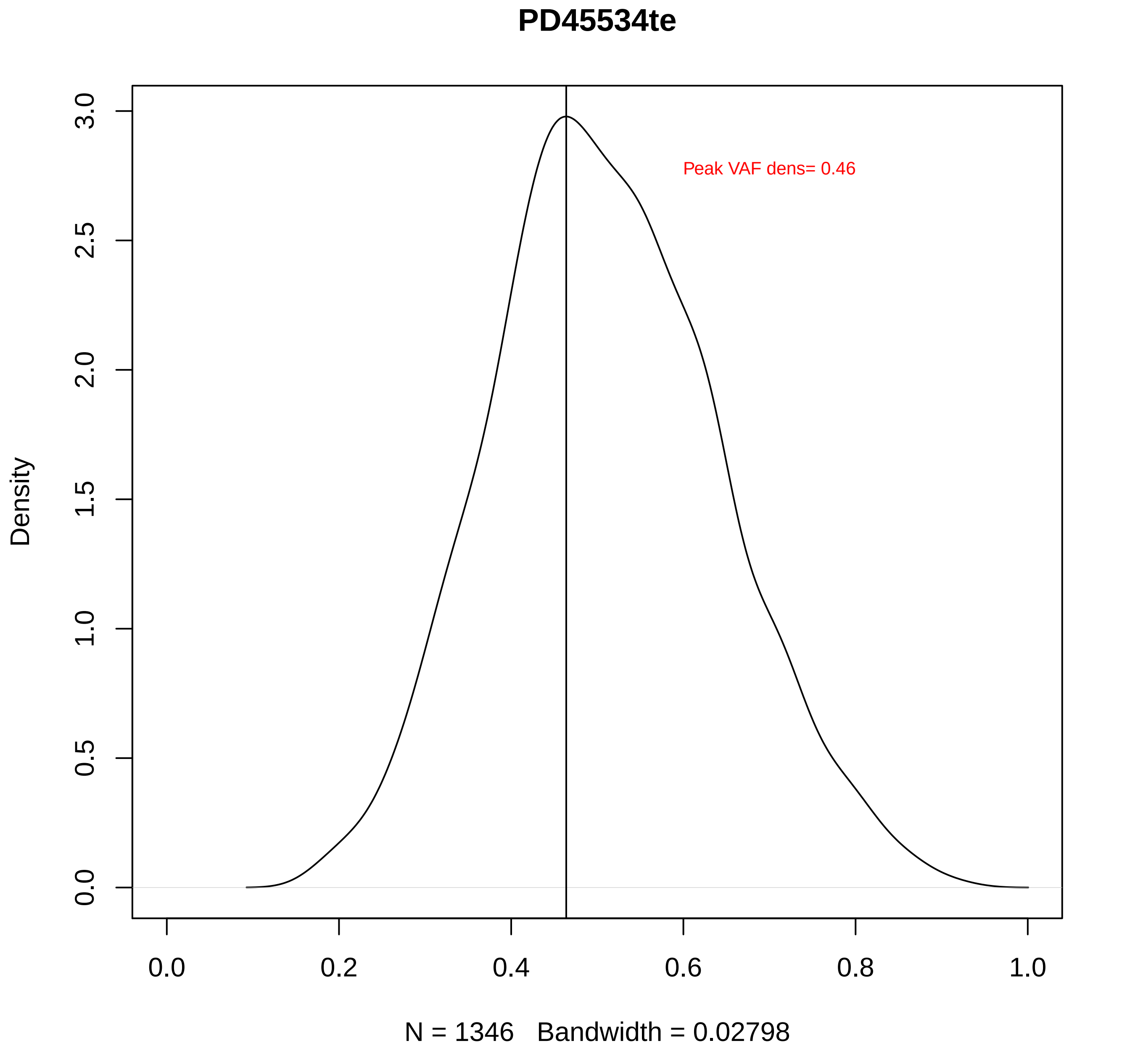

PD45534te
Peak VAF dens= 0.46
Density
3.0
2.5
2.0
1.5
1.0
0.5
0.0
0.0
0.2
0.4
0.6
0.8
1.0
N = 1346   Bandwidth = 0.02798

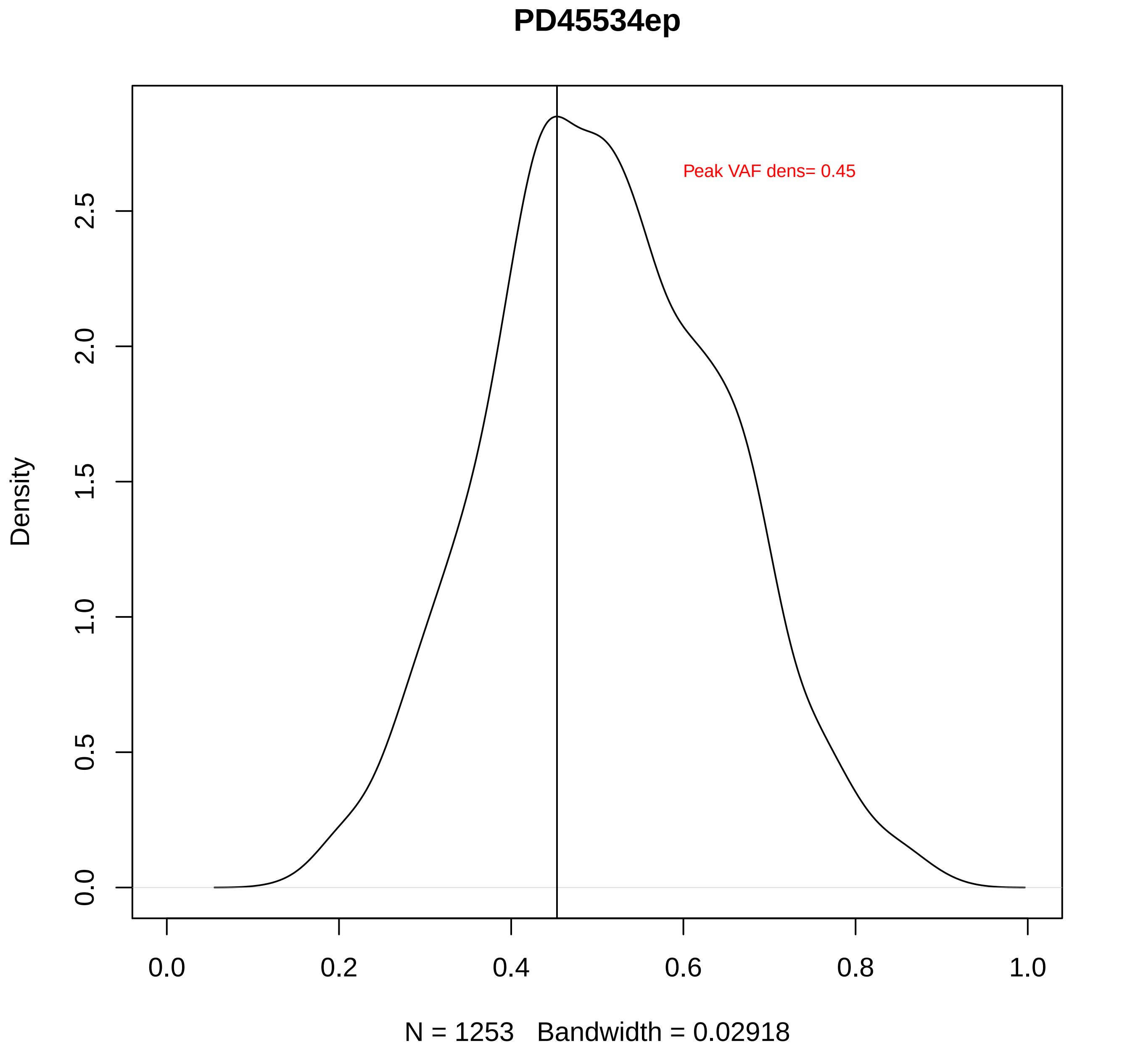

PD45534ep
Peak VAF dens= 0.45
Density
0.0
0.5
1.0
1.5
2.0
2.5
0.0
0.2
0.4
0.6
0.8
1.0
N = 1253   Bandwidth = 0.02918

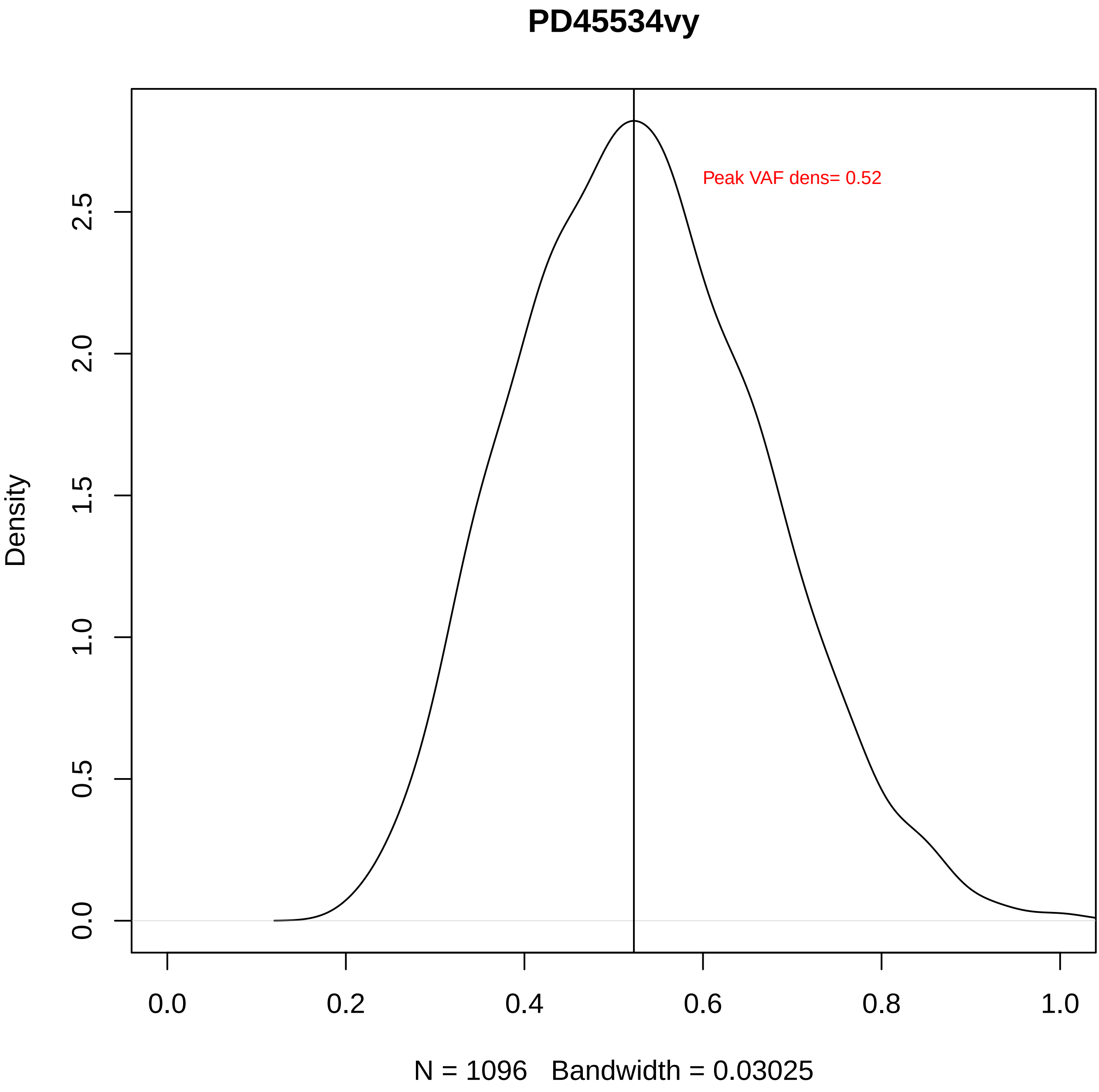
PD45534vy
Peak VAF dens= 0.52
Density
0.0
0.5
1.0
1.5
2.0
2.5
0.0
0.2
0.4
0.6
0.8
1.0
N = 1096   Bandwidth = 0.03025

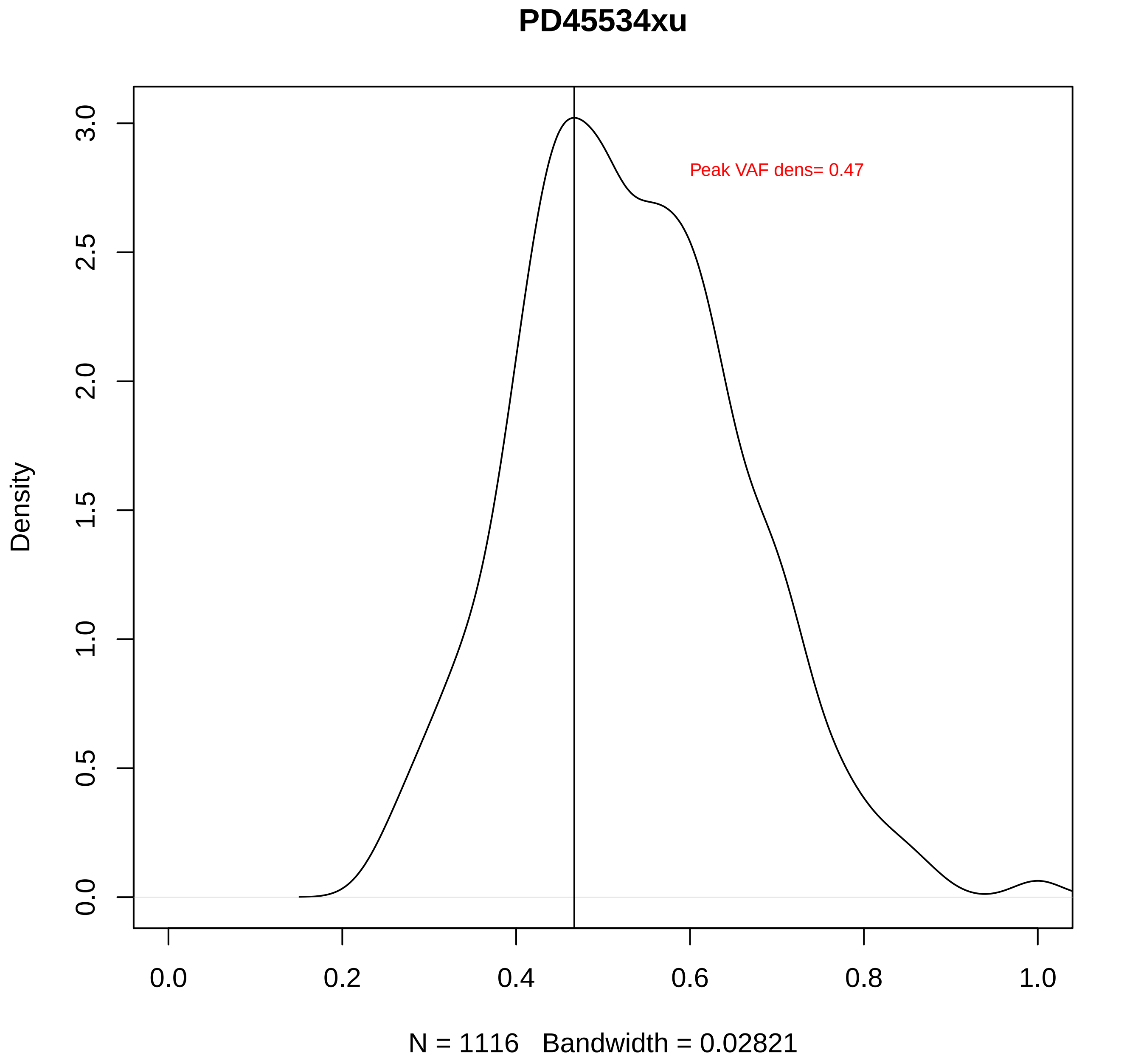
PD45534xu
Peak VAF dens= 0.47
Density
0.0
0.5
1.0
1.5
2.0
2.5
3.0
0.0
0.2
0.4
0.6
0.8
1.0
N = 1116   Bandwidth = 0.02821

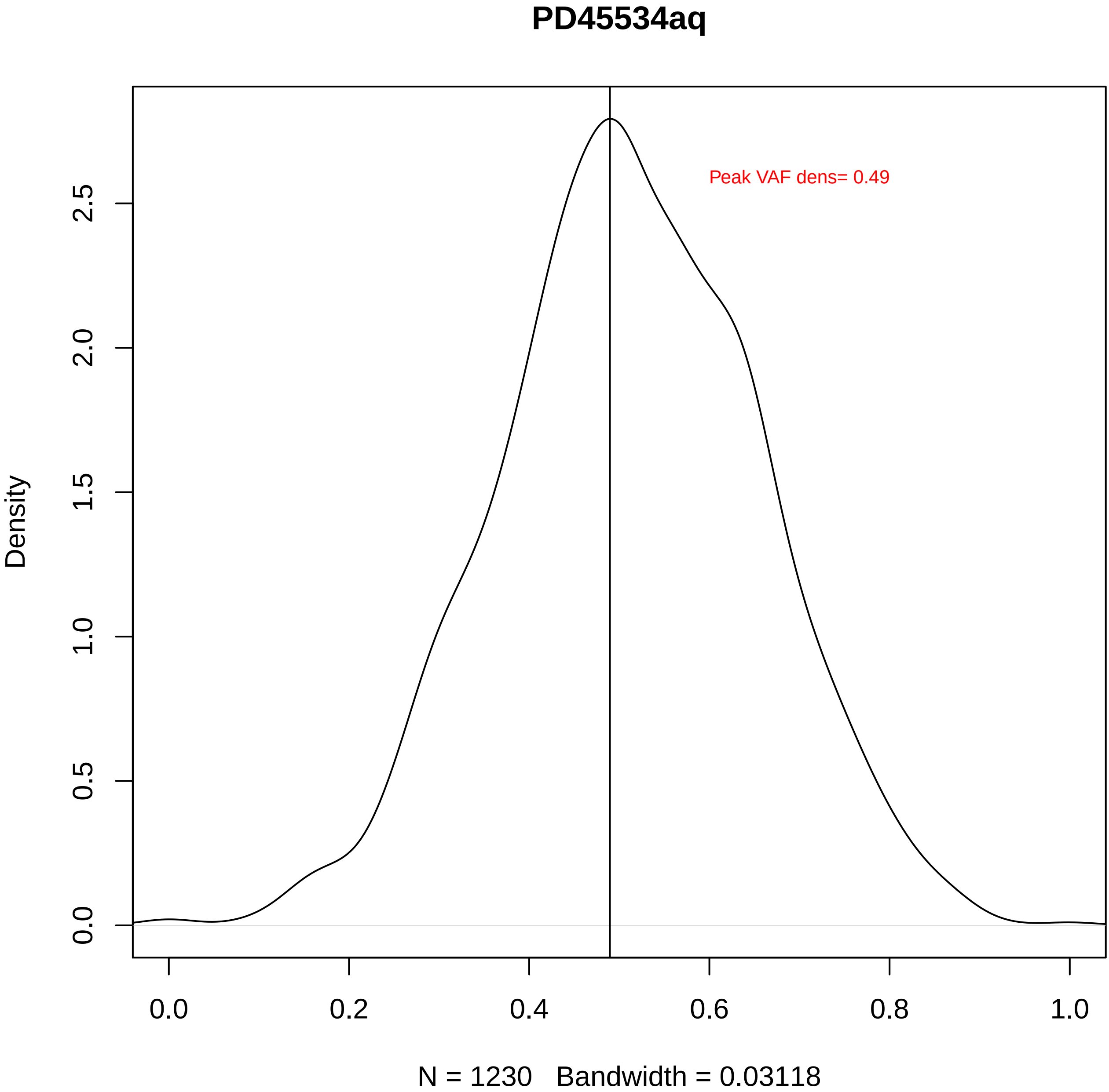
PD45534aq
Peak VAF dens= 0.49
Density
0.0
0.5
1.0
1.5
2.0
2.5
0.0
0.2
0.4
0.6
0.8
1.0
N = 1230 Bandwidth = 0.03118

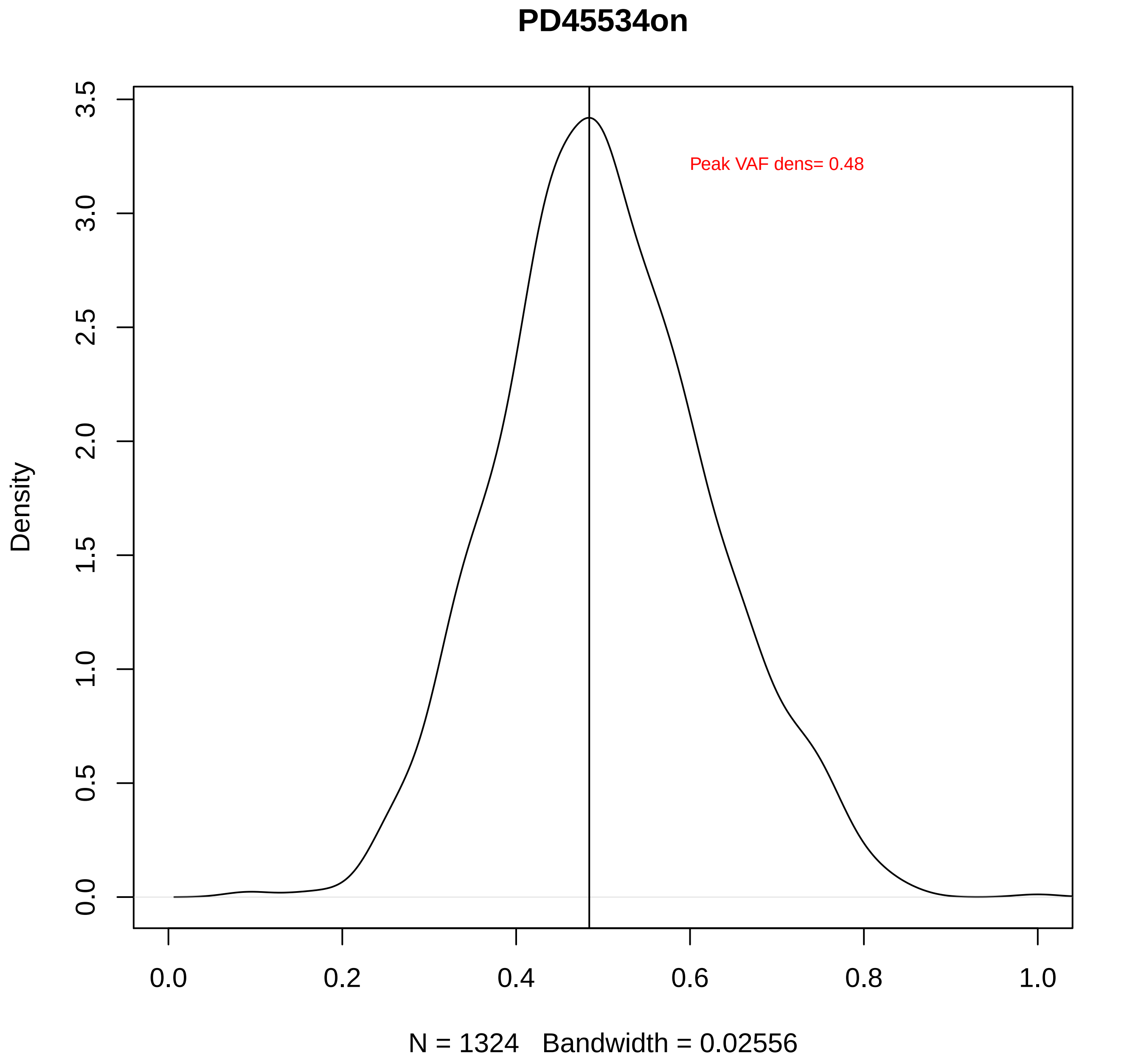
PD45534on
Peak VAF dens= 0.48
Density
0.0
0.5
1.0
1.5
2.0
2.5
3.0
3.5
0.0
0.2
0.4
0.6
0.8
1.0
N = 1324 Bandwidth = 0.02556

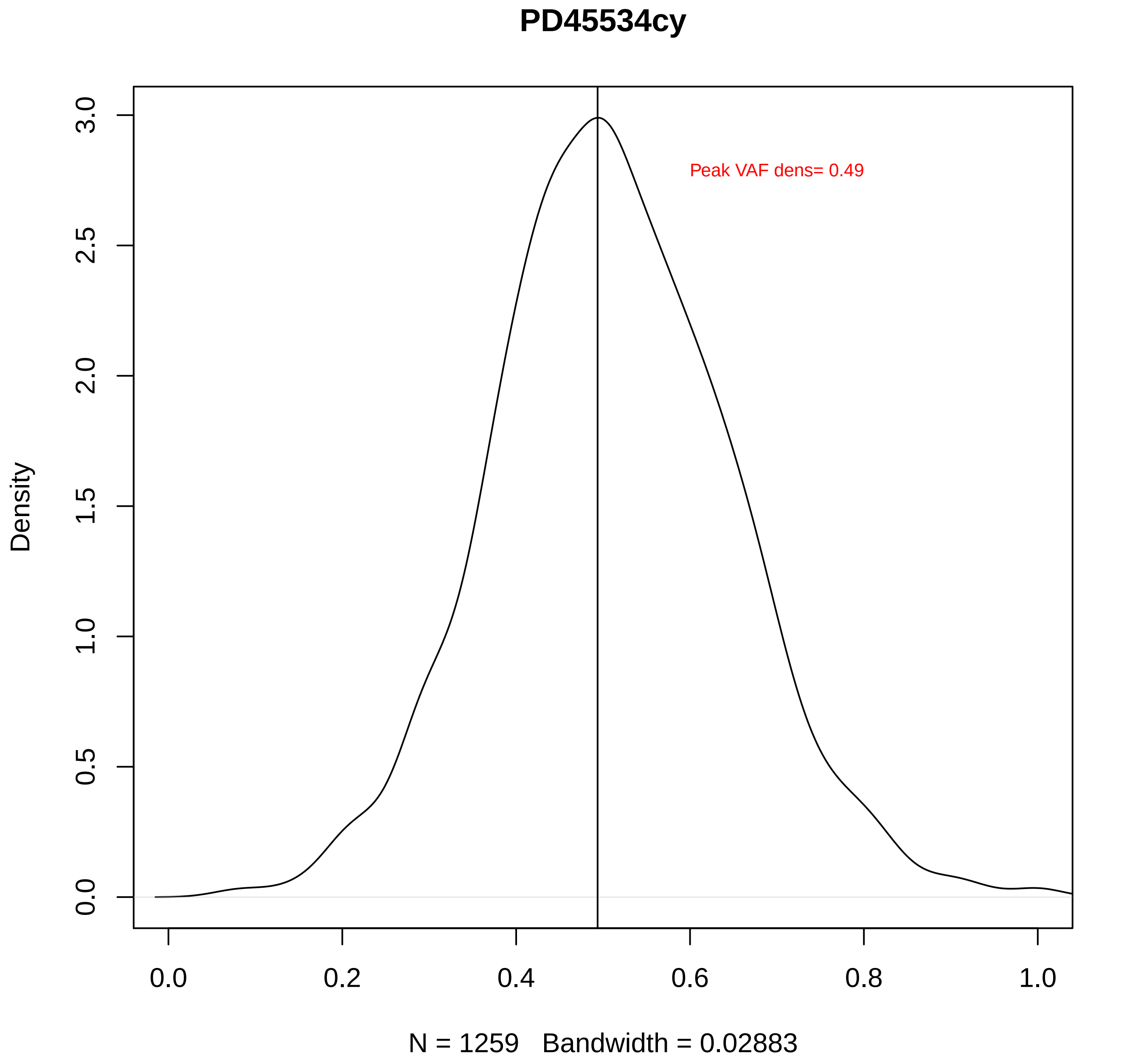
PD45534cy
Peak VAF dens= 0.49
Density
0.0
0.5
1.0
1.5
2.0
2.5
3.0
0.0
0.2
0.4
0.6
0.8
1.0
N = 1259   Bandwidth = 0.02883

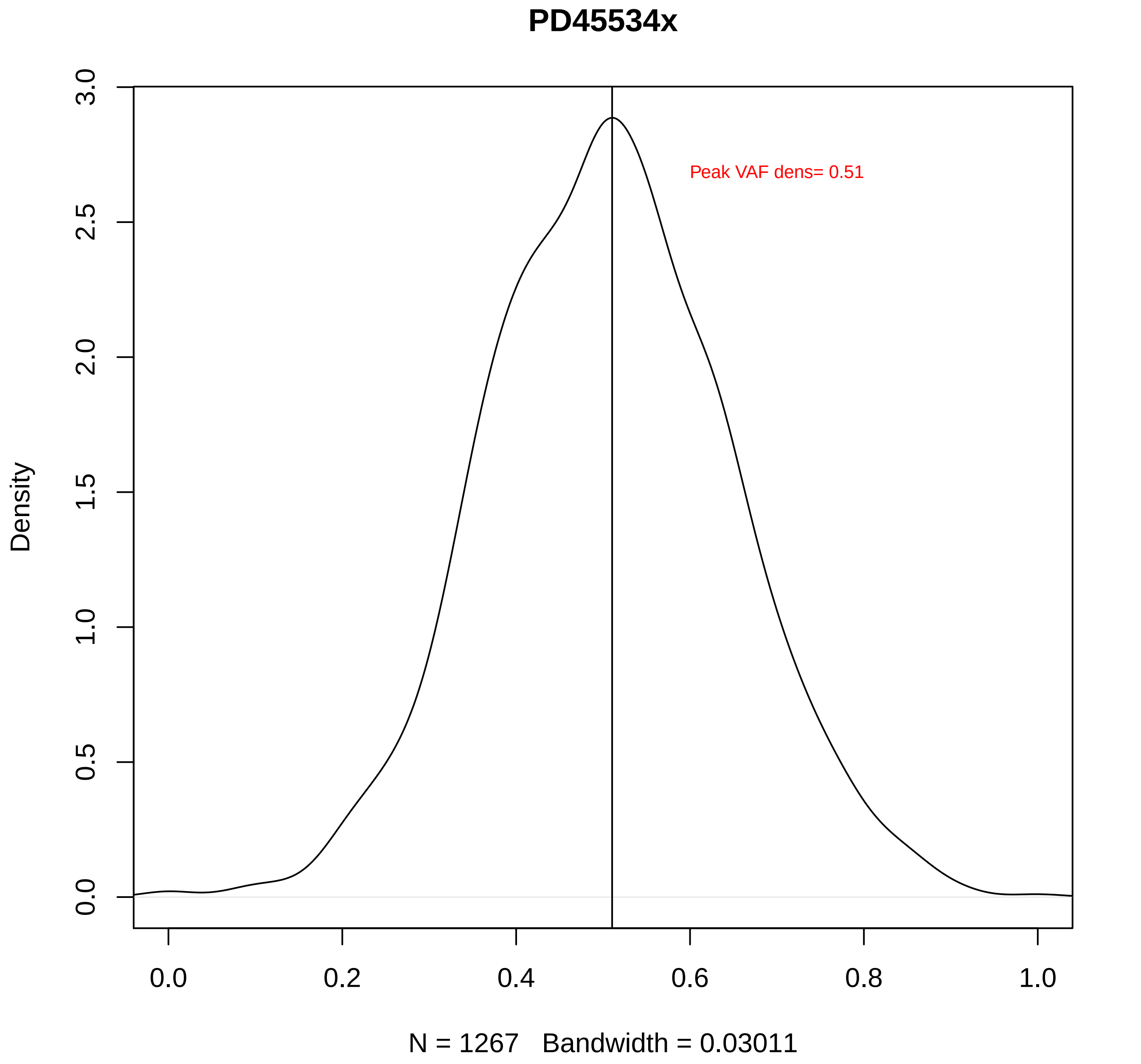
PD45534x
Peak VAF dens= 0.51
Density
0.0
0.5
1.0
1.5
2.0
2.5
3.0
0.0
0.2
0.4
0.6
0.8
1.0
N = 1267   Bandwidth = 0.03011

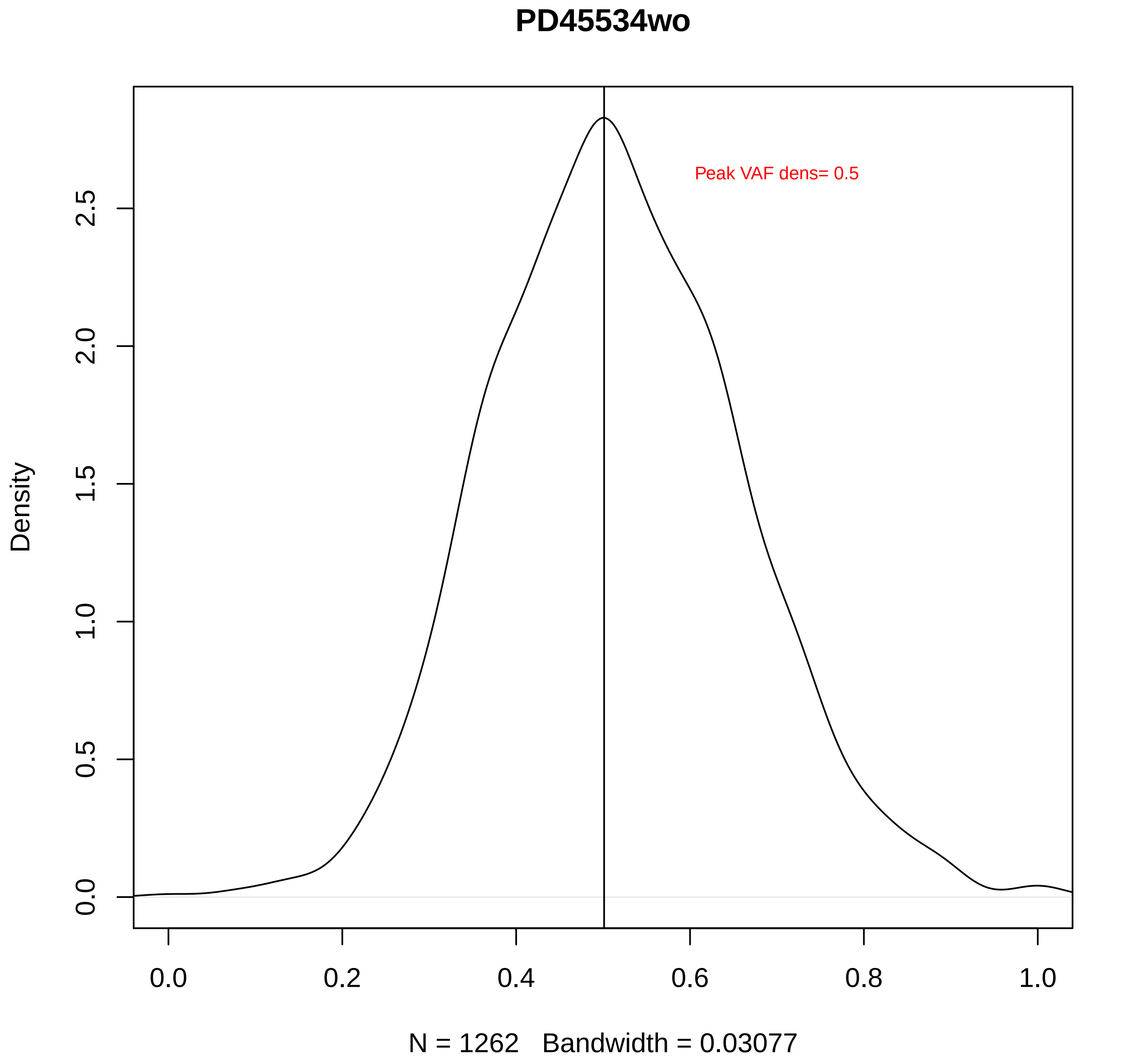
PD45534wo
Peak VAF dens= 0.5
Density
0.0
0.5
1.0
1.5
2.0
2.5
0.0
0.2
0.4
0.6
0.8
1.0
N = 1262   Bandwidth = 0.03077

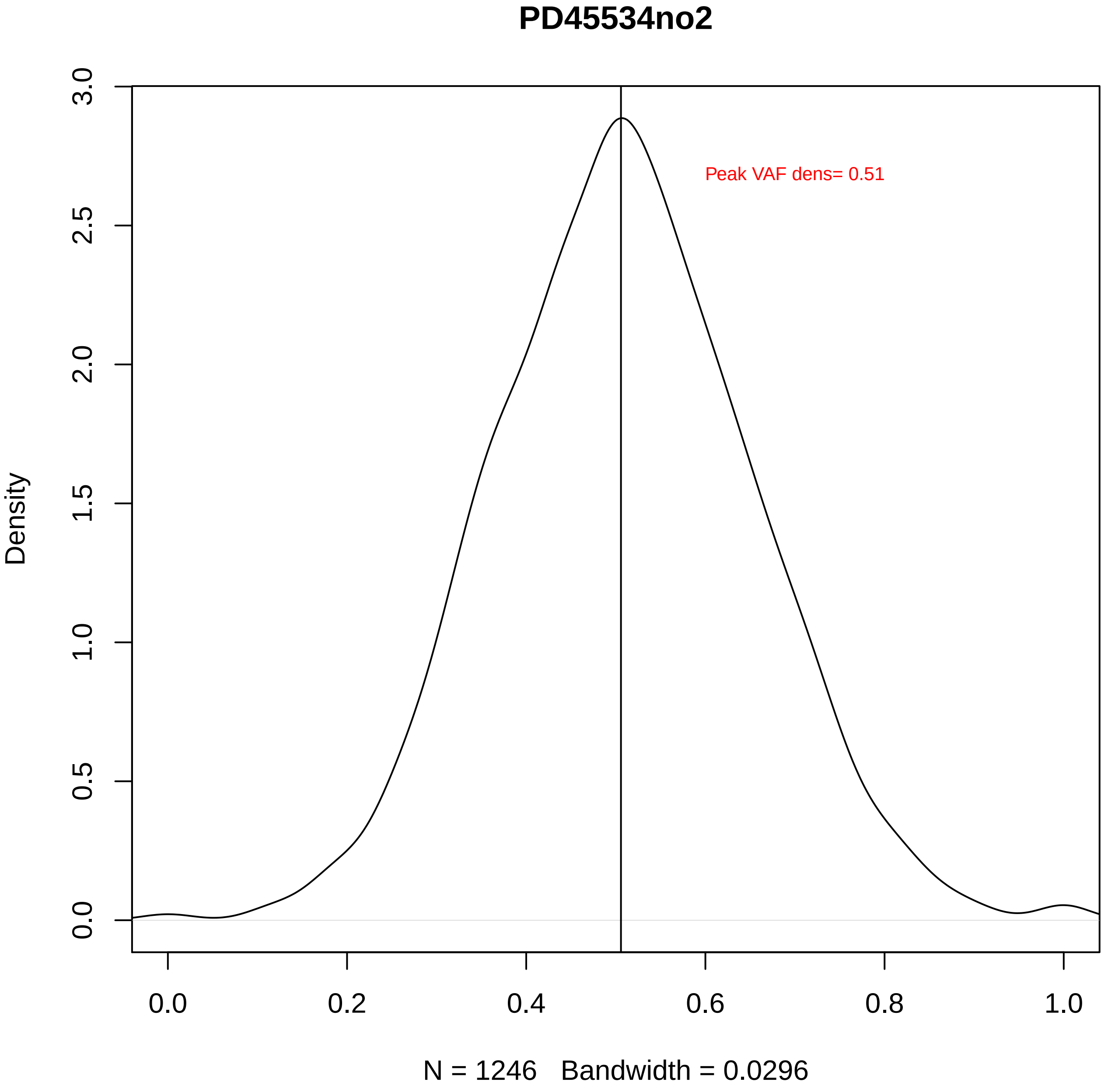
PD45534no2
Density
3.0
2.5
2.0
1.5
1.0
0.5
0.0
Peak VAF dens= 0.51
0.0
0.2
0.4
0.6
0.8
1.0
N = 1246   Bandwidth = 0.0296

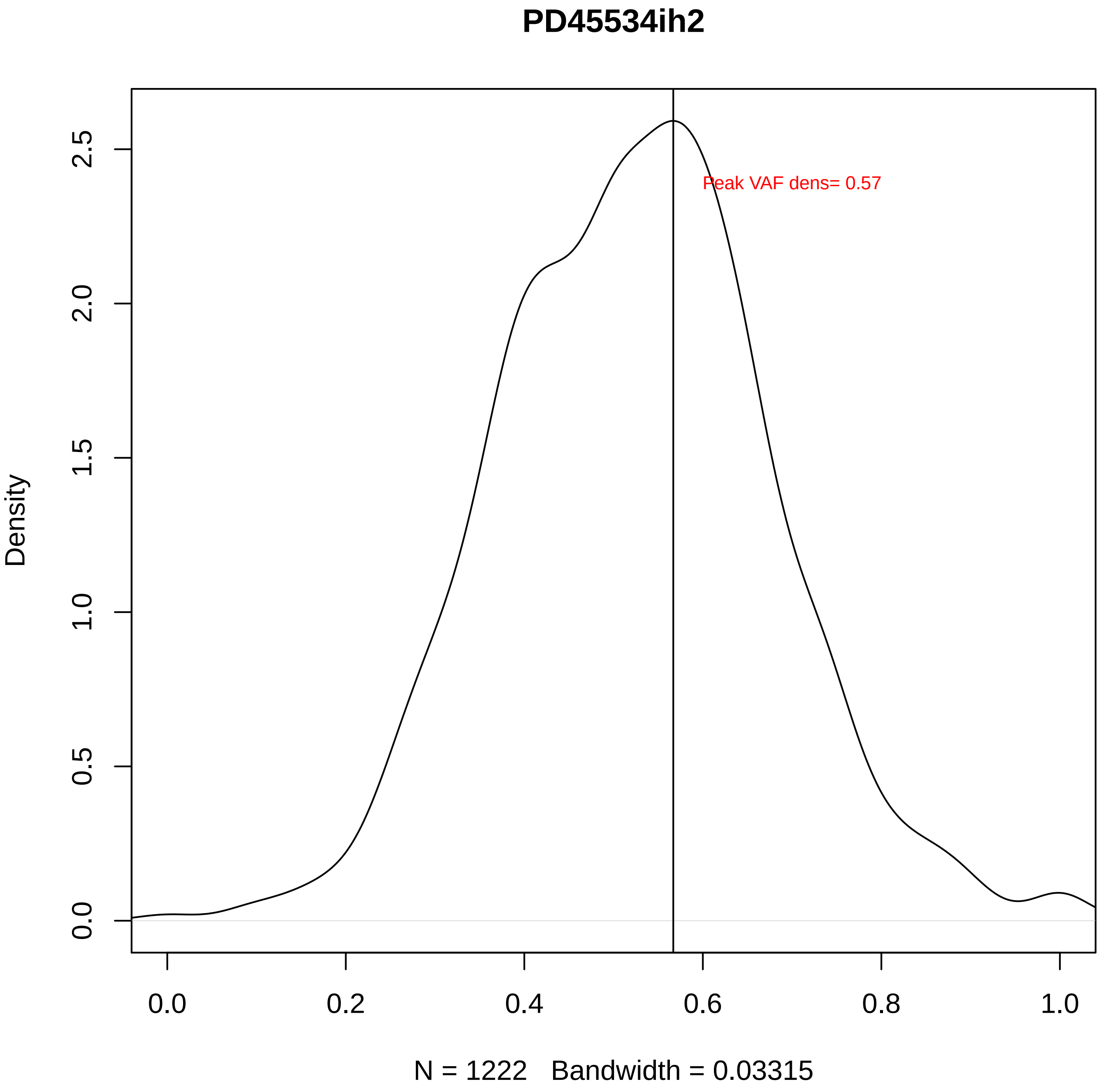
PD45534ih2
Peak VAF dens= 0.57
Density
0.0
0.5
1.0
1.5
2.0
2.5
0.0
0.2
0.4
0.6
0.8
1.0
N = 1222   Bandwidth = 0.03315

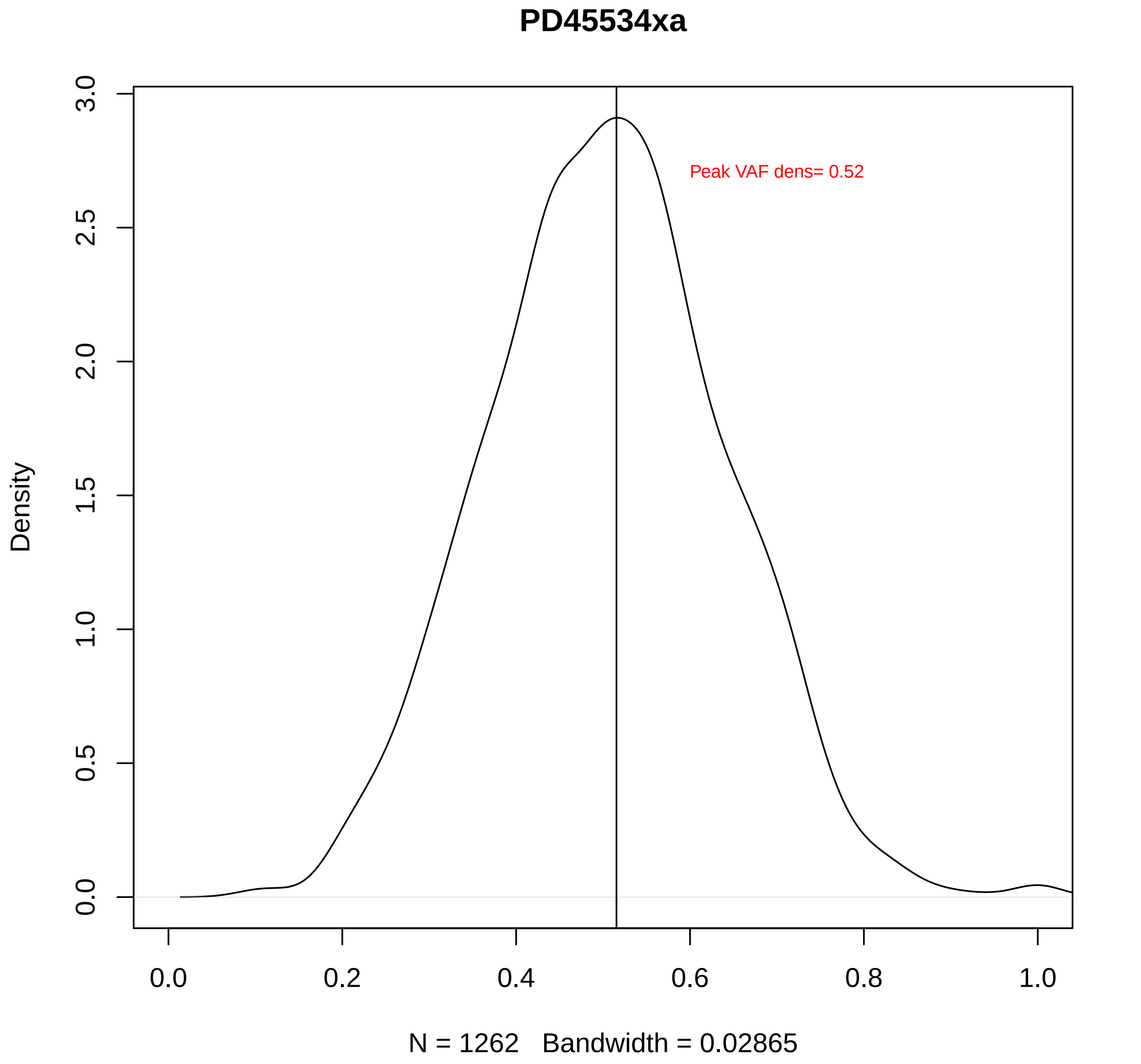
PD45534xa
Peak VAF dens= 0.52
Density
3.0
2.5
2.0
1.5
1.0
0.5
0.0
0.0
0.2
0.4
0.6
0.8
1.0
N = 1262 Bandwidth = 0.02865

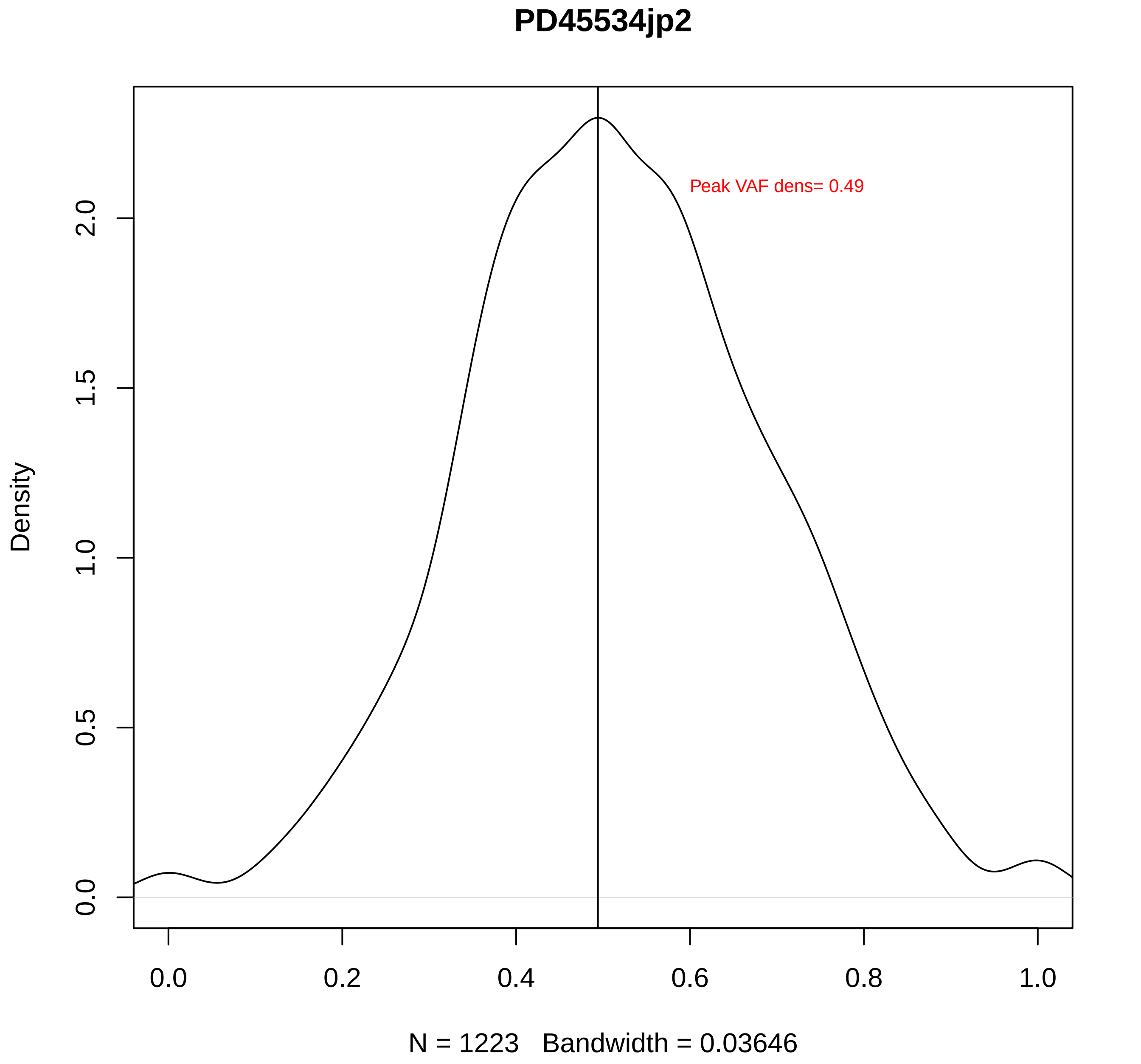
PD45534jp2
Peak VAF dens= 0.49
Density
0.0
0.5
1.0
1.5
2.0
0.0
0.2
0.4
0.6
0.8
1.0
N = 1223 Bandwidth = 0.03646

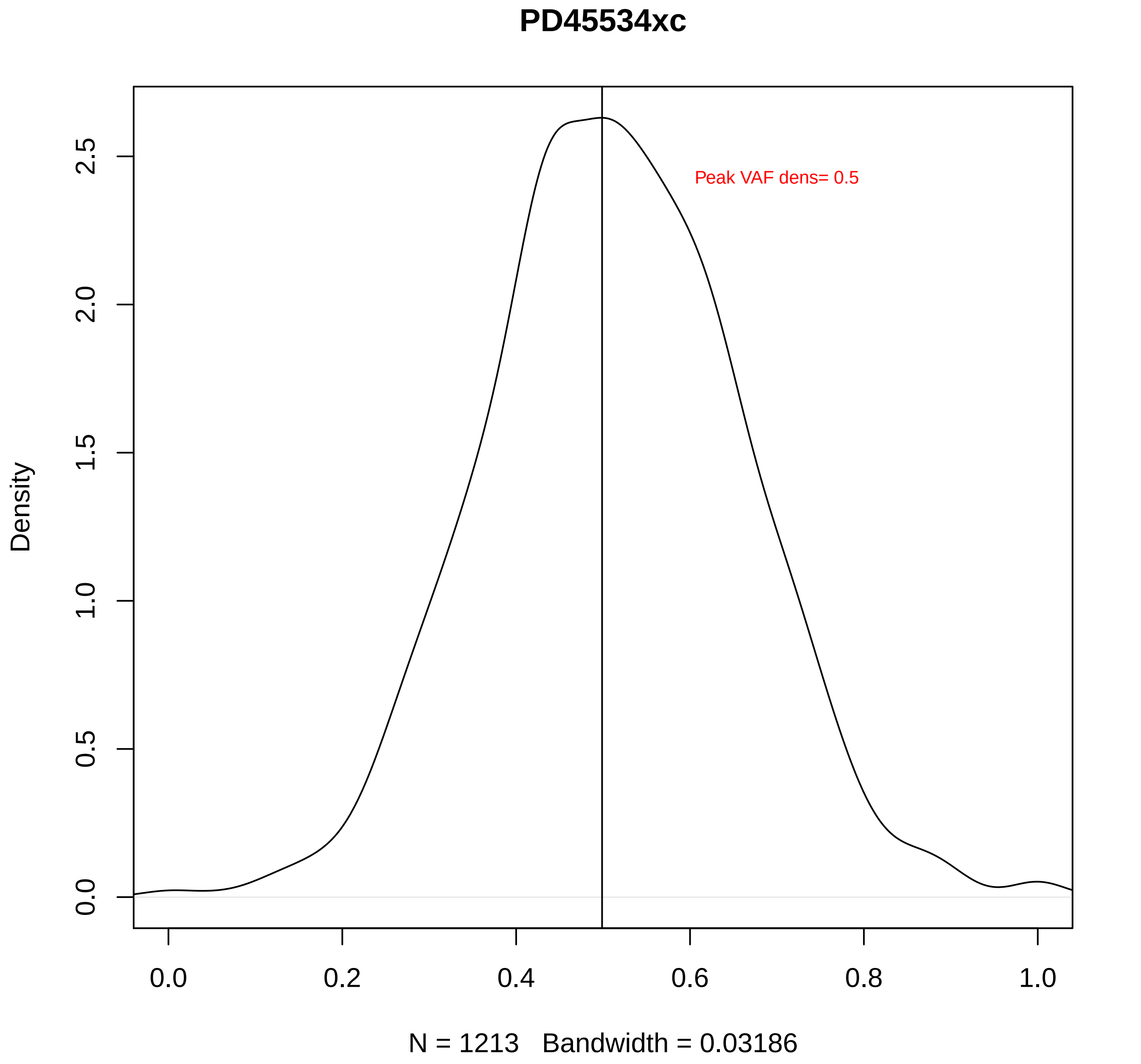

PD45534xc
Peak VAF dens= 0.5
Density
0.0
0.5
1.0
1.5
2.0
2.5
0.0
0.2
0.4
0.6
0.8
1.0
N = 1213 Bandwidth = 0.03186

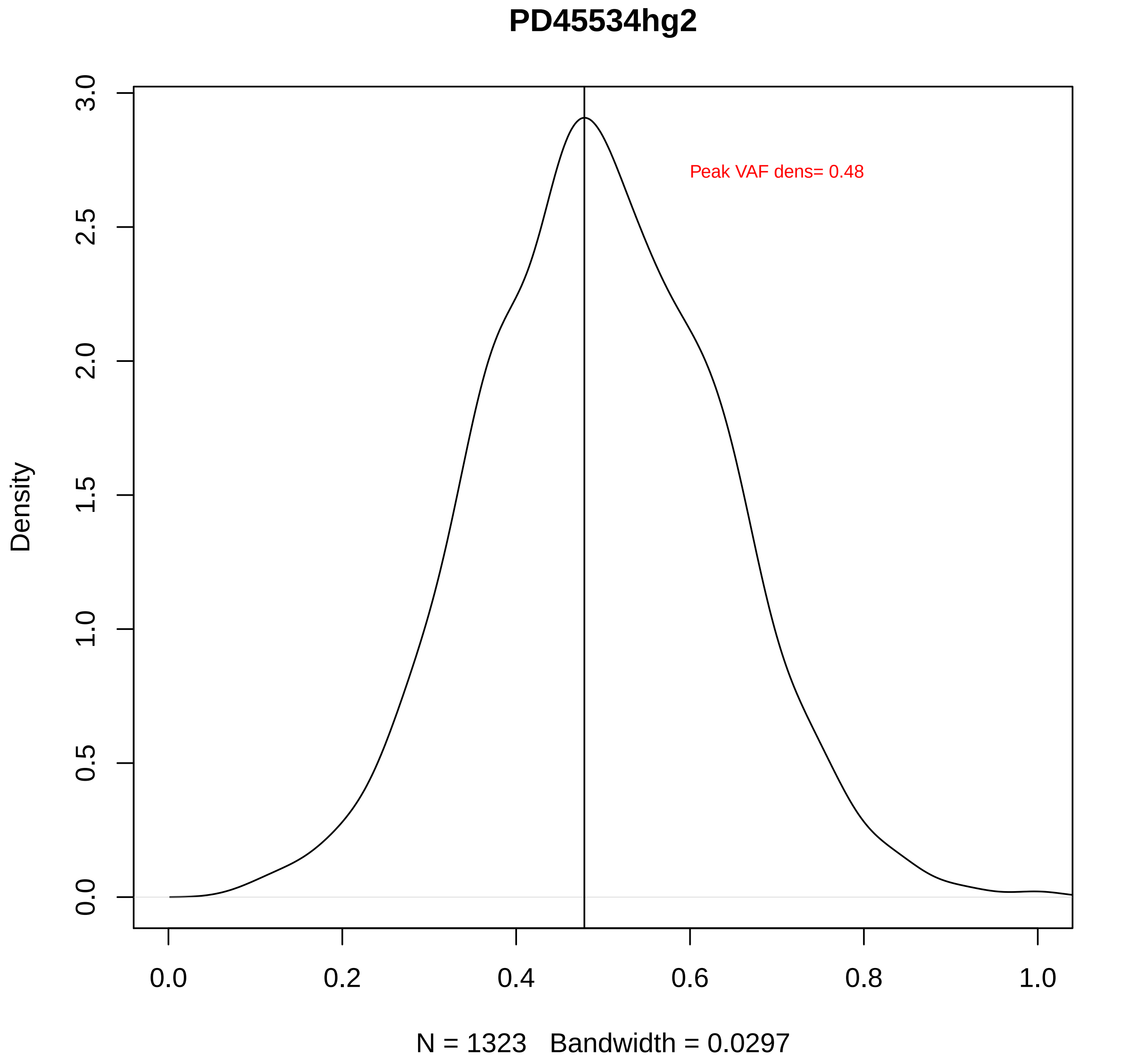

PD45534hg2
Peak VAF dens= 0.48
Density
0.0
0.5
1.0
1.5
2.0
2.5
3.0
0.0
0.2
0.4
0.6
0.8
1.0
N = 1323 Bandwidth = 0.0297

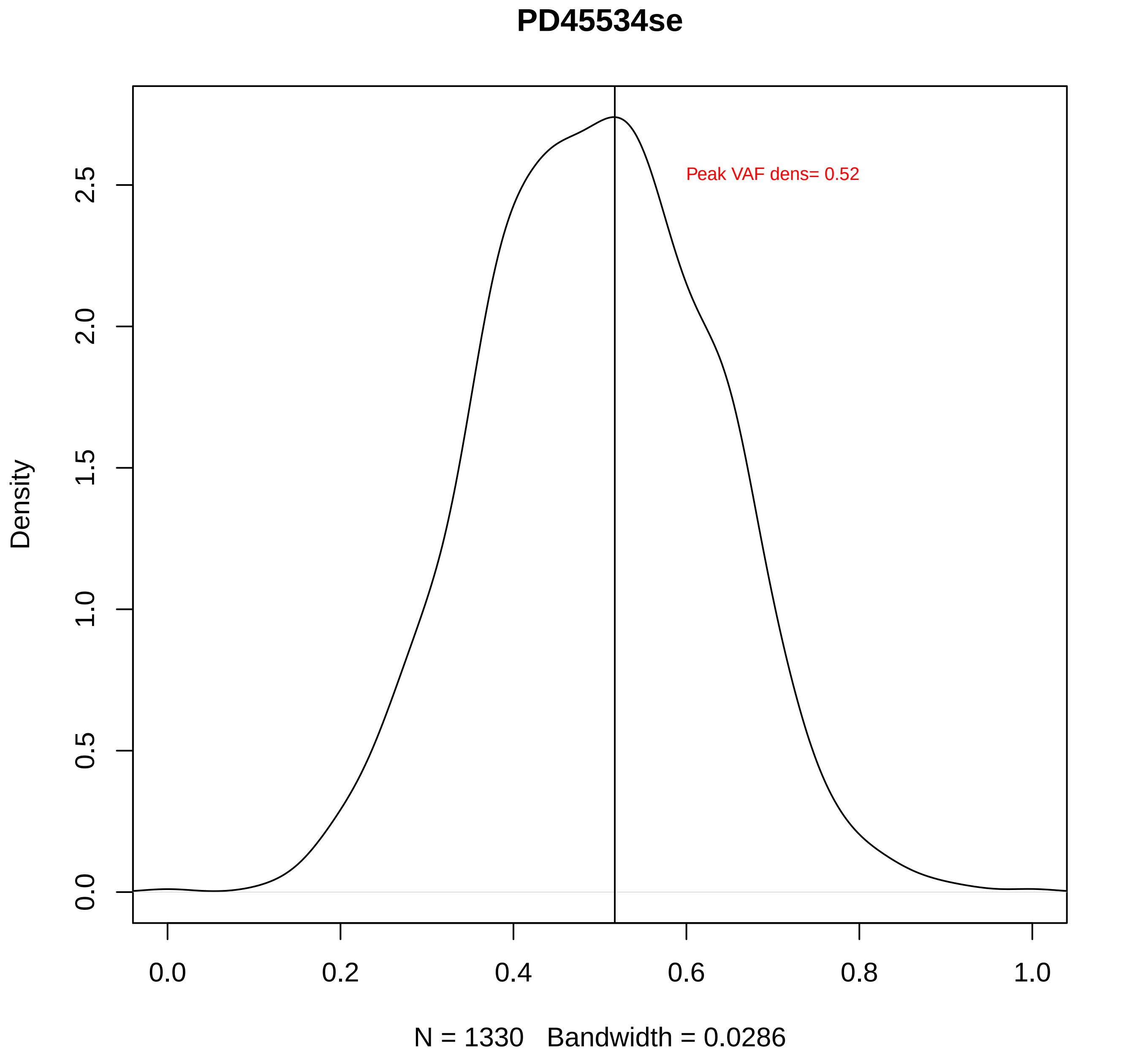
PD45534se
Peak VAF dens= 0.52
Density
0.0
0.5
1.0
1.5
2.0
2.5
0.0
0.2
0.4
0.6
0.8
1.0
N = 1330   Bandwidth = 0.0286

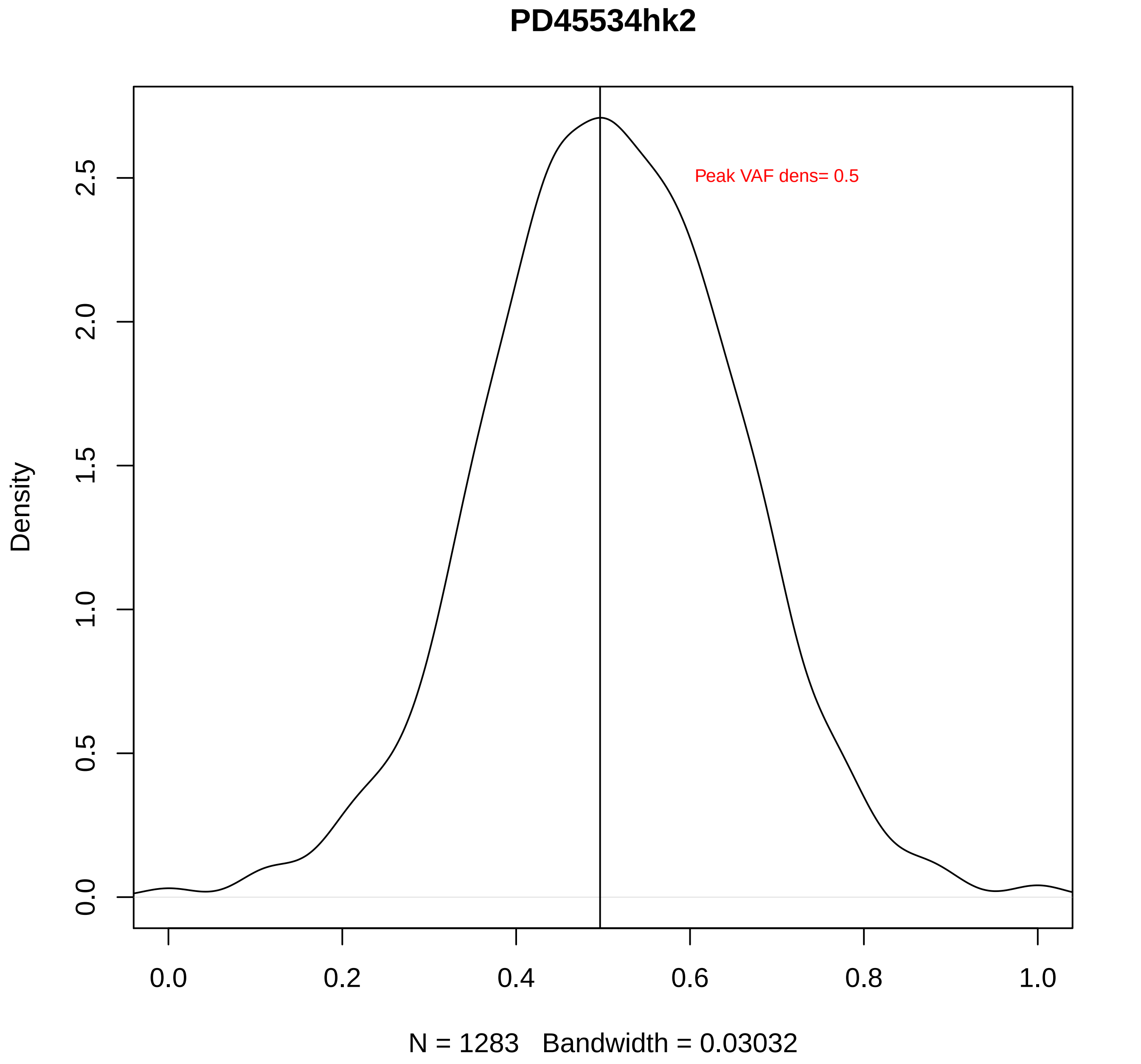

PD45534hk2
Peak VAF dens= 0.5
Density
0.0
0.5
1.0
1.5
2.0
2.5
0.0
0.2
0.4
0.6
0.8
1.0
N = 1283   Bandwidth = 0.03032

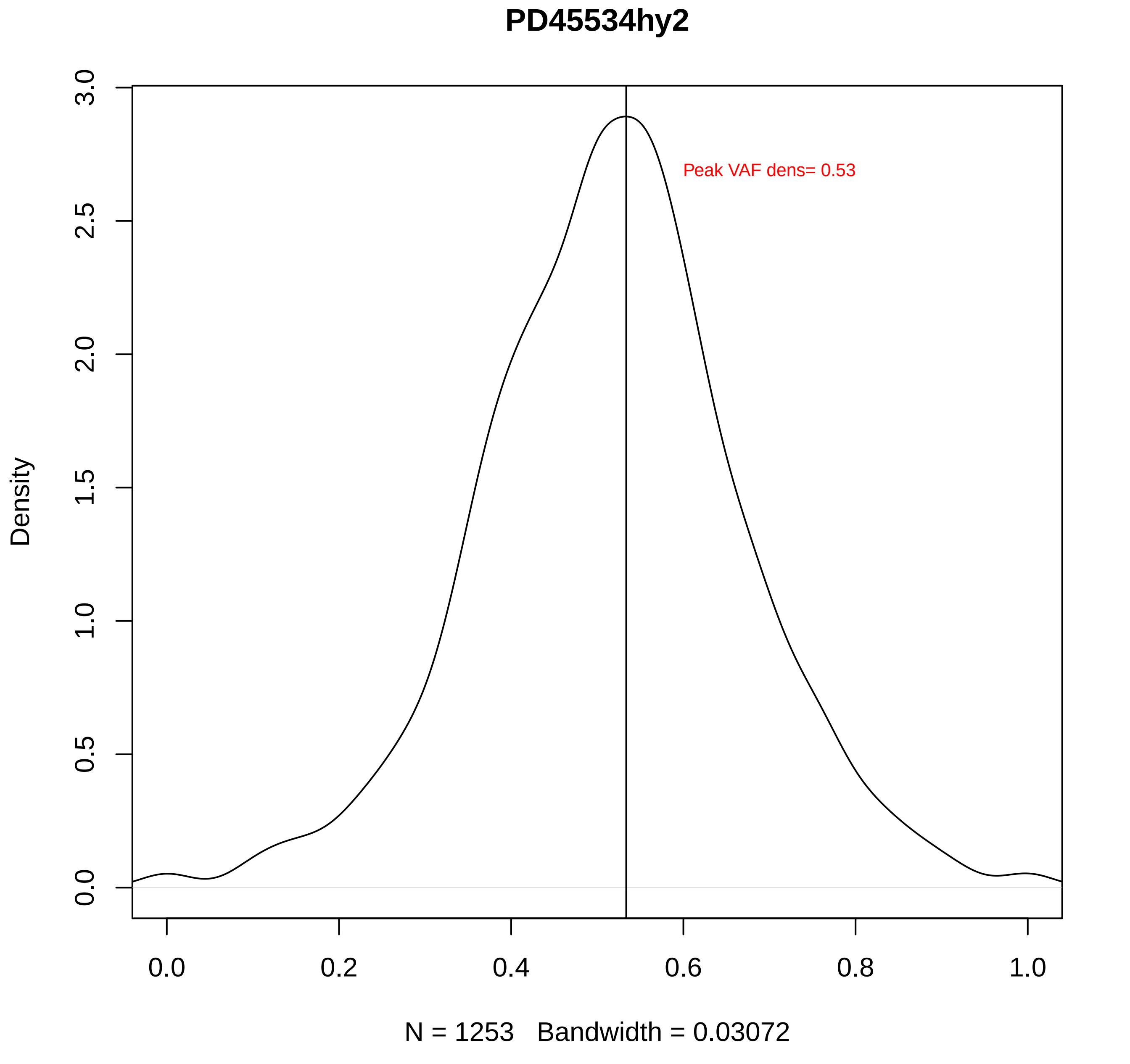
PD45534hy2
Peak VAF dens= 0.53
Density
0.0
0.5
1.0
1.5
2.0
2.5
3.0
0.0
0.2
0.4
0.6
0.8
1.0
N = 1253 Bandwidth = 0.03072

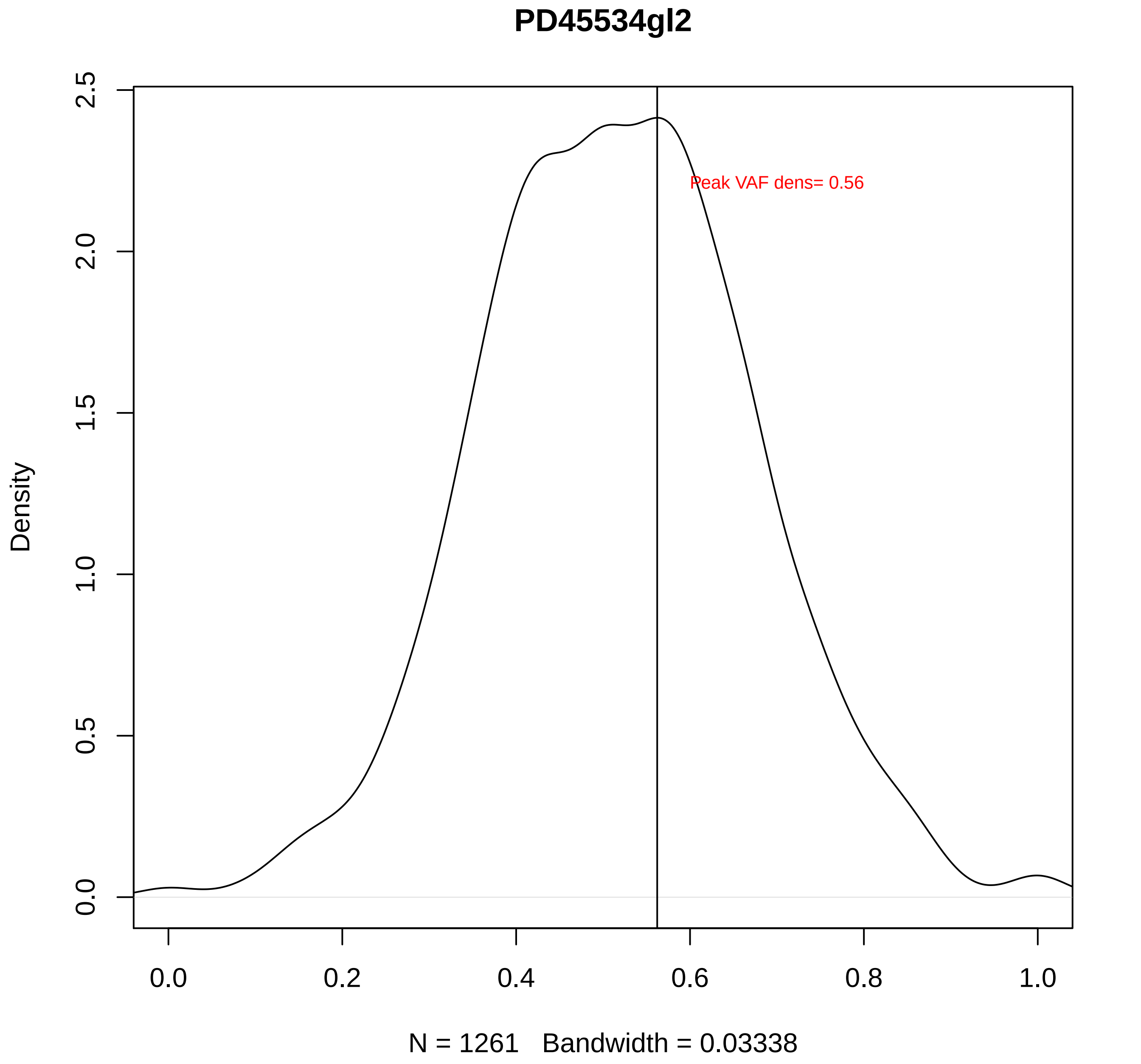
PD45534gl2
Peak VAF dens= 0.56
Density
0.0
0.5
1.0
1.5
2.0
2.5
0.0
0.2
0.4
0.6
0.8
1.0
N = 1261   Bandwidth = 0.03338

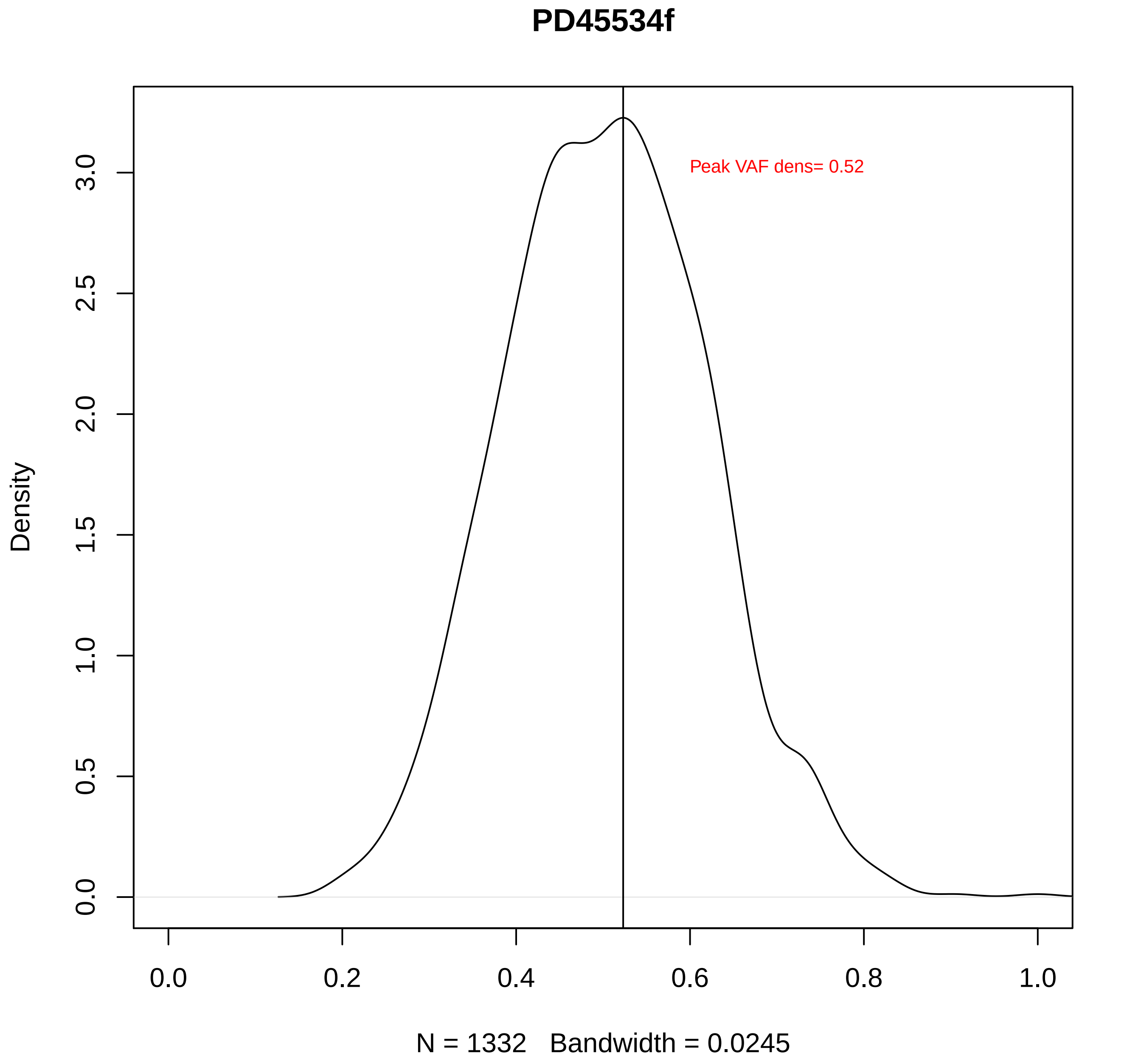

PD45534f
Peak VAF dens= 0.52
Density
0.0
0.5
1.0
1.5
2.0
2.5
3.0
0.0
0.2
0.4
0.6
0.8
1.0
N = 1332   Bandwidth = 0.0245

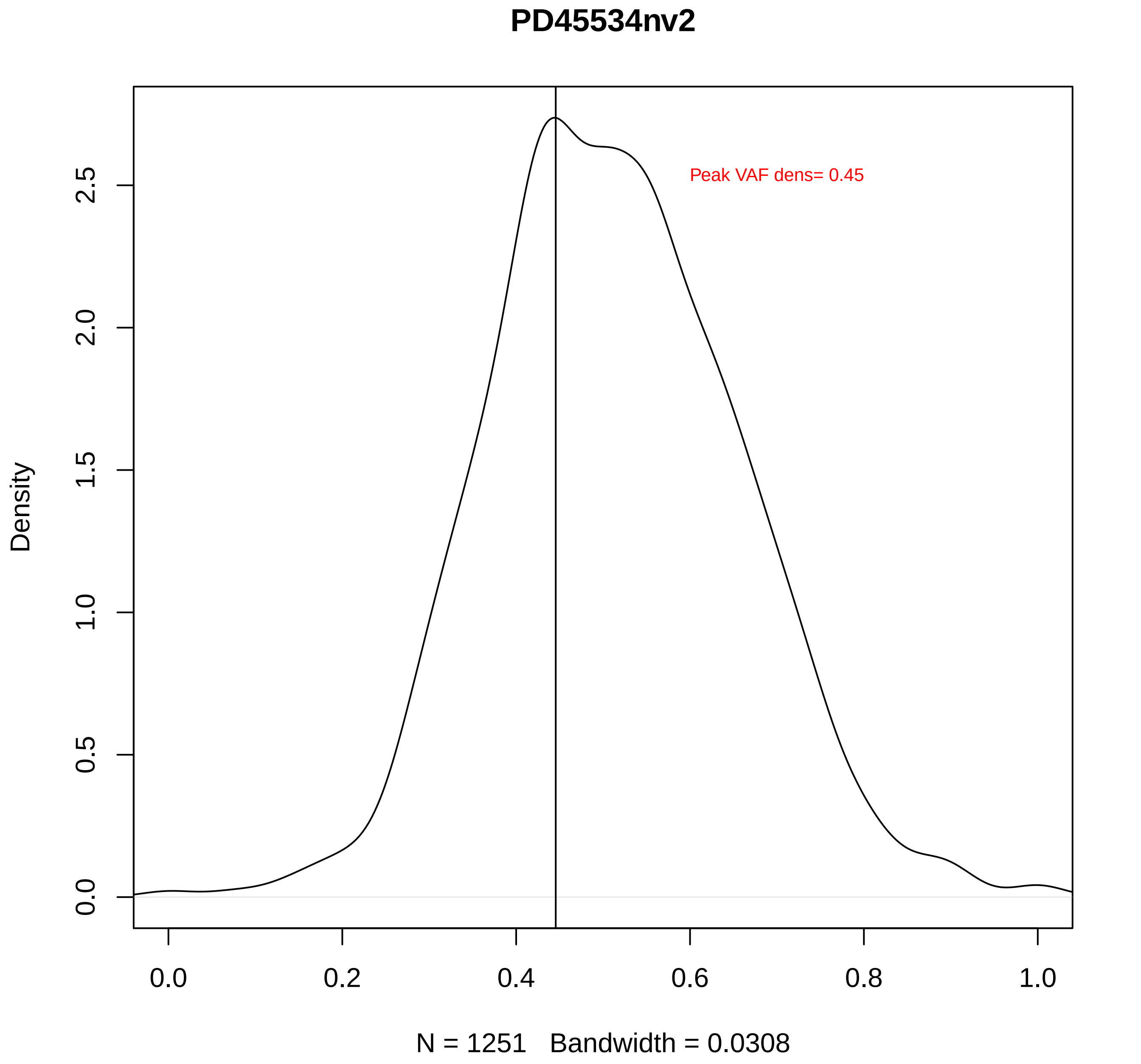
PD45534nv2
Peak VAF dens= 0.45
Density
0.0
0.5
1.0
1.5
2.0
2.5
0.0
0.2
0.4
0.6
0.8
1.0
N = 1251   Bandwidth = 0.0308

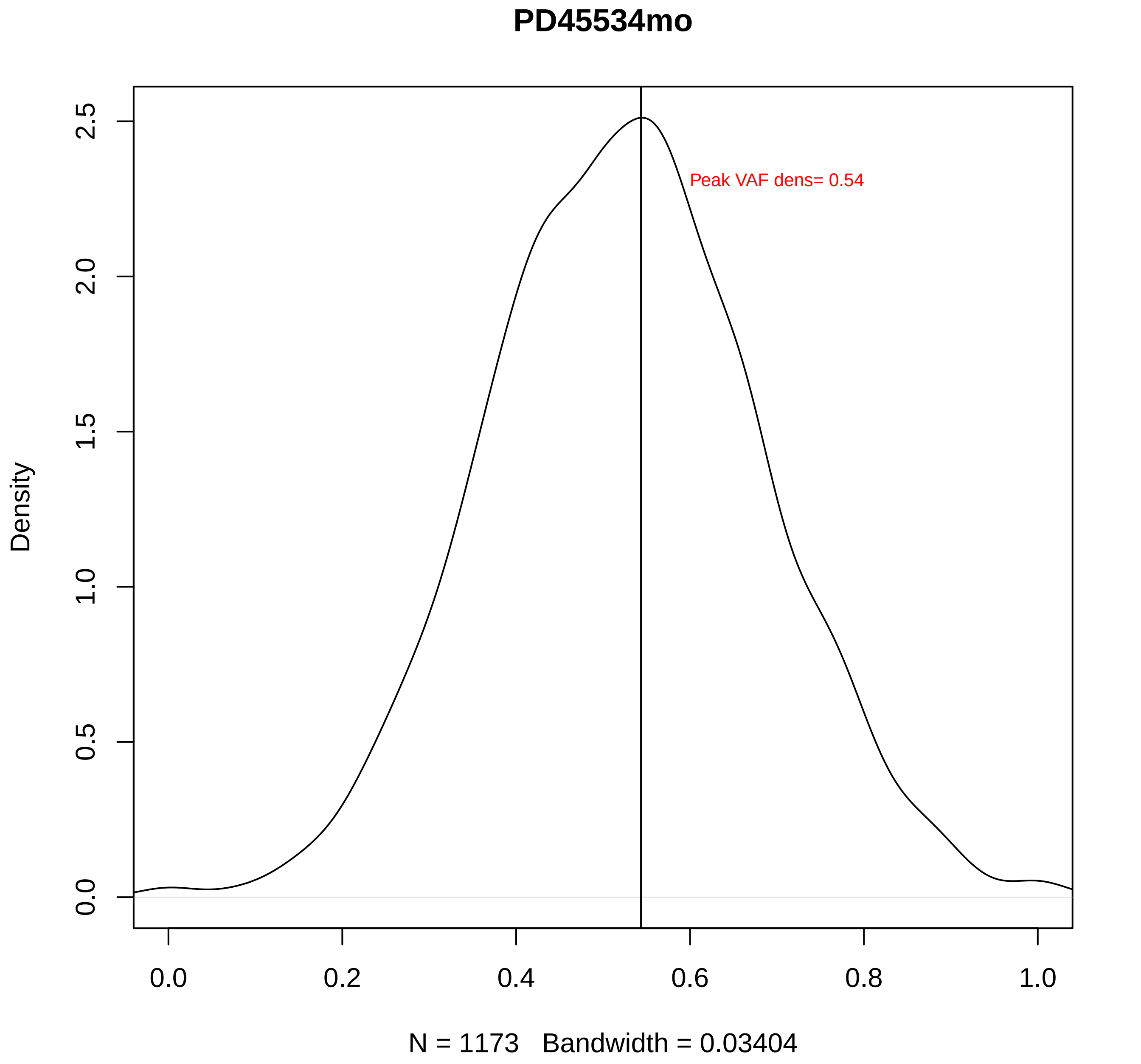

PD45534mo
Peak VAF dens= 0.54
Density
0.0
0.5
1.0
1.5
2.0
2.5
0.0
0.2
0.4
0.6
0.8
1.0
N = 1173   Bandwidth = 0.03404

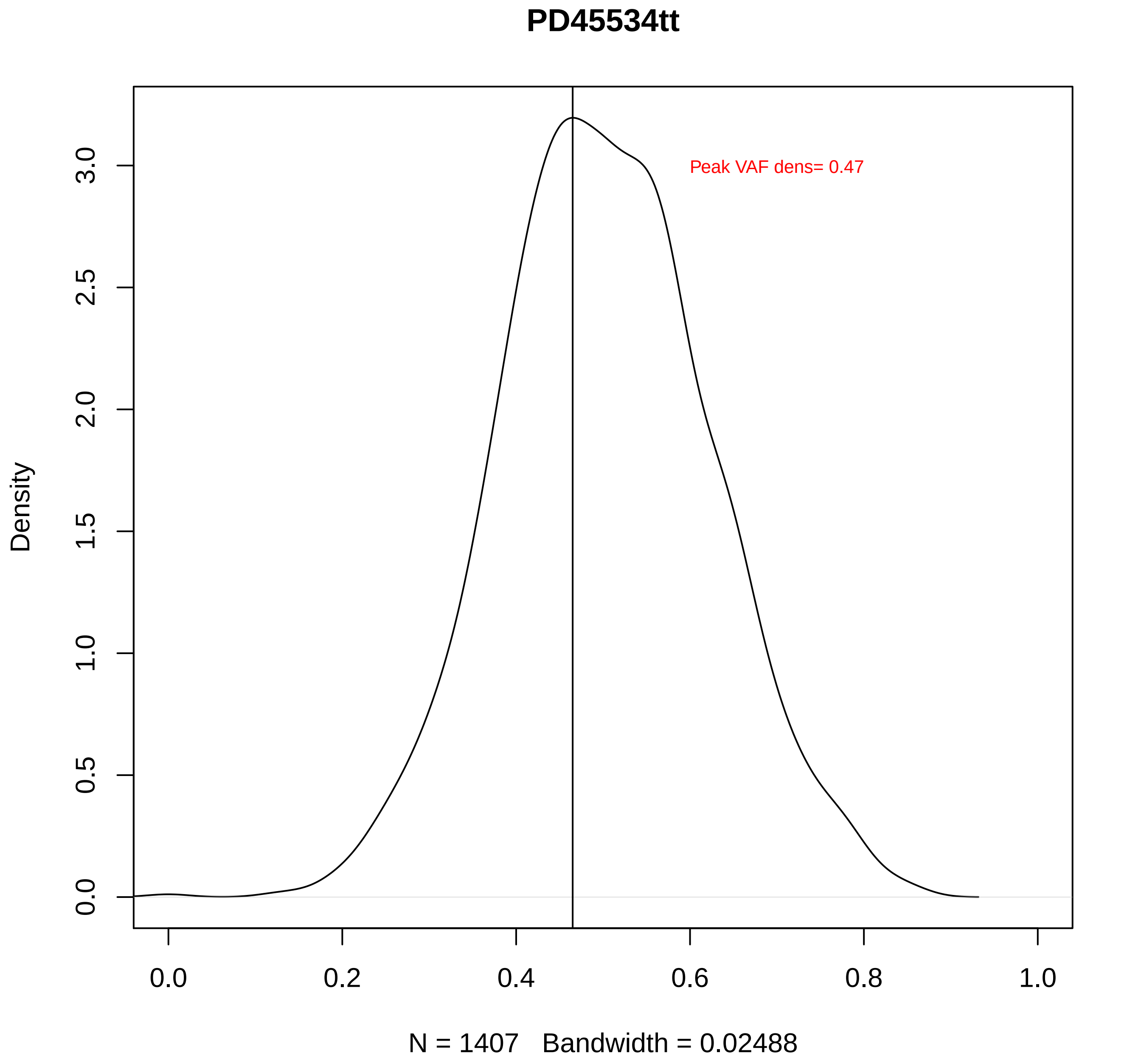
PD45534tt
Peak VAF dens= 0.47
Density
0.0
0.5
1.0
1.5
2.0
2.5
3.0
0.0
0.2
0.4
0.6
0.8
1.0
N = 1407   Bandwidth = 0.02488

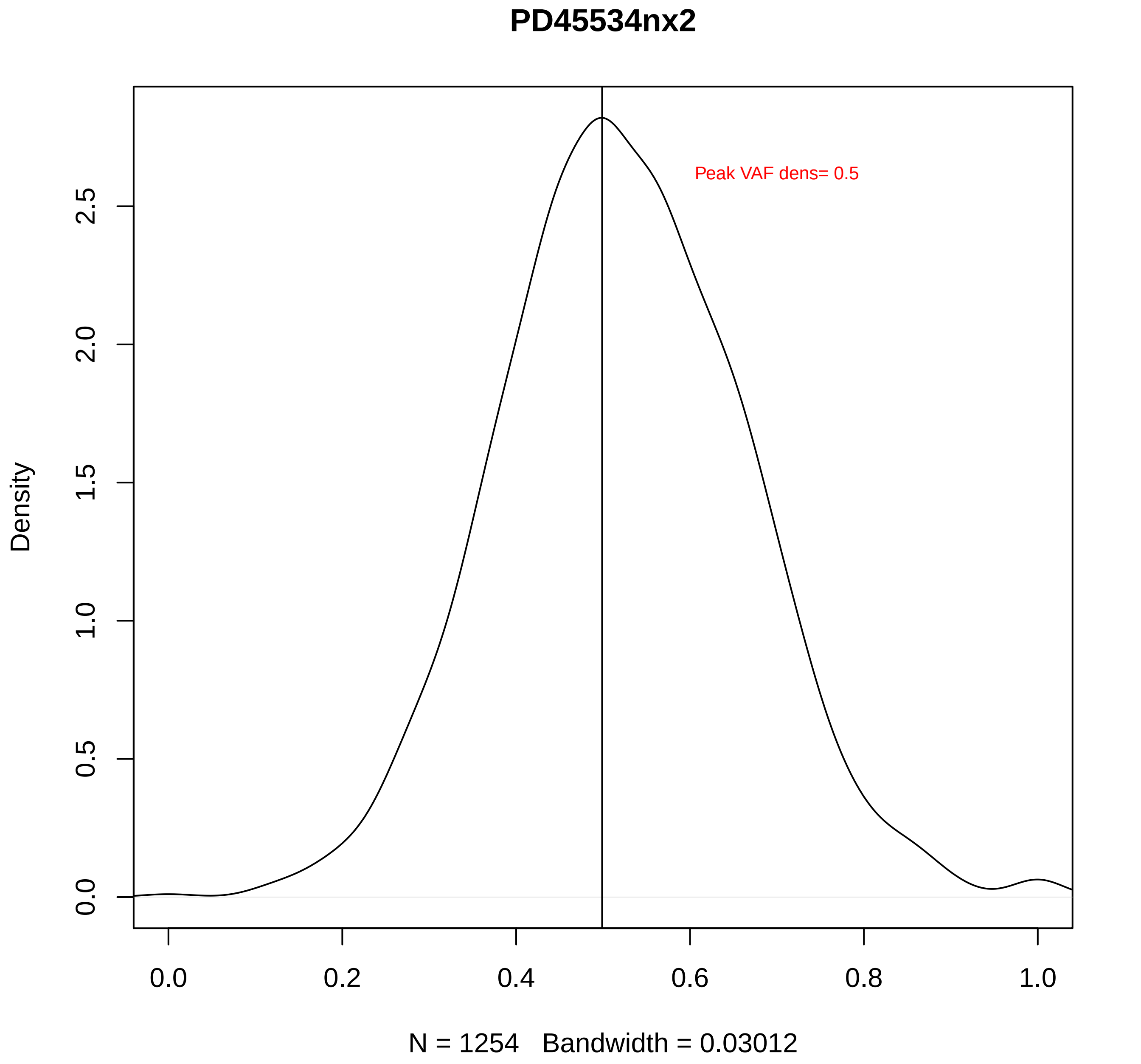
PD45534nx2
Peak VAF dens= 0.5
Density
0.0
0.5
1.0
1.5
2.0
2.5
0.0
0.2
0.4
0.6
0.8
1.0
N = 1254 Bandwidth = 0.03012

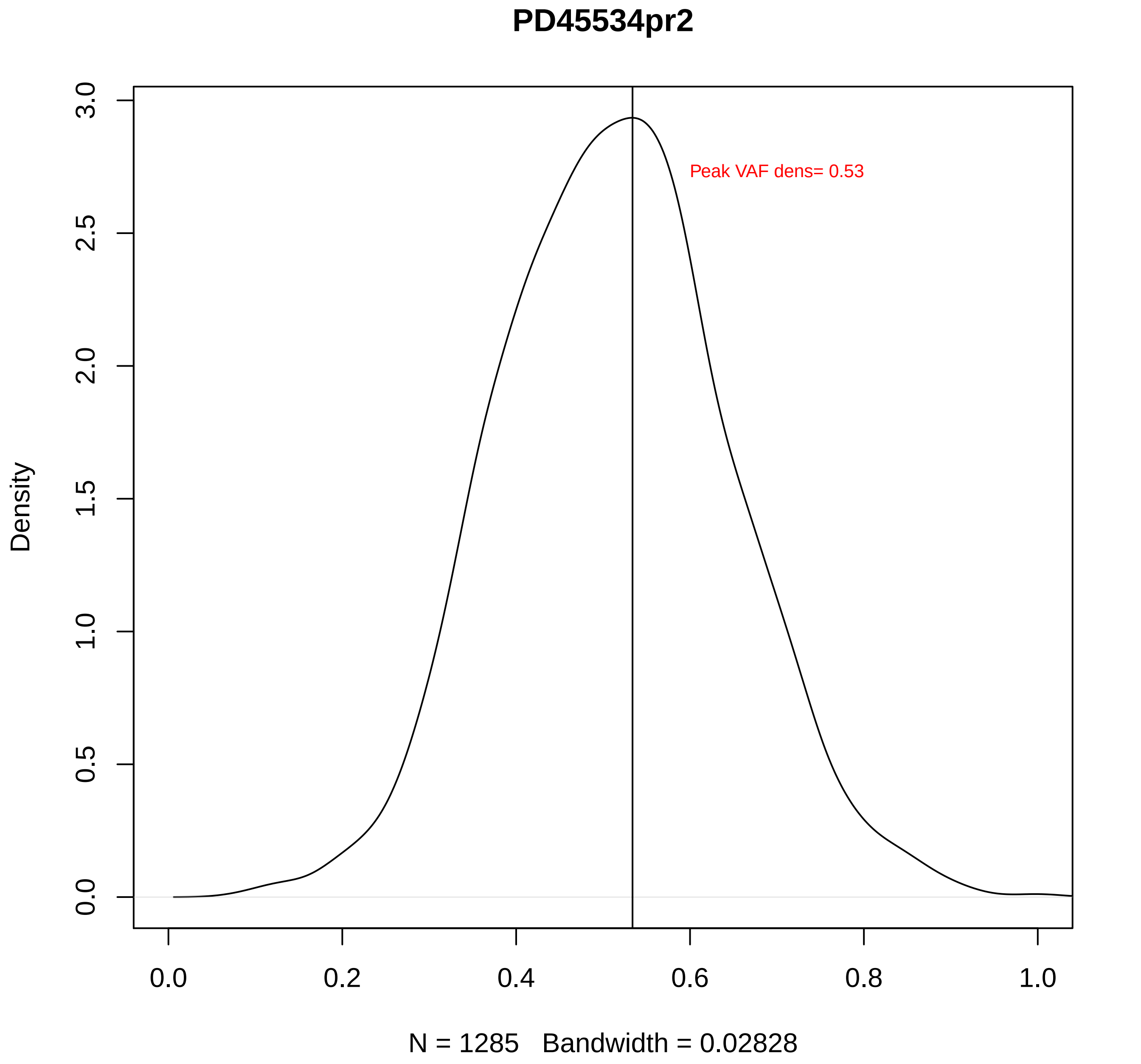
PD45534pr2
Peak VAF dens= 0.53
Density
0.0
0.5
1.0
1.5
2.0
2.5
3.0
0.0
0.2
0.4
0.6
0.8
1.0
N = 1285   Bandwidth = 0.02828

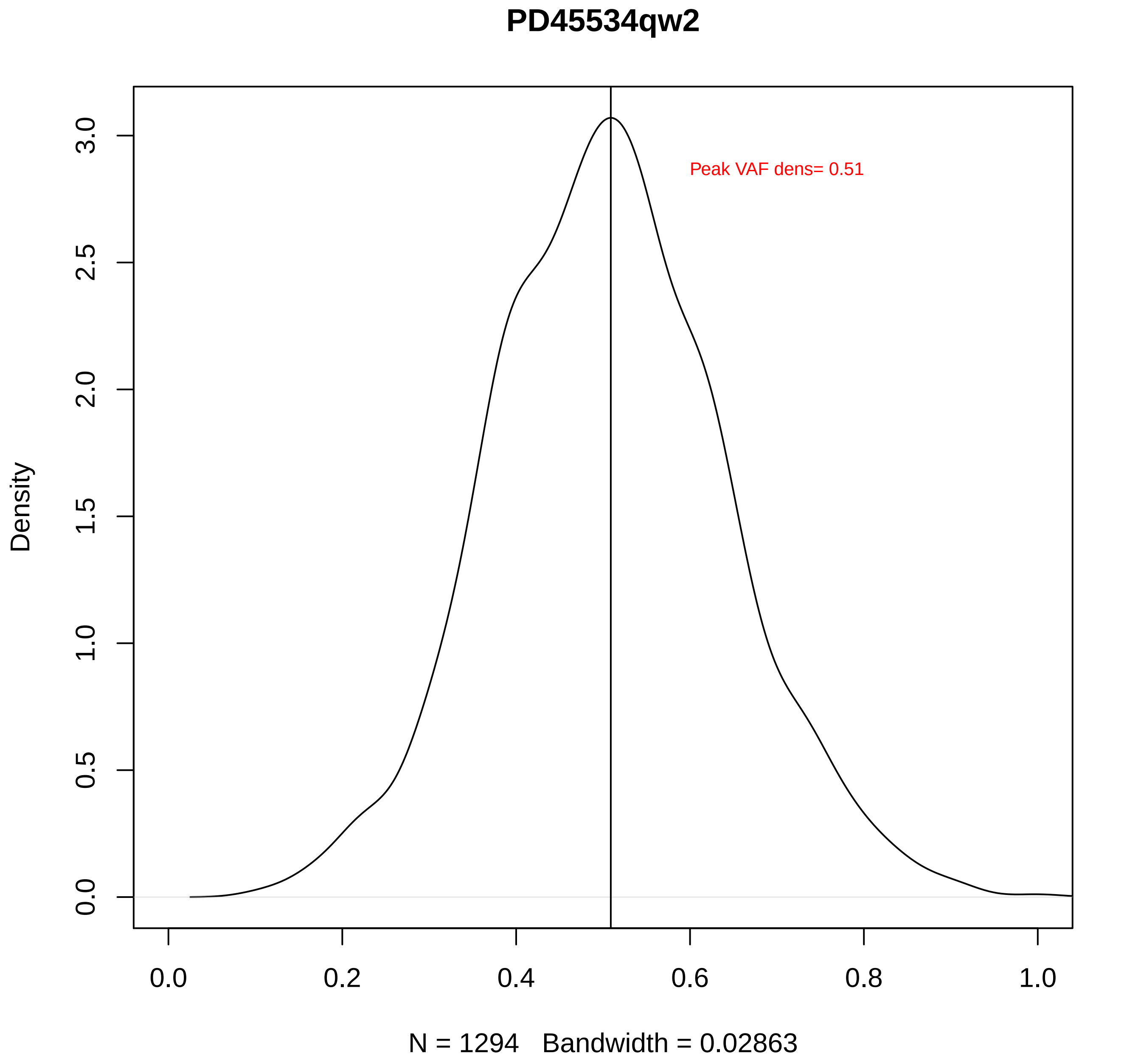
PD45534qw2
Peak VAF dens= 0.51
Density
0.0
0.5
1.0
1.5
2.0
2.5
3.0
0.0
0.2
0.4
0.6
0.8
1.0
N = 1294   Bandwidth = 0.02863

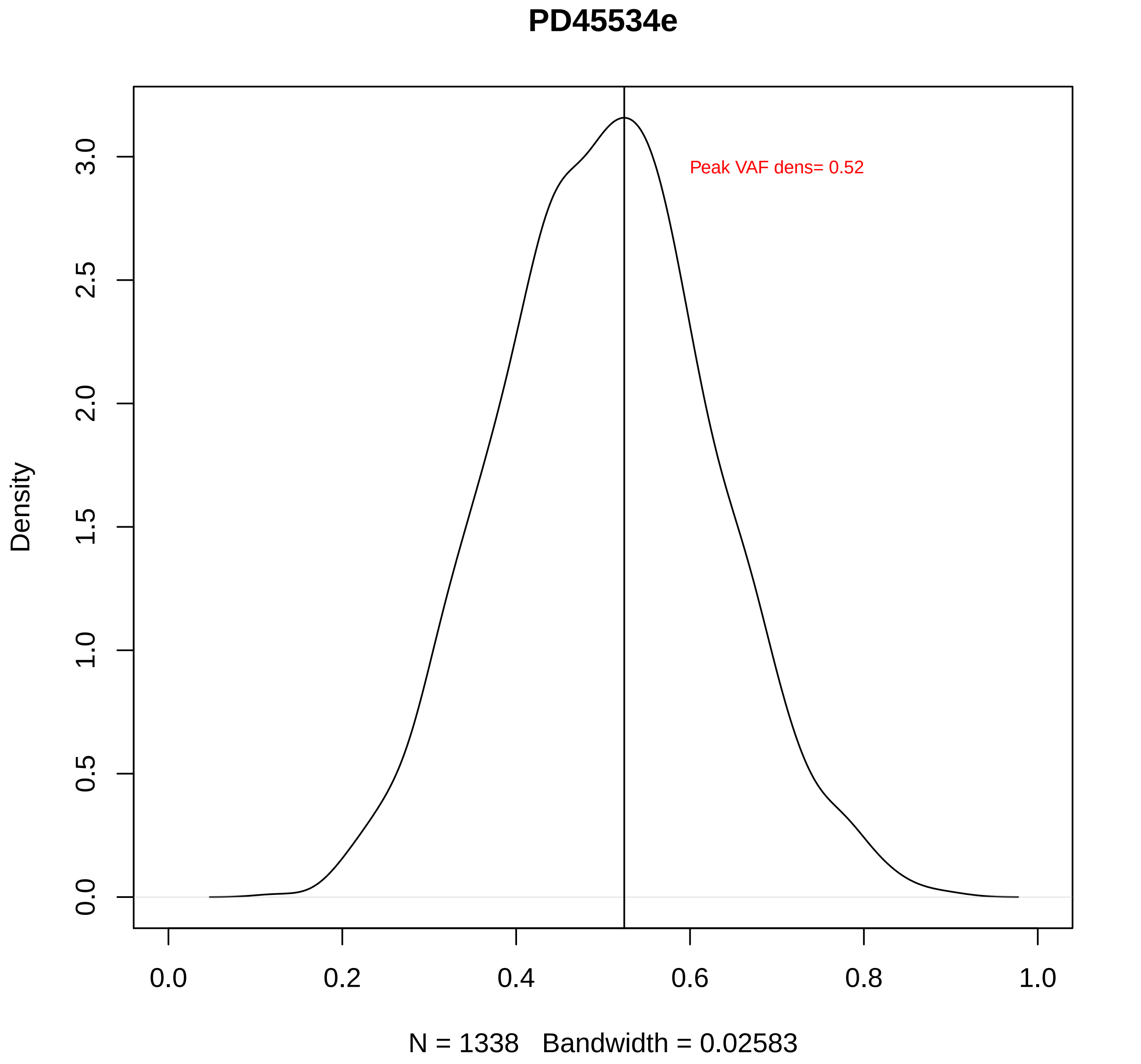
PD45534e
Peak VAF dens= 0.52
Density
0.0
0.5
1.0
1.5
2.0
2.5
3.0
0.0
0.2
0.4
0.6
0.8
1.0
N = 1338   Bandwidth = 0.02583

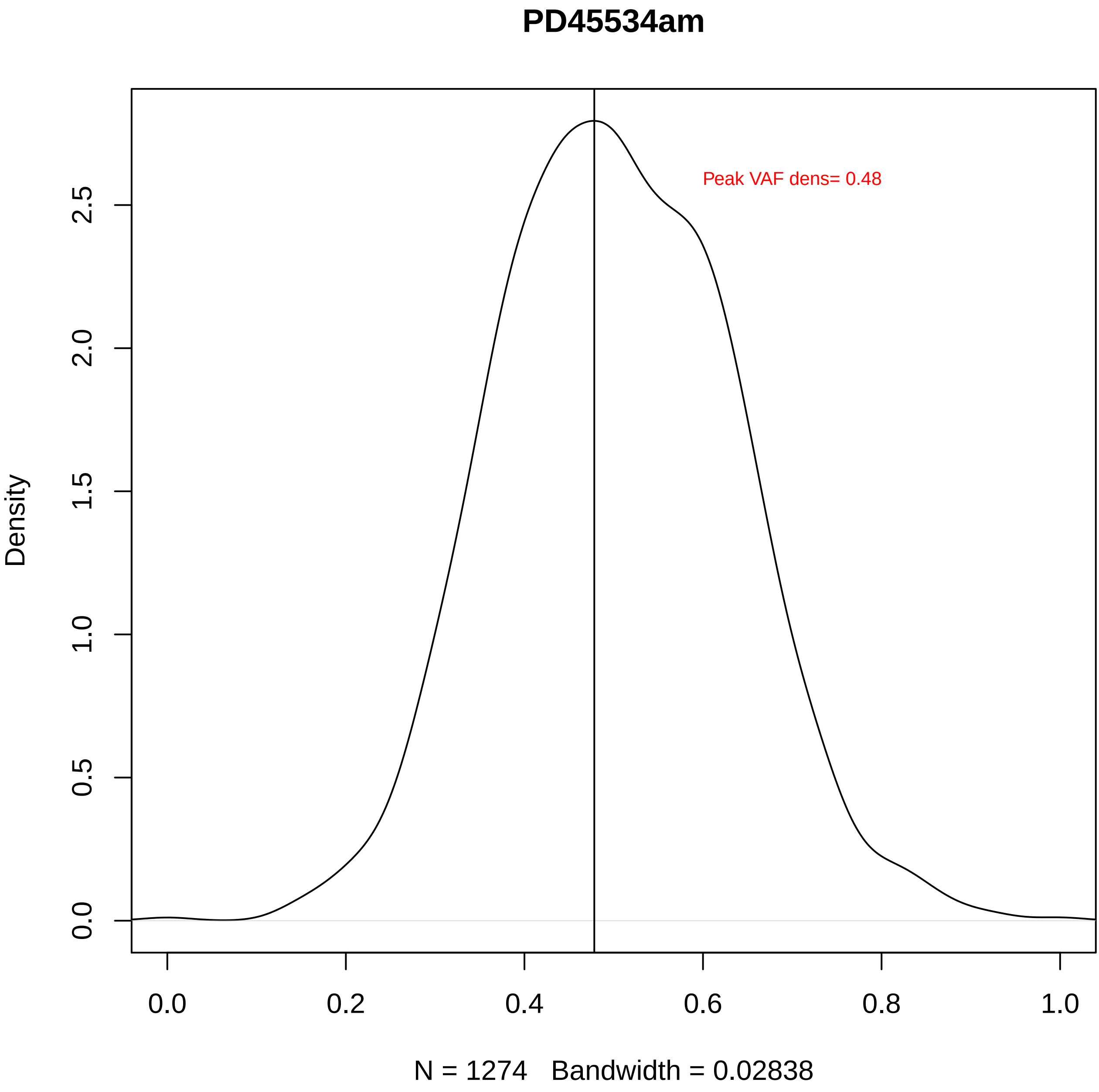

PD45534am
Peak VAF dens= 0.48
Density
0.0
0.5
1.0
1.5
2.0
2.5
0.0
0.2
0.4
0.6
0.8
1.0
N = 1274 Bandwidth = 0.02838

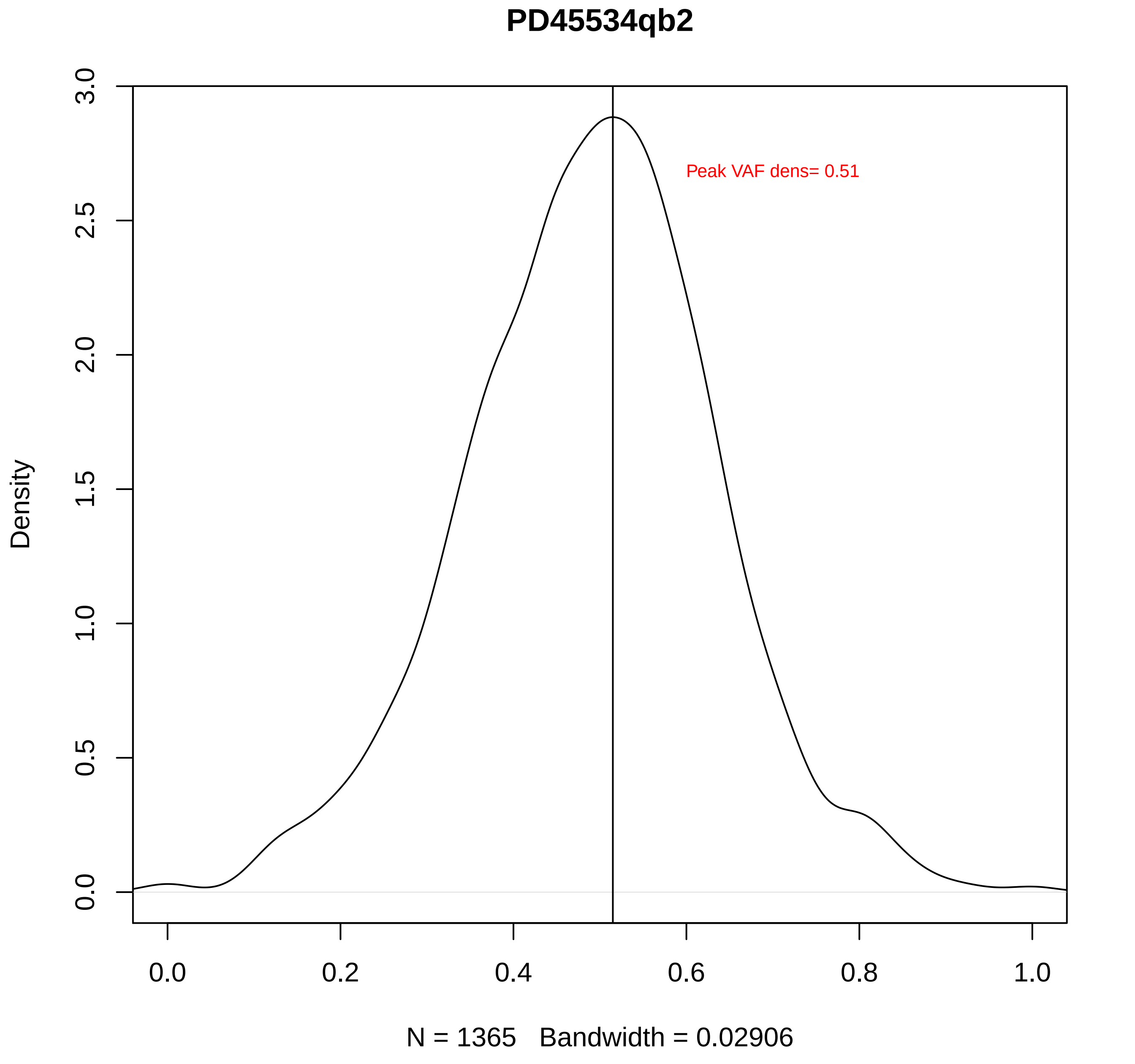

PD45534qb2
Peak VAF dens= 0.51
Density
0.0
0.5
1.0
1.5
2.0
2.5
3.0
0.0
0.2
0.4
0.6
0.8
1.0
N = 1365   Bandwidth = 0.02906

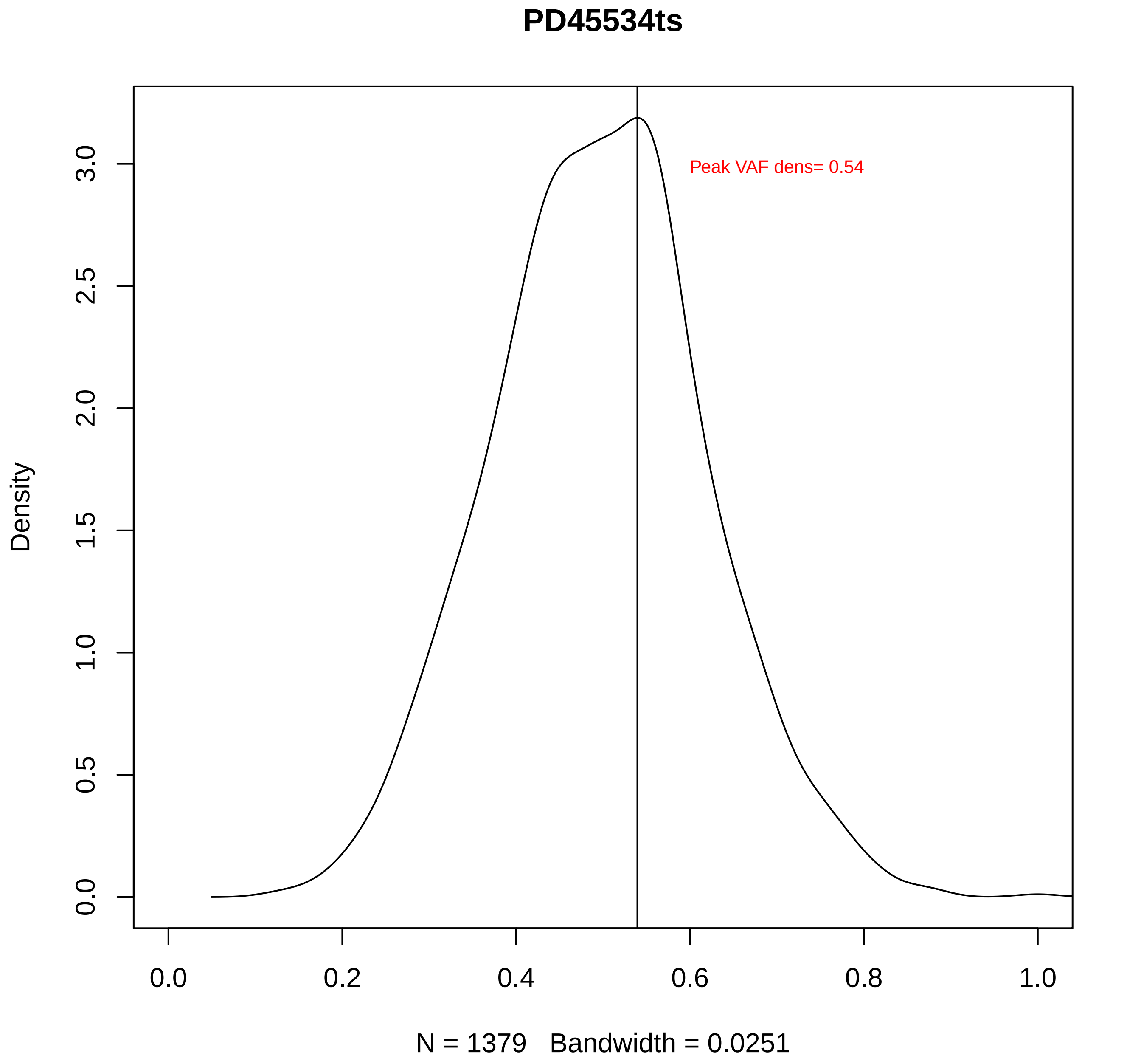
PD45534ts
Peak VAF dens= 0.54
Density
0.0
0.5
1.0
1.5
2.0
2.5
3.0
0.0
0.2
0.4
0.6
0.8
1.0
N = 1379 Bandwidth = 0.0251

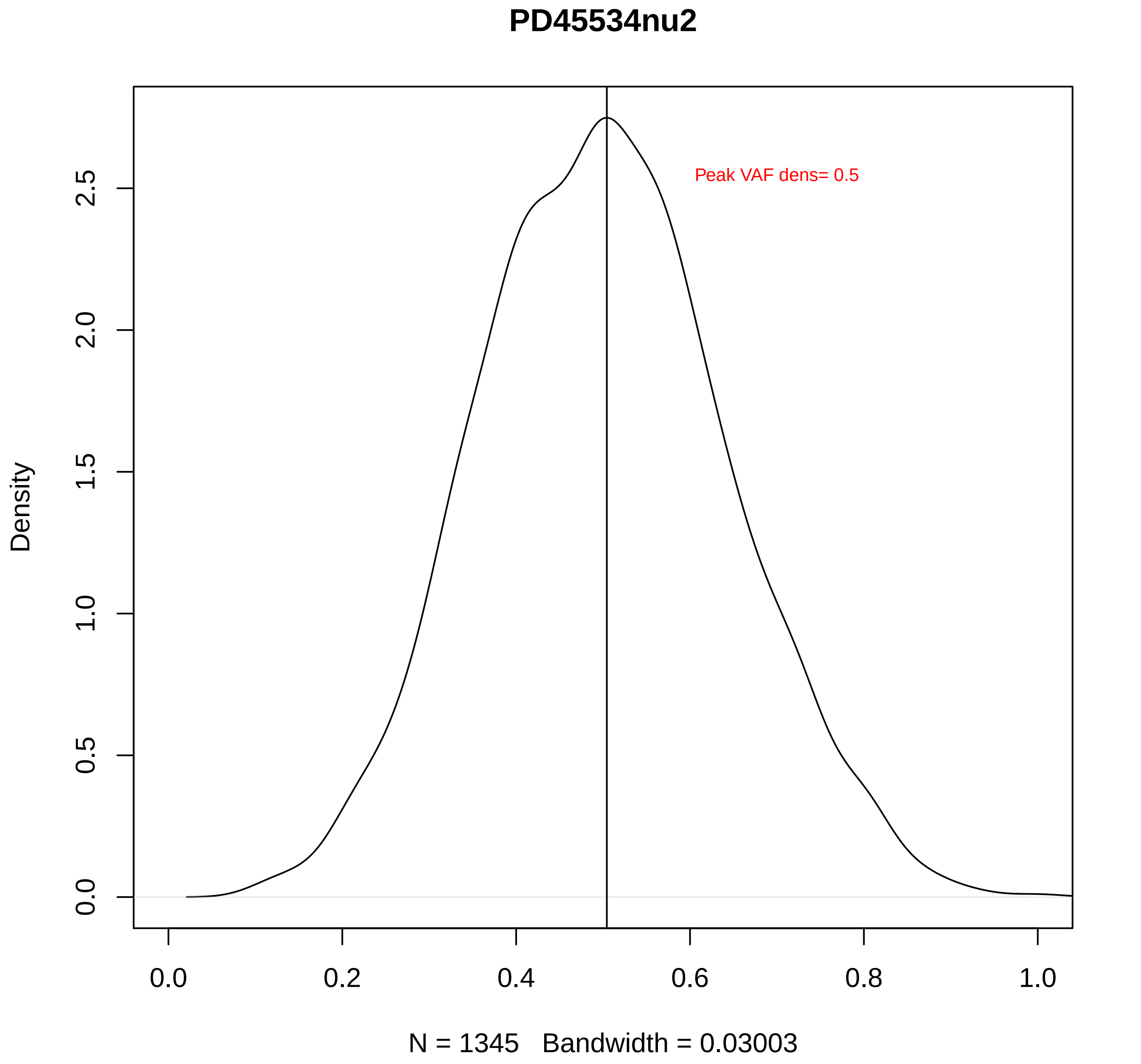

PD45534nu2
Peak VAF dens= 0.5
Density
0.0
0.5
1.0
1.5
2.0
2.5
0.0
0.2
0.4
0.6
0.8
1.0
N = 1345 Bandwidth = 0.03003

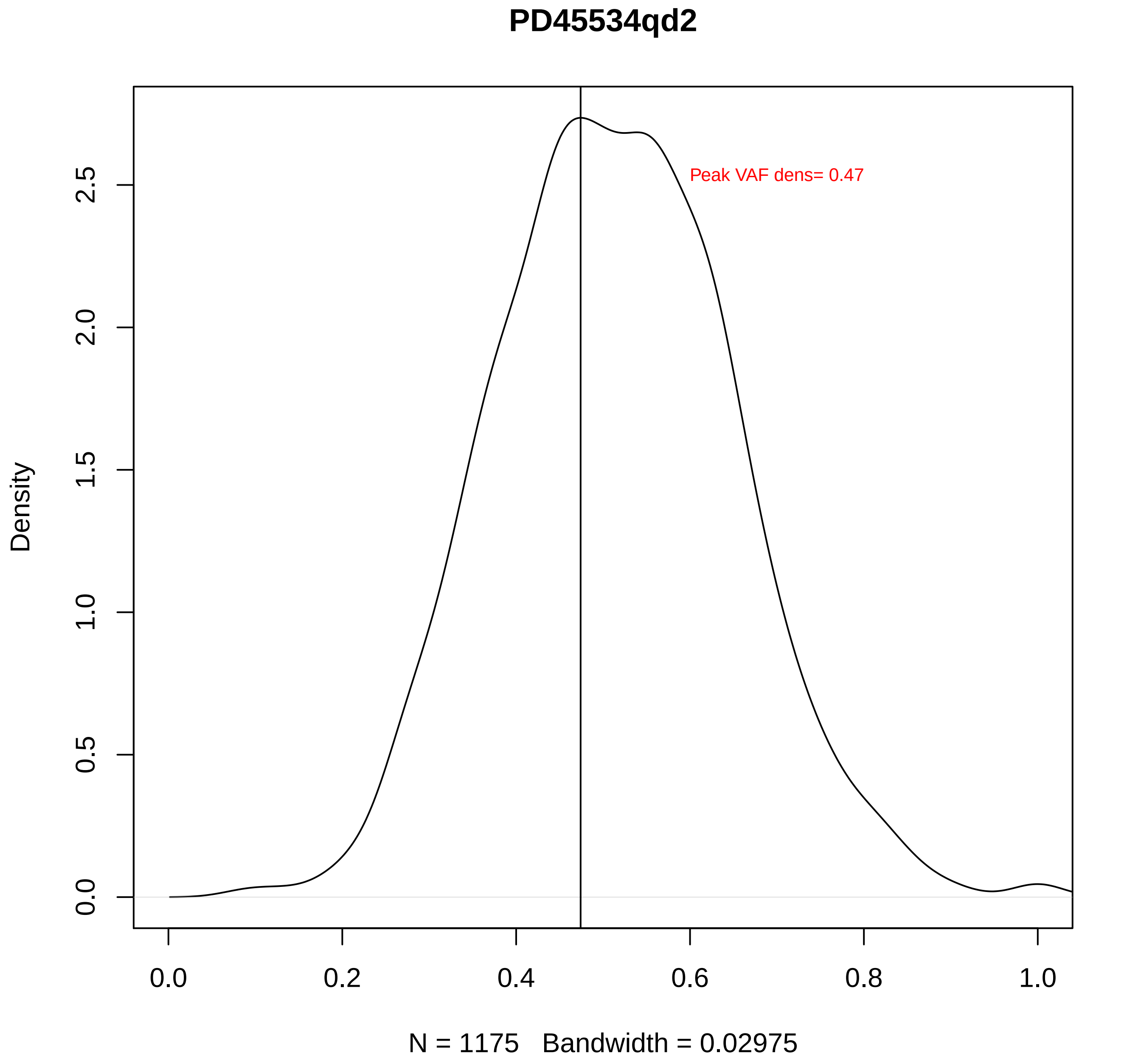

PD45534qd2
Peak VAF dens= 0.47
Density
0.0
0.5
1.0
1.5
2.0
2.5
0.0
0.2
0.4
0.6
0.8
1.0
N = 1175   Bandwidth = 0.02975

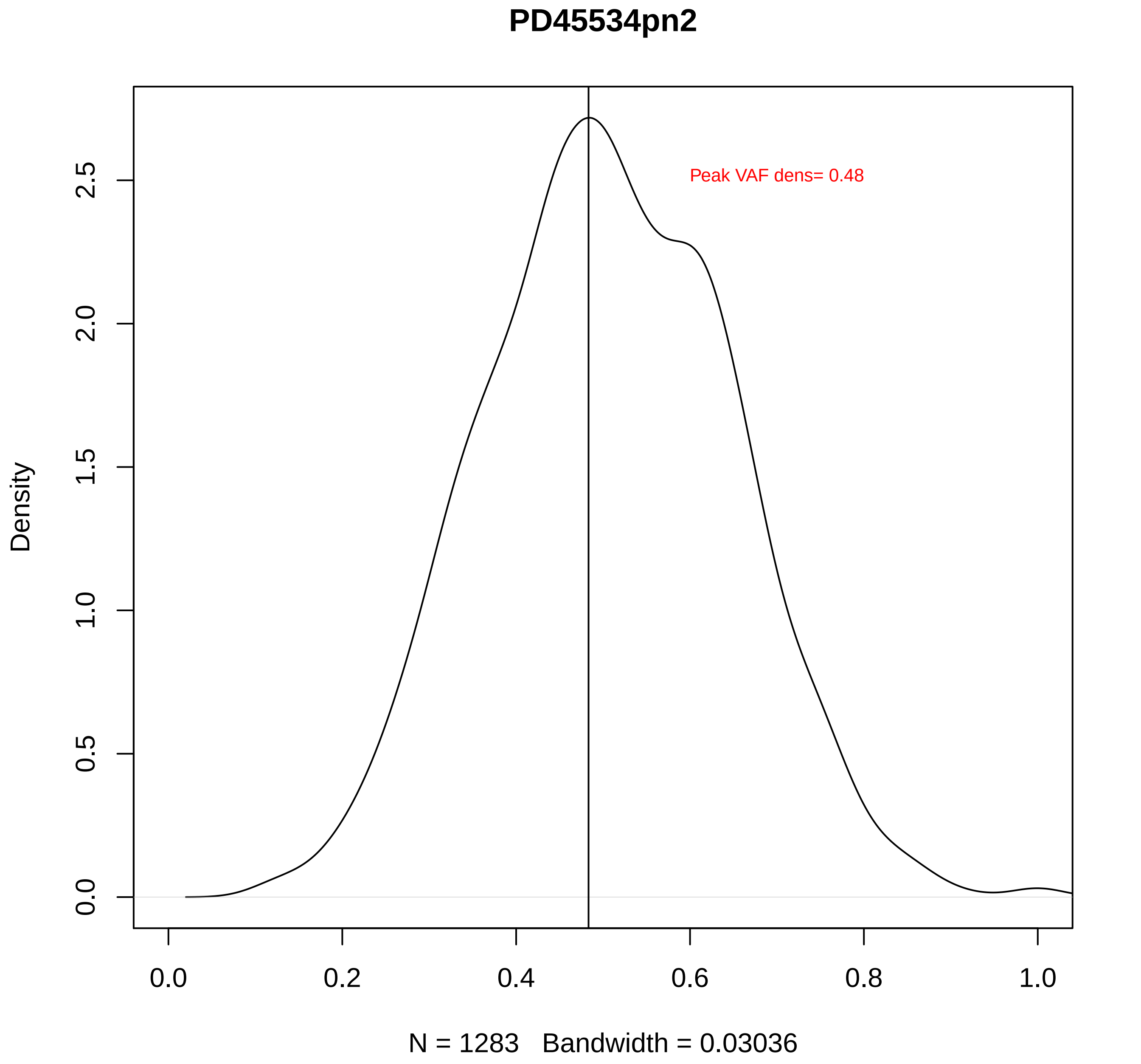
PD45534pn2
Peak VAF dens= 0.48
Density
0.0
0.5
1.0
1.5
2.0
2.5
0.0
0.2
0.4
0.6
0.8
1.0
N = 1283   Bandwidth = 0.03036

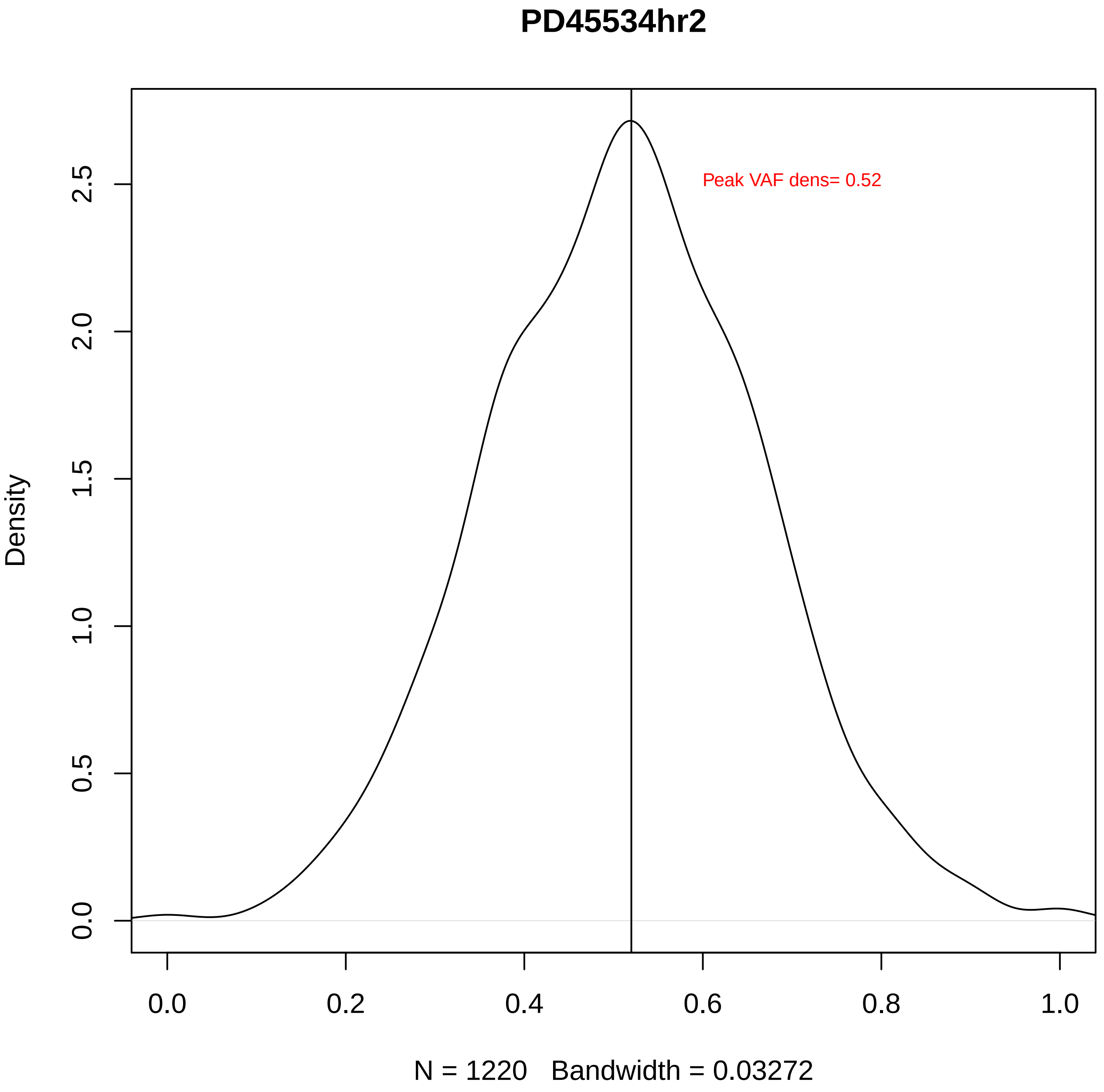
PD45534hr2
Peak VAF dens= 0.52
Density
0.0
0.5
1.0
1.5
2.0
2.5
0.0
0.2
0.4
0.6
0.8
1.0
N = 1220   Bandwidth = 0.03272

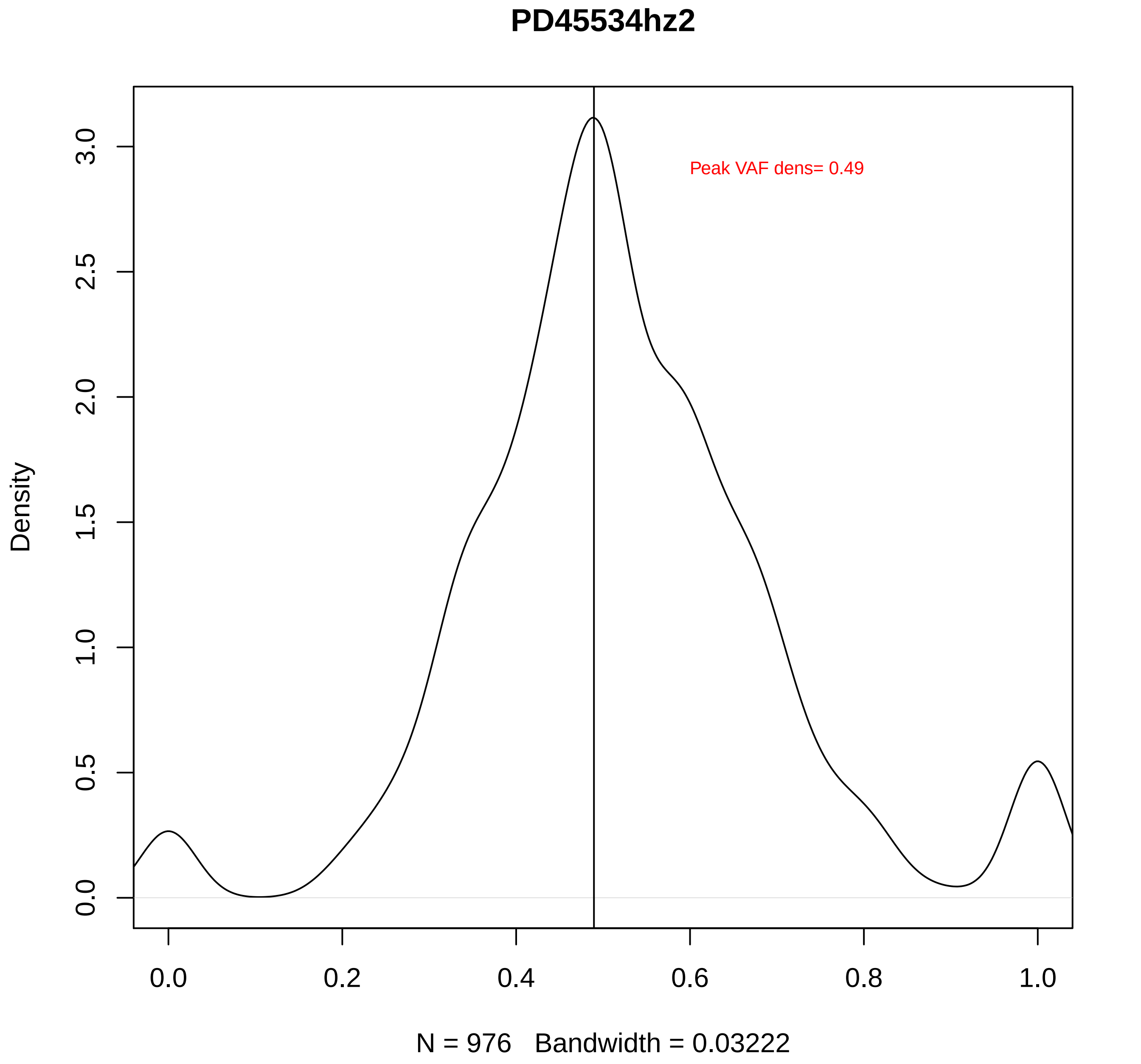

PD45534hz2
Peak VAF dens= 0.49
Density
0.0
0.5
1.0
1.5
2.0
2.5
3.0
0.0
0.2
0.4
0.6
0.8
1.0
N = 976   Bandwidth = 0.03222

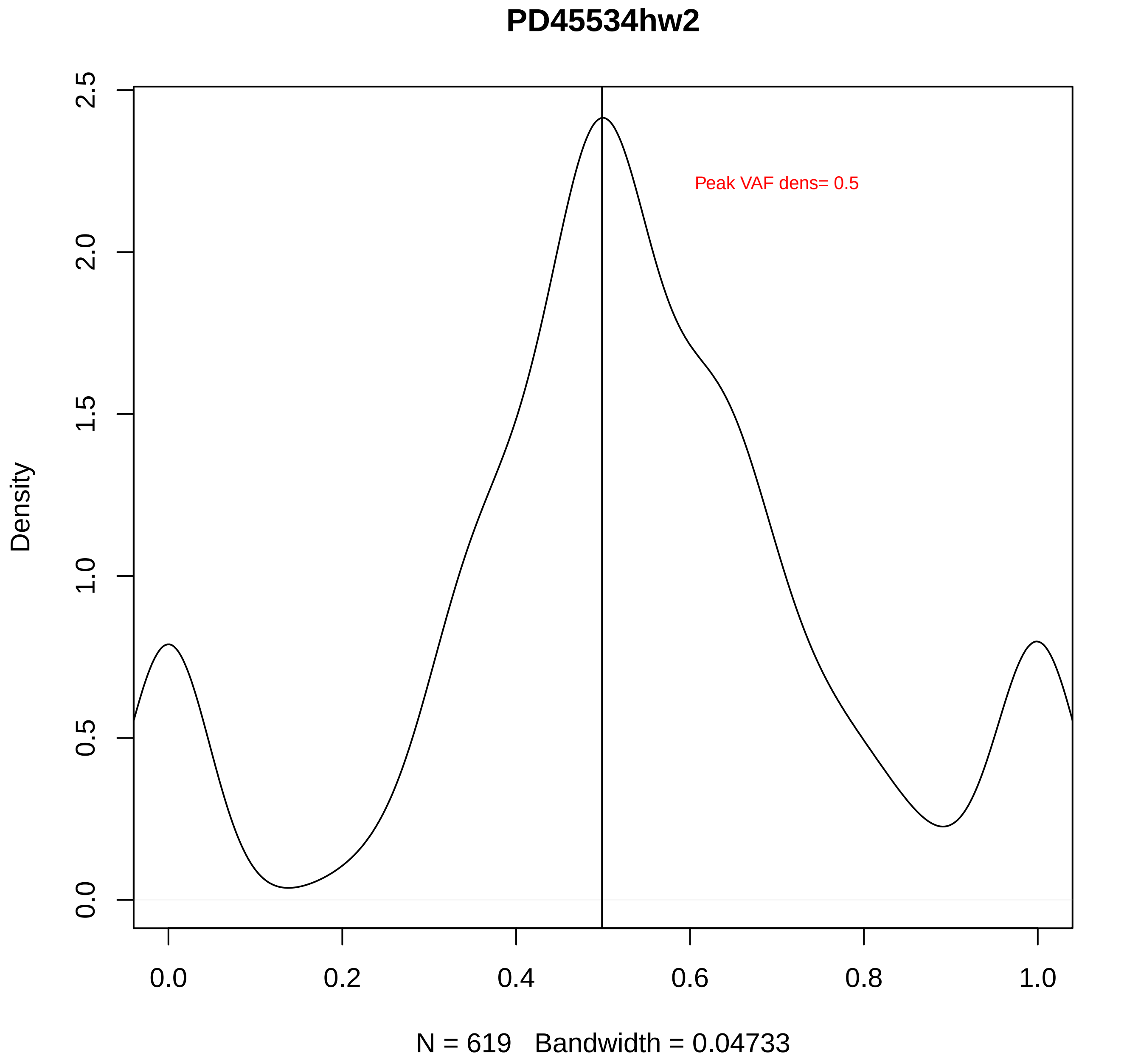
PD45534hw2
Peak VAF dens= 0.5
Density
0.0
0.5
1.0
1.5
2.0
2.5
0.0
0.2
0.4
0.6
0.8
1.0
N = 619   Bandwidth = 0.04733

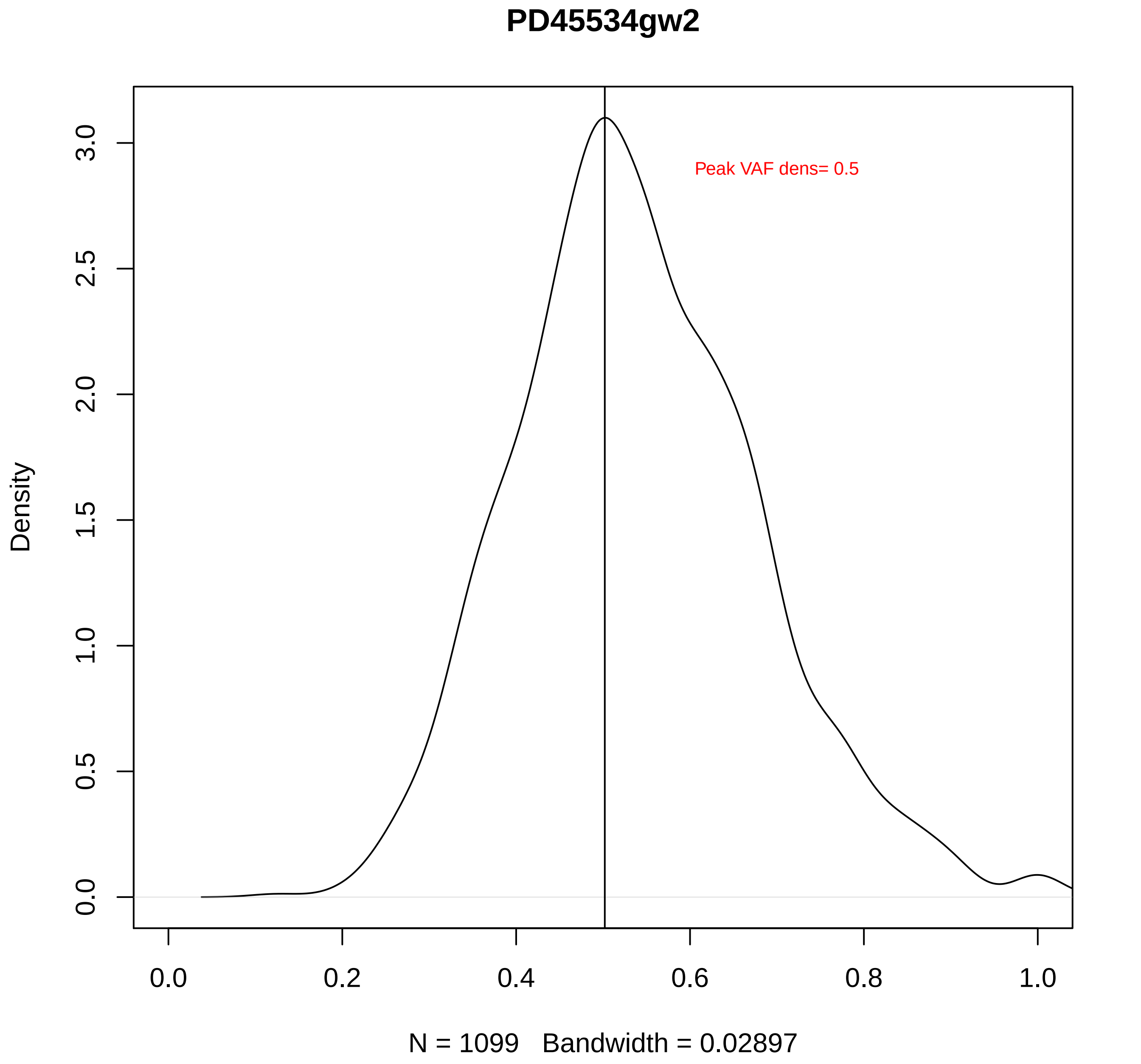

PD45534gw2
Peak VAF dens= 0.5
Density
0.0
0.5
1.0
1.5
2.0
2.5
3.0
0.0
0.2
0.4
0.6
0.8
1.0
N = 1099   Bandwidth = 0.02897

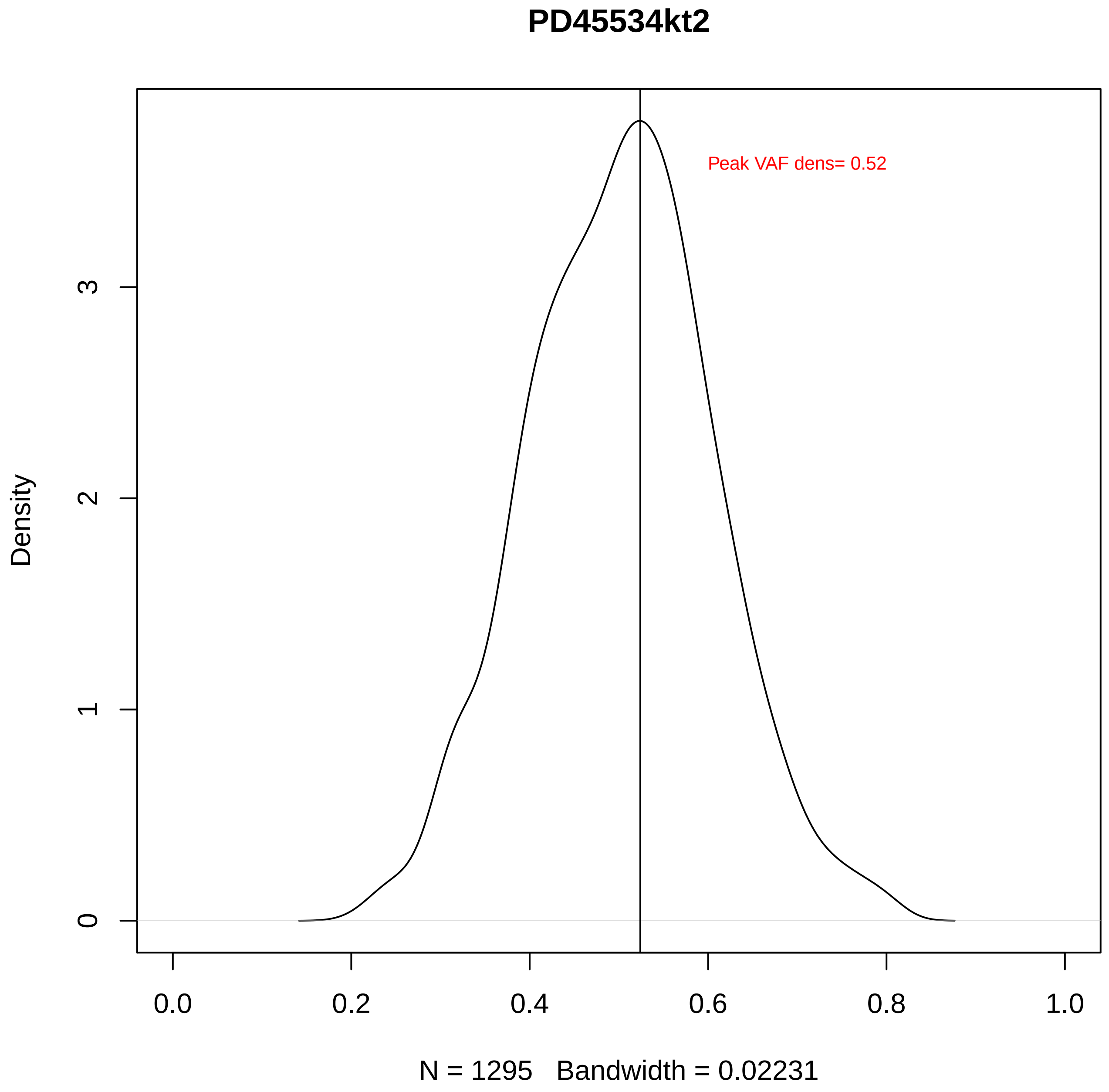

PD45534kt2
Peak VAF dens= 0.52
Density
0
1
2
3
0.0
0.2
0.4
0.6
0.8
1.0
N = 1295 Bandwidth = 0.02231

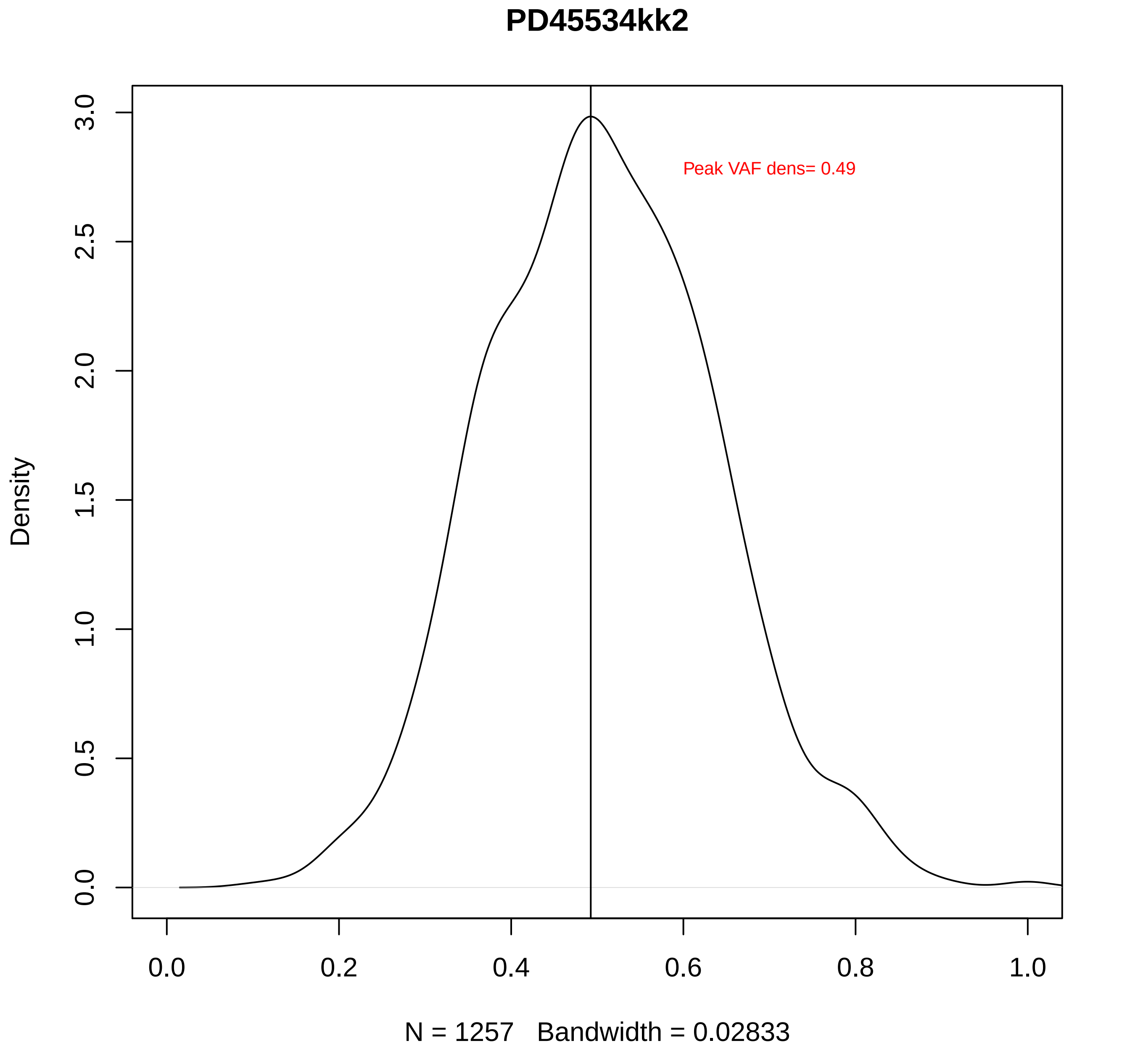
PD45534kk2
Peak VAF dens= 0.49
Density
0.0
0.5
1.0
1.5
2.0
2.5
3.0
0.0
0.2
0.4
0.6
0.8
1.0
N = 1257   Bandwidth = 0.02833

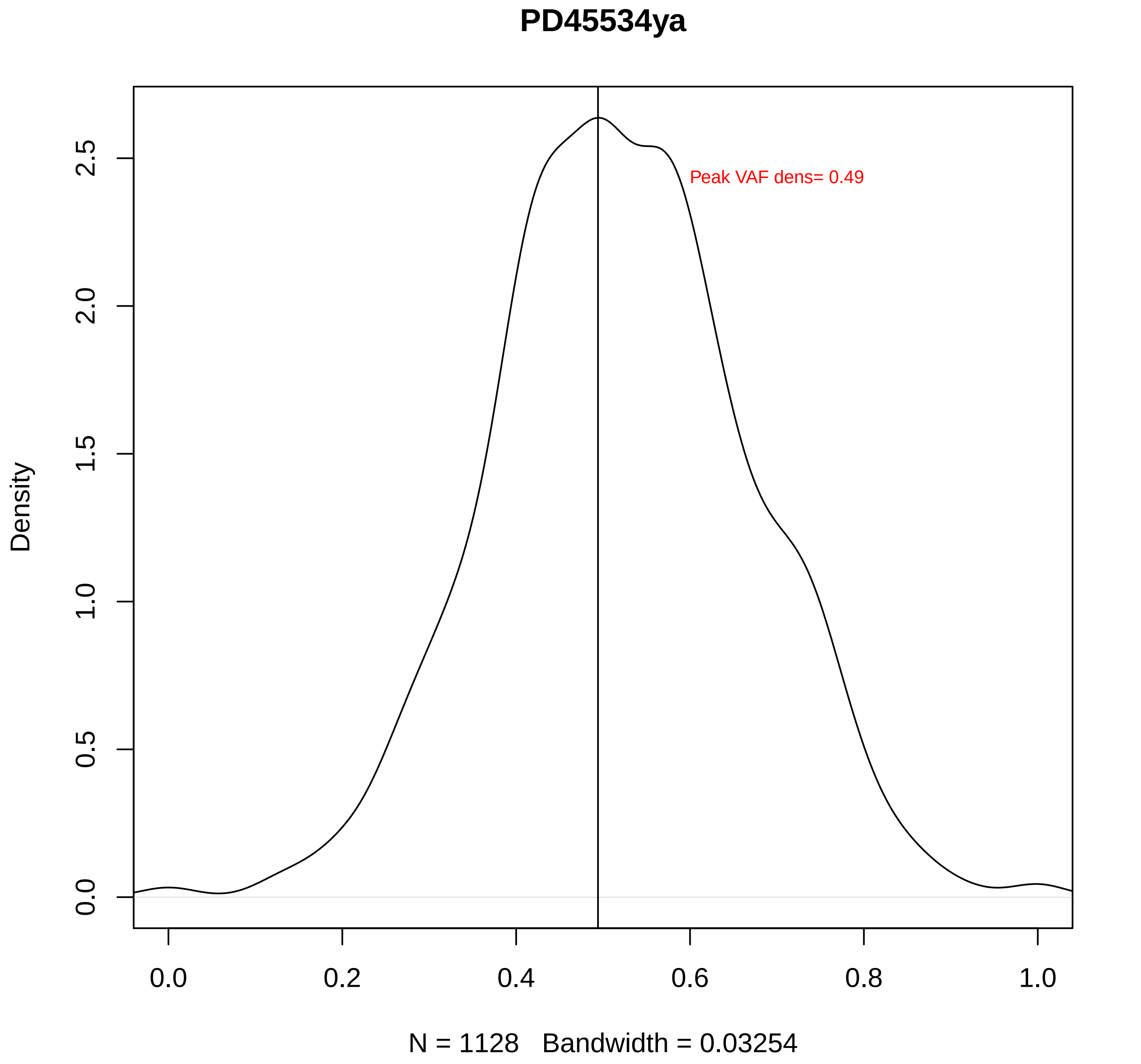
PD45534ya
Peak VAF dens= 0.49
Density
0.0
0.5
1.0
1.5
2.0
2.5
0.0
0.2
0.4
0.6
0.8
1.0
N = 1128   Bandwidth = 0.03254

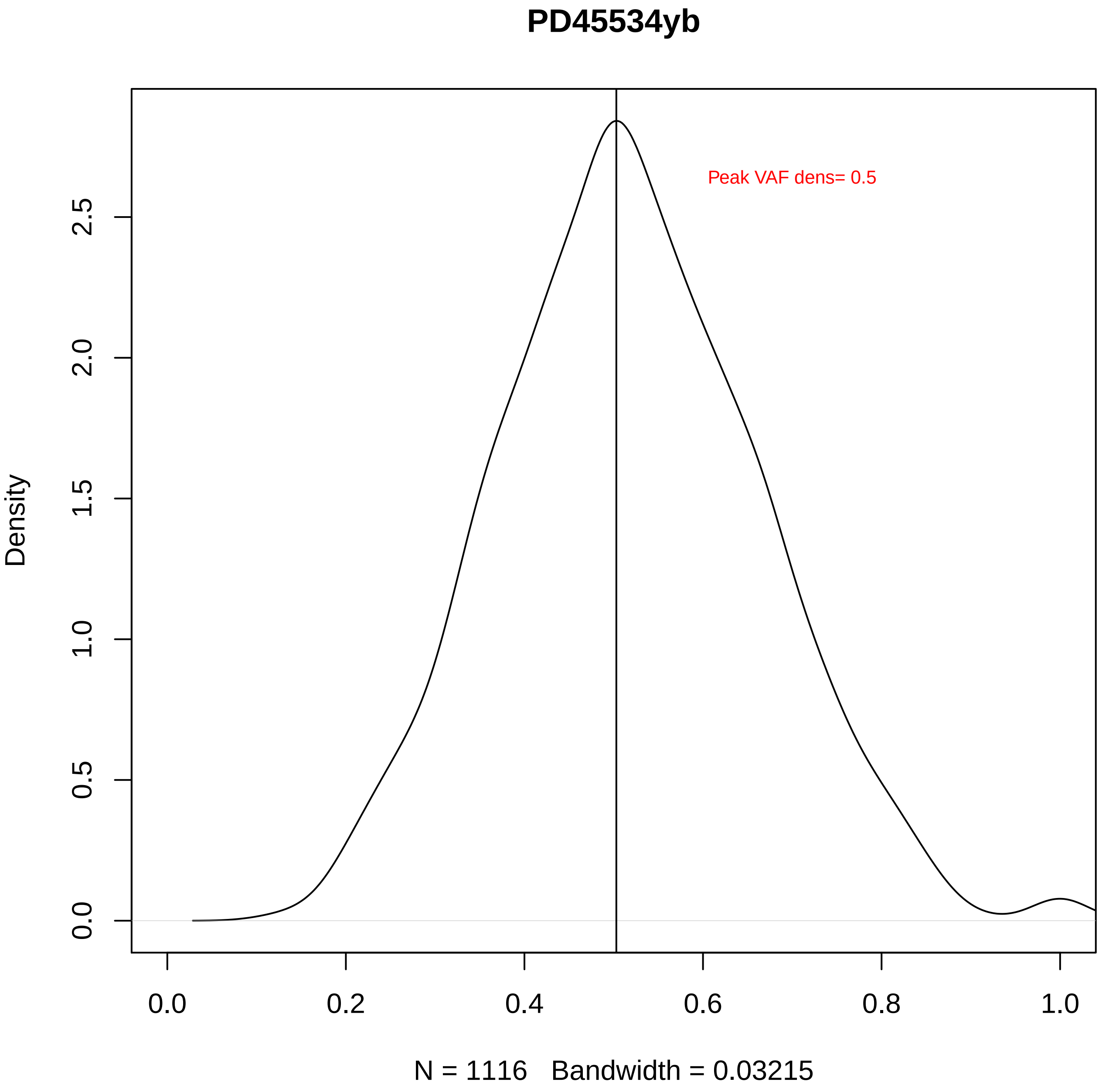

PD45534yb
Peak VAF dens= 0.5
Density
0.0
0.5
1.0
1.5
2.0
2.5
0.0
0.2
0.4
0.6
0.8
1.0
N = 1116 Bandwidth = 0.03215

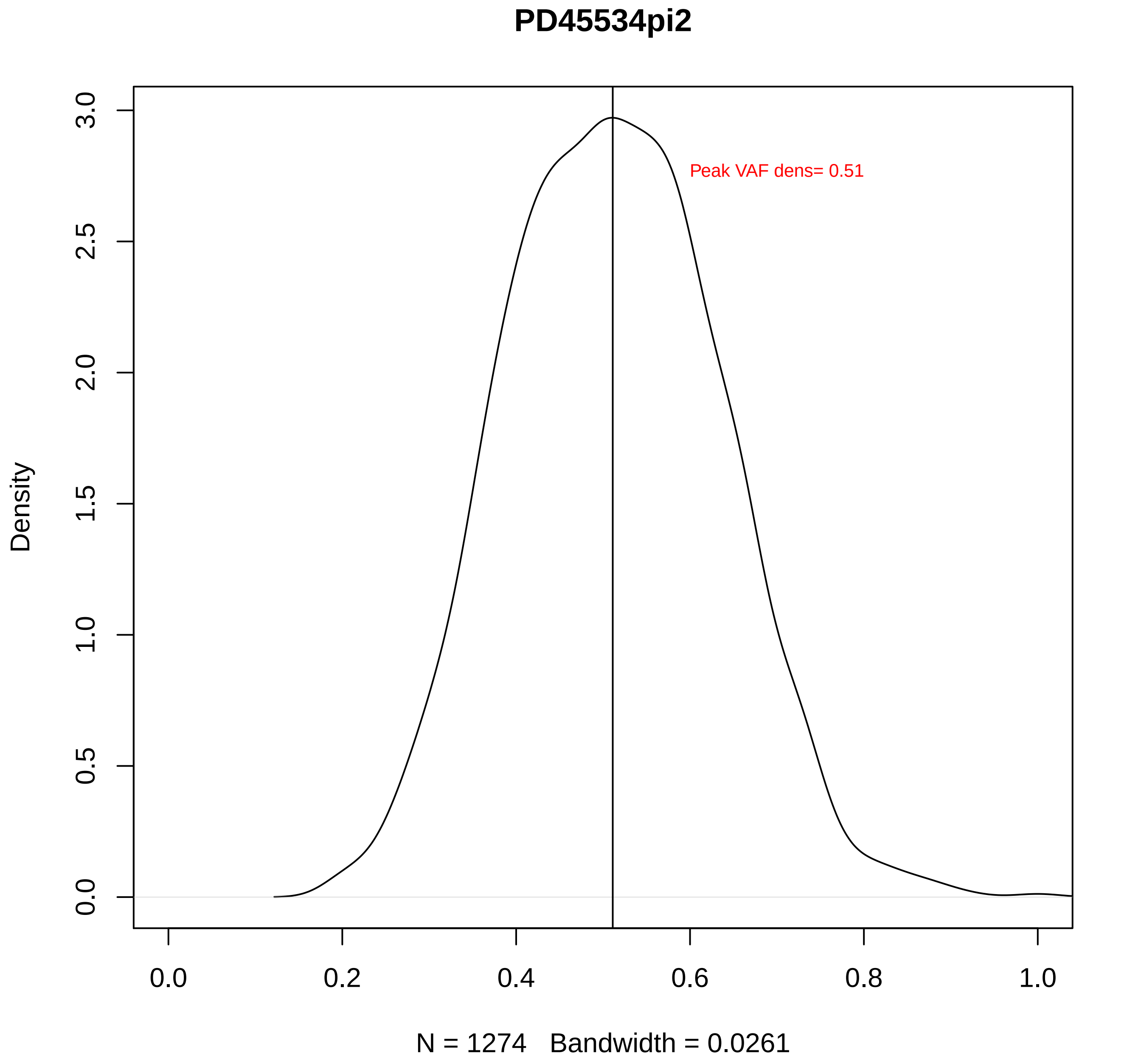
PD45534pi2
Peak VAF dens= 0.51
Density
3.0
2.5
2.0
1.5
1.0
0.5
0.0
0.0
0.2
0.4
0.6
0.8
1.0
N = 1274   Bandwidth = 0.0261

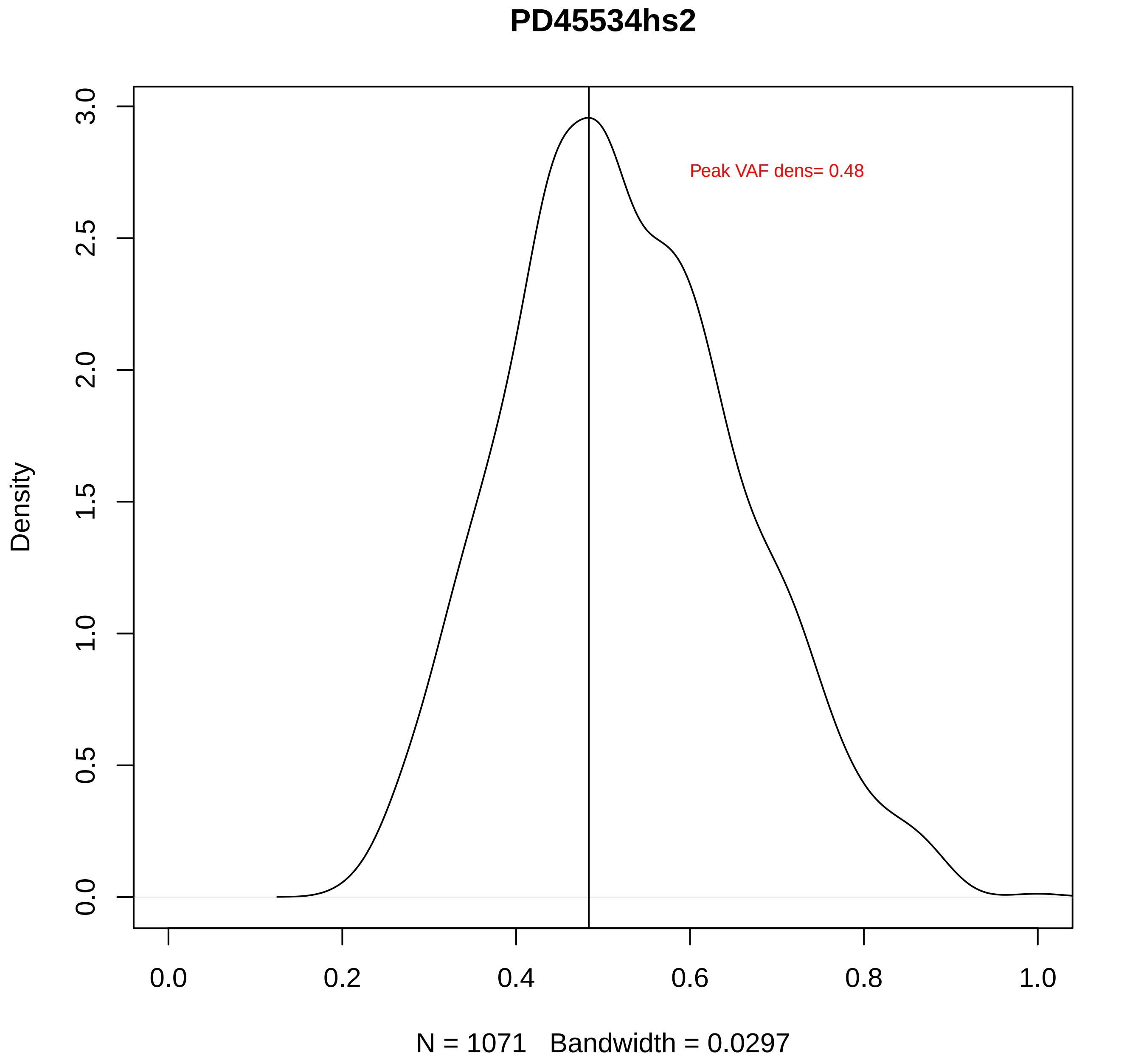
PD45534hs2
Peak VAF dens= 0.48
Density
0.0
0.5
1.0
1.5
2.0
2.5
3.0
0.0
0.2
0.4
0.6
0.8
1.0
N = 1071   Bandwidth = 0.0297

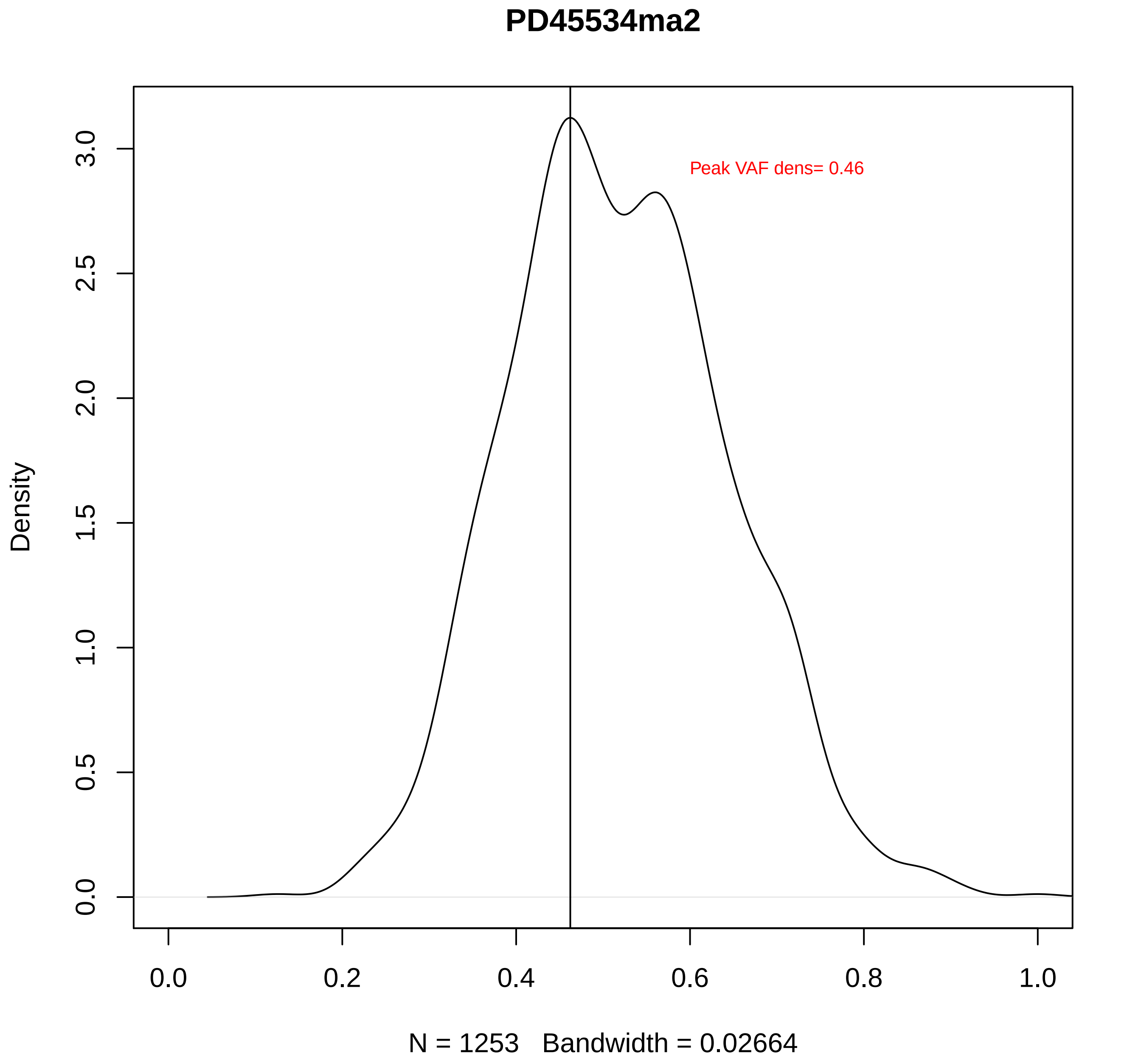
PD45534ma2
Peak VAF dens= 0.46
Density
0.0
0.5
1.0
1.5
2.0
2.5
3.0
0.0
0.2
0.4
0.6
0.8
1.0
N = 1253   Bandwidth = 0.02664

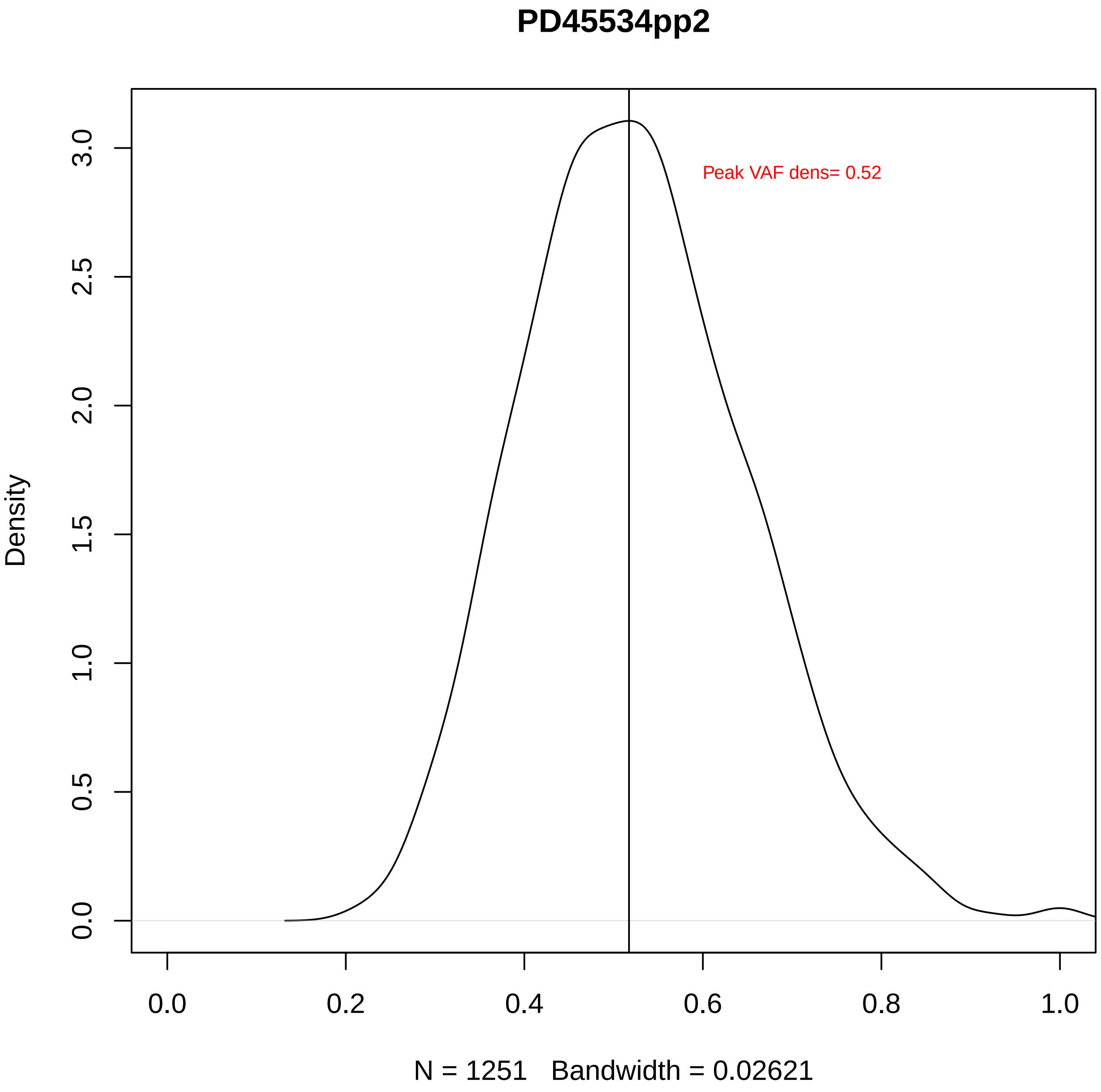

PD45534pp2
Peak VAF dens= 0.52
Density
0.0
0.5
1.0
1.5
2.0
2.5
3.0
0.0
0.2
0.4
0.6
0.8
1.0
N = 1251 Bandwidth = 0.02621

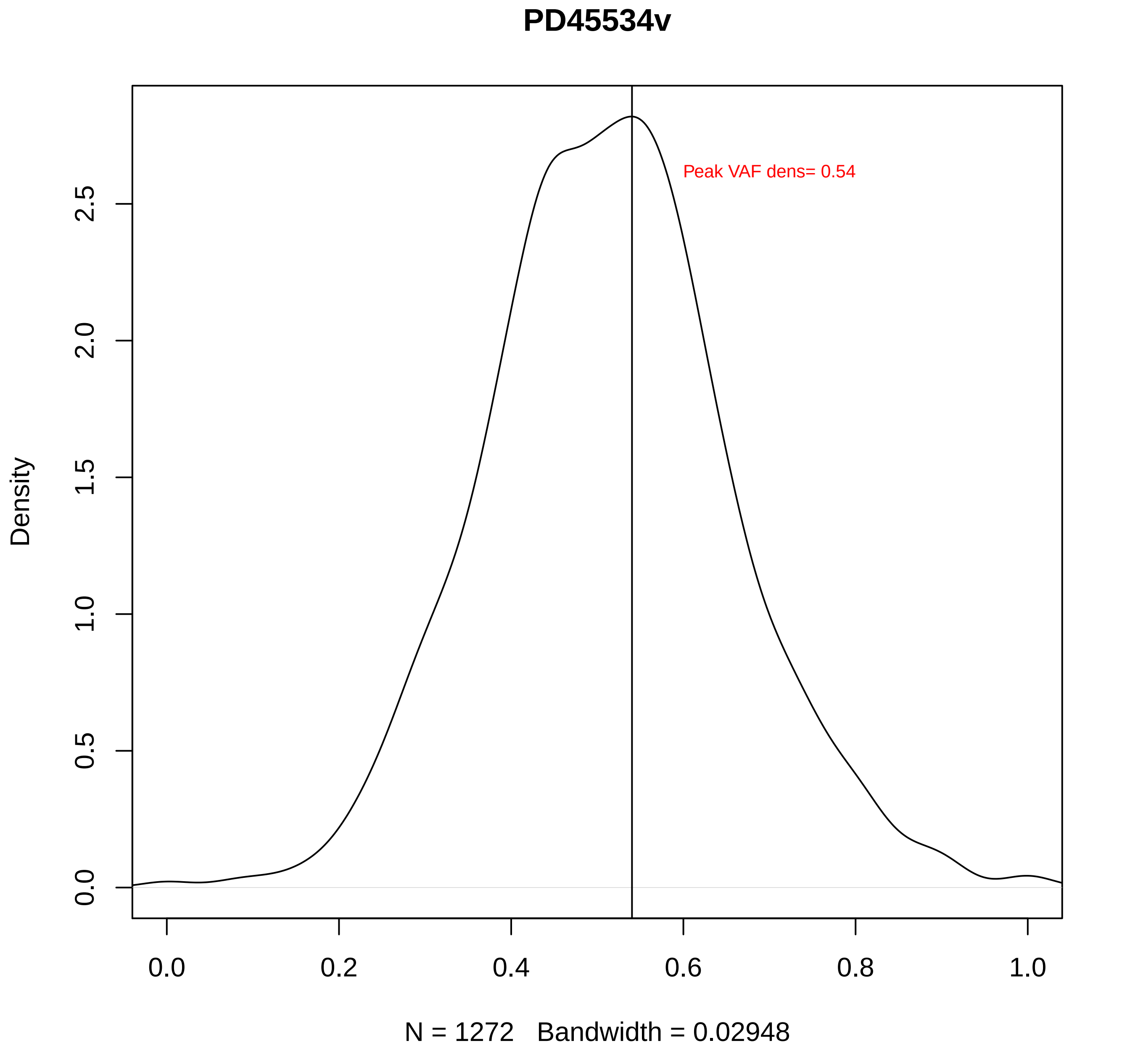
PD45534v
Peak VAF dens= 0.54
Density
0.0
0.5
1.0
1.5
2.0
2.5
0.0
0.2
0.4
0.6
0.8
1.0
N = 1272   Bandwidth = 0.02948

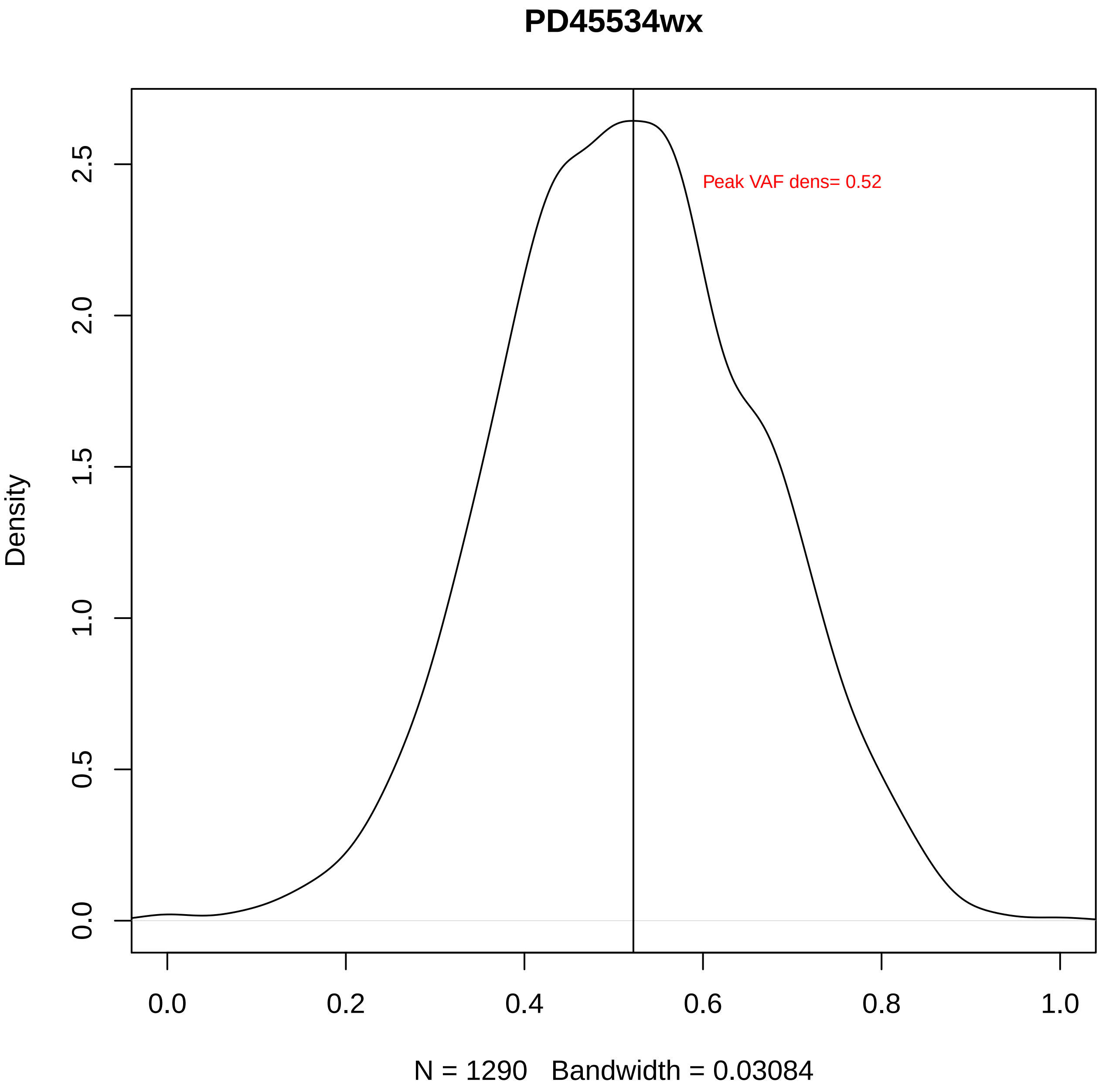
PD45534wx
Peak VAF dens= 0.52
Density
0.0
0.5
1.0
1.5
2.0
2.5
0.0
0.2
0.4
0.6
0.8
1.0
N = 1290 Bandwidth = 0.03084

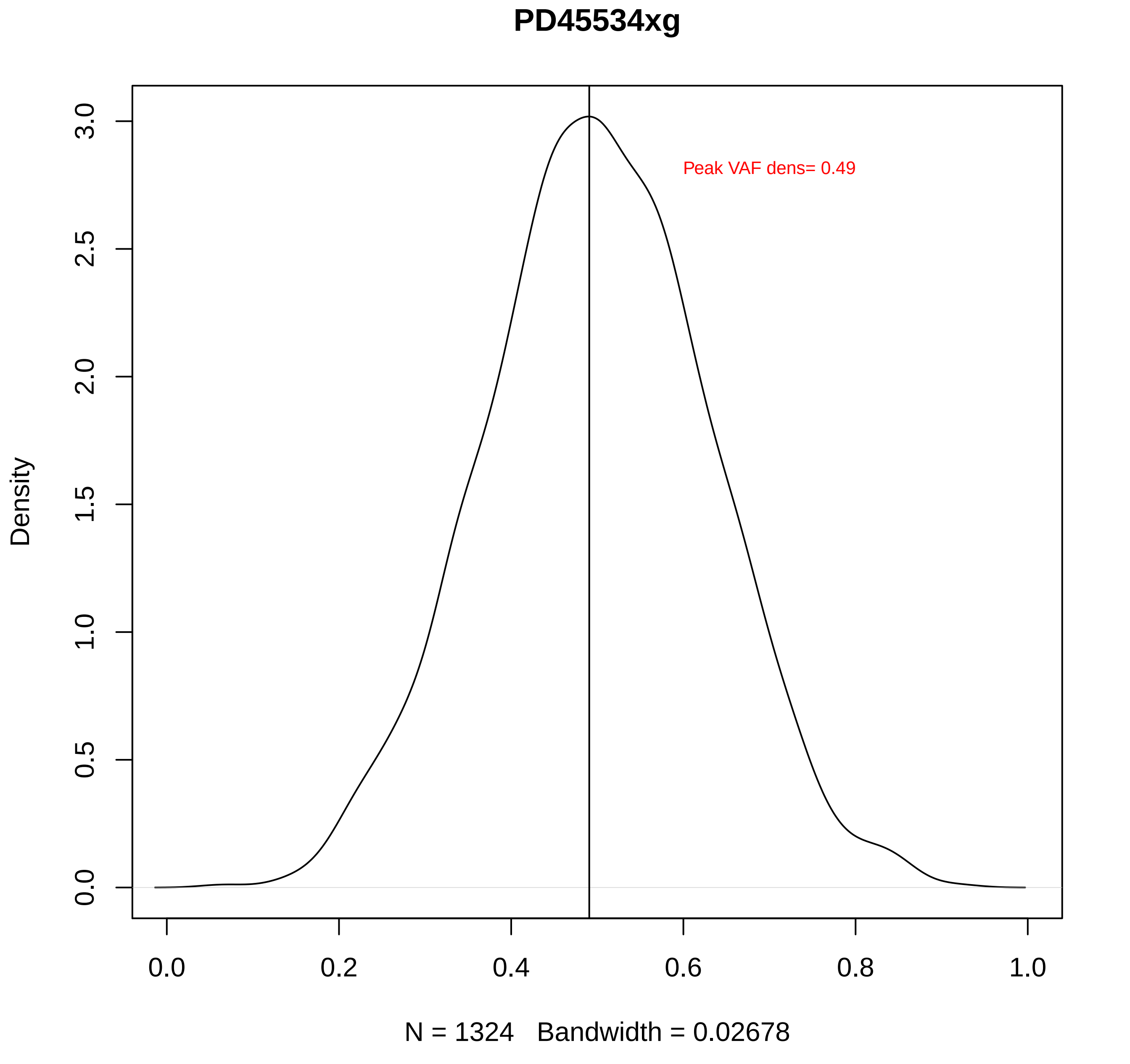

PD45534xg
Peak VAF dens= 0.49
Density
0.0
0.5
1.0
1.5
2.0
2.5
3.0
0.0
0.2
0.4
0.6
0.8
1.0
N = 1324   Bandwidth = 0.02678

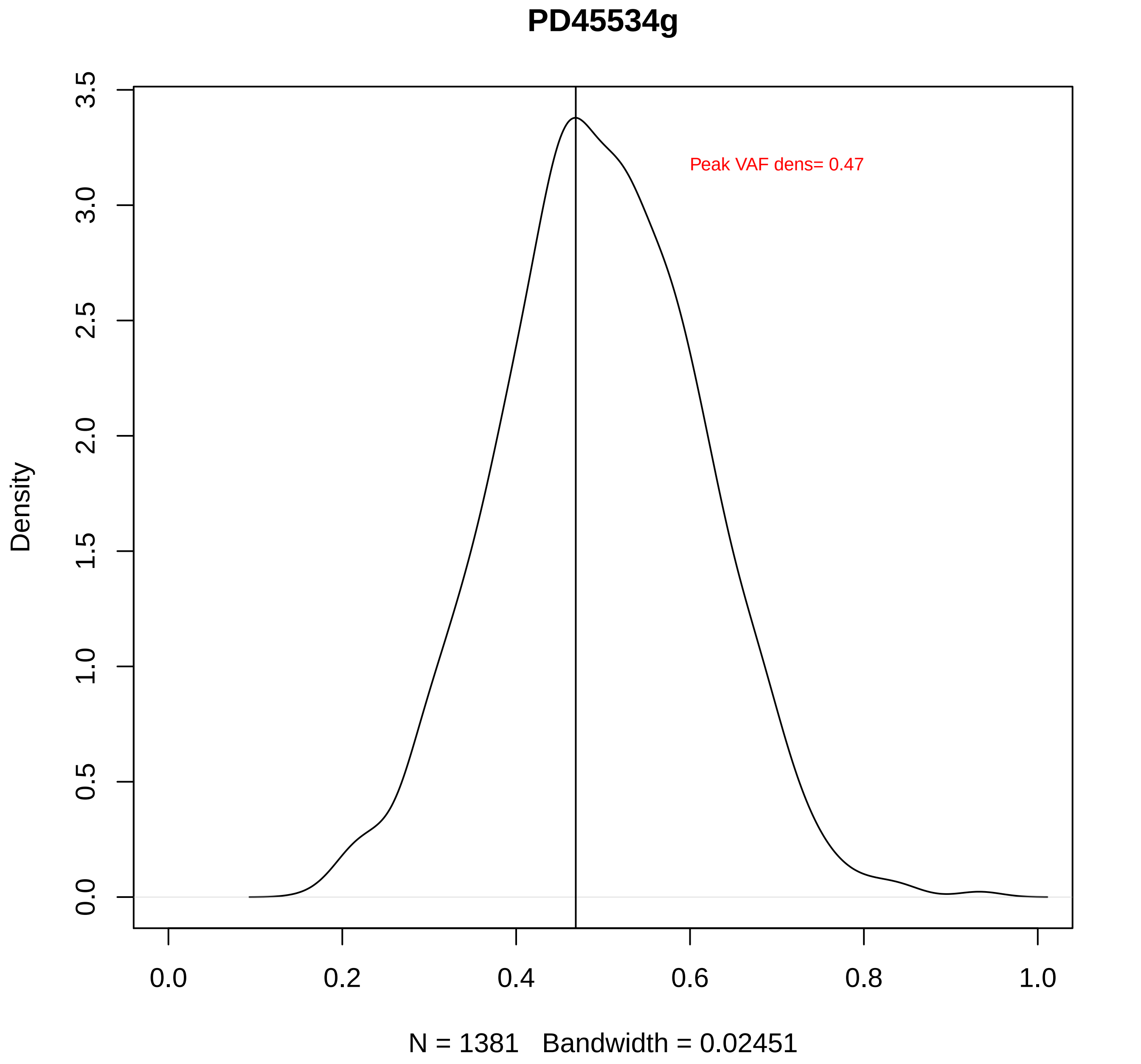

PD45534g
Peak VAF dens= 0.47
Density
0.0
0.5
1.0
1.5
2.0
2.5
3.0
3.5
0.0
0.2
0.4
0.6
0.8
1.0
N = 1381   Bandwidth = 0.02451

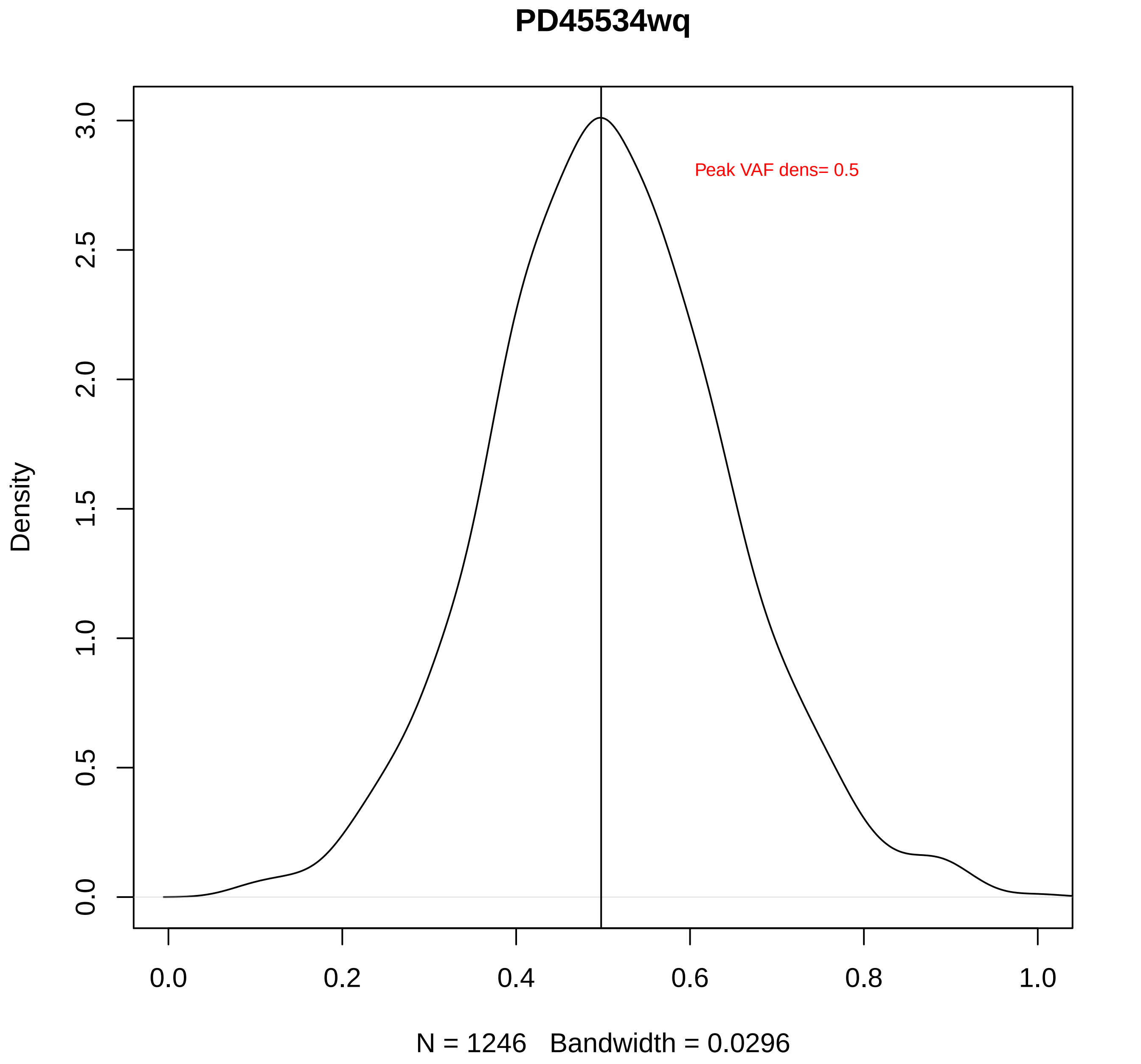
PD45534wq
Peak VAF dens= 0.5
Density
0.0
0.5
1.0
1.5
2.0
2.5
3.0
0.0
0.2
0.4
0.6
0.8
1.0
N = 1246   Bandwidth = 0.0296

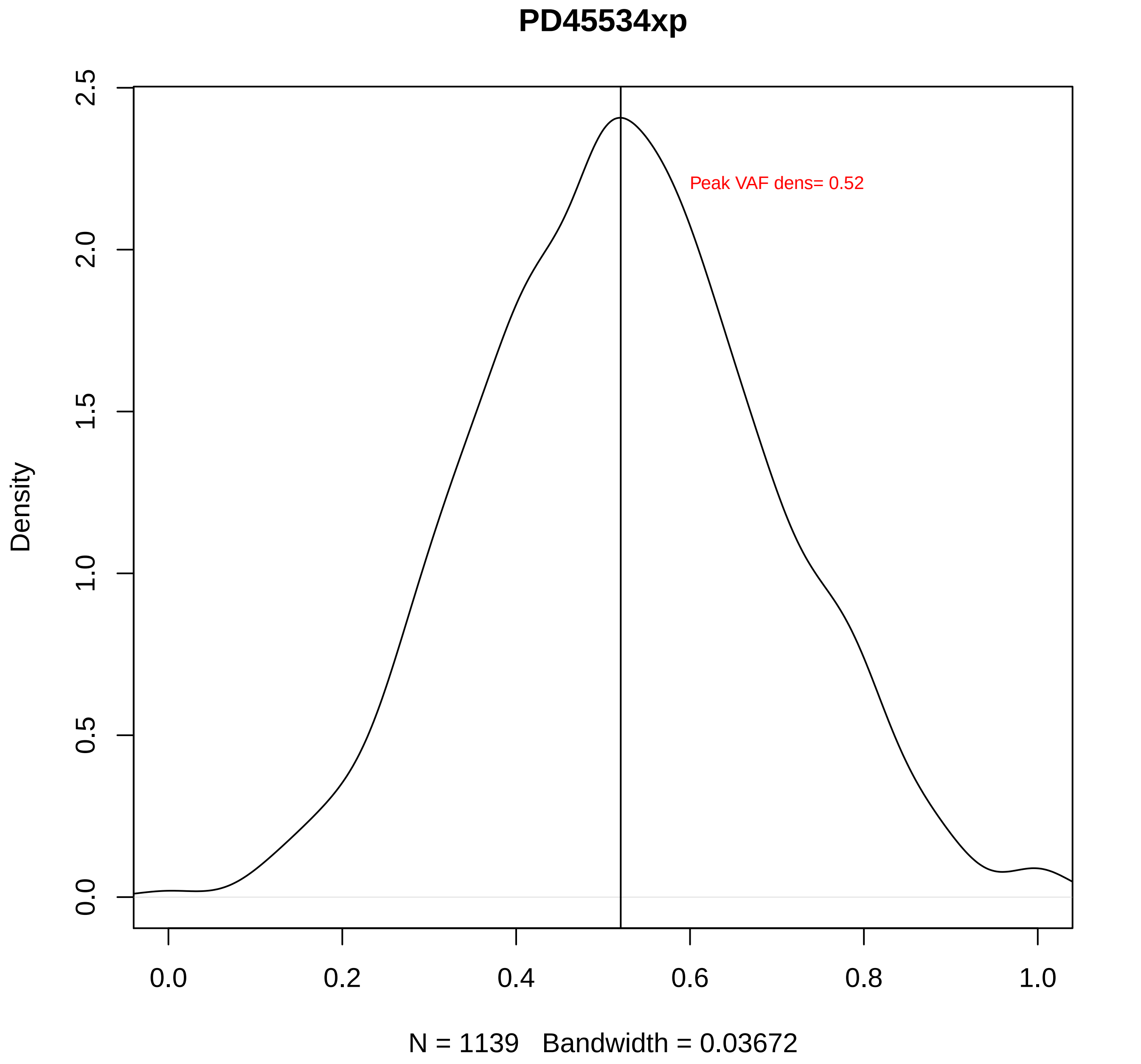

PD45534xp
Peak VAF dens= 0.52
Density
0.0
0.5
1.0
1.5
2.0
2.5
0.0
0.2
0.4
0.6
0.8
1.0
N = 1139   Bandwidth = 0.03672

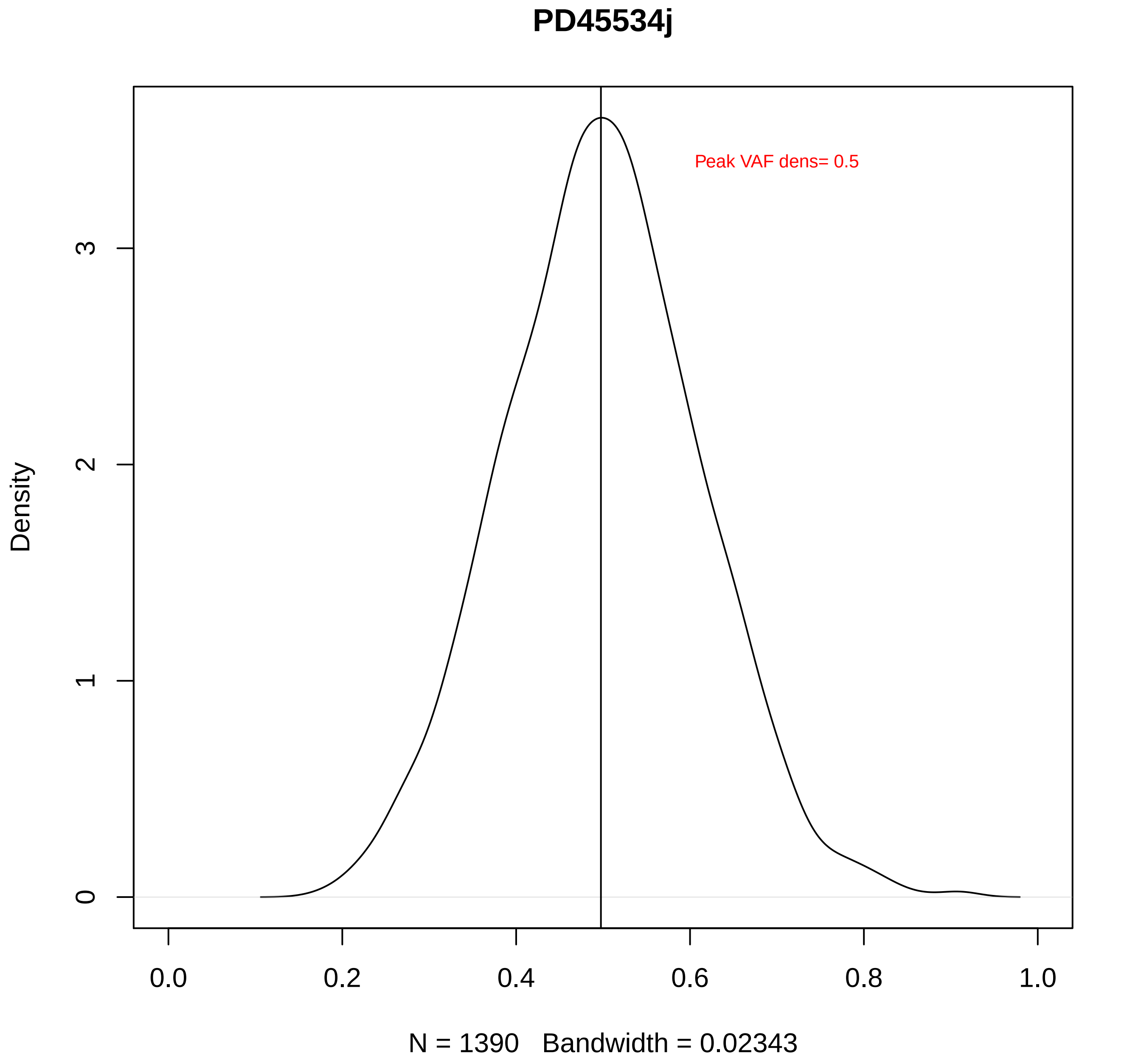
PD45534j
Peak VAF dens= 0.5
Density
0
1
2
3
0.0
0.2
0.4
0.6
0.8
1.0
N = 1390   Bandwidth = 0.02343

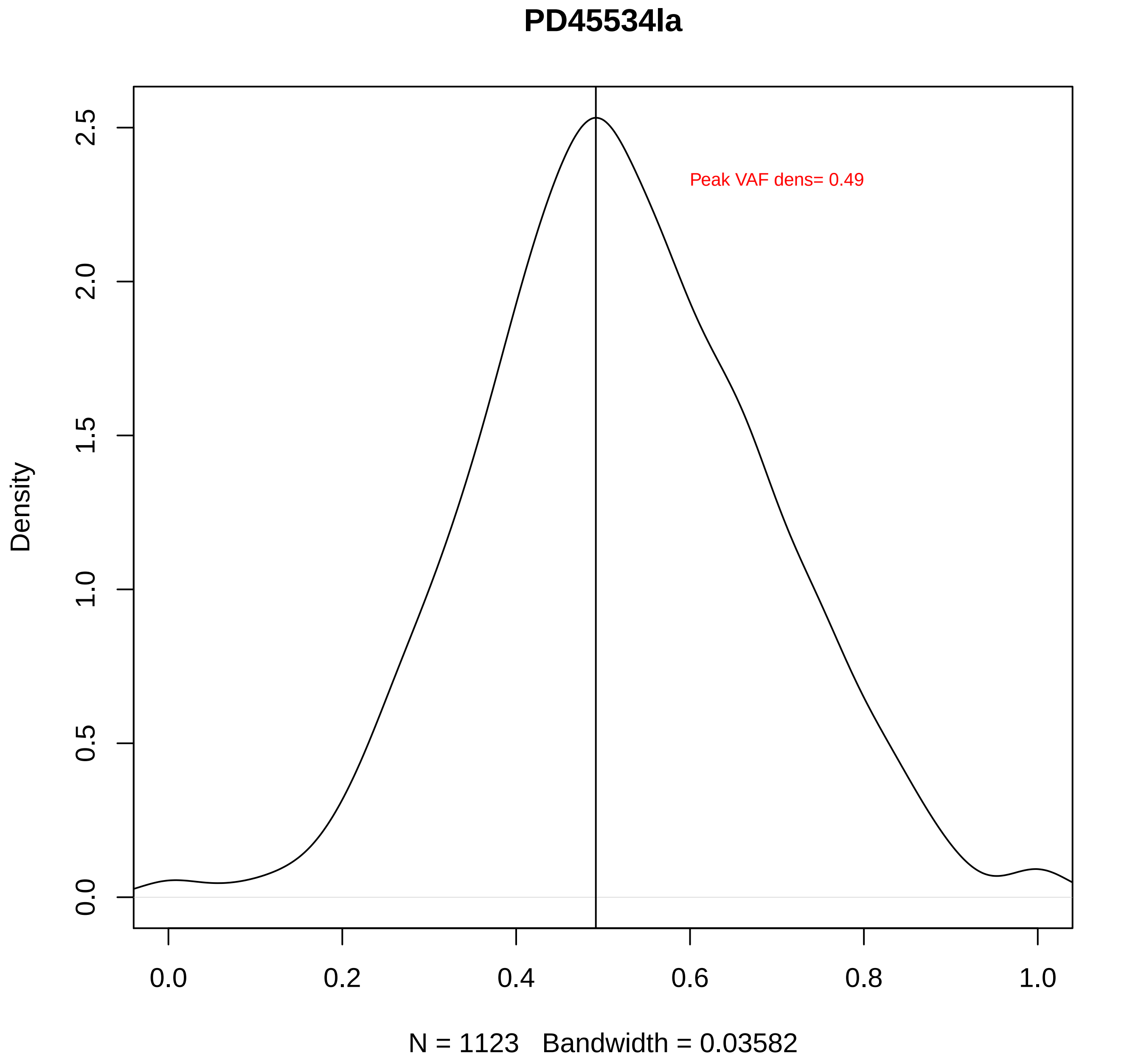

PD45534la
Peak VAF dens= 0.49
Density
2.5
2.0
1.5
1.0
0.5
0.0
0.0
0.2
0.4
0.6
0.8
1.0
N = 1123 Bandwidth = 0.03582

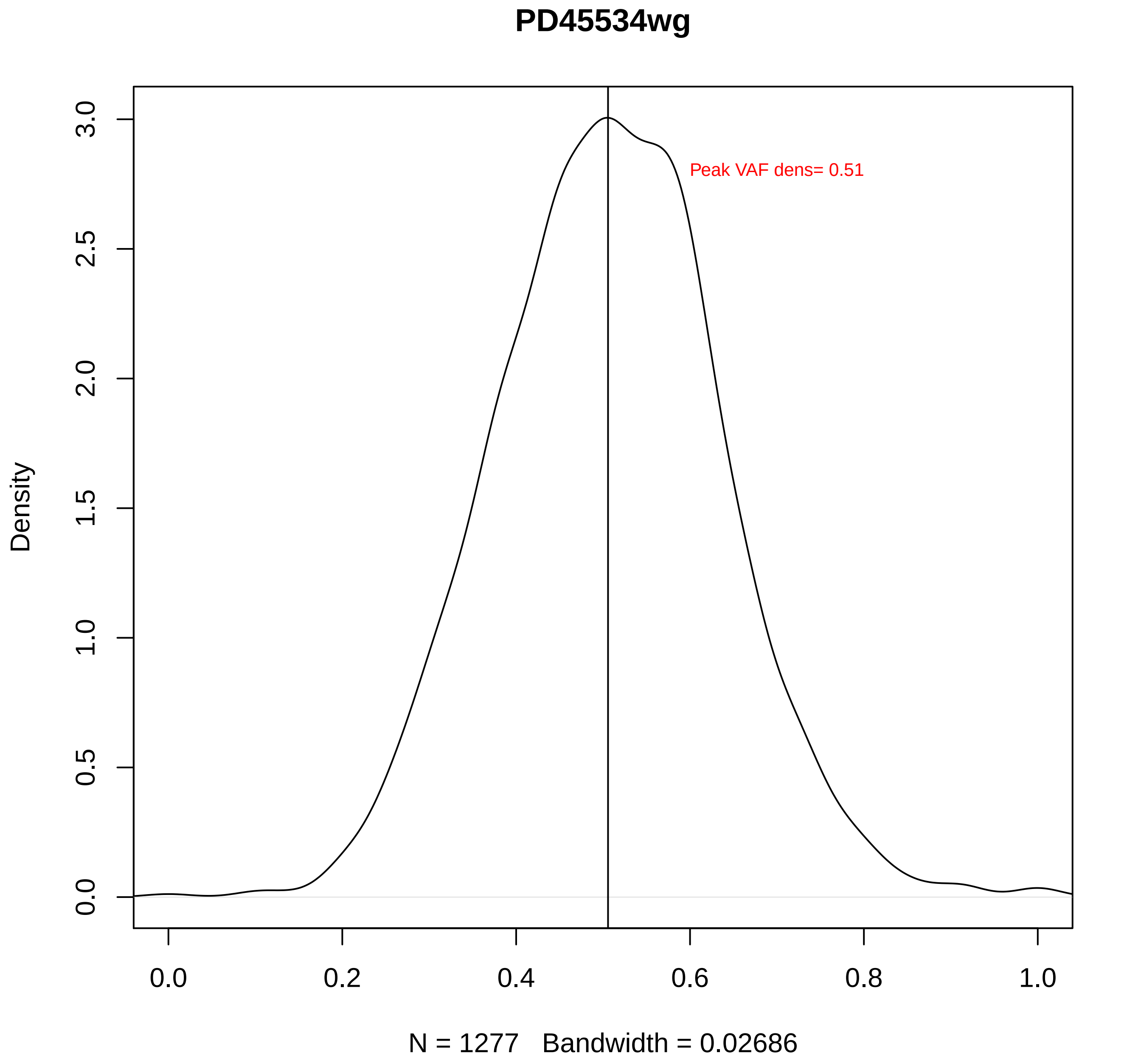
PD45534wg
Peak VAF dens= 0.51
Density
0.0
0.5
1.0
1.5
2.0
2.5
3.0
0.0
0.2
0.4
0.6
0.8
1.0
N = 1277 Bandwidth = 0.02686

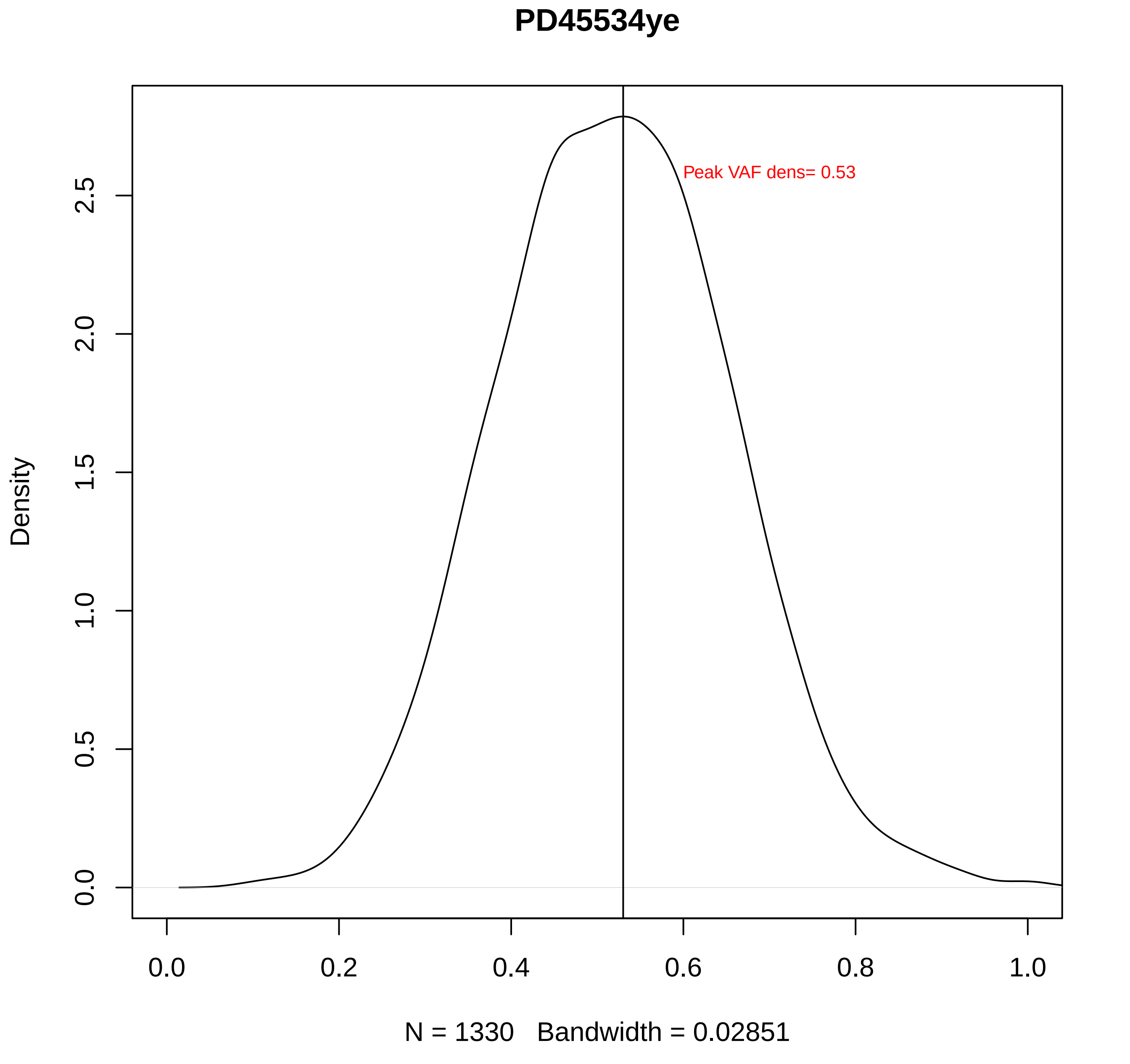

PD45534ye
Peak VAF dens= 0.53
Density
0.0
0.5
1.0
1.5
2.0
2.5
0.0
0.2
0.4
0.6
0.8
1.0
N = 1330   Bandwidth = 0.02851

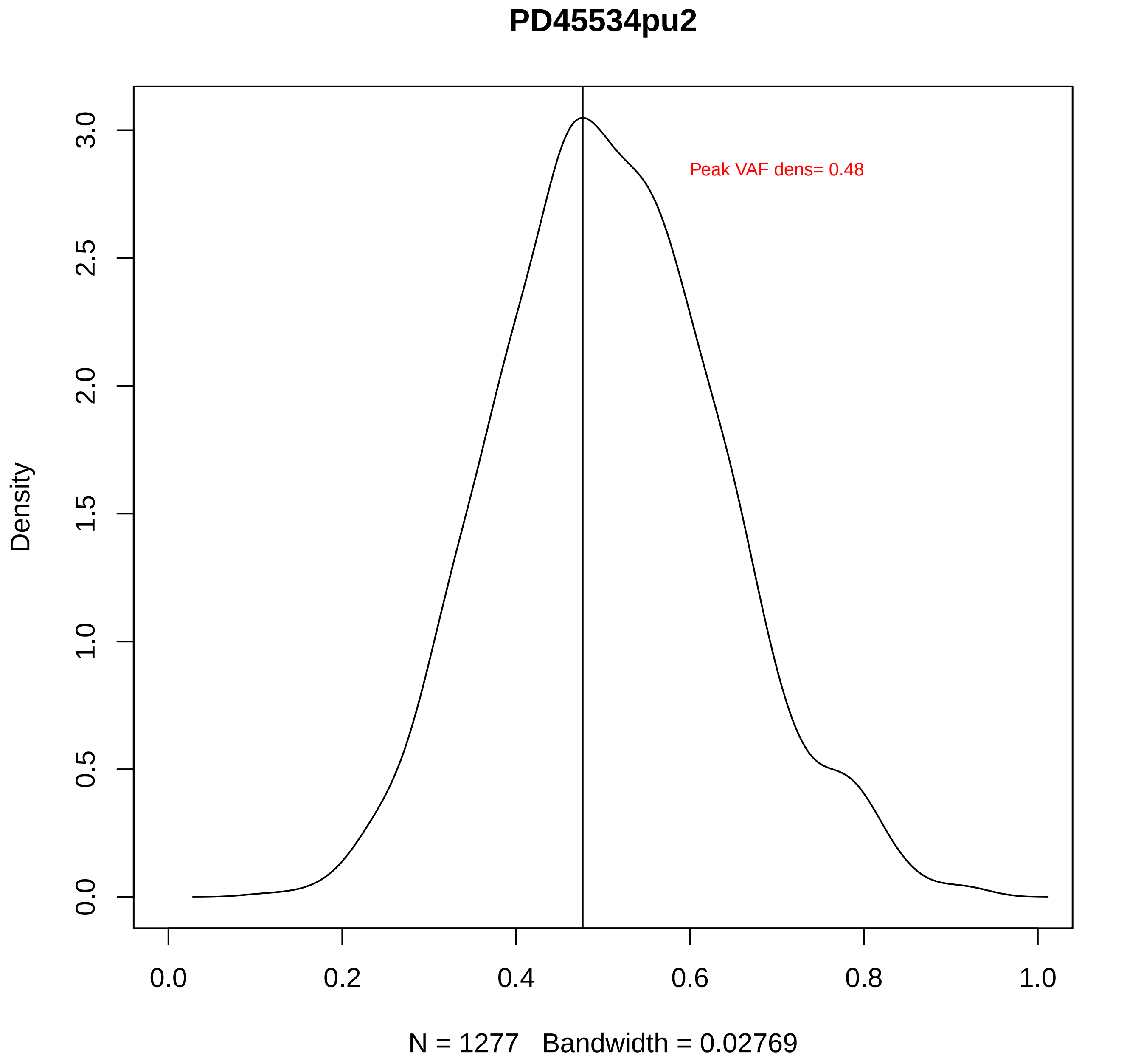
PD45534pu2
Peak VAF dens= 0.48
Density
0.0
0.5
1.0
1.5
2.0
2.5
3.0
0.0
0.2
0.4
0.6
0.8
1.0
N = 1277   Bandwidth = 0.02769

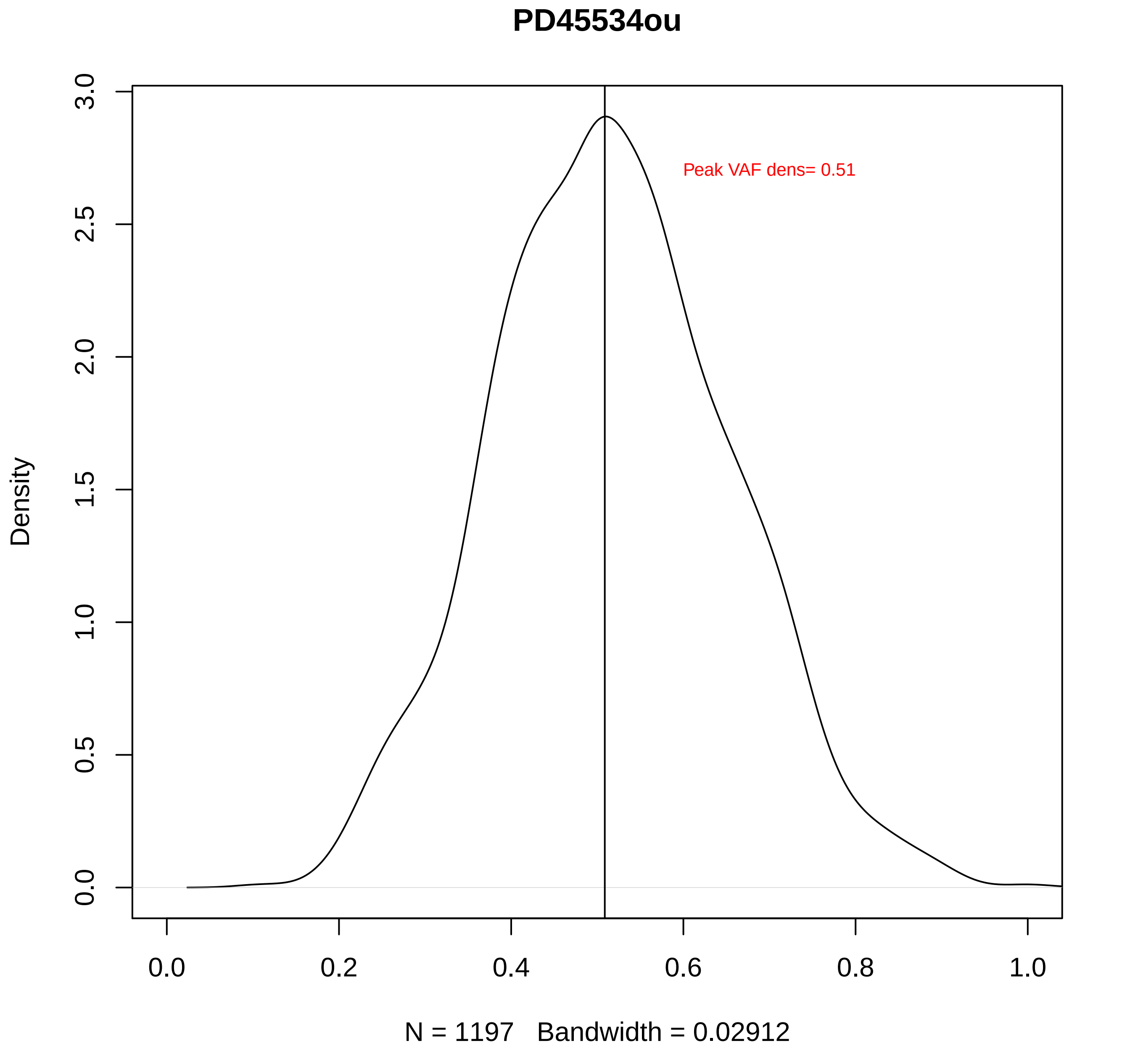
PD45534ou
Peak VAF dens= 0.51
Density
0.0
0.5
1.0
1.5
2.0
2.5
3.0
0.0
0.2
0.4
0.6
0.8
1.0
N = 1197   Bandwidth = 0.02912

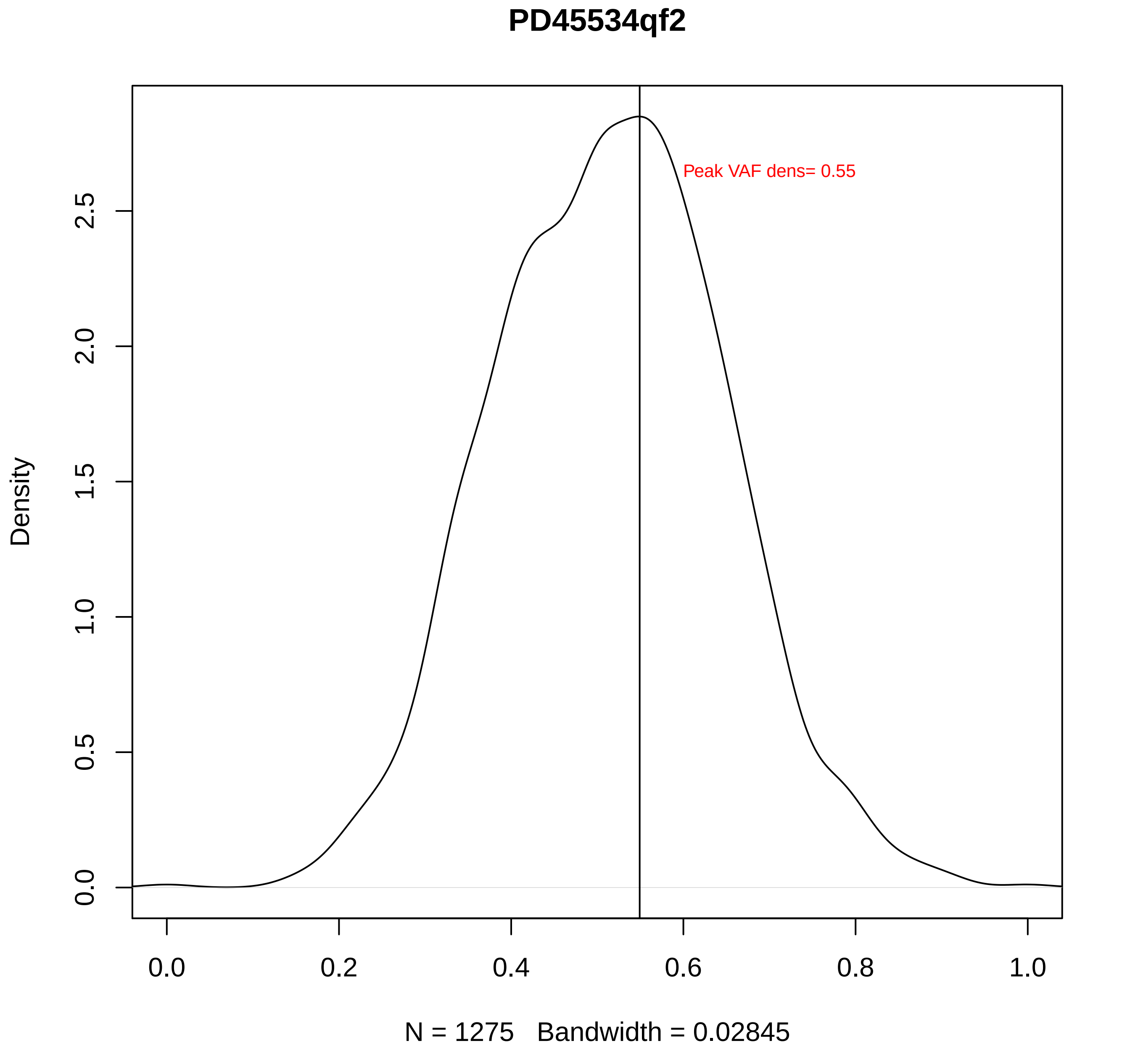
PD45534qf2
Peak VAF dens= 0.55
Density
0.0
0.5
1.0
1.5
2.0
2.5
0.0
0.2
0.4
0.6
0.8
1.0
N = 1275   Bandwidth = 0.02845

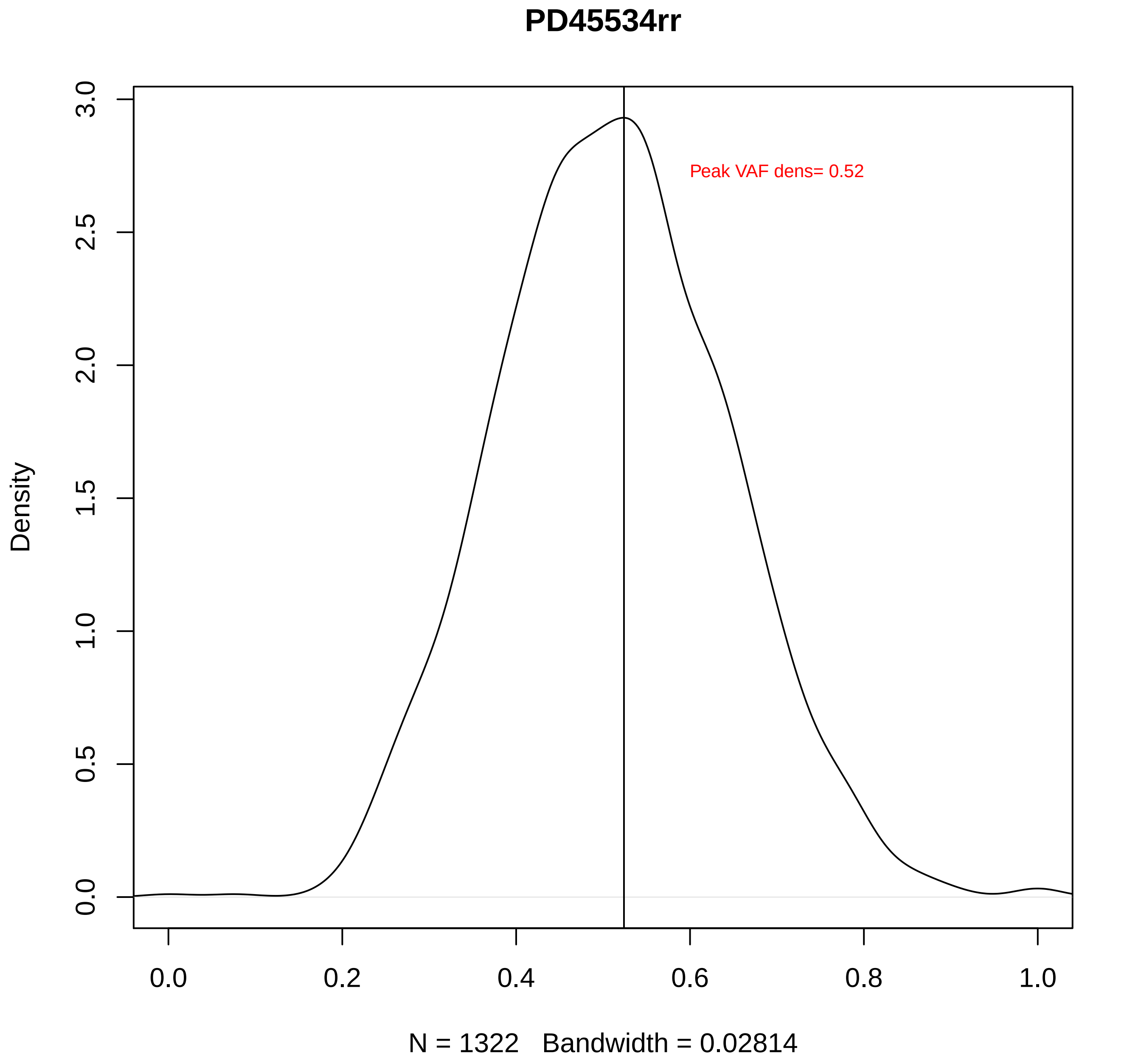

PD45534rr
Peak VAF dens= 0.52
Density
0.0
0.5
1.0
1.5
2.0
2.5
3.0
0.0
0.2
0.4
0.6
0.8
1.0
N = 1322   Bandwidth = 0.02814

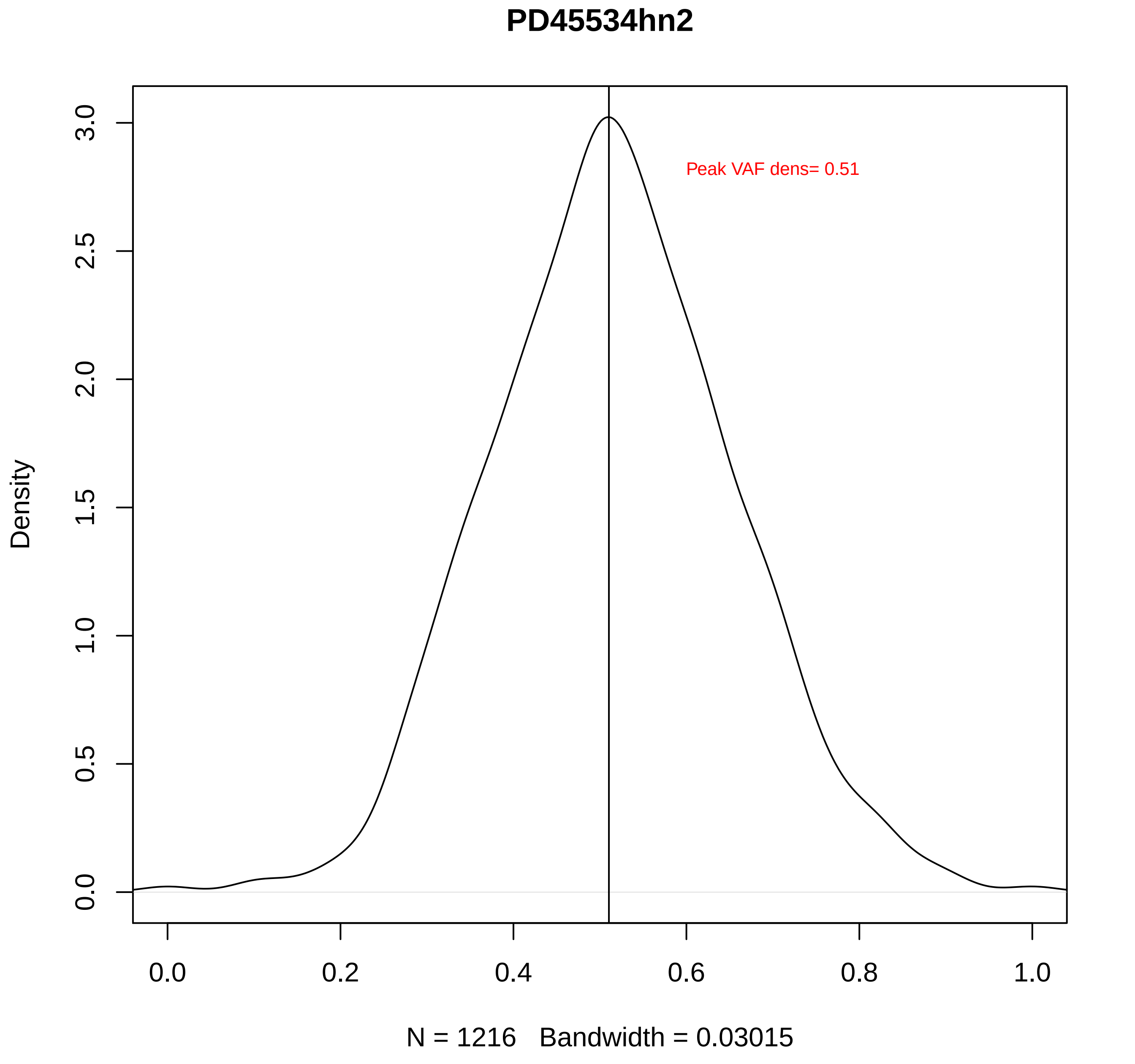
PD45534hn2
Peak VAF dens= 0.51
Density
0.0
0.5
1.0
1.5
2.0
2.5
3.0
0.0
0.2
0.4
0.6
0.8
1.0
N = 1216 Bandwidth = 0.03015

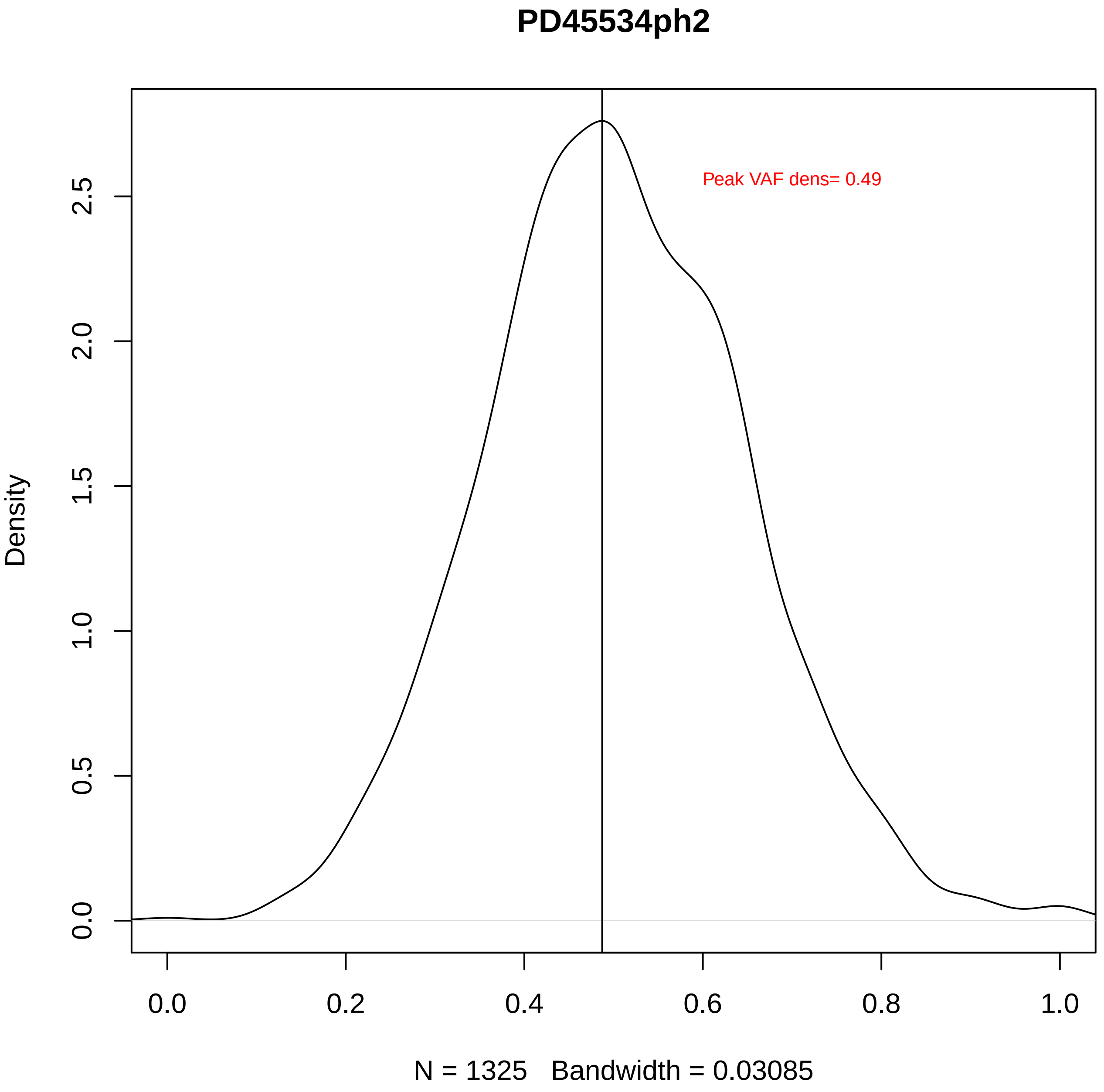

PD45534ph2
Peak VAF dens= 0.49
Density
0.0
0.5
1.0
1.5
2.0
2.5
0.0
0.2
0.4
0.6
0.8
1.0
N = 1325 Bandwidth = 0.03085

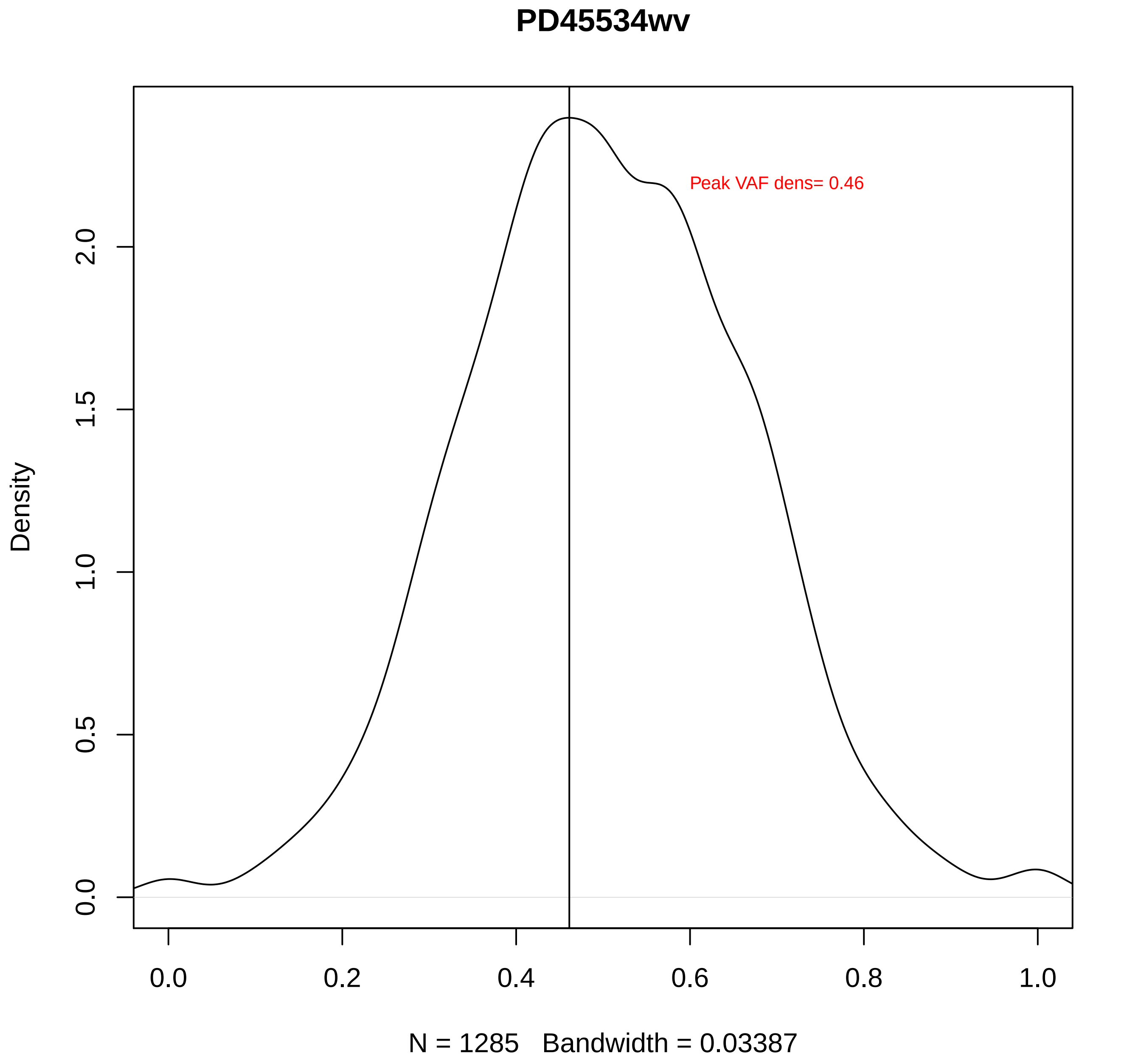
PD45534wv
Peak VAF dens= 0.46
Density
0.0
0.5
1.0
1.5
2.0
0.0
0.2
0.4
0.6
0.8
1.0
N = 1285   Bandwidth = 0.03387

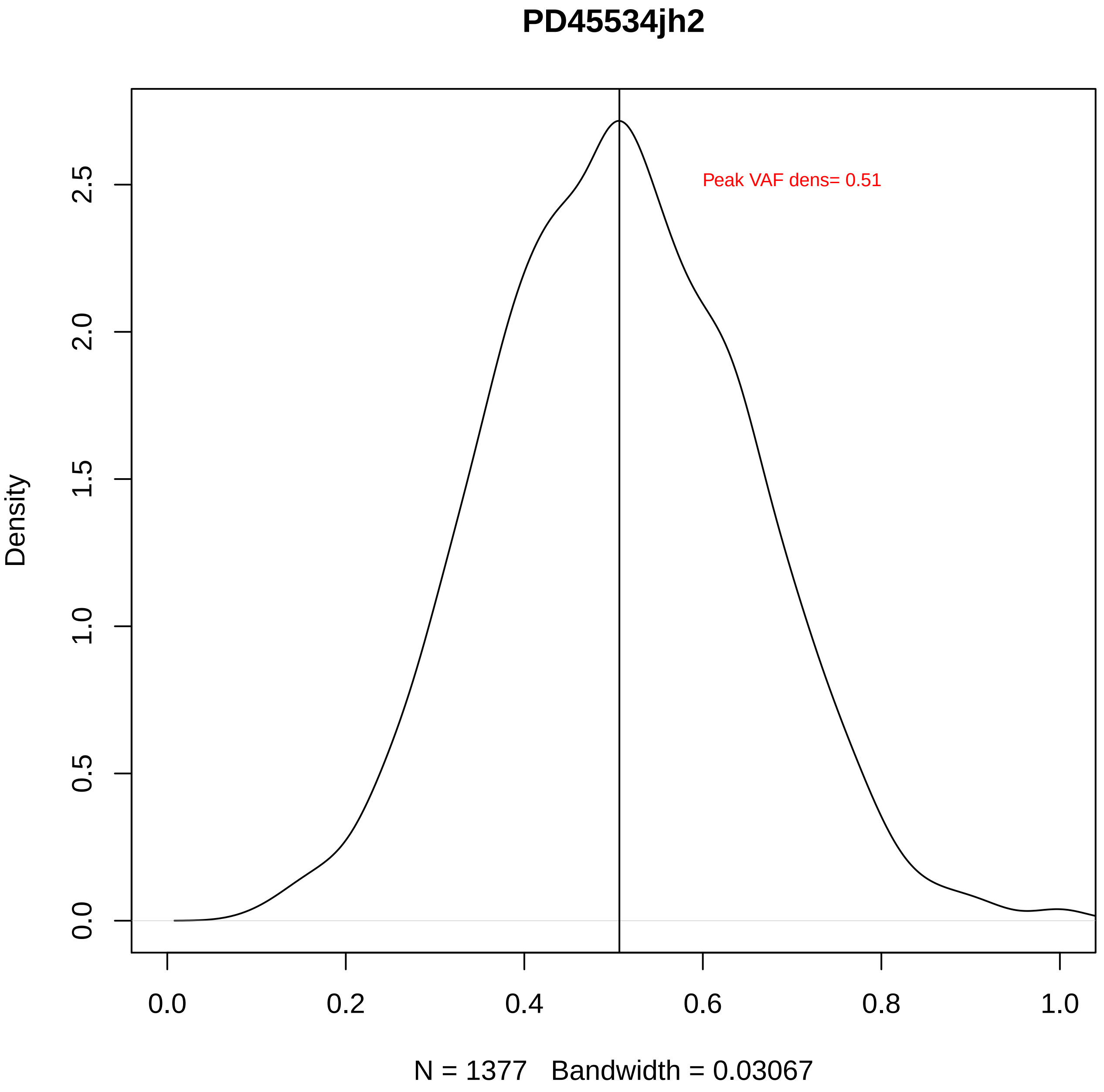
PD45534jh2
Peak VAF dens= 0.51
Density
0.0
0.5
1.0
1.5
2.0
2.5
0.0
0.2
0.4
0.6
0.8
1.0
N = 1377   Bandwidth = 0.03067

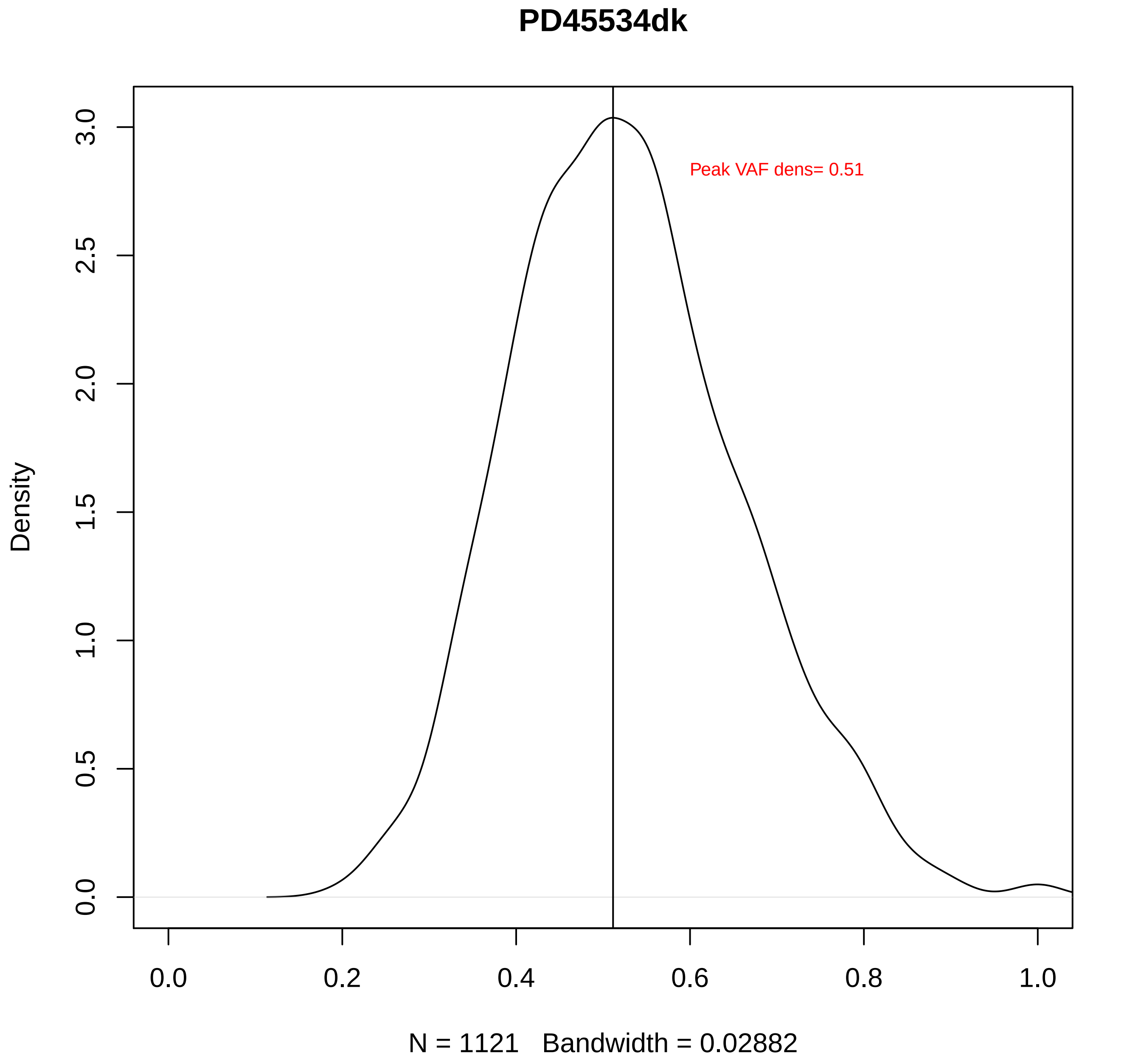
PD45534dk
Peak VAF dens= 0.51
Density
0.0
0.5
1.0
1.5
2.0
2.5
3.0
0.0
0.2
0.4
0.6
0.8
1.0
N = 1121 Bandwidth = 0.02882

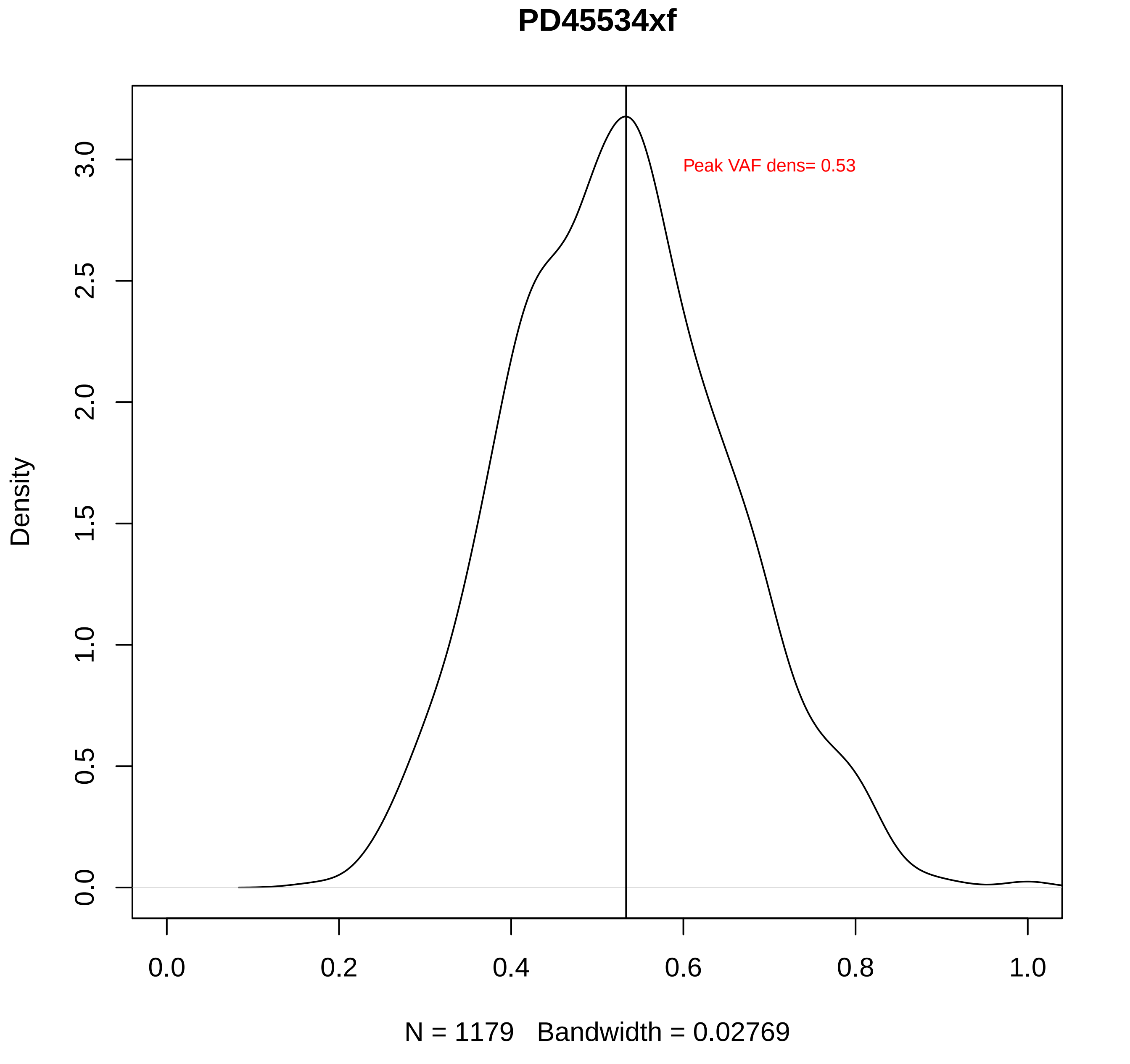
PD45534xf
Peak VAF dens= 0.53
Density
0.0
0.5
1.0
1.5
2.0
2.5
3.0
0.0
0.2
0.4
0.6
0.8
1.0
N = 1179   Bandwidth = 0.02769

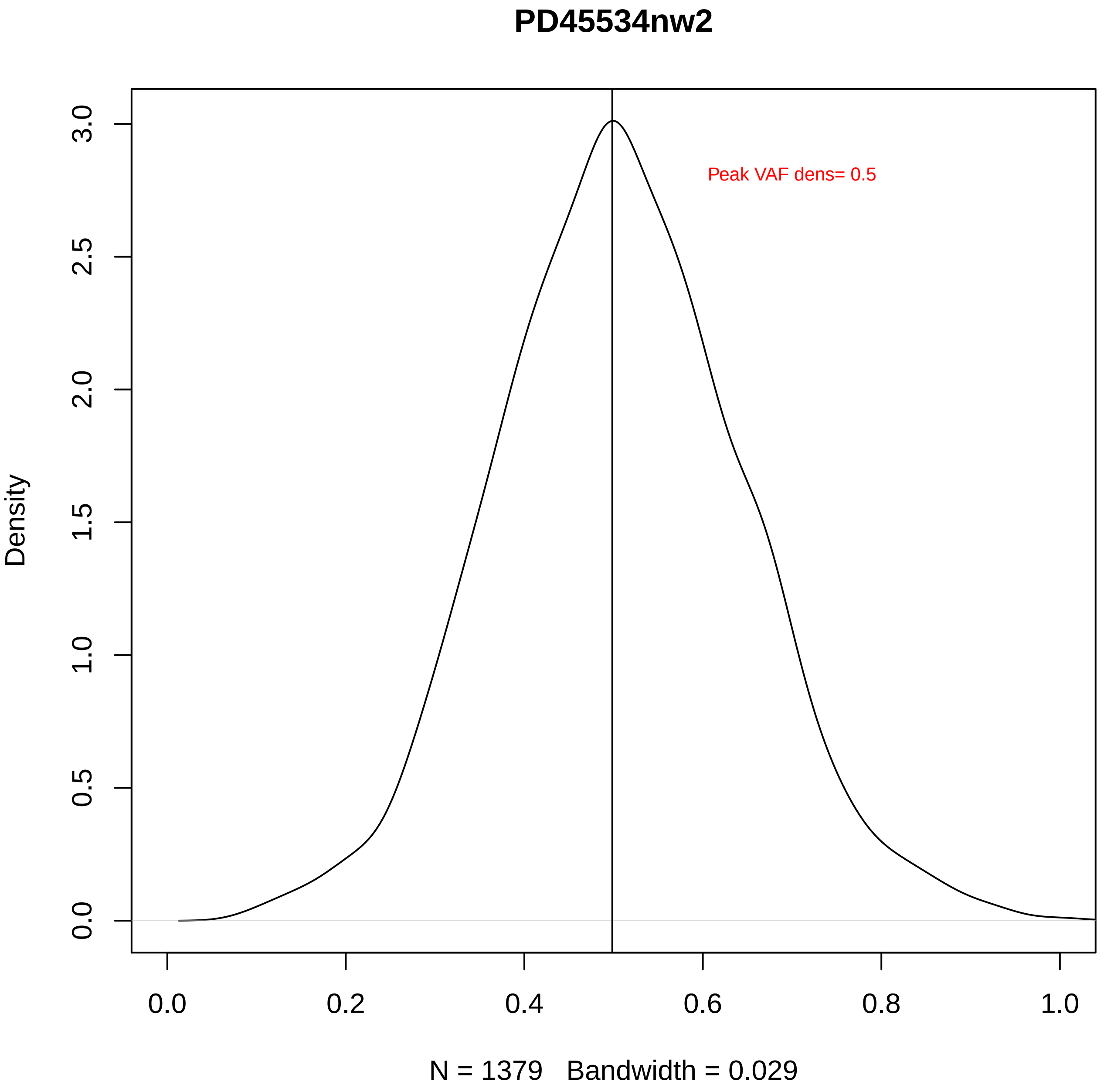
PD45534nw2
Peak VAF dens= 0.5
Density
3.0
2.5
2.0
1.5
1.0
0.5
0.0
0.0
0.2
0.4
0.6
0.8
1.0
N = 1379 Bandwidth = 0.029

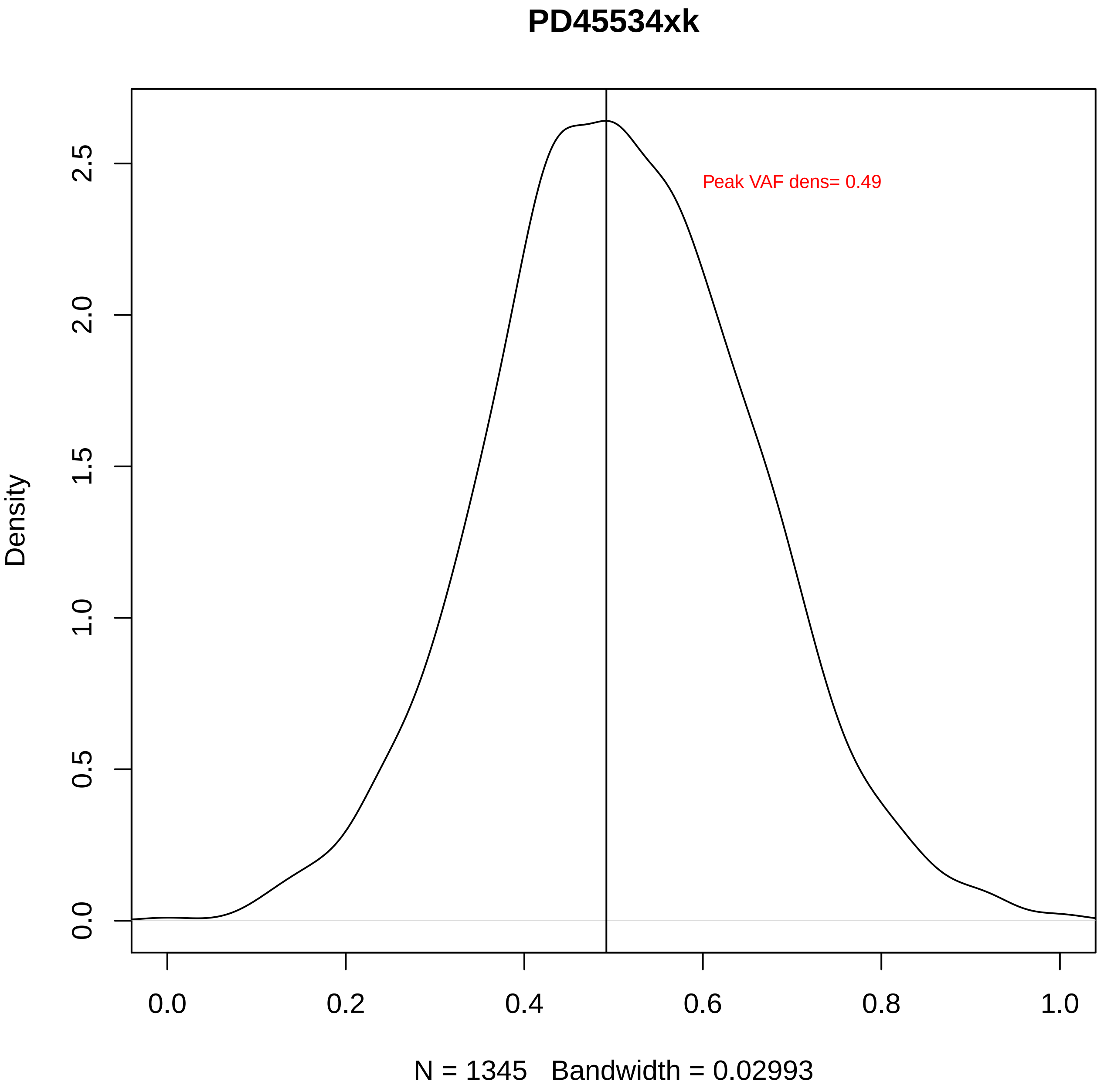
PD45534xk
Peak VAF dens= 0.49
Density
0.0
0.5
1.0
1.5
2.0
2.5
0.0
0.2
0.4
0.6
0.8
1.0
N = 1345 Bandwidth = 0.02993

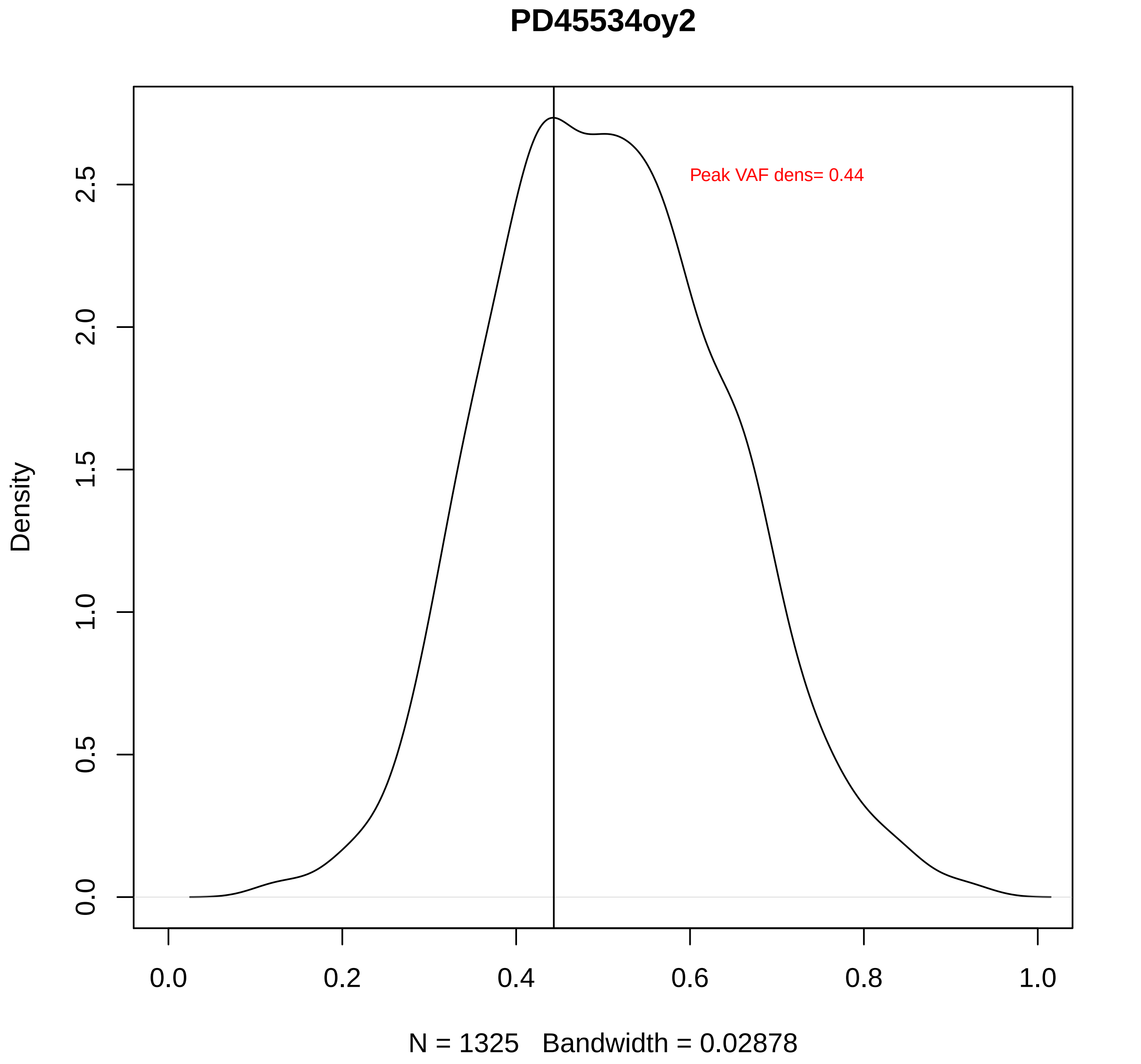
PD45534oy2
Peak VAF dens= 0.44
Density
0.0
0.5
1.0
1.5
2.0
2.5
0.0
0.2
0.4
0.6
0.8
1.0
N = 1325 Bandwidth = 0.02878

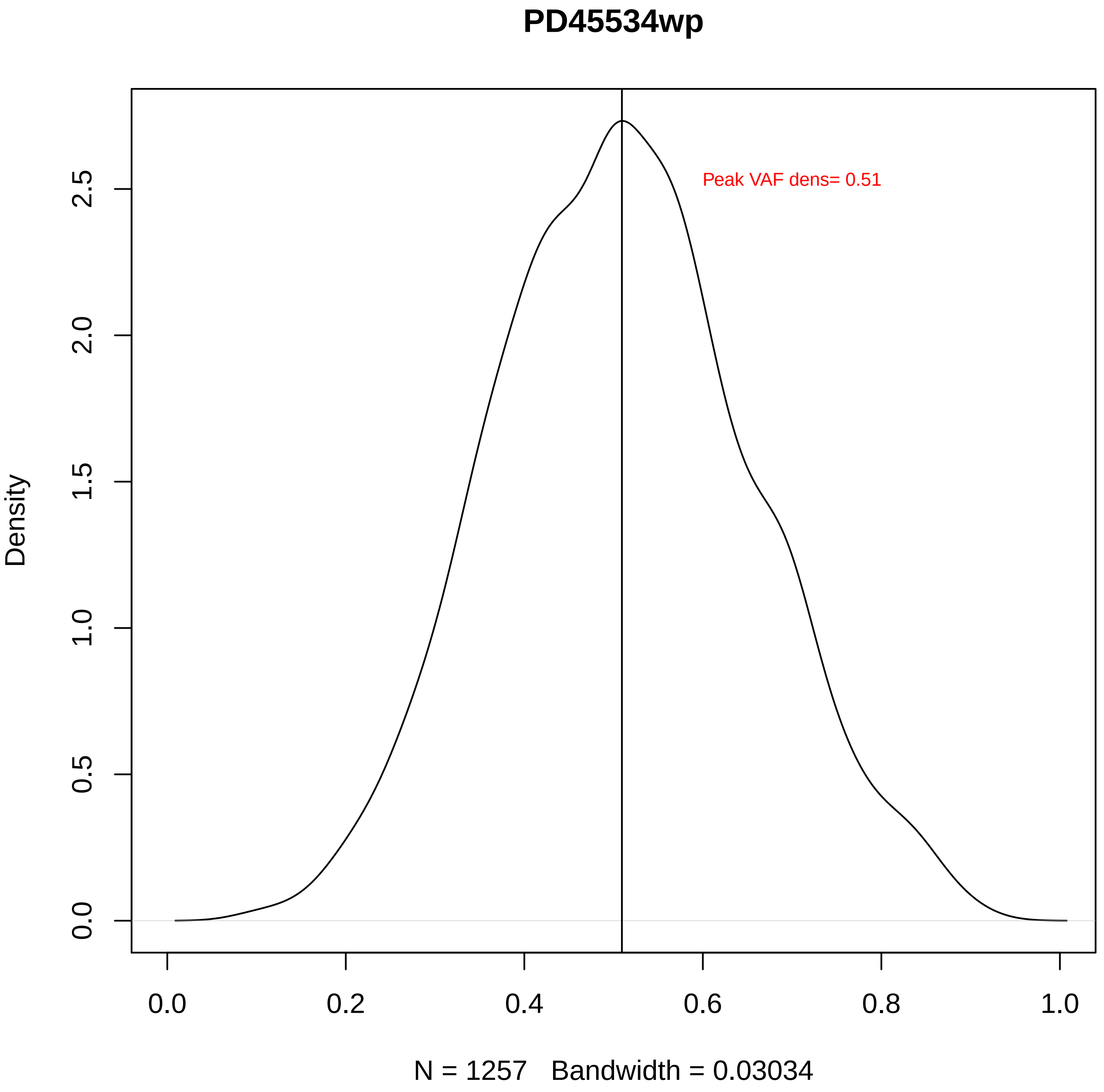
PD45534wp
Peak VAF dens= 0.51
Density
0.0
0.5
1.0
1.5
2.0
2.5
0.0
0.2
0.4
0.6
0.8
1.0
N = 1257   Bandwidth = 0.03034

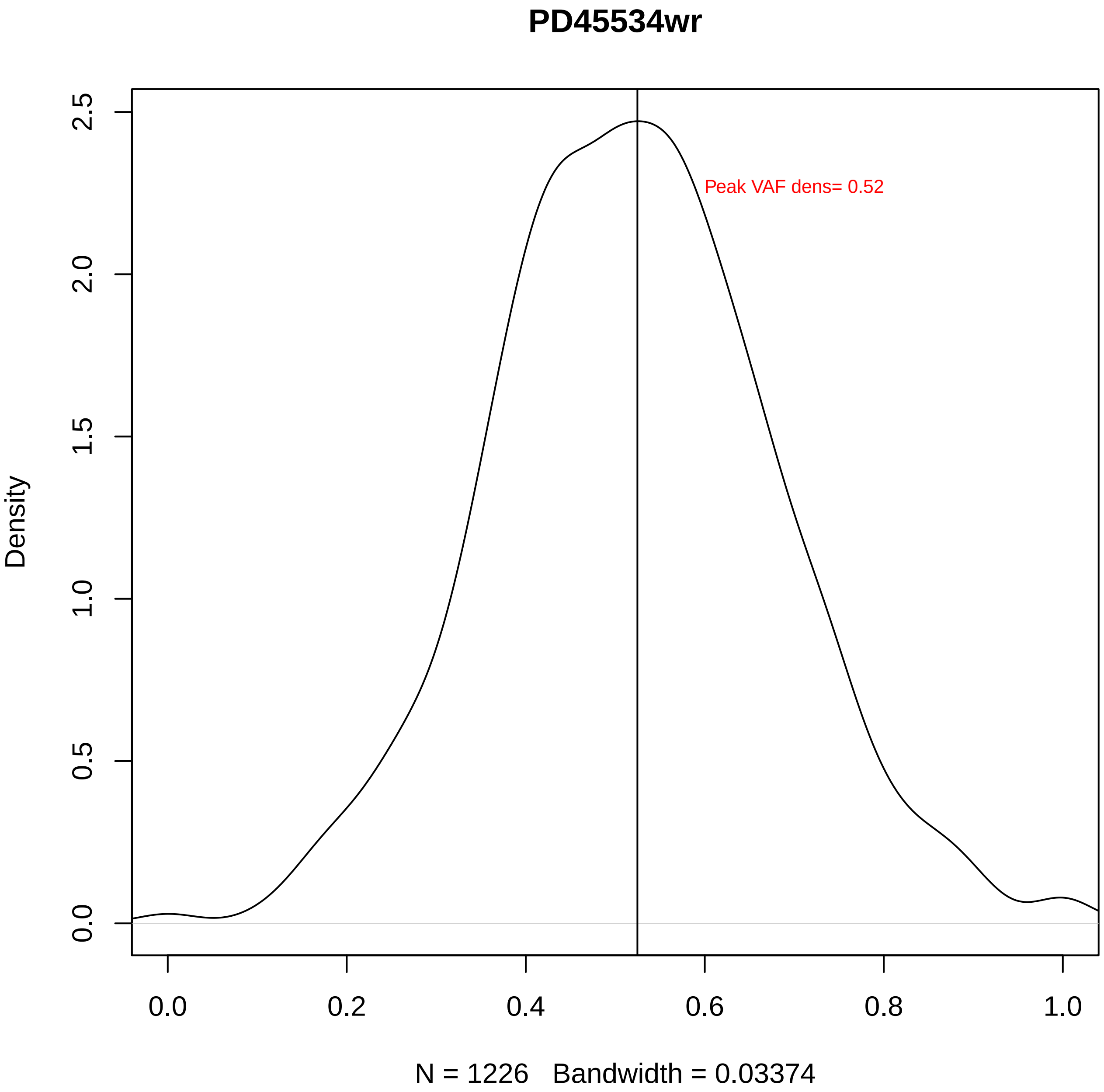
PD45534wr
Peak VAF dens= 0.52
Density
0.0
0.5
1.0
1.5
2.0
2.5
0.0
0.2
0.4
0.6
0.8
1.0
N = 1226   Bandwidth = 0.03374

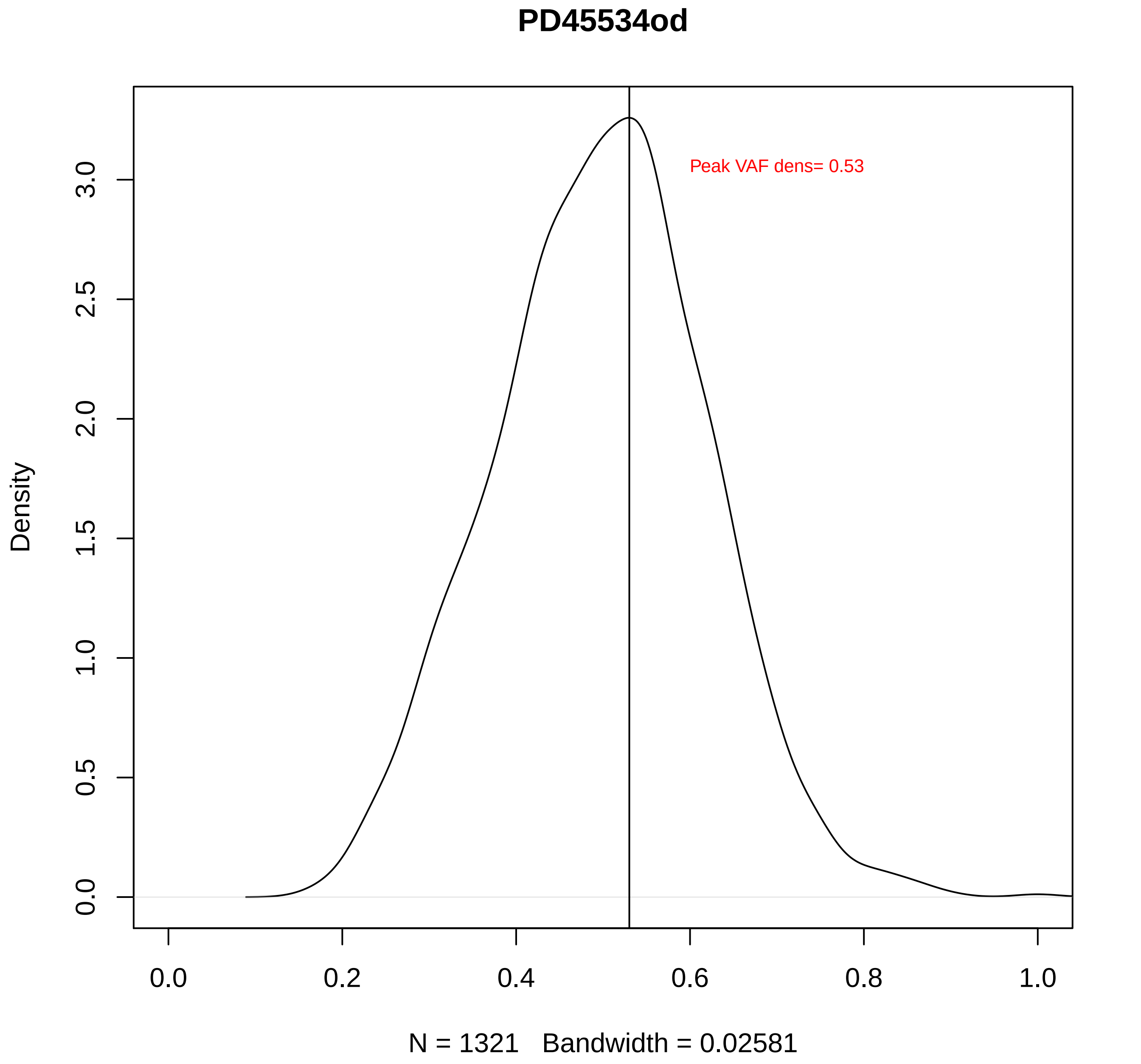
PD45534od
Peak VAF dens= 0.53
Density
0.0
0.5
1.0
1.5
2.0
2.5
3.0
0.0
0.2
0.4
0.6
0.8
1.0
N = 1321   Bandwidth = 0.02581

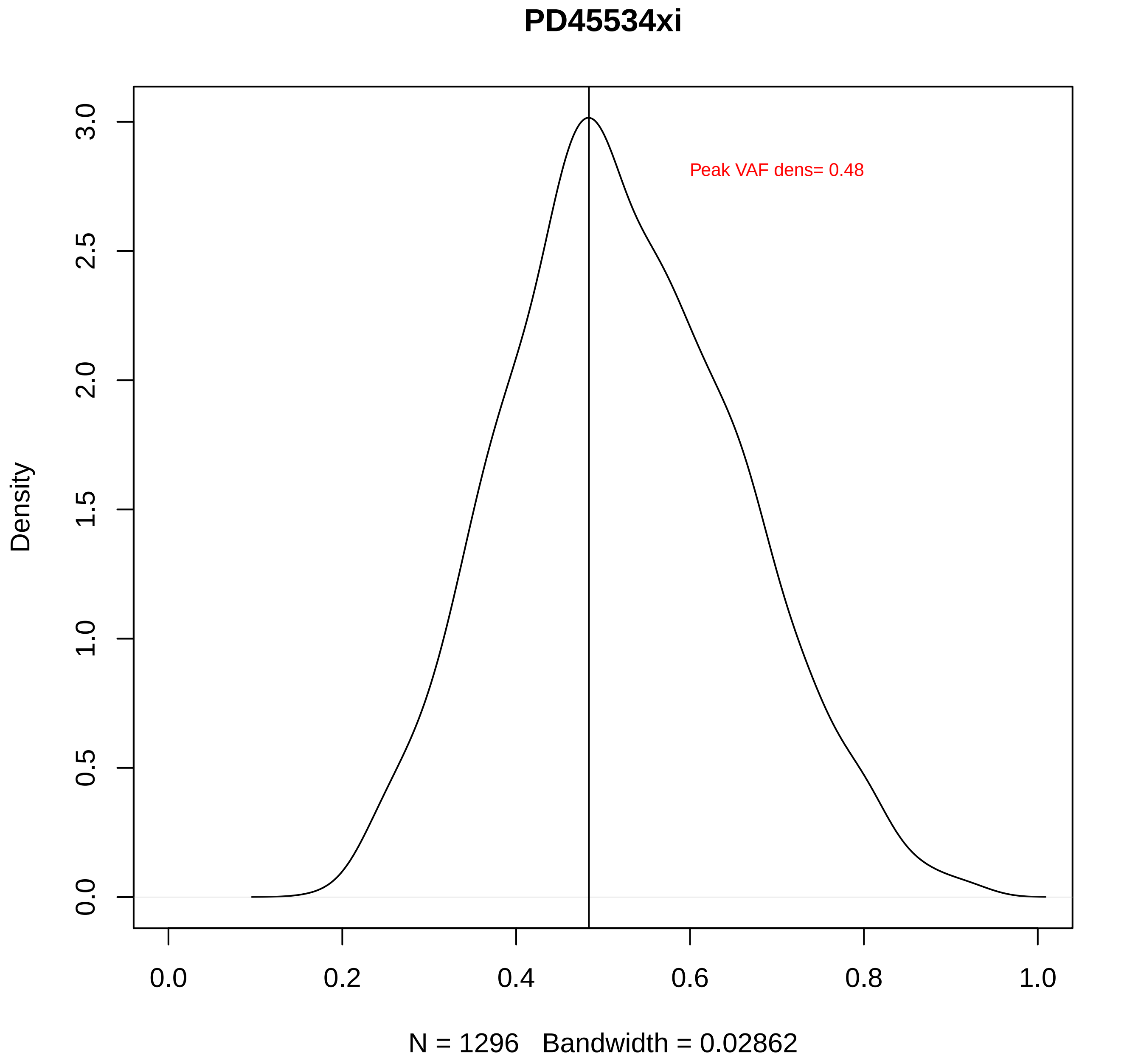

PD45534xi
Peak VAF dens= 0.48
Density
0.0
0.5
1.0
1.5
2.0
2.5
3.0
0.0
0.2
0.4
0.6
0.8
1.0
N = 1296 Bandwidth = 0.02862

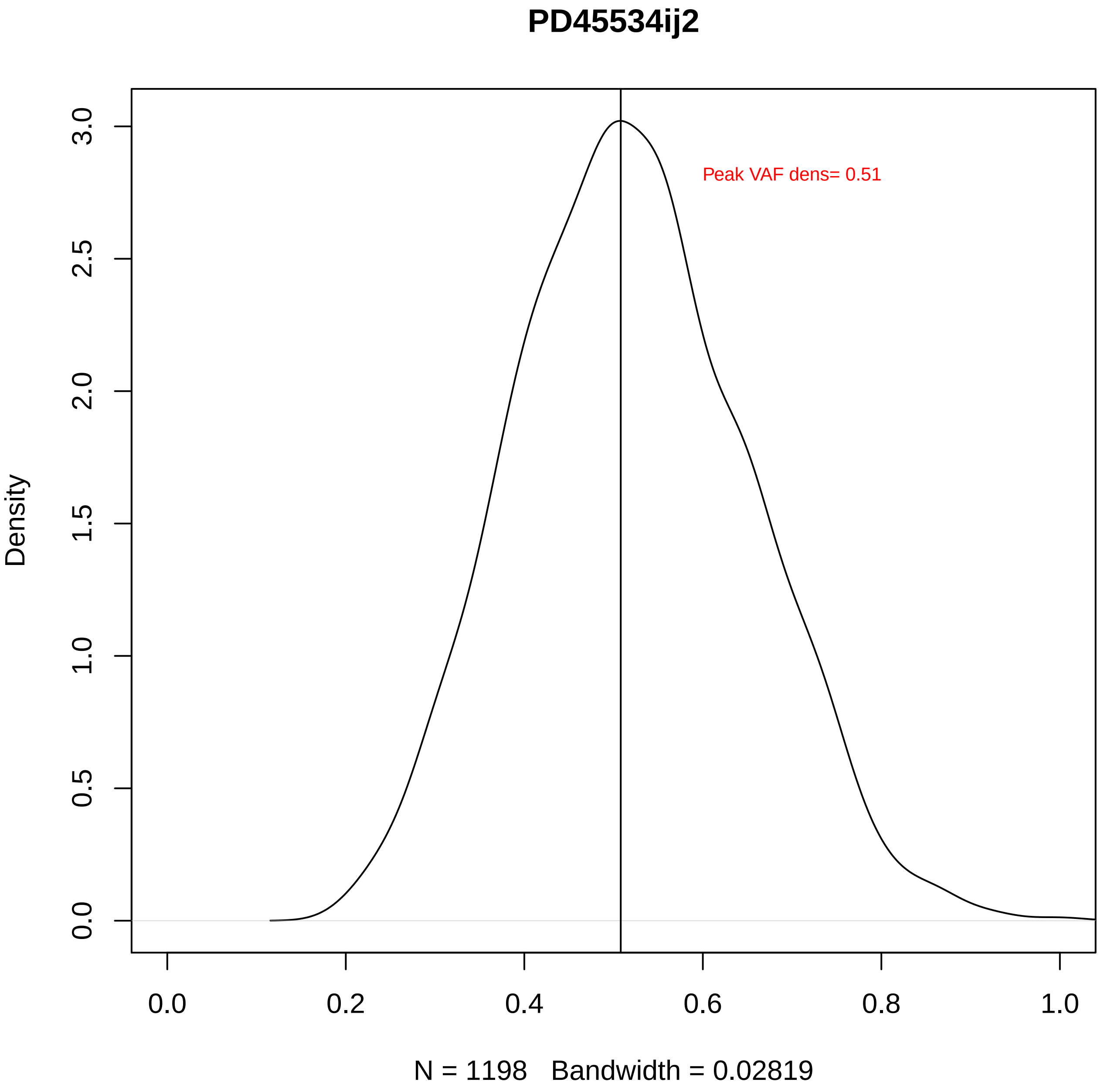
PD45534ij2
Peak VAF dens= 0.51
Density
0.0
0.5
1.0
1.5
2.0
2.5
3.0
0.0
0.2
0.4
0.6
0.8
1.0
N = 1198   Bandwidth = 0.02819

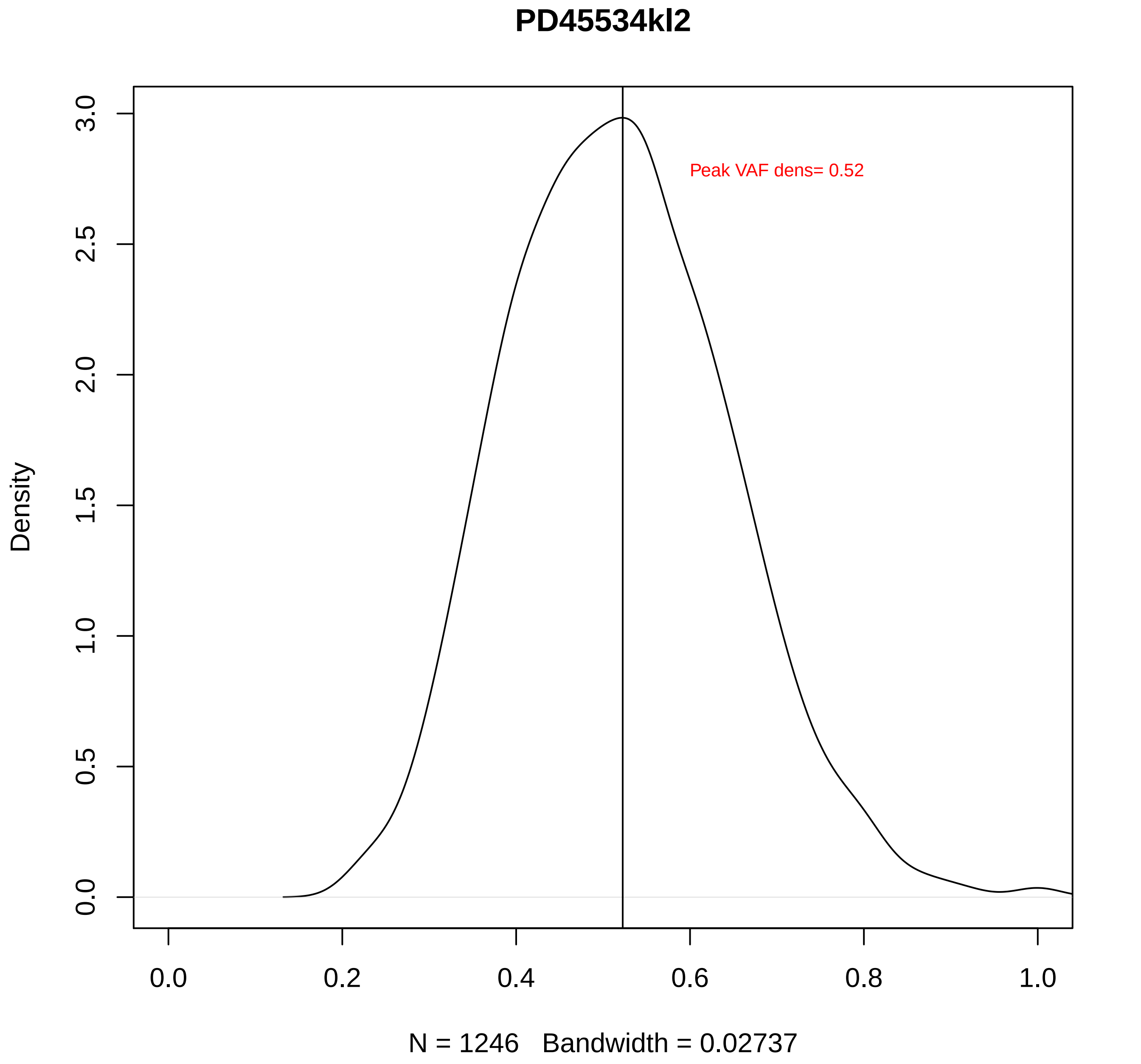
PD45534kl2
Peak VAF dens= 0.52
Density
0.0
0.5
1.0
1.5
2.0
2.5
3.0
0.0
0.2
0.4
0.6
0.8
1.0
N = 1246   Bandwidth = 0.02737

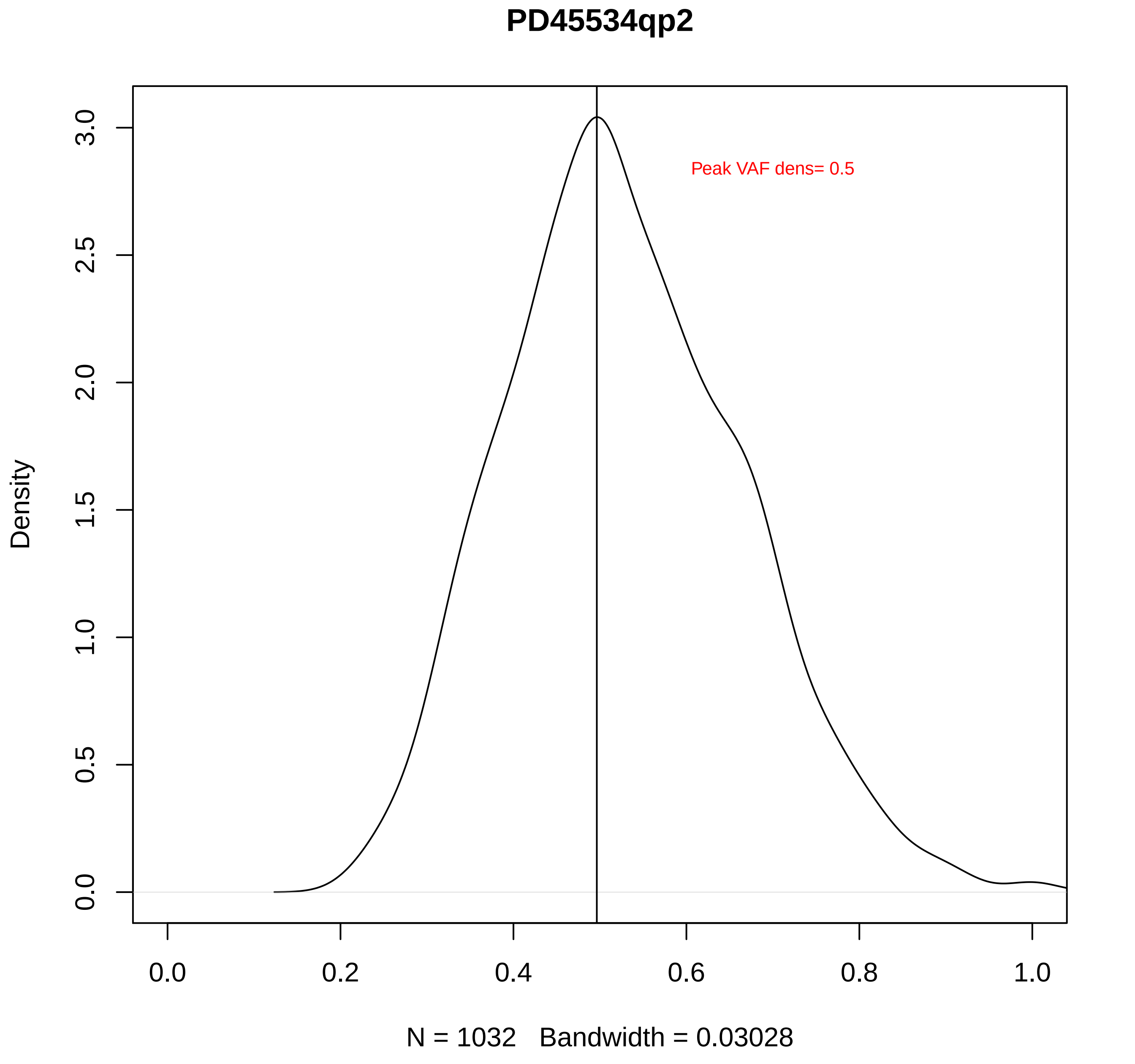
PD45534qp2
Peak VAF dens= 0.5
Density
0.0
0.5
1.0
1.5
2.0
2.5
3.0
0.0
0.2
0.4
0.6
0.8
1.0
N = 1032   Bandwidth = 0.03028

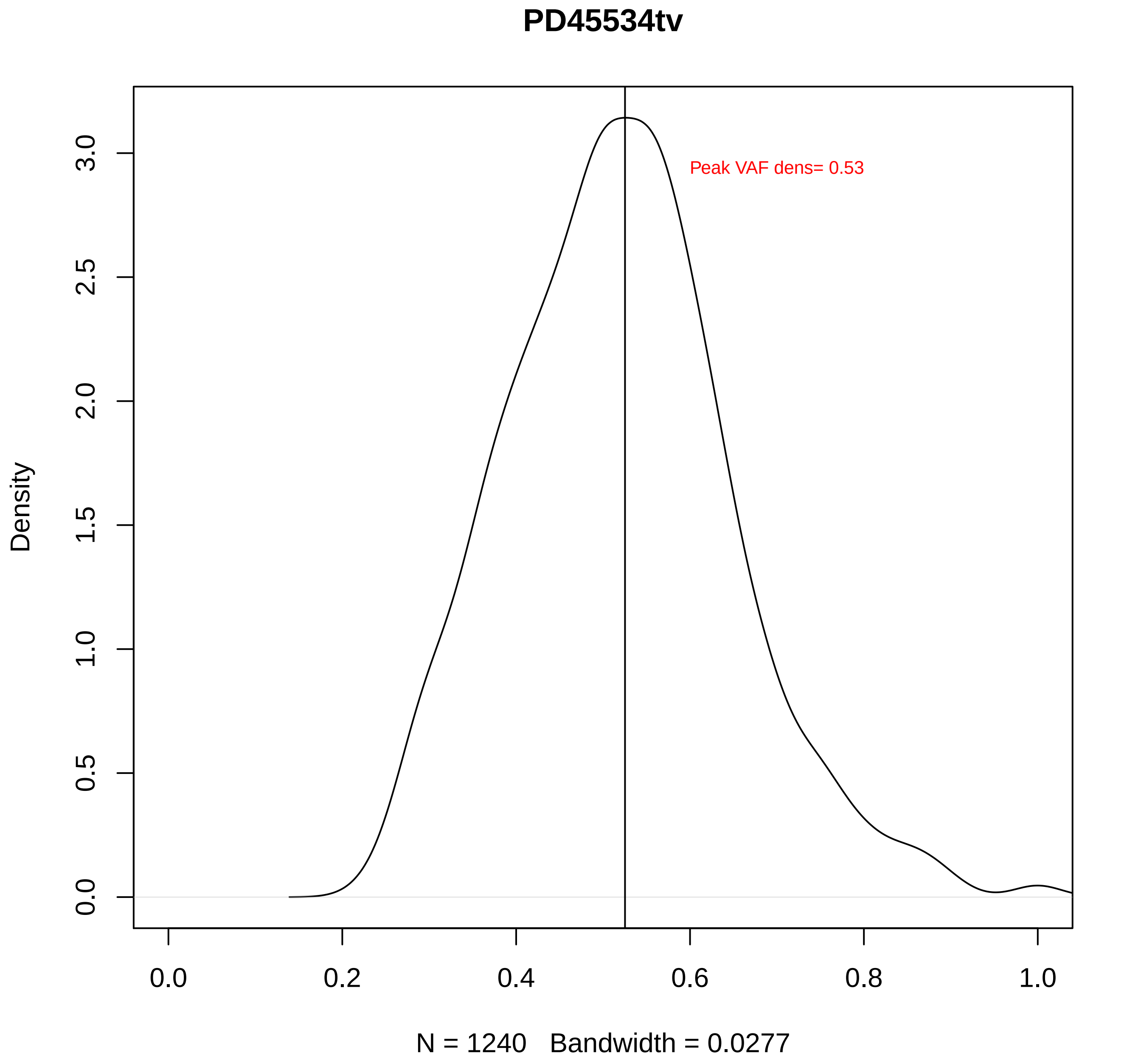
PD45534tv
Peak VAF dens= 0.53
Density
0.0
0.5
1.0
1.5
2.0
2.5
3.0
0.0
0.2
0.4
0.6
0.8
1.0
N = 1240   Bandwidth = 0.0277

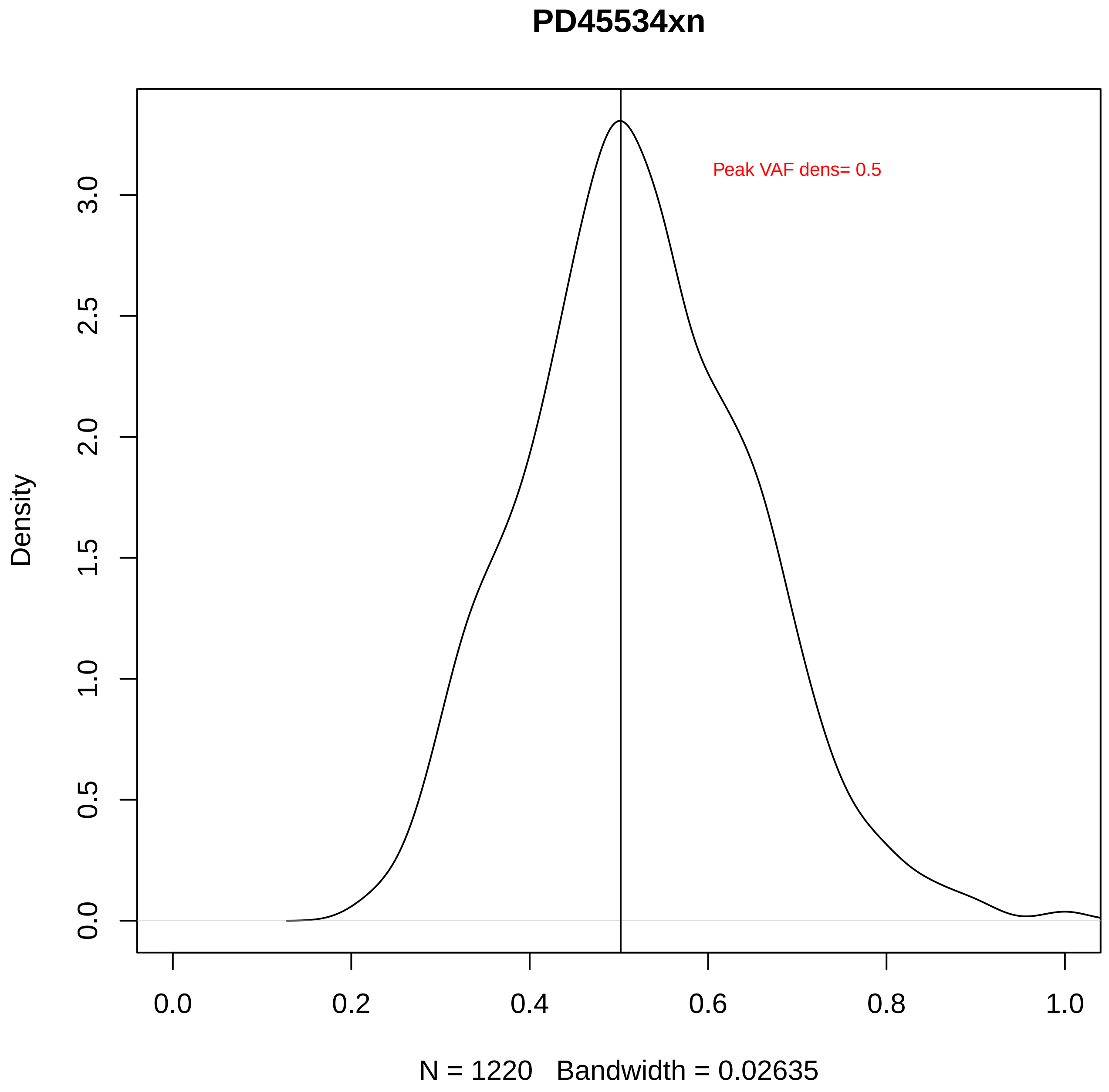

PD45534xn
Peak VAF dens= 0.5
Density
0.0
0.5
1.0
1.5
2.0
2.5
3.0
0.0
0.2
0.4
0.6
0.8
1.0
N = 1220   Bandwidth = 0.02635

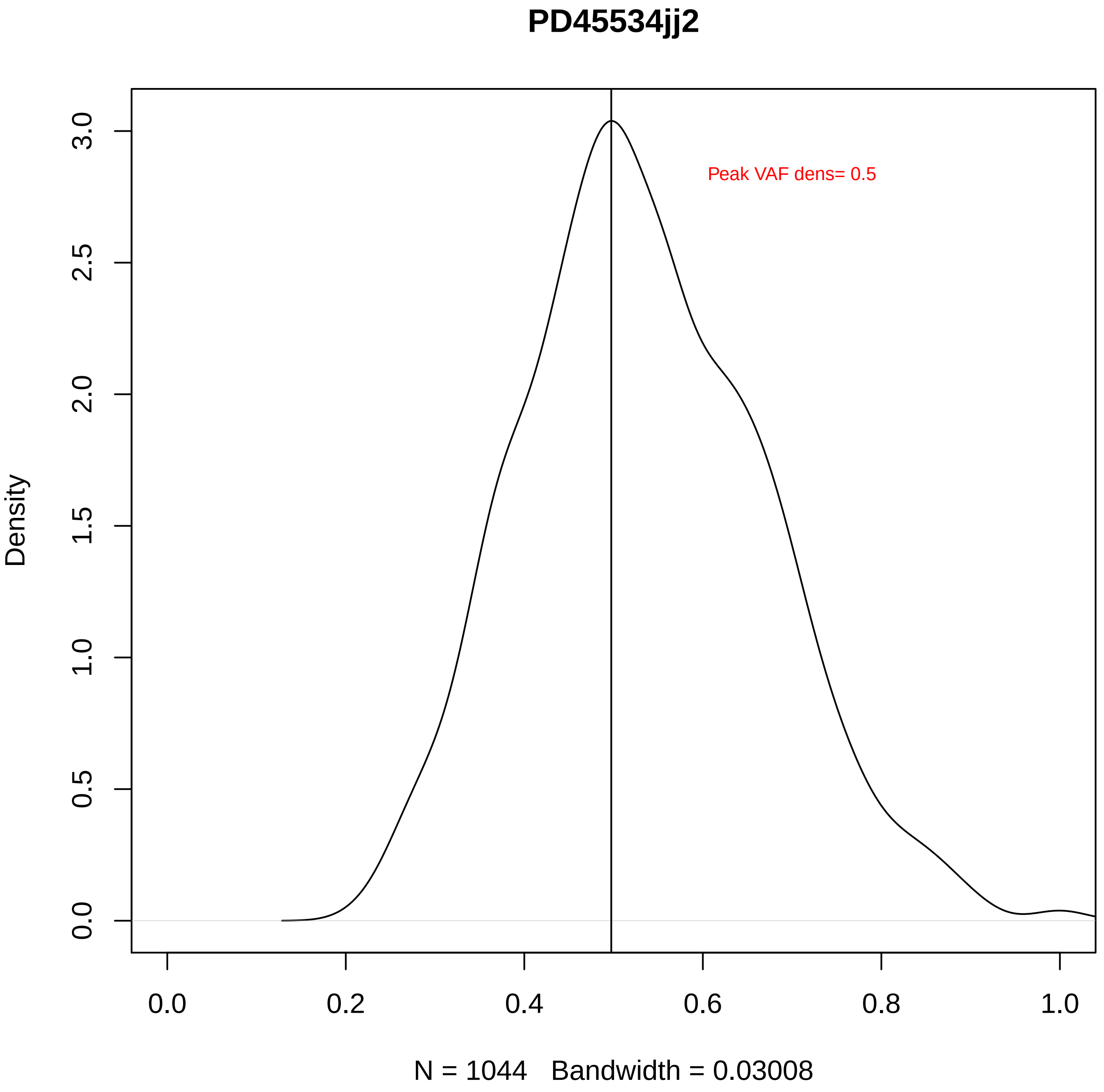

PD45534jj2
Peak VAF dens= 0.5
Density
0.0
0.5
1.0
1.5
2.0
2.5
3.0
0.0
0.2
0.4
0.6
0.8
1.0
N = 1044   Bandwidth = 0.03008

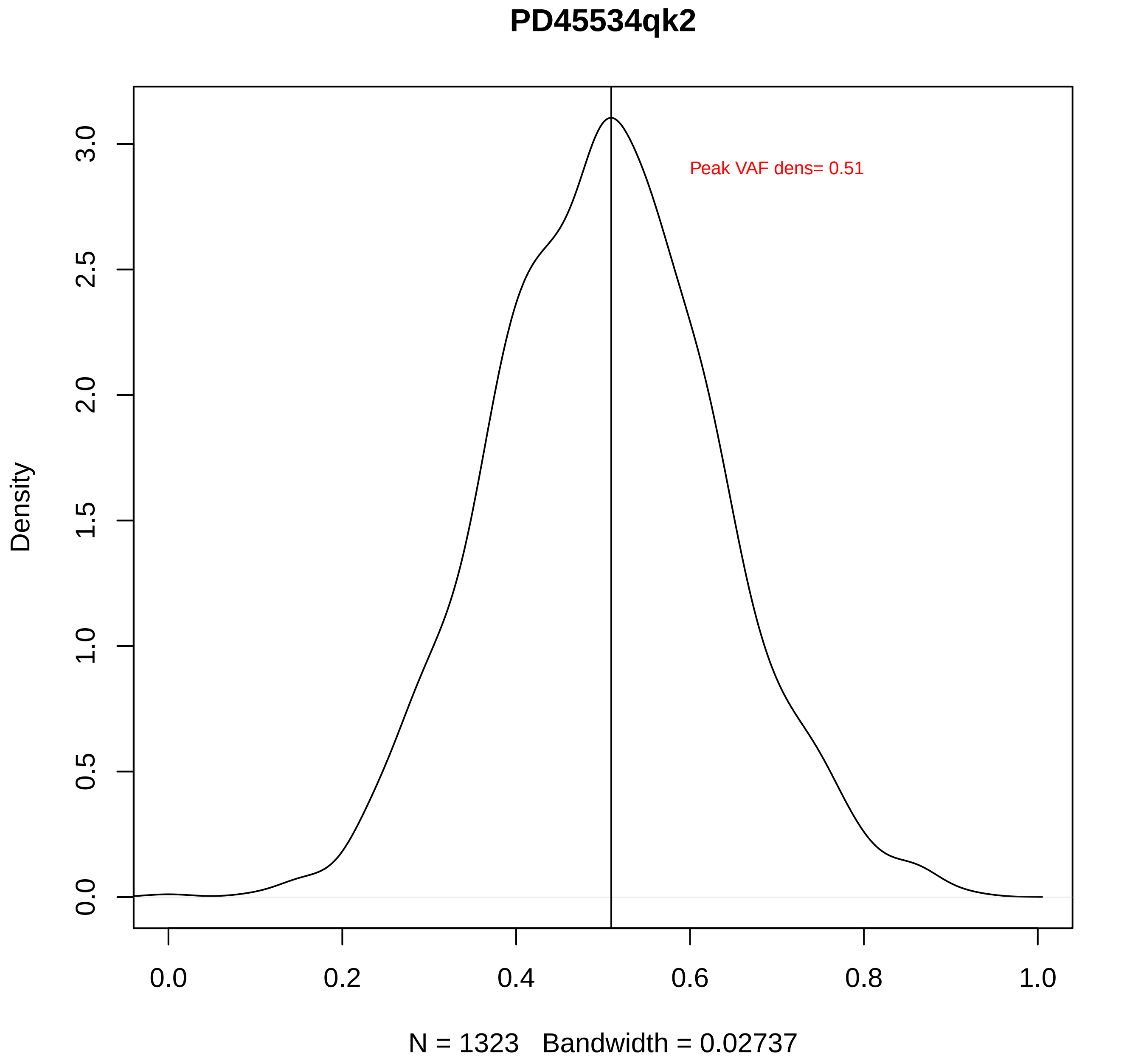

PD45534qk2
Peak VAF dens= 0.51
Density
0.0
0.5
1.0
1.5
2.0
2.5
3.0
0.0
0.2
0.4
0.6
0.8
1.0
N = 1323 Bandwidth = 0.02737

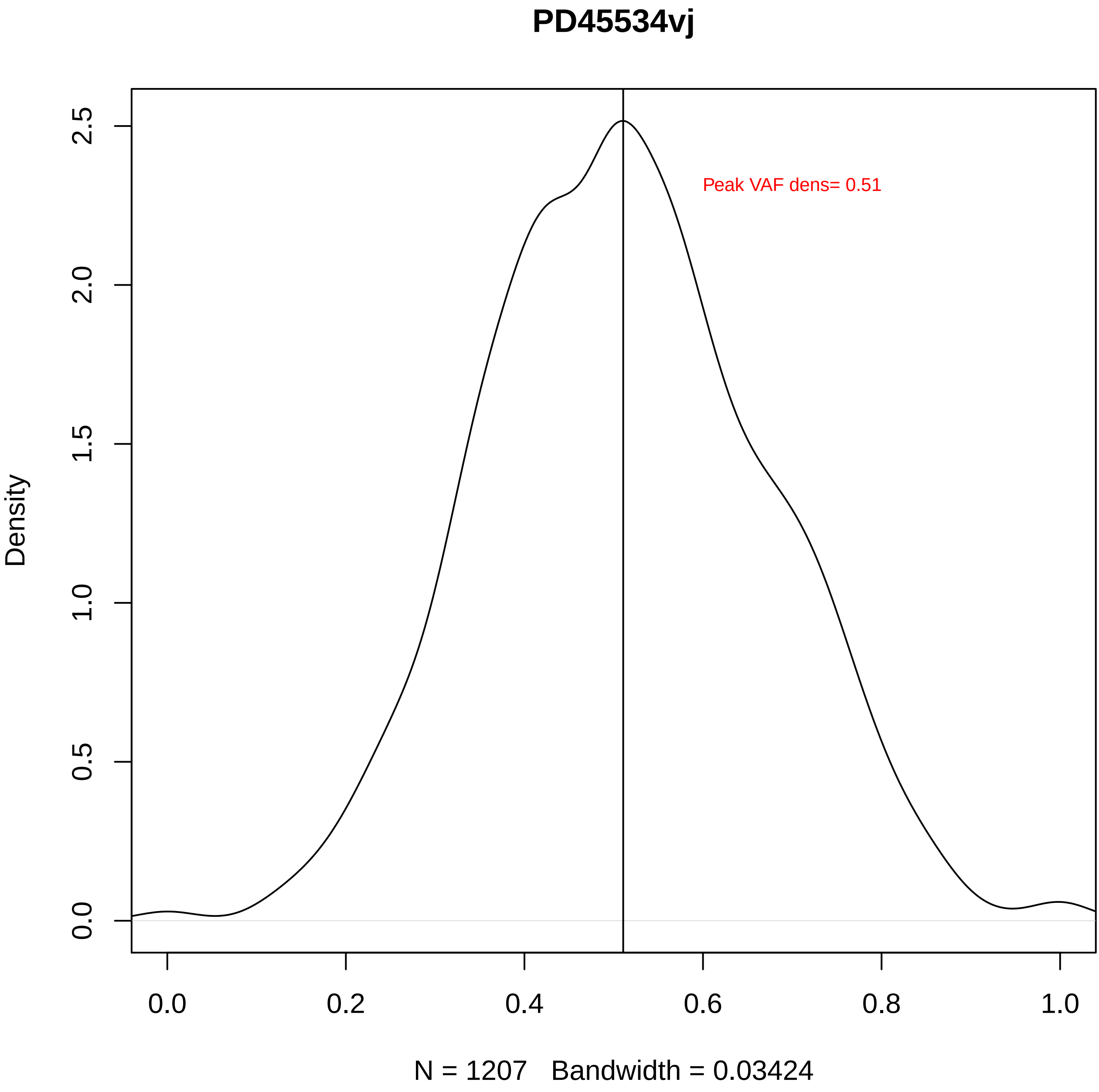
PD45534vj
Peak VAF dens= 0.51
Density
0.0
0.5
1.0
1.5
2.0
2.5
0.0
0.2
0.4
0.6
0.8
1.0
N = 1207   Bandwidth = 0.03424

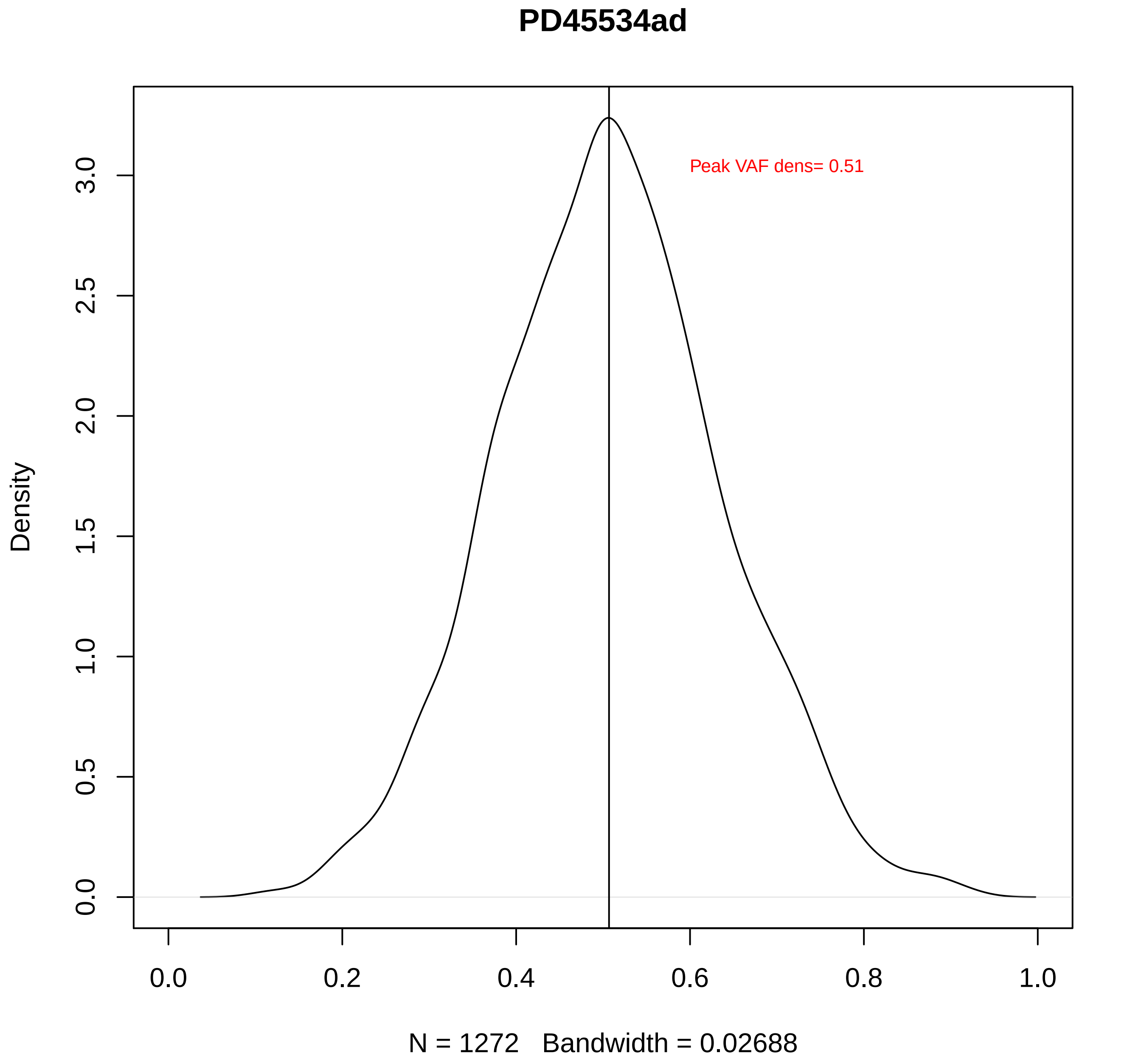
PD45534ad
Peak VAF dens= 0.51
Density
0.0
0.5
1.0
1.5
2.0
2.5
3.0
0.0
0.2
0.4
0.6
0.8
1.0
N = 1272   Bandwidth = 0.02688

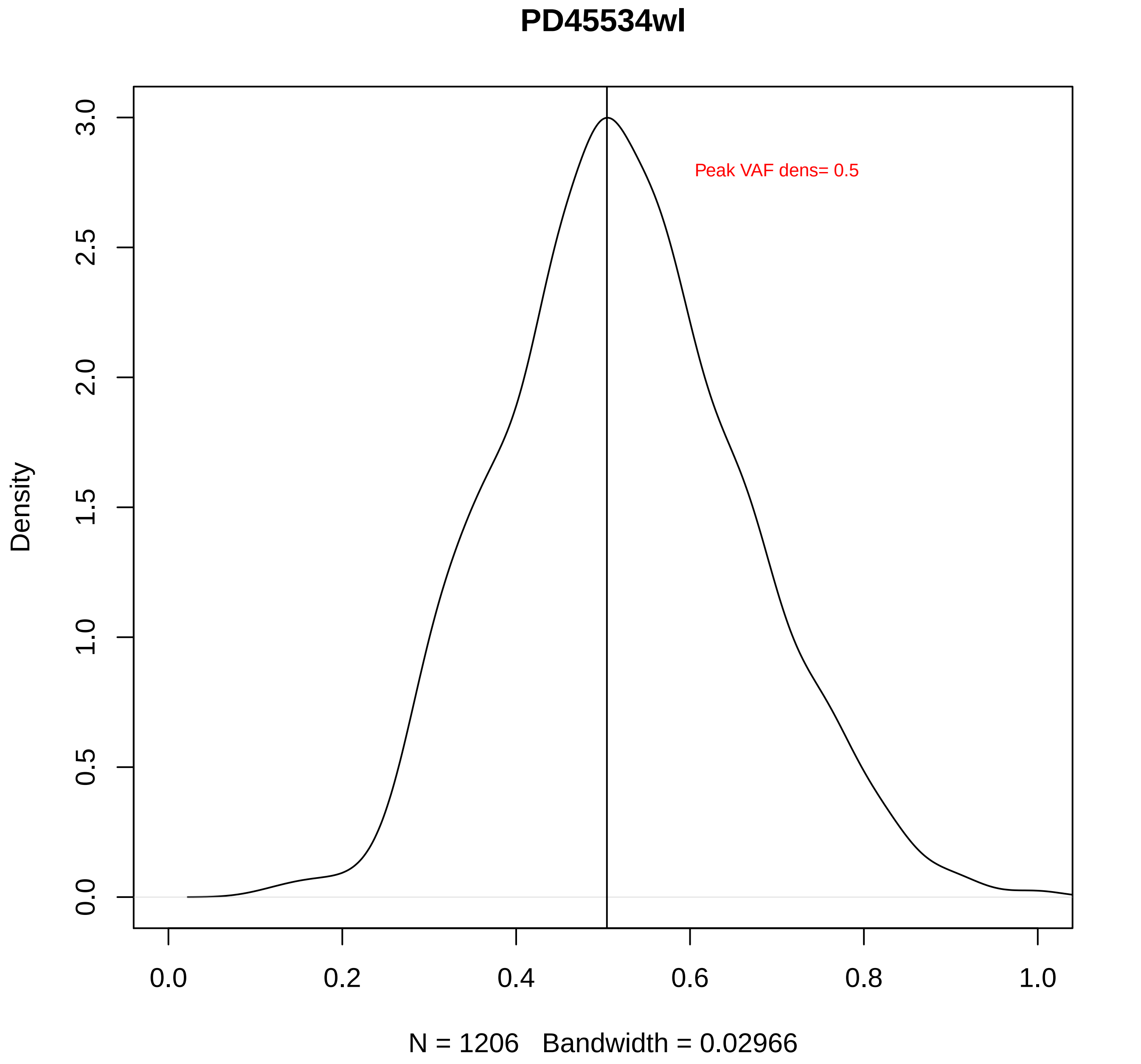

PD45534wl
Peak VAF dens= 0.5
Density
0.0
0.5
1.0
1.5
2.0
2.5
3.0
0.0
0.2
0.4
0.6
0.8
1.0
N = 1206 Bandwidth = 0.02966

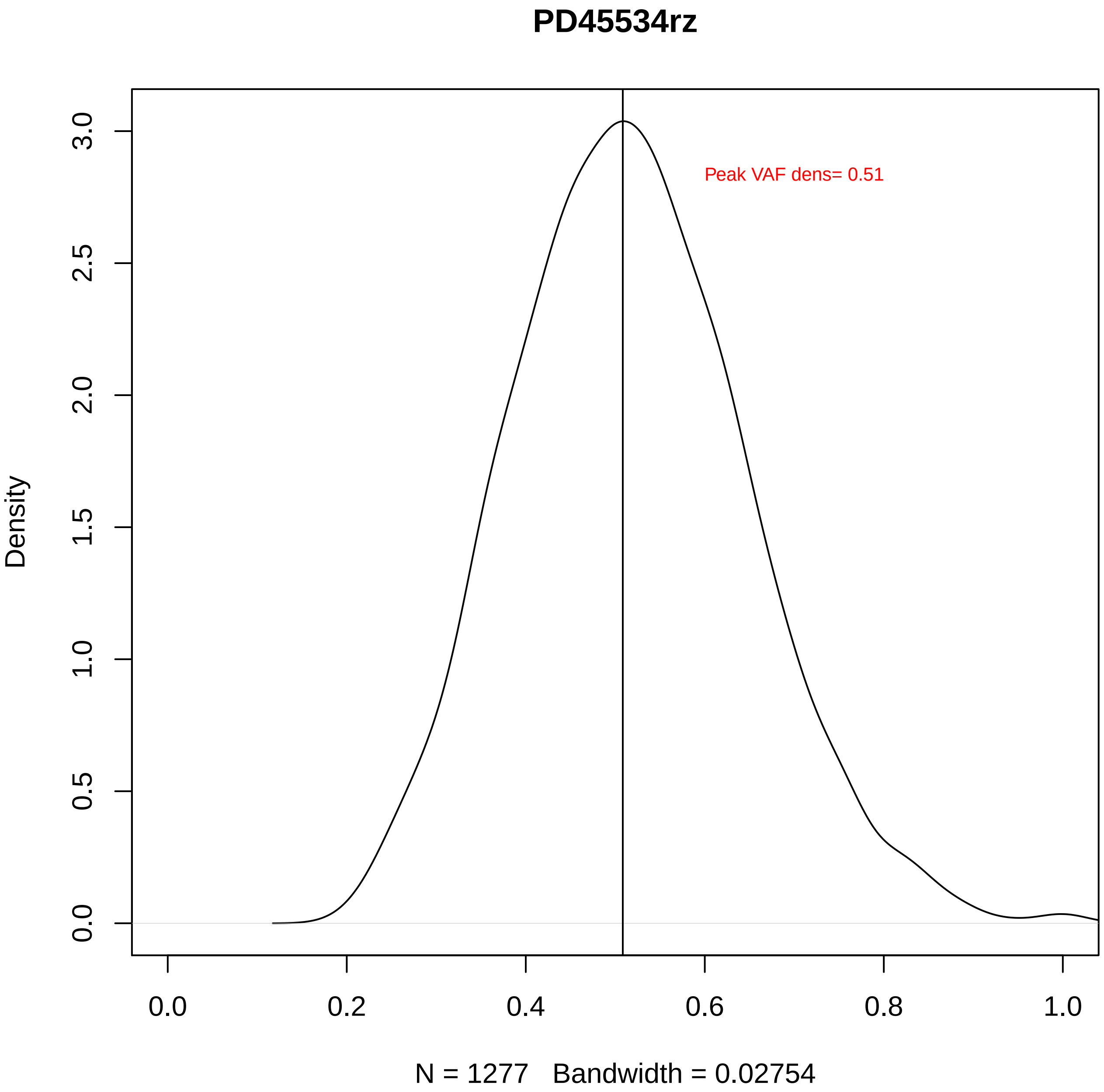

PD45534rz
Peak VAF dens= 0.51
Density
0.0
0.5
1.0
1.5
2.0
2.5
3.0
0.0
0.2
0.4
0.6
0.8
1.0
N = 1277   Bandwidth = 0.02754

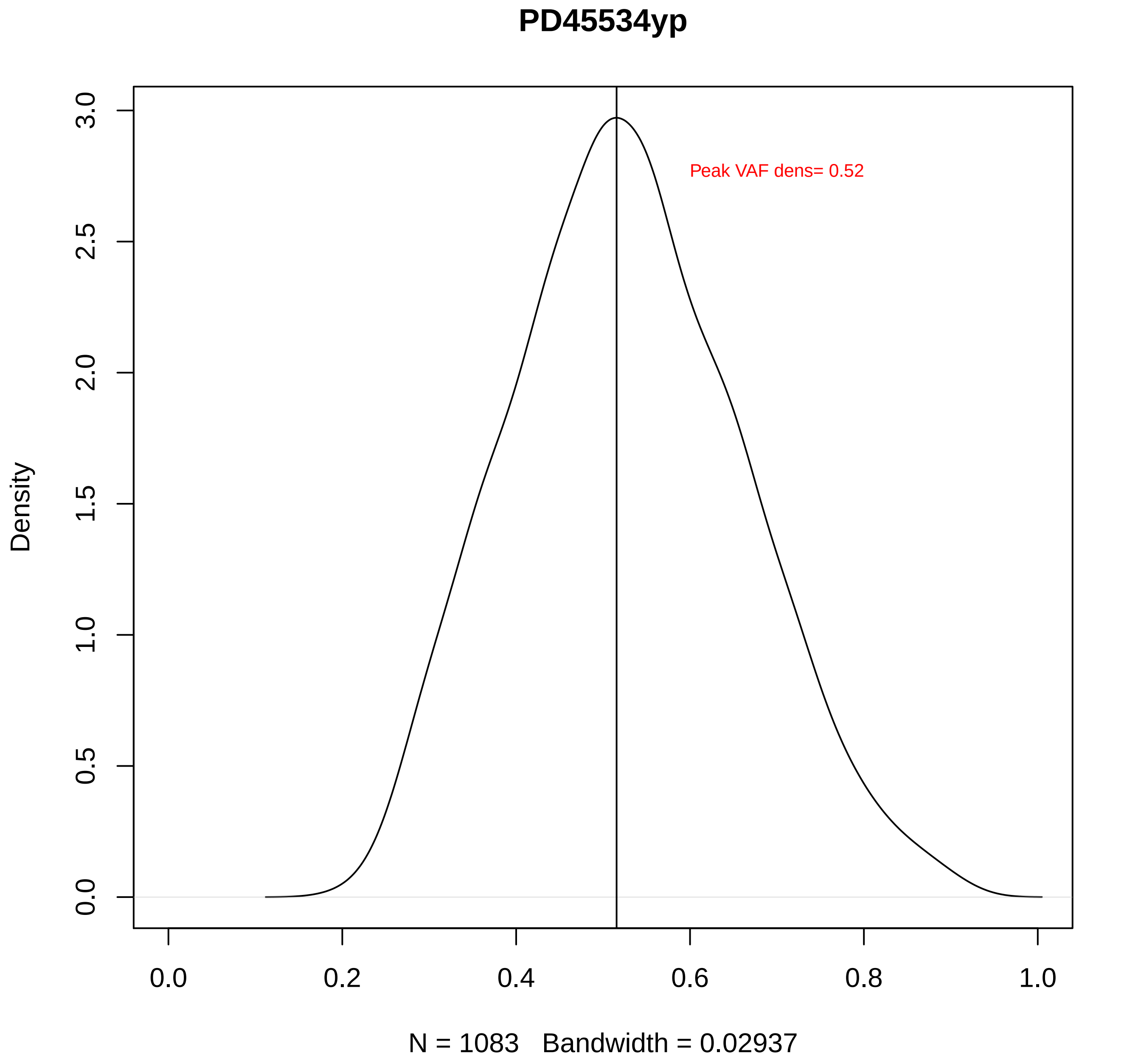
PD45534yp
Peak VAF dens= 0.52
Density
0.0
0.5
1.0
1.5
2.0
2.5
3.0
0.0
0.2
0.4
0.6
0.8
1.0
N = 1083   Bandwidth = 0.02937

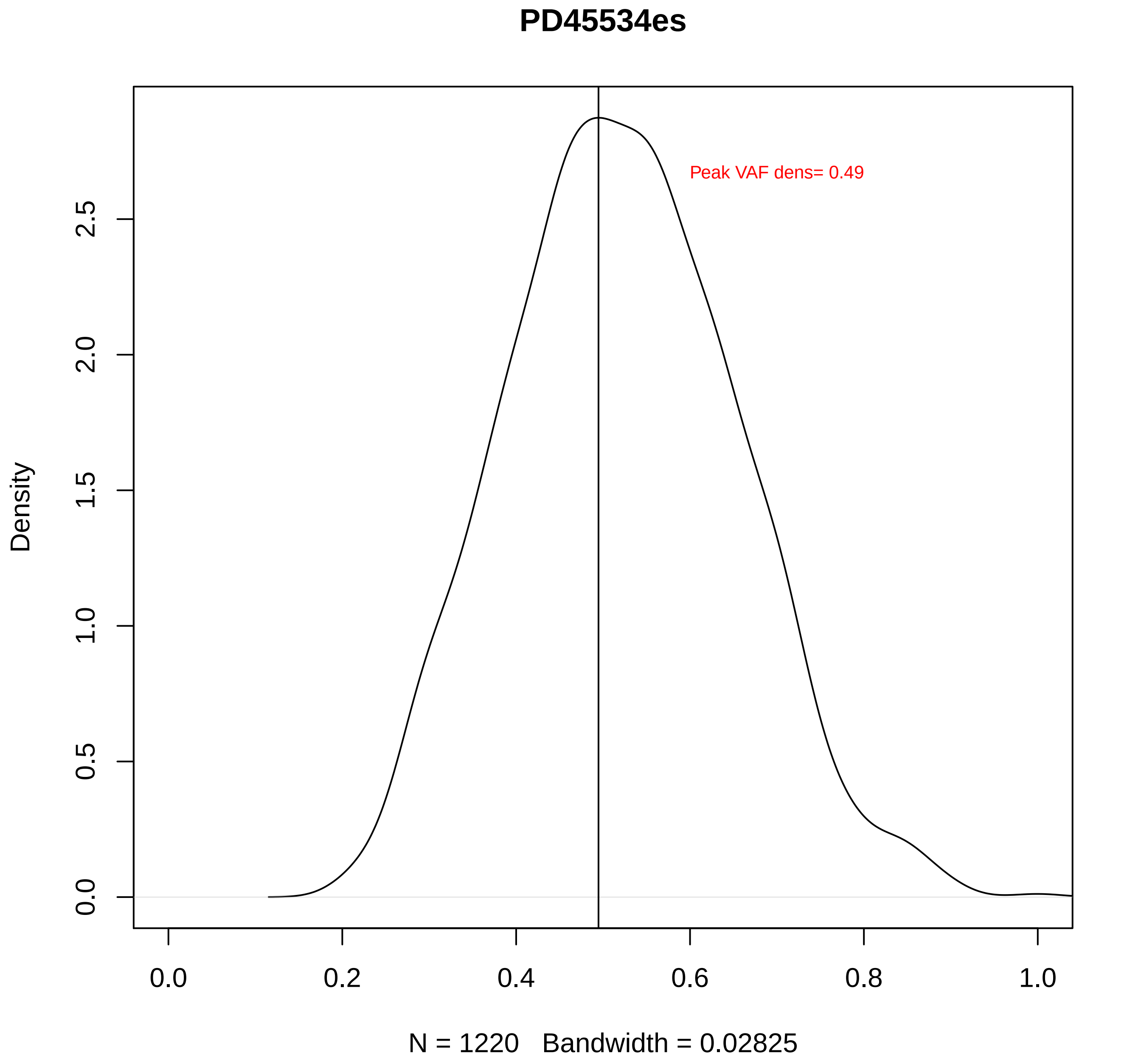
PD45534es
Peak VAF dens= 0.49
Density
0.0
0.5
1.0
1.5
2.0
2.5
0.0
0.2
0.4
0.6
0.8
1.0
N = 1220   Bandwidth = 0.02825

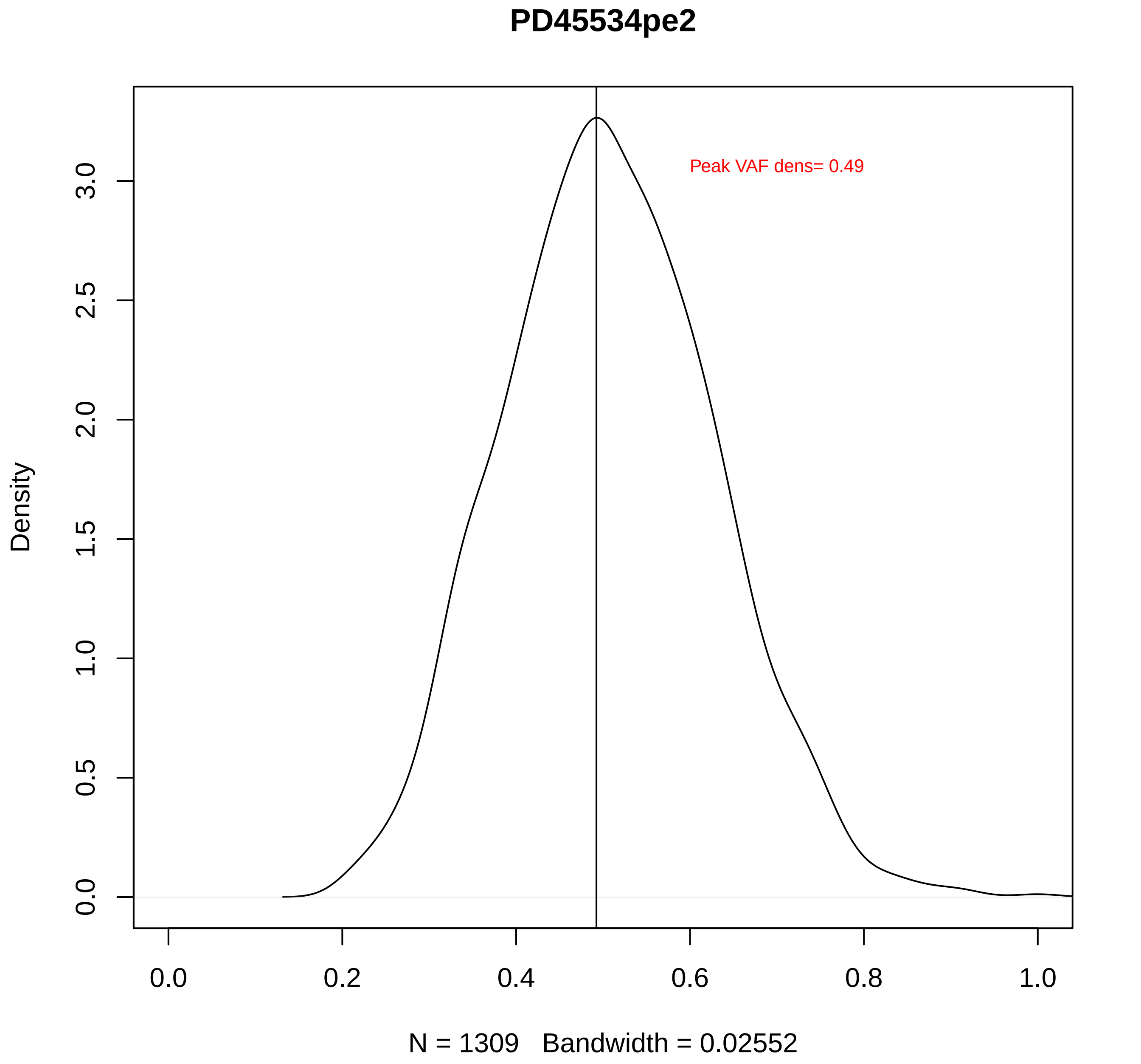
PD45534pe2
Peak VAF dens= 0.49
Density
0.0
0.5
1.0
1.5
2.0
2.5
3.0
0.0
0.2
0.4
0.6
0.8
1.0
N = 1309   Bandwidth = 0.02552

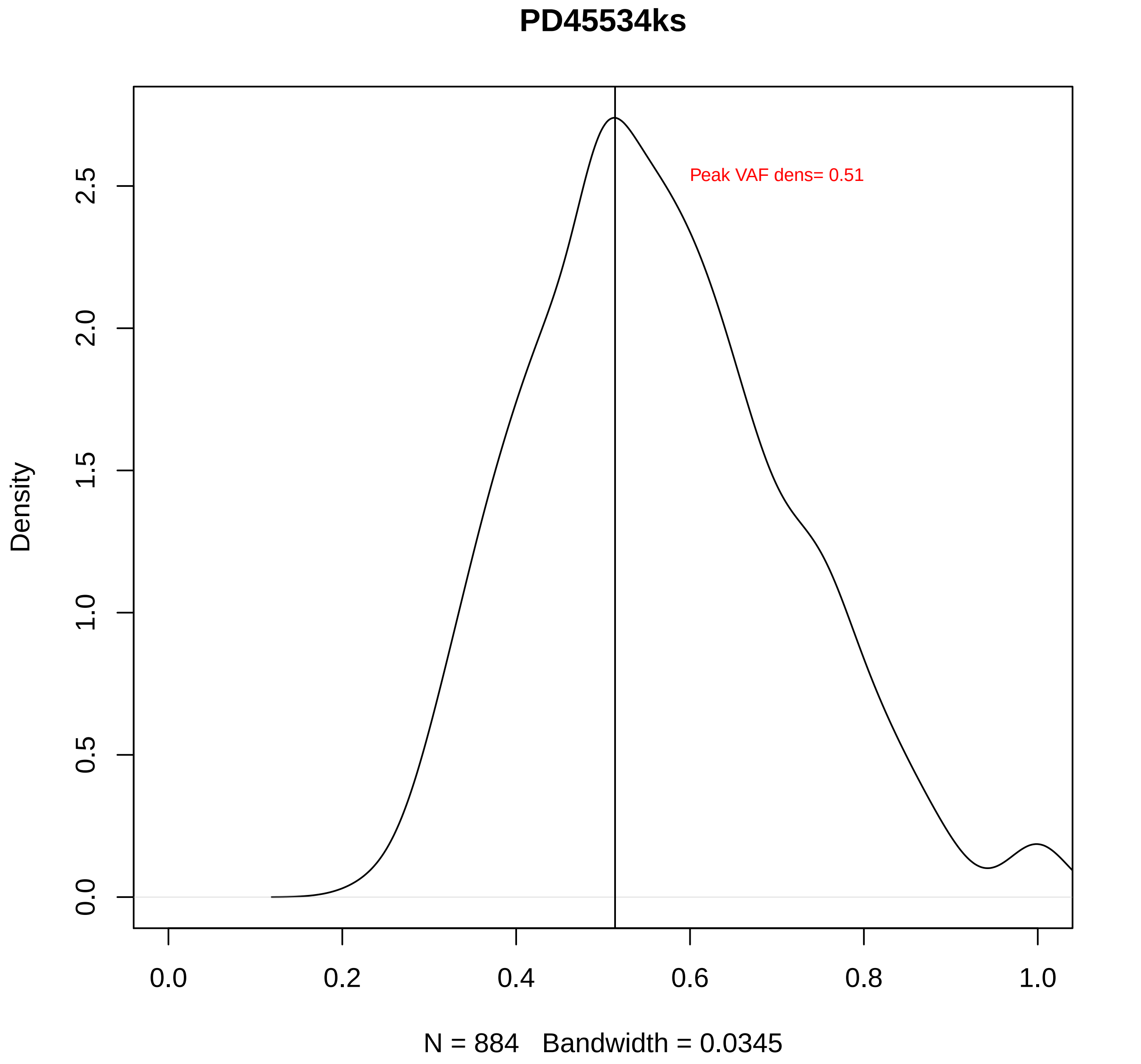
PD45534ks
Peak VAF dens= 0.51
Density
0.0
0.5
1.0
1.5
2.0
2.5
0.0
0.2
0.4
0.6
0.8
1.0
N = 884   Bandwidth = 0.0345

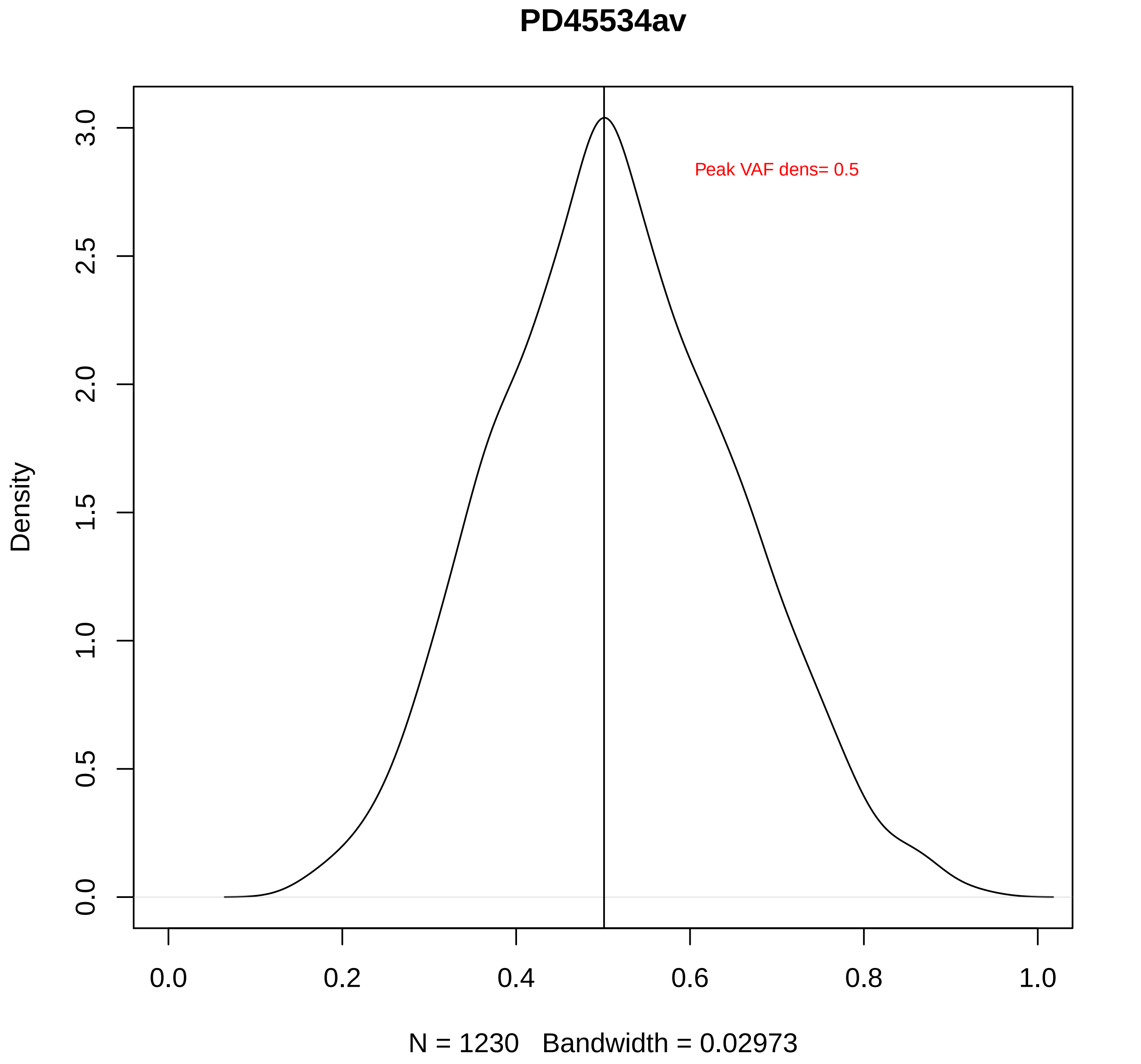

PD45534av
Peak VAF dens= 0.5
Density
0.0
0.5
1.0
1.5
2.0
2.5
3.0
0.0
0.2
0.4
0.6
0.8
1.0
N = 1230   Bandwidth = 0.02973

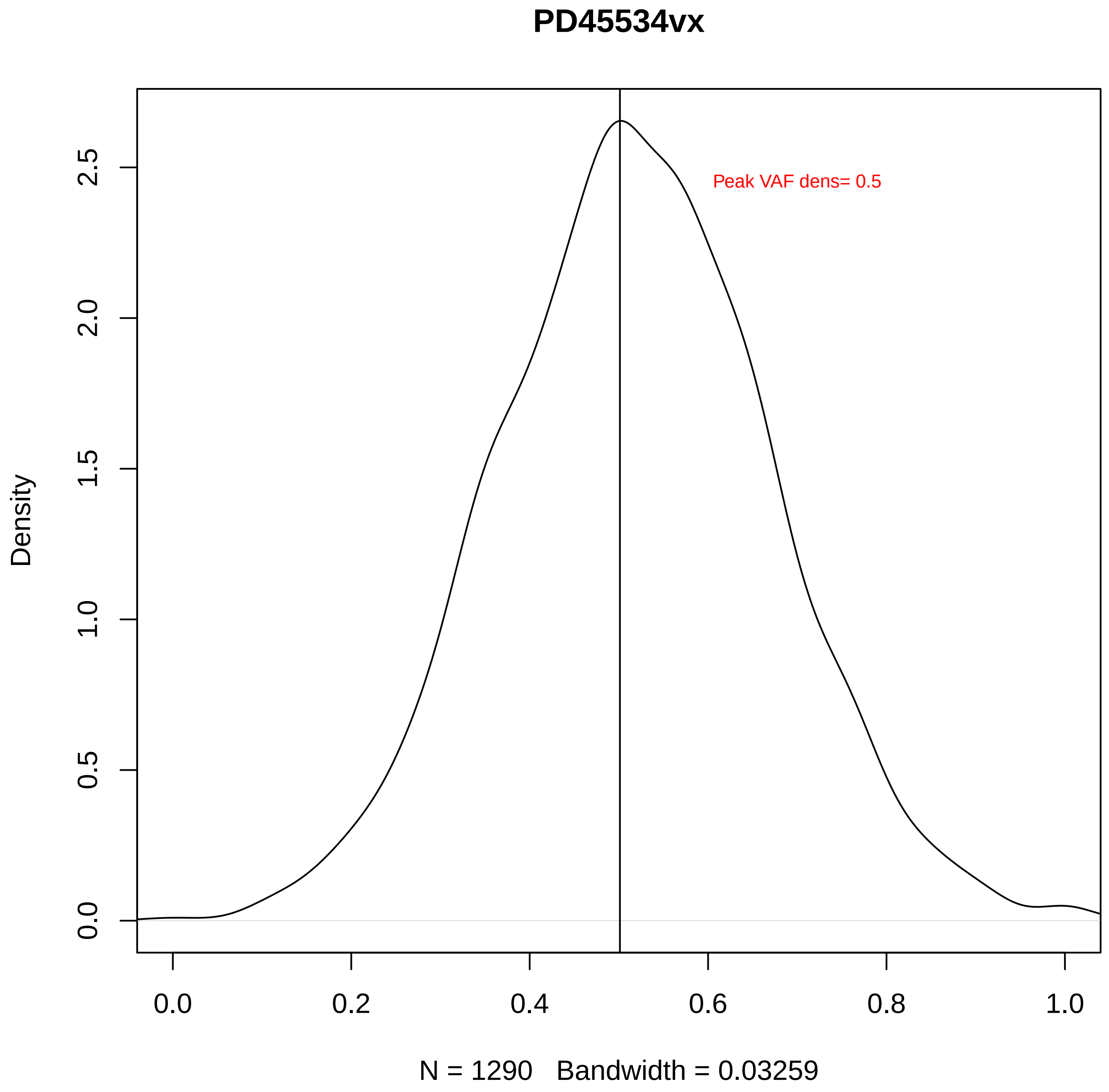
PD45534vx
Peak VAF dens= 0.5
Density
0.0
0.5
1.0
1.5
2.0
2.5
0.0
0.2
0.4
0.6
0.8
1.0
N = 1290   Bandwidth = 0.03259

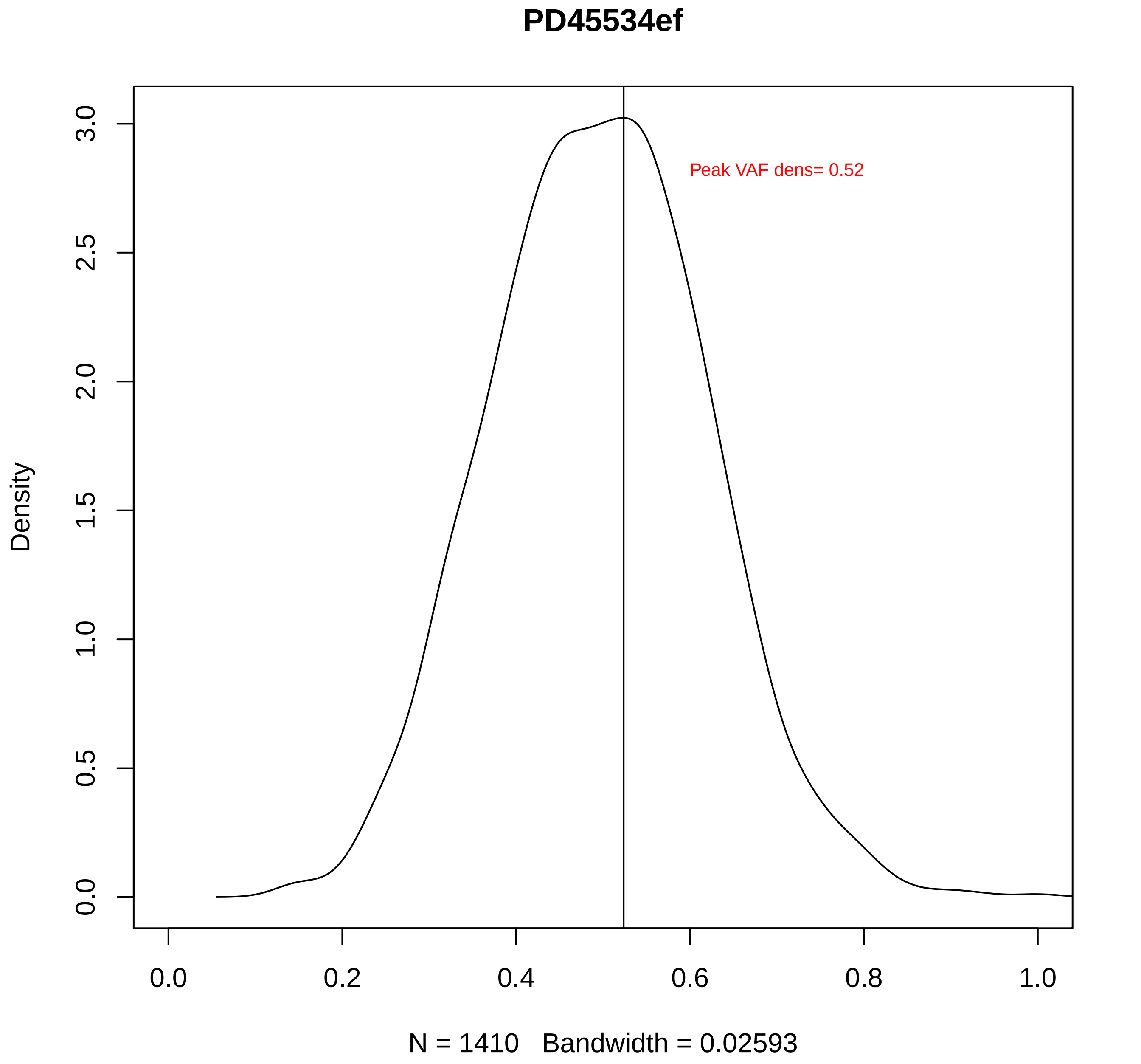
PD45534ef
Peak VAF dens= 0.52
Density
3.0
2.5
2.0
1.5
1.0
0.5
0.0
0.0
0.2
0.4
0.6
0.8
1.0
N = 1410   Bandwidth = 0.02593

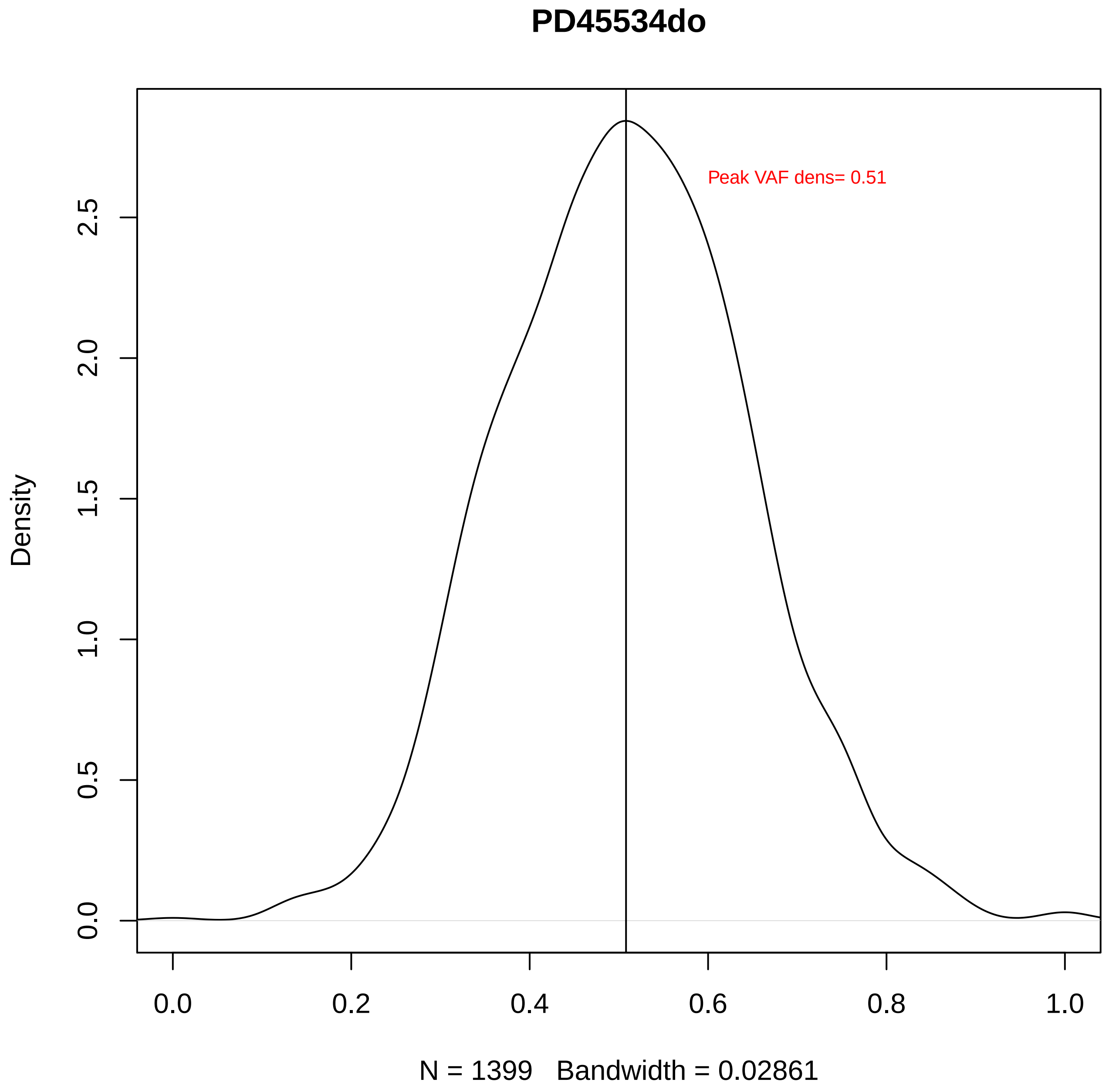
PD45534do
Peak VAF dens= 0.51
Density
0.0
0.5
1.0
1.5
2.0
2.5
0.0
0.2
0.4
0.6
0.8
1.0
N = 1399   Bandwidth = 0.02861

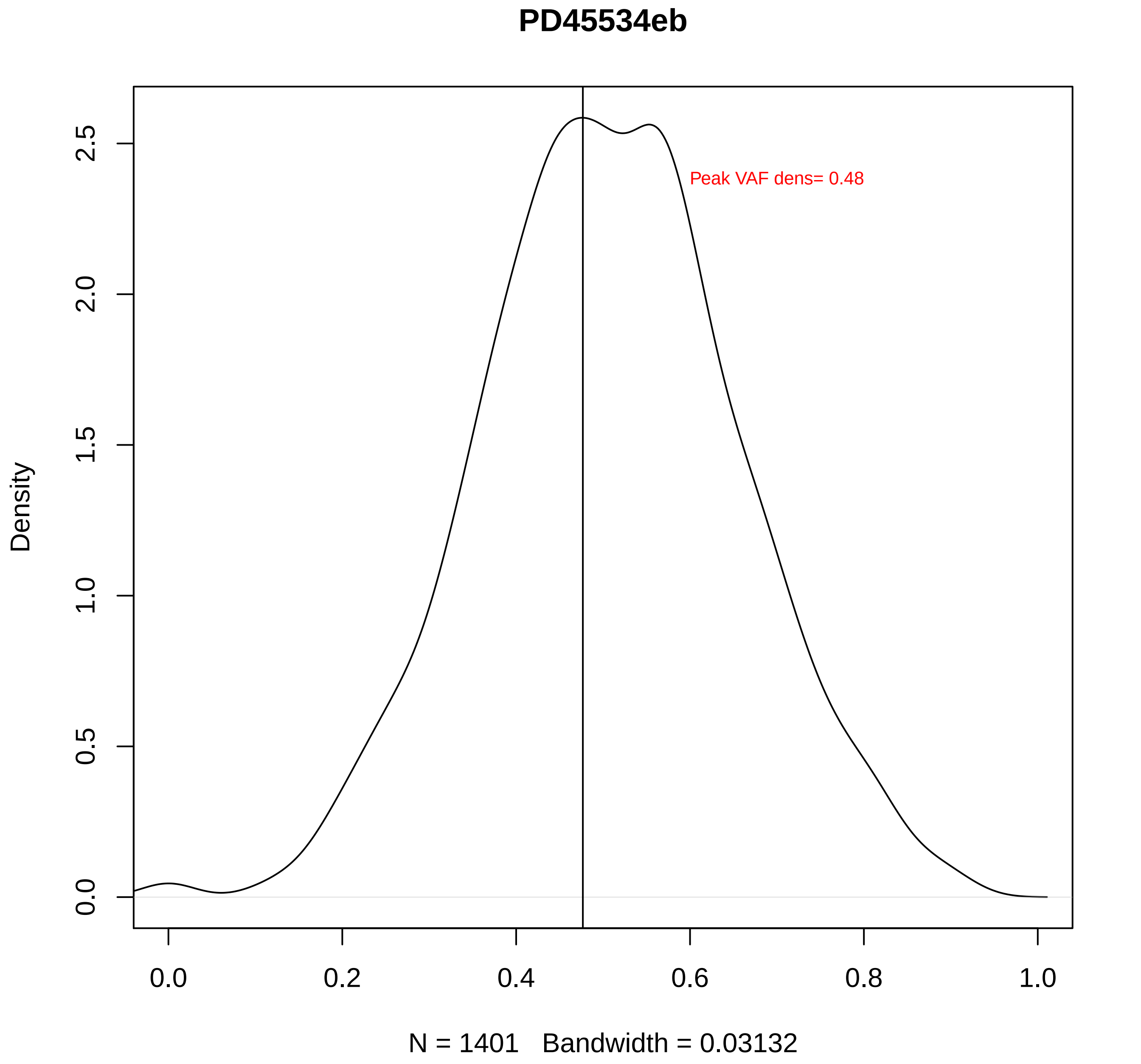

PD45534eb
Peak VAF dens= 0.48
Density
0.0
0.5
1.0
1.5
2.0
2.5
0.0
0.2
0.4
0.6
0.8
1.0
N = 1401   Bandwidth = 0.03132

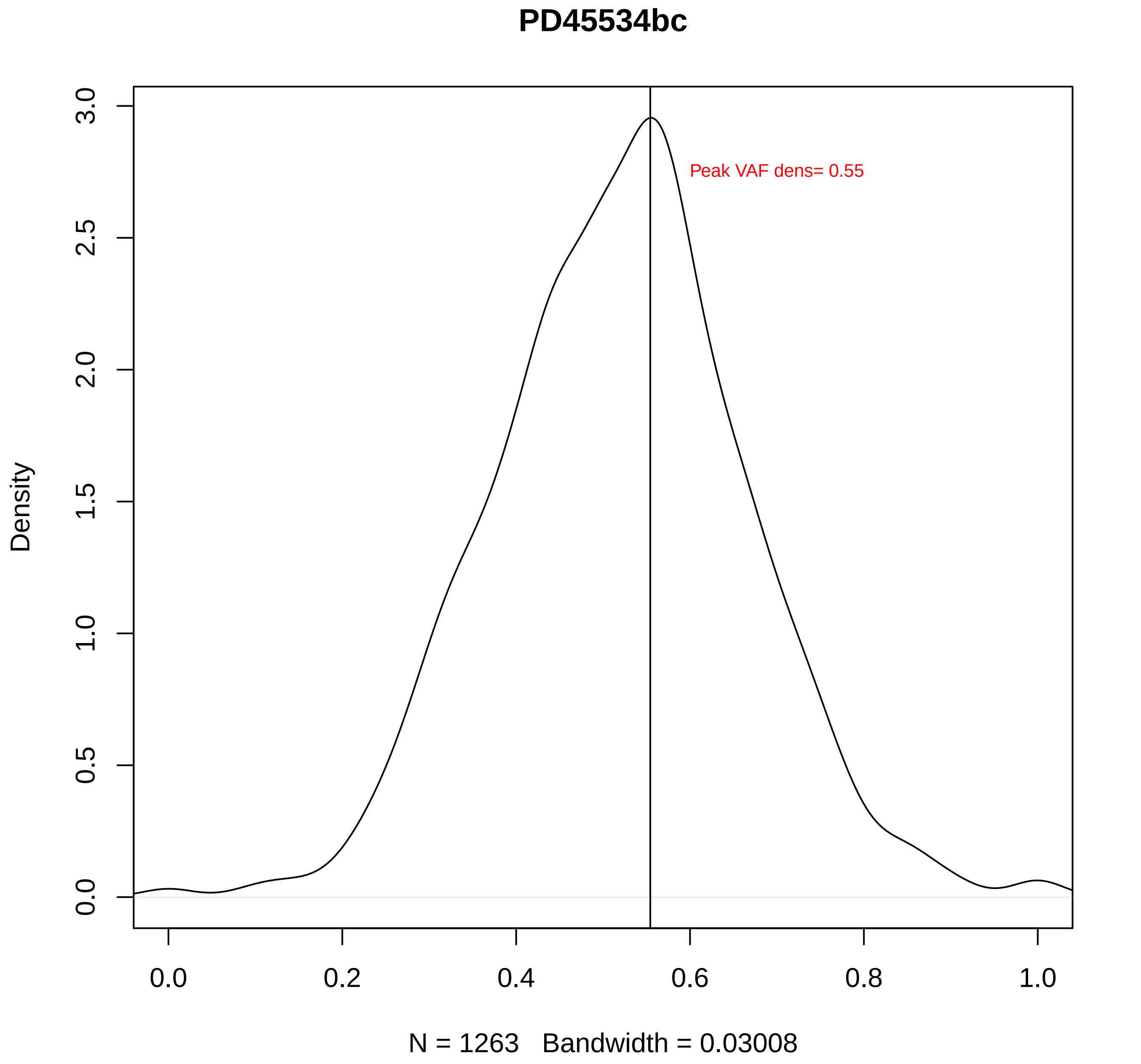
PD45534bc
Density
3.0
2.5
2.0
1.5
1.0
0.5
0.0
Peak VAF dens= 0.55
0.0
0.2
0.4
0.6
0.8
1.0
N = 1263   Bandwidth = 0.03008

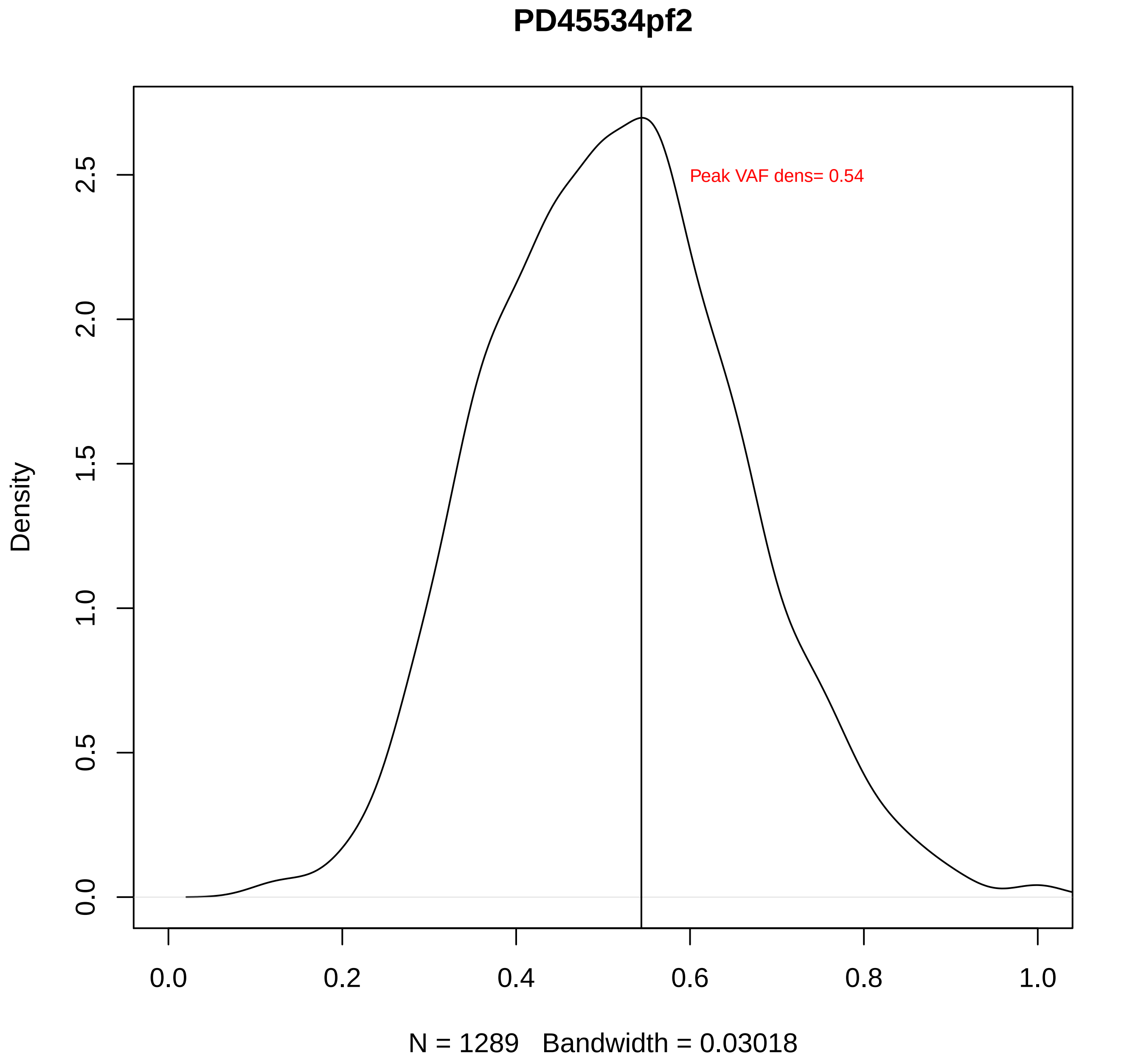
PD45534pf2
Peak VAF dens= 0.54
Density
0.0
0.5
1.0
1.5
2.0
2.5
0.0
0.2
0.4
0.6
0.8
1.0
N = 1289   Bandwidth = 0.03018

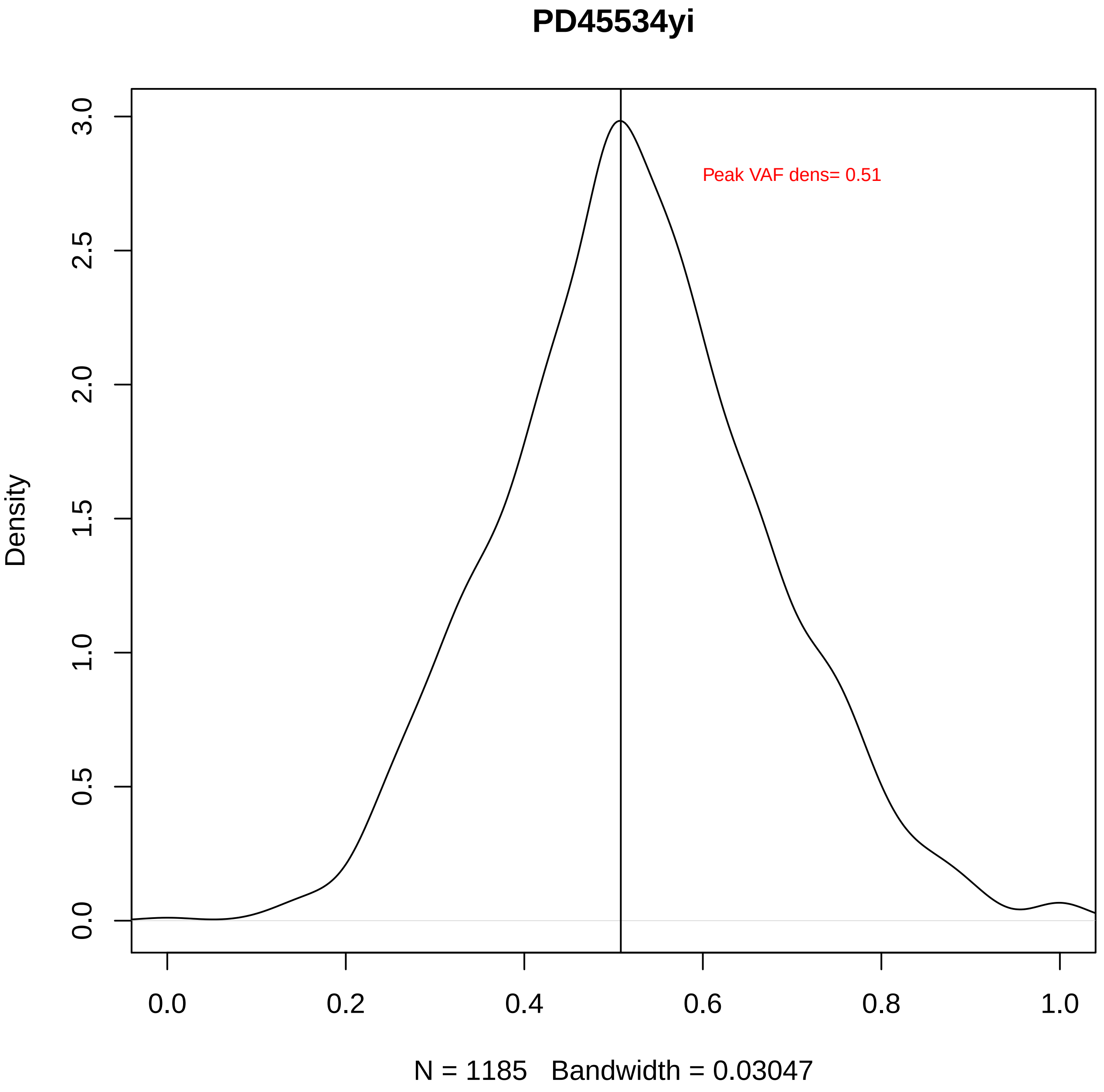

PD45534yi
Peak VAF dens= 0.51
Density
3.0
2.5
2.0
1.5
1.0
0.5
0.0
0.0
0.2
0.4
0.6
0.8
1.0
N = 1185   Bandwidth = 0.03047

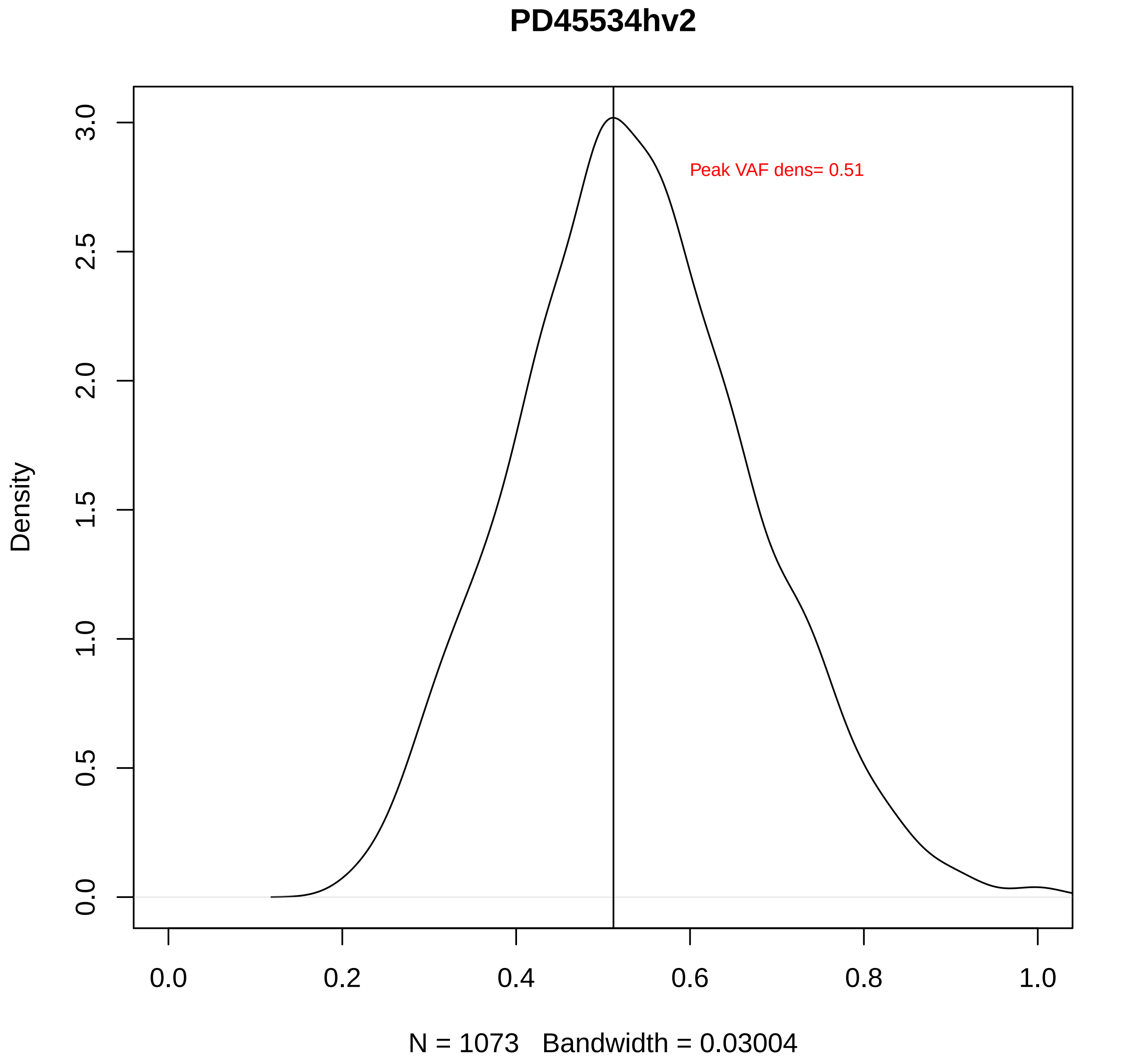

PD45534hv2
Peak VAF dens= 0.51
Density
0.0
0.5
1.0
1.5
2.0
2.5
3.0
0.0
0.2
0.4
0.6
0.8
1.0
N = 1073   Bandwidth = 0.03004

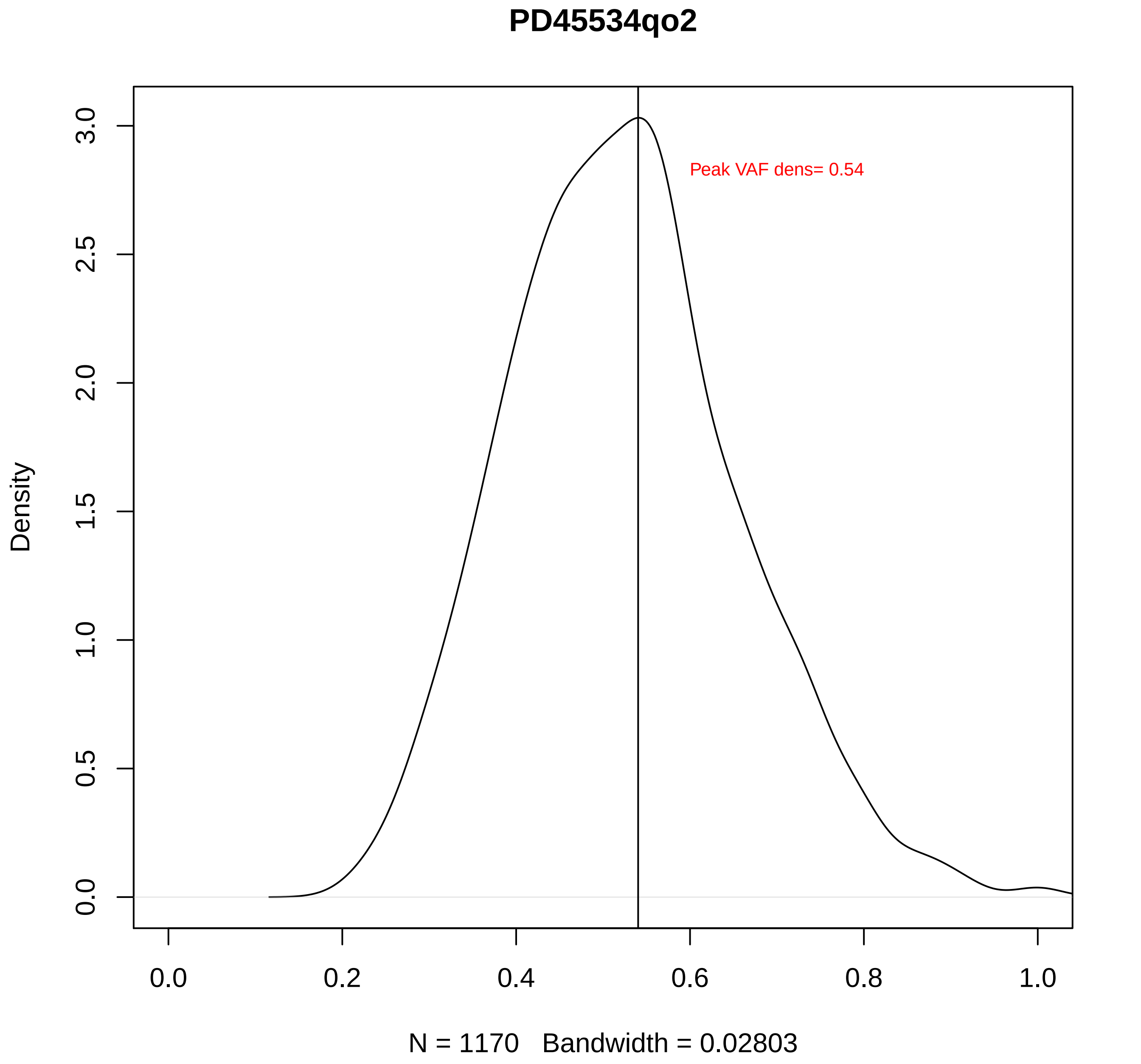

PD45534qo2
Peak VAF dens= 0.54
Density
0.0
0.5
1.0
1.5
2.0
2.5
3.0
0.0
0.2
0.4
0.6
0.8
1.0
N = 1170 Bandwidth = 0.02803

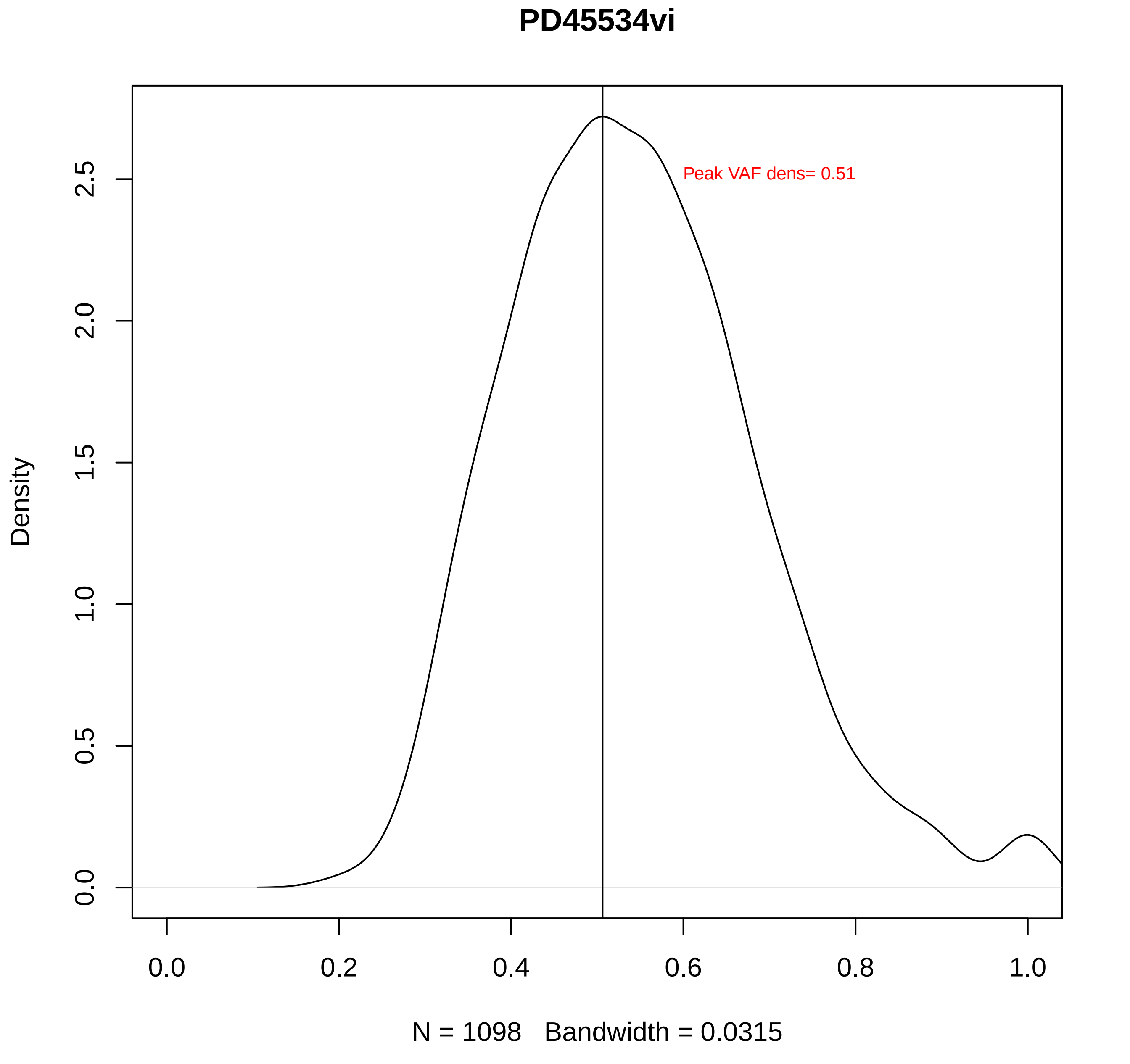
PD45534vi
Peak VAF dens= 0.51
Density
0.0
0.5
1.0
1.5
2.0
2.5
0.0
0.2
0.4
0.6
0.8
1.0
N = 1098   Bandwidth = 0.0315

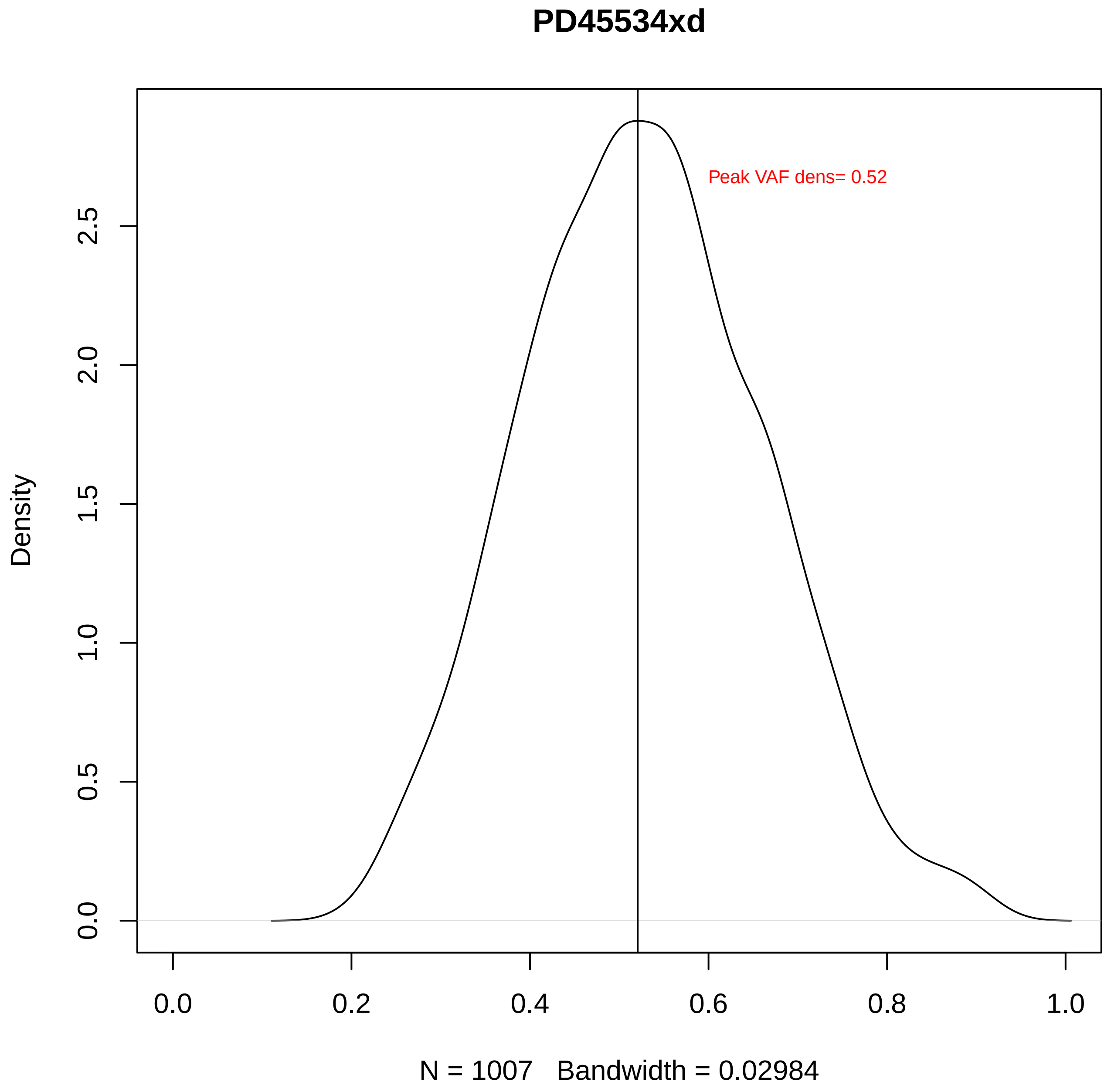

PD45534xd
Peak VAF dens= 0.52
Density
0.0
0.5
1.0
1.5
2.0
2.5
0.0
0.2
0.4
0.6
0.8
1.0
N = 1007   Bandwidth = 0.02984

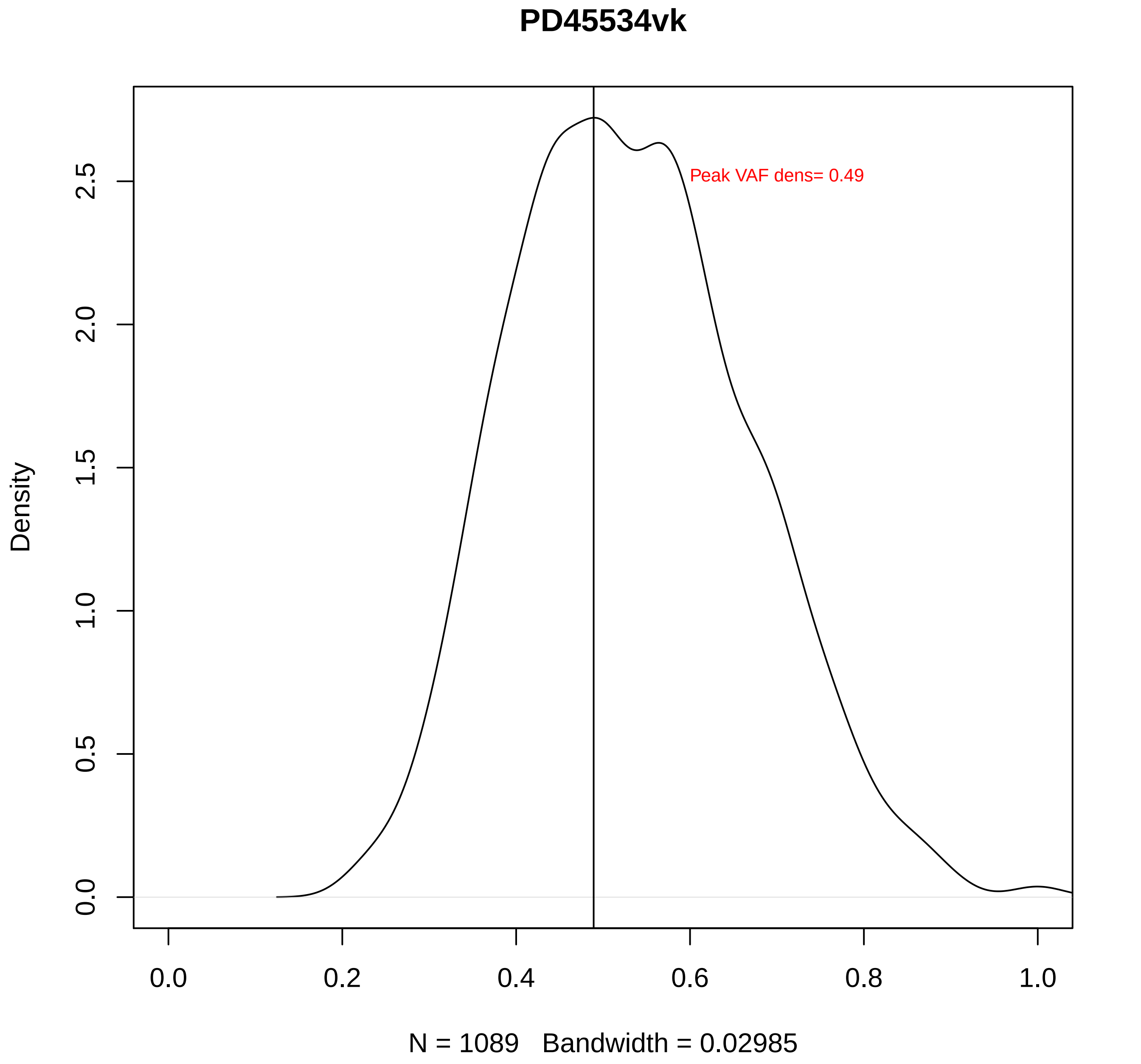

PD45534vk
Peak VAF dens= 0.49
Density
0.0
0.5
1.0
1.5
2.0
2.5
0.0
0.2
0.4
0.6
0.8
1.0
N = 1089 Bandwidth = 0.02985

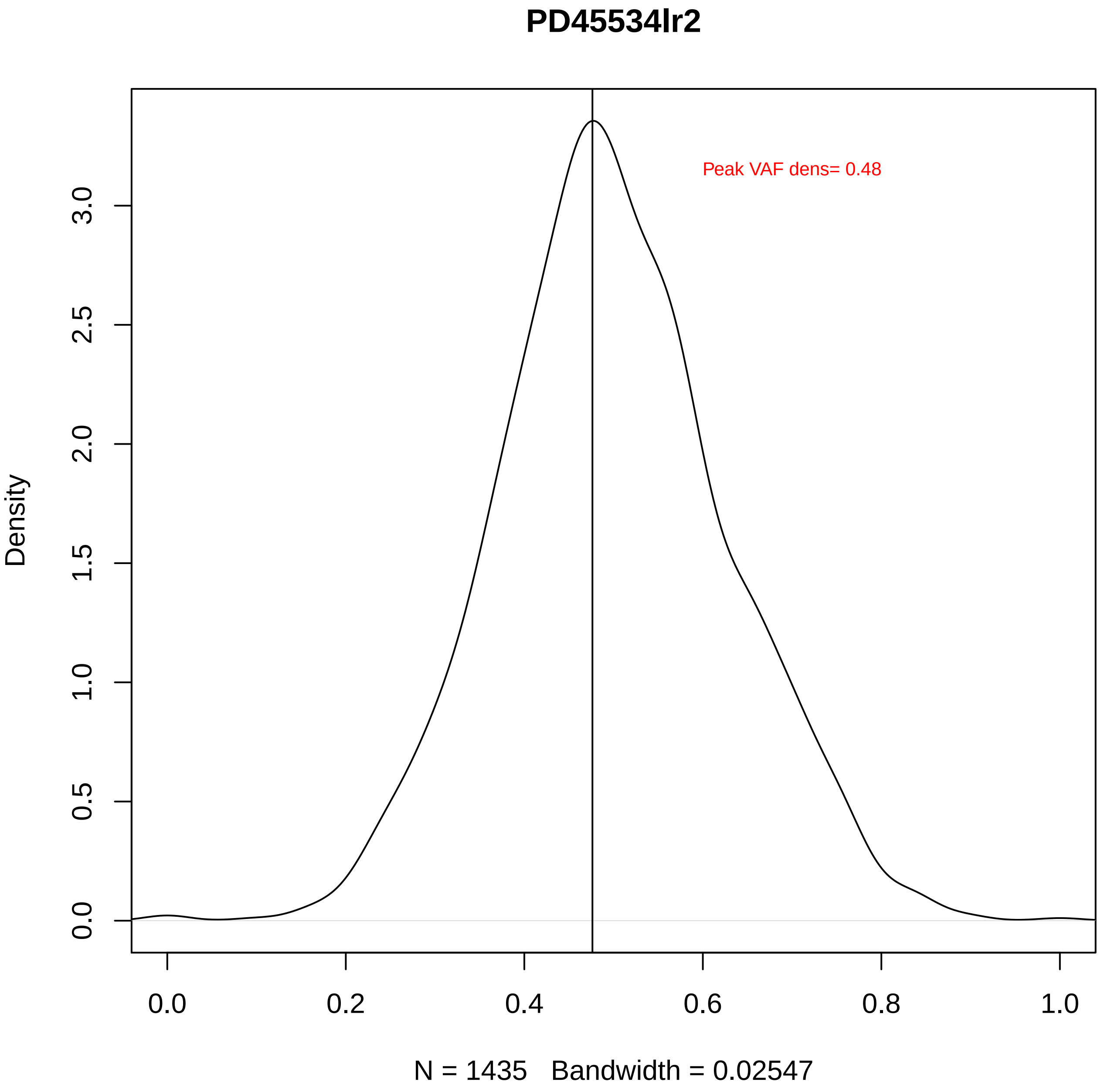

PD45534lr2
Peak VAF dens= 0.48
Density
0.0
0.5
1.0
1.5
2.0
2.5
3.0
0.0
0.2
0.4
0.6
0.8
1.0
N = 1435   Bandwidth = 0.02547

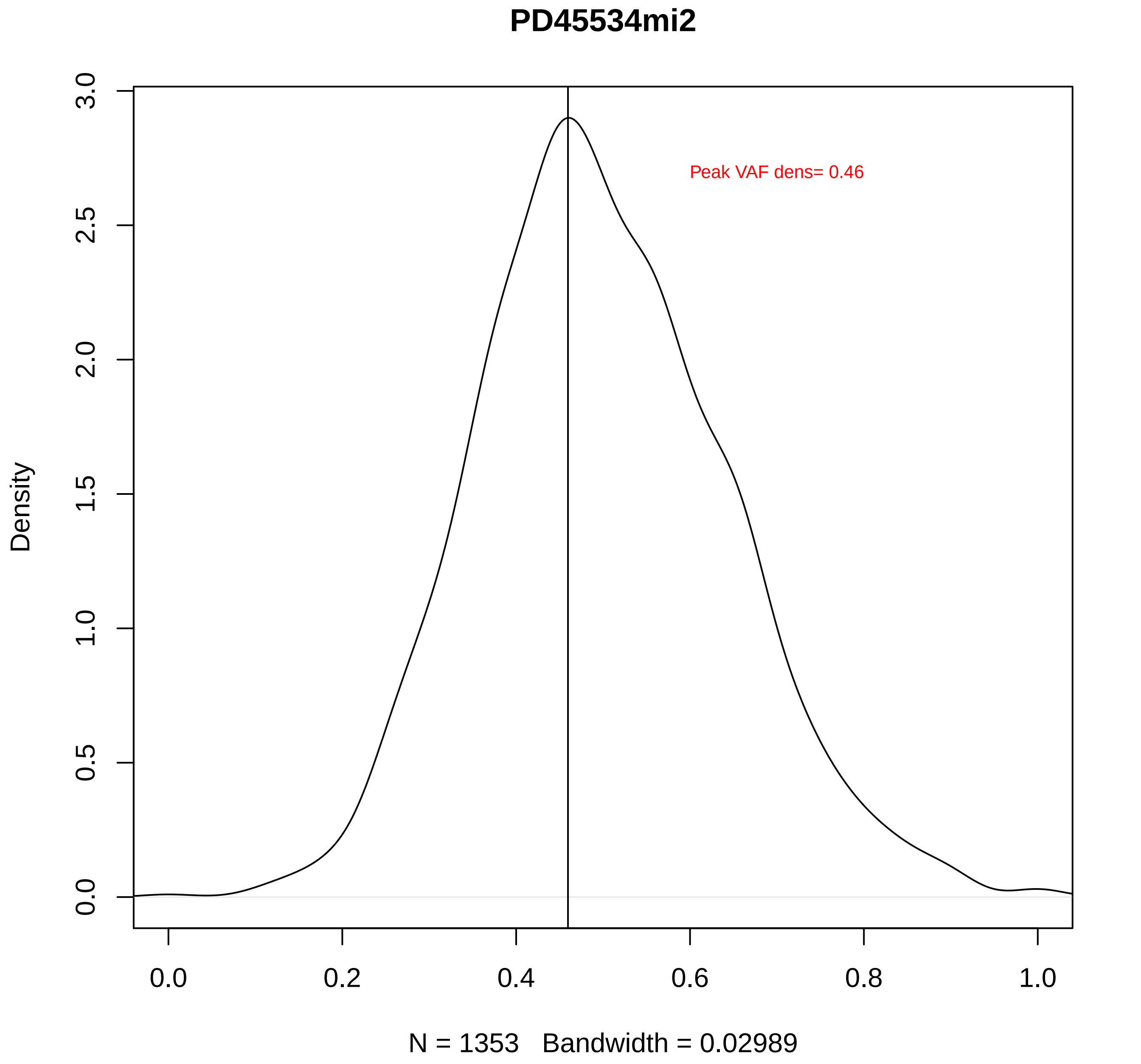
PD45534mi2
Peak VAF dens= 0.46
Density
0.0
0.5
1.0
1.5
2.0
2.5
3.0
0.0
0.2
0.4
0.6
0.8
1.0
N = 1353   Bandwidth = 0.02989

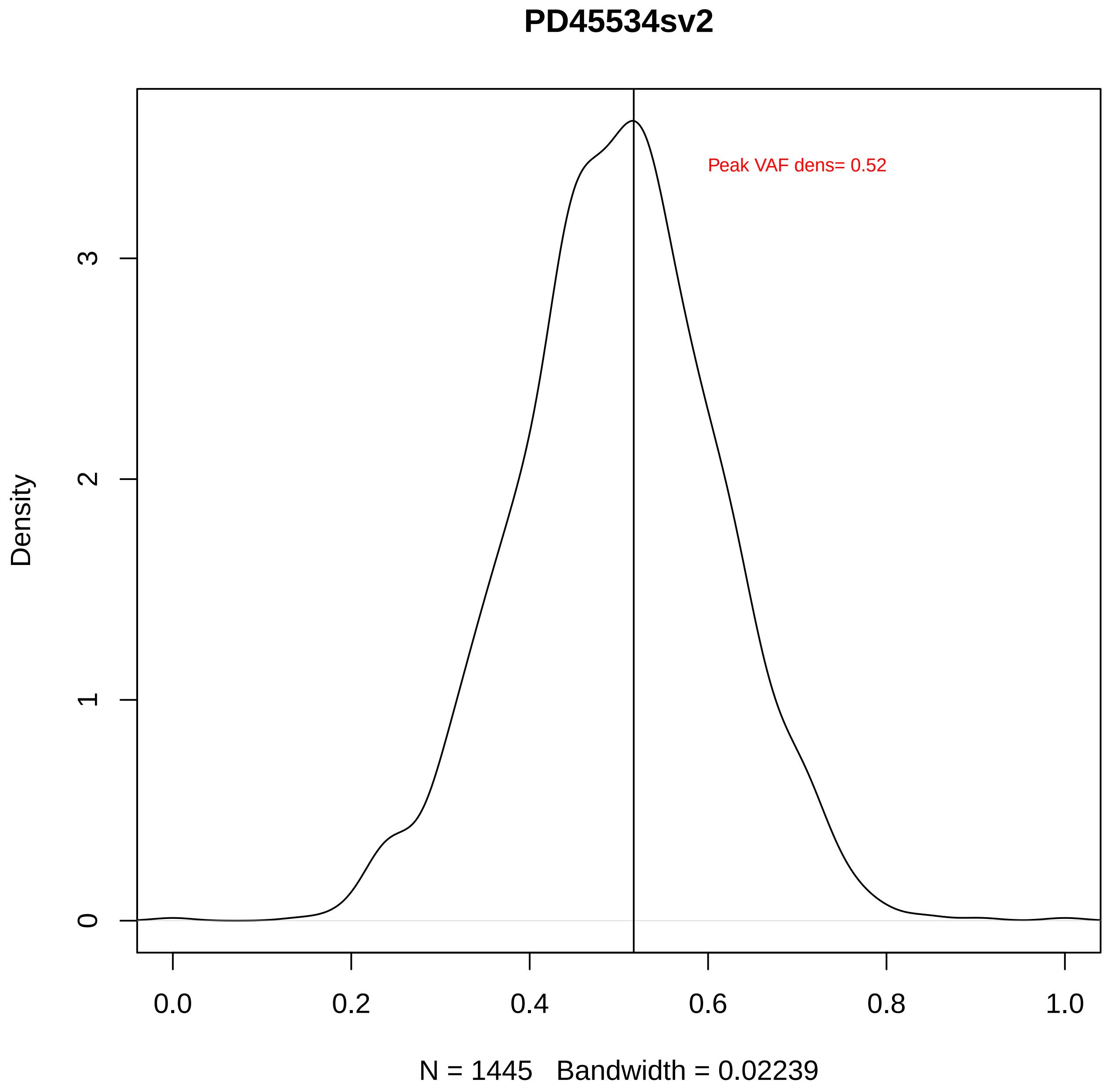
PD45534sv2
Peak VAF dens= 0.52
Density
0
1
2
3
0.0
0.2
0.4
0.6
0.8
1.0
N = 1445   Bandwidth = 0.02239

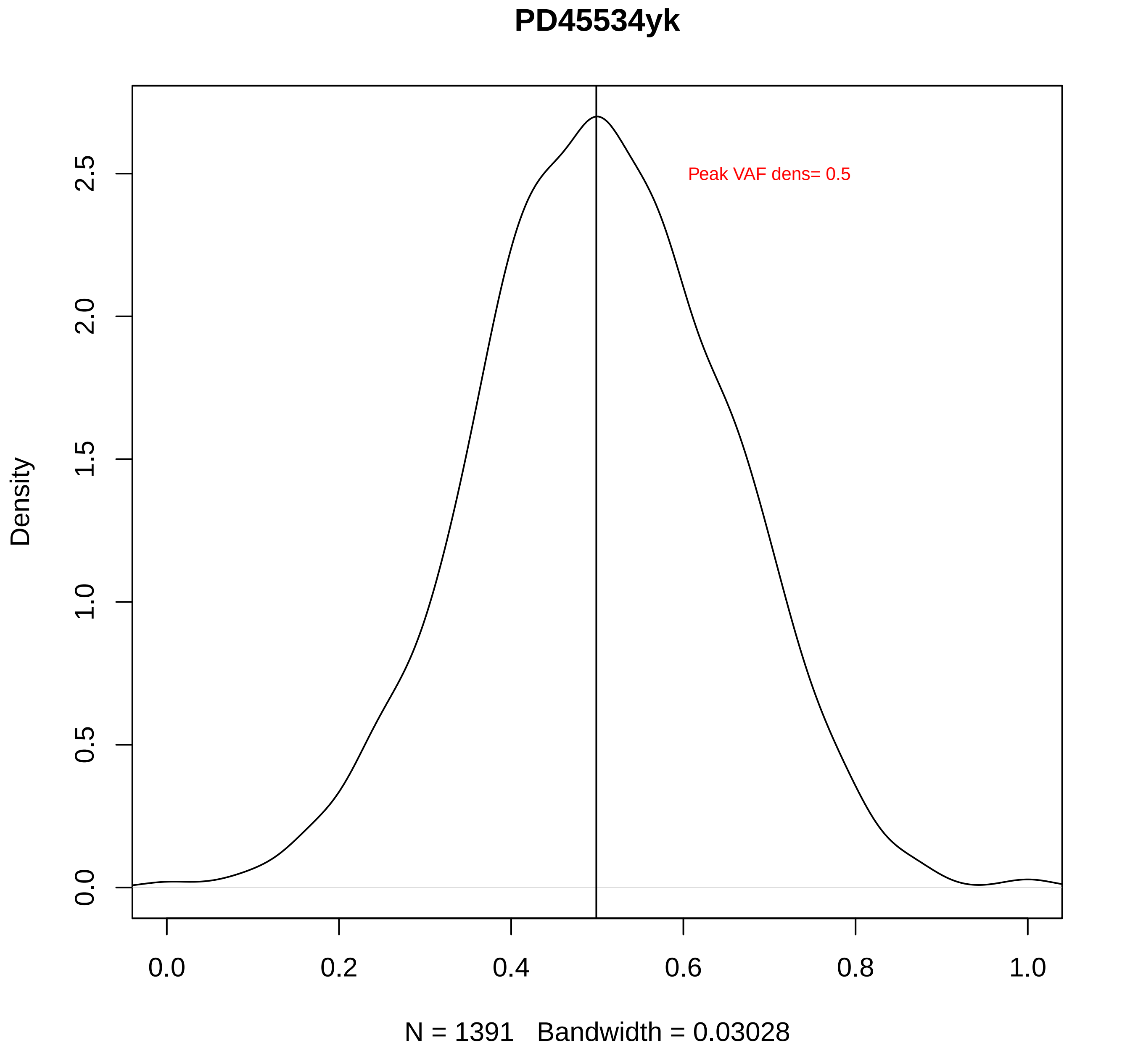

PD45534yk
Peak VAF dens= 0.5
Density
0.0
0.5
1.0
1.5
2.0
2.5
0.0
0.2
0.4
0.6
0.8
1.0
N = 1391   Bandwidth = 0.03028

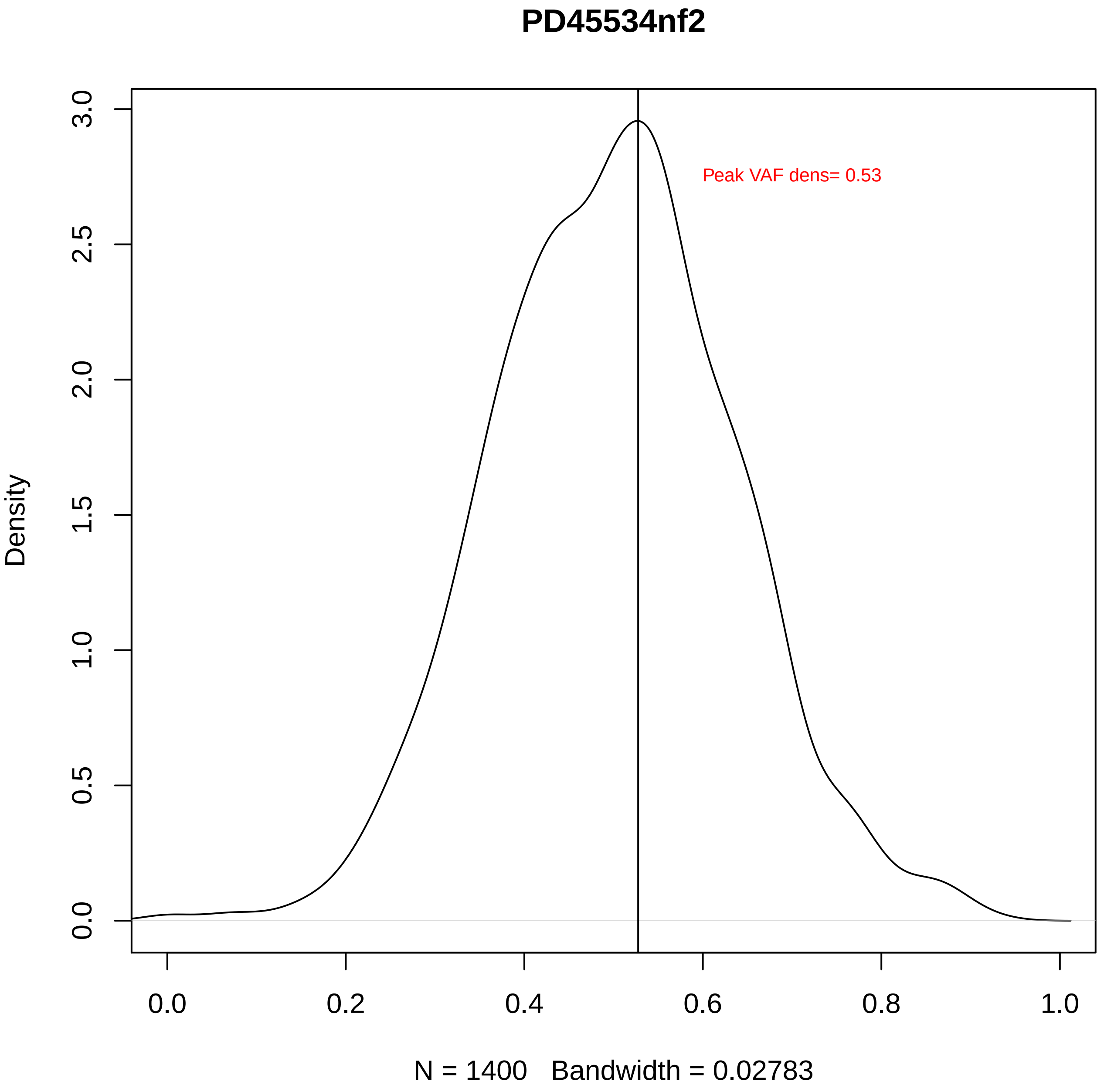

PD45534nf2
Peak VAF dens= 0.53
Density
0.0
0.5
1.0
1.5
2.0
2.5
3.0
0.0
0.2
0.4
0.6
0.8
1.0
N = 1400   Bandwidth = 0.02783

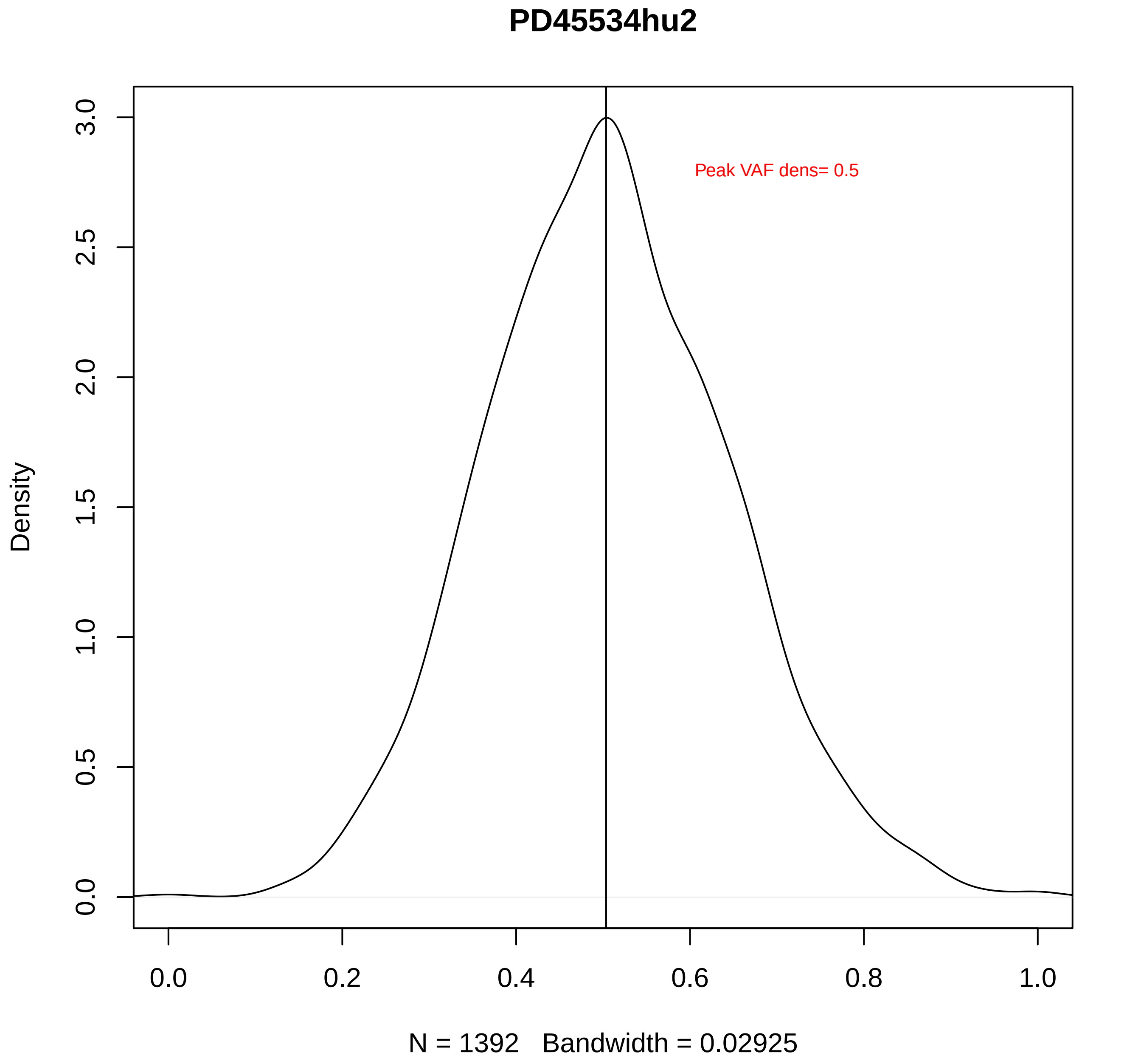
PD45534hu2
Peak VAF dens= 0.5
Density
0.0
0.5
1.0
1.5
2.0
2.5
3.0
0.0
0.2
0.4
0.6
0.8
1.0
N = 1392 Bandwidth = 0.02925

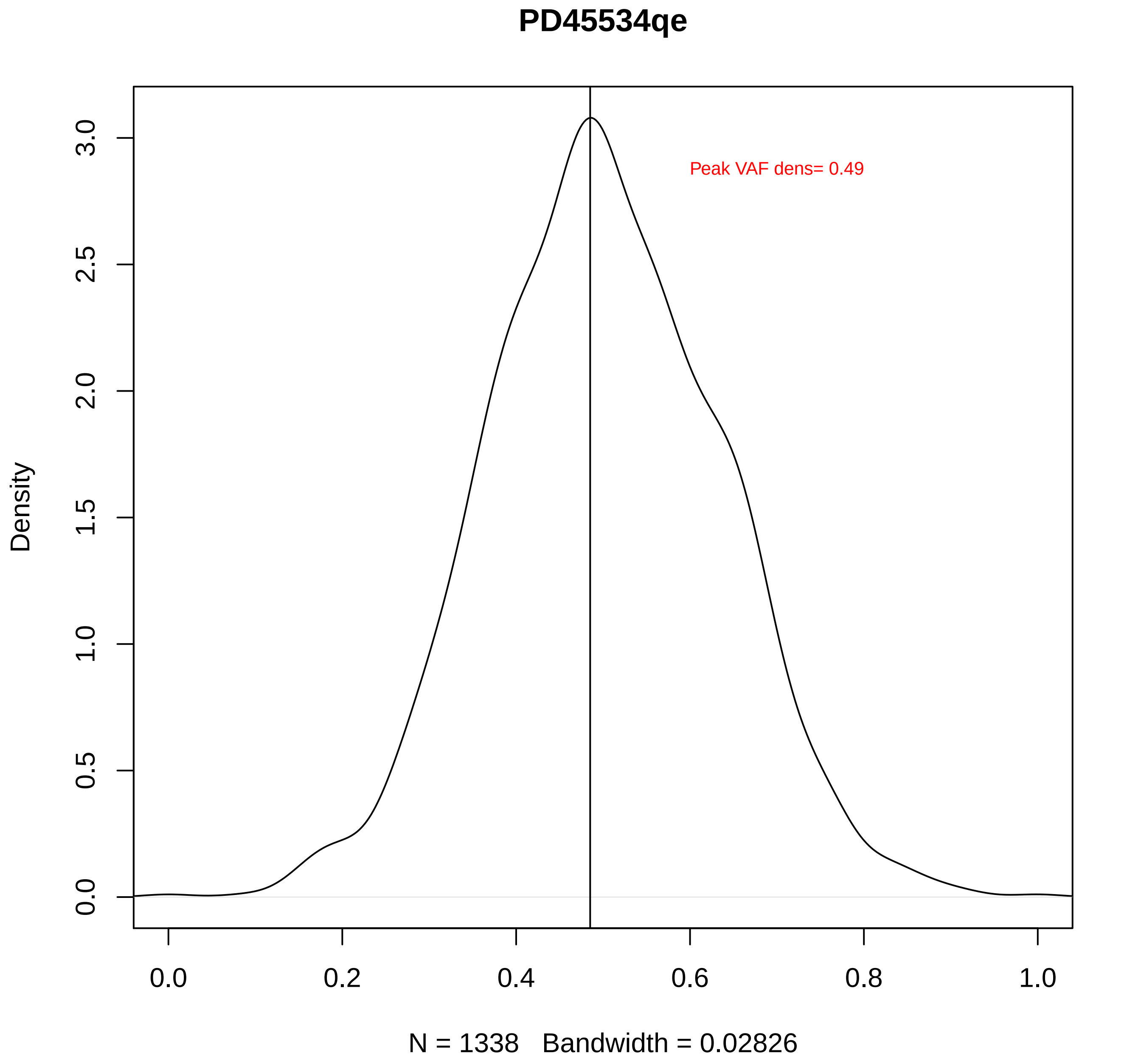
PD45534qe
Peak VAF dens= 0.49
Density
0.0
0.5
1.0
1.5
2.0
2.5
3.0
0.0
0.2
0.4
0.6
0.8
1.0
N = 1338   Bandwidth = 0.02826

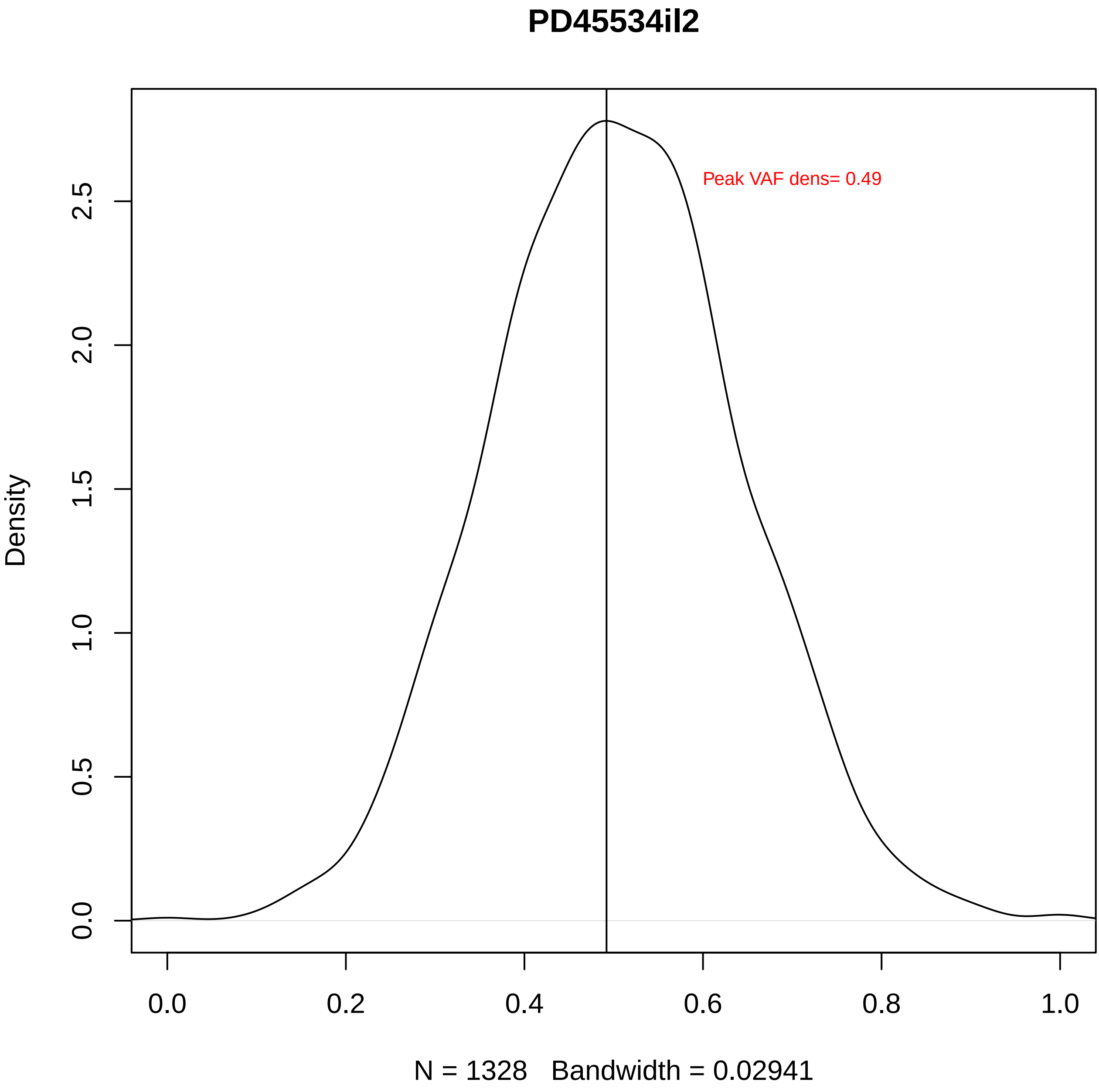
PD45534il2
Peak VAF dens= 0.49
Density
0.0
0.5
1.0
1.5
2.0
2.5
0.0
0.2
0.4
0.6
0.8
1.0
N = 1328   Bandwidth = 0.02941

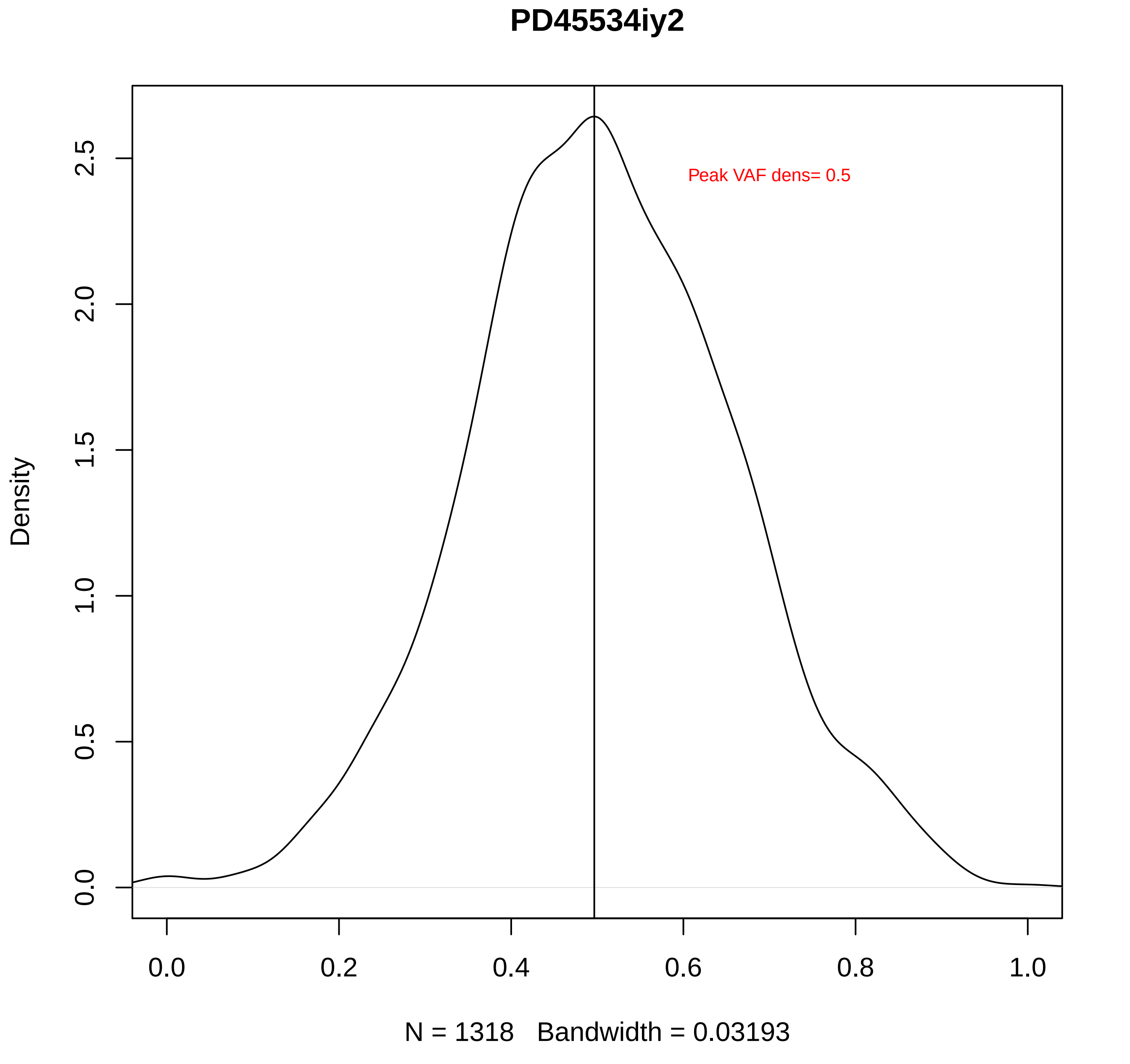

PD45534iy2
Density
2.5
2.0
1.5
1.0
0.5
0.0
Peak VAF dens= 0.5
0.0
0.2
0.4
0.6
0.8
1.0
N = 1318   Bandwidth = 0.03193

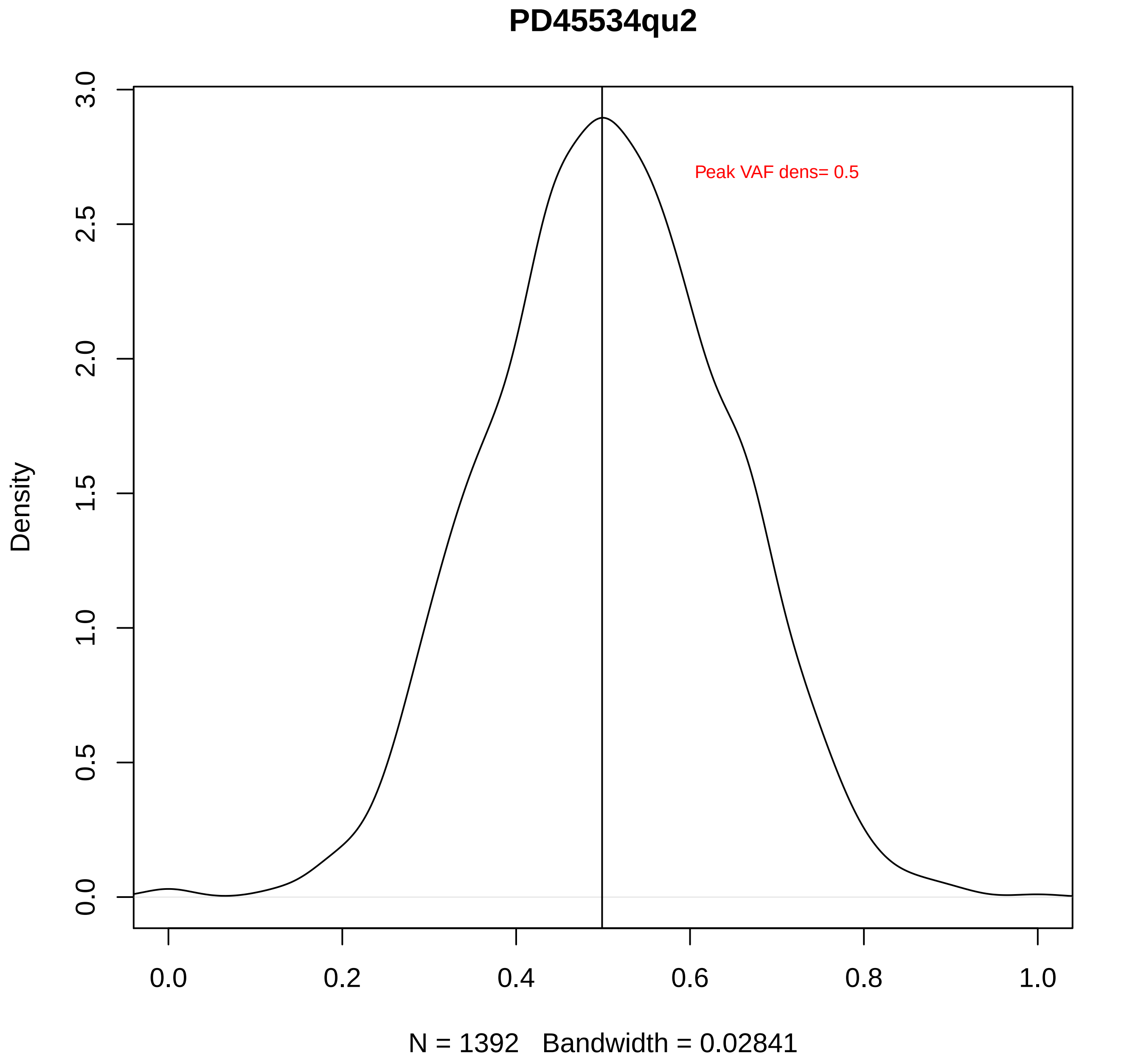
PD45534qu2
Density
3.0
2.5
2.0
1.5
1.0
0.5
0.0
Peak VAF dens= 0.5
0.0
0.2
0.4
0.6
0.8
1.0
N = 1392   Bandwidth = 0.02841

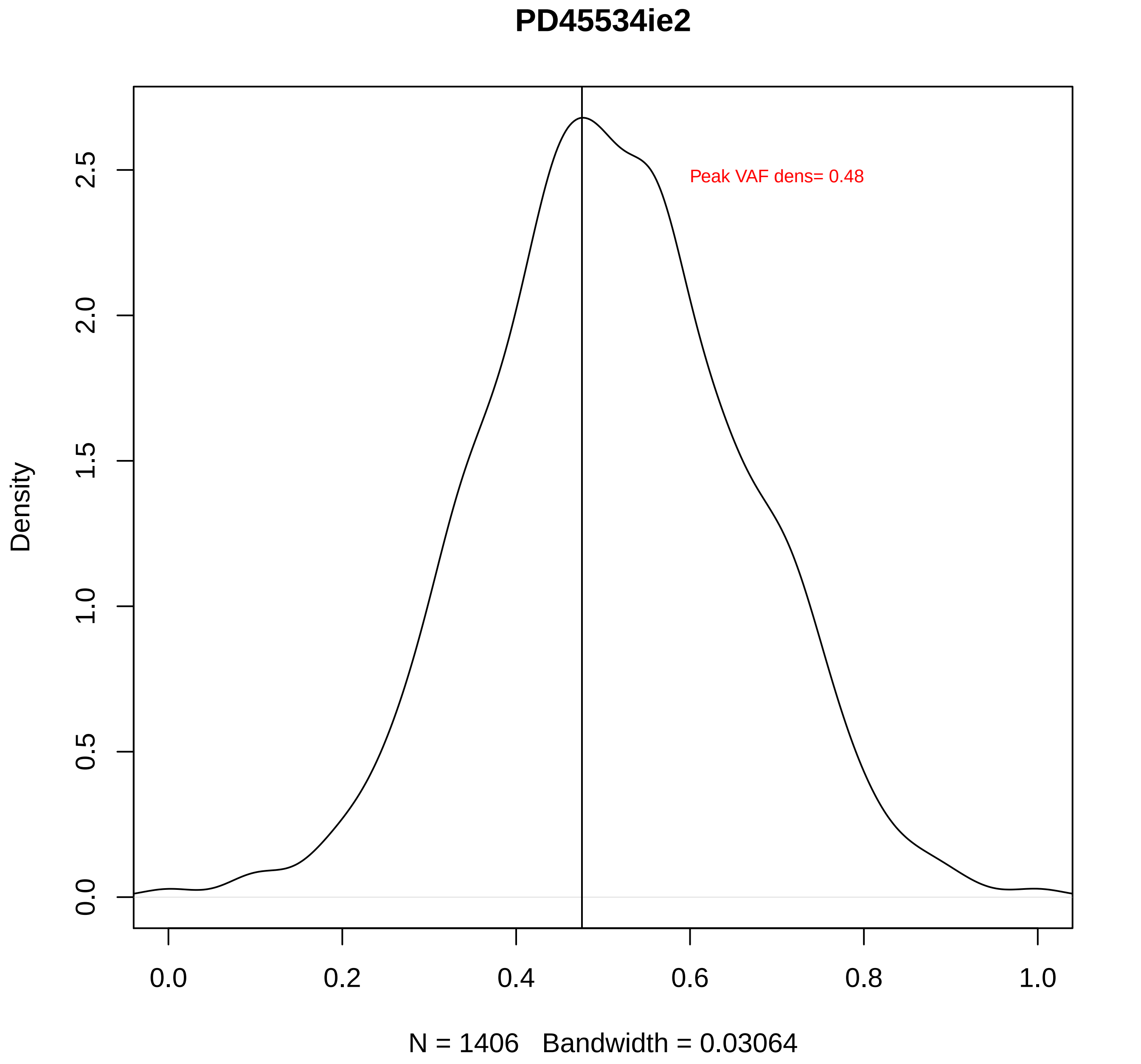
PD45534ie2
Peak VAF dens= 0.48
Density
0.0
0.5
1.0
1.5
2.0
2.5
0.0
0.2
0.4
0.6
0.8
1.0
N = 1406   Bandwidth = 0.03064

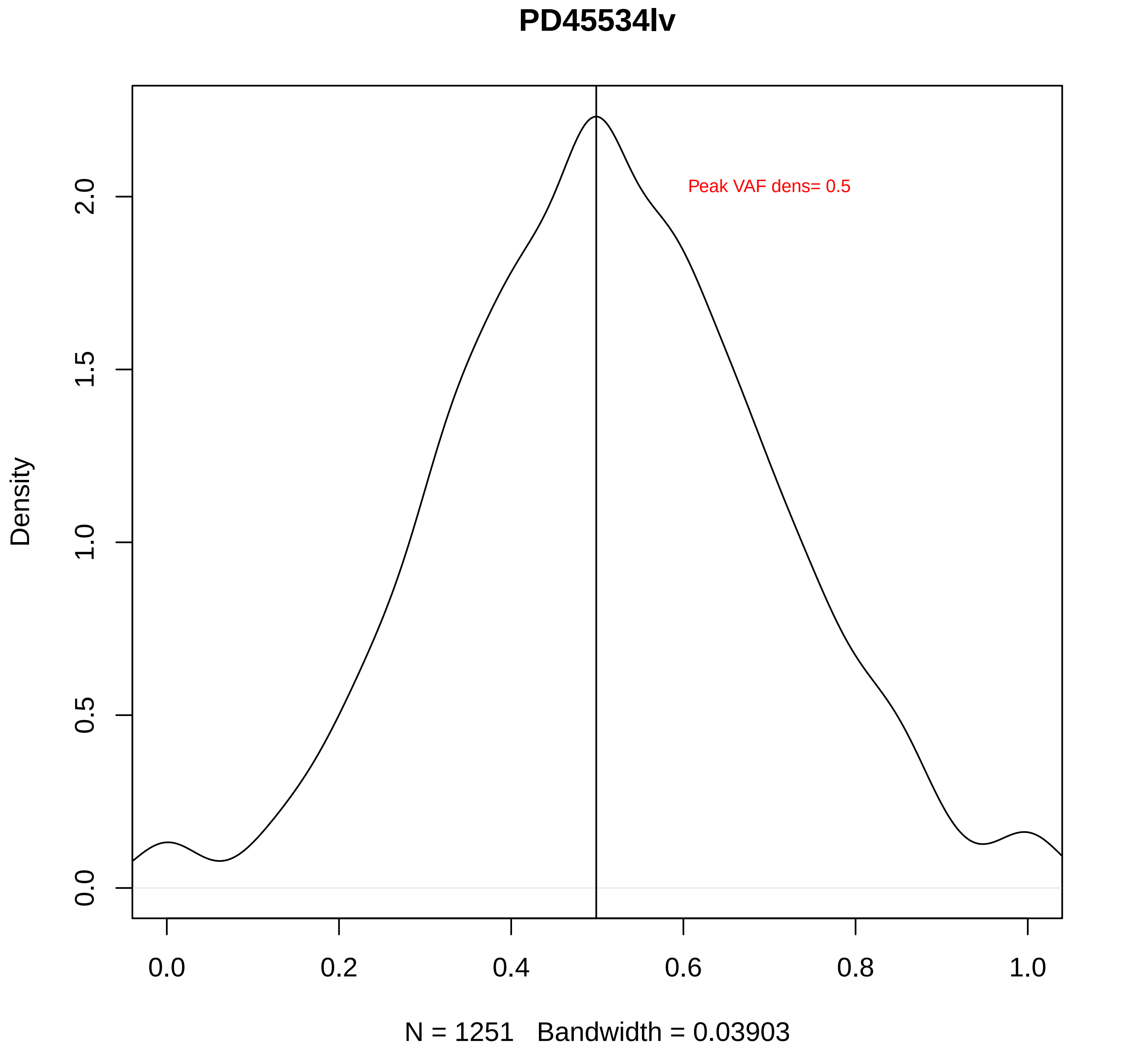
PD45534lv
Peak VAF dens= 0.5
Density
0.0
0.5
1.0
1.5
2.0
0.0
0.2
0.4
0.6
0.8
1.0
N = 1251   Bandwidth = 0.03903

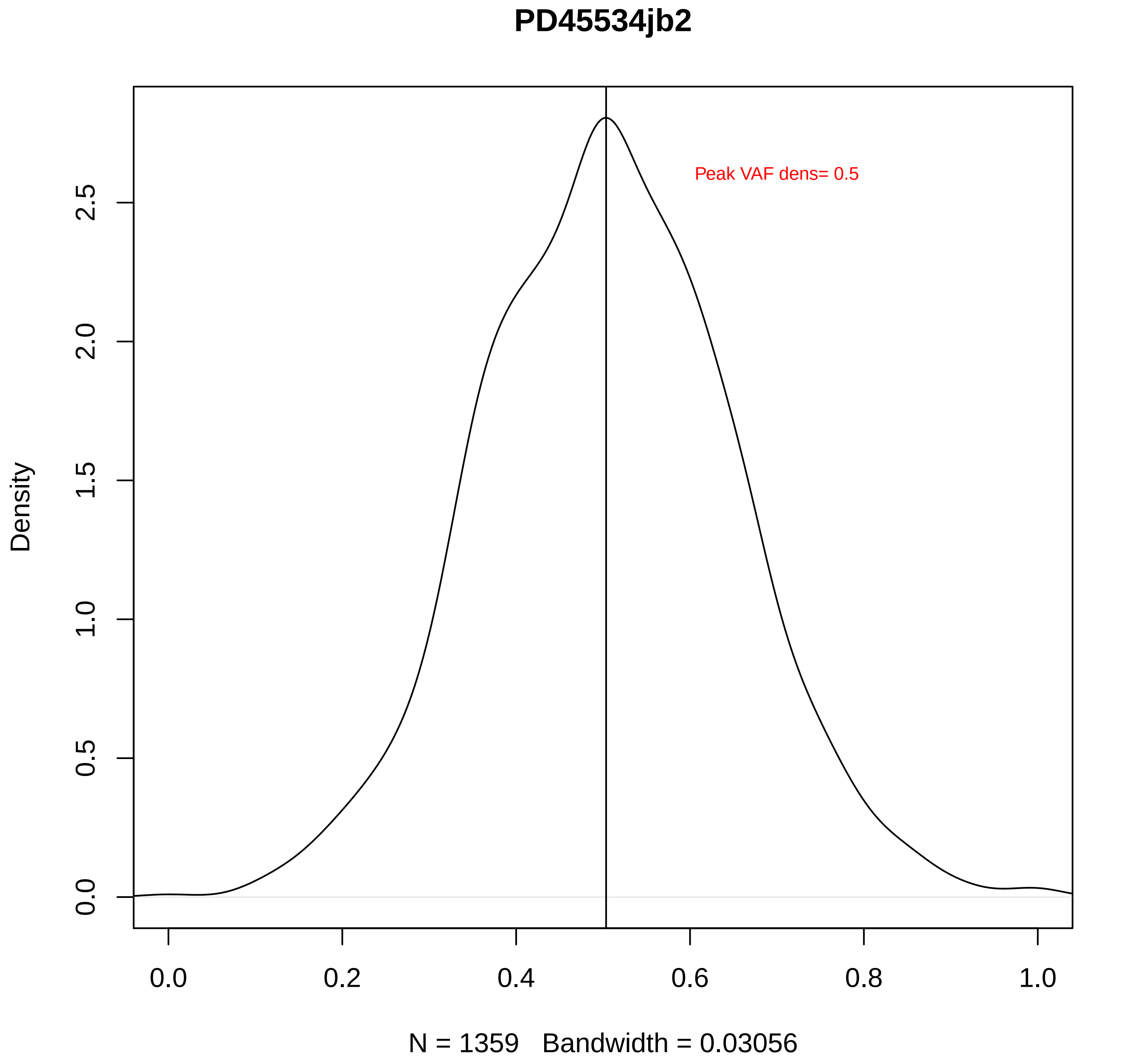
PD45534jb2
Peak VAF dens= 0.5
Density
0.0
0.5
1.0
1.5
2.0
2.5
0.0
0.2
0.4
0.6
0.8
1.0
N = 1359   Bandwidth = 0.03056

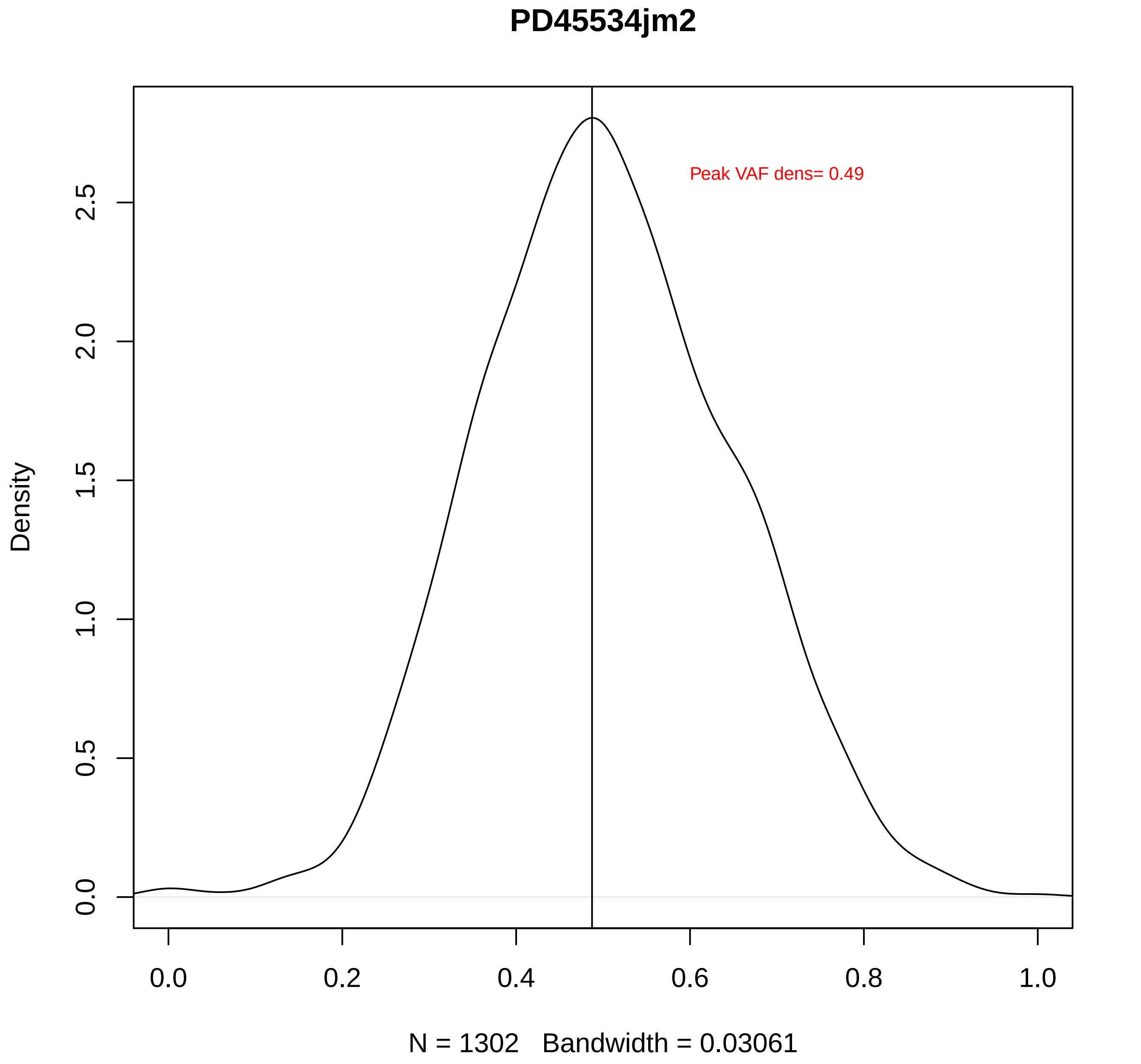
PD45534jm2
Peak VAF dens= 0.49
Density
0.0
0.5
1.0
1.5
2.0
2.5
0.0
0.2
0.4
0.6
0.8
1.0
N = 1302   Bandwidth = 0.03061

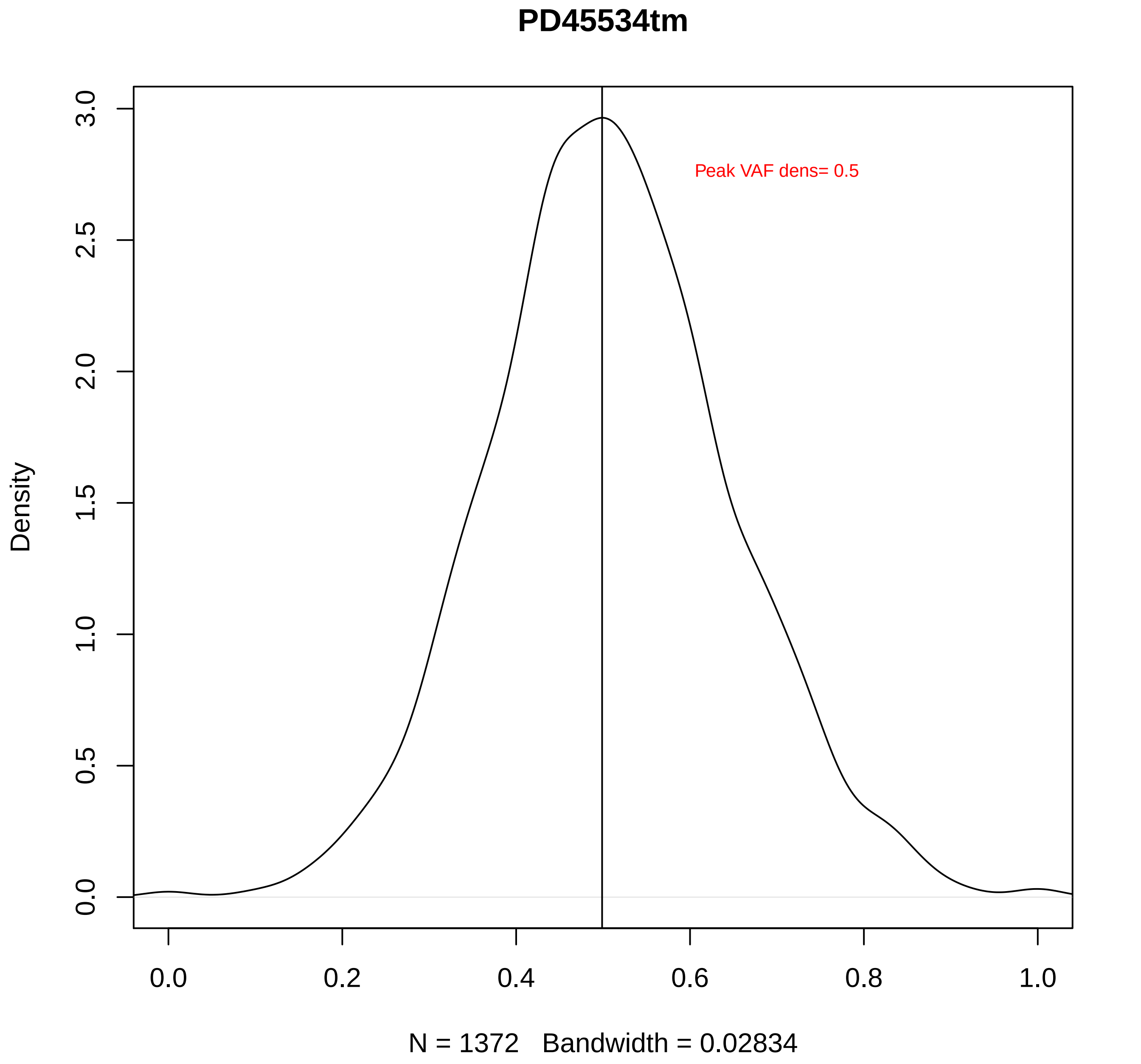

PD45534tm
Peak VAF dens= 0.5
Density
3.0
2.5
2.0
1.5
1.0
0.5
0.0
0.0
0.2
0.4
0.6
0.8
1.0
N = 1372   Bandwidth = 0.02834

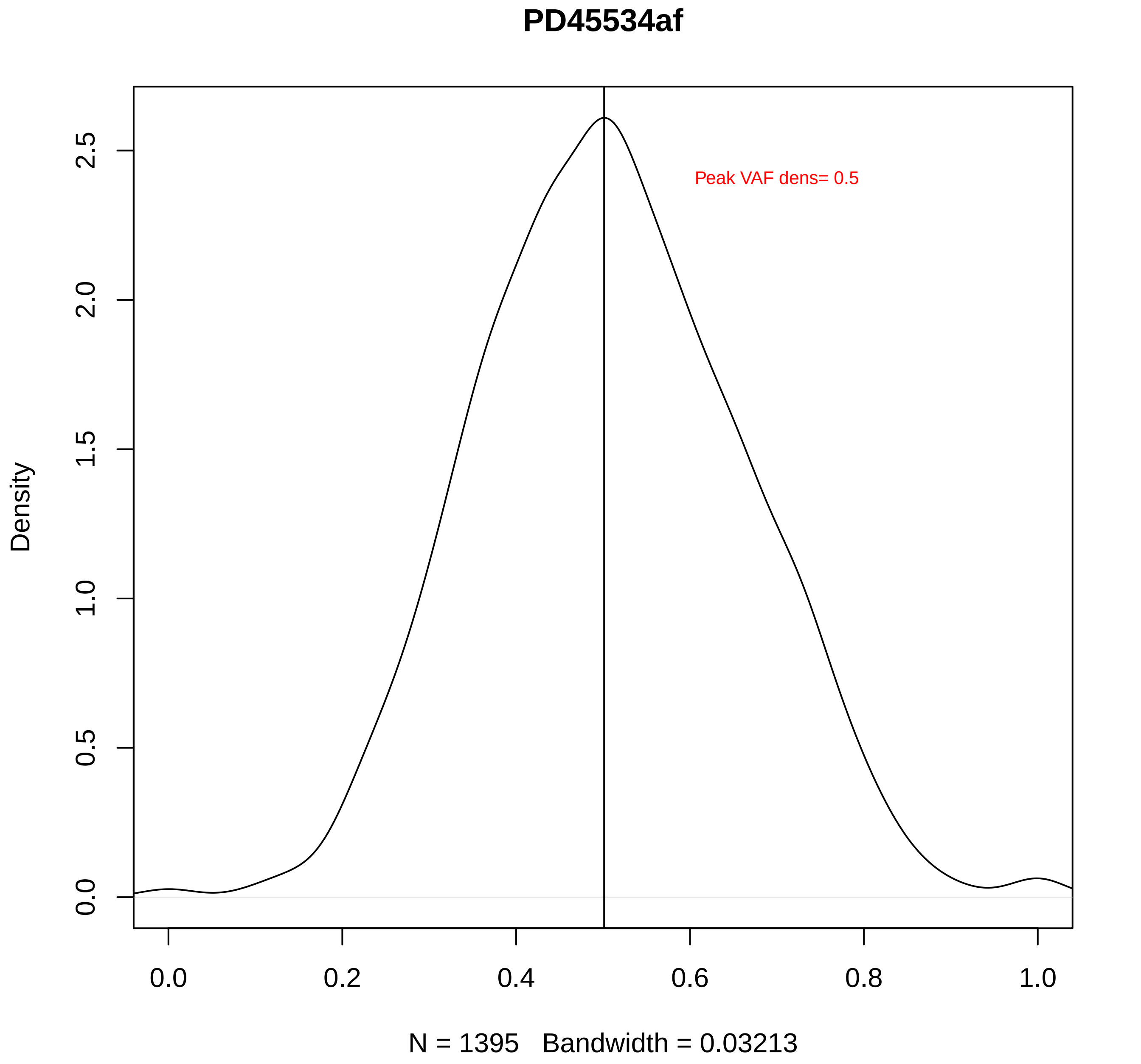

PD45534af
Peak VAF dens= 0.5
Density
0.0
0.5
1.0
1.5
2.0
2.5
0.0
0.2
0.4
0.6
0.8
1.0
N = 1395 Bandwidth = 0.03213

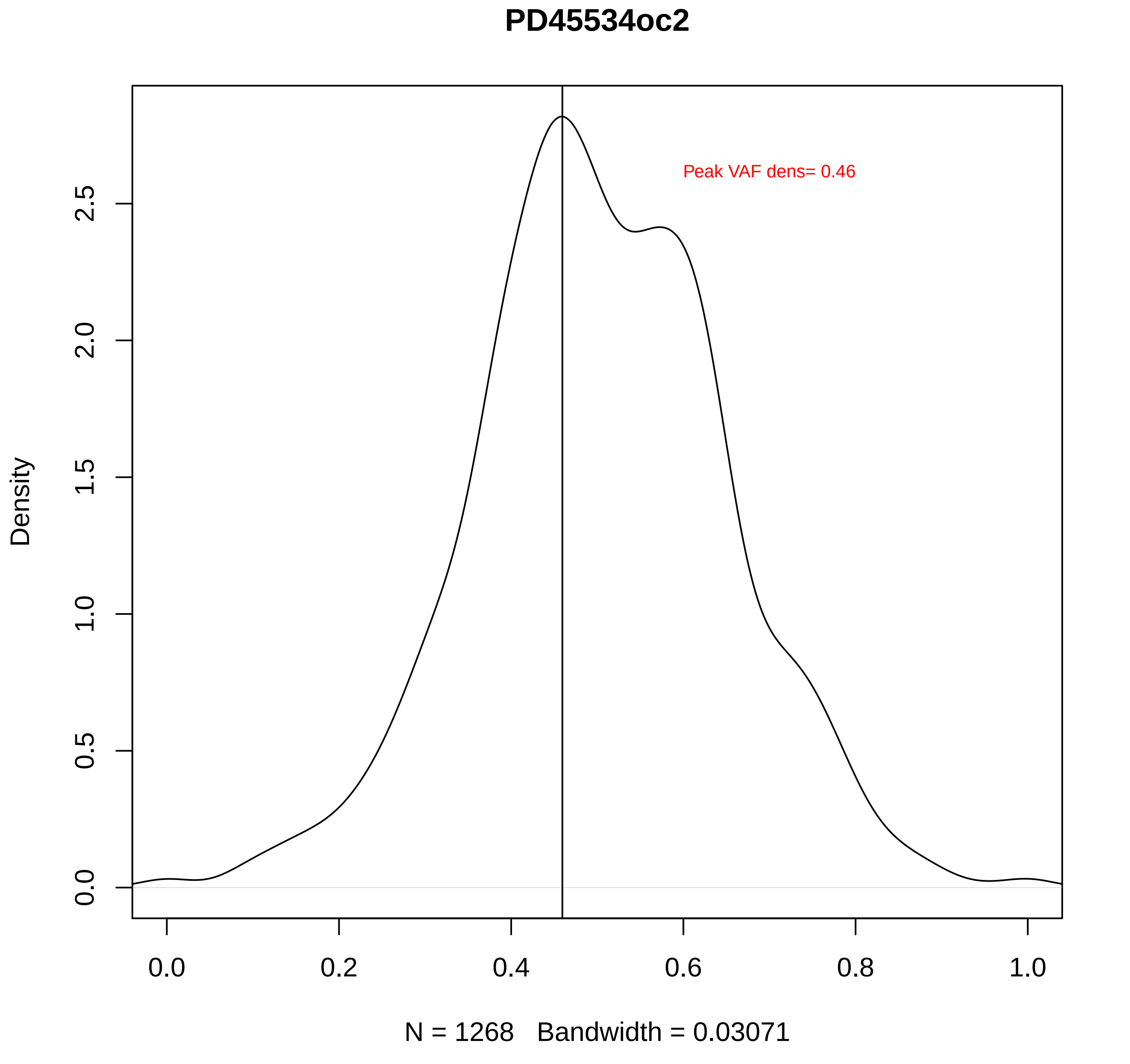
PD45534oc2
Peak VAF dens= 0.46
Density
0.0
0.5
1.0
1.5
2.0
2.5
0.0
0.2
0.4
0.6
0.8
1.0
N = 1268   Bandwidth = 0.03071

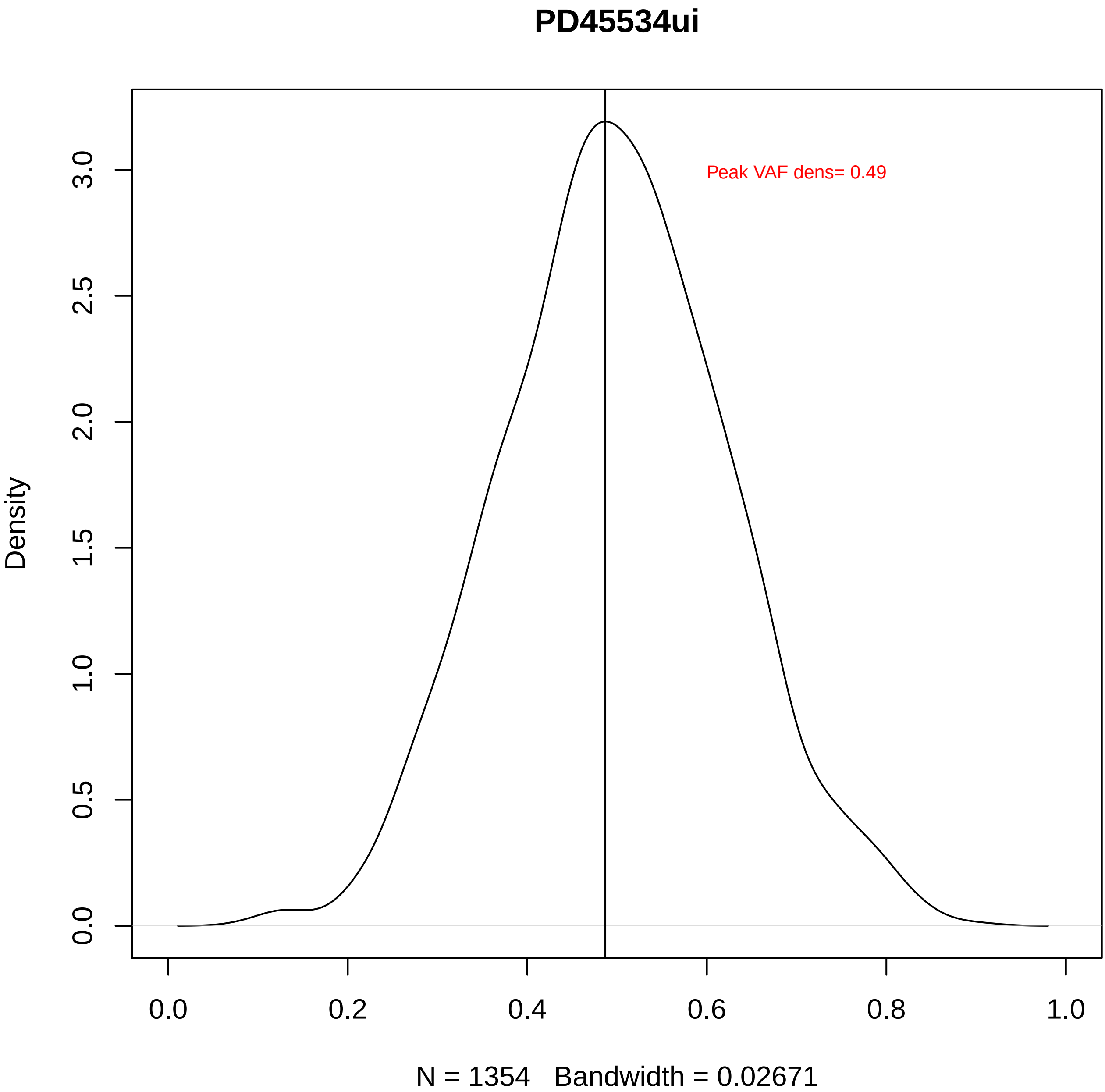
PD45534ui
Peak VAF dens= 0.49
Density
0.0
0.5
1.0
1.5
2.0
2.5
3.0
0.0
0.2
0.4
0.6
0.8
1.0
N = 1354   Bandwidth = 0.02671

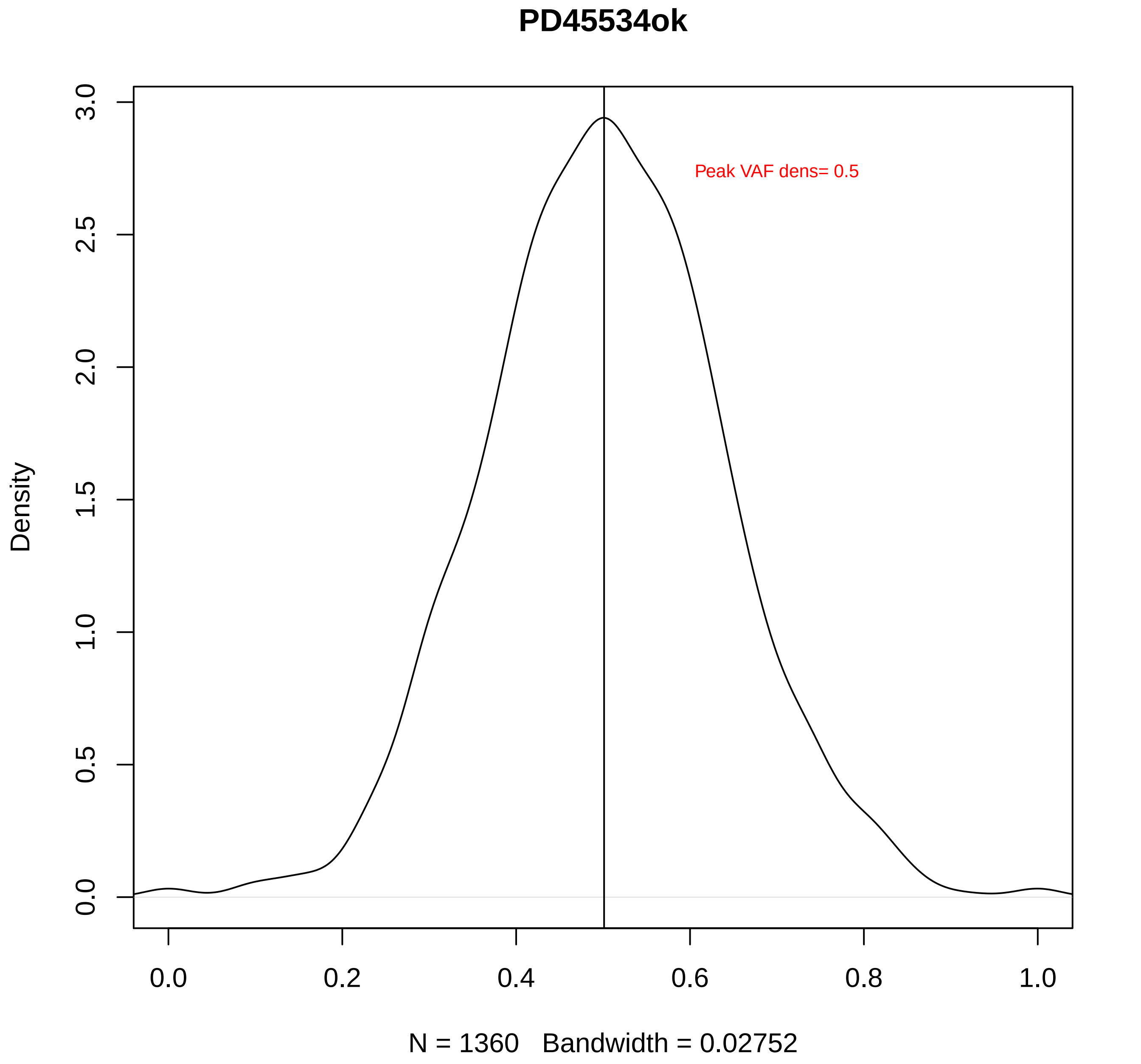
PD45534ok
Peak VAF dens= 0.5
Density
0.0
0.5
1.0
1.5
2.0
2.5
3.0
0.0
0.2
0.4
0.6
0.8
1.0
N = 1360   Bandwidth = 0.02752

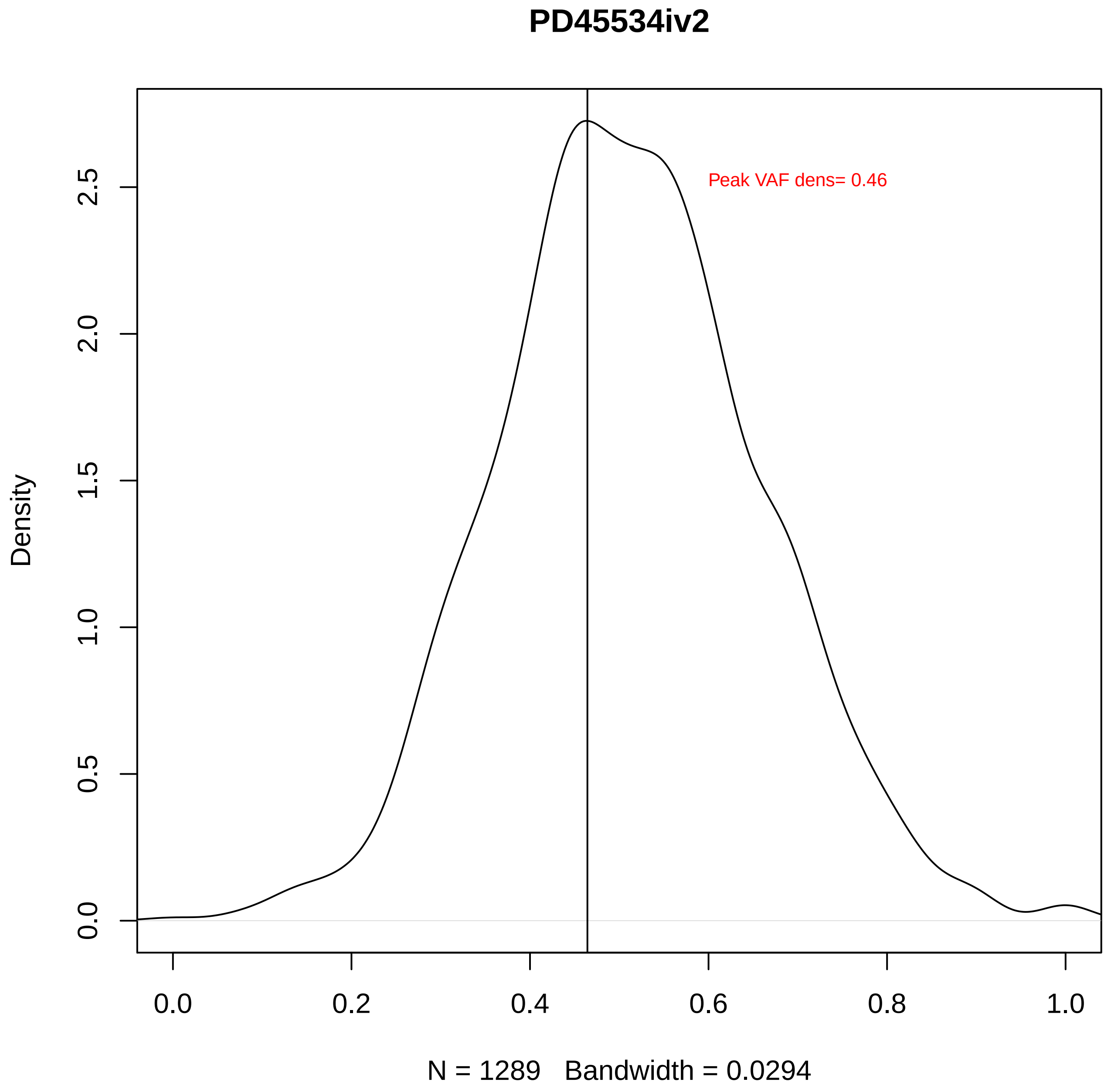
PD45534iv2
Peak VAF dens= 0.46
Density
0.0
0.5
1.0
1.5
2.0
2.5
0.0
0.2
0.4
0.6
0.8
1.0
N = 1289   Bandwidth = 0.0294

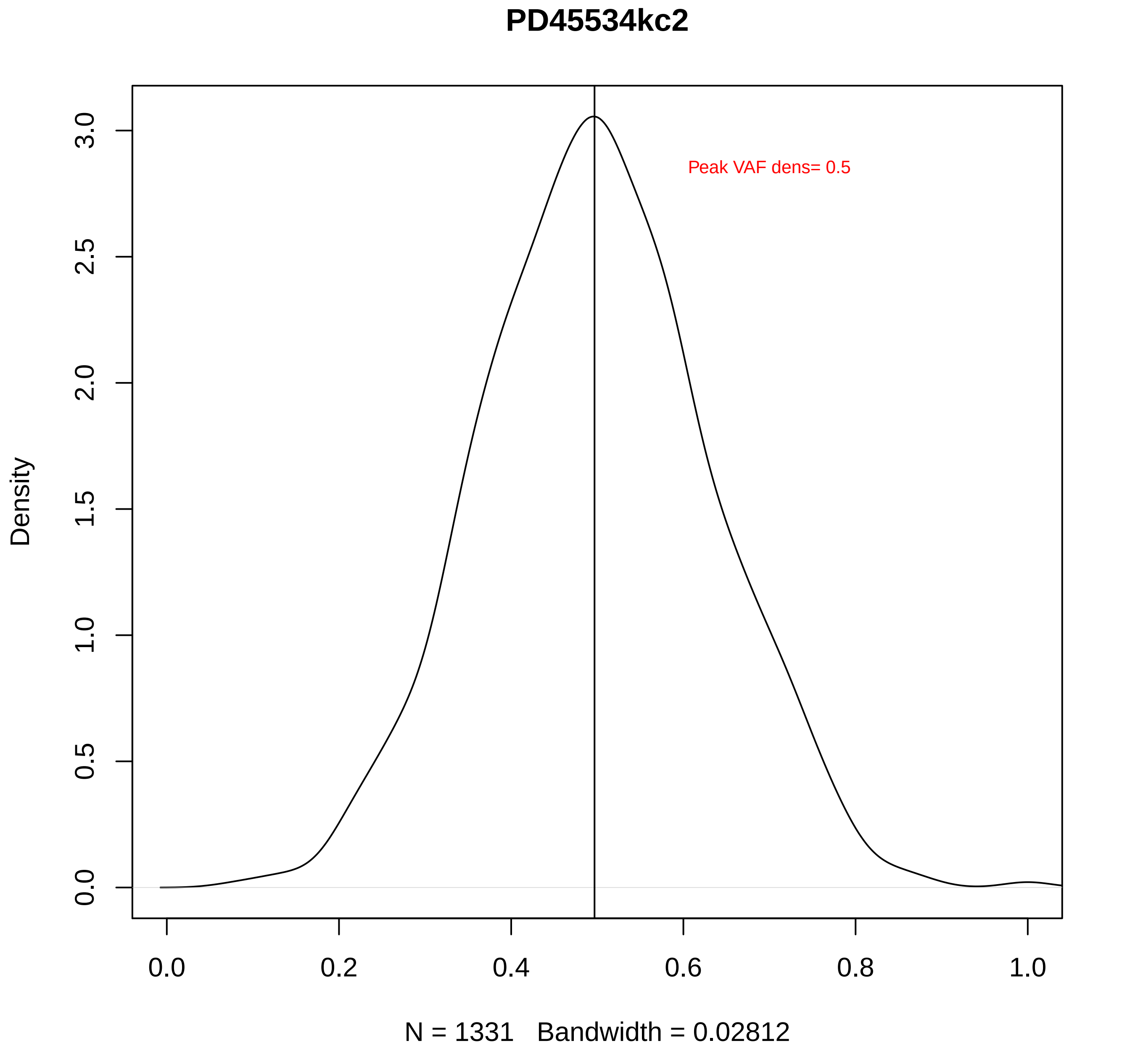
PD45534kc2
Peak VAF dens= 0.5
Density
0.0
0.5
1.0
1.5
2.0
2.5
3.0
0.0
0.2
0.4
0.6
0.8
1.0
N = 1331 Bandwidth = 0.02812

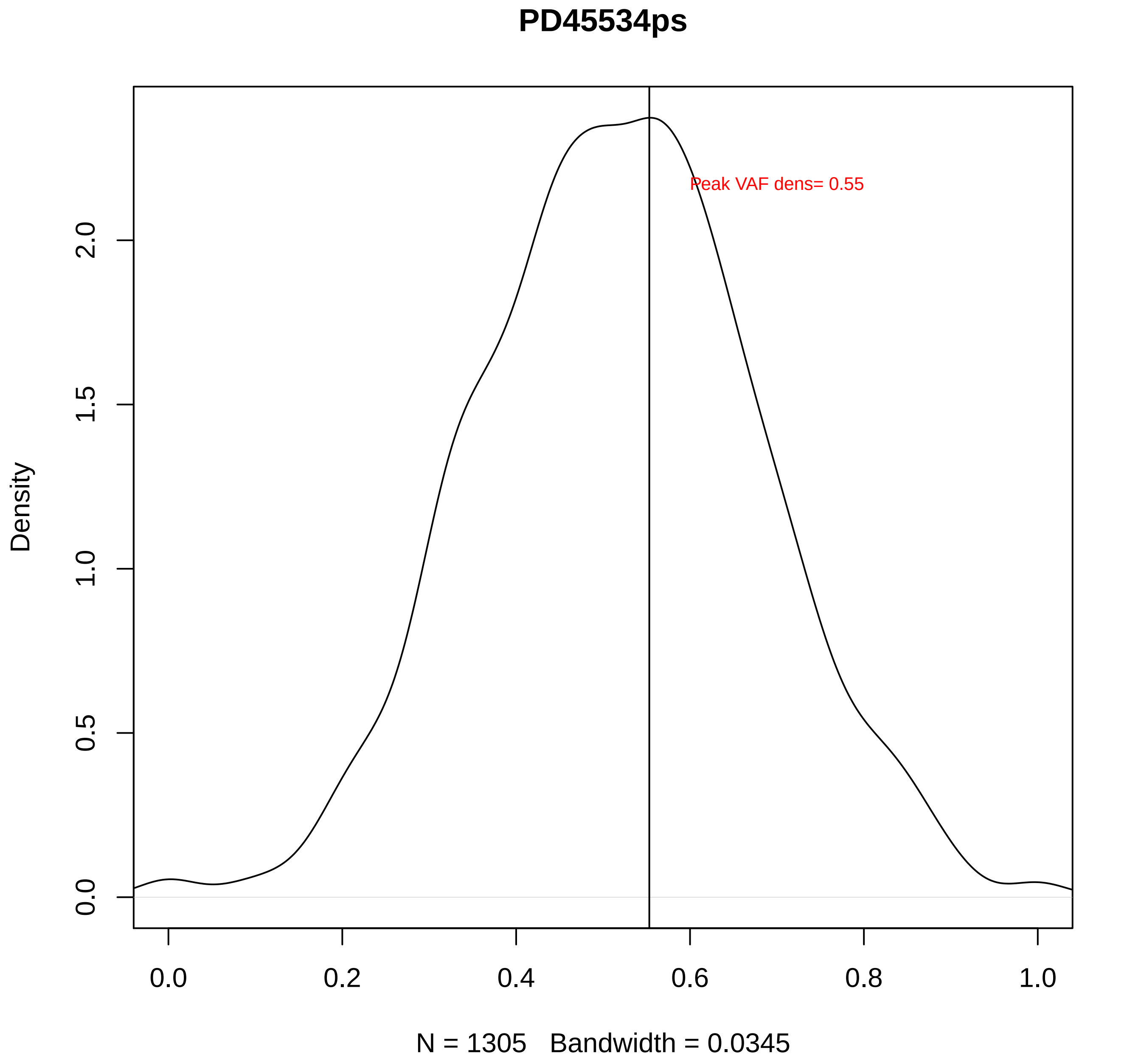
PD45534ps
Peak VAF dens= 0.55
Density
0.0
0.5
1.0
1.5
2.0
0.0
0.2
0.4
0.6
0.8
1.0
N = 1305   Bandwidth = 0.0345

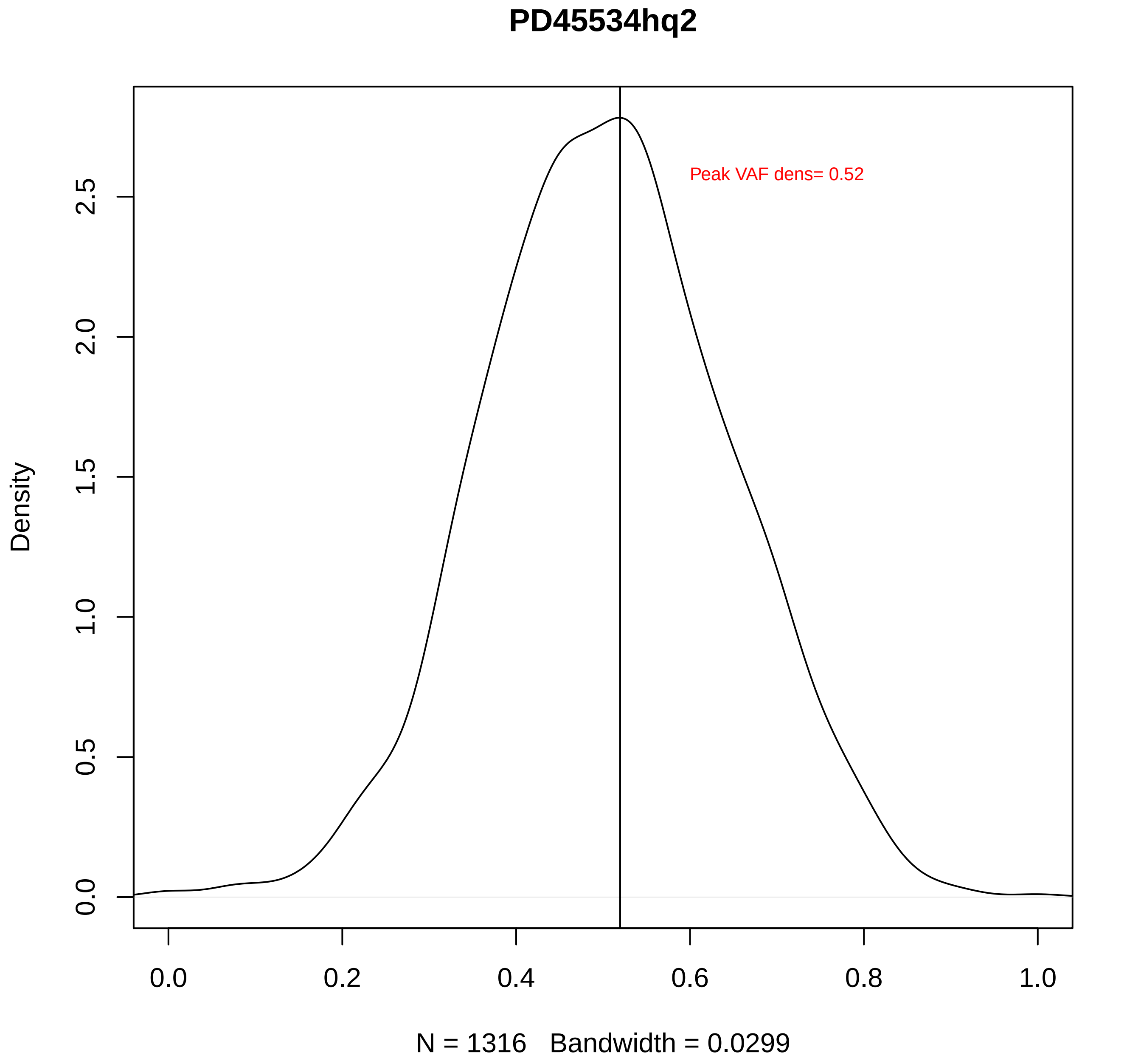

PD45534hq2
Peak VAF dens= 0.52
Density
0.0
0.5
1.0
1.5
2.0
2.5
0.0
0.2
0.4
0.6
0.8
1.0
N = 1316 Bandwidth = 0.0299

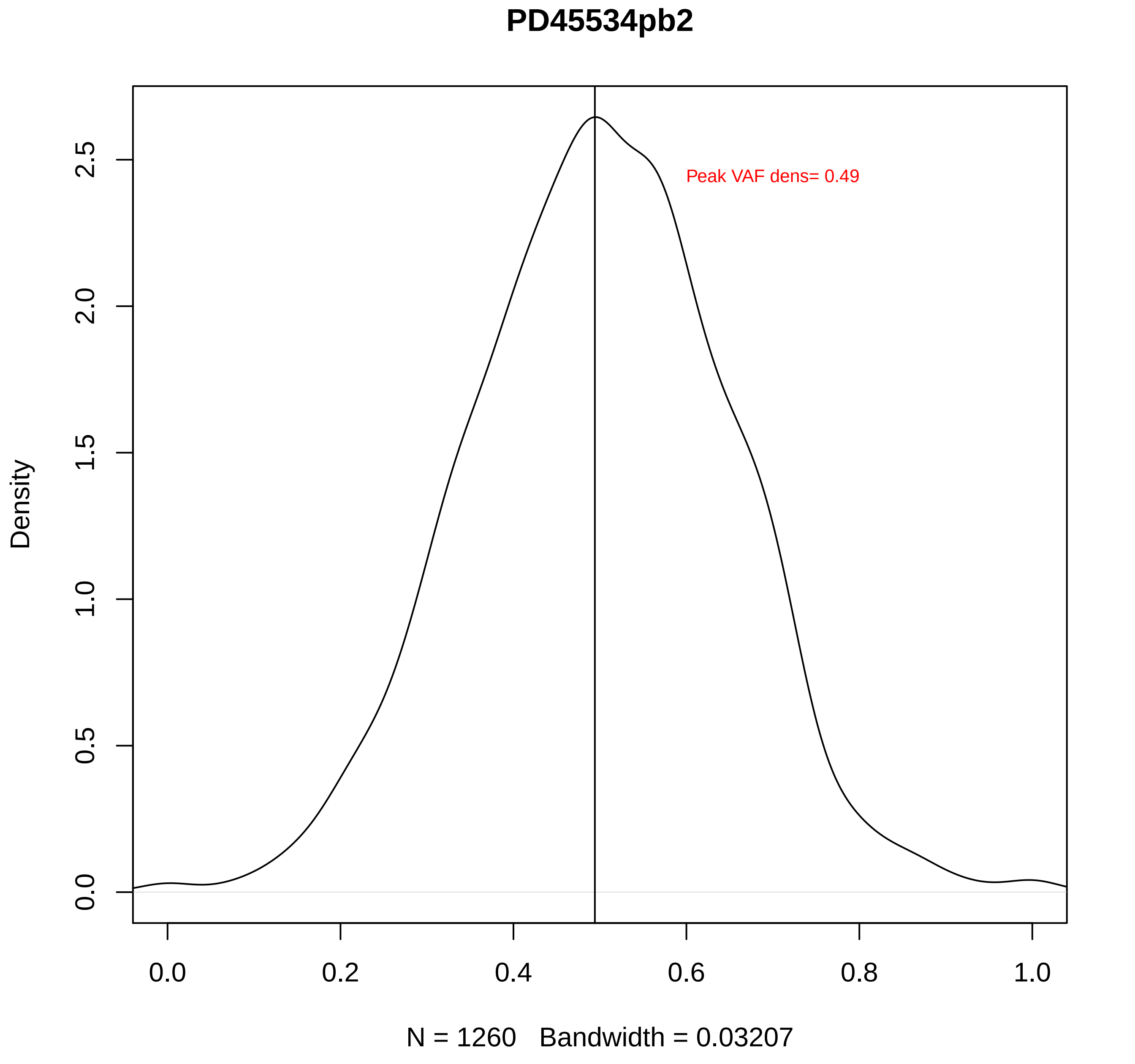
PD45534pb2
Peak VAF dens= 0.49
Density
0.0
0.5
1.0
1.5
2.0
2.5
0.0
0.2
0.4
0.6
0.8
1.0
N = 1260   Bandwidth = 0.03207

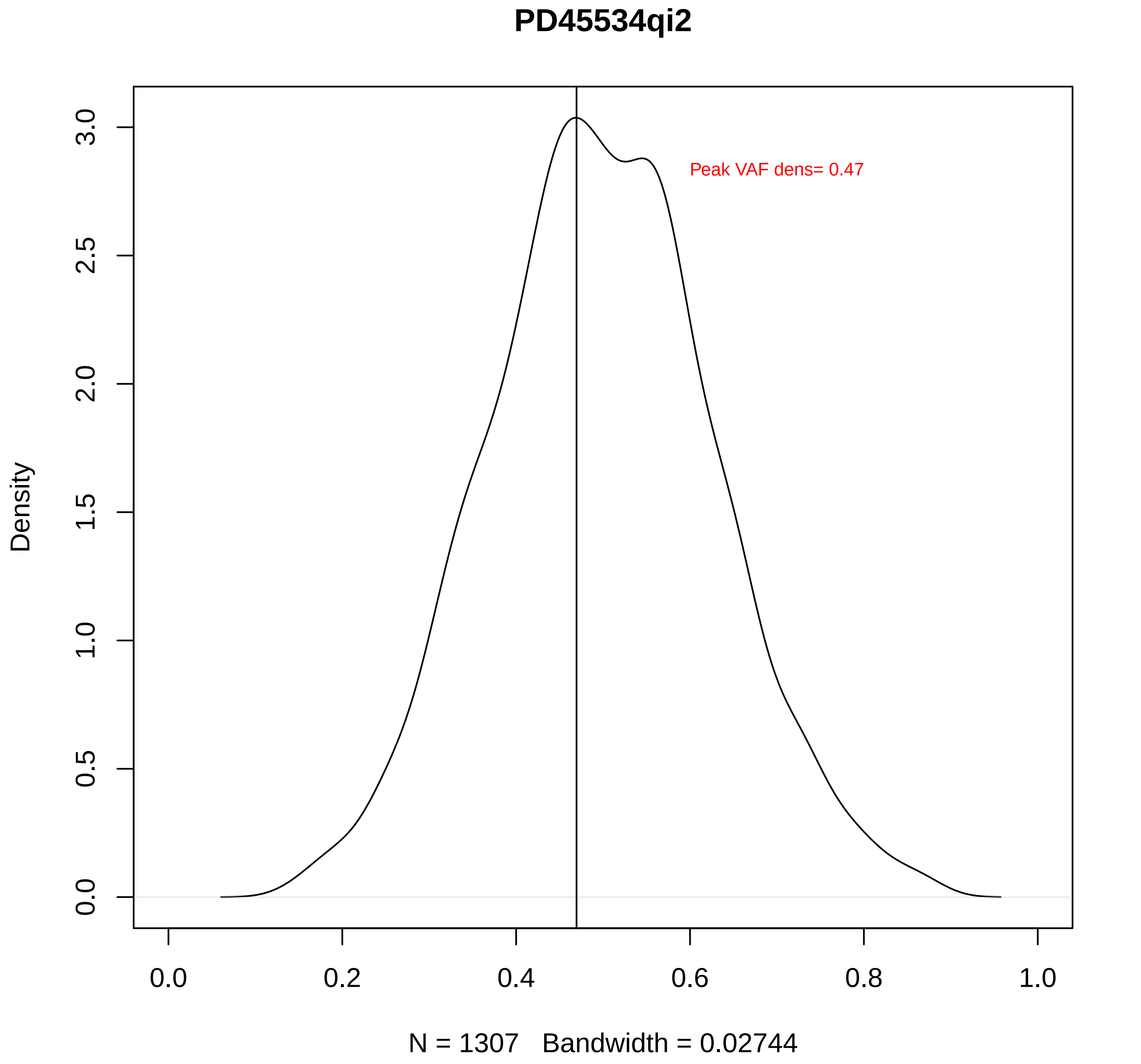
PD45534qi2
Peak VAF dens= 0.47
Density
3.0
2.5
2.0
1.5
1.0
0.5
0.0
0.0
0.2
0.4
0.6
0.8
1.0
N = 1307 Bandwidth = 0.02744

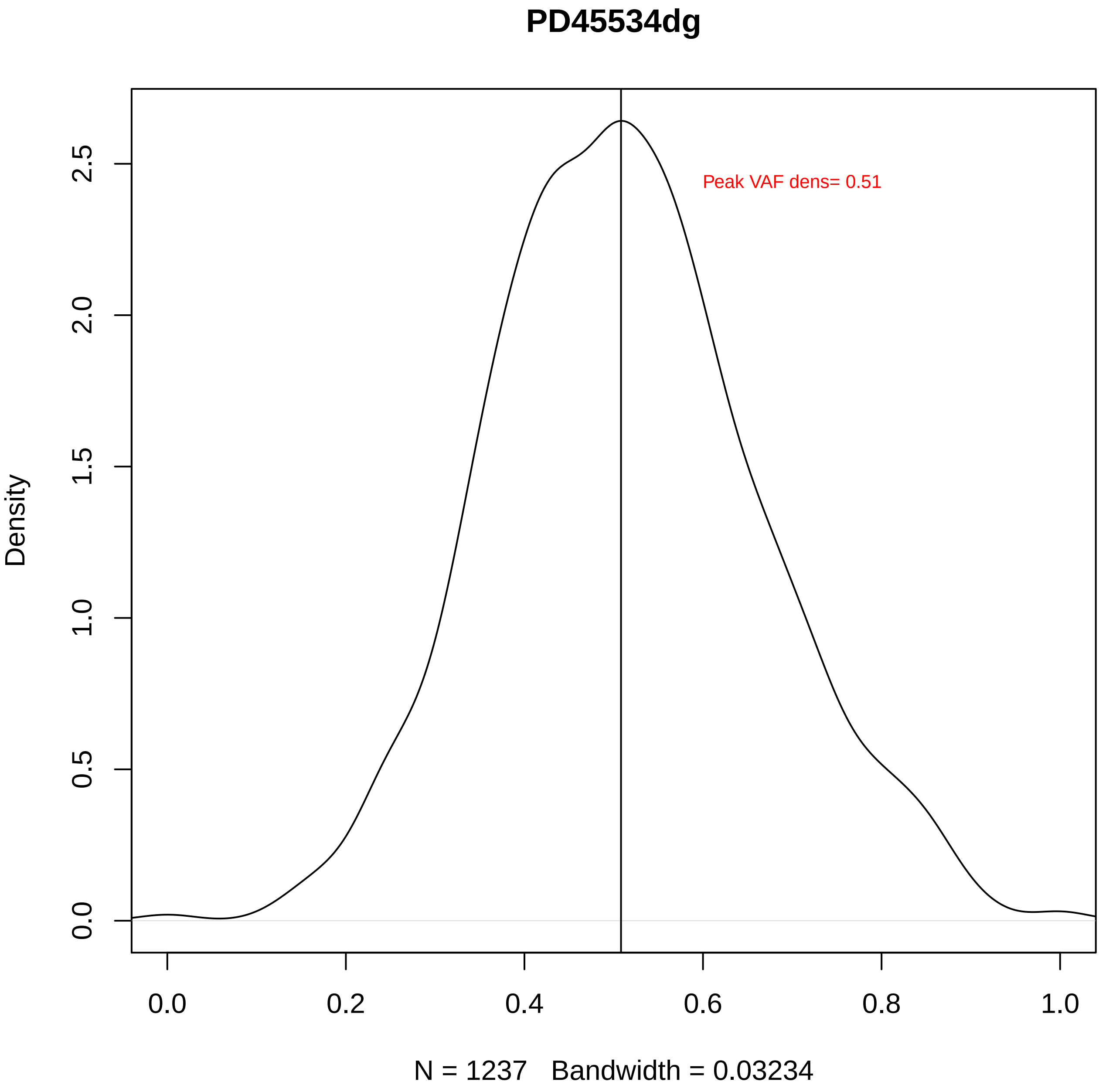
PD45534dg
Peak VAF dens= 0.51
Density
0.0
0.5
1.0
1.5
2.0
2.5
0.0
0.2
0.4
0.6
0.8
1.0
N = 1237 Bandwidth = 0.03234

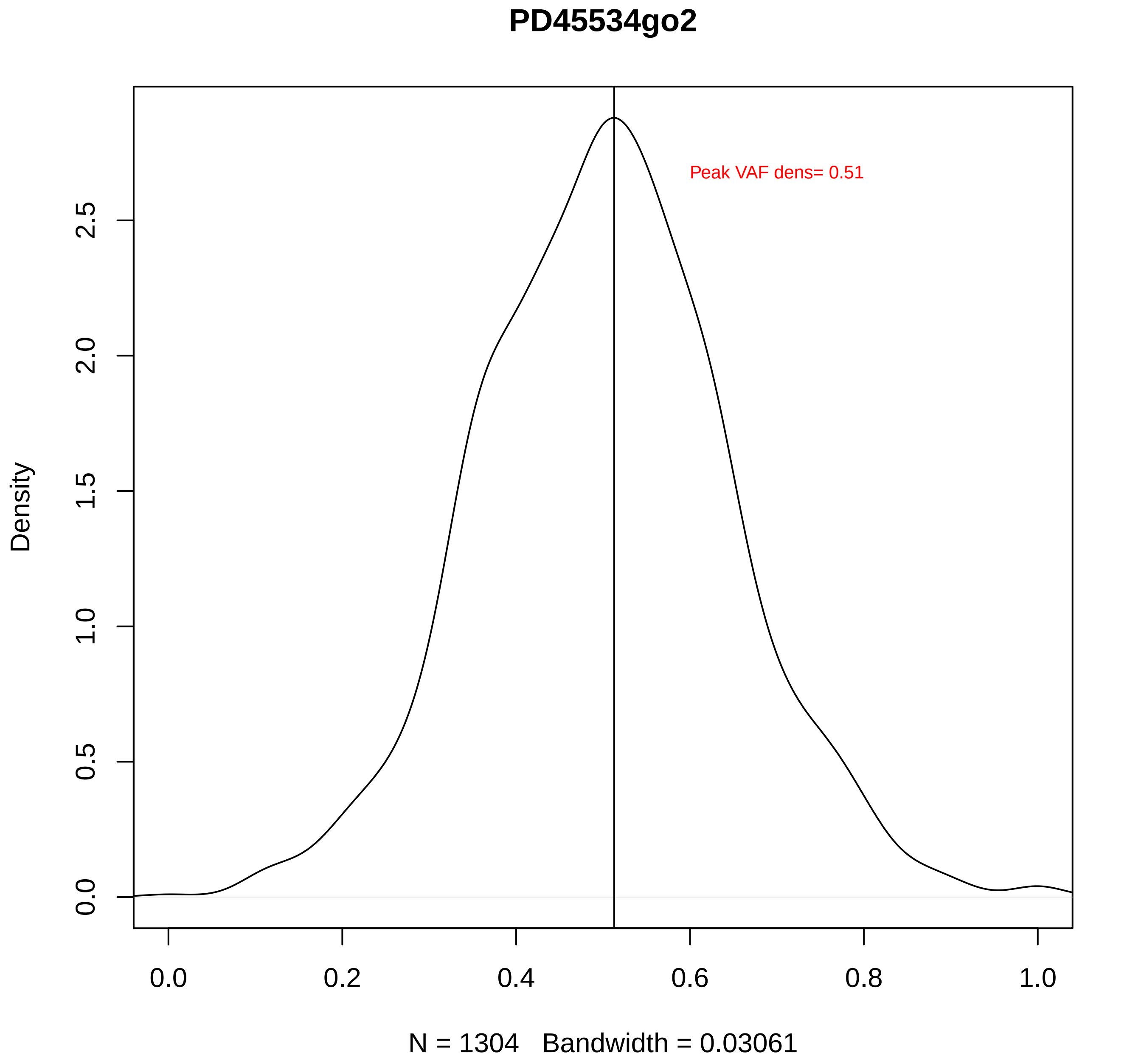

PD45534go2
Peak VAF dens= 0.51
Density
0.0
0.5
1.0
1.5
2.0
2.5
0.0
0.2
0.4
0.6
0.8
1.0
N = 1304   Bandwidth = 0.03061

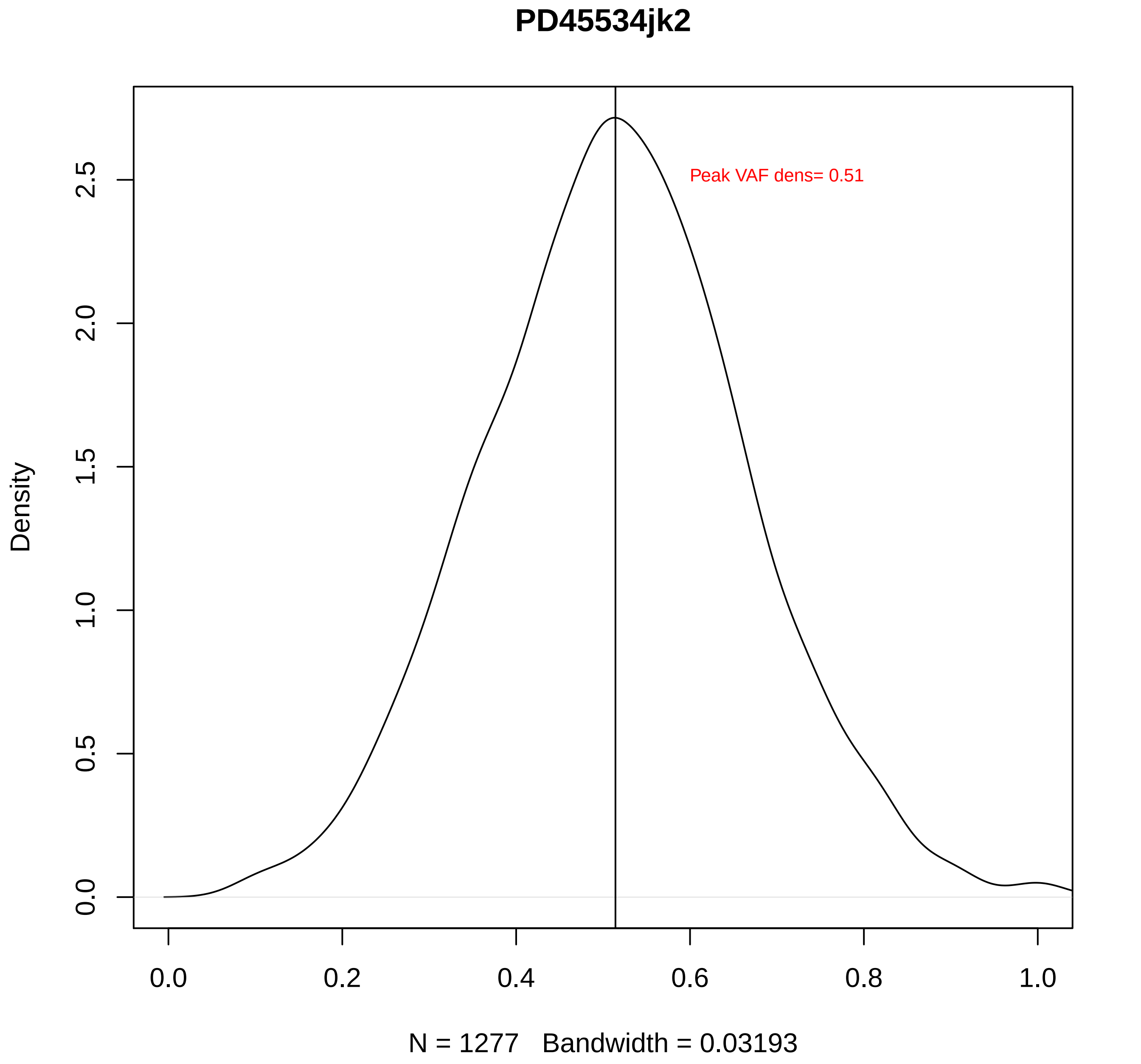
PD45534jk2
Peak VAF dens= 0.51
Density
0.0
0.5
1.0
1.5
2.0
2.5
0.0
0.2
0.4
0.6
0.8
1.0
N = 1277   Bandwidth = 0.03193

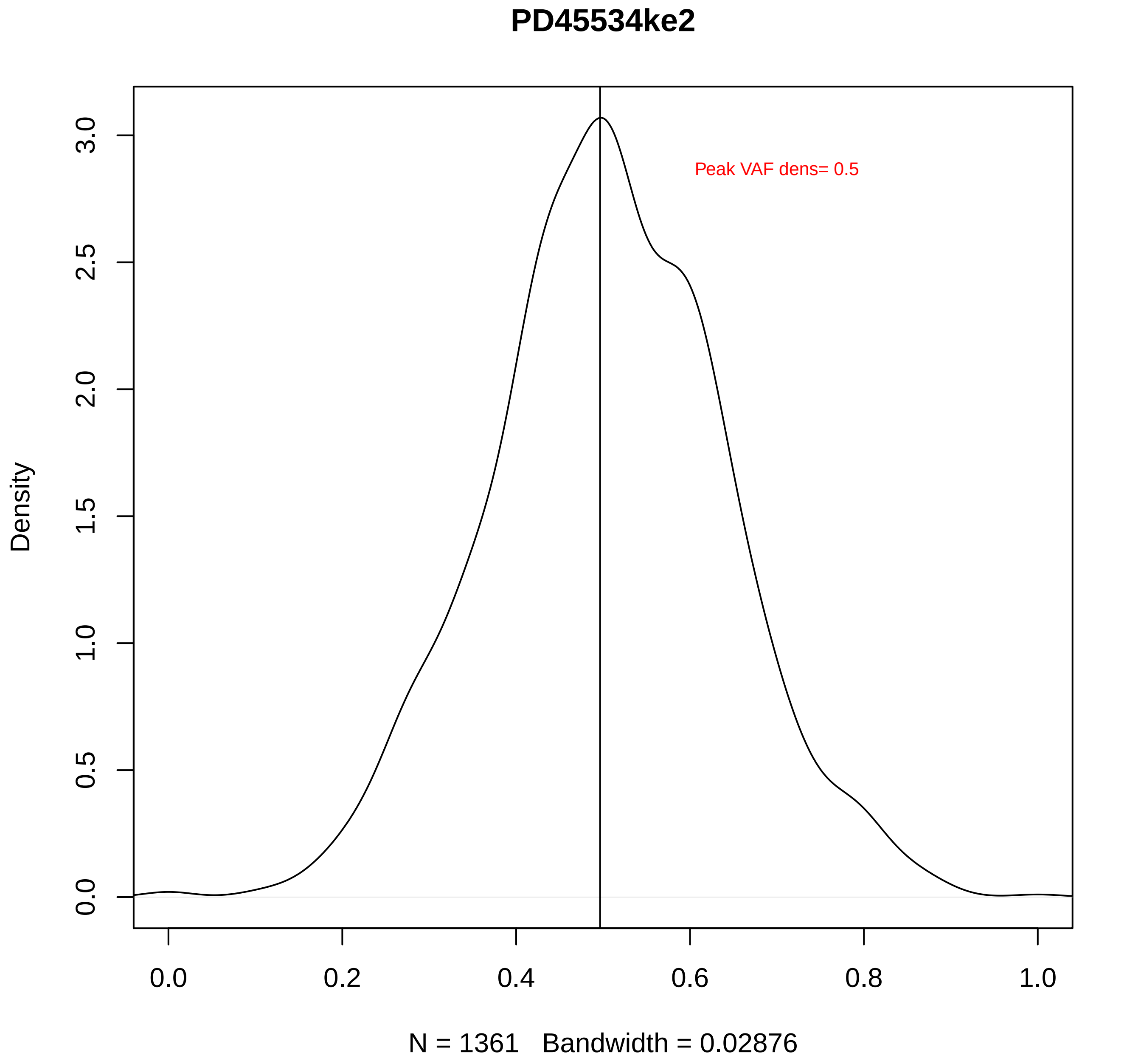
PD45534ke2
Peak VAF dens= 0.5
Density
0.0
0.5
1.0
1.5
2.0
2.5
3.0
0.0
0.2
0.4
0.6
0.8
1.0
N = 1361   Bandwidth = 0.02876

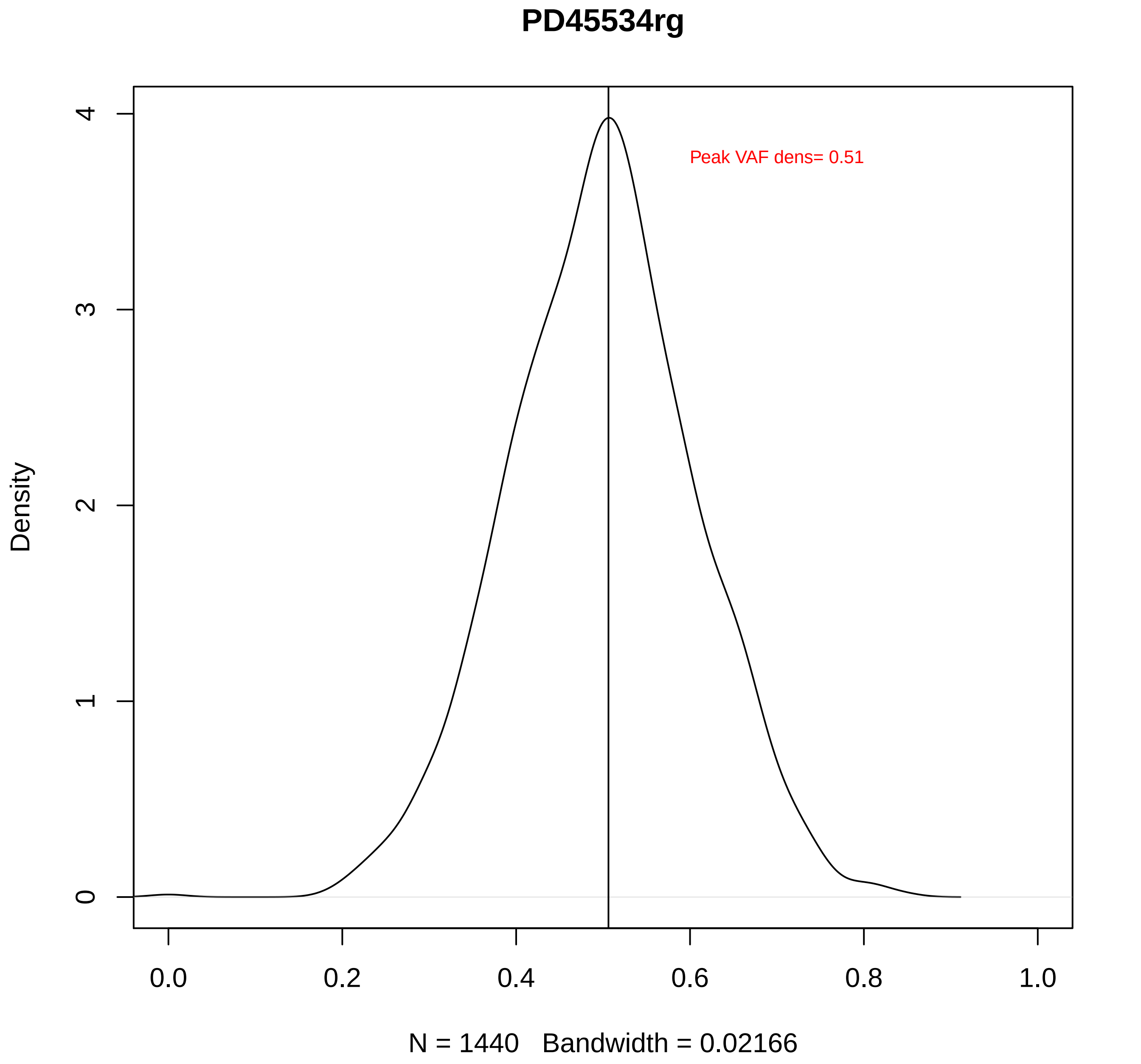

PD45534rg
Peak VAF dens= 0.51
Density
0
1
2
3
4
0.0
0.2
0.4
0.6
0.8
1.0
N = 1440   Bandwidth = 0.02166

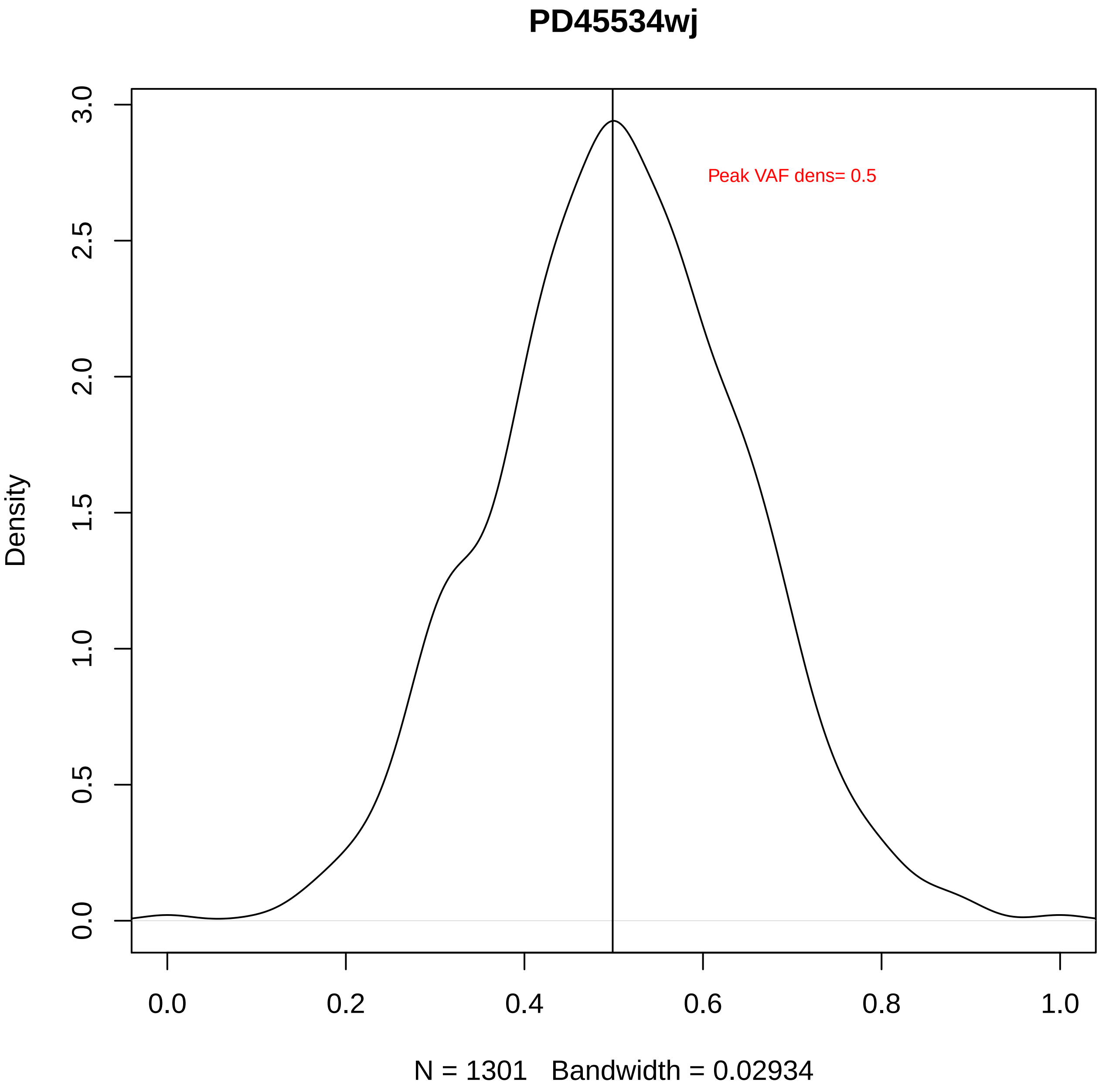
PD45534wj
Peak VAF dens= 0.5
Density
0.0
0.5
1.0
1.5
2.0
2.5
3.0
0.0
0.2
0.4
0.6
0.8
1.0
N = 1301   Bandwidth = 0.02934

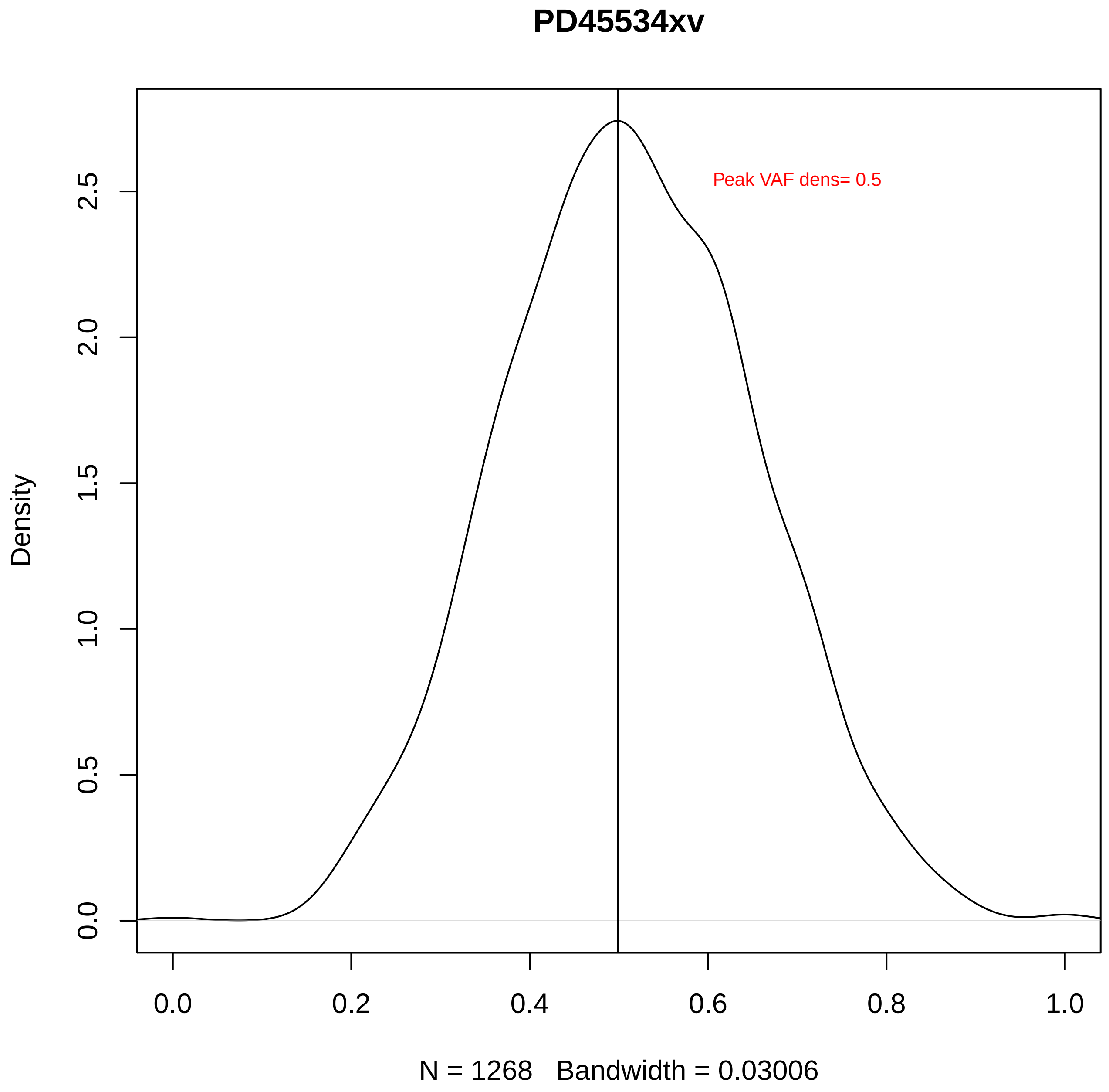

PD45534xv
Peak VAF dens= 0.5
Density
0.0
0.5
1.0
1.5
2.0
2.5
0.0
0.2
0.4
0.6
0.8
1.0
N = 1268 Bandwidth = 0.03006

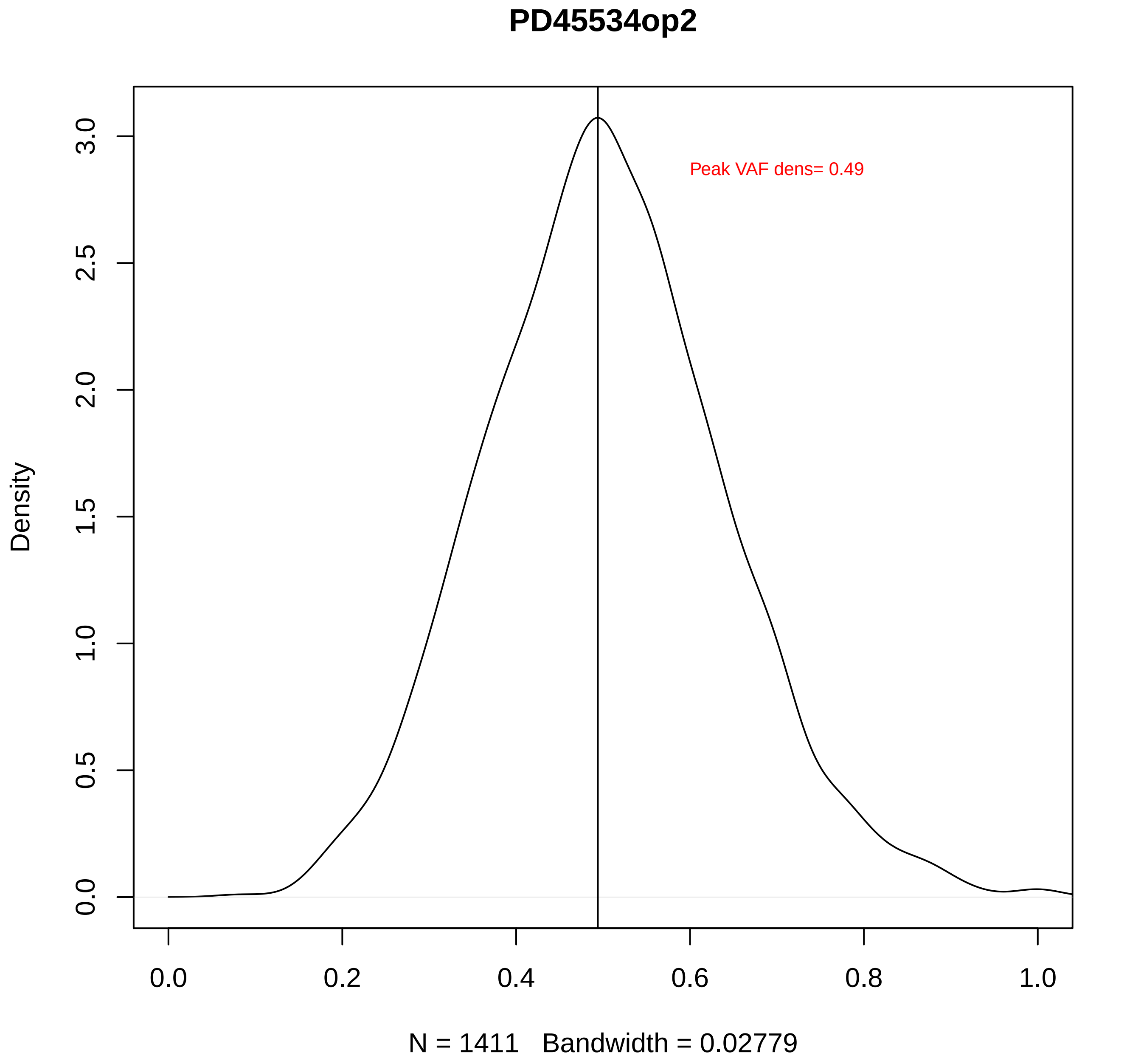

PD45534op2
Peak VAF dens= 0.49
Density
3.0
2.5
2.0
1.5
1.0
0.5
0.0
0.0
0.2
0.4
0.6
0.8
1.0
N = 1411   Bandwidth = 0.02779

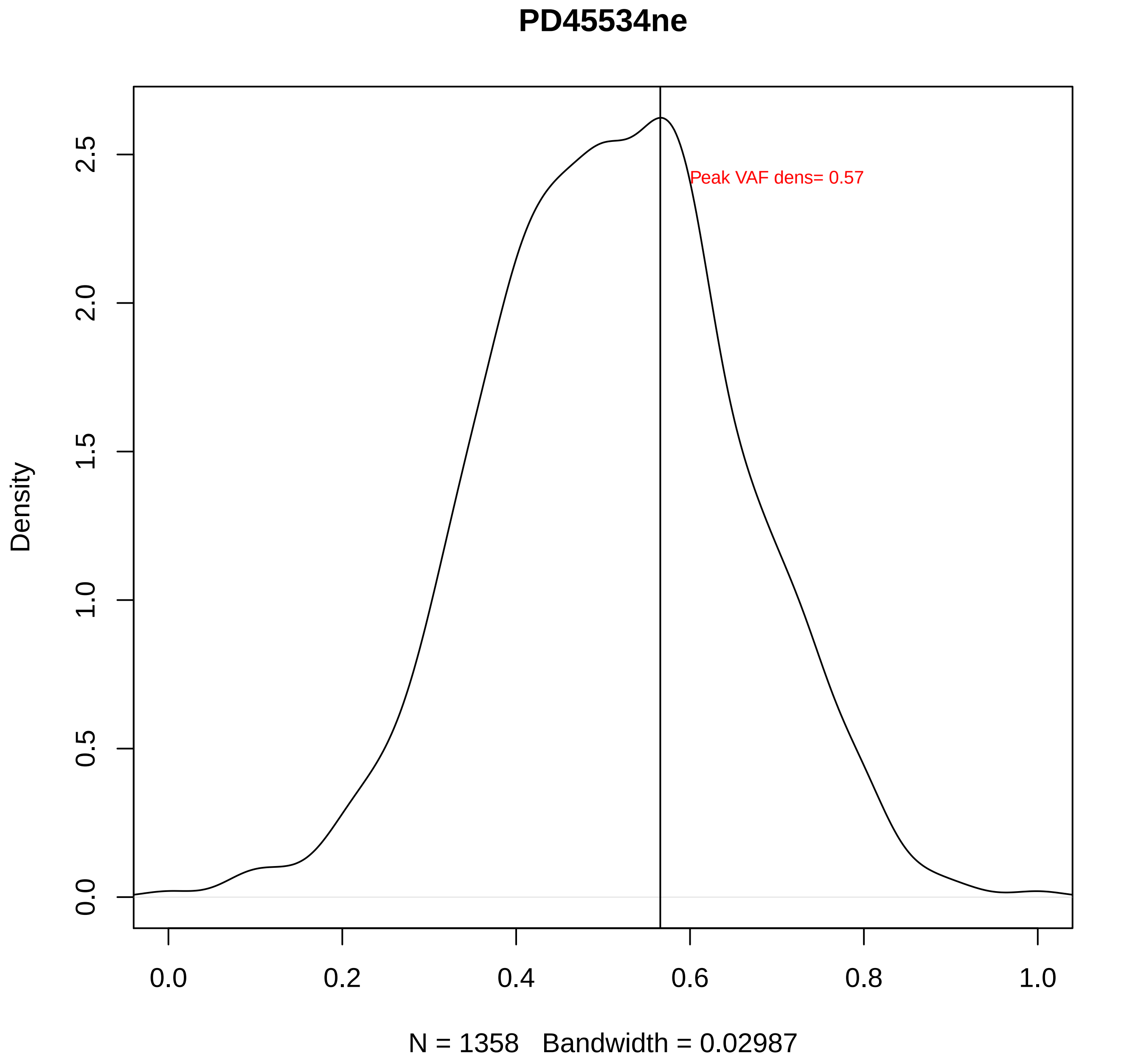
PD45534ne
Peak VAF dens= 0.57
Density
0.0
0.5
1.0
1.5
2.0
2.5
0.0
0.2
0.4
0.6
0.8
1.0
N = 1358   Bandwidth = 0.02987

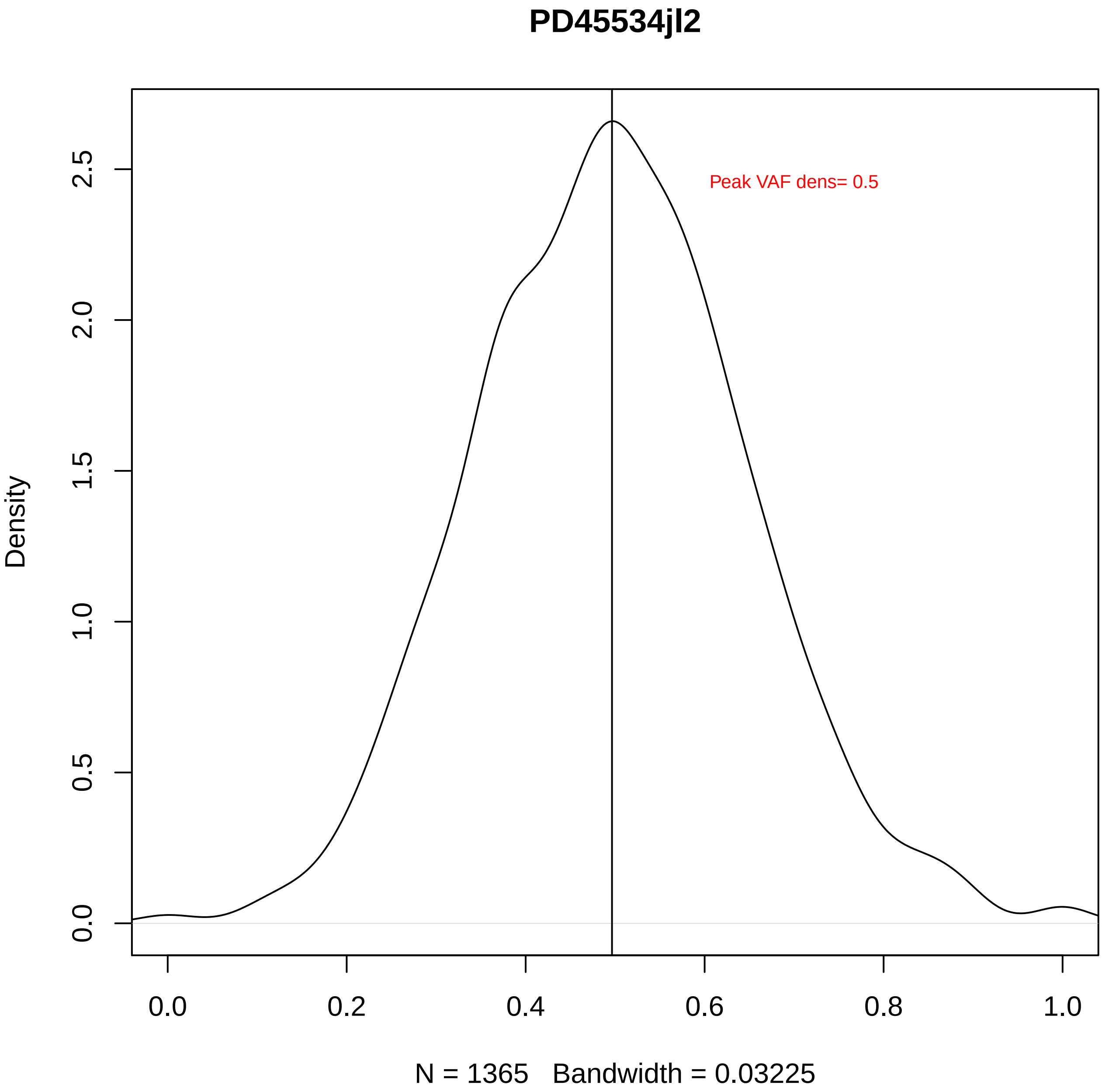

PD45534jl2
Peak VAF dens= 0.5
Density
0.0
0.5
1.0
1.5
2.0
2.5
0.0
0.2
0.4
0.6
0.8
1.0
N = 1365   Bandwidth = 0.03225

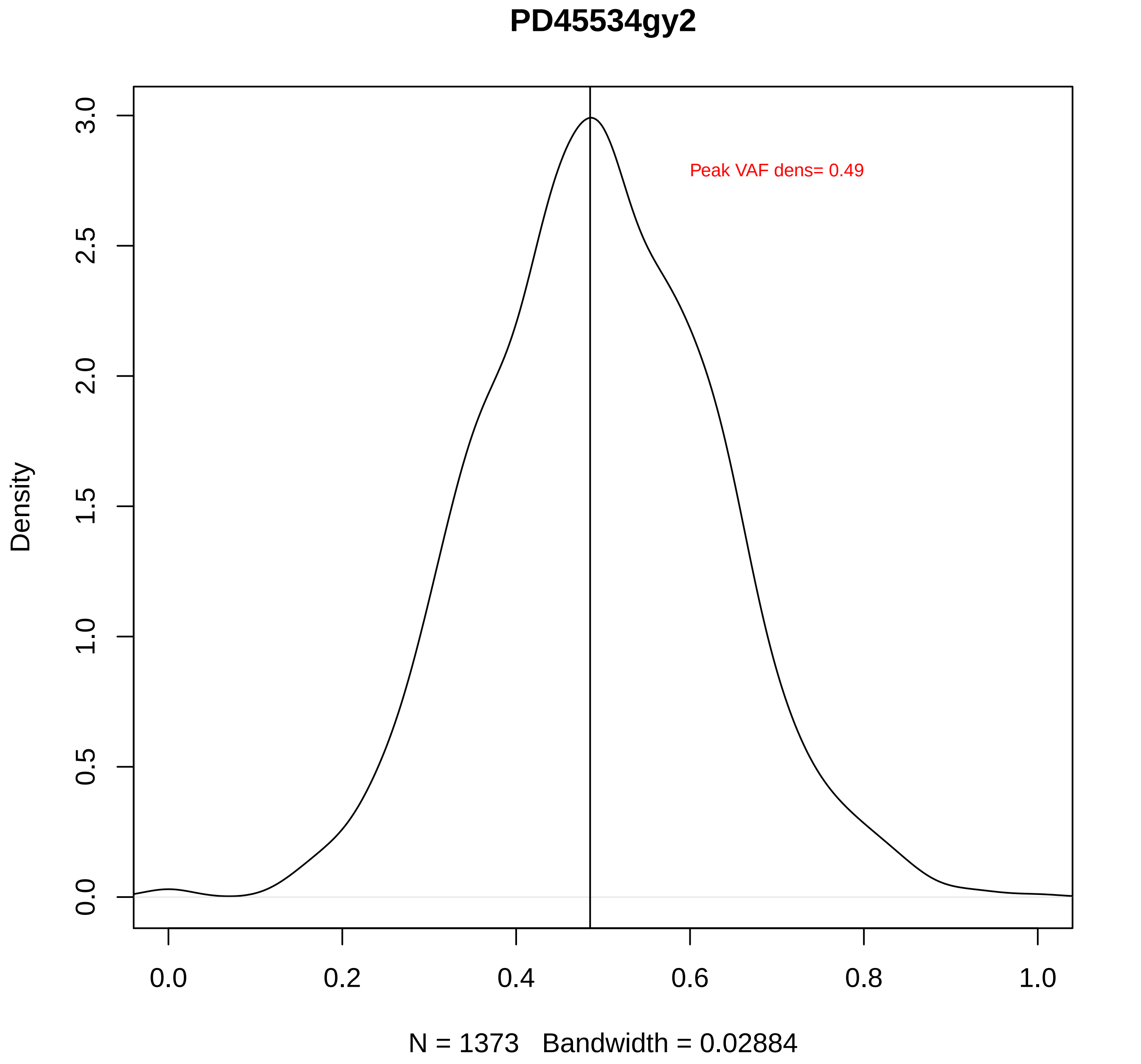

PD45534gy2
Peak VAF dens= 0.49
Density
0.0
0.5
1.0
1.5
2.0
2.5
3.0
0.0
0.2
0.4
0.6
0.8
1.0
N = 1373   Bandwidth = 0.02884

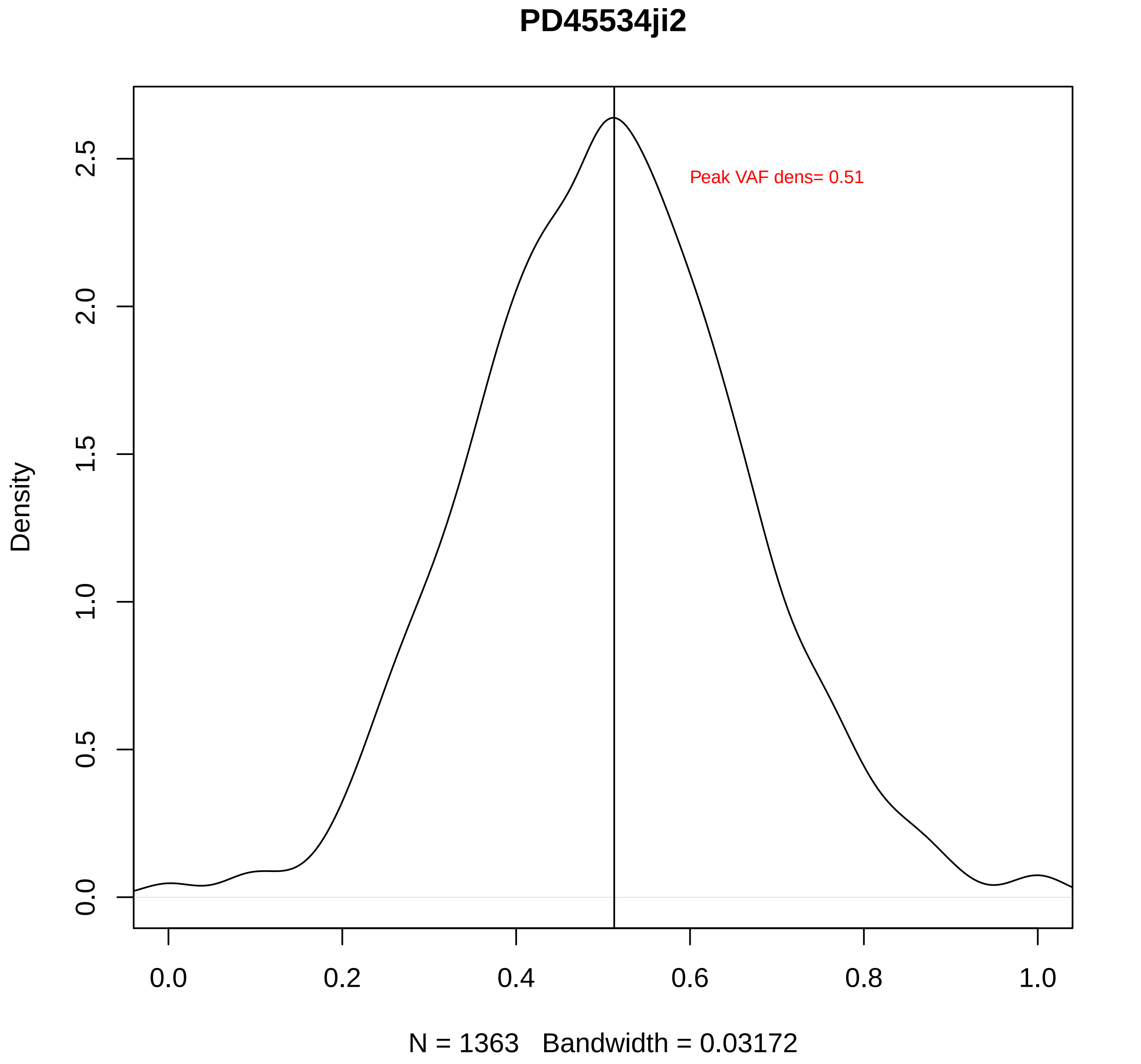

PD45534ji2
Peak VAF dens= 0.51
Density
0.0
0.5
1.0
1.5
2.0
2.5
0.0
0.2
0.4
0.6
0.8
1.0
N = 1363 Bandwidth = 0.03172

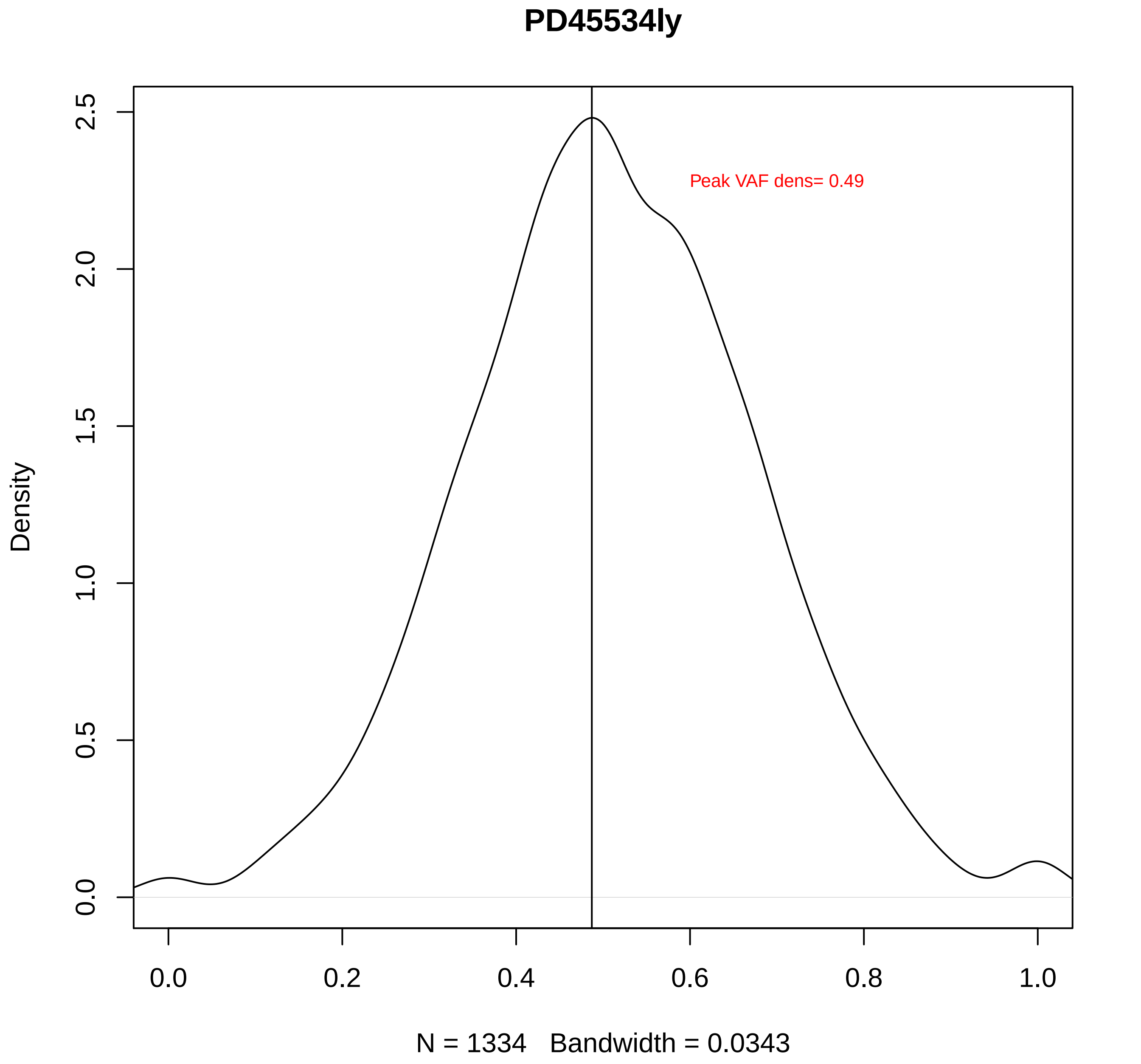
PD45534ly
Peak VAF dens= 0.49
Density
0.0
0.5
1.0
1.5
2.0
2.5
0.0
0.2
0.4
0.6
0.8
1.0
N = 1334   Bandwidth = 0.0343

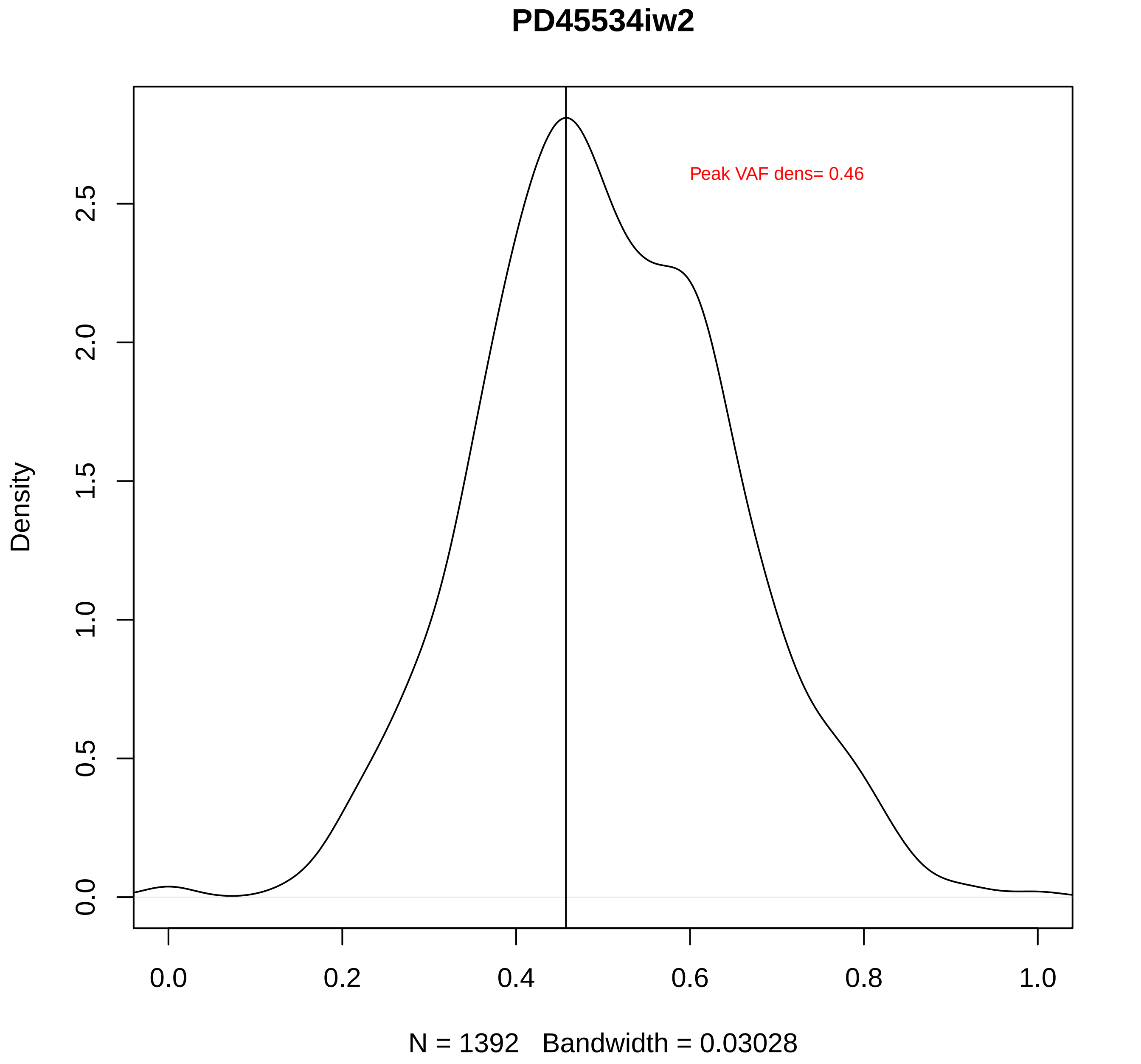
PD45534iw2
Peak VAF dens= 0.46
Density
0.0
0.5
1.0
1.5
2.0
2.5
0.0
0.2
0.4
0.6
0.8
1.0
N = 1392   Bandwidth = 0.03028

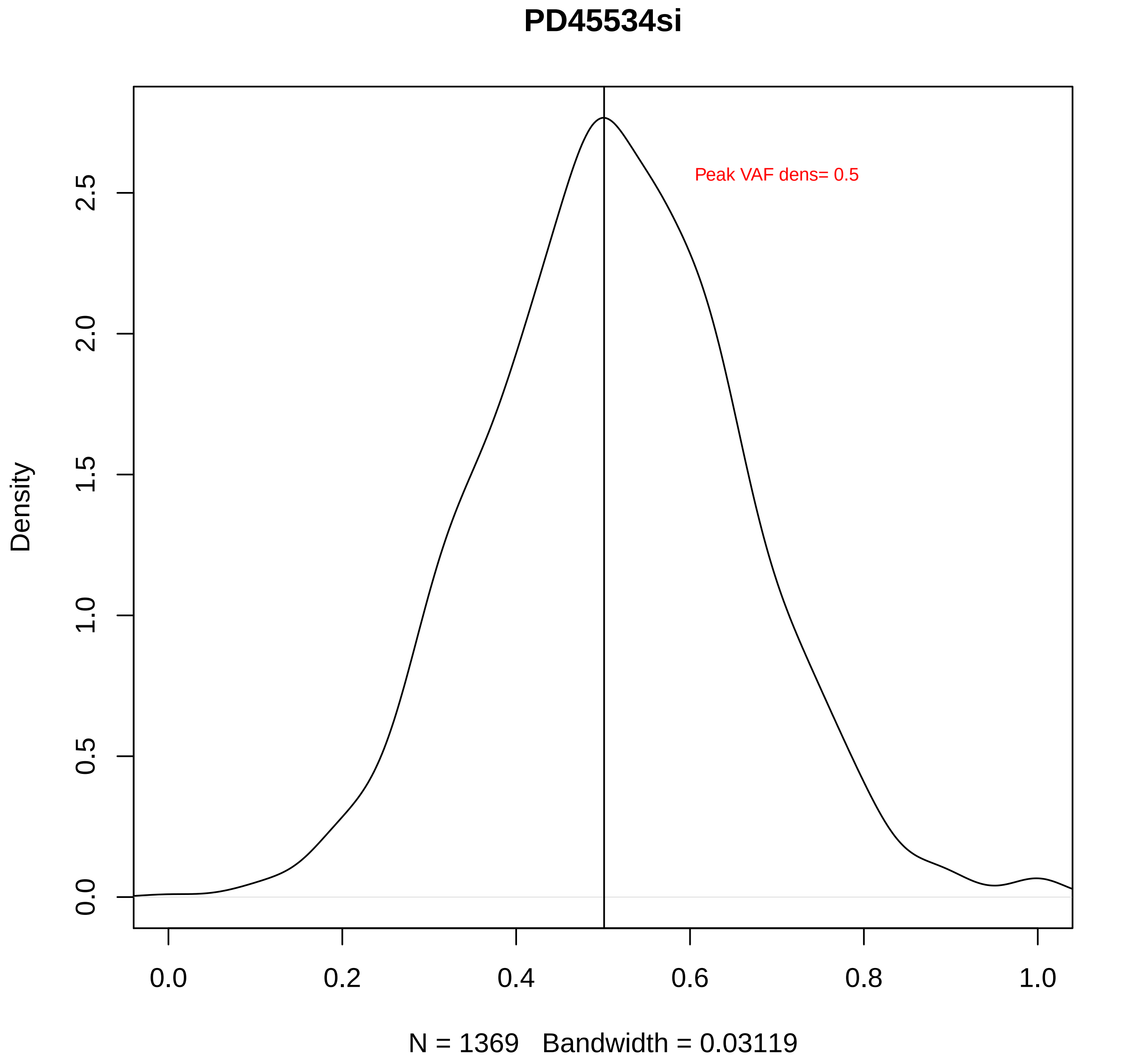
PD45534si
Peak VAF dens= 0.5
Density
0.0
0.5
1.0
1.5
2.0
2.5
0.0
0.2
0.4
0.6
0.8
1.0
N = 1369   Bandwidth = 0.03119

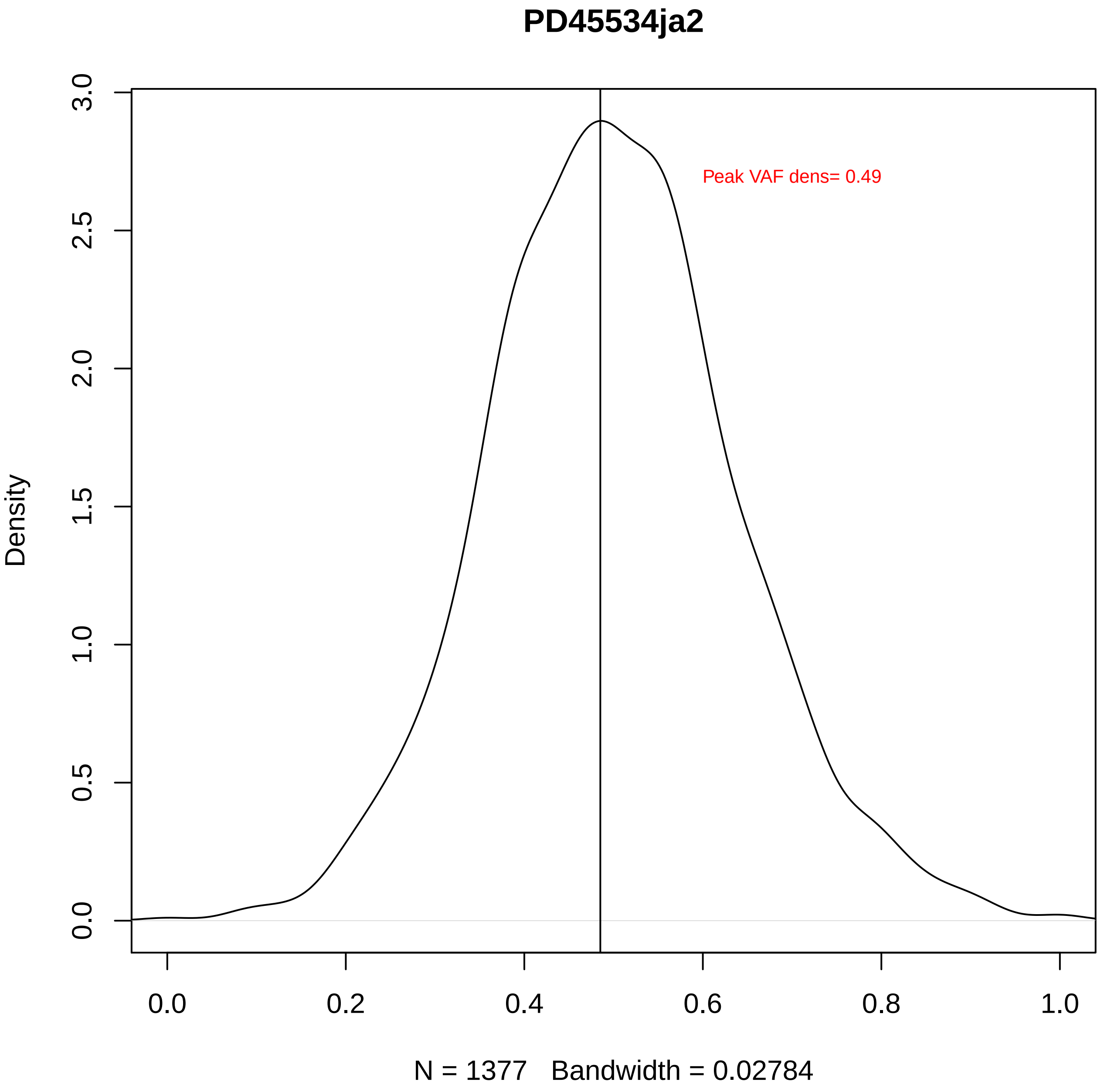
PD45534ja2
Peak VAF dens= 0.49
Density
0.0
0.5
1.0
1.5
2.0
2.5
3.0
0.0
0.2
0.4
0.6
0.8
1.0
N = 1377   Bandwidth = 0.02784

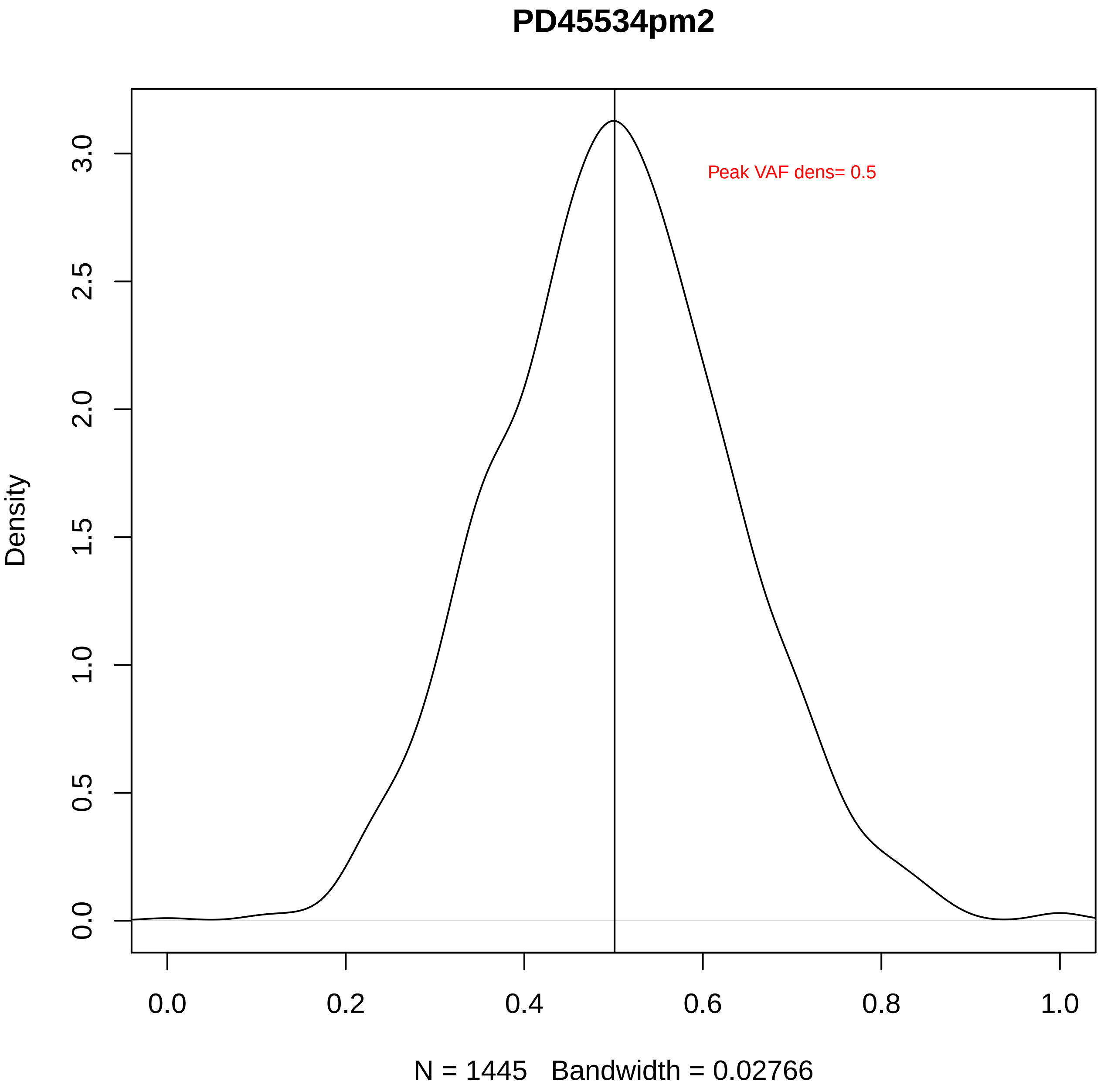

PD45534pm2
Peak VAF dens= 0.5
Density
0.0
0.5
1.0
1.5
2.0
2.5
3.0
0.0
0.2
0.4
0.6
0.8
1.0
N = 1445 Bandwidth = 0.02766

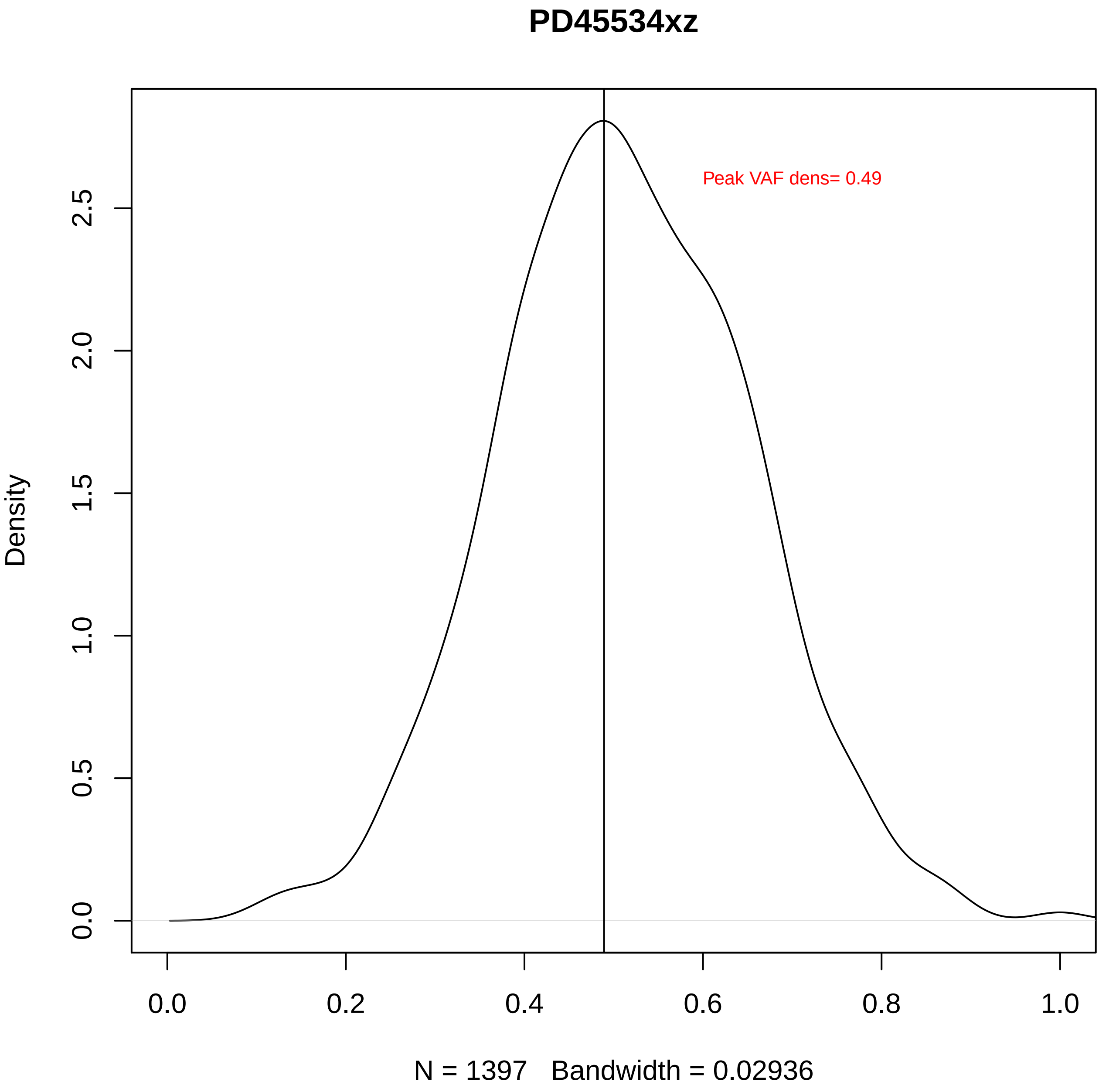

PD45534xz
Peak VAF dens= 0.49
Density
0.0
0.5
1.0
1.5
2.0
2.5
0.0
0.2
0.4
0.6
0.8
1.0
N = 1397   Bandwidth = 0.02936

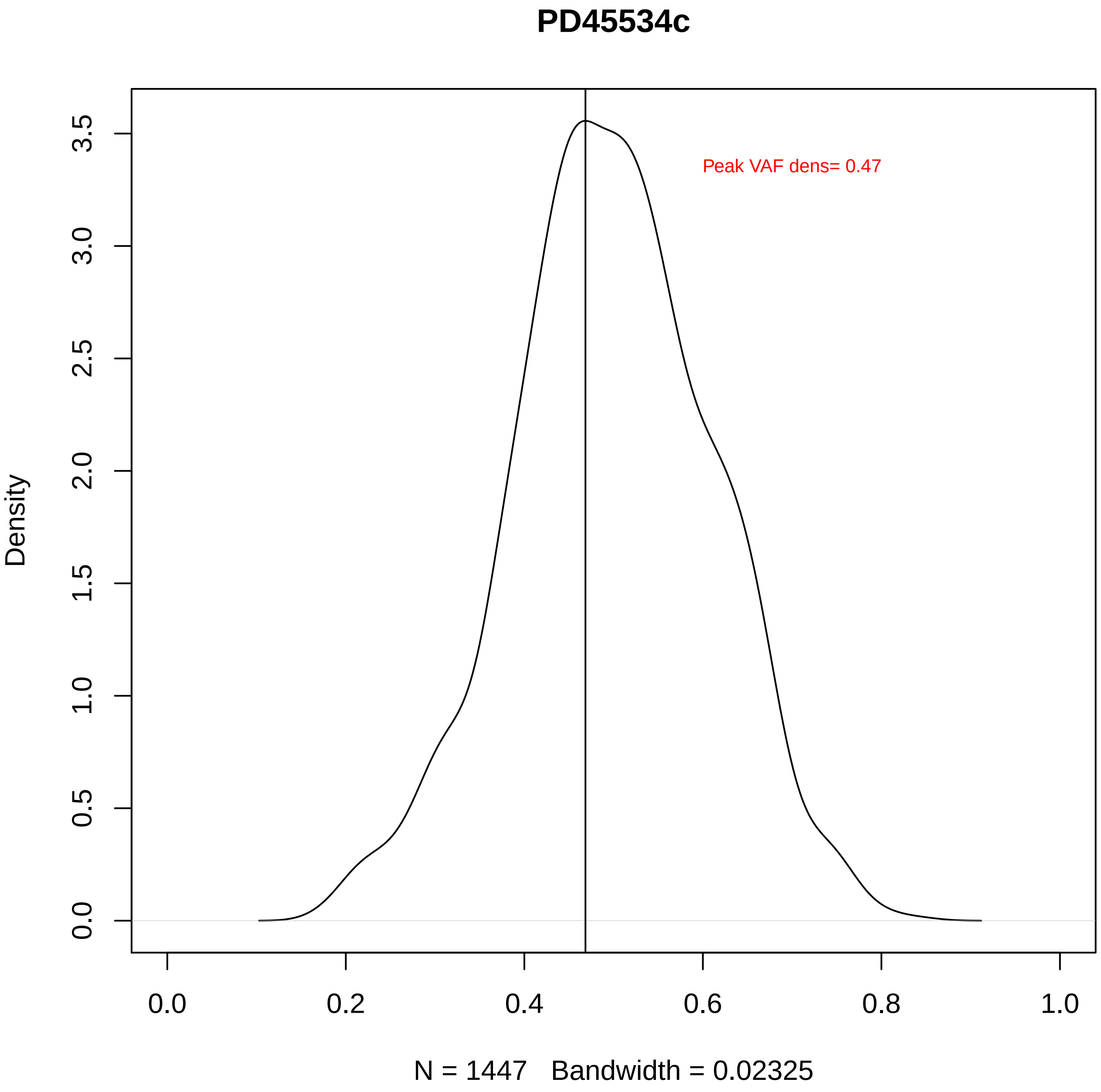
PD45534c
Peak VAF dens= 0.47
Density
0.0
0.5
1.0
1.5
2.0
2.5
3.0
3.5
0.0
0.2
0.4
0.6
0.8
1.0
N = 1447 Bandwidth = 0.02325

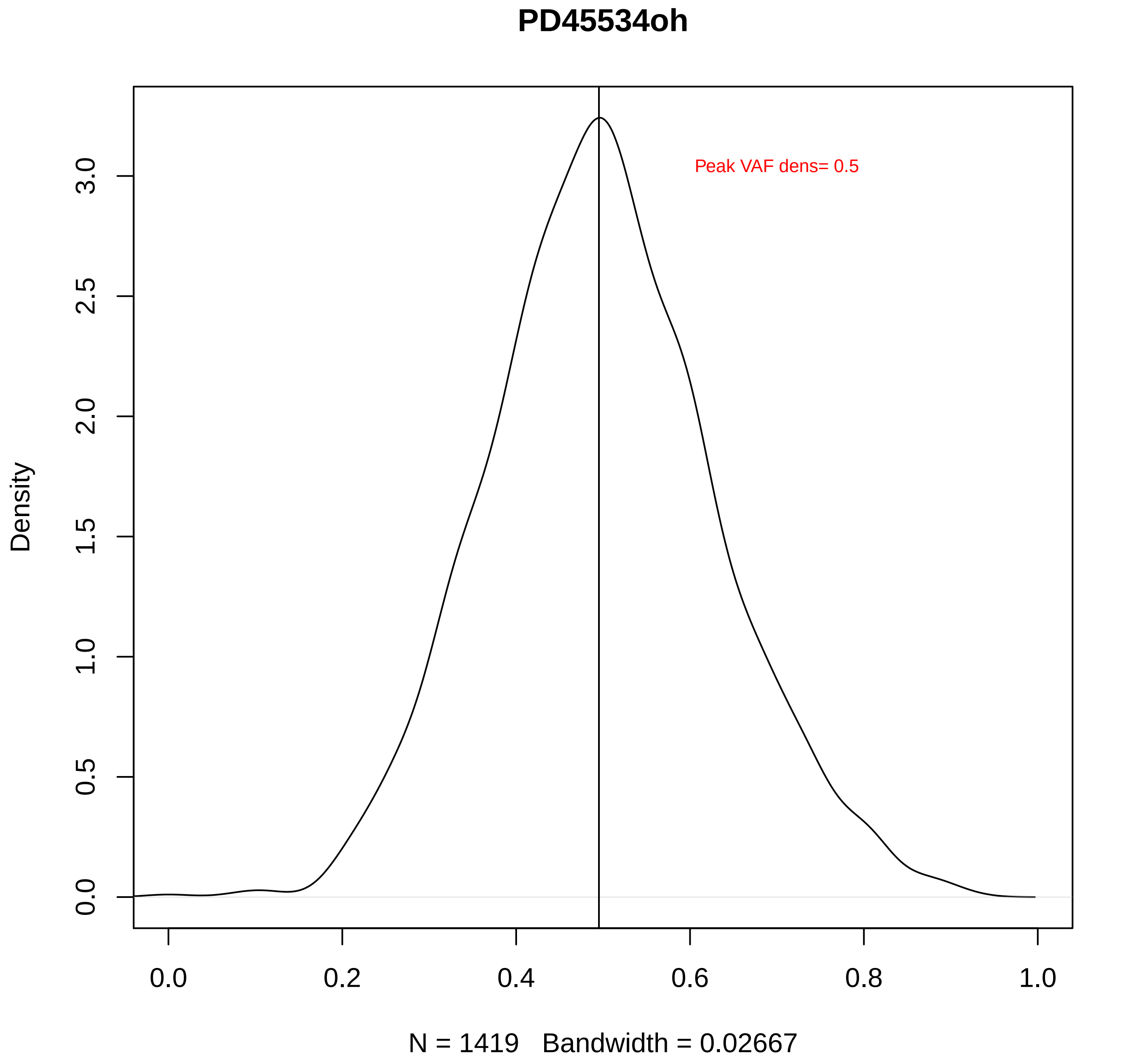
PD45534oh
Peak VAF dens= 0.5
Density
0.0
0.5
1.0
1.5
2.0
2.5
3.0
0.0
0.2
0.4
0.6
0.8
1.0
N = 1419   Bandwidth = 0.02667

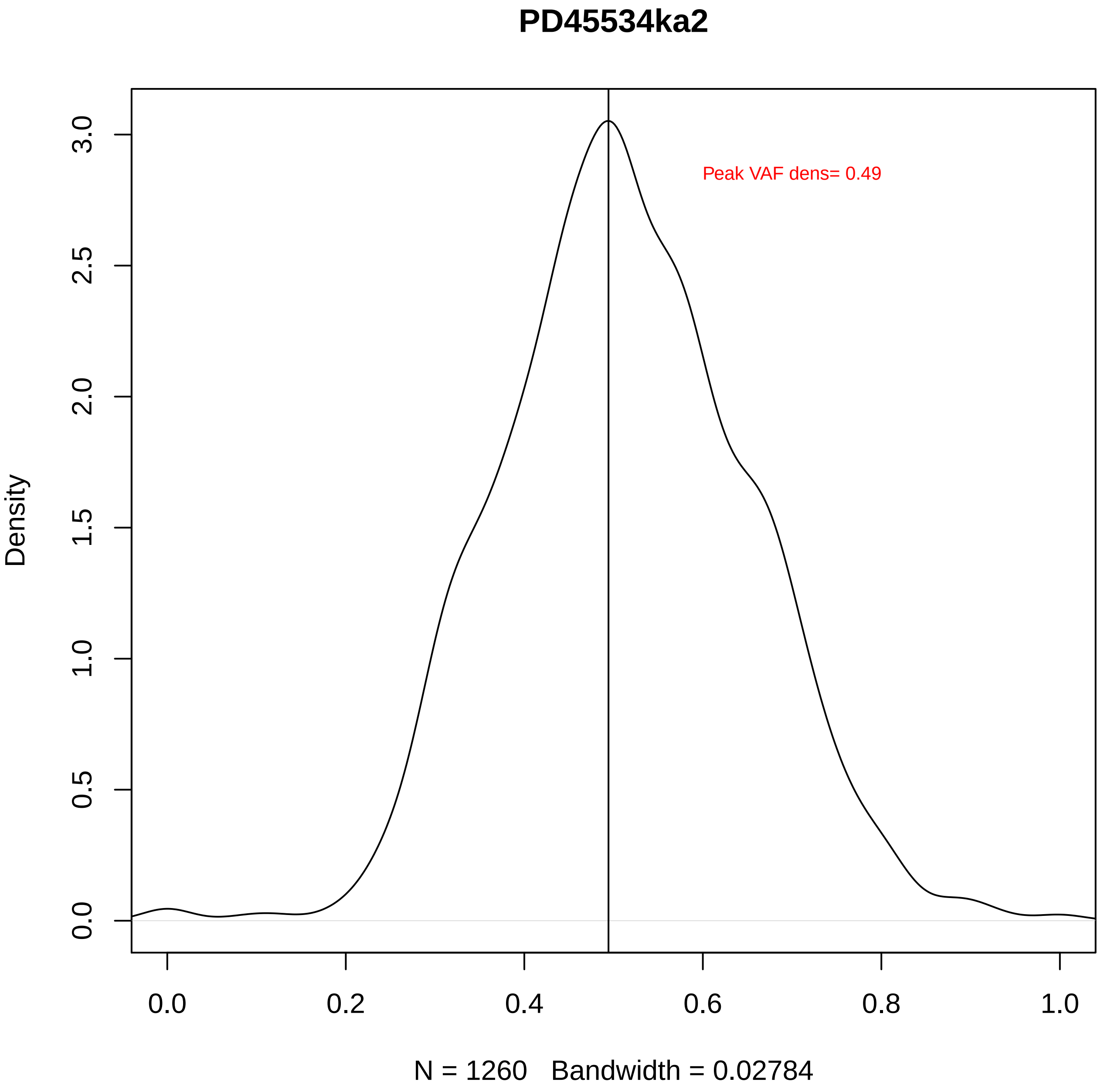
PD45534ka2
Peak VAF dens= 0.49
Density
0.0
0.5
1.0
1.5
2.0
2.5
3.0
0.0
0.2
0.4
0.6
0.8
1.0
N = 1260   Bandwidth = 0.02784

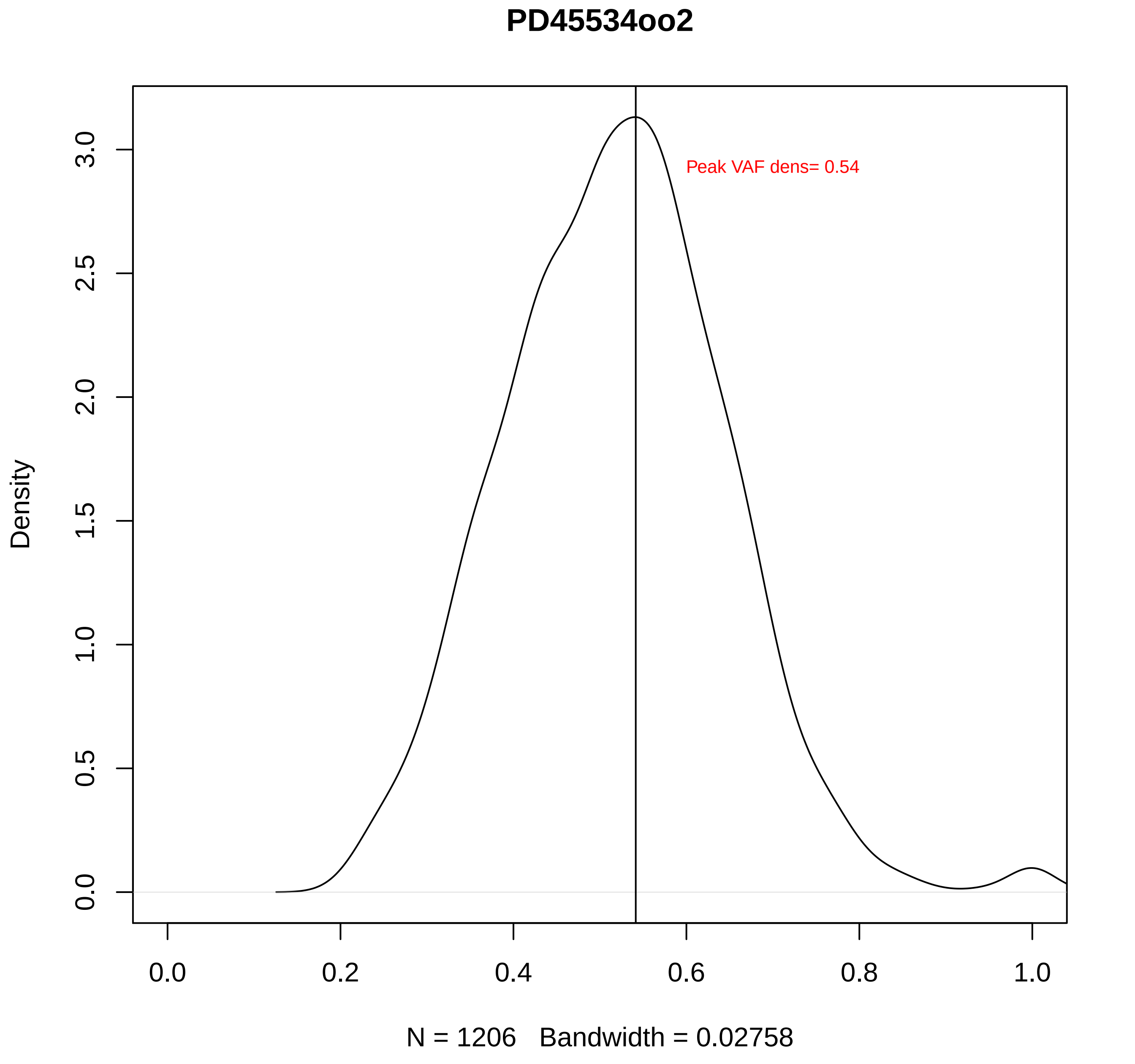

PD45534oo2
Peak VAF dens= 0.54
Density
0.0
0.5
1.0
1.5
2.0
2.5
3.0
0.0
0.2
0.4
0.6
0.8
1.0
N = 1206 Bandwidth = 0.02758

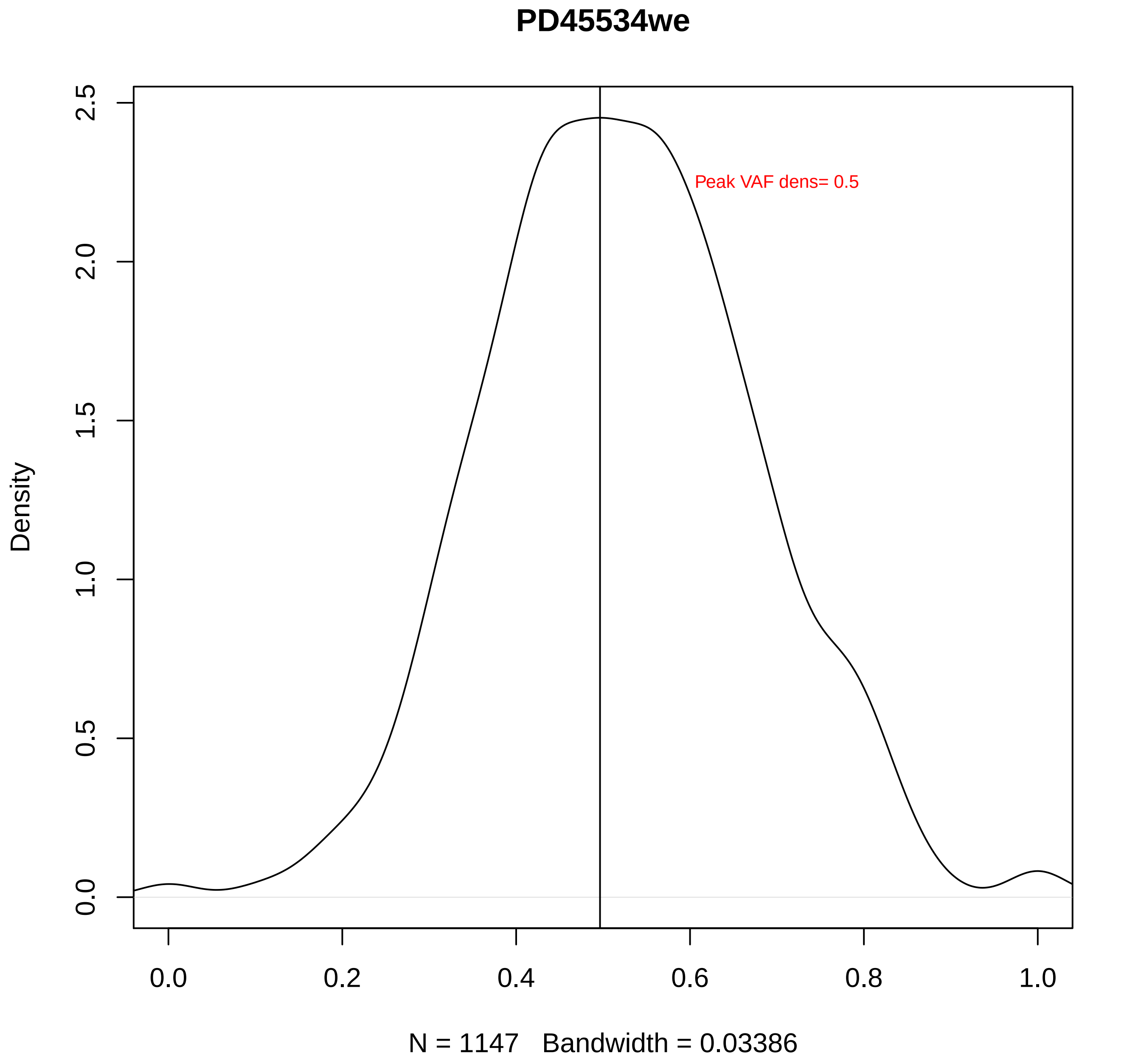

PD45534we
Peak VAF dens= 0.5
Density
0.0
0.5
1.0
1.5
2.0
2.5
0.0
0.2
0.4
0.6
0.8
1.0
N = 1147   Bandwidth = 0.03386

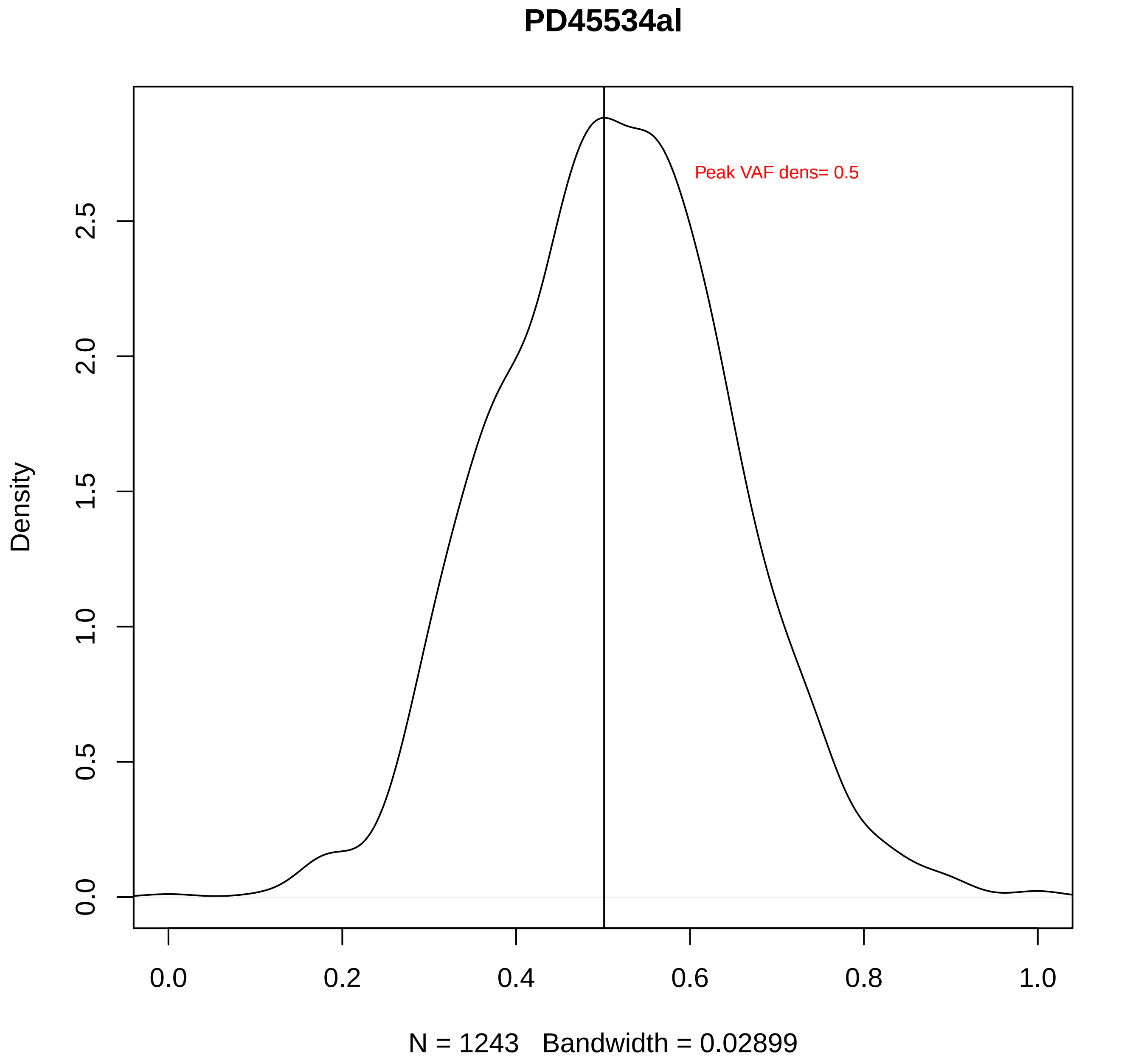
PD45534al
Peak VAF dens= 0.5
Density
0.0
0.5
1.0
1.5
2.0
2.5
0.0
0.2
0.4
0.6
0.8
1.0
N = 1243   Bandwidth = 0.02899

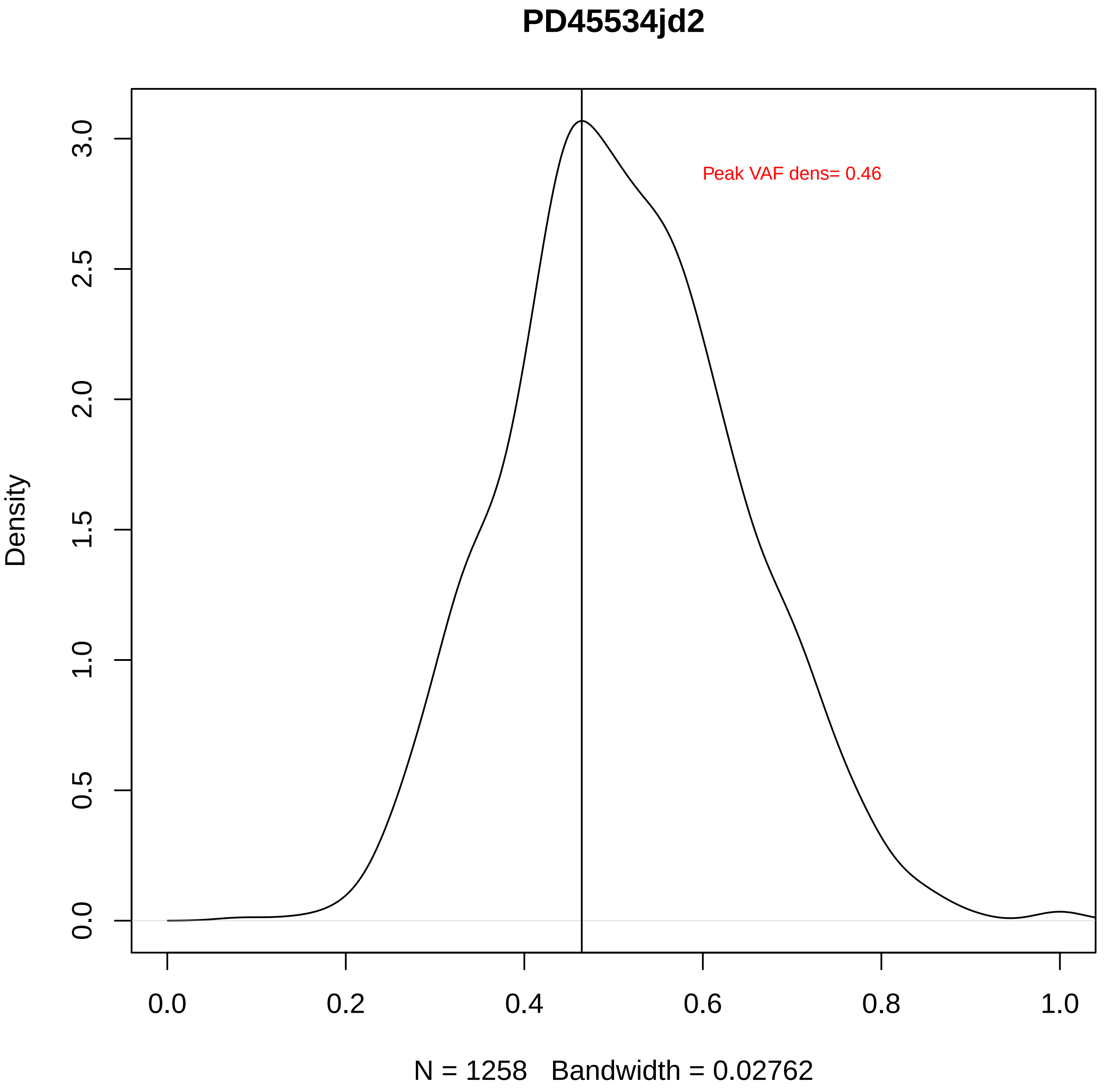

PD45534jd2
Peak VAF dens= 0.46
Density
3.0
2.5
2.0
1.5
1.0
0.5
0.0
0.0
0.2
0.4
0.6
0.8
1.0
N = 1258   Bandwidth = 0.02762

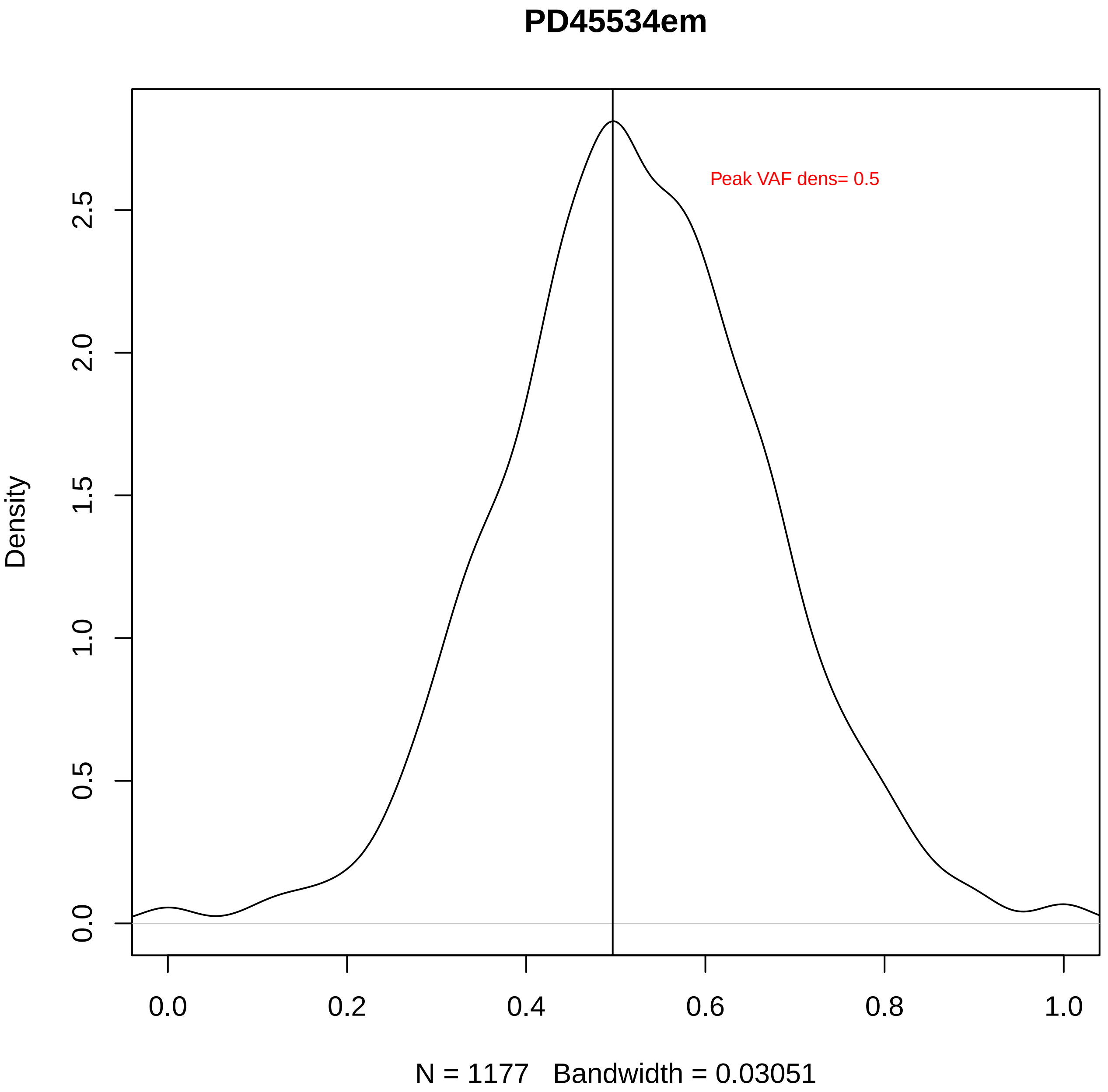
PD45534em
Peak VAF dens= 0.5
Density
0.0
0.5
1.0
1.5
2.0
2.5
0.0
0.2
0.4
0.6
0.8
1.0
N = 1177   Bandwidth = 0.03051

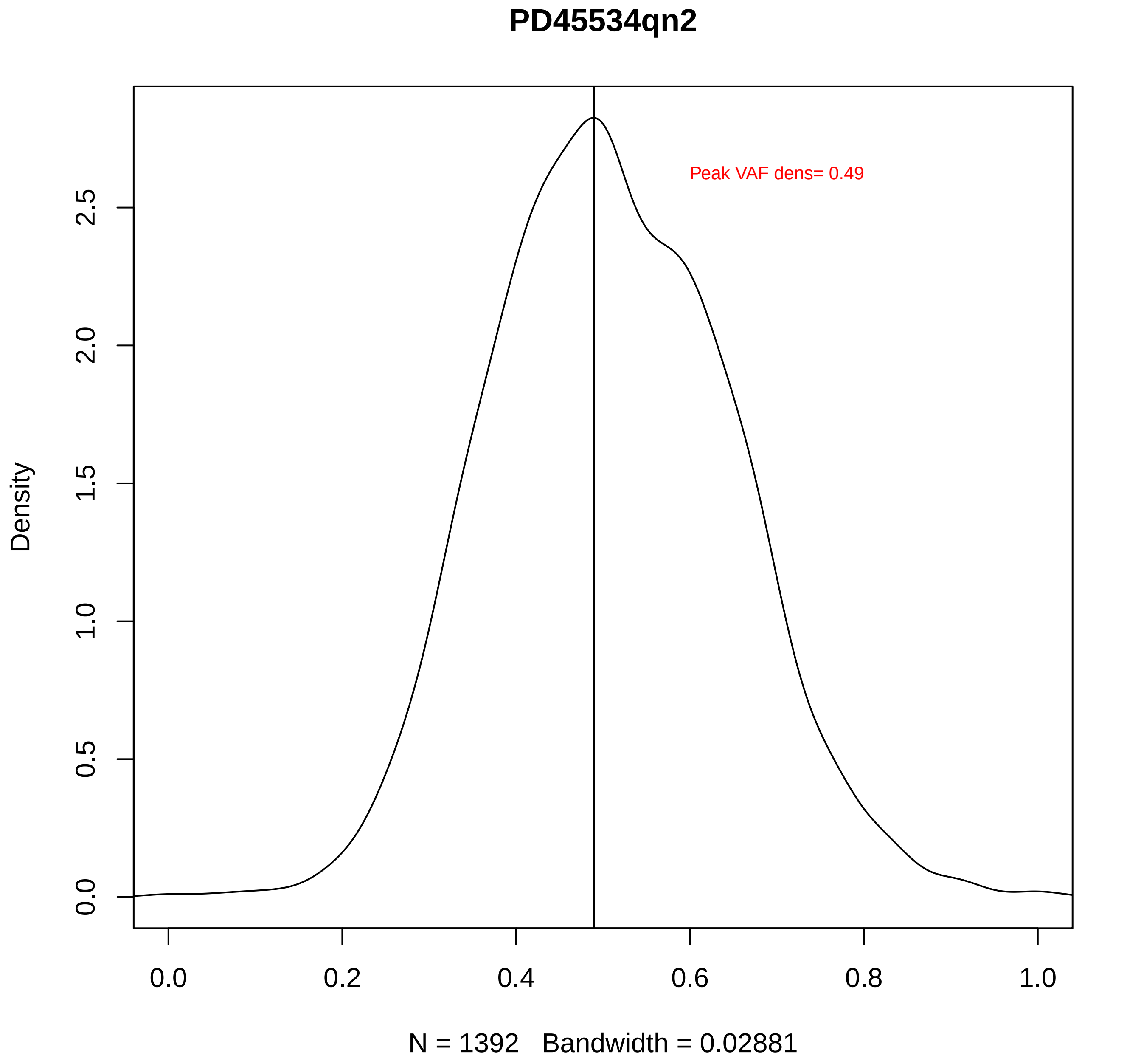
PD45534qn2
Peak VAF dens= 0.49
Density
0.0
0.5
1.0
1.5
2.0
2.5
0.0
0.2
0.4
0.6
0.8
1.0
N = 1392   Bandwidth = 0.02881

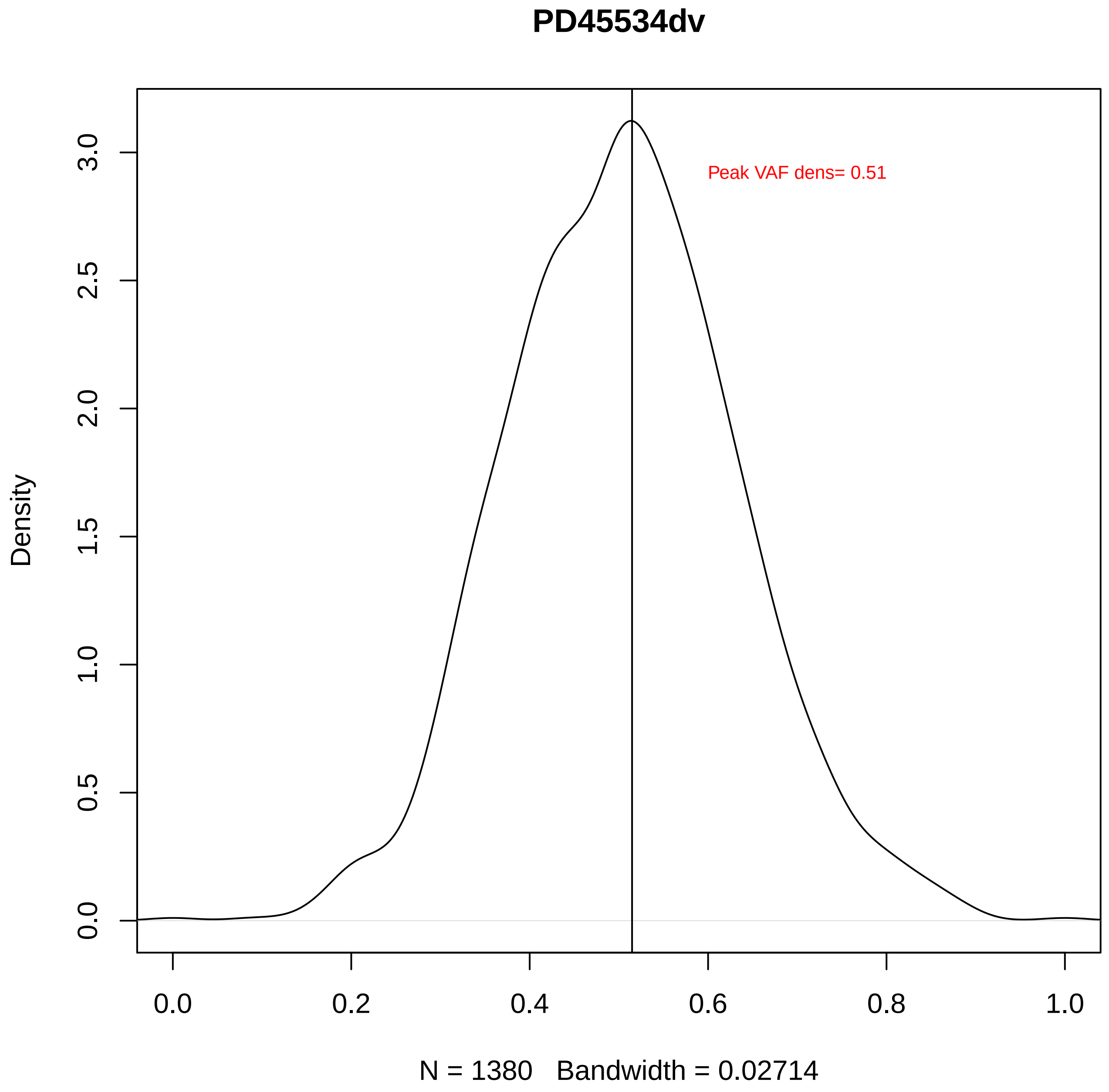
PD45534dv
Peak VAF dens= 0.51
Density
0.0
0.5
1.0
1.5
2.0
2.5
3.0
0.0
0.2
0.4
0.6
0.8
1.0
N = 1380   Bandwidth = 0.02714

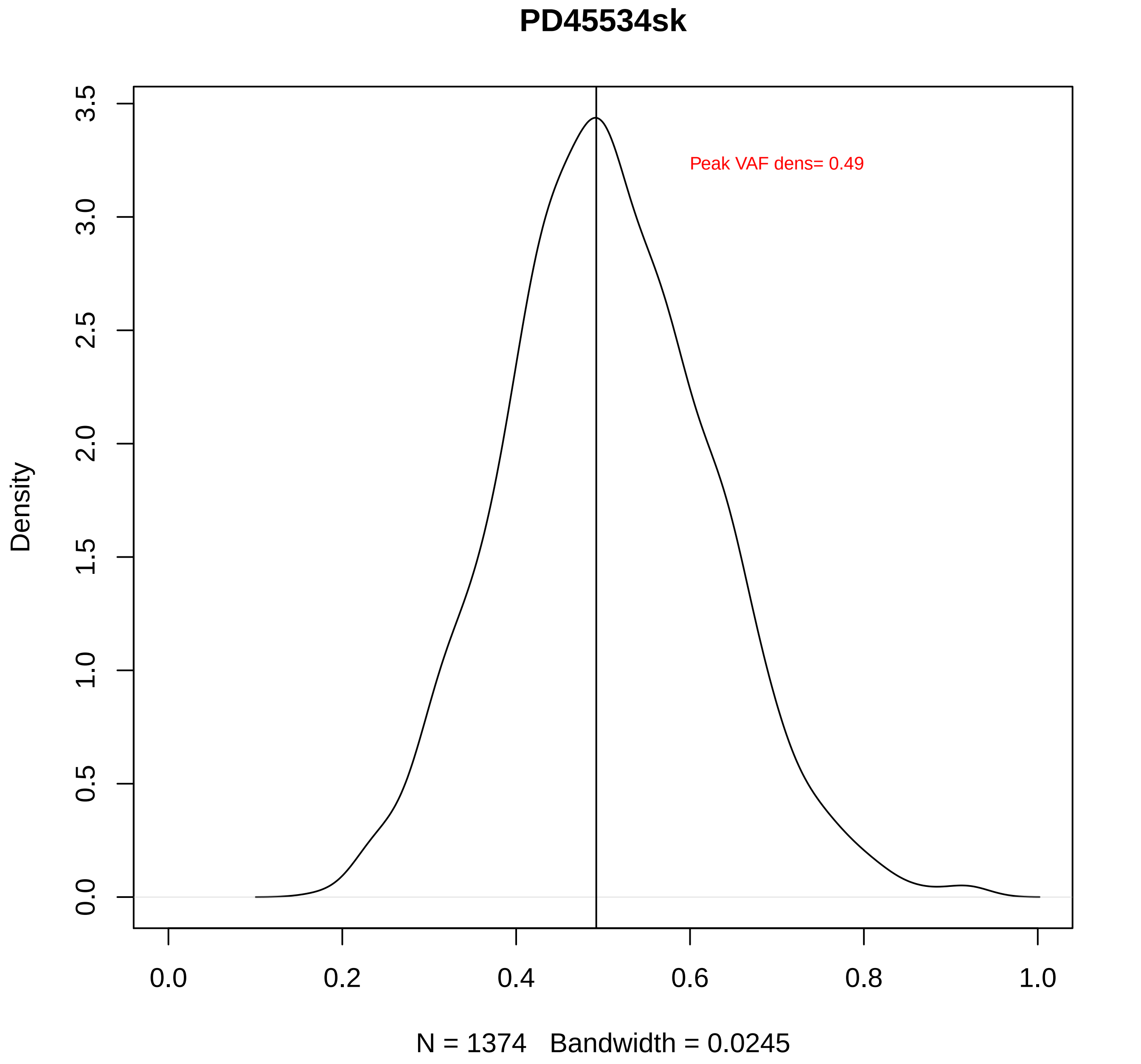
PD45534sk
Peak VAF dens= 0.49
Density
0.0
0.5
1.0
1.5
2.0
2.5
3.0
3.5
0.0
0.2
0.4
0.6
0.8
1.0
N = 1374   Bandwidth = 0.0245

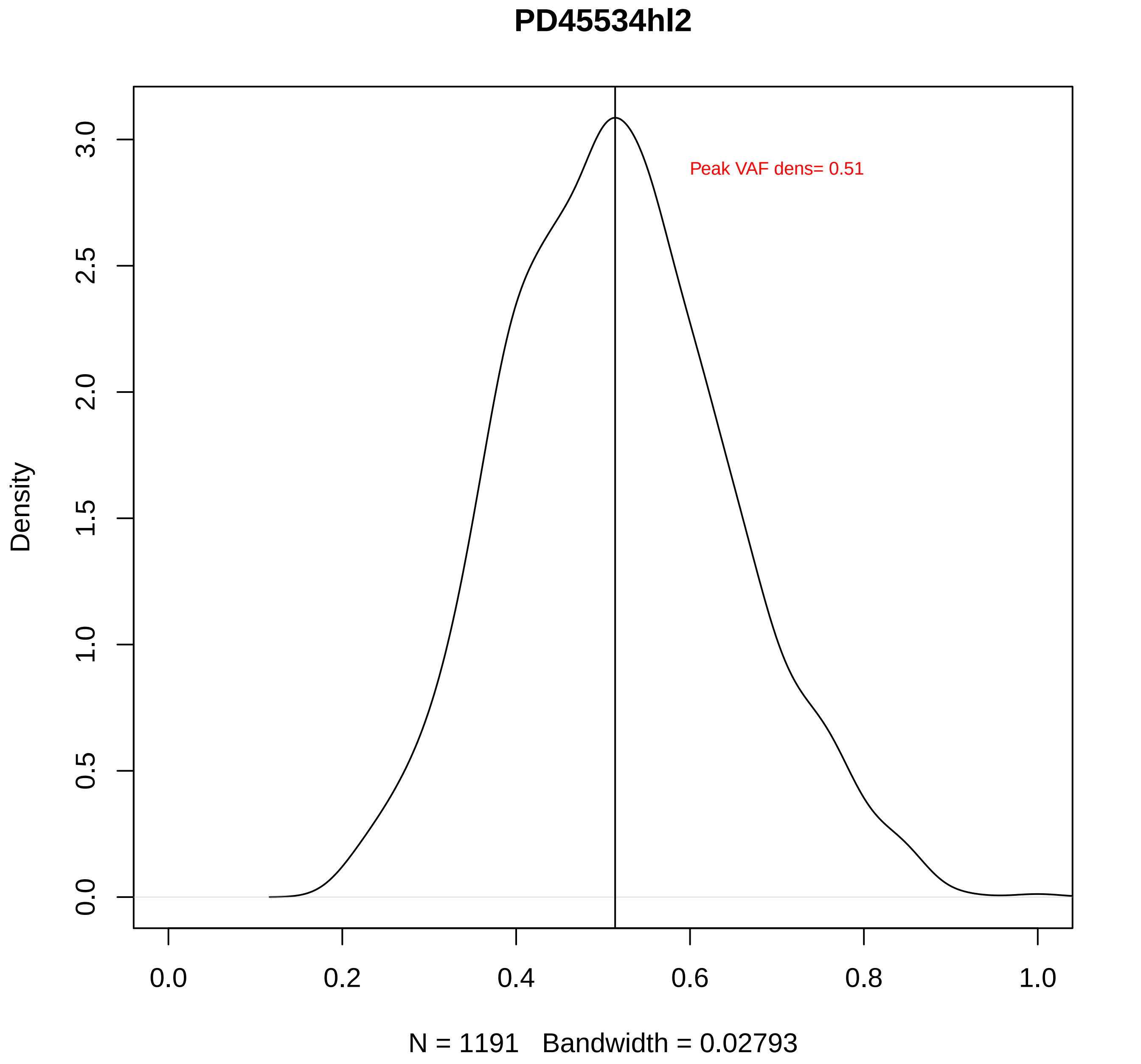

PD45534hl2
Peak VAF dens= 0.51
Density
0.0
0.5
1.0
1.5
2.0
2.5
3.0
0.0
0.2
0.4
0.6
0.8
1.0
N = 1191   Bandwidth = 0.02793

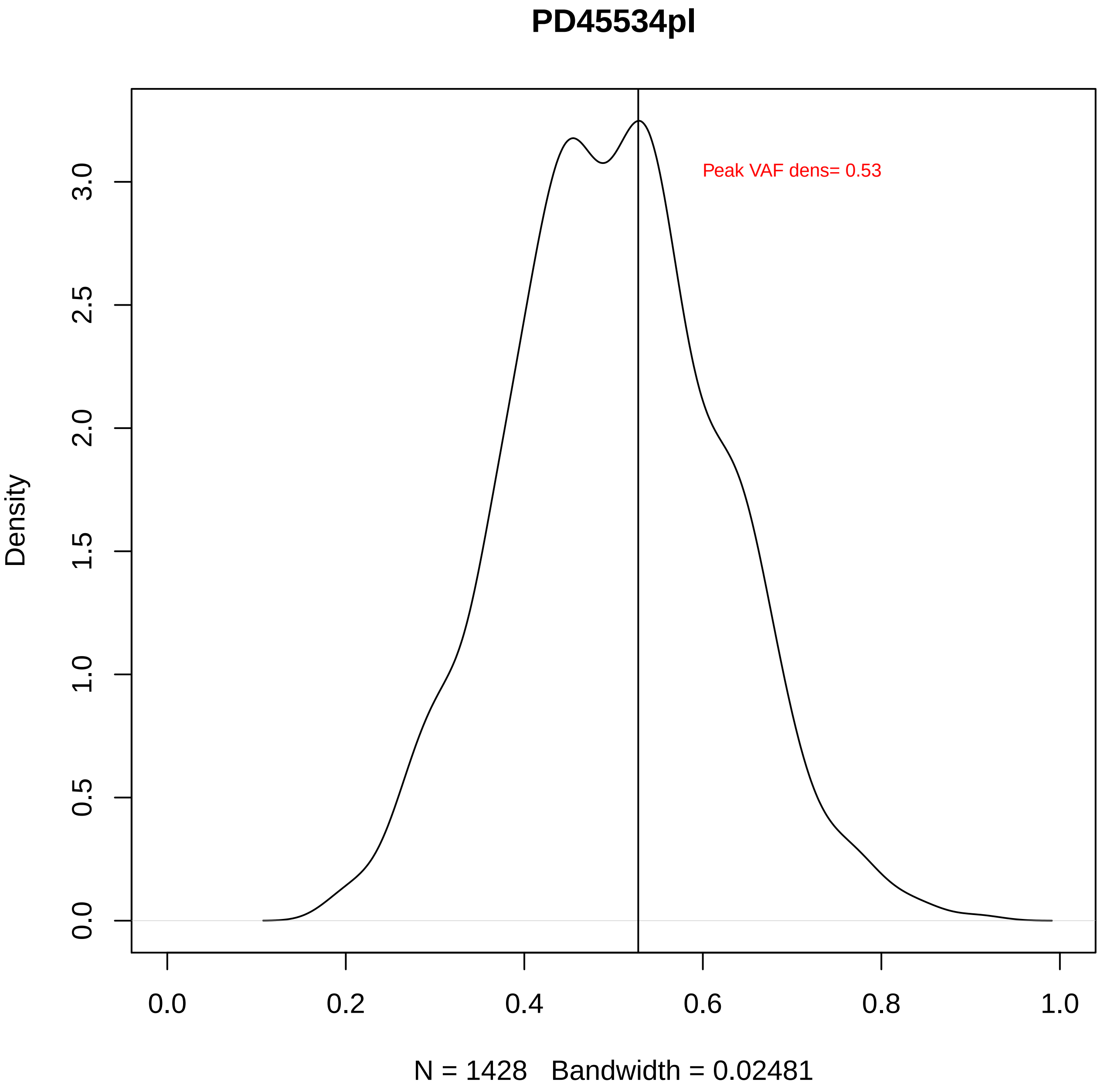

PD45534pl
Peak VAF dens= 0.53
Density
0.0
0.5
1.0
1.5
2.0
2.5
3.0
0.0
0.2
0.4
0.6
0.8
1.0
N = 1428   Bandwidth = 0.02481

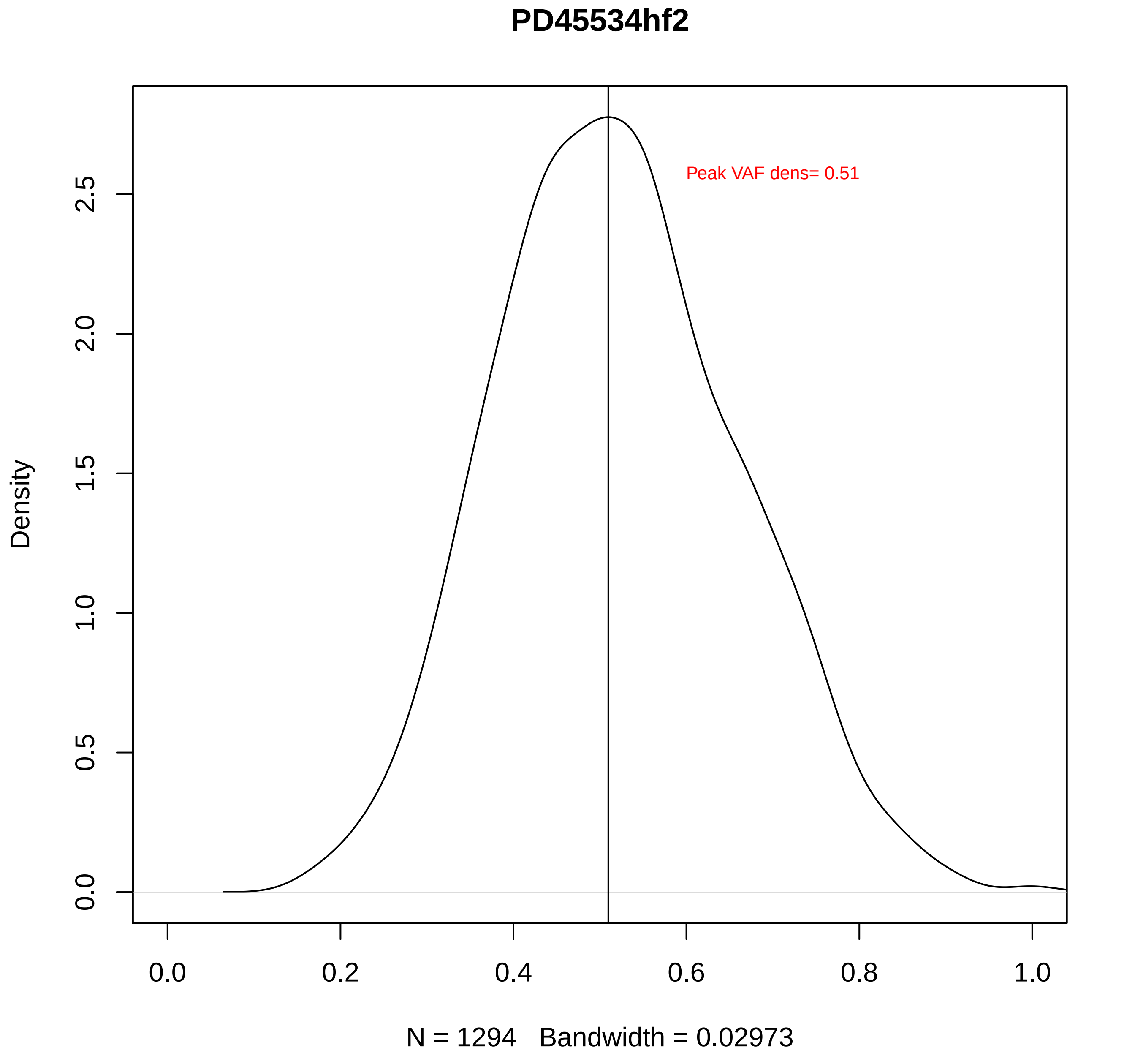
PD45534hf2
Peak VAF dens= 0.51
Density
0.0
0.5
1.0
1.5
2.0
2.5
0.0
0.2
0.4
0.6
0.8
1.0
N = 1294   Bandwidth = 0.02973

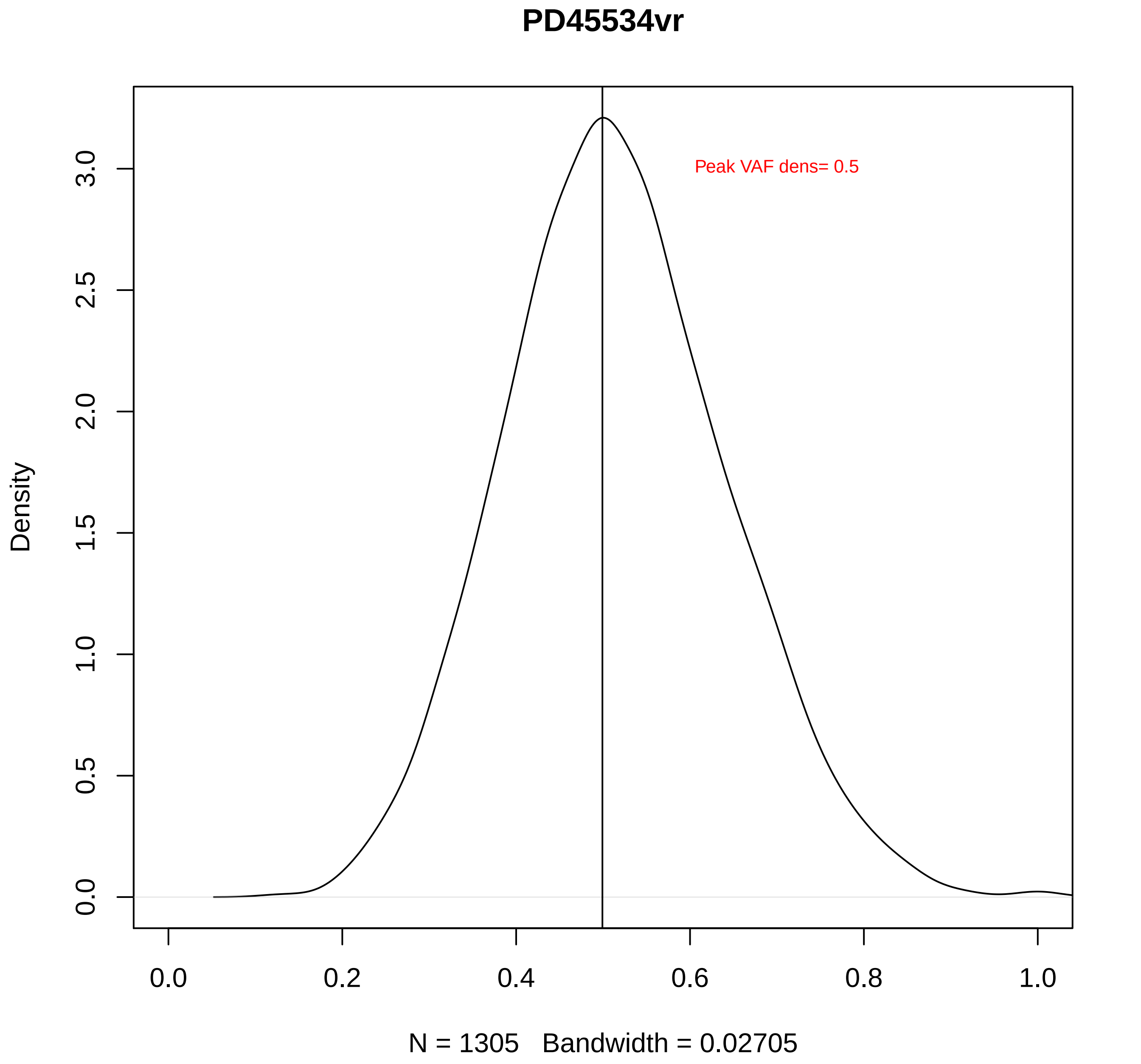
PD45534vr
Peak VAF dens= 0.5
Density
0.0
0.5
1.0
1.5
2.0
2.5
3.0
0.0
0.2
0.4
0.6
0.8
1.0
N = 1305   Bandwidth = 0.02705

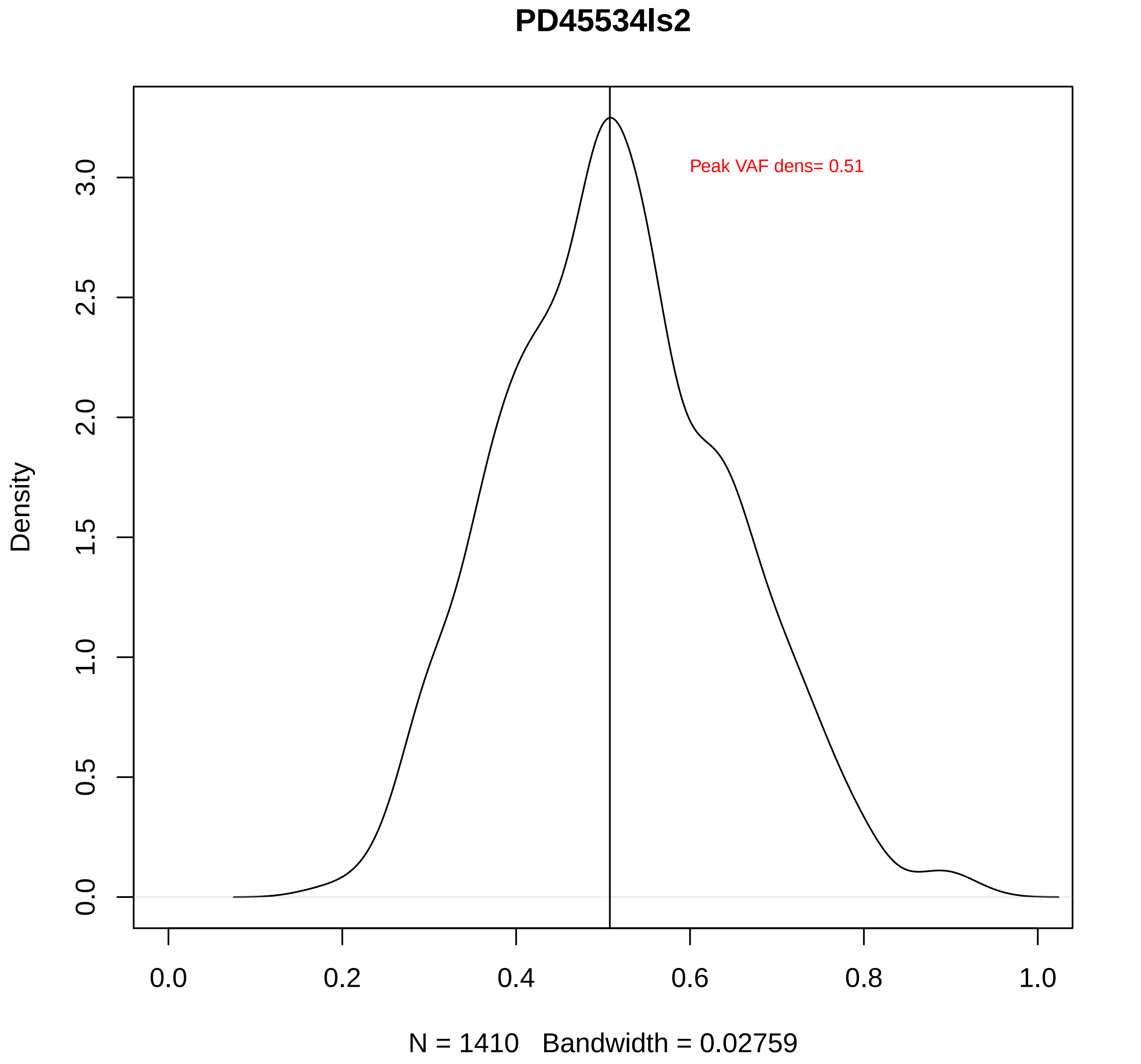

PD45534ls2
Peak VAF dens= 0.51
Density
0.0
0.5
1.0
1.5
2.0
2.5
3.0
0.0
0.2
0.4
0.6
0.8
1.0
N = 1410   Bandwidth = 0.02759

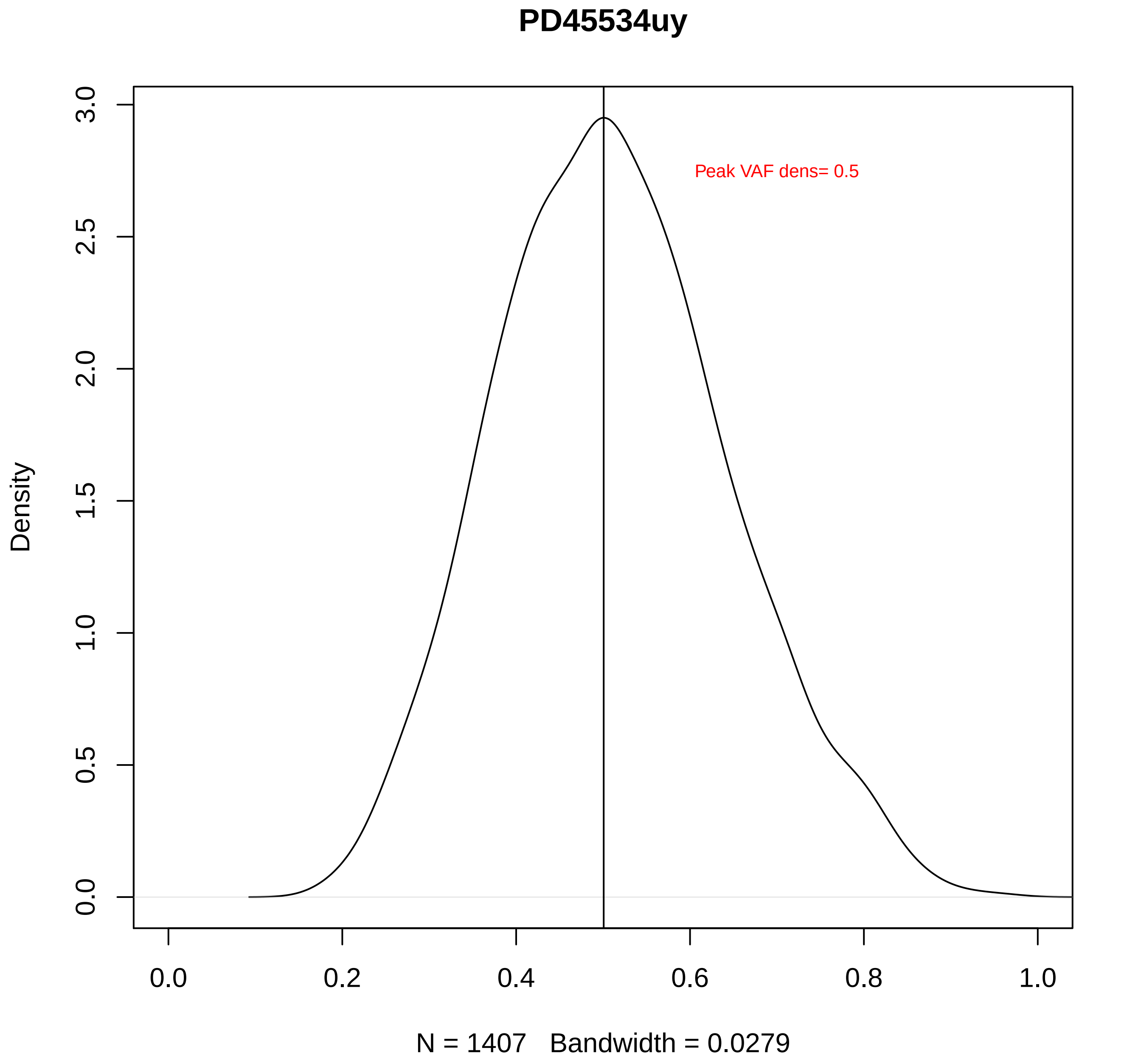
PD45534uy
Peak VAF dens= 0.5
Density
0.0
0.5
1.0
1.5
2.0
2.5
3.0
0.0
0.2
0.4
0.6
0.8
1.0
N = 1407   Bandwidth = 0.0279

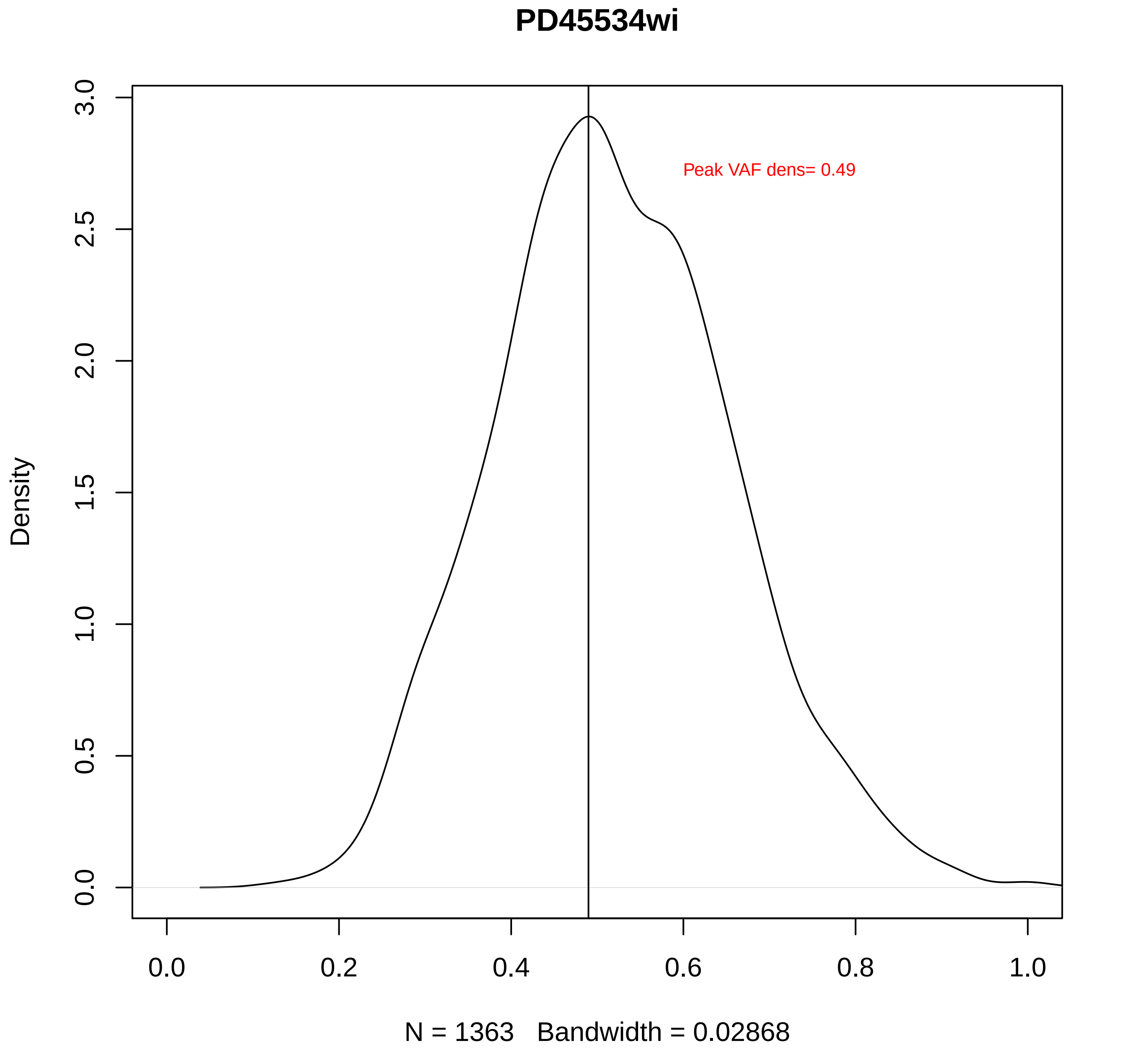
PD45534wi
Peak VAF dens= 0.49
Density
0.0
0.5
1.0
1.5
2.0
2.5
3.0
0.0
0.2
0.4
0.6
0.8
1.0
N = 1363   Bandwidth = 0.02868

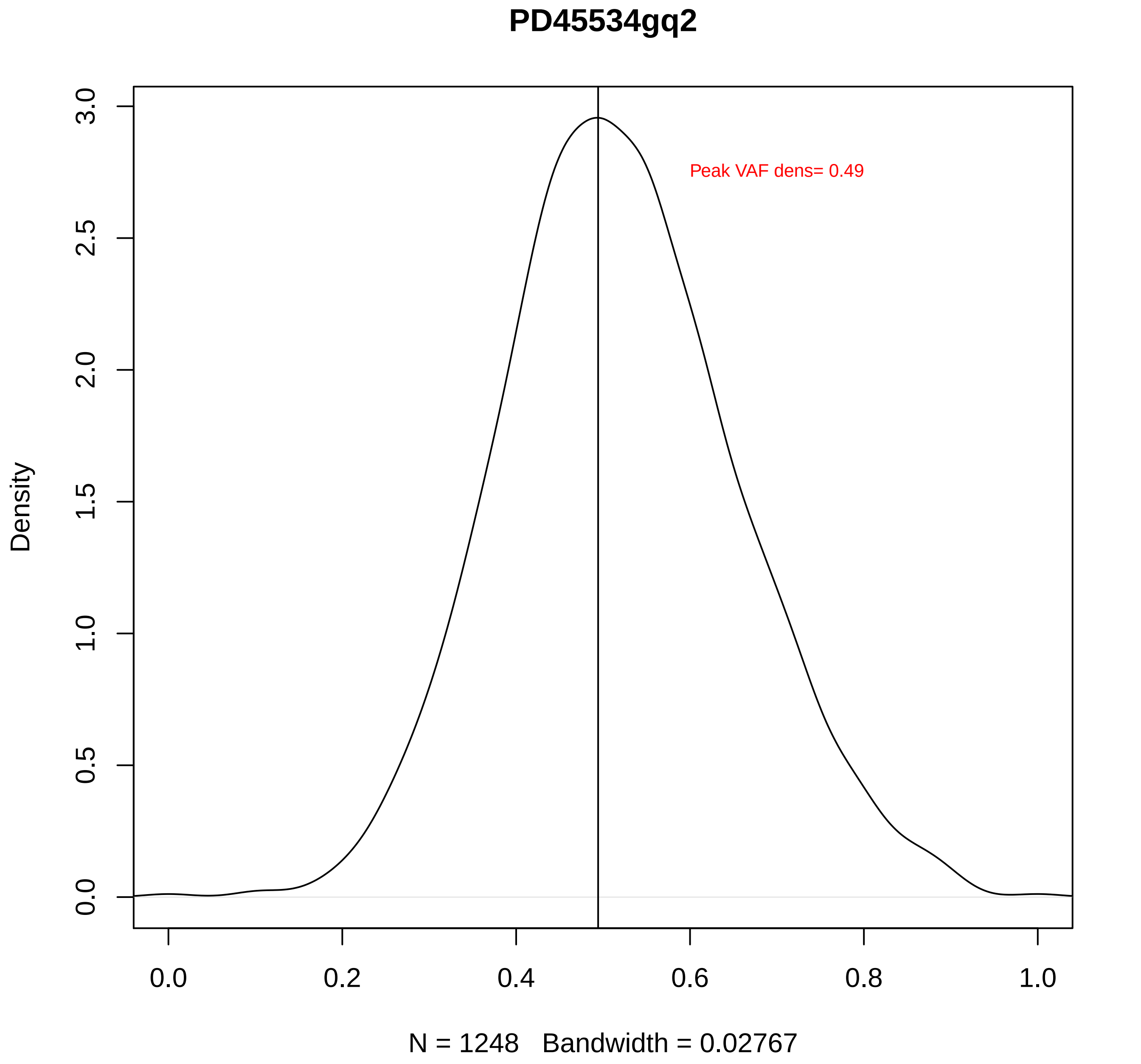
PD45534gq2
Peak VAF dens= 0.49
Density
0.0
0.5
1.0
1.5
2.0
2.5
3.0
0.0
0.2
0.4
0.6
0.8
1.0
N = 1248   Bandwidth = 0.02767

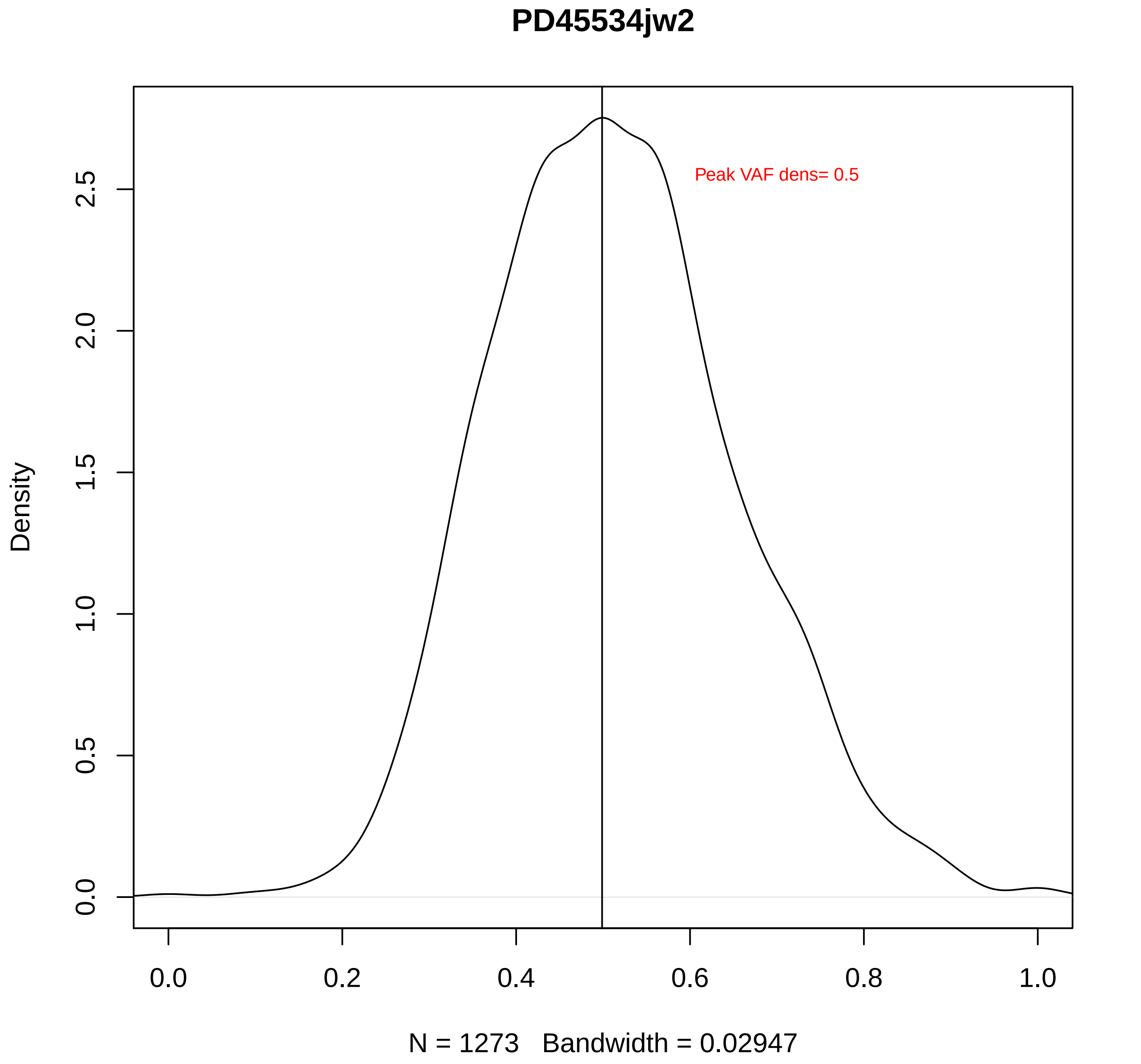
PD45534jw2
Peak VAF dens= 0.5
Density
0.0
0.5
1.0
1.5
2.0
2.5
0.0
0.2
0.4
0.6
0.8
1.0
N = 1273   Bandwidth = 0.02947

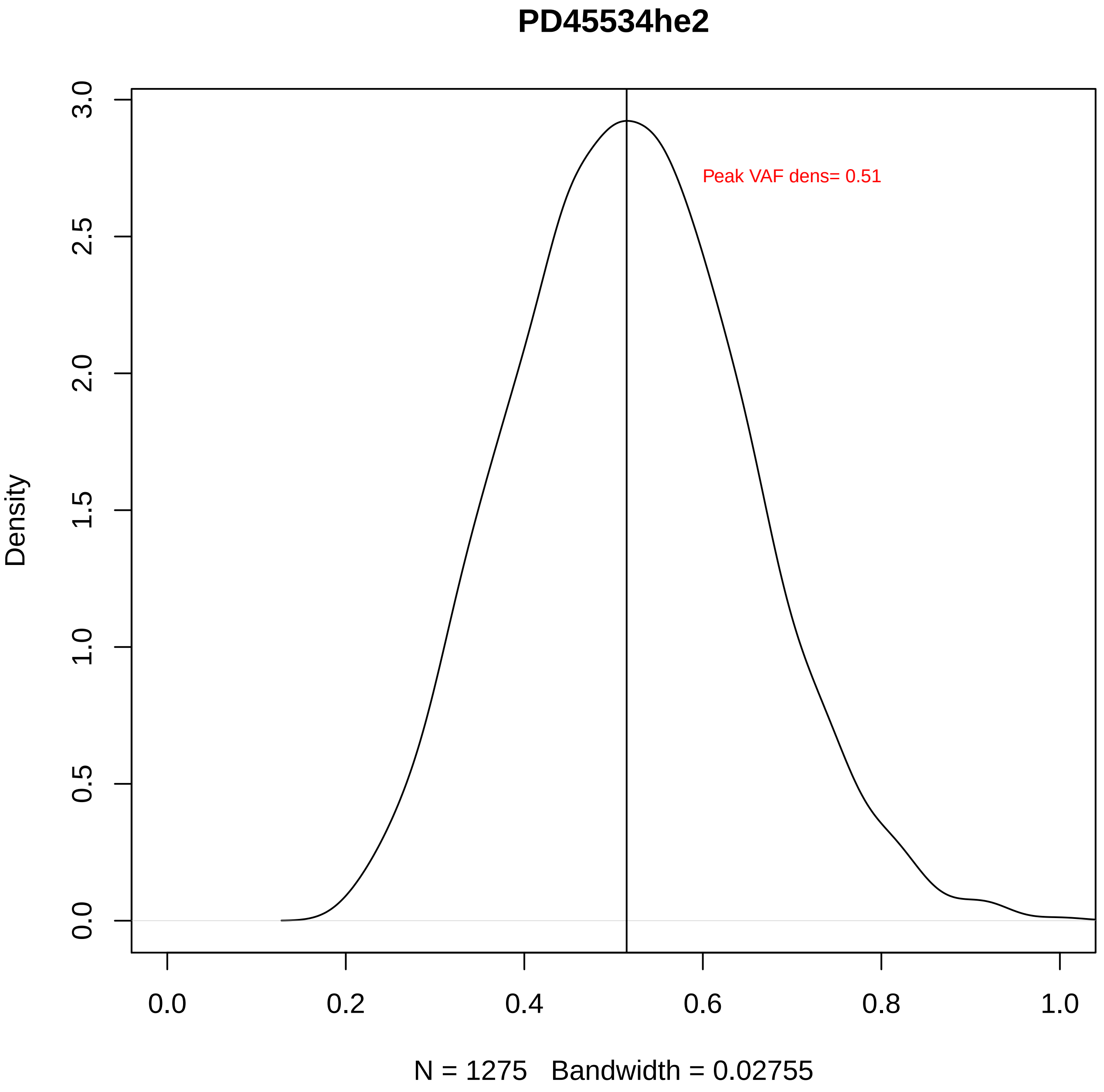
PD45534he2
Peak VAF dens= 0.51
Density
0.0
0.5
1.0
1.5
2.0
2.5
3.0
0.0
0.2
0.4
0.6
0.8
1.0
N = 1275   Bandwidth = 0.02755

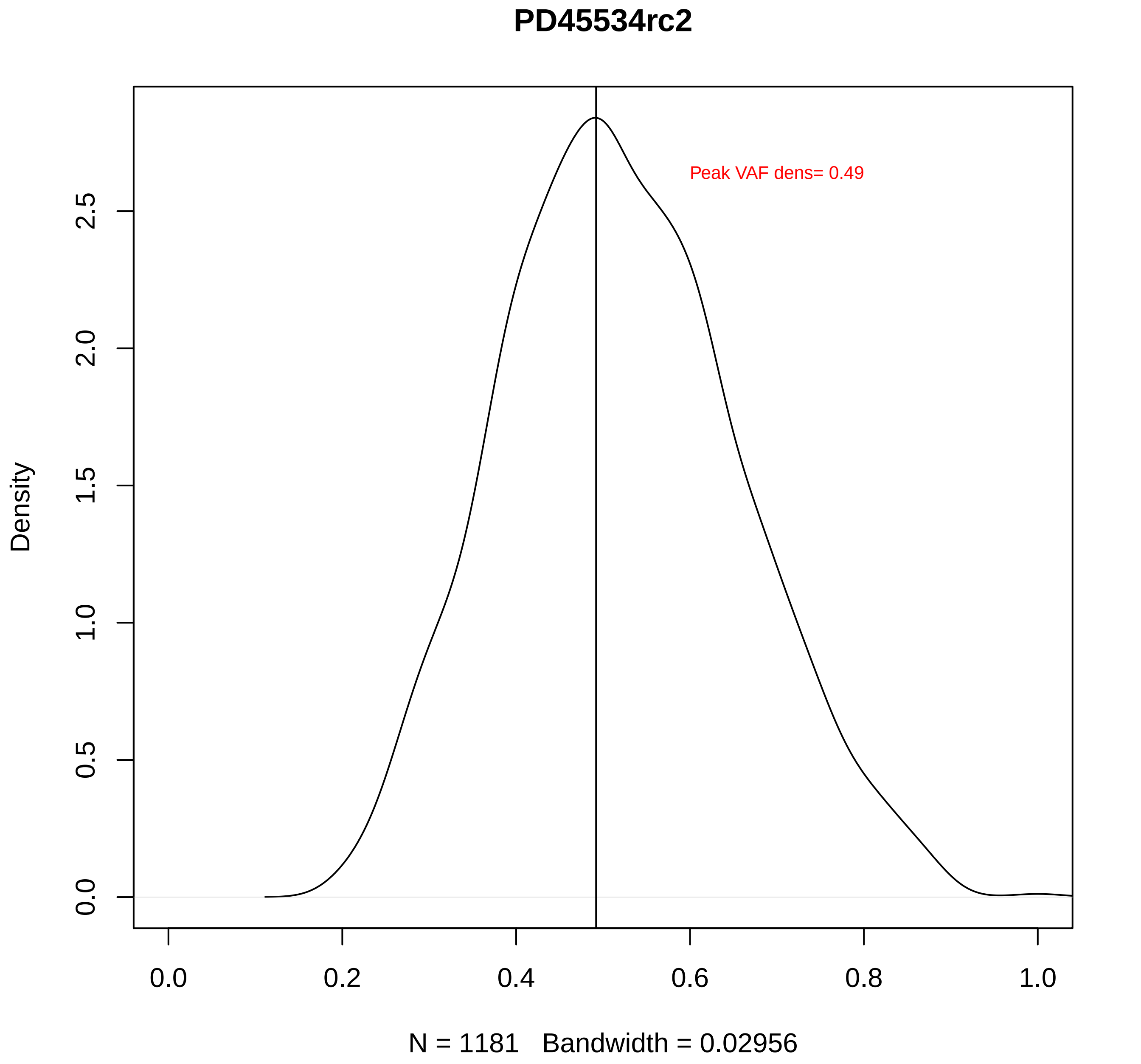

PD45534rc2
Peak VAF dens= 0.49
Density
0.0
0.5
1.0
1.5
2.0
2.5
0.0
0.2
0.4
0.6
0.8
1.0
N = 1181   Bandwidth = 0.02956

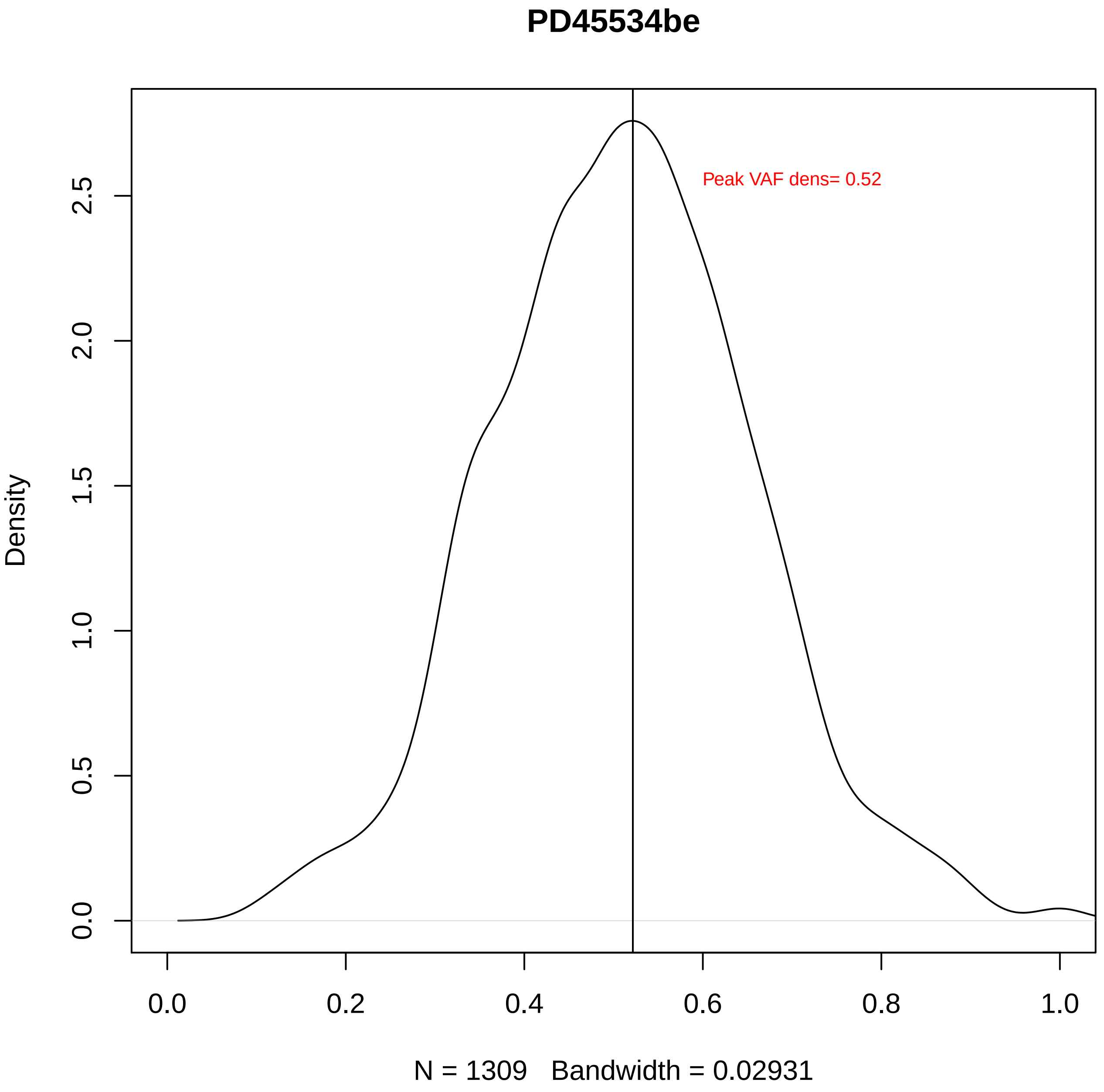
PD45534be
Peak VAF dens= 0.52
Density
0.0
0.5
1.0
1.5
2.0
2.5
0.0
0.2
0.4
0.6
0.8
1.0
N = 1309   Bandwidth = 0.02931

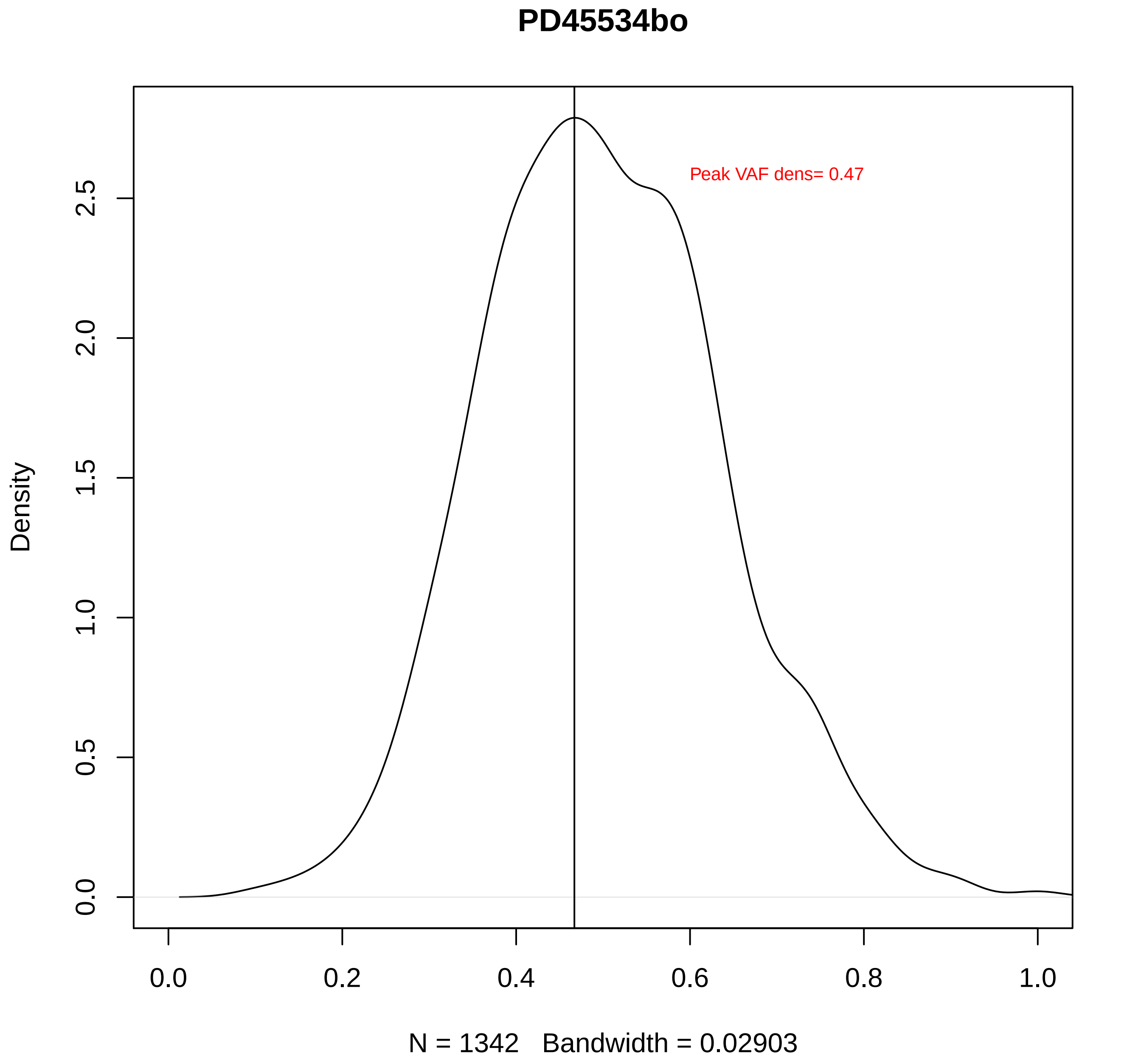
PD45534bo
Peak VAF dens= 0.47
Density
0.0
0.5
1.0
1.5
2.0
2.5
0.0
0.2
0.4
0.6
0.8
1.0
N = 1342   Bandwidth = 0.02903

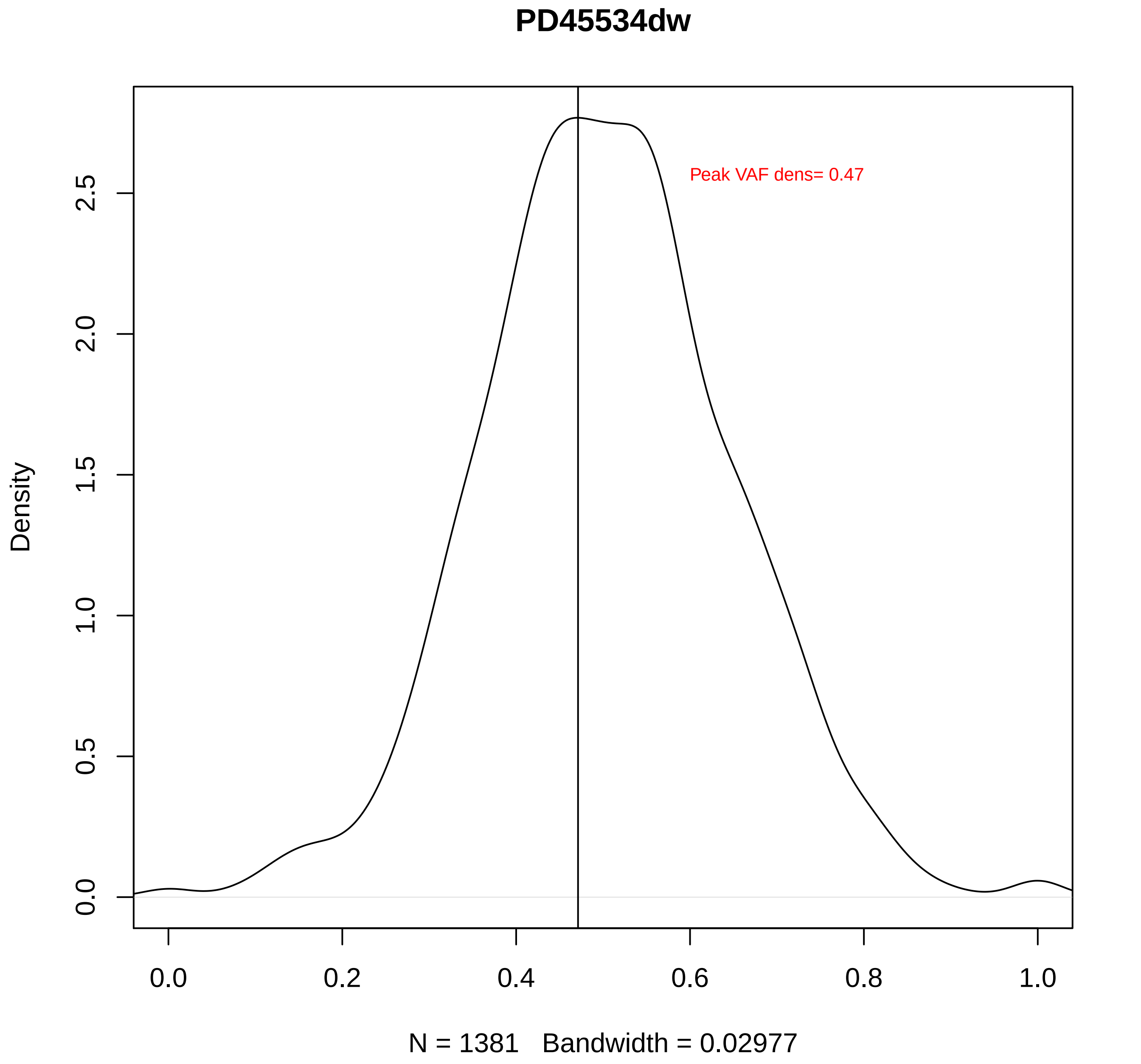
PD45534dw
Peak VAF dens= 0.47
Density
0.0
0.5
1.0
1.5
2.0
2.5
0.0
0.2
0.4
0.6
0.8
1.0
N = 1381   Bandwidth = 0.02977

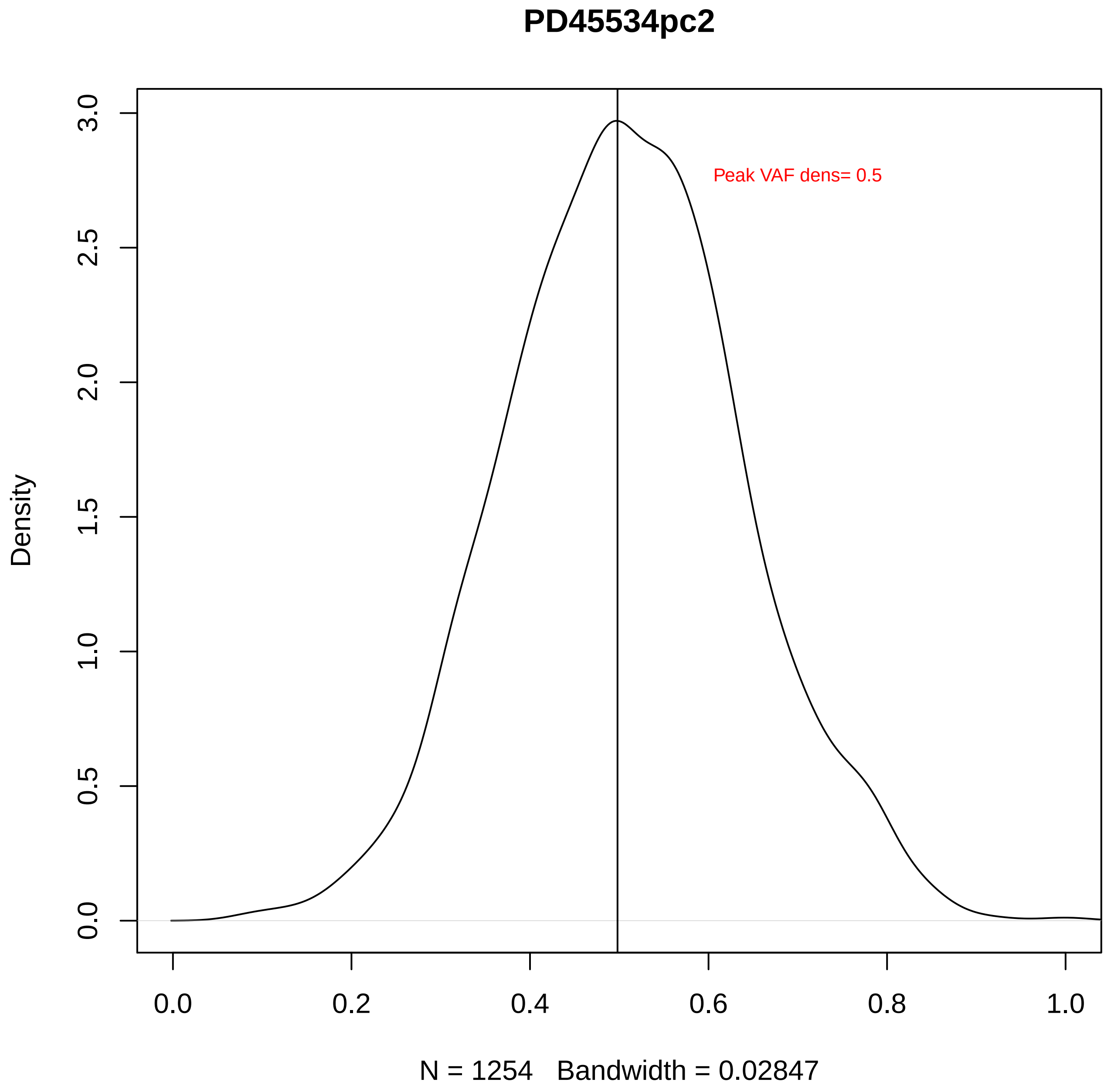
PD45534pc2
Peak VAF dens= 0.5
Density
0.0
0.5
1.0
1.5
2.0
2.5
3.0
0.0
0.2
0.4
0.6
0.8
1.0
N = 1254   Bandwidth = 0.02847

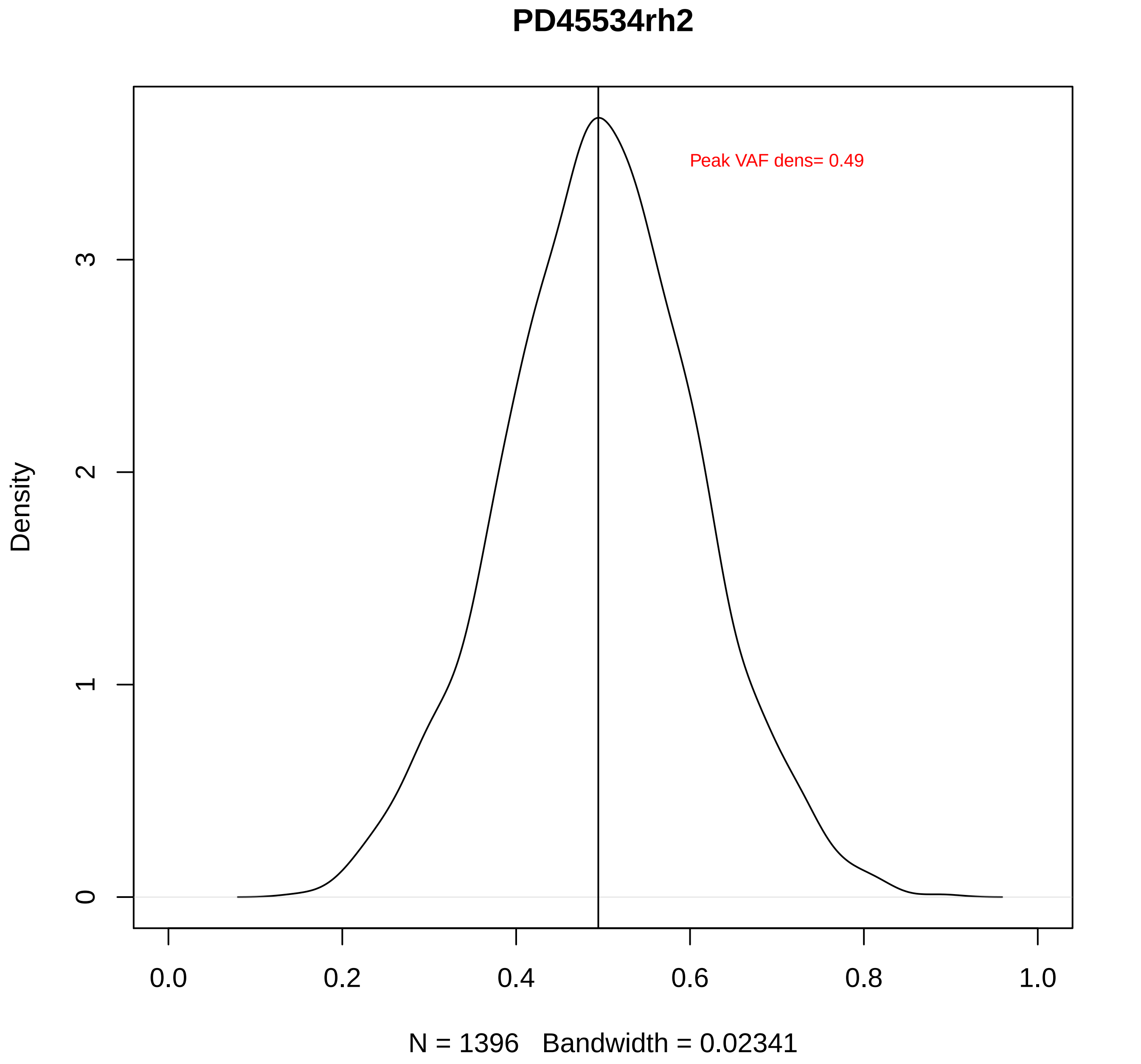
PD45534rh2
Peak VAF dens= 0.49
Density
0
1
2
3
0.0
0.2
0.4
0.6
0.8
1.0
N = 1396   Bandwidth = 0.02341

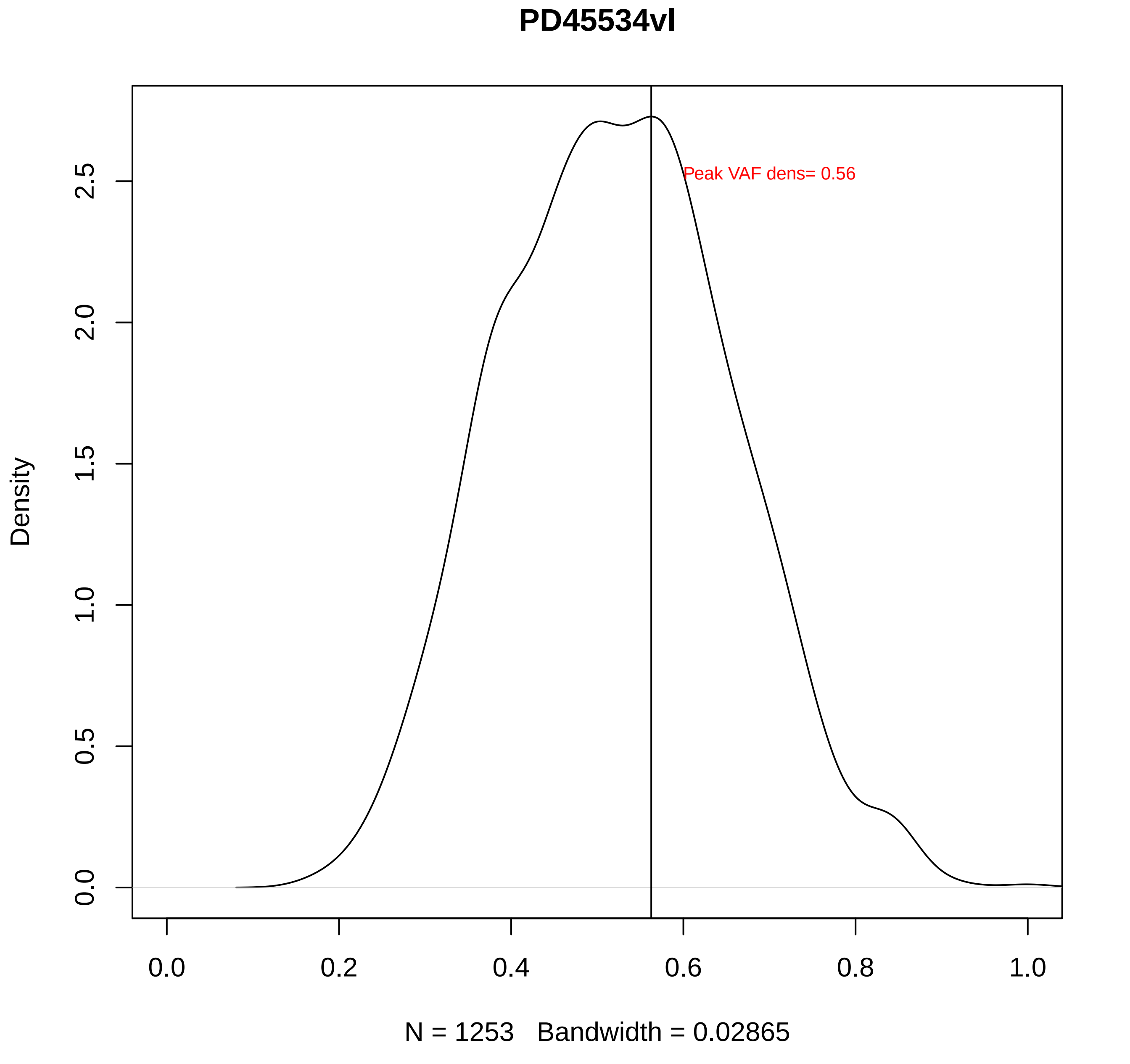

PD45534vl
Peak VAF dens= 0.56
Density
0.0
0.5
1.0
1.5
2.0
2.5
0.0
0.2
0.4
0.6
0.8
1.0
N = 1253   Bandwidth = 0.02865

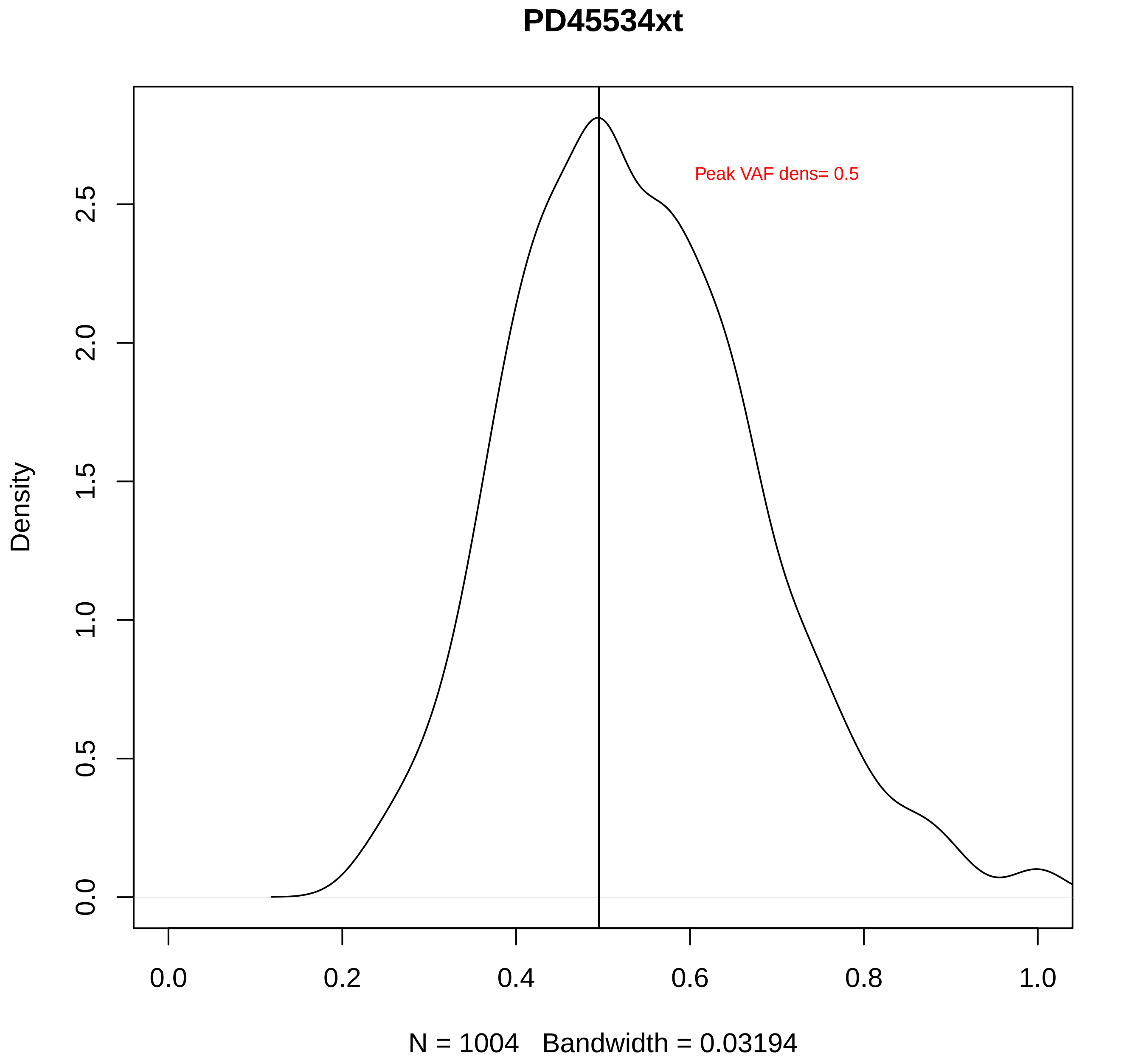
PD45534xt
Peak VAF dens= 0.5
Density
0.0
0.5
1.0
1.5
2.0
2.5
0.0
0.2
0.4
0.6
0.8
1.0
N = 1004   Bandwidth = 0.03194

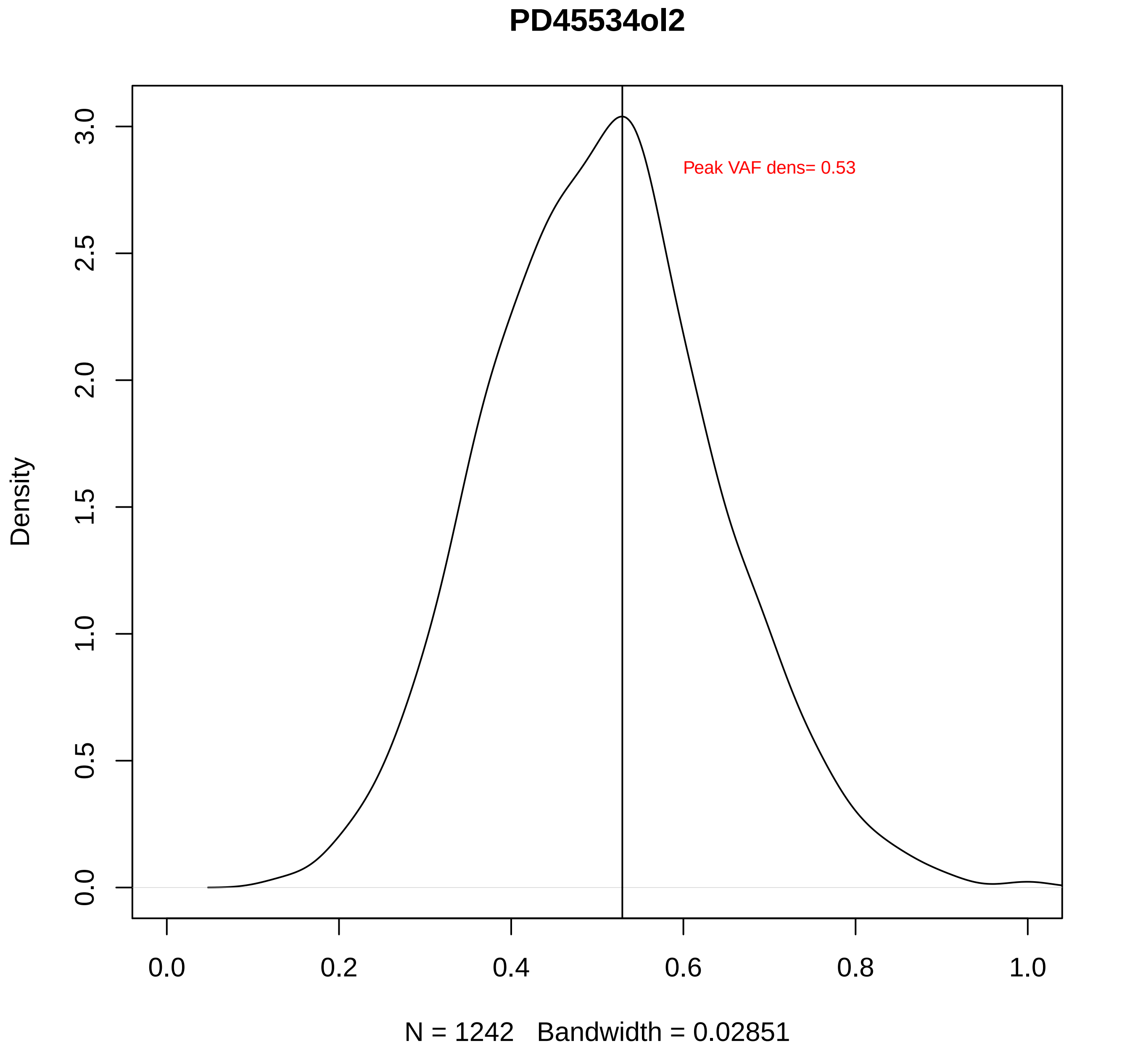

PD45534ol2
Peak VAF dens= 0.53
Density
0.0
0.5
1.0
1.5
2.0
2.5
3.0
0.0
0.2
0.4
0.6
0.8
1.0
N = 1242 Bandwidth = 0.02851

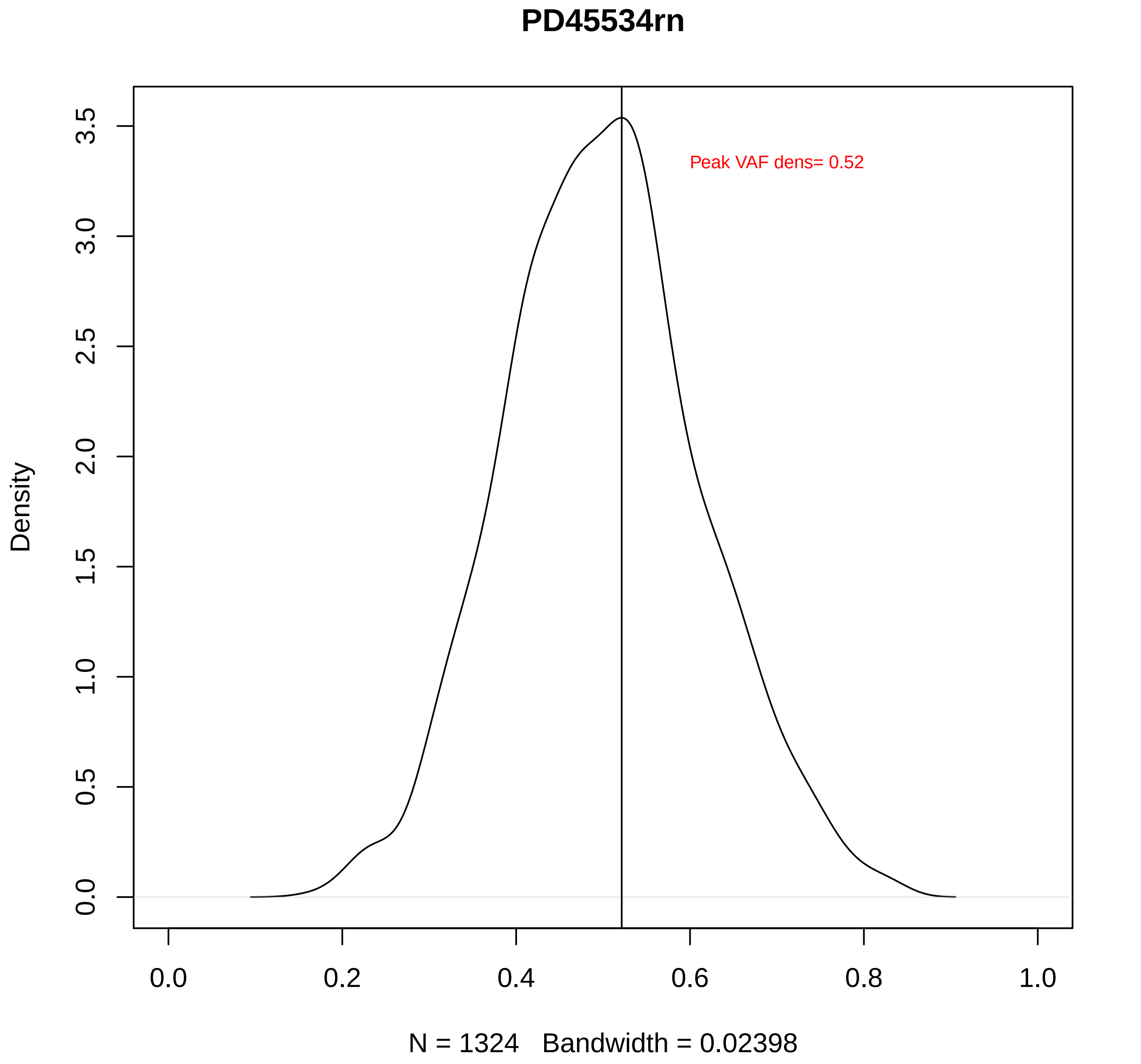
PD45534rn
Peak VAF dens= 0.52
Density
0.0
0.5
1.0
1.5
2.0
2.5
3.0
3.5
0.0
0.2
0.4
0.6
0.8
1.0
N = 1324   Bandwidth = 0.02398

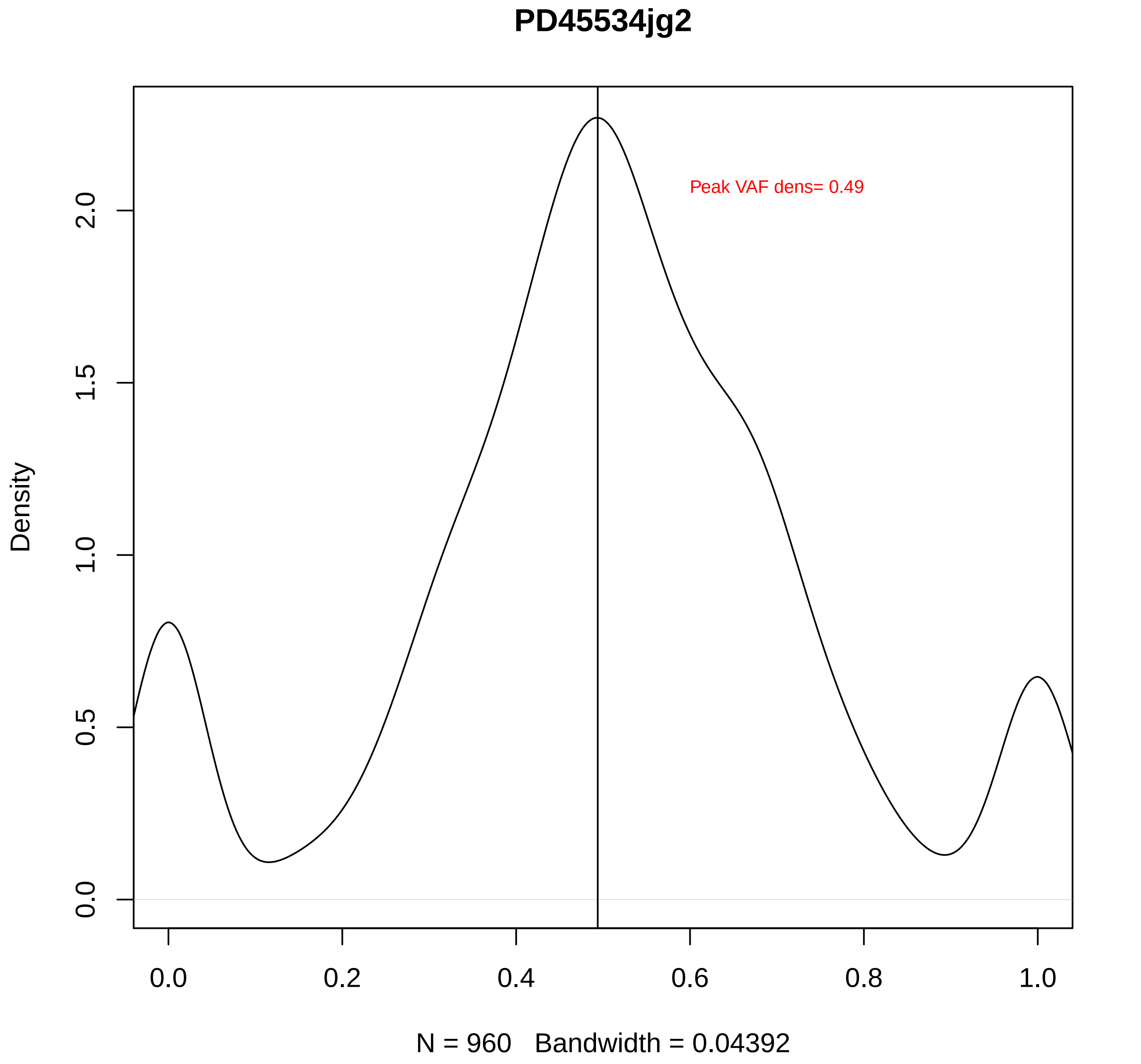

PD45534jg2
Peak VAF dens= 0.49
Density
0.0
0.5
1.0
1.5
2.0
0.0
0.2
0.4
0.6
0.8
1.0
N = 960   Bandwidth = 0.04392

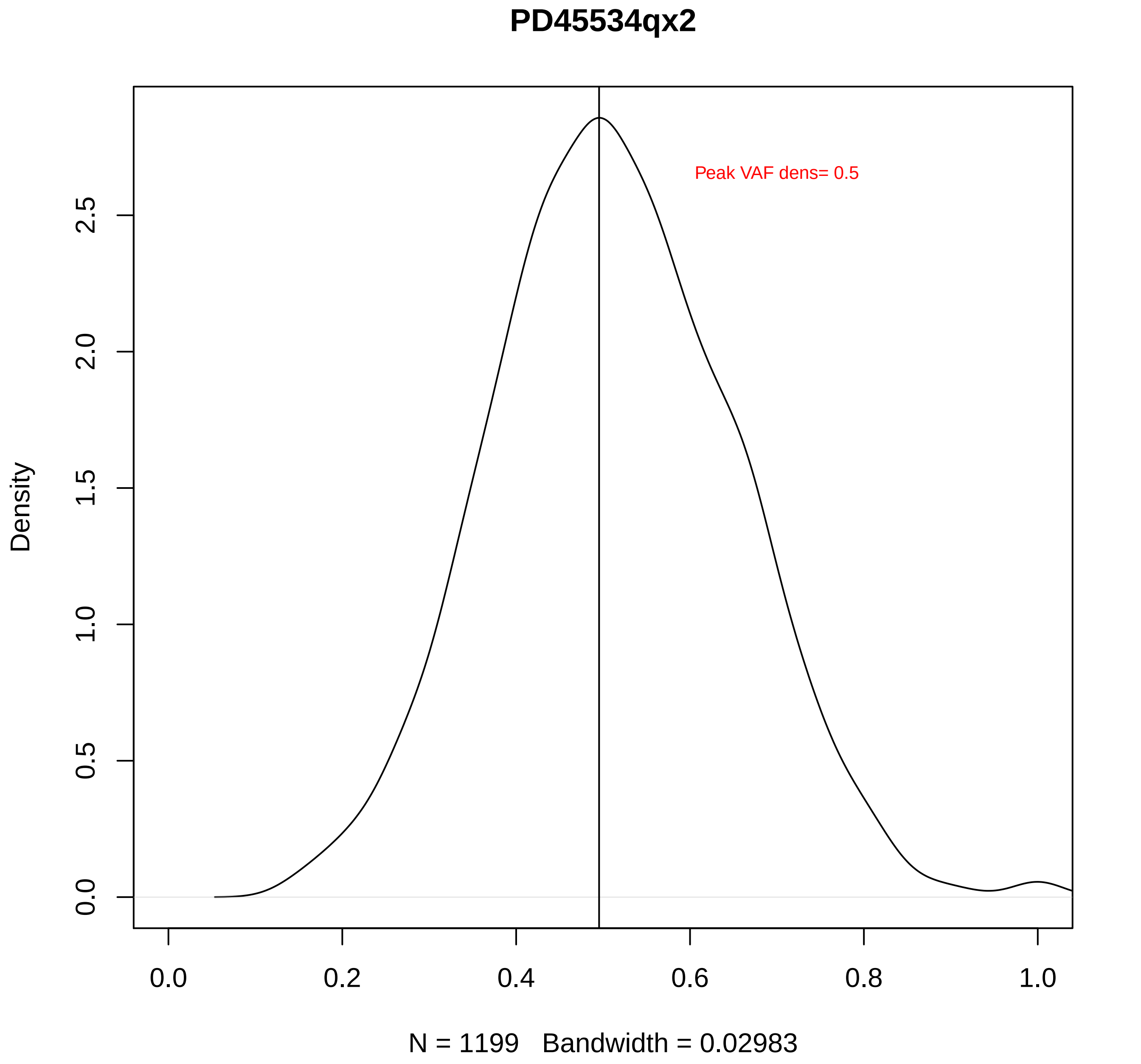

PD45534qx2
Peak VAF dens= 0.5
Density
0.0
0.5
1.0
1.5
2.0
2.5
0.0
0.2
0.4
0.6
0.8
1.0
N = 1199   Bandwidth = 0.02983

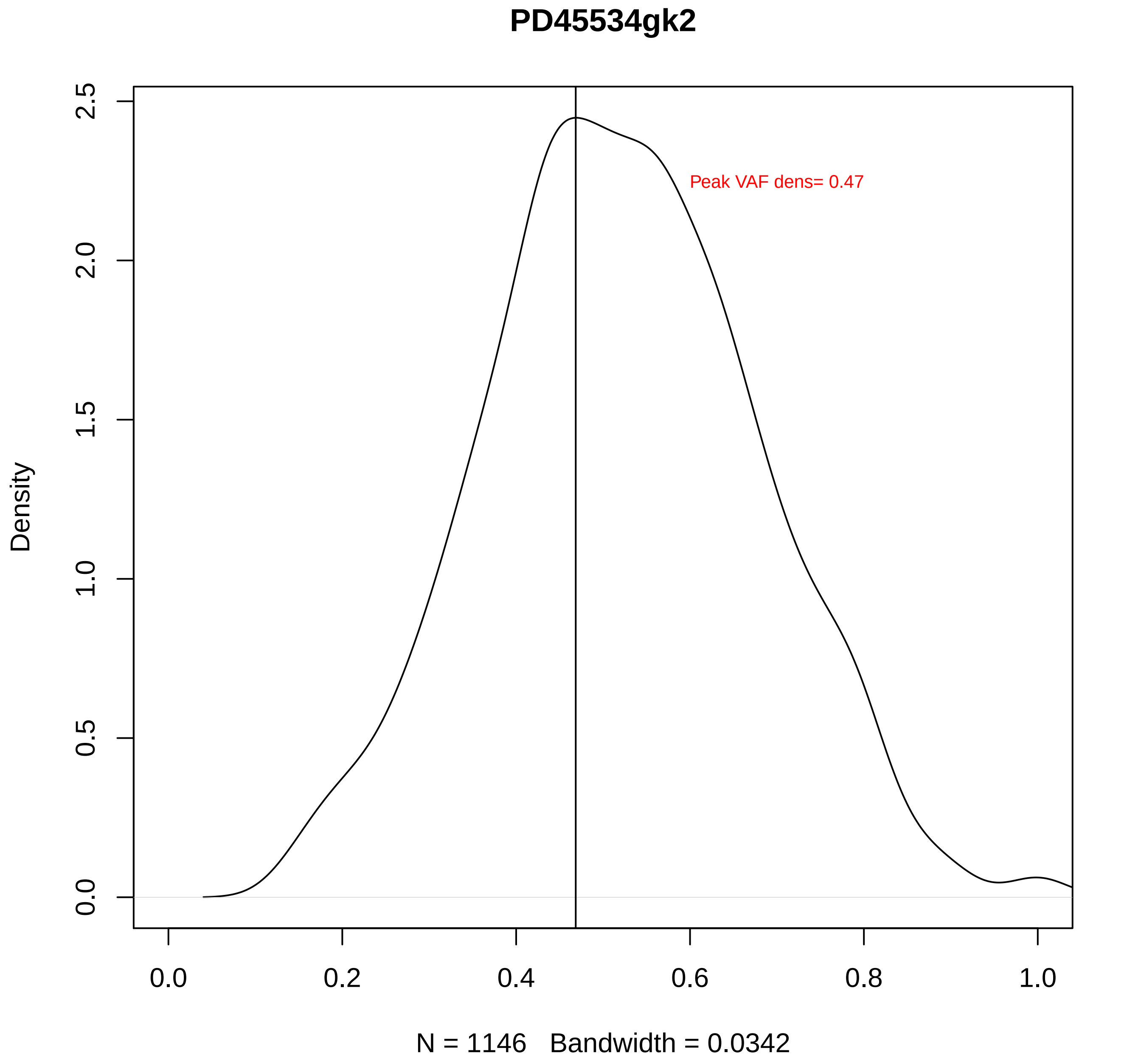

PD45534gk2
Peak VAF dens= 0.47
Density
0.0
0.5
1.0
1.5
2.0
2.5
0.0
0.2
0.4
0.6
0.8
1.0
N = 1146   Bandwidth = 0.0342

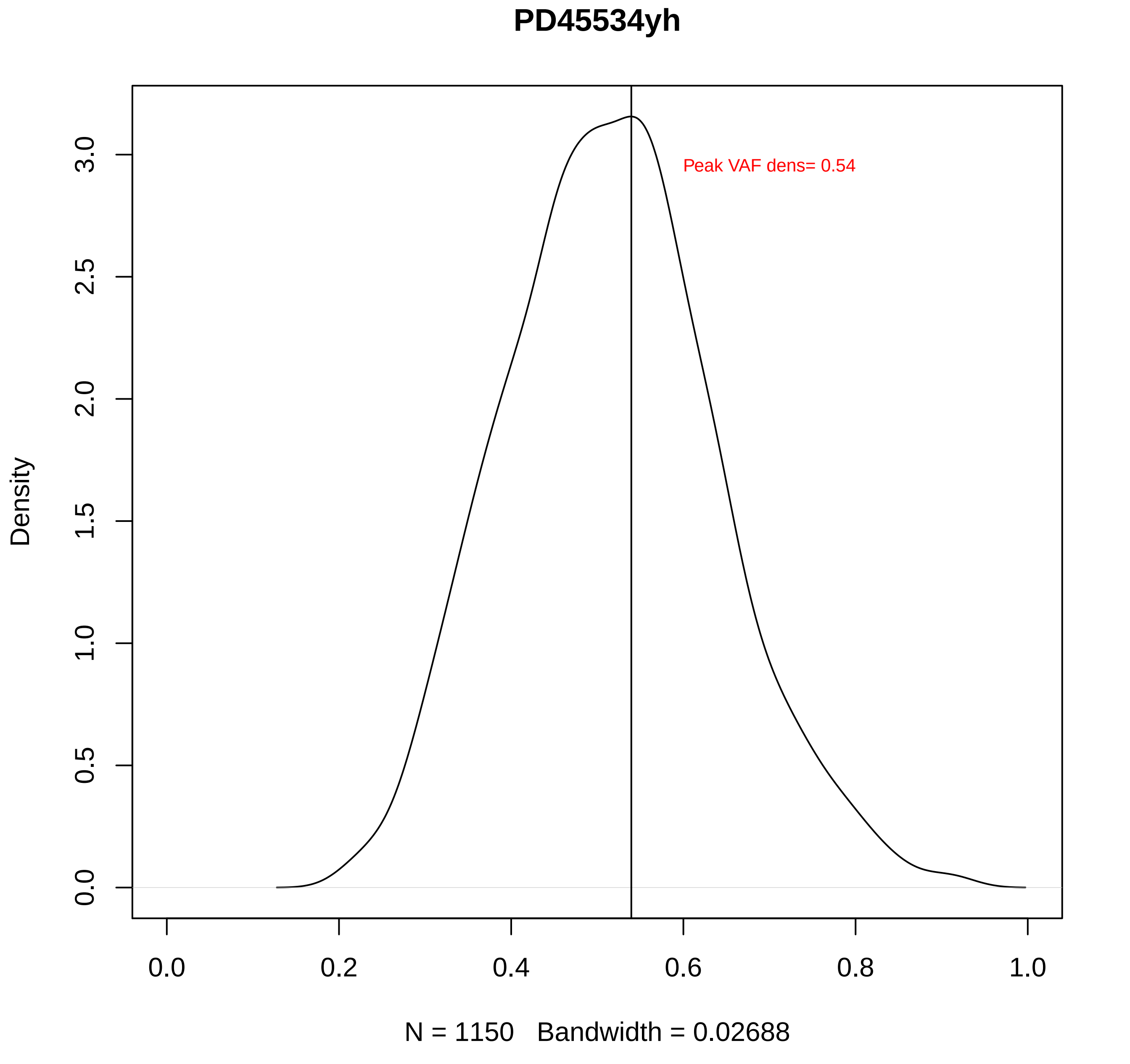

PD45534yh
Peak VAF dens= 0.54
Density
0.0
0.5
1.0
1.5
2.0
2.5
3.0
0.0
0.2
0.4
0.6
0.8
1.0
N = 1150   Bandwidth = 0.02688

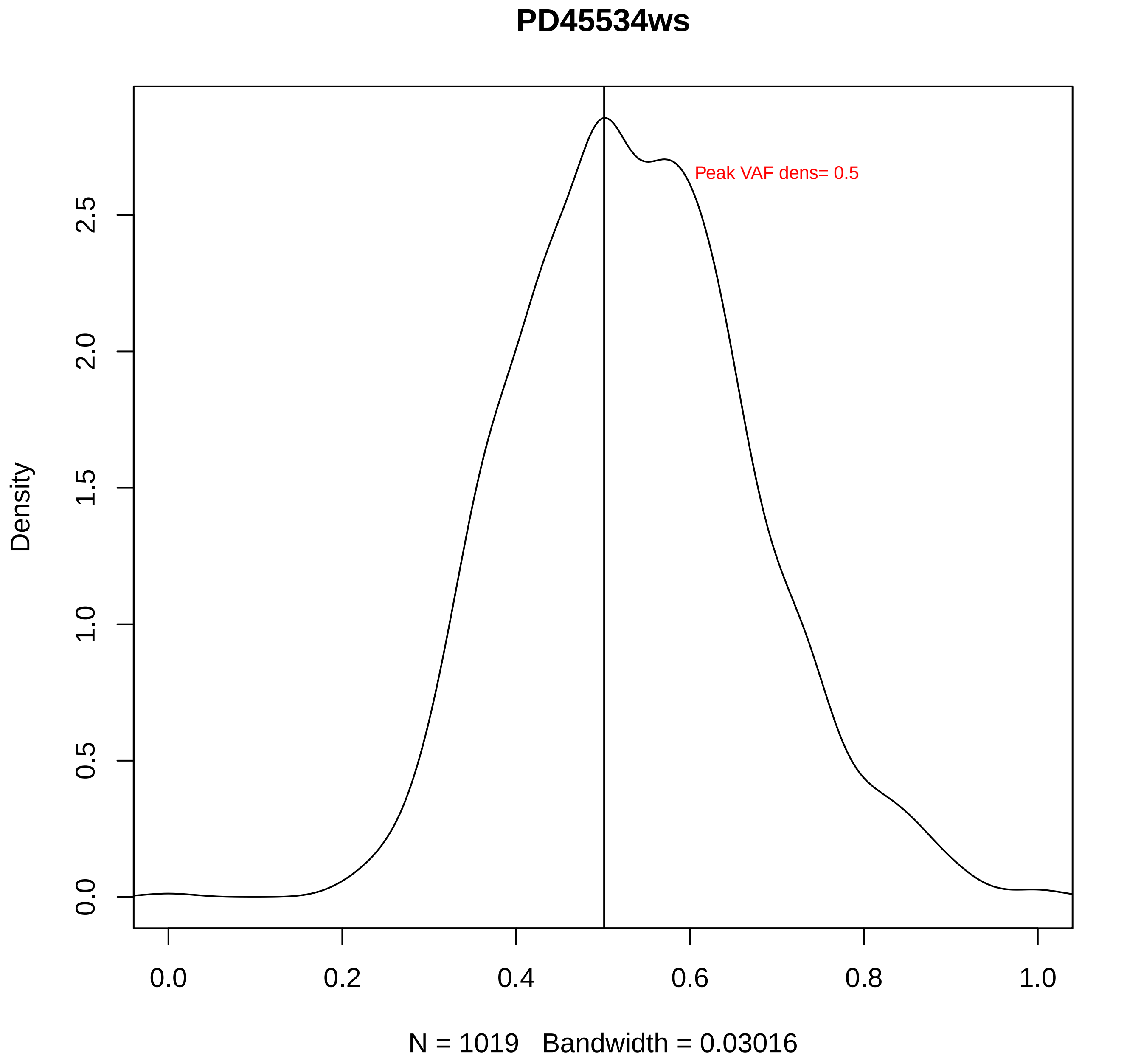
PD45534ws
Peak VAF dens= 0.5
Density
0.0
0.5
1.0
1.5
2.0
2.5
0.0
0.2
0.4
0.6
0.8
1.0
N = 1019   Bandwidth = 0.03016

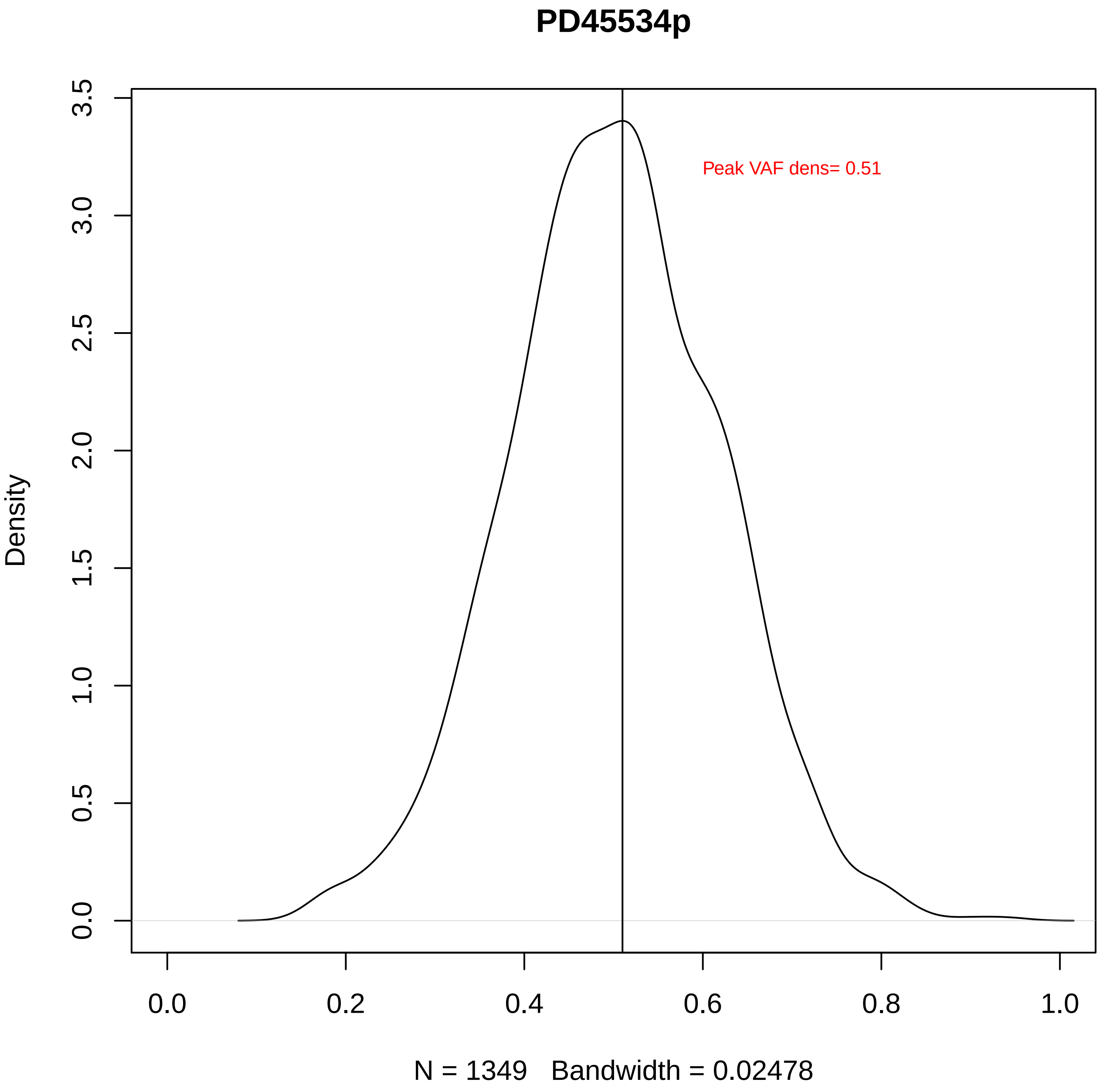
PD45534p
Peak VAF dens= 0.51
Density
0.0
0.5
1.0
1.5
2.0
2.5
3.0
3.5
0.0
0.2
0.4
0.6
0.8
1.0
N = 1349   Bandwidth = 0.02478

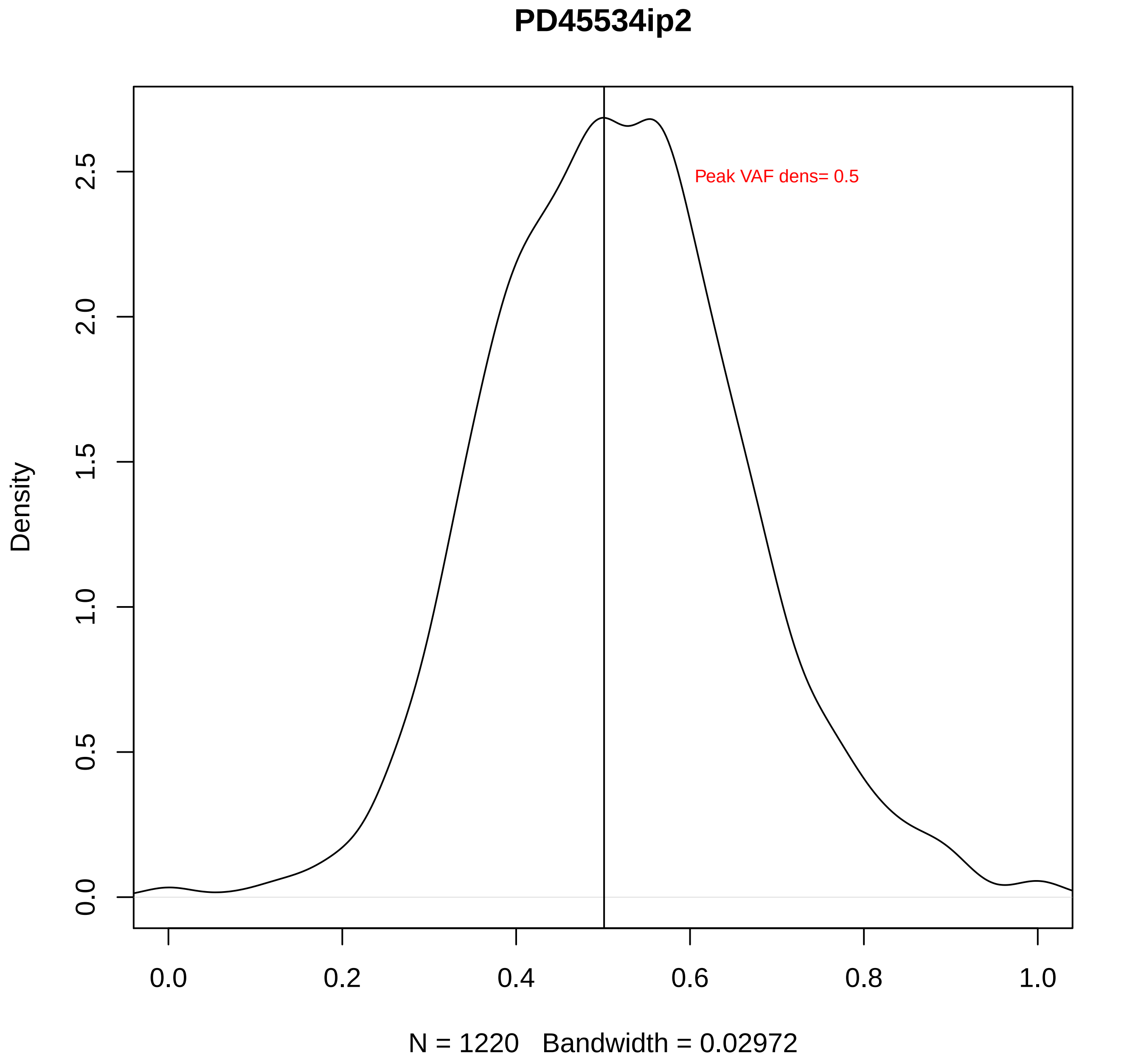
PD45534ip2
Peak VAF dens= 0.5
Density
0.0
0.5
1.0
1.5
2.0
2.5
0.0
0.2
0.4
0.6
0.8
1.0
N = 1220   Bandwidth = 0.02972

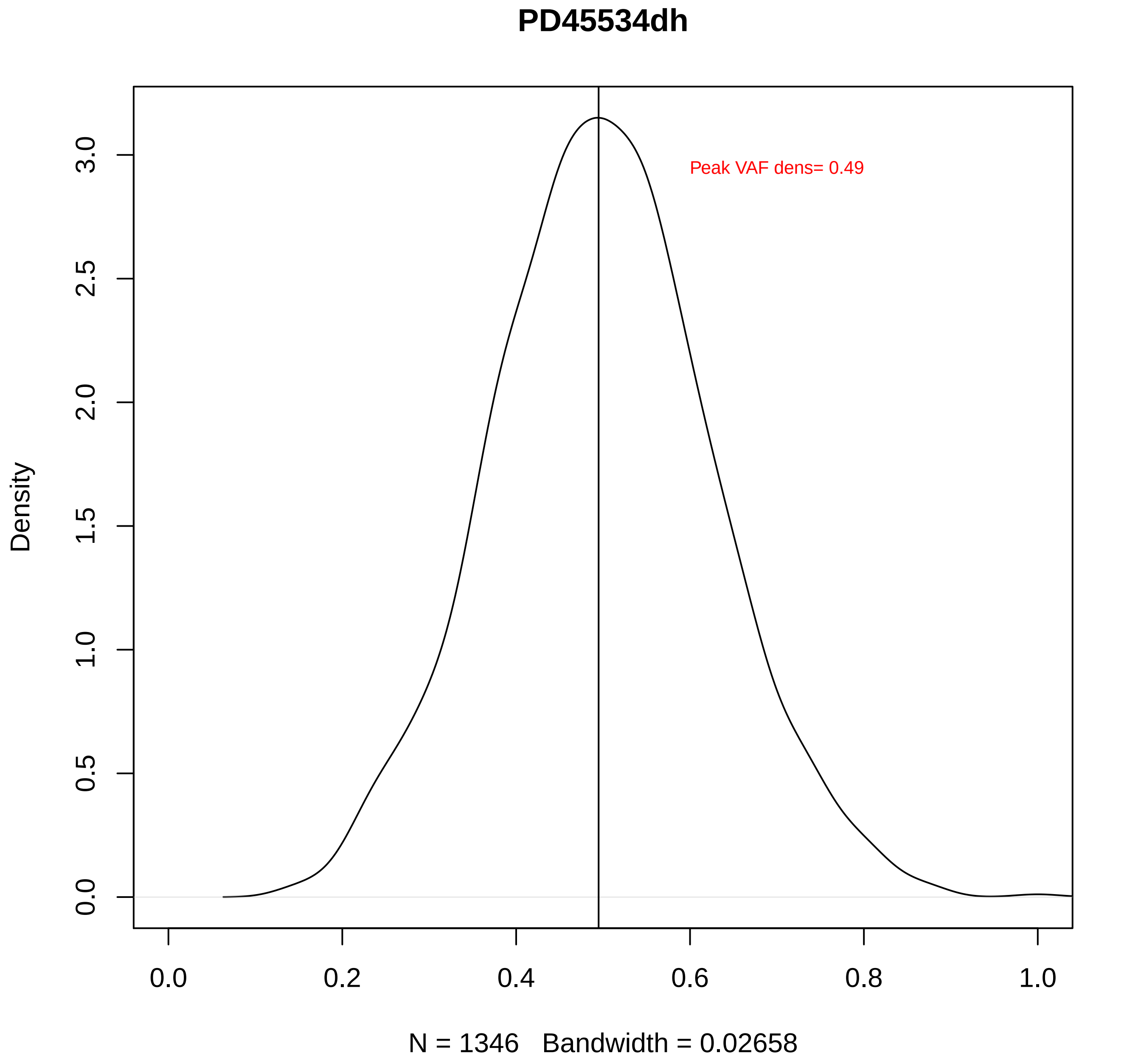

PD45534dh
Peak VAF dens= 0.49
Density
0.0
0.5
1.0
1.5
2.0
2.5
3.0
0.0
0.2
0.4
0.6
0.8
1.0
N = 1346 Bandwidth = 0.02658

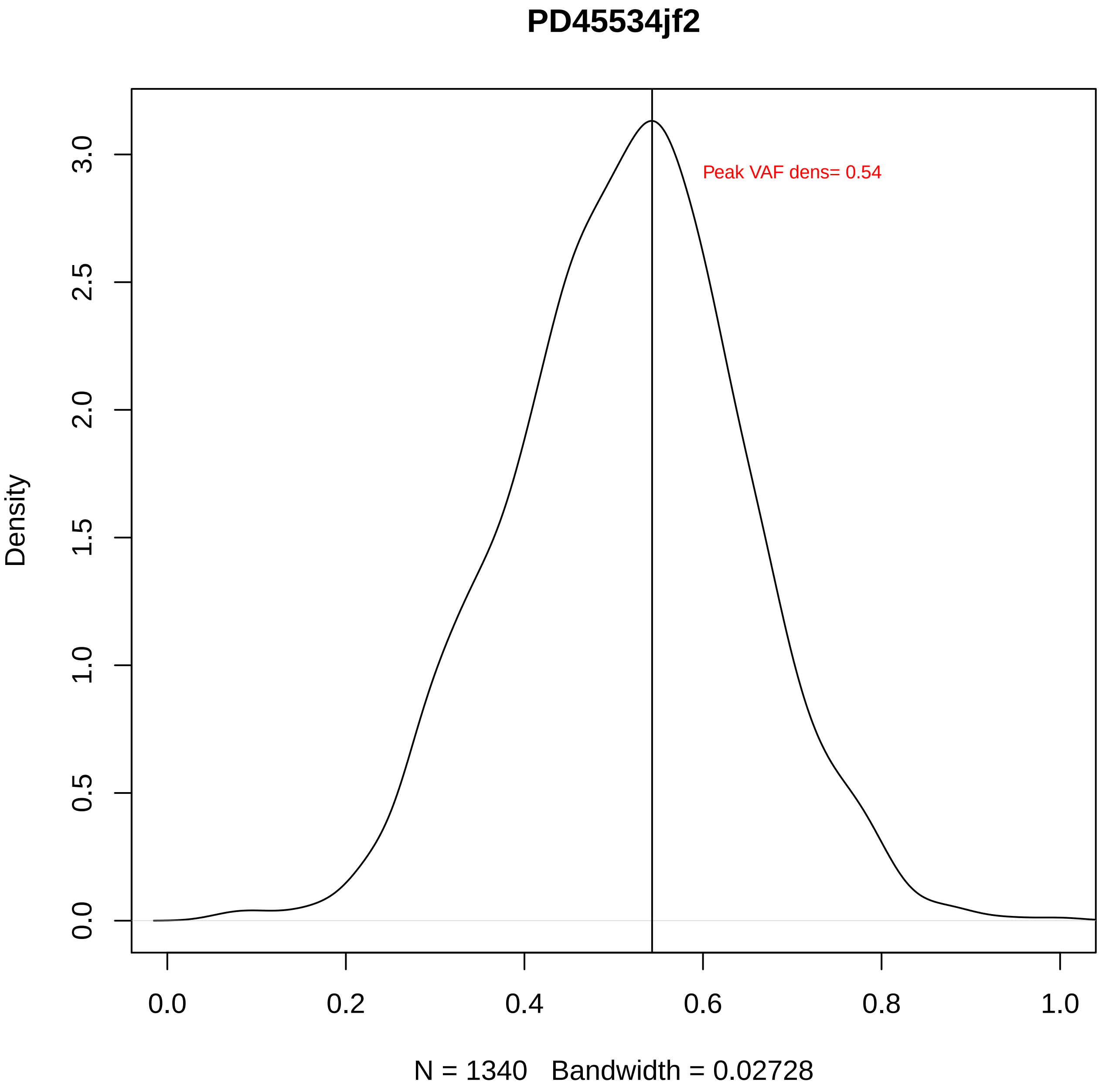
PD45534jf2
Peak VAF dens= 0.54
Density
0.0
0.5
1.0
1.5
2.0
2.5
3.0
0.0
0.2
0.4
0.6
0.8
1.0
N = 1340   Bandwidth = 0.02728

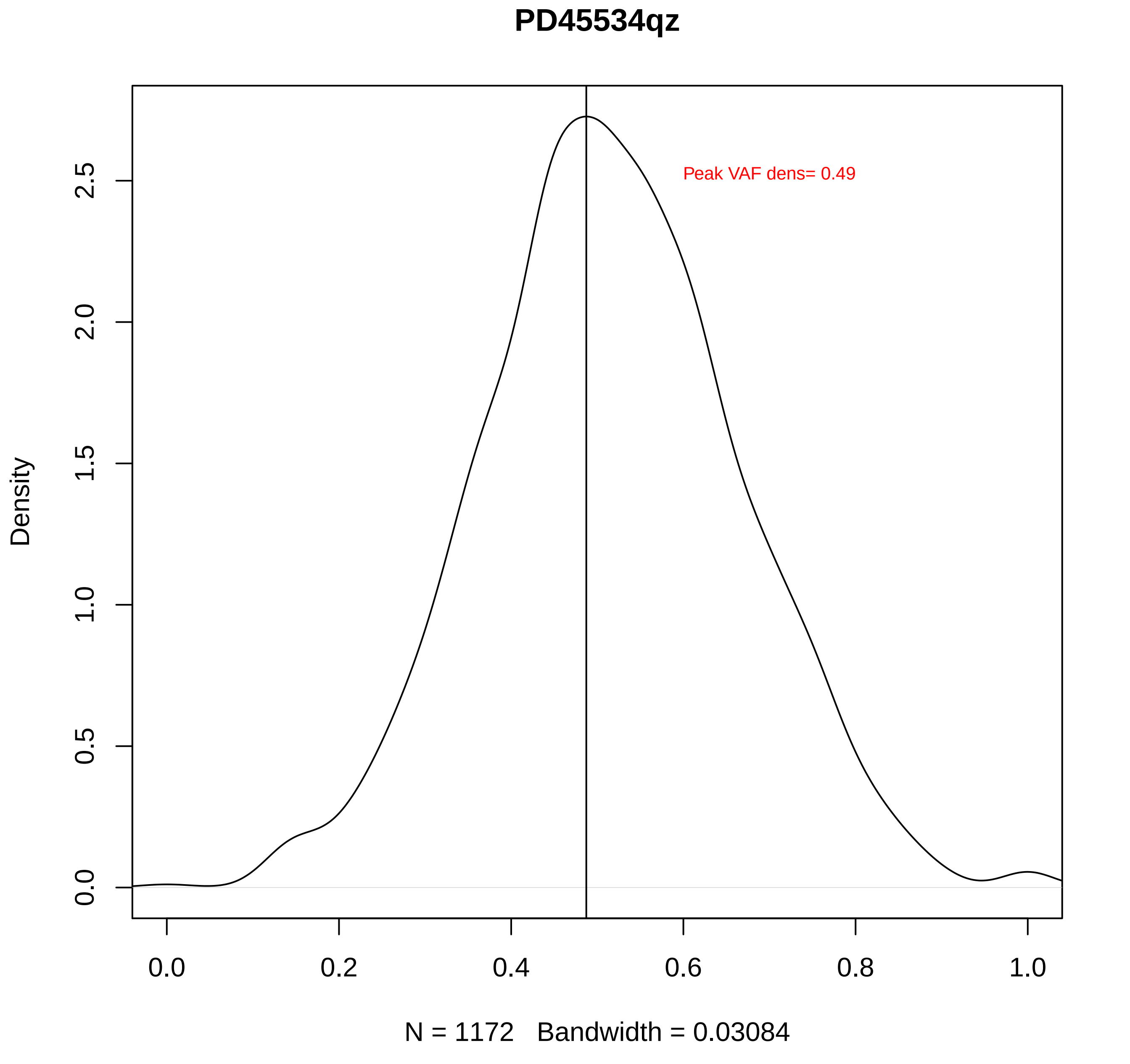

PD45534qz
Peak VAF dens= 0.49
Density
0.0
0.5
1.0
1.5
2.0
2.5
0.0
0.2
0.4
0.6
0.8
1.0
N = 1172 Bandwidth = 0.03084

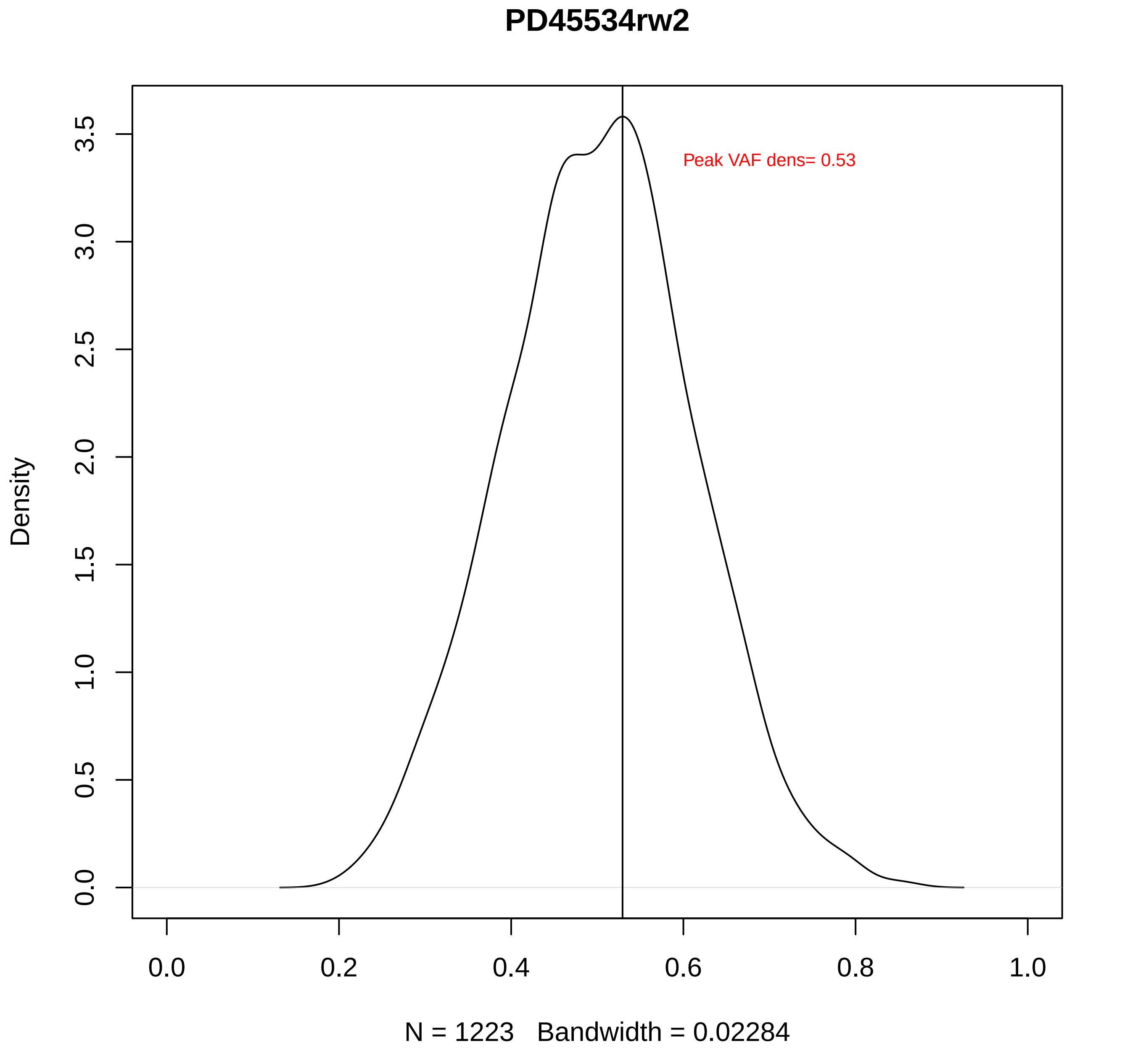
PD45534rw2
Peak VAF dens= 0.53
Density
0.0
0.5
1.0
1.5
2.0
2.5
3.0
3.5
0.0
0.2
0.4
0.6
0.8
1.0
N = 1223   Bandwidth = 0.02284

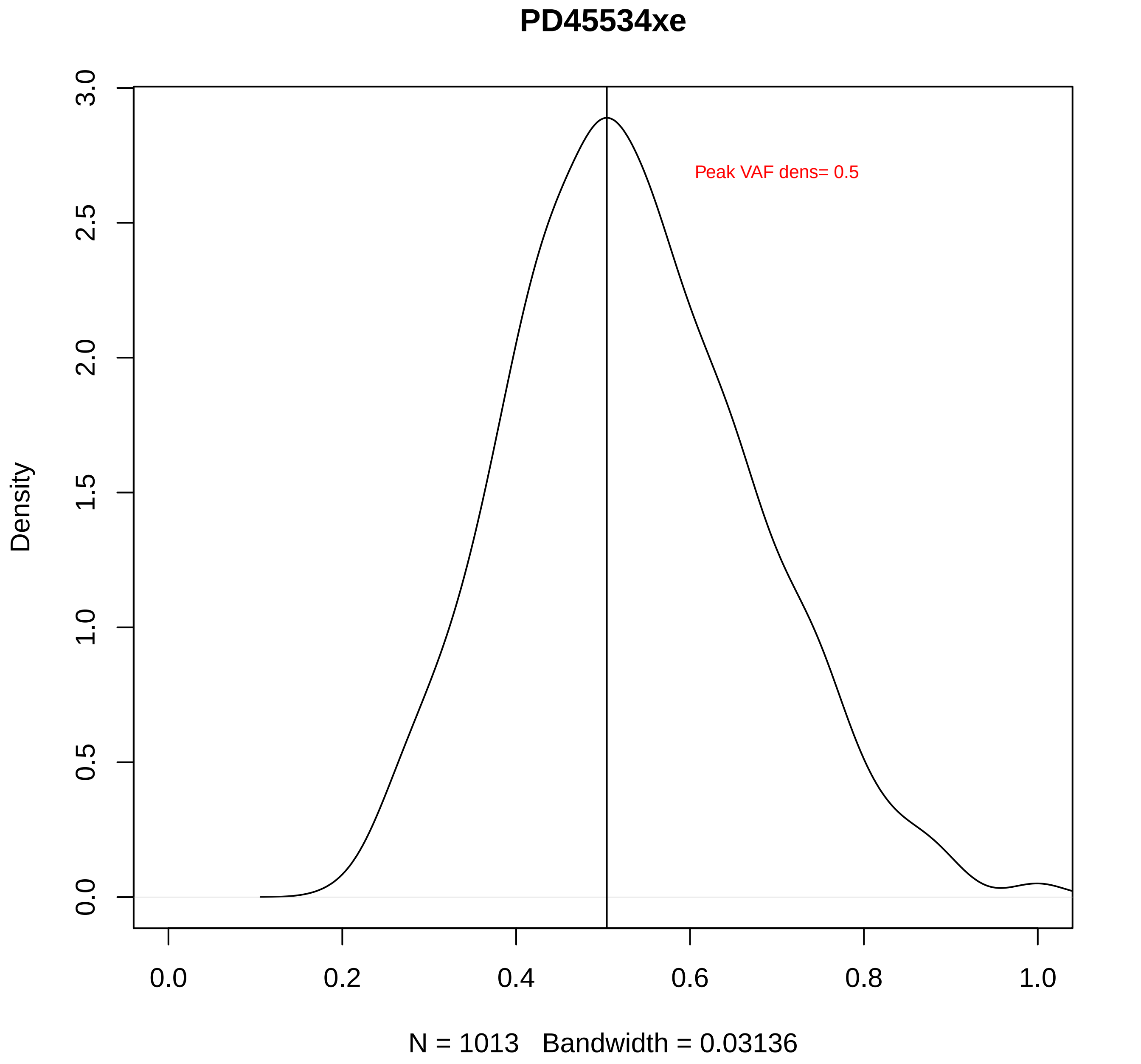
PD45534xe
Peak VAF dens= 0.5
Density
0.0
0.5
1.0
1.5
2.0
2.5
3.0
0.0
0.2
0.4
0.6
0.8
1.0
N = 1013 Bandwidth = 0.03136

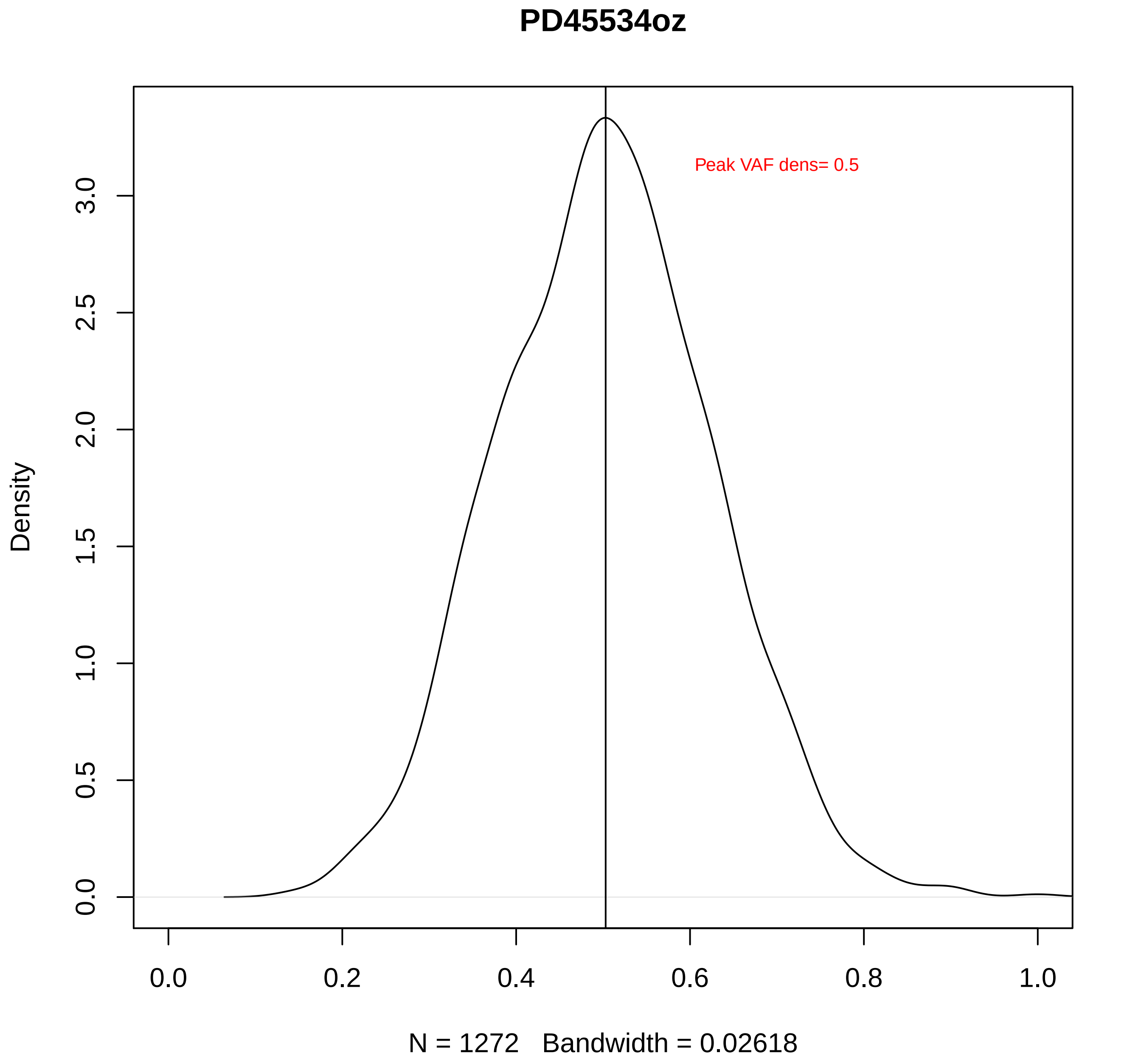

PD45534oz
Peak VAF dens= 0.5
Density
0.0
0.5
1.0
1.5
2.0
2.5
3.0
0.0
0.2
0.4
0.6
0.8
1.0
N = 1272   Bandwidth = 0.02618

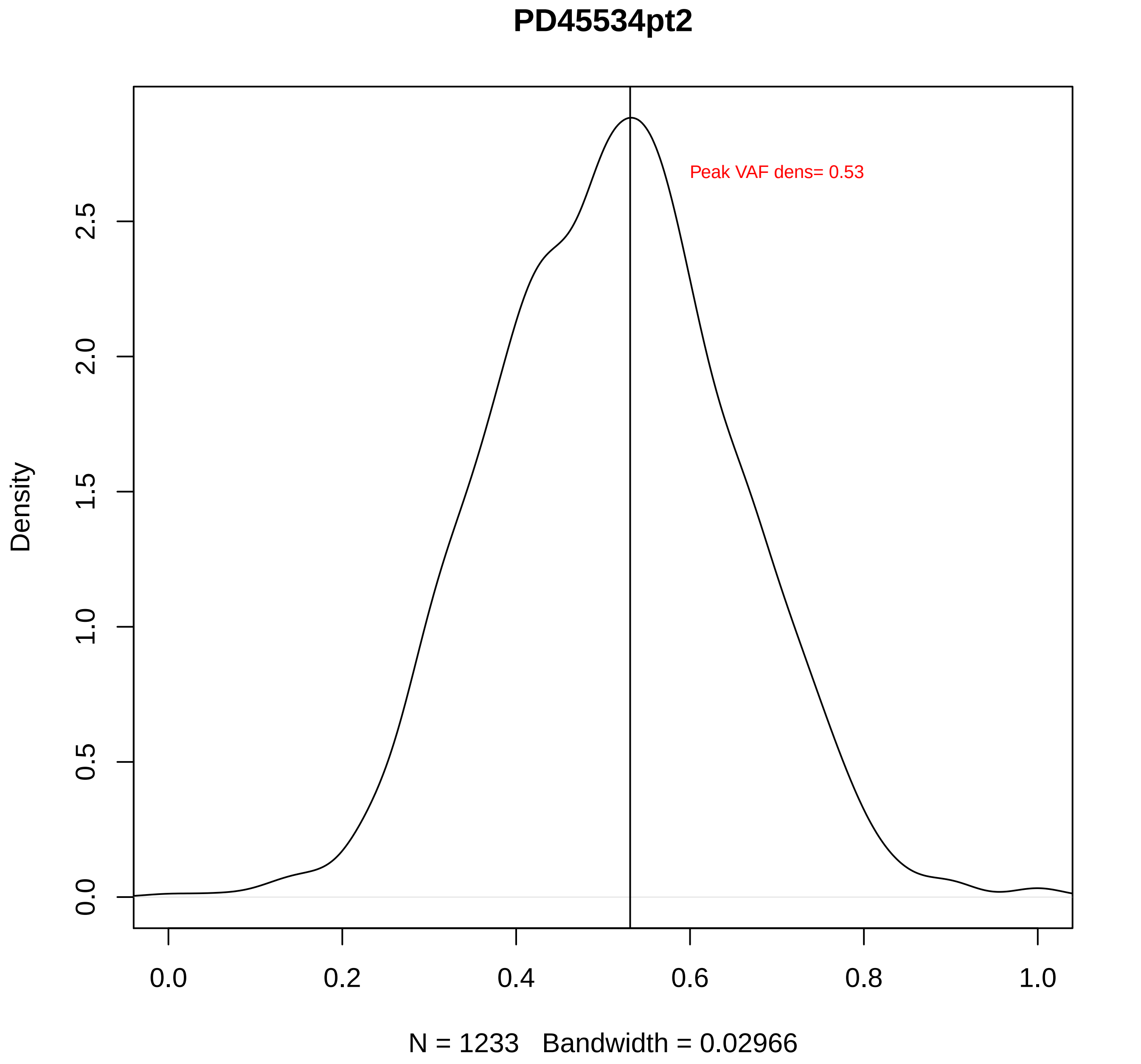
PD45534pt2
Peak VAF dens= 0.53
Density
0.0
0.5
1.0
1.5
2.0
2.5
0.0
0.2
0.4
0.6
0.8
1.0
N = 1233   Bandwidth = 0.02966

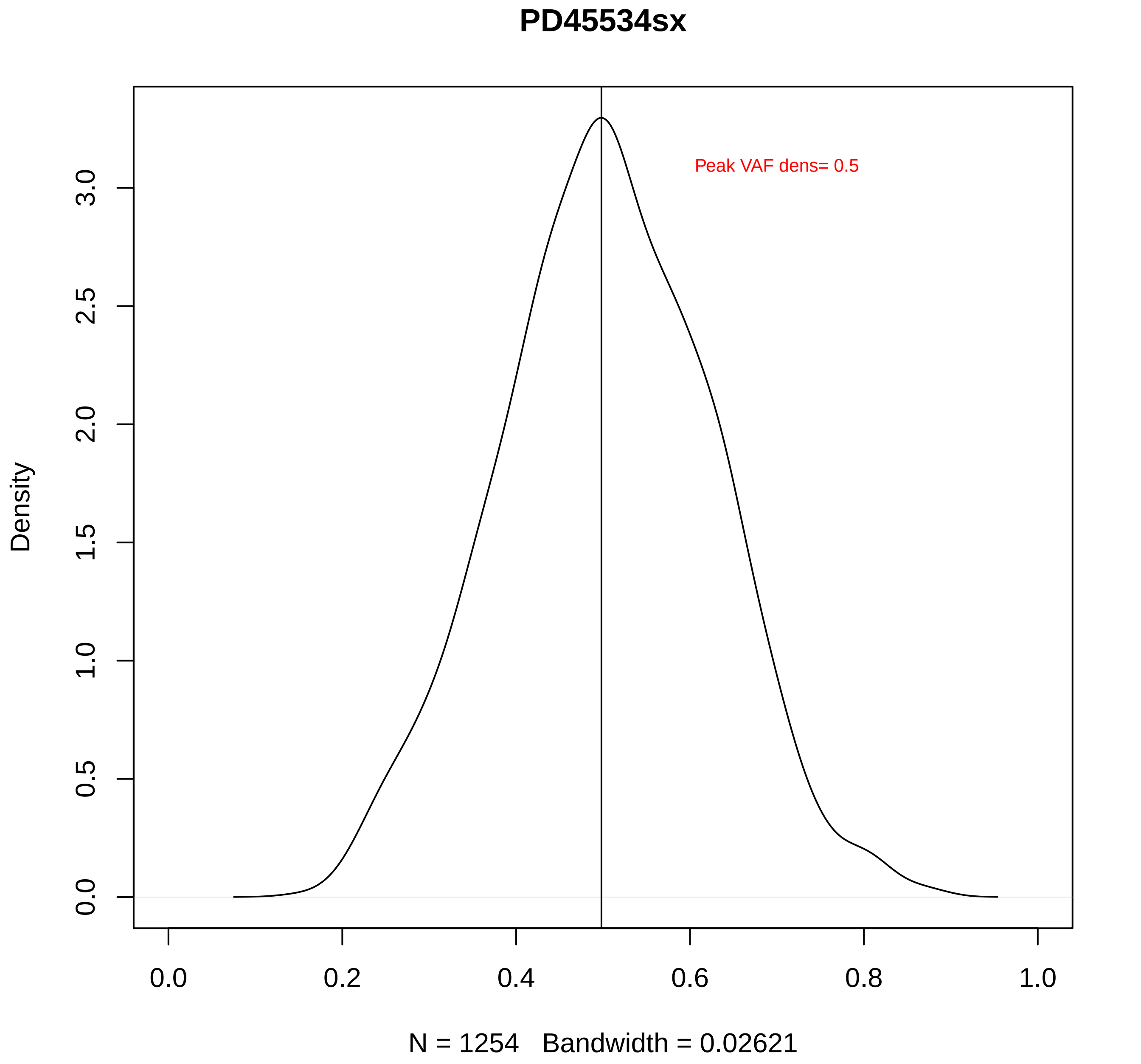
PD45534sx
Peak VAF dens= 0.5
Density
0.0
0.5
1.0
1.5
2.0
2.5
3.0
0.0
0.2
0.4
0.6
0.8
1.0
N = 1254 Bandwidth = 0.02621

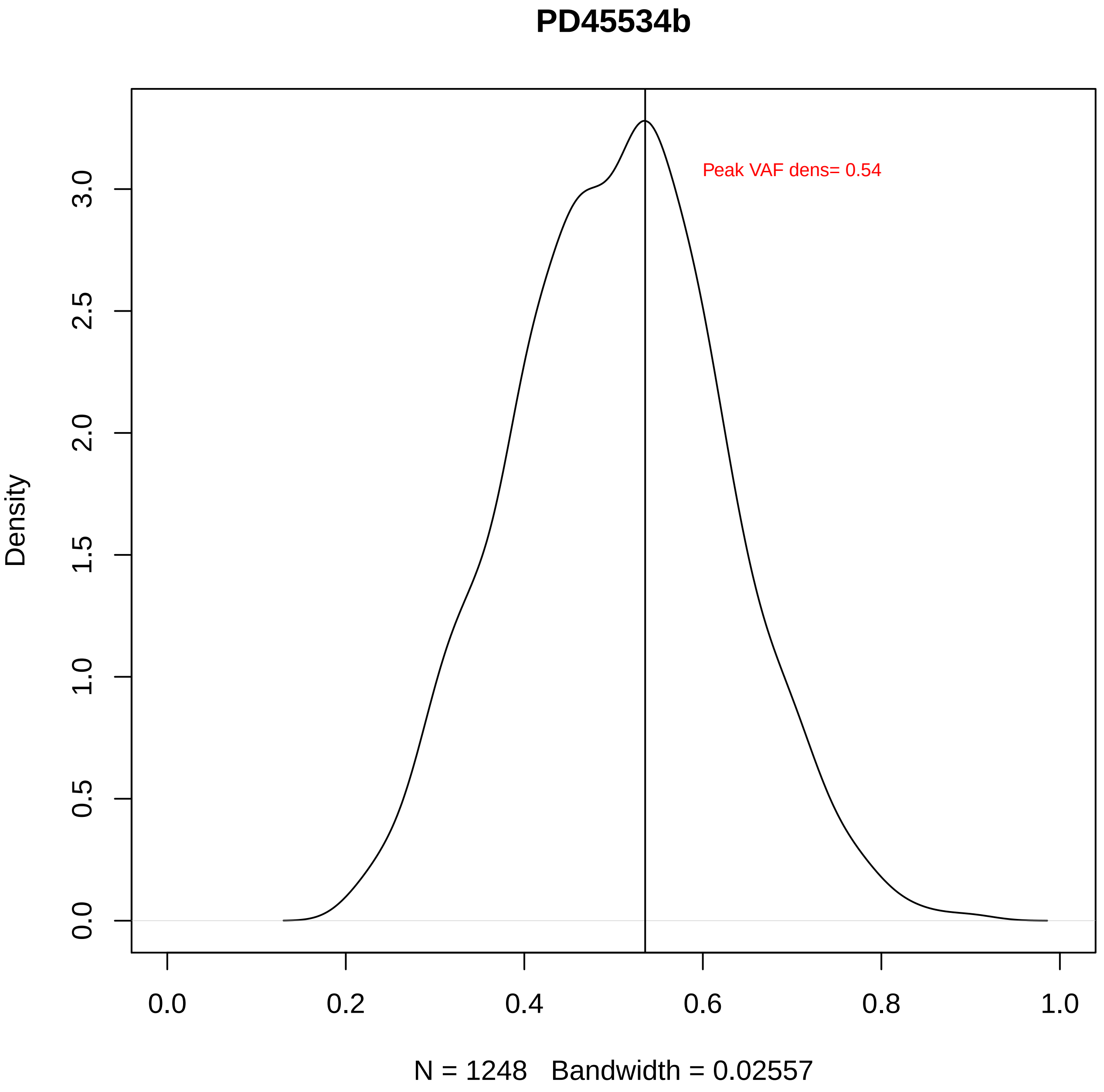

PD45534b
Peak VAF dens= 0.54
Density
0.0
0.5
1.0
1.5
2.0
2.5
3.0
0.0
0.2
0.4
0.6
0.8
1.0
N = 1248   Bandwidth = 0.02557

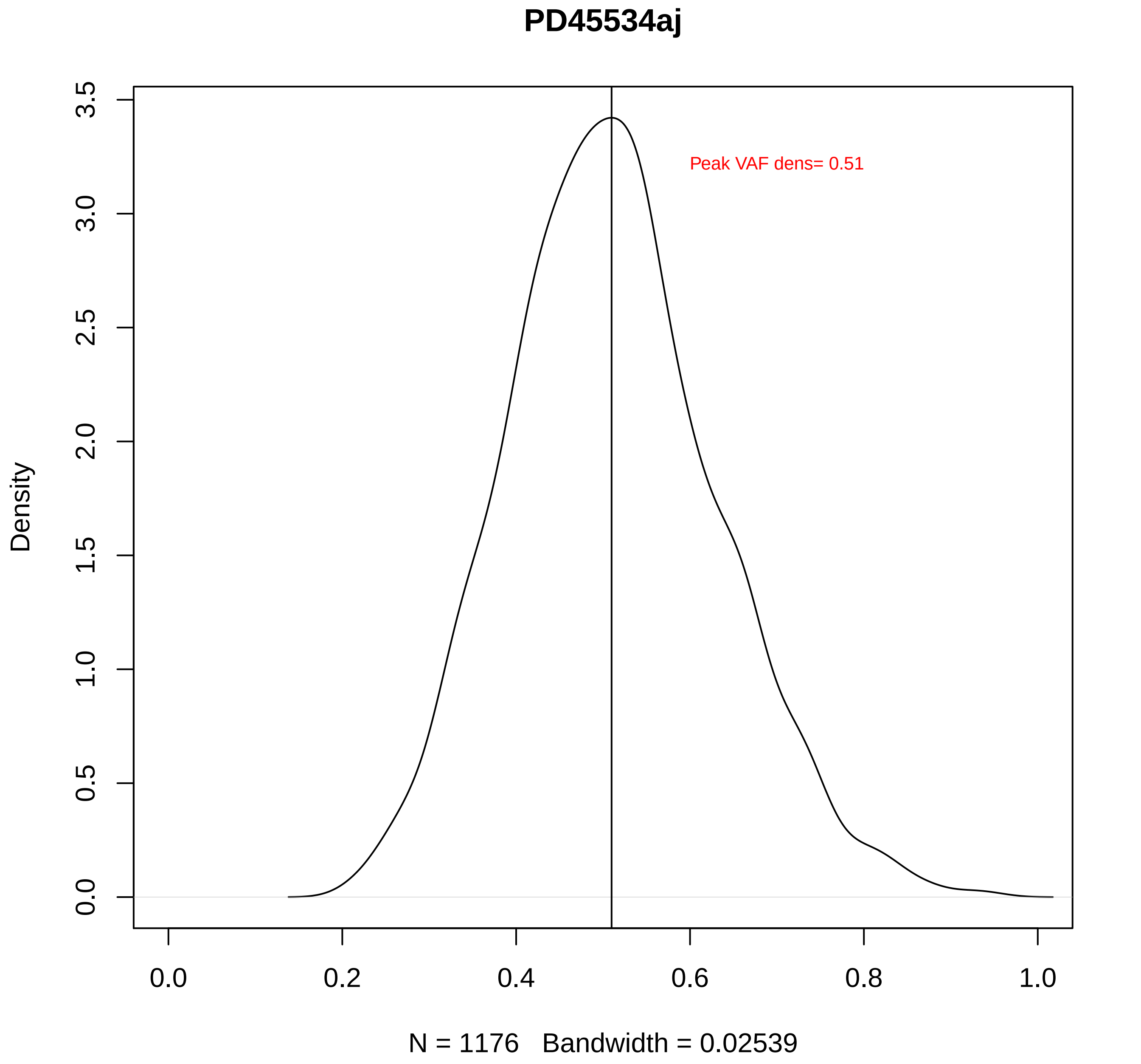
PD45534aj
Peak VAF dens= 0.51
Density
0.0
0.5
1.0
1.5
2.0
2.5
3.0
3.5
0.0
0.2
0.4
0.6
0.8
1.0
N = 1176 Bandwidth = 0.02539

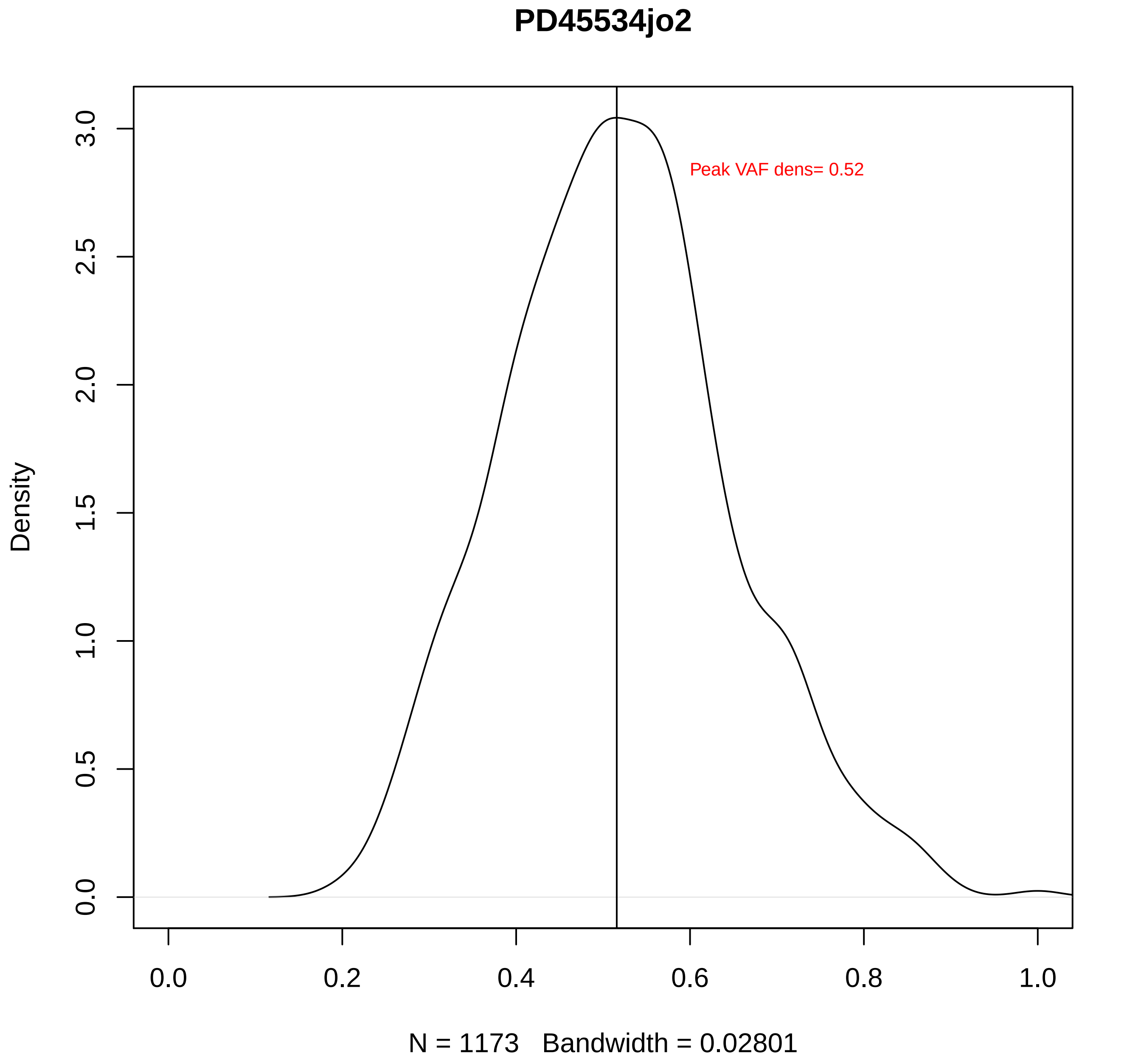
PD45534jo2
Peak VAF dens= 0.52
Density
0.0
0.5
1.0
1.5
2.0
2.5
3.0
0.0
0.2
0.4
0.6
0.8
1.0
N = 1173 Bandwidth = 0.02801

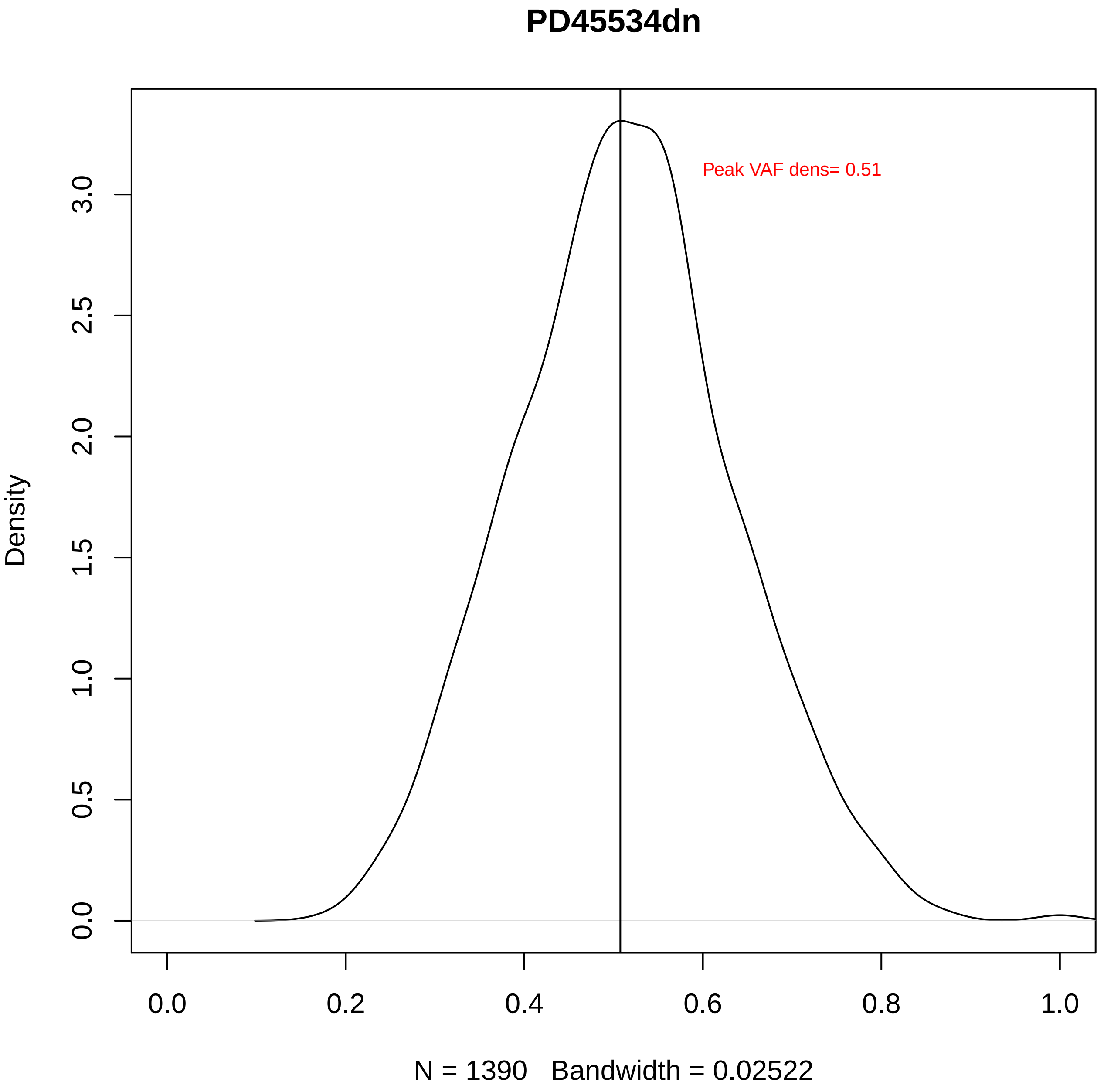

PD45534dn
Peak VAF dens= 0.51
Density
0.0
0.5
1.0
1.5
2.0
2.5
3.0
0.0
0.2
0.4
0.6
0.8
1.0
N = 1390   Bandwidth = 0.02522

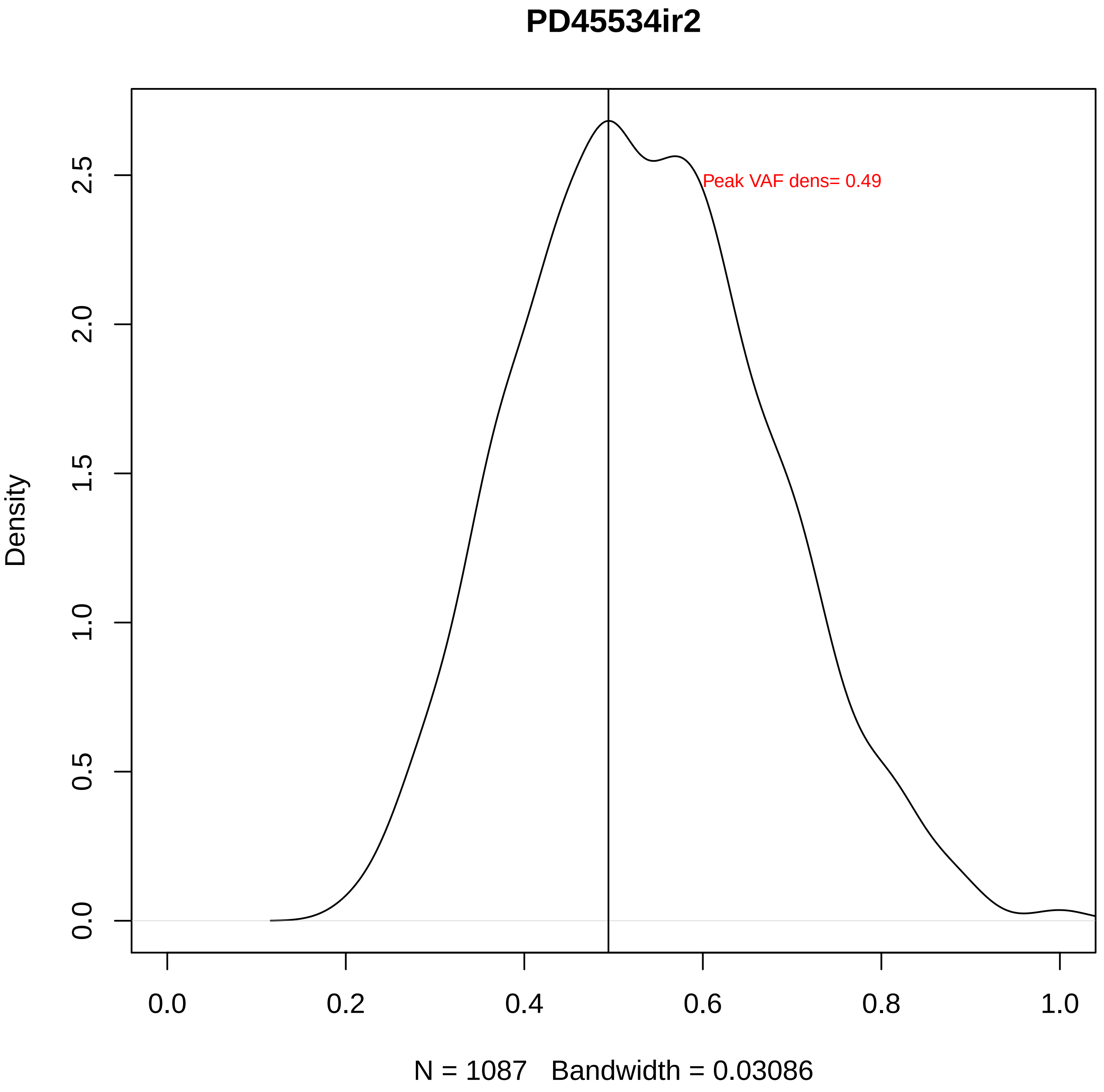
PD45534ir2
Peak VAF dens= 0.49
Density
0.0
0.5
1.0
1.5
2.0
2.5
0.0
0.2
0.4
0.6
0.8
1.0
N = 1087 Bandwidth = 0.03086

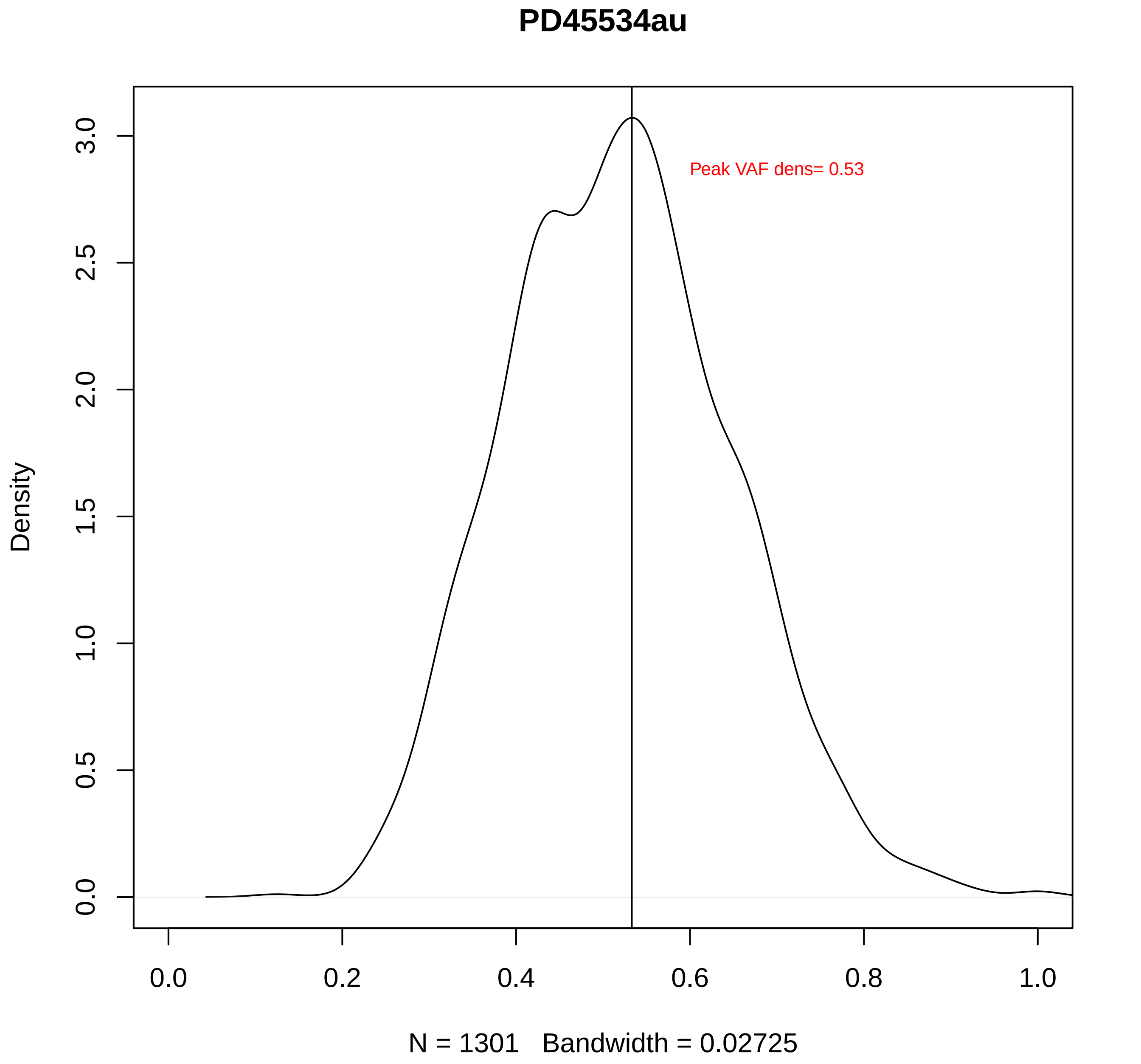
PD45534au
Peak VAF dens= 0.53
Density
0.0
0.5
1.0
1.5
2.0
2.5
3.0
0.0
0.2
0.4
0.6
0.8
1.0
N = 1301 Bandwidth = 0.02725

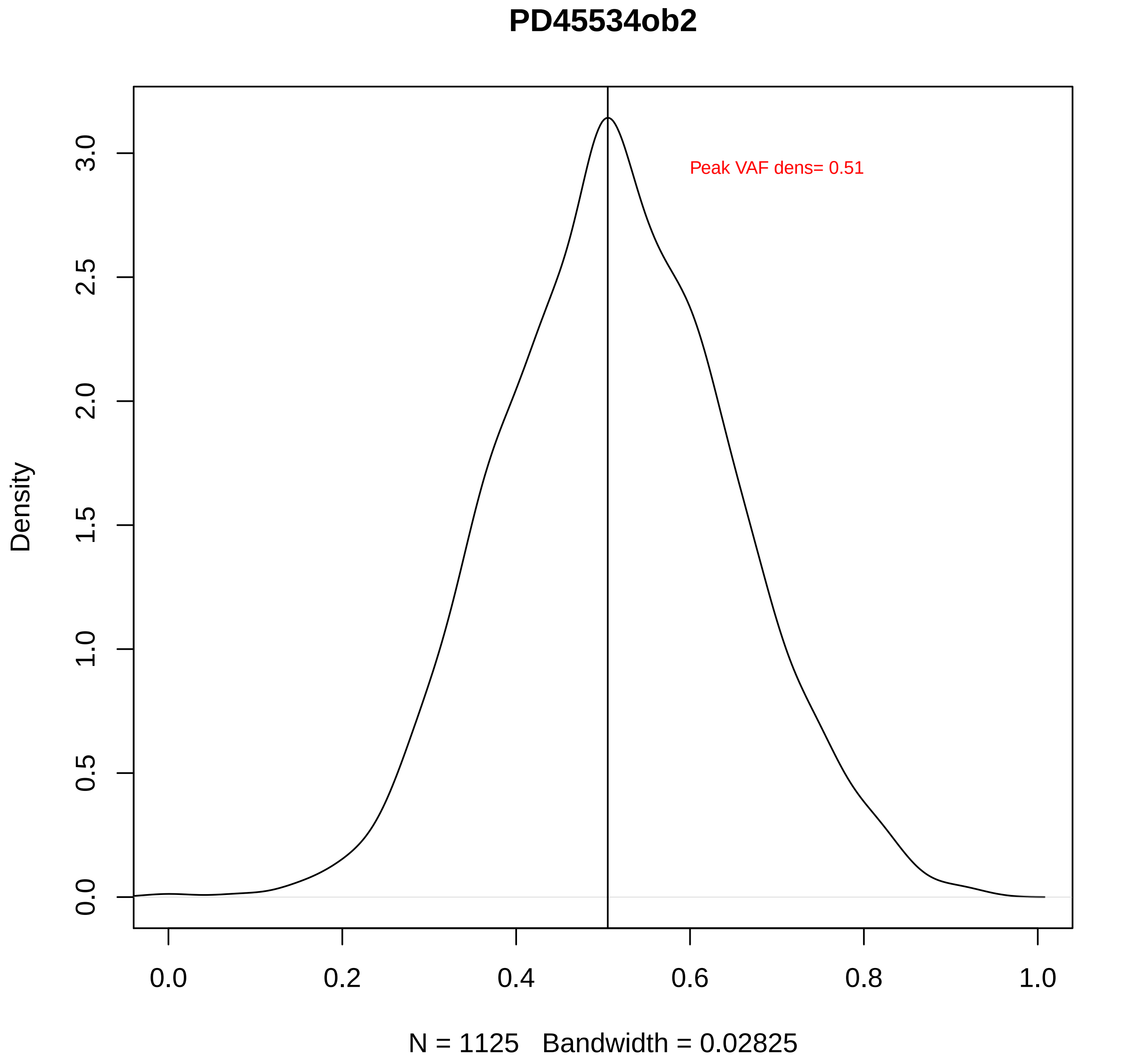
PD45534ob2
Peak VAF dens= 0.51
Density
0.0
0.5
1.0
1.5
2.0
2.5
3.0
0.0
0.2
0.4
0.6
0.8
1.0
N = 1125   Bandwidth = 0.02825

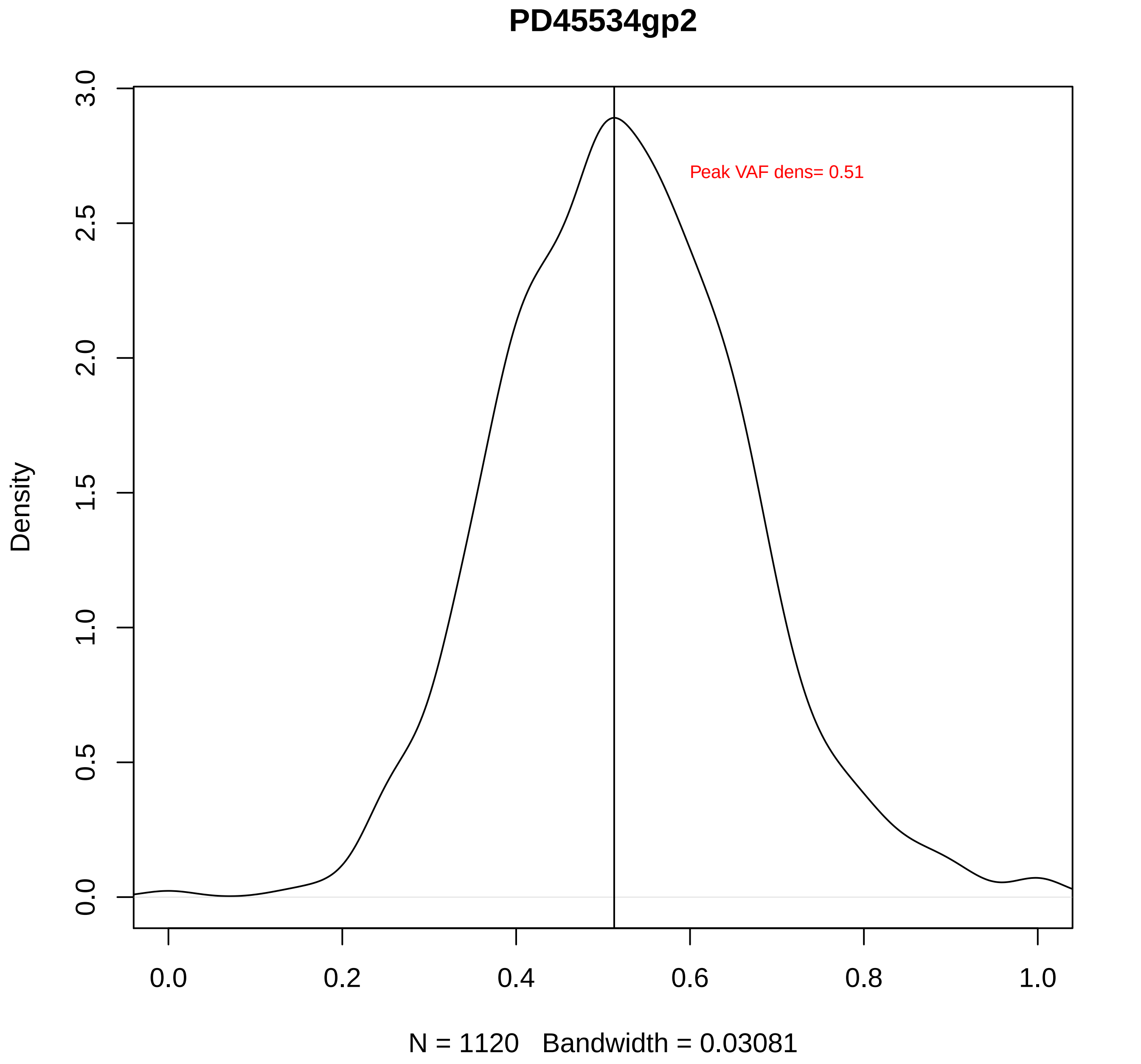
PD45534gp2
Peak VAF dens= 0.51
Density
0.0
0.5
1.0
1.5
2.0
2.5
3.0
0.0
0.2
0.4
0.6
0.8
1.0
N = 1120   Bandwidth = 0.03081

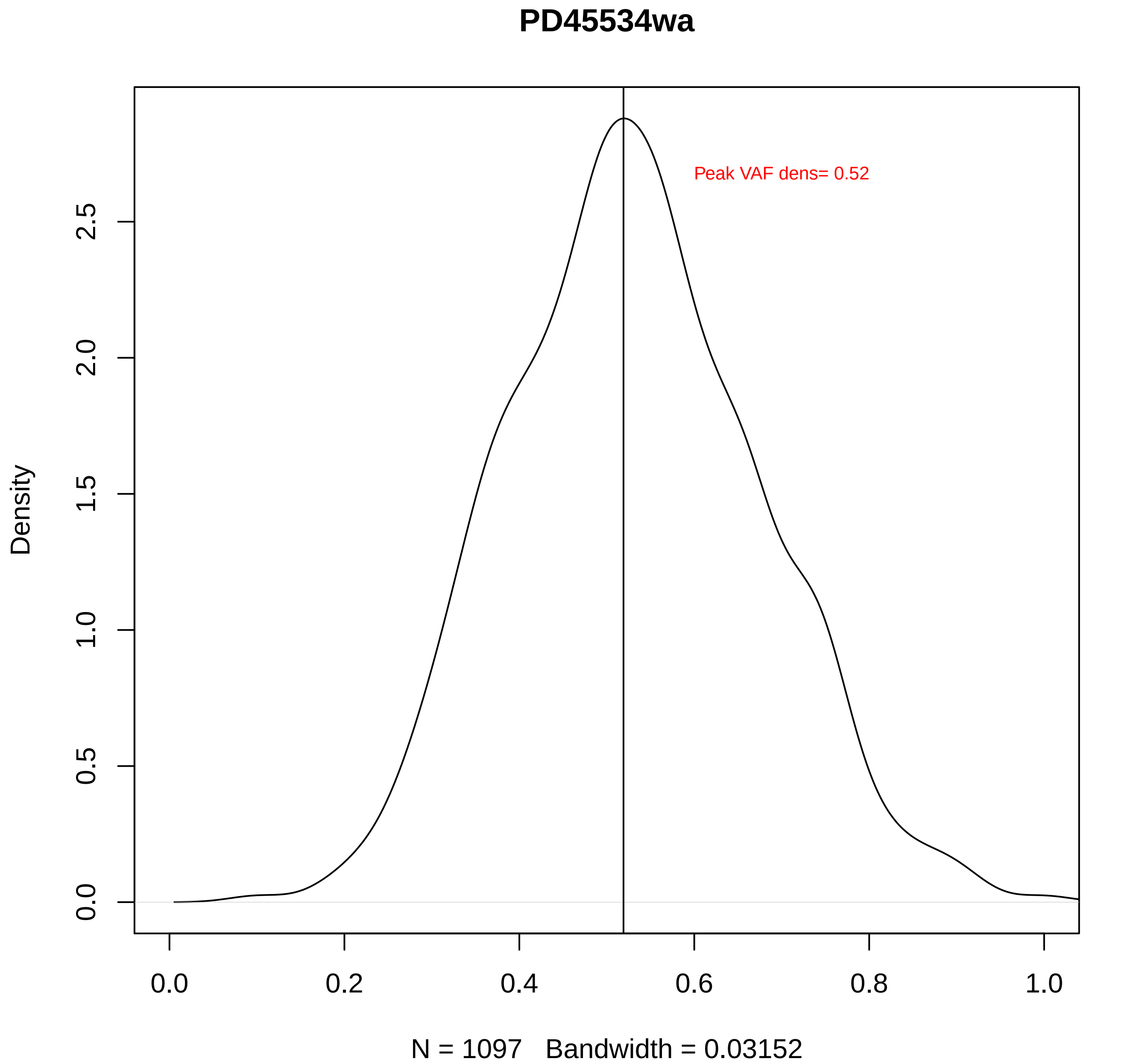

PD45534wa
Peak VAF dens= 0.52
Density
0.0
0.5
1.0
1.5
2.0
2.5
0.0
0.2
0.4
0.6
0.8
1.0
N = 1097 Bandwidth = 0.03152

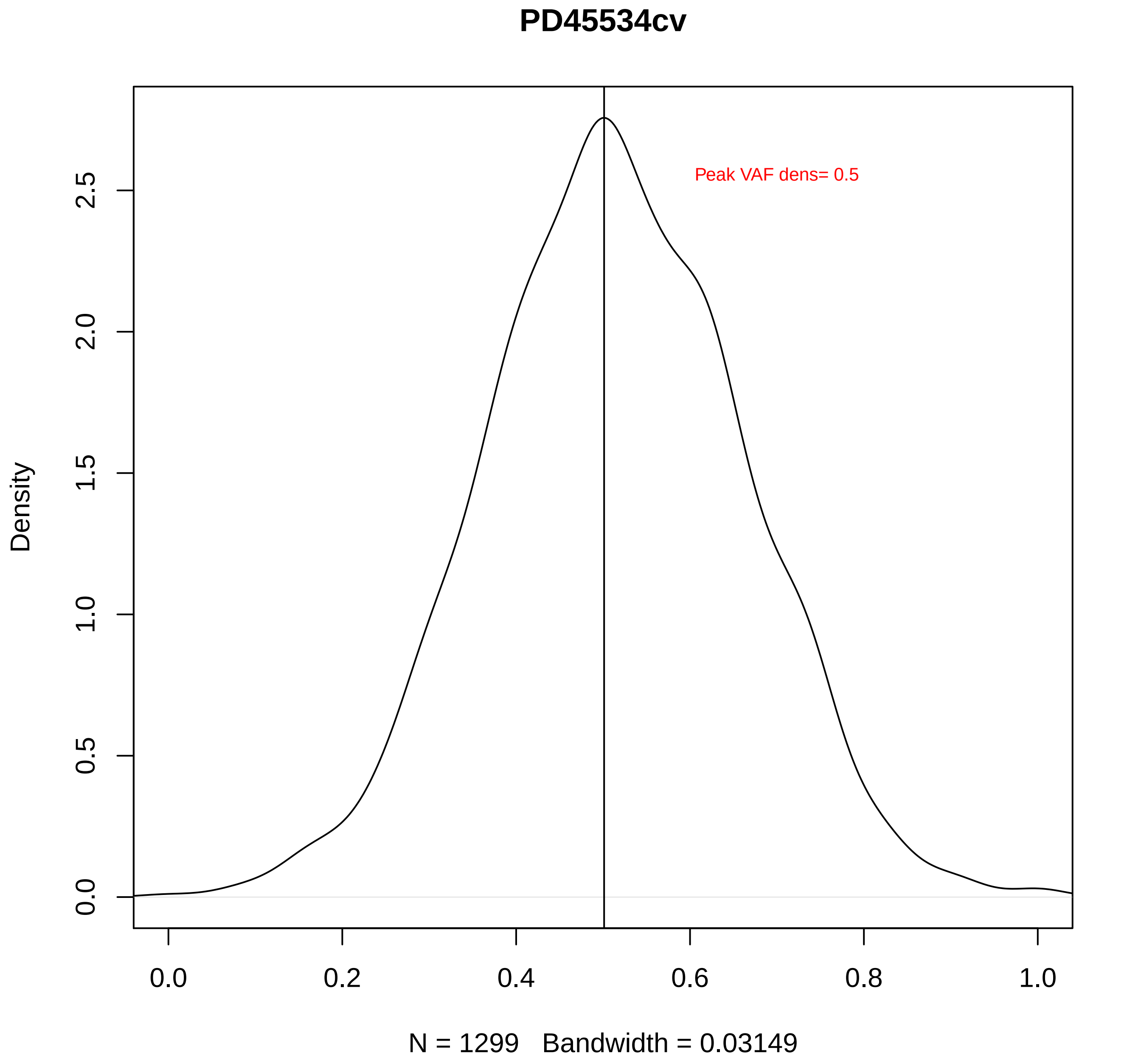
PD45534cv
Peak VAF dens= 0.5
Density
0.0
0.5
1.0
1.5
2.0
2.5
0.0
0.2
0.4
0.6
0.8
1.0
N = 1299   Bandwidth = 0.03149

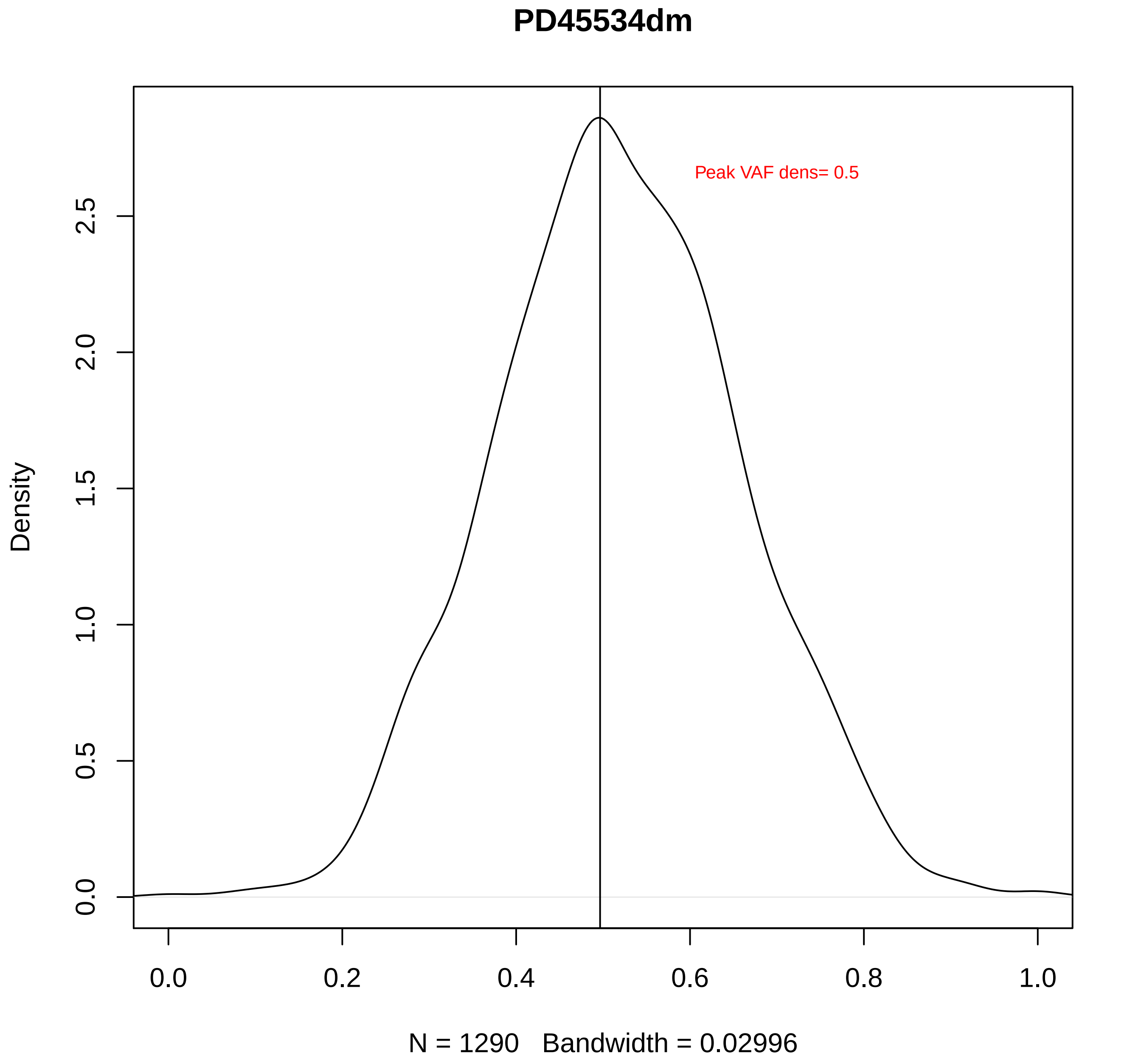
PD45534dm
Peak VAF dens= 0.5
Density
0.0
0.5
1.0
1.5
2.0
2.5
0.0
0.2
0.4
0.6
0.8
1.0
N = 1290   Bandwidth = 0.02996

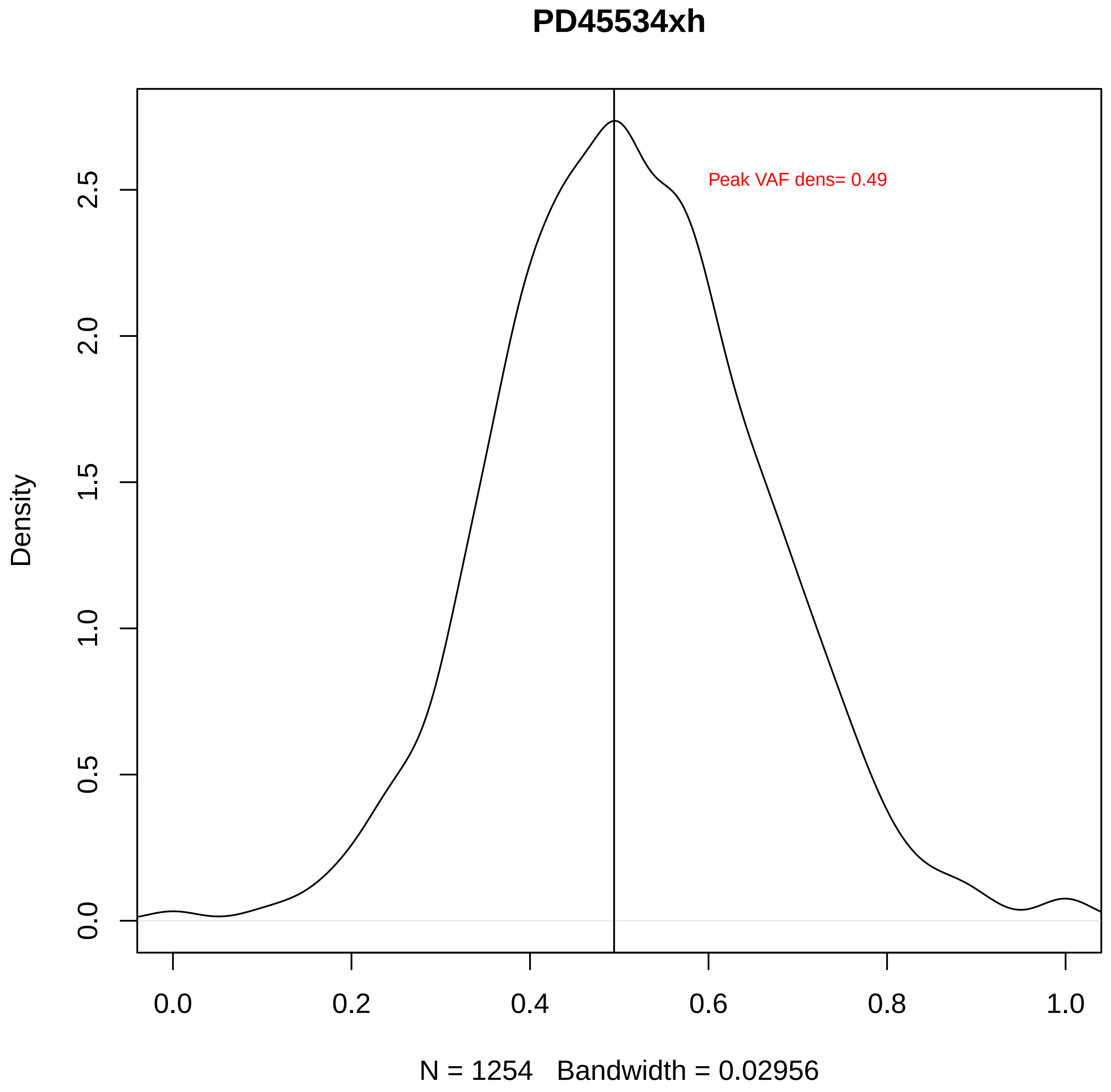

PD45534xh
Peak VAF dens= 0.49
Density
0.0
0.5
1.0
1.5
2.0
2.5
0.0
0.2
0.4
0.6
0.8
1.0
N = 1254 Bandwidth = 0.02956

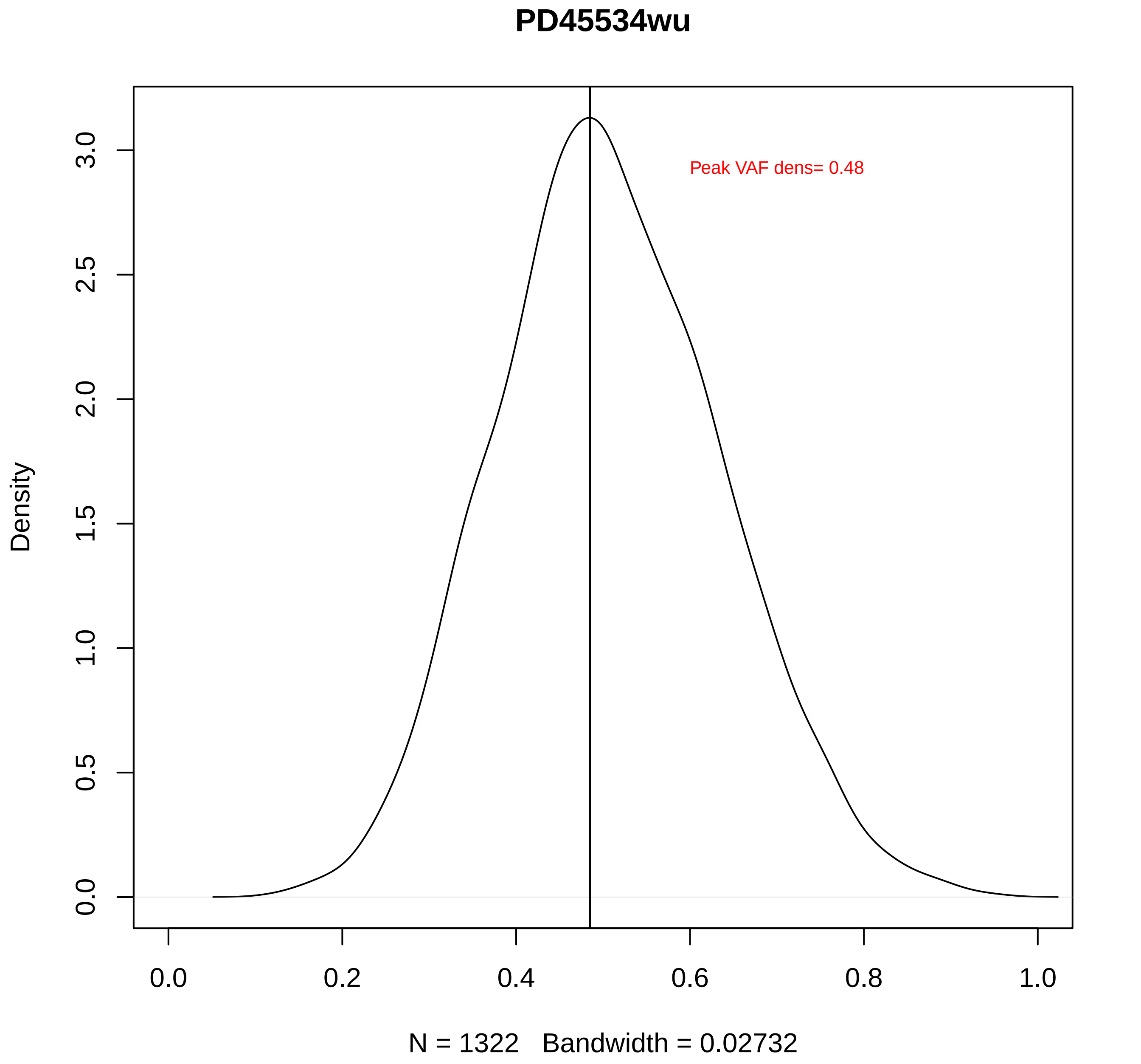
PD45534wu
Peak VAF dens= 0.48
Density
3.0
2.5
2.0
1.5
1.0
0.5
0.0
0.0
0.2
0.4
0.6
0.8
1.0
N = 1322   Bandwidth = 0.02732

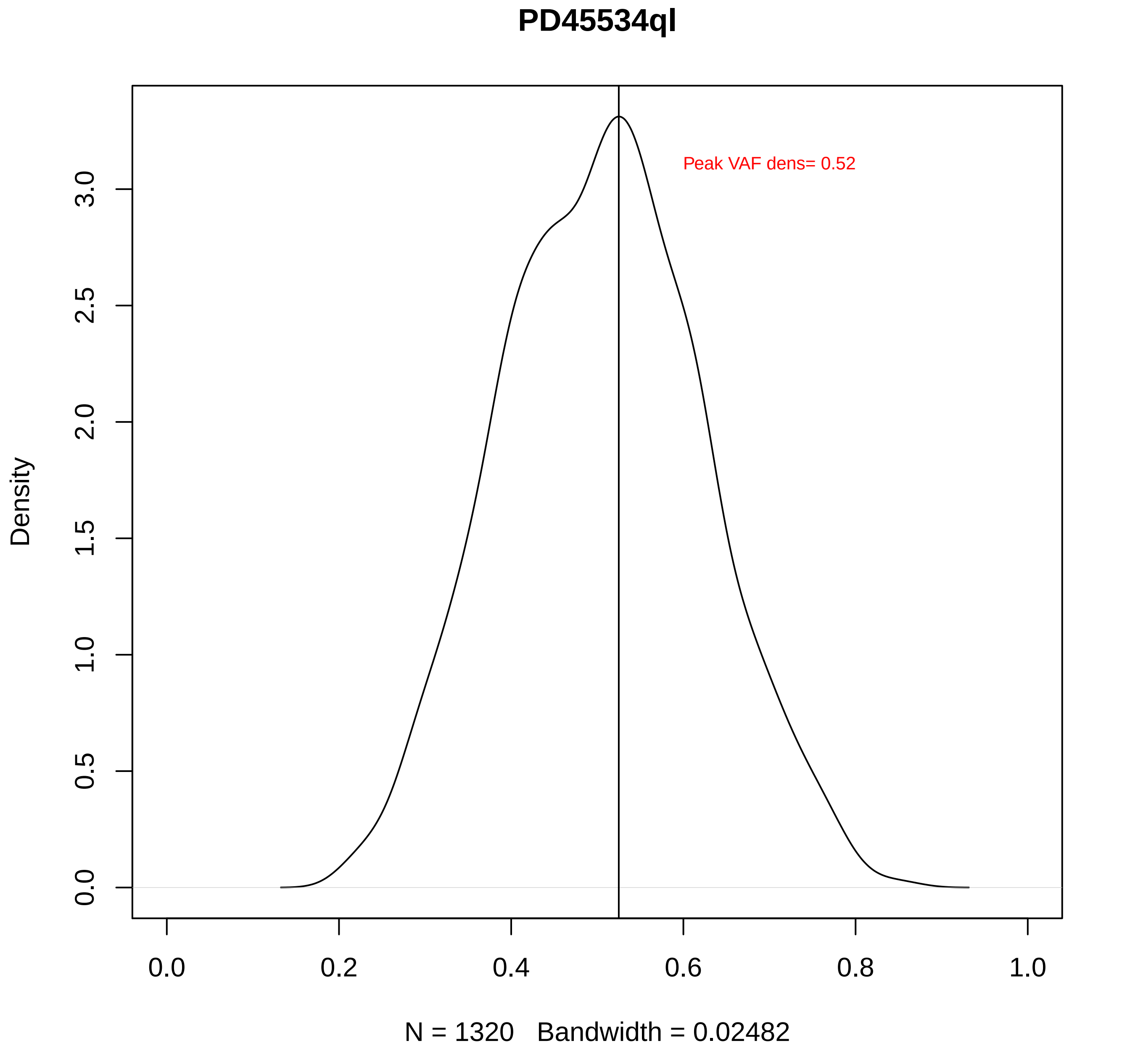
PD45534ql
Peak VAF dens= 0.52
Density
0.0
0.5
1.0
1.5
2.0
2.5
3.0
0.0
0.2
0.4
0.6
0.8
1.0
N = 1320   Bandwidth = 0.02482

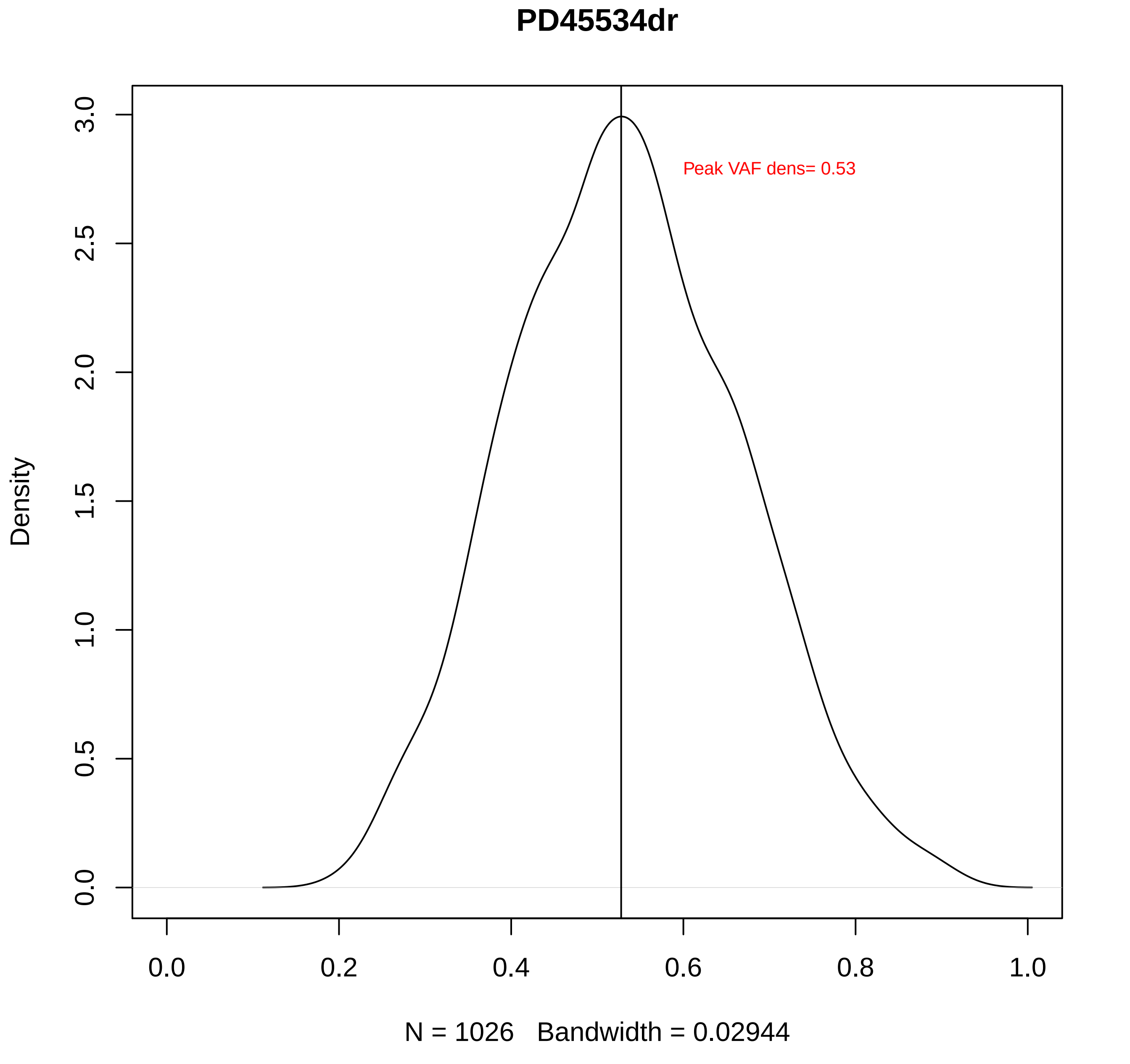

PD45534dr
Peak VAF dens= 0.53
Density
3.0
2.5
2.0
1.5
1.0
0.5
0.0
0.0
0.2
0.4
0.6
0.8
1.0
N = 1026 Bandwidth = 0.02944

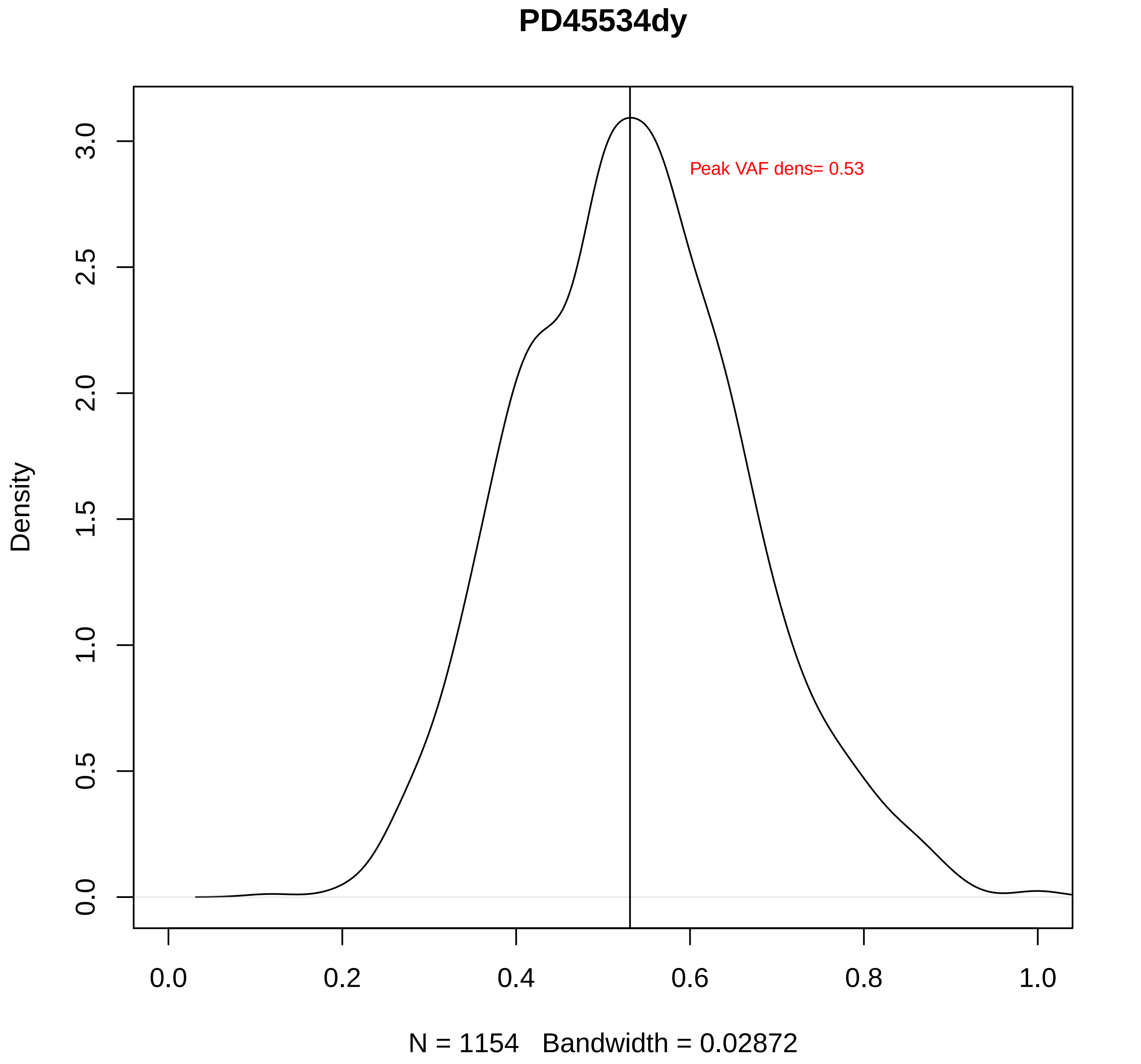
PD45534dy
Peak VAF dens= 0.53
Density
0.0
0.5
1.0
1.5
2.0
2.5
3.0
0.0
0.2
0.4
0.6
0.8
1.0
N = 1154   Bandwidth = 0.02872

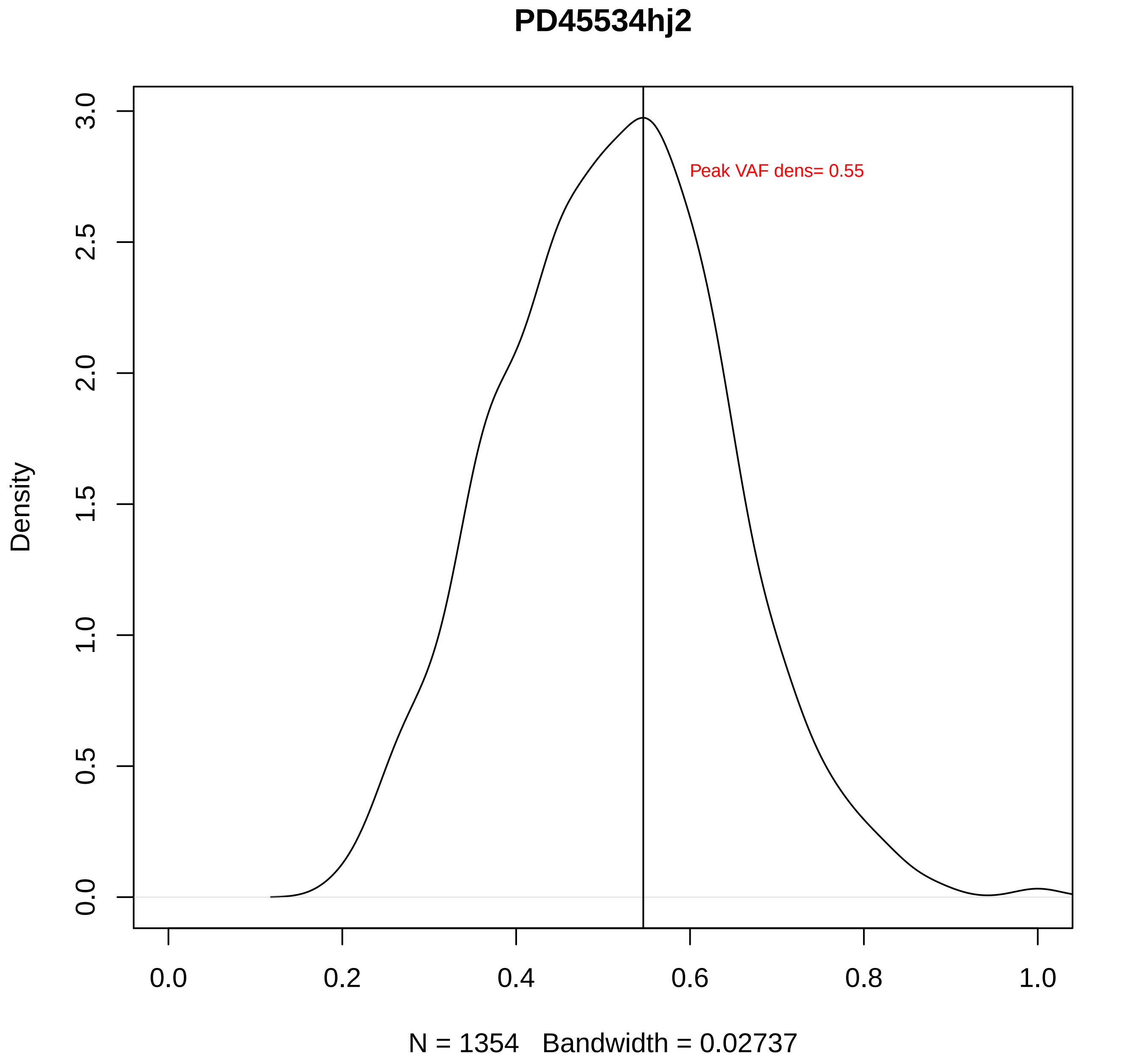

PD45534hj2
Peak VAF dens= 0.55
Density
3.0
2.5
2.0
1.5
1.0
0.5
0.0
0.0
0.2
0.4
0.6
0.8
1.0
N = 1354 Bandwidth = 0.02737

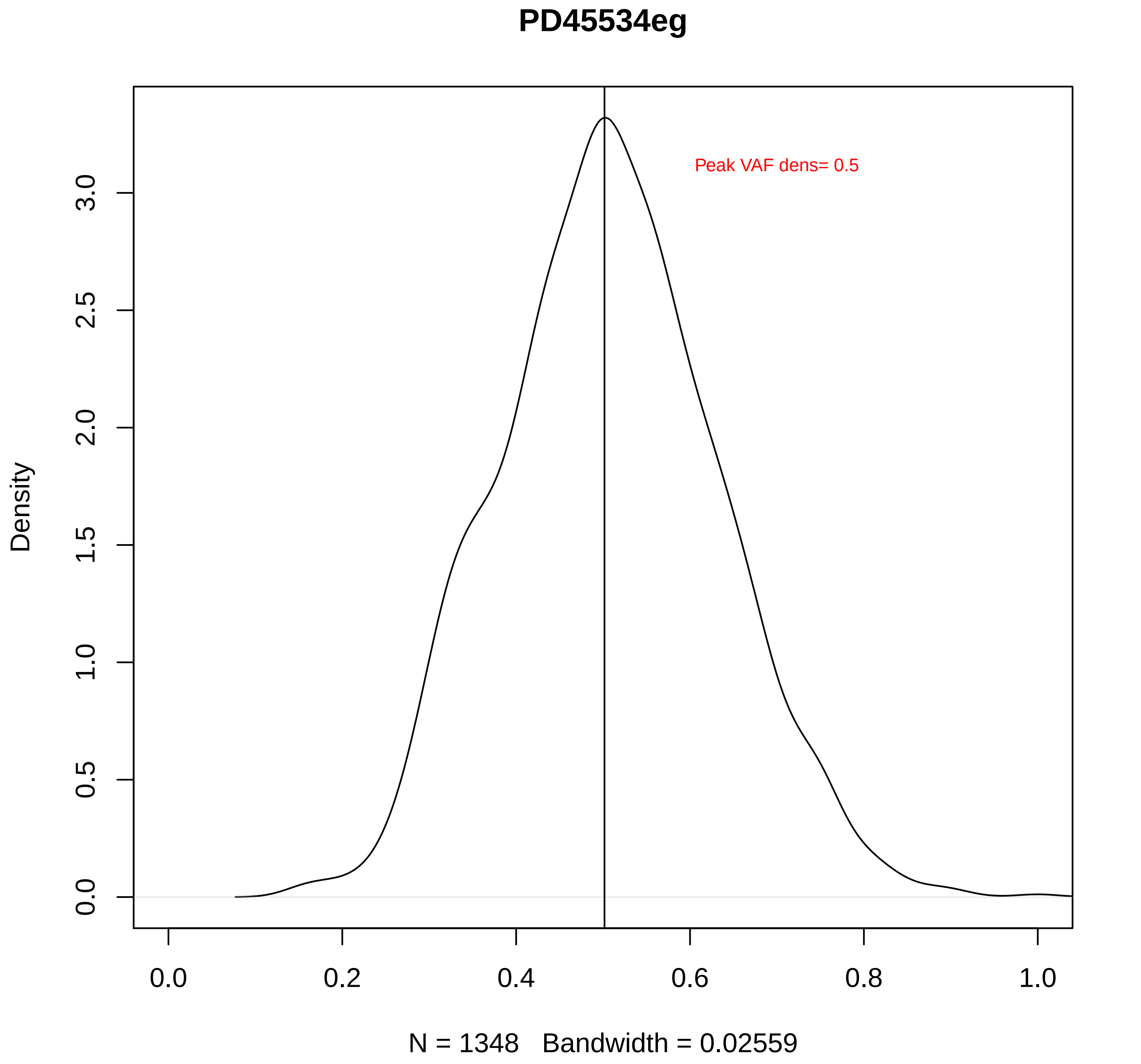
PD45534eg
Peak VAF dens= 0.5
Density
0.0
0.5
1.0
1.5
2.0
2.5
3.0
0.0
0.2
0.4
0.6
0.8
1.0
N = 1348 Bandwidth = 0.02559

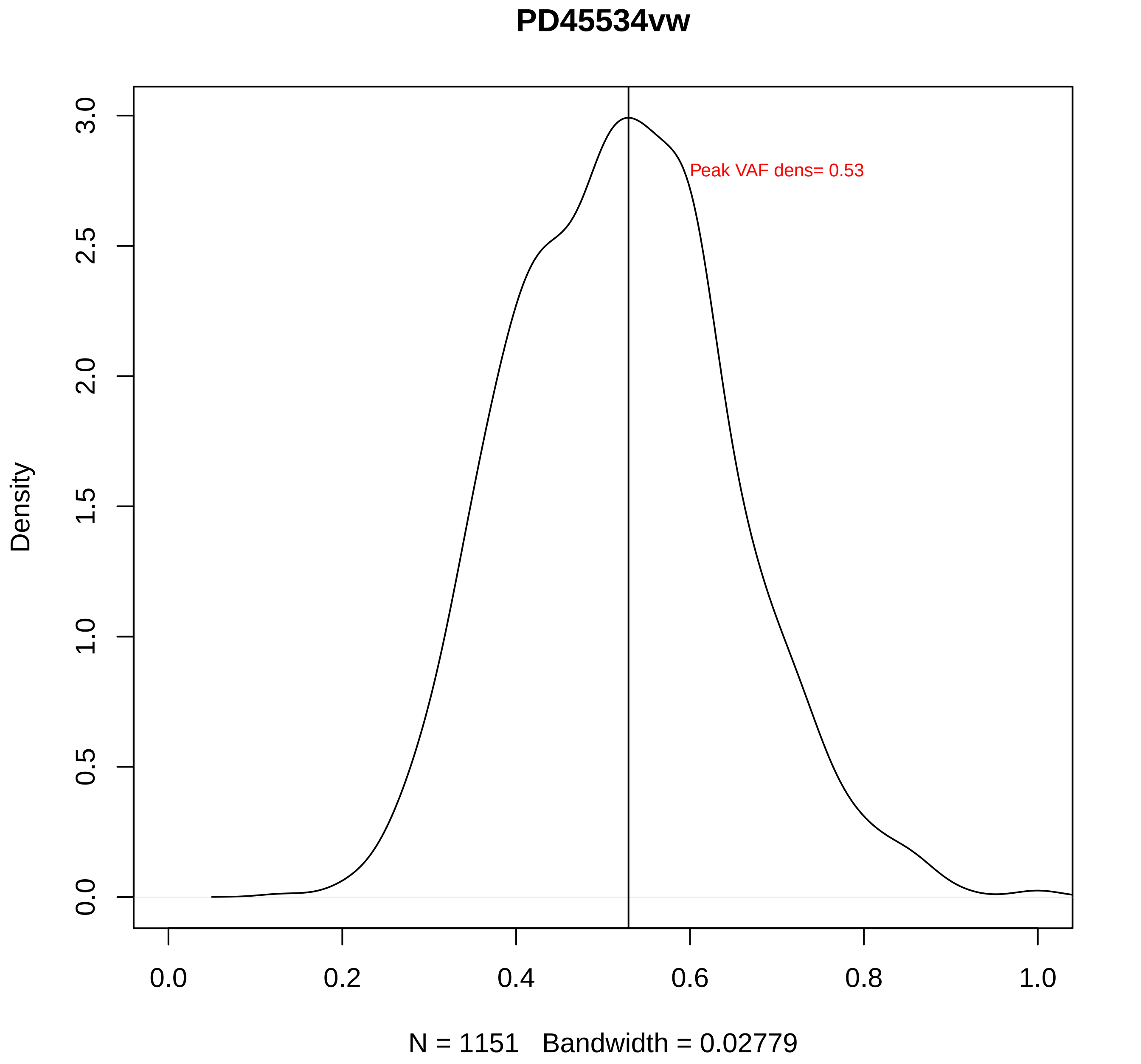

PD45534vw
Peak VAF dens= 0.53
Density
0.0
0.5
1.0
1.5
2.0
2.5
3.0
0.0
0.2
0.4
0.6
0.8
1.0
N = 1151 Bandwidth = 0.02779

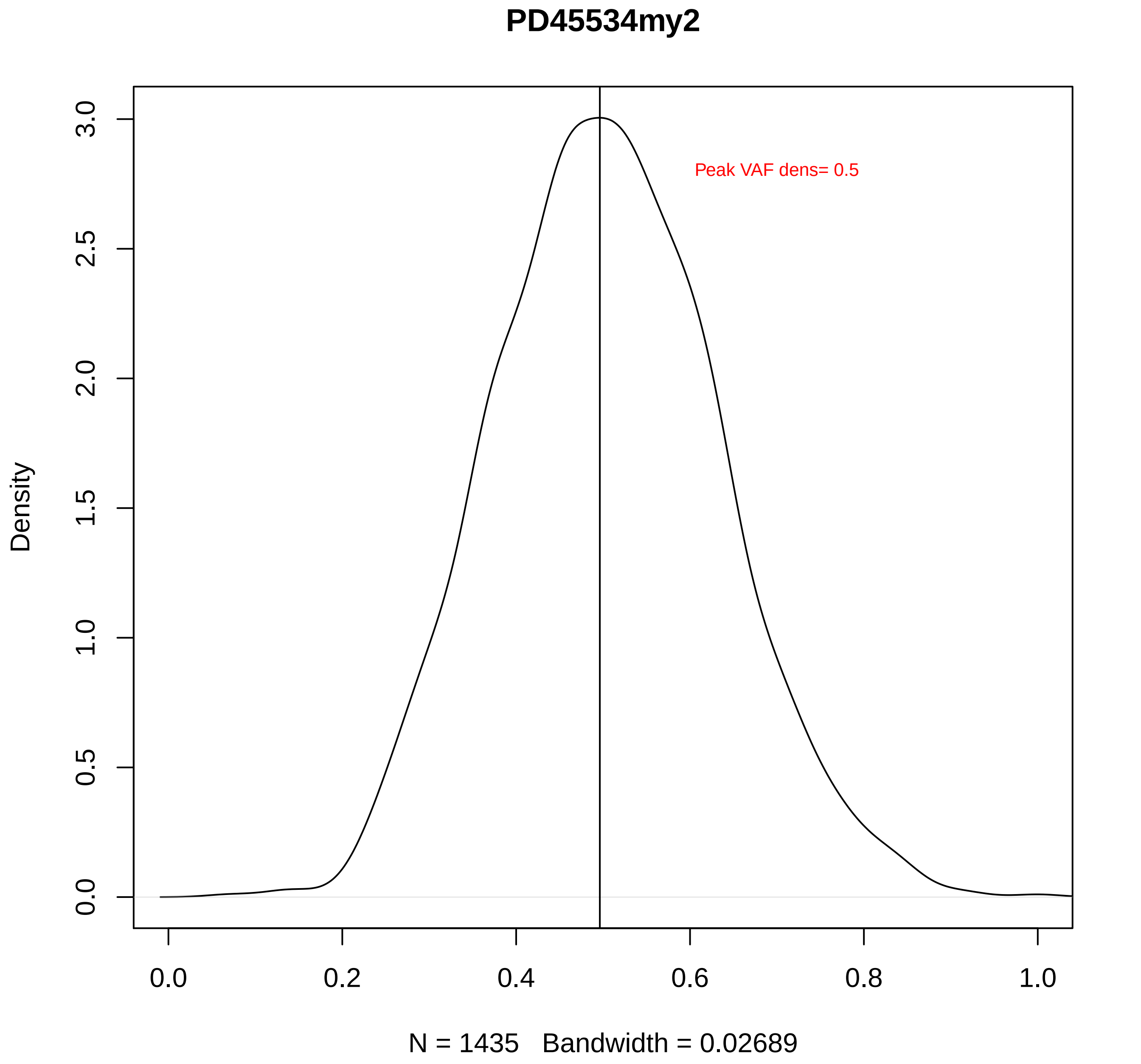
PD45534my2
Peak VAF dens= 0.5
Density
3.0
2.5
2.0
1.5
1.0
0.5
0.0
0.0
0.2
0.4
0.6
0.8
1.0
N = 1435   Bandwidth = 0.02689

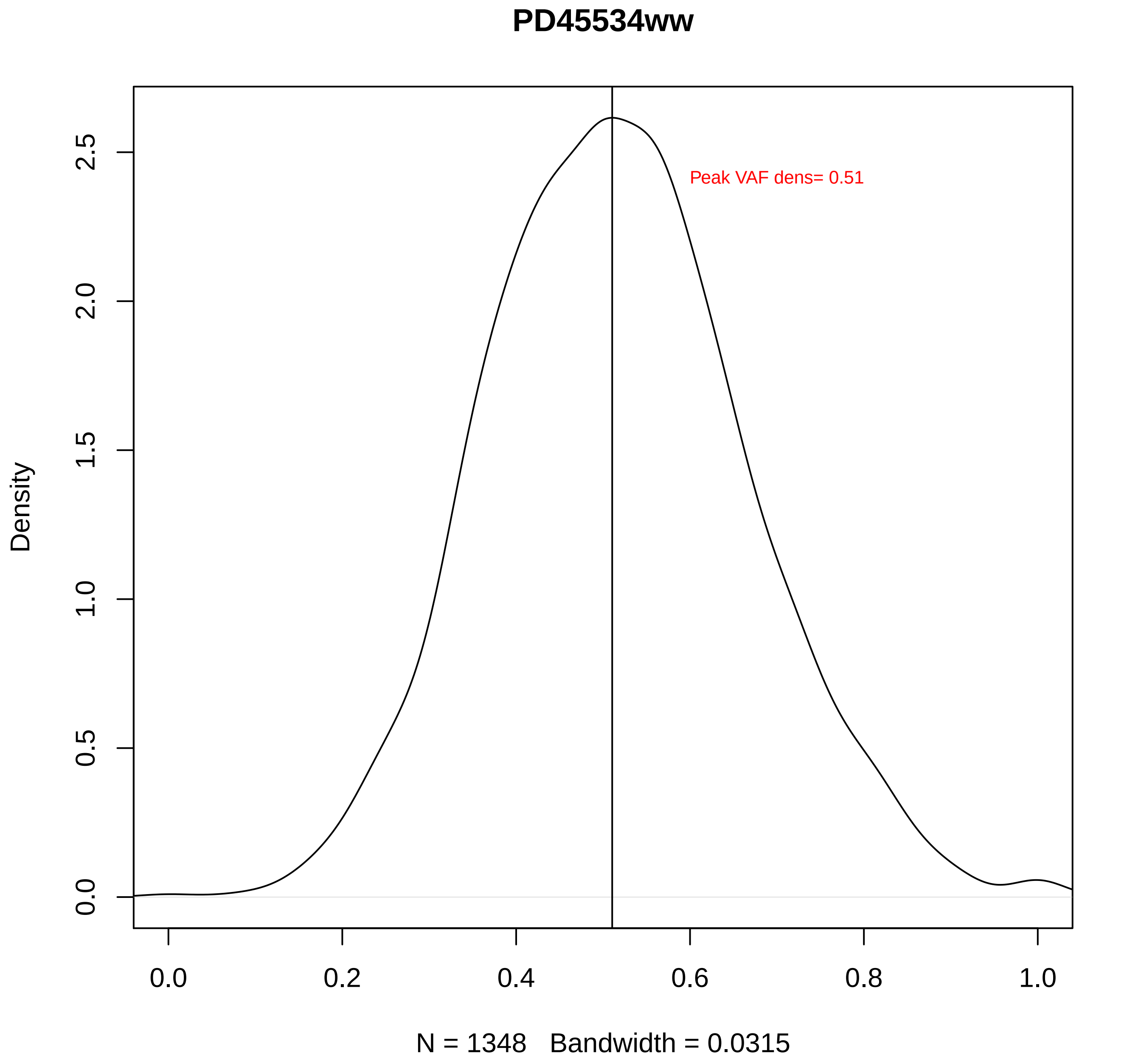
PD45534ww
Peak VAF dens= 0.51
Density
0.0
0.5
1.0
1.5
2.0
2.5
0.0
0.2
0.4
0.6
0.8
1.0
N = 1348   Bandwidth = 0.0315

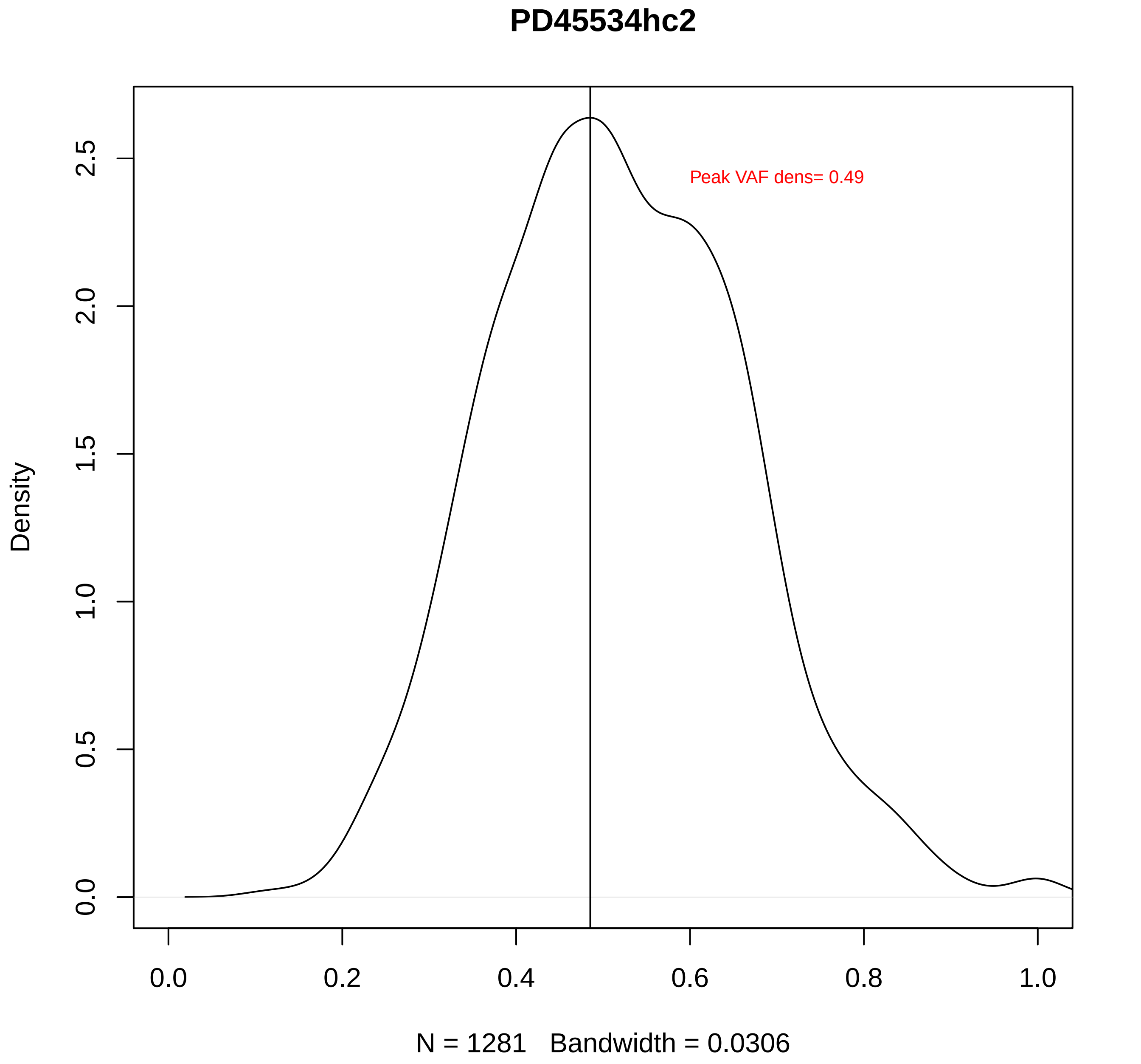
PD45534hc2
Peak VAF dens= 0.49
Density
0.0
0.5
1.0
1.5
2.0
2.5
0.0
0.2
0.4
0.6
0.8
1.0
N = 1281 Bandwidth = 0.0306

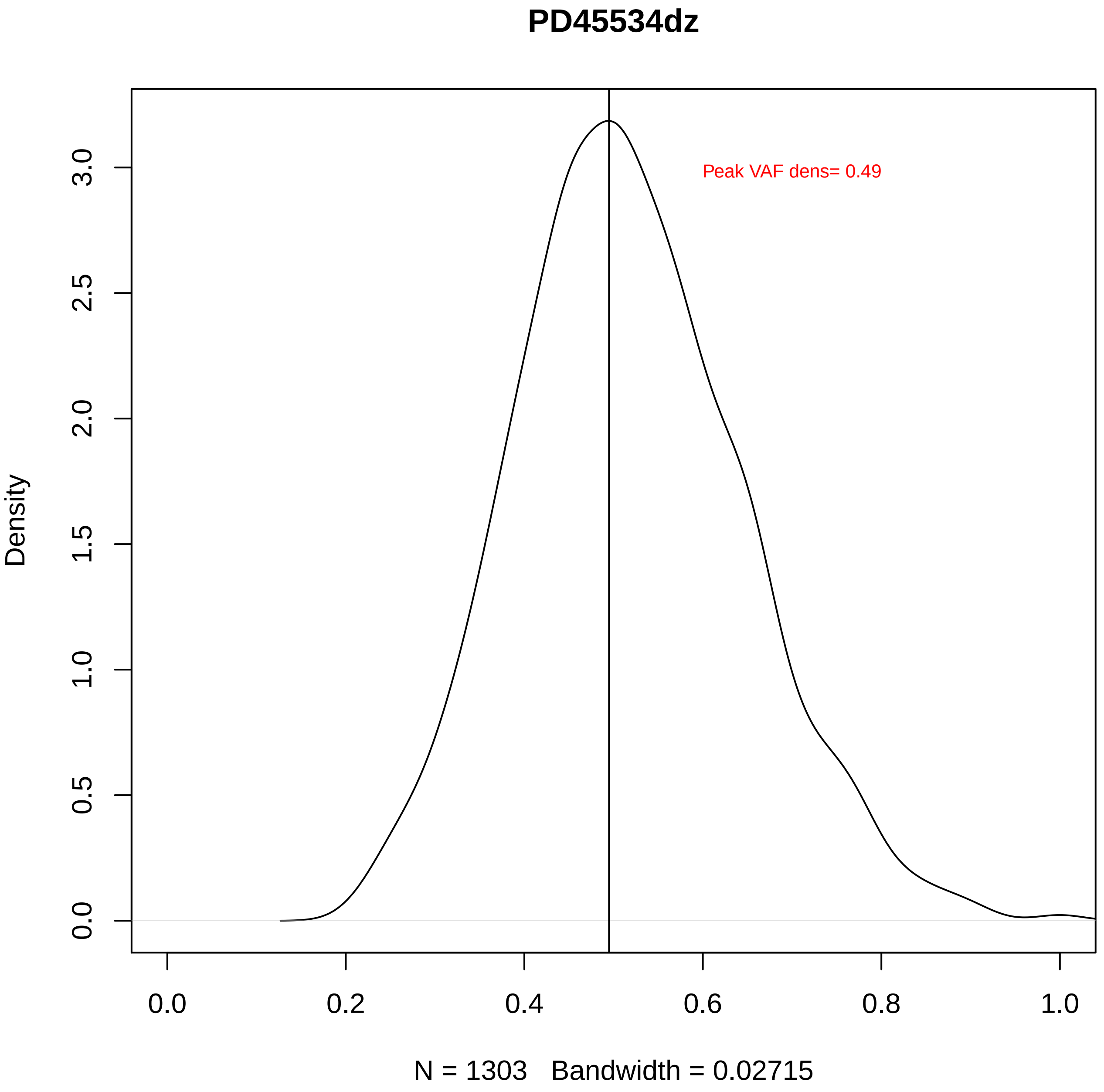
PD45534dz
Peak VAF dens= 0.49
Density
0.0
0.5
1.0
1.5
2.0
2.5
3.0
0.0
0.2
0.4
0.6
0.8
1.0
N = 1303 Bandwidth = 0.02715

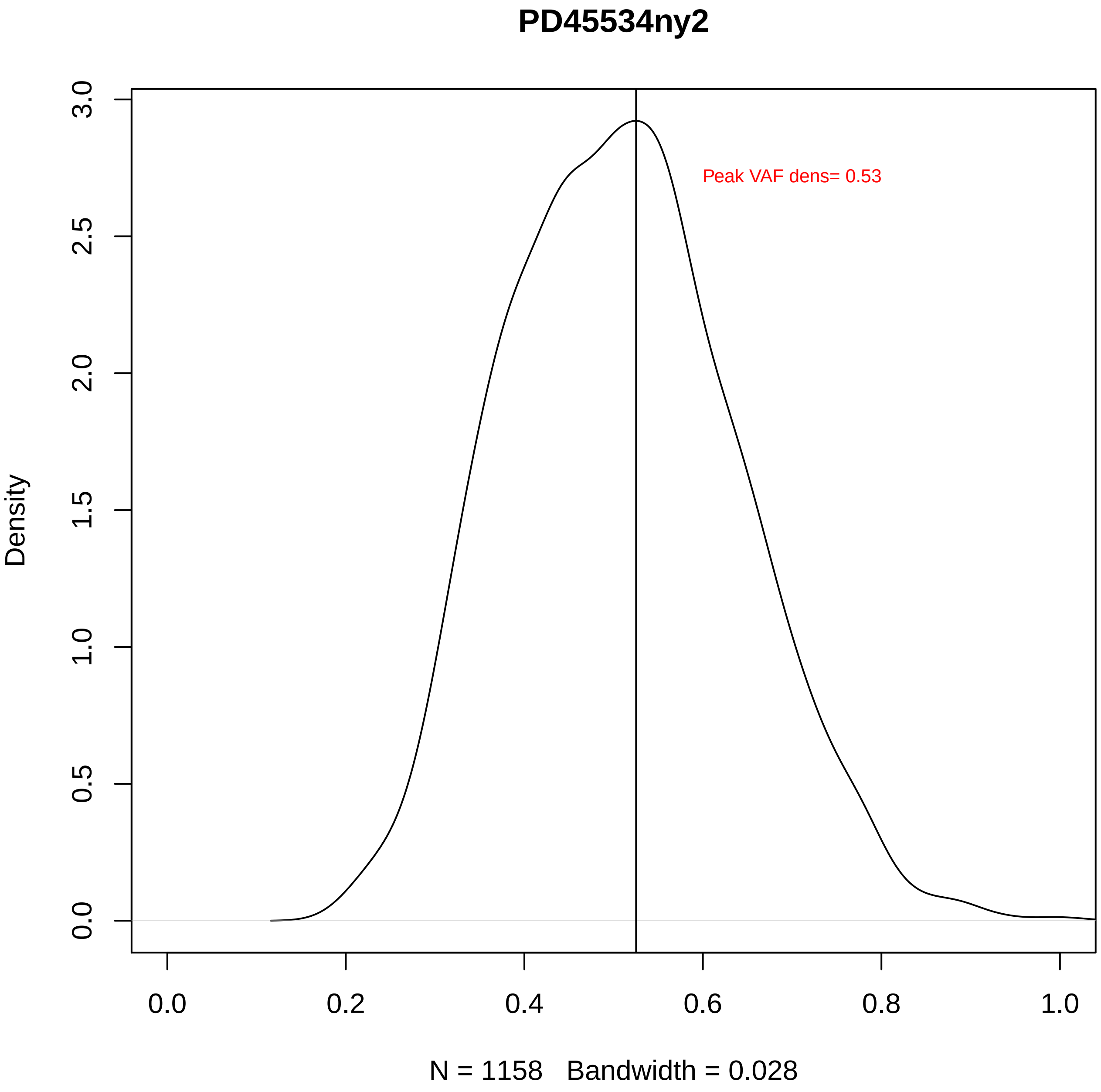

PD45534ny2
Peak VAF dens= 0.53
Density
0.0
0.5
1.0
1.5
2.0
2.5
3.0
0.0
0.2
0.4
0.6
0.8
1.0
N = 1158 Bandwidth = 0.028

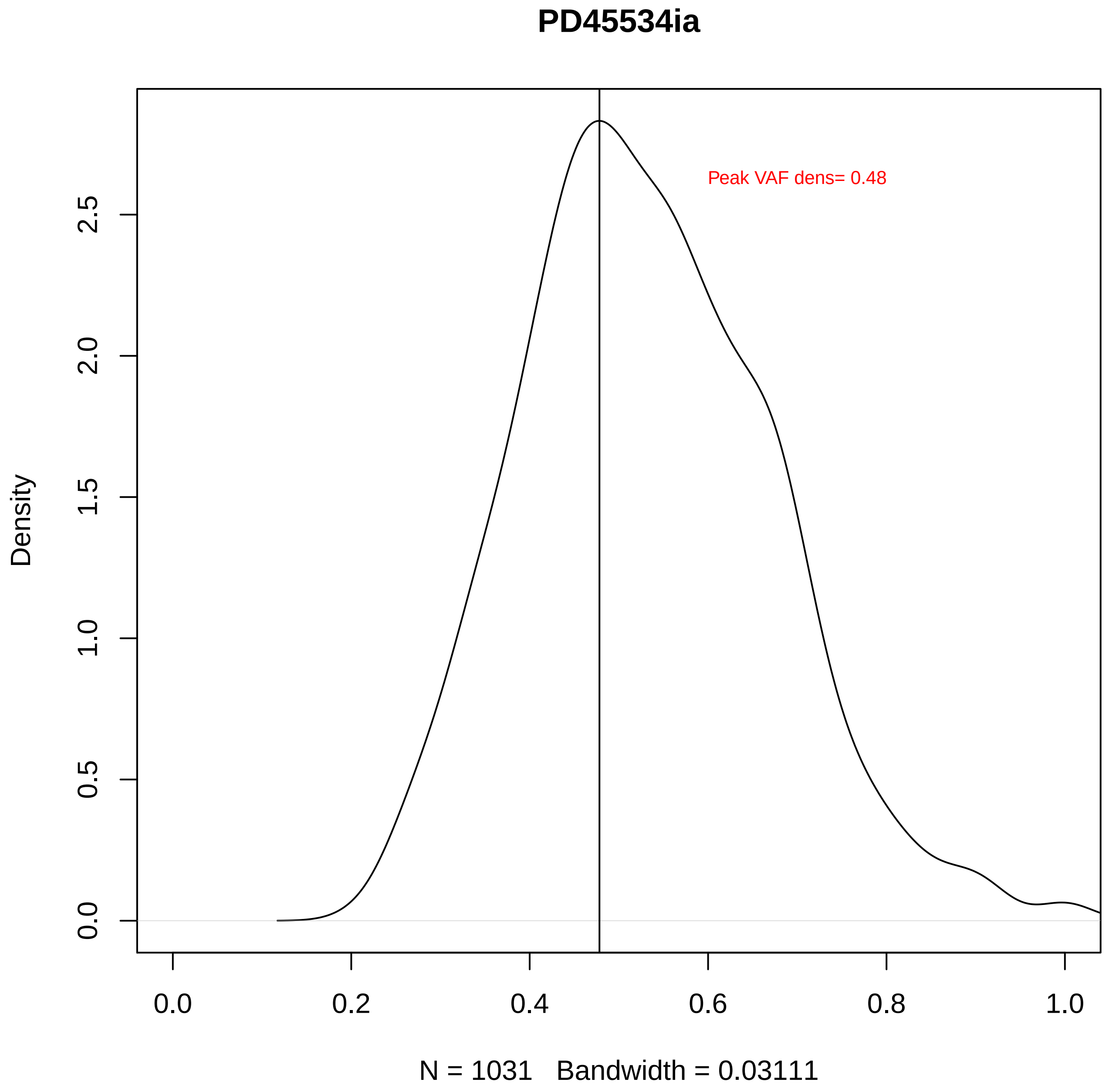
PD45534ia
Peak VAF dens= 0.48
Density
0.0
0.5
1.0
1.5
2.0
2.5
0.0
0.2
0.4
0.6
0.8
1.0
N = 1031   Bandwidth = 0.03111

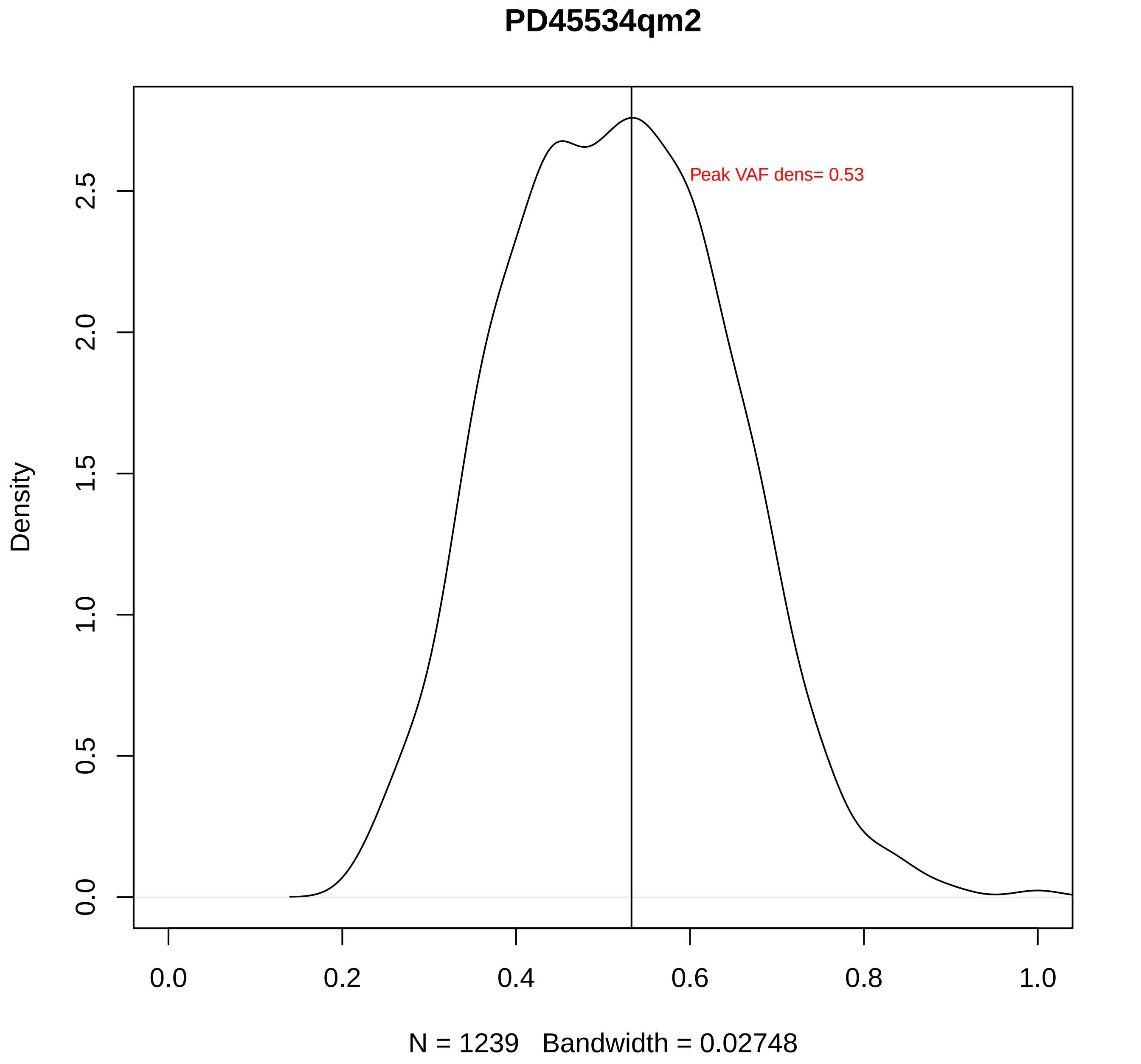
PD45534qm2
Peak VAF dens= 0.53
Density
0.0
0.5
1.0
1.5
2.0
2.5
0.0
0.2
0.4
0.6
0.8
1.0
N = 1239   Bandwidth = 0.02748

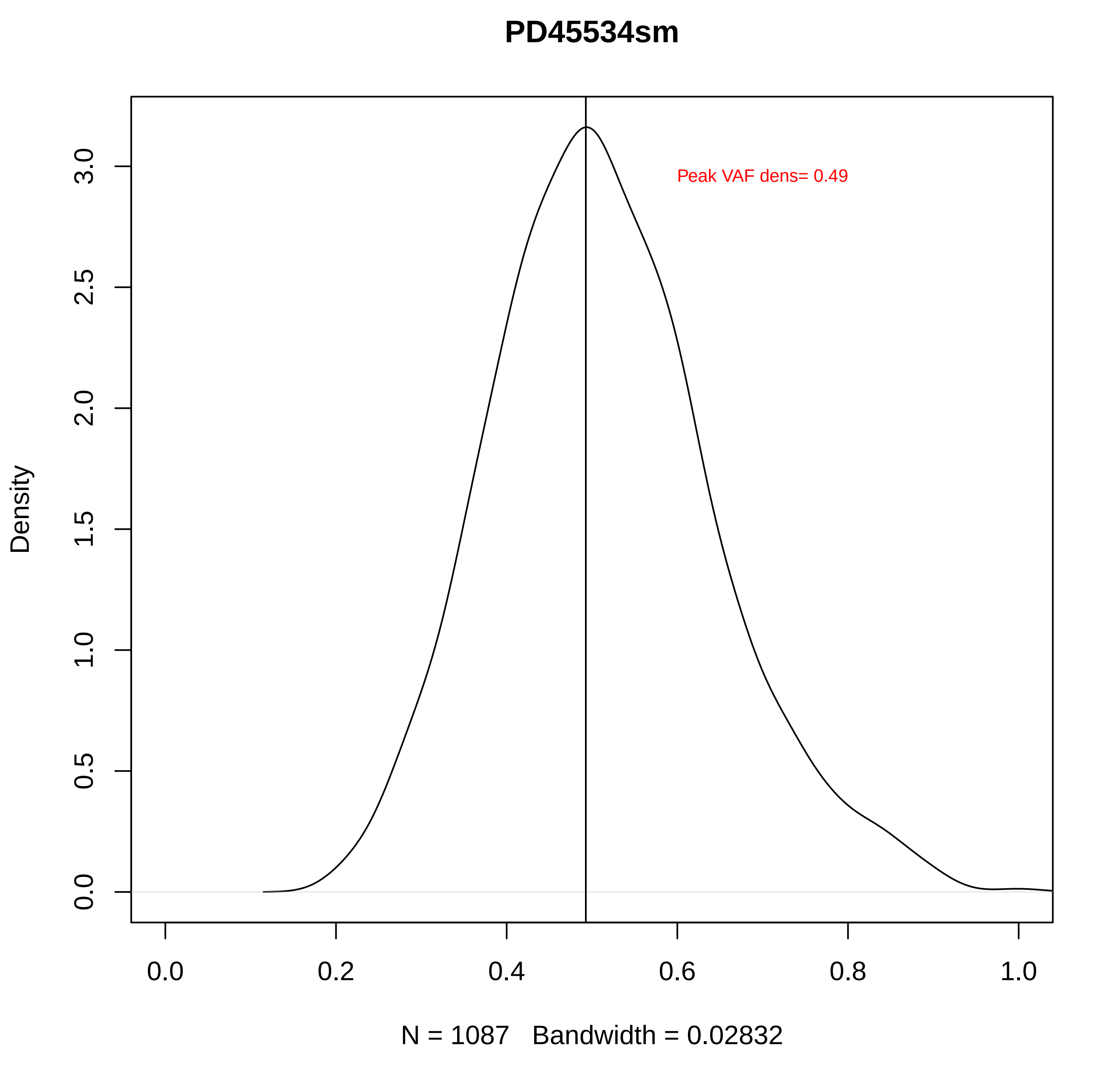
PD45534sm
Peak VAF dens= 0.49
Density
0.0
0.5
1.0
1.5
2.0
2.5
3.0
0.0
0.2
0.4
0.6
0.8
1.0
N = 1087   Bandwidth = 0.02832

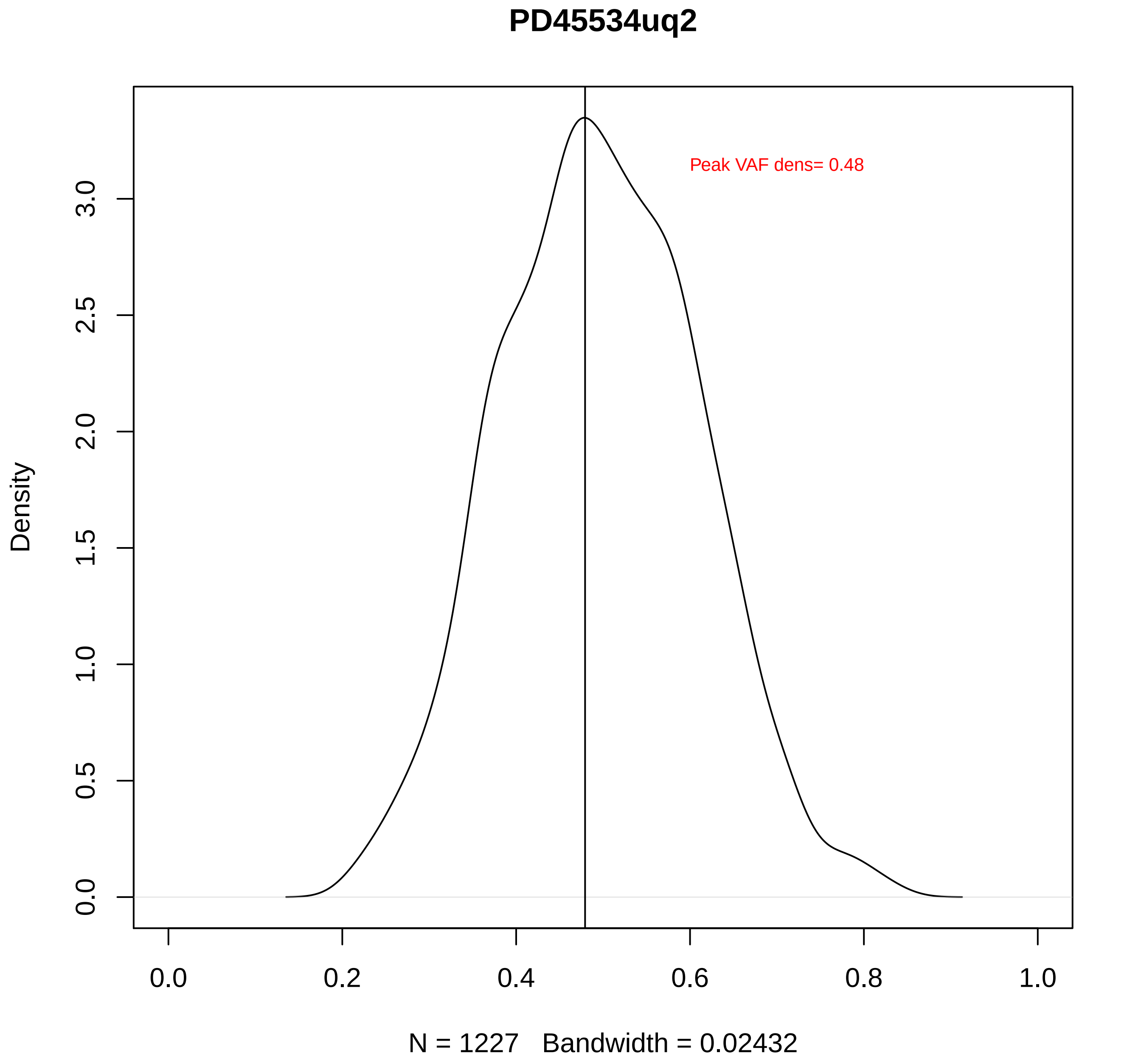
PD45534uq2
Peak VAF dens= 0.48
Density
0.0
0.5
1.0
1.5
2.0
2.5
3.0
0.0
0.2
0.4
0.6
0.8
1.0
N = 1227   Bandwidth = 0.02432

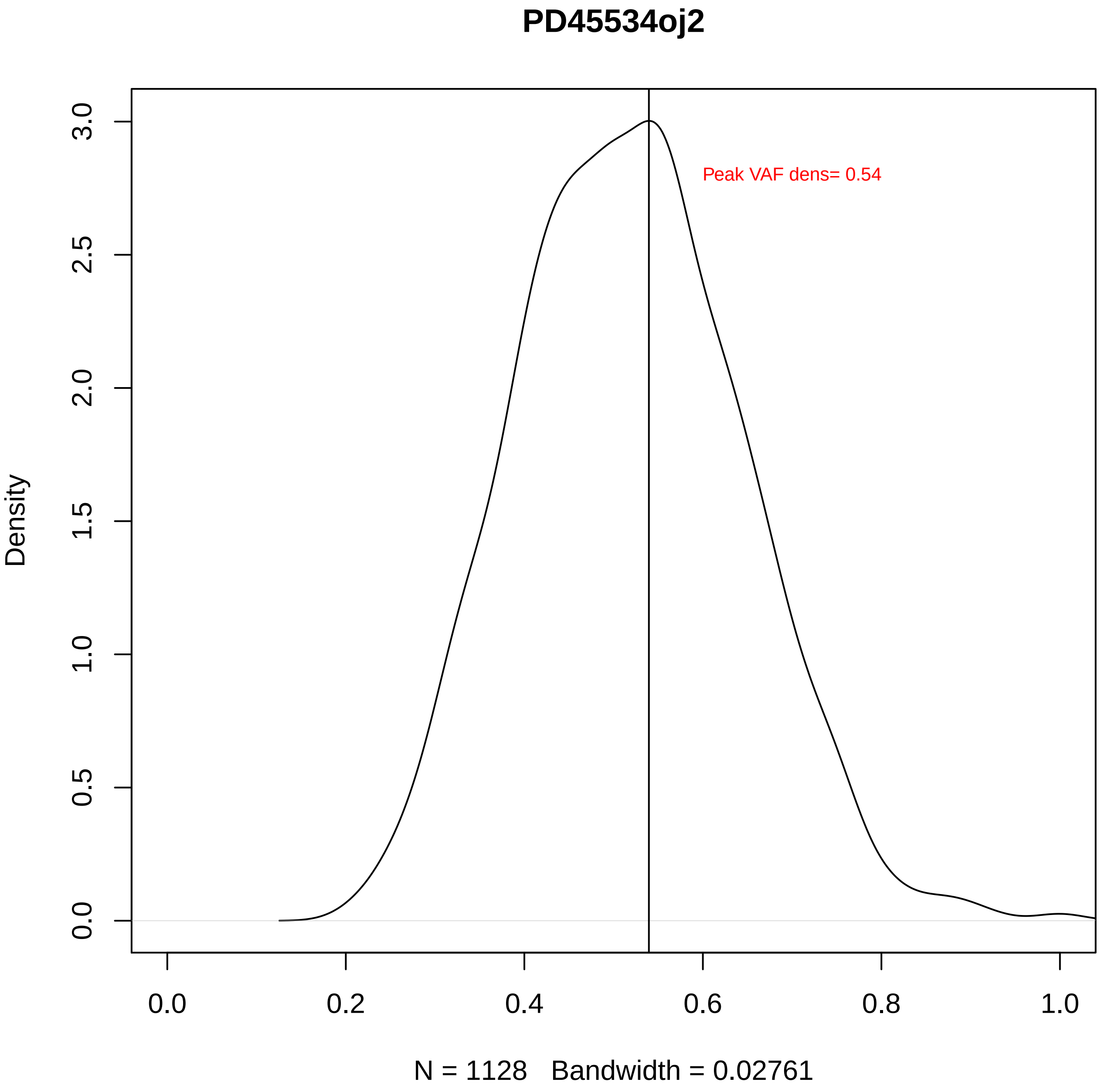

PD45534oj2
Peak VAF dens= 0.54
Density
3.0
2.5
2.0
1.5
1.0
0.5
0.0
0.0
0.2
0.4
0.6
0.8
1.0
N = 1128   Bandwidth = 0.02761

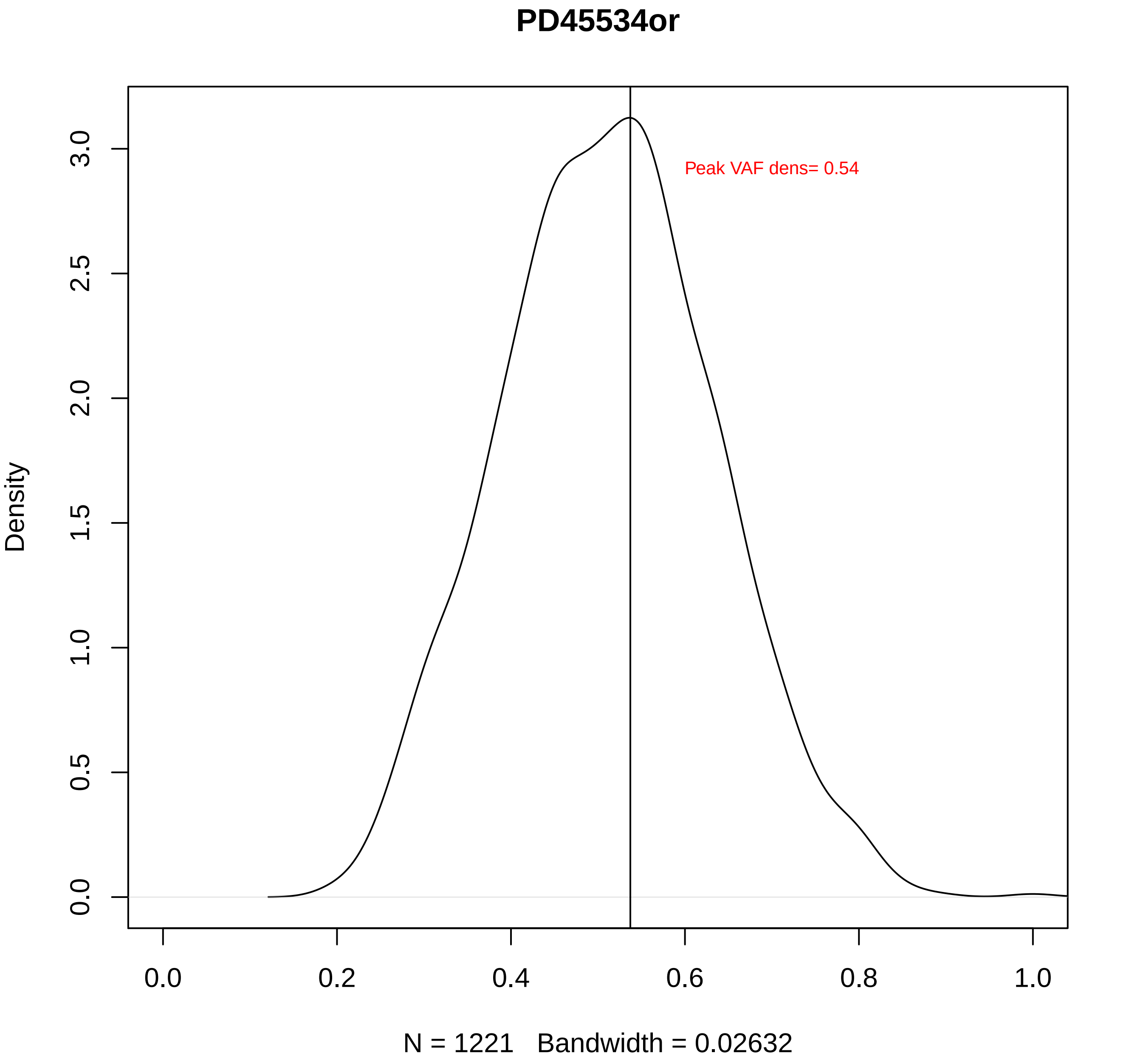

PD45534or
Peak VAF dens= 0.54
Density
0.0
0.5
1.0
1.5
2.0
2.5
3.0
0.0
0.2
0.4
0.6
0.8
1.0
N = 1221   Bandwidth = 0.02632

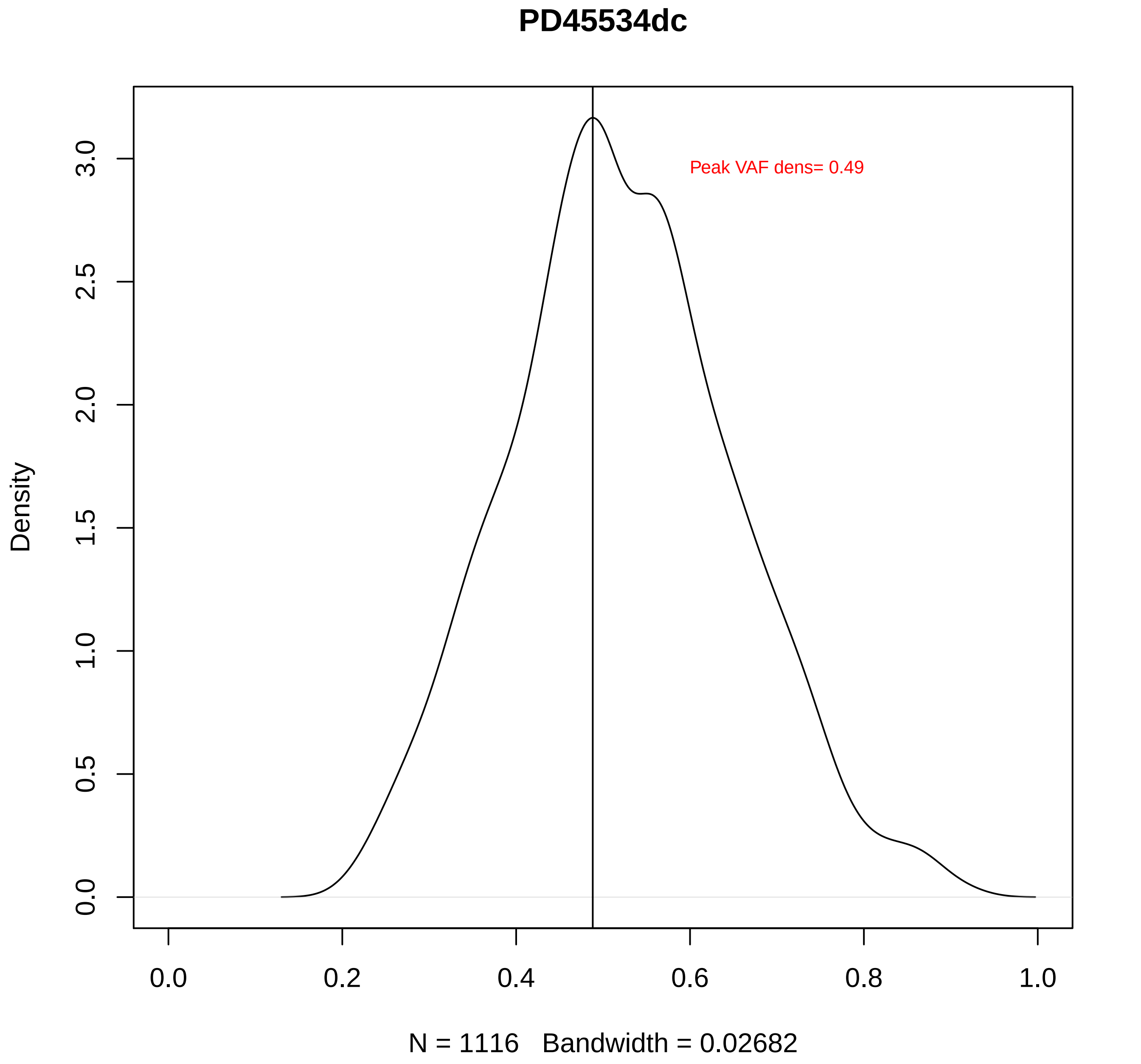
PD45534dc
Peak VAF dens= 0.49
Density
0.0
0.5
1.0
1.5
2.0
2.5
3.0
0.0
0.2
0.4
0.6
0.8
1.0
N = 1116   Bandwidth = 0.02682

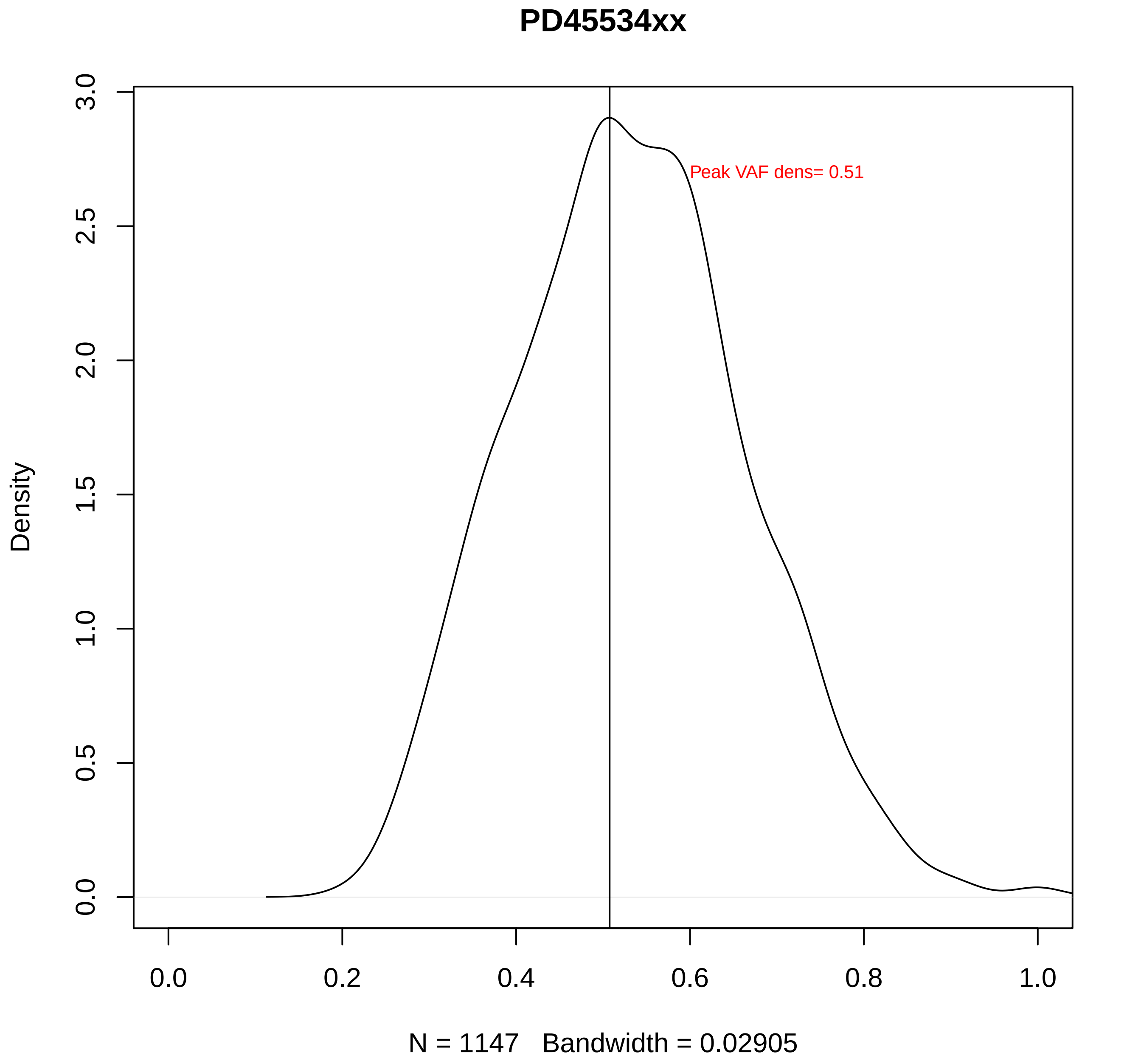
PD45534xx
Peak VAF dens= 0.51
Density
0.0
0.5
1.0
1.5
2.0
2.5
3.0
0.0
0.2
0.4
0.6
0.8
1.0
N = 1147   Bandwidth = 0.02905

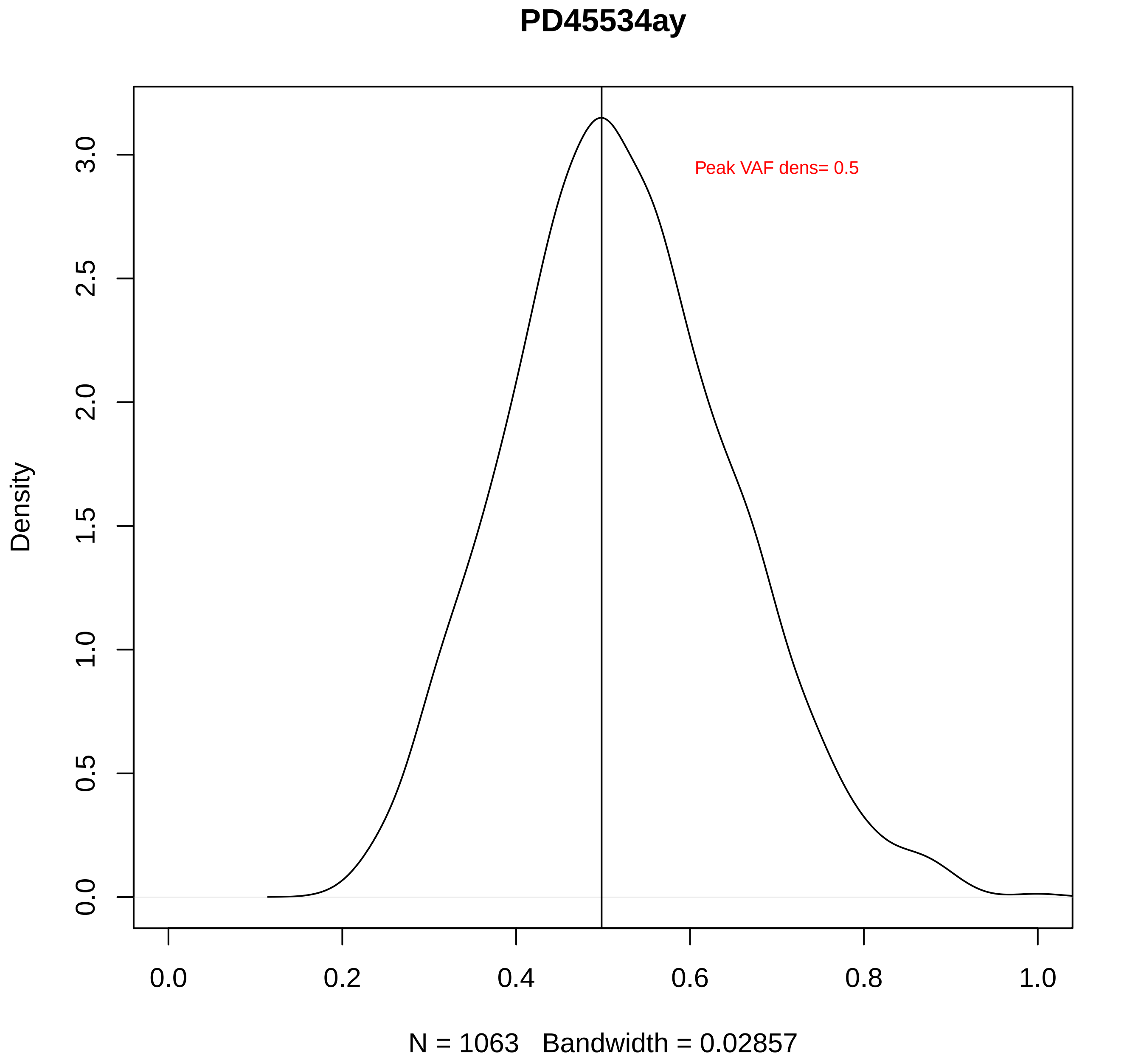
PD45534ay
Peak VAF dens= 0.5
Density
0.0
0.5
1.0
1.5
2.0
2.5
3.0
0.0
0.2
0.4
0.6
0.8
1.0
N = 1063   Bandwidth = 0.02857

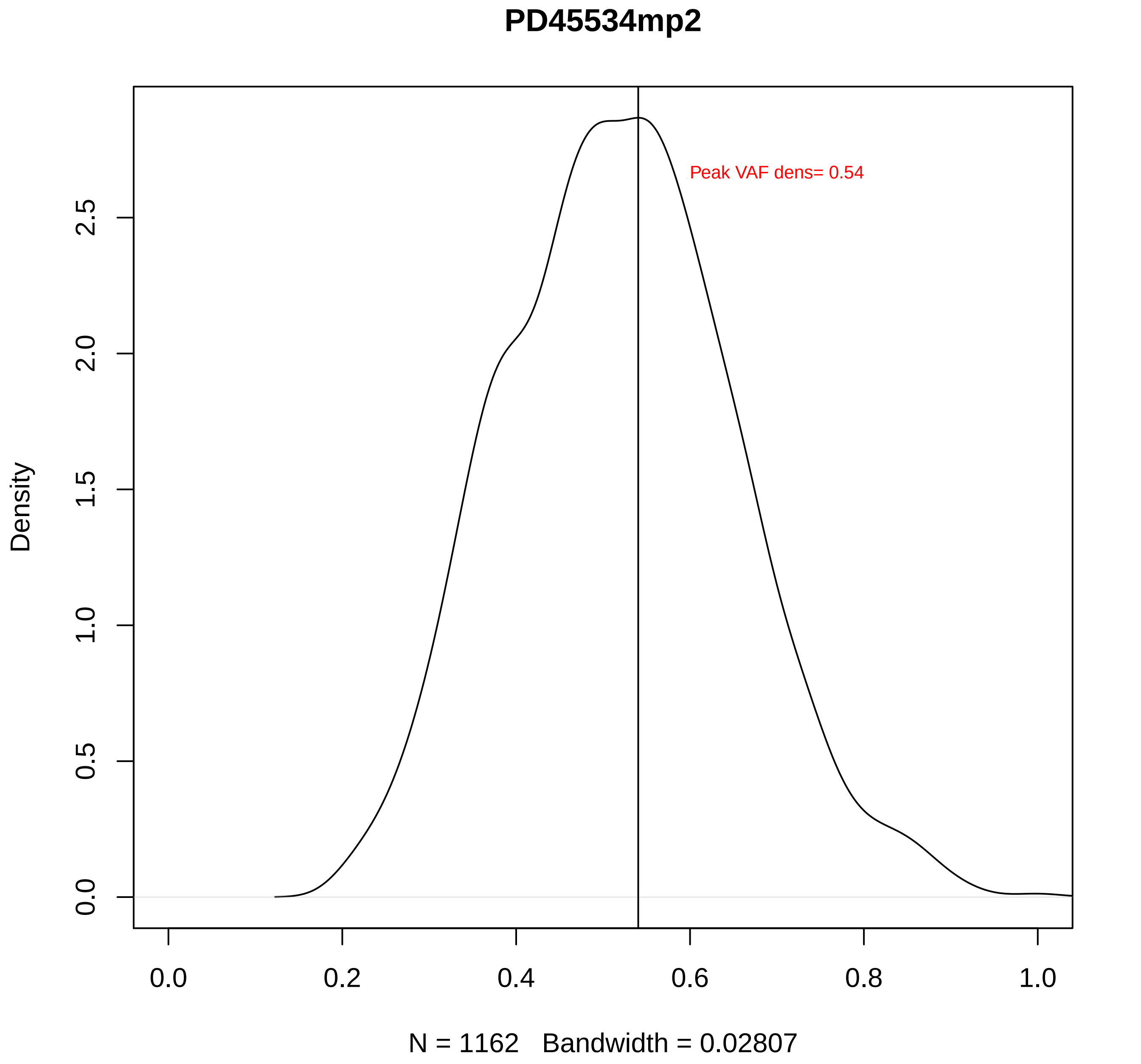
PD45534mp2
Peak VAF dens= 0.54
Density
0.0
0.5
1.0
1.5
2.0
2.5
0.0
0.2
0.4
0.6
0.8
1.0
N = 1162   Bandwidth = 0.02807

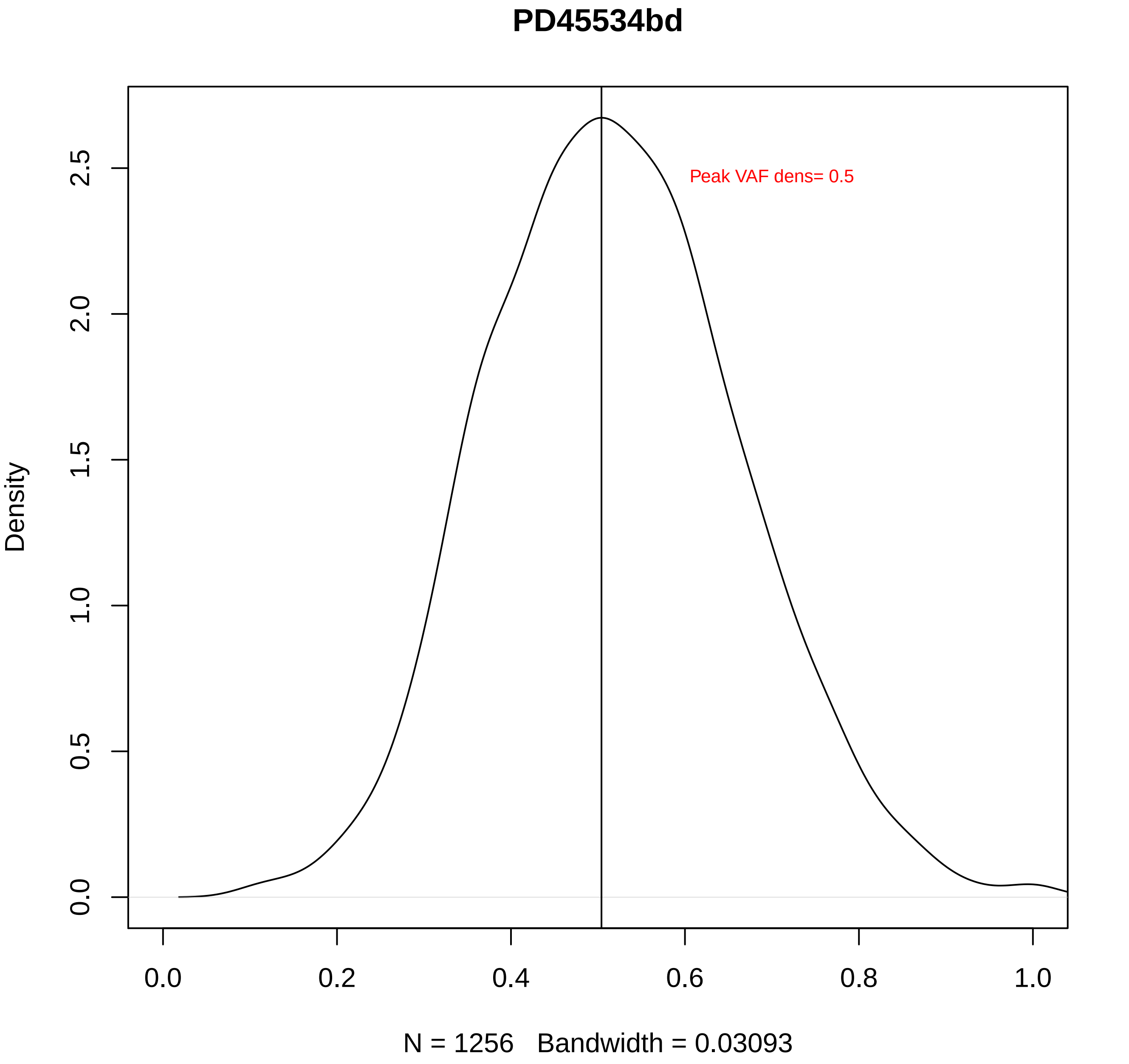
PD45534bd
Peak VAF dens= 0.5
Density
0.0
0.5
1.0
1.5
2.0
2.5
0.0
0.2
0.4
0.6
0.8
1.0
N = 1256   Bandwidth = 0.03093

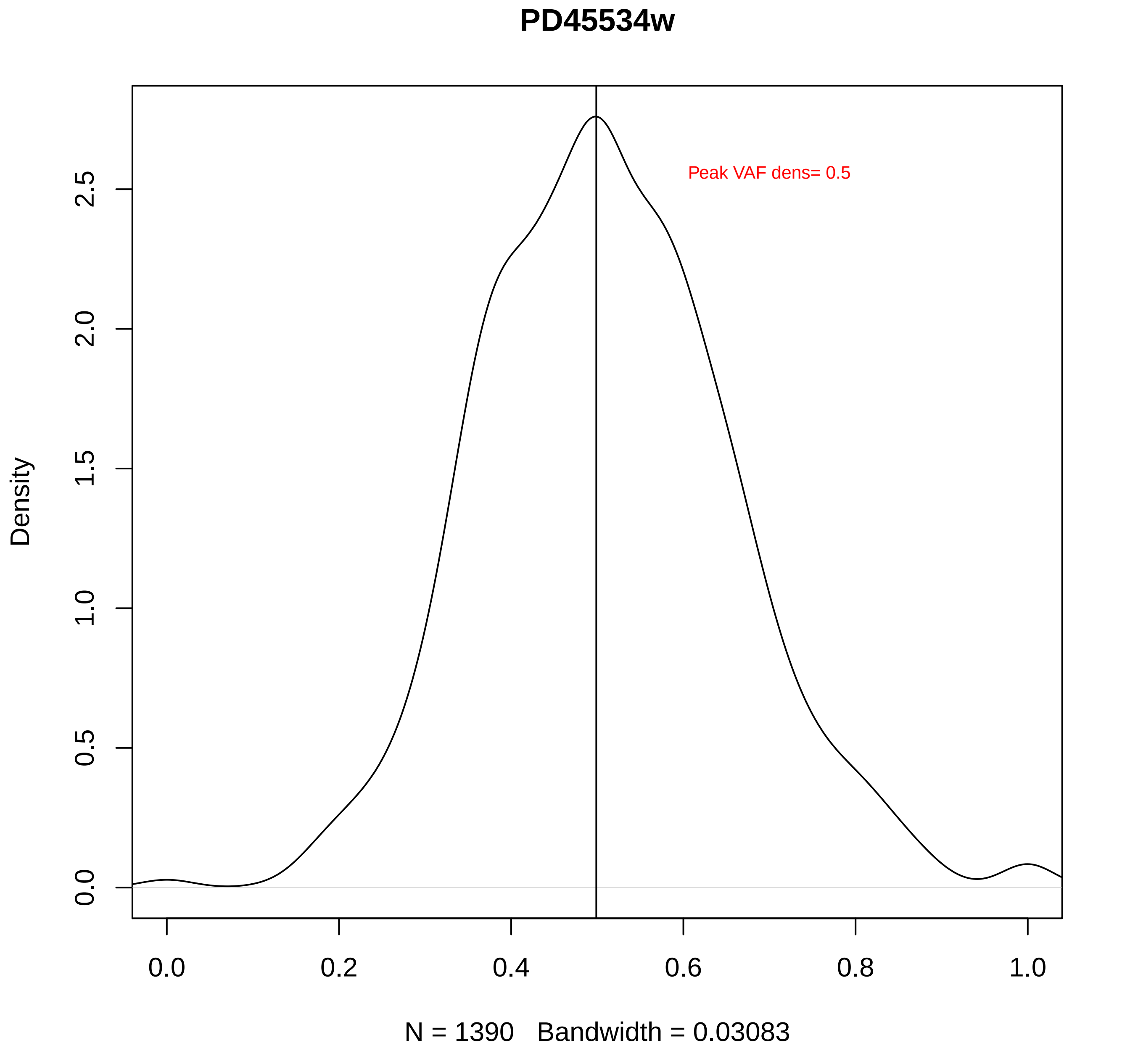
PD45534w
Peak VAF dens= 0.5
Density
0.0
0.5
1.0
1.5
2.0
2.5
0.0
0.2
0.4
0.6
0.8
1.0
N = 1390   Bandwidth = 0.03083

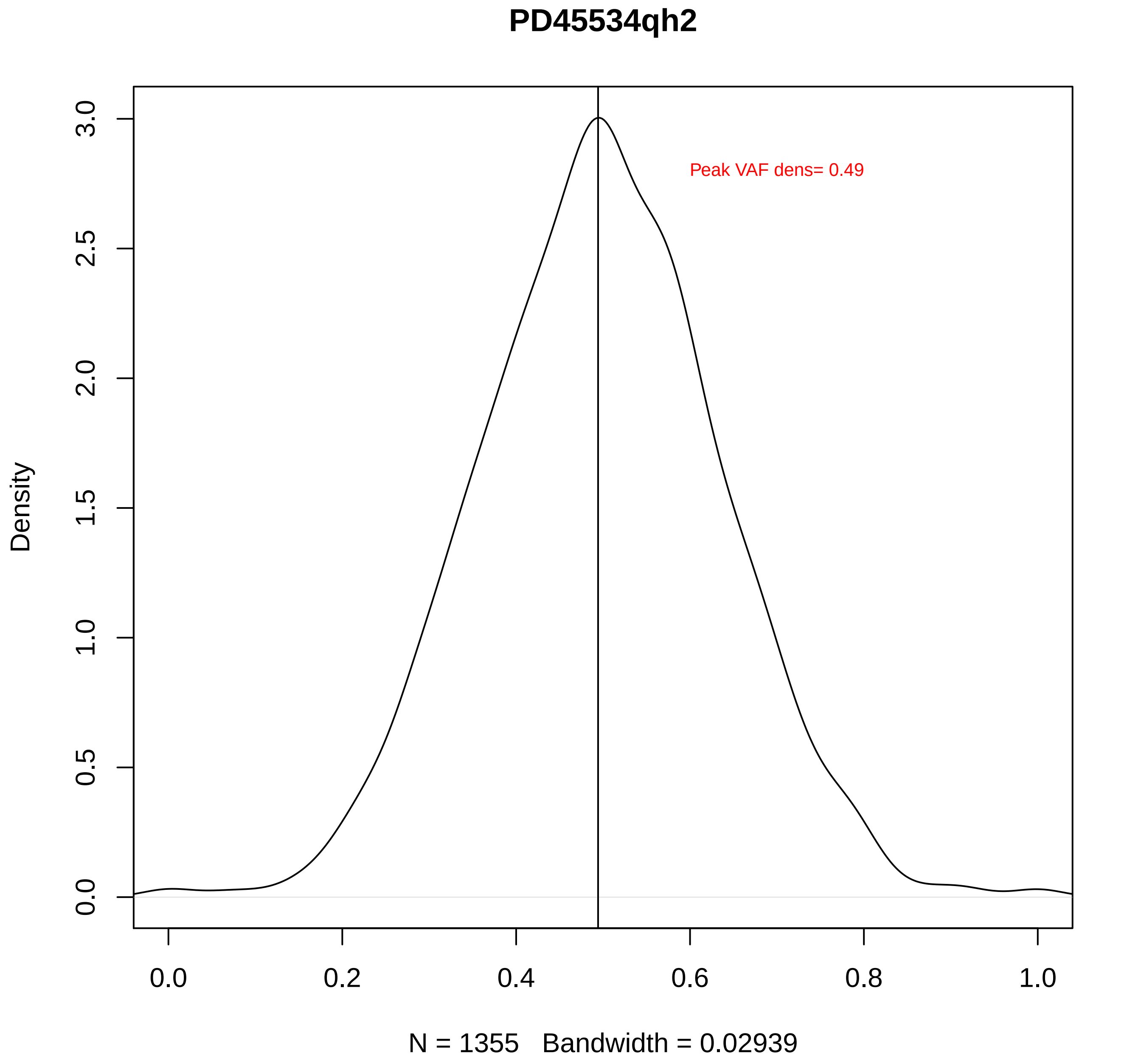

PD45534qh2
Peak VAF dens= 0.49
Density
0.0
0.5
1.0
1.5
2.0
2.5
3.0
0.0
0.2
0.4
0.6
0.8
1.0
N = 1355   Bandwidth = 0.02939

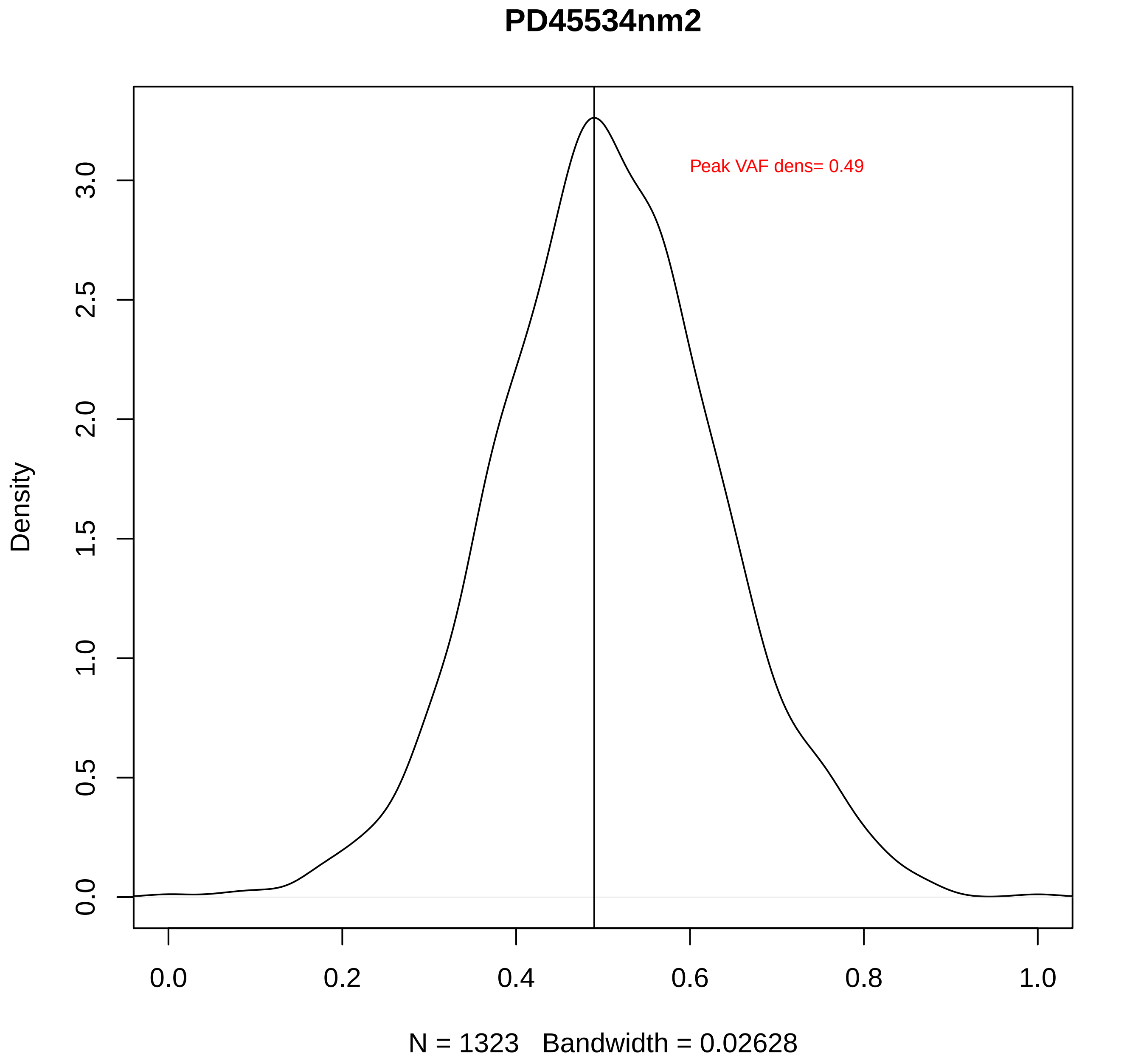
PD45534nm2
Peak VAF dens= 0.49
Density
0.0
0.5
1.0
1.5
2.0
2.5
3.0
0.0
0.2
0.4
0.6
0.8
1.0
N = 1323   Bandwidth = 0.02628

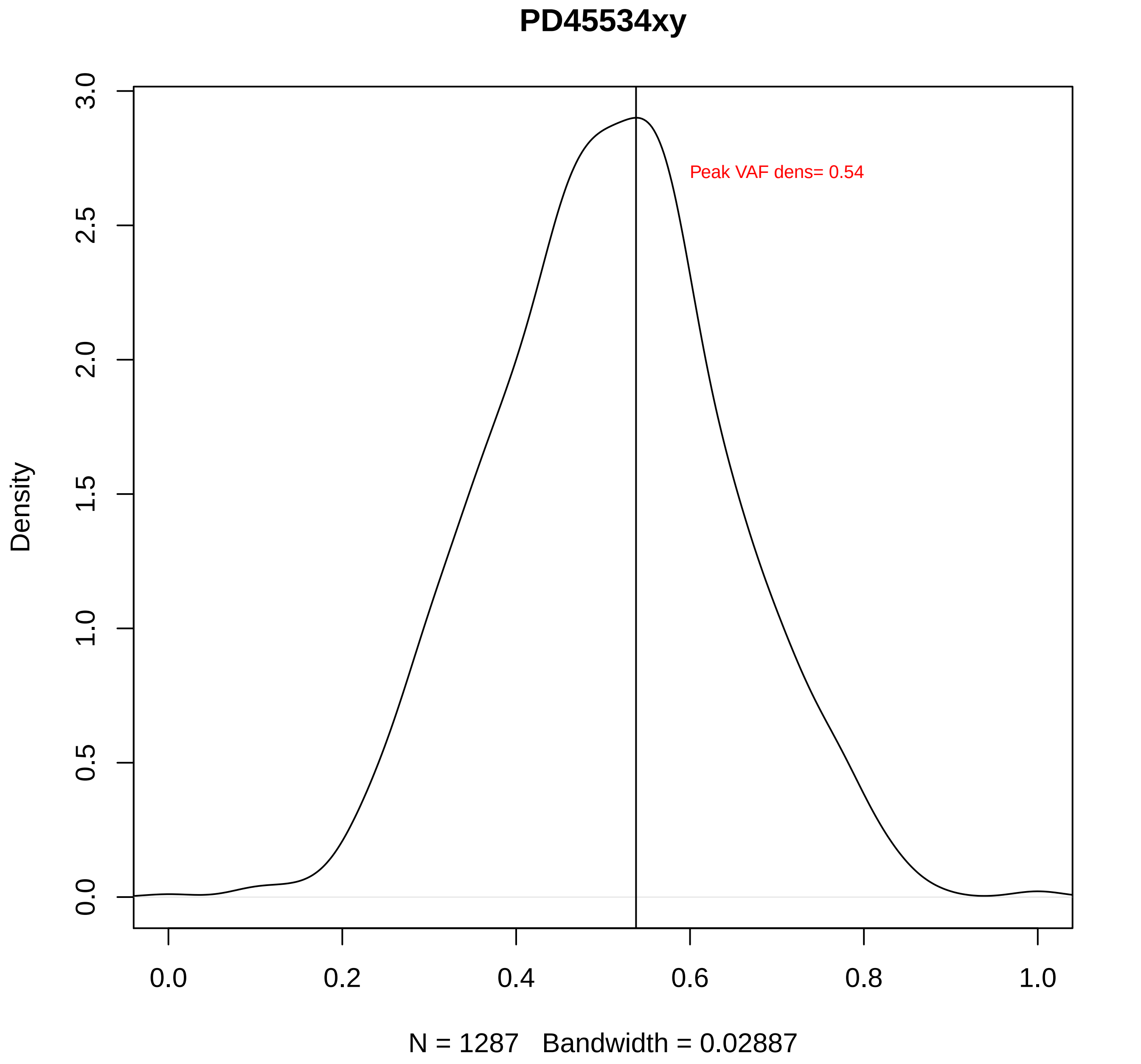

PD45534xy
Peak VAF dens= 0.54
Density
0.0
0.5
1.0
1.5
2.0
2.5
3.0
0.0
0.2
0.4
0.6
0.8
1.0
N = 1287   Bandwidth = 0.02887

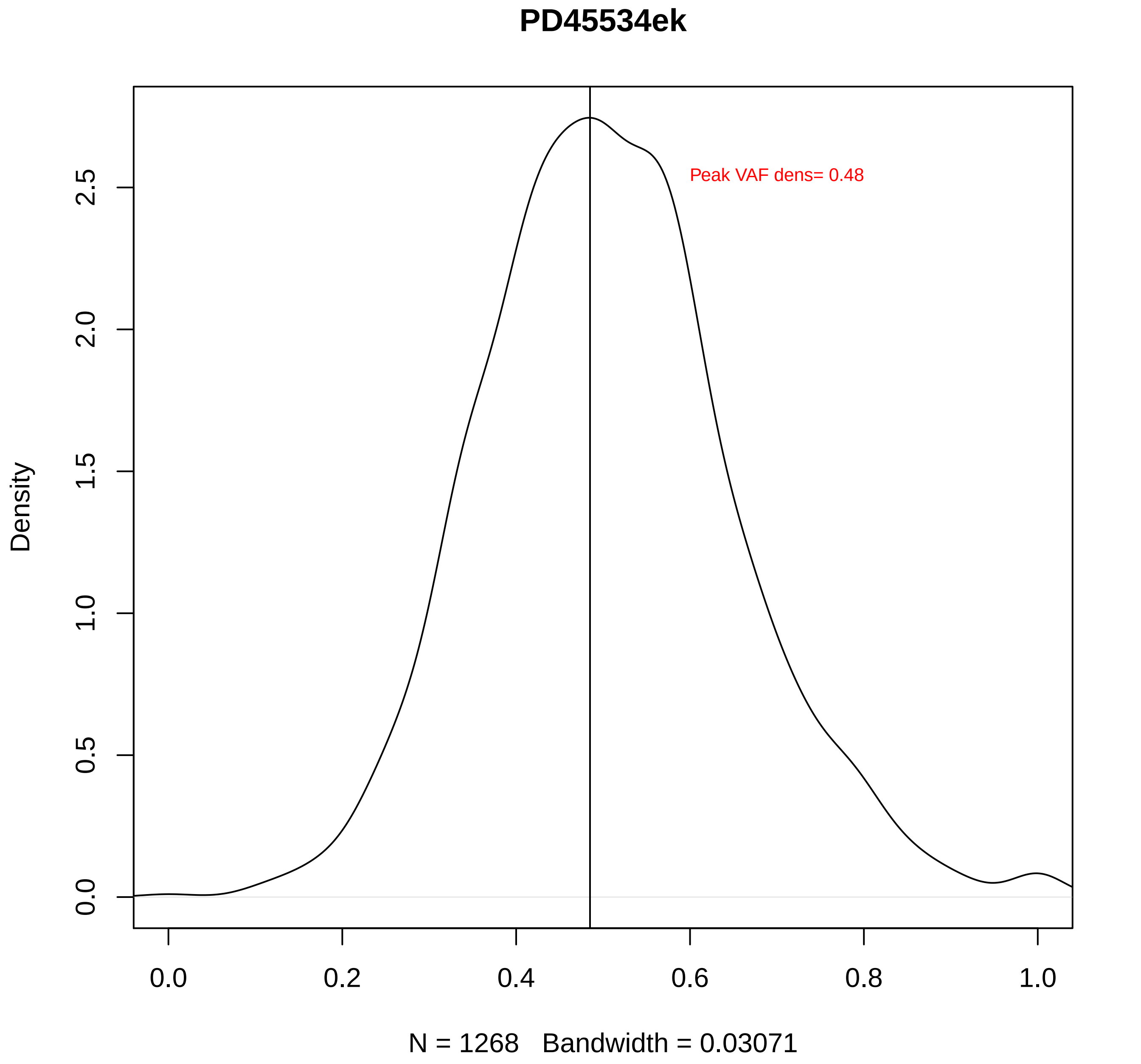
PD45534ek
Peak VAF dens= 0.48
Density
0.0
0.5
1.0
1.5
2.0
2.5
0.0
0.2
0.4
0.6
0.8
1.0
N = 1268   Bandwidth = 0.03071

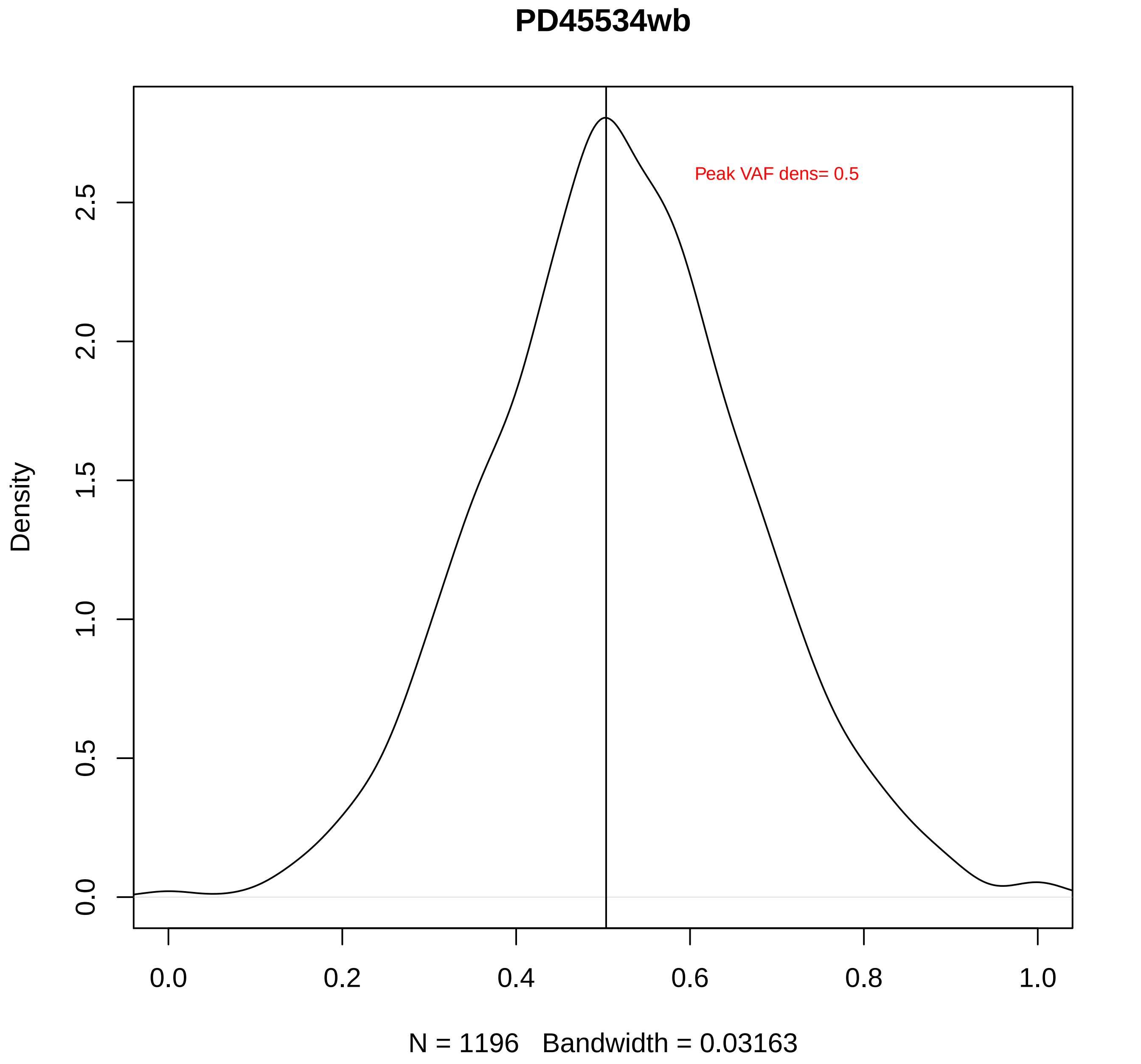
PD45534wb
Peak VAF dens= 0.5
Density
0.0
0.5
1.0
1.5
2.0
2.5
0.0
0.2
0.4
0.6
0.8
1.0
N = 1196   Bandwidth = 0.03163

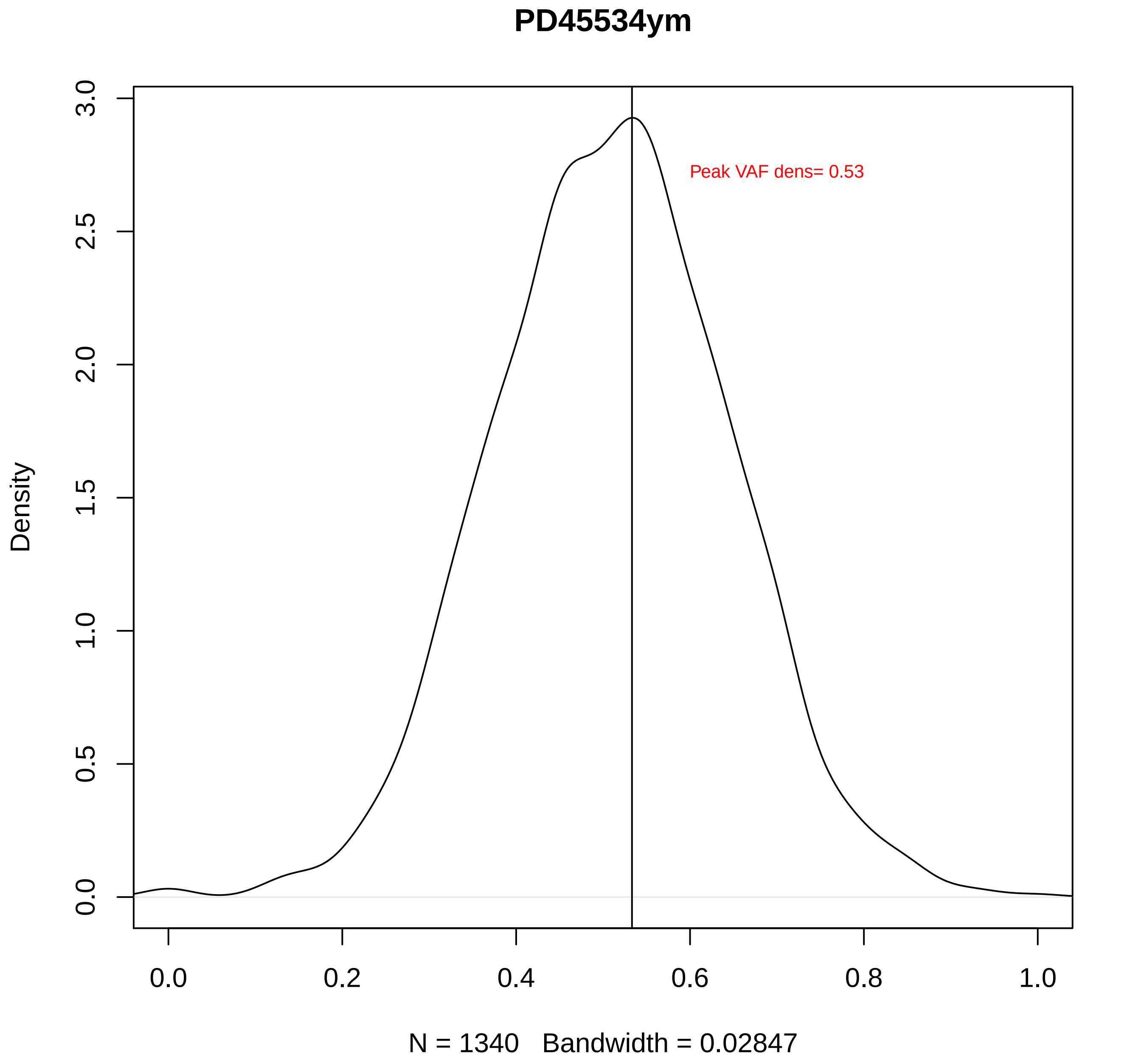
PD45534ym
Peak VAF dens= 0.53
Density
0.0
0.5
1.0
1.5
2.0
2.5
3.0
0.0
0.2
0.4
0.6
0.8
1.0
N = 1340   Bandwidth = 0.02847

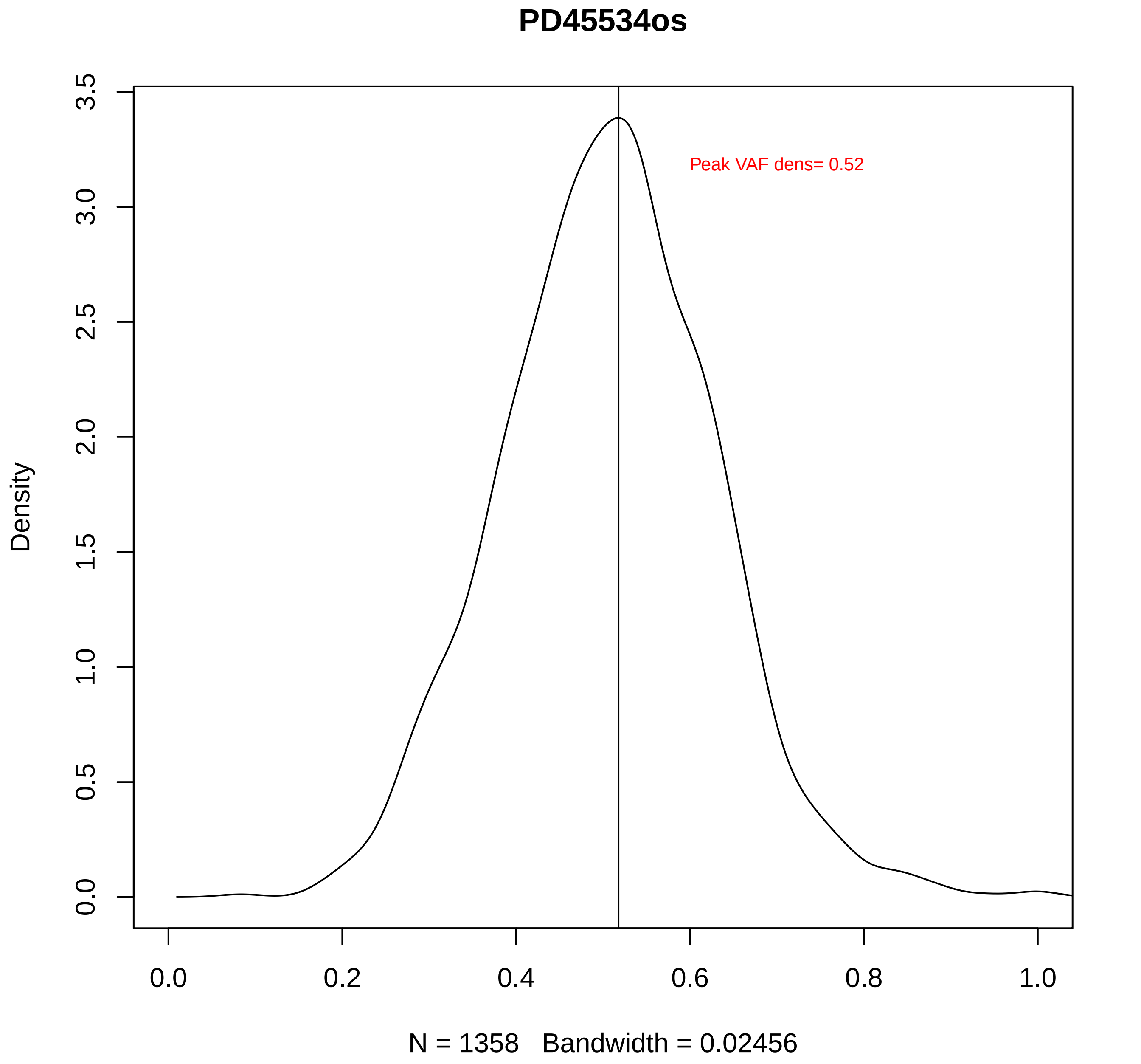
PD45534os
Peak VAF dens= 0.52
Density
0.0
0.5
1.0
1.5
2.0
2.5
3.0
3.5
0.0
0.2
0.4
0.6
0.8
1.0
N = 1358 Bandwidth = 0.02456

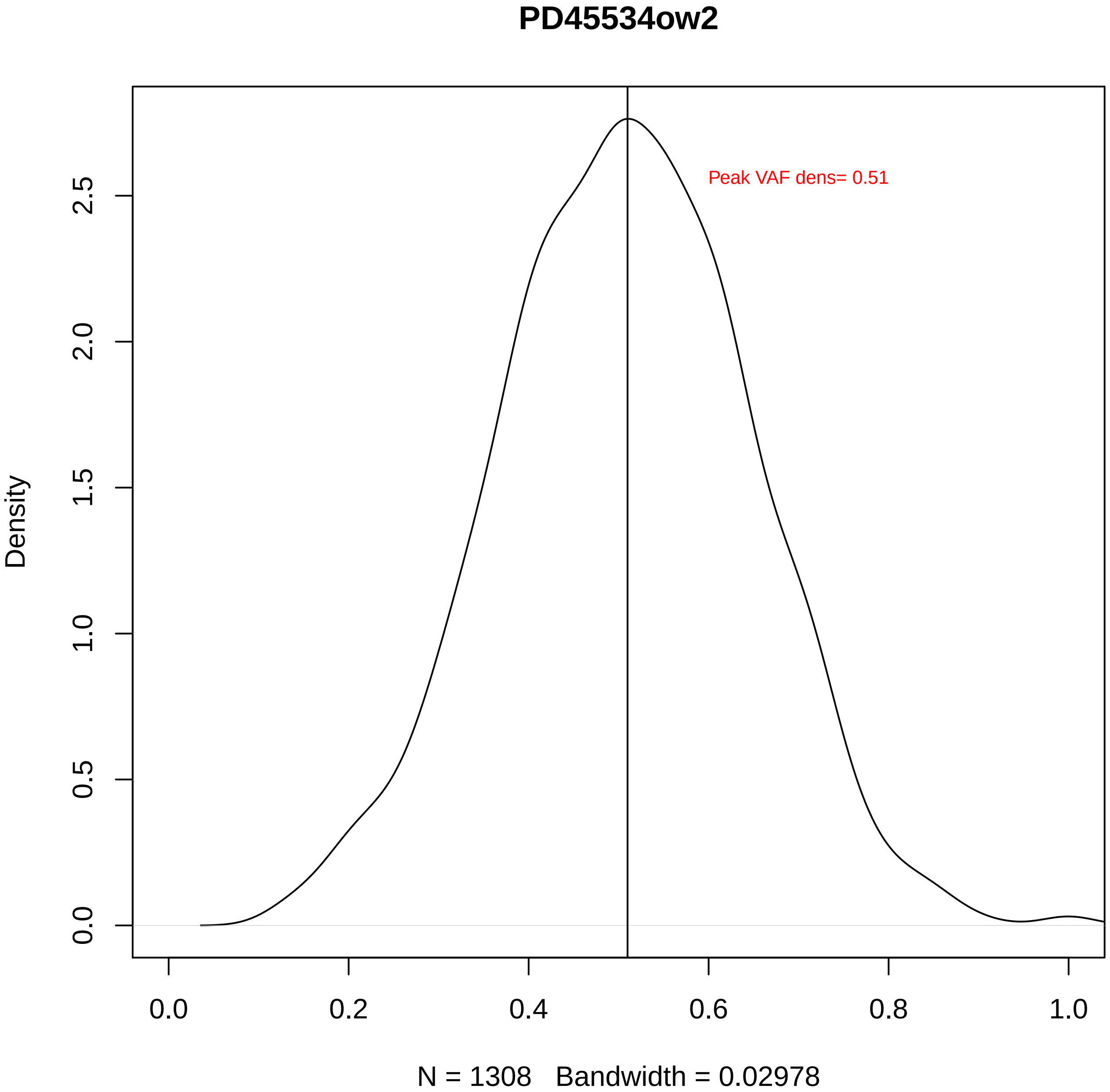
PD45534ow2
Peak VAF dens= 0.51
Density
0.0
0.5
1.0
1.5
2.0
2.5
0.0
0.2
0.4
0.6
0.8
1.0
N = 1308   Bandwidth = 0.02978

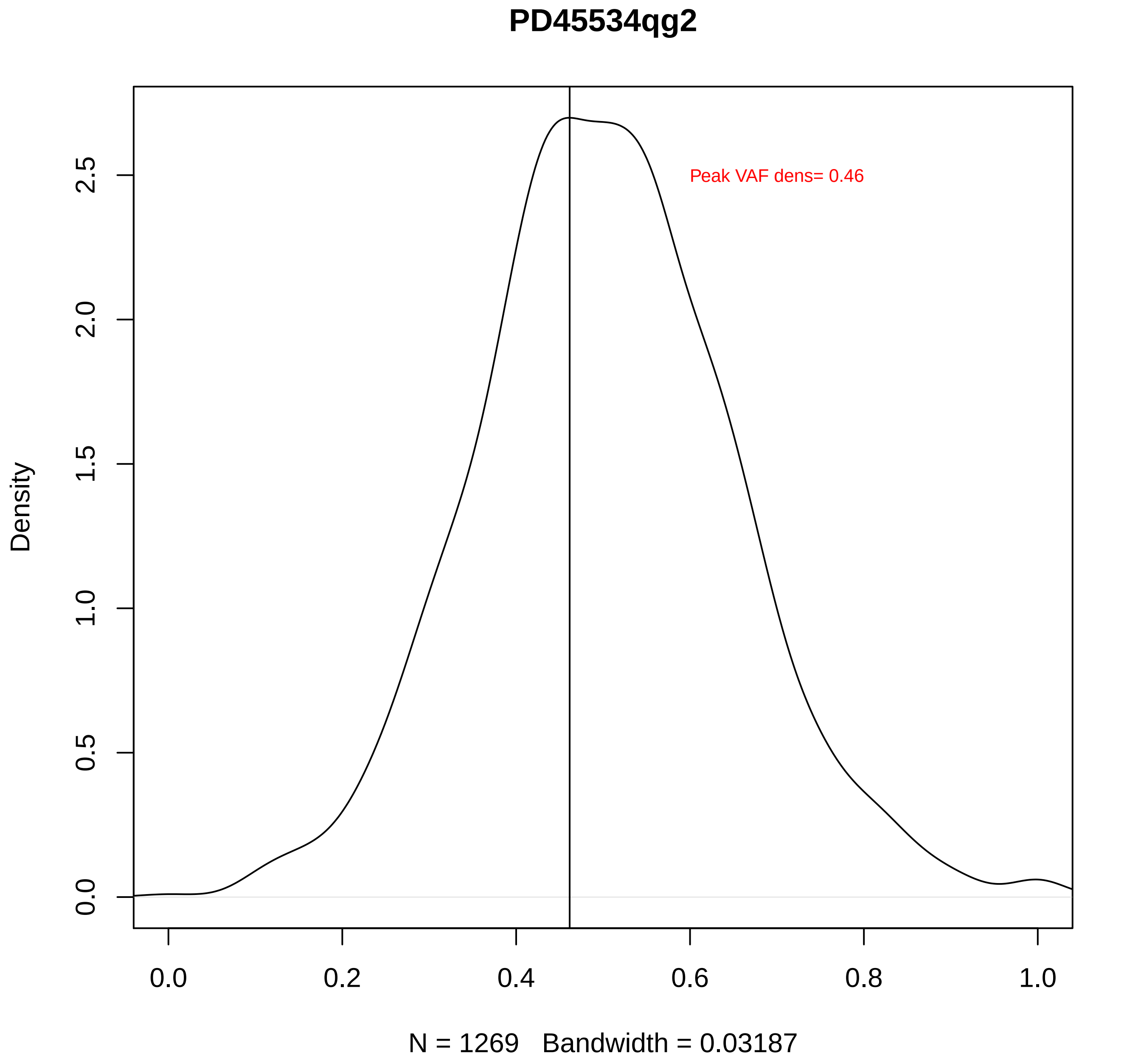
PD45534qg2
Peak VAF dens= 0.46
Density
0.0
0.5
1.0
1.5
2.0
2.5
0.0
0.2
0.4
0.6
0.8
1.0
N = 1269   Bandwidth = 0.03187

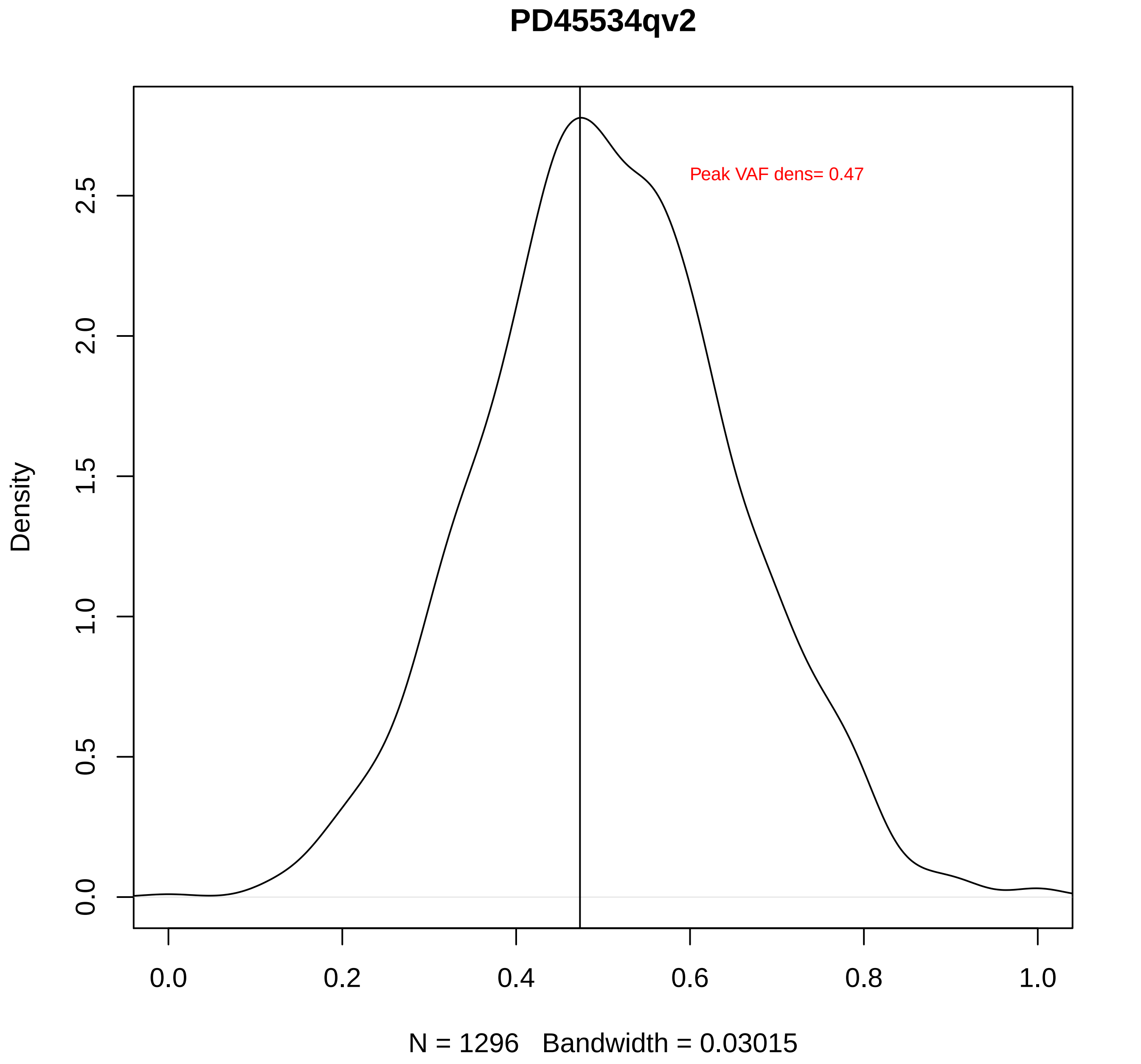

PD45534qv2
Peak VAF dens= 0.47
Density
0.0
0.5
1.0
1.5
2.0
2.5
0.0
0.2
0.4
0.6
0.8
1.0
N = 1296 Bandwidth = 0.03015

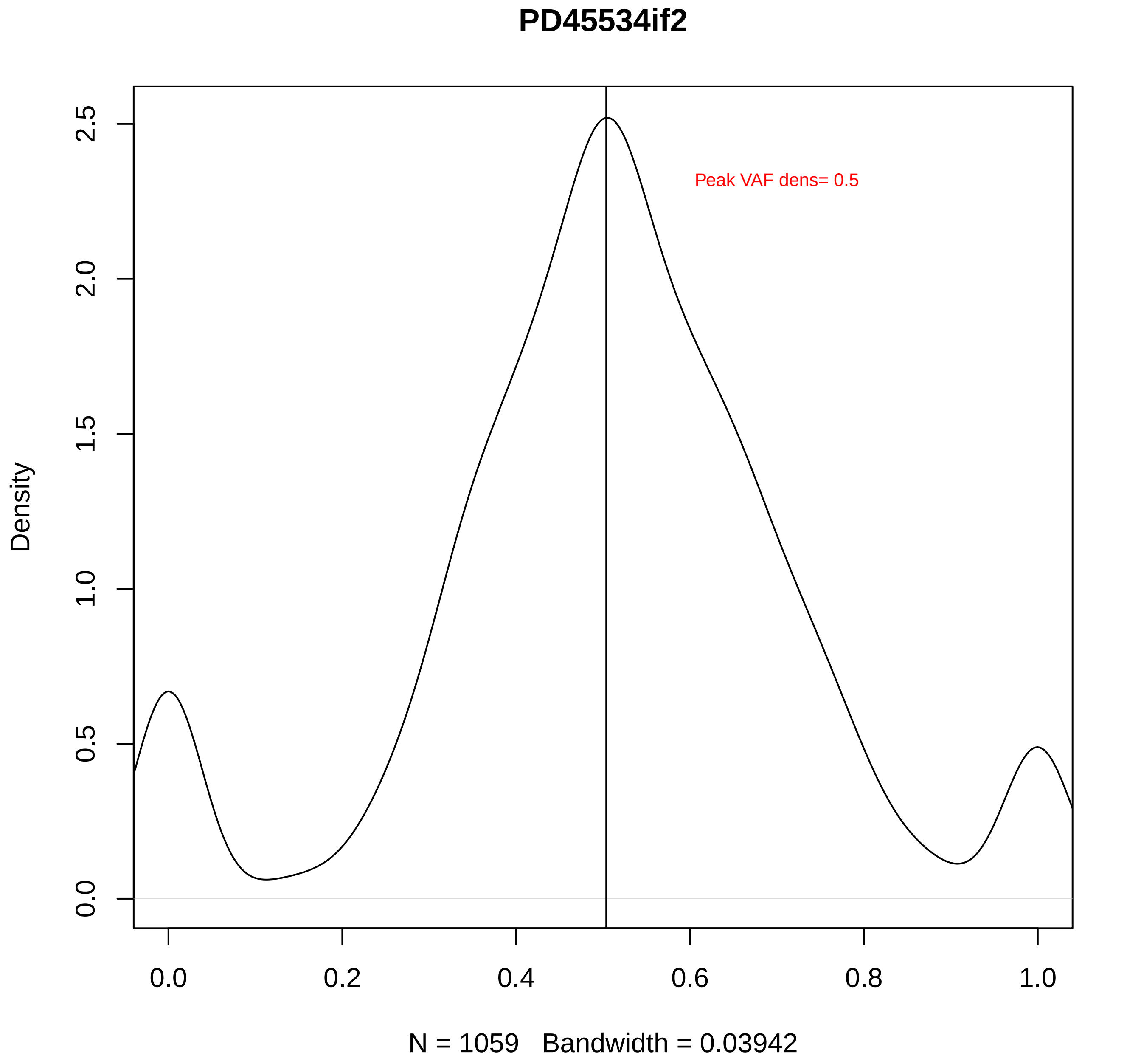

PD45534if2
Peak VAF dens= 0.5
Density
2.5
2.0
1.5
1.0
0.5
0.0
0.0
0.2
0.4
0.6
0.8
1.0
N = 1059   Bandwidth = 0.03942

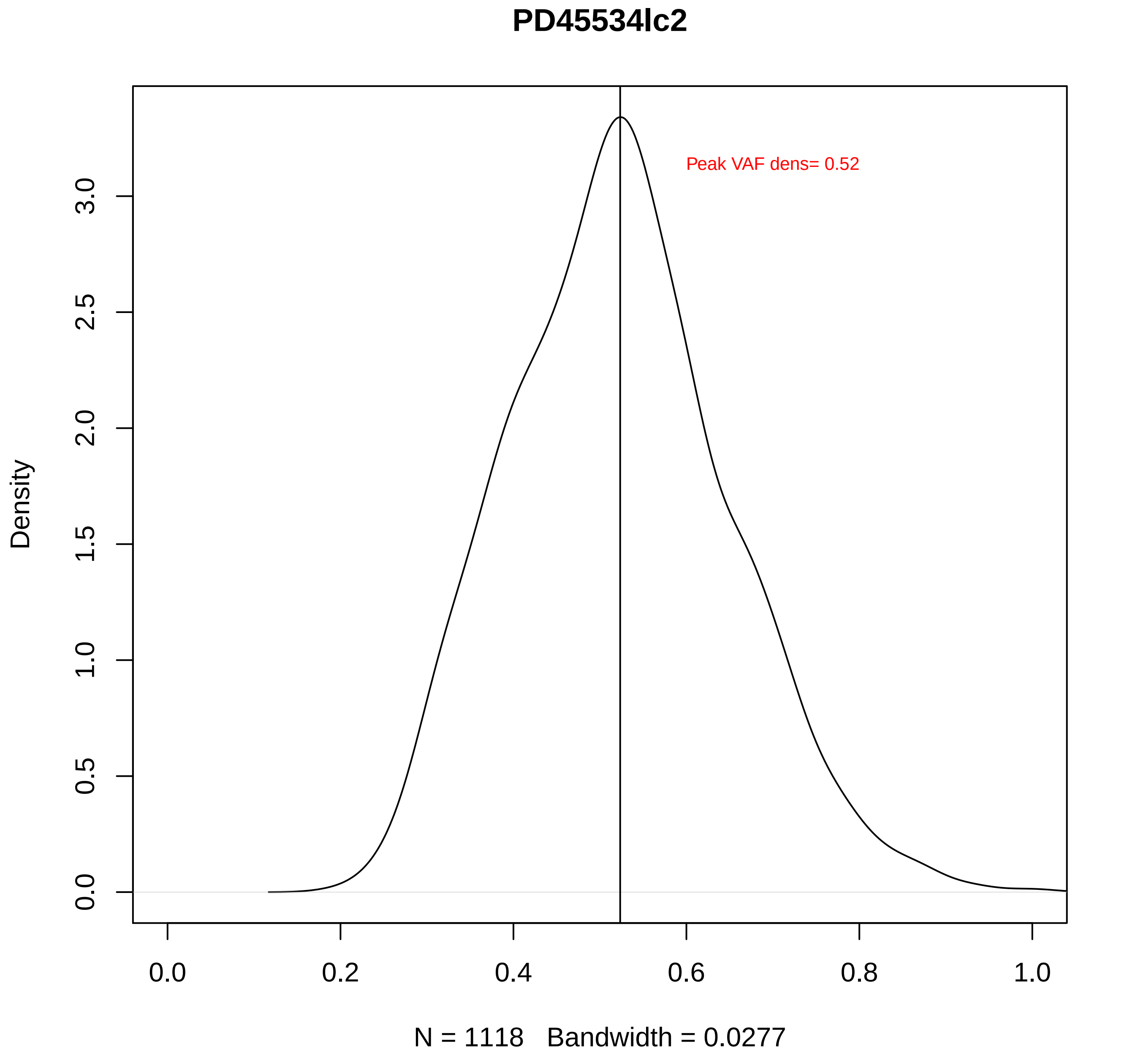

PD45534lc2
Peak VAF dens= 0.52
Density
0.0
0.5
1.0
1.5
2.0
2.5
3.0
0.0
0.2
0.4
0.6
0.8
1.0
N = 1118   Bandwidth = 0.0277

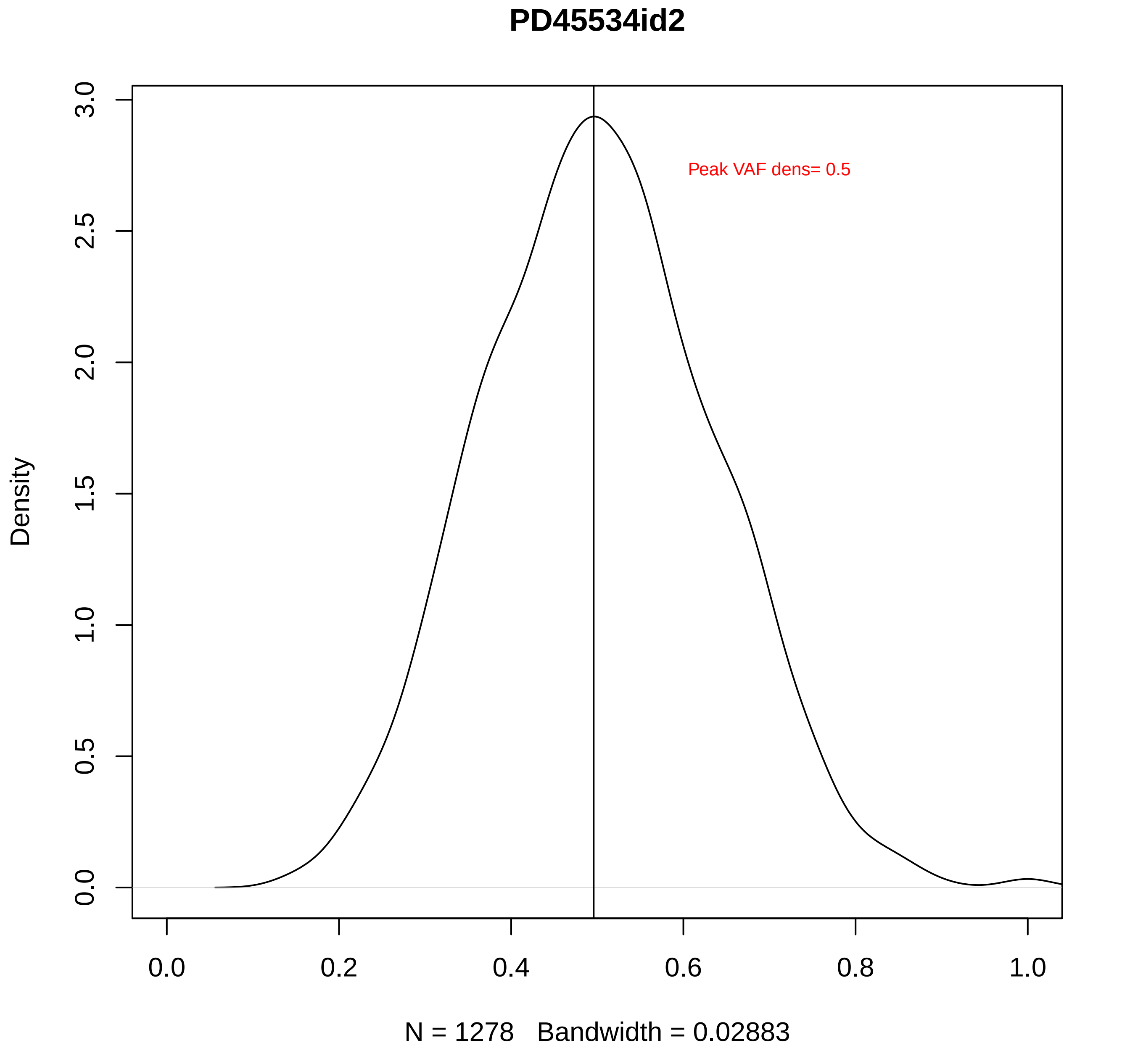

PD45534id2
Peak VAF dens= 0.5
Density
0.0
0.5
1.0
1.5
2.0
2.5
3.0
0.0
0.2
0.4
0.6
0.8
1.0
N = 1278   Bandwidth = 0.02883

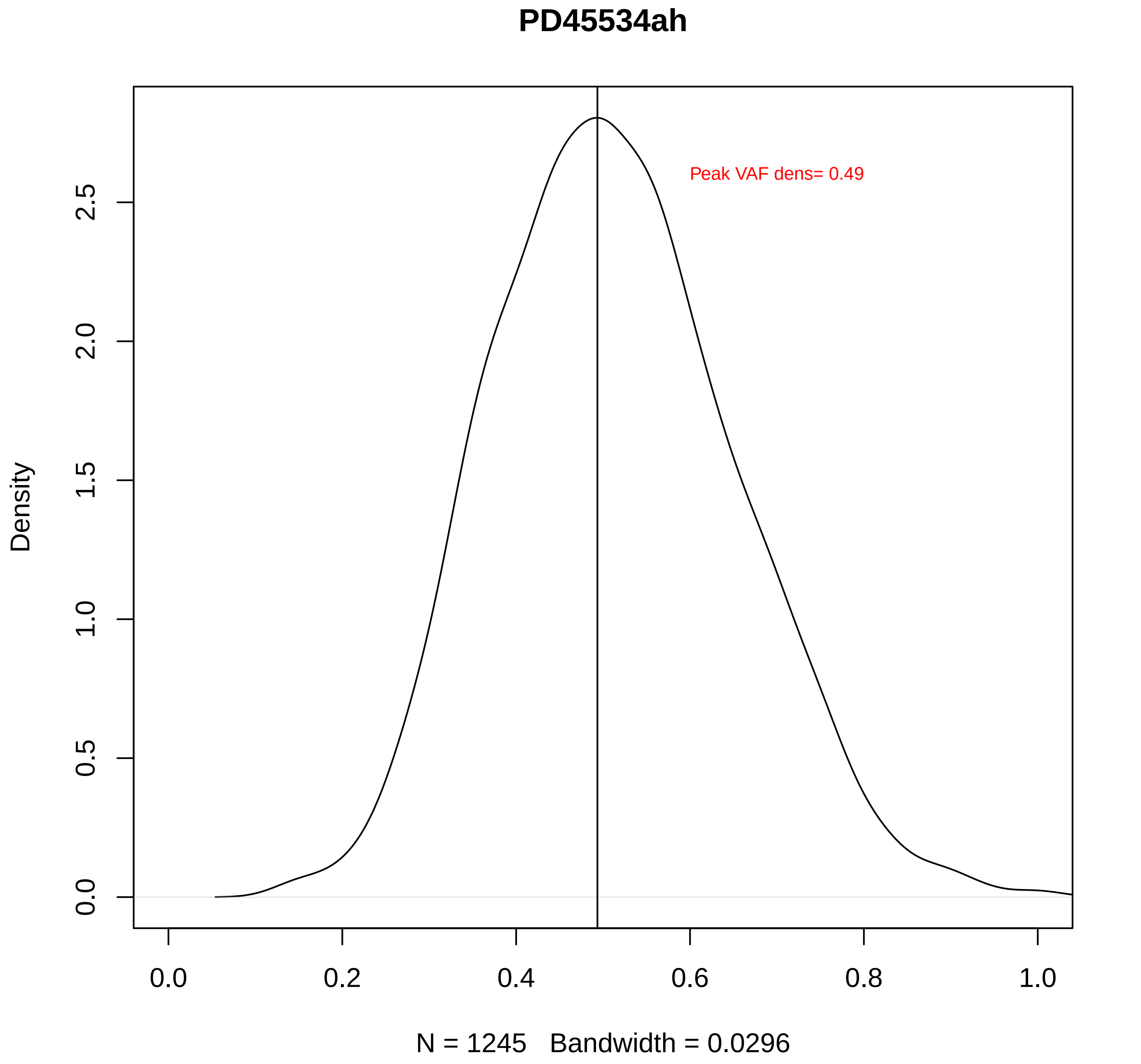
PD45534ah
Peak VAF dens= 0.49
Density
0.0
0.5
1.0
1.5
2.0
2.5
0.0
0.2
0.4
0.6
0.8
1.0
N = 1245   Bandwidth = 0.0296

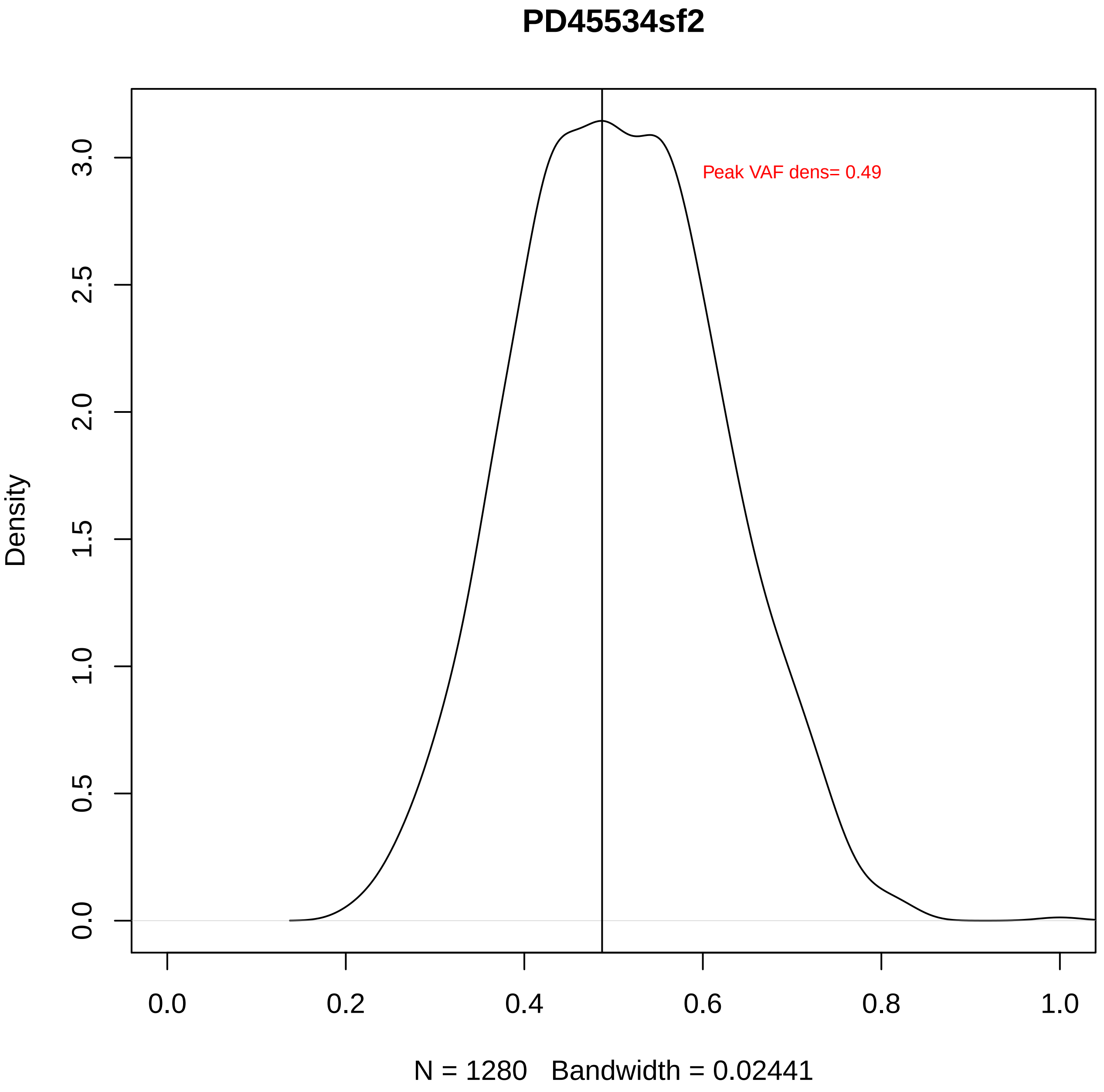

PD45534sf2
Peak VAF dens= 0.49
Density
0.0
0.5
1.0
1.5
2.0
2.5
3.0
0.0
0.2
0.4
0.6
0.8
1.0
N = 1280 Bandwidth = 0.02441

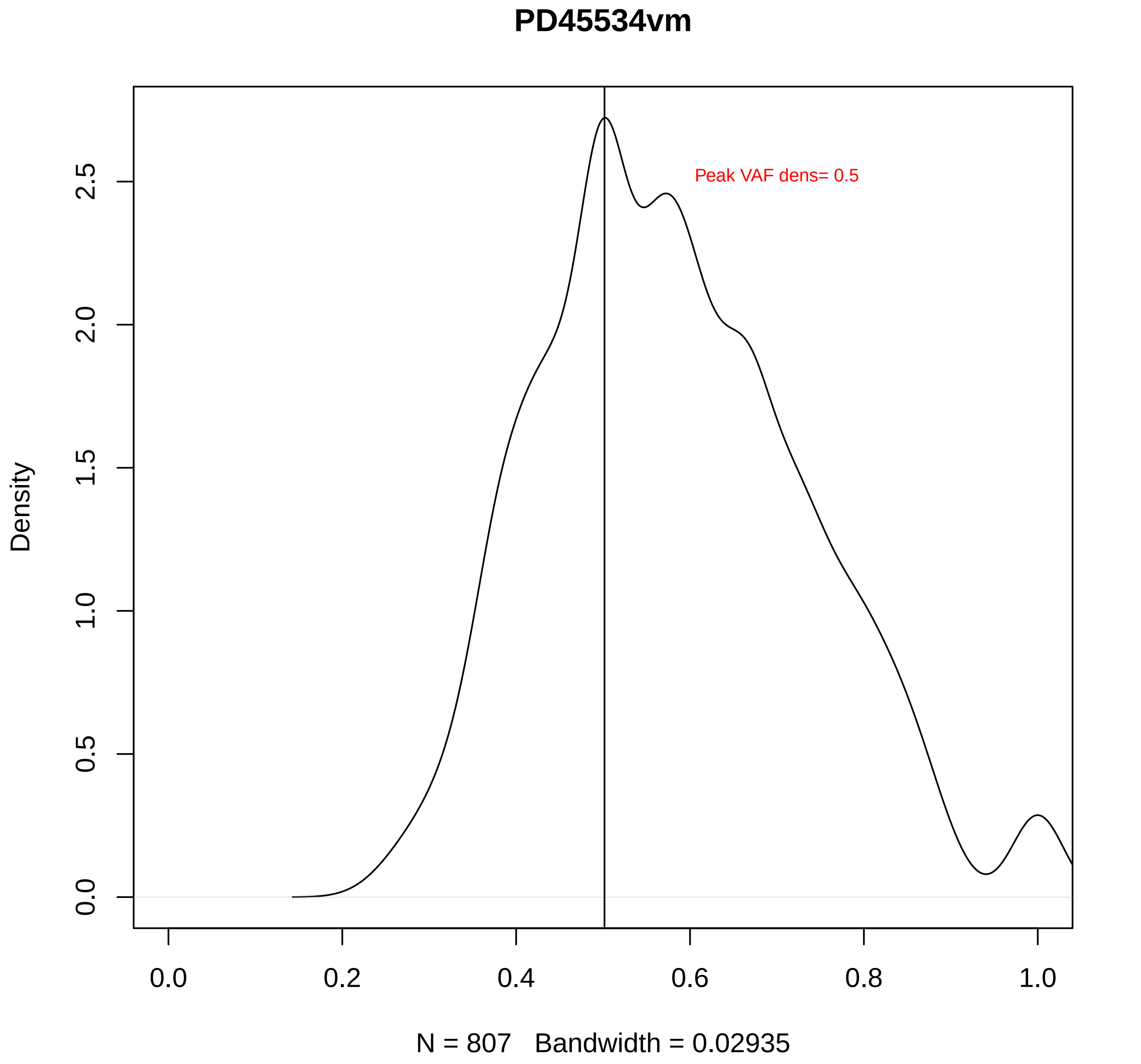
PD45534vm
Peak VAF dens= 0.5
Density
0.0
0.5
1.0
1.5
2.0
2.5
0.0
0.2
0.4
0.6
0.8
1.0
N = 807   Bandwidth = 0.02935

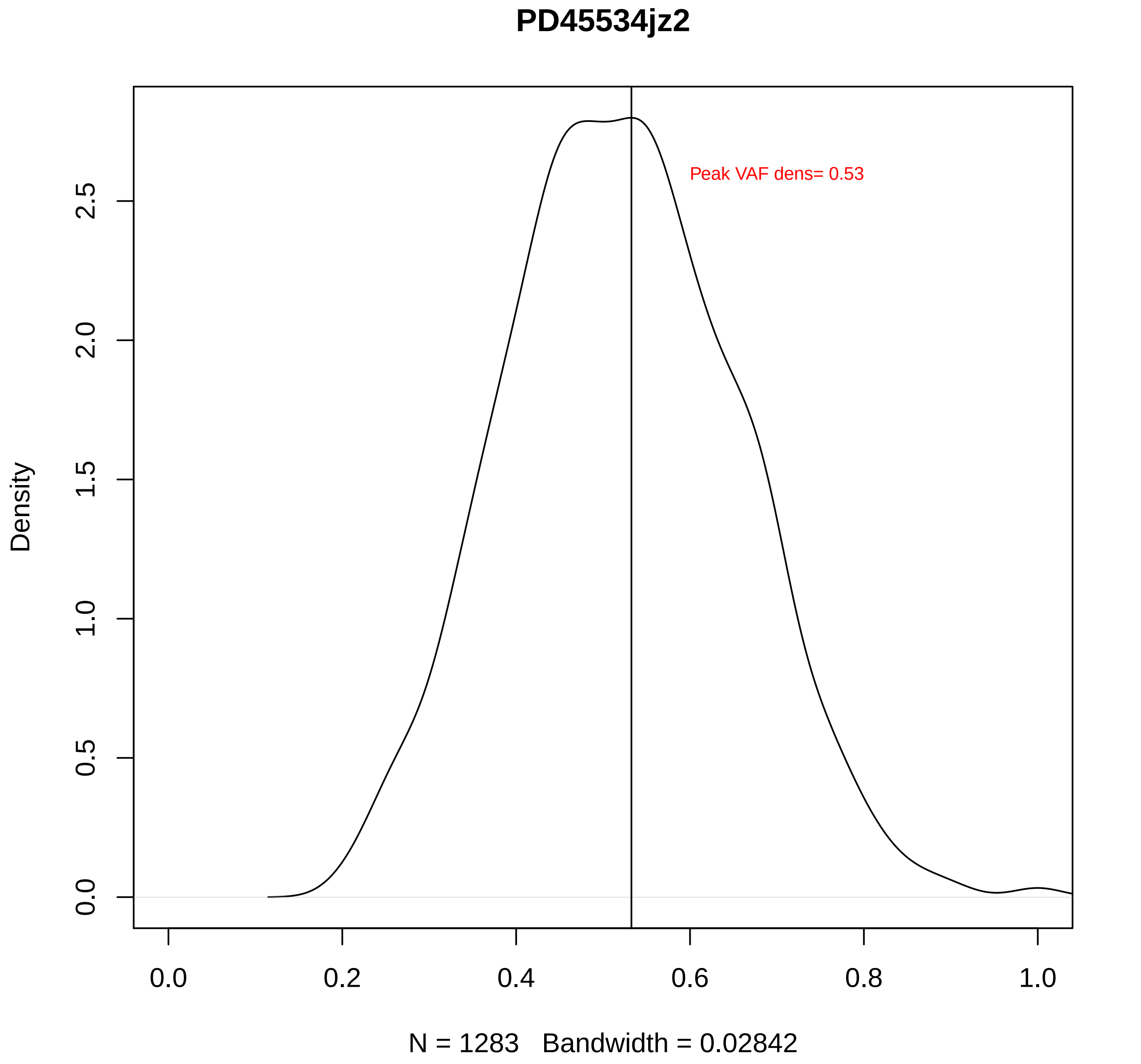

PD45534jz2
Peak VAF dens= 0.53
Density
0.0
0.5
1.0
1.5
2.0
2.5
0.0
0.2
0.4
0.6
0.8
1.0
N = 1283   Bandwidth = 0.02842

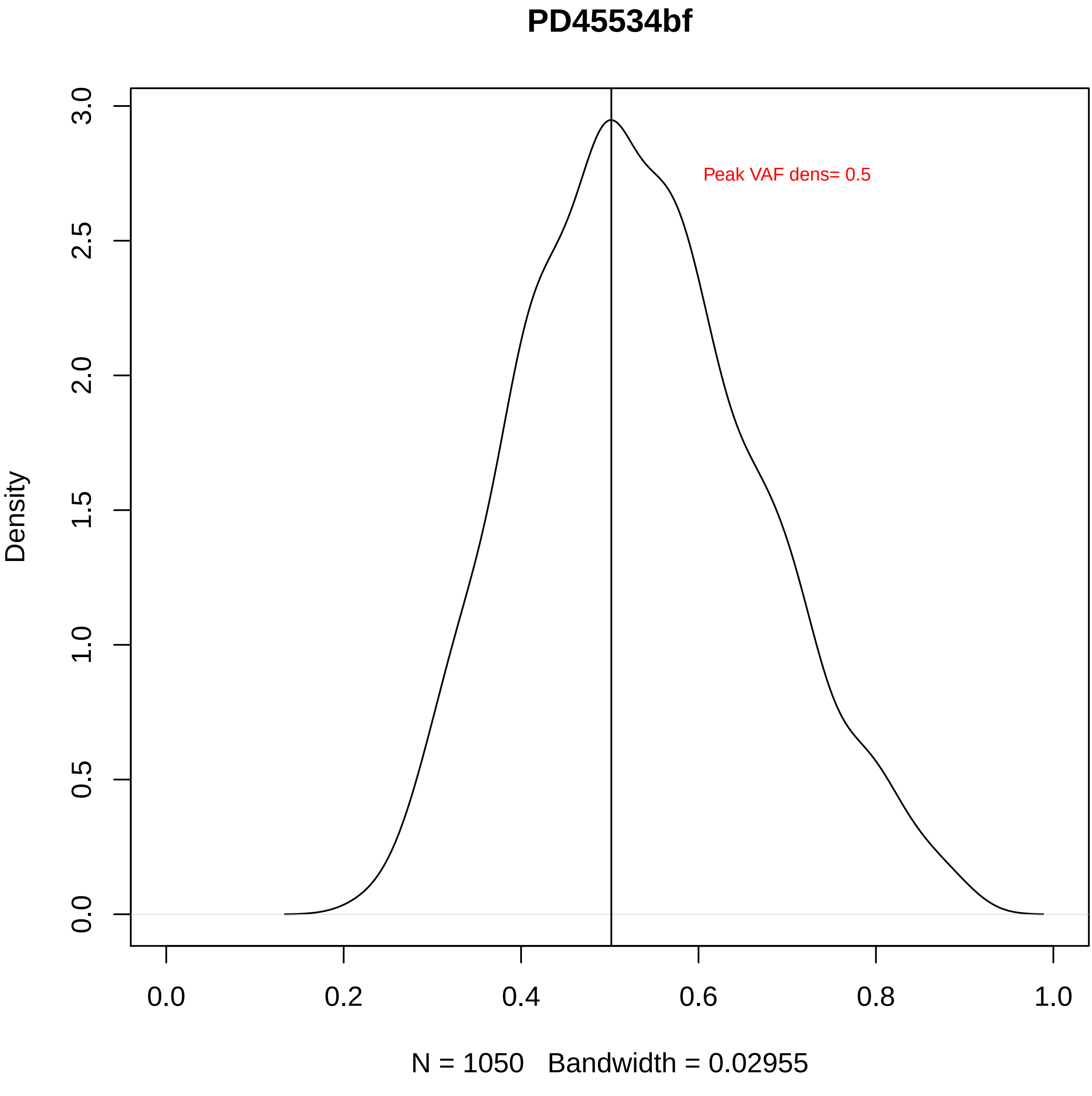

PD45534bf
Peak VAF dens= 0.5
Density
0.0
0.5
1.0
1.5
2.0
2.5
3.0
0.0
0.2
0.4
0.6
0.8
1.0
N = 1050 Bandwidth = 0.02955

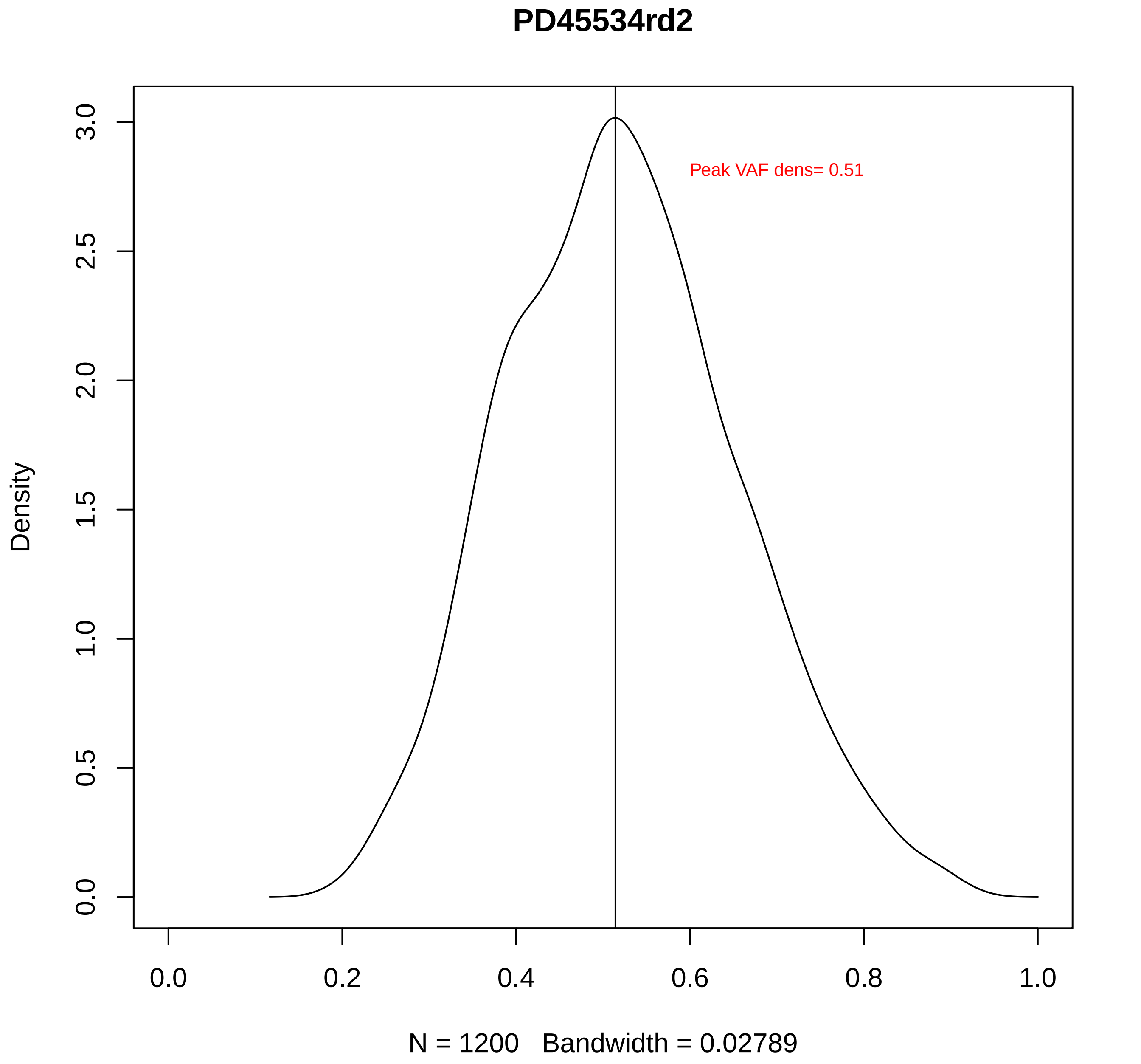
PD45534rd2
Peak VAF dens= 0.51
Density
0.0
0.5
1.0
1.5
2.0
2.5
3.0
0.0
0.2
0.4
0.6
0.8
1.0
N = 1200   Bandwidth = 0.02789

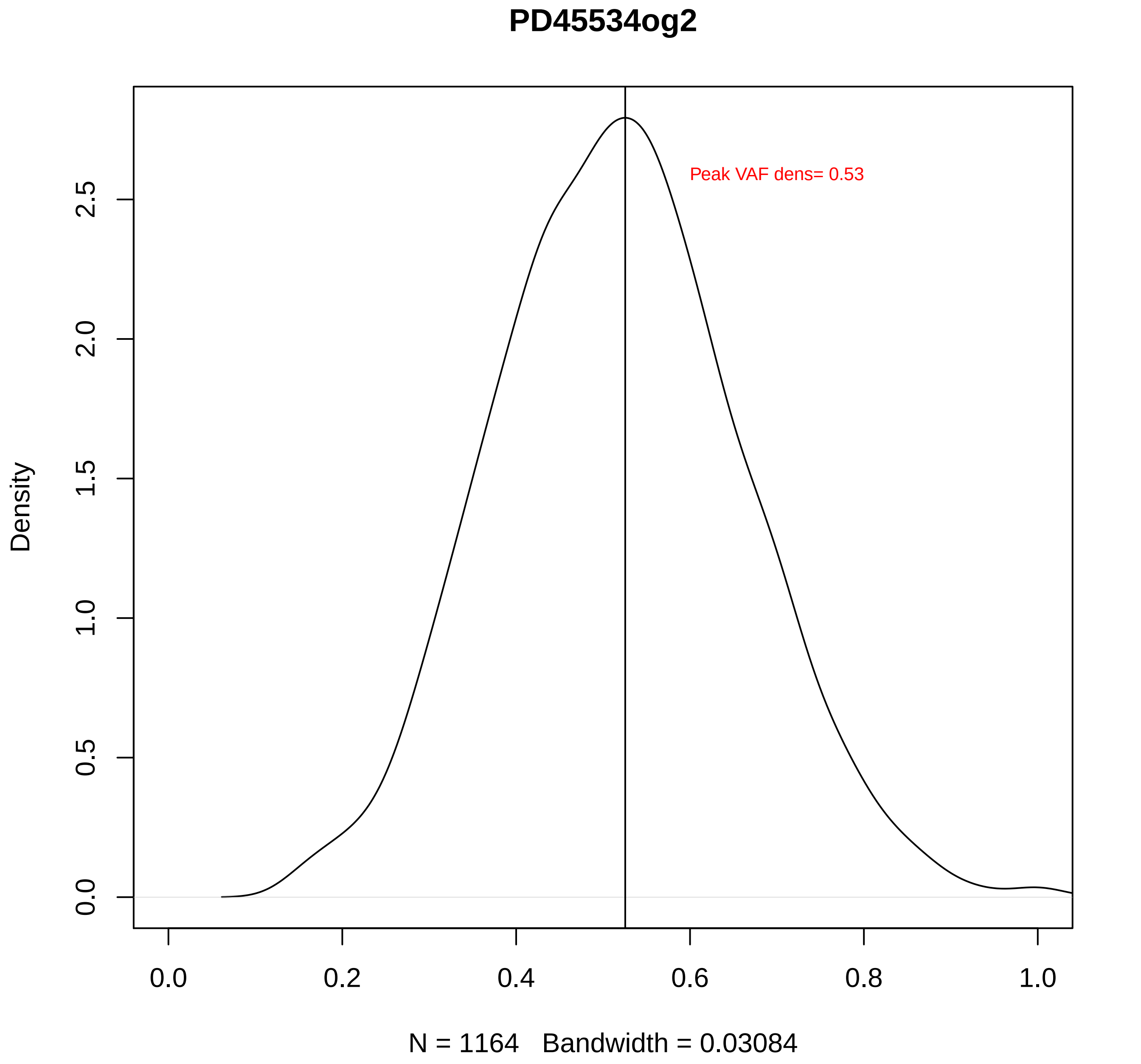

PD45534og2
Peak VAF dens= 0.53
Density
0.0
0.5
1.0
1.5
2.0
2.5
0.0
0.2
0.4
0.6
0.8
1.0
N = 1164   Bandwidth = 0.03084

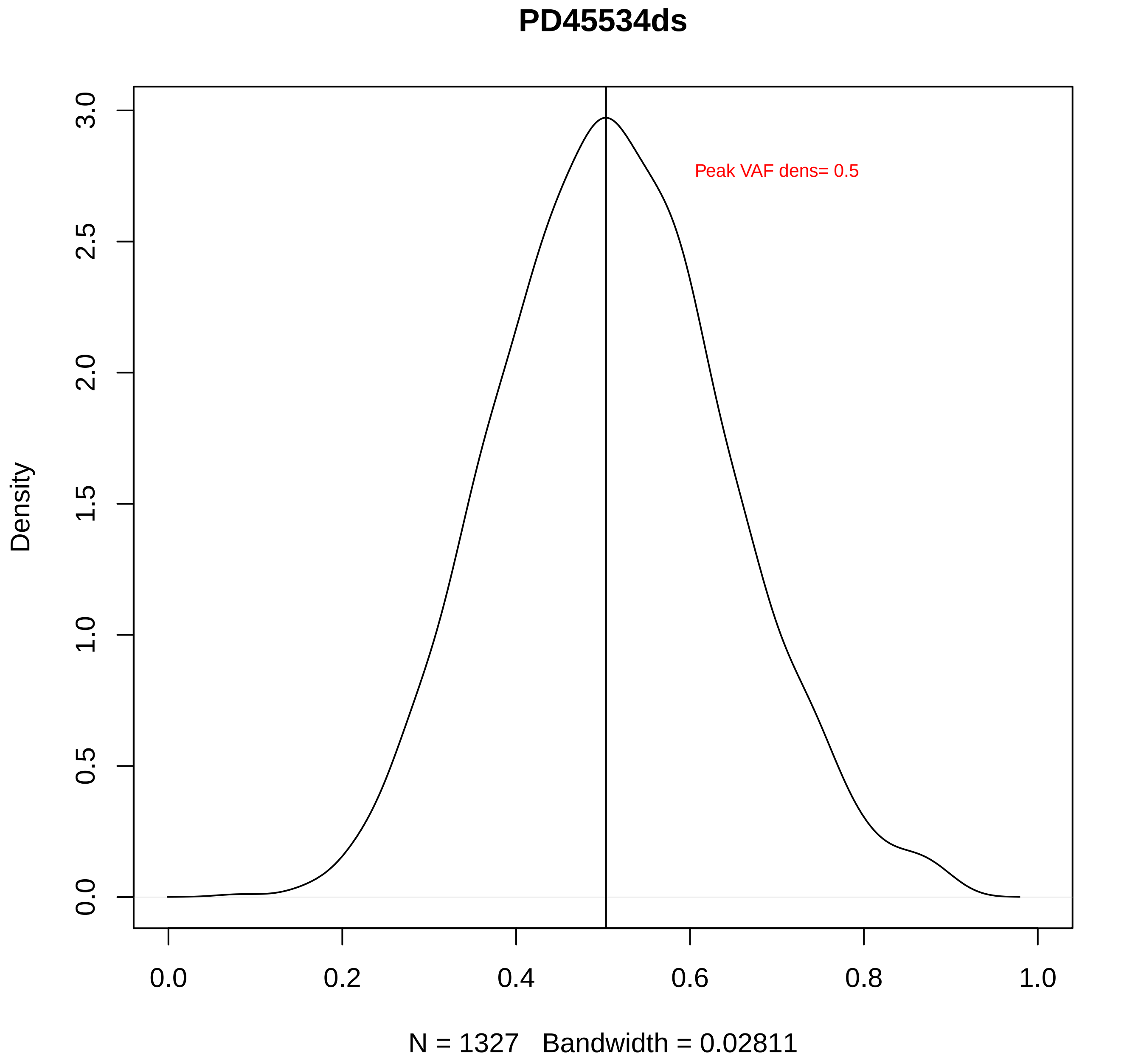

PD45534ds
Peak VAF dens= 0.5
Density
0.0
0.5
1.0
1.5
2.0
2.5
3.0
0.0
0.2
0.4
0.6
0.8
1.0
N = 1327   Bandwidth = 0.02811

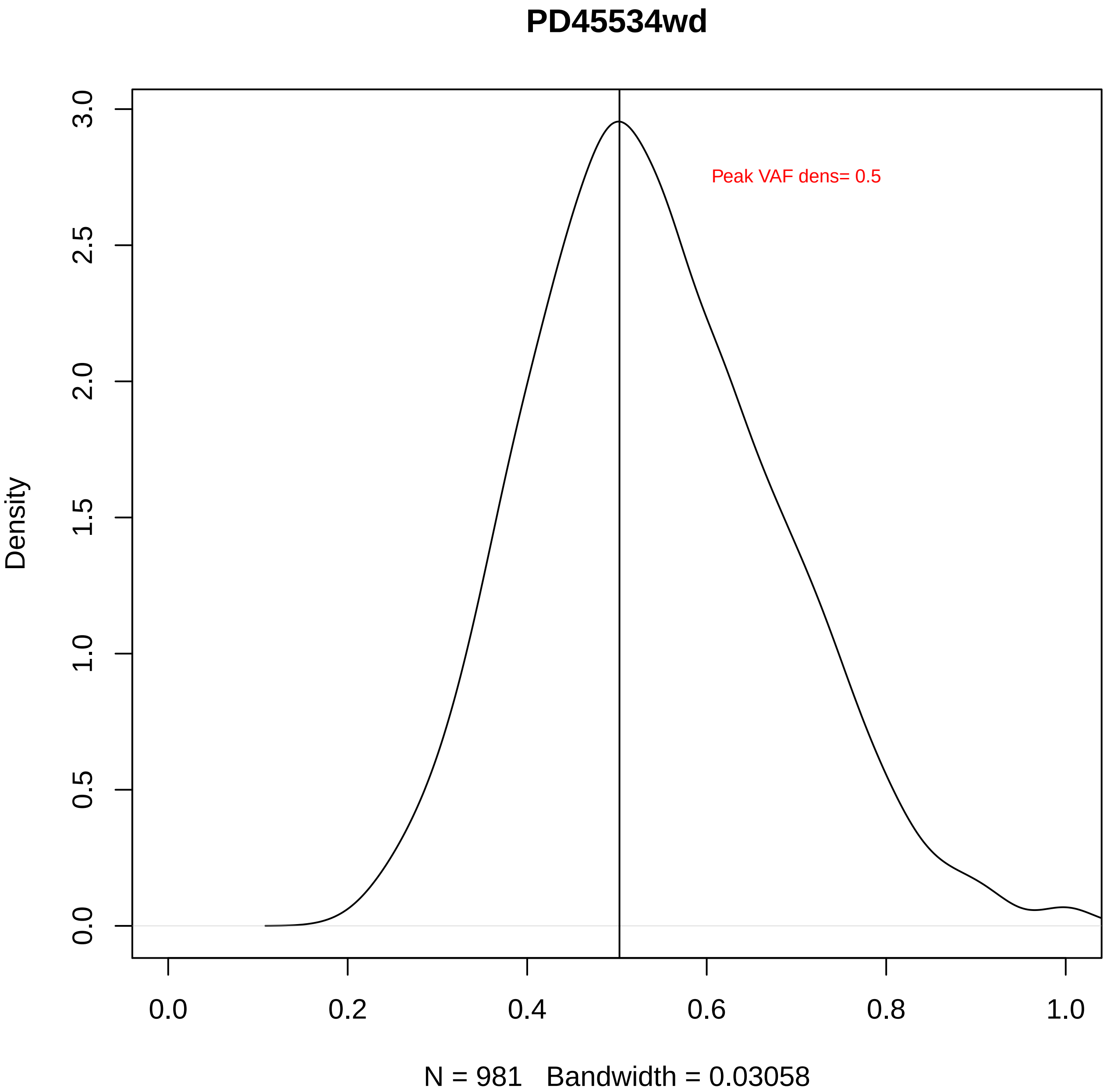
PD45534wd
Peak VAF dens= 0.5
Density
0.0
0.5
1.0
1.5
2.0
2.5
3.0
0.0
0.2
0.4
0.6
0.8
1.0
N = 981   Bandwidth = 0.03058

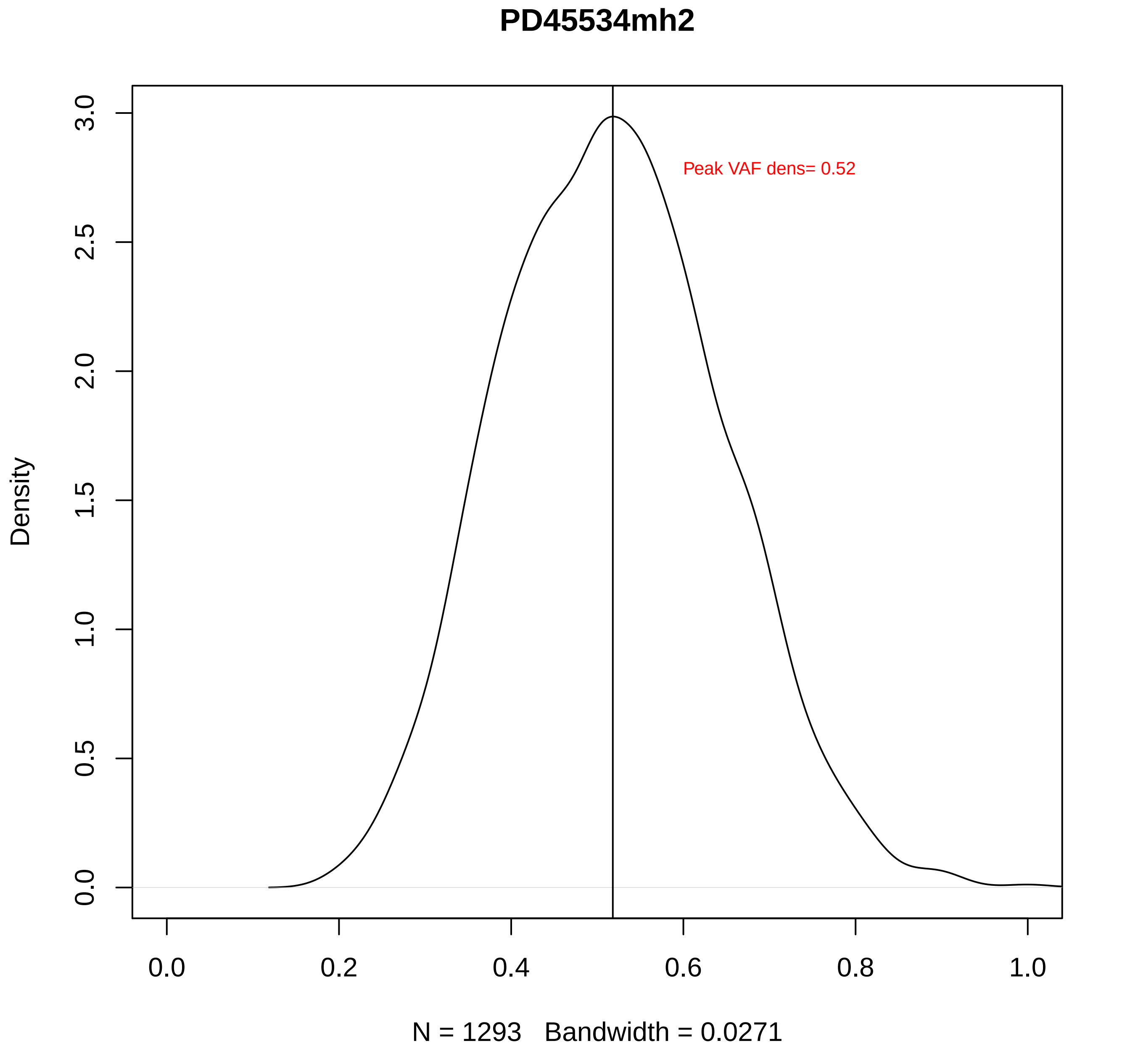

PD45534mh2
Peak VAF dens= 0.52
Density
3.0
2.5
2.0
1.5
1.0
0.5
0.0
0.0
0.2
0.4
0.6
0.8
1.0
N = 1293   Bandwidth = 0.0271

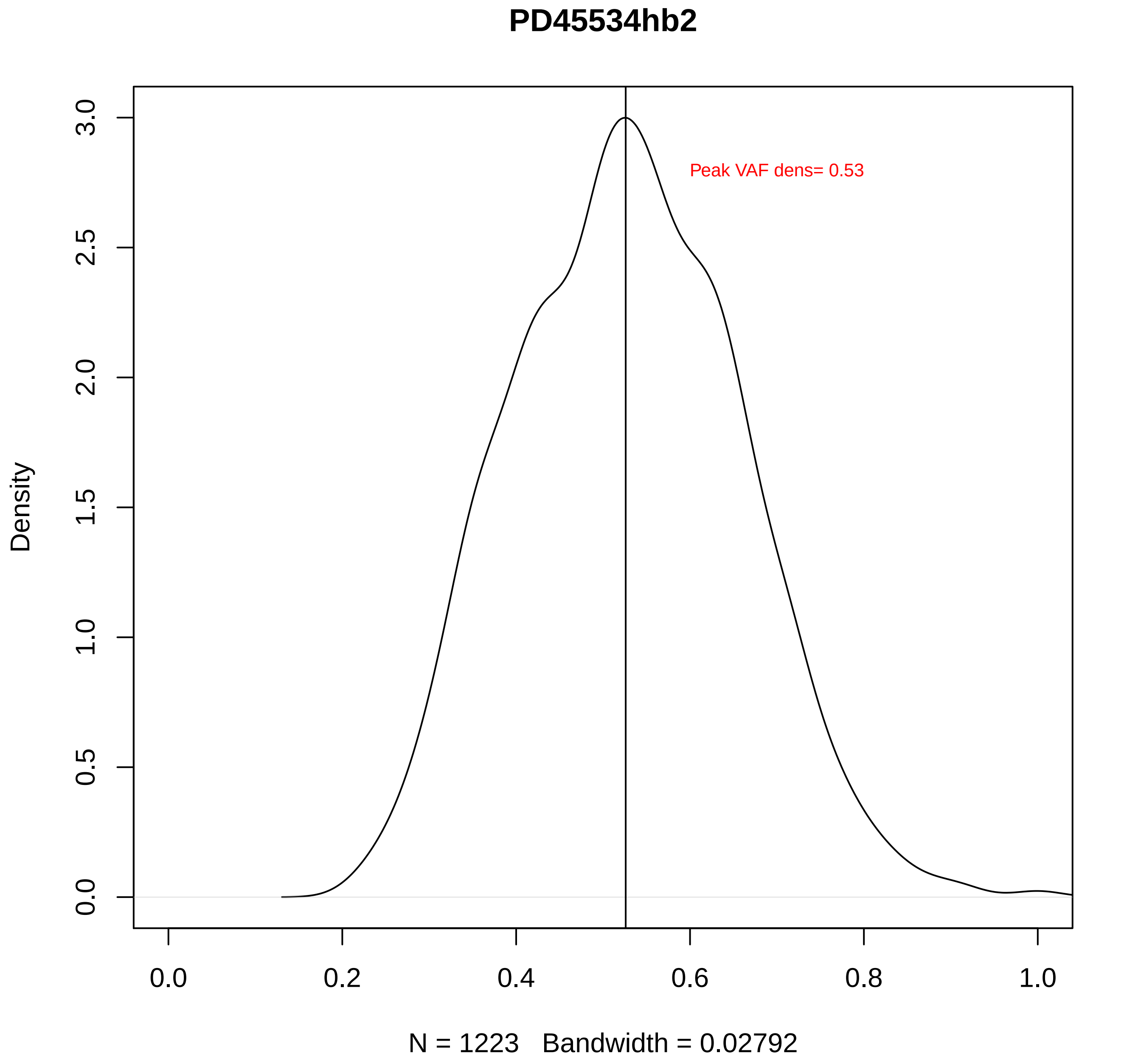
PD45534hb2
Peak VAF dens= 0.53
Density
0.0
0.5
1.0
1.5
2.0
2.5
3.0
0.0
0.2
0.4
0.6
0.8
1.0
N = 1223   Bandwidth = 0.02792

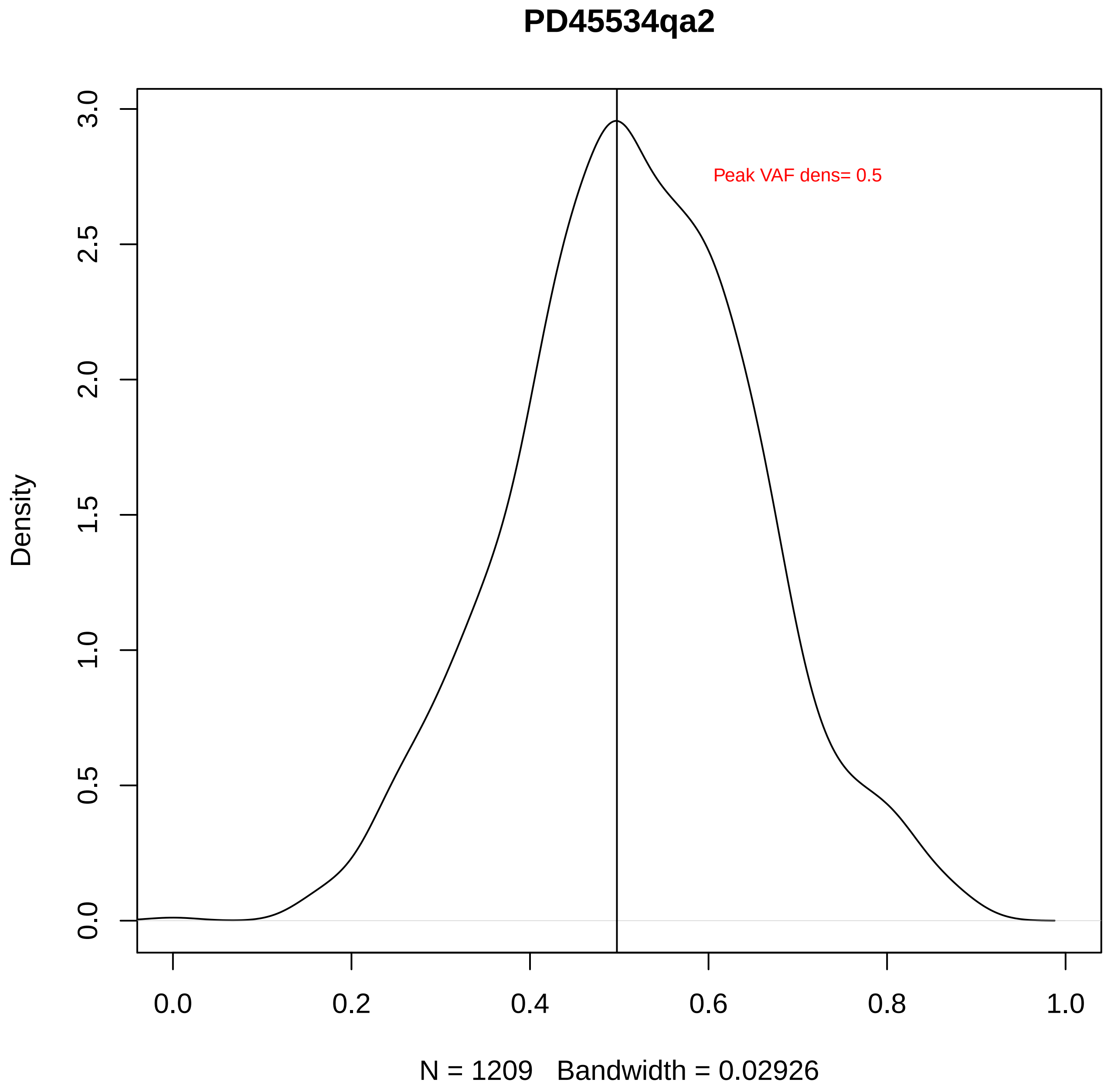
PD45534qa2
Peak VAF dens= 0.5
Density
3.0
2.5
2.0
1.5
1.0
0.5
0.0
0.0
0.2
0.4
0.6
0.8
1.0
N = 1209   Bandwidth = 0.02926

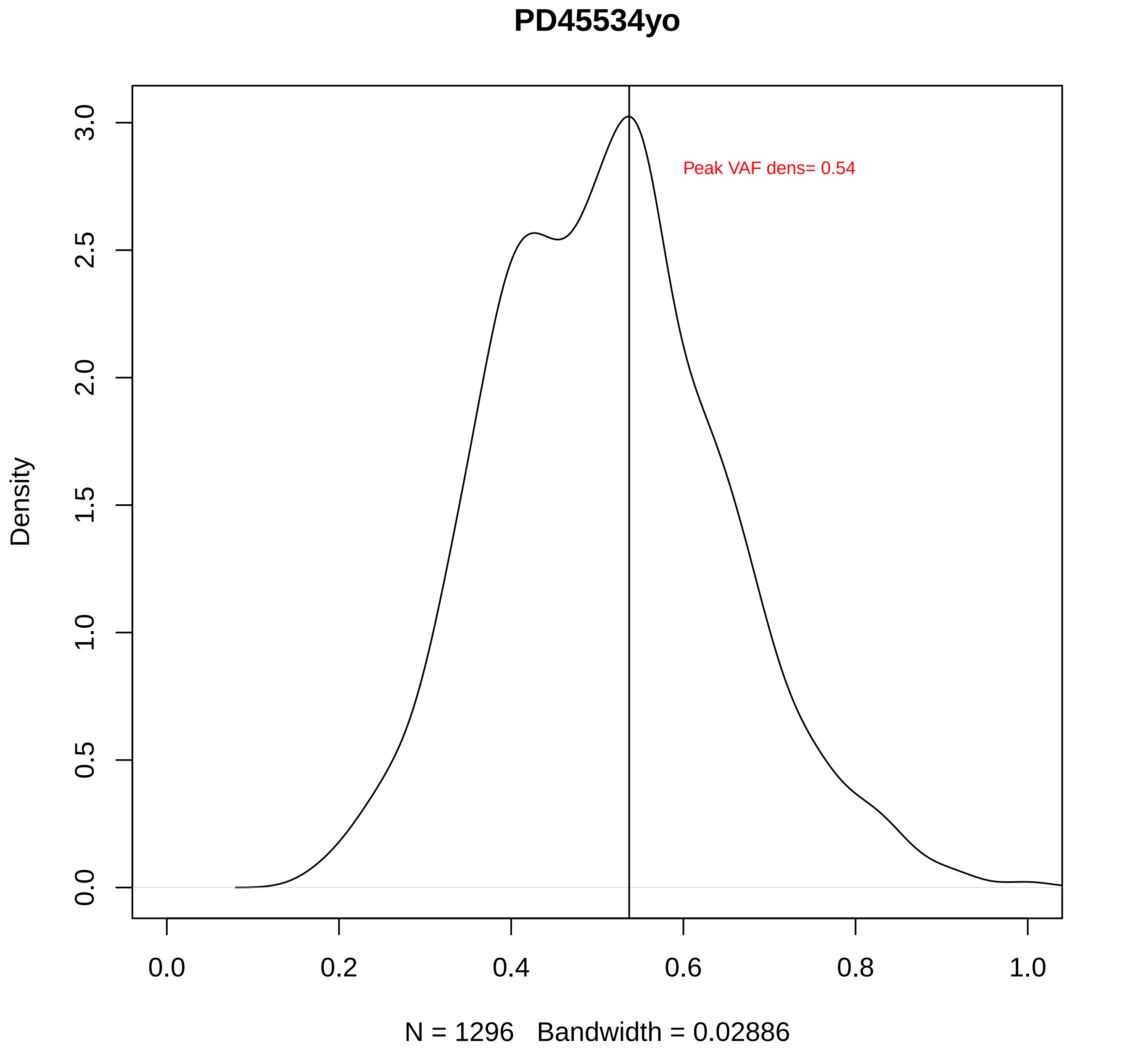
PD45534yo
Peak VAF dens= 0.54
Density
0.0
0.5
1.0
1.5
2.0
2.5
3.0
0.0
0.2
0.4
0.6
0.8
1.0
N = 1296   Bandwidth = 0.02886

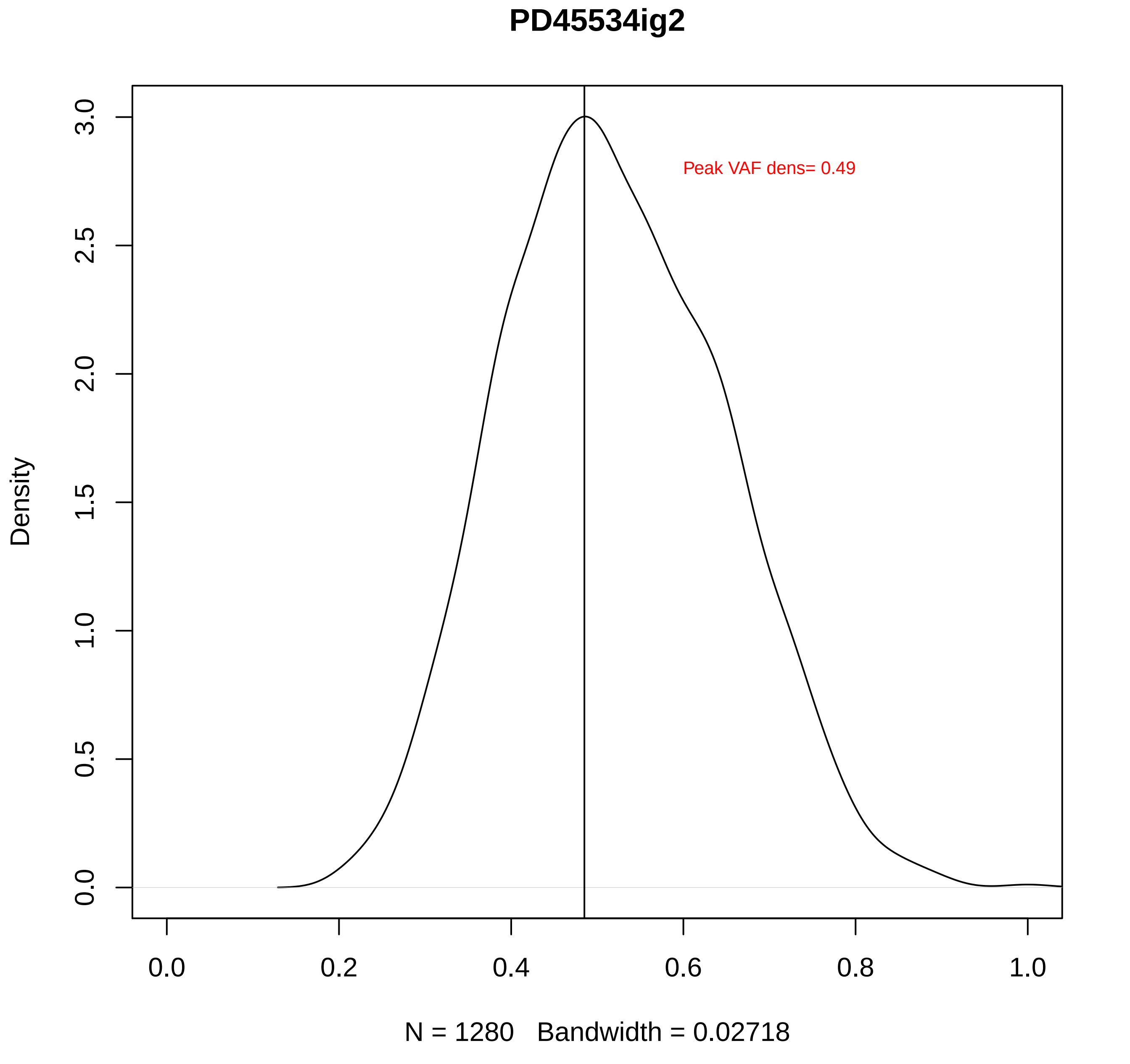
PD45534ig2
Peak VAF dens= 0.49
Density
0.0
0.5
1.0
1.5
2.0
2.5
3.0
0.0
0.2
0.4
0.6
0.8
1.0
N = 1280   Bandwidth = 0.02718

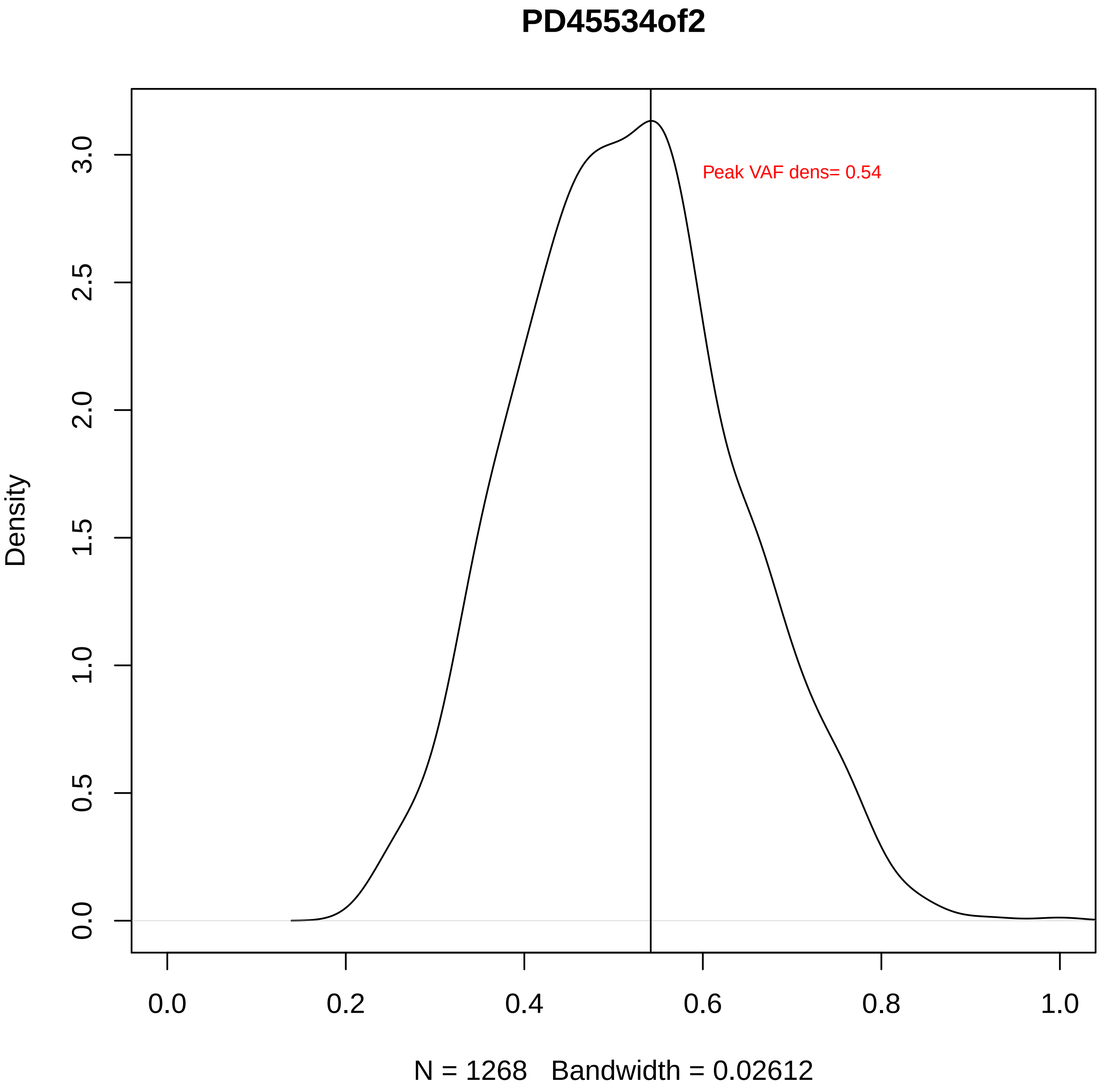
PD45534of2
Peak VAF dens= 0.54
Density
0.0
0.5
1.0
1.5
2.0
2.5
3.0
0.0
0.2
0.4
0.6
0.8
1.0
N = 1268 Bandwidth = 0.02612

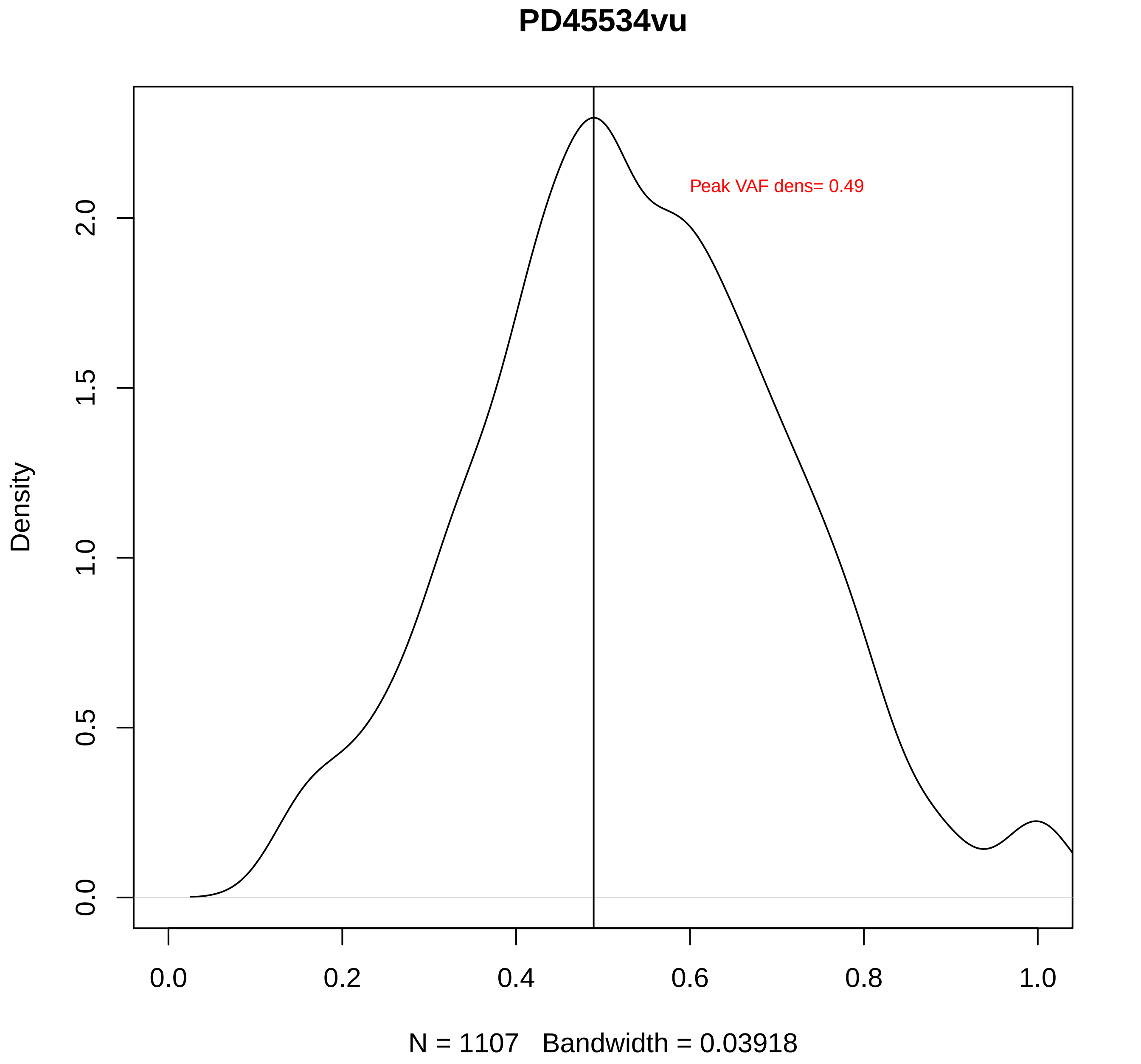

PD45534vu
Peak VAF dens= 0.49
Density
0.0
0.5
1.0
1.5
2.0
0.0
0.2
0.4
0.6
0.8
1.0
N = 1107   Bandwidth = 0.03918

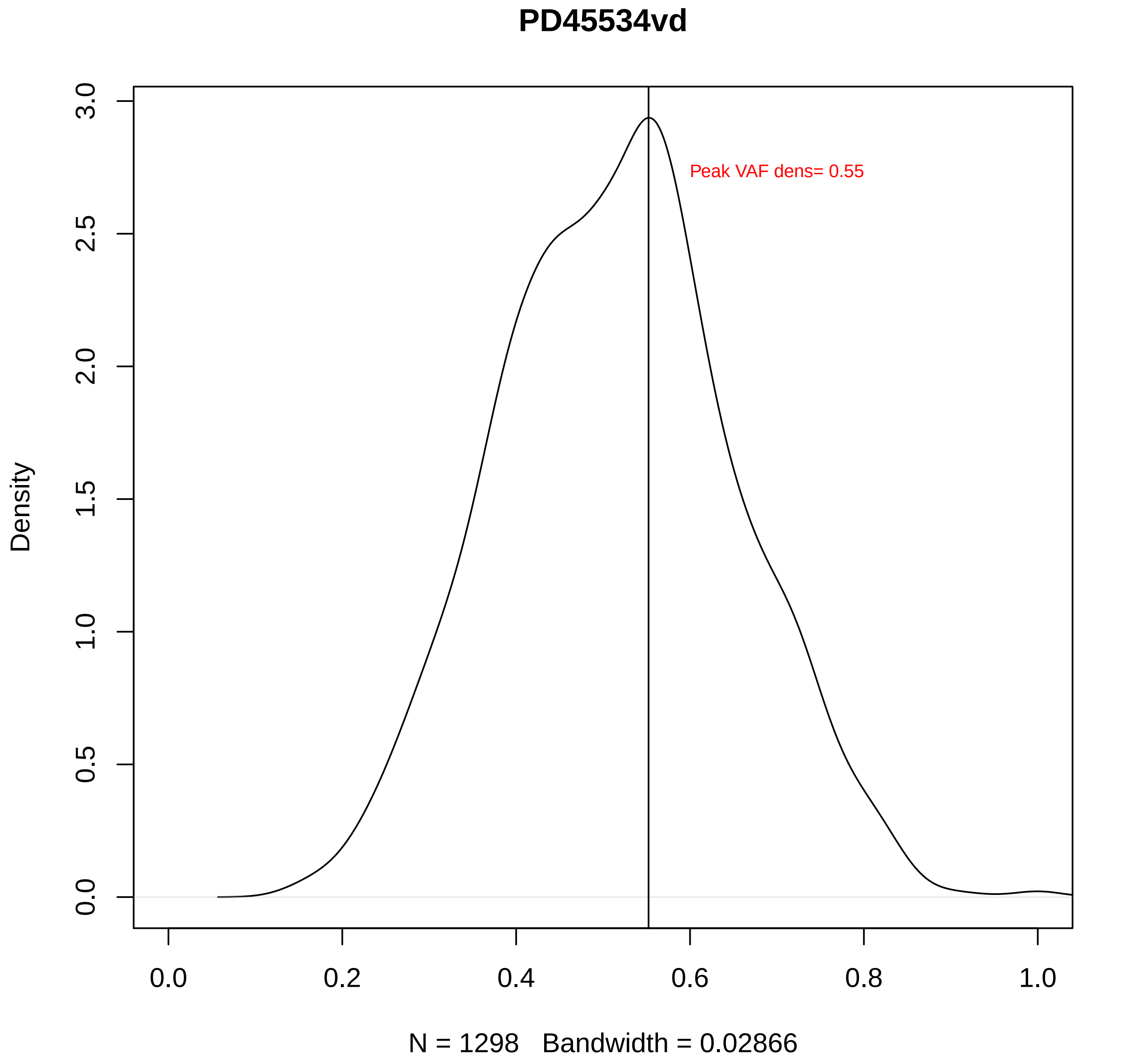
PD45534vd
Peak VAF dens= 0.55
Density
0.0
0.5
1.0
1.5
2.0
2.5
3.0
0.0
0.2
0.4
0.6
0.8
1.0
N = 1298   Bandwidth = 0.02866

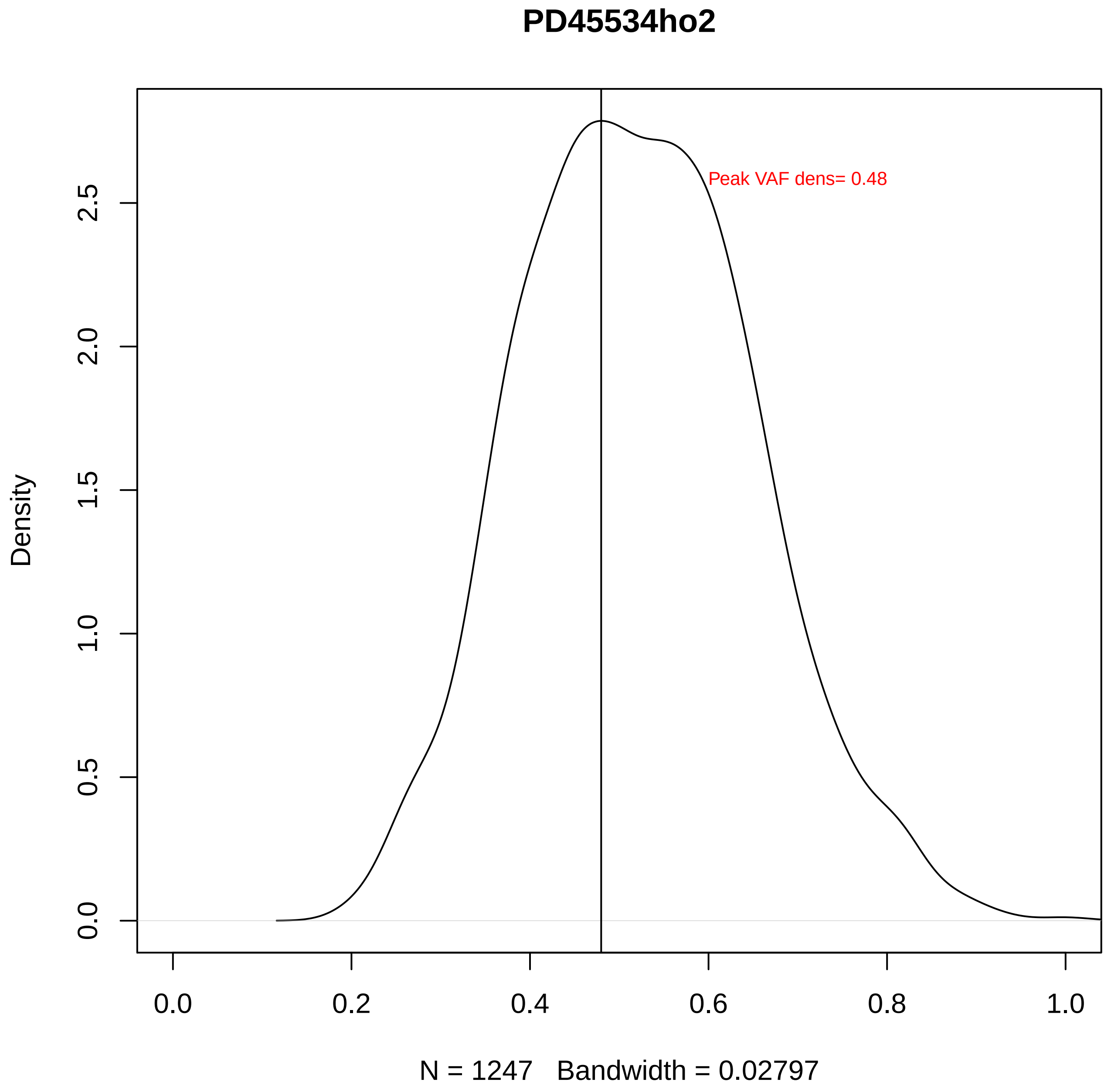

PD45534ho2
Peak VAF dens= 0.48
Density
0.0
0.5
1.0
1.5
2.0
2.5
0.0
0.2
0.4
0.6
0.8
1.0
N = 1247   Bandwidth = 0.02797

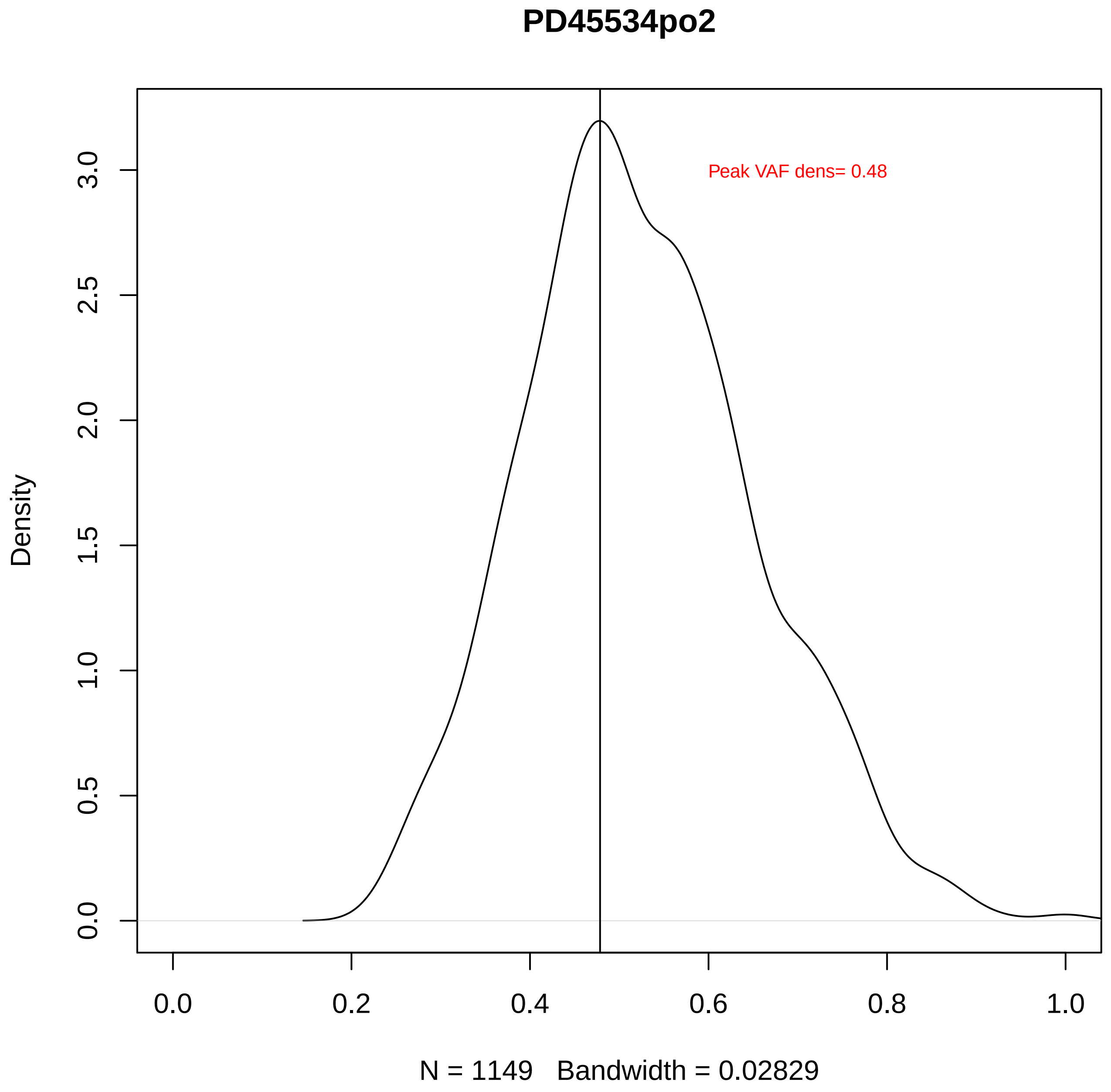
PD45534po2
Peak VAF dens= 0.48
Density
0.0
0.5
1.0
1.5
2.0
2.5
3.0
0.0
0.2
0.4
0.6
0.8
1.0
N = 1149 Bandwidth = 0.02829

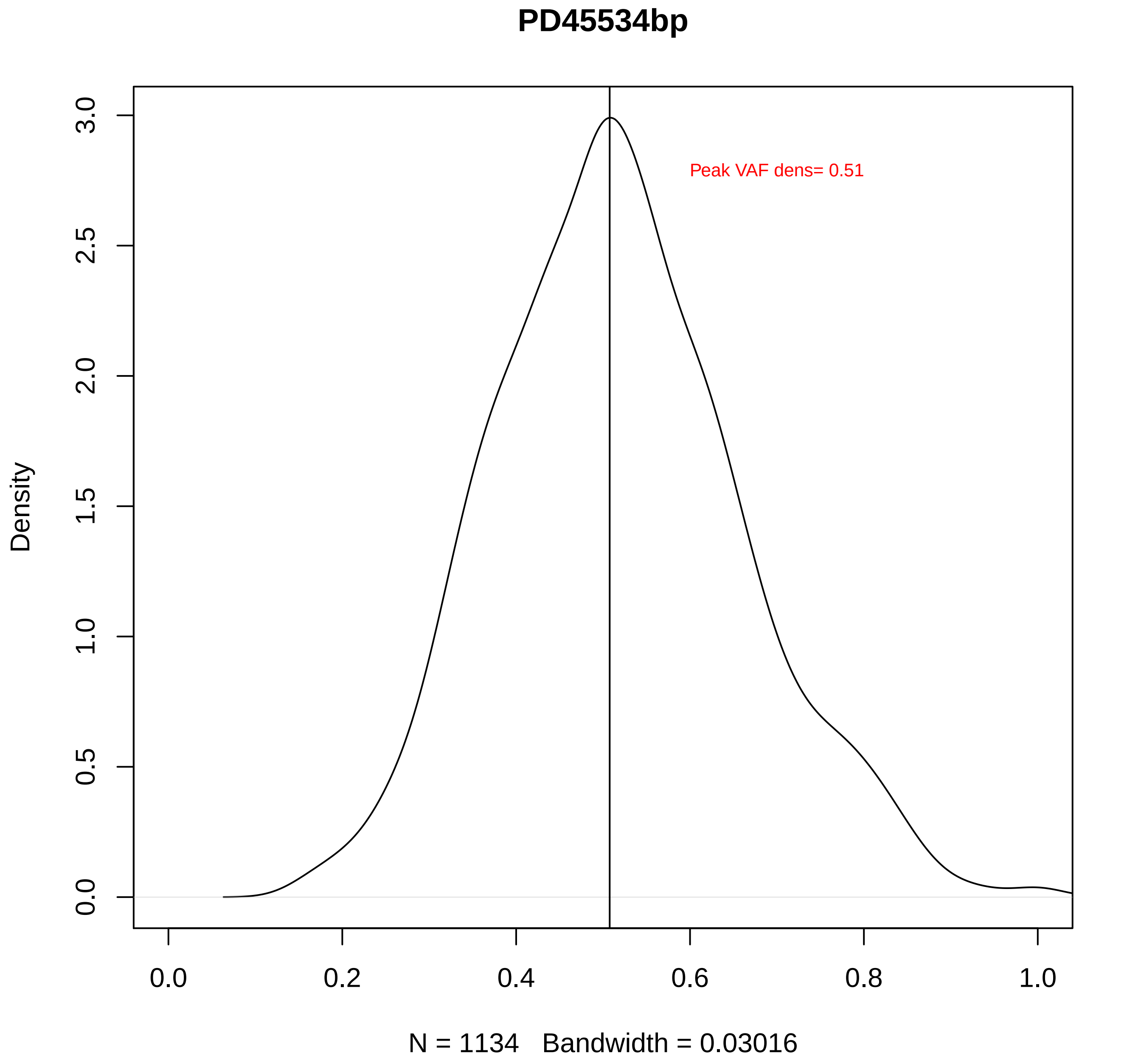

PD45534bp
Peak VAF dens= 0.51
Density
0.0
0.5
1.0
1.5
2.0
2.5
3.0
0.0
0.2
0.4
0.6
0.8
1.0
N = 1134 Bandwidth = 0.03016

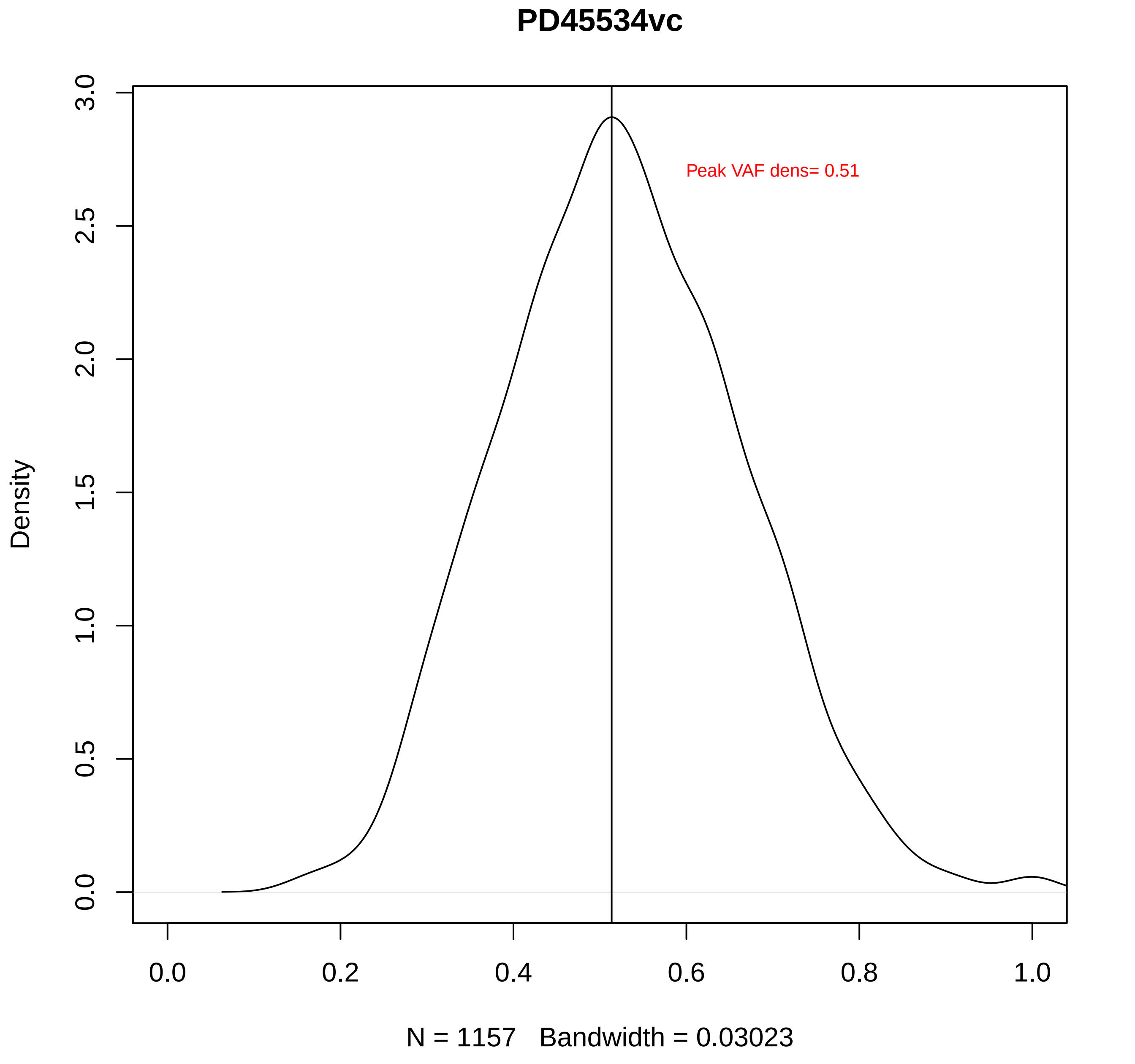
PD45534vc
Peak VAF dens= 0.51
Density
0.0
0.5
1.0
1.5
2.0
2.5
3.0
0.0
0.2
0.4
0.6
0.8
1.0
N = 1157   Bandwidth = 0.03023

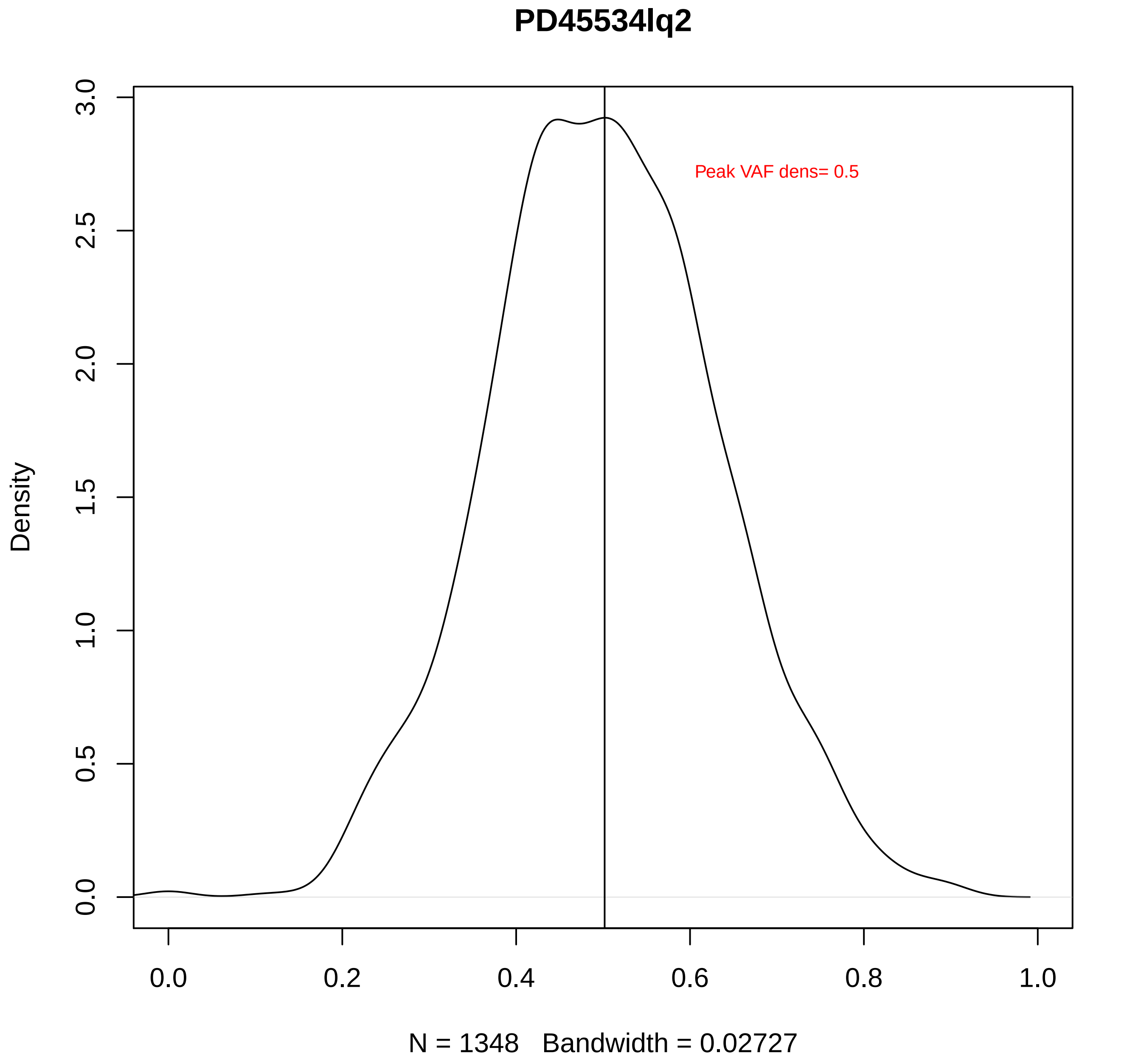
PD45534lq2
Peak VAF dens= 0.5
Density
0.0
0.5
1.0
1.5
2.0
2.5
3.0
0.0
0.2
0.4
0.6
0.8
1.0
N = 1348   Bandwidth = 0.02727

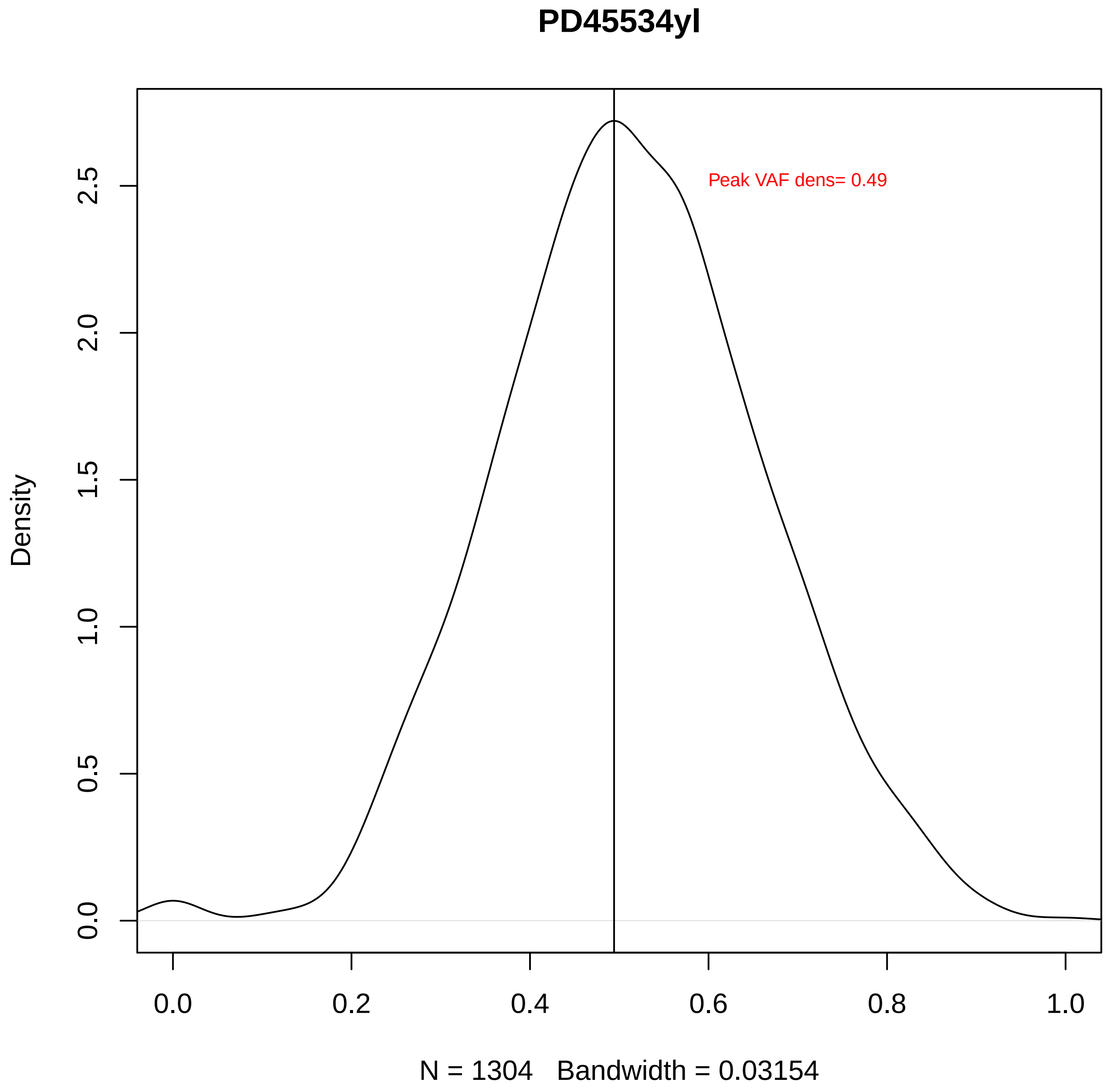

PD45534yl
Peak VAF dens= 0.49
Density
0.0
0.5
1.0
1.5
2.0
2.5
0.0
0.2
0.4
0.6
0.8
1.0
N = 1304   Bandwidth = 0.03154

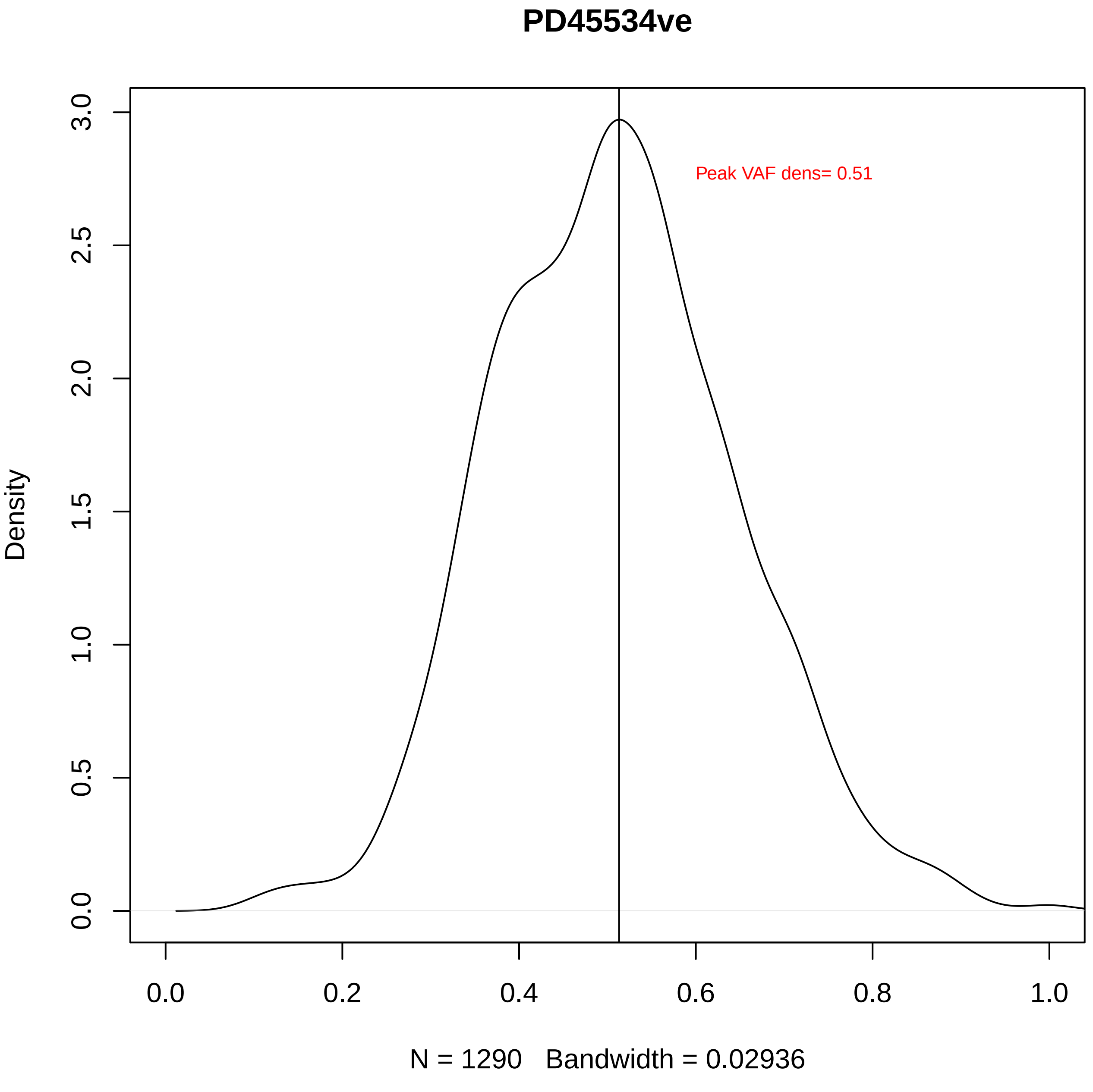
PD45534ve
Peak VAF dens= 0.51
Density
3.0
2.5
2.0
1.5
1.0
0.5
0.0
0.2
0.4
0.6
0.8
1.0
N = 1290   Bandwidth = 0.02936

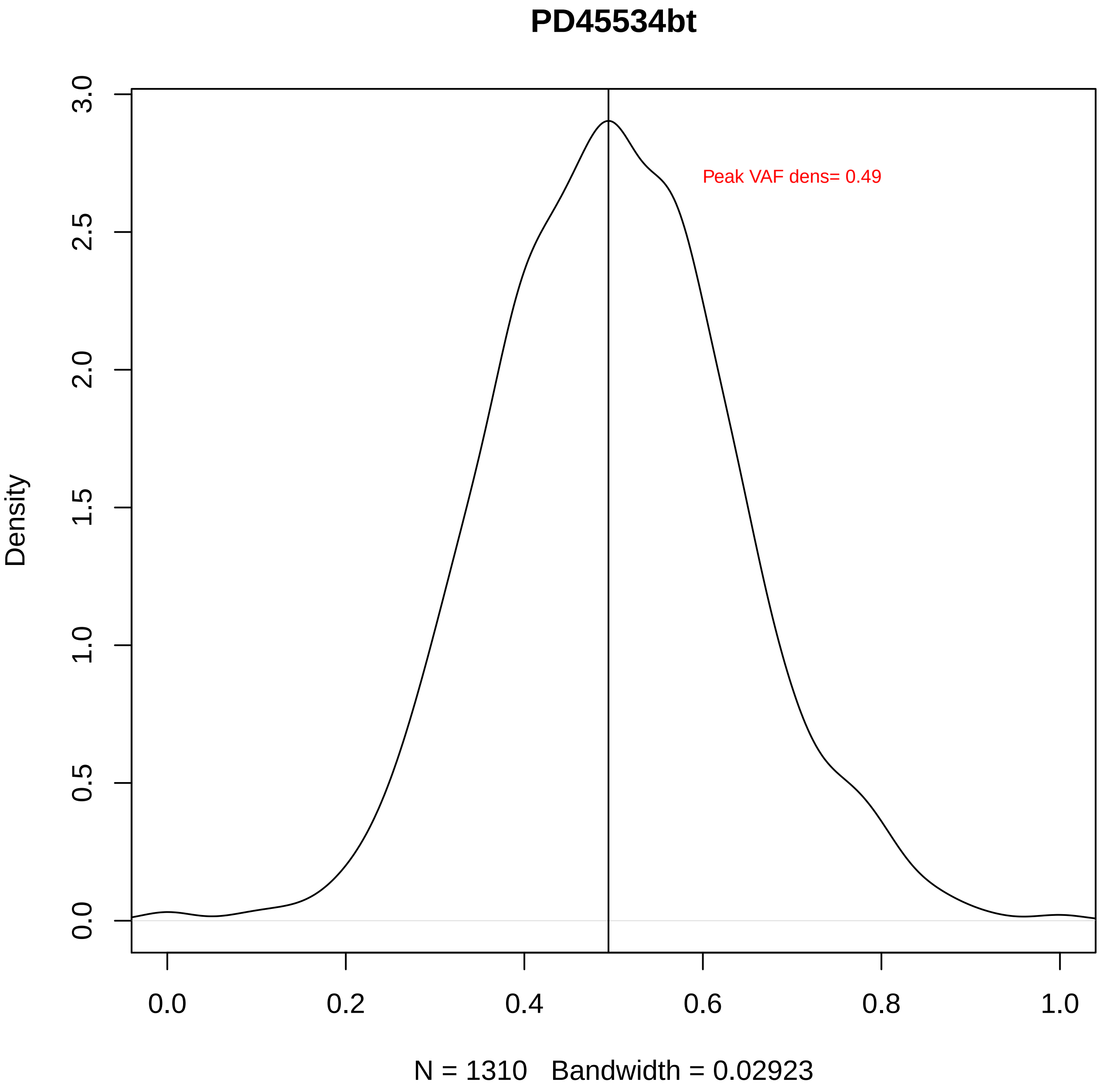
PD45534bt
Peak VAF dens= 0.49
Density
0.0
0.5
1.0
1.5
2.0
2.5
3.0
0.0
0.2
0.4
0.6
0.8
1.0
N = 1310   Bandwidth = 0.02923

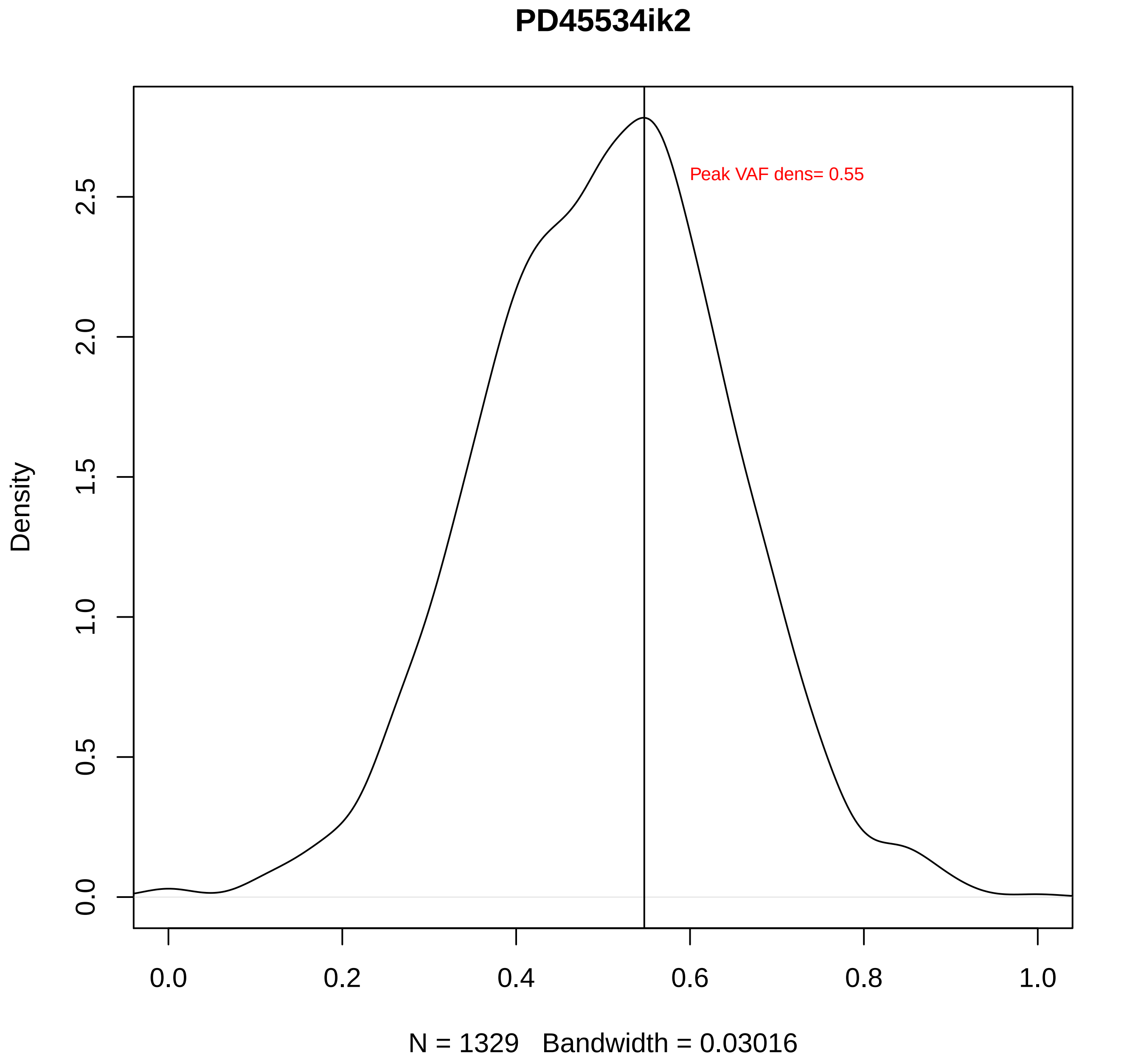
PD45534ik2
Peak VAF dens= 0.55
Density
0.0
0.5
1.0
1.5
2.0
2.5
0.0
0.2
0.4
0.6
0.8
1.0
N = 1329   Bandwidth = 0.03016

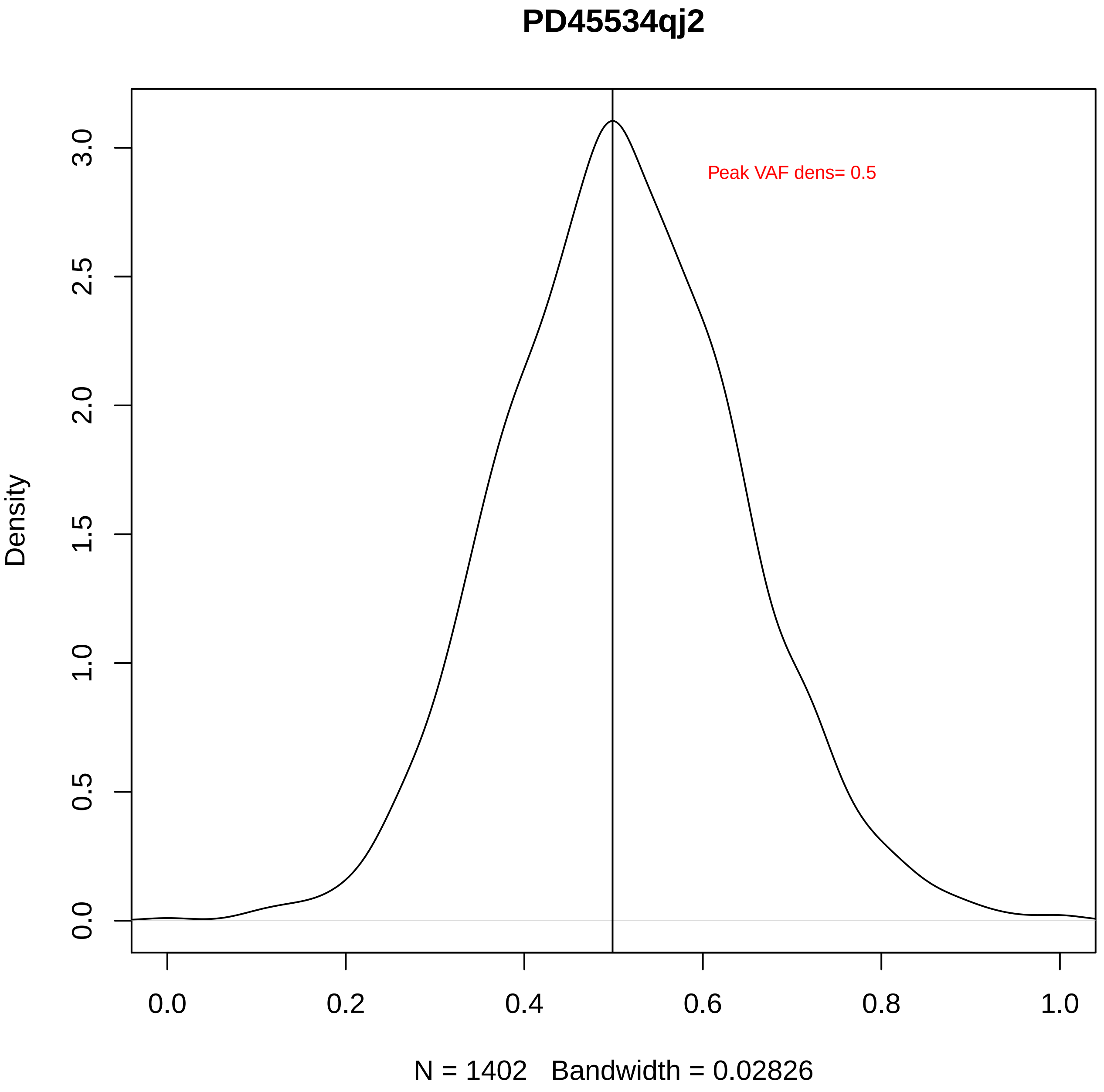
PD45534qj2
Peak VAF dens= 0.5
Density
0.0
0.5
1.0
1.5
2.0
2.5
3.0
0.0
0.2
0.4
0.6
0.8
1.0
N = 1402   Bandwidth = 0.02826

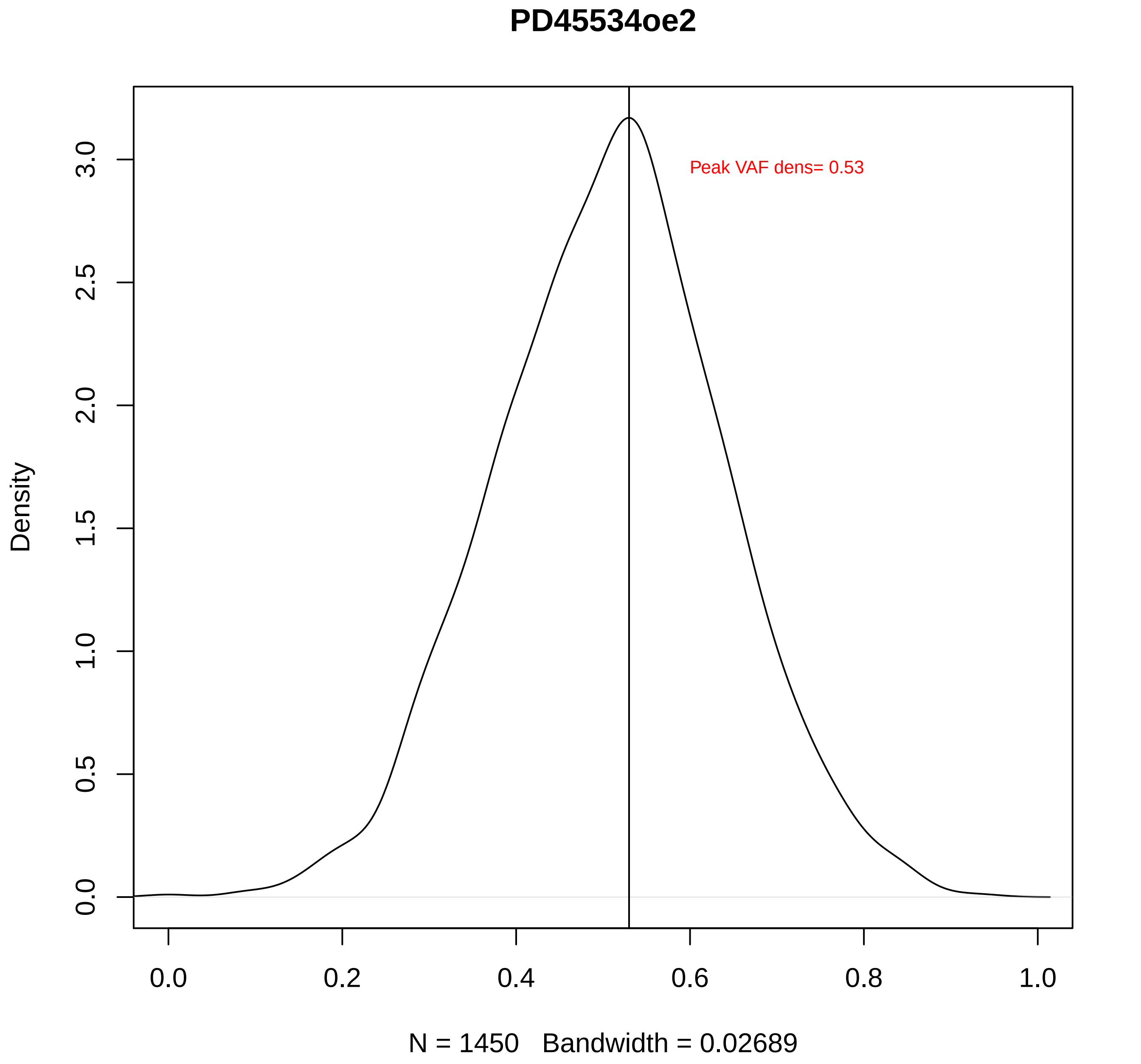
PD45534oe2
Peak VAF dens= 0.53
Density
0.0
0.5
1.0
1.5
2.0
2.5
3.0
0.0
0.2
0.4
0.6
0.8
1.0
N = 1450   Bandwidth = 0.02689

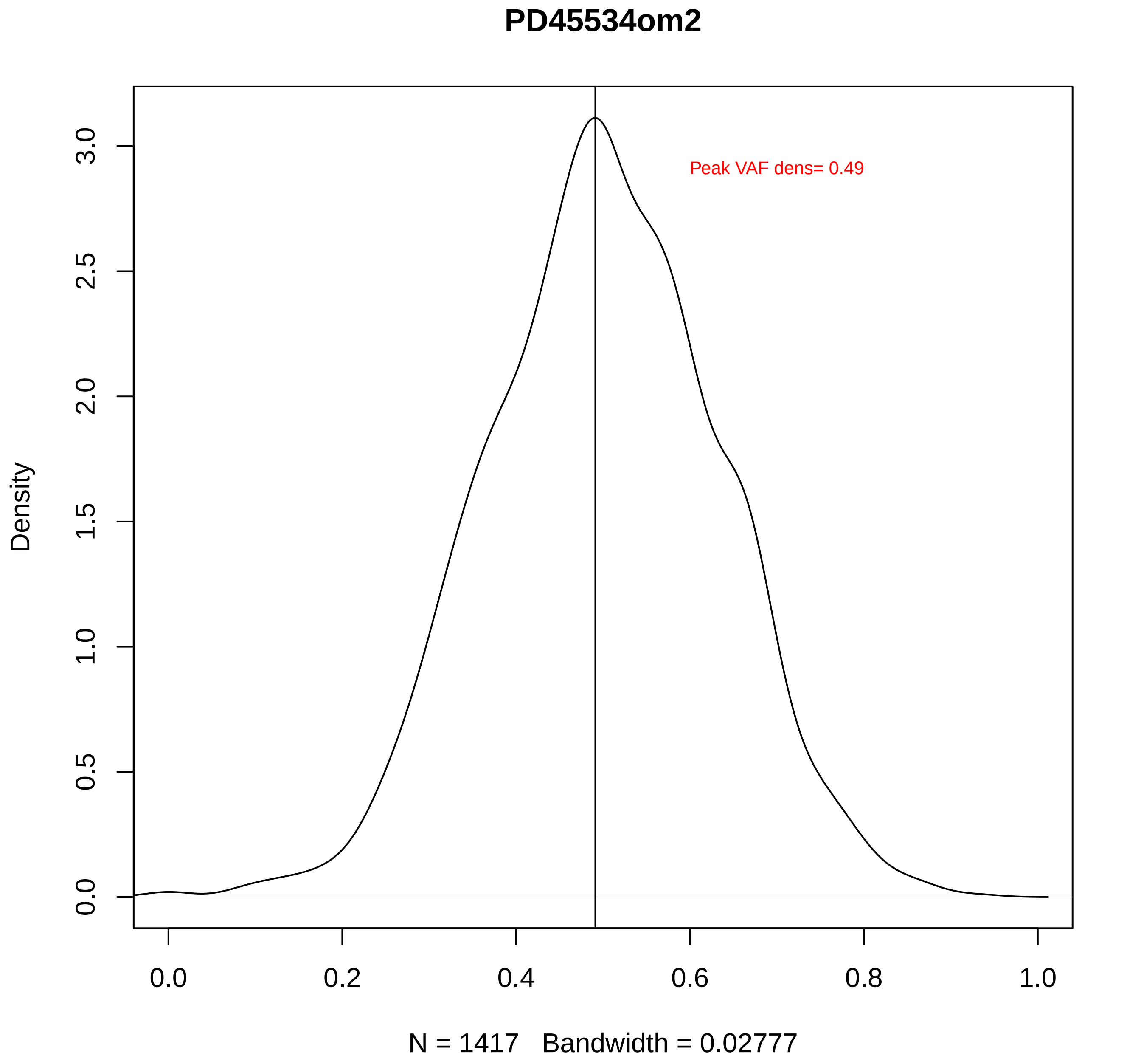
PD45534om2
Peak VAF dens= 0.49
Density
0.0
0.5
1.0
1.5
2.0
2.5
3.0
0.0
0.2
0.4
0.6
0.8
1.0
N = 1417   Bandwidth = 0.02777

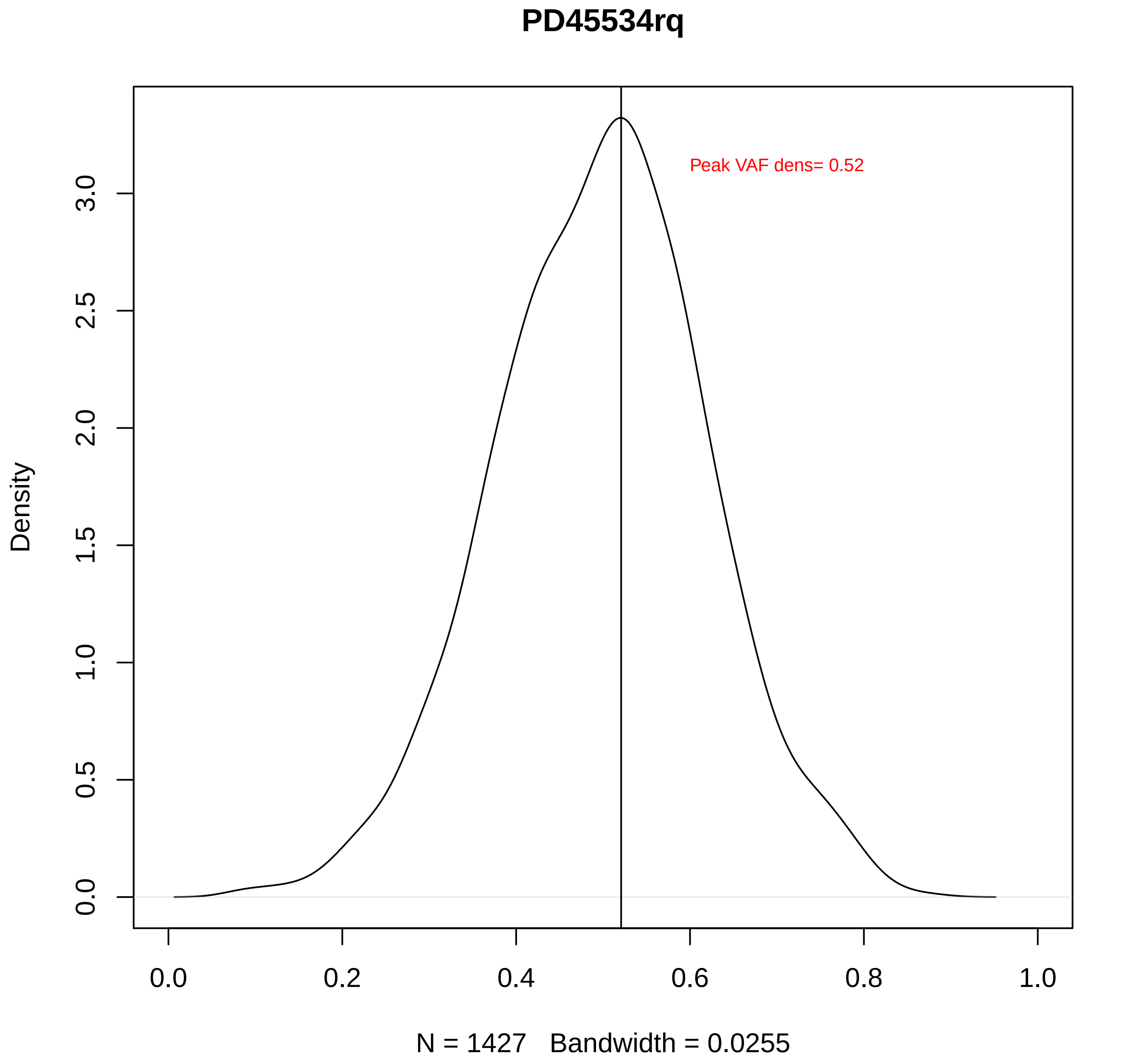

PD45534rq
Peak VAF dens= 0.52
Density
0.0
0.5
1.0
1.5
2.0
2.5
3.0
0.0
0.2
0.4
0.6
0.8
1.0
N = 1427   Bandwidth = 0.0255

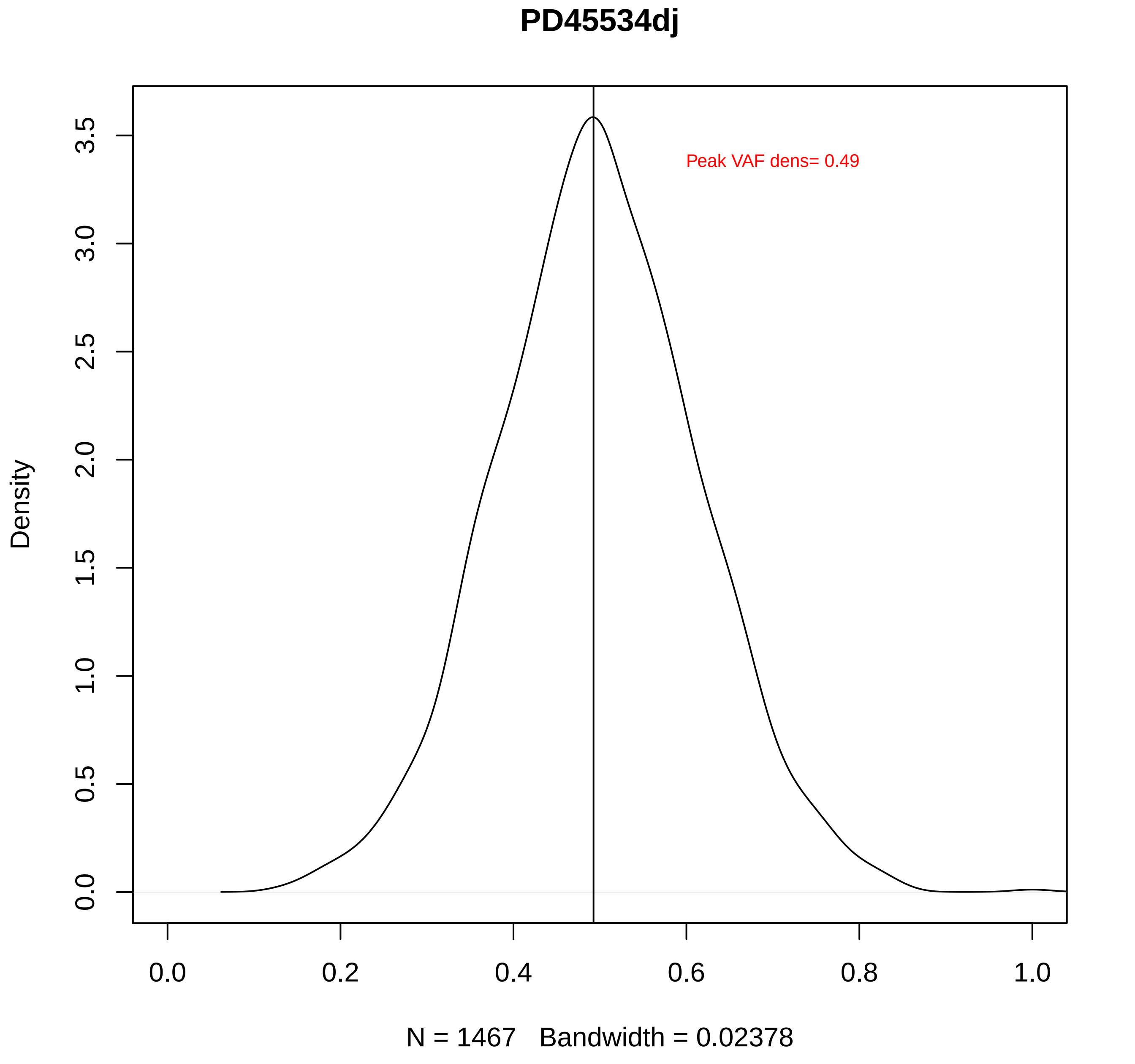
PD45534dj
Peak VAF dens= 0.49
Density
0.0
0.5
1.0
1.5
2.0
2.5
3.0
3.5
0.0
0.2
0.4
0.6
0.8
1.0
N = 1467   Bandwidth = 0.02378

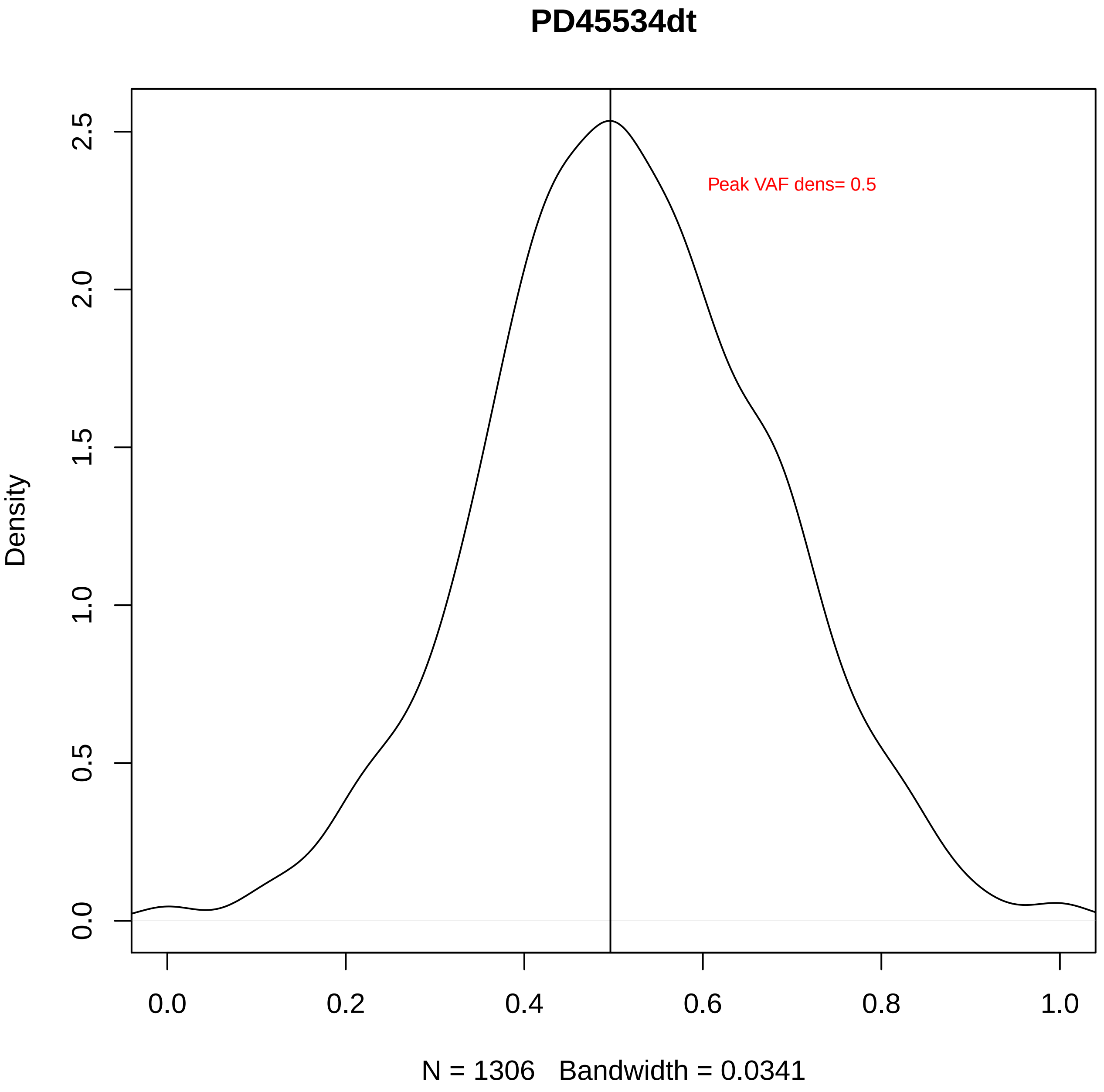
PD45534dt
Peak VAF dens= 0.5
Density
0.0
0.5
1.0
1.5
2.0
2.5
0.0
0.2
0.4
0.6
0.8
1.0
N = 1306 Bandwidth = 0.0341

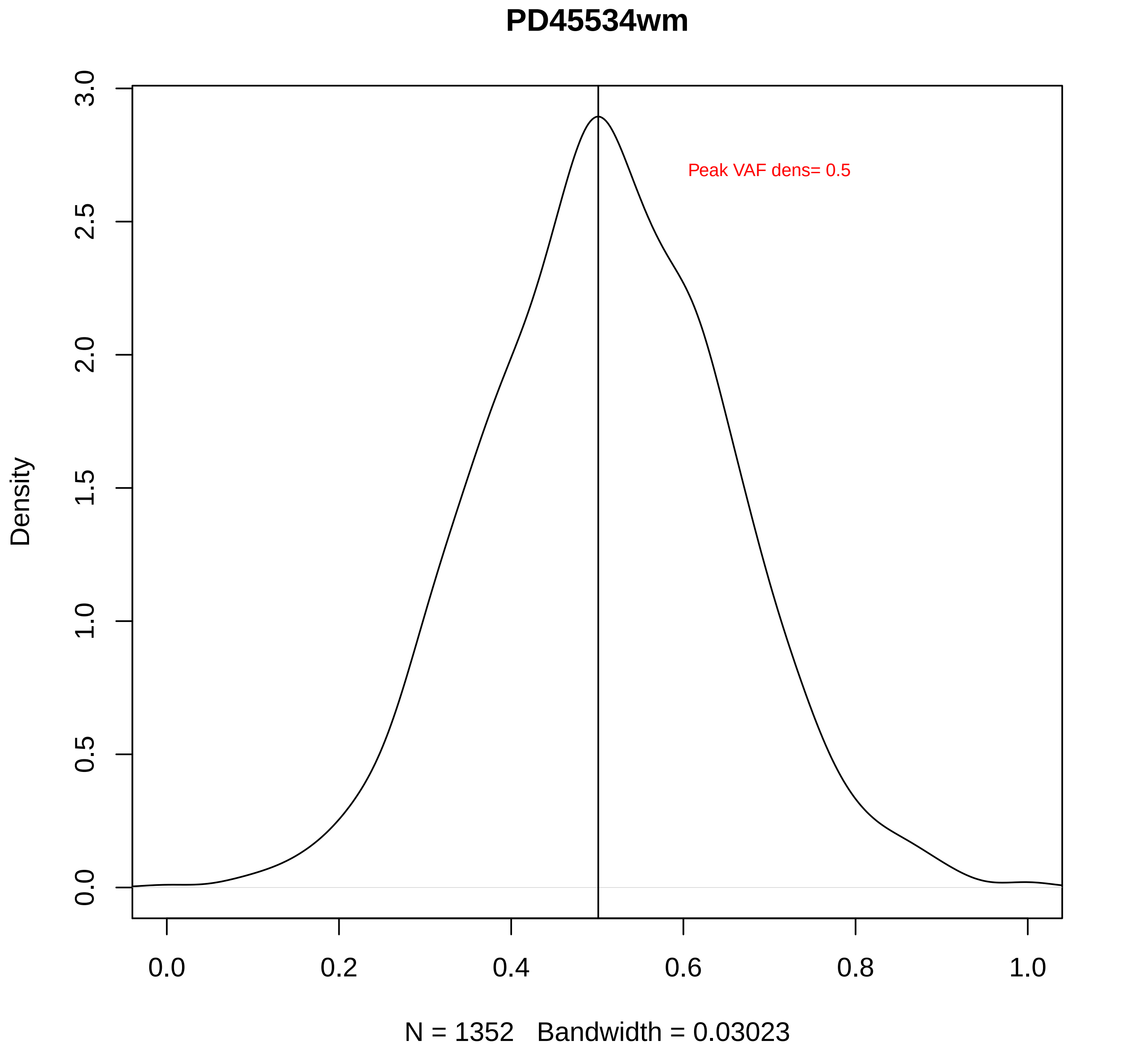

PD45534wm
Peak VAF dens= 0.5
Density
0.0
0.5
1.0
1.5
2.0
2.5
3.0
0.0
0.2
0.4
0.6
0.8
1.0
N = 1352   Bandwidth = 0.03023

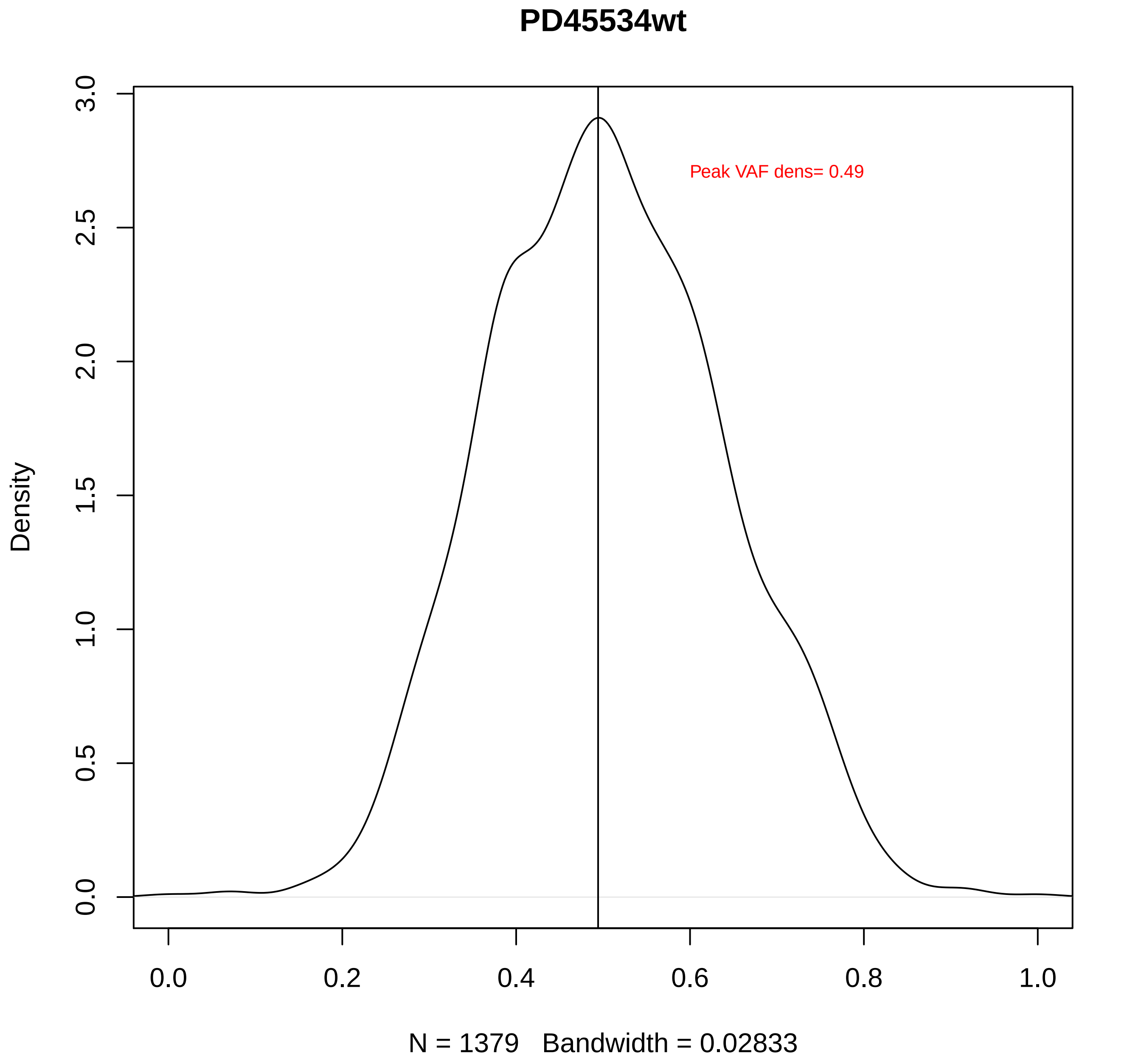
PD45534wt
Peak VAF dens= 0.49
Density
0.0
0.5
1.0
1.5
2.0
2.5
3.0
0.0
0.2
0.4
0.6
0.8
1.0
N = 1379   Bandwidth = 0.02833

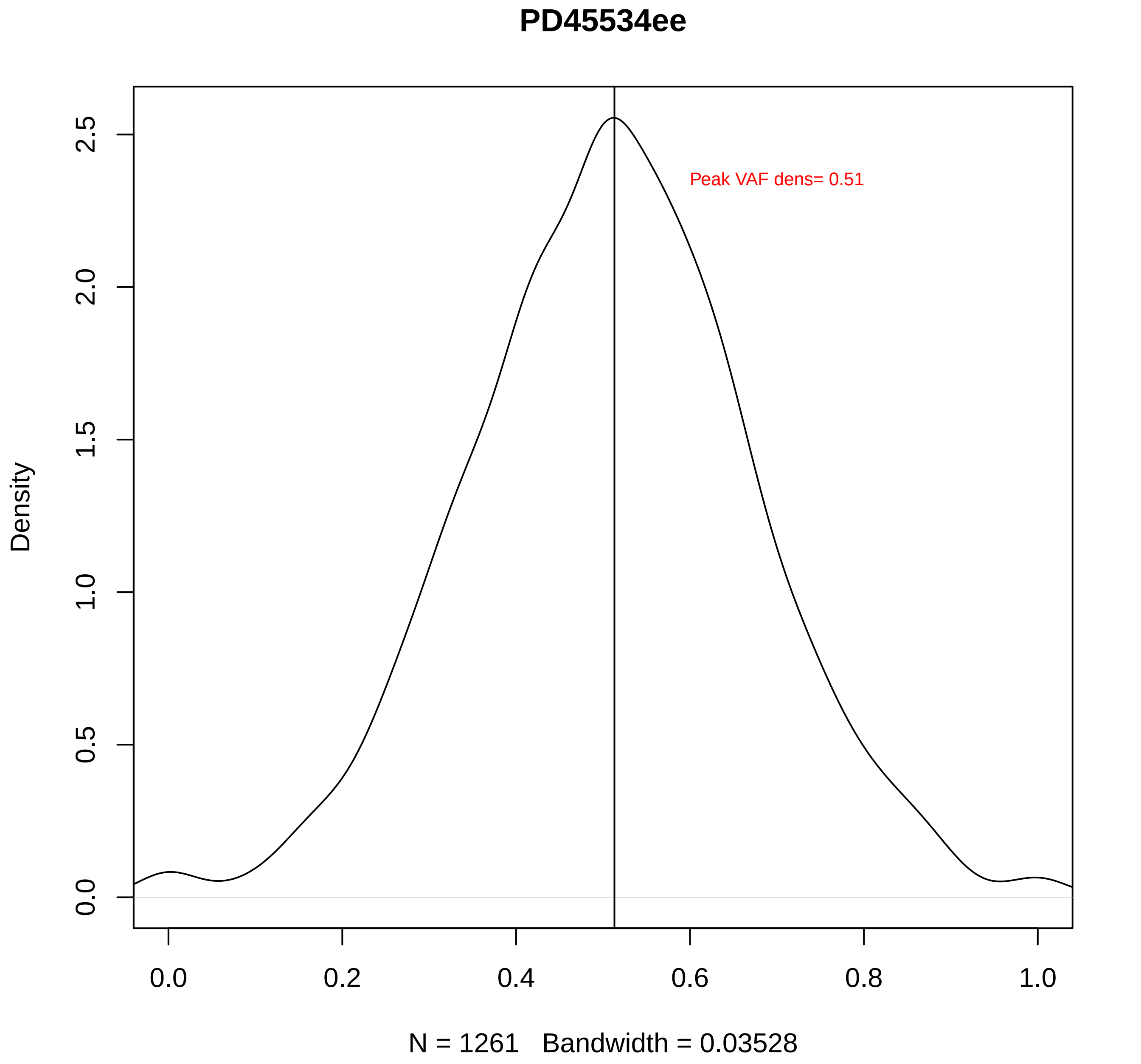
PD45534ee
Peak VAF dens= 0.51
Density
0.0
0.5
1.0
1.5
2.0
2.5
0.0
0.2
0.4
0.6
0.8
1.0
N = 1261 Bandwidth = 0.03528

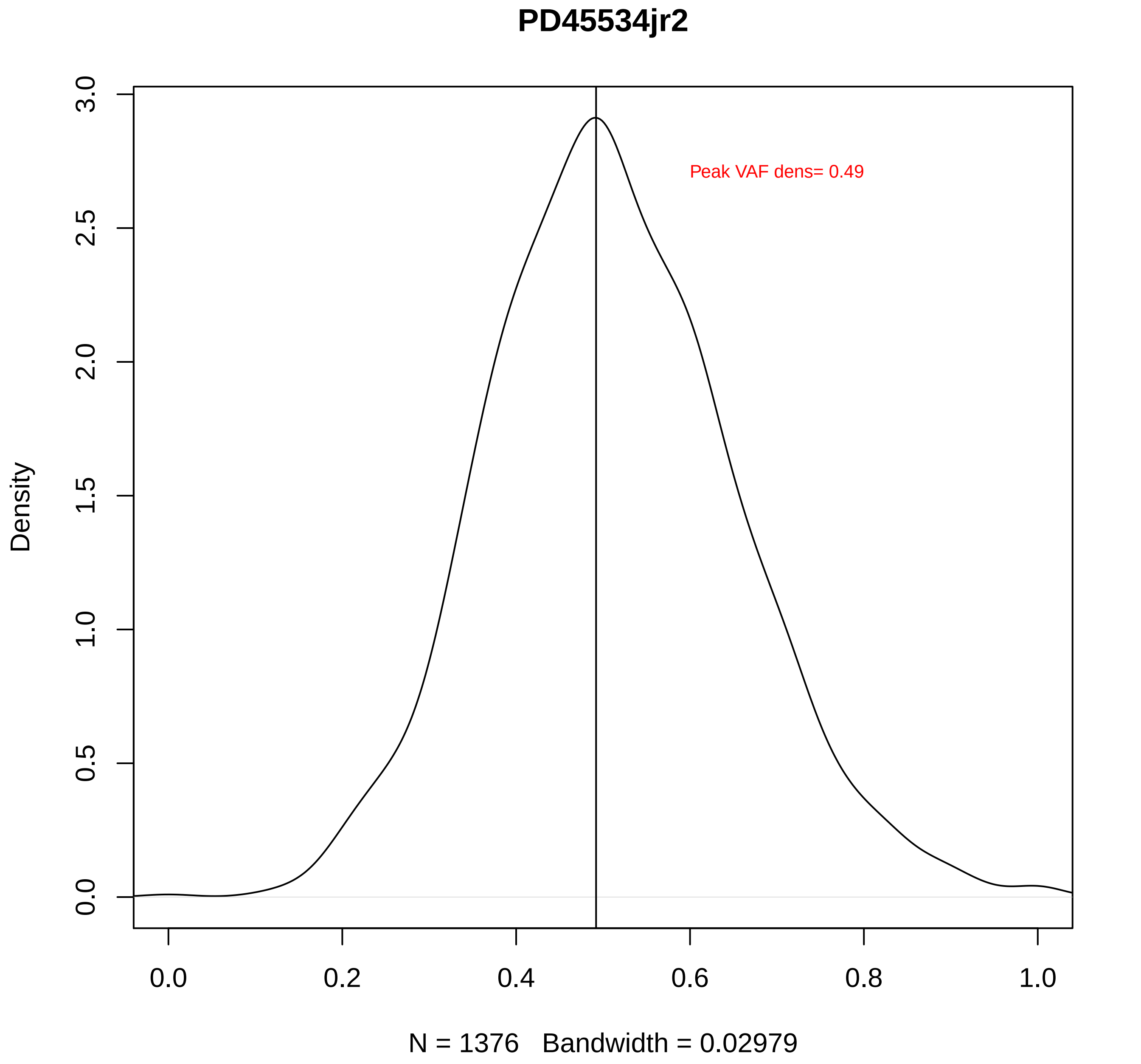
PD45534jr2
Peak VAF dens= 0.49
Density
0.0
0.5
1.0
1.5
2.0
2.5
3.0
0.0
0.2
0.4
0.6
0.8
1.0
N = 1376   Bandwidth = 0.02979

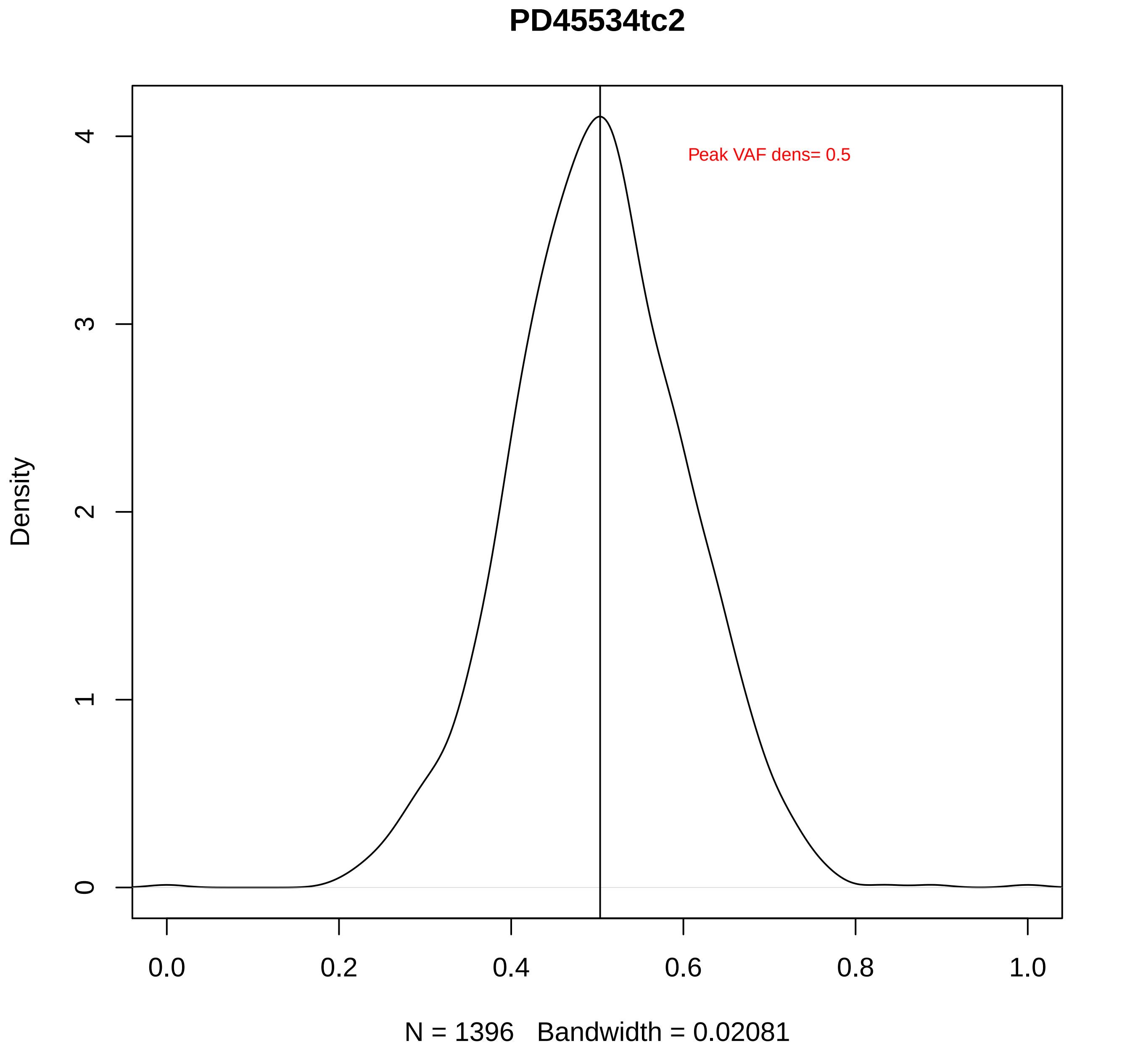
PD45534tc2
Peak VAF dens= 0.5
Density
0
1
2
3
4
0.0
0.2
0.4
0.6
0.8
1.0
N = 1396   Bandwidth = 0.02081

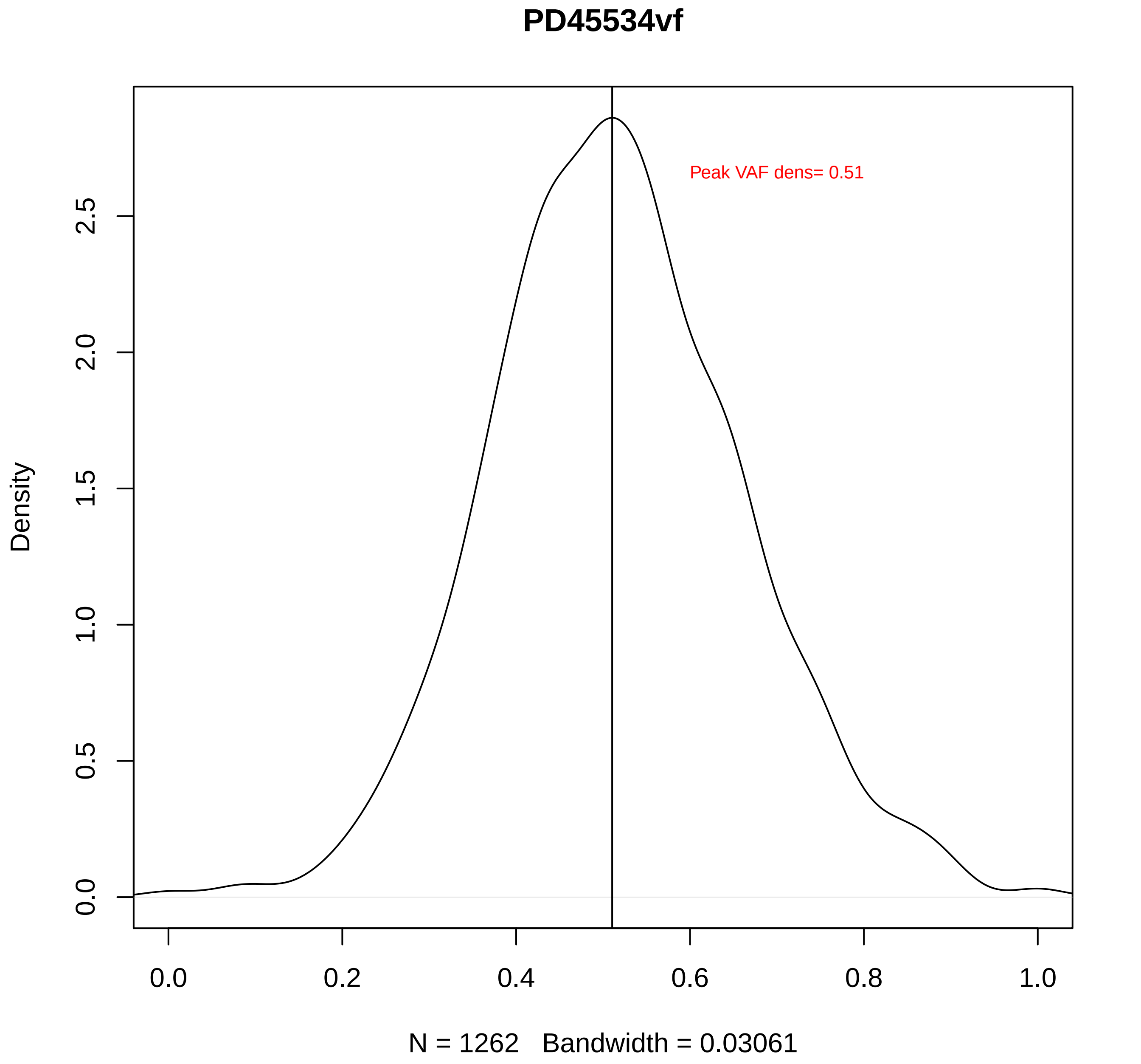

PD45534vf
Peak VAF dens= 0.51
Density
0.0
0.5
1.0
1.5
2.0
2.5
0.0
0.2
0.4
0.6
0.8
1.0
N = 1262   Bandwidth = 0.03061

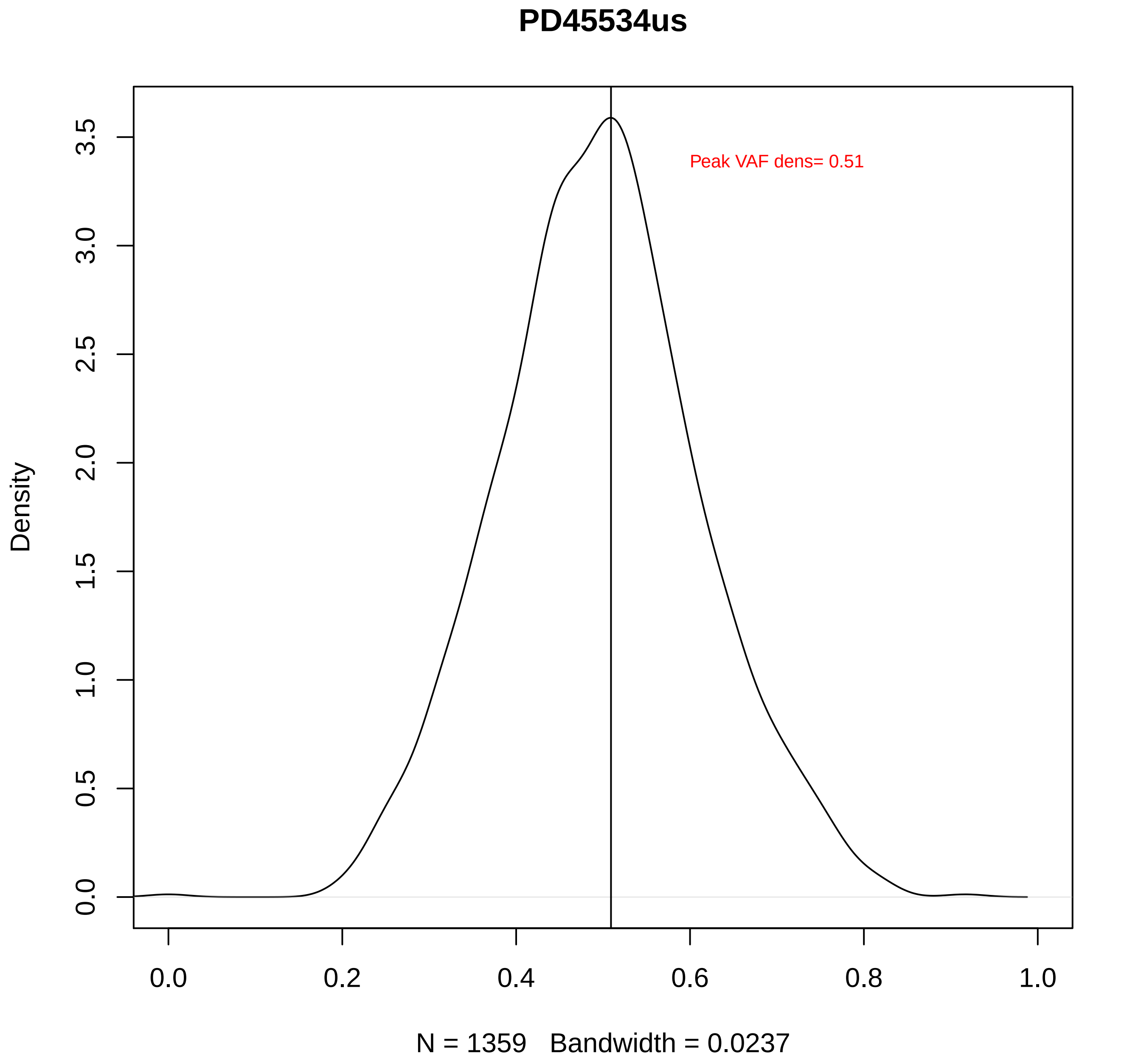

PD45534us
Peak VAF dens= 0.51
Density
0.0
0.5
1.0
1.5
2.0
2.5
3.0
3.5
0.0
0.2
0.4
0.6
0.8
1.0
N = 1359   Bandwidth = 0.0237

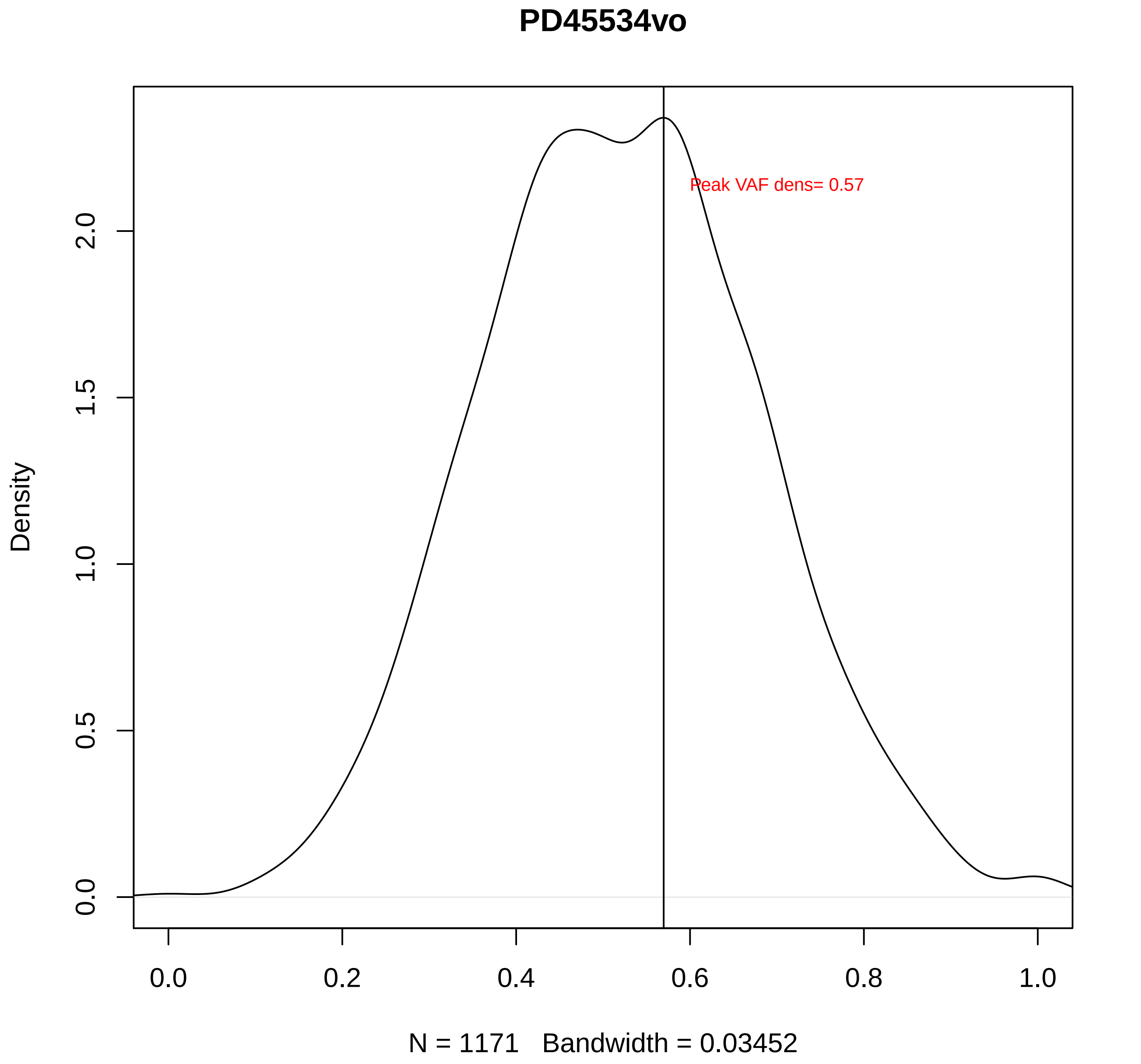
PD45534vo
Peak VAF dens= 0.57
Density
0.0
0.5
1.0
1.5
2.0
0.0
0.2
0.4
0.6
0.8
1.0
N = 1171 Bandwidth = 0.03452

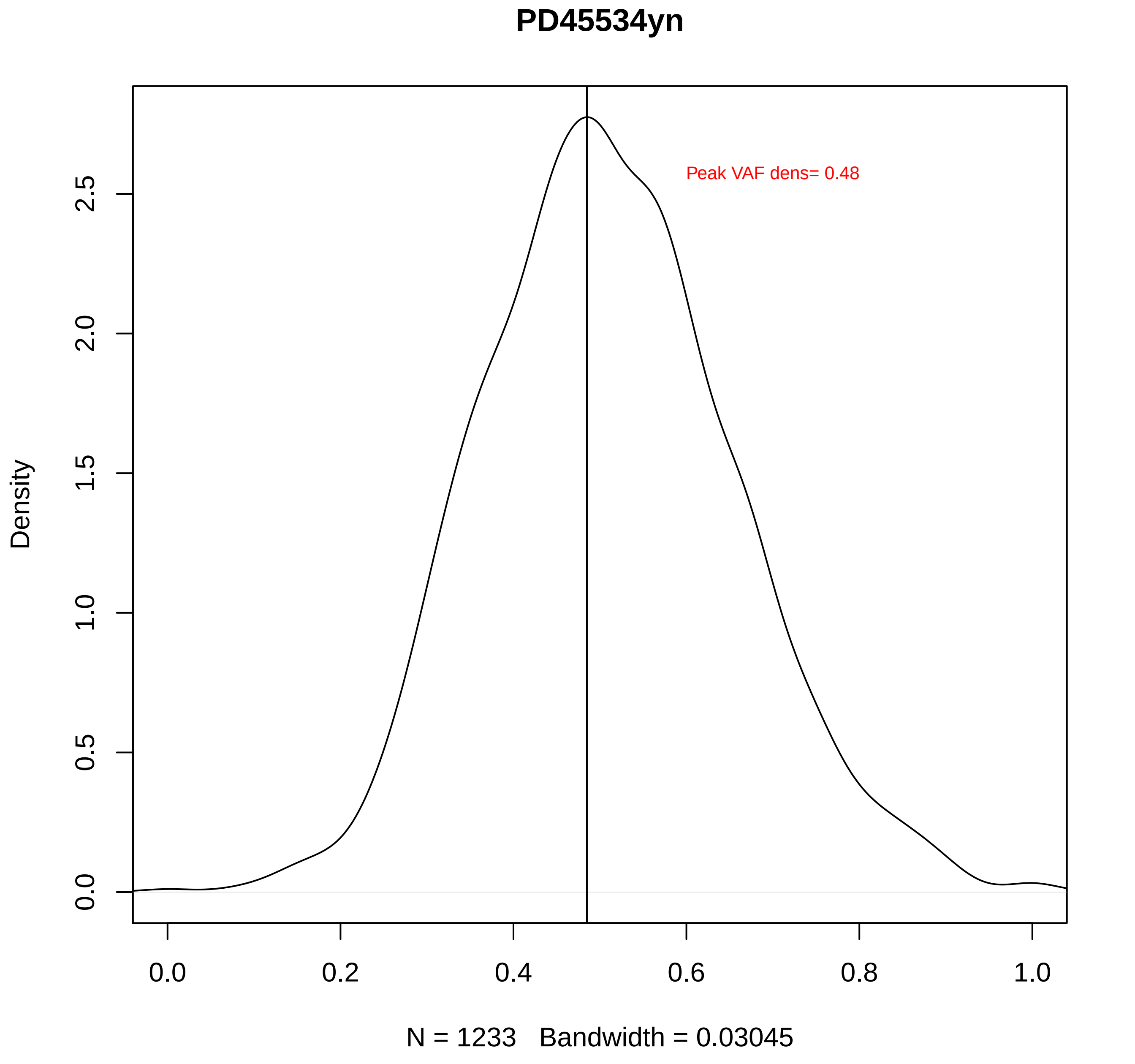
PD45534yn
Peak VAF dens= 0.48
Density
0.0
0.5
1.0
1.5
2.0
2.5
0.0
0.2
0.4
0.6
0.8
1.0
N = 1233   Bandwidth = 0.03045

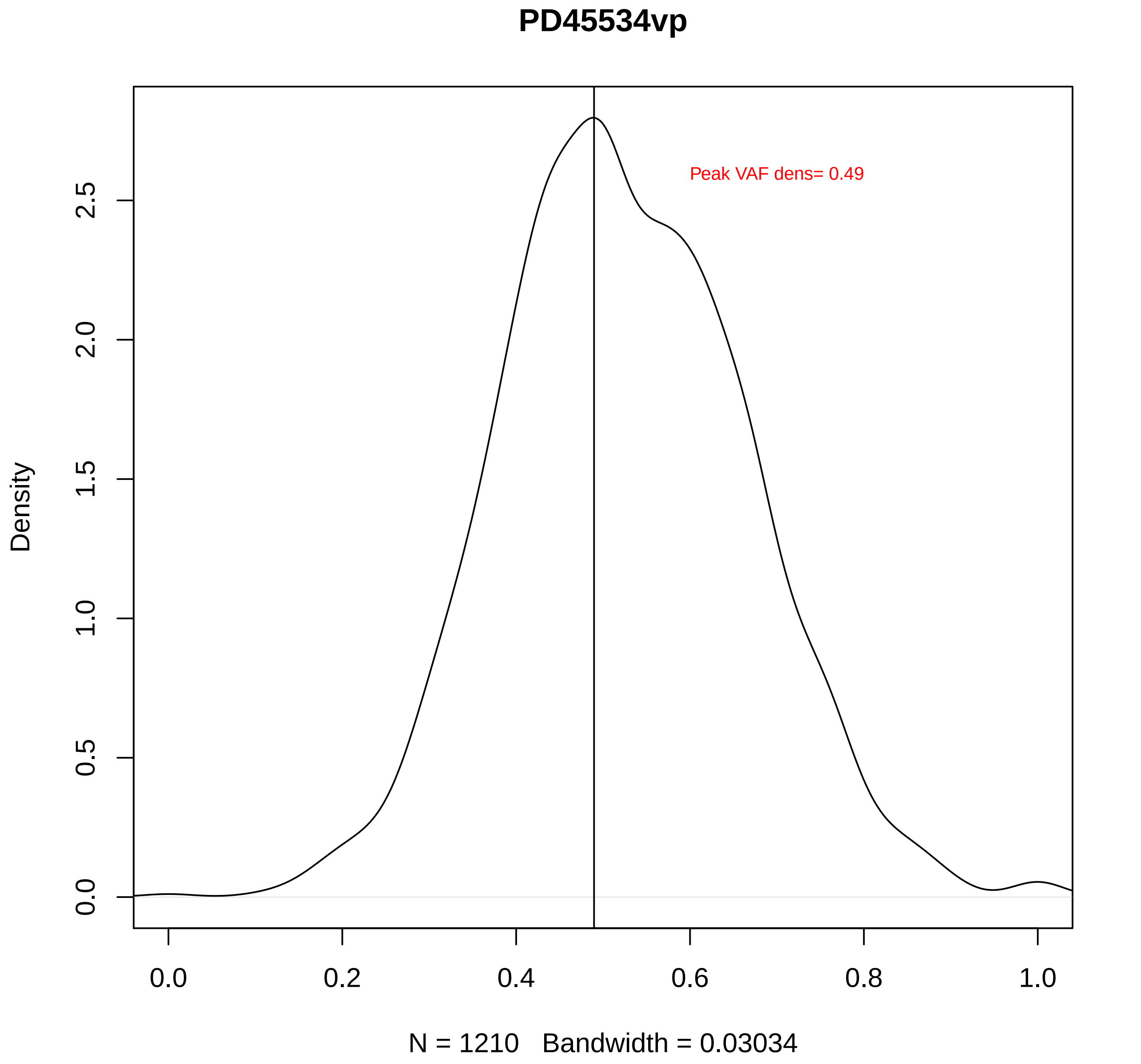

PD45534vp
Peak VAF dens= 0.49
Density
0.0
0.5
1.0
1.5
2.0
2.5
0.0
0.2
0.4
0.6
0.8
1.0
N = 1210   Bandwidth = 0.03034

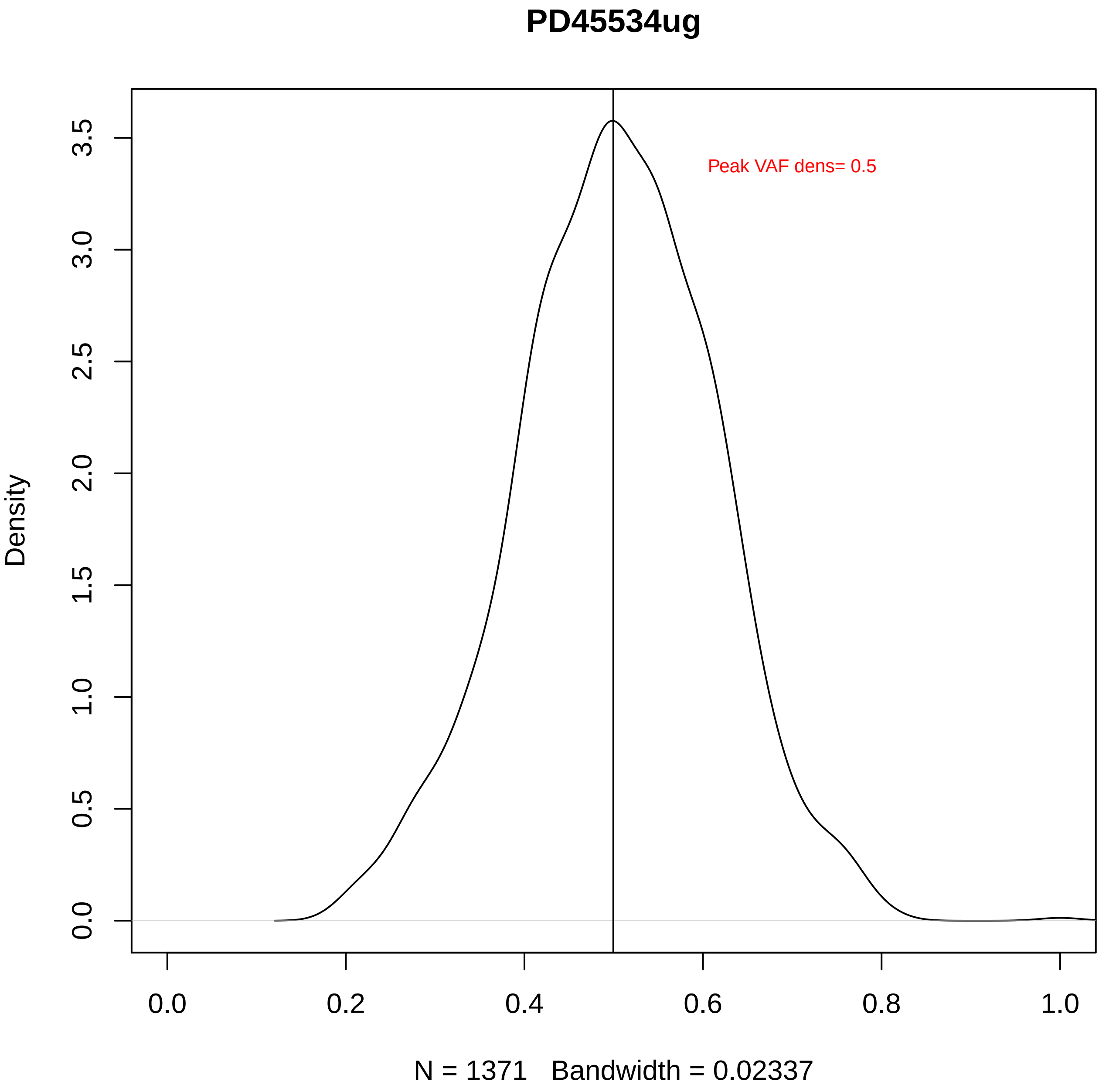
PD45534ug
Peak VAF dens= 0.5
Density
0.0
0.5
1.0
1.5
2.0
2.5
3.0
3.5
0.0
0.2
0.4
0.6
0.8
1.0
N = 1371   Bandwidth = 0.02337

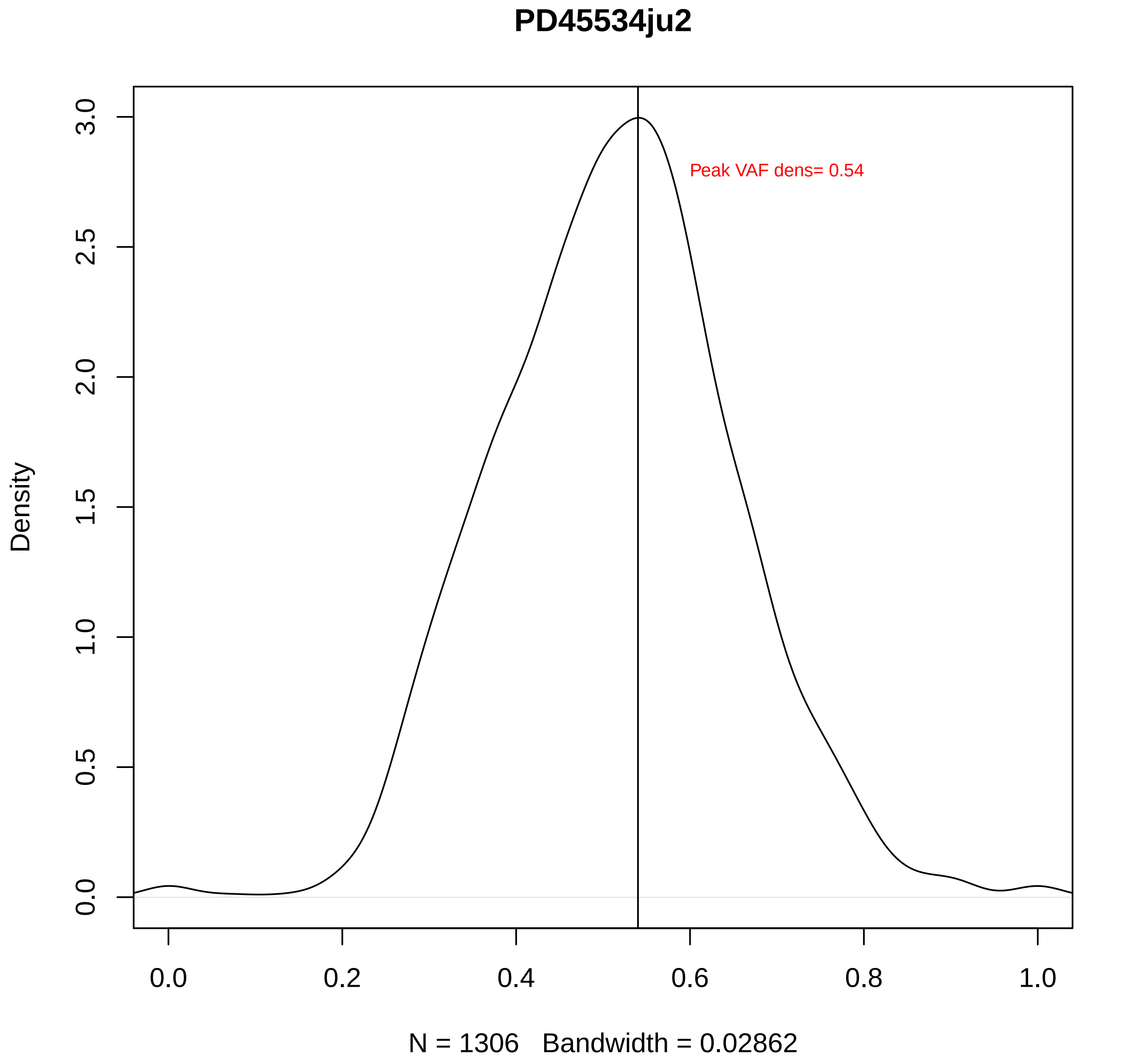

PD45534ju2
Peak VAF dens= 0.54
Density
0.0
0.5
1.0
1.5
2.0
2.5
3.0
0.0
0.2
0.4
0.6
0.8
1.0
N = 1306   Bandwidth = 0.02862

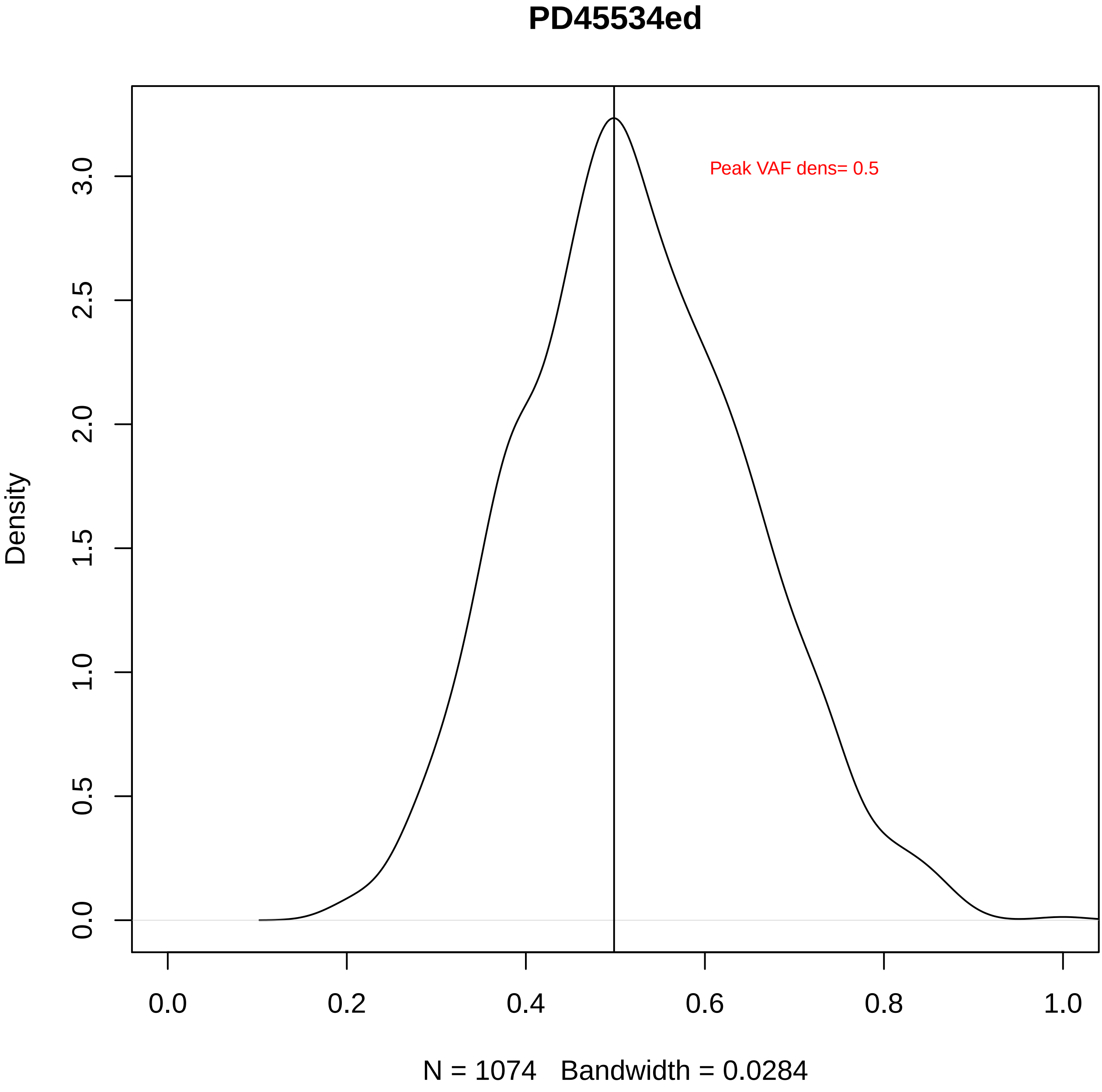

PD45534ed
Peak VAF dens= 0.5
Density
0.0
0.5
1.0
1.5
2.0
2.5
3.0
0.0
0.2
0.4
0.6
0.8
1.0
N = 1074   Bandwidth = 0.0284

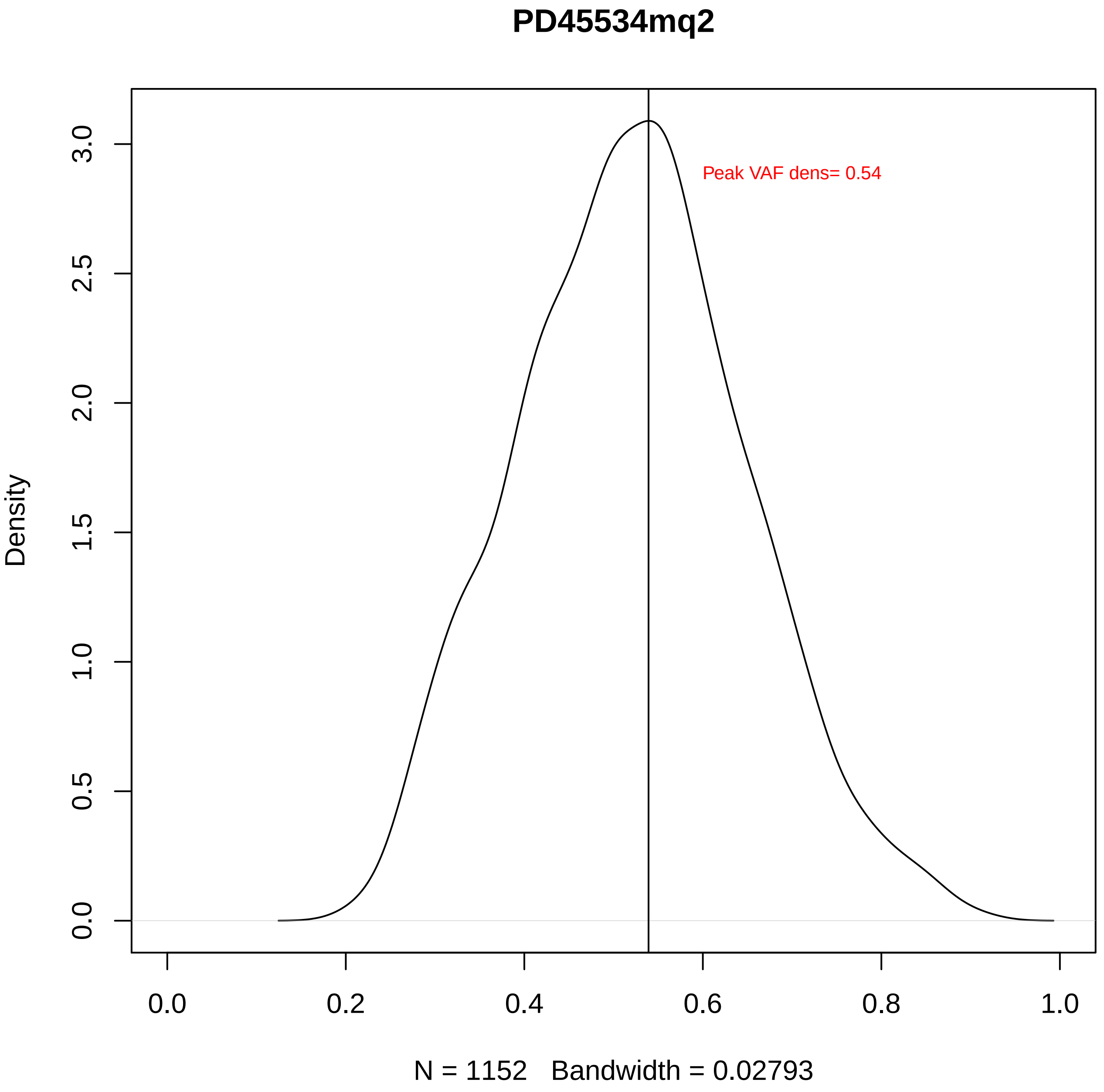
PD45534mq2
Peak VAF dens= 0.54
Density
0.0
0.5
1.0
1.5
2.0
2.5
3.0
0.0
0.2
0.4
0.6
0.8
1.0
N = 1152   Bandwidth = 0.02793

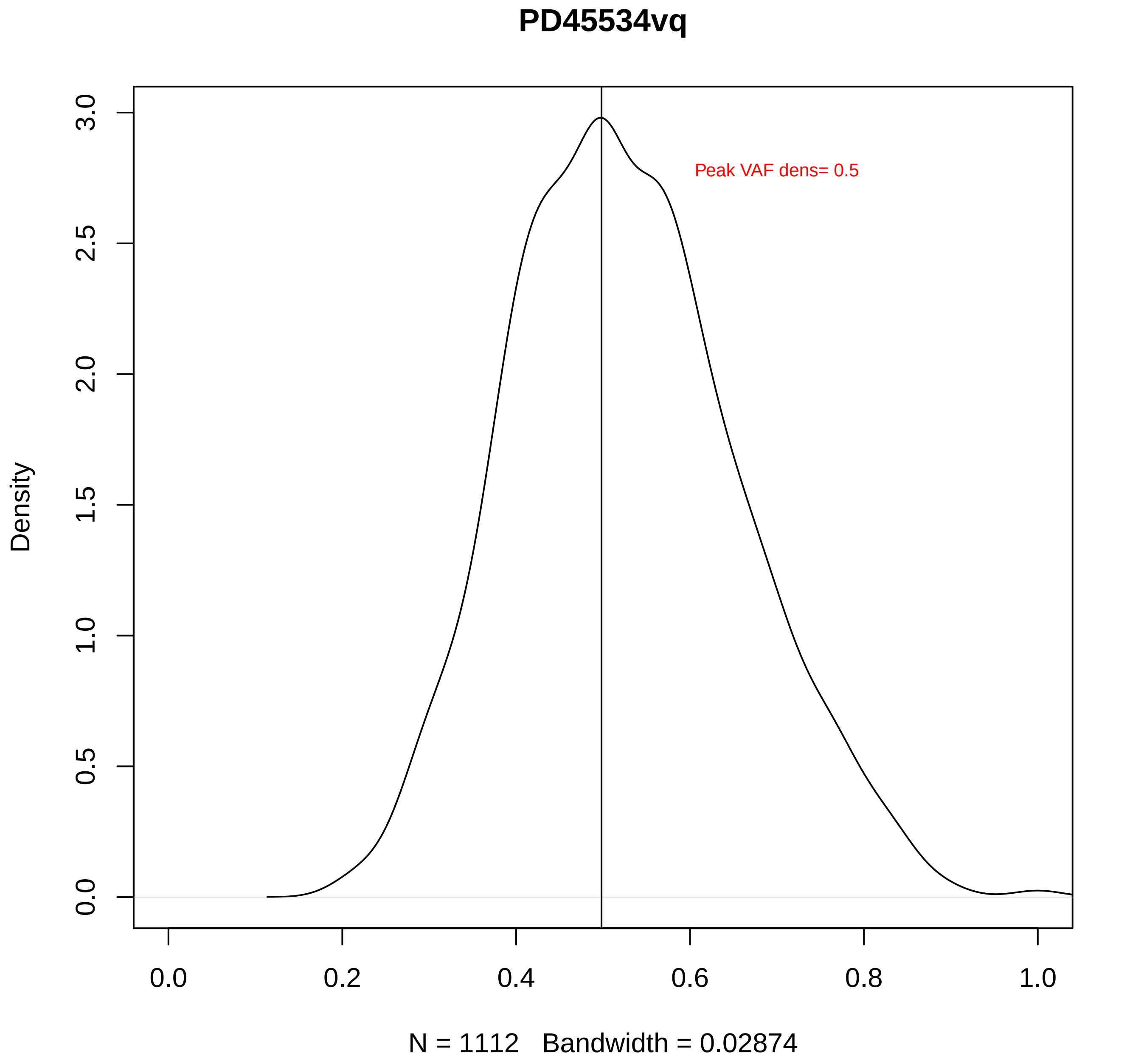
PD45534vq
Peak VAF dens= 0.5
Density
3.0
2.5
2.0
1.5
1.0
0.5
0.0
0.0
0.2
0.4
0.6
0.8
1.0
N = 1112   Bandwidth = 0.02874

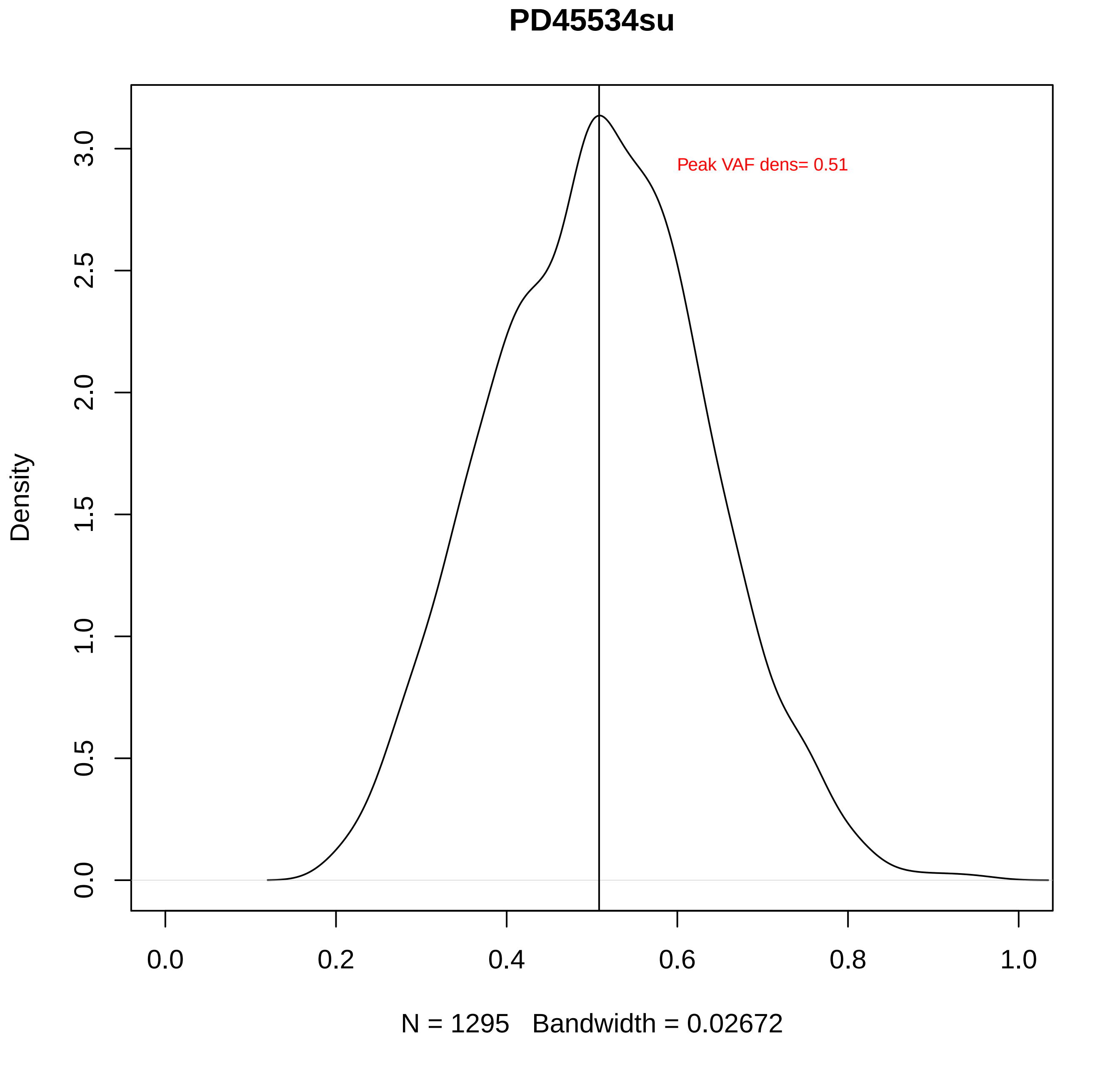

PD45534su
Peak VAF dens= 0.51
Density
0.0
0.5
1.0
1.5
2.0
2.5
3.0
0.0
0.2
0.4
0.6
0.8
1.0
N = 1295   Bandwidth = 0.02672

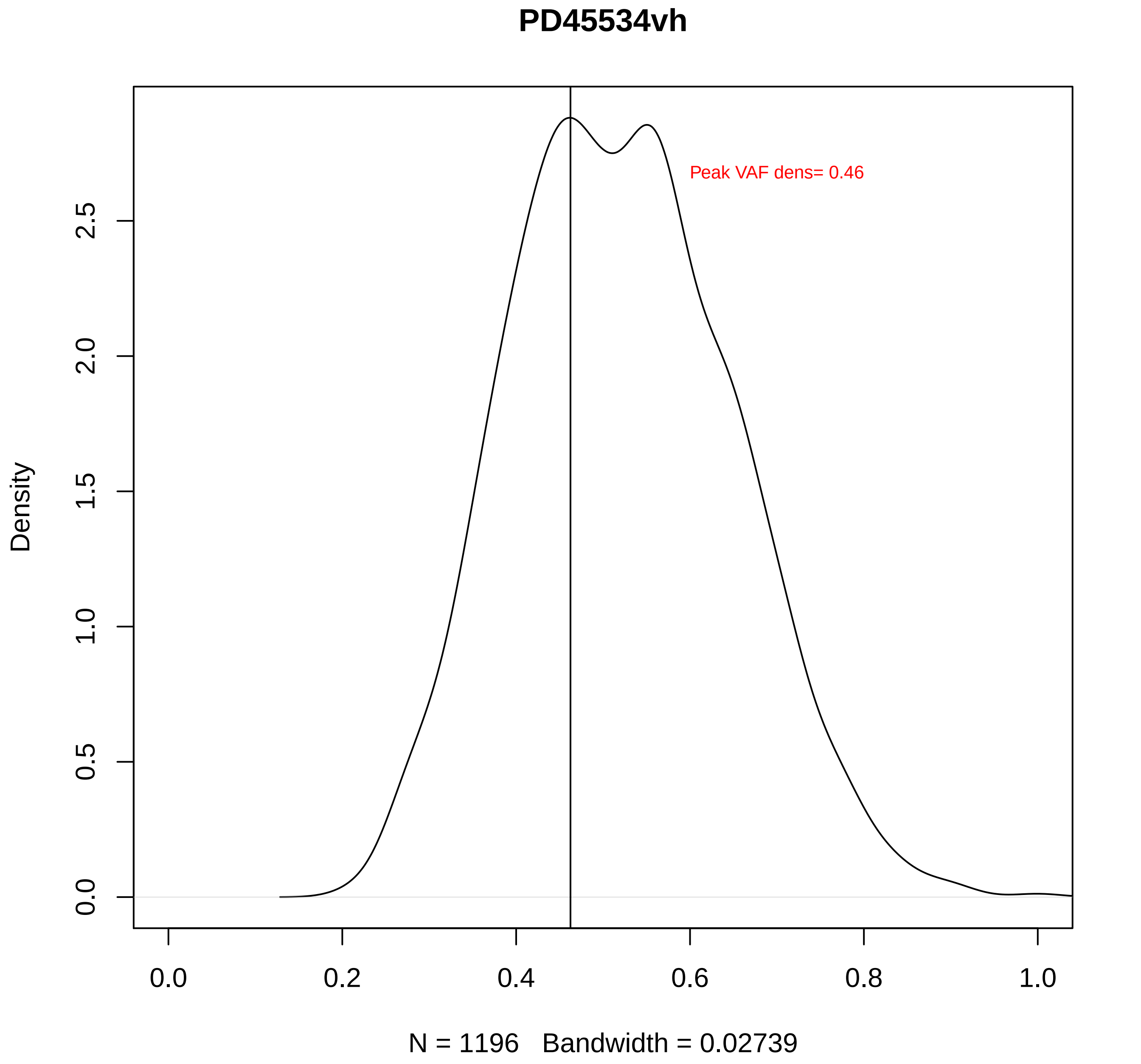
PD45534vh
Peak VAF dens= 0.46
Density
0.0
0.5
1.0
1.5
2.0
2.5
0.0
0.2
0.4
0.6
0.8
1.0
N = 1196   Bandwidth = 0.02739

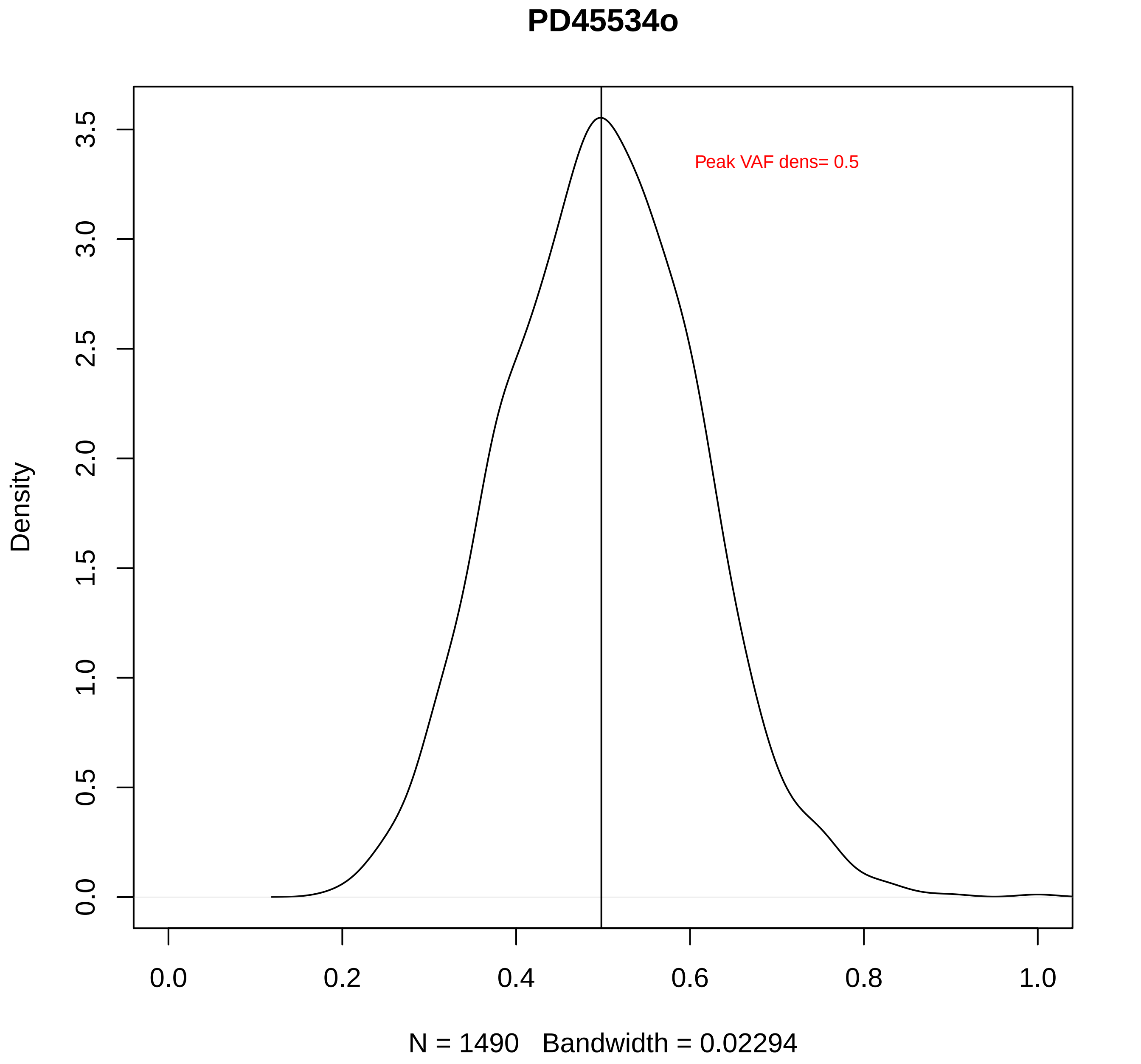

PD45534o
Peak VAF dens= 0.5
Density
0.0
0.5
1.0
1.5
2.0
2.5
3.0
3.5
0.0
0.2
0.4
0.6
0.8
1.0
N = 1490   Bandwidth = 0.02294

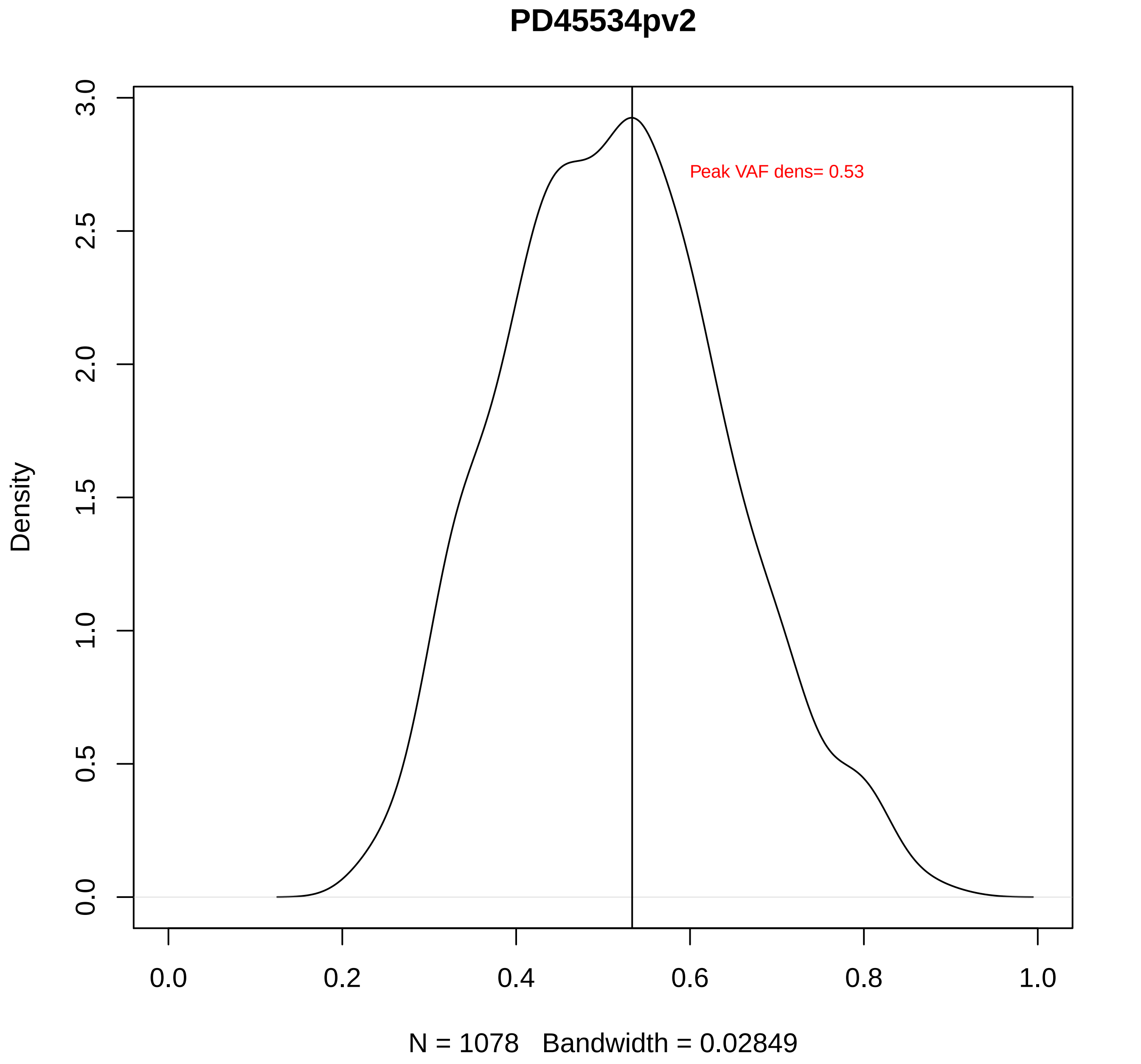
PD45534pv2
Peak VAF dens= 0.53
Density
0.0
0.5
1.0
1.5
2.0
2.5
3.0
0.0
0.2
0.4
0.6
0.8
1.0
N = 1078 Bandwidth = 0.02849

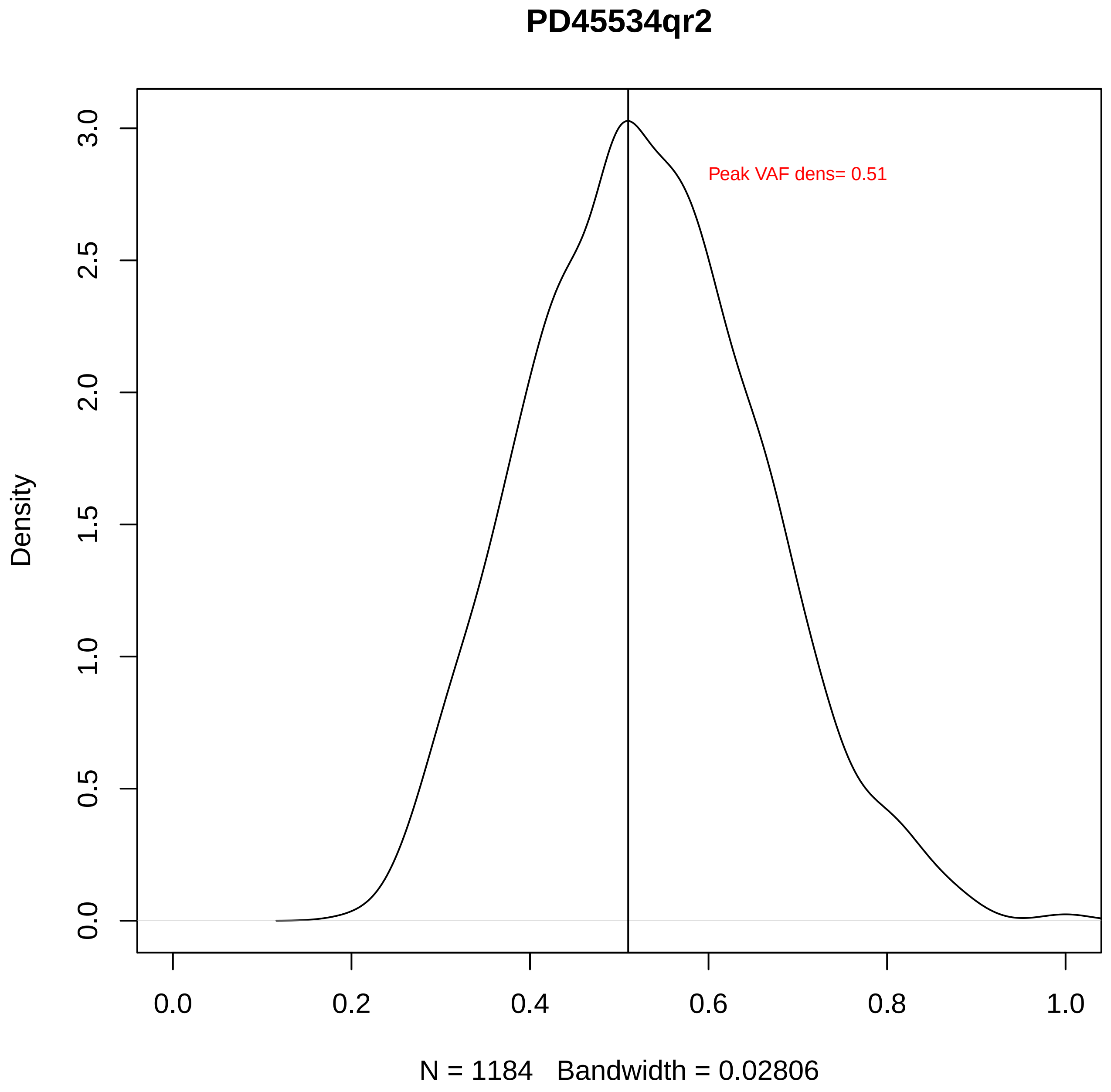
PD45534qr2
Peak VAF dens= 0.51
Density
0.0
0.5
1.0
1.5
2.0
2.5
3.0
0.0
0.2
0.4
0.6
0.8
1.0
N = 1184 Bandwidth = 0.02806

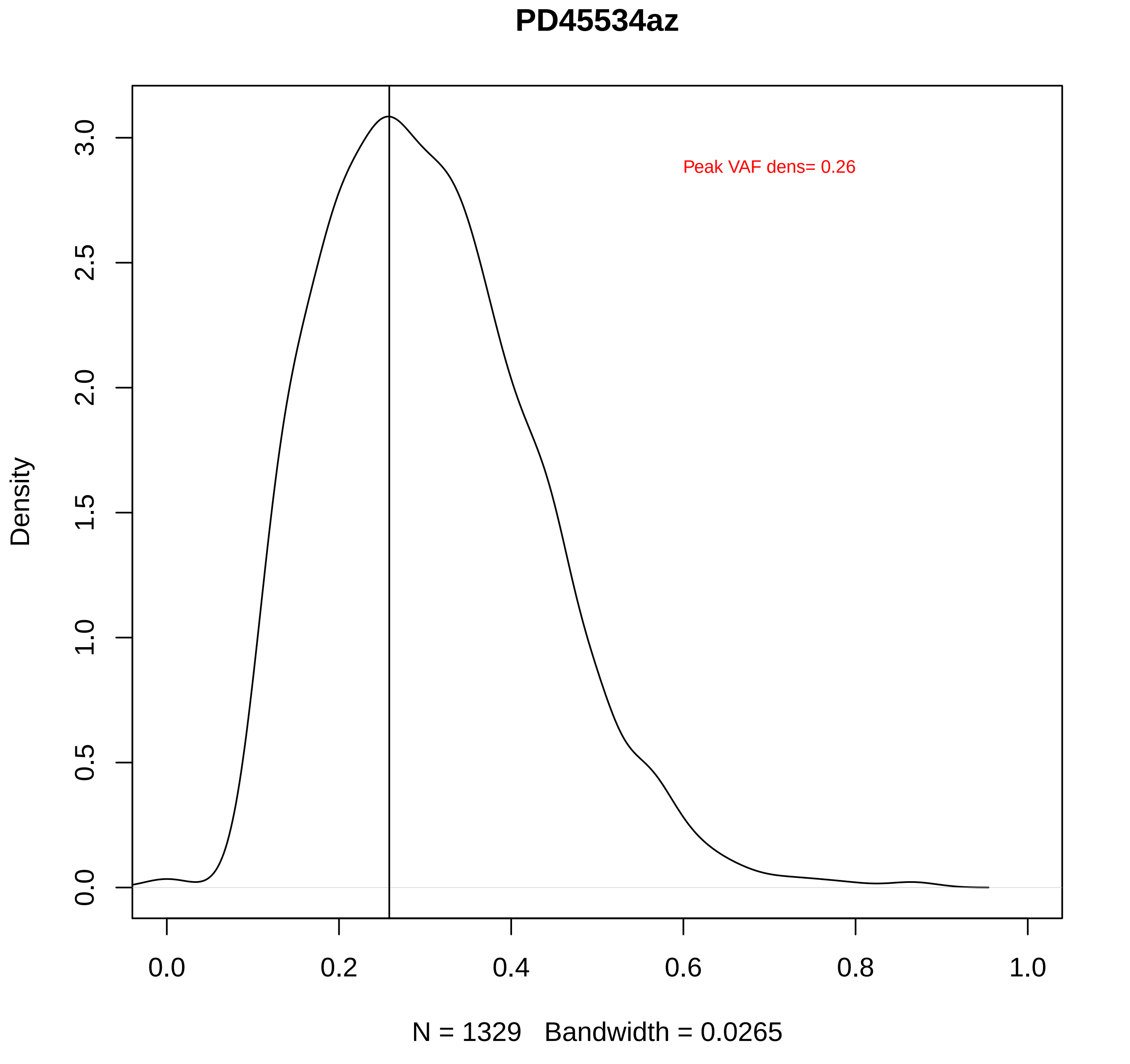

PD45534az
Peak VAF dens= 0.26
Density
0.0
0.5
1.0
1.5
2.0
2.5
3.0
0.0
0.2
0.4
0.6
0.8
1.0
N = 1329   Bandwidth = 0.0265

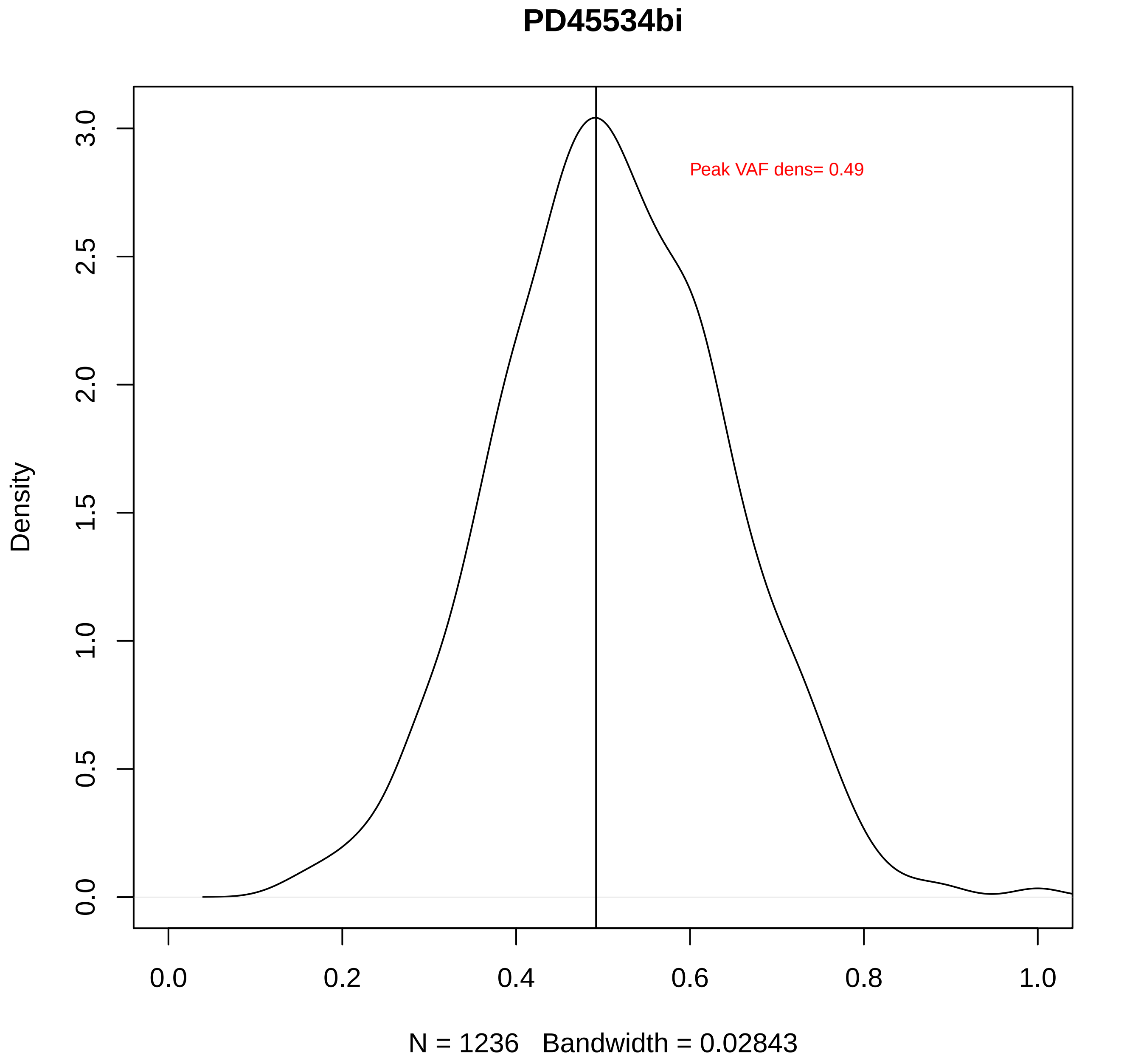
PD45534bi
Peak VAF dens= 0.49
Density
3.0
2.5
2.0
1.5
1.0
0.5
0.0
0.0
0.2
0.4
0.6
0.8
1.0
N = 1236   Bandwidth = 0.02843

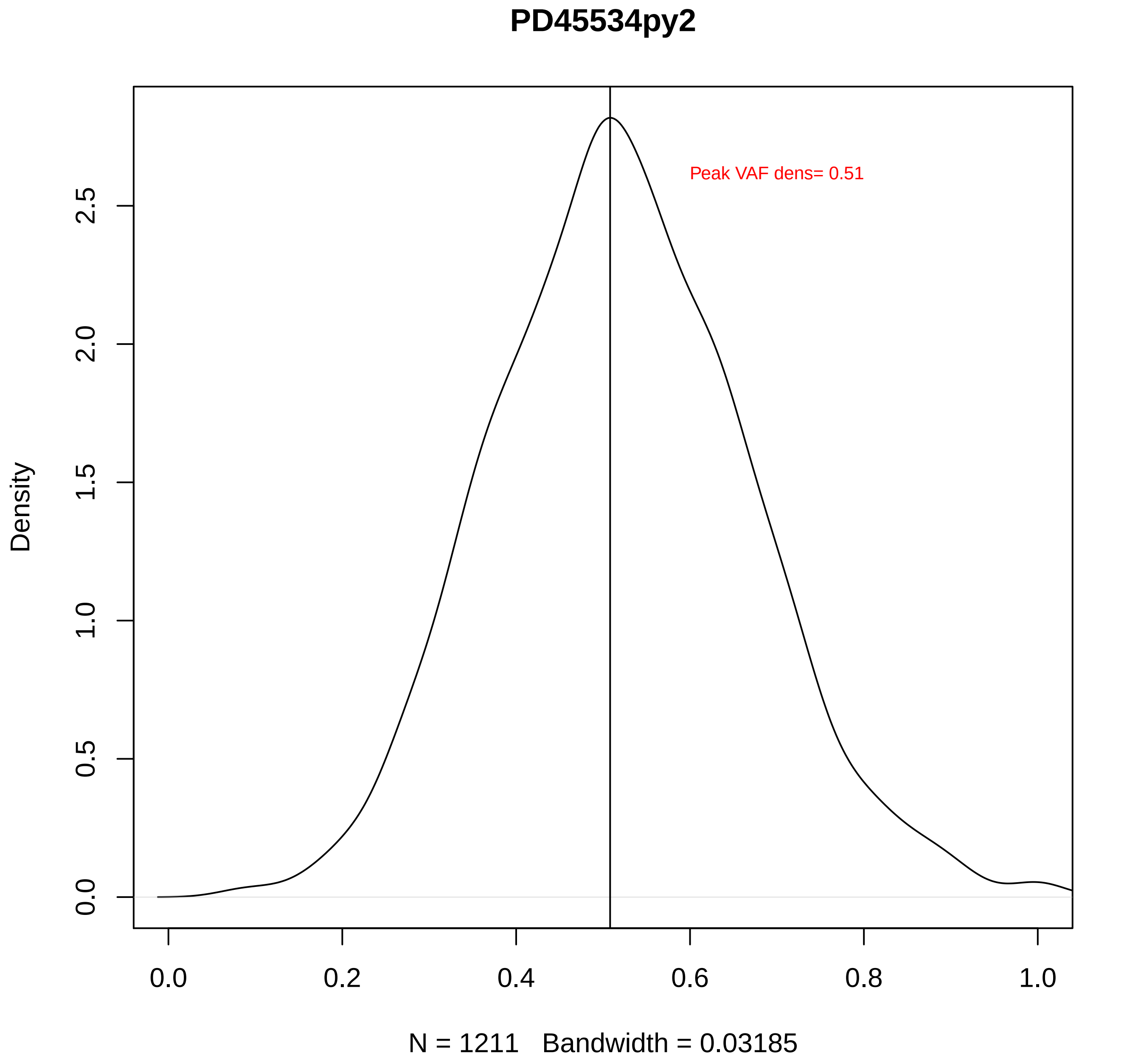
PD45534py2
Peak VAF dens= 0.51
Density
0.0
0.5
1.0
1.5
2.0
2.5
0.0
0.2
0.4
0.6
0.8
1.0
N = 1211   Bandwidth = 0.03185

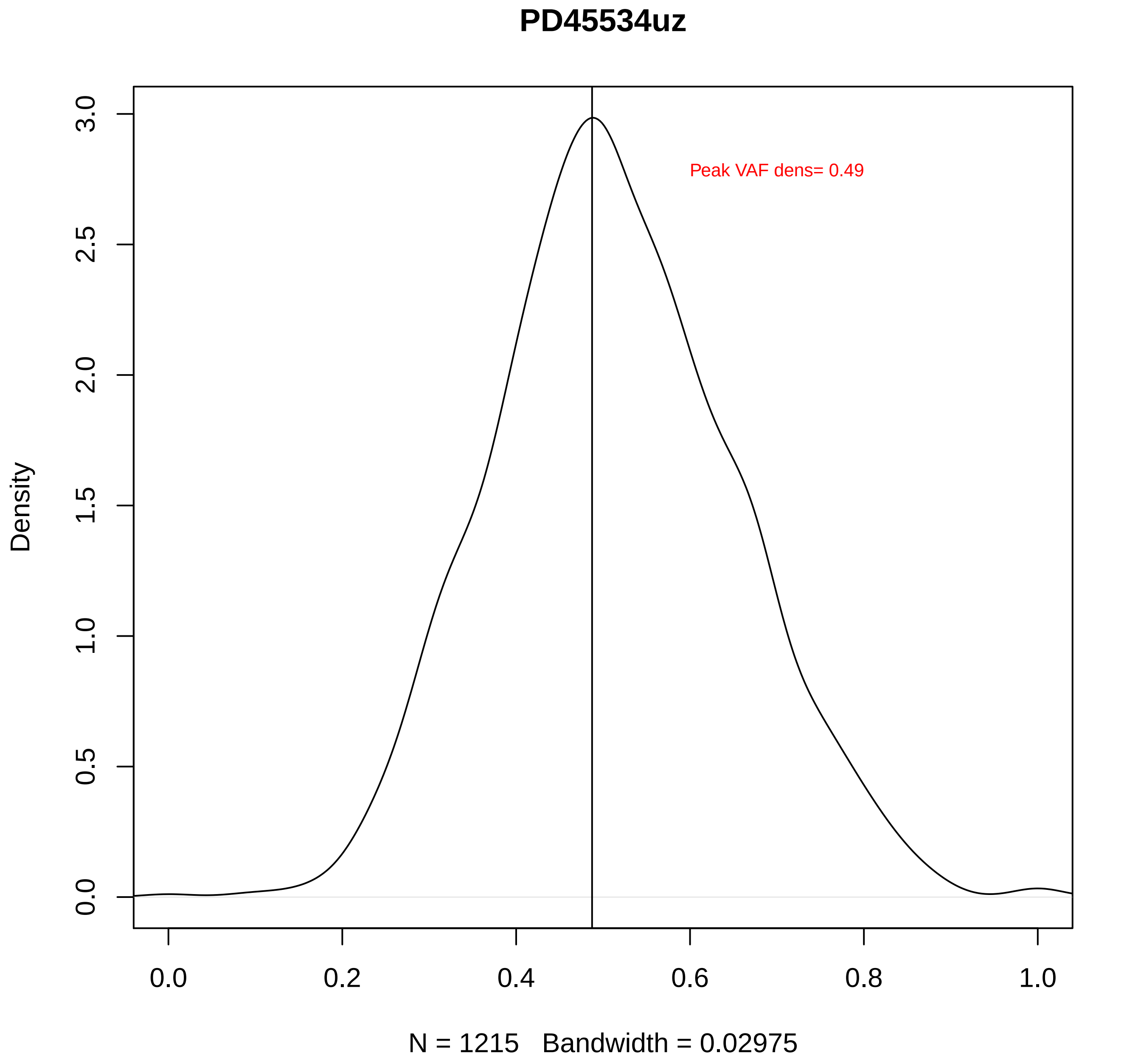
PD45534uz
Peak VAF dens= 0.49
Density
0.0
0.5
1.0
1.5
2.0
2.5
3.0
0.0
0.2
0.4
0.6
0.8
1.0
N = 1215   Bandwidth = 0.02975

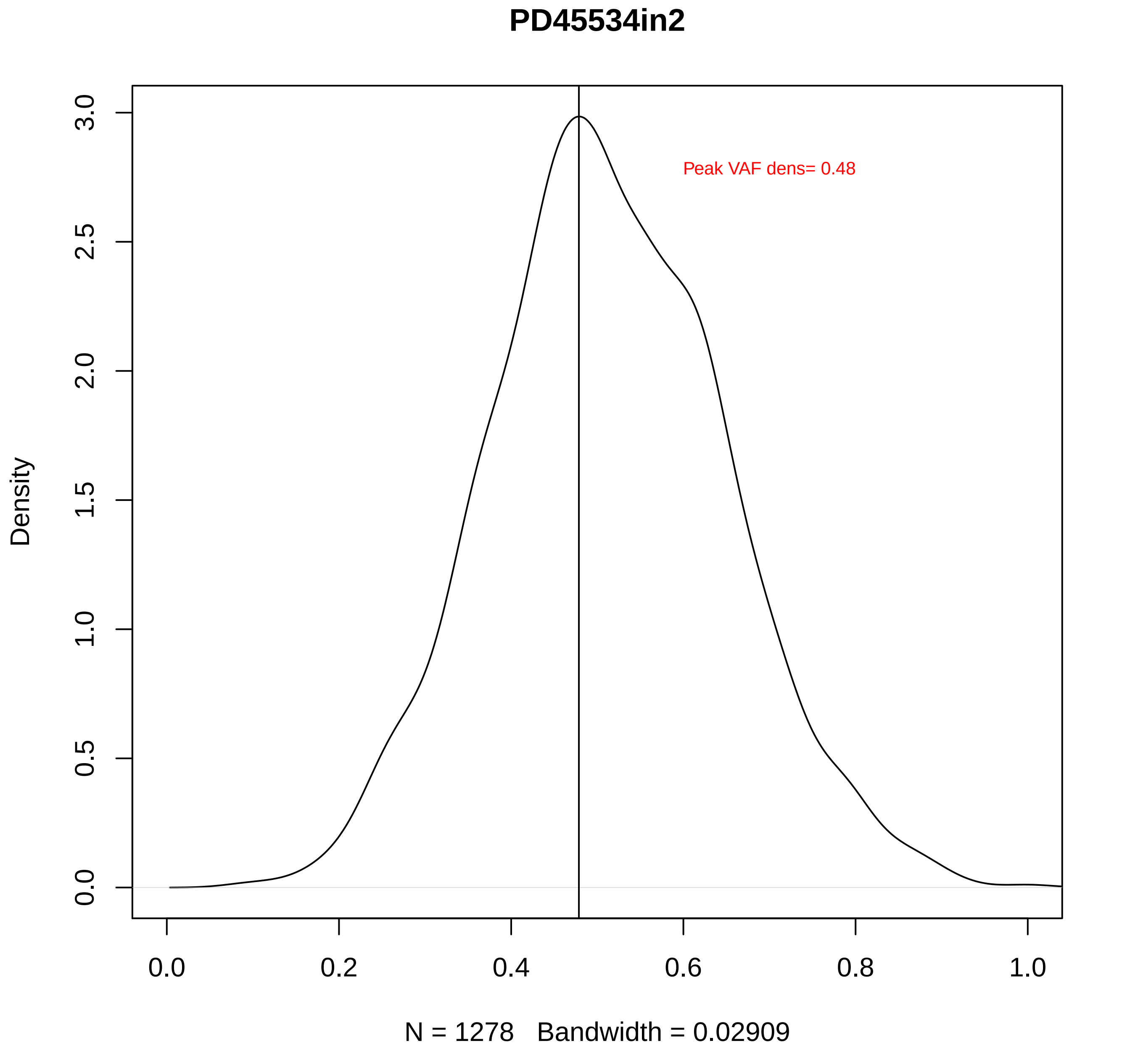

PD45534in2
Peak VAF dens= 0.48
Density
0.0
0.5
1.0
1.5
2.0
2.5
3.0
0.0
0.2
0.4
0.6
0.8
1.0
N = 1278   Bandwidth = 0.02909

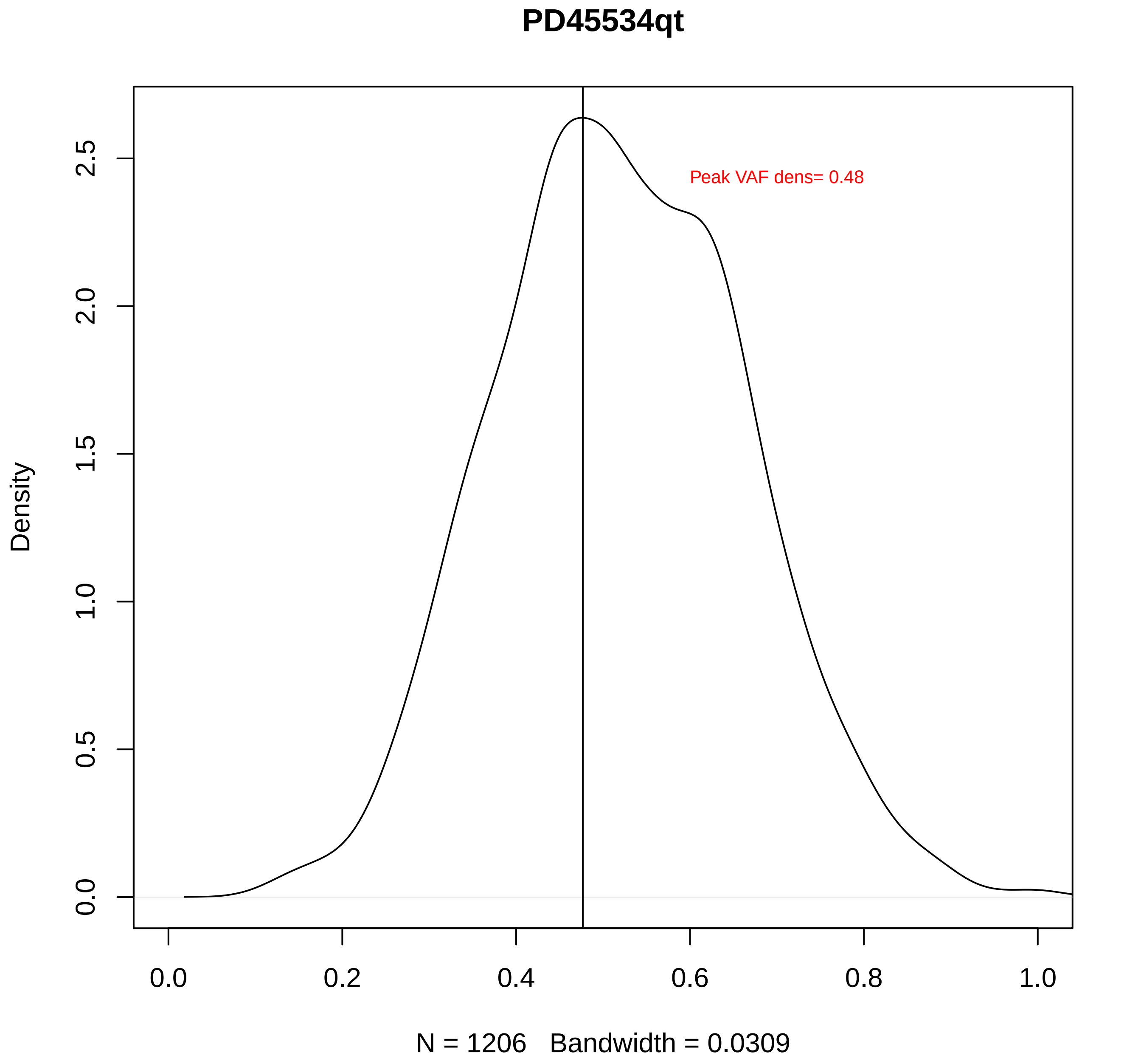

PD45534qt
Peak VAF dens= 0.48
Density
0.0
0.5
1.0
1.5
2.0
2.5
0.0
0.2
0.4
0.6
0.8
1.0
N = 1206   Bandwidth = 0.0309

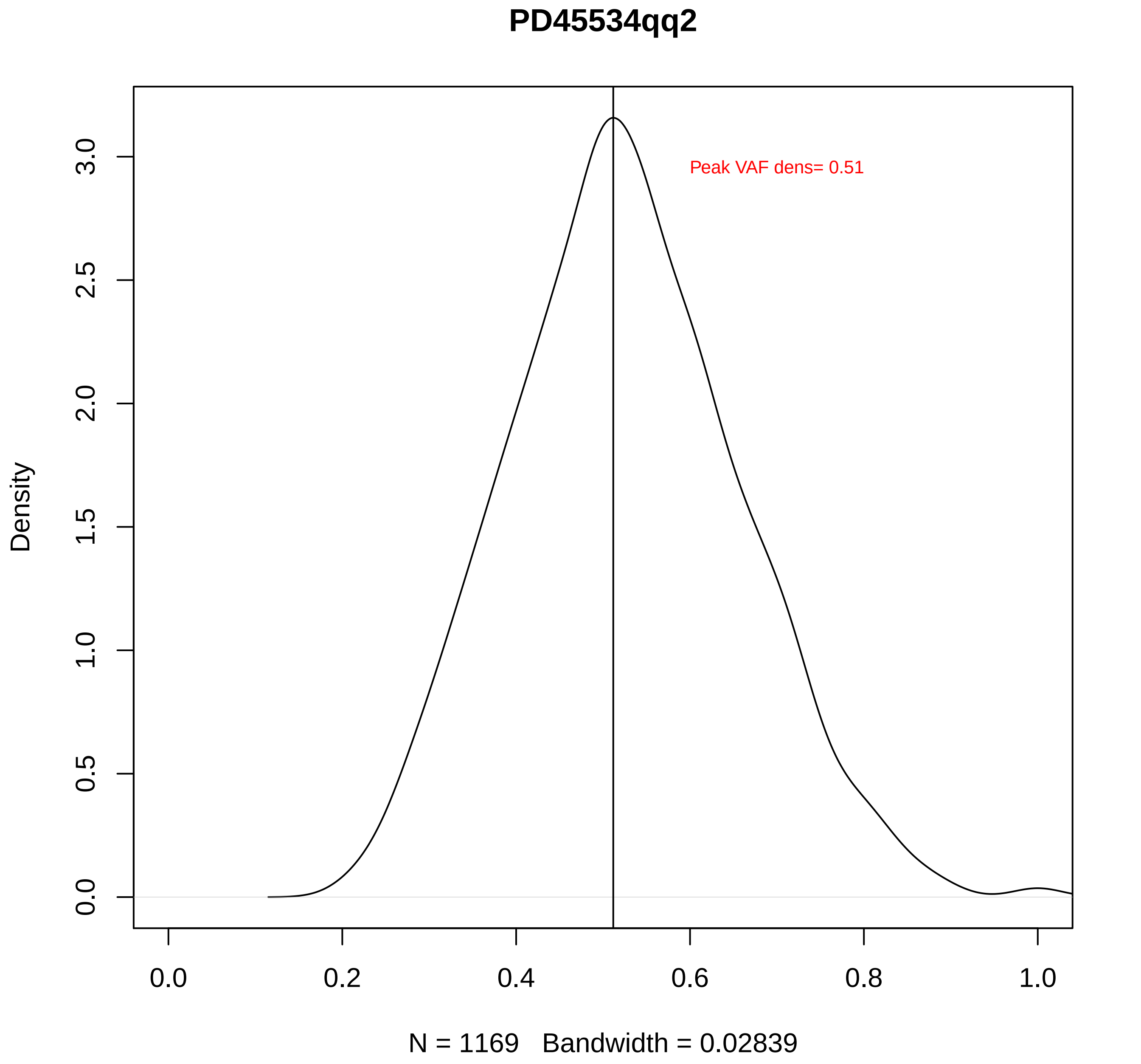

PD45534qq2
Peak VAF dens= 0.51
Density
3.0
2.5
2.0
1.5
1.0
0.5
0.0
0.0
0.2
0.4
0.6
0.8
1.0
N = 1169 Bandwidth = 0.02839

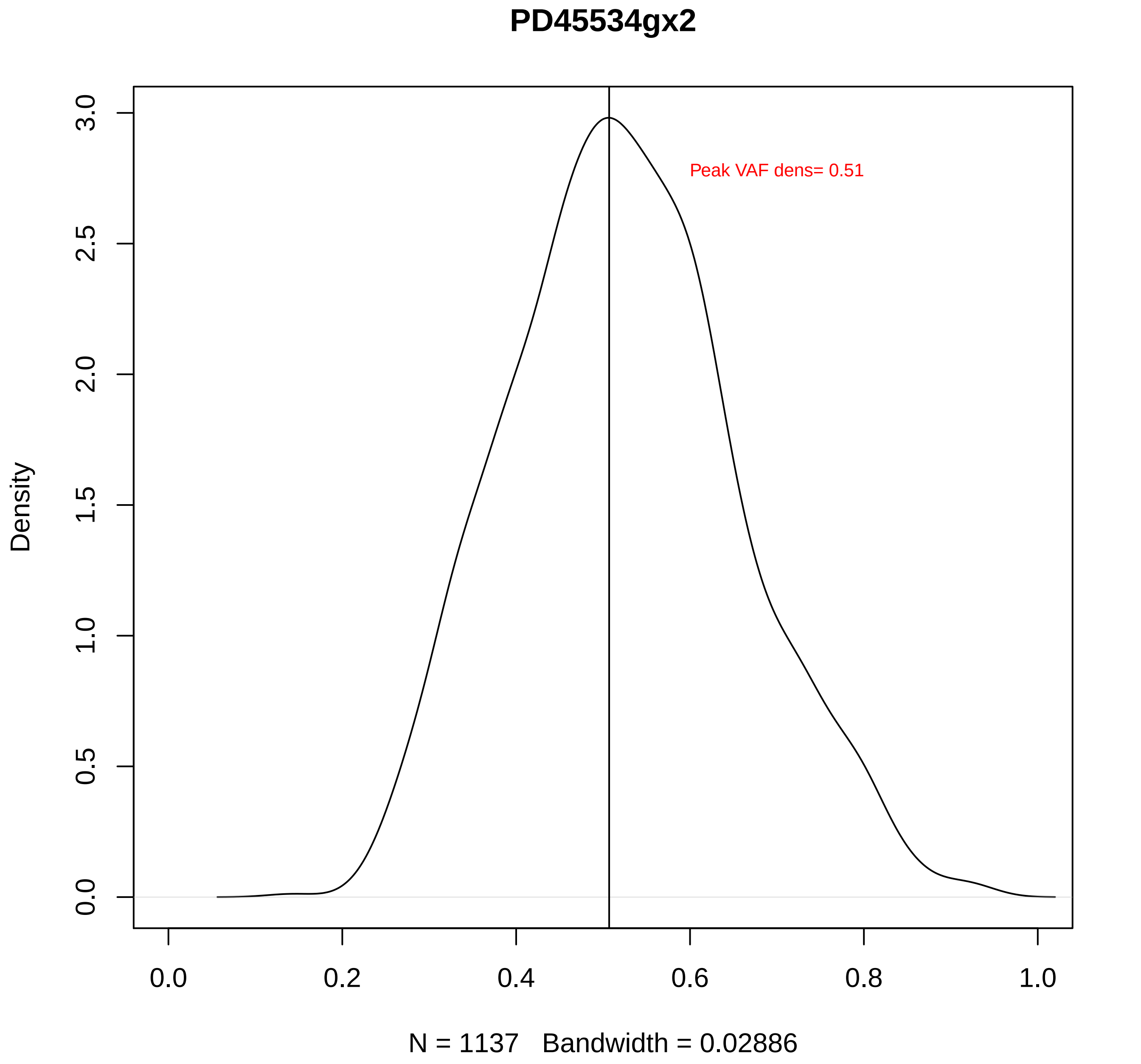

PD45534gx2
Peak VAF dens= 0.51
Density
0.0
0.5
1.0
1.5
2.0
2.5
3.0
0.0
0.2
0.4
0.6
0.8
1.0
N = 1137 Bandwidth = 0.02886

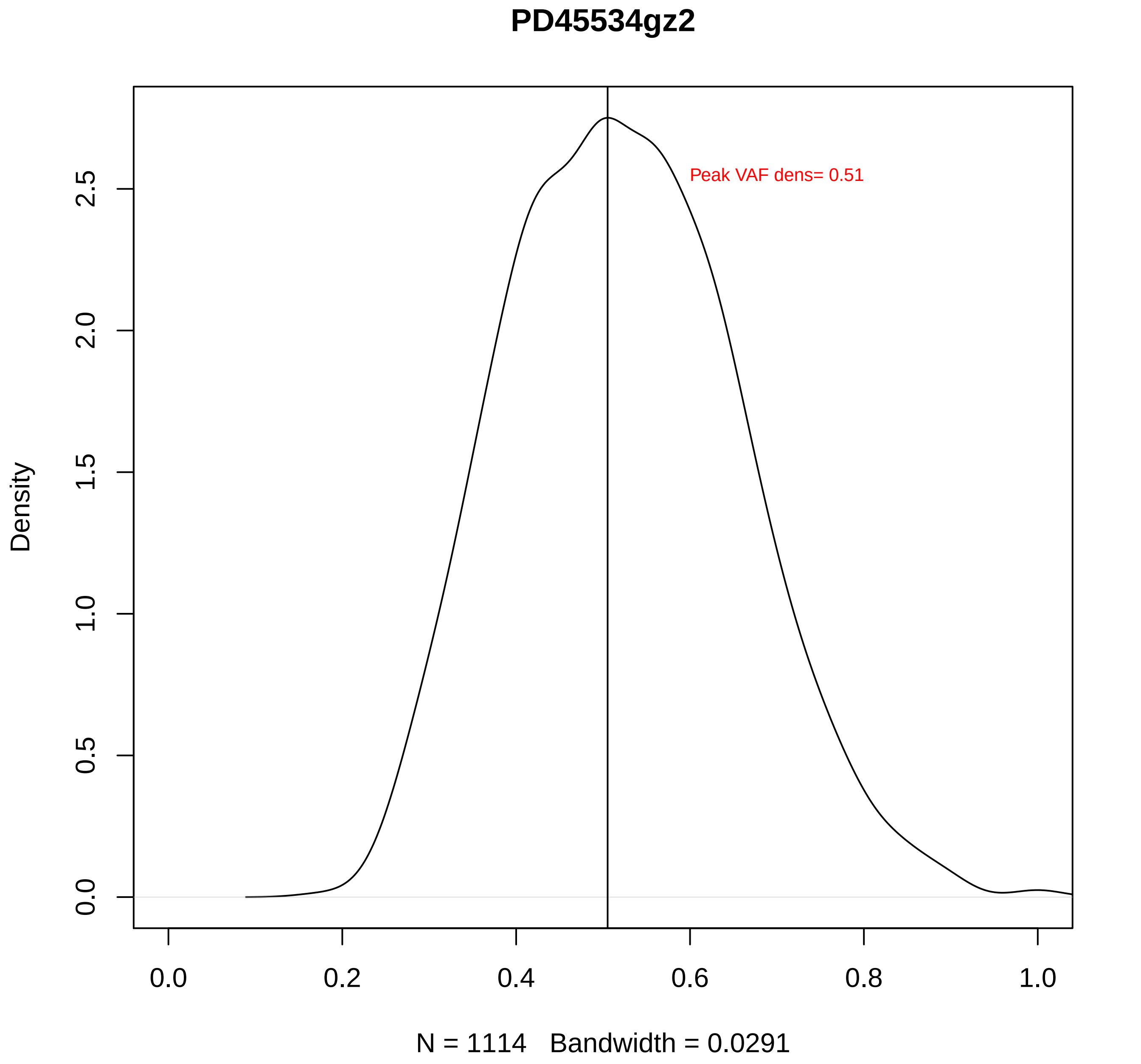
PD45534gz2
Peak VAF dens= 0.51
Density
0.0
0.5
1.0
1.5
2.0
2.5
0.0
0.2
0.4
0.6
0.8
1.0
N = 1114   Bandwidth = 0.0291

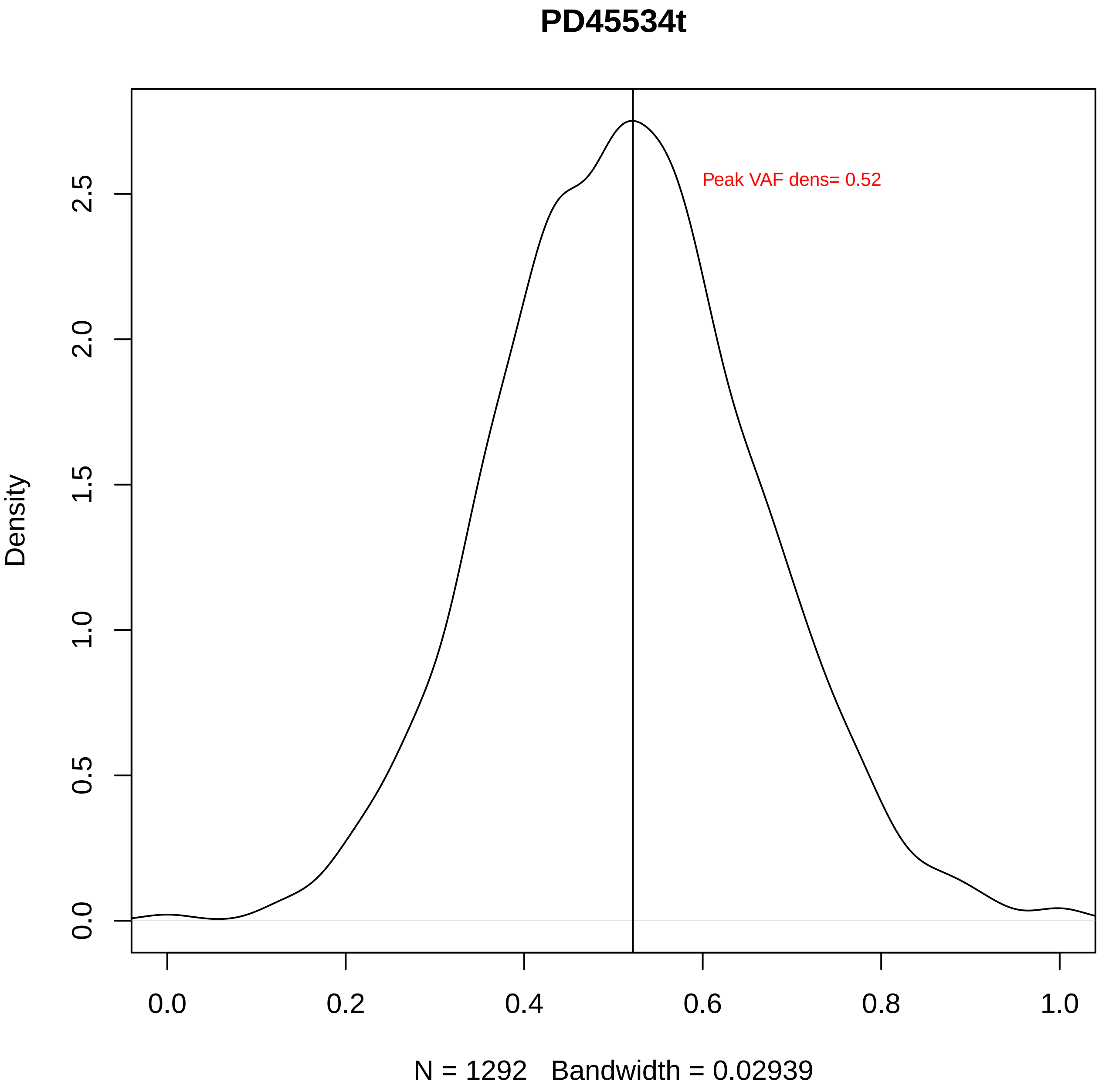
PD45534t
Peak VAF dens= 0.52
Density
0.0
0.5
1.0
1.5
2.0
2.5
0.0
0.2
0.4
0.6
0.8
1.0
N = 1292   Bandwidth = 0.02939

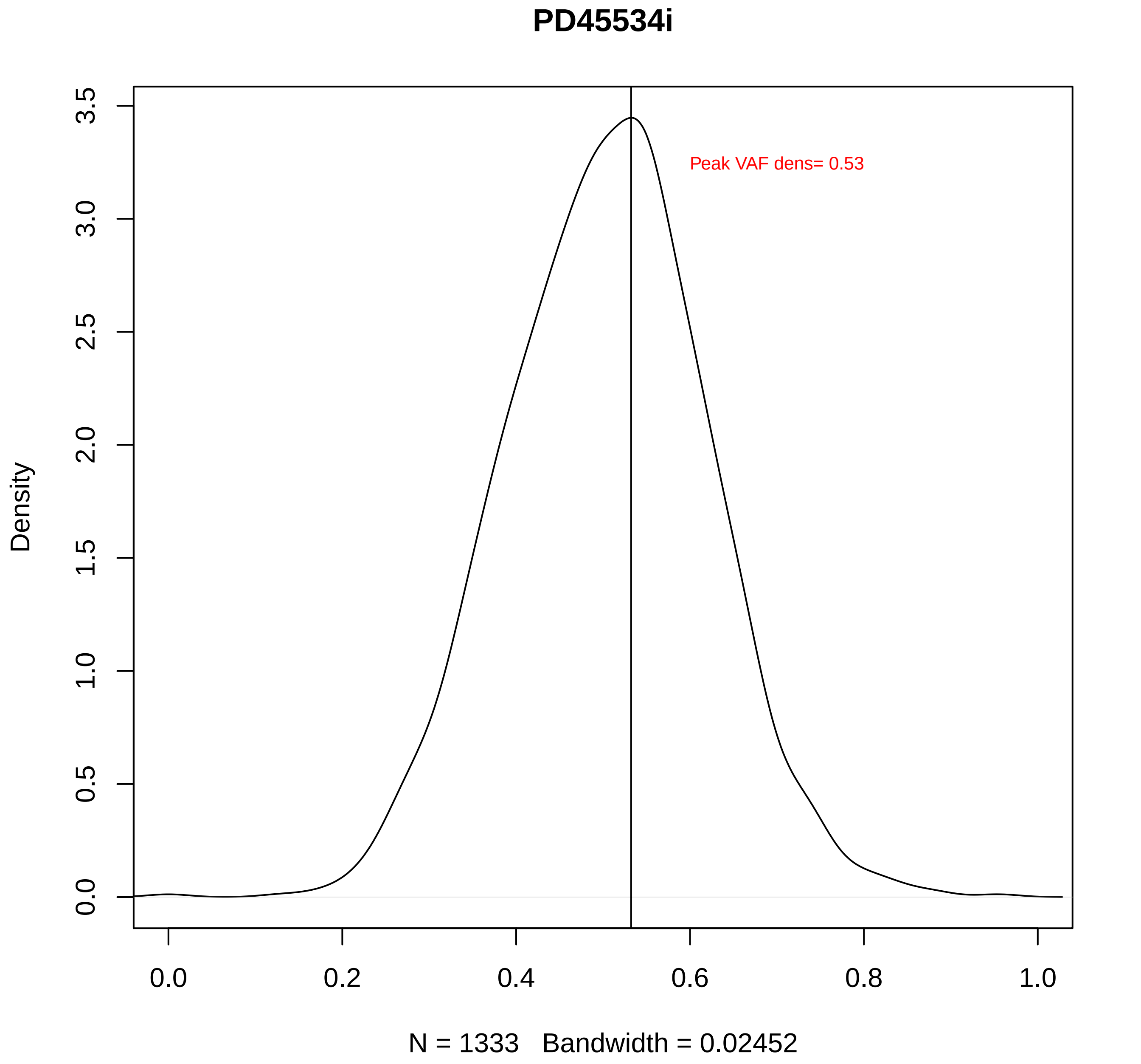

PD45534i
Peak VAF dens= 0.53
Density
0.0
0.5
1.0
1.5
2.0
2.5
3.0
3.5
0.0
0.2
0.4
0.6
0.8
1.0
N = 1333   Bandwidth = 0.02452

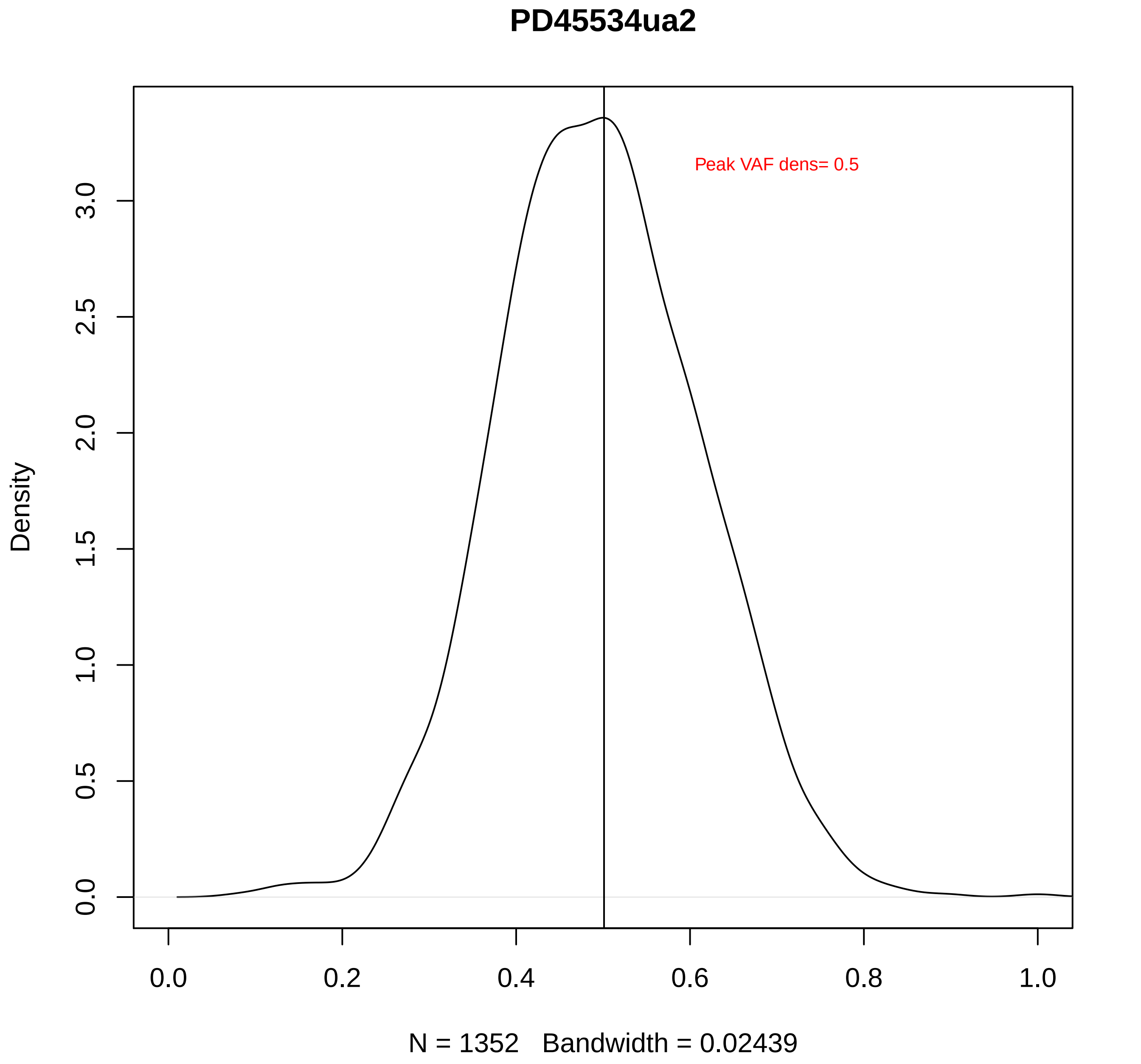
PD45534ua2
Peak VAF dens= 0.5
Density
0.0
0.5
1.0
1.5
2.0
2.5
3.0
0.0
0.2
0.4
0.6
0.8
1.0
N = 1352   Bandwidth = 0.02439

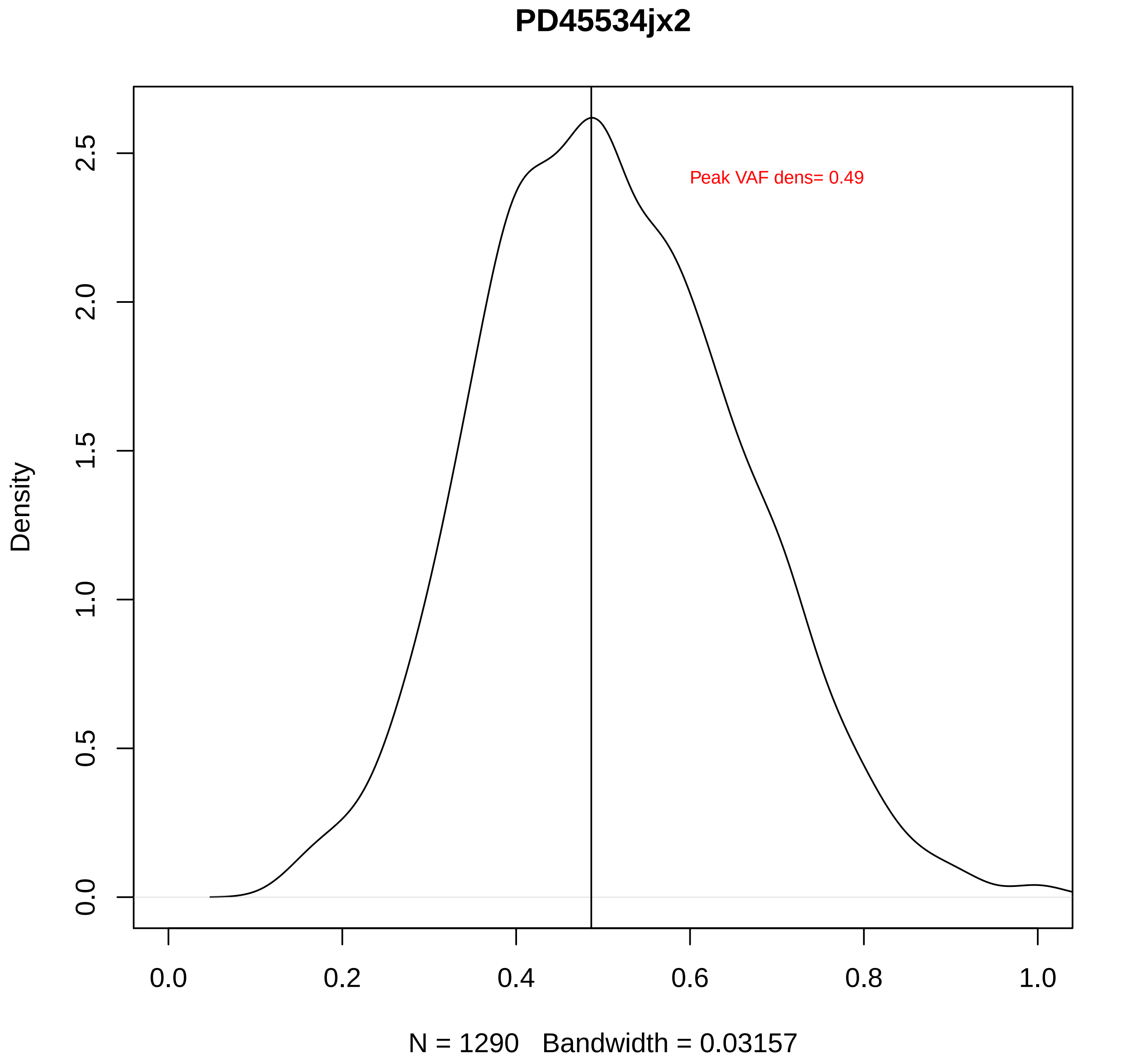
PD45534jx2
Peak VAF dens= 0.49
Density
0.0
0.5
1.0
1.5
2.0
2.5
0.0
0.2
0.4
0.6
0.8
1.0
N = 1290   Bandwidth = 0.03157

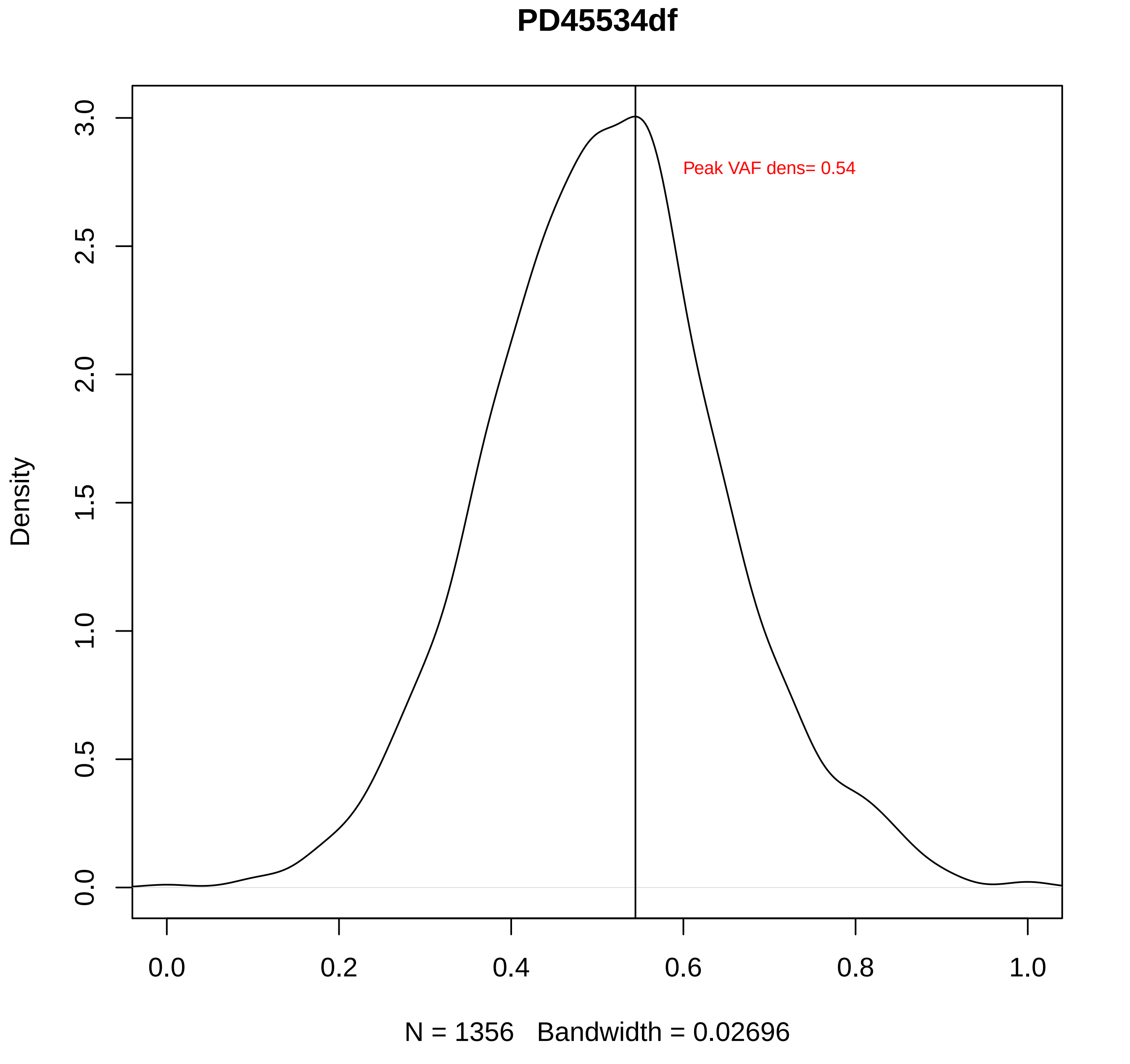
PD45534df
Peak VAF dens= 0.54
Density
0.0
0.5
1.0
1.5
2.0
2.5
3.0
0.0
0.2
0.4
0.6
0.8
1.0
N = 1356   Bandwidth = 0.02696

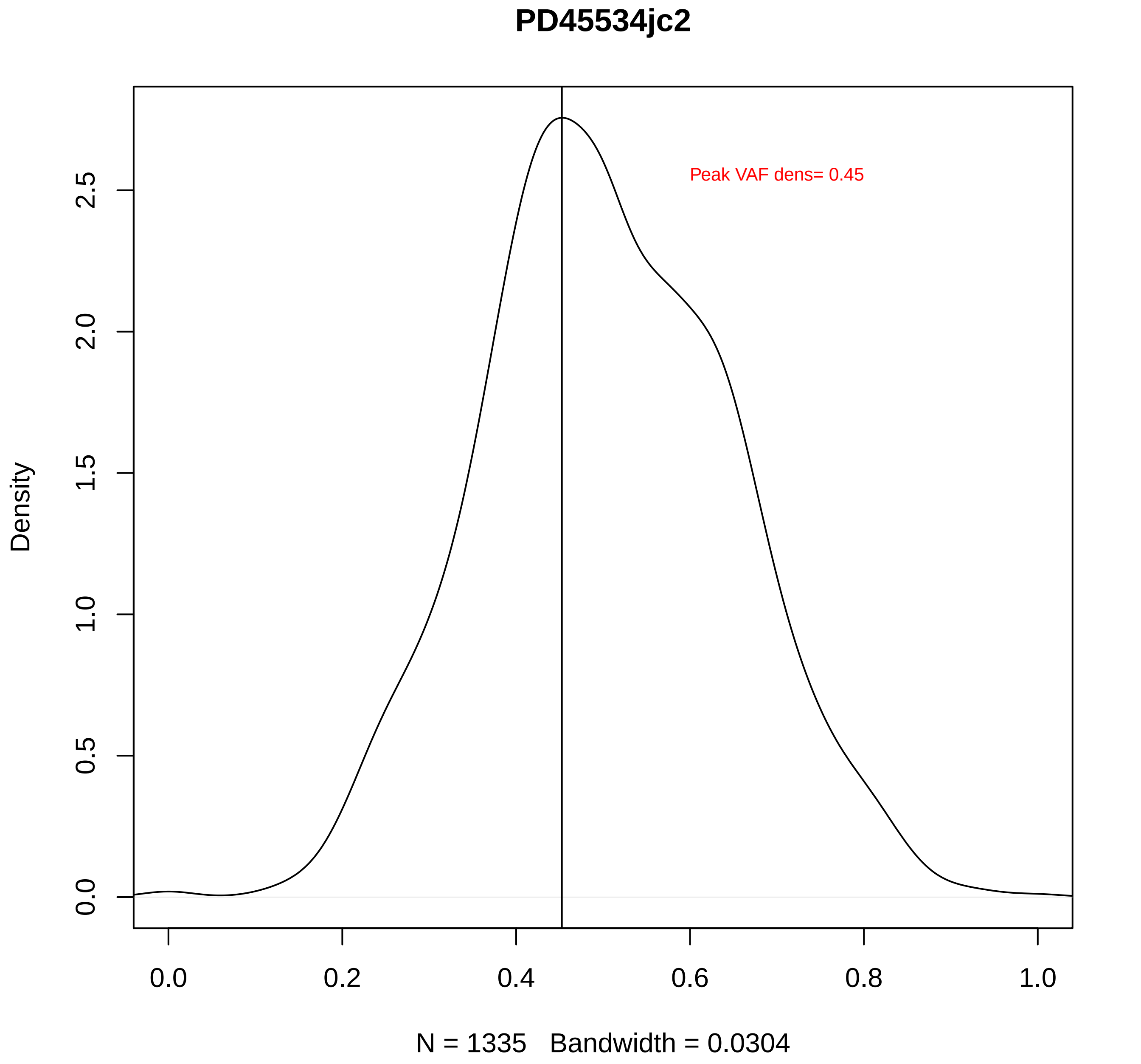
PD45534jc2
Peak VAF dens= 0.45
Density
0.0
0.5
1.0
1.5
2.0
2.5
0.0
0.2
0.4
0.6
0.8
1.0
N = 1335   Bandwidth = 0.0304

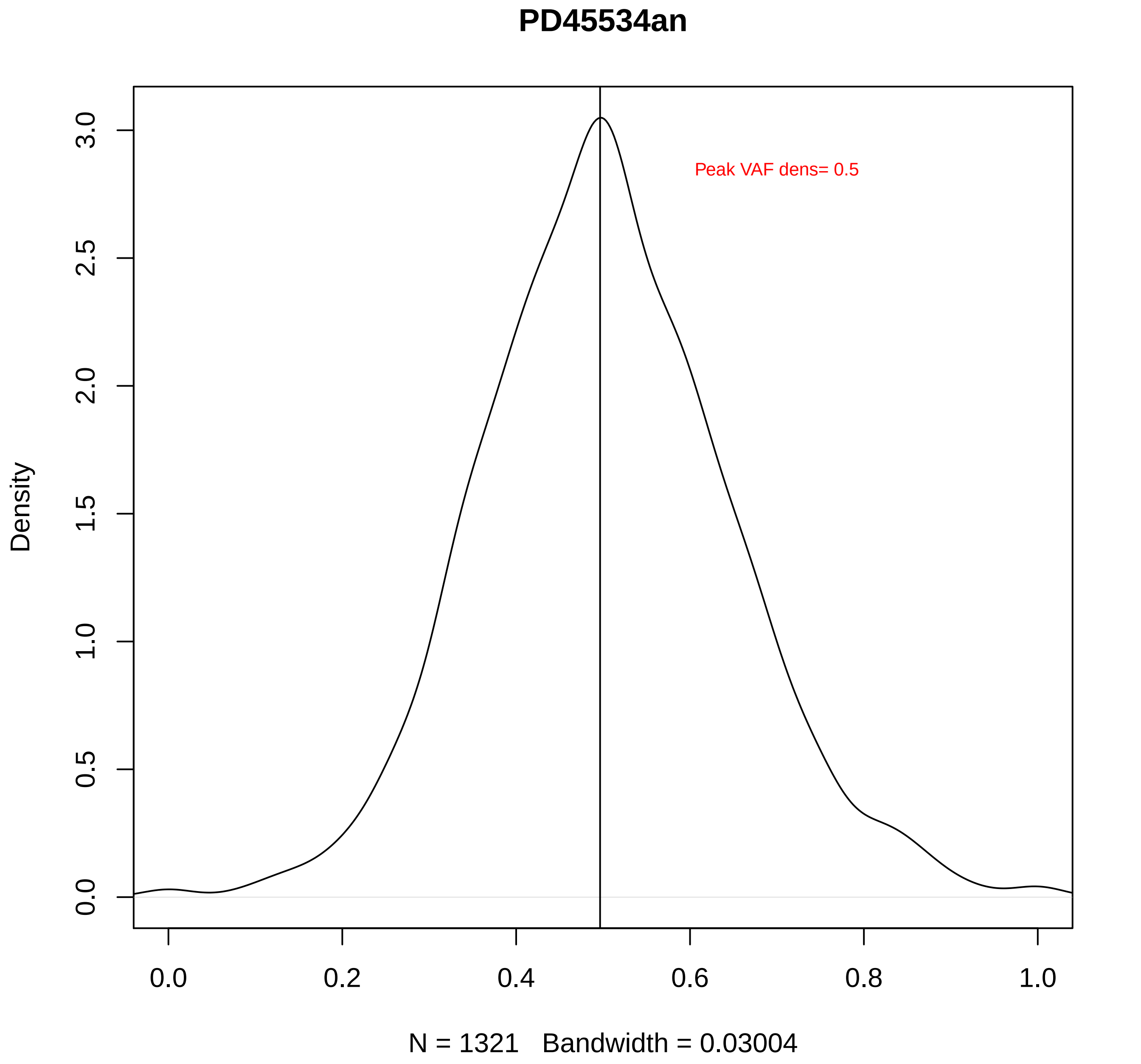

PD45534an
Peak VAF dens= 0.5
Density
0.0
0.5
1.0
1.5
2.0
2.5
3.0
0.0
0.2
0.4
0.6
0.8
1.0
N = 1321 Bandwidth = 0.03004

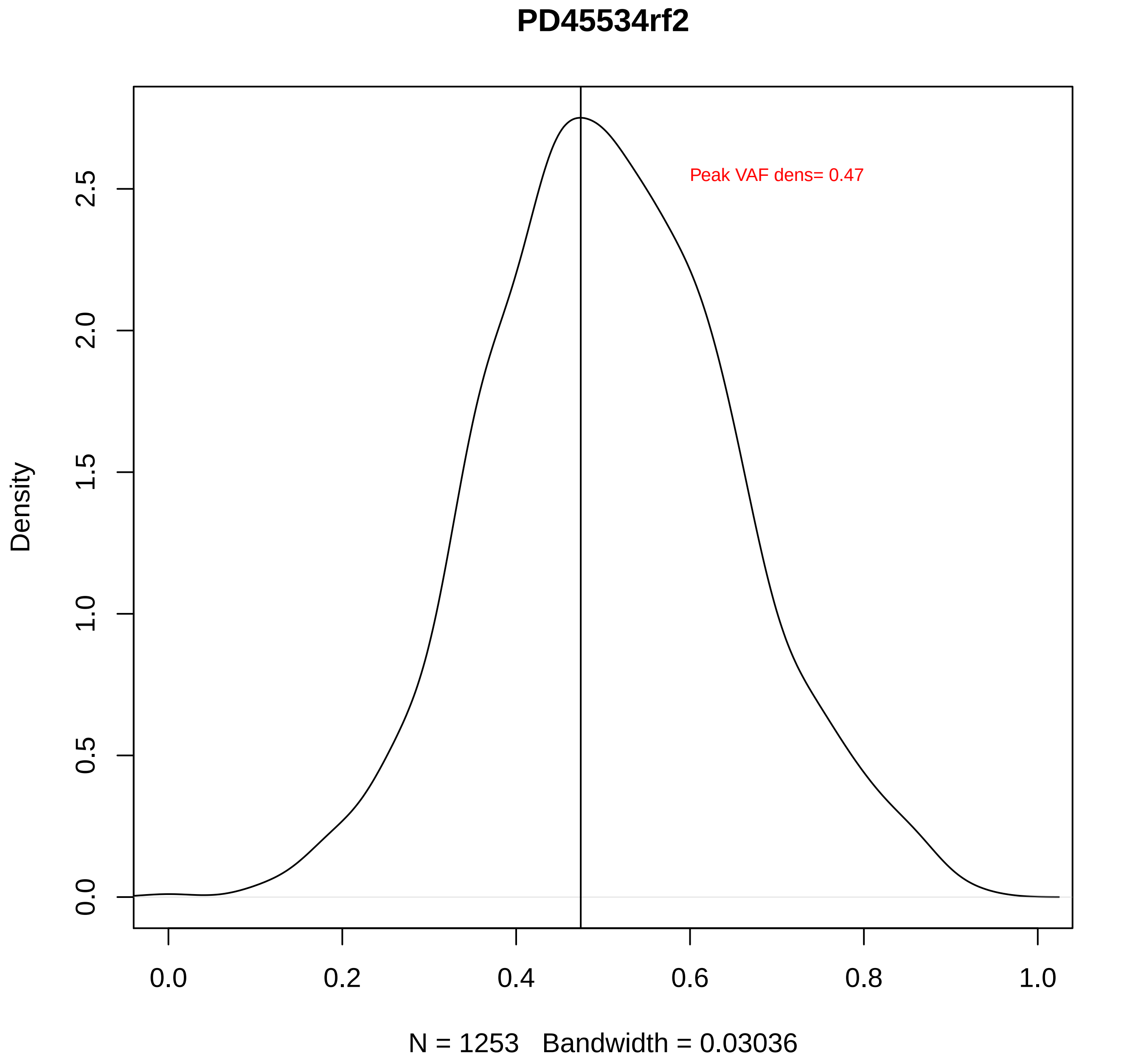

PD45534rf2
Peak VAF dens= 0.47
Density
0.0
0.5
1.0
1.5
2.0
2.5
0.0
0.2
0.4
0.6
0.8
1.0
N = 1253   Bandwidth = 0.03036

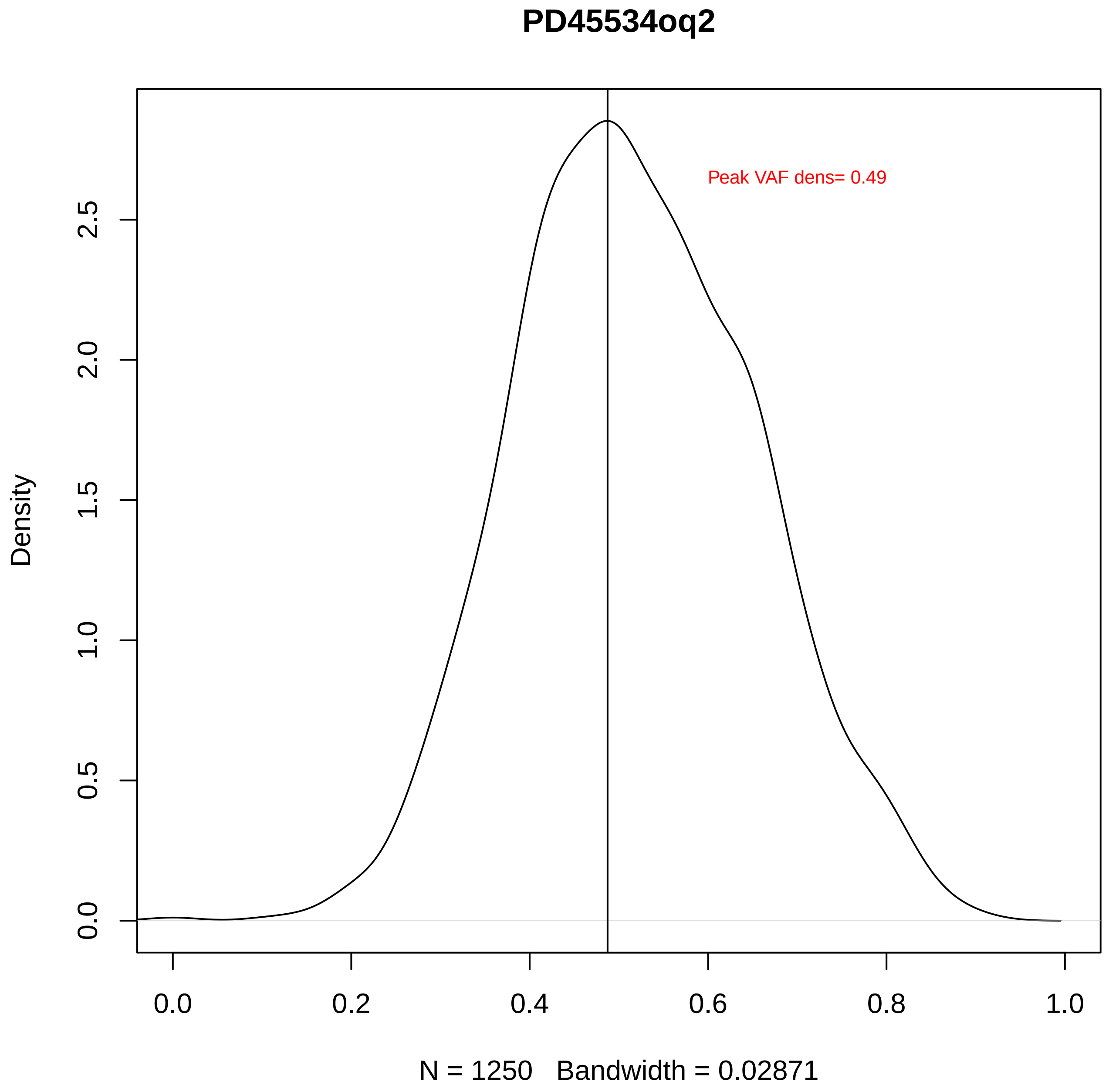

PD45534oq2
Peak VAF dens= 0.49
Density
0.0
0.5
1.0
1.5
2.0
2.5
0.0
0.2
0.4
0.6
0.8
1.0
N = 1250 Bandwidth = 0.02871

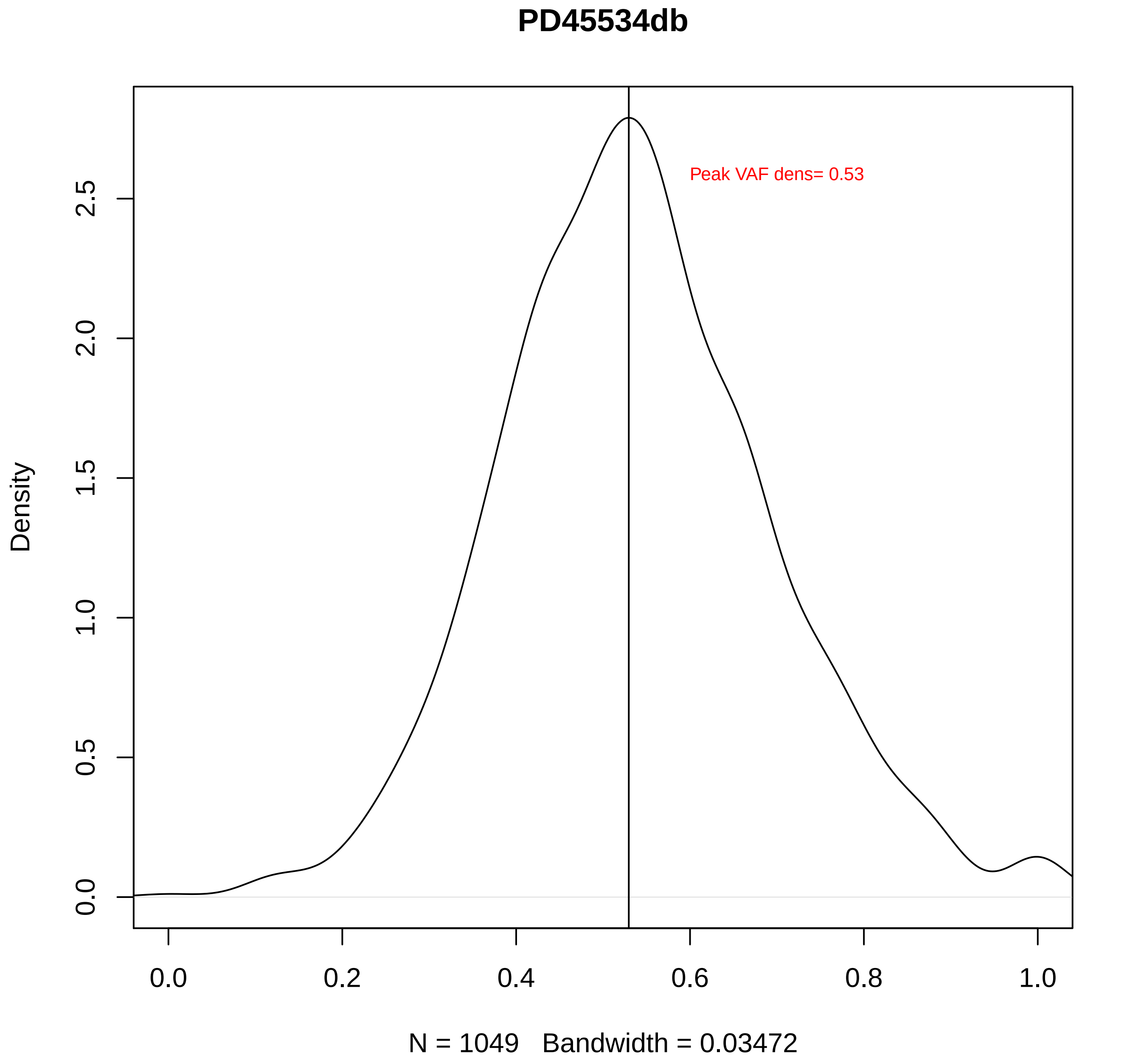

PD45534db
Peak VAF dens= 0.53
Density
0.0
0.5
1.0
1.5
2.0
2.5
0.0
0.2
0.4
0.6
0.8
1.0
N = 1049   Bandwidth = 0.03472

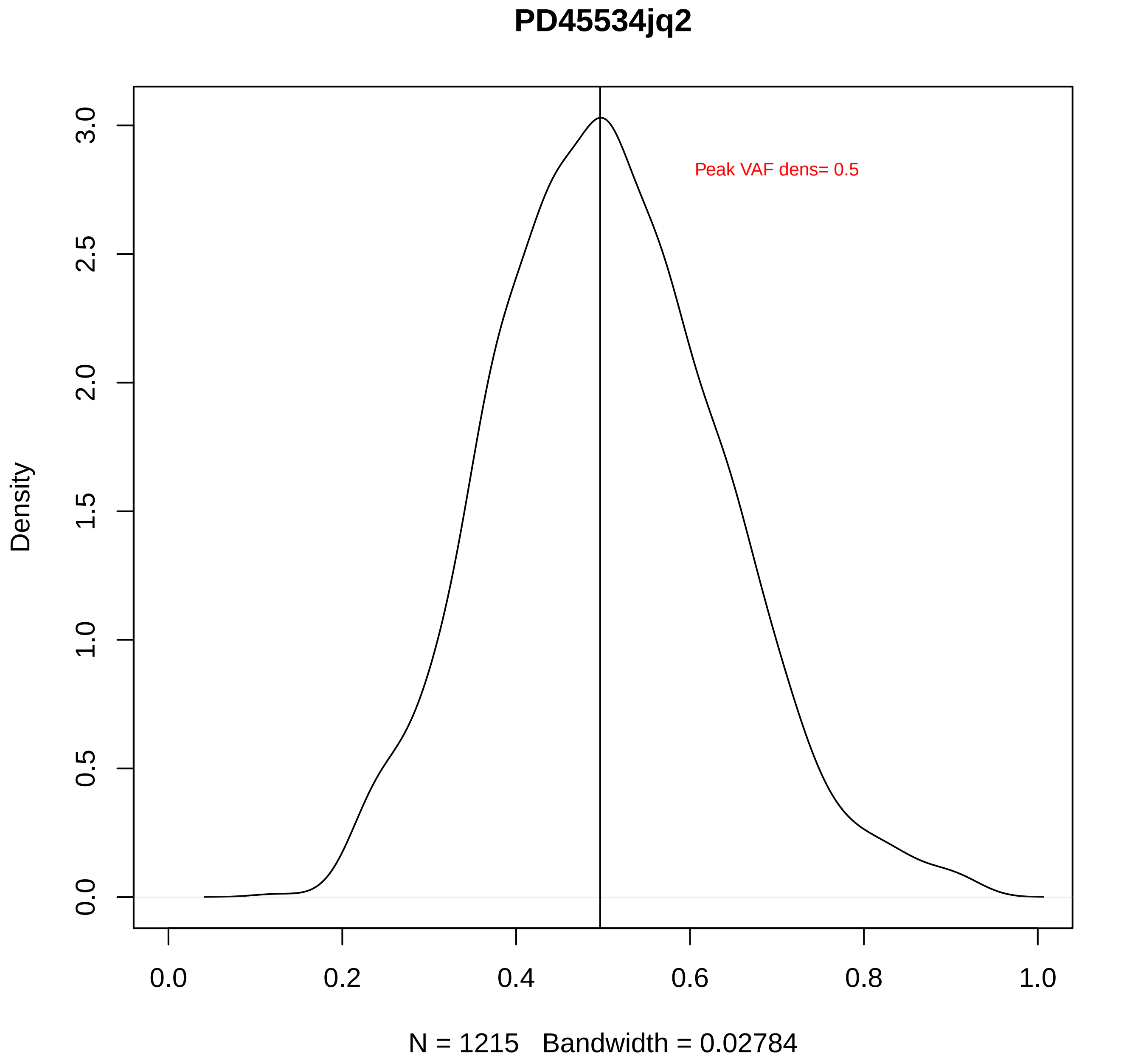
PD45534jq2
Peak VAF dens= 0.5
Density
0.0
0.5
1.0
1.5
2.0
2.5
3.0
0.0
0.2
0.4
0.6
0.8
1.0
N = 1215 Bandwidth = 0.02784

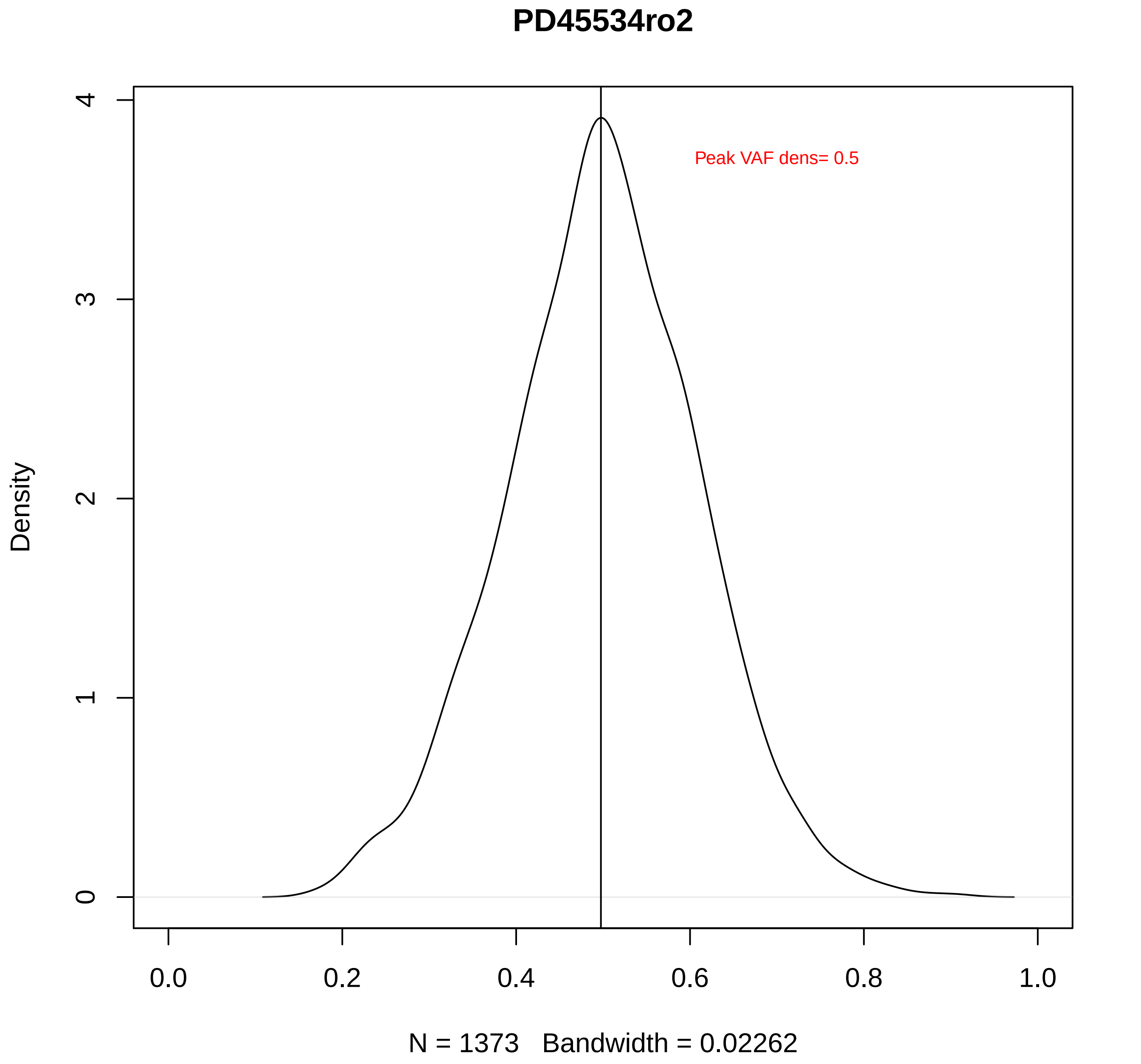

PD45534ro2
Peak VAF dens= 0.5
Density
0
1
2
3
4
0.0
0.2
0.4
0.6
0.8
1.0
N = 1373 Bandwidth = 0.02262

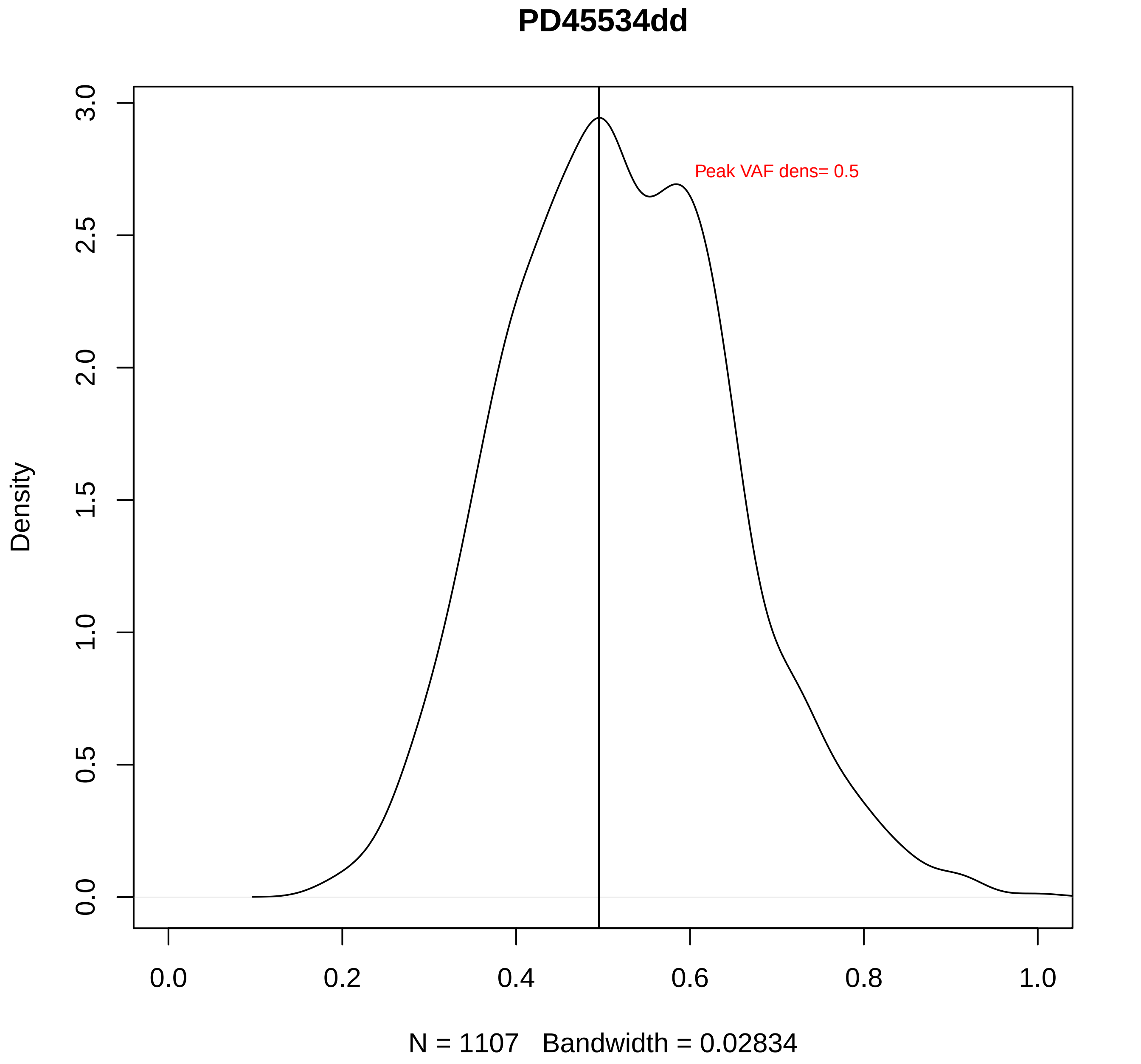
PD45534dd
Peak VAF dens= 0.5
Density
0.0
0.5
1.0
1.5
2.0
2.5
3.0
0.0
0.2
0.4
0.6
0.8
1.0
N = 1107   Bandwidth = 0.02834

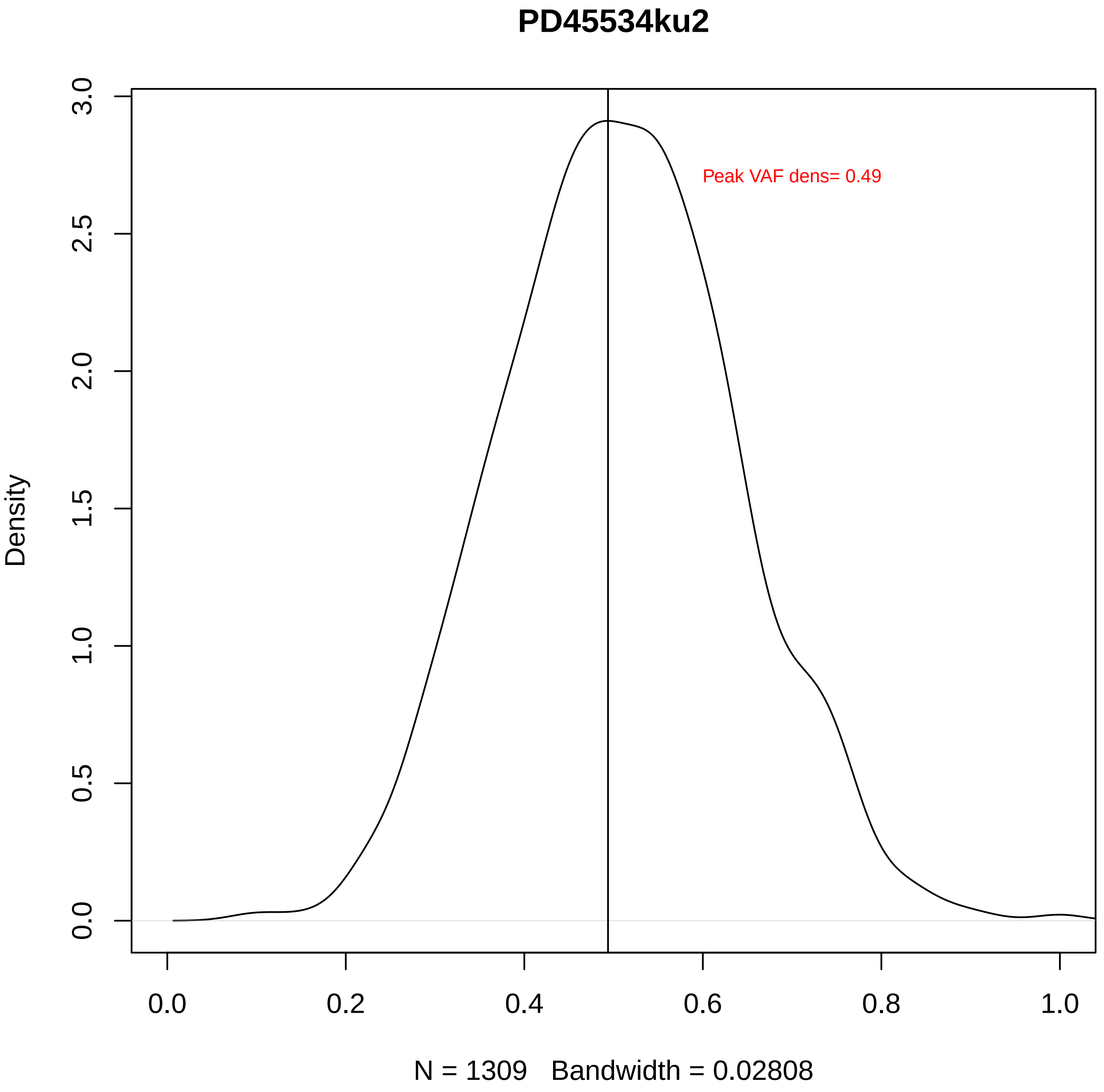
PD45534ku2
Peak VAF dens= 0.49
Density
0.0
0.5
1.0
1.5
2.0
2.5
3.0
0.0
0.2
0.4
0.6
0.8
1.0
N = 1309   Bandwidth = 0.02808

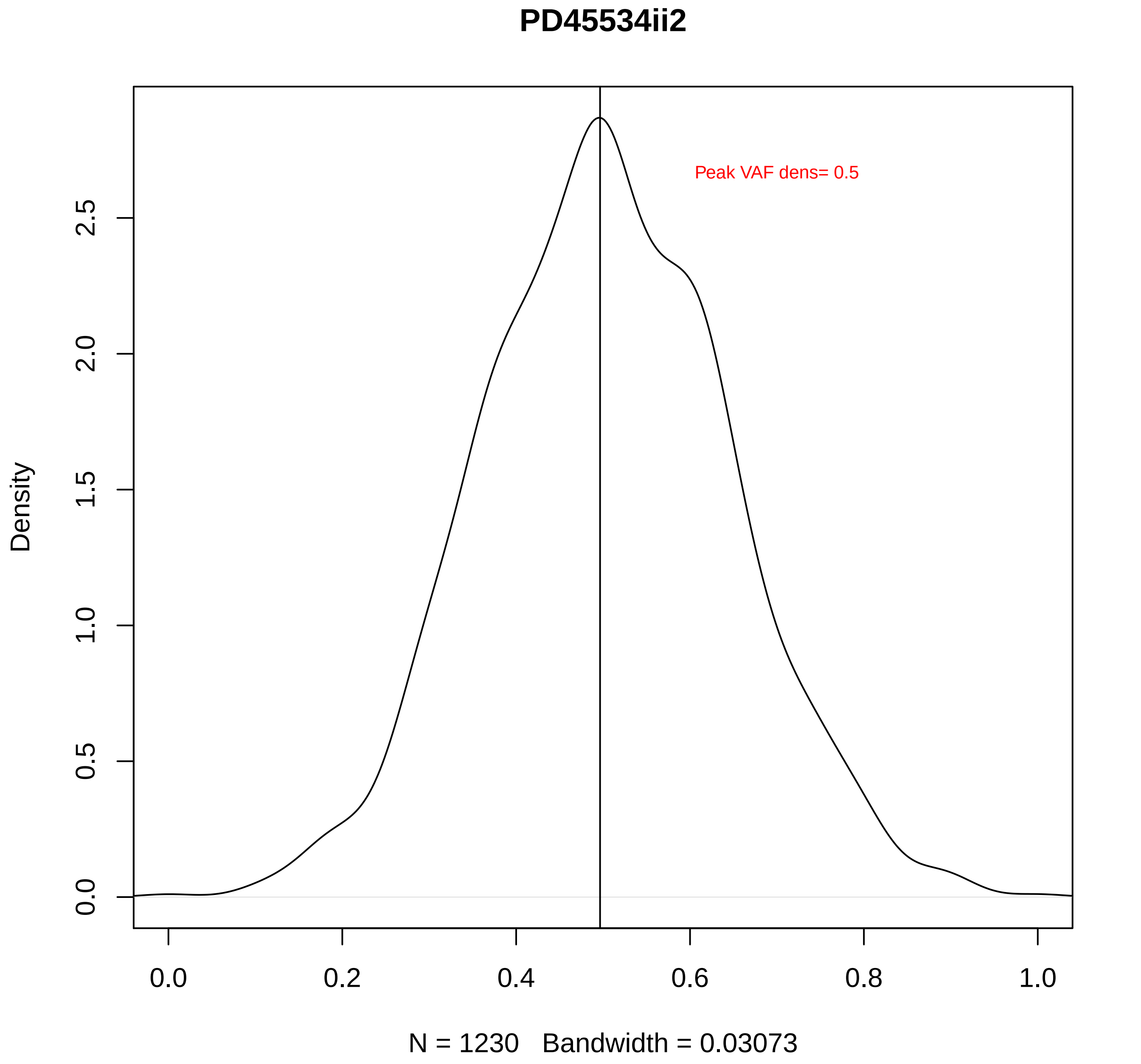
PD45534ii2
Peak VAF dens= 0.5
Density
0.0
0.5
1.0
1.5
2.0
2.5
0.0
0.2
0.4
0.6
0.8
1.0
N = 1230   Bandwidth = 0.03073

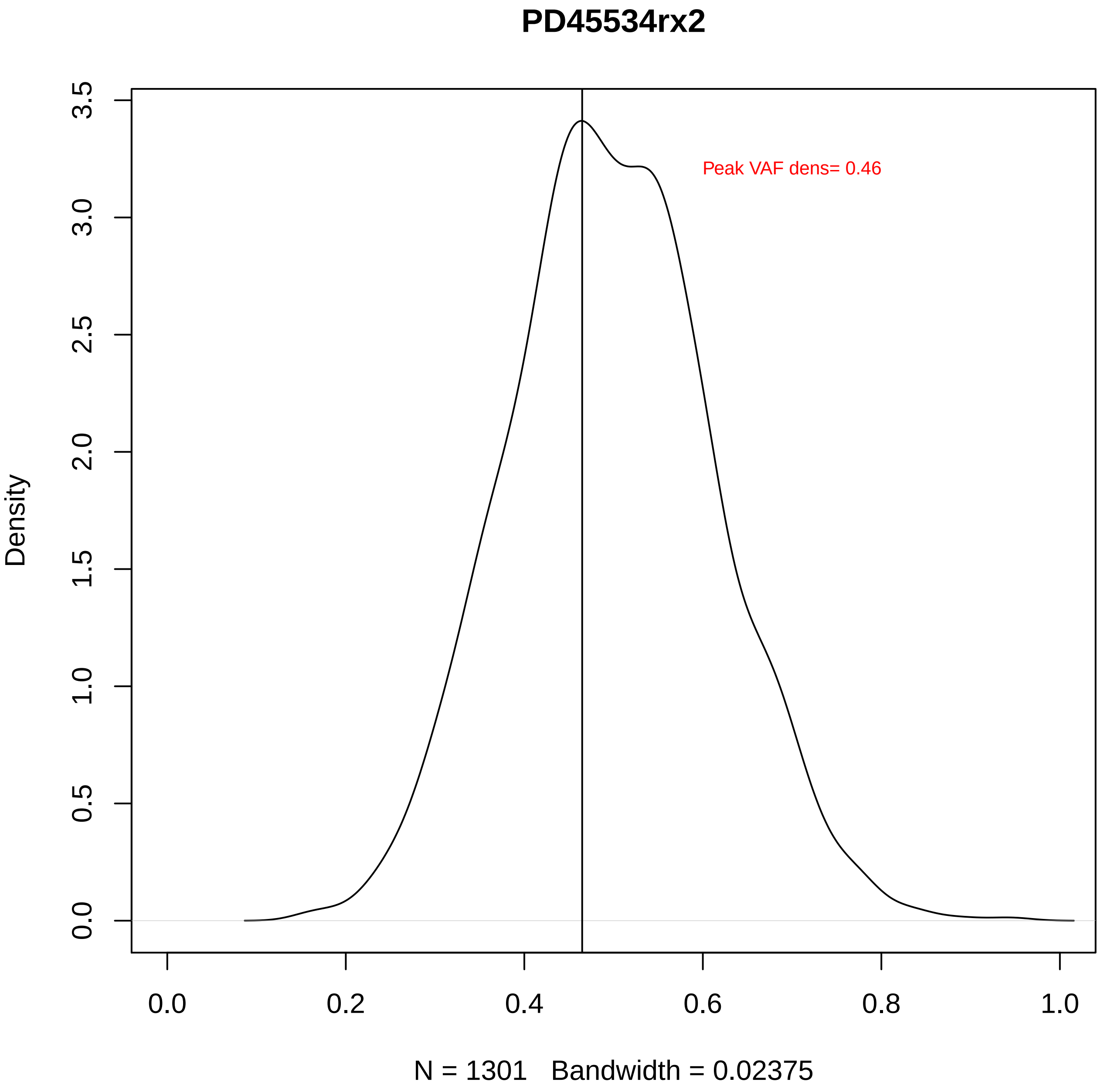
PD45534rx2
Peak VAF dens= 0.46
Density
0.0
0.5
1.0
1.5
2.0
2.5
3.0
3.5
0.0
0.2
0.4
0.6
0.8
1.0
N = 1301   Bandwidth = 0.02375

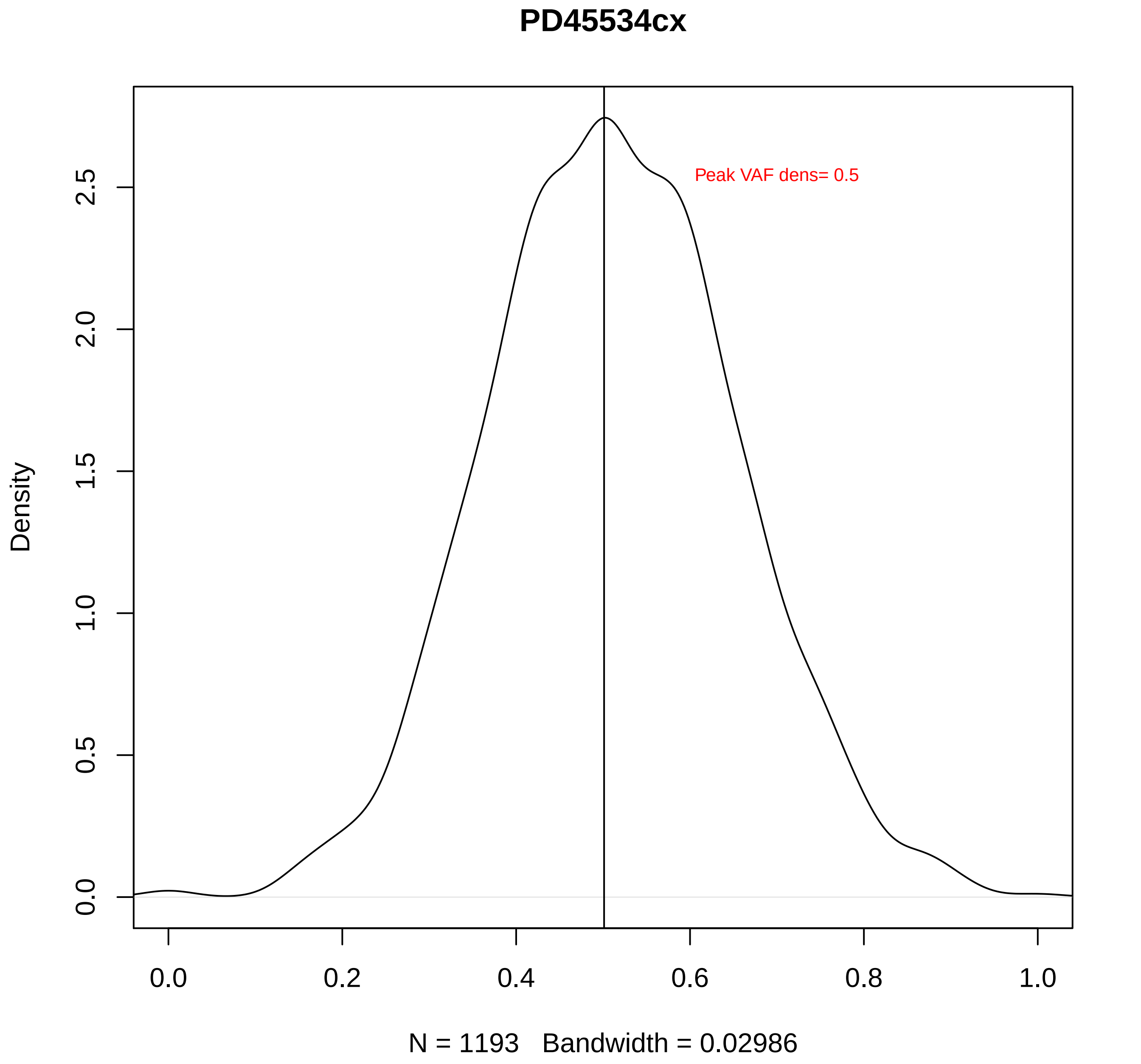

PD45534cx
Peak VAF dens= 0.5
Density
0.0
0.5
1.0
1.5
2.0
2.5
0.0
0.2
0.4
0.6
0.8
1.0
N = 1193   Bandwidth = 0.02986

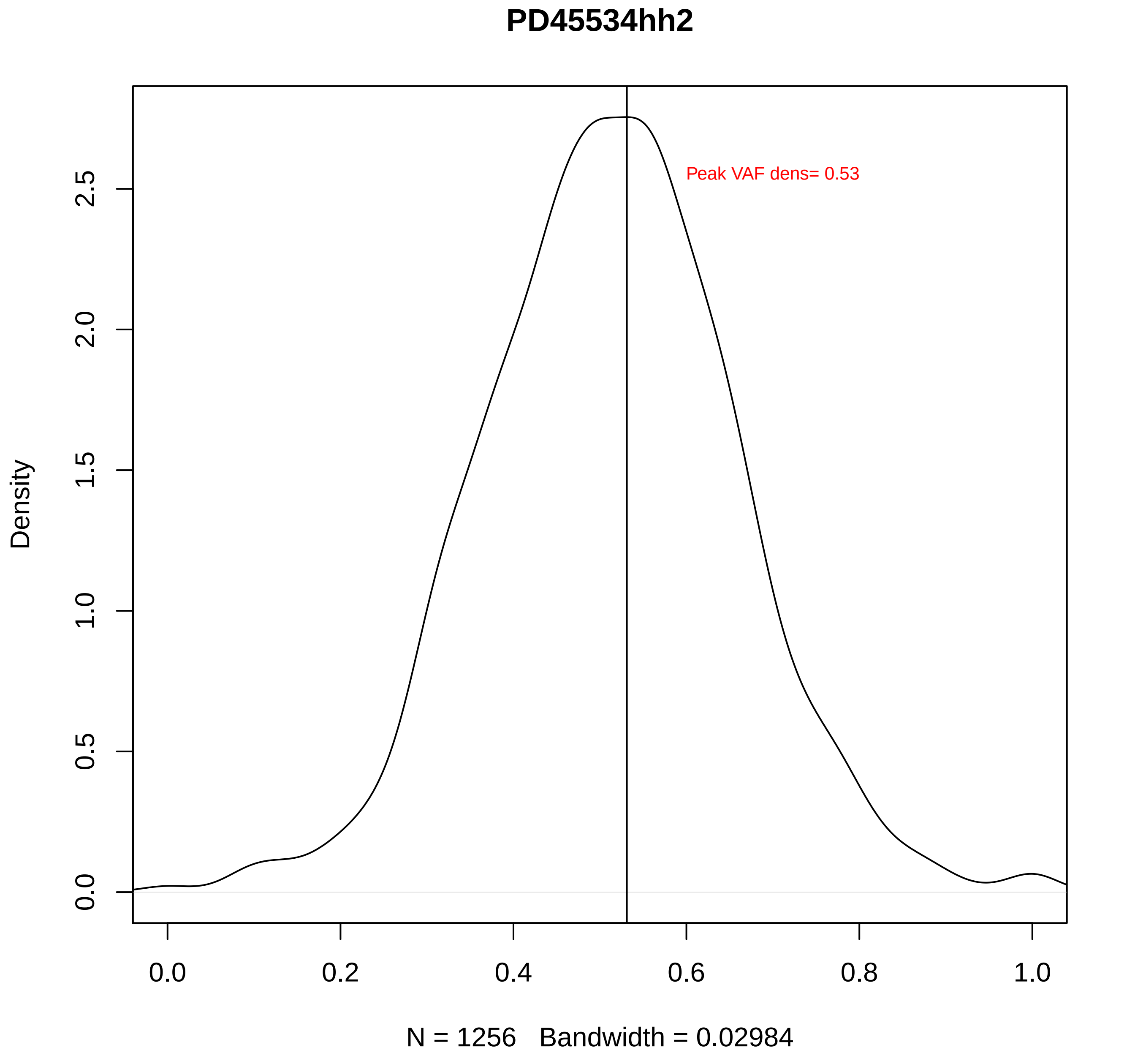

PD45534hh2
Peak VAF dens= 0.53
Density
0.0
0.5
1.0
1.5
2.0
2.5
0.0
0.2
0.4
0.6
0.8
1.0
N = 1256   Bandwidth = 0.02984

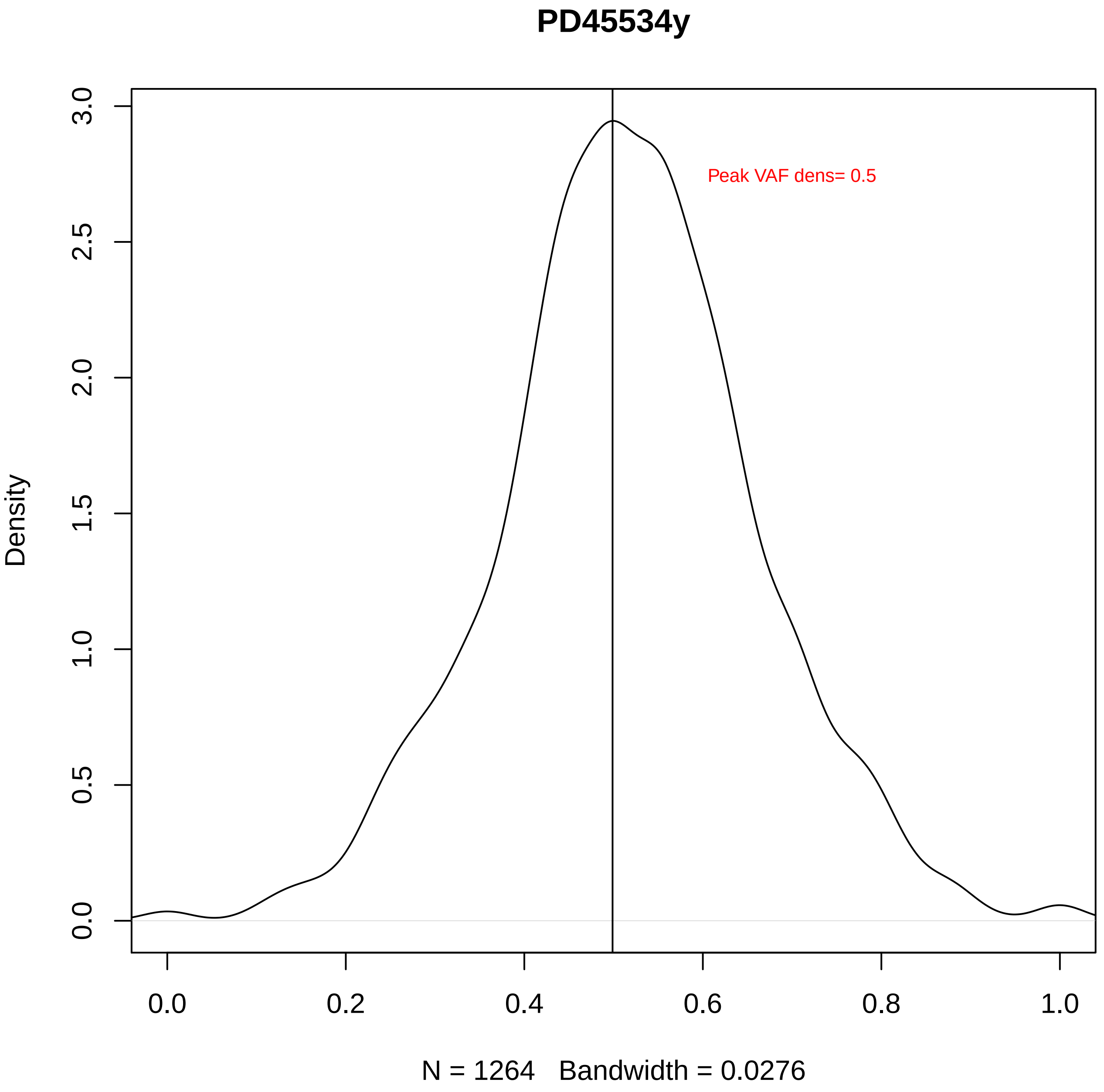

PD45534y
Peak VAF dens= 0.5
Density
0.0
0.5
1.0
1.5
2.0
2.5
3.0
0.0
0.2
0.4
0.6
0.8
1.0
N = 1264 Bandwidth = 0.0276

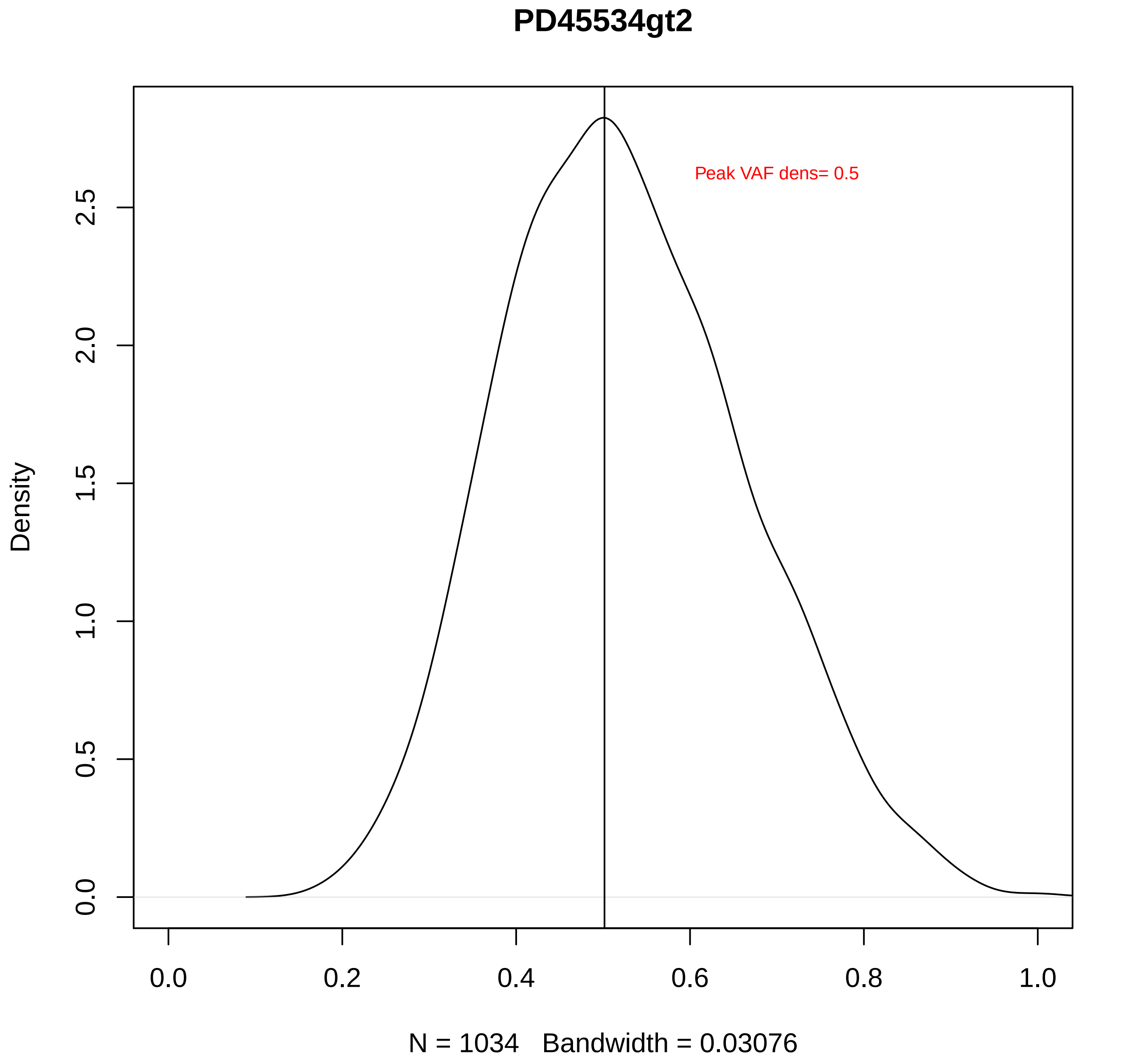
PD45534gt2
Peak VAF dens= 0.5
Density
0.0
0.5
1.0
1.5
2.0
2.5
0.0
0.2
0.4
0.6
0.8
1.0
N = 1034   Bandwidth = 0.03076

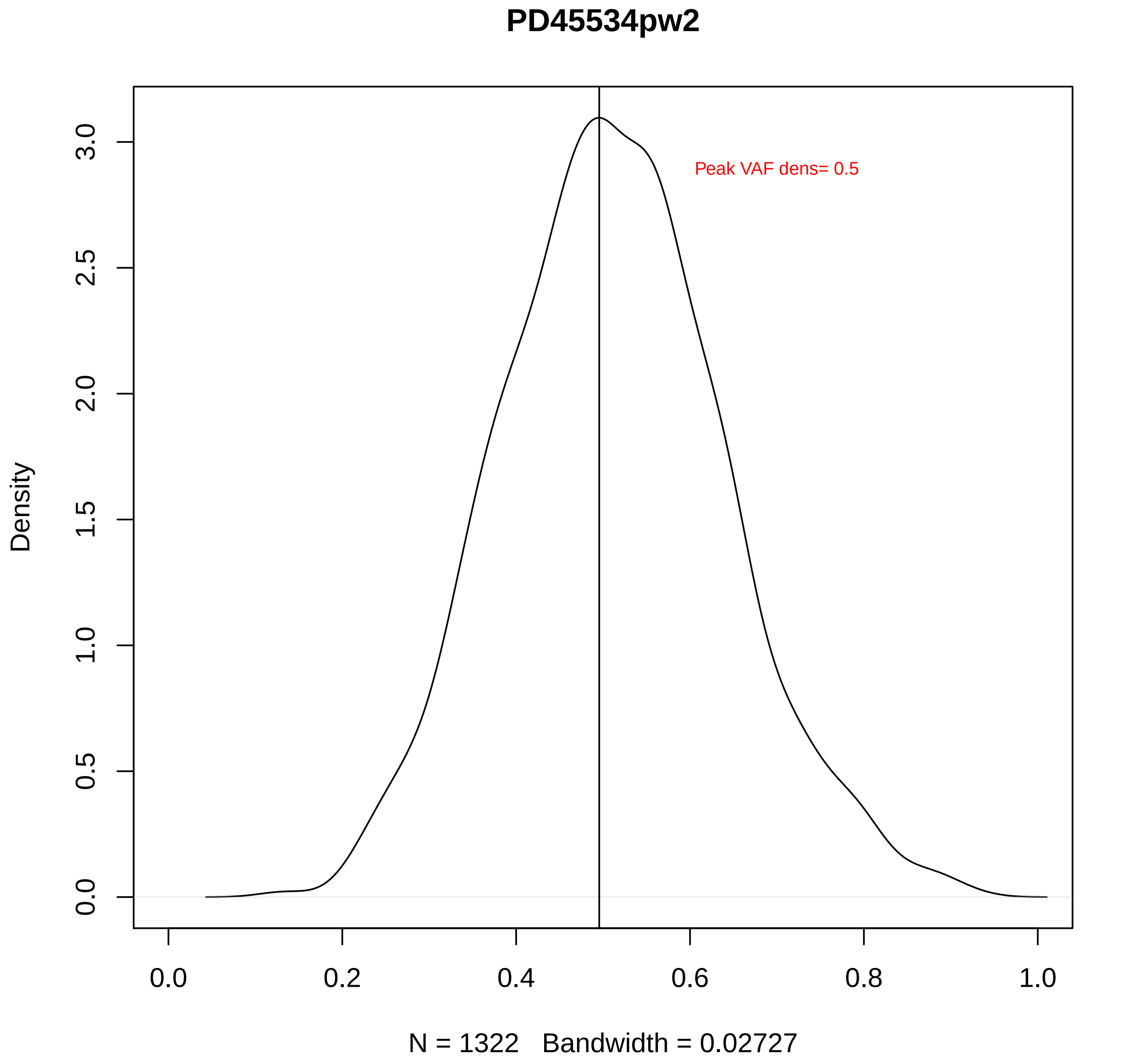
PD45534pw2
Peak VAF dens= 0.5
Density
0.0
0.5
1.0
1.5
2.0
2.5
3.0
0.0
0.2
0.4
0.6
0.8
1.0
N = 1322   Bandwidth = 0.02727

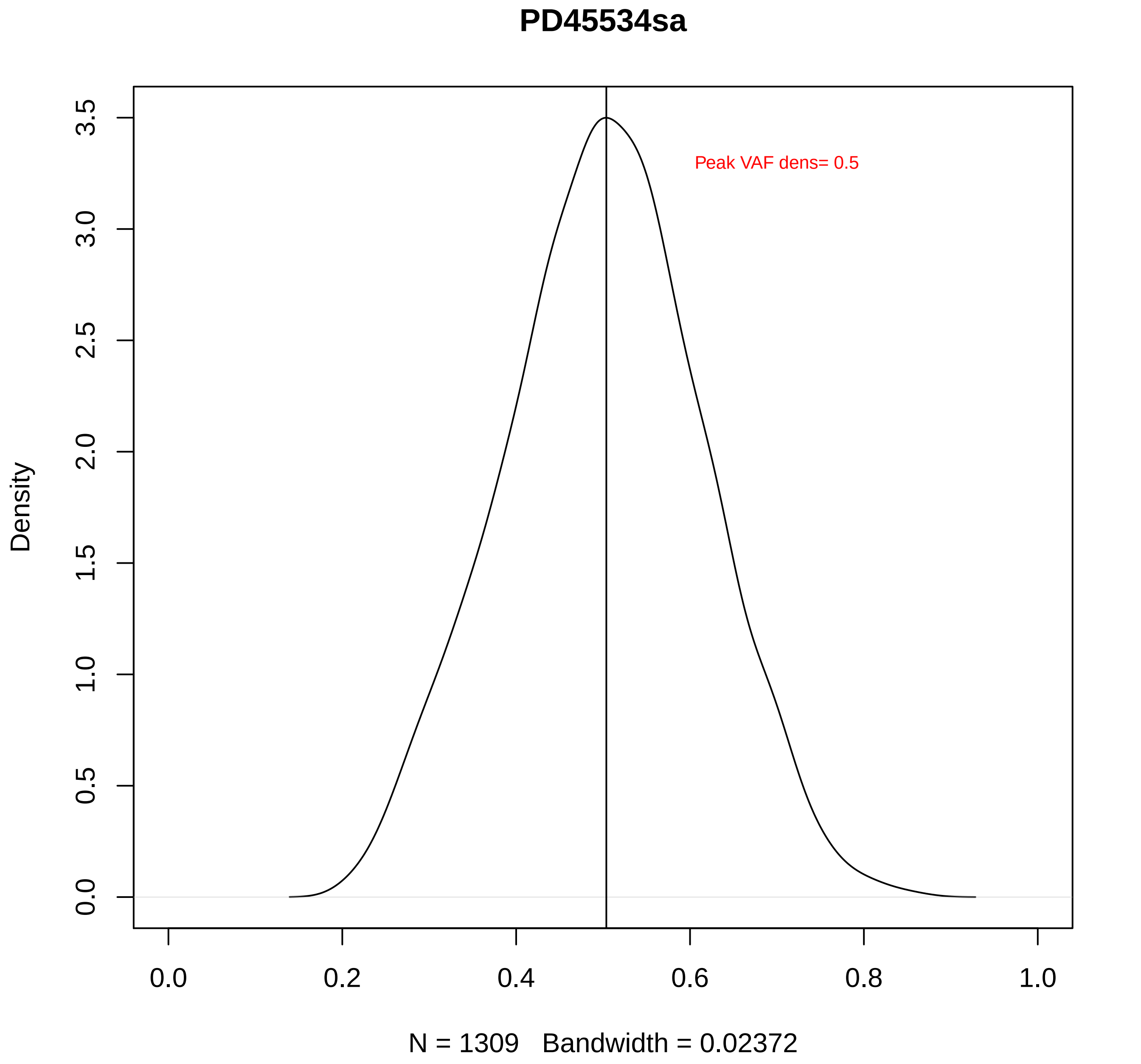
PD45534sa
Peak VAF dens= 0.5
Density
0.0
0.5
1.0
1.5
2.0
2.5
3.0
3.5
0.0
0.2
0.4
0.6
0.8
1.0
N = 1309   Bandwidth = 0.02372

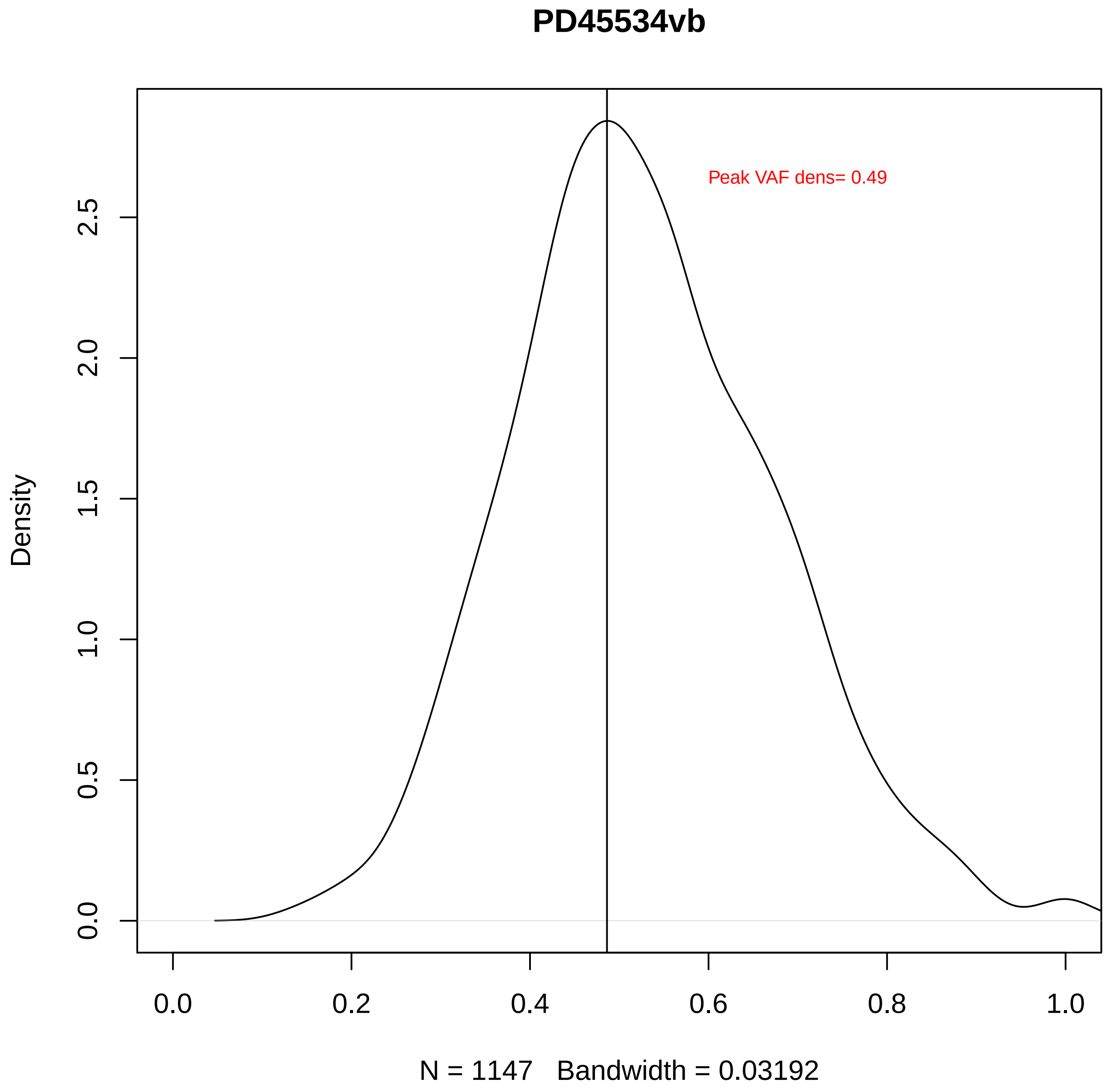

PD45534vb
Peak VAF dens= 0.49
Density
0.0
0.5
1.0
1.5
2.0
2.5
0.0
0.2
0.4
0.6
0.8
1.0
N = 1147 Bandwidth = 0.03192

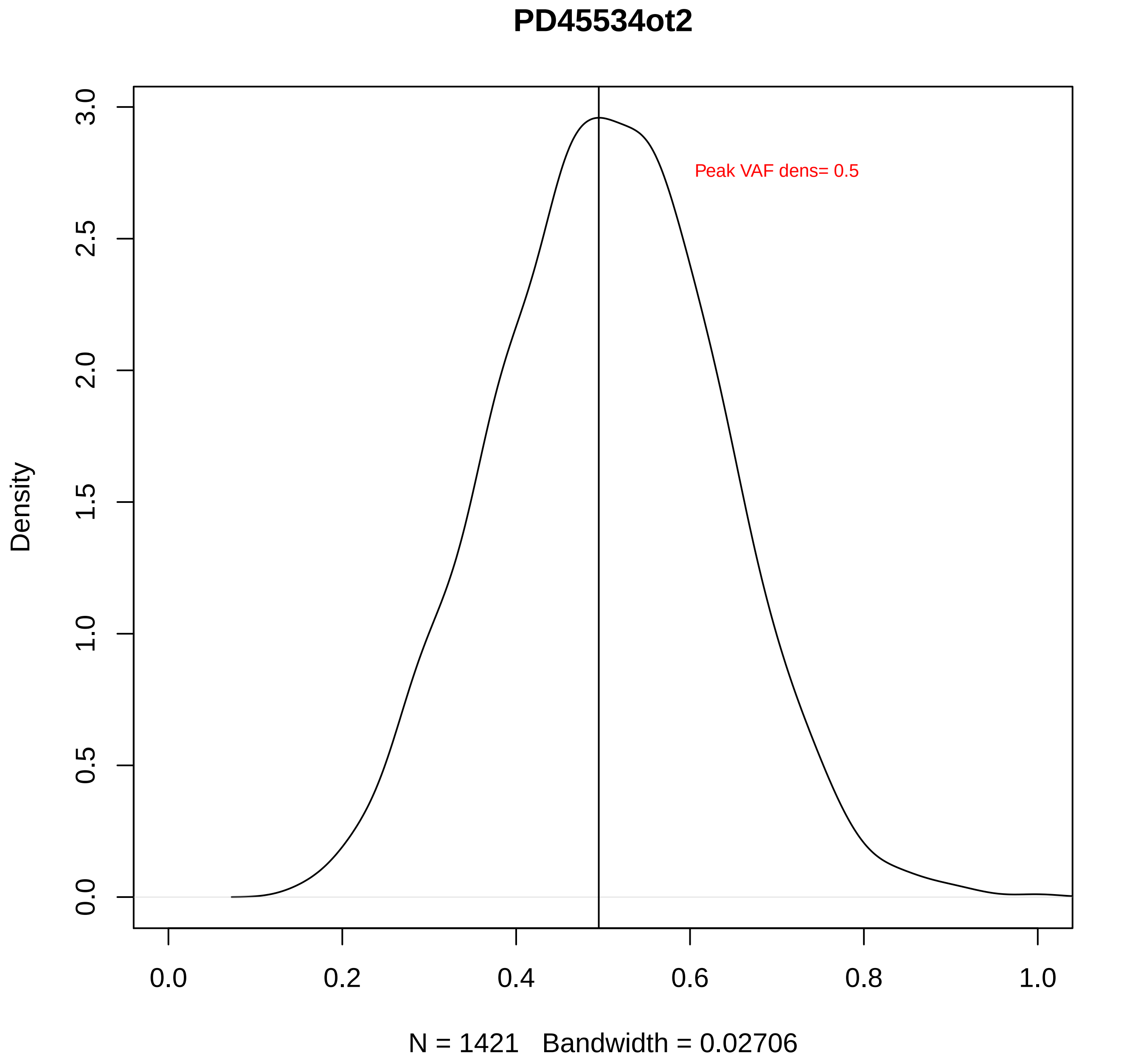
PD45534ot2
Peak VAF dens= 0.5
Density
0.0
0.5
1.0
1.5
2.0
2.5
3.0
0.0
0.2
0.4
0.6
0.8
1.0
N = 1421   Bandwidth = 0.02706

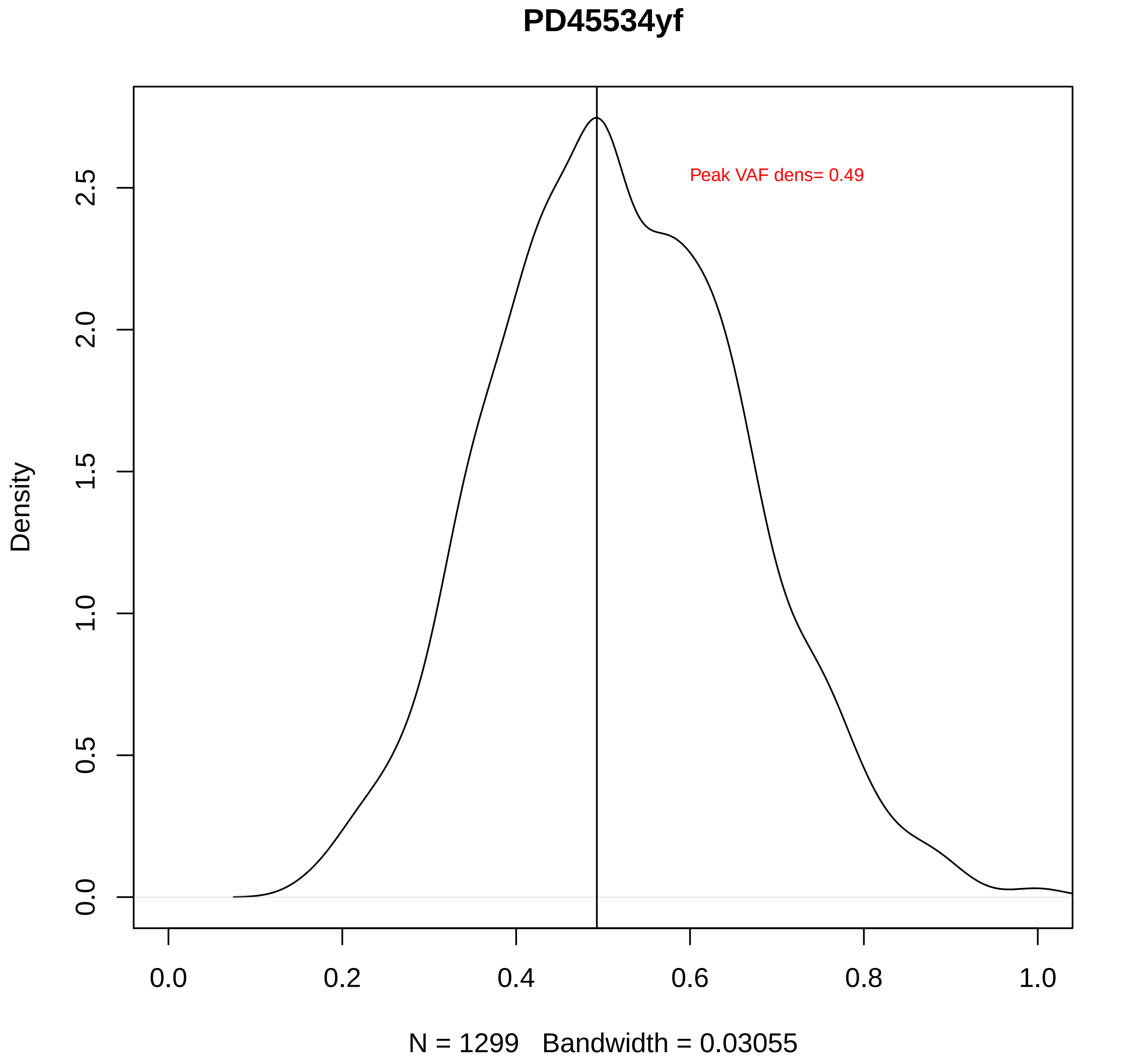
PD45534yf
Peak VAF dens= 0.49
Density
0.0
0.5
1.0
1.5
2.0
2.5
0.0
0.2
0.4
0.6
0.8
1.0
N = 1299   Bandwidth = 0.03055

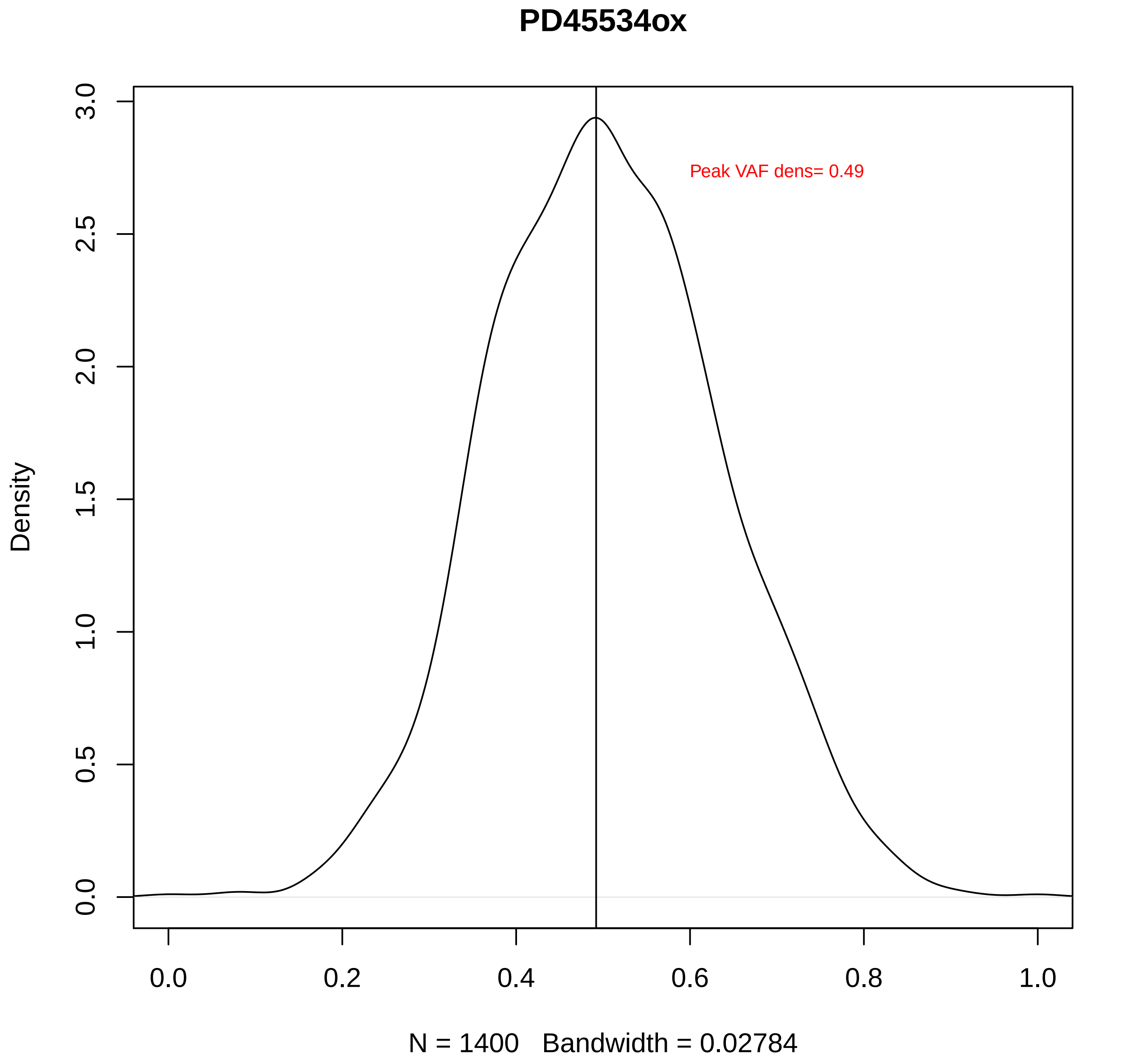
PD45534ox
Peak VAF dens= 0.49
Density
0.0
0.5
1.0
1.5
2.0
2.5
3.0
0.0
0.2
0.4
0.6
0.8
1.0
N = 1400   Bandwidth = 0.02784

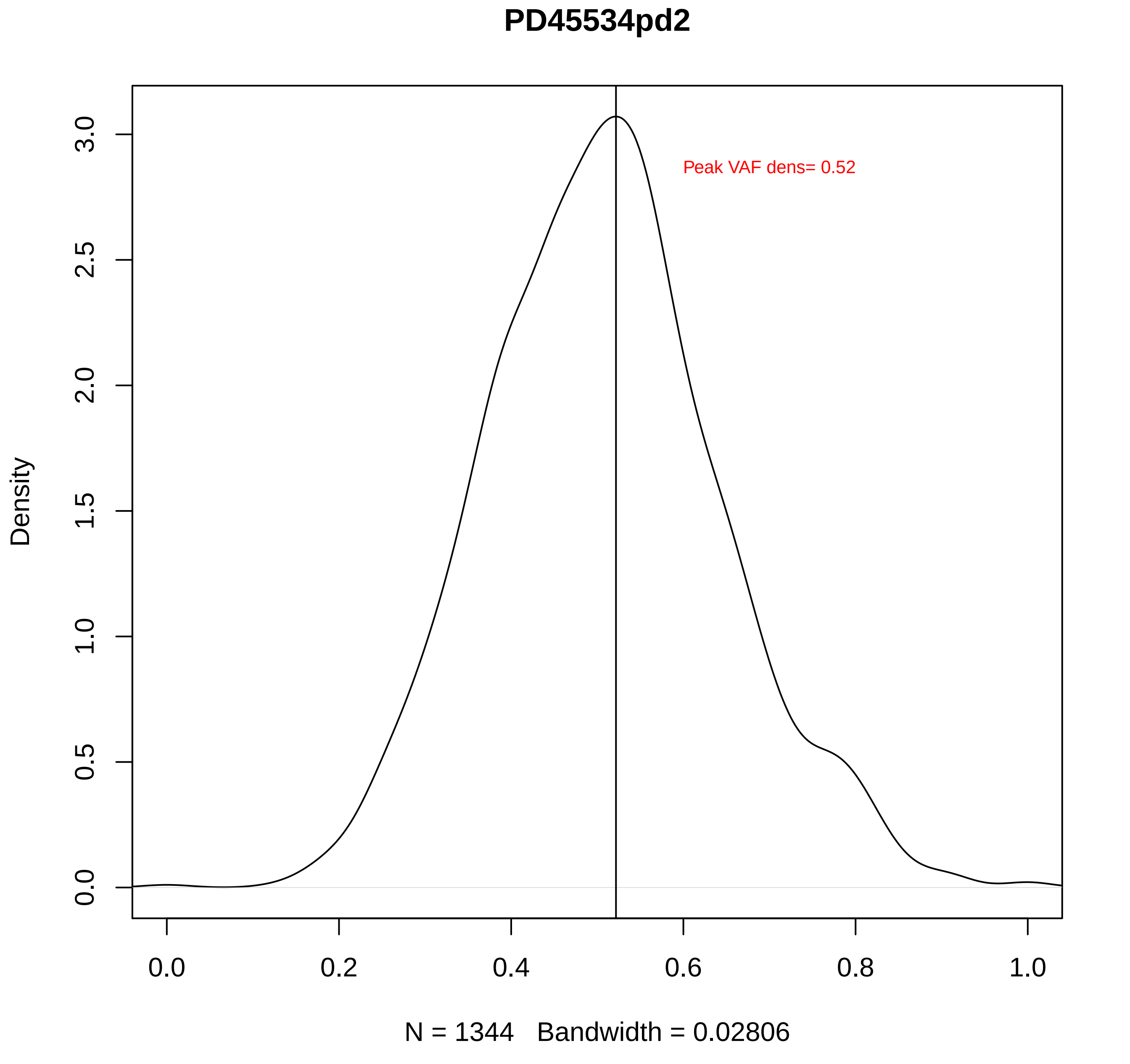
PD45534pd2
Peak VAF dens= 0.52
Density
0.0
0.5
1.0
1.5
2.0
2.5
3.0
0.0
0.2
0.4
0.6
0.8
1.0
N = 1344   Bandwidth = 0.02806

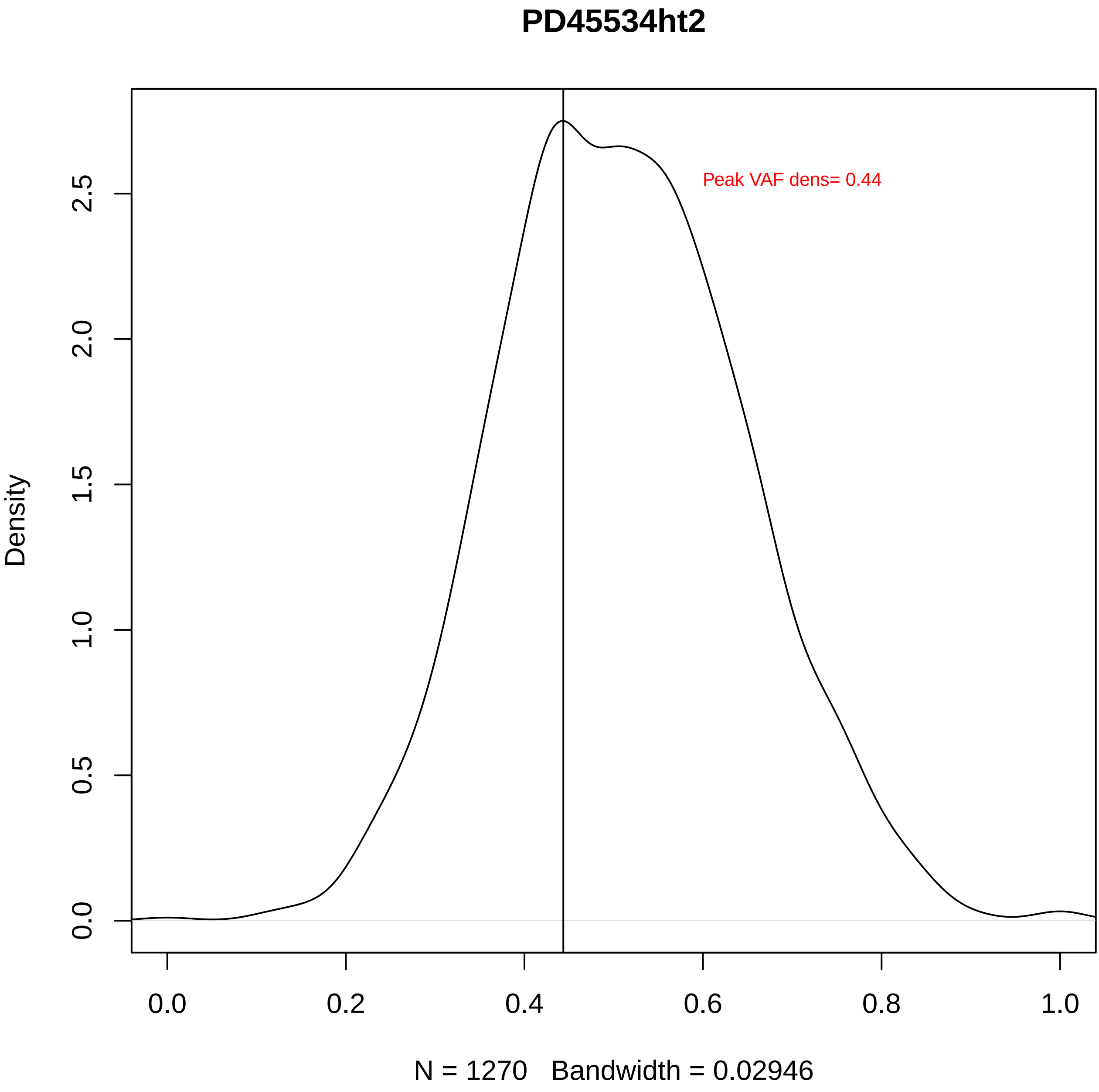
PD45534ht2
Peak VAF dens= 0.44
Density
0.0
0.5
1.0
1.5
2.0
2.5
0.0
0.2
0.4
0.6
0.8
1.0
N = 1270 Bandwidth = 0.02946

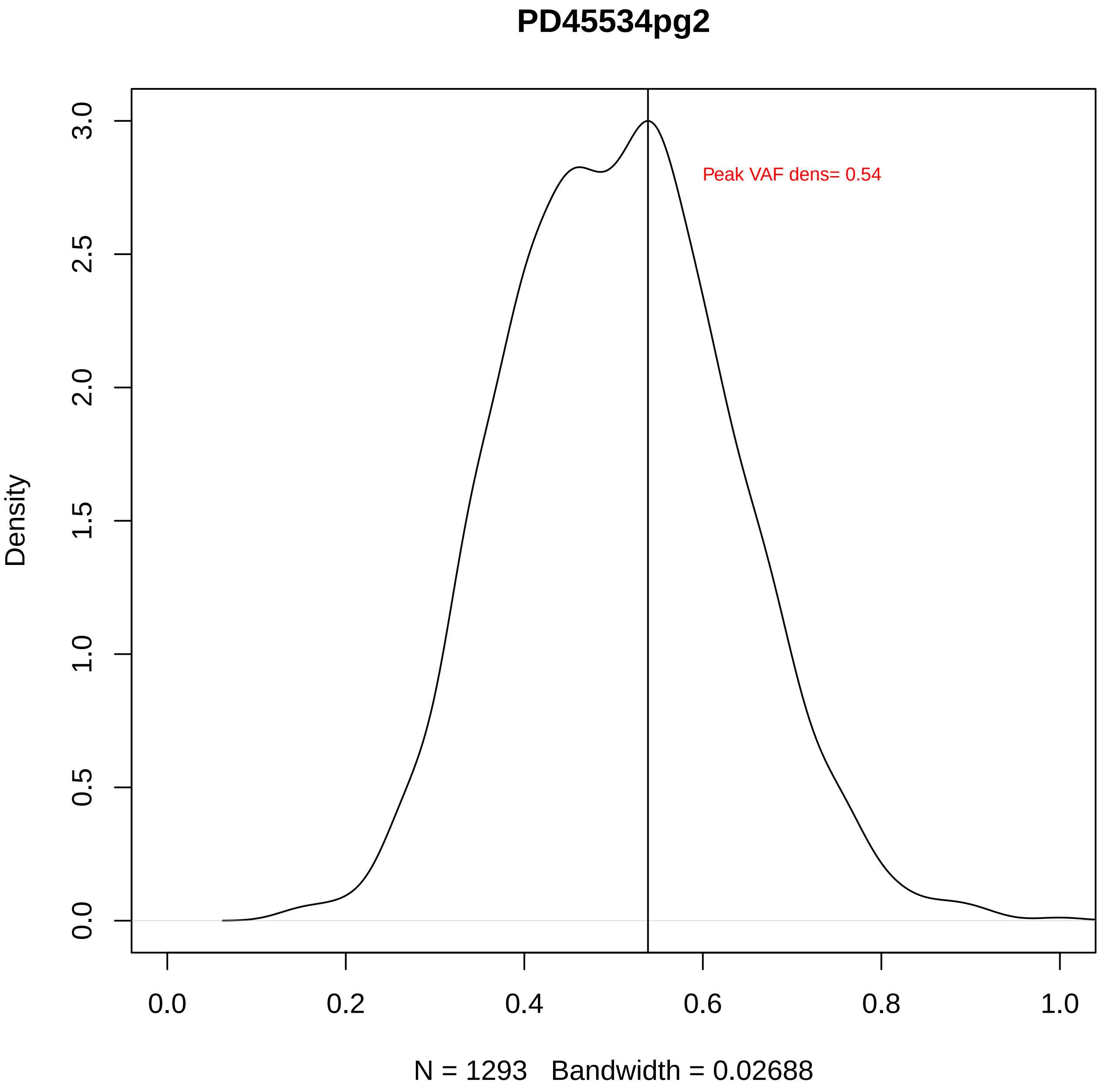
PD45534pg2
Peak VAF dens= 0.54
Density
0.0
0.5
1.0
1.5
2.0
2.5
3.0
0.0
0.2
0.4
0.6
0.8
1.0
N = 1293   Bandwidth = 0.02688

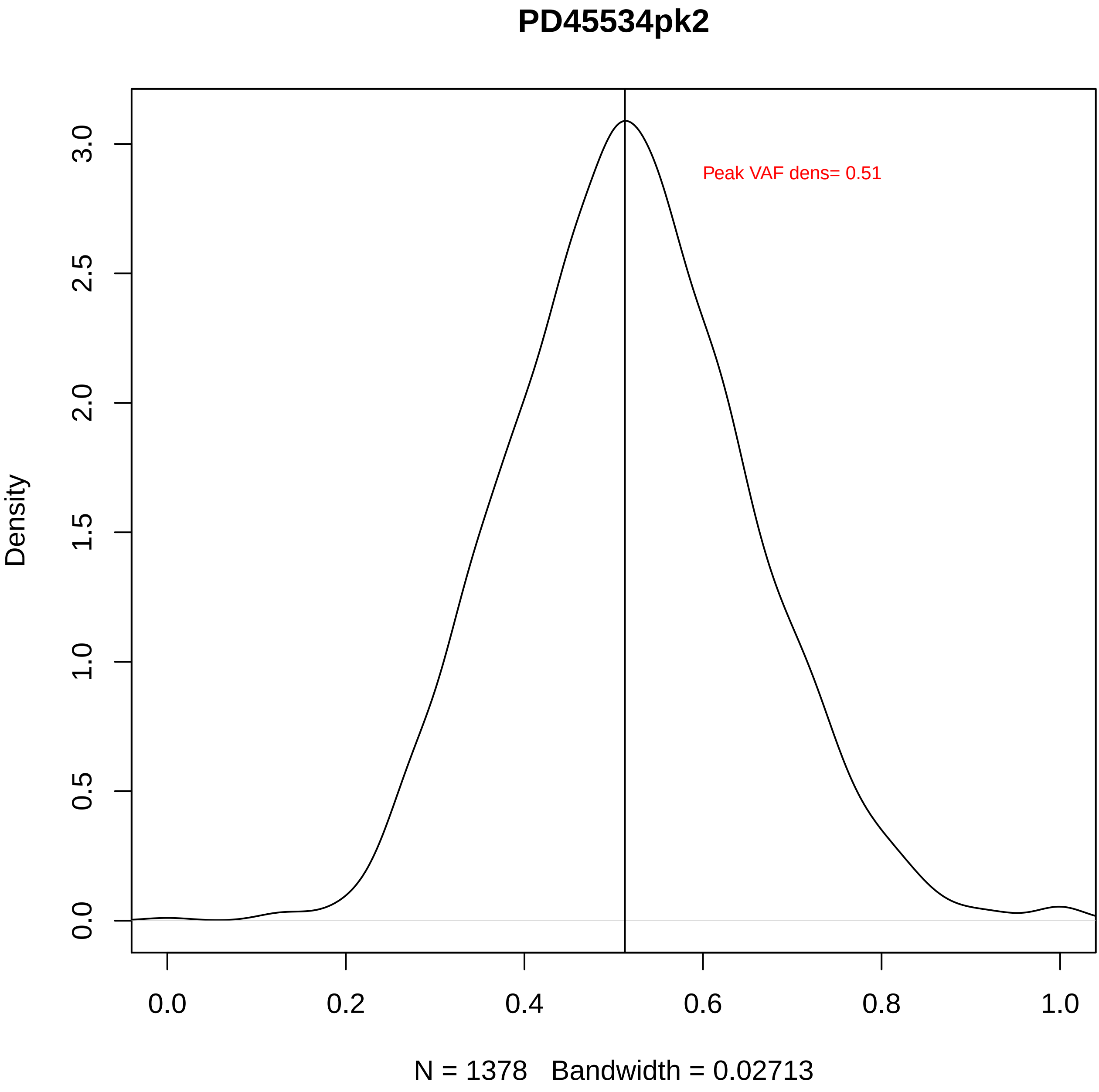

PD45534pk2
Peak VAF dens= 0.51
Density
0.0
0.5
1.0
1.5
2.0
2.5
3.0
0.0
0.2
0.4
0.6
0.8
1.0
N = 1378   Bandwidth = 0.02713

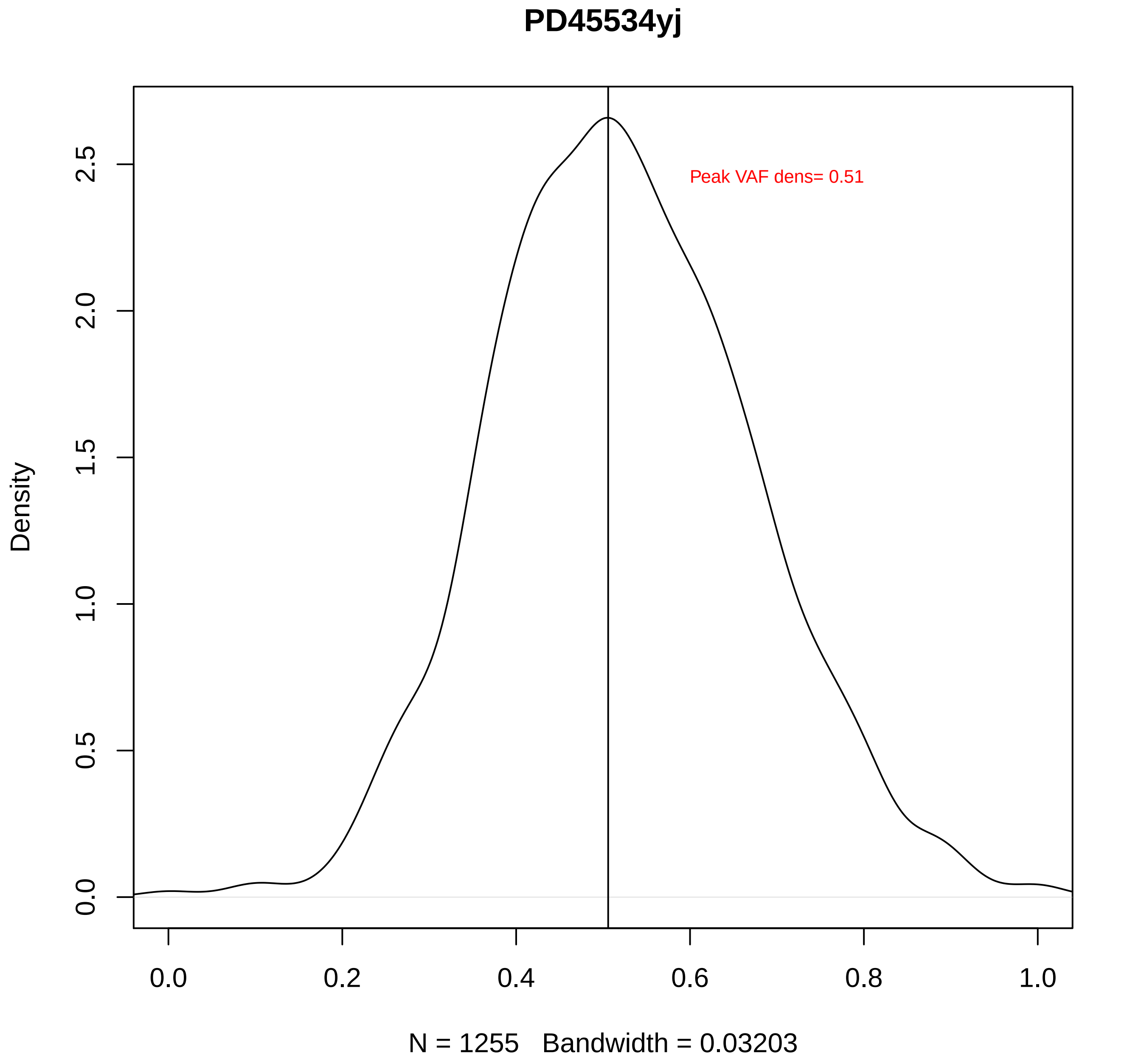
PD45534yj
Peak VAF dens= 0.51
Density
0.0
0.5
1.0
1.5
2.0
2.5
0.0
0.2
0.4
0.6
0.8
1.0
N = 1255   Bandwidth = 0.03203

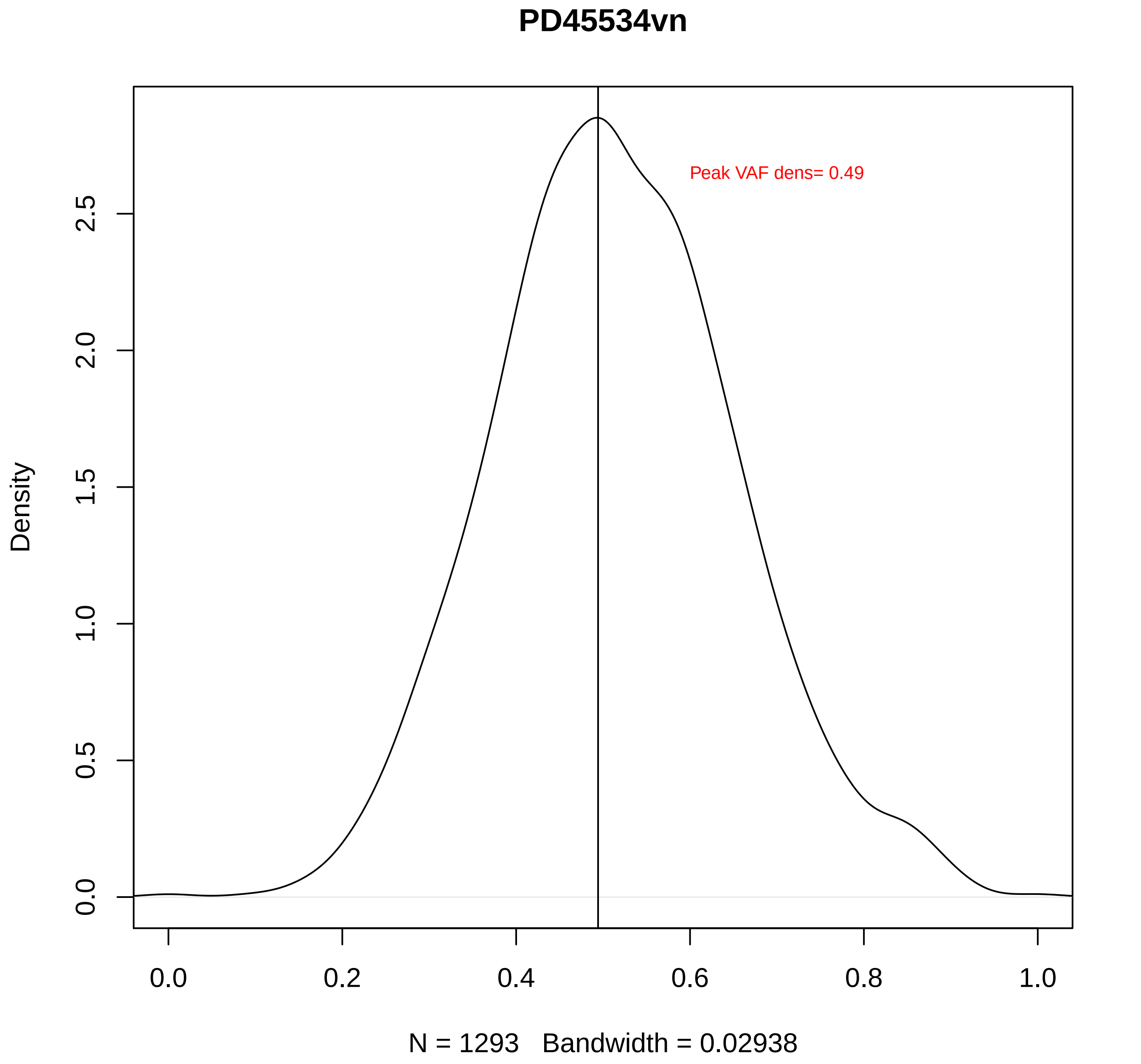

PD45534vn
Peak VAF dens= 0.49
Density
0.0
0.5
1.0
1.5
2.0
2.5
0.0
0.2
0.4
0.6
0.8
1.0
N = 1293   Bandwidth = 0.02938

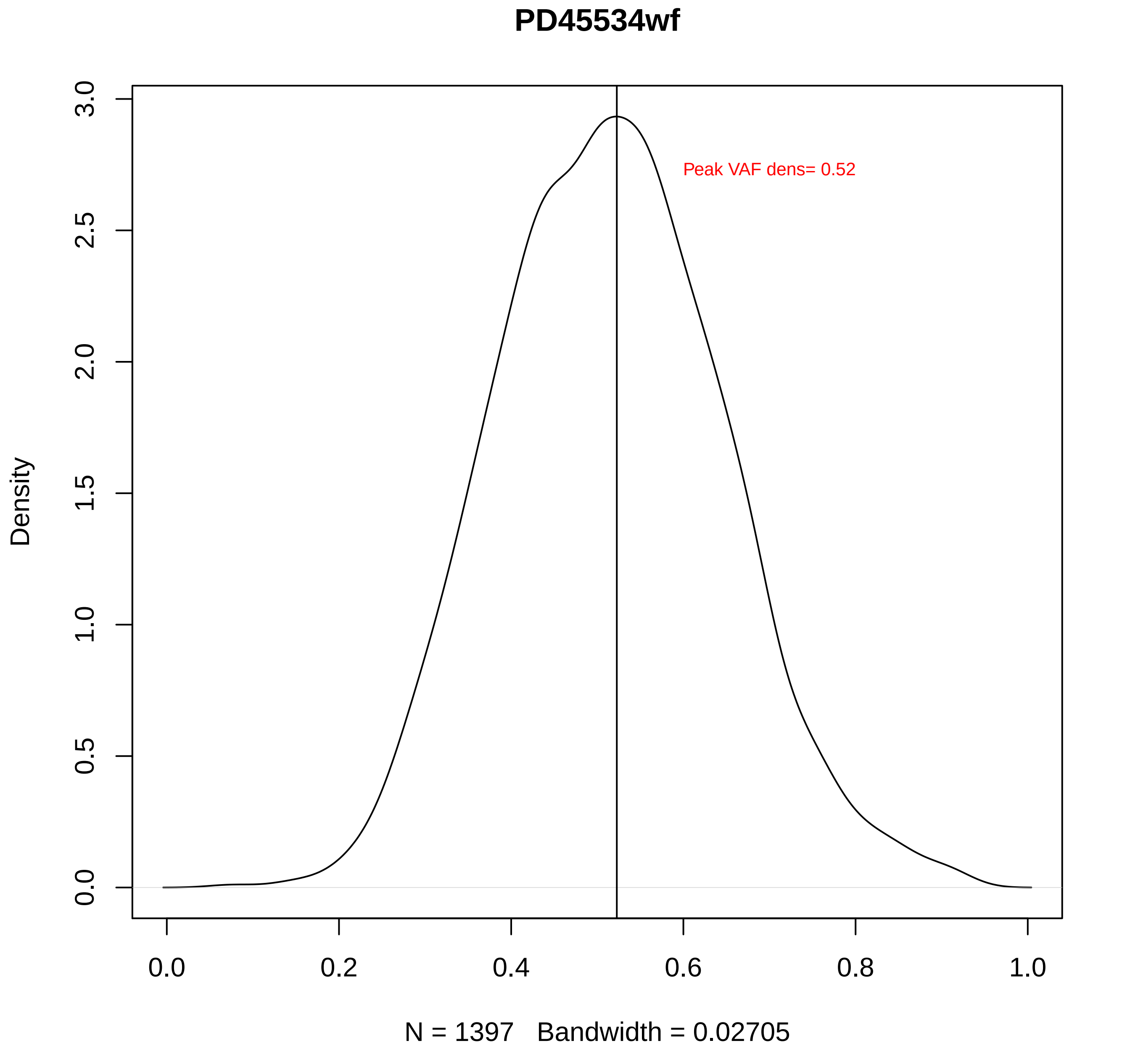
PD45534wf
Peak VAF dens= 0.52
Density
0.0
0.5
1.0
1.5
2.0
2.5
3.0
0.0
0.2
0.4
0.6
0.8
1.0
N = 1397   Bandwidth = 0.02705

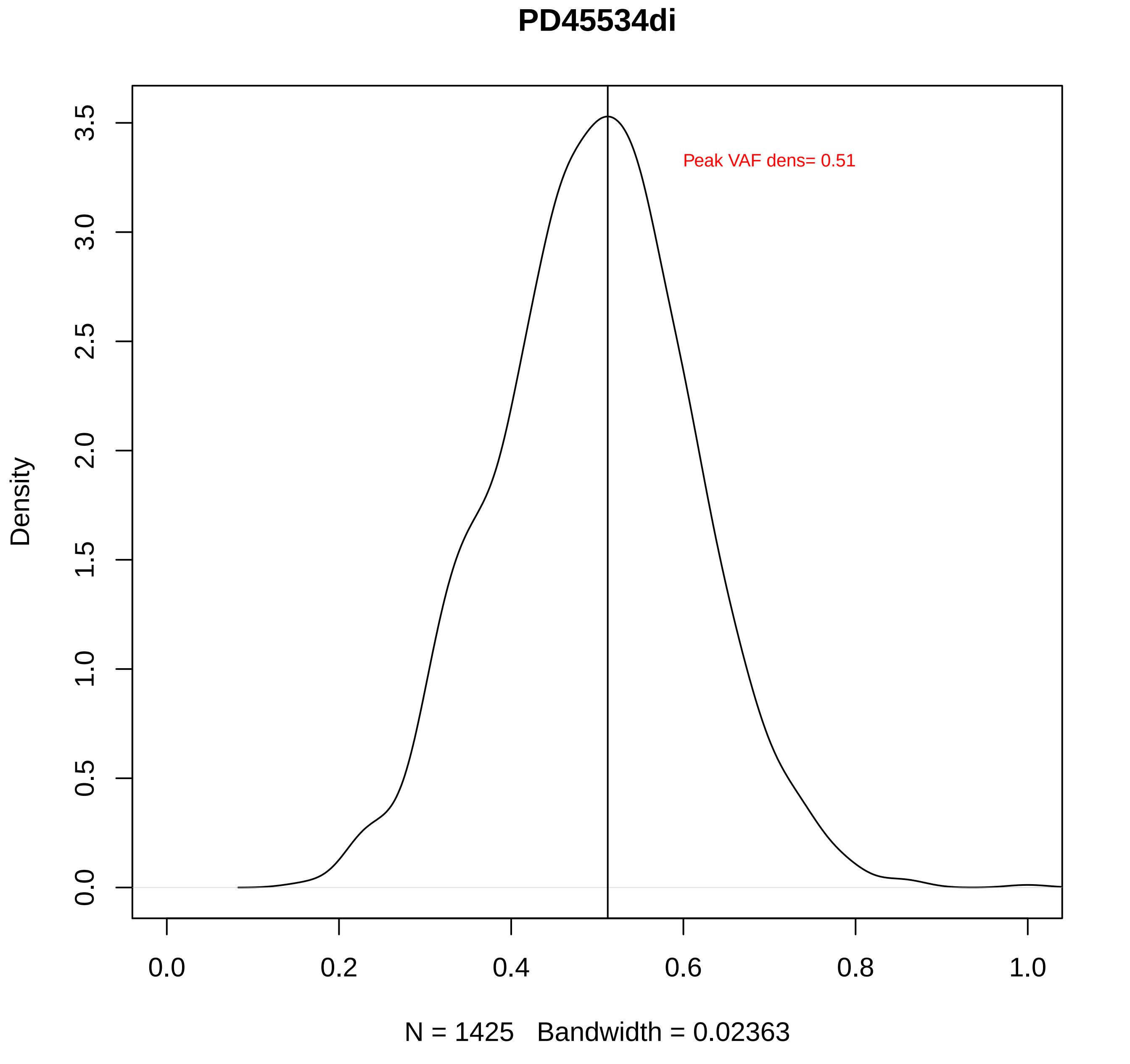
PD45534di
Peak VAF dens= 0.51
Density
0.0
0.5
1.0
1.5
2.0
2.5
3.0
3.5
0.0
0.2
0.4
0.6
0.8
1.0
N = 1425   Bandwidth = 0.02363

PD45534wc

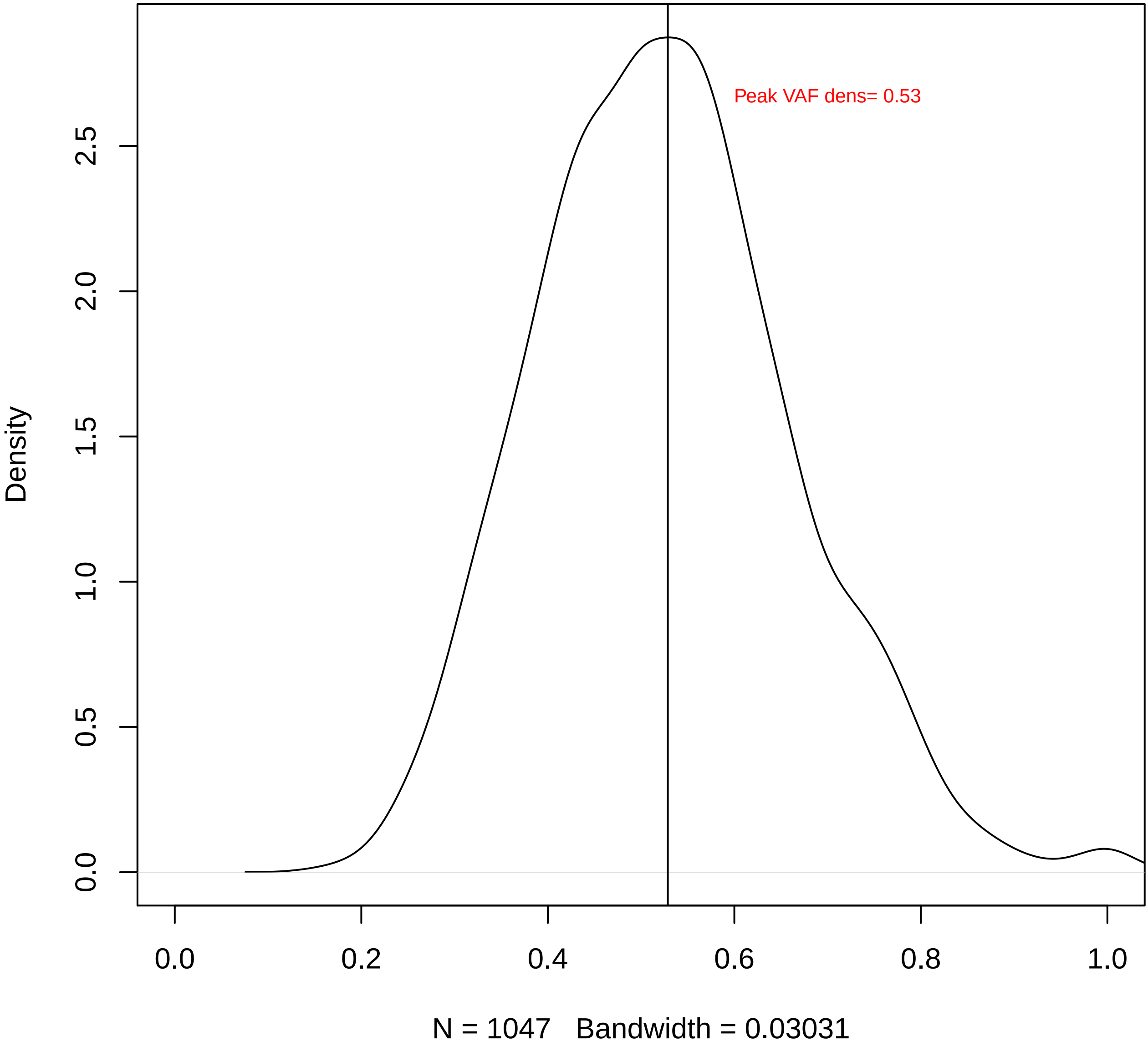
Peak VAF dens= 0.53
Density
0.0
0.5
1.0
1.5
2.0
2.5
0.0
0.2
0.4
0.6
0.8
1.0
N = 1047   Bandwidth = 0.03031

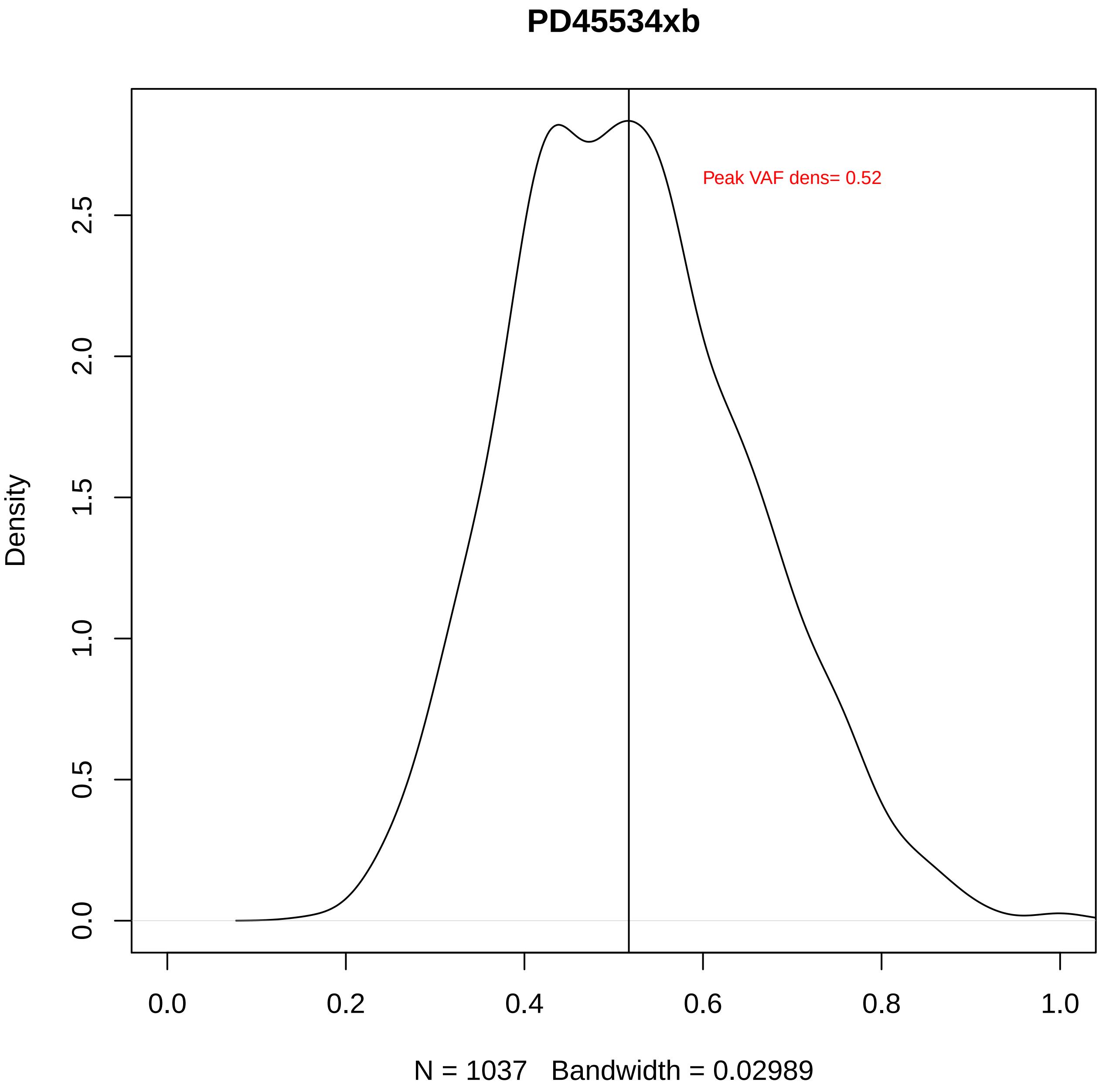
PD45534xb
Peak VAF dens= 0.52
Density
0.0
0.5
1.0
1.5
2.0
2.5
0.0
0.2
0.4
0.6
0.8
1.0
N = 1037   Bandwidth = 0.02989

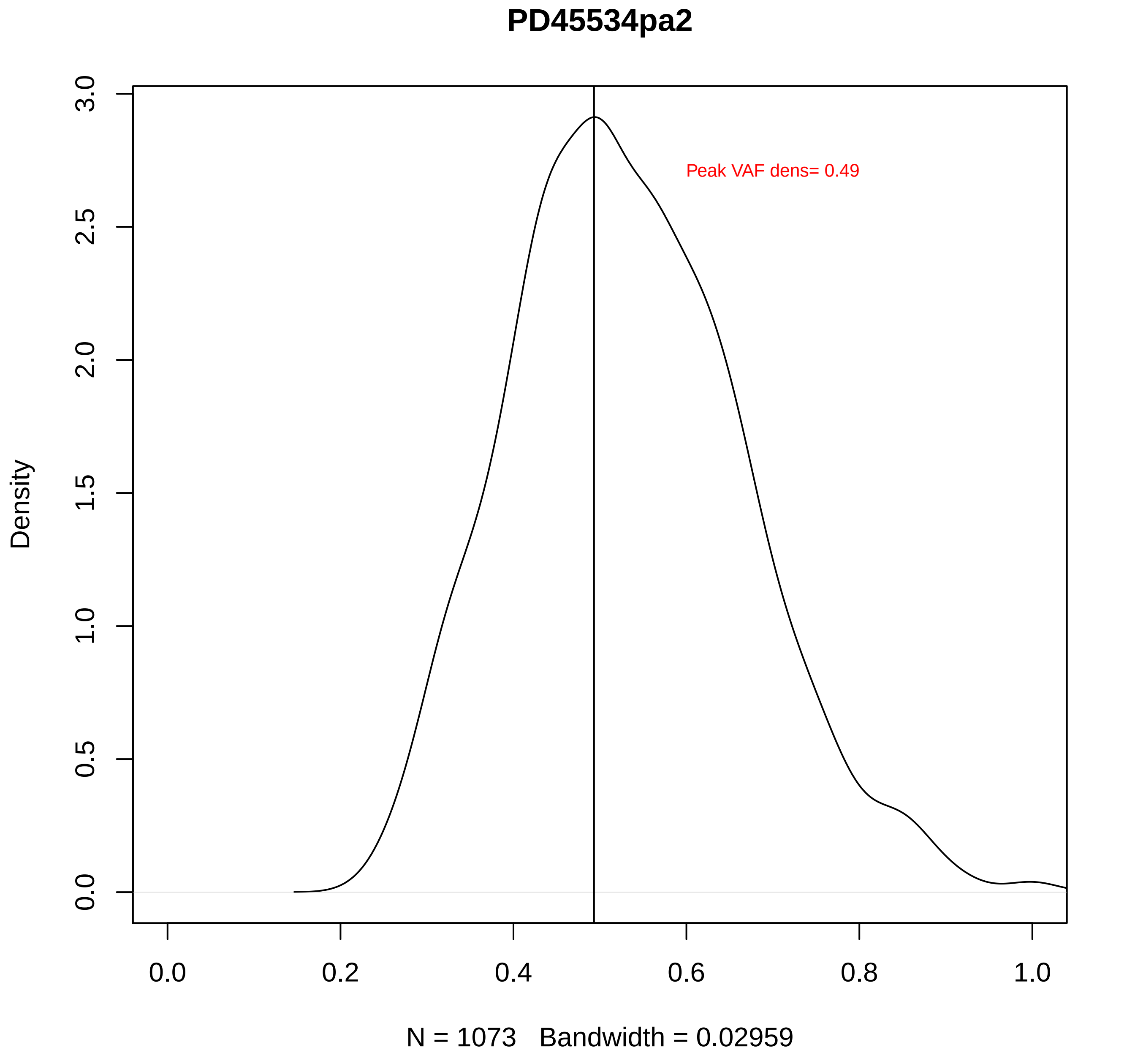

PD45534pa2
Peak VAF dens= 0.49
Density
0.0
0.5
1.0
1.5
2.0
2.5
3.0
0.0
0.2
0.4
0.6
0.8
1.0
N = 1073 Bandwidth = 0.02959

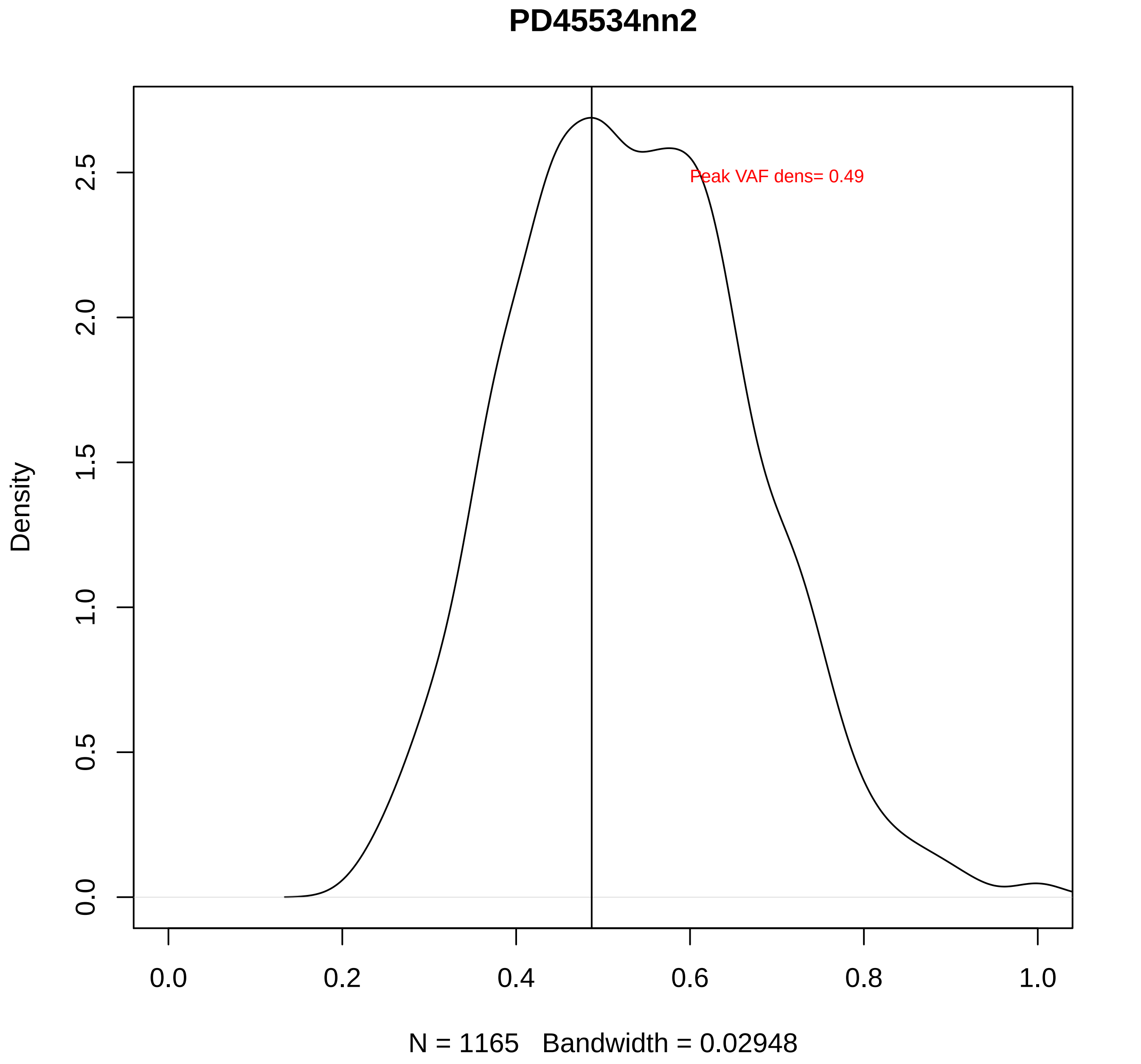
PD45534nn2
Peak VAF dens= 0.49
Density
0.0
0.5
1.0
1.5
2.0
2.5
0.0
0.2
0.4
0.6
0.8
1.0
N = 1165   Bandwidth = 0.02948

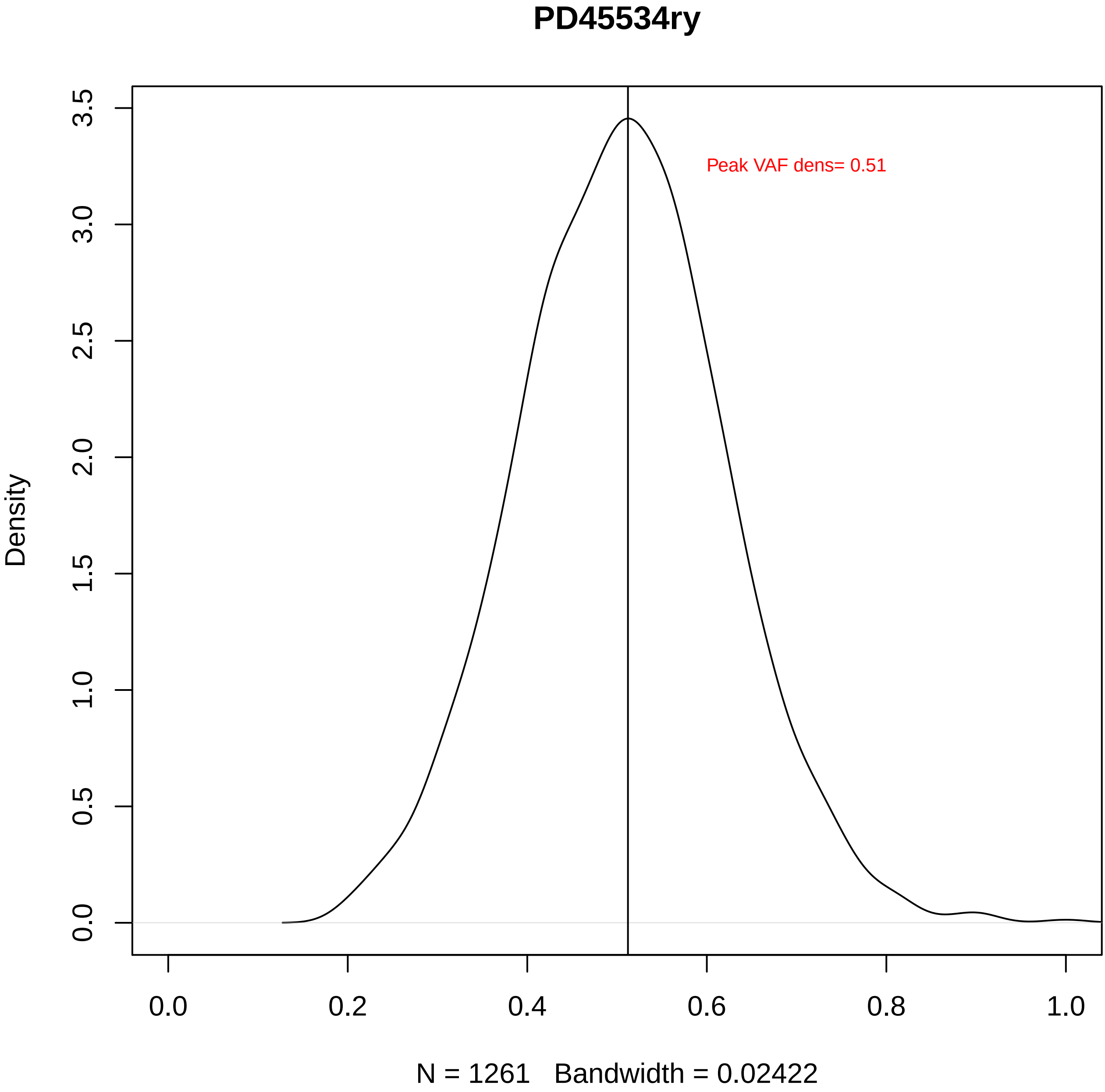
PD45534ry
Peak VAF dens= 0.51
Density
0.0
0.5
1.0
1.5
2.0
2.5
3.0
3.5
0.0
0.2
0.4
0.6
0.8
1.0
N = 1261   Bandwidth = 0.02422

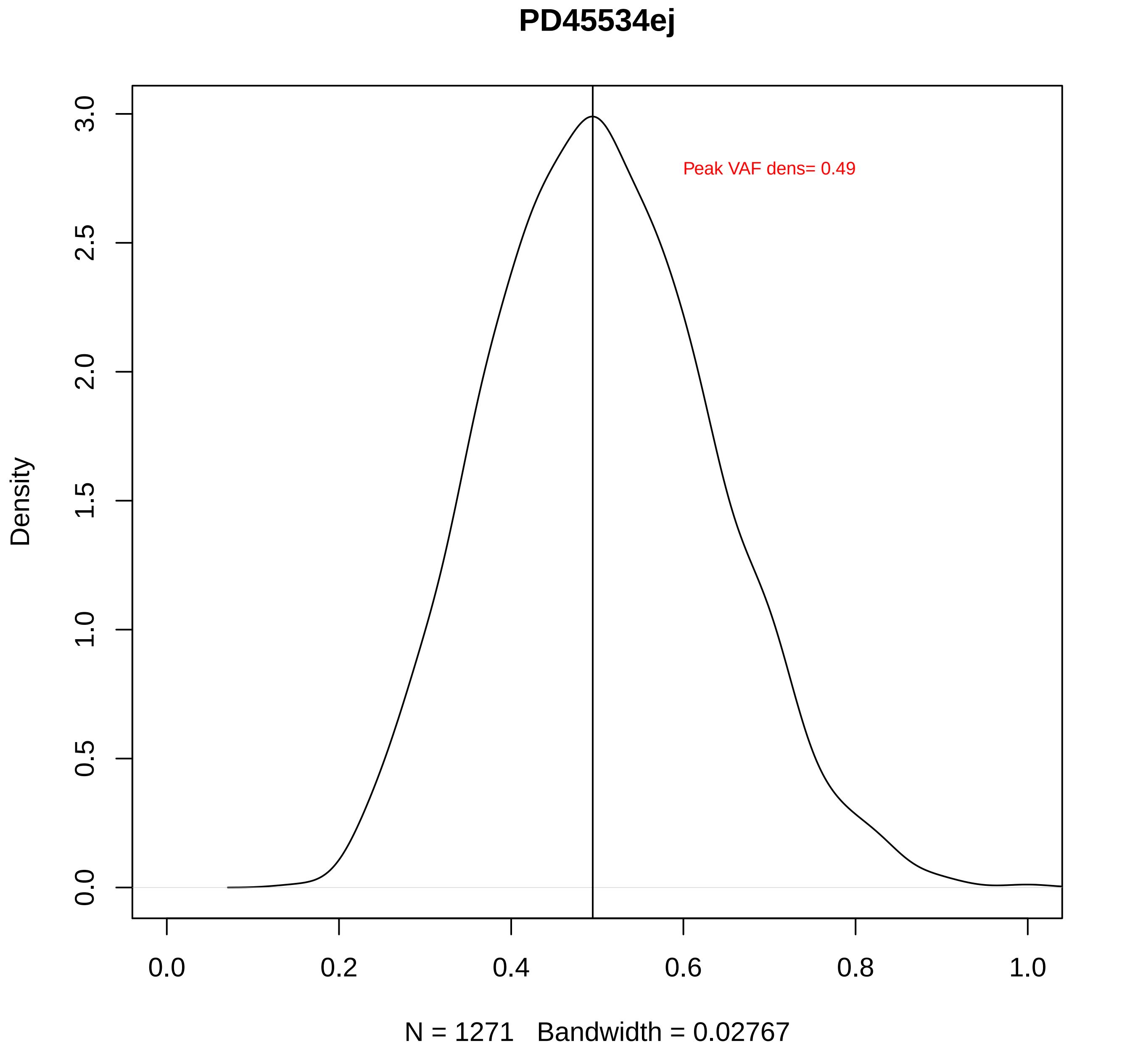
PD45534ej
Peak VAF dens= 0.49
Density
3.0
2.5
2.0
1.5
1.0
0.5
0.0
0.0
0.2
0.4
0.6
0.8
1.0
N = 1271   Bandwidth = 0.02767
