# Supplementary material for: Clonal dynamics of haematopoiesis across the human lifespan
Source: Nature. 2022 Jun 1;606(7913):343–50. doi: 10.1038/s41586-022-04786-y (PMC9177428; doi:10.1038/s41586-022-04786-y)
Supplement: Supplementary file 4 — HTMLs of notebooks outlining key statistical analyses presented in the manuscript, including analysis of phylogenetic trees. [file 41586_2022_4786_MOESM4_ESM.zip › Supplementary_code/SNV_indel_analysis/CB001_sample_vaf_plots.pdf]

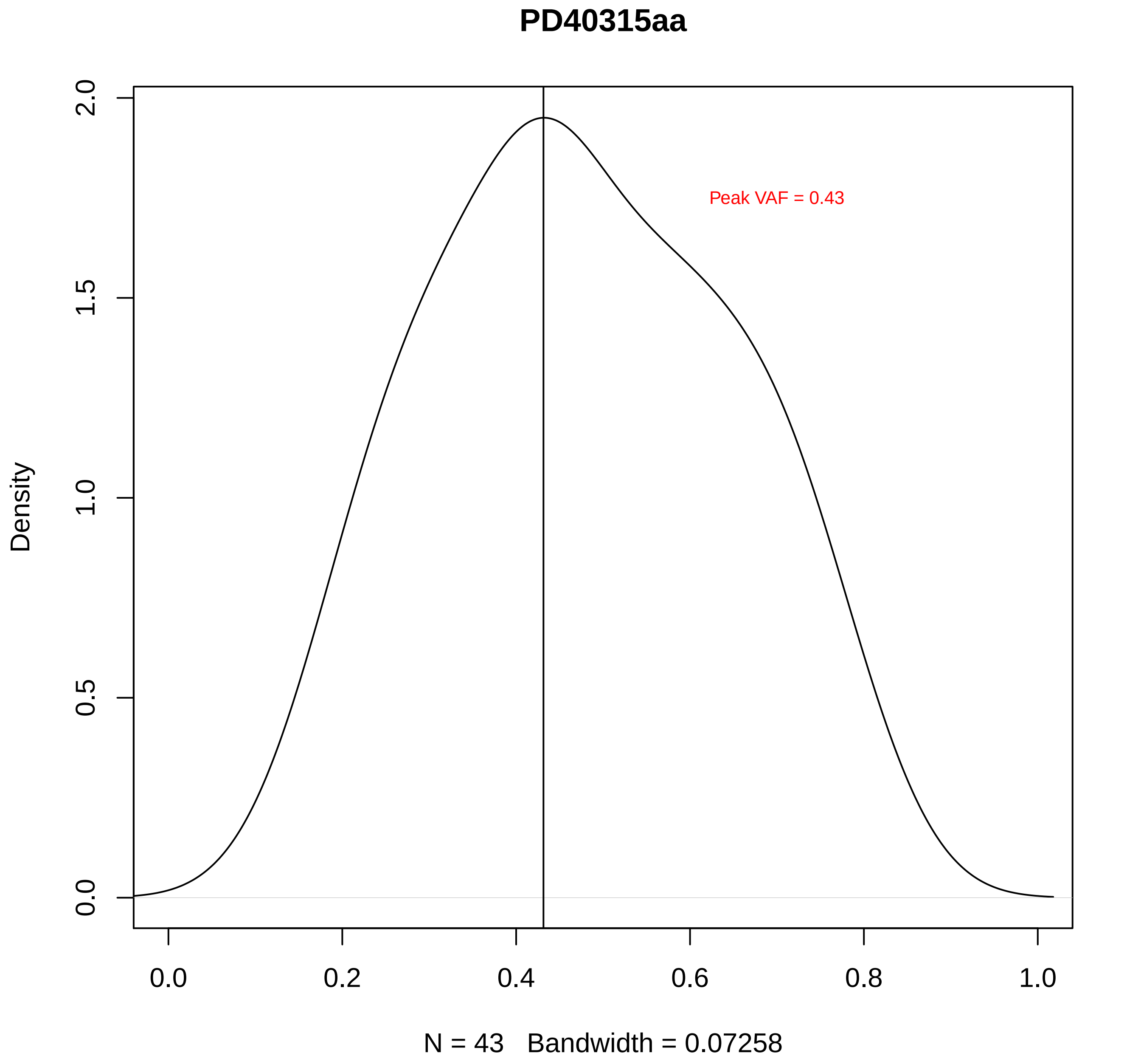
PD40315aa
Peak VAF = 0.43
Density
0.0
0.5
1.0
1.5
2.0
0.0
0.2
0.4
0.6
0.8
1.0
N = 43   Bandwidth = 0.07258

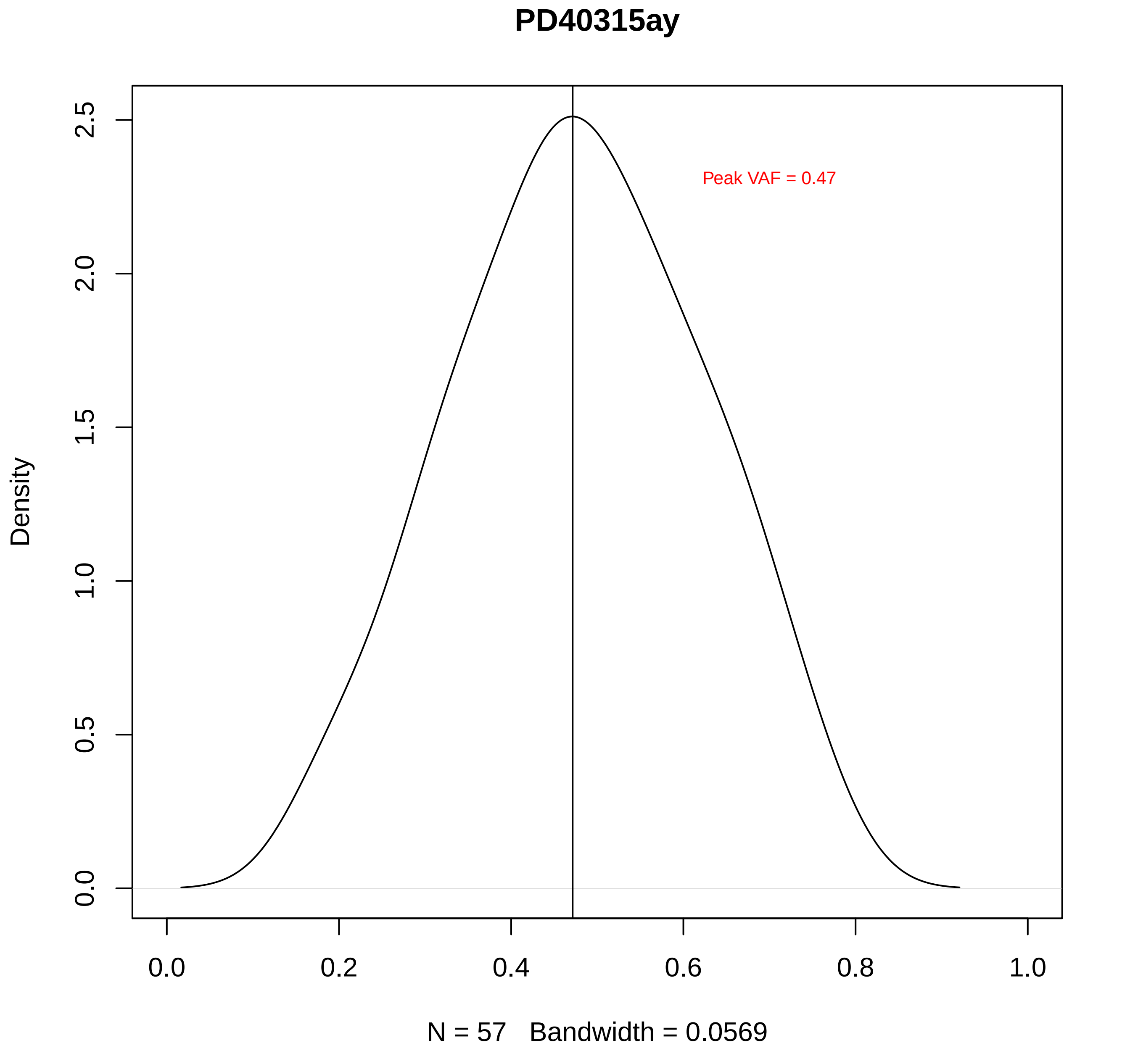

PD40315ay
Peak VAF = 0.47
Density
0.0
0.5
1.0
1.5
2.0
2.5
0.0
0.2
0.4
0.6
0.8
1.0
N = 57   Bandwidth = 0.0569

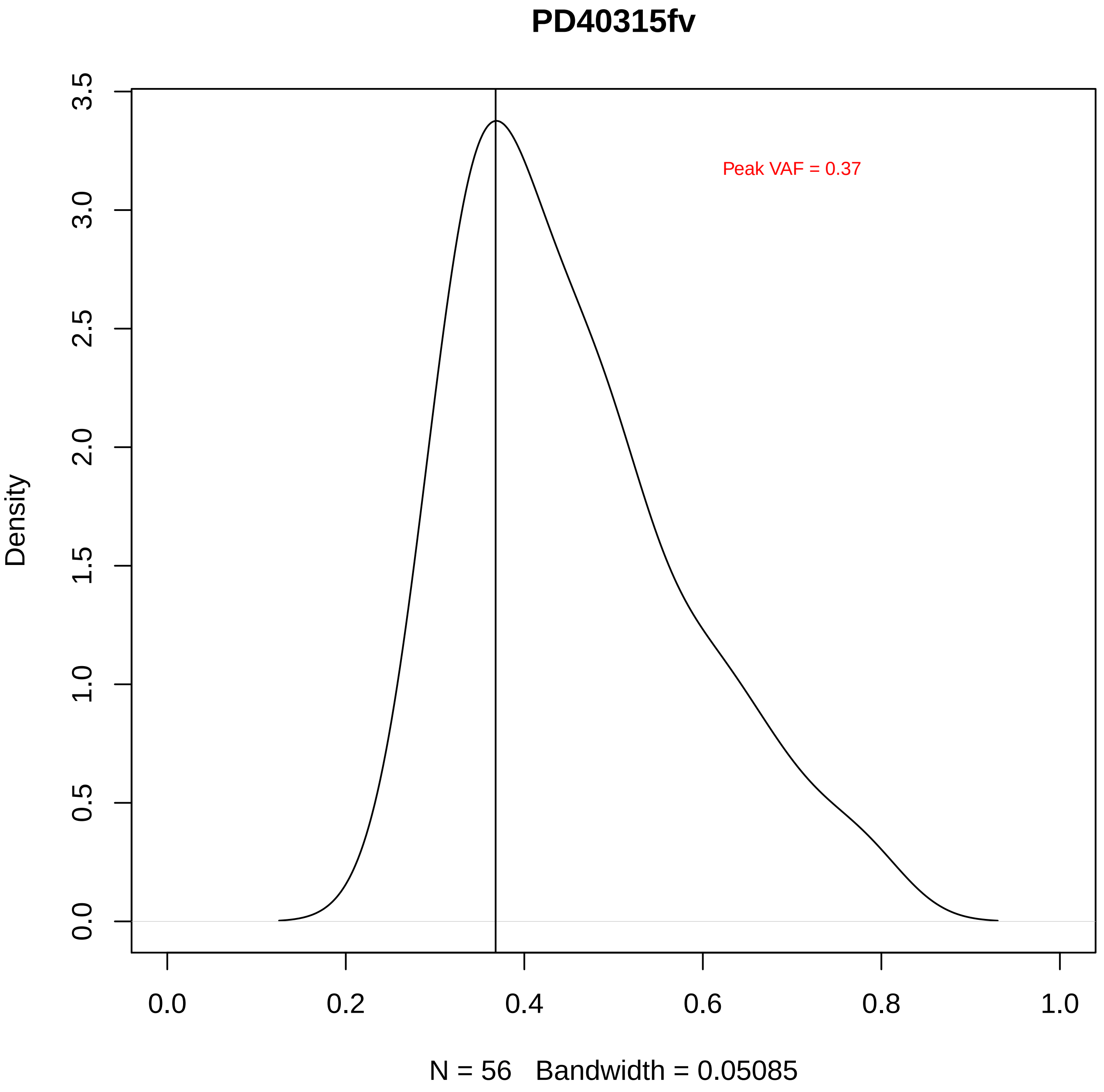
PD40315fv
Peak VAF = 0.37
Density
0.0
0.5
1.0
1.5
2.0
2.5
3.0
3.5
0.0
0.2
0.4
0.6
0.8
1.0
N = 56 Bandwidth = 0.05085

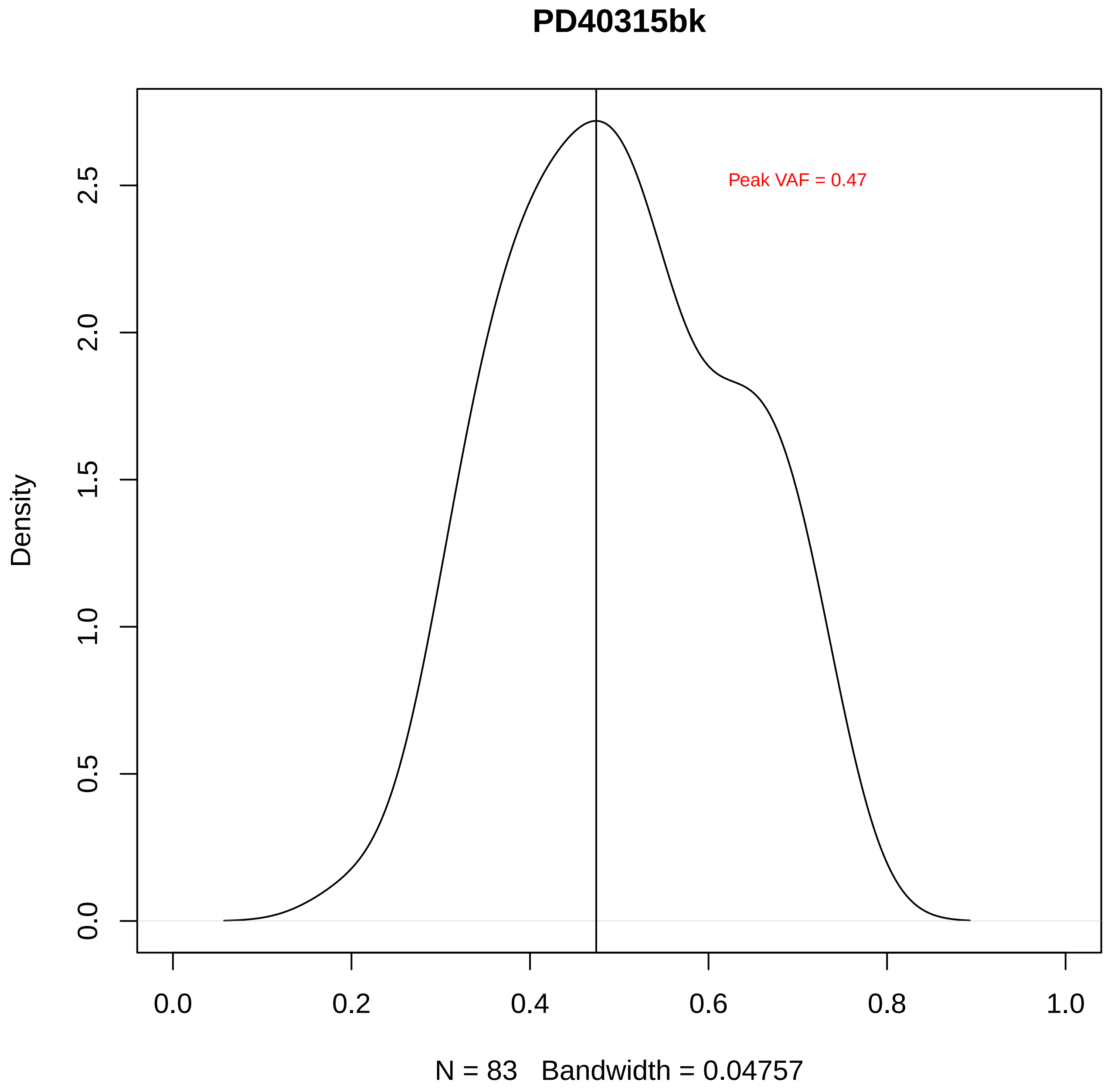
PD40315bk
Peak VAF = 0.47
Density
0.0
0.5
1.0
1.5
2.0
2.5
0.0
0.2
0.4
0.6
0.8
1.0
N = 83   Bandwidth = 0.04757

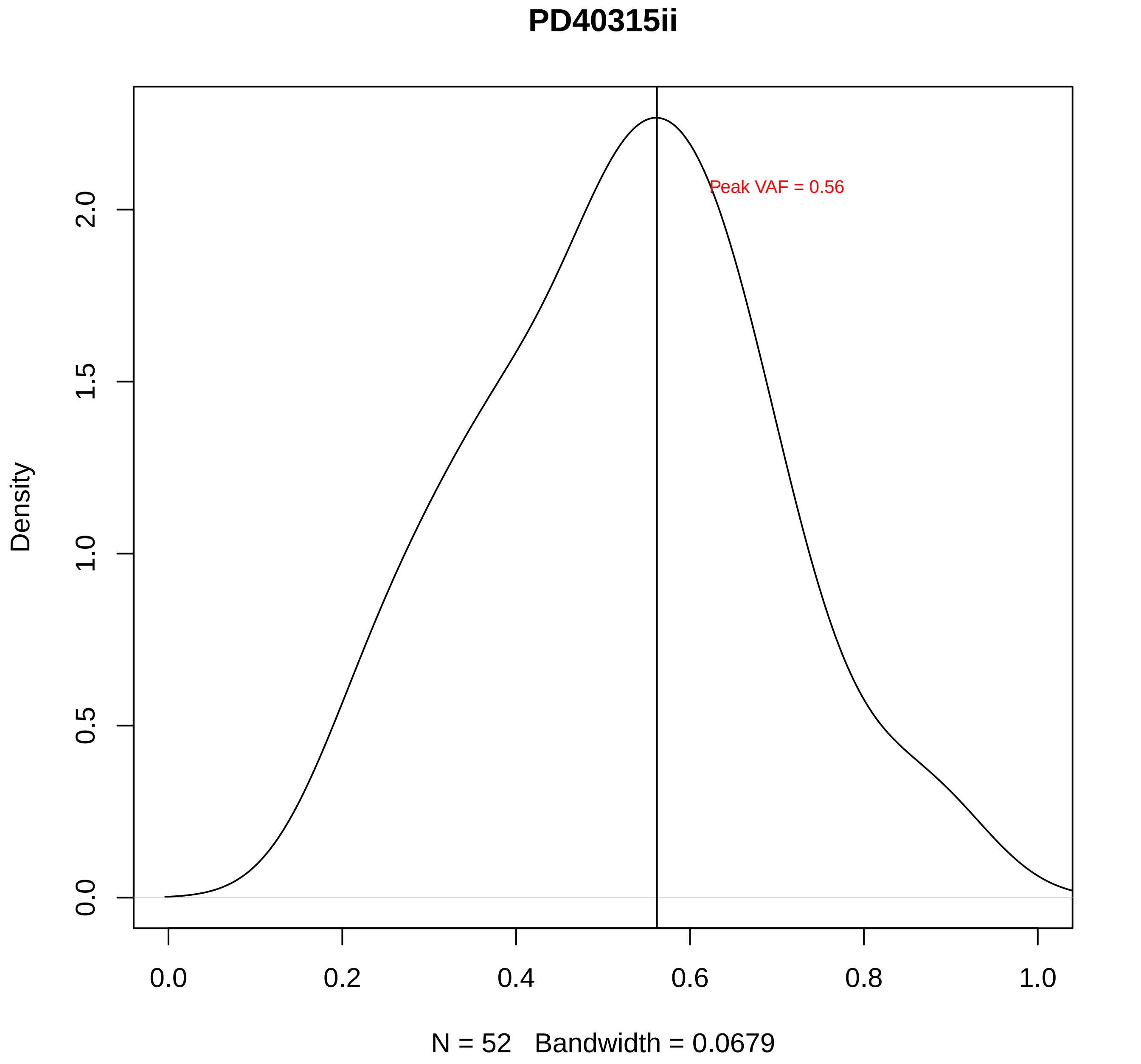
PD40315ii
Peak VAF = 0.56
Density
0.0
0.5
1.0
1.5
2.0
0.0
0.2
0.4
0.6
0.8
1.0
N = 52 Bandwidth = 0.0679

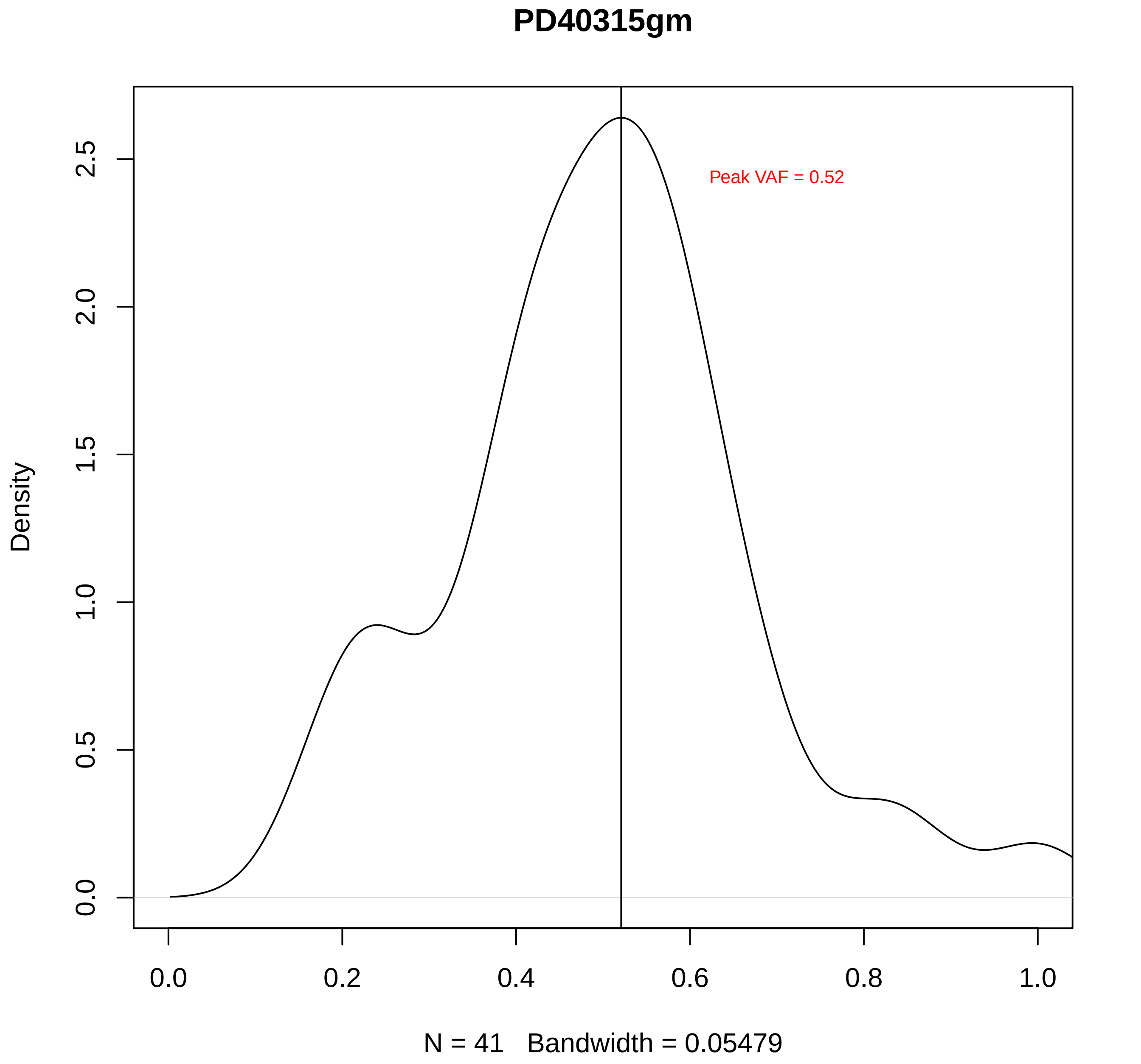
PD40315gm
Peak VAF = 0.52
Density
0.0
0.5
1.0
1.5
2.0
2.5
0.0
0.2
0.4
0.6
0.8
1.0
N = 41 Bandwidth = 0.05479

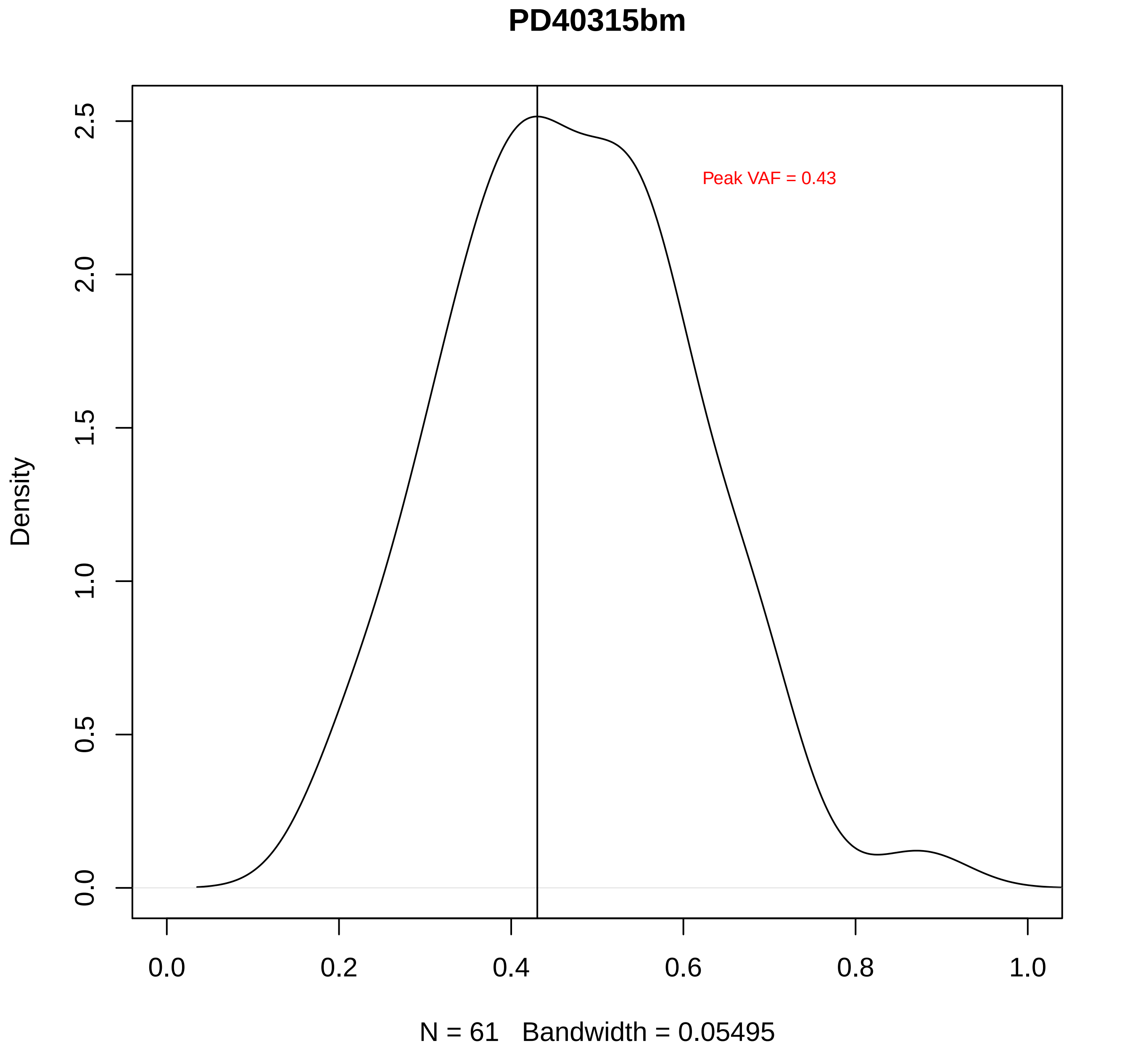

PD40315bm
Peak VAF = 0.43
Density
0.0
0.5
1.0
1.5
2.0
2.5
0.0
0.2
0.4
0.6
0.8
1.0
N = 61   Bandwidth = 0.05495

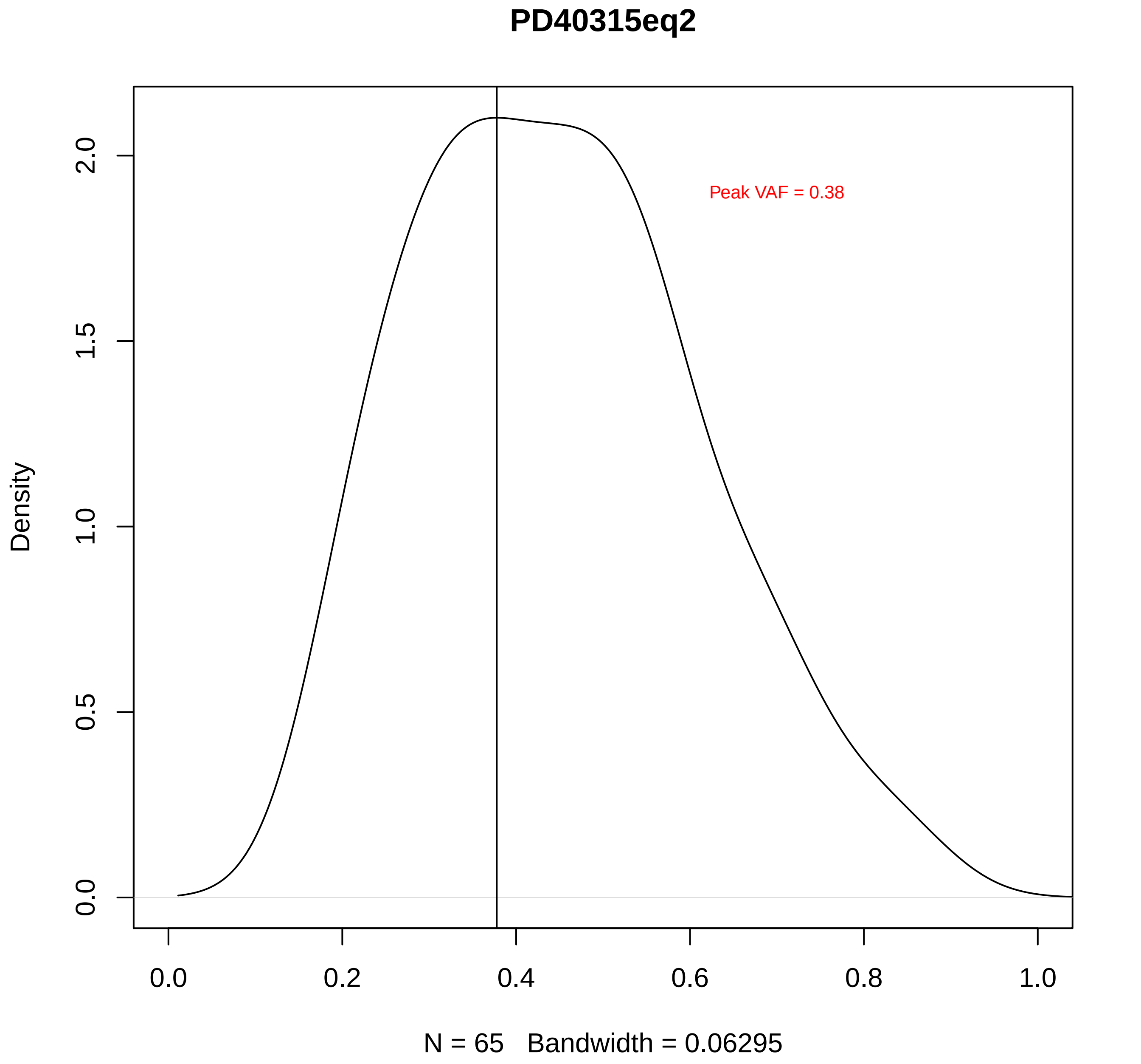
PD40315eq2
Peak VAF = 0.38
Density
0.0
0.5
1.0
1.5
2.0
0.0
0.2
0.4
0.6
0.8
1.0
N = 65   Bandwidth = 0.06295

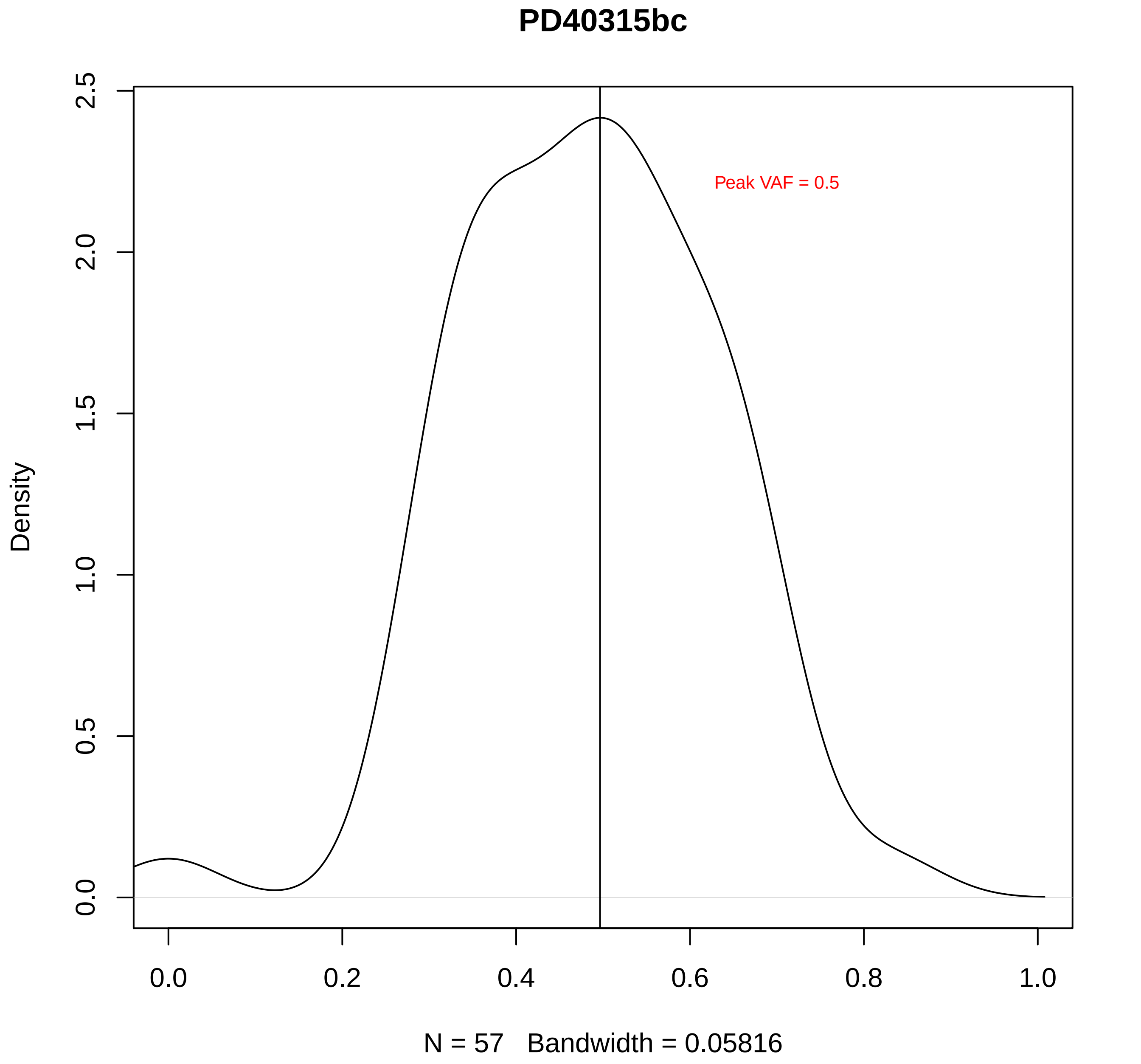

PD40315bc
Peak VAF = 0.5
Density
0.0
0.5
1.0
1.5
2.0
2.5
0.0
0.2
0.4
0.6
0.8
1.0
N = 57   Bandwidth = 0.05816

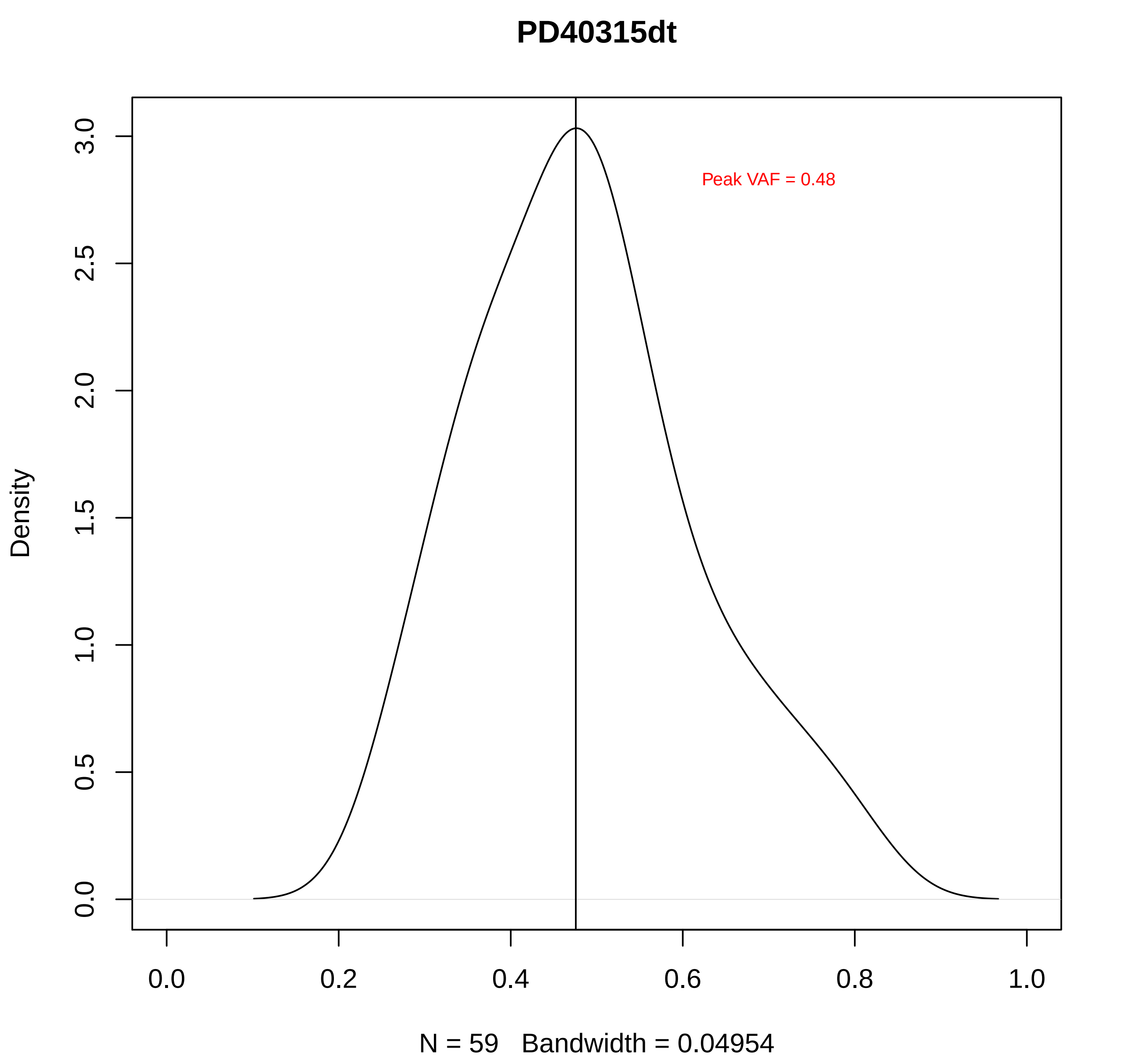
PD40315dt
Peak VAF = 0.48
Density
0.0
0.5
1.0
1.5
2.0
2.5
3.0
0.0
0.2
0.4
0.6
0.8
1.0
N = 59   Bandwidth = 0.04954

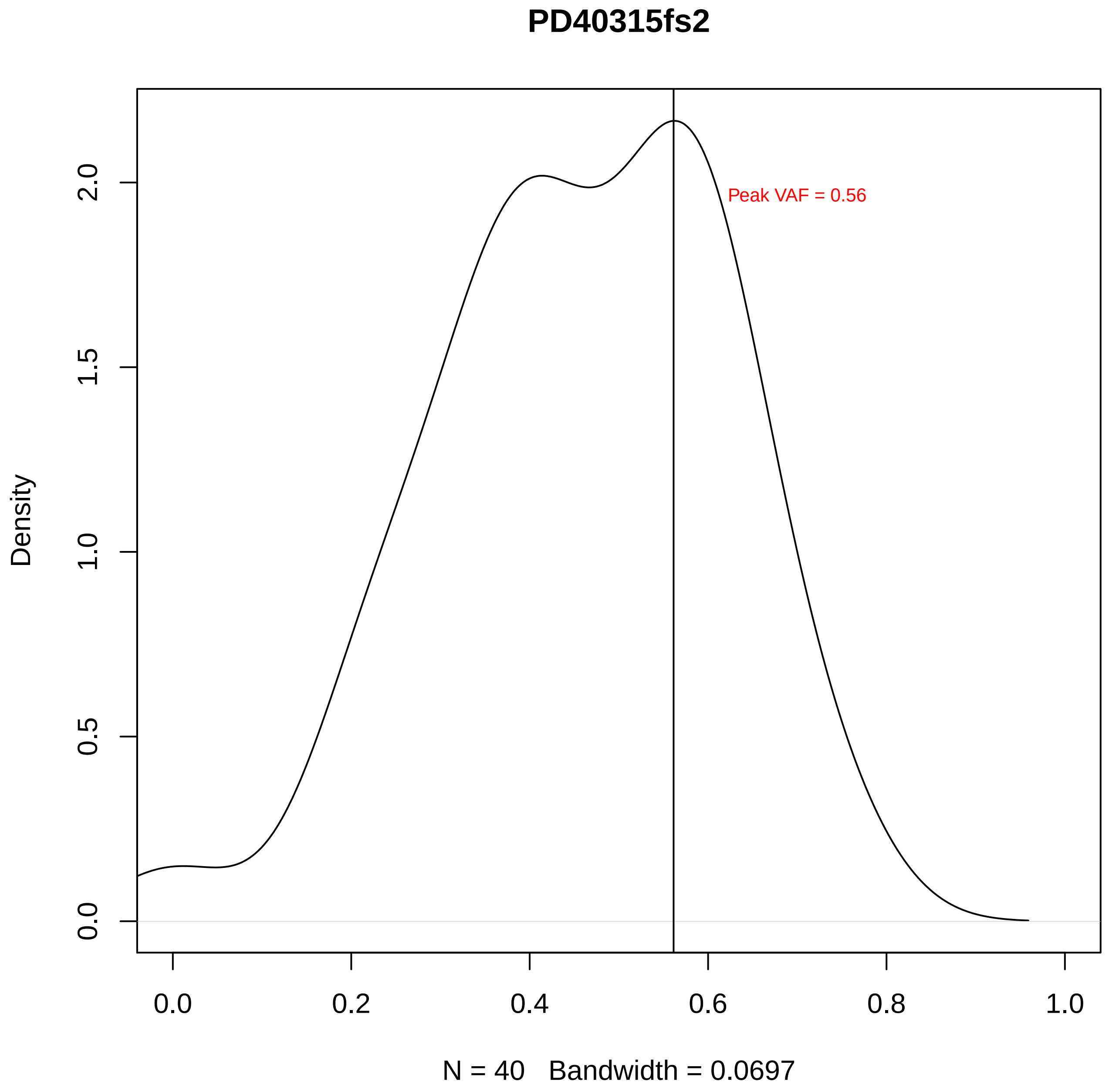
PD40315fs2
Peak VAF = 0.56
Density
0.0
0.5
1.0
1.5
2.0
0.0
0.2
0.4
0.6
0.8
1.0
N = 40   Bandwidth = 0.0697

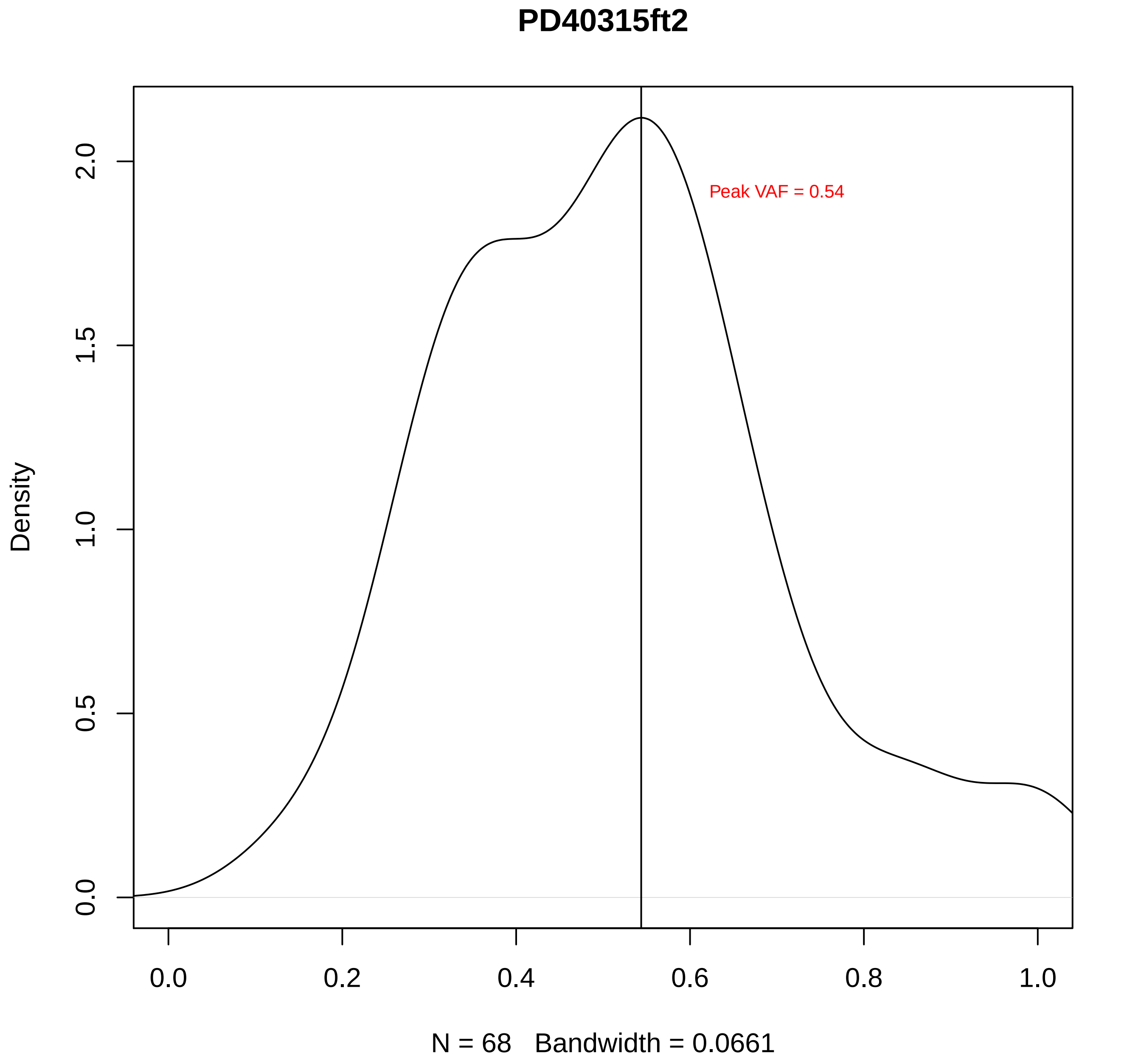
PD40315ft2
Peak VAF = 0.54
Density
0.0
0.5
1.0
1.5
2.0
0.0
0.2
0.4
0.6
0.8
1.0
N = 68 Bandwidth = 0.0661

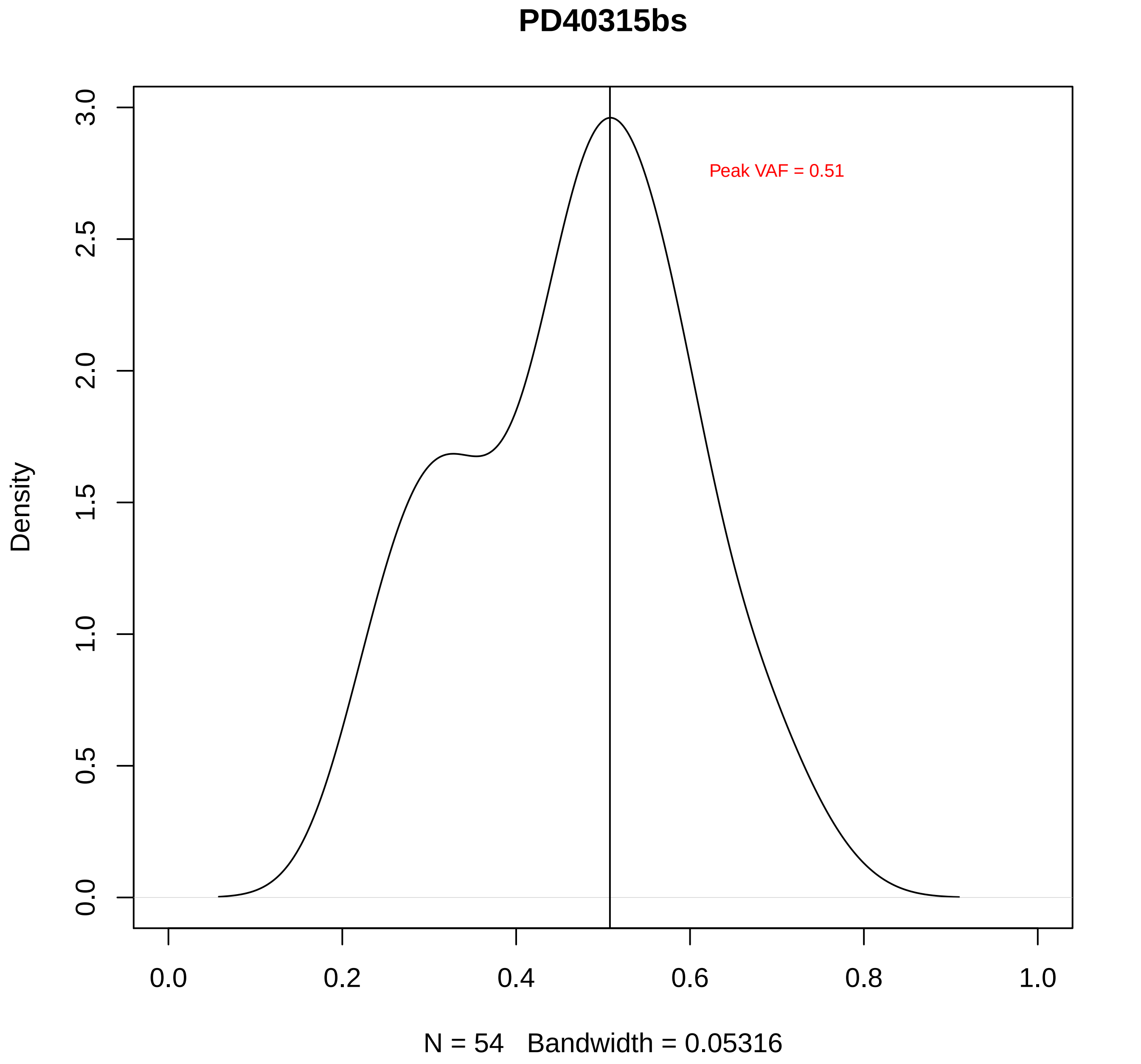

PD40315bs
Peak VAF = 0.51
Density
3.0
2.5
2.0
1.5
1.0
0.5
0.0
0.0
0.2
0.4
0.6
0.8
1.0
N = 54 Bandwidth = 0.05316

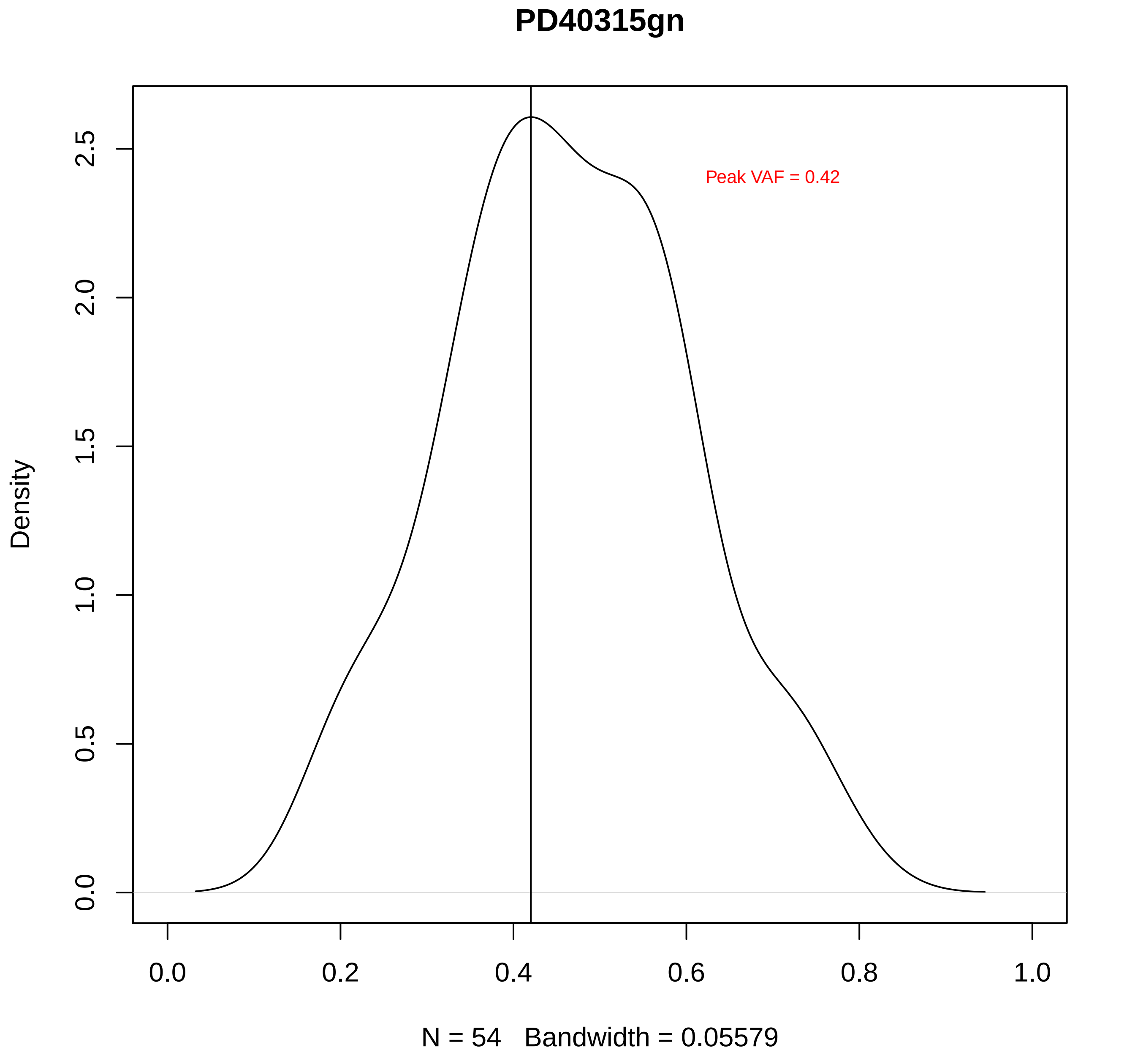
PD40315gn
Peak VAF = 0.42
Density
0.0
0.5
1.0
1.5
2.0
2.5
0.0
0.2
0.4
0.6
0.8
1.0
N = 54   Bandwidth = 0.05579

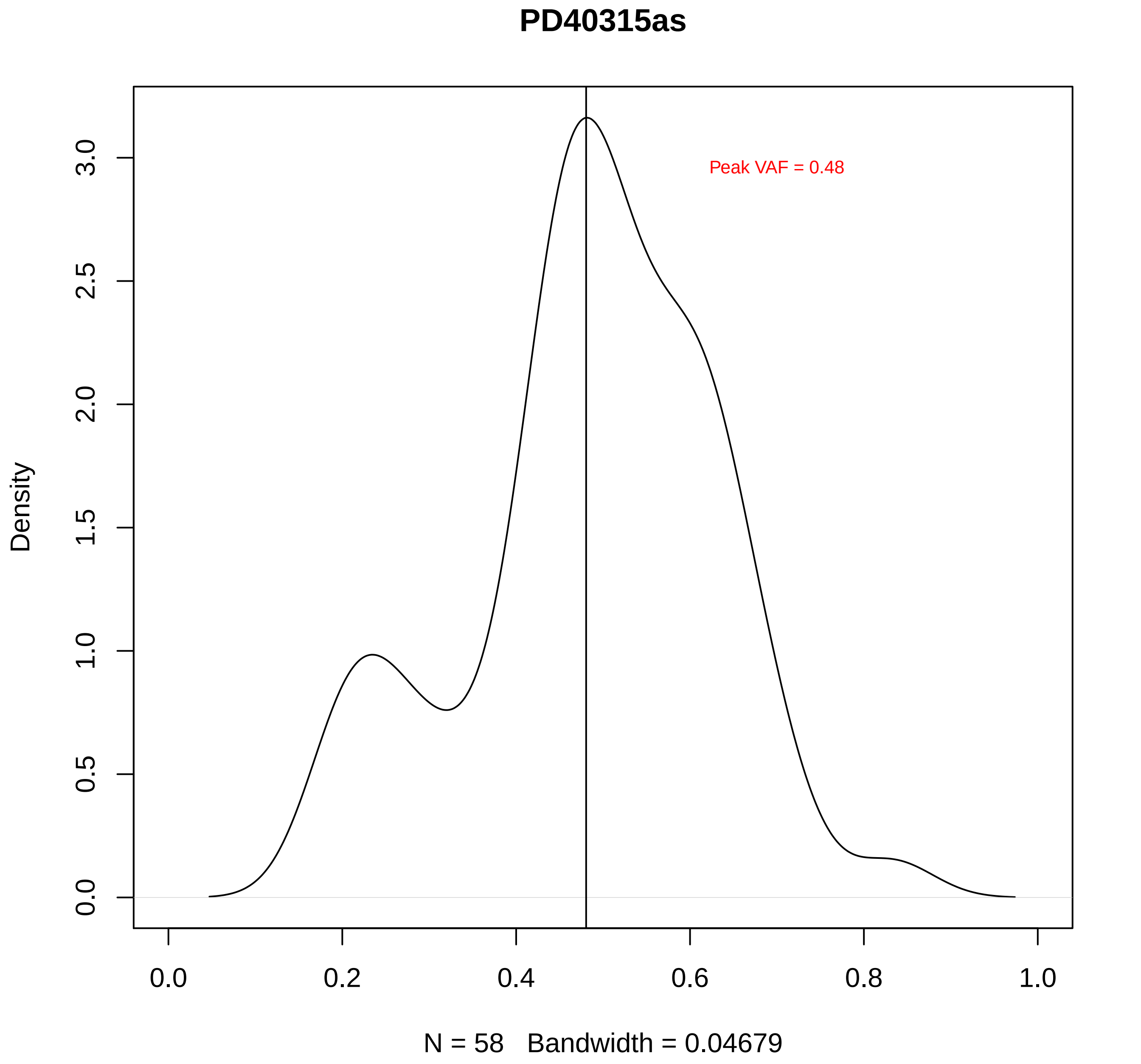

PD40315as
Peak VAF = 0.48
Density
0.0
0.5
1.0
1.5
2.0
2.5
3.0
0.0
0.2
0.4
0.6
0.8
1.0
N = 58 Bandwidth = 0.04679

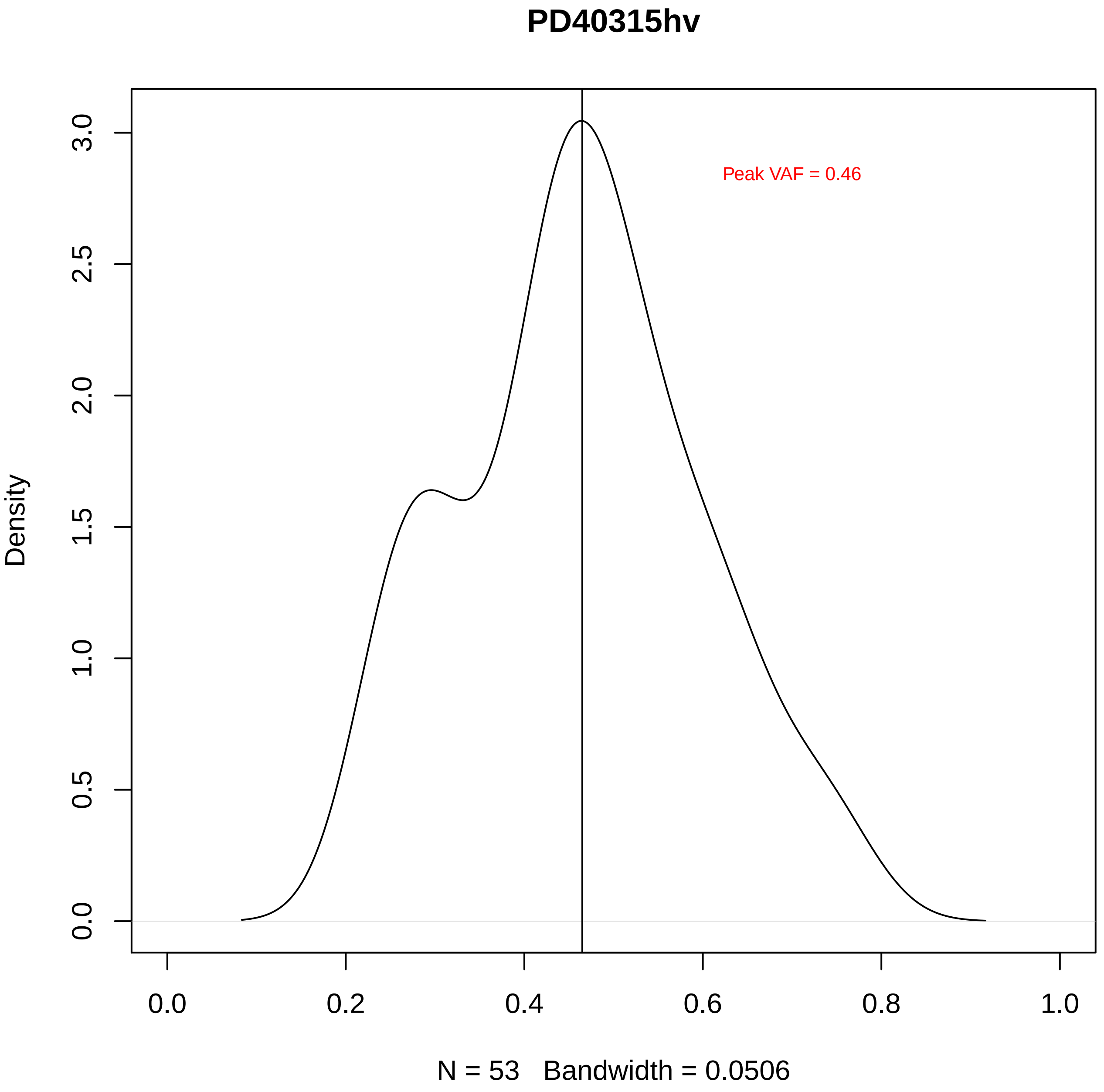
PD40315hv
Peak VAF = 0.46
Density
0.0
0.5
1.0
1.5
2.0
2.5
3.0
0.0
0.2
0.4
0.6
0.8
1.0
N = 53   Bandwidth = 0.0506

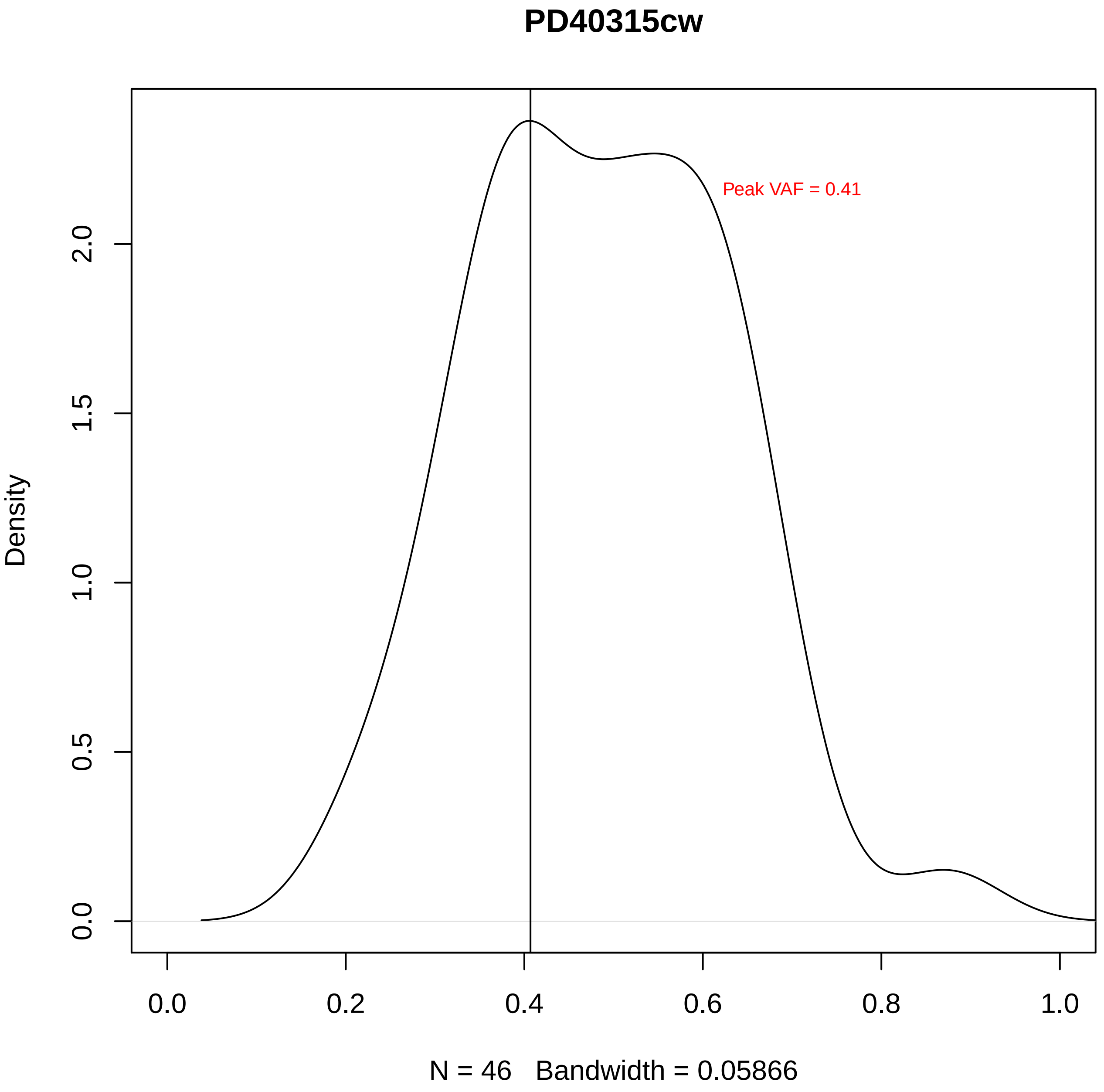

PD40315cw
Peak VAF = 0.41
Density
0.0
0.5
1.0
1.5
2.0
0.0
0.2
0.4
0.6
0.8
1.0
N = 46 Bandwidth = 0.05866

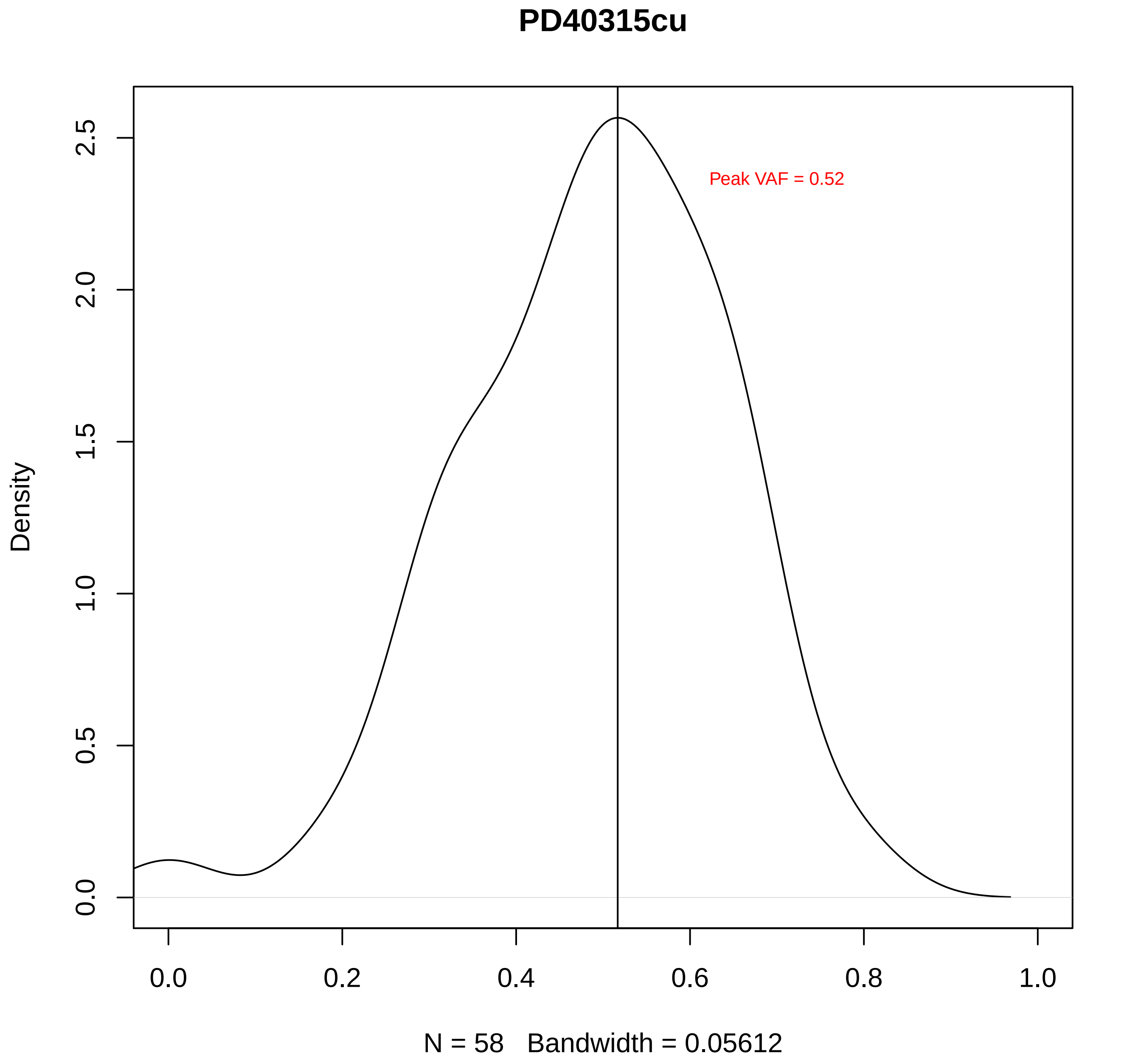
PD40315cu
Peak VAF = 0.52
Density
0.0
0.5
1.0
1.5
2.0
2.5
0.0
0.2
0.4
0.6
0.8
1.0
N = 58   Bandwidth = 0.05612

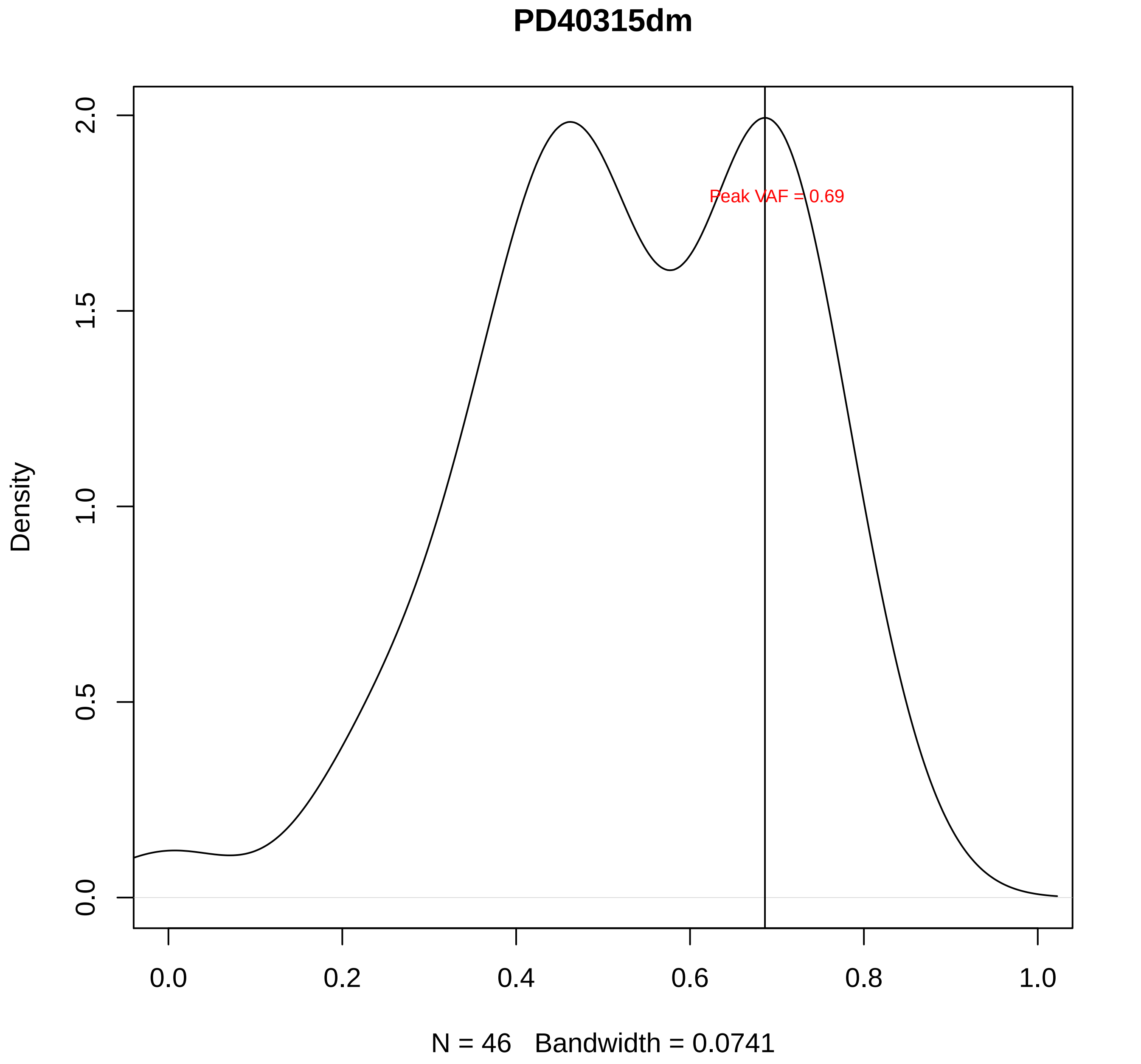
PD40315dm
Density
2.0
1.5
1.0
0.5
0.0
Peak VAF = 0.69
0.0
0.2
0.4
0.6
0.8
1.0
N = 46 Bandwidth = 0.0741

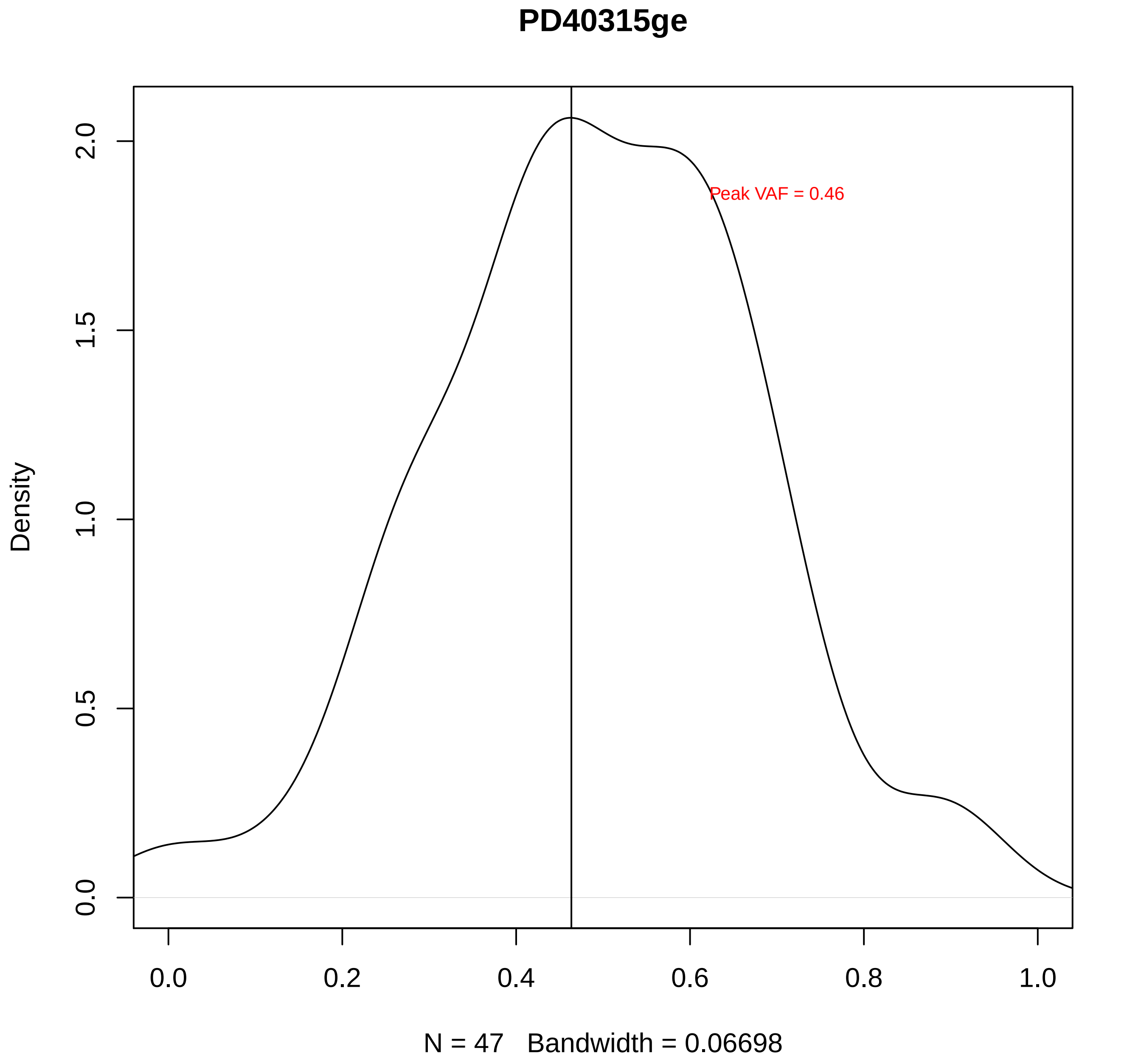

PD40315ge
Peak VAF = 0.46
Density
0.0
0.5
1.0
1.5
2.0
0.0
0.2
0.4
0.6
0.8
1.0
N = 47 Bandwidth = 0.06698

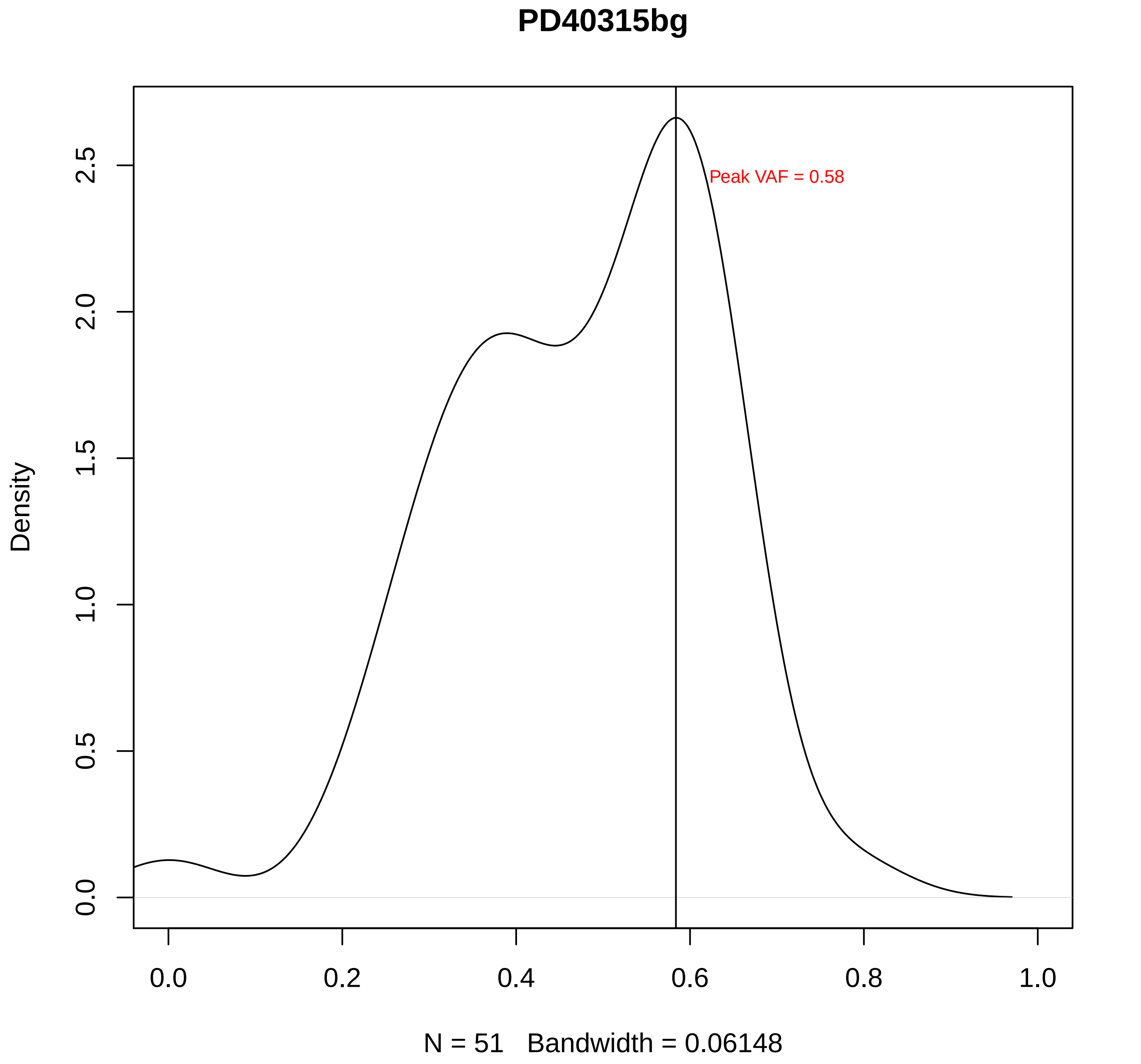
PD40315bg
Density
Peak VAF = 0.58
0.0
0.5
1.0
1.5
2.0
2.5
0.0
0.2
0.4
0.6
0.8
1.0
N = 51   Bandwidth = 0.06148

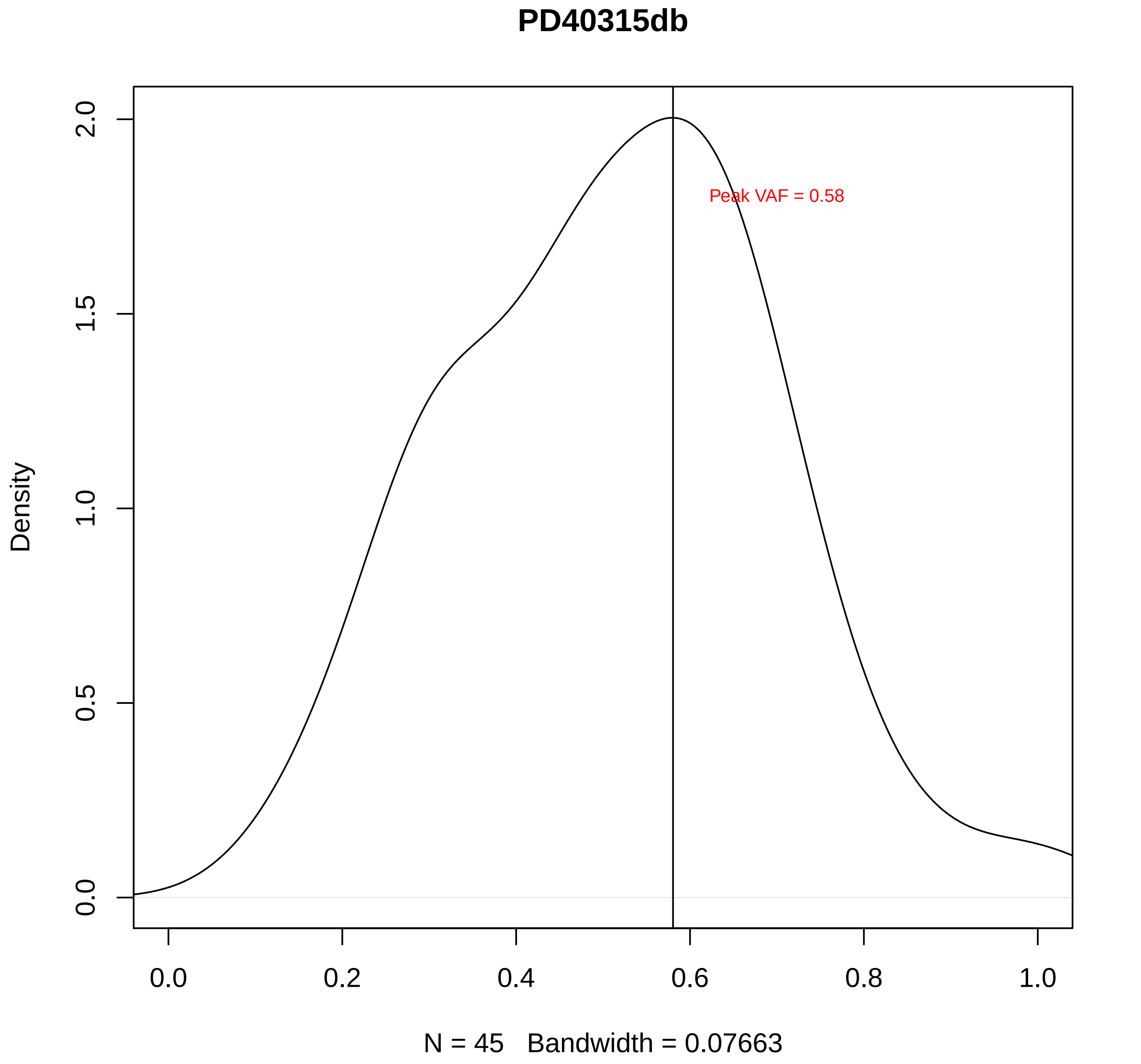

PD40315db
Peak VAF = 0.58
Density
0.0
0.5
1.0
1.5
2.0
0.0
0.2
0.4
0.6
0.8
1.0
N = 45 Bandwidth = 0.07663

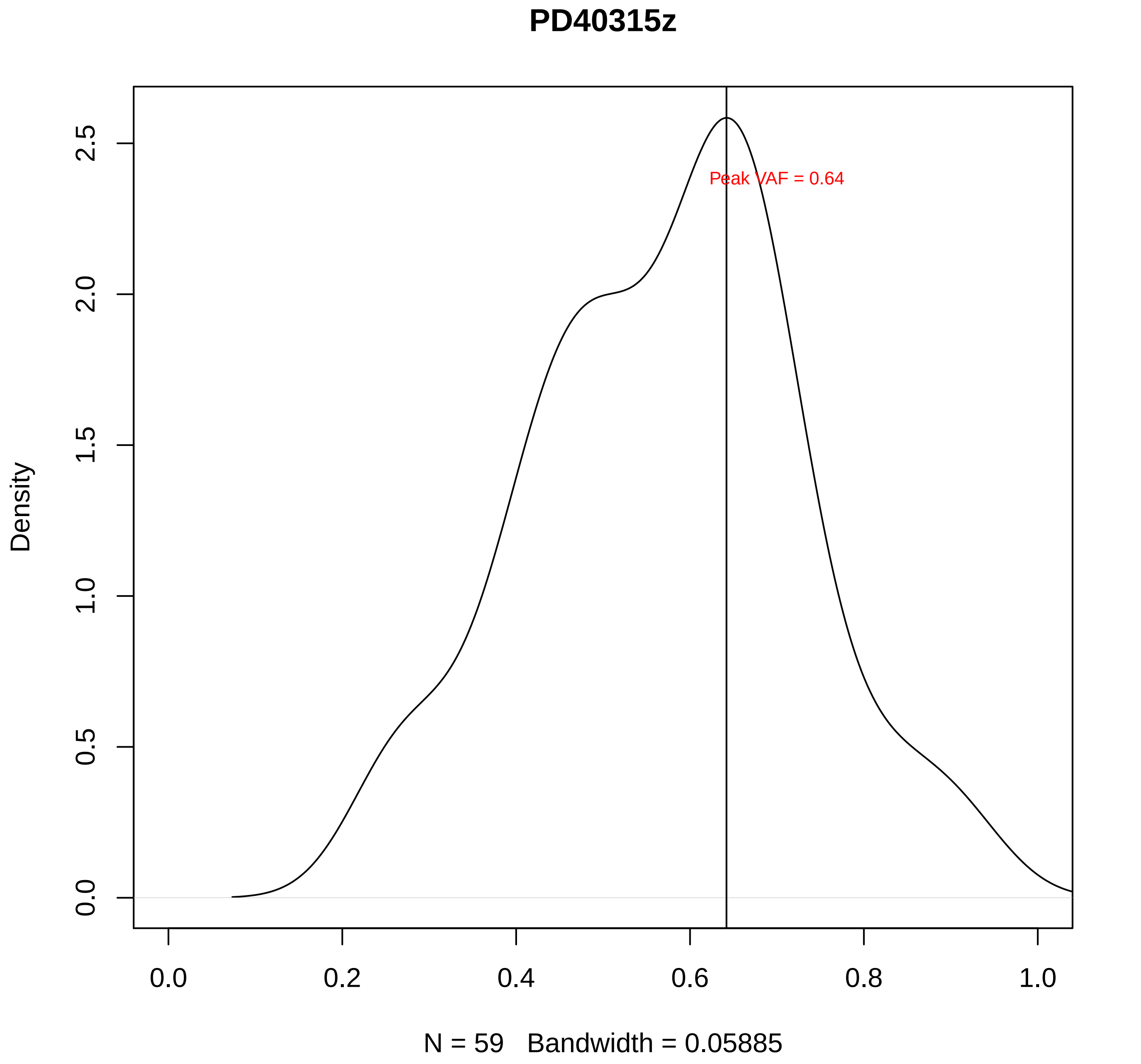
PD40315z
Peak VAF = 0.64
Density
0.0
0.5
1.0
1.5
2.0
2.5
0.0
0.2
0.4
0.6
0.8
1.0
N = 59   Bandwidth = 0.05885

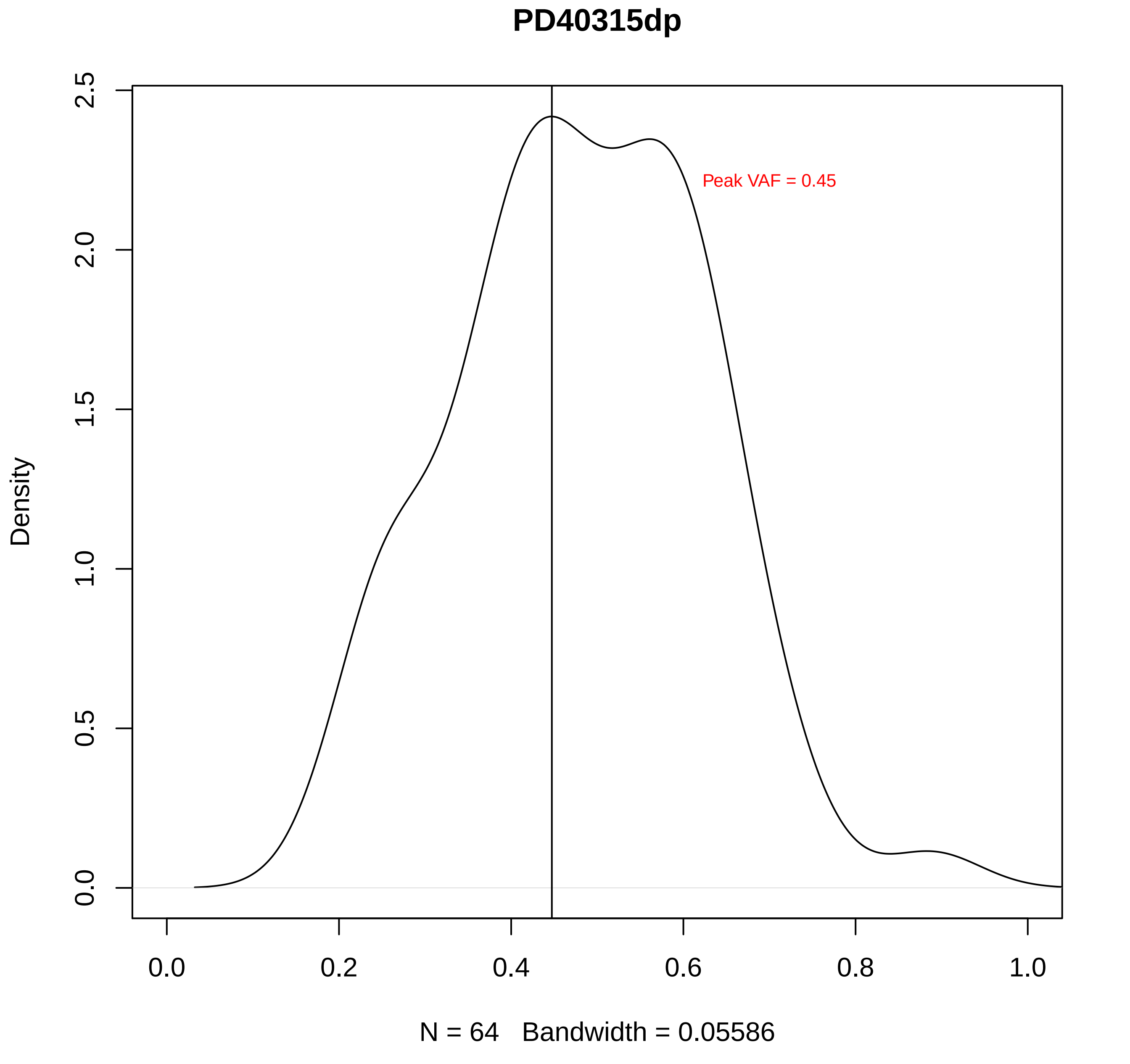
PD40315dp
Peak VAF = 0.45
Density
0.0
0.5
1.0
1.5
2.0
2.5
0.0
0.2
0.4
0.6
0.8
1.0
N = 64   Bandwidth = 0.05586

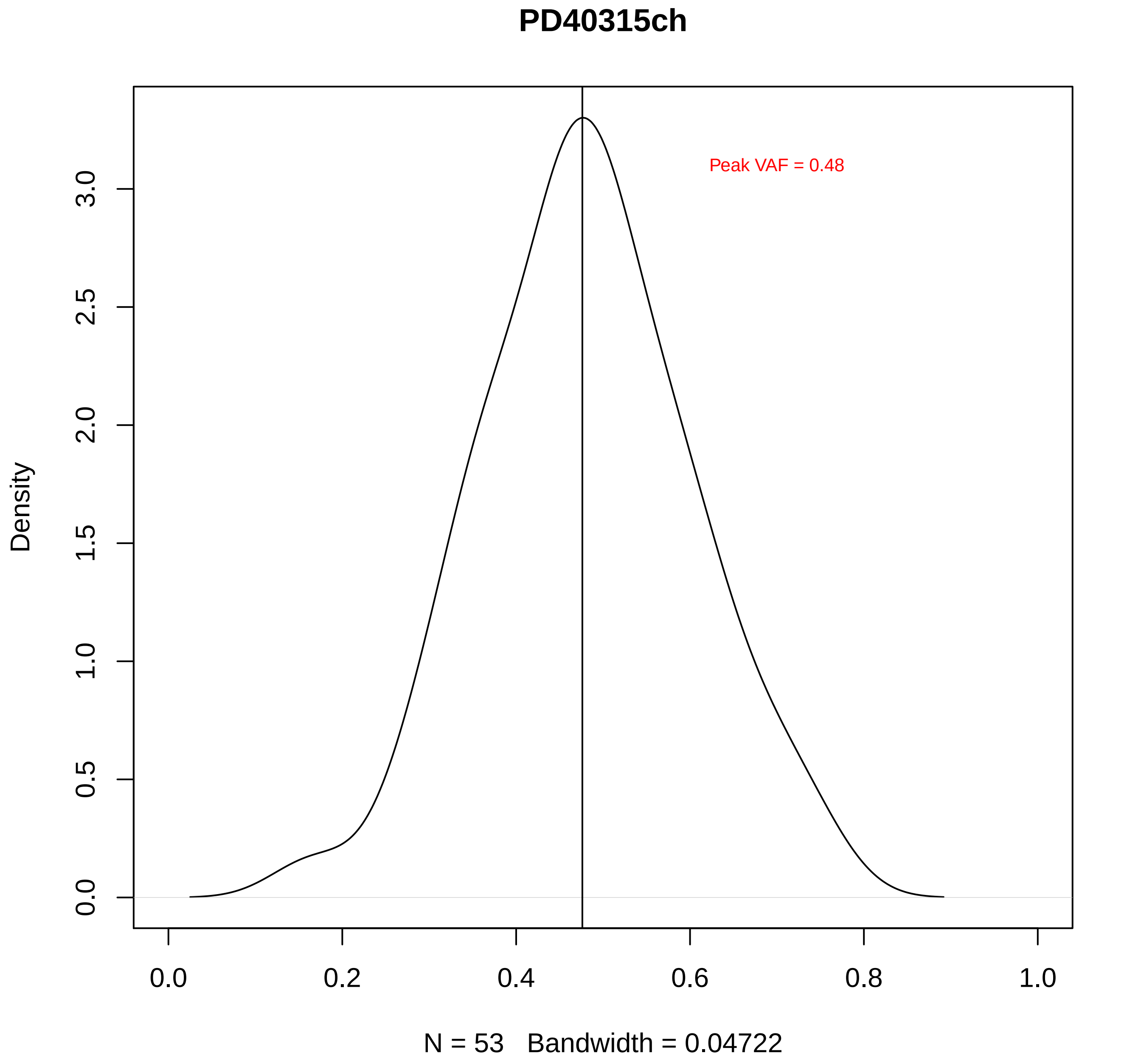
PD40315ch
Peak VAF = 0.48
Density
0.0
0.5
1.0
1.5
2.0
2.5
3.0
0.0
0.2
0.4
0.6
0.8
1.0
N = 53 Bandwidth = 0.04722

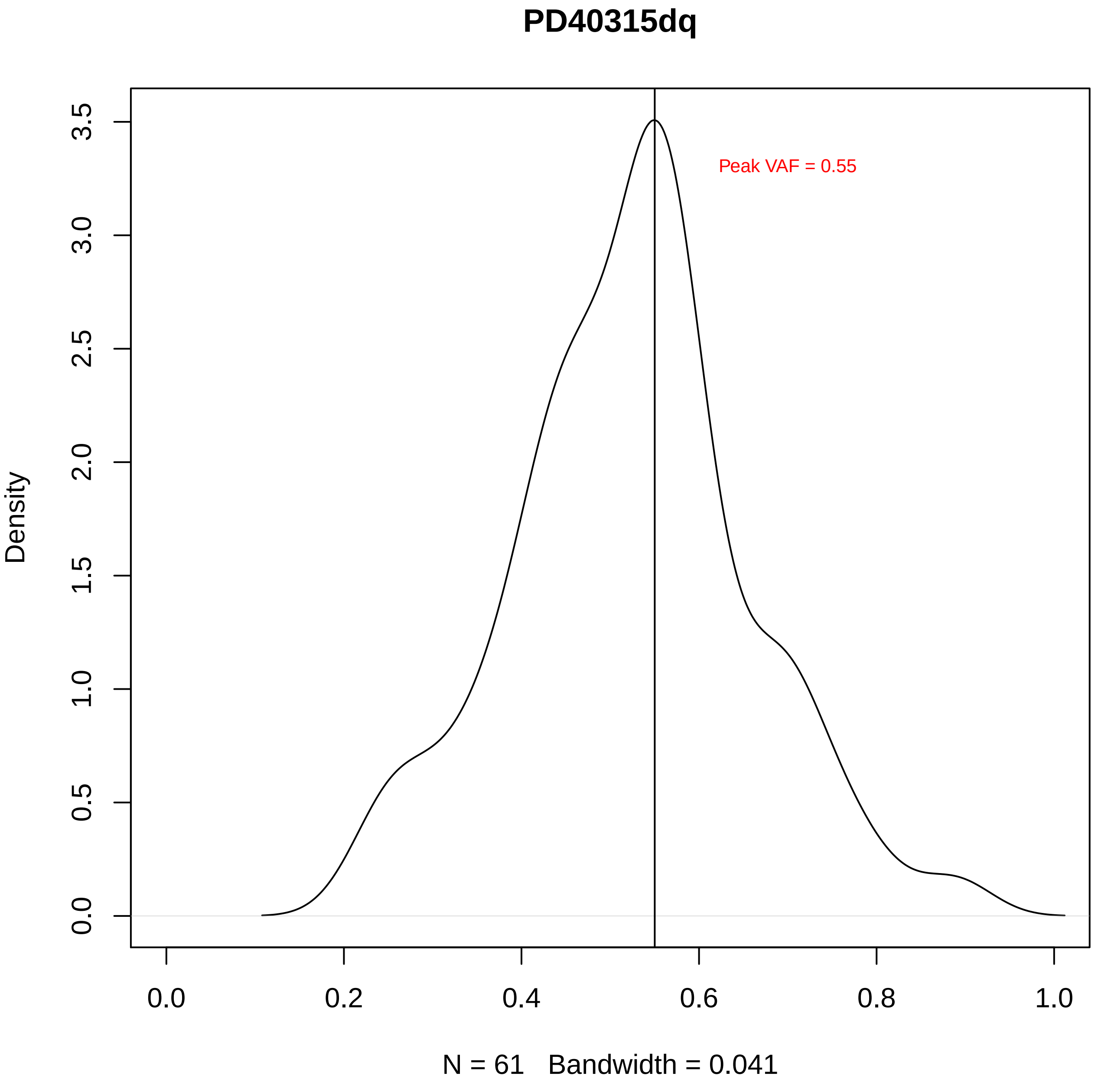
PD40315dq
Peak VAF = 0.55
Density
0.0
0.5
1.0
1.5
2.0
2.5
3.0
3.5
0.0
0.2
0.4
0.6
0.8
1.0
N = 61   Bandwidth = 0.041

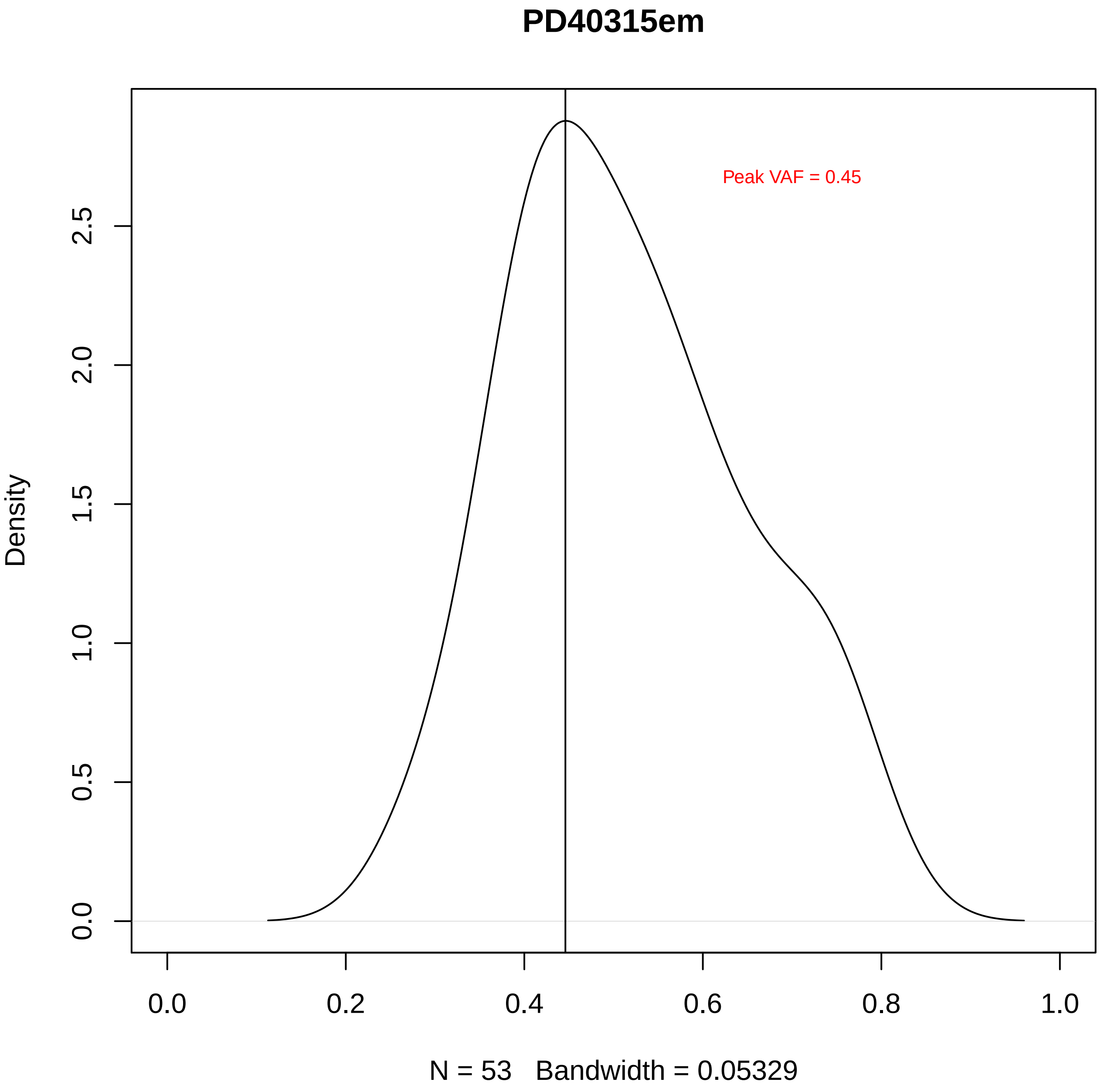

PD40315em
Peak VAF = 0.45
Density
0.0
0.5
1.0
1.5
2.0
2.5
0.0
0.2
0.4
0.6
0.8
1.0
N = 53   Bandwidth = 0.05329

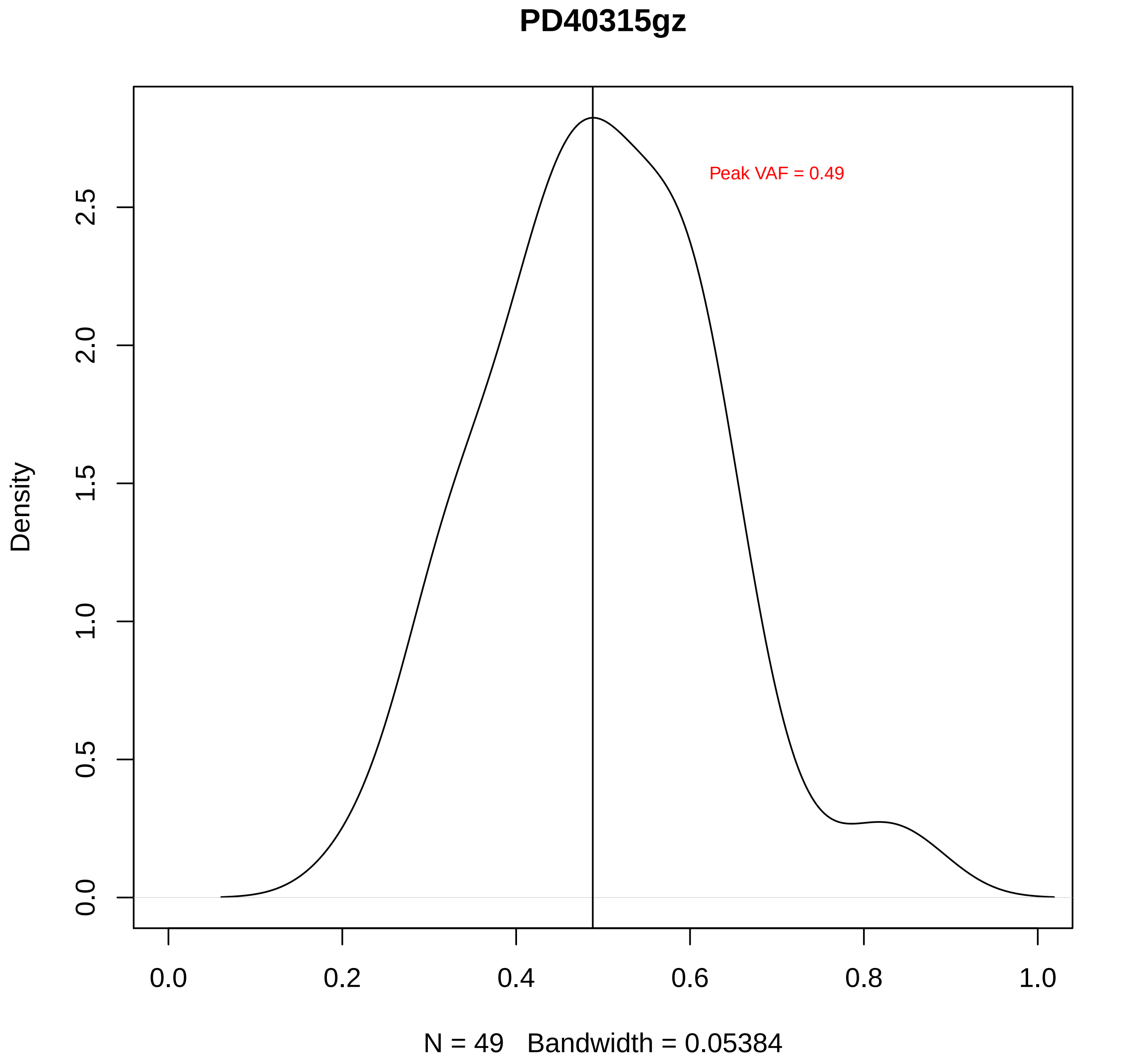
PD40315gz
Peak VAF = 0.49
Density
0.0
0.5
1.0
1.5
2.0
2.5
0.0
0.2
0.4
0.6
0.8
1.0
N = 49   Bandwidth = 0.05384

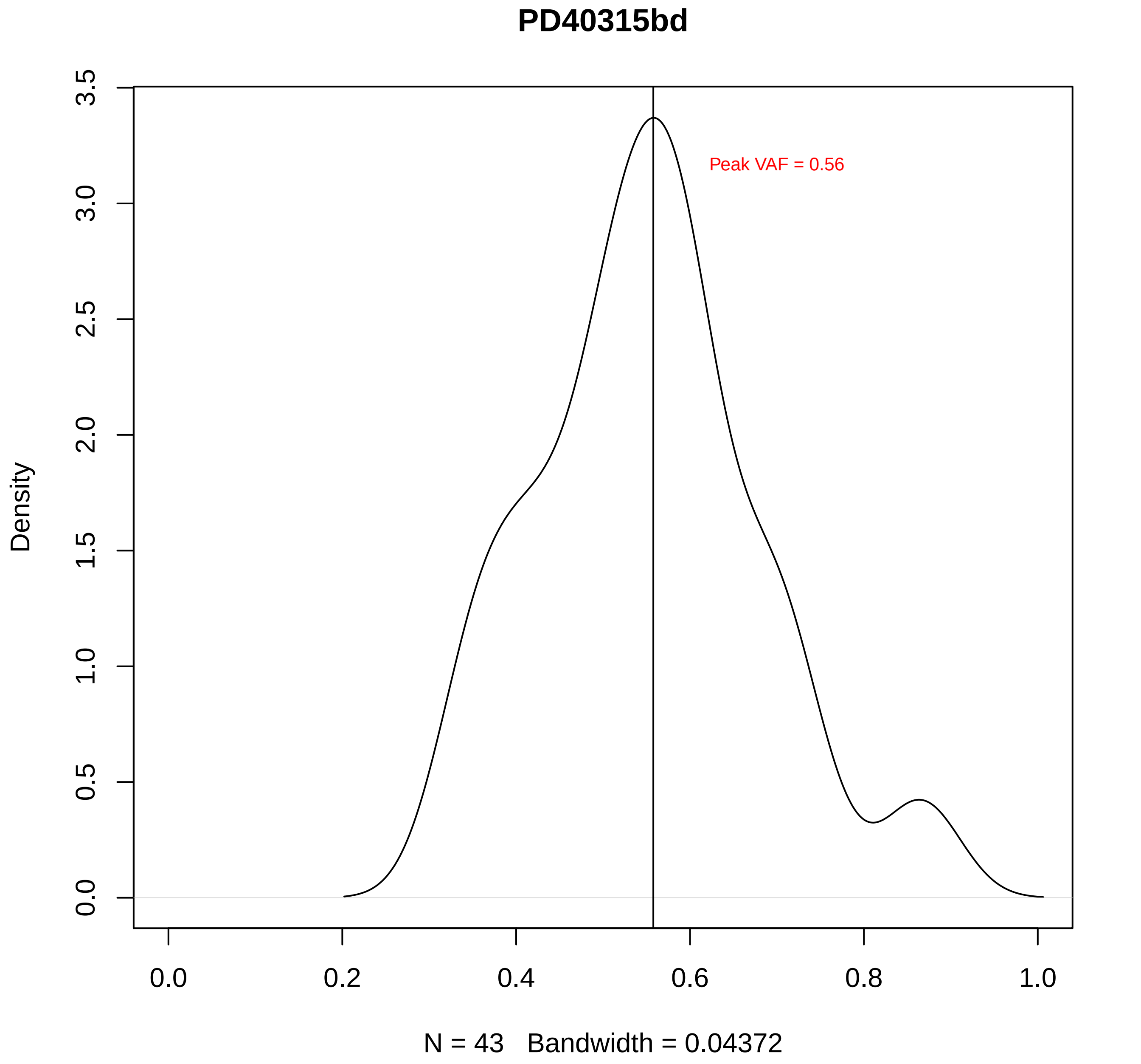
PD40315bd
Peak VAF = 0.56
Density
0.0
0.5
1.0
1.5
2.0
2.5
3.0
3.5
0.0
0.2
0.4
0.6
0.8
1.0
N = 43   Bandwidth = 0.04372

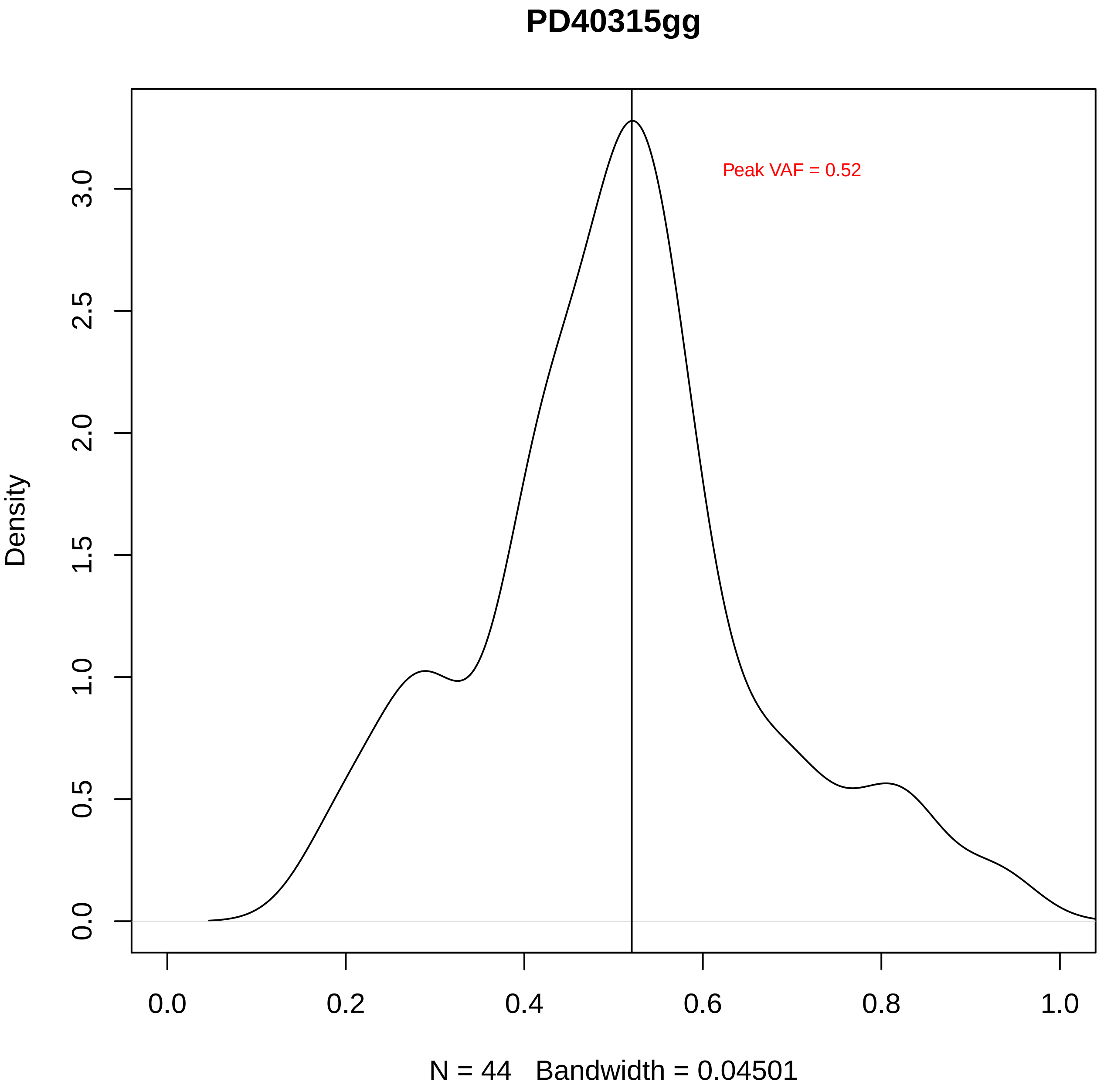
PD40315gg
Peak VAF = 0.52
Density
0.0
0.5
1.0
1.5
2.0
2.5
3.0
0.0
0.2
0.4
0.6
0.8
1.0
N = 44   Bandwidth = 0.04501

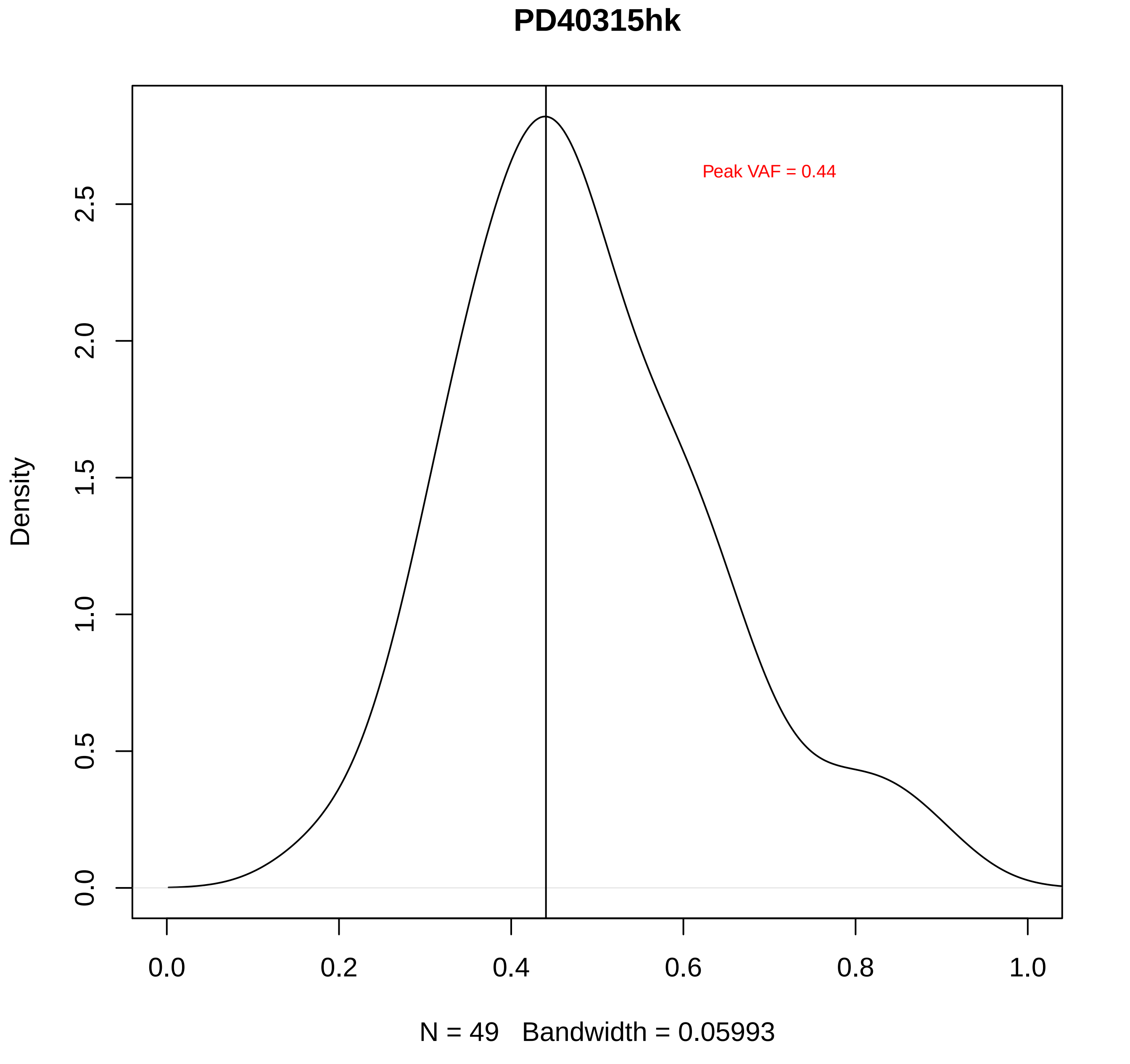
PD40315hk
Peak VAF = 0.44
Density
0.0
0.5
1.0
1.5
2.0
2.5
0.0
0.2
0.4
0.6
0.8
1.0
N = 49   Bandwidth = 0.05993

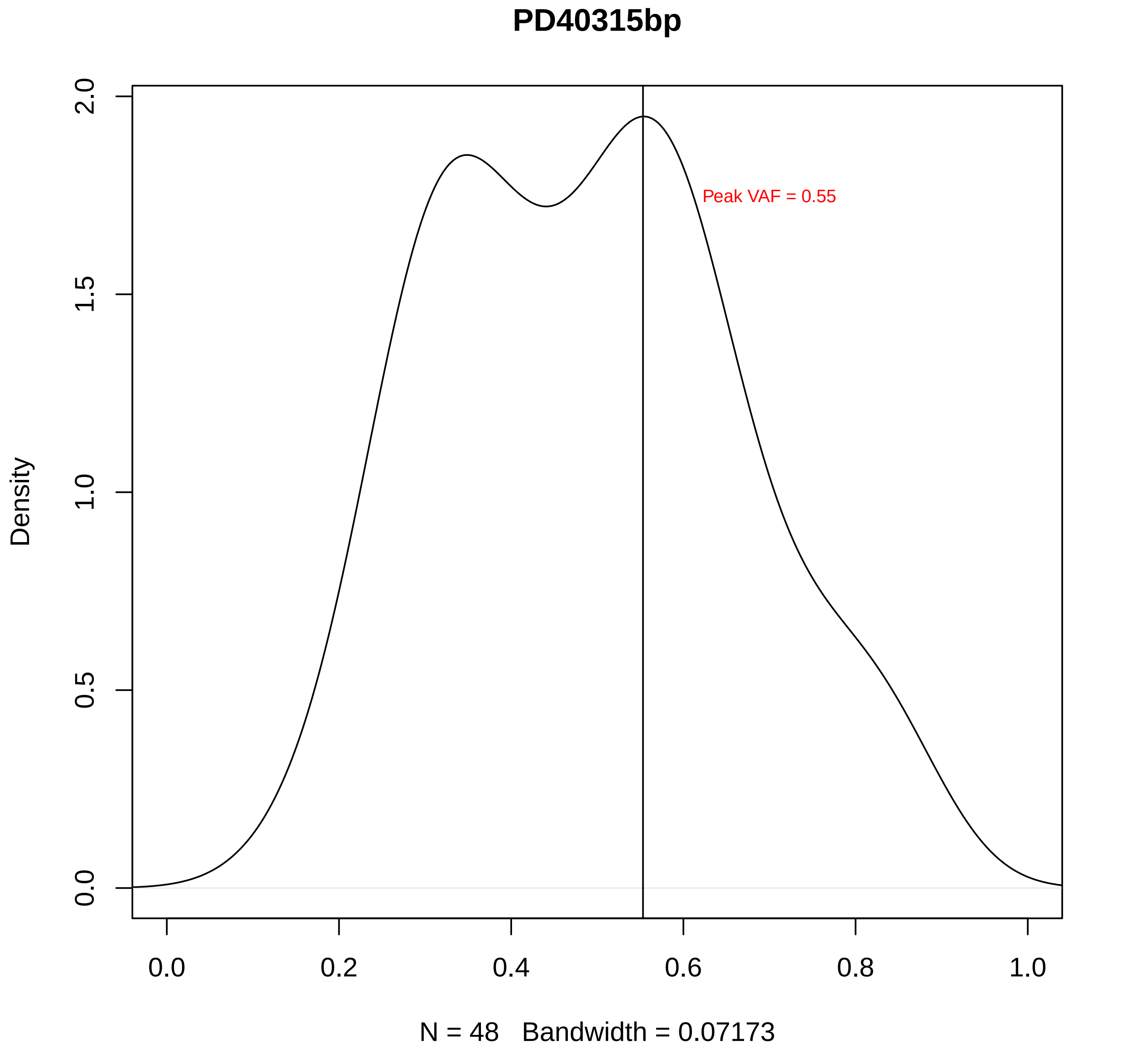
PD40315bp
Peak VAF = 0.55
Density
0.0
0.5
1.0
1.5
2.0
0.0
0.2
0.4
0.6
0.8
1.0
N = 48   Bandwidth = 0.07173

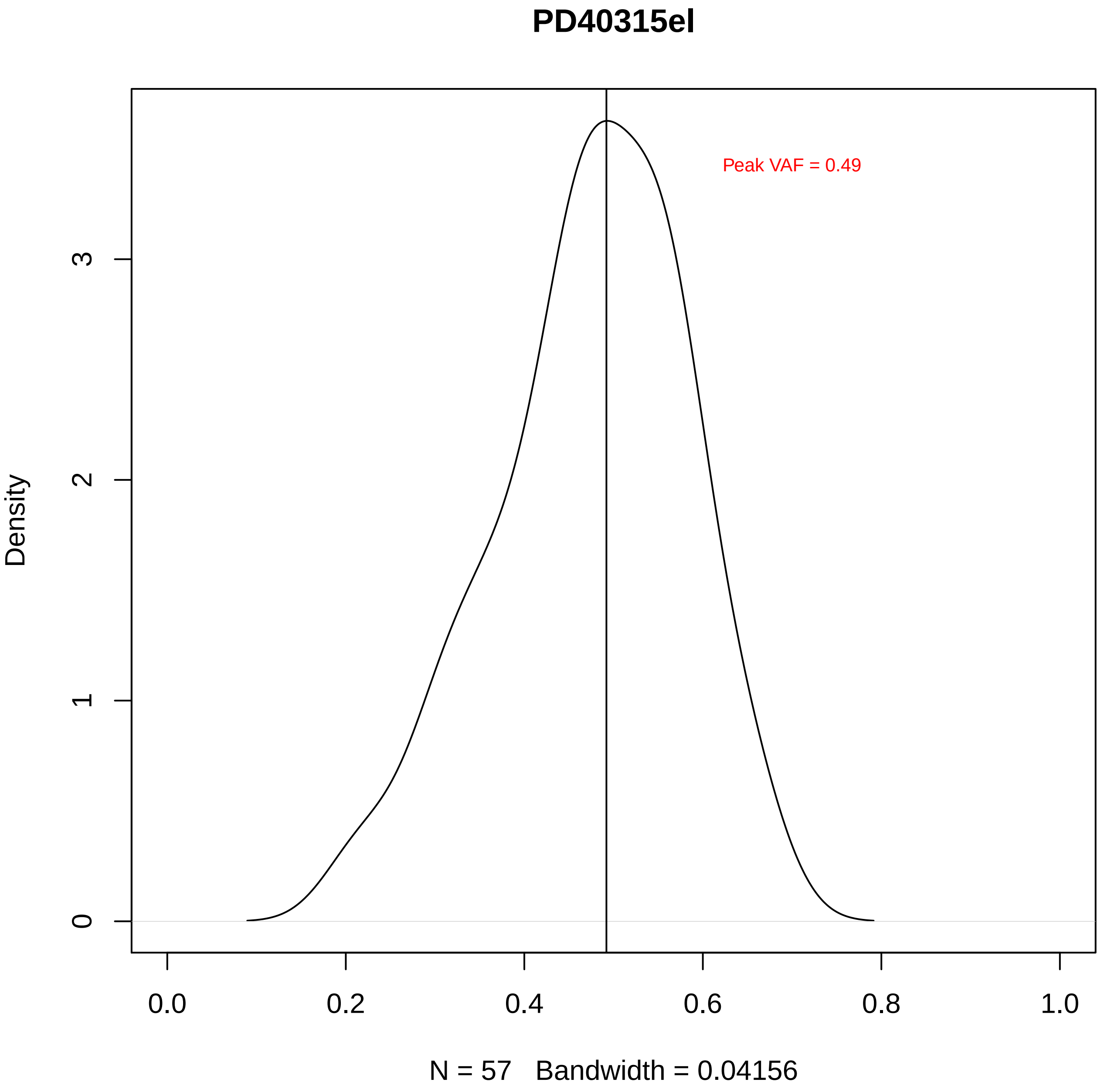
PD40315el
Peak VAF = 0.49
Density
0
1
2
3
0.0
0.2
0.4
0.6
0.8
1.0
N = 57   Bandwidth = 0.04156

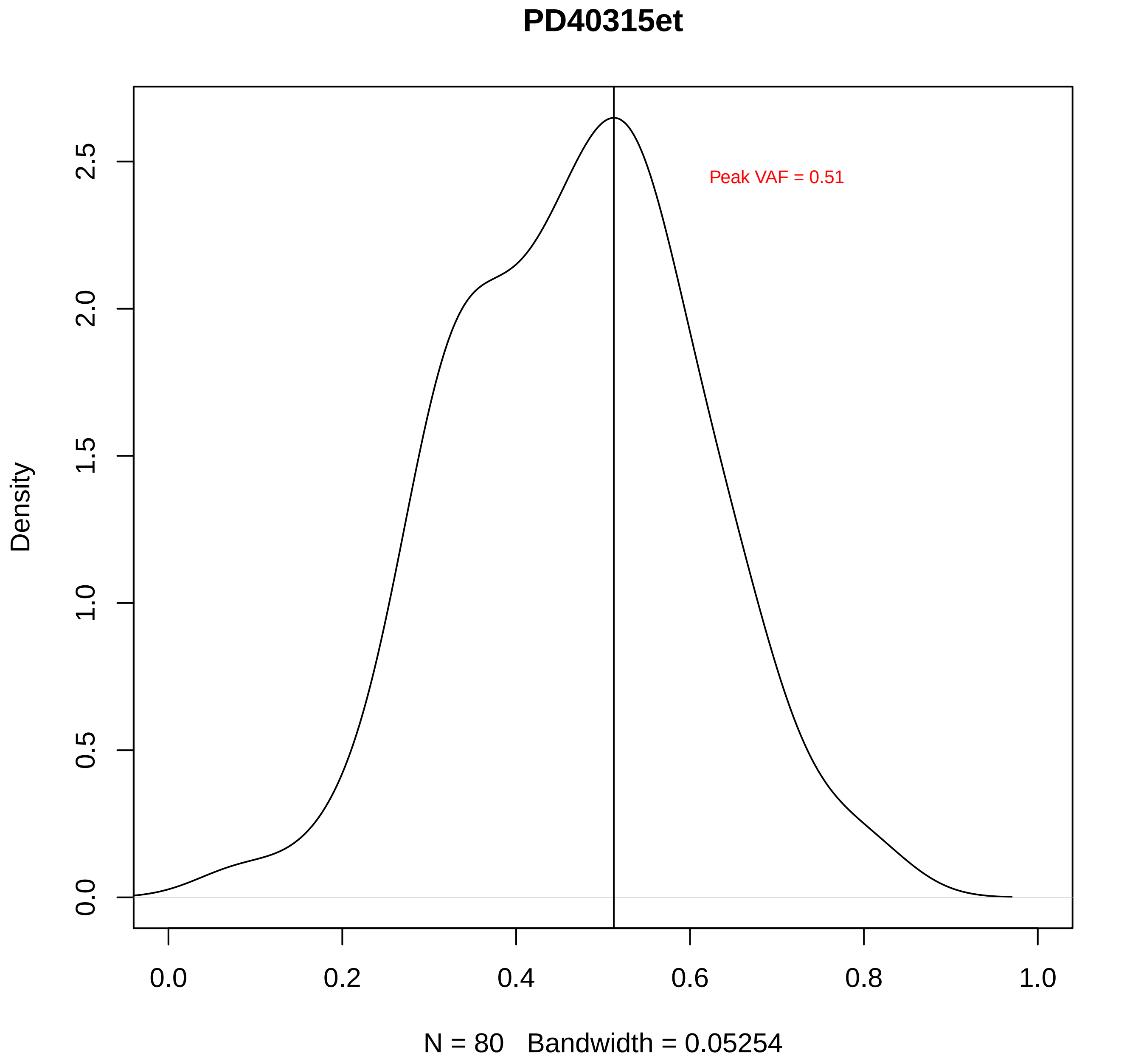
PD40315et
Peak VAF = 0.51
Density
0.0
0.5
1.0
1.5
2.0
2.5
0.0
0.2
0.4
0.6
0.8
1.0
N = 80 Bandwidth = 0.05254

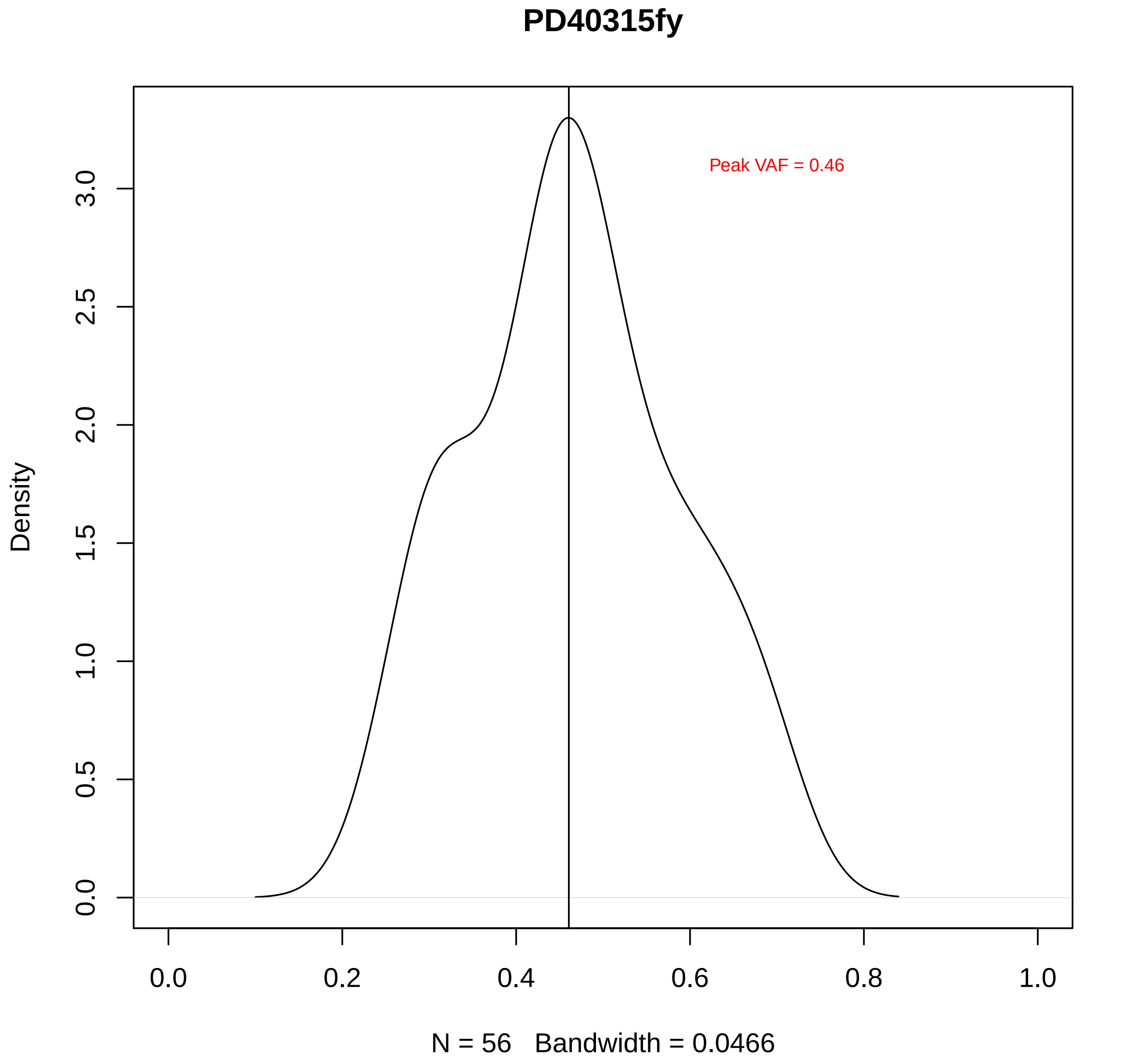
PD40315fy
Peak VAF = 0.46
Density
0.0
0.5
1.0
1.5
2.0
2.5
3.0
0.0
0.2
0.4
0.6
0.8
1.0
N = 56   Bandwidth = 0.0466

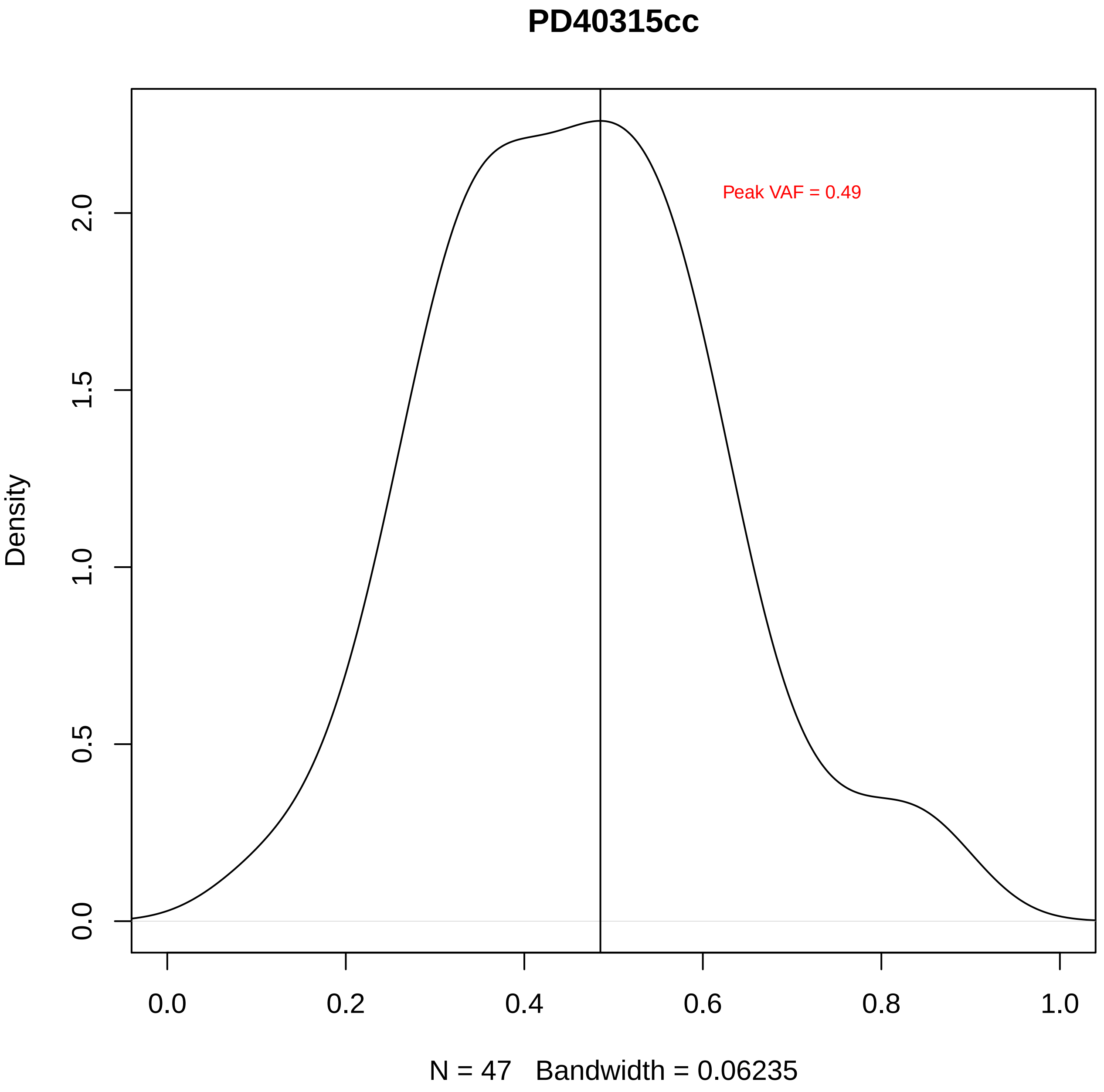
PD40315cc
Peak VAF = 0.49
Density
0.0
0.5
1.0
1.5
2.0
0.0
0.2
0.4
0.6
0.8
1.0
N = 47 Bandwidth = 0.06235

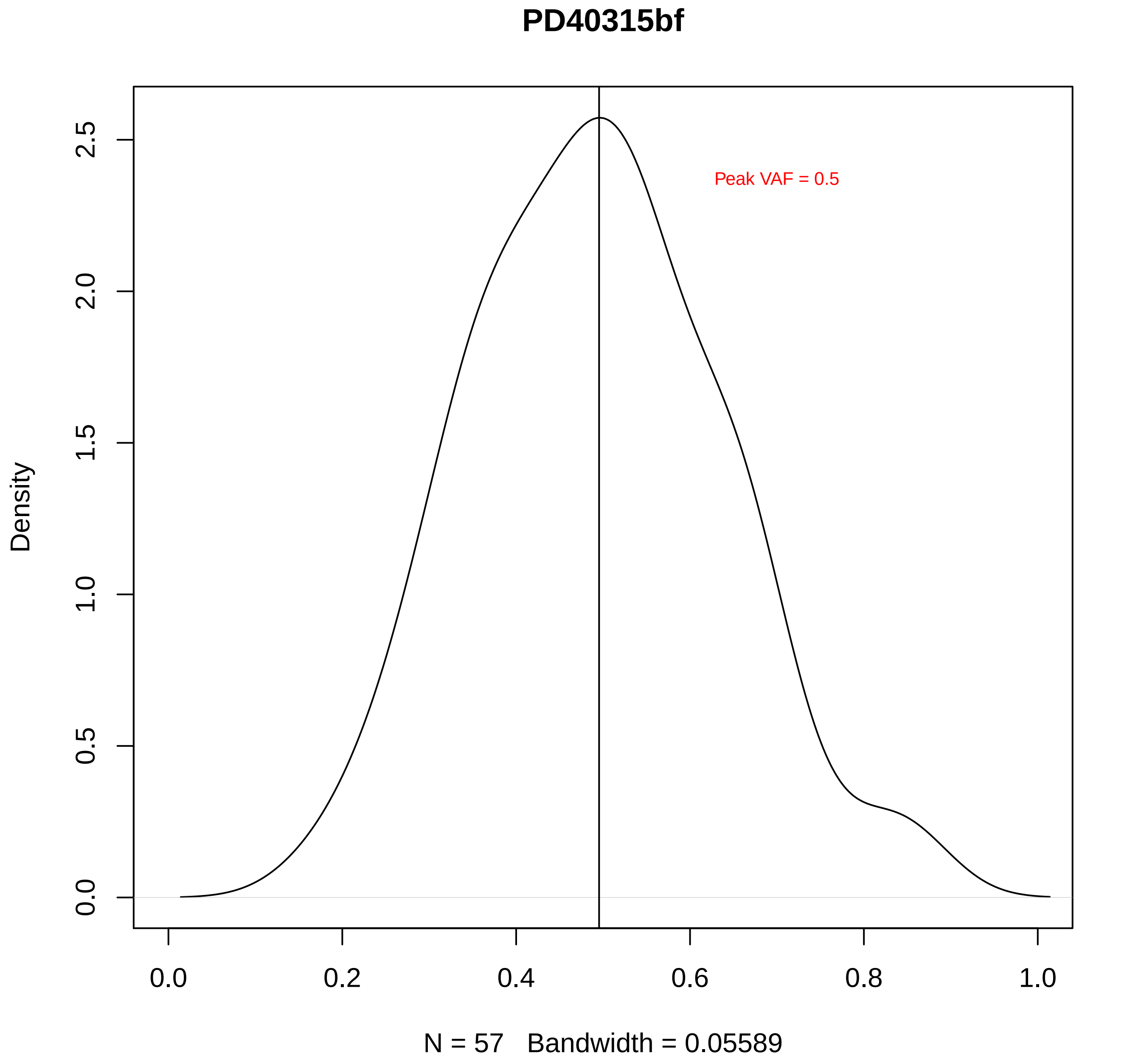

PD40315bf
Peak VAF = 0.5
Density
0.0
0.5
1.0
1.5
2.0
2.5
0.0
0.2
0.4
0.6
0.8
1.0
N = 57   Bandwidth = 0.05589

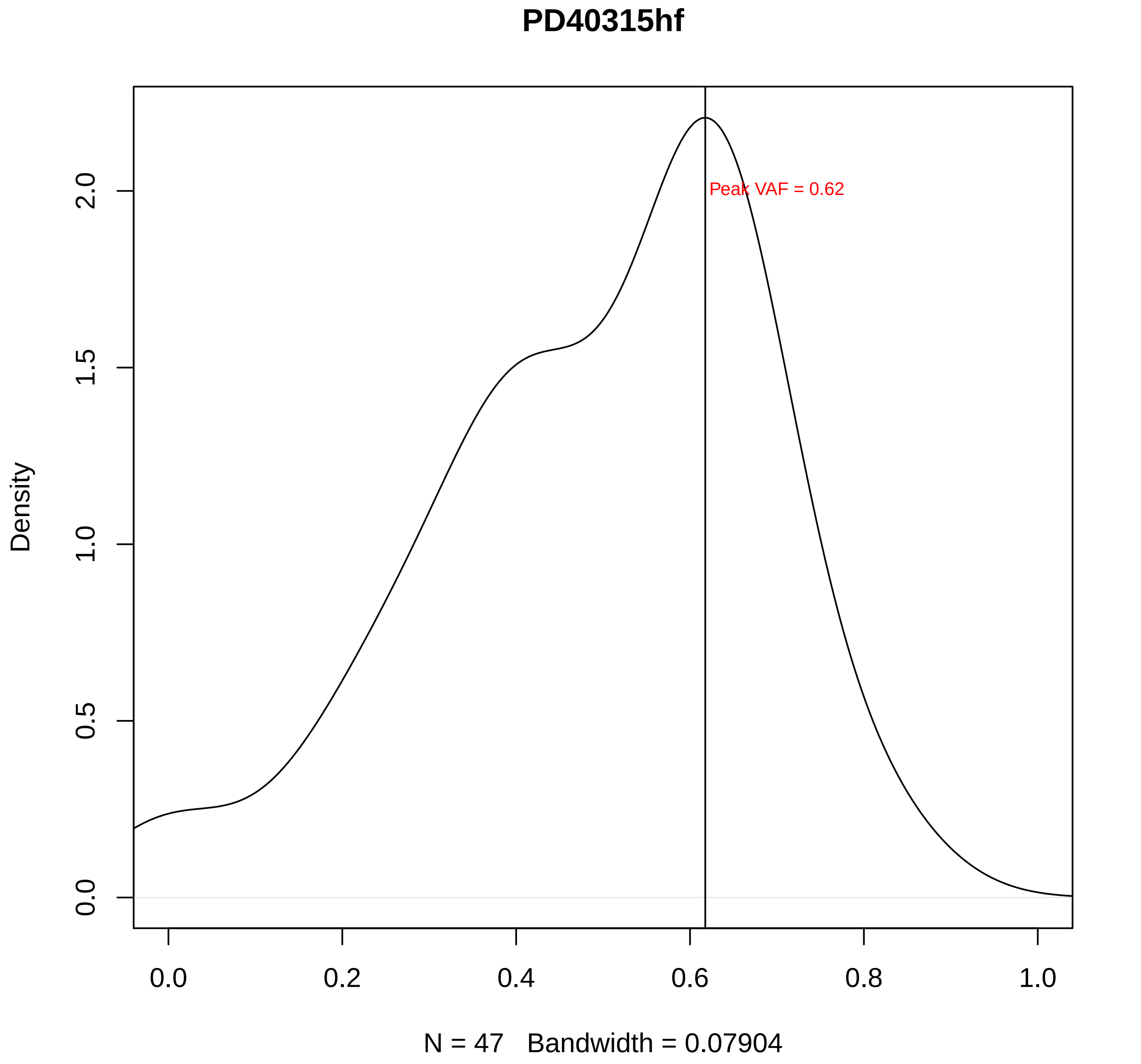

PD40315hf
Peak VAF = 0.62
Density
0.0
0.5
1.0
1.5
2.0
0.0
0.2
0.4
0.6
0.8
1.0
N = 47   Bandwidth = 0.07904

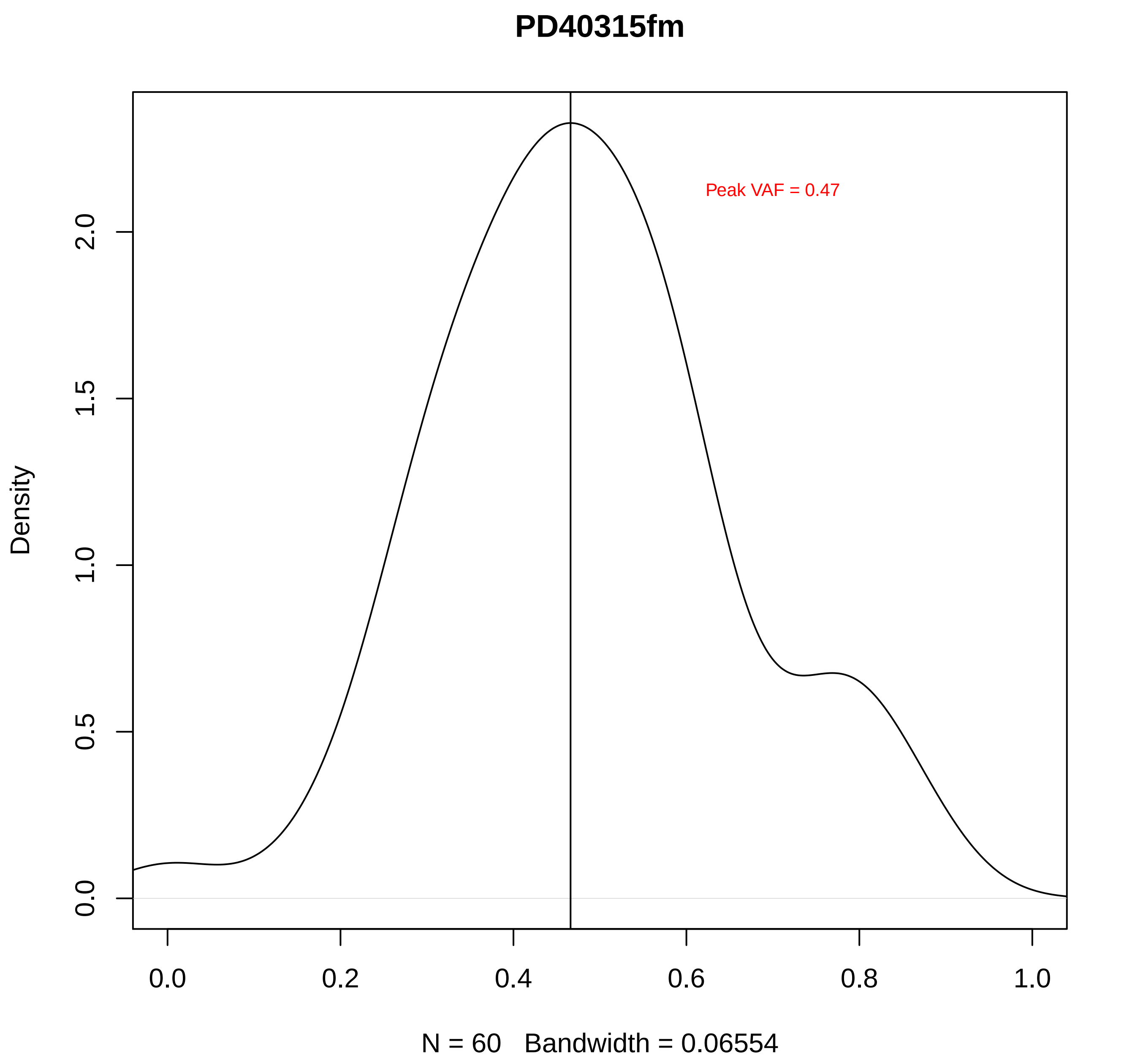
PD40315fm
Peak VAF = 0.47
Density
0.0
0.5
1.0
1.5
2.0
0.0
0.2
0.4
0.6
0.8
1.0
N = 60   Bandwidth = 0.06554

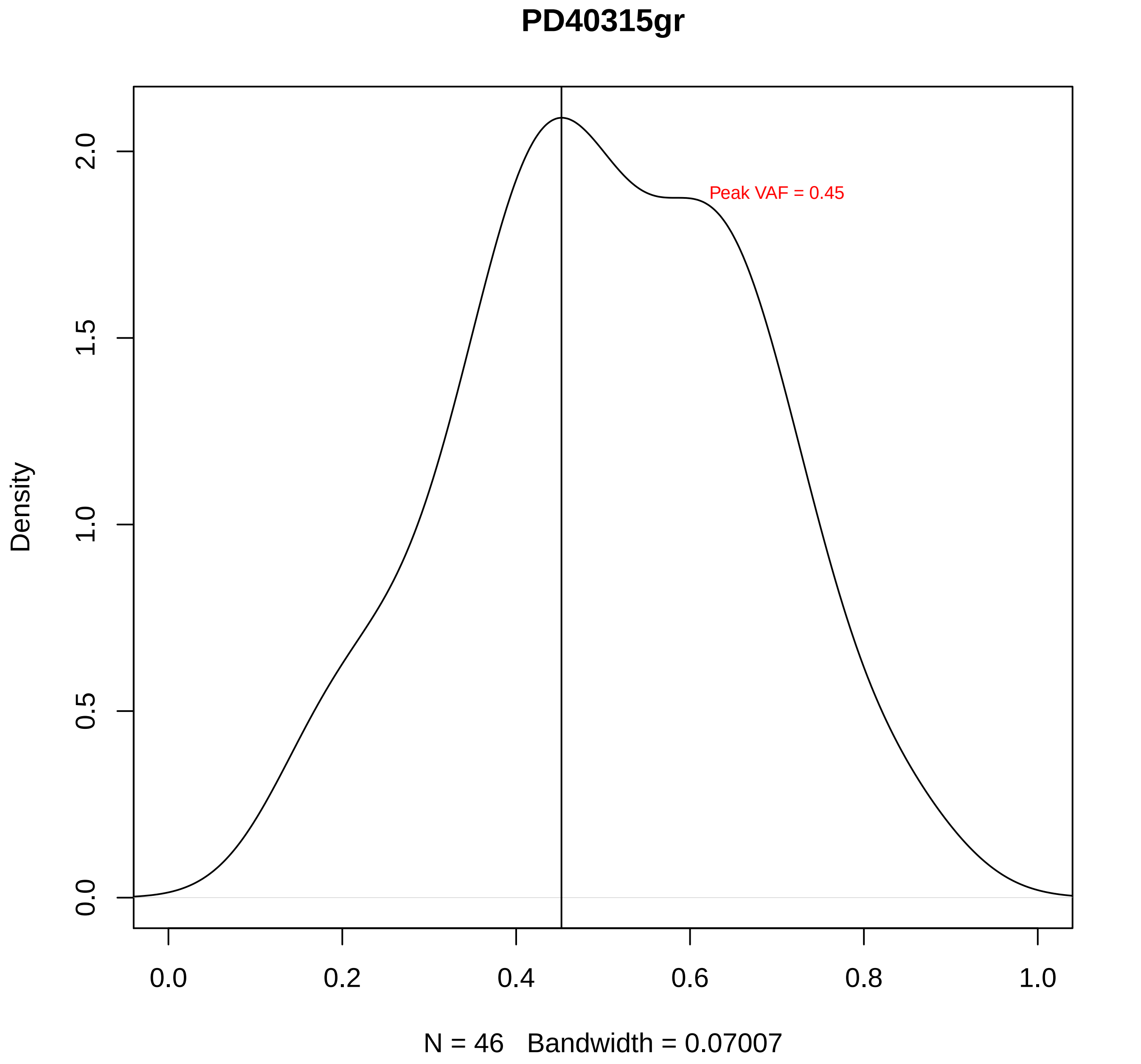
PD40315gr
Peak VAF = 0.45
Density
0.0
0.5
1.0
1.5
2.0
0.0
0.2
0.4
0.6
0.8
1.0
N = 46   Bandwidth = 0.07007

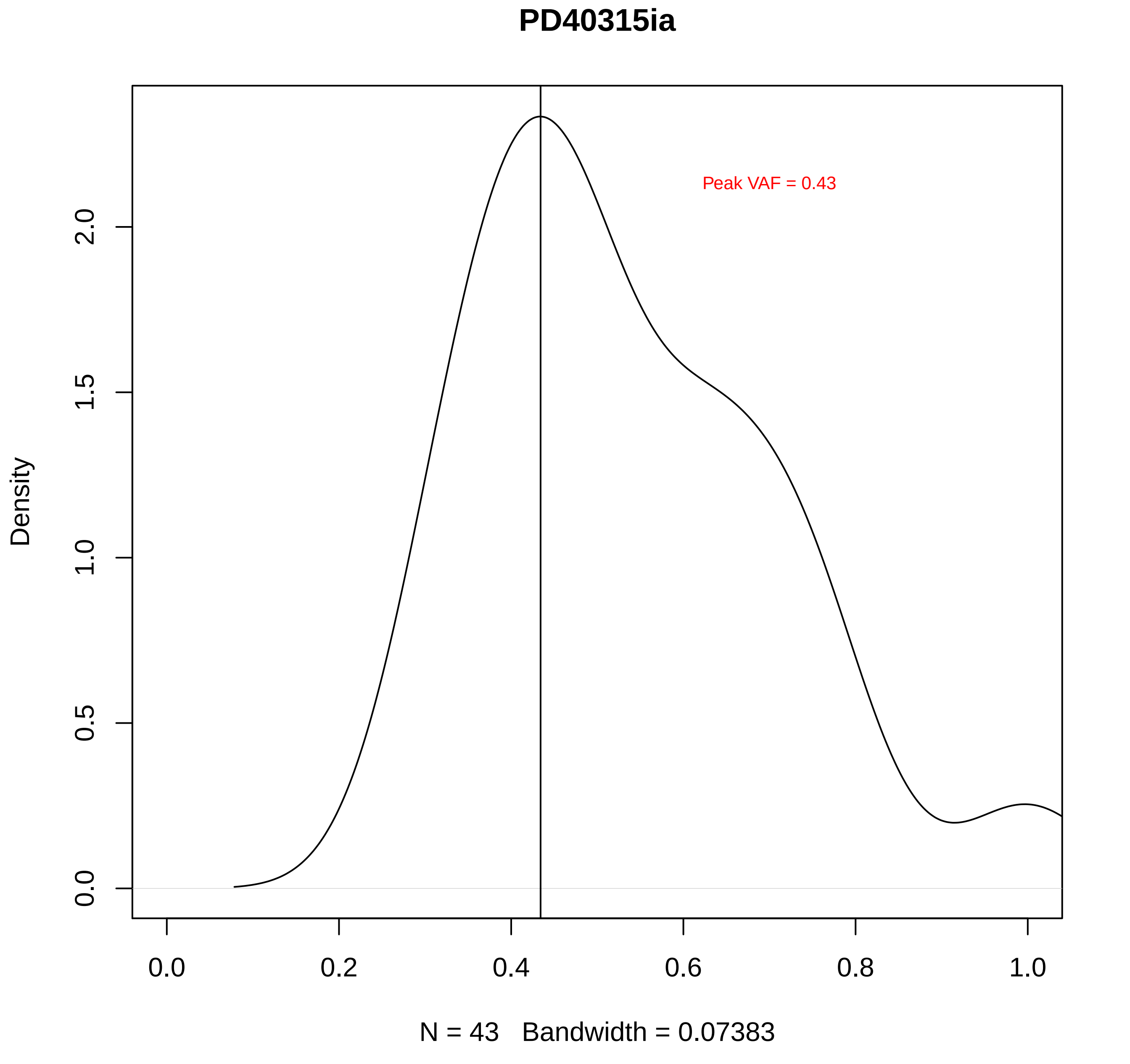
PD40315ia
Peak VAF = 0.43
Density
0.0
0.5
1.0
1.5
2.0
0.0
0.2
0.4
0.6
0.8
1.0
N = 43   Bandwidth = 0.07383

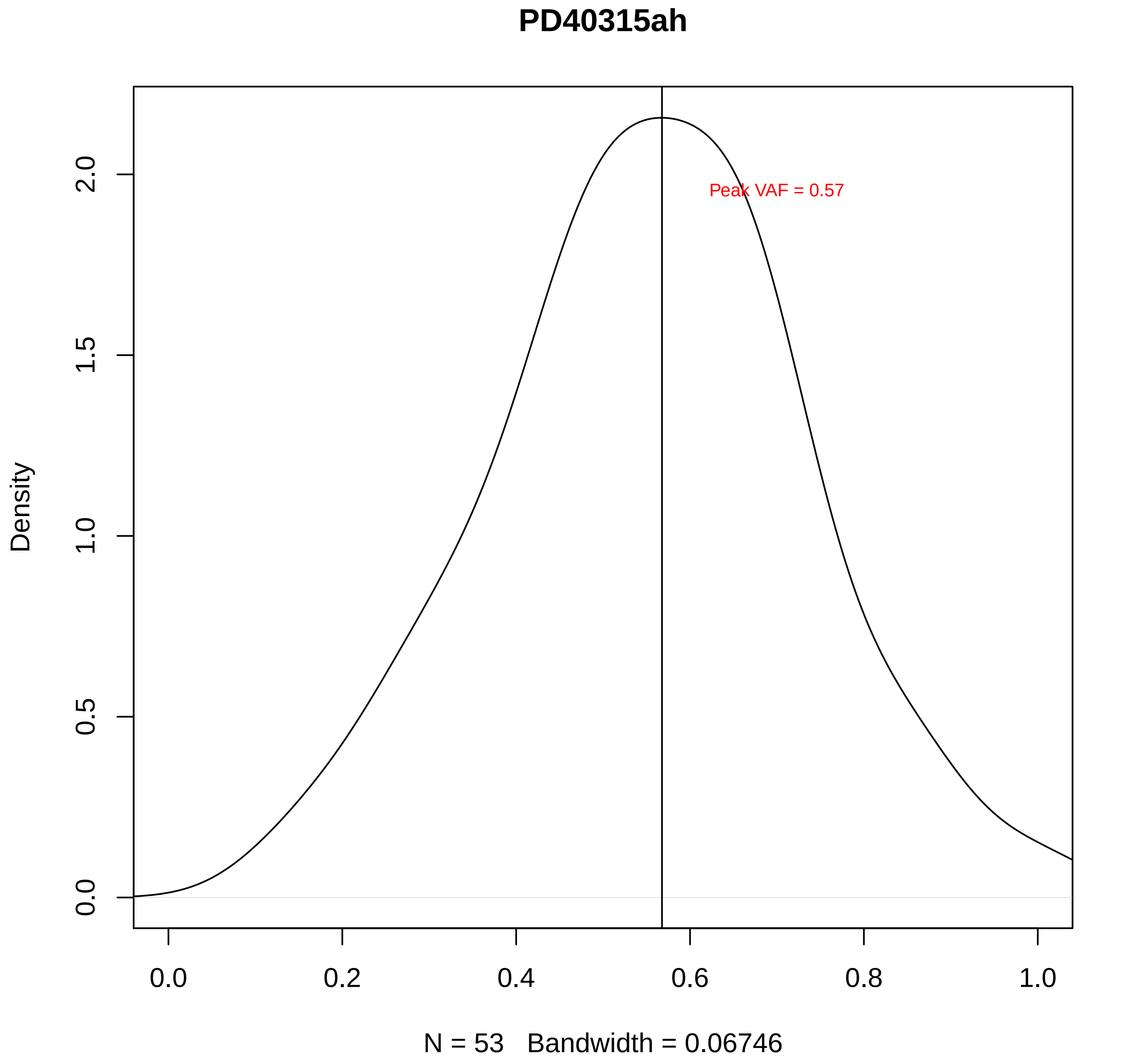
PD40315ah
Peak VAF = 0.57
Density
0.0
0.5
1.0
1.5
2.0
0.0
0.2
0.4
0.6
0.8
1.0
N = 53   Bandwidth = 0.06746

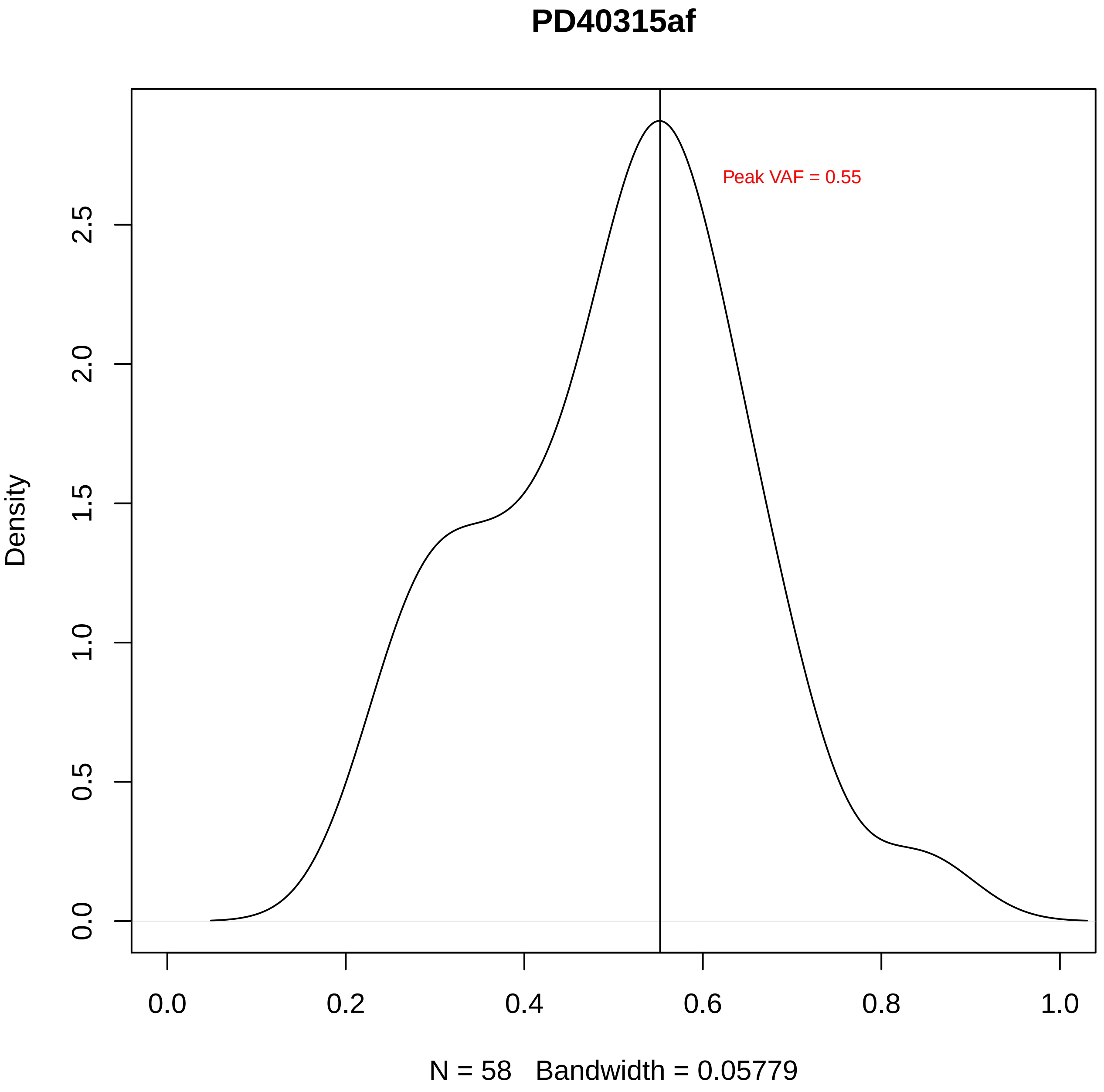
PD40315af
Peak VAF = 0.55
Density
0.0
0.5
1.0
1.5
2.0
2.5
0.0
0.2
0.4
0.6
0.8
1.0
N = 58   Bandwidth = 0.05779

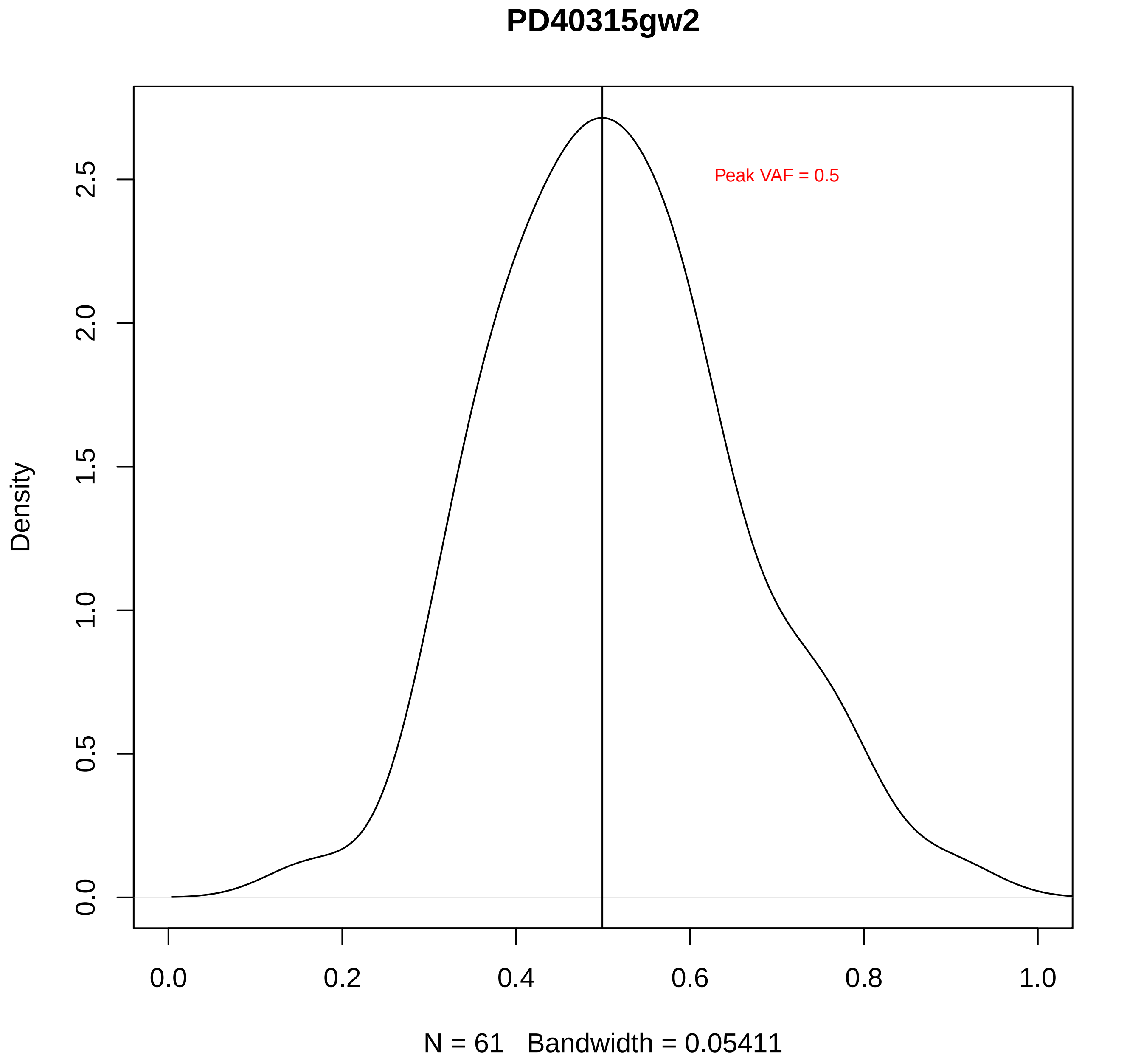
PD40315gw2
Peak VAF = 0.5
Density
0.0
0.5
1.0
1.5
2.0
2.5
0.0
0.2
0.4
0.6
0.8
1.0
N = 61   Bandwidth = 0.05411

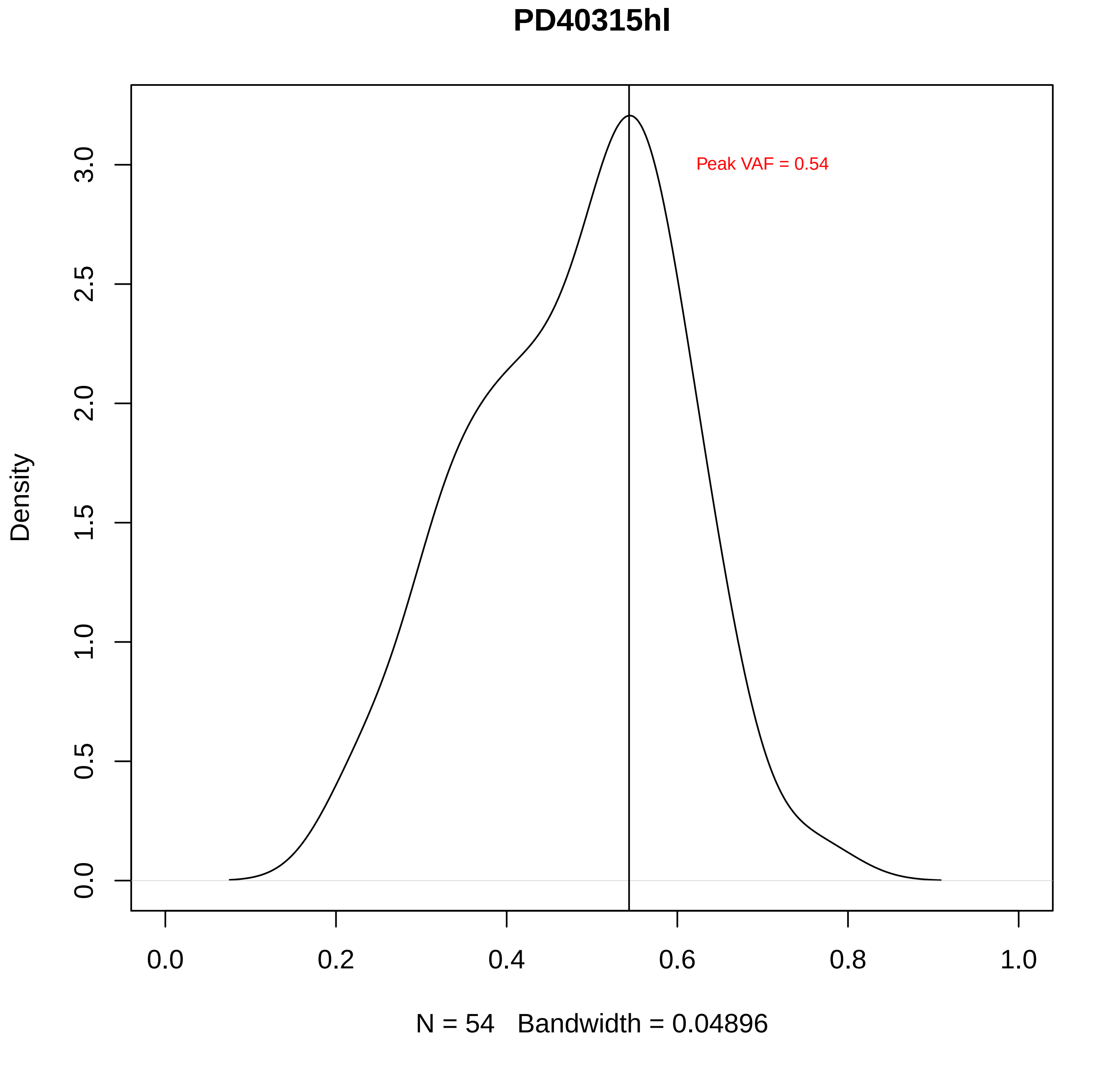
PD40315hl
Peak VAF = 0.54
Density
0.0
0.5
1.0
1.5
2.0
2.5
3.0
0.0
0.2
0.4
0.6
0.8
1.0
N = 54   Bandwidth = 0.04896

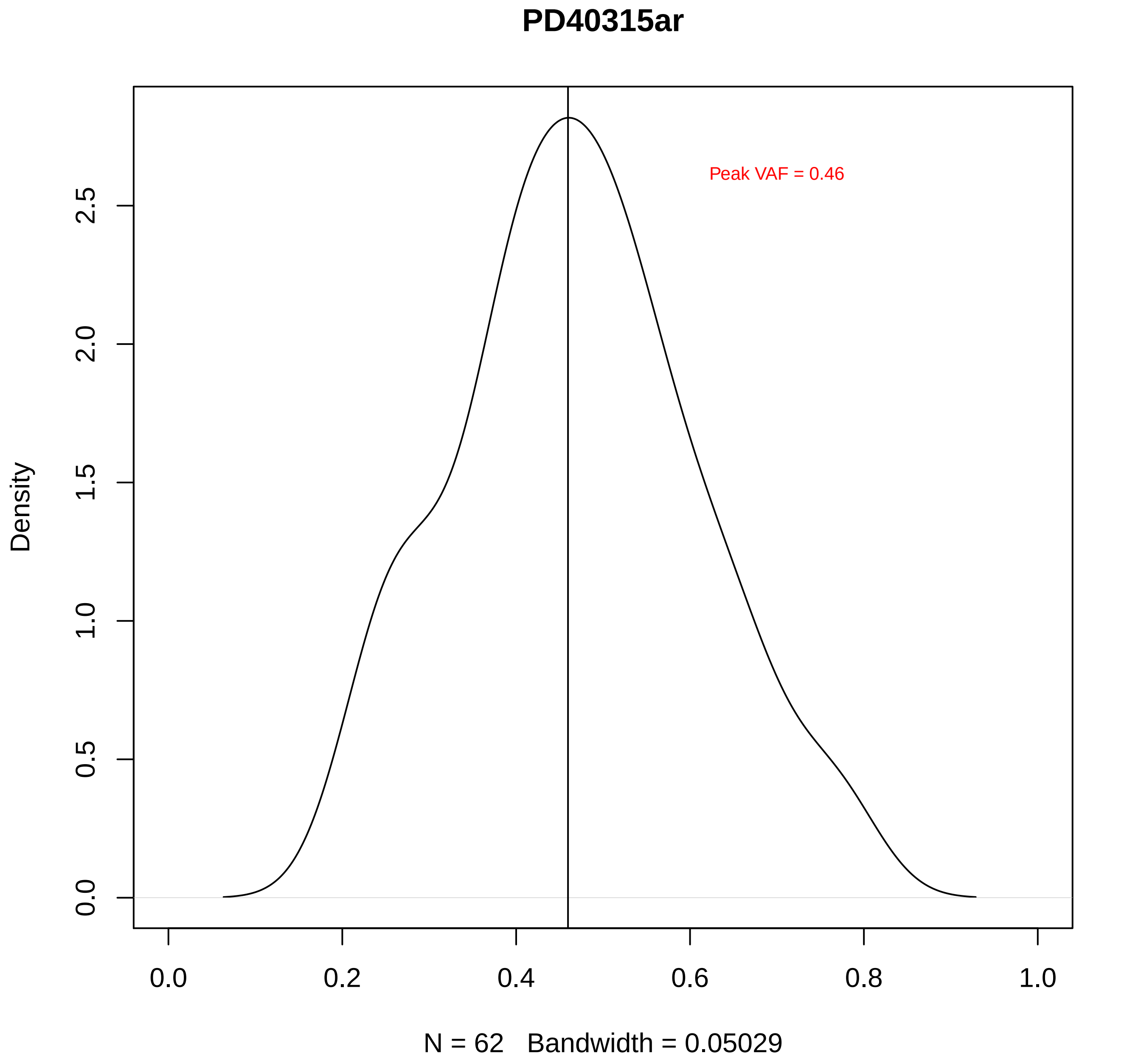
PD40315ar
Peak VAF = 0.46
Density
0.0
0.5
1.0
1.5
2.0
2.5
0.0
0.2
0.4
0.6
0.8
1.0
N = 62   Bandwidth = 0.05029

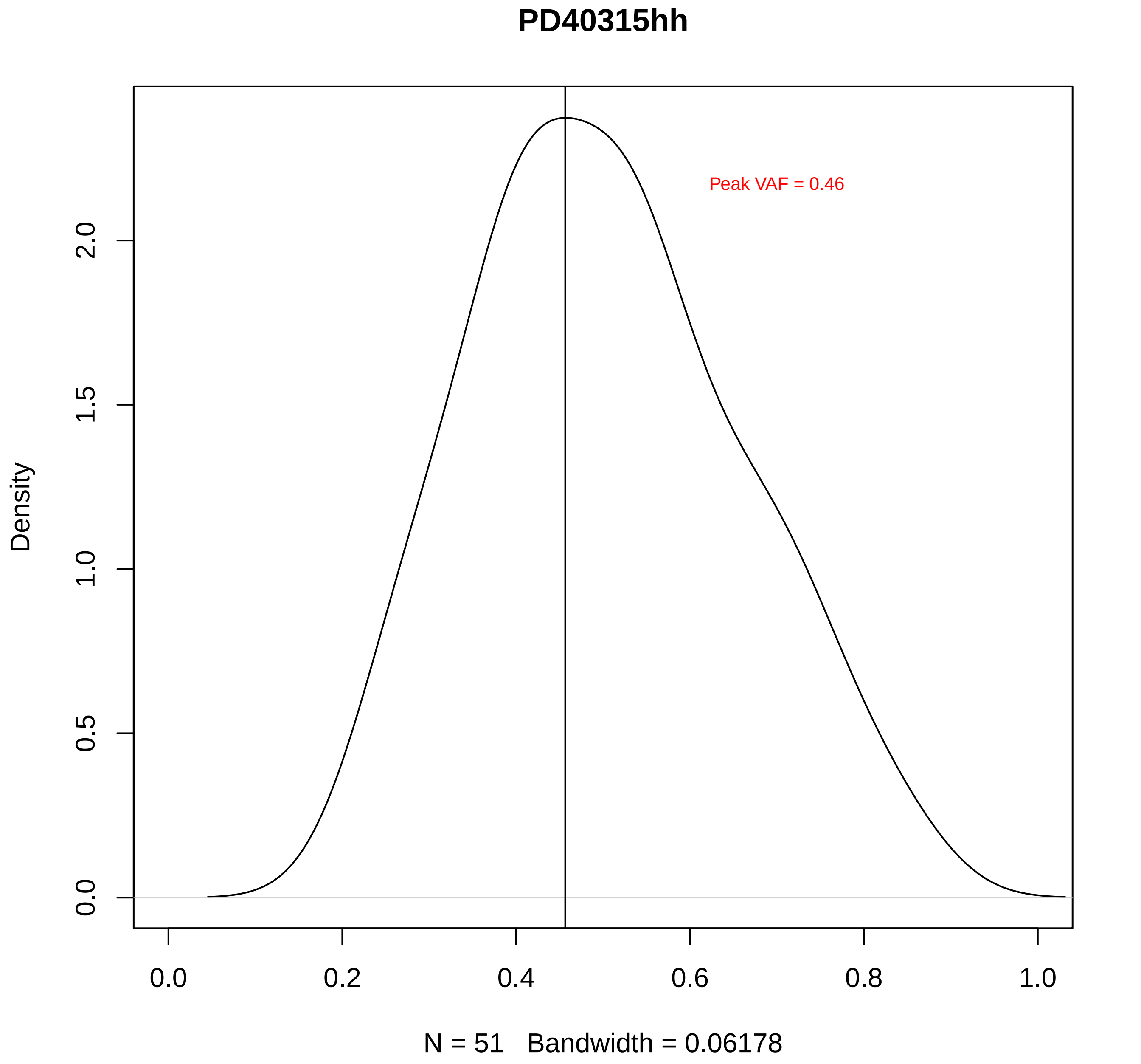

PD40315hh
Peak VAF = 0.46
Density
0.0
0.5
1.0
1.5
2.0
0.0
0.2
0.4
0.6
0.8
1.0
N = 51 Bandwidth = 0.06178

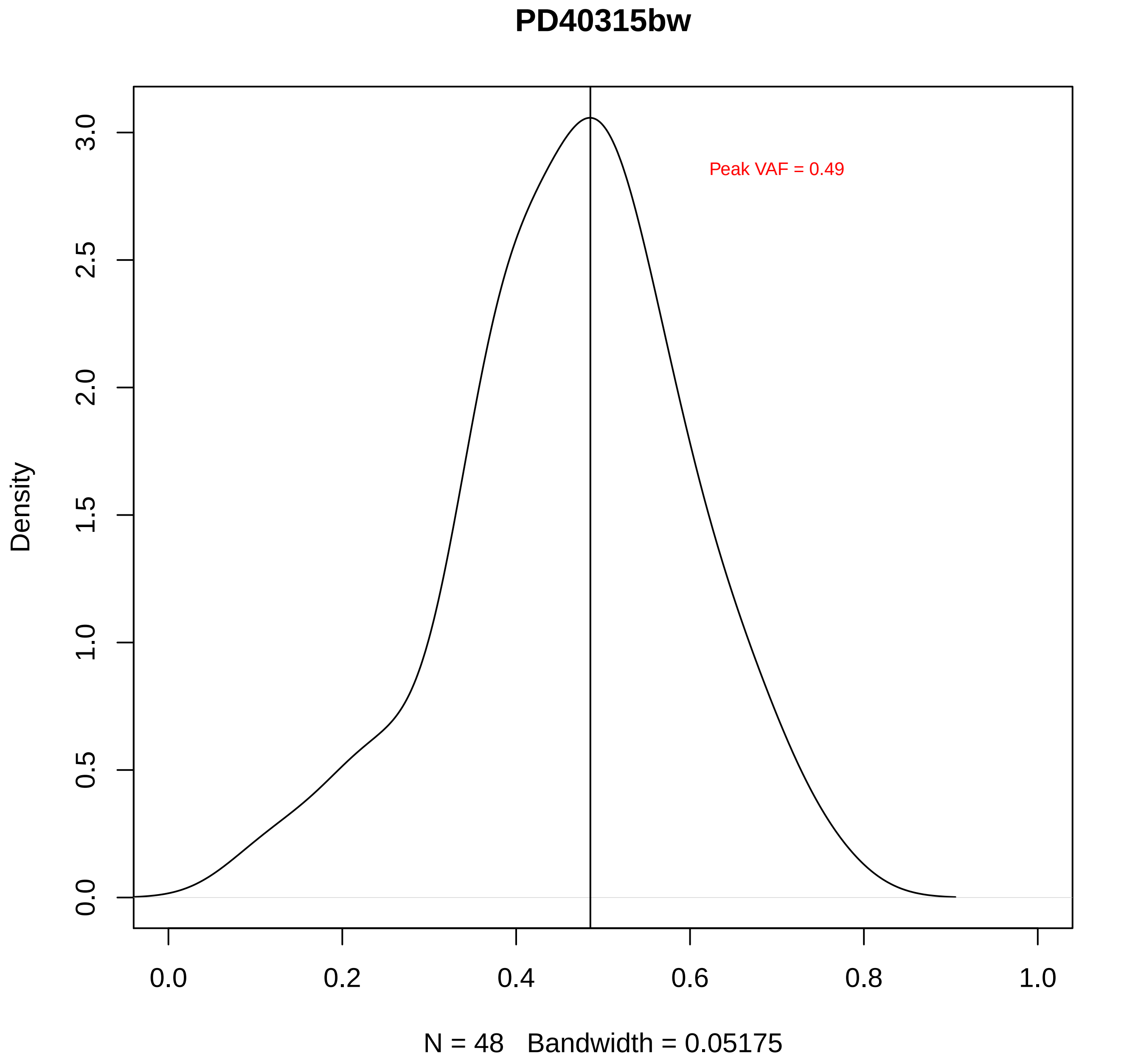
PD40315bw
Peak VAF = 0.49
Density
3.0
2.5
2.0
1.5
1.0
0.5
0.0
0.0
0.2
0.4
0.6
0.8
1.0
N = 48   Bandwidth = 0.05175

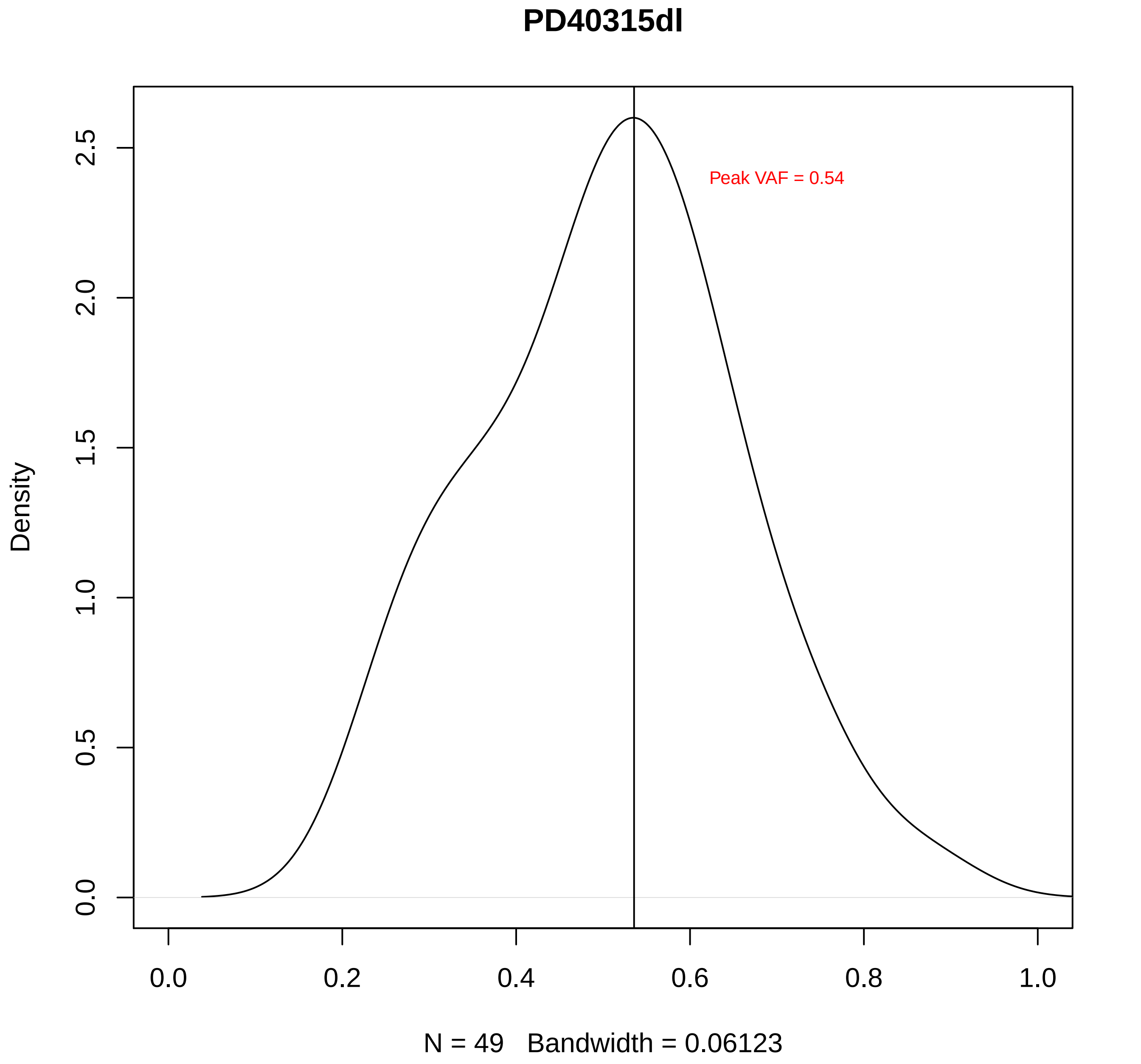

PD40315dl
Peak VAF = 0.54
Density
0.0
0.5
1.0
1.5
2.0
2.5
0.0
0.2
0.4
0.6
0.8
1.0
N = 49 Bandwidth = 0.06123

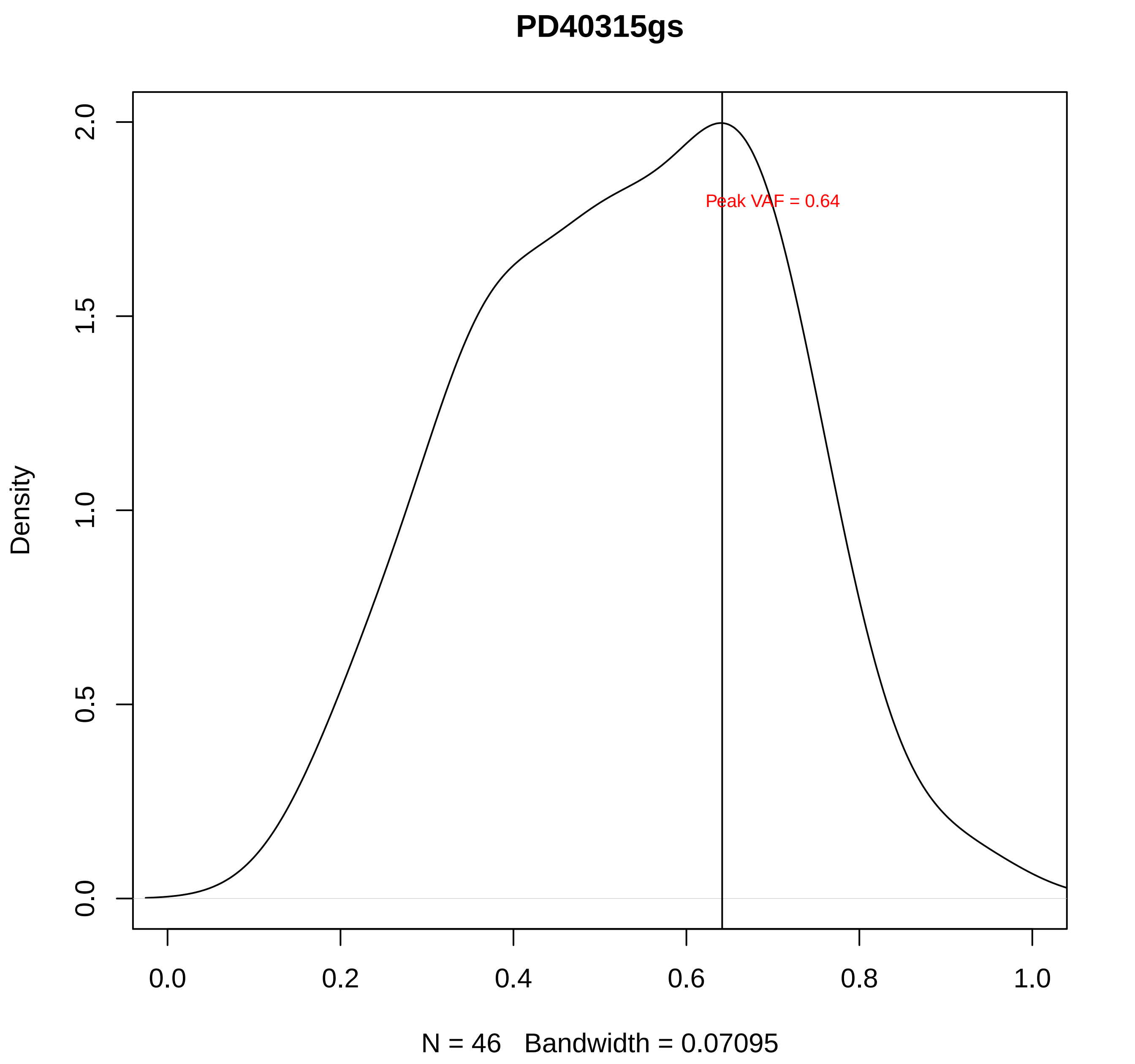

PD40315gs
Peak VAF = 0.64
Density
0.0
0.5
1.0
1.5
2.0
0.0
0.2
0.4
0.6
0.8
1.0
N = 46 Bandwidth = 0.07095

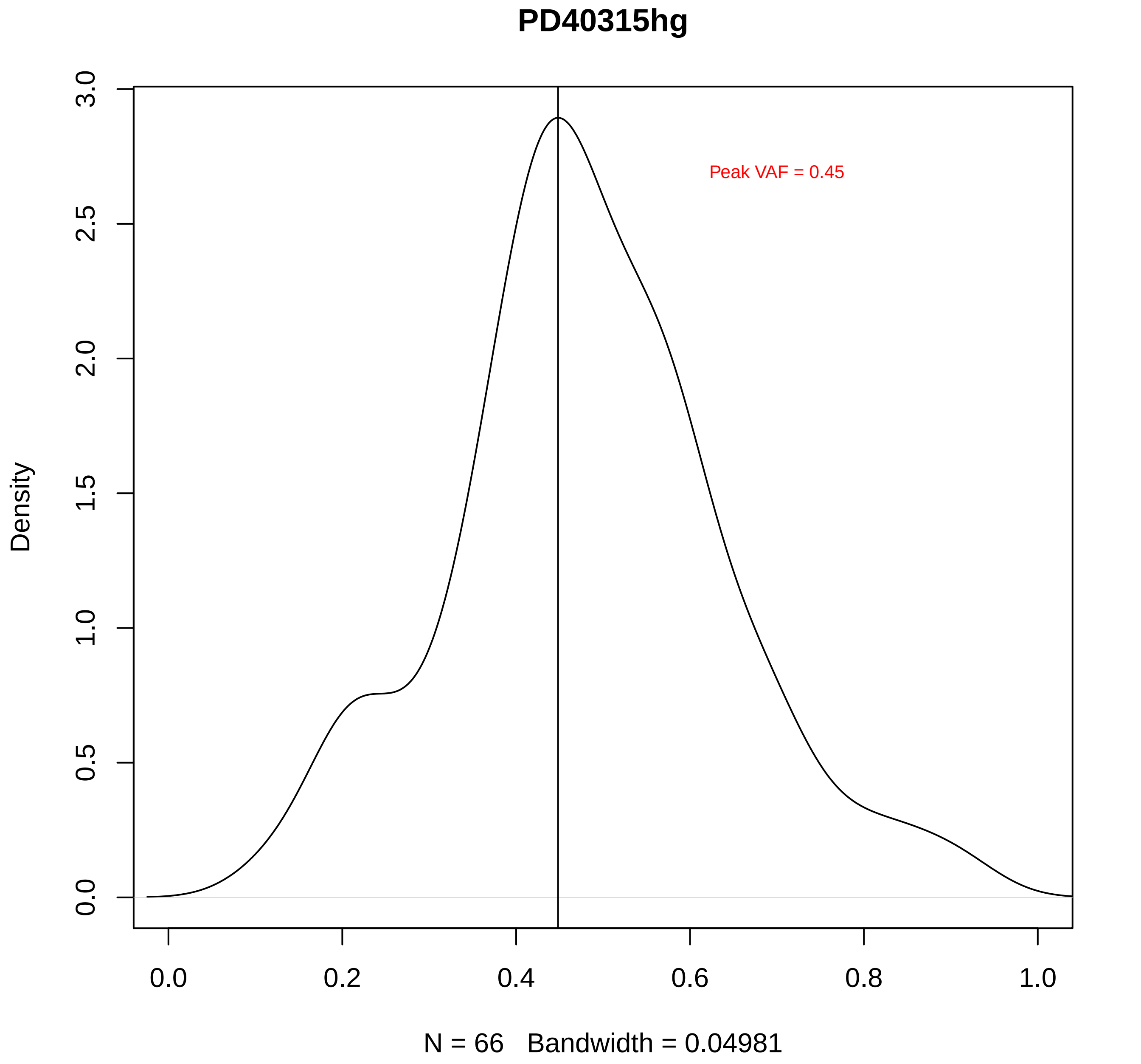

PD40315hg
Peak VAF = 0.45
Density
0.0
0.5
1.0
1.5
2.0
2.5
3.0
0.0
0.2
0.4
0.6
0.8
1.0
N = 66   Bandwidth = 0.04981

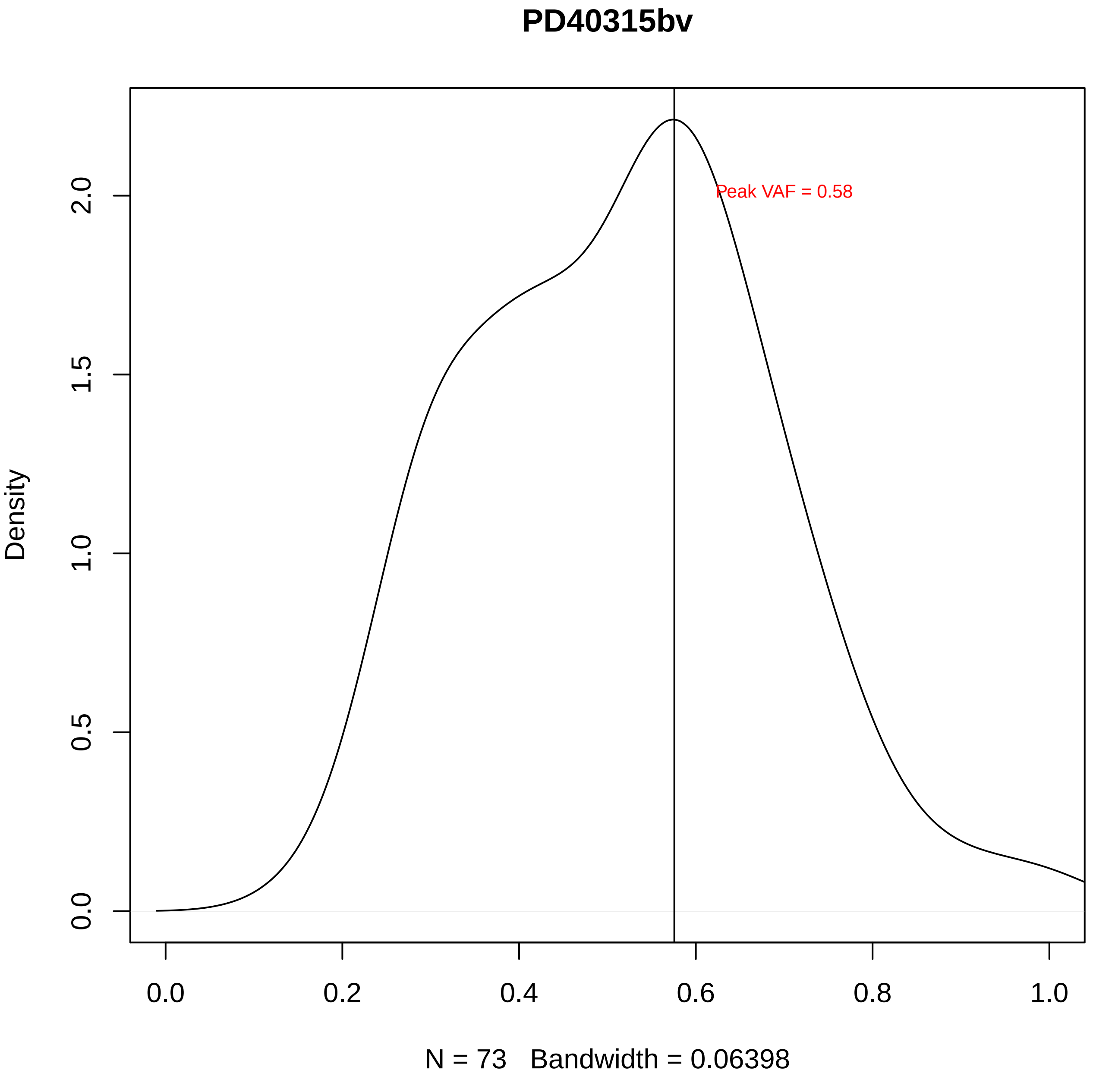
PD40315bv
Peak VAF = 0.58
Density
0.0
0.5
1.0
1.5
2.0
0.0
0.2
0.4
0.6
0.8
1.0
N = 73   Bandwidth = 0.06398

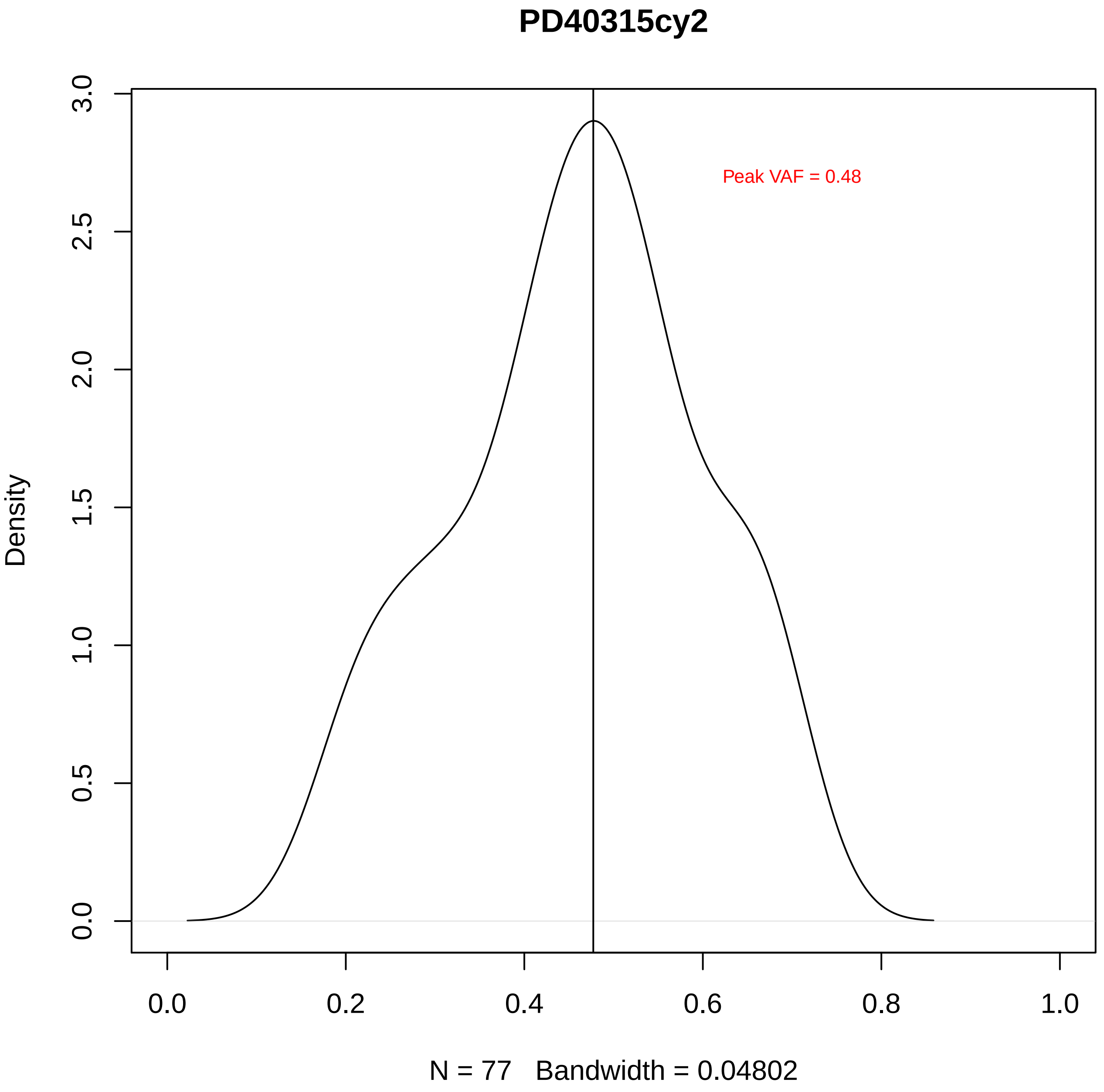

PD40315cy2
Peak VAF = 0.48
Density
0.0
0.5
1.0
1.5
2.0
2.5
3.0
0.0
0.2
0.4
0.6
0.8
1.0
N = 77   Bandwidth = 0.04802

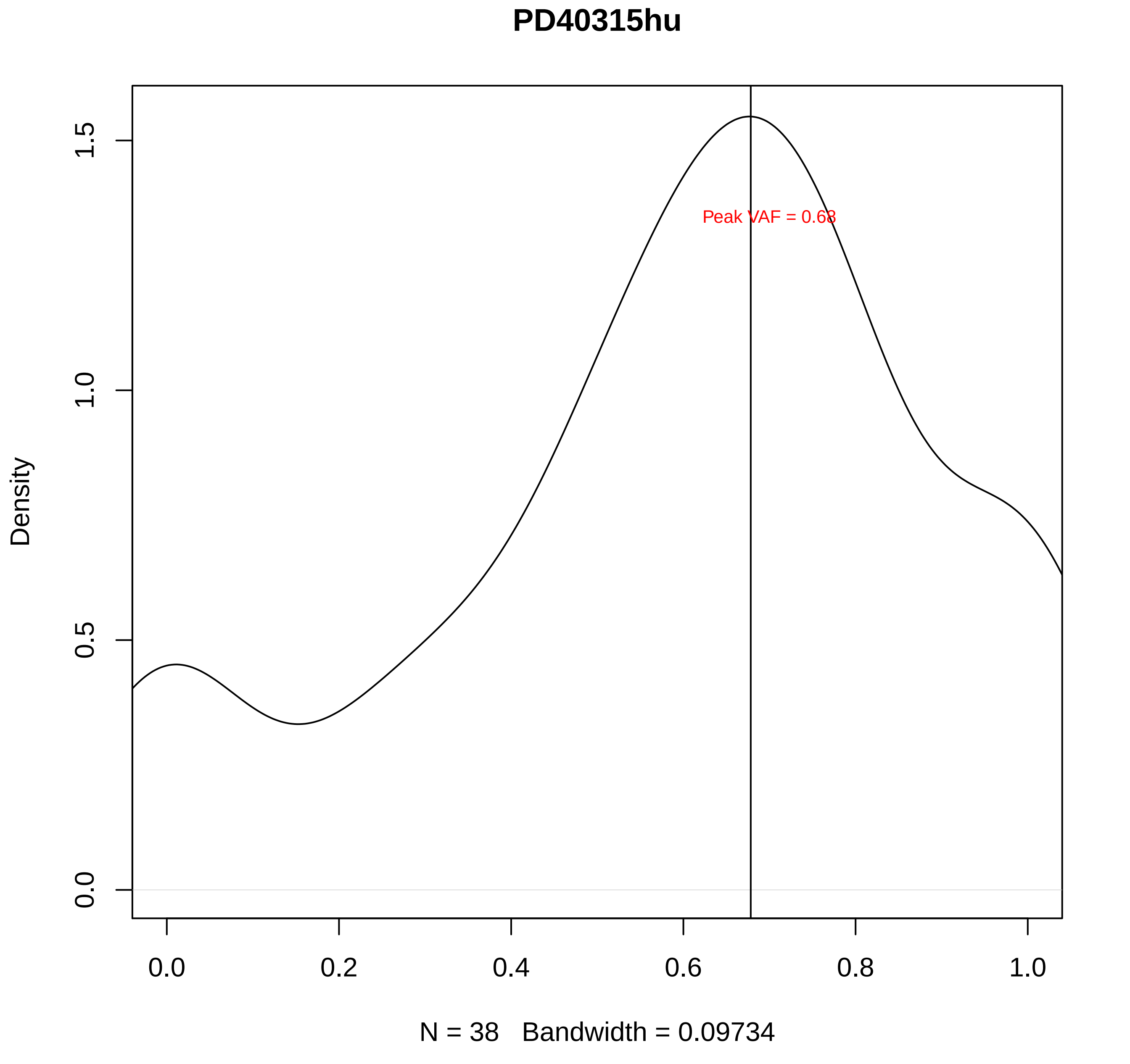
PD40315hu
Peak VAF = 0.68
Density
0.0
0.5
1.0
1.5
0.0
0.2
0.4
0.6
0.8
1.0
N = 38   Bandwidth = 0.09734

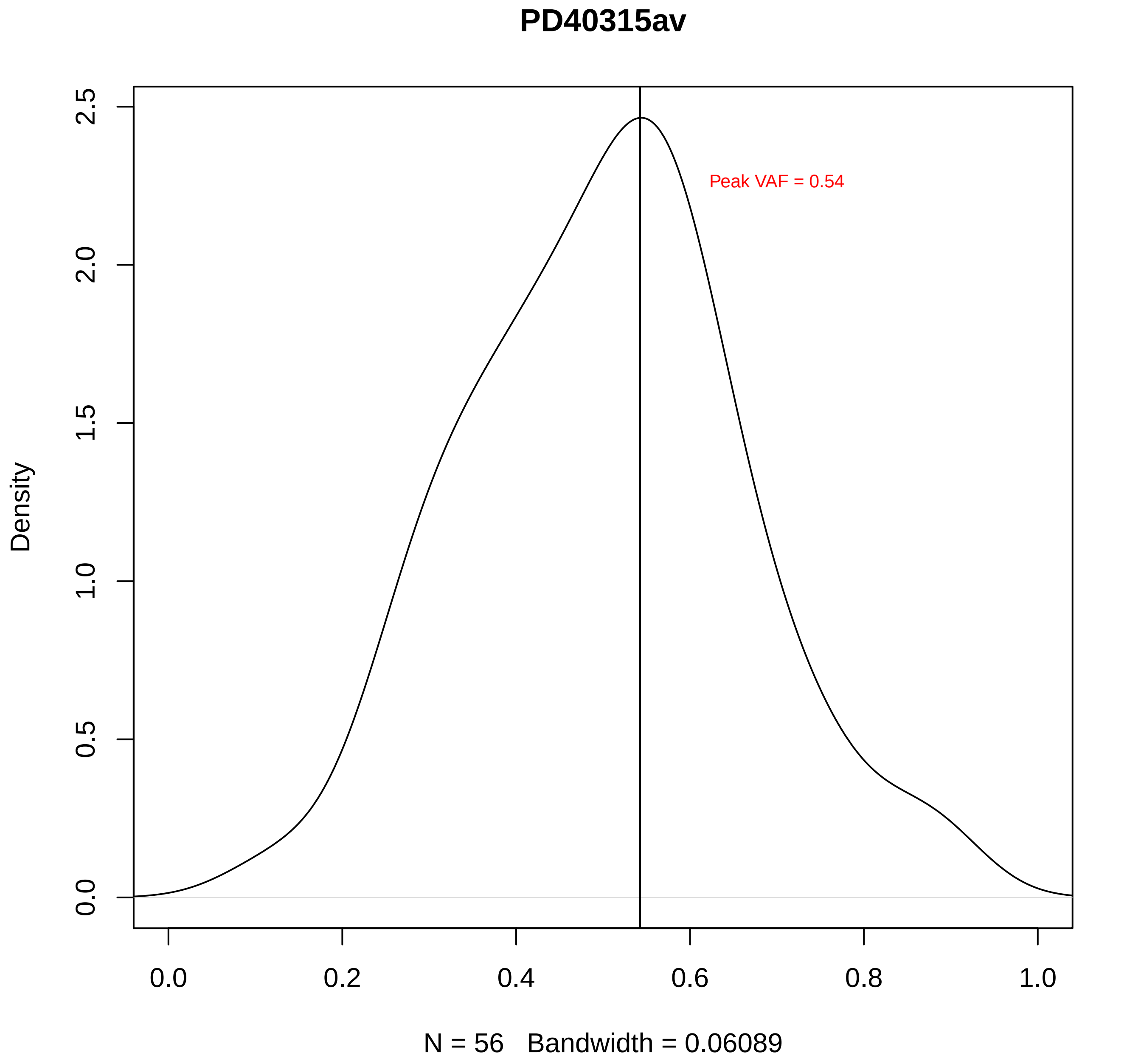
PD40315av
Peak VAF = 0.54
Density
N = 56 Bandwidth = 0.06089
0.0
0.5
1.0
1.5
2.0
2.5
0.0
0.2
0.4
0.6
0.8
1.0

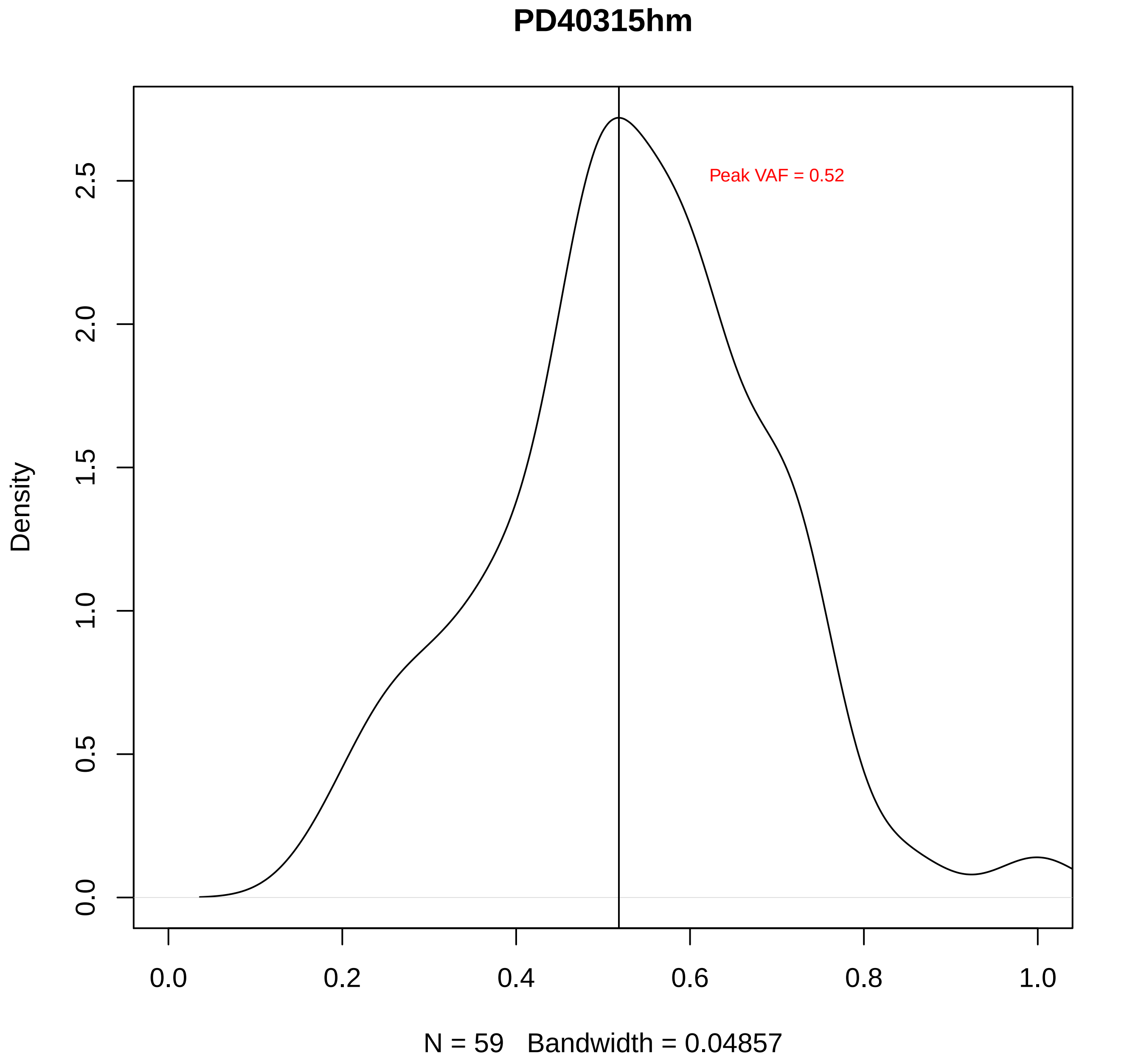
PD40315hm
Peak VAF = 0.52
Density
0.0
0.5
1.0
1.5
2.0
2.5
0.0
0.2
0.4
0.6
0.8
1.0
N = 59   Bandwidth = 0.04857

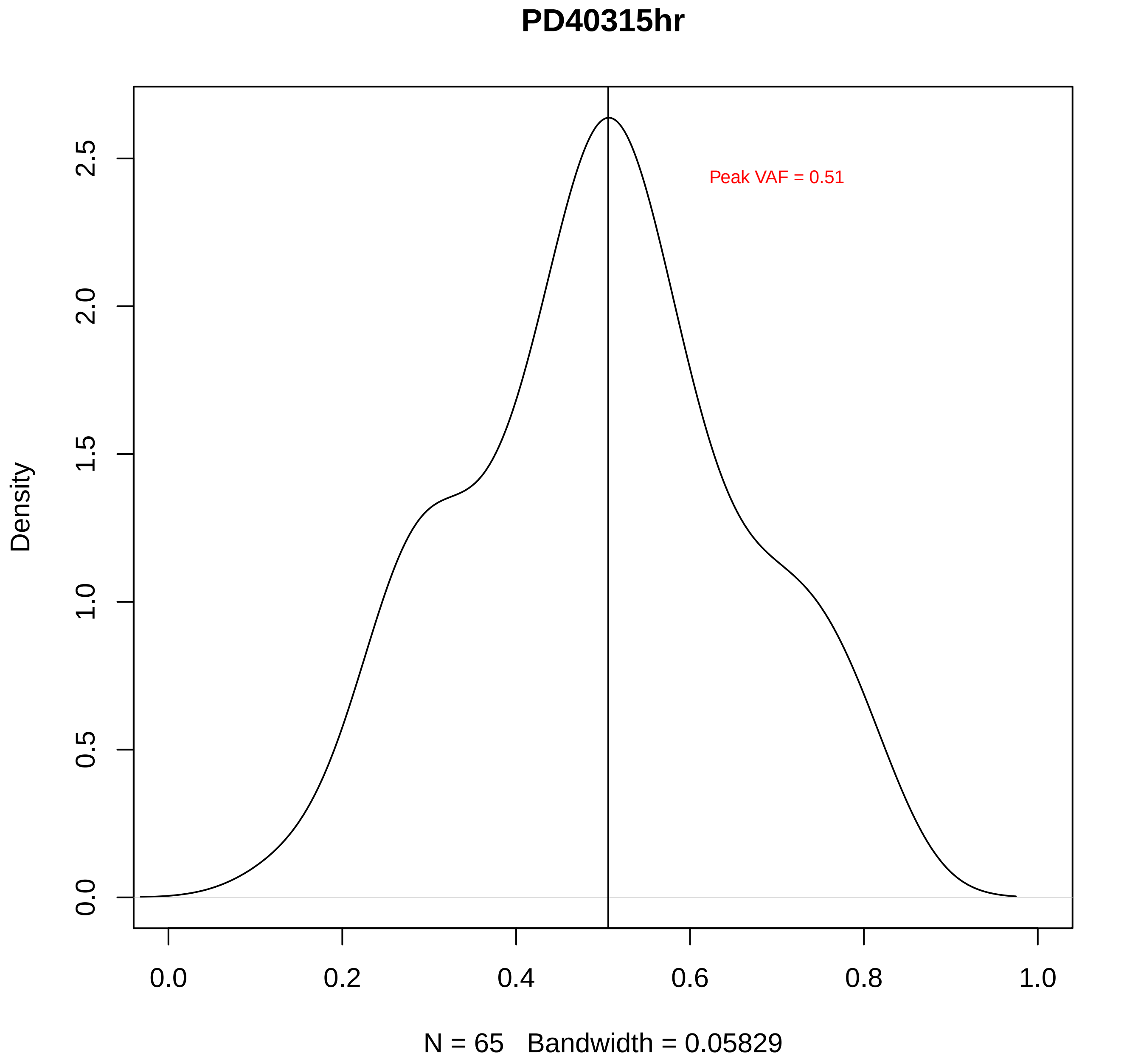
PD40315hr
Peak VAF = 0.51
Density
0.0
0.5
1.0
1.5
2.0
2.5
0.0
0.2
0.4
0.6
0.8
1.0
N = 65   Bandwidth = 0.05829

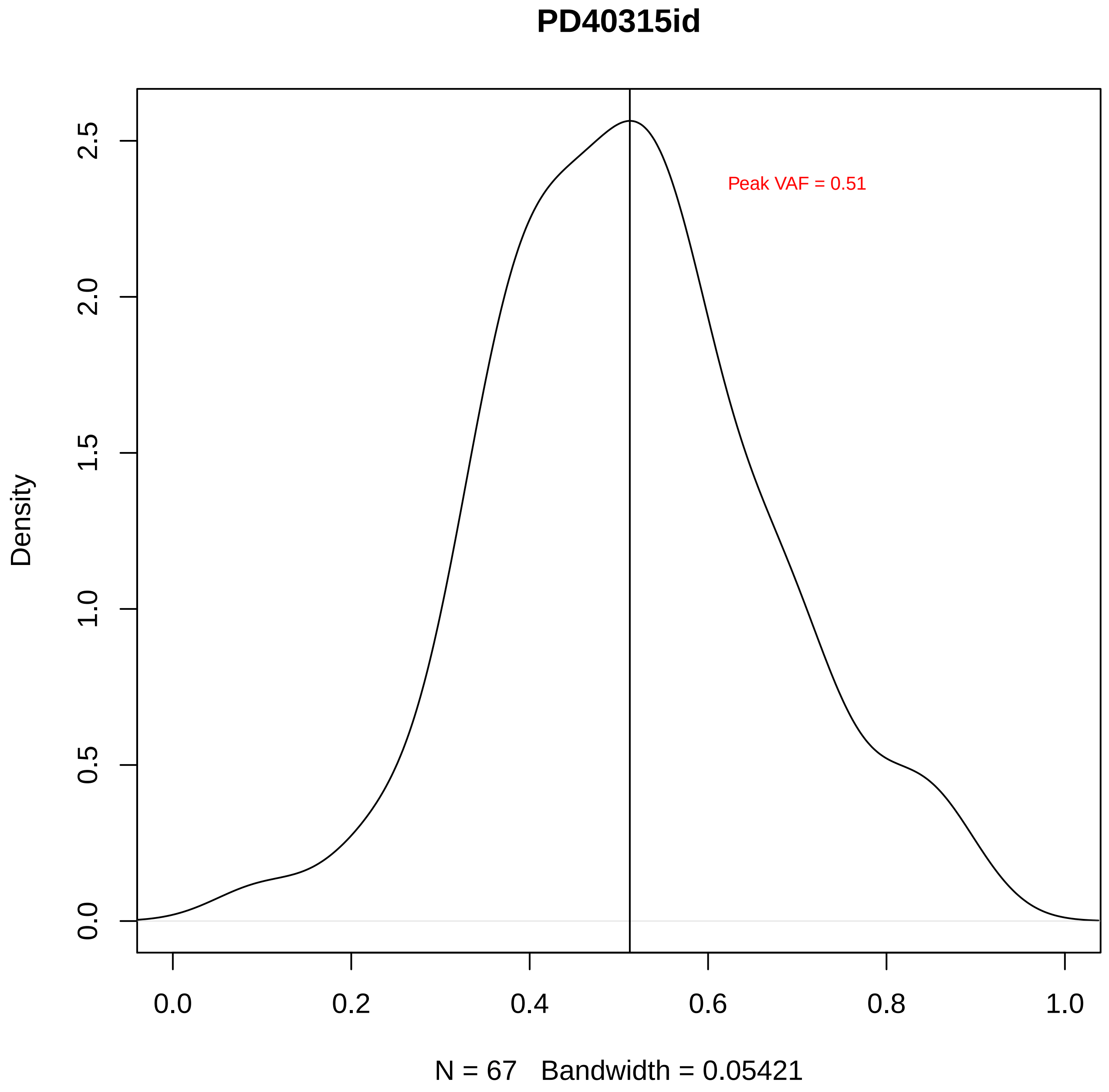

PD40315id
Peak VAF = 0.51
Density
0.0
0.5
1.0
1.5
2.0
2.5
0.0
0.2
0.4
0.6
0.8
1.0
N = 67   Bandwidth = 0.05421

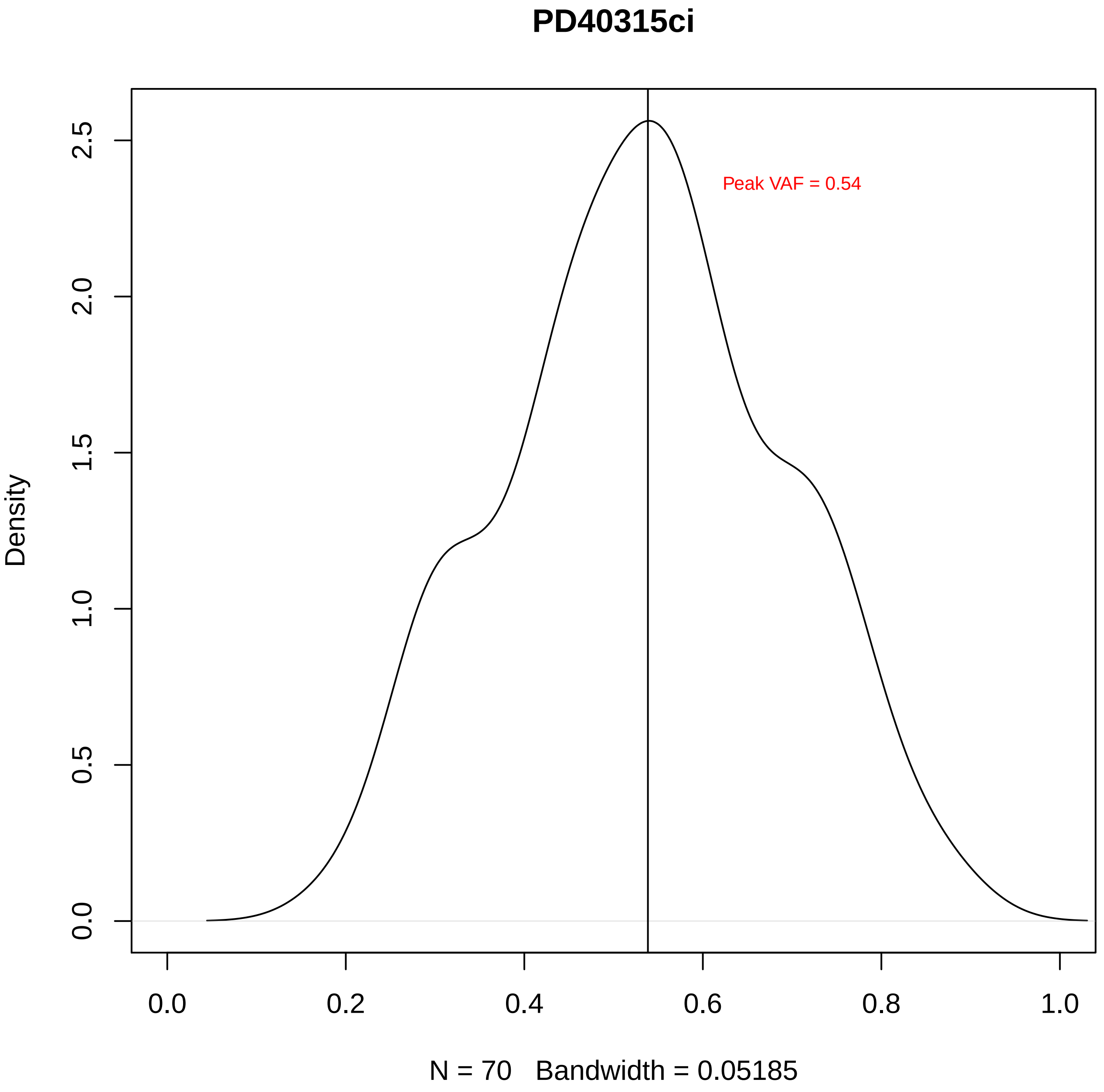
PD40315ci
Peak VAF = 0.54
Density
0.0
0.5
1.0
1.5
2.0
2.5
0.0
0.2
0.4
0.6
0.8
1.0
N = 70 Bandwidth = 0.05185

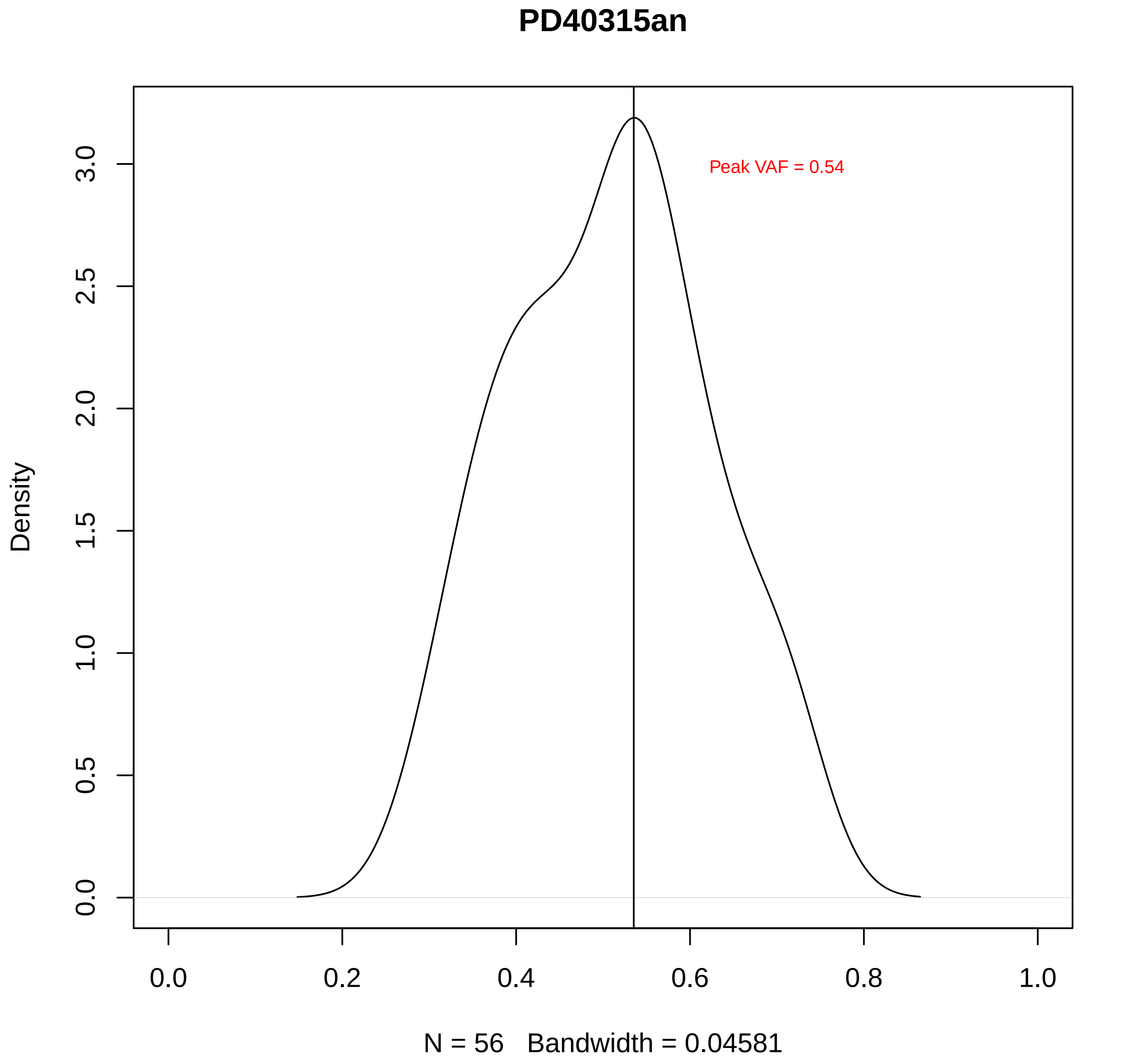
PD40315an
Peak VAF = 0.54
Density
0.0
0.5
1.0
1.5
2.0
2.5
3.0
0.0
0.2
0.4
0.6
0.8
1.0
N = 56   Bandwidth = 0.04581

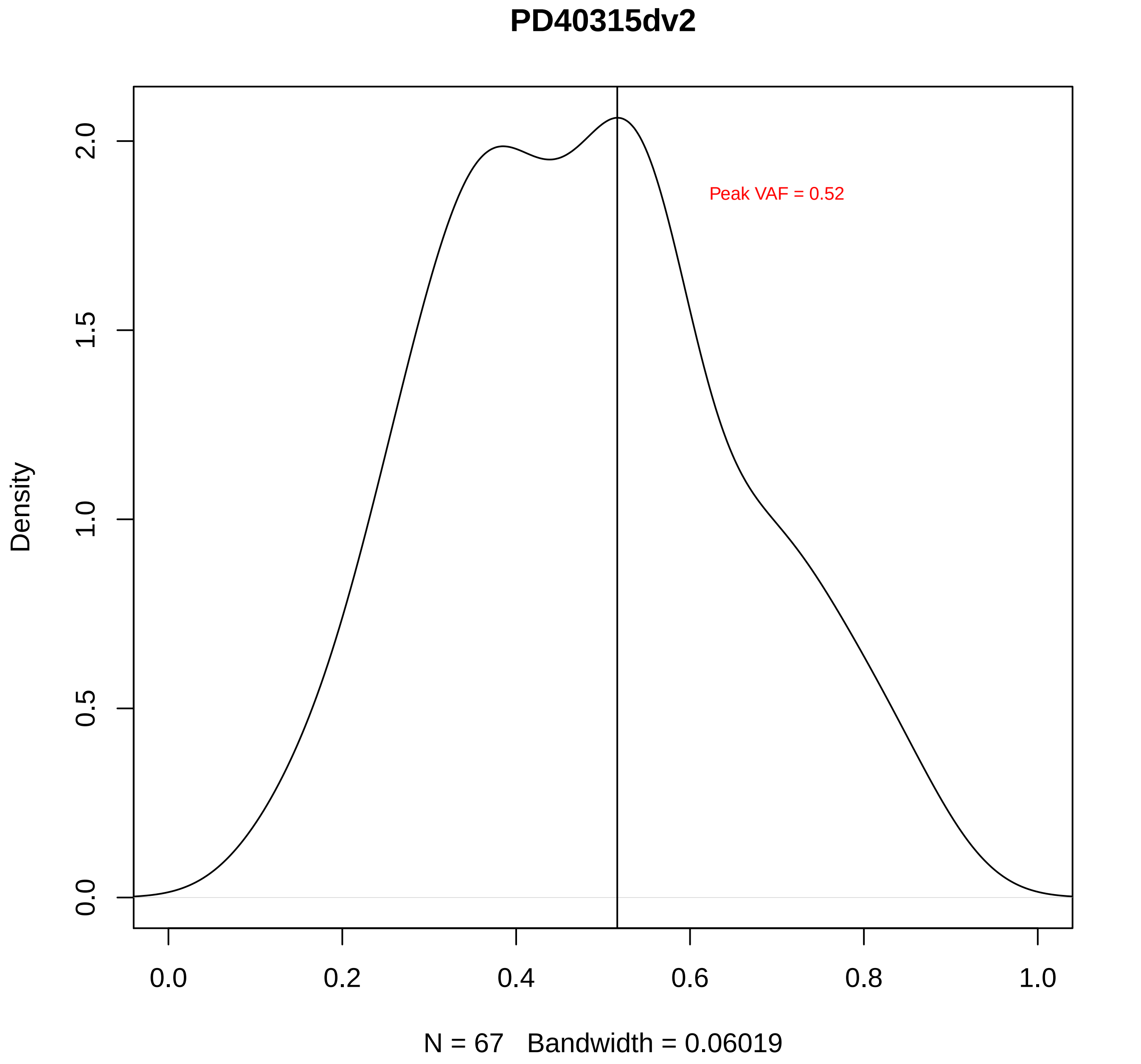
PD40315dv2
Peak VAF = 0.52
Density
0.0
0.5
1.0
1.5
2.0
0.0
0.2
0.4
0.6
0.8
1.0
N = 67   Bandwidth = 0.06019

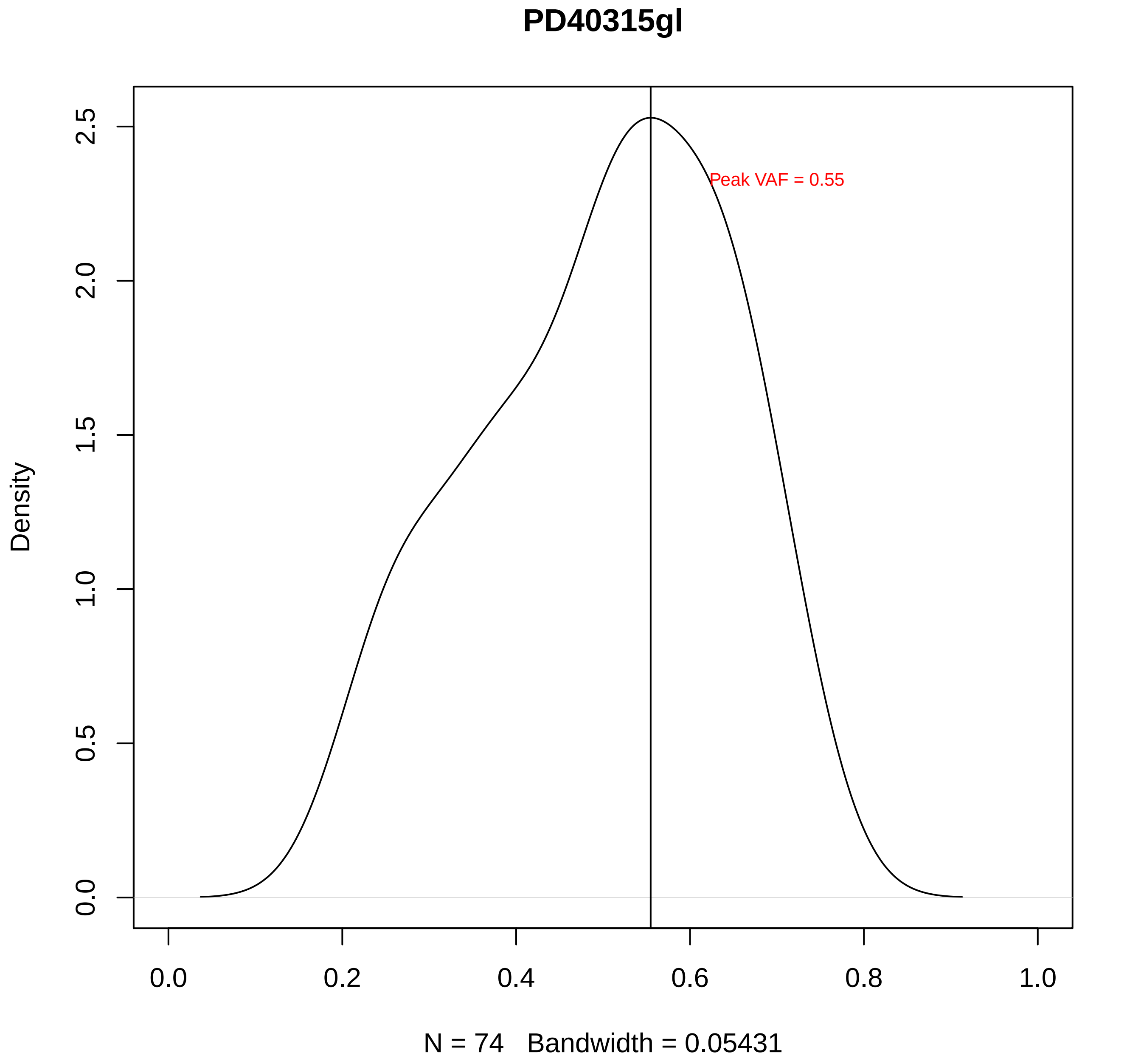

PD40315gl
Peak VAF = 0.55
Density
2.5
2.0
1.5
1.0
0.5
0.0
0.0
0.2
0.4
0.6
0.8
1.0
N = 74   Bandwidth = 0.05431

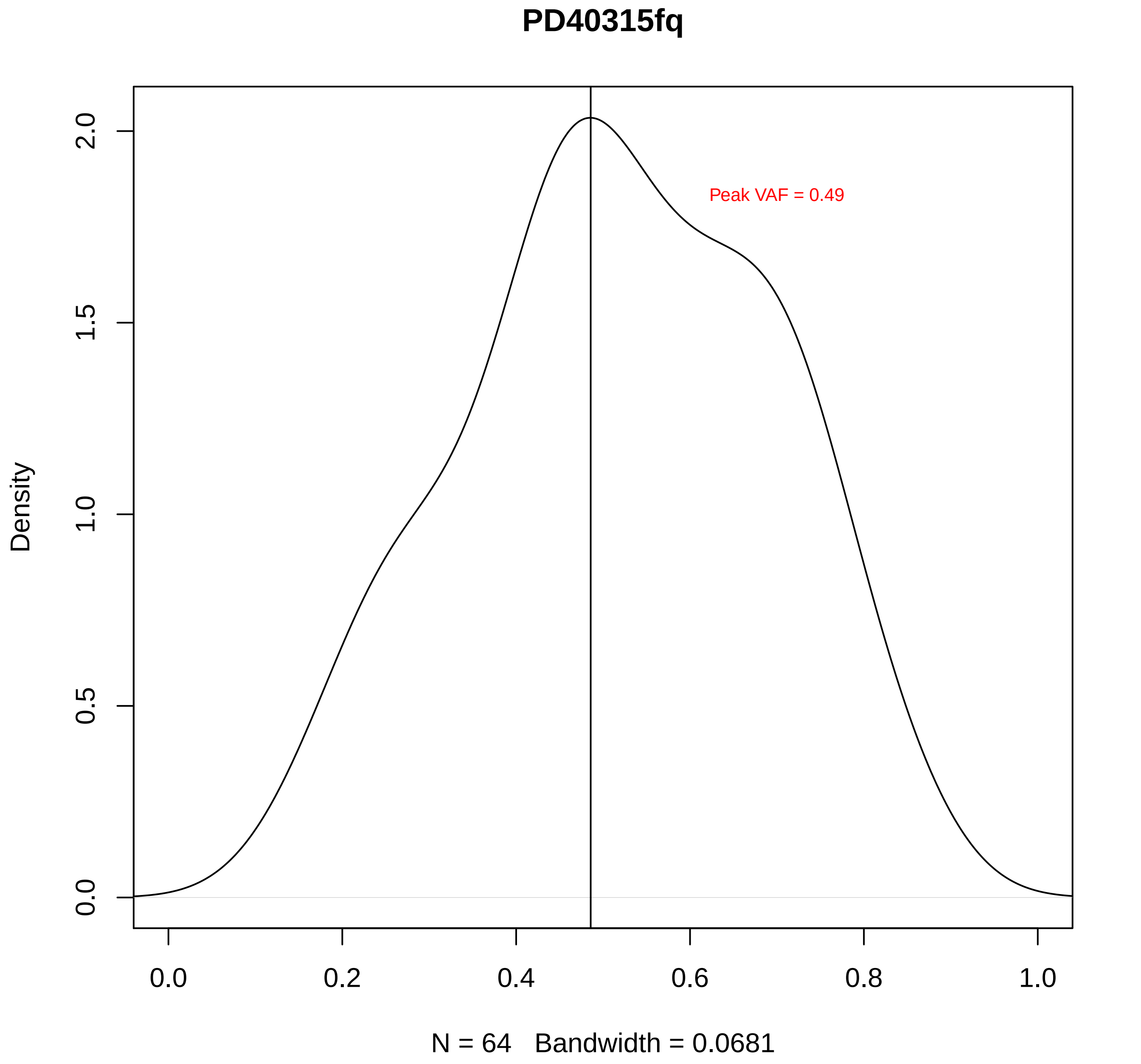
PD40315fq
Peak VAF = 0.49
Density
0.0
0.5
1.0
1.5
2.0
0.0
0.2
0.4
0.6
0.8
1.0
N = 64   Bandwidth = 0.0681

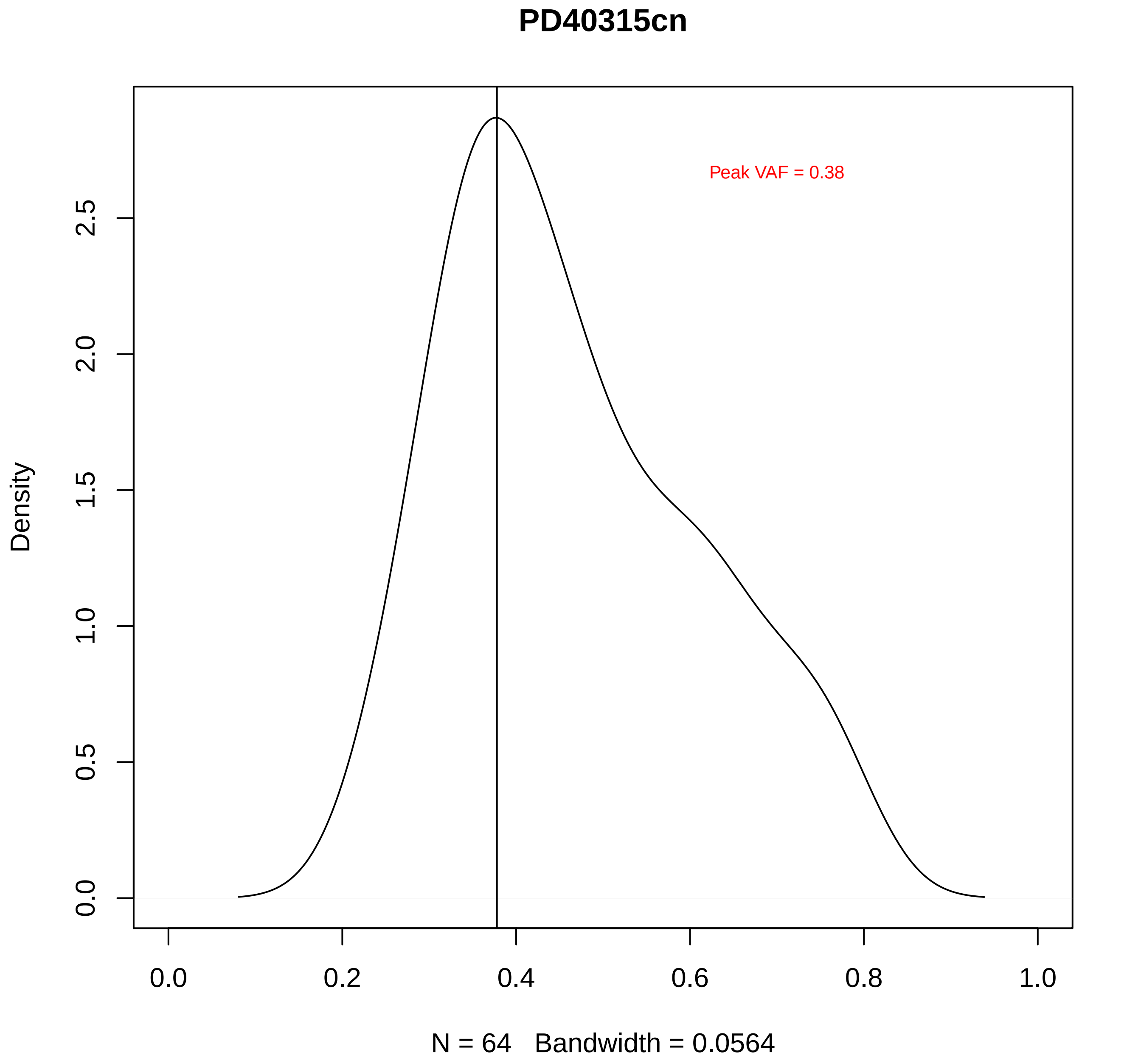
PD40315cn
Peak VAF = 0.38
Density
0.0
0.5
1.0
1.5
2.0
2.5
0.0
0.2
0.4
0.6
0.8
1.0
N = 64 Bandwidth = 0.0564

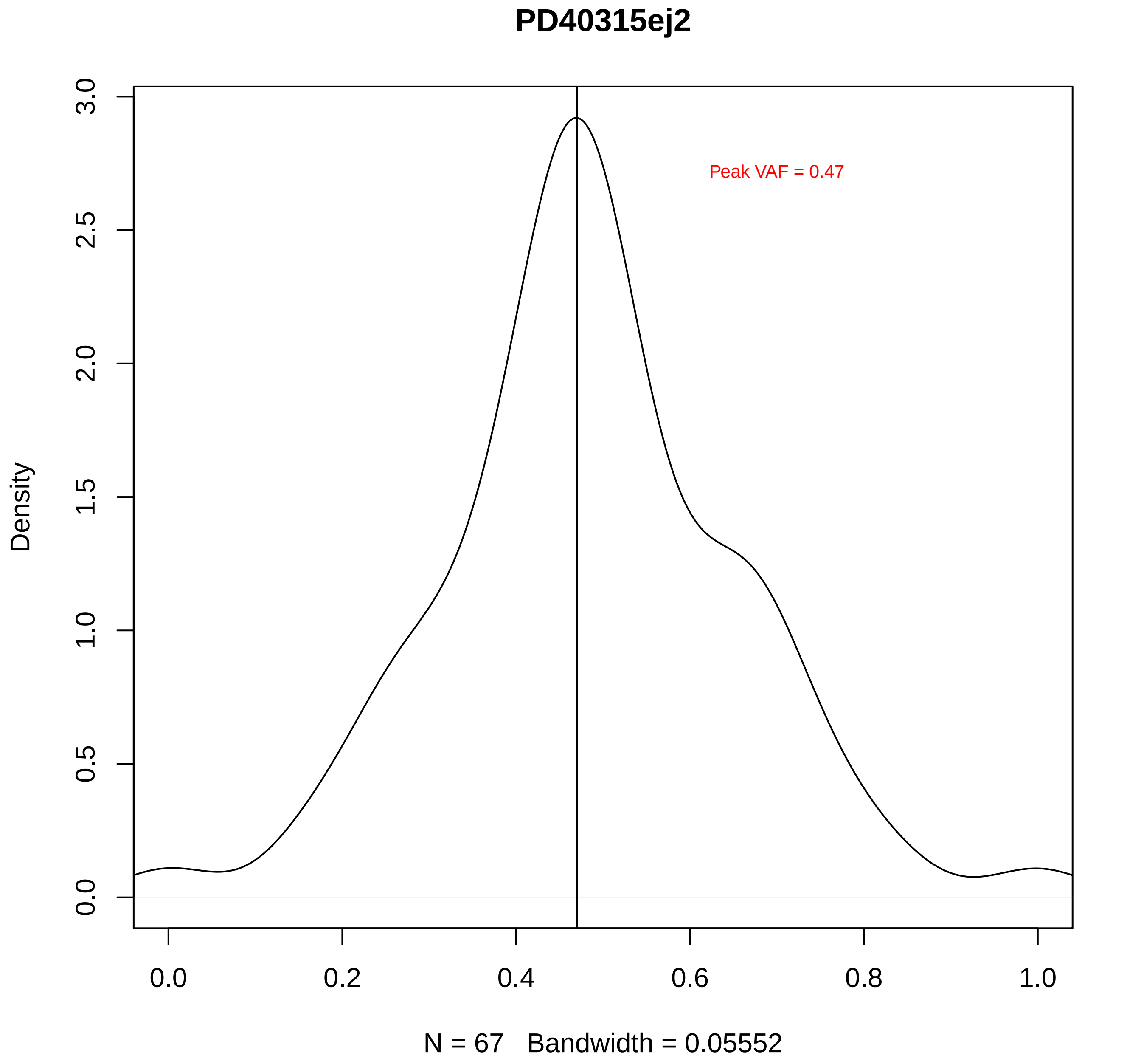

PD40315ej2
Peak VAF = 0.47
Density
3.0
2.5
2.0
1.5
1.0
0.5
0.0
0.0
0.2
0.4
0.6
0.8
1.0
N = 67   Bandwidth = 0.05552

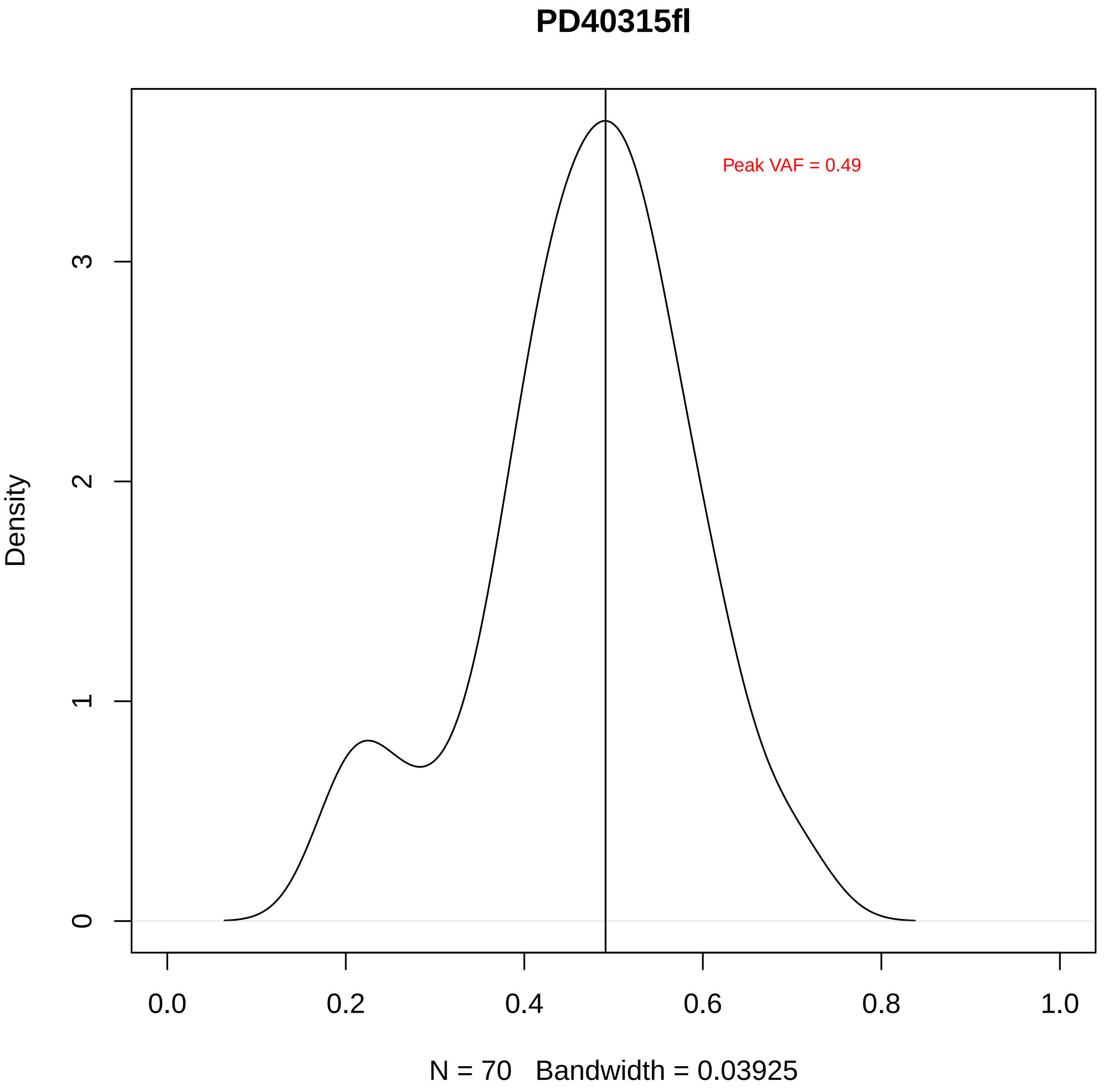

PD40315fl
Peak VAF = 0.49
Density
0
1
2
3
0.0
0.2
0.4
0.6
0.8
1.0
N = 70 Bandwidth = 0.03925

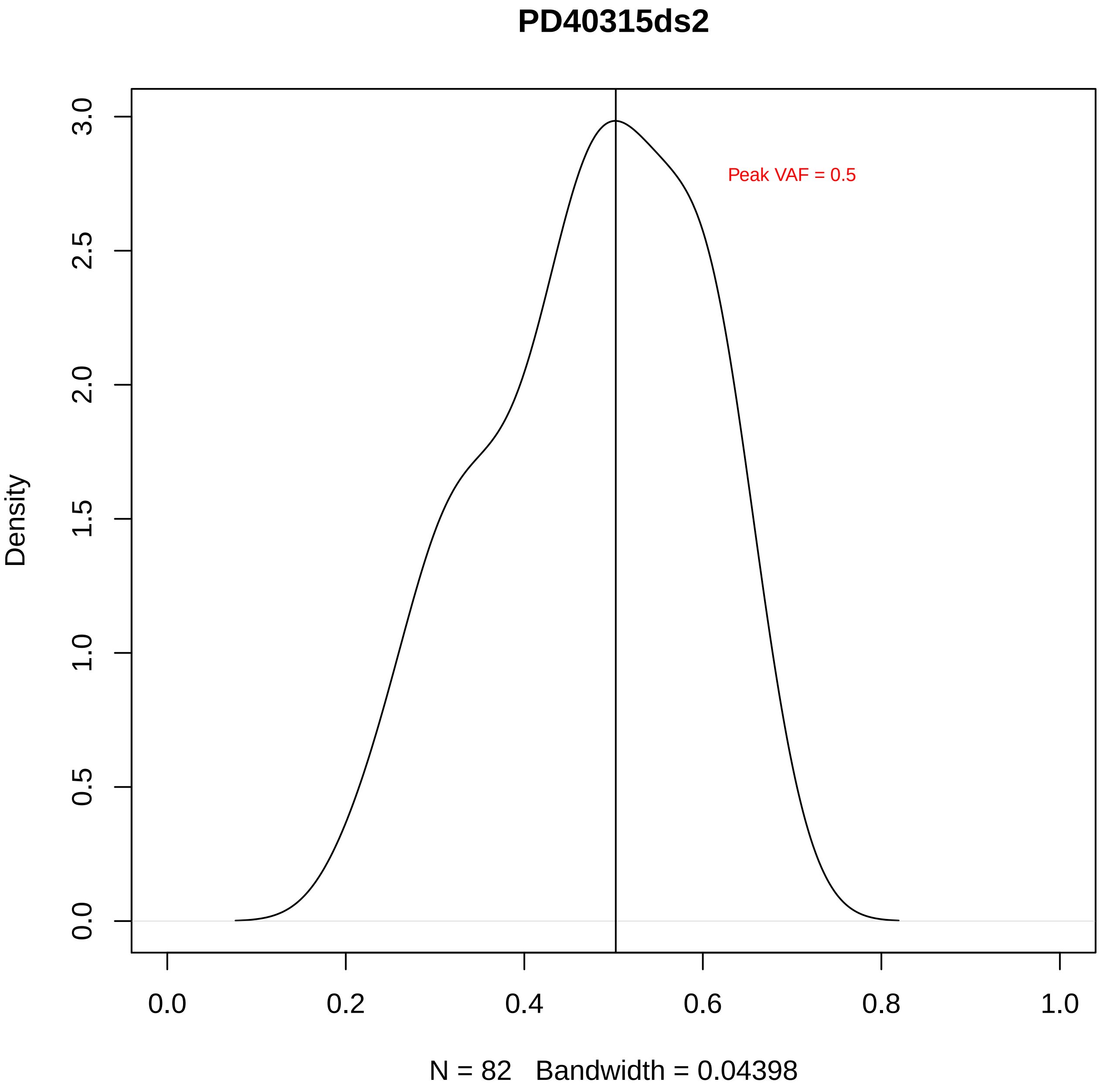

PD40315ds2
Peak VAF = 0.5
Density
0.0
0.5
1.0
1.5
2.0
2.5
3.0
0.0
0.2
0.4
0.6
0.8
1.0
N = 82 Bandwidth = 0.04398

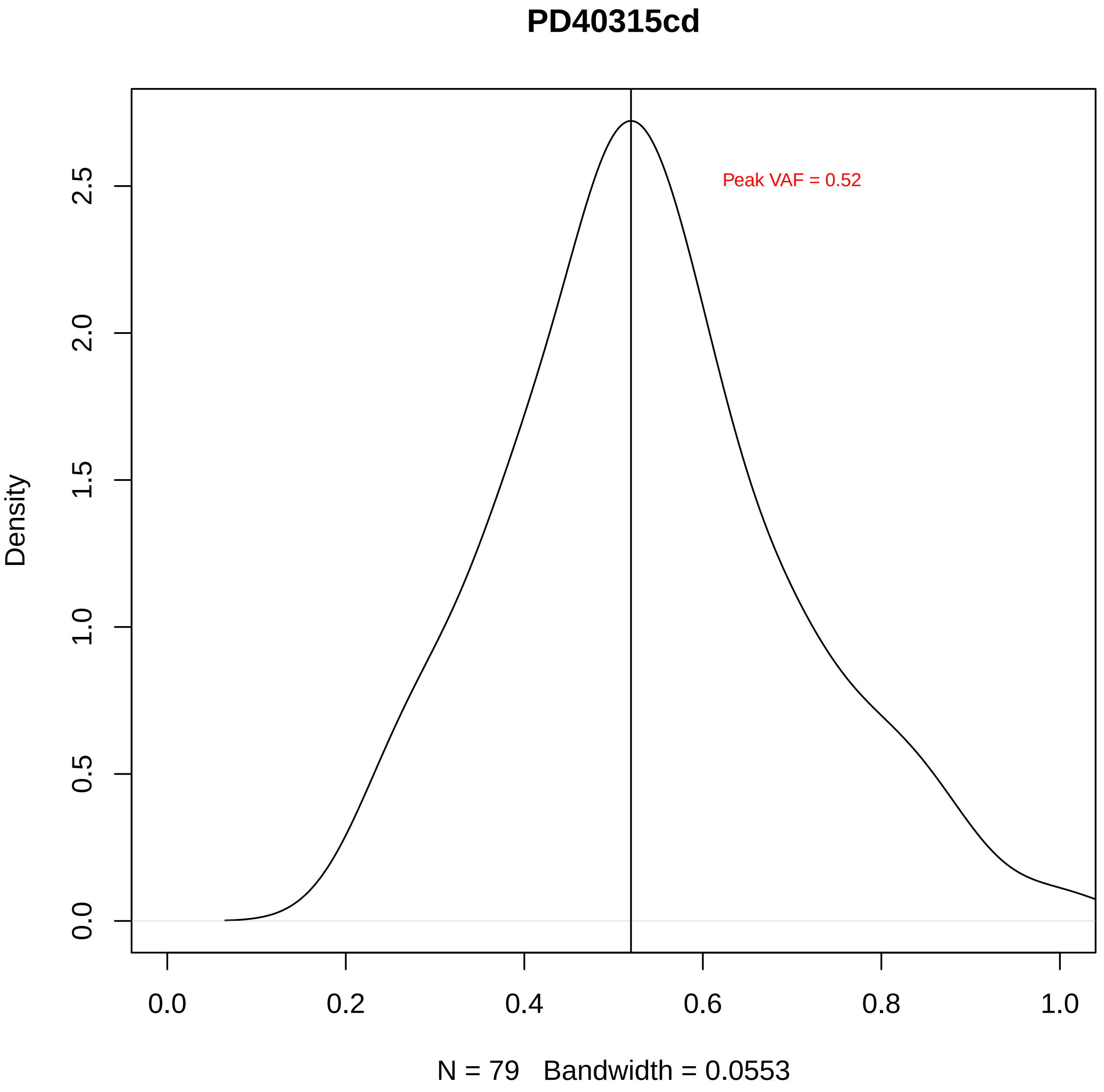
PD40315cd
Peak VAF = 0.52
Density
0.0
0.5
1.0
1.5
2.0
2.5
0.0
0.2
0.4
0.6
0.8
1.0
N = 79   Bandwidth = 0.0553

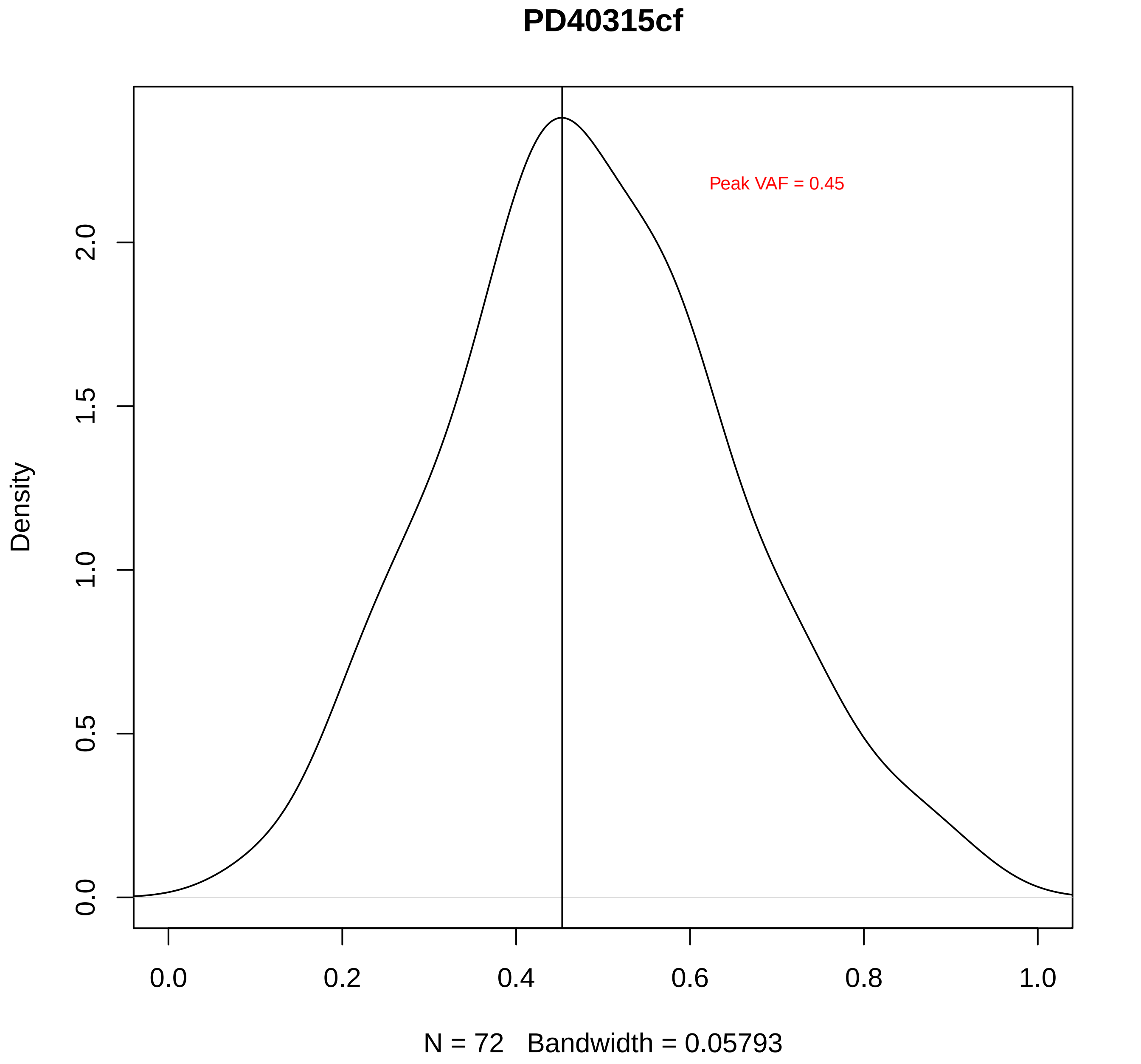

PD40315cf
Peak VAF = 0.45
Density
0.0
0.5
1.0
1.5
2.0
0.0
0.2
0.4
0.6
0.8
1.0
N = 72   Bandwidth = 0.05793

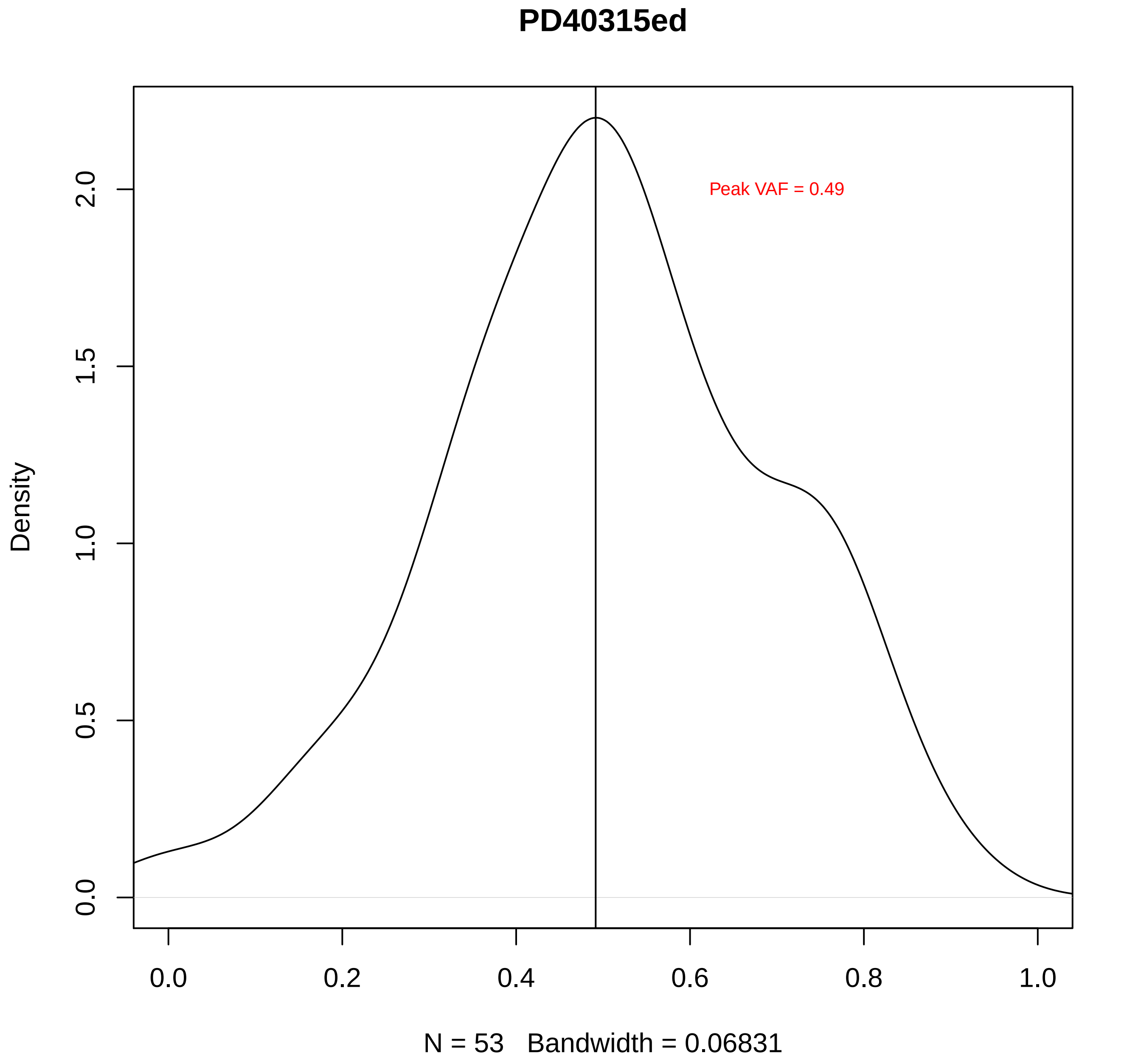

PD40315ed
Peak VAF = 0.49
Density
0.0
0.5
1.0
1.5
2.0
0.0
0.2
0.4
0.6
0.8
1.0
N = 53 Bandwidth = 0.06831

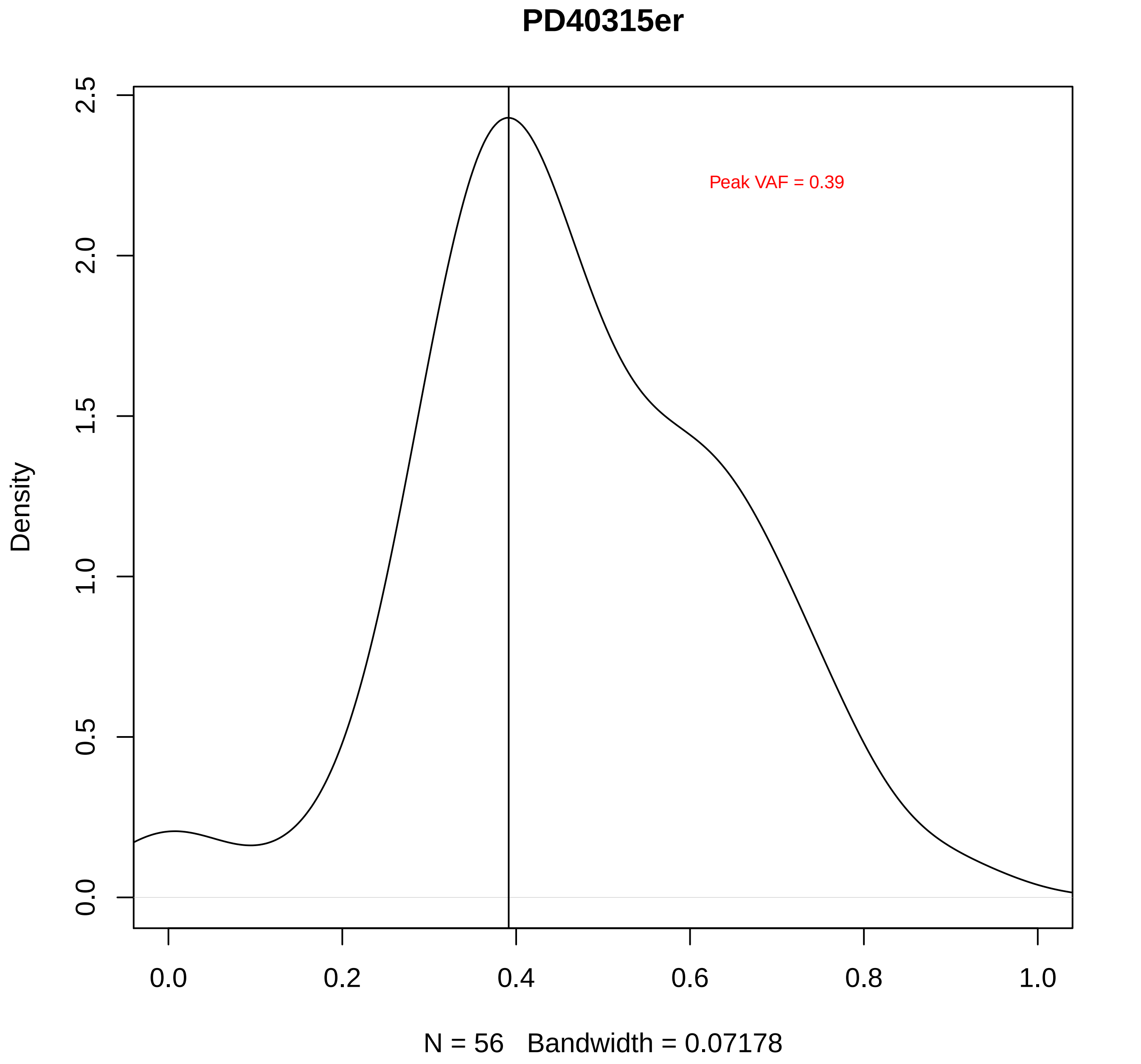
PD40315er
Peak VAF = 0.39
Density
0.0
0.5
1.0
1.5
2.0
2.5
0.0
0.2
0.4
0.6
0.8
1.0
N = 56   Bandwidth = 0.07178

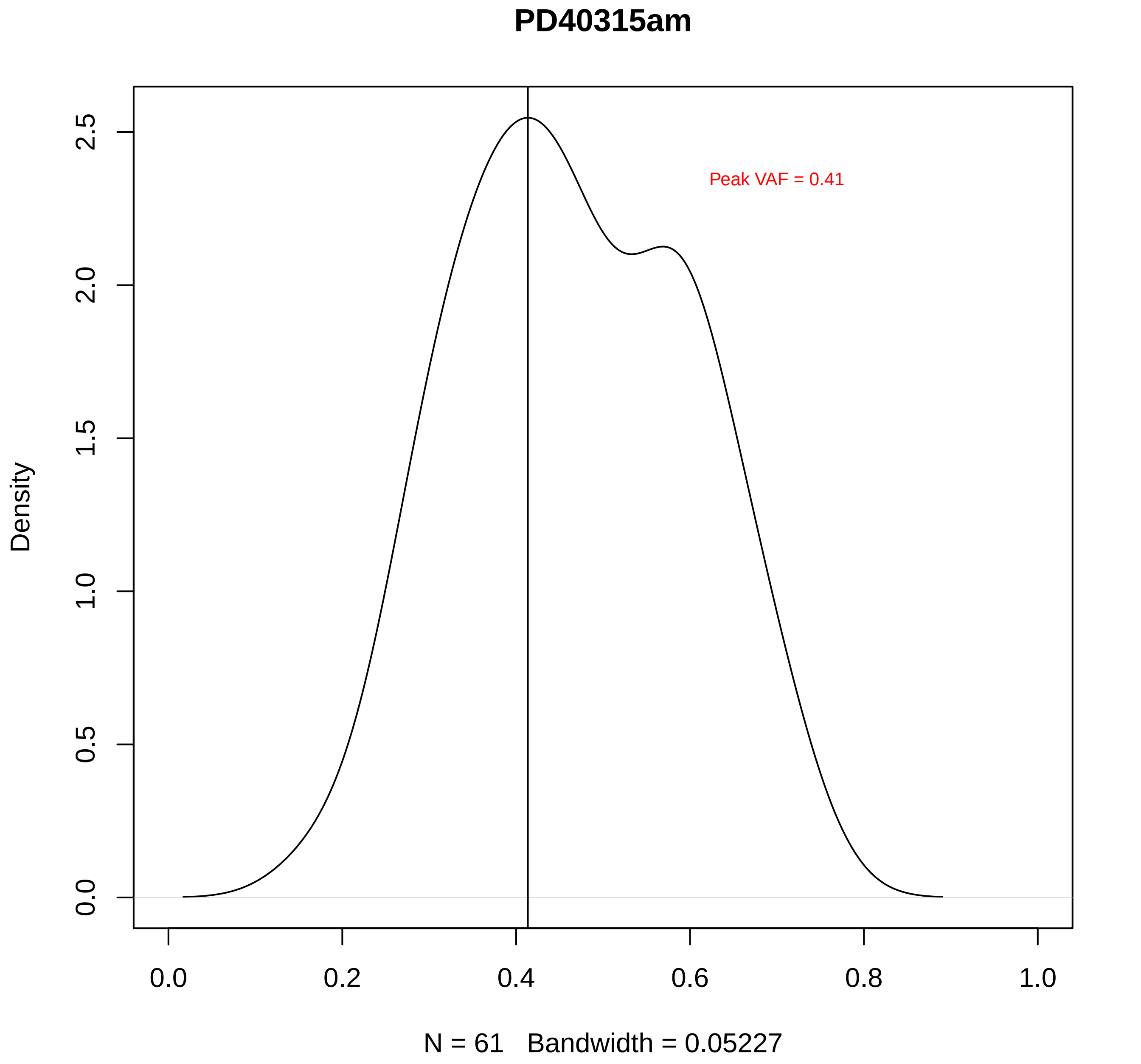
PD40315am
Peak VAF = 0.41
Density
0.0
0.5
1.0
1.5
2.0
2.5
0.0
0.2
0.4
0.6
0.8
1.0
N = 61 Bandwidth = 0.05227

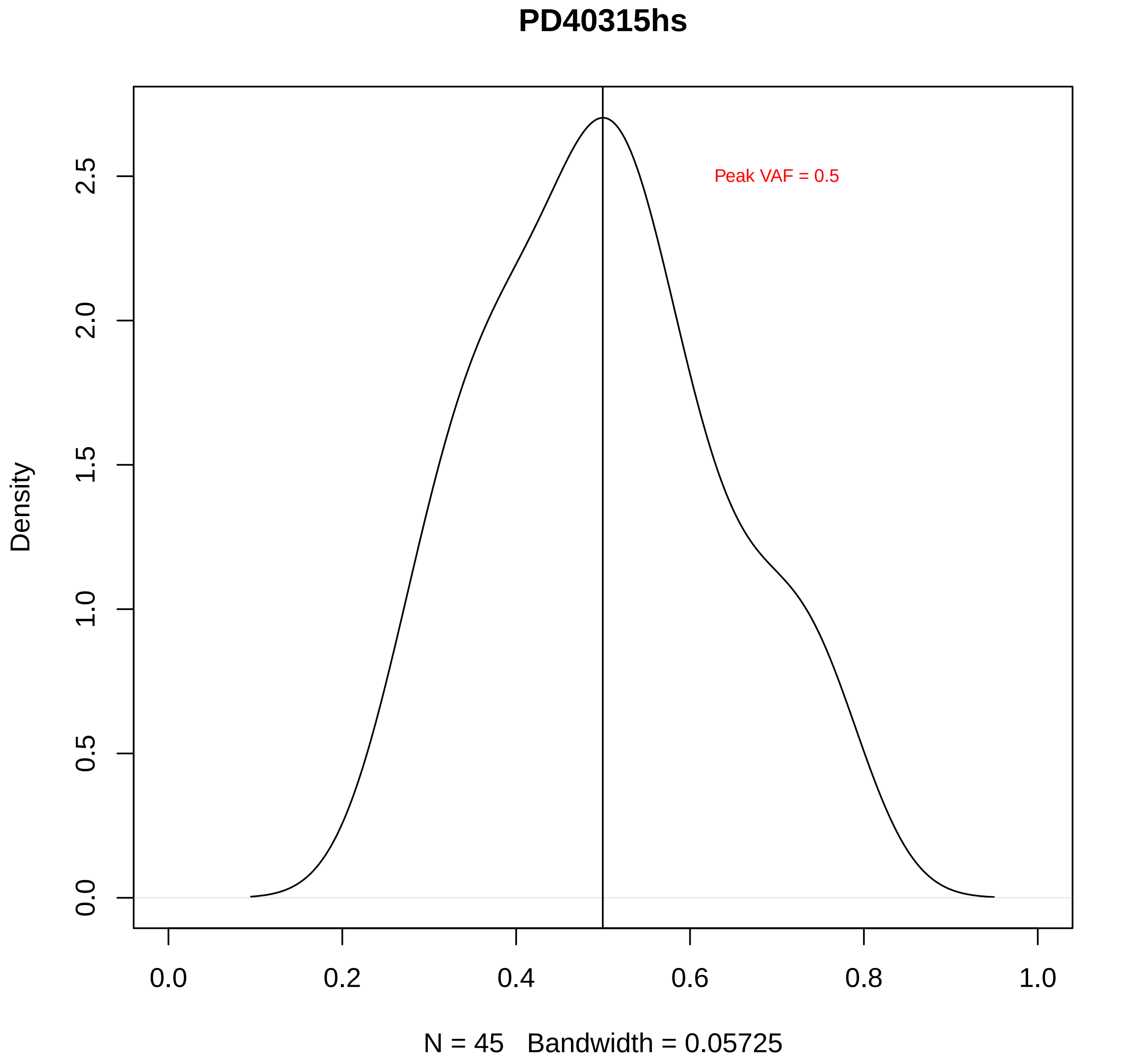

PD40315hs
Peak VAF = 0.5
Density
0.0
0.5
1.0
1.5
2.0
2.5
0.0
0.2
0.4
0.6
0.8
1.0
N = 45   Bandwidth = 0.05725

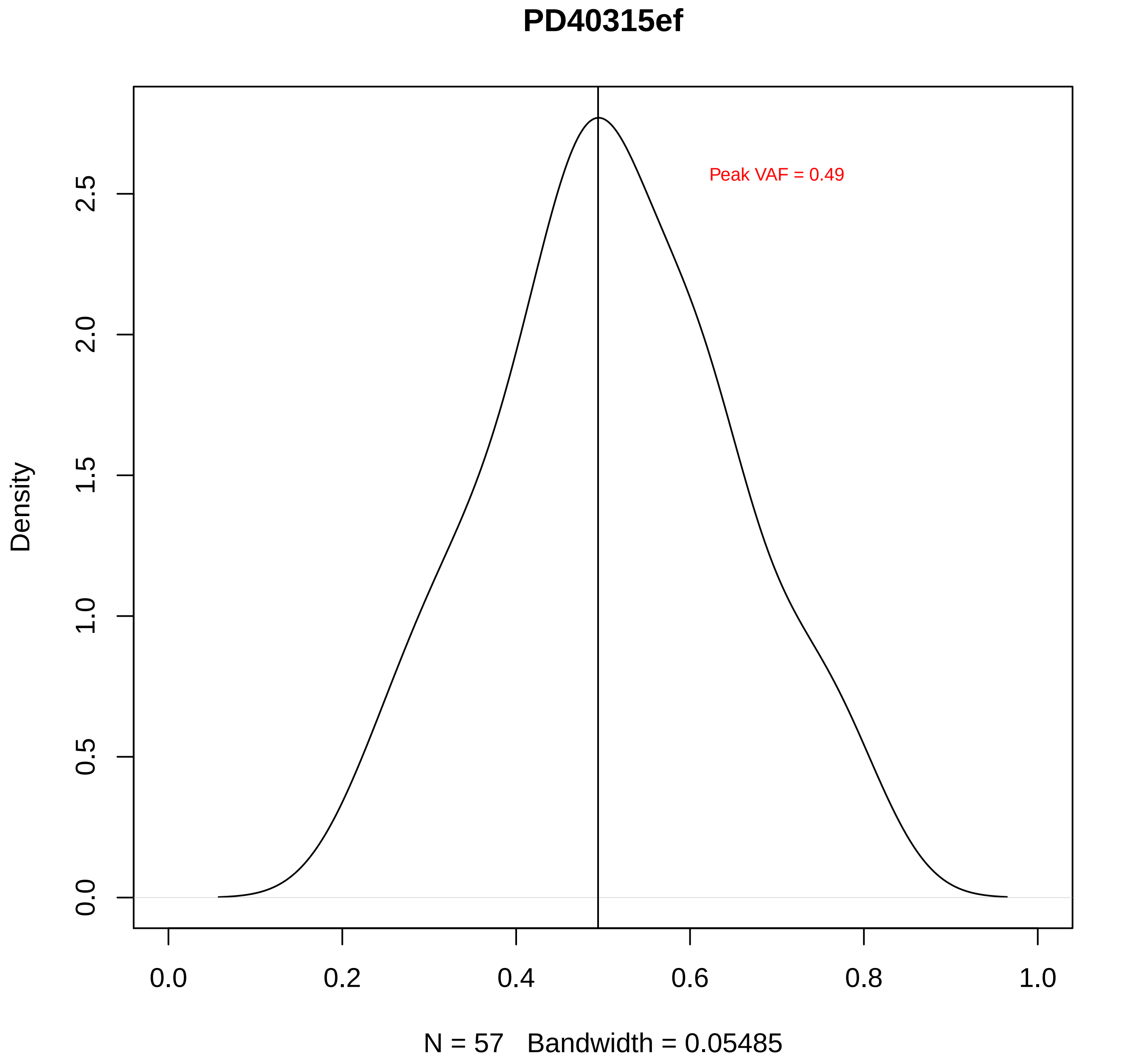

PD40315ef
Peak VAF = 0.49
Density
0.0
0.5
1.0
1.5
2.0
2.5
0.0
0.2
0.4
0.6
0.8
1.0
N = 57   Bandwidth = 0.05485

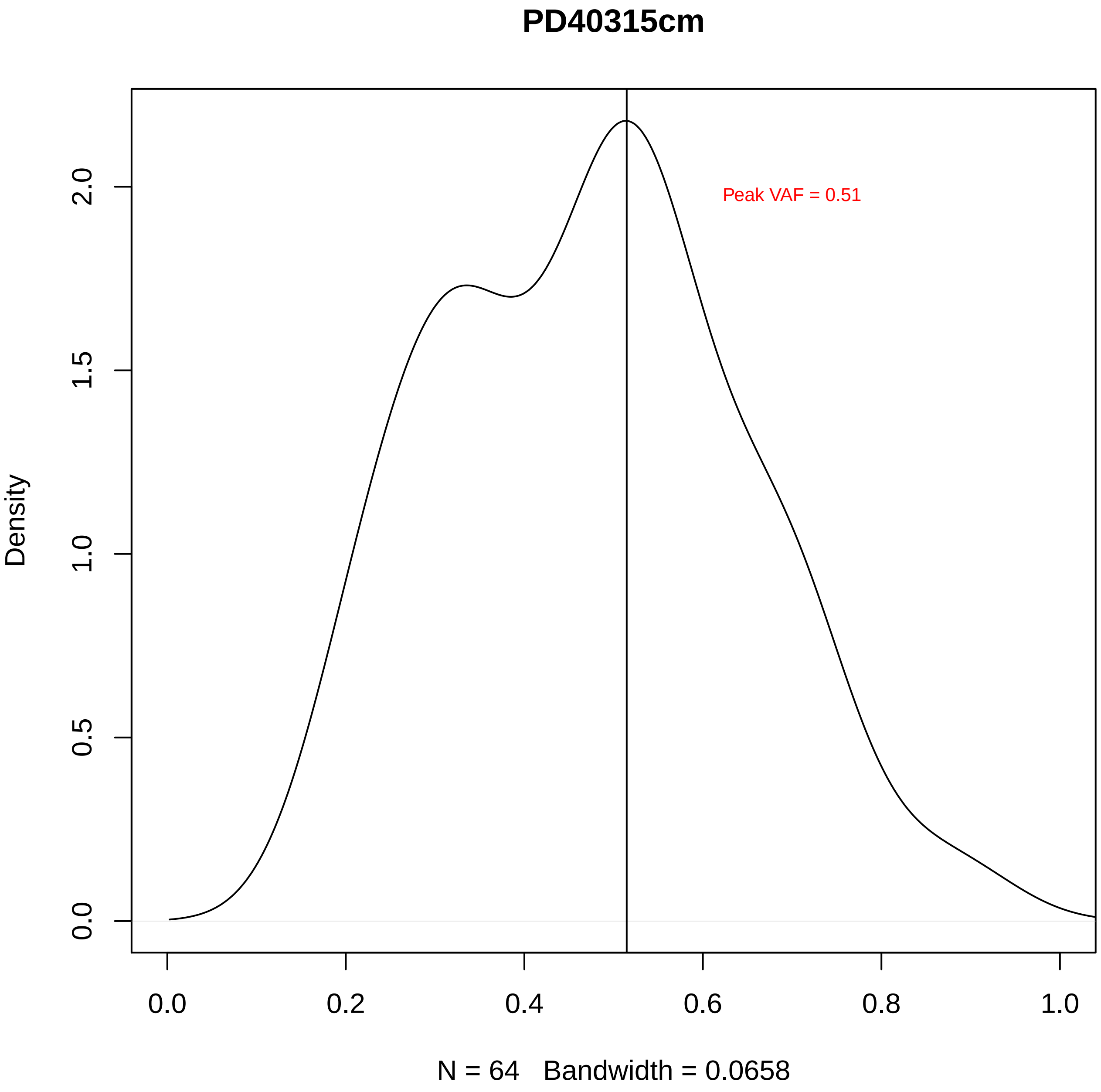
PD40315cm
Peak VAF = 0.51
Density
0.0
0.5
1.0
1.5
2.0
0.0
0.2
0.4
0.6
0.8
1.0
N = 64   Bandwidth = 0.0658

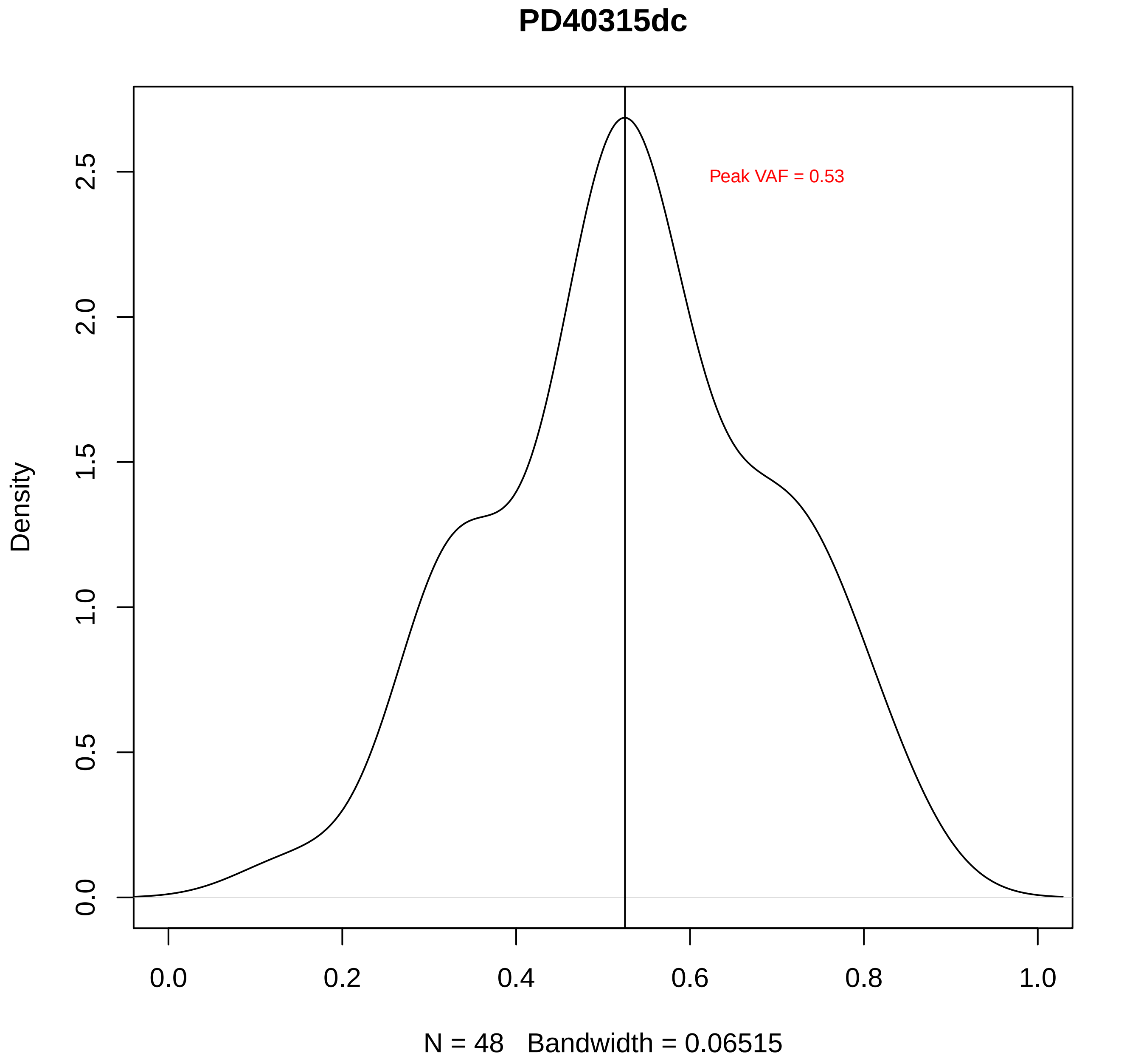
PD40315dc
Peak VAF = 0.53
Density
0.0
0.5
1.0
1.5
2.0
2.5
0.0
0.2
0.4
0.6
0.8
1.0
N = 48 Bandwidth = 0.06515

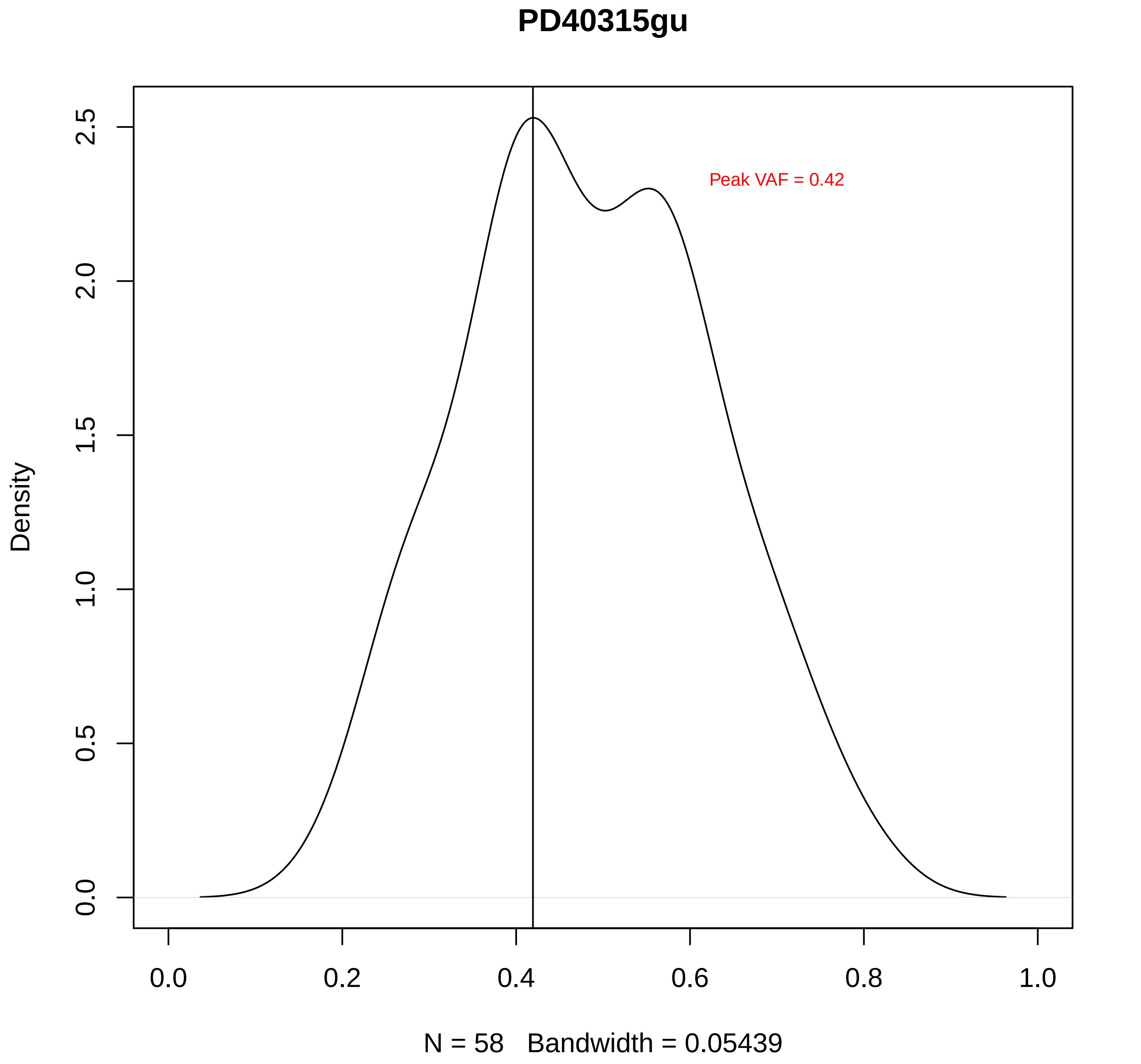
PD40315gu
Peak VAF = 0.42
Density
0.0
0.5
1.0
1.5
2.0
2.5
0.0
0.2
0.4
0.6
0.8
1.0
N = 58   Bandwidth = 0.05439

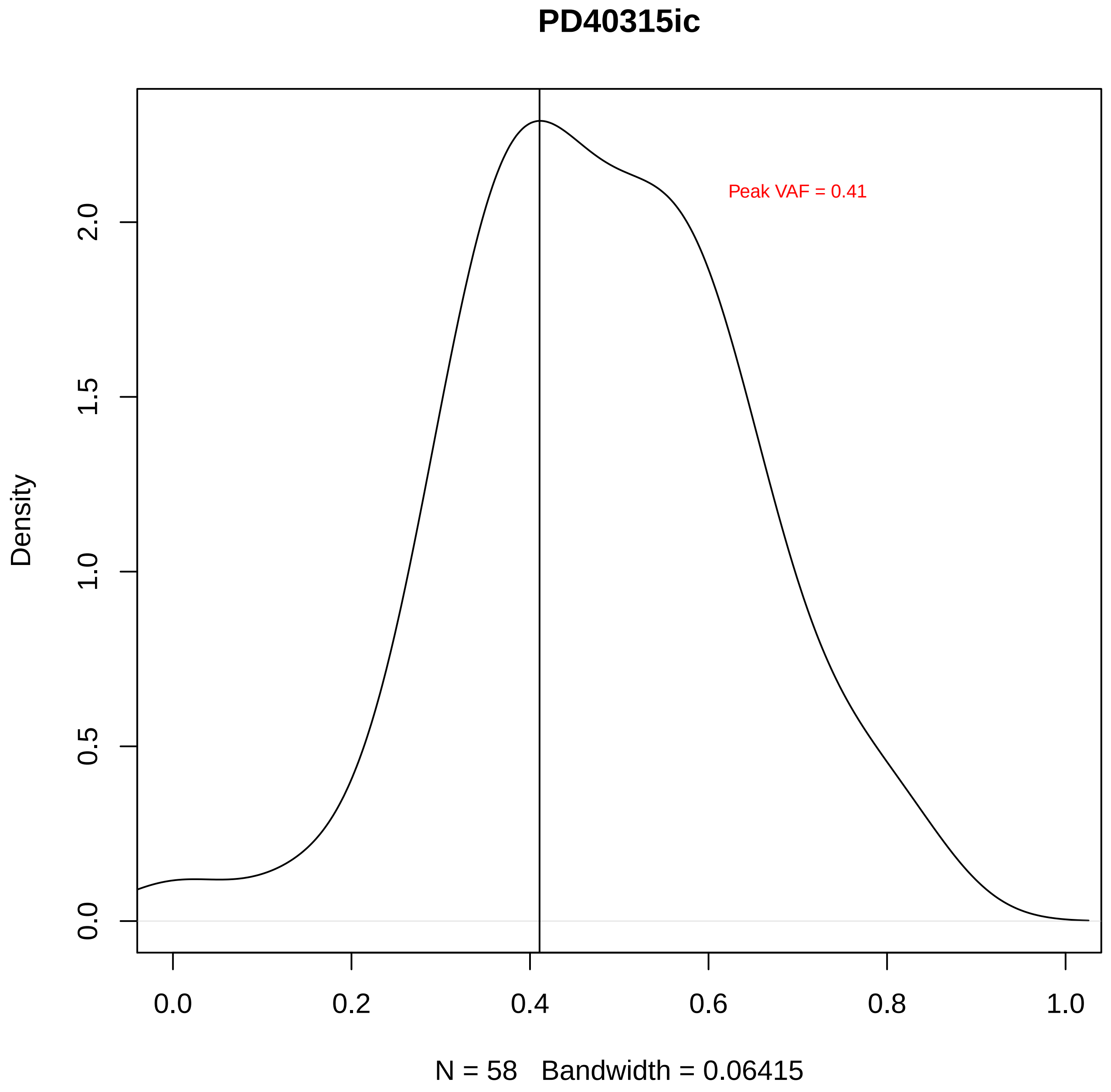
PD40315ic
Peak VAF = 0.41
Density
0.0
0.5
1.0
1.5
2.0
0.0
0.2
0.4
0.6
0.8
1.0
N = 58 Bandwidth = 0.06415

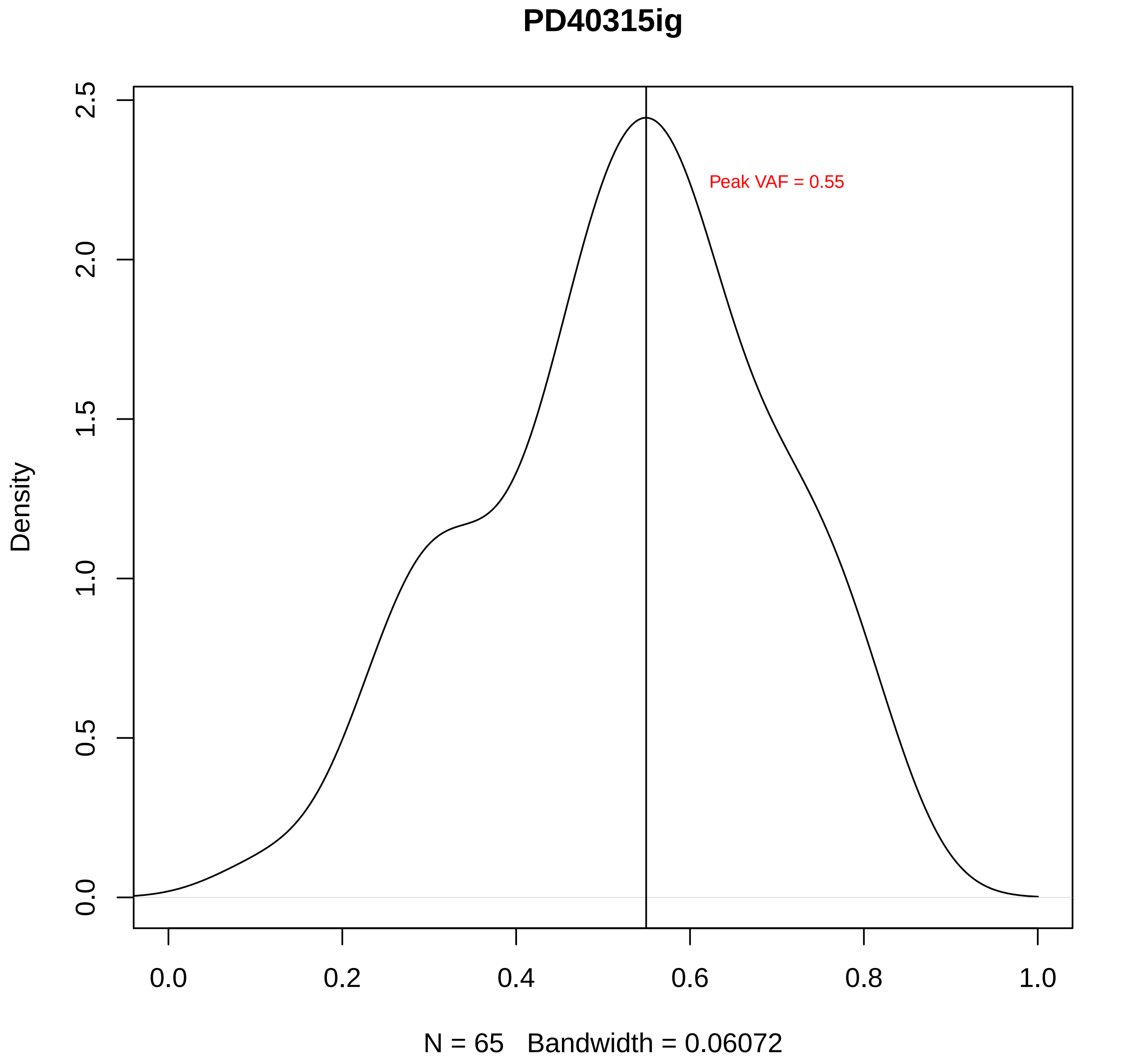
PD40315ig
Peak VAF = 0.55
Density
0.0
0.5
1.0
1.5
2.0
2.5
0.0
0.2
0.4
0.6
0.8
1.0
N = 65   Bandwidth = 0.06072

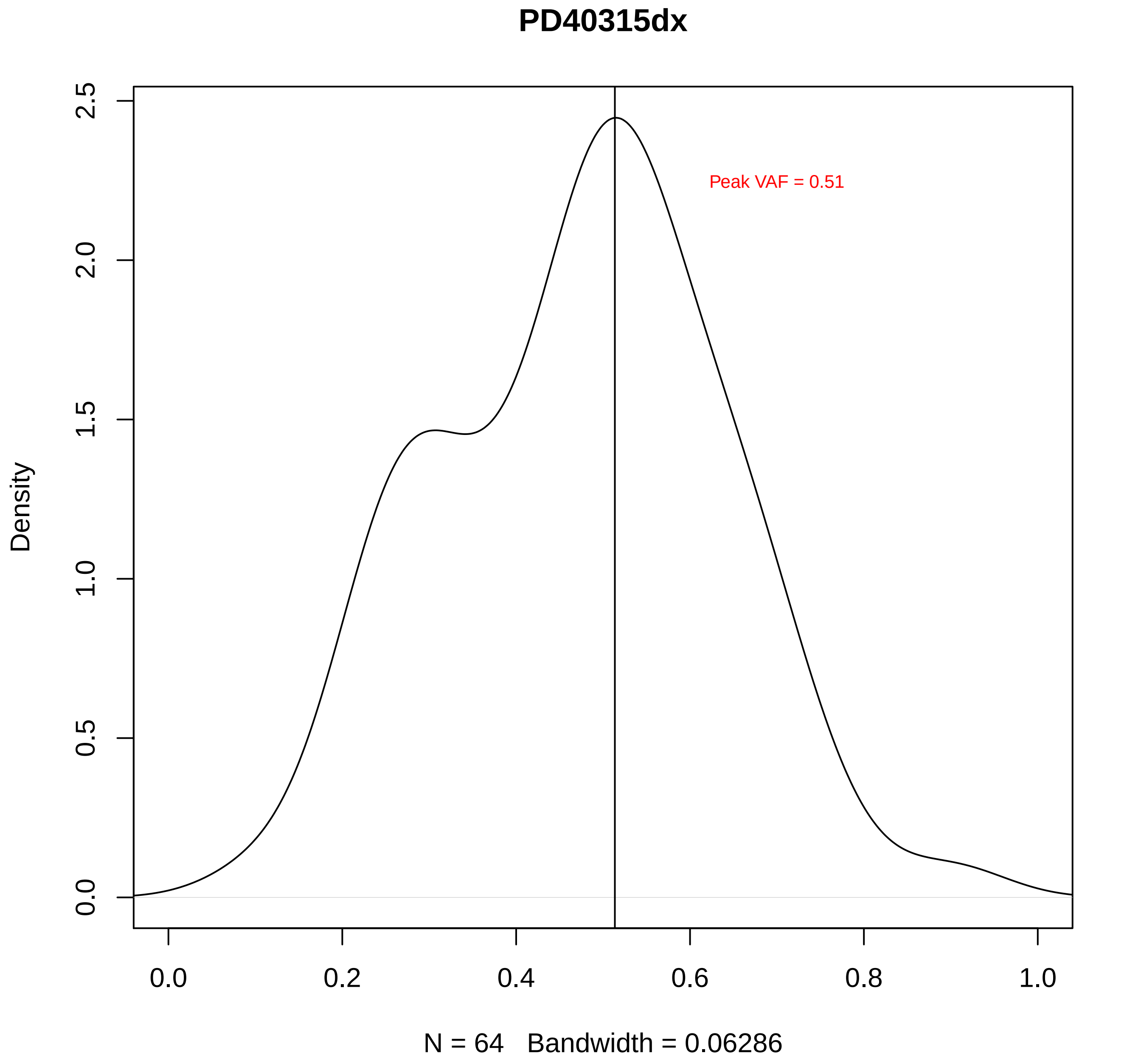

PD40315dx
Peak VAF = 0.51
Density
0.0
0.5
1.0
1.5
2.0
2.5
0.0
0.2
0.4
0.6
0.8
1.0
N = 64 Bandwidth = 0.06286

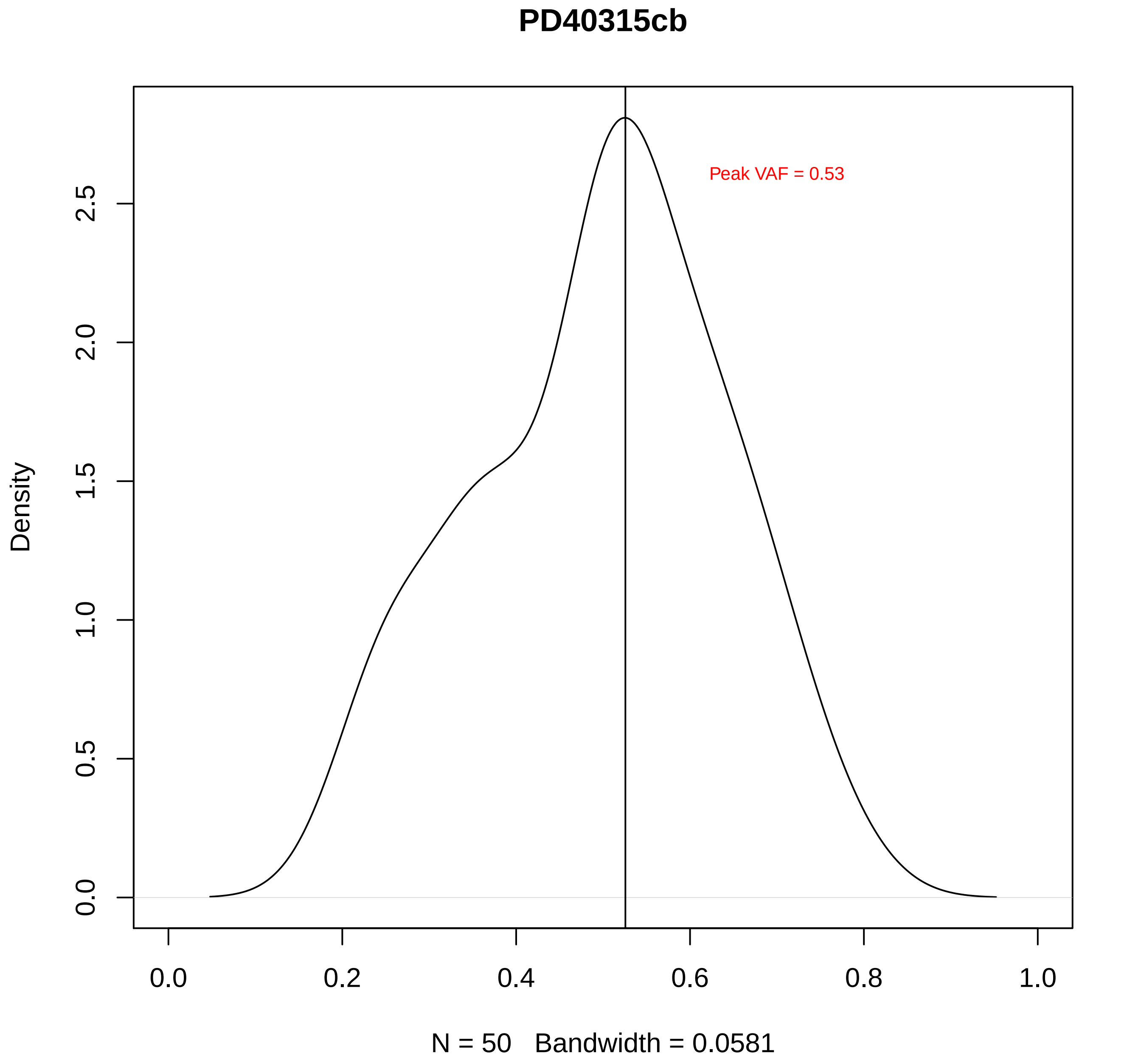

PD40315cb
Peak VAF = 0.53
Density
0.0
0.5
1.0
1.5
2.0
2.5
0.0
0.2
0.4
0.6
0.8
1.0
N = 50   Bandwidth = 0.0581

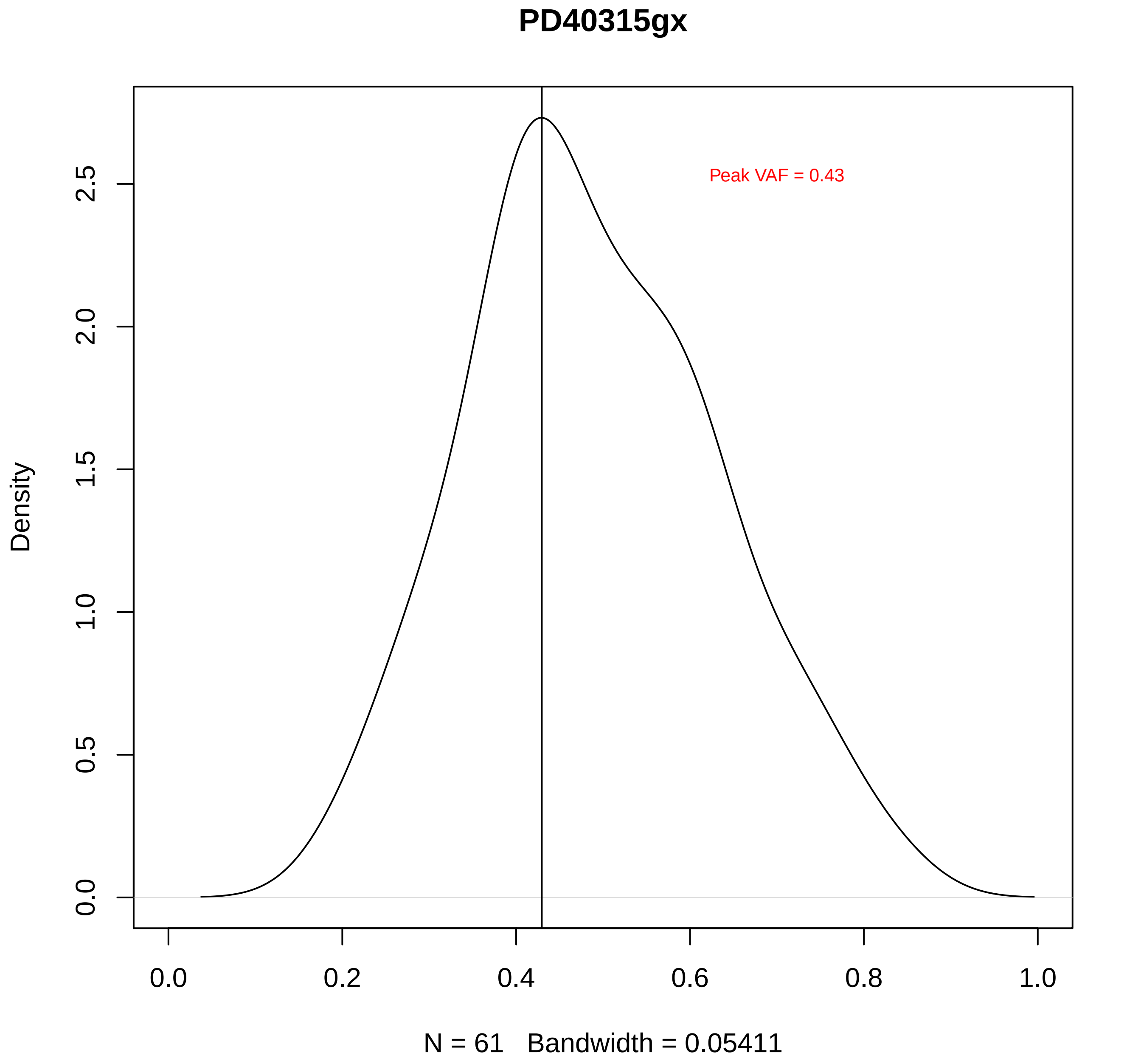
PD40315gx
Peak VAF = 0.43
Density
0.0
0.5
1.0
1.5
2.0
2.5
0.0
0.2
0.4
0.6
0.8
1.0
N = 61   Bandwidth = 0.05411

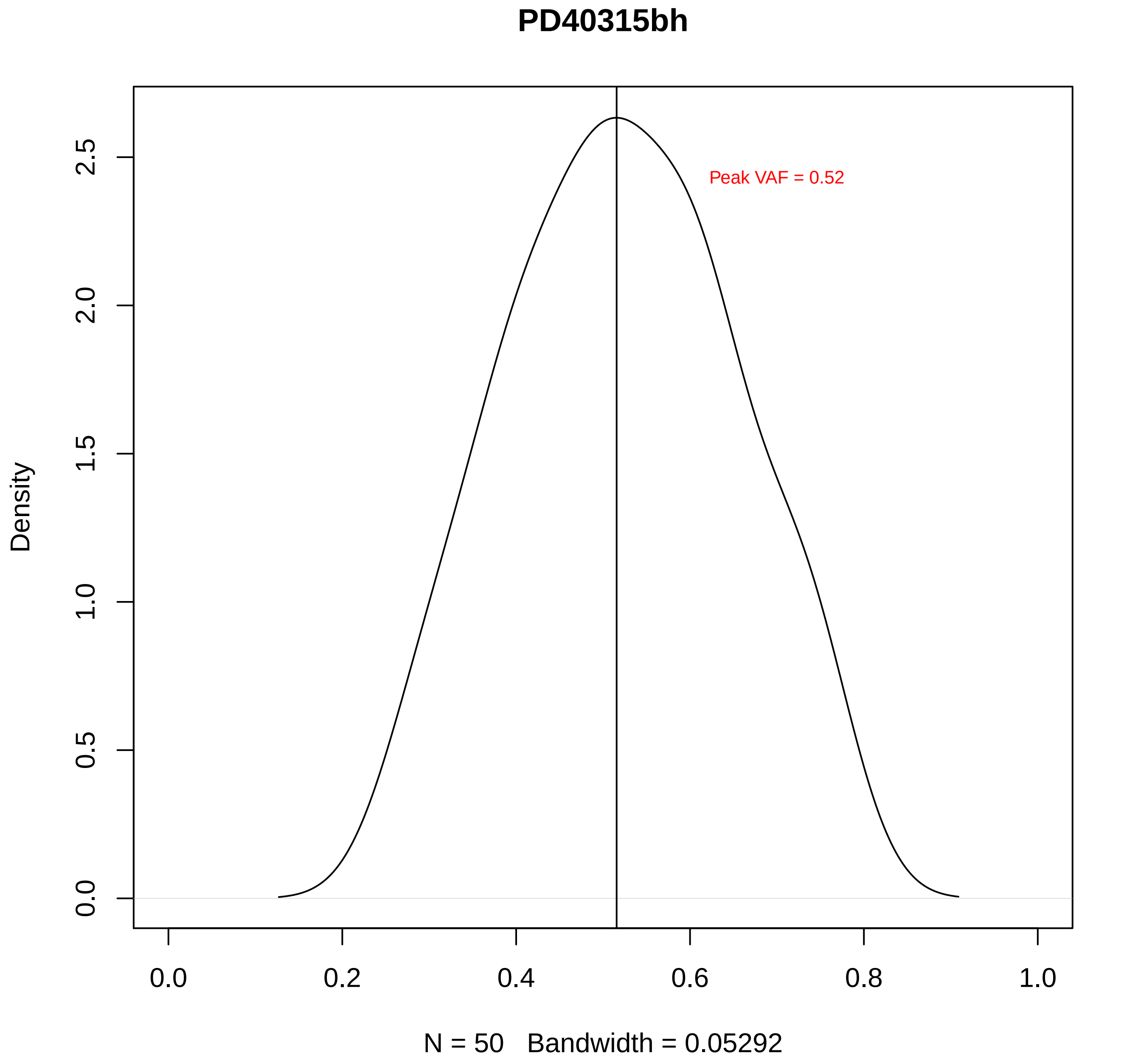
PD40315bh
Peak VAF = 0.52
Density
0.0
0.5
1.0
1.5
2.0
2.5
0.0
0.2
0.4
0.6
0.8
1.0
N = 50   Bandwidth = 0.05292

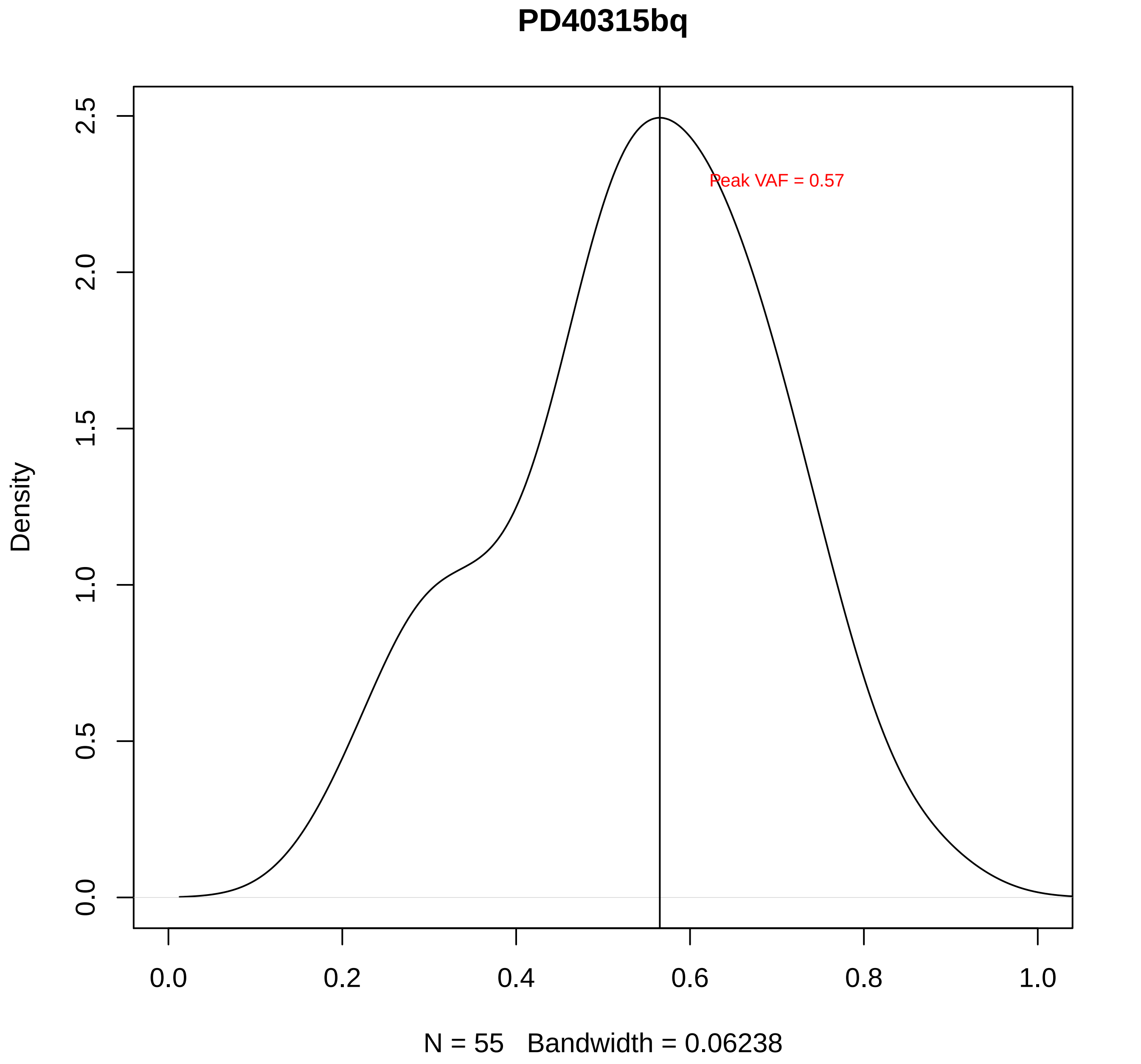

PD40315bq
Peak VAF = 0.57
Density
2.5
2.0
1.5
1.0
0.5
0.0
0.0
0.2
0.4
0.6
0.8
1.0
N = 55 Bandwidth = 0.06238

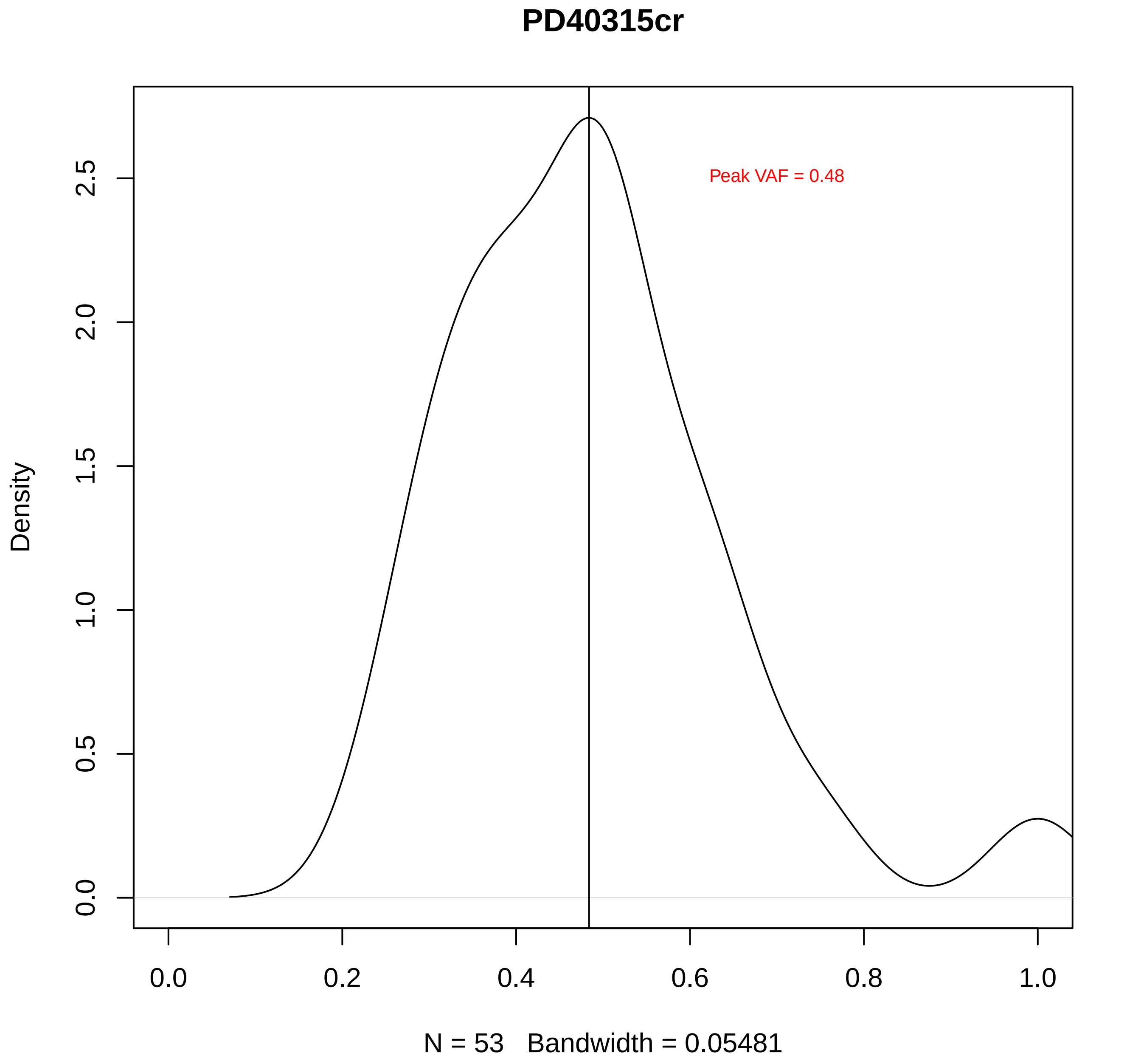

PD40315cr
Peak VAF = 0.48
Density
0.0
0.5
1.0
1.5
2.0
2.5
0.0
0.2
0.4
0.6
0.8
1.0
N = 53 Bandwidth = 0.05481

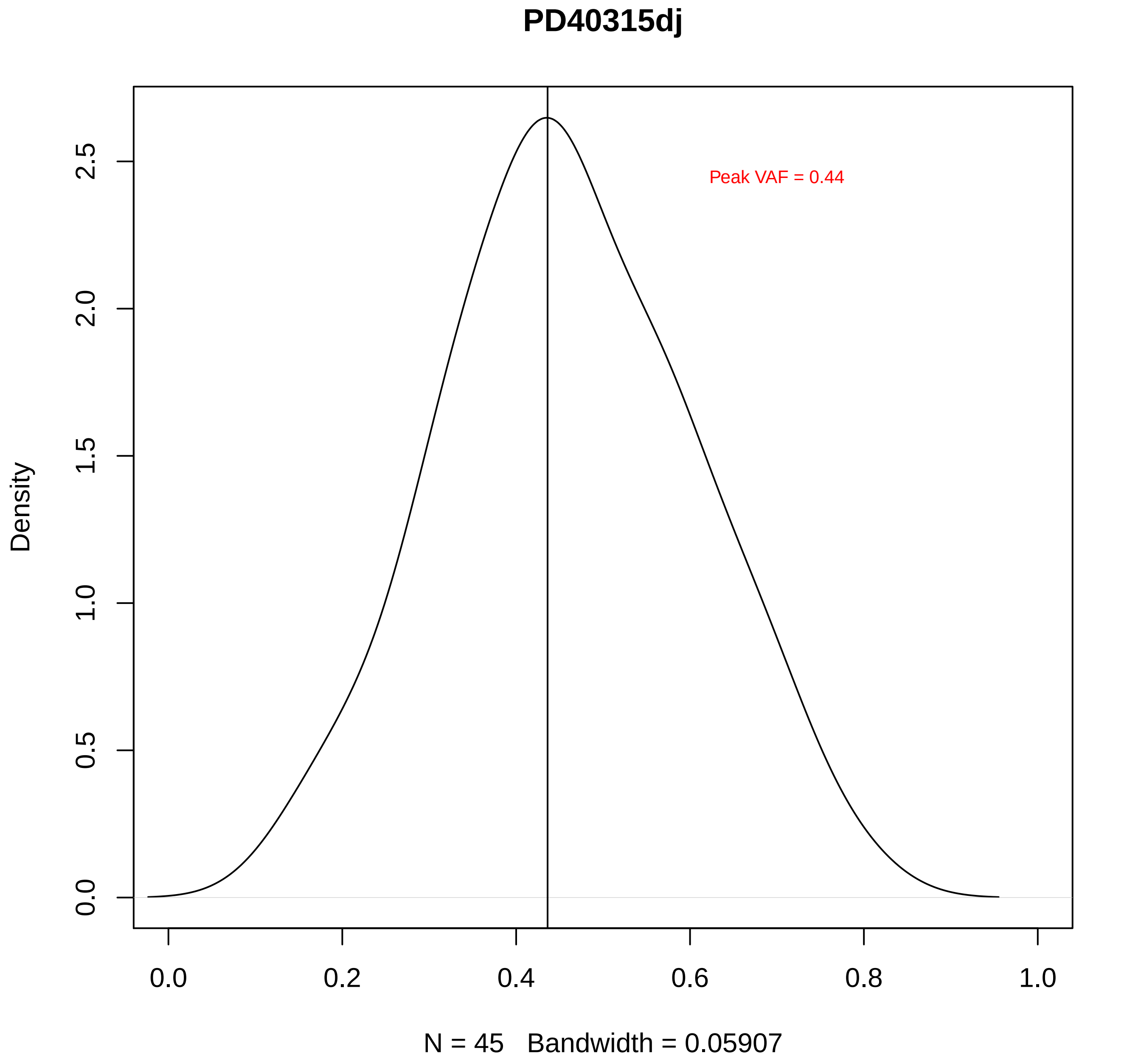
PD40315dj
Peak VAF = 0.44
Density
0.0
0.5
1.0
1.5
2.0
2.5
0.0
0.2
0.4
0.6
0.8
1.0
N = 45   Bandwidth = 0.05907

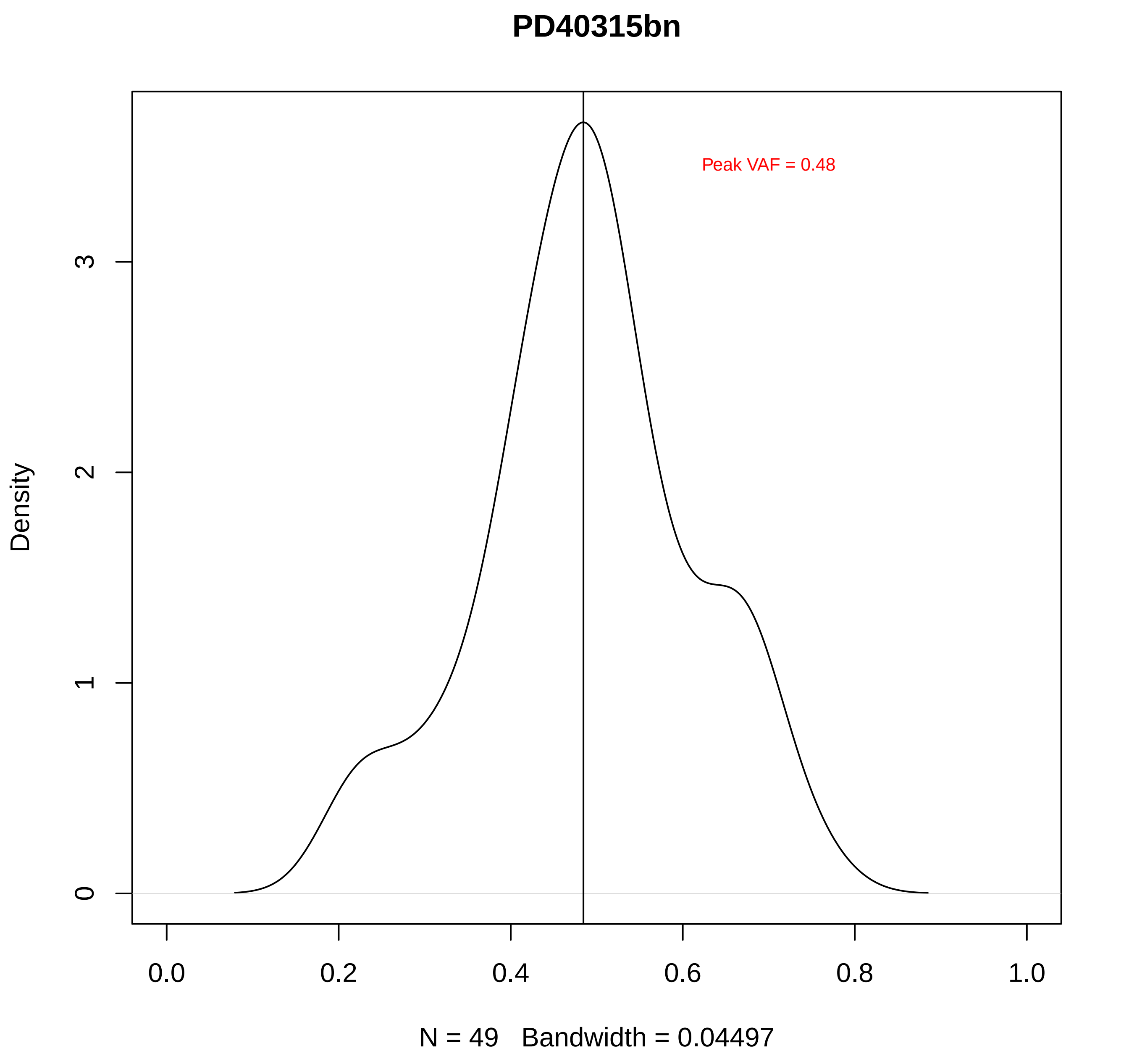
PD40315bn
Peak VAF = 0.48
Density
0
1
2
3
0.0
0.2
0.4
0.6
0.8
1.0
N = 49 Bandwidth = 0.04497

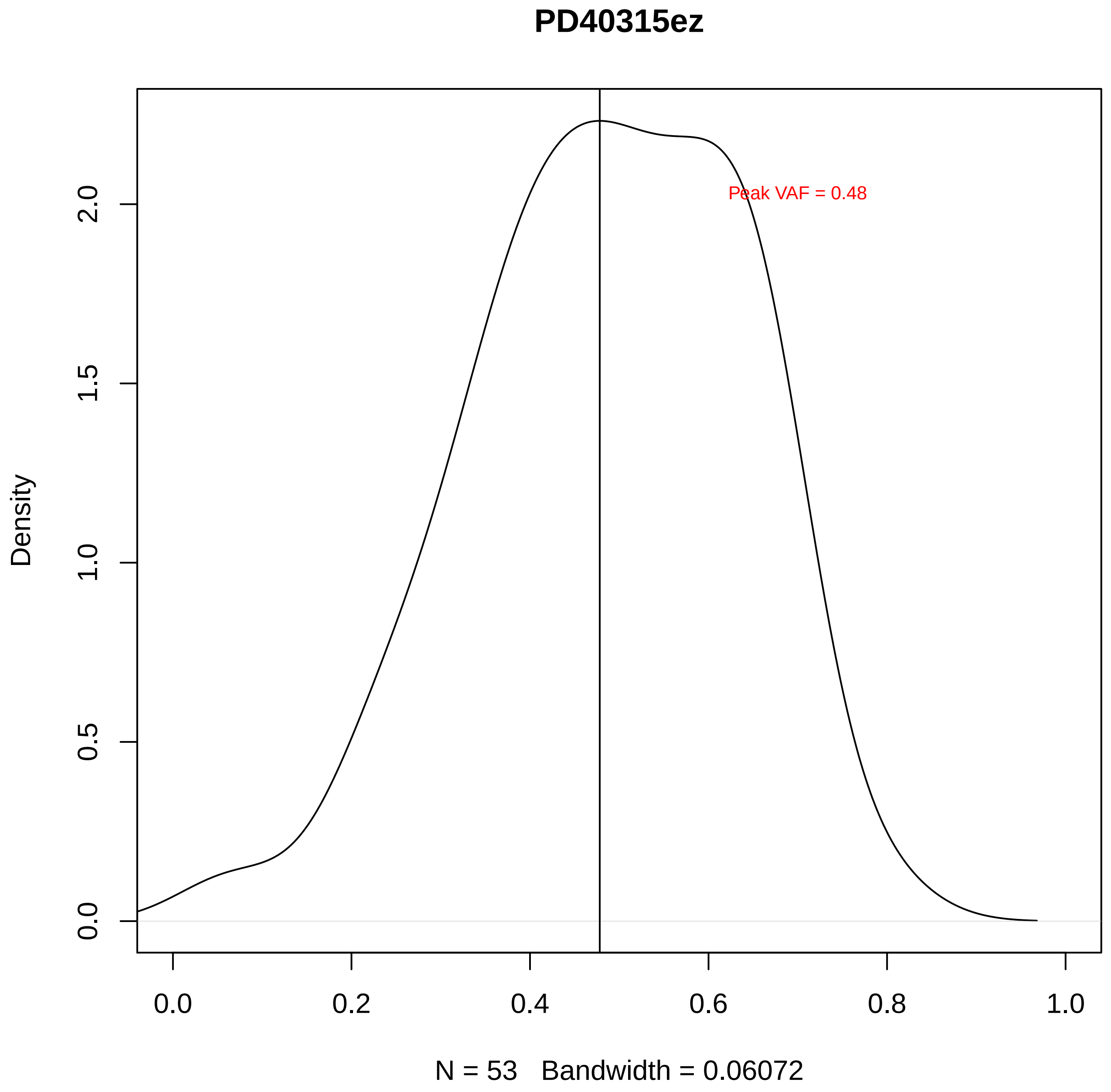

PD40315ez
Peak VAF = 0.48
Density
0.0
0.5
1.0
1.5
2.0
0.0
0.2
0.4
0.6
0.8
1.0
N = 53   Bandwidth = 0.06072

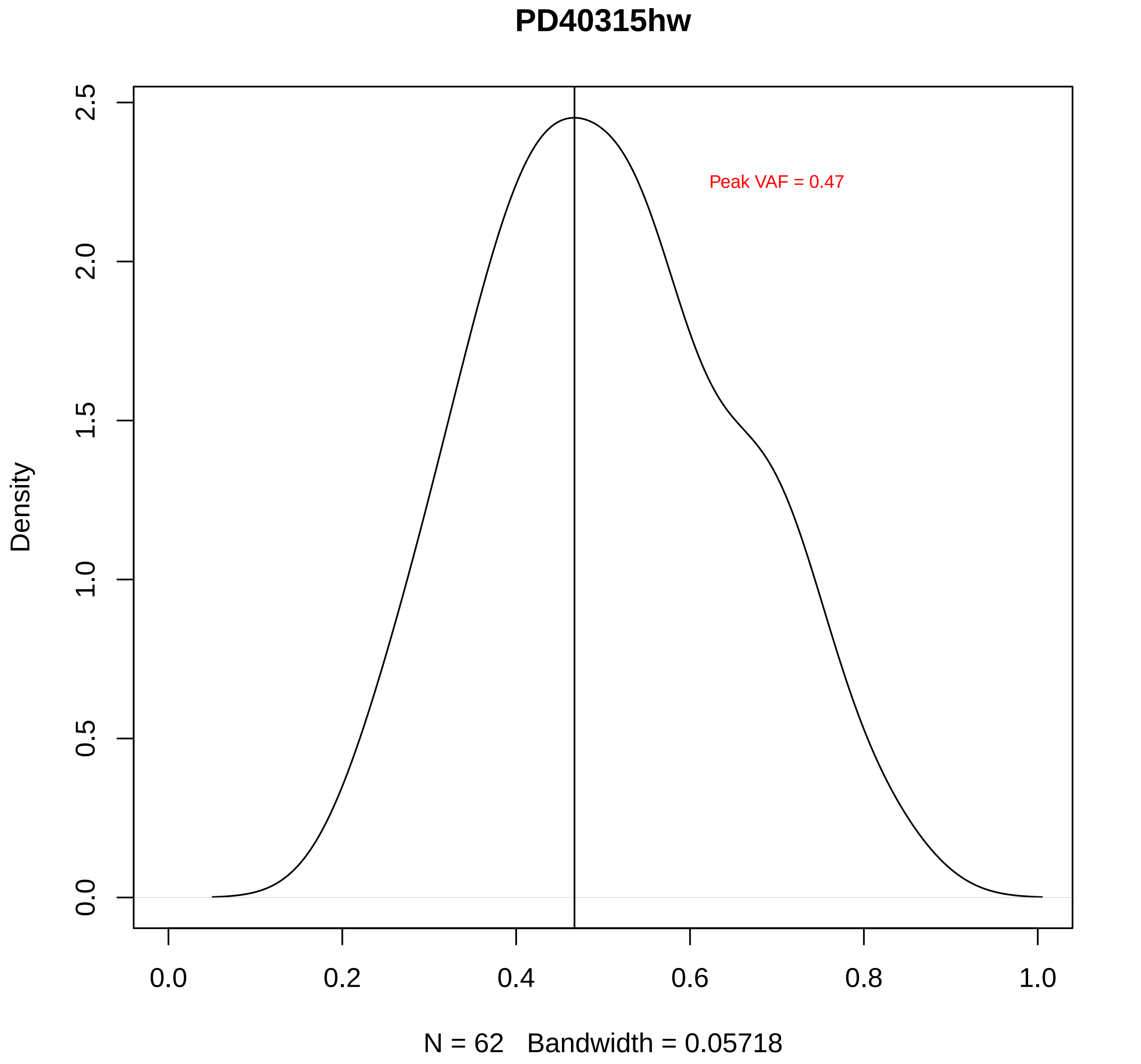
PD40315hw
Peak VAF = 0.47
Density
N = 62   Bandwidth = 0.05718
0.0
0.2
0.4
0.6
0.8
1.0
0.5
1.5
2.0
2.5

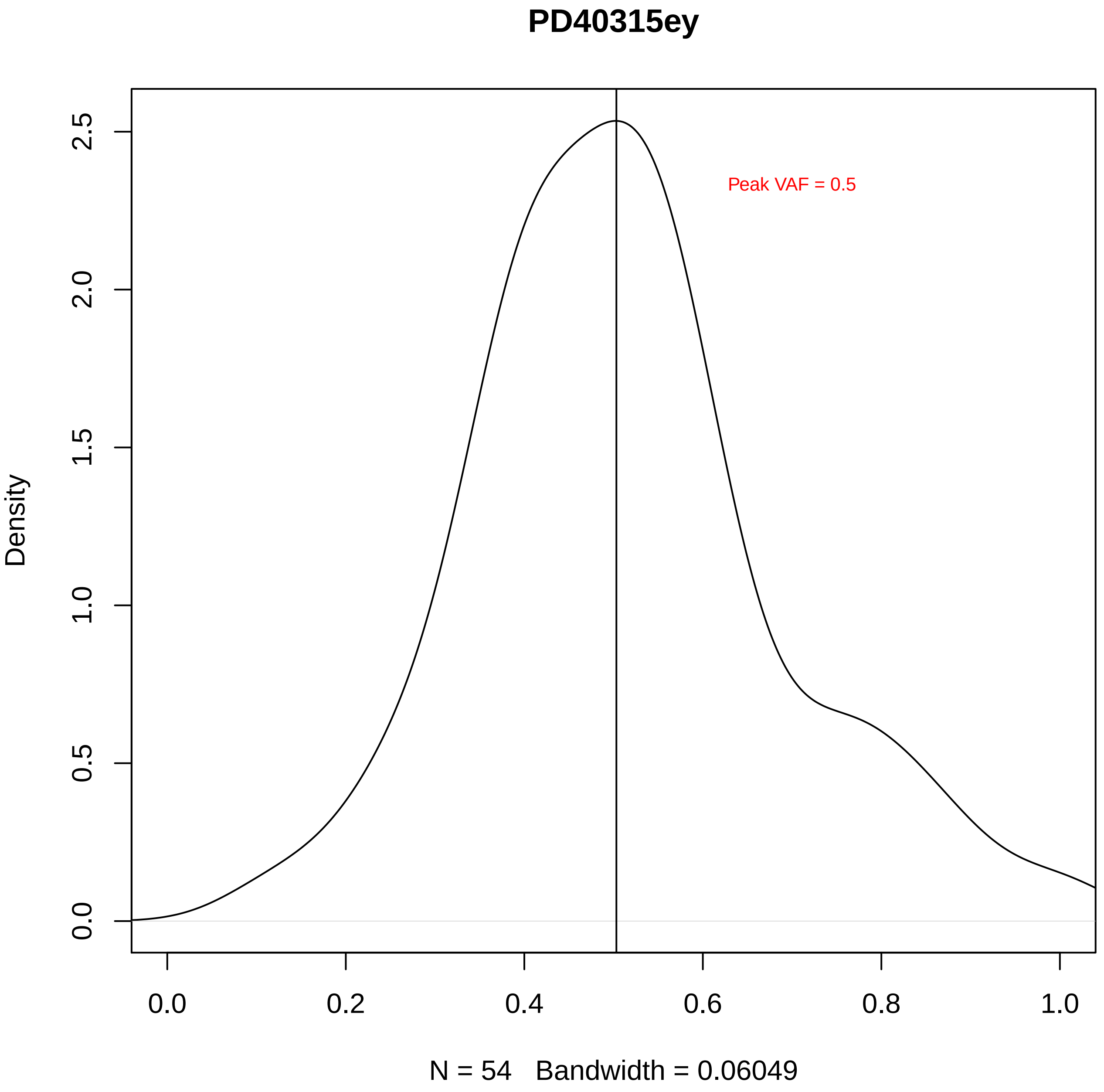
PD40315ey
Peak VAF = 0.5
Density
0.0
0.5
1.0
1.5
2.0
2.5
0.0
0.2
0.4
0.6
0.8
1.0
N = 54 Bandwidth = 0.06049

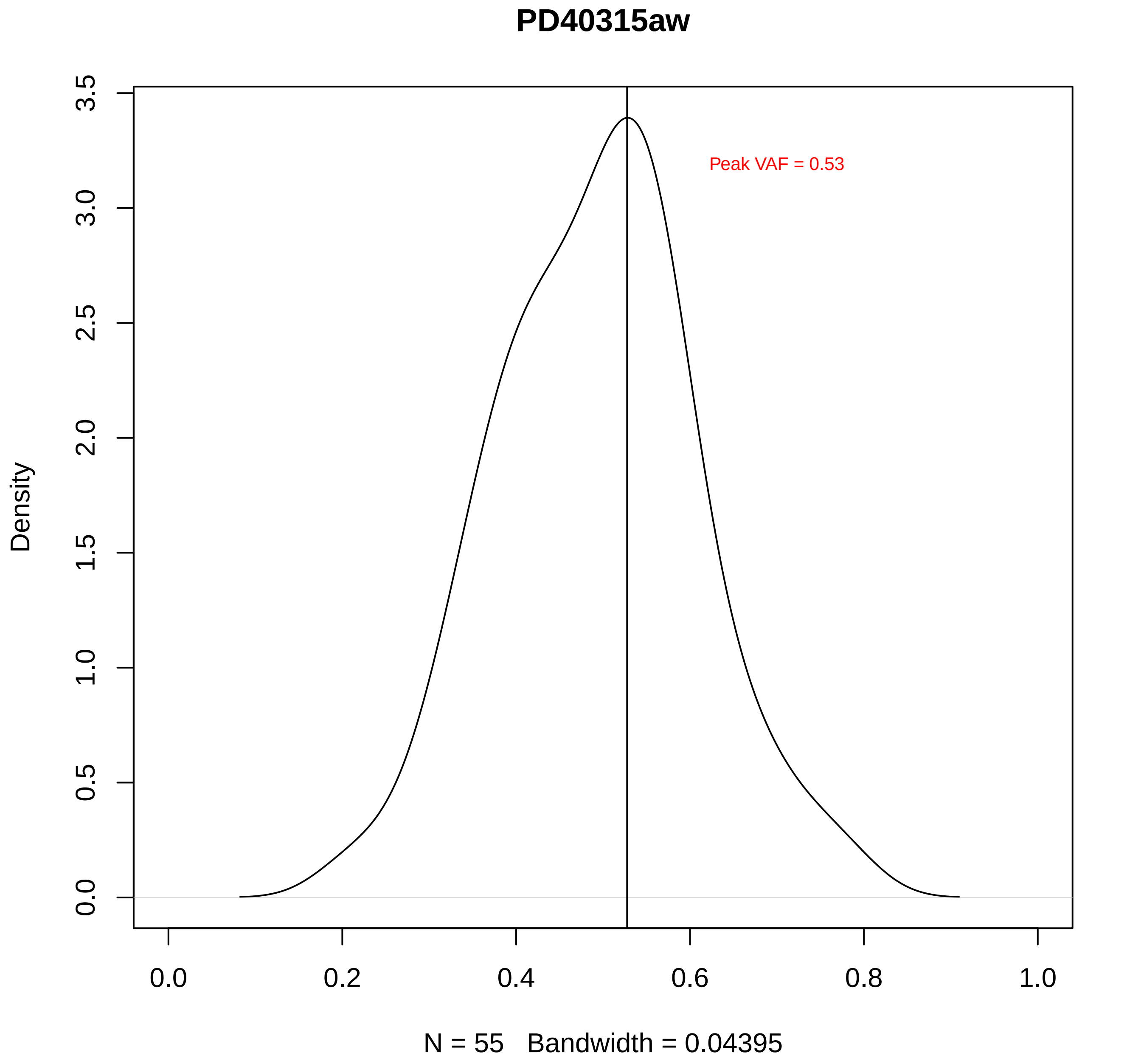
PD40315aw
Density
3.5
3.0
2.5
2.0
1.5
1.0
0.5
0.0
Peak VAF = 0.53
0.0
0.2
0.4
0.6
0.8
1.0
N = 55   Bandwidth = 0.04395

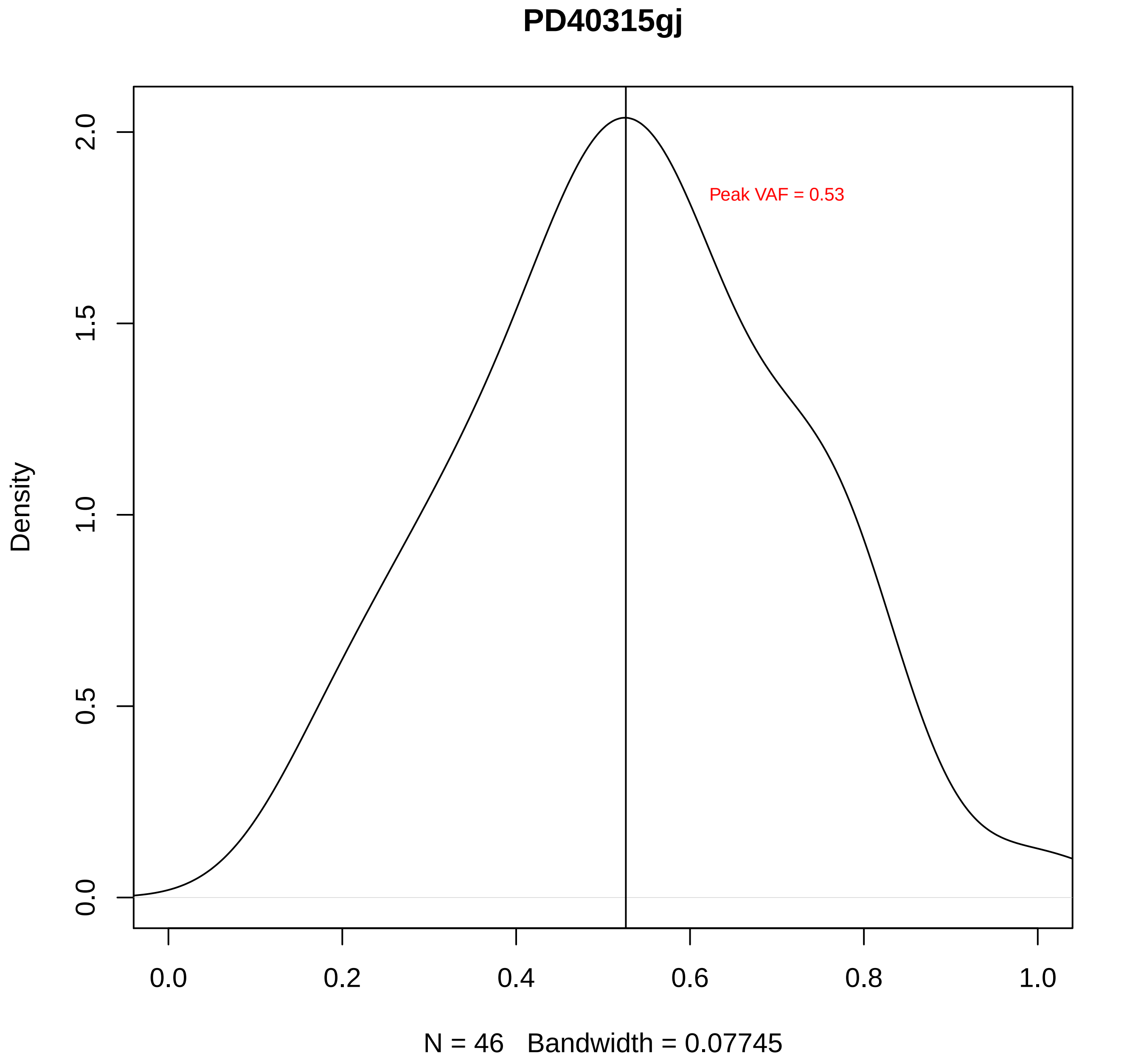
PD40315gj
Peak VAF = 0.53
Density
2.0
1.5
1.0
0.5
0.0
0.0
0.2
0.4
0.6
0.8
1.0
N = 46   Bandwidth = 0.07745

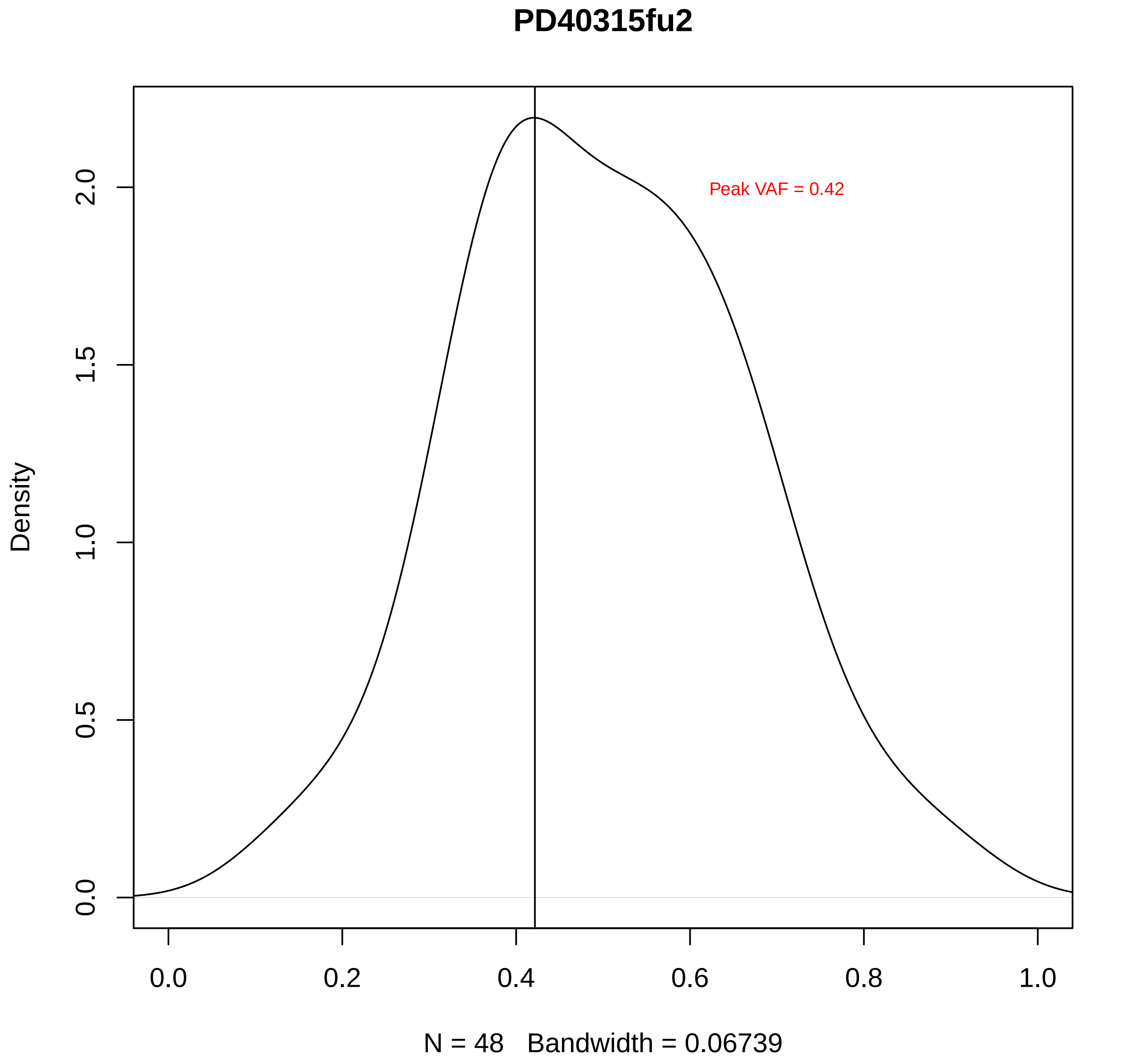
PD40315fu2
Peak VAF = 0.42
Density
0.0
0.5
1.0
1.5
2.0
0.0
0.2
0.4
0.6
0.8
1.0
N = 48   Bandwidth = 0.06739

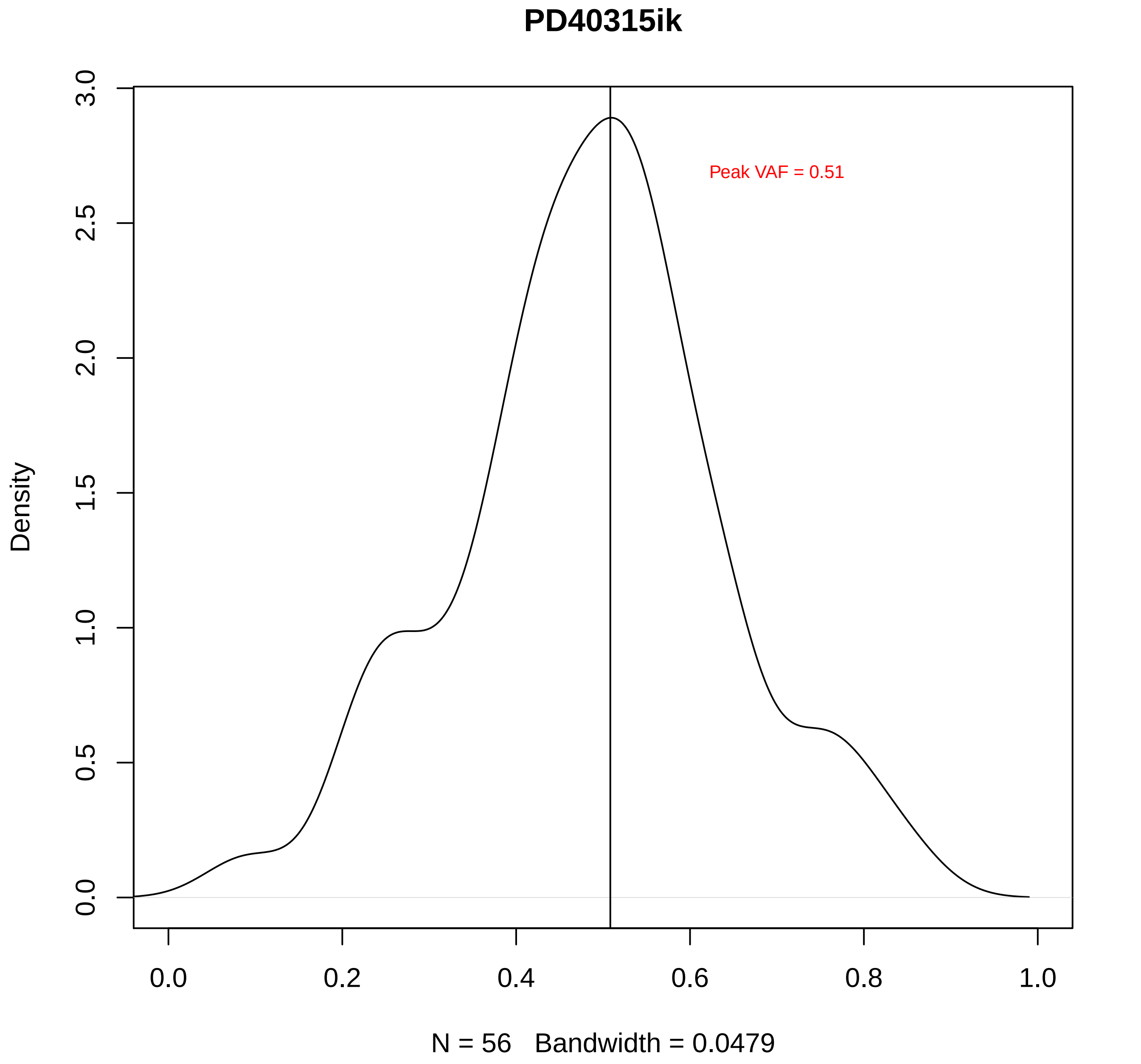
PD40315ik
Peak VAF = 0.51
Density
0.0
0.5
1.0
1.5
2.0
2.5
3.0
0.0
0.2
0.4
0.6
0.8
1.0
N = 56 Bandwidth = 0.0479

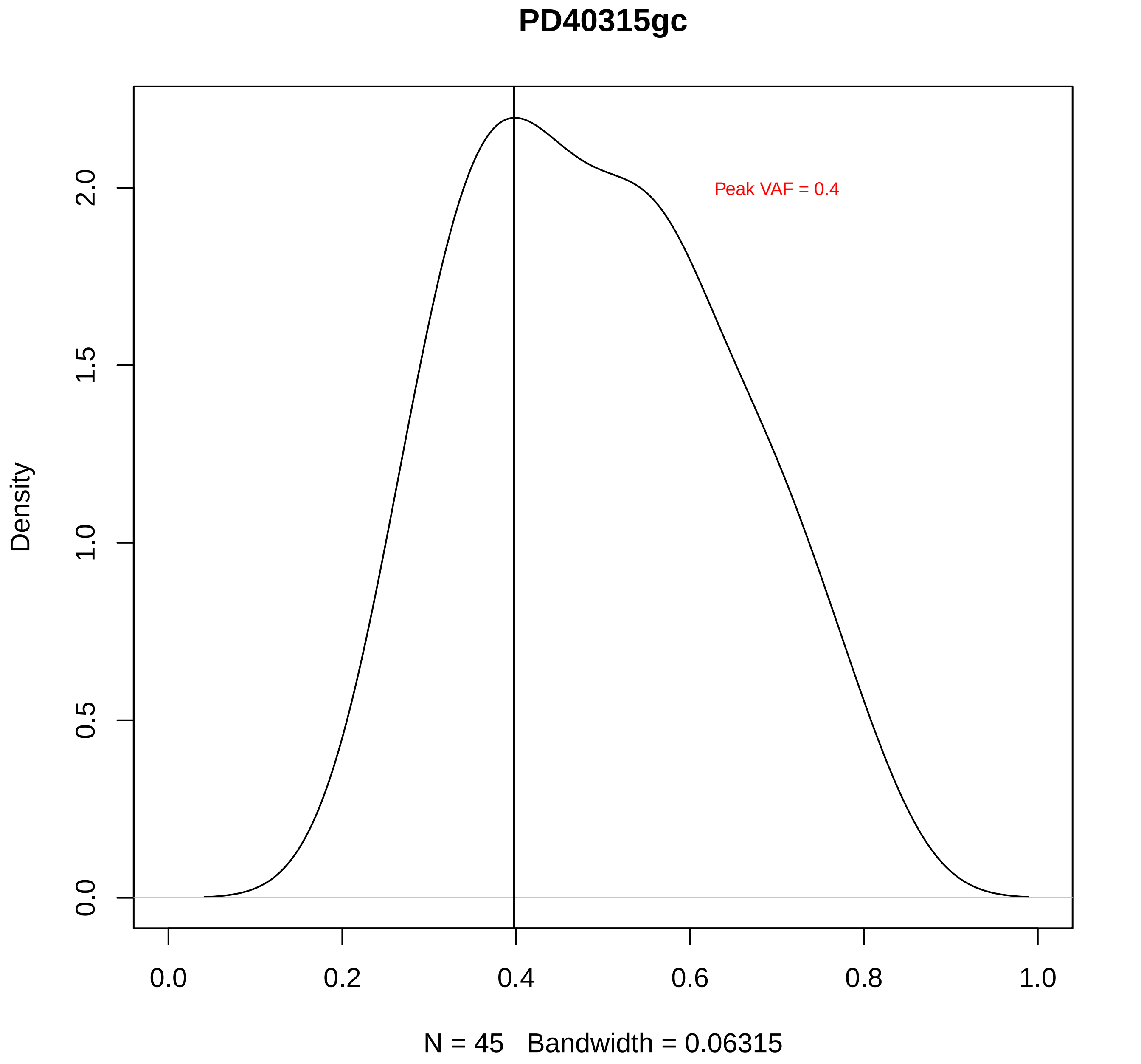

PD40315gc
Peak VAF = 0.4
Density
0.0
0.5
1.0
1.5
2.0
0.0
0.2
0.4
0.6
0.8
1.0
N = 45 Bandwidth = 0.06315

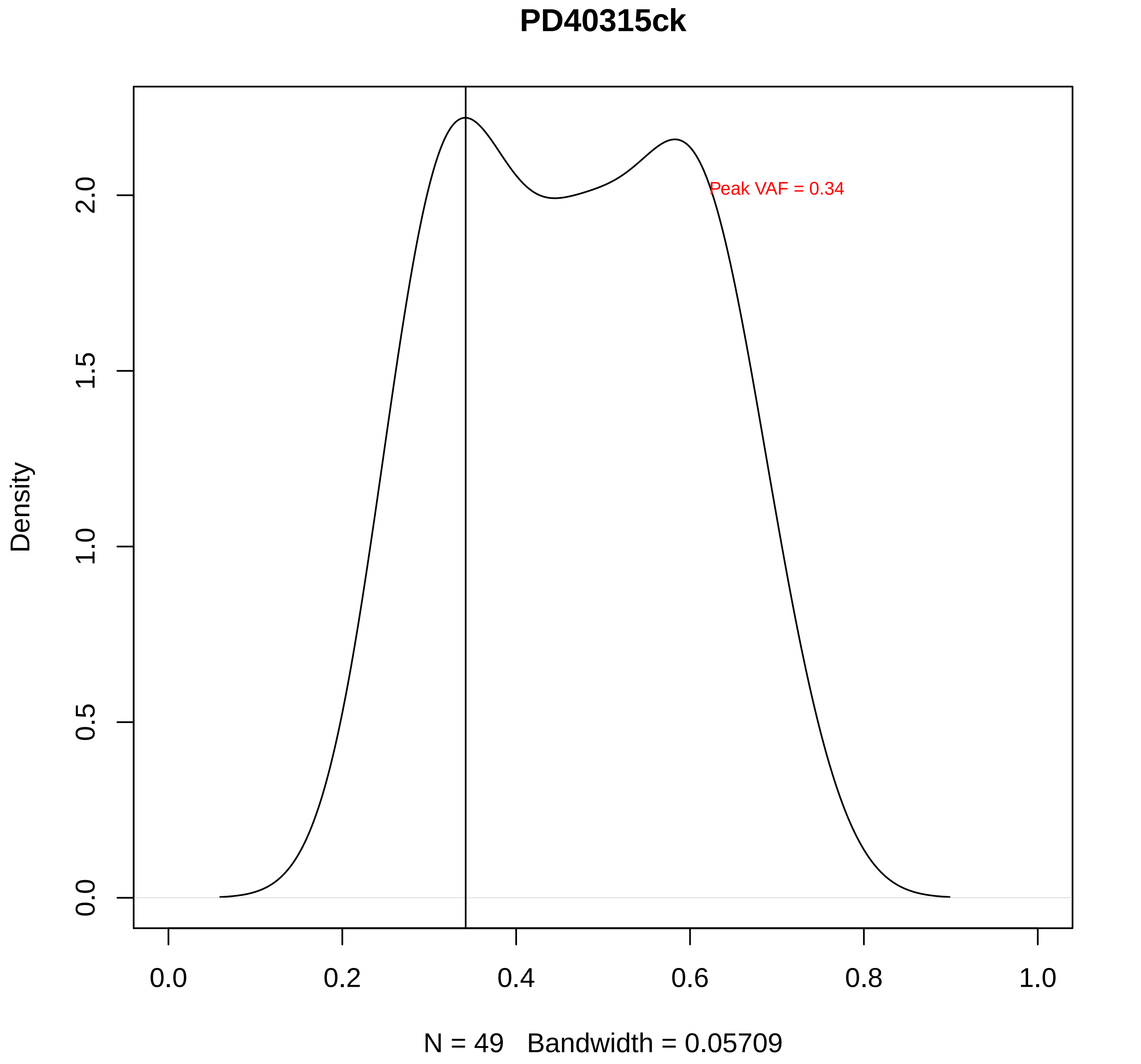

PD40315ck
Peak VAF = 0.34
Density
0.0
0.5
1.0
1.5
2.0
0.0
0.2
0.4
0.6
0.8
1.0
N = 49   Bandwidth = 0.05709

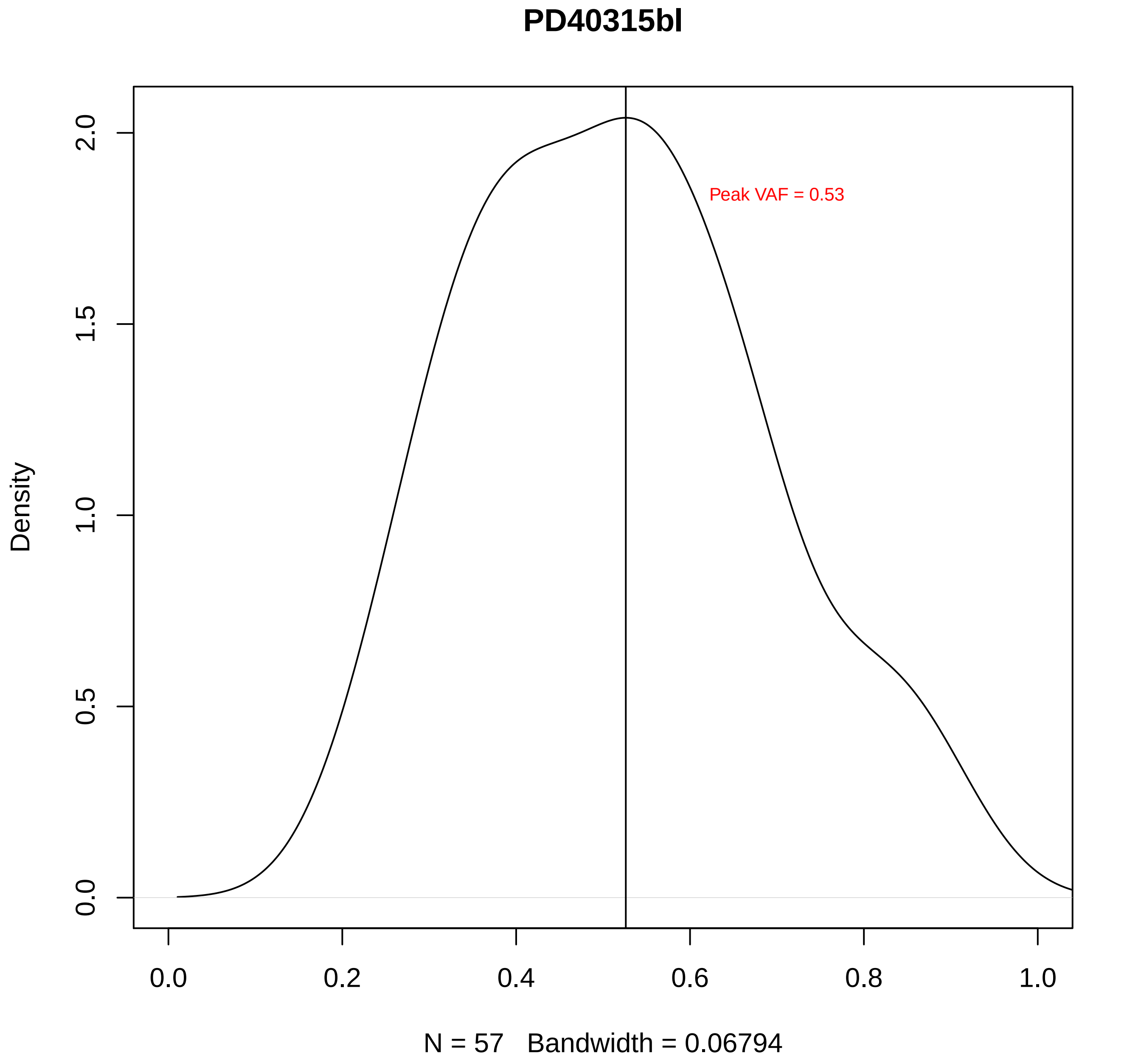

PD40315bl
Peak VAF = 0.53
Density
2.0
1.5
1.0
0.5
0.0
0.2
0.4
0.6
0.8
1.0
N = 57   Bandwidth = 0.06794

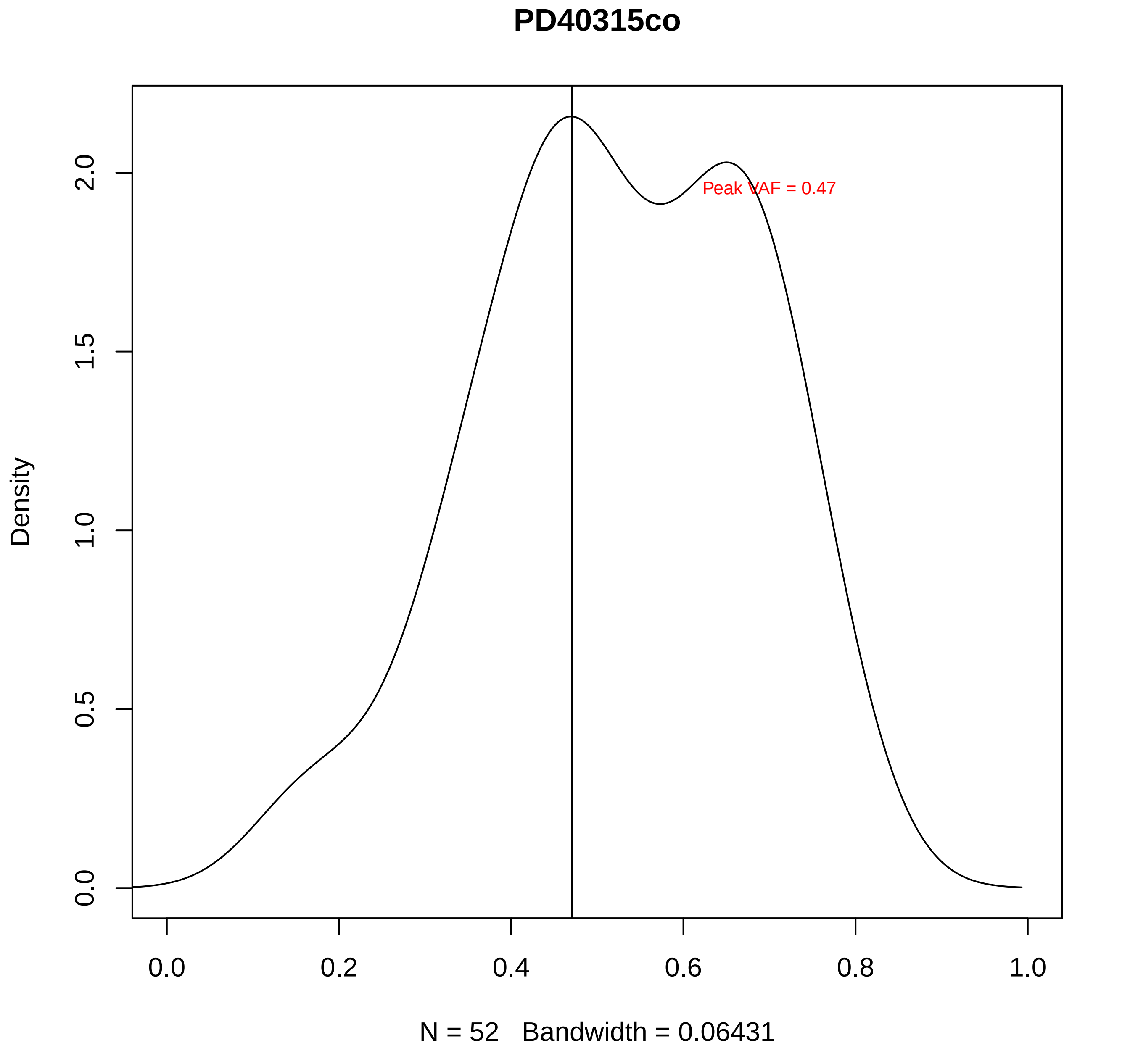
PD40315co
Peak VAF = 0.47
Density
0.0
0.5
1.0
1.5
2.0
0.0
0.2
0.4
0.6
0.8
1.0
N = 52   Bandwidth = 0.06431

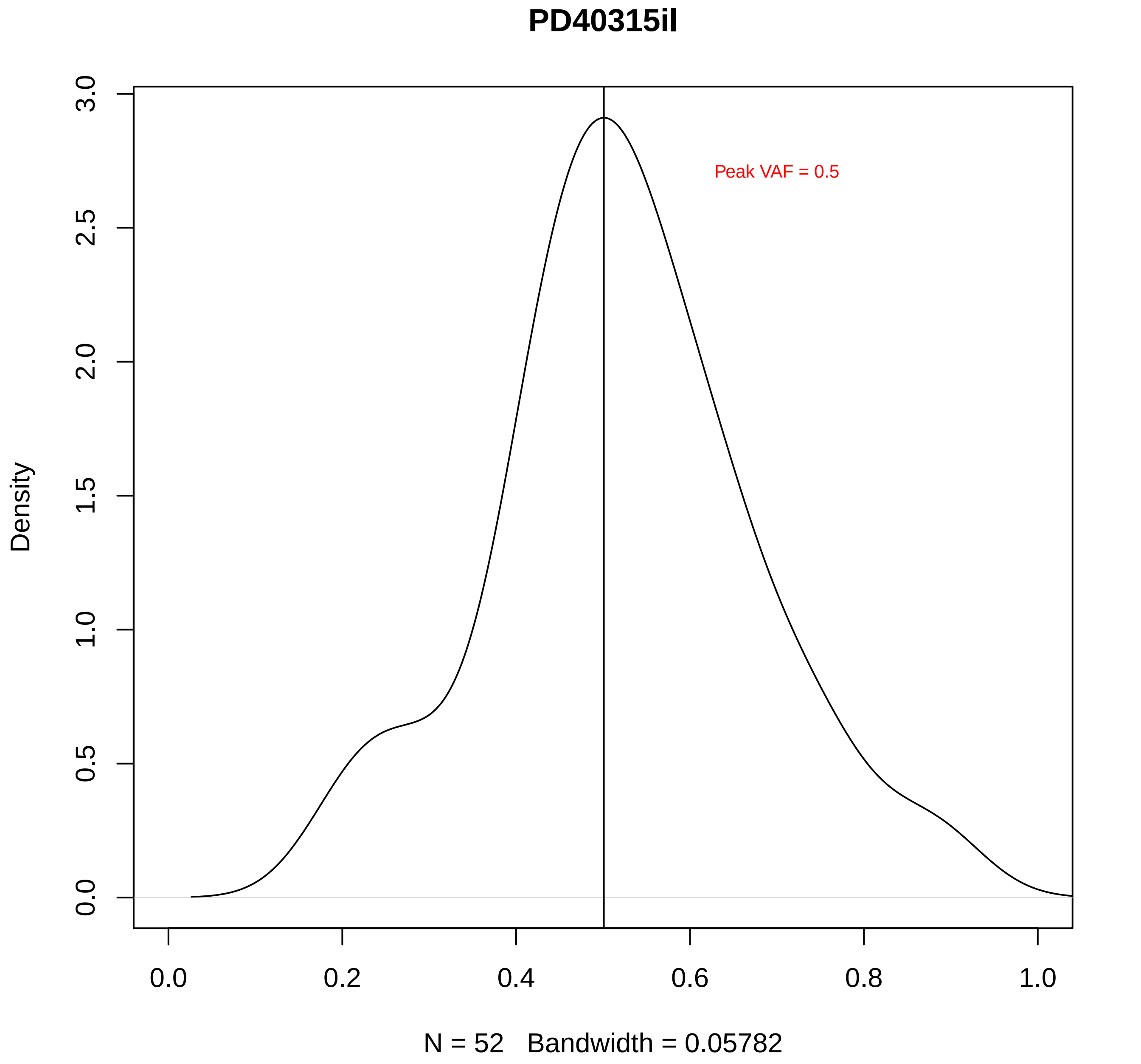
PD40315il
Peak VAF = 0.5
Density
0.0
0.5
1.0
1.5
2.0
2.5
3.0
0.0
0.2
0.4
0.6
0.8
1.0
N = 52   Bandwidth = 0.05782

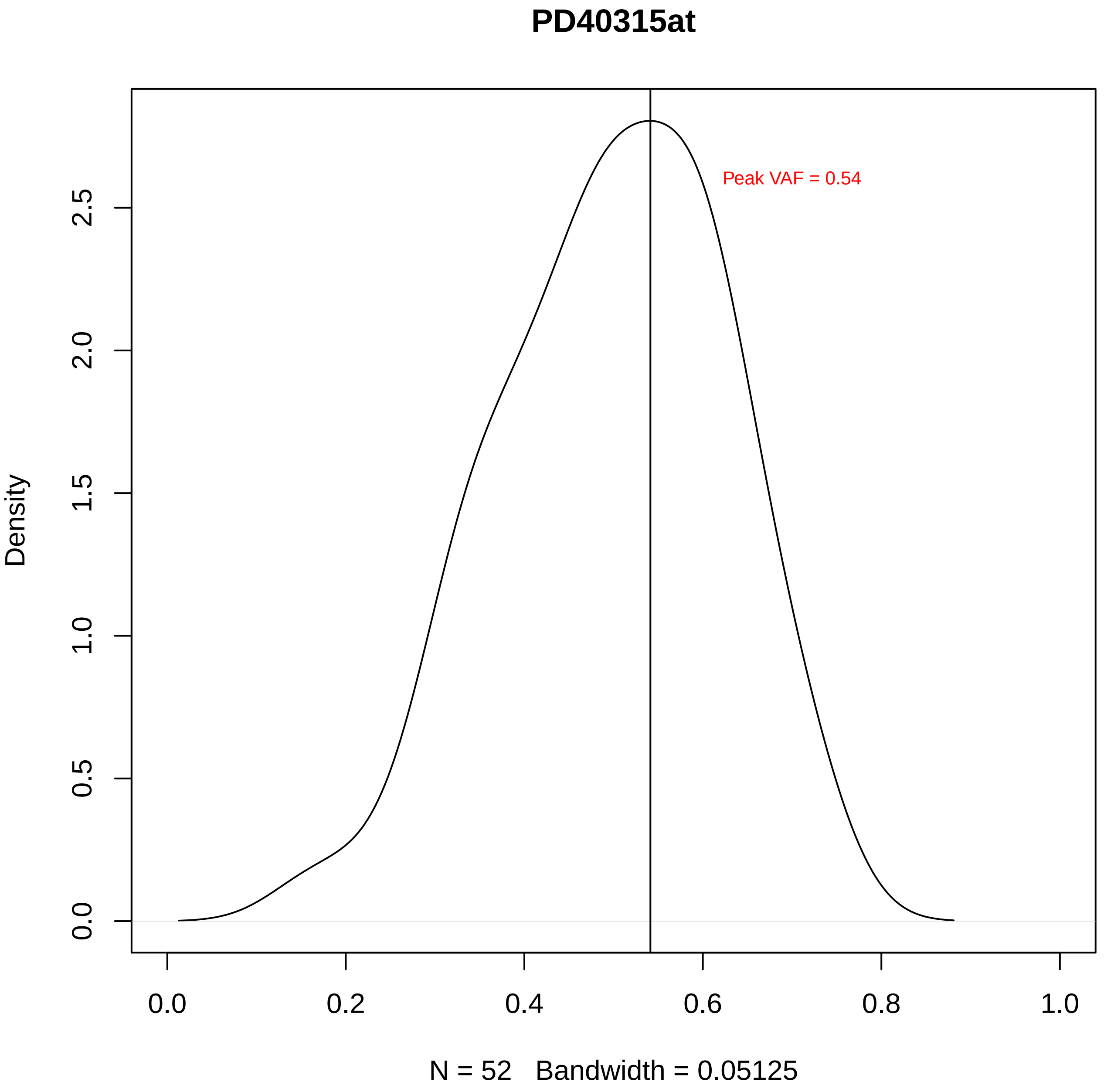
PD40315at
Density
2.5
2.0
1.5
1.0
0.5
0.0
Peak VAF = 0.54
0.0
0.2
0.4
0.6
0.8
1.0
N = 52   Bandwidth = 0.05125

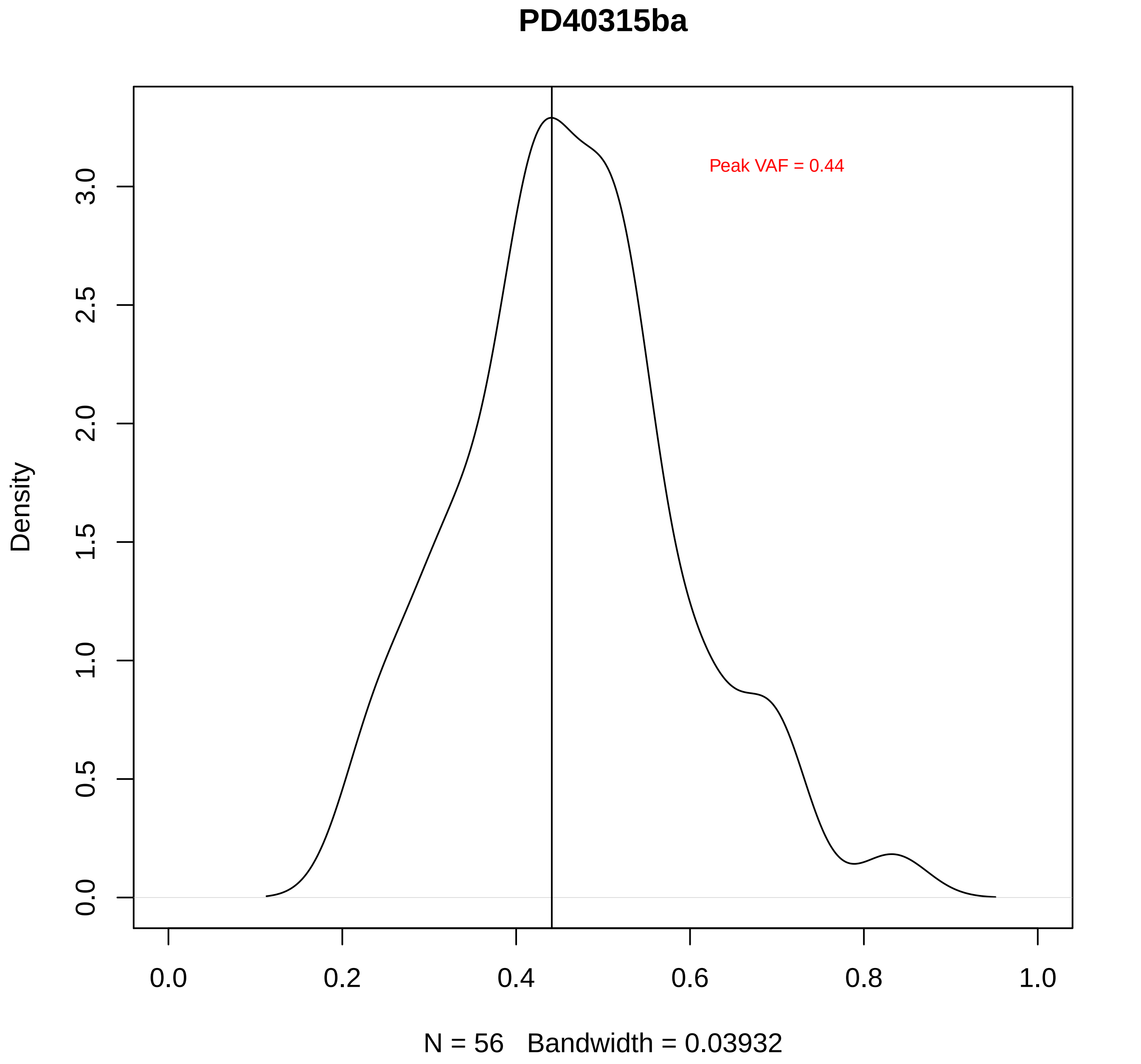

PD40315ba
Peak VAF = 0.44
Density
0.0
0.5
1.0
1.5
2.0
2.5
3.0
0.0
0.2
0.4
0.6
0.8
1.0
N = 56 Bandwidth = 0.03932

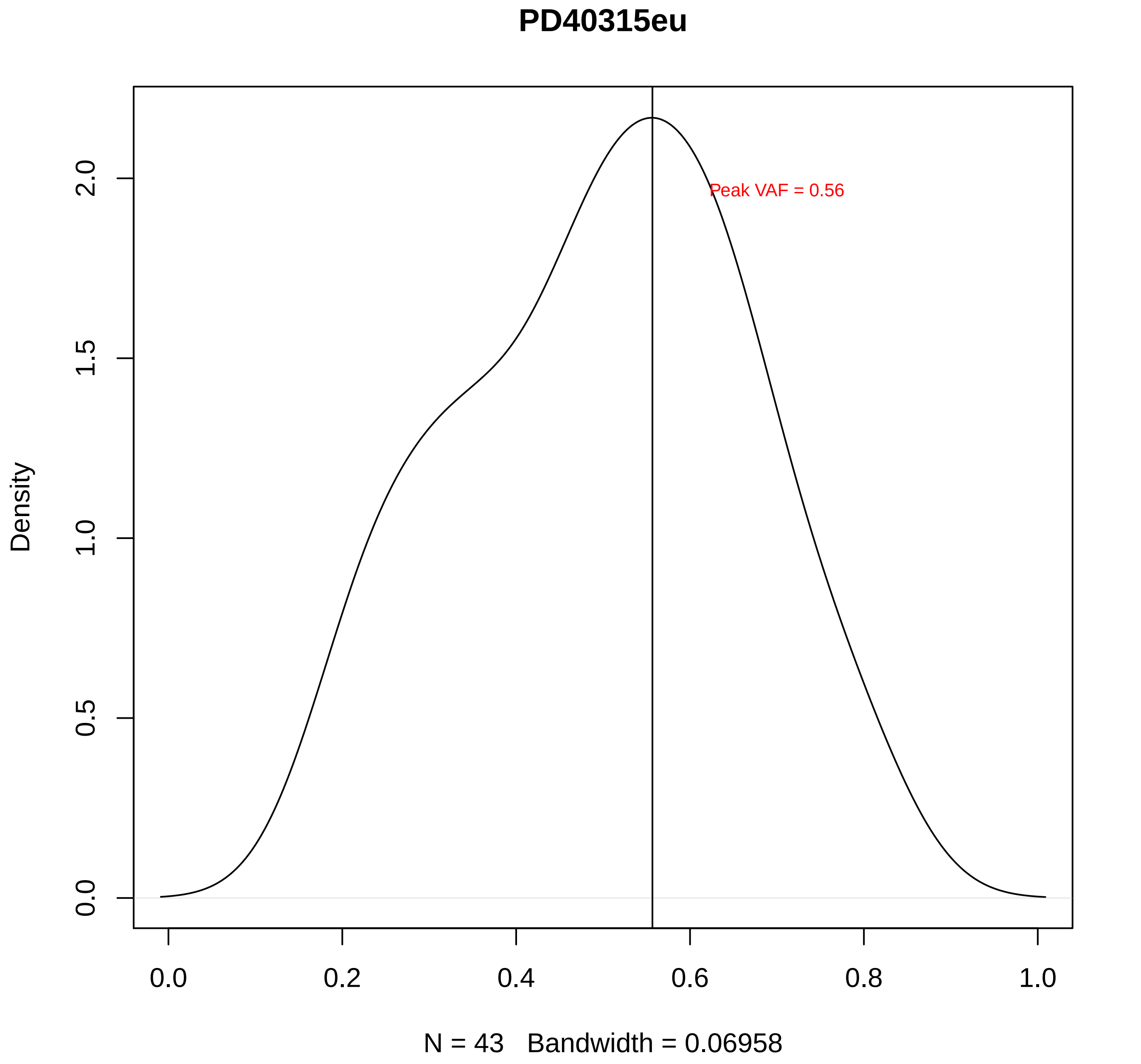

PD40315eu
Peak VAF = 0.56
Density
0.0
0.5
1.0
1.5
2.0
0.0
0.2
0.4
0.6
0.8
1.0
N = 43   Bandwidth = 0.06958

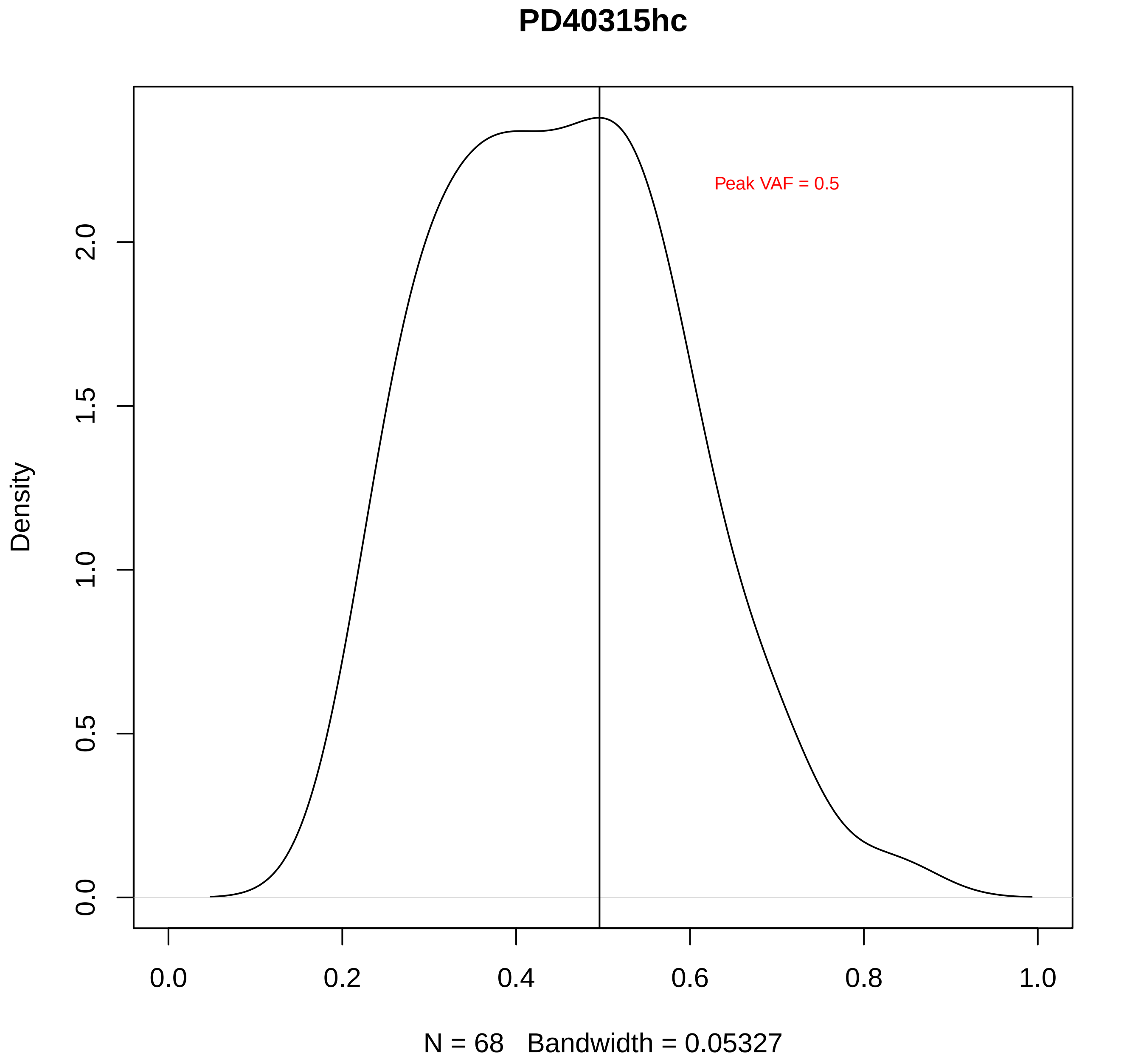
PD40315hc
Peak VAF = 0.5
Density
0.0
0.5
1.0
1.5
2.0
0.0
0.2
0.4
0.6
0.8
1.0
N = 68   Bandwidth = 0.05327

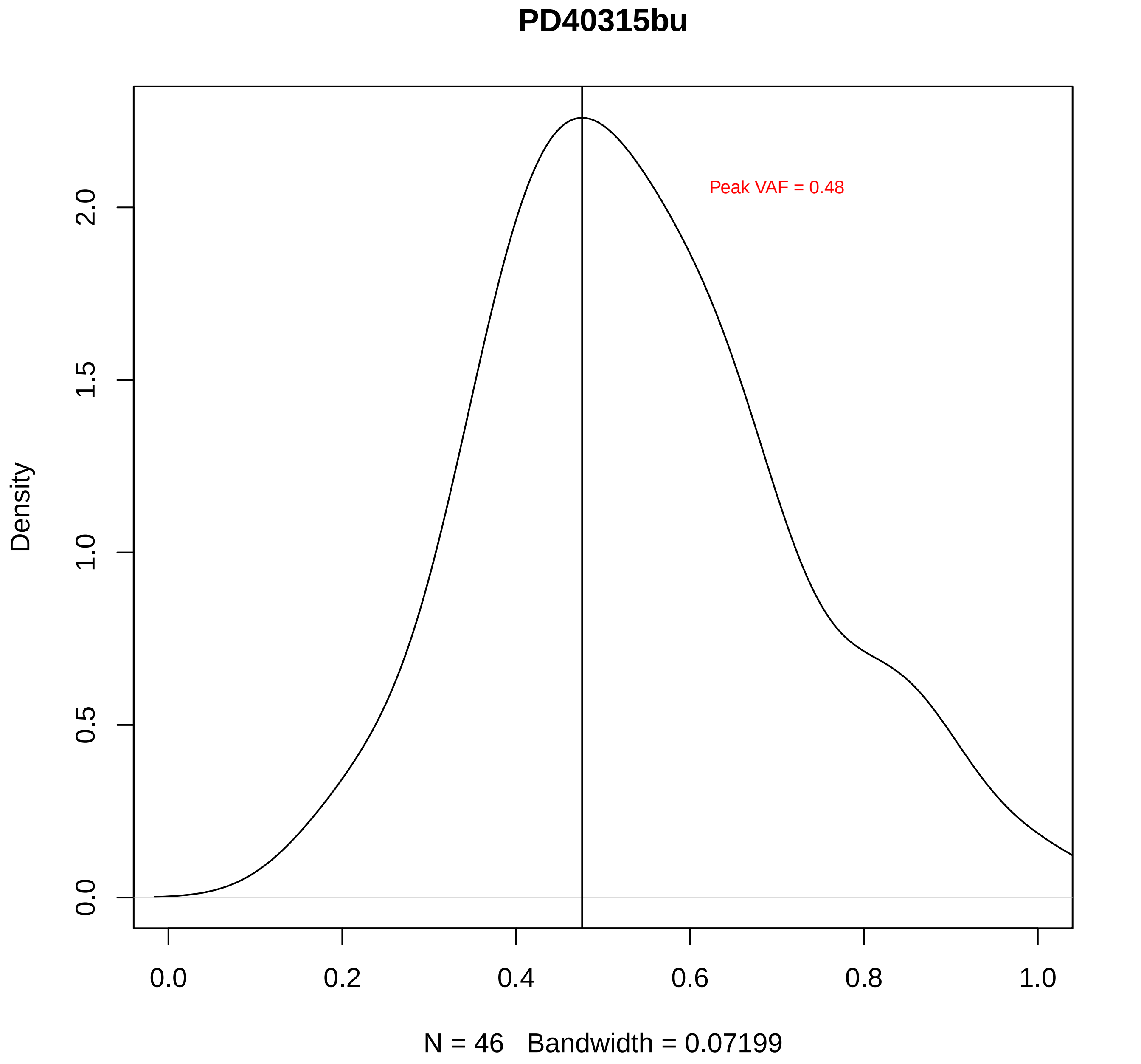
PD40315bu
Peak VAF = 0.48
Density
0.0
0.5
1.0
1.5
2.0
0.0
0.2
0.4
0.6
0.8
1.0
N = 46 Bandwidth = 0.07199

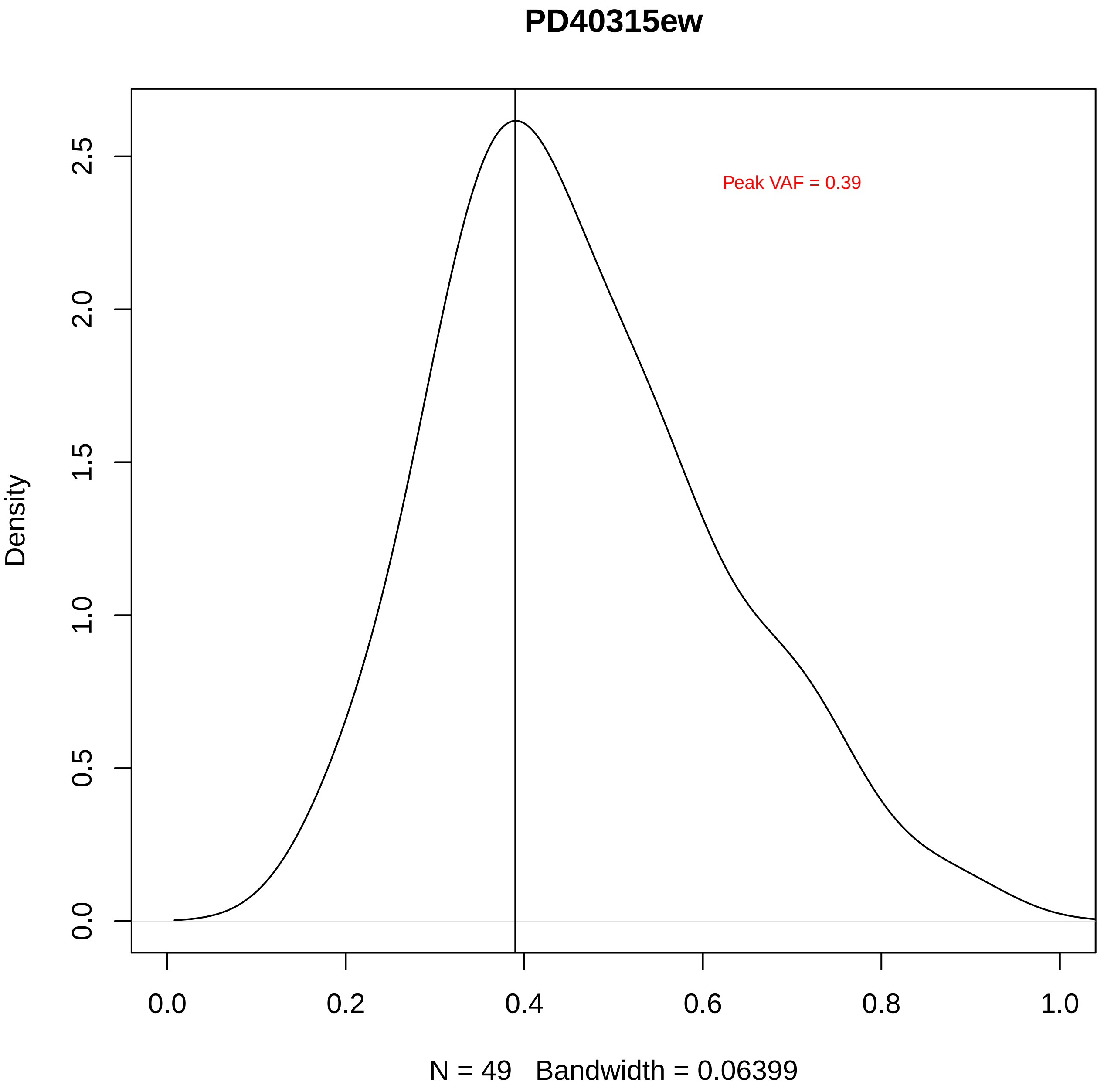
PD40315ew
Peak VAF = 0.39
Density
0.0
0.5
1.0
1.5
2.0
2.5
0.0
0.2
0.4
0.6
0.8
1.0
N = 49   Bandwidth = 0.06399

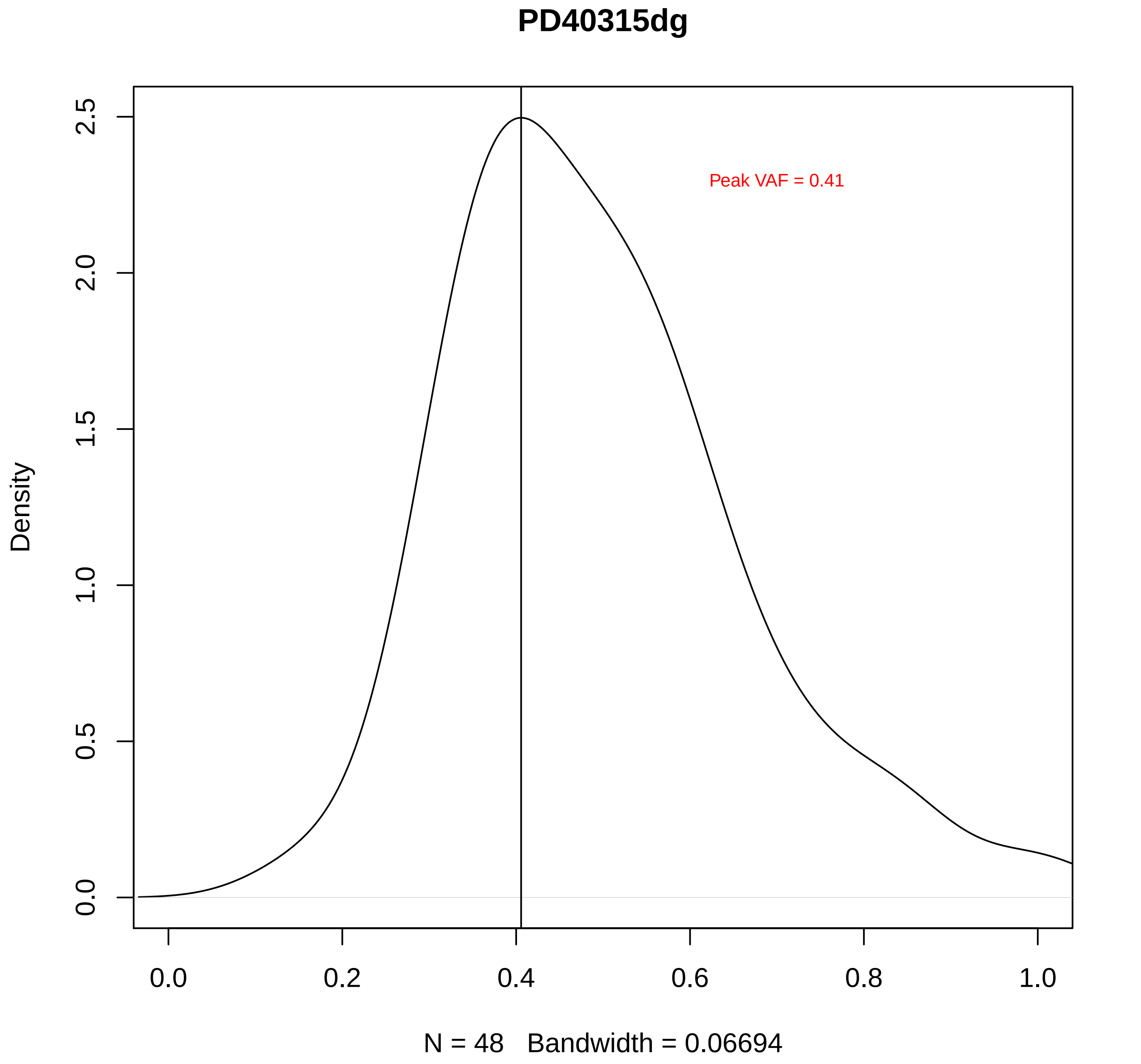

PD40315dg
Peak VAF = 0.41
Density
0.0
0.5
1.0
1.5
2.0
2.5
0.0
0.2
0.4
0.6
0.8
1.0
N = 48   Bandwidth = 0.06694

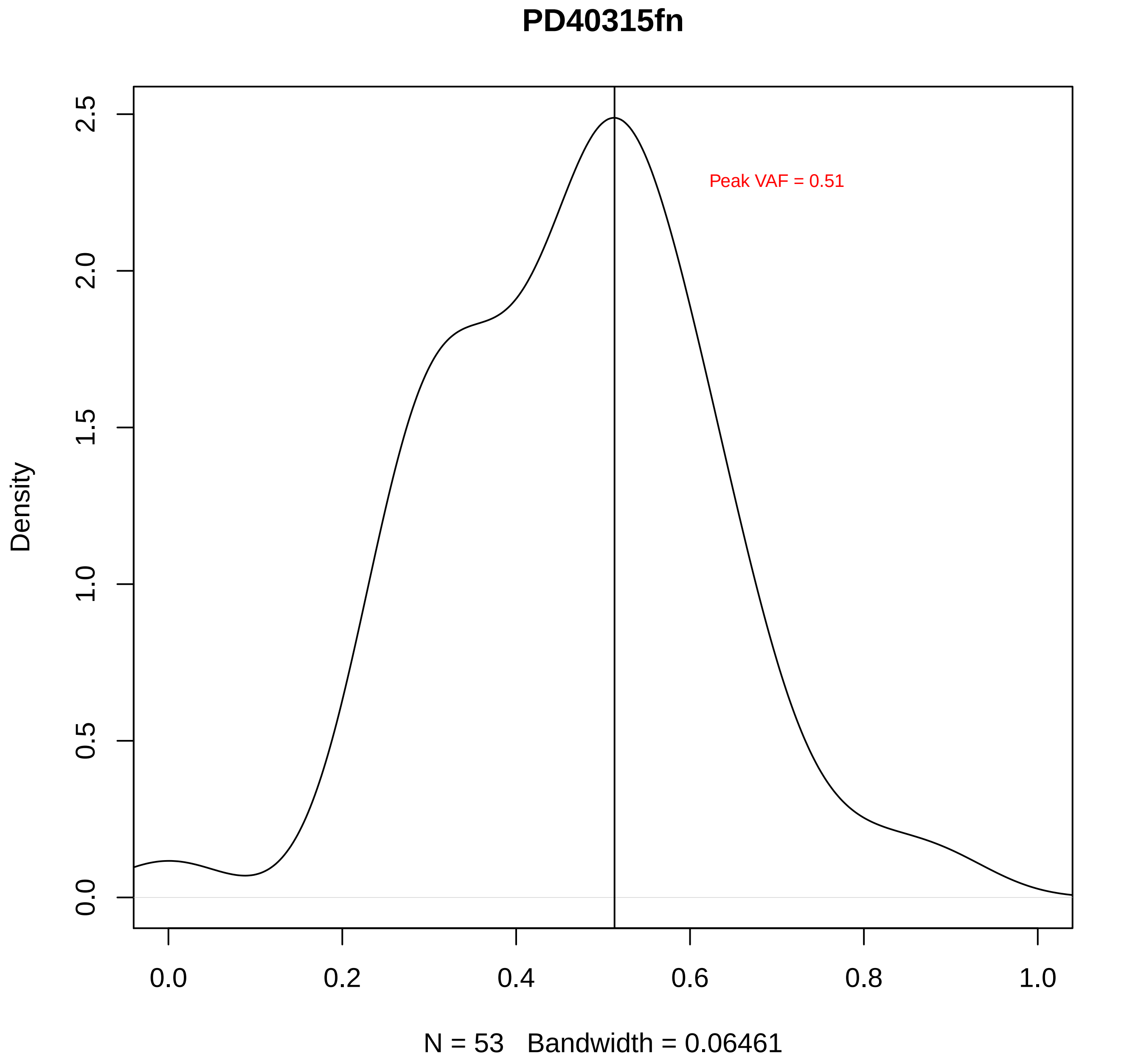
PD40315fn
Peak VAF = 0.51
Density
2.5
2.0
1.5
1.0
0.5
0.0
0.0
0.2
0.4
0.6
0.8
1.0
N = 53 Bandwidth = 0.06461

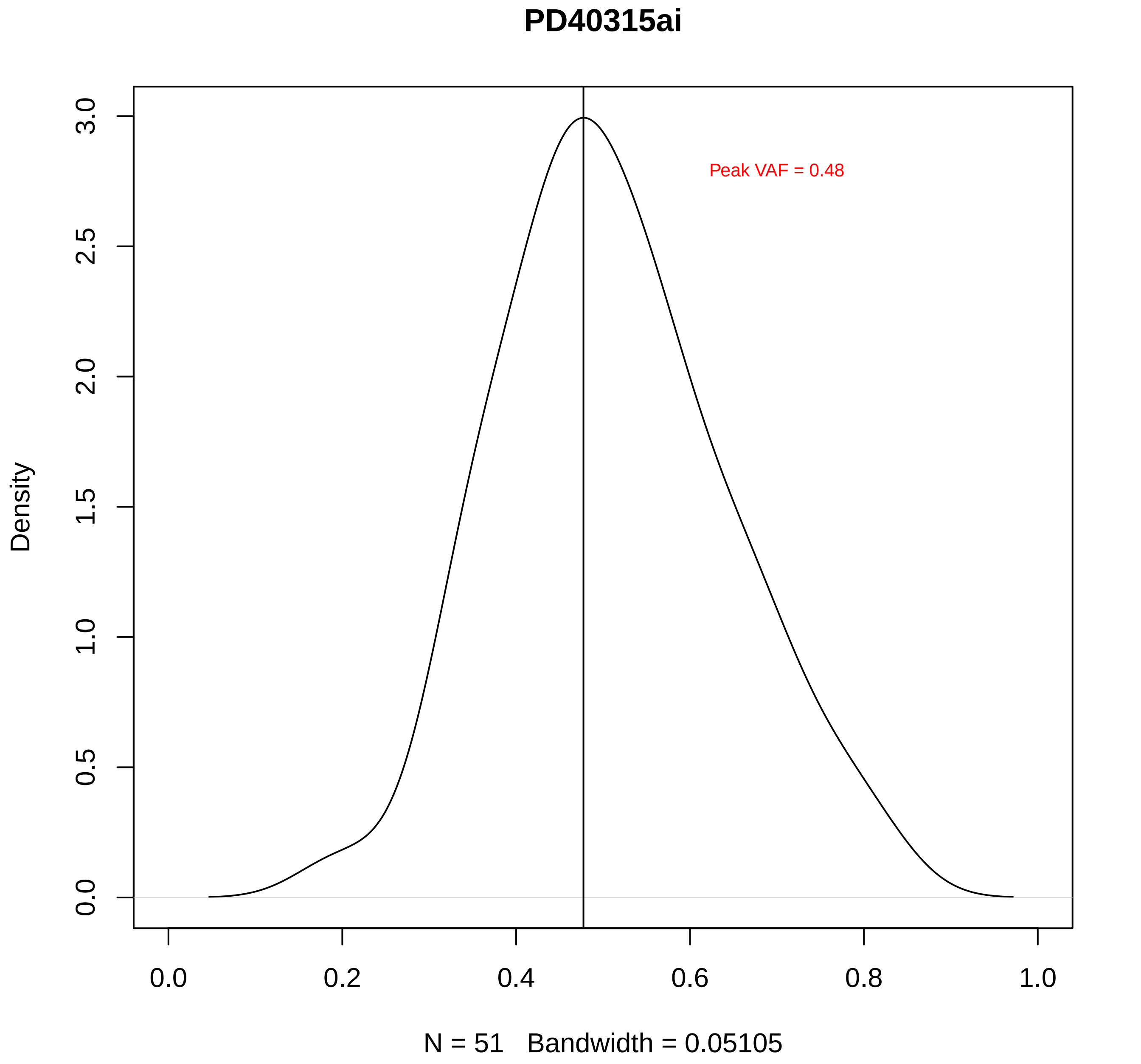
PD40315ai
Peak VAF = 0.48
Density
0.0
0.5
1.0
1.5
2.0
2.5
3.0
0.0
0.2
0.4
0.6
0.8
1.0
N = 51   Bandwidth = 0.05105

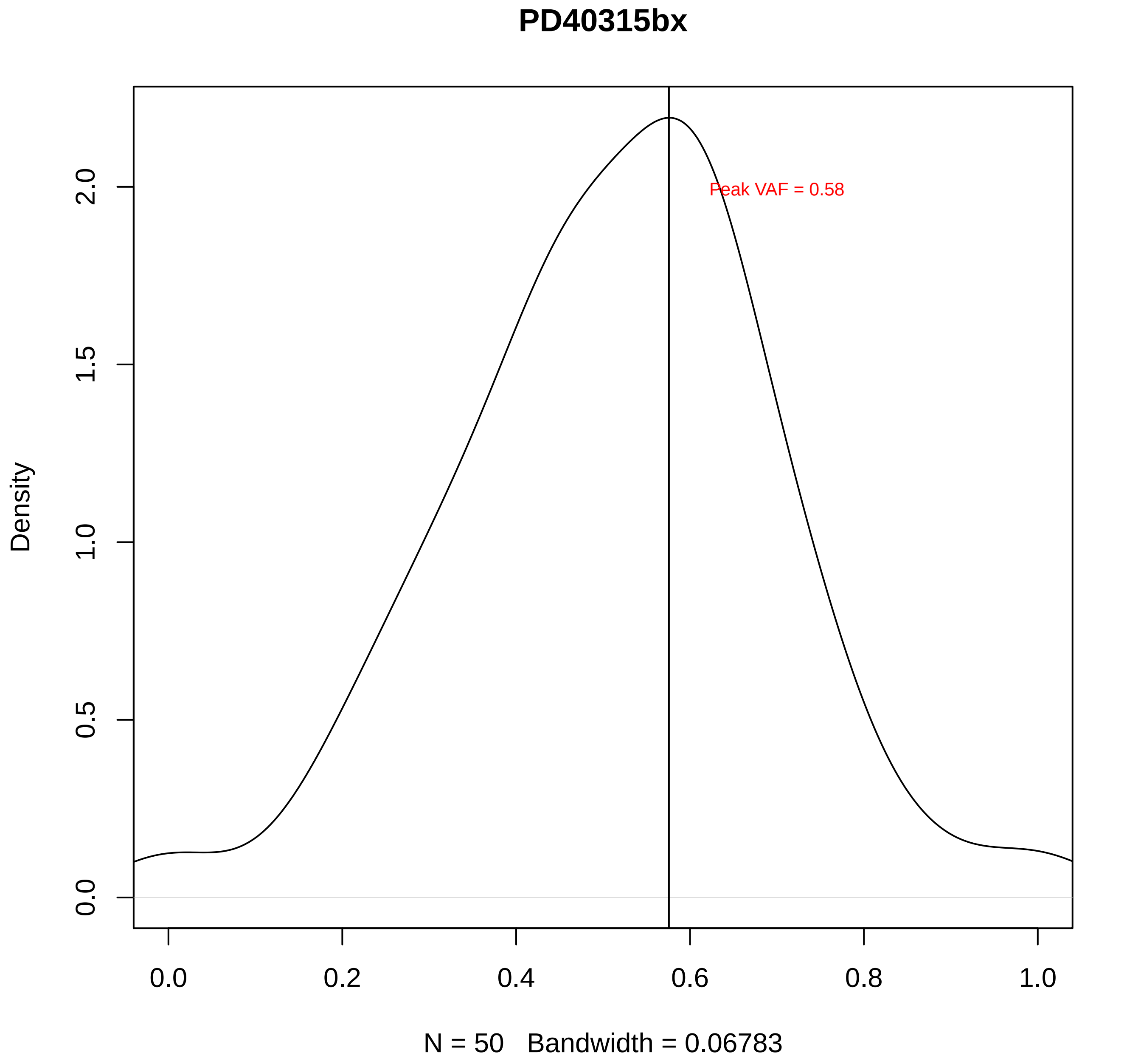
PD40315bx
Peak VAF = 0.58
Density
2.0
1.5
1.0
0.5
0.0
0.0
0.2
0.4
0.6
0.8
1.0
N = 50   Bandwidth = 0.06783

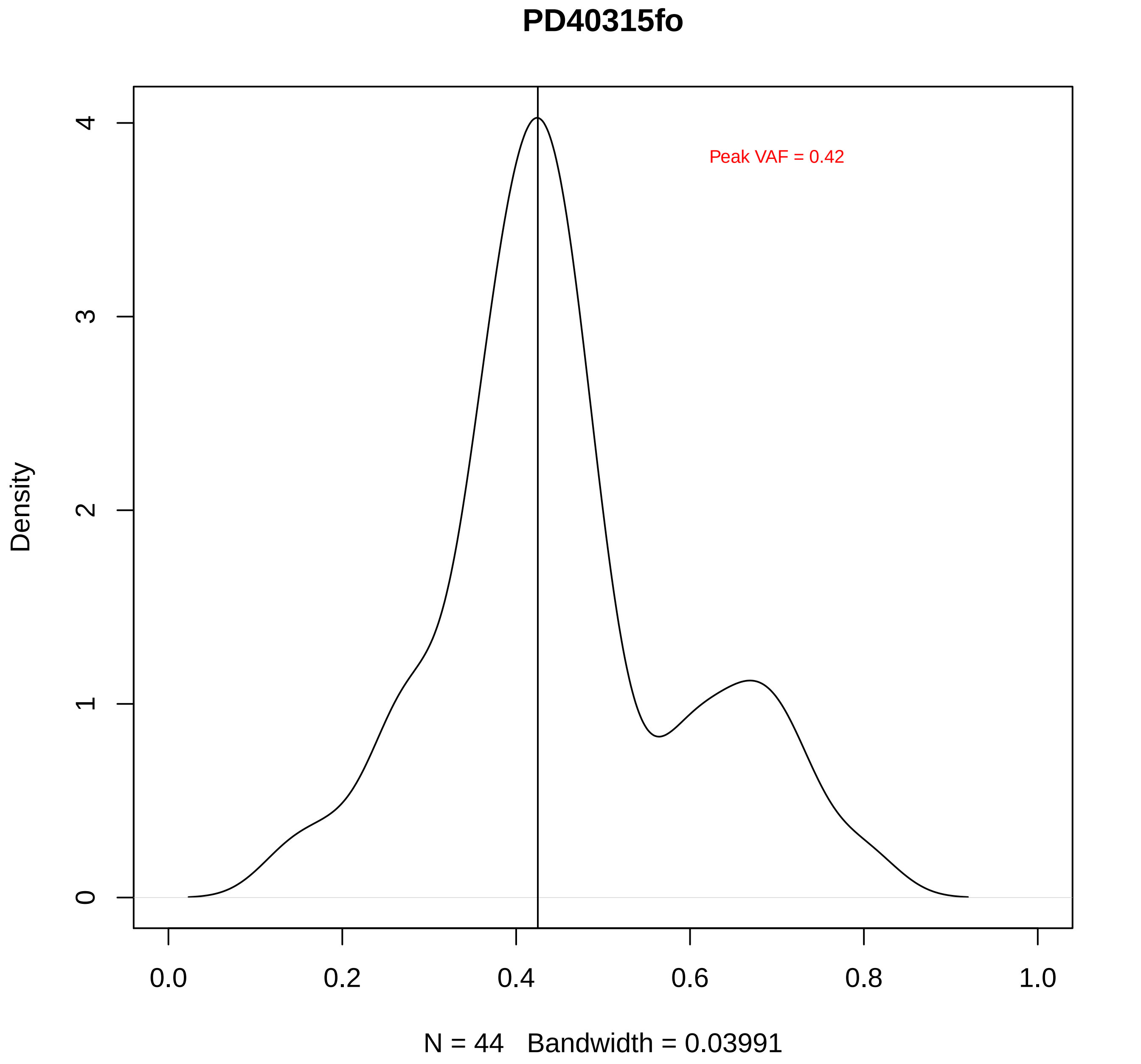
PD40315fo
Peak VAF = 0.42
Density
0
1
2
3
4
0.0
0.2
0.4
0.6
0.8
1.0
N = 44   Bandwidth = 0.03991

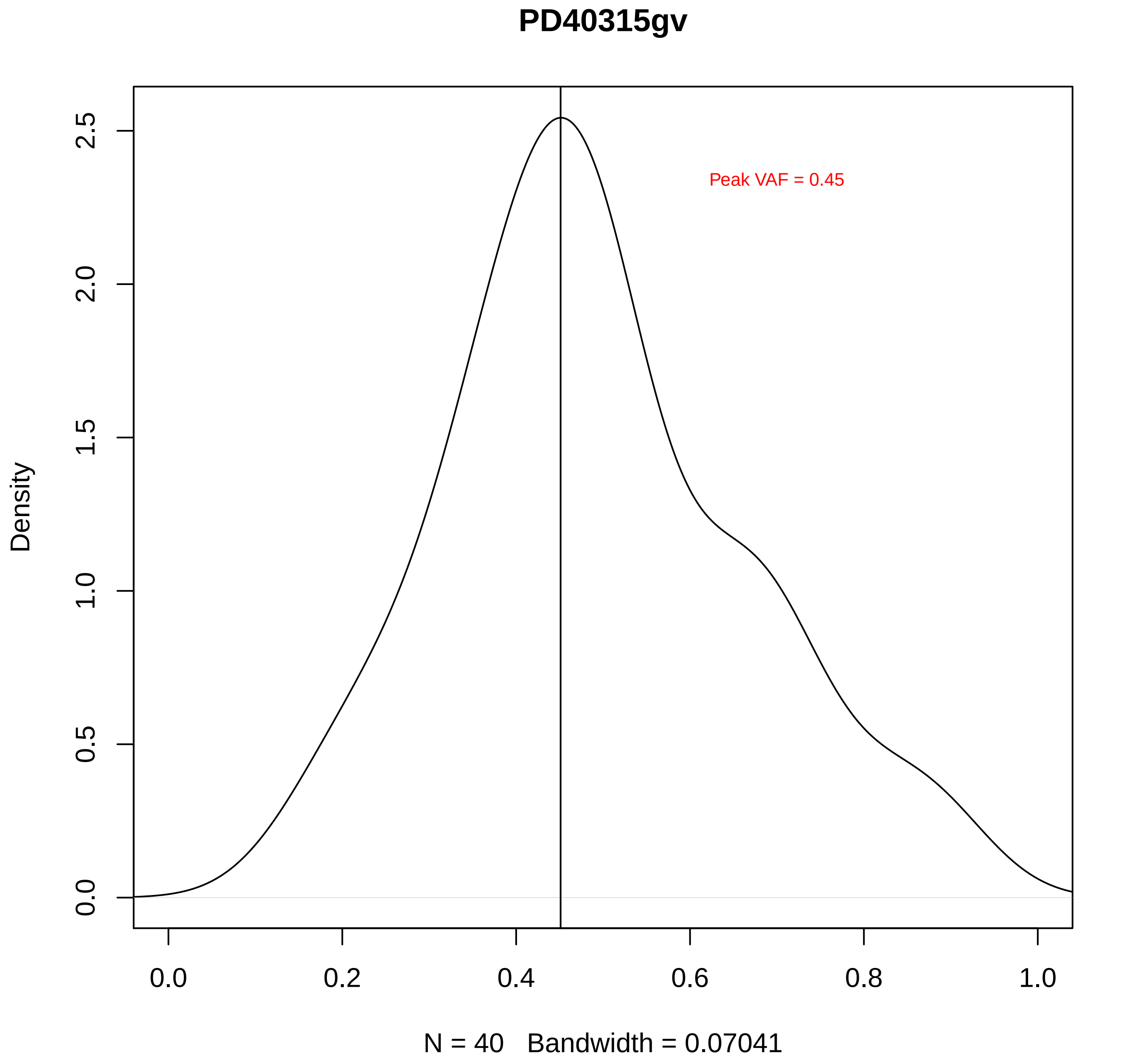

PD40315gv
Peak VAF = 0.45
Density
0.0
0.5
1.0
1.5
2.0
2.5
0.0
0.2
0.4
0.6
0.8
1.0
N = 40 Bandwidth = 0.07041

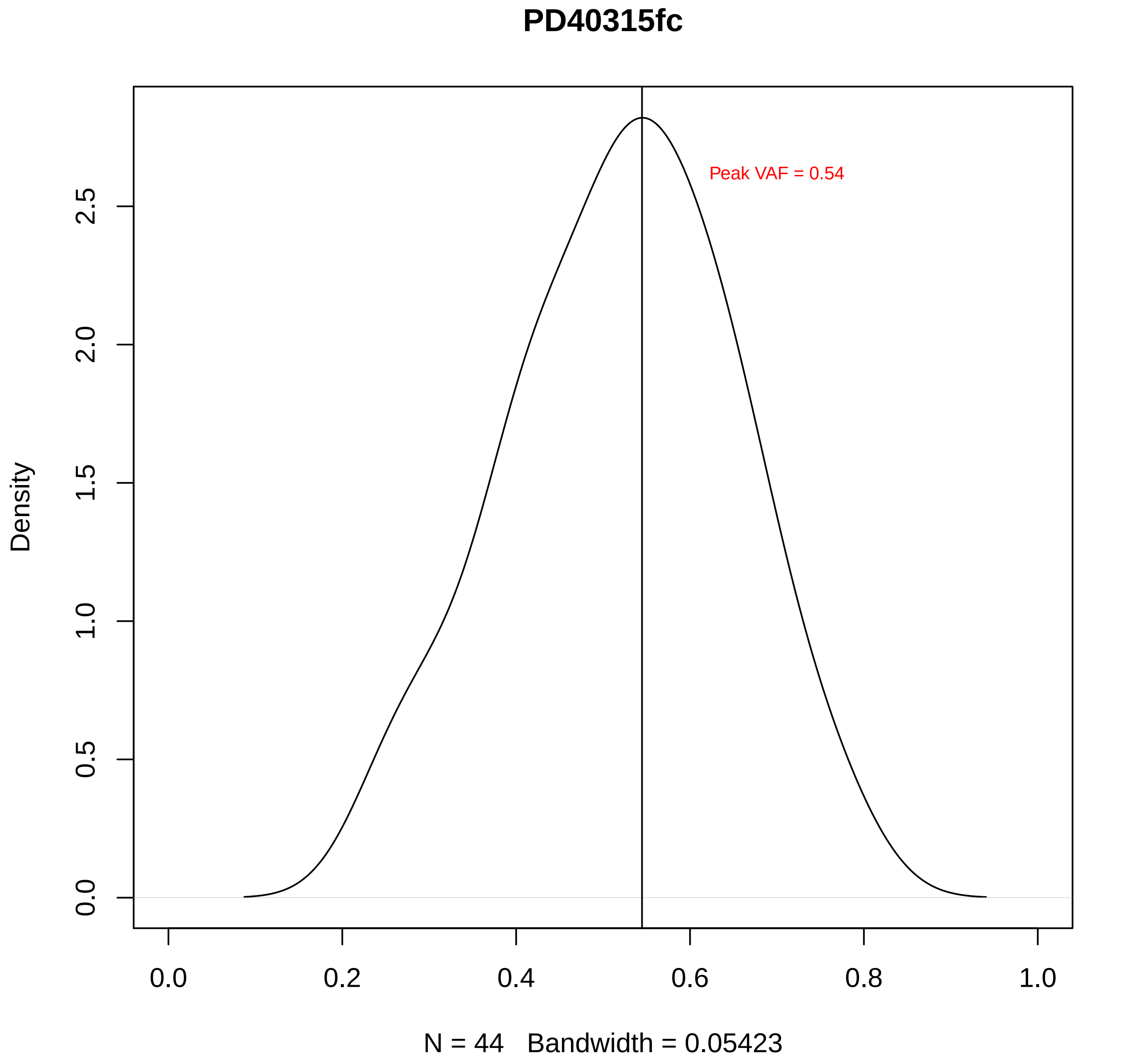
PD40315fc
Peak VAF = 0.54
Density
0.0
0.5
1.0
1.5
2.0
2.5
0.0
0.2
0.4
0.6
0.8
1.0
N = 44   Bandwidth = 0.05423

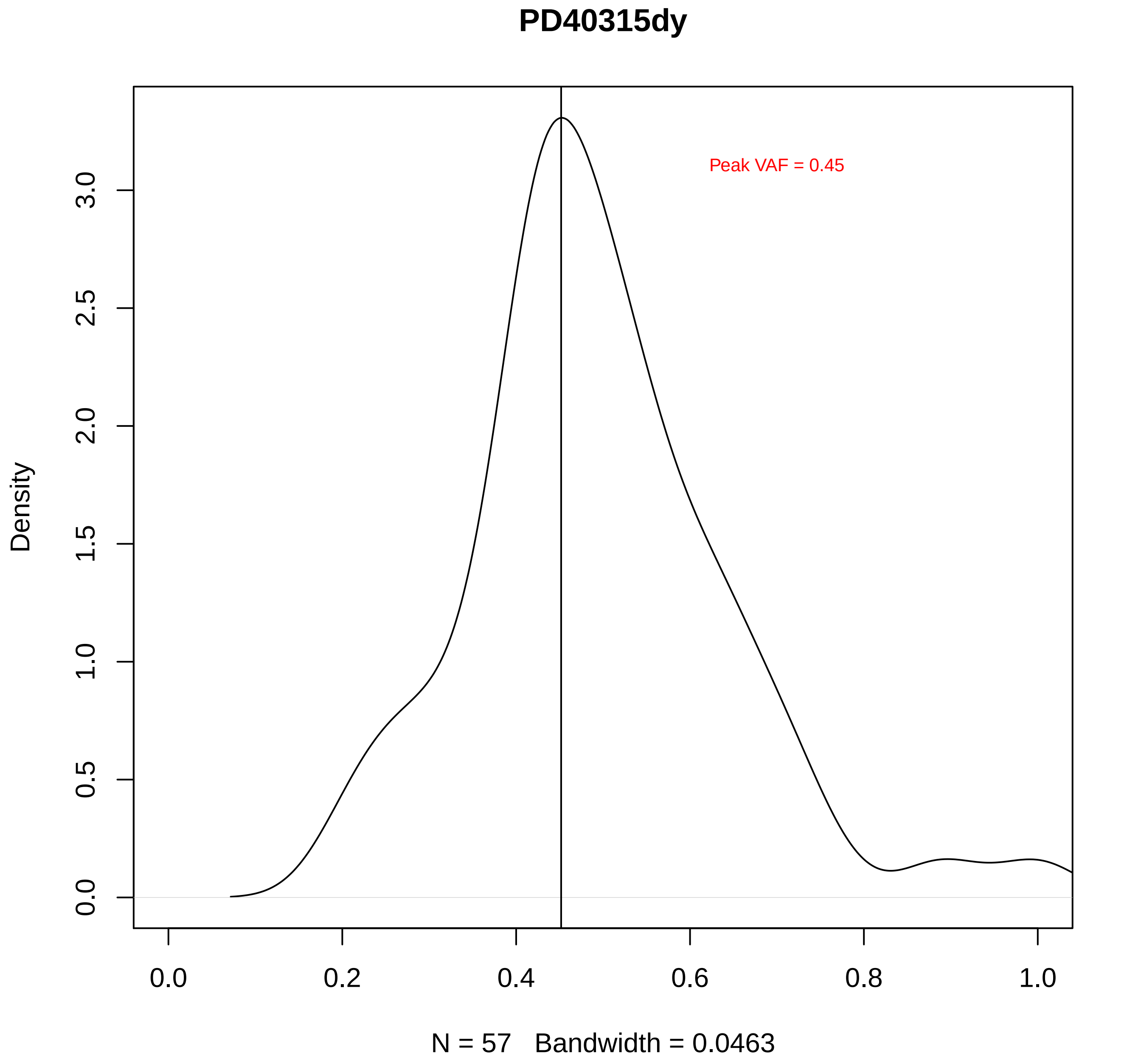
PD40315dy
Peak VAF = 0.45
Density
0.0
0.5
1.0
1.5
2.0
2.5
3.0
0.0
0.2
0.4
0.6
0.8
1.0
N = 57 Bandwidth = 0.0463

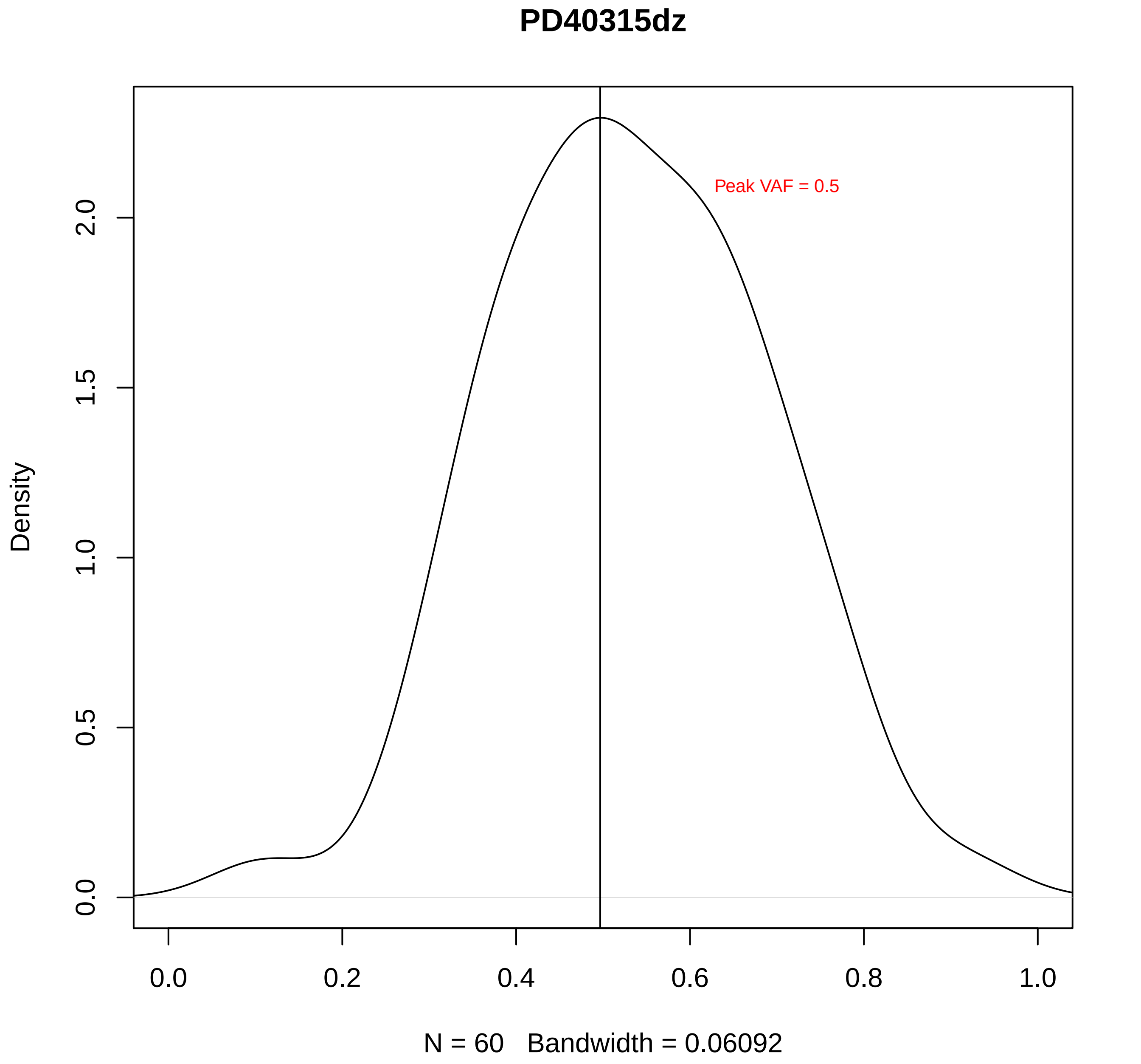
PD40315dz
Peak VAF = 0.5
Density
0.0
0.5
1.0
1.5
2.0
0.0
0.2
0.4
0.6
0.8
1.0
N = 60 Bandwidth = 0.06092

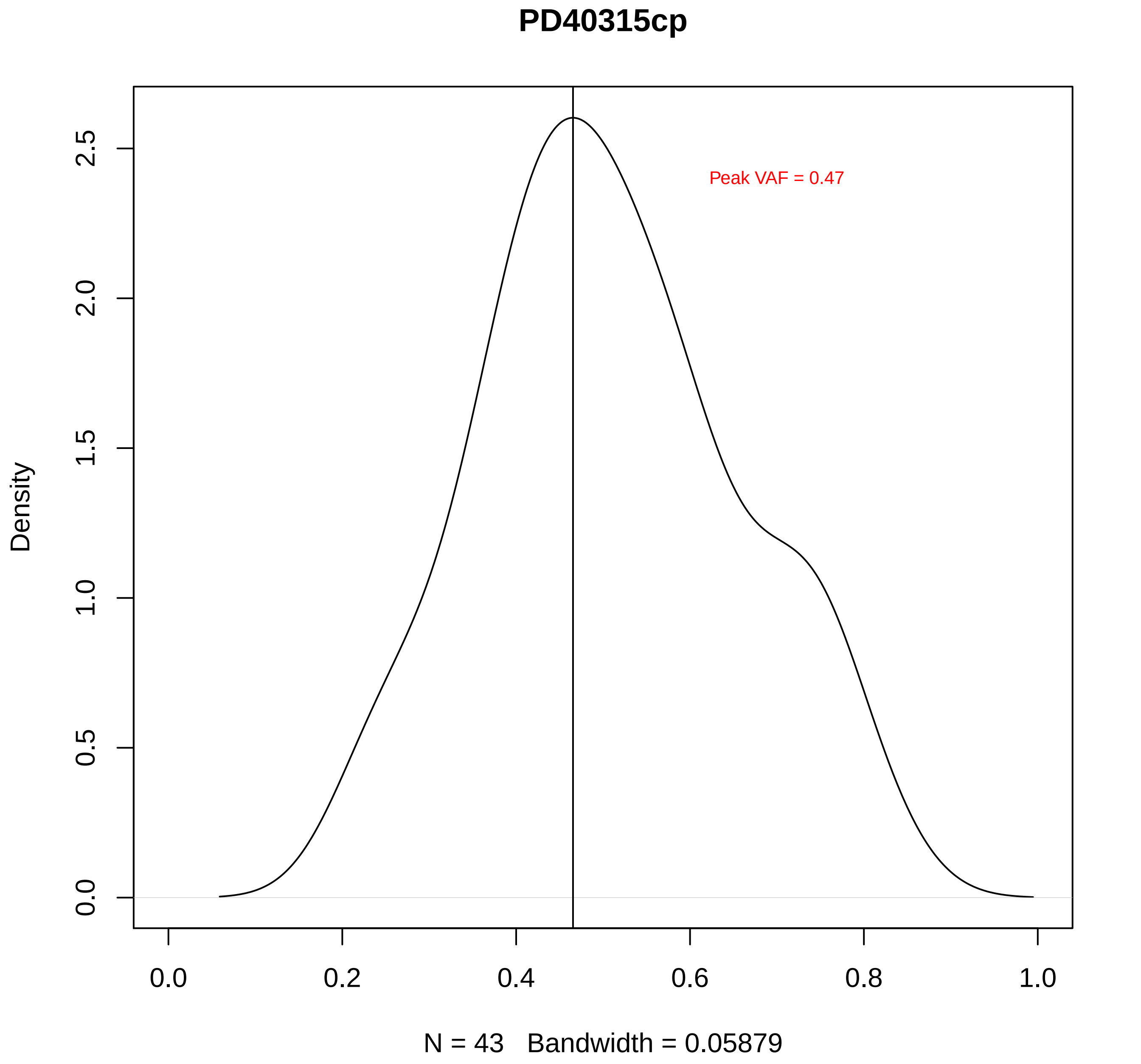

PD40315cp
Peak VAF = 0.47
Density
0.0
0.5
1.0
1.5
2.0
2.5
0.0
0.2
0.4
0.6
0.8
1.0
N = 43   Bandwidth = 0.05879

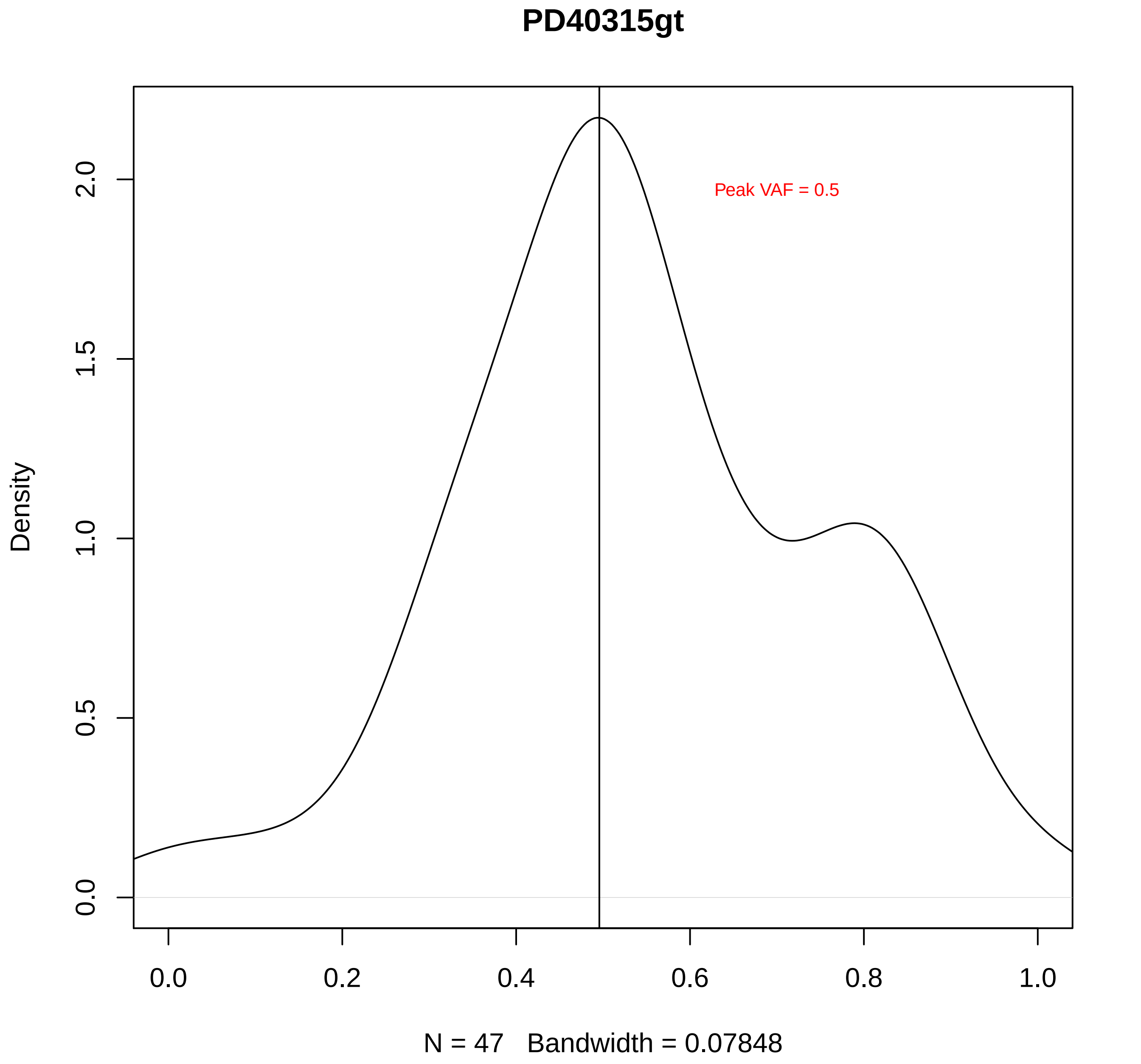
PD40315gt
Peak VAF = 0.5
Density
0.0
0.5
1.0
1.5
2.0
0.0
0.2
0.4
0.6
0.8
1.0
N = 47 Bandwidth = 0.07848

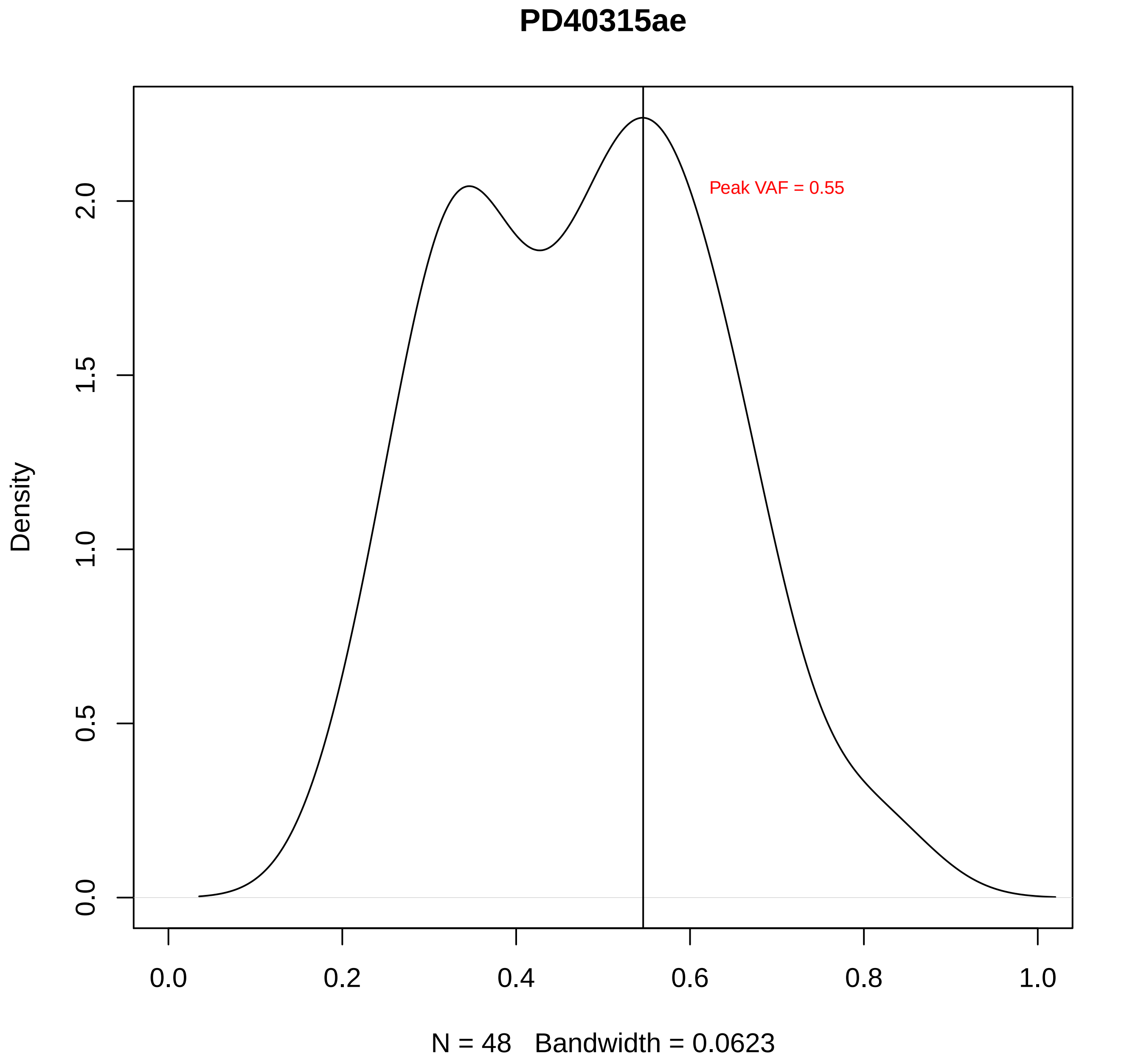

PD40315ae
Peak VAF = 0.55
Density
0.0
0.5
1.0
1.5
2.0
0.0
0.2
0.4
0.6
0.8
1.0
N = 48   Bandwidth = 0.0623

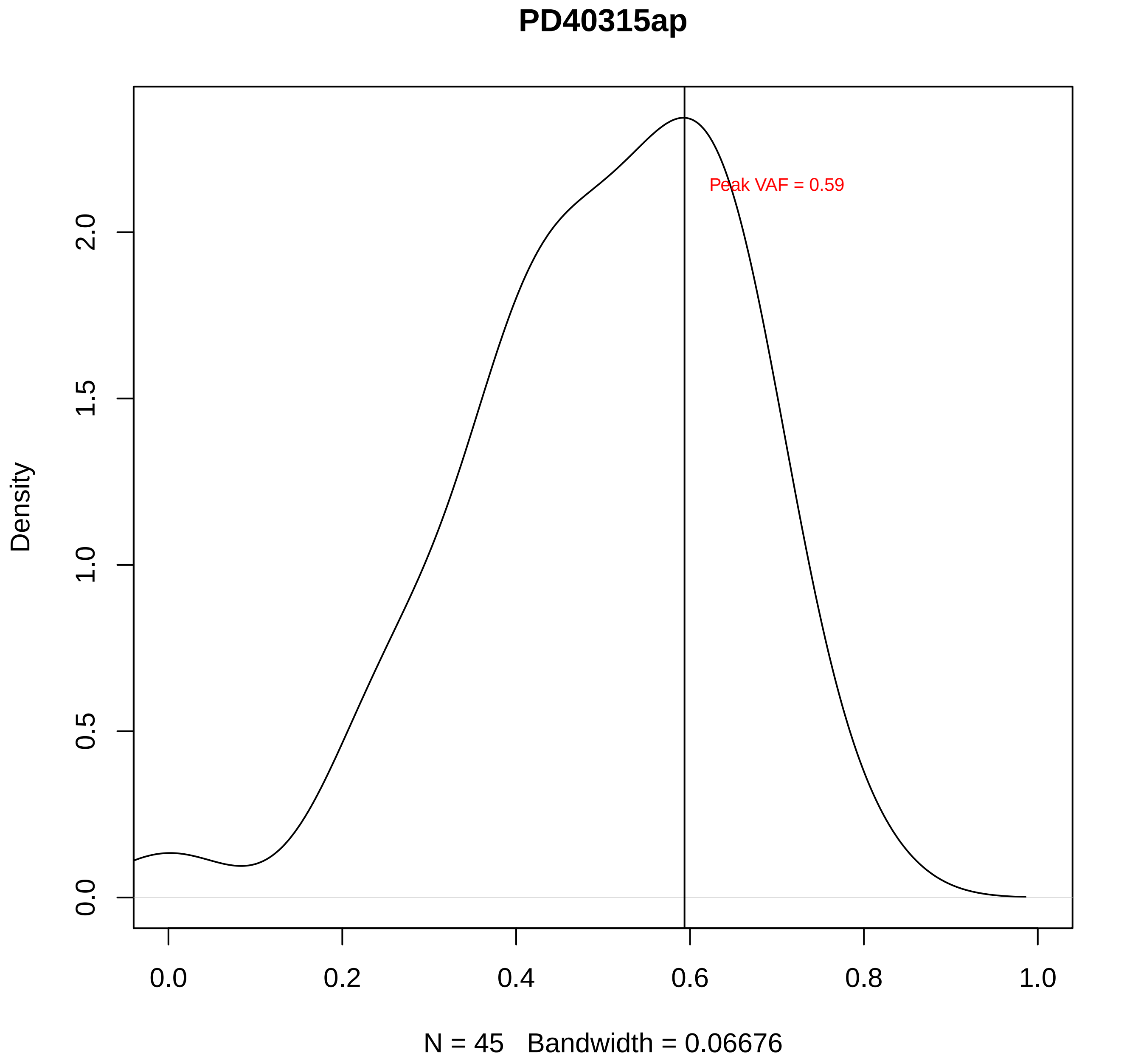

PD40315ap
Peak VAF = 0.59
Density
0.0
0.5
1.0
1.5
2.0
0.0
0.2
0.4
0.6
0.8
1.0
N = 45   Bandwidth = 0.06676

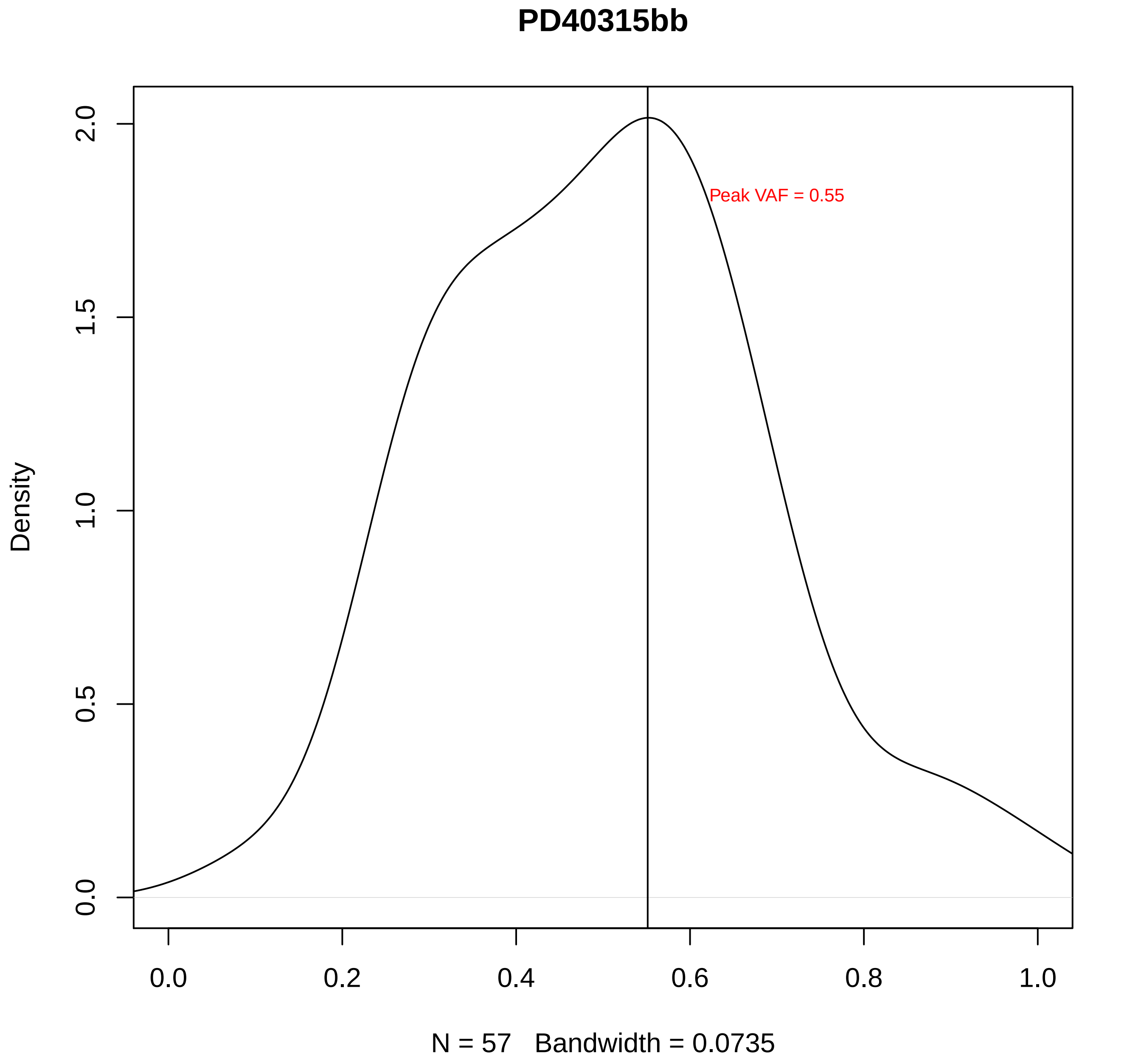
PD40315bb
Density
0.0
0.5
1.0
1.5
2.0
Peak VAF = 0.55
0.0
0.2
0.4
0.6
0.8
1.0
N = 57   Bandwidth = 0.0735

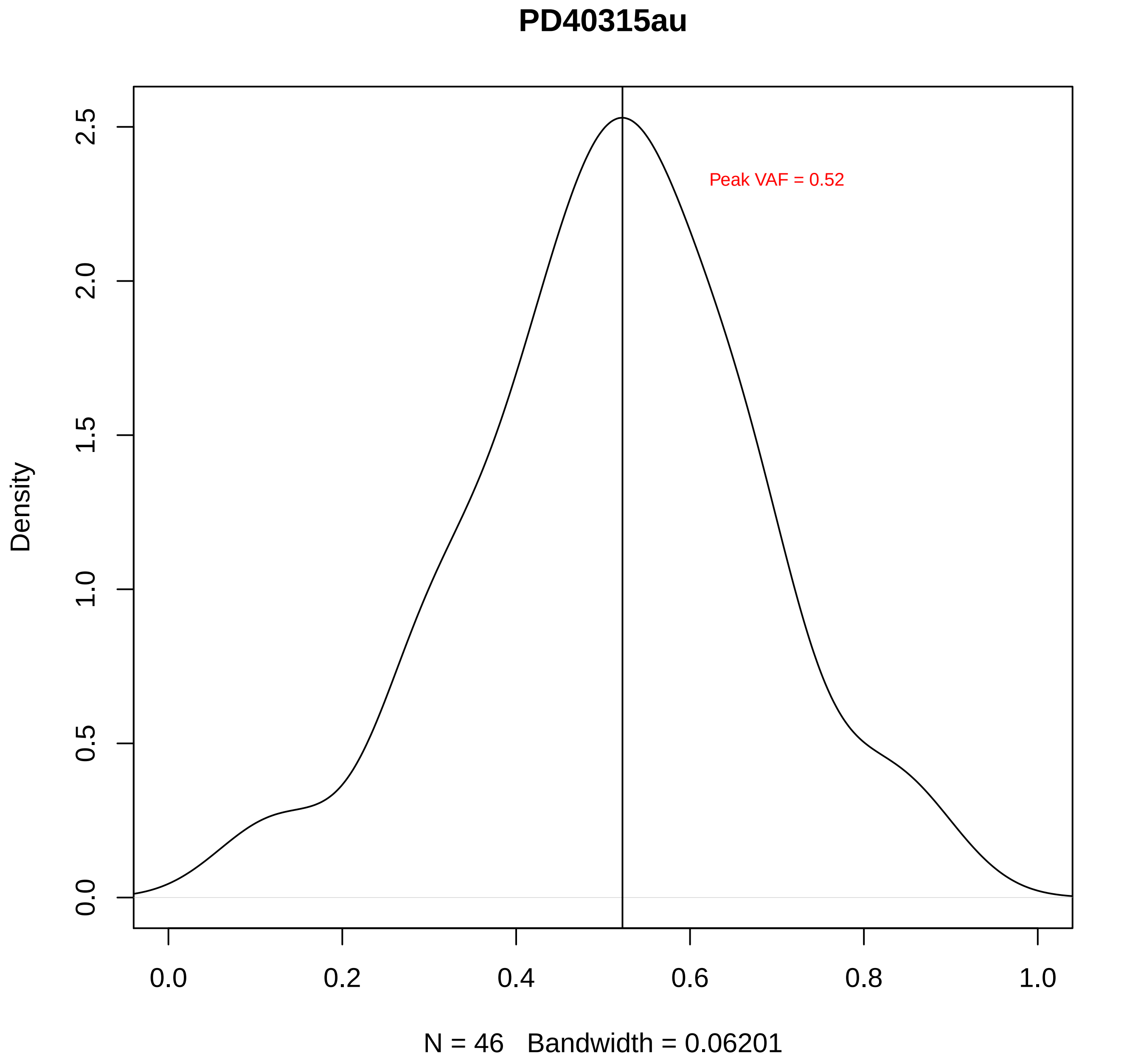
PD40315au
Peak VAF = 0.52
Density
0.0
0.5
1.0
1.5
2.0
2.5
0.0
0.2
0.4
0.6
0.8
1.0
N = 46   Bandwidth = 0.06201

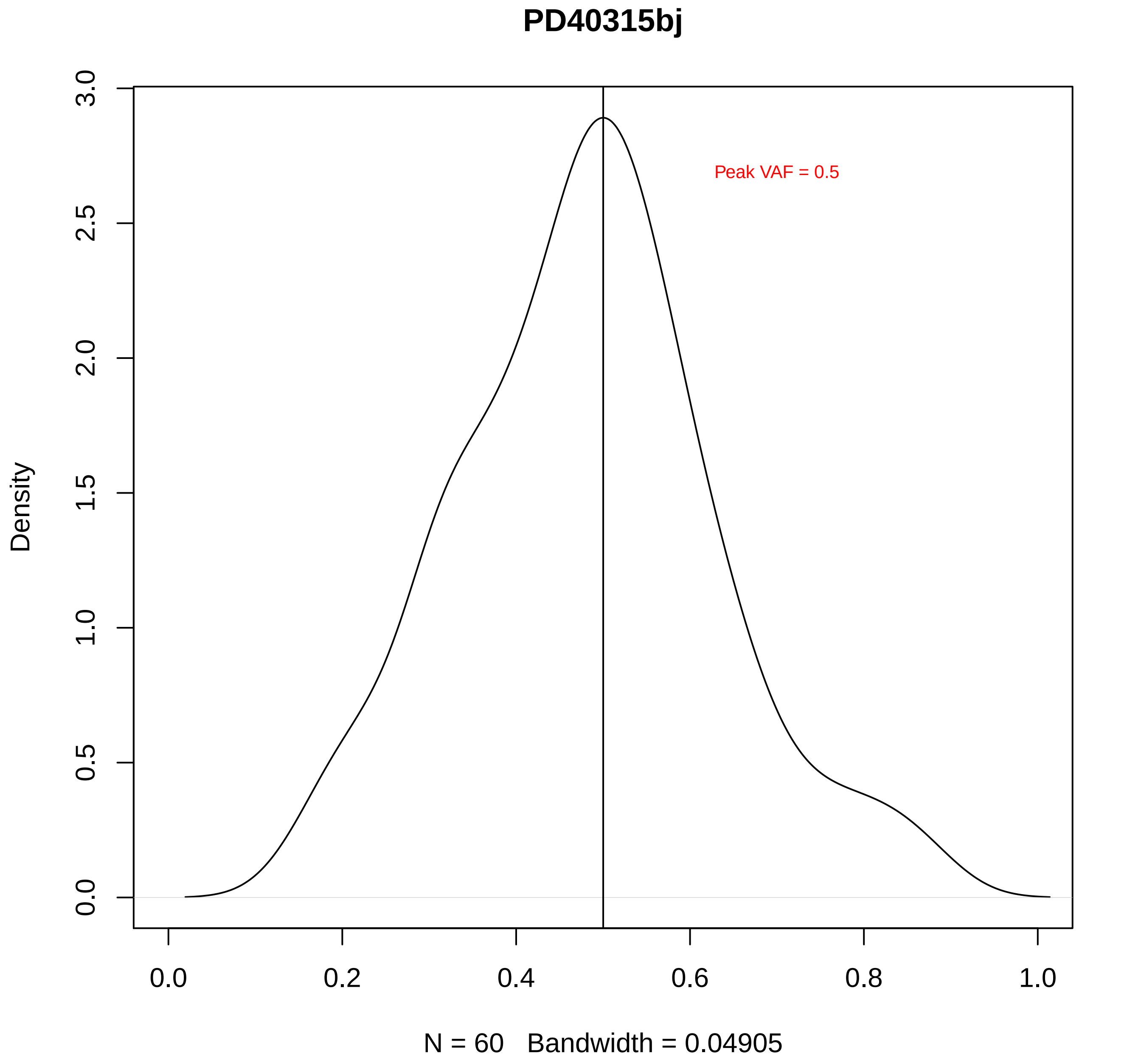
PD40315bj
Peak VAF = 0.5
Density
0.0
0.5
1.0
1.5
2.0
2.5
3.0
0.0
0.2
0.4
0.6
0.8
1.0
N = 60   Bandwidth = 0.04905

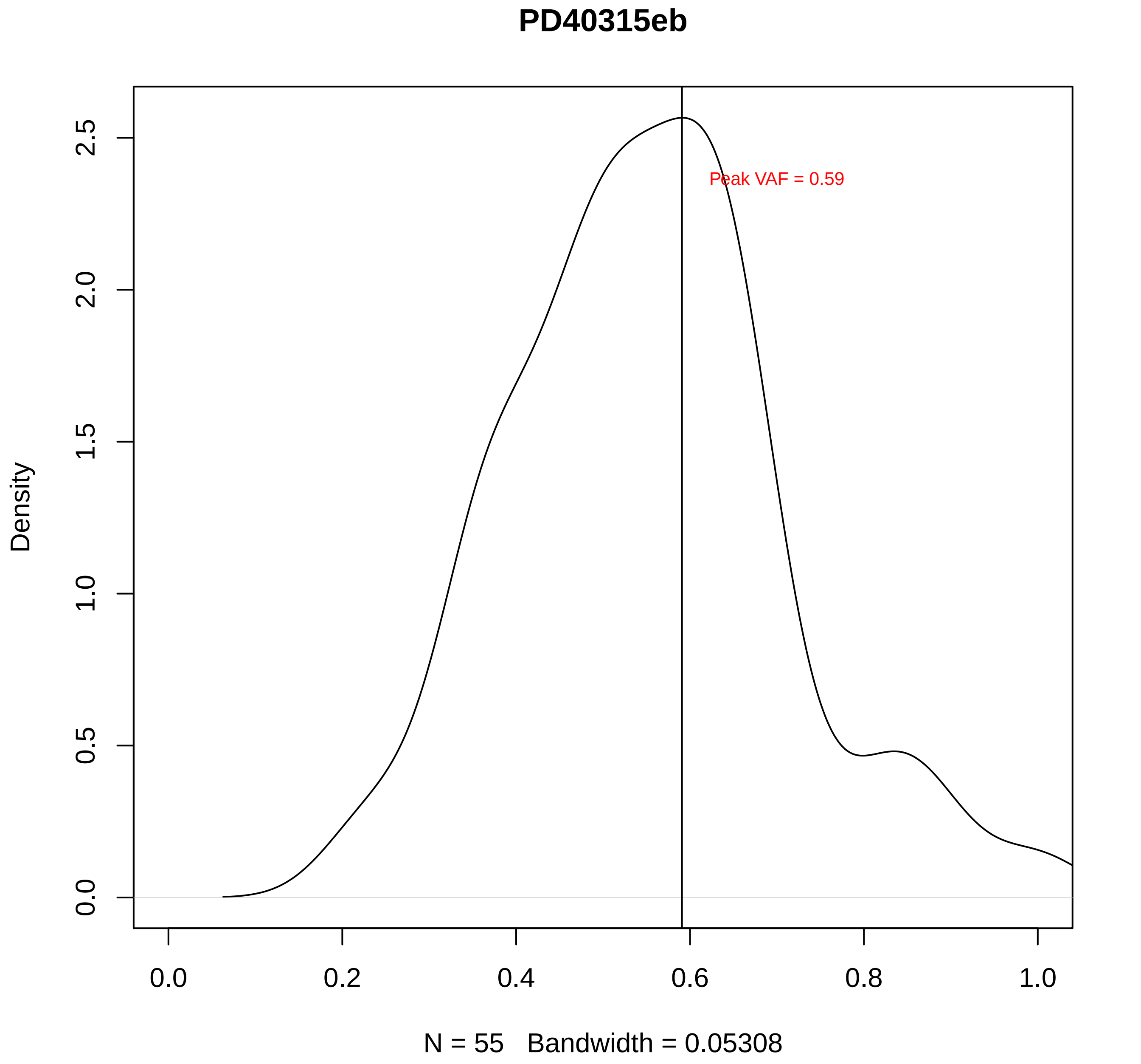
PD40315eb
Peak VAF = 0.59
Density
2.5
2.0
1.5
1.0
0.5
0.0
0.0
0.2
0.4
0.6
0.8
1.0
N = 55   Bandwidth = 0.05308

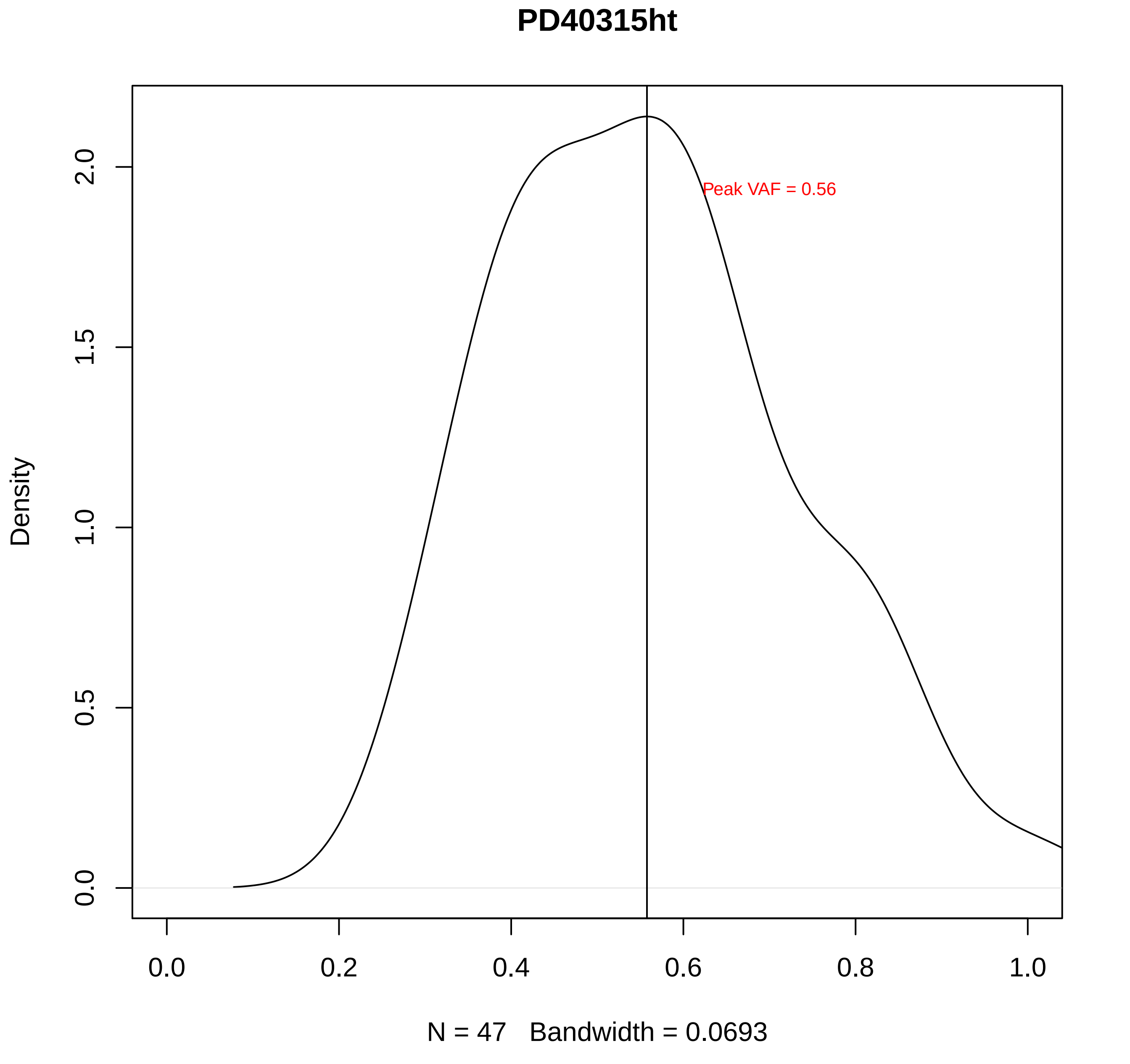
PD40315ht
Peak VAF = 0.56
Density
0.0
0.5
1.0
1.5
2.0
0.0
0.2
0.4
0.6
0.8
1.0
N = 47   Bandwidth = 0.0693

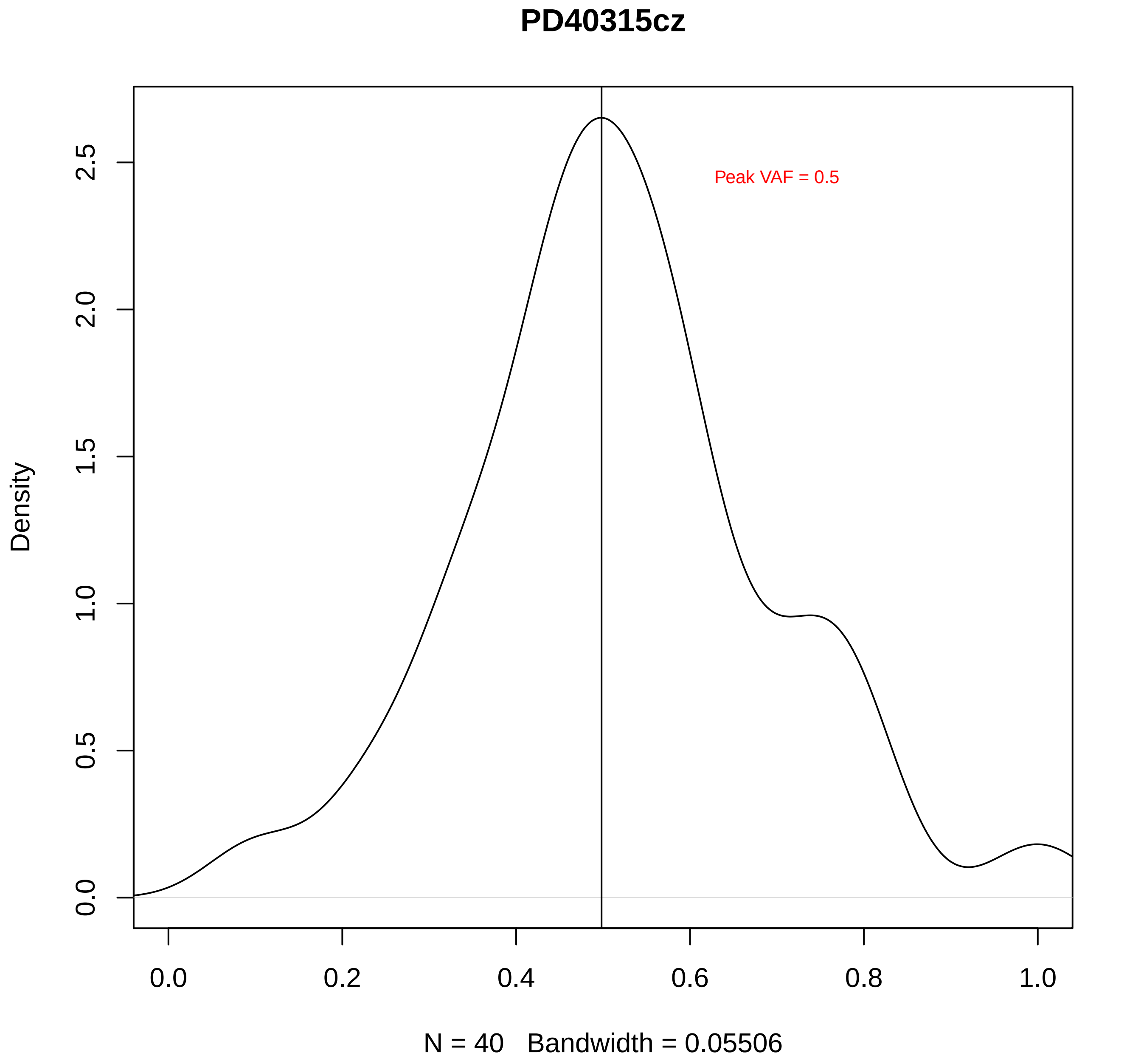

PD40315cz
Peak VAF = 0.5
Density
0.0
0.5
1.0
1.5
2.0
2.5
0.0
0.2
0.4
0.6
0.8
1.0
N = 40 Bandwidth = 0.05506

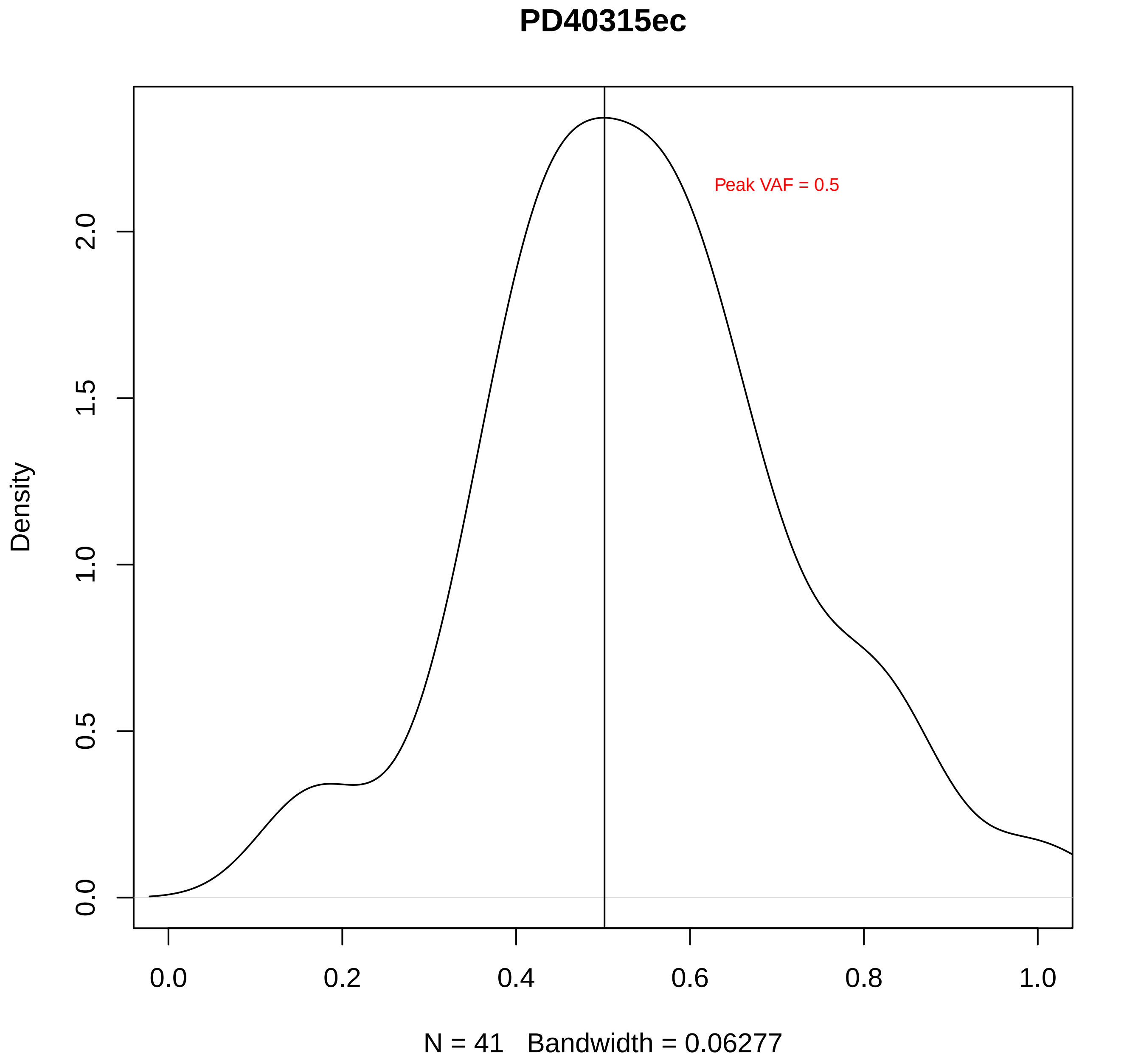

PD40315ec
Peak VAF = 0.5
Density
0.0
0.5
1.0
1.5
2.0
0.0
0.2
0.4
0.6
0.8
1.0
N = 41   Bandwidth = 0.06277

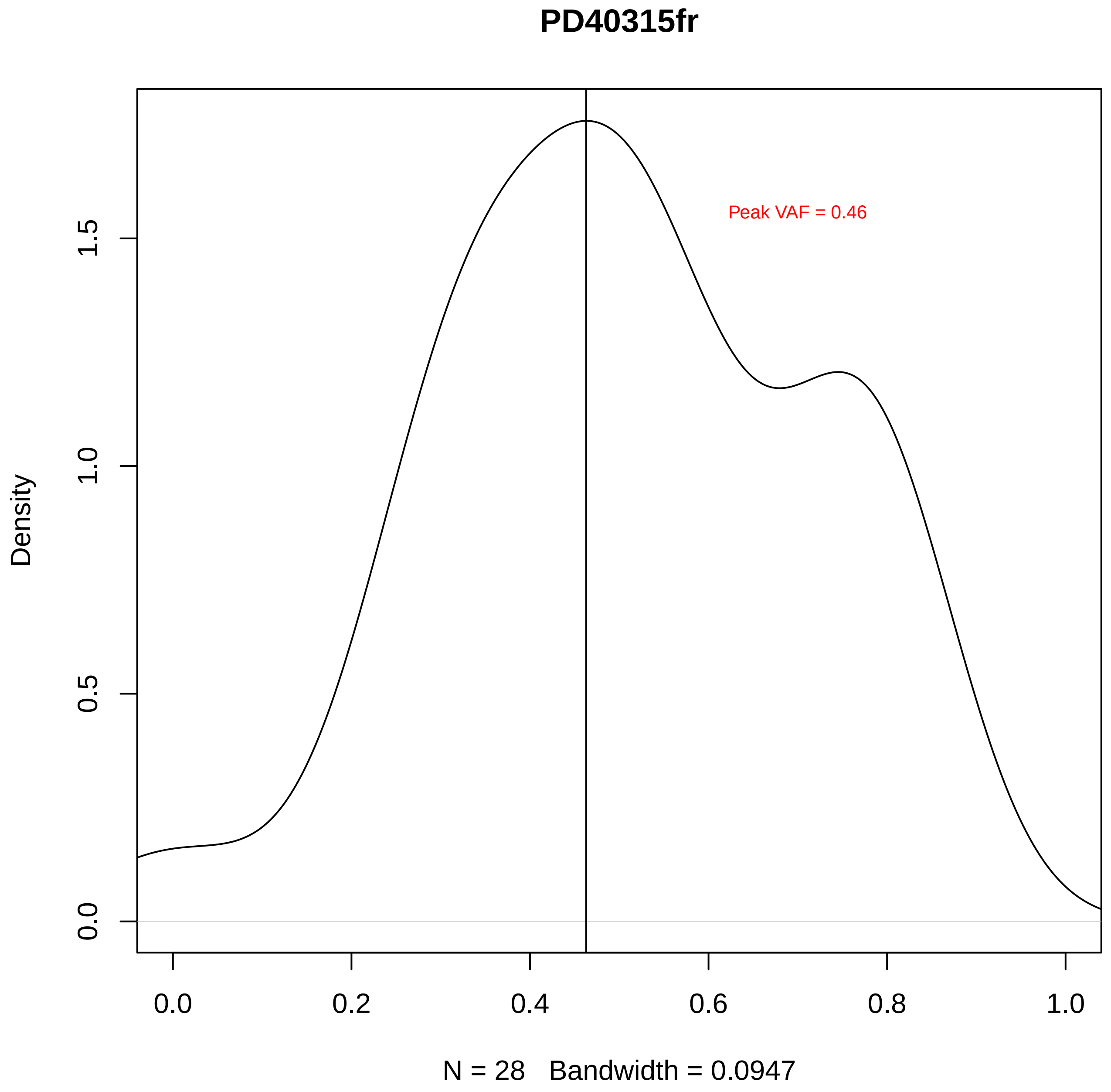

PD40315fr
Peak VAF = 0.46
Density
0.0
0.5
1.0
1.5
0.0
0.2
0.4
0.6
0.8
1.0
N = 28   Bandwidth = 0.0947

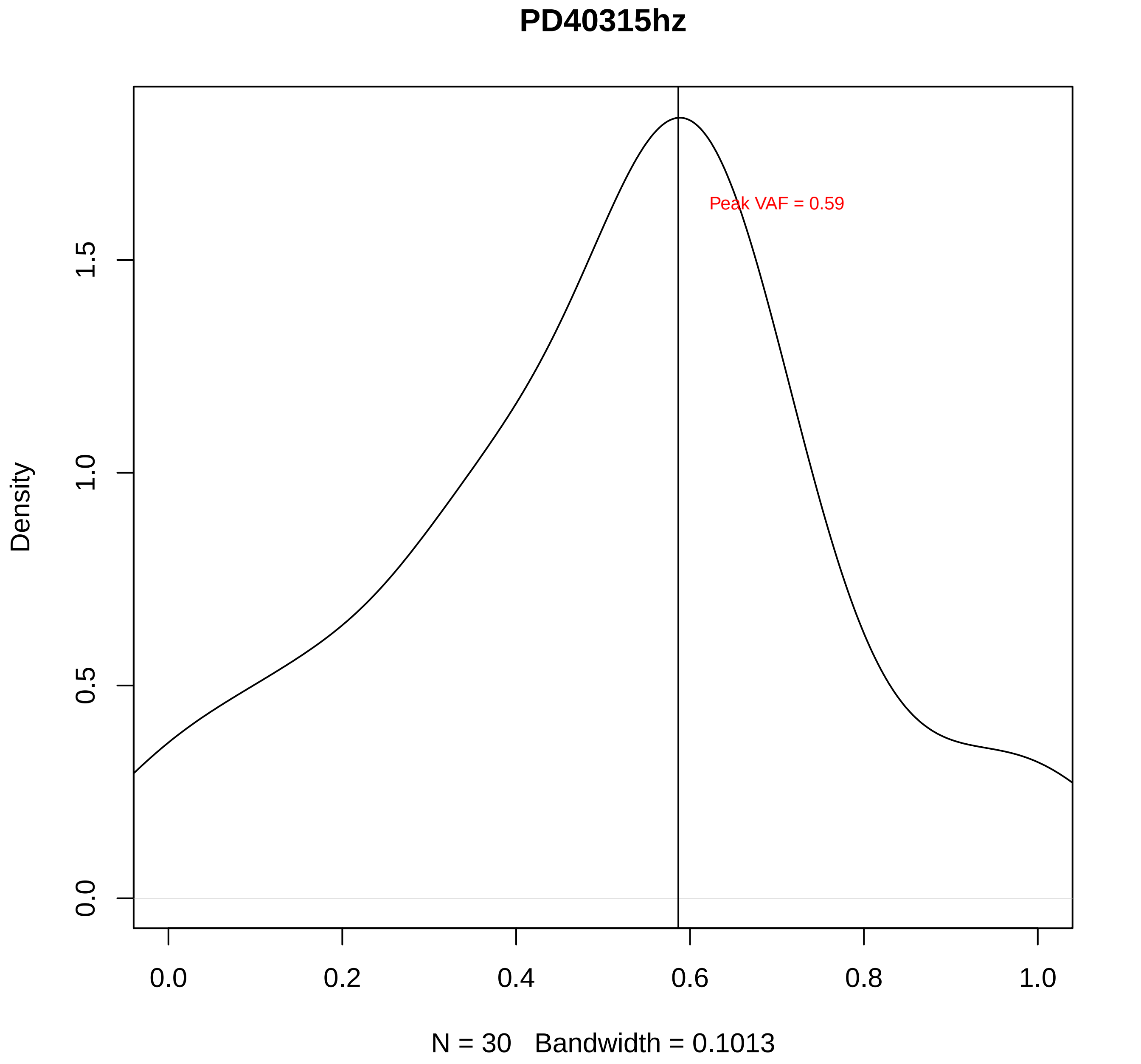

PD40315hz
Peak VAF = 0.59
Density
0.0
0.5
1.0
1.5
0.0
0.2
0.4
0.6
0.8
1.0
N = 30 Bandwidth = 0.1013

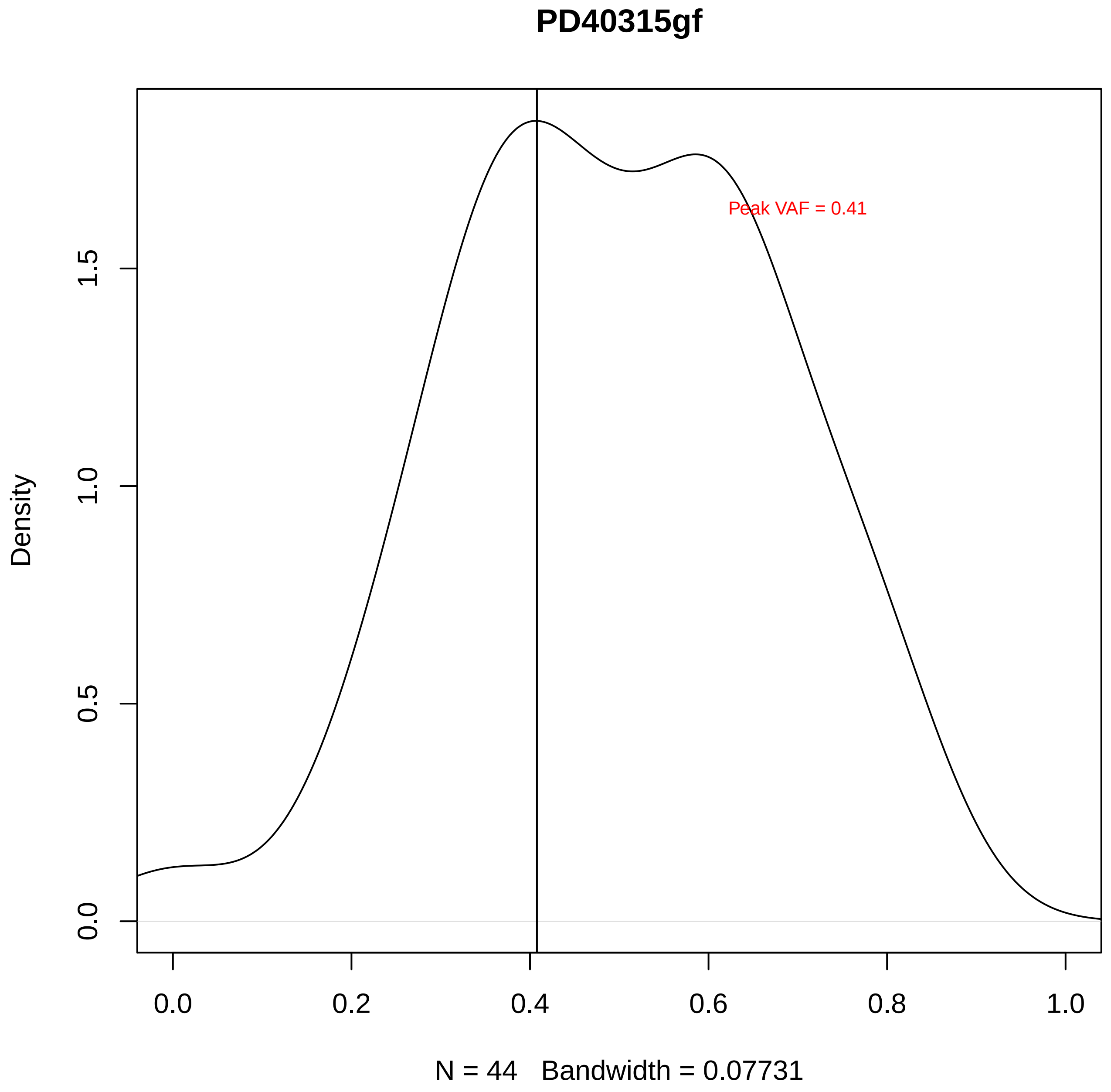
PD40315gf
Peak VAF = 0.41
Density
0.0
0.5
1.0
1.5
0.0
0.2
0.4
0.6
0.8
1.0
N = 44 Bandwidth = 0.07731

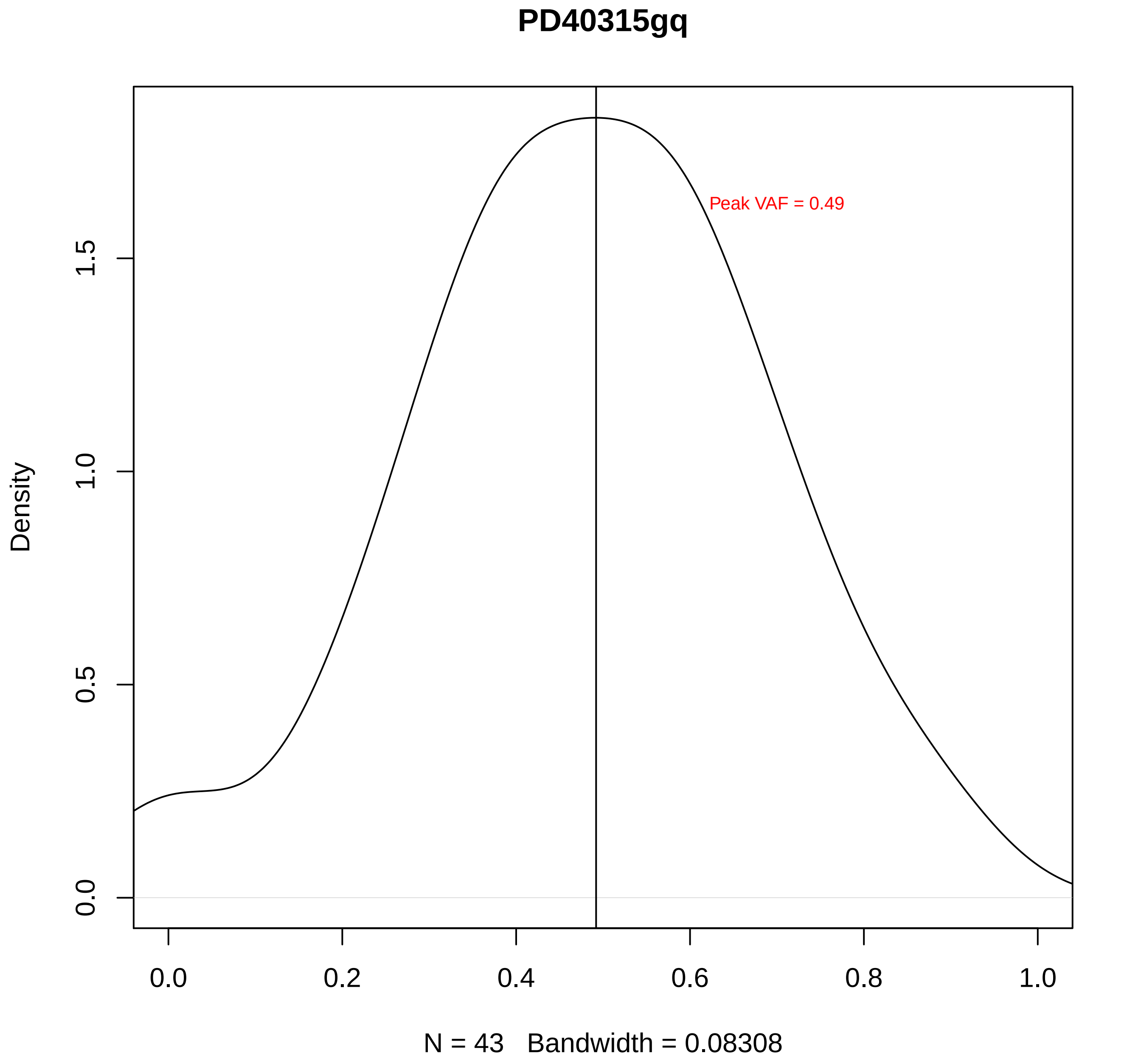

PD40315gq
Peak VAF = 0.49
Density
0.0
0.5
1.0
1.5
0.0
0.2
0.4
0.6
0.8
1.0
N = 43   Bandwidth = 0.08308

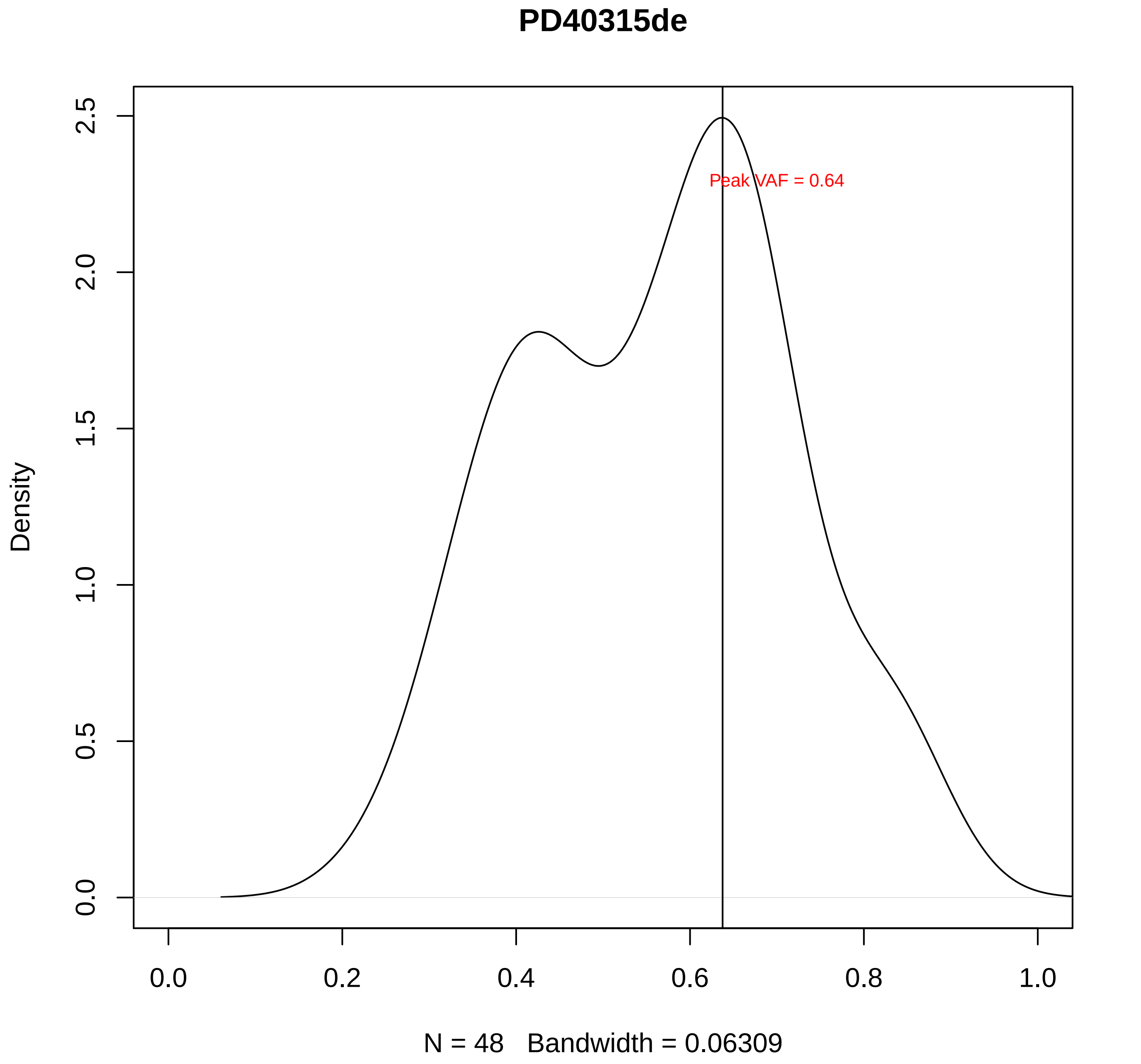
PD40315de
Peak VAF = 0.64
Density
0.0
0.5
1.0
1.5
2.0
2.5
0.0
0.2
0.4
0.6
0.8
1.0
N = 48   Bandwidth = 0.06309

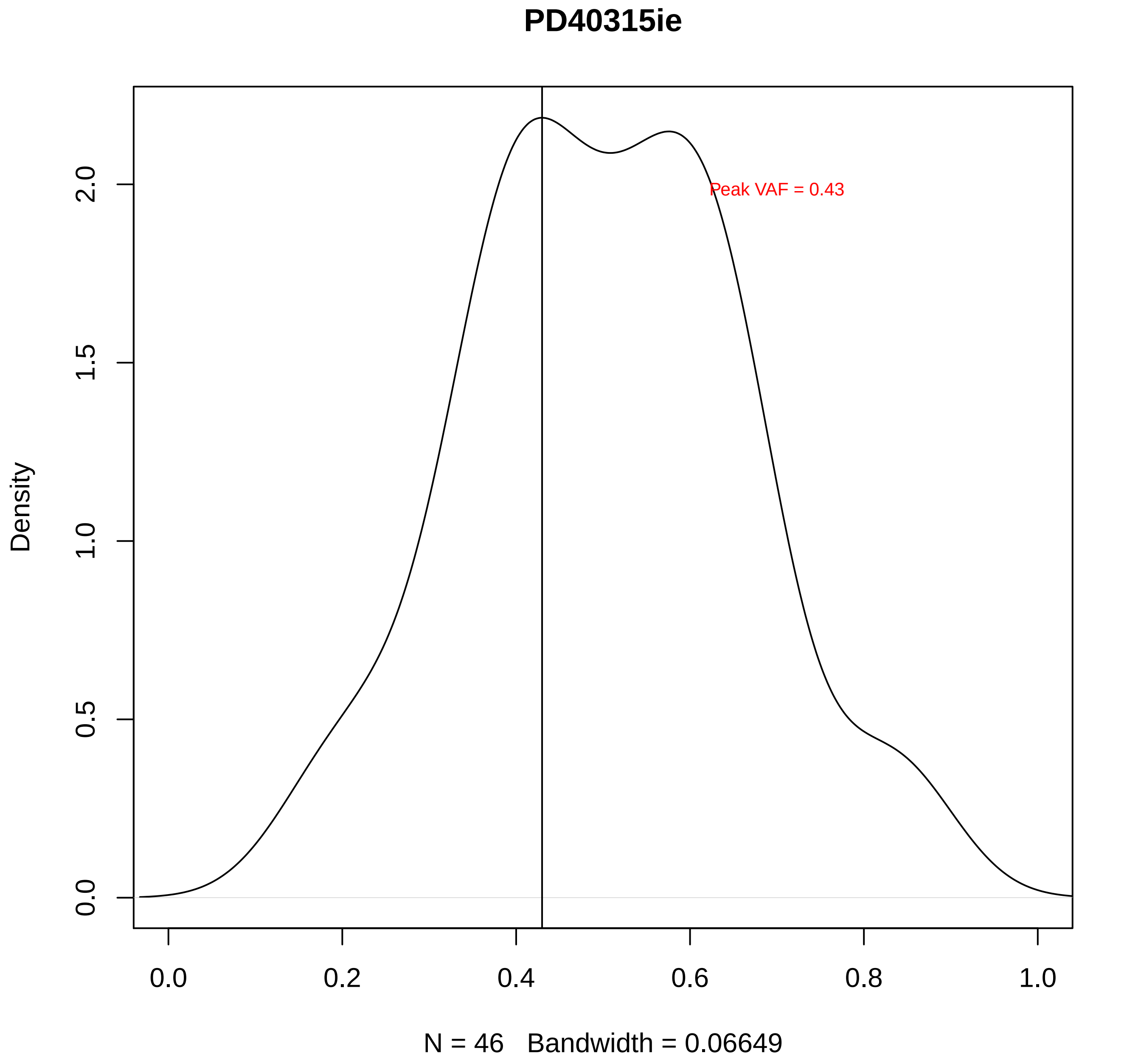
PD40315ie
Peak VAF = 0.43
Density
0.0
0.5
1.0
1.5
2.0
0.0
0.2
0.4
0.6
0.8
1.0
N = 46   Bandwidth = 0.06649

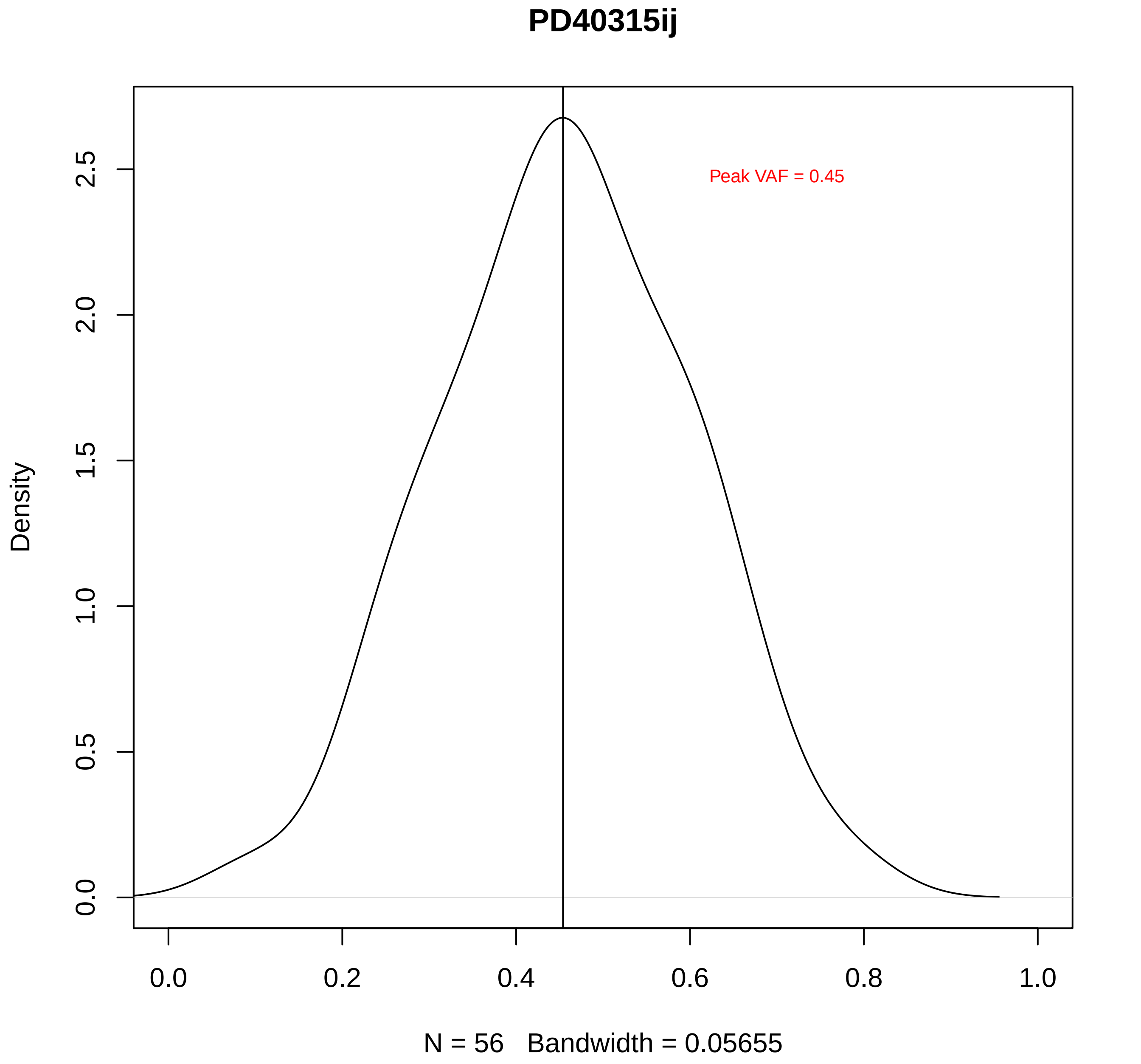
PD40315ij
Peak VAF = 0.45
Density
0.0
0.5
1.0
1.5
2.0
2.5
0.0
0.2
0.4
0.6
0.8
1.0
N = 56 Bandwidth = 0.05655

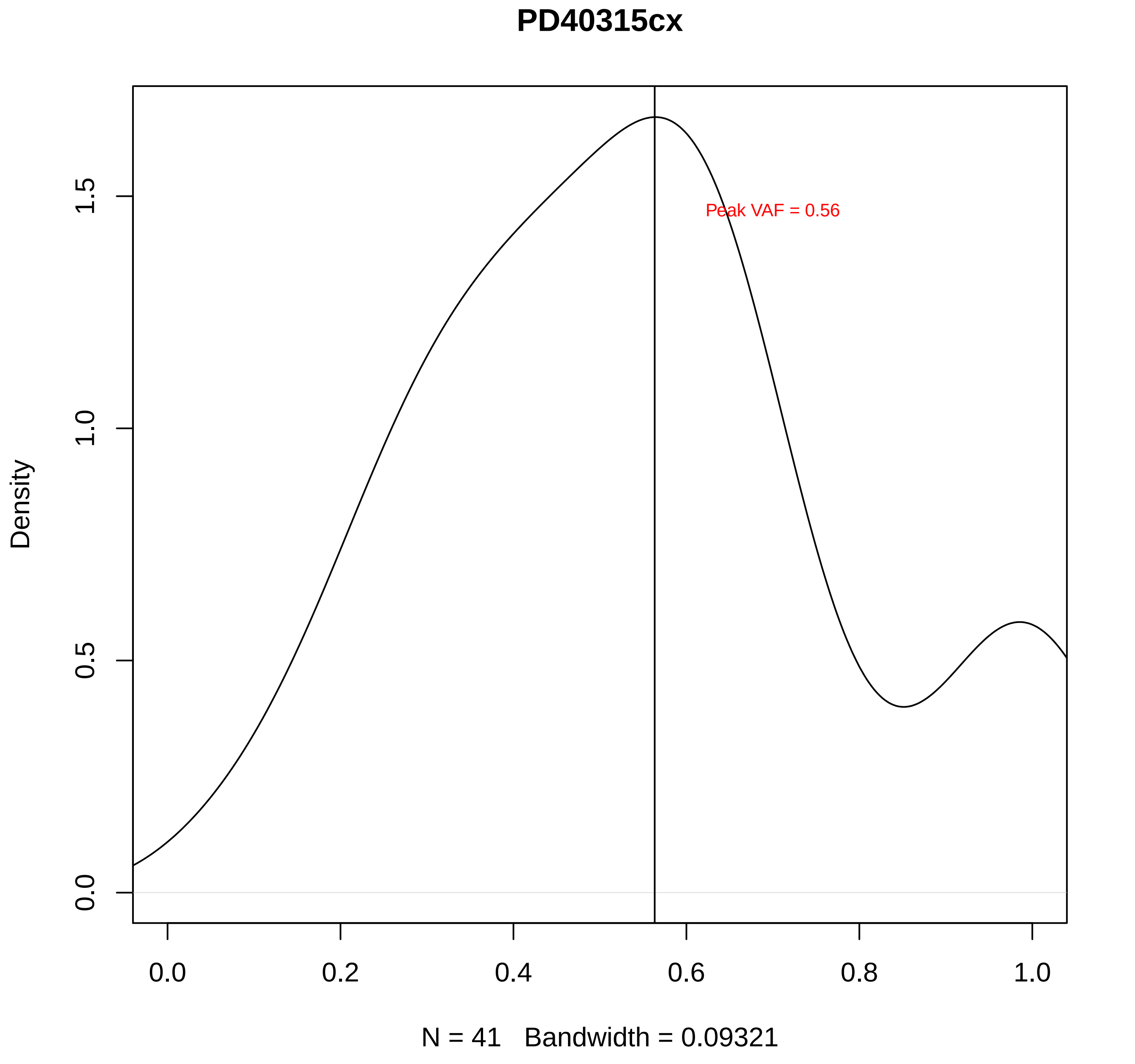

PD40315cx
Peak VAF = 0.56
Density
0.0
0.5
1.0
1.5
0.0
0.2
0.4
0.6
0.8
1.0
N = 41 Bandwidth = 0.09321

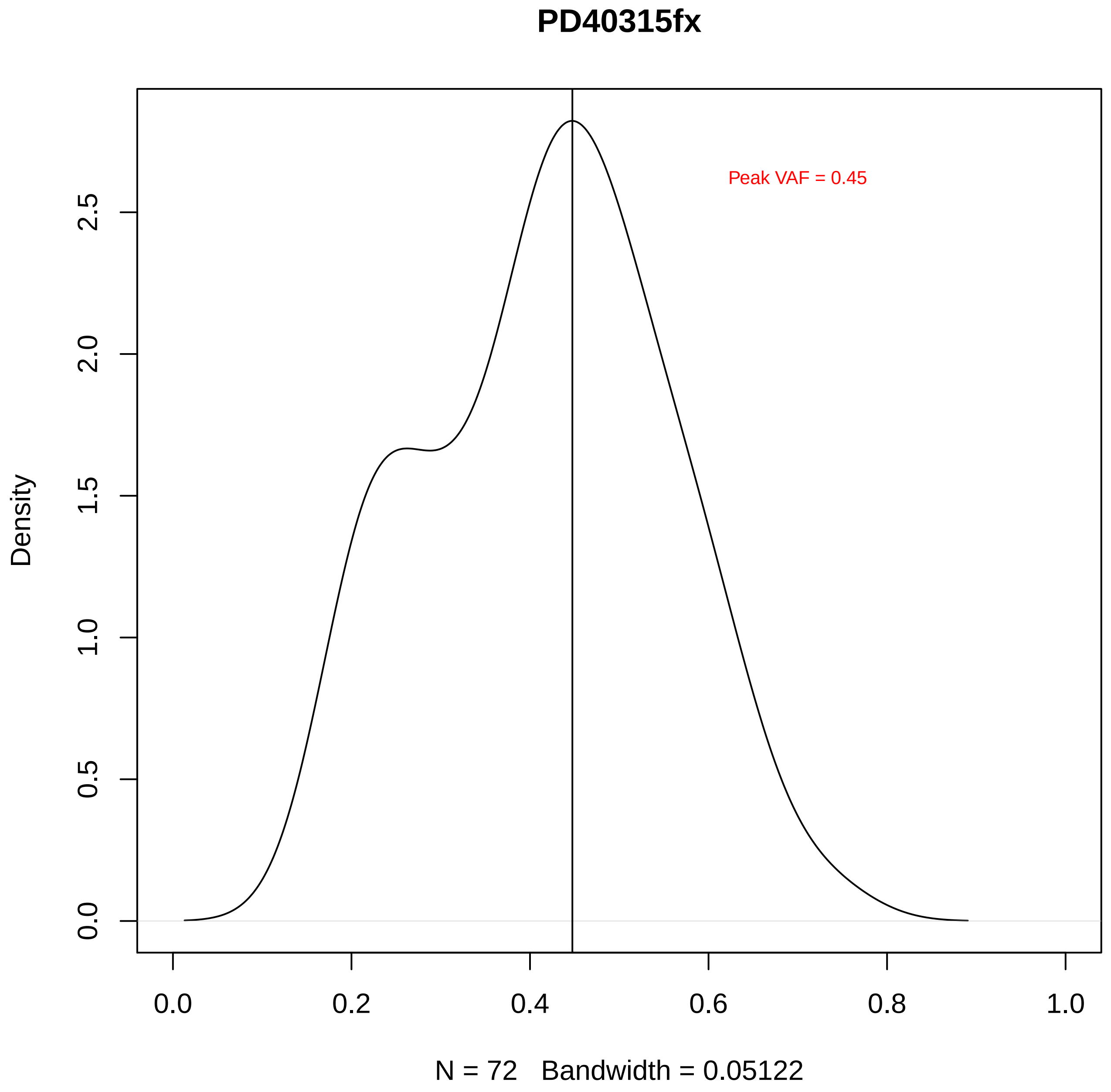

PD40315fx
Peak VAF = 0.45
Density
0.0
0.5
1.0
1.5
2.0
2.5
0.0
0.2
0.4
0.6
0.8
1.0
N = 72 Bandwidth = 0.05122

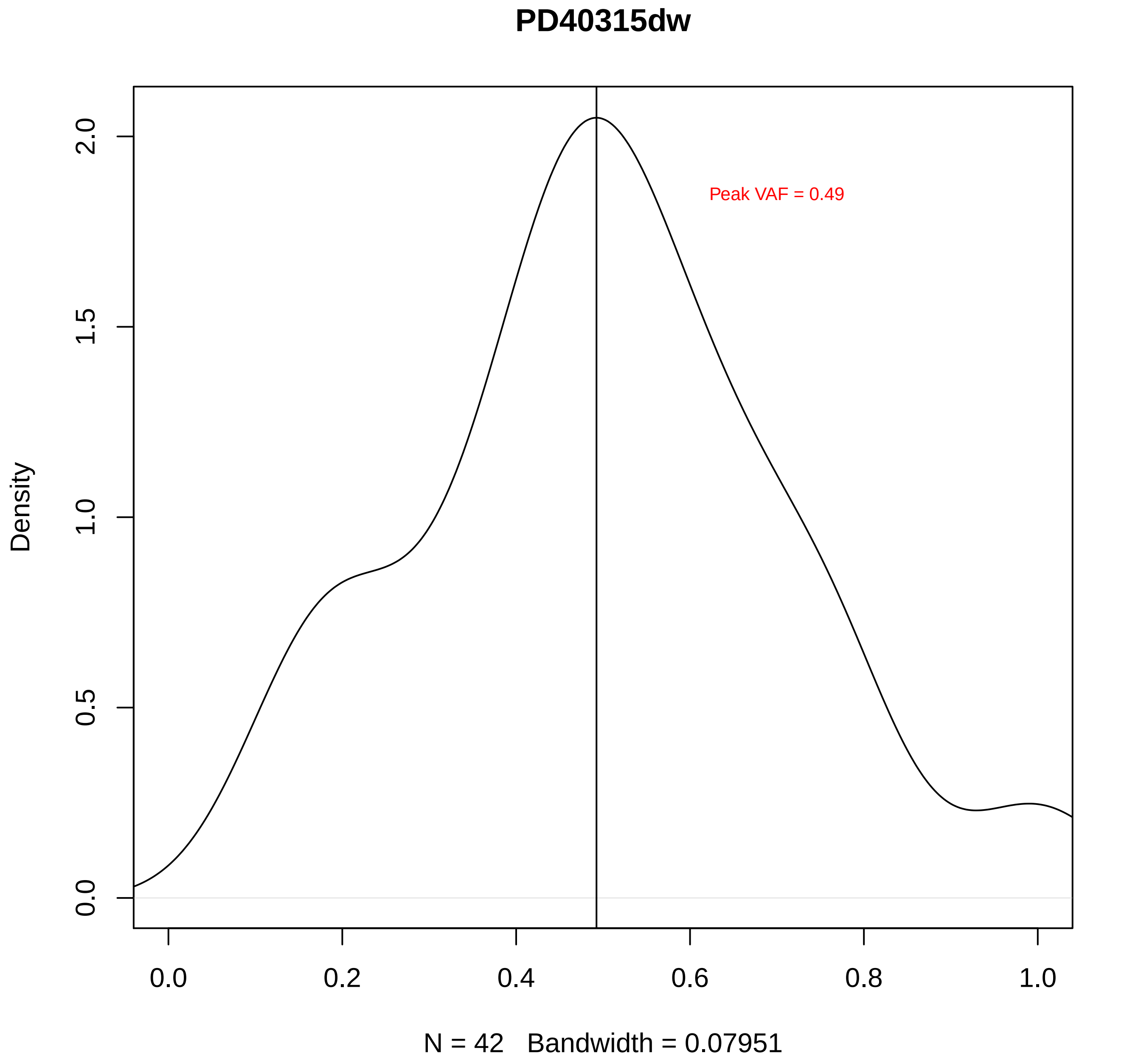

PD40315dw
Peak VAF = 0.49
Density
0.0
0.5
1.0
1.5
2.0
0.0
0.2
0.4
0.6
0.8
1.0
N = 42 Bandwidth = 0.07951

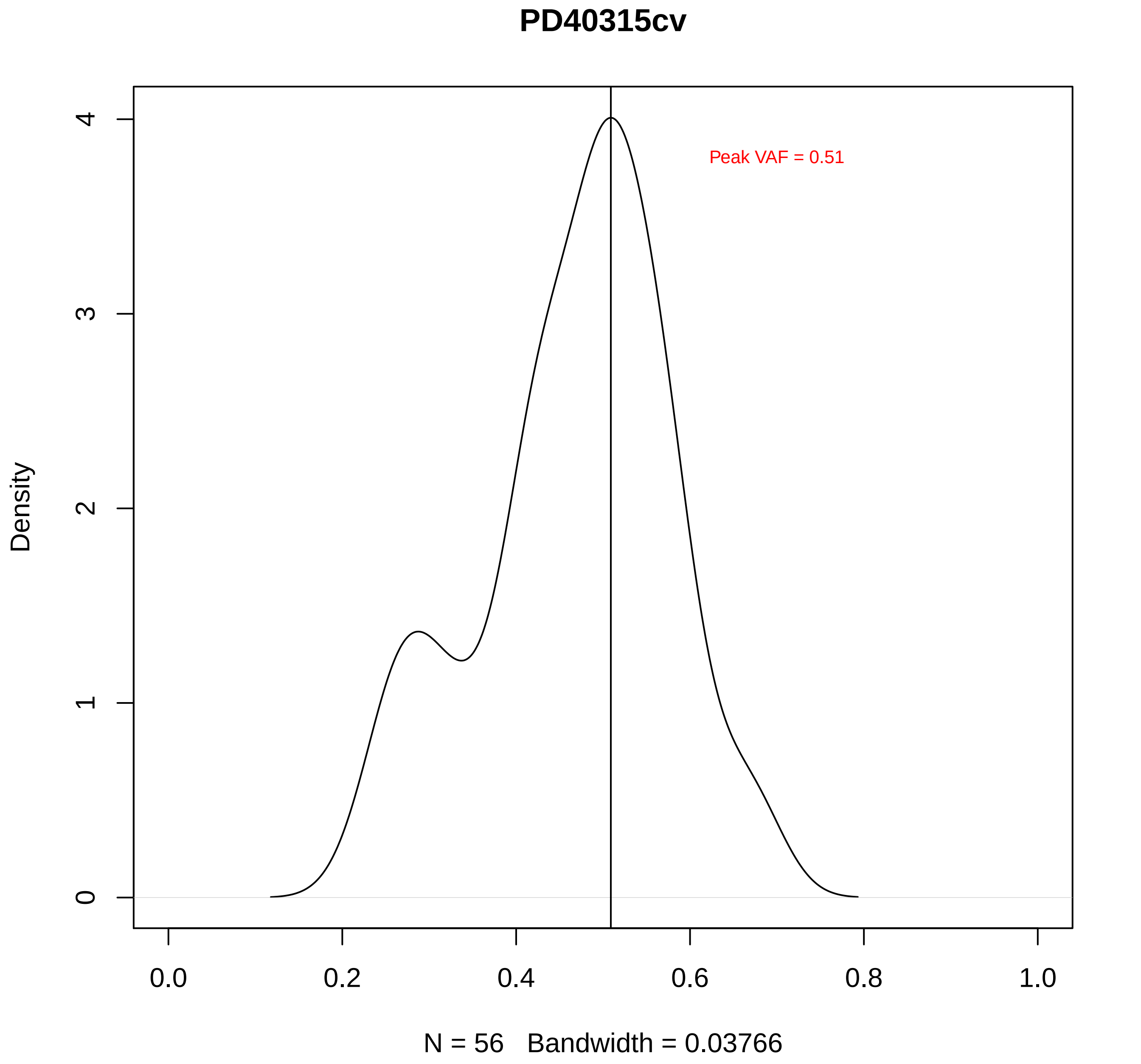
PD40315cv
Peak VAF = 0.51
Density
0
1
2
3
4
0.0
0.2
0.4
0.6
0.8
1.0
N = 56   Bandwidth = 0.03766

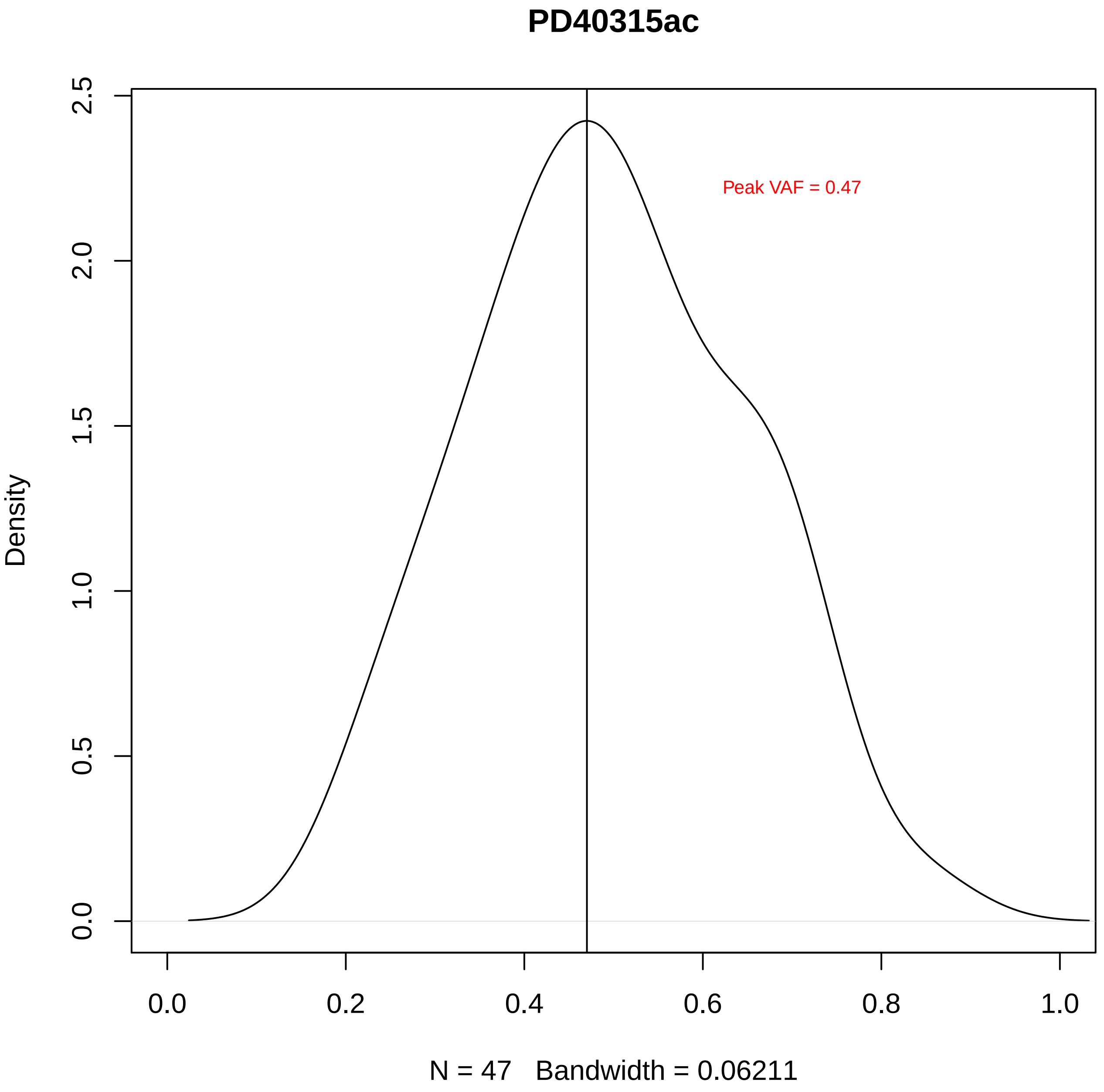

PD40315ac
Peak VAF = 0.47
Density
0.0
0.5
1.0
1.5
2.0
2.5
0.0
0.2
0.4
0.6
0.8
1.0
N = 47   Bandwidth = 0.06211

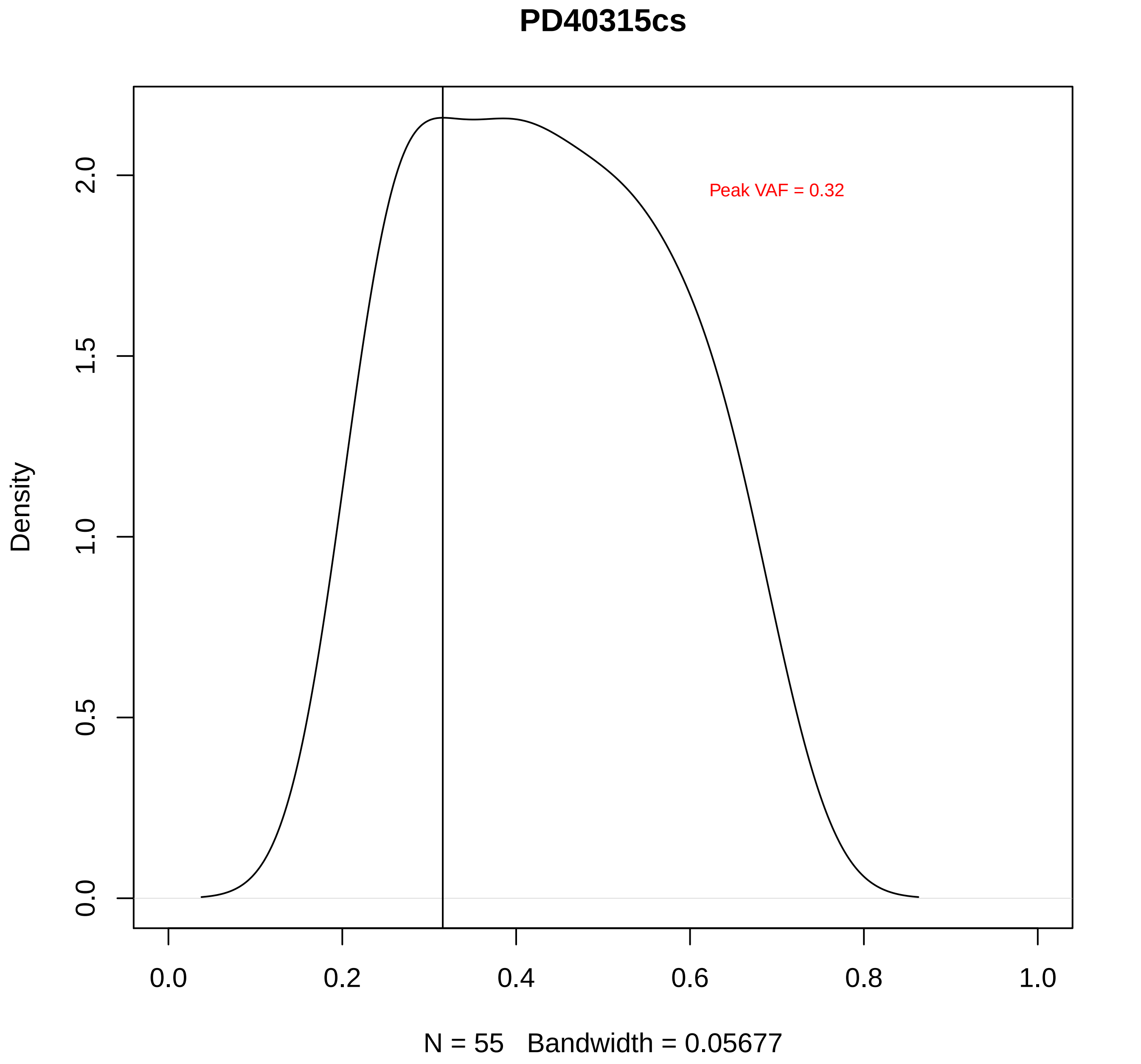

PD40315cs
Peak VAF = 0.32
Density
0.0
0.5
1.0
1.5
2.0
0.0
0.2
0.4
0.6
0.8
1.0
N = 55 Bandwidth = 0.05677

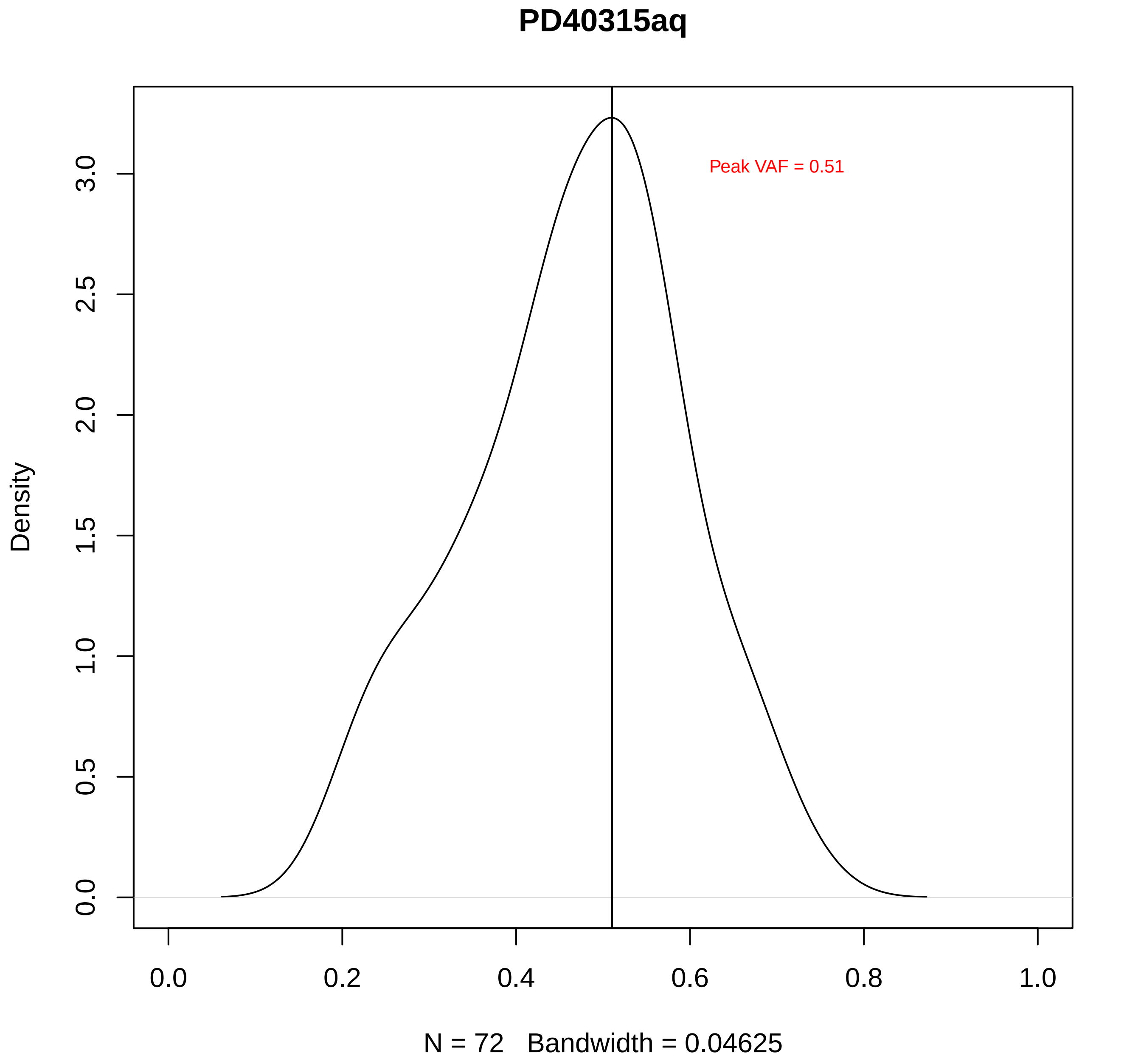
PD40315aq
Peak VAF = 0.51
Density
0.0
0.5
1.0
1.5
2.0
2.5
3.0
0.0
0.2
0.4
0.6
0.8
1.0
N = 72   Bandwidth = 0.04625

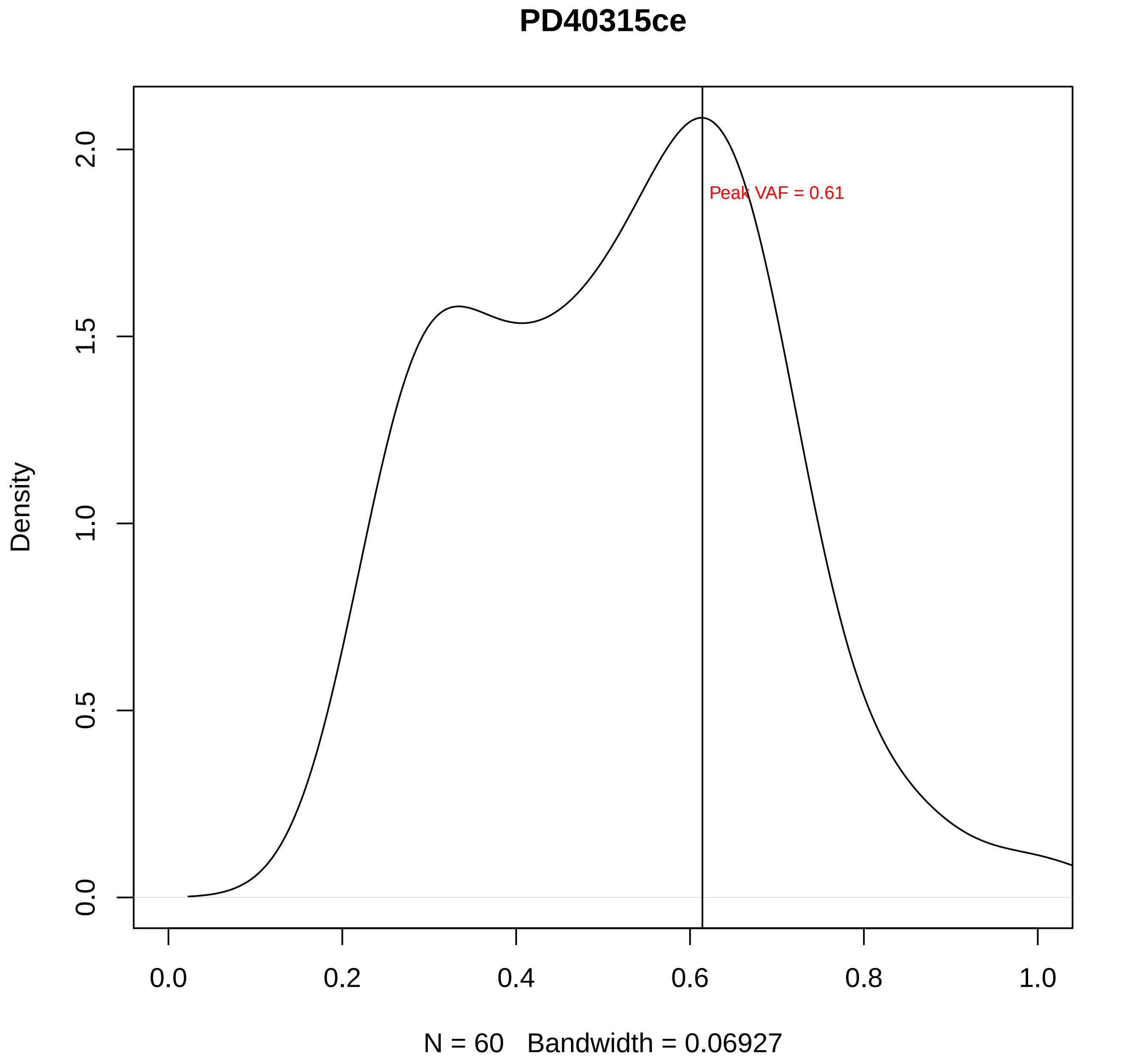
PD40315ce
Peak VAF = 0.61
Density
0.0
0.5
1.0
1.5
2.0
0.0
0.2
0.4
0.6
0.8
1.0
N = 60   Bandwidth = 0.06927

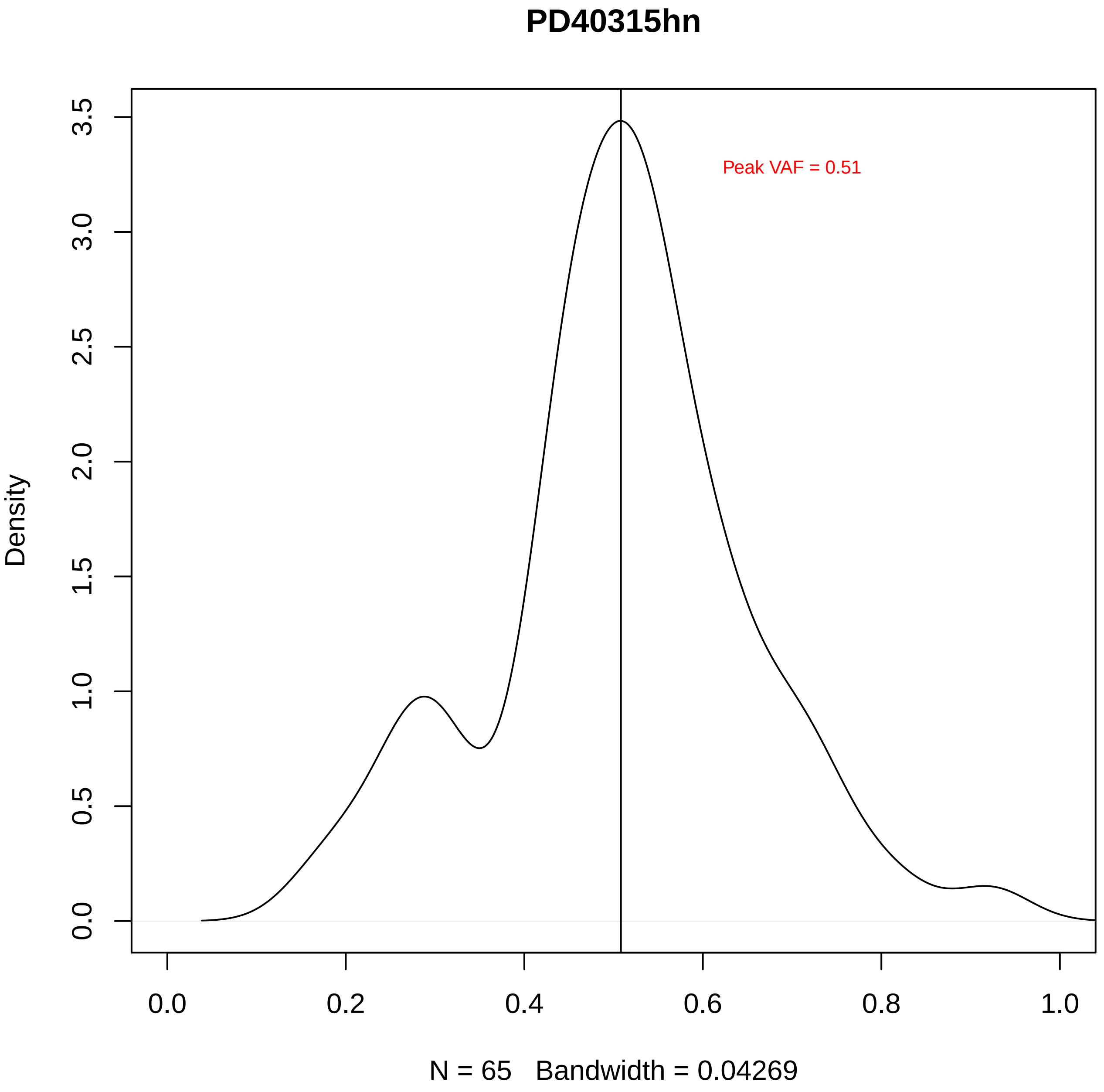

PD40315hn
Peak VAF = 0.51
Density
3.5
3.0
2.5
2.0
1.5
1.0
0.5
0.0
0.0
0.2
0.4
0.6
0.8
1.0
N = 65   Bandwidth = 0.04269

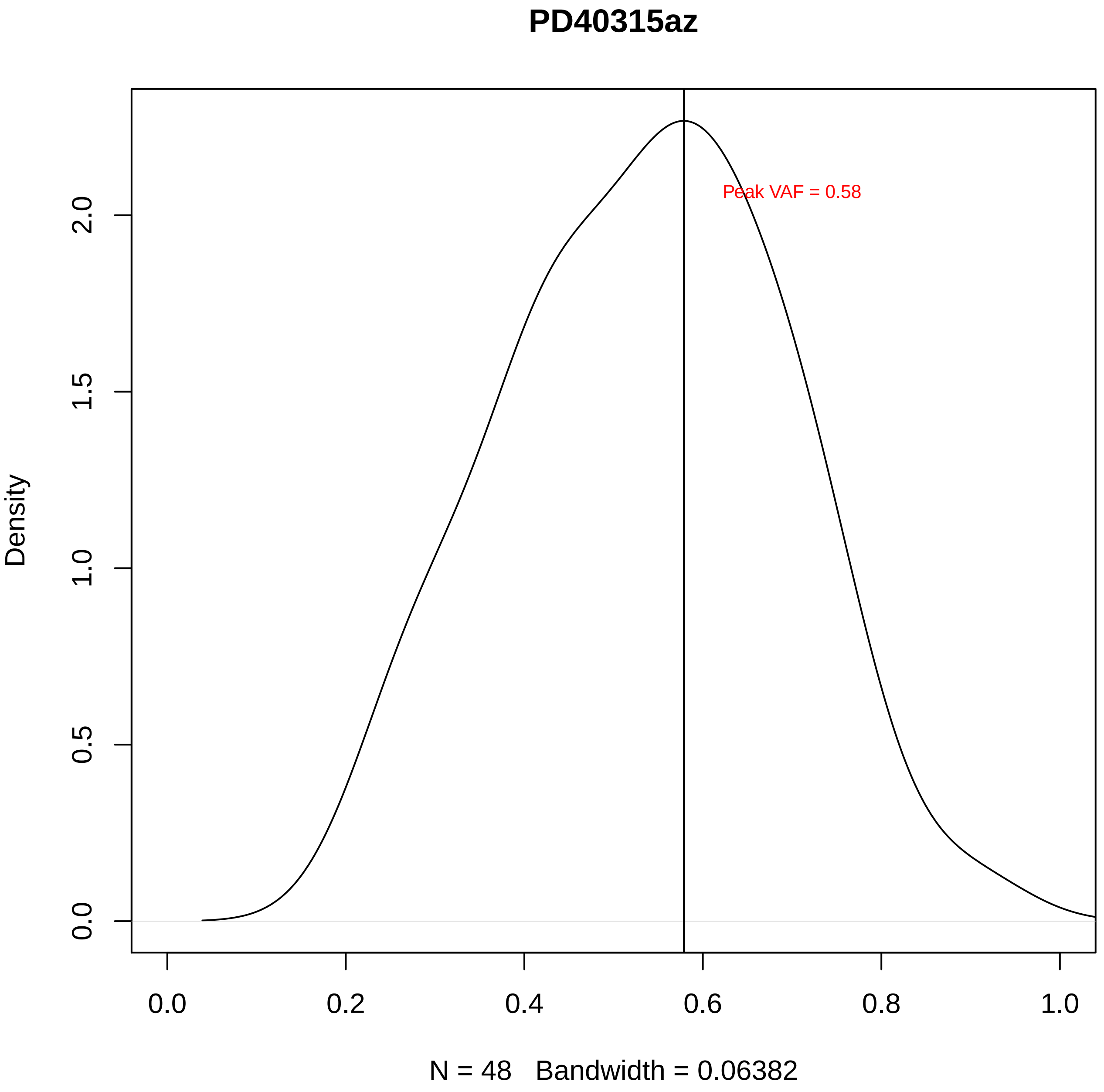
PD40315az
Peak VAF = 0.58
Density
0.0
0.5
1.0
1.5
2.0
0.0
0.2
0.4
0.6
0.8
1.0
N = 48 Bandwidth = 0.06382

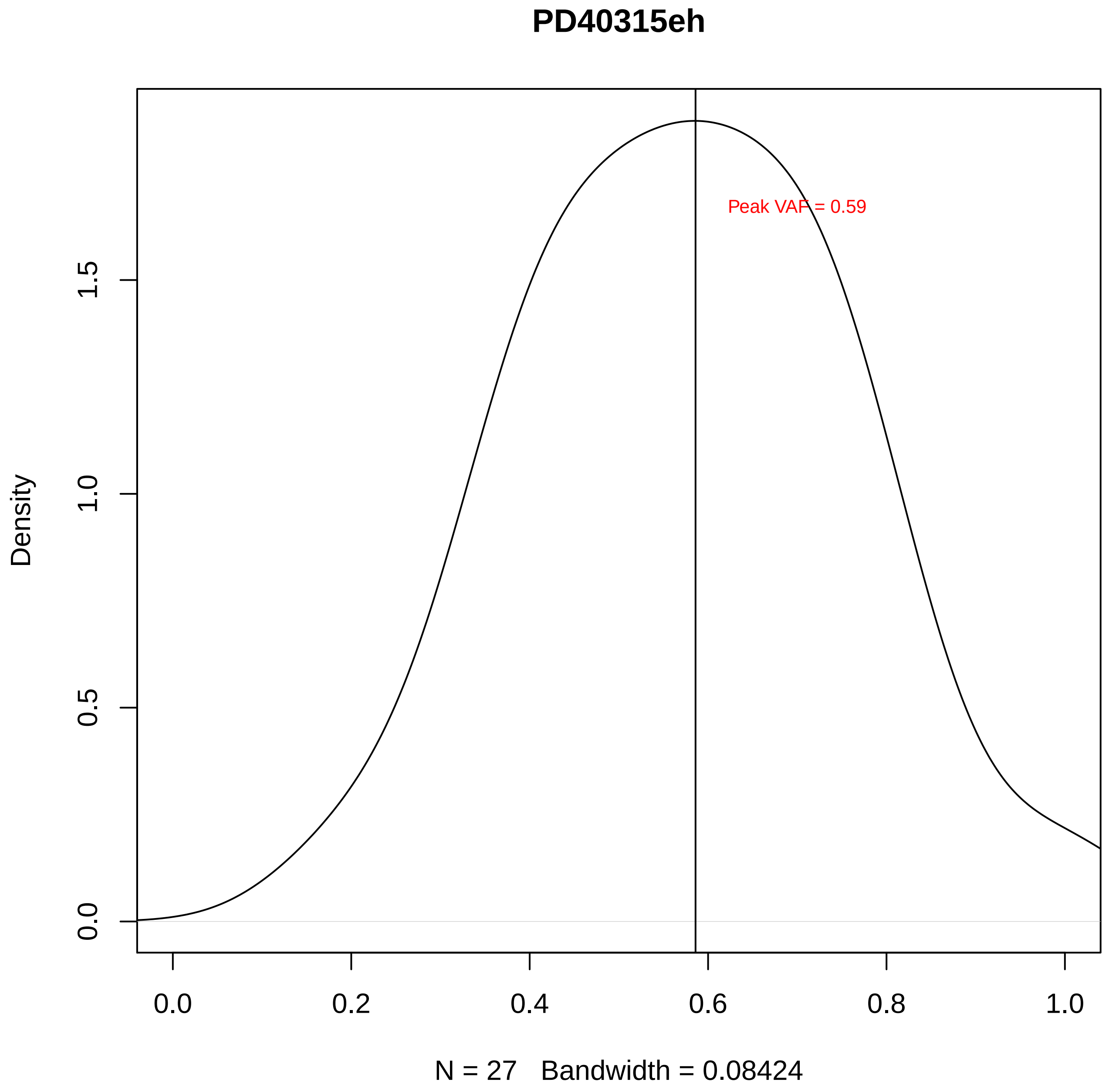
PD40315eh
Peak VAF = 0.59
Density
0.0
0.5
1.0
1.5
0.0
0.2
0.4
0.6
0.8
1.0
N = 27   Bandwidth = 0.08424

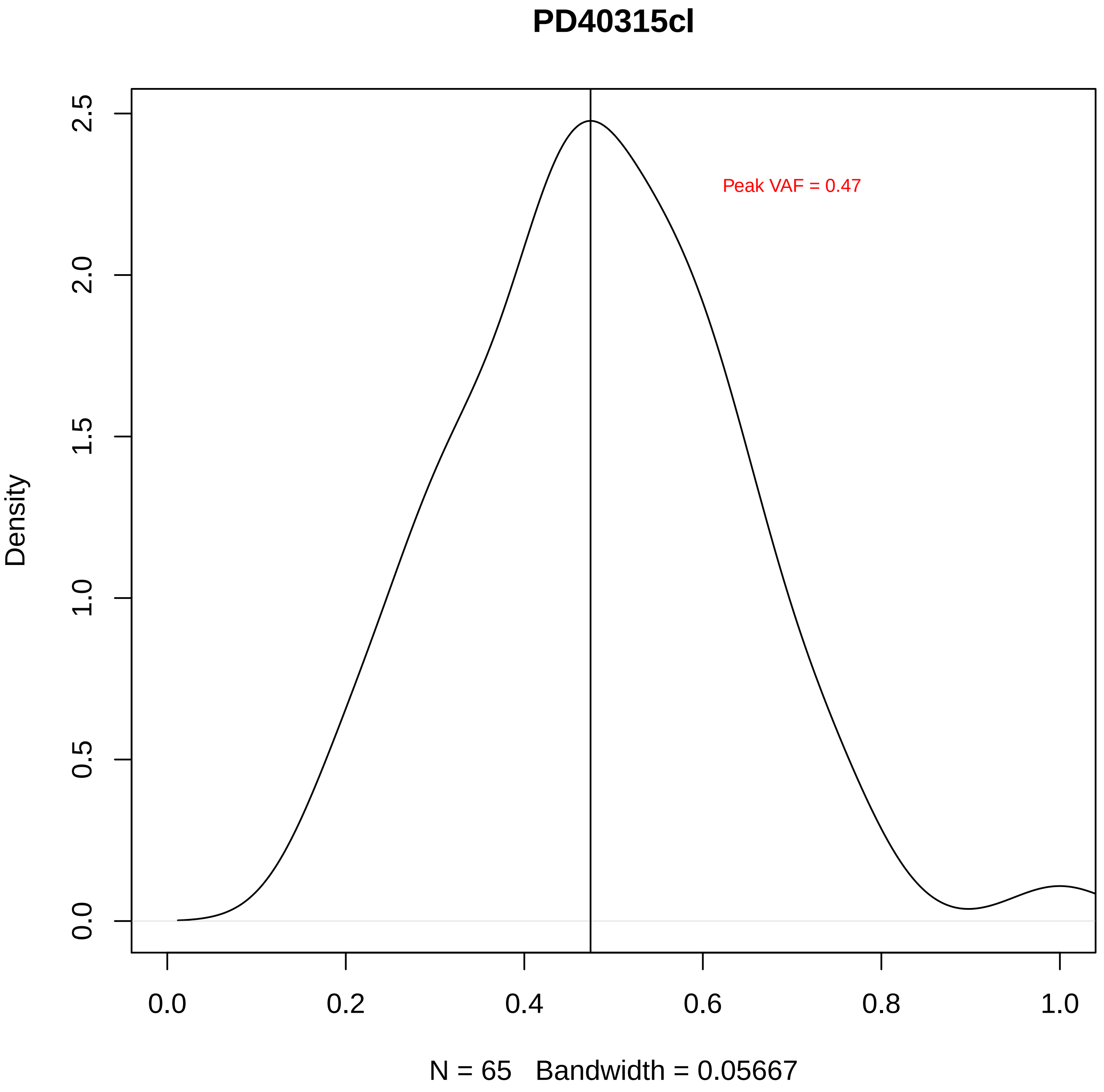
PD40315cl
Peak VAF = 0.47
Density
0.0
0.5
1.0
1.5
2.0
2.5
0.0
0.2
0.4
0.6
0.8
1.0
N = 65   Bandwidth = 0.05667

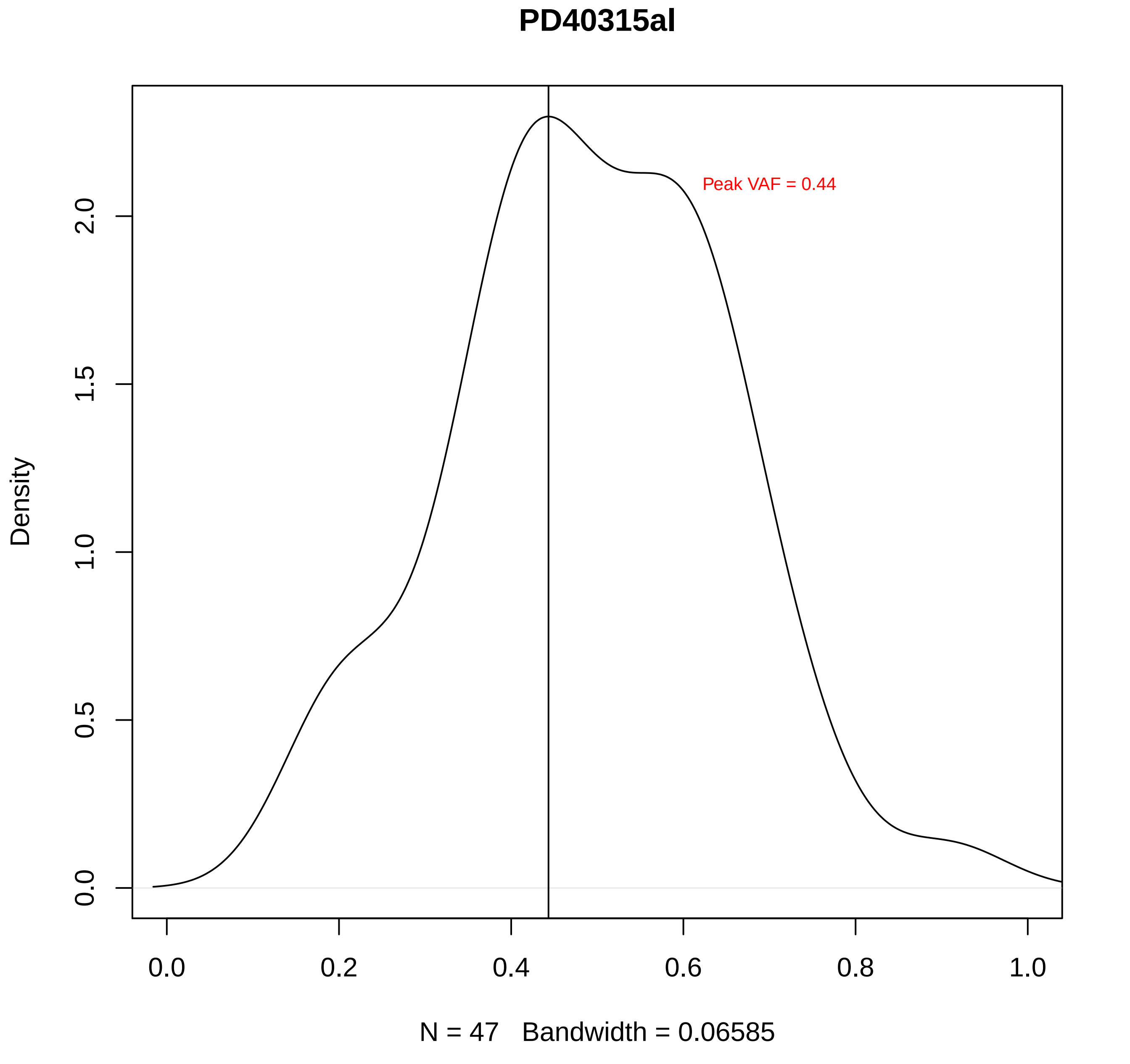
PD40315al
Peak VAF = 0.44
Density
0.0
0.5
1.0
1.5
2.0
0.0
0.2
0.4
0.6
0.8
1.0
N = 47   Bandwidth = 0.06585

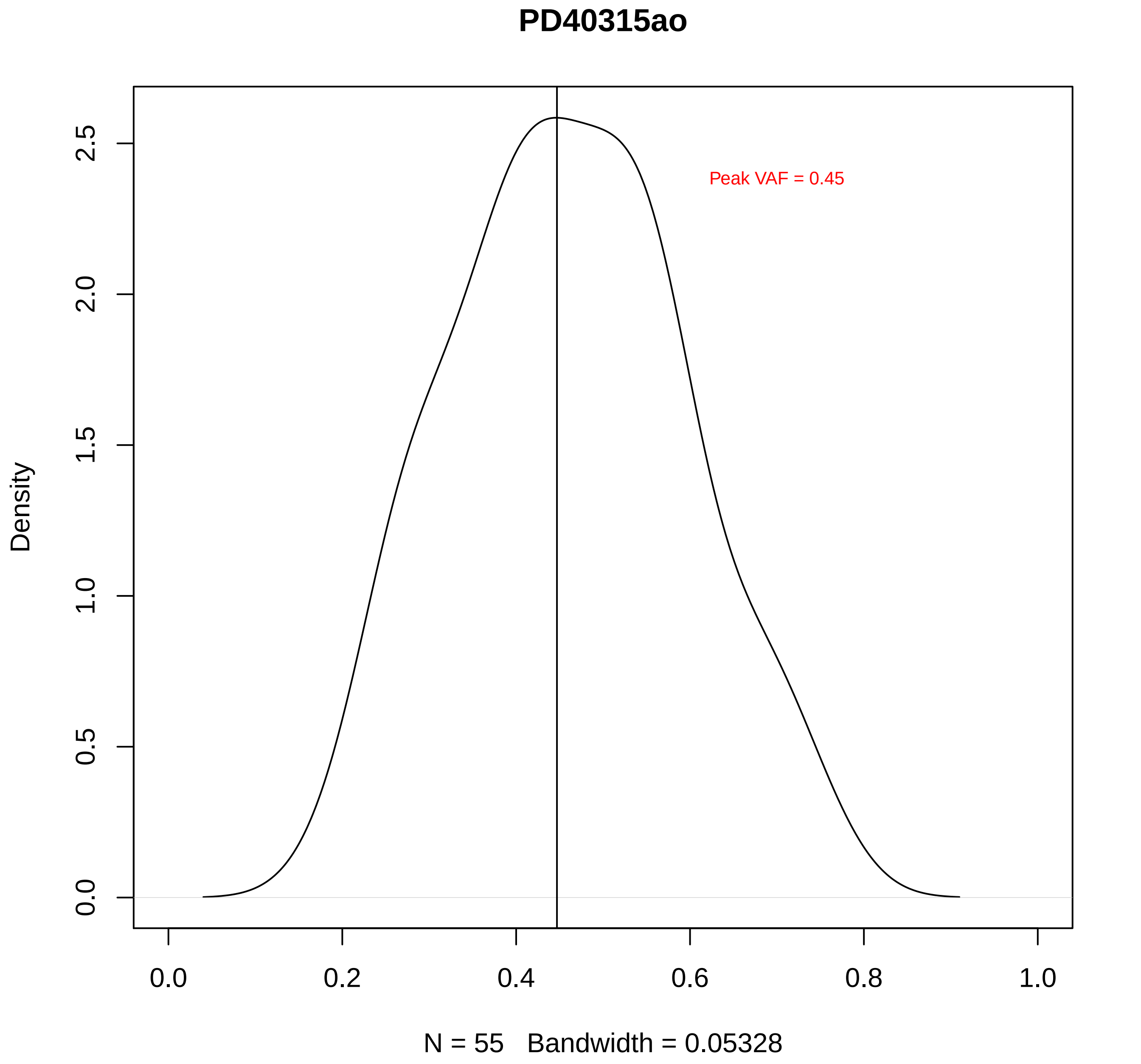
PD40315ao
Peak VAF = 0.45
Density
0.0
0.5
1.0
1.5
2.0
2.5
0.0
0.2
0.4
0.6
0.8
1.0
N = 55 Bandwidth = 0.05328

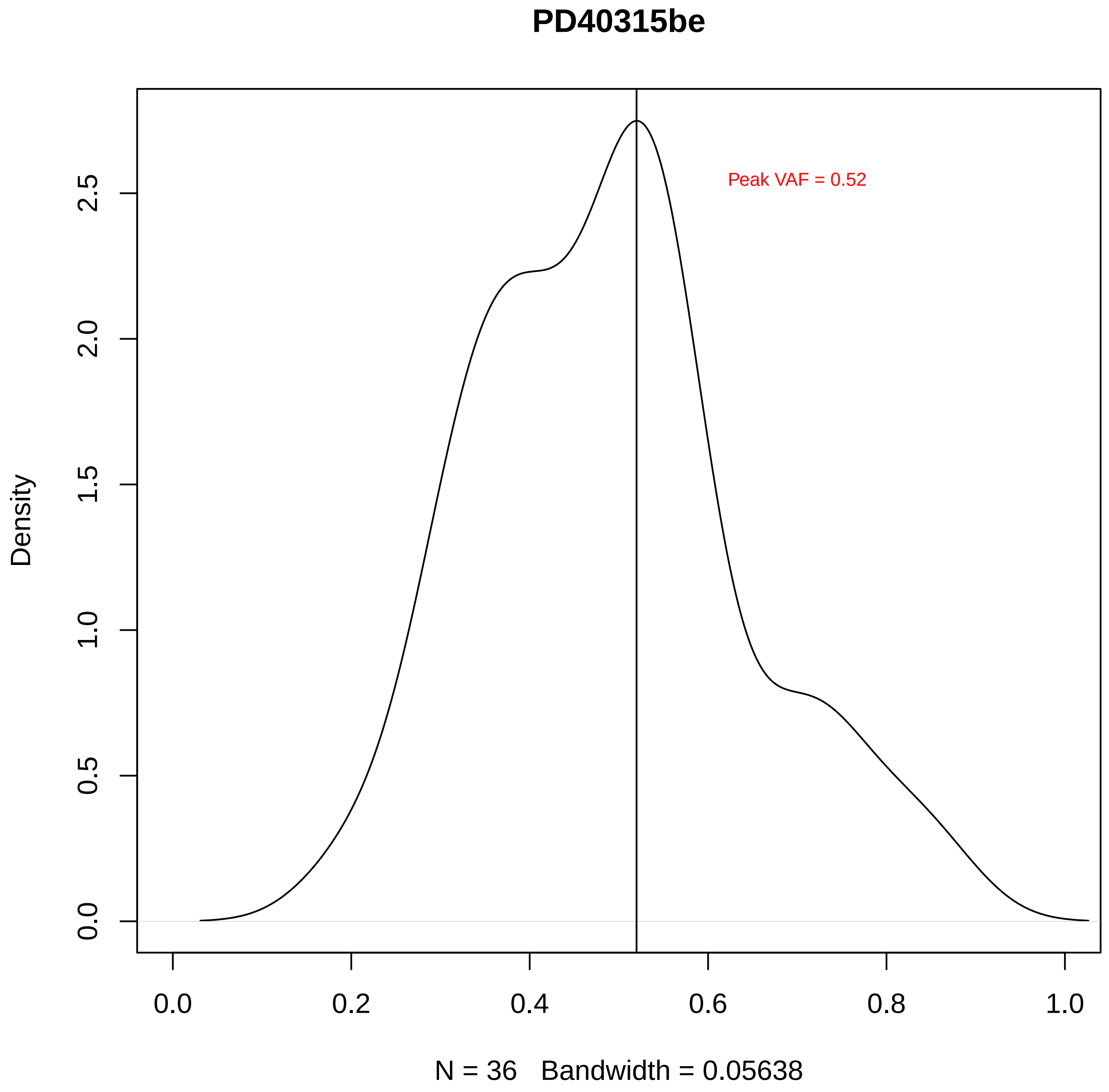

PD40315be
Peak VAF = 0.52
Density
0.0
0.5
1.0
1.5
2.0
2.5
0.0
0.2
0.4
0.6
0.8
1.0
N = 36   Bandwidth = 0.05638

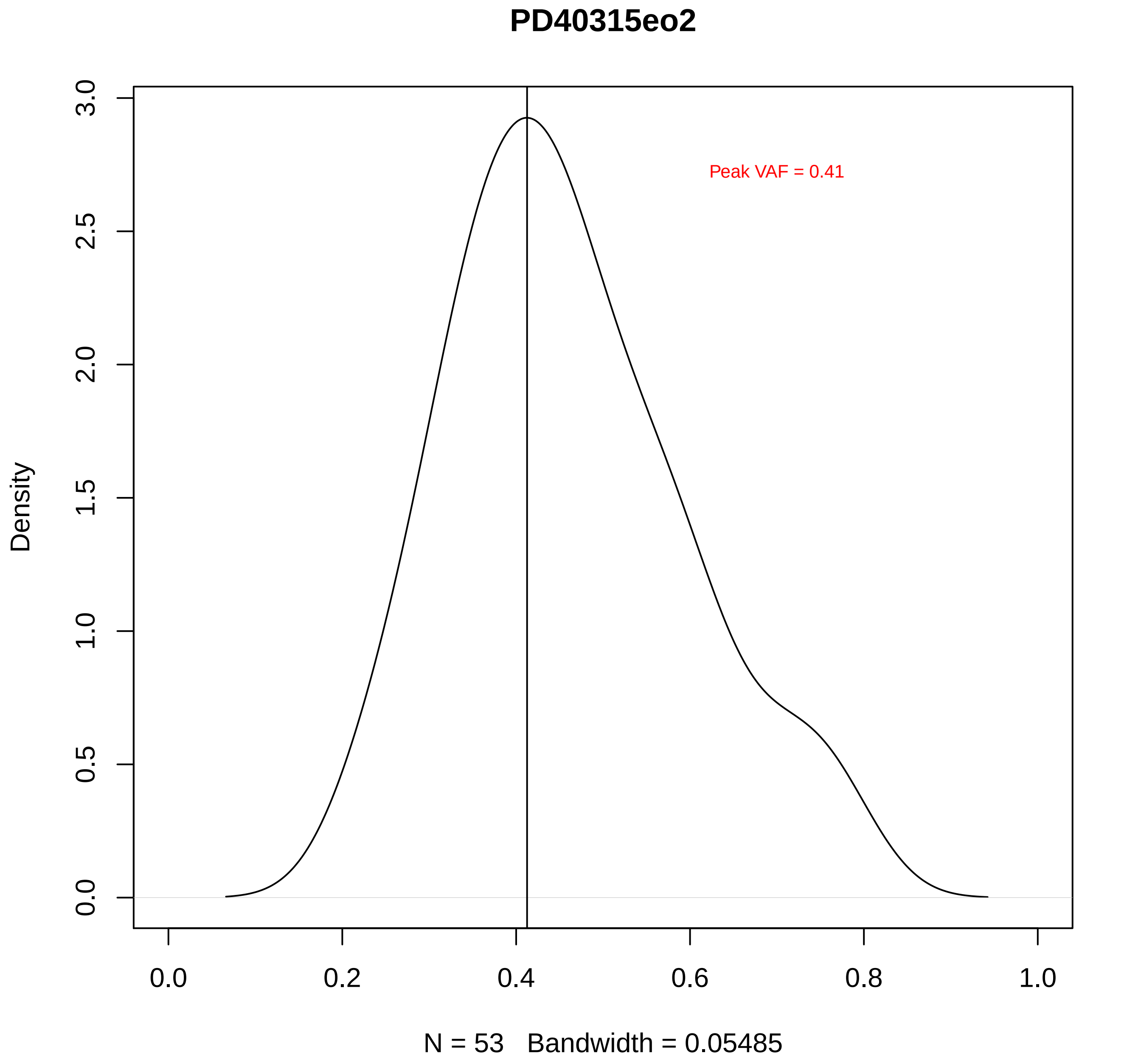
PD40315eo2
Peak VAF = 0.41
Density
0.0
0.5
1.0
1.5
2.0
2.5
3.0
0.0
0.2
0.4
0.6
0.8
1.0
N = 53   Bandwidth = 0.05485

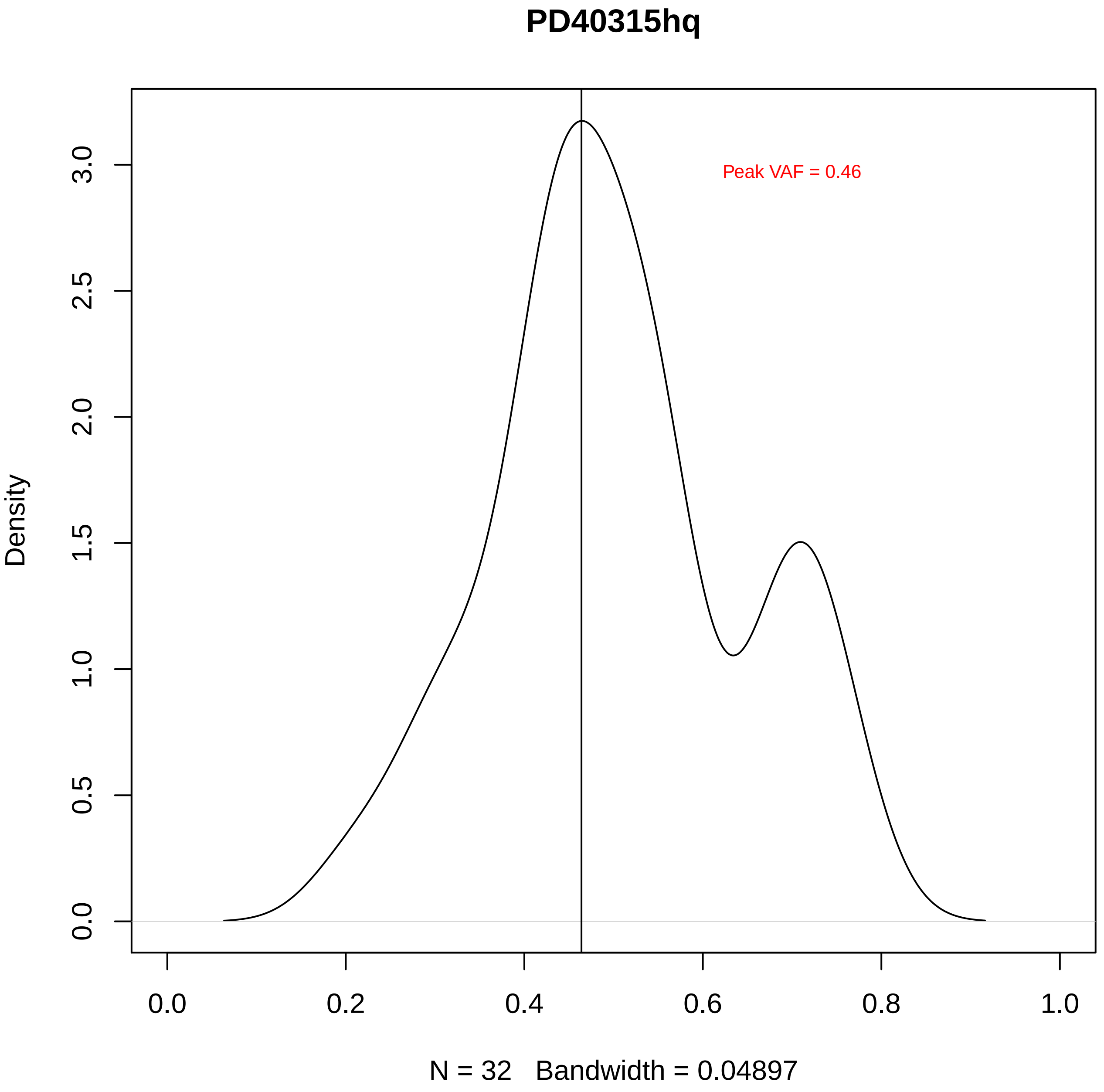
PD40315hq
Peak VAF = 0.46
Density
0.0
0.5
1.0
1.5
2.0
2.5
3.0
0.0
0.2
0.4
0.6
0.8
1.0
N = 32 Bandwidth = 0.04897

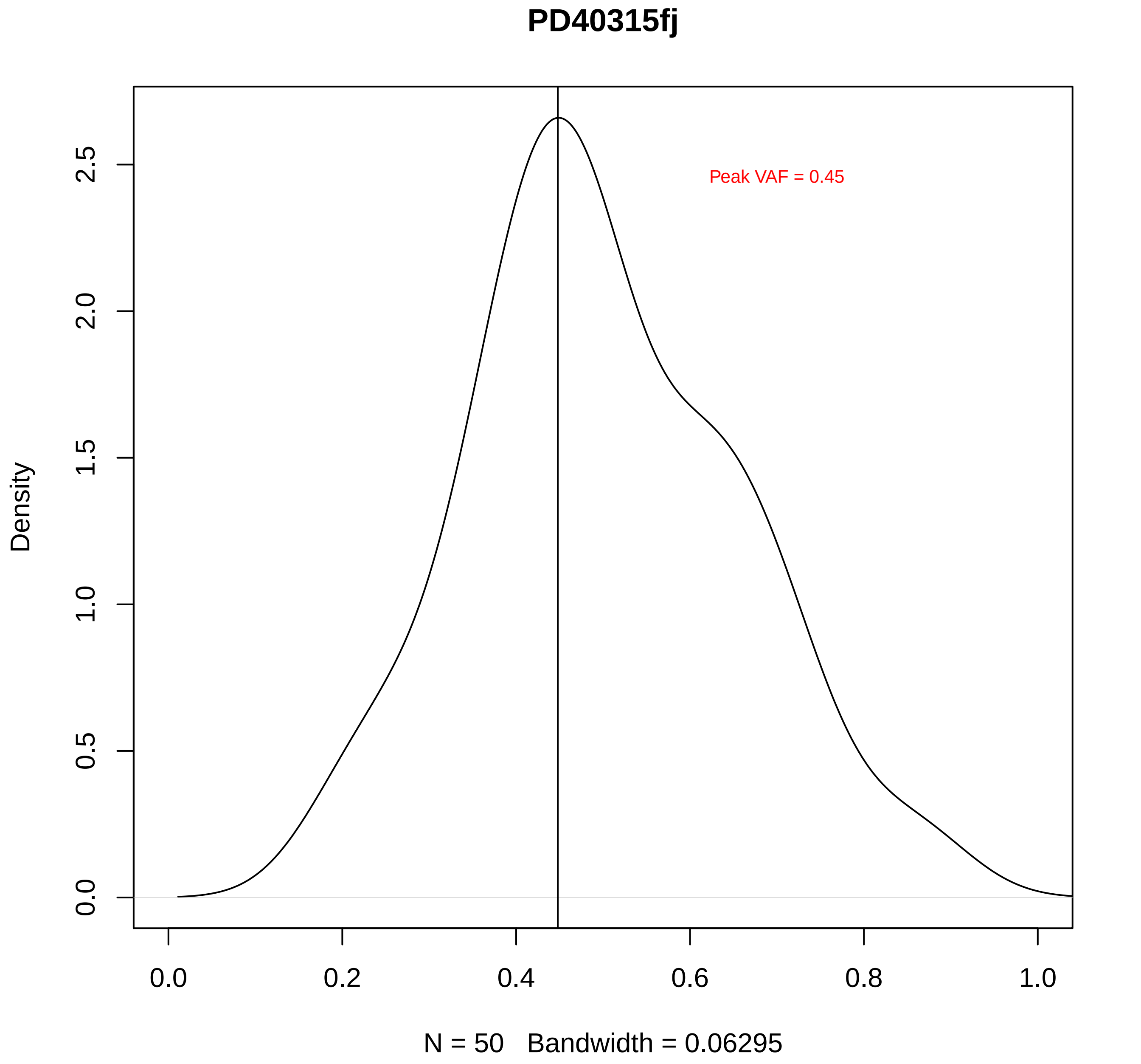
PD40315fj
Peak VAF = 0.45
Density
0.0
0.5
1.0
1.5
2.0
2.5
0.0
0.2
0.4
0.6
0.8
1.0
N = 50   Bandwidth = 0.06295

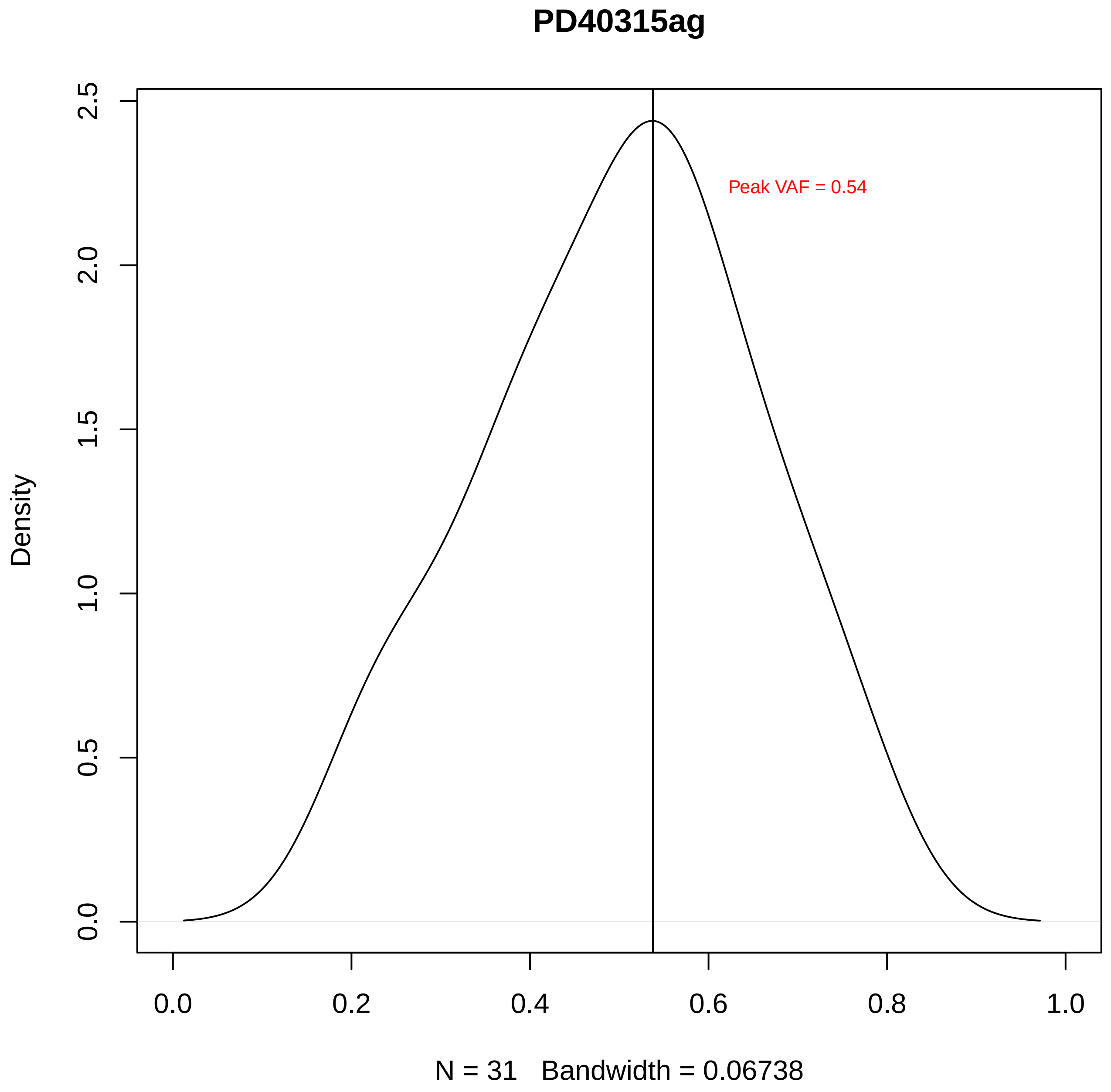
PD40315ag
Peak VAF = 0.54
Density
0.0
0.5
1.0
1.5
2.0
2.5
0.0
0.2
0.4
0.6
0.8
1.0
N = 31   Bandwidth = 0.06738

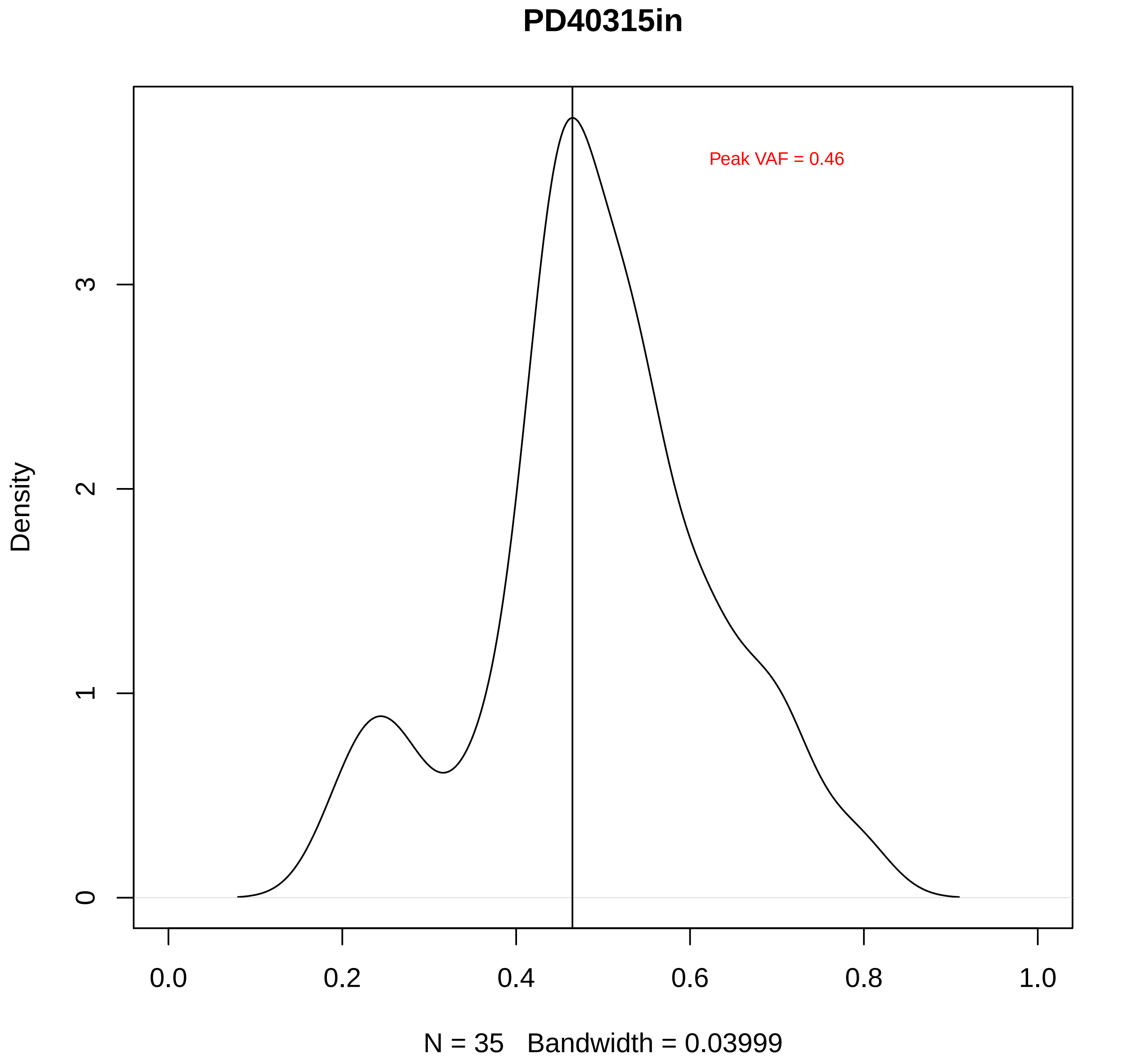
PD40315in
Peak VAF = 0.46
Density
0
1
2
3
0.0
0.2
0.4
0.6
0.8
1.0
N = 35   Bandwidth = 0.03999

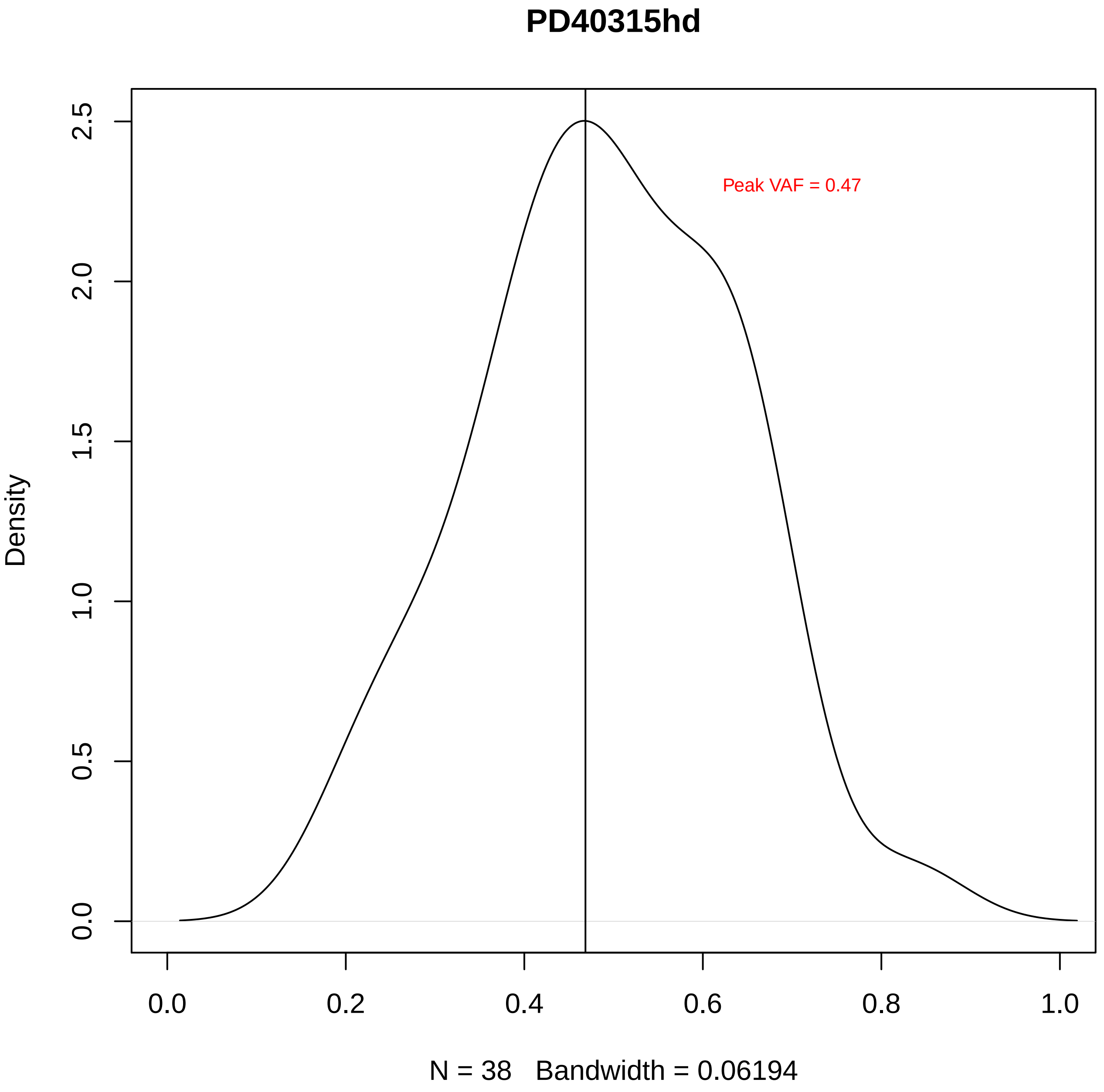
PD40315hd
Peak VAF = 0.47
Density
0.0
0.5
1.0
1.5
2.0
2.5
0.0
0.2
0.4
0.6
0.8
1.0
N = 38   Bandwidth = 0.06194

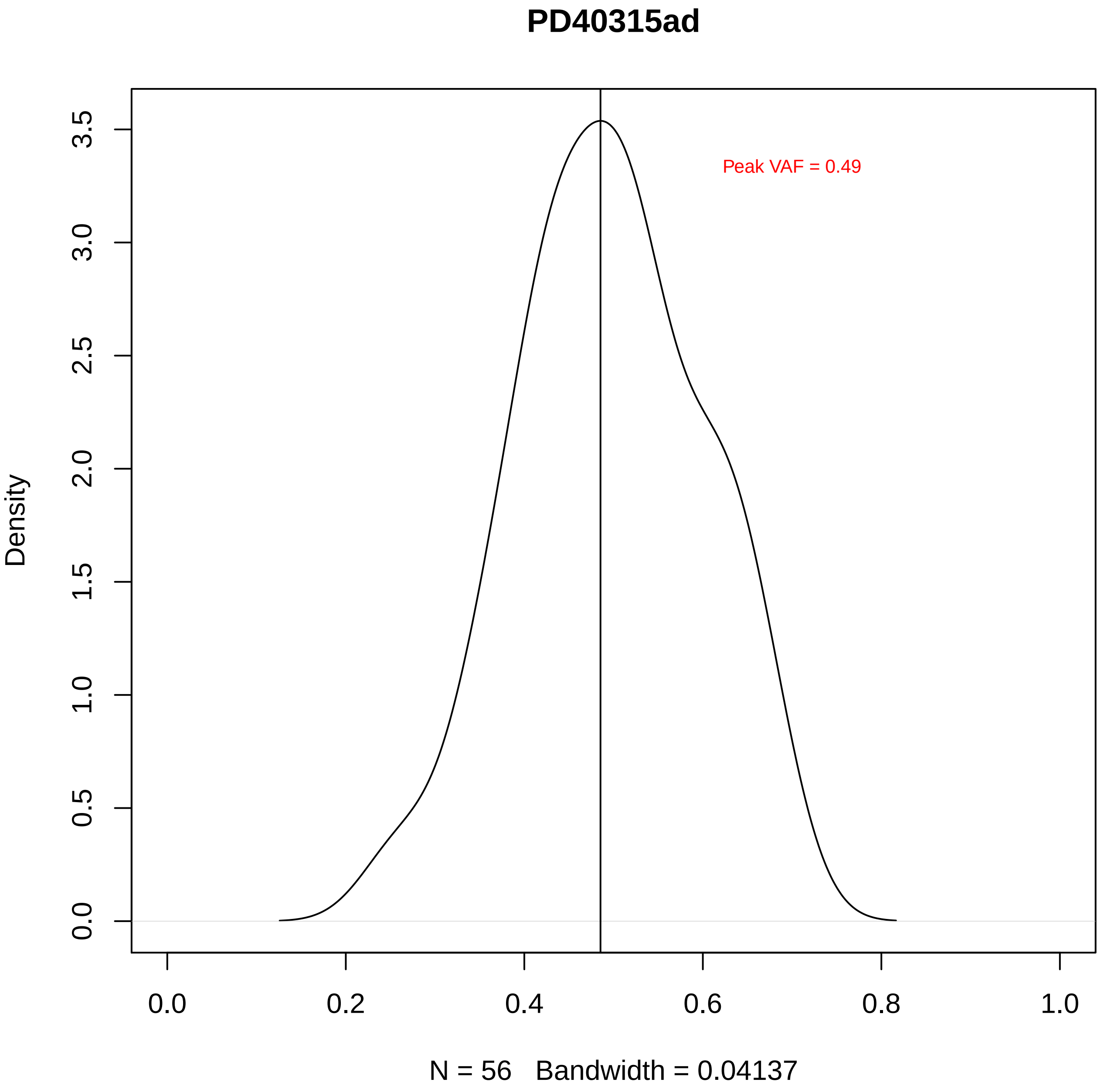
PD40315ad
Peak VAF = 0.49
Density
0.0
0.5
1.0
1.5
2.0
2.5
3.0
3.5
0.0
0.2
0.4
0.6
0.8
1.0
N = 56   Bandwidth = 0.04137

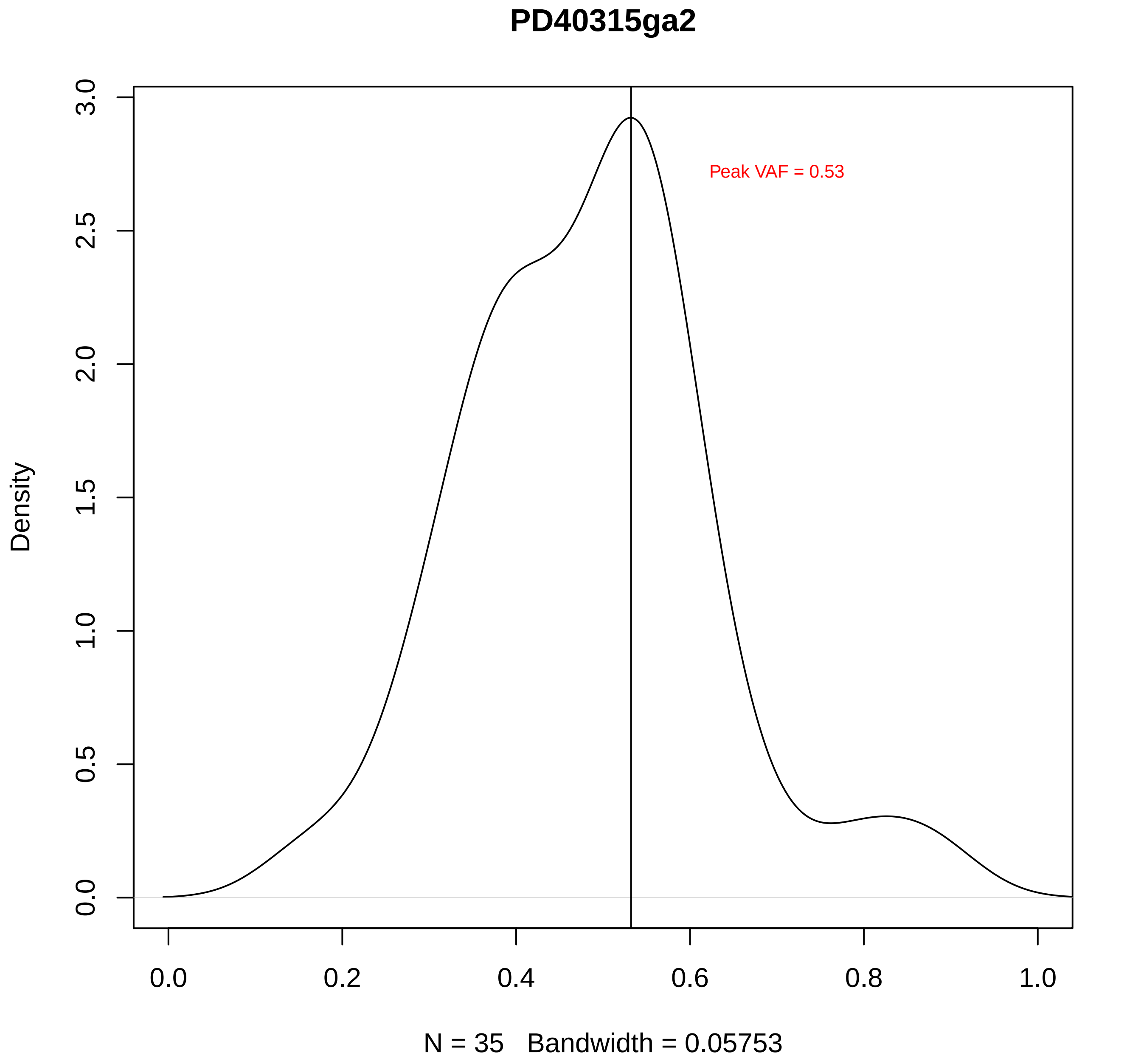
PD40315ga2
Peak VAF = 0.53
Density
0.0
0.5
1.0
1.5
2.0
2.5
3.0
0.0
0.2
0.4
0.6
0.8
1.0
N = 35 Bandwidth = 0.05753

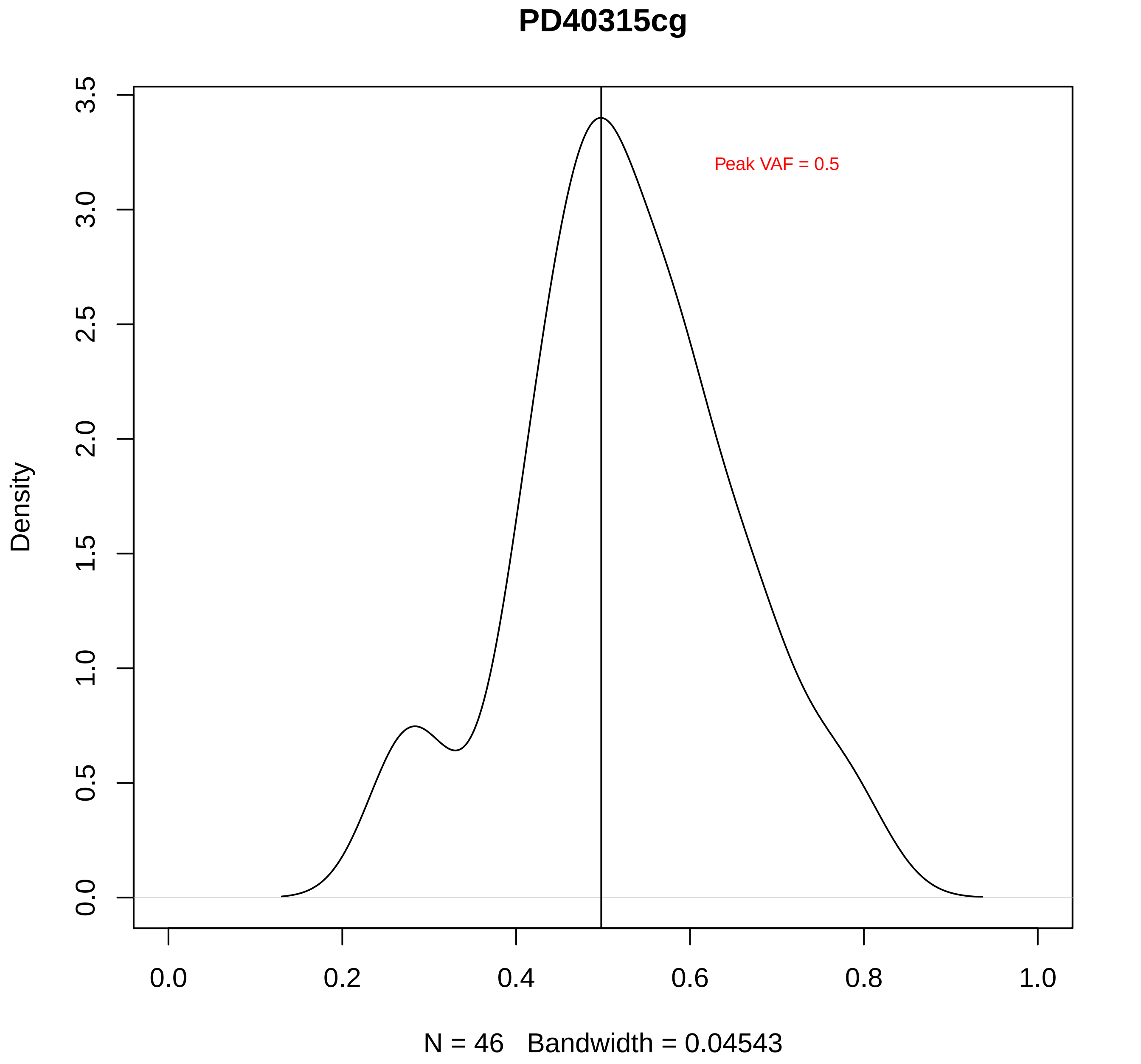
PD40315cg
Peak VAF = 0.5
Density
0.0
0.5
1.0
1.5
2.0
2.5
3.0
3.5
0.0
0.2
0.4
0.6
0.8
1.0
N = 46 Bandwidth = 0.04543

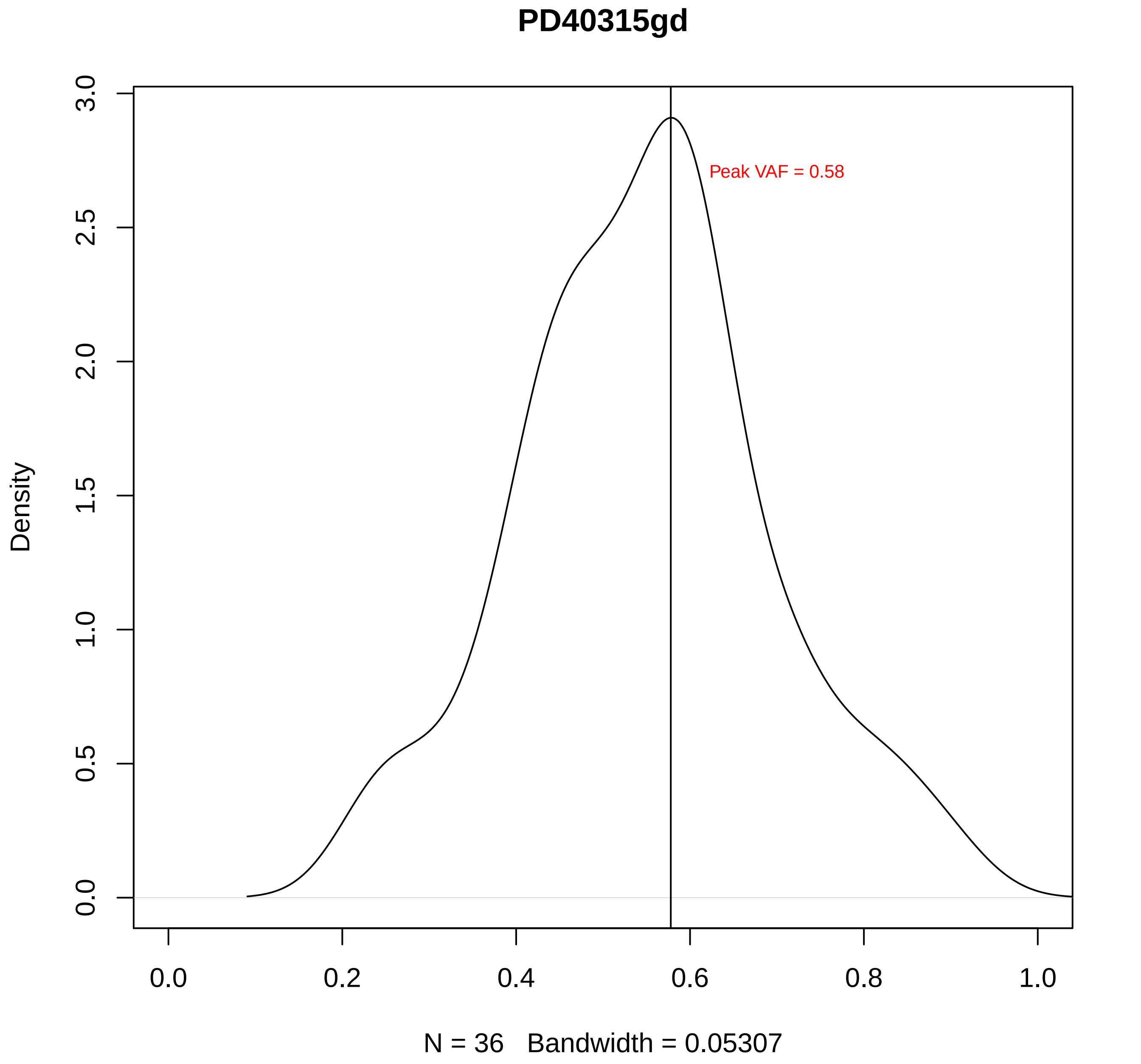
PD40315gd
Peak VAF = 0.58
Density
0.0
0.5
1.0
1.5
2.0
2.5
3.0
0.0
0.2
0.4
0.6
0.8
1.0
N = 36   Bandwidth = 0.05307

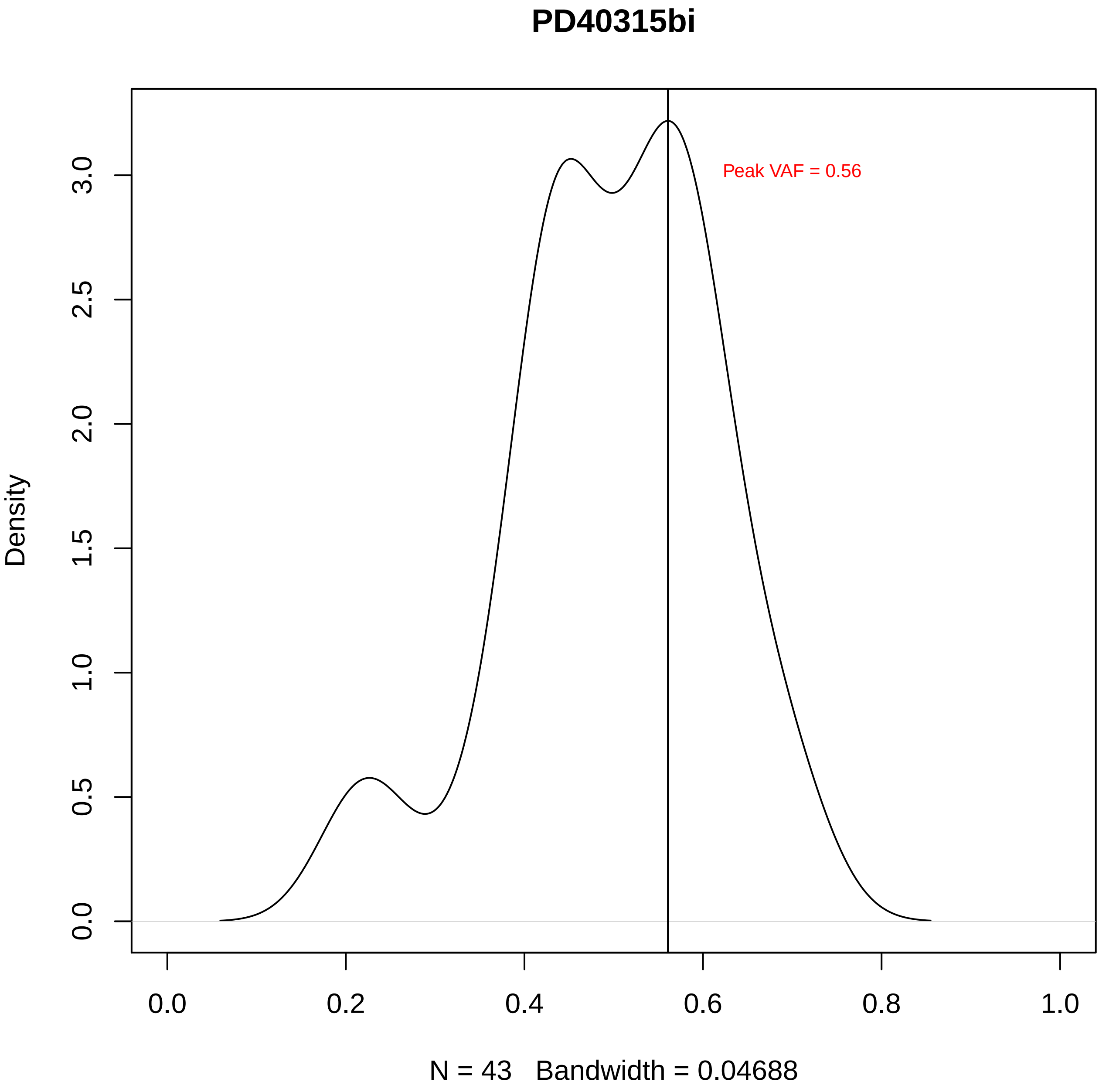
PD40315bi
Peak VAF = 0.56
Density
0.0
0.5
1.0
1.5
2.0
2.5
3.0
0.0
0.2
0.4
0.6
0.8
1.0
N = 43 Bandwidth = 0.04688

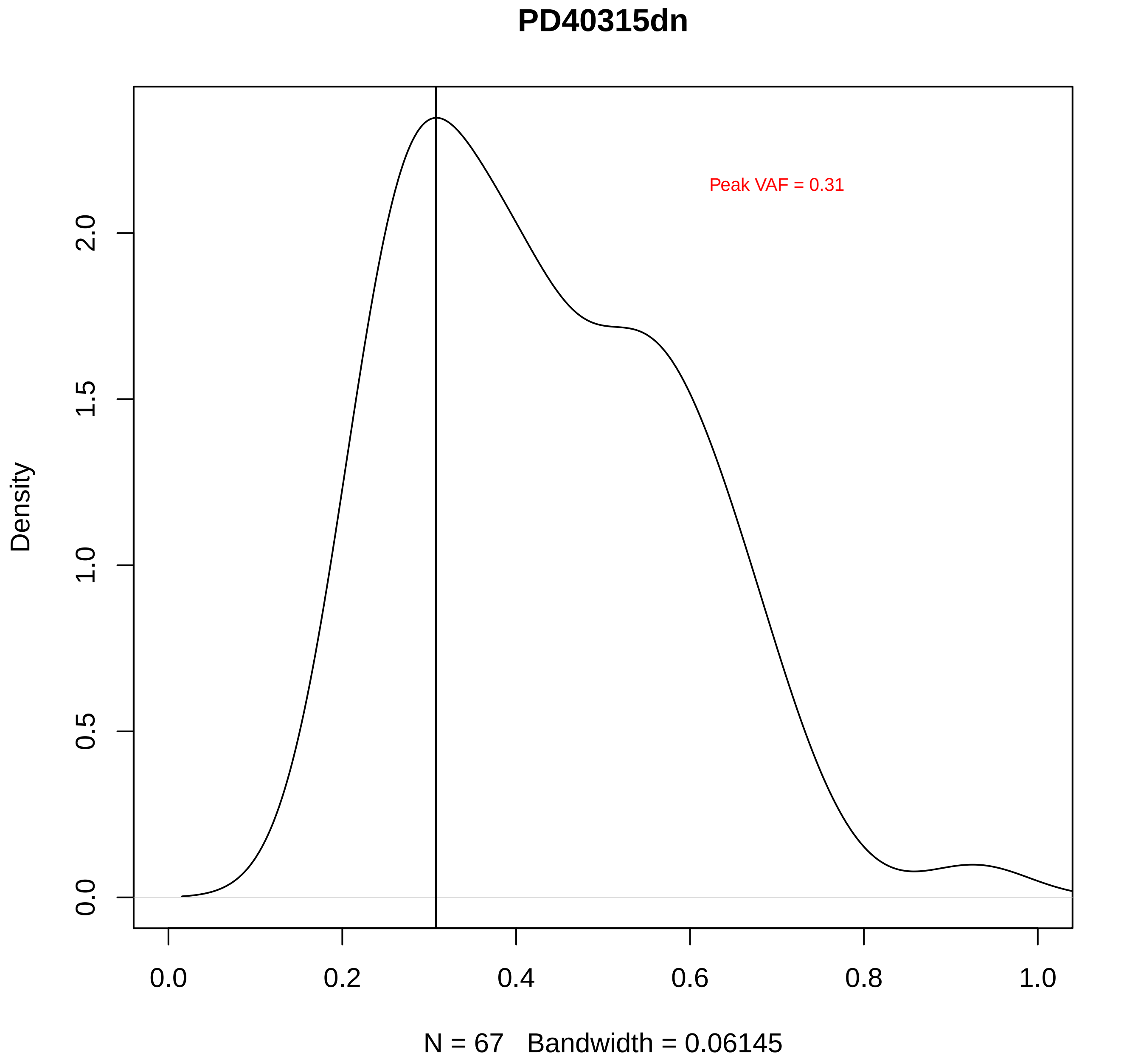
PD40315dn
Peak VAF = 0.31
Density
0.0
0.5
1.0
1.5
2.0
0.0
0.2
0.4
0.6
0.8
1.0
N = 67   Bandwidth = 0.06145

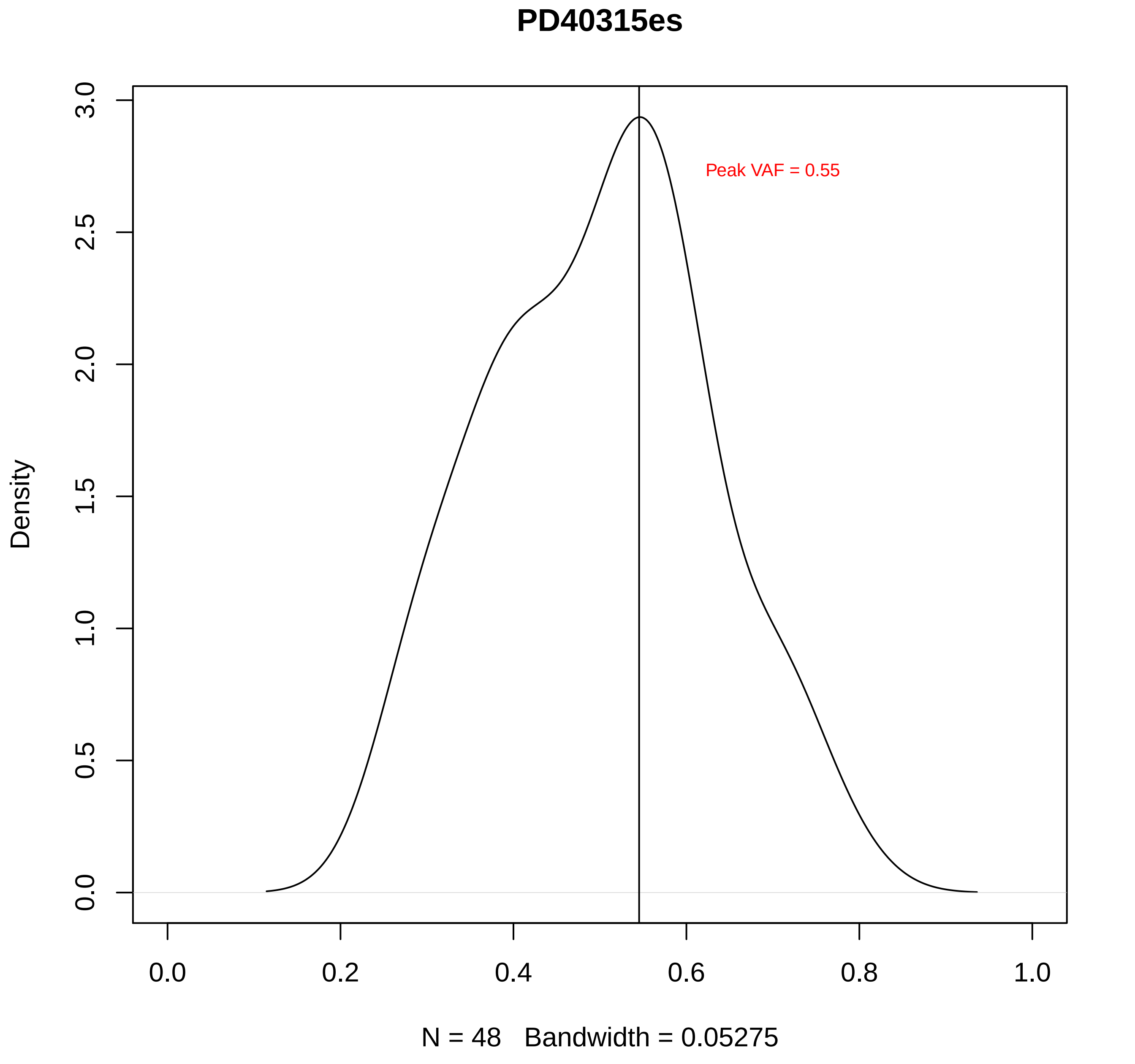

PD40315es
Peak VAF = 0.55
Density
3.0
2.5
2.0
1.5
1.0
0.5
0.0
0.0
0.2
0.4
0.6
0.8
1.0
N = 48   Bandwidth = 0.05275

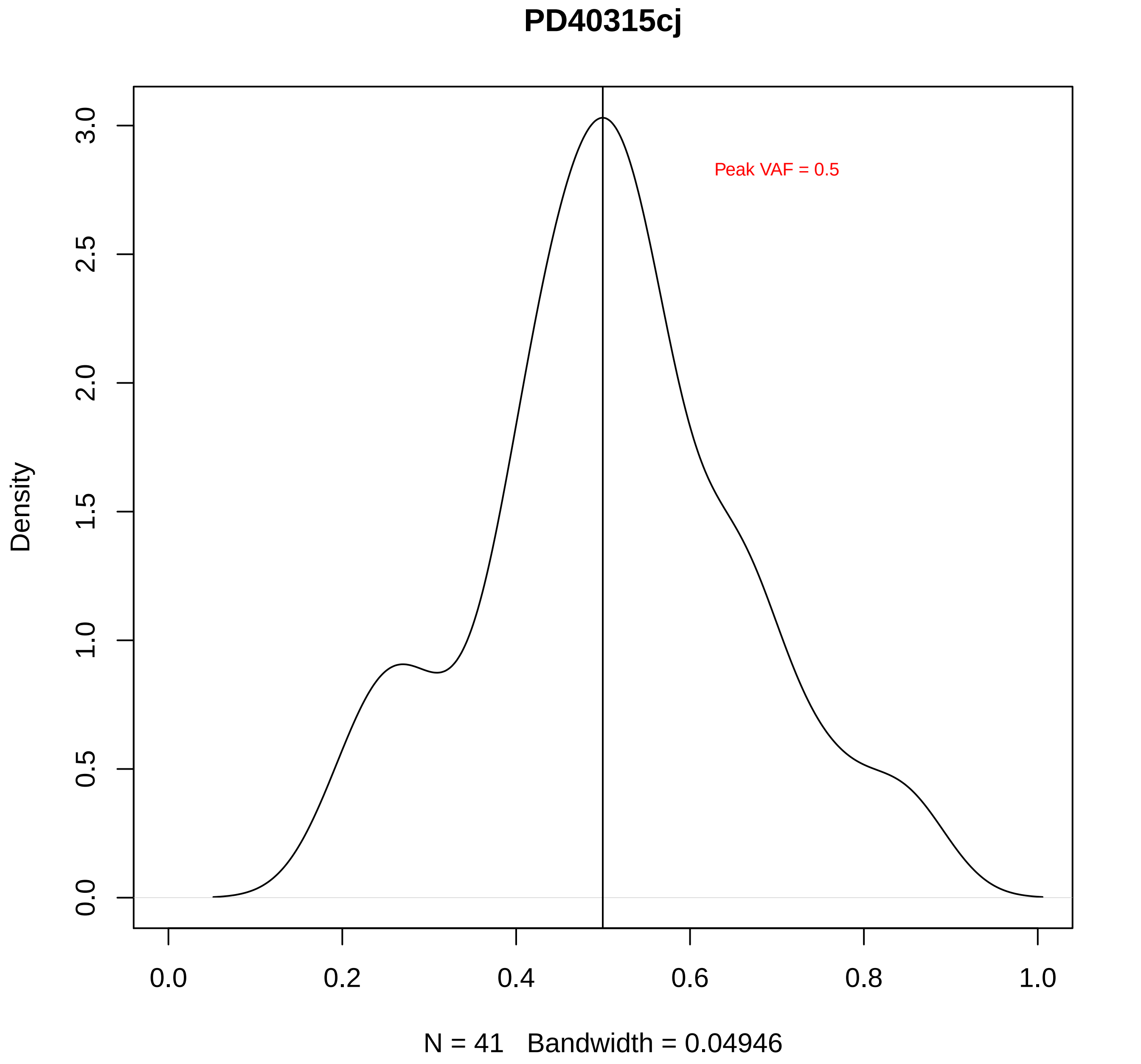
PD40315cj
Peak VAF = 0.5
Density
0.0
0.5
1.0
1.5
2.0
2.5
3.0
0.0
0.2
0.4
0.6
0.8
1.0
N = 41   Bandwidth = 0.04946

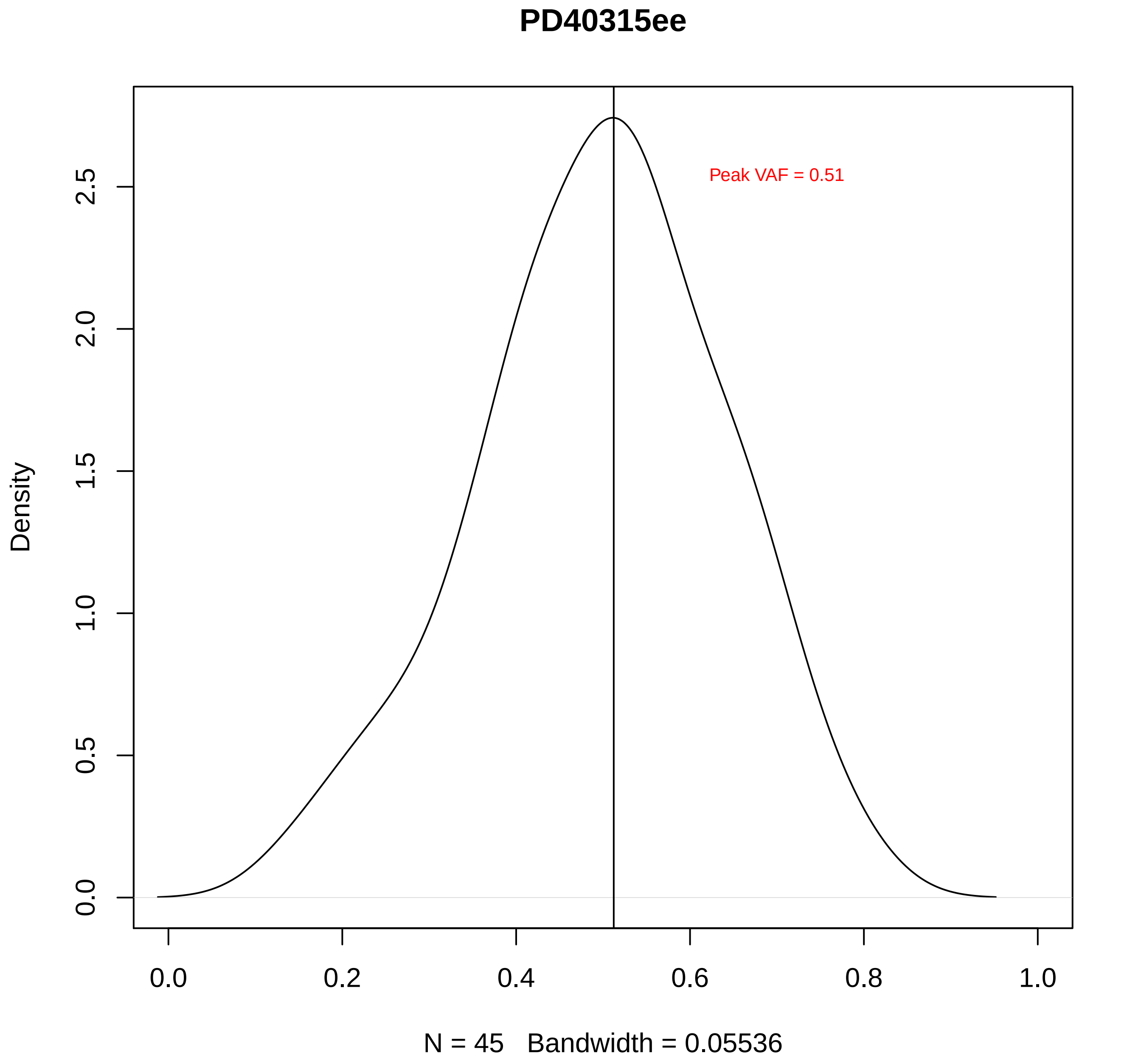
PD40315ee
Peak VAF = 0.51
Density
0.0
0.5
1.0
1.5
2.0
2.5
0.0
0.2
0.4
0.6
0.8
1.0
N = 45   Bandwidth = 0.05536

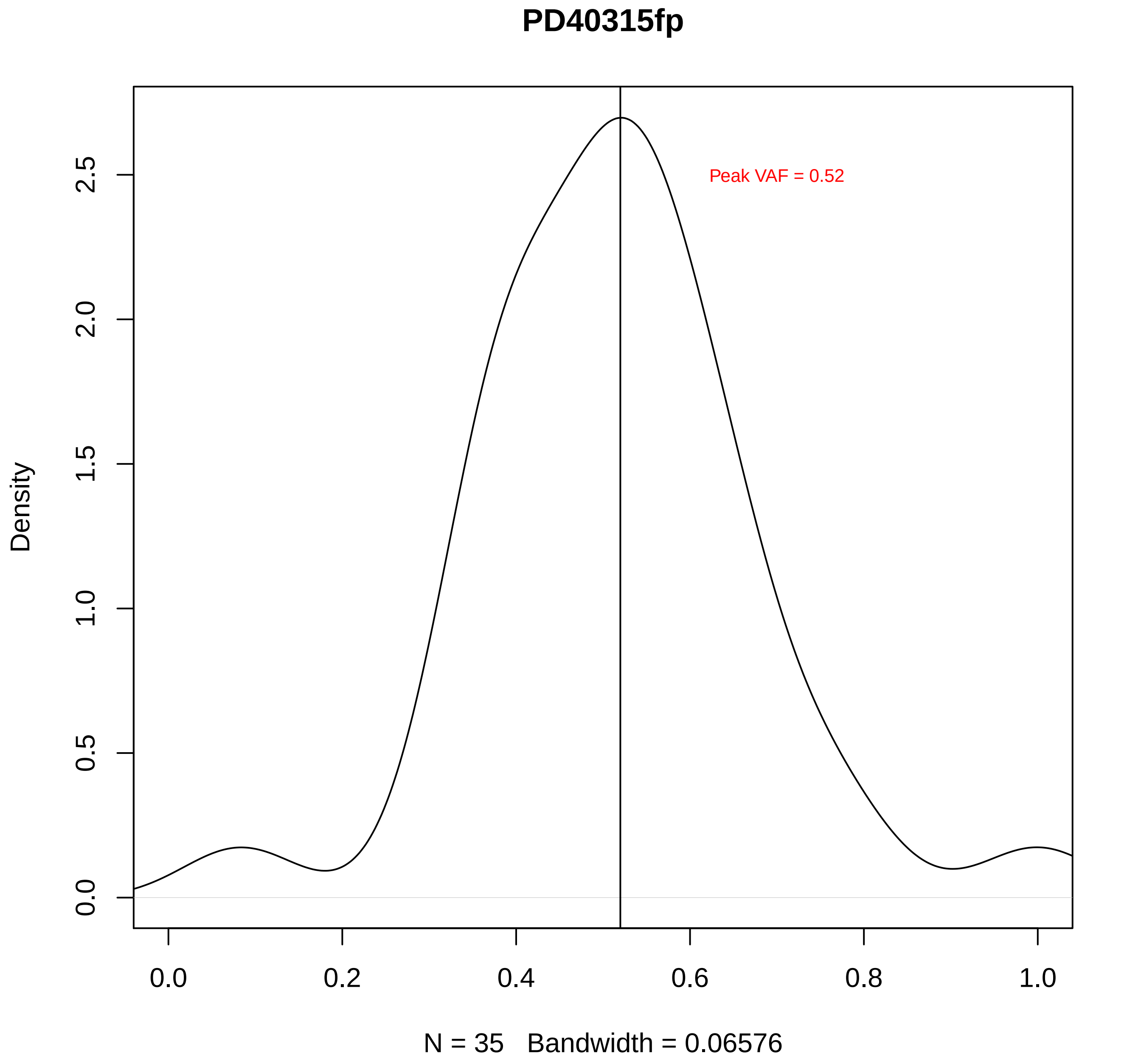

PD40315fp
Peak VAF = 0.52
Density
0.0
0.5
1.0
1.5
2.0
2.5
0.0
0.2
0.4
0.6
0.8
1.0
N = 35   Bandwidth = 0.06576

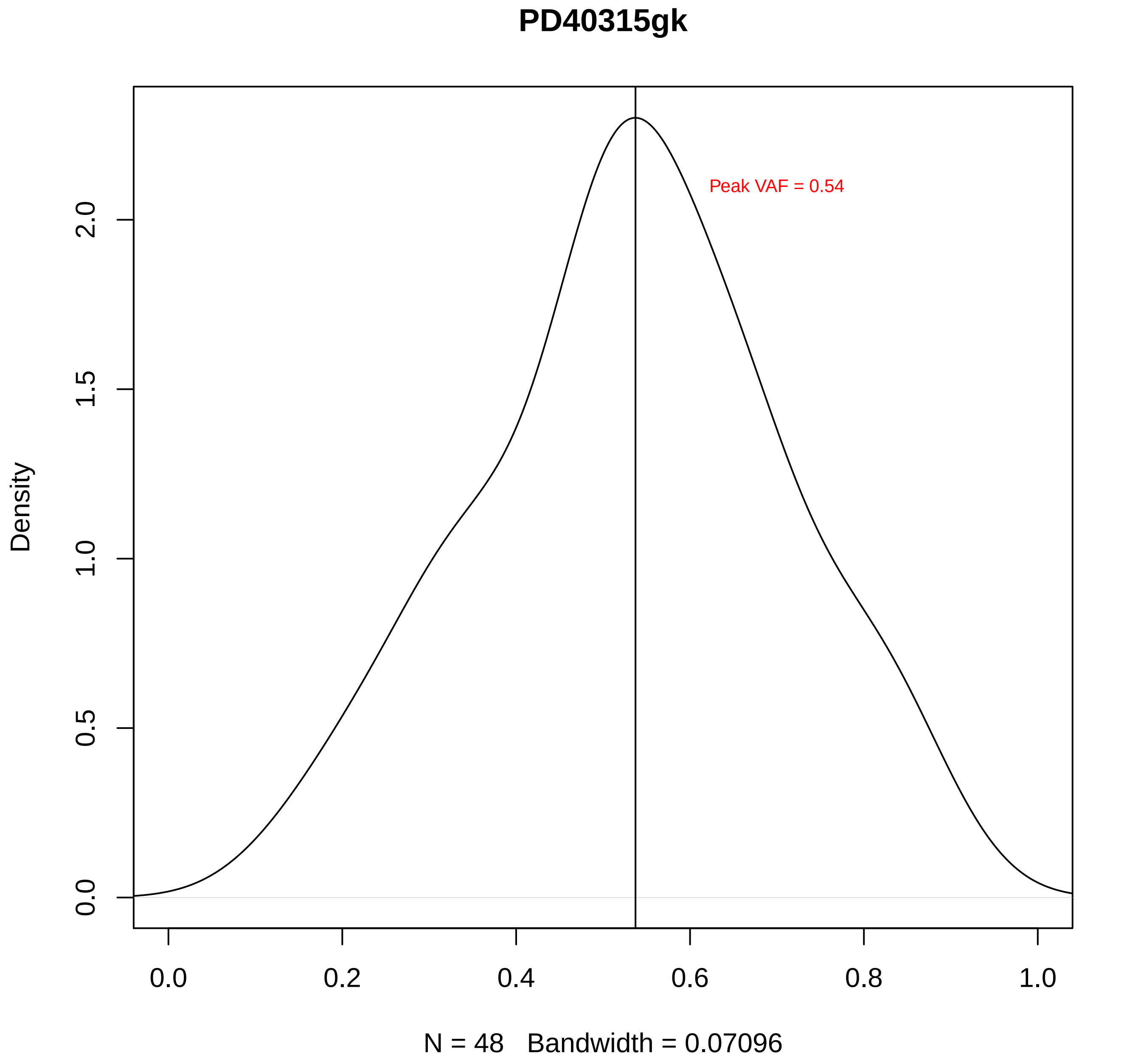

PD40315gk
Peak VAF = 0.54
Density
0.0
0.5
1.0
1.5
2.0
0.0
0.2
0.4
0.6
0.8
1.0
N = 48   Bandwidth = 0.07096

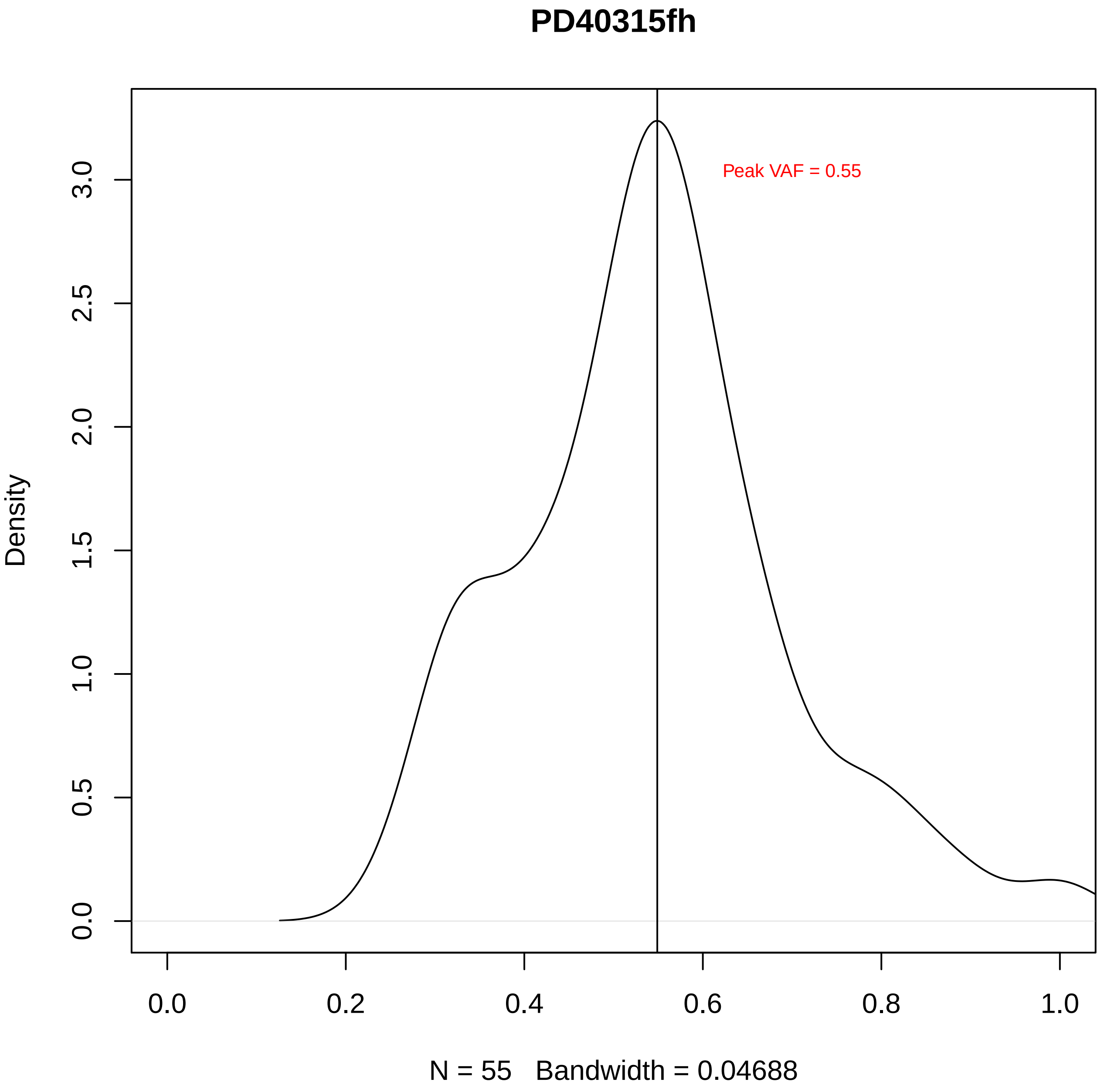
PD40315fh
Peak VAF = 0.55
Density
0.0
0.5
1.0
1.5
2.0
2.5
3.0
0.0
0.2
0.4
0.6
0.8
1.0
N = 55 Bandwidth = 0.04688

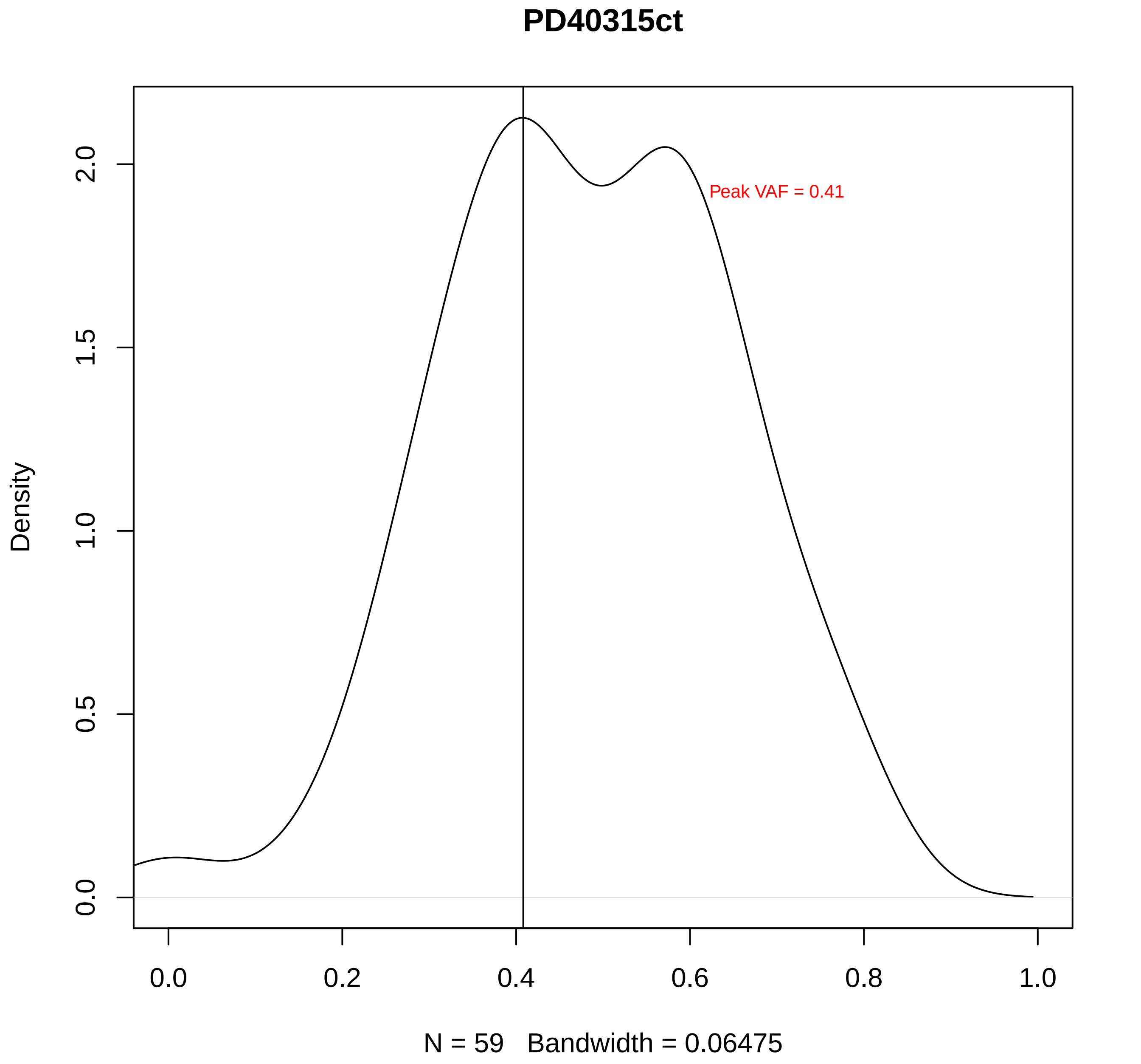
PD40315ct
Peak VAF = 0.41
Density
0.0
0.5
1.0
1.5
2.0
0.0
0.2
0.4
0.6
0.8
1.0
N = 59   Bandwidth = 0.06475

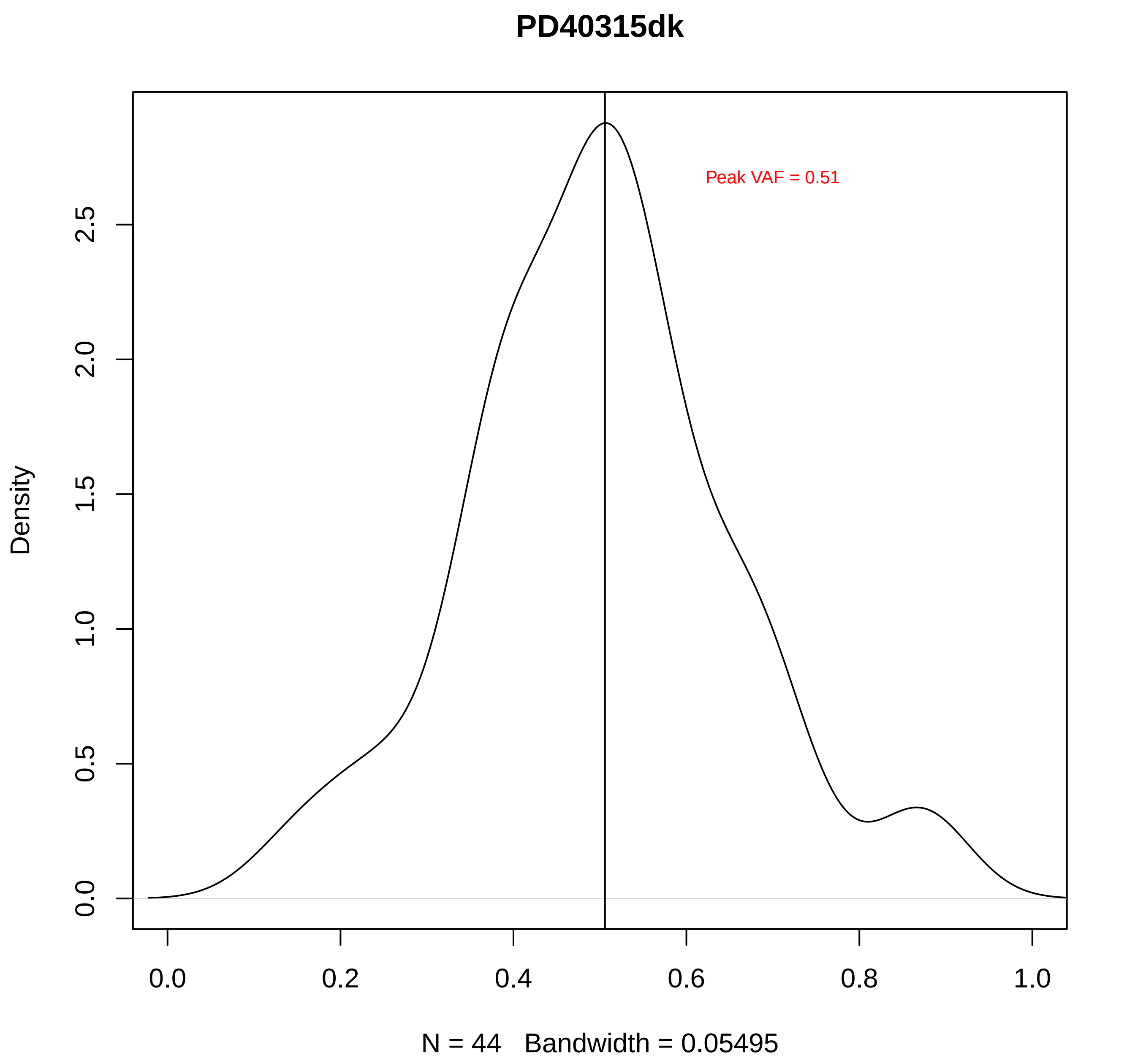
PD40315dk
Peak VAF = 0.51
Density
0.0
0.5
1.0
1.5
2.0
2.5
0.0
0.2
0.4
0.6
0.8
1.0
N = 44   Bandwidth = 0.05495

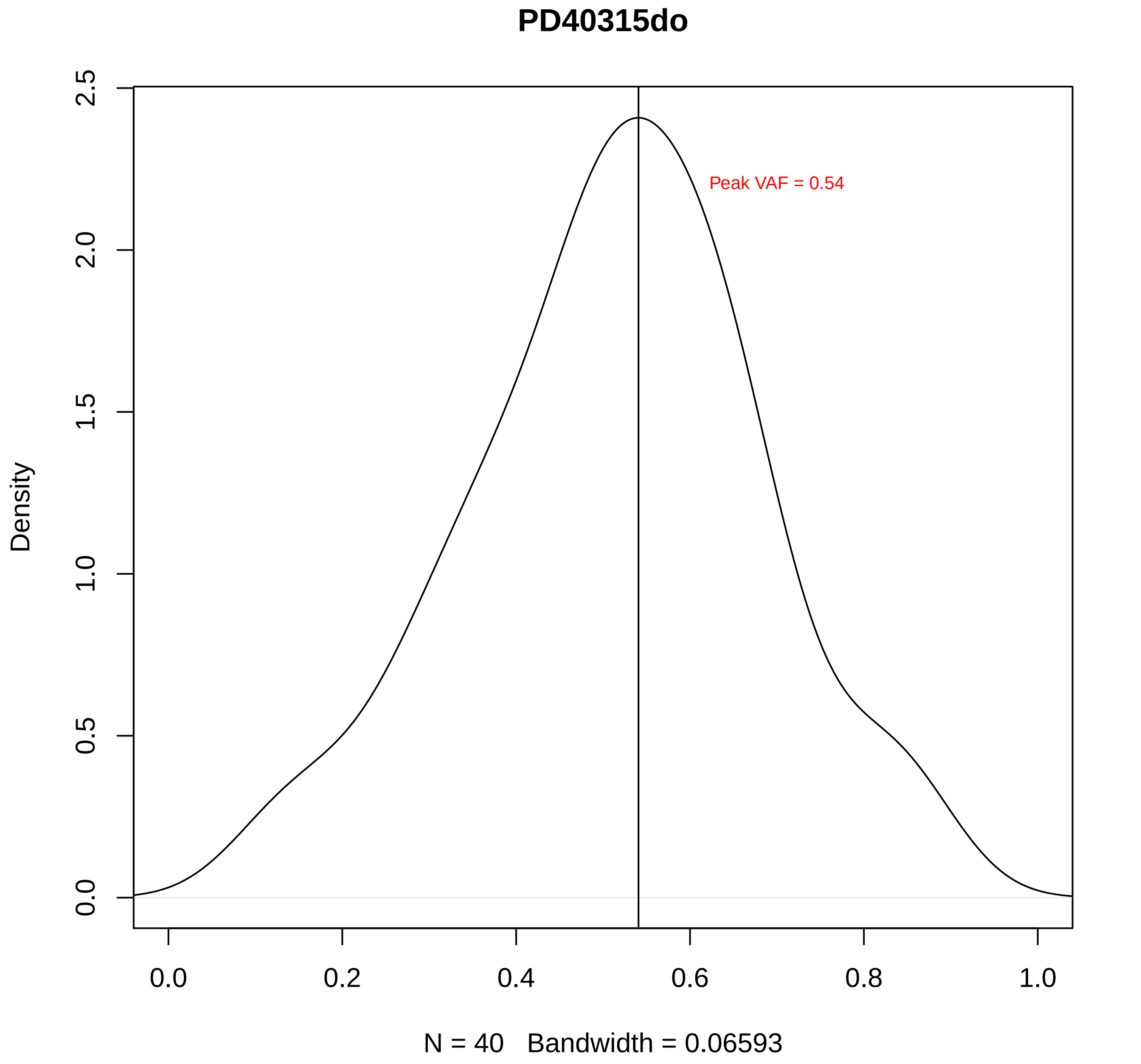

PD40315do
Peak VAF = 0.54
Density
0.0
0.5
1.0
1.5
2.0
2.5
0.0
0.2
0.4
0.6
0.8
1.0
N = 40 Bandwidth = 0.06593

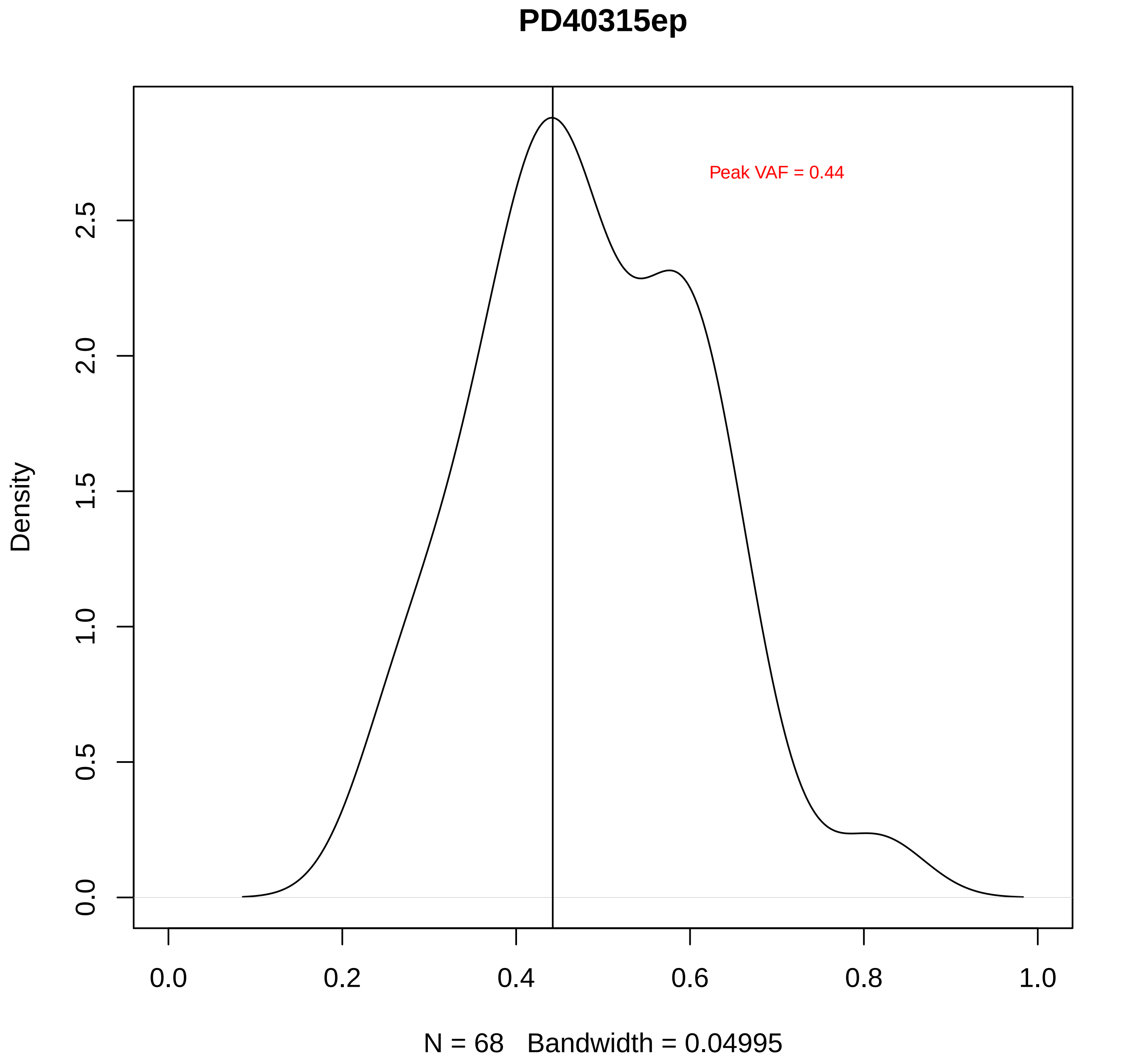
PD40315ep
Peak VAF = 0.44
Density
0.0
0.5
1.0
1.5
2.0
2.5
0.0
0.2
0.4
0.6
0.8
1.0
N = 68   Bandwidth = 0.04995

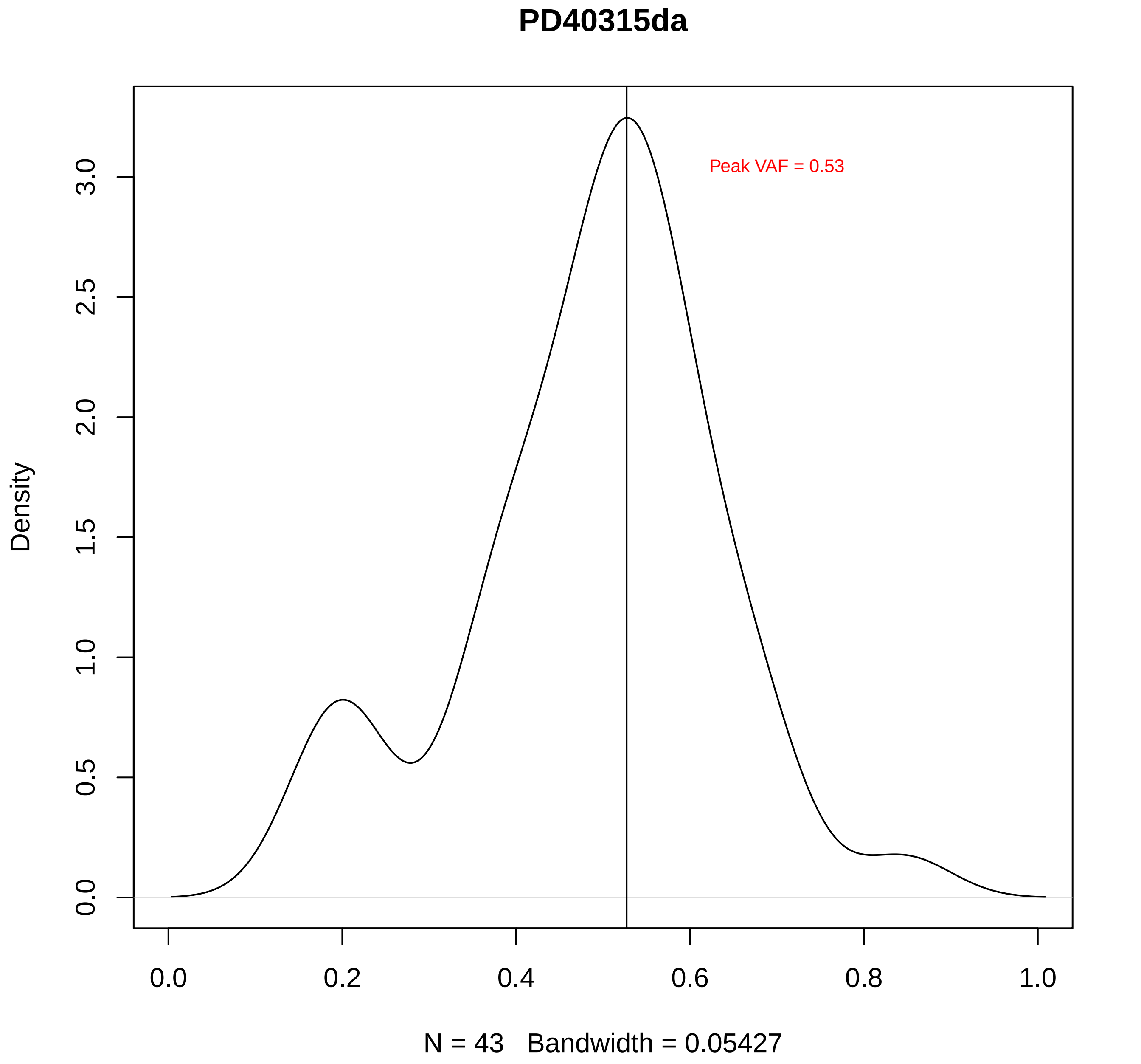
PD40315da
Peak VAF = 0.53
Density
0.0
0.5
1.0
1.5
2.0
2.5
3.0
0.0
0.2
0.4
0.6
0.8
1.0
N = 43 Bandwidth = 0.05427

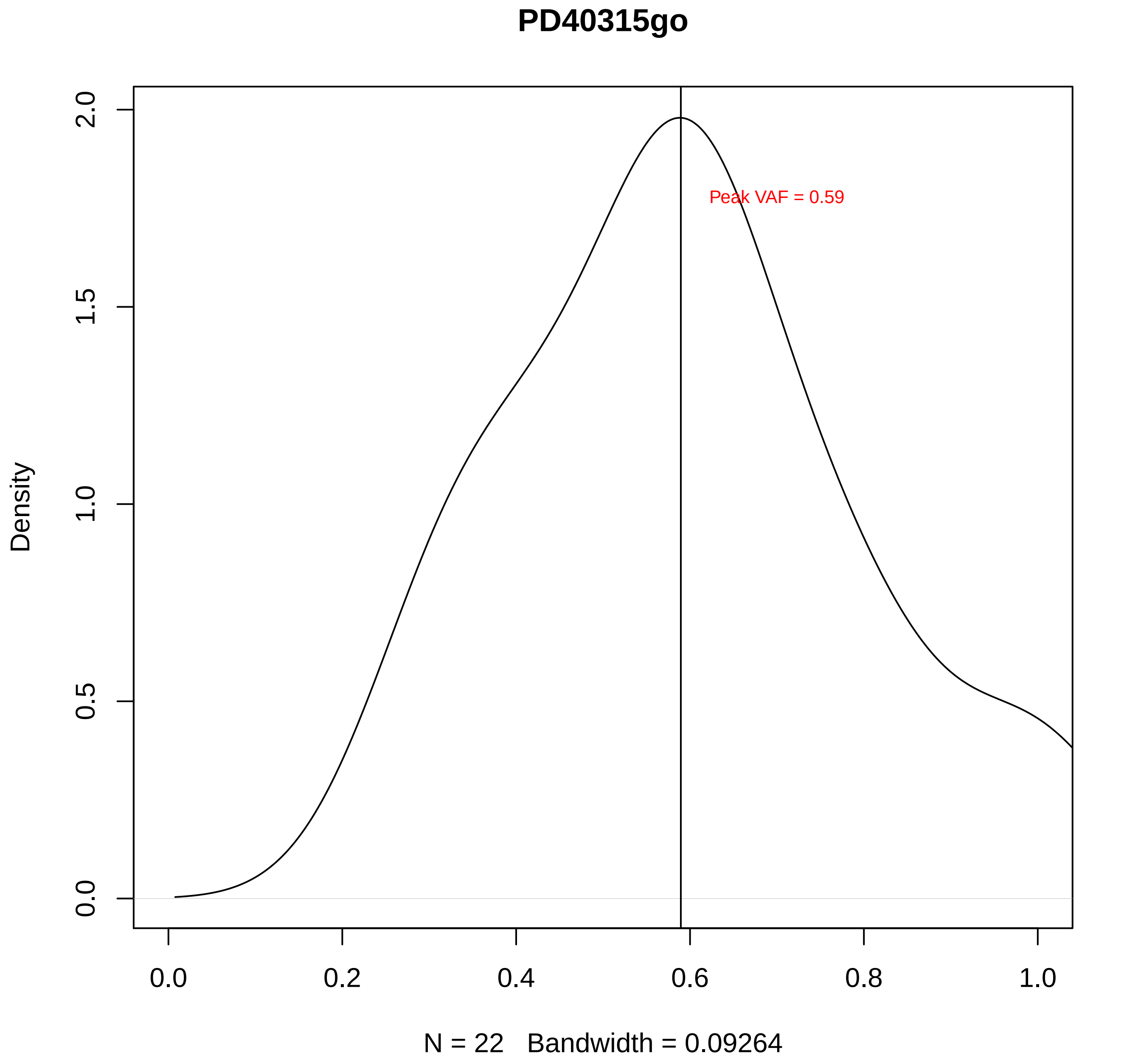
PD40315go
Peak VAF = 0.59
Density
0.0
0.5
1.0
1.5
2.0
0.0
0.2
0.4
0.6
0.8
1.0
N = 22   Bandwidth = 0.09264

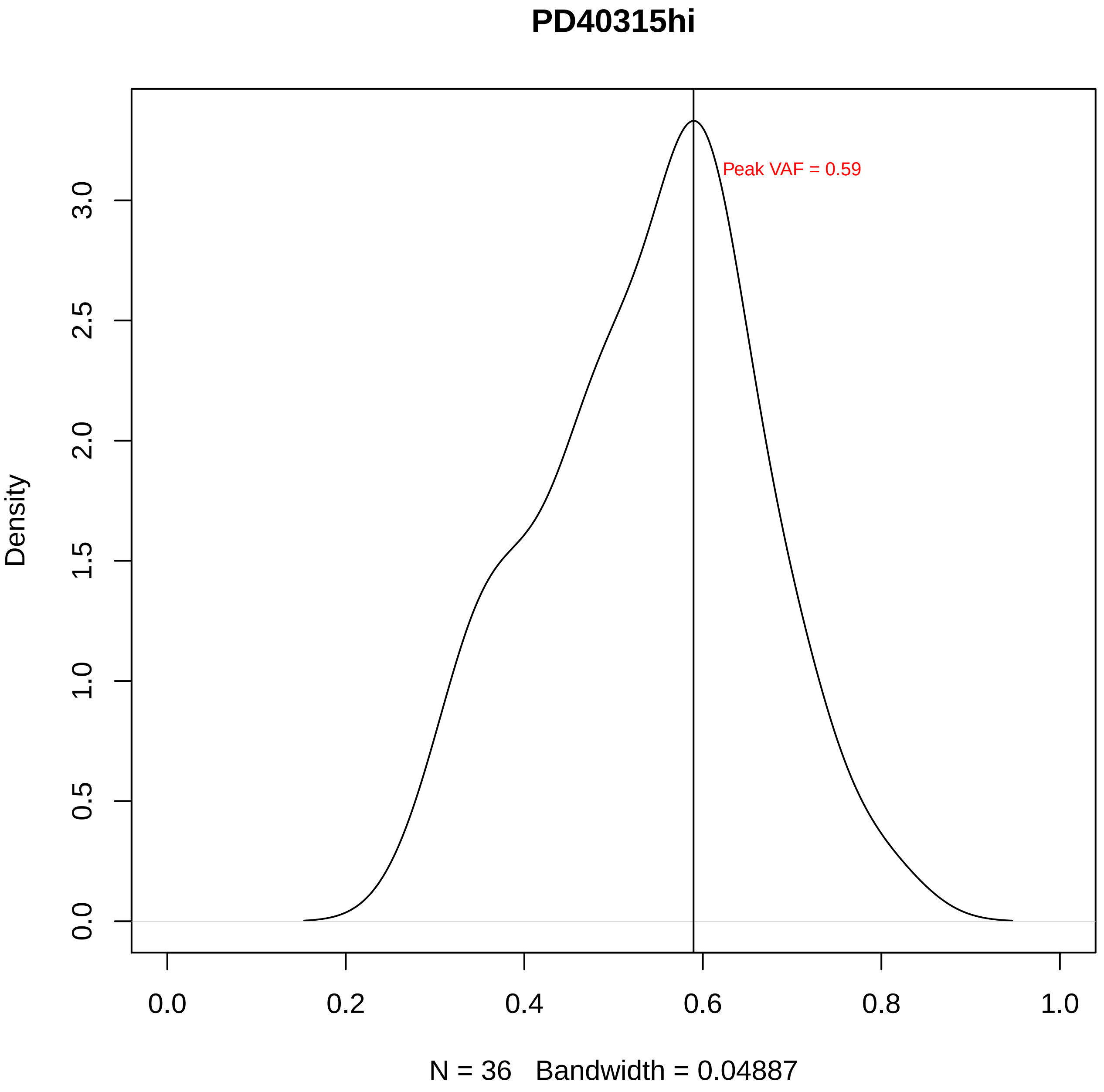
PD40315hi
Peak VAF = 0.59
Density
0.0
0.5
1.0
1.5
2.0
2.5
3.0
0.0
0.2
0.4
0.6
0.8
1.0
N = 36   Bandwidth = 0.04887

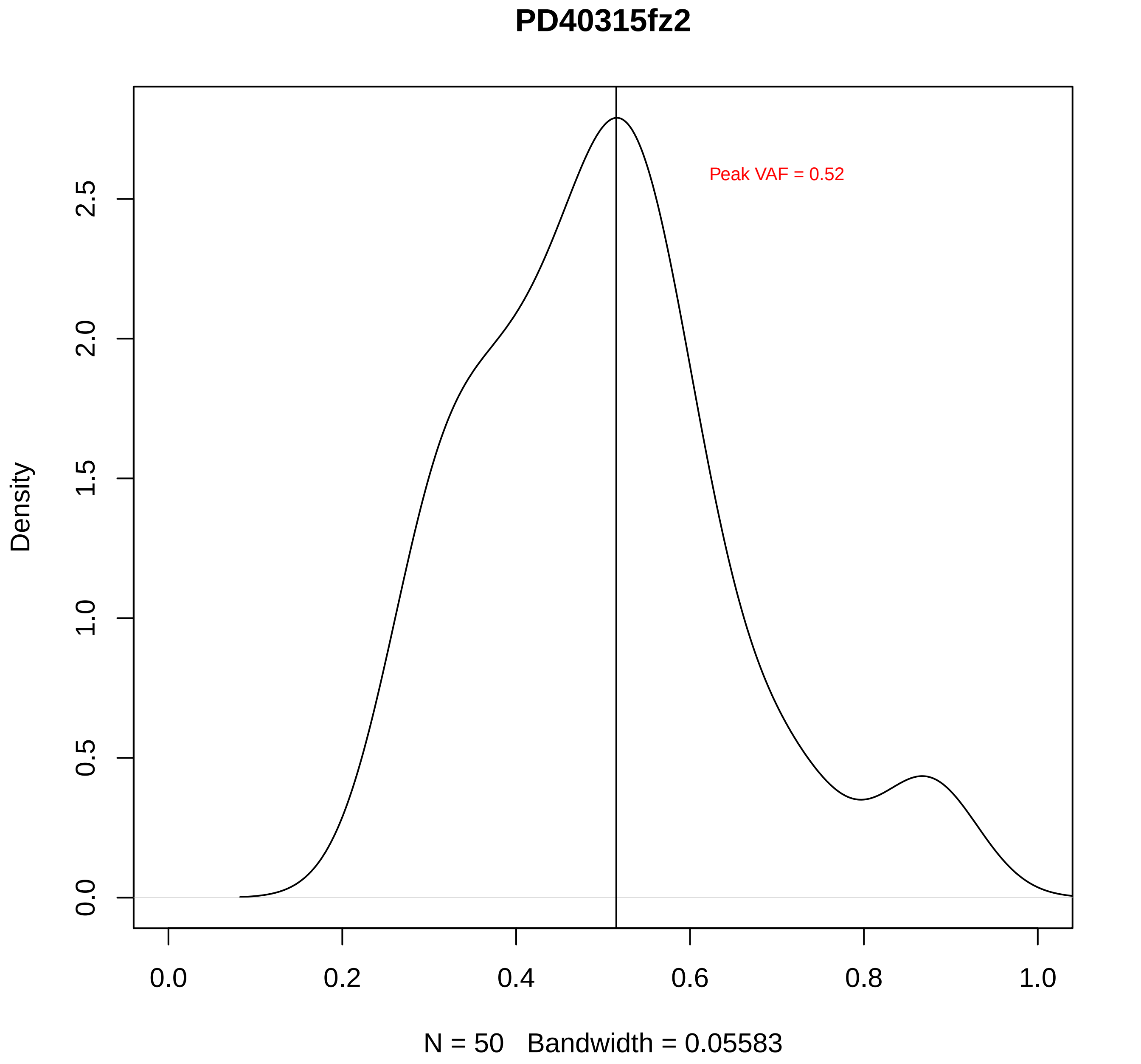
PD40315fz2
Peak VAF = 0.52
Density
0.0
0.5
1.0
1.5
2.0
2.5
0.0
0.2
0.4
0.6
0.8
1.0
N = 50 Bandwidth = 0.05583

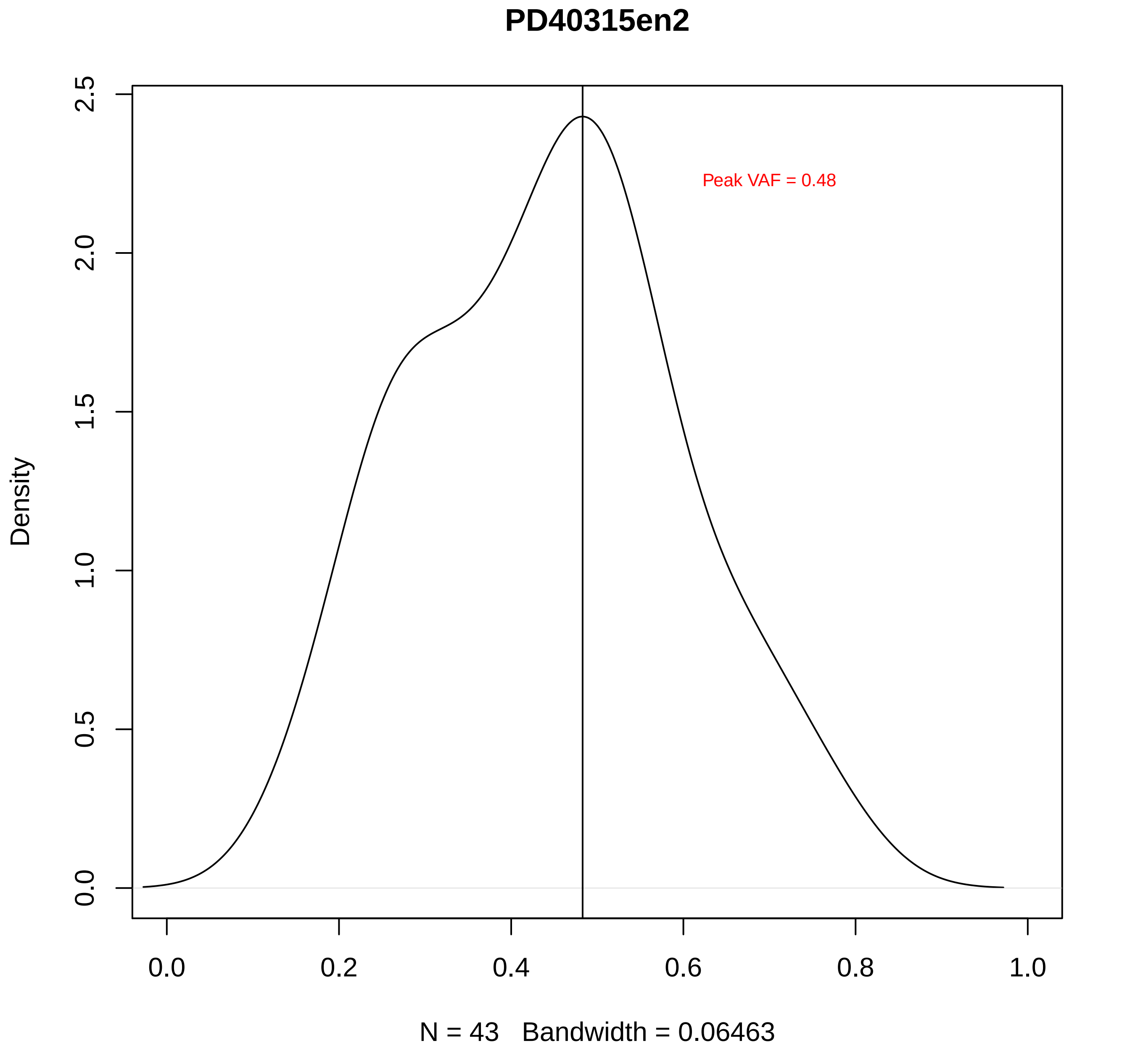
PD40315en2
Peak VAF = 0.48
Density
0.0
0.5
1.0
1.5
2.0
2.5
0.0
0.2
0.4
0.6
0.8
1.0
N = 43   Bandwidth = 0.06463

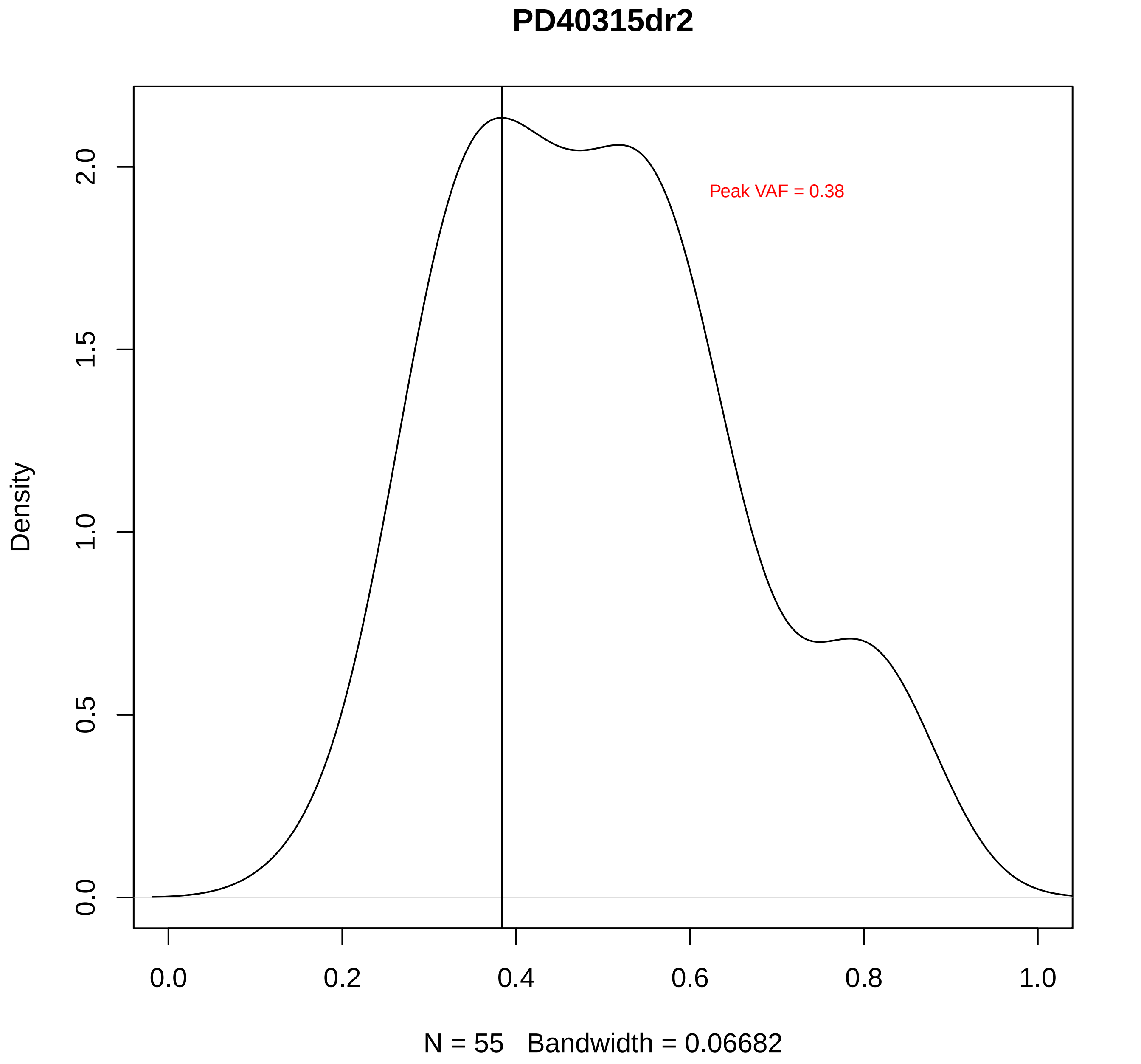
PD40315dr2
Peak VAF = 0.38
Density
0.0
0.5
1.0
1.5
2.0
0.0
0.2
0.4
0.6
0.8
1.0
N = 55   Bandwidth = 0.06682

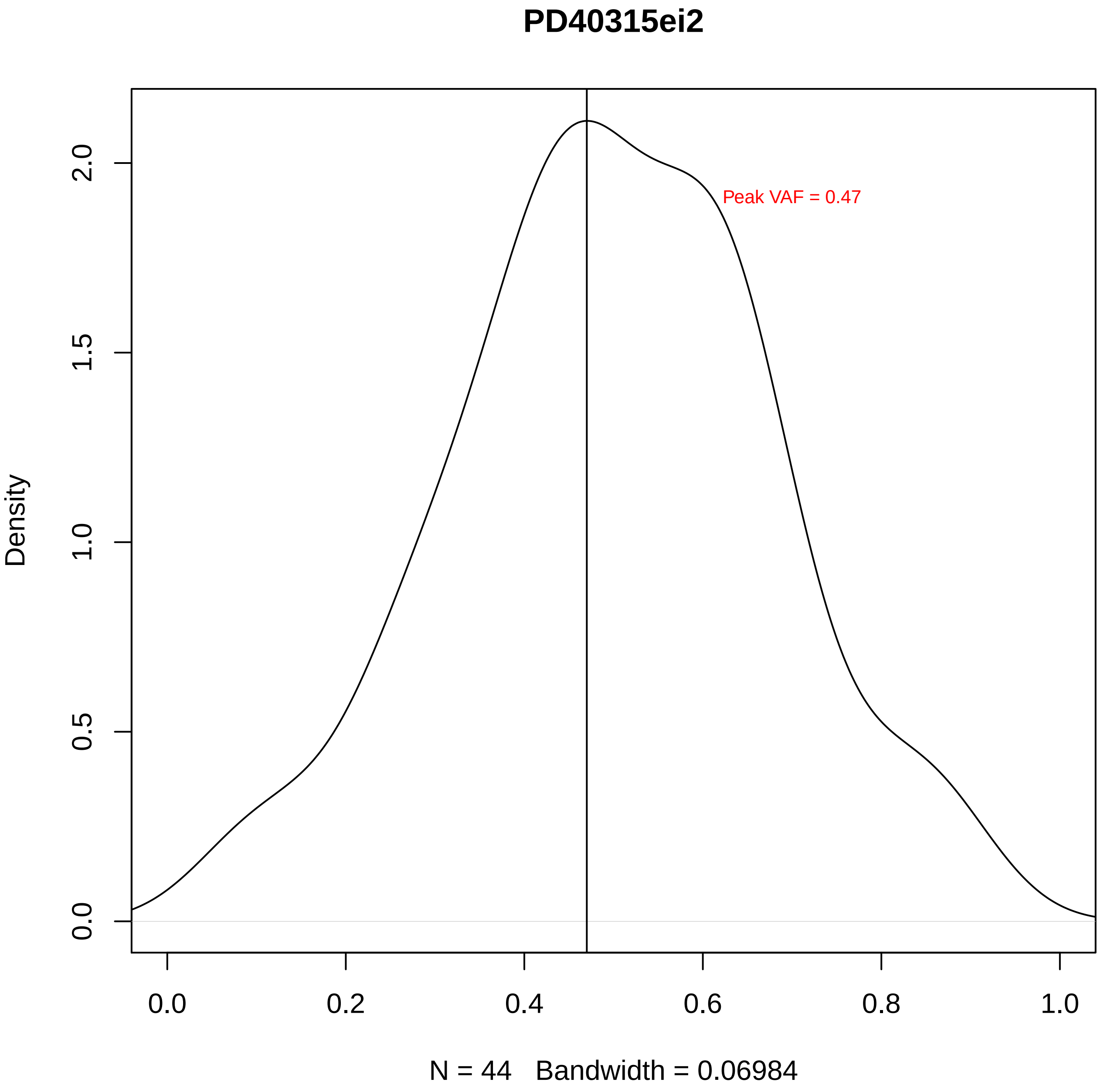
PD40315ei2
Peak VAF = 0.47
Density
0.0
0.5
1.0
1.5
2.0
0.0
0.2
0.4
0.6
0.8
1.0
N = 44   Bandwidth = 0.06984

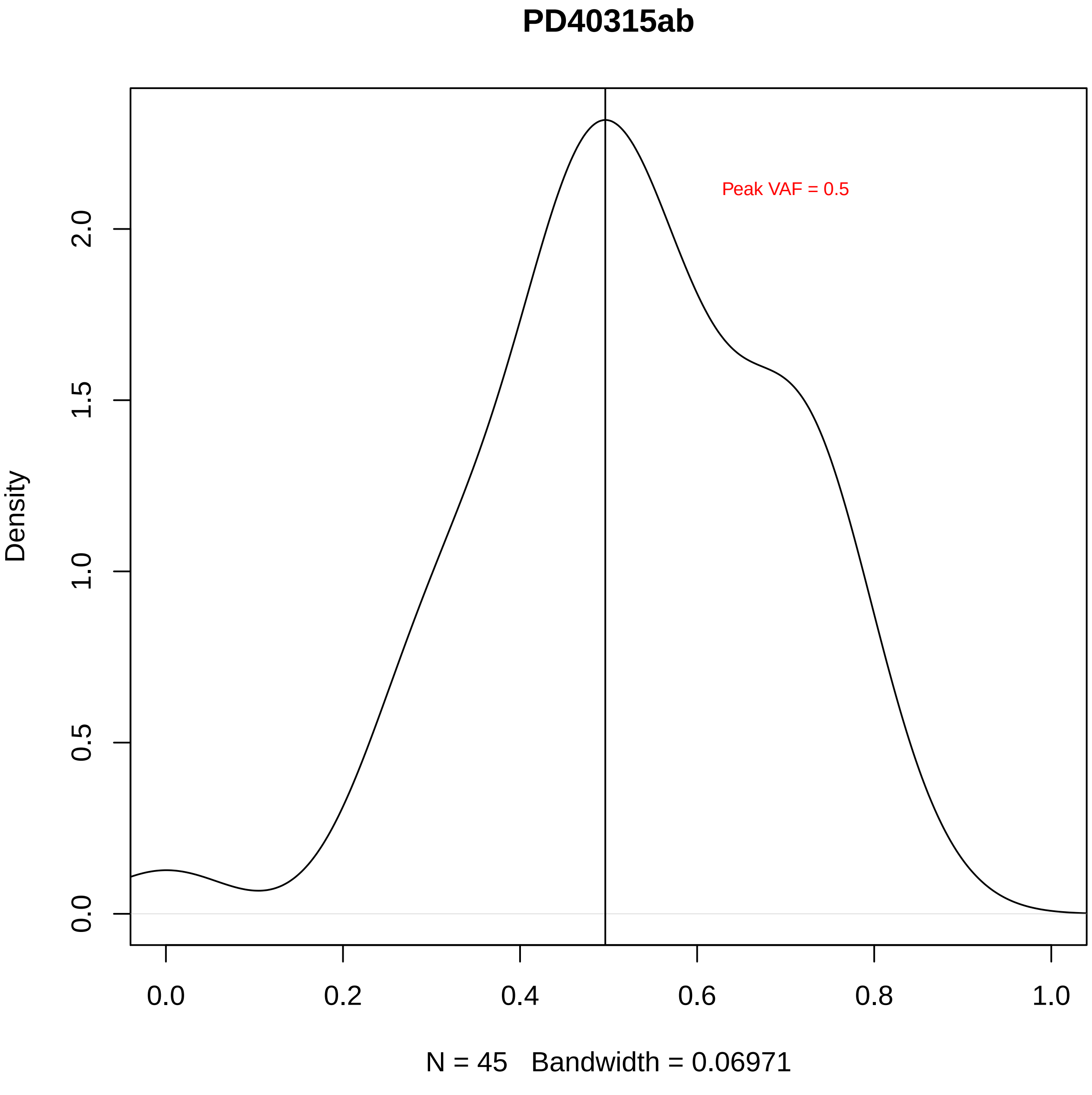

PD40315ab
Peak VAF = 0.5
Density
0.0
0.5
1.0
1.5
2.0
0.0
0.2
0.4
0.6
0.8
1.0
N = 45 Bandwidth = 0.06971

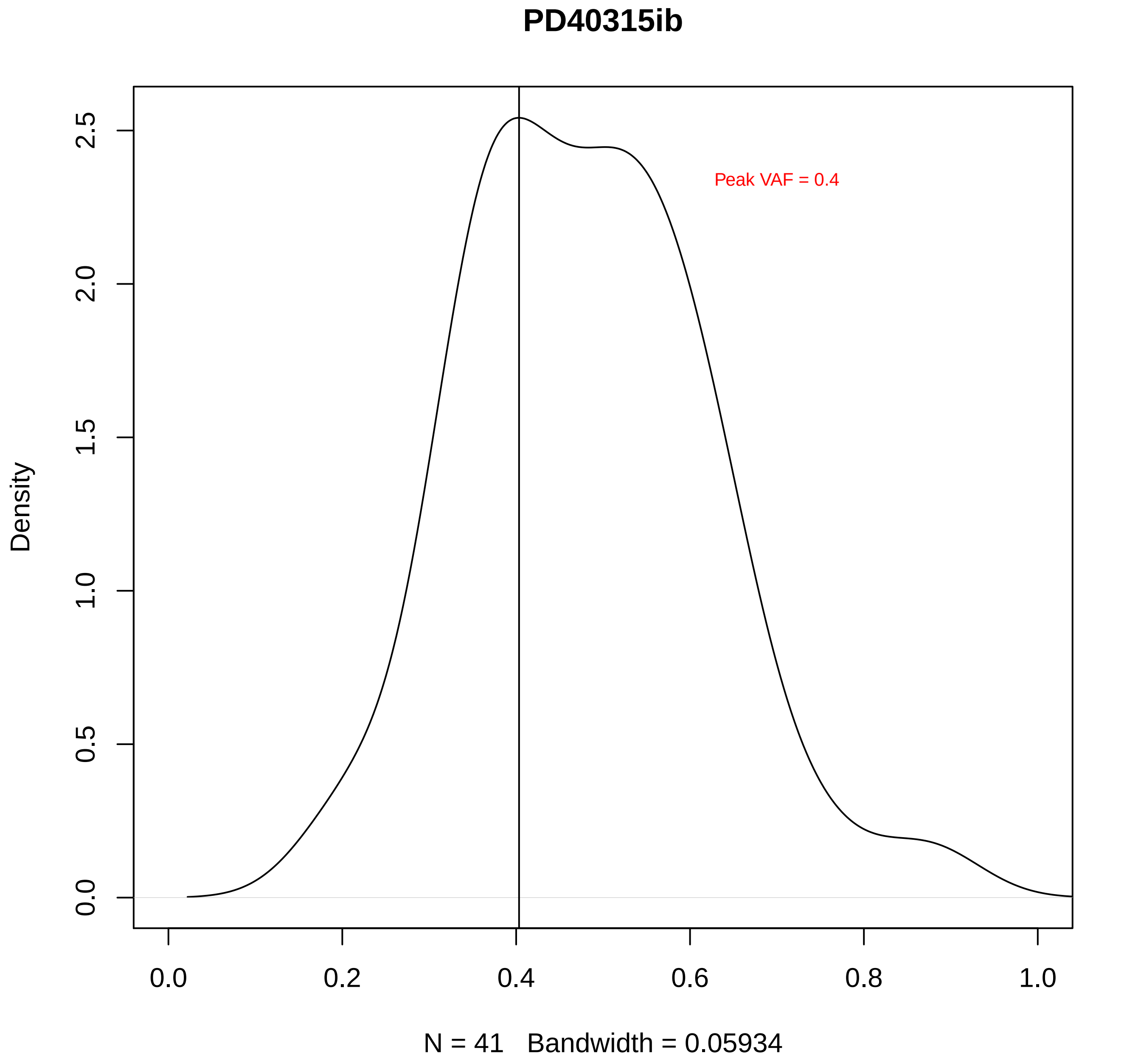

PD40315ib
Peak VAF = 0.4
Density
0.0
0.5
1.0
1.5
2.0
2.5
0.0
0.2
0.4
0.6
0.8
1.0
N = 41 Bandwidth = 0.05934

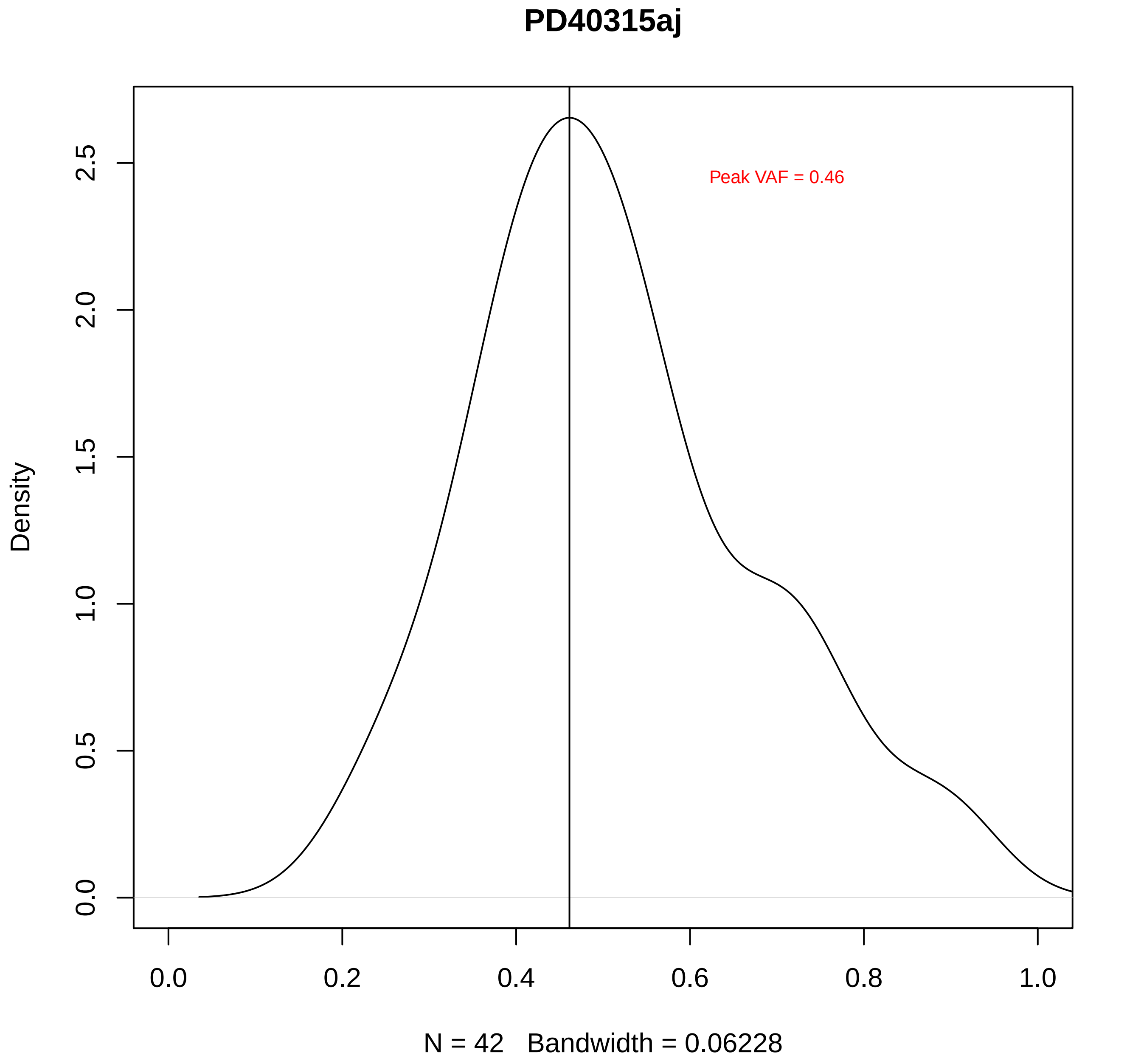

PD40315aj
Peak VAF = 0.46
Density
0.0
0.5
1.0
1.5
2.0
2.5
0.0
0.2
0.4
0.6
0.8
1.0
N = 42 Bandwidth = 0.06228

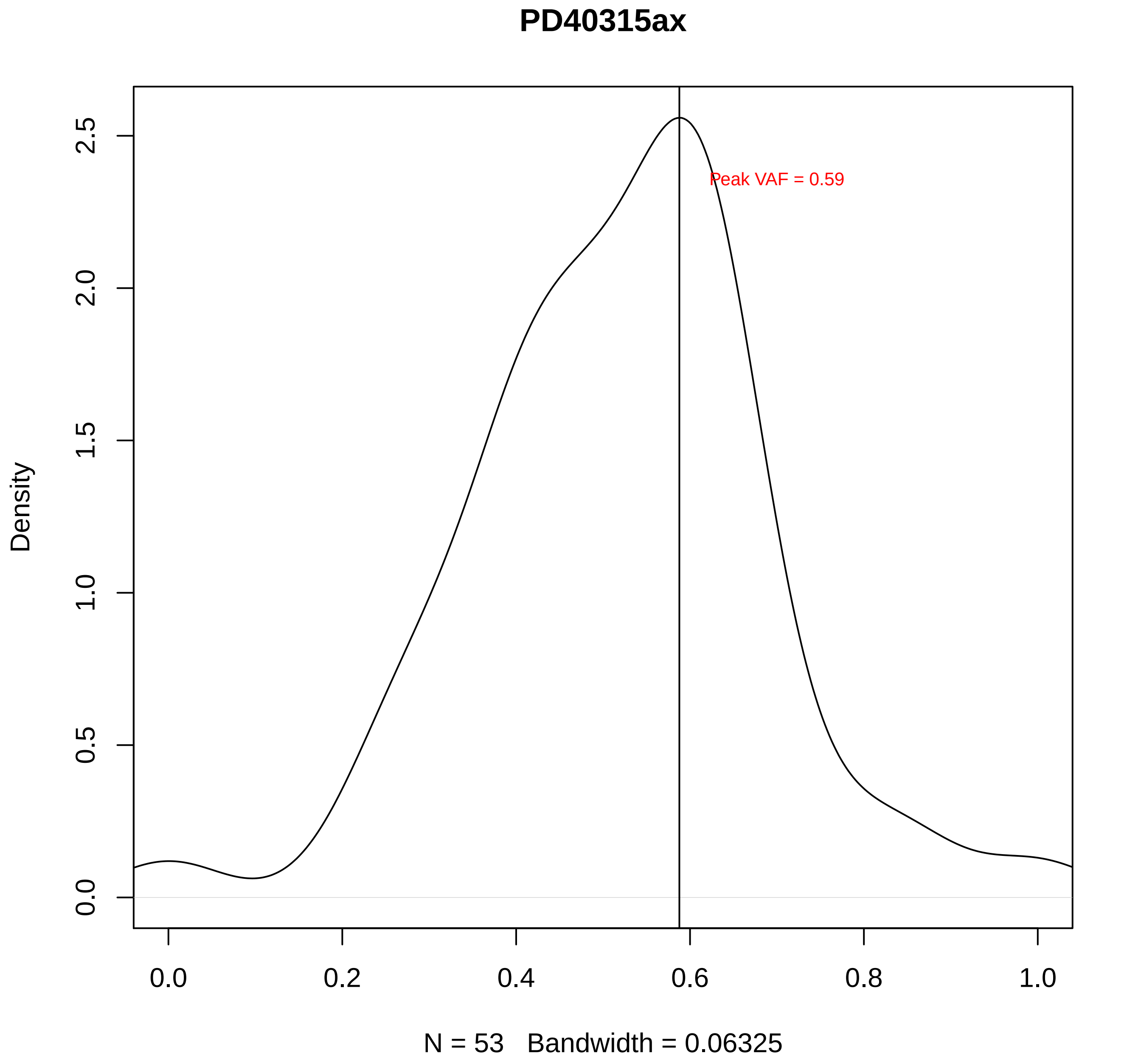

PD40315ax
Peak VAF = 0.59
Density
0.0
0.5
1.0
1.5
2.0
2.5
0.0
0.2
0.4
0.6
0.8
1.0
N = 53   Bandwidth = 0.06325

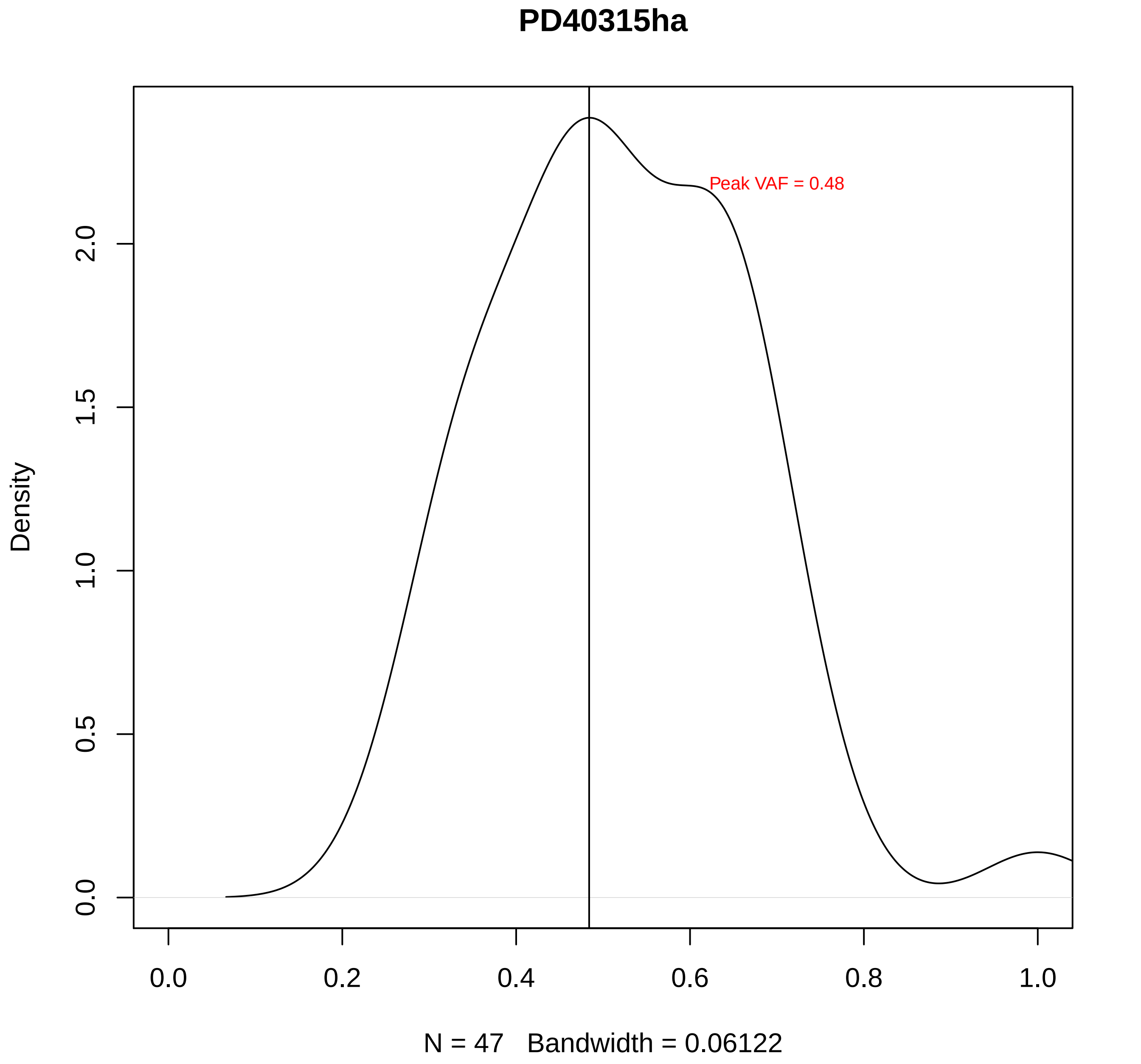
PD40315ha
Peak VAF = 0.48
Density
0.0
0.5
1.0
1.5
2.0
0.0
0.2
0.4
0.6
0.8
1.0
N = 47   Bandwidth = 0.06122

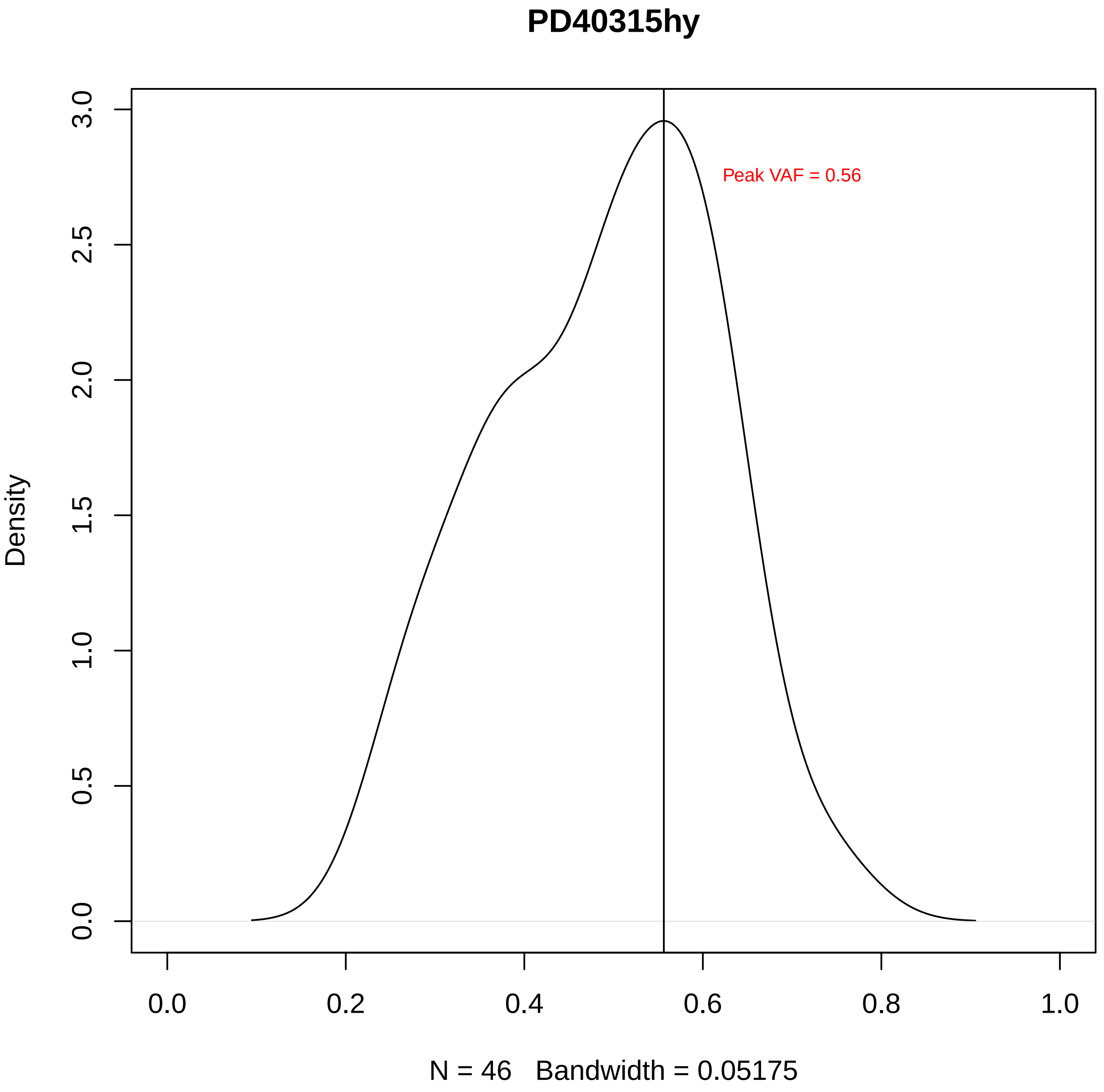
PD40315hy
Peak VAF = 0.56
Density
0.0
0.5
1.0
1.5
2.0
2.5
3.0
0.0
0.2
0.4
0.6
0.8
1.0
N = 46   Bandwidth = 0.05175

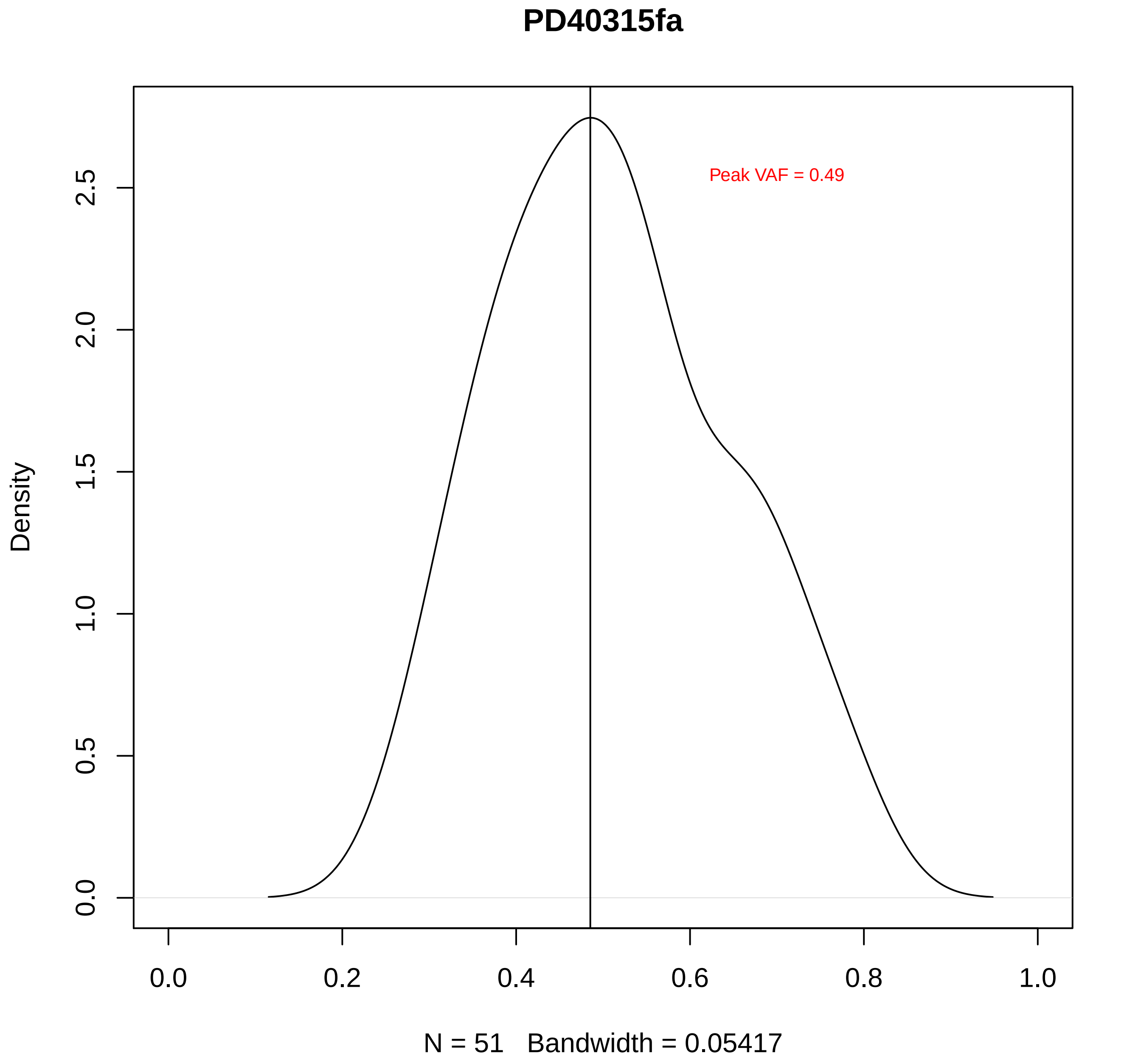
PD40315fa
Peak VAF = 0.49
Density
0.0
0.5
1.0
1.5
2.0
2.5
0.0
0.2
0.4
0.6
0.8
1.0
N = 51   Bandwidth = 0.05417

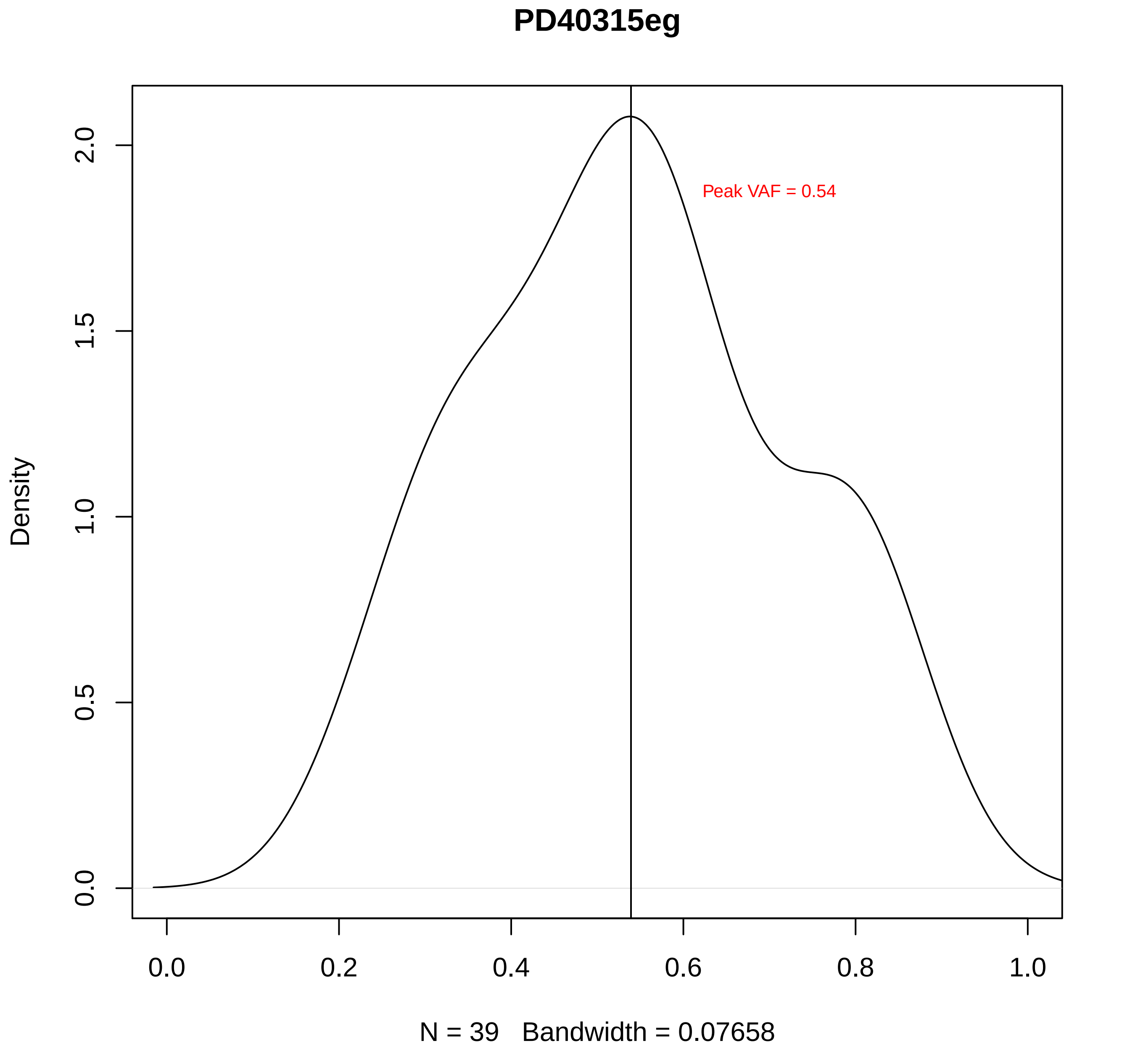
PD40315eg
Peak VAF = 0.54
Density
0.0
0.5
1.0
1.5
2.0
0.0
0.2
0.4
0.6
0.8
1.0
N = 39   Bandwidth = 0.07658

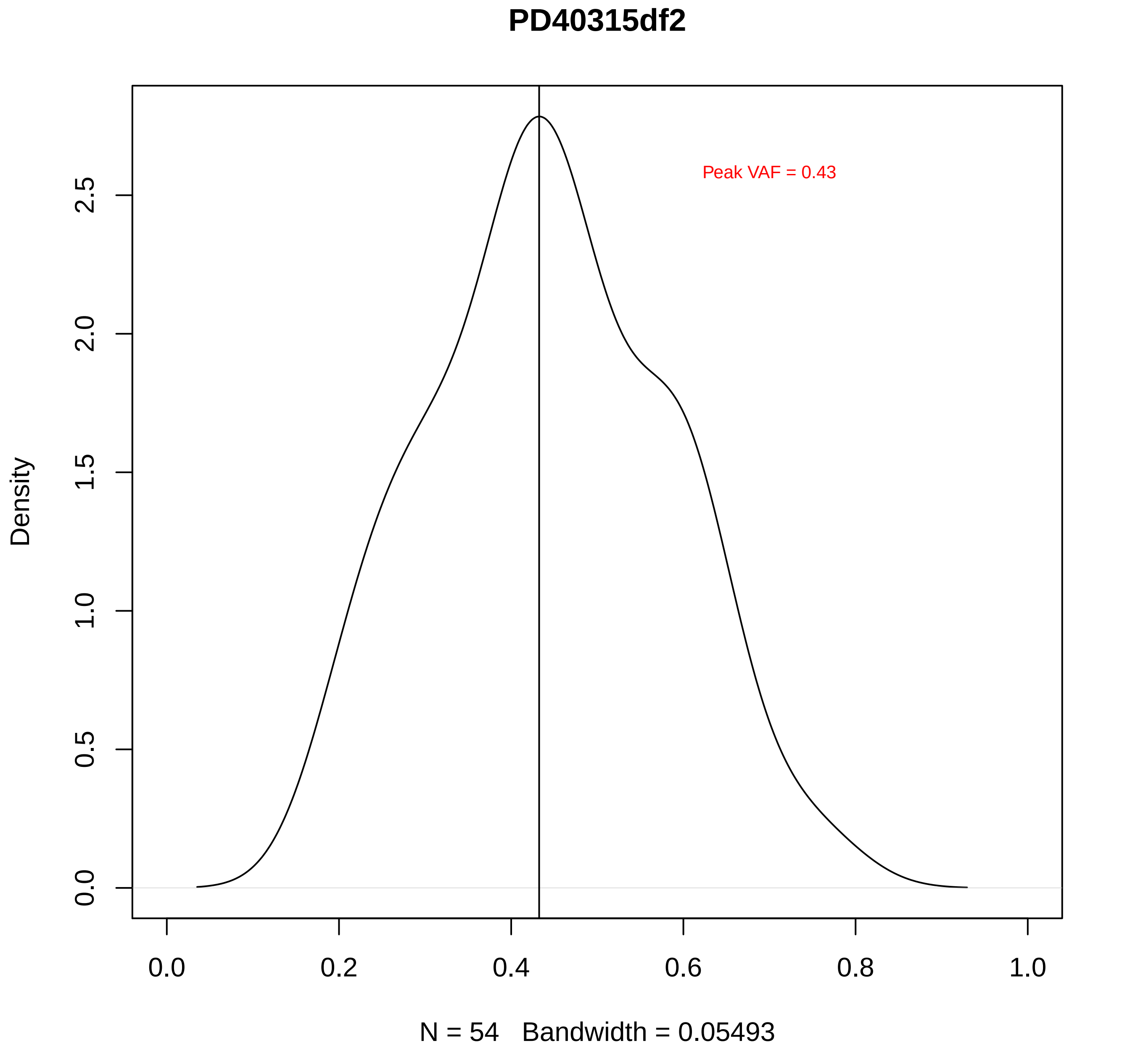
PD40315df2
Peak VAF = 0.43
Density
0.0
0.5
1.0
1.5
2.0
2.5
0.0
0.2
0.4
0.6
0.8
1.0
N = 54   Bandwidth = 0.05493

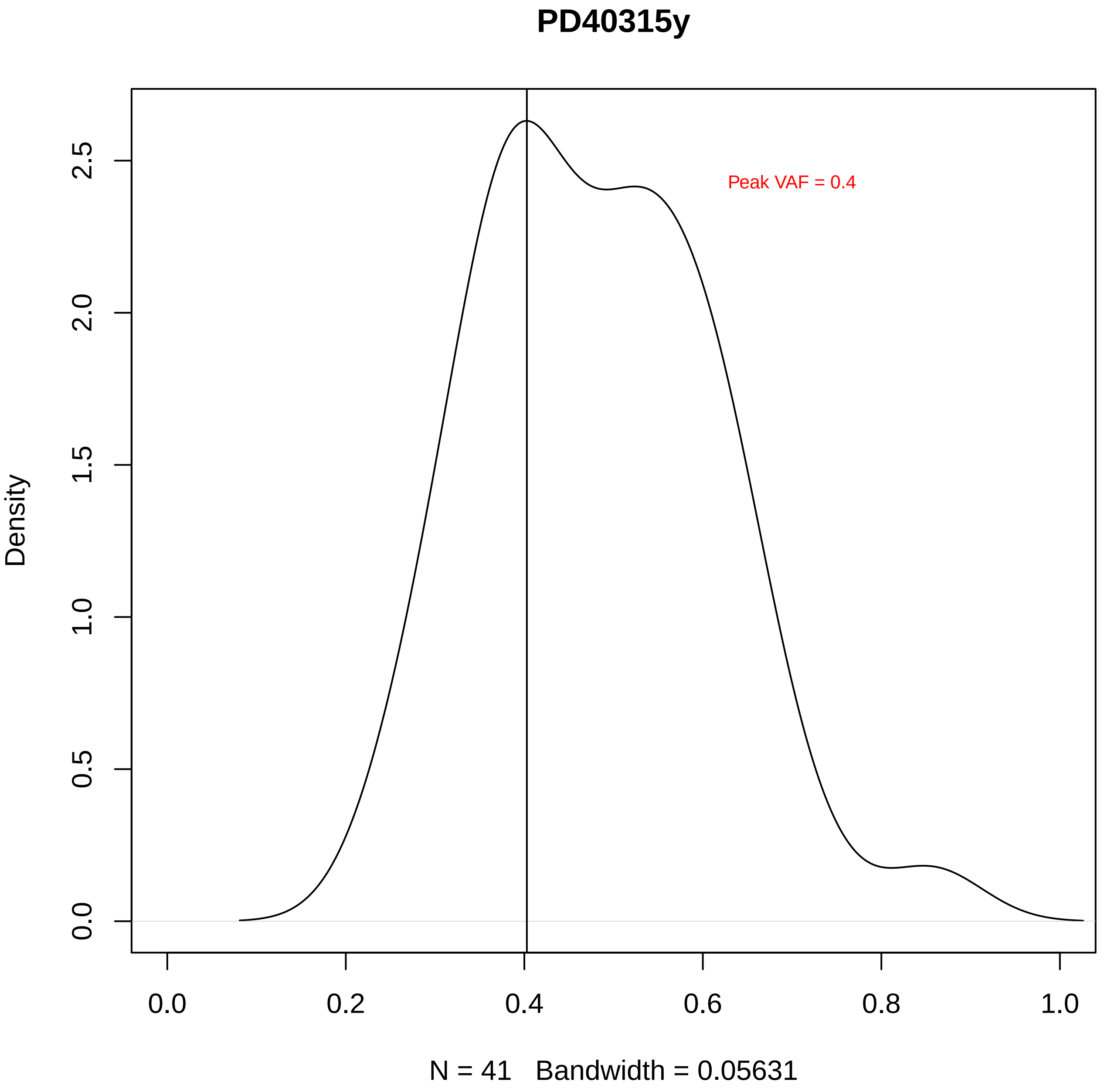
PD40315y
Peak VAF = 0.4
Density
0.0
0.5
1.0
1.5
2.0
2.5
0.0
0.2
0.4
0.6
0.8
1.0
N = 41 Bandwidth = 0.05631

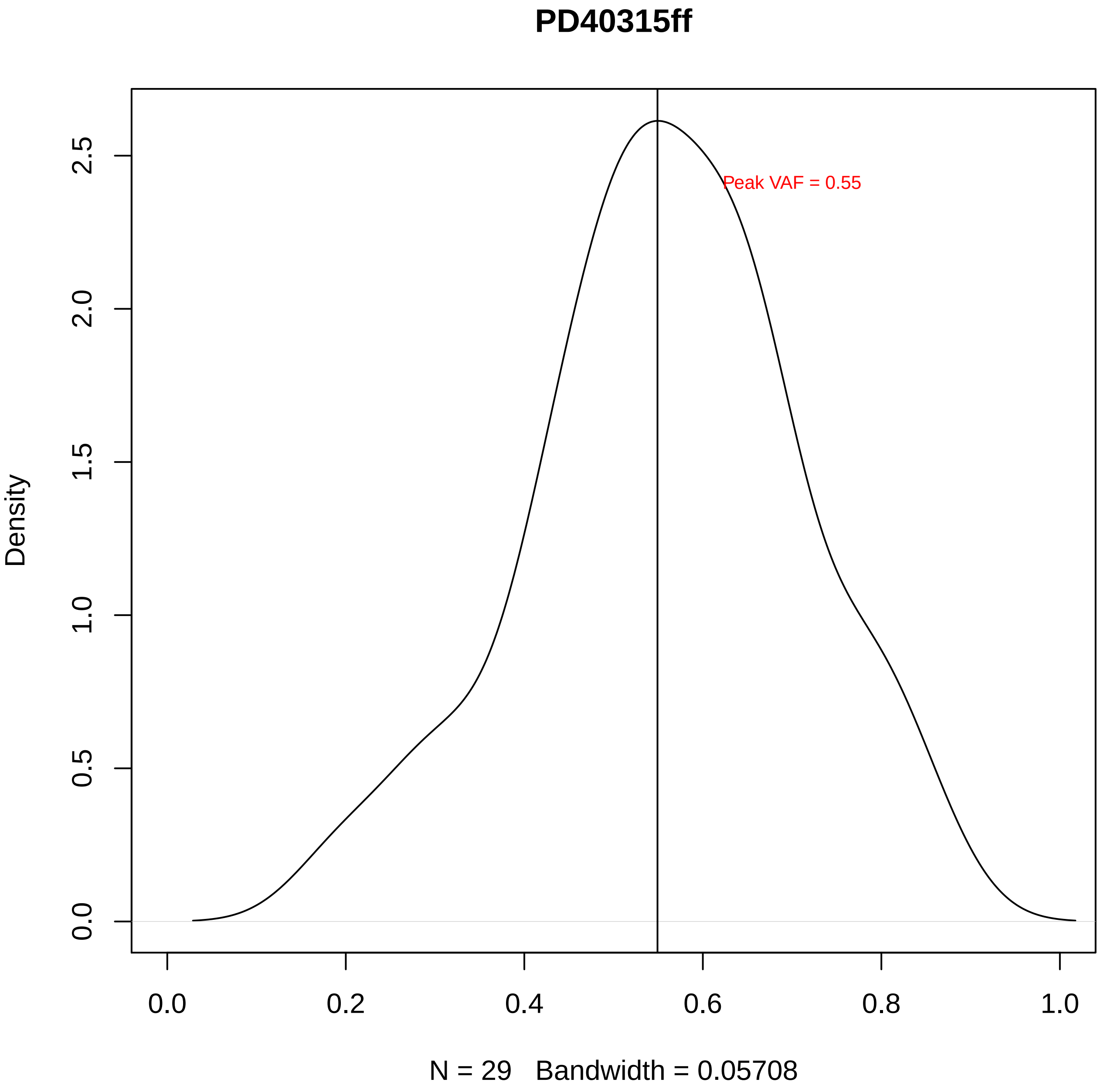
PD40315ff
Peak VAF = 0.55
Density
0.0
0.5
1.0
1.5
2.0
2.5
0.0
0.2
0.4
0.6
0.8
1.0
N = 29   Bandwidth = 0.05708

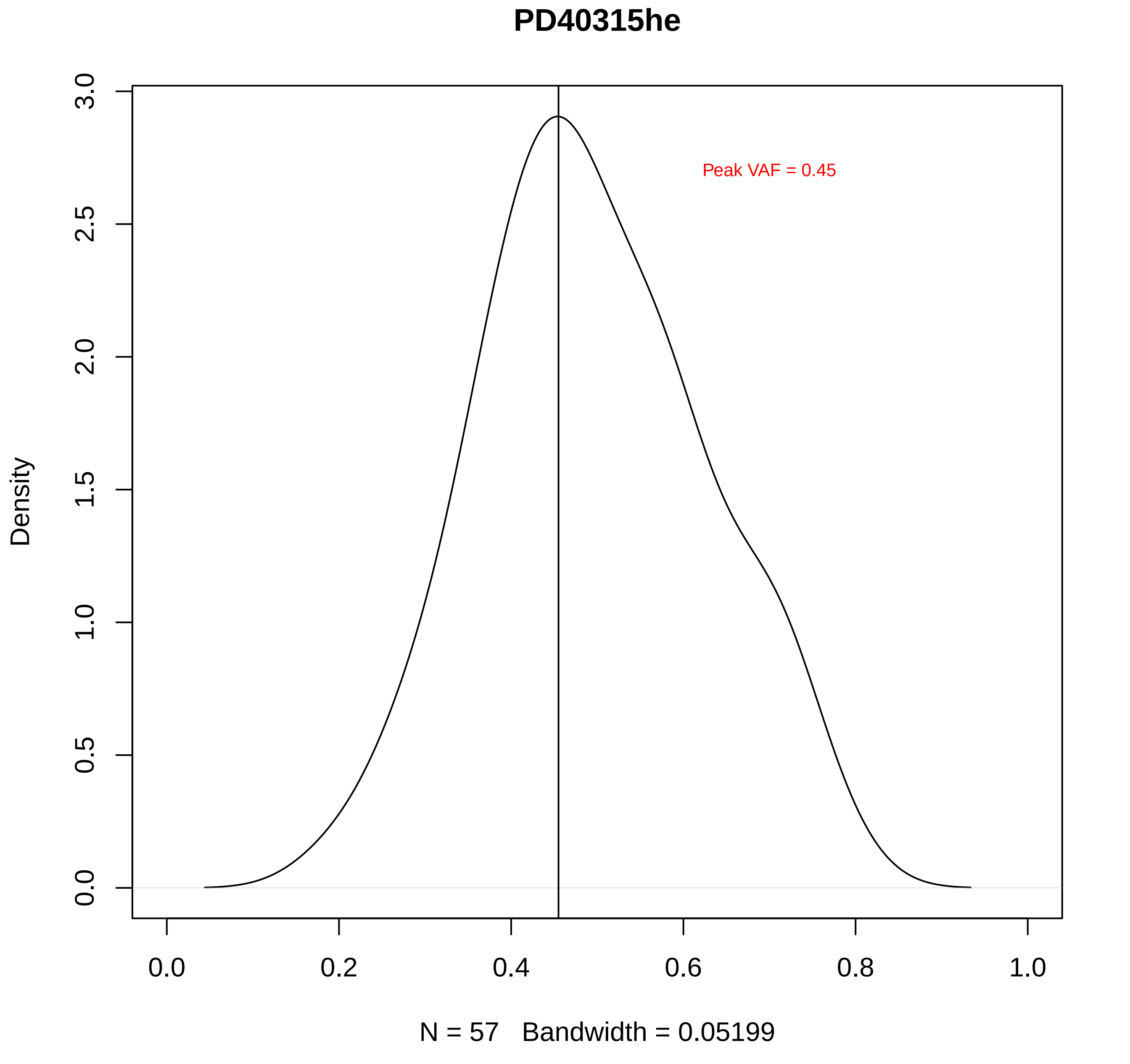
PD40315he
Peak VAF = 0.45
Density
0.0
0.5
1.0
1.5
2.0
2.5
3.0
0.0
0.2
0.4
0.6
0.8
1.0
N = 57 Bandwidth = 0.05199

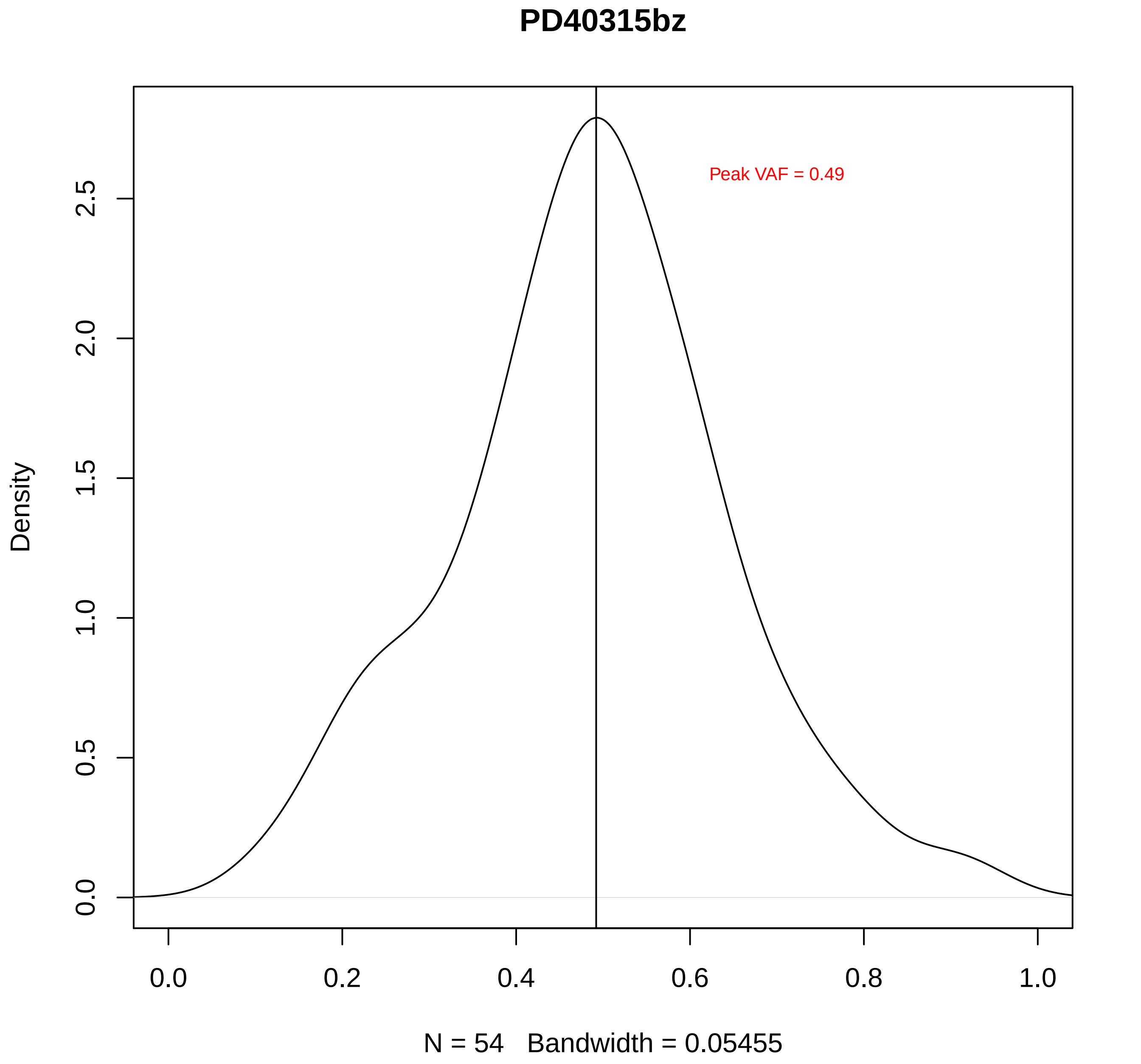

PD40315bz
Peak VAF = 0.49
Density
0.0
0.5
1.0
1.5
2.0
2.5
0.0
0.2
0.4
0.6
0.8
1.0
N = 54 Bandwidth = 0.05455

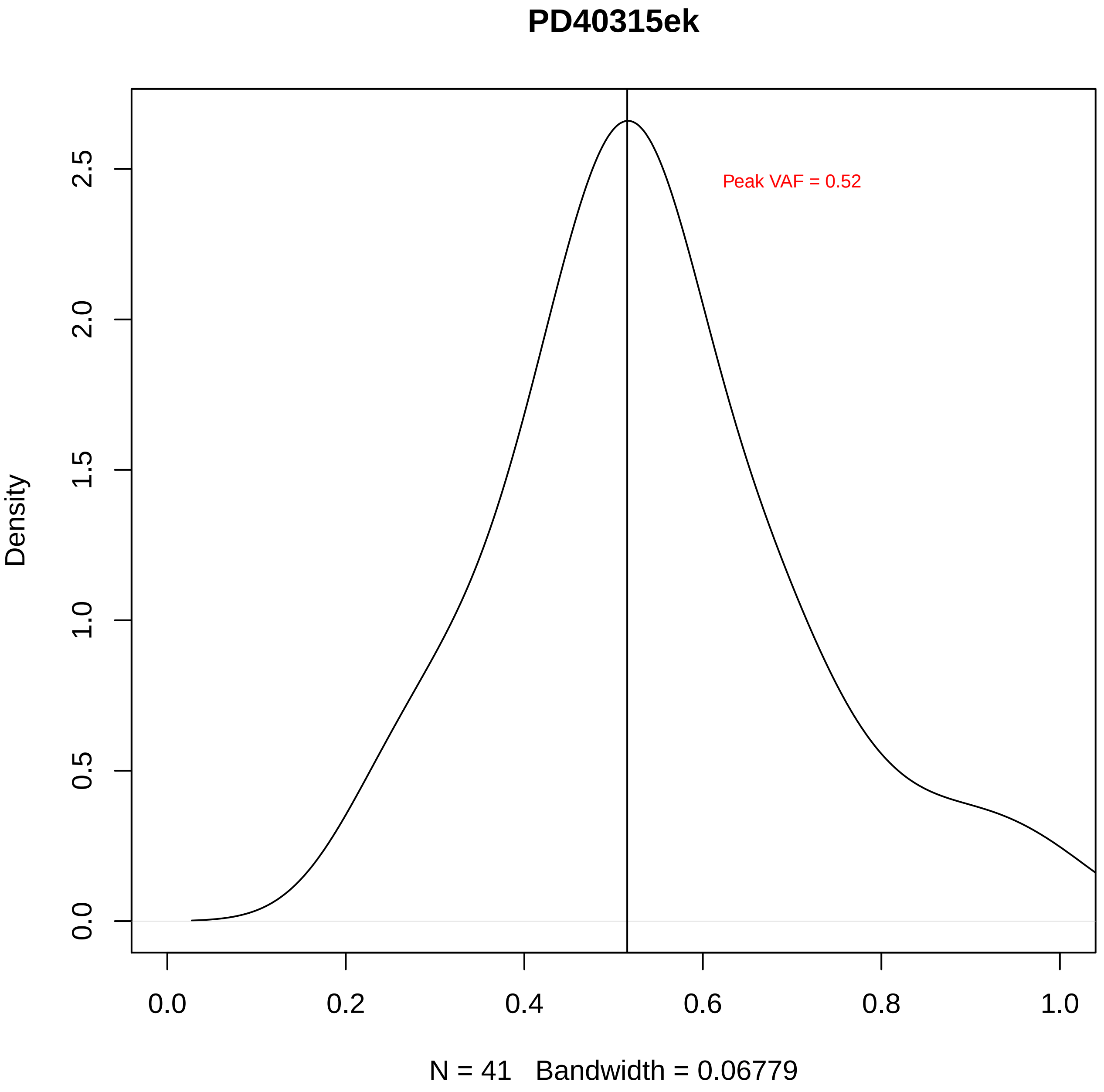

PD40315ek
Peak VAF = 0.52
Density
0.0
0.5
1.0
1.5
2.0
2.5
0.0
0.2
0.4
0.6
0.8
1.0
N = 41   Bandwidth = 0.06779

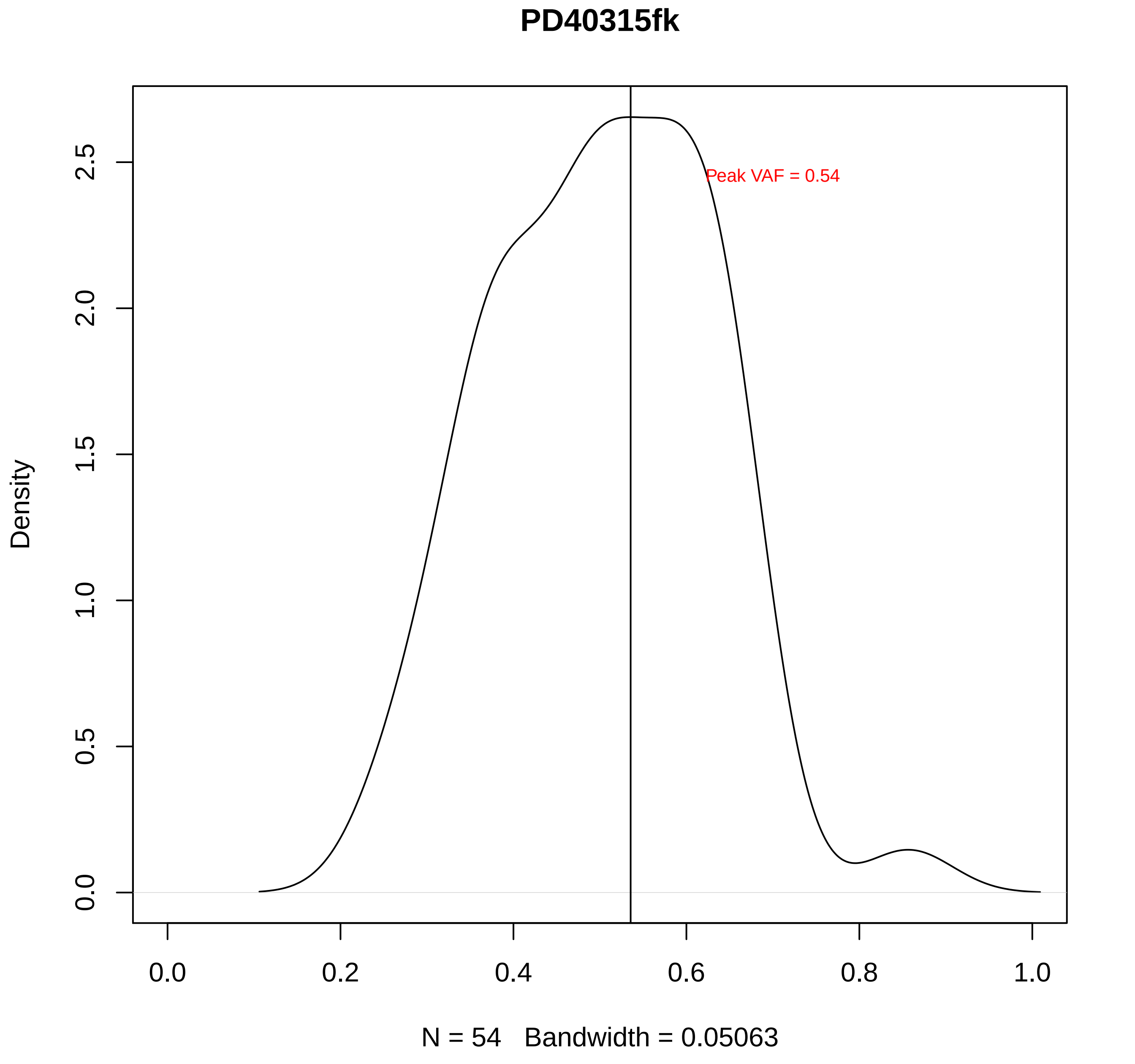

PD40315fk
Peak VAF = 0.54
Density
0.0
0.5
1.0
1.5
2.0
2.5
0.0
0.2
0.4
0.6
0.8
1.0
N = 54 Bandwidth = 0.05063

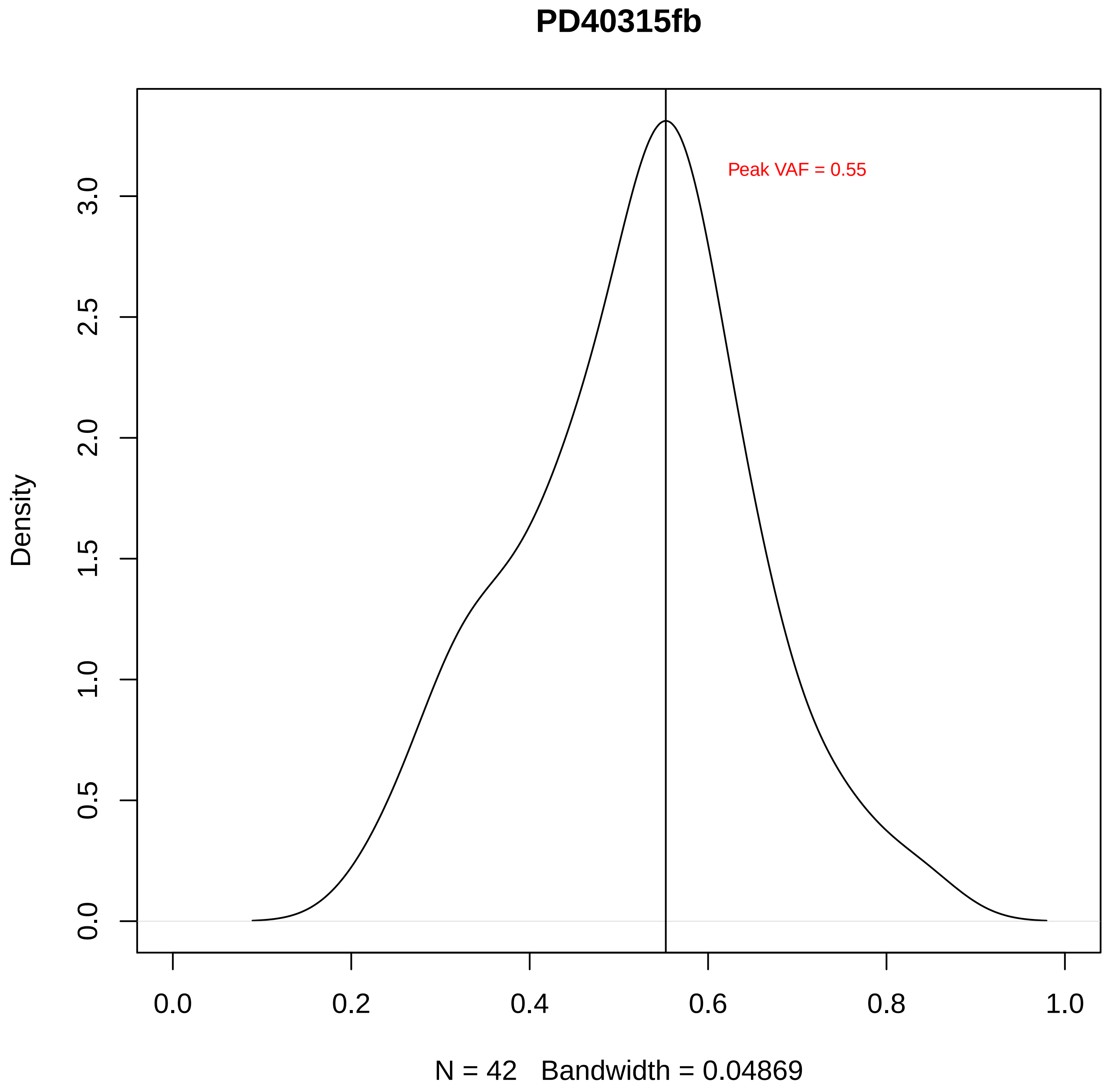
PD40315fb
Peak VAF = 0.55
Density
0.0
0.5
1.0
1.5
2.0
2.5
3.0
0.0
0.2
0.4
0.6
0.8
1.0
N = 42   Bandwidth = 0.04869

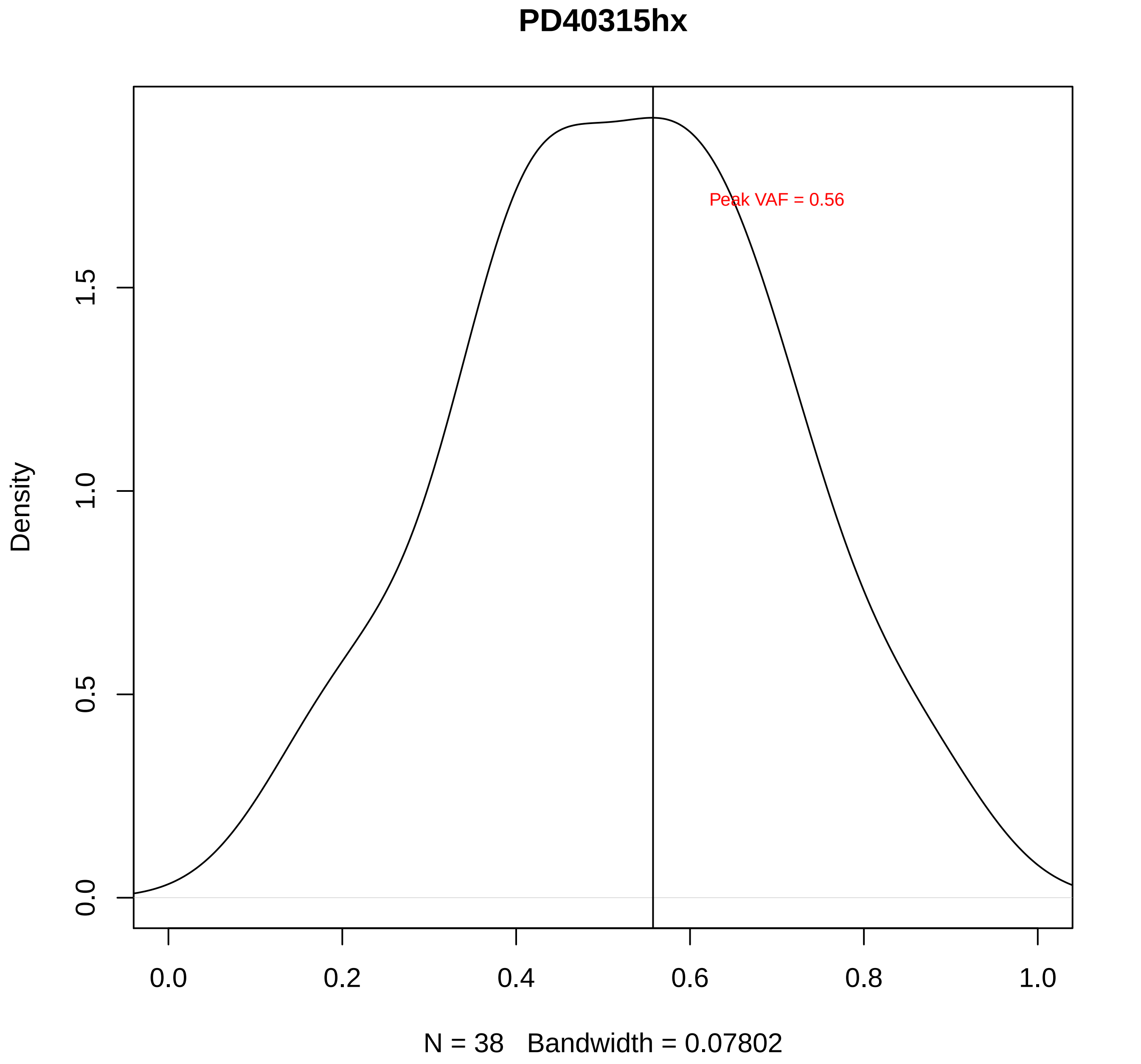
PD40315hx
Peak VAF = 0.56
Density
0.0
0.5
1.0
1.5
0.0
0.2
0.4
0.6
0.8
1.0
N = 38   Bandwidth = 0.07802

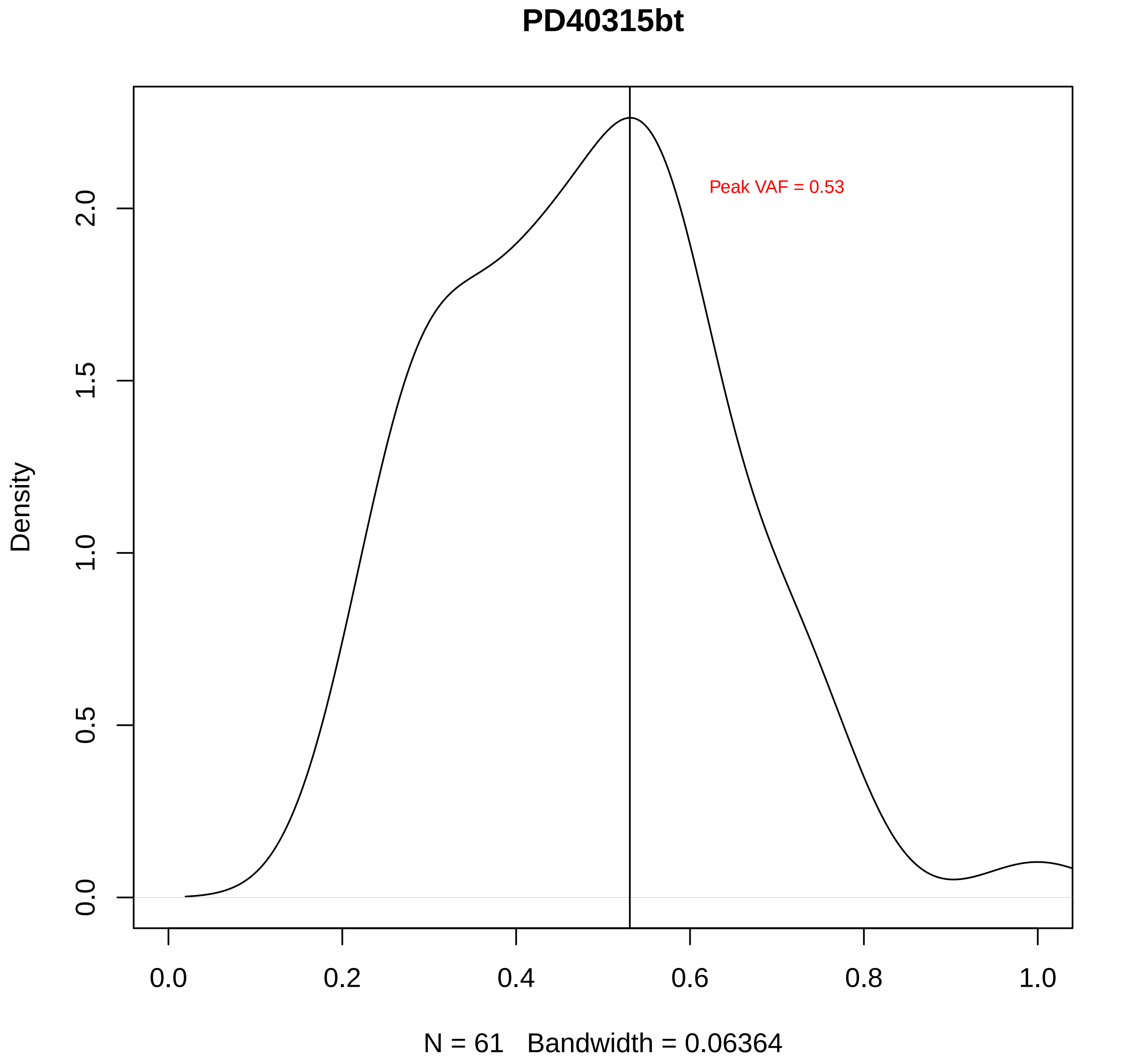
PD40315bt
Peak VAF = 0.53
Density
0.0
0.5
1.0
1.5
2.0
0.0
0.2
0.4
0.6
0.8
1.0
N = 61   Bandwidth = 0.06364

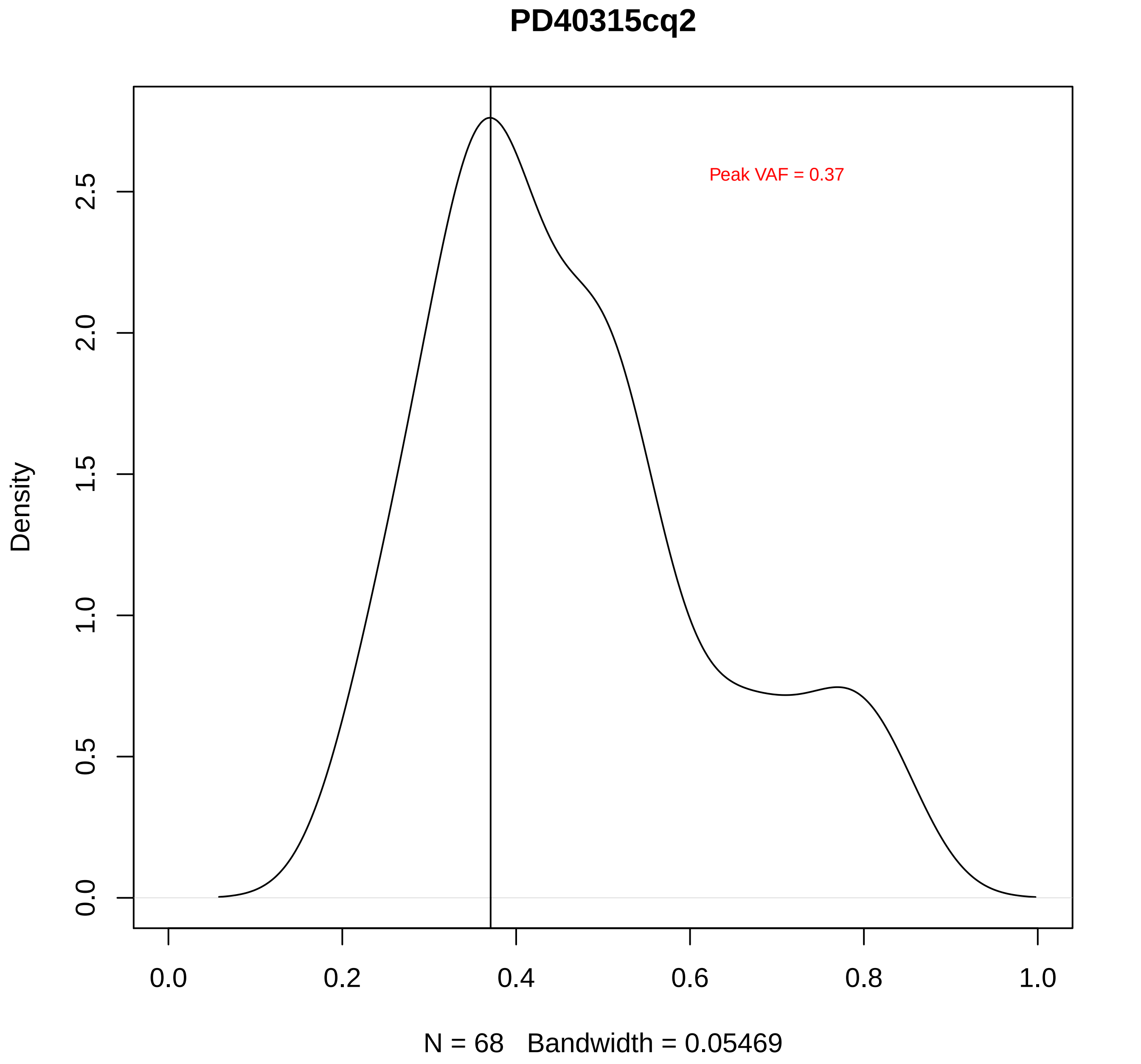

PD40315cq2
Peak VAF = 0.37
Density
0.0
0.5
1.0
1.5
2.0
2.5
0.0
0.2
0.4
0.6
0.8
1.0
N = 68 Bandwidth = 0.05469

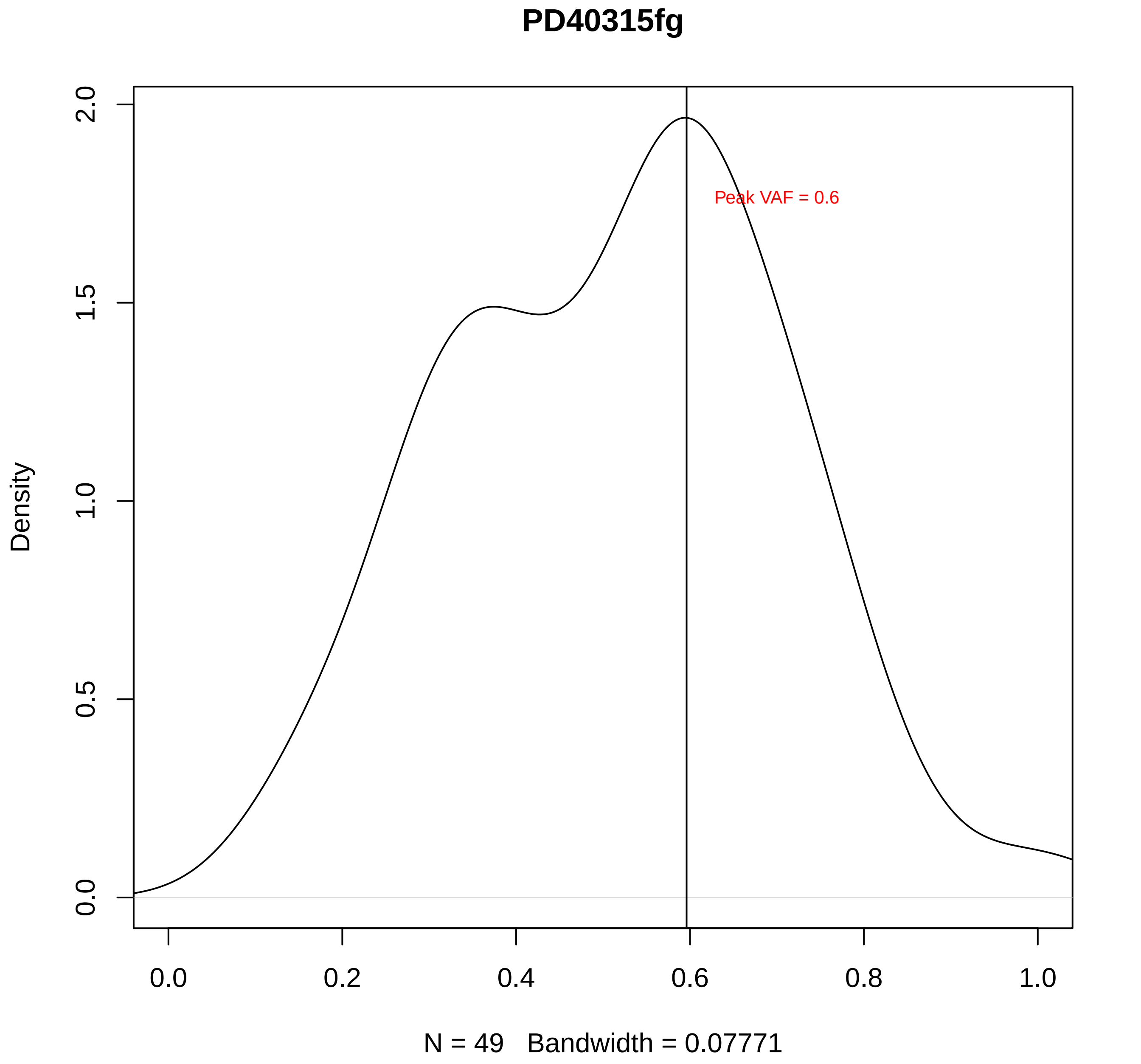
PD40315fg
Density
2.0
1.5
1.0
0.5
0.0
Peak VAF = 0.6
0.0
0.2
0.4
0.6
0.8
1.0
N = 49   Bandwidth = 0.07771

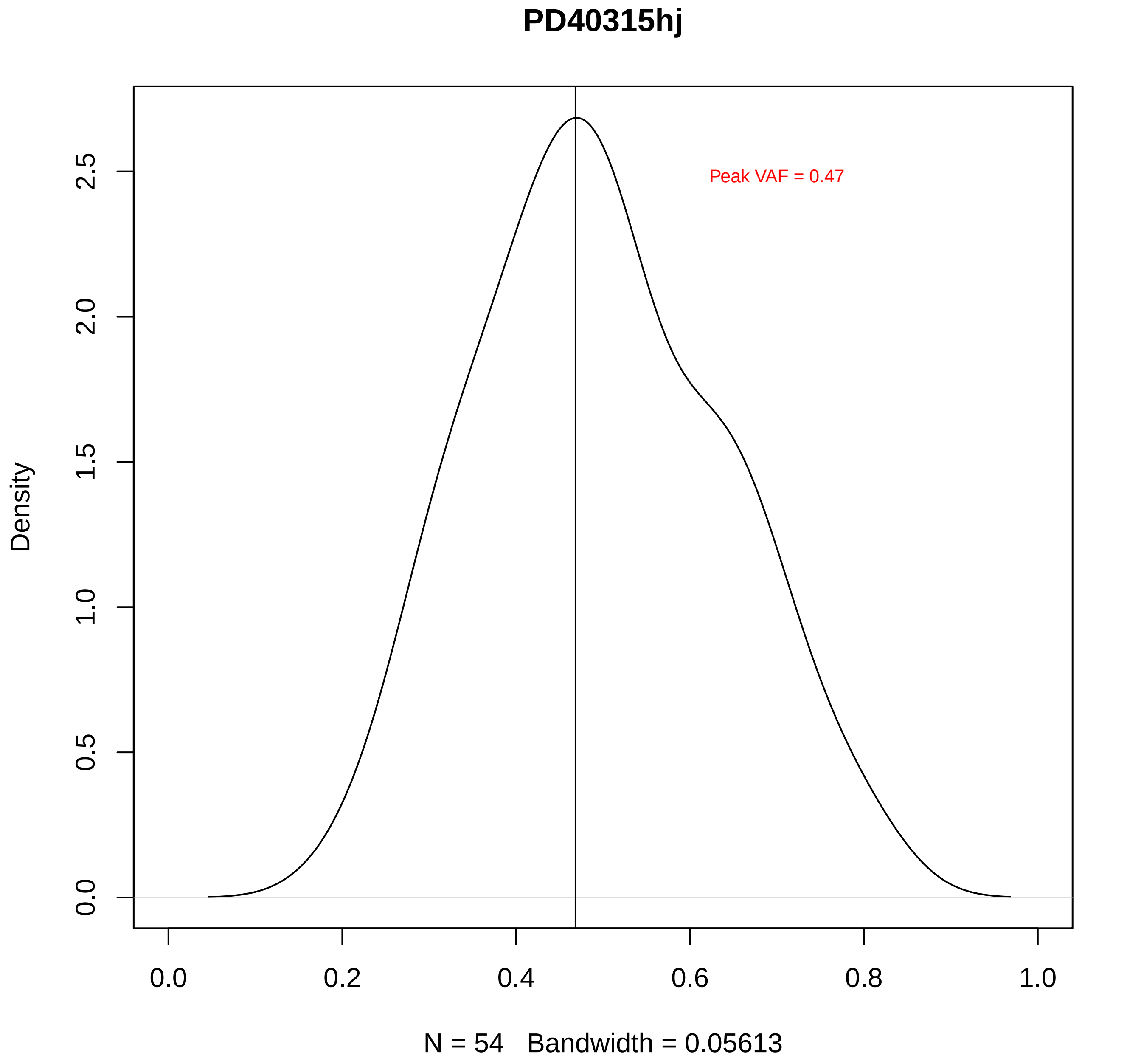

PD40315hj
Peak VAF = 0.47
Density
0.0
0.5
1.0
1.5
2.0
2.5
0.0
0.2
0.4
0.6
0.8
1.0
N = 54   Bandwidth = 0.05613

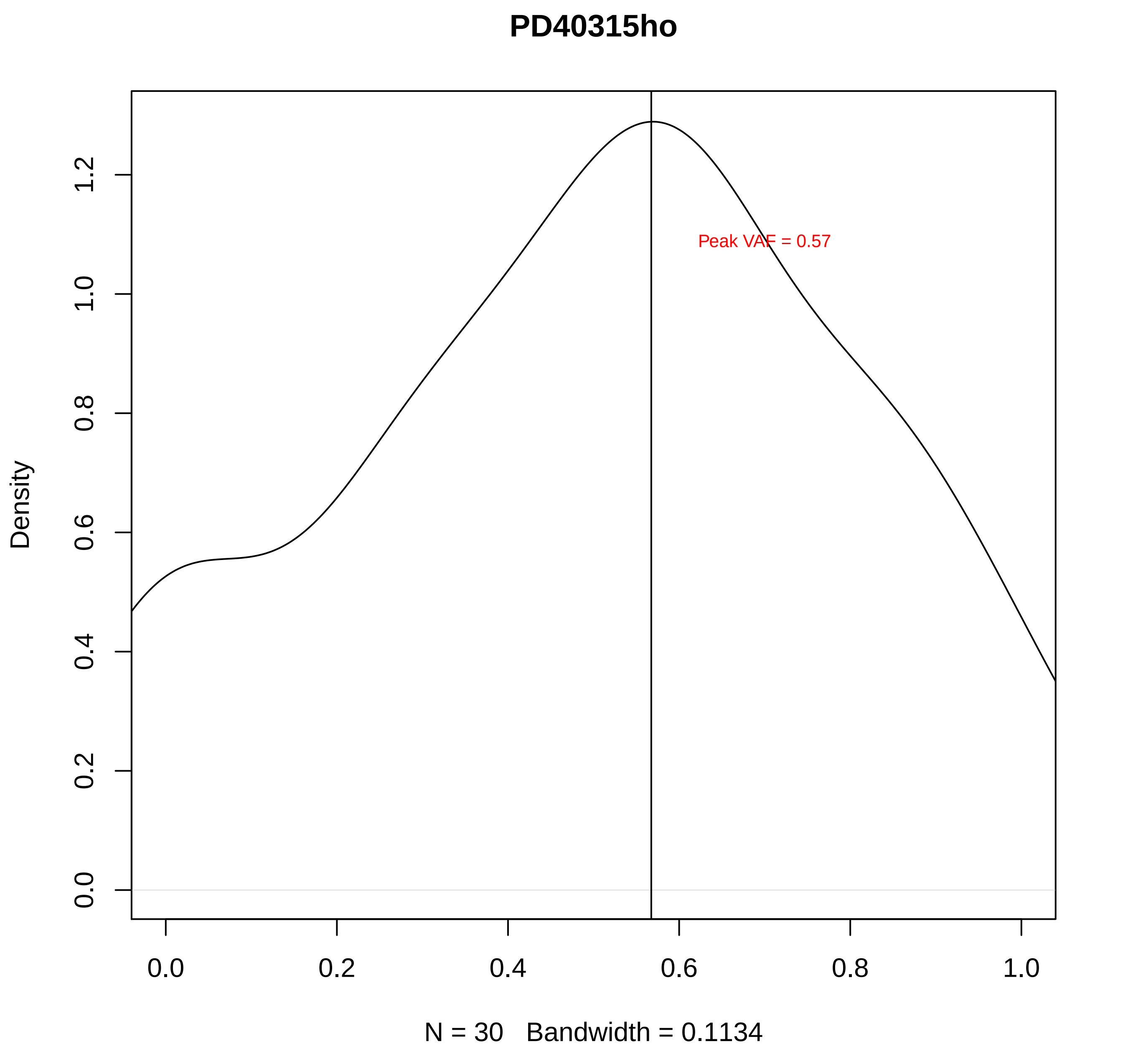
PD40315ho
Peak VAF = 0.57
Density
0.0
0.2
0.4
0.6
0.8
1.0
1.2
N = 30   Bandwidth = 0.1134

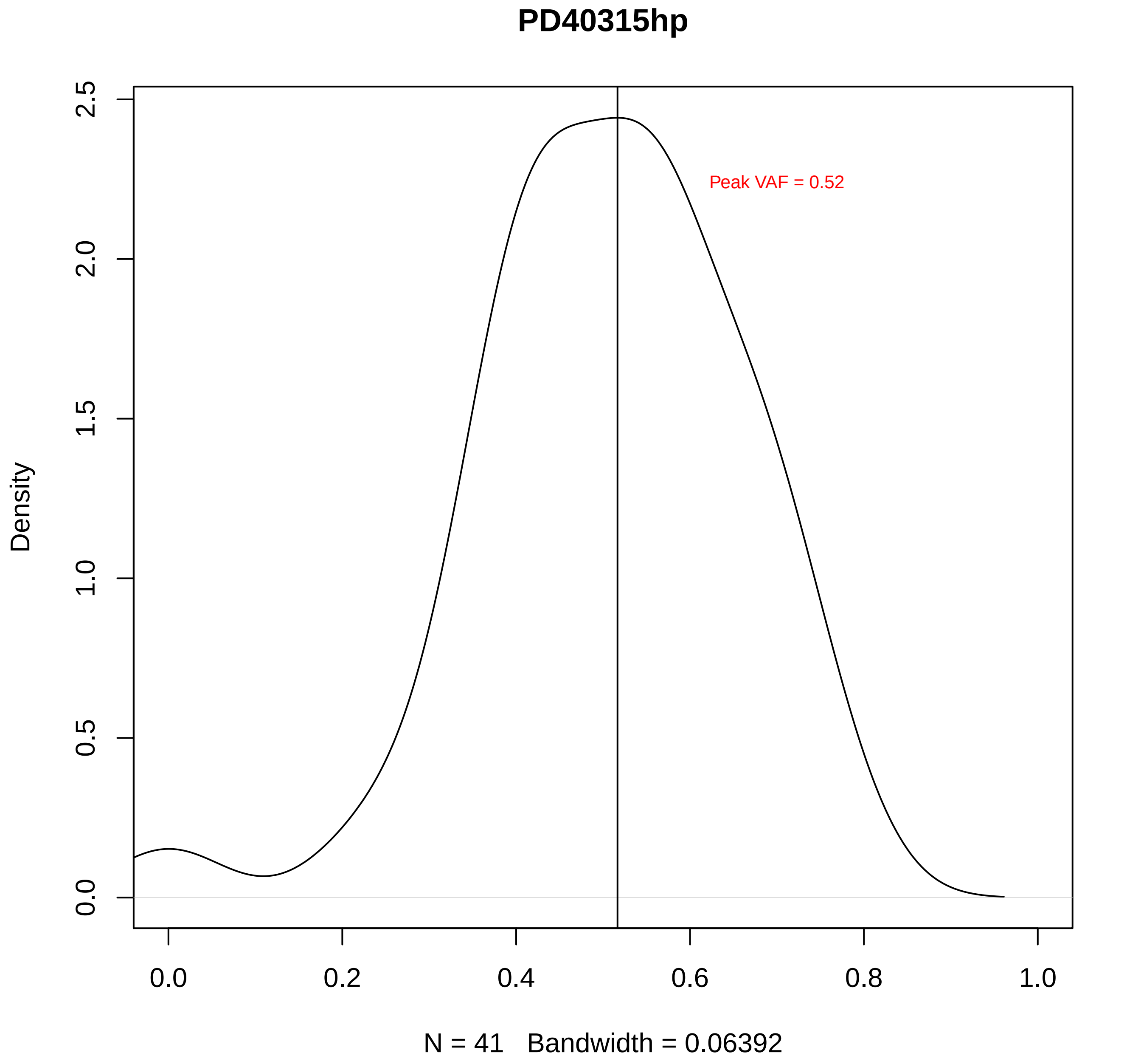
PD40315hp
Peak VAF = 0.52
Density
0.0
0.5
1.0
1.5
2.0
2.5
0.0
0.2
0.4
0.6
0.8
1.0
N = 41   Bandwidth = 0.06392

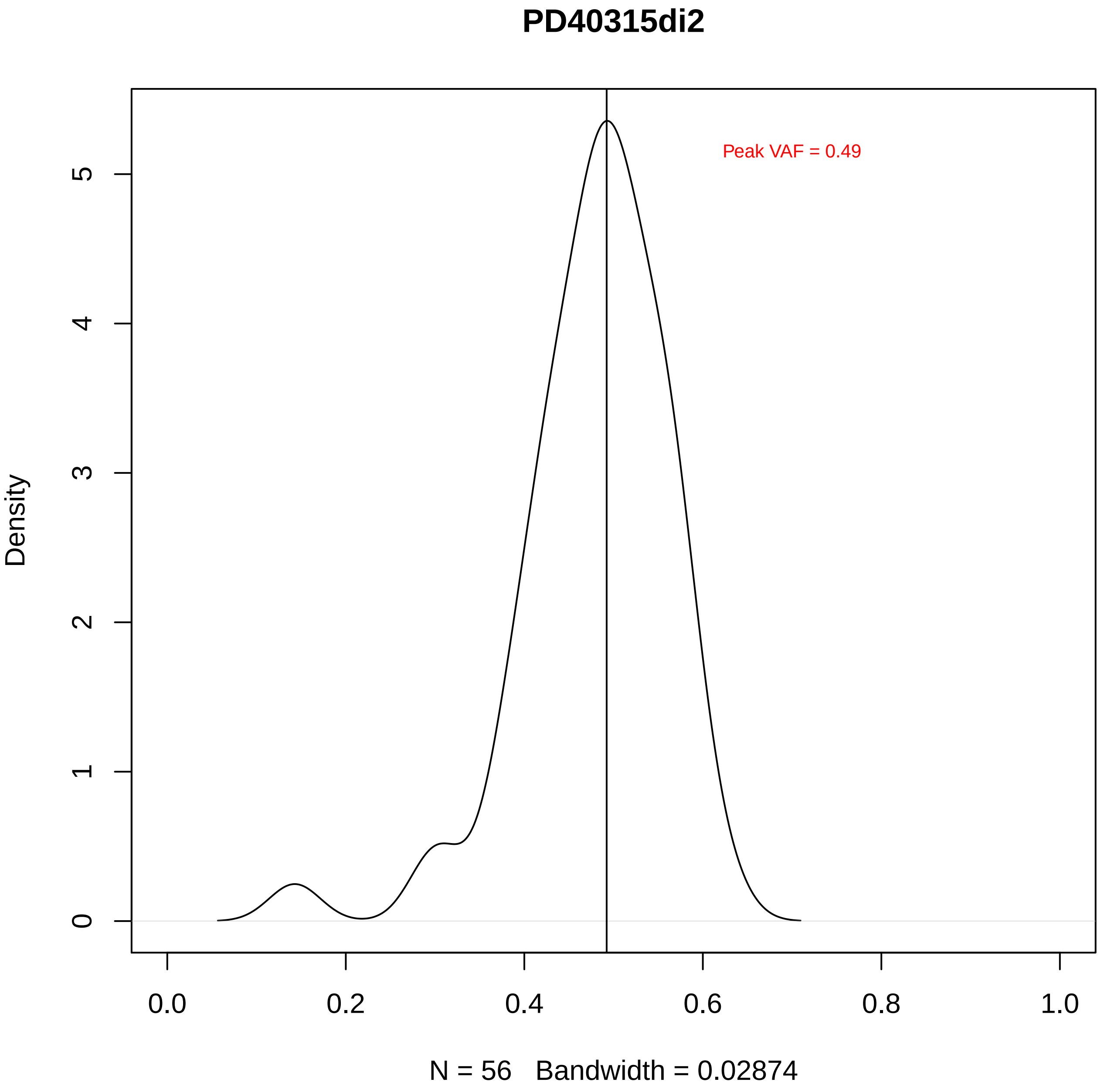
PD40315di2
Peak VAF = 0.49
Density
0
1
2
3
4
5
0.0
0.2
0.4
0.6
0.8
1.0
N = 56 Bandwidth = 0.02874

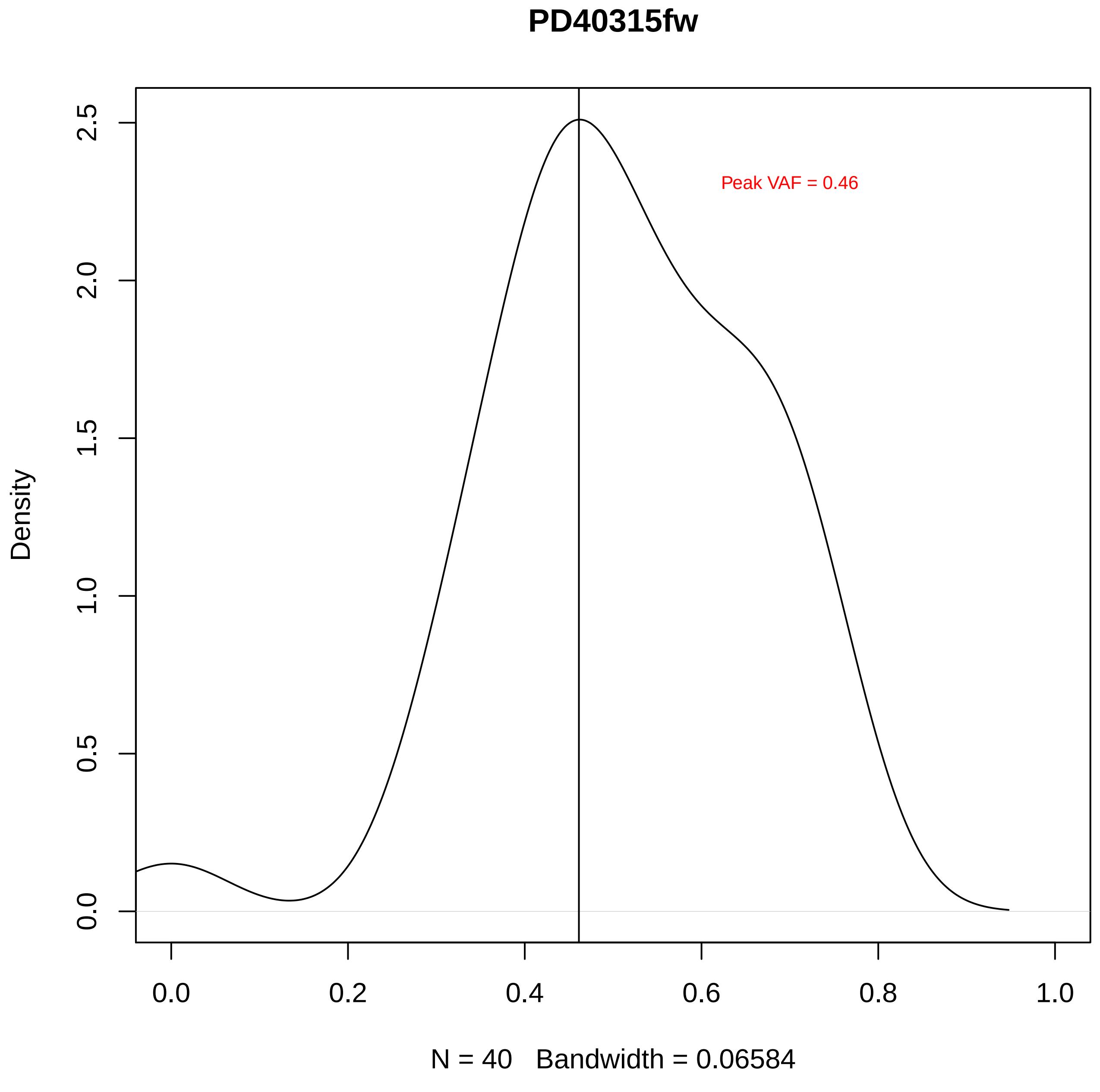
PD40315fw
Peak VAF = 0.46
Density
0.0
0.5
1.0
1.5
2.0
2.5
0.0
0.2
0.4
0.6
0.8
1.0
N = 40 Bandwidth = 0.06584

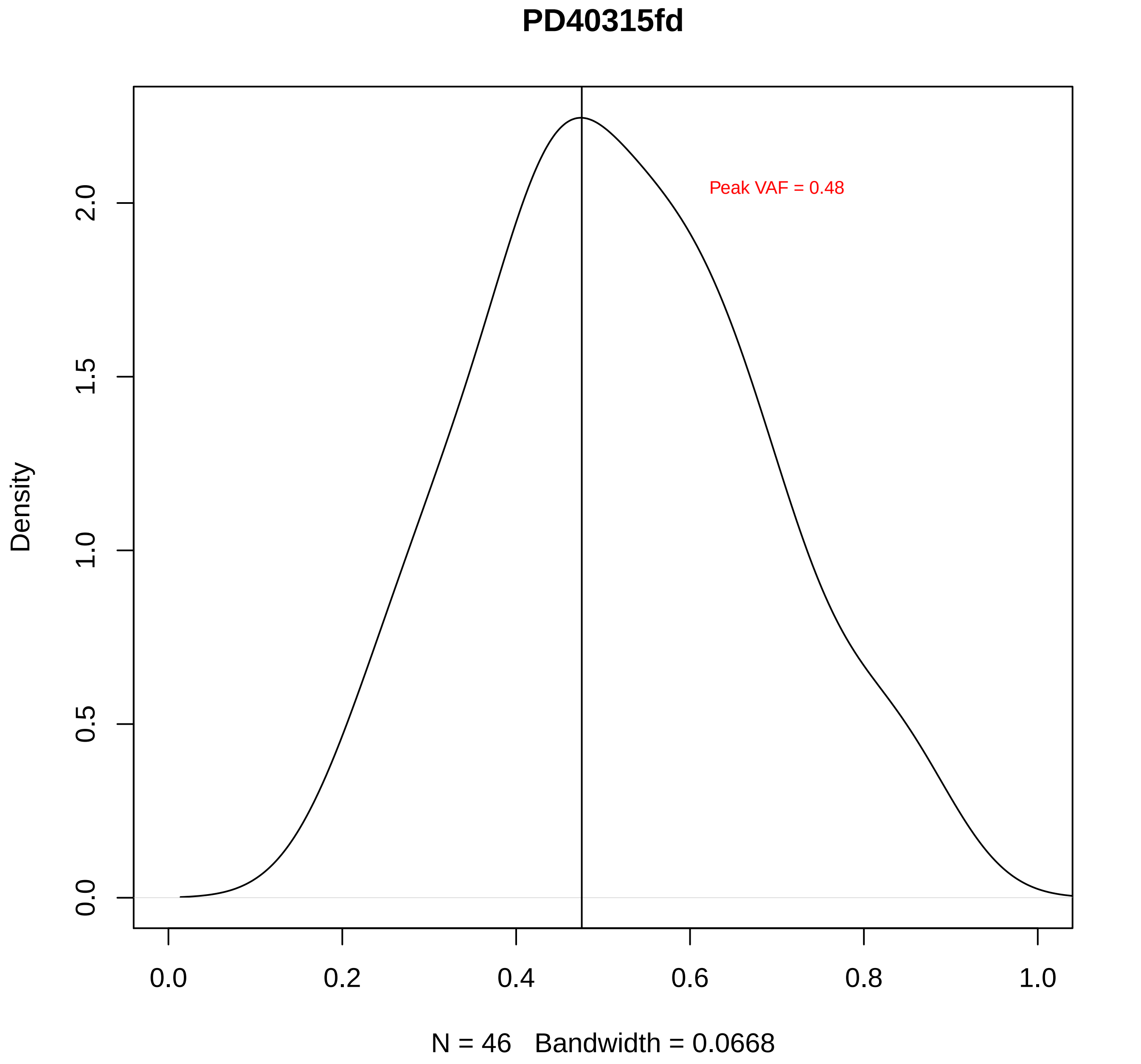
PD40315fd
Peak VAF = 0.48
Density
0.0
0.5
1.0
1.5
2.0
0.0
0.2
0.4
0.6
0.8
1.0
N = 46   Bandwidth = 0.0668

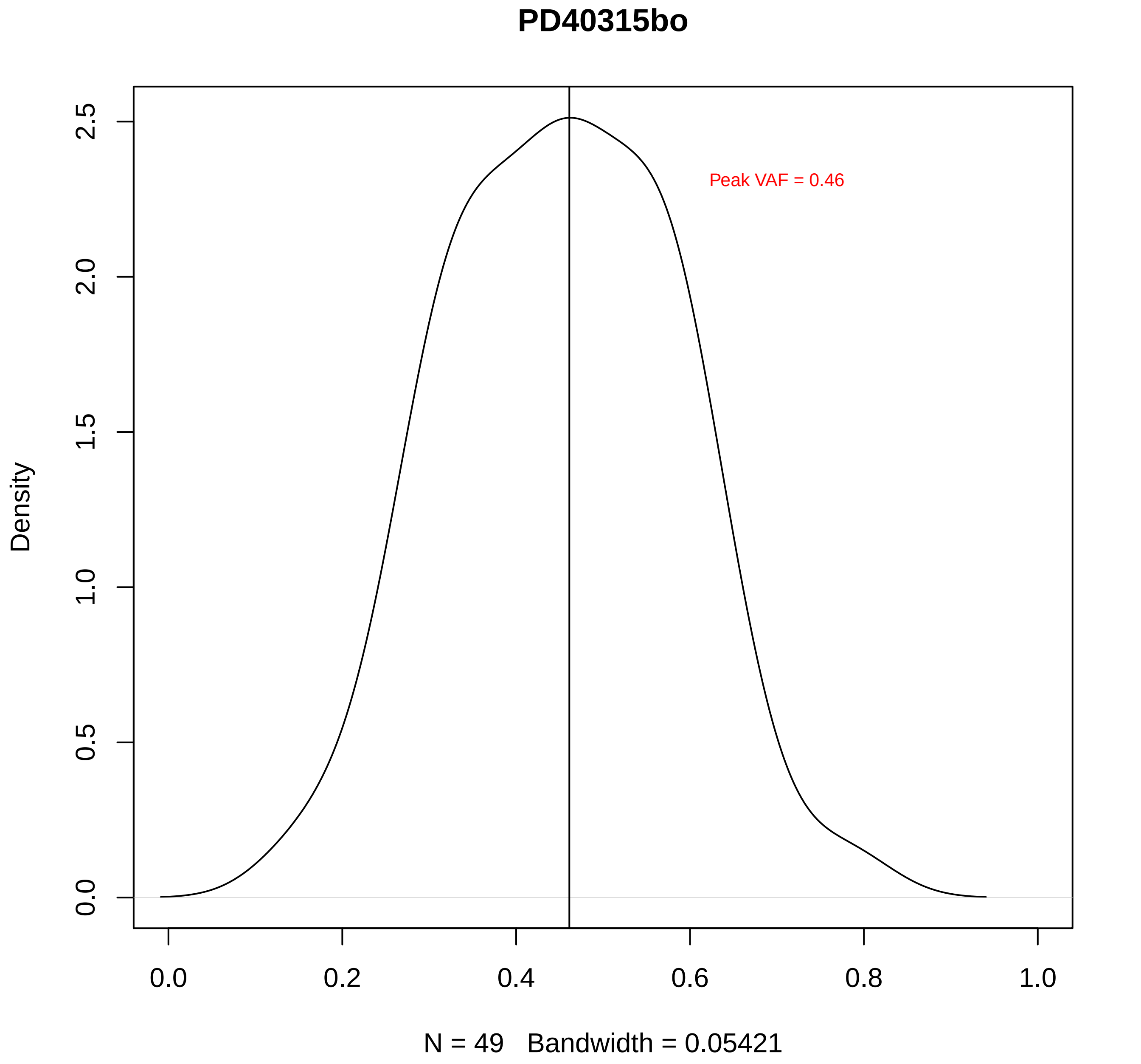

PD40315bo
Peak VAF = 0.46
Density
0.0
0.5
1.0
1.5
2.0
2.5
0.0
0.2
0.4
0.6
0.8
1.0
N = 49 Bandwidth = 0.05421

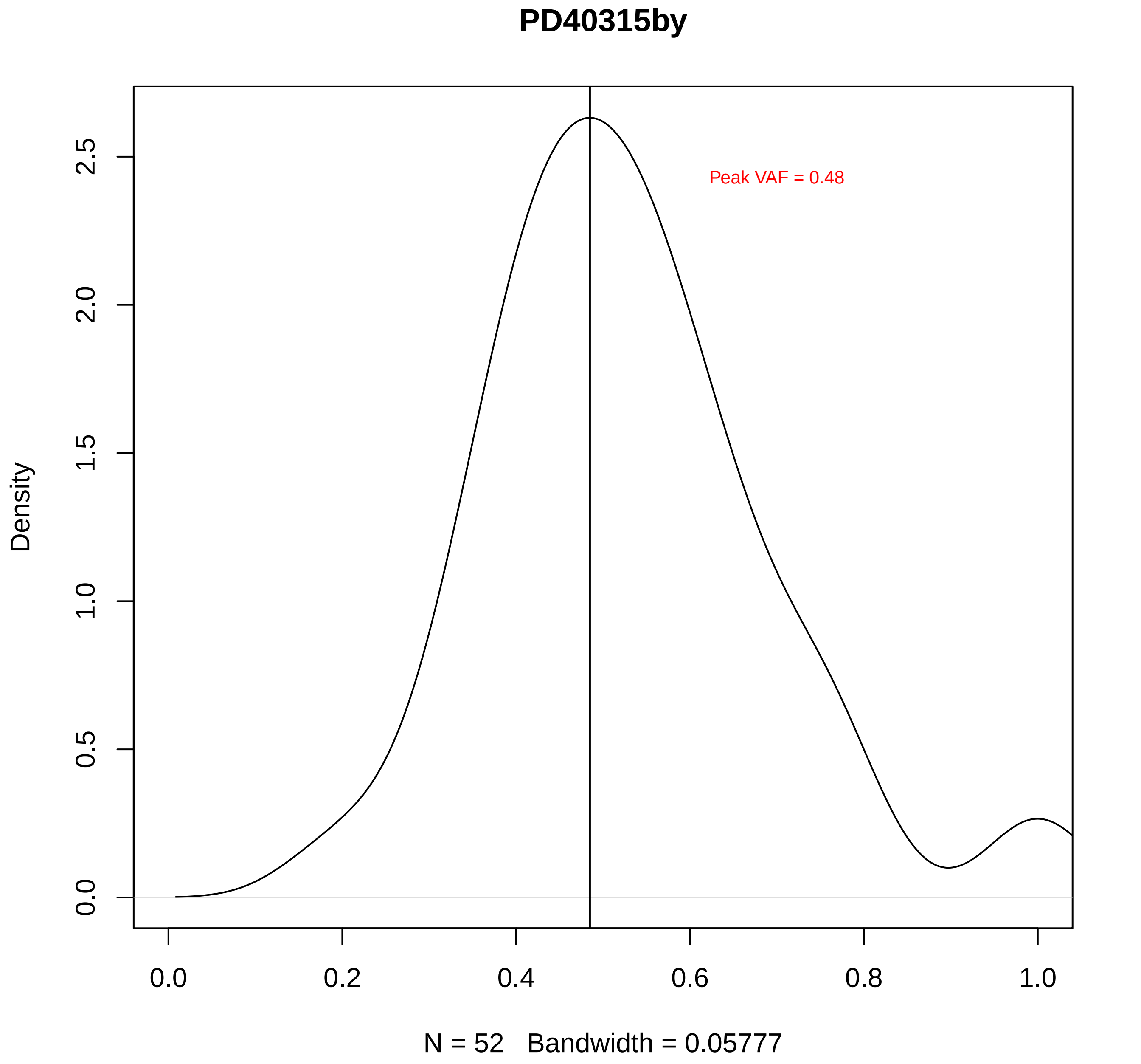
PD40315by
Peak VAF = 0.48
Density
0.0
0.5
1.0
1.5
2.0
2.5
0.0
0.2
0.4
0.6
0.8
1.0
N = 52   Bandwidth = 0.05777

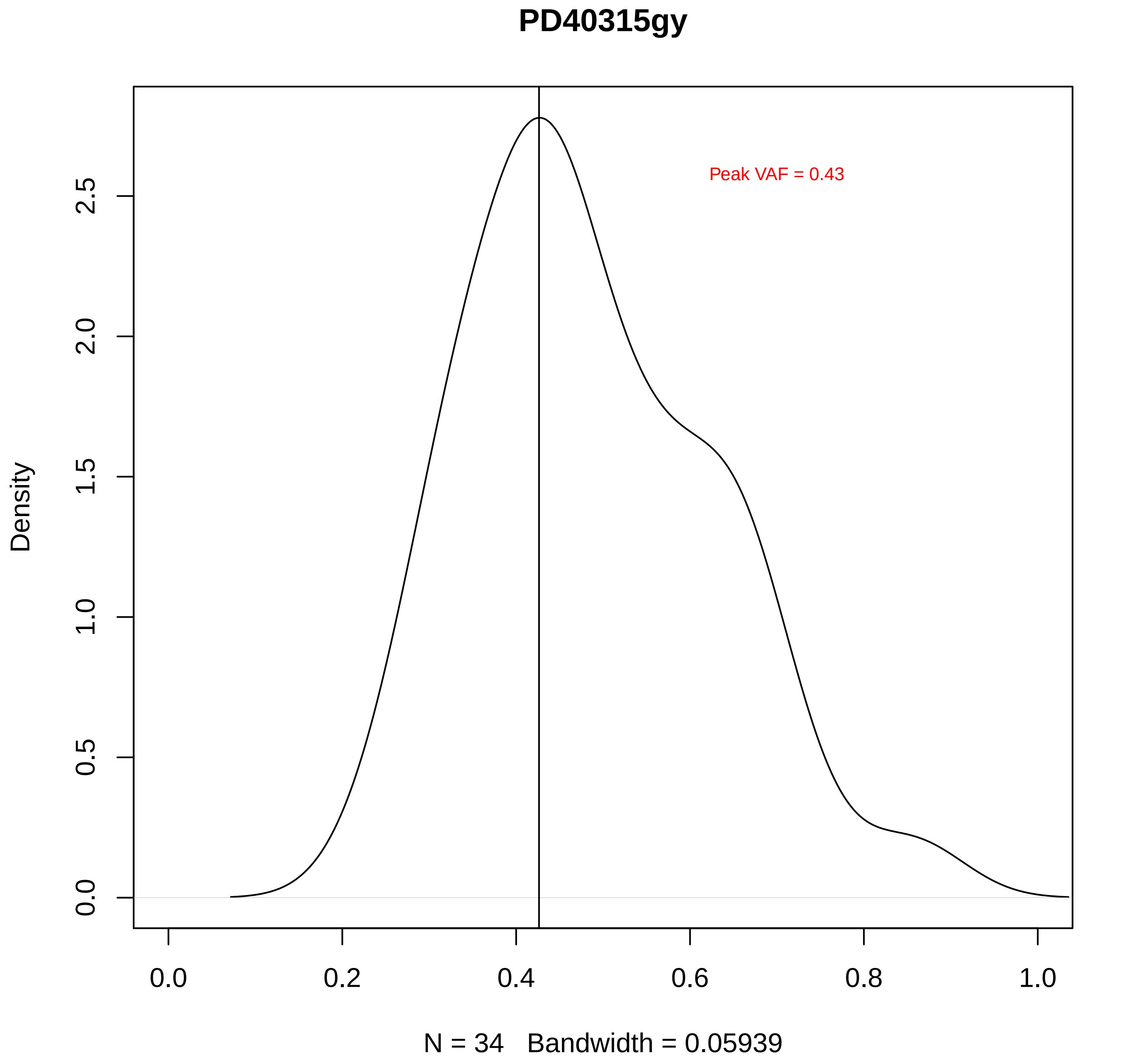
PD40315gy
Peak VAF = 0.43
Density
0.0
0.5
1.0
1.5
2.0
2.5
0.0
0.2
0.4
0.6
0.8
1.0
N = 34   Bandwidth = 0.05939

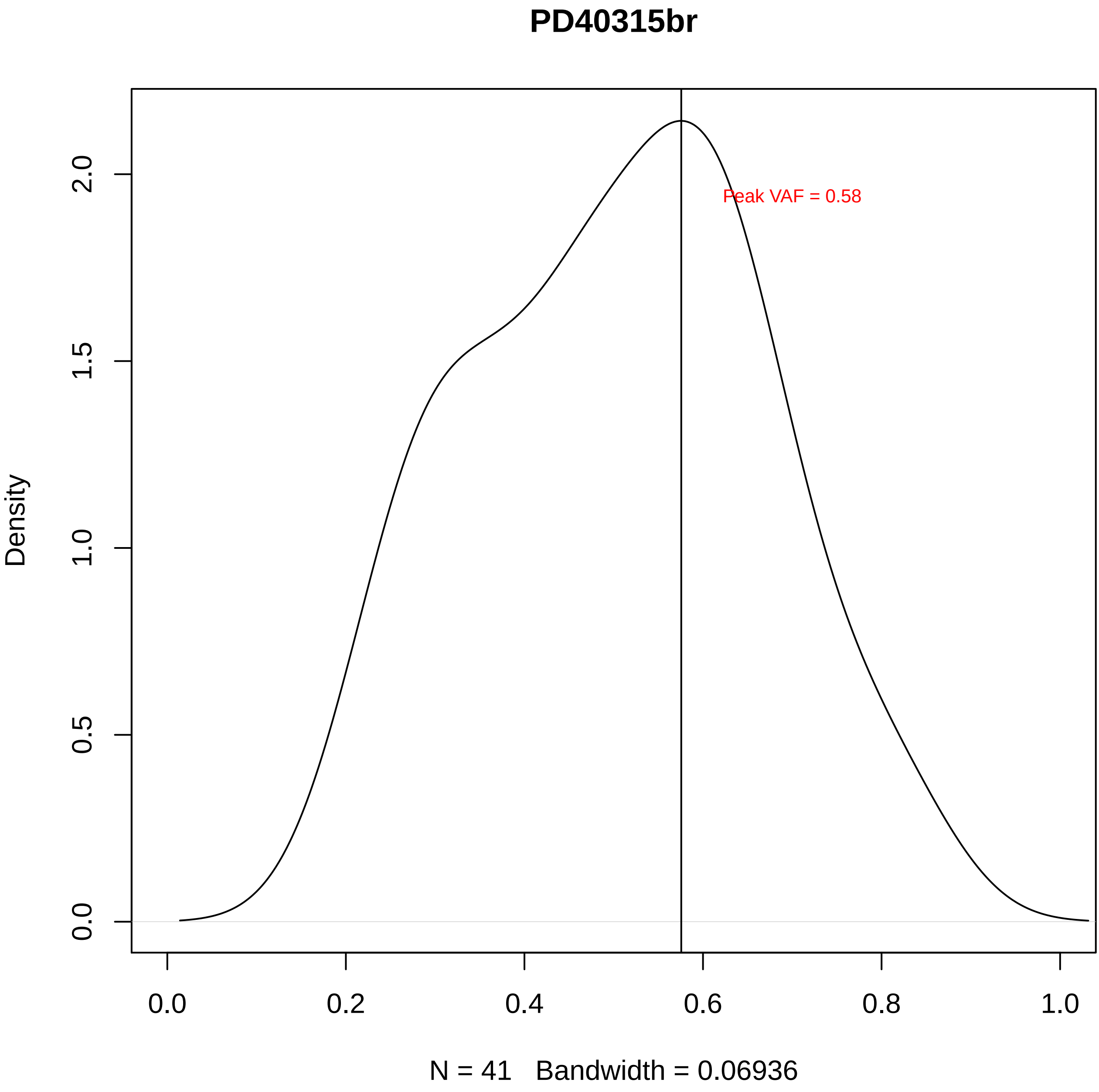
PD40315br
Peak VAF = 0.58
Density
0.0
0.5
1.0
1.5
2.0
0.0
0.2
0.4
0.6
0.8
1.0
N = 41   Bandwidth = 0.06936

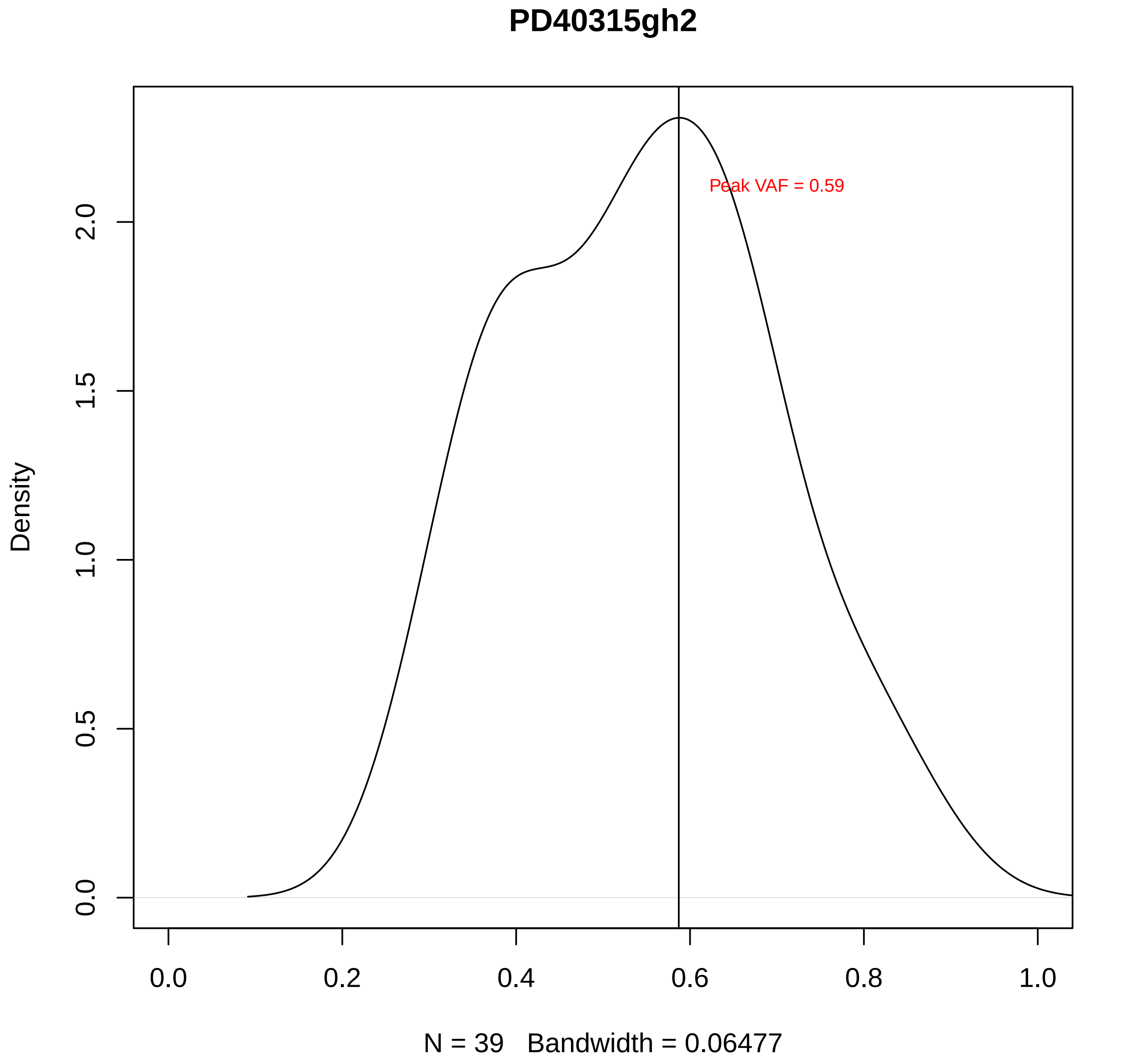
PD40315gh2
Peak VAF = 0.59
Density
0.0
0.5
1.0
1.5
2.0
0.0
0.2
0.4
0.6
0.8
1.0
N = 39 Bandwidth = 0.06477

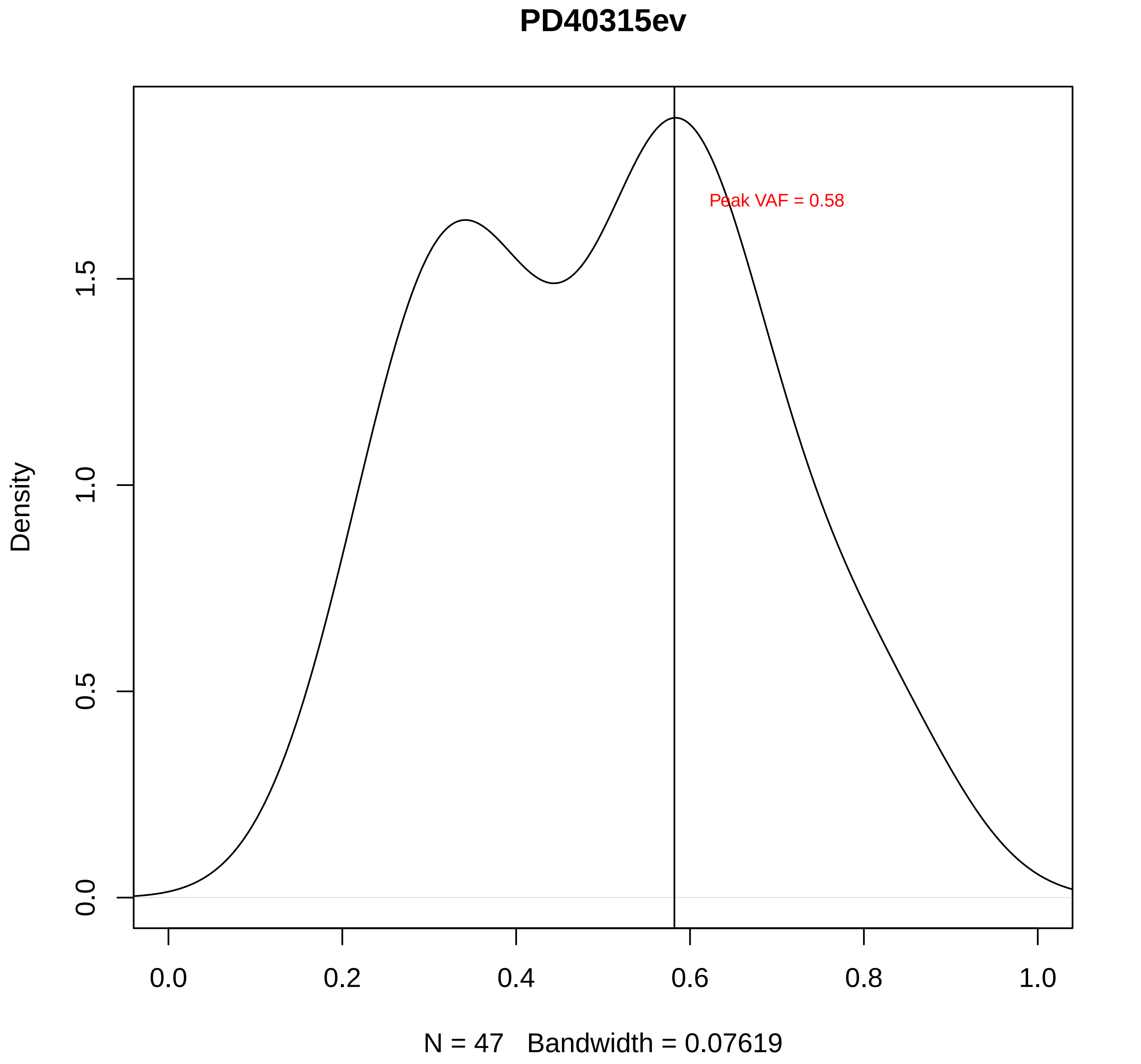

PD40315ev
Peak VAF = 0.58
Density
0.0
0.5
1.0
1.5
0.0
0.2
0.4
0.6
0.8
1.0
N = 47   Bandwidth = 0.07619

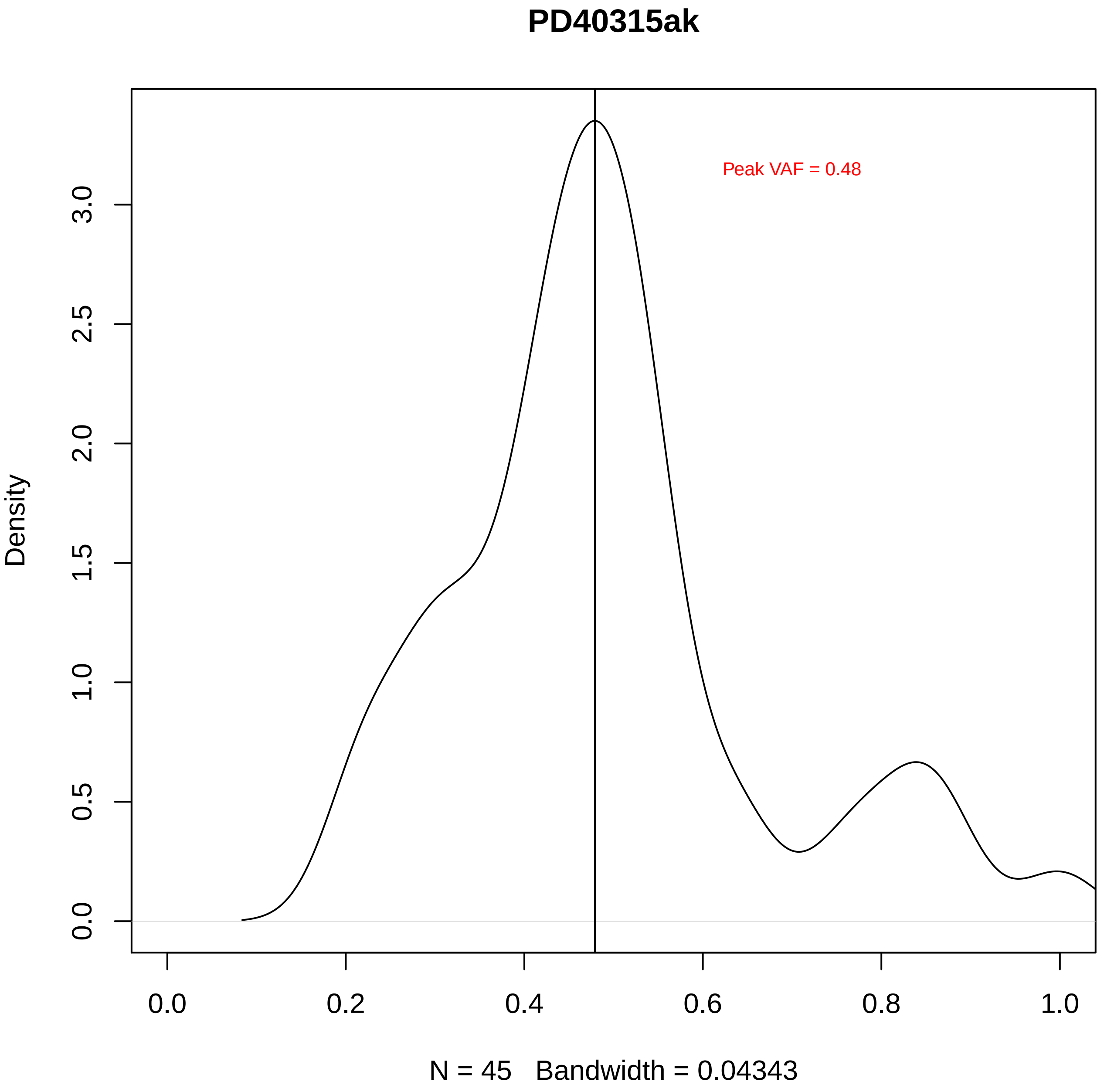
PD40315ak
Peak VAF = 0.48
Density
0.0
0.5
1.0
1.5
2.0
2.5
3.0
0.0
0.2
0.4
0.6
0.8
1.0
N = 45   Bandwidth = 0.04343

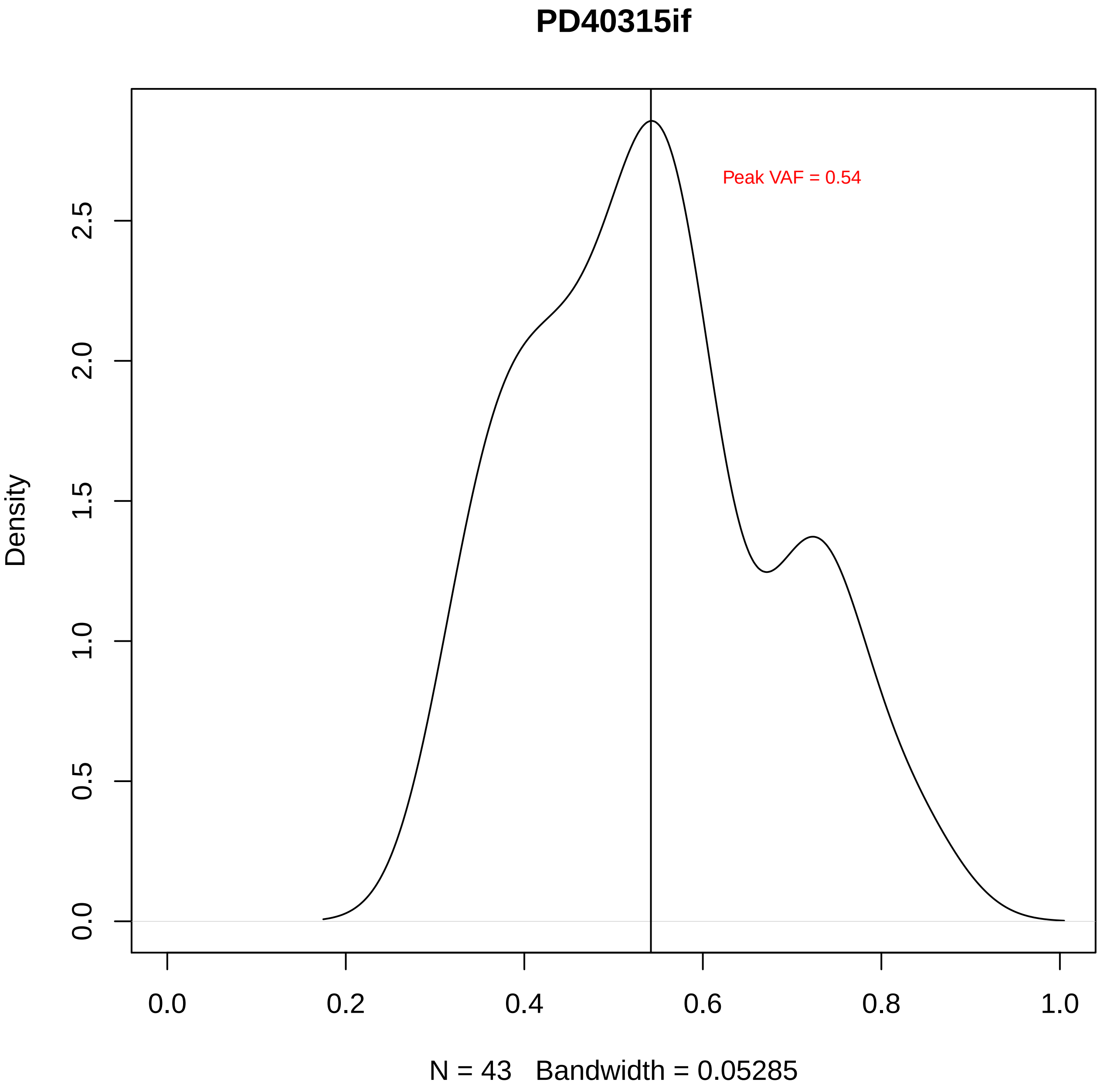
PD40315if
Peak VAF = 0.54
Density
0.0
0.5
1.0
1.5
2.0
2.5
0.0
0.2
0.4
0.6
0.8
1.0
N = 43   Bandwidth = 0.05285

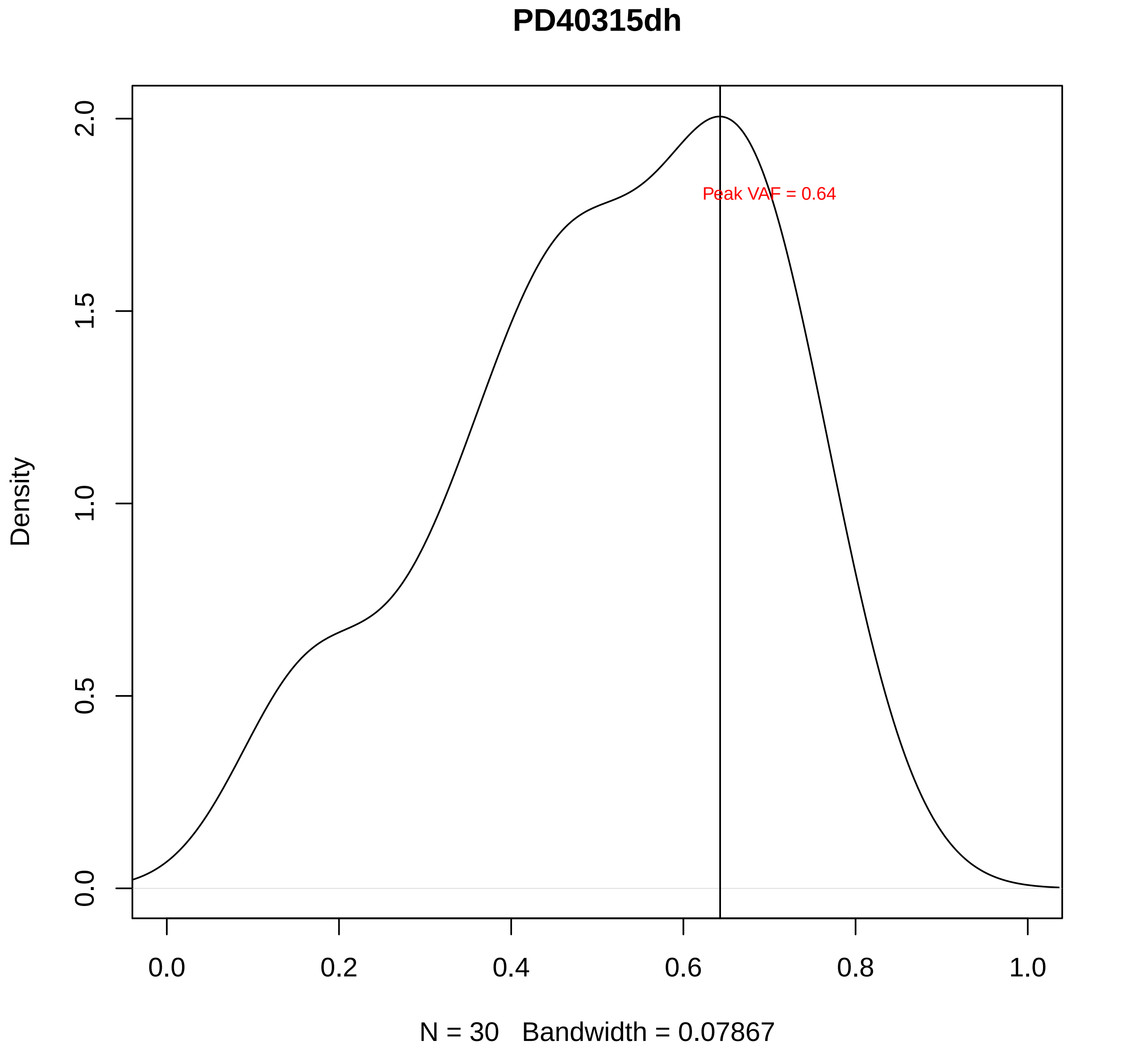
PD40315dh
Peak VAF = 0.64
Density
0.0
0.5
1.0
1.5
2.0
0.0
0.2
0.4
0.6
0.8
1.0
N = 30   Bandwidth = 0.07867

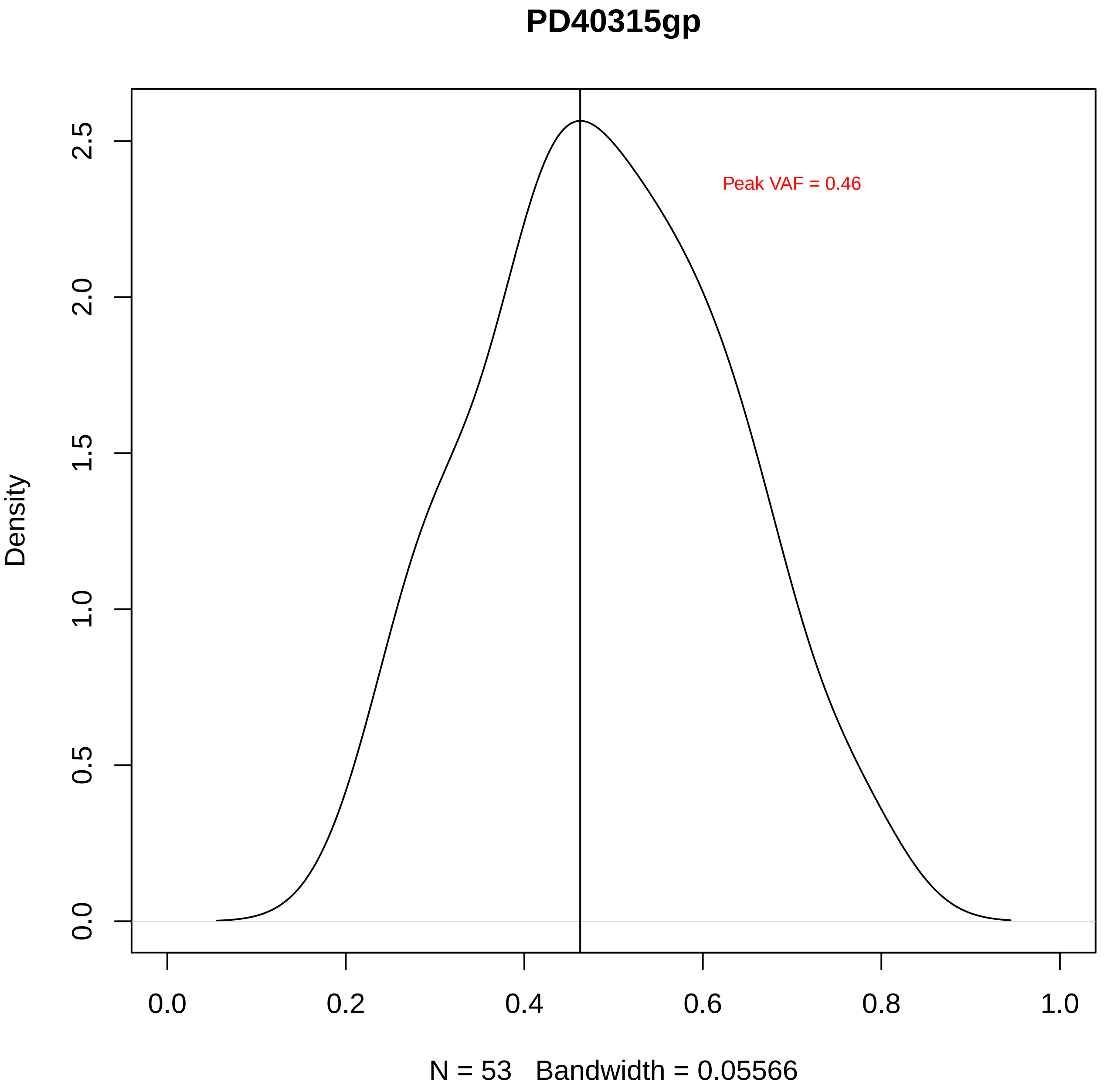

PD40315gp
Peak VAF = 0.46
Density
0.0
0.5
1.0
1.5
2.0
2.5
0.0
0.2
0.4
0.6
0.8
1.0
N = 53 Bandwidth = 0.05566

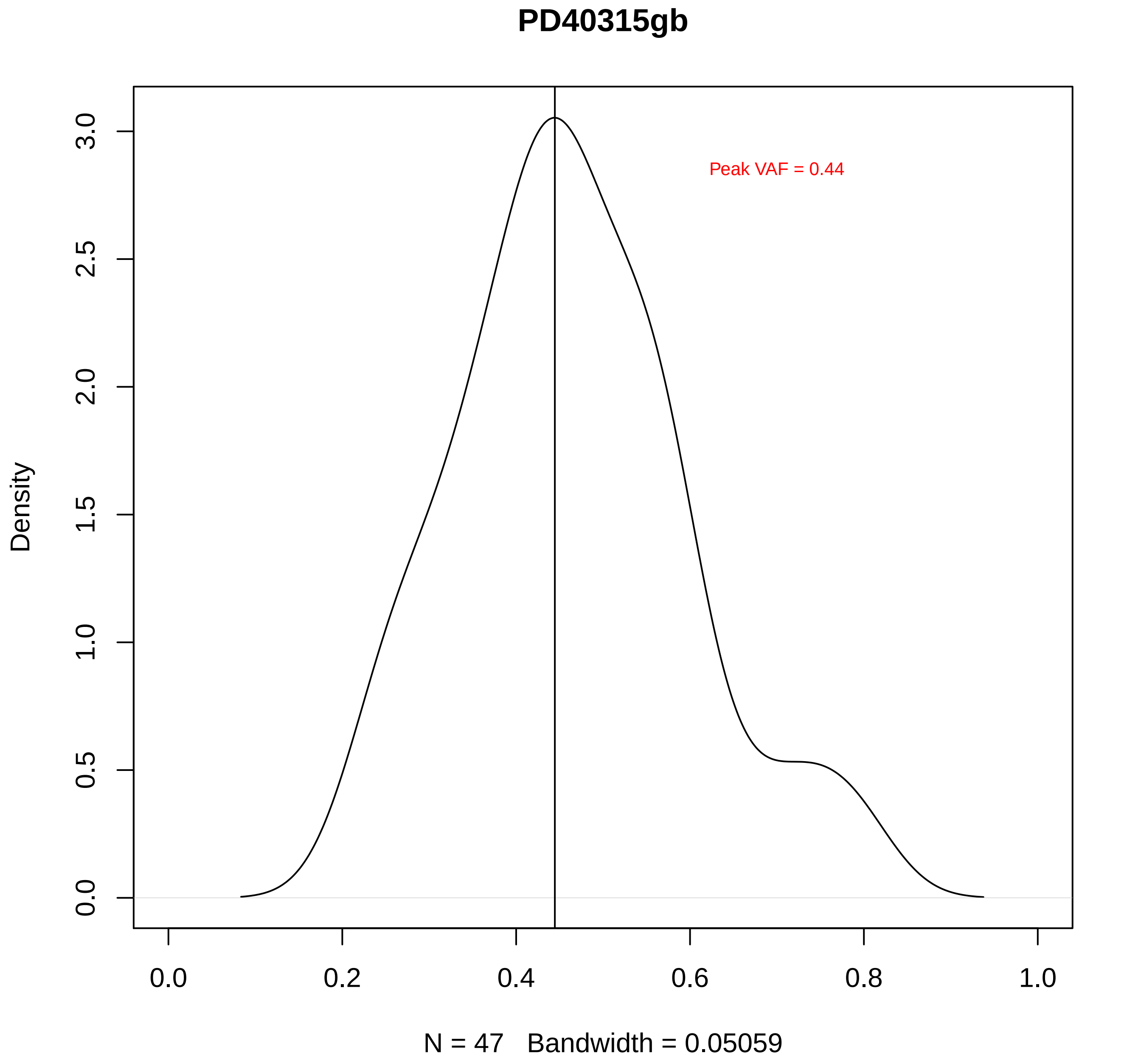
PD40315gb
Peak VAF = 0.44
Density
3.0
2.5
2.0
1.5
1.0
0.5
0.0
0.0
0.2
0.4
0.6
0.8
1.0
N = 47   Bandwidth = 0.05059

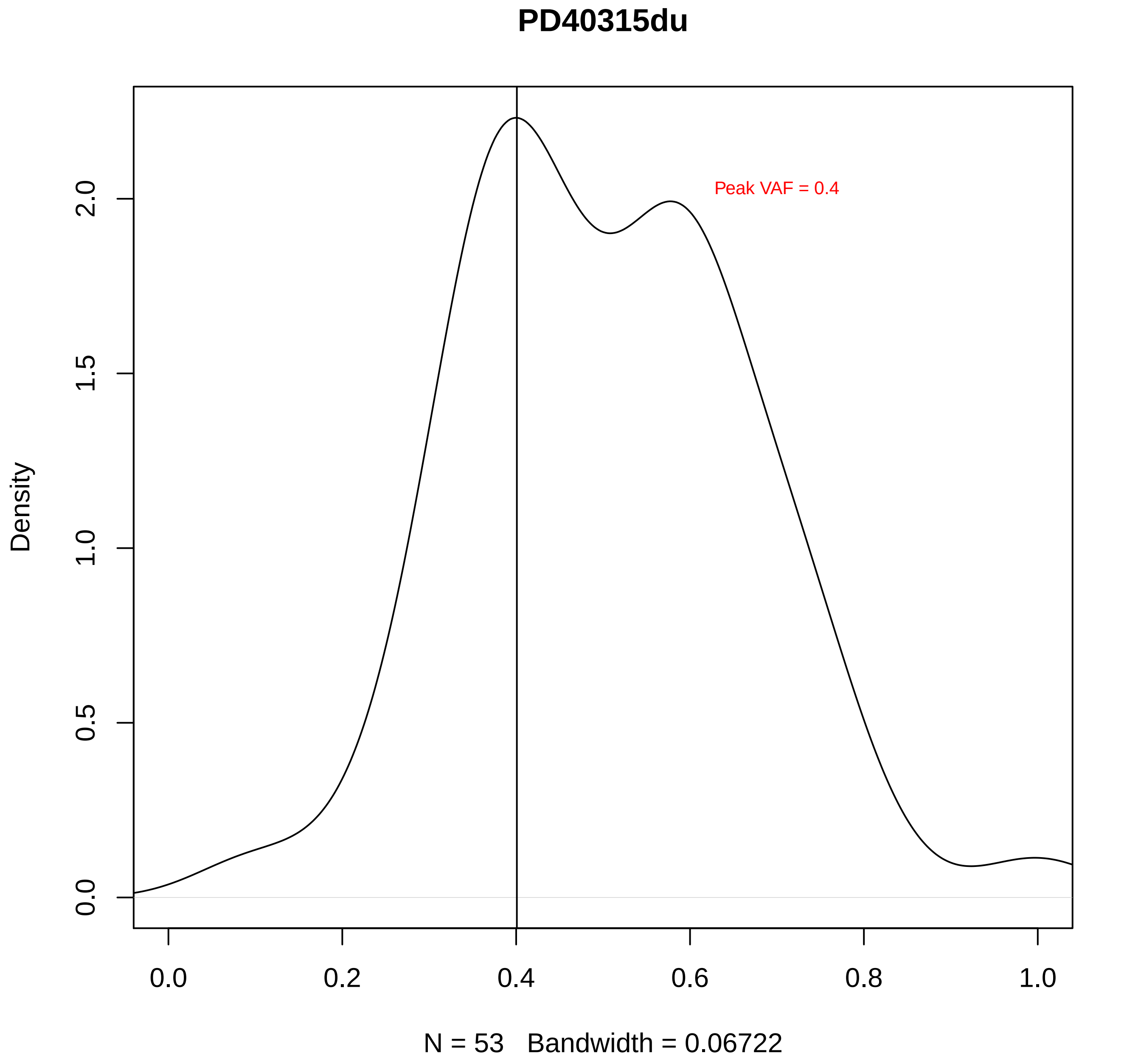
PD40315du
Peak VAF = 0.4
Density
2.0
1.5
1.0
0.5
0.0
0.0
0.2
0.4
0.6
0.8
1.0
N = 53   Bandwidth = 0.06722

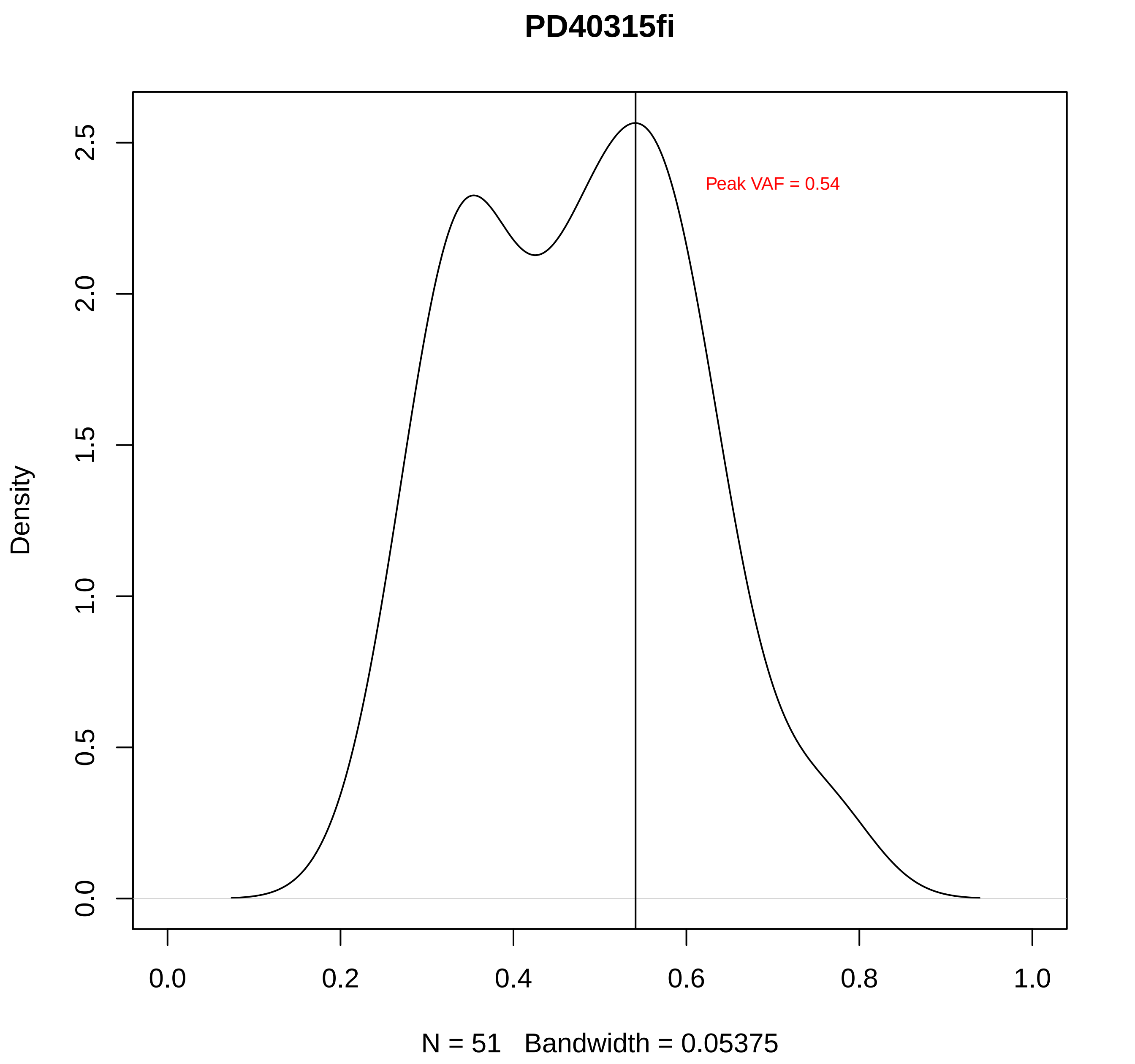
PD40315fi
Peak VAF = 0.54
Density
0.0
0.5
1.0
1.5
2.0
2.5
0.0
0.2
0.4
0.6
0.8
1.0
N = 51   Bandwidth = 0.05375

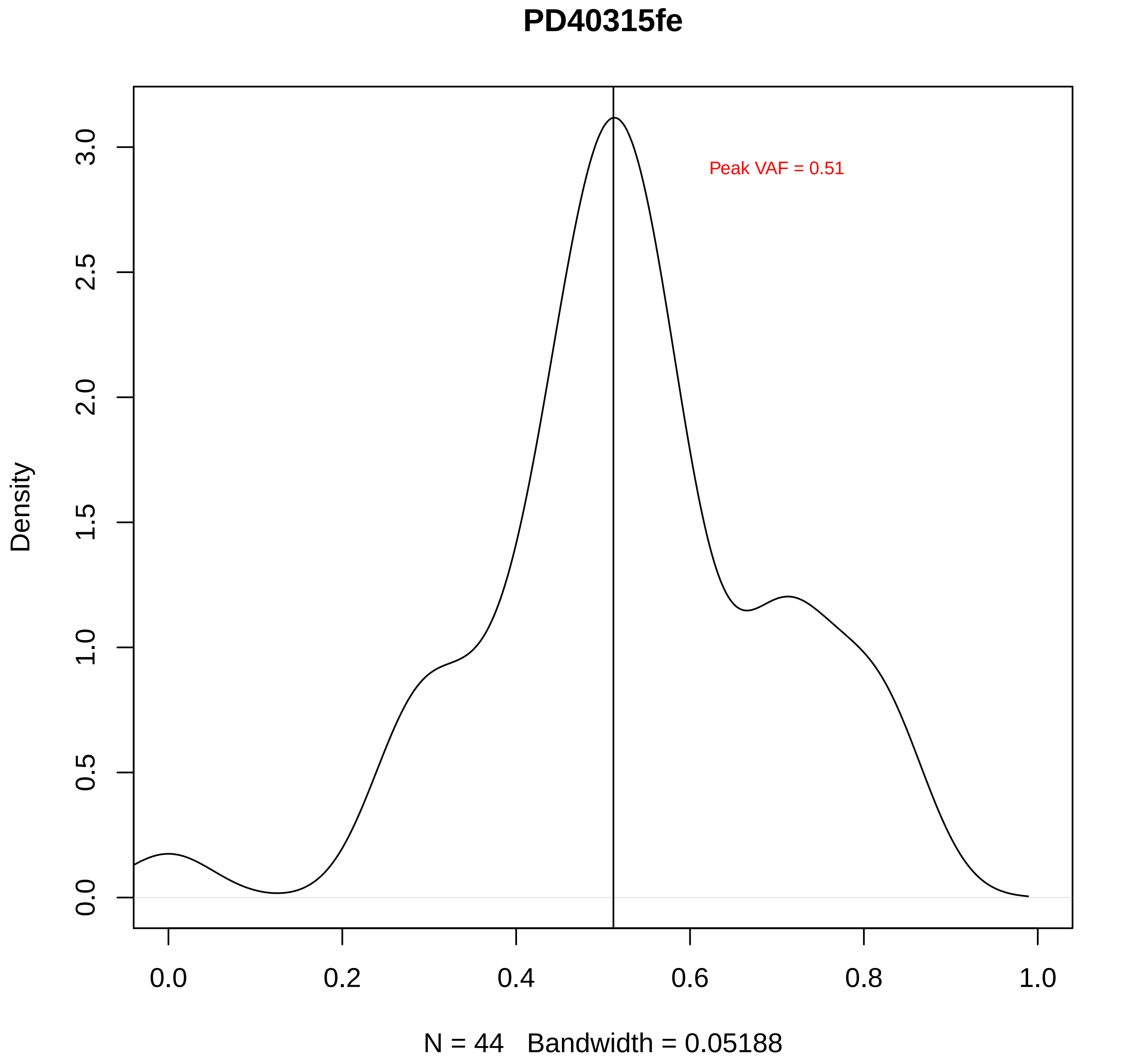
PD40315fe
Peak VAF = 0.51
Density
3.0
2.5
2.0
1.5
1.0
0.5
0.0
0.0
0.2
0.4
0.6
0.8
1.0
N = 44   Bandwidth = 0.05188

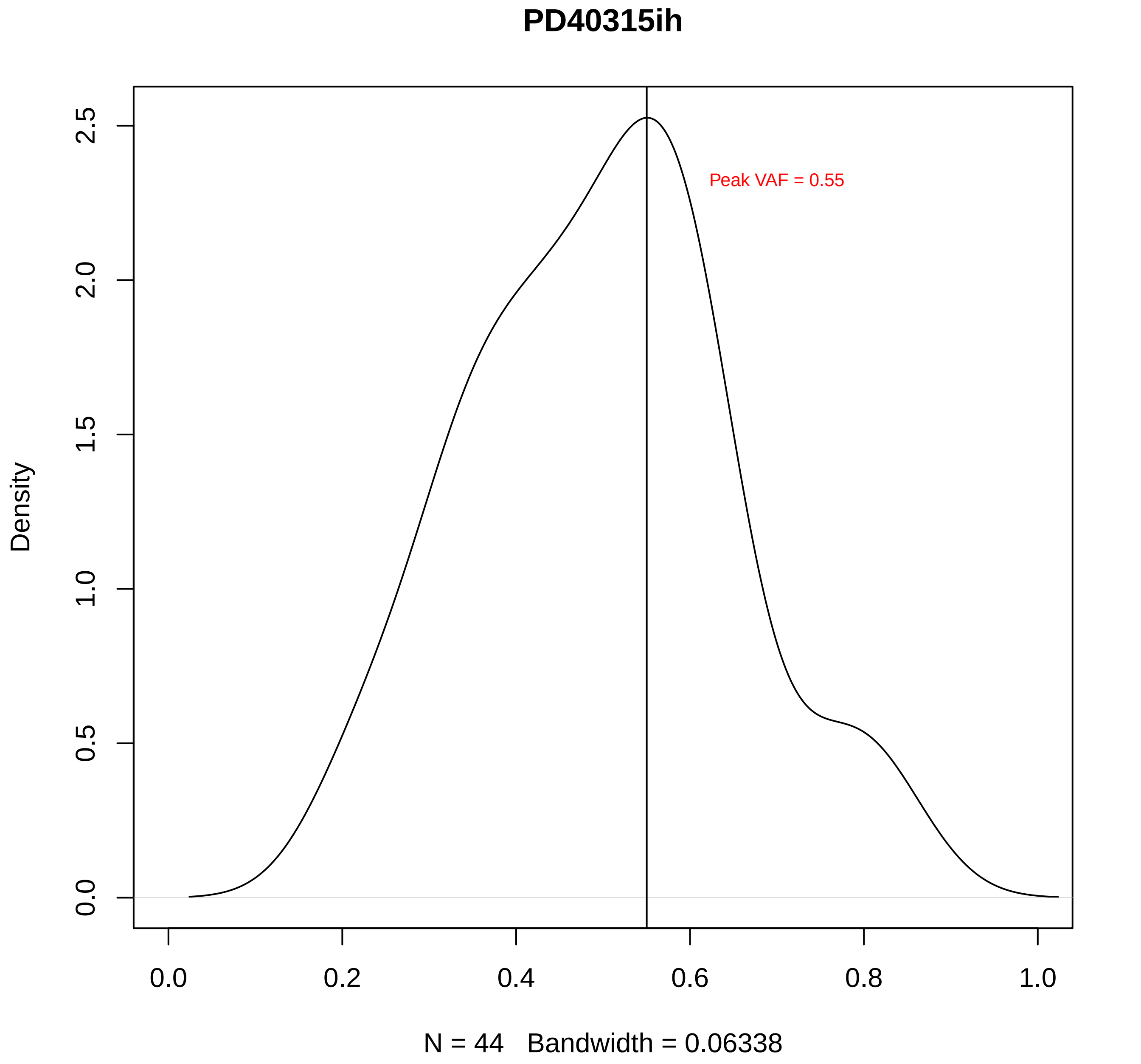
PD40315ih
Peak VAF = 0.55
Density
0.0
0.5
1.0
1.5
2.0
2.5
0.0
0.2
0.4
0.6
0.8
1.0
N = 44   Bandwidth = 0.06338

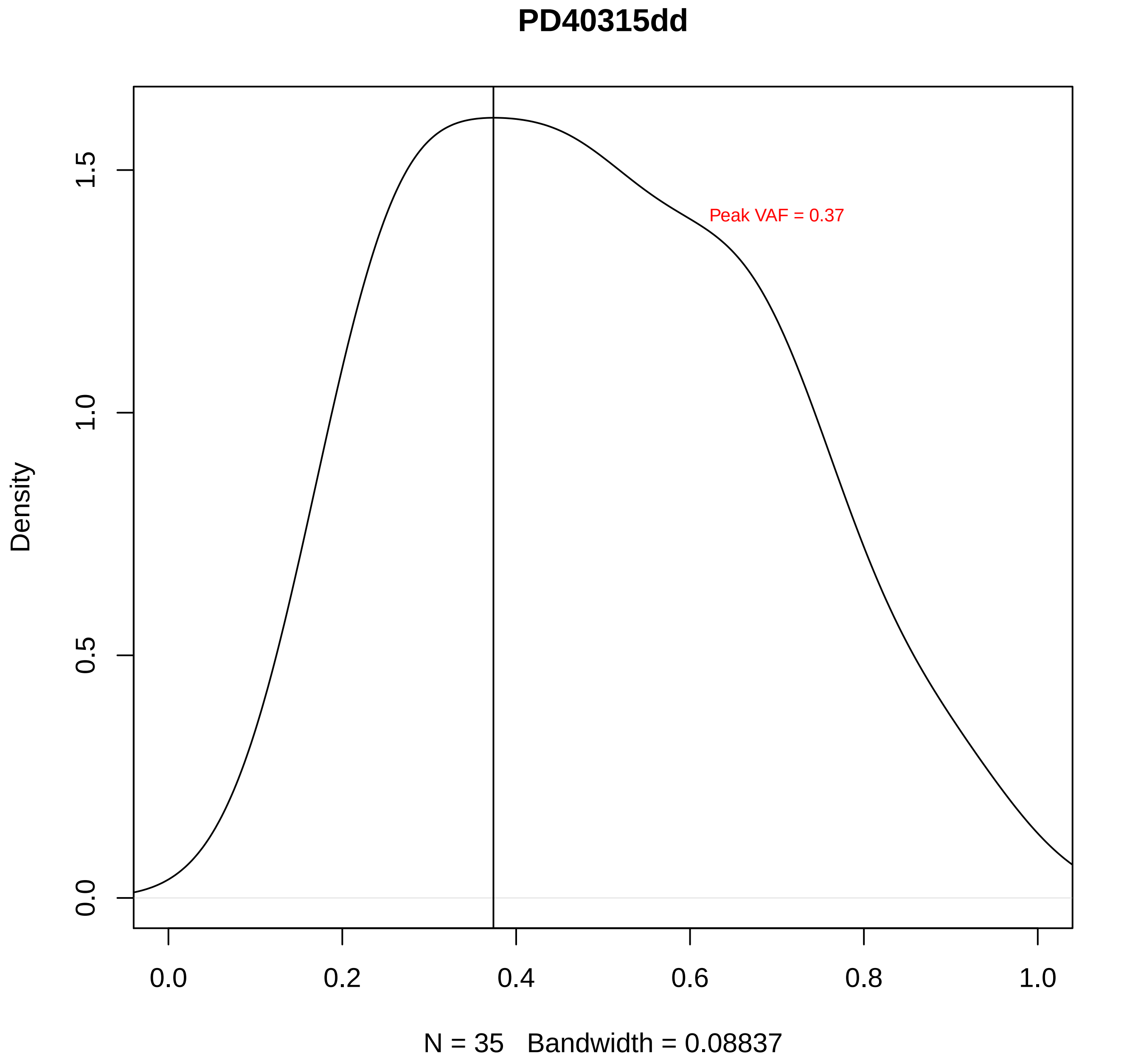
PD40315dd
Peak VAF = 0.37
Density
0.0
0.5
1.0
1.5
0.0
0.2
0.4
0.6
0.8
1.0
N = 35   Bandwidth = 0.08837

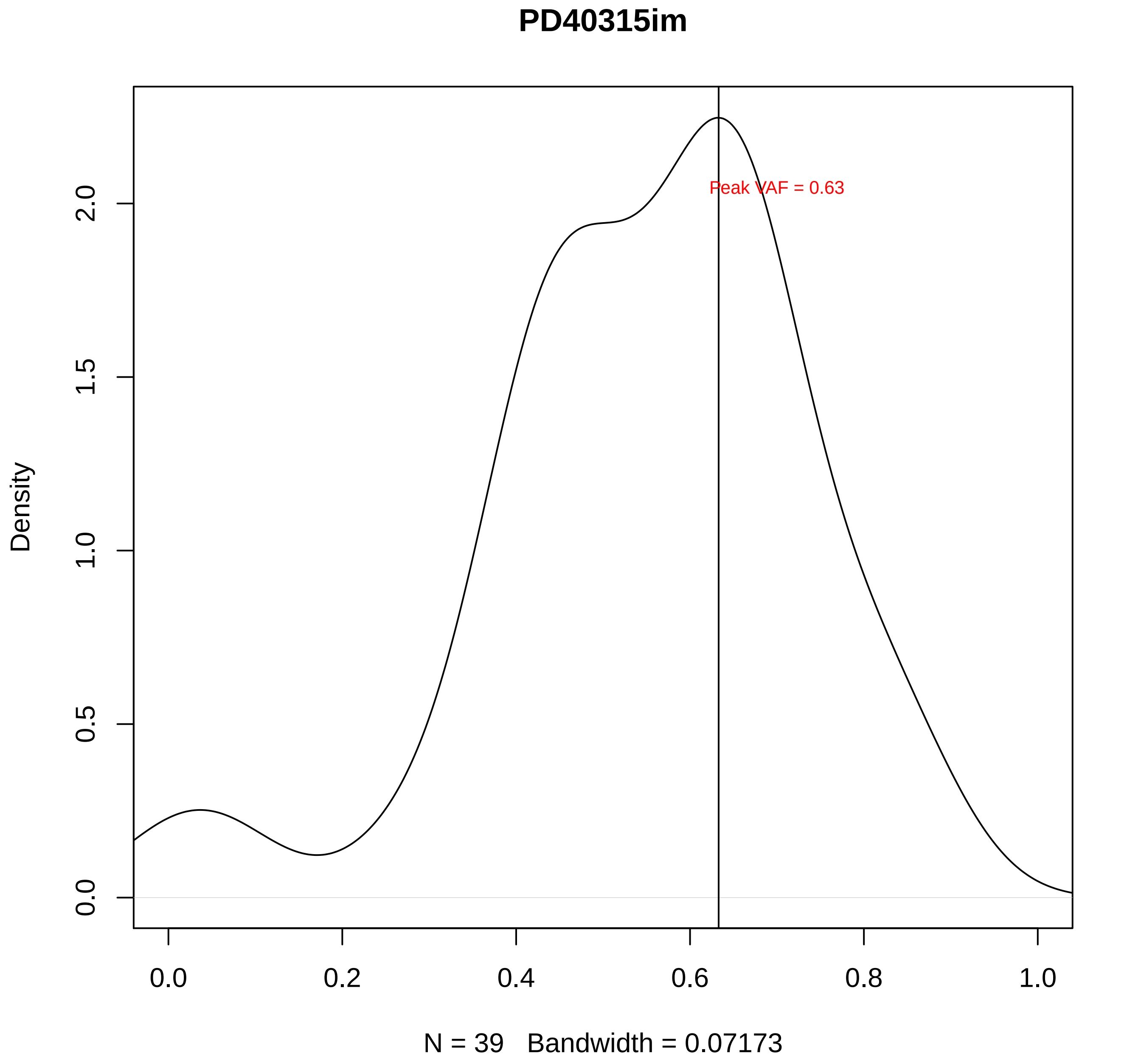
PD40315im
Peak VAF = 0.63
Density
0.0
0.5
1.0
1.5
2.0
0.0
0.2
0.4
0.6
0.8
1.0
N = 39   Bandwidth = 0.07173

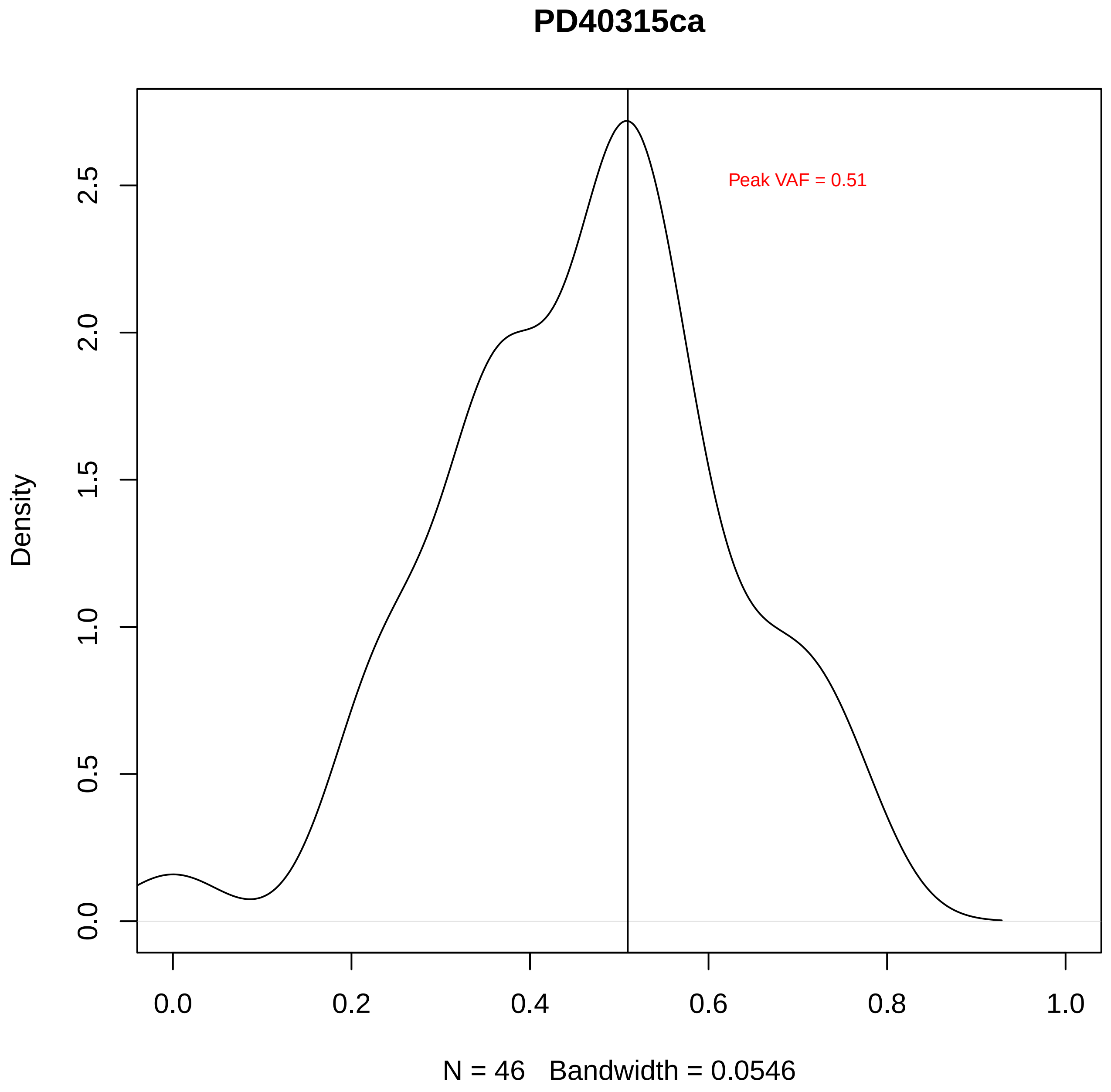

PD40315ca
Peak VAF = 0.51
Density
0.0
0.5
1.0
1.5
2.0
2.5
0.0
0.2
0.4
0.6
0.8
1.0
N = 46 Bandwidth = 0.0546

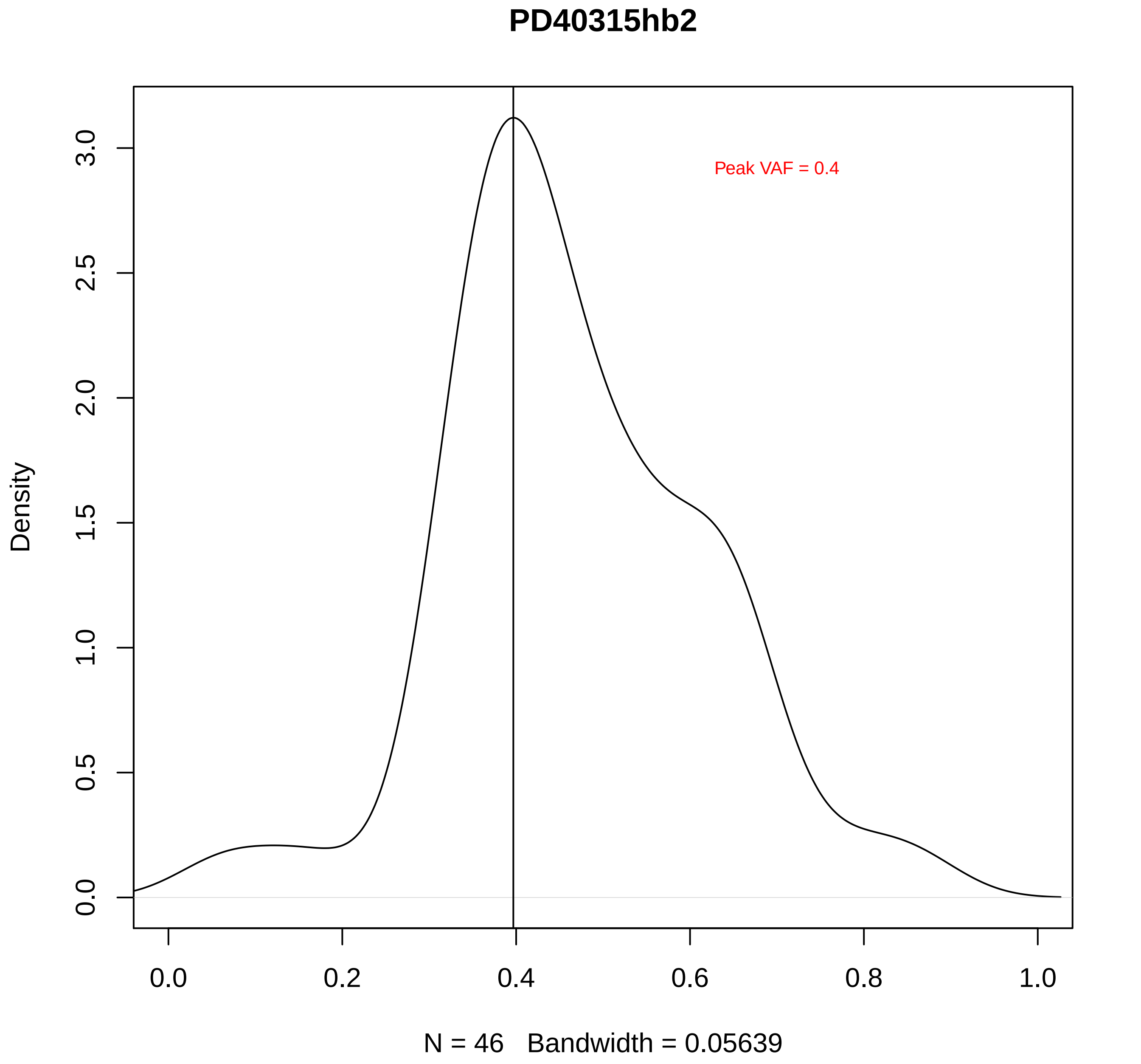
PD40315hb2
Peak VAF = 0.4
Density
0.0
0.5
1.0
1.5
2.0
2.5
3.0
0.0
0.2
0.4
0.6
0.8
1.0
N = 46   Bandwidth = 0.05639

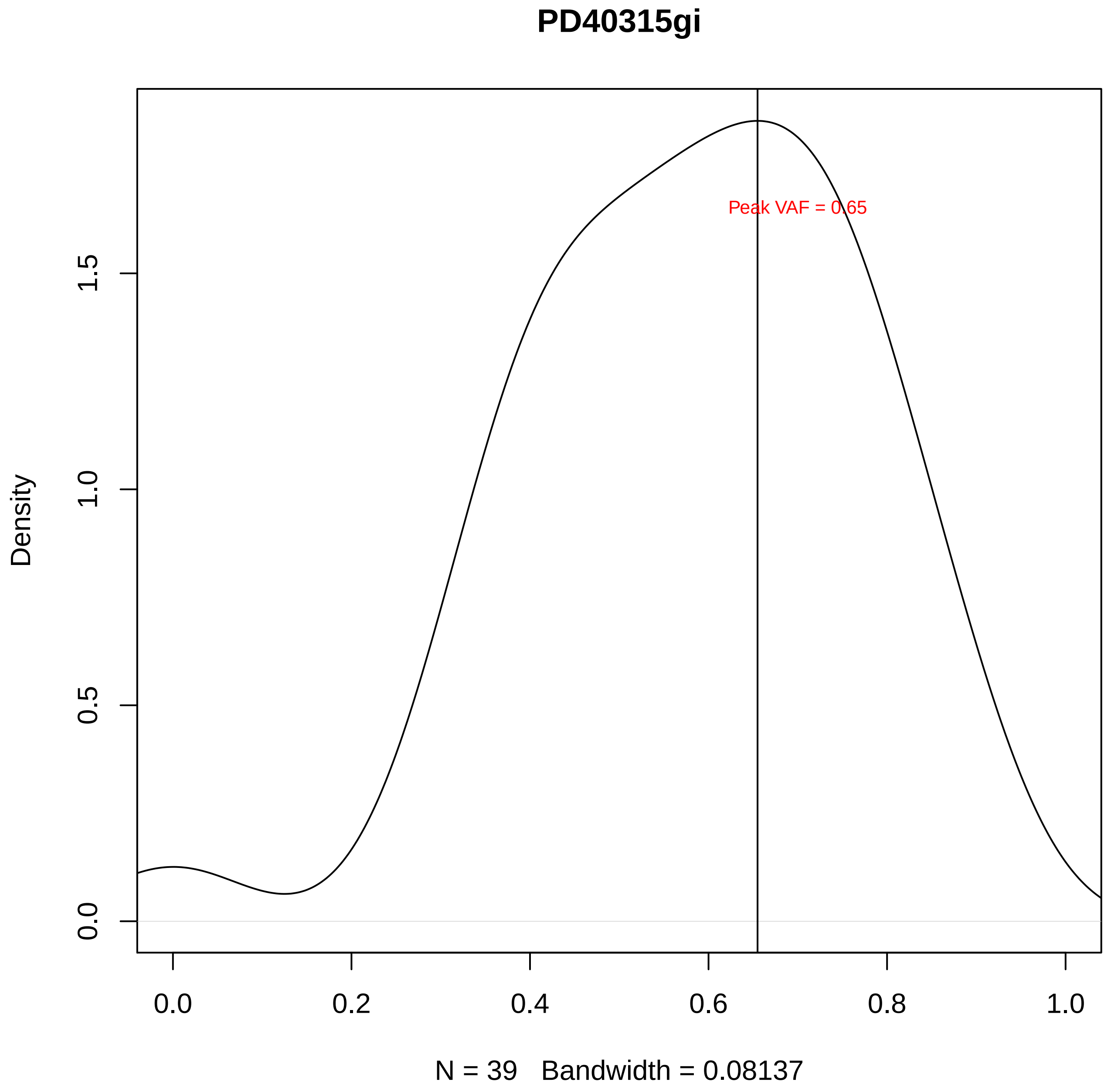
PD40315gi
Peak VAF = 0.65
Density
0.0
0.5
1.0
1.5
0.0
0.2
0.4
0.6
0.8
1.0
N = 39 Bandwidth = 0.08137

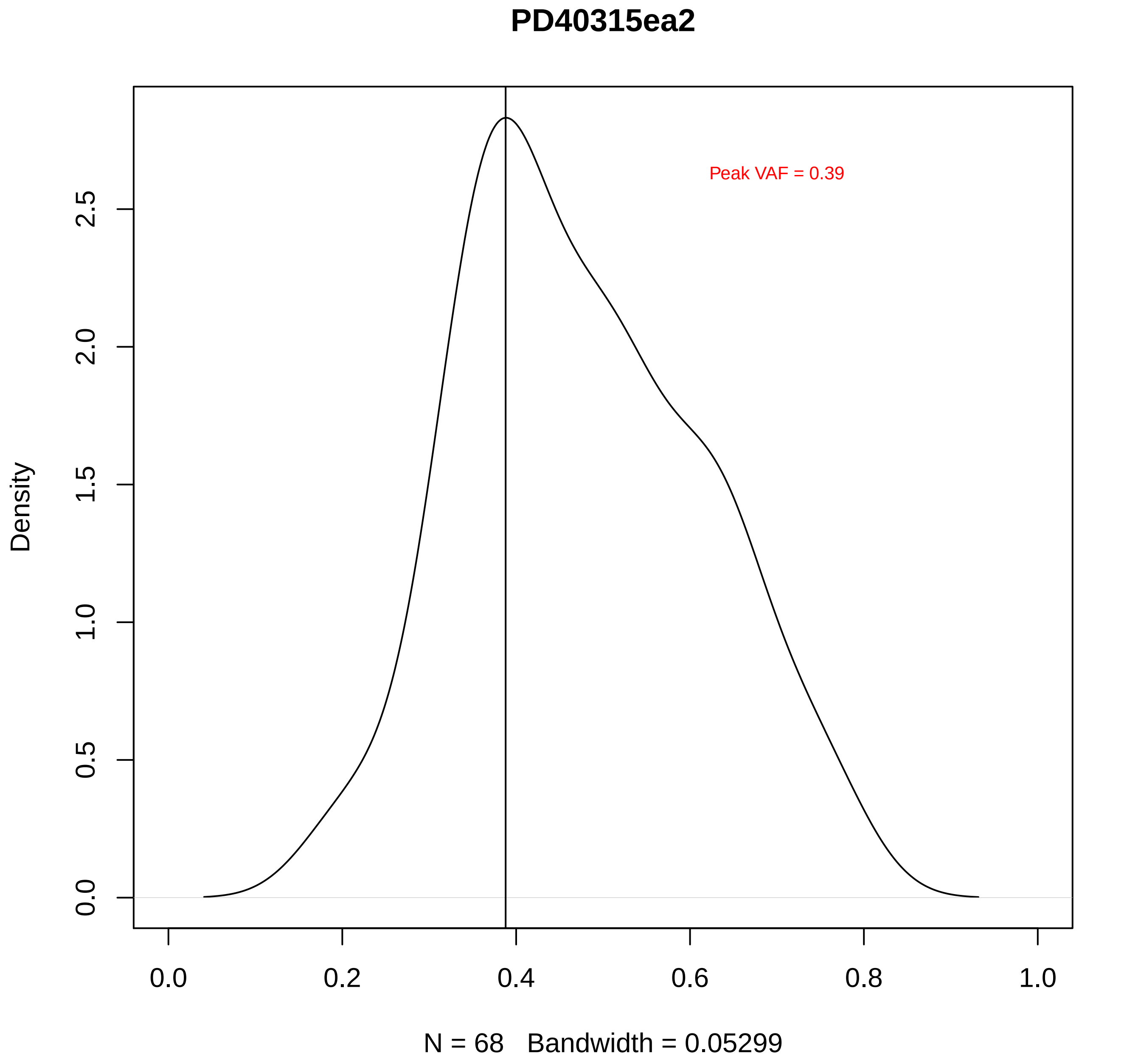
PD40315ea2
Peak VAF = 0.39
Density
0.0
0.5
1.0
1.5
2.0
2.5
0.0
0.2
0.4
0.6
0.8
1.0
N = 68 Bandwidth = 0.05299
