# Supplementary material for: Clonal dynamics of haematopoiesis across the human lifespan
Source: Nature. 2022 Jun 1;606(7913):343–50. doi: 10.1038/s41586-022-04786-y (PMC9177428; doi:10.1038/s41586-022-04786-y)
Supplement: Supplementary file 4 — HTMLs of notebooks outlining key statistical analyses presented in the manuscript, including analysis of phylogenetic trees. [file 41586_2022_4786_MOESM4_ESM.zip › Supplementary_code/Phylogeny_analysis/KX001_phylogeny_analysis.html]

Phylogeny analysis with non-clonal samples removed


# Phylogeny analysis with non-clonal samples removed

#### Emily Mitchell

### Summary

This script explores visualisation of the HSC/MPP phylogeny in different ways.  
1. Set file paths and working directory  
2. Source functions  
3. Load files  
4. Visualisation of phylogeny (including with steps in branch length adjustment) 5. Run phylodyn to visualise population trajectory predicted by pattern of coalescent events in the phylogeny  
6. Variant visualisation on the phylogeny  
7. Final phylogenies used in figures

##### Load packages

```
suppressMessages(library(stringr))
suppressMessages(library(ggtree))
suppressMessages(library(ape))
suppressMessages(library(seqinr))
suppressMessages(library(data.table))
suppressMessages(library(phytools))
suppressMessages(library(devtools))
suppressMessages(library(MCMCglmm))
suppressMessages(library(phangorn))
suppressMessages(library(spam))
suppressMessages(library(INLA))
suppressMessages(library(phylodyn))
suppressMessages(library(ggplot2))
```

### 1. Set file paths and working directory

```
PDID ="PD40521"
ID = "KX001" #Edit
Iteration = "KX001_4" #Edit
Run_ID = "KX001_4_01" #Edit
filtering_ID = "standard_rho01" #Edit
mutlim = 800 #Edit
age = 29 #Edit
age_breaks = c(0,10,20,30) #Edit
mut_breaks = c(0,100,200,300,400,500,600) #Edit
setwd = paste0("~/Documents/PhD/Sequencing_results/DNA_seq/",ID,"/",Iteration,"/trees/vaf_cut/") 
mats_and_param_file = paste0("~/Documents/PhD/Sequencing_results/DNA_seq/",ID,"/",Iteration,"/trees/vaf_cut/output/mats_and_params_", Run_ID)
tree_file_path = paste0("~/Documents/PhD/Sequencing_results/DNA_seq/",ID,"/",Iteration,"/trees/vaf_cut/output/tree_", Run_ID,"_",filtering_ID, ".tree")
file_annot = paste0("~/Documents/PhD/Sequencing_results/DNA_seq/",ID,"/",Iteration,"/trees/vaf_cut/output/annotated_mut_set_", Run_ID,"_",filtering_ID)
sensitivity_df = paste0("~/Documents/PhD/Sequencing_results/DNA_seq/",ID,"/",Iteration,"/trees/vaf_cut/output/",Iteration,"_sensitivity")
XY_cn = paste0("~/Documents/PhD/Sequencing_results/DNA_seq/",ID,"/",Iteration,"/ascat/XY_cn/",ID,"_meanCoverageXY.tsv")
XY_final = paste0("~/Documents/PhD/Sequencing_results/DNA_seq/",ID,"/",Iteration,"/trees/vaf_cut/driver_info/",Iteration,"_y_loss.csv")
CN_file = paste0("~/Documents/PhD/Sequencing_results/DNA_seq/",ID,"/",Iteration,"/ascat/",Iteration,"_ascat_summary.csv")
SV_file = "~/Documents/PhD/Sequencing_results/DNA_seq/XX_Summary/gridss/gridss_all_cut.csv"
mut_burden = paste0("~/Documents/PhD/Sequencing_results/DNA_seq/",ID,"/",Iteration,"/subs/vaf_cut/mutation_burden/",Iteration,"_sub_dep_adj.txt")
final_gene_file = paste0("~/Documents/PhD/Sequencing_results/DNA_seq/",ID,"/",Iteration,"/trees/vaf_cut/driver_info/",Iteration,"_driver_genes_final.csv")
mp_boot_file = paste0("~/Documents/PhD/Sequencing_results/DNA_seq/",ID,"/",Iteration,"/trees/vaf_cut/output/Filter_", Run_ID,"_",filtering_ID, ".fa.treefile")
info_nodes_file <- paste0("~/Documents/PhD/Sequencing_results/DNA_seq/",ID,"/",Iteration,"/trees/vaf_cut/driver_info/",Iteration,"_info_nodes.csv")
```

### 2. Source functions

```
function_files=list.files('~/Documents/Bioinformatics/CGP/Functions/', full.names = TRUE, pattern = ".R")
suppressMessages(sapply(function_files, source))
```

```
##         /Users/em16/Documents/Bioinformatics/CGP/Functions//filters_parallel_functions.R
## value   ?                                                                               
## visible FALSE                                                                           
##         /Users/em16/Documents/Bioinformatics/CGP/Functions//targeted_analysis_functions.R
## value   ?                                                                                
## visible FALSE                                                                            
##         /Users/em16/Documents/Bioinformatics/CGP/Functions//tree_functions.R
## value   ?                                                                   
## visible FALSE
```

### 3. Load files

##### Import myeloid malignancy gene list

```
chip_drivers = read.csv("~/Documents/Bioinformatics/CGP/Filtering/chip_drivers.csv", stringsAsFactors = FALSE, header = FALSE)
chip_drivers <- chip_drivers$V1
```

##### Import top clonal haem gene list

```
top_genes = read.csv("~/Documents/Bioinformatics/CGP/Filtering/final_gene_list.csv", stringsAsFactors = FALSE, header = FALSE)
top_genes <- top_genes$V1
```

##### Import cancer census gene list

```
cancer_drivers = read.table("~/Documents/Bioinformatics/CGP/Filtering/cancer_census.csv", stringsAsFactors = FALSE, header = TRUE, sep = ",")
cancer_drivers <- cancer_drivers$Gene.Symbol
```

##### Import dnds lists

```
dnds_genes_1500 = read.csv("~/Documents/Bioinformatics/CGP/Filtering/dnds_genes_1500.csv", stringsAsFactors = FALSE, header = FALSE)
dnds_genes_1500 <- dnds_genes_1500$V1
```

##### Import variant annotation list

```
top_variants = read.csv("~/Documents/Bioinformatics/CGP/Filtering/top_genes_annotation_EM_PC.csv", stringsAsFactors = FALSE, header = TRUE)
oncogenic_variants <- top_variants$variant_ID[top_variants$PC == "Oncogenic"]
possible_oncogenic_variants <- top_variants$variant_ID[top_variants$PC == "Possible_oncogenic"]
```

##### Import expanded clade list

```
expanded_clades = read.csv(paste0("~/Documents/PhD/Sequencing_results/DNA_seq/",ID,"/",Iteration,"/trees/vaf_cut/driver_info/",Iteration,"_expanded_clades_cut.csv"), stringsAsFactors = FALSE, header = TRUE)
```

##### Load data files for relevant individual

```
load(mats_and_param_file)
load(file_annot)
tree <- read.tree(tree_file_path)
mpboot_tree <- read.tree(mp_boot_file)
sensitivity <- read.table(sensitivity_df,stringsAsFactors = FALSE, header = TRUE)
XY <- read.table(XY_cn,stringsAsFactors = FALSE, header = TRUE)
XY_final <- read.csv(XY_final, stringsAsFactors = FALSE, header = TRUE)
CN <- read.csv(CN_file, stringsAsFactors = FALSE, header = TRUE)
SV <- read.csv(SV_file,stringsAsFactors = FALSE, header = TRUE)
muts <- read.table(mut_burden,stringsAsFactors = FALSE, header = TRUE)
info_nodes <- read.csv(info_nodes_file,stringsAsFactors = FALSE, header = TRUE)
```

##### Calculate mean muts

```
meanmut = mean(muts$Number_mutations_adj_as)
meanmut
```

```
## [1] 562.7629
```

##### Re-label the key objects to ensure compatibility with tree plotting functions

```
details <- filtered_muts$COMB_mats.tree.build$mat
details$Type <- as.character(details$Type)
details$Gene <- as.character(details$Gene)
details$variant_ID <- paste(details$Gene, details$Protein, sep = " ")
details$Type[details$Type == ""] <- "no_annotation"
mtr <- filtered_muts$COMB_mats.tree.build$NV
dep <- filtered_muts$COMB_mats.tree.build$NR
```

### 4. Visualisation of phylogeny

##### Plot raw mpboot tree

```
mpboot_tree <- drop.tip(mpboot_tree,"Ancestral")
mpboot_tree$edge.length <- rep(1,nrow(mpboot_tree$edge))
mpboot_tree <- phytools::force.ultrametric(mpboot_tree,method="extend")

mpboot = plot_tree_em(mpboot_tree,cex.label = 0)
```

```
## scale= 1
```

##### Plot tree with unadjusted branch lengths

```
plot_tree_em(tree,cex.label = 0)
```

```
## scale= 100
```

```
## 
## Phylogenetic tree with 407 tips and 406 internal nodes.
## 
## Tip labels:
##   PD40521aa, PD40521bp, PD40521gl, PD40521gq, PD40521eu, PD40521hv, ...
## Node labels:
##   , 100, 100, 1, 0, 0, ...
## 
## Rooted; includes branch lengths.
```

##### Make and save coverage corrected tree

```
tree_adj <- get_corrected_tree(tree, details, sensitivity, include_SNVs = TRUE, include_indels = FALSE, get_edge_from_tree = FALSE)

write.tree(tree_adj, paste0("~/Documents/PhD/Sequencing_results/DNA_seq/",ID,"/",Iteration,"/trees/vaf_cut/tree_files/",Iteration,"_adj.tree"))
```

##### Plot coverage adjusted tree

```
plot_tree_em(tree_adj,cex.label = 0)
```

```
## scale= 100
```

```
## 
## Phylogenetic tree with 407 tips and 406 internal nodes.
## 
## Tip labels:
##   PD40521aa, PD40521bp, PD40521gl, PD40521gq, PD40521eu, PD40521hv, ...
## Node labels:
##   , 100, 100, 1, 0, 0, ...
## 
## Rooted; includes branch lengths.
```

##### Make and save tree ultrametric (use corrected tree)

```
tree_ultra <- make.ultrametric.tree(tree_adj)
write.tree(tree_ultra, paste0("~/Documents/PhD/Sequencing_results/DNA_seq/",ID,"/",Iteration,"/trees/vaf_cut/tree_files/",Iteration,"_ultra.tree"))
```

##### Create ultrametric tree with age as x axis

```
tree_ultra_age <- tree_ultra
tree_ultra_age$edge.length <- tree_ultra_age$edge.length*age
```

##### Plot ultrametric age tree

```
plot_tree_em(tree_ultra_age,cex.label = 0)
```

```
## scale= 1
```

```
## 
## Phylogenetic tree with 407 tips and 406 internal nodes.
## 
## Tip labels:
##   PD40521aa, PD40521bp, PD40521gl, PD40521gq, PD40521eu, PD40521hv, ...
## Node labels:
##   , 100, 100, 1, 0, 0, ...
## 
## Rooted; includes branch lengths.
```

##### Create ultrametric tree with muts as x axis

```
tree_ultra_mut <- tree_ultra
tree_ultra_mut$edge.length <- tree_ultra_mut$edge.length*meanmut
```

##### Plot ultrametric tree with muts as x axis

```
plot_tree_em(tree_ultra_mut,cex.label = 0)
```

```
## scale= 100
```

```
## 
## Phylogenetic tree with 407 tips and 406 internal nodes.
## 
## Tip labels:
##   PD40521aa, PD40521bp, PD40521gl, PD40521gq, PD40521eu, PD40521hv, ...
## Node labels:
##   , 100, 100, 1, 0, 0, ...
## 
## Rooted; includes branch lengths.
```

### 5. Run phylodyn

##### Run phylodyn on tree with age on x axis (not adjusted for increased mutation rate before birth)

```
tree.BNPR.bespoke <- BNPR(tree_ultra_age)
plot_BNPR(tree.BNPR.bespoke, ylim = c(1E+0, 1E+9))
```

##### Ultrametric tree with age adjusted for increased mutation rate before birth on x axis

```
tree_phylo <- tree_ultra
age_adj = age + 3.3 # 55 (number of mutations in cord blood ie at birth) / 16.8 (number of mutations accumulated per year in adult life)
tree_phylo$edge.length <- tree_phylo$edge.length*age_adj
```

##### Run phylodyn putting on Nt confidence limits and time of birth

```
tree.BNPR.bespoke <- BNPR(tree_phylo)
plot_BNPR(tree.BNPR.bespoke, ylim = c(1E+0, 1E+9))
abline(h= 95000, col = "red")
abline(h= 48000, col = "grey")
abline(h= 234000, col = "grey")
abline(v= (age), col = "red")
```

### 6. Variant visualisation on the phylogeny

##### Add copy number and structural variants to details matrix

```
details <- rbind(details, info_nodes)
```

##### Annotate details matrix to allow plotting of driver mutations

```
details$protein_coding_mutation <- ifelse(details$Type %in% c("protein_coding:exon:CDS:substitution:codon_variant:non_synonymous_codon",

"protein_coding:exon:CDS:insertion:frameshift_variant",
                                                  "protein_coding:exon:CDS:substitution:codon_variant:stop_gained",
                                                  "protein_coding:exon:CDS:substitution:codon_variant:initiator_codon_change",
                                                  
"protein_coding:exon:CDS:deletion:frameshift_variant",
                                                  "protein_coding:exon:CDS:deletion:inframe_variant:inframe_codon_loss"),
                              "Protein coding mutation", "no")
details$splice_variant <- ifelse(grepl(pattern = "splice", x = details$Type),
                              "Splice variant", "no")
details$exon_UTR <- ifelse(grepl(pattern = "UTR", x = details$Type) & grepl(pattern = "exon", x = details$Type),
                                 "UTR exon", "no")
details$intron_UTR <- ifelse(grepl(pattern = "UTR", x = details$Type) & grepl(pattern = "intron", x = details$Type),
                           "UTR intron", "no")

details$protein_coding_chip_variant = ifelse(details$Gene %in% chip_drivers & details$protein_coding_mutation == "Protein coding mutation",
                                  "Protein coding variant in driver gene", "no")
details$dnds_variant_1500 = ifelse(details$Gene %in% dnds_genes_1500 & details$protein_coding_mutation == "Protein coding mutation",
                                  "Dnds gene", "no")

details$cancer_drivers = ifelse(details$Gene %in% cancer_drivers & details$protein_coding_mutation == "Protein coding mutation",
                                  "Cancer gene", "no")

details$top_genes = ifelse(details$Gene %in% top_genes & details$protein_coding_mutation == "Protein coding mutation",
                                  "Top gene", "no")


details$protein_coding_chip_variant_splice = ifelse(details$Gene %in% chip_drivers & details$splice_variant == "Splice variant",
                                  "Protein coding variant in driver gene", "no")

details$dnds_variant_1500_splice = ifelse(details$Gene %in% dnds_genes_1500 & details$splice_variant == "Splice variant",
                                  "Dnds gene", "no")

details$cancer_drivers_splice = ifelse(details$Gene %in% cancer_drivers & details$splice_variant == "Splice variant",
                                  "Cancer gene", "no")


details$top_genes_splice = ifelse(details$Gene %in% top_genes & details$splice_variant == "Splice variant",
                                  "Top gene", "no")

details$expanded_cancer_drivers = ifelse(details$Gene %in% cancer_drivers & details$protein_coding_mutation == "Protein coding mutation" & details$node > length(tree$tip.label),
                                  "Expanded cancer gene", "no")

details$oncogenic = ifelse(details$variant_ID %in% oncogenic_variants, #& details$node < length(tree$tip.label),
                                  "Oncogenic variant" , "no")

details$possible_oncogenic = ifelse(details$variant_ID %in% possible_oncogenic_variants, #& details$node < length(tree$tip.label),
                                  "Possible oncogenic variant" , "no")

details$expanded_cancer_drivers = ifelse(details$Gene %in% cancer_drivers & details$protein_coding_mutation == "Protein coding mutation" & details$node > length(tree$tip.label),
                                  "Expanded cancer gene", "no")
#To plot labels last
details$final = ifelse((details$variant_ID %in% oncogenic_variants) | (details$variant_ID %in% possible_oncogenic_variants) | (details$Mut_type == "CN"),
                                  "Final", "no")

details$SV = ifelse((details$Mut_type == "SV"),
                    "SV", "no")

details$PB = ifelse((details$Mut_type == "PB_HSC"),
                    "PB HSC", "no")

details$clade = ifelse((details$Mut_type == "Expanded_clade" ),
                    "Expanded clade", "no")
```

##### Save table of coding mutations in driver genes

```
driver <- details[details$protein_coding_chip_variant == "Protein coding variant in driver gene" | details$protein_coding_chip_variant_splice == "Protein coding variant in driver gene",]
driver <- driver[,1:15]
driver$Donor <- ID
write.csv(driver, paste0("~/Documents/PhD/Sequencing_results/DNA_seq/",ID,"/",Iteration,"/trees/vaf_cut/driver_info/",Iteration,"_driver_genes.csv"),row.names = FALSE)
```

##### Save table of coding mutations in cancer genes

```
cancer <- details[details$cancer_drivers == "Cancer gene" | details$cancer_drivers_splice == "Cancer gene",]
cancer <- cancer[,1:15]
cancer$Donor <- ID
write.csv(cancer, paste0("~/Documents/PhD/Sequencing_results/DNA_seq/",ID,"/",Iteration,"/trees/vaf_cut/driver_info/",Iteration,"_cancer_genes.csv"),row.names = FALSE)
```

##### Save table of coding mutations in top genes

```
top <- details[details$top_genes == "Top gene" | details$top_genes_splice == "Top gene",]
top <- top[,1:15]
top$Donor <- ID
write.csv(top, paste0("~/Documents/PhD/Sequencing_results/DNA_seq/",ID,"/",Iteration,"/trees/vaf_cut/driver_info/",Iteration,"_top_genes.csv"), row.names = FALSE)
```

##### Save table of coding mutations in top dnds hits

```
top <- details[details$dnds_variant_1500 == "Dnds gene" | details$dnds_variant_1500_splice == "Dnds gene",]
top <- top[,1:15]
top$Donor <- ID
write.csv(top, paste0("~/Documents/PhD/Sequencing_results/DNA_seq/",ID,"/",Iteration,"/trees/vaf_cut/driver_info/",Iteration,"_dnds_genes.csv"), row.names = FALSE)
```

##### Plot tree with drivers

```
tree_em =plot_tree_em(tree_ultra_age, cex.label = 0)
```

```
## scale= 1
```

```
plot_tree_labels(tree_em,
                 details = details,
                 type="label",
                 query.field = "protein_coding_chip_variant",
                 data.frame(value="Protein coding variant in driver gene",col="red",pch = 17,stringsAsFactors = FALSE),
                 label.field = "variant_ID",
                 cex.label = 2.0)
```

##### Labelling expanded clades with non-synonymous mutations

```
node_height_cutoff=0
prop_cutoff=0.005

min_total_samples=round(prop_cutoff*length(tree_ultra_mut$tip.label))

clonal_expansion_nodes=sapply(1:nrow(tree_ultra_mut$edge), function(i) {
  node=tree_ultra_mut$edge[i,2]
  if(node <= length(tree_ultra_mut$tip.label)) {
    result_1=FALSE
  } else {
    samples = extract.clade(node=node,tree_ultra_mut)$tip.label
    result_1= length(samples)>=min_total_samples
  }
  result_2=nodeHeights(tree_ultra_mut)[tree_ultra_mut$edge[,2]==node,2] > node_height_cutoff
  if(result_1 & result_2) {
    ancestors=get_ancestral_nodes(node,tree_ultra_mut$edge)
    ancestors = ancestors[ancestors != node]
    result_3=TRUE
    #result_3=all(nodeHeights(tree)[tree$edge[,2] %in% ancestors,2] < node_height_cutoff) #Can add this in if want to exclude downstream nodes from initial expansion
    if(result_3) {
      return(node)
    } else {
      return(NA)
    }
  } else {
    return(NA)
  }
})

clonal_expansion_nodes<-clonal_expansion_nodes[!is.na(clonal_expansion_nodes)]
```

##### Plot tree showing non-synonymous mutations on shared branches

```
#Plot the selected branches on the tree
tree=plot_tree(tree_ultra_mut,cex.label = 0)
```

```
## scale= 200
```

```
invisible(add_annotation(tree,
               details,
               matrices = NULL,
               annot_function = highlight_nodes,
               nodes=clonal_expansion_nodes
))
details$coding_expansion_node=ifelse(details$protein_coding_mutation=="Protein coding mutation" & details$node %in% clonal_expansion_nodes,1,0)
plot_tree_labels(tree,
                 details = details,
                 type = "label",
                 query.field = "coding_expansion_node",
                 data.frame(value="1",col="black",lwd = 2,stringsAsFactors = FALSE),
                 label.field = "variant_ID",
                 cex.label = 1.5)
```

##### Plot tree showing variants in top 1500 dNdS hits on shared branches

```
#Plot the selected branches on the tree
tree=plot_tree(tree_ultra_mut,cex.label = 0)
```

```
## scale= 200
```

```
invisible(add_annotation(tree,
               details,
               matrices = NULL,
               annot_function = highlight_nodes,
               nodes=clonal_expansion_nodes
))
details$coding_expansion_node=ifelse(details$dnds_variant_1500 =="Dnds gene" & details$node %in% clonal_expansion_nodes,1,0)
plot_tree_labels(tree,
                 details = details,
                 type = "label",
                 query.field = "coding_expansion_node",
                 data.frame(value="1",col="black",lwd = 2,stringsAsFactors = FALSE),
                 label.field = "variant_ID",
                 cex.label = 1.5)
```

##### Plot tree showing mutations in cancer genes on shared branches

```
#Plot the selected branches on the tree
tree=plot_tree(tree_ultra_mut,cex.label = 0)
```

```
## scale= 200
```

```
invisible(add_annotation(tree,
               details,
               matrices = NULL,
               annot_function = highlight_nodes,
               nodes=clonal_expansion_nodes
))
```

```
details$coding_expansion_node=ifelse(details$cancer_drivers == "Cancer gene" & details$node %in% clonal_expansion_nodes,1,0)
plot_tree_labels(tree,
                 details = details,
                 type = "label",
                 query.field = "coding_expansion_node",
                 data.frame(value="1",col="black",lwd = 2,stringsAsFactors = FALSE),
                 label.field = "variant_ID",
                 cex.label = 1.5)
```

### 7. Final phylogenies used in figures

##### Phylogeny with heatmap information below used in main figures

```
  tree= tree_ultra_age
  ##Set up heat map
  nodes_driver= details$node[(details$variant_ID %in% oncogenic_variants) | (details$variant_ID %in% possible_oncogenic_variants)]
  nodes_clade= expanded_clades$node
  
  labels=c("Known Driver","Expanded Clade")
  hm=matrix("white",ncol=length(tree$tip.label),nrow=2)
  colnames(hm)=tree$tip.label
  rownames(hm)=labels
  ## In this case we populate the colours based on the membership of the clade (could be signature contributions or whatever).
  ## Could also maybe use rownames as labels (on the LHS of the plot?).
  tips_driver = NULL
  for(i in 1:length(nodes_driver)){
  tips_driver <- c(tips_driver, get_samples_in_clade(nodes_driver[i],tree))
  }
  hm[1,match(tips_driver,tree$tip.label)]="red"
  
  tips_clade = NULL
  for(i in 1:length(nodes_clade)){
  tips_clade <- c(tips_clade, get_samples_in_clade(nodes_clade[i],tree))
  }
  hm[2,match(tips_clade,tree$tip.label)]="blue"

  
  ## plot basic tree (need to keep the returned tree)
  tree =plot_tree(tree_ultra_age, cex.label = 5, cex.terminal.dots = 0, vspace.reserve = 0.2)
```

```
## scale= 10
```

```
plot_tree_lines(tree,
                 details = details,
                 type = "line",
                 query.field = "clade",
                  col = "blue",
                 data.frame(value="Expanded clade",stringsAsFactors = FALSE))

plot_tree_lines(tree,
                 details = details,
                 type = "line",
                 query.field = "oncogenic",
                 col = "red",
                 data.frame(value="Oncogenic variant", stringsAsFactors = FALSE))

plot_tree_lines(tree,
                 details = details,
                 type = "line",
                 query.field = "possible_oncogenic",
                  col = "orange",
                 data.frame(value="Possible oncogenic variant",stringsAsFactors = FALSE))

plot_tree_lines(tree,
                 details = details,
                 type = "line",
                 query.field = "Mut_type",
                 col = "blue",
                 lty = 2,
                 data.frame(value="CN",stringsAsFactors = FALSE))

plot_tree_labels(tree,
                 details = details,
                 type = "label",
                 query.field = "final",
                 data.frame(value="Final",stringsAsFactors = FALSE, col = 0, pch = 17),
                 label.field = "variant_ID",
                 cex.label = 4.0)
  tree=add_heatmap(tree,heatmap=hm,cex.label = 5)
```

##### Phylogeny with structural variant information

```
tree= tree_ultra_age
##Set up heat map
  nodes_SV= info_nodes$node[info_nodes$Mut_type == "SV"]
  labels="Structural variant"
  hm=matrix("white",ncol=length(tree$tip.label),nrow=1)
  colnames(hm)=tree$tip.label
  rownames(hm)=labels
  ## In this case we populate the colours based on the membership of the clade (could be signature contributions or whatever).
  ## Could also maybe use rownames as labels (on the LHS of the plot?).
  tips_SV = NULL
  for(i in 1:length(nodes_SV)){
  tips_SV <- c(tips_SV, get_samples_in_clade(nodes_SV[i],tree))
  }
  hm[1,match(tips_SV,tree$tip.label)]="red"

  tree =plot_tree(tree_ultra_age, cex.label = 5, cex.terminal.dots = 0)
```

```
## scale= 10
```

```
plot_tree_lines(tree,
                 details = details,
                 type = "line",
                 query.field = "SV",
                 col = "red",
                 data.frame(value="SV", stringsAsFactors = FALSE))
plot_tree_labels(tree,
                 details = details,
                 type = "label",
                 query.field = "SV",
                 data.frame(value="SV",stringsAsFactors = FALSE, col = 0, pch = 17),
                 label.field = "variant_ID",
                 cex.label = 4.0)
tree=add_heatmap(tree,heatmap=hm,cex.label = 5)
```
