# Supplementary material for: Clonal dynamics of haematopoiesis across the human lifespan
Source: Nature. 2022 Jun 1;606(7913):343–50. doi: 10.1038/s41586-022-04786-y (PMC9177428; doi:10.1038/s41586-022-04786-y)
Supplement: Supplementary file 4 — HTMLs of notebooks outlining key statistical analyses presented in the manuscript, including analysis of phylogenetic trees. [file 41586_2022_4786_MOESM4_ESM.zip › Supplementary_code/Other_analysis/estimating_driver_acquisition_rate.html]

Estimating driver mutation acquisition rate


# Estimating driver mutation acquisition rate

#### Emily Mitchell

Script to estimate driver mutation acquisition rate based on the rate of accumulation of non-synonymous mutations (as observed in our data) and the dNdS results which estimate that 1 in 12 to 1 in 34 non-synonymous mutations in the dataset are drivers.

Linear regression estimate of non-synonymous driver accumulation rate per year in HSC/MPPs (using only samples with mean depth > 14) = 0.12 (0.49-0.55)

```
driver.sim=vector(mode = "list",length=500000)
for (i in 1:500000) {
non_syn_mut_per_year <- runif(1,0.11,0.13)
fraction_drivers <- runif(1,0.029,0.083) # 0.029 = 1 in 34 and 0.083 = 1 in 12
driver_rate <- non_syn_mut_per_year*fraction_drivers
driver.sim[i] <- driver_rate
}
```

```
driver.sim <- as.numeric(driver.sim)
driver.sim_95CI=quantile(driver.sim,probs=c(0.025,0.975))
driver.sim_95CI
```

```
##        2.5%       97.5% 
## 0.003615412 0.010039064
```

```
mean(driver.sim)
```

```
## [1] 0.006723907
```
