# Supplementary material for: Clonal dynamics of haematopoiesis across the human lifespan
Source: Nature. 2022 Jun 1;606(7913):343–50. doi: 10.1038/s41586-022-04786-y (PMC9177428; doi:10.1038/s41586-022-04786-y)
Supplement: Supplementary file 4 — HTMLs of notebooks outlining key statistical analyses presented in the manuscript, including analysis of phylogenetic trees. [file 41586_2022_4786_MOESM4_ESM.zip › Supplementary_code/Other_analysis/shannon_diversity.html]

Shannon Diversity Index Calculations


# Shannon Diversity Index Calculations

#### Emily Mitchell

### Summary

This script calculates a Shannon Diversity Index for each phylogeny and plots the results.

1. Open packages
2. Calculate Shannon Diversity Indices
3. Put results into a matrix
4. Plot results

##### Load packages

```
suppressMessages(library(ggtree))
```

```
## Warning: package 'ggtree' was built under R version 4.0.3
```

```
suppressMessages(library(ape))
suppressMessages(library(stringr))
suppressMessages(library(tidyr))
suppressMessages(library(phytools))
suppressMessages(library(ggplot2))
suppressMessages(library(phangorn))
suppressMessages(library(RColorBrewer))
suppressMessages(library(vegan))
```

### 1. Source functions

```
function_files=list.files('~/Documents/Bioinformatics/CGP/Functions/', full.names = TRUE, pattern = ".R")
suppressMessages(sapply(function_files, source))
```

```
##         /Users/em16/Documents/Bioinformatics/CGP/Functions//filters_parallel_functions.R
## value   ?                                                                               
## visible FALSE                                                                           
##         /Users/em16/Documents/Bioinformatics/CGP/Functions//targeted_analysis_functions.R
## value   ?                                                                                
## visible FALSE                                                                            
##         /Users/em16/Documents/Bioinformatics/CGP/Functions//tree_functions.R
## value   ?                                                                   
## visible FALSE
```

### 2. Calculating Shannon Diversity Indices for each phylogeny

##### KX001 Shannon Diversity Index

```
ID = "KX001" #Edit
Iteration = "KX001_4" #Edit
Run_ID = "KX001_4_01" #Edit
filtering_ID = "standard_rho01" #Edit

tree_file_path = paste0("~/Documents/PhD/Sequencing_results/DNA_seq/",ID,"/",Iteration,"/trees/vaf_cut/output/tree_", Run_ID,"_",filtering_ID, ".tree")
tree <- read.tree(tree_file_path)

time_point = 100
nodeheights <- nodeHeights(tree)
clades_post_cutoff=tree$edge[,2][nodeheights[,1] < time_point & !nodeheights[,2] < time_point]
clade_sizes=sapply(clades_post_cutoff,function(node) {length(getTips(tree,node))})
input=matrix(NA,nrow=length(clades_post_cutoff),ncol=2)
input[,1] <- clades_post_cutoff
input[,2] <- clade_sizes
shannon <- diversity(input, index = "shannon", MARGIN = 1, base = exp(1))
KX001_shannon_index <- sum(shannon)
KX001_shannon_index
```

```
## [1] 22.04154
```

##### KX002 Shannon Diversity Index

```
ID = "KX002" #Edit
Iteration = "KX002_2" #Edit
Run_ID = "KX002_2_01" #Edit
filtering_ID = "standard_rho01" #Edit

tree_file_path = paste0("~/Documents/PhD/Sequencing_results/DNA_seq/",ID,"/",Iteration,"/trees/vaf_cut/output/tree_", Run_ID,"_",filtering_ID, ".tree")
tree <- read.tree(tree_file_path)

time_point = 100
nodeheights <- nodeHeights(tree)
clades_post_cutoff=tree$edge[,2][nodeheights[,1] < time_point & !nodeheights[,2] < time_point]
clade_sizes=sapply(clades_post_cutoff,function(node) {length(getTips(tree,node))})
input=matrix(NA,nrow=length(clades_post_cutoff),ncol=2)
input[,1] <- clades_post_cutoff
input[,2] <- clade_sizes
shannon <- diversity(input, index = "shannon", MARGIN = 1, base = exp(1))
KX002_shannon_index <- sum(shannon)
KX002_shannon_index
```

```
## [1] 22.09882
```

##### SX001 Shannon Diversity Index

```
ID = "SX001" #Edit
Iteration = "SX001_5" #Edit
Run_ID = "SX001_5_01" #Edit
filtering_ID = "standard_rho01" #Edit

tree_file_path = paste0("~/Documents/PhD/Sequencing_results/DNA_seq/",ID,"/",Iteration,"/trees/vaf_cut/output/tree_", Run_ID,"_",filtering_ID, ".tree")
tree <- read.tree(tree_file_path)

time_point = 100
nodeheights <- nodeHeights(tree)
clades_post_cutoff=tree$edge[,2][nodeheights[,1] < time_point & !nodeheights[,2] < time_point]
clade_sizes=sapply(clades_post_cutoff,function(node) {length(getTips(tree,node))})
input=matrix(NA,nrow=length(clades_post_cutoff),ncol=2)
input[,1] <- clades_post_cutoff
input[,2] <- clade_sizes
shannon <- diversity(input, index = "shannon", MARGIN = 1, base = exp(1))
SX001_shannon_index <- sum(shannon)
SX001_shannon_index
```

```
## [1] 21.28248
```

##### AX001 Shannon Diversity Index

```
ID = "AX001" #Edit
Iteration = "AX001_4" #Edit
Run_ID = "AX001_4_01" #Edit
filtering_ID = "standard_rho01" #Edit

tree_file_path = paste0("~/Documents/PhD/Sequencing_results/DNA_seq/",ID,"/",Iteration,"/trees/vaf_cut/output/tree_", Run_ID,"_",filtering_ID, ".tree")
tree <- read.tree(tree_file_path)

time_point = 100
nodeheights <- nodeHeights(tree)
clades_post_cutoff=tree$edge[,2][nodeheights[,1] < time_point & !nodeheights[,2] < time_point]
clade_sizes=sapply(clades_post_cutoff,function(node) {length(getTips(tree,node))})
input=matrix(NA,nrow=length(clades_post_cutoff),ncol=2)
input[,1] <- clades_post_cutoff
input[,2] <- clade_sizes
shannon <- diversity(input, index = "shannon", MARGIN = 1, base = exp(1))
AX001_shannon_index <- sum(shannon)
AX001_shannon_index
```

```
## [1] 20.91973
```

##### KX007 Shannon Diversity Index

```
ID = "KX007" #Edit
Iteration = "KX007_2" #Edit
Run_ID = "KX007_2_01" #Edit
filtering_ID = "standard_rho01" #Edit

tree_file_path = paste0("~/Documents/PhD/Sequencing_results/DNA_seq/",ID,"/",Iteration,"/trees/vaf_cut/output/tree_", Run_ID,"_",filtering_ID, ".tree")
tree <- read.tree(tree_file_path)

time_point = 100
nodeheights <- nodeHeights(tree)
clades_post_cutoff=tree$edge[,2][nodeheights[,1] < time_point & !nodeheights[,2] < time_point]
clade_sizes=sapply(clades_post_cutoff,function(node) {length(getTips(tree,node))})
input=matrix(NA,nrow=length(clades_post_cutoff),ncol=2)
input[,1] <- clades_post_cutoff
input[,2] <- clade_sizes
shannon <- diversity(input, index = "shannon", MARGIN = 1, base = exp(1))
KX007_shannon_index <- sum(shannon)
KX007_shannon_index
```

```
## [1] 11.02397
```

##### KX008 Shannon Diversity Index

```
ID = "KX008" #Edit
Iteration = "KX008_2" #Edit
Run_ID = "KX008_2_01" #Edit
filtering_ID = "standard_rho01" #Edit

tree_file_path = paste0("~/Documents/PhD/Sequencing_results/DNA_seq/",ID,"/",Iteration,"/trees/vaf_cut/output/tree_", Run_ID,"_",filtering_ID, ".tree")
tree <- read.tree(tree_file_path)

time_point = 100
nodeheights <- nodeHeights(tree)
clades_post_cutoff=tree$edge[,2][nodeheights[,1] < time_point & !nodeheights[,2] < time_point]
clade_sizes=sapply(clades_post_cutoff,function(node) {length(getTips(tree,node))})
input=matrix(NA,nrow=length(clades_post_cutoff),ncol=2)
input[,1] <- clades_post_cutoff
input[,2] <- clade_sizes
shannon <- diversity(input, index = "shannon", MARGIN = 1, base = exp(1))
KX008_shannon_index <- sum(shannon)
KX008_shannon_index
```

```
## [1] 13.12317
```

##### KX004 Shannon Diversity Index

```
ID = "KX004" #Edit
Iteration = "KX004_4" #Edit
Run_ID = "KX004_4_01" #Edit
filtering_ID = "standard_rho01" #Edit

tree_file_path = paste0("~/Documents/PhD/Sequencing_results/DNA_seq/",ID,"/",Iteration,"/trees/vaf_cut/output/tree_", Run_ID,"_",filtering_ID, ".tree")
tree <- read.tree(tree_file_path)

time_point = 100
nodeheights <- nodeHeights(tree)
clades_post_cutoff=tree$edge[,2][nodeheights[,1] < time_point & !nodeheights[,2] < time_point]
clade_sizes=sapply(clades_post_cutoff,function(node) {length(getTips(tree,node))})
input=matrix(NA,nrow=length(clades_post_cutoff),ncol=2)
input[,1] <- clades_post_cutoff
input[,2] <- clade_sizes
shannon <- diversity(input, index = "shannon", MARGIN = 1, base = exp(1))
KX004_shannon_index <- sum(shannon)
KX004_shannon_index
```

```
## [1] 4.957865
```

##### KX003 Shannon Diversity Index

```
ID = "KX003" #Edit
Iteration = "KX003_5" #Edit
Run_ID = "KX003_5_01" #Edit
filtering_ID = "standard_rho01" #Edit

tree_file_path = paste0("~/Documents/PhD/Sequencing_results/DNA_seq/",ID,"/",Iteration,"/trees/vaf_cut/output/tree_", Run_ID,"_",filtering_ID, ".tree")
tree <- read.tree(tree_file_path)

time_point = 100
nodeheights <- nodeHeights(tree)
clades_post_cutoff=tree$edge[,2][nodeheights[,1] < time_point & !nodeheights[,2] < time_point]
clade_sizes=sapply(clades_post_cutoff,function(node) {length(getTips(tree,node))})
input=matrix(NA,nrow=length(clades_post_cutoff),ncol=2)
input[,1] <- clades_post_cutoff
input[,2] <- clade_sizes
shannon <- diversity(input, index = "shannon", MARGIN = 1, base = exp(1))
KX003_shannon_index <- sum(shannon)
KX003_shannon_index
```

```
## [1] 6.304215
```

### 3. Put results into a matrix

```
data <- matrix(NA, nrow = 8, ncol = 3)
shannon_indices <- c(KX001_shannon_index, KX002_shannon_index, SX001_shannon_index, AX001_shannon_index, KX007_shannon_index, KX008_shannon_index, KX004_shannon_index, KX003_shannon_index)
data[,3] <- shannon_indices
data[,1] <- c("KX001", "KX002", "SX001", "AX001", "KX007", "KX008", "KX004", "KX003")
data[,2] <- c(29,38,48,63,75,76,77,81)
colnames(data) <- c("donor_id", "age", "shannon_index")
data <- as.data.frame(data)
data$age <- as.numeric(data$age)
data$shannon_index <- as.numeric(data$shannon_index)
data$donor_id <- factor(data$donor_id, levels = c("KX001", "KX002","SX001","AX001", "KX007","KX008","KX004","KX003"))
```

### 4. Plot results

```
ggplot(data)+
  theme_bw()+
  labs(x="Age (years)", y="Shannon Diversity Index")+
  xlim(0,100)+
  ylim(0,25)+
  theme(text=element_text(size=14))+
  scale_color_manual(values = brewer.pal(10,"Paired")[c(3,4,7,8,5,6,9,10)])+
  geom_point(aes(x = age, y = shannon_index, col = donor_id), size = 3)
```
