# Supplementary material for: Clonal dynamics of haematopoiesis across the human lifespan
Source: Nature. 2022 Jun 1;606(7913):343–50. doi: 10.1038/s41586-022-04786-y (PMC9177428; doi:10.1038/s41586-022-04786-y)
Supplement: Supplementary file 4 — HTMLs of notebooks outlining key statistical analyses presented in the manuscript, including analysis of phylogenetic trees. [file 41586_2022_4786_MOESM4_ESM.zip › Supplementary_code/Other_analysis/Summary_CN_SV_final.html]

Copy number and structural variant analysis


# Copy number and structural variant analysis

#### Emily Mitchell

### Summary

This script performs visualisation of copy number abberations (CNAs) and structural variants (SVs).

##### Open libraries

```
suppressMessages(library(dplyr))
suppressMessages(library(ggplot2))
suppressMessages(library(RColorBrewer))
```

```
setwd("~/Documents/PhD/Sequencing_results/DNA_seq/XX_summary/telomere_mutation")
```

### 1. Load matrices of copy number / structural variant type per sample number for each donor

```
SV_summ <- read.csv("~/Documents/PhD/Sequencing_results/DNA_seq/XX_summary/telomere_mutation/data/SV_summ.csv", stringsAsFactors = F, row.names = 1)
CN_summ <- read.csv("~/Documents/PhD/Sequencing_results/DNA_seq/XX_summary/telomere_mutation/data/CN_summ.csv", stringsAsFactors = F, row.names = 1)
```

##### Cut to rows of interest

```
SV_summ <- as.matrix(SV_summ[6:9,],)
CN_summ <- as.matrix(CN_summ[6:9,])
```

### 2. Structural variant barplot

```
barplot(SV_summ, beside = FALSE, names.arg = c("CB001","CB002","KX001","KX002","SX001","AX001","KX007","KX008","KX004","KX003"), col = brewer.pal(10,"PRGn")[c(2,4,8,10)], ylim = c(0,0.04), cex.names = 0.5, legend.text = T, args.legend = list(x= "topleft"), main = "Number of structural variant events \nexpressed as a fraction of total sample number")
```

### 3. Autosomal CNA barplot

```
barplot(CN_summ[1:2,], beside = FALSE,names.arg = c("CB001","CB002","KX001","KX002","SX001","AX001","KX007","KX008","KX004","KX003"), col = brewer.pal(10,"GnBu")[c(9,7)], ylim = c(0,0.008), cex.names = 0.8, legend.text = TRUE,args.legend = list(x= "topleft"), main = "Number of autosomal CNA events \nexpressed as a fraction of total sample number")
```

```
## Warning in brewer.pal(10, "GnBu"): n too large, allowed maximum for palette GnBu is 9
## Returning the palette you asked for with that many colors
```

### 4. Barplot of Y loss in male individuals

```
barplot(CN_summ[3:4,c(3:7,10)], beside = FALSE,col = brewer.pal(10,"GnBu")[c(9,7)], ylim = c(0,0.2), legend.text = TRUE, args.legend = list(x= "topleft"), cex.names = 1, main = "Number of independent Y loss events \nexpressed as a fraction of total sample number")
```

```
## Warning in brewer.pal(10, "GnBu"): n too large, allowed maximum for palette GnBu is 9
## Returning the palette you asked for with that many colors
```
