# Supplementary material for: Clonal dynamics of haematopoiesis across the human lifespan
Source: Nature. 2022 Jun 1;606(7913):343–50. doi: 10.1038/s41586-022-04786-y (PMC9177428; doi:10.1038/s41586-022-04786-y)
Supplement: Supplementary file 4 — HTMLs of notebooks outlining key statistical analyses presented in the manuscript, including analysis of phylogenetic trees. [file 41586_2022_4786_MOESM4_ESM.zip › Supplementary_code/Other_analysis/all_DNDScv_final.html]

DNDScv for haematopoietic colony samples


# DNDScv for haematopoietic colony samples

#### Emily Mitchell

### Summary

This script performs dN/dS analysis on different subsets of the mutation dataset. The whole dataset includes unique mutations (SNVs and small indels) from all 10 donors. It is also broken down into ‘cord blood’ (CB001 and CB002), ‘young’ (KX001, KX002, SX001, AX001) and ‘old’ datasets (KX007, KX008, KX004, KX003). We used the R packed dndscv to perform the analysis.

1. Analysis for all old individuals - all variants: all\_old
2. Analysis all young individuals - all variants: all\_young
3. Analysis all cord blood - all variants: all\_CB
4. Analysis all individuals and all variants
5. Analysis for all old individuals - private variants: private\_old
6. Analysis for all old individuals - shared variants: shared\_old

##### Open libraries

```
suppressMessages(library(stringr))
suppressMessages(library(ape))
suppressMessages(library(seqinr))
suppressMessages(library(ggtree))
```

```
## Warning: package 'ggtree' was built under R version 4.0.3
```

```
suppressMessages(library(tidyr))
suppressMessages(library(dplyr))
suppressMessages(library(ggplot2))
suppressMessages(library(dndscv))
```

```
## Warning: replacing previous import 'Biostrings::translate' by
## 'seqinr::translate' when loading 'dndscv'
```

```
suppressMessages(library(phytools))
suppressMessages(library(ggplot2))
suppressMessages(library(forestplot))
```

##### Set file paths and working directory

```
ID = "All" #Edit
Iteration = "All" #Edit
```

##### Load files

```
all_old <- read.table("~/Documents/PhD/Sequencing_results/DNA_seq/XX_Summary/dNdS/final/All_dnds_mutset_old_all.txt",stringsAsFactors = F, header = T)

all_young <- read.table( "~/Documents/PhD/Sequencing_results/DNA_seq/XX_Summary/dNdS/final/All_dnds_mutset_young_all.txt",stringsAsFactors = F, header = T)

all_CB <- read.table("~/Documents/PhD/Sequencing_results/DNA_seq/XX_Summary/dNdS/final/All_dnds_mutset_CB_all.txt",  stringsAsFactors = F, header = T)

all <- read.table("~/Documents/PhD/Sequencing_results/DNA_seq/XX_Summary/dNdS/final/All_dnds_all.txt", stringsAsFactors = F, header = T)
```

##### Load files - shared muts

```
shared_old <- read.table("~/Documents/PhD/Sequencing_results/DNA_seq/XX_Summary/dNdS/final/All_dnds_mutset_old_shared.txt",  stringsAsFactors = F, header = T)


shared_young <- read.table("~/Documents/PhD/Sequencing_results/DNA_seq/XX_Summary/dNdS/final/All_dnds_mutset_young_shared.txt", stringsAsFactors = F, header = T)


shared_CB <- read.table( "~/Documents/PhD/Sequencing_results/DNA_seq/XX_Summary/dNdS/final/All_dnds_mutset_CB_shared.txt",  stringsAsFactors = F, header = T)

shared <- read.table("~/Documents/PhD/Sequencing_results/DNA_seq/XX_Summary/dNdS/final/All_dnds_shared.txt", stringsAsFactors = F, header = T)
```

##### Load files - private muts

```
private_old <- read.table("~/Documents/PhD/Sequencing_results/DNA_seq/XX_Summary/dNdS/final/All_dnds_mutset_old_private.txt",  stringsAsFactors = F, header = T)

private_young <- read.table("~/Documents/PhD/Sequencing_results/DNA_seq/XX_Summary/dNdS/final/All_dnds_mutset_young_private.txt",  stringsAsFactors = F, header = T)

private_CB <- read.table("~/Documents/PhD/Sequencing_results/DNA_seq/XX_Summary/dNdS/final/All_dnds_mutset_CB_private.txt",  stringsAsFactors = F, header = T)

private <- read.table("~/Documents/PhD/Sequencing_results/DNA_seq/XX_Summary/dNdS/final/All_dnds_private.txt", stringsAsFactors = F, header = T)
```

##### Import the myeloid malignancy panel list

```
chip_drivers = read.csv("~/Documents/Bioinformatics/CGP/Filtering/chip_drivers.csv", stringsAsFactors = FALSE, header = FALSE)
chip_drivers <- chip_drivers$V1
```

##### Import cancer gene list

```
cancer_drivers = read.csv("~/Documents/Bioinformatics/CGP/Filtering/martincorena2017_369drivers.csv", stringsAsFactors = FALSE, header = FALSE)
cancer_drivers <- cancer_drivers$V1
```

### 1. Analysis for all old individuals - all variants: all\_old

##### a) Global DNDS all\_old

```
#Run dndscv
dndscvout=dndscv(all_old,outp=1,max_muts_per_gene_per_sample = 100, max_coding_muts_per_sample = 400000)
```

```
## [1] Loading the environment...
```

```
## [2] Annotating the mutations...
```

```
## Warning in dndscv(all_old, outp = 1, max_muts_per_gene_per_sample = 100, :
## Mutations observed in contiguous sites within a sample. Please annotate or
## remove dinucleotide or complex substitutions for best results.
```

```
## Warning in dndscv(all_old, outp = 1, max_muts_per_gene_per_sample = 100, :
## Same mutations observed in different sampleIDs. Please verify that these are
## independent events and remove duplicates otherwise.
```

```
##     68% ...
```

```
## [3] Estimating global rates...
```

```
dndscvout$globaldnds
```

```
##      name      mle     cilow   cihigh
## wmis wmis 1.051490 1.0120589 1.092458
## wnon wnon 1.036599 0.9360658 1.147929
## wspl wspl 1.137474 1.0160294 1.273435
## wtru wtru 1.079523 0.9980574 1.167639
## wall wall 1.053140 1.0141525 1.093625
```

##### b) Per gene analysis all\_old

```
dndscvout=dndscv(all_old, max_muts_per_gene_per_sample = 100, max_coding_muts_per_sample = 1000000)
```

```
## [1] Loading the environment...
```

```
## [2] Annotating the mutations...
```

```
## Warning in dndscv(all_old, max_muts_per_gene_per_sample = 100,
## max_coding_muts_per_sample = 1e+06): Mutations observed in contiguous sites
## within a sample. Please annotate or remove dinucleotide or complex substitutions
## for best results.
```

```
## Warning in dndscv(all_old, max_muts_per_gene_per_sample = 100,
## max_coding_muts_per_sample = 1e+06): Same mutations observed in different
## sampleIDs. Please verify that these are independent events and remove duplicates
## otherwise.
```

```
##     68% ...
```

```
## [3] Estimating global rates...
```

```
## [4] Running dNdSloc...
```

```
## [5] Running dNdScv...
```

```
##     Regression model for substitutions (theta = 7.07).
```

```
##     Regression model for indels (theta = 1.13)
```

```
sel_cv = dndscvout$sel_cv
print(head(sel_cv, n= 30L), digits = 3)
```

```
##            gene_name n_syn n_mis n_non n_spl n_ind wmis_cv wnon_cv wspl_cv
## 5236          DNMT3A     1    15     1     3     2   30.59    90.0    90.0
## 19644         ZNF318     0     2     4     1     5    2.41    90.2    90.2
## 1467           ARID2     1     2     2     2     0    2.34    55.1    55.1
## 3490           CDK13     0     1     1     0     3    1.66    23.0    23.0
## 16680          SRCAP     0     5     2     1     1    3.25    29.1    29.1
## 17380           TET2     1     5     2     0     1    4.71    26.5    26.5
## 15166          RTDR1     0     5     0     0     0   22.47     0.0     0.0
## 3037            CBFB     0     2     0     0     1   26.01     0.0     0.0
## 4382         CSNK1A1     0     3     0     1     0   24.02    66.6    66.6
## 4537          CTNNA3     0     3     0     0     2    3.55     0.0     0.0
## 9729           LPHN2     0    10     1     0     0    7.73     7.3     7.3
## 9038        KIAA1919     0     3     1     0     0   17.21   100.7   100.7
## 10918         MUM1L1     1     0     0     0     2    0.00     0.0     0.0
## 16589         SPHKAP     0     6     0     0     2    3.20     0.0     0.0
## 4994            DFFA     0     0     0     1     1    0.00   112.0   112.0
## 8707           ITPKA     0     0     0     0     2    0.00     0.0     0.0
## 10071           MANF     1     3     0     0     0   40.11     0.0     0.0
## 2160          BTN3A2     0     0     0     1     1    0.00    99.4    99.4
## 14637         RNF165     0     5     0     0     0   15.19     0.0     0.0
## 18311          TRUB2     0     2     0     0     1   14.87     0.0     0.0
## 18162          TREM1     1     0     2     0     0    0.00   119.3   119.3
## 16632          SPPL3     0     4     0     0     0   21.59     0.0     0.0
## 10172           MAS1     0     0     2     0     0    0.00    98.6    98.6
## 16262           SMC3     0     2     0     0     2    4.97     0.0     0.0
## 7014          GDF5OS     0     0     2     0     0    0.00    96.0    96.0
## 12361      OVCH1-AS1     0     4     0     0     0   20.04     0.0     0.0
## 18765           UROS     0     0     1     1     0    0.00   104.5   104.5
## 7876       HIST1H2AC     0     3     0     0     0   32.45     0.0     0.0
## 504           ACSM2B     0     4     2     0     0    6.50    24.6    24.6
## 13888 PTGES3L-AARSD1     0     0     1     1     0    0.00    79.3    79.3
##       wind_cv  pmis_cv ptrunc_cv pallsubs_cv  pind_cv  qmis_cv qtrunc_cv
## 5236    40.69 1.02e-13  3.07e-07    1.11e-16 2.08e-03 2.05e-09  0.003080
## 19644   53.26 3.20e-01  1.61e-08    1.10e-07 3.49e-06 8.20e-01  0.000324
## 1467     0.00 3.29e-01  2.24e-06    1.21e-05 1.00e+00 8.20e-01  0.015017
## 3490    48.92 6.57e-01  4.00e-02    1.15e-01 1.70e-04 8.20e-01  0.971820
## 16680    6.52 6.17e-02  2.73e-04    5.26e-04 1.34e-01 8.20e-01  0.608634
## 17380   14.35 1.47e-02  3.04e-03    1.21e-03 6.54e-02 8.20e-01  0.971820
## 15166    0.00 1.60e-05  8.68e-01    8.86e-05 1.00e+00 1.61e-01  0.971820
## 3037   128.66 3.29e-03  8.85e-01    1.31e-02 7.72e-03 8.20e-01  0.971820
## 4382     0.00 4.89e-04  1.22e-02    1.41e-04 1.00e+00 7.83e-01  0.971820
## 4537    74.58 1.02e-01  6.83e-01    2.35e-01 6.45e-04 8.20e-01  0.971820
## 9729     0.00 6.96e-05  1.44e-01    2.24e-04 1.00e+00 3.62e-01  0.971820
## 9038     0.00 1.33e-03  7.75e-03    2.36e-04 1.00e+00 8.20e-01  0.971820
## 10918   68.38 1.79e-01  7.18e-01    3.82e-01 7.64e-04 8.20e-01  0.971820
## 16589   36.92 4.94e-02  6.24e-01    1.21e-01 2.51e-03 8.20e-01  0.971820
## 4994    63.50 6.30e-01  6.89e-03    2.27e-02 1.55e-02 8.20e-01  0.971820
## 8707    81.65 4.12e-01  8.55e-01    7.04e-01 5.40e-04 8.20e-01  0.971820
## 10071    0.00 9.48e-05  9.09e-01    4.86e-04 1.00e+00 3.62e-01  0.971820
## 2160    52.20 6.68e-01  7.86e-03    2.63e-02 1.88e-02 8.20e-01  0.971820
## 14637    0.00 1.04e-04  7.89e-01    5.02e-04 1.00e+00 3.62e-01  0.971820
## 18311   72.35 1.05e-02  8.84e-01    3.72e-02 1.36e-02 8.20e-01  0.971820
## 18162    0.00 5.28e-01  1.29e-04    5.10e-04 1.00e+00 8.20e-01  0.608634
## 16632    0.00 1.08e-04  8.57e-01    5.42e-04 1.00e+00 3.62e-01  0.971820
## 10172    0.00 3.61e-01  2.01e-04    5.74e-04 1.00e+00 8.20e-01  0.608634
## 16262   35.73 9.12e-02  7.31e-01    2.23e-01 2.67e-03 8.20e-01  0.971820
## 7014     0.00 3.66e-01  2.13e-04    6.11e-04 1.00e+00 8.20e-01  0.608634
## 12361    0.00 1.45e-04  8.43e-01    7.09e-04 1.00e+00 4.16e-01  0.971820
## 18765    0.00 6.11e-01  1.78e-04    7.50e-04 1.00e+00 8.20e-01  0.608634
## 7876     0.00 1.96e-04  9.45e-01    9.72e-04 1.00e+00 4.93e-01  0.971820
## 504      0.00 9.50e-03  3.70e-03    1.00e-03 1.00e+00 8.20e-01  0.971820
## 13888    0.00 4.79e-01  3.18e-04    1.10e-03 1.00e+00 8.20e-01  0.638977
##       qallsubs_cv pglobal_cv qglobal_cv
## 5236     2.23e-12   0.00e+00   0.00e+00
## 19644    1.11e-03   1.14e-11   1.14e-07
## 1467     8.13e-02   1.50e-04   1.00e+00
## 3490     9.90e-01   2.32e-04   1.00e+00
## 16680    8.77e-01   7.44e-04   1.00e+00
## 17380    9.90e-01   8.26e-04   1.00e+00
## 15166    4.45e-01   9.15e-04   1.00e+00
## 3037     9.90e-01   1.03e-03   1.00e+00
## 4382     5.67e-01   1.39e-03   1.00e+00
## 4537     9.90e-01   1.48e-03   1.00e+00
## 9729     6.78e-01   2.11e-03   1.00e+00
## 9038     6.78e-01   2.21e-03   1.00e+00
## 10918    9.90e-01   2.67e-03   1.00e+00
## 16589    9.90e-01   2.76e-03   1.00e+00
## 4994     9.90e-01   3.15e-03   1.00e+00
## 8707     9.90e-01   3.37e-03   1.00e+00
## 10071    8.77e-01   4.20e-03   1.00e+00
## 2160     9.90e-01   4.26e-03   1.00e+00
## 14637    8.77e-01   4.32e-03   1.00e+00
## 18311    9.90e-01   4.36e-03   1.00e+00
## 18162    8.77e-01   4.38e-03   1.00e+00
## 16632    8.77e-01   4.62e-03   1.00e+00
## 10172    8.77e-01   4.86e-03   1.00e+00
## 16262    9.90e-01   5.01e-03   1.00e+00
## 7014     8.77e-01   5.13e-03   1.00e+00
## 12361    9.42e-01   5.85e-03   1.00e+00
## 18765    9.42e-01   6.15e-03   1.00e+00
## 7876     9.90e-01   7.71e-03   1.00e+00
## 504      9.90e-01   7.91e-03   1.00e+00
## 13888    9.90e-01   8.60e-03   1.00e+00
```

```
write.csv(sel_cv, "sel_cv_all_old.csv",  row.names = F, quote=F)
```

##### c) Significant genes all\_old

```
signif_genes = sel_cv[sel_cv$qglobal_cv<0.1,c("gene_name", "qglobal_cv")]
rownames(signif_genes) = NULL
print(signif_genes)
```

```
##   gene_name   qglobal_cv
## 1    DNMT3A 0.000000e+00
## 2    ZNF318 1.140268e-07
```

##### d) Estimation number of drivers

```
wmis = dndscvout$globaldnds$mle[1]
nmis = (sum(dndscvout$annotmuts$impact =="Missense"))
ndrivers_all_old = (wmis-1)/wmis*nmis
ndrivers_all_old
```

```
## [1] 469.4172
```

```
wmis = dndscvout$globaldnds$cilow[1]
nmis = (sum(dndscvout$annotmuts$impact =="Missense"))
ndrivers_all_old_cilow = (wmis-1)/wmis*nmis
ndrivers_all_old_cilow
```

```
## [1] 114.2193
```

```
wmis = dndscvout$globaldnds$cihigh[1]
nmis = (sum(dndscvout$annotmuts$impact =="Missense"))
ndrivers_all_old_cihigh = (wmis-1)/wmis*nmis
ndrivers_all_old_cihigh
```

```
## [1] 811.295
```

##### Forest plot

```
dnds <- structure(list(
    mean  = c(NA,dndscvout$globaldnds$mle[1],dndscvout$globaldnds$mle[2],dndscvout$globaldnds$mle[4],dndscvout$globaldnds$mle[5]), 
    lower = c(NA,dndscvout$globaldnds$cilow[1],dndscvout$globaldnds$cilow[2],dndscvout$globaldnds$cilow[4],dndscvout$globaldnds$cilow[5]),
    upper = c(NA,dndscvout$globaldnds$cihigh[1],dndscvout$globaldnds$cihigh[2],dndscvout$globaldnds$cihigh[4],dndscvout$globaldnds$cihigh[5])),
    .Names = c("mean", "lower", "upper"), 
    row.names = c(NA,-1L),
    class = "data.frame")

tabletext<-
  c("name","missense", "nonsense","truncating", "all")

forestplot(tabletext, 
           dnds,new_page = FALSE,
           boxsize = 0.15,
           is.summary=FALSE,
           clip=c(0.8,1.2),
           xticks = c(0.8,0.9,1.0,1.1,1.2),
           xlog=FALSE, 
           col=fpColors(box="royalblue",line="darkblue", summary="royalblue"))
```

##### e) Removing annotated drivers from dataset all\_old

```
all_old_annot <- dndscvout$annotmuts
all_old_annot_no_drivers <- all_old_annot[!all_old_annot$gene %in% chip_drivers ,]
```

##### f) Annotated drivers removed all\_old

```
dndscvout=dndscv(all_old_annot_no_drivers, outp = 1, max_muts_per_gene_per_sample = 100, max_coding_muts_per_sample = 1000000)
```

```
## [1] Loading the environment...
```

```
## [2] Annotating the mutations...
```

```
## Warning in dndscv(all_old_annot_no_drivers, outp = 1,
## max_muts_per_gene_per_sample = 100, : Mutations observed in contiguous sites
## within a sample. Please annotate or remove dinucleotide or complex substitutions
## for best results.
```

```
## Warning in dndscv(all_old_annot_no_drivers, outp = 1,
## max_muts_per_gene_per_sample = 100, : Same mutations observed in different
## sampleIDs. Please verify that these are independent events and remove duplicates
## otherwise.
```

```
##     69% ...
```

```
## [3] Estimating global rates...
```

```
dndscvout$globaldnds
```

```
##      name      mle     cilow   cihigh
## wmis wmis 1.047166 1.0077414 1.088132
## wnon wnon 1.023352 0.9233631 1.134168
## wspl wspl 1.115701 0.9952990 1.250669
## wtru wtru 1.062607 0.9817256 1.150151
## wall wall 1.047865 1.0089184 1.088315
```

##### g) Number of drivers with annotated drivers removed

```
wmis = dndscvout$globaldnds$mle[1]
nmis = (sum(dndscvout$annotmuts$impact =="Missense"))
ndrivers_all_old = (wmis-1)/wmis*nmis
ndrivers_all_old
```

```
## [1] 427.0356
```

##### h) Cancer gene set analysis all\_old

```
dndscvout=dndscv(all_old,max_muts_per_gene_per_sample = 100, max_coding_muts_per_sample = 1000000, gene_list = cancer_drivers)
```

```
## [1] Loading the environment...
```

```
## [2] Annotating the mutations...
```

```
## Warning in dndscv(all_old, max_muts_per_gene_per_sample = 100,
## max_coding_muts_per_sample = 1e+06, : Mutations observed in contiguous sites
## within a sample. Please annotate or remove dinucleotide or complex substitutions
## for best results.
```

```
## Warning in dndscv(all_old, max_muts_per_gene_per_sample = 100,
## max_coding_muts_per_sample = 1e+06, : Same mutations observed in different
## sampleIDs. Please verify that these are independent events and remove duplicates
## otherwise.
```

```
## [3] Estimating global rates...
```

```
## [4] Running dNdSloc...
```

```
## [5] Running dNdScv...
```

```
##     Regression model for substitutions (theta = 1.93).
```

```
##     Regression model for indels (theta = 0.931)
```

```
sel_cv = dndscvout$sel_cv
print(head(sel_cv, n= 30L), digits = 3)
```

```
##     gene_name n_syn n_mis n_non n_spl n_ind wmis_cv wnon_cv wspl_cv wind_cv
## 99     DNMT3A     1    15     1     3     2   21.09    61.4    61.4   27.88
## 20      ARID2     1     2     2     2     0    1.83    44.3    44.3    0.00
## 47       CBFB     0     2     0     0     1   22.55     0.0     0.0   67.70
## 332      TET2     1     5     2     0     1    4.36    28.0    28.0    6.29
## 304      SMC3     0     2     0     0     2    5.37     0.0     0.0   20.90
## 116      EZH2     0     0     0     2     0    0.00    64.2    64.2    0.00
## 158     HNF1A     1     5     0     1     0   10.12    28.4    28.4    0.00
## 240      PAX5     0     4     0     0     0   23.42     0.0     0.0    0.00
## 110     ERBB2     0     0     1     0     1    0.00    22.5    22.5   10.13
## 215     MXRA5     0     9     0     0     0   11.94     0.0     0.0    0.00
## 39      BRCA1     1     0     0     1     1    0.00    12.7    12.7    6.75
## 303     SMC1A     0     3     0     0     1    7.16     0.0     0.0   10.31
## 38       BRAF     0     4     0     0     0   15.52     0.0     0.0    0.00
## 306    SMTNL2     1     0     0     0     1    0.00     0.0     0.0   27.55
## 25     ATP1B1     0     1     1     0     0    8.59    81.1    81.1    0.00
## 21     ARID5B     0     2     0     0     1    5.48     0.0     0.0   10.70
## 322      SUFU     0     0     0     0     1    0.00     0.0     0.0   26.24
## 213     MUC17     1     8     0     0     1    3.81     0.0     0.0    2.83
## 7        ADNP     0     4     0     0     0   11.79     0.0     0.0    0.00
## 16   ARHGAP35     1     0     1     0     0    0.00    22.9    22.9    0.00
## 358    ZBTB20     0     1     1     0     0    3.34    83.8    83.8    0.00
## 270     RAD21     0     0     0     1     0    0.00    49.7    49.7    0.00
## 24     ATP1A1     0     0     0     0     1    0.00     0.0     0.0   12.43
## 28      AXIN1     0     2     1     0     0    5.90    41.3    41.3    0.00
## 37      BMPR2     0     0     0     0     1    0.00     0.0     0.0   12.25
## 108     EPHA2     0     1     0     0     1    2.47     0.0     0.0   13.03
## 362   ZFP36L2     0     3     0     0     0   12.52     0.0     0.0    0.00
## 176    KANSL1     0     1     0     0     1    2.67     0.0     0.0   11.51
## 256     PPM1D     0     1     1     0     0    4.14    54.4    54.4    0.00
## 214      MUC6     0     0     1     0     0    0.00    20.8    20.8    0.00
##      pmis_cv ptrunc_cv pallsubs_cv pind_cv  qmis_cv qtrunc_cv qallsubs_cv
## 99  9.01e-08  7.66e-06    3.76e-09 0.00462 3.33e-05   0.00283    1.39e-06
## 20  5.52e-01  2.79e-05    1.29e-04 1.00000 7.64e-01   0.00516    2.38e-02
## 47  1.22e-02  8.83e-01    4.20e-02 0.01455 6.47e-01   0.95742    9.52e-01
## 332 6.22e-02  4.60e-03    8.89e-03 0.13655 7.64e-01   0.42523    4.70e-01
## 304 1.60e-01  7.47e-01    3.31e-01 0.00784 7.64e-01   0.95742    9.52e-01
## 116 4.97e-01  1.46e-03    3.13e-03 1.00000 7.64e-01   0.18054    3.30e-01
## 158 3.37e-03  3.70e-02    3.56e-03 1.00000 3.12e-01   0.95742    3.30e-01
## 240 1.22e-03  8.68e-01    5.04e-03 1.00000 2.26e-01   0.95742    3.73e-01
## 110 4.03e-01  5.80e-02    8.27e-02 0.08953 7.64e-01   0.95742    9.52e-01
## 215 2.16e-03  8.08e-01    7.59e-03 1.00000 2.66e-01   0.95742    4.68e-01
## 39  1.93e-01  9.02e-02    6.84e-02 0.12841 7.64e-01   0.95742    9.52e-01
## 303 6.49e-02  7.83e-01    1.63e-01 0.08811 7.64e-01   0.95742    9.52e-01
## 38  4.92e-03  7.96e-01    1.67e-02 1.00000 3.64e-01   0.95742    7.71e-01
## 306 3.70e-01  8.22e-01    6.56e-01 0.03498 7.64e-01   0.95742    9.52e-01
## 25  1.64e-01  1.41e-02    2.83e-02 1.00000 7.64e-01   0.86964    9.52e-01
## 21  1.55e-01  8.38e-01    3.47e-01 0.08518 7.64e-01   0.95742    9.52e-01
## 322 5.54e-01  8.48e-01    8.27e-01 0.03666 7.64e-01   0.95742    9.52e-01
## 213 6.18e-02  7.14e-01    1.48e-01 0.25872 7.64e-01   0.95742    9.52e-01
## 7   1.16e-02  8.44e-01    3.85e-02 1.00000 6.47e-01   0.95742    9.52e-01
## 16  2.11e-01  4.69e-02    4.32e-02 1.00000 7.64e-01   0.95742    9.52e-01
## 358 4.12e-01  1.36e-02    4.42e-02 1.00000 7.64e-01   0.86964    9.52e-01
## 270 5.36e-01  2.43e-02    5.35e-02 1.00000 7.64e-01   0.95742    9.52e-01
## 24  4.39e-01  8.01e-01    7.24e-01 0.07426 7.64e-01   0.95742    9.52e-01
## 28  1.38e-01  2.98e-02    5.49e-02 1.00000 7.64e-01   0.95742    9.52e-01
## 37  4.47e-01  8.09e-01    7.33e-01 0.07527 7.64e-01   0.95742    9.52e-01
## 108 5.34e-01  8.09e-01    7.90e-01 0.07111 7.64e-01   0.95742    9.52e-01
## 362 1.75e-02  9.16e-01    5.84e-02 1.00000 7.64e-01   0.95742    9.52e-01
## 176 5.01e-01  8.12e-01    7.65e-01 0.07972 7.64e-01   0.95742    9.52e-01
## 256 3.39e-01  2.19e-02    6.21e-02 1.00000 7.64e-01   0.95742    9.52e-01
## 214 3.08e-01  6.33e-02    6.34e-02 1.00000 7.64e-01   0.95742    9.52e-01
##     pglobal_cv qglobal_cv
## 99    4.47e-10   1.66e-07
## 20    1.28e-03   2.37e-01
## 47    5.14e-03   6.34e-01
## 332   9.37e-03   8.66e-01
## 304   1.81e-02   9.99e-01
## 116   2.12e-02   9.99e-01
## 158   2.37e-02   9.99e-01
## 240   3.17e-02   9.99e-01
## 110   4.37e-02   9.99e-01
## 215   4.46e-02   9.99e-01
## 39    5.04e-02   9.99e-01
## 303   7.52e-02   9.99e-01
## 38    8.49e-02   9.99e-01
## 306   1.10e-01   9.99e-01
## 25    1.29e-01   9.99e-01
## 21    1.34e-01   9.99e-01
## 322   1.36e-01   9.99e-01
## 213   1.63e-01   9.99e-01
## 7     1.64e-01   9.99e-01
## 16    1.79e-01   9.99e-01
## 358   1.82e-01   9.99e-01
## 270   2.10e-01   9.99e-01
## 24    2.11e-01   9.99e-01
## 28    2.14e-01   9.99e-01
## 37    2.15e-01   9.99e-01
## 108   2.18e-01   9.99e-01
## 362   2.24e-01   9.99e-01
## 176   2.32e-01   9.99e-01
## 256   2.35e-01   9.99e-01
## 214   2.38e-01   9.99e-01
```

##### i) Cancer gene set analysis excluding CHIP drivers all\_old

```
dndscvout=dndscv(all_old,max_muts_per_gene_per_sample = 100, max_coding_muts_per_sample = 1000000, gene_list = setdiff(cancer_drivers, chip_drivers))
```

```
## [1] Loading the environment...
```

```
## [2] Annotating the mutations...
```

```
## Warning in dndscv(all_old, max_muts_per_gene_per_sample = 100,
## max_coding_muts_per_sample = 1e+06, : Mutations observed in contiguous sites
## within a sample. Please annotate or remove dinucleotide or complex substitutions
## for best results.
```

```
## Warning in dndscv(all_old, max_muts_per_gene_per_sample = 100,
## max_coding_muts_per_sample = 1e+06, : Same mutations observed in different
## sampleIDs. Please verify that these are independent events and remove duplicates
## otherwise.
```

```
## [3] Estimating global rates...
```

```
## [4] Running dNdSloc...
```

```
## [5] Running dNdScv...
```

```
##     Regression model for substitutions (theta = 2.49).
```

```
##     Regression model for indels (theta = 1.04)
```

```
sel_cv = dndscvout$sel_cv
print(head(sel_cv), digits = 3)
```

```
##     gene_name n_syn n_mis n_non n_spl n_ind wmis_cv wnon_cv wspl_cv wind_cv
## 37       CBFB     0     2     0     0     1   18.24     0.0     0.0   65.55
## 122     HNF1A     1     5     0     1     0    9.81    24.0    24.0    0.00
## 236      SMC3     0     2     0     0     2    4.11     0.0     0.0   20.24
## 167     MXRA5     0     9     0     0     0    8.83     0.0     0.0    0.00
## 83      ERBB2     0     0     1     0     1    0.00    16.1    16.1    9.81
## 30      BRCA1     1     0     0     1     1    0.00    10.9    10.9    6.54
##     pmis_cv ptrunc_cv pallsubs_cv pind_cv qmis_cv qtrunc_cv qallsubs_cv
## 37  0.01298    0.8630     0.04423 0.01503   0.739     0.949       0.940
## 122 0.00252    0.0425     0.00279 1.00000   0.369     0.949       0.819
## 236 0.19441    0.7034     0.37887 0.00796   0.739     0.949       0.940
## 167 0.00242    0.7727     0.00842 1.00000   0.369     0.949       0.940
## 83  0.33699    0.0735     0.09590 0.09266   0.739     0.949       0.940
## 30  0.16983    0.1022     0.07307 0.13301   0.739     0.949       0.940
##     pglobal_cv qglobal_cv
## 37     0.00553      0.999
## 122    0.01923      0.999
## 236    0.02052      0.999
## 167    0.04867      0.999
## 83     0.05086      0.999
## 30     0.05475      0.999
```

### 2. Analysis all young individuals - all variants: all\_young

##### a) Global dnds all\_young

```
#Run dndscv
dndscvout=dndscv(all_young,outp=1, max_muts_per_gene_per_sample = 100, max_coding_muts_per_sample = 1000000)
```

```
## [1] Loading the environment...
```

```
## [2] Annotating the mutations...
```

```
## Warning in dndscv(all_young, outp = 1, max_muts_per_gene_per_sample = 100, :
## Mutations observed in contiguous sites within a sample. Please annotate or
## remove dinucleotide or complex substitutions for best results.
```

```
## Warning in dndscv(all_young, outp = 1, max_muts_per_gene_per_sample = 100, :
## Same mutations observed in different sampleIDs. Please verify that these are
## independent events and remove duplicates otherwise.
```

```
##     97% ...
```

```
## [3] Estimating global rates...
```

```
dndscvout$globaldnds
```

```
##      name       mle     cilow   cihigh
## wmis wmis 1.0696793 1.0219438 1.119645
## wnon wnon 1.0521100 0.9359022 1.182747
## wspl wspl 0.9944432 0.8641016 1.144446
## wtru wtru 1.0282236 0.9366619 1.128736
## wall wall 1.0665457 1.0195627 1.115694
```

##### b) Per gene analysis all\_young

```
dndscvout=dndscv(all_young,max_muts_per_gene_per_sample = 100, max_coding_muts_per_sample = 1000000)
```

```
## [1] Loading the environment...
```

```
## [2] Annotating the mutations...
```

```
## Warning in dndscv(all_young, max_muts_per_gene_per_sample = 100,
## max_coding_muts_per_sample = 1e+06): Mutations observed in contiguous sites
## within a sample. Please annotate or remove dinucleotide or complex substitutions
## for best results.
```

```
## Warning in dndscv(all_young, max_muts_per_gene_per_sample = 100,
## max_coding_muts_per_sample = 1e+06): Same mutations observed in different
## sampleIDs. Please verify that these are independent events and remove duplicates
## otherwise.
```

```
##     97% ...
```

```
## [3] Estimating global rates...
```

```
## [4] Running dNdSloc...
```

```
## [5] Running dNdScv...
```

```
##     Regression model for substitutions (theta = 6.04).
```

```
## Warning in theta.ml(Y, mu, sum(w), w, limit = control$maxit, trace =
## control$trace > : iteration limit reached
```

```
## Warning in theta.ml(Y, mu, sum(w), w, limit = control$maxit, trace =
## control$trace > : iteration limit reached
```

```
##     Regression model for indels (theta = 97.4)
```

```
sel_cv = dndscvout$sel_cv
print(head(sel_cv, n=30L), digits = 3)
```

```
##        gene_name n_syn n_mis n_non n_spl n_ind wmis_cv wnon_cv wspl_cv wind_cv
## 5467        ECE2     1     2     2     1     0    5.43    78.1    78.1     0.0
## 3786       CHRNG     0     0     0     0     2    0.00     0.0     0.0   198.1
## 7931    HIST2H3D     0     3     0     0     0   98.87     0.0     0.0     0.0
## 1623        ASPM     0     7     1     0     1    7.90    10.2    10.2    14.8
## 5416     DYNC1H1     0     4     1     2     1    3.23    24.2    24.2    11.0
## 3822       CILP2     0     3     1     0     1    6.54    45.4    45.4    44.1
## 15600       SETX     0     6     0     0     1    8.62     0.0     0.0    19.0
## 7589      GTF2E1     0     4     0     0     0   27.25     0.0     0.0     0.0
## 12936      PIAS4     0     0     2     0     0    0.00   165.6   165.6     0.0
## 4654      CYB5R4     0     1     0     1     1    5.95    45.7    45.7    98.3
## 16985      SUSD5     0     0     1     0     1    0.00    49.8    49.8    81.5
## 12673     PDCD10     0     0     1     1     0    0.00   141.8   141.8     0.0
## 17342      TEAD2     0     0     1     0     1    0.00    39.1    39.1   113.5
## 9654        LIPG     0     3     0     0     1    7.48     0.0     0.0   102.4
## 124   AC003102.1     0     0     0     0     1    0.00     0.0     0.0  1386.9
## 4675      CYP1A2     0     5     0     0     0   14.46     0.0     0.0     0.0
## 3723       CHEK2     0     2     0     0     1   10.66     0.0     0.0    87.4
## 19211       XCR1     0     2     0     0     1    7.40     0.0     0.0   153.6
## 15867    SLC17A4     0     1     1     0     1    2.71    23.0    23.0   103.0
## 19883     ZNF668     0     2     0     0     1   10.20     0.0     0.0    79.8
## 17311      TCP11     0     2     0     0     1    7.12     0.0     0.0    99.3
## 16288     SMIM15     0     0     0     0     1    0.00     0.0     0.0   684.2
## 4617       CXCR4     0     1     0     0     1   13.12     0.0     0.0   143.7
## 8488        IL7R     0     3     0     0     0   28.66     0.0     0.0     0.0
## 10092     MAP3K1     0     0     1     0     1    0.00    33.3    33.3    33.9
## 11111     NAPRT1     0     2     1     0     0   15.45    87.1    87.1     0.0
## 18591     UBE2L6     0     1     0     1     0   27.91   205.0   205.0     0.0
## 14345       RCN1     0     1     0     0     1    9.15     0.0     0.0   154.6
## 17293       TCF4     0     0     1     1     0    0.00    60.7    60.7     0.0
## 7914    HIST1H4D     0     0     0     0     1    0.00     0.0     0.0   493.4
##        pmis_cv ptrunc_cv pallsubs_cv  pind_cv qmis_cv qtrunc_cv qallsubs_cv
## 5467  7.73e-02  1.33e-05    2.70e-05 1.00e+00   0.844     0.267       0.423
## 3786  4.66e-01  8.04e-01    7.45e-01 5.11e-05   0.844     0.978       0.997
## 7931  7.16e-06  9.73e-01    4.21e-05 1.00e+00   0.144     0.979       0.423
## 1623  5.72e-04  1.01e-01    1.21e-03 6.55e-02   0.844     0.978       0.997
## 5416  9.46e-02  5.34e-04    1.30e-03 8.65e-02   0.844     0.978       0.997
## 3822  2.31e-02  1.91e-02    6.80e-03 2.24e-02   0.844     0.978       0.997
## 15600 7.38e-04  7.23e-01    2.98e-03 5.14e-02   0.844     0.978       0.997
## 7589  5.14e-05  8.86e-01    2.70e-04 1.00e+00   0.516     0.978       0.997
## 12936 5.61e-01  7.17e-05    2.98e-04 1.00e+00   0.844     0.664       0.997
## 4654  1.82e-01  1.89e-02    2.98e-02 1.01e-02   0.844     0.978       0.997
## 16985 4.01e-01  1.72e-02    3.84e-02 1.22e-02   0.844     0.978       0.997
## 12673 7.26e-01  9.92e-05    4.70e-04 1.00e+00   0.844     0.664       0.997
## 17342 5.07e-01  2.25e-02    5.70e-02 8.77e-03   0.844     0.978       0.997
## 9654  1.61e-02  7.89e-01    5.24e-02 9.72e-03   0.844     0.978       0.997
## 124   8.88e-01  9.76e-01    9.90e-01 7.21e-04   0.904     0.980       0.997
## 4675  1.64e-04  8.30e-01    7.87e-04 1.00e+00   0.844     0.978       0.997
## 3723  2.16e-02  8.36e-01    6.95e-02 1.14e-02   0.844     0.978       0.997
## 19211 4.46e-02  8.80e-01    1.31e-01 6.49e-03   0.844     0.978       0.997
## 15867 4.16e-01  4.07e-02    9.69e-02 9.66e-03   0.844     0.978       0.997
## 19883 2.36e-02  9.10e-01    7.66e-02 1.25e-02   0.844     0.978       0.997
## 17311 4.81e-02  8.16e-01    1.37e-01 1.00e-02   0.844     0.978       0.997
## 16288 8.36e-01  9.42e-01    9.76e-01 1.46e-03   0.867     0.978       0.997
## 4617  7.63e-02  9.32e-01    2.07e-01 6.93e-03   0.844     0.978       0.997
## 8488  3.19e-04  8.89e-01    1.51e-03 1.00e+00   0.844     0.978       0.997
## 10092 3.99e-01  2.69e-02    5.65e-02 2.91e-02   0.844     0.978       0.997
## 11111 1.02e-02  9.24e-03    1.65e-03 1.00e+00   0.844     0.978       0.997
## 18591 3.28e-02  3.61e-03    1.74e-03 1.00e+00   0.844     0.978       0.997
## 14345 1.14e-01  8.99e-01    2.84e-01 6.45e-03   0.844     0.978       0.997
## 17293 4.50e-01  5.89e-04    1.83e-03 1.00e+00   0.844     0.978       0.997
## 7914  6.77e-01  9.60e-01    9.16e-01 2.02e-03   0.844     0.978       0.997
##       pglobal_cv qglobal_cv
## 5467    0.000312          1
## 3786    0.000425          1
## 7931    0.000466          1
## 1623    0.000825          1
## 5416    0.001137          1
## 3822    0.001491          1
## 15600   0.001497          1
## 7589    0.002491          1
## 12936   0.002717          1
## 4654    0.002744          1
## 16985   0.004060          1
## 12673   0.004075          1
## 17342   0.004296          1
## 9654    0.004366          1
## 124     0.005882          1
## 4675    0.006409          1
## 3723    0.006437          1
## 19211   0.006861          1
## 15867   0.007463          1
## 19883   0.007589          1
## 17311   0.010405          1
## 16288   0.010768          1
## 4617    0.010826          1
## 8488    0.011347          1
## 10092   0.012175          1
## 11111   0.012196          1
## 18591   0.012775          1
## 14345   0.013362          1
## 17293   0.013385          1
## 7914    0.013515          1
```

```
write.csv(sel_cv, "sel_cv_all_young.csv",  row.names = F, quote=F)
```

##### c) Significant genes all\_young

```
signif_genes = sel_cv[sel_cv$qglobal_cv<0.1, c("gene_name","qglobal_cv")]
rownames(signif_genes) = NULL
print(signif_genes)
```

```
## [1] gene_name  qglobal_cv
## <0 rows> (or 0-length row.names)
```

##### d) Estimation number of drivers all\_young

```
wmis = dndscvout$globaldnds$mle[1]
nmis = (sum(dndscvout$annotmuts$impact =="Missense"))
ndrivers_all_young = (wmis-1)/wmis*nmis
ndrivers_all_young
```

```
## [1] 440.8699
```

```
wmis = dndscvout$globaldnds$cilow[1]
nmis = (sum(dndscvout$annotmuts$impact =="Missense"))
ndrivers_all_young_cilow = (wmis-1)/wmis*nmis
ndrivers_all_young_cilow
```

```
## [1] 145.3264
```

```
wmis = dndscvout$globaldnds$cihigh[1]
nmis = (sum(dndscvout$annotmuts$impact =="Missense"))
ndrivers_all_young_cihigh = (wmis-1)/wmis*nmis
ndrivers_all_young_cihigh
```

```
## [1] 723.2245
```

##### Forest plot

```
dnds <- structure(list(
    mean  = c(NA,dndscvout$globaldnds$mle[1],dndscvout$globaldnds$mle[2],dndscvout$globaldnds$mle[4],dndscvout$globaldnds$mle[5]), 
    lower = c(NA,dndscvout$globaldnds$cilow[1],dndscvout$globaldnds$cilow[2],dndscvout$globaldnds$cilow[4],dndscvout$globaldnds$cilow[5]),
    upper = c(NA,dndscvout$globaldnds$cihigh[1],dndscvout$globaldnds$cihigh[2],dndscvout$globaldnds$cihigh[4],dndscvout$globaldnds$cihigh[5])),
    .Names = c("mean", "lower", "upper"), 
    row.names = c(NA,-1L),
    class = "data.frame")

tabletext<-
  c("name","missense", "nonsense","truncating", "all")

forestplot(tabletext, 
           dnds,new_page = FALSE,
           boxsize = 0.15,
           is.summary=FALSE,
           clip=c(0.8,1.2),
           xticks = c(0.8,0.9,1.0,1.1,1.2),
           xlog=FALSE, 
           col=fpColors(box="royalblue",line="darkblue", summary="royalblue"))
```

##### e) Removing annotated drivers from dataset all\_young

```
all_young_annot <- dndscvout$annotmuts
all_young_annot_no_drivers <- all_young_annot[!all_young_annot$gene %in% chip_drivers ,]
```

##### f) Annotated drivers removed all\_young

```
dndscvout=dndscv(all_young_annot_no_drivers, outp = 1, max_muts_per_gene_per_sample = 100, max_coding_muts_per_sample = 1000000)
```

```
## [1] Loading the environment...
```

```
## [2] Annotating the mutations...
```

```
## Warning in dndscv(all_young_annot_no_drivers, outp = 1,
## max_muts_per_gene_per_sample = 100, : Mutations observed in contiguous sites
## within a sample. Please annotate or remove dinucleotide or complex substitutions
## for best results.
```

```
## Warning in dndscv(all_young_annot_no_drivers, outp = 1,
## max_muts_per_gene_per_sample = 100, : Same mutations observed in different
## sampleIDs. Please verify that these are independent events and remove duplicates
## otherwise.
```

```
##     98% ...
```

```
## [3] Estimating global rates...
```

```
dndscvout$globaldnds
```

```
##      name       mle     cilow   cihigh
## wmis wmis 1.0703115 1.0224112 1.120456
## wnon wnon 1.0545235 0.9377278 1.185866
## wspl wspl 0.9950613 0.8643760 1.145505
## wtru wtru 1.0298494 0.9379078 1.130804
## wall wall 1.0672749 1.0201241 1.116605
```

### 3. Analysis all cord blood - all variants: all\_CB

##### a) Global dnds all\_CB

```
#Run dndscv
dndscvout=dndscv(all_CB,outp=1, max_muts_per_gene_per_sample = 100, max_coding_muts_per_sample = 1000000)
```

```
## [1] Loading the environment...
```

```
## [2] Annotating the mutations...
```

```
## Warning in dndscv(all_CB, outp = 1, max_muts_per_gene_per_sample = 100, :
## Mutations observed in contiguous sites within a sample. Please annotate or
## remove dinucleotide or complex substitutions for best results.
```

```
## [3] Estimating global rates...
```

```
dndscvout$globaldnds
```

```
##      name       mle      cilow   cihigh
## wmis wmis 1.0508702 0.79890188 1.382308
## wnon wnon 1.3709405 0.70961334 2.648594
## wspl wspl 0.2174520 0.02876884 1.643631
## wtru wtru 0.9846501 0.52041663 1.862999
## wall wall 1.0557537 0.80500909 1.384600
```

##### b) Per gene analysis all\_CB

```
dndscvout=dndscv(all_CB,max_muts_per_gene_per_sample = 100, max_coding_muts_per_sample = 1000000)
```

```
## [1] Loading the environment...
```

```
## [2] Annotating the mutations...
```

```
## Warning in dndscv(all_CB, max_muts_per_gene_per_sample = 100,
## max_coding_muts_per_sample = 1e+06): Mutations observed in contiguous sites
## within a sample. Please annotate or remove dinucleotide or complex substitutions
## for best results.
```

```
## [3] Estimating global rates...
```

```
## [4] Running dNdSloc...
```

```
## [5] Running dNdScv...
```

```
##     Regression model for substitutions (theta = 0.207).
```

```
sel_cv = dndscvout$sel_cv
print(head(sel_cv, n= 30L), digits = 3)
```

```
##          gene_name n_syn n_mis n_non n_spl wmis_cv wnon_cv wspl_cv  pmis_cv
## 4067          COA1     0     0     1     0     0.0   10391   10391 9.63e-01
## 4657          CYBB     0     0     1     0     0.0    6506    6506 9.61e-01
## 16283       SMIM10     0     1     0     0  5904.0       0       0 8.85e-05
## 7231         GNG11     0     1     0     0  5860.6       0       0 8.92e-05
## 16461        SOX17     0     0     1     0     0.0    3261    3261 8.73e-01
## 12110        OR4N2     0     0     1     0     0.0    2535    2535 8.98e-01
## 8807         KCNA4     0     0     1     0     0.0    2332    2332 8.66e-01
## 5825         ERCC5     0     0     1     0     0.0    2222    2222 9.16e-01
## 2686       C5orf30     0     1     0     0  1706.4       0       0 3.31e-04
## 2183     C10orf105     0     1     0     0  1704.4       0       0 3.32e-04
## 18290        TRPC5     0     2     0     0   109.4       0       0 3.50e-04
## 2501       C1orf53     0     1     0     0  1436.4       0       0 3.98e-04
## 12390       P2RY13     0     1     0     0  1405.1       0       0 4.08e-04
## 10609        MOXD1     0     0     1     0     0.0    1361    1361 9.05e-01
## 19781       ZNF541     0     2     0     0    87.9       0       0 5.28e-04
## 8632          IRS4     0     2     0     0    85.8       0       0 5.59e-04
## 2478      C1orf195     0     1     0     0   876.2       0       0 6.76e-04
## 13957       PTPRZ1     0     2     0     0    70.3       0       0 8.40e-04
## 3919        CLEC5A     0     1     0     0   709.9       0       0 8.48e-04
## 2823       C9orf66     0     1     0     0   643.8       0       0 9.42e-04
## 3626        CEP290     0     0     0     1     0.0     587     587 8.75e-01
## 10358        METRN     0     1     0     0   587.8       0       0 1.04e-03
## 2074        BPIFA3     0     1     0     0   570.6       0       0 1.07e-03
## 698          AGAP1     0     0     1     0     0.0     541     541 7.83e-01
## 13706        PRR19     0     1     0     0   506.6       0       0 1.22e-03
## 15976      SLC26A6     0     1     0     0   432.6       0       0 1.45e-03
## 12184       OR5B17     0     1     0     0   413.9       0       0 1.52e-03
## 2893           CAD     0     0     1     0     0.0     393     393 7.82e-01
## 14720 RP1-228P16.5     0     1     0     0   345.4       0       0 1.85e-03
## 14498         RHCE     0     1     0     0   345.2       0       0 1.85e-03
##       ptrunc_cv pallsubs_cv qmis_cv qtrunc_cv qallsubs_cv
## 4067   4.87e-05    0.000261   0.994     0.802           1
## 4657   7.99e-05    0.000417   0.994     0.802           1
## 16283  9.98e-01    0.000460   0.896     1.000           1
## 7231   9.97e-01    0.000464   0.896     1.000           1
## 16461  1.66e-04    0.000823   0.994     0.837           1
## 12110  2.17e-04    0.001063   0.994     0.837           1
## 8807   2.37e-04    0.001149   0.994     0.837           1
## 5825   2.50e-04    0.001216   0.994     0.837           1
## 2686   9.94e-01    0.001592   0.994     1.000           1
## 2183   9.96e-01    0.001594   0.994     1.000           1
## 18290  9.58e-01    0.001666   0.994     1.000           1
## 2501   9.93e-01    0.001891   0.994     1.000           1
## 12390  9.94e-01    0.001933   0.994     1.000           1
## 10609  4.22e-04    0.001982   0.994     1.000           1
## 19781  9.55e-01    0.002443   0.994     1.000           1
## 8632   9.66e-01    0.002583   0.994     1.000           1
## 2478   9.91e-01    0.003099   0.994     1.000           1
## 13957  9.48e-01    0.003750   0.994     1.000           1
## 3919   9.85e-01    0.003823   0.994     1.000           1
## 2823   9.92e-01    0.004215   0.994     1.000           1
## 3626   1.04e-03    0.004564   0.994     1.000           1
## 10358  9.91e-01    0.004617   0.994     1.000           1
## 2074   9.83e-01    0.004755   0.994     1.000           1
## 698    1.14e-03    0.004826   0.994     1.000           1
## 13706  9.92e-01    0.005355   0.994     1.000           1
## 15976  9.88e-01    0.006268   0.994     1.000           1
## 12184  9.85e-01    0.006550   0.994     1.000           1
## 2893   1.61e-03    0.006647   0.994     1.000           1
## 14720  9.89e-01    0.007845   0.994     1.000           1
## 14498  9.83e-01    0.007851   0.994     1.000           1
```

```
write.csv(sel_cv, "sel_cv_all_CB.csv",  row.names = F, quote=F)
```

##### c) Significant genes all\_CB

```
signif_genes = sel_cv[sel_cv$qallsubs_cv <0.1, c("gene_name","qallsubs_cv")]
rownames(signif_genes) = NULL
print(signif_genes)
```

```
## [1] gene_name   qallsubs_cv
## <0 rows> (or 0-length row.names)
```

##### d) Estimation number of drivers all\_CB

```
wmis = dndscvout$globaldnds$mle[1]
nmis = (sum(dndscvout$annotmuts$impact =="Missense"))
ndrivers_CB = (wmis-1)/wmis*nmis
ndrivers_CB
```

```
## [1] 8.810206
```

```
wmis = dndscvout$globaldnds$cilow[1]
nmis = (sum(dndscvout$annotmuts$impact =="Missense"))
ndrivers_CB_cilow = (wmis-1)/wmis*nmis
ndrivers_CB_cilow
```

```
## [1] -45.81271
```

```
wmis = dndscvout$globaldnds$cihigh[1]
nmis = (sum(dndscvout$annotmuts$impact =="Missense"))
ndrivers_CB_cihigh = (wmis-1)/wmis*nmis
ndrivers_CB_cihigh
```

```
## [1] 50.33612
```

##### e) Removing annotated drivers all\_CB

```
all_CB_annot <- dndscvout$annotmuts
all_CB_annot_no_drivers <- all_CB_annot[!all_CB_annot$gene %in% chip_drivers ,]
```

##### f) Annotated drivers removed all\_CB

```
dndscvout=dndscv(all_CB_annot_no_drivers, outp = 1, max_muts_per_gene_per_sample = 100, max_coding_muts_per_sample = 1000000)
```

```
## [1] Loading the environment...
```

```
## [2] Annotating the mutations...
```

```
## [3] Estimating global rates...
```

```
dndscvout$globaldnds
```

```
##      name       mle      cilow   cihigh
## wmis wmis 1.0508702 0.79890188 1.382308
## wnon wnon 1.3709405 0.70961334 2.648594
## wspl wspl 0.2174520 0.02876884 1.643631
## wtru wtru 0.9846501 0.52041663 1.862999
## wall wall 1.0557537 0.80500909 1.384600
```

##### g) Cancer gene set analysis all\_CB

```
dndscvout=dndscv(all_CB,max_muts_per_gene_per_sample = 100, max_coding_muts_per_sample = 1000000, gene_list = cancer_drivers)
```

```
## [1] Loading the environment...
```

```
## [2] Annotating the mutations...
```

```
## Warning in dndscv(all_CB, max_muts_per_gene_per_sample = 100,
## max_coding_muts_per_sample = 1e+06, : Mutations observed in contiguous sites
## within a sample. Please annotate or remove dinucleotide or complex substitutions
## for best results.
```

```
## [3] Estimating global rates...
```

```
## Warning: glm.fit: fitted rates numerically 0 occurred
```

```
## [4] Running dNdSloc...
```

```
## [5] Running dNdScv...
```

```
## Warning in theta.ml(Y, mu, sum(w), w, limit = control$maxit, trace =
## control$trace > : iteration limit reached
```

```
## Warning in theta.ml(Y, mu, sum(w), w, limit = control$maxit, trace =
## control$trace > : iteration limit reached
```

```
##     Regression model for substitutions (theta = 1.22e+03).
```

```
sel_cv = dndscvout$sel_cv
print(head(sel_cv), digits = 3)
```

```
##     gene_name n_syn n_mis n_non n_spl wmis_cv wnon_cv wspl_cv pmis_cv ptrunc_cv
## 236     OR4N2     0     0     1     0       0   14706   14706 0.96257  3.38e-05
## 309     SOX17     0     0     1     0       0    5775    5775 0.92269  9.06e-05
## 6      ACVR2B     0     1     0     0     229       0       0 0.00289  9.81e-01
## 296   SLC26A3     0     1     0     0     224       0       0 0.00296  9.81e-01
## 318    STAT5B     0     1     0     0     136       0       0 0.00510  9.77e-01
## 13      AMER1     0     1     0     0     136       0       0 0.00510  9.91e-01
##     pallsubs_cv qmis_cv qtrunc_cv qallsubs_cv
## 236    0.000185   0.977    0.0125      0.0683
## 309    0.000469   0.977    0.0168      0.0867
## 6      0.011815   0.472    1.0000      0.9995
## 296    0.012081   0.472    1.0000      0.9995
## 318    0.019795   0.472    1.0000      0.9995
## 13     0.019804   0.472    1.0000      0.9995
```

##### h) Cancer gene set analysis excluding CHIP drivers all\_CB

```
dndscvout=dndscv(all_CB,max_muts_per_gene_per_sample = 100, max_coding_muts_per_sample = 1000000, gene_list = setdiff(cancer_drivers, chip_drivers))
```

```
## [1] Loading the environment...
```

```
## [2] Annotating the mutations...
```

```
## Warning in dndscv(all_CB, max_muts_per_gene_per_sample = 100,
## max_coding_muts_per_sample = 1e+06, : Mutations observed in contiguous sites
## within a sample. Please annotate or remove dinucleotide or complex substitutions
## for best results.
```

```
## [3] Estimating global rates...
```

```
## Warning: glm.fit: fitted rates numerically 0 occurred
```

```
## [4] Running dNdSloc...
```

```
## [5] Running dNdScv...
```

```
## Warning in theta.ml(Y, mu, sum(w), w, limit = control$maxit, trace =
## control$trace > : iteration limit reached
```

```
## Warning in theta.ml(Y, mu, sum(w), w, limit = control$maxit, trace =
## control$trace > : iteration limit reached
```

```
##     Regression model for substitutions (theta = 1.52e+03).
```

```
sel_cv = dndscvout$sel_cv
print(head(sel_cv), digits = 3)
```

```
##     gene_name n_syn n_mis n_non n_spl wmis_cv wnon_cv wspl_cv pmis_cv ptrunc_cv
## 181     OR4N2     0     0     1     0       0   11481   11481 0.95805  4.39e-05
## 241     SOX17     0     0     1     0       0    4771    4771 0.91340  1.11e-04
## 5      ACVR2B     0     1     0     0     182       0       0 0.00371  9.79e-01
## 228   SLC26A3     0     1     0     0     178       0       0 0.00380  9.78e-01
## 11      AMER1     0     1     0     0     109       0       0 0.00654  9.90e-01
## 247    STAT5B     0     1     0     0     108       0       0 0.00656  9.75e-01
##     pallsubs_cv qmis_cv qtrunc_cv qallsubs_cv
## 181    0.000236   0.974    0.0129      0.0693
## 241    0.000566   0.974    0.0163      0.0830
## 5      0.014846   0.480    1.0000      0.9994
## 228    0.015168   0.480    1.0000      0.9994
## 11     0.024794   0.480    1.0000      0.9994
## 247    0.024827   0.480    1.0000      0.9994
```

### 4. Analysis all individuals and all variants

##### a) Global dnds all

```
#Run dndscv
dndscvout=dndscv(all,outp=1, max_muts_per_gene_per_sample = 100, max_coding_muts_per_sample = 1000000)
```

```
## [1] Loading the environment...
```

```
## [2] Annotating the mutations...
```

```
## Warning in dndscv(all, outp = 1, max_muts_per_gene_per_sample = 100,
## max_coding_muts_per_sample = 1e+06): Mutations observed in contiguous sites
## within a sample. Please annotate or remove dinucleotide or complex substitutions
## for best results.
```

```
## Warning in dndscv(all, outp = 1, max_muts_per_gene_per_sample = 100,
## max_coding_muts_per_sample = 1e+06): Same mutations observed in different
## sampleIDs. Please verify that these are independent events and remove duplicates
## otherwise.
```

```
##     40% ...
```

```
##     79% ...
```

```
## [3] Estimating global rates...
```

```
dndscvout$globaldnds
```

```
##      name      mle     cilow   cihigh
## wmis wmis 1.058930 1.0285178 1.090242
## wnon wnon 1.046679 0.9697056 1.129762
## wspl wspl 1.071039 0.9809454 1.169407
## wtru wtru 1.056907 0.9955836 1.122007
## wall wall 1.058618 1.0286068 1.089506
```

```
write.csv(dndscvout$annotmuts, "dnds_annotmuts_all.csv",  row.names = F, quote=F)
```

##### b) Per gene analysis all

```
dndscvout=dndscv(all,max_muts_per_gene_per_sample = 100, max_coding_muts_per_sample = 1000000)
```

```
## [1] Loading the environment...
```

```
## [2] Annotating the mutations...
```

```
## Warning in dndscv(all, max_muts_per_gene_per_sample = 100,
## max_coding_muts_per_sample = 1e+06): Mutations observed in contiguous sites
## within a sample. Please annotate or remove dinucleotide or complex substitutions
## for best results.
```

```
## Warning in dndscv(all, max_muts_per_gene_per_sample = 100,
## max_coding_muts_per_sample = 1e+06): Same mutations observed in different
## sampleIDs. Please verify that these are independent events and remove duplicates
## otherwise.
```

```
##     40% ...
```

```
##     79% ...
```

```
## [3] Estimating global rates...
```

```
## [4] Running dNdSloc...
```

```
## [5] Running dNdScv...
```

```
##     Regression model for substitutions (theta = 7.72).
```

```
##     Regression model for indels (theta = 2.81)
```

```
sel_cv = dndscvout$sel_cv
print(head(sel_cv, n= 30L), digits = 3)
```

```
##       gene_name n_syn n_mis n_non n_spl n_ind wmis_cv wnon_cv wspl_cv wind_cv
## 5236     DNMT3A     1    17     1     3     2   20.15   51.46   51.46   25.45
## 19644    ZNF318     0     3     4     1     5    2.11   50.83   50.83   37.99
## 7931   HIST2H3D     0     3     1     0     0   53.93 1020.52 1020.52    0.00
## 3490      CDK13     0     3     1     0     3    2.90   13.07   13.07   34.10
## 3037       CBFB     0     3     0     0     1   21.81    0.00    0.00   90.84
## 18765      UROS     0     0     1     2     0    0.00   88.93   88.93    0.00
## 3822      CILP2     2     7     1     0     2    4.18   12.75   12.75   22.77
## 3723      CHEK2     0     4     0     1     1    8.38   19.34   19.34   41.48
## 16680     SRCAP     0     6     2     1     2    2.49   18.15   18.15    8.53
## 1467      ARID2     1     3     2     2     0    2.12   32.20   32.20    0.00
## 18591    UBE2L6     0     1     0     2     0   11.06  168.93  168.93    0.00
## 16589    SPHKAP     0    10     0     0     2    3.27    0.00    0.00   24.37
## 4537     CTNNA3     1     5     0     0     2    3.09    0.00    0.00   46.80
## 18746     UQCC1     0     2     1     0     1    8.56   28.71   28.71   55.49
## 9004   KIAA1217     5    15     1     0     2    2.60    2.67    2.67   19.45
## 2898      CADPS     1     8     0     0     2    2.49    0.00    0.00   31.87
## 504      ACSM2B     0     5     3     0     0    4.77   20.98   20.98    0.00
## 18770     USH1C     2     0     0     2     1    0.00    6.71    6.71   21.21
## 11903     OPRD1     0     0     1     0     1    0.00   40.23   40.23   58.34
## 3786      CHRNG     0     1     0     0     2    1.57    0.00    0.00   70.39
## 10918    MUM1L1     1     0     0     0     2    0.00    0.00    0.00   36.99
## 16262      SMC3     0     3     0     0     2    4.43    0.00    0.00   26.21
## 2984      CARD6     0     1     2     0     1    1.14   32.89   32.89   14.61
## 17293      TCF4     0     1     2     1     0    1.40   38.83   38.83    0.00
## 4994       DFFA     0     1     0     1     1    4.58   59.22   59.22   44.20
## 8707      ITPKA     0     0     0     0     2    0.00    0.00    0.00   49.64
## 14544     RIMS2     1    10     1     0     1    4.09    3.30    3.30   18.95
## 7914   HIST1H4D     0     2     0     0     1   11.30    0.00    0.00   87.88
## 19285      YRDC     1     0     0     1     1    0.00   79.08   79.08   35.05
## 7482      GRAP2     0     3     0     0     1    9.14    0.00    0.00   36.43
##        pmis_cv ptrunc_cv pallsubs_cv  pind_cv  qmis_cv qtrunc_cv qallsubs_cv
## 5236  1.80e-12  2.76e-06    9.21e-15 3.83e-03 3.61e-08   0.02773    1.85e-10
## 19644 3.07e-01  2.52e-07    1.60e-06 2.84e-06 7.99e-01   0.00506    1.07e-02
## 7931  3.92e-05  6.19e-04    8.53e-07 1.00e+00 3.93e-01   0.88901    8.56e-03
## 3490  1.58e-01  7.48e-02    9.11e-02 2.30e-04 7.99e-01   0.95527    9.72e-01
## 3037  6.20e-04  8.45e-01    2.78e-03 1.09e-02 7.99e-01   0.95527    9.72e-01
## 18765 5.08e-01  8.38e-06    3.57e-05 1.00e+00 7.99e-01   0.05612    1.79e-01
## 3822  8.11e-03  7.57e-02    8.81e-03 4.74e-03 7.99e-01   0.95527    9.72e-01
## 3723  3.62e-03  4.83e-02    2.92e-03 2.37e-02 7.99e-01   0.95527    9.72e-01
## 16680 1.13e-01  1.08e-03    2.72e-03 2.89e-02 7.99e-01   0.95527    9.72e-01
## 1467  3.00e-01  1.83e-05    8.99e-05 1.00e+00 7.99e-01   0.09168    3.31e-01
## 18591 9.02e-02  6.32e-05    9.89e-05 1.00e+00 7.99e-01   0.25384    3.31e-01
## 16589 1.63e-02  5.25e-01    3.95e-02 4.16e-03 7.99e-01   0.95527    9.72e-01
## 4537  6.42e-02  5.68e-01    1.44e-01 1.18e-03 7.99e-01   0.95527    9.72e-01
## 18746 3.12e-02  3.10e-02    1.17e-02 1.78e-02 7.99e-01   0.95527    9.72e-01
## 9004  1.43e-02  4.09e-01    4.37e-02 6.39e-03 7.99e-01   0.95527    9.72e-01
## 2898  7.42e-02  4.17e-01    1.26e-01 2.49e-03 7.99e-01   0.95527    9.72e-01
## 504   1.44e-02  6.97e-04    4.08e-04 1.00e+00 7.99e-01   0.93372    9.72e-01
## 18770 3.15e-02  4.81e-02    8.97e-03 4.57e-02 7.99e-01   0.95527    9.72e-01
## 11903 1.86e-01  2.13e-02    2.59e-02 1.69e-02 7.99e-01   0.95527    9.72e-01
## 3786  6.89e-01  7.11e-01    8.59e-01 5.30e-04 8.15e-01   0.95527    9.72e-01
## 10918 1.06e-01  6.53e-01    2.48e-01 1.86e-03 7.99e-01   0.95527    9.72e-01
## 16262 5.72e-02  6.50e-01    1.43e-01 3.62e-03 7.99e-01   0.95527    9.72e-01
## 2984  9.04e-01  1.96e-03    8.23e-03 6.54e-02 9.36e-01   0.95527    9.72e-01
## 17293 7.64e-01  1.07e-04    5.52e-04 1.00e+00 8.50e-01   0.35944    9.72e-01
## 4994  2.38e-01  1.38e-02    2.65e-02 2.23e-02 7.99e-01   0.95527    9.72e-01
## 8707  3.12e-01  8.21e-01    5.85e-01 1.05e-03 7.99e-01   0.95527    9.72e-01
## 14544 3.60e-03  3.34e-01    1.23e-02 5.09e-02 7.99e-01   0.95527    9.72e-01
## 7914  1.79e-02  9.46e-01    6.04e-02 1.13e-02 7.99e-01   0.95890    9.72e-01
## 19285 4.50e-01  9.94e-03    2.62e-02 2.80e-02 7.99e-01   0.95527    9.72e-01
## 7482  8.03e-03  7.65e-01    2.80e-02 2.69e-02 7.99e-01   0.95527    9.72e-01
##       pglobal_cv qglobal_cv
## 5236    1.33e-15   2.68e-11
## 19644   1.23e-10   1.24e-06
## 7931    1.28e-05   8.55e-02
## 3490    2.47e-04   1.00e+00
## 3037    3.47e-04   1.00e+00
## 18765   4.02e-04   1.00e+00
## 3822    4.63e-04   1.00e+00
## 3723    7.33e-04   1.00e+00
## 16680   8.21e-04   1.00e+00
## 1467    9.28e-04   1.00e+00
## 18591   1.01e-03   1.00e+00
## 16589   1.60e-03   1.00e+00
## 4537    1.65e-03   1.00e+00
## 18746   1.98e-03   1.00e+00
## 9004    2.57e-03   1.00e+00
## 2898    2.84e-03   1.00e+00
## 504     3.59e-03   1.00e+00
## 18770   3.61e-03   1.00e+00
## 11903   3.83e-03   1.00e+00
## 3786    3.96e-03   1.00e+00
## 10918   4.01e-03   1.00e+00
## 16262   4.44e-03   1.00e+00
## 2984    4.59e-03   1.00e+00
## 17293   4.69e-03   1.00e+00
## 4994    4.98e-03   1.00e+00
## 8707    5.16e-03   1.00e+00
## 14544   5.25e-03   1.00e+00
## 7914    5.65e-03   1.00e+00
## 19285   6.02e-03   1.00e+00
## 7482    6.18e-03   1.00e+00
```

```
write.csv(sel_cv, "sel_cv_all.csv",  row.names = F, quote=F)
```

##### c) Significant genes all

```
signif_genes = sel_cv[sel_cv$qglobal_cv<0.1, c("gene_name","qglobal_cv")]
rownames(signif_genes) = NULL
print(signif_genes)
```

```
##   gene_name   qglobal_cv
## 1    DNMT3A 2.676659e-11
## 2    ZNF318 1.240366e-06
## 3  HIST2H3D 8.550180e-02
```

##### d) Estimation number of drivers all

```
wmis = dndscvout$globaldnds$mle[1]
nmis = (sum(dndscvout$annotmuts$impact =="Missense"))
ndrivers_all = (wmis-1)/wmis*nmis
ndrivers_all
```

```
## [1] 920.2401
```

```
wmis = dndscvout$globaldnds$cilow[1]
nmis = (sum(dndscvout$annotmuts$impact =="Missense"))
ndrivers_all_cilow = (wmis-1)/wmis*nmis
ndrivers_all_cilow
```

```
## [1] 458.4955
```

```
wmis = dndscvout$globaldnds$cihigh[1]
nmis = (sum(dndscvout$annotmuts$impact =="Missense"))
ndrivers_all_cihigh = (wmis-1)/wmis*nmis
ndrivers_all_cihigh
```

```
## [1] 1368.723
```

##### Forest plot

```
dnds <- structure(list(
    mean  = c(NA,dndscvout$globaldnds$mle[1],dndscvout$globaldnds$mle[2],dndscvout$globaldnds$mle[4],dndscvout$globaldnds$mle[5]), 
    lower = c(NA,dndscvout$globaldnds$cilow[1],dndscvout$globaldnds$cilow[2],dndscvout$globaldnds$cilow[4],dndscvout$globaldnds$cilow[5]),
    upper = c(NA,dndscvout$globaldnds$cihigh[1],dndscvout$globaldnds$cihigh[2],dndscvout$globaldnds$cihigh[4],dndscvout$globaldnds$cihigh[5])),
    .Names = c("mean", "lower", "upper"), 
    row.names = c(NA,-1L),
    class = "data.frame")

tabletext<-
  c("name","missense", "nonsense","truncating", "all")

forestplot(tabletext, 
           dnds,new_page = FALSE,
           boxsize = 0.15,
           is.summary=FALSE,
           clip=c(0.8,1.2),
           xticks = c(0.8,0.9,1.0,1.1,1.2),
           xlog=FALSE, 
           col=fpColors(box="royalblue",line="darkblue", summary="royalblue"))
```

##### e) Removing annotated drivers all

```
all_annot <- dndscvout$annotmuts
all_annot_no_drivers <- all_annot[!all_annot$gene %in% chip_drivers ,]
```

##### f) Annotated drivers removed all

```
dndscvout=dndscv(all_annot_no_drivers, outp = 1, max_muts_per_gene_per_sample = 100, max_coding_muts_per_sample = 1000000)
```

```
## [1] Loading the environment...
```

```
## [2] Annotating the mutations...
```

```
## Warning in dndscv(all_annot_no_drivers, outp = 1, max_muts_per_gene_per_sample
## = 100, : Mutations observed in contiguous sites within a sample. Please annotate
## or remove dinucleotide or complex substitutions for best results.
```

```
## Warning in dndscv(all_annot_no_drivers, outp = 1, max_muts_per_gene_per_sample =
## 100, : Same mutations observed in different sampleIDs. Please verify that these
## are independent events and remove duplicates otherwise.
```

```
##     40% ...
```

```
##     80% ...
```

```
## [3] Estimating global rates...
```

```
dndscvout$globaldnds
```

```
##      name      mle     cilow   cihigh
## wmis wmis 1.056669 1.0262162 1.088026
## wnon wnon 1.040178 0.9632693 1.123227
## wspl wspl 1.058595 0.9688839 1.156613
## wtru wtru 1.047912 0.9867384 1.112878
## wall wall 1.055845 1.0258068 1.086763
```

##### g) Cancer gene set analysis all

```
dndscvout=dndscv(all,max_muts_per_gene_per_sample = 100, max_coding_muts_per_sample = 1000000, gene_list = cancer_drivers)
```

```
## [1] Loading the environment...
```

```
## [2] Annotating the mutations...
```

```
## Warning in dndscv(all, max_muts_per_gene_per_sample = 100,
## max_coding_muts_per_sample = 1e+06, : Mutations observed in contiguous sites
## within a sample. Please annotate or remove dinucleotide or complex substitutions
## for best results.
```

```
## Warning in dndscv(all, max_muts_per_gene_per_sample = 100,
## max_coding_muts_per_sample = 1e+06, : Same mutations observed in different
## sampleIDs. Please verify that these are independent events and remove duplicates
## otherwise.
```

```
## [3] Estimating global rates...
```

```
## [4] Running dNdSloc...
```

```
## [5] Running dNdScv...
```

```
##     Regression model for substitutions (theta = 2.02).
```

```
##     Regression model for indels (theta = 0.977)
```

```
sel_cv = dndscvout$sel_cv
print(head(sel_cv), digits = 3)
```

```
##     gene_name n_syn n_mis n_non n_spl n_ind wmis_cv wnon_cv wspl_cv wind_cv
## 99     DNMT3A     1    17     1     3     2   15.66    40.7    40.7   25.35
## 47       CBFB     0     3     0     0     1   20.19     0.0     0.0   61.55
## 20      ARID2     1     3     2     2     0    1.83    28.1    28.1    0.00
## 304      SMC3     0     3     0     0     2    5.04     0.0     0.0   19.00
## 215     MXRA5     0    12     0     0     0   11.10     0.0     0.0    0.00
## 332      TET2     1     5     2     0     1    2.97    17.3    17.3    5.72
##      pmis_cv ptrunc_cv pallsubs_cv pind_cv  qmis_cv qtrunc_cv qallsubs_cv
## 99  7.08e-07  0.000036    9.27e-08 0.00540 0.000262    0.0133    3.43e-05
## 47  4.43e-03  0.844688    1.63e-02 0.01599 0.537549    0.9384    7.53e-01
## 20  5.00e-01  0.000150    6.32e-04 1.00000 0.738350    0.0278    1.17e-01
## 304 1.23e-01  0.680216    2.46e-01 0.00915 0.738350    0.9384    9.24e-01
## 215 1.35e-03  0.762408    4.41e-03 1.00000 0.249617    0.9384    5.11e-01
## 332 1.67e-01  0.011899    3.34e-02 0.14864 0.738350    0.5754    9.24e-01
##     pglobal_cv qglobal_cv
## 99    1.12e-08   4.15e-06
## 47    2.41e-03   4.46e-01
## 20    5.28e-03   6.52e-01
## 304   1.60e-02   9.99e-01
## 215   2.83e-02   9.99e-01
## 332   3.13e-02   9.99e-01
```

##### h) Cancer gene set analysis excluding CHIP drivers all

```
dndscvout=dndscv(all,max_muts_per_gene_per_sample = 100, max_coding_muts_per_sample = 1000000, gene_list = setdiff(cancer_drivers, chip_drivers))
```

```
## [1] Loading the environment...
```

```
## [2] Annotating the mutations...
```

```
## Warning in dndscv(all, max_muts_per_gene_per_sample = 100,
## max_coding_muts_per_sample = 1e+06, : Mutations observed in contiguous sites
## within a sample. Please annotate or remove dinucleotide or complex substitutions
## for best results.
```

```
## Warning in dndscv(all, max_muts_per_gene_per_sample = 100,
## max_coding_muts_per_sample = 1e+06, : Same mutations observed in different
## sampleIDs. Please verify that these are independent events and remove duplicates
## otherwise.
```

```
## [3] Estimating global rates...
```

```
## [4] Running dNdSloc...
```

```
## [5] Running dNdScv...
```

```
##     Regression model for substitutions (theta = 2.41).
```

```
##     Regression model for indels (theta = 1.1)
```

```
sel_cv = dndscvout$sel_cv
print(head(sel_cv), digits = 3)
```

```
##     gene_name n_syn n_mis n_non n_spl n_ind wmis_cv wnon_cv wspl_cv wind_cv
## 37       CBFB     0     3     0     0     1   17.03    0.00    0.00   59.59
## 236      SMC3     0     3     0     0     2    4.10    0.00    0.00   18.40
## 167     MXRA5     0    12     0     0     0    8.74    0.00    0.00    0.00
## 83      ERBB2     1     0     1     0     1    0.00    6.52    6.52    8.92
## 196      POT1     0     1     2     0     0    2.47   43.63   43.63    0.00
## 52     CDKN1A     0     0     0     0     1    0.00    0.00    0.00   56.30
##     pmis_cv ptrunc_cv pallsubs_cv pind_cv qmis_cv qtrunc_cv qallsubs_cv
## 37  0.00472    0.8270     0.01728 0.01652   0.692     0.929       0.922
## 236 0.14783    0.6416     0.27983 0.00927   0.723     0.929       0.922
## 167 0.00171    0.7357     0.00554 1.00000   0.501     0.929       0.922
## 83  0.13885    0.1793     0.09283 0.10127   0.723     0.929       0.922
## 196 0.50750    0.0023     0.00958 1.00000   0.729     0.674       0.922
## 52  0.55390    0.8710     0.82959 0.01747   0.729     0.929       0.922
##     pglobal_cv qglobal_cv
## 37     0.00262      0.766
## 236    0.01805      0.999
## 167    0.03430      0.999
## 83     0.05328      0.999
## 196    0.05411      0.999
## 52     0.07585      0.999
```

### 5. Analysis for all old individuals - private variants: private\_old

##### a) Global DNDS private\_old

```
#Run dndscv
dndscvout=dndscv(private_old,outp=1,max_muts_per_gene_per_sample = 100, max_coding_muts_per_sample = 400000)
```

```
## [1] Loading the environment...
```

```
## [2] Annotating the mutations...
```

```
## Warning in dndscv(private_old, outp = 1, max_muts_per_gene_per_sample = 100, :
## Mutations observed in contiguous sites within a sample. Please annotate or
## remove dinucleotide or complex substitutions for best results.
```

```
## Warning in dndscv(private_old, outp = 1, max_muts_per_gene_per_sample = 100, :
## Same mutations observed in different sampleIDs. Please verify that these are
## independent events and remove duplicates otherwise.
```

```
##     76% ...
```

```
## [3] Estimating global rates...
```

```
dndscvout$globaldnds
```

```
##      name      mle     cilow   cihigh
## wmis wmis 1.048644 1.0072503 1.091738
## wnon wnon 1.036566 0.9306812 1.154497
## wspl wspl 1.138057 1.0109303 1.281170
## wtru wtru 1.080105 0.9944411 1.173149
## wall wall 1.050561 1.0096229 1.093160
```

##### b) Per gene analysis private\_old

```
dndscvout=dndscv(private_old, max_muts_per_gene_per_sample = 100, max_coding_muts_per_sample = 1000000)
```

```
## [1] Loading the environment...
```

```
## [2] Annotating the mutations...
```

```
## Warning in dndscv(private_old, max_muts_per_gene_per_sample = 100,
## max_coding_muts_per_sample = 1e+06): Mutations observed in contiguous sites
## within a sample. Please annotate or remove dinucleotide or complex substitutions
## for best results.
```

```
## Warning in dndscv(private_old, max_muts_per_gene_per_sample = 100,
## max_coding_muts_per_sample = 1e+06): Same mutations observed in different
## sampleIDs. Please verify that these are independent events and remove duplicates
## otherwise.
```

```
##     76% ...
```

```
## [3] Estimating global rates...
```

```
## [4] Running dNdSloc...
```

```
## [5] Running dNdScv...
```

```
##     Regression model for substitutions (theta = 6.46).
```

```
##     Regression model for indels (theta = 1.63)
```

```
sel_cv = dndscvout$sel_cv
print(head(sel_cv, n= 30L), digits = 3)
```

```
##            gene_name n_syn n_mis n_non n_spl n_ind wmis_cv wnon_cv wspl_cv
## 19644         ZNF318     0     1     3     1     3    1.34    80.6    80.6
## 3490           CDK13     0     1     1     0     3    1.88    26.1    26.1
## 1467           ARID2     1     2     2     2     0    2.57    60.7    60.7
## 5236          DNMT3A     0     6     1     0     0   16.36    29.9    29.9
## 16680          SRCAP     0     5     2     1     1    3.67    32.9    32.9
## 15166          RTDR1     0     5     0     0     0   24.95     0.0     0.0
## 9038        KIAA1919     0     3     1     0     0   19.35   112.8   112.8
## 10918         MUM1L1     1     0     0     0     2    0.00     0.0     0.0
## 4537          CTNNA3     0     2     0     0     2    2.66     0.0     0.0
## 4994            DFFA     0     0     0     1     1    0.00   126.9   126.9
## 8707           ITPKA     0     0     0     0     2    0.00     0.0     0.0
## 16262           SMC3     0     2     0     0     2    5.55     0.0     0.0
## 10071           MANF     1     3     0     0     0   45.46     0.0     0.0
## 18162          TREM1     1     0     2     0     0    0.00   131.9   131.9
## 10172           MAS1     0     0     2     0     0    0.00   119.0   119.0
## 7014          GDF5OS     0     0     2     0     0    0.00   115.3   115.3
## 9729           LPHN2     0     9     0     0     0    8.01     0.0     0.0
## 12361      OVCH1-AS1     0     4     0     0     0   22.36     0.0     0.0
## 18765           UROS     0     0     1     1     0    0.00   123.4   123.4
## 8242           HYOU1     2     5     1     0     0    9.28    18.9    18.9
## 7482           GRAP2     0     2     0     0     1   11.50     0.0     0.0
## 13888 PTGES3L-AARSD1     0     0     1     1     0    0.00    87.7    87.7
## 4098          COL1A2     0     4     0     3     0    3.59    24.0    24.0
## 16603         SPINK2     0     1     0     0     1   15.28     0.0     0.0
## 8649            ISM1     0     3     0     0     1    7.01     0.0     0.0
## 6618            FMO1     0     0     1     0     1    0.00    20.9    20.9
## 11627          NR5A1     0     1     1     0     1    2.30    41.4    41.4
## 9867          LRRTM4     0     7     0     0     0    8.90     0.0     0.0
## 15388           SCOC     0     1     0     0     1   11.95     0.0     0.0
## 7931        HIST2H3D     0     0     1     0     0    0.00  2522.9  2522.9
##       wind_cv  pmis_cv ptrunc_cv pallsubs_cv  pind_cv qmis_cv qtrunc_cv
## 19644   38.41 7.95e-01  6.09e-07    3.83e-06 2.42e-04   0.845    0.0122
## 3490    60.45 5.89e-01  3.51e-02    9.97e-02 6.59e-05   0.829    0.9744
## 1467     0.00 2.86e-01  1.64e-06    8.64e-06 1.00e+00   0.829    0.0164
## 5236     0.00 2.17e-05  3.02e-02    2.11e-05 1.00e+00   0.218    0.9744
## 16680    7.76 4.41e-02  2.01e-04    3.33e-04 1.17e-01   0.829    0.5755
## 15166    0.00 1.10e-05  8.74e-01    6.23e-05 1.00e+00   0.218    0.9744
## 9038     0.00 9.92e-04  6.90e-03    1.67e-04 1.00e+00   0.829    0.9744
## 10918   82.98 2.00e-01  7.31e-01    4.17e-01 4.52e-04   0.829    0.9744
## 4537    90.59 2.77e-01  7.01e-01    5.07e-01 3.80e-04   0.829    0.9744
## 4994    73.33 6.52e-01  6.06e-03    2.05e-02 1.35e-02   0.829    0.9744
## 8707    89.14 4.39e-01  8.63e-01    7.31e-01 3.93e-04   0.829    0.9744
## 16262   43.22 7.58e-02  7.45e-01    1.93e-01 1.61e-03   0.829    0.9744
## 10071    0.00 6.76e-05  9.15e-01    3.54e-04 1.00e+00   0.453    0.9744
## 18162    0.00 5.48e-01  1.07e-04    4.36e-04 1.00e+00   0.829    0.4996
## 10172    0.00 4.06e-01  1.40e-04    4.42e-04 1.00e+00   0.829    0.4996
## 7014     0.00 4.10e-01  1.49e-04    4.74e-04 1.00e+00   0.829    0.4996
## 9729     0.00 1.26e-04  6.28e-01    4.97e-04 1.00e+00   0.508    0.9744
## 12361    0.00 1.04e-04  8.51e-01    5.19e-04 1.00e+00   0.508    0.9744
## 18765    0.00 6.39e-01  1.30e-04    5.68e-04 1.00e+00   0.829    0.4996
## 8242     0.00 8.02e-04  4.93e-02    7.76e-04 1.00e+00   0.829    0.9744
## 7482    63.69 1.82e-02  8.30e-01    5.96e-02 1.55e-02   0.829    0.9744
## 13888    0.00 5.03e-01  2.66e-04    9.54e-04 1.00e+00   0.829    0.5928
## 4098     0.00 6.79e-02  5.23e-04    1.00e-03 1.00e+00   0.829    0.8198
## 16603  177.36 6.39e-02  9.27e-01    1.79e-01 5.61e-03   0.829    0.9744
## 8649    56.91 1.84e-02  7.98e-01    5.94e-02 1.73e-02   0.829    0.9744
## 6618    78.05 3.57e-01  4.50e-02    8.15e-02 1.27e-02   0.829    0.9744
## 11627   55.80 4.88e-01  2.09e-02    5.91e-02 1.77e-02   0.829    0.9744
## 9867     0.00 2.53e-04  7.60e-01    1.13e-03 1.00e+00   0.725    0.9744
## 15388  195.25 8.41e-02  9.09e-01    2.23e-01 5.10e-03   0.829    0.9744
## 7931     0.00 8.29e-01  2.39e-04    1.14e-03 1.00e+00   0.864    0.5928
##       qallsubs_cv pglobal_cv qglobal_cv
## 19644      0.0770   2.02e-08   0.000407
## 3490       0.9934   8.49e-05   0.732339
## 1467       0.0868   1.09e-04   0.732339
## 5236       0.1413   2.48e-04   1.000000
## 16680      0.8688   4.33e-04   1.000000
## 15166      0.3129   6.66e-04   1.000000
## 9038       0.6729   1.62e-03   1.000000
## 10918      0.9934   1.81e-03   1.000000
## 4537       0.9934   1.84e-03   1.000000
## 4994       0.9934   2.54e-03   1.000000
## 8707       0.9934   2.63e-03   1.000000
## 16262      0.9934   2.83e-03   1.000000
## 10071      0.8688   3.17e-03   1.000000
## 18162      0.8688   3.81e-03   1.000000
## 10172      0.8688   3.86e-03   1.000000
## 7014       0.8688   4.11e-03   1.000000
## 9729       0.8688   4.28e-03   1.000000
## 12361      0.8688   4.44e-03   1.000000
## 18765      0.8775   4.81e-03   1.000000
## 8242       0.9934   6.33e-03   1.000000
## 7482       0.9934   7.38e-03   1.000000
## 13888      0.9934   7.59e-03   1.000000
## 4098       0.9934   7.91e-03   1.000000
## 16603      0.9934   7.93e-03   1.000000
## 8649       0.9934   8.11e-03   1.000000
## 6618       0.9934   8.14e-03   1.000000
## 11627      0.9934   8.20e-03   1.000000
## 9867       0.9934   8.77e-03   1.000000
## 15388      0.9934   8.85e-03   1.000000
## 7931       0.9934   8.88e-03   1.000000
```

```
write.csv(sel_cv, "sel_cv_private_old.csv",  row.names = F, quote=F)
```

##### c) Significant genes private\_old

```
signif_genes = sel_cv[sel_cv$qglobal_cv<0.1,c("gene_name", "qglobal_cv")]
rownames(signif_genes) = NULL
print(signif_genes)
```

```
##   gene_name   qglobal_cv
## 1    ZNF318 0.0004066815
```

##### d) Estimation number of drivers

```
wmis = dndscvout$globaldnds$mle[1]
nmis = (sum(dndscvout$annotmuts$impact =="Missense"))
ndrivers_private_old = (wmis-1)/wmis*nmis
ndrivers_private_old
```

```
## [1] 399.72
```

```
wmis = dndscvout$globaldnds$cilow[1]
nmis = (sum(dndscvout$annotmuts$impact =="Missense"))
ndrivers_private_old_cilow = (wmis-1)/wmis*nmis
ndrivers_private_old_cilow
```

```
## [1] 62.02639
```

```
wmis = dndscvout$globaldnds$cihigh[1]
nmis = (sum(dndscvout$annotmuts$impact =="Missense"))
ndrivers_private_old_cihigh = (wmis-1)/wmis*nmis
ndrivers_private_old_cihigh
```

```
## [1] 724.0837
```

##### e) Removing annotated drivers from dataset private\_old

```
private_old_annot <- dndscvout$annotmuts
private_old_annot_no_drivers <- private_old_annot[!private_old_annot$gene %in% chip_drivers ,]
```

##### f) Annotated drivers removed private\_old

```
dndscvout=dndscv(private_old_annot_no_drivers, outp = 1, max_muts_per_gene_per_sample = 100, max_coding_muts_per_sample = 1000000)
```

```
## [1] Loading the environment...
```

```
## [2] Annotating the mutations...
```

```
## Warning in dndscv(private_old_annot_no_drivers, outp = 1,
## max_muts_per_gene_per_sample = 100, : Mutations observed in contiguous sites
## within a sample. Please annotate or remove dinucleotide or complex substitutions
## for best results.
```

```
## Warning in dndscv(private_old_annot_no_drivers, outp = 1,
## max_muts_per_gene_per_sample = 100, : Same mutations observed in different
## sampleIDs. Please verify that these are independent events and remove duplicates
## otherwise.
```

```
##     77% ...
```

```
## [3] Estimating global rates...
```

```
dndscvout$globaldnds
```

```
##      name      mle     cilow   cihigh
## wmis wmis 1.045600 1.0041837 1.088725
## wnon wnon 1.021652 0.9165414 1.138816
## wspl wspl 1.129783 1.0028100 1.272834
## wtru wtru 1.067940 0.9826939 1.160580
## wall wall 1.046760 1.0058276 1.089359
```

##### g) Number of drivers with annotated drivers removed

```
wmis = dndscvout$globaldnds$mle[1]
nmis = (sum(dndscvout$annotmuts$impact =="Missense"))
ndrivers_private_old = (wmis-1)/wmis*nmis
ndrivers_private_old
```

```
## [1] 372.3972
```

##### h) Cancer gene set analysis private\_old

```
dndscvout=dndscv(private_old,max_muts_per_gene_per_sample = 100, max_coding_muts_per_sample = 1000000, gene_list = cancer_drivers)
```

```
## [1] Loading the environment...
```

```
## [2] Annotating the mutations...
```

```
## Warning in dndscv(private_old, max_muts_per_gene_per_sample = 100,
## max_coding_muts_per_sample = 1e+06, : Mutations observed in contiguous sites
## within a sample. Please annotate or remove dinucleotide or complex substitutions
## for best results.
```

```
## Warning in dndscv(private_old, max_muts_per_gene_per_sample = 100,
## max_coding_muts_per_sample = 1e+06, : Same mutations observed in different
## sampleIDs. Please verify that these are independent events and remove duplicates
## otherwise.
```

```
## [3] Estimating global rates...
```

```
## [4] Running dNdSloc...
```

```
## [5] Running dNdScv...
```

```
##     Regression model for substitutions (theta = 1.28).
```

```
##     Regression model for indels (theta = 0.931)
```

```
sel_cv = dndscvout$sel_cv
print(head(sel_cv, n= 30L), digits = 3)
```

```
##     gene_name n_syn n_mis n_non n_spl n_ind wmis_cv wnon_cv wspl_cv wind_cv
## 20      ARID2     1     2     2     2     0    2.20    57.9    57.9    0.00
## 99     DNMT3A     0     6     1     0     0   46.63    84.9    84.9    0.00
## 304      SMC3     0     2     0     0     2   13.85     0.0     0.0   20.90
## 158     HNF1A     1     5     0     1     0   12.05    35.9    35.9    0.00
## 215     MXRA5     0     9     0     0     0   33.16     0.0     0.0    0.00
## 110     ERBB2     0     0     1     0     1    0.00    62.1    62.1   10.13
## 303     SMC1A     0     3     0     0     1   19.09     0.0     0.0   10.31
## 240      PAX5     0     3     0     0     0   45.44     0.0     0.0    0.00
## 25     ATP1B1     0     1     1     0     0   21.86   213.1   213.1    0.00
## 332      TET2     1     3     1     0     1    3.18    20.5    20.5    6.29
## 21     ARID5B     0     2     0     0     1   14.55     0.0     0.0   10.70
## 358    ZBTB20     0     1     1     0     0    8.86   255.0   255.0    0.00
## 7        ADNP     0     4     0     0     0   30.78     0.0     0.0    0.00
## 306    SMTNL2     1     0     0     0     1    0.00     0.0     0.0   27.55
## 47       CBFB     0     2     0     0     0   57.71     0.0     0.0    0.00
## 322      SUFU     0     0     0     0     1    0.00     0.0     0.0   26.24
## 270     RAD21     0     0     0     1     0    0.00   133.8   133.8    0.00
## 16   ARHGAP35     1     0     1     0     0    0.00    31.7    31.7    0.00
## 38       BRAF     0     3     0     0     0   30.25     0.0     0.0    0.00
## 256     PPM1D     0     1     1     0     0   10.76   143.5   143.5    0.00
## 108     EPHA2     0     1     0     0     1    6.66     0.0     0.0   13.03
## 282      RPL5     0     2     0     0     0   43.12     0.0     0.0    0.00
## 176    KANSL1     0     1     0     0     1    7.05     0.0     0.0   11.51
## 340     TOP2A     0     4     0     0     0   22.62     0.0     0.0    0.00
## 213     MUC17     1     7     0     0     1    4.10     0.0     0.0    2.83
## 116      EZH2     0     0     0     1     0    0.00    83.9    83.9    0.00
## 29      AXIN2     0     3     0     0     0   26.00     0.0     0.0    0.00
## 39      BRCA1     1     0     0     1     0    0.00    16.8    16.8    0.00
## 210      MSH2     0     1     0     1     0    8.62    93.5    93.5    0.00
## 24     ATP1A1     0     0     0     0     1    0.00     0.0     0.0   12.43
##      pmis_cv ptrunc_cv pallsubs_cv pind_cv qmis_cv qtrunc_cv qallsubs_cv
## 20  0.479419  2.14e-05    9.23e-05 1.00000   0.858   0.00792      0.0341
## 99  0.000606  2.40e-02    1.21e-03 1.00000   0.210   0.98182      0.2239
## 304 0.092038  8.44e-01    2.18e-01 0.00784   0.858   0.98182      0.9822
## 158 0.003676  3.15e-02    3.96e-03 1.00000   0.404   0.98182      0.3958
## 215 0.001137  8.91e-01    4.28e-03 1.00000   0.210   0.98182      0.3958
## 110 0.618536  3.38e-02    6.11e-02 0.08953   0.858   0.98182      0.9822
## 303 0.034682  8.71e-01    9.73e-02 0.08811   0.850   0.98182      0.9822
## 240 0.004362  9.18e-01    1.64e-02 1.00000   0.404   0.98182      0.9822
## 25  0.102800  8.77e-03    1.77e-02 1.00000   0.858   0.98182      0.9822
## 332 0.247813  5.86e-02    1.30e-01 0.13655   0.858   0.98182      0.9822
## 21  0.085274  9.04e-01    2.19e-01 0.08518   0.858   0.98182      0.9822
## 358 0.247579  7.20e-03    2.39e-02 1.00000   0.858   0.98182      0.9822
## 7   0.006833  9.09e-01    2.41e-02 1.00000   0.455   0.98182      0.9822
## 306 0.417002  8.39e-01    7.08e-01 0.03498   0.858   0.98182      0.9822
## 47  0.007376  9.28e-01    2.68e-02 1.00000   0.455   0.98182      0.9822
## 322 0.717219  9.06e-01    9.31e-01 0.03666   0.858   0.98182      0.9822
## 270 0.702316  1.46e-02    3.65e-02 1.00000   0.858   0.98182      0.9822
## 16  0.265973  3.61e-02    3.77e-02 1.00000   0.858   0.98182      0.9822
## 38  0.012164  8.73e-01    3.86e-02 1.00000   0.598   0.98182      0.9822
## 256 0.207174  1.35e-02    3.87e-02 1.00000   0.858   0.98182      0.9822
## 108 0.317630  8.84e-01    5.88e-01 0.07111   0.858   0.98182      0.9822
## 282 0.012920  9.15e-01    4.37e-02 1.00000   0.598   0.98182      0.9822
## 176 0.302648  8.86e-01    5.70e-01 0.07972   0.858   0.98182      0.9822
## 340 0.016204  8.38e-01    4.56e-02 1.00000   0.644   0.98182      0.9822
## 213 0.090278  7.56e-01    2.08e-01 0.25872   0.858   0.98182      0.9822
## 116 0.677997  2.44e-02    5.42e-02 1.00000   0.858   0.98182      0.9822
## 29  0.017414  9.08e-01    5.60e-02 1.00000   0.644   0.98182      0.9822
## 39  0.245505  7.30e-02    6.29e-02 1.00000   0.858   0.98182      0.9822
## 210 0.253948  2.16e-02    6.35e-02 1.00000   0.858   0.98182      0.9822
## 24  0.638598  8.77e-01    8.88e-01 0.07426   0.858   0.98182      0.9822
##     pglobal_cv qglobal_cv
## 20    0.000949      0.351
## 99    0.009339      1.000
## 304   0.012592      1.000
## 158   0.025844      1.000
## 215   0.027619      1.000
## 110   0.033944      1.000
## 303   0.049357      1.000
## 240   0.083611      1.000
## 25    0.088909      1.000
## 332   0.089477      1.000
## 21    0.092778      1.000
## 358   0.113311      1.000
## 7     0.113790      1.000
## 306   0.116353      1.000
## 47    0.123795      1.000
## 322   0.149448      1.000
## 270   0.157418      1.000
## 16    0.161294      1.000
## 38    0.164062      1.000
## 256   0.164592      1.000
## 108   0.174483      1.000
## 282   0.180586      1.000
## 176   0.185826      1.000
## 340   0.186329      1.000
## 213   0.211128      1.000
## 116   0.212052      1.000
## 29    0.217343      1.000
## 39    0.236846      1.000
## 210   0.238477      1.000
## 24    0.245309      1.000
```

##### i) Cancer gene set analysis excluding CHIP drivers private\_old

```
dndscvout=dndscv(private_old,max_muts_per_gene_per_sample = 100, max_coding_muts_per_sample = 1000000, gene_list = setdiff(cancer_drivers, chip_drivers))
```

```
## [1] Loading the environment...
```

```
## [2] Annotating the mutations...
```

```
## Warning in dndscv(private_old, max_muts_per_gene_per_sample = 100,
## max_coding_muts_per_sample = 1e+06, : Mutations observed in contiguous sites
## within a sample. Please annotate or remove dinucleotide or complex substitutions
## for best results.
```

```
## Warning in dndscv(private_old, max_muts_per_gene_per_sample = 100,
## max_coding_muts_per_sample = 1e+06, : Same mutations observed in different
## sampleIDs. Please verify that these are independent events and remove duplicates
## otherwise.
```

```
## [3] Estimating global rates...
```

```
## [4] Running dNdSloc...
```

```
## [5] Running dNdScv...
```

```
##     Regression model for substitutions (theta = 1.53).
```

```
##     Regression model for indels (theta = 1.04)
```

```
sel_cv = dndscvout$sel_cv
print(head(sel_cv), digits = 3)
```

```
##     gene_name n_syn n_mis n_non n_spl n_ind wmis_cv wnon_cv wspl_cv wind_cv
## 236      SMC3     0     2     0     0     2    8.26     0.0     0.0   20.24
## 122     HNF1A     1     5     0     1     0   11.48    30.7    30.7    0.00
## 167     MXRA5     0     9     0     0     0   19.20     0.0     0.0    0.00
## 83      ERBB2     0     0     1     0     1    0.00    34.1    34.1    9.81
## 235     SMC1A     0     3     0     0     1   11.49     0.0     0.0    9.99
## 20     ATP1B1     0     1     1     0     0   13.37   115.5   115.5    0.00
##     pmis_cv ptrunc_cv pallsubs_cv pind_cv qmis_cv qtrunc_cv qallsubs_cv
## 236 0.11831    0.7929     0.26346 0.00796   0.805      0.97       0.971
## 122 0.00314    0.0357     0.00356 1.00000   0.460      0.97       0.758
## 167 0.00141    0.8521     0.00517 1.00000   0.413      0.97       0.758
## 83  0.51490    0.0460     0.07609 0.09266   0.805      0.97       0.971
## 235 0.04318    0.8256     0.11610 0.09118   0.805      0.97       0.971
## 20  0.12564    0.0120     0.02334 1.00000   0.805      0.97       0.971
##     pglobal_cv qglobal_cv
## 236     0.0150          1
## 122     0.0237          1
## 167     0.0324          1
## 83      0.0420          1
## 235     0.0587          1
## 20      0.1110          1
```

### 6. Analysis for all old individuals - shared variants: shared\_old

##### a) Global DNDS shared\_old

```
#Run dndscv
dndscvout=dndscv(shared_old,outp=1,max_muts_per_gene_per_sample = 100, max_coding_muts_per_sample = 400000)
```

```
## [1] Loading the environment...
```

```
## [2] Annotating the mutations...
```

```
## Warning in dndscv(shared_old, outp = 1, max_muts_per_gene_per_sample = 100, :
## Mutations observed in contiguous sites within a sample. Please annotate or
## remove dinucleotide or complex substitutions for best results.
```

```
## Warning in dndscv(shared_old, outp = 1, max_muts_per_gene_per_sample = 100, :
## Same mutations observed in different sampleIDs. Please verify that these are
## independent events and remove duplicates otherwise.
```

```
## [3] Estimating global rates...
```

```
dndscvout$globaldnds
```

```
##      name      mle     cilow   cihigh
## wmis wmis 1.077572 0.9544637 1.216559
## wnon wnon 1.037664 0.7558524 1.424547
## wspl wspl 1.129915 0.7775586 1.641944
## wtru wtru 1.074002 0.8362290 1.379384
## wall wall 1.076722 0.9552314 1.213665
```

##### b) Per gene analysis shared\_old

```
dndscvout=dndscv(shared_old, max_muts_per_gene_per_sample = 100, max_coding_muts_per_sample = 1000000)
```

```
## [1] Loading the environment...
```

```
## [2] Annotating the mutations...
```

```
## Warning in dndscv(shared_old, max_muts_per_gene_per_sample = 100,
## max_coding_muts_per_sample = 1e+06): Mutations observed in contiguous sites
## within a sample. Please annotate or remove dinucleotide or complex substitutions
## for best results.
```

```
## Warning in dndscv(shared_old, max_muts_per_gene_per_sample = 100,
## max_coding_muts_per_sample = 1e+06): Same mutations observed in different
## sampleIDs. Please verify that these are independent events and remove duplicates
## otherwise.
```

```
## [3] Estimating global rates...
```

```
## [4] Running dNdSloc...
```

```
## [5] Running dNdScv...
```

```
## Warning in theta.ml(Y, mu, sum(w), w, limit = control$maxit, trace =
## control$trace > : iteration limit reached
```

```
## Warning in theta.ml(Y, mu, sum(w), w, limit = control$maxit, trace =
## control$trace > : iteration limit reached
```

```
##     Regression model for substitutions (theta = 10.5).
```

```
##     Regression model for indels (theta = 0.36)
```

```
sel_cv = dndscvout$sel_cv
print(head(sel_cv, n= 30L), digits = 3)
```

```
##             gene_name n_syn n_mis n_non n_spl n_ind wmis_cv wnon_cv wspl_cv
## 5236           DNMT3A     1     9     0     3     2  108.07   423.2   423.2
## 19644          ZNF318     0     1     1     0     2    6.59    95.9    95.9
## 4382          CSNK1A1     0     2     0     1     0   85.20   361.1   361.1
## 4968          DENND2C     0     0     0     1     1    0.00   159.9   159.9
## 2516             C1QB     1     1     0     1     0   46.26  1268.6  1268.6
## 14753 RP11-1396O13.13     0     2     0     0     0  149.03     0.0     0.0
## 6718            FREM1     0     2     0     0     1   14.77     0.0     0.0
## 16608          SPINK8     0     0     0     0     1    0.00     0.0     0.0
## 19039            WBP5     0     0     0     0     1    0.00     0.0     0.0
## 12603          PCDHB7     0     1     0     0     1   15.31     0.0     0.0
## 3539         CDRT15L2     0     2     0     0     0  104.54     0.0     0.0
## 17380            TET2     0     2     1     0     0   16.79   109.2   109.2
## 18593           UBE2N     0     0     0     0     1    0.00     0.0     0.0
## 3037             CBFB     0     0     0     0     1    0.00     0.0     0.0
## 13336            POT1     0     1     1     0     0   27.34   251.5   251.5
## 4108           COL3A1     1     3     0     0     0   26.79     0.0     0.0
## 4399           CSRNP3     0     1     1     0     0   23.16   271.8   271.8
## 9077            KIF4B     0     1     0     0     1   13.78     0.0     0.0
## 766              AIG1     0     0     0     0     1    0.00     0.0     0.0
## 13705           PRR18     0     0     1     0     0    0.00  1801.8  1801.8
## 18997          VSTM2B     0     0     0     0     1    0.00     0.0     0.0
## 6483            FFAR1     0     2     0     0     0   72.17     0.0     0.0
## 13104          PLCXD2     0     0     0     0     1    0.00     0.0     0.0
## 18311           TRUB2     0     0     0     0     1    0.00     0.0     0.0
## 2160           BTN3A2     0     0     0     0     1    0.00     0.0     0.0
## 643           ADIPOR2     0     0     0     0     1    0.00     0.0     0.0
## 2034            BMP15     0     0     0     0     1    0.00     0.0     0.0
## 15465           SEC62     0     0     0     0     1    0.00     0.0     0.0
## 5147             DMTN     0     0     0     0     1    0.00     0.0     0.0
## 19800          ZNF562     0     0     0     0     1    0.00     0.0     0.0
##       wind_cv  pmis_cv ptrunc_cv pallsubs_cv  pind_cv  qmis_cv qtrunc_cv
## 5236    374.7 7.55e-15  5.54e-08    0.00e+00 5.25e-05 1.52e-10   0.00111
## 19644   150.0 1.57e-01  7.94e-03    1.17e-02 3.17e-04 9.41e-01   0.98906
## 4382      0.0 2.46e-04  1.86e-03    1.14e-05 1.00e+00 9.41e-01   0.98906
## 4968    184.1 7.32e-01  4.52e-03    1.67e-02 5.38e-03 9.41e-01   0.98906
## 2516      0.0 1.77e-02  4.78e-04    1.47e-04 1.00e+00 9.41e-01   0.98906
## 14753     0.0 7.60e-05  9.59e-01    3.98e-04 1.00e+00 7.64e-01   0.98906
## 6718     78.4 9.63e-03  8.83e-01    3.46e-02 1.25e-02 9.41e-01   0.98906
## 16608  1745.4 9.18e-01  9.65e-01    9.94e-01 5.72e-04 9.41e-01   0.98906
## 19039  1629.0 9.15e-01  9.78e-01    9.94e-01 6.13e-04 9.41e-01   0.98906
## 12603   215.4 6.14e-02  9.46e-01    1.73e-01 4.60e-03 9.41e-01   0.98906
## 3539      0.0 1.60e-04  9.60e-01    8.04e-04 1.00e+00 9.41e-01   0.98906
## 17380     0.0 7.40e-03  6.87e-03    8.49e-04 1.00e+00 9.41e-01   0.98906
## 18593  1118.0 8.82e-01  9.69e-01    9.88e-01 8.93e-04 9.41e-01   0.98906
## 3037    909.8 8.59e-01  9.48e-01    9.82e-01 1.10e-03 9.41e-01   0.98906
## 13336     0.0 3.21e-02  2.76e-03    1.25e-03 1.00e+00 9.41e-01   0.98906
## 4108      0.0 2.76e-04  8.54e-01    1.31e-03 1.00e+00 9.41e-01   0.98906
## 4399      0.0 3.86e-02  2.53e-03    1.35e-03 1.00e+00 9.41e-01   0.98906
## 9077    138.5 6.91e-02  9.16e-01    1.90e-01 7.12e-03 9.41e-01   0.98906
## 766     695.3 8.60e-01  9.46e-01    9.82e-01 1.43e-03 9.41e-01   0.98906
## 13705     0.0 7.86e-01  3.30e-04    1.52e-03 1.00e+00 9.41e-01   0.98906
## 18997   598.1 7.95e-01  9.55e-01    9.65e-01 1.67e-03 9.41e-01   0.98906
## 6483      0.0 3.49e-04  9.68e-01    1.67e-03 1.00e+00 9.41e-01   0.98906
## 13104   559.0 8.40e-01  9.57e-01    9.78e-01 1.78e-03 9.41e-01   0.98906
## 18311   515.2 8.15e-01  9.49e-01    9.71e-01 1.93e-03 9.41e-01   0.98906
## 2160    510.6 8.38e-01  9.45e-01    9.77e-01 1.95e-03 9.41e-01   0.98906
## 643     442.0 8.26e-01  9.44e-01    9.74e-01 2.25e-03 9.41e-01   0.98906
## 2034    435.2 8.19e-01  9.49e-01    9.72e-01 2.29e-03 9.41e-01   0.98906
## 15465   427.6 8.34e-01  9.45e-01    9.76e-01 2.33e-03 9.41e-01   0.98906
## 5147    421.3 8.06e-01  9.35e-01    9.67e-01 2.36e-03 9.41e-01   0.98906
## 19800   400.6 8.25e-01  9.50e-01    9.74e-01 2.48e-03 9.41e-01   0.98906
##       qallsubs_cv pglobal_cv qglobal_cv
## 5236        0.000   0.00e+00      0.000
## 19644       0.999   5.02e-05      0.504
## 4382        0.114   1.41e-04      0.945
## 4968        0.999   9.24e-04      1.000
## 2516        0.983   1.44e-03      1.000
## 14753       0.999   3.51e-03      1.000
## 6718        0.999   3.77e-03      1.000
## 16608       0.999   4.82e-03      1.000
## 19039       0.999   5.12e-03      1.000
## 12603       0.999   6.49e-03      1.000
## 3539        0.999   6.53e-03      1.000
## 17380       0.999   6.85e-03      1.000
## 18593       0.999   7.09e-03      1.000
## 3037        0.999   8.44e-03      1.000
## 13336       0.999   9.58e-03      1.000
## 4108        0.999   1.00e-02      1.000
## 4399        0.999   1.03e-02      1.000
## 9077        0.999   1.03e-02      1.000
## 766         0.999   1.07e-02      1.000
## 13705       0.999   1.14e-02      1.000
## 18997       0.999   1.20e-02      1.000
## 6483        0.999   1.23e-02      1.000
## 13104       0.999   1.28e-02      1.000
## 18311       0.999   1.37e-02      1.000
## 2160        0.999   1.38e-02      1.000
## 643         0.999   1.56e-02      1.000
## 2034        0.999   1.58e-02      1.000
## 15465       0.999   1.61e-02      1.000
## 5147        0.999   1.62e-02      1.000
## 19800       0.999   1.70e-02      1.000
```

```
write.csv(sel_cv, "sel_cv_shared_old.csv",  row.names = F, quote=F)
```

##### c) Significant genes shared\_old

```
signif_genes = sel_cv[sel_cv$qglobal_cv<0.1,c("gene_name", "qglobal_cv")]
rownames(signif_genes) = NULL
print(signif_genes)
```

```
##   gene_name qglobal_cv
## 1    DNMT3A          0
```

##### d) Estimation number of drivers

```
wmis = dndscvout$globaldnds$mle[1]
nmis = (sum(dndscvout$annotmuts$impact =="Missense"))
ndrivers_shared_old = (wmis-1)/wmis*nmis
ndrivers_shared_old
```

```
## [1] 69.75622
```

```
wmis = dndscvout$globaldnds$cilow[1]
nmis = (sum(dndscvout$annotmuts$impact =="Missense"))
ndrivers_shared_old_cilow = (wmis-1)/wmis*nmis
ndrivers_shared_old_cilow
```

```
## [1] -46.22978
```

```
wmis = dndscvout$globaldnds$cihigh[1]
nmis = (sum(dndscvout$annotmuts$impact =="Missense"))
ndrivers_shared_old_cihigh = (wmis-1)/wmis*nmis
ndrivers_shared_old_cihigh
```

```
## [1] 172.4913
```

##### e) Removing annotated drivers from dataset shared\_old

```
shared_old_annot <- dndscvout$annotmuts
shared_old_annot_no_drivers <- shared_old_annot[!shared_old_annot$gene %in% chip_drivers ,]
```

##### f) Annotated drivers removed shared\_old

```
dndscvout=dndscv(shared_old_annot_no_drivers, outp = 1, max_muts_per_gene_per_sample = 100, max_coding_muts_per_sample = 1000000)
```

```
## [1] Loading the environment...
```

```
## [2] Annotating the mutations...
```

```
## Warning in dndscv(shared_old_annot_no_drivers, outp = 1,
## max_muts_per_gene_per_sample = 100, : Mutations observed in contiguous sites
## within a sample. Please annotate or remove dinucleotide or complex substitutions
## for best results.
```

```
## Warning in dndscv(shared_old_annot_no_drivers, outp = 1,
## max_muts_per_gene_per_sample = 100, : Same mutations observed in different
## sampleIDs. Please verify that these are independent events and remove duplicates
## otherwise.
```

```
## [3] Estimating global rates...
```

```
dndscvout$globaldnds
```

```
##      name       mle     cilow   cihigh
## wmis wmis 1.0615658 0.9393350 1.199702
## wnon wnon 1.0387078 0.7541534 1.430629
## wspl wspl 0.9738339 0.6530125 1.452273
## wtru wtru 1.0129764 0.7826319 1.311116
## wall wall 1.0580208 0.9376829 1.193802
```

##### g) Number of drivers with annotated drivers removed

```
wmis = dndscvout$globaldnds$mle[1]
nmis = (sum(dndscvout$annotmuts$impact =="Missense"))
ndrivers_shared_old = (wmis-1)/wmis*nmis
ndrivers_shared_old
```

```
## [1] 54.63158
```

##### h) Cancer gene set analysis shared\_old

```
dndscvout=dndscv(shared_old,max_muts_per_gene_per_sample = 100, max_coding_muts_per_sample = 1000000, gene_list = cancer_drivers)
```

```
## [1] Loading the environment...
```

```
## [2] Annotating the mutations...
```

```
## Warning in dndscv(shared_old, max_muts_per_gene_per_sample = 100,
## max_coding_muts_per_sample = 1e+06, : Mutations observed in contiguous sites
## within a sample. Please annotate or remove dinucleotide or complex substitutions
## for best results.
```

```
## Warning in dndscv(shared_old, max_muts_per_gene_per_sample = 100,
## max_coding_muts_per_sample = 1e+06, : Same mutations observed in different
## sampleIDs. Please verify that these are independent events and remove duplicates
## otherwise.
```

```
## [3] Estimating global rates...
```

```
## [4] Running dNdSloc...
```

```
## [5] Running dNdScv...
```

```
##     Regression model for substitutions (theta = 10.8).
```

```
sel_cv = dndscvout$sel_cv
print(head(sel_cv, n= 30L), digits = 3)
```

```
##     gene_name n_syn n_mis n_non n_spl wmis_cv wnon_cv wspl_cv  pmis_cv
## 99     DNMT3A     1     9     0     3   82.30   267.3   267.3 8.12e-14
## 253      POT1     0     1     1     0   19.75   165.9   165.9 4.61e-02
## 332      TET2     0     2     1     0   12.60    46.7    46.7 1.33e-02
## 48        CBL     0     2     0     0   25.76     0.0     0.0 3.01e-03
## 116      EZH2     0     0     0     1    0.00   111.7   111.7 7.18e-01
## 28      AXIN1     0     0     1     0    0.00   100.4   100.4 6.55e-01
## 214      MUC6     0     0     1     0    0.00    51.1    51.1 4.69e-01
## 34       BCOR     0     2     0     0   12.09     0.0     0.0 1.45e-02
## 23        ATM     0     0     0     1    0.00    29.1    29.1 5.02e-01
## 258     PPP6C     0     1     0     0   32.18     0.0     0.0 2.67e-02
## 80     CREBBP     0     2     0     0    8.24     0.0     0.0 3.14e-02
## 190     KMT2D     0     0     1     0    0.00    15.8    15.8 3.16e-01
## 44       CALR     0     1     0     0   24.49     0.0     0.0 3.62e-02
## 240      PAX5     0     1     0     0   23.12     0.0     0.0 3.86e-02
## 100    EEF1A1     0     1     0     0   22.89     0.0     0.0 3.91e-02
## 10        ALB     1     1     0     0   19.80     0.0     0.0 4.57e-02
## 130     FOXA2     0     1     0     0   17.19     0.0     0.0 5.39e-02
## 296   SLC26A3     0     1     0     0   15.72     0.0     0.0 5.95e-02
## 38       BRAF     0     1     0     0   15.38     0.0     0.0 6.10e-02
## 347      TSHR     0     1     0     0   14.43     0.0     0.0 6.55e-02
## 85       CTCF     0     1     0     0   14.16     0.0     0.0 6.69e-02
## 362   ZFP36L2     0     1     0     0   13.13     0.0     0.0 7.28e-02
## 338   TNFAIP3     0     1     0     0   13.02     0.0     0.0 7.35e-02
## 49       CBLB     0     1     0     0   12.20     0.0     0.0 7.91e-02
## 217     MYOCD     0     1     0     0   11.90     0.0     0.0 8.13e-02
## 123     FGFR1     0     1     0     0   11.86     0.0     0.0 8.16e-02
## 244    PDGFRA     0     1     0     0   11.15     0.0     0.0 8.75e-02
## 77     COL2A1     0     1     0     0    6.53     0.0     0.0 1.58e-01
## 11        ALK     0     1     0     0    6.21     0.0     0.0 1.68e-01
## 70       CHD4     0     1     0     0    5.73     0.0     0.0 1.83e-01
##     ptrunc_cv pallsubs_cv  qmis_cv qtrunc_cv qallsubs_cv
## 99   2.27e-07     0.00000 3.00e-11  8.38e-05       0.000
## 253  4.34e-03     0.00255 8.79e-01  6.96e-01       0.402
## 332  1.76e-02     0.00326 8.79e-01  9.81e-01       0.402
## 48   8.93e-01     0.01215 5.58e-01  9.81e-01       0.991
## 116  6.69e-03     0.02356 8.79e-01  6.96e-01       0.991
## 28   7.53e-03     0.02519 8.79e-01  6.96e-01       0.991
## 214  1.59e-02     0.04104 8.79e-01  9.81e-01       0.991
## 34   8.65e-01     0.04939 8.79e-01  9.81e-01       0.991
## 23   2.98e-02     0.07376 8.79e-01  9.81e-01       0.991
## 258  9.38e-01     0.08547 8.79e-01  9.81e-01       0.991
## 80   8.01e-01     0.09516 8.79e-01  9.81e-01       0.991
## 190  5.91e-02     0.09723 8.79e-01  9.81e-01       0.991
## 44   9.20e-01     0.11088 8.79e-01  9.81e-01       0.991
## 240  9.27e-01     0.11725 8.79e-01  9.81e-01       0.991
## 100  9.29e-01     0.11840 8.79e-01  9.81e-01       0.991
## 10   8.99e-01     0.13469 8.79e-01  9.81e-01       0.991
## 130  9.41e-01     0.15544 8.79e-01  9.81e-01       0.991
## 296  9.14e-01     0.16839 8.79e-01  9.81e-01       0.991
## 38   8.92e-01     0.17113 8.79e-01  9.81e-01       0.991
## 347  9.14e-01     0.18227 8.79e-01  9.81e-01       0.991
## 85   9.02e-01     0.18508 8.79e-01  9.81e-01       0.991
## 362  9.45e-01     0.19953 8.79e-01  9.81e-01       0.991
## 338  9.02e-01     0.19992 8.79e-01  9.81e-01       0.991
## 49   8.89e-01     0.21169 8.79e-01  9.81e-01       0.991
## 217  8.83e-01     0.21619 8.79e-01  9.81e-01       0.991
## 123  8.84e-01     0.21697 8.79e-01  9.81e-01       0.991
## 244  8.80e-01     0.22935 8.79e-01  9.81e-01       0.991
## 77   8.53e-01     0.36302 8.79e-01  9.81e-01       0.991
## 11   8.59e-01     0.37928 8.79e-01  9.81e-01       0.991
## 70   8.31e-01     0.40205 8.79e-01  9.81e-01       0.991
```

##### i) Cancer gene set analysis excluding CHIP drivers shared\_old

```
dndscvout=dndscv(shared_old,max_muts_per_gene_per_sample = 100, max_coding_muts_per_sample = 1000000, gene_list = setdiff(cancer_drivers, chip_drivers))
```

```
## [1] Loading the environment...
```

```
## [2] Annotating the mutations...
```

```
## Warning in dndscv(shared_old, max_muts_per_gene_per_sample = 100,
## max_coding_muts_per_sample = 1e+06, : Mutations observed in contiguous sites
## within a sample. Please annotate or remove dinucleotide or complex substitutions
## for best results.
```

```
## Warning in dndscv(shared_old, max_muts_per_gene_per_sample = 100,
## max_coding_muts_per_sample = 1e+06, : Same mutations observed in different
## sampleIDs. Please verify that these are independent events and remove duplicates
## otherwise.
```

```
## [3] Estimating global rates...
```

```
## [4] Running dNdSloc...
```

```
## [5] Running dNdScv...
```

```
##     Regression model for substitutions (theta = 2.37).
```

```
sel_cv = dndscvout$sel_cv
print(head(sel_cv), digits = 3)
```

```
##     gene_name n_syn n_mis n_non n_spl wmis_cv wnon_cv wspl_cv pmis_cv ptrunc_cv
## 196      POT1     0     1     1     0    36.9   405.3   405.3  0.0300   0.00214
## 22      AXIN1     0     0     1     0     0.0   166.6   166.6  0.7542   0.00566
## 166      MUC6     0     0     1     0     0.0    94.0    94.0  0.5949   0.01063
## 18        ATM     0     0     0     1     0.0    54.7    54.7  0.6220   0.01936
## 200     PPP6C     0     1     0     0    56.7     0.0     0.0  0.0186   0.95882
## 99      FOXA2     0     1     0     0    40.7     0.0     0.0  0.0268   0.95021
##     pallsubs_cv qmis_cv qtrunc_cv qallsubs_cv
## 196     0.00129   0.917     0.628       0.378
## 22      0.01999   0.917     0.829       0.995
## 166     0.03004   0.917     0.985       0.995
## 18      0.05277   0.917     0.985       0.995
## 200     0.06247   0.917     0.985       0.995
## 99      0.08596   0.917     0.985       0.995
```

##### Forest plot for estimated number of mutations

```
dnds <- structure(list(
    mean  = c(NA,ndrivers_CB,ndrivers_all_young,ndrivers_all_old,ndrivers_all,ndrivers_shared_old,ndrivers_private_old), 
    lower = c(NA,ndrivers_CB_cilow,ndrivers_all_young_cilow,ndrivers_all_old_cilow,ndrivers_all_cilow,ndrivers_shared_old_cilow,ndrivers_private_old_cilow),
    upper = c(NA,ndrivers_CB_cihigh,ndrivers_all_young_cihigh,ndrivers_all_old_cihigh,ndrivers_all_cihigh,ndrivers_shared_old_cihigh,ndrivers_private_old_cihigh)),
    .Names = c("mean", "lower", "upper"), 
    row.names = c(NA,-1L),
    class = "data.frame")

tabletext<-
  c("name","cord blood", "young","old", "all", "shared old", "private old")

forestplot(tabletext, 
           dnds,new_page = FALSE,
           boxsize = 0.15,
           is.summary=FALSE,
           clip=c(-200,1600),
           xticks = c(-400,0,400,800,1200,1600),
           xlog=FALSE, 
           col=fpColors(box="royalblue",line="darkblue", summary="royalblue"))
```
