# Supplementary material for: Clonal dynamics of haematopoiesis across the human lifespan
Source: Nature. 2022 Jun 1;606(7913):343–50. doi: 10.1038/s41586-022-04786-y (PMC9177428; doi:10.1038/s41586-022-04786-y)
Supplement: Supplementary file 4 — HTMLs of notebooks outlining key statistical analyses presented in the manuscript, including analysis of phylogenetic trees. [file 41586_2022_4786_MOESM4_ESM.zip › Supplementary_code/Other_analysis/phylofit_summary.html]

Phylofit and expanded clade summary plots


# Phylofit and expanded clade summary plots

#### Emily Mitchell

### Summary

Script to plot phylofit results and visualise distribution of expanded clades.

##### Open packages

```
suppressMessages(library(ggplot2))
suppressMessages(library(gplots))
suppressMessages(library(tidyverse))
suppressMessages(library(gridExtra))
suppressMessages(library(gtable))
suppressMessages(library(RColorBrewer))
```

##### Read in table of selection coefficients, and expanded clade information

```
sel <- read.csv("~/Documents/PhD/Sequencing_results/DNA_seq/XX_Summary/phylofit/clade_size_5/selection_coeff_all.csv", stringsAsFactors = F)
summary <- read.csv("~/Documents/PhD/Sequencing_results/DNA_seq/XX_Summary/phylofit/clade_0.01/expanded_clade_summary.csv", stringsAsFactors = F, header = T)
clades <- read.csv("~/Documents/PhD/Sequencing_results/DNA_seq/XX_Summary/phylofit/clade_0.01/expanded_clades.csv", stringsAsFactors = F, header = T)
```

##### Change selection coefficient to ‘fitness effect’

```
sel$fitness_effect <- sel$S*100
sel$fitness_effect_lb <- sel$S_lb*100
sel$fitness_effect_ub <- sel$S_ub*100
```

```
sel$donor_ID <- factor(sel$donor_ID, levels = c("KX001","KX002","AX001","KX007","KX008","KX004","KX003"))
```

##### Plot of fitness effects for donors age > 70

```
ggplot(sel[sel$donor_ID %in% c("KX007","KX008","KX004","KX003"),]) +
  theme_bw()+
  scale_fill_manual(values = brewer.pal(10,"Paired")[c(5,6,9,10)])+
  geom_bar(aes(x= reorder(variant_ID,fitness_effect), y = fitness_effect,fill= donor_ID), stat="identity")+
  geom_errorbar(aes(x= variant_ID, ymin= fitness_effect_lb, ymax= fitness_effect_ub), width = 0.3, , size = 0.5)+
  theme(axis.text.x = element_text(angle = 90, vjust = 0.5, hjust=1))+
  ylim(0,40)+
  labs(y = "Fitness effect (s) (% per year)", x = "Clade ID")
```

##### Plot of fitness effects for all donors

```
ggplot(sel) +
  theme_classic()+
  geom_bar(aes(reorder(variant_ID, S), S, fill = donor_ID), stat="identity", alpha = 2)+
  scale_fill_manual(values = brewer.pal(10,"Paired")[c(3,4,8,5,6,9,10)])+
  geom_errorbar(aes(x= variant_ID, ymin= S_lb, ymax= S_ub), width = 0.4, colour = "black", size = 0.5)+
  theme(axis.text.x = element_text(angle = 90, vjust = 0.5, hjust=1))+
  ylim(0,0.5)+
  labs(y = "S (Fitness effect)", x = "Driver ID")
```

##### Plot of expanded clade size distributions for all donors

```
ggplot(clades,
       aes(x = donor_info, y= cf, fill = cf))+
  geom_bar(stat = "identity", color = "light grey")+
  theme_bw()+
  ylim(0,60)+
  labs(x="Donor age and sex", y="Clonal fraction of expanded clades (%)")+
  scale_fill_distiller(palette ="YlOrRd", direction = 1)
```

```
## Warning: Removed 1 rows containing missing values (geom_bar).
```
