# Supplementary material for: Clonal dynamics of haematopoiesis across the human lifespan
Source: Nature. 2022 Jun 1;606(7913):343–50. doi: 10.1038/s41586-022-04786-y (PMC9177428; doi:10.1038/s41586-022-04786-y)
Supplement: Supplementary file 4 — HTMLs of notebooks outlining key statistical analyses presented in the manuscript, including analysis of phylogenetic trees. [file 41586_2022_4786_MOESM4_ESM.zip › Supplementary_code/Other_analysis/gamma_distribution_info.html]

Gamma distribution and probability of fixation


# Gamma distribution and probability of fixation

#### Nick Williams and Emily Mitchell

### Summary

This script explores aspects of the gamma distribution of driver mutation selection coefficients.

1. Example gamma distributions with varying shape and rate
2. The relationship between gamma distribution parameters and selected clone properties

```
suppressMessages(library("RColorBrewer"))
suppressMessages(library("truncdist"))
suppressMessages(library("ggplot2"))
```

```
setwd("~/Documents/PhD/Sequencing_results/DNA_seq/XX_summary/driver_modelling/gamma_dist")
```

### 1. Example gamma distributions with varying shape and rate

```
plot(NULL,xlim=c(0,0.3),ylim=c(0,25000000),ylab="Frequency",xlab="Selection Coeficient",main="Gamma distributions with varying shape and rate")
shapes = c(0.5,2.5,1.5,1.5,1.5)
rates = c(35,35,15,70,35)
cols=RColorBrewer::brewer.pal(length(shapes),"Paired")
for(i in 1:length(shapes)){
data = (rgamma(1000000, shape = shapes[i], rate = rates[i]))
dens = density(data)
lines(dens$x,length(data)*dens$y,type="l", col = cols[i], lwd = 2)
}
legend("topright",legend = sprintf("%3.2g",shapes),col=cols,lwd=2)
```

### 2. The relationship between gamma distribution parameters and selected clone properties

```
rates=seq(5,120,1)
##threshold=0.1
get_mean_prob_extinction=function(rate,shape,niter=1e4){
  s=rtrunc(n=niter,a=0.05, b=Inf,"gamma",shape = shape , rate=rate)
  mean(s/(1+s))
}

plot(NULL,xlim=c(0,120),ylim=c(0.05,0.3),ylab="Mean Fixation Probability",xlab="rate",main="Fixation Prob. vs Rate")
shapes=c(10**seq(-4,0,0.5),2)
N=1e4
cols=RColorBrewer::brewer.pal(length(shapes),"Paired")
for(i in 1:length(shapes)){
  lines(rates,sapply(rates,get_mean_prob_extinction,shape=shapes[i],niter=N),col=cols[i],lwd=2)
}
legend("topright",legend = sprintf("%3.2g",shapes),col=cols,lwd=2)
```

As a function of age (ignoring development and multiple drivers) would expect each tree to have T×driver\_per\_year×p¯fix fixed drivers in the population tree. Given the inverse relationship between p¯fix and rate we expect a positive relationship between rate and driver\_per\_year. This makes intuitive sense - a high rate leads to lower selective coefficients and so more driver events are required to provide the selective clades apparent in the trees.

Let us assume a sampled clade size of 5 out of 200 samples is required to detect a clade, then for a population size of 100,000 this corresponds to a population minimum detectable clade size of 2500.

There are some more analytic results available for the homogeneous birth-death process (Tavere):

```
alpha=function(t,s) (exp(s*t)-1)/((1+s)*exp(s*t)-1)
beta=function(t,s) (1+s)*alpha(t,s)
##probability distribution of clone size as a function of time and selective coefficient (conditional on size>=1)
pmf_clone_is_size_m=function(t,s,m){(1-beta(t,s))*beta(t,s)**(m-1)}
## sanity check it is normalised correctly for t=20 and s=0.1
print(sum(pmf_clone_is_size_m(20,0.1,1:1e5)))
```

```
## [1] 1
```

```
plot(NULL,xlim=c(0,80),ylim=c(0,1),main="Probability Clone Detectable (>2500) vs Time",xlab="Time(Years)",ylab="P")
fitness=seq(0.05,0.25,0.05)
sz=seq(1,2500)
t=seq(0,80)
cols=RColorBrewer::brewer.pal(length(fitness),"Paired")
for(i in 1:length(fitness)){
  lines(t,sapply(t,function(t) 1-sum(pmf_clone_is_size_m(t,s=fitness[i],m=sz))),col=cols[i],lwd=2)
}
legend("topleft",legend = sprintf("%3.2f",fitness),col=cols,lwd=2)
```
