# Supplementary material for: Clonal dynamics of haematopoiesis across the human lifespan
Source: Nature. 2022 Jun 1;606(7913):343–50. doi: 10.1038/s41586-022-04786-y (PMC9177428; doi:10.1038/s41586-022-04786-y)
Supplement: Supplementary file 4 — HTMLs of notebooks outlining key statistical analyses presented in the manuscript, including analysis of phylogenetic trees. [file 41586_2022_4786_MOESM4_ESM.zip › Supplementary_code/Other_analysis/Summary_telomere_mutation_all.html]

Mutation burden and telomere length analysis


# Mutation burden and telomere length analysis

#### Emily Mitchell

### Summary

This script performs analysis, visualisation and linear regression of mutation burden and telomere length across samples from the whole dataset.  
1. Data visualisation  
2. Calculation mean sequencing depth  
3. Calculation mean telomere length in cord blood  
4. Linear models  
5. Shapiro Wilks test of normality on telomere length data per individual  
6. qqplots of telomere length data per individual  
7. Density plots of telomere data per individual  
8. Calculating proportion of ‘outlying’ telomere lengths per individual  
9. Linear regression of proportion outliers with age

##### Open libraries

```
suppressMessages(library(dplyr))
suppressMessages(library(lme4))
suppressMessages(library(stringr))
suppressMessages(library(ggplot2))
suppressMessages(library(stats))
suppressMessages(library(RColorBrewer))
suppressMessages(library(ggpubr))
```

```
setwd("~/Documents/PhD/Sequencing_results/DNA_seq/XX_summary/telomere_mutation")
```

##### Load colony efficiency file

```
col_efficiency <- read.csv("~/Documents/PhD/Lab_work/Colony_efficiency/Colony_efficiency_cut.csv", stringsAsFactors = F)
```

##### Load matrix of mutation burdens, indel burdens and telomere lengths per sample

```
summ_cut <- read.csv("~/Documents/PhD/Sequencing_results/DNA_seq/XX_summary/telomere_mutation/data/Summary_cut.csv",  stringsAsFactors = F)
```

```
summ_cut$donor_id <- factor(summ_cut$donor_id, levels = c("CB001", "CB002", "KX001", "KX002","SX001","AX001", "KX007","KX008","KX004","KX003"))
col_efficiency$donor_id <- factor(col_efficiency$donor_id, levels = c("CB001", "CB002", "KX001", "KX002","SX001","AX001", "KX007","KX008","KX004","KX003"))
```

### 1. Data visualisation

```
ggplot(col_efficiency)+
  theme_bw()+
  scale_fill_manual(values = brewer.pal(10,"Paired")[c(1:4,7,8,5,6,9,10)])+
  labs(y="Colony efficiency", x="Donor")+
  theme(text=element_text(size=12))+
  ylim(0,1.0)+
  geom_col(aes(group = donor_id, y= efficiency, x = donor_id, fill = donor_id), width = 0.9)+
  labs(title = "Colony Efficiency")
```

```
ggplot(summ_cut[summ_cut$platform == "hiseq" & summ_cut$cell_type == "HSC",])+
  theme_bw()+
  labs(x="Age (years)", y="Telomere  lenght (bp)")+
  xlim(-10,100)+
  ylim(0,25000)+
  theme(text=element_text(size=12))+
  guides(fill="none")+
  scale_color_manual(values = brewer.pal(10,"Paired")[c(1,3,4,7,8,9,10)])+
  geom_jitter(aes(group = donor_id, x = age, y =tel_length, col = donor_id), width = 1)+
geom_boxplot(aes(group = donor_id, x = age, y =tel_length), outlier.alpha = 0 )+
  labs(title = "Telomere length - all data included")
```

```
ggplot(summ_cut[summ_cut$platform == "hiseq" & summ_cut$cell_type == "HSC",])+
  theme_bw()+
  labs(x="Age (years)", y="Telomere length (bp)")+
  xlim(-10,100)+
  ylim(0,15000)+
  theme(text=element_text(size=12))+
  guides(fill="none")+
  scale_color_manual(values = brewer.pal(10,"Paired")[c(1,3,4,7,8,9,10)])+
  geom_jitter(aes(group = donor_id, x = age, y =tel_length, col = donor_id), width = 1)+
  geom_boxplot(aes(group = donor_id, x = age, y =tel_length), outlier.alpha = 0 )+
  labs(title = "Telomere length - y-axis truncated at 15000bp")
```

```
ggplot(summ_cut[summ_cut$cell_type == "HSC",])+
  theme_bw()+
  labs(x="Age (years)", y="Number SNVs")+
  xlim(-10,100)+
  ylim(0,2000)+
  theme(text=element_text(size=12))+
  guides(fill="none")+
  scale_color_manual(values = brewer.pal(10,"Paired")[c(1:4,7,8,5,6,9,10)])+
  geom_smooth(data = (summ_cut[summ_cut$cell_type == "HSC",]), method = "lm",aes(x = age, y =sub_adj), size = 0.5, colour = "grey", se = T)+
  geom_jitter(aes(group = donor_id, x = age, y =sub_adj, col = donor_id))+
  labs(title ="Number of single nucleotide variants")
```

```
## `geom_smooth()` using formula 'y ~ x'
```

```
ggplot(summ_cut[summ_cut$cell_type == "HSC",])+
  theme_bw()+
  labs(x="Age (years)", y="Number indels")+
  xlim(-10,100)+
  ylim(0,100)+
  theme(text=element_text(size=12))+
  geom_smooth(data = (summ_cut[summ_cut$cell_type == "HSC",]), method = "lm",aes(x = age, y =indel_adj), size = 0.5, colour = "grey", se = T)+
  guides(fill="none")+
  scale_color_manual(values = brewer.pal(10,"Paired")[c(1:4,7,8,5,6,9,10)])+
  geom_jitter(aes(group = donor_id, x = age, y =indel_adj, col = donor_id))+
  labs("Number of indels")
```

```
## `geom_smooth()` using formula 'y ~ x'
```

```
ggplot(summ_cut[summ_cut$mean_depth > 14,])+
  theme_classic()+
  labs(x="Age (years)", y="Number non-synonymous mutations")+
  xlim(-10,100)+
  ylim(0,100)+
  theme(text=element_text(size=12))+
  guides(fill="none")+
  scale_color_manual(values = brewer.pal(10,"Paired")[c(1:4,7,8,5,6,9,10)])+
  geom_smooth(data = (summ_cut[summ_cut$mean_depth > 14,]), method = "lm",aes(x = age, y =number_non_syn), size = 0.5, colour = "grey", se = T)+
  geom_jitter(aes(group = donor_id, x = age, y =number_non_syn, col = donor_id), width = 0.5)+
  labs(title ="Number of non-synonymous variants")
```

```
## `geom_smooth()` using formula 'y ~ x'
```

```
## Warning: Removed 125 rows containing missing values (geom_point).
```

```
ggplot(subset(summ_cut, summ_cut$donor_id %in% c("CB002","SX001","AX001","KX004")))+
  theme_bw()+
  labs(x="Age (years)", y="Number indels")+
  xlim(-10,100)+
  ylim(0,100)+
  theme(text=element_text(size=12))+
geom_smooth(data = (subset(summ_cut, summ_cut$donor_id %in% c("CB002","SX001","AX001","KX004") & summ_cut$cell_type == "HSC")), method = "lm",aes(x = age, y =indel_adj), size = 0.5, colour = "red", se = T)+
  geom_smooth(data = (subset(summ_cut, summ_cut$donor_id %in% c("CB002","SX001","AX001","KX004") & summ_cut$cell_type == "Progenitor")), method = "lm",aes(x = age, y =indel_adj), size = 0.5, colour = "blue", se = T)+
  labs(title = "Number of indels in progenitors (blue) and HSC/MPPs (red)")
```

```
## `geom_smooth()` using formula 'y ~ x'
## `geom_smooth()` using formula 'y ~ x'
```

```
ggplot(subset(summ_cut, summ_cut$donor_id %in% c("CB002","SX001","AX001","KX004")))+
  theme_bw()+
  labs(x="Age (years)", y="Number SNVs")+
  xlim(-10,100)+
  ylim(0,2000)+
  theme(text=element_text(size=12))+
  geom_smooth(data = (subset(summ_cut, summ_cut$donor_id %in% c("CB002","SX001","AX001","KX004") & summ_cut$cell_type == "HSC")), method = "lm",aes(x = age, y =sub_adj), size = 0.5, colour = "red", se = T)+
  geom_smooth(data = (subset(summ_cut, summ_cut$donor_id %in% c("CB002","SX001","AX001","KX004") & summ_cut$cell_type == "Progenitor")), method = "lm",aes(x = age, y =sub_adj), size = 0.5, colour = "blue", se = T)+
  labs(title = "Number of SNVs in progenitors (blue) and HSC/MPPs (red)")
```

```
## `geom_smooth()` using formula 'y ~ x'
## `geom_smooth()` using formula 'y ~ x'
```

### 2. Calculation mean sequencing depth

```
mean(summ_cut$mean_depth)
```

```
## [1] 14.13358
```

### 3. Calculation mean telomere length in cord blood

```
mean_tel_CB001 <- mean(summ_cut$tel_length[summ_cut$donor_id == "CB001"])
mean_tel_CB001
```

```
## [1] 6946.516
```

### 4. Linear models

##### Linear model to assess the effect of age on the accumulation of SNVs in HSC/MPPs

```
age.mut <- lmer(sub_adj ~ age + (age | donor_id), data = summ_cut[summ_cut$cell_type == "HSC",], REML = F)
age.mut
```

```
## Linear mixed model fit by maximum likelihood  ['lmerMod']
## Formula: sub_adj ~ age + (age | donor_id)
##    Data: summ_cut[summ_cut$cell_type == "HSC", ]
##       AIC       BIC    logLik  deviance  df.resid 
##  37678.95  37715.70 -18833.48  37666.95      3368 
## Random effects:
##  Groups   Name        Std.Dev. Corr
##  donor_id (Intercept)  0.6415      
##           age          0.3974  0.88
##  Residual             63.9678      
## Number of obs: 3374, groups:  donor_id, 10
## Fixed Effects:
## (Intercept)          age  
##       54.56        16.83  
## optimizer (nloptwrap) convergence code: 0 (OK) ; 0 optimizer warnings; 1 lme4 warnings
```

```
summary(age.mut)
```

```
## Linear mixed model fit by maximum likelihood  ['lmerMod']
## Formula: sub_adj ~ age + (age | donor_id)
##    Data: summ_cut[summ_cut$cell_type == "HSC", ]
## 
##      AIC      BIC   logLik deviance df.resid 
##  37679.0  37715.7 -18833.5  37667.0     3368 
## 
## Scaled residuals: 
##     Min      1Q  Median      3Q     Max 
## -4.4624 -0.5091 -0.0147  0.4903  4.1829 
## 
## Random effects:
##  Groups   Name        Variance  Std.Dev. Corr
##  donor_id (Intercept)    0.4115  0.6415      
##           age            0.1579  0.3974  0.88
##  Residual             4091.8787 63.9678      
## Number of obs: 3374, groups:  donor_id, 10
## 
## Fixed effects:
##             Estimate Std. Error t value
## (Intercept)  54.5628     2.8373   19.23
## age          16.8323     0.1551  108.54
## 
## Correlation of Fixed Effects:
##     (Intr)
## age -0.336
## optimizer (nloptwrap) convergence code: 0 (OK)
## Model failed to converge with max|grad| = 1.07699 (tol = 0.002, component 1)
```

##### Linear model to assess the effect of age on the accumulation of indels in HSC/MPPs

```
age.indel <- lmer(indel_adj ~ age + (age | donor_id), data = summ_cut[summ_cut$cell_type == "HSC",], REML = F)
age.indel
```

```
## Linear mixed model fit by maximum likelihood  ['lmerMod']
## Formula: indel_adj ~ age + (age | donor_id)
##    Data: summ_cut[summ_cut$cell_type == "HSC", ]
##       AIC       BIC    logLik  deviance  df.resid 
##  22110.33  22147.08 -11049.17  22098.33      3368 
## Random effects:
##  Groups   Name        Std.Dev. Corr 
##  donor_id (Intercept) 1.4271        
##           age         0.0798   -0.98
##  Residual             6.3588        
## Number of obs: 3374, groups:  donor_id, 10
## Fixed Effects:
## (Intercept)          age  
##      3.2883       0.7071  
## optimizer (nloptwrap) convergence code: 0 (OK) ; 0 optimizer warnings; 2 lme4 warnings
```

```
summary(age.indel)
```

```
## Linear mixed model fit by maximum likelihood  ['lmerMod']
## Formula: indel_adj ~ age + (age | donor_id)
##    Data: summ_cut[summ_cut$cell_type == "HSC", ]
## 
##      AIC      BIC   logLik deviance df.resid 
##  22110.3  22147.1 -11049.2  22098.3     3368 
## 
## Scaled residuals: 
##     Min      1Q  Median      3Q     Max 
## -4.1395 -0.4755 -0.0186  0.5354  4.5450 
## 
## Random effects:
##  Groups   Name        Variance  Std.Dev. Corr 
##  donor_id (Intercept)  2.036731 1.4271        
##           age          0.006367 0.0798   -0.98
##  Residual             40.434389 6.3588        
## Number of obs: 3374, groups:  donor_id, 10
## 
## Fixed effects:
##             Estimate Std. Error t value
## (Intercept)  3.28828    0.93376   3.522
## age          0.70707    0.02757  25.650
## 
## Correlation of Fixed Effects:
##     (Intr)
## age -0.756
## optimizer (nloptwrap) convergence code: 0 (OK)
## unable to evaluate scaled gradient
## Model failed to converge: degenerate  Hessian with 2 negative eigenvalues
```

##### Linear model to assess effect of age on telomere length including only the ‘young adult’ individuals

```
age.tel <- lmer(tel_length ~ age + (age | donor_id), data = subset(summ_cut, summ_cut$platform == "hiseq" & !summ_cut$donor_id %in% c("CB001", "KX003", "KX004") & summ_cut$cell_type == "HSC"), REML = F)
age.tel
```

```
## Linear mixed model fit by maximum likelihood  ['lmerMod']
## Formula: tel_length ~ age + (age | donor_id)
##    Data: 
## subset(summ_cut, summ_cut$platform == "hiseq" & !summ_cut$donor_id %in%  
##     c("CB001", "KX003", "KX004") & summ_cut$cell_type == "HSC")
##       AIC       BIC    logLik  deviance  df.resid 
## 17992.773 18023.027 -8990.387 17980.773      1138 
## Random effects:
##  Groups   Name        Std.Dev. Corr 
##  donor_id (Intercept) 598.742       
##           age           9.665  -1.00
##  Residual             623.289       
## Number of obs: 1144, groups:  donor_id, 4
## Fixed Effects:
## (Intercept)          age  
##     5068.64       -47.82  
## optimizer (nloptwrap) convergence code: 0 (OK) ; 0 optimizer warnings; 1 lme4 warnings
```

```
summary(age.tel)
```

```
## Linear mixed model fit by maximum likelihood  ['lmerMod']
## Formula: tel_length ~ age + (age | donor_id)
##    Data: 
## subset(summ_cut, summ_cut$platform == "hiseq" & !summ_cut$donor_id %in%  
##     c("CB001", "KX003", "KX004") & summ_cut$cell_type == "HSC")
## 
##      AIC      BIC   logLik deviance df.resid 
##  17992.8  18023.0  -8990.4  17980.8     1138 
## 
## Scaled residuals: 
##     Min      1Q  Median      3Q     Max 
## -2.4018 -0.6403 -0.0954  0.4822  7.2163 
## 
## Random effects:
##  Groups   Name        Variance  Std.Dev. Corr 
##  donor_id (Intercept) 358492.54 598.742       
##           age             93.42   9.665  -1.00
##  Residual             388489.63 623.289       
## Number of obs: 1144, groups:  donor_id, 4
## 
## Fixed effects:
##             Estimate Std. Error t value
## (Intercept) 5068.644    353.658  14.332
## age          -47.824      5.845  -8.182
## 
## Correlation of Fixed Effects:
##     (Intr)
## age -0.992
## optimizer (nloptwrap) convergence code: 0 (OK)
## boundary (singular) fit: see ?isSingular
```

##### Linear model to assess effect of age on telomere length including all adult individuals

```
age.tel <- lmer(tel_length ~ age + (age | donor_id), data = subset(summ_cut, summ_cut$platform == "hiseq" & !summ_cut$donor_id %in% c("CB001") & summ_cut$cell_type == "HSC"), REML = F)
age.tel
```

```
## Linear mixed model fit by maximum likelihood  ['lmerMod']
## Formula: tel_length ~ age + (age | donor_id)
##    Data: 
## subset(summ_cut, summ_cut$platform == "hiseq" & !summ_cut$donor_id %in%  
##     c("CB001") & summ_cut$cell_type == "HSC")
##       AIC       BIC    logLik  deviance  df.resid 
##  20434.98  20466.01 -10211.49  20422.98      1298 
## Random effects:
##  Groups   Name        Std.Dev. Corr 
##  donor_id (Intercept) 593.74        
##           age          20.16   -1.00
##  Residual             603.51        
## Number of obs: 1304, groups:  donor_id, 6
## Fixed Effects:
## (Intercept)          age  
##     4512.38       -30.81  
## optimizer (nloptwrap) convergence code: 0 (OK) ; 0 optimizer warnings; 1 lme4 warnings
```

```
summary(age.tel)
```

```
## Linear mixed model fit by maximum likelihood  ['lmerMod']
## Formula: tel_length ~ age + (age | donor_id)
##    Data: 
## subset(summ_cut, summ_cut$platform == "hiseq" & !summ_cut$donor_id %in%  
##     c("CB001") & summ_cut$cell_type == "HSC")
## 
##      AIC      BIC   logLik deviance df.resid 
##  20435.0  20466.0 -10211.5  20423.0     1298 
## 
## Scaled residuals: 
##     Min      1Q  Median      3Q     Max 
## -2.4814 -0.6193 -0.1033  0.4804  7.4619 
## 
## Random effects:
##  Groups   Name        Variance Std.Dev. Corr 
##  donor_id (Intercept) 352523.8 593.74        
##           age            406.6  20.16   -1.00
##  Residual             364219.2 603.51        
## Number of obs: 1304, groups:  donor_id, 6
## 
## Fixed effects:
##             Estimate Std. Error t value
## (Intercept) 4512.382    267.883  16.845
## age          -30.809      9.003  -3.422
## 
## Correlation of Fixed Effects:
##     (Intr)
## age -0.993
## optimizer (nloptwrap) convergence code: 0 (OK)
## boundary (singular) fit: see ?isSingular
```

##### Linear model to assess the effect of age and cell type on the accumulation of SNVs

```
age.cell.type <- lmer(sub_adj ~ age + cell_type + (age | donor_id) + (cell_type | donor_id), data = subset(summ_cut, summ_cut$donor_id %in% c("CB002","SX001","AX001","KX004")), REML = F)
age.cell.type
```

```
## Linear mixed model fit by maximum likelihood  ['lmerMod']
## Formula: sub_adj ~ age + cell_type + (age | donor_id) + (cell_type | donor_id)
##    Data: subset(summ_cut, summ_cut$donor_id %in% c("CB002", "SX001", "AX001",  
##     "KX004"))
##       AIC       BIC    logLik  deviance  df.resid 
## 17394.194 17447.744 -8687.097 17374.194      1554 
## Random effects:
##  Groups     Name                Std.Dev. Corr 
##  donor_id   (Intercept)         16.4177       
##             age                  0.2034  -1.00
##  donor_id.1 (Intercept)         10.4668       
##             cell_typeProgenitor 39.9909  -0.93
##  Residual                       62.1517       
## Number of obs: 1564, groups:  donor_id, 4
## Fixed Effects:
##         (Intercept)                  age  cell_typeProgenitor  
##               35.55                16.93                32.39  
## optimizer (nloptwrap) convergence code: 0 (OK) ; 0 optimizer warnings; 1 lme4 warnings
```

```
summary(age.cell.type)
```

```
## Linear mixed model fit by maximum likelihood  ['lmerMod']
## Formula: sub_adj ~ age + cell_type + (age | donor_id) + (cell_type | donor_id)
##    Data: subset(summ_cut, summ_cut$donor_id %in% c("CB002", "SX001", "AX001",  
##     "KX004"))
## 
##      AIC      BIC   logLik deviance df.resid 
##  17394.2  17447.7  -8687.1  17374.2     1554 
## 
## Scaled residuals: 
##     Min      1Q  Median      3Q     Max 
## -3.9144 -0.4066  0.0111  0.5050  3.2502 
## 
## Random effects:
##  Groups     Name                Variance  Std.Dev. Corr 
##  donor_id   (Intercept)         2.695e+02 16.4177       
##             age                 4.136e-02  0.2034  -1.00
##  donor_id.1 (Intercept)         1.096e+02 10.4668       
##             cell_typeProgenitor 1.599e+03 39.9909  -0.93
##  Residual                       3.863e+03 62.1517       
## Number of obs: 1564, groups:  donor_id, 4
## 
## Fixed effects:
##                     Estimate Std. Error t value
## (Intercept)          35.5452    14.5463   2.444
## age                  16.9312     0.2059  82.227
## cell_typeProgenitor  32.3889    21.0481   1.539
## 
## Correlation of Fixed Effects:
##             (Intr) age   
## age         -0.911       
## cll_typPrgn -0.287 -0.046
## optimizer (nloptwrap) convergence code: 0 (OK)
## boundary (singular) fit: see ?isSingular
```

##### Linear model to assess the effect of age and cell type on the accumulation of indels

```
age.cell.type.indel <- lmer(indel_adj ~ age + cell_type + (age | donor_id) + (cell_type | donor_id), data = subset(summ_cut, summ_cut$donor_id %in% c("CB002","SX001","AX001","KX004")), REML = F)
age.cell.type.indel
```

```
## Linear mixed model fit by maximum likelihood  ['lmerMod']
## Formula: indel_adj ~ age + cell_type + (age | donor_id) + (cell_type |  
##     donor_id)
##    Data: subset(summ_cut, summ_cut$donor_id %in% c("CB002", "SX001", "AX001",  
##     "KX004"))
##       AIC       BIC    logLik  deviance  df.resid 
##  9913.536  9967.086 -4946.768  9893.536      1554 
## Random effects:
##  Groups     Name                Std.Dev. Corr 
##  donor_id   (Intercept)         0.007185      
##             age                 0.026109 -0.99
##  donor_id.1 (Intercept)         0.042272      
##             cell_typeProgenitor 0.270893 0.85 
##  Residual                       5.700129      
## Number of obs: 1564, groups:  donor_id, 4
## Fixed Effects:
##         (Intercept)                  age  cell_typeProgenitor  
##              3.0520               0.7343               0.6391  
## optimizer (nloptwrap) convergence code: 0 (OK) ; 0 optimizer warnings; 2 lme4 warnings
```

```
summary(age.cell.type.indel)
```

```
## Linear mixed model fit by maximum likelihood  ['lmerMod']
## Formula: indel_adj ~ age + cell_type + (age | donor_id) + (cell_type |  
##     donor_id)
##    Data: subset(summ_cut, summ_cut$donor_id %in% c("CB002", "SX001", "AX001",  
##     "KX004"))
## 
##      AIC      BIC   logLik deviance df.resid 
##   9913.5   9967.1  -4946.8   9893.5     1554 
## 
## Scaled residuals: 
##     Min      1Q  Median      3Q     Max 
## -4.0498 -0.4778 -0.0354  0.4971  4.4072 
## 
## Random effects:
##  Groups     Name                Variance  Std.Dev. Corr 
##  donor_id   (Intercept)         5.162e-05 0.007185      
##             age                 6.817e-04 0.026109 -0.99
##  donor_id.1 (Intercept)         1.787e-03 0.042272      
##             cell_typeProgenitor 7.338e-02 0.270893 0.85 
##  Residual                       3.249e+01 5.700129      
## Number of obs: 1564, groups:  donor_id, 4
## 
## Fixed effects:
##                     Estimate Std. Error t value
## (Intercept)          3.05199    0.31648   9.644
## age                  0.73428    0.01612  45.555
## cell_typeProgenitor  0.63913    0.46481   1.375
## 
## Correlation of Fixed Effects:
##             (Intr) age   
## age         -0.316       
## cll_typPrgn -0.278  0.059
## optimizer (nloptwrap) convergence code: 0 (OK)
## unable to evaluate scaled gradient
## Model failed to converge: degenerate  Hessian with 1 negative eigenvalues
```

##### Linear model to assess the effect of age on the accumulation of non-synonymous mutations (depth cut off)

```
age.non_syn.depth <- lmer(number_non_syn ~ age + (age | donor_id), data = subset(summ_cut, mean_depth > 14), REML = F)
```

```
## Warning in checkConv(attr(opt, "derivs"), opt$par, ctrl = control$checkConv, :
## unable to evaluate scaled gradient
```

```
## Warning in checkConv(attr(opt, "derivs"), opt$par, ctrl = control$checkConv, :
## Model failed to converge: degenerate Hessian with 1 negative eigenvalues
```

```
age.non_syn.depth
```

```
## Linear mixed model fit by maximum likelihood  ['lmerMod']
## Formula: number_non_syn ~ age + (age | donor_id)
##    Data: subset(summ_cut, mean_depth > 14)
##       AIC       BIC    logLik  deviance  df.resid 
##  6825.370  6857.071 -3406.685  6813.370      1450 
## Random effects:
##  Groups   Name        Std.Dev. Corr 
##  donor_id (Intercept) 0.55846       
##           age         0.01031  -0.88
##  Residual             2.49925       
## Number of obs: 1456, groups:  donor_id, 10
## Fixed Effects:
## (Intercept)          age  
##      0.2315       0.1229  
## optimizer (nloptwrap) convergence code: 0 (OK) ; 0 optimizer warnings; 2 lme4 warnings
```

```
summary(age.non_syn.depth)
```

```
## Linear mixed model fit by maximum likelihood  ['lmerMod']
## Formula: number_non_syn ~ age + (age | donor_id)
##    Data: subset(summ_cut, mean_depth > 14)
## 
##      AIC      BIC   logLik deviance df.resid 
##   6825.4   6857.1  -3406.7   6813.4     1450 
## 
## Scaled residuals: 
##     Min      1Q  Median      3Q     Max 
## -3.0653 -0.5817 -0.1314  0.5353  3.4542 
## 
## Random effects:
##  Groups   Name        Variance  Std.Dev. Corr 
##  donor_id (Intercept) 0.3118791 0.55846       
##           age         0.0001063 0.01031  -0.88
##  Residual             6.2462526 2.49925       
## Number of obs: 1456, groups:  donor_id, 10
## 
## Fixed effects:
##             Estimate Std. Error t value
## (Intercept) 0.231523   0.330038   0.702
## age         0.122937   0.005556  22.128
## 
## Correlation of Fixed Effects:
##     (Intr)
## age -0.900
## optimizer (nloptwrap) convergence code: 0 (OK)
## unable to evaluate scaled gradient
## Model failed to converge: degenerate  Hessian with 1 negative eigenvalues
```

### 5. Shapiro Wilks test of normality on telomere length data per individual

```
shapiro.test(summ_cut$tel_length[summ_cut$donor_id == "CB001" & summ_cut$cell_type == "HSC" & summ_cut$platform == "hiseq"])
```

```
## 
##  Shapiro-Wilk normality test
## 
## data:  summ_cut$tel_length[summ_cut$donor_id == "CB001" & summ_cut$cell_type == "HSC" & summ_cut$platform == "hiseq"]
## W = 0.79028, p-value = 1.058e-15
```

```
shapiro.test(summ_cut$tel_length[summ_cut$donor_id == "KX001" & summ_cut$cell_type == "HSC" & summ_cut$platform == "hiseq"])
```

```
## 
##  Shapiro-Wilk normality test
## 
## data:  summ_cut$tel_length[summ_cut$donor_id == "KX001" & summ_cut$cell_type == "HSC" & summ_cut$platform == "hiseq"]
## W = 0.97028, p-value = 2.297e-07
```

```
shapiro.test(summ_cut$tel_length[summ_cut$donor_id == "KX002" & summ_cut$cell_type == "HSC" & summ_cut$platform == "hiseq"])
```

```
## 
##  Shapiro-Wilk normality test
## 
## data:  summ_cut$tel_length[summ_cut$donor_id == "KX002" & summ_cut$cell_type == "HSC" & summ_cut$platform == "hiseq"]
## W = 0.86813, p-value < 2.2e-16
```

```
shapiro.test(summ_cut$tel_length[summ_cut$donor_id == "SX001" & summ_cut$cell_type == "HSC" & summ_cut$platform == "hiseq"])
```

```
## 
##  Shapiro-Wilk normality test
## 
## data:  summ_cut$tel_length[summ_cut$donor_id == "SX001" & summ_cut$cell_type == "HSC" & summ_cut$platform == "hiseq"]
## W = 0.91815, p-value = 1.847e-08
```

```
shapiro.test(summ_cut$tel_length[summ_cut$donor_id == "AX001" & summ_cut$cell_type == "HSC" & summ_cut$platform == "hiseq"])
```

```
## 
##  Shapiro-Wilk normality test
## 
## data:  summ_cut$tel_length[summ_cut$donor_id == "AX001" & summ_cut$cell_type == "HSC" & summ_cut$platform == "hiseq"]
## W = 0.95725, p-value = 3.08e-05
```

```
shapiro.test(summ_cut$tel_length[summ_cut$donor_id == "KX004" & summ_cut$cell_type == "HSC" & summ_cut$platform == "hiseq"])
```

```
## 
##  Shapiro-Wilk normality test
## 
## data:  summ_cut$tel_length[summ_cut$donor_id == "KX004" & summ_cut$cell_type == "HSC" & summ_cut$platform == "hiseq"]
## W = 0.93659, p-value = 0.0009823
```

```
shapiro.test(summ_cut$tel_length[summ_cut$donor_id == "KX003" & summ_cut$cell_type == "HSC" & summ_cut$platform == "hiseq"])
```

```
## 
##  Shapiro-Wilk normality test
## 
## data:  summ_cut$tel_length[summ_cut$donor_id == "KX003" & summ_cut$cell_type == "HSC" & summ_cut$platform == "hiseq"]
## W = 0.97375, p-value = 0.08012
```

### 6. qqplots of telomere length data per individual

```
ggqqplot(summ_cut$tel_length[summ_cut$donor_id == "CB001" & summ_cut$cell_type == "HSC" & summ_cut$platform == "hiseq"])
```

```
ggqqplot(summ_cut$tel_length[summ_cut$donor_id == "KX001" & summ_cut$cell_type == "HSC" & summ_cut$platform == "hiseq"])
```

```
ggqqplot(summ_cut$tel_length[summ_cut$donor_id == "KX002" & summ_cut$cell_type == "HSC" & summ_cut$platform == "hiseq"])
```

```
ggqqplot(summ_cut$tel_length[summ_cut$donor_id == "SX001" & summ_cut$cell_type == "HSC" & summ_cut$platform == "hiseq"])
```

```
ggqqplot(summ_cut$tel_length[summ_cut$donor_id == "AX001" & summ_cut$cell_type == "HSC" & summ_cut$platform == "hiseq"])
```

```
ggqqplot(summ_cut$tel_length[summ_cut$donor_id == "KX004" & summ_cut$cell_type == "HSC" & summ_cut$platform == "hiseq"])
```

```
ggqqplot(summ_cut$tel_length[summ_cut$donor_id == "KX003" & summ_cut$cell_type == "HSC" & summ_cut$platform == "hiseq"])
```

### 7. Density plots of telomere data per individual

```
ggdensity(summ_cut$tel_length[summ_cut$donor_id == "CB001" & summ_cut$cell_type == "HSC" & summ_cut$platform == "hiseq"])
```

```
ggdensity(summ_cut$tel_length[summ_cut$donor_id == "KX001" & summ_cut$cell_type == "HSC" & summ_cut$platform == "hiseq"])
```

```
ggdensity(summ_cut$tel_length[summ_cut$donor_id == "KX002" & summ_cut$cell_type == "HSC" & summ_cut$platform == "hiseq"])
```

```
ggdensity(summ_cut$tel_length[summ_cut$donor_id == "SX001" & summ_cut$cell_type == "HSC" & summ_cut$platform == "hiseq"])
```

```
ggdensity(summ_cut$tel_length[summ_cut$donor_id == "AX001" & summ_cut$cell_type == "HSC" & summ_cut$platform == "hiseq"])
```

```
ggdensity(summ_cut$tel_length[summ_cut$donor_id == "KX004" & summ_cut$cell_type == "HSC" & summ_cut$platform == "hiseq"])
```

```
ggdensity(summ_cut$tel_length[summ_cut$donor_id == "KX003" & summ_cut$cell_type == "HSC" & summ_cut$platform == "hiseq"])
```

### 8. Calculating proportion of ‘outlying’ telomere lengths per individual

#####- note all outliers have unexpectedly long rather than short telomeres

```
CB001_norm <- summ_cut[summ_cut$platform == "hiseq" & summ_cut$donor_id == "CB001", ]
CB001_outliers <- boxplot.stats(CB001_norm$tel_length)$out
CB001 <- (length(CB001_outliers)/length(CB001_norm$tel_length))*100
CB001
```

```
## [1] 6.965174
```

```
KX001_norm <- summ_cut[summ_cut$platform == "hiseq" & summ_cut$donor_id == "KX001", ]
KX001_outliers <- boxplot.stats(KX001_norm$tel_length)$out
KX001 <- (length(KX001_outliers)/length(KX001_norm$tel_length))*100
KX001
```

```
## [1] 2.457002
```

```
KX002_norm <- summ_cut[summ_cut$platform == "hiseq" & summ_cut$donor_id == "KX002", ]
KX002_outliers <- boxplot.stats(KX002_norm$tel_length)$out
KX002 <- (length(KX002_outliers)/length(KX002_norm$tel_length))*100
KX002
```

```
## [1] 5.263158
```

```
SX001_norm <- summ_cut[summ_cut$platform == "hiseq" & summ_cut$donor_id == "SX001", ]
SX001_outliers <- boxplot.stats(SX001_norm$tel_length)$out
SX001 <- (length(SX001_outliers)/length(SX001_norm$tel_length))*100
SX001
```

```
## [1] 0.5586592
```

```
AX001_norm <- summ_cut[summ_cut$platform == "hiseq" & summ_cut$donor_id == "AX001", ]
AX001_outliers <- boxplot.stats(AX001_norm$tel_length)$out
AX001 <- (length(AX001_outliers)/length(AX001_norm$tel_length))*100
AX001
```

```
## [1] 1.685393
```

```
KX004_norm <- summ_cut[summ_cut$platform == "hiseq" & summ_cut$donor_id == "KX004", ]
KX004_outliers <- boxplot.stats(KX004_norm$tel_length)$out
KX004 <- (length(KX004_outliers)/length(KX004_norm$tel_length))*100
KX004
```

```
## [1] 0
```

```
KX003_norm <- summ_cut[summ_cut$platform == "hiseq" & summ_cut$donor_id == "KX003", ]
KX003_outliers <- boxplot.stats(KX003_norm$tel_length)$out
KX003 <- (length(KX003_outliers)/length(KX003_norm$tel_length))*100
KX003
```

```
## [1] 1.176471
```

```
all_out <- rbind(CB001, KX001, KX002, SX001, AX001, KX004, KX003)
age <- c(0,29,38,48,63,77,81)
all_out <- cbind(all_out, age)
colnames(all_out) <- c("outliers", "age")
all_out <- as.data.frame(all_out)
```

### 9. Plot of percentage HSC/MPPs with outlying telomere lengths

```
ggplot(data= all_out)+
theme_bw()+
  labs(x="Age (years)", y="Percentage HSC/MPPs with outlying telomere lengths")+
  xlim(-10,100)+
  ylim(0,10)+
  theme(text=element_text(size=12))+
  geom_point(data = all_out, aes(x = age, y = all_out$outliers))+
 geom_smooth(data = all_out, method = lm, aes(x = age, y = outliers), se = FALSE)
```

```
## Warning: Use of `all_out$outliers` is discouraged. Use `outliers` instead.
```

```
## `geom_smooth()` using formula 'y ~ x'
```

```
pdf("~/Documents/PhD/Sequencing_results/DNA_seq/XX_summary/telomere_mutation/plots/tel_outliers.pdf", useDingbats = FALSE, width = 10, height = 8)
ggplot(data= all_out)+
theme_bw()+
  labs(x="Age (years)", y="Percentage HSC/MPPs with outlying telomere lengths")+
  xlim(-10,100)+
  scale_y_continuous(breaks = c(0,2,4,6,8))+
  theme(text=element_text(size=12))+
  geom_point(data = all_out, aes(x = age, y = all_out$outliers))+
 geom_smooth(data = all_out, method = lm, aes(x = age, y = outliers), se = FALSE)
```

```
## Warning: Use of `all_out$outliers` is discouraged. Use `outliers` instead.
```

```
## `geom_smooth()` using formula 'y ~ x'
```

```
dev.off()
```

```
## quartz_off_screen 
##                 2
```

### 10. Linear regression of proportion outliers with age

```
outliers <- all_out$outliers
lm_out <- lm(outliers ~ age)
summary(lm_out)
```

```
## 
## Call:
## lm(formula = outliers ~ age)
## 
## Residuals:
##       1       2       3       4       5       6       7 
##  0.7734 -1.5566  1.9255 -2.0279  0.2255 -0.4084  1.0685 
## 
## Coefficients:
##             Estimate Std. Error t value Pr(>|t|)   
## (Intercept)  6.19181    1.22117   5.070  0.00387 **
## age         -0.07511    0.02227  -3.373  0.01983 * 
## ---
## Signif. codes:  0 '***' 0.001 '**' 0.01 '*' 0.05 '.' 0.1 ' ' 1
## 
## Residual standard error: 1.562 on 5 degrees of freedom
## Multiple R-squared:  0.6947, Adjusted R-squared:  0.6336 
## F-statistic: 11.37 on 1 and 5 DF,  p-value: 0.01983
```
