# Supplementary material for: Clonal dynamics of haematopoiesis across the human lifespan
Source: Nature. 2022 Jun 1;606(7913):343–50. doi: 10.1038/s41586-022-04786-y (PMC9177428; doi:10.1038/s41586-022-04786-y)
Supplement: Supplementary file 4 — HTMLs of notebooks outlining key statistical analyses presented in the manuscript, including analysis of phylogenetic trees. [file 41586_2022_4786_MOESM4_ESM.zip › Supplementary_code/Other_analysis/loss_of_Y_simulations.html]

Monte Carlo tests for clone size of loss-of-Y


# Monte Carlo tests for clone size of loss-of-Y

#### Peter Campbell

We observe a series of phylogenetic trees from male individuals in which some clades have lost the Y chromosome. By eye, these clades seem to be larger than clades that have not lost Y. To test this formally, we use a randomisation / Monte Carlo test to define the null expected distribution of clade size. For each Monte Carlo iteration, we draw branches of the phylogenetic tree at random - one random branch for each observed instance of Y-loss. These branches are sampled (with replacement) from the set of all extant branches at the matched time-point in that individual, and the eventual clade size of that draw measured. For each simulation, the geometric mean (to allow for the log-normality of observed clade sizes) of clade sizes is calculated. We can then compare the distribution of geometric means from the Monte Carlo draws with the observed geometric mean.

```
suppressMessages(library(ape))
suppressMessages(library(phytools))
suppressMessages(library(phangorn))
suppressMessages(library(lattice))
set.seed(28)
```

```
# Read in trees and plot them
tree_files <- c("KX002_2_ultra.tree", "AX001_4_ultra.tree", "KX003_5_ultra.tree", "KX007_2_ultra.tree", "SX001_5_ultra.tree")
trees <- list()
for (i in tree_files) {
    sample_name <- strsplit(i, "_")[[1]][1]
    trees[[sample_name]] <- read.tree(i)
    plot(trees[[sample_name]], show.tip.label=FALSE, direction = "downwards", main=sample_name)
}
```

```
# Generate list of all branch start and end points in molecular time
samples <- names(trees)
branch_starts_ends <- list()
for (j in samples) {
    curr_tree <- trees[[j]]
    branches <- curr_tree$edge
    branches <- cbind(branches,rep(0, nrow(branches)))
    for (i in 1:nrow(branches)) {
        curr_ancests <- Ancestors(curr_tree, branches[i,2], "all")
        curr_edges <- which(branches[,2] %in% curr_ancests)
        branches[i,3] <- sum(curr_tree$edge.length[curr_edges])
    }
    branches <- cbind(branches, branches[,3] + curr_tree$edge.length)
    branch_starts_ends[[j]] <- branches
}
head(branch_starts_ends[["SX001"]])
```

```
##      [,1] [,2]        [,3]        [,4]
## [1,]  363  364 0.000000000 0.003386608
## [2,]  364  365 0.003386608 0.005657900
## [3,]  365  366 0.005657900 0.006794511
## [4,]  366  367 0.006794511 0.014734775
## [5,]  367  368 0.014734775 0.015318616
## [6,]  368  369 0.015318616 0.017638928
```

```
# Read in loss-of-Y data
branches_lost_Y <- data.frame(Sample = character(), Branch = numeric(), stringsAsFactors = FALSE)
files <- list.files()
files <- files[grepl(pattern = "y_loss_cut.csv", files)]
for (j in samples) {
    y_cn <- read.table(files[grepl(pattern = j, files)][1], header=TRUE, stringsAsFactors = FALSE, sep=",")
    colonies_lost_Y <- y_cn[y_cn$cnY == "loss Y", "id"]
    curr_tree <- trees[[j]]
    
    # For each colony with loss-of-Y, we need to find the most ancestral branch where all descendants have lost Y
    unique_branches <- c()
    accounted_colonies <- c()
    for (k in colonies_lost_Y) {
        if (k %in% accounted_colonies) {next} # Already accounted for
        accounted_colonies <- append(accounted_colonies, k)
        index_colony <- which(curr_tree$tip.label == k)
        index_edge <- which(curr_tree$edge[,2] == index_colony)
        all_y <- TRUE
        while (all_y) {
            # Find all sisters of current node
            curr_node = curr_tree$edge[index_edge,1]
            curr_descendants <- unlist(Descendants(curr_tree, curr_node, type = "tips"))
            curr_desc_names <- curr_tree$tip.label[curr_descendants]
            if (!all(curr_desc_names %in% colonies_lost_Y) | 
                   branch_starts_ends[[j]][index_edge, 3] < 0.1) {
                unique_branches <- append(unique_branches, index_edge)
                all_y <- FALSE
            } else {
                accounted_colonies <- unique(append(accounted_colonies, curr_desc_names))
                index_edge <- which(curr_tree$edge[,2] == curr_node)
            }
        }
    }
    branches_lost_Y <- rbind(branches_lost_Y, data.frame(Sample = rep(j, length(unique_branches)),
                                                        Branch = unique_branches))
}
head(branches_lost_Y)
```

```
##   Sample Branch
## 1  KX002    564
## 2  AX001    453
## 3  AX001    139
## 4  AX001    700
## 5  AX001    456
## 6  AX001    532
```

```
# Function to measure geometric mean of clade size for given set of branches
geom_mean_clades <- function(trees, branches) {
    clade_sizes <- c()
    for (i in 1:nrow(branches)) {
        Size <- length(unlist(Descendants(trees[[branches[i,1]]], trees[[branches[i,1]]]$edge[branches[i,2],2], "tips"))) /
                    length(trees[[branches[i,1]]]$tip.label)
        clade_sizes <- append(clade_sizes, Size)
    }
    return(exp(mean(log(clade_sizes))))
}
obs_geom_mean <- geom_mean_clades(trees, branches_lost_Y)
print(obs_geom_mean)
```

```
## [1] 0.004127494
```

```
# Function to generate random draw of branches
draw_random_branches <- function(branches_lost_Y, branch_starts_ends) {
    random_branches <- c()
    for (i in 1:nrow(branches_lost_Y)) {
        # Find random branch of the same age
        # First take a random age from the range
        curr_samp <- branches_lost_Y[i,1]
        curr_branch <- branches_lost_Y[i,2]
        curr_start_ends <- branch_starts_ends[[curr_samp]]
        curr_start <- max(0.1,curr_start_ends[curr_branch,3])
        curr_end <- curr_start_ends[curr_branch,4]
        rand_age <- runif(1, curr_start, curr_end)
        
        # Now find all branches that span that age and draw one at random
        same_age_branches <- which(curr_start_ends[,3] <= rand_age & 
                                    curr_start_ends[,4] >= rand_age)
        random_branches <- append(random_branches,sample(same_age_branches, 1))
    }
    out <- branches_lost_Y
    out$Branch <- random_branches
    return(out)
}
```

```
iters <- 2000
null_geom_means <- rep(0,iters)
for (i in 1:iters) {
    curr_draw <- draw_random_branches(branches_lost_Y, branch_starts_ends)
    null_geom_means[i] <- geom_mean_clades(trees, curr_draw)
}
```

```
hist(null_geom_means, breaks = 20, probability = TRUE, 
     #xlim=c(min(c(null_geom_means, obs_geom_mean)), max(c(null_geom_means, obs_geom_mean))))
     xlim= c(0.0025, 0.0045),
     xlab = "Null geometric means",
     main = "")
abline(v = obs_geom_mean, col="blue")
```

```
print(sum(null_geom_means > obs_geom_mean) / iters)
```

```
## [1] 0
```
