# Supplementary material for: Clonal dynamics of haematopoiesis across the human lifespan
Source: Nature. 2022 Jun 1;606(7913):343–50. doi: 10.1038/s41586-022-04786-y (PMC9177428; doi:10.1038/s41586-022-04786-y)
Supplement: Supplementary file 4 — HTMLs of notebooks outlining key statistical analyses presented in the manuscript, including analysis of phylogenetic trees. [file 41586_2022_4786_MOESM4_ESM.zip › Supplementary_code/Other_analysis/driver_modelling_parameter_estimates_PPC.html]

Driver modelling parameter estimates


# Driver modelling parameter estimates

#### Emily Mitchell

### Summary

Script to explore the posterior distributions of parameter estimates derived from population modelling incorporating driver mutations.

```
suppressMessages(library(ggplot2))
```

```
data <- read.csv("~/Documents/PhD/Sequencing_results/DNA_seq/XX_summary/driver_modelling/PPC_output/data/Hierarchical_ABC_tol_0.02.csv", stringsAsFactors = FALSE)
```

```
dens_drivers <- density(data[,"number_drivers_per_year"])
dens_rate <- density(data[,"gamma_rate"])
dens_shape <- density(data[,"gamma_shape"])

peak_drivers = dens_drivers$x[which.max(dens_drivers$y)]
peak_rate = dens_rate$x[which.max(dens_rate$y)]
peak_shape = dens_shape$x[which.max(dens_shape$y)]

drivers_result_95CI=quantile(data[,"number_drivers_per_year"],probs=c(0.025,0.975))
rate_result_95CI=quantile(data[,"gamma_rate"],probs=c(0.025,0.975))
shape_result_95CI=quantile(data[,"gamma_shape"],probs=c(0.025,0.975))
```

```
#Print peak values
peak_drivers
```

```
## [1] 197.5005
```

```
#Print peak values
peak_rate
```

```
## [1] 32.99249
```

```
#Print peak values
peak_shape
```

```
## [1] 0.7341109
```

```
#Print 95 CI values
drivers_result_95CI
```

```
##     2.5%    97.5% 
## 112.8587 199.9948
```

```
#Print 95 CI values
rate_result_95CI
```

```
##     2.5%    97.5% 
## 30.43289 39.21962
```

```
#Print 95 CI values
shape_result_95CI
```

```
##      2.5%     97.5% 
## 0.3548763 1.6042894
```

```
ggplot(data, aes(x=number_drivers_per_year)) + geom_density()+ theme_classic()+
    theme(axis.text.x = element_text(size=16), axis.text.y = element_text(size=16),axis.title.x = element_text(size=24),axis.title.y = element_text(size=24))
```

```
ggplot(data, aes(x=gamma_shape)) + geom_density() + theme_classic()+
    theme(axis.text.x = element_text(size=16), axis.text.y = element_text(size=16),axis.title.x = element_text(size=24),axis.title.y = element_text(size=24))
```

```
ggplot(data, aes(x= gamma_rate)) + geom_density() + theme_classic()+
    theme(axis.text.x = element_text(size=16), axis.text.y = element_text(size=16),axis.title.x = element_text(size=24),axis.title.y = element_text(size=24))
```

```
ggplot(data, aes(x= gamma_rate, y = number_drivers_per_year)) + geom_density_2d() + theme_classic()+
    theme(axis.text.x = element_text(size=16), axis.text.y = element_text(size=16),axis.title.x = element_text(size=24),axis.title.y = element_text(size=24))
```

```
ggplot(data, aes(x= gamma_shape, y = number_drivers_per_year)) + geom_density_2d() + theme_classic()+
    theme(axis.text.x = element_text(size=16), axis.text.y = element_text(size=16),axis.title.x = element_text(size=24),axis.title.y = element_text(size=24))
```

```
ggplot(data, aes(x= gamma_shape, y = gamma_rate)) + geom_density_2d() + theme_classic() +
    theme(axis.text.x = element_text(size=16), axis.text.y = element_text(size=16),axis.title.x = element_text(size=24),axis.title.y = element_text(size=24))
```

### Creating plot of gamma distribution with 95CI

```
sel_coeff <- seq(from = 0.05, to = 0.35, by = 0.001)

sel <- matrix(NA, nrow = length(data$number_drivers_per_year), ncol = 301)
colnames(sel) <- sel_coeff

#data <- cbind(data, sel)

for (i in sel_coeff) {
  sel[,as.character(i)] <- dgamma(i, shape = data$gamma_shape, rate = data$gamma_rate)
}
med <- apply(sel, 2, median)
qu <- apply(sel, 2, function(x) quantile(x, probs=c(0.025,0.975)))
plot(names(med), med, pch=19, cex=.2, xlim = c(0.05,0.3), ylab = "Density", xlab = "Fitness effect (%)") #ylim=c(0,max(qu)))
polygon(as.numeric(c(colnames(qu), rev(colnames(qu)))), c(qu[1,], rev(qu[2,])), col="grey", border=NA)
lines(names(med), med)
```

```
#segments(as.numeric(colnames(qu)), qu[1,], as.numeric(colnames(qu)), qu[2,], col="grey", lwd=0.4)
```

```
ggplot(data, aes(x=number_drivers_per_year)) + geom_density() + theme_classic()+
  geom_vline(xintercept = peak_drivers)
```
