# Supplementary material for: Clonal dynamics of haematopoiesis across the human lifespan
Source: Nature. 2022 Jun 1;606(7913):343–50. doi: 10.1038/s41586-022-04786-y (PMC9177428; doi:10.1038/s41586-022-04786-y)
Supplement: Supplementary file 4 — HTMLs of notebooks outlining key statistical analyses presented in the manuscript, including analysis of phylogenetic trees. [file 41586_2022_4786_MOESM4_ESM.zip › Supplementary_code/Other_analysis/lolliplots.html]

Lolliplots


# Lolliplots

#### Margarete Fabre, adapted by Emily Mitchell

Script to make lolliplot figures for variants in DNMT3A, ZNF318 and HIST2H3D.

```
suppressMessages(library(Gviz))
```

```
## Warning: package 'Gviz' was built under R version 4.0.3
```

```
## Warning: package 'S4Vectors' was built under R version 4.0.3
```

```
## Warning: package 'BiocGenerics' was built under R version 4.0.3
```

```
## Warning: package 'IRanges' was built under R version 4.0.3
```

```
## Warning: package 'GenomicRanges' was built under R version 4.0.3
```

```
## Warning: package 'GenomeInfoDb' was built under R version 4.0.3
```

```
suppressMessages(library(rtracklayer))
```

```
## Warning: package 'rtracklayer' was built under R version 4.0.3
```

```
suppressMessages(library(trackViewer))
```

```
## Warning: package 'trackViewer' was built under R version 4.0.3
```

```
suppressMessages(library(dplyr))
```

```
variants <- read.csv("~/Documents/PhD/Sequencing_results/DNA_seq/XX_Summary/lolliplots/lolliplot_variants.csv", stringsAsFactors = F)
variants$score1 <- 1
variants$color <- as.character(variants$color)
```

```
domains <- read.table("~/Documents/PhD/Sequencing_results/DNA_seq/XX_Summary/lolliplots/domains.txt", stringsAsFactors = F, header = T)
```

```
df = variants %>% filter(gene == "DNMT3A")
  sample.gr <- GRanges("DNMT3A", IRanges(df$pos, width=1))
  sample.gr$color <- df$color
  sample.gr$score <- as.numeric(df$score1)
  sample.gr$SNPsideID <- df$SNPsideID
 
  df2 = domains %>% filter(gene=="DNMT3A")
  features <- GRanges("DNMT3A", IRanges(df2$start, width=df2$length, names=df2$description))
  features$fill <- df2$fillColour
  features$height <- df2$fillHeight
 
  sample.gr.labelSize <- sample.gr
  sample.gr.labelSize$label.parameter.gp <- gpar(cex=1.4)
 
  xaxis <- c(1, max(df2$stop))
  yaxis <- c(0, max(df$score))

lolliplot(sample.gr.labelSize, features, xaxis=xaxis, yaxis=yaxis, ylab="Recurrence", dashline.col="white", cex=1)
```

```
df = variants %>% filter(gene == "ZNF318")
  sample.gr <- GRanges("ZNF318", IRanges(df$pos, width=1))
  sample.gr$color <- df$color
  sample.gr$score <- as.numeric(df$score1)
  sample.gr$SNPsideID <- df$SNPsideID
 
  df2 = domains %>% filter(gene=="ZNF318")
  features <- GRanges("ZNF318", IRanges(df2$start, width=df2$length, names=df2$description))
  features$fill <- df2$fillColour
  features$height <- df2$fillHeight
 
  sample.gr.labelSize <- sample.gr
  sample.gr.labelSize$label.parameter.gp <- gpar(cex=1.4)
 
  xaxis <- c(1, max(df2$stop))
  yaxis <- c(0, max(df$score))

lolliplot(sample.gr.labelSize, features, xaxis=xaxis, yaxis=yaxis, ylab="Recurrence", dashline.col="white", cex=1)
```

```
df = variants %>% filter(gene == "HIST2H3D")
  sample.gr <- GRanges("HIST2H3D", IRanges(df$pos, width=1))
  sample.gr$color <- df$color
  sample.gr$score <- as.numeric(df$score1)
  sample.gr$SNPsideID <- df$SNPsideID
 
  df2 = domains %>% filter(gene=="HIST2H3D")
  features <- GRanges("HIST2H3D", IRanges(df2$start, width=df2$length, names=df2$description))
  features$fill <- df2$fillColour
  features$height <- df2$fillHeight
 
  sample.gr.labelSize <- sample.gr
  sample.gr.labelSize$label.parameter.gp <- gpar(cex=1.4)
 
  xaxis <- c(1, max(df2$stop))
  yaxis <- c(0, max(df$score))

lolliplot(sample.gr.labelSize, features, xaxis=xaxis, yaxis=yaxis, ylab="Recurrence", dashline.col="white", cex=1)
```
