# Supplementary material for: Clonal dynamics of haematopoiesis across the human lifespan
Source: Nature. 2022 Jun 1;606(7913):343–50. doi: 10.1038/s41586-022-04786-y (PMC9177428; doi:10.1038/s41586-022-04786-y)
Supplement: Supplementary file 4 — HTMLs of notebooks outlining key statistical analyses presented in the manuscript, including analysis of phylogenetic trees. [file 41586_2022_4786_MOESM4_ESM.zip › Supplementary_code/Other_analysis/estimating_N.html]

Estimating N (HSC population size)


# Estimating N (HSC population size)

#### Emily Mitchell

Script to estimate the HSC population size based on parameter estimates from our data and previously published work.

```
N.sim=vector(mode = "list",length=100000)
for (i in 1:100000) {
tel_loss_bp_per_year <- runif(1,30,40) # range covering estimated HSC telomere loss per year from our own data (30 bp) and granulocyte telomere loss per year across many individuals (38 bp) from published data
tel_loss_division <- runif(1,30,100) # range of published HSC telomere loss in bp per cell division
symm_division_rate <- runif(1,0.8,1) # proportion of cell divisions that are symmetric
cell_division <- tel_loss_bp_per_year/tel_loss_division # number cell divisions per year
symm_division <- cell_division*symm_division_rate # number symmetric cell divsions per year
mean_generation_time <- 1/symm_division
Nt <- runif(1, 50000, 250000) # HSC Nt as estimated from our dataset
N <- Nt / mean_generation_time
N.sim[i] <- N
}
```

```
N.sim <- as.numeric(N.sim)
N.sim_95CI=quantile(N.sim,probs=c(0.025,0.975))
N.sim_95CI
```

```
##      2.5%     97.5% 
##  22363.54 195274.22
```

```
mean(N.sim)
```

```
## [1] 81100.22
```
